# RBAD: The first database dedicated alterations of blood RNA in individuals with Alzheimer’s disease and their clinical relevance

**DOI:** 10.4103/NRR.NRR-D-24-01165

**Published:** 2025-03-25

**Authors:** Tingting Duan, Jinyu Chu, Jinquan Li, Shiyao Pan, Dan Liu, Guirong Cheng, Yu Luo, Wen Zhou, Zhiming Wang, Wei Tan, Qiong Wu, Yan Zeng, Feifei Hu

**Affiliations:** 1 Department of Internal Medicine, Wuhan Asia General Hospital, Wuhan University of Science and Technology, Wuhan, Hubei Province, China; 2 Brain Science and Advanced Technology Institute, School of Medicine, Wuhan University of Science and Technology, Wuhan, Hubei Province, China; 3 School of Public Health, Wuhan University of Science and Technology, Wuhan, Hubei Province, China; 4 Community Health Service Center, Geriatric Hospital, Wuhan University of Science and Technology, Wuhan, Hubei Province, China

**Keywords:** Alzheimer’s disease, biomarker, blood, clinical relevance, erythroid cells, *GZMK*^+^ *CD8*^+^ T cells, high throughput sequencing, mild cognitive impairment, olfactory transduction, RNA

## Abstract

Alzheimer’s disease-associated transcriptomic landscapes have been defined in brain tissue. However, changes in blood RNA and their clinical relevance remain poorly understood. In this study, we developed an RNA profile based on 1468 blood samples from both human and mouse studies, which include bulk RNA sequencing (RNA-seq), microRNA-seq, and single-cell RNA-seq data. We developed a comprehensive analysis pipeline that conducted over 11 million comparisons and correlations to identify more than 20,000 blood features. With these findings, we established a blood RNA database related to Alzheimer’s disease, RNAs in Blood of AD (RBAD, http://www.bioinform.cn/RBAD/). Using RBAD, we initially validated well-established Alzheimer’s disease-related pathways, including olfactory transduction. We then observed a decrease in both the proportion and functionality of erythroid cells, likely attributed to their elevated *CD45* levels and interactions with *GZMK*^+^ *CD8*^+^ T cells. Furthermore, we identified 449 blood RNAs linked to patients’ overall survival, along with two mRNAs (*H4C3* and *CTU1*) associated with cognitive decline. In summary, RBAD is the first web-based analysis platform dedicated to investigating blood RNA changes in Alzheimer’s disease, and provides valuable insights into potential peripheral biomarkers and pathogenic mechanisms related to Alzheimer’s disease.

## Graphical abstract

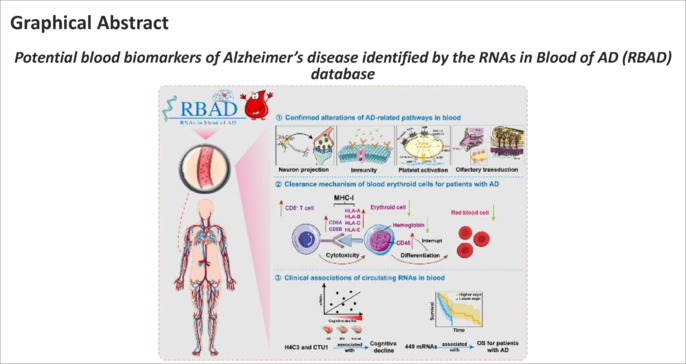

## Introduction

With the rapidly growing aging population, the incidence of Alzheimer’s disease (AD) is predicted to increase. AD currently accounts for 60%–80% of all dementia cases (No authors listed, 2023) and imposes a heavy burden on patients, their families, caregivers, and society as a whole (Scheltens et al., 2016). Early detection of individuals at high risk of developing AD enables effective intervention. To achieve this, cerebrospinal fluid and imaging biomarkers corresponding to the natural pathological course of AD are essential (Teunissen et al., 2022; Lyu et al., 2025). However, the high cost, invasiveness, and shortage of qualified professionals limit the use of such biomarkers in large-scale populations. Various peripheral AD biomarkers, including P-tau181 (Thijssen et al., 2020; Zhao et al., 2025), P-tau217 (Palmqvist et al., 2020), P-tau231 (Mielke et al., 2021), amyloid-beta (Aβ)42 (Ashton et al., 2023; Abyadeh et al., 2024), Aβ_40_/Aβ_42_ ratio (Vergallo et al., 2019), and neurofilament light-chain protein, have demonstrated diagnostic utility (Nakamura et al., 2018; Schindler et al., 2019; Keshavan et al., 2021). However, these biomarkers remain in the preliminary stages of development and are not yet ready for clinical implementation, particularly for large-scale screening.

Blood-based circulating nucleic acids, especially cell-free RNA or RNA from peripheral blood mononuclear cells (PBMCs), represent alternative markers for the noninvasive monitoring of organ-specific dynamic pathological changes (Koh et al., 2014; Munchel et al., 2020; Shigemizu et al., 2020; Toden et al., 2020; Larson et al., 2021) and are particularly useful in diagnosing neurodegeneration (Shigemizu et al., 2020; Toden et al., 2020). The various transcripts provide multidimensional insights regarding gene expression, alternative splicing, genetic mutation, and epigenetic modifications (Gimelbrant et al., 2007), making RNAs more informative markers than proteins. Indeed, several studies have reported the potential of blood RNAs in distinguishing patients with AD from control individuals (Leidinger et al., 2013; Feng et al., 2018; Shigemizu et al., 2020; Toden et al., 2020). Tan et al. (2017) reported higher levels of TREM2 mRNA in the blood of patients with AD than in controls. Furthermore, Toden et al. (2020) selected 1658 differentially expressed genes (DEGs) in blood to construct an AD classifier with an area under the curve of 0.83. Blood microRNAs (miRNAs) have also been associated with cognitive decline (Satoh et al., 2015; Islam et al., 2021), demonstrating relatively high areas under the curve for distinguishing AD from its prodromal stage, mild cognitive impairment (MCI) (Yang et al., 2018).

Despite this recent progress, gaps remain in understanding blood RNA characteristics in AD and MCI. In particular, reanalysis, evaluation, and comparison of relevant studies are limited. Although existing studies provide insights into blood RNAs in AD, the processing and data analysis pipelines are distinct between studies. Notably, most data are confidential and cannot be accessed or shared without proper authorization (Shigemizu et al., 2020; Wu et al., 2020). Moreover, the available evidence is typically one-sided, originating from a single category of high-throughput sequencing data (Shigemizu et al., 2020; Toden et al., 2020), including bulk RNA sequencing (RNA-seq) (Toden et al., 2020), miRNA-seq (Satoh et al., 2015; Islam et al., 2021), or single-cell RNA (scRNA)-seq (Gate et al., 2020) data. Considering the heterogeneity of AD, the lack of comprehensive clinical features presents another major hurdle in existing studies. Previous studies also lack stratified analyses that consider variations in blood RNA regarding patient sex, age, apolipoprotein E (*ApoE*) genotype, and overall survival (OS). Additionally, although the widespread use and high demand for sequencing technology have accelerated the emergence of multidomain databases, there is a notable dearth of tools and databases specifically designed to identify blood RNA characteristics in AD and MCI. The SCAD-Brain (Li et al., 2023), SC2Disease (Zhao et al., 2021), ssREAD (Wang et al., 2024), and The Alzheimer’s Cell Atlas (Zhao et al., 2021) databases have helped enhance scRNA investigations in brain tissue and advance the understanding of AD, and the Variant Effect Predictor for AD (Rangaswamy et al., 2020) and Alz-Disc (Kulandaisamy et al., 2023) tools help discriminate neutral AD-related mutations. However, these databases and platforms do not include blood-based RNAs. Hence, an urgent need exists to integrate multisource studies on blood RNAs into a widely accessible database using a unified workflow and clinically stratified reports. Such a tool would facilitate the exploration and identification of peripheral AD biomarkers for the early diagnosis and characterization of pathogenic mechanisms while informing equitable policy and strategy development for precision diagnosis and prognosis.

Thus, in the present study, we constructed the RNAs in Blood of AD (RBAD) repository to provide a centralized database comprising AD-associated peripheral biomarkers and features related to AD development.

## Methods

### Data collection

Public blood RNA-seq data (bulk RNA-seq, miRNA-seq, and scRNA-seq) from patients with AD or MCI and healthy individuals were collected from the Gene Expression Omnibus (GEO: https://www.ncbi.nlm.nih.gov/geo/) and AD Knowledge Portal–a repository for multiomics data on AD and aging–before September 2023. The patients involved in the GEO and AD Knowledge Portal database have obtained ethical approval. Our study is based on these open source data, so there are no ethical issues. The samples comprised whole blood, plasma, serum, and PBMCs. “Alzheimer’s disease,” “mild cognitive impairment,” and “RNA-seq” keywords from the Medical Subject Headings database were used to build a search formula for GEO dataset searching. The information for each sample was manually confirmed according to the metadata and related published studies. Although different diagnostic criteria were used across studies, all of the studies used standard evaluation methods (**Additional Table 1**). Irrelevant samples (e.g., ambiguous disease status) were discarded. In total, 12 datasets with 1426 human and 42 mouse samples were collected (**Additional Table 2**).

**Additional Table 1:** Diagnostic criteria of AD, MCI, and control in each data set or cohort that has been collected in RBAD

Dataset	RNA type	AD	MCI	Control	Cohort
MCSA	mRNA		(1) Cognition concern by the participant, informant, coordinator, or physician; (2) Impairment in at least one neuropsychological domain; (3) Essentially normal functional activities, as determined by the Clinical Dementia Rating (CDR) and the Functional Activities Questionnaire; (4) Absence of dementia, diagnosed using the Diagnostic and Statistical Manual of Mental Disorders, Fourth Edition	Not meeting the above criteria for MCI; Not diagnosed with dementia.	MCSA
ACOM	mRNA	National Institute of Neurological and Communicative Disorders and Stroke (NINCDS) and the Alzheimer’s Disease and Related Disorders Association (ADRDA): Meet clinical criteria for probable or possible AD dementia, and the neuropathological examination, using widely accepted criteria, demonstrates the presence of the AD		Not meeting the above criteria for AD; Not diagnosed with dementia.	ACOM
Emory	mRNA		Subjective memory concerns; Montreal Cognitive Assessment (MoCA) <26; CDR = 0.5; Functional Assessment Questionnaire (FAQ) <9.	MoCA >26; CDR = 0.	Emory
ROSMAP	mRNA	A clinical diagnosis of cognitive status is rendered at every assessment based on a three-stage process including: (1) Cognitive Testing: Participants complete a standardized cognitive test battery, scored by computer using a decision tree to assess impairment levels across five cognitive domains. (2) Neuropsychologist's Judgment: A neuropsychologist, blinded to demographic information, reviews the test scores and clinical data to assess the presence of cognitive impairment and dementia. (3) Clinician's Diagnostic Classification: A clinician (such as a neurologist, geriatrician, or geriatric nurse practitioner) examines the participant and all data to provide a final diagnosis.	Final consensus cognitive diagnosis value is 2 (MCI (One impaired domain) and no other cause of CI) or 3 (MCI (One impaired domain) and another cause of CI). If the final consensus cognitive diagnosis value is NA, clinical diagnosis of cognitive status is 2 (MCI and no other condition contributing to CI) or 3 (MCI and another condition contributing to CI).	Final consensus cognitive diagnosis value is 1 (No cognitive impairment (No impaired domains)). If the final consensus cognitive diagnosis value is NA, clinical diagnosis of cognitive status is 1 (NCI: No cognitive impairment).	ROSMAP
		Final consensus cognitive diagnosis value is 4 (AD and no other cause of CI (NINCDS probable AD)) or 5 (Alzheimer ’s dementia and another cause of CI (NINCDS possible			
SRP310421	mRNA	Participants diagnosed with Late-Onset Alzheimer's Disease (LOAD) were identified through neurology specialists and recruited according to the Global Deterioration Scale (GDS) and CDR criteria	—	Not have any cognitive or behavioral changes attributable to psychiatric or neurological disorders.	
SRP223445	mRNA	National Alzheimer Coordinating Center (NACC): The diagnosis involved multiple neuropsychological tests, including the MMSE, MoCA, animal naming tests, and Craft Story Recall tests		No clinical signs of cognitive decline or dementia; Normal performance in neuropsychological testing battery.	
SRP330776	scRNA	Meet National Institute on Aging and the Alzheimer’s Association (NIA-AA) probable AD criteria and have positive amyloid PET; Mini_-_menta1 state examination (MMSE) < 26'		Not meeting the above criteria for AD; MMSE > 26; CDR = 0.	
SRP309935	scRNA	Meets the NINCDS-ADRDA and DSM-IV criteria for AD; Brain imaging (CT or MRI) consistent with a diagnosis of AD.	_	Not meeting the above criteria for AD.	
SRP215507	scRNA	Study subjects underwent a battery of neuropsychological assessments to determine group status, including: cognitive examination, evaluation of cerebellar function, deep tendon reflexes, sensory input, and motor function. The MoCA examination was used to test study subjects for cognitive impairment. The MoCA assesses several cognitive domains: short-term memory recall (5 points), visuospatial abilities (4), executive functions (4), attention (1), concentration (3), working memory (1), language (6) and orientation to time and space (6). MoCA < 26.	MoCA < 26.	MoCA > 26.	Stanford Alzheimer’s Disease Research Center (ADRC)
SRP022043	miRNA	Patients fulfilled the following criteria of the NINCDS-ADRDA: MMSE > 14 and < 26; Cognitive deficits in multiple areas; Progressive memory and cognitive decline; No loss of consciousness; No systemic or brain diseases causing cognitive decline.	MMSE > 22 and < 28; Not demented, with memory complaints.	No cognitive impairments or neurological disorders. Normal cognitive test results, free from diseases affecting cognition.	
SRP325058	mRNA	3xTg-AD mice		C57BL/6 wild type (WT) mice	
SRP268520	miRNA			C57BL/6 WT mice	
SRP268687	miRNA			C57BL/6 WT mice	

**Additional Table 2:** Characteristics of samples collected in RBAD

Data type	Organism	Characteristics	Sample group			
			ALL	AD	MCT	Control
**Bulk RNA-seq**	**Human**	**Sample size**	1332	205	280	847
		**Sex (N=1332)**				
		Male	494	62	111	321
		Female	838	143	169	526
		**Age [ mean ± SD (range)]**				
		Age at last follow up (N=365)	82.5±5.6 (65.3-90.0)	84.3±4.5 (69.7-89.7)	85.0±3.0 (75.2-89.6)	81.3±6.1 (65.3-90.0)
		Age at diagnosis (N=71)	84.2±4.8 (71.0-89.9)	84.5±4.8 (71.0-89.9)	84.0±4.8 (76.7-89.3)	_-_
		Age at sampling (N=1185)	72.3±10.6 (50-95)	80.3±8.1 (54-94)	73.9±10.5 (50-90)	70.4±10.3 (50.7-95)
		**ApoE genotype (N=1028)**				
		E2E2	7	0	3	4
		E2E3	130	10	16	104
		E2E4	35	5	5	25
		E3E3	595	89	112	394
		E3E4	235	42	41	152
		E4E4	26	9	1	16
		**MMSE [mean ± SD (range)]**				
		MMSE at last follow up (N=608)	24.0±7.6 (0-30)	13.4±8.0 (0-28)	25.7±3.0 (13-30)	28.2±1.9 (14-30)
		MMSE at diagnosis (N=126)	20.1±5.2 (5-30)	19.9±5.2 (5-30)	22.6±4.5 (15-28)	-
	**Mouse**	**Sample size**	42	27	0	15
		Age (months) [ mean ± SD (range)]	5.6±1.0 (3-6)	5.7±0.9 (3-6)	-	5.4±1.2 (3-6)
**miRNA-seq**	**Human**	**Sample size**	70	48	0	22
		**Sex**				
		Male	34	22	0	12
		Female	36	26	0	10
		**Age (years) [ mean ± SD (range)]**				
		Age at sampling (N=70)	69.3±7.8 (51-83)	69.43±7.9 (51-83)	-	69.0±7.6 (59-81)
**scRNA-seq**	**Human**	**Sample size**	24	11	2	11
		**Age (years) [mean ± SD (range)]**				
		Ase at samplins (N=74)	72.8±8.1 (60-90)	74.1±9.2 (60-90)	-	70.5±4.8 (65-77)

### Data preprocessing

For bulk RNA-seq data processing, Trim Galore (Altos Labs, Cambridge, UK) and Trimmomatic (version 0.39, The Usadel lab, Düsseldorf, Germany) (Bolger et al., 2014) with default parameters were used to remove adapter sequences and trim reads from the TruSeq-based and Smart-seq2-based libraries, respectively. FastQC (v0.11.9, Babraham Bioinformatics, Babraham, UK) was used for quality control. Hisat2 (v2.2.1, Johns Hopkins University, Baltimore, USA) (Kim et al., 2019) aligned clean reads to reference genomes. HTseq-count and featureCounts (Liao et al., 2014) were used to generate the expression matrix for TruSeq-based and Smart-seq2-based bulk RNA-seq data, respectively. Gene expression levels were normalized using the transcripts per million (TPM) method. For single-cell data, CellRanger software (v.6.1.2, 10x Genomics, Pleasanton, CA, USA) was used to acquire gene counts by aligning reads to the reference genome (GRCh38 for humans and GRCm39 for mice).

For clinical data, we excluded samples lacking information on gender, age, education, *ApoE* genotype, Mini-Mental State Examination (MMSE) score, and Braak stage in the following analyses: survival analysis; gene expression differential analysis by sex, *ApoE* genotype, or Braak stage; and correlation analyses between gene expression and age, MMSE score, and education years. When age was treated as a covariate, missing values were imputed with the median. For race and *ApoE* genotype, missing values were categorized separately.

### Gene expression analysis

Several R packages were used to perform gene expression analyses. For RNA-seq data, DESeq2 (Love et al., 2014) was used to compute DEGs according to a false discovery rate (FDR) ≤ 0.05 and fold change (FC) > 1.5. Datasets with available sex, age, race, and *ApoE* genotype were included as covariates in differential gene expression analysis to control for potential confounding factors. The sva (Leek et al., 2012) package was used to remove batch effects across projects and to perform data integration (**Additional Figure 1**). The expression trend cluster of mRNA from control to MCI to AD was obtained by Fuzzy C-Means (FCM) clustering with Mfuzz (Kumar and Futschik, 2007) package. The Limma package (Ritchie et al., 2015) was used to analyze plasma proteome data from Hubei Memory & Aging Cohort Study (HMACS) (ChiCTR, www.chictr.org.cn; Registration number: ChiCTR1800019164), which was approved by the ethics committee at Wuhan University of Science and Technology (protocol code: 201845; approved on October 22, 2018), Wuhan, China. Written informed consents were obtained from all participants, or in the case of cognitively impaired persons, from a proxy (usually a guardian or a family member), and were kept in the HMACS study administrative office at Wuhan University of Science and Technology. The Genotype-Tissue Expression Project (GTEx) and Human Protein Atlas (HPA) databases were used to verify the tissue and cell-type specificity of genes.

The Seurat (Hao et al., 2021) package was used to perform cell quality control, clustering, and differential scRNA-seq expression analysis. Highly variable genes were identified by mean expression and gene variance. Principal component analysis and t-distributed stochastic neighbor embedding were applied using highly variable genes to reduce dimensionality. Cell clusters were identified by a shared nearest-neighbor modularity optimization method based on the original Louvain algorithm. Cell types (CTs) were then annotated by cross-validating the CT-specifically expressed genes, pre-existing cell annotations from published studies, and known cell markers from the CellMarker databases (Hu et al., 2023). The CellChat (Jin et al., 2021) R package was used for cell communication analysis. ClusterProfiler (Yu et al., 2012; Wu et al., 2021) and DOSE (Yu et al., 2015) were used to perform Gene Set Enrichment Analysis (GSEA) based on the Kyoto Encyclopedia of Genes and Genomes (KEGG) (Kanehisa and Goto, 2000) and Gene Ontology (GO) (Gene Ontology Consortium, 2015) databases. The GO database includes molecular function, biological process, and cellular component terms.

For differentially expressed miRNAs, pathway enrichment analyses, including over-representation analysis, were performed using miRPath (Vlachos et al., 2015) and miEAA (Kern et al., 2020). miRPath annotated miRNAs indirectly by deriving GO and pathway terms from the target genes of the miRNAs. miEAA compiled enriched terms from 19 data sources: GO, NPInter, miRTarBase, miRWalk, miRPathDB, miRBase, Mammal ncRNA-Disease Repository, TransmiR, KEGG, Human MicroRNA Disease Database, HumanCellularmiRNome, SM2miR, miRGeneDB, curated literature, the tool for annotations of human miRNAs, RNALocate, Tissue Atlas, miRandola, and isomiRdb. The ImmuCellAI (Miao et al., 2020) tool was used to estimate the immune cell abundances based on the mRNA expression of immune cell markers. CATT (Chen et al., 2020) software and the immunarch (Nazarov et al., 2022) R package predicted T-cell receptors using RNA-seq data. For additional details on the processing methods, please refer to the folded “help” page in each result panel on the RBAD website (http://www.bioinform.cn/RBAD/).

### Clinical association analysis

Gene expression and clinical data were merged by sample ID for correlation and group comparison analyses. Clinical data included age, sex, race, OS, *ApoE* genotype, educational background, and cognitive function as determined by MMSE (**Additional Table 2**). To reduce confounding effects, partial correlation analysis was performed using Spearman’s method to calculate the correlation between gene expression and continuous clinical features, by adjusting for available covariates, including gender, age, race, or *ApoE* genotype. The two-sided nonparametric Wilcoxon test was used to evaluate differences in blood RNA expression levels between sex and *ApoE* genotype groups.

Survival analysis was conducted solely for the ROSMAP dataset, which includes follow-up time information. The healthy individuals and patients with unclear follow-up times were excluded. The OS time was calculated as the difference between the age at AD diagnosis and the age at the last follow-up or age at death. To identify blood RNAs associated with OS, the samples were grouped based on the median expression level of each RNA. The single-variable survival difference between groups was evaluated by the log-rank test. The Cox proportional hazard regression method was used to screen blood RNAs with expression associated with OS independent of age, sex, *ApoE* genotype, and education years. The Kaplan–Meier method was used to draw survival curves. To identify risk or protective genes for OS after AD diagnosis, univariate and multivariate Cox proportional hazard regression models were used, which provided the hazard ratio of the higher expression group with the lower expression group as the reference. Genes with a hazard ratio > 1 were considered risk genes, whereas those with a hazard ratio < 1 were regarded as protective genes.

### Statistical analysis

The Pearson chi-square test was used to analyze differences in blood CTs between AD patients and healthy controls. Partial correlation analysis was performed using Spearman’s method to assess the associations between blood RNAs and continuous variables (age, education, and MMSE score). The two-sided nonparametric Wilcoxon test was used to evaluate differences in blood RNA expression levels between categorical variables. All analyses were performed for AD, MCI, control, and total samples. *P*-values in statistical analyses were adjusted for multiple comparisons using the FDR method. A difference was considered significant when FDR < 0.05. All statistical analysis results were calculated by R.

### Web-based analysis platform construction

The user interface and back-end of RBAD were written in the R/shiny (Chang et al., 2022) framework. The document-oriented database MongoDB was used to store the RBAD data. Result visualization was primarily performed using the DT (Xie et al., 2022) and ggplot2 (Wickham, 2016) R packages to provide publication-quality figures. The URLs of databases, software, and R packages can be found in **Additional Table 3**.

**Additional Table 3:** The URLs of databases, softwares, and R packages

Database/R package	URLs
SCAD-Brain	https://www.bioinform.cn/SCAD-Brain/
SC2Disease	http://www.easybioai.com/sc2disease/
ssREAD	https://bmblx.bmi.osumc.edu/ssread/
TACA	https://taca.lerner.ccf.org/
VEPAD	http://web.iitm.ac.in/bioinfo2/vepad/
Alz-Disc	https://web.iitm.ac.in/bioinfo2/alzdisc/
GTEx	https://www.gtexportal.org/
HPA	https://www.proteinatlas.org/
GEO	https://www.ncbi.nlm.nih.gov/geo/
AD Knowledge Portal	https://adknowledgeportal.synapse.org/
FastQC	https://www.bioinformatics.babraham.ac.uk/projects/fastqc/
Hisat2	http://daehwankimlab.github.io/hisat2/
DESeq2	https://bioconductor.org/packages/release/bioc/html/DESeq2.html
sva	https://bioconductor.org/packages/release/bioc/html/sva.html
Limma	https://bioconductor.org/packages/release/bioc/html/limma.html
Seurat	https://satij alab.org/seurat/
CellMarker	http://117.50.127.228/CellMarker/
CellChat	https://github.com/jinworks/CellChat
ClusterProfiler	https://bioconductor.org/packages/release/bioc/html/clusterProfiler.html
DOSE	https://bioconductor.org/packages/release/bioc/html/DOSE.html
miRPath	https://dianalab.e-ce.uth.gr/html/mirpathv3/index.php?R=mirpath
miEAA	https://ccb-compute2.cs.uni-saarland.de/mieaa2/
GO	http://geneontology.org/
NPInter	http://bigdata.ibp.ac.cn/npinter4/
miRTarBase	https://mirtarbase.cuhk.edu.cn/~miRTarBase/miRTarBase_2019/php/index.php
miRWalk	http://mirwalk.umm.uni-heidelberg.de/
miRPathDB	https://mpd.bioinf.uni-sb.de/
miRBase	https://www.mirbase.org/
MNDR	http://www.rna-society.org/mndr/
TransmiR	http://www.cuilab.cn/transmir
KEGG	https://www.genome.jp/kegg/
HMDD	http://www.cuilab.cn/hmdd
HumanCellularmiRNome	https://genome.ucsc.edu/cgi-bin/hgHubConnect
SM2miR	http://www.jianglab.cn/SM2miR/
miRGeneDB	https://www.mirgenedb.org/
TAM	http://www.lirmed.com/tam2/
RNALocate	http://www.rna-society.org/rnalocate/
TissueAtlas	https://www.ccb.uni-saarland.de/tissueatlas/
miRandola	http://mirandola.iit.cnr.it/
isomiRdb	https://ccb-compute.cs.uni-saarland.de/isomirdb
ImmuCellAI	https://guolab.wchscu.cn/ImmuCellAI/#!/
immunarch	https://immunarch.com/
DT	https://rstudio.github.io/DT/
ggplot2	https://ggplot2.tidyverse.org/

## Results

### Overview of RBAD

**Figure 1:**
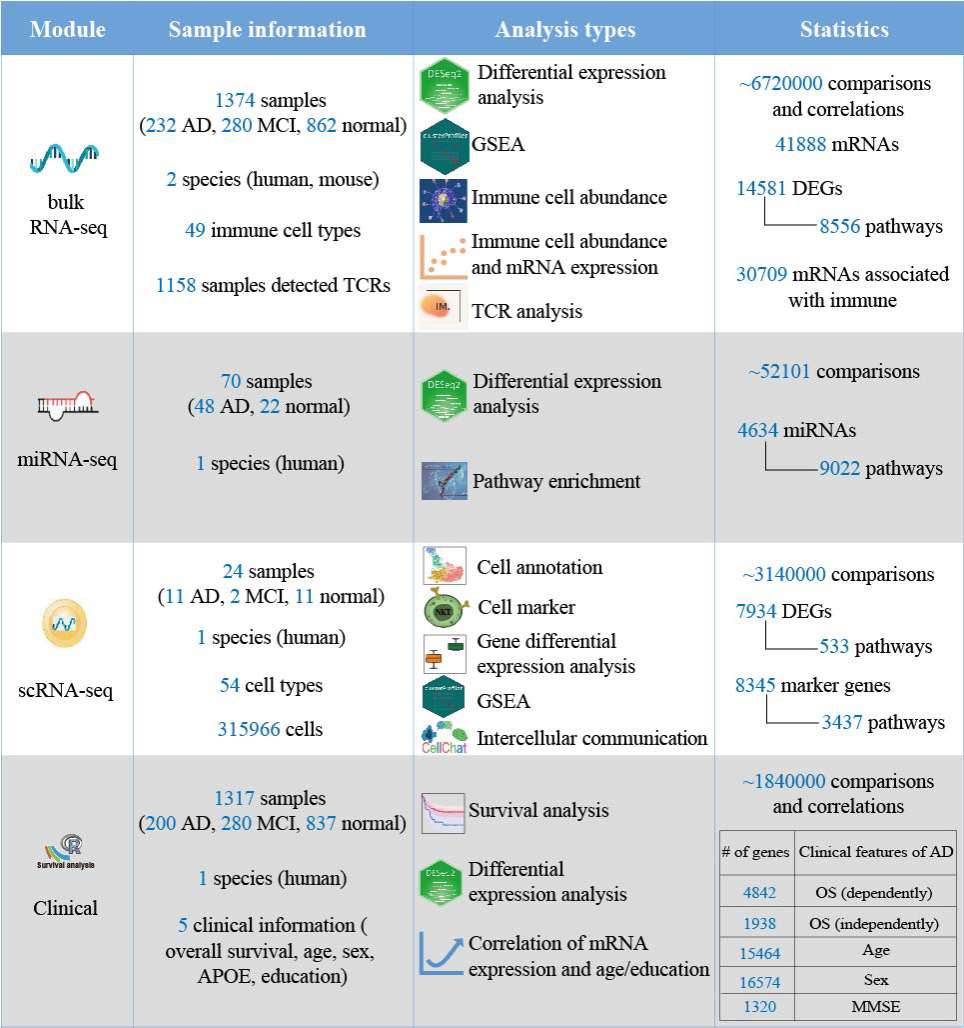
An overview of data and analysis types provided by RBAD. The sample information, analysis types, and statistical information of the bulk RNA-seq, miRNA-seq, scRNA-seq, and Clinical modules. AD: Alzheimer’s disease; *APOE*: apolipoprotein E; DEG: differentially expressed gene; GSEA: gene set enrichment analysis; MCI: mild cognitive impairment; miRNA: microRNA; MMSE: Mini-Mental State Examination; OS: overall survival; RBAD: RNAs in Blood of AD, http://www.bioinform.cn/RBAD/; RNA-seq: RNA sequence; scRNA: single-cell RNA; TCR: T cell receptors.

RBAD comprises blood RNA characteristics associated with AD extracted from 1374 bulk RNA-seq, 70 miRNA-seq, and 24 scRNA-seq samples. It provides pertinent details for human-based datasets as much as possible, specifically age, sex, race, *ApoE* status, diagnosis, MMSE score, Braak stages, and survival times. Using a uniform pipeline, approximately 11.2 million comparisons and correlations were conducted, enabling a comprehensive analysis of RNA in blood samples related to AD. Differential expression and pathway enrichment analyses were performed across the three sequencing data types. The immune cell abundance, T-cell receptor, and clinical association analyses were conducted for bulk RNA-seq data, whereas cell marker, cell annotation, and intercellular communication analyses were designed for scRNA-seq data. Together, over 20,000 disease-associated DEGs, 17,000 pathways, and 22,000 clinical characteristic-associated genes were detected (**Figure 1** and **Additional Tables 4–6**). For detailed operational procedures, please refer to the Supplementary Information section (**Additional Figure 2** and **Additional File 1**) and the online database.

**Figure 2:**
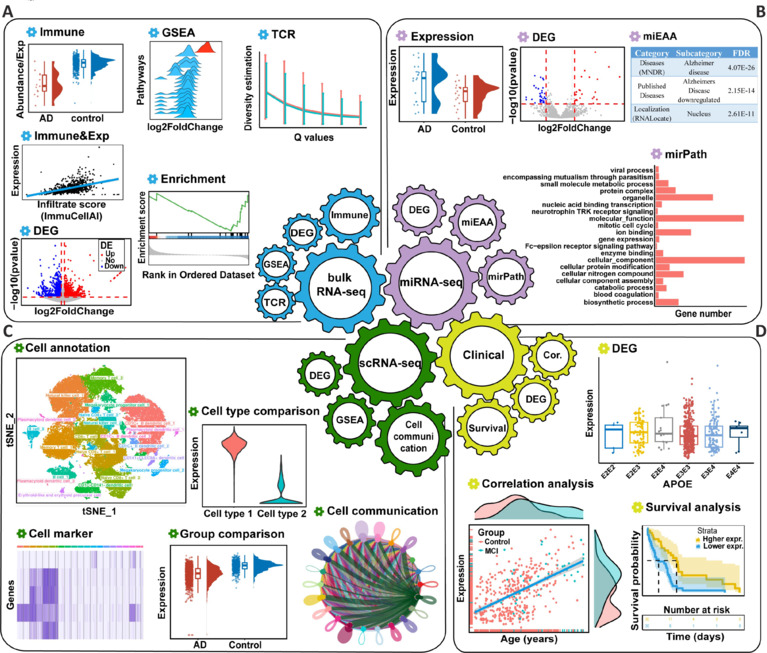
The architecture of RBAD. (A–D) Representative results provided by the bulk RNA-seq module (A), miRNA-seq module (B), scRNA-seq module (C), and clinical module (D). AD: Alzheimer’s disease; *APOE*: apolipoprotein E; DEG: differentially expressed gene; GSEA: gene set enrichment analysis; miRNA: microRNA; RBAD: RNAs in blood of AD, http://www.bioinform.cn/RBAD/; RNA-seq: RNA sequence; scRNA: single-cell RNA; TCR: T cell receptors.

RBAD provides four panels: bulk RNA-seq, miRNA-seq, scRNA-seq, and Clinical. The bulk RNA-seq panel comprises five modules (**Figure 2A**): (1) DEG, which identifies DEGs between AD, MCI, and control groups; (2) GSEA, which offers pathway enrichment analysis for DEGs; (3) Immune abundance, which presents the abundance data of 24 immune CTs and their differences between disease groups; (4) Immune and Expression, which explores correlations between gene expression and immune cell abundance; and (5) T-cell receptor, which compares the CDR3 and V(D)J genes between disease groups. The miRNA-seq panel contains three modules (**Figure 2B**): (1) DEmiRNA, which identifies differentially expressed miRNAs between the AD and control groups; (2) Pathway enrichment by miEAA (Kern et al., 2020); and (3) Pathway enrichment by miRPath (Vlachos et al., 2015). The scRNA-seq panel has five modules with popular visual analytic tools (**Figure 2C**): (1) Cell annotation, for CT annotation; (2) Cell marker, which identifies the CT-specific expressed genes and performs GSEA; (3) Comparison between CTs, which calculates DEGs between any two CTs and performs GSEA; (4) DEG, which calculates the DEGs for each CT in AD, MCI, and control samples and performs GSEA; and (5) Cell communication, which estimates the strength and number of interactions between CTs. The Clinical panel assesses the association between gene expression and clinical characteristics, presented in three modules (**Figure 2D**): (1) Survival analysis, which compares OS between groups defined by median gene expression; (2) Difference analysis, which compares gene expression across categorical variables, such as sex, *ApoE* genotypes, and Braak stages; and (3) Correlation analysis, which calculates correlations between gene expression and continuous variables, including age at AD onset, age at sampling, MMSE score at last visit, and years of education. All analytical results are displayed as publication-quality figures (**Figure 2**) via a user-friendly and login-free website, which eliminates the need for extensive programming knowledge. Only protein-coding genes are presented in the bulk RNA-seq, scRNA-seq, and Clinical panels.

### Olfactory-related biomarkers in blood associated with the cognitive decline continuum

**Figure 3:**
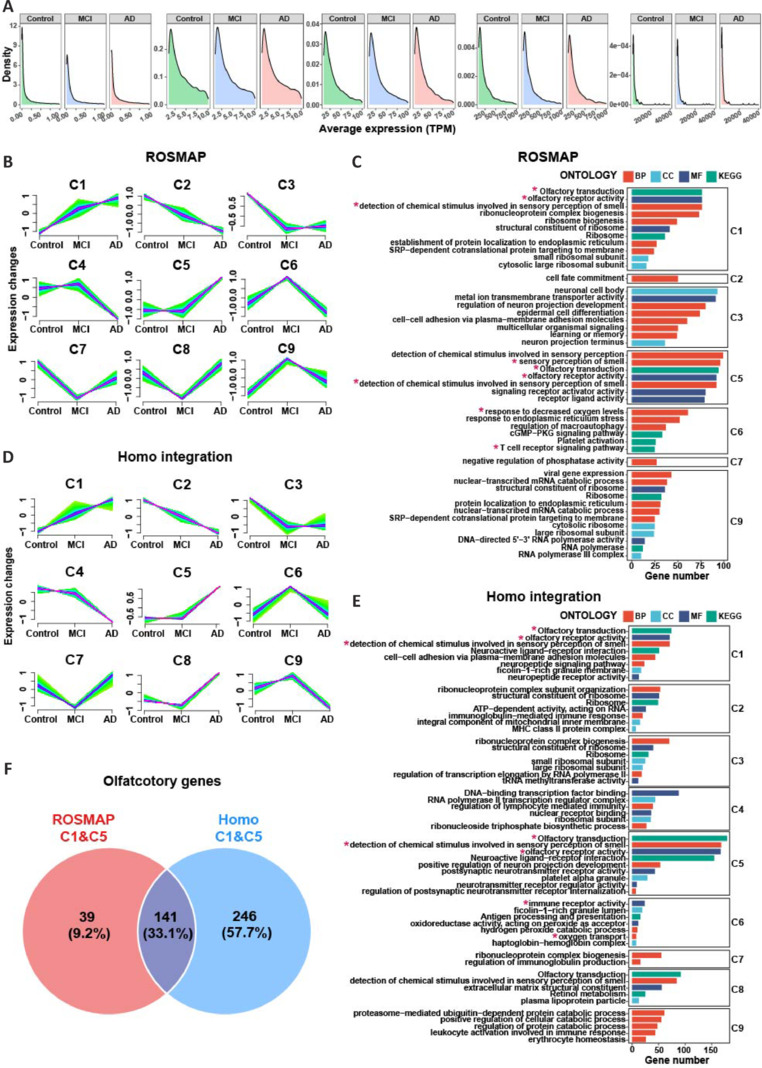
Distribution of blood mRNAs from healthy to MCI to AD individuals and their related BPs. (A) The distribution of blood mRNA expression in normal, MCI, and AD. The density plot was separated into five sections (0–1, 1–10, 10–100, 100–1000, and > 1000) by the average expression of each gene in all samples. (B) The FCM clustering results from ROSMAP dataset in RBAD and (C) their representative GO terms and KEGG pathways. (D) The FCM clustering results for Homo integration dataset in RBAD and (E) their representative GO terms and KEGG pathways. *: Pathways consistently found by the same cluster in two datasets. A complete list of clustered genes and their associated pathways are shown in Additional Table 2. (F) The overlap between olfactory-related genes in C1&C5 clusters of ROSMAP and Homo datasets. AD: Alzheimer’s disease; BP: biological process; CC: cellular component; GO: Gene Ontology; KEGG: Kyoto Encyclopedia of Genes and Genomes; MCI: mild cognitive impairment; MF: molecular function; RBAD: RNAs in Blood of AD, http://www.bioinform.cn/RBAD/.

A case study was conducted to demonstrate the capability of RBAD in identifying blood biomarkers for AD. Using the ROSMAP dataset (PBMC; AD: 150, MCI: 137, Control: 322; Smart-seq2), we compared the expression profiles of 19 355 blood mRNAs between AD, MCI, and healthy control samples. Approximately 9.1% of the mRNAs were highly expressed (TPM value > 10), whereas 58.2% were expressed at low levels (TPM = 0–1; **Figure 3A**). FCM clustering was used to perform a series of tests from healthy individuals to those with MCI and AD. We observed seven expression patterns across nine clusters (C1–C9; **Figure 3B** and **Additional Table 7**). Each cluster contained a balanced number of genes (**Additional Figure 3A**). C1 and C2 contained blood mRNAs with consistently increasing or decreasing expression when progressing from healthy to MCI to AD. C3 included blood mRNAs with similar expression changes in both MCI and AD. C4 and C5 showed specific changes in AD, whereas changes of C6–C9 were specific to MCI.

To further investigate the functional role of each cluster, we performed pathway enrichment analysis (**Figure 3C** and **Additional Table 8**). The top-ranked KEGG pathways and GO terms enriched by C1 suggested dysfunction in ribosomal function and translational initiation. Notably, both C1 and C5 indicated abnormal olfactory transduction (**Additional Figure 3B**). C2 was associated with cell fate commitment inactivation. C6 was enriched in T-cell receptors, autophagy, endoplasmic reticulum, and platelet activation. C7 was associated with phosphatase dysfunction, C8 with neuron projection dysregulation, and C9 with ribosome and RNA polymerase.

To validate these results, an external dataset (Homo, an integrated dataset in RBAD with 723 specimens from five independent datasets; AD: 55, MCI: 143, control: 525) was analyzed using FCM clustering (**Figure 3D** and **Additional Table 9**). The results showed that 11% of genes had similar expression trends in the ROSMAP and Homo datasets (**Additional Figure 3C** and **Additional Table 10**). Several pathways, including olfactory transduction, were repeated in the external dataset (**Figure 3E**, **F** and **Additional Table 11**). Additionally, three scRNA-seq datasets indicated that olfactory-related genes were expressed at low levels in blood cells (**Additional Table 12**).

Exosomes are recognized as key mediators of brain-peripheral communication (Han et al., 2023), and we focused on neuronally derived exosomes to elucidate the potential mechanism underlying the upregulation of olfactory-related mRNA in blood. Due to the lack of relevant datasets from AD models, we used a traumatic brain injury (TBI) model, as TBI is a well-established risk factor for AD (Brett et al., 2022) and shares pathological mechanisms with AD, including tau pathology (Smith et al., 2013; Zhao et al., 2023) and Aβ deposition (Johnson et al., 2012). Thus, we analyzed an RNA-seq dataset (GSE254880) that characterizes mRNA in neuronally derived exosomes from the serum of patients with severe TBI. Our analysis revealed the upregulation of 21 olfactory-related genes in these exosomes compared with that in normal samples (**Additional Table 13** and **Additional Figure 3D**). This suggests that neuronal damage in the olfactory neuroepithelium and brain following a TBI induces the release of olfactory-related mRNAs into the blood through exosomes. Moreover, the proteomic analysis of 199 plasma samples (normal: 137, MCI: 36, dementia: 38) from our self-constructed HMACS (**Additional file 2**) indicated that the protein products of olfactory-related genes were not significantly elevated in patients with MCI or dementia (**Additional Table 14**). These findings suggest that the elevated release of olfactory-related mRNAs, rather than proteins, into the blood may be a key mechanism in AD.

**Additional Table 13:** Differential expression analysis of olfaction-related genes using RNA-seq in neuronally-derived exosomes extracted from the serum of traumatic brain injury patients.

Method	R package limma				
Description	Data set: GSE254880, a RNA-seq dataset of neuronal exosomes extracted from traumatic brain injury (TBI) patients. Olfactory-related genes were collected from KEGG hsa04740. The genes significantly upregulated in TBI were remained in this table.				

**Symbol**	**LogFC**	**AveExpr**	**t**	**P value**	**B**
OR1B1	2.917236821	1.670782694	4.162449806	0.000508694	-0.253881374
OR52E4	2.11076958	2.187958006	3.747966153	0.001322508	-0.995961666
OR2A4	2.978052171	2.339359154	3.73597919	0.001359498	-1.017499494
OR4K13	2.289608047	2.135711039	3.689219174	0.001513873	-1.101524151
OR5AS1	2.974596071	1.65992642	3.124631436	0.005479197	-2.11045291
OR51B4	2.215499379	1.669083753	3.025029333	0.006849416	-2.285779085
OR7D2	1.983910931	2.338854731	3.000221725	0.007239242	-2.329243355
OR10Q1	1.921571976	1.133414373	2.976067246	0.007639365	-2.371478087
OR4K3	2.683954282	2.248570797	2.926453649	0.008529247	-2.457949968
OR2A7	1.912679996	2.180468897	2.703534655	0.013911403	-2.841070783
OR4F5	1.829709446	2.264584755	2.591090783	0.017730649	-3.030312933
OR51M1	1.680171207	2.477455375	2.577515598	0.018253732	-3.052949975
OR2H2	1.707570515	1.849183264	2.527750698	0.020298389	-3.135520863
OR5AU1	2.101353528	3.012311363	2.297903318	0.0328433	-3.507509669
OR5M1	1.75184731	1.923132033	2.268816924	0.034864673	-3.553365187
OR51I1	1.881936864	1.483271819	2.240618291	0.036933081	-3.597535459
OR52J3	2.034502078	1.716762409	2.221257394	0.038417825	-3.627695718
OR51B2	1.602778083	0.974017426	2.202489577	0.039909098	-3.656800055
OR8H1	2.217159987	1.588184895	2.175511637	0.042145582	-3.698404272
OR8D1	2.130798424	1.633330132	2.170028915	0.042613854	-3.706825534
OR2B3	1.935393969	2.479827749	2.165095993	0.043039217	-3.714392418

### Erythroid dysfunction in the blood of patients with Alzheimer’s disease

**Figure 4:**
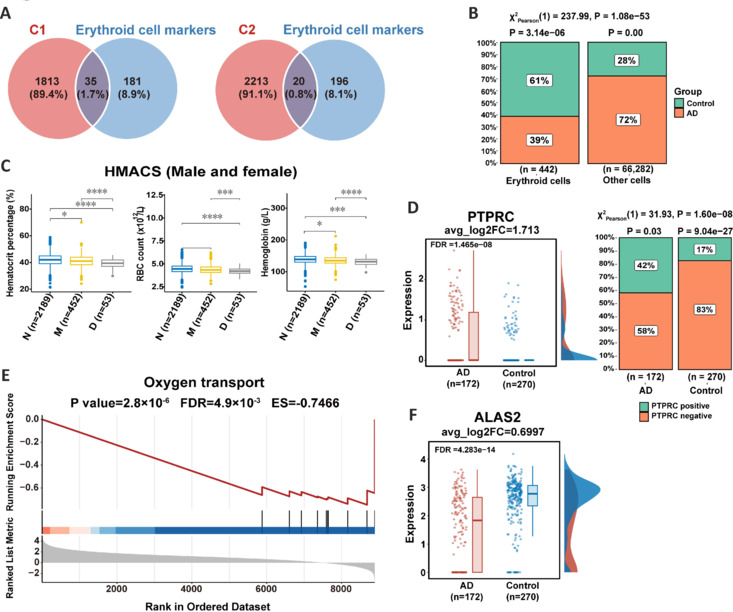
Erythroid dysfunction in the blood of patients with AD. (A) The overlapped genes between C1/C2 clusters from ROSMAP and cell markers of erythroid cells (scRNA-seq dataset collected in RBAD: SRP309935). (B) The percentage of erythroid cells in AD and healthy controls was evaluated and compared using a chi-square test. (C) Comparison of hematocrit percentage, RBC count, and hemoglobin levels between normal (N; *n* = 2189), MCI (M; *n* = 452), and dementia (D; *n* = 53) individuals based on the laboratory data collected from our self-constructed HMACS cohort using the Wilcoxon rank sum test. (D) The expression of *PTPRC* (*CD45*) in erythroid cells was compared between AD and control individuals (left). Chi-square test was applied to determine whether the percentage of erythroid cells that expressed *CD45* was associated with disease status (right). (E) The GSEA of oxygen transport pathway enriched by DEGs between erythroid cells from patients with AD and from normal individuals. (F) The expression of *ALAS2*, responsible for heme synthesis, was compared between erythroid cells from patients with AD and from controls. AD: Alzheimer’s disease; DEG: differentially expressed gene; GSEA: gene set enrichment analysis; MACS: Hubei Memory & Aging Cohort Study; MCI: mild cognitive impairment; RBAD: RNAs in Blood of AD, http://www.bioinform.cn/RBAD/; RBC: red blood cell; scRNA-seq: single-cell RNA sequence.

Molecules that change along the cognitive decline continuum can provide insights for AD biomarker discovery. Thus, we focused on mRNAs that were consistently upregulated (C1) or downregulated (C2) (**Figure 3B**). Analysis of scRNA-seq data showed that erythroid cell markers predominantly overlapped with C1 and C2 (**Figure 4A** and **Additional Tables 15** and **16**). Further analysis of scRNA-seq data (SRP309935) indicated that the erythroid cell proportions in the blood of patients with AD were lower than those in healthy individuals (**Figure 4B**). Similarly, laboratory data for the 2694 participants in the HMACS cohort showed that both the proportion (defined by hematocrit percentage) and the number of red blood cells (RBCs) were lower in patients with MCI, and even more so in those with dementia, compared with healthy individuals (**Figure 4C**), independent of sex (**Additional Figure 4A**). Together, these findings suggest a reduction in RBC proportion in patients with cognitive decline.

**Additional Table 16:** Summary of the overlapped genes

Cell type (detected in scRNA-seq data)	Gene cluster (detected in bulk RNA-seq data)	Overlap between cell markers and gene cluster
B cell 1	1	IL32
B cell 2	1	KLRB1, S100A4
CD4+ T cell	1	KLRB1
CD8+ T cell 2	1	IL32
Erythroid cell	1	BTF3, CLIC1, EEF1A1, GYPC, IL32, NPM1, RPL12, RPL15, RPL17, RPL 18, RPL22, RPL23, RPL24, RPL29, RPL34, RPL36, RPL7, RPL7A, RPL9, RPS10, RPS13, RPS19, RPS2, RPS24, RPS27A, RPS28, RPS3A, S100A4, SRGN, SRP 14, TPT1
Erythroid cell	2	CA1, TRIM58
Megakaryocyte 1	2	PF4
Megakaryocyte_2	1	IL32, KLRB 1, MYO 1F, NPM1, RPL 18, RPL23, RPL24, RPL29, RPL34, RPS10, RPS13, RPS19, RPS24, S100A4, UQCRB
Megakaryocyte 2	2	ITGA2B, PARVB, PF4, TREML1
Monocyte 1	1	IL32, NFKBIA, S100A4, S100A8
Monocyte 1	2	HLA-B, HLA-DRA
Monocyte_2	1	CTSW, IL32, NPC2, RPS19, S100A4
Monocyte 3	1	NFKBIA, RPL 15, S100A8
Monocyte 3	2	MT-CO3, PF4
Naive CD8+ T cell 1	1	RPL18, RPL36, RPS2, RPS27A
Naive CD8+ T cell 2	1	RPL36, RPS27A, RPS28, S100A4
Natural killer cell 1	1	CTSW
Natural killer cell 2	1	CTSW
Natural killer cell_2	2	CCL4, CD7, HOPX

To clarify the mechanism underlying erythrocytopenia, we analyzed plasma proteomic data from the HMACS cohort (*n* = 199, AD: 26, MCI: 36, normal: 137) to evaluate RBC destruction. A quality marker panel that identifies specific biomarkers of intravascular hemolysis, as described in a previously published study (Geyer et al., 2019), was applied. The chi-square test indicated that hemolysis was not associated with disease status (**Additional Figure 4B**). Additionally, the level of plasma-free hemoglobin (*HBB*, *HBA1*, and *HBD*) was lower in patients than in healthy individuals (**Additional Figure 4C**). A differential expression analysis of erythroid-associated gene expression was also performed using scRNA-seq data of PBMCs (SRP309935). The transcription of *EPB41*, a gene associated with mechanical resilience in erythroid cells (Salomao et al., 2008), did not significantly differ between patients with AD and healthy controls (**Additional Figure 4D**). Overall, these findings suggest that RBC destruction does not differ between patients with AD and healthy controls.

We hypothesized that RBC generation may be reduced in patients with AD. *CD45* reportedly arrests erythroid cells at undifferentiated stages, interrupting their differentiation into mature erythrocytes (Long et al., 2022). We found that the expression of *CD45* (*PTPRC*) was significantly upregulated in erythroid cells (**Figure 4D**, log2FC = 1.7, FDR = 1.47 × 10^–8^) and that *CD45*^+^ erythroid cells were more abundant in patients with AD than in healthy individuals (AD: 42% *vs*. healthy: 17%, *P* = 1.6 × 10^–8^, chi-square test), suggesting impaired erythropoiesis in the blood of patients with AD. Furthermore, it is well-established that during erythroid cell differentiation, genes encoding hemoglobin are increasingly transcribed (Brand et al., 2004; Dzierzak and Philipsen, 2013). However, analysis of PBMC scRNA-seq from erythroid cells and plasma proteome data from AD and healthy samples indicated decreased oxygen transport capacity (**Figure 4E** and **Additional Figure 4C**). Additionally, the expression of *ALAS2* (**Figure 4F**), which is involved in heme synthesis, was downregulated in erythroid cells from patients with AD compared with that in cells from healthy individuals. Overall, these findings suggest that hematopoiesis is suppressed in the blood of patients with AD.

**Figure 5:**
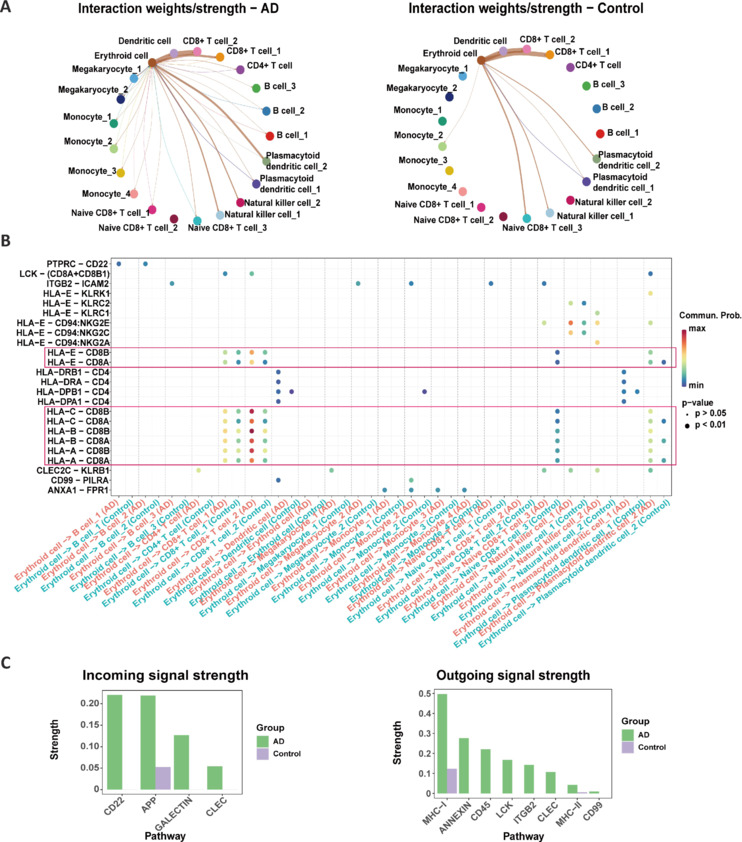
The cell communication analysis for blood erythroid cells in AD patients and healthy controls. (A) The interaction strength between erythroid cells and other cell types in AD and control. (B) The signal pathway sourced from the erythroid cell. The *x*-axis represents the direction of information flow from erythroid cells to other cell types. The *y*-axis shows the ligand–receptor pairs. The circle colors indicate the communication probability. (C) The incoming and outgoing signal strength of each pathway in the erythroid cell. AD: Alzheimer’s disease.

Cell communication analysis was conducted to investigate the potential mechanism underlying the decrease in erythroid cell proportion in the blood of individuals with AD. We observed certain nascent interactions between erythroid cells and other CTs in AD (**Figure 5A**), although the total number of cell interactions was lower than in healthy individuals (**Additional Figure 4F**). For example, interactions between erythroid cells and B cells or monocytes mediated by *PTPRC* were detected only in patients with AD (**Figure 5A** and **B**). Overall, the outgoing and incoming signals of erythroid cells were primarily conducted by C-type lectin receptors and major histocompatibility complex-I pathways, respectively (**Figure 5C**). The outgoing signals from erythroid cells to a cluster of *CD8*^+^ T cells highly expressing *GZMK* (Duan et al., 2023) were transduced through the pairing of human leukocyte antigen (HLA) class I (*HLA-A*, *HLA-B*, *HLA-C*, and *HLA-E*) ligands with *CD8B* and *CD8A* receptors in the major histocompatibility complex-I pathway (**Figure 5B**). The expression of *HLA-A*, *HLA-B*, *HLA-C*, and *HLA-E* on erythroid cells (**Additional Figure 5A**) and *CD8A* and *CD8B* on *GZMK*^+^ *CD8*^+^ T cells (**Additional Figure 5B**) was upregulated in patients with AD compared with that in healthy individuals. Together, these results suggest a potential mechanism of erythroid cell clearance through *GZMK*^+^ *CD8*^+^ T cells.

### Blood mRNAs are prognostic factors for the overall survival of patients with Alzheimer’s disease

**Figure 6:**
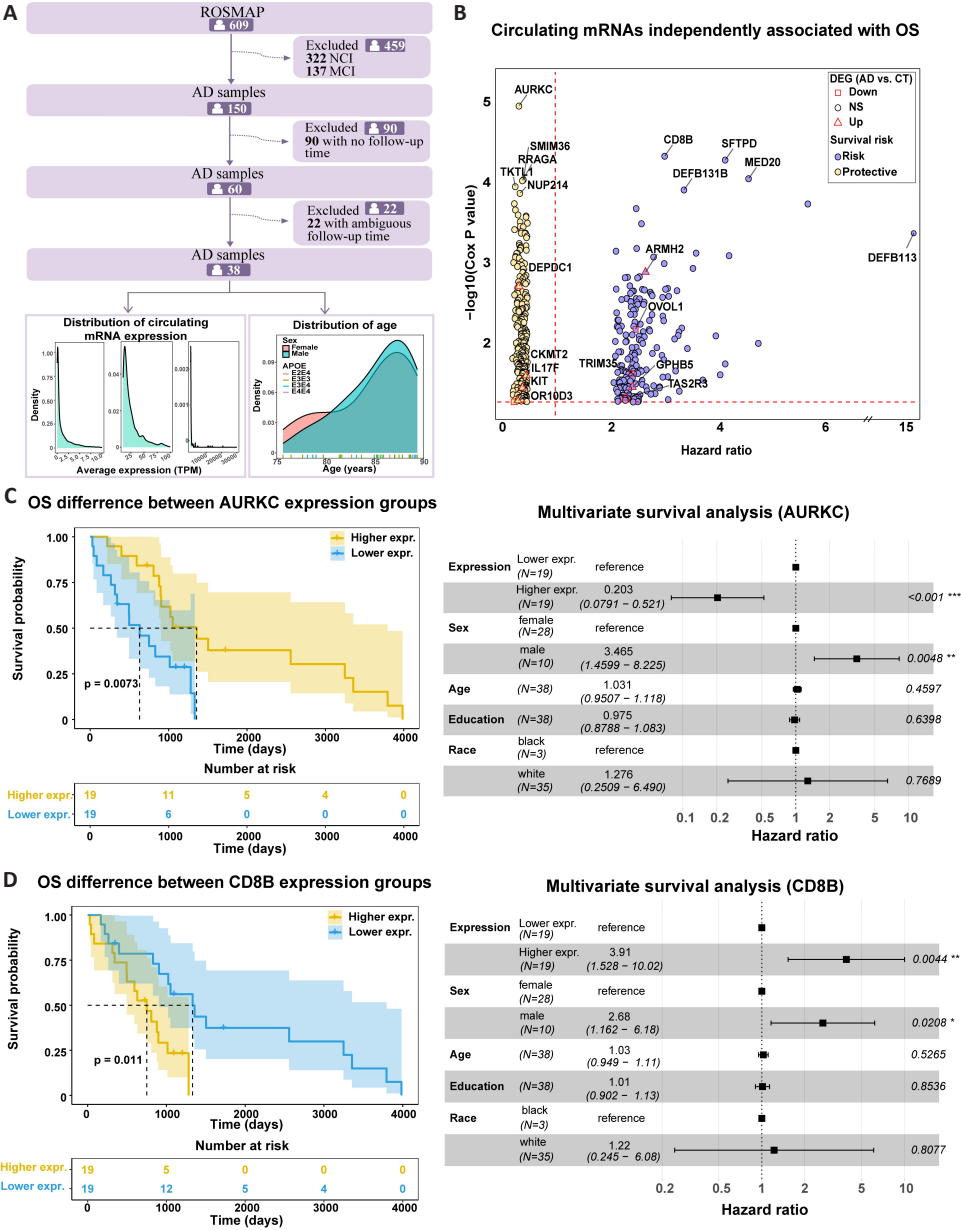
The association between blood mRNA expression and AD OS. (A) The inclusion and exclusion criteria that determined the AD population from the ROSMAP cohort. A total of 38 AD patients were used to determine the association between blood mRNA expression and AD OS. The distribution of blood mRNA expression and age at the last follow-up of these 38 patients was displayed by density plot. (B) The relationship between hazard ratio and *P* value of multivariate Cox hazard regression model for each of the 449 blood mRNAs that were significantly and independently associated with AD OS. The red triangles and squares indicate blood mRNAs that were up- and down-regulated, respectively, in AD compared with healthy controls. Detailed survival result are shown in Additional Table 4. (C, D) The survival difference and hazard ratio of *AURKC* (C) and *CD8B* (D). The lower expression of *AURKC*/*CD8B* was set as the reference group. The multivariate Cox proportional hazard regression model was used to determine the hazard ratio of the group with higher *AURKC*/*CD8B* expression. Sex, age, education level, and race were set as covariates. AD: Alzheimer’s disease; OS: overall survival; RBAD: RNAs in Blood of AD, http://www.bioinform.cn/RBAD/.

To examine the effects of blood mRNAs on AD prognosis, the association between blood mRNA expression and OS was assessed in 38 patients with definite follow-up data from the ROSMAP cohort (**Figure 6A**). The average age of these 38 patients was 84.80 years, with a similar age distribution between men and women (**Figure 6A**). We used univariate and multivariate Cox proportional hazard regression, the log-rank test, and the Kaplan–Meier method to establish associations between blood mRNA expression and patient OS. We identified 449 blood mRNAs that were associated with OS independent of sex, age, race, and education (*P* ≤ 0.05, multivariate Cox proportional-hazard regression; **Additional Table 17** and **Figure 6B**), including 257 risk (high expression associated with high risk of death) and 192 protective (high expression associated with low risk of death) mRNAs. Functional enrichment analyses primarily identified olfactory, immune response, and metabolic-related pathways (**Additional Table 18**). *AURKC* (**Figure 6C**) and *CD8B* (**Figure 6D**) encoded the most significant protective and risk mRNAs, respectively. Of these OS-associated genes, one protective (*KIT*) and three risk (*TAS2R3*, *ARMH2*, and *GPHB5*) genes were significantly down- and up-regulated in AD, respectively (**Figure 6B** and **Additional Table 17**). According to the GTEx and HPA databases, *AURKC* and *ARMH2* were specifically expressed in the testis, *KIT* was specifically expressed in the breast, and *CD8B* was specifically expressed in the thymus (**Additional Figure 6**).

### Blood mRNAs are associated with future cognitive decline

**Figure 7:**
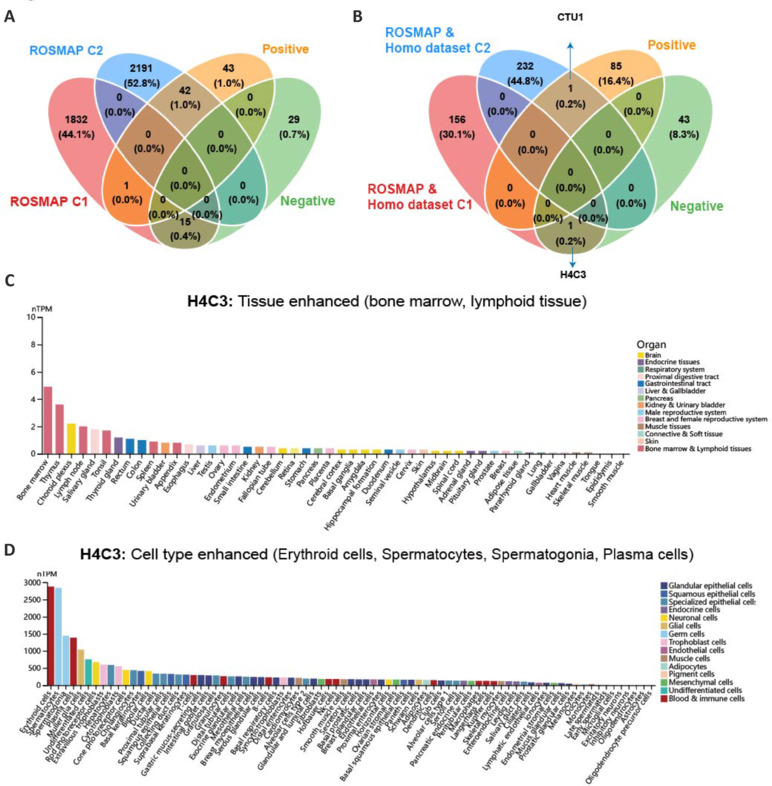
Overlap between DEGs and OS-related genes and their tissue and cell-type specificity. (A) Overlap between genes in C1/C2 cluster of ROSMAP and genes with positive or negative correlation to MMSE. (B) Overlapped genes between C1/C2 clusters where ROSMAP and Homo datasets overlapped and genes with positive or negative correlation with MMSE. (C, D) Expression of *H4C3* across various tissues (C) and cell types from the HPA database (D). DEG: Differentially expressed gene; HPA: Human Protein Atlas; MMSE: Mini-Mental State Examination; OS: overall survival.

To explore blood biomarkers with predictive power for future cognitive decline, a Spearman correlation analysis was performed between blood mRNA expression (at sampling) and the MMSE score (at the last follow-up) for the total population (**Additional Table 19**) and AD population (**Additional Table 20**) from the ROSMAP cohort, with average interval times of 3.89 ± 3.65 and 2.45 ± 3.03 years, respectively. We identified 86 positive (correlation coefficient ≥ 0.2 and FDR ≤ 0.05) and 44 negative (correlation coefficient ≤ –0.2 and FDR ≤ 0.05) correlations between the expression of blood mRNAs and the MMSE score within the total sample population (*n* = 609, healthy: 322, MCI: 137, AD: 150; **Additional Table 6**). Among them, 57 genes (43%) overlapped with C1/C2 in the ROSMAP dataset (**Figure 7A**). *H4C3* and *CTU1*, which also exhibited consistent up- and down-regulation in MCI and AD compared with those in healthy controls in the Homo dataset (**Figure 7B**), were negatively and positively correlated, respectively, with MMSE score after 3.89 ± 3.65 years of follow-up. The HPA and GTEx databases consistently indicated enhanced expression of *H4C3* (Tessadori et al., 2022) in the bone marrow and lymphoid tissue (**Figure 7C**). The HPA scRNA-seq data from normal tissue also showed enhanced expression of *H4C3* in erythroid cells (**Figure 7D**). In contrast, *CTU1*, a gene involved in the regulation of tRNA binding and nucleotidyltransferase activity (Schlieker et al., 2008), showed non-tissue specificity but with enhanced expression in type I alveolar cells (**Additional Figure 7**). These results highlighted the capacity of RBAD to explore predictors of future cognitive decline within peripheral tissue.

## Discussion

We developed RBAD (http://www.bioinform.cn/RBAD/), a unique repository of blood RNA characteristics associated with AD. RBAD offers four panels, called bulk RNA-seq, miRNA-seq, scRNA-seq, and Clinical, which enable diverse analytical activities. Identifying disturbances in mRNAs from blood samples of patients with AD and MCI can help elucidate the peripheral mechanism of AD pathogenesis and inform possible future directions for early diagnosis using circulating nucleic acids. Moreover, the case studies demonstrated the pivotal role of disrupted homeostasis in olfactory transduction and erythroid cells in AD progression. Validation studies using external datasets further underscored the capacity of RBAD to search for potential biomarkers and novel features in the blood of patients with AD. Overall, our data-driven analysis of blood RNA sequence data provides a foundation for future studies to investigate peripheral molecular pathogenesis and identify novel diagnostic biomarkers.

The RBAD database offers a unique perspective on the role of blood RNA in AD and MCI, differentiating itself from previous efforts that primarily focused on brain tissue-based next-generation sequencing data (Jiang et al., 2020; Zhou et al., 2022; Li et al., 2023) or single-omics approaches (Leidinger et al., 2013; Feng et al., 2018; Shigemizu et al., 2020; Toden et al., 2020). The shift from brain tissue to blood-based analysis is noteworthy because peripheral biomarkers have the potential to provide less invasive and more accessible means for early detection, which is particularly important for AD. The use of blood RNA profiling in RBAD thus represents a significant step forward in exploring a more practical and scalable alternative for AD research. Furthermore, our study integrates a comprehensive RNA dataset derived from human and mouse blood, combining bulk RNA-seq, miRNA-seq, and scRNA-seq data. This multi-layered approach provides a more holistic view of the changes in blood RNA, uncovering subtle biological alterations that may be missed in traditional single-omics studies. One of the most significant contributions of the RBAD platform is its ability to identify novel associations between blood RNAs and the clinical features of AD. Although previous studies have identified changes in specific genes or pathways in AD (Toden et al., 2020), linking these findings to clinical features remains a major challenge. The RBAD database fills this gap by highlighting the potential of blood RNAs as AD biomarkers and determining their multifaceted clinical relevance, thus paving the way for more accessible diagnostic testing.

We identified eight expression tendencies of RNA in the blood that are associated with well-documented AD pathways, including mitochondrion dysfunction (Lin and Beal, 2006), platelet activation (Sevush et al., 1998), neuron projection (Sun et al., 2022), immunity (Yang et al., 2023), and olfactory transduction (Dan et al., 2021; Hu et al., 2022) in two datasets. Olfactory decline is an early symptom in patients with AD (Hu et al., 2022). However, although most research has focused on the association between AD and the clinical symptoms of olfactory decline (Hu et al., 2022), corresponding molecular evidence, particularly in the blood, is lacking. Using multiple transcriptomic datasets, our study verified the elevated olfactory-related molecules in the blood of patients with AD or MCI. Additionally, an RNA-seq study of blood exosomes from TBI patients detected increased olfactory-related mRNAs (Bhomia et al., 2024). A recent study has suggested an important role for the blood–olfactory barrier (BOB)—a functional blood–brain barrier extension—in preventing the exchange of circulating factors and pathogens between the periphery and brain through the “nose-to-brain” axis (Wellford and Moseman, 2024). Our findings suggest the destruction of the blood–olfactory barrier in AD, potentially causing the release of olfactory-related mRNA into the blood through extracellular vesicles. These findings indicate the clinical value of olfactory molecular biomarkers in the blood for diagnosing AD.

Hypoxia is widely accepted as a trigger of chronic brain disorders, including dementia (Burtscher et al., 2021; Xu et al., 2024). Moreover, anemia is a common cause of brain hypoxia, making it a potential risk factor for dementia in late life (Chaparro and Suchdev, 2019). Indeed, indicators of erythrocyte size, count, and oxygen-carrying function also play essential roles in dementia (Qiang et al., 2023). In this study, an integrated exploration of scRNA-seq, bulk RNA-seq, proteome, and laboratory data from blood showed that the proportion of erythroid cells and erythrocytes in patients with AD or all-cause dementia was lower than that in healthy individuals. This is consistent with previous large-scale epidemiological studies (Faux et al., 2014; Qiang et al., 2023). However, the mechanism of erythrocytopenia in AD remains unclear. Our analyses indicated a significant decrease in oxygen transport and heme synthesis in AD. Notably, *CD45*, which interrupts erythroid differentiation into mature erythrocytes (Long et al., 2022), was upregulated in erythroid cells. Additionally, because cytotoxic *CD8*^+^ T cells contribute to immunity to endogenous antigens (Jung et al., 2002), increased interactions between erythroid cells and *GZMK*^+^ *CD8*^+^ T cells in patients with AD, mainly attributed to the major histocompatibility complex-I pathway, may also serve as a mechanism of erythrocytopenia. Together, these results suggest that erythroid lineage cells are arrested, preventing their differentiation into mature erythrocytes in patients with AD, potentially resulting in anemia and brain hypoxia, which are widely accepted risk factors for AD (Burtscher et al., 2021). Hence, erythroid dysfunction might act as a potential indicator of AD or early AD.

The association between blood RNA and AD mortality remains unclear. Although AD primarily affects the brain, several pathological processes have systemic manifestations detectable in the blood. For example, metabolic, neuroinflammation, and immune system dysregulation provide information regarding overall health that can significantly shape the disease trajectory and patient survival (Zheng et al., 2024). Our survival analysis indicated that the expression levels of 449 blood mRNAs were independently associated with the OS of patients with AD. These genes were associated with the olfactory system, metabolic process, and immune response. Of these, only 10 were differentially expressed, and none were correlated with the MMSE score. These results underscore the limited prognostic utility of disease-state-related genes in predicting AD outcomes, and emphasize the value of blood molecules reflecting overall health in predicting the long-term quality of life, especially for chronic diseases such as AD. This further highlights the importance of comprehensive supportive care and management of comorbidities for patients with AD.

Importantly, our results highlighted the capacity of RBAD to explore predictors of future cognitive decline within peripheral tissues. We found that *H4C3* and *CTU1*, genes that were consistently up- and down-regulated, respectively, in MCI and AD compared with those in healthy controls in the Homo dataset, were correlated with MMSE score after 3.89 ± 3.65 years of follow-up. The protein encoded by *H4C3*, a member of the histone H4 family that constitutes a core component of the nucleosome, plays a pivotal role in transcriptional regulation, DNA repair, and DNA replication (Peng et al., 2021). Additionally, *H4C3* is involved in amyloid fibril formation (Tessadori et al., 2022). Our findings suggest the enhanced expression of *H4C3* in erythroid cells, which supports our previous finding regarding the importance of erythroid dysfunction in patients with AD.

There are several potential limitations in this study that warrant careful consideration. First, the small sample size in several datasets may introduce potential bias in the comparative analysis owing to sample imbalance. This limitation is particularly pertinent in our survival analysis, where the average age of participants was 84.8 years, restricting the generalizability of our findings to younger populations with AD. Consequently, future studies should aim to include larger and more balanced cohorts to enhance the robustness and applicability of the results. Second, the integration of multiomics data is an emerging trend in biomedical research. Our study is limited by the lack of other omics data types, such as proteomics, metabolomics, and epigenomics, which are crucial for a comprehensive understanding of AD pathogenesis. Expanding the database to incorporate these additional data types in future studies will provide a more complete view of the molecular mechanisms underlying AD and facilitate the identification of novel biomarkers and therapeutic targets.

In conclusion, the RBAD analysis platform is a new resource for investigating blood-based biomarkers of AD, filling an important gap in the field of AD. Its capacity to identify novel RNA features associated with clinical aspects of AD offers possibilities for early-stage diagnosis, disease progression monitoring, and the development of more accessible diagnostic tools. However, its full potential will be realized through continued validation, expansion, and integration with other molecular data types. As such, RBAD is not only a tool for the present but also a platform that can evolve with the growing field of Alzheimer’s research. The RBAD database will be maintained for at least 5 years and further developed by expanding our analytical pipelines and including more data.

## Additional files:

***Additional Figure 1:*** *Batch effects removal of bulk RNA-seq data-sets.*

Additional Figure 1 Batch effects removal of bulk RNA-seq datasets.
(A, B) PCA diagram of bulk RNA-seq data from ACOM, Emory, MCSA, SRP223445, and SRP310421 datasets before (A) and after batch effects removal (B). ACOM: The ACOM Study; Emory: the Vascular Contributors to Prodromal Alzheimer’s disease (Emory_Vascular) study; MCSA: Mayo Clinic Study of Aging; PCA: principal component analysis; RNA-seq: RNA sequence.

***Additional Figure 2:*** *An overview of RBAD web interface.*

Additional Figure 2 An overview of RBAD web interface.
(A) The sidebar panel and welcome page of RBAD. Created with Adobe Illustrator 2022. The logo of RBAD was created by Canva (https://www.canva.cn/). (B) An example of drop-down lists on RBAD. (C) The sub-modules of bulk RNA-seq, miRNA-seq, scRNA-seq, and clinical modules. (D) The results of the cell communication sub-module of the scRNA-seq module. miRNA: microRNA; RBAD: RNAs in Blood of AD, http://www.bioinform.cn/RBAD/; RNA-seq: RNA sequence; scRNA: single-cell RNA.

***Additional Figure 3:*** *Validation of gene clusters and olfactory transduction pathway using external datasets.*

Additional Figure 3 Validation of gene clusters and olfactory transduction pathway using external datasets.
(A) The number of genes in each cluster determined by fuzzy c-means clustering. (B) The overlap of olfactory-related genes in Figure 3F was pathway mapped to the olfactory transduction KEGG pathway (hsa04740). The olfactory receptors that were detected in both ROSMAP and Homo integrated data sets were highlighted in pink. Created from https://www.genome.jp/pathway/hsa04740. (C) The overlap between genes in the C1-C5 clusters from ROSMAP and Homo data-sets. (D) The overlap between up-regulated genes (*P* value < 0.05 & log2FC > 1) found in GEO data-set GSE254880 and olfactory-transduction-related genes collected from KEGG hsa04740. FC: Fold change; GEO: Gene Expression Omnibus; KEGG: Kyoto Encyclopedia of Genes and Genomes.

***Additional Figure 4:*** *Validation of the RBC dysfunction in AD using HMACS cohort and single cell RNA-seq data.*

Additional Figure 4 Validation of the RBC dysfunction in AD using HMACS cohort and single cell RNA-seq data.
(A) Comparison of hematocrit percentage, RBC count, and hemoglobin levels between normal (N, *n =* 2189), MCI (M, *n* = 452), and dementia (D, *n* = 53) patients, stratified by sex. The laboratory data used in this study were collected from our self-constructed HMACS cohort (*n* = 2694). The comparison between groups was performed using the Wilcoxon rank sum test. (B) Chi-square test was used to verify whether hemolysis in HMACS cohort was related to disease status. (C) Comparison of plasma hemoglobin between control (*n* = 137), MCI (*n* = 36), and AD (*n* = 26) patients. The proteome data used in this study were collected from our self-constructed HMACS cohort (*n* = 199). The comparison between groups was performed using the Wilcoxon rank sum test. (D) The expression of *EPB41*, which is associated with mechanical resilience in erythroid cells, was compared between individuals with AD and control. (E) The total number and strength of cell interactions in the blood of healthy controls and AD patients. AD: Alzheimer’s disease; HMACS: Hubei Memory & Aging Cohort Study; MCI: mild cognitive impairment; RBC: red blood cell; RNA-seq: RNA sequence.

***Additional Figure 5:*** *Comparison of expressions of HLA-A, HLA-B, HLA-C, HLA-E, CD8A, and CD8B in erythroid cells between AD and control based on scRNA-seq dataset (SRP309935) from RBAD.*

Additional Figure 5 Comparison of expressions of *HLA-A, HLA-B, HLA-C, HLA-E, CD8A*, and *CD8B* in erythroid cells between AD and control based on scRNA-seq dataset (SRP309935) from RBAD.
(A) The expression of *HLA-A*, *HLA-B, HLA-C,* and *HLA-E* genes in erythroid cells was compared between individuals with AD and control. (B) The expression of *CD8A* and *CD8B* genes in CD8^+^ T cells was compared between individuals with AD and control. AD: Alzheimer’s disease; HLA: human leukocyte antigen; RBAD: RNAs in Blood of AD, http://www.bioinform.cn/RBAD/; scRNA-seq: single-cell RNA sequence.

***Additional Figure 6:*** *Tissue and sex specificity of survival-related genes.*

Additional Figure 6 Tissue and sex specificity of survival-related genes.
(A) Tissue specificity and cell type specificity of *ARMH2, AURKC, CD8B, GPHB5, KIT,* and *TAS2R3* from the HPA database. Created with WPS Office 12.1.0.19302. (B) Association of *ARMH2, AURKC, CD8B, GPHB5, KIT,*and *TAS2R3* with OS in female, male, and total population. (C) Expression of *ARMH2, AURKC, CD8B, GPHB5, KIT,* and *TAS2R3* across various tissues from the HPA database. HPA: Human Protein Atlas; OS: overall survival.

***Additional Figure 7:*** *Tissue and cell type specificity of CTU1.*

Additional Figure 7 Tissue and cell type specificity of *CTU1*.
(A) Tissue specificity and (B) cell type specificity of *CTU1* from the HPA database. HPA: Human Protein Atlas.

***Additional Table 1:*** *Diagnostic criteria of AD, MCI, and control in each dataset or cohort that has been collected in RBAD.*

***Additional Table 2:*** *Characteristics of samples collected in RBAD.*

***Additional Table 3:*** *The URLs of databases, softwares, and R packages.*

***Additional Table 4:*** *Disease-associated DEGs detected in RBAD.*

Additional Table 4 Disease-associated DEGs detected in RBAD

***Additional Table 5:*** *Disease-associated pathways enriched by disease-associated DEGs detected in RBAD.*

Additional Table 5 Disease-associated pathways enriched by disease-associated DEGs detected in RBAD

***Additional Table 6:*** *Clinical-associated genes detected in RBAD.*

Additional Table 6 Clinical-associated genes detected in RBAD

***Additional Table 7:*** *Time-series analysis of the expression of circulating mRNA using ROSMAP cohort dataset.*

Additional Table 7 Time-Series analysis of the expression of circulatingmRNA using ROSMAP cohort dataset.

***Additional Table 8:*** *Pathway enrichment (GO and KEGG) of each cluster found in ROSMAP cohort dataset.*

Additional Table 8 Pathway enrichment (GO and KEGG) of each cluster found in ROSMAP cohort dataset.

***Additional Table 9:*** *Time-series analysis of the expression of circulating mRNA using Homo dataset (integrated ACOM, Emory, SRP310421, and SRP223445 datasets).*

Additional Table 9 Time-Series analysis of the expression of circulating mRNAusing Homo dataset (integrated ACOM, Emory, SRP310421, and SRP223445datasets).

***Additional Table 10:*** *The overlapped genes between clusters identified from ROSMAP and Homo datasets.*

Additional Table 10 The overlapped genes between clustersidentified from ROSMAP and Homo datasets.

***Additional Table 11:*** *Pathway enrichment of each cluster (GO and KEGG) found in Homo dataset.*

Additional Table 11 Pathway enrichment of each cluster (GO and KEGG) found in Homo dataset.

***Additional Table 12:*** *The expression of olfactory-related gene in blood cells from AD and control evaluated by scRNA-seq.*

Additional Table 12 The expression of olfactory-related gene inblood cells from AD and control evaluated by scRNA-seq.

***Additional Table 13:*** *Differential expression analysis of olfaction-related genes using RNA-seq in neuronally-derived exosomes extracted from the serum of traumatic brain injury patients.*

***Additional Table 14:*** *Differential expression analysis of olfactory-related genes using HMACS plasma proteome data.*

Additional Table 14 Differential expression analysis of olfactory-related genes using HMACS plasma proteome data.

***Additional Table 15:*** *The overlapped genes between gene clusters (from bulk RNA-seq) and cell markers and differentially expressed genes (from scRNA-seq).*

Additional Table 15 The overlapped genes between gene clusters (from bulk RNA-seq) and cell markers and differentially expressed genes (from scRNA-seq)

***Additional Table 16:*** *Summary of the overlapped genes.*

***Additional Table 17:*** *Circulating mRNAs that significantly associated with OS independent of sex, age, ApoE genotype, education, and race.*

Additional Table 17 Circulating mRNAs that significantly associated with OS independent of sex, age, ApoE genotype, education, and race.

***Additional Table 18:*** *Pathway enrichment of OS-related circulating mRNAs.*

Additional Table 18 Pathway enrichment of OS-related circulating mRNAs.

***Additional Table 19:*** *Correlation between RNA expression and cognitive function in total population (N = 608; healthy: 322, MCI: 137, and AD: 149).*

Additional Table 19 Correlation between RNA expression and cognitivefunction in total population (N = 608; healthy: 322, MCI: 137, and AD: 149)

***Additional Table 20:*** *Correlation between RNA expression and cognitive function in AD population (N = 149).*

Additional Table 20 Correlation between RNA expression and cognitive function in AD population (N = 149)

***Additional file 1:*** *Tutorial of RBAD.*

Additional file 1 Tutorial of RBAD

***Additional file 2:*** *Methods for detailed information about the participants.*

Additional file 2 Methods for detailed information about the participants

## Data Availability

*Processed data are available online http://www.bioinform.cn/RBAD/. Raw data are accessible on GEO and Synapse databases. Due to privacy or ethical restrictions, the proteomic data from HMACS individuals that support the findings of this study are available from the corresponding author upon responsible request*.
