# Supplementary figures and images for: RBAD: The first database dedicated alterations of blood RNA in individuals with Alzheimer’s disease and their clinical relevance
Source: Neural Regen Res. 2025 Mar 25;21(6):2553–62. doi: 10.4103/NRR.NRR-D-24-01165 (PMC13211806; doi:10.4103/NRR.NRR-D-24-01165)

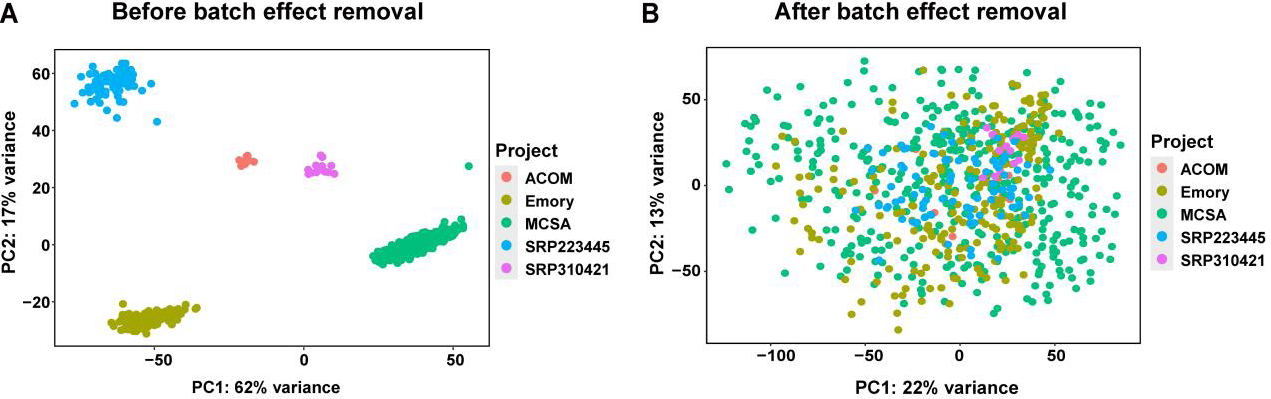

Supplement: Supplementary file 1 [file NRR-21-2553_Suppl1.tif]

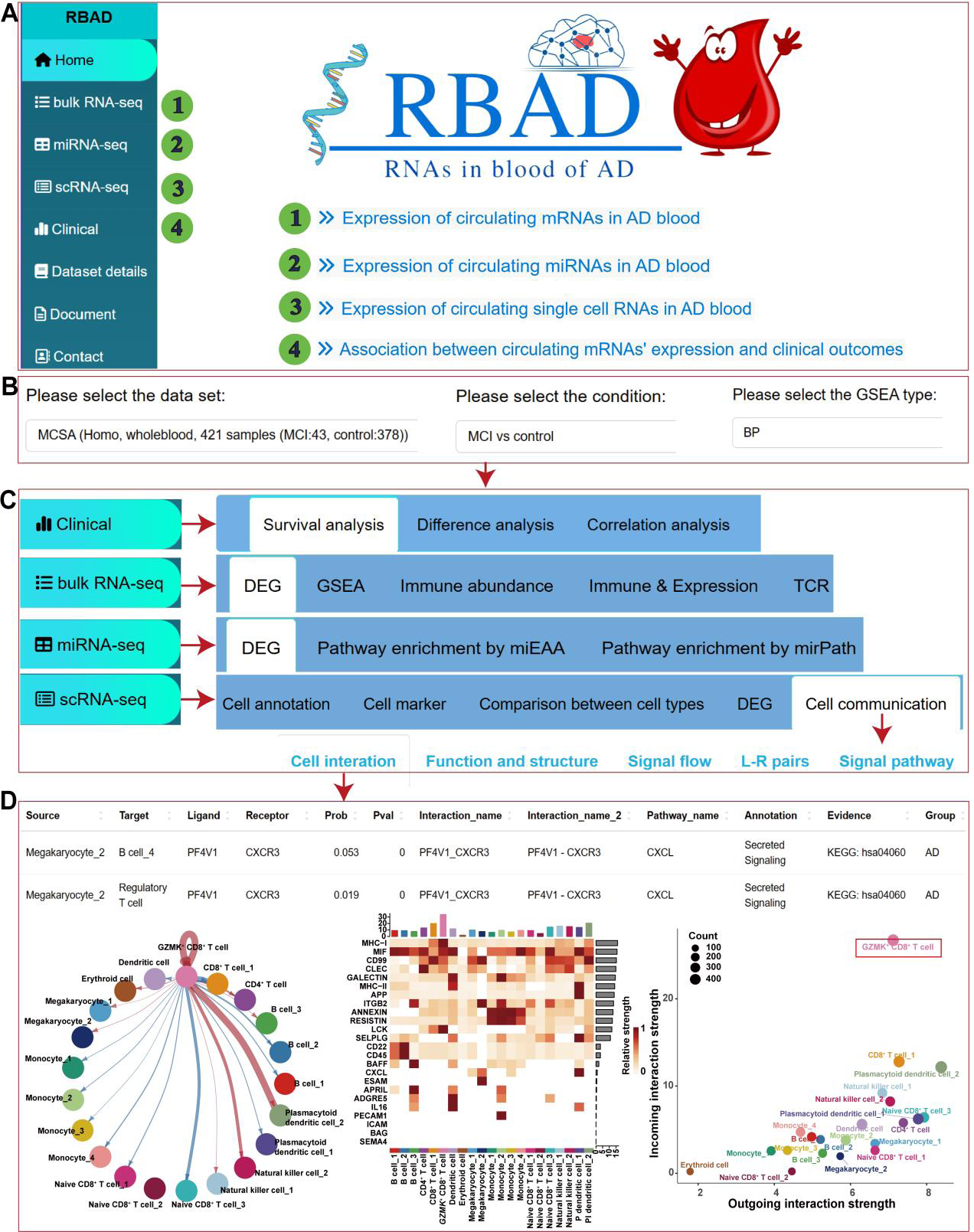

Supplement: Supplementary file 4 [file NRR-21-2553_Suppl2.tif]

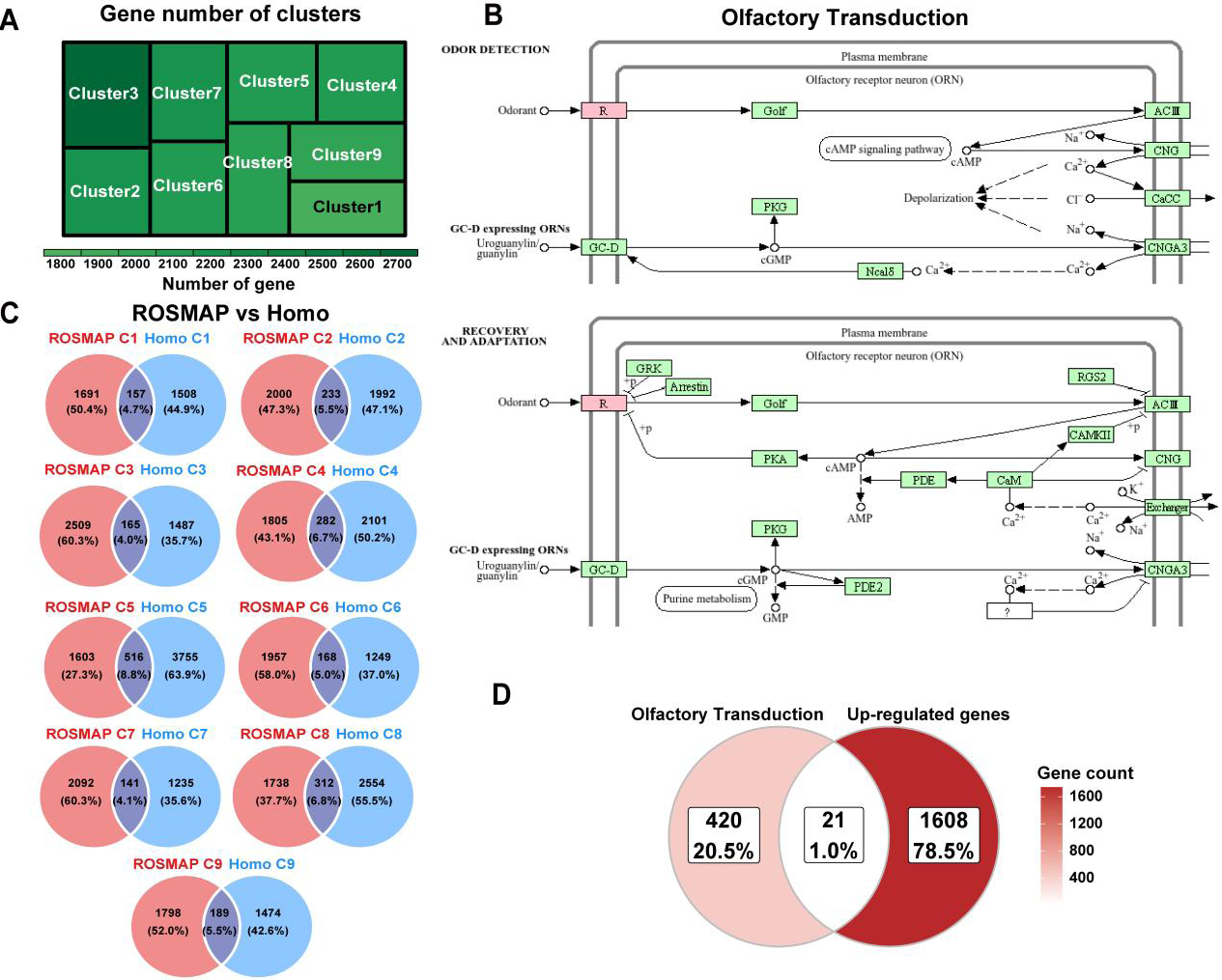

Supplement: Supplementary file 7 [file NRR-21-2553_Suppl3.tif]

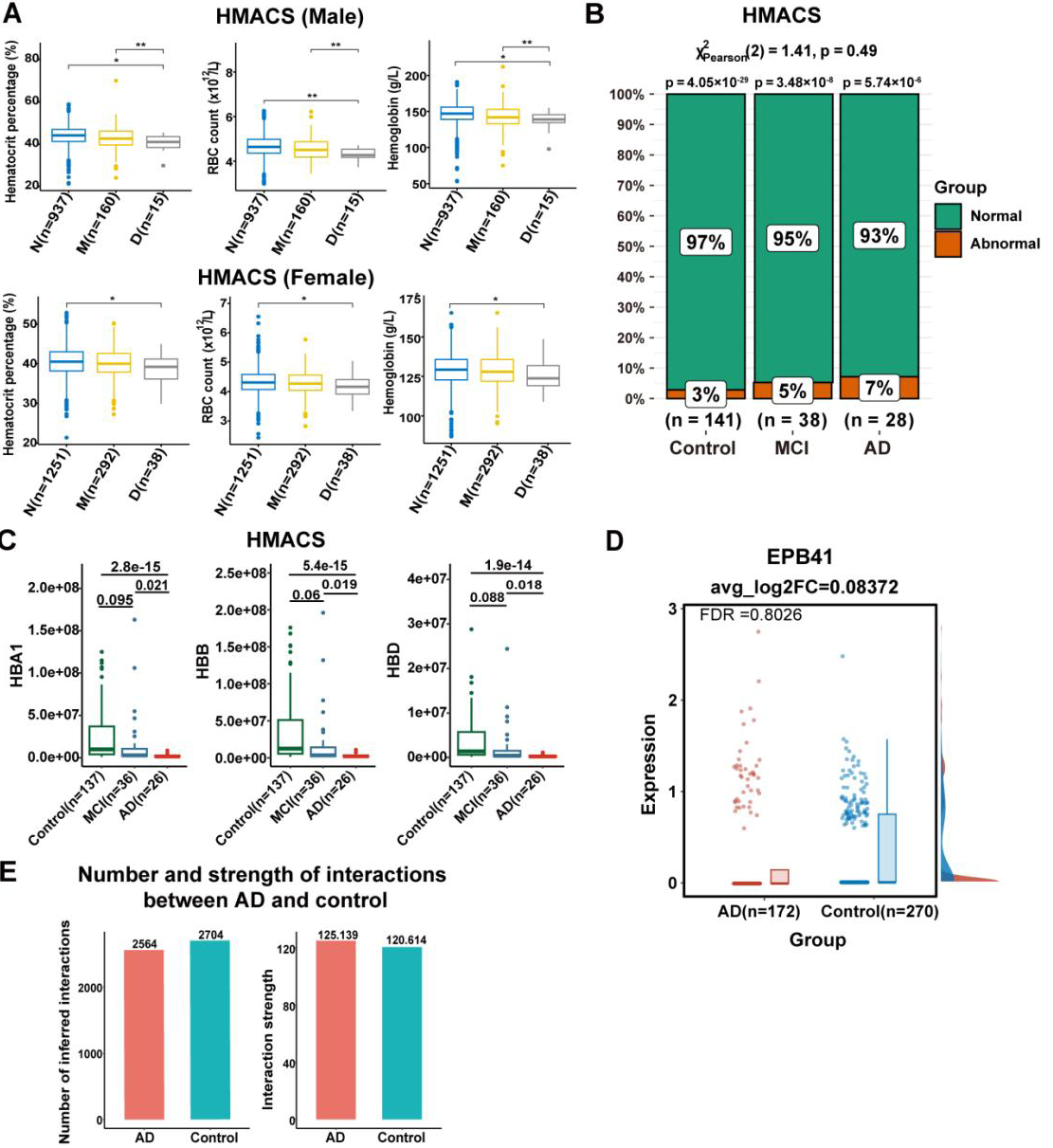

Supplement: Supplementary file 16 [file NRR-21-2553_Suppl4.tif]

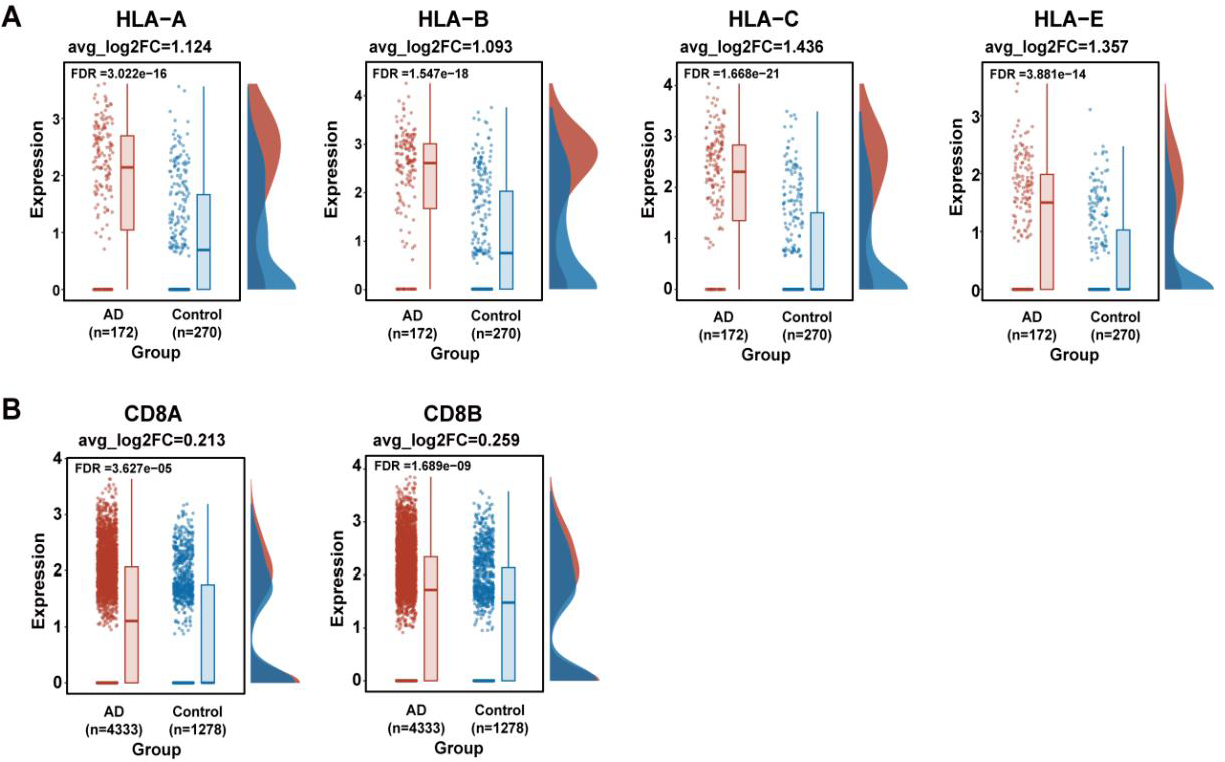

Supplement: Supplementary file 17 [file NRR-21-2553_Suppl5.tif]

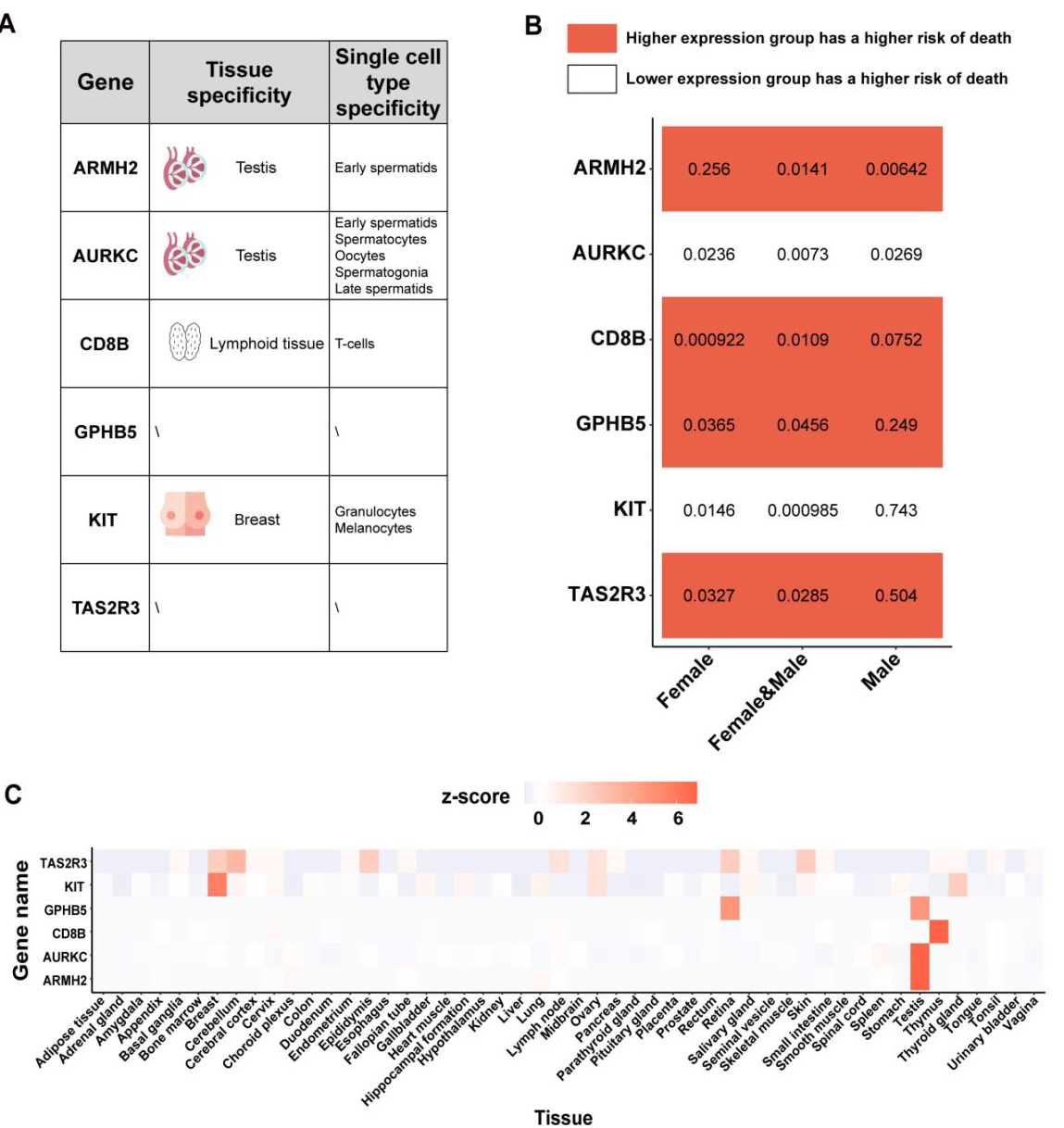

Supplement: Supplementary file 20 [file NRR-21-2553_Suppl6.tif]

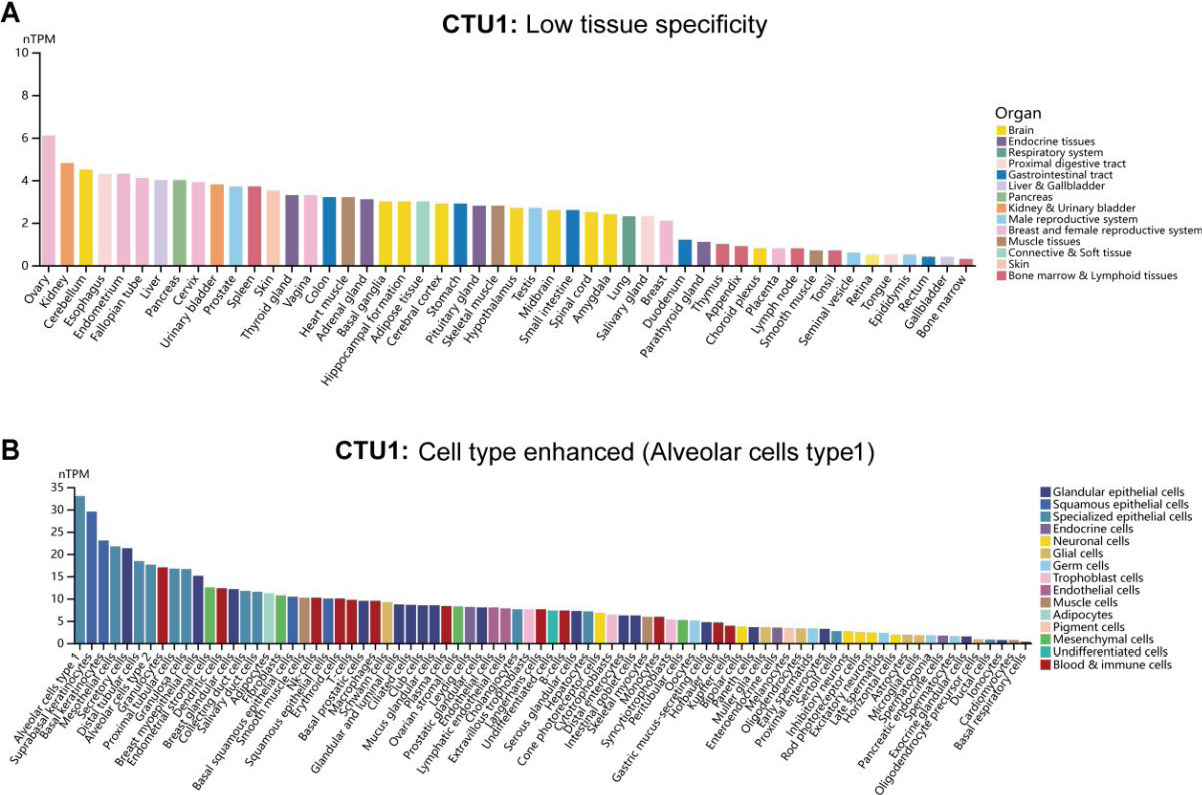

Supplement: Supplementary file 23 [file NRR-21-2553_Suppl7.tif]
