# Supplementary material for: RBAD: The first database dedicated alterations of blood RNA in individuals with Alzheimer’s disease and their clinical relevance
Source: Neural Regen Res. 2025 Mar 25;21(6):2553–62. doi: 10.4103/NRR.NRR-D-24-01165 (PMC13211806; doi:10.4103/NRR.NRR-D-24-01165)
Supplement: Supplementary file 5 [file NRR-21-2553_Suppl16.pdf]

Additional file 1 Tutorial of RBAD

Four functional panels are presented by RBAD:

- Bulk RNA-Seq
- miRNA-seq
- scRNA-seq
- Clinical

Example result illustration of RBAD

Users can click on the sidebar panel to access either the bulk RNA-seq, miRNA-seq, scRNA-seq, or clinical modules (Additional Figure 2A). All datasets are displayed in ready-to-analyze formats comprising project, species, and sample size. Users can further define additional parameters to decide the analysis program (Additional Figure 2B). For example, clicking the “scRNA-seq” panel to get five modules (Additional Figure 2C), then clicking the “Cell communication” module and “Cell interaction” sub-module in turn (Additional Figure 2C) to require a detailed report about the corresponding selection. Both panels provide plenty of tables and publish-level figures that are available for download and personalized processing (Additional Figure 2D).

Results presentation

Bulk RNA-seq

The bulk RNA-seq module of RBAD performs mRNA expression, immune abundance, and TCR analysis between AD/MCI and normal samples (Figure 1), including five sub-modules:

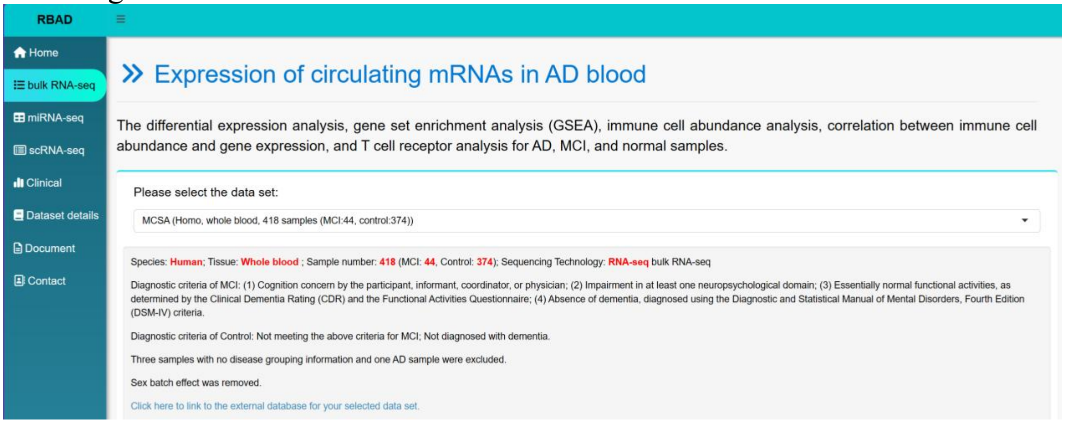

Figure 1 The bulk RNA-seq module analysis page of RBAD.

DEG

This sub-module calculates the differentially expressed genes (DEGs) between AD/MCI and normal samples (Figure 2).

By selecting the 'ACOM' and 'AD vs control' in the DropDownLists, users can get the volcano plot summarizing the distribution of DEGs between AD and normal samples of ACOM.

By clicking 'APOC2' in the result table, users can get the rain cloud plot showing the differential expression of the APOC2 gene between AD and normal samples of ACOM (FDR = 1.269e-29).

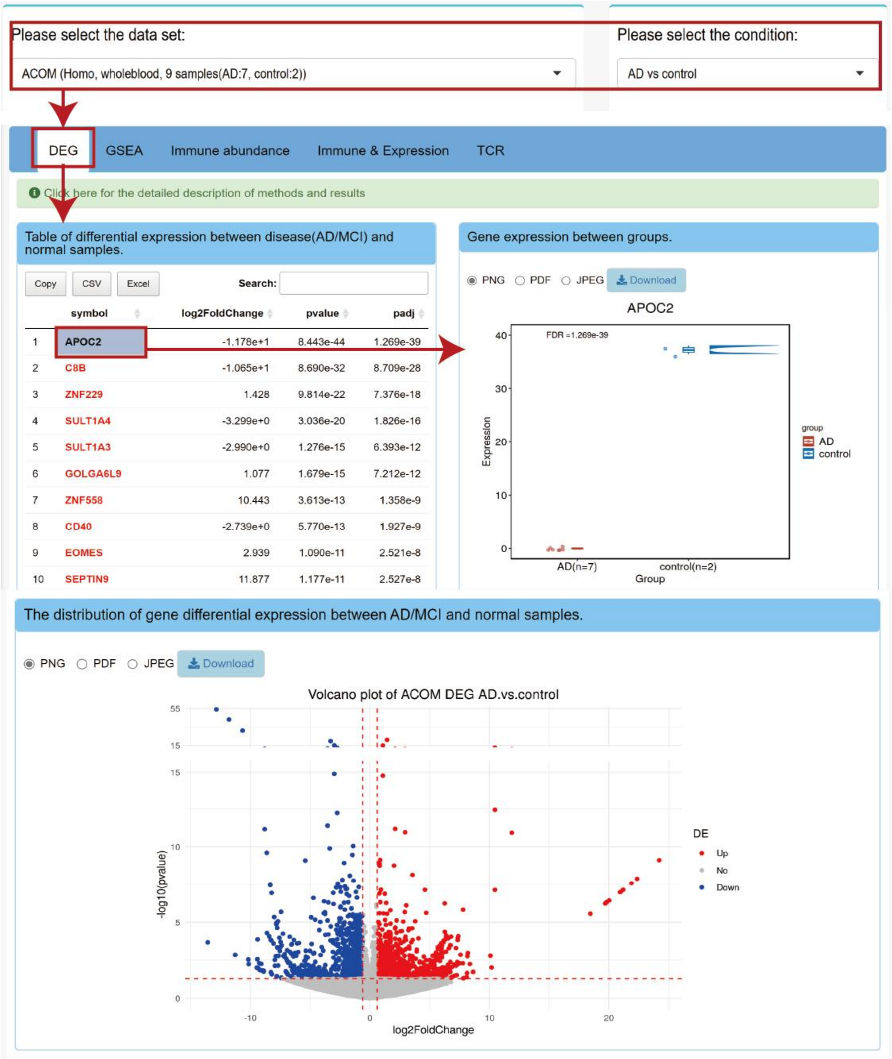

Figure 2 The "DEG" submodule analysis page of the bulk RNA-seq module.

GSEA

This sub-module analyzes the Gene Set Enrichment Analysis (GSEA) of DEGs between AD/MCI and normal blood samples (Figure 3).

By selecting the 'GO' and 'BP' in the DropDownLists, users can get the Ridge plot which shows the fold change distribution core enrichment genes from the top 20 (P-value < 0.05, arranged by P-value) enrichment pathways. By clicking 'GO:0097006' in the result table, the user can get a graphical view of the enrichment score of the gene set of this pathway.

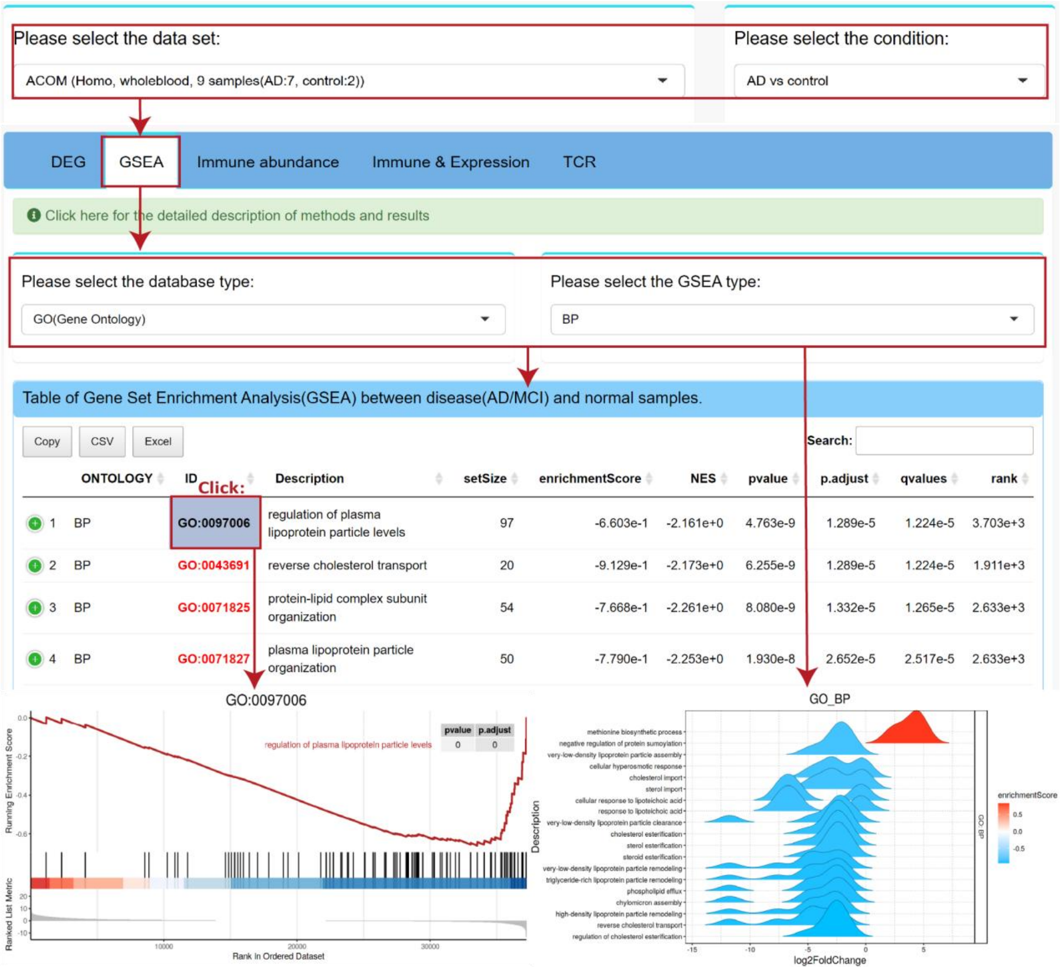

Figure 3 The "GSEA" submodule analysis page of the bulk RNA-seq module.

Immune abundance

This sub-module provides the immune cells' abundance between AD/MCI and normal samples from ImmuCellAI (Figure 4). The estimates of the abundance of immune cells were based on gene set signature.

By clicking 'NK' in the result table, users can get the rain cloud plot showing the difference in immune abundance of NK cells between AD and normal samples (FDR = 0.056).

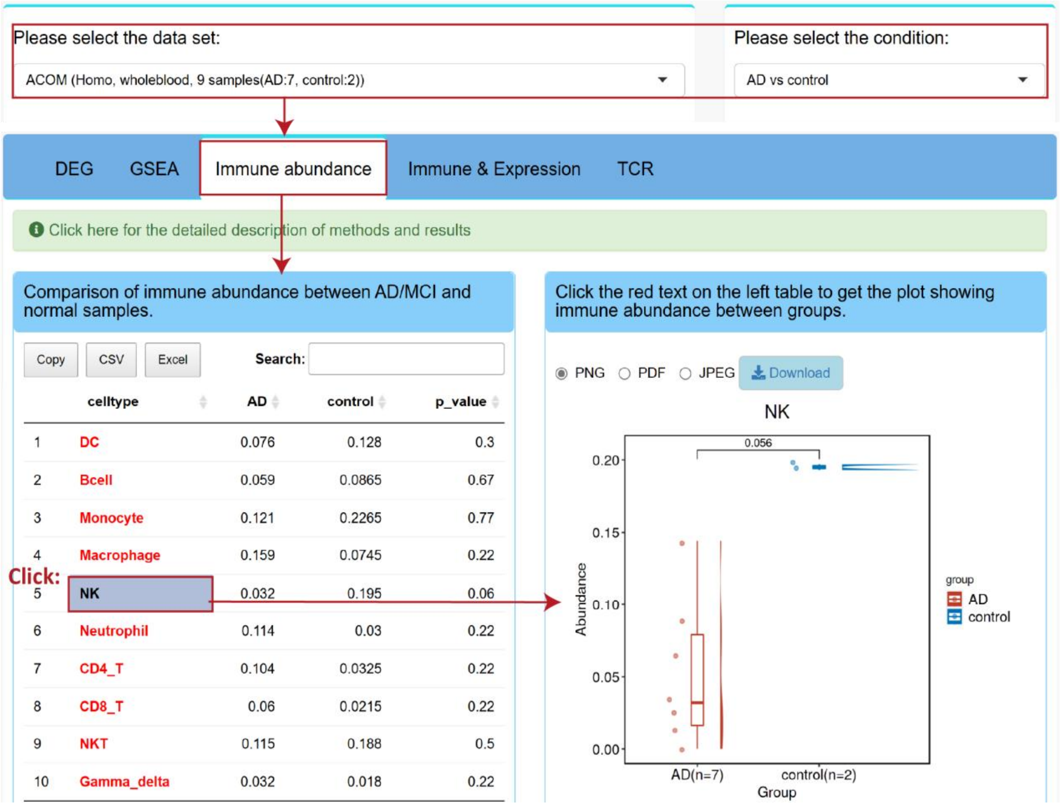

Figure 4 The "Immune abundance" submodule analysis page of the bulk RNA-seq module.

Immune & expression

This sub-module estimates the association between gene expression and immune cells' abundance (Figure 5).

By selecting the 'DC' in the cell type DropDownList and clicking 'A1CF' in the result table, users can get the scatter plot showing the expression of the A1CF gene correlated with DC cells abundance.

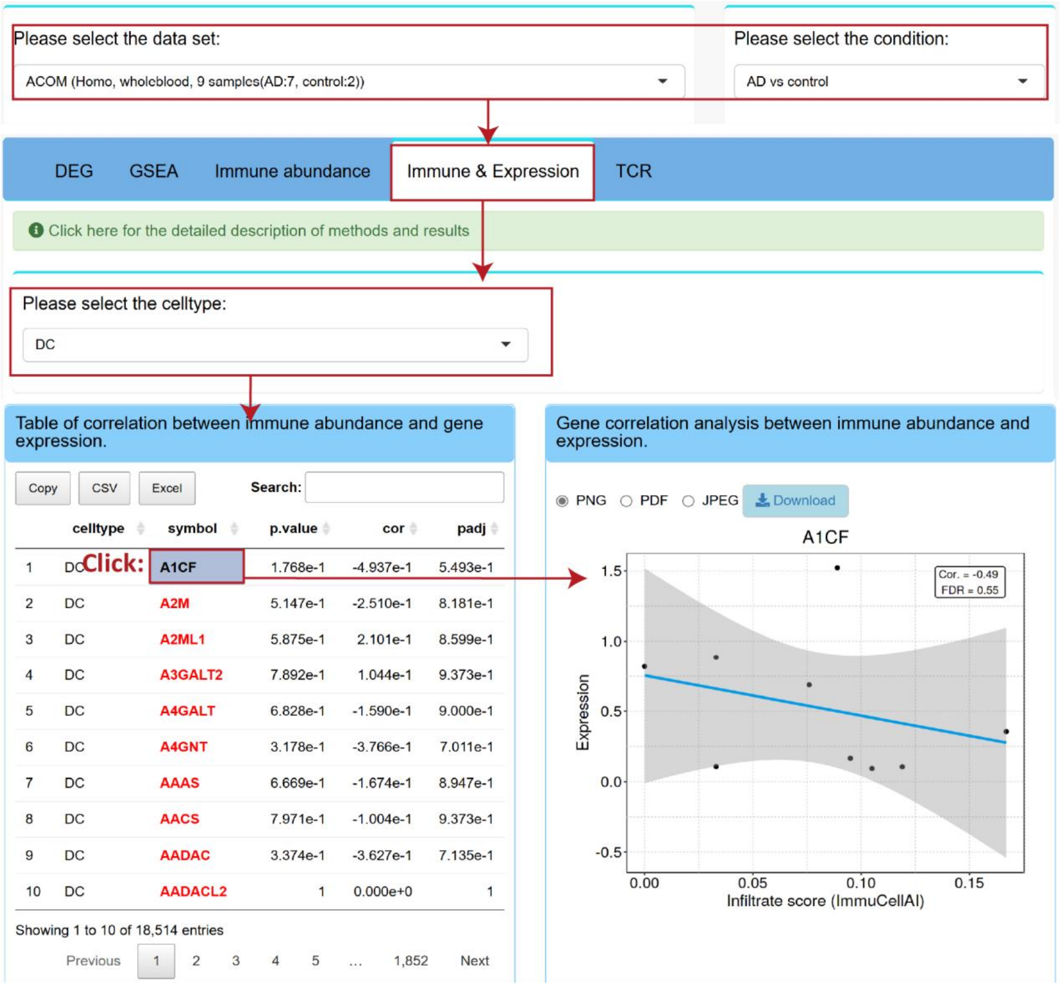

Figure 5 The "GSEA" submodule analysis page of the bulk RNA-seq module.

TCR

This sub-module is used to estimate the usage of V(D)J genes and CDR3 amino acid in AD/MCI and normal samples.

In this sub-module, we provide four types of TCR analysis visualization results: Exploratory analysis, Clonality analysis, Gene usage analysis, and Diversity estimation.

Exploratory analysis

The Exploratory analysis is used to compute basic statistics, such as number of clones or distributions of lengths and counts (Figure 6).

By selecting 'Homo' and 'AD vs control' in the DropDownLists and clicking 'Exploratory analysis', users can obtain plots of the relative abundance, rare clonal fraction, and top clonal fraction of Homo between the AD/MCI and control samples.

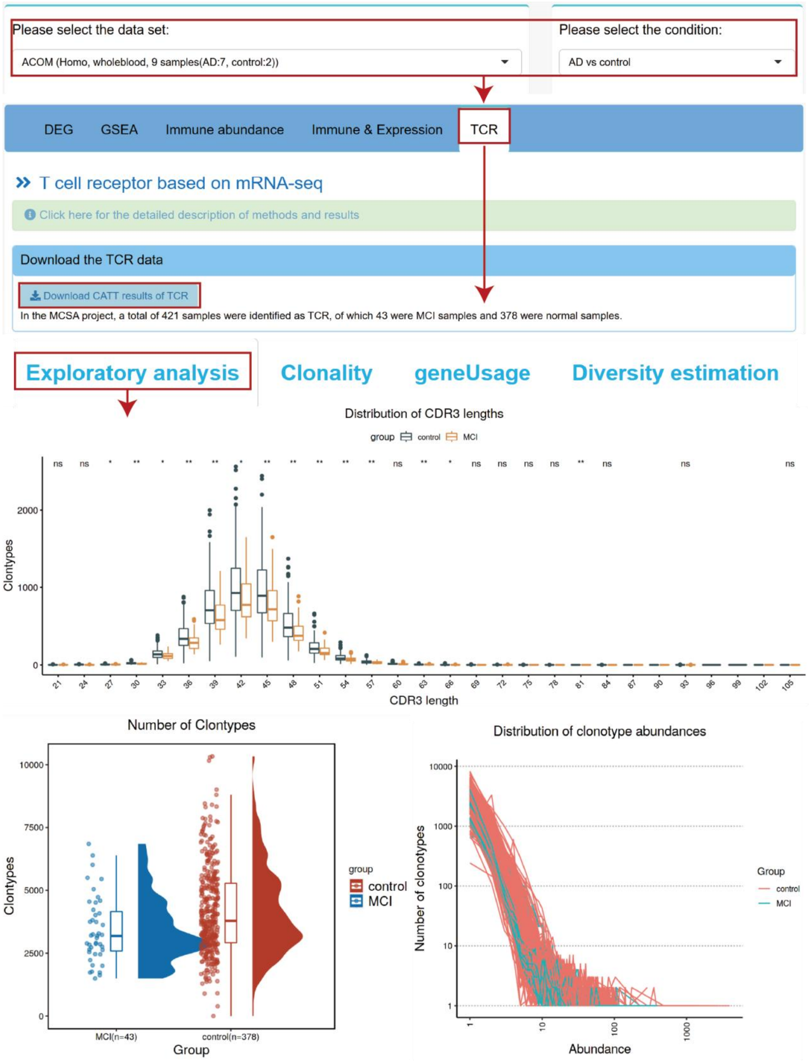

Figure 6 The representative results of "Exploratory Analysis" of the "TCR" submodule.

Clonality

The Clonality is used to compute the clonality of repertoires (Figure 7).

By clicking 'Clonality', users can obtain plots of the CDR3 lengths, number of clonotypes, and clonotype abundances of Homo between the AD/MCI and control samples.

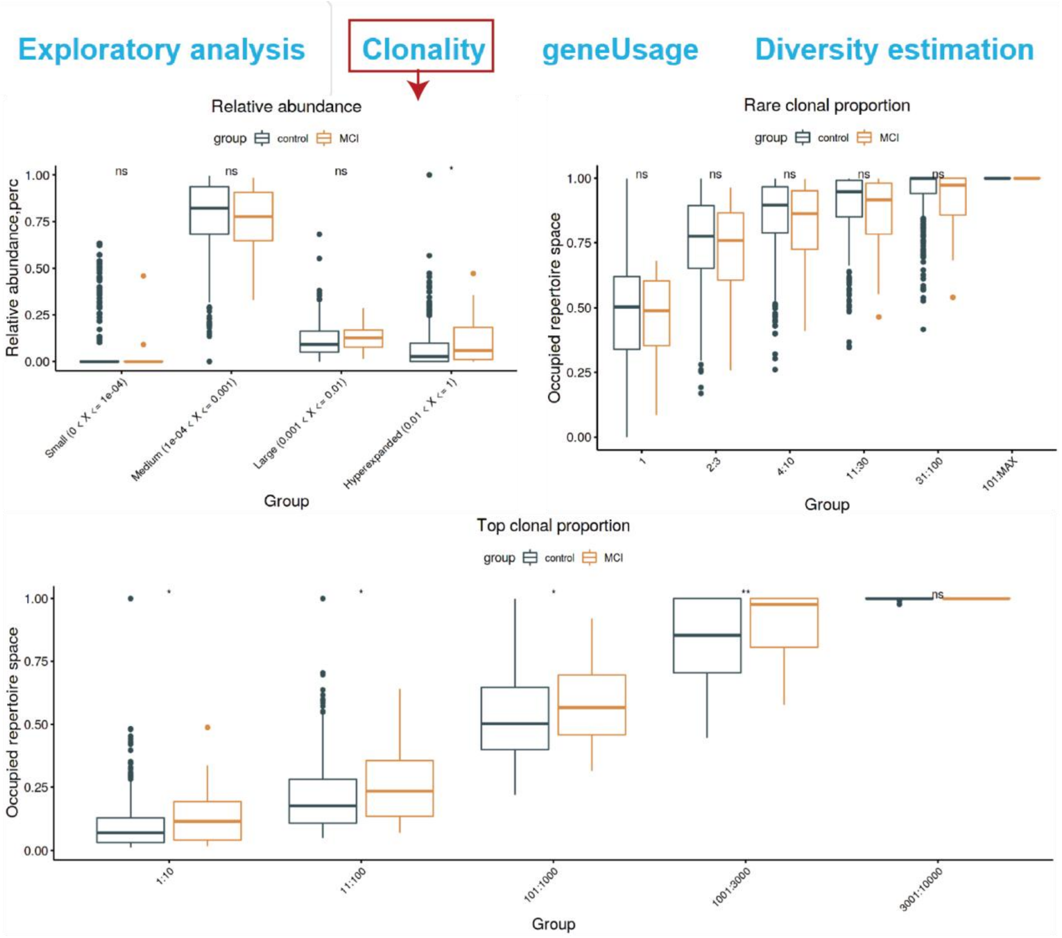

Figure 7 The representative results of "Clonality" analysis of the "TCR" submodule.

GeneUsage

The GeneUsage is used to analyze the distributions of V or J genes (Figure 8).

By clicking 'Diversity estimation', users can obtain plots of the K-means clustering and TRBV/TRBD/TRBJ gene usage of Homo between the AD/MCI and control samples.

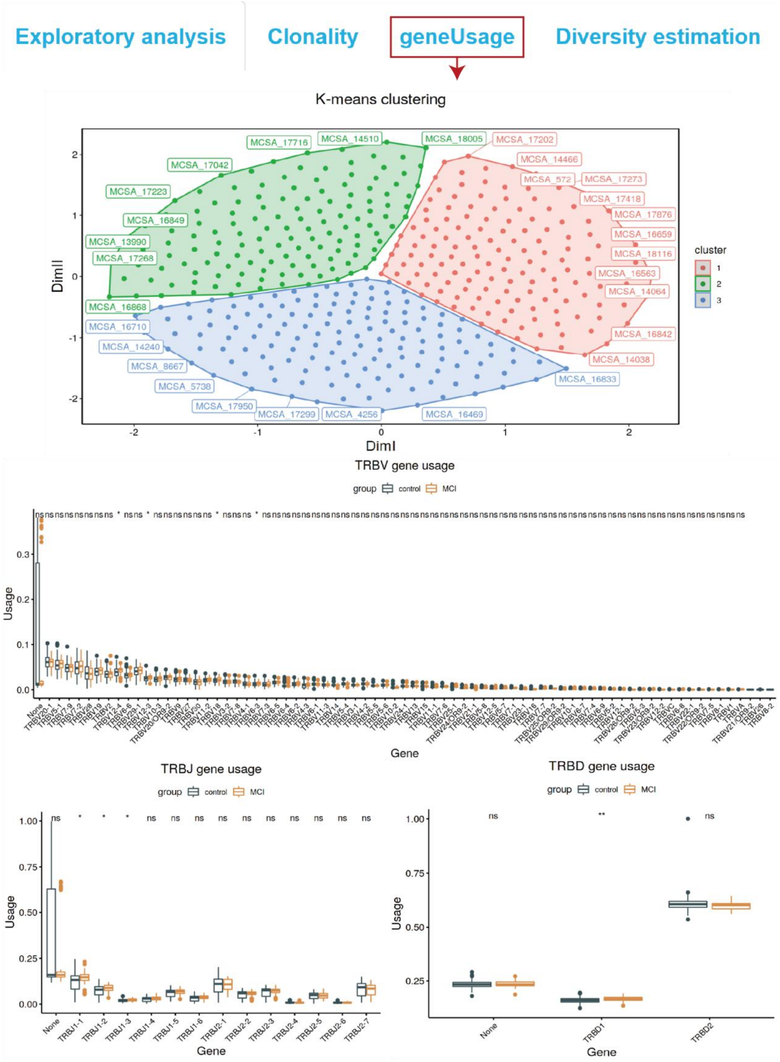

Figure 8 The representative results of “geneUsage” analysis of the "TCR" submodule.

Diversity estimation

The Diversity estimation is used to estimate the diversity of repertoires (Figure 9). By clicking on 'Diversity Estimation', the user can obtain plots of the estimated repertoire diversity between AD/MCI and control samples by chao1/hill number of Homo.

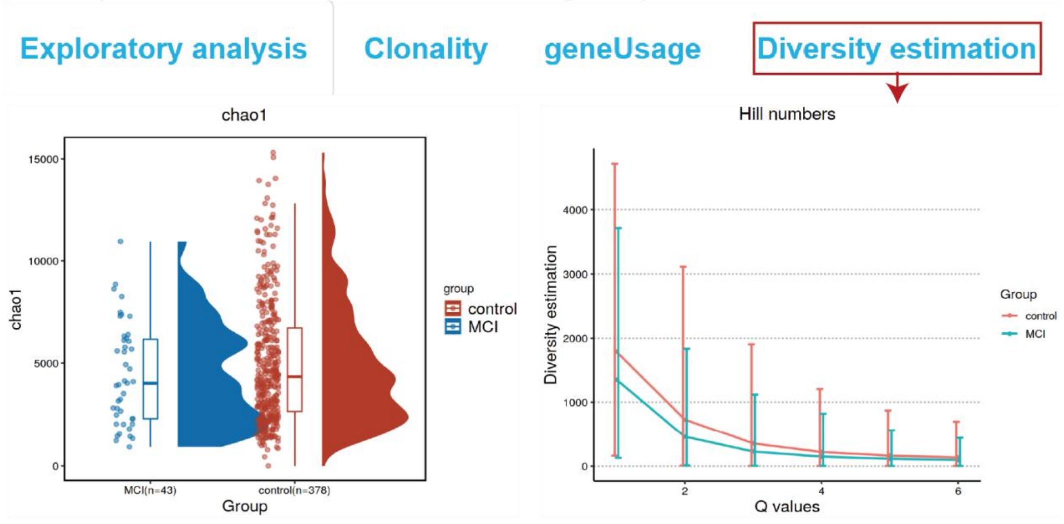

Figure 9 The representative results of “Diversity estimation” analysis of the "TCR" submodule.

miRNA-seq

The miRNA-seq module of RBAD performs miRNA expression, and pathway enrichment between AD and normal samples (or between different age groups of mouse), including three sub-modules (Figure 10):

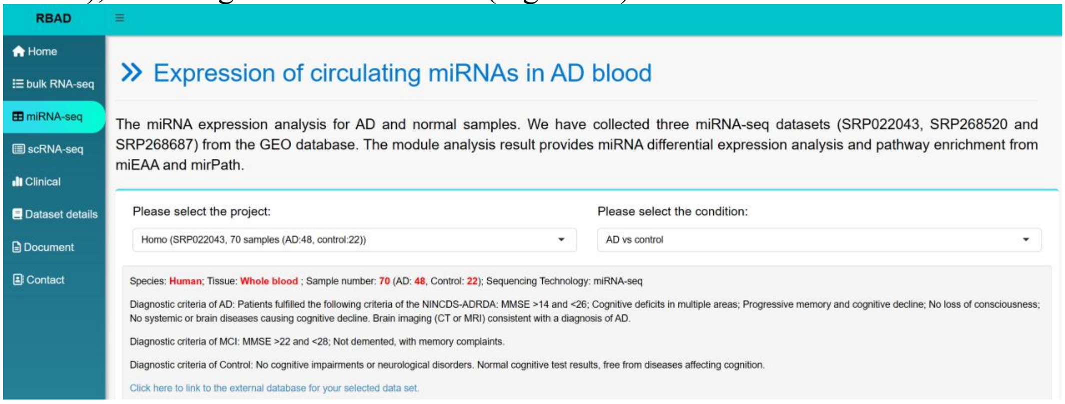

Figure 10 The miRNA-seq module analysis page of RBAD.

DEG

This sub-module analyzes the miRNA differential expression between AD and normal samples (or between different age groups of mouse) (Figure 11).

By selecting the 'Homo' and 'AD vs control' in the DropDownLists, users can get the volcano plot summarizing the distribution of differential miRNA expression between AD and normal samples of Homo.

By clicking 'hsa-miR-99b-5p' in the result table, users can get the rain cloud plot showing the differential expression of hsa-miR-99b-5p between AD and normal samples of Homo (FDR = 2.410e-4).

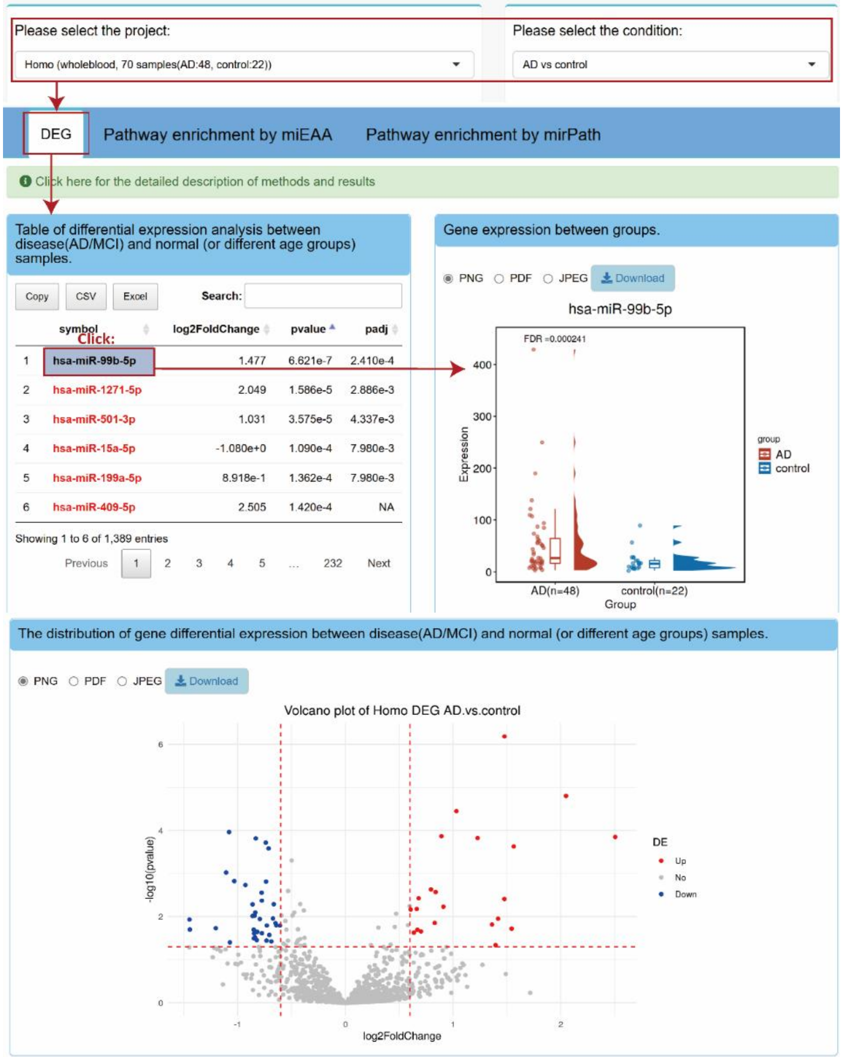

Figure 11 The "DEG" submodule analysis page of the miRNA-seq module.

Pathway enrichment by miEAA

This sub-module analyzes the pathway enrichment between AD and normal samples (or between the different age groups of the mouse) from miEAA (Figure 12).

Through selecting the 'GO' in the DropDownList, users can get the Ridge plot which shows the expression distribution of the core enrichment genes of the top 20 (P-value < 0.05, arranged by P.value) enrichment pathways.

By clicking 'endocrine pancreas development GO0031018' in the result table, the user can get a graphical view of the enrichment score of the gene set of this pathway.

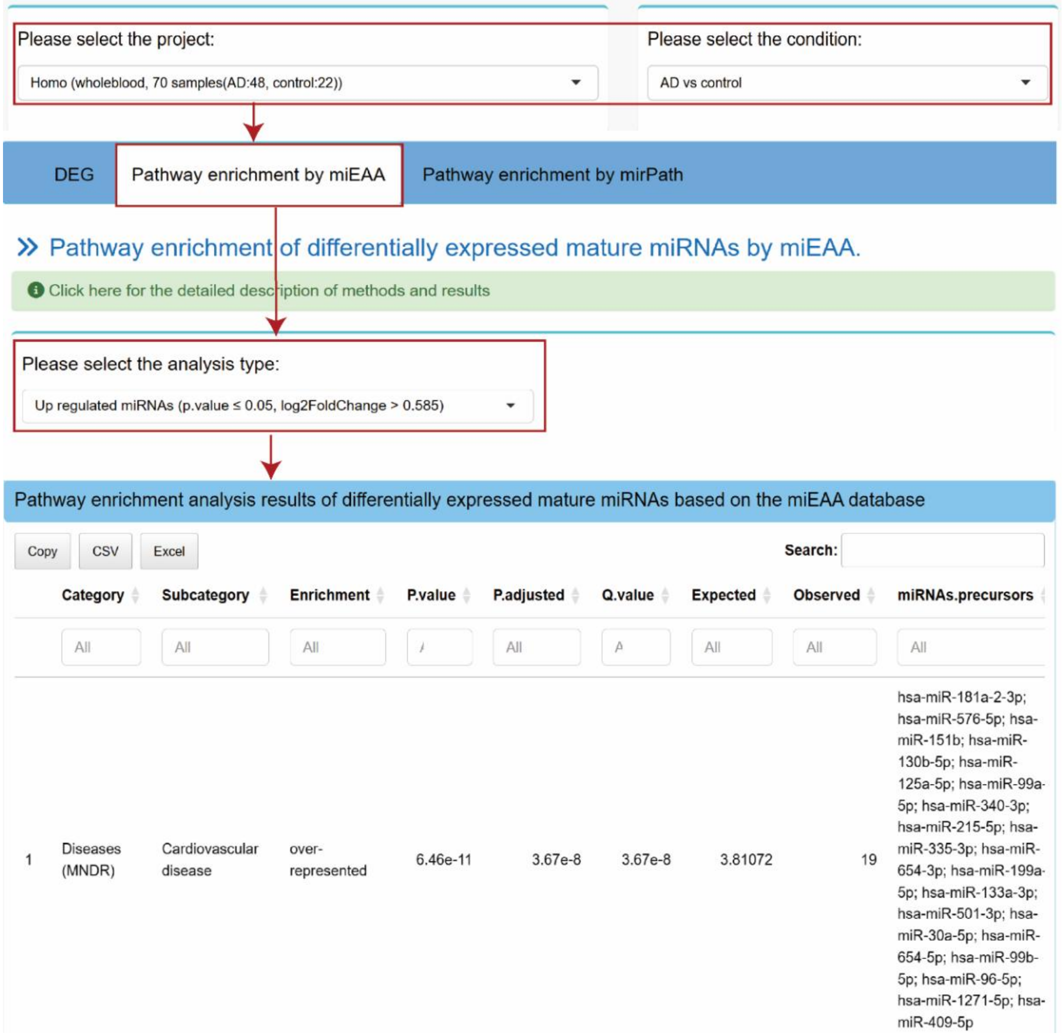

Figure 12 The “Pathway enrichment by miEAA” submodule analysis page of the miRNA-seq module.

Pathway enrichment by mirPath

This sub-module analyzes the pathway enrichment between AD and normal samples (or between the different age groups of the mouse) from mirPath (Figure 13). Through selecting the ‘GO-up’ in the DropDownList, users can get the bar plot showing the top 20 (P-value ≤ 0.05) pathways from the table of GO-up results.

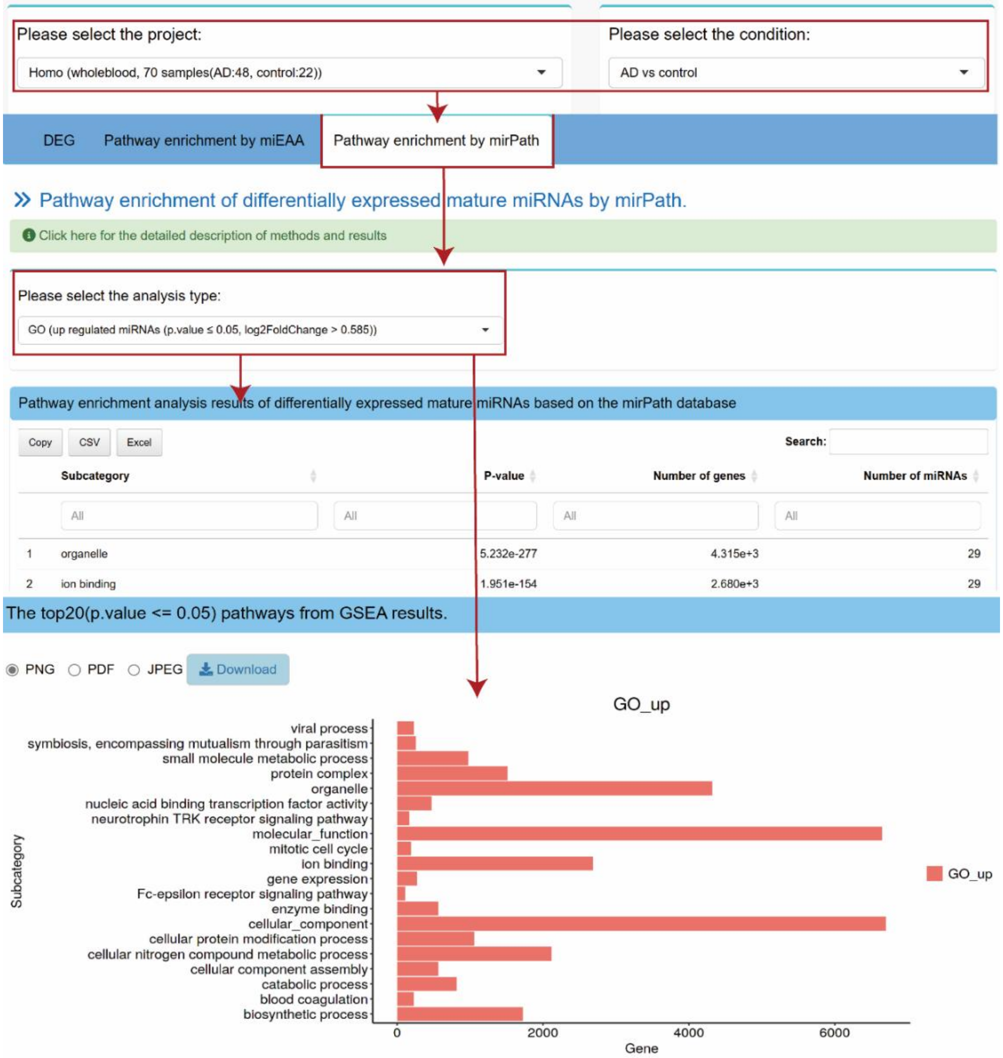

Figure 13 The "Pathway enrichment by mirPath" submodule analysis page of the miRNA-seq module.

scRNA-seq

The scRNA-seq module of RBAD provides cell marker analysis, DEGs, and GSEA for AD blood scRNA-seq data, including five sub-modules (Figure 14):

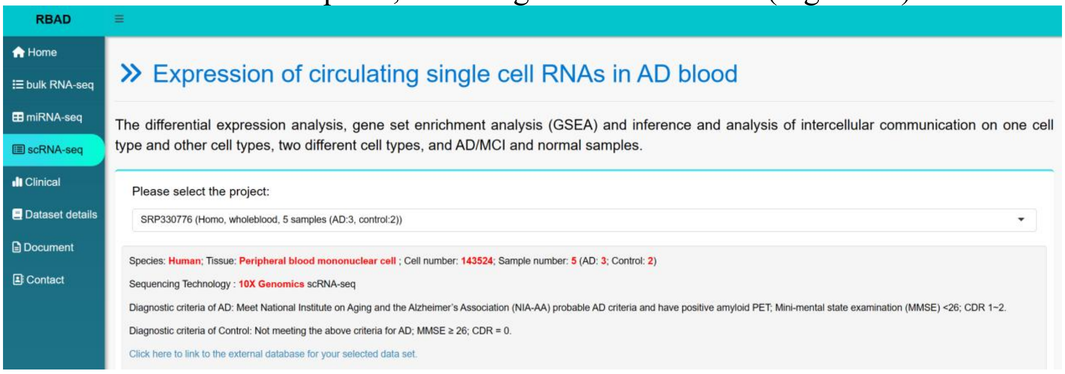

Figure 14 The scRNA-seq module analysis page of RBAD.

Cell annotation

This sub-module annotates the cell type of scRNA-seq data in blood. By selecting 'SRP309935' in the DropDownLists and clicking 'Cell annotation', users can obtain three types of plots: the tSNE scatter diagram, pie chart, and stacked column chart (Figure 15).

The tSNE scatter diagram summarizes the clustering display of tsne after cluster analysis. Each color represents a cell group identified after cluster analysis, and scattered points represent each cell. The subgroups close to each other represent the connection between the two.

The pie chart summarizes the proportion of different cell types in the project. Different sectors represent different cell types. When you put the mouse on different sectors, you can see the cell names, cell numbers, and the proportion of different cell types in this project.

The stacked column chart summarizes the proportion of different cell types in each sample. This is a stacked histogram of percentages. Each layer of the column represents the percentage of each cell type in each sample. When you put the mouse on different levels of the column, you can see the cell names, cell numbers, and the proportion of different cell types in this sample.

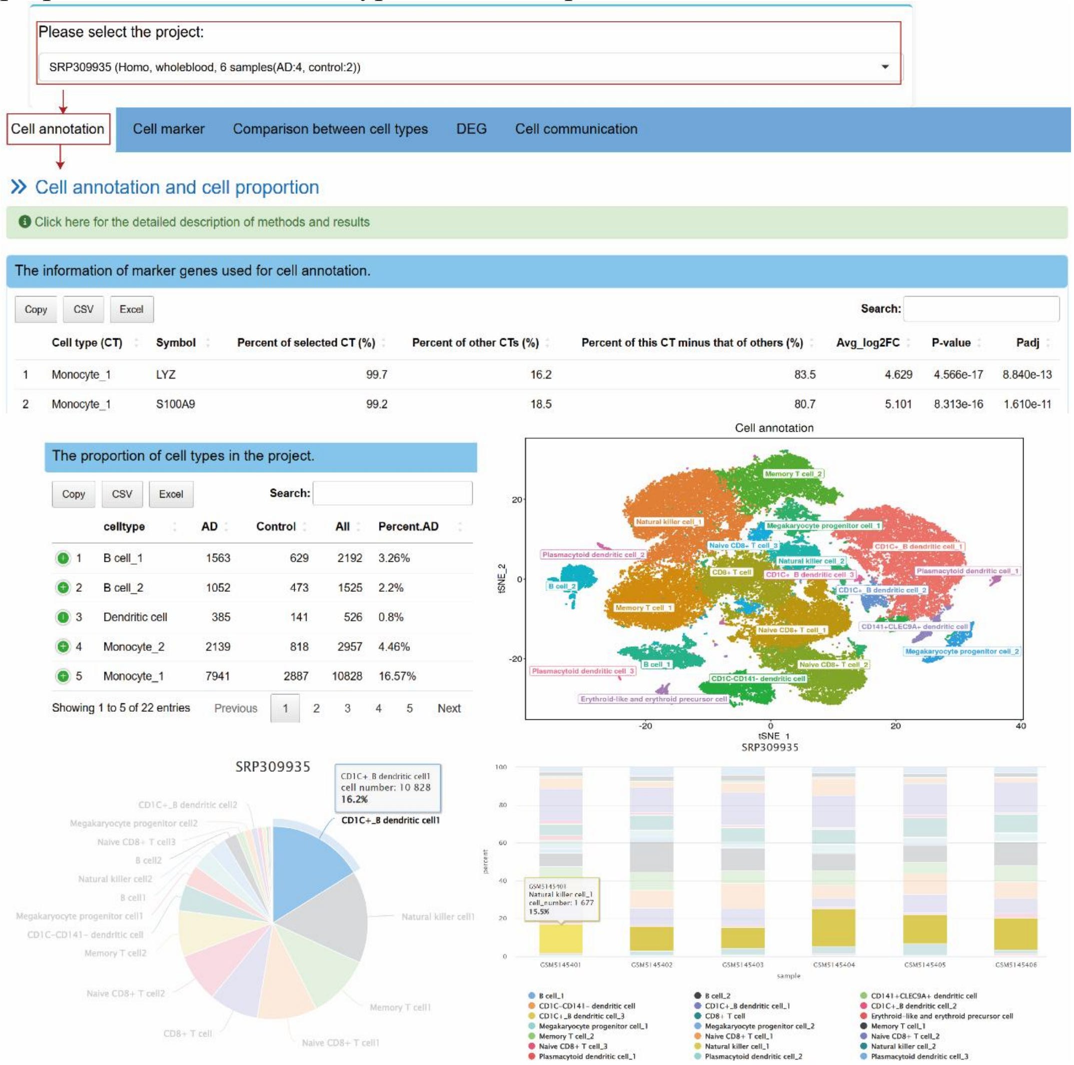

Figure 15 The “Cell annotation” submodule analysis page of the scRNA-seq module.

Cell marker

DEG

This sub-module of cell markers calculates the specific expressed genes of one cell type and other cell types (Figure 16).

By selecting 'B cell_1' in the DropDownList and then clicking 'CD79A' in the result table, users can get the feature plot and violin plot of CD79A gene expression between different cell types in B cell_1.

By selecting '|avg_log2FC|' and 'top5' in the DropDownLists, users can get the bubble plot and heatmap summarize the expression distribution of the first five genes in each cluster with |avg_log2FC| as the screening criterion in B cell_1.

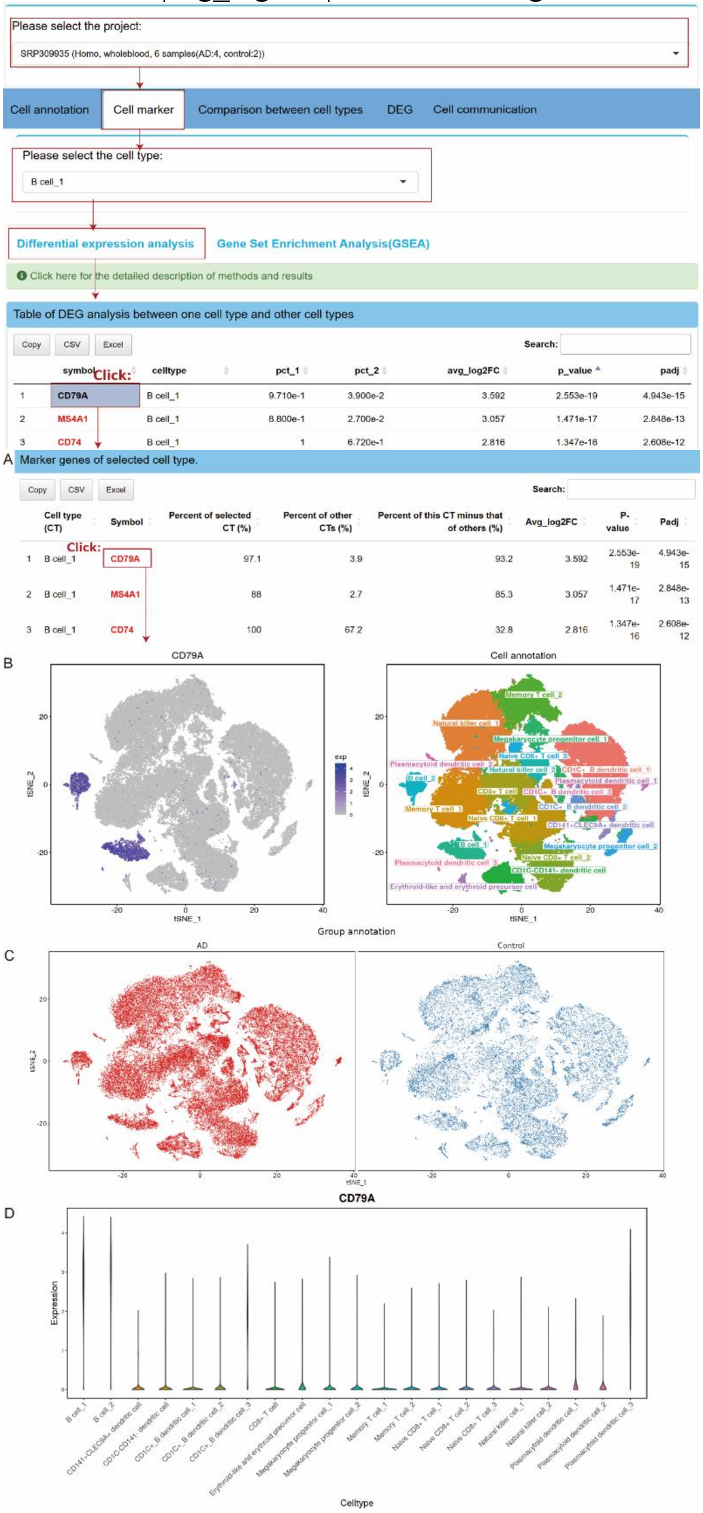

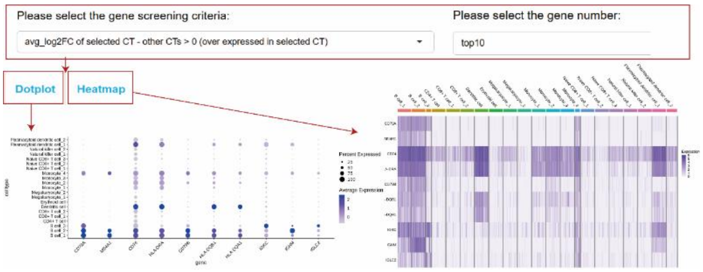

Figure 16 The representative results of “Differentional expression analysis” of the "Cell marker" submodule.

GSEA

This sub-module analyzes the GSEA of the specific expressed genes between one cell type and other cell types (Figure 17).

By selecting 'B cell_1', 'GO', and 'BP' in the DropDownLists, users can get the ridge plot showing the distribution of core-enriched genes of the first 20 ($p < 0.05$, arranged by p-value) enrichment pathways of the specifically expressed genes of B cell_1.

By clicking 'GO:0045047' in the result table, the user can get a graphical view of the enrichment score of the gene set of this pathway.

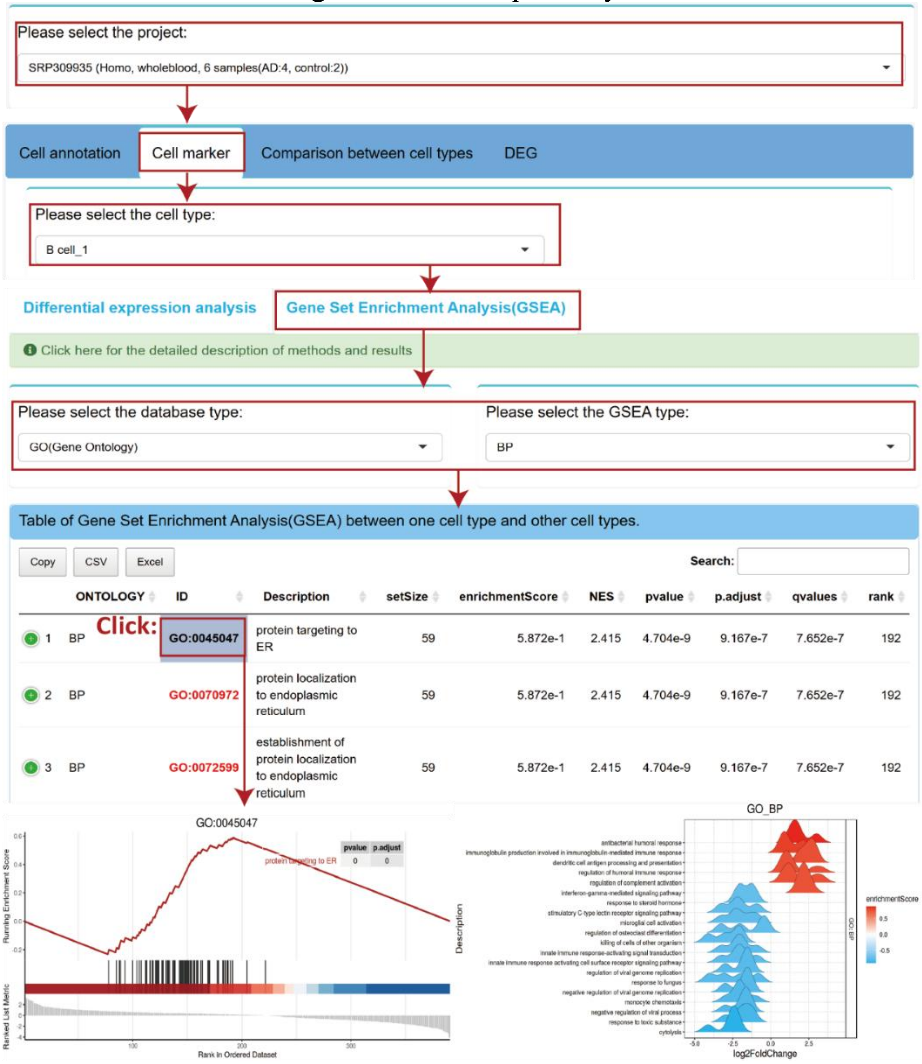

Figure 17 The representative results of “GSEA” of the "Cell marker" submodule.

Comparison between cell types

DEG

This sub-module of comparison between cell types module calculates the DEGs between a selected cell type and another cell type (Figure 18).

By selecting 'B cell_1' and 'Plasmacytoid dendritic cell_3' in the DropDownLists and then clicking 'DLGAP5' in the result table, users can get the feature plot and violin plot of DLGAP5 gene differential expression between B cell_1 and Plasmacytoid dendritic cell_3.

Through select '|avg_log2FC|' and 'top5' in the DropDownLists, users can get the bubble plot and heatmap summarize the expression distribution of the first five differentially expressed genes in B cell_1 and Plasmacytoid dendritic cell_3 with |avg_log2FC| as the screening criterion.

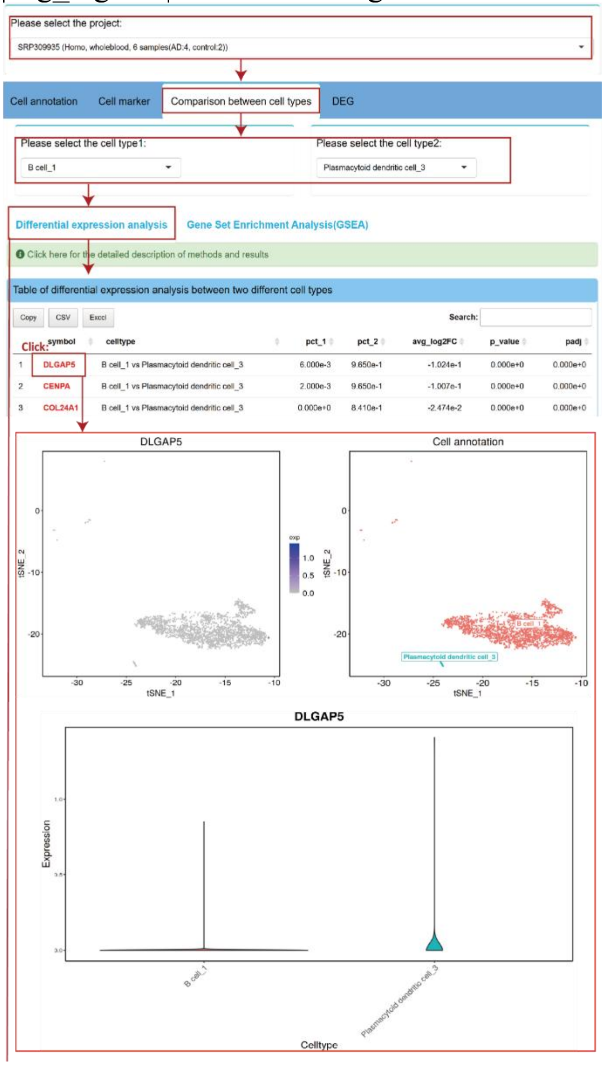

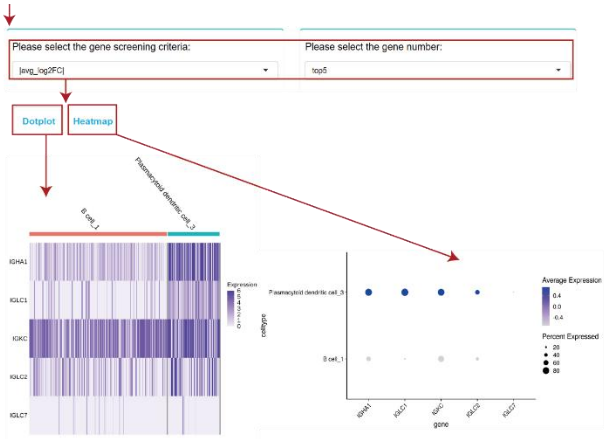

Fitgure 18 The representative results of “Differentional expression analysis” of the "Comparison between cell types" submodule.

GSEA

This sub-module analyzes the GSEA of the DEGs between the selected cell type and another cell type (Figure 19).

By selecting 'B cell_1' and 'Plasmacytoid dendritic cell_3', 'GO', and 'BP' in the DropDownLists, users can get the ridge plot showing the distribution of core-enriched genes of the first 20 ($P < 0.05$, arranged by p-value) enrichment pathways of differentially expressed genes between B cell _1 and plasmacytoid dendritic cell _3.

By clicking 'GO:0030449' in the result table, the user can get a graphical view of the enrichment score of the gene set of this pathway.

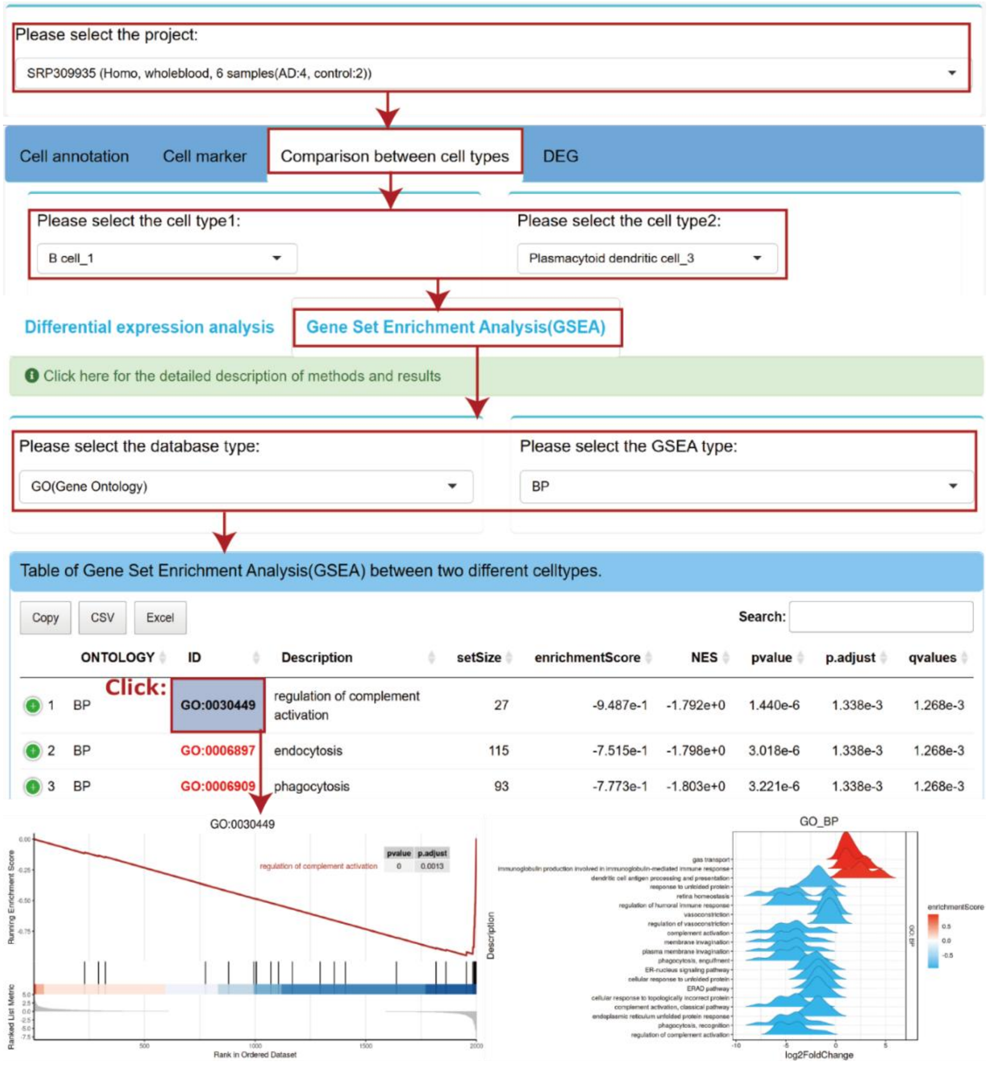

Figure 19 The representative results of “GSEA” of the "Comparison between cell types" submodule.

DEG

DEG

This sub-module analyzes the differential expression between AD/MCI and normal samples in each cell type (Figure 20).

By selecting 'B cell_1' and 'AD vs control' in the DropDownLists and then clicking 'RPS3A' in the result table, users can get the rain cloud plot showing the differential expression of RPS3A gene between AD and normal sample in B cell_1 (FDR = 1.061e-123).

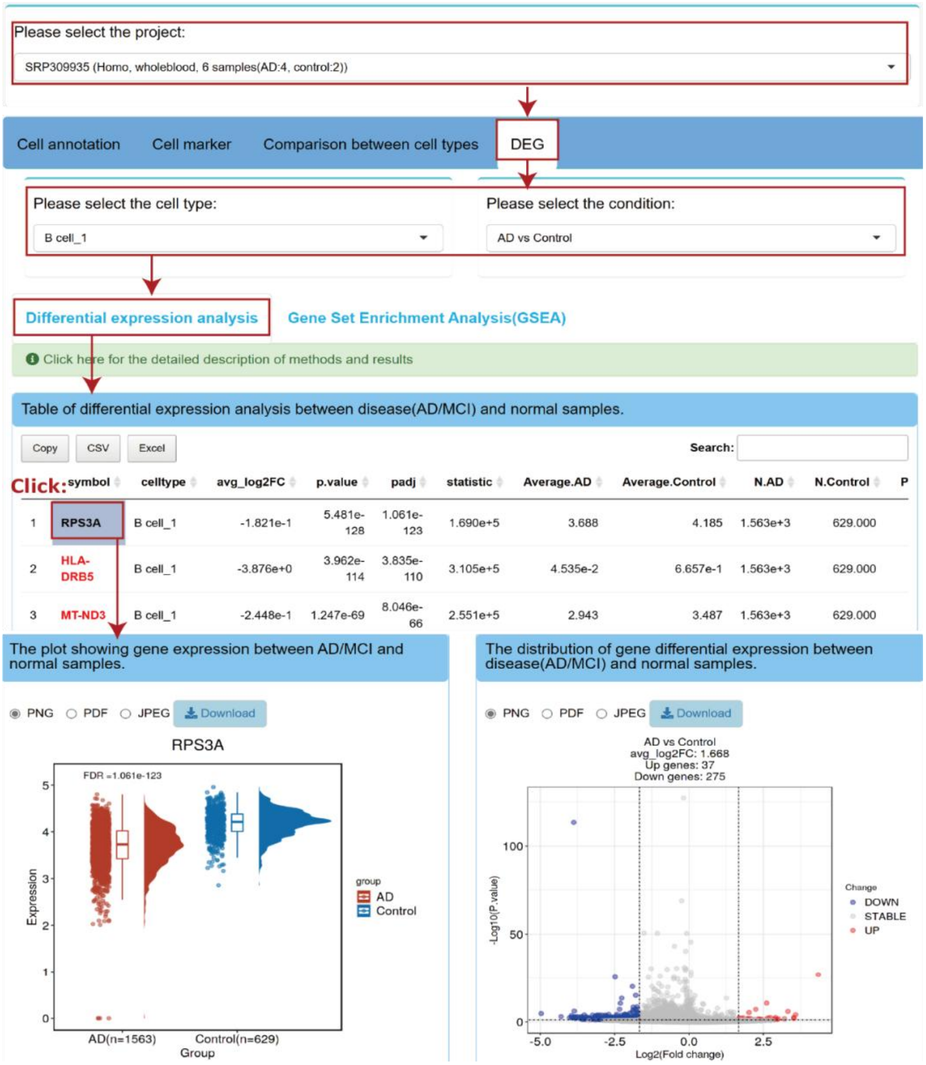

Figure 20 The representative results of "Differentional expression analysis" of the "DEG" submodule.

GSEA

This sub-module analyzes the GSEA of the DEGs between AD/MCI and normal samples in each cell type (Figure 21).

By selecting 'B cell_1' and 'AD vs control', 'GO' and 'BP' in the DropDownLists, users can get the ridge plot showing the distribution of core-enriched genes of the first 20 (P-value < 0.05, arranged by p-value) enrichment pathways of differentially expressed genes between AD/MCI and normal samples in B cell_1.

By clicking 'GO:0001906' in the result table, the user can get a graphical view of the enrichment score of the gene set of this pathway.

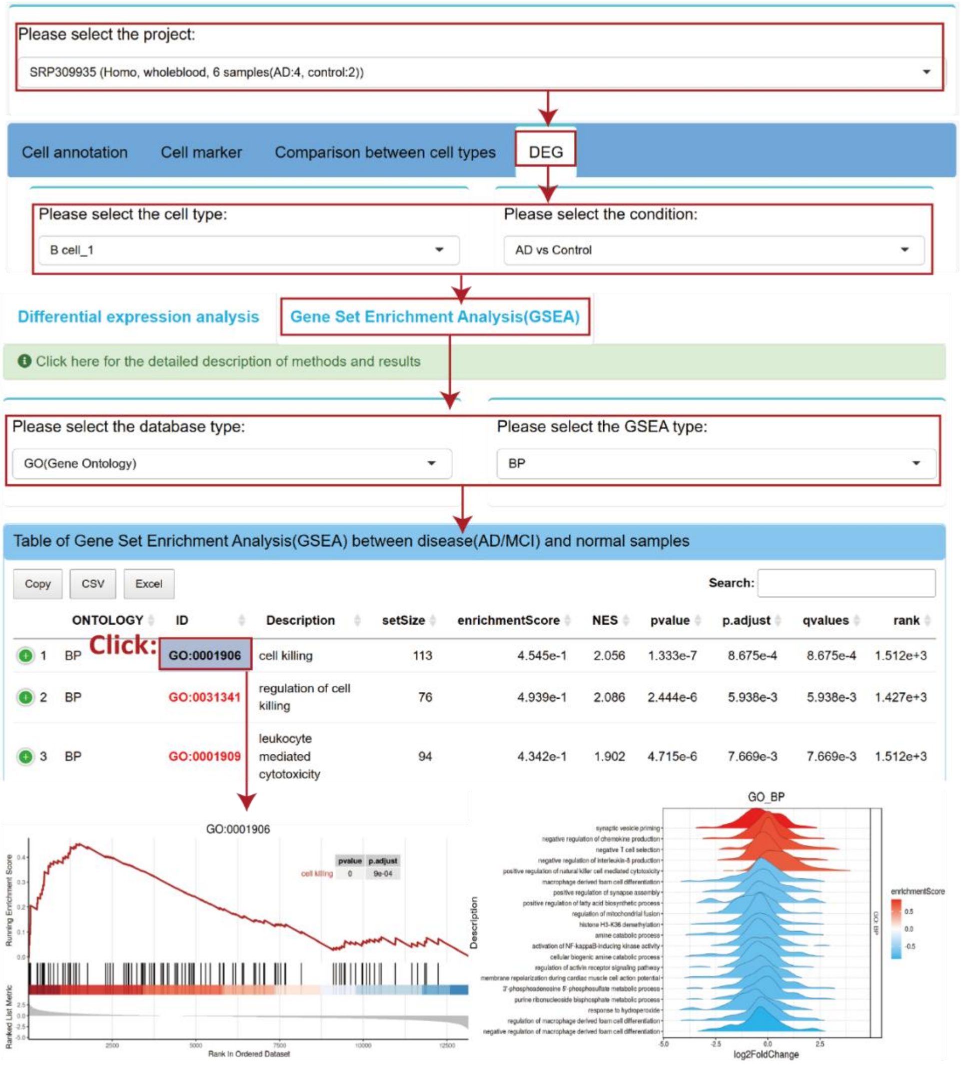

Figure 21 The representative results of “GSEA” of the "DEG" submodule.

Cell communication

Cell interaction

This sub-module calculates the general principle of cell communication (Figure 22). By selecting 'AD vs control' in the DropDownList, users can get a table of prediction results of the intercellular communication network, a histogram showing the total number of interactions and interaction strength for each group, a circle plot showing the total number of interactions for each group, a circle plot showing the total interaction strength for each group, and a scatter diagram showing the comparison of outgoing and incoming interaction strength in 2D space.

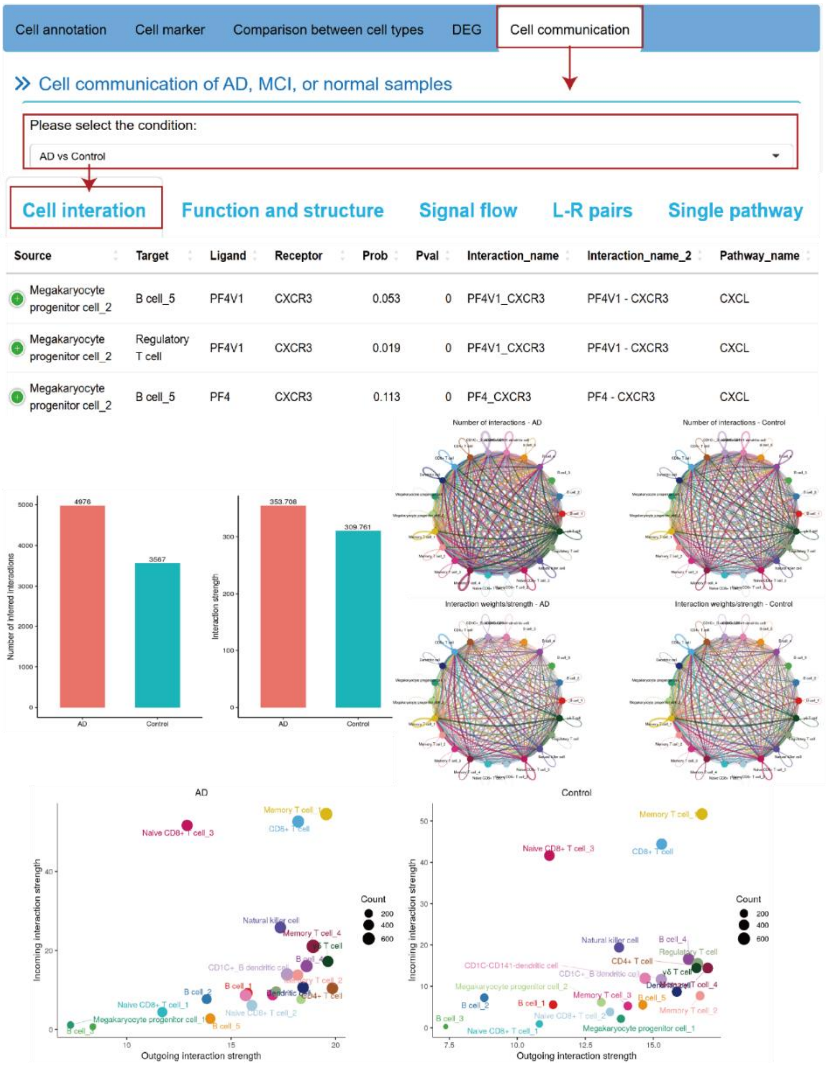

Figure 22 The representative results of “Cell interaction” of the "Cell communication" submodule.

Function and structure

This sub-module calculates the joint multiple learning and classification of inferred communication networks based on their functional and topological similarities (Figure 23).

In this sub-module, users can get plots to identify signal groups according to their functional/structural similarity.

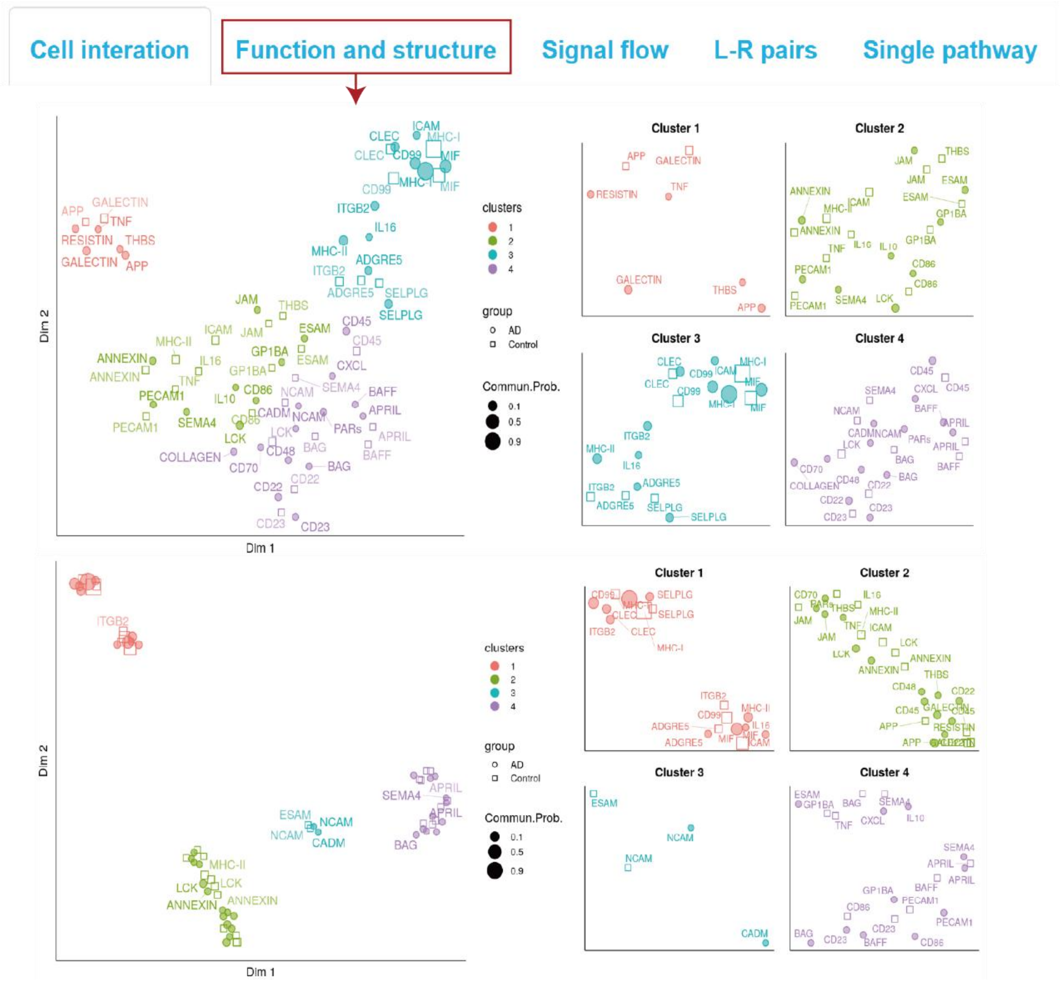

Figure 23 The representative results of "Function and structure" of the "Cell communication" submodule.

Signal flow

This sub-module identifies and visualizes conservative and environment-specific signal pathways. In this sub-module, the rankNet function was used to compare the overall signal flow of each signaling pathway (Figure 24).

In this sub-module, users can get the histogram to compare the overall information flow of each signal pathway and the heatmaps to compare incoming/outgoing/overall signals associated with each cell population.

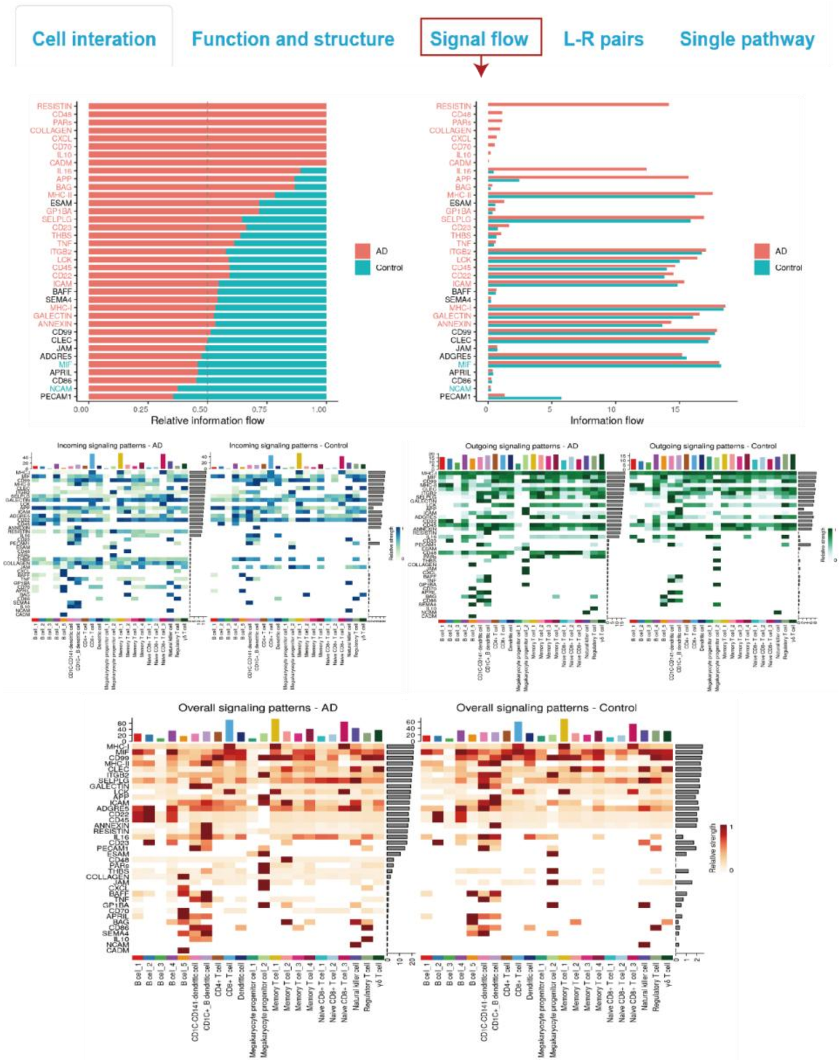

Figure 24 The representative results of “Signal flow” of the "Cell communication" submodule.

L-R pairs

This sub-module compares the communication probability of ligand-receptor pair regulation from some cell groups to other cell groups (Figure 25).

By selecting 'B cell_1' in the DropDownList, users can get the plot to identify the up-regulated and down-regulated signal ligand-receptor pairs in B cell_1.

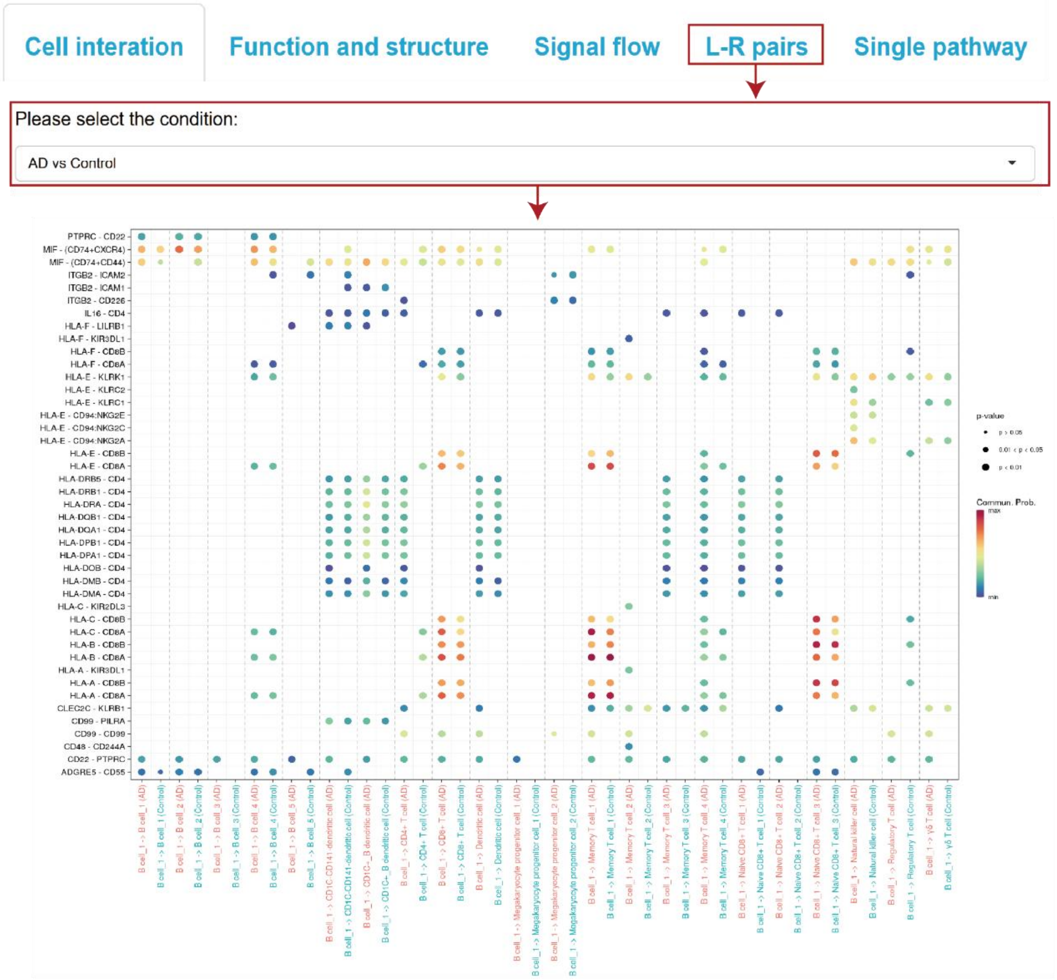

Figure 25 The representative results of “L-R pairs” of the "Cell communication" submodule.

Signal pathway

This sub-module compares the distribution of signal gene expression between different data sets (Figure 26).

By selecting 'MHC-I' in the DropDownList, users can get the violin plot showing the gene distribution and a table showing the differentially expressed genes between AD and normal samples of the MHC-1 signal pathway.

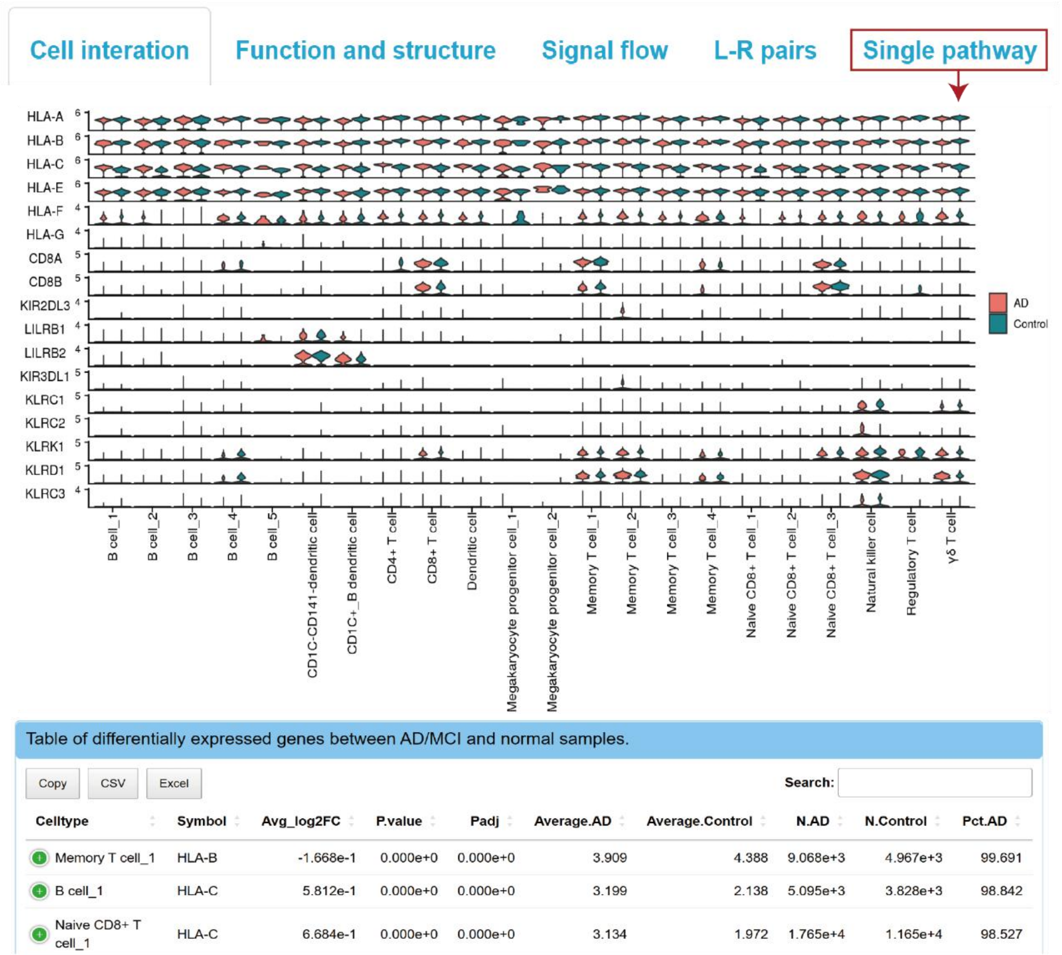

Figure 26 The representative results of “Signal pathway” of the "Cell communication" submodule.

Clinical

The Clinical module of RBAD performs the association between blood mRNAs' expression and overall survival, sex, APOE genotype, Braak stage, age, education, and MMSE, including three sub-modules (Figure 27):

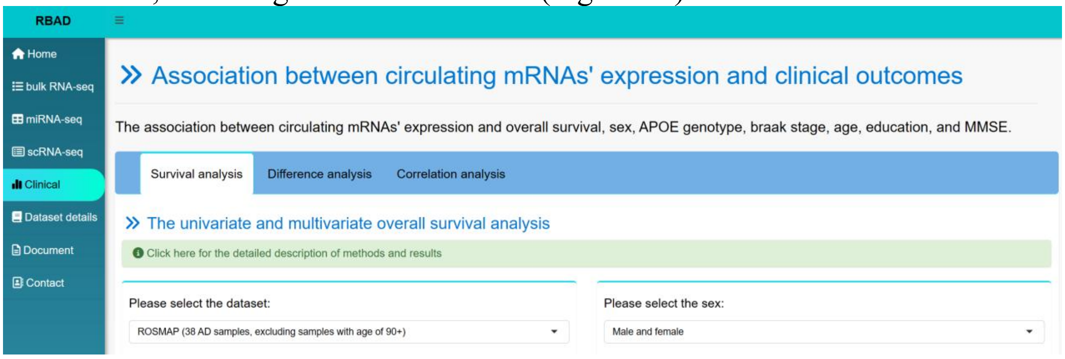

Figure 27 The Clinical module analysis page of RBAD.

Survival analysis

This sub-module analyzes the association between blood mRNAs' expression and survival time.

Through selecting the dataset and sex in the DropDownLists, users can get the table that presents detailed information about the Gene symbol, Hazard Ratio, Cox P value, Logrank P value, and Higher risk of death (Figure 28).
By clicking 'TSPAN6' in the result table, users can get the Kaplan-Meier curve, univariate forest plot, and multivariate forest plot.

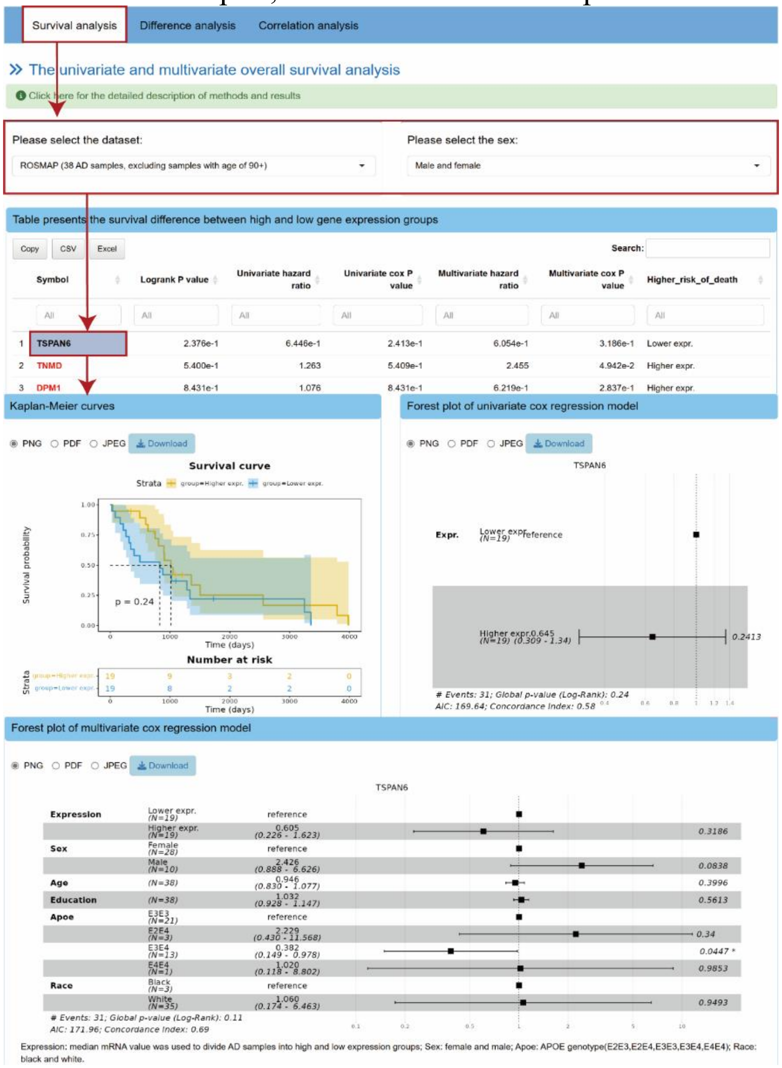

Figure 28 The “Survival analysis” submodule analysis page of the Clinical module.

Difference analysis
This sub-module calculates the differentially expressed genes for sex, APOE genotype, and Braak stage (Figure 29).
Through selecting the clinical outcome and dataset in the DropDownList, users can get a table of the proportion of sex in the project and a table of difference analysis results.
By clicking 'IL7R' in the result table, the user can get a rain cloud plot showing the differential expression of the IL7R gene between female AD and female normal samples of Homo (FDR = 5.399e-114).

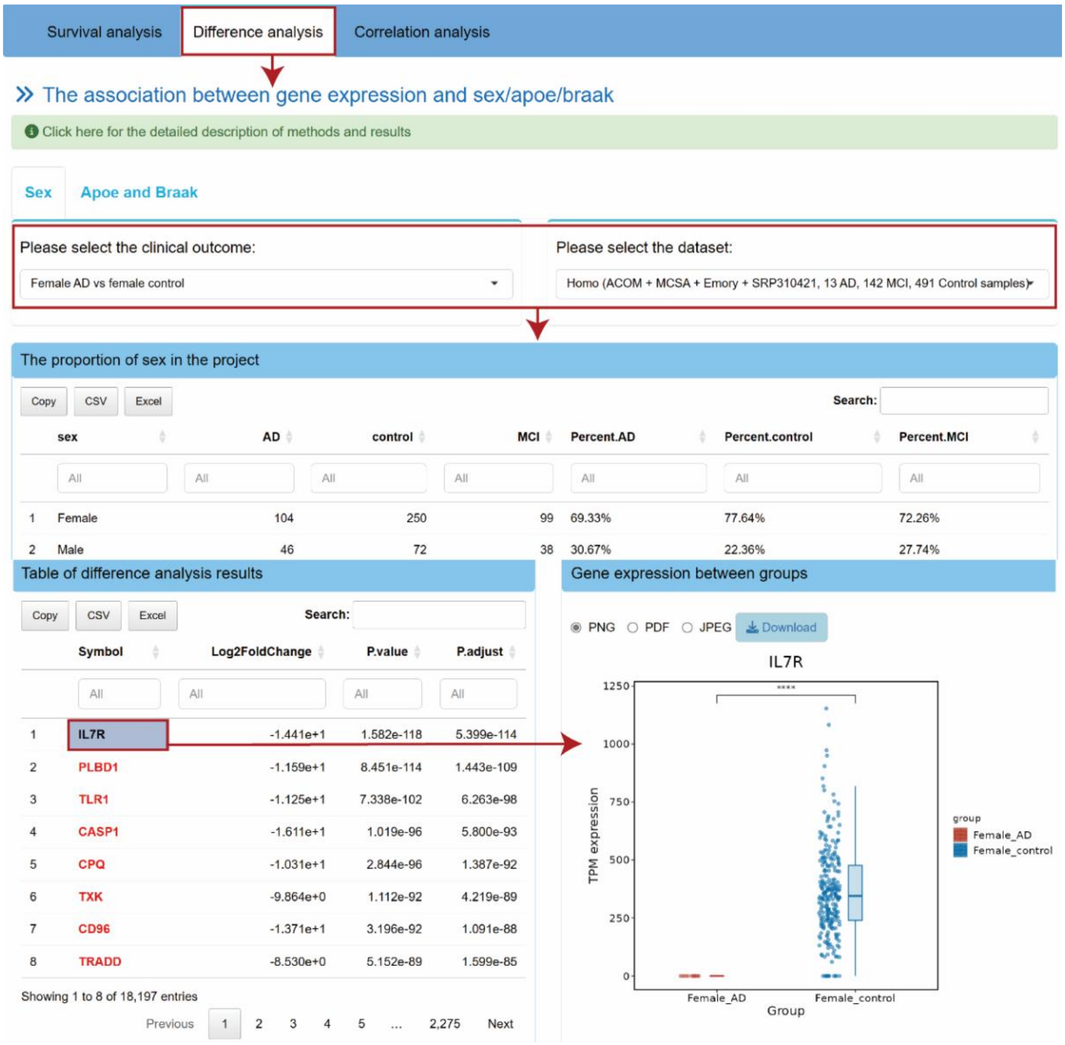

Figure 29 The "Difference analysis" submodule analysis page of the Clinical module.

Correlation analysis

This sub-module is designed to estimate the association between gene expression and age/MMSE/education (Figure 30).

Through selecting the clinical outcome and dataset in the DropDownList and clicking 'SOX18' in the result table, users can get the scatter plot showing the expression of the SOX18 gene correlated with age at sampling.

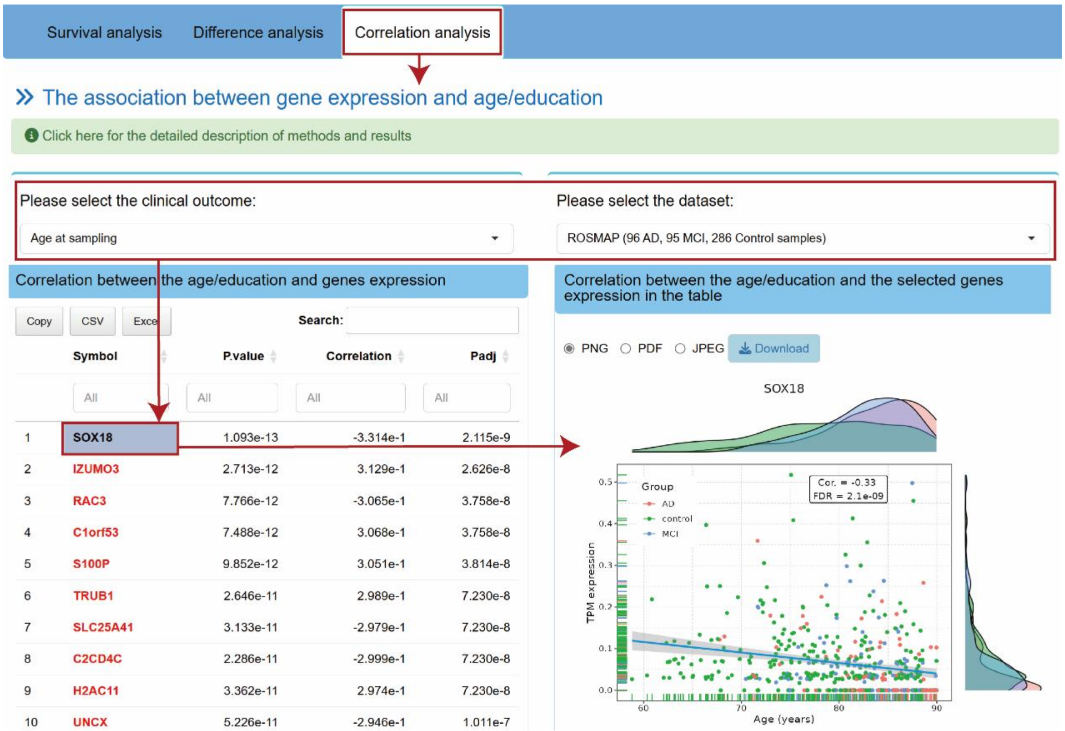

Figure 30 The "Correlation analysis" submodule analysis page of the Clinical module.
