# Supplementary material for: RBAD: The first database dedicated alterations of blood RNA in individuals with Alzheimer’s disease and their clinical relevance
Source: Neural Regen Res. 2025 Mar 25;21(6):2553–62. doi: 10.4103/NRR.NRR-D-24-01165 (PMC13211806; doi:10.4103/NRR.NRR-D-24-01165)
Supplement: Supplementary file 6 [file NRR-21-2553_Suppl4.pdf]

**Additional Table 7. Time-Series analysis of the expression of circulating mRNA using ROSMAP cohort dataset.**	
Method	Time-Series Analysis by Mfuzz R package
Description	Data set: ROSMAP cohort in RBAD.<br>Clusters of circulating mRNA expression trend from control to MCI to AD.<br>●Control/MCI/AD: Normalized average expression of each gene in control, MCI, and AD groups. ●Cluster: range from 1 to 9, corresponding to Figure 3B.

Symbol	Control	MCI	AD	Cluster
AADAC	-1.117547338	0.307147039	0.810400299	1
AADACL2	-0.97788147	-0.042858919	1.02074039	1
AAMP	-1.135071198	0.383932683	0.751138516	1
AANAT	-1.077948847	0.180477963	0.897470884	1
AARSD1	-1.137669623	0.739951357	0.397718266	1
ABCB10	-1.15444268	0.59835365	0.55608903	1
ABCC5	-1.123247264	0.329811882	0.793435383	1
ABHD13	-1.15001619	0.664991829	0.485024361	1
ABHD18	-1.138645244	0.73550101	0.403144234	1
ABHD4	-1.116141483	0.301806927	0.814334556	1
ABL2	-1.154631056	0.566345398	0.588285657	1
ABRACL	-1.133731946	0.377158553	0.756573393	1
ACAD8	-1.152304703	0.640537154	0.511767549	1
ACAP2	-1.106579384	0.267612337	0.838967046	1
ACMSD	-1.112485723	0.28832191	0.824163814	1
ACOT1	-1.092489722	0.222439896	0.870049826	1
ACOT12	-1.150103075	0.485904534	0.664198541	1
ACR	-0.995031148	-0.009864717	1.004895866	1
ACSF3	-1.123089295	0.329159563	0.793929731	1
ACSM1	-1.145079612	0.701359491	0.443720122	1
ACSS1	-1.145637815	0.447776922	0.697860893	1
ACTC1	-0.924333883	-0.137170322	1.061504205	1
ACTL6A	-1.082153045	0.192208941	0.889944105	1
ACTR5	-1.115157965	0.298123716	0.817034249	1
ACTR6	-1.03409415	0.072089811	0.962004339	1
ADAMTSL4	-1.016715754	0.034314834	0.98240092	1
ADAT3	-1.152254769	0.511075791	0.641178978	1
ADD3	-1.037753139	0.080354828	0.95739831	1
ADGRG7	-1.154399282	0.554358382	0.6000409	1
ADH1B	-1.149860135	0.666397288	0.483462846	1
ADI1	-1.15440494	0.55457669	0.59982825	1
ADK	-0.928647604	-0.129991114	1.058638719	1
ADPRM	-1.152840974	0.519690714	0.63315026	1
ADRM1	-1.152579377	0.636875033	0.515704344	1
ADSS1	-1.144627927	0.704109948	0.440517979	1
ADSS2	-0.930434132	-0.126998473	1.057432605	1
ADTRP	-1.152745876	0.518211871	0.634534005	1
AEBP2	-1.114131893	0.294325403	0.81980649	1
AFM	-1.100839022	0.248566757	0.852272265	1
AGBL5	-1.152689268	0.635341152	0.517348116	1
AGFG2	-1.153753304	0.536379927	0.617373377	1

AGGF1	-1.154415752	0.599416056	0.554999696	1
AGRP	-1.141513488	0.420057646	0.721455842	1
AGT	-0.968210243	-0.060804681	1.029014925	1
AGTPBP1	-1.154509567	0.559068404	0.595441163	1
AGTR1	-1.131750835	0.367494382	0.764256453	1
AGTR2	-1.103254914	0.256464035	0.846790879	1
AGXT2	-1.007993247	0.016182919	0.991810328	1
AIFM1	-1.13760449	0.740243248	0.397361242	1
AIP	-1.152325055	0.51205148	0.640273574	1
AKAP9	-1.120097811	0.317076336	0.803021476	1
AKIP1	-0.938752779	-0.112909979	1.051662758	1
AKR7A2	-0.983444288	-0.032327471	1.015771759	1
ALAS1	-0.977436905	-0.043693815	1.02113072	1
ALDH18A1	-1.149408324	0.479072691	0.670335634	1
ALDH1B1	-1.138033223	0.399723117	0.738310106	1
ALDH9A1	-1.138321925	0.736992416	0.401329509	1
ALG9	-1.136440263	0.391082767	0.745357496	1
ALKAL1	-1.130487142	0.36153025	0.768956892	1
ALLC	-1.141335018	0.418957976	0.722377042	1
ALPK2	-1.126083698	0.341791699	0.784291999	1
ALX1	-1.077003263	0.177879975	0.899123288	1
AMD1	-1.146368127	0.453267059	0.693101068	1
AMMECR1L	-1.069388558	0.157459492	0.911929066	1
ANAPC10	-1.115631788	0.299892873	0.815738915	1
ANAPC4	-1.148533618	0.677479678	0.47105394	1
ANG	-1.146432153	0.692673135	0.453759018	1
ANGPT1	-1.152242333	0.510904567	0.641337766	1
ANGPTL3	-0.936762674	-0.116303851	1.053066525	1
ANGPTL4	-1.102045732	0.252490783	0.849554948	1
ANKFY1	-1.137143788	0.742290067	0.394853721	1
ANKRD10	-1.117909386	0.308537137	0.80937225	1
ANKRD33B	-1.065107384	0.14634171	0.918765674	1
ANKRD34C	-0.983328868	-0.032547596	1.015876464	1
ANKRD7	-0.902602846	-0.172382052	1.074984898	1
ANO5	-0.970817424	-0.056010854	1.026828278	1
ANOS1	-1.102084181	0.252616482	0.849467699	1
ANP32E	-0.988832082	-0.021973672	1.010805754	1
ANPEP	-1.139987461	0.410866609	0.729120852	1
AOX1	-1.150392708	0.661495035	0.488897673	1
AP1G1	-1.109160818	0.276512594	0.832648224	1
AP3B2	-1.146665503	0.69109806	0.455567443	1
AP5M1	-1.085730973	0.202437535	0.883293439	1
AP5S1	-1.122683632	0.327491227	0.795192404	1
APBB1IP	-1.14950199	0.479967656	0.669534334	1
APCS	-1.154538603	0.560522365	0.594016239	1
APLP1	-1.096136269	0.233641737	0.862494532	1
APOA1	-1.094182736	0.22760255	0.866580186	1
APP	-1.072581992	0.165918342	0.90666365	1
ARFRP1	-0.963827351	-0.068792806	1.032620156	1
ARG2	-1.132639587	0.760859441	0.371780146	1
ARHGAP18	-0.920914857	-0.142814375	1.063729232	1
ARHGEF1	-1.152996529	0.522191236	0.630805293	1
ARL2BP	-1.13864195	0.40312566	0.73551629	1
ARL6IP4	-1.152689517	0.635337623	0.517351894	1
ARMC5	-1.105711567	0.264669303	0.841042264	1

ARMCX1	-0.983241679	-0.032713834	1.015955513	1
ARMCX2	-1.034293942	0.072538118	0.961755823	1
ARMCX5	-0.96328276	-0.069779279	1.033062039	1
ARMH2	-1.028784357	0.060297409	0.968486948	1
ARPC5L	-1.1475007	0.68524747	0.46225323	1
ARRDC3	-0.923635537	-0.138326419	1.061961956	1
ASB14	-1.145638063	0.447778754	0.697859309	1
ASB17	-1.131164972	0.364710997	0.766453975	1
ASPDH	-1.153600982	0.62043054	0.533170441	1
ASPN	-1.132762875	0.760381957	0.372380919	1
ASRGL1	-1.145870764	0.449504654	0.69636611	1
ASZ1	-1.12848657	0.352374115	0.776112456	1
ATF6	-1.154038394	0.610879772	0.543158623	1
ATG4C	-1.099599048	0.244575917	0.855023131	1
ATP4A	-0.984896913	-0.029551115	1.014448028	1
ATP5F1B	-1.152520987	0.637673291	0.514847696	1
ATP5MC3	-1.038135134	0.081224464	0.95691067	1
ATP5MF-PTCD1	-1.154205659	0.606376912	0.547828748	1
ATP6AP2	-1.111587604	0.285091478	0.826496126	1
ATP6V0C	-1.009157811	0.018574395	0.990583416	1
ATP6V1E1	-1.136077195	0.746914126	0.38916307	1
AWAT2	-1.137926769	0.738792713	0.399134056	1
AZI2	-1.110035892	0.279581495	0.830454397	1
B3GALT4	-1.10062675	0.247880626	0.852746124	1
B3GLCT	-1.152667449	0.517018349	0.6356491	1
B3GNT8	-0.937748504	-0.114624511	1.052373016	1
BAAT	-1.149890156	0.483761357	0.666128799	1
BABAM1	-1.149641268	0.668328471	0.481312798	1
BAG5	-1.10126959	0.24996227	0.85130732	1
BAP1	-1.154247185	0.60514282	0.549104365	1
BARD1	-1.128112875	0.350699916	0.77741296	1
BBC3	-1.142712948	0.715076129	0.427636819	1
BCL6	-1.101279802	0.24999543	0.851284372	1
BCLAF3	-1.023752054	0.04933003	0.974422024	1
BEGAIN	-1.06925512	0.157109196	0.912145925	1
BET1	-0.950219011	-0.093056732	1.043275743	1
BEX2	-1.024398203	0.050727281	0.973670922	1
BFSP2	-1.005665891	0.011429763	0.994236127	1
BMERB1	-1.14107129	0.72372583	0.41734546	1
BMI1	-1.083134282	0.194990893	0.888143389	1
BMP5	-1.057717678	0.127704274	0.930013404	1
BMS1	-1.124007986	0.332974439	0.791033546	1
BNIP1	-0.902570467	-0.172433384	1.075003851	1
BOC	-1.128735074	0.353493524	0.77524155	1
BOD1	-1.141209724	0.723019674	0.41819005	1
BOLA2B	-1.154686916	0.582200845	0.572486071	1
BPIFB6	-1.090784529	0.217304116	0.873480413	1
BPNT2	-1.15397548	0.612419986	0.541555493	1
BRI3	-1.09337876	0.225142859	0.868235901	1
BRWD1	-1.076458471	0.17638967	0.9000688	1
BSDC1	-1.135043588	0.751252685	0.383790903	1
BTC	-1.023395497	0.048560358	0.974835139	1
BTF3	-1.002350858	0.004718414	0.997632444	1
BTG4	-1.132540772	0.371299747	0.761241025	1
BTN3A1	-0.95722845	-0.080657866	1.037886315	1

BTN3A3	-1.149447606	0.670000511	0.479447095	1
BUD31	-1.134204143	0.754680647	0.379523496	1
C10orf88	-1.132540935	0.371300538	0.761240397	1
C10orf99	-1.101800502	0.251690056	0.850110446	1
C11orf1	-1.154498086	0.558524027	0.595974059	1
C11orf80	-0.920136137	-0.144094257	1.064230395	1
C11orf98	-1.152645642	0.635955183	0.516690458	1
C12orf29	-1.134189787	0.754738559	0.379451229	1
C12orf54	-0.922386276	-0.140390329	1.062776605	1
C12orf57	-1.126114481	0.341924628	0.784189853	1
C12orf76	-1.152566588	0.515515748	0.63705084	1
C14orf39	-1.143901238	0.435504772	0.708396465	1
C16orf78	-1.15193148	0.506753026	0.645178453	1
C16orf82	-1.128996259	0.354675382	0.774320877	1
C16orf87	-1.153812296	0.616122078	0.537690218	1
C17orf100	-1.152699659	0.63519388	0.517505779	1
C19orf33	-0.90812415	-0.163580941	1.071705092	1
C1GALT1C1	-0.906477517	-0.166215746	1.072693263	1
C1orf174	-1.10443751	0.26039117	0.84404634	1
C1orf216	-1.038601614	0.082288204	0.95631341	1
C1orf52	-0.906925125	-0.165500371	1.072425496	1
C1QA	-1.113198913	0.290909532	0.82228938	1
C1QBP	-1.149950325	0.665588009	0.484362316	1
C1QC	-0.961644579	-0.072738708	1.034383287	1
C20orf144	-1.078876116	0.183039762	0.895836354	1
C21orf91	-1.115879965	0.300823419	0.815056546	1
C3orf62	-1.031863027	0.067106357	0.964756669	1
C4BPA	-1.15009229	0.664297443	0.485794847	1
C4orf33	-0.892646558	-0.188016214	1.080662772	1
C4orf46	-1.030917506	0.065006955	0.965910551	1
C5orf15	-1.151428573	0.500486758	0.650941815	1
C5orf49	-1.153665143	0.534494013	0.61917113	1
C6orf118	-1.154695699	0.574452697	0.580243002	1
C6orf120	-1.133771132	0.756417265	0.377353868	1
C6orf62	-1.154682598	0.571766896	0.582915702	1
C7orf31	-1.147803811	0.464769835	0.683033976	1
C7orf61	-1.015381708	0.031508125	0.983873582	1
C8B	-1.006733753	0.01360636	0.993127393	1
C8orf76	-1.008882809	0.018008872	0.990873937	1
C9orf131	-1.138994359	0.405122947	0.733871412	1
CALCOCO1	-1.154571091	0.562312359	0.592258733	1
CAMK1	-1.072572667	0.165893426	0.906679241	1
CAMSAP1	-1.129481148	0.356884249	0.7725969	1
CAPN8	-1.080220065	0.186778093	0.893441971	1
CARD19	-1.064134287	0.143848566	0.920285721	1
CARS1	-1.05838395	0.129357558	0.929026392	1
CARTPT	-1.082931951	0.194415847	0.888516104	1
CASP4	-1.149496682	0.479916731	0.669579951	1
CASP5	-0.941612181	-0.108007243	1.049619424	1
CAV3	-1.137135873	0.742324963	0.394810911	1
CBLL2	-1.096647303	0.235236573	0.861410731	1
CBR3	-1.133435102	0.757750668	0.375684434	1
CCDC153	-1.024253722	0.050414576	0.973839146	1
CCDC172	-1.019123924	0.03941324	0.979710684	1
CCDC178	-1.119269934	0.313817737	0.805452198	1

CCDC181	-0.905113459	-0.168391916	1.073505375	1
CCDC182	-0.892387437	-0.188419153	1.08080659	1
CCDC192	-1.085055729	0.200489053	0.884566676	1
CCDC195	-1.154607973	0.564642148	0.589965824	1
CCDC197	-1.04362518	0.093869997	0.949755183	1
CCDC47	-0.964272271	-0.067985889	1.032258159	1
CCDC59	-1.149961288	0.484472208	0.66548908	1
CCER1	-1.152839393	0.63317355	0.519665843	1
CCK	-1.146187135	0.694301194	0.451885941	1
CCL18	-0.963556367	-0.069283833	1.0328402	1
CCN4	-1.081478762	0.190307124	0.891171637	1
CCNI	-1.04968002	0.108152008	0.941528012	1
CCNI2	-1.028465933	0.059597544	0.96886839	1
CCNL2	-1.106890535	0.268673415	0.83821712	1
CCNY	-1.154693444	0.580852087	0.573841357	1
CCP110	-0.923930773	-0.137837869	1.061768642	1
CCR3	-1.12189041	0.324256737	0.797633673	1
CCR9	-0.899991134	-0.17651219	1.076503324	1
CCT6A	-1.110547142	0.281387128	0.829160014	1
CD101	-1.139890089	0.410295619	0.72959447	1
CD163	-1.041927597	0.089929969	0.951997628	1
CD19	-0.917033345	-0.14917353	1.066206876	1
CD200R1L	-1.140856568	0.724813253	0.416043315	1
CD209	-1.018335682	0.037739873	0.980595809	1
CD276	-1.153483103	0.53083351	0.622649593	1
CD58	-1.068107628	0.154107013	0.914000615	1
CD63	-1.144546539	0.704598304	0.439948235	1
CDADC1	-1.089701488	0.214074383	0.875627105	1
CDAN1	-1.152365684	0.639743818	0.512621867	1
CDC14A	-1.151606656	0.502648821	0.648957835	1
CDC25C	-1.151571783	0.64935098	0.502220802	1
CDC26	-1.069527101	0.157823456	0.911703645	1
CDCA7	-1.045116567	0.09735419	0.947762377	1
CDH9	-1.023970311	0.049801641	0.97416867	1
CDK16	-0.97209845	-0.053643699	1.025742149	1
CDK2AP2	-0.963664728	-0.06908752	1.032752248	1
CDK8	-1.138896571	0.404566658	0.734329913	1
CDKAL1	-1.15020516	0.66325624	0.48694892	1
CDKN2A	-1.149664568	0.481539541	0.668125027	1
CDO1	-1.127140445	0.346393307	0.780747139	1
CDV3	-0.906560328	-0.166083444	1.072643772	1
CEACAM18	-1.121576466	0.322986481	0.798589985	1
CEBPZ	-0.903101494	-0.171591106	1.0746926	1
CEBPZOS	-1.12529779	0.338419852	0.786877937	1
CEMIP	-1.132498858	0.761402581	0.371096277	1
CENPV	-1.017837866	0.036685349	0.981152518	1
CEP192	-1.154582006	0.562962955	0.591619051	1
CEP295	-1.150581841	0.490904481	0.65967736	1
CEP350	-1.152089053	0.508827632	0.643261421	1
CEP68	-0.978945544	-0.040856576	1.019802119	1
CERS3	-1.146889418	0.689563173	0.457326244	1
CES1	-1.122576222	0.327051118	0.795525104	1
CETN3	-1.154308305	0.55109169	0.603216616	1
CETP	-0.925920704	-0.134537043	1.060457747	1
CFAP298	-1.076080162	0.175357556	0.900722606	1

CFAP53	-1.130265056	0.360497054	0.769768002	1
CFAP97	-1.1020618	0.25254331	0.84951849	1
CFL2	-1.15440364	0.599877302	0.554526338	1
CGRRF1	-1.143013031	0.713425183	0.429587848	1
CHAMP1	-1.034492975	0.072985062	0.961507913	1
CHCHD1	-1.099886564	0.245497621	0.854388942	1
CHCHD3	-1.13306676	0.373868419	0.759198341	1
CHD2	-1.009353758	0.018977647	0.990376111	1
CHIC1	-1.153731298	0.535901404	0.617829894	1
CHIC2	-0.914713395	-0.152950236	1.067663631	1
CHN2	-1.150020905	0.664948984	0.485071921	1
CHRAC1	-1.153474215	0.530661875	0.62281234	1
CHRNB1	-1.129321422	0.356154488	0.773166934	1
CHST4	-1.10957899	0.277975751	0.831603239	1
CIAO1	-1.144354086	0.438609478	0.705744608	1
CISH	-1.141288036	0.418669634	0.722618402	1
CKM	-0.904840232	-0.16882711	1.073667342	1
CKS2	-1.100975258	0.249007759	0.851967498	1
CLCNKB	-1.153871084	0.614831992	0.539039092	1
CLDN17	-1.110490321	0.281185978	0.829304343	1
CLDN18	-0.990539227	-0.018660372	1.009199599	1
CLDN20	-1.135608419	0.386709925	0.748898493	1
CLDN34	-1.153894859	0.539597862	0.614296998	1
CLEC11A	-1.152814809	0.519280239	0.633534569	1
CLEC1B	-0.97955909	-0.039699436	1.019258526	1
CLEC4A	-1.12321215	0.329666752	0.793545398	1
CLIC1	-1.109460763	0.277561459	0.831899304	1
CLK3	-1.016734421	0.034354196	0.982380225	1
CLNS1A	-1.137285354	0.741664404	0.39562095	1
CLPB	-1.124459608	0.334868986	0.789590623	1
CMIP	-1.146141205	0.694603537	0.451537667	1
CMTM2	-0.900072649	-0.176383598	1.076456247	1
CMTM4	-1.06020108	0.133893205	0.926307875	1
CMTM8	-1.132648531	0.760824853	0.371823679	1
CNGA1	-1.141775725	0.720089558	0.421686166	1
CNIH1	-1.100068222	0.246081104	0.853987118	1
CNN2	-1.141973817	0.422926612	0.719047205	1
CNOT2	-0.930520957	-0.126852737	1.057373695	1
CNTNAP3C	-1.145209601	0.7005549	0.444654701	1
COG7	-1.078246176	0.181297869	0.896948307	1
COL4A5	-0.918286387	-0.147126206	1.065412592	1
COMMD4	-1.142510758	0.71617562	0.426335138	1
COPS9	-0.972929604	-0.052103661	1.025033265	1
COPZ1	-0.999954643	-0.0000907	1.00004535	1
COQ6	-1.149336212	0.478388804	0.670947408	1
COX6A1	-1.154612413	0.564951763	0.58966065	1
COX7A1	-1.120262088	0.317727164	0.802534923	1
COX7A2L	-1.154558682	0.561604905	0.592953777	1
CPEB4	-1.148189908	0.468052743	0.680137165	1
CPED1	-1.143967982	0.708009513	0.435958469	1
CPHXL	-1.150091665	0.664303163	0.485788502	1
CPNE6	-1.009388877	0.019049947	0.99033893	1
CPO	-1.116358136	0.302624016	0.813734119	1
CPQ	-0.988115466	-0.023359767	1.011475233	1
CRABP2	-1.152627544	0.636207913	0.516419631	1

CRAT	-1.149856598	0.666428865	0.483427733	1
CRLF1	-1.132680824	0.371980915	0.760699908	1
CRNKL1	-1.146557573	0.454727962	0.691829612	1
CRNN	-1.120100231	0.317085913	0.803014318	1
CROCC	-1.106366731	0.266888951	0.83947778	1
CRTAM	-1.041075574	0.087962664	0.95311291	1
CRYAB	-1.010410057	0.021155819	0.989254238	1
CSF2	-1.150534509	0.660136319	0.49039819	1
CSN3	-0.895918329	-0.182911472	1.078829801	1
CSNK1E	-1.150876659	0.494122972	0.656753687	1
CSNK1G3	-0.969251103	-0.058894664	1.028145767	1
CSRNP2	-0.89670936	-0.181672496	1.078381856	1
CSTA	-1.137479553	0.396678162	0.740801392	1
CSTB	-1.154381671	0.600690178	0.553691493	1
CSTF3	-1.104090287	0.259233807	0.84485648	1
CT45A5	-0.909724343	-0.161012179	1.070736522	1
CTAGE15	-1.119634672	0.315249041	0.804385631	1
CTAGE6	-1.110101511	0.27981272	0.830288791	1
CTAGE9	-1.1545131	0.595273922	0.559239179	1
CTIF	-1.149301864	0.478064602	0.671237262	1
CTNNBL1	-1.150287297	0.662489802	0.487797496	1
CTSW	-1.125004942	0.33717402	0.787830922	1
CUX1	-1.085968328	0.203124496	0.882843831	1
CWF19L2	-0.899518707	-0.177257051	1.076775758	1
CWH43	-0.945230951	-0.101757084	1.046988035	1
CXCL13	-1.147793354	0.683111202	0.464682151	1
CXCL14	-1.074600206	0.171341364	0.903258842	1
CYB561	-1.006580012	0.013292548	0.993287464	1
CYB561D1	-1.153810127	0.616168833	0.537641294	1
CYLC1	-0.926247469	-0.133993691	1.06024116	1
CYLC2	-1.127217516	0.346732056	0.78048546	1
CYP1B1	-0.916772486	-0.149599083	1.066371569	1
CYP27A1	-1.136911728	0.743309492	0.393602236	1
CYP2C18	-0.921341799	-0.142111787	1.063453586	1
CYP2D6	-1.148840932	0.473805436	0.675035496	1
CYP2J2	-1.116455035	0.302990147	0.813464889	1
CYTL1	-0.970968981	-0.055731204	1.026700185	1
DAG1	-1.115651977	0.29996847	0.815683507	1
DAOA	-1.108040191	0.272621444	0.835418747	1
DAZAP1	-1.015321612	0.031381982	0.98393963	1
DAZL	-1.034182586	0.072288208	0.961894378	1
DBNDD2	-1.154686601	0.582256587	0.572430014	1
DBNL	-1.154381239	0.600705859	0.55367538	1
DBP	-0.975631946	-0.047073482	1.022705428	1
DCAF13	-1.020554503	0.042461712	0.978092791	1
DDIT3	-1.048729799	0.105886279	0.94284352	1
DDN	-1.024440098	0.050817986	0.973622112	1
DDX27	-1.037869226	0.080618971	0.957250255	1
DDX31	-1.144725569	0.703521197	0.441204372	1
DDX46	-1.153576573	0.620899716	0.532676857	1
DDX52	-1.075806293	0.174611782	0.901194511	1
DDX53	-1.050166905	0.109316566	0.940850339	1
DEFA5	-0.910607721	-0.159590589	1.07019831	1
DEFB105B	-1.142436293	0.425858293	0.716578	1
DEFB112	-1.151528924	0.501697918	0.649831006	1

DEFB125	-1.12707531	0.346107359	0.780967951	1
DEFB127	-1.093872324	0.226651126	0.867221198	1
DEFB129	-1.03413144	0.072173459	0.961957981	1
DEFB4B	-1.098765305	0.241915413	0.856849892	1
DEPDC1	-1.001162964	0.002329999	0.998832964	1
DGKZ	-1.135867685	0.388063192	0.747804493	1
DHRS11	-1.135348101	0.385359714	0.749988387	1
DHRS4	-1.108983286	0.275893271	0.833090015	1
DHTKD1	-1.151268331	0.652679104	0.498589227	1
DHX29	-1.076165943	0.17559139	0.900574553	1
DHX9	-1.151907765	0.645461982	0.506445782	1
DIRAS3	-0.922654369	-0.139947867	1.062602235	1
DKK4	-1.072566066	0.165875791	0.906690275	1
DLEU7	-1.133674866	0.376874349	0.756800517	1
DLL4	-1.139053793	0.733591953	0.40546184	1
DMP1	-1.147606316	0.684482009	0.463124307	1
DMRT3	-1.097972389	0.239401835	0.858570553	1
DNAJB6	-1.134984476	0.383487674	0.751496802	1
DNAJC12	-0.917524685	-0.148371366	1.06589605	1
DNAJC25	-1.014094565	0.028811817	0.985282748	1
DNAJC3	-1.154099289	0.544783193	0.609316096	1
DNAJC5	-1.147164864	0.459522885	0.687641979	1
DNAJC5B	-1.144412332	0.705398926	0.439013406	1
DNAJC9	-1.137749172	0.739593995	0.398155177	1
DNASE2B	-1.084221427	0.198093383	0.886128043	1
DOK5	-1.146023502	0.450649178	0.695374324	1
DONSON	-1.055035949	0.121101489	0.93393446	1
DPP9	-1.144189153	0.706717706	0.437471447	1
DRAM2	-1.087104042	0.206426523	0.880677519	1
DSC2	-0.998798833	-0.002398021	1.001196854	1
DSG1	-1.050636594	0.11044234	0.940194254	1
DSPP	-1.15412766	0.608560002	0.545567658	1
DST	-1.144080483	0.707354269	0.436726214	1
DYNAP	-1.033865111	0.071576294	0.962288817	1
DYNC1LI1	-1.054437329	0.119638685	0.934798644	1
DYNLT2	-1.149913107	0.665922945	0.483990163	1
DZIP3	-1.141408592	0.721998116	0.419410476	1
ECHS1	-1.154693162	0.580920972	0.57377219	1
EDNRB	-0.898760766	-0.178450657	1.077211423	1
EED	-1.153684697	0.534905311	0.618779386	1
EEF1A1	-1.146789497	0.45653848	0.690251017	1
EEF1E1	-1.093239076	0.224717004	0.868522072	1
EEF1E1-BLOC1S5	-1.017035726	0.034989882	0.982045844	1
EEFSEC	-1.011765312	0.023961277	0.987804035	1
EFNA5	-1.147201479	0.687383754	0.459817725	1
EHMT2	-1.071138459	0.162076523	0.909061936	1
EID3	-0.988560271	-0.022499742	1.011060014	1
EIF2D	-1.101308519	0.250088694	0.851219825	1
EIF2S3B	-0.977666156	-0.043263403	1.020929559	1
EIF3A	-1.147357158	0.461079009	0.686278149	1
EIF3B	-1.136238701	0.746223845	0.390014856	1
EIF3C	-0.894638381	-0.184912274	1.079550655	1
EIF3D	-1.031150858	0.065524396	0.965626461	1
EIF3G	-1.142854067	0.428551434	0.714302633	1
EIF3J	-1.150266683	0.487583823	0.66268286	1

EIF4A1	-1.123313172	0.330084486	0.793228686	1
EIF4EBP1	-1.145418034	0.446165955	0.699252078	1
EIF4G2	-1.039276499	0.083830644	0.955445855	1
EIF5A2	-0.944233669	-0.103484662	1.047718332	1
ELAVL4	-0.897397904	-0.180592516	1.07799042	1
ELOA2	-0.949123106	-0.094976855	1.04409996	1
ELOA3CP	-1.072659104	0.166124427	0.906534677	1
ELOF1	-1.124467534	0.334902347	0.789565186	1
EMC9	-1.116309354	0.302439857	0.813869497	1
EME1	-1.138670818	0.73538232	0.403288498	1
EML4	-1.084306283	0.198336457	0.885969827	1
ENHO	-1.118455891	0.310647346	0.807808546	1
ENTHD1	-0.983474118	-0.03227057	1.015744688	1
ENTPD2	-0.964153081	-0.068202142	1.032355222	1
ERCC8	-1.138941326	0.404821059	0.734120267	1
ERH	-1.136441409	0.745352558	0.391088851	1
ERICH4	-1.098686012	0.241663327	0.857022685	1
ERMN	-0.969945938	-0.057616801	1.027562739	1
ERP29	-1.132878044	0.372943536	0.759934508	1
ETDA	-1.152454617	0.638567242	0.513887375	1
ETDC	-1.154530532	0.560106063	0.594424468	1
EVC2	-1.05087808	0.111022045	0.939856035	1
EVI2A	-1.024477949	0.050899946	0.973578003	1
EVI2B	-1.153106624	0.62907793	0.524028694	1
EVPL	-1.153544687	0.62150478	0.532039908	1
EXOC1L	-0.984610424	-0.03009955	1.014709974	1
EXOC3	-1.069458671	0.157643652	0.911815019	1
EXOSC2	-0.983707818	-0.031824615	1.015532433	1
EXTL2	-1.046778707	0.101262887	0.94551582	1
EZH1	-1.10523164	0.263051895	0.842179745	1
F12	-1.15258051	0.636859432	0.515721078	1
F5	-1.004710742	0.009489016	0.995221726	1
F9	-0.939950731	-0.110859781	1.050810512	1
FABP3	-0.905922413	-0.167102046	1.073024459	1
FABP7	-1.0216405	0.044785899	0.976854601	1
FAM110A	-1.152024913	0.644048839	0.507976075	1
FAM133A	-0.946289464	-0.099919129	1.046208592	1
FAM149B1	-1.032030586	0.067479172	0.964551414	1
FAM161B	-1.002916783	0.005859315	0.997057468	1
FAM169A	-1.067420091	0.152316861	0.915103231	1
FAM174A	-1.143486721	0.432716063	0.710770658	1
FAM209A	-0.977504792	-0.043566387	1.021071179	1
FAM209B	-1.002260677	0.00453679	0.997723886	1
FAM20B	-0.9017	-0.173812175	1.075512175	1
FAM210B	-1.058202837	0.128907633	0.929295205	1
FAM216B	-1.15367819	0.618910166	0.534768024	1
FAM228A	-0.904529174	-0.169322278	1.073851451	1
FAM24B	-1.082930722	0.194412356	0.888518366	1
FAM50B	-1.150102238	0.485896016	0.664206222	1
FAM72C	-0.947996661	-0.096945404	1.044942065	1
FAM72D	-1.09048931	0.2164213	0.87406801	1
FAM76B	-1.015693978	0.032163998	0.98352998	1
FAM8A1	-1.131638945	0.764678652	0.366960293	1
FAM98A	-1.144409092	0.438990911	0.705418181	1
FANCA	-0.911548863	-0.158073272	1.069622135	1

FANCF	-0.932148486	-0.124115895	1.056264382	1
FANCI	-1.151289679	0.652450156	0.498839522	1
FARS2	-1.089299978	0.21288325	0.876416728	1
FASN	-1.153268114	0.52683955	0.626428564	1
FASTKD3	-0.968041104	-0.061114583	1.029155687	1
FBXO28	-0.998403893	-0.003184608	1.001588501	1
FBXO33	-1.143252406	0.431161077	0.712091329	1
FBXO46	-1.067181156	0.151696241	0.915484915	1
FBXW7	-1.143301736	0.711814542	0.431487194	1
FCGR2B	-0.89237811	-0.188433654	1.080811763	1
FCMR	-0.921460192	-0.141916845	1.063377037	1
FCRL4	-1.14007871	0.728675422	0.411403288	1
FCRL6	-1.153197079	0.627611994	0.525585085	1
FEM1C	-1.087769549	0.208373141	0.879396408	1
FETUB	-1.029597826	0.062089031	0.967508795	1
FFAR4	-1.022455949	0.046536815	0.975919134	1
FGF10	-1.084883692	0.199993993	0.884889699	1
FGF16	-1.101415101	0.250435037	0.850980063	1
FGF9	-1.030332366	0.063711411	0.966620955	1
FGFBP1	-0.991328675	-0.017122747	1.008451422	1
FGFR1OP2	-1.146847732	0.689850707	0.456997025	1
FIS1	-1.106029463	0.265744614	0.840284849	1
FITM1	-1.115507291	0.299427082	0.816080208	1
FLRT1	-1.154544943	0.593688348	0.560856595	1
FMC1-LUC7L2	-1.05494162	0.12087072	0.9340709	1
FMO5	-1.153304408	0.527492191	0.625812217	1
FN3KRP	-1.017480445	0.035929311	0.981551134	1
FNDC10	-0.936307343	-0.117078265	1.053385608	1
FOLH1	-0.895045493	-0.184276409	1.079321903	1
FOXE1	-1.091110956	0.218282411	0.872828545	1
FOXR2	-0.910619153	-0.159572176	1.070191329	1
FPGT	-1.0678116	0.153335452	0.914476148	1
FSD1L	-0.99148753	-0.016812923	1.008300453	1
FSD2	-1.145499284	0.698739889	0.446759395	1
FSTL4	-1.108027404	0.272577291	0.835450113	1
FTMT	-1.073330527	0.167922555	0.905407972	1
FTSJ1	-1.136379405	0.390759757	0.745619648	1
FUCA1	-1.123899506	0.332521279	0.791378228	1
FUOM	-0.995682056	-0.008580667	1.004262722	1
FUT1	-1.154210549	0.547976131	0.606234418	1
FUT8	-1.144805538	0.70303665	0.441768888	1
FXYD7	-1.108235887	0.273297869	0.834938018	1
FZR1	-0.997717973	-0.004548536	1.00226651	1
GABRB1	-1.018576628	0.038250906	0.980325722	1
GAGE12B	-1.153918167	0.540153549	0.613764618	1
GAL3ST4	-0.980270047	-0.038356202	1.018626249	1
GALM	-1.108538347	0.274345882	0.834192465	1
GALNT13	-1.105985299	0.265595035	0.840390264	1
GANC	-1.153460627	0.623059971	0.530400655	1
GAS2	-0.980882898	-0.037196263	1.018079161	1
GATAD2A	-1.117014925	0.305114008	0.811900917	1
GBX1	-1.060119885	0.133689695	0.926430189	1
GCG	-1.026332892	0.054930048	0.971402844	1
GCHFR	-1.08961997	0.213832281	0.875787689	1
GCK	-1.079517382	0.184819729	0.894697653	1

GDF9	-0.999806162	-0.000387564	1.000193726	1
GDI2	-1.153395978	0.624219441	0.529176537	1
GFRA1	-1.154485215	0.557931554	0.596553661	1
GGTA1	-1.154025991	0.611189153	0.542836837	1
GGTLC3	-1.000792956	0.001587803	0.999205153	1
GIMAP1-GIMAP5	-1.047450198	0.102849748	0.944600451	1
GINM1	-0.938202492	-0.113849921	1.052052413	1
GIPR	-0.954112089	-0.086195756	1.040307845	1
GLMN	-0.946055811	-0.100325216	1.046381027	1
GLRB	-0.959326624	-0.076905931	1.036232555	1
GLRX2	-1.147036085	0.68854484	0.458491245	1
GLRX5	-1.145233507	0.444827238	0.700406269	1
GLS2	-0.986127998	-0.027189476	1.013317474	1
GLUL	-1.152795037	0.633823143	0.518971894	1
GMPPB	-1.147695617	0.683829978	0.463865639	1
GNA15	-1.153110799	0.629011233	0.524099566	1
GNAS	-1.135428215	0.385774349	0.749653867	1
GNG13	-1.148886508	0.474219284	0.674667224	1
GNMT	-0.99540264	-0.009132173	1.004534812	1
GNRH1	-0.90994218	-0.160661854	1.070604034	1
GOLM2	-1.143499052	0.710700736	0.432798316	1
GORAB	-1.132262278	0.762311161	0.369951116	1
GPATCH1	-1.153028508	0.522718855	0.630309653	1
GPATCH4	-0.929699971	-0.128229667	1.057929637	1
GPER1	-1.004855111	0.009781988	0.995073122	1
GPHB5	-0.968969341	-0.059412205	1.028381547	1
GPKOW	-1.139211288	0.406362783	0.732848505	1
GPR152	-1.148997967	0.475237948	0.67376002	1
GPR160	-1.110961253	0.28285667	0.828104583	1
GPR171	-0.966498736	-0.063934464	1.030433199	1
GPR18	-1.144479577	0.704998492	0.439481085	1
GPR37	-1.151963061	0.644798896	0.507164165	1
GPR52	-1.013778216	0.028150873	0.985627342	1
GPR82	-1.089749683	0.214217586	0.875532097	1
GPX1	-1.154694892	0.574220201	0.580474691	1
GPX2	-1.141090736	0.72362688	0.417463855	1
GPX4	-1.110497817	0.281212507	0.82928531	1
GPX5	-1.139011226	0.733792162	0.405219064	1
GRIA1	-0.910362027	-0.159986228	1.070348255	1
GRIN2A	-1.042518737	0.091298891	0.951219846	1
GRIPAP1	-1.151247732	0.498348425	0.652899308	1
GRK1	-1.152795025	0.633823315	0.51897171	1
GRM8	-1.053018612	0.116187662	0.93683095	1
GRXCR2	-1.151760436	0.504564555	0.647195881	1
GSTA5	-0.964928368	-0.066794346	1.031722714	1
GSTT2B	-1.149165082	0.672381882	0.4767832	1
GTF2A1L	-1.129076572	0.355039905	0.774036667	1
GTF2A2	-1.147484451	0.685364695	0.462119756	1
GTF2F1	-1.127050029	0.345996455	0.781053574	1
GTF2H2	-1.043146014	0.092755103	0.950390911	1
GTF2H4	-1.148232126	0.679814875	0.468417251	1
GTF3C3	-1.147774534	0.683250035	0.464524498	1
GTF3C6	-1.061399413	0.136906024	0.924493389	1
GTSF1L	-1.151793891	0.64680621	0.504987681	1
GUCA1B	-1.142031625	0.423290306	0.718741319	1

GUCY2F	-1.107991472	0.272453248	0.835538225	1
GXYLT2	-1.154688011	0.572685935	0.582002076	1
GYPB	-1.078510138	0.182026975	0.896483164	1
GYPC	-0.959814758	-0.076030318	1.035845076	1
H1-10	-1.139476061	0.731588982	0.407887079	1
H1-2	-1.125077604	0.337482611	0.787594993	1
H1-3	-1.09599277	0.233195041	0.862797729	1
H1-4	-1.133994976	0.755522142	0.378472834	1
H2AC11	-1.149349253	0.6708371	0.478512153	1
H2AC18	-1.136959797	0.393860839	0.743098957	1
H2AC19	-1.152946353	0.631573365	0.521372988	1
H2AJ	-1.151024253	0.495778966	0.655245287	1
H2AL1Q	-1.068090305	0.154061831	0.914028475	1
H2AP	-1.154549742	0.593435634	0.561114108	1
H2AW	-1.038788267	0.082714385	0.956073882	1
H2BC21	-0.94259557	-0.106313843	1.048909414	1
H2BE1	-1.049661293	0.108107263	0.941554029	1
H2BW1	-1.041344528	0.088582943	0.952761584	1
H3-3B	-1.153853135	0.615230705	0.53862243	1
H3-5	-1.154685228	0.57219299	0.582492238	1
H4C3	-1.132222232	0.36975783	0.762464402	1
HAGH	-1.154654036	0.568352438	0.586301598	1
HAT1	-1.142766193	0.714784878	0.427981315	1
HAUS3	-0.92935324	-0.128810463	1.058163702	1
HAUS7	-1.151924255	0.506659295	0.645264961	1
HAVCR2	-1.152478587	0.638245982	0.514232605	1
HBEGF	-1.031002602	0.065195599	0.965807003	1
HBS1L	-1.051222352	0.111849559	0.939372792	1
HCCS	-1.027019072	0.056427629	0.970591443	1
HCN3	-1.088973087	0.211915931	0.877057156	1
HDDC2	-0.976113749	-0.046172909	1.022286658	1
HDHD2	-0.911138609	-0.158735039	1.069873648	1
HEATR5A	-1.144190566	0.706709404	0.437481162	1
HEBP1	-1.070100758	0.159333362	0.910767395	1
HELB	-1.152587454	0.636763728	0.515823726	1
HENMT1	-1.154332115	0.602425249	0.551906866	1
HEPN1	-1.142723892	0.427707569	0.715016323	1
HERPUD1	-0.984044769	-0.031181132	1.015225901	1
HEY2	-1.071467292	0.162949015	0.908518277	1
HINT2	-1.148443692	0.678182487	0.470261204	1
HINT3	-0.985314989	-0.028750003	1.014064993	1
HLA-DQA2	-1.151197747	0.653430807	0.49776694	1
HMBS	-1.140783534	0.415602569	0.725180966	1
HMCES	-1.149841703	0.483280016	0.666561688	1
HMGCLL1	-0.905514111	-0.167753336	1.073267447	1
HMMR	-1.132658191	0.371870704	0.760787487	1
HMOX1	-0.981435516	-0.036148682	1.017584198	1
HNF1B	-1.09544388	0.231490965	0.863952915	1
HNMT	-0.973782741	-0.05051944	1.024302181	1
HNRNPA1L2	-0.913435827	-0.155022425	1.068458253	1
HNRNPA1P48	-1.090250841	0.215709531	0.87454131	1
HNRNPH1	-1.034159747	0.072236965	0.961922782	1
HNRNPH3	-1.055041206	0.121114354	0.933926853	1
HNRNPM	-1.154468992	0.597259729	0.557209263	1
HOXA5	-1.108568141	0.274449284	0.834118857	1

HP	-0.901463397	-0.174186543	1.07564994	1
HPCAL1	-1.142201025	0.717840465	0.42436056	1
HPR	-1.153510665	0.53136966	0.622141005	1
HPRT1	-0.991588999	-0.016614949	1.008203948	1
HPSE2	-0.913927136	-0.154226167	1.068153303	1
HS3ST5	-1.149765377	0.482526407	0.66723897	1
HS6ST2	-0.935795726	-0.117947483	1.053743209	1
HSCB	-1.116246643	0.302203267	0.814043376	1
HSFY1	-1.145229547	0.444798641	0.700430905	1
HSP90AA1	-1.153499864	0.622341025	0.531158839	1
HSPA1A	-1.152452253	0.63859883	0.513853423	1
HSPA1B	-1.151763884	0.647155828	0.504608056	1
HTATIP2	-1.080363575	0.187179084	0.893184491	1
HTR6	-1.127858587	0.349566854	0.778291733	1
HYLS1	-0.917911869	-0.147738676	1.065650545	1
ICE1	-1.147224681	0.460004912	0.68721977	1
ID1	-1.121982968	0.324632298	0.79735067	1
ID2	-1.133445811	0.375737446	0.757708365	1
IDH3B	-1.153235716	0.626971971	0.526263745	1
IDI1	-0.981718304	-0.035612005	1.017330309	1
IER2	-1.124532536	0.33517613	0.789356406	1
IFITM10	-0.924221215	-0.137356955	1.06157817	1
IFNA17	-1.150526657	0.490314465	0.660212192	1
IFNA5	-1.004018387	0.00808581	0.995932577	1
IFNG	-1.046992498	0.101767626	0.945224872	1
IFNGR2	-1.10435143	0.260103911	0.84424752	1
IFRD1	-1.14434755	0.438564216	0.705783334	1
IFT88	-1.008275826	0.016762397	0.991513429	1
IGF1	-0.964040805	-0.068405791	1.032446596	1
IGF2BP3	-1.094132222	0.227447573	0.86668465	1
IGHV3OR16-17	-1.137943563	0.738716696	0.399226867	1
IGIP	-1.153214633	0.52589235	0.627322283	1
IL11RA	-1.147141306	0.459333543	0.687807763	1
IL13RA2	-1.08396236	0.197352101	0.886610259	1
IL17F	-1.105052543	0.262450146	0.842602397	1
IL22RA1	-1.043241177	0.092976347	0.950264829	1
IL22RA2	-1.033349229	0.070421292	0.962927937	1
IL23A	-0.982739287	-0.033670946	1.016410233	1
IL27RA	-0.992070619	-0.015674485	1.007745105	1
IL32	-1.04003349	0.08556565	0.95446784	1
IL33	-1.066728423	0.150522398	0.916206025	1
IL37	-1.136966945	0.743067618	0.393899327	1
ILDR2	-1.088576495	0.210745281	0.877831214	1
ILVBL	-1.027669334	0.057850231	0.969819102	1
ING2	-1.153094331	0.523820537	0.629273794	1
ING5	-0.929570651	-0.128446336	1.058016987	1
INHBE	-1.150958992	0.655916115	0.495042876	1
INIP	-1.036285075	0.077024715	0.959260359	1
INPP5B	-1.119694042	0.315482665	0.804211377	1
INPPL1	-1.15369294	0.618613091	0.535079849	1
INSL5	-1.132072472	0.369036394	0.763036079	1
INTS6	-1.154230473	0.548584351	0.605646122	1
IPMK	-1.059325696	0.1317033	0.927622396	1
IQCG	-0.968954295	-0.059439833	1.028394127	1
IRAK1	-1.048628443	0.10564515	0.942983293	1

ISM1	-1.061248371	0.136525314	0.924723057	1
ISOC1	-1.06202256	0.138479675	0.923542885	1
ITGA10	-1.141926891	0.42263195	0.71929494	1
ITGA7	-1.132430317	0.761666387	0.37076393	1
ITGB1	-1.136573206	0.744783092	0.391790114	1
ITGB1BP1	-0.959424233	-0.076730923	1.036155156	1
ITGB3BP	-1.148129032	0.680599932	0.4675291	1
JAK2	-0.982059053	-0.034964781	1.017023835	1
JRKL	-1.154119727	0.545346425	0.608773302	1
KANSL1	-1.000521555	0.001043928	0.999477627	1
KAT2A	-1.150912704	0.65638818	0.494524524	1
KAT2B	-1.153712749	0.535502182	0.618210567	1
KAT6A	-1.1522929	0.640689498	0.511603402	1
KATNBL1	-1.0790898	0.183632125	0.895457675	1
KATNIP	-1.152658222	0.516879411	0.635778811	1
KAZN	-1.148670989	0.676395372	0.472275617	1
KBTBD8	-1.079055204	0.18353617	0.895519034	1
KCNA1	-1.154700276	0.578024744	0.576675532	1
KCNB2	-1.130505897	0.361617704	0.768888193	1
KCNE2	-1.011661401	0.023745743	0.987915659	1
KCNH5	-1.11577695	0.300436832	0.815340118	1
KCNIP4	-1.079614051	0.185088651	0.8945254	1
KCNJ13	-1.009684723	0.019659334	0.990025389	1
KCNK1	-1.046782111	0.10127092	0.945511191	1
KCTD12	-1.154461088	0.556866423	0.597594665	1
KCTD4	-1.092243934	0.221695717	0.870548217	1
KDM1A	-1.14594065	0.695913486	0.450027164	1
KDM3B	-0.908545366	-0.162905563	1.07145093	1
KDM4B	-1.15470052	0.577170187	0.577530332	1
KIAA1191	-1.150366598	0.661742627	0.488623971	1
KIF15	-1.088588501	0.210780671	0.877807829	1
KIF20A	-1.132903811	0.759834213	0.373069598	1
KIF27	-1.145095792	0.44383612	0.701259671	1
KIF2B	-1.021980187	0.045514666	0.976465521	1
KIN	-1.130971405	0.363798418	0.767172987	1
KIR3DL1	-1.081730928	0.191017426	0.890713502	1
KLF9	-1.144783173	0.441610798	0.703172375	1
KLHL15	-1.134588932	0.381469368	0.753119563	1
KLHL23	-1.107575959	0.271021961	0.836553998	1
KLRB1	-1.125101031	0.337582178	0.787518853	1
KLRC4	-1.151381003	0.499918872	0.651462131	1
KLRC4-KLRK1	-1.141856121	0.422188527	0.719667594	1
KLRF2	-1.082466018	0.193094401	0.889371617	1
KLRG1	-0.925612282	-0.13504955	1.060661832	1
KLRK1	-1.149830624	0.483170275	0.666660349	1
KMT2C	-1.074278908	0.170473912	0.903804997	1
KMT2E	-1.15460313	0.590290362	0.564312769	1
KPNA2	-1.091760573	0.220236095	0.871524477	1
KRBOX5	-1.152080346	0.643368902	0.508711444	1
KRCC1	-1.111684545	0.285438679	0.826245867	1
KRT10	-0.947167036	-0.098391967	1.045559002	1
KRT28	-1.141740305	0.421465313	0.720274992	1
KRTAP11-1	-0.952448634	-0.089135037	1.041583671	1
KRTAP13-1	-1.137025927	0.394217147	0.74280878	1
KRTAP15-1	-1.07537693	0.173444943	0.901931987	1

KRTAP19-5	-1.105318264	0.263343298	0.841974966	1
KRTAP19-8	-0.98063127	-0.03767275	1.01830402	1
KRTAP2-1	-0.898188655	-0.179350462	1.077539118	1
KRTAP2-3	-0.99970205	-0.000595635	1.000297684	1
KRTAP20-2	-1.152504856	0.514613009	0.637891847	1
KRTAP20-4	-1.131516811	0.366378666	0.765138145	1
KRTAP21-1	-1.14469243	0.703721369	0.440971061	1
KRTAP22-2	-1.033769112	0.071361192	0.96240792	1
KRTAP25-1	-0.914613563	-0.153112356	1.067725919	1
KRTAP29-1	-1.122241934	0.325685668	0.796556267	1
KRTAP3-2	-0.974728199	-0.048759678	1.023487876	1
KRTAP5-2	-1.154566379	0.592526473	0.562039906	1
KRTAP7-1	-0.968258591	-0.060716072	1.028974664	1
LALBA	-1.035910424	0.076177898	0.959732526	1
LAMB2	-1.11913639	0.313295368	0.805841022	1
LAMP5	-1.05217383	0.114143141	0.938030689	1
LAMTOR3	-1.153704178	0.618385228	0.535318951	1
LAMTOR4	-1.154522438	0.594824114	0.559698324	1
LAMTOR5	-0.893028177	-0.187422428	1.080450605	1
LCE3C	-1.154683447	0.582782625	0.571900822	1
LCE3E	-1.123591927	0.331240362	0.792351565	1
LCE6A	-1.081073276	0.189167276	0.891906	1
LECT2	-1.13887753	0.404458528	0.734419002	1
LEMD1	-1.141402327	0.419371903	0.722030424	1
LEPROT	-0.995972426	-0.008007062	1.003979488	1
LEUTX	-1.069468183	0.157668642	0.911799542	1
LFNG	-1.046825257	0.101372748	0.945452509	1
LGALS13	-1.154532243	0.560193505	0.594338738	1
LGALS3BP	-1.152240371	0.641362776	0.510877595	1
LHCGR	-1.073738557	0.169018584	0.904719973	1
LHX8	-1.05521529	0.12154051	0.933674781	1
LILRA1	-1.154303805	0.603363435	0.550940369	1
LIMCH1	-0.913386651	-0.15510208	1.068488732	1
LIN54	-1.026095886	0.054413639	0.971682247	1
LIPI	-1.107369599	0.270313262	0.837056337	1
LMBRD2	-1.130428562	0.361257303	0.769171259	1
LMNB2	-1.122722164	0.327649278	0.795072886	1
LNX1	-1.004577024	0.009217774	0.99535925	1
LOXL3	-1.138926013	0.734192037	0.404733976	1
LPXN	-1.153242456	0.626859444	0.526383012	1
LRCH2	-1.143376284	0.711394996	0.431981288	1
LRRC10	-0.942904997	-0.105780232	1.048685229	1
LRRC17	-0.921327666	-0.142135055	1.063462721	1
LRRC18	-1.1382579	0.737285765	0.400972135	1
LRRC28	-1.075454901	0.173656624	0.901798277	1
LRRC39	-1.021153417	0.043742398	0.977411019	1
LRRC40	-1.147166014	0.459532131	0.687633883	1
LRRC47	-1.071801532	0.163837455	0.907964077	1
LRRC57	-1.142343504	0.717077504	0.425266	1
LRRC72	-1.014819227	0.030328433	0.984490794	1
LRRFIP1	-1.15453173	0.56016721	0.594364519	1
LRWD1	-0.931285852	-0.125567694	1.056853546	1
LSG1	-1.05819063	0.128877321	0.929313309	1
LSM1	-1.131850103	0.763880877	0.367969225	1
LSM10	-0.918343631	-0.147032549	1.06537618	1

LSM3	-1.13424187	0.379713531	0.754528339	1
LSM4	-0.908860358	-0.162400136	1.071260494	1
LTA4H	-1.152385647	0.639481767	0.51290388	1
LUC7L2	-1.064688957	0.145268171	0.919420786	1
LXN	-1.031700956	0.066745976	0.96495498	1
LY6G6C	-0.957970176	-0.079333689	1.037303865	1
LYAR	-0.929768619	-0.128114624	1.057883244	1
LYG1	-1.144390144	0.43885941	0.705530734	1
LYPD4	-0.964848095	-0.066940235	1.03178833	1
LYPLA1	-1.08137485	0.19001475	0.8913601	1
LYZ	-0.909129592	-0.161967878	1.07109747	1
MAD2L1BP	-1.100165164	0.246392841	0.853772323	1
MAD2L2	-0.978710891	-0.041298626	1.020009517	1
MAGEB16	-1.139450637	0.407740181	0.731710455	1
MAGEB3	-1.105259146	0.263144401	0.842114745	1
MAGEB4	-0.988532866	-0.022552761	1.011085627	1
MAGEB5	-0.911574505	-0.158031892	1.069606397	1
MAGEB6	-1.145249155	0.44494028	0.700308875	1
MAGEE2	-1.146136124	0.69463693	0.451499194	1
MAGEH1	-1.078905592	0.18312143	0.895784162	1
MAL2	-1.15360415	0.533234898	0.620369252	1
MAMSTR	-1.092518932	0.222528423	0.869990508	1
MAN2B1	-1.15403229	0.542999901	0.61103239	1
MANEA	-1.044235493	0.09529322	0.948942273	1
MAP1LC3B	-1.065220719	0.146632881	0.918587839	1
MAP1LC3B2	-1.130477591	0.361485727	0.768991864	1
MAP3K12	-1.149059167	0.673257859	0.475801309	1
MAP3K14	-0.988269864	-0.023061362	1.011331226	1
MAP3K2	-0.952516923	-0.0890146	1.041531523	1
MAP3K7CL	-1.129466505	0.356817259	0.772649246	1
MAP9	-1.148035264	0.68130824	0.466727024	1
MAPKAP1	-1.108989001	0.275913191	0.83307581	1
MARCOL	-0.92625576	-0.133979899	1.060235659	1
MARF1	-0.905789618	-0.167313927	1.073103545	1
MAT2A	-1.024017805	0.049904313	0.974113492	1
MATN2	-1.018515334	0.038120865	0.980394469	1
MBD4	-1.153642533	0.53402315	0.619619383	1
MBOAT7	-0.931422828	-0.125337346	1.056760174	1
MBTPS1	-1.154698492	0.575466558	0.579231934	1
MCCD1	-1.114201093	0.294580179	0.819620914	1
MCMDC2	-1.133803605	0.756287756	0.377515849	1
MCOLN1	-1.145680054	0.697591424	0.44808863	1
MCTS2P	-1.123856576	0.332342143	0.791514433	1
MCUB	-1.142948544	0.429166613	0.713781931	1
MDM4	-1.015053816	0.030820172	0.984233645	1
MED10	-1.050466532	0.11003446	0.940432072	1
MED12	-1.139008836	0.733803396	0.405205439	1
MED14	-0.92511396	-0.13587691	1.06099087	1
MED17	-1.147173255	0.687582865	0.459590391	1
MED31	-0.982320393	-0.034467984	1.016788377	1
MED7	-1.154396987	0.554270399	0.600126588	1
MEIG1	-1.070797068	0.16117236	0.909624708	1
METRNL	-1.126974671	0.345666151	0.78130852	1
METTL18	-1.011073548	0.022527756	0.988545791	1
METTL24	-1.148514277	0.47088298	0.677631297	1

METTL2B	-1.070323927	0.159922016	0.910401911	1
MFAP4	-1.080365003	0.187183076	0.893181927	1
MFSD2A	-1.041113942	0.088051111	0.953062832	1
MGMT	-1.149248121	0.67168882	0.477559301	1
MGRN1	-1.101548234	0.250868105	0.850680129	1
MGST1	-0.950536991	-0.092498685	1.043035676	1
MIB1	-1.004475831	0.009012583	0.995463248	1
MICAL2	-0.924141552	-0.13748889	1.061630442	1
MICOS13	-1.150876882	0.494125458	0.656751425	1
MINPP1	-1.139667578	0.408997353	0.730670225	1
MIS18A	-1.059515499	0.132177342	0.927338157	1
MKKS	-1.145793738	0.448931018	0.696862721	1
MLPH	-1.154568339	0.592415678	0.562152662	1
MMAA	-1.143997377	0.436158703	0.707838674	1
MMGT1	-0.984045254	-0.031180205	1.015225459	1
MMP10	-1.153048288	0.523047668	0.63000062	1
MMP3	-0.938828231	-0.112781011	1.051609242	1
MMRN1	-1.150766452	0.657860065	0.492906387	1
MOAP1	-1.127955499	0.349998092	0.777957407	1
MOCS2	-0.935634892	-0.118220533	1.053855424	1
MORC3	-0.935120365	-0.119093401	1.054213766	1
MOSMO	-0.930069493	-0.127610218	1.057679711	1
MOSPD1	-1.073390678	0.168083972	0.905306706	1
MPHOSPH6	-1.011039825	0.022457955	0.98858187	1
MRAS	-1.147862204	0.465260634	0.68260157	1
MRFAP1L1	-1.133685601	0.756757831	0.37692777	1
MRGPRG	-1.051981208	0.113678036	0.938303172	1
MRGPRX2	-1.143572265	0.433287558	0.710284708	1
MROH7	-1.131460314	0.765350217	0.366110097	1
MRPL1	-1.053346611	0.11698356	0.936363051	1
MRPL17	-1.080475938	0.187493288	0.89298265	1
MRPL22	-1.152467548	0.514073388	0.63839416	1
MRPL30	-1.144022798	0.707690719	0.436332079	1
MRPL35	-1.051420069	0.112325373	0.939094696	1
MRPL36	-0.981674009	-0.035696095	1.017370104	1
MRPL38	-1.024436391	0.050809959	0.973626432	1
MRPL41	-1.050999377	0.11131346	0.939685917	1
MRPL43	-1.148328151	0.469250568	0.679077583	1
MRPL51	-1.098273252	0.240353697	0.857919555	1
MRPS15	-1.138326338	0.401354165	0.736972173	1
MRPS17	-1.151464559	0.650545563	0.500918996	1
MRPS18C	-1.058455427	0.129535229	0.928920198	1
MRPS22	-1.110665762	0.281807426	0.828858336	1
MRPS31	-1.133280303	0.37491948	0.758360823	1
MRPS6	-1.026365624	0.055001401	0.971364222	1
MRPS9	-1.104922629	0.262014265	0.842908363	1
MS4A13	-1.13589537	0.388208206	0.747687165	1
MS4A18	-1.152936227	0.631726984	0.521209242	1
MS4A3	-0.919437354	-0.145241003	1.064678357	1
MSANTD2	-1.100832651	0.248546145	0.852286506	1
MSL2	-1.029753954	0.062433504	0.96732045	1
MSX1	-1.152419372	0.513382999	0.639036374	1
MT1B	-1.142734865	0.427778535	0.71495633	1
MTARC2	-0.991502899	-0.01678294	1.008285839	1
MTERF1	-1.068414611	0.154908393	0.913506218	1

MTF2	-1.109409345	0.277381435	0.832027911	1
MTFR1	-1.138041545	0.399769238	0.738272307	1
MTPAP	-1.135164721	0.750751115	0.384413605	1
MUS81	-1.153434119	0.623539057	0.529895062	1
MXD1	-1.078469403	0.181914384	0.896555019	1
MYBPC1	-1.048535093	0.105423162	0.943111931	1
MYBPH	-1.119660458	0.315350489	0.804309969	1
MYDGF	-1.147717521	0.464048152	0.683669369	1
MYH1	-1.051315189	0.112072922	0.939242266	1
MYH4	-1.071022229	0.161768505	0.909253724	1
MYL6B	-1.099853797	0.245392468	0.854461329	1
MYO1F	-1.137045111	0.394320625	0.742724485	1
N6AMT1	-1.133364801	0.375336715	0.758028085	1
NAAA	-0.918574527	-0.146654672	1.065229199	1
NADSYN1	-1.152526781	0.637594573	0.514932209	1
NAGA	-1.148587802	0.471534252	0.677053551	1
NAP1L2	-0.997013486	-0.005946507	1.002959993	1
NARS1	-1.092717891	0.223131924	0.869585967	1
NAT10	-0.957396431	-0.080358183	1.037754613	1
NAXE	-1.149073161	0.475930528	0.673142632	1
NBEAL2	-0.894144295	-0.185683322	1.079827617	1
NCAPH	-1.032957573	0.069545917	0.963411656	1
NCEH1	-0.967065572	-0.062899401	1.029964973	1
NCF4	-1.152443398	0.513726419	0.638716979	1
NCKAP5	-1.061747524	0.137784527	0.923962997	1
NCR3	-1.137687014	0.397813706	0.739873308	1
NDE1	-1.134049603	0.755302849	0.378746754	1
NDUFA4	-1.123846335	0.332299429	0.791546906	1
NDUFAB1	-1.154488462	0.596409145	0.558079316	1
NDUFAF7	-0.947607156	-0.097624903	1.045232059	1
NDUFB3	-1.038536543	0.082139702	0.956396841	1
NDUFB6	-1.154206106	0.547842166	0.606363939	1
NECAB2	-1.146342514	0.693271757	0.453070757	1
NECTIN2	-0.997277073	-0.005423791	1.002700864	1
NEIL3	-1.15086185	0.493958524	0.656903325	1
NEK1	-1.044703083	0.096386049	0.948317034	1
NEK6	-1.146472922	0.692399708	0.454073214	1
NEU4	-1.153757176	0.617292504	0.536464672	1
NEURL1	-1.066436861	0.149767892	0.916668969	1
NFIL3	-1.154609984	0.589828534	0.564781449	1
NFKB2	-1.137480743	0.396684657	0.740796086	1
NFKBIA	-1.103213612	0.256327625	0.846885987	1
NFX1	-0.931151088	-0.125794254	1.056945342	1
NFYB	-0.917116237	-0.149038258	1.066154494	1
NIFK	-1.108507776	0.274239813	0.834267963	1
NIPSNAP2	-1.080455489	0.18743609	0.893019399	1
NIT2	-1.054091425	0.118795237	0.935296188	1
NKIRAS1	-1.154698646	0.579159894	0.575538751	1
NKTR	-1.12217966	0.32543201	0.79674765	1
NLRX1	-1.154378522	0.553574221	0.600804301	1
NMD3	-0.967991353	-0.061205714	1.029197067	1
NME1-NME2	-1.126308574	0.342764299	0.783544275	1
NME2	-1.117075393	0.305344245	0.811731148	1
NMT1	-1.137585591	0.740327828	0.397257763	1
NOL12	-1.148715148	0.472671138	0.67604401	1

NOL3	-1.152366465	0.639733588	0.512632877	1
NOM1	-1.113490133	0.291971968	0.821518165	1
NOP10	-1.153190533	0.62771959	0.525470943	1
NOP56	-1.153591128	0.620620572	0.532970557	1
NOS1AP	-1.039350342	0.083999662	0.955350681	1
NOTCH3	-0.972531076	-0.052842503	1.025373579	1
NOX3	-1.153503859	0.531236706	0.622267153	1
NPAT	-1.11082499	0.282372422	0.828452568	1
NPC2	-1.120843529	0.320042196	0.800801334	1
NPM1	-1.132619508	0.760937058	0.37168245	1
NPM3	-1.138206126	0.737522511	0.400683616	1
NPSR1	-0.924622082	-0.136692718	1.061314801	1
NPY1R	-1.065832203	0.148206748	0.917625455	1
NR1D2	-1.01023067	0.02078539	0.98944528	1
NR2C2AP	-1.151964693	0.507185477	0.644779217	1
NRSN2	-1.024037281	0.049946422	0.974090859	1
NSMCE3	-1.151098686	0.654472474	0.496626212	1
NSUN6	-0.923016538	-0.139349745	1.062366283	1
NUDCD2	-1.153669187	0.534578765	0.619090422	1
NUDT2	-1.018080266	0.037198603	0.980881663	1
NUDT5	-1.048912335	0.106320802	0.942591534	1
NUDT7	-1.154472896	0.597092144	0.557380753	1
NUDT9	-1.126670612	0.344337591	0.782333021	1
NUMA1	-1.009837218	0.019973669	0.989863549	1
NUMB	-1.147625606	0.463284067	0.684341539	1
NUP107	-1.154698405	0.57927137	0.575427035	1
NUP37	-0.923546583	-0.138473559	1.062020142	1
OAZ3	-1.020323805	0.0419691	0.978354705	1
OCEL1	-1.142798819	0.428192757	0.714606061	1
OGA	-1.123616135	0.331340962	0.792275173	1
OGG1	-1.14470128	0.441033333	0.703667947	1
OLA1	-0.950204994	-0.093081322	1.043286316	1
OLFML2B	-1.145084233	0.701330994	0.443753239	1
OMA1	-1.105288304	0.263242486	0.842045817	1
OOSP1	-1.041918269	0.089908395	0.952009874	1
OOSP4A	-1.055457184	0.122133229	0.933323956	1
OOSP4B	-0.936682284	-0.116440632	1.053122916	1
OPA3	-1.146824301	0.456812335	0.690011966	1
OPLAH	-1.088304451	0.209944106	0.878360345	1
OPRD1	-1.147713545	0.683698542	0.464015003	1
OPRPN	-1.154132117	0.545692623	0.608439494	1
OR10AD1	-1.136214893	0.389889074	0.746325819	1
OR10G6	-0.990980517	-0.017801288	1.008781805	1
OR10J5	-1.151082556	0.654640659	0.496441897	1
OR10K2	-1.144796897	0.441707786	0.70308911	1
OR10X1	-1.074037516	0.169823224	0.904214292	1
OR13C3	-0.919824862	-0.144605284	1.064430146	1
OR13C4	-0.938315573	-0.113656861	1.051972434	1
OR13C9	-1.103055534	0.255805995	0.847249539	1
OR13H1	-1.016223582	0.0332779	0.982945682	1
OR14A2	-1.020891586	0.043182188	0.977709399	1
OR14C36	-0.968692672	-0.059920033	1.028612705	1
OR14I1	-1.085184852	0.200860984	0.884323868	1
OR14L1P	-1.00809542	0.016392383	0.991703037	1
OR1C1	-1.003373056	0.006780595	0.996592461	1

OR1E1	-1.149225251	0.477344995	0.671880256	1
OR1L1	-1.154057105	0.543649697	0.610407409	1
OR1L3	-1.151652074	0.503209746	0.648442328	1
OR1L4	-1.125501358	0.339289228	0.78621213	1
OR1L6	-1.130704743	0.362546829	0.768157914	1
OR2AJ1	-0.912055087	-0.157255946	1.069311033	1
OR2AK2	-1.129137222	0.355315534	0.773821688	1
OR2B6	-0.961222713	-0.073498897	1.034721609	1
OR2F1	-1.02873979	0.060199407	0.968540384	1
OR2M2	-1.153297771	0.625925534	0.527372237	1
OR2M7	-1.029115686	0.061026501	0.968089185	1
OR2T1	-1.098831596	0.24212629	0.856705307	1
OR2T29	-1.138191663	0.737588574	0.400603089	1
OR2T6	-1.147540369	0.462579686	0.684960682	1
OR4A15	-1.10027023	0.246730985	0.853539245	1
OR4C11	-0.981377585	-0.036258574	1.017636159	1
OR4C12	-1.041178296	0.088199489	0.952978807	1
OR4C13	-1.154376076	0.553483457	0.600892619	1
OR4C3	-0.937367841	-0.115273397	1.052641238	1
OR4C5	-1.054906483	0.120784784	0.934121699	1
OR4D2	-1.038534811	0.08213575	0.956399061	1
OR4D5	-1.152603475	0.516061208	0.636542267	1
OR4F4	-1.043422301	0.093397683	0.950024618	1
OR4F6	-0.921595772	-0.141693546	1.063289317	1
OR4K5	-1.079204376	0.18395006	0.895254316	1
OR4N5	-1.014174787	0.028979534	0.985195253	1
OR51A4	-1.047415578	0.102767822	0.944647756	1
OR51F2	-0.949388802	-0.094511781	1.043900582	1
OR51G2	-1.108340278	0.273659227	0.834681051	1
OR51I1	-0.968551058	-0.060179828	1.028730886	1
OR51I2	-1.006211943	0.012541862	0.99367008	1
OR51Q1	-1.127951434	0.349979986	0.777971447	1
OR52A1	-0.922905241	-0.139533601	1.062438841	1
OR52E8	-1.153974026	0.541519277	0.612454749	1
OR52I1	-1.025328988	0.052745643	0.972583345	1
OR5AR1	-0.954871344	-0.08485032	1.039721664	1
OR5B2	-1.124529945	0.335165211	0.789364734	1
OR5B3	-1.154700452	0.577736492	0.57696396	1
OR5BS1P	-1.146542158	0.454608491	0.691933667	1
OR5C1	-0.937675944	-0.114748241	1.052424185	1
OR5D14	-1.076834015	0.177416483	0.899417532	1
OR5F1	-1.113665686	0.292614078	0.821051608	1
OR5K2	-1.149976098	0.484620848	0.66535525	1
OR5T1	-1.144700682	0.441029126	0.703671556	1
OR5W2	-1.139698072	0.409174744	0.730523328	1
OR6B1	-1.152316652	0.511934114	0.640382539	1
OR6C65	-1.144589348	0.440247642	0.704341705	1
OR6C68	-0.924084683	-0.137583062	1.061667745	1
OR6N1	-0.98981465	-0.020068616	1.009883265	1
OR6S1	-1.078109647	0.1809212	0.897188447	1
OR6V1	-1.154668222	0.58481556	0.569852663	1
OR7G1	-1.107937709	0.272267729	0.83566998	1
OR7G3	-1.15465895	0.585816593	0.568842357	1
OR8B12	-1.08747956	0.207523844	0.879955715	1
OR8G1	-0.946974255	-0.098727709	1.045701965	1

OR8J1	-1.145831982	0.696616445	0.449215537	1
OR8K3	-1.060768964	0.135318787	0.925450177	1
OR8K5	-1.137947363	0.738699486	0.399247877	1
OR8S1	-1.148129441	0.68059683	0.467532611	1
OR8U3	-1.132463669	0.370925589	0.76153808	1
OR9A2	-1.144456116	0.439317757	0.70513836	1
OR9A4	-1.154689032	0.572880313	0.581808719	1
OR9G4	-1.154363522	0.553023038	0.601340484	1
ORC1	-1.135902761	0.747655829	0.388246932	1
ORC5	-1.154143286	0.546007898	0.608135388	1
OSR2	-1.012630194	0.025758057	0.986872136	1
OTUB2	-1.061709912	0.137689536	0.924020376	1
OTULIN	-1.067195875	0.151734451	0.915461425	1
OVOL2	-1.144194438	0.706686654	0.437507784	1
OXR1	-1.15457866	0.562760473	0.591818188	1
P2RX5-TAX1BP3	-1.037041306	0.078737756	0.95830355	1
P2RY1	-0.952580227	-0.088902936	1.041483163	1
P2RY10	-1.064208056	0.14403714	0.920170916	1
P2RY11	-1.154446928	0.556265933	0.598180995	1
P3R3URF	-1.131932616	0.36836465	0.763567965	1
PAAF1	-1.106317469	0.26672158	0.839595889	1
PAF1	-0.945959203	-0.100493056	1.046452259	1
PAFAH1B3	-1.114726988	0.296522915	0.818204072	1
PAGE1	-1.120724698	0.319567593	0.801157104	1
PAGE2B	-1.151223407	0.653158466	0.498064942	1
PAIP2B	-0.965128148	-0.066431138	1.031559286	1
PANK2	-1.10199475	0.25232418	0.849670571	1
PAPLN	-1.153133189	0.628651968	0.524481221	1
PCCB	-0.921604868	-0.141678561	1.06328343	1
PCDH1	-0.983460752	-0.032296067	1.015756818	1
PCDHB8	-0.937626837	-0.114831966	1.052458804	1
PCDHGA11	-1.149245016	0.671714833	0.477530183	1
PCDHGA2	-1.137542224	0.397020517	0.740521706	1
PCDHGA3	-1.152847468	0.519793022	0.633054445	1
PCDHGA7	-1.135113034	0.75096535	0.384147684	1
PCNA	-1.140143189	0.411783466	0.728359723	1
PDRG1	-0.983725965	-0.031789975	1.01551594	1
PDSS2	-1.152605752	0.516095024	0.636510728	1
PDZD8	-0.972242105	-0.053377758	1.025619863	1
PEAK1	-1.154219641	0.605967488	0.548252154	1
PEDS1	-1.051747449	0.113114137	0.938633312	1
PERCC1	-1.142326962	0.717166336	0.425160626	1
PFDN2	-1.078386382	0.181684997	0.896701384	1
PFDN4	-1.150287898	0.48780373	0.662484168	1
PFKP	-1.090255344	0.21572296	0.874532384	1
PGAM4	-1.126722619	0.34456436	0.782158259	1
PGBD2	-0.917532189	-0.148359108	1.065891297	1
PGM1	-1.137739417	0.739637866	0.398101551	1
PGM2	-0.938268057	-0.113737989	1.052006047	1
PHB	-1.079995899	0.18615244	0.893843458	1
PHF23	-1.127352954	0.347328408	0.780024546	1
PHF5A	-1.063456362	0.142118859	0.921337504	1
PHTF1	-1.14691954	0.689354879	0.457564661	1
PIAS2	-1.122760527	0.327806724	0.794953804	1
PIBF1	-1.094584431	0.228837049	0.865747382	1

PIGF	-1.150587472	0.4909649	0.659622571	1
PIGP	-1.014270666	0.029180039	0.985090626	1
PIRT	-1.142975114	0.429340039	0.713635075	1
PITHD1	-1.148272965	0.468770934	0.679502031	1
PLA2G4A	-1.145504207	0.698708778	0.446795428	1
PLA2G6	-0.925331899	-0.135515175	1.060847074	1
PLAAT2	-1.119685563	0.315449286	0.804236276	1
PLAC1	-1.087237802	0.206817072	0.88042073	1
PLBD1	-1.149120745	0.47637109	0.672749655	1
PLCB2	-1.1376473	0.740051466	0.397595834	1
PLEK2	-1.132538506	0.761249762	0.371288744	1
PLEKHD1	-1.129822489	0.358450939	0.77137155	1
PLEKHF2	-1.074652925	0.171483849	0.903169076	1
PLLP	-1.154581486	0.562931267	0.591650219	1
PLN	-1.142716083	0.427657081	0.715059002	1
PLOD1	-1.062160541	0.13882877	0.92333177	1
PLSCR4	-0.908419415	-0.163107573	1.071526988	1
PLSCR5	-1.015897373	0.032591561	0.983305811	1
PLTP	-1.146576553	0.454875202	0.691701351	1
PMF1-BGLAP	-1.147228655	0.460036995	0.68719166	1
PNMA1	-1.087396587	0.207281147	0.88011544	1
PNMA8B	-0.90961366	-0.16119012	1.07080378	1
PNO1	-1.136429342	0.745404578	0.391024764	1
POLA1	-1.154679764	0.571341323	0.583338441	1
POLR1D	-1.124864909	0.336580285	0.788284624	1
POLR1H	-1.055722493	0.122784077	0.932938416	1
POLR2D	-0.969789167	-0.057905313	1.027694481	1
POLR2J	-0.91798304	-0.147622322	1.065605362	1
POMGNT1	-1.082268308	0.19253484	0.889733468	1
PON3	-1.081428646	0.190166092	0.891262554	1
PPDPFL	-1.154676704	0.583763418	0.570913287	1
PPFIA4	-1.134194183	0.379473358	0.754720826	1
PPP1CA	-1.068465381	0.155041054	0.913424327	1
PPP1R12C	-0.991331928	-0.017116404	1.008448332	1
PPP1R16B	-1.022143199	0.0458647	0.9762785	1
PPP1R26	-1.150723318	0.49243468	0.658288639	1
PPP2R1B	-1.150410719	0.661323783	0.489086936	1
PPP2R2D	-0.895440393	-0.183659148	1.079099541	1
PPP2R3C	-1.151120963	0.654239549	0.496881415	1
PPP2R5A	-0.976871264	-0.044754672	1.021625936	1
PPP3R1	-1.078787072	0.182793143	0.895993929	1
PPP3R2	-1.146767944	0.456369179	0.690398765	1
PPP4R3A	-1.152295253	0.511636097	0.640659156	1
PRAMEF12	-1.154531665	0.560163889	0.594367776	1
PRDM5	-1.01655457	0.033975057	0.982579514	1
PRDM7	-1.138522952	0.402455853	0.736067099	1
PRDX4	-1.018427331	0.037934207	0.980493124	1
PRELID3B	-1.15062296	0.659276368	0.491346592	1
PREPL	-1.025313832	0.052712723	0.972601108	1
PRG3	-1.148373078	0.678730576	0.469642502	1
PRICKLE2	-0.975938573	-0.046500475	1.022439048	1
PRKAR2B	-1.133297086	0.375002292	0.758294795	1
PRKCSH	-1.038363882	0.081745846	0.956618036	1
PRKDC	-1.150366367	0.488621547	0.66174482	1
PRKG2	-1.054970304	0.120940882	0.934029422	1

PRMT1	-1.147828059	0.464973401	0.682854657	1
PRMT3	-1.035794511	0.075916149	0.959878362	1
PROM1	-1.016640969	0.034157163	0.982483806	1
PRORP	-1.140887804	0.416232152	0.724655652	1
PROX2	-1.065991294	0.148617031	0.917374263	1
PRPF39	-1.153580822	0.532762412	0.62081841	1
PRR32	-1.111122558	0.283430803	0.827691755	1
PRRX1	-0.997825672	-0.004334565	1.002160237	1
PRSS3	-0.940806154	-0.109392435	1.050198589	1
PRSS50	-1.014127822	0.02888134	0.985246482	1
PRSS53	-1.139610028	0.730946995	0.408663033	1
PRSS58	-0.975962138	-0.046456419	1.022418557	1
PSCA	-1.152637664	0.516570926	0.636066738	1
PSKH2	-1.08333455	0.195560802	0.887773747	1
PSMA5	-1.087764018	0.208356927	0.879407091	1
PSMB5	-1.098386793	0.240713512	0.857673282	1
PSMC5	-0.902047289	-0.17326236	1.075309649	1
PSRC1	-0.962558873	-0.071088478	1.03364735	1
PSTPIP1	-0.936215623	-0.117234167	1.053449789	1
PTCD1	-1.040253482	0.086070846	0.954182636	1
PTGER4	-0.909140218	-0.161950813	1.071091031	1
PTGES3L-AARSD1	-1.153909996	0.539957827	0.613952169	1
PTGFR	-1.067317363	0.152049934	0.915267428	1
PTH	-0.988003548	-0.02357599	1.011579538	1
PTH2	-1.132909491	0.759812095	0.373097396	1
PTN	-1.081414932	0.190127506	0.891287426	1
PTP4A1	-1.139097999	0.405714289	0.73338371	1
PTPN4	-1.154225499	0.605794171	0.548431328	1
PTPN7	-1.137073304	0.742600506	0.394472798	1
PTPRN2	-0.969537851	-0.058367583	1.027905434	1
PUS3	-0.951033218	-0.091626992	1.04266021	1
PVR	-1.132189055	0.762591239	0.369597816	1
PYDC2	-1.151699519	0.503800025	0.647899493	1
PYGL	-1.116054899	0.30148097	0.814573929	1
QSER1	-1.008590872	0.017409064	0.991181808	1
R3HCC1	-1.122260756	0.325762375	0.796498381	1
RAB12	-1.15469992	0.576315242	0.578384679	1
RAB1A	-1.153740787	0.617633694	0.536107093	1
RAB2A	-1.145734698	0.697241788	0.44849291	1
RAB32	-0.963232602	-0.069870069	1.033102671	1
RAB44	-1.051812995	0.113272196	0.938540799	1
RAB7A	-1.101978337	0.252270558	0.849707779	1
RABGGTB	-1.154070195	0.610072792	0.543997402	1
RABL2A	-1.119975322	0.316591977	0.803383345	1
RABL2B	-1.154476945	0.596916824	0.557560121	1
RAC2	-1.134598545	0.753080343	0.381518202	1
RAD51	-1.131961604	0.368503728	0.763457876	1
RAD51AP1	-0.928273265	-0.130616727	1.058889992	1
RAD9A	-1.14504165	0.701593345	0.443448305	1
RAG1	-0.935545715	-0.118371886	1.053917602	1
RAG2	-0.987950276	-0.023678886	1.011629162	1
RAMAC	-1.089167446	0.212490807	0.87667664	1
RANBP1	-1.139346739	0.732205692	0.407141048	1
RANGAP1	-1.14473024	0.441237292	0.703492949	1
RAP1GDS1	-1.133484337	0.375928273	0.757556065	1

RAP2B	-1.133225297	0.374648276	0.75857702	1
RAPGEF6	-1.131293549	0.365319092	0.765974456	1
RASA2	-1.153065107	0.629736356	0.523328751	1
RASGRF2	-0.961676499	-0.072681158	1.034357656	1
RAVER2	-1.05371835	0.117887014	0.935831336	1
RBBP9	-1.148143806	0.467655969	0.680487836	1
RBM11	-0.944254948	-0.103447843	1.047702791	1
RBM15	-1.027449731	0.057369425	0.970080306	1
RBM25	-1.120114167	0.317141071	0.802973096	1
RBM48	-1.09710097	0.236657721	0.860443249	1
RBMX	-1.152639668	0.63603873	0.516600938	1
RBP5	-0.928338331	-0.130508022	1.058846353	1
RCOR1	-1.065149134	0.14644895	0.918700184	1
RD3L	-0.987096525	-0.025325844	1.012422369	1
RDH13	-1.150229561	0.487200228	0.663029332	1
RDH14	-0.964297364	-0.067940353	1.032237716	1
REEP3	-1.15132721	0.499281401	0.652045809	1
RELA	-1.078118474	0.180945544	0.89717293	1
REP15	-0.964734575	-0.067146497	1.031881072	1
REPS2	-1.044341559	0.095540928	0.948800631	1
REXO2	-1.154613369	0.589593941	0.565019428	1
RFC1	-1.131430161	0.365966882	0.765463279	1
RFLNB	-1.026578212	0.055465034	0.971113178	1
RGS4	-1.005835582	0.011775158	0.994060425	1
RGSL1	-0.928901522	-0.129566471	1.058467993	1
RHOB	-1.11825417	0.309866764	0.808387407	1
RHOH	-1.153179735	0.627896533	0.525283202	1
RHOU	-1.109425142	0.277436732	0.83198841	1
RHOXF1	-1.022813899	0.047306964	0.975506934	1
RIOK1	-1.016673128	0.03422496	0.982448168	1
RIOK2	-1.104364839	0.260148643	0.844216196	1
RIT1	-1.071964316	0.164270739	0.907693577	1
RMDN3	-0.908067321	-0.163672019	1.071739339	1
RMI1	-1.065464713	0.147260297	0.918204416	1
RNASE11	-1.13155717	0.366570707	0.764986463	1
RNASE4	-1.143877767	0.708532226	0.435345541	1
RNASE6	-1.133458329	0.375799435	0.757658894	1
RNASE7	-1.153328726	0.527934109	0.625394616	1
RNF10	-1.153791867	0.616560103	0.537231764	1
RNF103-CHMP3	-0.96778122	-0.061590491	1.029371712	1
RNF133	-1.151694083	0.503732171	0.647961912	1
RNF148	-1.153537617	0.531899832	0.621637785	1
RNF149	-1.137199467	0.742044332	0.395155136	1
RNF150	-1.134170593	0.379354638	0.754815955	1
RNF151	-0.946486154	-0.099577113	1.046063267	1
RNF175	-1.041843249	0.08973491	0.95210834	1
RNF186	-1.145859024	0.69644195	0.449417074	1
RNF19B	-1.134695599	0.382011825	0.752683774	1
RNLS	-0.904799619	-0.168891778	1.073691397	1
ROPN1L	-1.03793859	0.080776857	0.957161733	1
RPL10L	-1.133799785	0.377496787	0.756302997	1
RPL12	-1.148074162	0.681015067	0.467059095	1
RPL15	-1.131368235	0.365673025	0.76569521	1
RPL17	-1.128344433	0.351736037	0.776608396	1
RPL17-C18orf32	-1.033125184	0.069920379	0.963204804	1

RPL18	-1.107905898	0.272158004	0.835747894	1
RPL22	-1.130964951	0.363768047	0.767196903	1
RPL23	-1.05371975	0.11789042	0.935829331	1
RPL24	-1.15412723	0.545555637	0.608571593	1
RPL26L1	-1.143852327	0.435173139	0.708679188	1
RPL29	-1.018653295	0.0384136	0.980239695	1
RPL34	-1.035815088	0.075962607	0.959852481	1
RPL36	-1.152585831	0.515799723	0.636786108	1
RPL7	-1.152055782	0.64367112	0.508384661	1
RPL7A	-1.04943378	0.107563974	0.941869806	1
RPL9	-0.990310385	-0.019105447	1.009415832	1
RPS10	-1.151892173	0.645647711	0.506244461	1
RPS13	-1.061992867	0.138404581	0.923588286	1
RPS19	-1.135810066	0.3877617	0.748048366	1
RPS2	-1.154393466	0.600257391	0.554136075	1
RPS24	-1.127610451	0.348465953	0.779144499	1
RPS27A	-0.894080469	-0.185782873	1.079863343	1
RPS27AP5	-0.921886406	-0.141214659	1.063101065	1
RPS28	-1.152308527	0.640487726	0.511820801	1
RPS3A	-1.00838926	0.016995158	0.991394102	1
RPS4Y2	-0.920621265	-0.143297159	1.063918423	1
RPSAP58	-1.049781353	0.108394177	0.941387175	1
RRAGB	-1.09294524	0.223822614	0.869122626	1
RRAGC	-1.149365175	0.670702228	0.478662947	1
RRM2B	-1.133435248	0.757750092	0.375685156	1
RRP7A	-1.094820428	0.229564068	0.86525636	1
RTCB	-1.132939416	0.759695507	0.37324391	1
RTKN	-0.96629778	-0.064301062	1.030598842	1
RTL10	-1.126770486	0.344773249	0.781997237	1
RTL5	-1.152506207	0.514632632	0.637873575	1
RTN4	-1.081954958	0.191649408	0.89030555	1
RTP1	-1.154693962	0.573971881	0.580722081	1
RTP4	-1.007969001	0.016133222	0.991835779	1
RTP5	-1.154700068	0.576447163	0.578252905	1
RTRAF	-0.894371911	-0.185328206	1.079700116	1
RUSC1	-0.933933745	-0.121102699	1.055036443	1
RWDD3	-1.154008738	0.542394037	0.611614701	1
S100A4	-1.13290465	0.373073705	0.759830945	1
S100A7L2	-1.14846101	0.470413448	0.678047562	1
S100A8	-1.148618243	0.676813263	0.471804979	1
S1PR4	-1.051826166	0.11330396	0.938522205	1
SAC3D1	-1.151986849	0.507475369	0.64451148	1
SACM1L	-1.144281456	0.43810729	0.706174166	1
SALL1	-1.133584336	0.757160009	0.376424327	1
SAMD9	-1.032082432	0.067594575	0.964487857	1
SAP18	-1.151327704	0.499287233	0.652040472	1
SAPCD1	-1.152396861	0.639334043	0.513062818	1
SARS1	-1.148816597	0.675231508	0.473585089	1
SAT2	-1.151990743	0.507526425	0.644464318	1
SBSPON	-1.150072343	0.664480033	0.48559231	1
SCAMP1	-1.148783734	0.675495526	0.473288208	1
SCAMP4	-1.091213475	0.218590126	0.872623349	1
SCAND1	-0.98007223	-0.038730201	1.018802432	1
SCAP	-1.094288912	0.227928491	0.866360421	1
SCARB2	-1.123813349	0.332161887	0.791651462	1

SCD	-0.91283829	-0.15598978	1.068828071	1
SCD5	-1.141412885	0.419436914	0.72197597	1
SCG5	-1.089934838	0.21476818	0.875166657	1
SCGB2A1	-0.944601068	-0.102848678	1.047449746	1
SCGB3A2	-1.150261761	0.662728887	0.487532874	1
SCHIP1	-0.984496412	-0.030317686	1.014814098	1
SCOC	-1.12992295	0.358913919	0.771009031	1
SCP2	-1.152530665	0.514988914	0.637541751	1
SDHC	-1.114713992	0.296474768	0.818239224	1
SDR42E2	-1.122205038	0.325535355	0.796669684	1
SEC11A	-1.154426826	0.598985587	0.555441239	1
SEC31A	-1.133027298	0.373674709	0.759352589	1
SEC61G	-1.058116891	0.128694257	0.929422634	1
SEC62	-1.064785302	0.145515158	0.919270145	1
SEL1L	-0.931553213	-0.125118019	1.056671232	1
SEL1L2	-1.154689898	0.581637825	0.573052073	1
SELENOF	-1.152380354	0.512828987	0.639551367	1
SELL	-0.987152422	-0.025218133	1.012370555	1
SEMA4G	-1.094097702	0.227341698	0.866756004	1
SENP3	-1.149617773	0.481084649	0.668533124	1
SENP5	-1.064728046	0.145368362	0.919359684	1
SENP6	-1.131131456	0.364552737	0.766578719	1
SERHL2	-1.098179697	0.240057462	0.858122234	1
SERPINA9	-1.142293789	0.424949514	0.717344275	1
SERPINB10	-1.149197909	0.672108564	0.477089345	1
SERPINB7	-0.921934525	-0.141135345	1.063069869	1
SERPINE2	-0.990987725	-0.017787246	1.008774971	1
SERPINI2	-1.153521628	0.531584605	0.621937024	1
SESN1	-1.138368351	0.401589061	0.73677929	1
SETD4	-0.943431871	-0.104870769	1.04830264	1
SF1	-1.143503317	0.710676541	0.432826776	1
SFMBT2	-1.118510206	0.310857862	0.807652344	1
SFPQ	-0.961564483	-0.072883098	1.034447582	1
SFXN1	-1.151465225	0.50092702	0.650538205	1
SGF29	-0.945399956	-0.101463929	1.046863885	1
SGO1	-1.150469907	0.660758307	0.4897116	1
SGSM3	-1.153732197	0.617811345	0.535920852	1
SH3D19	-1.14881412	0.675251434	0.473562687	1
SH3KBP1	-1.154260142	0.549514069	0.604746073	1
SHB	-1.133832609	0.377660626	0.756171982	1
SHMT2	-0.970575898	-0.05645629	1.027032188	1
SIAH1	-1.097748197	0.238694035	0.859054162	1
SIGLECL1	-0.935172611	-0.119004814	1.054177424	1
SIT1	-1.143743661	0.709304824	0.434438838	1
SKA3	-1.117096736	0.305425547	0.811671188	1
SKP1	-1.093122255	0.224361187	0.868761068	1
SLAIN1	-0.929100974	-0.129232752	1.058333726	1
SLBP	-1.134949234	0.751642141	0.383307093	1
SLC10A2	-1.020951047	0.043309364	0.977641682	1
SLC10A5	-1.151181123	0.653606687	0.497574437	1
SLC12A3	-1.153557692	0.532298648	0.621259044	1
SLC1A1	-1.154698172	0.575324614	0.579373558	1
SLC22A3	-1.10861158	0.2746001	0.83401148	1
SLC22A5	-1.1396796	0.409067269	0.730612331	1
SLC23A1	-1.154695202	0.574307335	0.580387867	1

SLC23A2	-1.150350557	0.661894345	0.488456213	1
SLC23A3	-1.148031826	0.466697719	0.681334107	1
SLC25A11	-1.022887265	0.047464935	0.97542233	1
SLC25A22	-1.130109373	0.359775356	0.770334017	1
SLC25A27	-0.992350574	-0.01512722	1.007477794	1
SLC27A1	-1.005951282	0.012010761	0.993940521	1
SLC2A4RG	-1.101517638	0.250768536	0.850749102	1
SLC2A9	-1.139827858	0.729896242	0.409931617	1
SLC35B1	-0.943565257	-0.104640353	1.048205611	1
SLC37A1	-1.112775304	0.289370158	0.823405146	1
SLC37A3	-1.152549602	0.515266101	0.637283501	1
SLC38A2	-1.151383912	0.651430425	0.499953487	1
SLC39A10	-1.147279978	0.686827839	0.460452139	1
SLC39A11	-0.914940556	-0.152581223	1.067521779	1
SLC39A2	-1.097193776	0.236949069	0.860244707	1
SLC39A3	-1.116803178	0.304309093	0.812494085	1
SLC39A5	-1.027718368	0.057957641	0.969760727	1
SLC3A2	-1.106409025	0.267032708	0.839376317	1
SLC41A2	-1.149956873	0.665528937	0.484427936	1
SLC41A3	-1.045136468	0.097400829	0.947735639	1
SLC51B	-1.154647522	0.586906258	0.567741264	1
SLC66A3	-1.119035188	0.312900103	0.806135085	1
SLC6A12	-1.106155325	0.266171235	0.83998409	1
SLC7A13	-1.152472338	0.514142424	0.638329914	1
SLC7A5	-1.146385403	0.453399623	0.69298578	1
SLC9B2	-1.082954627	0.194480258	0.888474369	1
SLCO4C1	-1.154507868	0.59552103	0.558986839	1
SLF2	-1.031392721	0.066061184	0.965331537	1
SLFN11	-1.137917274	0.738835675	0.399081599	1
SLFN12	-1.137194744	0.742065195	0.395129549	1
SLFN13	-1.154376758	0.553508726	0.600868032	1
SLFNL1	-1.065252514	0.146714595	0.918537919	1
SLMAP	-1.038773473	0.082680595	0.956092878	1
SLU7	-1.120989499	0.320626229	0.80036327	1
SLURP2	-1.019494457	0.040201397	0.979293061	1
SLX4	-1.150691113	0.658607028	0.492084085	1
SMAD1	-0.990562399	-0.018615289	1.009177688	1
SMAD2	-1.154611608	0.56489505	0.589716558	1
SMARCA5	-1.109201596	0.276655005	0.832546591	1
SMARCB1	-1.114947321	0.297340321	0.817606999	1
SMC5	-0.899663046	-0.177029548	1.076692594	1
SMG8	-1.113014269	0.290237668	0.8227766	1
SMIM11	-1.012950895	0.026425593	0.986525302	1
SMIM13	-0.937013218	-0.115877402	1.05289062	1
SMIM2	-1.154360169	0.601458688	0.552901481	1
SMIM29	-1.139775586	0.730149176	0.40962641	1
SMNDC1	-1.144214718	0.706567428	0.43764729	1
SMPD1	-1.139583209	0.408507437	0.731075772	1
SMPX	-1.131989363	0.36863698	0.763352382	1
SMR3A	-1.103814031	0.258315567	0.845498464	1
SNAI1	-1.049924647	0.108736812	0.941187835	1
SNAPC2	-1.143188025	0.712451582	0.430736443	1
SNCG	-1.145073708	0.443677816	0.701395892	1
SNIP1	-1.111623938	0.285221569	0.826402369	1
SNRNP40	-0.928561966	-0.130134282	1.058696248	1

SNTG2	-1.066281779	0.149367029	0.91691475	1
SNUPN	-0.907924061	-0.163901567	1.071825628	1
SNX18	-1.120777948	0.319780175	0.800997772	1
SNX7	-0.989668594	-0.020352131	1.010020725	1
SOCS5	-1.099275839	0.243542391	0.855733448	1
SOSTDC1	-1.098601101	0.24139356	0.857207542	1
SOWAHD	-0.897866495	-0.179856716	1.077723211	1
SP140L	-1.139754504	0.730251045	0.409503459	1
SPAG9	-1.016540884	0.033946214	0.98259467	1
SPANXN5	-1.046513123	0.100636505	0.945876618	1
SPATA24	-0.898003678	-0.179641181	1.077644859	1
SPATA48	-0.999550046	-0.000899301	1.000449347	1
SPINK1	-0.995754244	-0.00843811	1.004192354	1
SPINK6	-1.045680661	0.098677666	0.947002995	1
SPINT3	-1.125291458	0.338392856	0.786898602	1
SPINT4	-1.104964386	0.262154309	0.842810076	1
SPO11	-1.030193203	0.063403704	0.966789499	1
SPRR1A	-1.109871115	0.279001543	0.830869572	1
SPRY3	-0.921877102	-0.141229994	1.063107096	1
SPTY2D1	-1.152151345	0.642486957	0.509664388	1
SPTY2D1OS	-1.114224991	0.29466821	0.81955678	1
SRBD1	-1.110888618	0.282598457	0.828290161	1
SRCAP	-1.146884259	0.45728546	0.689598799	1
SREBF1	-1.145141495	0.700977202	0.444164293	1
SREK1IP1	-1.133936966	0.755754653	0.378182313	1
SRGN	-1.085933714	0.20302425	0.882909464	1
SRP14	-0.917963317	-0.147654569	1.065617885	1
SRP19	-0.928236518	-0.130678114	1.058914632	1
SRSF1	-0.89832973	-0.179128674	1.077458405	1
SRSF7	-1.039414132	0.084145707	0.955268424	1
SSBP3	-1.154428403	0.555504829	0.598923574	1
SST	-1.143410587	0.432209156	0.711201431	1
SSTR4	-0.953315803	-0.087604213	1.040920017	1
ST6GALNAC3	-1.090407141	0.216175913	0.874231228	1
ST7L	-1.132761774	0.372375546	0.760386228	1
STAG1	-1.147189205	0.687470392	0.459718813	1
STC2	-1.154346855	0.601922235	0.55242462	1
STEAP1	-0.962524384	-0.071150795	1.033675179	1
STH	-1.113662255	0.292601519	0.821060736	1
STK17A	-1.132514036	0.7613441	0.371169936	1
STK31	-1.087301902	0.207004354	0.880297548	1
STMN2	-1.013992472	0.028598442	0.98539403	1
STRA8	-1.131952791	0.368461437	0.763491354	1
STRADB	-1.153676413	0.534730594	0.618945818	1
STRC	-1.138602376	0.402902655	0.735699721	1
STRIT1	-0.986054648	-0.027330413	1.01338506	1
STX18	-1.073957114	0.16960669	0.904350424	1
STX5	-1.03734801	0.079433945	0.957914065	1
STX6	-1.0778021	0.180073829	0.897728271	1
STYK1	-1.140393777	0.727125296	0.41326848	1
SUCLG2	-1.152876398	0.520250935	0.632625463	1
SUGP1	-1.132564381	0.761149945	0.371414436	1
SULT1C3	-0.934079637	-0.120855941	1.054935578	1
SULT1E1	-1.105427997	0.263712773	0.841715223	1
SUMO1	-1.140716721	0.415200308	0.725516413	1

SUPV3L1	-1.153467492	0.622935032	0.53053246	1
SURF4	-1.126296013	0.342709879	0.783586134	1
SUSD1	-1.108737989	0.275039343	0.833698646	1
SUZ12	-1.040634471	0.086946814	0.953687658	1
SVBP	-1.133383675	0.757953659	0.375430016	1
SWI5	-1.052762814	0.11556777	0.937195043	1
SYCE1L	-1.130582724	0.361976265	0.768606459	1
SYCE2	-1.056844191	0.125544612	0.931299579	1
SYCP2	-1.138937535	0.404799499	0.734138037	1
SYCP2L	-0.944940665	-0.102260348	1.047201013	1
SYNE1	-1.003141466	0.006312821	0.996828645	1
SYNGR2	-1.153779363	0.61682571	0.536953653	1
SYS1-DBNDD2	-1.135352947	0.385384774	0.749968174	1
SYTL5	-1.118508591	0.3108516	0.807656991	1
TAAR8	-1.135513187	0.386214995	0.749298192	1
TAAR9	-0.955073979	-0.084490831	1.03956481	1
TAB1	-1.060360773	0.134293694	0.926067079	1
TACR1	-1.15467955	0.571310514	0.583369037	1
TACR3	-1.06426911	0.144193263	0.920075847	1
TAF1A	-1.134402881	0.380526384	0.753876498	1
TAF4B	-1.152607848	0.516126168	0.636481679	1
TAF7L	-1.154680714	0.583200066	0.571480649	1
TAFA1	-1.153250788	0.626719951	0.526530837	1
TANGO6	-1.15080046	0.493280041	0.657520419	1
TARS3	-1.154269006	0.604471217	0.54979779	1
TAS2R10	-1.140041899	0.411186599	0.7288553	1
TAS2R50	-1.139735249	0.409391237	0.730344012	1
TAS2R8	-1.097101049	0.236657969	0.86044308	1
TAS2R9	-1.04934972	0.107363377	0.941986343	1
TASOR	-1.153827122	0.615800958	0.538026164	1
TASP1	-1.036650886	0.077852737	0.958798149	1
TBC1D3F	-0.962716962	-0.070802761	1.033519724	1
TBCA	-1.151393177	0.500063832	6.51E-01	1
TBCC	-1.110767626	0.282168767	0.828598859	1
TBCD	-1.018427335	0.037934215	0.98049312	1
TCEAL4	-1.123032919	0.328927127	0.794105792	1
TCEAL7	-0.981325443	-0.036357468	1.017682912	1
TCEAL8	-1.142715855	0.427655607	0.715060247	1
TCEAL9	-1.058421199	0.129450142	0.928971058	1
TCF12	-1.151417003	0.651068731	0.500348272	1
TCF19	-0.938601574	-0.113168366	1.05176994	1
TECTB	-1.154327246	0.602589133	0.551738113	1
TEFM	-0.915615075	-0.151484491	1.067099566	1
TESK2	-1.152847007	0.633061252	0.519785754	1
TEX10	-1.035222167	0.07462542	0.960596747	1
TEX12	-1.024679841	0.051337297	0.973342543	1
TEX38	-1.15401462	0.542544383	0.611470237	1
TFAP2C	-1.154472551	0.597107028	0.557365523	1
TFB2M	-1.1537641	0.536616664	0.617147436	1
TFCP2	-1.150085543	0.664359247	0.485726296	1
TFDP3	-0.910085733	-0.16043091	1.070516643	1
TFF1	-1.145345754	0.699705658	0.445640096	1
TFRC	-1.108394034	0.273845453	0.834548581	1
TGFBRAP1	-1.151211581	0.653284117	0.497927464	1
TGIF1	-1.090666759	0.216951717	0.873715042	1

TGIF2LX	-1.143776805	0.709114366	0.434662439	1
TGIF2LY	-0.910871383	-0.159165799	1.070037182	1
THAP10	-1.141815783	0.719879492	0.421936292	1
THAP11	-1.079811328	0.185637936	0.894173391	1
THAP7	-1.095946821	0.233052112	0.862894709	1
THEM6	-0.995278451	-0.009377149	1.0046556	1
THOC1	-1.15469748	0.579650306	0.575047174	1
THRA	-1.153347959	0.528286337	0.625061622	1
TIAL1	-0.911747938	-0.157751955	1.069499892	1
TIGD2	-0.981702577	-0.035641863	1.01734444	1
TIMM8B	-1.153949329	0.540909311	0.613040018	1
TIMM9	-1.144562586	0.704502188	0.440060398	1
TIMMDC1	-1.143558439	0.710363391	0.433195047	1
TKTL2	-0.898514951	-0.178837392	1.077352343	1
TLCD4-RWDD3	-0.899083411	-0.177942768	1.077026179	1
TLE1	-1.0944184	0.228326346	0.866092054	1
TLX1	-1.072796722	0.166492434	0.906304288	1
TM9SF1	-1.146981197	0.458054033	0.688927163	1
TMEM101	-1.080099281	0.186440876	0.893658404	1
TMEM102	-1.138381296	0.736719806	0.40166149	1
TMEM123	-1.154653057	0.586395093	0.568257963	1
TMEM128	-1.150918513	0.494589404	0.656329109	1
TMEM138	-0.984386219	-0.030528449	1.014914669	1
TMEM147	-1.14683982	0.456934633	0.689905187	1
TMEM178A	-1.078066082	0.180801073	0.897265008	1
TMEM192	-1.135725441	0.387319674	0.748405767	1
TMEM200A	-1.130227023	0.360320547	0.769906475	1
TMEM240	-0.951584464	-0.090657462	1.042241926	1
TMEM247	-0.953281338	-0.087665114	1.040946453	1
TMEM254	-1.137080916	0.742567016	0.3945139	1
TMEM256	-1.144711948	0.441108434	0.703603514	1
TMEM258	-1.154672076	0.570314864	0.584357213	1
TMEM41B	-1.047100836	0.102023574	0.945077261	1
TMEM47	-0.908800633	-0.162495993	1.071296626	1
TMEM50B	-0.89781387	-0.179939383	1.077753253	1
TMEM51	-0.942929109	-0.105738634	1.048667743	1
TMEM87B	-1.069267599	0.157141942	0.912125656	1
TMEM9B	-1.090295686	0.215843292	0.874452394	1
TMOD4	-1.136664131	0.744388845	0.392275286	1
TMPRSS11D	-1.15458135	0.562923032	0.591658319	1
TNFRSF17	-1.068284148	0.154567662	0.913716486	1
TNFRSF1A	-1.059354067	0.131774131	0.927579936	1
TNNC2	-1.044672437	0.09631436	0.948358077	1
TOMM22	-1.132076967	0.369058015	0.763018952	1
TOMM40L	-1.142185244	0.424260569	0.717924675	1
TOMT	-1.029347965	0.061538158	0.967809807	1
TP53INP1	-1.014635285	0.029943127	0.984692158	1
TP53TG5	-1.142110561	0.718322383	0.423788178	1
TPCN1	-1.136336133	0.39053039	0.745805743	1
TPK1	-1.037540189	0.0798706	0.957669589	1
TPRG1	-1.131454183	0.366080972	0.765373211	1
TPRKB	-0.948804004	-0.09553503	1.044339035	1
TPT1	-1.087865772	0.208655321	0.879210452	1
TRAM1L1	-1.126065193	0.341711821	0.784353372	1
TRAPPC5	-1.150093124	0.485803331	0.664289793	1

TREML2	-0.956788101	-0.081442887	1.038230988	1
TREX2	-1.092949986	0.223837043	0.869112943	1
TRIM23	-1.059301348	0.13164252	0.927658828	1
TRIM34	-1.153557056	0.621271092	0.532285964	1
TRIM51	-0.907284547	-0.164925478	1.072210025	1
TRIM77	-1.082574216	0.193400921	0.889173296	1
TRMT10C	-0.910305835	-0.160076685	1.070382521	1
TRPC4AP	-1.154632703	0.566477062	0.588155641	1
TRPC5OS	-1.084327539	0.198397366	0.885930173	1
TRPC6	-1.151942144	0.645050538	0.506891606	1
TRPM5	-0.91979478	-0.144654652	1.064449432	1
TRUB1	-0.947180855	-0.098367894	1.045548749	1
TSC1	-1.154361934	0.601396553	0.55296538	1
TSC22D2	-1.058894679	0.130628402	0.928266276	1
TSC22D4	-1.088569027	0.210723267	0.87784576	1
TSEN34	-1.004940912	0.009956169	0.994984743	1
TSEN54	-1.093568687	0.225722598	0.867846089	1
TSHB	-1.150938743	0.494815744	0.656122999	1
TSPAN5	-0.959840262	-0.075984541	1.035824803	1
TSPYL5	-1.140616626	0.41459934	0.726017286	1
TTC13	-1.153732426	0.535925814	0.617806612	1
TTC31	-0.952207065	-0.089560926	1.041767991	1
TTLL12	-0.920648606	-0.143252212	1.063900817	1
TTLL5	-1.037676584	0.080180703	0.957495881	1
TUBA4A	-1.137153908	0.742245437	0.394908471	1
TUBB2A	-1.022022857	0.045606273	0.976416585	1
TUBB2B	-1.150728535	0.492491602	0.658236933	1
TUBB3	-1.109236572	0.276777198	0.832459374	1
TUBG2	-1.153814863	0.61606667	0.537748194	1
TUSC1	-1.140610675	0.726047	0.414563675	1
TUSC2	-1.154671101	0.584475996	0.570195105	1
TVP23A	-1.095832639	0.232697158	0.863135481	1
TVP23B	-1.10357725	0.257530336	0.846046913	1
TVP23C	-1.067329372	0.152081131	0.91524824	1
TVP23C-CDRT4	-1.154493526	0.596181493	0.558312033	1
TWF1	-1.058038949	0.128500827	0.929538122	1
TWIST2	-0.984111869	-0.031052918	1.015164787	1
TWNK	-1.153916558	0.613801625	0.540114934	1
TXLNA	-1.137487324	0.396720578	0.740766745	1
TXNDC12	-1.133246679	0.374753662	0.758493017	1
TXNDC15	-1.154642186	0.567267883	0.587374303	1
TXNL4B	-0.977409136	-0.043745932	1.021155069	1
TYMS	-1.147838879	0.682774534	0.465064345	1
UBD	-1.15467567	0.570774834	0.583900836	1
UBE2B	-1.09176029	0.220235243	0.871525047	1
UBE2C	-0.905251789	-0.168171496	1.073423284	1
UBE2D3	-1.129561784	0.357253467	0.772308317	1
UBE2E1	-1.146204523	0.694186505	0.452018017	1
UBE3D	-0.911410401	-0.158296682	1.069707082	1
UBLCP1	-1.129995525	0.359248924	0.770746602	1
UCKL1	-1.129764909	0.358185968	0.771578941	1
UFSP1	-1.105806225	0.264989157	0.840817067	1
UGGT2	-1.07388242	0.169405619	0.904476801	1
UGT1A3	-1.033658104	0.071112558	0.962545546	1
UGT2A1	-1.039626823	0.08463293	0.954993893	1

UGT2A2	-1.074054493	0.169868957	0.904185536	1
UGT2A3	-0.917210448	-0.148884483	1.066094932	1
UGT2B15	-0.901395918	-0.174293281	1.075689199	1
UMODL1	-1.153214912	0.62731766	0.525897252	1
UNC45A	-1.143809715	0.708924934	0.434884781	1
UNC93A	-1.083816176	0.19693436	0.886881816	1
UPF2	-0.945665363	-0.101003328	1.046668691	1
UQCRB	-1.141414826	0.721965952	0.419448875	1
UROD	-1.044686573	0.096347425	0.948339147	1
USF2	-0.916753115	-0.149630675	1.06638379	1
USO1	-1.138982356	0.405054581	0.733927775	1
USP1	-1.053930864	0.118404175	0.935526689	1
USP12	-1.153572008	0.532585131	0.620986877	1
USP17L10	-1.154647136	0.567706206	0.58694093	1
USP17L12	-1.144462938	0.439365232	0.705097706	1
USP17L17	-1.154156741	0.546391836	0.607764904	1
USP17L20	-1.137266839	0.741746396	0.395520442	1
USP17L21	-1.076039262	0.175246107	0.900793155	1
USP17L26	-1.148439227	0.470221986	0.678217241	1
USP17L27	-1.111643299	0.285290908	0.826352391	1
USP17L30	-1.14973851	0.667476052	0.482262458	1
USP38	-1.154647497	0.567738927	0.58690857	1
USP39	-1.127747693	0.349074274	0.778673419	1
USP47	-1.036779482	0.078144094	0.958635388	1
USP51	-1.154273296	0.604337174	0.549936122	1
USPL1	-1.055183237	0.121462017	0.933721219	1
UST	-1.140720591	0.725497008	0.415223583	1
UTP14A	-1.154658807	0.568827622	0.585831184	1
UTS2R	-0.938256445	-0.113757815	1.05201426	1
VCAN	-1.145207154	0.700570098	0.444637056	1
VCX	-1.085907447	0.20294819	0.882959257	1
VCX2	-0.972053708	-0.053726508	1.025780216	1
VEGFA	-0.963963626	-0.068545749	1.032509374	1
VGLL2	-1.15466784	0.584859485	0.569808355	1
VIPAS39	-1.154644678	0.567486104	0.587158574	1
VMA21	-0.97941935	-0.03996315	1.019382501	1
VMAC	-1.0678361	0.153399261	0.914436838	1
VMO1	-0.978924559	-0.040896119	1.019820678	1
VNN2	-1.134003422	0.75548826	0.378515162	1
VPS13B	-1.142414344	0.425717998	0.716696346	1
VPS18	-1.138201611	0.400658472	0.737543139	1
VPS26C	-1.153273223	0.626342288	0.526930936	1
VPS41	-1.138962682	0.734020113	0.404942569	1
VPS4A	-1.1504296	0.661143853	0.489285747	1
VSIG4	-0.901904366	-0.173488676	1.075393042	1
VSTM1	-0.985702699	-0.028006254	1.013708952	1
VSTM5	-1.154313888	0.603033288	0.551280601	1
VSX1	-1.145724653	0.697306149	0.448418504	1
VTA1	-1.136312725	0.390406419	0.745906307	1
VWA5B2	-1.087256702	0.206872285	0.880384417	1
VWC2L	-1.152336452	0.512211	0.640125452	1
VXN	-1.117783886	0.308054571	0.809729316	1
WASHC3	-0.95618332	-0.082519697	1.038703017	1
WDR38	-1.122418277	0.326405159	0.796013118	1
WDR75	-1.076924542	0.177664339	0.899260203	1

WDR83OS	-1.153003547	0.63069694	0.522306607	1
WFDC10B	-1.153753667	0.617365808	0.536387859	1
WFDC9	-1.046946229	0.10165835	0.945287879	1
WIPI2	-1.088308911	0.20995723	0.878351681	1
WNT11	-1.136915838	0.39362433	0.743291507	1
WNT7A	-0.926180754	-0.134104657	1.060285411	1
WNT9B	-1.088066328	0.209244052	0.878822276	1
XAGE2	-0.9216022	-0.141682957	1.063285157	1
XCL1	-1.154196023	0.547540347	0.606655676	1
XCL2	-1.083661452	0.19649264	0.887168812	1
XKR3	-0.947332749	-0.098103245	1.045435994	1
XPO4	-1.117700666	0.30773499	0.809965676	1
XRCC5	-1.138669958	0.403283645	0.735386313	1
XXYLT1	-1.154604221	0.564386262	0.59021796	1
YBEY	-1.079109348	0.183686353	0.895422995	1
YJU2B	-1.109731746	0.278511773	0.831219973	1
YY2	-0.918828636	-0.146238598	1.065067233	1
ZBED1	-1.12933561	0.356219228	0.773116382	1
ZBTB16	-0.987359304	-0.024819339	1.012178643	1
ZBTB2	-1.135762826	0.748247989	0.387514838	1
ZBTB26	-0.911405044	-0.158305324	1.069710368	1
ZBTB7B	-1.145575652	0.447319428	0.698256224	1
ZBTB8OS	-1.116971481	0.304948697	0.812022784	1
ZC3H12B	-1.153458982	0.53036914	0.623089842	1
ZC3H12C	-1.138512945	0.736113315	0.40239963	1
ZC3H15	-1.071384301	0.162728668	0.908655633	1
ZC3H4	-1.137908735	0.738874297	0.399034438	1
ZC3HAV1L	-1.150357045	0.661833024	0.488524021	1
ZC4H2	-1.068415413	0.154910489	0.913504924	1
ZCCHC8	-1.148587187	0.677058399	0.471528788	1
ZDHHC21	-1.146426873	0.692708496	0.453718377	1
ZFAND4	-0.897906859	-0.179793304	1.077700163	1
ZFAND6	-1.002824604	0.005673348	0.997151256	1
ZFHX2	-0.920477083	-0.143534143	1.064011226	1
ZFP1	-1.134648587	0.752876004	0.381772583	1
ZFP37	-1.075140552	0.172803781	0.90233677	1
ZFP41	-1.151817894	0.505292696	0.646525197	1
ZFP57	-0.995382507	-0.009171893	1.0045544	1
ZFP62	-1.153399676	0.624153927	0.529245749	1
ZFP64	-1.073012737	0.16707065	0.905942087	1
ZFYVE9	-1.136540831	0.391617636	0.744923195	1
ZG16	-1.136245486	0.390050716	0.74619477	1
ZG16B	-1.152443845	0.638711024	0.513732821	1
ZGLP1	-1.069782981	0.15849638	0.911286601	1
ZHX1-C8orf76	-1.148504762	0.677705794	0.470798968	1
ZIM2	-1.152792561	0.518933391	0.63385917	1
ZMAT2	-1.149270119	0.671504275	0.477765844	1
ZMAT4	-1.154258216	0.604805424	0.549452792	1
ZMYND19	-1.057061682	0.126081528	0.930980155	1
ZNF10	-1.138092088	0.738042494	0.400049594	1
ZNF135	-0.984843732	-0.029652953	1.014496686	1
ZNF143	-1.147016684	0.688680148	0.458336536	1
ZNF165	-1.154661538	0.569111943	0.585549596	1
ZNF181	-1.138811636	0.734726851	0.404084785	1
ZNF217	-1.010351344	0.021034554	0.98931679	1

ZNF25	-1.152391352	0.639406667	0.512984685	1
ZNF256	-1.119267703	0.313809001	0.805458702	1
ZNF263	-1.028411701	0.059478428	0.968933274	1
ZNF281	-1.146435255	0.453782901	0.692652355	1
ZNF287	-1.154505561	0.595628926	0.558876634	1
ZNF318	-1.00340723	0.006849649	0.996557581	1
ZNF32	-0.906195844	-0.166665597	1.072861441	1
ZNF322	-0.905966222	-0.167032133	1.072998355	1
ZNF324	-1.138255895	0.737294944	0.400960951	1
ZNF331	-1.151304043	0.652295679	0.499008364	1
ZNF33A	-1.152434247	0.513595403	0.638838844	1
ZNF350	-1.14133625	0.418965542	0.722370708	1
ZNF367	-1.009492016	0.01926233	0.990229686	1
ZNF385B	-0.939638681	-0.111394357	1.051033038	1
ZNF407	-1.139809113	0.40982211	0.729987003	1
ZNF408	-0.910954802	-0.159031356	1.069986157	1
ZNF438	-1.152347174	0.63998575	0.512361425	1
ZNF445	-1.011337754	0.023074886	0.988262868	1
ZNF471	-0.99171216	-0.016374575	1.008086735	1
ZNF473	-0.925430082	-0.135352156	1.060782238	1
ZNF48	-0.99439248	-0.01112226	1.00551474	1
ZNF490	-1.074455983	0.17095179	0.903504193	1
ZNF510	-1.149262328	0.671569684	0.477692644	1
ZNF57	-1.153934092	0.540537891	0.613396202	1
ZNF600	-1.153858625	0.615109208	0.538749418	1
ZNF615	-1.07839475	0.181708112	0.896686637	1
ZNF622	-0.892550556	-0.188165522	1.080716078	1
ZNF625	-0.991767891	-0.016265776	1.008033667	1
ZNF677	-1.142882708	0.714145022	0.428737686	1
ZNF678	-1.142245408	0.71760332	0.424642088	1
ZNF689	-1.15216757	0.642283615	0.509883956	1
ZNF705B	-1.016789983	0.034471372	0.982318611	1
ZNF711	-1.152978977	0.63107528	0.521903698	1
ZNF735	-1.032394057	0.068288687	0.964105371	1
ZNF736	-1.076956521	0.177751925	0.899204596	1
ZNF750	-1.056415687	0.124488358	0.931927329	1
ZNF763	-1.089840573	0.214487776	0.875352797	1
ZNF79	-1.009381913	0.019035609	0.990346303	1
ZNF800	-0.983646005	-0.031942596	1.015588601	1
ZNF804B	-1.126822319	0.344999632	0.781822688	1
ZNF844	-1.03776051	0.080371597	0.957388913	1
ZNF846	-1.062905832	0.140718486	0.922187346	1
ZNF852	-1.153374124	0.624604667	0.528769457	1
ZNF879	-1.146000451	0.695524609	0.450475842	1
ZNF90	-1.13580977	0.748049618	0.387760152	1
ZP2	-1.153669605	0.53458753	0.619082075	1
ZSWIM7	-1.096368224	0.234364837	0.862003387	1
A1BG	0.883896082	0.201515856	-1.085411938	2
AACS	0.978395208	0.04189289	-1.020288098	2
AAR2	0.994276676	0.011350028	-1.005626704	2
AARD	0.80367702	0.3161987	-1.11987572	2
AARS2	1.123129928	-0.329327214	-0.793802715	2
ABCB7	1.005609102	-0.011314216	-0.994294887	2
ABCC11	0.988394451	0.022820479	-1.01121493	2
ABCC6	0.984017394	0.031233433	-1.015250827	2

ABCD4	0.817622006	0.297319786	-1.114941792	2
ABHD11	0.993251571	0.013362928	-1.006614499	2
ABI2	1.061686739	-0.137631021	-0.924055718	2
ABLIM1	1.132131089	-0.369318502	-0.762812586	2
ABRA	1.081347547	-0.18993796	-0.891409588	2
ACAD11	1.073459456	-0.168268607	-0.905190849	2
ACBD6	0.799972091	0.32114743	-1.121119521	2
ACIN1	1.004273121	-0.008601735	-0.995671386	2
ACKR1	1.119883504	-0.316229414	-0.80365409	2
ACLY	1.022208962	-0.046005967	-0.976202996	2
ACOT4	1.057743411	-0.127768031	-0.92997538	2
ACOT6	0.866837665	0.227220515	-1.09405818	2
ACOT8	1.110155834	-0.280004258	-0.830151576	2
ACP1	0.905857239	0.167206042	-1.07306328	2
ACSL4	0.868862605	0.224209974	-1.093072578	2
ACSL6	1.070313317	-0.159894013	-0.910419304	2
ADAM28	1.099476792	-0.244184655	-0.855292136	2
ADAMTS1	1.107887218	-0.272093587	-0.835793631	2
ADAMTS10	1.041022049	-0.0878393	-0.953182748	2
ADAMTS15	1.123351739	-0.330244125	-0.793107614	2
ADAMTSL3	1.017466978	-0.035900842	-0.981566136	2
ADAT2	0.924906933	0.136220379	-1.061127312	2
ADCK5	0.985788625	0.027841312	-1.013629937	2
ADCY10	1.082009343	-0.191802958	-0.890206385	2
ADCY2	0.807538142	0.311011743	-1.118549885	2
ADCY7	1.075284814	-0.17319498	-0.902089834	2
ADGRF1	1.116858656	-0.304519782	-0.812338873	2
ADGRG3	1.117832177	-0.308240167	-0.80959201	2
ADGRG5	1.119247628	-0.313730421	-0.805517207	2
ADGRL1	0.930382548	0.127085044	-1.057467591	2
ADH1C	0.961459963	0.073071477	-1.03453144	2
ADIG	0.896664364	0.181743022	-1.078407387	2
ADNP	1.014299752	-0.029240878	-0.985058874	2
ADORA2A	1.11243327	-0.288132385	-0.824300885	2
ADPRS	1.111730515	-0.285603446	-0.826127069	2
ADRA1B	0.846769423	0.256494806	-1.103264229	2
AFG3L2	1.039518814	-0.084385457	-0.955133357	2
AGAP2	0.926620496	0.133372954	-1.059993449	2
AGAP3	1.063630467	-0.142562524	-0.921067943	2
AGBL2	0.985241224	0.028891418	-1.014132642	2
AGL	0.975056497	0.048147606	-1.023204103	2
AGMAT	0.920925565	0.142796762	-1.063722327	2
AGO2	1.115345403	-0.298822409	-0.816522994	2
AGPAT1	1.042075976	-0.090273266	-0.95180271	2
AGTRAP	1.038484731	-0.082021485	-0.956463245	2
AGXT	0.829718064	0.28060912	-1.110327184	2
AHCYL2	1.109594502	-0.278030144	-0.831564358	2
AHSA1	0.831917998	0.277535293	-1.109453291	2
AICDA	1.098664665	-0.241595487	-0.857069178	2
AIF1L	1.101371977	-0.250294866	-0.851077111	2
AIMP1	0.960456673	0.074877269	-1.035333943	2
AIPL1	0.980494394	0.037931805	-1.018426199	2
AK9	1.105362739	-0.263493004	-0.841869736	2
AKNA	0.922520019	0.14016963	-1.062689649	2
AKR1B10	1.11983941	-0.316055458	-0.803783953	2

ALDH1L2	0.803206629	0.316828543	-1.120035172	2
ALG10	0.983872235	0.031510699	-1.015382934	2
ALG12	1.12025553	-0.317701158	-0.802554373	2
ALKAL2	0.884170505	0.201095817	-1.085266322	2
ALKBH1	1.124446747	-0.334814853	-0.789631894	2
ALOX5AP	1.123308355	-0.33006455	-0.793243805	2
AMER3	0.980681914	0.037576875	-1.018258789	2
AMIGO2	0.831962508	0.27747299	-1.109435498	2
AMMECR1	0.80355245	0.316365541	-1.11991799	2
AMTN	1.122602541	-0.327158896	-0.795443645	2
ANAPC1	1.014416939	-0.029486055	-0.984930884	2
ANAPC5	0.845139128	0.258829652	-1.10396878	2
ANAPC7	0.87278681	0.218345007	-1.091131817	2
ANGEL1	1.047253264	-0.102383886	-0.944869378	2
ANGPT4	1.056070479	-0.123638951	-0.932431528	2
ANGPTL6	1.126601074	-0.344034684	-0.782566391	2
ANK1	0.971054402	0.05557354	-1.026627942	2
ANK3	1.055506202	-0.122253418	-0.933252784	2
ANKK1	0.945610781	0.101098075	-1.046708856	2
ANKMY1	0.990673276	0.018399524	-1.009072801	2
ANKRD12	1.12359105	-0.331236717	-0.792354333	2
ANKRD30B	0.89122752	0.190220441	-1.081447961	2
ANKRD30BL	1.130991478	-0.363892892	-0.767098586	2
ANKRD55	1.113743798	-0.292900189	-0.820843609	2
ANKRD62	0.846054863	0.257518949	-1.103573812	2
ANKRD63	1.026785654	-0.055917784	-0.97086787	2
ANKRD9	0.873720948	0.216942843	-1.090663792	2
ANKS1A	1.122083068	-0.325039009	-0.797044059	2
ANLN	1.037225131	-0.079154922	-0.958070209	2
ANP32A	1.110800038	-0.282283824	-0.828516214	2
ANXA1	1.086470461	-0.204581345	-0.881889115	2
AP1AR	0.938760004	0.112897631	-1.051657635	2
AP1M2	0.904287346	0.169707031	-1.073994376	2
AP3D1	1.041209824	-0.088272196	-0.952937628	2
AP3M2	0.910423143	0.159887832	-1.070310975	2
APBA3	0.856597845	0.242282988	-1.098880833	2
APBB1	1.110186921	-0.280113915	-0.830073006	2
APC	0.861971213	0.234412185	-1.096383398	2
APH1A	0.967877537	0.061414149	-1.029291686	2
APOBEC3H	1.118670546	-0.311480159	-0.807190387	2
APOD	1.128719387	-0.353422716	-0.775296671	2
APOE	0.824825874	0.287406116	-1.112231991	2
APOL4	1.013230515	-0.027008186	-0.986222329	2
AQP12A	1.029948015	-0.062861948	-0.967086067	2
AQP12B	1.091863215	-0.220545622	-0.871317593	2
AQP5	0.981557659	0.035916929	-1.017474588	2
AQP6	0.84568921	0.258042551	-1.103731761	2
ARAP1	0.949152957	0.094924617	-1.044077574	2
ARC	1.098166207	-0.240014768	-0.858151439	2
ARFGAP2	0.95261412	0.088843145	-1.041457265	2
ARHGAP15	0.935387417	0.118640483	-1.054027901	2
ARHGAP33	0.933481051	0.121867872	-1.055348923	2
ARHGAP8	1.12307171	-0.329087041	-0.793984669	2
ARHGDIG	1.105536528	-0.264078571	-0.841457957	2
ARHGEF10	0.884718574	0.200256293	-1.084974867	2

ARHGEF25	1.00391927	-0.007885173	-0.996034097	2
ARHGEF3	0.83935848	0.267057978	-1.106416458	2
ARL14	0.798374279	0.323273159	-1.121647438	2
ARL14EP	0.863806991	0.231706414	-1.095513405	2
ARL8B	1.126021179	-0.341521927	-0.784499252	2
ARPC1A	0.983306989	0.032589316	-1.015896305	2
ARPC1B	0.933657073	0.121570437	-1.05522751	2
ARPC2	1.032606077	-0.0687614	-0.963844676	2
ARV1	1.070244097	-0.159711368	-0.910532729	2
ARX	0.808548255	0.309649741	-1.118197996	2
AS3MT	1.0320567	-0.067537296	-0.964519404	2
ASB1	1.095604859	-0.231989995	-0.863614865	2
ASB10	0.929126656	0.129189771	-1.058316427	2
ASB15	1.127552682	-0.348210315	-0.779342368	2
ASB18	1.038033357	-0.080992635	-0.957040722	2
ASCL2	1.0628083	-0.140470794	-0.922337506	2
ASIC4	0.913644179	0.154684848	-1.068329028	2
ASMTL	1.008714248	-0.017662482	-0.991051766	2
ASPG	1.071458403	-0.162925409	-0.908532994	2
ASPH	1.131040314	-0.364122898	-0.766917416	2
ASPHD2	1.072378701	-0.165375459	-0.907003241	2
ASXL3	0.85665233	0.242203543	-1.098855873	2
ATE1	0.839947664	0.266222891	-1.106170554	2
ATF5	1.028256773	-0.059138268	-0.969118504	2
ATF7	1.040826922	-0.087389802	-0.95343712	2
ATG14	0.827627433	0.283520223	-1.111147657	2
ATG2B	0.948593376	0.095903237	-1.044496614	2
ATOH7	0.936042621	0.117528139	-1.053570761	2
ATP10D	0.942764143	0.106023182	-1.048787326	2
ATP1A3	1.108012588	-0.272526137	-0.835486451	2
ATP2B1	1.095368916	-0.231258789	-0.864110126	2
ATP2B2	1.106670553	-0.267922915	-0.838747637	2
ATP5F1E	0.956348098	0.082226465	-1.038574563	2
ATP5MC2	0.873534367	0.217223093	-1.09075746	2
ATP6V0A2	0.944897075	0.102335891	-1.047232965	2
ATP6V0A4	1.067639589	-0.152887673	-0.914751916	2
ATP7B	1.100731253	-0.248218258	-0.852512995	2
ATXN7L3B	1.095858954	-0.232778934	-0.86308002	2
AURKAIP1	1.115752624	-0.300345611	-0.815407012	2
AURKB	0.922434034	0.140311528	-1.062745562	2
AUTS2	0.894583447	0.184998038	-1.079581485	2
AVEN	1.077580328	-0.179463743	-0.898116586	2
AVL9	1.058268142	-0.129069819	-0.929198322	2
AVPI1	0.89675376	0.181602898	-1.078356658	2
AZGP1	1.125575582	-0.339606907	-0.785968675	2
B3GALT6	0.947115327	0.098482036	-1.045597363	2
B3GNTL1	1.064301719	-0.144276669	-0.92002505	2
B4GALNT1	1.020468495	-0.042278013	-0.978190482	2
B4GALNT4	0.902382787	0.172730861	-1.075113648	2
B4GALT2	0.971086172	0.055514892	-1.026601064	2
B9D2	1.038260152	-0.081509357	-0.956750794	2
BACH1	1.124791002	-0.336267436	-0.788523566	2
BAG6	1.069622919	-0.158075335	-0.911547584	2
BAK1	1.114334228	-0.295070909	-0.819263319	2
BAMBI	1.126898041	-0.345330697	-0.781567344	2

BCAN	1.018412961	-0.037903732	-0.980509229	2
BCAR1	1.089891583	-0.214639491	-0.875252092	2
BCAT1	0.996496525	0.006970509	-1.003467034	2
BCL11A	1.130435079	-0.361287652	-0.769147427	2
BEND4	1.132473145	-0.370971541	-0.761501604	2
BEST3	1.088497379	-0.210512129	-0.87798525	2
BGN	1.101453847	-0.250561024	-0.850892823	2
BHLHE22	1.050381134	-0.109829755	-0.94055138	2
BICC1	1.006390232	-0.012905379	-0.993484853	2
BICRA	1.113902974	-0.293483989	-0.820418986	2
BID	0.87757561	0.211132025	-1.088707636	2
BIRC5	1.096257918	-0.234020804	-0.862237114	2
BIRC7	1.085028785	-0.200411482	-0.884617303	2
BLCAP	0.918395949	0.146946942	-1.065342892	2
BMT2	1.062633934	-0.140028274	-0.92260566	2
BNIP5	0.867986236	0.225514214	-1.09350045	2
BORCS7-ASMT	1.048638473	-0.105669008	-0.942969466	2
BRK1	1.068319547	-0.154660091	-0.913659456	2
BRS3	1.030168552	-0.063349214	-0.966819338	2
BTBD1	1.132819597	-0.372657842	-0.760161755	2
BTBD11	1.024311287	-0.050539146	-0.973772141	2
BTBD18	0.956423391	0.082092439	-1.03851583	2
BTN1A1	0.895598741	0.183411507	-1.079010248	2
BZW2	1.122871674	-0.328263362	-0.794608312	2
C11orf21	0.868542502	0.224686594	-1.093229096	2
C11orf65	1.007623586	-0.015425643	-0.992197943	2
C11orf68	0.877336062	0.211494315	-1.088830377	2
C11orf86	1.11215339	-0.28712293	-0.82503046	2
C13orf46	0.899445249	0.17737281	-1.076818058	2
C14orf132	1.021315894	-0.044090284	-0.97722561	2
C15orf48	1.033083698	-0.069827673	-0.963256025	2
C15orf61	1.126880603	-0.345254416	-0.781626187	2
C15orf62	1.132236117	-0.369824829	-0.762411288	2
C16orf90	1.122348078	-0.326118529	-0.79622955	2
C17orf113	0.917616937	0.148220664	-1.065837601	2
C17orf114	0.92546518	0.135293873	-1.060759053	2
C17orf75	1.131847371	-0.367956145	-0.763891226	2
C1QL1	1.06942877	-0.157565105	-0.911863665	2
C1QTNF1	1.048649878	-0.105696137	-0.942953742	2
C20orf141	1.100220505	-0.246570914	-0.853649591	2
C22orf39	0.915364524	0.151892049	-1.067256573	2
C2CD2L	1.013178054	-0.026898842	-0.986279212	2
C2CD4B	1.112722146	-0.289177485	-0.823544661	2
C2orf78	1.099236918	-0.243418117	-0.855818801	2
C2orf81	0.848169436	0.2544847	-1.102654137	2
C3orf70	1.026438923	-0.05516122	-0.971277703	2
C3orf80	0.839200417	0.267281874	-1.106482291	2
C3orf84	1.025664602	-0.053475039	-0.972189562	2
C6orf132	1.073499341	-0.168375711	-0.90512363	2
C6orf141	1.129088413	-0.355093695	-0.773994718	2
C6orf89	0.9710354	0.055608616	-1.026644015	2
C8G	1.080326091	-0.187074314	-0.893251777	2
C8orf33	0.886350007	0.197752253	-1.08410226	2
C8orf48	1.069437603	-0.157588307	-0.911849296	2
CA1	0.897408634	0.180575674	-1.077984308	2

CA11	1.114039173	-0.29398434	-0.820054833	2
CABLES1	1.07219977	-0.164898134	-0.907301636	2
CACNA1A	0.810625316	0.306842485	-1.117467801	2
CACNG8	0.998601861	0.002790439	-1.001392299	2
CADM4	1.069355219	-0.157371949	-0.91198327	2
CALB1	1.105744893	-0.264781883	-0.840963011	2
CALCRL	0.922337457	0.140470874	-1.062808331	2
CALHM5	1.131114707	-0.364473688	-0.766641018	2
CALM1	0.916814944	0.149529835	-1.066344779	2
CAMK1G	1.099484568	-0.244209531	-0.855275037	2
CAMK2D	1.005607834	-0.011311635	-0.994296199	2
CAMK2N2	1.097480461	-0.237850413	-0.859630048	2
CAMSAP3	1.131502668	-0.366311406	-0.765191261	2
CAPG	1.013423501	-0.027410586	-0.986012915	2
CAPN11	1.095463742	-0.231552502	-0.86391124	2
CAPZA1	1.12069122	-0.319434024	-0.801257196	2
CARMIL2	1.130013446	-0.359331713	-0.770681732	2
CARMIL3	1.130015117	-0.359339438	-0.770675679	2
CARNS1	1.114482306	-0.295617582	-0.818864724	2
CASP14	1.035851163	-0.076044064	-0.959807099	2
CASP9	0.88572156	0.198717734	-1.084439293	2
CASR	0.877214684	0.211677825	-1.088892509	2
CASTOR2	0.806831702	0.311963037	-1.118794739	2
CATSPER2	0.898198163	0.179335518	-1.07753368	2
CATSPERZ	0.985595286	0.028212384	-1.01380767	2
CBFA2T3	1.130416912	-0.361203056	-0.769213856	2
CBLC	0.977200799	0.044136823	-1.021337622	2
CBLIF	1.129531931	-0.35711671	-0.77241522	2
CBX8	0.942581586	0.10633795	-1.048919536	2
CCDC102A	1.114364947	-0.29518424	-0.819180707	2
CCDC103	0.850458614	0.251187805	-1.101646419	2
CCDC112	0.890089337	0.19198416	-1.082073498	2
CCDC113	1.08400939	-0.197486581	-0.886522809	2
CCDC125	0.867900168	0.225642194	-1.093542362	2
CCDC136	1.073481827	-0.168328677	-0.90515315	2
CCDC15	0.916290545	0.150384701	-1.066675246	2
CCDC157	1.012962819	-0.026450426	-0.986512393	2
CCDC166	1.039963253	-0.085404448	-0.954558805	2
CCDC180	0.972989411	0.051992717	-1.024982128	2
CCDC184	1.026504097	-0.055303357	-0.97120074	2
CCDC185	1.090296817	-0.215846664	-0.874450153	2
CCDC188	0.939728364	0.111240758	-1.050969122	2
CCDC194	1.101529907	-0.250808461	-0.850721447	2
CCDC198	0.839561044	0.266770961	-1.106332006	2
CCDC27	1.014607699	-0.029885361	-0.984722338	2
CCDC38	1.070118948	-0.159381318	-0.910737631	2
CCDC40	1.099526533	-0.244343798	-0.855182736	2
CCDC74A	1.022936662	-0.047571317	-0.975365345	2
CCDC77	1.095521032	-0.231730057	-0.863790975	2
CCDC85A	1.028066462	-0.058720686	-0.969345775	2
CCDC88C	1.078012291	-0.180652793	-0.897359498	2
CCDC9	0.902737167	0.172169069	-1.074906237	2
CCDC92B	1.039309793	-0.083906844	-0.955402949	2
CCKAR	1.077614035	-0.179556417	-0.898057618	2
CCL27	1.0112963	-0.02298901	-0.98830729	2

CCL4	1.08548518	-0.201727277	-0.883757903	2
CCL5	1.076506196	-0.176520037	-0.899986159	2
CCN1	1.108777264	-0.275175928	-0.833601336	2
CCN2	1.075105697	-0.172709313	-0.902396384	2
CCND1	0.822570818	0.2905215	-1.113092318	2
CCND2	1.102884471	-0.255242333	-0.847642138	2
CCNP	0.96999748	0.057521921	-1.0275194	2
CCNT1	1.109475993	-0.277614802	-0.831861191	2
CCR5	1.12842504	-0.352097698	-0.776327342	2
CCR7	1.084987544	-0.200292775	-0.884694769	2
CD177	1.117860037	-0.30834729	-0.809512747	2
CD247	0.997797422	0.004390698	-1.00218812	2
CD300A	0.928172363	0.130785274	-1.058957637	2
CD38	1.092350946	-0.222019556	-0.87033139	2
CD3D	0.904378099	0.169562661	-1.073940761	2
CD46	0.854501799	0.245333672	-1.099835471	2
CD47	1.006792784	-0.013726893	-0.993065891	2
CD7	1.060512293	-0.134673972	-0.925838321	2
CD72	0.996625764	0.006714656	-1.003340421	2
CD81	0.995678179	0.008588322	-1.004266501	2
CD93	1.122423689	-0.326427271	-0.795996418	2
CD99	1.109760768	-2.79E-01	-0.831147063	2
CDC25A	0.885393656	0.199221045	-1.084614701	2
CDC25B	0.87583851	0.213755648	-1.089594157	2
CDC27	1.097541383	-0.238042217	-0.859499166	2
CDC42BPB	1.02245296	-0.046530387	-0.975922573	2
CDCA5	0.848743949	0.253658468	-1.102402417	2
CDCP2	1.02945087	-0.061764973	-0.967685897	2
CDH1	1.086992689	-0.206101664	-0.880891025	2
CDH22	1.017134232	-0.035197845	-0.981936386	2
CDH23	0.933751679	0.121410529	-1.055162208	2
CDHR3	0.933572435	0.121713469	-1.055285904	2
CDHR5	1.032227028	-0.067916546	-0.964310482	2
CDK18	0.874755658	0.215386986	-1.090142644	2
CDK20	0.796276828	0.326055904	-1.122332732	2
CDKL5	0.811956013	0.305039275	-1.116995288	2
CDSN	0.990140236	0.019436184	-1.00957642	2
CDX2	1.088777077	-0.211336953	-0.877440123	2
CEACAM1	1.07351035	-0.168405278	-0.905105072	2
CEACAM5	1.132081256	-0.369078648	-0.763002608	2
CEACAM8	1.087442506	-0.207415443	-0.880027062	2
CELF6	0.980060825	0.038751759	-1.018812584	2
CELSR1	1.042514023	-0.091287962	-0.951226061	2
CEMIP2	1.093471403	-0.225425541	-0.868045861	2
CENPK	1.020879537	-0.04315642	-0.977723117	2
CEP170B	1.093428682	-0.225295164	-0.868133518	2
CEP63	1.019002361	-0.039154881	-0.97984748	2
CFAP107	0.829396763	0.281057148	-1.110453911	2
CFAP46	1.092515109	-0.222516837	-0.869998272	2
CFAP54	1.024642591	-0.051256581	-0.97338601	2
CFAP58	0.885978296	0.198323447	-1.084301743	2
CFAP65	1.108572368	-0.274463958	-0.83410841	2
CFAP70	1.069966073	-0.158978446	-0.910987627	2
CFAP74	1.079873032	-0.185809877	-0.894063155	2
CFAP99	1.081065867	-0.189146475	-0.891919392	2

CFB	1.127024709	-0.345885429	-0.78113928	2
CFHR1	0.854729927	0.245002171	-1.099732098	2
CFHR2	1.130848874	-0.363222514	-0.76762636	2
CFI	1.050779711	-0.110785828	-0.939993883	2
CGB1	1.055134904	-0.121343682	-0.933791222	2
CGB2	1.078237796	-0.18127474	-0.896963056	2
CGB5	1.030100008	-0.063197727	-0.966902281	2
CGB7	1.067979622	-0.153773235	-0.914206387	2
CGB8	1.131505049	-0.366322728	-0.76518232	2
CGGBP1	1.124761257	-0.336141627	-0.78861963	2
CH25H	1.068157331	-0.154236675	-0.913920656	2
CHAF1A	1.122899634	-0.328378351	-0.794521284	2
CHCHD10	1.050377213	-0.109820356	-0.940556857	2
CHCHD6	0.981009365	0.036956662	-1.017966027	2
CHIT1	0.903897461	0.17032696	-1.074224421	2
CHRFAM7A	0.809431556	0.308457005	-1.11788856	2
CHRNE	0.979682042	0.039467322	-1.019149364	2
CHST1	0.959116704	0.077282165	-1.036398869	2
CHST2	0.9320388	0.124300646	-1.056339446	2
CHST8	1.114122153	-0.294289557	-0.819832595	2
CHTF18	1.117159683	-0.305665464	-0.811494219	2
CIBAR1	0.806401309	0.312542104	-1.118943413	2
CILP2	0.961007826	0.073885813	-1.034893639	2
CKMT1A	0.939194681	0.112154336	-1.051349017	2
CKMT1B	0.887993368	0.195222241	-1.083215609	2
CLCN4	1.112974607	-0.29009353	-0.822881077	2
CLDN3	0.842459543	0.26265358	-1.105113123	2
CLEC18A	1.125964464	-0.341277429	-0.784687035	2
CLEC18C	1.092341817	-0.221991922	-0.870349895	2
CLEC3B	0.950576445	0.092429415	-1.04300586	2
CLEC4C	1.123098743	-0.329198538	-0.793900205	2
CLEC4G	0.84527229	0.258639179	-1.103911469	2
CLEC6A	0.903865921	0.170377089	-1.07424301	2
CLIP3	1.01220433	-0.024872701	-0.987331628	2
CLK2	1.126632844	-0.344173032	-0.782459813	2
CLMP	0.891473766	0.189838363	-1.081312129	2
CLPP	1.033282852	-0.070272844	-0.963010008	2
CLRN3	1.10288916	-0.255257772	-0.847631388	2
CLTCL1	0.980156003	0.038571843	-1.018727846	2
CMKLR1	1.070142771	-0.159444128	-0.910698643	2
CMTM7	1.065492037	-0.147330606	-0.91816143	2
CNIH2	1.121598706	-0.323076285	-0.798522421	2
CNN3	1.128339617	-0.351714443	-0.776625174	2
CNPY2	1.123842909	-0.332285142	-0.791557768	2
CNPY3	0.954610738	0.0853124	-1.039923138	2
CNRIP1	1.126417747	-0.343237751	-0.783179996	2
CNTLN	1.120870358	-0.320149454	-0.800720905	2
CNTNAP3	1.098654373	-0.241562785	-0.857091588	2
COA3	1.080721532	-0.188180801	-0.892540731	2
COL14A1	0.948128248	0.096715709	-1.044843958	2
COL20A1	1.106343918	-0.266811431	-0.839532487	2
COL27A1	0.994730807	0.010456382	-1.005187189	2
COL2A1	1.107199679	-0.269730763	-0.837468916	2
COL5A1	1.126278348	-0.342633365	-0.783644983	2
COL5A3	0.840143443	0.265945222	-1.106088665	2

COL6A1	1.098374401	-0.240674226	-0.857700176	2
COL6A2	0.847804477	0.255009152	-1.102813629	2
COL8A1	1.098286143	-0.24039453	-0.857891613	2
COLEC12	1.07785061	-0.180207385	-0.897643225	2
COMMD5	1.131667329	-0.367095664	-0.764571665	2
COMMD7	1.083838737	-0.196998807	-0.886839931	2
COQ9	0.9734866	0.051069752	-1.024556352	2
CORIN	0.937034706	0.115840817	-1.052875522	2
CORO1C	0.94711842	0.098476649	-1.045595069	2
CORO2B	0.829370623	0.281093588	-1.110464211	2
COX17	1.131886278	-0.368142505	-0.763743773	2
COX4I2	0.886070035	0.198182512	-1.084252547	2
COX6A2	1.128489667	-0.352388035	-0.776101633	2
COX6B2	1.007919808	-0.016032404	-0.991887404	2
CPEB2	1.113702233	-0.292747912	-0.820954321	2
CPEB3	0.874819716	0.21529057	-1.090110286	2
CPT1C	0.869235846	0.223653893	-1.092889739	2
CPZ	0.958093524	0.079113251	-1.037206775	2
CRACR2B	1.121665757	-0.3233472	-0.798318557	2
CRADD	0.830065297	0.280124673	-1.11018997	2
CREB3L3	0.968954423	0.059439598	-1.02839402	2
CRISP2	1.105367839	-0.263510175	-0.841857665	2
CROCC2	1.087784363	-0.208416571	-0.879367792	2
CRYAA	1.094734863	-0.229300325	-0.865434538	2
CRYZ	0.888436127	0.194539273	-1.082975401	2
CSH1	0.844114871	0.260293327	-1.104408198	2
CSH2	1.069915101	-0.158844194	-0.911070907	2
CSMD2	1.076897091	-0.177589167	-0.899307924	2
CSNK1G2	0.804255616	0.31542336	-1.119678976	2
CST8	0.99593147	0.008087997	-1.004019467	2
CT47A5	0.876492104	0.212769458	-1.089261562	2
CTDNEP1	0.983697614	0.031844093	-1.015541707	2
CTDSP1	1.04280265	-0.091957528	-0.950845122	2
CTDSPL	1.125933354	-0.341143411	-0.784789943	2
CTF1	0.868932769	0.224105466	-1.093038235	2
CTNND1	1.112171083	-0.287186653	-0.82498443	2
CTPS2	0.917847231	0.147844335	-1.065691566	2
CTRB2	0.975814761	0.046731904	-1.022546665	2
CTRC	1.104429361	-0.260363966	-0.844065395	2
CTSE	1.102289079	-0.253287042	-0.849002037	2
CTU1	1.054784007	-0.120485352	-0.934298656	2
CTXND2	1.131446362	-0.366043821	-0.765402541	2
CUBN	1.028345767	-0.059333641	-0.969012127	2
CUL3	0.890689055	0.191055312	-1.081744367	2
CUL5	1.089108157	-0.212315359	-0.876792798	2
CUL7	0.920850168	0.142920776	-1.063770944	2
CUX2	1.096792385	-0.235690502	-0.861101882	2
CXCL3	1.074608439	-0.171363614	-0.903244825	2
CXorf49	0.926560043	0.133473583	-1.060033626	2
CXorf49B	1.016983532	-0.034879719	-0.982103813	2
CYB5R4	0.827513447	0.283678665	-1.111192112	2
CYB5RL	1.061959091	-0.138319175	-0.923639916	2
CYBA	0.955190043	0.084284847	-1.03947489	2
CYLD	1.062461301	-0.139590524	-0.922870777	2
CYP11A1	1.117878095	-0.308416747	-0.809461348	2

CYP11B1	1.067541038	-0.152631307	-0.914909731	2
CYP21A2	0.829508591	0.280901239	-1.11040983	2
CYP27C1	1.119474075	-0.314617991	-0.804856083	2
CYP2E1	1.099472205	-0.244169985	-0.85530222	2
CYP2W1	1.11228406	-0.287593846	-0.824690214	2
CYP4A11	1.074497594	-0.171064158	-0.903433436	2
CYP4X1	1.083723428	-0.196669522	-0.887053906	2
CYREN	1.032411482	-0.068327523	-0.964083959	2
CYSRT1	0.860385579	0.236742359	-1.097127938	2
CYTH2	1.071146744	-0.162098486	-0.909048257	2
CZIB	0.968055398	0.061088398	-1.029143796	2
D2HGDH	1.086564505	-0.204854738	-0.881709767	2
DACT1	1.046029242	-0.099497066	-0.946532176	2
DAPK1	0.953048267	0.088076833	-1.041125099	2
DBNDD1	1.065031599	-0.146147104	-0.918884496	2
DBX1	1.068006718	-0.15384387	-0.914162848	2
DCAF10	1.03354356	-0.070856112	-0.962687448	2
DCAF11	0.950564058	0.092451164	-1.043015222	2
DCAF12L2	0.96468166	0.067242622	-1.031924282	2
DCAF15	1.045512888	-0.098283712	-0.947229176	2
DCK	1.122798405	-0.327962258	-0.794836147	2
DCTN6	1.037849079	-0.08057312	-0.957275959	2
DCUN1D4	1.073103203	-0.167313011	-0.905790192	2
DCUN1D5	0.986209989	0.027031903	-1.013241893	2
DDOST	1.122230419	-0.325638748	-0.796591672	2
DDX60	0.978777492	0.041173188	-1.01995068	2
DEFB108B	1.12619689	-0.34228082	-0.78391607	2
DEFB119	1.093858181	-0.226607831	-0.867250351	2
DEFB130B	0.975227854	0.047827923	-1.023055777	2
DENND2A	0.854223153	0.24573841	-1.099961563	2
DENND2C	0.804186161	0.315516468	-1.119702629	2
DENND4B	0.844309556	0.260015312	-1.104324867	2
DENND4C	0.946960144	0.098752279	-1.045712424	2
DEPDC5	1.065020816	-0.146119419	-0.918901397	2
DERL3	0.954234858	0.085978369	-1.040213226	2
DET1	1.050543646	-0.110219374	-0.940324273	2
DGAT2	1.079795992	-0.185595213	-0.894200779	2
DGKG	0.795911017	0.326540344	-1.122451361	2
DGKH	0.91805418	0.147506002	-1.065560182	2
DGUOK	0.881137846	0.205725957	-1.086863803	2
DHDH	1.022697652	-0.047056747	-0.975640905	2
DHRS9	0.867123386	0.226796373	-1.093919759	2
DHX33	1.101769609	-0.251589305	-0.850180304	2
DIAPH1	1.092574744	-0.222697628	-0.869877115	2
DIO3	0.950325559	0.09286979	-1.043195348	2
DIRAS1	1.074130897	-0.170074833	-0.904056064	2
DISC1	0.876336831	0.21300385	-1.089340681	2
DKC1	0.942003233	0.107334299	-1.049337532	2
DLG3	0.914197917	0.153786977	-1.067984894	2
DLK1	0.977045733	0.044427625	-1.021473358	2
DLK2	0.897972858	0.179689608	-1.077662466	2
DLX4	0.989488593	0.020701377	-1.01018997	2
DLX6	0.962355793	0.071455341	-1.033811134	2
DMBX1	0.972798742	0.052346353	-1.025145095	2
DMRTA2	0.937658831	0.114777419	-1.05243625	2

DMRTC1	1.047518346	-0.103011047	-0.944507299	2
DMRTC1B	1.095400272	-0.231355887	-0.864044385	2
DNAH17	1.111589874	-0.285099602	-0.826490272	2
DNAJB12	1.081513175	-0.190403996	-0.891109179	2
DNAJC24	1.087856604	-0.208628426	-0.879228178	2
DNAJC5G	1.092186384	-0.221521663	-0.870664721	2
DNAJC6	1.121650302	-0.323284733	-0.798365569	2
DNAL4	1.055008562	-0.121034479	-0.933974083	2
DNER	1.122769306	-0.327842763	-0.794926543	2
DNM1	1.085886224	-0.202886748	-0.882999476	2
DNMT3B	1.117794242	-0.308094363	-0.80969988	2
DOC2B	1.043962524	-0.094656225	-0.949306299	2
DOCK3	1.110848451	-0.282455749	-0.828392702	2
DPEP2NB	1.057735978	-0.127749615	-0.929986363	2
DPF2	1.079074606	-0.183589981	-0.895484625	2
DPP8	1.022861996	-0.047410521	-0.975451475	2
DPPA4	0.932805864	0.123007718	-1.055813582	2
DPY19L1	0.861456765	0.235168893	-1.096625658	2
DPYSL4	1.069888792	-0.158774913	-0.911113879	2
DRD4	0.83644114	0.271181101	-1.107622241	2
DTD2	0.947831902	0.097232904	-1.045064806	2
DTX2	0.842636409	0.262401703	-1.105038112	2
DTX3L	0.896530982	0.18195205	-1.078483032	2
DUOX1	1.039846057	-0.085135571	-0.954710486	2
DUS2	0.995726032	0.008493826	-1.004219858	2
DUS3L	1.100289186	-0.246792026	-0.85349716	2
DUSP18	1.034867038	-0.073825967	-0.961041071	2
DUSP19	1.130719129	-0.362614185	-0.768104944	2
DUSP9	1.117127855	-0.305544131	-0.811583723	2
DYDC1	1.048902384	-0.106297105	-0.94260528	2
DYNLT2B	1.131380266	-0.365730085	-0.765650181	2
E2F1	1.033335463	-0.070390502	-0.962944961	2
EBAG9	1.006392572	-0.012910152	-0.99348242	2
EBF1	0.803027411	0.317068393	-1.120095804	2
ECE2	0.813562366	0.302857603	-1.116419969	2
ECI2	0.947594154	0.097647574	-1.045241728	2
EEA1	1.097127467	-0.236740882	-0.860386585	2
EFCAB11	1.073086973	-0.16726952	-0.905817452	2
EFCAB14	1.017240898	-0.035423116	-0.981817782	2
EFCAB3	0.974128084	0.049877162	-1.024005246	2
EFCC1	1.088481031	-0.210463969	-0.878017063	2
EFNB1	0.826124124	0.28560753	-1.111731654	2
EFS	1.056496634	-0.12468773	-0.931808904	2
EFTUD2	0.983072555	0.03303618	-1.016108735	2
EGLN3	0.802796018	0.317377974	-1.120173993	2
EHBP1	1.075878556	-0.174808448	-0.901070108	2
EHD1	1.036436571	-0.077367487	-0.959069084	2
EHD3	1.089471667	-0.213392183	-0.876079484	2
EIF1B	0.947901563	0.09711136	-1.045012923	2
EIF2AK1	0.954514167	0.085483557	-1.039997724	2
EIF2AK3	1.086391638	-0.204352334	-0.882039304	2
EIF2B3	1.100169665	-0.246407321	-0.853762344	2
EIF2B4	1.015282397	-0.031299681	-0.983982716	2
EIF3H	1.123156149	-0.32943545	-0.7937207	2
EIF4E1B	1.080768769	-0.188313153	-0.892455615	2

EIF4E2	0.959477345	0.076635678	-1.036113023	2
EIF4EBP2	1.015359745	-0.031462021	-0.983897724	2
ELK4	0.894971634	0.184391806	-1.07936344	2
EMC8	0.871978602	0.219556265	-1.091534867	2
EML1	0.840928791	0.264830487	-1.105759278	2
EML5	1.120397817	-0.318265965	-0.802131852	2
EMSY	1.105176013	-0.26286489	-0.842311124	2
EMX1	1.110517239	-0.281281255	-0.829235984	2
EMX2	0.948629563	0.095839991	-1.044469554	2
ENPP2	0.914806878	0.152798399	-1.067605276	2
ENSA	1.098262142	-0.240318504	-0.857943638	2
EOLA1	0.876666474	0.21250616	-1.089172634	2
EPB41L4B	1.132057818	-0.368965915	-0.763091902	2
EPB41L5	1.131167204	-0.36472154	-0.766445664	2
EPCAM	1.090505078	-0.216468406	-0.874036672	2
EPHB1	1.026511862	-0.055320294	-0.971191568	2
EPS8	1.006246999	-0.012613323	-0.993633676	2
EPS8L2	1.075961796	-0.175035088	-0.900926707	2
EPX	0.888283485	0.194774792	-1.083058277	2
ERAS	1.092427997	-0.222252884	-0.870175113	2
ERBB2	0.883867035	0.201560304	-1.085427339	2
ERBB3	1.125460509	-0.339114552	-0.786345957	2
EREG	1.119448637	-0.314518157	-0.80493048	2
ERGIC1	1.130083201	-0.359654235	-0.770428965	2
ERICH2	1.006845448	-0.013834446	-0.993011003	2
ERICH5	1.07993173	-0.185973502	-0.893958229	2
ERLIN2	0.81174222	0.305329232	-1.117071452	2
ERVMER34-1	0.914564793	0.153191542	-1.067756335	2
ESAM	0.844054575	0.260379414	-1.104433989	2
ESCO1	0.898222638	0.179297043	-1.077519681	2
ESCO2	1.015875096	-0.03254472	-0.983330377	2
ESX1	1.128166121	-0.350937797	-0.777228323	2
ETV2	0.977210268	0.044119063	-1.02132933	2
ETV5	0.830123729	0.280043124	-1.110166853	2
EVL	0.908504551	0.162971031	-1.071475582	2
EWSR1	1.12628782	-0.342674391	-0.78361343	2
EXD3	1.015971155	-0.032746734	-0.983224421	2
EXOSC7	0.916856502	0.149462048	-1.06631855	2
EXOSC9	0.895866771	0.182992162	-1.078858933	2
EXT2	0.851932893	0.24905782	-1.100990712	2
EYA3	0.989907483	0.019888353	-1.009795836	2
F13A1	0.940879051	0.109267261	-1.050146312	2
F8	0.935037239	0.119234329	-1.054271567	2
FA2H	1.018384388	-0.037843143	-0.980541246	2
FAAP100	0.819165384	0.295205258	-1.114370643	2
FAM104A	1.03409439	-0.072090348	-0.962004041	2
FAM107A	1.091139432	-0.218367859	-0.872771573	2
FAM110C	1.100218568	-0.24656468	-0.853653888	2
FAM126A	0.826014125	0.285760065	-1.11177419	2
FAM131B	1.12388562	-0.332463324	-0.791422296	2
FAM131C	0.989611116	0.020463672	-1.010074788	2
FAM151A	1.111578659	-0.285059457	-0.826519202	2
FAM163B	1.116406004	-0.302804833	-0.813601172	2
FAM167A	1.129509861	-0.357015657	-0.772494204	2
FAM181A	1.040659131	-0.087003557	-0.953655574	2

FAM183A	1.064142387	-0.143869267	-0.92027312	2
FAM193A	0.887439382	0.196075985	-1.083515367	2
FAM214B	1.052999864	-0.116142205	-0.936857659	2
FAM221B	1.09249951	-0.222469558	-0.870029952	2
FAM227B	0.948466633	0.096124715	-1.044591348	2
FAM43B	0.962893839	0.070482959	-1.033376798	2
FAM71D	0.821280262	0.292299446	-1.113579708	2
FAM72A	1.080123796	-0.186509302	-0.893614494	2
FAM90A16P	0.939050658	0.112400695	-1.051451352	2
FAM90A18P	1.07806892	-0.180808899	-0.897260021	2
FAM90A22P	1.129864074	-0.358642483	-0.771221592	2
FAM90A23P	1.013794884	-0.028185681	-0.985609203	2
FAM90A7P	1.129971133	-0.359136278	-0.770834855	2
FAM90A9P	0.992900685	0.014050561	-1.006951246	2
FAM9B	0.92793982	0.131173576	-1.059113395	2
FANCD2OS	0.864685028	0.230409213	-1.095094241	2
FARSA	1.078141232	-0.181008313	-0.897132919	2
FATE1	0.950623623	0.092346577	-1.0429702	2
FBF1	0.907045427	0.165307995	-1.072353422	2
FBLIM1	1.131436373	-0.365996381	-0.765439993	2
FBLL1	1.043366764	-0.093268458	-0.950098306	2
FBXL14	1.03256851	-0.068677616	-0.963890895	2
FBXL19	1.043083932	-0.092610813	-0.950473118	2
FBXL6	0.971550116	0.054657898	-1.026208013	2
FBXL8	0.81969099	0.29448398	-1.114174971	2
FBXO10	0.823236781	0.289602617	-1.112839398	2
FBXO11	1.110501075	-0.281224038	-0.829277037	2
FBXO17	1.073556044	-0.168528018	-0.905028026	2
FBXO27	1.084136345	-0.1978498	-0.886286546	2
FBXO34	1.076963184	-0.177770177	-0.899193007	2
FBXO36	0.915809519	0.151168056	-1.066977575	2
FBXO41	1.030554392	-0.064202664	-0.966351728	2
FBXO43	1.095430179	-0.231448522	-0.863981657	2
FBXO6	0.947661422	0.097530271	-1.045191693	2
FBXO7	0.937770411	0.114587151	-1.052357563	2
FBXO8	0.990144434	0.019428026	-1.00957246	2
FBXW4	0.938346098	0.113604739	-1.051950837	2
FCF1	1.052498389	-0.114927721	-0.937570669	2
FCGR2A	1.05084353	-0.110939067	-0.939904463	2
FDFT1	0.963851501	0.068749029	-1.03260053	2
FDX2	0.827166618	0.284160583	-1.111327201	2
FEZ2	0.889585149	0.192764244	-1.082349393	2
FEZF1	1.128761612	-0.353613357	-0.775148255	2
FEZF2	1.07782347	-0.180132658	-0.897690812	2
FGF21	1.042484902	-0.09122045	-0.951264452	2
FGF3	1.036131496	-0.076677437	-0.95945406	2
FGF4	1.061048185	-0.136021162	-0.925027023	2
FGFR2	0.931865313	0.124592769	-1.056458082	2
FGFR3	1.038836314	-0.082824141	-0.956012173	2
FGGY	1.094071292	-0.227260715	-0.866810577	2
FIBCD1	1.016139829	-0.033101615	-0.983038214	2
FJX1	1.130458849	-0.361398385	-0.769060464	2
FKBP11	0.906591203	0.166034111	-1.072625314	2
FKBP1C	0.994768016	0.010383109	-1.005151125	2
FLOT1	1.120916294	-0.320333188	-0.800583107	2

FLT4	0.950834207	0.091976704	-1.042810911	2
FLVCR2	1.067054233	-0.151366879	-0.915687355	2
FMNL3	1.097806742	-0.238878747	-0.858927995	2
FNDC3A	1.099888711	-0.245504514	-0.854384197	2
FNDC4	1.0226885	-0.047037051	-0.975651448	2
FNDC5	0.926660071	0.133307069	-1.05996714	2
FNDC7	0.871307119	0.22056129	-1.091868409	2
FNTB	1.095230208	-0.230829541	-0.864400667	2
FOXA2	0.96237721	0.071416661	-1.03379387	2
FOXB1	0.896000257	0.182783237	-1.078783494	2
FOXB2	1.118951463	-0.312573488	-0.806377975	2
FOXC2	1.120211213	-0.317525457	-0.802685756	2
FOXE3	0.972121162	0.053601661	-1.025722823	2
FOXF2	1.071180809	-0.162188803	-0.908992006	2
FOXG1	1.131291193	-0.365307939	-0.765983255	2
FOXI2	1.117330129	-0.306316026	-0.811014103	2
FOXI3	0.835518058	0.27248164	-1.107999698	2
FOXK1	0.90681596	0.165674899	-1.072490859	2
FOXL2NB	1.129389221	-0.356463991	-0.77292523	2
FOXL3	1.063395967	-0.141965045	-0.921430922	2
FOXM1	0.985413122	0.028561829	-1.013974951	2
FOXN4	1.09131294	-0.218888892	-0.872424048	2
FOXS1	1.055163709	-0.121414203	-0.933749506	2
FPGT-TNNI3K	1.048473626	-0.10527704	-0.943196586	2
FRMD4B	1.077111248	-0.178175937	-0.898935311	2
FRMPD1	0.831457495	0.278179623	-1.109637117	2
FRS3	0.869357412	0.223472696	-1.092830108	2
FTCD	1.089894424	-0.214647941	-0.875246483	2
FUBP1	0.819555962	0.294669334	-1.114225296	2
FUBP3	0.8049061	0.314550874	-1.119456974	2
FUNDC2	0.922889872	0.139558985	-1.062448857	2
FUT10	1.05308112	-0.116339248	-0.936741872	2
FUT2	1.086106075	-0.203523664	-0.882582411	2
FUZ	1.031035999	-0.06526965	-0.965766349	2
FXYD2	1.035905154	-0.076165996	-0.959739158	2
FZD10	1.123641929	-0.331448199	-0.792193731	2
FZD7	0.956498775	0.081958226	-1.038457001	2
FZD8	1.01483013	-0.030351281	-0.98447885	2
GAA	1.048195393	-0.104616096	-0.943579297	2
GABRB3	0.918974989	0.145998862	-1.064973851	2
GABRG3	1.050130242	-0.109228787	-0.940901455	2
GADL1	1.112097292	-0.286920965	-0.825176328	2
GAGE10	1.052549415	-0.115051171	-0.937498244	2
GAL3ST3	0.973553842	0.050944835	-1.024498677	2
GALK1	1.045257583	-0.097684749	-0.947572834	2
GALNT4	1.04761506	-0.103240039	-0.944375021	2
GALNT5	0.995536711	0.008867601	-1.004404312	2
GALNT6	0.835942694	0.271883608	-1.107826302	2
GALR1	1.052074063	-0.113902194	-0.938171869	2
GAREM2	0.899352735	0.177518573	-1.076871308	2
GARRE1	1.121969547	-0.324577812	-0.797391735	2
GAS8	1.013964139	-0.028539238	-0.985424901	2
GATA5	1.09929106	-0.243591001	-0.855700059	2
GATB	0.856392814	0.242581881	-1.098974695	2
GBP4	0.914898605	0.152649384	-1.067547989	2

GBP6	1.125594578	-0.339688268	-0.785906309	2
GCAT	0.953911631	0.086550574	-1.040462205	2
GCC2	1.090277135	-0.215787955	-0.87448918	2
GCGR	1.077562237	-0.17941401	-0.898148227	2
GCKR	0.862212813	0.23405658	-1.096269393	2
GCNT1	0.881859322	0.204626767	-1.086486089	2
GDF3	0.962003023	0.072092186	-1.034095209	2
GDF7	0.997214403	0.005548107	-1.002762511	2
GDPD1	0.938925921	0.112613998	-1.051539919	2
GDPGP1	0.805216671	0.314134007	-1.119350677	2
GEMIN4	0.845105086	0.258878338	-1.103983424	2
GEN1	1.044328185	-0.095509687	-0.948818498	2
GET1	0.863895365	0.231575941	-1.095471306	2
GET4	0.999303308	0.001391931	-1.000695239	2
GFAP	1.109591503	-0.278019627	-0.831571875	2
GFI1	1.13105586	-0.36419616	-0.7668597	2
GFI1B	1.120140849	-0.317246706	-0.802894143	2
GFRA3	0.913696387	0.154600238	-1.068296625	2
GFRAL	0.984256404	0.030776664	-1.015033068	2
GFUS	1.113681741	-0.292672865	-0.821008876	2
GGA3	0.842277823	0.262912294	-1.105190117	2
GGT5	1.044479332	-0.095862844	-0.948616488	2
GGT6	1.079669902	-0.185244091	-0.89442581	2
GHDC	1.119760355	-0.315743822	-0.804016533	2
GIMAP2	0.997446495	0.005087596	-1.002534092	2
GINS2	1.084352066	-0.198467657	-0.885884409	2
GJA10	1.086485031	-0.204623693	-0.881861339	2
GK3P	0.924240325	0.137325302	-1.061565628	2
GLI2	0.832046458	0.277355469	-1.109401927	2
GLMP	1.09693948	-0.236151261	-0.860788219	2
GLO1	1.070968823	-0.16162704	-0.909341784	2
GLP1R	1.049372327	-0.107417317	-0.94195501	2
GLRX	0.877303914	0.211542924	-1.088846837	2
GLYCTK	0.988521522	0.022574706	-1.011096228	2
GMPS	0.838117669	0.268814032	-1.106931701	2
GNAI1	0.960474408	0.074845389	-1.035319796	2
GNAL	0.978710407	0.041299537	-1.020009945	2
GNAO1	1.101476326	-0.250634134	-0.850842191	2
GNB2	1.066435827	-0.149765216	-0.91667061	2
GNG10	0.828446734	0.282380543	-1.110827277	2
GNG4	1.132348411	-0.370367395	-0.761981016	2
GNG8	0.907037537	0.165320612	-1.07235815	2
GNGT1	1.100722711	-0.248190651	-0.852532061	2
GOLGA8B	0.944923192	0.10229063	-1.047213822	2
GOLGA8H	0.970565851	0.056474813	-1.027040664	2
GOLGA8O	0.865965001	0.228514637	-1.094479638	2
GOLPH3	1.107231219	-0.269838811	-0.837392408	2
GOLPH3L	1.10849821	-0.274206628	-0.834291582	2
GOLT1A	1.079297294	-0.184208056	-0.895089238	2
GOT2	1.096187607	-0.233801665	-0.862385942	2
GPATCH11	1.06744543	-0.152382723	-0.915062708	2
GPBAR1	0.903493752	0.170968372	-1.074462124	2
GPBP1L1	1.113328708	-0.291382635	-0.821946073	2
GPC3	0.933560215	0.121734117	-1.055294333	2
GPR141	0.999443575	0.001111922	-1.000555497	2

GPR142	1.107714213	-0.271497554	-0.836216659	2
GPR155	1.101652497	-0.251207605	-0.850444892	2
GPR161	1.041028537	-0.087854254	-0.953174283	2
GPR20	1.117399974	-0.306583003	-0.810816971	2
GPR4	1.070381677	-0.160074458	-0.910307219	2
GPR75	0.849364889	0.252764574	-1.102129464	2
GPR83	1.037569306	-0.079936784	-0.957632522	2
GPRIN1	1.08859071	-0.210787186	-0.877803524	2
GPSM1	1.110825729	-0.282375046	-0.828450683	2
GPT	0.93217548	0.124070421	-1.056245901	2
GPX8	1.08685657	-0.205704881	-0.881151689	2
GRB10	1.120627855	-0.319181375	-0.80144648	2
GREB1	0.955568968	0.083611953	-1.039180922	2
GREP1	1.087927884	-0.208837565	-0.87909032	2
GRIK4	0.82640825	0.28521341	-1.11162166	2
GRK5	0.989196928	0.021266904	-1.010463832	2
GRK6	0.867079572	0.226861426	-1.093940999	2
GRK7	0.930653001	0.126631053	-1.057284053	2
GRWD1	1.131886122	-0.368141759	-0.763744363	2
GSC	0.969127299	0.059122113	-1.028249412	2
GSDMD	1.132508654	-0.371143817	-0.761364837	2
GSTM2	1.120006594	-0.316715564	-0.803291031	2
GSTM4	0.949354693	0.0945715	-1.043926194	2
GSTO2	1.091901504	-0.220661143	-0.871240361	2
GSX2	1.037063307	-0.078787668	-0.958275639	2
GTF2E2	1.118363375	-0.310289099	-0.808074276	2
GTF2F2	1.106167565	-0.266212752	-0.839954813	2
GTPBP6	0.902725835	0.17218704	-1.074912875	2
GZMH	0.861094003	0.23570208	-1.096796083	2
H2AC6	0.886798757	0.197062153	-1.08386091	2
H2BW2	0.911039745	0.158894432	-1.069934177	2
H3C10	1.000811462	-0.001624904	-0.999186558	2
H3C15	1.113999394	-0.293838125	-0.820161269	2
H3C6	0.988750682	0.022131258	-1.01088194	2
H4C9	0.901188831	0.174620762	-1.075809593	2
H6PD	0.937312475	0.115367728	-1.052680204	2
HAAO	1.094604212	-0.228897937	-0.865706275	2
HAND1	0.960447741	0.074893327	-1.035341068	2
HAND2	1.024774028	-0.051541439	-0.973232589	2
HAP1	1.093900335	-0.226736892	-0.867163444	2
HAPLN2	0.983547081	0.032131369	-1.01567845	2
HAS3	1.073751899	-0.169054465	-0.904697434	2
HBP1	1.096395767	-0.234450784	-0.861944983	2
HBZ	0.93427702	0.120521968	-1.054798988	2
HDAC1	1.006363321	-0.012850498	-0.993512823	2
HDAC3	1.131108801	-0.364445824	-0.766662978	2
HDDC3	1.127432387	-0.34767879	-0.779753597	2
HEATR4	1.030470829	-0.064017725	-0.966453104	2
HERC2	0.924812911	0.136376318	-1.061189229	2
HERPUD2	0.929636296	0.128336357	-1.057972654	2
HES2	1.098516899	-0.241126225	-0.857390674	2
HES5	1.115228956	-0.298388163	-0.816840793	2
HEXD	1.07754862	-0.17937658	-0.89817204	2
HGFAC	0.879180774	0.208700346	-1.08788112	2
HHLA1	1.02489317	-0.051799765	-0.973093405	2

HID1	0.919240555	0.145563662	-1.064804218	2
HIKESHI	1.015723492	-0.032226023	-0.983497469	2
HIP1	1.078740726	-0.182664833	-0.896075893	2
HIP1R	1.003554	-0.007146303	-0.996407697	2
HIRA	1.120439328	-0.318430944	-0.802008383	2
HKDC1	1.028116824	-0.058831164	-0.96928566	2
HLA-A	1.059989427	-0.133362881	-0.926626546	2
HLA-B	0.96406056	0.068369963	-1.032430523	2
HLA-DRA	0.830814046	0.279079129	-1.109893175	2
HLA-F	0.9423764	0.106691581	-1.049067981	2
HLX	1.110811782	-0.282325521	-0.828486261	2
HM13	1.105329351	-0.263380614	-0.841948738	2
HMGB3	1.093121691	-0.22435947	-0.868762221	2
HMGN1	0.806867063	0.311915444	-1.118782507	2
HMGN4	1.090985807	-0.217907073	-0.873078734	2
HMGXB3	1.09317656	-0.224526552	-0.868650008	2
HMX3	0.892000121	0.189021071	-1.081021192	2
HNRNPR	0.90323392	0.171380925	-1.074614845	2
HNRNPUL2	0.835174215	0.272965586	-1.108139801	2
HOATZ	1.066883939	-0.15092531	-0.915958629	2
HOGA1	1.028599934	-0.059891966	-0.968707968	2
HOMEZ	0.864520417	0.230652558	-1.095172975	2
HOPX	0.938155471	0.113930184	-1.052085655	2
HOXA1	1.127938438	-0.349922121	-0.778016317	2
HOXA10	1.068595132	-0.155380248	-0.913214883	2
HOXB1	0.949595966	0.094148962	-1.043744928	2
HOXB4	1.113497012	-0.291997105	-0.821499906	2
HOXB8	1.113149847	-0.290730864	-0.822418982	2
HOXC4	1.113792809	-0.293079833	-0.820712976	2
HOXC5	0.978132908	0.042386281	-1.02051919	2
HOXD10	1.102947007	-0.255448293	-0.847498714	2
HOXD13	1.077399703	-0.178967436	-0.898432267	2
HPCAL4	1.04651635	-0.100644111	-0.945872239	2
HPD	1.09213135	-0.221355287	-0.870776063	2
HPF1	1.048189558	-0.104602243	-0.943587315	2
HPGDS	0.796559564	0.325681299	-1.122240862	2
HPS3	1.008385581	-0.016987608	-0.991397973	2
HPS6	0.956428718	0.082082956	-1.038511674	2
HRG	1.110927312	-0.282735988	-0.828191324	2
HRH2	0.976704748	0.04506667	-1.021771418	2
HRH3	1.100126254	-0.246267688	-0.853858566	2
HRK	0.997318263	0.005342071	-1.002660334	2
HS3ST4	0.883329857	0.202381865	-1.085711722	2
HSD17B1	0.853134	0.247318587	-1.100452586	2
HSD3B2	1.07298068	-0.166984799	-0.905995881	2
HSP90AB1	1.093546723	-0.225655512	-0.867891211	2
HSPA13	0.959874847	0.075922458	-1.035797305	2
HSPB11	1.093972895	-0.226959138	-0.867013757	2
HSPB2-C11orf52	1.088579822	-0.210755089	-0.877824734	2
HSPB6	0.945539023	0.101222619	-1.046761643	2
HSPG2	1.093207763	-0.2246216	-0.868586163	2
HTN1	1.117760299	-0.307963958	-0.809796341	2
HTR3C	0.884811421	0.200113988	-1.084925408	2
HTT	1.132461723	-0.370916153	-0.761545569	2
HUNK	1.066145657	-0.149015438	-0.917130219	2

IARS1	0.798532842	0.323062434	-1.121595276	2
IDH2	1.131977371	-0.368579407	-0.763397964	2
IFRD2	0.975015582	0.048223915	-1.023239497	2
IFT43	1.121970842	-0.324583069	-0.797387773	2
IFT81	1.126272124	-0.342606413	-0.783665712	2
IGF2BP2	0.9832256	0.032744487	-1.015970087	2
IGFBP2	1.091532719	-0.219549802	-0.871982917	2
IGFBP5	1.085729621	-0.202433624	-0.883295997	2
IGFL3	1.124780005	-0.336220918	-0.788559087	2
IGSF10	1.129259784	-0.355873441	-0.773386343	2
IGSF11	1.088932001	-0.211794508	-0.877137494	2
IGSF9B	1.011532065	-0.023477571	-0.988054495	2
IKZF1	1.111509477	-0.284811919	-0.826697558	2
IL11	0.848774832	0.253614033	-1.102388864	2
IL15RA	0.949877999	0.093654739	-1.043532738	2
IL17D	0.965473844	0.065802223	-1.031276066	2
IL17REL	0.797423163	0.324536111	-1.121959274	2
IL20RB	1.090997976	-0.217943555	-0.873054422	2
IL27	0.96886838	0.059597561	-1.028465941	2
IL2RG	0.959321342	0.076915399	-1.036236741	2
IL3RA	0.920110804	0.144135859	-1.064246664	2
IL5RA	1.106623818	-0.267763676	-0.838860143	2
IL6R	1.124121869	-0.333450966	-0.790670903	2
INHA	1.090068722	-0.215166758	-0.874901964	2
INKA2	0.798801283	0.322705572	-1.121506854	2
INO80C	0.850241219	0.251501444	-1.101742662	2
INPP5E	1.128231682	-0.351231004	-0.777000678	2
INS	1.090493044	-0.216432454	-0.87406059	2
INS-IGF2	1.015730826	-0.032241436	-0.98348939	2
INTS6L	1.005618625	-0.01133359	-0.994285035	2
INTU	1.115001703	-0.297542388	-0.817459314	2
INVS	0.801263043	0.319426221	-1.120689264	2
IPCEF1	0.845875714	0.257775524	-1.103651238	2
IPO7	1.025559724	-0.053247013	-0.972312711	2
IPPK	0.870000809	0.222513051	-1.09251386	2
IQSEC2	0.891535887	0.189741948	-1.081277835	2
IRAG2	0.803528257	0.316397939	-1.119926196	2
IRF4	1.020333191	-0.041989135	-0.978344056	2
IRF6	1.087948448	-0.208897918	-0.87905053	2
IRS1	0.857015284	0.241674125	-1.09868941	2
IRX2	0.845192737	0.258752976	-1.103945712	2
IRX5	1.086061576	-0.203394673	-0.882666903	2
ITGA1	1.12172464	-0.323585321	-0.798139319	2
ITGA2B	1.114079481	-0.294132566	-0.819946915	2
ITGB7	1.087023511	-0.20619156	-0.880831951	2
ITIH1	1.021661103	-0.044830075	-0.976831027	2
ITPKA	1.084396485	-0.198594986	-0.885801499	2
ITPKC	0.846299967	0.257167792	-1.103467758	2
IVD	0.983117808	0.032949944	-1.016067752	2
IVL	1.104169963	-0.259499062	-0.844670901	2
IWS1	0.984834882	0.0296699	-1.014504782	2
IZUMO1	0.925457335	0.135306901	-1.060764236	2
IZUMO2	1.086513892	-0.204707582	-0.88180631	2
JAM3	0.848609571	0.253851794	-1.102461365	2
JPT1	1.020154859	-0.041608596	-0.978546263	2

JTB	1.086524618	-0.204738765	-0.881785854	2
JUND	1.059153675	-0.131274038	-0.927879637	2
KANTR	1.099299328	-0.24361741	-0.855681918	2
KAT8	1.078829427	-0.182910436	-0.895918991	2
KATNB1	0.993085916	0.013687649	-1.006773565	2
KCNA7	1.124062203	-0.3332012	-0.790861002	2
KCND3	0.831415629	0.278238177	-1.109653806	2
KCNG1	1.114262616	-0.294806858	-0.819455758	2
KCNH3	1.095254099	-0.230903442	-0.864350657	2
KCNJ10	1.096227503	-0.233925994	-0.862301509	2
KCNJ9	0.925269791	0.135618279	-1.06088807	2
KCNK4	1.046839679	-0.101406789	-0.94543289	2
KCNMA1	0.985865331	0.027694035	-1.013559366	2
KCNMB1	1.038670202	-0.082444773	-0.956225429	2
KCNN3	1.121178449	-0.32138395	-0.7997945	2
KCNS1	0.934054207	0.120898958	-1.054953165	2
KCNT1	0.87806242	0.210395298	-1.088457718	2
KCTD18	1.106107699	-0.266009744	-0.840097955	2
KDELR1	1.077022987	-0.177934022	-0.899088966	2
KHNYN	0.958826209	0.077802497	-1.036628705	2
KIAA1671	0.799308328	0.322031119	-1.121339447	2
KIAA2012	1.041149597	-0.088133314	-0.953016283	2
KIAA2013	1.070134669	-0.159422765	-0.910711904	2
KIF12	0.862480039	0.233663084	-1.096143123	2
KIF17	0.930366124	0.127112604	-1.057478728	2
KIF1A	0.914492317	0.153309205	-1.067801522	2
KIF23	0.997712316	0.004559775	-1.002272091	2
KIF2C	0.858443454	0.239587758	-1.098031212	2
KIF3A	1.015699105	-0.032174773	-0.983524332	2
KIF4A	0.956572243	0.081827401	-1.038399644	2
KIFAP3	1.060223463	-0.133949319	-0.926274144	2
KIFC1	0.874855372	0.215236898	-1.09009227	2
KIFC2	0.826881687	0.284556295	-1.111437983	2
KLC2	0.907842047	0.16403295	-1.071874997	2
KLC3	1.086663927	-0.205143951	-0.881519976	2
KLF8	1.077503557	-0.179252733	-0.898250824	2
KLHL13	1.075907095	-0.174886139	-0.901020955	2
KLHL20	1.118656563	-0.31142584	-0.807230723	2
KLHL34	1.041534844	-0.089022269	-0.952512575	2
KLHL35	0.989290666	0.021085201	-1.010375867	2
KLK11	0.87255758	0.21868873	-1.09124631	2
KLK5	1.064531538	-0.144864874	-0.919666664	2
KLK7	0.941813597	0.10766071	-1.049474307	2
KLLN	1.099630695	-0.244677263	-0.854953432	2
KLRC2	1.046289258	-0.10010906	-0.946180197	2
KMT2A	1.04943769	-0.107573304	-0.941864385	2
KPNA6	1.084330328	-0.198405358	-0.88592497	2
KREMEN2	1.004642507	-0.009350591	-0.995291916	2
KRIT1	0.91462923	0.153086915	-1.067716146	2
KRT18	1.128378839	-0.351890347	-0.776488492	2
KRT27	0.930889505	0.126233831	-1.057123336	2
KRT34	1.019675991	-0.040587896	-0.979088095	2
KRT39	1.075728825	-0.174401043	-0.901327782	2
KRT40	1.122143004	-0.325282808	-0.796860196	2
KRT72	1.043305444	-0.093125811	-0.950179633	2

KRT73	0.86493423	0.230040684	-1.094974914	2
KRT8	0.965106289	0.066470888	-1.031577177	2
KRTAP1-4	0.828106018	0.282854673	-1.110960691	2
KRTAP10-2	1.001654198	-0.003316645	-0.998337552	2
KRTAP10-3	0.81375824	0.302591207	-1.116349447	2
KRTAP10-5	1.038255982	-0.081499853	-0.956756129	2
KRTAP10-7	0.884640869	0.200375373	-1.085016241	2
KRTAP12-3	0.806966328	0.311781828	-1.118748156	2
KRTAP4-16	1.098151424	-0.239967985	-0.858183439	2
KRTAP4-5	1.108451364	-0.27404417	-0.834407194	2
KRTAP4-8	0.946784378	0.099058252	-1.045842629	2
KRTAP5-11	1.118482649	-0.310751039	-0.807731611	2
KRTAP9-3	1.111732152	-0.285609315	-0.826122837	2
KRTAP9-6	0.948423221	0.096200561	-1.044623782	2
LACTB	1.095827869	-0.232682336	-0.863145533	2
LAMA5	0.863529311	0.232116242	-1.095645554	2
LAMB1	0.941864257	0.107573525	-1.049437782	2
LAMB4	1.131451117	-0.366066404	-0.765384713	2
LAMP2	1.046359615	-0.100274775	-0.94608484	2
LAMP3	1.031130204	-0.06547858	-0.965651624	2
LAMTOR1	1.00353316	-0.007104171	-0.996428988	2
LAP3	0.965249064	0.066211221	-1.031460285	2
LAPTM4B	0.919275061	0.145507099	-1.064782159	2
LARGE2	0.898255738	0.179245008	-1.077500746	2
LCE1A	1.00293146	-0.005888929	-0.997042531	2
LCOR	1.064756736	-0.145441913	-0.919314823	2
LDAH	1.128224735	-0.351199919	-0.777024816	2
LDHC	1.13264697	-0.371816077	-0.760830893	2
LEFTY1	1.131831704	-0.367881146	-0.763950558	2
LEPROTL1	1.129543211	-0.357168376	-0.772374835	2
LGALS14	1.112568097	-0.28861976	-0.823948337	2
LGALS7	0.958069844	0.079155574	-1.037225419	2
LGR4	0.991593424	0.016606313	-1.008199738	2
LHFPL5	1.123893234	-0.332495101	-0.791398134	2
LHX2	1.081461111	-0.190257448	-0.891203663	2
LHX3	1.058043839	-0.12851296	-0.929530879	2
LHX6	1.111024173	-0.283080509	-0.827943665	2
LIG3	0.840249483	0.265794791	-1.106044274	2
LILRA4	0.983290829	0.032620128	-1.015910957	2
LILRB5	1.061023648	-0.135959402	-0.925064246	2
LIMS3	1.022116335	-0.045807001	-0.976309334	2
LIMS4	0.926083646	0.134266146	-1.060349792	2
LINGO1	0.847342618	0.255672393	-1.103015011	2
LIPG	0.907626081	0.164378817	-1.072004898	2
LIPT1	0.91457206	0.153179743	-1.067751803	2
LLCFC1	1.011442849	-0.023292651	-0.988150199	2
LMAN1L	0.996467073	0.007028801	-1.003495874	2
LMF1	1.1007425	-0.248254615	-0.852487885	2
LMO4	1.044199438	-0.095209041	-0.948990396	2
LONRF2	0.92067763	0.143204495	-1.063882124	2
LPAR2	1.056740255	-0.125288218	-0.931452037	2
LPO	0.894802018	0.18465675	-1.079458768	2
LRFN1	1.035916409	-0.076191417	-0.959724992	2
LRP8	0.957339379	0.080459978	-1.037799357	2
LRRC10B	0.904564938	0.169265361	-1.073830299	2

LRRC14	1.130341408	-0.36085177	-0.769489638	2
LRRC37B	0.814171304	0.302029159	-1.116200463	2
LRRC41	0.916865038	0.149448124	-1.066313162	2
LRRC61	1.071047341	-0.161835036	-0.909212305	2
LRRC8E	0.834887017	0.2733696	-1.108256617	2
LRRC9	1.044461013	-0.095820029	-0.948640983	2
LRRK1	0.800035357	0.321063156	-1.121098513	2
LSMEM1	1.122803877	-0.327984736	-0.794819141	2
LSS	1.052362026	-0.114597941	-0.937764085	2
LTK	1.076570288	-0.176695168	-0.89987512	2
LTO1	0.805282376	0.314045787	-1.119328164	2
LUZP2	1.130254602	-0.360448525	-0.769806077	2
LVRN	0.823036573	0.289878961	-1.112915535	2
LY6D	1.095539154	-0.231786236	-0.863752918	2
LY6G6F	1.102419955	-0.253715974	-0.848703981	2
LY6L	1.109053916	-0.276139533	-0.832914383	2
LY9	1.012334145	-0.025142454	-0.987191691	2
LYPD1	0.906452374	0.166255911	-1.072708285	2
LYPD3	0.992387728	0.015054556	-1.007442285	2
LYPD5	1.067173242	-0.151675697	-0.915497545	2
LYPD6B	1.078236337	-0.181270713	-0.896965624	2
LYRM9	1.093965771	-0.226937314	-0.867028458	2
MAB21L1	1.067253267	-0.151883463	-0.915369804	2
MAB21L2	0.983745793	0.031752123	-1.015497916	2
MAB21L4	1.122498052	-0.326731237	-0.795766814	2
MACF1	0.861449862	0.235179042	-1.096628904	2
MACROD1	1.115843403	-0.300686157	-0.815157246	2
MAFA	1.039645283	-0.084675237	-0.954970046	2
MAGEA4	1.125554874	-0.339518239	-0.786036635	2
MAGEA9B	0.868386673	0.22491852	-1.093305193	2
MAGEB10	1.116691544	-0.303885567	-0.812805977	2
MAGEC3	0.857676236	0.240709196	-1.098385432	2
MAGEL2	0.802710188	0.31749278	-1.120202968	2
MAK	0.815004229	0.300894724	-1.115898953	2
MAN1C1	1.082494113	-0.193173974	-0.88932014	2
MAN2A2	0.835975228	0.271837773	-1.107813	2
MANBAL	1.042106826	-0.090344669	-0.951762157	2
MANSC1	1.124547709	-0.335240075	-0.789307635	2
MAP1LC3C	1.070446368	-0.160245279	-0.910201089	2
MAP3K1	1.123119803	-0.329285428	-0.793834375	2
MAP3K21	1.130923441	-0.36357282	-0.767350621	2
MAP3K3	0.904715859	0.169025134	-1.073740993	2
MAP7D2	1.114202994	-0.294587183	-0.819615812	2
MAPK15	1.06343341	-0.142060399	-0.921373011	2
MAPK7	0.828423881	0.282412352	-1.110836233	2
MAPKAPK5	1.039037283	-0.083283448	-0.955753834	2
MAPRE2	1.024044847	-0.049962781	-0.974082066	2
MARCHF8	0.81231737	0.304548968	-1.116866338	2
MARK2	1.059504721	-0.132150412	-0.927354309	2
MARK4	1.071705813	-0.163582859	-0.908122954	2
MAS1	0.941698368	0.10785898	-1.049557347	2
MBD2	1.065879464	-0.148328596	-0.917550868	2
MBD3L2B	1.081607476	-0.190669548	-0.890937928	2
MC5R	1.106277195	-0.266584806	-0.839692389	2
MCM5	0.89472699	0.184773919	-1.079500909	2

MCM9	1.068225965	-0.15441578	-0.913810185	2
MCTP1	1.128437429	-0.35215333	-0.776284099	2
MDFIC	0.882738961	0.203284648	-1.086023609	2
MEAK7	1.124696939	-0.335869779	-0.788827159	2
MED12L	1.04889372	-0.106276473	-0.942617247	2
MED27	1.009269262	-0.018803728	-0.990465535	2
MED28	0.985540641	0.028317228	-1.013857869	2
MEIOB	1.130051546	-0.359507824	-0.770543722	2
MEIOSIN	1.082776246	-0.193973816	-0.88880243	2
MELTF	1.011292356	-0.022980841	-0.988311515	2
METTL1	1.047985799	-0.104118724	-0.943867075	2
METTL14	0.918264667	0.147161738	-1.065426405	2
METTL17	0.923774153	0.138097079	-1.061871231	2
MEX3D	1.039931895	-0.085332493	-0.954599402	2
MFSD14A	1.094284086	-0.227913669	-0.866370416	2
MFSD5	0.828033887	0.282955015	-1.110988902	2
MGARP	0.948398519	0.096243715	-1.044642234	2
MGAT2	0.996486268	0.00699081	-1.003477078	2
MGAT4B	1.077393968	-0.178951688	-0.89844228	2
MGAT5B	0.839039933	0.26750914	-1.106549073	2
MGST3	0.899157919	0.177825438	-1.076983357	2
MIA-RAB4B	1.08284186	-0.194160034	-0.888681826	2
MIA3	0.960243837	0.075259779	-1.035503616	2
MICAL3	1.083883472	-0.197126618	-0.886756853	2
MINAR2	1.102415941	-0.253702813	-0.848713129	2
MITF	1.129961735	-0.359092892	-0.770868843	2
MKI67	1.104297508	-0.259924079	-0.844373429	2
MLC1	1.087966009	-0.208949465	-0.879016544	2
MLYCD	1.020656948	-0.042680588	-0.97797636	2
MMD2	0.816321439	0.299097706	-1.115419145	2
MMP1	0.938669477	0.11305234	-1.051721817	2
MMP14	0.890196457	0.191818329	-1.082014786	2
MMP24OS	1.11049112	-0.281188805	-0.829302314	2
MMP25	1.017830239	-0.036669206	-0.981161033	2
MNX1	0.849157944	0.253062593	-1.102220536	2
MOCS3	1.0738368	-0.169282854	-0.904553946	2
MOGAT2	0.928956989	0.12947368	-1.058430668	2
MON2	1.120482429	-0.318602342	-0.801880087	2
MORC2	0.815721782	0.29991625	-1.115638032	2
MORC4	1.009648843	-0.019585398	-0.990063445	2
MORN5	1.131047994	-0.364159088	-0.766888906	2
MOV10L1	0.924601734	0.136726448	-1.061328183	2
MPI	1.131888703	-0.368154125	-0.763734578	2
MPO	1.108606562	-0.274582675	-0.834023887	2
MPPE1	0.892489049	0.188261168	-1.080750217	2
MPRIP	0.992535984	0.014764531	-1.007300516	2
MPZL3	1.057151203	-0.126302683	-0.93084852	2
MRGPRE	1.114993961	-0.297513614	-0.817480347	2
MRPL2	1.055014989	-0.121050204	-0.933964785	2
MRPL3	1.065279318	-0.146783493	-0.918495825	2
MRPL33	0.857082762	0.241575665	-1.098658426	2
MRPL4	0.931869665	0.124585442	-1.056455108	2
MRPL50	0.980966545	0.037037797	-1.018004342	2
MRPS11	1.119778873	-0.31581679	-0.803962083	2
MRTFA	1.043396403	-0.09333742	-0.950058983	2

MS4A14	0.82699547	0.284398295	-1.111393765	2
MSANTD1	0.983470493	0.032277485	-1.015747978	2
MSH4	1.082162968	-0.192236986	-0.889925982	2
MSH5	1.016130516	-0.033082016	-0.9830485	2
MSH5-SAPCD1	0.814689019	0.301324208	-1.116013227	2
MSH6	0.943905342	0.104052562	-1.047957904	2
MSLNL	0.925006925	0.136054507	-1.061061431	2
MST1	0.948469205	0.096120221	-1.044589426	2
MT-CO3	0.935845803	0.117862448	-1.05370825	2
MT-CYB	0.803736338	0.316119243	-1.119855581	2
MT-ND1	1.095303729	-0.231057004	-0.864246725	2
MT-ND4L	1.132520719	-0.371202379	-0.76131834	2
MT1H	1.131147365	-0.364627849	-0.766519517	2
MTAP	0.913758658	0.154499307	-1.068257965	2
MTBP	1.081285788	-0.189764307	-0.891521481	2
MTCL1	1.021398826	-0.04426793	-0.977130896	2
MTG2	0.838768017	0.267894072	-1.106662089	2
MTHFR	0.836048389	0.27173469	-1.107783079	2
MTMR11	1.037667128	-0.0801592	-0.957507929	2
MTTP	1.001633281	-0.003274605	-0.998358676	2
MUC21	0.942868355	0.105843442	-1.048711796	2
MUC3A	1.101484468	-0.25066062	-0.850823848	2
MUC4	1.054459701	-0.119693282	-0.934766419	2
MVB12A	0.964772296	0.067077966	-1.031850262	2
MVP	0.799131926	0.322265821	-1.121397747	2
MYADML2	1.06120183	-0.136408059	-0.924793771	2
MYBPHL	1.040187898	-0.085920189	-0.954267708	2
MYCBPAP	1.101190024	-0.249704003	-0.85148602	2
MYD88	1.058728559	-0.130214711	-0.928513848	2
MYL10	0.929204533	0.129059423	-1.058263956	2
MYLK	0.848565117	0.253915739	-1.102480857	2
MYLK3	0.956075455	0.082711586	-1.038787041	2
MYO18B	1.099763007	-0.245101262	-0.854661745	2
MYOF	0.906593074	0.166031121	-1.072624195	2
MYOM1	0.969744977	0.057986618	-1.027731595	2
MYZAP	0.848069305	0.254628622	-1.102697927	2
MZT2B	1.028230575	-0.059080768	-0.969149807	2
NABP2	0.934871357	0.119515477	-1.054386834	2
NADK	0.939775445	0.111160111	-1.050935556	2
NAE1	0.934780441	0.119669525	-1.054449966	2
NANOG	1.038725684	-0.082571456	-0.956154228	2
NANOGP8	0.946659373	0.099275784	-1.045935156	2
NAPSA	0.940133288	0.110546868	-1.050680156	2
NASP	1.059321289	-0.131692299	-0.927628991	2
NAT1	1.017164952	-0.035262716	-0.981902236	2
NBAS	1.12168867	-0.323439836	-0.798248834	2
NBPF4	1.125986092	-0.341370643	-0.78461545	2
NCAPG	0.865509344	0.229189571	-1.094698915	2
NCKAP1	1.103263261	-0.256491608	-0.846771653	2
NCKAP1L	1.077592133	-0.179496197	-0.898095936	2
NCKIPSD	1.093408373	-0.225233195	-0.868175177	2
NCOA6	0.960068474	0.075574793	-1.035643267	2
NCR1	1.083140276	-0.195007941	-0.888132335	2
NDST2	1.048395979	-0.10509251	-0.943303469	2
NDUFA9	1.061241012	-0.13650677	-0.924734241	2

NDUFV3	1.109727159	-0.278495665	-0.831231494	2
NEFH	1.060545755	-0.134757989	-0.925787765	2
NEGR1	0.87688146	0.212181418	-1.089062878	2
NEIL2	1.122508695	-0.326774769	-0.795733926	2
NEK3	1.097443561	-0.237734284	-0.859709277	2
NEK5	0.988154333	0.02328466	-1.011438994	2
NELFA	1.131692543	-0.367215986	-0.764476557	2
NELL2	1.094645529	-0.229025145	-0.865620384	2
NEMP1	1.081937727	-0.191600768	-0.890336959	2
NEURL1B	1.114976318	-0.29744805	-0.817528268	2
NEUROD2	1.09016416	-0.215451107	-0.874713053	2
NFIB	1.020620856	-0.042603468	-0.978017388	2
NGEF	1.131755106	-0.367514792	-0.764240314	2
NGF	0.898029152	0.179601151	-1.077630303	2
NIBAN3	0.939747162	0.111208559	-1.050955721	2
NIN	0.935561046	0.118345869	-1.053906915	2
NIPAL4	1.069921127	-0.158860063	-0.911061064	2
NIPSNAP3B	1.119080175	-0.313075746	-0.806004429	2
NKAIN2	1.06757907	-0.152730227	-0.914848843	2
NKX2-1	1.113231898	-0.2910297	-0.822202198	2
NKX2-2	0.82705941	0.284309495	-1.111368905	2
NKX2-3	1.108196714	-0.273162363	-0.835034351	2
NKX2-6	1.03761974	-0.080051445	-0.957568295	2
NKX2-8	1.043155832	-0.092777924	-0.950377908	2
NKX3-2	0.953172567	0.087857286	-1.041029853	2
NLRP1	1.105292406	-0.263256286	-0.842036119	2
NLRP12	0.94801381	0.096915474	-1.044929283	2
NMNAT2	1.119094596	-0.31313207	-0.805962526	2
NMRAL1	0.873499853	0.217274925	-1.090774777	2
NMT2	1.119294742	-0.313914874	-0.805379868	2
NNAT	1.108026442	-0.272573969	-0.835452473	2
NOLC1	1.064313037	-0.144305619	-0.920007417	2
NOS1	0.913441308	0.155013547	-1.068454855	2
NOSTRIN	1.034139469	-0.07219147	-0.961947998	2
NOTCH2NLR	0.932372297	0.123738784	-1.05611108	2
NOTCH4	0.877356931	0.211462758	-1.08881969	2
NOTO	0.925125042	0.135858521	-1.060983562	2
NOTUM	1.130663156	-0.362352217	-0.768310939	2
NPAS1	1.111103555	-0.283363115	-0.82774044	2
NPBWR2	0.979894777	0.039065531	-1.018960308	2
NPEPPS	1.07161542	-0.163342552	-0.908272868	2
NPIPA1	1.129006153	-0.354720263	-0.774285891	2
NPIPA5	1.049906615	-0.108693684	-0.941212931	2
NPIPB2	0.956952679	0.081149586	-1.038102266	2
NPR3	0.927774861	0.131448908	-1.059223769	2
NPTN	1.034754774	-0.073573469	-0.961181305	2
NR2E1	0.840801022	0.265011942	-1.105812964	2
NR2F2	0.934297283	0.120487675	-1.054784958	2
NRARP	1.129283842	-0.355983101	-0.773300741	2
NRAS	0.887651741	0.195748824	-1.083400565	2
NRDE2	0.9148613	0.152709991	-1.067571291	2
NREP	1.088006033	-0.209066972	-0.878939061	2
NRN1	0.936773502	0.116285427	-1.053058928	2
NSUN4	0.956077269	0.08270836	-1.038785629	2
NTMT2	1.053780185	-0.11803744	-0.935742745	2

NUCB2	0.966469713	0.06398742	-1.030457133	2
NUDT11	1.000843331	-0.001688802	-0.999154529	2
NUDT15	0.804500896	0.31509447	-1.119595366	2
NUDT16L1	1.032410173	-0.068324605	-0.964085568	2
NUDT4	0.934147166	0.120741698	-1.054888864	2
NUDT4B	0.861151481	0.235617621	-1.096769102	2
NUDT6	1.108932183	-0.275715199	-0.833216984	2
NUDT8	1.081876489	-0.19142795	-0.89044854	2
NUFIP1	0.987115019	0.025290209	-1.012405228	2
NUP133	1.101783053	-0.251633147	-0.850149906	2
NUP54	1.122327583	-0.326034897	-0.796292686	2
NUP85	0.823816618	0.288801784	-1.112618402	2
NUPR1	0.822575237	0.290515406	-1.113090643	2
NUTM2G	0.925017605	0.136036787	-1.061054392	2
NXF2	0.873915232	0.216650925	-1.090566157	2
NXF2B	0.824195692	0.288277836	-1.112473528	2
NXNL1	0.956519454	0.081921404	-1.038440859	2
NXNL2	1.001721238	-0.003451411	-0.998269827	2
OASL	0.981210033	0.036576313	-1.017786345	2
OCLN	1.129125935	-0.355264218	-0.773861718	2
OCRL	1.080152632	-0.186589797	-0.893562835	2
ODAD1	0.945169946	0.101862874	-1.047032821	2
ODAPH	0.921611427	0.141667758	-1.063279185	2
ODF2L	1.01773217	-0.03646168	-0.98127049	2
ODF3L2	1.115577846	-0.299690971	-0.815886875	2
OLFM1	1.072095933	-0.164621349	-0.907474584	2
OLIG1	1.106038534	-0.265775343	-0.84026319	2
ONECUT3	1.066194277	-0.149140992	-0.917053285	2
OPN1LW	1.131338997	-0.365534404	-0.765804593	2
OPN1MW	1.080100442	-0.186444117	-0.893656325	2
OPN1MW3	1.025107453	-0.052264653	-0.972842801	2
OPRM1	1.096914728	-0.23607369	-0.860841038	2
OR10A3	0.911294418	0.158483773	-1.069778191	2
OR10G3	1.049732563	-0.108277564	-0.941454999	2
OR10H1	0.883483092	0.202147589	-1.085630682	2
OR1F1	0.849794088	0.252146164	-1.101940252	2
OR1G1	1.111530033	-0.284885452	-0.826644581	2
OR1L8	0.905658886	0.167522463	-1.073181349	2
OR1M1	1.083582481	-0.196267357	-0.887315125	2
OR2A1	0.976388728	0.045658414	-1.022047142	2
OR2A42	1.083710864	-0.196633659	-0.887077205	2
OR2AP1	1.005066963	-0.010212144	-0.994854819	2
OR2T3	1.055612819	-0.122514932	-0.933097887	2
OR3A3	1.031588367	-0.066495752	-0.965092615	2
OR4D1	1.013467179	-0.027501696	-0.985965483	2
OR4E2	0.868646449	0.224531851	-1.0931783	2
OR4M2B	1.122872453	-0.328266566	-0.794605887	2
OR51G1	0.97546	0.047394603	-1.022854603	2
OR51M1	0.891517136	0.189771051	-1.081288187	2
OR5A1	1.094815751	-0.229549646	-0.865266105	2
OR6C74	1.03339601	-0.070525936	-0.962870074	2
OR7A10	0.943300793	0.105097131	-1.048397924	2
OR7A17	0.990224175	0.019273042	-1.009497218	2
OR7D2	1.12386601	-0.332381498	-0.791484512	2
OSBP2	0.911074981	0.158837626	-1.069912607	2

OSBPL11	0.969740494	0.057994865	-1.027735359	2
OSBPL5	1.111199785	-0.28370602	-0.827493765	2
OSCAR	1.022575283	-0.046793463	-0.975781821	2
OSR1	1.094979201	-0.230053918	-0.864925283	2
OTOGL	1.002101948	-0.004217235	-0.997884713	2
OTOP2	0.882559662	0.203558391	-1.086118053	2
OTOS	1.004863235	-0.009798478	-0.995064756	2
OTP	1.12224064	-0.325680391	-0.796560249	2
OTUD7B	1.036654535	-0.077861003	-0.958793533	2
OVOL1	1.065780738	-0.148074095	-0.917706642	2
OVOL3	0.797820805	0.324008315	-1.12182912	2
P2RX2	1.104449753	-0.260432043	-0.84401771	2
P2RX3	1.079795546	-0.185593971	-0.894201575	2
P2RY12	0.941413694	0.108348584	-1.049762278	2
P2RY14	1.096818191	-0.235771297	-0.861046893	2
P3H2	1.089968857	-0.214869421	-0.875099436	2
P3H4	1.10101595	-0.249139581	-0.851876368	2
PABPC1L2A	1.036975316	-0.078588073	-0.958387243	2
PABPC1L2B	1.065644799	-0.147723878	-0.917920921	2
PAEP	1.125715647	-0.3402074	-0.785508247	2
PAICS	0.918828894	0.146238175	-1.065067068	2
PALM3	1.028163527	-0.058933631	-0.969229895	2
PAM16	0.805364112	0.313936033	-1.119300145	2
PAN3	1.043318848	-0.093156989	-0.950161859	2
PAPOLG	1.125495148	-0.339262668	-0.78623248	2
PARN	1.132541537	-0.371303465	-0.761238072	2
PARP15	0.986295261	0.026867988	-1.013163249	2
PARVB	1.110262131	-0.280379358	-0.829882773	2
PASK	1.059636105	-0.132478785	-0.92715732	2
PATE2	1.108661598	-0.274773836	-0.833887762	2
PAX7	0.874815088	0.215297536	-1.090112624	2
PBLD	1.044170863	-0.095142337	-0.949028526	2
PCDH11X	1.122147517	-0.32530117	-0.796846346	2
PCDH11Y	1.040509209	-0.086658663	-0.953850545	2
PCDHA4	0.986815725	0.025866674	-1.012682399	2
PCDHB1	1.084431417	-0.198695147	-0.88573627	2
PCDHB15	1.122063007	-0.324957457	-0.79710555	2
PCDHB2	1.001126433	-0.002256685	-0.998869748	2
PCDHB9	0.963469044	0.069441993	-1.032911037	2
PCED1A	1.081022949	-0.189026003	-0.891996947	2
PCMTD2	0.839537623	0.266804152	-1.106341775	2
PCNX2	0.886616084	0.197343142	-1.083959226	2
PCSK1N	1.131796828	-0.367714271	-0.764082558	2
PCSK4	0.888183494	0.194929036	-1.08311253	2
PCSK9	1.075429376	-0.173587316	-0.90184206	2
PCYOX1L	0.84369946	0.260886248	-1.104585708	2
PCYT1B	0.991376619	0.017029253	-1.008405872	2
PDAP1	1.087196227	-0.206695645	-0.880500582	2
PDCD1LG2	0.819816698	0.294311386	-1.114128085	2
PDCD2	0.880763873	0.206295147	-1.08705902	2
PDCD6IP	1.122509147	-0.326776618	-0.795732529	2
PDE10A	1.117982883	-0.308820088	-0.809162794	2
PDE4DIP	0.953196232	0.08781548	-1.041011712	2
PDLIM4	1.014856488	-0.030406513	-0.984449975	2
PDLIM5	1.12081286	-0.319919631	-0.800893228	2

PDPR	1.078397347	-0.181715288	-0.896682059	2
PDSS1	0.813455314	0.303003164	-1.116458478	2
PEDS1-UBE2V1	0.987430429	0.024682183	-1.012112611	2
PER2	1.055007998	-0.121033099	-0.933974899	2
PEX7	0.860640922	0.236367548	-1.097008471	2
PF4	1.112087182	-0.286884579	-0.825202604	2
PFKFB3	1.004543255	-0.009149293	-0.995393962	2
PFKFB4	0.819132564	0.295250278	-1.114382841	2
PFKL	0.903197357	0.171438962	-1.074636319	2
PGBD4	1.096650969	-0.235248037	-0.861402933	2
PGK2	1.118182064	-0.309588218	-0.808593846	2
PGRMC2	1.067799465	-0.153303849	-0.914495616	2
PHC1	1.114373716	-0.295216599	-0.819157117	2
PHC2	0.941373704	0.108417338	-1.049791041	2
PHF19	1.127603741	-0.348436244	-0.779167497	2
PHOX2B	1.107495545	-0.27074563	-0.836749915	2
PHPT1	0.858603511	0.239353617	-1.097957129	2
PI15	0.945752177	0.100852605	-1.046604783	2
PIANP	1.061689299	-0.137637485	-0.924051814	2
PIGR	0.881020265	0.205904958	-1.086925223	2
PIGU	0.822434029	0.290710119	-1.113144148	2
PIK3CG	1.119347448	-0.31412135	-0.805226098	2
PIK3R6	0.983958002	0.031346889	-1.015304891	2
PIKFYVE	1.025610274	-0.053356909	-0.972253365	2
PIM2	1.076250294	-0.175821437	-0.900428857	2
PINX1	0.980872959	0.03721509	-1.018088049	2
PITPNC1	0.935901052	0.117768617	-1.05366967	2
PIWIL3	1.084291274	-0.198293451	-0.885997822	2
PKD1	1.079532407	-0.184861516	-0.894670891	2
PKDCC	1.016323535	-0.033488349	-0.982835186	2
PLA2G1B	0.875253649	0.214637147	-1.089890795	2
PLA2G2D	0.963063087	0.07017682	-1.033239907	2
PLA2G3	1.116828026	-0.304403443	-0.812424584	2
PLA2G4E	1.067070429	-0.151408893	-0.915661535	2
PLAC8L1	1.062291686	-0.139160792	-0.923130894	2
PLAGL2	0.877130978	0.211804355	-1.088935334	2
PLCD1	0.817652853	0.297277573	-1.114930426	2
PLCD4	0.84087109	0.264912438	-1.105783528	2
PLCZ1	0.814695527	0.301315344	-1.11601087	2
PLEKHA2	1.049506048	-0.107736488	-0.94176956	2
PLEKHA3	0.959298734	0.076955929	-1.036254662	2
PLEKHA5	1.128266732	-0.351387895	-0.776878837	2
PLEKHA7	1.08391165	-0.197207147	-0.886704503	2
PLEKHF1	1.032746696	-0.06907513	-0.963671566	2
PLEKHG5	0.97288863	0.052179659	-1.025068289	2
PLEKHJ1	0.86104537	0.235773536	-1.096818905	2
PLEKHM1	0.994058762	0.011778426	-1.005837187	2
PLET1	0.866050464	0.228387986	-1.09443845	2
PLPBP	1.001754077	-0.003517433	-0.998236644	2
PLPP4	0.945867178	0.100652902	-1.046520079	2
PLPP6	0.890437038	0.191445765	-1.081882803	2
PLSCR3	1.062794462	-0.14043566	-0.922358801	2
PLVAP	1.114473889	-0.295586485	-0.818887404	2
PLXNB2	0.895775964	0.183134257	-1.078910221	2
PMEPA1	1.10961655	-0.278107473	-0.831509077	2

PMF1	1.075544512	-0.173900024	-0.901644488	2
PNCK	0.910328758	0.160039785	-1.070368543	2
PNLDC1	1.129828502	-0.358478627	-0.771349875	2
PNMA3	0.965720381	0.065353371	-1.031073752	2
PNMA8A	1.124964971	-0.337004413	-0.787960558	2
PNPLA6	0.954692745	0.085167025	-1.03985977	2
POC1B-GALNT4	1.105186912	-0.262901521	-0.842285391	2
PODNL1	0.927554765	0.131816119	-1.059370884	2
PODXL2	0.87800081	0.210488574	-1.088489384	2
POGLUT1	1.012713205	-0.025930778	-0.986782427	2
POGLUT3	0.990323861	0.019079246	-1.009403107	2
POLD3	0.797110671	0.324950665	-1.122061337	2
POLR1B	1.032403813	-0.068310429	-0.964093384	2
POLR3B	1.079879519	-0.185827959	-0.894051561	2
POM121L12	0.806142179	0.312890565	-1.119032744	2
POMK	1.042820706	-0.091999442	-0.950821265	2
PON1	1.095947436	-0.233054024	-0.862893412	2
POP4	0.808223494	0.310087867	-1.11831136	2
POTEH	1.105808218	-0.264995897	-0.840812321	2
POTEI	0.98160716	0.035822983	-1.017430143	2
POTEM	1.049547472	-0.107835397	-0.941712075	2
POU2F1	1.121380409	-0.322196006	-0.799184404	2
POU3F3	0.854008018	0.246050765	-1.100058783	2
PPARA	0.949372075	0.094541068	-1.043913143	2
PPARGC1A	0.842219856	0.262994805	-1.105214661	2
PPARGC1B	1.055762778	-0.122882973	-0.932879805	2
PPIE	1.07190002	-0.164099556	-0.907800464	2
PPIL2	1.091818867	-0.22041186	-0.871407008	2
PPM1A	1.102841842	-0.255101998	-0.847739844	2
PPM1F	0.885374902	0.199249821	-1.084624724	2
PPP1R11	1.077238883	-0.178525992	-0.89871289	2
PPP1R13B	1.104671718	-0.261173882	-0.843497836	2
PPP1R13L	0.971265772	0.055183257	-1.026449029	2
PPP1R14C	1.088474476	-0.210444659	-0.878029817	2
PPP1R14D	1.130506423	-0.361620159	-0.768886265	2
PPP1R16A	1.106076686	-0.265904623	-0.840172063	2
PPP1R32	0.919423672	0.145263439	-1.064687111	2
PPP1R35	0.877938834	0.210582392	-1.088521226	2
PPP1R9A	1.03115282	-0.065528751	-0.96562407	2
PPP2R3B	1.080399993	-0.187280897	-0.893119096	2
PPP4R1	1.065935264	-0.148472496	-0.917462768	2
PPP4R3B	0.869431989	0.223361518	-1.092793507	2
PPP6R3	0.91303224	0.155675922	-1.068708162	2
PPT2-EGFL8	1.062810643	-0.140476744	-0.922333899	2
PRAC2	1.040403193	-0.0864149	-0.953988292	2
PRAMEF1	0.817731636	0.297169754	-1.11490139	2
PRAMEF18	0.906552181	0.166096461	-1.072648642	2
PRAMEF2	0.830084961	0.28009723	-1.110182192	2
PRCD	1.034067342	-0.072029683	-0.962037659	2
PRDM12	1.093054358	-0.224154526	-0.868899832	2
PRDM4	1.090959135	-0.217827124	-0.873132011	2
PRDX5	0.867705917	0.225930969	-1.093636886	2
PREX2	1.123634343	-0.331416656	-0.792217687	2
PRH1	0.915639078	0.151445436	-1.067084514	2
PRKAA1	1.129623542	-0.357536616	-0.772086926	2

PRKACG	1.06663775	-0.150287632	-0.916350118	2
PRKAG1	0.84661645	0.256714156	-1.103330607	2
PRKCH	1.01827897	-0.037619651	-0.980659319	2
PRKCI	0.950499678	0.09256419	-1.043063868	2
PRMT8	1.045936847	-0.09927976	-0.946657087	2
PROCR	1.115256682	-0.298491506	-0.816765177	2
PROSER3	1.02589619	-0.053978864	-0.971917326	2
PRR23C	1.009579192	-0.019441895	-0.990137297	2
PRRG3	1.115552356	-0.299595612	-0.815956745	2
PRRT1	1.080278164	-0.18694039	-0.893337774	2
PRRX2	0.960814499	0.074233736	-1.035048236	2
PRSS22	1.018695325	-0.038502809	-0.980192516	2
PRSS36	0.93418461	0.120678345	-1.054862954	2
PRSS55	1.030710758	-0.064548879	-0.966161879	2
PRXL2B	0.832965858	0.276067365	-1.109033223	2
PSEN2	1.104110198	-0.259300078	-0.84481012	2
PSG3	1.071828262	-0.163908576	-0.907919686	2
PSG6	1.0690342	-0.156529785	-0.912504414	2
PSMA3	1.117475764	-0.306872963	-0.810602801	2
PSMB10	0.922593677	0.140048054	-1.062641732	2
PSMB8	0.806586012	0.312293645	-1.118879656	2
PSORS1C1	1.055710576	-0.122754825	-0.932955751	2
PSPN	1.08340315	-0.195756189	-0.887646961	2
PSTPIP2	1.062769505	-0.140372305	-0.9223972	2
PTBP2	1.089824534	-0.214440085	-0.875384449	2
PTCD2	0.907028789	0.165334604	-1.072363392	2
PTCHD4	1.131801364	-0.367735966	-0.764065398	2
PTCRA	1.068594672	-0.155379046	-0.913215626	2
PTGER1	1.062901474	-0.140707416	-0.922194058	2
PTGR2	1.044955261	-0.096976306	-0.947978955	2
PTHLH	1.033265676	-0.070234438	-0.963031238	2
PTMS	1.077546636	-0.179371127	-0.898175509	2
PTPN13	1.128250126	-0.351313549	-0.776936577	2
PTPN23	1.114267943	-0.294826493	-0.81944145	2
PTPN3	1.115197194	-0.29826982	-0.816927374	2
PTPN6	1.0626672	-0.140112669	-0.922554531	2
PTPRA	0.92405252	0.137636316	-1.061688836	2
PTRHD1	0.918412575	0.146919737	-1.065332311	2
PVALEF	1.122932924	-0.328515315	-0.794417609	2
PXDC1	1.102283438	-0.253268563	-0.849014875	2
PXDN	0.803131452	0.316929162	-1.120060613	2
PYDC1	1.106937089	-0.268832443	-0.838104646	2
PYM1	1.105920114	-0.265374374	-0.840545741	2
QPCT	0.848661281	0.253777406	-1.102438687	2
QRICH2	1.036675545	-0.077908595	-0.95876695	2
RAB11B	1.048374859	-0.105042327	-0.943332531	2
RAB22A	1.114816068	-0.296853145	-0.817962923	2
RAB36	0.882585056	0.203519627	-1.086104682	2
RAB39A	1.116732557	-0.304041099	-0.812691458	2
RAB3A	0.864560875	0.230592755	-1.095153631	2
RAB3B	1.116565107	-0.303406562	-0.813158545	2
RAB4B	1.099344046	-0.24376027	-0.855583776	2
RAB6D	0.817546506	0.297423096	-1.114969602	2
RAB8A	1.119052915	-0.312969302	-0.806083612	2
RABEP1	1.06043476	-0.134479349	-0.925955411	2

RABGAP1	0.867278824	0.226565542	-1.093844366	2
RABL3	0.938758566	0.112900088	-1.051658654	2
RAC3	1.099041419	-0.242794498	-0.856246921	2
RAD51C	0.824889205	0.287318464	-1.112207669	2
RAD9B	0.966911837	0.063180273	-1.030092109	2
RADIL	1.101476094	-0.25063338	-0.850842714	2
RAF1	0.836508225	0.271086508	-1.107594734	2
RAI2	1.073181897	-0.167523932	-0.905657965	2
RALB	0.826161804	0.285555273	-1.111717077	2
RALBP1	0.87846705	0.209782447	-1.088249497	2
RALGAPA1	0.916102362	0.15069125	-1.066793612	2
RAP1B	1.059190466	-0.131365818	-0.927824648	2
RASGRP3	1.024494423	-0.050935622	-0.973558801	2
RASL10B	0.804270819	0.315402977	-1.119673797	2
RASSF9	1.104144527	-0.259414362	-0.844730165	2
RAVER1	0.893304199	0.18699268	-1.080296879	2
RBCK1	1.094183686	-0.227605464	-0.866578222	2
RBKS	1.121168848	-0.321345398	-0.79982345	2
RBM10	0.888007173	0.195200954	-1.083208128	2
RBM23	0.971666059	0.054443569	-1.026109628	2
RBM26	1.076937945	-0.177701046	-0.899236899	2
RBM28	0.807504384	0.311057225	-1.118561609	2
RBM33	0.98076176	0.037425692	-1.018187452	2
RBM41	1.113139977	-0.290694937	-0.822445041	2
RBMXL2	1.050641805	-0.110454843	-0.940186963	2
RBP1	0.882456217	0.203716284	-1.086172501	2
RBPJL	1.008673554	-0.017578885	-0.991094669	2
RBPMS	0.987451659	0.024641237	-1.012092896	2
RBSN	1.122109676	-0.325147215	-0.796962461	2
RCAN2	0.989122196	0.021411733	-1.010533928	2
RCOR3	0.873668287	0.217021952	-1.090690239	2
RD3	0.802302352	0.318038091	-1.120340443	2
RDH10	1.088840377	-0.211523845	-0.877316532	2
RDH8	0.836958361	0.270451531	-1.107409893	2
REEP2	1.120954845	-0.320487471	-0.800467374	2
REG4	1.075541267	-0.173891206	-0.901650061	2
RELB	1.132609826	-0.371635356	-0.76097447	2
REN	1.029156523	-0.061116425	-0.968040099	2
REXO5	1.037548991	-0.079890606	-0.957658385	2
RFNG	1.118501061	-0.310822407	-0.807678654	2
RFPL1	0.90629746	0.166503338	-1.072800798	2
RFTN1	0.855403579	0.244022512	-1.099426091	2
RGS16	1.095847998	-0.232744884	-0.863103114	2
RGS7	1.081083063	-0.189194755	-0.891888309	2
RGS8	0.961338856	0.073289688	-1.034628544	2
RGS9BP	0.995676872	0.008590902	-1.004267774	2
RHCE	1.088747667	-0.211250149	-0.877497517	2
RHEBL1	1.008087308	-0.016375751	-0.991711557	2
RHO	1.058410658	-0.12942394	-0.928986718	2
RHOBTB3	0.850212482	0.251542893	-1.101755376	2
RHOC	0.846762006	0.256505443	-1.103267449	2
RHPN2	0.88539411	0.199220347	-1.084614458	2
RIBC1	0.945116542	0.101955472	-1.047072015	2
RIC8B	0.79982704	0.321340616	-1.121167656	2
RIIAD1	0.960911008	0.074060074	-1.034971082	2

RILP	1.041667136	-0.089327851	-0.952339285	2
RILPL2	1.060944678	-0.135760681	-0.925183997	2
RIMKLB	1.056511465	-0.124724268	-0.931787197	2
RIPPLY3	0.952317697	0.08936591	-1.041683607	2
RITA1	1.057675259	-0.12759919	-0.930076069	2
RNASET2	0.979830681	0.039186613	-1.019017294	2
RND3	1.1085811	-0.274494269	-0.834086831	2
RNF113A	0.975626597	0.047083474	-1.022710071	2
RNF122	1.013234232	-0.027015934	-0.986218298	2
RNF138	0.913375519	0.155120111	-1.06849563	2
RNF146	0.979049062	0.040661475	-1.019710537	2
RNF157	1.028980728	-0.060729419	-0.968251309	2
RNF182	1.125137445	-0.337737013	-0.787400432	2
RNF208	1.116254026	-0.302231114	-0.814022913	2
RNF216	1.105565411	-0.264175982	-0.841389429	2
RNF220	0.887538497	0.195923305	-1.083461802	2
RNF224	0.823952523	0.288613975	-1.112566498	2
RNF44	0.812992324	0.303632426	-1.11662475	2
RNF5	1.079089017	-0.183629954	-0.895459063	2
RNF6	1.072904639	-0.166781213	-0.906123426	2
ROM1	0.859893013	0.237464915	-1.097357927	2
RORB	1.130070722	-0.359596506	-0.770474216	2
RP2	0.844795581	0.259320862	-1.104116442	2
RP9	1.040795824	-0.087318197	-0.953477627	2
RPAP1	0.827555474	0.283620251	-1.111175725	2
RPL3	0.8022459	0.318113546	-1.120359446	2
RPL3L	0.960931936	0.07402241	-1.034954346	2
RPL7L1	0.983069506	0.033041991	-1.016111497	2
RPL8	1.117354736	-0.30641006	-0.810944676	2
RPLP1	1.058011904	-0.128433726	-0.929578178	2
RPRD1B	1.116356127	-0.30261643	-0.813739697	2
RPRM	1.036736609	-0.078046941	-0.958689668	2
RPS5	0.877479273	0.211277743	-1.088757016	2
RPTOR	1.065694393	-0.147851618	-0.917842775	2
RRAS	0.980512111	0.037898278	-1.018410389	2
RRM2	1.01644609	-0.03374648	-0.982699609	2
RS1	1.09712693	-0.236739197	-0.860387733	2
RSPRY1	1.009305044	-0.018877373	-0.990427671	2
RTBDN	1.042908641	-0.092203607	-0.950705034	2
RTEL1-TNFRSF6B	0.817895111	0.296945985	-1.114841096	2
RTF1	0.874970977	0.215062856	-1.090033833	2
RTP3	0.975300799	0.047691794	-1.022992593	2
RUSF1	1.092381121	-0.222110917	-0.870270204	2
RYR3	0.876743666	0.212389573	-1.089133239	2
S100A16	0.972700492	0.052528511	-1.025229003	2
S100A7	0.993986681	0.01192007	-1.00590675	2
SACS	0.936510414	0.116732983	-1.053243398	2
SALL3	1.106980699	-0.268981475	-0.837999224	2
SBK3	1.082146332	-0.192189966	-0.889956366	2
SBNO2	1.077932609	-0.180433226	-0.897499383	2
SCGB1C1	0.933008432	0.122665919	-1.055674352	2
SCGB1C2	0.932113496	0.124174836	-1.056288332	2
SCML4	0.819303549	0.295015715	-1.114319264	2
SCN3B	1.060200126	-0.133890814	-0.926309312	2
SCNM1	0.865517403	0.229177639	-1.094695042	2

SCNN1B	1.091224082	-0.218621977	-0.872602105	2
SCO2	1.065262008	-0.146738996	-0.918523011	2
SCPEP1	0.896755816	0.181599675	-1.078355491	2
SCTR	1.081662379	-0.190824228	-0.890838151	2
SCYGR2	1.035786693	-0.075898499	-0.959888194	2
SCYGR3	0.877566743	0.211145439	-1.088712182	2
SCYGR4	1.107431032	-0.270524094	-0.836906938	2
SCYGR6	0.821826962	0.29154672	-1.113373682	2
SDHB	0.804542012	0.315039326	-1.119581339	2
SDK1	1.100678016	-0.24804622	-0.852631795	2
SDR39U1	0.856804609	0.241981464	-1.098786073	2
SEC14L1	1.129693044	-0.357855654	-0.77183739	2
SEC14L4	1.10128723	-0.250019552	-0.851267678	2
SEC14L6	1.093764167	-0.226320138	-0.867444029	2
SEC23B	1.026551132	-0.055405955	-0.971145177	2
SECISBP2L	1.095978088	-0.233149367	-0.862828722	2
SECTM1	0.998170887	0.003648243	-1.001819131	2
SELENOO	1.121564733	-0.322939114	-0.798625618	2
SEMA3G	1.090222384	-0.215624676	-0.874597709	2
SEMA4A	1.012104361	-0.024665047	-0.987439314	2
SEPTIN12	1.049233647	-0.107086508	-0.94214714	2
SEPTIN14	1.126018913	-0.341512154	-0.784506759	2
SEPTIN2	0.870495474	0.221774502	-1.092269976	2
SEPTIN3	0.799141601	0.322252949	-1.12139455	2
SEPTIN4	1.049082701	-0.106726662	-0.94235604	2
SERGEF	1.109312497	-0.277042598	-0.832269898	2
SERINC2	1.128044092	-0.35039294	-0.777651152	2
SERPINB4	1.098832061	-0.242127769	-0.856704292	2
SERPING1	0.817517859	0.297462292	-1.11498015	2
SF3B3	0.982298247	0.034510097	-1.016808343	2
SFSWAP	1.067009575	-0.151251044	-0.915758531	2
SFT2D2	1.084607882	-0.199201469	-0.885406413	2
SGCA	0.925086597	0.135922315	-1.061008912	2
SGCE	0.935125206	0.119085193	-1.054210399	2
SGK2	1.011053505	-0.022486271	-0.988567234	2
SGPL1	1.037540605	-0.079871544	-0.957669061	2
SH2D2A	0.935266127	0.118846223	-1.05411235	2
SH3BP1	0.995404112	0.009129267	-1.004533379	2
SH3GLB2	1.091242951	-0.218678642	-0.872564309	2
SH3PXD2B	0.937198452	0.115561964	-1.052760416	2
SH3RF3	1.118070785	-0.309158843	-0.808911942	2
SH3TC1	0.968948814	0.059449896	-1.02839871	2
SH3TC2	1.086554573	-0.204825856	-0.881728716	2
SHANK2	1.100806653	-0.24846205	-0.852344603	2
SHC2	0.996815545	0.006338775	-1.00315432	2
SHC3	1.024501827	-0.050951656	-0.973550171	2
SHD	0.975421654	0.047466198	-1.022887852	2
SHF	0.854200941	0.245770665	-1.099971606	2
SHH	0.843698139	0.260888132	-1.104586272	2
SHISA5	0.957401285	0.080349521	-1.037750805	2
SHISA8	0.920356724	0.143731917	-1.064088641	2
SHOX	1.092275884	-0.221792379	-0.870483505	2
SIDT1	1.054698476	-0.120276342	-0.934422134	2
SIGLEC14	1.115167859	-0.29816056	-0.817007299	2
SIGLEC5	1.02696862	-0.056317392	-0.970651228	2

SIPA1L3	1.010603726	-0.021555977	-0.989047749	2
SIRT1	1.033604782	-0.070993166	-0.962611617	2
SIRT3	0.92980341	0.128056316	-1.057859726	2
SKIDA1	1.071563067	-0.163203426	-0.90835964	2
SLAIN2	0.932062832	0.124260171	-1.056323003	2
SLC12A7	1.096441874	-0.234594706	-0.861847168	2
SLC16A13	0.94080417	0.109395841	-1.050200011	2
SLC16A14	0.840236338	0.26581344	-1.106049778	2
SLC16A3	1.107087301	-0.26934604	-0.837741261	2
SLC17A7	1.103659007	-0.257801278	-0.845857729	2
SLC1A5	0.923183164	0.13907441	-1.062257574	2
SLC22A10	0.926883735	0.132934613	-1.059818348	2
SLC22A6	1.039675231	-0.084743877	-0.954931354	2
SLC24A3	0.952724458	0.088648461	-1.041372919	2
SLC24A4	1.058208606	-0.128921958	-0.929286648	2
SLC25A20	1.031969143	-0.067342436	-0.964626707	2
SLC25A36	0.810315344	0.307261996	-1.117577339	2
SLC25A41	1.010514535	-0.021371661	-0.989142874	2
SLC25A52	0.79990333	0.321239014	-1.121142345	2
SLC27A5	1.058191348	-0.128879104	-0.929312244	2
SLC28A1	0.952415246	0.089193915	-1.041609161	2
SLC29A2	1.132731497	-0.372227867	-0.76050363	2
SLC30A2	0.878419295	0.2098548	-1.088274095	2
SLC30A4	1.000341267	-0.000682884	-0.999658383	2
SLC30A6	0.852258434	0.248586777	-1.10084521	2
SLC35A2	1.10875201	-0.275088097	-0.833663913	2
SLC35A4	1.00164185	-0.003291827	-0.998350023	2
SLC35E1	1.078147851	-0.181026569	-0.897121281	2
SLC35E3	0.992805983	0.01423603	-1.007042013	2
SLC38A1	0.975009601	0.04823507	-1.023244671	2
SLC38A10	1.131359799	-0.365633018	-0.76572678	2
SLC39A1	0.85830502	0.239790215	-1.098095235	2
SLC39A4	0.810459886	0.307066399	-1.117526286	2
SLC39A6	1.062068208	-0.138595138	-0.923473069	2
SLC39A8	0.82626251	0.285415592	-1.111678103	2
SLC41A1	0.889137682	0.193455952	-1.082593634	2
SLC44A2	0.846162611	0.257364597	-1.103527208	2
SLC44A5	1.127034769	-0.345929537	-0.781105233	2
SLC5A2	1.081563867	-0.190546726	-0.891017141	2
SLC5A4	0.954070071	0.086270143	-1.040340214	2
SLC5A5	1.121067679	-0.320939505	-0.800128174	2
SLC5A8	1.129631914	-0.357575025	-0.772056889	2
SLC6A16	0.882905664	0.203030054	-1.085935719	2
SLC6A18	1.022601856	-0.046850627	-0.97575123	2
SLFN12L	1.026512675	-0.055322067	-0.971190608	2
SLFN5	1.113165574	-0.290788122	-0.822377452	2
SLK	1.072708778	-0.166257229	-0.906451549	2
SLURP1	1.113430114	-0.291752727	-0.821677387	2
SMARCA1	1.125005184	-0.337175046	-0.787830137	2
SMC1A	1.132836032	-0.372738143	-0.760097889	2
SMCR8	1.017530225	-0.036034554	-0.981495671	2
SMIM10L1	0.981909334	0.035249234	-1.017158568	2
SMIM10L2B	0.868935105	0.224101987	-1.093037092	2
SMIM38	1.12987275	-0.358682461	-0.771190289	2
SMIM44	0.801777985	0.318738722	-1.120516707	2

SMIM45	1.131895841	-0.368188337	-0.763707505	2
SMIM5	0.878534983	0.209679511	-1.088214494	2
SMIM7	0.808103642	0.3102495	-1.118353142	2
SMN1	0.973183518	0.051632526	-1.024816044	2
SMN2	0.904463706	0.169426456	-1.073890162	2
SMPD2	1.122636742	-0.327299015	-0.795337727	2
SMPD3	1.115827693	-0.300627199	-0.815200494	2
SMR3B	0.970107995	0.057318437	-1.027426432	2
SMTNL1	0.902450942	0.172622846	-1.075073788	2
SMTNL2	1.099996689	-0.245851238	-0.854145451	2
SMUG1	1.090477544	-0.216386153	-0.874091391	2
SMYD1	0.964100556	0.06829742	-1.032397976	2
SNAPC4	0.837751906	0.269330998	-1.107082904	2
SNN	1.079230381	-0.18402225	-0.895208131	2
SNRK	0.899065887	0.177970361	-1.077036249	2
SNRPD1	1.079016874	-0.183429879	-0.895586995	2
SNTA1	1.115461427	-0.29925566	-0.816205767	2
SNX10	1.029243497	-0.061307985	-0.967935512	2
SNX25	1.036063552	-0.076523864	-0.959539689	2
SNX30	1.016794335	-0.03448055	-0.982313785	2
SNX33	1.035861949	-0.076068421	-0.959793529	2
SORBS2	0.958154439	0.079004363	-1.037158802	2
SORD	0.922616938	0.140009658	-1.062626596	2
SORL1	1.118603727	-0.311220673	-0.807383054	2
SOX1	1.062457919	-0.139581954	-0.922875966	2
SOX11	1.119188559	-0.313499323	-0.805689236	2
SOX17	0.798115671	0.323616733	-1.121732404	2
SOX18	0.936268607	0.11714411	-1.053412717	2
SOX3	0.98221459	0.034669154	-1.016883744	2
SP7	0.969066051	0.059234611	-1.028300662	2
SPACA1	0.810587207	0.306894071	-1.117481279	2
SPACA4	1.016594468	-0.034059145	-0.982535323	2
SPACA7	0.925439933	0.135335798	-1.060775731	2
SPAG8	1.09741548	-0.237645933	-0.859769547	2
SPANXA2	1.116961231	-0.304909707	-0.812051525	2
SPART	1.130166741	-0.360041052	-0.770125689	2
SPATA31C2	1.026138714	-0.054506923	-0.97163179	2
SPATA45	1.026561082	-0.055427663	-0.97113342	2
SPATA6L	1.107021372	-0.269120527	-0.837900844	2
SPATC1	0.97127802	0.055160635	-1.026438655	2
SPCS3	0.836378458	0.271269477	-1.107647934	2
SPDYE10	1.126517003	-0.343668927	-0.782848076	2
SPDYE13	1.106305724	-0.266681689	-0.839624036	2
SPDYE14	1.116565815	-0.303409242	-0.813156573	2
SPDYE15	1.123833859	-0.332247401	-0.791586458	2
SPECC1L	0.806751587	0.312070856	-1.118822443	2
SPEG	1.080258233	-0.186884707	-0.893373526	2
SPEGNB	1.121811026	-0.323935016	-0.79787601	2
SPEM1	0.848837879	0.253523308	-1.102361187	2
SPEM2	0.808904092	0.309169442	-1.118073534	2
SPG11	1.075231061	-0.17304918	-0.902181881	2
SPG7	0.897835586	0.179905271	-1.077740857	2
SPHK2	0.912975126	0.155768359	-1.068743485	2
SPIB	1.0003298	-0.000659927	-0.999669873	2
SPIN3	0.805252067	0.314086483	-1.11933855	2

SPINDOC	1.1284865	-0.3523738	-0.7761127	2
SPIRE2	0.846739183	0.256538171	-1.103277355	2
SPOCD1	1.066799128	-0.15070554	-0.916093588	2
SPRYD3	0.819284538	0.295041798	-1.114326336	2
SPRYD4	1.125407303	-0.338887205	-0.786520098	2
SPZ1	1.060121026	-0.133692556	-0.92642847	2
SRF	1.045301391	-0.09778748	-0.947513911	2
SRM	0.931955759	0.124440487	-1.056396246	2
SRPRA	0.919630006	0.144925015	-1.064555021	2
SRPX2	1.104372622	-0.260174608	-0.844198013	2
SRSF4	1.126657532	-0.344280587	-0.782376945	2
SSBP2	0.986502344	0.026469756	-1.012972101	2
SSBP4	1.124716531	-0.335952558	-0.788763973	2
SSH3	1.012202495	-0.024868889	-0.987333605	2
SSR4	0.934318178	0.120452311	-1.054770488	2
SSRP1	1.121458041	-0.322508751	-0.79894929	2
SSTR5	0.947788947	0.097307841	-1.045096788	2
SSU72	1.118777014	-0.311894073	-0.806882941	2
SSU72P3	0.85292616	0.247619796	-1.100545956	2
ST3GAL3	0.977981109	0.042671663	-1.020652772	2
ST3GAL4	1.092952704	-0.223845309	-0.869107395	2
ST3GAL5	0.959828879	0.076004972	-1.035833851	2
ST3GAL6	1.015136822	-0.030994257	-0.984142565	2
ST6GALNAC1	1.06154099	-0.137263133	-0.924277858	2
ST6GALNAC6	0.972261224	0.053342358	-1.025603581	2
STARD6	1.086112402	-0.203542006	-0.882570395	2
STARD8	0.917857522	0.147827514	-1.065685036	2
STAT1	0.875352007	0.214488967	-1.089840973	2
STAT5A	0.944829875	0.102452334	-1.047282209	2
STEAP3	1.017908215	-0.03683426	-0.981073954	2
STIM2	1.102391242	-0.253621828	-0.848769414	2
STK11	0.923743354	0.13814804	-1.061891395	2
STK19	0.858324304	0.239762016	-1.09808632	2
STMN1	1.095295471	-0.231031447	-0.864264024	2
STON2	1.061061239	-0.136054023	-0.925007216	2
STPG3	1.117551763	-0.307163991	-0.810387772	2
STRADA	0.99798519	0.004017514	-1.002002704	2
STRN	0.882189595	0.204123103	-1.086312698	2
STRN4	1.050302777	-0.109641993	-0.940660784	2
SUB1	1.093622721	-0.225887681	-0.86773504	2
SUCLA2	1.111443055	-0.284574425	-0.82686863	2
SULT2A1	1.036865757	-0.078339649	-0.958526109	2
SUPT16H	0.815340936	0.300435717	-1.115776653	2
SUPT20H	0.904637753	0.169149466	-1.07378722	2
SUPT5H	0.812112672	0.304826746	-1.116939418	2
SUSD4	1.016952119	-0.034813426	-0.982138693	2
SV2C	0.967129209	0.062783104	-1.029912314	2
SVOP	1.036622097	-0.077787529	-0.958834568	2
SWSAP1	1.019268271	-0.03972016	-0.979548111	2
SYCE1	1.060062948	-0.133547036	-0.926515912	2
SYCE3	0.871510484	0.220257034	-1.091767519	2
SYN3	1.012829189	-0.026172182	-0.986657007	2
SYNGR1	0.964394072	0.067764831	-1.032158903	2
SYNGR3	1.050804721	-0.110845876	-0.939958845	2
SYNGR4	1.076009972	-0.17516631	-0.900843662	2

SYNPR	1.000431996	-0.000864552	-0.999567444	2
SYT13	1.123397519	-0.330433735	-0.792963784	2
TADA1	0.868828691	0.224260483	-1.093089174	2
TADA2B	1.091750764	-0.220206528	-0.871544236	2
TADA3	0.895607939	0.18339712	-1.079005059	2
TAF11L13	0.865052788	0.2298653	-1.094918088	2
TAF11L14	0.839812144	0.266415042	-1.106227186	2
TAF11L2	1.113532411	-0.292126492	-0.821405919	2
TAF11L3	1.047542784	-0.1030689	-0.944473884	2
TAF11L6	1.118301288	-0.310048914	-0.808252374	2
TAF11L9	1.032388055	-0.06827531	-0.964112745	2
TAF1B	1.131303336	-0.365365446	-0.765937891	2
TAF1C	1.116897075	-0.304665771	-0.812231304	2
TAF1L	1.007912997	-0.016018447	-0.99189455	2
TAF9	1.106110395	-0.266018883	-0.840091512	2
TAFA5	0.906949544	0.165461327	-1.07241087	2
TAL1	0.948745448	0.095637412	-1.044382861	2
TAMM41	0.979842955	0.039163429	-1.019006384	2
TANC2	0.946180657	0.100108262	-1.046288918	2
TAOK2	1.020922801	-0.043248947	-0.977673854	2
TAS2R30	1.076323291	-0.176020609	-0.900302682	2
TBC1D1	1.020438472	-0.042213901	-0.978224571	2
TBC1D19	1.014532364	-0.029727636	-0.984804727	2
TBC1D22B	1.046624972	-0.100900216	-0.945724756	2
TBC1D2B	0.843792581	0.260753371	-1.104545952	2
TBC1D3D	0.936662038	0.116475077	-1.053137115	2
TBC1D3G	0.915163803	0.152218402	-1.067382205	2
TBC1D3K	0.837183168	0.270134238	-1.107317406	2
TBC1D4	1.01508897	-0.030893893	-0.984195077	2
TBKBP1	0.992376247	0.015077012	-1.007453259	2
TBL1X	1.108730972	-0.275014948	-0.833716024	2
TCF21	1.128596467	-0.352868551	-0.775727916	2
TCF24	0.990513396	0.018710626	-1.009224021	2
TCF25	1.103309606	-0.256644743	-0.846664863	2
TCF3	0.963026794	0.070242478	-1.033269272	2
TCF7L2	0.944330903	0.103316398	-1.047647301	2
TCTE1	0.953612696	0.087079385	-1.040692081	2
TCTN3	1.117853317	-0.308321447	-0.809531869	2
TEAD4	0.97022327	0.05710613	-1.0273294	2
TECTA	0.940342821	0.110187559	-1.05053038	2
TEKT5	0.997980304	0.004027228	-1.002007532	2
TELO2	1.038332742	-0.081674842	-0.9566579	2
TENM1	0.978622514	0.041465044	-1.020087558	2
TENM4	0.879526562	0.208175581	-1.087702144	2
TENT4B	0.993826264	0.012235193	-1.006061457	2
TET2	0.970184799	0.057176991	-1.02736179	2
TEX11	1.129357027	-0.356316977	-0.773040049	2
TEX261	0.814099868	0.302126386	-1.116226254	2
TEX35	0.818968437	0.295475372	-1.114443809	2
TEX48	1.111832786	-0.285970301	-0.825862485	2
TF	1.1255039	-0.339300101	-0.786203799	2
TFDP2	1.023351375	-0.048465183	-0.974886193	2
TFF2	1.028305603	-0.059245458	-0.969060144	2
TGFB1I1	0.803026805	0.317069204	-1.120096009	2
TGIF2	1.015478988	-0.031712374	-0.983766614	2

THAP2	1.042941315	-0.092279487	-0.950661828	2
THEMIS2	1.106851056	-0.268538614	-0.838312443	2
THPO	0.840472978	0.265477649	-1.105950627	2
THRAP3	1.072627822	-0.166040814	-0.906587008	2
TIGD3	1.125099971	-0.337577671	-0.787522299	2
TIMM23B	1.044198774	-0.095207491	-0.948991283	2
TINCR	1.006006193	-0.012122606	-0.993883586	2
TIRAP	1.126732582	-0.344607826	-0.782124756	2
TK2	1.076347165	-0.176085768	-0.900261397	2
TLCD3A	0.924394715	0.137069535	-1.06146425	2
TLCD3B	0.957076864	0.080928195	-1.038005059	2
TLE4	1.050859886	-0.110978347	-0.939881539	2
TLE6	1.129183699	-0.355526955	-0.773656744	2
TLR4	1.033963659	-0.071797189	-0.96216647	2
TLR7	0.830417843	0.279632538	-1.110050381	2
TM4SF1	1.108902575	-0.275612069	-0.833290506	2
TMED6	1.109932751	-0.279218364	-0.830714387	2
TMED7-TICAM2	1.130534105	-0.361749296	-0.768784809	2
TMEM106C	1.026160607	-0.054554617	-0.971605991	2
TMEM119	0.903064211	0.17165027	-1.074714481	2
TMEM121	0.870813378	0.221299521	-1.092112899	2
TMEM145	0.947626971	0.097590351	-1.045217321	2
TMEM14C	1.112718364	-0.289163784	-0.823554581	2
TMEM17	0.940193499	0.110443635	-1.050637134	2
TMEM175	0.868511218	0.22473316	-1.093244378	2
TMEM176A	1.124411667	-0.334667262	-0.789744406	2
TMEM176B	1.075447918	-0.173637662	-0.901810256	2
TMEM191C	1.089586972	-0.213734319	-0.875852654	2
TMEM199	0.980584799	0.037760713	-1.018345511	2
TMEM200B	0.910966275	0.159012863	-1.069979138	2
TMEM208	1.036339582	-0.07714802	-0.959191562	2
TMEM210	0.977066178	0.044389291	-1.021455469	2
TMEM214	1.079818759	-0.18565864	-0.894160119	2
TMEM220	0.858358724	0.23971168	-1.098070404	2
TMEM233	1.106055734	-0.265833622	-0.840222112	2
TMEM238	1.016586814	-0.034043012	-0.982543802	2
TMEM248	0.797060683	0.325016963	-1.122077645	2
TMEM25	1.022555246	-0.046750361	-0.975804885	2
TMEM256-PLSCR3	1.069116284	-0.15674499	-0.912371294	2
TMEM270	0.988897656	0.021846697	-1.010744353	2
TMEM271	0.823022972	0.289897732	-1.112920704	2
TMEM35A	0.797341148	0.324644932	-1.121986079	2
TMEM43	0.834610944	0.273757788	-1.108368732	2
TMEM68	1.004078034	-0.00820658	-0.995871454	2
TMEM74B	1.112529806	-0.288481274	-0.824048533	2
TMEM79	0.884571041	0.200482366	-1.085053407	2
TMEM89	0.880068101	0.207353085	-1.087421186	2
TMUB1	1.037232844	-0.079172432	-0.958060412	2
TMX1	1.085036568	-0.200433888	-0.88460268	2
TNFAIP3	0.912212252	0.157002026	-1.069214278	2
TNFRSF11B	1.026315641	-0.054892446	-0.971423195	2
TNFRSF25	1.099434265	-0.244048646	-0.855385618	2
TNIP1	0.899871889	0.176700264	-1.076572153	2
TNN	1.085538136	-0.201880206	-0.88365793	2
TNNI3	0.873219357	0.217696034	-1.090915391	2

TNPO3	0.985152701	0.029061087	-1.014213788	2
TNS2	1.085060937	-0.200504049	-0.884556888	2
TNS3	1.122007439	-0.324731674	-0.797275765	2
TONSL	0.832716137	0.27641742	-1.109133556	2
TOP2B	0.815355661	0.300415637	-1.115771299	2
TOP3B	0.815446304	0.300292028	-1.115738331	2
TOR1AIP1	0.9659278	0.064975526	-1.030903326	2
TOR1B	1.090253699	-0.215718055	-0.874535644	2
TP53I11	0.836249787	0.27145086	-1.107700647	2
TPBGL	0.918401839	0.146937304	-1.065339143	2
TPCN2	0.86278713	0.233210661	-1.09599779	2
TPM1	0.985186061	0.028997152	-1.014183213	2
TPRG1L	0.867965895	0.225544462	-1.093510357	2
TPST2	1.116192551	-0.301999336	-0.814193214	2
TPTE	1.103458726	-0.257137899	-0.846320827	2
TRAF4	1.000217383	-0.000434909	-0.999782475	2
TRAM2	0.878547889	0.209659954	-1.088207843	2
TRAPPC1	0.85304534	0.24744709	-1.100492429	2
TRAPPC12	0.940994026	0.109069793	-1.050063819	2
TRAPPC13	0.960252012	0.075245091	-1.035497103	2
TRAPPC2	0.893447621	0.186769292	-1.080216913	2
TRAPPC8	1.126203008	-0.342307283	-0.783895726	2
TREH	0.861167785	0.235593662	-1.096761447	2
TREML1	0.995229922	0.009472853	-1.004702775	2
TREML4	1.086749469	-0.205392938	-0.88135653	2
TRIM14	1.101938436	-0.252140234	-0.849798203	2
TRIM2	0.898264734	0.179230865	-1.077495599	2
TRIM21	0.876447927	0.212836151	-1.089284078	2
TRIM22	0.799899471	0.321244155	-1.121143626	2
TRIM3	0.829139033	0.281416366	-1.110555399	2
TRIM33	1.114159404	-0.294426667	-0.819732737	2
TRIM39	0.931793257	0.124714067	-1.056507325	2
TRIM41	0.97132103	0.055081192	-1.026402221	2
TRIM58	1.076667303	-0.176960384	-0.89970692	2
TRIM6	0.910720174	0.159409441	-1.070129615	2
TRIM71	1.05990721	-0.133157021	-0.926750189	2
TRIM72	1.105849755	-0.265136346	-0.840713409	2
TRIP10	1.039155964	-0.08355486	-0.955601103	2
TRIP11	1.074401256	-0.170804046	-0.90359721	2
TRMO	0.830982891	0.278843182	-1.109826074	2
TRMT1	1.03685743	-0.07832077	-0.958536659	2
TROAP	0.971037319	0.055605072	-1.026642392	2
TRPV3	1.126614225	-0.344091941	-0.782522284	2
TRPV4	1.066865402	-0.150877267	-0.915988135	2
TSC22D1	0.945980937	0.100455301	-1.046436238	2
TSGA10	1.109463267	-0.277570229	-0.831893038	2
TSGA10IP	1.113037842	-0.29032337	-0.822714472	2
TSHZ3	1.017366455	-0.035688385	-0.981678071	2
TSPAN17	1.118790408	-0.311946184	-0.806844224	2
TSPAN3	0.96623537	0.064414879	-1.030650248	2
TSPAN33	0.950107835	0.093251745	-1.04335958	2
TSPAN4	1.101232259	-0.249841075	-0.851391184	2
TSPO	1.037939475	-0.080778871	-0.957160604	2
TSPY10	1.131551459	-0.366543525	-0.765007934	2
TSPY2	1.07291258	-0.16680247	-0.90611011	2

TSPY3	1.103611316	-0.257643207	-0.845968109	2
TSSK6	0.984591663	0.030135448	-1.014727112	2
TST	1.112917284	-0.289885315	-0.82303197	2
TTC21B	0.844109565	0.260300902	-1.104410468	2
TTC23	1.128513448	-0.35249495	-0.776018497	2
TTC38	0.923122093	0.139175336	-1.062297429	2
TTC39B	1.115183865	-0.298220172	-0.816963693	2
TTLL7	0.952825022	0.08847098	-1.041296002	2
TTPA	1.048149427	-0.104506978	-0.943642449	2
TUBA3D	0.9449989	0.102159414	-1.047158314	2
TUBG1	1.024779803	-0.051553957	-0.973225845	2
TUBGCP6	0.89723626	0.180846182	-1.078082442	2
TYK2	1.005371383	-0.010830747	-0.994540636	2
TYROBP	0.981613999	0.035810003	-1.017424002	2
U2AF2	1.041086823	-0.087988593	-0.95309823	2
UBA7	1.011323175	-0.023044683	-0.988278492	2
UBASH3B	1.081082609	-0.189193478	-0.89188913	2
UBE2E2	1.027561551	-0.057614201	-0.96994735	2
UBE2F-SCLY	0.869669102	0.223007937	-1.092677039	2
UBE2G1	0.961228107	0.073489182	-1.034717289	2
UBE2H	0.84047048	0.265481195	-1.105951675	2
UBE2L3	0.868384309	0.224922038	-1.093306347	2
UBE2R2	0.91189716	0.157511017	-1.069408177	2
UBE4A	0.994540378	0.010831255	-1.005371633	2
UBIAD1	0.878336363	0.209980434	-1.088316797	2
UBL4B	1.116265151	-0.302273075	-0.813992076	2
UBXN2A	1.131730473	-0.367397095	-0.764333378	2
UCP2	1.040599047	-0.086865309	-0.953733738	2
UCP3	1.129642716	-0.357624592	-0.772018124	2
UNC119B	0.949905357	0.093606781	-1.043512138	2
UNC13A	1.042957797	-0.092317769	-0.950640028	2
UNC80	1.120920034	-0.320348151	-0.800571883	2
UNCX	1.104670499	-0.261169804	-0.843500695	2
UPRT	1.105794752	-0.264950377	-0.840844375	2
UQCRC1	1.125312837	-0.33848402	-0.786828817	2
UQCRHL	0.988030855	0.023523241	-1.011554095	2
USF1	1.129910654	-0.358857205	-0.771053449	2
USH1G	1.124345848	-0.334390548	-0.7899553	2
USP54	0.978088256	0.042470239	-1.020558495	2
USP9Y	1.007324988	-0.014814586	-0.992510402	2
UTP23	0.868685891	0.224473126	-1.093159018	2
UTP25	0.879559628	0.208125384	-1.087685012	2
UTY	1.034257807	-0.072457011	-0.961800795	2
VAPB	1.107269366	-0.26996954	-0.837299826	2
VASH1	1.11447602	-0.295594358	-0.818881662	2
VAV1	0.942087643	0.107188965	-1.049276608	2
VCAM1	0.960517661	0.074767625	-1.035285286	2
VCPKMT	0.998723192	0.002548744	-1.001271936	2
VEPH1	1.110746679	-0.282094433	-0.828652246	2
VGF	0.931033967	0.125991101	-1.057025068	2
VIRMA	0.959994853	0.075707002	-1.035701855	2
VPS36	0.920306915	0.143813749	-1.064120664	2
VPS37B	1.023882412	-0.049611665	-0.974270747	2
VPS37C	1.079896702	-0.185875851	-0.894020851	2
VPS51	1.094479747	-0.228514972	-0.865964775	2

VSIG1	1.122500675	-0.326741966	-0.795758709	2
VWA1	0.839756889	0.266493375	-1.106250265	2
WAC	1.115926733	-0.30099908	-0.814927653	2
WARS1	1.097306652	-0.237303709	-0.860002943	2
WASF2	1.009102734	-0.018461094	-0.99064164	2
WDFY1	0.804181265	0.315523031	-1.119704296	2
WDR48	0.889453434	0.192967914	-1.082421347	2
WDR70	0.870324171	0.222030335	-1.092354506	2
WDR76	1.064473189	-0.14471547	-0.919757719	2
WDR83	1.096413883	-0.234507327	-0.861906556	2
WDR87	0.826366759	0.285270974	-1.111637733	2
WDTC1	0.804424675	0.315196685	-1.119621361	2
WEE1	1.027114222	-0.056635584	-0.970478638	2
WFIKKN1	0.809236319	0.308720775	-1.117957094	2
WNT10A	1.123228365	-0.329733762	-0.793494603	2
WNT2	1.021297031	-0.044049886	-0.977247145	2
WNT2B	0.819423916	0.294850554	-1.114274471	2
WNT3A	1.104933279	-0.26204998	-0.842883299	2
WNT5A	1.076600705	-0.176778303	-0.899822401	2
WNT6	0.888390987	0.194608929	-1.082999916	2
WWC3	0.945593342	0.101128344	-1.046721686	2
XAF1	1.099251441	-0.243464486	-0.855786955	2
XG	1.062026381	-0.138489337	-0.923537043	2
XIRP1	0.982708722	0.033729133	-1.016437855	2
XKR5	1.104461762	-0.260472141	-0.843989621	2
XNDC1N	1.075693522	-0.174305037	-0.901388485	2
XPC	1.04476018	-0.096519637	-0.948240543	2
XPNPEP2	0.811877093	0.305146321	-1.117023414	2
XPO5	1.111372235	-0.284321388	-0.827050847	2
XPR1	0.990204172	0.019311923	-1.009516095	2
XRCC3	1.127271851	-0.346971138	-0.780300713	2
XYLB	1.003269396	-0.006571177	-0.996698219	2
YAE1	1.045590929	-0.098466928	-0.947124001	2
YES1	0.805152222	0.314220529	-1.119372752	2
YIPF2	0.981218492	0.036560275	-1.017778767	2
YIPF3	0.827116131	0.284230714	-1.111346844	2
YIPF5	0.927640569	0.131672983	-1.059313552	2
YLPM1	1.05993344	-0.133222688	-0.926710752	2
YME1L1	1.033368111	-0.070463527	-0.962904584	2
YTHDF2	1.1175826	-0.307282156	-0.810300444	2
ZAR1	1.08968612	-0.214028733	-0.875657388	2
ZBED3	1.035088241	-0.074323802	-0.960764439	2
ZBTB14	1.058712993	-0.130175961	-0.928537031	2
ZBTB22	0.978199673	0.042260728	-1.020460401	2
ZBTB46	1.061685149	-0.137627006	-0.924058143	2
ZBTB8A	1.121289478	-0.321830106	-0.799459373	2
ZC3H12D	0.862976641	0.232931342	-1.095907983	2
ZC3H13	0.984796111	0.029744133	-1.014540244	2
ZC3H14	0.870627158	0.221577785	-1.092204943	2
ZC3H6	1.08496515	-0.200228332	-0.884736818	2
ZC3H7B	0.849954128	0.251915457	-1.101869585	2
ZCCHC17	1.044840611	-0.096707876	-0.948132735	2
ZDHHC24	1.063208336	-0.141487485	-0.921720851	2
ZFC3H1	1.053180045	-0.116579236	-0.936600809	2
ZFP90	1.098207482	-0.240145419	-0.858062063	2

ZFYVE28	0.993719998	0.012443863	-1.006163861	2
ZIC2	1.019926496	-0.041121637	-0.97880486	2
ZMYND12	0.980146602	0.038589616	-1.018736217	2
ZMYND15	1.099963099	-0.245743343	-0.854219755	2
ZNF124	0.926095163	0.134246996	-1.060342159	2
ZNF154	0.879571127	0.208107927	-1.087679054	2
ZNF197	1.023015121	-0.047740326	-0.975274794	2
ZNF213	1.055518964	-0.122284715	-0.933234249	2
ZNF219	1.121957205	-0.324527714	-0.797429491	2
ZNF22	0.984300852	0.030691686	-1.014992538	2
ZNF225	1.124923317	-0.336827778	-0.788095539	2
ZNF232	1.048150326	-0.104509113	-0.943641213	2
ZNF233	1.128414594	-0.352050802	-0.776363792	2
ZNF236	0.947772384	0.097336735	-1.045109118	2
ZNF24	0.956766163	0.081481975	-1.038248138	2
ZNF254	0.978904421	0.040934065	-1.019838486	2
ZNF283	1.110021137	-0.279529522	-0.830491615	2
ZNF304	0.918165627	0.147323743	-1.06548937	2
ZNF326	1.123218878	-0.329694554	-0.793524324	2
ZNF333	0.904621114	0.169175951	-1.073797065	2
ZNF34	1.087202173	-0.206713011	-0.880489163	2
ZNF341	0.809546553	0.308301603	-1.117848156	2
ZNF382	0.807661397	0.310845663	-1.11850706	2
ZNF384	1.1241873	-0.333725118	-0.790462182	2
ZNF385C	1.017791474	-0.036587167	-0.981204308	2
ZNF394	0.973313538	0.051391155	-1.024704692	2
ZNF398	0.987189097	0.025147454	-1.012336551	2
ZNF410	0.89050456	0.191341171	-1.081845731	2
ZNF419	1.015780103	-0.032345009	-0.983435093	2
ZNF430	0.964046282	0.068395858	-1.03244214	2
ZNF454	0.819288047	0.295036984	-1.114325031	2
ZNF461	1.13002089	-0.359366114	-0.770654776	2
ZNF497	1.019133515	-0.039433628	-0.979699887	2
ZNF506	0.86981875	0.222784707	-1.092603458	2
ZNF512B	1.070173576	-0.159525359	-0.910648218	2
ZNF517	1.120014931	-0.316748519	-0.803266413	2
ZNF526	1.098326662	-0.240522912	-0.85780375	2
ZNF532	1.025777639	-0.053720901	-0.972056737	2
ZNF534	1.066598048	-0.150184871	-0.916413176	2
ZNF549	0.951501557	0.090803359	-1.042304916	2
ZNF554	1.126884338	-0.345270752	-0.781613585	2
ZNF556	1.127308301	-0.347131643	-0.780176658	2
ZNF557	0.972289938	0.053289185	-1.025579124	2
ZNF578	0.87661649	0.212581643	-1.089198133	2
ZNF584	1.127966601	-0.350047539	-0.777919062	2
ZNF586	1.087987042	-0.209011213	-0.878975829	2
ZNF597	0.954588246	0.085352267	-1.039940513	2
ZNF599	1.119273364	-0.313831166	-0.805442198	2
ZNF653	0.872310708	0.21905875	-1.091369458	2
ZNF660	1.091846531	-0.220495294	-0.871351237	2
ZNF667	0.81846626	0.296163734	-1.114629994	2
ZNF695	0.89592959	0.182893848	-1.078823438	2
ZNF70	1.002003561	-0.004019239	-0.997984323	2
ZNF724	1.110804657	-0.282300222	-0.828504435	2
ZNF75A	1.029259696	-0.061343672	-0.967916025	2

ZNF774	0.976204244	0.046003632	-1.022207875	2
ZNF778	1.082124233	-0.192127512	-0.889996721	2
ZNF781	1.114484536	-0.295625824	-0.818858712	2
ZNF789	1.117709491	-0.307768863	-0.809940628	2
ZNF792	1.080101551	-0.186447212	-0.893654339	2
ZNF793	1.109653775	-0.27823807	-0.831415706	2
ZNF813	1.129874435	-0.358690227	-0.771184208	2
ZNF839	1.054066045	-0.118733403	-0.935332642	2
ZNF843	1.016006999	-0.032822129	-0.983184869	2
ZNF853	1.083983603	-0.197412839	-0.886570764	2
ZNF865	1.131182978	-0.364796066	-0.766386913	2
ZNF93	1.022180482	-0.045944783	-0.976235699	2
ZNHIT3	1.029571738	-0.06203149	-0.967540248	2
ZNRF1	1.129643261	-0.357627091	-0.77201617	2
ZSCAN22	0.808093981	0.310262528	-1.118356509	2
ZSCAN25	1.092064809	-0.221154212	-0.870910597	2
ZSCAN30	1.074851152	-0.172019978	-0.902831174	2
ZSCAN4	0.850962146	0.250460913	-1.10142306	2
ZSCAN5A	1.126275455	-0.342620836	-0.783654619	2
ZSCAN5B	1.126526372	-0.34370966	-0.782816711	2
ZSCAN5C	1.010812002	-0.021986594	-0.988825408	2
ZSCAN9	1.09049627	-0.216442093	-0.874054177	2
ZSWIM3	1.130942459	-0.363662245	-0.767280214	2
ZYG11A	1.070025759	-0.159135697	-0.910890062	2
ZZEF1	0.896135261	0.182571882	-1.078707143	2
A2ML1	1.151190504	-0.497683011	-0.653507493	3
AADACL4	1.151743277	-0.504348436	-0.647394841	3
AARS1	1.101683303	-0.85037533	-0.251307974	3
AASS	1.154525534	-0.594672334	-0.559853201	3
ABCA12	1.147430473	-0.685753098	-0.461677375	3
ABCA13	1.154487322	-0.596460003	-0.558027319	3
ABCA8	1.154274013	-0.604314709	-0.549959304	3
ABCB11	1.131657867	-0.764607339	-0.367050528	3
ABCB6	1.133638977	-0.376695838	-0.756943139	3
ABCC12	1.116938451	-0.812115381	-0.30482307	3
ABCC9	1.151780806	-0.504821912	-0.646958894	3
ABCG8	1.14830678	-0.469064597	-0.679242183	3
ABR	1.150508125	-0.49011717	-0.660390955	3
ACAA1	1.137419173	-0.74107031	-0.396348863	3
ACBD3	1.094441517	-0.866044101	-0.228397416	3
ACBD5	1.151542519	-0.501863409	-0.64967911	3
ACBD7	1.109661776	-0.831395631	-0.278266146	3
ACE2	1.146236509	-0.693975194	-0.452261315	3
ACER1	1.129169459	-0.7737073	-0.355462159	3
ACHE	1.131794749	-0.764090425	-0.367704323	3
ACOD1	1.153823579	-0.537945629	-0.61587795	3
ACOT7	1.098243038	-0.857985036	-0.240258003	3
ACOT9	1.13641515	-0.745465734	-0.390949417	3
ACP7	1.154664646	-0.569447758	-0.585216888	3
ACRBP	1.153956034	-0.61288211	-0.541073924	3
ACSL1	1.144410236	-0.70541138	-0.438998856	3
ACSM2B	1.138417703	-0.73655236	-0.401865344	3
ACSM5	1.131688819	-0.764490608	-0.367198212	3
ACTG2	1.105604488	-0.841296675	-0.264307813	3
ACTL7B	1.12698211	-0.781283369	-0.345698741	3

ACTL8	1.140357764	-0.727303444	-0.41305432	3
ACTR3B	1.150537814	-0.490433453	-0.660104361	3
ADA	1.144043063	-0.707572639	-0.436470424	3
ADA2	1.151356426	-0.499627015	-0.651729411	3
ADAD1	1.153394946	-0.529157242	-0.624237704	3
ADAD2	1.149950463	-0.665586759	-0.484363704	3
ADAM19	1.152956426	-0.52153634	-0.631420086	3
ADAM22	1.15435499	-0.552714918	-0.601640072	3
ADAMTS18	1.143160674	-0.430556385	-0.712604289	3
ADAMTS7	1.152616785	-0.51625916	-0.636357625	3
ADAMTSL1	1.138865504	-0.734475239	-0.404390265	3
ADAMTSL2	1.153002931	-0.630706459	-0.522296471	3
ADAMTSL5	1.153233714	-0.526228365	-0.62700535	3
ADARB1	1.152925305	-0.52103314	-0.631892165	3
ADCY5	1.14189053	-0.719486551	-0.422403979	3
ADGRA1	1.142532456	-0.716058112	-0.426474345	3
ADGRA2	1.110064246	-0.830382857	-0.279681389	3
ADGRD2	1.152455592	-0.513901386	-0.638554206	3
ADGRF3	1.146325733	-0.452942298	-0.693383435	3
ADGRL2	1.141758449	-0.421578413	-0.720180036	3
ADGRL3	1.154688961	-0.572866408	-0.581822553	3
ADORA1	1.145621437	-0.697965195	-0.447656242	3
ADPRH	1.148927517	-0.474592989	-0.674334528	3
ADRA2C	1.134375047	-0.38038565	-0.753989397	3
AFAP1L2	1.112010671	-0.825401329	-0.286609342	3
AFG1L	1.14472168	-0.441176972	-0.703544708	3
AGA	1.108270546	-0.834852742	-0.273417804	3
AGBL1	1.150437148	-0.661071802	-0.489365346	3
AGR2	1.133513943	-0.376075021	-0.757438922	3
AHDC1	1.103118454	-0.847104921	-0.256013532	3
AHSG	1.154700214	-0.576600966	-0.578099248	3
AJAP1	1.135161582	-0.38439745	-0.750764132	3
AK3	1.136097294	-0.389268882	-0.746828412	3
AK7	1.154012471	-0.611523085	-0.542489386	3
AKAP6	1.114703406	-0.818267854	-0.296435552	3
AKR1C1	1.150788917	-0.493153034	-0.657635883	3
AKR1C2	1.139249283	-0.732668512	-0.406580771	3
AKR1C8P	1.129548552	-0.772355708	-0.357192845	3
AKT1S1	1.142814751	-0.714518637	-0.428296114	3
AKT2	1.15108175	-0.65464905	-0.4964327	3
ALAS2	1.099812667	-0.85455215	-0.245260516	3
ALDH1L1	1.145246456	-0.444920779	-0.700325677	3
ALDH3B2	1.146413442	-0.453615062	-0.69279838	3
ALDH7A1	1.151450397	-0.500748623	-0.650701775	3
ALDOA	1.151987584	-0.64450259	-0.507484993	3
ALG8	1.139032406	-0.733692584	-0.405339822	3
ALKBH6	1.154309204	-0.603187188	-0.551122016	3
ALKBH8	1.152932622	-0.631781568	-0.521151054	3
ALOX12	1.134584033	-0.381444486	-0.753139547	3
ALOX12B	1.152957231	-0.631407815	-0.521549416	3
ALS2CL	1.13641092	-0.390926963	-0.745483957	3
AMBP	1.151453757	-0.650664742	-0.500789016	3
AMER2	1.151047787	-0.655001836	-0.496045952	3
AMH	1.154337206	-0.602252691	-0.552084515	3
AMOTL2	1.147173319	-0.459590904	-0.687582415	3

AMY1C	1.111067303	-0.827833277	-0.283234025	3
AMY2A	1.134091151	-0.755135837	-0.378955313	3
ANGEL2	1.106832622	-0.838356936	-0.268475686	3
ANK2	1.129012825	-0.774262296	-0.354750529	3
ANKFN1	1.150348864	-0.661910341	-0.488438523	3
ANKLE2	1.15138766	-0.65138955	-0.49999811	3
ANKRA2	1.153191771	-0.627699255	-0.525492516	3
ANKRD11	1.154634903	-0.587979585	-0.566655317	3
ANKRD18A	1.153583624	-0.532818904	-0.62076472	3
ANKRD2	1.151134352	-0.497035154	-0.654099198	3
ANKRD20A1	1.14822202	-0.679892124	-0.468329897	3
ANKRD20A3P	1.154396155	-0.554238586	-0.600157569	3
ANKRD20A4P	1.135151683	-0.750805184	-0.3843465	3
ANKRD23	1.154683725	-0.582738292	-0.571945433	3
ANKRD24	1.151330245	-0.499317234	-0.652013012	3
ANKRD30A	1.151206953	-0.653333223	-0.49787373	3
ANKRD34B	1.133313847	-0.37508502	-0.758228826	3
ANKRD36	1.147707474	-0.463964401	-0.683743073	3
ANKRD39	1.131804987	-0.764051689	-0.367753298	3
ANKRD45	1.103343457	-0.846586822	-0.256756634	3
ANKRD50	1.154696906	-0.574840011	-0.579856894	3
ANKRD61	1.149061372	-0.475821661	-0.673239712	3
ANKS1B	1.139307095	-0.406912932	-0.732394162	3
ANKS6	1.137776724	-0.73947	-0.398306724	3
ANO3	1.153408998	-0.623988345	-0.529420653	3
ANTXRL	1.154288562	-0.550434046	-0.603854516	3
ANXA13	1.154670582	-0.570132136	-0.584538446	3
AOC1	1.151518681	-0.50157346	-0.649945221	3
APBA2	1.150241231	-0.662920571	-0.487320661	3
APCDD1L	1.141993692	-0.718942124	-0.423051568	3
APLNR	1.152013901	-0.507830865	-0.644183036	3
APLP2	1.154389918	-0.600388462	-0.554001455	3
APOB	1.144342015	-0.705816116	-0.438525898	3
APOBEC1	1.105219513	-0.842208396	-0.263011117	3
APOBEC3D	1.154697544	-0.579626287	-0.575071256	3
APOC4	1.0937525	-0.867468049	-0.226284451	3
AQP1	1.136226225	-0.74627729	-0.389948935	3
AQP10	1.142486678	-0.426180789	-0.716305889	3
AQP2	1.143419599	-0.432269075	-0.711150524	3
AQP7	1.129925499	-0.770999822	-0.358925677	3
AQP9	1.142369217	-0.716939298	-0.425429919	3
AR	1.153850446	-0.538560374	-0.615290072	3
ARAP2	1.123245498	-0.793440919	-0.329804579	3
ARFGAP1	1.135602497	-0.386679114	-0.748923382	3
ARGFX	1.153737837	-0.617694796	-0.536043041	3
ARHGAP17	1.118983358	-0.806285486	-0.312697872	3
ARHGAP42	1.15094152	-0.494846858	-0.656094662	3
ARHGEF12	1.148236693	-0.679779941	-0.468456753	3
ARHGEF17	1.154560586	-0.592849184	-0.561711402	3
ARHGEF28	1.154038765	-0.543168277	-0.610870487	3
ARHGEF33	1.147203349	-0.459832801	-0.687370548	3
ARID2	1.149226214	-0.671872203	-0.477354011	3
ARID3C	1.153120231	-0.524260021	-0.62886021	3
ARID5B	1.138436436	-0.401970314	-0.736466122	3
ARIH1	1.143490527	-0.710749077	-0.43274145	3

ARL13B	1.153972425	-0.612493	-0.541479425	3
ARL16	1.134261442	-0.754449262	-0.37981218	3
ARL5A	1.146521888	-0.692070327	-0.454451562	3
ARL6	1.111341368	-0.827130208	-0.284211159	3
ARMC3	1.153201046	-0.62754668	-0.525654366	3
ARMCX4	1.110544588	-0.829166505	-0.281378083	3
ARMCX5-GPRASP2	1.095959694	-0.862867544	-0.23309215	3
ARNTL	1.146352561	-0.45314772	-0.69320484	3
ARPIN-AP3S2	1.14348459	-0.432701851	-0.710782739	3
ARPP21	1.099690724	-0.854821154	-0.24486957	3
ARR3	1.154665191	-0.585157078	-0.569508113	3
ARRB1	1.149249621	-0.477573378	-0.671676244	3
ARSL	1.152764935	-0.634259523	-0.518505412	3
ARVCF	1.122882701	-0.794573995	-0.328308705	3
ASAP3	1.133528851	-0.757379898	-0.376148953	3
ASB13	1.102975334	-0.84743371	-0.255541624	3
ASB3	1.10741105	-0.836955546	-0.270455505	3
ASF1A	1.146801886	-0.456635899	-0.690165987	3
ASH1L	1.145556046	-0.447175435	-0.69838061	3
ASIC1	1.153167329	-0.628099034	-0.525068295	3
ASPA	1.137490579	-0.396738352	-0.740752227	3
ASS1	1.154698228	-0.57534865	-0.579349578	3
ASTN2	1.153943957	-0.613166013	-0.540777944	3
ATAD3A	1.10479482	-0.843208868	-0.261585952	3
ATAD3C	1.152960766	-0.521606857	-0.631353909	3
ATCAY	1.13458502	-0.753135518	-0.381449502	3
ATG16L1	1.133970198	-0.755621501	-0.378348697	3
ATG9B	1.154256789	-0.604849312	-0.549407477	3
ATL1	1.135233089	-0.750467235	-0.384765855	3
ATN1	1.101659401	-0.850429305	-0.251230096	3
ATP10A	1.150744205	-0.658081416	-0.492662789	3
ATP10B	1.148295994	-0.468970845	-0.679325148	3
ATP12A	1.119000972	-0.806234389	-0.312766583	3
ATP1B2	1.153399001	-0.529233105	-0.624165896	3
ATP2C2	1.154684651	-0.582587973	-0.572096679	3
ATP5F1A	1.13621421	-0.38988547	-0.74632874	3
ATP5PD	1.14922652	-0.477356875	-0.671869645	3
ATP6V0D1	1.142370006	-0.71693505	-0.425434956	3
ATP6V1G2-DDX39B	1.144304276	-0.438264899	-0.706039377	3
ATP8A2	1.146277366	-0.693704643	-0.452572723	3
ATP9B	1.101897977	-0.849889845	-0.252008133	3
ATXN10	1.145122721	-0.444029395	-0.701093325	3
ATXN3	1.142914168	-0.713971655	-0.428942513	3
ATXN7	1.147583297	-0.462933937	-0.68464936	3
AVIL	1.154329726	-0.55182393	-0.602505796	3
AVPR1B	1.138467116	-0.402142357	-0.736324759	3
AWAT1	1.153458313	-0.623101998	-0.530356315	3
AXDND1	1.129515358	-0.772474534	-0.357040824	3
AXL	1.146901152	-0.689482085	-0.457419067	3
B3GALT9	1.14793892	-0.682030449	-0.46590847	3
B4GALNT2	1.140657451	-0.725813242	-0.414844208	3
B4GALT4	1.154699882	-0.578416393	-0.576283489	3
B4GALT5	1.133359019	-0.375308141	-0.758050878	3
BAG4	1.132060903	-0.76308015	-0.368980753	3
BAHCC1	1.148234409	-0.679797412	-0.468436997	3

BAIAP2	1.154645162	-0.567529089	-0.587116073	3
BANF2	1.154146767	-0.608039983	-0.546106784	3
BANK1	1.153950116	-0.613021526	-0.54092859	3
BARHL2	1.100148067	-0.853810224	-0.246337843	3
BARX2	1.144664469	-0.703889981	-0.440774488	3
BASP1	1.154080054	-0.54426165	-0.609818403	3
BBLN	1.153120328	-0.524261669	-0.628858658	3
BBOX1	1.150798404	-0.657541003	-0.493257401	3
BBS1	1.1377009	-0.397889938	-0.739810962	3
BBS5	1.153178931	-0.525269243	-0.627909688	3
BCAT2	1.146122024	-0.451392485	-0.694729538	3
BCCIP	1.149791805	-0.482786711	-0.667005095	3
BCL11B	1.133783734	-0.756367018	-0.377416716	3
BCL2	1.106569893	-0.838989871	-0.267580022	3
BCL6B	1.154502754	-0.595759297	-0.558743457	3
BCL7A	1.1052908	-0.842039916	-0.263250884	3
BCOR	1.137430167	-0.396408782	-0.741021385	3
BCS1L	1.149388717	-0.670502408	-0.478886309	3
BDH2	1.116231016	-0.814086676	-0.30214434	3
BDKRB1	1.147987125	-0.466317335	-0.68166979	3
BDNF	1.141805752	-0.421873621	-0.719932131	3
BECN2	1.148879997	-0.474160069	-0.674719928	3
BEND2	1.150955062	-0.65595631	-0.494998752	3
BEND6	1.130902821	-0.767426923	-0.363475897	3
BET1L	1.147069578	-0.458758759	-0.688310819	3
BFAR	1.152928174	-0.631848821	-0.521079353	3
BHLHA15	1.154267898	-0.604505744	-0.549762154	3
BHLHA9	1.136036928	-0.747085697	-0.388951231	3
BHLHE23	1.151412621	-0.65111673	-0.500295891	3
BICDL2	1.15323158	-0.526190668	-0.627040911	3
BIK	1.154549151	-0.593466963	-0.561082188	3
BLK	1.146769122	-0.690390695	-0.456378427	3
BLNK	1.142720564	-0.427686049	-0.715034514	3
BLZF1	1.144709949	-0.441094355	-0.703615594	3
BMF	1.154345216	-0.601978686	-0.55236653	3
BMP2K	1.13414815	-0.754906396	-0.379241754	3
BMP8A	1.114224011	-0.81955941	-0.2946646	3
BMPER	1.128906268	-0.774638714	-0.354267554	3
BNIPL	1.151149706	-0.653937889	-0.497211817	3
BOLL	1.137341153	-0.741417	-0.395924153	3
BORA	1.136757795	-0.392776258	-0.743981537	3
BORCS5	1.104037865	-0.844978477	-0.259059387	3
BPGM	1.130573422	-0.768640598	-0.361932824	3
BPHL	1.137583257	-0.740338269	-0.397244988	3
BPIFB1	1.132928266	-0.759738959	-0.373189307	3
BRAF	1.14508931	-0.701299669	-0.443789642	3
BRD2	1.154637723	-0.58774939	-0.566888334	3
BRD3	1.148134672	-0.680557152	-0.46757752	3
BRD3OS	1.149320567	-0.67107955	-0.478241017	3
BRDT	1.150465393	-0.489663805	-0.660801588	3
BRINP2	1.146964495	-0.689043208	-0.457921287	3
BTBD16	1.141553377	-0.721248995	-0.420304383	3
BTBD8	1.143241348	-0.712153286	-0.431088062	3
BTNL2	1.154603396	-0.564330653	-0.590272744	3
BTNL8	1.125114732	-0.787474307	-0.337640425	3

BUB1B	1.139624469	-0.730877599	-0.40874687	3
BVES	1.112476756	-0.824187254	-0.288289502	3
C10orf143	1.11930892	-0.805338518	-0.313970402	3
C10orf53	1.154501075	-0.595836842	-0.558664233	3
C10orf62	1.147328068	-0.460842368	-0.6864857	3
C10orf82	1.152776276	-0.634095534	-0.518680742	3
C11orf52	1.13952988	-0.731331456	-0.408198425	3
C11orf94	1.153122657	-0.628821283	-0.524301374	3
C12orf50	1.154681466	-0.583088274	-0.571593192	3
C14orf180	1.154689309	-0.581754771	-0.572934538	3
C16orf74	1.142600826	-0.715687098	-0.426913728	3
C16orf96	1.134718983	-0.382130924	-0.752588059	3
C17orf98	1.139200454	-0.732899781	-0.406300673	3
C18orf25	1.126490133	-0.782938	-0.343552133	3
C19orf12	1.127852213	-0.778313698	-0.349538515	3
C19orf67	1.149590042	-0.480816024	-0.668774018	3
C19orf84	1.140949993	-0.724341285	-0.416608708	3
C1orf100	1.152758161	-0.518400924	-0.634357237	3
C1orf109	1.128413775	-0.776366649	-0.352047127	3
C1orf232	1.153214055	-0.627331851	-0.525882204	3
C1orf68	1.154693091	-0.573755027	-0.580938064	3
C1QL4	1.153021609	-0.63041699	-0.522604619	3
C1QTNF4	1.15057966	-0.659698563	-0.490881097	3
C1QTNF6	1.138623619	-0.735601287	-0.403022332	3
C20orf202	1.153006987	-0.63064374	-0.522363247	3
C21orf58	1.147250518	-0.460213675	-0.687036842	3
C21orf62	1.154654239	-0.568372176	-0.586282063	3
C2CD4A	1.141169318	-0.723226201	-0.417943117	3
C2CD4D	1.136371811	-0.390719485	-0.745652326	3
C2CD5	1.148436921	-0.470201736	-0.678235185	3
C2orf15	1.153413036	-0.623916431	-0.529496605	3
C2orf50	1.127084994	-0.780935144	-0.34614985	3
C2orf69	1.151428275	-0.500483189	-0.650945086	3
C2orf72	1.137468413	-0.396617366	-0.740851047	3
C2orf88	1.152480317	-0.514257588	-0.638222729	3
C4orf17	1.120743174	-0.801101838	-0.319641335	3
C4orf50	1.152878642	-0.5202866	-0.632592042	3
C5orf63	1.146064007	-0.695109721	-0.450954286	3
C8orf34	1.154483959	-0.557874704	-0.596609255	3
C8orf89	1.137065933	-0.394433002	-0.742632931	3
C9orf116	1.154647646	-0.567752484	-0.586895162	3
CA4	1.150122453	-0.664020528	-0.486101925	3
CA5A	1.147505432	-0.685213304	-0.462292127	3
CA6	1.122345515	-0.796237447	-0.326108069	3
CABP2	1.154202308	-0.606474181	-0.547728126	3
CABP4	1.134679101	-0.752751263	-0.381927838	3
CACNA1C	1.150455963	-0.660891907	-0.489564056	3
CACNA1D	1.153995935	-0.542068953	-0.611926982	3
CACNA1E	1.152262785	-0.511186368	-0.641076416	3
CACNA1S	1.138575427	-0.402750936	-0.735824491	3
CACNA2D1	1.112339717	-0.824545092	-0.287794624	3
CACNG3	1.116734525	-0.812685962	-0.304048562	3
CACNG7	1.13493529	-0.751699606	-0.383235684	3
CADM2	1.154360834	-0.552925543	-0.601435291	3
CALD1	1.154697141	-0.579774203	-0.574922939	3

CALHM2	1.140330015	-0.412889474	-0.72744054	3
CAMK2G	1.1540743	-0.609967121	-0.544107179	3
CAMKV	1.144078691	-0.436713956	-0.707364735	3
CANX	1.10071151	-0.85255706	-0.248154451	3
CAPN15	1.143102375	-0.712929122	-0.430173252	3
CAPN3	1.145319132	-0.69987224	-0.445446892	3
CAPN6	1.117879912	-0.809456177	-0.308423735	3
CAPNS1	1.112805484	-0.823325888	-0.289479596	3
CAPRIN2	1.107793157	-0.836023751	-0.271769406	3
CAPS2	1.102322496	-0.848925981	-0.253396514	3
CASKIN1	1.154533867	-0.594257002	-0.560276865	3
CAVIN1	1.121487423	-0.798860216	-0.322627208	3
CBARP	1.138555928	-0.735914694	-0.402641234	3
CC2D1A	1.150824183	-0.493541614	-0.657282569	3
CCDC102B	1.108319071	-0.834733283	-0.273585788	3
CCDC122	1.131047309	-0.766891447	-0.364155862	3
CCDC141	1.145131464	-0.701039259	-0.444092206	3
CCDC150	1.149225315	-0.671879721	-0.477345594	3
CCDC152	1.107916322	-0.835722366	-0.272193956	3
CCDC158	1.153975198	-0.541548463	-0.612426735	3
CCDC159	1.154416929	-0.599370734	-0.555046195	3
CCDC179	1.152441731	-0.638739209	-0.513702522	3
CCDC18	1.117952883	-0.809248321	-0.308704562	3
CCDC24	1.13593469	-0.747520357	-0.388414333	3
CCDC28A	1.149051877	-0.475734044	-0.673317833	3
CCDC3	1.154698136	-0.579389139	-0.575308997	3
CCDC30	1.154580517	-0.591708008	-0.56287251	3
CCDC42	1.151516557	-0.649968883	-0.501547675	3
CCDC62	1.141131703	-0.723418155	-0.417713549	3
CCDC7	1.10627239	-0.839703899	-0.266568491	3
CCDC82	1.133068344	-0.759192147	-0.373876197	3
CCDC86	1.154194603	-0.547498069	-0.606696534	3
CCDC87	1.110161625	-0.830136943	-0.280024682	3
CCDC89	1.14947353	-0.479694921	-0.66977861	3
CCDC91	1.153157716	-0.524902351	-0.628255365	3
CCDC92	1.129846014	-0.771286736	-0.358559278	3
CCER2	1.142769483	-0.428002623	-0.71476686	3
CCHCR1	1.100240332	-0.8536056	-0.246634732	3
CCL16	1.149123702	-0.672725176	-0.476398525	3
CCL20	1.099034161	-0.856262796	-0.242771365	3
CCL25	1.142942928	-0.713812945	-0.429129984	3
CCL3	1.136885628	-0.743423677	-0.393461951	3
CCL8	1.147441837	-0.461770377	-0.68567146	3
CCM2	1.153021609	-0.630416994	-0.522604614	3
CCN5	1.150988996	-0.495380528	-0.655608468	3
CCNB1IP1	1.095546308	-0.863737891	-0.231808418	3
CCNF	1.137741163	-0.739630014	-0.39811115	3
CCNJ	1.127747687	-0.77867344	-0.349074247	3
CCNYL1	1.103903807	-0.845290085	-0.258613723	3
CD164L2	1.15409476	-0.609435097	-0.544659662	3
CD300LF	1.154673366	-0.584196992	-0.570476374	3
CD3G	1.149378088	-0.4787854	-0.670592687	3
CD4	1.149121996	-0.672739303	-0.476382693	3
CD55	1.135561287	-0.749096453	-0.386464834	3
CD83	1.144236511	-0.706439156	-0.437797355	3

CDC14B	1.15184458	-0.646211319	-0.505633261	3
CDC34	1.14240897	-0.716725306	-0.425683663	3
CDC42EP5	1.147176612	-0.459617408	-0.687559204	3
CDC5L	1.145269165	-0.445084969	-0.700184197	3
CDC6	1.151272348	-0.498636266	-0.652636082	3
CDC73	1.153426988	-0.529759936	-0.623667053	3
CDH11	1.134100211	-0.75509939	-0.379000821	3
CDH15	1.097533618	-0.859515852	-0.238017767	3
CDH16	1.14856987	-0.677194798	-0.471375072	3
CDH17	1.151505603	-0.650090778	-0.501414825	3
CDH26	1.15355081	-0.532161551	-0.62138926	3
CDH3	1.147337781	-0.686416452	-0.460921329	3
CDH7	1.149994653	-0.665187257	-0.484807396	3
CDHR1	1.136825989	-0.393141767	-0.743684222	3
CDIN1	1.144034096	-0.436409191	-0.707624905	3
CDIP1	1.102625393	-0.848235131	-0.254390262	3
CDK2AP1	1.125638638	-0.785761559	-0.33987708	3
CDK5R2	1.150380768	-0.488772404	-0.661608364	3
CDON	1.154685776	-0.582399492	-0.572286284	3
CDRT1	1.153894103	-0.614314139	-0.539579964	3
CDY2A	1.150451151	-0.660937957	-0.489513193	3
CDY2B	1.15292897	-0.631836787	-0.521092183	3
CDYL	1.150397791	-0.661446743	-0.488951048	3
CDYL2	1.154413734	-0.599493594	-0.55492014	3
CEBPE	1.150663338	-0.658880533	-0.491782805	3
CEL	1.135195403	-0.384571615	-0.750623788	3
CELA2A	1.132626872	-0.760908597	-0.371718275	3
CELA3A	1.144022011	-0.707695303	-0.436326708	3
CELA3B	1.152006016	-0.644278956	-0.50772706	3
CELF5	1.141985794	-0.718983893	-0.423001901	3
CEMP1	1.141777547	-0.421697539	-0.720080008	3
CENPB	1.154027854	-0.611142858	-0.542884996	3
CENPE	1.106737152	-0.83858719	-0.268149962	3
CENPI	1.135450654	-0.385890626	-0.749560028	3
CEP19	1.143633427	-0.433697421	-0.709936006	3
CEP55	1.12532338	-0.786794393	-0.338528987	3
CEP57L1	1.130067263	-0.770486753	-0.35958051	3
CEP72	1.112195035	-0.824922095	-0.28727294	3
CEP89	1.153359102	-0.624867587	-0.528491515	3
CERS6	1.154466826	-0.557114726	-0.597352099	3
CES3	1.110835069	-0.828426852	-0.282408216	3
CES4A	1.154611442	-0.564883395	-0.589728047	3
CFAP100	1.150485107	-0.489872687	-0.66061242	3
CFAP20DC	1.135886276	-0.38816056	-0.747725717	3
CFAP221	1.145045062	-0.701572345	-0.443472717	3
CFAP410	1.110515063	-0.829241512	-0.281273551	3
CFAP43	1.141268744	-0.722717376	-0.418551368	3
CFAP47	1.141407451	-0.722004	-0.419403451	3
CFAP95	1.154610977	-0.564850784	-0.589760193	3
CFC1	1.149787842	-0.482747632	-0.66704021	3
CFC1B	1.095439967	-0.863961123	-0.231478844	3
CFLAR	1.154693647	-0.573891943	-0.580801704	3
CGA	1.147466654	-0.461973724	-0.68549293	3
CGB3	1.152499845	-0.514540274	-0.637959571	3
CHD8	1.152001105	-0.507662497	-0.644338608	3

CHD9	1.149912974	-0.48398883	-0.665924143	3
CHERP	1.15082814	-0.65724282	-0.49358532	3
CHGA	1.136315972	-0.745892363	-0.390423609	3
CHGB	1.100544381	-0.852929668	-0.247614713	3
CHI3L1	1.154559628	-0.592901888	-0.56165774	3
CHKB	1.108821277	-0.833492226	-0.275329051	3
CHM	1.145393776	-0.445989256	-0.699404519	3
CHMP3	1.139158759	-0.406061824	-0.733096935	3
CHN1	1.151880615	-0.506095573	-0.645785042	3
CHP2	1.154358537	-0.601516008	-0.552842529	3
CHPT1	1.133566547	-0.757230544	-0.376336003	3
CHRD	1.152549079	-0.515258434	-0.637290645	3
CHRDL2	1.153660598	-0.619261636	-0.534398962	3
CHRM5	1.154642202	-0.567269278	-0.587372924	3
CHRNA2	1.152646335	-0.516700853	-0.635945482	3
CHRNA3	1.134477189	-0.380902526	-0.753574663	3
CHRNA7	1.152313269	-0.640426354	-0.511886916	3
CHST10	1.15123843	-0.652998531	-0.498239898	3
CHST3	1.137864256	-0.739075307	-0.398788949	3
CHST6	1.154697821	-0.579518363	-0.575179458	3
CIBAR2	1.152855682	-0.632932992	-0.51992269	3
CIDEA	1.154635419	-0.587937793	-0.566697627	3
CLASP1	1.152053425	-0.643700047	-0.508353378	3
CLCA2	1.138882615	-0.734395216	-0.404487399	3
CLCN3	1.145351325	-0.445680555	-0.69967077	3
CLDN12	1.137866191	-0.398799624	-0.739066568	3
CLDN15	1.147756894	-0.46437692	-0.683379974	3
CLDN16	1.122372157	-0.796155345	-0.326216811	3
CLDN19	1.153321142	-0.527795878	-0.625525264	3
CLDN22	1.153949745	-0.540919514	-0.613030231	3
CLDN23	1.147676974	-0.463710508	-0.683966467	3
CLEC14A	1.135659289	-0.748684517	-0.386974772	3
CLIC5	1.138609728	-0.735665663	-0.402944065	3
CLPTM1L	1.154468302	-0.557179107	-0.597289195	3
CLPX	1.154474207	-0.597035544	-0.557438663	3
CLU	1.147710216	-0.68372296	-0.463987256	3
CLUH	1.152648654	-0.635913007	-0.516735647	3
CLVS2	1.154545618	-0.59365303	-0.560892588	3
CMA1	1.105963777	-0.840441612	-0.265522166	3
CMC4	1.137616776	-0.740188238	-0.397428538	3
CMKLR2	1.15289264	-0.520509565	-0.632383075	3
CNDP1	1.143750706	-0.434486338	-0.709264369	3
CNGA3	1.145251239	-0.444955345	-0.700295895	3
CNGA4	1.154698383	-0.57928132	-0.575417063	3
CNIH4	1.10342211	-0.846405364	-0.257016745	3
CNKSR2	1.147374912	-0.686151264	-0.461223649	3
CNNM1	1.152710053	-0.517663883	-0.63504617	3
CNOT3	1.154484296	-0.596594345	-0.557889951	3
CNST	1.147154218	-0.45943728	-0.687716937	3
CNTN1	1.149164634	-0.672385607	-0.476779026	3
CNTN4	1.15296105	-0.631349577	-0.521611473	3
CNTNAP2	1.148308342	-0.469078178	-0.679230164	3
CNTNAP3B	1.14982844	-0.483148651	-0.666679788	3
COA7	1.103706434	-0.845747893	-0.257958541	3
COBL	1.148695856	-0.472498175	-0.676197681	3

COBLL1	1.151526836	-0.649854301	-0.501672536	3
COG6	1.15419272	-0.547442131	-0.606750589	3
COL12A1	1.141819856	-0.719858117	-0.421961739	3
COL19A1	1.108586769	-0.83407282	-0.274513949	3
COL23A1	1.151249538	-0.652880037	-0.498369501	3
COL24A1	1.097149272	-0.860339943	-0.236809329	3
COL28A1	1.137178223	-0.742138146	-0.395040076	3
COL9A1	1.127280528	-0.780271192	-0.347009335	3
COLGALT1	1.148214026	-0.468260834	-0.679953192	3
COPS3	1.14000736	-0.729023849	-0.410983511	3
CORO1A	1.122537708	-0.795644239	-0.326893469	3
CORO6	1.154578636	-0.591819608	-0.562759029	3
COX19	1.104932134	-0.842885994	-0.262046141	3
COX8C	1.153467201	-0.622940333	-0.530526868	3
CPA1	1.132420685	-0.761703423	-0.370717261	3
CPD	1.1001846	-0.853729229	-0.24645537	3
CPEB1	1.143678943	-0.709675813	-0.434003129	3
CPLANE1	1.134376574	-0.753983205	-0.380393369	3
CPLX2	1.141571944	-0.721152598	-0.420419347	3
CPNE5	1.152477801	-0.638256554	-0.514221247	3
CPNE7	1.124471069	-0.789553838	-0.334917231	3
CPSF4	1.13180938	-0.764035064	-0.367774316	3
CPT1A	1.13891792	-0.404687974	-0.734229946	3
CPXCR1	1.123381613	-0.79301377	-0.330367843	3
CPXM1	1.140660808	-0.414864359	-0.725796449	3
CPXM2	1.134482497	-0.753553079	-0.380929417	3
CRACD	1.132840743	-0.76007958	-0.372761163	3
CREB3L2	1.152679879	-0.517206009	-0.635473871	3
CREG2	1.153290373	-0.626051526	-0.527238847	3
CRELD1	1.147125121	-0.687921502	-0.459203618	3
CRHBP	1.149912502	-0.665928383	-0.483984118	3
CRLF2	1.125875646	-0.784980661	-0.340894985	3
CRTAC1	1.151651895	-0.648444361	-0.503207535	3
CRX	1.151861083	-0.646016441	-0.505844642	3
CRY1	1.151734608	-0.504239479	-0.647495128	3
CRYBA2	1.154699537	-0.576032877	-0.57866666	3
CRYGD	1.153158706	-0.524919411	-0.628239295	3
CRYGS	1.154422954	-0.599137096	-0.555285858	3
CSF2RA	1.128906627	-0.774637449	-0.354269178	3
CSGALNACT1	1.132962783	-0.759604408	-0.373358375	3
CSN2	1.146464982	-0.692453019	-0.454011963	3
CSNK1A1	1.127370077	-0.779966179	-0.347403899	3
CSTL1	1.114511483	-0.818786076	-0.295725407	3
CTAG1A	1.104107852	-0.844815585	-0.259292267	3
CTDSP2	1.153805868	-0.616260457	-0.537545411	3
CTLA4	1.147960877	-0.466094533	-0.681866344	3
CTNNBIP1	1.14062195	-0.725990694	-0.414631257	3
CTNND2	1.154591005	-0.56352205	-0.591068955	3
CTRB1	1.154061211	-0.61030283	-0.543758381	3
CTSC	1.142560374	-0.715906756	-0.426653618	3
CTSF	1.140890271	-0.416247076	-0.724643195	3
CTSZ	1.123201031	-0.793580221	-0.32962081	3
CTTN	1.108961712	-0.833143629	-0.275818082	3
CUL2	1.14076051	-0.415463847	-0.725296663	3
CUL9	1.135917811	-0.74759199	-0.388325821	3

CWF19L1	1.144644035	-0.440630995	-0.70401304	3
CX3CL1	1.154655715	-0.586138908	-0.568516807	3
CXCL1	1.154319099	-0.551458116	-0.602860982	3
CXCL11	1.147062514	-0.688360222	-0.458702293	3
CXCL12	1.142595555	-0.426879816	-0.715715739	3
CXCL17	1.099541335	-0.855150169	-0.244391166	3
CXCL2	1.154480541	-0.596759748	-0.557720793	3
CXCL8	1.105252063	-0.842131485	-0.263120579	3
CXorf51A	1.142149868	-0.42403664	-0.718113227	3
CXorf51B	1.15209422	-0.643197545	-0.508896675	3
CXorf65	1.145390947	-0.445968664	-0.699422282	3
CYBC1	1.151667036	-0.648271617	-0.503395419	3
CYFIP2	1.150521364	-0.490258072	-0.660263292	3
CYP1A2	1.131075795	-0.766785653	-0.364290143	3
CYP2B6	1.150835299	-0.657170846	-0.493664453	3
CYP3A4	1.105754688	-0.840939712	-0.264814975	3
CYP3A43	1.125493564	-0.786237674	-0.33925589	3
CYP4A22	1.149380459	-0.478807908	-0.670572551	3
CYP7B1	1.148649672	-0.6765645	-0.472085172	3
CYRIA	1.152485035	-0.514325772	-0.638159263	3
CYYR1	1.153044008	-0.630067662	-0.522976346	3
DAB2IP	1.148038225	-0.681285958	-0.466752267	3
DACT2	1.143394075	-0.711294646	-0.432099429	3
DACT3	1.133572819	-0.757205679	-0.37636714	3
DAPK3	1.148230778	-0.679825181	-0.468405597	3
DAZ1	1.154699846	-0.578445395	-0.576254451	3
DAZ2	1.154678835	-0.571208307	-0.583470529	3
DAZ3	1.154643391	-0.567372819	-0.587270571	3
DAZ4	1.154605416	-0.564467225	-0.590138192	3
DBT	1.097183242	-0.860267253	-0.23691599	3
DCAF4L1	1.147279049	-0.686834434	-0.460444616	3
DCAF8L2	1.146710973	-0.690788267	-0.455922706	3
DCC	1.154608446	-0.589933628	-0.564674819	3
DCHS2	1.154664979	-0.585180385	-0.569484594	3
DCLK1	1.129700727	-0.771809781	-0.357890947	3
DCLK2	1.09470481	-0.865497081	-0.229207729	3
DCN	1.154516997	-0.559429386	-0.595087612	3
DCTN2	1.100122768	-0.853866291	-0.246256478	3
DCTN5	1.127815084	-0.778441576	-0.349373508	3
DCXR	1.112407206	-0.824368955	-0.288038251	3
DDA1	1.151706661	-0.647817385	-0.503889276	3
DDB2	1.115082262	-0.817240308	-0.297841954	3
DDC	1.154408118	-0.554700238	-0.599707881	3
DDX18	1.121333715	-0.79932566	-0.322008055	3
DDX21	1.153230082	-0.627065846	-0.526164236	3
DDX25	1.149089035	-0.476077299	-0.673011735	3
DEFA1B	1.141741627	-0.720268076	-0.421473551	3
DEFA3	1.154666277	-0.585036504	-0.569629773	3
DEFA6	1.137012676	-0.742866973	-0.394145702	3
DEFB1	1.133695069	-0.376974898	-0.75672017	3
DEFB107A	1.112917494	-0.823031419	-0.289886075	3
DEFB124	1.150527021	-0.660208673	-0.490318348	3
DGCR6	1.109672367	-0.831369053	-0.278303315	3
DGCR8	1.15447371	-0.597057025	-0.557416685	3
DGKB	1.154685811	-0.582393441	-0.572292371	3

DGKD	1.103209847	-0.846894655	-0.256315191	3
DGKI	1.116472141	-0.813417317	-0.303054824	3
DHH	1.119412944	-0.805034815	-0.314378129	3
DHRS7B	1.146846573	-0.689858689	-0.456987884	3
DIO2	1.11679916	-0.812505319	-0.304293841	3
DIP2C	1.153088833	-0.523727689	-0.629361144	3
DIPK2B	1.141840297	-0.719750765	-0.422089532	3
DIS3	1.152755166	-0.51835478	-0.634400386	3
DIS3L	1.144225863	-0.437724017	-0.706501847	3
DISP2	1.142223031	-0.424500091	-0.71772294	3
DIXDC1	1.102478773	-0.84856987	-0.253908903	3
DLX3	1.153977627	-0.612368563	-0.541609064	3
DMAC2	1.142357849	-0.425357428	-0.717000421	3
DMAP1	1.134914794	-0.751784031	-0.383130763	3
DMBT1	1.154149266	-0.546177955	-0.60797131	3
DMD	1.153953897	-0.541021375	-0.612932521	3
DMRT1	1.154593266	-0.563666027	-0.590927238	3
DMRT2	1.143599605	-0.710128966	-0.433470639	3
DMWD	1.140986056	-0.416827433	-0.724158623	3
DNAAF1	1.132280728	-0.762240505	-0.370040223	3
DNAAF3	1.149799057	-0.482858256	-0.666940801	3
DNAAF8	1.11359081	-0.821250753	-0.292340056	3
DNAH1	1.140455421	-0.726819773	-0.413635649	3
DNAH10	1.154699629	-0.576095083	-0.578604546	3
DNAH11	1.154559898	-0.592887033	-0.561672865	3
DNAH5	1.15095899	-0.655916134	-0.495042856	3
DNAH7	1.152524308	-0.63762819	-0.514896118	3
DNAH9	1.14809312	-0.68087184	-0.46722128	3
DNAI3	1.108419831	-0.834484975	-0.273934856	3
DNAJC27	1.126446718	-0.783083187	-0.34336353	3
DNASE1L3	1.147818284	-0.682926985	-0.464891299	3
DNHD1	1.146915636	-0.689381898	-0.457533738	3
DNMT1	1.10012185	-0.853868325	-0.246253526	3
DOCK1	1.154612402	-0.56495104	-0.589661362	3
DOCK5	1.144833375	-0.702867477	-0.441965898	3
DPH1	1.144173667	-0.437365027	-0.70680864	3
DPH2	1.127259376	-0.780343149	-0.346916227	3
DPH6	1.145293403	-0.700032989	-0.445260414	3
DPP6	1.121206931	-0.799708596	-0.321498335	3
DPPA2	1.153520836	-0.531569041	-0.621951796	3
DPPA3	1.134872978	-0.751956115	-0.382916864	3
DPPA5	1.135103228	-0.384097273	-0.751005956	3
DPY19L2	1.124734042	-0.788707475	-0.336026567	3
DPY19L4	1.154556931	-0.561507628	-0.593049304	3
DPYSL3	1.111231902	-0.827411361	-0.283820541	3
DRC3	1.117411251	-0.810785122	-0.306626129	3
DRD1	1.145861606	-0.696425274	-0.449436332	3
DRD2	1.122572117	-0.795537808	-0.327034309	3
DRGX	1.135306433	-0.385144376	-0.750162057	3
DSC3	1.152926361	-0.631876218	-0.521050143	3
DSCAM	1.152085839	-0.643301119	-0.50878472	3
DSG2	1.151479858	-0.650376396	-0.501103462	3
DSG3	1.149222626	-0.671902199	-0.477320427	3
DTNA	1.147381817	-0.686101871	-0.461279946	3
DTNB	1.121739316	-0.798094614	-0.323644702	3

DTWD1	1.13775151	-0.739583478	-0.398168032	3
DUSP13	1.154295693	-0.60362594	-0.550669754	3
DUSP29	1.098781377	-0.856814849	-0.241966527	3
DXO	1.153468571	-0.622915362	-0.530553209	3
DYNC2I1	1.104651719	-0.843544737	-0.261106982	3
DYNC2I2	1.109061559	-0.832895368	-0.276166191	3
DZIP1	1.15282679	-0.633358931	-0.519467859	3
EARS2	1.146100899	-0.45123277	-0.694868129	3
EBF2	1.140244538	-0.727861911	-0.412382627	3
EBLN1	1.142281061	-0.424868581	-0.71741248	3
EBNA1BP2	1.151969233	-0.507244785	-0.644724448	3
ECEL1	1.131543908	-0.765036323	-0.366507585	3
ECM1	1.151661202	-0.503322963	-0.648338238	3
ECSCR	1.150036245	-0.485226826	-0.664809419	3
EDN3	1.113481086	-0.821542172	-0.291938915	3
EFCAB1	1.150018679	-0.485049465	-0.664969214	3
EFHC1	1.147856179	-0.4652099	-0.682646278	3
EFHD1	1.150644012	-0.659070251	-0.491573761	3
EFHD2	1.122929013	-0.79442979	-0.328499223	3
EFNB3	1.15462072	-0.589068129	-0.56555259	3
EGF	1.134211382	-0.379559949	-0.754651433	3
EGFL6	1.14053456	-0.726426457	-0.414108103	3
EGFL8	1.142093046	-0.718415469	-0.423677576	3
EGR1	1.141869734	-0.719596003	-0.422273731	3
EGR2	1.117051952	-0.811796982	-0.305254971	3
EHF	1.151734214	-0.504234535	-0.647499679	3
EIF1AX	1.14989014	-0.666128946	-0.483761194	3
EIF1AY	1.151398462	-0.500126841	-0.651271621	3
EIF2AK2	1.101716843	-0.850299564	-0.251417279	3
EIF2S3	1.154561588	-0.592793837	-0.561767751	3
EIF4ENIF1	1.142307246	-0.717272127	-0.425035118	3
EIF5	1.107159634	-0.837566012	-0.269593621	3
EIF5A	1.14456261	-0.440060563	-0.704502046	3
EIPR1	1.154492897	-0.596209927	-0.55828297	3
ELAPOR2	1.1515382	-0.501810793	-0.649727407	3
ELAVL3	1.102536427	-0.848438316	-0.254098111	3
ELF2	1.152678641	-0.635491349	-0.517187292	3
ELK3	1.137086214	-0.742543699	-0.394542516	3
ELMO2	1.139082064	-0.733458812	-0.405623252	3
ELN	1.149146542	-0.67253586	-0.476610682	3
ELOVL7	1.147156354	-0.459454456	-0.687701899	3
EMC1	1.139759901	-0.409534928	-0.730224973	3
EME2	1.151233144	-0.4981783	-0.653054845	3
EML3	1.153480259	-0.53077853	-0.622701729	3
EMP2	1.13962202	-0.73088937	-0.40873265	3
EN1	1.154617593	-0.589294663	-0.565322929	3
ENDOD1	1.109182961	-0.832593044	-0.276589917	3
ENDOU	1.149245734	-0.477536913	-0.671708821	3
ENDOV	1.154154743	-0.546334528	-0.607820214	3
ENKD1	1.14581823	-0.449113164	-0.696705067	3
ENKUR	1.14452161	-0.439774151	-0.704747458	3
ENPP1	1.12144514	-0.798988386	-0.322456754	3
ENPP5	1.133548234	-0.376245115	-0.757303119	3
ENTPD3	1.138484842	-0.402241829	-0.736243013	3
EP400	1.098961965	-0.856420635	-0.24254133	3

EPDR1	1.15319464	-0.62765211	-0.52554253	3
EPG5	1.151421407	-0.651020444	-0.500400963	3
EPHA10	1.133694542	-0.376972275	-0.756722267	3
EPHA4	1.137504787	-0.740688849	-0.396815938	3
EPHA6	1.122909982	-0.794489064	-0.328420918	3
EPHA7	1.133183639	-0.758740537	-0.374443103	3
EPHA8	1.151740363	-0.647428565	-0.504311798	3
EPHX2	1.153207541	-0.627439536	-0.525768005	3
EPHX4	1.154496769	-0.596034218	-0.558462551	3
EPM2A	1.154171992	-0.607339266	-0.546832725	3
EPYC	1.14994443	-0.484303276	-0.665641154	3
ERAP1	1.144910275	-0.702398767	-0.442511507	3
ERFE	1.151748721	-0.504416939	-0.647331782	3
ERG	1.154699401	-0.57875313	-0.575946271	3
ERI1	1.153763465	-0.536602709	-0.617160756	3
ERN1	1.139829925	-0.409943694	-0.729886231	3
ESM1	1.154621822	-0.588987199	-0.565634623	3
ESPN	1.139109611	-0.405780661	-0.73332895	3
ESRP1	1.151616832	-0.648842683	-0.502774149	3
ESRRG	1.147807656	-0.683005562	-0.464802094	3
ETNK2	1.151908091	-0.506450002	-0.64545809	3
ETNPPL	1.143849389	-0.708696153	-0.435153236	3
ETS2	1.148101461	-0.467292699	-0.680808762	3
ETV3L	1.15431097	-0.60312929	-0.55118168	3
EVI5L	1.107720703	-0.836200807	-0.271519896	3
EVPLL	1.14040298	-0.727079732	-0.413323248	3
EVX1	1.154698161	-0.57531998	-0.579378181	3
EXD2	1.123091795	-0.793921919	-0.329169875	3
EXOC3L2	1.154181591	-0.607068155	-0.547113436	3
EXOSC6	1.142053217	-0.423426345	-0.718626872	3
EYA4	1.14230403	-0.425014656	-0.717289374	3
F10	1.102727346	-0.848002005	-0.254725341	3
F11	1.117938485	-0.809289352	-0.308649133	3
F2R	1.148481488	-0.470593733	-0.677887756	3
F2RL1	1.139177558	-0.733008083	-0.406169475	3
F7	1.152773264	-0.518634132	-0.634139132	3
F8A1	1.137071732	-0.394464308	-0.742607424	3
F8A2	1.141994153	-0.423054465	-0.718939688	3
F8A3	1.140059692	-0.411291306	-0.728768386	3
FABP1	1.141381386	-0.722138371	-0.419243015	3
FAF2	1.144593685	-0.440278007	-0.704315677	3
FAHD2B	1.150417063	-0.66126337	-0.489153694	3
FAM107B	1.118837058	-0.806709306	-0.312127752	3
FAM120B	1.136961855	-0.393871919	-0.743089936	3
FAM124B	1.141006238	-0.416949959	-0.724056279	3
FAM135B	1.108531296	-0.834209882	-0.274321414	3
FAM153A	1.143370149	-0.431940572	-0.711429577	3
FAM153B	1.149165369	-0.476785874	-0.672379495	3
FAM166C	1.125303851	-0.786858153	-0.338445697	3
FAM177A1	1.143434306	-0.711067397	-0.432366909	3
FAM178B	1.149549845	-0.480427879	-0.669121966	3
FAM186A	1.153835331	-0.615621942	-0.53821339	3
FAM186B	1.151774862	-0.647028144	-0.504746717	3
FAM187B	1.15392235	-0.540254115	-0.613668235	3
FAM205A	1.153504495	-0.622255375	-0.53124912	3

FAM20A	1.152475074	-0.63829318	-0.514181894	3
FAM237A	1.154648733	-0.567851935	-0.586796799	3
FAM240B	1.146188106	-0.694294796	-0.45189331	3
FAM25A	1.154671145	-0.570200412	-0.584470732	3
FAM25C	1.128365679	-0.776534366	-0.351831314	3
FAM47A	1.097543425	-0.859494777	-0.238048647	3
FAM53B	1.153250917	-0.526533128	-0.626717789	3
FAM72B	1.13392244	-0.378109626	-0.755812814	3
FAM76A	1.154699354	-0.575917582	-0.578781772	3
FAM81A	1.137231309	-0.395327708	-0.741903601	3
FAM83C	1.153853549	-0.538631989	-0.61522156	3
FAM83F	1.144590032	-0.7043376	-0.440252432	3
FAM90A10P	1.143127228	-0.430336473	-0.712790755	3
FAM90A14P	1.142664842	-0.427326189	-0.715338652	3
FAM90A17P	1.150114542	-0.486021297	-0.664093245	3
FAM90A19P	1.15441604	-0.599404969	-0.555011071	3
FAM90A8P	1.112778088	-0.823397837	-0.289380251	3
FAM95C	1.146771721	-0.456398829	-0.690372892	3
FAM9A	1.138268074	-0.401028883	-0.737239192	3
FAM9C	1.15440696	-0.599751828	-0.554655132	3
FANCC	1.154508542	-0.559019168	-0.595489375	3
FANCD2	1.135295625	-0.750207071	-0.385088554	3
FANCG	1.140187655	-0.412046103	-0.728141552	3
FANCL	1.154667274	-0.5697432	-0.584924074	3
FANK1	1.141752923	-0.720208968	-0.421543954	3
FAP	1.117738991	-0.809856868	-0.307882122	3
FASTKD2	1.144539363	-0.704641262	-0.4398981	3
FAT4	1.098317021	-0.857824659	-0.240492362	3
FBLN5	1.141227493	-0.418298756	-0.722928737	3
FBN3	1.129069351	-0.774062239	-0.355007113	3
FBXL15	1.152068359	-0.508551799	-0.64351656	3
FBXL16	1.139928886	-0.72940597	-0.410522916	3
FBXL2	1.134084705	-0.378922947	-0.755161759	3
FBXO15	1.119436153	-0.804966979	-0.314469174	3
FBXO44	1.137374447	-0.396105283	-0.741269164	3
FBXO45	1.151240005	-0.498258272	-0.652981734	3
FBXW10	1.135824091	-0.387835047	-0.747989044	3
FBXW11	1.154118735	-0.608799856	-0.545318879	3
FBXW12	1.153663612	-0.619201647	-0.534461964	3
FCN3	1.147057464	-0.458661936	-0.688395528	3
FCRL5	1.138413689	-0.736570834	-0.401842854	3
FEN1	1.126859396	-0.781697711	-0.345161685	3
FERMT2	1.149997932	-0.665157531	-0.484840401	3
FEZ1	1.13705333	-0.742688351	-0.394364979	3
FGD1	1.10489321	-0.842977578	-0.261915631	3
FGD5	1.141566365	-0.420384793	-0.721181572	3
FGF1	1.149741215	-0.482289003	-0.667452212	3
FGF14	1.126647888	-0.78240932	-0.344238569	3
FGF19	1.147680837	-0.463742634	-0.683938203	3
FGF2	1.1434012	-0.711254432	-0.432146768	3
FGF6	1.13589396	-0.747693144	-0.388200816	3
FGFBP3	1.153909382	-0.613966234	-0.539943148	3
FHIT	1.154367459	-0.553166496	-0.601200963	3
FICD	1.154113599	-0.608937058	-0.545176541	3
FIGN	1.154100651	-0.544820448	-0.609280203	3

FILIP1L	1.15107429	-0.496347604	-0.654726686	3
FIZ1	1.148314373	-0.469130635	-0.679183737	3
FKBP1A	1.150668442	-0.658830343	-0.491838099	3
FKBP9	1.14582805	-0.696641787	-0.449186263	3
FKBPL	1.138767322	-0.40383385	-0.734933472	3
FLII	1.154085765	-0.609670091	-0.544415674	3
FLT1	1.123745236	-0.791867148	-0.331878088	3
FLT3	1.144786032	-0.703155035	-0.441630997	3
FLT3LG	1.138066833	-0.738157373	-0.39990946	3
FMR1	1.149708949	-0.667736114	-0.481972835	3
FN3K	1.12493925	-0.78804392	-0.33689533	3
FNBP1L	1.125053164	-0.787674385	-0.337378779	3
FOLR2	1.148340794	-0.67898006	-0.469360735	3
FOXA1	1.152508286	-0.637845437	-0.51466285	3
FOXD2	1.144862091	-0.702692683	-0.442169408	3
FOXD3	1.141698392	-0.42120433	-0.720494062	3
FOXD4	1.15468422	-0.572025718	-0.582658502	3
FOXD4L1	1.095427265	-0.86398777	-0.231439495	3
FOXD4L3	1.132663413	-0.760767285	-0.371896128	3
FOXD4L4	1.154178865	-0.547033475	-0.60714539	3
FOXD4L5	1.141564226	-0.72119268	-0.420371546	3
FOXF1	1.152144243	-0.642575747	-0.509568496	3
FOXO1	1.130503884	-0.768895567	-0.361608317	3
FPR3	1.142970033	-0.713663173	-0.42930686	3
FREM2	1.14918196	-0.672241467	-0.476940493	3
FREM3	1.154685874	-0.582382707	-0.572303167	3
FRG2	1.098593264	-0.857224594	-0.24136867	3
FRG2B	1.152755094	-0.518353674	-0.63440142	3
FRK	1.146385552	-0.692984782	-0.453400771	3
FRMD3	1.139401958	-0.731942724	-0.407459234	3
FRMD4A	1.147922081	-0.465765975	-0.682156106	3
FRMPD2	1.151626206	-0.648736426	-0.50288978	3
FRMPD3	1.116483269	-0.813386363	-0.303096906	3
FRRS1L	1.152601427	-0.516030802	-0.636570625	3
FSBP	1.151352405	-0.499579358	-0.651773047	3
FTCDNL1	1.154164648	-0.546619658	-0.60754499	3
FUT7	1.15023659	-0.487272752	-0.662963839	3
FUT9	1.150785847	-0.657666554	-0.493119294	3
FXN	1.150045757	-0.664722755	-0.485323002	3
FXYD4	1.147254415	-0.460245198	-0.687009217	3
FYCO1	1.15024286	-0.487337477	-0.662905383	3
FZD1	1.143144756	-0.430451685	-0.712693071	3
FZD5	1.133584843	-0.757158	-0.376426843	3
G3BP1	1.136974663	-0.393940884	-0.743033779	3
G6PC1	1.154026806	-0.611168917	-0.542857889	3
GABRA4	1.136136652	-0.746660406	-0.389476246	3
GABRD	1.095412846	-0.864018014	-0.231394832	3
GABRG2	1.150061911	-0.485486545	-0.664575365	3
GAD1	1.141161593	-0.723265651	-0.417895942	3
GAK	1.154244018	-0.54900512	-0.605238899	3
GAL	1.139954867	-0.729279585	-0.410675282	3
GAL3ST1	1.152811242	-0.633586739	-0.519224502	3
GALK2	1.140287082	-0.727652359	-0.412634724	3
GALNS	1.15417674	-0.607205471	-0.546971269	3
GALNT15	1.15204634	-0.508259441	-0.643786899	3

GALNT17	1.154195488	-0.606671057	-0.547524432	3
GALNTL5	1.151893943	-0.645626659	-0.506267283	3
GALNTL6	1.154265011	-0.549669546	-0.604595464	3
GALP	1.154651333	-0.568093974	-0.586557359	3
GALR3	1.154018508	-0.542644116	-0.611374392	3
GAN	1.141103974	-0.417544504	-0.723559471	3
GAREM1	1.143154779	-0.430517605	-0.712637174	3
GASK1A	1.143632555	-0.709940986	-0.433691569	3
GATA6	1.116717572	-0.812733311	-0.303984261	3
GATAD1	1.15415103	-0.546228314	-0.607922716	3
GATD1	1.094764789	-0.865372242	-0.229392546	3
GATM	1.144018395	-0.436302037	-0.707716358	3
GBA	1.137873842	-0.398841831	-0.739032011	3
GBF1	1.132493379	-0.761423686	-0.371069694	3
GCLC	1.153375947	-0.624572665	-0.528803282	3
GCLM	1.140286153	-0.727656938	-0.412629215	3
GCNT3	1.142778911	-0.428063706	-0.714715205	3
GCNT4	1.143655163	-0.433843336	-0.709811826	3
GDF5	1.137370429	-0.396083419	-0.741287011	3
GDPD2	1.133679103	-0.37689543	-0.756783672	3
GET3	1.138630923	-0.403063495	-0.735567427	3
GFPT2	1.108331424	-0.83470286	-0.273628564	3
GGN	1.147718714	-0.464058102	-0.683660612	3
GGT7	1.154340293	-0.552192834	-0.602147459	3
GH1	1.149084718	-0.673047353	-0.476037364	3
GHSR	1.112363029	-0.824484269	-0.28787876	3
GIP	1.15035612	-0.661841767	-0.488514353	3
GJA3	1.136745158	-0.744036558	-0.3927086	3
GJA4	1.153492429	-0.531014248	-0.622478181	3
GJA5	1.096287011	-0.862175499	-0.234111512	3
GJB1	1.133587937	-0.37644221	-0.757145727	3
GJB3	1.154513674	-0.595246642	-0.559267032	3
GJB6	1.142306169	-0.425028268	-0.717277902	3
GJC1	1.125706917	-0.785536984	-0.340169933	3
GJD2	1.111843675	-0.825834289	-0.286009387	3
GK	1.152058319	-0.643639973	-0.508418346	3
GLB1L3	1.147258005	-0.686983767	-0.460274238	3
GLI3	1.151283275	-0.498764366	-0.652518909	3
GLI4	1.151884717	-0.50614838	-0.645736337	3
GLIS1	1.120550318	-0.801677809	-0.318872509	3
GLIS3	1.150340228	-0.488348346	-0.661991882	3
GLRA2	1.148153063	-0.680417531	-0.467735532	3
GLT6D1	1.134133641	-0.379168809	-0.754964832	3
GLYATL1	1.127483698	-0.779578322	-0.347905375	3
GLYATL2	1.126121804	-0.784165541	-0.341956264	3
GMEB2	1.099218689	-0.855858762	-0.243359927	3
GMNC	1.15355444	-0.532233812	-0.621320629	3
GMPR	1.15443735	-0.55586911	-0.59856824	3
GNE	1.154242091	-0.548944891	-0.6052972	3
GNG7	1.147481594	-0.462096301	-0.685385293	3
GNPTAB	1.134599126	-0.381521152	-0.753077974	3
GNRH2	1.126238752	-0.783776815	-0.342461937	3
GOLGA6A	1.154181893	-0.547122328	-0.607059565	3
GOLGA6B	1.138763981	-0.403814942	-0.734949038	3
GOLGA6C	1.153499297	-0.531147804	-0.622351494	3

GOLGA6D	1.151311252	-0.49909323	-0.652218022	3
GOLGA7B	1.130918668	-0.767368287	-0.363550381	3
GOLGA8F	1.111818234	-0.825900155	-0.28591808	3
GOLGA8G	1.141732515	-0.72031574	-0.421416775	3
GOLGA8J	1.148332212	-0.469285946	-0.679046266	3
GOLIM4	1.113717848	-0.820912738	-0.292805109	3
GOSR1	1.152778269	-0.518711615	-0.634066654	3
GOT1L1	1.136960756	-0.393866002	-0.743094753	3
GP1BA	1.102890703	-0.847627851	-0.255262852	3
GP1BB	1.127113807	-0.780837483	-0.346276324	3
GP5	1.147710314	-0.463988074	-0.68372224	3
GPAA1	1.130173837	-0.770099903	-0.360073934	3
GPC1	1.14866281	-0.472202503	-0.676460306	3
GPC2	1.153822477	-0.615901858	-0.537920619	3
GPCPD1	1.153207654	-0.525769986	-0.627437668	3
GPN1	1.133197702	-0.758685358	-0.374512344	3
GPR25	1.140905748	-0.416340721	-0.724565027	3
GPR3	1.148981756	-0.475089201	-0.673892555	3
GPR45	1.148983935	-0.673874754	-0.475109181	3
GPR63	1.14401091	-0.707759932	-0.436250978	3
GPS2	1.147509585	-0.68518331	-0.462326275	3
GRAPL	1.15393097	-0.613468738	-0.540462232	3
GRIFIN	1.149296165	-0.671285261	-0.478010904	3
GRIN2B	1.154337503	-0.55209491	-0.602242593	3
GRIN3B	1.151123061	-0.654217582	-0.496905479	3
GRK2	1.143733911	-0.709360792	-0.434373119	3
GRM1	1.151676584	-0.503514135	-0.648162449	3
GRM2	1.120435988	-0.802018321	-0.318417666	3
GRN	1.132788036	-0.760284321	-0.372503714	3
GSC2	1.138058896	-0.39986544	-0.738193456	3
GSDMB	1.122681121	-0.795200188	-0.327480933	3
GSN	1.137613055	-0.397408153	-0.740204902	3
GSPT2	1.136330611	-0.390501139	-0.745829472	3
GSTM1	1.133841697	-0.37770601	-0.756135686	3
GSTP1	1.152760896	-0.518443082	-0.634317813	3
GSTT4	1.153103177	-0.523970249	-0.629132928	3
GSTZ1	1.154667266	-0.569742282	-0.584924984	3
GSX1	1.120323933	-0.802351384	-0.317972548	3
GTF2A1	1.127863797	-0.778273777	-0.34959002	3
GTF2E1	1.109332743	-0.832219339	-0.277113404	3
GTPBP1	1.147044977	-0.68848277	-0.458562207	3
GUCA1C	1.153418633	-0.529602064	-0.623816569	3
GUCA2B	1.149837357	-0.483236947	-0.66660041	3
GUCD1	1.149690799	-0.48179541	-0.667895389	3
GUCY1A2	1.131472209	-0.765305591	-0.366166618	3
GYS1	1.150692568	-0.492099899	-0.658592669	3
H1-7	1.124505337	-0.789443802	-0.335061535	3
H2AC1	1.154627075	-0.566033565	-0.588593511	3
H2AC14	1.138699718	-0.403451655	-0.735248063	3
H2BC1	1.148310434	-0.469096377	-0.679214057	3
H2BC10	1.153016665	-0.5225229	-0.630493765	3
H2BC13	1.149664458	-0.668125991	-0.481538466	3
H3C14	1.152796262	-0.518990951	-0.633805311	3
H4C7	1.15381935	-0.537849723	-0.615969628	3
H4C8	1.139466803	-0.407833578	-0.731633225	3

HABP2	1.143773345	-0.434639083	-0.709134263	3
HACE1	1.144491277	-0.439562606	-0.704928671	3
HAS2	1.154170476	-0.60738185	-0.546788626	3
HAX1	1.153477388	-0.622754304	-0.530723084	3
HBM	1.100642991	-0.852709912	-0.247933079	3
HBQ1	1.123561725	-0.792446829	-0.331114896	3
HCN1	1.154418314	-0.59931722	-0.555101094	3
HCN2	1.152111228	-0.509124393	-0.642986835	3
HDGFL3	1.15374733	-0.536249488	-0.617497842	3
HECTD4	1.140494125	-0.413866557	-0.726627569	3
HELZ	1.146375127	-0.45332076	-0.693054368	3
HEPH	1.154280671	-0.550175563	-0.604105108	3
HERC5	1.125862132	-0.78502529	-0.340836842	3
HERC6	1.112865051	-0.823169349	-0.289695702	3
HES1	1.134193661	-0.754722935	-0.379470726	3
HES4	1.153733664	-0.617781059	-0.535952605	3
HEXIM1	1.152322596	-0.640305485	-0.51201711	3
HEYL	1.140535411	-0.72642222	-0.41411319	3
HGD	1.140794781	-0.72512441	-0.415670371	3
HHIPL1	1.14740959	-0.46150665	-0.685902941	3
HHLA2	1.149534373	-0.669255518	-0.480278855	3
HIC2	1.153687015	-0.618732689	-0.534954326	3
HIF1AN	1.140658318	-0.725808904	-0.414849414	3
HIVEP1	1.146065235	-0.450963551	-0.695101685	3
HK3	1.152980071	-0.521921576	-0.631058495	3
HLA-DPB1	1.152878542	-0.52028501	-0.632593531	3
HLF	1.106318338	-0.839593805	-0.266724533	3
HLTF	1.102736016	-0.847982166	-0.25475385	3
HMCN2	1.102811561	-0.847809215	-0.255002346	3
HMGCL	1.138959275	-0.40492318	-0.734036095	3
HMGCR	1.138154178	-0.400394548	-0.73775963	3
HMGN2	1.148565154	-0.471333245	-0.677231909	3
HMX1	1.153207797	-0.525772483	-0.627435314	3
HNF1A	1.154468746	-0.59727023	-0.557198517	3
HNRNPCL2	1.151443819	-0.500669602	-0.650774217	3
HNRNPD	1.153257837	-0.626601617	-0.52665622	3
HNRNPH2	1.130306468	-0.769617086	-0.360689382	3
HNRNPUL2-BSCL2	1.1424157	-0.425726659	-0.716689041	3
HOMER3	1.144028316	-0.707658578	-0.436369738	3
HOXA11	1.138079993	-0.399982469	-0.738097524	3
HOXA13	1.147481594	-0.685385297	-0.462096297	3
HOXA2	1.154298331	-0.550757449	-0.603540882	3
HOXA6	1.146003041	-0.695507734	-0.450495307	3
HOXA7	1.154490217	-0.558159643	-0.596330573	3
HOXB13	1.148286454	-0.67939846	-0.468887994	3
HOXB5	1.150877683	-0.494134355	-0.656743328	3
HOXB6	1.15204198	-0.50820169	-0.64384029	3
HOXC12	1.124021378	-0.790990942	-0.333030435	3
HOXC6	1.154497206	-0.55848291	-0.596014295	3
HOXC8	1.147950808	-0.466009172	-0.681941636	3
HOXC9	1.151389423	-0.500019104	-0.651370319	3
HOXD1	1.122645328	-0.795311128	-0.327334199	3
HOXD8	1.150090077	-0.485772361	-0.664317716	3
HRAS	1.124724802	-0.78873729	-0.335987512	3
HRC	1.106133278	-0.840036808	-0.26609647	3

HRCT1	1.148714631	-0.676048135	-0.472666495	3
HSD17B13	1.154698737	-0.579115501	-0.575583236	3
HSD17B7	1.115122905	-0.81712971	-0.297993195	3
HSD3B7	1.14342524	-0.711118648	-0.432306592	3
HSFX4	1.132130409	-0.76281518	-0.369315228	3
HSPA12A	1.154608154	-0.58995349	-0.564654665	3
HSPA12B	1.146924572	-0.45760453	-0.689320042	3
HSPH1	1.147408116	-0.68591351	-0.461494606	3
HTD2	1.113988441	-0.820190563	-0.293797878	3
HTR1B	1.15268499	-0.635401659	-0.517283331	3
HTR2A	1.141702565	-0.720472267	-0.421230298	3
HTR3D	1.153751656	-0.536343896	-0.61740776	3
HYAL3	1.132277308	-0.762253602	-0.370023706	3
HYAL4	1.135010273	-0.383619952	-0.75139032	3
IARS2	1.149958809	-0.665511459	-0.48444735	3
ICAM1	1.154510876	-0.559131492	-0.595379384	3
IFIH1	1.154520286	-0.594928814	-0.559591472	3
IFITM5	1.148522119	-0.470952265	-0.677569854	3
IFT140	1.116991817	-0.811965751	-0.305026065	3
IFT20	1.154024586	-0.542800574	-0.611224011	3
IFT22	1.152490247	-0.638089067	-0.51440118	3
IFT27	1.124861286	-0.788296345	-0.33656494	3
IFT57	1.110689724	-0.828797332	-0.281892392	3
IGBP1P2	1.097967882	-0.858580288	-0.239387594	3
IGF2BP1	1.130900619	-0.767435067	-0.363465552	3
IGFL4	1.145669939	-0.697656013	-0.448013926	3
IGHMBP2	1.15305251	-0.523118096	-0.629934414	3
IGLL1	1.144705118	-0.441060346	-0.703644772	3
IGSF23	1.140215879	-0.412213002	-0.728002877	3
IHO1	1.123751193	-0.791848294	-0.331902899	3
IKBKE	1.145951894	-0.695840475	-0.45011142	3
IKBKG	1.151661031	-0.648340183	-0.503320848	3
IKZF2	1.143395605	-0.432109596	-0.711286009	3
IKZF4	1.115559823	-0.815936282	-0.299623541	3
IL10RB	1.139183854	-0.406205542	-0.732978311	3
IL12RB2	1.153030088	-0.522745048	-0.63028504	3
IL17B	1.146914345	-0.689390836	-0.457523509	3
IL17RC	1.139781495	-0.409660891	-0.730120604	3
IL1A	1.154395636	-0.554218767	-0.600176869	3
IL1RN	1.125709549	-0.785528322	-0.340181227	3
IL31RA	1.15087413	-0.494094874	-0.656779256	3
IL4I1	1.127703626	-0.778824832	-0.348878794	3
IL9R	1.145648663	-0.447856912	-0.697791751	3
ILDR1	1.141516309	-0.721441221	-0.420075089	3
IMMP1L	1.130895964	-0.767452285	-0.36344368	3
IMPG2	1.139951583	-0.410656014	-0.729295569	3
INHBA	1.154251751	-0.54924809	-0.605003661	3
INHBC	1.145574905	-0.447313941	-0.698260965	3
INMT	1.151315877	-0.652168159	-0.499147718	3
INO80D	1.135612833	-0.748879936	-0.386732898	3
INPP1	1.154631253	-0.566361063	-0.588270189	3
INSC	1.153906735	-0.614026751	-0.539879983	3
INSM1	1.14930089	-0.478055426	-0.671245464	3
INSYN1	1.146327349	-0.693372687	-0.452954661	3
IP6K3	1.140287062	-0.727652457	-0.412634605	3

IPO4	1.111234657	-0.82740429	-0.283830367	3
IQANK1	1.150220245	-0.663116039	-0.487104206	3
IQCF1	1.148619966	-0.676799646	-0.47182032	3
IQCF6	1.141567788	-0.420393606	-0.721174182	3
IQCJ-SCHIP1	1.143691291	-0.434086173	-0.709605119	3
IRF1	1.149729113	-0.482170298	-0.667558814	3
IRF5	1.125911375	-0.784862607	-0.341048768	3
IRX3	1.143778628	-0.709103882	-0.434674746	3
IRX6	1.135392567	-0.38558975	-0.749802817	3
ISG15	1.144769211	-0.703257028	-0.441512183	3
ISG20	1.154274129	-0.604311051	-0.549963079	3
ISL2	1.098116287	-0.858259478	-0.239856808	3
ISLR2	1.154009225	-0.542406469	-0.611602756	3
ISX	1.146389142	-0.692960807	-0.453428336	3
ISYNA1	1.149964217	-0.484501584	-0.665462633	3
ITGA8	1.10210589	-0.849418418	-0.252687472	3
ITGB2	1.154522432	-0.559698022	-0.59482441	3
ITGB3	1.145214662	-0.444691209	-0.700523453	3
ITGB4	1.154543876	-0.560799883	-0.593743993	3
ITGB6	1.152527726	-0.637581726	-0.514946001	3
ITGB8	1.15106715	-0.654800922	-0.496266228	3
ITIH3	1.142772445	-0.714750635	-0.42802181	3
ITIH5	1.153735316	-0.535988371	-0.617746944	3
ITPRIPL1	1.149068349	-0.475886075	-0.673182274	3
ITSN1	1.11760556	-0.810235392	-0.307370168	3
JAKMIP3	1.154193815	-0.606719159	-0.547474656	3
JAM2	1.153595042	-0.533049835	-0.620545206	3
JARID2	1.151947702	-0.644983774	-0.506963928	3
JCAD	1.15391103	-0.539982545	-0.613928485	3
JKAMP	1.154281074	-0.604092374	-0.5501887	3
JPH1	1.137783138	-0.739441118	-0.39834202	3
JUP	1.15406585	-0.543881599	-0.610184251	3
KALRN	1.154537797	-0.594057474	-0.560480323	3
KBTBD12	1.141713653	-0.720414342	-0.421299311	3
KCNA3	1.152828467	-0.633334307	-0.51949416	3
KCNA5	1.140858882	-0.416057296	-0.724801585	3
KCNB1	1.11474147	-0.81816489	-0.29657658	3
KCND1	1.099730149	-0.854734226	-0.244995923	3
KCND2	1.132891779	-0.759881053	-0.373010727	3
KCNE1	1.134683225	-0.381948828	-0.752734397	3
KCNG4	1.138225106	-0.400789336	-0.73743577	3
KCNH4	1.134443294	-0.38073087	-0.753712423	3
KCNH6	1.154675763	-0.58388865	-0.570787113	3
KCNJ18	1.139493576	-0.40798835	-0.731505227	3
KCNJ4	1.151414327	-0.65109805	-0.500316277	3
KCNJ6	1.151972578	-0.644684063	-0.507288515	3
KCNK15	1.154697995	-0.579447934	-0.575250061	3
KCNK2	1.121410344	-0.799093784	-0.32231656	3
KCNMB3	1.14055978	-0.414258927	-0.726300853	3
KCNMB4	1.099795951	-0.854589048	-0.245206904	3
KCNN1	1.151745393	-0.647370332	-0.504375061	3
KCNQ4	1.151184812	-0.653567702	-0.497617109	3
KCNQ5	1.137535321	-0.39698278	-0.740552541	3
KCNRG	1.13521803	-0.750529812	-0.384688218	3
KCNS2	1.148165644	-0.467843754	-0.680321891	3

KCNT2	1.114826111	-0.817935716	-0.296890396	3
KCNU1	1.151437844	-0.500597893	-0.650839951	3
KCNV2	1.152924739	-0.631900712	-0.521024027	3
KCTD11	1.14876278	-0.675663455	-0.473099325	3
KDM2A	1.154279644	-0.604137568	-0.550142075	3
KDM4F	1.141189377	-0.418065661	-0.723123716	3
KERA	1.152856529	-0.519936075	-0.632920454	3
KHDRBS2	1.09486443	-0.865164665	-0.229699765	3
KIAA1210	1.138494814	-0.736197007	-0.402297807	3
KIAA1217	1.140769389	-0.415517328	-0.725252061	3
KIAA1549	1.14925714	-0.477643935	-0.671613205	3
KIAA1958	1.138345255	-0.736885358	-0.401459898	3
KIF13B	1.152339086	-0.512247928	-0.640091158	3
KIF14	1.14453602	-0.704661265	-0.439874755	3
KIF21A	1.153024781	-0.630367669	-0.522657112	3
KIF21B	1.105496473	-0.841552948	-0.263943524	3
KIF5A	1.133932647	-0.3781607	-0.755771947	3
KIF5C	1.120491	-0.801854563	-0.318636436	3
KIF7	1.145807264	-0.696775685	-0.449031579	3
KIFC3	1.146598305	-0.69155415	-0.455044155	3
KIT	1.154645385	-0.587096434	-0.567548951	3
KITLG	1.099388538	-0.85548608	-0.243902458	3
KLB	1.118611232	-0.807361423	-0.31124981	3
KLF14	1.152438184	-0.638786452	-0.513651732	3
KLF15	1.15164277	-0.50309451	-0.64854826	3
KLF7	1.09659127	-0.861529876	-0.235061394	3
KLHDC3	1.126980415	-0.7812891	-0.345691316	3
KLHDC9	1.13606325	-0.746973568	-0.389089682	3
KLHL14	1.122429434	-0.795978694	-0.326450739	3
KLHL2	1.152745105	-0.634545074	-0.518200031	3
KLHL31	1.140873387	-0.416144971	-0.724728416	3
KLHL33	1.154286977	-0.550381936	-0.603905041	3
KLHL4	1.143752303	-0.709255197	-0.434497106	3
KLHL41	1.145788064	-0.696899211	-0.448888853	3
KLHL42	1.140442639	-0.726883188	-0.413559451	3
KLK12	1.135230621	-0.384753129	-0.750477492	3
KLK15	1.12778861	-0.778532695	-0.349255915	3
KLK6	1.132754476	-0.760414534	-0.372339943	3
KLK9	1.122332859	-0.796276437	-0.326056422	3
KMT5A	1.148816564	-0.675231776	-0.473584788	3
KNCN	1.152481648	-0.514276822	-0.638204827	3
KNDC1	1.150040637	-0.66476941	-0.485271227	3
KNTC1	1.141510352	-0.420038263	-0.721472089	3
KPRP	1.138089611	-0.738053764	-0.400035847	3
KPTN	1.150890869	-0.656609814	-0.494281054	3
KREMEN1	1.124513389	-0.789417934	-0.335095455	3
KRT13	1.14129119	-0.722602214	-0.418688977	3
KRT16	1.138450879	-0.402051286	-0.736399593	3
KRT19	1.145912996	-0.696092821	-0.449820175	3
KRT31	1.142576631	-0.426758105	-0.715818526	3
KRT33B	1.143870568	-0.435296738	-0.708573831	3
KRT36	1.143480691	-0.432675859	-0.710804833	3
KRT38	1.11676634	-0.812597069	-0.304169271	3
KRT75	1.097459125	-0.859675862	-0.237783264	3
KRT81	1.154268024	-0.549766225	-0.604501799	3

KRT83	1.149789753	-0.482766473	-0.66702328	3
KRTAP10-1	1.15468052	-0.583228651	-0.571451869	3
KRTAP10-12	1.136472224	-0.745219624	-0.3912526	3
KRTAP10-6	1.150641288	-0.659096953	-0.491544335	3
KRTAP12-1	1.153376086	-0.528805864	-0.624570222	3
KRTAP12-2	1.145236361	-0.444847844	-0.700388516	3
KRTAP16-1	1.135647685	-0.748733356	-0.386914328	3
KRTAP19-4	1.154508314	-0.559008184	-0.59550013	3
KRTAP21-3	1.15285707	-0.63291245	-0.51994462	3
KRTAP4-4	1.129352614	-0.773055782	-0.356296832	3
KRTAP4-6	1.135253159	-0.384869368	-0.750383791	3
KRTAP4-7	1.112619672	-0.823813292	-0.288806381	3
KRTAP5-4	1.153741968	-0.617609224	-0.536132744	3
KRTAP6-2	1.135259094	-0.750359108	-0.384899986	3
KRTAP6-3	1.130583092	-0.768605106	-0.361977986	3
KRTAP9-2	1.148611202	-0.471742308	-0.676868895	3
KRTAP9-4	1.147286703	-0.686780067	-0.460506636	3
KRTAP9-7	1.151108502	-0.654369934	-0.496738568	3
KRTAP9-8	1.104960125	-0.842820106	-0.262140019	3
KRTAP9-9	1.154669151	-0.569961393	-0.584707758	3
KY	1.152567015	-0.515522024	-0.63704499	3
L1CAM	1.142740105	-0.714927671	-0.427812434	3
LAD1	1.14233702	-0.425224686	-0.717112334	3
LAMA1	1.133435905	-0.757747498	-0.375688407	3
LAMA3	1.145740838	-0.697202431	-0.448538406	3
LAMA4	1.149035184	-0.475580186	-0.673454997	3
LAMC3	1.145954862	-0.450133663	-0.695821198	3
LARP4	1.121428344	-0.79903927	-0.322389073	3
LARP6	1.127277321	-0.780282103	-0.346995218	3
LAT	1.135143516	-0.750839044	-0.384304472	3
LAYN	1.154161886	-0.54653989	-0.607621996	3
LBHD2	1.132616063	-0.760950373	-0.37166569	3
LBX1	1.147909205	-0.465657127	-0.682252078	3
LCE1E	1.137834489	-0.398624828	-0.739209661	3
LCE1F	1.153364088	-0.528583593	-0.624780495	3
LCE2D	1.150708431	-0.658435983	-0.492272448	3
LCE3A	1.154645951	-0.567599501	-0.58704645	3
LCE3B	1.15398075	-0.61229365	-0.5416871	3
LCMT2	1.153977378	-0.612374548	-0.541602829	3
LCN12	1.153899896	-0.539717274	-0.614182623	3
LCNL1	1.151575607	-0.50226763	-0.649307977	3
LCTL	1.108698375	-0.833796742	-0.274901633	3
LDLRAD1	1.147130236	-0.459244661	-0.687885575	3
LEMD2	1.15456368	-0.592677648	-0.561886033	3
LEMD3	1.116393886	-0.813634839	-0.302759047	3
LENG9	1.106078478	-0.840167782	-0.265910696	3
LGALS7B	1.139027184	-0.405310043	-0.733717141	3
LGALSL	1.140654091	-0.414824045	-0.725830046	3
LGI3	1.113985493	-0.820198448	-0.293787045	3
LGR6	1.154348267	-0.601873496	-0.552474772	3
LHFPL3	1.115549166	-0.815965487	-0.299583679	3
LIFR	1.116008909	-0.814700941	-0.301307968	3
LIMA1	1.102691013	-0.848085118	-0.254605895	3
LIMK1	1.148342756	-0.678964922	-0.469377834	3
LIN28B	1.144891444	-0.702513728	-0.442377715	3

LINC02203	1.150009826	-0.484960201	-0.665049625	3
LINC02210-CRHR1	1.098637846	-0.857127568	-0.241510278	3
LIPM	1.144362076	-0.705697249	-0.438664828	3
LITAF	1.11863141	-0.807303257	-0.311328153	3
LMNTD1	1.150990627	-0.655591707	-0.49539892	3
LMX1A	1.148620959	-0.676791793	-0.471829166	3
LNPK	1.149012241	-0.67364316	-0.475369081	3
LPAR4	1.139383984	-0.732028378	-0.407355607	3
LPAR5	1.146585821	-0.454947165	-0.691638657	3
LPIN2	1.120114029	-0.802973504	-0.317140525	3
LRATD1	1.123876471	-0.791451327	-0.332425144	3
LRFN2	1.10627039	-0.83970869	-0.2665617	3
LRFN3	1.153268684	-0.526849731	-0.626418953	3
LRIF1	1.153386825	-0.529005628	-0.624381196	3
LRP2	1.110066489	-0.830377199	-0.27968929	3
LRP2BP	1.154628968	-0.588448235	-0.566180733	3
LRP4	1.124304492	-0.790087669	-0.334216823	3
LRP6	1.154147018	-0.608033096	-0.546113922	3
LRRC15	1.136979813	-0.743011191	-0.393968622	3
LRRC37A3	1.139442828	-0.731747744	-0.407695084	3
LRRC4	1.152878133	-0.632599617	-0.520278516	3
LRRC45	1.151519536	-0.649935695	-0.501583841	3
LRRC46	1.154472062	-0.557343964	-0.597128098	3
LRRC55	1.147841362	-0.46508523	-0.682756132	3
LRRC56	1.147372037	-0.686171827	-0.46120021	3
LRRC7	1.146536287	-0.691973268	-0.454563019	3
LRRC73	1.13713063	-0.742348074	-0.394782557	3
LRRC74B	1.154264113	-0.604623281	-0.549640832	3
LRRFIP2	1.140451946	-0.413614926	-0.726837019	3
LSM14A	1.154310652	-0.551170936	-0.603139716	3
LTBP1	1.140525199	-0.726473043	-0.414052156	3
LTBP2	1.148630012	-0.471909819	-0.676720192	3
LYL1	1.126666559	-0.782346632	-0.344319927	3
LYN	1.148817541	-0.473593629	-0.675223912	3
LYPD2	1.153060239	-0.523247246	-0.629812993	3
LYPD6	1.154514032	-0.595229581	-0.559284451	3
LZTR1	1.152877668	-0.520271124	-0.632606545	3
MAB21L3	1.150161693	-0.663658839	-0.486502854	3
MACC1	1.115621851	-0.815766182	-0.299855668	3
MACROD2	1.112616063	-0.823822747	-0.288793316	3
MAEA	1.153610583	-0.620244519	-0.533366064	3
MAG	1.130567996	-0.768660508	-0.361907488	3
MAGEA11	1.138111901	-0.737952298	-0.400159603	3
MAGEB18	1.097226146	-0.860175408	-0.237050738	3
MAGEC2	1.130996503	-0.767079953	-0.363916551	3
MAGEE1	1.145109528	-0.701174849	-0.443934679	3
MAGI1	1.147315586	-0.686574621	-0.460740965	3
MAGI3	1.146162761	-0.694461747	-0.451701014	3
MAJIN	1.149965126	-0.484510711	-0.665454416	3
MALL	1.154674832	-0.584010113	-0.570664719	3
MANBA	1.127578761	-0.779253074	-0.348325687	3
MAP1B	1.142047351	-0.718657972	-0.42338938	3
MAP2	1.153390337	-0.529071151	-0.624319186	3
MAP2K2	1.153778145	-0.616851479	-0.536926666	3
MAP3K11	1.1453726	-0.699537418	-0.445835182	3

MAP3K13	1.151796754	-0.505024	-0.646772754	3
MAP3K15	1.093940109	-0.867081408	-0.2268587	3
MAP3K20	1.154187857	-0.606889794	-0.547298063	3
MAPK1	1.115262844	-0.816748368	-0.298514475	3
MAPK8IP2	1.147673549	-0.68399152	-0.463682029	3
MAPKBP1	1.136715806	-0.392551528	-0.744164278	3
MAPRE3	1.15076791	-0.657845537	-0.492922373	3
MARCHF10	1.152166244	-0.50986598	-0.642300264	3
MARCHF6	1.124928209	-0.788079691	-0.336848518	3
MARCKS	1.141406941	-0.419400313	-0.722006628	3
MARK3	1.100019618	-0.854094715	-0.245924903	3
MARVELD2	1.153233245	-0.627013175	-0.52622007	3
MAS1L	1.143686589	-0.434054544	-0.709632045	3
MASP1	1.150883753	-0.656681898	-0.494201854	3
MATN4	1.094796864	-0.865305447	-0.229491416	3
MBTD1	1.143980719	-0.436045201	-0.707935518	3
MC4R	1.154538774	-0.594007521	-0.560531253	3
MCF2L	1.152656161	-0.635807747	-0.516848414	3
MCIDAS	1.149391898	-0.670475373	-0.478916525	3
MCM2	1.148169019	-0.68029622	-0.467872799	3
MCM8	1.152172505	-0.509950863	-0.642221642	3
MCOLN3	1.100016912	-0.854100704	-0.245916208	3
MDGA1	1.138216068	-0.400738984	-0.737477084	3
MDGA2	1.148417135	-0.678389005	-0.470028129	3
ME3	1.095327907	-0.864196073	-0.231131834	3
MECOM	1.152642593	-0.635997835	-0.516644758	3
MED13	1.145752041	-0.448621458	-0.697130582	3
MED21	1.151379181	-0.499897204	-0.651481977	3
MED30	1.153242372	-0.626860844	-0.526381528	3
MED6	1.147956042	-0.466053537	-0.681902505	3
MED8	1.096579109	-0.861555725	-0.235023384	3
MEF2B	1.136417398	-0.74545605	-0.390961348	3
MEGF10	1.144550087	-0.704577064	-0.439973023	3
MEGF6	1.154395715	-0.600173925	-0.55422179	3
MEIS1	1.12620598	-0.783885844	-0.342320135	3
MEIS3	1.149207324	-0.672030018	-0.477177306	3
METAP1	1.129454784	-0.772691134	-0.35676365	3
MFAP1	1.153659635	-0.619280786	-0.534378849	3
MFAP5	1.136462518	-0.391201008	-0.745261509	3
MFHAS1	1.118140989	-0.808711329	-0.30942966	3
MFN1	1.142594298	-0.715722573	-0.426871725	3
MFNG	1.150793574	-0.657589315	-0.493204259	3
MFSD2B	1.154397875	-0.554304403	-0.600093472	3
MGA	1.150118994	-0.664052334	-0.48606666	3
MGAM	1.111591058	-0.826487216	-0.285103843	3
MGAT4C	1.140359822	-0.727293271	-0.413066551	3
MGME1	1.132715687	-0.760564895	-0.372150793	3
MGST2	1.136439674	-0.391079638	-0.745360036	3
MICALL1	1.138844148	-0.404269097	-0.73457505	3
MIEN1	1.131427973	-0.76547148	-0.365956494	3
MINAR1	1.148434998	-0.470184854	-0.678250144	3
MINDY3	1.116997128	-0.81195085	-0.305046278	3
MIOX	1.141193625	-0.418091621	-0.723102004	3
MISP	1.134573936	-0.381393214	-0.753180722	3
MIXL1	1.149572128	-0.480642866	-0.668929262	3

MLF1	1.146976361	-0.458015583	-0.688960778	3
MLIP	1.148465828	-0.67800999	-0.470455838	3
MLLT1	1.13715785	-0.394929801	-0.742228049	3
MLLT11	1.151249983	-0.498374695	-0.652875288	3
MLST8	1.144719102	-0.703560287	-0.441158815	3
MLX	1.152257061	-0.641149672	-0.511107389	3
MLXIP	1.147563657	-0.684791917	-0.46277174	3
MMD	1.150717031	-0.658350897	-0.492366134	3
MMEL1	1.152378568	-0.639574825	-0.512803743	3
MMP11	1.138774139	-0.734901707	-0.403872432	3
MMP12	1.150803832	-0.657486663	-0.493317168	3
MMP15	1.10217065	-0.849271331	-0.252899319	3
MMP19	1.09936688	-0.855533645	-0.243833234	3
MMP23B	1.138504775	-0.736151033	-0.402353742	3
MMP26	1.128779308	-0.775086013	-0.353693295	3
MMRN2	1.138058766	-0.73819405	-0.399864716	3
MNS1	1.154675297	-0.583949623	-0.570725675	3
MNT	1.151380712	-0.651465303	-0.499915409	3
MOGAT3	1.137817439	-0.398530882	-0.739286556	3
MORN4	1.151931229	-0.50674977	-0.645181459	3
MPHOSPH10	1.146746912	-0.690542738	-0.456204174	3
MPIG6B	1.134473278	-0.38088271	-0.753590567	3
MPL	1.152505403	-0.637884447	-0.514620956	3
MPP4	1.154695278	-0.580366225	-0.574329052	3
MRC2	1.154232007	-0.548631708	-0.605600299	3
MRGPRF	1.154700207	-0.578107731	-0.576592476	3
MROH2B	1.137282074	-0.741678933	-0.39560314	3
MRPL12	1.153198195	-0.525604567	-0.627593628	3
MRPL13	1.149922877	-0.665835157	-0.48408772	3
MRPS21	1.139031048	-0.733698968	-0.405332081	3
MRPS23	1.145615572	-0.447613055	-0.698002517	3
MRPS30	1.14712337	-0.459189569	-0.687933801	3
MS4A10	1.154658189	-0.585893577	-0.568764612	3
MS4A5	1.148827153	-0.675146536	-0.473680618	3
MSI2	1.103787467	-0.845560077	-0.25822739	3
MSRB3	1.097329706	-0.859953525	-0.237376181	3
MSX2	1.148591697	-0.677022844	-0.471568853	3
MT-CO2	1.154673339	-0.584200364	-0.570472975	3
MT4	1.153257959	-0.526658387	-0.626599572	3
MTCP1	1.154495031	-0.558381757	-0.596113274	3
MTLN	1.1211929	-0.79975092	-0.32144198	3
MTMR12	1.134360113	-0.754049931	-0.380310183	3
MTMR14	1.150510977	-0.4901475	-0.660363476	3
MTPN	1.150546104	-0.660024138	-0.490521966	3
MTREX	1.150253375	-0.487446131	-0.662807244	3
MUC1	1.154685662	-0.57226679	-0.582418872	3
MUC22	1.139837394	-0.729850046	-0.409987348	3
MUC6	1.095666911	-0.863484395	-0.232182516	3
MUCL3	1.101541464	-0.850695392	-0.250846073	3
MXD4	1.141846368	-0.422127506	-0.719718862	3
MXRA5	1.146729169	-0.456065138	-0.690664031	3
MYBL2	1.152080586	-0.508714652	-0.643365934	3
MYBPC2	1.118095489	-0.808841375	-0.309254114	3
MYCN	1.154207824	-0.547893907	-0.606313917	3
MYCT1	1.140861864	-0.724786545	-0.416075319	3

MYEF2	1.151816867	-0.505279619	-0.646537247	3
MYH14	1.143062231	-0.713152274	-0.429909957	3
MYH15	1.148661459	-0.676471023	-0.472190436	3
MYH6	1.154681498	-0.583083382	-0.571598117	3
MYH7	1.141049865	-0.723834761	-0.417215105	3
MYL3	1.154269713	-0.604449172	-0.549820542	3
MYL6	1.149156687	-0.476705048	-0.672451639	3
MYL9	1.13630211	-0.39035022	-0.745951889	3
MYLK2	1.154399555	-0.554368884	-0.600030671	3
MYNN	1.153506561	-0.622217115	-0.531289446	3
MYO10	1.126457761	-0.783046271	-0.343411489	3
MYO15B	1.153031913	-0.630256596	-0.522775317	3
MYO16	1.128533031	-0.775950002	-0.352583029	3
MYO1B	1.135936374	-0.747513212	-0.388423162	3
MYO1G	1.154691494	-0.581303651	-0.573387843	3
MYO3A	1.113487279	-0.821525738	-0.29196154	3
MYO5B	1.09675184	-0.861188245	-0.235563595	3
MYO5C	1.140215561	-0.728004441	-0.41221112	3
MYO6	1.144835373	-0.441980052	-0.702855321	3
MYOC	1.151787631	-0.646879303	-0.504908328	3
MYOG	1.142940002	-0.429110902	-0.7138291	3
MYOM3	1.152449326	-0.638637906	-0.51381142	3
MYPN	1.152298335	-0.511678935	-0.640619399	3
MYPOP	1.15425436	-0.549330535	-0.604923826	3
MYRFL	1.130054503	-0.770533006	-0.359521497	3
MYT1	1.147165371	-0.687638409	-0.459526962	3
MYT1L	1.106735997	-0.838589974	-0.268146023	3
N4BP3	1.139853922	-0.729769937	-0.410083985	3
NAB1	1.098059924	-0.858381384	-0.239678541	3
NALCN	1.142018886	-0.718808794	-0.423210092	3
NANOS1	1.146528822	-0.454505223	-0.692023599	3
NANOS3	1.101202895	-0.851457125	-0.24974577	3
NANS	1.145214207	-0.444687931	-0.700526276	3
NAPG	1.125550901	-0.786049669	-0.339501232	3
NARS2	1.105612541	-0.841277556	-0.264334985	3
NAT8	1.135676449	-0.748612263	-0.387064186	3
NAV3	1.142665329	-0.427329332	-0.715335997	3
NBEA	1.141048263	-0.723842907	-0.417205356	3
NBPF26	1.143138255	-0.712729313	-0.430408942	3
NBPF3	1.140401197	-0.413312637	-0.72708856	3
NBPF6	1.154666762	-0.584981978	-0.569684784	3
NCALD	1.129837285	-0.771318212	-0.358519073	3
NCAPH2	1.115939762	-0.814891727	-0.301048035	3
NCBP2AS2	1.096990628	-0.860679029	-0.236311598	3
NCCRP1	1.118521642	-0.807619438	-0.310902204	3
NCMAP	1.154259406	-0.549490642	-0.604768764	3
NDNF	1.127207998	-0.7805178	-0.346690198	3
NDOR1	1.132266146	-0.762296348	-0.369969798	3
NDRG2	1.12660652	-0.782548127	-0.344058394	3
NDUFA11	1.14811762	-0.680686425	-0.467431194	3
NDUFA2	1.154582603	-0.591583263	-0.562999339	3
NDUFA7	1.121975922	-0.79737223	-0.324603692	3
NDUFAF1	1.151943667	-0.645032245	-0.506911423	3
NDUFB8	1.153524041	-0.621891997	-0.531632044	3
NEK10	1.153886483	-0.6144864	-0.539400083	3

NEO1	1.153966102	-0.541322473	-0.612643629	3
NEUROG2	1.15398945	-0.541905374	-0.612084075	3
NEUROG3	1.154414648	-0.554956139	-0.599458509	3
NFAT5	1.149839599	-0.483259161	-0.666580438	3
NFATC2IP	1.136795605	-0.743816771	-0.392978833	3
NFE2L1	1.147331629	-0.460871313	-0.686460316	3
NFIX	1.142495819	-0.716256451	-0.426239368	3
NFRKB	1.145494841	-0.698767965	-0.446726876	3
NGB	1.142006601	-0.423132778	-0.718873824	3
NHLH1	1.131651778	-0.764630291	-0.367021487	3
NICN1	1.152626541	-0.516404647	-0.636221894	3
NKAIN1	1.105873525	-0.840656783	-0.265216742	3
NKAIN3	1.118057819	-0.808948968	-0.30910885	3
NKAIN4	1.153316346	-0.625607677	-0.527708669	3
NKAPL	1.127921796	-0.778073757	-0.34984804	3
NKD2	1.144125755	-0.43703624	-0.707089515	3
NKRF	1.154008787	-0.542395308	-0.611613479	3
NKX3-1	1.152417796	-0.639057263	-0.513360533	3
NKX6-1	1.150655623	-0.65895632	-0.491699304	3
NLGN1	1.140014479	-0.728989125	-0.411025354	3
NLGN2	1.129793047	-0.77147763	-0.358315416	3
NLK	1.154670291	-0.570097097	-0.584573194	3
NLRC5	1.153151176	-0.628361432	-0.524789744	3
NLRP10	1.14377019	-0.434617787	-0.709152403	3
NLRP2	1.142252579	-0.424687616	-0.717564962	3
NLRP2B	1.150435847	-0.489351619	-0.661084227	3
NMB	1.15085546	-0.656967794	-0.493887666	3
NME6	1.152370583	-0.512690969	-0.639679614	3
NOA1	1.128269912	-0.77686778	-0.351402132	3
NOBOX	1.152123895	-0.509294453	-0.642829442	3
NOL10	1.145999615	-0.450469561	-0.695530054	3
NOL4	1.125647052	-0.785733902	-0.33991315	3
NOL4L	1.153150996	-0.524786646	-0.62836435	3
NOS3	1.136095606	-0.389259996	-0.74683561	3
NOX5	1.133232681	-0.374684664	-0.758548017	3
NOXA1	1.145728814	-0.448449318	-0.697279495	3
NOXRED1	1.146023626	-0.695373517	-0.450650109	3
NPAS4	1.146476359	-0.692376623	-0.454099736	3
NPC1L1	1.137451705	-0.740925485	-0.39652622	3
NPFF	1.154528127	-0.55998391	-0.594544217	3
NPHP3	1.154638654	-0.566966354	-0.5876723	3
NPHP3-ACAD11	1.15076	-0.657924333	-0.492835667	3
NPIPA3	1.134623733	-0.381646205	-0.752977528	3
NPIPB11	1.129254142	-0.773406409	-0.355847733	3
NPIPB6	1.125598088	-0.785894781	-0.339703308	3
NPM2	1.154435656	-0.555799698	-0.598635958	3
NPNT	1.112559749	-0.823970189	-0.28858956	3
NPPC	1.154377536	-0.60083995	-0.553537586	3
NPTXR	1.118390777	-0.807995616	-0.310395161	3
NPW	1.126191639	-0.783933528	-0.342258111	3
NPY4R2	1.152099221	-0.508963558	-0.643135663	3
NR1H4	1.118259126	-0.808373211	-0.309885915	3
NR1I2	1.13806286	-0.399887421	-0.738175439	3
NR2C1	1.154325228	-0.60265676	-0.551668467	3
NR2E3	1.139003712	-0.733827474	-0.405176238	3

NR5A1	1.154445324	-0.598246338	-0.556198986	3
NRG4	1.146105125	-0.69484042	-0.451264705	3
NRGN	1.15469689	-0.579862371	-0.574834519	3
NRN1L	1.134623586	-0.381645456	-0.75297813	3
NRP2	1.154699209	-0.575831908	-0.5788673	3
NRTN	1.153666775	-0.534528187	-0.619138588	3
NRXN3	1.153764148	-0.617146417	-0.536617731	3
NSG1	1.110121234	-0.830238985	-0.279882249	3
NSMAF	1.1470644	-0.688347034	-0.458717366	3
NSRP1	1.153432457	-0.529863539	-0.623568918	3
NSUN5	1.147587332	-0.684620047	-0.462967284	3
NT5C1A	1.154621227	-0.589030983	-0.565590244	3
NT5DC2	1.135579709	-0.74901911	-0.3865606	3
NTF3	1.154700508	-0.577578352	-0.577122156	3
NTF4	1.144045075	-0.707560905	-0.43648417	3
NTN4	1.150576687	-0.490849221	-0.659727466	3
NTNG2	1.137560543	-0.740439843	-0.3971207	3
NTRK2	1.154684418	-0.57205813	-0.582626287	3
NTRK3	1.115663172	-0.815652774	-0.300010398	3
NUAK1	1.148823671	-0.675174574	-0.473649097	3
NUAK2	1.153081309	-0.629480445	-0.523600864	3
NUDCD1	1.141738505	-0.720284409	-0.421454096	3
NUDT14	1.129070171	-0.774059337	-0.355010834	3
NUDT18	1.153280032	-0.626227062	-0.527052971	3
NUDT21	1.097912692	-0.858699451	-0.23921324	3
NUP210L	1.154635974	-0.566743239	-0.587892736	3
NUP214	1.115006914	-0.817445154	-0.297561761	3
NUP42	1.143683584	-0.434034339	-0.709649245	3
NUP43	1.134501683	-0.753475032	-0.381026651	3
NUP62	1.149584547	-0.480762884	-0.668821663	3
NUPR2	1.15315194	-0.628349059	-0.52480288	3
NUTM1	1.125697297	-0.785568644	-0.340128653	3
NUTM2F	1.153280428	-0.527060065	-0.626220363	3
NXF3	1.107149288	-0.837591089	-0.269558199	3
NXPH4	1.147466251	-0.685495828	-0.461970423	3
NYX	1.152164465	-0.509841884	-0.642322582	3
OAS3	1.10616547	-0.839959826	-0.266205644	3
OAZ2	1.149294061	-0.671302974	-0.477991087	3
OBSL1	1.154279235	-0.550128758	-0.604150477	3
OCIAD1	1.12425719	-0.790238936	-0.334018254	3
ODF4	1.151094453	-0.65451665	-0.496577803	3
OGDHL	1.154503008	-0.595747543	-0.558755465	3
OLR1	1.151440527	-0.500630086	-0.650810441	3
ONECUT1	1.1546076	-0.589991067	-0.564616533	3
OPCML	1.150413678	-0.661295611	-0.489118067	3
OPN1MW2	1.143398404	-0.432128189	-0.711270215	3
OPRK1	1.154697747	-0.579547571	-0.575150177	3
OR10P1	1.108462889	-0.83437876	-0.274084129	3
OR10Q1	1.110667439	-0.828854067	-0.281813372	3
OR10R2	1.154536642	-0.560420273	-0.594116369	3
OR11H6	1.095161737	-0.864543921	-0.230617816	3
OR12D2	1.142856632	-0.714288523	-0.428568109	3
OR1D5	1.105770815	-0.840901341	-0.264869475	3
OR1I1	1.150142107	-0.486302536	-0.663839571	3
OR1J4	1.153100197	-0.629180435	-0.523919761	3

OR1K1	1.154065369	-0.543868813	-0.610196556	3
OR2A25	1.136590519	-0.744708109	-0.39188241	3
OR2AE1	1.154016189	-0.5425846	-0.611431589	3
OR2G2	1.151388473	-0.500007787	-0.651380686	3
OR2H2	1.14903883	-0.475613776	-0.673425054	3
OR2J2	1.136562763	-0.744828301	-0.391734461	3
OR3A2	1.120164327	-0.80282464	-0.317339687	3
OR4A5	1.125157439	-0.787335374	-0.337822065	3
OR4C6	1.152470262	-0.514112498	-0.638357764	3
OR4D9	1.126240148	-0.783772168	-0.34246798	3
OR4E1	1.141328037	-0.722412935	-0.418915102	3
OR4F17	1.153631586	-0.619834664	-0.533796921	3
OR4K17	1.153828516	-0.538057888	-0.615770627	3
OR4M2	1.153669678	-0.53458906	-0.619080618	3
OR4N4C	1.129693644	-0.771835234	-0.357858411	3
OR4S2	1.129326156	-0.773150069	-0.356176087	3
OR51B2	1.152785993	-0.518831372	-0.633954621	3
OR51B4	1.151241361	-0.652967285	-0.498274076	3
OR51B5	1.102621784	-0.848243379	-0.254378405	3
OR51L1	1.154545327	-0.593668251	-0.560877077	3
OR51V1	1.147038363	-0.688528943	-0.45850942	3
OR52A5	1.102234417	-0.849126381	-0.253108036	3
OR52I2	1.126000906	-0.784566402	-0.341434504	3
OR52K1	1.095233448	-0.864393887	-0.230839561	3
OR52L1	1.114046961	-0.820033987	-0.294012974	3
OR52M1	1.152365192	-0.639750257	-0.512614936	3
OR52R1	1.116236441	-0.814071645	-0.302164796	3
OR56A5	1.135930817	-0.388394016	-0.747536801	3
OR5A2	1.121322975	-0.799358134	-0.321964841	3
OR5AC2	1.152780704	-0.518749344	-0.63403136	3
OR5AP2	1.144477377	-0.705011614	-0.439465763	3
OR5AS1	1.140513719	-0.413983565	-0.726530154	3
OR5AU1	1.149738843	-0.667473117	-0.482265726	3
OR5B12	1.154042855	-0.610767794	-0.54327506	3
OR5H14	1.11850133	-0.807677879	-0.310823451	3
OR5M9	1.137353029	-0.741364283	-0.395988746	3
OR6C4	1.138453597	-0.73638707	-0.402066527	3
OR6F1	1.138681209	-0.735334063	-0.403347146	3
OR6K3	1.112227455	-0.824837687	-0.287389768	3
OR6Y1	1.14776904	-0.464478515	-0.683290525	3
OR8A1	1.127111995	-0.780843624	-0.346268372	3
OR8B8	1.134344825	-0.754111879	-0.380232946	3
OR8K1	1.141351742	-0.419060729	-0.722291013	3
OR8U1	1.115962098	-0.814830122	-0.301131976	3
OR9I1	1.127134208	-0.780768295	-0.346365913	3
OR9Q2	1.150451141	-0.660938047	-0.489513094	3
ORM2	1.153513492	-0.622088492	-0.531425	3
OSBP	1.148004376	-0.681540391	-0.466463984	3
OSBPL2	1.119669862	-0.804282368	-0.315387494	3
OSBPL6	1.121901903	-0.797598558	-0.324303345	3
OTX1	1.152827099	-0.633354402	-0.519472697	3
OXA1L	1.145504599	-0.698706302	-0.446798297	3
OXCT2	1.139582171	-0.408501419	-0.731080752	3
OXT	1.153389401	-0.529053685	-0.624335717	3
P2RX6	1.154580087	-0.562846485	-0.591733602	3

PACRG	1.133309506	-0.375063592	-0.758245914	3
PACSIN1	1.121720177	-0.79815291	-0.323567267	3
PADI3	1.143278815	-0.711943235	-0.43133558	3
PADI4	1.146233452	-0.693995407	-0.452238045	3
PAFAH1B1	1.145809578	-0.449048789	-0.696760788	3
PAIP1	1.152092823	-0.508878007	-0.643214816	3
PALM	1.152865179	-0.632792232	-0.520072947	3
PALM2AKAP2	1.109089437	-0.832825989	-0.276263448	3
PANO1	1.154476734	-0.557550712	-0.596926021	3
PANX2	1.154629192	-0.588430863	-0.566198329	3
PAPPA	1.138282768	-0.737171904	-0.401110864	3
PAQR9	1.136956151	-0.743114937	-0.393841214	3
PARD3	1.105279202	-0.842067336	-0.263211866	3
PARD3B	1.142550635	-0.426591059	-0.715959576	3
PARS2	1.152885356	-0.632491922	-0.520393434	3
PARVA	1.151290509	-0.652441235	-0.498849275	3
PATE3	1.136289827	-0.390285215	-0.746004612	3
PATJ	1.146360913	-0.693149172	-0.453211741	3
PATL1	1.151742842	-0.647399877	-0.504342964	3
PATZ1	1.120055945	-0.803145249	-0.316910696	3
PAWR	1.145349997	-0.44567091	-0.699679087	3
PAX1	1.109350882	-0.832174027	-0.277176855	3
PAX4	1.154423181	-0.555294929	-0.599128251	3
PCARE	1.140559921	-0.726300149	-0.414259773	3
PCBP3	1.121306564	-0.799407742	-0.321898822	3
PCDH12	1.104163944	-0.844684926	-0.259479018	3
PCDH8	1.146488557	-0.454193908	-0.692294649	3
PCDH9	1.146107163	-0.694827051	-0.451280112	3
PCDHA1	1.151793691	-0.646808547	-0.504985145	3
PCDHA10	1.14550316	-0.698715395	-0.446787765	3
PCDHA11	1.142684711	-0.715230294	-0.427454417	3
PCDHA12	1.150642937	-0.659080789	-0.491562148	3
PCDHA13	1.152359085	-0.512528898	-0.639830187	3
PCDHA2	1.149727478	-0.482154279	-0.667573199	3
PCDHA3	1.151997049	-0.507609198	-0.644387851	3
PCDHA5	1.12954161	-0.772380566	-0.357161045	3
PCDHA7	1.138980822	-0.405045846	-0.733934976	3
PCDHA9	1.113541196	-0.821382586	-0.292158609	3
PCDHAC1	1.144486562	-0.704956816	-0.439529746	3
PCDHAC2	1.140061638	-0.411302764	-0.728758874	3
PCDHB3	1.154213032	-0.548051253	-0.606161779	3
PCDHB7	1.150430864	-0.489299071	-0.661131792	3
PCNT	1.122879261	-0.794584701	-0.32829456	3
PCP4L1	1.152059043	-0.643631083	-0.50842796	3
PCSK1	1.153379726	-0.528873481	-0.624506244	3
PCSK7	1.099922738	-0.854308995	-0.245613743	3
PDCD7	1.138343679	-0.736892594	-0.401451084	3
PDE11A	1.110222687	-0.829982565	-0.280240123	3
PDE4C	1.1511696	-0.497441256	-0.653728345	3
PDE6G	1.127690641	-0.778869422	-0.348821219	3
PDE7B	1.150016322	-0.664990628	-0.485025694	3
PDF	1.147051349	-0.688438259	-0.45861309	3
PDHX	1.149868164	-0.483542593	-0.666325571	3
PDP1	1.122490222	-0.795791003	-0.32669922	3
PDPK1	1.139259603	-0.406640023	-0.73261958	3

PDPN	1.144333908	-0.705864113	-0.438469795	3
PDXP	1.10832639	-0.834715259	-0.273611131	3
PDYN	1.096335287	-0.862073208	-0.234262079	3
PDZD11	1.154096627	-0.609386093	-0.544710533	3
PDZD4	1.130611744	-0.768499902	-0.362111841	3
PDZD7	1.154524581	-0.594719193	-0.559805388	3
PDZD9	1.116570666	-0.813143059	-0.303427607	3
PDZK1	1.146510639	-0.692146092	-0.454364547	3
PDZRN4	1.153923456	-0.540280762	-0.613642694	3
PEAR1	1.147701646	-0.463915848	-0.683785798	3
PEBP4	1.153013585	-0.522472041	-0.630541544	3
PEG10	1.146881898	-0.689615104	-0.457266794	3
PEG3	1.149548714	-0.480416978	-0.669131737	3
PEMT	1.146278802	-0.452583685	-0.693695117	3
PEX11A	1.154464818	-0.55702752	-0.597437299	3
PEX5L	1.098742733	-0.856899096	-0.241843637	3
PEX6	1.154599836	-0.564093331	-0.590506505	3
PFAS	1.100779282	-0.852405748	-0.248373535	3
PFN3	1.134490976	-0.380972386	-0.75351859	3
PGA4	1.15093546	-0.656156483	-0.494778978	3
PGA5	1.15451436	-0.595213912	-0.559300448	3
PGAP1	1.151937864	-0.645101905	-0.506835959	3
PGBD5	1.152161622	-0.642358237	-0.509803385	3
PGC	1.133696322	-0.376981134	-0.756715188	3
PGGT1B	1.137448321	-0.740940557	-0.396507765	3
PGK1	1.153860209	-0.538786114	-0.615074095	3
PGPEP1L	1.149553412	-0.480462263	-0.669091149	3
PGR	1.154698942	-0.575686423	-0.579012519	3
PHAX	1.1452975	-0.700007408	-0.445290092	3
PHEX	1.141876619	-0.719559776	-0.422316843	3
PHGDH	1.149082991	-0.673061596	-0.476021395	3
PHLDB1	1.146982808	-0.458066846	-0.688915962	3
PHYKPL	1.152335946	-0.512203918	-0.640132029	3
PI3	1.145696719	-0.697484917	-0.448211802	3
PIEZO1	1.148452429	-0.470337989	-0.67811444	3
PIEZO2	1.111249817	-0.82736538	-0.283884437	3
PIGC	1.151885197	-0.506154559	-0.645730638	3
PIGZ	1.145258253	-0.700252209	-0.445006044	3
PIK3CA	1.145802016	-0.448992551	-0.696809465	3
PIMREG	1.148485881	-0.470632443	-0.677853438	3
PIP4P2	1.150579718	-0.659698005	-0.490881713	3
PIP5K1A	1.13696929	-0.743057337	-0.393911954	3
PIP5K1B	1.150224567	-0.487148742	-0.663075824	3
PIPOX	1.14897102	-0.673980213	-0.474990807	3
PITPNM2	1.126397328	-0.783248192	-0.343149136	3
PITX1	1.11291283	-0.823043689	-0.289869141	3
PITX2	1.132536061	-0.761259191	-0.37127687	3
PIWIL1	1.128824983	-0.774925244	-0.353899739	3
PIWIL2	1.153388056	-0.624359464	-0.529028592	3
PKD1L1	1.114727732	-0.818202058	-0.296525674	3
PKHD1L1	1.148864121	-0.674848309	-0.474015812	3
PKIB	1.152159624	-0.509776344	-0.64238328	3
PKIG	1.101928006	-0.849821833	-0.252106173	3
PKN1	1.154584981	-0.563145388	-0.591439593	3
PKNOX2	1.144347935	-0.438566886	-0.705781049	3

PKP2	1.122975492	-0.794284939	-0.328690553	3
PLA2G2E	1.154054586	-0.543583176	-0.610471411	3
PLA2G4C	1.154181104	-0.547099136	-0.607081968	3
PLA2R1	1.122713703	-0.795099135	-0.327614568	3
PLAGL1	1.154010258	-0.542432827	-0.611577431	3
PLAT	1.143559277	-0.433200656	-0.710358622	3
PLCH2	1.154444906	-0.598263356	-0.55618155	3
PLCL1	1.152494078	-0.514456674	-0.638037405	3
PLCXD2	1.138956359	-0.73404977	-0.404906589	3
PLCXD3	1.134278212	-0.754381471	-0.379896741	3
PLD1	1.152773614	-0.518639553	-0.634134062	3
PLD5	1.127714398	-0.778787832	-0.348926567	3
PLD6	1.138096255	-0.400072726	-0.738023529	3
PLEKHA6	1.149522058	-0.480160406	-0.669361652	3
PLEKHG1	1.151109186	-0.496746401	-0.654362786	3
PLEKHH2	1.137163373	-0.742203681	-0.394959693	3
PLEKHO1	1.150506157	-0.490096243	-0.660409914	3
PLIN1	1.153753225	-0.617375039	-0.536378185	3
PLOD3	1.14682222	-0.690026274	-0.456795946	3
PLP1	1.149765012	-0.48252281	-0.667242202	3
PLS3	1.146756877	-0.690474545	-0.456282332	3
PLXNA1	1.15066991	-0.491854003	-0.658815907	3
PLXNB1	1.152121249	-0.509258899	-0.64286235	3
PMIS2	1.120502049	-0.801821655	-0.318680394	3
PMM2	1.153328047	-0.52792172	-0.625406327	3
PNLIPRP1	1.152233976	-0.641444232	-0.510789744	3
PNMA5	1.143958834	-0.708062625	-0.435896209	3
PNMA6E	1.142327097	-0.425161487	-0.71716561	3
PNMT	1.146280959	-0.452600148	-0.693680812	3
PNPLA4	1.145442382	-0.699098846	-0.446343537	3
PNPT1	1.153179903	-0.525286113	-0.627893789	3
POLG	1.147124937	-0.459202144	-0.687922793	3
POLQ	1.154693623	-0.580807641	-0.573885982	3
POLR2J2	1.104332019	-0.844292855	-0.260039164	3
POP1	1.13903093	-0.733699525	-0.405331405	3
POT1	1.131506555	-0.765176664	-0.366329891	3
POTEB	1.151963564	-0.507170734	-0.64479283	3
POTEB3	1.148316614	-0.679166473	-0.469150141	3
POTEC	1.151848723	-0.505686274	-0.646162449	3
POTED	1.149714057	-0.667691238	-0.482022819	3
POU2F3	1.138446954	-0.736417673	-0.402029281	3
POU3F2	1.15287053	-0.63271276	-0.520157769	3
POU4F3	1.144361832	-0.705698696	-0.438663136	3
PPCDC	1.127977345	-0.777881944	-0.350095401	3
PPCS	1.142894761	-0.714078631	-0.42881613	3
PPFIBP1	1.147950435	-0.466006011	-0.681944424	3
PPIB	1.153244351	-0.626827761	-0.52641659	3
PPIL6	1.150251247	-0.487424132	-0.662827115	3
PPM1H	1.11232679	-0.824578808	-0.287747982	3
PPM1L	1.153346305	-0.625090363	-0.528255941	3
PPP1CC	1.154565309	-0.592586577	-0.561978732	3
PPP1R36	1.14043243	-0.726933811	-0.413498619	3
PPP1R3B	1.147778311	-0.683222186	-0.464556125	3
PPP1R3D	1.147982124	-0.68170727	-0.466274854	3
PPP2R2A	1.144986418	-0.443053734	-0.701932684	3

PPP2R3A	1.130176242	-0.770091163	-0.360085079	3
PPP6C	1.151495353	-0.501290722	-0.650204632	3
PPRC1	1.150326451	-0.488204658	-0.662121793	3
PPT2	1.141016675	-0.417013352	-0.724003323	3
PPTC7	1.147866053	-0.465293057	-0.682572996	3
PPY	1.112331508	-0.824566503	-0.287765005	3
PQBP1	1.094951211	-0.864983691	-0.229967521	3
PRAC1	1.102131255	-0.84936082	-0.252770435	3
PRAF2	1.151405239	-0.651197528	-0.500207712	3
PRAMEF11	1.12304843	-0.794057368	-0.328991062	3
PRAMEF14	1.141432793	-0.419559574	-0.721873219	3
PRAMEF15	1.153862518	-0.538839689	-0.615022829	3
PRAMEF20	1.145992304	-0.45041463	-0.695577674	3
PRAMEF25	1.139063896	-0.73354439	-0.405519506	3
PRAMEF26	1.130422326	-0.769194063	-0.361228263	3
PRAMEF4	1.150834945	-0.657174414	-0.493660531	3
PRAMEF5	1.134225511	-0.754594401	-0.37963111	3
PRAMEF6	1.153813382	-0.616098649	-0.537714733	3
PRAMEF7	1.149780159	-0.482671919	-0.66710824	3
PRAMEF8	1.154502203	-0.558717422	-0.595784781	3
PRAMEF9	1.150161501	-0.663660612	-0.486500889	3
PRB1	1.153761927	-0.53656891	-0.617193017	3
PRB2	1.154536823	-0.560429649	-0.594107173	3
PRDM15	1.154391487	-0.554060895	-0.600330592	3
PRDM6	1.128574421	-0.775805135	-0.352769286	3
PRDM9	1.153906914	-0.614022661	-0.539884252	3
PRELID2	1.14476943	-0.703255698	-0.441513732	3
PRELP	1.143655119	-0.709812073	-0.433843047	3
PRICKLE1	1.147681588	-0.683932708	-0.46374888	3
PRIMA1	1.12652496	-0.782821437	-0.343703524	3
PRKCA	1.148888569	-0.474238041	-0.674650528	3
PRKCG	1.123897463	-0.791384713	-0.33251275	3
PRKD2	1.133174759	-0.374399387	-0.758775372	3
PRKG1	1.115457283	-0.816217108	-0.299240175	3
PRL	1.139566063	-0.731158034	-0.408408028	3
PRLR	1.135851417	-0.387978026	-0.747873391	3
PROB1	1.152435802	-0.513617653	-0.638818149	3
PROCA1	1.143338885	-0.711605664	-0.431733221	3
PROKR1	1.152873559	-0.52020584	-0.632667719	3
PROS1	1.129845344	-0.77128915	-0.358556194	3
PRPF6	1.134985109	-0.383490919	-0.75149419	3
PRPH	1.153773868	-0.536832022	-0.616941846	3
PRPS2	1.148199452	-0.468135045	-0.680064407	3
PRPSAP1	1.154696125	-0.580112772	-0.574583353	3
PRR15	1.136582055	-0.391837284	-0.744744771	3
PRR23B	1.110321275	-0.829733032	-0.280588243	3
PRR30	1.145553449	-0.447156377	-0.698397072	3
PRR35	1.149830862	-0.483172629	-0.666658233	3
PRRC2C	1.154585481	-0.563176254	-0.591409227	3
PRSS16	1.139565898	-0.408407071	-0.731158826	3
PRSS21	1.15424789	-0.605121393	-0.549126496	3
PRSS38	1.141941139	-0.422721369	-0.719219771	3
PRSS45P	1.153470892	-0.530597875	-0.622873017	3
PRSS48	1.113316293	-0.82197894	-0.291337353	3
PRSS56	1.135715983	-0.387270327	-0.748445656	3

PRSS57	1.152088296	-0.508817528	-0.643270768	3
PRX	1.117333433	-0.811004782	-0.306328652	3
PSAPL1	1.13868345	-0.735323652	-0.403359798	3
PSEN1	1.139561362	-0.408380784	-0.731180578	3
PSG7	1.107263589	-0.83731385	-0.269949739	3
PSG9	1.15090377	-0.494424828	-0.656478942	3
PSIP1	1.148853929	-0.674930637	-0.473923292	3
PSMB11	1.152893751	-0.520527295	-0.632366456	3
PSMC3	1.111257091	-0.827346706	-0.283910384	3
PSMC3IP	1.138595208	-0.40286229	-0.735732918	3
PSMD9	1.133643365	-0.756925709	-0.376717656	3
PTGDS	1.148530852	-0.677501378	-0.471029474	3
PTGIR	1.154679602	-0.583361694	-0.571317908	3
PTPN1	1.130969856	-0.767178727	-0.363791129	3
PTPN11	1.141330269	-0.722401463	-0.418928805	3
PTPN14	1.136997679	-0.742932803	-0.394064876	3
PTPRE	1.141746142	-0.720244457	-0.421501685	3
PTPRH	1.153279747	-0.626231893	-0.527047854	3
PTPRJ	1.152646826	-0.516708218	-0.635938607	3
PTPRN	1.137306804	-0.741569352	-0.395737451	3
PTPRO	1.154401353	-0.599963302	-0.554438051	3
PTPRQ	1.119925162	-0.803531307	-0.316393855	3
PTPRU	1.12380089	-0.791690935	-0.332109955	3
PUF60	1.123877112	-0.791449293	-0.332427818	3
PWP1	1.15411683	-0.60885083	-0.545266	3
PWWP2A	1.13442166	-0.380621379	-0.753800281	3
PWWP4	1.151682921	-0.648089897	-0.503593025	3
PXN	1.133981377	-0.755576682	-0.378404695	3
PYY	1.118345002	-0.808127001	-0.310218001	3
QTRT2	1.095957305	-0.862872586	-0.233084719	3
R3HDM1	1.154690254	-0.581565624	-0.57312463	3
RAB39B	1.143284302	-0.71191244	-0.431371862	3
RAB3C	1.154639003	-0.566995766	-0.587643237	3
RAB3IP	1.147795507	-0.683095305	-0.464700202	3
RAB42	1.096695524	-0.861308135	-0.235387389	3
RAB7B	1.152023783	-0.644062629	-0.507961154	3
RAB9B	1.134155189	-0.754878035	-0.379277154	3
RABEPK	1.154693185	-0.580915507	-0.573777678	3
RAD17	1.149115805	-0.476325271	-0.672790535	3
RAD54B	1.149363615	-0.670715446	-0.478648169	3
RAET1L	1.136361859	-0.745695137	-0.390666722	3
RAI1	1.133573784	-0.757201852	-0.376371932	3
RALGAPA2	1.144195464	-0.437514841	-0.706680624	3
RANBP17	1.150220707	-0.663111739	-0.487108968	3
RANBP3	1.110682982	-0.828814498	-0.281868484	3
RAP1GAP	1.140813526	-0.415783432	-0.725030094	3
RAPGEF1	1.154678975	-0.571228126	-0.583450849	3
RAPGEFL1	1.152118866	-0.642891981	-0.509226884	3
RAPSN	1.153136343	-0.62860115	-0.524535193	3
RARRES2	1.150274739	-0.662607472	-0.487667267	3
RASA4B	1.108734803	-0.833706536	-0.275028267	3
RASAL3	1.147140346	-0.687814513	-0.459325833	3
RASEF	1.095666288	-0.863485705	-0.232180582	3
RASGEF1C	1.1452351	-0.444838738	-0.700396362	3
RASL11A	1.14236021	-0.716987727	-0.425372483	3

RASSF8	1.136952922	-0.743129086	-0.393823837	3
RBFOX2	1.154695752	-0.580227094	-0.574468658	3
RBM14-RBM4	1.154301932	-0.550877659	-0.603424273	3
RBM27	1.139308099	-0.73238939	-0.406918709	3
RBM3	1.14566903	-0.448007216	-0.697661814	3
RBM4	1.152924764	-0.63190034	-0.521024424	3
RBM42	1.154027781	-0.542883096	-0.611144685	3
RBM4B	1.146365791	-0.45324914	-0.693116651	3
RBMS2	1.152354685	-0.512466979	-0.639887706	3
RBMS3	1.137208722	-0.742003442	-0.39520528	3
RBMXL3	1.143919596	-0.708290161	-0.435629435	3
RBMY1A1	1.153825404	-0.615838315	-0.537987088	3
RBMY1B	1.154616651	-0.589362015	-0.565254636	3
RBMY1D	1.154596345	-0.56386461	-0.590731735	3
RBMY1E	1.153685717	-0.534926867	-0.61875885	3
RBMY1F	1.154666434	-0.569647576	-0.585018858	3
RBMY1J	1.127574941	-0.779266158	-0.348308782	3
RBP4	1.146394634	-0.692924118	-0.453470517	3
RBPMS2	1.154683968	-0.571984674	-0.582699294	3
RDM1	1.139190516	-0.406243714	-0.732946801	3
RECK	1.152559468	-0.515410988	-0.63714848	3
RECQL4	1.152164503	-0.509842392	-0.642322111	3
RELCH	1.154655979	-0.586113052	-0.568542927	3
RELN	1.101367987	-0.851086087	-0.2502819	3
REM1	1.154081948	-0.544312661	-0.609769287	3
REPS1	1.148291477	-0.679359868	-0.468931609	3
RERE	1.134696202	-0.752681307	-0.382014894	3
RFESD	1.143985987	-0.707904901	-0.436081086	3
RFPL2	1.154006141	-0.542327867	-0.611678274	3
RFTN2	1.117895621	-0.809411453	-0.308484168	3
RFX1	1.137419387	-0.396350028	-0.741069358	3
RFX4	1.154631782	-0.588228529	-0.566403253	3
RGL3	1.143866284	-0.435267696	-0.708598587	3
RGS6	1.13325356	-0.758465973	-0.374787588	3
RGS7BP	1.126097891	-0.784244912	-0.341852979	3
RHBDD2	1.153017508	-0.630480683	-0.522536826	3
RHBDL1	1.150703569	-0.492219528	-0.658484041	3
RHD	1.1420508	-0.423411115	-0.718639685	3
RHEX	1.154312814	-0.55124415	-0.603068664	3
RHOD	1.153320201	-0.625541437	-0.527778765	3
RIMS1	1.15418824	-0.547309396	-0.606878844	3
RIMS2	1.153440636	-0.530018886	-0.62342175	3
RIMS3	1.148256055	-0.468624358	-0.679631697	3
RIPPLY1	1.150836342	-0.493675978	-0.657160364	3
RIPPLY2	1.154269636	-0.54981807	-0.604451567	3
RNASE10	1.149342491	-0.478448179	-0.670894312	3
RNASE12	1.112260534	-0.824751521	-0.287509013	3
RNASE8	1.151810472	-0.505198247	-0.646612224	3
RND1	1.14680747	-0.456679834	-0.690127637	3
RNF112	1.153639612	-0.619676939	-0.533962673	3
RNF13	1.152995182	-0.630826059	-0.522169123	3
RNF212	1.153397359	-0.52920237	-0.624194989	3
RNF24	1.144162308	-0.437287014	-0.706875294	3
RNF31	1.153786019	-0.537101471	-0.616684548	3
RNFT2	1.124928842	-0.788077643	-0.336851199	3

ROBO2	1.093751634	-0.867469832	-0.226281803	3
ROMO1	1.122855576	-0.794658395	-0.328197181	3
ROR1	1.115318698	-0.816595927	-0.298722771	3
RORC	1.150715044	-0.658370569	-0.492344475	3
RP1L1	1.149734154	-0.48221973	-0.667514424	3
RPAIN	1.154672863	-0.584259965	-0.570412898	3
RPGRIP1L	1.154699871	-0.576274987	-0.578424884	3
RPL10	1.14948623	-0.669669692	-0.479816538	3
RPL13A	1.112560885	-0.823967214	-0.288593671	3
RPP38	1.137825984	-0.398577962	-0.739248022	3
RPP40	1.139050192	-0.405441291	-0.733608901	3
RPS16	1.133195512	-0.374501559	-0.758693953	3
RPS27L	1.102144661	-0.849330372	-0.252814289	3
RPS6KA6	1.152379775	-0.512820811	-0.639558964	3
RPS8	1.147809589	-0.682991276	-0.464818313	3
RRAD	1.100191743	-0.853713389	-0.246478353	3
RRAS2	1.129502847	-0.772519297	-0.356983549	3
RRH	1.113651321	-0.821089829	-0.292561492	3
RSAD2	1.135598875	-0.386660275	-0.7489386	3
RSL1D1	1.140127867	-0.728434811	-0.411693056	3
RSPH10B2	1.119956075	-0.803440135	-0.31651594	3
RSPO4	1.145094305	-0.701268845	-0.44382546	3
RSRC1	1.14620808	-0.694163029	-0.452045051	3
RTL3	1.153590836	-0.532964631	-0.620626205	3
RTL4	1.14873297	-0.472831158	-0.675901812	3
RTL8A	1.144500935	-0.439629925	-0.704871009	3
RUBCNL	1.154589746	-0.591147263	-0.563442483	3
RXFP4	1.094406822	-0.866116064	-0.228290758	3
RYR2	1.133932303	-0.378158977	-0.755773326	3
S100A10	1.144325699	-0.705912692	-0.438413007	3
SAA2	1.148933671	-0.474649182	-0.674284489	3
SAG	1.154600473	-0.564135452	-0.590465021	3
SALL4	1.15029052	-0.662459574	-0.487830946	3
SAMD12	1.154638515	-0.58768383	-0.566954685	3
SAMD15	1.108804049	-0.833534943	-0.275269105	3
SAMD5	1.147529819	-0.685037035	-0.462492784	3
SAMD7	1.104375077	-0.844192277	-0.260182799	3
SANBR	1.154618245	-0.565370471	-0.589247774	3
SAR1A	1.147965255	-0.466131664	-0.681833591	3
SARDH	1.108410457	-0.834508089	-0.273902369	3
SARM1	1.154679277	-0.583408002	-0.571271276	3
SAXO2	1.152342927	-0.640041129	-0.512301798	3
SBF1	1.115383608	-0.816418602	-0.298965006	3
SBK2	1.140669829	-0.725751311	-0.414918518	3
SCAI	1.15124879	-0.652888022	-0.498360768	3
SCARA5	1.138840006	-0.404245606	-0.7345944	3
SCARF2	1.154045127	-0.543334531	-0.610710596	3
SCEL	1.116827304	-0.812426603	-0.304400701	3
SCG2	1.134754938	-0.752440764	-0.382314174	3
SCGB1A1	1.100519391	-0.852985319	-0.247534071	3
SCGB2B2	1.143272243	-0.711980103	-0.43129214	3
SCGB3A1	1.098542956	-0.85733402	-0.241208936	3
SCN10A	1.15199194	-0.644449806	-0.507542134	3
SCN11A	1.130293368	-0.769664843	-0.360628525	3
SCN1A	1.154391802	-0.554072861	-0.600318941	3

SCN4B	1.149992893	-0.66520321	-0.484789683	3
SCN5A	1.133832999	-0.756170423	-0.377662577	3
SCN8A	1.142979991	-0.429371893	-0.713608098	3
SCN9A	1.140475481	-0.41375529	-0.726720192	3
SCO1	1.1453069	-0.445358208	-0.699948692	3
SCRN1	1.137904435	-0.399010691	-0.738893744	3
SCRT1	1.1350127	-0.383632406	-0.751380295	3
SCT	1.152810851	-0.633592461	-0.51921839	3
SCUBE1	1.119423356	-0.805004387	-0.314418968	3
SCX	1.131704638	-0.764430916	-0.367273722	3
SCYGR7	1.147033787	-0.458472905	-0.688560881	3
SDC3	1.153906062	-0.614042117	-0.539863945	3
SDHAF4	1.106404711	-0.83938667	-0.267018041	3
SDR16C5	1.154202321	-0.606473784	-0.547728537	3
SEBOX	1.142028892	-0.423273096	-0.718755796	3
SELE	1.154157048	-0.546400666	-0.607756382	3
SELENOP	1.151612901	-0.502725706	-0.648887195	3
SEMA3B	1.154569765	-0.562235185	-0.59233458	3
SEMA3D	1.149198659	-0.672102314	-0.477096344	3
SEMA4F	1.150151776	-0.486401379	-0.663750397	3
SEMA5A	1.14391102	-0.708339834	-0.435571186	3
SEMA5B	1.154376108	-0.553484664	-0.600891444	3
SEMA6D	1.106885174	-0.838230066	-0.268655109	3
SEPTIN1	1.121906571	-0.797584294	-0.324322277	3
SEPTIN6	1.153596644	-0.533082333	-0.620514311	3
SERINC1	1.149869944	-0.483560286	-0.666309659	3
SERPINA10	1.145018795	-0.443284908	-0.701733887	3
SERPINA12	1.095627624	-0.863567011	-0.232060613	3
SERPINA6	1.134656684	-0.752842917	-0.381813766	3
SERPINB11	1.100551822	-0.852913095	-0.247638728	3
SERPINB12	1.148375997	-0.678707987	-0.46966801	3
SERPINB13	1.149911424	-0.665938059	-0.483973365	3
SERPINB6	1.124821597	-0.788424696	-0.336396901	3
SERTAD4	1.152660916	-0.516919937	-0.635740978	3
SESTD1	1.154266714	-0.604542573	-0.549724141	3
SETDB2	1.152869035	-0.52013407	-0.632734965	3
SEZ6	1.145486812	-0.44666814	-0.698818672	3
SEZ6L	1.121363134	-0.799236678	-0.322126457	3
SF3A2	1.154629222	-0.566200633	-0.588428588	3
SFRP1	1.139141365	-0.40596227	-0.733179096	3
SFRP5	1.145550777	-0.698414007	-0.44713677	3
SFTPA1	1.134297301	-0.754304265	-0.379993036	3
SFTPB	1.135739956	-0.387395423	-0.748344532	3
SFTPC	1.150066348	-0.485531523	-0.664534826	3
SGIP1	1.154695069	-0.574269706	-0.580425363	3
SGPP1	1.148477682	-0.470560199	-0.677917483	3
SH2B2	1.138454203	-0.402069926	-0.736384276	3
SH2D4B	1.152406413	-0.51319849	-0.639207923	3
SH2D5	1.153394574	-0.529150279	-0.624244295	3
SH3BGRL2	1.133452129	-0.757683398	-0.375768731	3
SHANK1	1.143521999	-0.432951495	-0.710570504	3
SHBG	1.142630851	-0.427107056	-0.715523795	3
SHC1	1.149072662	-0.673146744	-0.475925917	3
SHC4	1.151401935	-0.651233664	-0.500168271	3
SHCBP1L	1.139862428	-0.729728693	-0.410133735	3

SHFL	1.136652471	-0.744439468	-0.392213002	3
SHISA6	1.145346568	-0.69970056	-0.445646008	3
SHKBP1	1.14831265	-0.679197003	-0.469115646	3
SHOC2	1.134265105	-0.754434456	-0.379830649	3
SHPRH	1.15466782	-0.569806049	-0.584861771	3
SHROOM2	1.141230162	-0.418315086	-0.722915076	3
SHROOM4	1.132743692	-0.760456355	-0.372287336	3
SIAE	1.146573747	-0.454853424	-0.691720323	3
SIGLEC8	1.154546771	-0.59359256	-0.56095421	3
SIM1	1.14660756	-0.691491456	-0.455116104	3
SIM2	1.148380814	-0.469710123	-0.678670691	3
SIRPB1	1.116598372	-0.813065856	-0.303532517	3
SIRPG	1.148554349	-0.471237467	-0.677316882	3
SIRT6	1.15023089	-0.487213939	-0.663016951	3
SKA1	1.154367007	-0.553149995	-0.601217012	3
SLAMF7	1.149222404	-0.477318351	-0.671904053	3
SLC11A2	1.154464872	-0.557029859	-0.597435014	3
SLC12A1	1.14733214	-0.460875463	-0.686456676	3
SLC12A9	1.140249834	-0.412413987	-0.727835847	3
SLC13A3	1.146400155	-0.453512932	-0.692887223	3
SLC14A2	1.152285765	-0.640781393	-0.511504372	3
SLC15A1	1.142801635	-0.714590615	-0.42821102	3
SLC15A2	1.154148285	-0.60799829	-0.546149994	3
SLC16A11	1.143451387	-0.710970783	-0.432480604	3
SLC16A12	1.097523422	-0.859537761	-0.237985662	3
SLC16A9	1.154466095	-0.557082961	-0.597383134	3
SLC17A6	1.150300431	-0.662366553	-0.487933878	3
SLC17A8	1.124336287	-0.789985912	-0.334350375	3
SLC19A3	1.135736995	-0.748357026	-0.387379969	3
SLC1A3	1.100336837	-0.85339133	-0.246945507	3
SLC1A6	1.152613938	-0.636397172	-0.516216766	3
SLC1A7	1.154674392	-0.58406677	-0.570607622	3
SLC22A12	1.146354564	-0.693191492	-0.453163072	3
SLC22A14	1.1238676	-0.791479468	-0.332388131	3
SLC22A16	1.154633455	-0.566537681	-0.588095774	3
SLC22A23	1.153903012	-0.53979133	-0.614111682	3
SLC22A8	1.154228926	-0.548536669	-0.605692256	3
SLC24A1	1.152755772	-0.518364123	-0.63439165	3
SLC25A13	1.150758204	-0.657942213	-0.492815991	3
SLC25A21	1.149449839	-0.479468417	-0.669981422	3
SLC25A26	1.14595248	-0.695836668	-0.450115812	3
SLC25A29	1.108793696	-0.833560608	-0.275233088	3
SLC25A45	1.154396213	-0.6001554	-0.554240813	3
SLC25A48	1.148906757	-0.674503103	-0.474403654	3
SLC25A6	1.127846372	-0.778333822	-0.34951255	3
SLC26A3	1.122076294	-0.797064825	-0.32501147	3
SLC26A5	1.135585566	-0.748994514	-0.386591052	3
SLC26A9	1.146959185	-0.457879111	-0.689080074	3
SLC27A4	1.149768666	-0.482558761	-0.667209905	3
SLC28A2	1.133023274	-0.373654966	-0.759368308	3
SLC2A7	1.101791699	-0.850130354	-0.251661346	3
SLC30A8	1.154520821	-0.594902829	-0.559617992	3
SLC31A2	1.145173082	-0.700781545	-0.444391537	3
SLC34A3	1.154643656	-0.567396077	-0.587247579	3
SLC35D3	1.133486901	-0.757545926	-0.375940975	3

SLC35F3	1.149989019	-0.484750716	-0.665238303	3
SLC36A1	1.146418571	-0.692764066	-0.453654505	3
SLC36A2	1.128285291	-0.776814286	-0.351471005	3
SLC38A3	1.154467148	-0.557128776	-0.597338372	3
SLC38A4	1.153763219	-0.617165931	-0.536597287	3
SLC39A12	1.15460645	-0.564537704	-0.590068746	3
SLC39A14	1.147817643	-0.68293173	-0.464885913	3
SLC46A1	1.13823773	-0.400859681	-0.737378049	3
SLC4A10	1.138770148	-0.734920305	-0.403849842	3
SLC4A11	1.151568998	-0.649382276	-0.502186721	3
SLC5A11	1.152441842	-0.513704112	-0.638737729	3
SLC5A6	1.138016815	-0.738384608	-0.399632207	3
SLC6A1	1.15469918	-0.578883437	-0.575815743	3
SLC6A13	1.134100886	-0.379004214	-0.755096673	3
SLC6A15	1.137736303	-0.73965187	-0.398084433	3
SLC6A3	1.153516729	-0.531488421	-0.622028307	3
SLC6A4	1.154578273	-0.562737198	-0.591841074	3
SLC6A6	1.142192363	-0.424305666	-0.717886696	3
SLC6A7	1.144953403	-0.442818384	-0.702135019	3
SLC7A10	1.151745332	-0.504374295	-0.647371037	3
SLC7A11	1.151662345	-0.648325191	-0.503337154	3
SLC7A14	1.12709094	-0.780914994	-0.346175946	3
SLC7A4	1.124117157	-0.790685925	-0.333431232	3
SLC7A7	1.116356949	-0.813737416	-0.302619533	3
SLC7A9	1.153931708	-0.540480094	-0.613451613	3
SLC8A2	1.152038897	-0.643878018	-0.508160879	3
SLC9A3	1.149373212	-0.478739151	-0.670634061	3
SLC9A3R2	1.149811848	-0.66682728	-0.482984568	3
SLC9A8	1.122210489	-0.796652933	-0.325557556	3
SLC9C1	1.152105281	-0.643060601	-0.509044679	3
SLC9C2	1.154697569	-0.579616481	-0.575081089	3
SLCO3A1	1.134019394	-0.378595231	-0.755424163	3
SLCO5A1	1.150976421	-0.495238862	-0.65573756	3
SLIRP	1.154655332	-0.568479048	-0.586176284	3
SLIT1	1.138729022	-0.73511179	-0.403617233	3
SMAD9	1.153109116	-0.524070995	-0.629038121	3
SMARCAL1	1.119944191	-0.80347519	-0.316469001	3
SMDT1	1.153350819	-0.528338918	-0.625011901	3
SMG7	1.153816486	-0.616031613	-0.537784872	3
SMG9	1.152003146	-0.50768932	-0.644313826	3
SMIM23	1.137207283	-0.742009801	-0.395197482	3
SMIM32	1.154024812	-0.542806411	-0.611218401	3
SMIM36	1.134916972	-0.751775062	-0.38314191	3
SMIM9	1.142224059	-0.424506612	-0.717717448	3
SMLR1	1.131227658	-0.766220384	-0.365007273	3
SMO	1.153123789	-0.628803118	-0.524320671	3
SMOC1	1.140815307	-0.725021128	-0.415794179	3
SMS	1.15092621	-0.656250755	-0.494675455	3
SMURF1	1.102116573	-0.849394162	-0.252722411	3
SNAI2	1.151749258	-0.647325557	-0.504423701	3
SNAP25	1.140192093	-0.412072337	-0.728119756	3
SNCB	1.124099677	-0.790741634	-0.333358043	3
SNX16	1.147378216	-0.686127632	-0.461250584	3
SNX9	1.101616204	-0.850526811	-0.251089392	3
SOAT2	1.146556237	-0.454717596	-0.69183864	3

SOBP	1.141026555	-0.723953171	-0.417073383	3
SOCS2	1.154623021	-0.565724431	-0.588898589	3
SOCS7	1.153276638	-0.626284547	-0.52699209	3
SORBS1	1.153269348	-0.626407741	-0.526861607	3
SORBS3	1.153414551	-0.623889423	-0.529525129	3
SOWAHA	1.132783714	-0.760301096	-0.372482618	3
SOX21	1.152839006	-0.519659753	-0.633179253	3
SOX30	1.152368857	-0.512666613	-0.639702245	3
SOX5	1.097005466	-0.860647341	-0.236358125	3
SP3	1.120362961	-0.802235454	-0.318127507	3
SP5	1.154567504	-0.562104494	-0.592463009	3
SP6	1.097117822	-0.860407214	-0.236710608	3
SPACA3	1.148818434	-0.675216731	-0.473601703	3
SPAG11A	1.129839009	-0.771311995	-0.358527015	3
SPAG11B	1.136800894	-0.743793705	-0.39300719	3
SPAG17	1.152408464	-0.51322766	-0.639180804	3
SPAG4	1.134565167	-0.381348696	-0.753216472	3
SPAG7	1.15414248	-0.608157451	-0.545985028	3
SPANXN4	1.151898838	-0.50633046	-0.645568379	3
SPARC	1.106930995	-0.838119373	-0.268811622	3
SPATA13	1.152313937	-0.640417706	-0.511896231	3
SPATA18	1.15468656	-0.572422738	-0.582263821	3
SPATA2	1.133750688	-0.377251944	-0.756498744	3
SPATA20	1.151837022	-0.64630037	-0.505536652	3
SPATA21	1.146146147	-0.694571047	-0.451575099	3
SPATA31E1	1.14662968	-0.69134146	-0.45528822	3
SPATA33	1.153016634	-0.522522377	-0.630494257	3
SPATA5	1.105610414	-0.841282605	-0.264327809	3
SPATS2	1.148178577	-0.4679551	-0.680223478	3
SPC25	1.13821394	-0.400727131	-0.737486809	3
SPDYE11	1.14862646	-0.47187817	-0.67674829	3
SPDYE12	1.15363194	-0.619827712	-0.533804229	3
SPDYE17	1.154417455	-0.555067048	-0.599350407	3
SPDYE8	1.143256433	-0.431187673	-0.71206876	3
SPDYE9	1.140150228	-0.411825017	-0.728325211	3
SPECC1	1.153177092	-0.627939739	-0.525237353	3
SPECC1L-ADORA2A	1.149440533	-0.479379581	-0.670060951	3
SPHKAP	1.142327515	-0.71716337	-0.425164145	3
SPINK2	1.154330947	-0.602464646	-0.551866302	3
SPINK5	1.139107621	-0.733338337	-0.405769283	3
SPN	1.140212157	-0.41219098	-0.728021176	3
SPOCK2	1.151025073	-0.49578826	-0.655236813	3
SPPL2A	1.153276278	-0.526985646	-0.626290632	3
SPPL2C	1.145787574	-0.696902361	-0.448885213	3
SPPL3	1.143226333	-0.712237358	-0.430988975	3
SPRED3	1.135034761	-0.751289165	-0.383745596	3
SPRR1B	1.136438413	-0.745365476	-0.391072937	3
SPRYD7	1.139091757	-0.733413131	-0.405678626	3
SPSB3	1.149208498	-0.672020217	-0.477188281	3
SPSB4	1.143869457	-0.708580253	-0.435289204	3
SPTBN5	1.108738793	-0.833696654	-0.275042138	3
SRARP	1.149652304	-0.481420134	-0.668232171	3
SRCIN1	1.139796526	-0.409748613	-0.730047912	3
SRD5A3	1.152589992	-0.636728703	-0.515861288	3
SRRM1	1.146685198	-0.690963995	-0.455721203	3

SSMEM1	1.153126019	-0.628767308	-0.52435871	3
SSX4	1.149088749	-0.476074656	-0.673014093	3
SSX4B	1.14520213	-0.444600827	-0.700601303	3
ST3GAL2	1.145626422	-0.447692962	-0.69793346	3
ST8SIA1	1.149883991	-0.666184008	-0.483699983	3
ST8SIA2	1.133625239	-0.376627544	-0.756997695	3
ST8SIA5	1.116663035	-0.81288554	-0.303777495	3
ST8SIA6	1.144887929	-0.442352756	-0.702535173	3
STAB2	1.151028858	-0.495831141	-0.655197717	3
STAMBPL1	1.132314021	-0.762112921	-0.370201099	3
STARD3	1.123895122	-0.791392143	-0.332502979	3
STARD7	1.149645026	-0.668295693	-0.481349333	3
STBD1	1.114677093	-0.818338993	-0.2963381	3
STEAP2	1.154542496	-0.560726792	-0.593815704	3
STIL	1.116908238	-0.812200038	-0.3047082	3
STIMATE	1.123084875	-0.793943542	-0.329141333	3
STK39	1.14160036	-0.420595439	-0.72100492	3
STMN4	1.135451691	-0.749555692	-0.385895999	3
STOML1	1.149723889	-0.667604785	-0.482119104	3
STON1	1.132276665	-0.762256065	-0.370020601	3
STON1-GTF2A1L	1.150566974	-0.490745163	-0.659821811	3
STRA6	1.149996126	-0.48482222	-0.665173906	3
STRIP2	1.13194709	-0.763513006	-0.368434084	3
STX1B	1.140522378	-0.726487081	-0.414035297	3
STX7	1.152980817	-0.521933775	-0.631047042	3
STXBP5L	1.139308219	-0.732388823	-0.406919395	3
STXBP6	1.151332237	-0.499340762	-0.651991475	3
SUCO	1.150394912	-0.661474104	-0.488920808	3
SULF1	1.146270525	-0.693749993	-0.452520532	3
SUMF2	1.146847595	-0.68985165	-0.456995946	3
SUMO2	1.113962772	-0.8202592	-0.293703572	3
SUN2	1.154494948	-0.558377906	-0.596117042	3
SUOX	1.132014437	-0.763257025	-0.368757412	3
SUPT3H	1.153211582	-0.525838814	-0.627372767	3
SUPT6H	1.152406063	-0.639212555	-0.513193508	3
SURF6	1.134815941	-0.382625437	-0.752190504	3
SV2A	1.15442003	-0.599250795	-0.555169235	3
SVIL	1.143317286	-0.711727154	-0.431590132	3
SYCN	1.134290576	-0.379959108	-0.754331468	3
SYCP1	1.102990473	-0.847398957	-0.255591516	3
SYNE3	1.131974072	-0.763410503	-0.368563569	3
SYP	1.149600584	-0.48091806	-0.668682524	3
SYPL1	1.153430737	-0.623599809	-0.529830928	3
SYPL2	1.153433904	-0.529890986	-0.623542918	3
SYT10	1.138618655	-0.735624296	-0.402994359	3
SYT14	1.152964436	-0.631297886	-0.521666549	3
SYT16	1.149582452	-0.668839828	-0.480742624	3
SYT17	1.144209322	-0.70659916	-0.437610162	3
SYT8	1.133972491	-0.755612312	-0.378360179	3
SYTL1	1.149835764	-0.483221172	-0.666614592	3
SYTL4	1.121068501	-0.800125698	-0.320942804	3
TAAR6	1.137070113	-0.742614545	-0.394455568	3
TACO1	1.151535665	-0.501779933	-0.649755732	3
TADA2A	1.130549391	-0.76872876	-0.36182063	3
TAF11L4	1.139192563	-0.406255447	-0.732937116	3

TAF11L5	1.149849592	-0.48335823	-0.666491363	3
TAF11L8	1.126970007	-0.781324286	-0.345645721	3
TAF4	1.12400336	-0.791048259	-0.332955101	3
TAF6L	1.154582915	-0.591564494	-0.563018421	3
TANC1	1.147848584	-0.682702605	-0.465145979	3
TANGO2	1.122859276	-0.794646886	-0.328212389	3
TAS2R43	1.09425208	-0.866436688	-0.227815392	3
TAS2R46	1.153549624	-0.621411673	-0.53213795	3
TAS2R5	1.154433461	-0.555710026	-0.598723435	3
TBATA	1.153663042	-0.534450058	-0.619212984	3
TBC1D15	1.108909129	-0.833274234	-0.275634895	3
TBC1D17	1.149682902	-0.481718317	-0.667964585	3
TBC1D24	1.138530352	-0.736032913	-0.402497439	3
TBC1D26	1.131637748	-0.764683163	-0.366954585	3
TBC1D32	1.150929237	-0.494709322	-0.656219915	3
TBC1D8B	1.154700133	-0.576511756	-0.578188377	3
TBCCD1	1.154624045	-0.588822243	-0.565801802	3
TBR1	1.154393744	-0.554146663	-0.600247081	3
TBX18	1.15009735	-0.66425105	-0.485846301	3
TBX4	1.146045561	-0.695230304	-0.450815257	3
TBX5	1.112839964	-0.823235294	-0.28960467	3
TBXA2R	1.134730735	-0.752539932	-0.382190803	3
TBXT	1.14598376	-0.450350468	-0.695633292	3
TCEA2	1.143826733	-0.708826859	-0.434999874	3
TCEAL5	1.108812508	-0.833513968	-0.27529854	3
TCF20	1.153582205	-0.532790283	-0.620791922	3
TCIM	1.152044439	-0.508234262	-0.643810177	3
TCN1	1.151471369	-0.650470312	-0.501001057	3
TCP10L	1.150603437	-0.491136413	-0.659467024	3
TDP1	1.149791305	-0.667009526	-0.482781779	3
TDRD10	1.14968879	-0.481775794	-0.667912996	3
TDRD6	1.137999657	-0.399537192	-0.738462465	3
TEDC1	1.124959069	-0.787979689	-0.336979381	3
TEDDM1	1.15051829	-0.490225333	-0.660292956	3
TEK	1.135374147	-0.385494428	-0.749879719	3
TENM2	1.144730547	-0.703491096	-0.441239451	3
TERB1	1.150056087	-0.664628532	-0.485427555	3
TERB2	1.146374651	-0.693057543	-0.453317109	3
TERF2	1.153987327	-0.541851987	-0.61213534	3
TET1	1.136418859	-0.745449754	-0.390969106	3
TEX13B	1.153834968	-0.615629882	-0.538205086	3
TEX13D	1.099893901	-0.85437273	-0.245521171	3
TEX15	1.149825797	-0.666703301	-0.483122496	3
TEX19	1.129662789	-0.771946065	-0.357716725	3
TEX22	1.150412962	-0.489110529	-0.661302433	3
TEX264	1.113520329	-0.821438003	-0.292082325	3
TEX33	1.151262403	-0.498519861	-0.652742542	3
TEX37	1.145154714	-0.444259353	-0.700895361	3
TEX46	1.139668196	-0.730667248	-0.409000948	3
TFAP2D	1.128739148	-0.775227233	-0.353511915	3
TFDP1	1.152869278	-0.632731368	-0.520137909	3
TFPI	1.153651788	-0.619436501	-0.534215287	3
TGM5	1.154674484	-0.570619554	-0.58405493	3
TGM6	1.153093995	-0.523814859	-0.629279136	3
TH	1.148908844	-0.674486173	-0.474422671	3

THBS2	1.147404601	-0.461465894	-0.685938707	3
THBS4	1.136866386	-0.743507792	-0.393358594	3
THRB	1.099521242	-0.855194377	-0.244326864	3
TIFAB	1.136073005	-0.389141015	-0.74693199	3
TIGD5	1.154451457	-0.556456167	-0.59799529	3
TIGD6	1.127910424	-0.778112998	-0.349797426	3
TIMELESS	1.135216362	-0.75053674	-0.384679622	3
TIMM44	1.149595571	-0.668726044	-0.480869526	3
TKT	1.144986315	-0.701933313	-0.443053002	3
TLCD2	1.115653395	-0.815679616	-0.299973779	3
TLCD4	1.154521352	-0.594877043	-0.559644309	3
TLE2	1.154513886	-0.595236528	-0.559277358	3
TLK1	1.137721658	-0.398003953	-0.739717704	3
TLR10	1.140825169	-0.415853695	-0.724971474	3
TLX3	1.139275914	-0.406733711	-0.732542203	3
TMBIM1	1.144732409	-0.703479832	-0.441252577	3
TMC1	1.137604208	-0.740244511	-0.397359698	3
TMCO2	1.142207811	-0.71780424	-0.424403571	3
TMDD1	1.138249304	-0.737325104	-0.400924199	3
TMED1	1.106351268	-0.839514863	-0.266836405	3
TMED3	1.107961257	-0.835612284	-0.272348973	3
TMEM100	1.141809057	-0.42189427	-0.719914788	3
TMEM11	1.147192665	-0.687445981	-0.459746684	3
TMEM114	1.147527731	-0.462475589	-0.685052141	3
TMEM132E	1.147441929	-0.461771131	-0.685670798	3
TMEM139	1.154682321	-0.571723881	-0.582958439	3
TMEM150C	1.127149759	-0.780715537	-0.346434223	3
TMEM151A	1.136219151	-0.389911568	-0.746307583	3
TMEM186	1.105649888	-0.841188852	-0.264461037	3
TMEM187	1.151032609	-0.655158938	-0.495873672	3
TMEM190	1.15468236	-0.571730007	-0.582952354	3
TMEM204	1.137809891	-0.398489308	-0.739320583	3
TMEM205	1.112519975	-0.824074249	-0.288445727	3
TMEM209	1.097432334	-0.859733377	-0.237698957	3
TMEM229A	1.143924106	-0.435660075	-0.708264031	3
TMEM229B	1.146904016	-0.689462283	-0.457441732	3
TMEM231	1.153693577	-0.535093377	-0.6186002	3
TMEM232	1.154005912	-0.611683875	-0.542322037	3
TMEM234	1.153091167	-0.629324081	-0.523767087	3
TMEM235	1.146039848	-0.450772227	-0.695267621	3
TMEM239	1.146184514	-0.451866045	-0.694318469	3
TMEM241	1.145103331	-0.701213124	-0.443890207	3
TMEM249	1.150710253	-0.492292287	-0.658417966	3
TMEM251	1.151929997	-0.506733776	-0.64519622	3
TMEM255B	1.136435723	-0.745377073	-0.39105865	3
TMEM275	1.138558388	-0.402655073	-0.735903315	3
TMEM44	1.145366364	-0.699576517	-0.445789847	3
TMEM45A	1.147036144	-0.45849171	-0.688544434	3
TMEM52B	1.154144311	-0.608107332	-0.546036979	3
TMEM59L	1.153904363	-0.539823479	-0.614080884	3
TMEM74	1.140227933	-0.727943608	-0.412284324	3
TMEM88	1.150826025	-0.493561963	-0.657264062	3
TMEM88B	1.152691128	-0.517376312	-0.635314816	3
TMEM9	1.138818865	-0.404125755	-0.73469311	3
TMEM92	1.130312693	-0.769594387	-0.360718305	3

TMIGD3	1.135259539	-0.750357257	-0.384902282	3
TMPRSS11B	1.153702829	-0.535290176	-0.618412653	3
TMPRSS4	1.148282094	-0.468850144	-0.67943195	3
TMPRSS6	1.148125605	-0.467499692	-0.680625913	3
TMPRSS7	1.149408914	-0.670330615	-0.479078299	3
TMPRSS9	1.109756845	-0.831156922	-0.278599923	3
TMTC4	1.148821794	-0.675189689	-0.473632104	3
TMUB2	1.09573771	-0.863335419	-0.232402291	3
TNFAIP2	1.153535934	-0.621669381	-0.531866553	3
TNFRSF10B	1.101486667	-0.850818893	-0.250667774	3
TNFRSF11A	1.154367738	-0.601191032	-0.553176706	3
TNFRSF4	1.151461946	-0.65057441	-0.500887536	3
TNFRSF8	1.129550024	-0.77235044	-0.357199584	3
TNFRSF9	1.154504593	-0.55883063	-0.595673964	3
TNK1	1.094123685	-0.866702299	-0.227421385	3
TNK2	1.154446553	-0.556250278	-0.598196276	3
TNKS	1.116811265	-0.812471468	-0.304339797	3
TNKS2	1.154338671	-0.602202797	-0.552135874	3
TNNC1	1.154648619	-0.56784141	-0.586807209	3
TNPO2	1.132882993	-0.372967741	-0.759915251	3
TNR	1.15089623	-0.656555458	-0.494340771	3
TNS4	1.139047922	-0.733619586	-0.405428336	3
TNXB	1.143081121	-0.713047319	-0.430033802	3
TOLLIP	1.154698497	-0.575468915	-0.579229582	3
TOPAZ1	1.133657154	-0.756870921	-0.376786233	3
TOPBP1	1.144509681	-0.70481876	-0.439690921	3
TOX	1.121861734	-0.797721255	-0.324140479	3
TP53INP2	1.10728871	-0.837252861	-0.270035849	3
TP63	1.107621985	-0.836441766	-0.271180219	3
TPD52L2	1.151744495	-0.647380731	-0.504363764	3
TPH1	1.146623799	-0.691381363	-0.455242436	3
TPM3	1.124779197	-0.788561697	-0.3362175	3
TPM4	1.154661498	-0.585553854	-0.569107644	3
TPRN	1.135867687	-0.388063199	-0.747804487	3
TPRX1	1.109216703	-0.832508925	-0.276707778	3
TPSD1	1.137473711	-0.740827434	-0.396646277	3
TPST1	1.142354812	-0.717016742	-0.425338071	3
TPX2	1.129902133	-0.771084221	-0.358817912	3
TRAF2	1.137944262	-0.399230731	-0.738713531	3
TRAK1	1.152697622	-0.51747484	-0.635222781	3
TRAM1	1.149924929	-0.665816707	-0.484108222	3
TRAPPC10	1.152364829	-0.63975501	-0.512609819	3
TRAPPC2L	1.150413121	-0.66130092	-0.489112201	3
TRARG1	1.140231294	-0.412304221	-0.727927074	3
TRIM15	1.12636807	-0.783345858	-0.343022211	3
TRIM16	1.14752065	-0.462417303	-0.685103346	3
TRIM25	1.106262932	-0.839726554	-0.266536378	3
TRIM26	1.14113218	-0.417716456	-0.723415724	3
TRIM32	1.154428346	-0.555502543	-0.598925803	3
TRIM35	1.10654985	-0.839038065	-0.267511785	3
TRIM36	1.146433785	-0.453771584	-0.692662202	3
TRIM37	1.133360981	-0.375317839	-0.758043143	3
TRIM43B	1.138840201	-0.404246716	-0.734593485	3
TRIM49	1.154700474	-0.577015525	-0.577684948	3
TRIM49B	1.136243746	-0.390041522	-0.746202224	3

TRIM49C	1.153716953	-0.618124616	-0.535592337	3
TRIM54	1.146837133	-0.689923685	-0.456913448	3
TRIM55	1.139439691	-0.731762721	-0.407676969	3
TRIM61	1.126972813	-0.7813148	-0.345658014	3
TRIM73	1.135890821	-0.747706454	-0.388184367	3
TRIOBP	1.119620322	-0.804427721	-0.315192601	3
TRIT1	1.129301147	-0.773239139	-0.356062008	3
TRMT5	1.154435667	-0.598635523	-0.555800144	3
TRO	1.152117145	-0.509203787	-0.642913358	3
TRPA1	1.152438823	-0.638777935	-0.513660888	3
TRPM6	1.13960767	-0.730958324	-0.408649346	3
TRPV5	1.154617285	-0.565300554	-0.589316731	3
TSACC	1.138469283	-0.402154515	-0.736314768	3
TSBP1	1.150745861	-0.492680895	-0.658064966	3
TSEN2	1.154297133	-0.60357954	-0.550717593	3
TSGA13	1.122969918	-0.794302318	-0.3286676	3
TSPAN10	1.122532661	-0.795659842	-0.32687282	3
TSPAN11	1.147044131	-0.458555458	-0.688488673	3
TSPAN13	1.136872159	-0.393389597	-0.743482562	3
TSPAN15	1.154424887	-0.599061574	-0.555363313	3
TSPAN8	1.146107611	-0.694824114	-0.451283497	3
TSPAN9	1.154693587	-0.573876882	-0.580816705	3
TSPY1	1.142626675	-0.427080152	-0.715546523	3
TSPY4	1.138900856	-0.404591003	-0.734309853	3
TSPY8	1.151357901	-0.499644499	-0.651713402	3
TSPY9P	1.140285836	-0.412627335	-0.727658501	3
TSPYL6	1.126529167	-0.782807351	-0.343721816	3
TSR2	1.154497269	-0.55848585	-0.596011418	3
TSSK4	1.146644299	-0.455402095	-0.691242204	3
TSTD3	1.14955864	-0.669045965	-0.480512674	3
TTC6	1.152011139	-0.644216648	-0.507794492	3
TTC9	1.144619309	-0.440457551	-0.704161759	3
TTLL3	1.146106845	-0.694829137	-0.451277708	3
TTLL8	1.153124365	-0.524330488	-0.628793877	3
TTLL9	1.154560049	-0.592878726	-0.561681324	3
TUBA3C	1.121621777	-0.798452303	-0.323169474	3
TUBB1	1.14116677	-0.723239214	-0.417927556	3
TUBB6	1.151780017	-0.504811925	-0.646968092	3
TUBGCP3	1.14998261	-0.484686284	-0.665296326	3
TUT4	1.154681349	-0.583105745	-0.571575605	3
TXNDC5	1.148995203	-0.673782635	-0.475212568	3
TYRO3	1.151380406	-0.499911773	-0.651468633	3
U2AF1L4	1.125476366	-0.786294019	-0.339182348	3
UBA5	1.150539051	-0.660092395	-0.490446656	3
UBE2E3	1.145207339	-0.700568953	-0.444638385	3
UBE2N	1.137093332	-0.394580963	-0.742512369	3
UBE2U	1.149024674	-0.673541238	-0.475483436	3
UBE2Z	1.1082514	-0.834899855	-0.273351545	3
UBL3	1.154690397	-0.58153624	-0.573154157	3
UBTD1	1.148078127	-0.680985132	-0.467092995	3
UBTFL1	1.143002833	-0.429521158	-0.713481674	3
UCHL1	1.145147041	-0.444204166	-0.700942875	3
UCN2	1.153405161	-0.624056583	-0.529348577	3
UGCG	1.143163474	-0.430574809	-0.712588665	3
UGT1A6	1.110918259	-0.828214452	-0.282703808	3

UGT2B11	1.118324604	-0.808185514	-0.310139089	3
UGT2B4	1.119896314	-0.803616341	-0.316279974	3
UHRF2	1.138090135	-0.400038757	-0.738051378	3
UNC13B	1.148966123	-0.474945953	-0.67402017	3
UNC5D	1.143988579	-0.707889833	-0.436098746	3
UNC93B1	1.146380523	-0.453362166	-0.693018357	3
UNG	1.147653732	-0.684136354	-0.463517379	3
UPF1	1.106696788	-0.838684451	-0.268012337	3
UPK2	1.143672771	-0.433961646	-0.709711125	3
UPK3A	1.149107017	-0.672863215	-0.476243802	3
UPK3B	1.154601918	-0.564231572	-0.590370345	3
UQCC1	1.096310014	-0.862126765	-0.234183248	3
URGCP	1.106047814	-0.840241028	-0.265806786	3
USF3	1.117586349	-0.810289824	-0.307296524	3
USH1C	1.154608716	-0.589915236	-0.56469348	3
USH2A	1.141416524	-0.721957193	-0.419459331	3
USP13	1.154649991	-0.567968257	-0.586681735	3
USP15	1.102157605	-0.849300968	-0.252856637	3
USP17L2	1.109416508	-0.832010001	-0.277406507	3
USP25	1.145592998	-0.698146059	-0.44744694	3
USP29	1.13912185	-0.405850638	-0.733271212	3
UTF1	1.135391259	-0.749808277	-0.385582982	3
UTP20	1.14367538	-0.709696199	-0.433979182	3
VANGL1	1.154496551	-0.59604416	-0.558452391	3
VASH2	1.13252378	-0.761306541	-0.371217239	3
VEZF1	1.140721903	-0.415231475	-0.725490428	3
VGLL4	1.150328866	-0.662099033	-0.488229833	3
VHLL	1.152293831	-0.640677496	-0.511616335	3
VIL1	1.151340838	-0.651898406	-0.499442432	3
VILL	1.154615473	-0.565169699	-0.589445774	3
VIPR2	1.149817721	-0.483042616	-0.666775105	3
VIT	1.112909475	-0.823052517	-0.289856958	3
VKORC1L1	1.141215851	-0.418227527	-0.722988324	3
VLDLR	1.133261642	-0.758434204	-0.374827438	3
VN1R2	1.154091731	-0.609514415	-0.544577317	3
VPREB1	1.137436695	-0.396444366	-0.740992328	3
VPS26B	1.133769417	-0.377345316	-0.756424102	3
VPS28	1.154050686	-0.543480438	-0.610570248	3
VPS35L	1.138986224	-0.405076609	-0.733909615	3
VSTM2A	1.152907739	-0.520751005	-0.632156734	3
VWA2	1.146731561	-0.456083873	-0.690647688	3
VWA3B	1.145854946	-0.696468285	-0.449386661	3
VWA5B1	1.126986814	-0.781267467	-0.345719347	3
VWCE	1.145185031	-0.700707437	-0.444477594	3
WAS	1.096837007	-0.861006787	-0.23583022	3
WASHC1	1.129499114	-0.77253265	-0.356966464	3
WASL	1.154202384	-0.547730405	-0.606471978	3
WDFY4	1.138655477	-0.403201945	-0.735453532	3
WDR13	1.103440897	-0.846361994	-0.257078903	3
WDR37	1.146569381	-0.454819543	-0.691749838	3
WDR61	1.135363525	-0.385439482	-0.749924043	3
WDR64	1.151504309	-0.650105166	-0.501399142	3
WDR72	1.150227552	-0.487179514	-0.663048038	3
WDR93	1.151439703	-0.650819501	-0.500620202	3
WEE2	1.138935415	-0.734147975	-0.40478744	3

WFDC1	1.153320428	-0.625537529	-0.527782899	3
WIPF3	1.153312252	-0.527634331	-0.625677922	3
WIZ	1.153660722	-0.534401561	-0.619259162	3
WNK3	1.150113588	-0.486011575	-0.664102013	3
WNT4	1.134486517	-0.380949789	-0.753536728	3
WNT7B	1.112221514	-0.824853158	-0.287368355	3
WNT8A	1.150605665	-0.491160378	-0.659445287	3
WWTR1	1.15270764	-0.517627144	-0.635080496	3
XKR4	1.149792588	-0.482794425	-0.666998162	3
XKR7	1.15456934	-0.562210535	-0.592358804	3
XKRX	1.150700467	-0.492185783	-0.658514684	3
DC1N-ZNF705EP-ALG1	1.133243381	-0.374737403	-0.758505978	3
XRCC2	1.152846587	-0.519779144	-0.633067443	3
XRN1	1.110657468	-0.828879445	-0.281778024	3
YBX2	1.136197172	-0.3897955	-0.746401672	3
YBX3	1.135404724	-0.749752038	-0.385652686	3
YIF1B	1.152119817	-0.642880151	-0.509239667	3
YJU2	1.133128873	-0.75895523	-0.374173643	3
YKT6	1.153700569	-0.535242022	-0.618458547	3
YPEL2	1.153665162	-0.619170754	-0.534494408	3
YPEL4	1.148541153	-0.471120613	-0.67742054	3
YWHAE	1.120733622	-0.801130411	-0.319603211	3
ZAN	1.149173261	-0.672313865	-0.476859396	3
ZBED2	1.154548317	-0.561037246	-0.593511071	3
ZBED9	1.149247531	-0.671693762	-0.477553769	3
ZBTB34	1.149269994	-0.477764671	-0.671505323	3
ZBTB41	1.146021918	-0.695384662	-0.450637256	3
ZBTB7A	1.109110414	-0.832773766	-0.276336648	3
ZBTB7C	1.153845364	-0.615401996	-0.538443368	3
ZBTB8B	1.154176395	-0.607215203	-0.546961193	3
ZC3HC1	1.143564504	-0.710328883	-0.433235621	3
ZDHHC5	1.154289575	-0.550467398	-0.603822177	3
ZEB1	1.115801394	-0.815272874	-0.30052852	3
ZFP2	1.147316099	-0.686570968	-0.460745131	3
ZFP42	1.102400081	-0.848749273	-0.253650808	3
ZFTA	1.152554842	-0.63721183	-0.515343012	3
ZFYVE16	1.154598024	-0.590623912	-0.563974112	3
ZIC5	1.137158359	-0.394932556	-0.742225803	3
ZIK1	1.128529358	-0.775962852	-0.352566506	3
ZIM3	1.136849503	-0.743581553	-0.39326795	3
ZMYM1	1.15460977	-0.589843165	-0.564766605	3
ZNF106	1.118375789	-0.808038645	-0.310337144	3
ZNF114	1.13200012	-0.763311479	-0.368688642	3
ZNF157	1.152921464	-0.631950129	-0.520971335	3
ZNF19	1.132678228	-0.760709956	-0.371968272	3
ZNF2	1.149725725	-0.667588631	-0.482137094	3
ZNF20	1.15177691	-0.504772613	-0.647004297	3
ZNF221	1.154014913	-0.611463022	-0.54255189	3
ZNF227	1.138309918	-0.401262439	-0.737047479	3
ZNF260	1.14685244	-0.457034165	-0.689818275	3
ZNF264	1.107094005	-0.837725025	-0.26936898	3
ZNF282	1.132561997	-0.761159145	-0.371402852	3
ZNF296	1.1483423	-0.469373864	-0.678968436	3
ZNF3	1.154700423	-0.577797614	-0.576902809	3
ZNF302	1.130410522	-0.769237218	-0.361173304	3

ZNF311	1.108351615	-0.834653122	-0.273698493	3
ZNF366	1.150518075	-0.660295029	-0.490223046	3
ZNF396	1.147089788	-0.68816934	-0.458920449	3
ZNF425	1.154700048	-0.578271782	-0.576428265	3
ZNF444	1.138823749	-0.734670313	-0.404153436	3
ZNF462	1.14864919	-0.676568316	-0.472080874	3
ZNF469	1.142531172	-0.426466103	-0.716065069	3
ZNF470	1.110442494	-0.829425733	-0.281016761	3
ZNF486	1.143326843	-0.711673411	-0.431653433	3
ZNF521	1.149927294	-0.665795434	-0.484131861	3
ZNF528	1.150248216	-0.487392807	-0.662855409	3
ZNF536	1.130823807	-0.767718942	-0.363104865	3
ZNF544	1.149349162	-0.478511293	-0.670837869	3
ZNF558	1.153829771	-0.61574329	-0.538086481	3
ZNF561	1.129924693	-0.771002732	-0.358921961	3
ZNF563	1.143634245	-0.709931334	-0.433702911	3
ZNF564	1.105452221	-0.841657832	-0.263794389	3
ZNF565	1.147977409	-0.466234813	-0.681742596	3
ZNF572	1.128584976	-0.775768171	-0.352816805	3
ZNF573	1.1475547	-0.684856861	-0.462697839	3
ZNF576	1.128957737	-0.774457012	-0.354500725	3
ZNF592	1.150101534	-0.664212675	-0.48588886	3
ZNF596	1.148527756	-0.471002101	-0.677525655	3
ZNF611	1.153371288	-0.52871687	-0.624654419	3
ZNF614	1.111375092	-0.827043499	-0.284331593	3
ZNF626	1.15134324	-0.65187239	-0.499470851	3
ZNF652	1.143703331	-0.434167179	-0.709536152	3
ZNF655	1.143999066	-0.436170216	-0.70782885	3
ZNF665	1.133567777	-0.376342108	-0.757225669	3
ZNF697	1.154698684	-0.579141362	-0.575557322	3
ZNF704	1.150850794	-0.493835956	-0.657014838	3
ZNF705D	1.127000523	-0.781221104	-0.345779419	3
ZNF706	1.124187704	-0.790460891	-0.333726813	3
ZNF714	1.151888032	-0.645696943	-0.50619109	3
ZNF716	1.106778182	-0.83848827	-0.268289912	3
ZNF722P	1.136403066	-0.745517788	-0.390885278	3
ZNF723	1.153753563	-0.536385588	-0.617367975	3
ZNF737	1.1538591	-0.615098682	-0.538760418	3
ZNF749	1.154600645	-0.590453766	-0.564146879	3
ZNF761	1.131475398	-0.765293627	-0.366181771	3
ZNF764	1.149231847	-0.671825083	-0.477406764	3
ZNF782	1.098002933	-0.858504568	-0.239498365	3
ZNF785	1.129310839	-0.773204627	-0.356106211	3
ZNF787	1.14519908	-0.444578836	-0.700620244	3
ZNF790	1.154098319	-0.544756713	-0.609341606	3
ZNF791	1.149888077	-0.483740656	-0.666147422	3
ZNF821	1.141837875	-0.422074386	-0.719763489	3
ZNF831	1.134827381	-0.382683857	-0.752143524	3
ZNF837	1.15444047	-0.555997587	-0.598442884	3
ZNF841	1.145514467	-0.698643908	-0.446870559	3
ZNF850	1.146224594	-0.694053956	-0.452170638	3
ZNF98	1.150825062	-0.49355132	-0.657273742	3
ZNFX1	1.137460666	-0.3965751	-0.740885566	3
ZPLD1	1.154603147	-0.590289216	-0.564313931	3
ZPR1	1.153161709	-0.628190498	-0.52497121	3

ZSCAN10	1.154635051	-0.587967578	-0.566667474	3
ZSWIM4	1.107314532	-0.837190149	-0.270124383	3
ZXDB	1.144357426	-0.438632612	-0.705724814	3
ZYG11B	1.154095671	-0.544684476	-0.609411194	3
AATK	0.139112673	0.923160011	-1.062272685	4
ABCB5	0.405909623	0.73322254	-1.139132163	4
ABCC2	0.784869731	0.341039489	-1.12590922	4
ABCC3	0.480300849	0.669235809	-1.149536657	4
ABCC8	0.587508753	0.567131853	-1.154640605	4
ABHD3	0.576684671	0.578015612	-1.154700283	4
ABHD6	0.136582025	0.924688853	-1.061270878	4
ABI1	0.726978446	0.413444979	-1.140423425	4
ABI3	0.642447577	0.509706914	-1.152154491	4
ABL1	0.130832161	0.92814429	-1.058976451	4
ACACA	0.588533488	0.566094372	-1.15462786	4
ACACB	0.722089536	0.419301326	-1.141390862	4
ACAD9	0.597992619	0.556458903	-1.154451522	4
ACADSB	0.511872649	0.640439597	-1.152312246	4
ACD	0.646097105	0.505757153	-1.151854258	4
ACE	0.653076497	0.498154614	-1.151231111	4
ACER2	0.596992459	0.557482744	-1.154475203	4
ACO1	0.76176014	0.37064579	-1.132405929	4
ACO2	0.140035191	0.92260147	-1.062636661	4
ACOX2	0.198579476	0.885811599	-1.084391075	4
ACSF2	0.556447789	0.598003469	-1.154451259	4
ACTL10	0.131467775	0.927763555	-1.05923133	4
ACTR1B	0.526532045	0.626718812	-1.153250856	4
ACTR3	0.774444261	0.354517085	-1.128961347	4
ACYP2	0.6809638	0.467117151	-1.148080951	4
ADAM21	0.651591336	0.499777799	-1.151369134	4
ADAM23	0.245903682	0.854109331	-1.100013013	4
ADAM30	0.088550679	0.952779866	-1.041330545	4
ADAMTS12	0.255620759	0.847378587	-1.102999346	4
ADAMTS13	0.298487691	0.816767968	-1.115255659	4
ADAMTS14	0.131003846	0.92804148	-1.059045326	4
ADCK1	0.372506262	0.760282296	-1.132788557	4
ADCY6	0.576468085	0.578232004	-1.154700089	4
ADD1	0.462005113	0.685465368	-1.147470481	4
ADGRD1	0.29683147	0.817978754	-1.114810224	4
ADGRE3	0.764874216	0.366712794	-1.13158701	4
ADGRE5	0.395423656	0.741825345	-1.137249001	4
ADGRV1	0.257932791	0.845765879	-1.10369867	4
ADPRHL1	0.679574263	0.468689284	-1.148263548	4
ADRA1A	0.728254357	0.411910317	-1.140164674	4
ADRA1D	0.769821442	0.360428941	-1.130250382	4
ADRB3	0.073239135	0.961366916	-1.034606051	4
AFDN	0.125159061	0.931528817	-1.056687878	4
AGO1	0.7810213	0.34603826	-1.12705956	4
AGPAT3	0.722207128	0.41916091	-1.141368037	4
AGPS	0.150161438	0.916427555	-1.066588993	4
AHI1	0.782255506	0.344438181	-1.126693687	4
AHNAK2	0.092483363	0.950545718	-1.043029082	4
AHSP	0.565926852	0.588698836	-1.154625688	4
AIMP2	0.447050912	0.698488162	-1.145539074	4
AK4	0.44909812	0.696718088	-1.145816209	4

AK4P3	0.073891752	0.961004527	-1.034896279	4
AKAP12	0.119141715	0.935091869	-1.054233584	4
AKAP13	0.783860892	0.342352589	-1.126213482	4
AKNAD1	0.284168123	0.82716119	-1.111329314	4
AKR1D1	0.692975002	0.453412014	-1.146387016	4
AKR1E2	0.591470778	0.563113689	-1.154584467	4
ALG10B	0.249640094	0.851530232	-1.101170326	4
ALG13	0.362190976	0.768437698	-1.130628675	4
ALG14	0.749782679	0.38561471	-1.135397389	4
ALG5	0.311228642	0.807377138	-1.11860578	4
ALOX5	0.591705618	0.562874939	-1.154580558	4
ALPK1	0.351509482	0.776784399	-1.128293881	4
ALPK3	0.165849687	0.906706609	-1.072556296	4
ALPL	0.295460967	0.818978942	-1.114439909	4
ALS2	0.246087122	0.853982972	-1.100070094	4
ALX4	0.713835011	0.429103921	-1.142938932	4
ALYREF	0.213549908	0.875974926	-1.089524835	4
AMER1	0.639720913	0.51264652	-1.152367433	4
AMFR	0.112943839	0.938732967	-1.051676806	4
AMHR2	0.580943427	0.573749642	-1.154693069	4
AMIGO1	0.216933507	0.873727164	-1.09066067	4
AMZ2	0.600305197	0.554086978	-1.154392174	4
ANGPT2	0.33343657	0.790681861	-1.124118431	4
ANGPTL2	0.081769756	0.956604611	-1.038374367	4
ANGPTL8	0.381967165	0.752719662	-1.134686827	4
ANKAR	0.423626194	0.718458711	-1.142084904	4
ANKRD13D	0.73859165	0.399379517	-1.137971167	4
ANKRD22	0.466673259	0.681355697	-1.148028956	4
ANKRD35	0.275280418	0.833526882	-1.1088073	4
ANKRD36B	0.618091253	0.535627329	-1.153718582	4
ANKRD6	0.710734767	0.432758284	-1.143493051	4
ANKS4B	0.71632595	0.426157017	-1.142482967	4
ANO1	0.694203729	0.451998184	-1.146201913	4
ANTKMT	0.183208701	0.895728384	-1.078937085	4
ANXA4	0.743227821	0.393702563	-1.136930384	4
ANXA7	0.230722965	0.864472782	-1.095195747	4
AOC2	0.24639895	0.853768113	-1.100167063	4
AOC3	0.084743899	0.954931341	-1.03967524	4
AP1S3	0.714225298	0.428642827	-1.142868125	4
AP2M1	0.178340449	0.898830794	-1.077171243	4
AP4B1	0.19138076	0.890479004	-1.081859764	4
APC2	0.158673828	0.91117657	-1.069850398	4
APEX2	0.481710896	0.667971246	-1.149682142	4
APMAP	0.751040193	0.384054765	-1.135094959	4
APOF	0.52809217	0.625245203	-1.153337373	4
APOL1	0.422892485	0.719075901	-1.141968386	4
APOLD1	0.263923832	0.841566799	-1.105490631	4
APOM	0.127935543	0.929875462	-1.057811005	4
AQR	0.335489899	0.789117055	-1.124606954	4
ARAP3	0.349405541	0.778416753	-1.127822294	4
ARCN1	0.697202585	0.448538229	-1.145740814	4
AREG	0.288127739	0.824304244	-1.112431984	4
ARF3	0.542617445	0.611400024	-1.154017469	4
ARF5	0.769375872	0.360996699	-1.130372571	4
ARFGAP3	0.156198865	0.912709043	-1.068907908	4

ARHGAP10	0.22710783	0.866913588	-1.094021418	4
ARHGAP25	0.349627943	0.778244383	-1.127872325	4
ARHGAP29	0.153559997	0.914337803	-1.067897799	4
ARHGAP44	0.390558471	0.745782962	-1.136341434	4
ARHGEF11	0.245895154	0.854115205	-1.100010359	4
ARHGEF18	0.522026124	0.630960337	-1.152986461	4
ARHGEF4	0.223371518	0.869425282	-1.092796799	4
ARHGEF40	0.456234559	0.690516228	-1.146750787	4
ARHGEF6	0.550740036	0.603557771	-1.154297808	4
ARHGEF7	0.221043108	0.87098492	-1.092028028	4
ARL10	0.296552836	0.818182227	-1.114735062	4
ARL2-SNX15	0.590791949	0.563803452	-1.154595401	4
ARL9	0.624973112	0.528379935	-1.153353048	4
ARMC10	0.590119537	0.564486158	-1.154605695	4
ARMC8	0.724724088	0.416150157	-1.140874245	4
ARMH1	0.336353667	0.788457715	-1.124811381	4
ARPC4-TTLL3	0.392266646	0.744395868	-1.136662514	4
ARPC5	0.116728378	0.936513122	-1.0532415	4
ARSF	0.715982548	0.42656385	-1.142546398	4
ART1	0.549257679	0.604994376	-1.154252055	4
ASB12	0.374803438	0.758453337	-1.133256775	4
ASGR1	0.418418721	0.72282837	-1.141247091	4
ATG16L2	0.365591808	0.765759299	-1.131351107	4
ATG4A	0.28109916	0.829366626	-1.110465786	4
ATG4D	0.626731427	0.526518677	-1.153250104	4
ATP13A4	0.698536581	0.446994847	-1.145531428	4
ATP1B3	0.389628501	0.746537025	-1.136165526	4
ATP23	0.087120397	0.953589503	-1.0407099	4
ATP2B4	0.491059218	0.659537037	-1.150596255	4
ATP5IF1	0.489457689	0.660988207	-1.150445896	4
ATP6V0B	0.348713969	0.778952475	-1.127666444	4
ATP6V0E2	0.673932009	0.475044916	-1.148976925	4
ATP6V1A	0.418862799	0.722456719	-1.141319519	4
ATP6V1B2	0.641779828	0.510427709	-1.152207536	4
ATP6V1E2	0.539048964	0.614822543	-1.153871507	4
ATP6V1G1	0.484831346	0.665165686	-1.149997033	4
ATP8A1	0.510207925	0.641983494	-1.152191419	4
ATP8B1	0.542916242	0.61111282	-1.154029062	4
ATRIP	0.669094615	0.480458396	-1.149553011	4
ATRN	0.18303471	0.895839582	-1.078874292	4
ATXN1	0.546254347	0.607897594	-1.154151941	4
ATXN7L3	0.730682735	0.408982245	-1.139664979	4
AVP	0.584081992	0.570592281	-1.154674273	4
B3GAT1	0.443325795	0.701698721	-1.145024517	4
B3GAT3	0.169611515	0.904347391	-1.073958906	4
B4GALNT3	0.399245909	0.738701099	-1.137947007	4
B4GAT1	0.441816149	0.70299607	-1.144812219	4
BAIAP2L2	0.255760017	0.847281573	-1.10304159	4
BARHL1	0.495625645	0.65538506	-1.151010705	4
BATF2	0.700834814	0.444329674	-1.145164488	4
BAZ2B	0.499169503	0.652148222	-1.151317725	4
BBS2	0.449534022	0.696340677	-1.145874699	4
BBX	0.089059665	0.952491372	-1.041551037	4
BCL7C	0.440903353	0.70377945	-1.144682803	4
BCR	0.13667538	0.924632541	-1.061307921	4

BDH1	0.581694609	0.572995005	-1.154689614	4
BECN1	0.473405173	0.675391518	-1.148796692	4
BHMT	0.581358144	0.573333099	-1.154691243	4
BICDL1	0.785486364	0.34023593	-1.125722294	4
BIRC6	0.193697671	0.888981225	-1.082678897	4
BIVM	0.234122936	0.862167738	-1.096290674	4
BLOC1S3	0.478612862	0.670747027	-1.149359889	4
BMP7	0.554106035	0.600286641	-1.154392676	4
BMPR1A	0.733203167	0.4059331	-1.139136267	4
BNIP3L	0.33323516	0.790835159	-1.124070318	4
BOD1L2	0.699189406	0.446238591	-1.145427996	4
BOLA2-SMG1P6	0.456276796	0.690479376	-1.146756172	4
BORCS8	0.305326236	0.811744429	-1.117070665	4
BPNT1	0.490699735	0.659862996	-1.150562731	4
BPTF	0.492577493	0.658158908	-1.150736401	4
BRCA1	0.228040605	0.866284809	-1.094325414	4
BRI3BP	0.644483349	0.507505824	-1.151989172	4
BRME1	0.442048151	0.702796835	-1.144844986	4
BRMS1	0.327144236	0.795454725	-1.122598962	4
BROX	0.09139133	0.951167274	-1.042558604	4
BRSK2	0.251718617	0.850090641	-1.101809258	4
BSND	0.211925287	0.877050965	-1.088976252	4
BSX	0.311719507	0.807012622	-1.118732129	4
BTBD2	0.437184488	0.706962883	-1.144147371	4
BTBD3	0.480798606	0.668789635	-1.149588241	4
BTD	0.556303166	0.598144652	-1.154447818	4
BTF3L4	0.200130963	0.884800346	-1.084931309	4
C10orf90	0.338931163	0.786486431	-1.125417594	4
C10orf95	0.784681319	0.341284872	-1.125966191	4
C11orf54	0.288771506	0.82383853	-1.112610036	4
C12orf4	0.740745989	0.396745989	-1.137491978	4
C12orf75	0.23608365	0.860834256	-1.096917906	4
C15orf40	0.682911443	0.464908942	-1.147820385	4
C17orf50	0.735835712	0.40273729	-1.138573002	4
C19orf44	0.625326532	0.528006138	-1.15333267	4
C19orf71	0.526059336	0.627164795	-1.153224131	4
C1orf112	0.553533456	0.600843969	-1.154377425	4
C1orf162	0.6131989	0.540743652	-1.153942552	4
C1orf43	0.691347156	0.455281685	-1.14662884	4
C1orf50	0.596357071	0.558132555	-1.154489626	4
C1orf74	0.551451736	0.602867176	-1.154318912	4
C1QB	0.299182911	0.816259045	-1.115441956	4
C1QL2	0.58141806	0.573272902	-1.154690962	4
C1QTNF12	0.204598613	0.881877789	-1.086476402	4
C1RL	0.759823339	0.373083264	-1.132906603	4
C20orf173	0.585995718	0.56866145	-1.154657167	4
C20orf96	0.208866448	0.879071278	-1.087937726	4
C22orf15	0.769149405	0.361285133	-1.130434538	4
C2orf49	0.518502602	0.634262151	-1.152764753	4
C2orf68	0.085336503	0.95459714	-1.039933643	4
C2orf76	0.334549326	0.789834297	-1.124383623	4
C3AR1	0.42018445	0.721349545	-1.141533995	4
C4BPB	0.47159195	0.677002346	-1.148594296	4
C4orf47	0.21345609	0.876037122	-1.089493212	4
C6orf136	0.145279928	0.919413617	-1.064693545	4

C6orf52	0.256411472	0.846827529	-1.103239001	4
C8orf74	0.527168172	0.626118274	-1.153286446	4
CA13	0.460808161	0.686515697	-1.147323858	4
CA5B	0.484685857	0.665296711	-1.149982568	4
CAB39L	0.525890479	0.627324048	-1.153214526	4
CACFD1	0.548915388	0.605325758	-1.154241146	4
CACNA1B	0.283430899	0.827691686	-1.111122585	4
CACNA1H	0.199937928	0.884926268	-1.084864196	4
CACNB1	0.326956117	0.795596898	-1.122553016	4
CACNG6	0.103940212	0.943970316	-1.047910528	4
CACUL1	0.252760486	0.849367728	-1.102128214	4
CACYBP	0.787982363	0.336975881	-1.124958244	4
CALHM6	0.221991596	0.870350114	-1.09234171	4
CALM2	0.337162767	0.787839523	-1.125002291	4
CALM3	0.513914432	0.638542068	-1.1524565	4
CALML5	0.597112765	0.557359652	-1.154472418	4
CALML6	0.599416907	0.554998823	-1.15441573	4
CALU	0.465870777	0.68206369	-1.147934467	4
CAMK2B	0.280726387	0.829633982	-1.11036037	4
CAMKK2	0.167481151	0.905684787	-1.073165938	4
CAND1	0.205367867	0.88137299	-1.086740858	4
CANT1	0.535071517	0.61862103	-1.153692547	4
CAP1	0.249861532	0.851377029	-1.101238561	4
CAPN13	0.235930662	0.860938414	-1.096869076	4
CAPN5	0.213613712	0.875932625	-1.089546336	4
CAPRIN1	0.280282925	0.82995189	-1.110234814	4
CAPS	0.117206236	0.936232056	-1.053438292	4
CARD8	0.288696598	0.823892738	-1.112589336	4
CARD9	0.335492663	0.789114947	-1.124607609	4
CARF	0.458636607	0.688417687	-1.147054293	4
CARS2	0.079896193	0.957655256	-1.037551449	4
CASKIN2	0.556317871	0.598130298	-1.154448168	4
CASP10	0.666717179	0.483107058	-1.149824236	4
CASP7	0.188196754	0.892530472	-1.080727226	4
CASZ1	0.33867791	0.786680373	-1.125358283	4
CATSPER4	0.438434448	0.705894351	-1.144328799	4
CBL	0.389674198	0.746499989	-1.136174188	4
CBLN1	0.423187588	0.718827722	-1.142015311	4
CBWD1	0.074546159	0.960640821	-1.03518698	4
CBX1	0.395270396	0.741950341	-1.137220737	4
CC2D1B	0.48678704	0.663402369	-1.150189408	4
CCAR1	0.638435766	0.514028676	-1.152464443	4
CCDC12	0.779061295	0.348573427	-1.127634722	4
CCDC138	0.324804936	0.797220539	-1.122025475	4
CCDC201	0.387173379	0.748524015	-1.135697395	4
CCDC32	0.676358044	0.472317646	-1.148675689	4
CCDC50	0.119097696	0.935117832	-1.054215528	4
CCDC74B	0.133247979	0.926695562	-1.059943541	4
CCDC78	0.288754488	0.823850846	-1.112605334	4
CCDC81	0.51766218	0.635047761	-1.152709941	4
CCDC93	0.652753777	0.498507576	-1.151261353	4
CCDC97	0.564717357	0.589891704	-1.154609061	4
CCL21	0.407254878	0.732111626	-1.139366504	4
CCL22	0.433590337	0.710027125	-1.143617463	4
CCN3	0.598634095	0.555801608	-1.154435703	4

CCNB2	0.126075361	0.930983824	-1.057059186	4
CCND3	0.45857908	0.68846801	-1.14704709	4
CCNO	0.626112968	0.52717379	-1.153286758	4
CCNQ	0.086388734	0.954003076	-1.04039181	4
CCR2	0.240772104	0.857633169	-1.098405272	4
CCS	0.156627722	0.912443838	-1.06907156	4
CCSER2	0.195746305	0.887653376	-1.083399681	4
CCT3	0.724325206	0.416627963	-1.14095317	4
CD2	0.229121519	0.865555304	-1.094676823	4
CD248	0.645264511	0.506659782	-1.151924293	4
CD27	0.170931697	0.903516844	-1.074448541	4
CD34	0.72467212	0.416212422	-1.140884542	4
CD36	0.40158971	0.736778758	-1.138368467	4
CD5	0.119906523	0.934640535	-1.054547058	4
CD6	0.094473188	0.949410842	-1.043884031	4
CD86	0.115722408	0.937104244	-1.052826652	4
CDC14C	0.702009286	0.442964639	-1.144973926	4
CDC42	0.519964843	0.632893506	-1.152858349	4
CDCP1	0.314644203	0.804836549	-1.119480752	4
CDH6	0.341906988	0.784203409	-1.126110397	4
CDHR2	0.454036399	0.692431751	-1.14646815	4
CDIPT	0.303069668	0.813406399	-1.116476067	4
CDK13	0.455771828	0.690919849	-1.146691677	4
CDKN2AIPNL	0.32536317	0.79679958	-1.12216275	4
CDKN2D	0.660178831	0.490351279	-1.15053011	4
CDPF1	0.287733264	0.824589447	-1.112322711	4
CDR2L	0.203917467	0.882324381	-1.086241848	4
CEACAM19	0.368232691	0.763672404	-1.131905095	4
CEACAM20	0.778995944	0.348657831	-1.127653775	4
CEACAM21	0.664384938	0.4856978	-1.150082737	4
CENPC	0.257036496	0.846391584	-1.10342808	4
CENPO	0.65150024	0.499877265	-1.151377504	4
CENPT	0.412093324	0.728102319	-1.140195643	4
CEP128	0.535801202	0.617925457	-1.153726658	4
CEP135	0.12799351	0.92984088	-1.057834391	4
CEP164	0.650535084	0.500930423	-1.151465508	4
CEP250	0.366078091	0.765375486	-1.131453577	4
CEP43	0.701991597	0.442985214	-1.144976811	4
CEP57	0.426529618	0.716011449	-1.142541067	4
CEP83	0.442030117	0.702812323	-1.144842441	4
CERS2	0.763266487	0.368745463	-1.13201195	4
CERT1	0.165059315	0.907200895	-1.07226021	4
CES2	0.7471666	0.388851323	-1.136017923	4
CFAP141	0.706750222	0.437433395	-1.144183617	4
CFAP418	0.083469851	0.955648946	-1.039118798	4
CFAP57	0.556804329	0.597655313	-1.154459642	4
CFAP92	0.718433873	0.423655708	-1.142089581	4
CFAP97D2	0.72364056	0.417447488	-1.141088048	4
CFH	0.592241887	0.562329498	-1.154571385	4
CHAF1B	0.269410086	0.837695931	-1.107106017	4
CHCHD5	0.370491747	0.761882364	-1.132374111	4
CHEK1	0.183656311	0.895442208	-1.079098518	4
CHEK2	0.432644876	0.710831168	-1.143476044	4
CHFR	0.733509504	0.405561799	-1.139071303	4
CHKA	0.616041372	0.537774662	-1.153816034	4

CHMP4A	0.207330546	0.880082933	-1.087413479	4
CHMP4B	0.500554121	0.650880073	-1.151434194	4
CHMP7	0.291795994	0.821645968	-1.113441962	4
CHRNB2	0.47733723	0.671887191	-1.149224421	4
CHRND	0.264606447	0.841086509	-1.105692956	4
CHST14	0.435240767	0.708621543	-1.14386231	4
CIAPIN1	0.785062567	0.340788275	-1.125850842	4
CIB1	0.489586756	0.660871353	-1.15045811	4
CILP	0.676672891	0.471963097	-1.148635988	4
CINP	0.362736719	0.76800857	-1.130745289	4
CIZ1	0.163330376	0.908280463	-1.071610839	4
CKAP5	0.707936032	0.436044598	-1.14398063	4
CKLF	0.310557926	0.807874883	-1.118432809	4
CKLF-CMTM1	0.448329737	0.697382924	-1.145712662	4
CLCC1	0.088318677	0.9529113	-1.041229977	4
CLCN1	0.305437997	0.811662006	-1.117100003	4
CLDN6	0.738755627	0.399179336	-1.137934963	4
CLDN9	0.54789745	0.606310491	-1.154207941	4
CLDND1	0.218926268	0.87239911	-1.091325378	4
CLDND2	0.370373775	0.761975955	-1.13234973	4
CLEC18B	0.680918026	0.467168984	-1.14808701	4
CLEC19A	0.411108057	0.728920489	-1.140028546	4
CLEC2B	0.462660755	0.684889449	-1.147550203	4
CLK4	0.749960579	0.385394189	-1.135354768	4
CLN5	0.438402447	0.705921725	-1.144324173	4
CLN6	0.082835441	0.956005819	-1.03884126	4
CLSTN3	0.378681481	0.755355111	-1.134036592	4
CMTM1	0.282243873	0.828544912	-1.110788785	4
CMTM5	0.753779955	0.38064671	-1.134426666	4
CMTR1	0.571354505	0.583325349	-1.154679854	4
CNGB1	0.221793365	0.870482845	-1.09227621	4
CNKSR1	0.09918264	0.946712902	-1.045895542	4
CNOT11	0.585418847	0.569243928	-1.154662775	4
CNOT9	0.112645985	0.938907213	-1.051553198	4
CNPY1	0.551173831	0.603136907	-1.154310738	4
CNTN2	0.588649396	0.565976945	-1.154626341	4
CNTN5	0.720844673	0.420786482	-1.141631155	4
COA1	0.164481533	0.907561925	-1.072043458	4
COA4	0.210693549	0.877865396	-1.088558945	4
COL11A2	0.504540074	0.64721842	-1.151758495	4
COL18A1	0.571568656	0.583112647	-1.154681303	4
COL1A1	0.662292322	0.488016009	-1.150308331	4
COL4A2	0.347014684	0.780267058	-1.127281742	4
COLEC11	0.561588645	0.592969745	-1.15455839	4
COPB1	0.788126756	0.336786923	-1.124913679	4
COPS7A	0.608796124	0.545322751	-1.154118875	4
COQ8B	0.48564676	0.66443095	-1.150077709	4
COX10	0.340045493	0.78563242	-1.125677913	4
COX18	0.761223911	0.371321298	-1.132545209	4
CP	0.406953909	0.73236031	-1.13931422	4
CPA4	0.384168574	0.750948523	-1.135117097	4
CPA5	0.691612603	0.454977069	-1.146589671	4
CPE	0.783347807	0.343019679	-1.126367486	4
CPLX3	0.600661818	0.553720633	-1.15438245	4
CPNE1	0.197425808	0.88656233	-1.083988139	4

CPNE8	0.619487393	0.534161824	-1.153649217	4
CPOX	0.749915287	0.385450337	-1.135365624	4
CPSF3	0.130038654	0.928619169	-1.058657823	4
CRACDL	0.076925227	0.95931586	-1.036241087	4
CRAMP1	0.648106659	0.503574799	-1.151681458	4
CRBN	0.760556999	0.372160726	-1.132717725	4
CREBL2	0.49688156	0.654239416	-1.151120976	4
CREBRF	0.621378372	0.532173014	-1.153551387	4
CRELD2	0.670411996	0.478987356	-1.149399351	4
CRHR1	0.233870689	0.862339068	-1.096209757	4
CRIP1	0.70835282	0.435555958	-1.143908777	4
CRMP1	0.679090228	0.469236282	-1.14832651	4
CRYBA4	0.794654747	0.328202002	-1.122856749	4
CRYBB3	0.752957854	0.381670697	-1.134628551	4
CSAG1	0.748796674	0.38683596	-1.135632635	4
CSDE1	0.680934706	0.467150097	-1.148084802	4
CSE1L	0.503210585	0.648441557	-1.151652141	4
CSGALNACT2	0.290997751	0.822225378	-1.113223129	4
CSNK1D	0.504797571	0.646981312	-1.151778883	4
CSNK1G1	0.760143417	0.372680899	-1.132824317	4
CSNK2A3	0.380700882	0.753736488	-1.13443737	4
CSPG5	0.450258071	0.695713379	-1.14597145	4
CSPP1	0.525646792	0.627553821	-1.153200612	4
CSRP1	0.139995605	0.922625451	-1.062621056	4
CST4	0.55785103	0.596632405	-1.154483435	4
CST7	0.376367456	0.757205426	-1.133572883	4
CT47A10	0.137086796	0.924384297	-1.061471093	4
CT47A2	0.108567156	0.94128655	-1.049853706	4
CT47A6	0.231533756	0.863923935	-1.095457691	4
CTAG1B	0.235893591	0.86096365	-1.096857241	4
CTAG2	0.46202219	0.685450373	-1.147472563	4
CTDP1	0.49614254	0.654913744	-1.151056284	4
CTHRC1	0.281818377	0.828850474	-1.11066885	4
CTNNA1	0.59361058	0.560935848	-1.154546428	4
CTNNAL1	0.691084857	0.455582587	-1.146667444	4
CTTNBP2NL	0.468841336	0.679439743	-1.148281079	4
CTU2	0.24765032	0.852905094	-1.100555414	4
CTXN1	0.599902464	0.554500507	-1.154402972	4
CUTA	0.692444122	0.454022186	-1.146466307	4
CX3CR1	0.19577299	0.887636058	-1.083409048	4
CXCR3	0.739034256	0.398839089	-1.137873345	4
CXXC1	0.659669392	0.490913268	-1.15058266	4
CYB561D2	0.169531472	0.904397703	-1.073929176	4
CYB5D2	0.389372012	0.746744861	-1.136116873	4
CYB5R1	0.200977952	0.884247485	-1.085225437	4
CYC1	0.383904958	0.751160842	-1.135065801	4
CYFIP1	0.770181586	0.359969771	-1.130151357	4
CYP11B2	0.584167899	0.570505698	-1.154673597	4
CYP3A7-CYP3A51P	0.326781489	0.795728849	-1.122510338	4
CYP4F22	0.263684881	0.841734836	-1.105419717	4
CYP4F3	0.666462173	0.483390692	-1.149852865	4
CYP7A1	0.265451406	0.840491469	-1.105942875	4
CYSLTR2	0.519648108	0.633190158	-1.152838266	4
DAAM1	0.341979243	0.784147881	-1.126127124	4
DAO	0.567228823	0.587412911	-1.154641734	4

DAPK2	0.663582407	0.486587554	-1.150169962	4
DCAF4	0.170882957	0.903547531	-1.074430488	4
DCAF5	0.728342198	0.411804565	-1.140146763	4
DCAF7	0.69023042	0.456562079	-1.146792499	4
DCDC2B	0.290812311	0.822359906	-1.113172217	4
DCLRE1B	0.124440858	0.931955539	-1.056396397	4
DCLRE1C	0.244622587	0.854991035	-1.099613623	4
DCP1B	0.510540759	0.641675048	-1.152215807	4
DCST2	0.382995647	0.751892738	-1.134888385	4
DDR1	0.346137891	0.780944377	-1.127082268	4
DDT	0.57534751	0.579350715	-1.154698225	4
DDTL	0.357689049	0.771967712	-1.12965676	4
DDX11	0.29727442	0.817655157	-1.114929577	4
DDX39B	0.162861025	0.908573132	-1.071434156	4
DDX50	0.242939842	0.856147169	-1.099087011	4
DDX55	0.127460837	0.93015856	-1.057619397	4
DEFB130A	0.704007235	0.440637764	-1.144644999	4
DEFB4A	0.570893309	0.583783247	-1.154676556	4
DEGS1	0.43540403	0.708482361	-1.143886391	4
DENND5A	0.515554362	0.637014848	-1.15256921	4
DENR	0.244553296	0.855038687	-1.099591983	4
DEPTOR	0.567955056	0.586694793	-1.154649849	4
DEUP1	0.146641609	0.918582507	-1.065224116	4
DGKA	0.308619465	0.809311313	-1.117930778	4
DHCR7	0.124186923	0.93210632	-1.056293243	4
DHFR2	0.499888718	0.651489749	-1.151378468	4
DHODH	0.690925494	0.455765355	-1.146690849	4
DHRS13	0.707892719	0.436095363	-1.143988082	4
DHRS4L2	0.526296522	0.626941048	-1.15323757	4
DHRSX	0.093358728	0.950046833	-1.04340556	4
DHX15	0.769732308	0.360542546	-1.130274854	4
DHX30	0.692732509	0.453690777	-1.146423286	4
DHX35	0.150548298	0.916190125	-1.066738423	4
DHX37	0.098917877	0.946865025	-1.045782902	4
DHX40	0.464851163	0.68296234	-1.147813503	4
DICER1	0.379741589	0.754505849	-1.134247438	4
DIDO1	0.660146069	0.490387432	-1.1505335	4
DIMT1	0.562982299	0.591600025	-1.154582324	4
DIPK2A	0.439828304	0.704701064	-1.144529368	4
DIS3L2	0.134310542	0.926056947	-1.060367488	4
DLAT	0.453318244	0.693056555	-1.146374799	4
DLEC1	0.543735996	0.610324371	-1.154060366	4
DLX2	0.245096556	0.854664984	-1.099761539	4
DMKN	0.507625657	0.644372645	-1.151998302	4
DMRTB1	0.370968732	0.761503833	-1.132472565	4
DMTF1	0.158847695	0.911068735	-1.069916431	4
DNA2	0.146342764	0.918765031	-1.065107795	4
DNAAF5	0.512037983	0.640286106	-1.152324089	4
DNAAF9	0.132653527	0.92705246	-1.059705987	4
DNAI1	0.432458371	0.710989676	-1.143448048	4
DNAI2	0.769895575	0.360334443	-1.130230018	4
DNAJA2	0.2700824	0.837219888	-1.107302288	4
DNAJB8	0.289608394	0.823232597	-1.11284099	4
DNAJC10	0.748569864	0.38711665	-1.135686514	4
DNAJC14	0.57393254	0.580761268	-1.154693808	4

DNAJC21	0.221126773	0.870928953	-1.092055726	4
DNAJC22	0.376781696	0.756874546	-1.133656242	4
DNAJC25-GNG10	0.19225182	0.889916395	-1.082168216	4
DNALI1	0.250656378	0.850826786	-1.101483164	4
DNASE1L1	0.354505214	0.774453514	-1.128958728	4
DOCK10	0.731179064	0.408382613	-1.139561678	4
DOCK7	0.376369506	0.75720379	-1.133573295	4
DOCK8	0.242197852	0.856656233	-1.098854085	4
DPAGT1	0.461734839	0.685702657	-1.147437495	4
DPEP1	0.78630137	0.339172752	-1.125474122	4
DPF1	0.691085014	0.455582407	-1.146667421	4
DPM3	0.615580433	0.538256797	-1.153837229	4
DPP4	0.418369927	0.722869195	-1.141239121	4
DPYD	0.137916298	0.92388339	-1.061799688	4
DR1	0.165149379	0.907144595	-1.072293974	4
DRG1	0.330180099	0.793156175	-1.123336274	4
DRICH1	0.375494158	0.757902488	-1.133396646	4
DSCAML1	0.337070845	0.787909785	-1.124980629	4
DTX1	0.780156786	0.347157351	-1.127314137	4
DTYMK	0.283687926	0.827506784	-1.11119471	4
DUOXA2	0.660142169	0.490391735	-1.150533904	4
DUSP7	0.340086585	0.785600907	-1.125687492	4
DUT	0.293949598	0.820080125	-1.114029723	4
DUX4	0.363633731	0.767302665	-1.130936396	4
DUXA	0.716604257	0.425827169	-1.142431425	4
DVL2	0.450493039	0.6955097	-1.146002739	4
DYNC2LI1	0.12499274	0.931627672	-1.056620412	4
DYRK1B	0.220449388	0.871381923	-1.091831311	4
DYSF	0.491891991	0.658781423	-1.150673414	4
E2F3	0.762277906	0.369993056	-1.132270962	4
E2F8	0.716763218	0.425638714	-1.142401932	4
EAPP	0.530101533	0.623343444	-1.153444977	4
EBF4	0.11709897	0.936295163	-1.053394133	4
EBI3	0.125396777	0.931387491	-1.056784268	4
ECH1	0.226654617	0.867218847	-1.093873464	4
ECHDC2	0.248240815	0.852497416	-1.100738231	4
ECT2L	0.343387079	0.783065061	-1.12645214	4
EDN1	0.29932399	0.816155722	-1.115479712	4
EFHB	0.53525826	0.618443072	-1.153701332	4
EFHC2	0.708349359	0.435560016	-1.143909375	4
EFR3A	0.638841358	0.5135927	-1.152434058	4
EFR3B	0.38093111	0.753551721	-1.134482831	4
EGFL7	0.189633982	0.891605441	-1.081239423	4
EGLN1	0.404498302	0.734386233	-1.138884535	4
EIF2A	0.605192999	0.549052533	-1.154245533	4
EIF4H	0.553779002	0.600605007	-1.15438401	4
EIF5B	0.596176488	0.558317148	-1.154493637	4
ELANE	0.725035436	0.415777029	-1.140812465	4
ELAPOR1	0.294668642	0.819556466	-1.114225108	4
ELAVL1	0.16780845	0.905479544	-1.073287994	4
ELFN1	0.654601071	0.496485285	-1.151086356	4
ELL2	0.780660273	0.34650577	-1.127166043	4
ELP5	0.091710621	0.950985635	-1.042696256	4
EMD	0.083196349	0.955802836	-1.038999185	4
EN2	0.494934497	0.65601484	-1.150949336	4

ENO3	0.175289341	0.900765789	-1.076055129	4
ENOX2	0.250470047	0.850955822	-1.101425869	4
ENPP6	0.437130181	0.707009274	-1.144139455	4
ENPP7	0.418650476	0.722634436	-1.141284912	4
ENTPD8	0.702941618	0.441879562	-1.14482118	4
ENY2	0.56818772	0.586464603	-1.154652322	4
EOMES	0.761406243	0.371091664	-1.132497907	4
EPAS1	0.38040216	0.753976153	-1.134378313	4
EPB41	0.416691847	0.724271859	-1.140963706	4
EPHB3	0.305987761	0.811256407	-1.117244168	4
EPN3	0.277895943	0.831660283	-1.109556226	4
EPPK1	0.63360441	0.519205624	-1.152810033	4
EPS15	0.773863016	0.355262553	-1.129125569	4
EPS8L3	0.709964936	0.433663424	-1.14362836	4
ERCC6L2	0.641768381	0.51044006	-1.152208441	4
ERG28	0.639913498	0.512439213	-1.15235271	4
ERGIC2	0.424325557	0.717869944	-1.142195502	4
ERICH1	0.748967821	0.386624099	-1.13559192	4
ERLIN1	0.193149652	0.889335874	-1.082485526	4
ERMP1	0.52340052	0.629668868	-1.153069388	4
ESR2	0.57597701	0.57872244	-1.15469945	4
ESRP2	0.788517108	0.336275893	-1.124793001	4
ESYT1	0.210725973	0.877843972	-1.088569945	4
ESYT2	0.752911163	0.381728819	-1.134639982	4
ETFRF1	0.081717219	0.956634108	-1.038351327	4
ETS1	0.57023785	0.584433601	-1.154671451	4
ETV1	0.740356362	0.39722285	-1.137579212	4
EVA1B	0.415939431	0.724899939	-1.14083937	4
EVX2	0.404614077	0.734290841	-1.138904917	4
EXOC3L1	0.613019421	0.540930784	-1.153950205	4
EXOC3L4	0.518622975	0.634149567	-1.152772543	4
EXOC6	0.666200901	0.483681203	-1.149882104	4
EXOSC10	0.673937452	0.475038806	-1.148976259	4
EXOSC3	0.080437801	0.957351809	-1.03778961	4
EXOSC8	0.474849639	0.674105962	-1.148955601	4
EXPH5	0.550397571	0.603889882	-1.154287453	4
EXT1	0.655795864	0.495174871	-1.150970735	4
EXTL3	0.715543651	0.427083551	-1.142627202	4
EYS	0.165457624	0.906951859	-1.072409483	4
EZR	0.194243687	0.888627639	-1.082871326	4
FAAP24	0.637614617	0.514910689	-1.152525306	4
FABP6	0.185445035	0.894297039	-1.079742074	4
FAF1	0.424635698	0.717608703	-1.142244402	4
FAIM	0.692657464	0.453777028	-1.146434493	4
FAM102A	0.237176547	0.860089643	-1.09726619	4
FAM114A1	0.709624366	0.434063564	-1.14368793	4
FAM117B	0.525469036	0.627721387	-1.153190423	4
FAM118B	0.284067246	0.827233807	-1.111301052	4
FAM120A	0.560036027	0.594493128	-1.154529155	4
FAM120AOS	0.66749242	0.482244232	-1.149736652	4
FAM135A	0.739746709	0.397968494	-1.137715203	4
FAM13A	0.294504161	0.81967629	-1.114180451	4
FAM180B	0.14866592	0.917344323	-1.066010243	4
FAM181B	0.624903043	0.528454026	-1.153357069	4
FAM199X	0.711504674	0.431852148	-1.143356822	4

FAM222B	0.790941163	0.333095858	-1.124037021	4
FAM240C	0.567428692	0.587215335	-1.154644027	4
FAM241A	0.451703157	0.694459887	-1.146163044	4
FAM246A	0.389232654	0.74685776	-1.136090414	4
FAM246B	0.595471131	0.5590378	-1.154508931	4
FAM47E	0.663352003	0.486842837	-1.150194841	4
FAM47E-STBD1	0.335282359	0.789275381	-1.124557741	4
FAM71E1	0.18572628	0.894116755	-1.079843034	4
FAM83E	0.16789776	0.905423526	-1.073321285	4
FAM89B	0.709420969	0.434302454	-1.143723423	4
FAM90A1	0.22257968	0.869956161	-1.092535841	4
FAM90A26	0.128959316	0.929264334	-1.05822365	4
FANCE	0.079589976	0.957826722	-1.037416698	4
FAR2	0.307519773	0.810124802	-1.117644575	4
FASTKD5	0.084134639	0.955274659	-1.039409298	4
FAT2	0.186597768	0.893557719	-1.080155487	4
FBH1	0.408553604	0.731037565	-1.139591169	4
FBXL20	0.390879709	0.745522308	-1.136402016	4
FBXL22	0.727843424	0.41240487	-1.140248294	4
FBXL5	0.293539533	0.820378571	-1.113918104	4
FBXO2	0.150962259	0.915935936	-1.066898195	4
FBXO21	0.335328892	0.789239886	-1.124568778	4
FBXO24	0.278859889	0.830970937	-1.109830826	4
FBXO9	0.57928973	0.575408634	-1.154698364	4
FBXW2	0.450288287	0.695687189	-1.145975477	4
FBXW8	0.30543261	0.81166598	-1.117098589	4
FCHO2	0.64646474	0.505358303	-1.151823044	4
FDXR	0.610525645	0.543526802	-1.154052448	4
FECH	0.780155306	0.347159266	-1.127314572	4
FEM1B	0.626755864	0.526492781	-1.153248645	4
FERD3L	0.57325319	0.58143768	-1.15469087	4
FERMT1	0.579798363	0.574898711	-1.154697073	4
FEV	0.753223545	0.381339887	-1.134563432	4
FFAR2	0.63123313	0.521735543	-1.152968672	4
FGD2	0.695429218	0.450585868	-1.146015086	4
FGD3	0.237062044	0.860167701	-1.097229745	4
FGD4	0.6335499	0.51926386	-1.152813761	4
FGD6	0.297848781	0.817235315	-1.115084097	4
FGF22	0.272914027	0.835210857	-1.108124884	4
FGF8	0.731218483	0.408334974	-1.139553456	4
FGFRL1	0.746555592	0.38960559	-1.136161182	4
FHIP2B	0.314047787	0.805280887	-1.119328674	4
FHOD3	0.66825435	0.481395413	-1.149649764	4
FIBP	0.543861403	0.610203688	-1.154065091	4
FIGNL2	0.232591009	0.863207465	-1.095798474	4
FITM2	0.322936356	0.798627693	-1.12156405	4
FKBP10	0.143732497	0.920356371	-1.064088868	4
FKBP2	0.294101799	0.819969317	-1.114071116	4
FKBP3	0.732736885	0.406497971	-1.139234856	4
FKBP5	0.437482704	0.706708086	-1.14419079	4
FKRP	0.378539522	0.75546876	-1.134008282	4
FLAD1	0.627883687	0.525296833	-1.15318052	4
FLCN	0.640805671	0.511478207	-1.152283878	4
FLNC	0.710028796	0.433588373	-1.14361717	4
FMNL1	0.696774001	0.449033524	-1.145807525	4

FNBP1	0.409070472	0.730609679	-1.139680151	4
FNDC9	0.135678732	0.925233371	-1.060912103	4
FOXC1	0.703254677	0.441514922	-1.144769598	4
FOXD1	0.758626553	0.37458613	-1.133212683	4
FOXK2	0.182410031	0.896238621	-1.078648652	4
FOXL2	0.674823062	0.474044183	-1.148867245	4
FOXN3	0.640744003	0.511544667	-1.152288669	4
FOXP1	0.394593784	0.742501921	-1.137095705	4
FOXQ1	0.644736913	0.507231287	-1.1519682	4
FPR1	0.466954218	0.68110767	-1.148061888	4
FRAT1	0.696550831	0.449291324	-1.145842155	4
FRYL	0.238381686	0.859267449	-1.097649135	4
FSCN2	0.552318221	0.602025629	-1.15434385	4
FSD1	0.366020268	0.765421135	-1.131441403	4
FSTL3	0.488442411	0.661906826	-1.150349236	4
FTO	0.324631859	0.797351001	-1.12198286	4
G6PD	0.570405919	0.584266888	-1.154672807	4
GAB1	0.671452922	0.47782331	-1.149276232	4
GABBR1	0.440381276	0.704227151	-1.144608427	4
GABRA5	0.225433054	0.86804081	-1.093473864	4
GABRE	0.596138186	0.558356296	-1.154494482	4
GALNT2	0.658615567	0.49207468	-1.150690247	4
GALNT9	0.677209281	0.471358748	-1.14856803	4
GALT	0.429741325	0.713295161	-1.143036486	4
GAPDH	0.2272184	0.86683909	-1.09405749	4
GAPDHS	0.277793476	0.831733516	-1.109526991	4
GARS1	0.516612651	0.636027799	-1.15264045	4
GART	0.400981461	0.737278112	-1.138259572	4
GATA3	0.411616725	0.728498201	-1.140114925	4
GBA2	0.25189109	0.849971029	-1.101862119	4
GBP3	0.567035737	0.587603738	-1.154639476	4
GCN1	0.395402021	0.741842991	-1.137245012	4
GDAP2	0.185415735	0.894315817	-1.079731552	4
GDF2	0.127408009	0.930190054	-1.057598063	4
GDF6	0.491465767	0.659168244	-1.150634011	4
GDPD3	0.246797446	0.853493423	-1.100290869	4
GFM2	0.399668858	0.738354573	-1.138023431	4
GFRA2	0.084273296	0.95519655	-1.039469847	4
GID4	0.334486697	0.789882028	-1.124368725	4
GIGYF2	0.089881592	0.952025088	-1.04190668	4
GIPC1	0.745221514	0.391250272	-1.136471786	4
GIPC3	0.164709244	0.90741967	-1.072128914	4
GJB7	0.177542633	0.899337463	-1.076880096	4
GJC2	0.433376699	0.710208882	-1.143585581	4
GJD3	0.506402277	0.645502122	-1.151904399	4
GJD4	0.388096564	0.747777494	-1.135874058	4
GLB1L2	0.721821234	0.419621625	-1.141442858	4
GLG1	0.385630489	0.749769948	-1.135400437	4
GLIPR2	0.241307588	0.857266441	-1.098574029	4
GLIS2	0.190111156	0.891297964	-1.081409121	4
GLRX3	0.601255979	0.553109931	-1.154365909	4
GLT8D2	0.343205074	0.783205144	-1.126410218	4
GMPPA	0.532532127	0.621037239	-1.153569366	4
GNAT1	0.078459773	0.958458967	-1.03691874	4
GNB1L	0.269900489	0.837348729	-1.107249218	4

GNL3	0.459229707	0.687898665	-1.147128372	4
GNPDA1	0.725481652	0.415242001	-1.140723653	4
GNPNAT1	0.249802905	0.851417595	-1.101220499	4
GOLGA4	0.198361498	0.885953525	-1.084315023	4
GOLGA5	0.29522301	0.819152443	-1.114375453	4
GOLGA7	0.518739076	0.634040965	-1.152780042	4
GOLGA8A	0.434398319	0.709339332	-1.14373765	4
GOLGA8M	0.75603996	0.377825695	-1.133865655	4
GOLGA8N	0.237037089	0.860184712	-1.097221801	4
GOLGA8Q	0.456431376	0.690344489	-1.146775865	4
GPANK1	0.659824188	0.490742542	-1.15056673	4
GPC4	0.543292372	0.610751144	-1.154043517	4
GPNMB	0.785597254	0.340091348	-1.125688602	4
GPR101	0.622296827	0.531205428	-1.153502255	4
GPR139	0.621731682	0.53180093	-1.153532612	4
GPR153	0.775635113	0.352987835	-1.128622948	4
GPR173	0.470457261	0.678008729	-1.14846599	4
GPR180	0.647738488	0.503975028	-1.151713516	4
GPR182	0.332391116	0.791477199	-1.123868315	4
GPR32	0.477301065	0.671919492	-1.149220557	4
GPR62	0.596291504	0.558199583	-1.154491086	4
GPR89A	0.658585048	0.492108292	-1.15069334	4
GPR89B	0.795553197	0.327013946	-1.122567143	4
GPRC5B	0.353095448	0.775551379	-1.128646828	4
GPX3	0.667856897	0.481838291	-1.149695188	4
GRAMD1A	0.632538468	0.520343768	-1.152882236	4
GRAMD1C	0.525328806	0.627853556	-1.153182361	4
GRAMD2B	0.246300205	0.85383616	-1.100136365	4
GRAP2	0.393317736	0.743541041	-1.136858777	4
GRB7	0.742803128	0.394224085	-1.137027213	4
GRIN1	0.682936866	0.464880082	-1.147816948	4
GRINA	0.101381923	0.945447221	-1.046829144	4
GRIP1	0.576321487	0.57837844	-1.154699928	4
GSTCD	0.300135354	0.815561174	-1.115696527	4
GTF2H5	0.70942242	0.434300749	-1.14372317	4
GTF3C1	0.703708844	0.440985661	-1.144694505	4
GTF3C4	0.429539337	0.713466276	-1.143005613	4
GUCA1A	0.515678228	0.63689938	-1.152577609	4
GUCY1B1	0.696983422	0.44879154	-1.145774962	4
GZMM	0.370193712	0.76211878	-1.132312492	4
H1-0	0.750261033	0.385021631	-1.135282664	4
H2AB1	0.625605884	0.527710567	-1.153316451	4
H2AB2	0.53151814	0.622000104	-1.153518243	4
H2AB3	0.65164633	0.499717745	-1.151364076	4
H2AX	0.084710378	0.954950238	-1.039660615	4
H2BC15	0.664787958	0.485250643	-1.150038601	4
H2BC17	0.413626424	0.72682745	-1.140453874	4
H3C7	0.094585136	0.949346905	-1.043932041	4
HACL1	0.139097614	0.923169124	-1.062266738	4
HADH	0.493507023	0.657314027	-1.15082105	4
HARBI1	0.519064364	0.633736613	-1.152800977	4
HCFC1R1	0.162152094	0.90901487	-1.071166964	4
HCK	0.640141432	0.512193792	-1.152335223	4
HCRT	0.655977808	0.494975151	-1.150952959	4
HCRTR1	0.685087944	0.462434836	-1.14752278	4

HDGFL2	0.679325298	0.468970676	-1.148295974	4
HDX	0.327812035	0.794949786	-1.122761821	4
HEATR1	0.751831202	0.383072135	-1.134903337	4
HEATR3	0.463225779	0.684392792	-1.147618571	4
HEATR6	0.386156684	0.749345268	-1.135501953	4
HECTD3	0.720730399	0.420922692	-1.141653091	4
HELZ2	0.189832841	0.891477324	-1.081310165	4
HEMK1	0.290302247	0.822729785	-1.113032033	4
HEPHL1	0.481234311	0.668398881	-1.149633191	4
HERC1	0.142426271	0.921150744	-1.063577014	4
HEXIM2	0.432570236	0.710894607	-1.143464843	4
HGH1	0.221111864	0.870938927	-1.092050791	4
HGSNAT	0.622488382	0.531003492	-1.153491875	4
HHATL	0.373410184	0.759563171	-1.132973355	4
HIF3A	0.656950577	0.49390659	-1.150857167	4
HIGD1B	0.728654338	0.411428683	-1.140083021	4
HINFP	0.256700099	0.846626255	-1.103326354	4
HIPK3	0.213243105	0.876178292	-1.089421397	4
HIVEP2	0.264562759	0.841117259	-1.105680018	4
HIVEP3	0.360367345	0.769869764	-1.130237109	4
HK1	0.325747848	0.796509344	-1.122257192	4
HLA-DPA1	0.165125212	0.907159703	-1.072284915	4
HLA-DQB1	0.761057289	0.371531095	-1.132588384	4
HMBOX1	0.487714579	0.662564724	-1.150279303	4
HNRNPK	0.469295178	0.679038094	-1.148333272	4
HNRNPUL1	0.774952324	0.353864968	-1.128817292	4
HOOK3	0.363618393	0.767314741	-1.130933134	4
HOXA9	0.476053589	0.673032882	-1.149086472	4
HP1BP3	0.247190711	0.853222214	-1.100412925	4
HROB	0.521002311	0.631921078	-1.15292339	4
HSD11B2	0.745669991	0.390697714	-1.136367705	4
HSD17B12	0.384282589	0.750856674	-1.135139263	4
HSD17B3	0.712696935	0.430447129	-1.143144063	4
HSF4	0.78023121	0.347061065	-1.127292276	4
HSPA2	0.722859329	0.418381718	-1.141241047	4
HTR1D	0.453269157	0.693099244	-1.146368401	4
HTR4	0.791891767	0.331845689	-1.123737455	4
HUWE1	0.539459643	0.614429367	-1.15388901	4
HVCN1	0.321055282	0.800041268	-1.12109655	4
HYKK	0.449981597	0.695952969	-1.145934565	4
ICA1L	0.265031958	0.840786926	-1.105818885	4
IDE	0.198326976	0.885975998	-1.084302975	4
IDH3G	0.764667523	0.366974376	-1.131641899	4
IDNK	0.379125736	0.754999336	-1.134125072	4
IFNA4	0.478260093	0.671062495	-1.149322588	4
IFNAR1	0.299196175	0.816249332	-1.115445506	4
IFNGR1	0.184209596	0.895088253	-1.079297848	4
IFT80	0.722596132	0.418696244	-1.141292375	4
IGF2R	0.485677681	0.664403074	-1.150080756	4
IGSF9	0.091488166	0.951112194	-1.04260036	4
IKBIP	0.499938764	0.65144391	-1.151382675	4
IKBKB	0.267762409	0.838861037	-1.106623446	4
IL12RB1	0.286260888	0.825652828	-1.111913716	4
IL18BP	0.772280414	0.357289159	-1.129569573	4
IL21R	0.225770835	0.867813643	-1.093584478	4

IL4R	0.501523584	0.649990988	-1.151514572	4
IL6	0.155315584	0.913254819	-1.068570403	4
IMPDH1	0.152783637	0.914815965	-1.067599602	4
INAVA	0.69725761	0.44847462	-1.145732229	4
INCA1	0.795341167	0.327294465	-1.122635632	4
INF2	0.537560888	0.616245667	-1.153806556	4
INO80B-WBP1	0.380663737	0.753766293	-1.134430031	4
INO80E	0.793380241	0.329884614	-1.123264855	4
INPP5F	0.781647476	0.345226815	-1.126874291	4
INSIG1	0.795426627	0.32718141	-1.122608037	4
INTS10	0.408557796	0.731034096	-1.139591891	4
INTS12	0.40703791	0.732290911	-1.139328821	4
INTS7	0.16326355	0.908322143	-1.071585693	4
INTS9	0.280444469	0.829836101	-1.11028057	4
IPO9	0.277507547	0.831937821	-1.109445368	4
IQCC	0.648034711	0.503653026	-1.151687737	4
IQCD	0.593504242	0.561044204	-1.154548446	4
IQCE	0.743926773	0.392843594	-1.136770367	4
IQGAP2	0.255404659	0.847529102	-1.102933761	4
IRF2BP1	0.129570617	0.928899044	-1.05846966	4
IRF2BP2	0.690956525	0.455729769	-1.146686294	4
IRGC	0.214253574	0.875508219	-1.089761793	4
IRGM	0.095507343	0.948819839	-1.044327181	4
IRS4	0.697033855	0.448733256	-1.14576711	4
IRX1	0.720911428	0.420706903	-1.141618331	4
ITFG1	0.396042716	0.741320233	-1.137362949	4
ITFG2	0.624670995	0.528699348	-1.153370343	4
ITGA6	0.26608173	0.840047201	-1.106128931	4
ITGAL	0.367082566	0.764582017	-1.131664583	4
ITGAM	0.37826749	0.755686492	-1.133953982	4
ITIH4	0.343016645	0.783350141	-1.126366786	4
ITM2B	0.656254504	0.494671338	-1.150925842	4
ITM2C	0.183058963	0.895824083	-1.078883046	4
ITPR2	0.701514297	0.443540194	-1.145054491	4
ITPR3	0.136051462	0.92500876	-1.061060222	4
IYD	0.748528409	0.387167943	-1.135696352	4
JADE1	0.358587115	0.771264942	-1.129852057	4
JAK3	0.730440116	0.409275215	-1.139715331	4
JAML	0.19006407	0.891328314	-1.081392383	4
JDP2	0.772806257	0.356616297	-1.129422554	4
JMJD8	0.737513815	0.400694214	-1.13820803	4
KBTBD11	0.51049088	0.64172128	-1.15221216	4
KCNA10	0.214326251	0.875459993	-1.089786244	4
KCNF1	0.783923454	0.342271216	-1.12619467	4
KCNG3	0.34037954	0.785376202	-1.125755742	4
KCNJ5	0.359008732	0.770934768	-1.1299435	4
KCNK12	0.506205133	0.645683989	-1.151889122	4
KCNK18	0.741461068	0.395870154	-1.137331221	4
KCNK7	0.708676492	0.435176303	-1.143852795	4
KCNMB2	0.326096749	0.796245992	-1.122342742	4
KCNQ3	0.694408804	0.451761999	-1.146170803	4
KCNS3	0.630323316	0.522704315	-1.153027631	4
KCTD10	0.377501011	0.75629962	-1.133800631	4
KCTD13	0.791049493	0.332953479	-1.124002972	4
KCTD17	0.72484282	0.416007884	-1.140850703	4

KDM6A	0.475623451	0.673416429	-1.14903988	4
KDM8	0.164326744	0.907658602	-1.071985346	4
KDSR	0.610318116	0.543742496	-1.154060612	4
KIAA0319L	0.150977226	0.915926743	-1.066903969	4
KIAA0586	0.648501033	0.503145887	-1.15164692	4
KIAA0825	0.540915294	0.613034279	-1.153949573	4
KIAA0895	0.216987517	0.87369121	-1.090678727	4
KIAA0930	0.406073384	0.733087395	-1.139160778	4
KIAA1522	0.177078876	0.899631753	-1.07671063	4
KICS2	0.526003389	0.627217563	-1.153220952	4
KIF1C	0.076286212	0.959672163	-1.035958375	4
KIF25	0.595876597	0.558623615	-1.154500212	4
KIF2A	0.576037865	0.57866168	-1.154699545	4
KIF3C	0.074702576	0.96055384	-1.035256415	4
KIRREL3	0.769019804	0.361450155	-1.130469959	4
KISS1	0.741849588	0.395393933	-1.137243521	4
KISS1R	0.084492431	0.955073077	-1.039565508	4
KLF12	0.440724468	0.70393288	-1.144657348	4
KLF16	0.362403186	0.768270865	-1.130674051	4
KLHL12	0.214951901	0.875044665	-1.089996566	4
KLHL22	0.176726449	0.899855285	-1.076581733	4
KLHL36	0.755396652	0.378629595	-1.134026247	4
KLHL40	0.459509935	0.687653319	-1.147163254	4
KLK1	0.30997744	0.808305363	-1.118282803	4
KLK13	0.559337845	0.595177281	-1.154515127	4
KLK2	0.220736219	0.871190163	-1.091926381	4
KNOP1	0.753479857	0.38102064	-1.134500497	4
KPNA7	0.112639659	0.938910913	-1.051550572	4
KRAS	0.674770504	0.474103242	-1.148873746	4
KRT23	0.294951178	0.819350585	-1.114301764	4
KRT3	0.767587984	0.363271276	-1.13085926	4
KRT33A	0.085494258	0.954508129	-1.040002387	4
KRT4	0.382118546	0.752598008	-1.134716553	4
KRT6B	0.686962519	0.460298483	-1.147261001	4
KRT77	0.342830745	0.783493162	-1.126323908	4
KRT79	0.646772746	0.505024009	-1.151796755	4
KRT9	0.3259826	0.796332164	-1.122314764	4
KRTAP1-5	0.706211837	0.438063236	-1.144275073	4
KRTAP10-8	0.484946066	0.665062358	-1.150008423	4
KRTAP13-3	0.180486623	0.897465367	-1.07795199	4
KRTAP3-1	0.53870447	0.615152214	-1.153856684	4
KRTAP4-2	0.781431686	0.345506535	-1.126938221	4
KRTAP4-9	0.795101777	0.327611074	-1.122712852	4
KRTAP5-9	0.450918116	0.695141094	-1.14605921	4
KSR1	0.326369108	0.796040344	-1.122409451	4
KYAT3	0.292570149	0.821083537	-1.113653686	4
KYNU	0.531692152	0.621834943	-1.153527095	4
L3MBTL1	0.587665451	0.566973285	-1.154638736	4
LACTBL1	0.600607101	0.553776851	-1.154383952	4
LAG3	0.323266959	0.798378945	-1.121645904	4
LAMC1	0.743269628	0.393651207	-1.136920836	4
LAMC2	0.579534492	0.575163289	-1.15469778	4
LARP1	0.564908139	0.589703655	-1.154611794	4
LAS1L	0.581641568	0.573048312	-1.15468988	4
LATS2	0.36327076	0.767588391	-1.13085915	4

LBX2	0.283047912	0.827967101	-1.111015013	4
LCE1D	0.549424772	0.604832562	-1.154257334	4
LCN1	0.333643299	0.79052448	-1.124167779	4
LCN10	0.394085051	0.742916372	-1.137001423	4
LCN6	0.073882478	0.961009679	-1.034892157	4
LDB1	0.489000728	0.661401792	-1.15040252	4
LDLRAD4	0.168679748	0.904932767	-1.073612514	4
LDOC1	0.331425026	0.79221133	-1.123636356	4
LEKR1	0.643947954	0.508085223	-1.152033176	4
LGALS1	0.430450657	0.712693943	-1.1431446	4
LGALS3	0.675199615	0.473620946	-1.14882056	4
LHB	0.476627415	0.672520927	-1.149148341	4
LHPP	0.677205625	0.47136287	-1.148568495	4
LHX4	0.634672801	0.5180634	-1.152736201	4
LHX5	0.129261703	0.929083675	-1.058345377	4
LILRA2	0.080682848	0.957214444	-1.037897291	4
LILRB2	0.460110528	0.68712723	-1.147237758	4
LIMD1	0.207872023	0.87972649	-1.087598513	4
LINGO4	0.651958617	0.499376658	-1.151335275	4
LIPA	0.788235812	0.336644181	-1.124879993	4
LITAFD	0.6895283	0.457366166	-1.146894465	4
LIX1L	0.430752018	0.712438371	-1.143190389	4
LLPH	0.692938311	0.453454199	-1.14639251	4
LMLN2	0.615451417	0.538391698	-1.153843115	4
LMNB1	0.154564318	0.913718549	-1.068282867	4
LMNTD2	0.25733665	0.846182118	-1.103518768	4
LMO2	0.736466278	0.401970124	-1.138436402	4
LNX2	0.241997092	0.856793894	-1.098790986	4
LONP2	0.163977685	0.907876547	-1.071854232	4
LONRF1	0.159317451	0.91077727	-1.070094722	4
LOXL1	0.126468106	0.930750033	-1.057218139	4
LRBA	0.332294675	0.79155052	-1.123845195	4
LRCH3	0.559276958	0.59523692	-1.154513878	4
LRFN5	0.104956163	0.943382427	-1.04833859	4
LRIG1	0.457871235	0.689086958	-1.146958193	4
LRMDA	0.495366686	0.655621082	-1.150987768	4
LRRC1	0.633786492	0.519011062	-1.152797554	4
LRRC25	0.152268356	0.915133072	-1.067401428	4
LRRC26	0.696653769	0.449172423	-1.145826192	4
LRRC32	0.791212525	0.33273916	-1.123951685	4
LRRC36	0.62213544	0.531375525	-1.153510965	4
LRRC3C	0.086573253	0.953898815	-1.040472067	4
LRRC71	0.577098735	0.577601767	-1.154700502	4
LRRC74A	0.193325247	0.889222264	-1.082547511	4
LRRC75B	0.291399618	0.821933746	-1.113333364	4
LRRC8C	0.747887759	0.387960265	-1.135848024	4
LRRN4CL	0.664990069	0.485026315	-1.150016384	4
LSM7	0.396083032	0.741287327	-1.137370358	4
LSR	0.642678096	0.509457948	-1.152136044	4
LST1	0.396433744	0.741001002	-1.137434746	4
LTC4S	0.192231394	0.889929595	-1.082160989	4
LY6G6E	0.091131055	0.951315282	-1.042446337	4
LY6H	0.201933742	0.883622928	-1.08555667	4
LY96	0.692046556	0.45447886	-1.146525416	4
LYNX1	0.09857128	0.947064087	-1.045635366	4

LYPLA2	0.760348528	0.372422963	-1.132771492	4
LYST	0.243685317	0.855635269	-1.099320587	4
LZTS3	0.410794751	0.729180472	-1.139975224	4
M1AP	0.748084033	0.387717597	-1.135801631	4
MAGEA12	0.637111758	0.515450389	-1.152562147	4
MAGEA9	0.648154374	0.503522916	-1.15167729	4
MAGOHB	0.439060377	0.705358718	-1.144419095	4
MAML1	0.461177543	0.686191712	-1.147369255	4
MAN2C1	0.358658314	0.771209196	-1.12986751	4
MAOA	0.364152839	0.766893829	-1.131046668	4
MAP1LC3A	0.557584868	0.596892633	-1.154477501	4
MAP3K5	0.350795321	0.777338915	-1.128134236	4
MAP3K6	0.606299679	0.547908633	-1.154208312	4
MAP4K1	0.509654345	0.642496257	-1.152150602	4
MAP4K4	0.373215001	0.759718512	-1.132933513	4
MAP6D1	0.63972692	0.512640054	-1.152366974	4
MAP7D3	0.58491023	0.569757166	-1.154667396	4
MAPK10	0.365872805	0.765537539	-1.131410344	4
MAPK8IP1	0.780471577	0.346750024	-1.127221601	4
MAPK9	0.249083664	0.851915026	-1.10099869	4
MAPT	0.381995521	0.752696875	-1.134692397	4
MASP2	0.097606218	0.947617871	-1.045224089	4
MAST1	0.506863311	0.645076657	-1.151939968	4
MAT2B	0.087621829	0.953305835	-1.040927663	4
MATN1	0.649844659	0.501683042	-1.151527701	4
MAVS	0.157839915	0.911693449	-1.069533364	4
MAX	0.766131868	0.365119518	-1.131251386	4
MBD3L1	0.657858823	0.492907754	-1.150766577	4
MBD3L2	0.175833012	0.900421525	-1.076254537	4
MBNL1	0.739019456	0.398857166	-1.137876621	4
MBOAT2	0.771106647	0.358789275	-1.129895922	4
MCEMP1	0.693277565	0.453064077	-1.146341642	4
MCMBP	0.719316982	0.422605729	-1.141922711	4
MCPH1	0.302280004	0.813986984	-1.116266988	4
MCUR1	0.219233055	0.872194376	-1.091427431	4
MDFI	0.417824041	0.723325773	-1.141149814	4
MDM2	0.566468257	0.588164337	-1.154632594	4
MDP1	0.511299155	0.640971793	-1.152270947	4
MED18	0.78899517	0.335649637	-1.124644807	4
MED25	0.488518378	0.661838127	-1.150356505	4
MEF2C	0.32811299	0.794722103	-1.122835093	4
MEGF11	0.442947159	0.702024315	-1.144971474	4
MESD	0.631569366	0.52137725	-1.152946616	4
MESP2	0.366119332	0.765342926	-1.131462258	4
METTL21A	0.768570958	0.362021437	-1.130592394	4
METTL9	0.616624477	0.537164368	-1.153788844	4
MFAP2	0.45457954	0.69195888	-1.14653842	4
MFAP3L	0.724087745	0.41691229	-1.141000035	4
MFN2	0.453856035	0.692588717	-1.146444752	4
MIA	0.687116229	0.460123082	-1.147239311	4
MICAL1	0.740282362	0.39731339	-1.137595752	4
MIER2	0.229297484	0.865436457	-1.094733941	4
MIF4GD	0.456290699	0.690467245	-1.146757944	4
MIIP	0.728797631	0.411256075	-1.140053706	4
MINDY1	0.763311455	0.368688672	-1.132000126	4

MIX23	0.636665111	0.515929486	-1.152594596	4
MKS1	0.623063712	0.530396708	-1.153460421	4
MLH1	0.459361527	0.687783264	-1.14714479	4
MLLT6	0.753928803	0.380461185	-1.134389989	4
MMP9	0.713247509	0.429797566	-1.143045075	4
MMS19	0.532703014	0.620874859	-1.153577873	4
MMS22L	0.523567652	0.629511684	-1.153079336	4
MND1	0.734232251	0.404685177	-1.138917428	4
MOB1A	0.088909332	0.952576601	-1.041485933	4
MOB1B	0.715267058	0.427410913	-1.142677972	4
MOB2	0.537825406	0.615992871	-1.153818277	4
MOB3B	0.339550871	0.786011625	-1.125562496	4
MOCS1	0.741419724	0.395920815	-1.137340539	4
MORF4L1	0.236522174	0.860535596	-1.09705777	4
MPC2	0.109322348	0.940846972	-1.05016932	4
MPP7	0.121487901	0.933705906	-1.055193807	4
MPPED1	0.28094888	0.829474422	-1.110423302	4
MPV17	0.112172284	0.93918419	-1.051356474	4
MPZL1	0.125303915	0.931442705	-1.056746619	4
MRAP2	0.609739546	0.544343548	-1.154083094	4
MREG	0.788258627	0.336614317	-1.124872944	4
MRGPRX3	0.781410894	0.345533482	-1.126944376	4
MRNIP	0.172366654	0.902612558	-1.074979212	4
MROH2A	0.762293542	0.369973337	-1.132266879	4
MROH6	0.147575291	0.918011805	-1.065587096	4
MRPL21	0.589484398	0.565130529	-1.154614927	4
MRPL23	0.442759416	0.702185706	-1.144945122	4
MRPL37	0.466994415	0.681072179	-1.148066594	4
MRPL42	0.543430252	0.610618524	-1.154048777	4
MRPL48	0.760172738	0.372644032	-1.13281677	4
MRPS27	0.138662194	0.923432519	-1.062094713	4
MRPS7	0.45566535	0.691012697	-1.146678046	4
MRRF	0.270371837	0.837014833	-1.107386671	4
MS4A1	0.536719429	0.617049339	-1.153768767	4
MS4A4A	0.286448952	0.825517104	-1.111966056	4
MS4A7	0.557509696	0.596966114	-1.154475811	4
MSI1	0.482548828	0.667218829	-1.149767656	4
MSL3	0.473975557	0.67488413	-1.148859688	4
MSMO1	0.131475823	0.927758732	-1.059234555	4
MSS51	0.113876379	0.938186992	-1.052063371	4
MT-ATP6	0.184295016	0.895033585	-1.079328601	4
MT-ND4	0.676221143	0.472471765	-1.148692908	4
MT-ND5	0.563145593	0.591439392	-1.154584985	4
MTCH1	0.784192055	0.341921762	-1.126113817	4
MTDH	0.452213704	0.69401655	-1.146230254	4
MTERF2	0.158992147	0.910979127	-1.069971274	4
MTF1	0.411253855	0.728799474	-1.140053329	4
MTFP1	0.163435599	0.908214826	-1.071650425	4
MTHFS	0.566706399	0.587929128	-1.154635526	4
MTMR10	0.144248676	0.9200421	-1.064290775	4
MTMR2	0.437344885	0.70682585	-1.144170735	4
MTMR9	0.311365522	0.807275511	-1.118641033	4
MTRES1	0.071738312	0.962199084	-1.033937395	4
MTRNR2L8	0.105746339	0.942924643	-1.048670982	4
MTUS2	0.241709448	0.856991075	-1.098700523	4

MUC12	0.540248229	0.613673876	-1.153922105	4
MUC20	0.496952423	0.654174727	-1.15112715	4
MVD	0.729860434	0.409974816	-1.13983525	4
MX1	0.587823633	0.566813188	-1.15463682	4
MX2	0.504483073	0.647270899	-1.151753972	4
MXRA8	0.696752589	0.449058262	-1.145810851	4
MYCL	0.245976363	0.854059269	-1.100035633	4
MYEOV	0.431378076	0.711907166	-1.143285241	4
MYH7B	0.198696437	0.88573543	-1.084431867	4
MYL5	0.212125766	0.876918294	-1.08904406	4
MYL7	0.784652424	0.341322498	-1.125974922	4
MYLPF	0.427782554	0.714952932	-1.142735486	4
MYO18A	0.579108981	0.57558977	-1.154698751	4
MYO19	0.673511755	0.475516513	-1.149028268	4
MYO1A	0.787113396	0.3381122	-1.125225596	4
MYO1E	0.689804343	0.45705012	-1.146854462	4
MYO3B	0.10675293	0.942340794	-1.049093723	4
MYO5A	0.396038083	0.741324015	-1.137362098	4
MYOZ3	0.710910655	0.432551354	-1.143462009	4
MYRF	0.773321295	0.355956772	-1.129278067	4
MYRIP	0.663157178	0.487058643	-1.150215821	4
MZB1	0.717934849	0.424248487	-1.142183336	4
MZT2A	0.125563889	0.931288114	-1.056852004	4
N4BP2L2	0.744192317	0.392517043	-1.136709359	4
NAA20	0.266465753	0.839776374	-1.106242127	4
NAA25	0.292738867	0.820960896	-1.113699763	4
NAA80	0.352450433	0.776053114	-1.128503547	4
NACC2	0.219005725	0.872346092	-1.091351817	4
NANOGNB	0.311565362	0.807127112	-1.118692474	4
NAPEPLD	0.134760891	0.925786019	-1.06054691	4
NAT16	0.281341341	0.82919287	-1.110534211	4
NAV2	0.685146734	0.462367913	-1.147514647	4
NBPF11	0.274710807	0.833932648	-1.108643455	4
NBPF15	0.649105468	0.502488116	-1.151593585	4
NBR1	0.519420226	0.633403526	-1.152823752	4
NCAPD3	0.662939164	0.487300074	-1.150239237	4
NCAPG2	0.290903681	0.822293625	-1.113197306	4
NCBP2	0.71401122	0.428895773	-1.142906993	4
NCOA1	0.685582832	0.461871331	-1.147454162	4
NCSTN	0.550827292	0.603473134	-1.154300425	4
NDUFA3	0.64909649	0.502497891	-1.15159438	4
NDUFB9	0.39048558	0.745842094	-1.136327674	4
NDUFC2	0.200212651	0.88474705	-1.084959701	4
NDUFS1	0.61968514	0.533954056	-1.153639196	4
NDUFS6	0.496697547	0.654407373	-1.15110492	4
NECTIN1	0.435054219	0.708780545	-1.143834763	4
NECTIN4	0.67387778	0.475105784	-1.148983564	4
NEDD8-MDP1	0.759341071	0.373689175	-1.133030246	4
NEK8	0.597010202	0.557464591	-1.154474793	4
NEK9	0.082594399	0.956141332	-1.038735731	4
NENF	0.097094423	0.94791127	-1.045005693	4
NEUROD6	0.76331842	0.368679875	-1.131998295	4
NFE2L3	0.241188129	0.857348273	-1.098536402	4
NFIA	0.253330697	0.848971709	-1.102302406	4
NGFR	0.738579391	0.39939448	-1.137973871	4

NHSL1	0.129675666	0.928836241	-1.058511907	4
NIBAN1	0.239079468	0.858790862	-1.097870331	4
NID1	0.435950314	0.70801647	-1.143966784	4
NIP7	0.431651613	0.711674955	-1.143326569	4
NIPA2	0.286789912	0.825270962	-1.112060873	4
NIPAL2	0.336385009	0.788433778	-1.124818787	4
NKAP	0.746679008	0.389453288	-1.136132296	4
NLGN4X	0.283021289	0.827986242	-1.111007531	4
NLGN4Y	0.711802816	0.431501007	-1.143303823	4
NLRC3	0.32029289	0.800613331	-1.120906222	4
NLRC4	0.707066824	0.437062806	-1.14412963	4
NLRP11	0.675841819	0.472898661	-1.148740481	4
NMRK2	0.302788467	0.813613206	-1.116401673	4
NOL11	0.67580297	0.472942372	-1.148745341	4
NOP16	0.475454175	0.673567319	-1.149021494	4
NOPCHAP1	0.370940897	0.761525929	-1.132466826	4
NOTCH2	0.324944135	0.797115595	-1.12205973	4
NOXO1	0.266608159	0.839675913	-1.106284073	4
NPAP1	0.495183231	0.655788247	-1.150971478	4
NPAS3	0.61871348	0.534974487	-1.153687968	4
NPHS1	0.782454815	0.344179518	-1.126634334	4
NPRL2	0.195939041	0.887528282	-1.083467323	4
NPRL3	0.425059455	0.717251615	-1.14231107	4
NPTX2	0.672452834	0.476703708	-1.149156543	4
NQO2	0.671738873	0.477503273	-1.149242146	4
NR2F1	0.552808011	0.601549568	-1.154357579	4
NR2F6	0.133761372	0.926387114	-1.060148485	4
NR6A1	0.198999577	0.885537965	-1.084537542	4
NRBF2	0.712448872	0.430739638	-1.14318851	4
NRXN1	0.583188766	0.571492025	-1.154680791	4
NSD3	0.751015622	0.384085271	-1.135100894	4
NSF	0.212760254	0.8764982	-1.089258454	4
NSFL1C	0.601592966	0.552763373	-1.154356339	4
NSL1	0.460456893	0.686823671	-1.147280565	4
NSMCE1	0.542454443	0.611556661	-1.154011104	4
NSMCE2	0.62141557	0.532133847	-1.153549417	4
NSMCE4A	0.366481504	0.765056923	-1.131538427	4
NSMF	0.490533243	0.660013917	-1.15054716	4
NSUN2	0.777572506	0.350494308	-1.128066814	4
NSUN3	0.390612816	0.745738873	-1.136351689	4
NT5C2	0.545226404	0.608888997	-1.154115401	4
NUCB1	0.690040686	0.456779438	-1.146820124	4
NUDT1	0.516851472	0.635804893	-1.152656364	4
NUDT13	0.409506872	0.730248217	-1.139755089	4
NUDT16	0.43170507	0.711629567	-1.143334637	4
NUDT22	0.337767288	0.787377274	-1.125144563	4
NUGGC	0.092821576	0.950353034	-1.04317461	4
NUTM2A	0.09067181	0.951576311	-1.042248121	4
NUTM2B	0.095426653	0.94886598	-1.044292634	4
NUTM2D	0.270145726	0.83717503	-1.107320756	4
NWD1	0.571986584	0.582697396	-1.15468398	4
NXPE3	0.785458003	0.340272905	-1.125730907	4
NXT2	0.580989003	0.573703875	-1.154692878	4
OBP2A	0.681166077	0.466888064	-1.148054141	4
ODF3L1	0.754842263	0.379321803	-1.134164066	4

OLFML2A	0.271735735	0.836047647	-1.107783382	4
OLIG2	0.375785816	0.757669763	-1.133455579	4
OLIG3	0.672510954	0.476638589	-1.149149543	4
OR10H2	0.791019012	0.332993543	-1.124012555	4
OR10K1	0.767763892	0.363047739	-1.130811631	4
OR13J1	0.600472849	0.553914773	-1.154387622	4
OR1A1	0.777136936	0.351055516	-1.128192452	4
OR1S2	0.191760766	0.890233635	-1.081994401	4
OR2A5	0.793551194	0.329659105	-1.123210299	4
OR2L13	0.568519024	0.586136714	-1.154655738	4
OR2S2	0.712502753	0.43067611	-1.143178864	4
OR2T10	0.755358491	0.378677259	-1.13403575	4
OR2T34	0.096127858	0.948464834	-1.044592692	4
OR4P4	0.212168815	0.876889802	-1.089058616	4
OR52N2	0.723028521	0.418179473	-1.141207994	4
OR56A3	0.765207543	0.366290789	-1.131498332	4
OR5T3	0.365482298	0.765845704	-1.131328002	4
OR8B2	0.175659357	0.900531512	-1.076190869	4
ORC6	0.432940729	0.710579657	-1.143520387	4
OS9	0.268641612	0.83823961	-1.106881222	4
OSBPL10	0.282624427	0.828271499	-1.110895926	4
OSBPL9	0.772229665	0.35735407	-1.129583735	4
OSGIN1	0.103501512	0.944223931	-1.047725443	4
OSTF1	0.10777342	0.941748096	-1.049521516	4
OTUD3	0.660128813	0.490406472	-1.150535285	4
OTUD5	0.642882527	0.509237099	-1.152119626	4
OTUD6A	0.781887739	0.34491527	-1.126803009	4
OTUD6B	0.077160712	0.95918448	-1.036345192	4
OVCA2	0.344383606	0.782297562	-1.126681169	4
OXLD1	0.33068598	0.792772393	-1.123458373	4
P2RY13	0.294519168	0.819665359	-1.114184527	4
P2RY2	0.14784683	0.917845705	-1.065692534	4
P3H1	0.582419977	0.572265679	-1.154685656	4
P4HB	0.266548242	0.839718184	-1.106266426	4
P4HTM	0.58907167	0.565549001	-1.154620671	4
PACC1	0.34079615	0.785056523	-1.125852673	4
PADI2	0.737892293	0.400232781	-1.138125074	4
PAG1	0.199260756	0.885367776	-1.084628532	4
PAGR1	0.210908213	0.877723545	-1.088631758	4
PAK1	0.316755054	0.80326153	-1.120016585	4
PALD1	0.437613877	0.706595985	-1.144209862	4
PAM	0.436346636	0.707678296	-1.144024931	4
PANK4	0.644219696	0.507791193	-1.152010889	4
PAPOLA	0.44987641	0.696044103	-1.145920513	4
PAPSS2	0.373378248	0.759588591	-1.132966838	4
PAQR4	0.537566297	0.616240499	-1.153806796	4
PAQR5	0.73423483	0.404682048	-1.138916878	4
PAQR8	0.496782331	0.654329991	-1.151112322	4
PARD6A	0.611844669	0.542154652	-1.153999321	4
PARL	0.71747414	0.424795407	-1.142269548	4
PARM1	0.708412469	0.435486003	-1.143898472	4
PARP14	0.613882526	0.54003051	-1.153913035	4
PARP3	0.177832936	0.899153158	-1.076986094	4
PARP9	0.470781848	0.677720974	-1.148502822	4
PARVG	0.333187605	0.790871349	-1.124058953	4

PAX5	0.528036677	0.625297663	-1.15333434	4
PBX3	0.174231221	0.901435153	-1.075666373	4
PBX4	0.164288118	0.907682724	-1.071970842	4
PCBD2	0.556390538	0.598059362	-1.154449899	4
PCDHA8	0.452244754	0.69398958	-1.146234333	4
PCDHB11	0.21427993	0.87549073	-1.089770661	4
PCDHB12	0.303646881	0.812981685	-1.116628565	4
PCGF3	0.373842895	0.759218667	-1.133061563	4
PCGF5	0.468311643	0.679908265	-1.148219908	4
PCLAF	0.51759297	0.635112424	-1.152705394	4
PCOLCE	0.494540332	0.656373788	-1.15091412	4
PCYOX1	0.305137964	0.811883255	-1.117021219	4
PCYT2	0.135617264	0.925270402	-1.060887666	4
PDCD6-AHRR	0.409663992	0.730118035	-1.139782027	4
PDCL	0.786673253	0.338687208	-1.125360461	4
PDE8A	0.536544627	0.617216194	-1.153760821	4
PDGFRB	0.145090094	0.91952937	-1.064619464	4
PDHB	0.203649105	0.882500232	-1.086149337	4
PDK3	0.637770693	0.514743112	-1.152513805	4
PDLIM1	0.295735611	0.818778632	-1.114514244	4
PDP2	0.737523932	0.400681883	-1.138205815	4
PDS5B	0.563119143	0.591465413	-1.154584556	4
PDZK1IP1	0.658940732	0.491716479	-1.150657211	4
PEBP1	0.287815429	0.824530053	-1.112345482	4
PEX11B	0.549561827	0.604699813	-1.15426164	4
PEX11G	0.189869181	0.891453908	-1.081323089	4
PEX14	0.7919835	0.331724954	-1.123708454	4
PFKFB1	0.725027664	0.415786345	-1.140814009	4
PGGHG	0.415156373	0.725553042	-1.140709415	4
PGM2L1	0.450428871	0.695565329	-1.1459942	4
PHETA2	0.286389803	0.825559794	-1.111949597	4
PHF11	0.342229561	0.783955477	-1.126185037	4
PHF14	0.590563205	0.564035758	-1.154598963	4
PHF20L1	0.476277019	0.672833582	-1.149110601	4
PHF21A	0.662208605	0.488108627	-1.150317231	4
PHKA1	0.512652695	0.639715175	-1.15236787	4
PHKB	0.729260998	0.410697688	-1.139958686	4
PHLPP2	0.530231407	0.623220377	-1.153451784	4
PI4KB	0.772710962	0.356738273	-1.129449235	4
PIAS4	0.3395985	0.785975119	-1.125573619	4
PIDD1	0.643296724	0.50878947	-1.152086194	4
PIFO	0.077902268	0.958770484	-1.036672752	4
PIGL	0.2487585	0.852139775	-1.100898275	4
PIGM	0.548921059	0.605320269	-1.154241328	4
PIGN	0.723155113	0.418028121	-1.141183234	4
PIGO	0.786672984	0.33868756	-1.125360544	4
PIGQ	0.310535574	0.807891464	-1.118427038	4
PIK3AP1	0.178384167	0.898803016	-1.077187183	4
PIK3C2B	0.197149945	0.886741689	-1.083891635	4
PILRA	0.200673105	0.884446535	-1.08511964	4
PILRB	0.119016307	0.935165833	-1.05418214	4
PINK1	0.705761822	0.438589358	-1.144351181	4
PINLYP	0.762079003	0.370243863	-1.132322867	4
PIP4K2B	0.727151196	0.413237348	-1.140388544	4
PITRM1	0.549026458	0.605218243	-1.1542447	4

PJA2	0.191158915	0.890622197	-1.081781111	4
PKD2	0.114445989	0.937853177	-1.052299166	4
PKD2L2	0.606697498	0.547497071	-1.154194569	4
PKM	0.724966955	0.415859112	-1.140826066	4
PKMYT1	0.290452821	0.822620617	-1.113073438	4
PKNOX1	0.553559672	0.600818459	-1.154378131	4
PLA2G12A	0.526169632	0.627060756	-1.153230388	4
PLA2G2F	0.43210323	0.711291417	-1.143394647	4
PLA2G7	0.541526156	0.612448146	-1.153974302	4
PLAC8	0.708332373	0.435579935	-1.143912308	4
PLCB4	0.394837213	0.742303523	-1.137140736	4
PLCG1	0.790727623	0.33337645	-1.124104074	4
PLCL2	0.653915237	0.497236623	-1.15115186	4
PLD2	0.555303667	0.599119732	-1.154423399	4
PLEKHA4	0.569865941	0.584802396	-1.154668337	4
PLEKHG4	0.292709216	0.820982451	-1.113691667	4
PLEKHH1	0.765684315	0.365686831	-1.131371146	4
PLOD2	0.263478651	0.841879825	-1.105358476	4
PLPP1	0.779297932	0.34826773	-1.127565662	4
PLPP2	0.663963622	0.486165017	-1.150128639	4
PLPPR3	0.709330486	0.434408705	-1.143739191	4
PLSCR1	0.455477767	0.691176239	-1.146654006	4
PLXNA4	0.098749406	0.946961795	-1.0457112	4
PNMA6A	0.719632716	0.422230038	-1.141862754	4
PNPLA1	0.505025612	0.64677127	-1.151796881	4
PNPLA5	0.605454126	0.548782758	-1.154236884	4
PNPLA8	0.432632698	0.710841519	-1.143474217	4
PODN	0.182481984	0.896192674	-1.078674658	4
POFUT1	0.364083095	0.766948771	-1.131031866	4
POGZ	0.740534989	0.397004262	-1.137539251	4
POLA2	0.289728151	0.823145841	-1.112873992	4
POLDIP3	0.539976773	0.613934016	-1.153910789	4
POLE	0.513890612	0.63856423	-1.152454842	4
POLR1C	0.450989333	0.695079321	-1.146068654	4
POLR2C	0.387478571	0.748277311	-1.135755882	4
POLR2E	0.144568848	0.919847062	-1.06441591	4
POLR3D	0.463328454	0.684302507	-1.147630961	4
POMT1	0.126189562	0.930915855	-1.057105418	4
POMT2	0.383655567	0.751361648	-1.135017215	4
POTEG	0.686947093	0.460316083	-1.147263176	4
POU2F2	0.155163686	0.913348615	-1.068512301	4
POU3F1	0.782059608	0.344692343	-1.126751951	4
POU4F1	0.618142527	0.53557355	-1.153716078	4
POU4F2	0.434715979	0.709068755	-1.143784734	4
POU5F2	0.475107156	0.673876558	-1.148983714	4
PPIAL4A	0.790507155	0.333666054	-1.124173209	4
PPIAL4D	0.512982731	0.639408483	-1.152391214	4
PPIAL4E	0.614304992	0.539589514	-1.153894507	4
PPIAL4F	0.718016609	0.424151393	-1.142168002	4
PPIAL4G	0.569211645	0.585450829	-1.154662474	4
PPIL4	0.562858751	0.591721539	-1.15458029	4
PPIP5K2	0.385311701	0.750027113	-1.135338814	4
PPM1J	0.729063365	0.410935891	-1.139999256	4
PPOX	0.762153586	0.370149827	-1.132303412	4
PPP1R1B	0.299127828	0.816299382	-1.11542721	4

PPP1R2	0.429443781	0.713547213	-1.142990994	4
PPP1R2B	0.443893373	0.7012104	-1.145103772	4
PPP1R2C	0.221465149	0.870702543	-1.092167693	4
PPP1R37	0.072752139	0.961637129	-1.034389268	4
PPP1R3F	0.092987398	0.950258531	-1.043245929	4
PPP2R5D	0.45750922	0.689403321	-1.14691254	4
PPP2R5E	0.369264051	0.762855729	-1.13211978	4
PPP6R1	0.138168237	0.923731148	-1.061899385	4
PRAM1	0.296305712	0.818362634	-1.114668347	4
PRAMEF13	0.367877318	0.763953586	-1.131830905	4
PRAMEF19	0.703592302	0.441121502	-1.144713804	4
PRAP1	0.233785565	0.862396875	-1.096182439	4
PRCC	0.615250898	0.538601323	-1.153852221	4
PRDM10	0.279413983	0.830574344	-1.109988327	4
PREP	0.77360581	0.355592231	-1.129198041	4
PRKCB	0.6539686	0.497178185	-1.151146786	4
PRKD3	0.761982103	0.370366025	-1.132348128	4
PRLHR	0.262004429	0.842915266	-1.104919695	4
PRM1	0.617321687	0.536434093	-1.15375578	4
PRMT6	0.15230296	0.915111783	-1.067414743	4
PRND	0.468404633	0.679826033	-1.148230667	4
PROX1	0.126835157	0.93053143	-1.057366587	4
PROZ	0.583682773	0.570994528	-1.154677301	4
PRPF8	0.657279078	0.493545452	-1.15082453	4
PRR12	0.467861011	0.680306639	-1.14816765	4
PRR18	0.159502834	0.910662201	-1.070165035	4
PRR5	0.48160019	0.668070602	-1.149670791	4
PRR5-ARHGAP8	0.724005606	0.417010619	-1.141016225	4
PRR7	0.583327012	0.571352831	-1.154679843	4
PRRT1B	0.547796459	0.606408126	-1.154204585	4
PRSS27	0.660165317	0.490366191	-1.150531509	4
PRSS33	0.198121796	0.886109552	-1.084231348	4
PRTN3	0.758126504	0.375213324	-1.133339828	4
PSD	0.778687904	0.349055575	-1.127743479	4
PSD4	0.41731497	0.723751311	-1.14106628	4
PSMA2	0.755082123	0.37902238	-1.134104503	4
PSMA4	0.084946646	0.954817031	-1.039763677	4
PSMB2	0.240812137	0.85760576	-1.098417897	4
PSMB6	0.41105027	0.728968448	-1.140018718	4
PSMB9	0.439074782	0.705346386	-1.144421169	4
PSME1	0.1081446	0.94153232	-1.04967692	4
PSME3	0.172453044	0.902558066	-1.07501111	4
PTDSS1	0.083810632	0.955457123	-1.039267754	4
PTEN	0.106954369	0.942223861	-1.04917823	4
PTGS1	0.503025325	0.648611852	-1.151637177	4
PTK2	0.779042283	0.348597982	-1.127640265	4
PTK2B	0.61614036	0.537671088	-1.153811448	4
PTPDC1	0.073894512	0.961002994	-1.034897506	4
PTPRCAP	0.230023288	0.86494599	-1.094969278	4
PTPRS	0.446563862	0.698908688	-1.14547255	4
PTTG1IP	0.610472843	0.543581686	-1.15405453	4
PTX4	0.190435124	0.891089108	-1.081524232	4
PUDP	0.788827659	0.335869125	-1.124696784	4
PUM2	0.682632159	0.465225923	-1.147858082	4
PUS7L	0.513159257	0.639244396	-1.152403653	4

PXDNL	0.366565342	0.764990701	-1.131556043	4
PYCARD	0.225298314	0.868131401	-1.093429715	4
QDPR	0.300149286	0.815550959	-1.115700246	4
QPCTL	0.609685875	0.544399284	-1.154085159	4
QRFPR	0.70008961	0.44519472	-1.145284331	4
QSOX2	0.592565876	0.561999802	-1.154565678	4
R3HDM4	0.454973632	0.691615597	-1.146589229	4
RAB13	0.560964841	0.593582128	-1.154546969	4
RAB30	0.743615632	0.393226068	-1.1368417	4
RAB3D	0.761217826	0.371328961	-1.132546787	4
RAB3GAP2	0.218549942	0.87265015	-1.091200091	4
RAB41	0.687066159	0.460180221	-1.14724638	4
RAB43	0.569571761	0.585094	-1.154665761	4
RAB5B	0.481293498	0.668345785	-1.149639283	4
RAB6A	0.501026591	0.650446896	-1.151473487	4
RAB6C	0.433914177	0.709751531	-1.143665708	4
RAC1	0.269084635	0.837926239	-1.107010875	4
RAD21	0.537573399	0.616233713	-1.153807112	4
RAD50	0.131152122	0.927952671	-1.059104793	4
RAD51D	0.250901233	0.850657179	-1.101558412	4
RAET1G	0.639651383	0.512721353	-1.152372736	4
RALGPS1	0.411283342	0.728774997	-1.140058339	4
RAN	0.506592349	0.645326741	-1.15191909	4
RAP1A	0.498892433	0.65240175	-1.151294183	4
RAP1GAP2	0.424027248	0.718121135	-1.142148383	4
RAPGEF3	0.170659925	0.90368793	-1.074347856	4
RASGRP4	0.55006616	0.60421115	-1.154277309	4
RASIP1	0.194007399	0.888780682	-1.082788081	4
RBM12	0.599187659	0.555233997	-1.154421655	4
RBM39	0.551439039	0.602879501	-1.154318541	4
RBM43	0.158424296	0.911331292	-1.069755588	4
RBM5	0.662463753	0.487826322	-1.150290075	4
RBM6	0.273684333	0.834663193	-1.108347526	4
RBM7	0.097512038	0.947671877	-1.045183915	4
RBM8A	0.660215152	0.490311198	-1.15052635	4
RBMS1	0.548477527	0.605749476	-1.154227003	4
RBX1	0.128353627	0.929625989	-1.057979616	4
RC3H1	0.666966145	0.482830055	-1.149796199	4
RCAN1	0.246777913	0.853506891	-1.100284804	4
RCHY1	0.55801373	0.596473294	-1.154487023	4
RCOR2	0.225075246	0.868281346	-1.093356592	4
REEP1	0.573395205	0.581296323	-1.154691528	4
RELL1	0.382048294	0.752654467	-1.134702761	4
RELL2	0.78327913	0.343108931	-1.126388062	4
REST	0.125897168	0.93108986	-1.056987028	4
RFC4	0.617372506	0.53638084	-1.153753346	4
RFLNA	0.258695434	0.845232964	-1.103928398	4
RFPL4AL1	0.705446499	0.438957828	-1.144404327	4
RGL4	0.596213926	0.558278883	-1.154492808	4
RGP1	0.738492682	0.399500314	-1.137992996	4
RHBDF2	0.48136114	0.6682851	-1.14964624	4
RHNO1	0.578846511	0.575852734	-1.154699245	4
RHOT1	0.188525562	0.892318987	-1.080844549	4
RIMKLA	0.309953255	0.808323292	-1.118276547	4
RLBP1	0.124973554	0.931639074	-1.056612628	4

RMC1	0.703672554	0.441027962	-1.144700517	4
RMDN1	0.268789094	0.838135307	-1.106924401	4
RMDN2	0.093055612	0.95021965	-1.043275261	4
RNASE2	0.615843736	0.537981418	-1.153825154	4
RNASE3	0.52753815	0.625768796	-1.153306947	4
RNASEK	0.660984753	0.489461505	-1.150446258	4
RNASEK-C17orf49	0.767609864	0.363243475	-1.130853339	4
RND2	0.524856696	0.62829837	-1.153155066	4
RNF111	0.564828573	0.589782086	-1.154610659	4
RNF115	0.584842908	0.569825077	-1.154667985	4
RNF123	0.196717647	0.887022638	-1.083740286	4
RNF125	0.305690523	0.811475732	-1.117166255	4
RNF135	0.398120544	0.739622328	-1.137742872	4
RNF144A	0.366333279	0.765173989	-1.131507267	4
RNF165	0.417412734	0.723669607	-1.141082341	4
RNF167	0.471712516	0.676895339	-1.148607854	4
RNF207	0.614986039	0.538878134	-1.153864173	4
RNF215	0.411517791	0.728580353	-1.140098144	4
RNF225	0.43476314	0.709028576	-1.143791716	4
RNF227	0.528018226	0.625315105	-1.153333331	4
RNF38	0.721380725	0.420147256	-1.141527981	4
RNF41	0.528785542	0.624589449	-1.153374991	4
RNF8	0.549000195	0.605243666	-1.154243861	4
RNH1	0.736923925	0.401412928	-1.138336853	4
RNMT	0.118776373	0.93530731	-1.054083682	4
RNPEPL1	0.490610323	0.659944049	-1.150554372	4
ROGDI	0.584374834	0.570297099	-1.154671933	4
RPA2	0.634595925	0.518145638	-1.152741563	4
RPAP2	0.1614834	0.909431181	-1.070914581	4
RPAP3	0.288475306	0.82405285	-1.112528156	4
RPEL1	0.463069872	0.684529865	-1.147599737	4
RPH3AL	0.353839097	0.774972473	-1.12881157	4
RPL18A	0.56739825	0.587245431	-1.154643681	4
RPL38	0.138685602	0.923418363	-1.062103964	4
RPL41	0.090635686	0.951596837	-1.042232523	4
RPN2	0.288426369	0.824088252	-1.112514621	4
RPP14	0.750947345	0.384170036	-1.135117381	4
RPP25	0.50090336	0.650559901	-1.15146326	4
RPRD1A	0.474021178	0.674843534	-1.148864712	4
RPRML	0.152349398	0.915083212	-1.06743261	4
RPS14	0.095697992	0.948710797	-1.044408789	4
RPS26	0.690355552	0.456418699	-1.146774251	4
RPS6KB1	0.165121157	0.907162238	-1.072283395	4
RPS6KC1	0.756843363	0.376820726	-1.133664088	4
RRP15	0.139017339	0.923217695	-1.062235034	4
RRP1B	0.559636129	0.594885058	-1.154521187	4
RRP36	0.732091556	0.407279163	-1.139370719	4
RSAD1	0.375700052	0.757738205	-1.133438257	4
RSBN1	0.324846619	0.797189115	-1.122035734	4
RSF1	0.643510789	0.508558039	-1.152068828	4
RSKR	0.074672027	0.960570829	-1.035242856	4
RSPH6A	0.45527527	0.691352747	-1.146628016	4
RSPO3	0.512966502	0.639423567	-1.152390069	4
RTL1	0.372338379	0.760415777	-1.132754156	4
RTN1	0.322148101	0.79922041	-1.121368511	4

RUBCN	0.616146004	0.537665183	-1.153811186	4
RUNDC1	0.450264173	0.69570809	-1.145972263	4
RUNDC3A	0.161070703	0.909687942	-1.070758645	4
RUVBL1	0.698290554	0.447279689	-1.145570243	4
RYR1	0.294459251	0.819709004	-1.114168254	4
S100A11	0.672248635	0.476932464	-1.149181099	4
S100A14	0.524484975	0.628648433	-1.153133409	4
SAMD1	0.795688846	0.326834434	-1.12252328	4
SAMD14	0.575116953	0.57958071	-1.154697662	4
SAMD4A	0.542673504	0.611346147	-1.154019651	4
SAMD9L	0.397291019	0.740300647	-1.137591666	4
SAP25	0.761401501	0.371097637	-1.132499138	4
SAP30L	0.379248741	0.754900798	-1.134149539	4
SARAF	0.58237067	0.572315274	-1.154685944	4
SARS2	0.474718339	0.674222902	-1.148941242	4
SASH3	0.398371855	0.739416703	-1.137788559	4
SAV1	0.755566194	0.378417798	-1.133983993	4
SBF2	0.075495062	0.960112866	-1.035607928	4
SCAF8	0.156961674	0.912237223	-1.069198897	4
SCAMP3	0.102747507	0.944659485	-1.047406992	4
SCARB1	0.302957495	0.813488903	-1.116446398	4
SCLT1	0.177412927	0.899419789	-1.076832715	4
SCLY	0.071787205	0.962172	-1.033959206	4
SCN2B	0.68404076	0.463626056	-1.147666816	4
SCN4A	0.553119887	0.601246295	-1.154366182	4
SCRIB	0.146945416	0.918396882	-1.065342298	4
SCYL2	0.21467169	0.875230717	-1.089902407	4
SCYL3	0.390141443	0.746121204	-1.136262647	4
SDCBP2	0.246002553	0.854041229	-1.100043782	4
SDR42E1	0.732521805	0.406758407	-1.139280213	4
SDR9C7	0.625901816	0.527397345	-1.153299162	4
SEC13	0.227859801	0.866406743	-1.094266543	4
SEC14L5	0.422971992	0.719009044	-1.141981037	4
SEC23IP	0.366198399	0.765280497	-1.131478896	4
SEC61B	0.661113175	0.489319639	-1.150432814	4
SEC63	0.648520405	0.503124813	-1.151645218	4
SECISBP2	0.141687836	0.921599238	-1.063287074	4
SEH1L	0.440176886	0.704402353	-1.144579239	4
SELENBP1	0.355099668	0.77399006	-1.129089728	4
SELP	0.422295639	0.719577594	-1.141873233	4
SEMA3C	0.183124344	0.8957823	-1.078906644	4
SEMA4D	0.792972352	0.330422441	-1.123394792	4
SENP2	0.323263639	0.798381443	-1.121645082	4
SEPSECS	0.720311659	0.421421636	-1.141733296	4
SEPTIN11	0.07529848	0.960222297	-1.035520777	4
SERF1A	0.604767282	0.549492172	-1.154259454	4
SERF1B	0.493201306	0.657592	-1.150793306	4
SERPINA4	0.403570481	0.735150269	-1.138720751	4
SFXN5	0.409301703	0.730418176	-1.139719879	4
SGPP2	0.608960763	0.545151946	-1.154112709	4
SGTB	0.508565494	0.643503894	-1.152069389	4
SH2B1	0.515326311	0.637227394	-1.152553705	4
SH2D3C	0.080671255	0.957220943	-1.037892198	4
SH2D4A	0.593390364	0.56116023	-1.154550593	4
SH2D6	0.270230234	0.837115162	-1.107345396	4

SH2D7	0.716002422	0.426540311	-1.142542732	4
SH3BP4	0.219873529	0.871766716	-1.091640245	4
SH3BP5L	0.534325084	0.619331975	-1.153657059	4
SH3GLB1	0.474539868	0.674381828	-1.148921696	4
SH3YL1	0.666850452	0.482958787	-1.149809239	4
SHISA2	0.37822336	0.755721807	-1.133945167	4
SHISA3	0.773242582	0.356057598	-1.12930018	4
SHLD2	0.154754818	0.913601002	-1.068355819	4
SHOX2	0.729984936	0.409824603	-1.13980954	4
SHQ1	0.55537125	0.599053835	-1.154425085	4
SIGIRR	0.302648474	0.813716138	-1.116364612	4
SIMC1	0.609029481	0.545080645	-1.154110126	4
SIPA1	0.743057018	0.393912346	-1.136969363	4
SIPA1L2	0.69606868	0.449848041	-1.145916721	4
SIX2	0.325628811	0.79659917	-1.12222798	4
SIX3	0.78581782	0.339803697	-1.125621517	4
SIX5	0.202718234	0.883109769	-1.085828003	4
SIX6	0.717022763	0.425330929	-1.142353692	4
SKAP1	0.184940084	0.894620569	-1.079560653	4
SLAMF1	0.40742141	0.731973989	-1.139395399	4
SLAMF9	0.095666331	0.948728907	-1.044395238	4
SLC10A7	0.284956885	0.826593113	-1.111549998	4
SLC12A4	0.617518472	0.536227866	-1.153746338	4
SLC12A8	0.675642083	0.473123366	-1.148765449	4
SLC16A4	0.523807523	0.629286038	-1.153093561	4
SLC16A5	0.555512089	0.598916494	-1.154428583	4
SLC16A8	0.733751056	0.405268915	-1.139019971	4
SLC17A2	0.342085773	0.784066004	-1.126151777	4
SLC17A5	0.296726295	0.818055566	-1.114781861	4
SLC19A1	0.684088506	0.463571776	-1.147660282	4
SLC22A15	0.537426772	0.616373812	-1.153800584	4
SLC22A18	0.756328499	0.377464893	-1.133793392	4
SLC22A18AS	0.264735937	0.840995357	-1.105731294	4
SLC22A7	0.680944951	0.467138495	-1.148083446	4
SLC25A15	0.509798931	0.642362363	-1.152161293	4
SLC25A18	0.510053981	0.64212612	-1.1521801	4
SLC25A25	0.548363762	0.605859532	-1.154223294	4
SLC25A32	0.639092715	0.513322405	-1.15241512	4
SLC25A34	0.738342518	0.399683568	-1.138026086	4
SLC25A37	0.520601979	0.632296448	-1.152898427	4
SLC25A43	0.641584462	0.510638484	-1.152222946	4
SLC30A9	0.237428336	0.859917958	-1.097346294	4
SLC35A1	0.403559119	0.735159621	-1.13871874	4
SLC35A5	0.46594896	0.681994741	-1.147943701	4
SLC35B2	0.450225875	0.695741284	-1.145967158	4
SLC35B3	0.56476499	0.589844757	-1.154609747	4
SLC35E4	0.767136754	0.363844428	-1.130981182	4
SLC35F6	0.484595094	0.66537844	-1.149973533	4
SLC38A5	0.700710607	0.444473914	-1.14518452	4
SLC39A9	0.297048837	0.817819977	-1.114868814	4
SLC44A1	0.658164063	0.492571819	-1.150735882	4
SLC45A4	0.786038829	0.339515376	-1.125554205	4
SLC46A2	0.46072293	0.686590434	-1.147313365	4
SLC4A1AP	0.168626687	0.904966081	-1.073592768	4
SLC4A7	0.378882682	0.755194004	-1.134076686	4

SLC52A2	0.324392749	0.797531195	-1.121923944	4
SLC52A3	0.421121376	0.720563681	-1.141685057	4
SLC5A1	0.604029759	0.550253293	-1.154283052	4
SLC5A9	0.644050659	0.507974105	-1.152024764	4
SLC66A1	0.527137701	0.626147049	-1.153284751	4
SLC6A20	0.217834053	0.873127394	-1.090961447	4
SLC7A1	0.458987924	0.688110291	-1.147098214	4
SLCO2A1	0.39271686	0.744029842	-1.136746701	4
SLIT2	0.519243963	0.633568525	-1.152812488	4
SLX9	0.187893565	0.892725405	-1.08061897	4
SMAD3	0.430965334	0.712257416	-1.143222749	4
SMAD4	0.146778226	0.918499043	-1.065277269	4
SMAP2	0.644204043	0.507808132	-1.152012175	4
SMARCA4	0.593446321	0.561103219	-1.15454954	4
SMARCAD1	0.071643729	0.96225147	-1.0338952	4
SMG1	0.280866242	0.82953369	-1.110399933	4
SMIM10L2A	0.275766447	0.833180446	-1.108946893	4
SMIM17	0.550847964	0.60345308	-1.154301044	4
SMIM22	0.739311854	0.398499973	-1.137811827	4
SNAP91	0.670023632	0.479421269	-1.149444901	4
SNAPC1	0.689755811	0.457105692	-1.146861503	4
SNED1	0.304735941	0.812179594	-1.116915535	4
SNRNP200	0.473210318	0.67556478	-1.148775097	4
SNRNP70	0.222096103	0.870280125	-1.092376228	4
SNRPN	0.46913706	0.679178051	-1.148315111	4
SNX3	0.732692215	0.406552068	-1.139244282	4
SNX31	0.742028756	0.395174237	-1.137202993	4
SNX8	0.289370688	0.823404762	-1.11277545	4
SOS1	0.769343931	0.361037386	-1.130381317	4
SOX12	0.744219291	0.392483864	-1.136703155	4
SOX7	0.647647716	0.504073677	-1.151721392	4
SOX8	0.572463253	0.582223535	-1.154686788	4
SOX9	0.606664414	0.547531305	-1.154195719	4
SP8	0.511054514	0.641198711	-1.152253226	4
SP9	0.638292966	0.514182124	-1.15247509	4
SPAAR	0.62181053	0.531717871	-1.153528401	4
SPACA5	0.19622064	0.887345461	-1.0835661	4
SPACA5B	0.123556761	0.932480286	-1.056037047	4
SPAG5	0.647365732	0.504380059	-1.15174579	4
SPANXB1	0.088192551	0.952982736	-1.041175287	4
SPANXD	0.241190095	0.857346926	-1.098537021	4
SPATA31A6	0.147006592	0.918359495	-1.065366087	4
SPATA31D1	0.303401694	0.813162127	-1.116563821	4
SPATA6	0.24347333	0.855780881	-1.099254211	4
SPATA9	0.35227333	0.776190813	-1.128464143	4
SPCS2	0.592663483	0.561900451	-1.154563934	4
SPDEF	0.453721529	0.692705753	-1.146427282	4
SPDL1	0.616115216	0.537697399	-1.153812614	4
SPDYE3	0.281228536	0.829273809	-1.110502346	4
SPEF1	0.580265318	0.574430307	-1.154695624	4
SPOPL	0.199124482	0.885456581	-1.084581063	4
SPRING1	0.774556375	0.354373224	-1.128929599	4
SPSB1	0.418954601	0.722379868	-1.141334469	4
SPSB2	0.120639231	0.934207725	-1.054846957	4
SPTSSA	0.126062546	0.930991451	-1.057053997	4

SQSTM1	0.347192159	0.780129879	-1.127322038	4
SRD5A1	0.134845256	0.925735249	-1.060580504	4
SRFBP1	0.076975105	0.959288036	-1.036263141	4
SRGAP3	0.442114364	0.702739964	-1.144854328	4
SRMS	0.48928725	0.661142493	-1.150429742	4
SRP68	0.577380928	0.57731961	-1.154700538	4
SRPK1	0.310172056	0.80816107	-1.118333126	4
SRRM3	0.328862766	0.794154535	-1.1230173	4
SRSF9	0.462360433	0.685153305	-1.147513738	4
SS18L2	0.478618785	0.67074173	-1.149360514	4
SSBP1	0.429013522	0.713911544	-1.142925066	4
SSTR3	0.602689223	0.551635034	-1.154324257	4
SSU72P2	0.590686318	0.563910735	-1.154597054	4
SSU72P5	0.742987816	0.393997326	-1.136985142	4
SSU72P7	0.290340354	0.822702159	-1.113042513	4
SSX3	0.66126534	0.489151517	-1.150416857	4
ST14	0.71102315	0.432418981	-1.14344213	4
STAC2	0.749869157	0.385507521	-1.135376677	4
STAC3	0.322805225	0.798726332	-1.121531557	4
STAMBP	0.517214834	0.635465629	-1.152680463	4
STAP2	0.772057631	0.357574077	-1.129631707	4
STAR	0.153608315	0.914308028	-1.067916343	4
STARD10	0.297400492	0.817563026	-1.114963518	4
STARD9	0.214824172	0.875129481	-1.089953653	4
STAT3	0.692923911	0.453470754	-1.146394665	4
STAT5B	0.117598279	0.936001335	-1.053599614	4
STEEP1	0.252402312	0.84961635	-1.102018662	4
STK10	0.690907286	0.455786235	-1.146693521	4
STK11IP	0.515702217	0.636877016	-1.152579233	4
STK35	0.702312292	0.442612134	-1.144924427	4
STMN3	0.633312819	0.51951711	-1.152829929	4
STOM	0.391544313	0.744982747	-1.13652706	4
STOML3	0.615270195	0.538581152	-1.153851347	4
STRAP	0.149912498	0.916580277	-1.066492775	4
STT3B	0.696740517	0.449072209	-1.145812726	4
STUB1	0.703926007	0.440732482	-1.144658489	4
STX16-NPEPL1	0.101718972	0.945252926	-1.046971898	4
STXBP4	0.251521362	0.85022741	-1.101748772	4
SULF2	0.681205726	0.466843153	-1.148048879	4
SULT1C4	0.491432496	0.659198431	-1.150630927	4
SULT4A1	0.48862694	0.661739942	-1.150366882	4
SUPT20HL1	0.51557353	0.636996981	-1.152570511	4
SYDE2	0.63771831	0.514799359	-1.152517669	4
SYK	0.182232434	0.896352013	-1.078584447	4
SYMPK	0.158534425	0.911263013	-1.069797438	4
SYNE2	0.350057062	0.777911677	-1.127968739	4
SYNJ2	0.786905193	0.338384246	-1.125289438	4
SYNJ2BP	0.09686523	0.948042595	-1.044907826	4
SYNPO2L	0.644099554	0.507921201	-1.152020754	4
SYT15	0.158652061	0.911190069	-1.06984213	4
SYT5	0.59072266	0.563873827	-1.154596487	4
SYTL3	0.268410897	0.838402742	-1.106813639	4
TACC2	0.51106052	0.641193142	-1.152253661	4
TAF8	0.217407545	0.873411531	-1.090819076	4
TAF9B	0.716065222	0.426465922	-1.142531144	4

TAGLN2	0.180487632	0.897464725	-1.077952356	4
TANK	0.632202903	0.520701761	-1.152904664	4
TAP2	0.542251525	0.611751615	-1.154003139	4
TAS2R14	0.470574205	0.677905067	-1.148479272	4
TAX1BP1	0.073770641	0.961071802	-1.034842444	4
TAX1BP3	0.642378536	0.509781467	-1.152160003	4
TBC1D10C	0.288675648	0.823907898	-1.112583546	4
TBC1D2	0.763417198	0.368555112	-1.13197231	4
TBC1D3	0.574515267	0.580180639	-1.154695906	4
TBC1D30	0.624786692	0.528577041	-1.153363733	4
TBC1D31	0.630382766	0.522641044	-1.15302381	4
TBC1D3L	0.681416275	0.466604624	-1.148020899	4
TBC1D8	0.085483656	0.954514111	-1.039997767	4
TBC1D9	0.355617407	0.773586165	-1.129203571	4
TBC1D9B	0.263030454	0.84219481	-1.105225263	4
TBP	0.793047116	0.330323883	-1.123371	4
TBPL1	0.116588703	0.936595243	-1.053183946	4
TBRG1	0.508049764	0.643980729	-1.152030493	4
TBX1	0.45811909	0.688870286	-1.146989376	4
TBX10	0.412317284	0.727916217	-1.140233501	4
TBX3	0.758787029	0.374384758	-1.133171787	4
TCEA1	0.376285363	0.757270981	-1.133556345	4
TCEAL1	0.467601716	0.680535774	-1.14813749	4
TCF15	0.632815569	0.520048038	-1.152863606	4
TCL1B	0.790386319	0.333824742	-1.124211061	4
TCN2	0.572592928	0.582094579	-1.154687507	4
TCTN1	0.530108222	0.623337106	-1.153445328	4
TDRD9	0.716972815	0.425390169	-1.142362984	4
TEAD3	0.303000343	0.81345739	-1.116457732	4
TENM3	0.709438525	0.434281837	-1.143720362	4
TERT	0.225631036	0.867907673	-1.093538709	4
TES	0.201399842	0.883971891	-1.085371733	4
TEX13A	0.390120281	0.746138364	-1.136258645	4
TEX28	0.736628128	0.401773107	-1.138401235	4
TEX44	0.686067816	0.46131876	-1.147386576	4
TEX51	0.324856544	0.797181633	-1.122038177	4
TEX54	0.628635102	0.524499134	-1.153134236	4
TEX9	0.159492226	0.910668786	-1.070161012	4
TFAM	0.516528294	0.636106521	-1.152634815	4
TFAP2A	0.592899965	0.561659698	-1.154559663	4
TFAP2E	0.435612542	0.708304567	-1.143917109	4
TFE3	0.264697443	0.841022455	-1.105719898	4
TFIP11	0.631198291	0.521772659	-1.152970949	4
TGFB3	0.433346839	0.710234282	-1.143581121	4
TGFBR2	0.644061902	0.507961941	-1.152023842	4
TGFBR3L	0.641471833	0.510759974	-1.152231807	4
TGIF2-RAB5IF	0.092282942	0.95065986	-1.042942802	4
TGM2	0.468681732	0.679580945	-1.148262676	4
TGM7	0.6438448	0.508196811	-1.152041611	4
THADA	0.136637532	0.924655372	-1.061292904	4
THAP6	0.616608861	0.537180717	-1.153789578	4
THEM4	0.420476309	0.721104831	-1.141581139	4
THNSL2	0.536861002	0.616914177	-1.153775179	4
THY1	0.730447094	0.40926679	-1.139713884	4
TICRR	0.093531579	0.949948253	-1.043479832	4

TIGAR	0.684621925	0.462965148	-1.147587073	4
TIGIT	0.072354354	0.96185771	-1.034212064	4
TIMM21	0.419918469	0.721572493	-1.141490962	4
TIMM23B-AGAP6	0.193380465	0.889186533	-1.082566998	4
TIMM29	0.349908714	0.778026713	-1.127935427	4
TKTL1	0.110882301	0.93993759	-1.050819891	4
TLE7	0.19132646	0.890514056	-1.081840516	4
TLNRD1	0.401259055	0.737050257	-1.138309312	4
TLR1	0.601511582	0.55284708	-1.154358663	4
TM7SF2	0.454224426	0.692268083	-1.146492509	4
TM9SF4	0.466527799	0.681484076	-1.148011875	4
TMA16	0.527373611	0.625924236	-1.153297847	4
TMC4	0.56342549	0.591163986	-1.154589476	4
TMCO6	0.361314824	0.769126089	-1.130440913	4
TMED10	0.667924629	0.481762834	-1.149687463	4
TMED8	0.726306527	0.414252114	-1.140558641	4
TMED9	0.501191382	0.650295756	-1.151487138	4
TMEM106B	0.359054092	0.770899237	-1.129953329	4
TMEM126A	0.530789338	0.62269148	-1.153480819	4
TMEM131	0.141993352	0.921413731	-1.063407083	4
TMEM132A	0.498500401	0.652760339	-1.15126074	4
TMEM132C	0.585134271	0.569531126	-1.154665398	4
TMEM134	0.524675729	0.628468811	-1.153144541	4
TMEM141	0.232563591	0.863226057	-1.095789647	4
TMEM150A	0.537609183	0.616199518	-1.153808702	4
TMEM164	0.663642315	0.486521166	-1.150163481	4
TMEM168	0.289908597	0.823015099	-1.112923696	4
TMEM170B	0.627312714	0.525902496	-1.153215211	4
TMEM184C	0.132722867	0.927010843	-1.05973371	4
TMEM191B	0.46770755	0.680442258	-1.148149808	4
TMEM200C	0.793203149	0.330118161	-1.12332131	4
TMEM217	0.624336007	0.529053378	-1.153389385	4
TMEM245	0.468769545	0.679503259	-1.148272805	4
TMEM273	0.077081266	0.959228809	-1.036310075	4
TMEM39B	0.28689069	0.825198191	-1.11208888	4
TMEM54	0.152653466	0.914896093	-1.067549558	4
TMEM86A	0.339767163	0.785845829	-1.125612992	4
TMEM94	0.231978187	0.863622866	-1.095601053	4
TMEM95	0.259758368	0.844489429	-1.104247796	4
TMEM98	0.320538179	0.800429332	-1.120967511	4
TMPRSS12	0.178346572	0.898826904	-1.077173476	4
TMTC1	0.147786389	0.917882681	-1.06566907	4
TNFAIP8L2	0.080708718	0.957199939	-1.037908657	4
TNFRSF10C	0.135371431	0.925418474	-1.060789905	4
TNFRSF14	0.762918155	0.369185257	-1.132103412	4
TNFSF10	0.459019861	0.68808234	-1.147102201	4
TNFSF12	0.300547268	0.815259123	-1.115806391	4
TNP2	0.552521601	0.601827983	-1.154349583	4
TNRC6B	0.443046446	0.70193895	-1.144985396	4
TOGARAM1	0.225617054	0.867917076	-1.09353413	4
TOGARAM2	0.64400241	0.508026308	-1.152028718	4
TOM1L2	0.777530429	0.350548538	-1.128078967	4
TOP1	0.306928673	0.810561644	-1.117490318	4
TP53TG3B	0.07426323	0.960798107	-1.035061337	4
TP53TG3E	0.366681848	0.764898664	-1.131580512	4

TP53TG3F	0.228688592	0.865847596	-1.094536189	4
TP73	0.15555526	0.913106784	-1.068662043	4
TPD52L1	0.107253981	0.942049884	-1.049303864	4
TPGS1	0.761811778	0.370580712	-1.13239249	4
TPH2	0.349845994	0.778075343	-1.127921337	4
TPM2	0.776184444	0.352281523	-1.128465967	4
TPPP	0.103286184	0.944348361	-1.047634544	4
TPPP2	0.758852382	0.374302736	-1.133155119	4
TPPP3	0.680865652	0.467228287	-1.148093939	4
TRA2A	0.246518343	0.853685827	-1.10020417	4
TRAF1	0.428005596	0.714764346	-1.142769942	4
TRAFD1	0.21221423	0.876859741	-1.089073971	4
TRAK2	0.35362514	0.775139081	-1.128764221	4
TRAP1	0.606523261	0.547677352	-1.154200612	4
TRAPPC11	0.398607137	0.739224142	-1.137831279	4
TRAPPC2B	0.338472944	0.786837296	-1.12531024	4
TRHDE	0.472427847	0.676260156	-1.148688004	4
TRIM46	0.496134893	0.654920718	-1.151055611	4
TRIM47	0.176569576	0.899954752	-1.076524328	4
TRIM5	0.291975499	0.8215156	-1.113491099	4
TRIM50	0.380389224	0.75398653	-1.134375754	4
TRIM52	0.381724443	0.752914678	-1.134639122	4
TRIM74	0.24989833	0.851351567	-1.101249897	4
TRIM8	0.259280776	0.844823623	-1.104104399	4
TRIML2	0.621418861	0.532130382	-1.153549243	4
TRMT10B	0.519173693	0.633634294	-1.152807988	4
TRMT2B	0.56851237	0.586143301	-1.15465567	4
TRMT44	0.287697664	0.824615179	-1.112312843	4
TRMT61A	0.215361722	0.874772444	-1.090134166	4
TRPV2	0.076895596	0.959332388	-1.036227985	4
TRUB2	0.082304124	0.956304465	-1.038608589	4
TSEN15	0.14303479	0.92078084	-1.06381563	4
TSG101	0.44365564	0.701414973	-1.145070613	4
TSKU	0.661121997	0.489309893	-1.15043189	4
TSNAX-DISC1	0.395127242	0.742067076	-1.137194318	4
TSNAXIP1	0.408920615	0.730733762	-1.139654377	4
TSPEAR	0.726360747	0.41418701	-1.140547757	4
TSPOAP1	0.178174184	0.898936425	-1.077110609	4
TTBK2	0.757485526	0.376016641	-1.133502167	4
TTC5	0.179554845	0.898058618	-1.077613463	4
TTC7B	0.678547114	0.469849647	-1.148396762	4
TTF1	0.199758604	0.88504322	-1.084801824	4
TTK	0.446027911	0.699371174	-1.145399085	4
TTLL10	0.685778448	0.461648494	-1.147426942	4
TTLL11	0.236821783	0.860331456	-1.097153239	4
TTYH1	0.329127353	0.793954132	-1.123081485	4
TUBB8	0.278319677	0.831357353	-1.10967703	4
TUG1	0.781467252	0.345460438	-1.12692769	4
TUT1	0.563344437	0.591243748	-1.154588185	4
TUT7	0.20092523	0.884281915	-1.085207145	4
TXNDC9	0.262866497	0.842309994	-1.105176492	4
TYW1	0.46185201	0.685599794	-1.147451804	4
TYW3	0.627812191	0.525372695	-1.153184887	4
TYW5	0.73414973	0.40478531	-1.13893504	4
UBA1	0.787352497	0.33779968	-1.125152177	4

UBA6	0.516699594	0.635946656	-1.152646251	4
UBAP1L	0.730825449	0.408809868	-1.139635317	4
UBAP2L	0.730676659	0.408989582	-1.139666241	4
UBASH3A	0.255271352	0.847621932	-1.102893284	4
UBE2O	0.13080402	0.928161139	-1.058965159	4
UBE2Q1	0.649347961	0.50222409	-1.151572051	4
UBE4B	0.319292936	0.801362906	-1.120655841	4
UBL7	0.482017006	0.667696457	-1.149713463	4
UBXN11	0.537747942	0.61606691	-1.153814852	4
UBXN6	0.347274759	0.780066024	-1.127340783	4
UCHL5	0.31349024	0.805695996	-1.119186236	4
UEVLD	0.318908946	0.801650523	-1.120559469	4
UHMK1	0.596732239	0.557748929	-1.154481168	4
UHRF1BP1L	0.206279059	0.880774446	-1.087053506	4
UIMC1	0.572480015	0.582206867	-1.154686882	4
ULBP3	0.174716183	0.901128475	-1.075844658	4
UNC50	0.204386283	0.882017042	-1.086403325	4
UNC5A	0.115971304	0.936958061	-1.052929365	4
USHBP1	0.682160424	0.465761078	-1.147921502	4
USP17L8	0.634308016	0.518453559	-1.152761575	4
USP19	0.653371691	0.497831634	-1.151203326	4
USP3	0.456401873	0.690370235	-1.146772108	4
USP37	0.149714616	0.916701642	-1.066416257	4
USP6NL	0.510212029	0.641979691	-1.152191721	4
UVRAG	0.746312759	0.389905183	-1.136217943	4
VAC14	0.131503673	0.927742043	-1.059245715	4
VAMP5	0.727183128	0.413198964	-1.140382092	4
VAPA	0.124889892	0.93168879	-1.056578683	4
VARS2	0.578733136	0.575966297	-1.154699433	4
VASP	0.278122627	0.831498243	-1.10962087	4
VAT1	0.714477074	0.428345248	-1.142822322	4
VCL	0.283240478	0.827828638	-1.111069115	4
VHL	0.717005288	0.425351656	-1.142356943	4
VPS11	0.575208173	0.57948972	-1.154697892	4
VPS25	0.649011383	0.502590536	-1.151601918	4
VPS39	0.301952837	0.814227375	-1.116180212	4
VPS52	0.150925696	0.915958392	-1.066884088	4
VPS54	0.782081062	0.344664511	-1.126745573	4
VRK3	0.496750723	0.654358841	-1.151109563	4
VSIG10L	0.709234264	0.434521682	-1.143755947	4
VSNL1	0.422579705	0.719338857	-1.141918562	4
VTI1B	0.673512553	0.475515618	-1.149028171	4
WASHC2C	0.755424065	0.378595353	-1.134019418	4
WBP1	0.499104621	0.652207599	-1.151312219	4
WDFY2	0.764508894	0.367175078	-1.131683972	4
WDR11	0.413784607	0.726695788	-1.140480395	4
WDR17	0.329818068	0.793430692	-1.123248761	4
WDR24	0.334210427	0.790092542	-1.124302969	4
WDR4	0.094603557	0.949336383	-1.04393994	4
WDR5	0.249375621	0.851713157	-1.101088778	4
WDR62	0.466770259	0.681270075	-1.148040334	4
WDR7	0.168796159	0.904859669	-1.073655828	4
WDR82	0.345967669	0.781075796	-1.127043465	4
WFDC3	0.33569861	0.788957798	-1.124656408	4
WFDC8	0.41953332	0.721895213	-1.141428533	4

WFIKKN2	0.479197378	0.670224043	-1.149421422	4
WIPF1	0.118013771	0.935756686	-1.053770457	4
WNK1	0.695177879	0.450875704	-1.146053583	4
WNT3	0.735293903	0.403395951	-1.138689854	4
WSB2	0.37636395	0.757208226	-1.133572176	4
WWC2	0.594835907	0.559686289	-1.154522196	4
XKR6	0.44325202	0.701762172	-1.145014192	4
XKR8	0.781670287	0.345197241	-1.126867528	4
XPO6	0.737989019	0.400114817	-1.138103836	4
XRN2	0.347244337	0.780089543	-1.12733388	4
XYLT1	0.685625461	0.461822774	-1.147448235	4
YIPF6	0.179658984	0.897992348	-1.077651332	4
YPEL1	0.37198224	0.760698856	-1.132681095	4
YWHAB	0.456314839	0.690446182	-1.14676102	4
YY1AP1	0.464253077	0.683488998	-1.147742075	4
ZADH2	0.070665333	0.962792981	-1.033458314	4
ZBED5	0.158495943	0.911286872	-1.069782815	4
ZBED8	0.689105662	0.457849836	-1.146955498	4
ZBP1	0.766549751	0.36458949	-1.131139241	4
ZBTB20	0.660509911	0.489985858	-1.15049577	4
ZBTB32	0.631786452	0.521145847	-1.152932299	4
ZBTB33	0.191425738	0.890449967	-1.081875706	4
ZBTB37	0.574988008	0.579709313	-1.154697321	4
ZC3H10	0.215193774	0.874884018	-1.090077793	4
ZC3H18	0.729338914	0.410603761	-1.139942674	4
ZC3H3	0.165525529	0.90690939	-1.07243492	4
ZC3H7A	0.584230935	0.57044216	-1.154673095	4
ZCCHC14	0.600995254	0.553377966	-1.15437322	4
ZDHHC14	0.769288125	0.361108468	-1.130396592	4
ZDHHC2	0.129200717	0.929120116	-1.058320833	4
ZDHHC8	0.375495952	0.757901057	-1.133397008	4
ZDHHC9	0.743034636	0.393939832	-1.136974467	4
ZEB2	0.363401416	0.767485553	-1.130886969	4
ZFAT	0.794608297	0.328263382	-1.122871679	4
ZFHX4	0.688993133	0.457978571	-1.146971704	4
ZFP69	0.110207402	0.940331252	-1.050538654	4
ZFPM1	0.661829005	0.488528465	-1.150357469	4
ZFR	0.749112519	0.38644494	-1.135557459	4
ZFX	0.206465777	0.880651715	-1.087117492	4
ZFYVE19	0.206075206	0.88090841	-1.086983616	4
ZFYVE26	0.51633044	0.636291128	-1.152621567	4
ZHX3	0.707682062	0.436342222	-1.144024285	4
ZIC1	0.702606993	0.442269159	-1.144876152	4
ZIC3	0.144311176	0.920004033	-1.064315209	4
ZIC4	0.456016146	0.690706766	-1.146722912	4
ZKSCAN1	0.472716872	0.676003373	-1.148720244	4
ZKSCAN7	0.255849307	0.847219361	-1.103068668	4
ZMAT3	0.366129978	0.765334521	-1.131464498	4
ZMPSTE24	0.658445515	0.492261952	-1.150707467	4
ZMYM4	0.426424705	0.716100016	-1.142524721	4
ZNF100	0.490382981	0.660150102	-1.150533083	4
ZNF133	0.353493251	0.775241763	-1.128735014	4
ZNF134	0.32321738	0.798416255	-1.121633634	4
ZNF141	0.095538932	0.948801773	-1.044340705	4
ZNF142	0.635636044	0.517032333	-1.152668377	4

ZNF148	0.414775972	0.725870108	-1.14064608	4
ZNF16	0.602929703	0.551387322	-1.154317025	4
ZNF174	0.351756996	0.776592112	-1.128349107	4
ZNF182	0.710824709	0.432652475	-1.143477184	4
ZNF189	0.533725222	0.619902882	-1.153628105	4
ZNF195	0.217479746	0.873363441	-1.090843187	4
ZNF234	0.636283644	0.516338461	-1.152622105	4
ZNF235	0.690888615	0.455807645	-1.14669626	4
ZNF248	0.31730126	0.802853365	-1.120154625	4
ZNF26	0.436880053	0.707222906	-1.144102959	4
ZNF274	0.41187884	0.728280504	-1.140159344	4
ZNF280C	0.173807501	0.901702953	-1.075510454	4
ZNF319	0.390576968	0.745767957	-1.136344924	4
ZNF362	0.419128068	0.722234629	-1.141362696	4
ZNF397	0.518283939	0.634466624	-1.152750563	4
ZNF414	0.715388901	0.427266721	-1.142655622	4
ZNF417	0.205838088	0.881064193	-1.086902281	4
ZNF428	0.782048695	0.344706499	-1.126755194	4
ZNF43	0.371246808	0.761283062	-1.13252987	4
ZNF432	0.321163355	0.799960136	-1.12112349	4
ZNF436	0.622678731	0.530802783	-1.153481514	4
ZNF439	0.33255105	0.791355588	-1.123906638	4
ZNF440	0.739890154	0.397793107	-1.137683261	4
ZNF485	0.770963588	0.358971938	-1.129935526	4
ZNF496	0.337566459	0.787530874	-1.125097333	4
ZNF512	0.100393451	0.946016538	-1.046409989	4
ZNF518A	0.78588257	0.339719236	-1.125601806	4
ZNF518B	0.447096116	0.69844912	-1.145545237	4
ZNF543	0.518814628	0.633970286	-1.152784914	4
ZNF559	0.268720523	0.838183805	-1.106904327	4
ZNF559-ZNF177	0.594207616	0.560327228	-1.154534844	4
ZNF567	0.140660249	0.922222656	-1.062882904	4
ZNF579	0.662497888	0.487788546	-1.150286435	4
ZNF581	0.713340553	0.429687747	-1.1430283	4
ZNF585B	0.203226389	0.882777113	-1.086003501	4
ZNF606	0.277429226	0.831993772	-1.109422998	4
ZNF616	0.662699356	0.487565563	-1.150264919	4
ZNF618	0.195169098	0.888027833	-1.083196931	4
ZNF638	0.185172528	0.894471663	-1.079644191	4
ZNF641	0.533926487	0.619711375	-1.153637862	4
ZNF648	0.11758743	0.936007721	-1.053595151	4
ZNF662	0.664932174	0.485090579	-1.150022754	4
ZNF664	0.727588844	0.412711119	-1.140299963	4
ZNF718	0.594098563	0.560438428	-1.154536992	4
ZNF727	0.532183348	0.621368558	-1.153551906	4
ZNF738	0.246440976	0.85373915	-1.100180126	4
ZNF75D	0.313678899	0.805555564	-1.119234463	4
ZNF77	0.350053392	0.777914523	-1.127967915	4
ZNF777	0.330117888	0.793203355	-1.123321244	4
ZNF91	0.472134914	0.676520329	-1.148655243	4
ZNHIT6	0.090574272	0.95163173	-1.042206002	4
ZNRD2	0.506549288	0.645366477	-1.151915765	4
ZNRF4	0.728195335	0.411981365	-1.1401767	4
ZSCAN18	0.196868657	0.886924514	-1.083793171	4
ZSWIM5	0.181460726	0.896844446	-1.078305172	4

ZSWIM9	0.507284825	0.644687471	-1.151972296	4
A1CF	-0.879663328	-0.207967936	1.087631264	5
A4GALT	-0.818797126	-0.295710258	1.114507384	5
AADACL3	-0.185611929	-0.894190063	1.079801992	5
AASDH	-0.6096833	-0.544401958	1.154085258	5
AATF	-0.881941079	-0.204502116	1.086443196	5
ABCA10	-0.86927385	-0.223597251	1.092871101	5
ABCA2	-0.788576232	-0.336198465	1.124774697	5
ABCA6	-0.510476595	-0.64173452	1.152211114	5
ABCA7	-0.810399163	-0.307148577	1.117547739	5
ABCB1	-0.652945474	-0.498297932	1.151243406	5
ABCD1	-0.72858153	-0.411516373	1.140097903	5
ABCD2	-0.160433657	-0.910084026	1.070517682	5
ABCF1	-0.230335985	-0.864734554	1.095070539	5
ABCF3	-0.412641852	-0.727646432	1.140288284	5
ABCG1	-0.616128711	-0.537683277	1.153811988	5
ABITRAM	-0.637616811	-0.514908335	1.152525145	5
ABTB1	-0.427529101	-0.715167175	1.142696276	5
ACADM	-0.233774871	-0.862404136	1.096179007	5
ACADVL	-0.361170696	-0.769239266	1.130409961	5
ACAN	-0.507908116	-0.644111647	1.152019762	5
ACAT1	-0.37672705	-0.756918204	1.133645254	5
ACAT2	-0.345284545	-0.781602946	1.126887491	5
ACCSL	-0.632604514	-0.52027329	1.152877804	5
ACKR2	-0.509658541	-0.642492371	1.152150912	5
ACOT11	-0.476766775	-0.672396543	1.149163318	5
ACOT13	-0.164260378	-0.907700047	1.071960425	5
ACOXL	-0.312137886	-0.806701775	1.118839661	5
ACSBG2	-0.259418254	-0.844727442	1.104145696	5
ACSL5	-0.639990541	-0.512356267	1.152346807	5
ACSM4	-0.393417731	-0.743459665	1.136877397	5
ACTBL2	-0.554183693	-0.600211024	1.154394717	5
ACTN1	-0.351683286	-0.776649381	1.128332666	5
ACTR3C	-0.649434495	-0.502129853	1.151564348	5
ACTRT3	-0.85886728	-0.238967622	1.097834902	5
ACVR1	-0.80051969	-0.320417732	1.120937422	5
ACVR2A	-0.809628232	-0.30819121	1.117819442	5
ADAL	-0.512060945	-0.640264786	1.152325732	5
ADAM18	-0.43158774	-0.711729184	1.143316925	5
ADAM2	-0.152423419	-0.915037667	1.067461086	5
ADAM20	-0.821749064	-0.291654014	1.113403077	5
ADAM7	-0.673484264	-0.475547354	1.149031618	5
ADAM9	-0.72420157	-0.416776012	1.140977582	5
ADAMDEC1	-0.646741837	-0.505057562	1.151799399	5
ADAMTS19	-0.694338519	-0.451842953	1.146181472	5
ADAMTS5	-0.706541454	-0.437677679	1.144219133	5
ADAT1	-0.154888478	-0.91351851	1.068406988	5
ADCYAP1	-0.279758215	-0.830327832	1.110086047	5
ADCYAP1R1	-0.813006787	-0.303612776	1.116619562	5
ADGB	-0.314197357	-0.805169483	1.119366841	5
ADGRL4	-0.718219198	-0.423910763	1.142129961	5
ADH1A	-0.265778797	-0.840260756	1.106039553	5
ADIPOQ	-0.56422336	-0.590378434	1.154601795	5
ADM5	-0.330918701	-0.792595768	1.123514469	5
AEN	-0.297106999	-0.817777486	1.114884485	5

AFAP1L1	-0.884253503	-0.200968736	1.085222239	5
AFMID	-0.670010733	-0.479435677	1.14944641	5
AGBL3	-0.571086461	-0.583591506	1.154677967	5
AGPAT2	-0.148144971	-0.917663266	1.065808237	5
AHCTF1	-0.417705692	-0.723424723	1.141130415	5
AKAP11	-0.835962643	-0.271855503	1.107818146	5
AKAP14	-0.414023067	-0.726497264	1.140520331	5
AKAP17A	-0.561356985	-0.593197215	1.1545542	5
AKAP3	-0.237715242	-0.859722267	1.097437509	5
AKR1C3	-0.596360534	-0.558129014	1.154489548	5
AKR1C4	-0.564007682	-0.590590854	1.154598536	5
AKR7A3	-0.203853821	-0.882366092	1.086219913	5
ALAD	-0.14817933	-0.917642237	1.065821566	5
ALG2	-0.622748045	-0.530729685	1.15347773	5
ALK	-0.688139019	-0.458955096	1.147094116	5
ALKBH7	-0.864168462	-0.231172622	1.095341083	5
ALOXE3	-0.384262149	-0.750873141	1.13513529	5
AMBRA1	-0.141363815	-0.921795903	1.063159718	5
AMELX	-0.653209633	-0.498008961	1.151218594	5
AMPD1	-0.583365209	-0.571314369	1.154679577	5
ANAPC11	-0.808987705	-0.309056544	1.11804425	5
ANAPC13	-0.133965301	-0.926264536	1.060229837	5
ANKH	-0.699130269	-0.446307124	1.145437392	5
ANKMY2	-0.359983949	-0.770170468	1.130154417	5
ANKRD27	-0.368149404	-0.763738313	1.131887717	5
ANKRD34A	-0.422450835	-0.719447172	1.141898007	5
ANKRD60	-0.525424345	-0.627763511	1.153187856	5
ANKS3	-0.836504237	-0.271092132	1.107596369	5
ANO10	-0.444860747	-0.7003774	1.145238147	5
APOA5	-0.458190217	-0.688808097	1.146998313	5
APOBEC2	-0.779943956	-0.347432638	1.127376595	5
APOBEC3A	-0.540191112	-0.613728619	1.153919731	5
APOBEC3B	-0.276127956	-0.832922641	1.109050597	5
APOBEC3G	-0.280082209	-0.830095725	1.110177934	5
APOC3	-0.806818271	-0.311981114	1.118799384	5
APPBP2	-0.860992672	-0.235850956	1.096843628	5
AQP11	-0.26499296	-0.840814389	1.105807349	5
ARFIP2	-0.562208977	-0.592360336	1.154569313	5
ARG1	-0.31399232	-0.805322195	1.119314515	5
ARHGAP22	-0.223422481	-0.869391097	1.092813578	5
ARHGAP39	-0.150529595	-0.916201607	1.066731202	5
ARHGEF16	-0.841992559	-0.263318266	1.105310825	5
ARHGEF2	-0.396314307	-0.741098524	1.137412831	5
ARHGEF35	-0.135615792	-0.925271289	1.060887081	5
ARHGEF5	-0.390108312	-0.746148069	1.136256381	5
ARID3B	-0.487649797	-0.662623256	1.150273053	5
ARID4B	-0.406224063	-0.732963024	1.139187086	5
ARL14EPL	-0.668693612	-0.480905694	1.149599307	5
ARL4C	-0.814522699	-0.301550741	1.11607344	5
ARL6IP5	-0.848840291	-0.253519837	1.102360128	5
ARL8A	-0.374838463	-0.758425415	1.133263878	5
ASAH2B	-0.652501867	-0.498782997	1.151284863	5
ASB4	-0.252940926	-0.849242438	1.102183364	5
ASB9	-0.611365116	-0.542653767	1.154018884	5
ASCL1	-0.32852045	-0.794413721	1.122934171	5

ASCL3	-0.796226612	-0.326122419	1.122349032	5
ASIC5	-0.892019001	-0.18899174	1.081010742	5
ASMT	-0.616079529	-0.537734738	1.153814268	5
ASNS	-0.413727348	-0.726743449	1.140470798	5
ASTL	-0.186292742	-0.893753452	1.080046194	5
ATF2	-0.218479015	-0.872697451	1.091176466	5
ATF3	-0.484846058	-0.665152436	1.149998494	5
ATF7-NPFF	-0.367888673	-0.763944604	1.131833277	5
ATG101	-0.211568851	-0.877286765	1.088855616	5
ATG2A	-0.567266395	-0.587375774	1.154642169	5
ATG4B	-0.799644539	-0.321583621	1.12122816	5
ATG5	-0.549375296	-0.604880478	1.154255774	5
ATIC	-0.674665012	-0.474221768	1.148886781	5
ATOH1	-0.399742019	-0.738294615	1.138036634	5
ATOH8	-0.160808285	-0.909851138	1.070659424	5
ATP11C	-0.817857046	-0.296998095	1.114855141	5
ATP13A2	-0.271555839	-0.836175305	1.107731144	5
ATP5MGL	-0.132345069	-0.927237545	1.059582615	5
ATP5MK	-0.459260225	-0.68787195	1.147132175	5
ATP6V1B1	-0.241272605	-0.857290406	1.098563011	5
ATP6V1D	-0.324022532	-0.797810097	1.121832629	5
ATP8B4	-0.660295179	-0.490222881	1.150518059	5
ATPAF2	-0.447580677	-0.698030497	1.145611174	5
ATRNL1	-0.494845672	-0.656095742	1.150941414	5
ATRX	-0.723219589	-0.417951024	1.141170613	5
ATXN2L	-0.845422792	-0.258423853	1.103846645	5
AZIN1	-0.227039519	-0.866959609	1.093999128	5
B3GALNT2	-0.372757837	-0.760082225	1.132840062	5
B3GNT2	-0.619525517	-0.534121771	1.153647289	5
BAD	-0.466944107	-0.681116597	1.148060705	5
BAG2	-0.229836216	-0.865072446	1.094908662	5
BAZ1A	-0.143082136	-0.920752047	1.063834183	5
BCAS2	-0.786570846	-0.338820941	1.125391787	5
BCAS3	-0.351999819	-0.776403417	1.128403236	5
BCDIN3D	-0.151542604	-0.915579357	1.067121961	5
BCHE	-0.445002893	-0.700254923	1.145257817	5
BCL3	-0.644171759	-0.507843069	1.152014828	5
BCLAF1	-0.316230631	-0.803653181	1.119883812	5
BCORL1	-0.393046484	-0.74376174	1.136808223	5
BEND5	-0.54681924	-0.607352288	1.154171528	5
BEST2	-0.244682682	-0.854949705	1.099632387	5
BEX1	-0.729572357	-0.410322287	1.139894643	5
BHLHE40	-0.662754768	-0.487504225	1.150258992	5
BIN1	-0.545627279	-0.608502509	1.154129788	5
BIRC2	-0.267588718	-0.838983729	1.106572447	5
BLOC1S1	-0.283040811	-0.827972206	1.111013017	5
BLOC1S5	-0.695344265	-0.450683844	1.146028109	5
BMP2	-0.146533378	-0.918648618	1.065181996	5
BOLA1	-0.866577233	-0.227606931	1.094184164	5
BPIFA2	-0.318584628	-0.801893348	1.120477976	5
BRD8	-0.272081372	-0.835802303	1.107883675	5
BRICD5	-0.85645914	-0.242485202	1.098944343	5
BRSK1	-0.405680253	-0.733411789	1.139092042	5
BST1	-0.745493094	-0.390915705	1.136408799	5
BTBD10	-0.485428698	-0.664627502	1.1500562	5

BTG2	-0.778720856	-0.349013035	1.127733891	5
BUB1	-0.312090652	-0.806736876	1.118827528	5
BUD13	-0.205160136	-0.881509354	1.086669489	5
BZW1	-0.303333573	-0.813212251	1.116545824	5
C10orf120	-0.419205771	-0.722169561	1.141375331	5
C10orf71	-0.875737487	-0.213907975	1.089645462	5
C11orf16	-0.670459319	-0.478934468	1.149393787	5
C12orf71	-0.163014662	-0.908477348	1.07149201	5
C12orf73	-0.55207077	-0.602266043	1.154336813	5
C14orf28	-0.602910627	-0.551406974	1.154317601	5
C17orf107	-0.62904691	-0.524061657	1.153108566	5
C17orf58	-0.272770677	-0.835312721	1.108083398	5
C18orf54	-0.393181086	-0.743652232	1.136833318	5
C1GALT1C1L	-0.822760015	-0.290260547	1.113020562	5
C1orf105	-0.765573319	-0.365827473	1.131400792	5
C1orf116	-0.142651278	-0.921014001	1.063665278	5
C1orf198	-0.605168689	-0.549077645	1.154246333	5
C1orf53	-0.870903943	-0.221164158	1.092068101	5
C1QL3	-0.130615586	-0.928273948	1.058889534	5
C1QTNF7	-0.83731459	-0.269948694	1.107263284	5
C20orf85	-0.533966993	-0.619672828	1.153639821	5
C2CD2	-0.830948316	-0.278891503	1.10983982	5
C2CD6	-0.552847765	-0.601510917	1.154358682	5
C3orf22	-0.321661739	-0.79958586	1.121247599	5
C3orf33	-0.484245048	-0.665693564	1.149938612	5
C4A	-0.513705352	-0.638736576	1.152441928	5
C4B	-0.375866746	-0.757605173	1.133471919	5
C4orf45	-0.789583572	-0.334878233	1.124461805	5
C4orf48	-0.63782308	-0.514686858	1.152509938	5
C4orf51	-0.176471551	-0.900016897	1.076488447	5
C5orf34	-0.343949113	-0.782632303	1.126581416	5
C5orf51	-0.888905015	-0.193815388	1.082720403	5
C6	-0.529809314	-0.623620282	1.153429597	5
C7orf25	-0.816203023	-0.299259407	1.11546243	5
C7orf33	-0.650967335	-0.500458913	1.151426248	5
C8A	-0.451938409	-0.694255635	1.146194044	5
C8orf58	-0.316834471	-0.8032022	1.120036671	5
C9orf152	-0.269988841	-0.837286156	1.107274997	5
C9orf40	-0.740459074	-0.397097167	1.13755624	5
CA8	-0.30351369	-0.813079711	1.116593401	5
CA9	-0.185113135	-0.894509715	1.07962285	5
CABCOCO1	-0.470718675	-0.677776987	1.148495662	5
CABP5	-0.512273158	-0.640067727	1.152340885	5
CABS1	-0.840860548	-0.26492741	1.105787957	5
CACNG5	-0.726610016	-0.413887641	1.140497657	5
CADM3	-0.240419636	-0.857874432	1.098294067	5
CADPS2	-0.184133776	-0.895136771	1.079270547	5
CALCR	-0.770523794	-0.359533251	1.130057045	5
CAMK2A	-0.168605156	-0.904979599	1.073584755	5
CAMP	-0.275085574	-0.83366571	1.108751284	5
CAMTA2	-0.79124818	-0.332692283	1.123940462	5
CAPN2	-0.276642167	-0.832555754	1.109197921	5
CAPN9	-0.294268157	-0.81984818	1.114116337	5
CAPNS2	-0.242594086	-0.85638444	1.098978526	5
CAPZA3	-0.166774767	-0.906127463	1.072902231	5

CARD10	-0.483156206	-0.666672997	1.149829203	5
CARD11	-0.677398608	-0.471145338	1.148543946	5
CATIP	-0.549477815	-0.604781188	1.154259003	5
CATSPERE	-0.581494424	-0.573196175	1.154690599	5
CAV1	-0.232764489	-0.863089817	1.095854306	5
CBR4	-0.836902133	-0.270530874	1.107433007	5
CBX6	-0.661481446	-0.488912693	1.150394139	5
CBY3	-0.606720524	-0.547473243	1.154193768	5
CCDC105	-0.264969867	-0.840830651	1.105800518	5
CCDC124	-0.33965846	-0.785929159	1.125587619	5
CCDC160	-0.627874261	-0.525306835	1.153181096	5
CCDC171	-0.672771611	-0.476346481	1.149118092	5
CCDC175	-0.846974938	-0.256200026	1.103174964	5
CCDC183	-0.60559833	-0.548633743	1.154232073	5
CCDC54	-0.86557721	-0.22908908	1.09466629	5
CCDC69	-0.773717394	-0.355449222	1.129166615	5
CCDC85C	-0.204290851	-0.882079618	1.086370469	5
CCIN	-0.600450734	-0.553937491	1.154388224	5
CCL11	-0.650303964	-0.501182433	1.151486397	5
CCL14	-0.521138509	-0.631793335	1.152931844	5
CCL15-CCL14	-0.321936591	-0.799379362	1.121315953	5
CCL19	-0.565256475	-0.589360202	1.154616677	5
CCL24	-0.377785858	-0.756071824	1.133857682	5
CCL26	-0.775004073	-0.35379852	1.128802593	5
CCL28	-0.468385873	-0.679842624	1.148228497	5
CCNJL	-0.800824447	-0.32001137	1.120835817	5
CCNK	-0.330706027	-0.79275718	1.123463207	5
CCNL1	-0.200947108	-0.884267628	1.085214736	5
CD160	-0.37014438	-0.762157906	1.132302285	5
CD200	-0.503322345	-0.648338806	1.151661152	5
CD226	-0.305530084	-0.811594085	1.117124169	5
CD320	-0.424965884	-0.717330479	1.142296363	5
CD5L	-0.733275837	-0.405845033	1.13912087	5
CD70	-0.554921343	-0.599492421	1.154413764	5
CD79A	-0.68534437	-0.462142899	1.14748727	5
CD8B2	-0.16762014	-0.90559764	1.07321778	5
CD9	-0.784978269	-0.340898102	1.125876371	5
CDA	-0.379205405	-0.754935516	1.134140921	5
CDC20	-0.583416214	-0.571263006	1.154679219	5
CDC23	-0.441170643	-0.703550139	1.144720781	5
CDCA4	-0.612669844	-0.541295155	1.153964999	5
CDCA7L	-0.383854751	-0.751201273	1.135056024	5
CDH24	-0.237512426	-0.85986061	1.097373036	5
CDK11B	-0.548278389	-0.605942112	1.154220501	5
CDK2	-0.159464343	-0.910686095	1.070150438	5
CDKL3	-0.708462606	-0.4354272	1.143889806	5
CDKN2B	-0.602712191	-0.551611378	1.154323569	5
CDR1	-0.850711549	-0.250822748	1.101534298	5
CDR2	-0.679428882	-0.468853612	1.148282494	5
CDRT4	-0.726159987	-0.414428048	1.140588035	5
CDS2	-0.27732842	-0.832065779	1.109394198	5
CDX1	-0.145541614	-0.919254006	1.06479562	5
CDX4	-0.210765998	-0.877817525	1.088583523	5
CEACAM16	-0.384107119	-0.750998025	1.135105144	5
CELA1	-0.874097078	-0.216377604	1.090474682	5

CELA2B	-0.704250487	-0.440354055	1.144604542	5
CELF3	-0.130013332	-0.928634315	1.058647648	5
CENPA	-0.694204861	-0.45199688	1.146201741	5
CENPN	-0.461203791	-0.686168685	1.147372476	5
CENPU	-0.487276961	-0.662960038	1.150236998	5
CENPVL1	-0.23847551	-0.85920339	1.097678899	5
CENPVL2	-0.67383709	-0.475151453	1.148988543	5
CENPVL3	-0.796266568	-0.326069495	1.122336063	5
CEP131	-0.642396854	-0.509761687	1.152158541	5
CEP170	-0.683643353	-0.464077712	1.147721065	5
CEP41	-0.50585665	-0.646005369	1.15186202	5
CEP95	-0.385326641	-0.750015063	1.135341704	5
CER1	-0.140482352	-0.9223305	1.062812852	5
CERS4	-0.608383216	-0.545750976	1.154134192	5
CERS5	-0.822734309	-0.290296007	1.113030316	5
CETN1	-0.448152425	-0.697536262	1.145688687	5
CFAP20	-0.836767051	-0.270721457	1.107488508	5
CFAP210	-0.586032658	-0.568624138	1.154656795	5
CFAP276	-0.754136525	-0.380202216	1.13433874	5
CFHR4	-0.762432387	-0.369798214	1.132230602	5
CFTR	-0.300967852	-0.814950569	1.115918421	5
CHD4	-0.556566834	-0.59788724	1.154454074	5
CHD6	-0.471531631	-0.677055876	1.148587507	5
CHI3L2	-0.536147406	-0.617595236	1.153742642	5
CHIA	-0.216264355	-0.874172407	1.090436762	5
CHMP1B	-0.503097879	-0.648545163	1.151643042	5
CHORDC1	-0.420954234	-0.720703934	1.141658168	5
CHRM2	-0.64051946	-0.511786613	1.152306072	5
CHRNB3	-0.244345076	-0.855181856	1.099526933	5
CHRNG	-0.807966984	-0.310433763	1.118400747	5
CHST15	-0.787856062	-0.337141131	1.124997193	5
CHST7	-0.680426108	-0.467725826	1.148151934	5
CHST9	-0.232373489	-0.863354945	1.095728434	5
CHUK	-0.434979567	-0.708844164	1.143823731	5
CHURC1	-0.55489124	-0.599521759	1.154412999	5
CHURC1-FNTB	-0.721502785	-0.420001641	1.141504425	5
CIAO2A	-0.882823267	-0.203155904	1.08597917	5
CIB3	-0.238482565	-0.859198572	1.097681137	5
CKAP2	-0.891492705	-0.18980897	1.081301675	5
CKAP4	-0.459096899	-0.688014915	1.147111814	5
CLBA1	-0.817536084	-0.297437356	1.11497344	5
CLC	-0.437539164	-0.706659837	1.144199001	5
CLCF1	-0.367469248	-0.764276327	1.131745575	5
CLCN2	-0.541708239	-0.612273356	1.153981595	5
CLDN14	-0.833531824	-0.275273483	1.108805307	5
CLDN2	-0.487107241	-0.663113299	1.150220539	5
CLDN24	-0.724053584	-0.416953186	1.14100677	5
CLDN25	-0.365925817	-0.765495695	1.131421512	5
CLDN7	-0.681468606	-0.466545329	1.148013935	5
CLEC9A	-0.81153473	-0.305610548	1.117145279	5
CLHC1	-0.594669222	-0.559856376	1.154525597	5
CLIC3	-0.307062503	-0.810462765	1.117525268	5
CLNK	-0.479992263	-0.669512291	1.149504554	5
CLP1	-0.539119836	-0.614754704	1.153874541	5
CLPTM1	-0.602604064	-0.551722737	1.154326801	5

CLRN2	-0.542669238	-0.611350247	1.154019486	5
CLUAP1	-0.555756214	-0.598678379	1.154434593	5
CMAS	-0.481356805	-0.66828899	1.149645794	5
CMC2	-0.847398584	-0.255592053	1.102990636	5
CNBD1	-0.367246065	-0.76445278	1.131698845	5
CNFN	-0.589949095	-0.564659124	1.154608219	5
CNIH3	-0.687079465	-0.460165037	1.147244502	5
CNOT8	-0.804976994	-0.314455733	1.119432727	5
CNR1	-0.851626494	-0.249500929	1.101127423	5
CNR2	-0.179003121	-0.898409575	1.077412696	5
CNTN6	-0.569086972	-0.58557433	1.154661302	5
COA6	-0.710771693	-0.432714845	1.143486538	5
COG1	-0.557323024	-0.597148562	1.154471586	5
COG3	-0.564020367	-0.590578361	1.154598729	5
COIL	-0.690617104	-0.456118932	1.146736036	5
COL3A1	-0.704909327	-0.439585191	1.144494518	5
COL6A3	-0.23852386	-0.859170375	1.097694235	5
COL6A5	-0.514123922	-0.638347132	1.152471054	5
COL6A6	-0.302865402	-0.813556631	1.116422033	5
COLGALT2	-0.238994042	-0.858849229	1.097843272	5
COMMD8	-0.215141504	-0.874918739	1.090060243	5
COPA	-0.319892943	-0.800913236	1.12080618	5
COPRS	-0.350154352	-0.777836224	1.127990576	5
COPS6	-0.770070308	-0.360111671	1.130181979	5
COPS8	-0.764822402	-0.366778375	1.131600777	5
CORO1B	-0.444921285	-0.700325241	1.145246526	5
CORO2A	-0.568962507	-0.585697607	1.154660114	5
CPA6	-0.542333107	-0.61167324	1.154006347	5
CPB1	-0.737778644	-0.400371364	1.138150009	5
CPLANE2	-0.193884265	-0.888860418	1.082744683	5
CPLX1	-0.576374751	-0.578325238	1.154699989	5
CPLX4	-0.596205541	-0.558287453	1.154492994	5
CPNE4	-0.659672862	-0.490909441	1.150582303	5
CPSF7	-0.211257434	-0.877492701	1.088750135	5
CREB3L4	-0.327785048	-0.794970199	1.122755247	5
CRH	-0.244629025	-0.854986608	1.099615633	5
CRIPT	-0.296940674	-0.817898991	1.114839664	5
CRISP1	-0.643372882	-0.508707141	1.152080023	5
CRISP3	-0.505527879	-0.646308456	1.151836335	5
CRK	-0.151481778	-0.915616742	1.06709852	5
CRLF3	-0.821849264	-0.291516	1.113365263	5
CRP	-0.367288447	-0.764419275	1.131707722	5
CRTC3	-0.139769238	-0.922762559	1.062531797	5
CRY2	-0.505273866	-0.646542548	1.151816415	5
CRYBG2	-0.507368618	-0.644610083	1.151978701	5
CRYGA	-0.768945242	-0.361545081	1.130490323	5
CRYGN	-0.823599772	-0.289101363	1.112701136	5
CSAD	-0.719349606	-0.422566917	1.141916523	5
CSF1	-0.606544261	-0.547655625	1.154199886	5
CSF3	-0.196655015	-0.887063331	1.083718345	5
CSN1S1	-0.362669405	-0.768061515	1.13073092	5
CSRNP1	-0.168559599	-0.9050082	1.073567799	5
CSRNP3	-0.867432064	-0.226337913	1.093769977	5
CT45A1	-0.859755284	-0.237666842	1.097422126	5
CT45A2	-0.824012489	-0.288531095	1.112543584	5

CT45A3	-0.854221137	-0.245741337	1.099962474	5
CT45A6	-0.178460441	-0.898754548	1.077214989	5
CT45A7	-0.419480161	-0.721939744	1.141419905	5
CT45A8	-0.572554341	-0.582132954	1.154687295	5
CT45A9	-0.170484217	-0.903798512	1.074282729	5
CT47A4	-0.606693455	-0.547501254	1.15419471	5
CT47A7	-0.570462971	-0.584210289	1.15467326	5
CT55	-0.606617005	-0.54758036	1.154197365	5
CT83	-0.748010756	-0.387808203	1.135818959	5
CTAGE1	-0.423657864	-0.718432058	1.142089923	5
CTBS	-0.587566501	-0.567073419	1.15463992	5
CTSA	-0.310670612	-0.807791284	1.118461896	5
CTSK	-0.625412236	-0.527915469	1.153327704	5
CTTNBP2	-0.368204312	-0.763694863	1.131899175	5
CTXN3	-0.826212038	-0.285485601	1.111697639	5
CWC15	-0.176230071	-0.900169955	1.076400025	5
CWC25	-0.652468964	-0.498818964	1.151287928	5
CXCR1	-0.604681242	-0.549580999	1.154262241	5
CXorf58	-0.375304991	-0.758053391	1.133358381	5
CXXC4	-0.799294902	-0.322048985	1.121343886	5
CYB5A	-0.205818873	-0.881076815	1.086895688	5
CYB5R3	-0.180738784	-0.897304703	1.078043488	5
CYBRD1	-0.396179823	-0.741208316	1.137388139	5
CYP2C19	-0.267926893	-0.838744827	1.10667172	5
CYP2C8	-0.588863356	-0.565760138	1.154623494	5
CYP2F1	-0.855025301	-0.244572762	1.099598063	5
CYP39A1	-0.212390887	-0.876742796	1.089133683	5
CYP3A5	-0.427313895	-0.715349041	1.142662936	5
CYP46A1	-0.469618544	-0.678751792	1.148370336	5
CYP4V2	-0.189090171	-0.891955639	1.08104581	5
CYP4Z1	-0.43348892	-0.710113413	1.143602333	5
CYP51A1	-0.50653159	-0.645382808	1.151914398	5
CYRIB	-0.742921795	-0.394078392	1.137000188	5
CYS1	-0.843036391	-0.261831812	1.104868203	5
DAGLA	-0.26953043	-0.837610747	1.107141176	5
DAW1	-0.718227346	-0.423901084	1.14212843	5
DAZAP2	-0.793601232	-0.329593088	1.12319432	5
DBN1	-0.800425643	-0.320543096	1.120968739	5
DBX2	-0.233965223	-0.862274865	1.096240088	5
DCAF8L1	-0.386547234	-0.749029905	1.135577139	5
DCLK3	-0.860340008	-0.236809233	1.097149241	5
DCST1	-0.675753567	-0.472997953	1.14875152	5
DCSTAMP	-0.671841379	-0.477388521	1.1492299	5
DCUN1D2	-0.654715612	-0.496359744	1.151075355	5
DCUN1D3	-0.34473277	-0.782028443	1.126761213	5
DDIT4L	-0.212067029	-0.876957168	1.089024197	5
DDX43	-0.498026662	-0.653193454	1.151220116	5
DDX5	-0.489805272	-0.660673478	1.150478749	5
DDX60L	-0.344540222	-0.782176863	1.126717085	5
DEDD2	-0.771341388	-0.358489469	1.129830857	5
DEF6	-0.321347535	-0.799821845	1.12116938	5
DEF8	-0.552671104	-0.601682664	1.154353768	5
DEFB105A	-0.303927467	-0.812775128	1.116702595	5
DEFB106A	-0.202881404	-0.883002975	1.085884378	5
DEFB113	-0.858396705	-0.239656134	1.098052838	5

DEFB114	-0.650480155	-0.500990324	1.151470479	5
DEFB126	-0.84305455	-0.26180593	1.10486048	5
DEFB128	-0.808930348	-0.309133992	1.11806434	5
DEFB131A	-0.537122365	-0.616664593	1.153786958	5
DEFB131B	-0.768318112	-0.362343093	1.130661205	5
DEFB135	-0.368821406	-0.76320635	1.132027755	5
DENND1A	-0.451439282	-0.694688926	1.146128208	5
DENND1C	-0.141689537	-0.921598205	1.063287742	5
DESI1	-0.270612765	-0.836844094	1.107456859	5
DFFA	-0.706428146	-0.437810235	1.144238381	5
DGAT1	-0.799905766	-0.32123577	1.121141536	5
DGKK	-0.300304649	-0.815437049	1.115741698	5
DGKQ	-0.190814904	-0.890844166	1.08165907	5
DGLUCY	-0.145941531	-0.919009982	1.064951512	5
DHX38	-0.607419204	-0.54674994	1.154169144	5
DKK2	-0.415244388	-0.725479662	1.14072405	5
DLGAP5	-0.638464027	-0.513998306	1.152462333	5
DMAC2L	-0.530632337	-0.622840345	1.153472682	5
DMGDH	-0.168598222	-0.904983952	1.073582175	5
DMRTA1	-0.810003757	-0.307683491	1.117687248	5
DNAH12	-0.696182519	-0.449716628	1.145899147	5
DNAJB11	-0.150684656	-0.91610641	1.066791066	5
DNAJB2	-0.559799523	-0.594724941	1.154524464	5
DNAJB4	-0.311912343	-0.806869367	1.11878171	5
DNAJC19	-0.423559137	-0.718515139	1.142074276	5
DNAJC2	-0.780188305	-0.347116574	1.12730488	5
DNAJC30	-0.5742478	-0.580447191	1.154694991	5
DNLZ	-0.271774563	-0.836020091	1.107794654	5
DNMT3L	-0.200035557	-0.884862586	1.084898143	5
DNTT	-0.771912656	-0.357759436	1.129672093	5
DOC2A	-0.702449406	-0.442452577	1.144901983	5
DOHH	-0.574344168	-0.580351162	1.15469533	5
DOK4	-0.245227841	-0.854574638	1.099802479	5
DOLK	-0.440047413	-0.704513316	1.144560729	5
DPH7	-0.681384477	-0.466640652	1.148025129	5
DPP3	-0.583047354	-0.571634384	1.154681737	5
DPT	-0.845320907	-0.258569627	1.103890534	5
DRC1	-0.686318175	-0.461033378	1.147351553	5
DSCC1	-0.482808198	-0.666985786	1.149793984	5
DSN1	-0.846840939	-0.256392238	1.103233177	5
DTL	-0.189504119	-0.89168909	1.081193209	5
DUSP11	-0.773471039	-0.355764928	1.129235967	5
DUSP22	-0.87309532	-0.217882185	1.090977505	5
DUSP26	-0.415452373	-0.725306233	1.140758605	5
DUSP3	-0.328762375	-0.794230557	1.122992931	5
DUXB	-0.818162341	-0.296580071	1.114742412	5
DYNC1I1	-0.332720615	-0.791226631	1.123947246	5
DYNC1LI2	-0.701058889	-0.444069401	1.14512829	5
DYNLL2	-0.783432667	-0.342909382	1.126342049	5
DYNLT3	-0.807866124	-0.310569733	1.118435857	5
DYNLT5	-0.530715197	-0.622761782	1.153476979	5
DZIP1L	-0.763703493	-0.368193406	1.131896899	5
E2F5	-0.167584792	-0.905619805	1.073204597	5
E2F7	-0.482077224	-0.667642389	1.149719614	5
EAF1	-0.881392292	-0.205338467	1.086730759	5

EBLN2	-0.658105661	-0.492636103	1.150741764	5
EBP	-0.495714334	-0.655304211	1.151018545	5
ECD	-0.817871964	-0.296977673	1.114849637	5
EDA2R	-0.84368628	-0.260905053	1.104591333	5
EDARADD	-0.637146609	-0.515412995	1.152559605	5
EDF1	-0.424693858	-0.717559703	1.142253562	5
EDN2	-0.150199119	-0.916404434	1.066603553	5
EDNRA	-0.275278981	-0.833527906	1.108806887	5
EEF1AKMT2	-0.329235444	-0.793872245	1.123107689	5
EEF1B2	-0.740013233	-0.397642595	1.137655828	5
EEPD1	-0.399930634	-0.738140016	1.13807065	5
EFCAB10	-0.566695615	-0.58793978	1.154635395	5
EFCAB13	-0.668252024	-0.481398006	1.14965003	5
EFCAB2	-0.46903226	-0.6792708	1.14830306	5
EFCAB7	-0.663044171	-0.487183796	1.150227967	5
EFEMP1	-0.458230099	-0.688773224	1.147003323	5
EFNA1	-0.590416672	-0.56418454	1.154601212	5
EGLN2	-0.252333694	-0.849663968	1.101997662	5
EID2	-0.283714842	-0.827487418	1.11120226	5
EIF3CL	-0.72809144	-0.412106418	1.140197858	5
EIF3K	-0.48544821	-0.664609916	1.150058127	5
EIF4B	-0.757778495	-0.375649561	1.133428056	5
ELK1	-0.238531155	-0.859165394	1.097696549	5
ELP4	-0.75476163	-0.379422436	1.134184066	5
EMC4	-0.417024909	-0.723993669	1.141018577	5
EMC6	-0.478946756	-0.670448324	1.14939508	5
EMP3	-0.659901298	-0.490657484	1.150558782	5
ENC1	-0.593730051	-0.560814093	1.154544144	5
ENG	-0.431311991	-0.711963256	1.143275246	5
ENOSF1	-0.30784951	-0.809880988	1.117730497	5
ENTPD6	-0.503364065	-0.648300447	1.151664512	5
ENTPD7	-0.647720405	-0.503994681	1.151715086	5
EPC2	-0.375923389	-0.757559962	1.133483352	5
EPGN	-0.421188613	-0.720507253	1.141695866	5
EPHA1	-0.274808521	-0.83386306	1.108671581	5
EPHB6	-0.836734028	-0.270768042	1.10750207	5
EPO	-0.286268445	-0.825647374	1.11191582	5
EPPIN-WFDC6	-0.587294509	-0.567348604	1.154643114	5
EQTN	-0.691197582	-0.455453284	1.146650866	5
ERBB4	-0.505133594	-0.646671792	1.151805386	5
ERBIN	-0.157875373	-0.911671482	1.069546855	5
ERCC6L	-0.462049851	-0.685426083	1.147475934	5
ERF	-0.451819567	-0.694358824	1.146178391	5
ERGIC3	-0.853527925	-0.246747405	1.100275329	5
ERICH6B	-0.743373081	-0.393524114	1.136897196	5
ERMARD	-0.184239225	-0.895069291	1.079308516	5
ERP27	-0.615235213	-0.538617718	1.153852931	5
ERVH48-1	-0.789399438	-0.335119707	1.124519145	5
ERVV-1	-0.504090803	-0.647631955	1.151722759	5
ERVW-1	-0.328202102	-0.794654672	1.122856773	5
ESD	-0.711433632	-0.431935798	1.14336943	5
ESF1	-0.635100414	-0.517605825	1.152706239	5
ESS2	-0.454013308	-0.692451848	1.146465156	5
ESYT3	-0.280359115	-0.829897282	1.110256397	5
ETAA1	-0.743860813	-0.392924689	1.136785503	5

ETFBKMT	-0.755877885	-0.378028298	1.133906182	5
EYA2	-0.139363607	-0.923008148	1.062371754	5
F13B	-0.657583812	-0.493210313	1.150794125	5
F2RL2	-0.799810684	-0.321362397	1.121173082	5
FABP12	-0.309707544	-0.808505417	1.118212962	5
FABP4	-0.707973322	-0.436000891	1.143974213	5
FADD	-0.355871762	-0.773387654	1.129259415	5
FADS1	-0.395278616	-0.741943637	1.137222254	5
FADS2	-0.26933404	-0.837749753	1.107083793	5
FADS3	-0.19235386	-0.88985045	1.08220431	5
FAM104B	-0.265431264	-0.84050566	1.105936924	5
FAM111B	-0.161632369	-0.909338466	1.070970836	5
FAM117A	-0.314518772	-0.804930022	1.119448794	5
FAM131A	-0.410303096	-0.72958827	1.139891366	5
FAM151B	-0.725921167	-0.414714698	1.140635866	5
FAM168A	-0.444862023	-0.7003763	1.145238324	5
FAM170A	-0.530840545	-0.622642921	1.153483467	5
FAM171A1	-0.331003543	-0.792531365	1.123534908	5
FAM171B	-0.564826446	-0.589784182	1.154610629	5
FAM185A	-0.677613176	-0.470903414	1.14851659	5
FAM217A	-0.878572465	-0.209622712	1.088195177	5
FAM220A	-0.492715711	-0.658033332	1.150749044	5
FAM237B	-0.710351415	-0.433209129	1.143560544	5
FAM24A	-0.672173789	-0.477016296	1.149190085	5
FAM3C	-0.799368415	-0.32195116	1.121319574	5
FAM43A	-0.200136954	-0.884796437	1.084933391	5
FAM47C	-0.570661684	-0.584013124	1.154674808	5
FAM83G	-0.546397449	-0.607759487	1.154156936	5
FAM83H	-0.448120204	-0.697564123	1.145684327	5
FASLG	-0.741940263	-0.395282754	1.137223017	5
FAT3	-0.768354633	-0.362296641	1.130651274	5
FBL	-0.637268944	-0.515281723	1.152550667	5
FBP1	-0.322195044	-0.799185126	1.121380171	5
FBRSL1	-0.556092993	-0.598349782	1.154442775	5
FBXO31	-0.560535041	-0.594003805	1.154538846	5
FBXO32	-0.823933507	-0.288640256	1.112573763	5
FBXO40	-0.14456849	-0.91984728	1.064415771	5
FGA	-0.483131825	-0.666694915	1.149826739	5
FGF7	-0.841599408	-0.263877467	1.105476874	5
FGG	-0.62153784	-0.532005092	1.153542932	5
FH	-0.238403889	-0.85925229	1.097656179	5
FHIP2A	-0.351953684	-0.776439272	1.128392955	5
FHL5	-0.5115048	-0.640780996	1.152285796	5
FHOD1	-0.879738007	-0.207854533	1.08759254	5
FIGLA	-0.494303721	-0.656589183	1.150892904	5
FKBP14	-0.858828083	-0.239024993	1.097853076	5
FKBP1B	-0.819210184	-0.295143804	1.114353988	5
FKBP4	-0.887190511	-0.196459232	1.083649744	5
FMN2	-0.48571016	-0.664373794	1.150083954	5
FMO1	-0.664983006	-0.485034155	1.150017161	5
FMO2	-0.514725235	-0.637787342	1.152512577	5
FMO3	-0.580308197	-0.574387281	1.154695478	5
FNDC1	-0.251813532	-0.850024819	1.101838351	5
FNDC8	-0.678021887	-0.470442415	1.148464303	5
FOLR3	-0.520401979	-0.632483913	1.152885892	5

FOSL1	-0.535898262	-0.617832891	1.153731152	5
FOXI1	-0.134972336	-0.925658762	1.060631098	5
FOXJ3	-0.751065318	-0.384023571	1.135088889	5
FOXO4	-0.25631256	-0.846896489	1.10320905	5
FOXP2	-0.251466405	-0.850265509	1.101731914	5
FRMD6	-0.380118337	-0.754203791	1.134322128	5
FRMD7	-0.564879375	-0.589732009	1.154611384	5
FRMD8	-0.860644141	-0.236362823	1.097006964	5
FRMPD4	-0.696651106	-0.449175499	1.145826605	5
FRZB	-0.453857208	-0.692587696	1.146444904	5
FSHB	-0.827289946	-0.28398925	1.111279196	5
FSHR	-0.669718876	-0.479761621	1.149480497	5
FSTL5	-0.860759167	-0.236193924	1.096953092	5
FTSJ3	-0.768424237	-0.362208101	1.130632338	5
FUS	-0.717044431	-0.425305229	1.14234966	5
FUT4	-0.674580605	-0.474316594	1.1488972	5
FXYD3	-0.592663384	-0.561900552	1.154563936	5
FXYD5	-0.229254943	-0.865465191	1.094720135	5
FYB2	-0.643806342	-0.50823841	1.152044753	5
FZD2	-0.593817879	-0.560724575	1.154542454	5
GAB2	-0.527946719	-0.625382697	1.153329417	5
GABARAPL2	-0.5145824	-0.637920348	1.152502748	5
GABRA6	-0.798264997	-0.323418362	1.121683359	5
GABRQ	-0.685562817	-0.461894128	1.147456944	5
GADD45B	-0.736079191	-0.402441143	1.138520334	5
GAGE12C	-0.146815819	-0.918476074	1.065291893	5
GAGE12D	-0.207661547	-0.879865069	1.087526617	5
GAGE12E	-0.317814351	-0.802469716	1.120284068	5
GAGE12F	-0.430542766	-0.712615838	1.143158604	5
GAGE12G	-0.323008423	-0.798573477	1.121581901	5
GAGE12H	-0.348997209	-0.778733115	1.127730324	5
GAGE12J	-0.297786416	-0.817280916	1.115067332	5
GALNT16	-0.34048835	-0.785292723	1.125781073	5
GALR2	-0.813201062	-0.303348781	1.116549842	5
GAPT	-0.66936501	-0.480156659	1.149521669	5
GAS2L2	-0.147261105	-0.918203922	1.065465027	5
GAS6	-0.851916826	-0.249081062	1.100997887	5
GATA1	-0.575181337	-0.579516489	1.154697826	5
GBGT1	-0.347550876	-0.779852525	1.127403401	5
GBP7	-0.270535035	-0.836899185	1.107434219	5
GCFC2	-0.701538886	-0.443511611	1.145050497	5
GCH1	-0.140988719	-0.922023467	1.063012186	5
GCNT7	-0.710747427	-0.432743392	1.143490819	5
GDE1	-0.440249394	-0.704340204	1.144589598	5
GDF11	-0.386436607	-0.749119248	1.135555855	5
GEMIN5	-0.845408751	-0.258443944	1.103852695	5
GET1-SH3BGR	-0.841893801	-0.263458769	1.10535257	5
GGA2	-0.604494643	-0.549773611	1.154268254	5
GGACT	-0.785738051	-0.339907739	1.12564579	5
GGTLC1	-0.361104501	-0.769291239	1.13039574	5
GGTLC2	-0.420229675	-0.72131163	1.141541305	5
GHRHR	-0.645940883	-0.505926586	1.151867469	5
GID8	-0.797184787	-0.32485236	1.122037147	5
GIMAP5	-0.269674668	-0.837508633	1.107183301	5
GIMD1	-0.860620507	-0.236397521	1.097018028	5

GJA8	-0.596722975	-0.557758404	1.154481379	5
GJA9	-0.788088536	-0.336836943	1.124925479	5
GJB2	-0.726201414	-0.414378315	1.140579729	5
GJB4	-0.516548851	-0.636087337	1.152636189	5
GJE1	-0.428108085	-0.714677674	1.142785759	5
GK2	-0.817369809	-0.297664829	1.115034638	5
GKN1	-0.885995699	-0.198296713	1.084292412	5
GKN2	-0.889183209	-0.193385602	1.082568811	5
GLB1L	-0.534930074	-0.618755794	1.153685868	5
GLDC	-0.623122482	-0.530334702	1.153457184	5
GLDN	-0.647467858	-0.504269108	1.151736966	5
GLYATL3	-0.656597947	-0.494294092	1.150892039	5
GLYR1	-0.644593557	-0.50738651	1.151980067	5
GNA14	-0.407076508	-0.732259019	1.139335528	5
GNAI3	-0.217903119	-0.873081369	1.090984488	5
GNAT3	-0.680398682	-0.467756861	1.148155543	5
GNLY	-0.366326744	-0.765179149	1.131505893	5
GNPTG	-0.712978255	-0.430115288	1.143093543	5
GNRHR	-0.699215802	-0.446207999	1.145423801	5
GON7	-0.402965895	-0.735647708	1.138613603	5
GPA33	-0.482449839	-0.667307751	1.149757591	5
GPATCH2L	-0.174328288	-0.901373784	1.075702072	5
GPBP1	-0.858048334	-0.240165487	1.098213821	5
GPC5	-0.695940678	-0.449995781	1.145936459	5
GPD1L	-0.300452409	-0.815328694	1.115781103	5
GPR119	-0.701564978	-0.443481281	1.145046259	5
GPR137B	-0.610484415	-0.543569659	1.154054074	5
GPR15	-0.197600763	-0.886448548	1.084049311	5
GPR158	-0.592579436	-0.561986001	1.154565437	5
GPR176	-0.751423219	-0.383579085	1.135002304	5
GPR22	-0.571078694	-0.583599217	1.154677911	5
GPR26	-0.883518753	-0.20209306	1.085611813	5
GPR27	-0.200056873	-0.884848681	1.084905554	5
GPR33	-0.358931426	-0.770995319	1.129926745	5
GPR65	-0.193882157	-0.888861783	1.08274394	5
GPR68	-0.708543886	-0.435331863	1.143875749	5
GPR87	-0.762070694	-0.370254339	1.132325033	5
GPRASP2	-0.315106907	-0.804491622	1.119598529	5
GPRC6A	-0.806356902	-0.312601829	1.118958731	5
GRAP	-0.324176903	-0.797693816	1.121870719	5
GRB14	-0.586707754	-0.567941954	1.154649708	5
GREM2	-0.350316248	-0.777710647	1.128026895	5
GRID1	-0.86512146	-0.229763696	1.094885156	5
GRIK2	-0.175313017	-0.900750801	1.076063818	5
GRIN2C	-0.874477803	-0.215805071	1.090282873	5
GRK4	-0.298909059	-0.816459561	1.11536862	5
GRP	-0.759914247	-0.372969003	1.132883251	5
GRXCR1	-0.853708795	-0.246485019	1.100193814	5
GSR	-0.201825065	-0.883693979	1.085519044	5
GSS	-0.519699472	-0.633142058	1.15284153	5
GSTA4	-0.151448981	-0.915636899	1.06708588	5
GSTM5	-0.52413342	-0.628979371	1.153112791	5
GSTO1	-0.758538595	-0.374696484	1.133235079	5
GTF2H2C	-0.785219454	-0.34058384	1.125803294	5
GTF3C5	-0.850698657	-0.250841359	1.101540016	5

GTPBP4	-0.380979435	-0.753512932	1.134492367	5
GYPA	-0.541455941	-0.612515539	1.153971481	5
GYPE	-0.644748415	-0.507218832	1.151967247	5
GYS2	-0.787603848	-0.337471031	1.125074879	5
GZMK	-0.292174617	-0.821370957	1.113545574	5
H1-6	-0.723263226	-0.417898842	1.141162067	5
H2AC16	-0.56530959	-0.58930782	1.154617409	5
H2AC21	-0.240574135	-0.857768689	1.098342825	5
H2AC7	-0.846101515	-0.257452122	1.103553637	5
H2BC14	-0.891059313	-0.190481331	1.081540644	5
H2BC3	-0.472143021	-0.67651313	1.148656151	5
H2BC5	-0.632370159	-0.520523344	1.152893503	5
H2BC8	-0.854555065	-0.245256281	1.099811346	5
H2BC9	-0.769769564	-0.360495063	1.130264627	5
H4C1	-0.843848419	-0.260673684	1.104522103	5
H4C14	-0.154364591	-0.91384176	1.068206352	5
H4C5	-0.799874533	-0.321277367	1.121151901	5
HAMP	-0.641110788	-0.511149312	1.1522601	5
HAPLN1	-0.797877934	-0.323932461	1.121810395	5
HARS2	-0.543707344	-0.610351941	1.154059285	5
HASPIN	-0.856361793	-0.242627094	1.098988887	5
HAUS1	-0.449475216	-0.696391603	1.145866819	5
HAUS4	-0.51772691	-0.634987279	1.152714189	5
HCRTR2	-0.867247987	-0.226611341	1.093859328	5
HDAC4	-0.628976892	-0.524136054	1.153112946	5
HDGFL1	-0.389000433	-0.747045851	1.136046284	5
HDHD5	-0.51140783	-0.640870969	1.1522788	5
HEATR5B	-0.421509383	-0.720237994	1.141747377	5
HEBP2	-0.768023548	-0.362717677	1.130741225	5
HELT	-0.356580139	-0.772834504	1.129414642	5
HEPACAM2	-0.645206611	-0.506722519	1.151929129	5
HES6	-0.765584364	-0.365813479	1.131397843	5
HESX1	-0.408049175	-0.731454917	1.139504092	5
HEXA	-0.513014434	-0.639379016	1.15239345	5
HHAT	-0.358247807	-0.771530546	1.129778353	5
HIBADH	-0.614518272	-0.539366797	1.153885069	5
HIGD1A	-0.722226809	-0.419137406	1.141364215	5
HIGD1C	-0.325077836	-0.797014781	1.122092617	5
HIRIP3	-0.574065989	-0.580628333	1.154694323	5
HLA-DQB2	-0.730626667	-0.409049957	1.139676624	5
HLA-DRB1	-0.254688445	-0.84802768	1.102716124	5
HLA-DRB5	-0.742511896	-0.394581544	1.137093439	5
HMG20A	-0.815132732	-0.300719573	1.115852305	5
HMGA1	-0.216527805	-0.873997153	1.090524959	5
HNRNPA1	-0.531410158	-0.622102577	1.153512734	5
HNRNPC	-0.493312406	-0.657490994	1.150803399	5
HOMER2	-0.612284947	-0.541696165	1.153981112	5
HORMAD2	-0.778947016	-0.348721018	1.127668035	5
HPGD	-0.775402794	-0.353286376	1.12868917	5
HPN	-0.265594842	-0.8403904	1.105985242	5
HRURF	-0.5503695	-0.603917098	1.154286599	5
HSD11B1	-0.602730879	-0.55159213	1.154323009	5
HSD17B6	-0.738426638	-0.399580915	1.138007554	5
HSDL1	-0.755644784	-0.378319605	1.133964389	5
HSF2	-0.84690076	-0.256306434	1.103207194	5

HSFX1	-0.768196352	-0.362497948	1.130694301	5
HSFX2	-0.794350305	-0.328604219	1.122954523	5
HSFX3	-0.806267424	-0.312722161	1.118989585	5
HSFY2	-0.563066388	-0.59151731	1.154583698	5
HSPA14	-0.622931589	-0.530536092	1.153467681	5
HSPA5	-0.82282783	-0.290166994	1.112994824	5
HSPA6	-0.493595427	-0.657233627	1.150829055	5
HSPB3	-0.701165509	-0.443945531	1.14511104	5
HSPB7	-0.267172059	-0.839277948	1.106450007	5
HSPB9	-0.840826919	-0.264975167	1.105802086	5
HTR2B	-0.300958983	-0.814957077	1.11591606	5
IAPP	-0.465918373	-0.682021717	1.147940089	5
IBTK	-0.723066672	-0.418133863	1.141200535	5
ID3	-0.191592285	-0.890342437	1.081934722	5
IDI2	-0.274996179	-0.833729395	1.108725574	5
IDUA	-0.40121761	-0.73708428	1.13830189	5
IFI6	-0.580641005	-0.57405327	1.154694274	5
IFIT2	-0.227067715	-0.866940614	1.094008329	5
IFITM3	-0.264042103	-0.84148361	1.105525713	5
IFNA14	-0.75407117	-0.380283702	1.134354872	5
IFNA2	-0.470804042	-0.677701295	1.148505337	5
IFNA21	-0.1691238	-0.904653878	1.073777678	5
IFNB1	-0.29212966	-0.821403617	1.113533277	5
IFNE	-0.823506924	-0.289229604	1.112736529	5
IFNL1	-0.325013338	-0.797063416	1.122076754	5
IFNLR1	-0.288546716	-0.824001187	1.112547903	5
IFNW1	-0.34305986	-0.783316889	1.12637675	5
IFT172	-0.867293046	-0.226544419	1.093837465	5
IGDCC3	-0.794834919	-0.32796388	1.1227988	5
IGFL1	-0.776545731	-0.351816687	1.128362418	5
IGFL2	-0.337068733	-0.787911399	1.124980132	5
IGLON5	-0.746636505	-0.389505742	1.136142247	5
IL13	-0.743539905	-0.393319132	1.136859037	5
IL13RA1	-0.701900628	-0.443091015	1.144991643	5
IL17C	-0.231743617	-0.86378179	1.095525406	5
IL17RD	-0.234635861	-0.861819195	1.096455055	5
IL18	-0.719319646	-0.42260256	1.141922206	5
IL1B	-0.478546468	-0.67080641	1.149352878	5
IL1F10	-0.814123308	-0.302094485	1.116217793	5
IL1R2	-0.762387563	-0.369854754	1.132242317	5
IL2	-0.295687807	-0.818813503	1.11450131	5
IL22	-0.704458177	-0.440111752	1.144569929	5
IL25	-0.578951593	-0.575747464	1.154699057	5
IL3	-0.555555718	-0.598873944	1.154429662	5
IL4	-0.396054125	-0.741310921	1.137365046	5
ILF2	-0.617456955	-0.53629234	1.153749295	5
ILF3	-0.812792181	-0.303904305	1.116696487	5
IMMT	-0.40999685	-0.729842169	1.13983902	5
IMPDH2	-0.789882737	-0.334485767	1.124368504	5
IMPG1	-0.696112521	-0.449797434	1.145909955	5
INCENP	-0.676382615	-0.472289981	1.148672596	5
INKA1	-0.176601745	-0.899934356	1.076536102	5
INPP5D	-0.386048789	-0.749432368	1.135481157	5
INSL4	-0.743905106	-0.392870233	1.13677534	5
INTS2	-0.67831543	-0.470111173	1.148426603	5

IPO5	-0.740688313	-0.396816594	1.137504907	5
IPP	-0.616298474	-0.537505624	1.153804098	5
IQCF2	-0.816368499	-0.299033437	1.115401936	5
IQCF3	-0.597720785	-0.556737292	1.154458077	5
IQCF5	-0.225024327	-0.868315568	1.093339895	5
IQCM	-0.261657503	-0.843158678	1.104816181	5
IQCN	-0.663614171	-0.486552355	1.150166526	5
IQUB	-0.195613436	-0.887739596	1.083353032	5
IRAK1BP1	-0.382558091	-0.752244659	1.13480275	5
IRAK4	-0.816906476	-0.298298386	1.115204861	5
IREB2	-0.72328184	-0.417876581	1.141158421	5
IRF8	-0.215159487	-0.874906794	1.090066281	5
IRGQ	-0.681029588	-0.46704265	1.148072238	5
IRS2	-0.739949699	-0.397720293	1.137669992	5
ISCA1	-0.807366609	-0.311242823	1.118609433	5
ISCA2	-0.624501044	-0.528878977	1.153380021	5
ITGA9	-0.675902588	-0.472830285	1.148732873	5
ITGAD	-0.492695548	-0.658051652	1.150747201	5
ITIH2	-0.597282918	-0.557185531	1.154468449	5
ITM2A	-0.16046919	-0.910061941	1.070531131	5
ITPK1	-0.73901908	-0.398857625	1.137876705	5
ITPRID2	-0.33564704	-0.788997152	1.124644192	5
IVNS1ABP	-0.836493735	-0.271106941	1.107600676	5
JAGN1	-0.525727975	-0.627477279	1.153205255	5
JMJD1C	-0.568995678	-0.585664754	1.154660432	5
JMJD4	-0.147993459	-0.917755988	1.065749447	5
JMJD6	-0.196388376	-0.887236532	1.083624908	5
JPH3	-0.812117934	-0.304819606	1.11693754	5
JUN	-0.67615969	-0.472540939	1.148700629	5
JUNB	-0.59254941	-0.562016561	1.154565971	5
KAAG1	-0.828467615	-0.282351477	1.110819092	5
KARS1	-0.590089751	-0.564516388	1.154606138	5
KAT6B	-0.447294043	-0.698278154	1.145572197	5
KATNA1	-0.701372738	-0.443704726	1.145077464	5
KAZALD1	-0.880357032	-0.206913921	1.087270953	5
KCNA2	-0.475964888	-0.673111991	1.149076879	5
KCNA6	-0.608003745	-0.546144342	1.154148086	5
KCNAB2	-0.327058954	-0.795519182	1.122578136	5
KCNC2	-0.883632089	-0.201919731	1.08555182	5
KCNIP2	-0.167777094	-0.905499211	1.073276305	5
KCNJ16	-0.421950141	-0.719867858	1.141818	5
KCNJ2	-0.55727135	-0.59719906	1.154470409	5
KCTD5	-0.335292657	-0.789267527	1.124560183	5
KCTD7	-0.259206571	-0.844875532	1.104082103	5
KCTD8	-0.517799683	-0.634919277	1.15271896	5
KCTD9	-0.229600362	-0.865231836	1.094832199	5
KDF1	-0.204445278	-0.881978355	1.086423633	5
KDM5D	-0.521496581	-0.631457396	1.152953977	5
KEAP1	-0.481222081	-0.668409852	1.149631932	5
KHDC3L	-0.595505323	-0.559002879	1.154508203	5
KIF19	-0.780584116	-0.346604358	1.127188474	5
KIF20B	-0.289286235	-0.823465919	1.112752154	5
KIR2DL1	-0.882531312	-0.203601666	1.086132978	5
KIR3DL3	-0.290444472	-0.822626671	1.113071143	5
KIRREL1	-0.570181932	-0.584489061	1.154670993	5

KIZ	-0.143443577	-0.920532189	1.063975766	5
KL	-0.843719595	-0.260857519	1.104577114	5
KLF1	-0.279429885	-0.830562958	1.109992843	5
KLF18	-0.336586243	-0.788280073	1.124866316	5
KLHL9	-0.370935691	-0.761530061	1.132465752	5
KLK10	-0.196271392	-0.887312504	1.083583896	5
KLK3	-0.136240499	-0.924894803	1.061135302	5
KLKB1	-0.861552977	-0.235027424	1.096580402	5
KRT2	-0.25226858	-0.849709152	1.101977732	5
KRT32	-0.803222308	-0.316807556	1.120029864	5
KRT5	-0.192909263	-0.889491367	1.08240063	5
KRT6C	-0.390374017	-0.745932588	1.136306605	5
KRT82	-0.569245975	-0.585416819	1.154662794	5
KRTAP10-11	-0.199584681	-0.885156626	1.084741307	5
KRTAP13-2	-0.826262808	-0.28541518	1.111677987	5
KRTAP19-1	-0.2407134	-0.857673358	1.098386758	5
KRTAP19-2	-0.647110803	-0.504656955	1.151767757	5
KRTAP19-7	-0.677433025	-0.471106537	1.148539563	5
KRTAP2-2	-0.845009974	-0.25901435	1.104024325	5
KRTAP2-4	-0.638897012	-0.51353286	1.152429872	5
KRTAP20-3	-0.74759233	-0.388325401	1.135917731	5
KRTAP21-2	-0.585626159	-0.569034645	1.154660805	5
KRTAP24-1	-0.649096383	-0.502498007	1.15159439	5
KRTAP26-1	-0.479711538	-0.669763728	1.149475267	5
KRTAP3-3	-0.675801664	-0.47294384	1.148745505	5
KRTAP5-3	-0.65862349	-0.492065953	1.150689444	5
KRTAP5-7	-0.414050005	-0.726474834	1.140524839	5
KRTAP5-8	-0.674530107	-0.474373321	1.148903428	5
KRTAP6-1	-0.355684497	-0.77353381	1.129218307	5
KRTDAP	-0.268162174	-0.838578559	1.106740733	5
L3MBTL2	-0.805084244	-0.314311782	1.119396026	5
LARS1	-0.15442399	-0.913805121	1.068229111	5
LAT2	-0.445565704	-0.699769803	1.145335508	5
LAX1	-0.514192065	-0.638283714	1.152475779	5
LBH	-0.231948986	-0.863642652	1.095591638	5
LBP	-0.511820489	-0.640488016	1.152308504	5
LCA5	-0.765024567	-0.366522468	1.131547035	5
LCE2B	-0.786742993	-0.338596124	1.125339117	5
LCE3D	-0.284063112	-0.827236782	1.111299894	5
LCK	-0.576498105	-0.578202014	1.154700119	5
LCLAT1	-0.174785523	-0.901084611	1.075870134	5
LCMT1	-0.212634084	-0.876581763	1.089215846	5
LCORL	-0.857486468	-0.240986352	1.09847282	5
LDB2	-0.143152349	-0.920709346	1.063861695	5
LENG1	-0.566171638	-0.588457213	1.154628851	5
LENG8	-0.673568936	-0.475452361	1.149021297	5
LEO1	-0.873151302	-0.217798174	1.090949476	5
LEPR	-0.281508673	-0.829072788	1.11058146	5
LETM1	-0.23392573	-0.862301688	1.096227418	5
LETM2	-0.638385887	-0.514082278	1.152468165	5
LETMD1	-0.235945192	-0.860928522	1.096873715	5
LGALS12	-0.312510695	-0.806424661	1.118935356	5
LHX1	-0.478641643	-0.670721284	1.149362927	5
LIMS2	-0.360542417	-0.76973241	1.130274826	5
LIN9	-0.517256908	-0.635426337	1.152683244	5

LINC00672	-0.523375746	-0.629692164	1.153067911	5
LINC02218	-0.819761552	-0.294387105	1.114148657	5
LINGO2	-0.666765385	-0.483053429	1.149818814	5
LINGO3	-0.58473251	-0.569936428	1.154668939	5
LIPC	-0.862989485	-0.232912407	1.095901893	5
LIPN	-0.152351999	-0.915081611	1.067433611	5
LLGL2	-0.435283897	-0.708584776	1.143868674	5
LMBR1L	-0.231344725	-0.864051942	1.095396667	5
LMBRD1	-0.629999205	-0.523049173	1.153048379	5
LMCD1	-0.241131282	-0.85738721	1.098518492	5
LMO1	-0.223142019	-0.869579198	1.092721217	5
LMOD1	-0.409847191	-0.729966216	1.139813407	5
LMOD3	-0.576814251	-0.577886121	1.154700373	5
LMX1B	-0.320486443	-0.800468145	1.120954588	5
LNP1	-0.328850816	-0.794163584	1.1230144	5
LONRF3	-0.297527622	-0.817470108	1.11499773	5
LPIN3	-0.58538351	-0.569279595	1.154663106	5
LPP	-0.872041857	-0.219461527	1.091503384	5
LRAT	-0.186673134	-0.893509346	1.08018248	5
LRIG3	-0.227504733	-0.866646122	1.094150856	5
LRIT2	-0.503396399	-0.648270716	1.151667115	5
LRRC30	-0.326477116	-0.795958773	1.122435889	5
LRRC3B	-0.384050121	-0.751043934	1.135094055	5
LRRC52	-0.709150018	-0.434620587	1.143770605	5
LRRC53	-0.55096473	-0.603339801	1.154304531	5
LRRC70	-0.688662348	-0.45835689	1.147019238	5
LRRD1	-0.588083213	-0.5665504	1.154633613	5
LRRTM3	-0.887123527	-0.196562353	1.083685881	5
LRTM1	-0.708110961	-0.435839544	1.143950505	5
LRTOMT	-0.505467187	-0.646364394	1.151831582	5
LSM5	-0.585970623	-0.568686796	1.15465742	5
LTB4R	-0.684167575	-0.463481881	1.147649456	5
LTBP4	-0.794553111	-0.328336299	1.12288941	5
LUZP4	-0.288130839	-0.824302003	1.112432842	5
LY6K	-0.868775469	-0.224339742	1.093115211	5
LYRM7	-0.656335344	-0.494582555	1.1509179	5
LYSMD1	-0.374904912	-0.758372438	1.13327735	5
LYSMD2	-0.343765122	-0.782774003	1.126539125	5
LYZL1	-0.825727361	-0.286157595	1.111884956	5
LYZL2	-0.697087659	-0.44867107	1.145758729	5
LYZL4	-0.590094319	-0.564511751	1.15460607	5
MACO1	-0.469948825	-0.678459262	1.148408086	5
MADD	-0.891222047	-0.19022893	1.081450977	5
MAFF	-0.35726902	-0.772296158	1.129565178	5
MAFK	-0.578337331	-0.576362645	1.154699976	5
MAGEB1	-0.223338276	-0.869447578	1.092785854	5
MAGED1	-0.161509669	-0.909414833	1.070924502	5
MAGED4	-0.301438454	-0.814605145	1.116043599	5
MAGED4B	-0.36920847	-0.762899765	1.132108235	5
MAIP1	-0.55456371	-0.599840895	1.154404605	5
MALT1	-0.72125328	-0.420299271	1.141552552	5
MAMLD1	-0.155926861	-0.912877177	1.068804039	5
MAP2K1	-0.823147859	-0.289725365	1.112873225	5
MAP3K9	-0.334382901	-0.789961127	1.124344028	5
MAP7D1	-0.809011908	-0.309023863	1.118035771	5

MAPK6	-0.841114666	-0.264566443	1.105681109	5
MAPKAPK2	-0.339212774	-0.786270708	1.125483482	5
MARCHF11	-0.779725489	-0.347715131	1.127440619	5
MARCHF2	-0.354479686	-0.774473409	1.128953095	5
MARCHF9	-0.550267348	-0.604016133	1.154283482	5
MARCKSL1	-0.496182014	-0.654877739	1.151059753	5
MARS1	-0.307794848	-0.809921412	1.11771626	5
MARVELD3	-0.367330027	-0.764386403	1.13171643	5
MAST4	-0.282836293	-0.82811923	1.110955522	5
MAT1A	-0.817008046	-0.298159539	1.115167585	5
MBIP	-0.297155017	-0.817742403	1.11489742	5
MBL2	-0.193180854	-0.889315689	1.082496542	5
MC1R	-0.681506682	-0.466502183	1.148008865	5
MCAT	-0.404590008	-0.734310673	1.138900681	5
MCHR1	-0.250076736	-0.851228102	1.101304837	5
MCM10	-0.556581885	-0.597872544	1.154454428	5
MCRIP1	-0.253618457	-0.848771757	1.102390214	5
MCRIP2	-0.729540569	-0.410360619	1.139901189	5
MCTP2	-0.596862193	-0.557616006	1.154478199	5
MCU	-0.861626762	-0.234918917	1.096545679	5
MDC1	-0.168658827	-0.904945902	1.073604729	5
MDFIC2	-0.646673876	-0.505131331	1.151805207	5
MDH2	-0.529624876	-0.623794966	1.153419842	5
MDN1	-0.341457058	-0.784549079	1.126006137	5
MED26	-0.131469209	-0.927762696	1.059231904	5
MED29	-0.568379428	-0.586274886	1.154654314	5
MED9	-0.603151434	-0.551158861	1.154310295	5
MEDAG	-0.649547824	-0.50200642	1.151554244	5
MEF2D	-0.407850676	-0.731619086	1.139469762	5
MEIKIN	-0.732805407	-0.406414983	1.13922039	5
MEIOC	-0.467726599	-0.680425425	1.148152024	5
MERTK	-0.362298465	-0.768353198	1.130651664	5
METTL22	-0.759462855	-0.373536207	1.132999062	5
METTL25B	-0.808133701	-0.310208966	1.118342666	5
METTL6	-0.682375692	-0.465516909	1.147892601	5
MEX3A	-0.688025647	-0.459084638	1.147110284	5
MFSD10	-0.319586194	-0.801143164	1.120729358	5
MFSD6L	-0.28174557	-0.828902743	1.110648313	5
MID1	-0.813345053	-0.303153063	1.116498117	5
MIER3	-0.863529943	-0.23211531	1.095645253	5
MIF	-0.256998701	-0.846417954	1.103416655	5
MIOS	-0.339100284	-0.786356887	1.125457171	5
MKNK1	-0.263320432	-0.841991037	1.105311469	5
MKRN1	-0.4916858	-0.658968575	1.150654375	5
MKRN2	-0.848584598	-0.253887717	1.102472316	5
MKRN3	-0.616241328	-0.53756543	1.153806758	5
MLH3	-0.809560351	-0.308282956	1.117843306	5
MMP16	-0.228495509	-0.865977909	1.094473418	5
MMP20	-0.596818791	-0.557660402	1.154479193	5
MMP21	-0.500795803	-0.650658519	1.151454322	5
MMP8	-0.72726823	-0.413096657	1.140364886	5
MMUT	-0.659636335	-0.490949722	1.150586057	5
MOB3C	-0.539391447	-0.614494669	1.153886116	5
MOGS	-0.874722411	-0.215437024	1.090159435	5
MORN2	-0.632988805	-0.519863104	1.15285191	5

MOS	-0.172132884	-0.902759985	1.074892869	5
MPC1	-0.589275711	-0.565342146	1.154617857	5
MPND	-0.771667537	-0.35807275	1.129740288	5
MR1	-0.156282129	-0.912657564	1.068939693	5
MRE11	-0.384055422	-0.751039664	1.135095086	5
MRFAP1	-0.71888706	-0.42311704	1.1420041	5
MRLN	-0.773634004	-0.355556099	1.129190103	5
MRPL11	-0.88293958	-0.202978249	1.085917828	5
MRPL15	-0.490616251	-0.659938675	1.150554926	5
MRPL32	-0.677575592	-0.470945794	1.148521387	5
MRPL46	-0.689686102	-0.457185509	1.146871611	5
MRPL49	-0.482836915	-0.66695998	1.149796895	5
MRPL52	-0.650375574	-0.501104358	1.151479932	5
MRPL9	-0.844845609	-0.259249348	1.104094957	5
MRPS2	-0.275636463	-0.833273116	1.108909579	5
MRPS36	-0.57466633	-0.580030056	1.154696386	5
MS4A8	-0.367531679	-0.76422696	1.131758639	5
MSANTD3-TMEFF1	-0.752399818	-0.382365108	1.134764926	5
MSANTD5	-0.228035038	-0.866288564	1.094323602	5
MSGN1	-0.144477445	-0.91990275	1.064380195	5
MSH3	-0.214632871	-0.875256487	1.089889358	5
MSLN	-0.485664347	-0.664415095	1.150079442	5
MSRA	-0.533754161	-0.619875349	1.15362951	5
MSRB2	-0.581833646	-0.572855257	1.154688903	5
MT-ATP8	-0.547035316	-0.607143611	1.154178928	5
MT1E	-0.422574327	-0.719343377	1.141917704	5
MT3	-0.522566052	-0.630453225	1.153019277	5
MTIF2	-0.761471729	-0.371009175	1.132480904	5
MTNR1A	-0.841924003	-0.263415803	1.105339806	5
MTRNR2L11	-0.412891585	-0.727438785	1.14033037	5
MTRNR2L13	-0.756909216	-0.376738301	1.133647517	5
MTRNR2L3	-0.650202713	-0.501292814	1.151495526	5
MTRNR2L4	-0.759611176	-0.373349871	1.132961047	5
MTRNR2L6	-0.691227021	-0.455419513	1.146646534	5
MTX1	-0.377935149	-0.755952405	1.133887554	5
MTX3	-0.244135747	-0.855325754	1.099461501	5
MUC13	-0.639466883	-0.512919895	1.152386778	5
MUC15	-0.596651224	-0.557831784	1.154483009	5
MUC7	-0.416476041	-0.724452057	1.140928098	5
MUL1	-0.20992386	-0.878373709	1.08829757	5
MUSTN1	-0.563929553	-0.590667789	1.154597342	5
MYBPC3	-0.4473407	-0.698237847	1.145578547	5
MYF5	-0.627279554	-0.525937659	1.153217213	5
MYF6	-0.455884174	-0.690821873	1.146706047	5
MYH11	-0.475921645	-0.673150554	1.149072199	5
MYH2	-0.662223536	-0.488092109	1.150315645	5
MYL1	-0.537369633	-0.616428401	1.153798034	5
MYLIP	-0.746917543	-0.38915885	1.136076394	5
MYLK4	-0.317373935	-0.802799038	1.120172973	5
MYOZ2	-0.721091717	-0.420491946	1.141583663	5
MZT1	-0.564329785	-0.590273598	1.154603383	5
NAA11	-0.854747991	-0.244975916	1.099723907	5
NAA30	-0.445669861	-0.699679992	1.145349852	5
NAB2	-0.22051608	-0.871337342	1.091853422	5
NACA2	-0.399849219	-0.738206752	1.138055971	5

NACAD	-0.700286061	-0.444966757	1.145252818	5
NANP	-0.142204816	-0.92128529	1.063490106	5
NAPB	-0.50352274	-0.648154536	1.151677276	5
NAT2	-0.821626345	-0.291823014	1.11344936	5
NAXD	-0.354693471	-0.774306777	1.129000247	5
NBPF19	-0.557176479	-0.597291763	1.154468242	5
NCAM1	-0.287245146	-0.824942174	1.11218732	5
NCBP2L	-0.511612545	-0.640681013	1.152293558	5
NCK2	-0.520943043	-0.631976662	1.152919705	5
NCKAP5L	-0.770209109	-0.35993467	1.130143779	5
NCL	-0.219266751	-0.872171884	1.091438636	5
NCOA3	-0.385739967	-0.749681611	1.135421578	5
NCOR2	-0.638705296	-0.513738978	1.152444275	5
NCR3LG1	-0.433790319	-0.709856948	1.143647267	5
NDUFA8	-0.879978287	-0.207489551	1.087467839	5
NDUFAF4	-0.702943217	-0.441877701	1.144820917	5
NDUFB7	-0.688683166	-0.458333085	1.147016251	5
NDUFS4	-0.619896435	-0.533731998	1.153628434	5
NDUFV1	-0.158940618	-0.911011094	1.069951712	5
NEBL	-0.141005593	-0.922013232	1.063018825	5
NECAB1	-0.237567129	-0.859823299	1.097390429	5
NECTIN3	-0.800146165	-0.320915535	1.1210617	5
NEFM	-0.162176869	-0.908999439	1.071176308	5
NEK4	-0.844806189	-0.259305698	1.104111887	5
NEMF	-0.45639144	-0.690379339	1.14677078	5
NEPRO	-0.757572254	-0.37590799	1.133480244	5
NET1	-0.665632535	-0.484312851	1.149945386	5
NETO2	-0.283211031	-0.827849813	1.111060844	5
NEUROD4	-0.152909545	-0.91473845	1.067647994	5
NEXMIF	-0.635742504	-0.516918304	1.152660807	5
NFKBIB	-0.616932394	-0.536841922	1.153774316	5
NFKBID	-0.235226168	-0.861417808	1.096643976	5
NFKBIL1	-0.881543083	-0.205108745	1.086651828	5
NIBAN2	-0.134270629	-0.92608095	1.060351579	5
NID2	-0.625502618	-0.52781984	1.153322458	5
NKX2-5	-0.425399262	-0.716965148	1.14236441	5
NKX6-3	-0.459718243	-0.687470891	1.147189135	5
NLE1	-0.468208493	-0.679999471	1.148207963	5
NLRP6	-0.191557895	-0.890364642	1.081922537	5
NLRP9	-0.373990381	-0.759101208	1.13309159	5
NME1	-0.873556164	-0.217190359	1.090746523	5
NME5	-0.670731827	-0.478629856	1.149361683	5
NNT	-0.182761835	-0.89601393	1.078775764	5
NOD1	-0.327904494	-0.794879846	1.12278434	5
NOMO1	-0.468346363	-0.679877563	1.148223926	5
NOP53	-0.827381397	-0.28386218	1.111243577	5
NPDC1	-0.858802335	-0.239062677	1.097865012	5
NPHS2	-0.143115643	-0.92073167	1.063847313	5
NPPA	-0.884383242	-0.200770051	1.085153293	5
NPS	-0.588343668	-0.566286646	1.154630314	5
NPY	-0.656492715	-0.494409699	1.150902413	5
NPY2R	-0.501803588	-0.64973402	1.151537608	5
NPY5R	-0.300171856	-0.815534413	1.115706269	5
NQO1	-0.341261274	-0.784699441	1.125960715	5
NR1H2	-0.699598487	-0.445764373	1.14536286	5

NR5A2	-0.247031159	-0.853332261	1.100363421	5
NRCAM	-0.437726559	-0.706499673	1.144226232	5
NRG3	-0.289313105	-0.823446461	1.112759566	5
NRL	-0.388887594	-0.747137229	1.136024824	5
NRM	-0.250043756	-0.851250927	1.101294683	5
NRSN1	-0.372157413	-0.760559633	1.132717046	5
NT5C1B-RDH14	-0.66707584	-0.482707979	1.149783819	5
NTAN1	-0.78044417	-0.346785495	1.127229665	5
NTMT1	-0.20430534	-0.882070117	1.086375458	5
NTN3	-0.44154148	-0.703231879	1.14477336	5
NTN5	-0.375627118	-0.757796403	1.133423522	5
NTPCR	-0.860273241	-0.236907204	1.097180445	5
NTS	-0.693274053	-0.453068116	1.146342169	5
NTSR2	-0.802570124	-0.317680095	1.120250219	5
NUDC	-0.506113577	-0.645768437	1.151882014	5
NUP153	-0.706156314	-0.438128166	1.14428448	5
NUP35	-0.583408565	-0.571270708	1.154679273	5
NUP62CL	-0.783350683	-0.343015941	1.126366624	5
NUP93	-0.668356252	-0.48128183	1.149638082	5
NUP98	-0.142077394	-0.921362689	1.063440083	5
NUS1	-0.561005018	-0.5935427	1.154547718	5
NVL	-0.781385871	-0.345565912	1.126951783	5
NXPH1	-0.365249656	-0.766029227	1.131278883	5
NXPH2	-0.852091784	-0.248827943	1.100919727	5
OARD1	-0.592244163	-0.562327183	1.154571345	5
OAS2	-0.565465582	-0.58915396	1.154619542	5
OBSCN	-0.765961528	-0.365335481	1.131297009	5
OCA2	-0.137257367	-0.924281339	1.061538705	5
OCIAD2	-0.510773211	-0.64145956	1.152232771	5
OCSTAMP	-0.348314586	-0.779261667	1.127576252	5
ODAD2	-0.310490735	-0.807924725	1.11841546	5
ODAM	-0.854718423	-0.245018892	1.099737314	5
ODC1	-0.597432995	-0.557031925	1.15446492	5
ODF2	-0.178530534	-0.898710004	1.077240538	5
ODF3	-0.3139539	-0.805350807	1.119304707	5
ODR4	-0.446473636	-0.698986565	1.145460202	5
OFD1	-0.413361636	-0.727047793	1.140409429	5
OIP5	-8.85E-01	-0.200180998	1.0849487	5
OLFM3	-0.831300115	-0.278399716	1.109699832	5
OMD	-0.316641129	-0.803346632	1.119987761	5
OOEP	-0.838428686	-0.2683742	1.106802886	5
OPN4	-0.735155825	-0.403563731	1.138719556	5
OPN5	-0.305432276	-0.811666226	1.117098502	5
OPRL1	-0.256091545	-0.847050551	1.103142096	5
OR10A2	-0.421990808	-0.719833699	1.141824507	5
OR10A6	-0.524281757	-0.628839749	1.153121507	5
OR10C1	-0.84526074	-0.258655702	1.103916441	5
OR10D3	-0.798895863	-0.322579804	1.121475667	5
OR10G2	-0.531282912	-0.622223315	1.153506226	5
OR10S1	-0.868960762	-0.224063768	1.09302453	5
OR10V1	-0.628344135	-0.524808109	1.153152244	5
OR10Z1	-0.737730894	-0.400429584	1.138160479	5
OR11A1	-0.72865903	-0.411423032	1.140082062	5
OR11H12	-0.329710035	-0.793512589	1.123222624	5
OR11H2	-0.177704663	-0.899234603	1.076939266	5

OR11H4	-0.710987761	-0.432460625	1.143448386	5
OR13C2	-0.883913854	-0.201488661	1.085402515	5
OR13C5	-0.43510951	-0.708733421	1.143842931	5
OR13F1	-0.518620036	-0.634152317	1.152772353	5
OR14J1	-0.615124413	-0.538733526	1.153857939	5
OR14K1	-0.526663744	-0.626594516	1.15325826	5
OR1E2	-0.411494292	-0.728599864	1.140094156	5
OR1Q1	-0.556293368	-0.598154215	1.154447584	5
OR2A12	-0.425718574	-0.71669586	1.142414434	5
OR2A2	-0.776511857	-0.35186028	1.128372137	5
OR2AG2	-0.257274001	-0.846225844	1.103499845	5
OR2B3	-0.417617755	-0.723498238	1.141115993	5
OR2D2	-0.670528765	-0.47885685	1.149385615	5
OR2I1P	-0.624836244	-0.528524653	1.153360897	5
OR2J3	-0.485069461	-0.6649512	1.150020661	5
OR2K2	-0.87730642	-0.211539134	1.088845554	5
OR2L5	-0.531659824	-0.621865629	1.153525453	5
OR2M3	-0.422451342	-0.719446746	1.141898088	5
OR2T11	-0.424011892	-0.718134063	1.142145955	5
OR2T12	-0.161461227	-0.909444979	1.070906206	5
OR2T27	-0.64644265	-0.505382275	1.151824925	5
OR2T4	-0.820142651	-0.293863702	1.114006354	5
OR2T8	-0.677002137	-0.471592185	1.148594322	5
OR2W3	-0.70938865	-0.434340407	1.143729056	5
OR3A1	-0.482615785	-0.667158675	1.149774459	5
OR4A16	-0.865563152	-0.229109898	1.094673049	5
OR4A47	-0.72736742	-0.412977398	1.140344818	5
OR4C16	-0.569229564	-0.585433077	1.154662641	5
OR4D10	-0.341216836	-0.784733564	1.1259504	5
OR4D11	-0.195175906	-0.888023418	1.083199324	5
OR4F5	-0.183112957	-0.895789577	1.078902534	5
OR4K1	-0.849247751	-0.252933275	1.102181026	5
OR4K14	-0.612049946	-0.541940915	1.153990861	5
OR4L1	-0.346100146	-0.780973521	1.127073666	5
OR4M1	-0.387989639	-0.747863997	1.135853636	5
OR4Q3	-0.701728802	-0.443290821	1.145019623	5
OR4S1	-0.529485688	-0.623926769	1.153412456	5
OR4X2	-0.242033942	-0.856768628	1.09880257	5
OR51B6	-0.828539607	-0.282251258	1.110790865	5
OR51C1P	-0.26030882	-0.84410402	1.10441284	5
OR51F1	-0.675574148	-0.47319978	1.148773928	5
OR51T1	-0.338368298	-0.7869174	1.125285697	5
OR52D1	-0.612012392	-0.541980021	1.153992413	5
OR52E2	-0.888107653	-0.195046007	1.08315366	5
OR52E4	-0.617837971	-0.535892935	1.153730906	5
OR52E5	-0.649702017	-0.501838453	1.151540471	5
OR52E6	-0.598015963	-0.556434992	1.154450955	5
OR52K2	-0.363822194	-0.767154263	1.130976457	5
OR52N5	-0.231953195	-0.8636398	1.095592995	5
OR52W1	-0.363051692	-0.767760782	1.130812474	5
OR56A1	-0.801745027	-0.318782739	1.120527767	5
OR56B2P	-0.693667698	-0.452615238	1.146282937	5
OR5D13	-0.376094177	-0.757423629	1.133517806	5
OR5D16	-0.868567582	-0.22464926	1.093216842	5
OR5H15	-0.209844182	-0.878426303	1.088270485	5

OR5H2	-0.748926372	-0.386675413	1.135601785	5
OR5I1	-0.738367571	-0.399652996	1.138020568	5
OR5J2	-0.64041823	-0.511895667	1.152313897	5
OR5K1	-0.767951716	-0.362808998	1.130760714	5
OR5L1	-0.62171587	-0.531817586	1.153533455	5
OR5M1	-0.763816777	-0.368050243	1.13186702	5
OR5M3	-0.619235048	-0.534426886	1.153661934	5
OR5P3	-0.578391556	-0.576308356	1.154699912	5
OR5T2	-0.433653945	-0.709973002	1.143626947	5
OR5V1	-0.213603537	-0.87593937	1.089542908	5
OR6B2	-0.275444949	-0.833409628	1.108854577	5
OR6B3	-0.569393983	-0.585270174	1.154664157	5
OR6C2	-0.397842513	-0.739849749	1.137692262	5
OR6C76	-0.882110019	-0.204244483	1.086354502	5
OR6P1	-0.559780705	-0.594743383	1.154524088	5
OR6T1	-0.547985251	-0.606225599	1.15421085	5
OR6X1	-0.870242271	-0.222152622	1.092394893	5
OR7A5	-0.442078607	-0.702770677	1.144849283	5
OR7E24	-0.268442111	-0.838380674	1.106822785	5
OR7G2	-0.527341535	-0.625954535	1.15329607	5
OR8D1	-0.440477144	-0.70414496	1.144622104	5
OR8D2	-0.385373267	-0.749977455	1.135350722	5
OR8D4	-0.639922434	-0.512429591	1.152352026	5
OR8J3	-0.257347045	-0.846174863	1.103521907	5
OR9G1	-0.254299549	-0.848298227	1.102597777	5
OR9K2	-0.617189918	-0.536572157	1.153762075	5
OR9Q1	-0.129976739	-0.928656202	1.058632942	5
ORMDL2	-0.76569146	-0.365677777	1.131369237	5
OSBPL7	-0.364544937	-0.766584866	1.131129803	5
OSCP1	-0.574583421	-0.580112704	1.154696126	5
OSGIN2	-0.581852281	-0.572836525	1.154688806	5
OTOF	-0.515469176	-0.637094248	1.152563424	5
OTOP1	-0.602200537	-0.5521382	1.154338737	5
OTOR	-0.74248822	-0.394610597	1.137098817	5
OTUD7A	-0.291307022	-0.822000954	1.113307976	5
P2RY4	-0.506179143	-0.645707962	1.151887105	5
P2RY8	-0.337407219	-0.78765264	1.125059859	5
P4HA2	-0.395890957	-0.741444091	1.137335048	5
PABIR1	-0.703674293	-0.441025936	1.144700229	5
PABPC1	-0.708532773	-0.435344899	1.143877672	5
PABPC3	-0.185778362	-0.894083362	1.079861724	5
PABPC4	-0.679155756	-0.46916225	1.148318006	5
PABPC5	-0.412462831	-0.727795249	1.14025808	5
PABPN1L	-0.541844862	-0.612142181	1.153987043	5
PACSIN3	-0.362905353	-0.767875917	1.13078127	5
PAGE4	-0.397263777	-0.740322913	1.13758669	5
PAK1IP1	-0.858858726	-0.238980141	1.097838868	5
PAK5	-0.722032426	-0.419369514	1.141401939	5
PALMD	-0.501752012	-0.649781358	1.151533371	5
PALS2	-0.290341783	-0.822701123	1.113042906	5
PANK1	-0.823772176	-0.288863191	1.112635367	5
PANX3	-0.140385341	-0.922389299	1.06277464	5
PAPOLB	-0.474835332	-0.674118705	1.148954037	5
PATL2	-0.408347134	-0.731208421	1.139555555	5
PAX2	-0.51719656	-0.635482694	1.152679254	5

PAXX	-0.258506373	-0.845365119	1.103871492	5
PBXIP1	-0.831183629	-0.278562585	1.109746214	5
PC	-0.611562688	-0.542448171	1.154010858	5
PCBP2	-0.236851394	-0.860311276	1.09716267	5
PCCA	-0.374372953	-0.758796435	1.133169388	5
PCDH15	-0.463497583	-0.684153765	1.147651348	5
PCDH20	-0.881579777	-0.205052835	1.086632611	5
PCGF1	-0.822685643	-0.290363135	1.113048778	5
PCGF2	-0.31874264	-0.801775051	1.120517691	5
PCP4	-0.632506799	-0.520377559	1.152884359	5
PCTP	-0.374897801	-0.758378108	1.133275909	5
PDCD2L	-0.230643928	-0.864526256	1.095170184	5
PDE12	-0.764223414	-0.367536163	1.131759578	5
PDE1B	-0.579917795	-0.574778932	1.154696727	5
PDE6C	-0.506159641	-0.64572595	1.151885591	5
PDE6H	-0.700186503	-0.445082292	1.145268795	5
PDGFB	-0.770625838	-0.359403043	1.130028881	5
PDGFC	-0.523873718	-0.629223758	1.153097476	5
PDGFD	-0.698013356	-0.447600513	1.145613869	5
PDLIM2	-0.136420645	-0.924786181	1.061206826	5
PDZD3	-0.435689638	-0.708238819	1.143928457	5
PEA15	-0.76359383	-0.368331972	1.131925802	5
PECR	-0.326880667	-0.795653913	1.122534579	5
PELI2	-0.555610881	-0.598820142	1.154431023	5
PELI3	-0.780998232	-0.346068138	1.127066371	5
PEPD	-0.256955854	-0.846447848	1.103403702	5
PERM1	-0.218208596	-0.872877757	1.091086353	5
PET117	-0.294764527	-0.819486603	1.11425113	5
PEX2	-0.788999728	-0.335643665	1.124643392	5
PFN2	-0.56138932	-0.593165468	1.154554788	5
PFN4	-0.645023319	-0.506921092	1.151944411	5
PGAM2	-0.246540555	-0.853670517	1.100211072	5
PGAM5	-0.521067995	-0.631859474	1.152927469	5
PGAP4	-0.864678254	-0.230419229	1.095097483	5
PGLYRP1	-0.672678501	-0.476450837	1.149129338	5
PGRMC1	-0.760019493	-0.372836704	1.132856197	5
PHC3	-0.355173503	-0.773932474	1.129105977	5
PHF10	-0.582689006	-0.571995026	1.154684032	5
PHF2	-0.789165116	-0.335426904	1.12459202	5
PHGR1	-0.703226165	-0.441548137	1.144774302	5
PHKG2	-0.150785427	-0.916044534	1.066829961	5
PHLDA2	-0.666404689	-0.483454617	1.149859306	5
PHLDB2	-0.535485462	-0.618226506	1.153711969	5
PHOSPHO2	-0.599657913	-0.55475152	1.154409433	5
PHOX2A	-0.852730232	-0.247903646	1.100633878	5
PHRF1	-0.871091469	-0.220883804	1.091975273	5
PHYHIP	-0.427815134	-0.714925388	1.142740522	5
PHYHIPL	-0.323230294	-0.798406536	1.121636831	5
PI4K2A	-0.444184923	-0.700959442	1.145144364	5
PI4KA	-0.352685966	-0.775869943	1.128555909	5
PIAS1	-0.523745813	-0.629344095	1.153089907	5
PIGBOS1	-0.881711253	-0.204852473	1.086563726	5
PIH1D2	-0.305529993	-0.811594152	1.117124145	5
PIK3C3	-0.780590353	-0.346596285	1.127186638	5
PIK3R1	-0.846026014	-0.257560272	1.103586286	5

PIK3R2	-0.757909034	-0.375485953	1.133394987	5
PITPNB	-0.728734278	-0.411332394	1.140066671	5
PITX3	-0.674307073	-0.474623821	1.148930894	5
PJA1	-0.432089083	-0.711303435	1.143392517	5
PJVK	-0.223941641	-0.86904274	1.092984381	5
PKIA	-0.481785454	-0.667904326	1.149689779	5
PKP3	-0.14175221	-0.921560157	1.063312366	5
PLA2G10	-0.192271486	-0.889903687	1.082175173	5
PLA2G15	-0.746162569	-0.39009043	1.136252999	5
PLA2G2A	-0.530606915	-0.622864447	1.153471362	5
PLAC9	-0.505204231	-0.646606711	1.151810942	5
PLCB3	-0.739810645	-0.397890325	1.137700971	5
PLD3	-0.498478522	-0.652780346	1.151258869	5
PLEK	-0.289004183	-0.823670123	1.112674306	5
PLEKHA1	-0.790469259	-0.333715823	1.124185082	5
PLEKHG4B	-0.484121395	-0.665804852	1.149926247	5
PLEKHN1	-0.592399243	-0.562169386	1.154568629	5
PLK1	-0.652131409	-0.499187874	1.151319283	5
PLP2	-0.81696452	-0.298219041	1.115183562	5
PLPPR2	-0.267200703	-0.839257725	1.106458429	5
PLPPR4	-0.129724159	-0.928807247	1.058531407	5
PLPPR5	-0.262585249	-0.84250753	1.105092779	5
PLS1	-0.638477925	-0.513983369	1.152461294	5
PMAIP1	-0.324275823	-0.797619294	1.121895117	5
PMCH	-0.860585074	-0.236449541	1.097034615	5
PMFBP1	-0.317384172	-0.802791385	1.120175557	5
PMVK	-0.846815952	-0.256428076	1.103244028	5
PNLIPRP3	-0.50649477	-0.645416782	1.151911553	5
PNMA2	-0.518566641	-0.634202258	1.152768899	5
PNN	-0.700364426	-0.444875806	1.145240232	5
PNRC2	-0.845692	-0.258038557	1.103730558	5
POC1B	-0.299191889	-0.816252471	1.115444359	5
POF1B	-0.738663711	-0.399291552	1.137955262	5
POLDIP2	-0.771913073	-0.357758903	1.129671976	5
POLE4	-0.781533739	-0.345374258	1.126907998	5
POLI	-0.399854348	-0.738202548	1.138056896	5
POLR1G	-0.879990777	-0.207470575	1.087461352	5
POLR2G	-0.367017731	-0.764633259	1.13165099	5
POLR3A	-0.53285063	-0.620734566	1.153585196	5
PON2	-0.768130675	-0.362581467	1.130712141	5
POP5	-0.815647748	-0.300017255	1.115665003	5
POPDC2	-0.638573048	-0.513881134	1.152454182	5
POR	-0.375677699	-0.757756043	1.133433742	5
POSTN	-0.562190497	-0.592378497	1.154568994	5
POTEE	-0.701840924	-0.443160446	1.14500137	5
POTEJ	-0.663986473	-0.486139682	1.150126155	5
POU2AF1	-0.544021485	-0.610049611	1.154071096	5
POU3F4	-0.573035227	-0.581654588	1.154689815	5
POU6F1	-0.252841045	-0.849311795	1.102152839	5
PPARG	-0.671139907	-0.478173507	1.149313414	5
PPFIA1	-0.699189679	-0.446238274	1.145427953	5
PPFIA2	-0.179449603	-0.898125582	1.077575185	5
PPFIA3	-0.61548043	-0.538361363	1.153841793	5
PPM1G	-0.693501378	-0.452806613	1.146307991	5
PPM1K	-0.834902466	-0.273347873	1.108250339	5

PPM1N	-0.823679838	-0.288990762	1.1126706	5
PPP1R18	-0.823571054	-0.28914103	1.112712085	5
PPP1R27	-0.607273108	-0.546901235	1.154174342	5
PPP1R3A	-0.294743889	-0.819501641	1.11424553	5
PPP1R3G	-0.857029071	-0.241654009	1.09868308	5
PPP1R42	-0.434053023	-0.70963334	1.143686363	5
PPP2CA	-0.317822633	-0.802463522	1.120286155	5
PPP4R2	-0.83092016	-0.278930852	1.109851012	5
PPP4R3C	-0.495838843	-0.655190694	1.151029537	5
PRADC1	-0.691594616	-0.454997713	1.146592329	5
PRAMEF10	-0.501856761	-0.649685213	1.151541973	5
PRAMEF33	-0.516997853	-0.635668236	1.152666089	5
PRDX6	-0.676010881	-0.472708422	1.148719303	5
PRICKLE4	-0.630151314	-0.522887344	1.153038658	5
PRIM1	-0.403187627	-0.735465311	1.138652938	5
PRKAA2	-0.436225047	-0.707782061	1.144007108	5
PRKACB	-0.548655614	-0.605577167	1.154232781	5
PRKAG2	-0.70290515	-0.441922029	1.144827179	5
PRM3	-0.184078386	-0.895172213	1.079250599	5
PRNP	-0.215188598	-0.874887457	1.090076055	5
PRODH2	-0.265051198	-0.840773377	1.105824575	5
PROSER1	-0.1735201	-0.901884518	1.075404618	5
PROSER2	-0.855858962	-0.243359635	1.099218597	5
PRPF3	-0.578096017	-0.5766042	1.154700217	5
PRPF31	-0.64700793	-0.504768667	1.151776598	5
PRPF38B	-0.440765723	-0.703897498	1.144663221	5
PRPS1L1	-0.607888555	-0.546263714	1.154152269	5
PRR11	-0.171770362	-0.902988525	1.074758887	5
PRR23D1	-0.639370964	-0.513023097	1.152394061	5
PRR23D2	-0.63622821	-0.516397877	1.152626087	5
PRR29	-0.171905886	-0.902903101	1.074808986	5
PRR3	-0.444281061	-0.700876671	1.145157732	5
PRR33	-0.366602212	-0.764961576	1.131563788	5
PRR4	-0.840976704	-0.264762433	1.105739136	5
PRRG2	-0.23524413	-0.86140559	1.09664972	5
PRRT3	-0.823330116	-0.289473759	1.112803875	5
PRSS41	-0.197946804	-0.886223429	1.084170234	5
PRUNE2	-0.272484527	-0.835516007	1.108000534	5
PSD3	-0.442410474	-0.702485582	1.144896056	5
PSG11	-0.257875538	-0.845805867	1.103681404	5
PSG2	-0.854505803	-0.245327855	1.099833658	5
PSMA8	-0.411085084	-0.728939555	1.140024639	5
PSMB4	-0.539339951	-0.614543977	1.153883928	5
PSMC2	-0.343515917	-0.782965882	1.126481799	5
PSMD11	-0.143526222	-0.920481903	1.064008125	5
PSMD12	-0.65823985	-0.492488391	1.150728241	5
PSMG1	-0.743016535	-0.393962059	1.136978594	5
PSPC1	-0.781074997	-0.345968704	1.127043701	5
PTF1A	-0.453162041	-0.693192388	1.146354429	5
PTGS2	-0.46077331	-0.686546258	1.147319568	5
PTH1R	-0.134583502	-0.925892754	1.060476256	5
PTK7	-0.38983161	-0.746372401	1.136204011	5
PTP4A3	-0.285110391	-0.826482497	1.111592888	5
PTPN22	-0.521598717	-0.631361549	1.152960265	5
PTPN9	-0.184753506	-0.894740062	1.079493568	5

PTRH2	-0.405870664	-0.733254687	1.139125352	5
PURG	-0.566762816	-0.587873396	1.154636212	5
PUS7	-0.610817311	-0.543223574	1.154040884	5
PWWP3A	-0.772853616	-0.356555673	1.129409288	5
QRFP	-0.336740507	-0.78816222	1.124902727	5
QRICH1	-0.823097347	-0.289795085	1.112892432	5
QSOX1	-0.134171757	-0.926140407	1.060312164	5
R3HCC1L	-0.856161303	-0.24291925	1.099080553	5
RAB11FIP3	-0.317041138	-0.803047779	1.120088917	5
RAB15	-0.260129349	-0.844229707	1.104359056	5
RAB17	-0.529258559	-0.624141801	1.15340036	5
RAB19	-0.738733744	-0.399206054	1.137939797	5
RAB20	-0.447461552	-0.698133433	1.145594985	5
RAB34	-0.784172438	-0.341947288	1.126119727	5
RAB6B	-0.47971903	-0.669757019	1.149476049	5
RAB9A	-0.28478619	-0.826716094	1.111502284	5
RABAC1	-0.226850155	-0.867087164	1.093937319	5
RAD51AP2	-0.31544354	-0.804240563	1.119684103	5
RALY	-0.484902571	-0.665101535	1.150004106	5
RALYL	-0.794344972	-0.328611263	1.122956234	5
RAMACL	-0.797198621	-0.32483401	1.122032631	5
RAMP2	-0.708110647	-0.435839912	1.143950559	5
RANBP6	-0.29381351	-0.820179186	1.113992695	5
RANBP9	-0.130556057	-0.928309581	1.058865637	5
RANGRF	-0.655123203	-0.495912862	1.151036065	5
RAP2A	-0.61067175	-0.543374918	1.154046668	5
RAP2C	-0.490875351	-0.659703774	1.150579124	5
RAPGEF2	-0.188969207	-0.892033506	1.081002712	5
RAPGEF4	-0.835948756	-0.271875068	1.107823824	5
RARRES1	-0.780281702	-0.346995736	1.127277439	5
RASA1	-0.48623875	-0.663897113	1.150135862	5
RASA3	-0.851609495	-0.249525506	1.101135001	5
RASAL1	-0.373615442	-0.759399775	1.133015217	5
RASSF6	-0.152762769	-0.914828811	1.06759158	5
RASSF7	-0.756409936	-0.377363035	1.133772971	5
RBBP4	-0.732769617	-0.40645833	1.139227947	5
RBBP6	-0.784379282	-0.341678097	1.126057379	5
RBBP7	-0.568009258	-0.586641173	1.154650431	5
RBFA	-0.468195598	-0.680010871	1.14820647	5
RBIS	-0.497898723	-0.653310382	1.151209106	5
RBL1	-0.617732263	-0.536003763	1.153736026	5
RBM14	-0.299756775	-0.815838655	1.11559543	5
RBM20	-0.690411301	-0.456354813	1.146766114	5
RBM44	-0.40321295	-0.735444478	1.138657427	5
RBMXL1	-0.835366063	-0.2726956	1.108061663	5
RCC1	-0.843773147	-0.260781104	1.104554251	5
RCCD1	-0.774334197	-0.354658294	1.128992491	5
RCL1	-0.599397989	-0.555018233	1.154416221	5
REELD1	-0.537332843	-0.616463547	1.15379639	5
REG1A	-0.237869699	-0.859616889	1.097486588	5
RERG	-0.25943643	-0.844714725	1.104151155	5
RESP18	-0.726954883	-0.413473296	1.140428179	5
RETN	-0.655695432	-0.495285096	1.150980527	5
REV1	-0.610492298	-0.543561465	1.154053763	5
RFPL3	-0.663534835	-0.486640269	1.150175104	5

RFWD3	-0.32601792	-0.796305502	1.122323422	5
RFX3	-0.533934809	-0.619703456	1.153638265	5
RFXAP	-0.564881691	-0.589729727	1.154611417	5
RGMA	-0.482217222	-0.667516677	1.149733898	5
RGPD1	-0.257807206	-0.845853589	1.103660795	5
RGPD2	-0.18074167	-0.897302865	1.078044534	5
RGS17	-0.199089382	-0.885479453	1.084568835	5
RGS2	-0.13530721	-0.925457148	1.060764359	5
RGS21	-0.769140957	-0.361295891	1.130436848	5
RGS9	-0.654722098	-0.496352634	1.151074732	5
RHBG	-0.430862508	-0.712344648	1.143207156	5
RHEB	-0.492190142	-0.658510726	1.150700868	5
RHOQ	-0.831973954	-0.277456968	1.109430922	5
RHOXF2	-0.370506412	-0.761870729	1.132377141	5
RHOXF2B	-0.816728449	-0.298541697	1.115270145	5
RIF1	-0.693029774	-0.453349038	1.146378812	5
RIN3	-0.707700104	-0.436321082	1.144021187	5
RIPOR3	-0.783682663	-0.342584371	1.126267034	5
RLN1	-0.647999138	-0.5036917	1.151690839	5
RNASE13	-0.852121195	-0.248785386	1.100906581	5
RNASE9	-0.447804902	-0.697836708	1.14564161	5
RNF103	-0.716109411	-0.426413576	1.142522986	5
RNF126	-0.200662009	-0.884453779	1.085115787	5
RNF139	-0.638139259	-0.514347261	1.152486521	5
RNF141	-0.28808137	-0.824337776	1.112419146	5
RNF152	-0.374168764	-0.758959117	1.133127881	5
RNF19A	-0.438255763	-0.706047191	1.144302954	5
RPA1	-0.537900445	-0.615921143	1.153821588	5
RPL10A	-0.813983376	-0.302284913	1.11626829	5
RPL14	-0.551362728	-0.602953576	1.154316303	5
RPL30	-0.5545744	-0.599830481	1.154404881	5
RPL36AL	-0.814298669	-0.301855784	1.116154453	5
RPL39L	-0.83264318	-0.276519663	1.109162842	5
RPL5	-0.670305124	-0.479106783	1.149411907	5
RPLP0	-0.66496738	-0.485051501	1.15001888	5
RPP21	-0.530435123	-0.623027301	1.153462424	5
RPP30	-0.878707509	-0.209418035	1.088125544	5
RPS29	-0.192519382	-0.889743461	1.082262843	5
RPS3	-0.661754073	-0.488611316	1.150365389	5
RPS4X	-0.738459657	-0.399540619	1.138000276	5
RPS6KL1	-0.878675282	-0.209466883	1.088142166	5
RPS9	-0.308696156	-0.809254544	1.1179507	5
RPSA	-0.758413685	-0.374853176	1.133266861	5
RPTN	-0.881702538	-0.204865756	1.086568294	5
RPUSD4	-0.6978734	-0.447762451	1.145635852	5
RRP12	-0.606071151	-0.54814497	1.15421612	5
RRS1	-0.52565764	-0.627543594	1.153201233	5
RSPH3	-0.473982362	-0.674878076	1.148860437	5
RTCA	-0.304688633	-0.812214457	1.11690309	5
RTL8B	-0.829964649	-0.280265121	1.11022977	5
RTTN	-0.364422972	-0.766680986	1.131103958	5
RUFY4	-0.197806316	-0.886314836	1.084121152	5
RUNX1	-0.269044948	-0.837954319	1.106999266	5
RUVBL2	-0.866724036	-0.227389133	1.094113169	5
RXRG	-0.535864086	-0.617865485	1.153729571	5

RXYLT1	-0.500788692	-0.650665039	1.151453731	5
S100A1	-0.623885759	-0.529528997	1.153414756	5
S100A13	-0.633789674	-0.519007661	1.152797335	5
S100A6	-0.858101926	-0.240087149	1.098189075	5
S100A7A	-0.212036627	-0.876977288	1.089013914	5
S100P	-0.76760169	-0.363253861	1.130855551	5
S1PR1	-0.158696721	-0.911162373	1.069859094	5
S1PR5	-0.535830818	-0.617897213	1.153728031	5
SAA2-SAA4	-0.464531617	-0.683243767	1.147775384	5
SAA4	-0.579829276	-0.574867709	1.154696985	5
SAMD11	-0.587372249	-0.567269961	1.15464221	5
SAMD13	-0.464901264	-0.682918207	1.147819471	5
SARNP	-0.737529561	-0.400675022	1.138204583	5
SART1	-0.413830178	-0.726657853	1.140488031	5
SATB1	-0.726442782	-0.414088498	1.14053128	5
SCAF4	-0.862754295	-0.233259047	1.096013341	5
SCAPER	-0.756793179	-0.376883533	1.133676712	5
SCARA3	-0.678345565	-0.47007716	1.148422726	5
SCG3	-0.709042517	-0.434746777	1.143789294	5
SCGB1D1	-0.1821987	-0.896373549	1.078572248	5
SCGB1D2	-0.24879099	-0.852117323	1.100908312	5
SCGB2A2	-0.598247846	-0.556197441	1.154445287	5
SCN1B	-0.177783067	-0.899184823	1.07696789	5
SCP2D1	-0.466428377	-0.681571812	1.148000189	5
SCRT2	-0.367067843	-0.764593654	1.131661497	5
SDAD1	-0.681578332	-0.466420989	1.14799932	5
SDC2	-0.622684727	-0.53079646	1.153481187	5
SDF2L1	-0.34871242	-0.778953674	1.127666095	5
SDF4	-0.615587215	-0.538249704	1.153836919	5
SEC22A	-0.479151887	-0.670264758	1.149416645	5
SELENON	-0.305633954	-0.811517464	1.117151418	5
SEMA6A	-0.730942672	-0.408668256	1.139610928	5
SEMG1	-0.437129817	-0.707009585	1.144139402	5
SEMG2	-0.758748717	-0.374432837	1.133181554	5
SERAC1	-0.362057045	-0.768542971	1.130600017	5
SERPINA3	-0.422789222	-0.719162725	1.141951947	5
SERPINA7	-0.498900261	-0.652394588	1.15129485	5
SERPINC1	-0.548643483	-0.605588905	1.154232388	5
SERPINE1	-0.434140325	-0.709559015	1.143699341	5
SERPINE3	-0.321072145	-0.80002861	1.121100754	5
SERTAD1	-0.54417307	-0.609903688	1.154076757	5
SERTAD3	-0.54293212	-0.611097555	1.154029675	5
SETSIP	-0.668548861	-0.481067102	1.149615963	5
SF3A3	-0.190904444	-0.8907864	1.081690844	5
SFMBT1	-0.188065897	-0.892614614	1.080680512	5
SFR1	-0.436875654	-0.707226662	1.144102317	5
SFTA2	-0.239990877	-0.858167781	1.098158658	5
SFXN2	-0.496668742	-0.654433661	1.151102404	5
SFXN4	-0.782166329	-0.34455389	1.126720219	5
SGCD	-0.243824515	-0.855539636	1.099364151	5
SGK1	-0.806773143	-0.312041847	1.11881499	5
SGMS1	-0.713744637	-0.429210657	1.142955294	5
SGSH	-0.784637263	-0.34134224	1.125979503	5
SH3BP5	-0.319321035	-0.801341854	1.120662889	5
SHCBP1	-0.562815074	-0.591764493	1.154579567	5

SHLD1	-0.134884595	-0.925711572	1.060596168	5
SHMT1	-0.865288433	-0.229516599	1.094805032	5
SI	-0.465301342	-0.682565695	1.147867037	5
SIAH3	-0.731339239	-0.408189016	1.139528255	5
SIDT2	-0.605721868	-0.548506063	1.154227931	5
SIGLEC1	-0.543092818	-0.610943049	1.154035867	5
SIGLEC15	-0.709290134	-0.434456085	1.14374622	5
SIK3	-0.581079739	-0.573612751	1.154692491	5
SIRPA	-0.325354301	-0.79680627	1.122160571	5
SLC10A6	-0.354772802	-0.774244932	1.129017734	5
SLC12A2	-0.580524063	-0.574170649	1.154694712	5
SLC12A5	-0.590064015	-0.564542506	1.154606521	5
SLC15A5	-0.510465204	-0.641745077	1.152210281	5
SLC16A7	-0.42974374	-0.713293115	1.143036854	5
SLC17A1	-0.439200411	-0.705238834	1.144439245	5
SLC17A3	-0.725870925	-0.414774991	1.140645916	5
SLC18A2	-0.15174077	-0.915457539	1.06719831	5
SLC18A3	-0.550078337	-0.604199347	1.154277684	5
SLC18B1	-0.81103087	-0.306293314	1.117324184	5
SLC22A9	-0.828060432	-0.28291809	1.110978522	5
SLC24A2	-0.378046378	-0.755863419	1.133909797	5
SLC25A3	-0.75842355	-0.374840803	1.133264352	5
SLC25A31	-0.877050259	-0.211926354	1.088976613	5
SLC25A33	-0.468019281	-0.680166745	1.148186026	5
SLC25A40	-0.833245388	-0.275675358	1.108920746	5
SLC25A47	-0.290214791	-0.822793184	1.113007975	5
SLC25A53	-0.838720423	-0.26796143	1.106681853	5
SLC26A2	-0.196121386	-0.887409906	1.083531292	5
SLC26A7	-0.199283631	-0.885352868	1.084636499	5
SLC27A6	-0.781837695	-0.34498017	1.126817865	5
SLC2A13	-0.474829616	-0.674123796	1.148953412	5
SLC2A4	-0.48324432	-0.666593781	1.149838101	5
SLC2A5	-0.132532744	-0.927124943	1.059657687	5
SLC30A10	-0.534308521	-0.619347743	1.153656265	5
SLC30A3	-0.401660718	-0.73672044	1.138381158	5
SLC35F4	-0.881920292	-0.204533811	1.086454103	5
SLC35G4	-0.591908689	-0.562668435	1.154577124	5
SLC35G5	-0.71528478	-0.427389942	1.142674722	5
SLC37A4	-0.422551476	-0.719362584	1.141914061	5
SLC44A3	-0.159286538	-0.910796456	1.070082994	5
SLC48A1	-0.345770385	-0.781228077	1.126998462	5
SLC4A3	-0.550417422	-0.603870635	1.154288057	5
SLC5A10	-0.52432119	-0.628802629	1.153123819	5
SLC5A3	-0.199754067	-0.885046179	1.084800246	5
SLC6A14	-0.43057577	-0.71258785	1.14316362	5
SLC6A19	-0.657011005	-0.49384017	1.150851175	5
SLC7A3	-0.471400817	-0.677171955	1.148572772	5
SLC7A6OS	-0.491675727	-0.658977717	1.150653444	5
SLC9A1	-0.584294408	-0.570378178	1.154672585	5
SLC9A5	-0.840897615	-0.264874766	1.105772381	5
SLCO1B1	-0.522315179	-0.630688889	1.153004067	5
SLCO1B3-SLCO1B7	-0.602541611	-0.55178705	1.154328661	5
SLFN14	-0.208243534	-0.879481798	1.087725332	5
SLN	-0.345512622	-0.781426989	1.126939612	5
SLX1A	-0.854634117	-0.245141412	1.099775529	5

SLX1B	-0.872360008	-0.21898487	1.091344878	5
SMAD6	-0.724083479	-0.416917397	1.141000876	5
SMC4	-0.770895233	-0.359059203	1.129954436	5
SMCO1	-0.655717845	-0.495260499	1.150978343	5
SMCO2	-0.172784852	-0.902348716	1.075133568	5
SMIM21	-0.840063353	-0.266058822	1.106122175	5
SMIM27	-0.731213809	-0.408340622	1.139554431	5
SMIM33	-0.303263485	-0.813263819	1.116527304	5
SMIM34	-0.48266925	-0.667110639	1.149779888	5
SMIM41	-0.594213423	-0.560321306	1.154534729	5
SMIM42	-0.704665857	-0.439869395	1.144535253	5
SMIM43	-0.372115269	-0.76059313	1.132708399	5
SMIM6	-0.682244105	-0.46566617	1.147910275	5
SNAP29	-0.709223153	-0.434534728	1.143757881	5
SNCA	-0.833947345	-0.274690168	1.108637514	5
SNORC	-0.353425847	-0.775294234	1.128720081	5
SNRNP25	-0.450856122	-0.695194863	1.146050985	5
SNRNP27	-0.603306239	-0.550999322	1.154305561	5
SNRNP48	-0.726140975	-0.414450872	1.140591846	5
SNRPB2	-0.623485425	-0.529951676	1.153437101	5
SNRPC	-0.631815462	-0.521114919	1.152930381	5
SNRPD3	-0.623402908	-0.530038773	1.153441682	5
SNRPE	-0.588754502	-0.565870447	1.154624949	5
SNRPF	-0.172964426	-0.902235381	1.075199807	5
SNX21	-0.427480275	-0.71520844	1.142688716	5
SOD1	-0.669212868	-0.480326449	1.149539317	5
SOD3	-0.457111847	-0.689750436	1.146862283	5
SOS2	-0.513938835	-0.638519363	1.152458198	5
SOX10	-0.776328559	-0.352096132	1.128424691	5
SOX14	-0.193531806	-0.88908859	1.082620395	5
SP100	-0.173393691	-0.901964356	1.075358047	5
SP4	-0.337608625	-0.787498627	1.125107252	5
SPACA6	-0.385312723	-0.750026289	1.135339012	5
SPACA9	-0.17975399	-0.897931882	1.077685872	5
SPAG6	-0.172441656	-0.902565249	1.075006905	5
SPAM1	-0.827115878	-0.284231065	1.111346943	5
SPANXN1	-0.424214605	-0.717963381	1.142177986	5
SPANXN2	-0.401313646	-0.737005439	1.138319085	5
SPATA1	-0.838790388	-0.26786241	1.106652798	5
SPATA12	-0.65487133	-0.496189041	1.151060371	5
SPATA31A3	-0.763752221	-0.368131828	1.13188405	5
SPATA31D4	-0.429093524	-0.713843814	1.142937338	5
SPATA32	-0.874723494	-0.215435393	1.090158887	5
SPATA4	-0.499717256	-0.651646778	1.151364035	5
SPEM3	-0.225448231	-0.868030605	1.093478836	5
SPEN	-0.687904061	-0.459223543	1.147127604	5
SPI1	-0.418026317	-0.723156622	1.141182938	5
SPIC	-0.661330296	-0.489079739	1.150410035	5
SPINK14	-0.377768024	-0.756086088	1.133854112	5
SPINK7	-0.452450883	-0.693810508	1.146261391	5
SPINK9	-0.526617886	-0.626637798	1.153255684	5
SPOCK1	-0.456479373	-0.690302603	1.146781975	5
SPOCK3	-0.352603428	-0.775934138	1.128537565	5
SPOP	-0.712732203	-0.430405533	1.143137736	5
SPRTN	-0.617730793	-0.536005304	1.153736097	5

SPRY1	-0.49073236	-0.659833418	1.150565779	5
SPTA1	-0.866370782	-0.227913127	1.094283909	5
SPTBN1	-0.882531414	-0.20360151	1.086132924	5
SPTLC1	-0.174016759	-0.901570715	1.075587474	5
SREBF2	-0.353073411	-0.775568528	1.128641938	5
SRRT	-0.221136181	-0.870922659	1.09205884	5
SRXN1	-0.767248221	-0.363702877	1.130951098	5
SSC4D	-0.480208647	-0.669318429	1.149527075	5
SSU72P8	-0.60665599	-0.547540022	1.154196012	5
SSX2	-0.432834946	-0.710669595	1.143504541	5
SSX2B	-0.503040489	-0.648597915	1.151638403	5
ST13	-0.875191922	-0.214730127	1.089922049	5
STAM2	-0.298873543	-0.816485562	1.115359105	5
STARD4	-0.560562946	-0.593976434	1.15453938	5
STC1	-0.49511464	-0.655850738	1.150965378	5
STEAP1B	-0.137732762	-0.923994266	1.061727028	5
STK16	-0.196650149	-0.887066492	1.083716641	5
STK26	-0.399686568	-0.738340059	1.138026627	5
STK4	-0.42727237	-0.715384128	1.142656498	5
STPG1	-0.275653754	-0.833260789	1.108914544	5
STUM	-0.43896827	-0.705437561	1.144405831	5
STX19	-0.822698289	-0.290345692	1.113043981	5
STX2	-0.584787966	-0.569880495	1.154668461	5
STX8	-0.670194646	-0.479230223	1.149424869	5
SUCNR1	-0.456153255	-0.69058716	1.146740416	5
SUGCT	-0.144978352	-0.919597493	1.064575845	5
SUMO4	-0.663047767	-0.487179814	1.150227581	5
SUSD5	-0.539811508	-0.614092353	1.15390386	5
SUSD6	-0.751727945	-0.383200466	1.134928411	5
SUV39H2	-0.366626235	-0.764942598	1.131568833	5
SYNM	-0.488474296	-0.661877992	1.150352288	5
SYNPO	-0.303595664	-0.813019381	1.116615045	5
SYT6	-0.502379755	-0.649205001	1.151584755	5
SZRD1	-0.694736071	-0.451384958	1.146121029	5
TAAR1	-0.72249488	-0.418817212	1.141312092	5
TAAR2	-0.502754105	-0.648861101	1.151615206	5
TAAR5	-0.619053361	-0.534617681	1.153671041	5
TAC1	-0.50732528	-0.644650109	1.151975389	5
TAC3	-0.452384122	-0.69386851	1.146252632	5
TAF11L11	-0.329003498	-0.794047949	1.123051447	5
TAF13	-0.879602522	-0.208060261	1.087662783	5
TAFA4	-0.491524955	-0.659114538	1.150639493	5
TAL2	-0.605303835	-0.548938037	1.154241872	5
TAMALIN	-0.428494349	-0.714350932	1.142845282	5
TAPBPL	-0.851466265	-0.249732558	1.101198823	5
TARBP1	-0.35263906	-0.775906425	1.128545485	5
TARS2	-0.287538892	-0.824729929	1.112268821	5
TAS1R2	-0.130112313	-0.928575108	1.058687421	5
TAS2R13	-0.466645669	-0.681380049	1.148025718	5
TAS2R16	-0.648389006	-0.503267745	1.151656751	5
TAS2R3	-0.349752594	-0.778147754	1.127900348	5
TAS2R38	-0.885177156	-0.199553191	1.084730347	5
TAS2R40	-0.886593092	-0.197378501	1.083971594	5
TAS2R41	-0.229908176	-0.865023806	1.094931982	5
TASL	-0.484441041	-0.665517139	1.14995818	5

TAT	-0.392633949	-0.744097262	1.136731211	5
TATDN1	-0.689373505	-0.457543344	1.146916849	5
TBC1D10A	-0.142818512	-0.920912342	1.063730854	5
TBC1D23	-0.300707562	-0.815141544	1.115849105	5
TBC1D25	-0.305364682	-0.811716077	1.117080759	5
TBC1D3B	-0.684248708	-0.46338963	1.147638338	5
TBL2	-0.403256099	-0.735408977	1.138665076	5
TBX22	-0.534899125	-0.61878528	1.153684404	5
TCAIM	-0.646789719	-0.505005584	1.151795303	5
TCAP	-0.573343103	-0.581348186	1.154691289	5
TCF4	-0.17070222	-0.903661309	1.074363528	5
TCFL5	-0.395689509	-0.741608469	1.137297978	5
TCP1	-0.545282941	-0.6088345	1.15411744	5
TCP11X1	-0.696280524	-0.449603478	1.145884002	5
TCP11X2	-0.840829309	-0.264971773	1.105801082	5
TDO2	-0.81974405	-0.294411135	1.114155185	5
TEKT4	-0.507839692	-0.64417488	1.152014571	5
TENT5A	-0.796817464	-0.325339461	1.122156925	5
TENT5D	-0.861772608	-0.234704395	1.096477003	5
TERF2IP	-0.641272532	-0.510974914	1.152247446	5
TEX14	-0.517799193	-0.634919735	1.152718928	5
TEX26	-0.533262908	-0.620342617	1.153605525	5
TEX30	-0.609337248	-0.544761237	1.154098485	5
TEX43	-0.820389567	-0.29352442	1.113913988	5
TEX47	-0.675997533	-0.472723443	1.148720976	5
TEX55	-0.645059807	-0.506881565	1.151941372	5
TFAP2B	-0.875913542	-0.213642493	1.089556035	5
TFF3	-0.439391498	-0.705075214	1.144466712	5
TFG	-0.205221835	-0.881468855	1.08669069	5
TFPI2	-0.765932226	-0.365372626	1.131304852	5
TGFA	-0.292871289	-0.820864622	1.113735911	5
THEMIS	-0.743468785	-0.393406525	1.13687531	5
THNSL1	-0.641707341	-0.510505919	1.15221326	5
THOC2	-0.686728959	-0.460564934	1.147293893	5
THOP1	-0.375363885	-0.758006413	1.133370298	5
THRSP	-0.75266739	-0.382032213	1.134699603	5
THSD1	-0.175480468	-0.900644789	1.076125257	5
THTPA	-0.489866098	-0.660618388	1.150484486	5
THUMPD2	-0.178803469	-0.898536518	1.077339986	5
TIAM2	-0.845373277	-0.258494701	1.103867978	5
TIFA	-0.842449158	-0.262668367	1.105117525	5
TIMD4	-0.58184025	-0.572848618	1.154688869	5
TIMM10	-0.698517944	-0.447016428	1.145534371	5
TIMP3	-0.563782019	-0.590813051	1.154595069	5
TJP1	-0.466892886	-0.68116182	1.148054706	5
TJP3	-0.276131807	-0.832919894	1.109051701	5
TLCD1	-0.734926875	-0.403841863	1.138768738	5
TLL1	-0.419702413	-0.721753546	1.141455958	5
TM4SF18	-0.814775734	-0.301206078	1.115981811	5
TM4SF4	-0.625820353	-0.527483579	1.153303932	5
TMCO1	-0.427932586	-0.714826082	1.142758668	5
TMEFF1	-0.787627187	-0.337440507	1.125067695	5
TMEM104	-0.287048916	-0.82508392	1.112132836	5
TMEM107	-0.564371727	-0.590232279	1.154604006	5
TMEM120A	-0.144960763	-0.919608215	1.064568978	5

TMEM125	-0.382585477	-0.752222638	1.134808114	5
TMEM126B	-0.722433374	-0.418890687	1.141324061	5
TMEM130	-0.611087915	-0.542942147	1.154030062	5
TMEM132D	-0.728046438	-0.412160579	1.140207017	5
TMEM160	-0.247458998	-0.853037122	1.100496121	5
TMEM167B	-0.165189824	-0.90711931	1.072309134	5
TMEM170A	-0.255107383	-0.847736095	1.102843478	5
TMEM182	-0.683100201	-0.464694643	1.147794844	5
TMEM184A	-0.610253408	-0.543809738	1.154063147	5
TMEM196	-0.720947806	-0.420663534	1.14161134	5
TMEM198	-0.449345055	-0.69650431	1.145849365	5
TMEM225	-0.194000002	-0.888785473	1.082785474	5
TMEM242	-0.872044675	-0.219457306	1.091501981	5
TMEM244	-0.284472238	-0.826942223	1.111414461	5
TMEM252	-0.655905569	-0.495054453	1.150960022	5
TMEM26	-0.637136725	-0.515423601	1.152560326	5
TMEM266	-0.1485388	-0.917422169	1.06596097	5
TMEM267	-0.496469917	-0.654615093	1.15108501	5
TMEM37	-0.504226614	-0.647506969	1.151733583	5
TMEM60	-0.700836525	-0.444327686	1.145164212	5
TMEM62	-0.589200926	-0.565417967	1.154618894	5
TMEM67	-0.861749555	-0.234738306	1.096487862	5
TMEM70	-0.250040199	-0.851253389	1.101293588	5
TMEM81	-0.83601796	-0.271777566	1.107795525	5
TMEM91	-0.720893727	-0.420728005	1.141621732	5
TMF1	-0.374106374	-0.759008817	1.133115191	5
TMIE	-0.866737462	-0.227369211	1.094106674	5
TMIGD1	-0.454302616	-0.692200012	1.146502628	5
TMPO	-0.179257121	-0.898248032	1.077505154	5
TMPRSS11A	-0.778706897	-0.349031056	1.127737953	5
TMPRSS11E	-0.807247879	-0.311402735	1.118650615	5
TMPRSS13	-0.704595656	-0.439951326	1.144546981	5
TMPRSS5	-0.246477921	-0.853713687	1.100191608	5
TMSB4Y	-0.595314648	-0.559197595	1.154512243	5
TNC	-0.151135111	-0.915829758	1.066964869	5
TNFRSF10A	-0.649815724	-0.501714569	1.151530293	5
TNFRSF13B	-0.679963031	-0.468249706	1.148212737	5
TNFRSF18	-0.694732064	-0.451389575	1.146121639	5
TNFSF18	-0.521990355	-0.630993921	1.152984276	5
TNFSF4	-0.41746058	-0.723629618	1.141090198	5
TNIK	-0.302658272	-0.813708935	1.116367207	5
TNNI2	-0.348897805	-0.778810108	1.127707913	5
TNNT2	-0.801115356	-0.3196233	1.120738655	5
TNS1	-0.461921054	-0.685539176	1.14746023	5
TOB2	-0.41691248	-0.724087586	1.141000066	5
TOE1	-0.329747572	-0.793484134	1.123231707	5
TOMM70	-0.3808861	-0.753587847	1.134473947	5
TOPORS	-0.264631733	-0.84106871	1.105700443	5
TOR1AIP2	-0.458530533	-0.688510476	1.147041009	5
TP53BP1	-0.340663439	-0.785158373	1.125821812	5
TP53BP2	-0.827201723	-0.284111817	1.11131354	5
TP53I13	-0.272541845	-0.835475293	1.108017138	5
TPBG	-0.641041274	-0.511224254	1.152265528	5
TPTEP2-CSNK1E	-0.649361195	-0.502209679	1.151570874	5
TRADD	-0.330638894	-0.792808123	1.123447018	5

TRAF6	-0.884251755	-0.200971413	1.085223168	5
TRAPPC3L	-0.245994835	-0.854046545	1.100041381	5
TRAT1	-0.78850746	-0.336288526	1.124795987	5
TRIB2	-0.834844396	-0.273429542	1.108273937	5
TRIM39-RPP21	-0.753528319	-0.380960265	1.134488585	5
TRIM42	-0.59931363	-0.555104777	1.154418407	5
TRIM48	-0.428644835	-0.714223598	1.142868434	5
TRIM59	-0.83971353	-0.266554839	1.106268369	5
TRIM64C	-0.404436632	-0.734437041	1.138873673	5
TRIP12	-0.526469799	-0.626777552	1.153247351	5
TRMT2A	-0.721901667	-0.419525615	1.141427282	5
TRPC3	-0.539399442	-0.614487014	1.153886455	5
TRPC4	-0.487944258	-0.662357171	1.150301429	5
TRPM1	-0.61835703	-0.535348535	1.153705565	5
TSN	-0.649611013	-0.501937591	1.151548604	5
TSNAX	-0.619881602	-0.533747589	1.153629191	5
TSPAN1	-0.689189829	-0.457753536	1.146943364	5
TSPAN12	-0.612414082	-0.541561644	1.153975726	5
TSPAN16	-0.213639095	-0.875915795	1.08955489	5
TSPAN19	-0.481555615	-0.668110603	1.149666218	5
TSPAN2	-0.7351762	-0.403538976	1.138715175	5
TSPYL4	-0.534620661	-0.619050522	1.153671183	5
TSR3	-0.859284437	-0.238356802	1.09764124	5
TSTD1	-0.869202557	-0.223703504	1.092906061	5
TTC26	-0.373313061	-0.759640473	1.132953534	5
TTC29	-0.828899372	-0.281750266	1.110649638	5
TTC39A	-0.163525178	-0.908158942	1.07168412	5
TTC8	-0.326644862	-0.795832067	1.122476929	5
TTLL6	-0.534936065	-0.618750087	1.153686152	5
TTR	-0.527990017	-0.62534177	1.153331788	5
TUBA1B	-0.513123695	-0.639277455	1.15240115	5
TUBA8	-0.785520009	-0.340192066	1.125712074	5
TUBB4A	-0.279542327	-0.830482445	1.110024772	5
TUBD1	-0.637351896	-0.515192699	1.152544595	5
TUBGCP5	-0.316578367	-0.80339351	1.119971877	5
TULP3	-0.482285987	-0.667454921	1.149740908	5
TXLNG	-0.639834051	-0.512524738	1.152358789	5
TXNDC17	-0.317673145	-0.802575322	1.120248466	5
TXNRD1	-0.408565657	-0.731027589	1.139593246	5
TXNRD2	-0.78006687	-0.347273664	1.127340534	5
TYRP1	-0.794875186	-0.327910653	1.12278584	5
UBC	-0.333474579	-0.790652928	1.124127507	5
UBE2J1	-0.199147065	-0.885441866	1.084588931	5
UBE2L6	-0.65787589	-0.492888974	1.150764864	5
UBE3A	-0.399752555	-0.73828598	1.138038535	5
UBL4A	-0.210487399	-0.878001586	1.088488985	5
UBN1	-0.362805105	-0.767954778	1.130759884	5
UCHL3	-0.293980598	-0.820057557	1.114038155	5
UCN	-0.63502266	-0.517689045	1.152711705	5
UGT1A10	-0.705881847	-0.438449065	1.144330912	5
UGT1A4	-0.424928739	-0.717361783	1.142290523	5
UGT1A8	-0.616628293	-0.537160372	1.153788665	5
UGT1A9	-0.857164076	-0.241456998	1.098621074	5
UGT2B17	-0.462386285	-0.685130595	1.14751688	5
UGT3A1	-0.608478913	-0.545651747	1.154130661	5

UHRF1	-0.707304424	-0.43678459	1.144089015	5
UNC5C	-0.781808452	-0.345018092	1.126826544	5
UQCR10	-0.444864112	-0.700374501	1.145238613	5
UQCRFS1	-0.246432371	-0.853745081	1.100177451	5
URAD	-0.851471055	-0.249725634	1.10119669	5
USP17L1	-0.524801897	-0.628349986	1.153151883	5
USP17L22	-0.650344214	-0.501138551	1.151482764	5
USP17L3	-0.428617116	-0.714247055	1.142864171	5
USP26	-0.735307345	-0.403379615	1.13868696	5
USP53	-0.273435244	-0.834840341	1.108275585	5
UTP11	-0.287545361	-0.824725254	1.112270615	5
UTP14C	-0.528092244	-0.625245133	1.153337377	5
UTP3	-0.810723745	-0.306709232	1.117432976	5
UTS2	-0.759840855	-0.37306125	1.132902105	5
VAMP2	-0.149851426	-0.916617737	1.066469163	5
VASN	-0.414733896	-0.72590517	1.140639066	5
VCX3A	-0.805247437	-0.314092699	1.119340136	5
VCX3B	-0.573523717	-0.581168386	1.154692103	5
VCY1B	-0.256116176	-0.847033384	1.10314956	5
VDR	-0.48512938	-0.664897217	1.150026597	5
VEGFD	-0.410896998	-0.729095637	1.139992635	5
VEZT	-0.764102119	-0.367689538	1.131791657	5
VGLL1	-0.808273015	-0.310021073	1.118294088	5
VIPR1	-0.533266641	-0.620339068	1.153605709	5
VMP1	-0.836532827	-0.271051817	1.107584643	5
VN1R1	-0.748455127	-0.38725861	1.135713737	5
VN1R4	-0.416610811	-0.724339529	1.14095034	5
VPREB3	-0.812675806	-0.304062355	1.11673816	5
VPS37D	-0.187544363	-0.892949833	1.080494196	5
VSIG10L2	-0.282726808	-0.828197922	1.110924729	5
VTCN1	-0.130974088	-0.928059301	1.05903339	5
VWF	-0.426206574	-0.716284128	1.142490702	5
WARS2	-0.874048412	-0.216450758	1.090499171	5
WASF3	-0.687714725	-0.459439807	1.147154532	5
WBP11	-0.83610394	-0.271656412	1.107760351	5
WDR35	-0.530691731	-0.622784032	1.153475763	5
WDR74	-0.842032156	-0.263261925	1.105294082	5
WFDC12	-0.345111273	-0.781736592	1.126847865	5
WFS1	-0.657343359	-0.493474768	1.150818127	5
WHRN	-0.302657575	-0.813709447	1.116367022	5
WIPI1	-0.613509631	-0.540419575	1.153929207	5
WNT10B	-0.601177507	-0.553190611	1.154368118	5
WNT16	-0.594401668	-0.56012932	1.154530988	5
WNT9A	-0.396020768	-0.741338148	1.137358915	5
WRN	-0.515572362	-0.636998069	1.152570431	5
WTAP	-0.169380942	-0.904492309	1.073873251	5
WWP2	-0.362819916	-0.767943128	1.130763044	5
XIRP2	-0.725778437	-0.414885971	1.140664408	5
XPA	-0.809066373	-0.308950313	1.118016685	5
XRCC4	-0.26959567	-0.837564562	1.107160232	5
YDJC	-0.597611122	-0.556849574	1.154460696	5
YPEL5	-0.148179087	-0.917642386	1.065821472	5
YRDC	-0.403049148	-0.735579229	1.138628377	5
YTHDF3	-0.503070734	-0.648570114	1.151640848	5
ZBED4	-0.445335912	-0.699967912	1.145303824	5

ZBTB1	-0.705758054	-0.438593763	1.144351817	5
ZBTB10	-0.651155493	-0.500253588	1.151409081	5
ZBTB39	-0.639463858	-0.51292315	1.152387008	5
ZBTB40	-0.4318772	-0.711483399	1.143360598	5
ZBTB42	-0.356945831	-0.772548775	1.129494606	5
ZBTB5	-0.383237594	-0.751698069	1.134935663	5
ZC2HC1B	-0.451745239	-0.694423354	1.146168593	5
ZC3H12A	-0.360346046	-0.769886472	1.130232519	5
ZCCHC10	-0.616019881	-0.537797147	1.153817028	5
ZCCHC12	-0.818469403	-0.296159428	1.114628831	5
ZDHHC13	-0.472582182	-0.676123048	1.14870523	5
ZDHHC7	-0.810962042	-0.30638654	1.117348582	5
ZFAND1	-0.650946756	-0.500481367	1.151428123	5
ZFAND2B	-0.788153538	-0.33675187	1.124905409	5
ZFHX3	-0.708095374	-0.435857817	1.143953191	5
ZKSCAN2	-0.756996537	-0.376628993	1.133625531	5
ZKSCAN8P1	-0.564605186	-0.590002249	1.154607435	5
ZMIZ1	-0.195922869	-0.887538779	1.083461649	5
ZMYM5	-0.343003979	-0.783359887	1.126363866	5
ZMYM6	-0.559775267	-0.594748713	1.15452398	5
ZNF101	-0.373571132	-0.759435052	1.133006184	5
ZNF112	-0.508131615	-0.643905069	1.152036685	5
ZNF136	-0.272064727	-0.83581412	1.107878848	5
ZNF140	-0.725711361	-0.414966449	1.14067781	5
ZNF184	-0.698006071	-0.447608943	1.145615014	5
ZNF202	-0.70214526	-0.442806471	1.14495173	5
ZNF212	-0.822202577	-0.291029178	1.113231755	5
ZNF214	-0.647394601	-0.504348696	1.151743297	5
ZNF223	-0.877591476	-0.211108025	1.088699501	5
ZNF229	-0.500954269	-0.650513218	1.151467487	5
ZNF230	-0.449407161	-0.696450534	1.145857695	5
ZNF239	-0.277211223	-0.832149483	1.109360706	5
ZNF251	-0.150542786	-0.916193509	1.066736295	5
ZNF324B	-0.524438873	-0.62869184	1.153130712	5
ZNF329	-0.320168114	-0.800706911	1.120875025	5
ZNF33B	-0.546920586	-0.607254419	1.154175005	5
ZNF343	-0.418755197	-0.722546789	1.141301986	5
ZNF347	-0.802173939	-0.318209721	1.12038366	5
ZNF35	-0.735710243	-0.402889861	1.138600104	5
ZNF385A	-0.671042641	-0.478282299	1.14932494	5
ZNF385D	-0.424492225	-0.717729566	1.142221791	5
ZNF404	-0.712116417	-0.431131512	1.143247929	5
ZNF433	-0.378676893	-0.755358784	1.134035677	5
ZNF441	-0.755770928	-0.378161974	1.133932902	5
ZNF443	-0.161978717	-0.909122843	1.07110156	5
ZNF45	-0.765462328	-0.365968087	1.131430415	5
ZNF451	-0.403508089	-0.73520162	1.138709709	5
ZNF479	-0.835277247	-0.272820601	1.108097848	5
ZNF511	-0.610155735	-0.543911228	1.154066963	5
ZNF511-PRAP1	-0.690839828	-0.455863587	1.146703415	5
ZNF514	-0.655057689	-0.495984706	1.151042395	5
ZNF524	-0.751942125	-0.382934254	1.13487638	5
ZNF527	-0.187130929	-0.893215418	1.080346348	5
ZNF589	-0.267500866	-0.839045776	1.106546642	5
ZNF593	-0.776560369	-0.351797849	1.128358218	5

ZNF593OS	-0.616385485	-0.537414555	1.153800039	5
ZNF598	-0.797790488	-0.324048566	1.121839054	5
ZNF607	-0.381683589	-0.752947498	1.134631087	5
ZNF613	-0.225677518	-0.86787641	1.093553928	5
ZNF620	-0.445639868	-0.699705855	1.145345723	5
ZNF621	-0.54997611	-0.604298422	1.154274532	5
ZNF623	-0.212176384	-0.876884792	1.089061175	5
ZNF627	-0.157478009	-0.9119176	1.069395609	5
ZNF669	-0.710563802	-0.432959378	1.143523179	5
ZNF680	-0.406409131	-0.732810239	1.13921937	5
ZNF699	-0.540702775	-0.613238099	1.153940875	5
ZNF701	-0.739550759	-0.398208023	1.137758782	5
ZNF707	-0.701887852	-0.443105873	1.144993725	5
ZNF728	-0.574901922	-0.579795161	1.154697082	5
ZNF771	-0.755349122	-0.378688962	1.134038083	5
ZNF776	-0.13056623	-0.928303491	1.058869722	5
ZNF780A	-0.554810395	-0.599600543	1.154410938	5
ZNF780B	-0.301985683	-0.814203245	1.116188928	5
ZNF786	-0.146112721	-0.918905486	1.065018207	5
ZNF80	-0.363387038	-0.76749687	1.130883909	5
ZNF823	-0.497878826	-0.653328566	1.151207392	5
ZNF860	-0.622161183	-0.531348395	1.153509578	5
ZNF891	-0.633773015	-0.519025464	1.152798479	5
ZNHIT1	-0.536361358	-0.617391097	1.153752455	5
ZP3	-0.710555258	-0.432969426	1.143524684	5
ZRANB1	-0.778423985	-0.349396208	1.127820193	5
ZSCAN16	-0.804344855	-0.315303716	1.119648571	5
ZSWIM2	-0.45267467	-0.693616051	1.146290721	5
ZWINT	-0.80935891	-0.30855516	1.11791407	5
ZYX	-0.85791997	-0.240353092	1.098273061	5
AAAS	-0.346529396	1.12717142	-0.780642023	6
AAGAB	-0.533430632	1.153613743	-0.620183111	6
AAMDC	-0.303827805	1.116676308	-0.812848503	6
AASDHPPT	-0.401057277	1.138273164	-0.737215888	6
ABAT	-0.603444242	1.154301317	-0.550857075	6
ABCA5	-0.125880577	1.056980308	-0.931099731	6
ABCA9	-0.53678973	1.153771954	-0.616982224	6
ABCB8	-0.37700129	1.13370037	-0.75669908	6
ABCC1	-0.014142035	1.006996016	-0.992853981	6
ABHD16A	-0.535308347	1.153703681	-0.618395335	6
ABHD5	0.050588507	0.973745586	-1.024334094	6
ABHD8	-0.289359484	1.11277236	-0.823412876	6
ABLIM2	-0.501317358	1.151497555	-0.650180197	6
ABO	0.01848085	0.990631489	-1.009112339	6
ACAP1	-0.204124071	1.086313031	-0.88218896	6
ACER3	0.042210176	0.978226551	-1.020436728	6
ACOX1	-0.296326193	1.114673878	-0.818347685	6
ACP2	-0.209602662	1.088188357	-0.878585695	6
ACP3	-0.492303894	1.150711319	-0.658407425	6
ACP5	-0.028127291	1.013766922	-0.985639632	6
ACSM3	-0.162806255	1.071413528	-0.908607273	6
ACSS2	-0.060372769	1.028818622	-0.968445853	6
ACSS3	-0.3707428	1.132425956	-0.761683156	6
ACTB	-0.363355708	1.130877239	-0.767521531	6
ACTN3	-0.519573767	1.152833537	-0.63325977	6

ACTN4	-0.525540957	1.15319455	-0.627653592	6
ACTR10	0.002963713	0.99851485	-1.001478562	6
ACTR2	-0.366367758	1.131514517	-0.765146759	6
ACTRT1	-0.025495333	1.012503882	-0.98700855	6
ACTRT2	-0.623003329	1.153463741	-0.530460412	6
ACVR1B	-0.519466455	1.152826701	-0.633360246	6
ACVRL1	-0.12401816	1.056224661	-0.932206501	6
ADAM10	-0.618324455	1.153707165	-0.53538271	6
ADAM15	-0.261720901	1.104835105	-0.843114204	6
ADAM8	-0.244215343	1.099486385	-0.855271042	6
ADAMTS8	-0.21639028	1.090478926	-0.874088645	6
ADCY1	-0.29353695	1.113917401	-0.820380451	6
ADGRB1	-0.488807748	1.150384138	-0.66157639	6
ADGRB2	0.030179599	0.984568589	-1.014748188	6
ADRA2A	-0.00098071	1.000489994	-0.999509284	6
ADRB1	0.039593943	0.979614975	-1.019208919	6
AEBP1	-0.56564321	1.154621937	-0.588978727	6
AFAP1	-0.586157947	1.15465552	-0.568497573	6
AFF1	-0.144226577	1.064282136	-0.920055558	6
AFF2	-0.388777484	1.136003871	-0.747226387	6
AFF3	0.038578651	0.980152401	-1.018731053	6
AFTPH	-0.434700124	1.143782386	-0.709082263	6
AGAP1	-0.419037466	1.141347956	-0.722310491	6
AGAP4	-0.074160897	1.035015878	-0.960854981	6
AGAP5	-0.398400266	1.13779372	-0.739393454	6
AGAP6	-0.163822428	1.071795884	-0.907973455	6
AGAP9	0.015565501	0.992126389	-1.007691889	6
AGER	-0.588221487	1.154631871	-0.566410384	6
AGFG1	-0.215867918	1.090303941	-0.874436023	6
AGPAT4	-0.524238187	1.153118949	-0.628880762	6
AGRN	-0.476104153	1.149091937	-0.672987784	6
AHCYL1	-0.55413041	1.154393317	-0.600262908	6
AHNAK	-0.194533467	1.082973357	-0.88843989	6
AHRR	-0.098985187	1.045811543	-0.946826356	6
AIDA	-0.438209451	1.14429625	-0.7060868	6
AJM1	-0.16277114	1.071400301	-0.908629161	6
AK2	-0.378528392	1.134006061	-0.75547767	6
AKAP10	-0.194270883	1.082880904	-0.888610022	6
AKAP5	-0.466804464	1.148044345	-0.681239881	6
AKAP7	-0.558634801	1.15450045	-0.595865648	6
AKT1	-0.433059508	1.143538167	-0.710478658	6
AKT3	-0.43944057	1.14447376	-0.70503319	6
AKTIP	-0.11941519	1.054345725	-0.934930535	6
ALDH1A3	-0.449321613	1.14584622	-0.696524607	6
ALDH3A1	-0.335232475	1.124545906	-0.789313431	6
ALDH3A2	-0.499972195	1.151385484	-0.651413289	6
ALDH5A1	-0.615328245	1.153848714	-0.53852047	6
ALG1	-0.479969992	1.149502233	-0.669532241	6
ALG6	-0.328910589	1.123028906	-0.794118317	6
ALOX15	-0.383168502	1.134922167	-0.751753666	6
ALOX15B	-0.581482895	1.154690654	-0.573207759	6
ALPP	0.068224344	0.964140842	-1.032365186	6
ALX3	-0.352045968	1.128413517	-0.776367549	6
AMT	-0.242757011	1.099029658	-0.856272647	6
AMZ1	-0.365555479	1.131343443	-0.765787964	6

ANKHD1	-0.517171552	1.1526776	-0.635506048	6
ANKHD1-EIF4EBP3	-0.42656314	1.142546288	-0.715983148	6
ANKRD31	-0.627907936	1.153179038	-0.525271102	6
ANKRD37	-0.430196502	1.143105916	-0.712909415	6
ANKRD42	-0.43837512	1.144320221	-0.705945101	6
ANKRD52	0.015138594	0.992344758	-1.007483352	6
ANKZF1	-0.155948098	1.06881215	-0.912864052	6
ANO7	-0.230747673	1.095203737	-0.864456064	6
AOAH	-0.576475473	1.154700097	-0.578224624	6
AP1G2	-0.011374505	1.005638734	-0.994264229	6
AP1M1	-0.379698805	1.134238948	-0.754540143	6
AP3S2	-0.452447414	1.146260936	-0.693813522	6
AP4S1	-0.532759611	1.153580683	-0.620821073	6
APAF1	-0.612385375	1.153976926	-0.541591551	6
APBA1	-0.091105422	1.042435277	-0.951329856	6
APBB2	-0.607468919	1.154167369	-0.546698451	6
APBB3	-0.512438985	1.152352694	-0.639913709	6
APEX1	-0.002663667	1.001329173	-0.998665506	6
APH1B	-0.275273227	1.108805233	-0.833532006	6
API5	-0.313624901	1.119220662	-0.805595762	6
APLF	-0.28768894	1.112310425	-0.824621484	6
APOL3	-0.460367749	1.147269559	-0.686901809	6
APOL6	-0.325664694	1.122236787	-0.796572093	6
ARF1	-0.268593517	1.106867137	-0.838273621	6
ARFGEF1	0.012380664	0.993752186	-1.00613285	6
ARFGEF2	-0.466954513	1.148061923	-0.68110741	6
ARFIP1	-0.525474776	1.153190753	-0.627715977	6
ARGLU1	-0.599028105	1.154425742	-0.555397637	6
ARHGAP24	-0.467340065	1.148106989	-0.680766924	6
ARHGAP30	0.03745066	0.980748575	-1.018199235	6
ARHGAP32	0.012947653	0.993463306	-1.006410959	6
ARHGAP9	-0.339974008	1.125661245	-0.785687237	6
ARHGDIA	-0.213359799	1.089460749	-0.876100949	6
ARHGEF10L	-0.165561082	1.072448236	-0.906887154	6
ARHGEF15	-0.330583166	1.123433575	-0.79285041	6
ARHGEF39	-0.198852892	1.084486417	-0.885633524	6
ARID1B	-0.345582158	1.126955493	-0.781373335	6
ARL11	-0.378303206	1.133961115	-0.755657909	6
ARL17A	-0.268062353	1.106711459	-0.838649106	6
ARL17B	-0.345044602	1.12683261	-0.781788009	6
ARL6IP6	-0.063730211	1.030340866	-0.966610656	6
ARMC12	-0.288038434	1.112407257	-0.824368822	6
ARMS2	-0.332372235	1.123863789	-0.791491555	6
ARPC3	-0.172034538	1.074856532	-0.902821994	6
ARPC4	-0.145867047	1.064922487	-0.91905544	6
ARSD	-0.17705721	1.076702708	-0.899645498	6
ARSH	-0.110355504	1.050600399	-0.940244896	6
ART5	-0.446103178	1.145409419	-0.69930624	6
ARTN	0.007589496	0.996183652	-1.003773148	6
ASAP1	-0.078998148	1.037156064	-0.958157916	6
ASB8	-0.078969811	1.037143578	-0.958173767	6
ASCC3	-0.320889595	1.121055229	-0.800165634	6
ASDURF	-0.138760384	1.062133518	-0.923373134	6
ASF1B	-0.319383027	1.120678434	-0.801295407	6
ASIC3	-0.04084007	1.019794373	-0.978954302	6

ASL	-0.573060849	1.154689942	-0.581629093	6
ASNSD1	-0.561046538	1.15454849	-0.593501951	6
ASXL1	-0.194909955	1.083105819	-0.888195865	6
ASXL2	-0.065075795	1.030948563	-0.965872767	6
ATAD1	-0.527634697	1.153312273	-0.625677575	6
ATAT1	-0.335861215	1.124694911	-0.788833696	6
ATF4	-0.447967039	1.145663588	-0.697696549	6
ATG13	-0.177314756	1.076796846	-0.89948209	6
ATL3	-0.200533309	1.085071099	-0.88453779	6
ATM	-0.474193117	1.148883631	-0.674690514	6
ATMIN	-0.021151837	1.010408129	-0.989256292	6
ATOX1	-0.325129534	1.122105329	-0.796975795	6
ATP11B	-0.370004928	1.132273421	-0.762268492	6
ATP1A4	-0.278637867	1.109767646	-0.83112978	6
ATP1B1	0.054183183	0.97180687	-1.025990054	6
ATP1B4	-0.083399005	1.039087819	-0.955688814	6
ATP2A1	-0.514337075	1.152485816	-0.638148742	6
ATP5F1D	-0.274855594	1.108685127	-0.833829534	6
ATP5MC1	-0.600747333	1.154380096	-0.553632763	6
ATP5MF	-0.287690669	1.112310904	-0.824620235	6
ATP5PO	-0.561119585	1.154549843	-0.593430258	6
ATP6V0E1	-0.542379159	1.154008154	-0.611628995	6
ATP6V1C2	-0.564168254	1.154600967	-0.590432713	6
ATP6V1H	-0.027152103	1.01329955	-0.986147446	6
ATXN2	-0.361107556	1.130396396	-0.769288841	6
ATXN7L1	-0.395963064	1.137348308	-0.741385243	6
AUH	-0.411542869	1.140102398	-0.72855953	6
AUNIP	-0.541903021	1.153989356	-0.612086336	6
AURKC	-0.094432212	1.043866455	-0.949434243	6
B9D1	-0.30365207	1.116629935	-0.812977865	6
BABAM2	-0.306670808	1.117422932	-0.810752124	6
BACH2	-0.418586784	1.141274523	-0.722687739	6
BANP	-0.050686736	1.024379474	-0.973692738	6
BATF3	-0.25064729	1.10148037	-0.85083308	6
BAZ2A	-0.605717728	1.15422807	-0.548510342	6
BBIP1	-0.204339757	1.086387308	-0.882047551	6
BBS4	-0.326149002	1.122355545	-0.796206543	6
BCAP29	-0.169051764	1.073750895	-0.904699131	6
BCKDHA	-0.419263763	1.141384758	-0.722120995	6
BCL10	-0.491359588	1.150624166	-0.659264578	6
BCL2L12	-0.497145291	1.151143928	-0.653998636	6
BCL2L14	-0.353682003	1.128776809	-0.775094805	6
BCL9L	-0.58832722	1.154630525	-0.566303305	6
BCO1	-0.028412513	1.013903484	-0.985490971	6
BEAN1	-0.242226732	1.098863159	-0.856636427	6
BEND7	-0.463984492	1.147709884	-0.683725393	6
BHMT2	-0.476828323	1.149169926	-0.672341603	6
BICD1	-0.355898344	1.129265248	-0.773366904	6
BIN2	-0.017957561	1.008857845	-0.990900284	6
BIVM-ERCC5	-0.174091022	1.075614799	-0.901523777	6
BMPR2	-0.478150657	1.149310991	-0.671160334	6
BNC2	-0.1063246	1.04891393	-0.94258933	6
BNIP2	-0.300400829	1.11576735	-0.815366521	6
BOK	-0.301100811	1.115953806	-0.814852995	6
BOLA2	-0.48691605	1.150201964	-0.663285914	6

BOLA3	-0.343113376	1.126389086	-0.78327571	6
BPIFC	-0.609539305	1.154090779	-0.544551475	6
BRAP	-0.390578294	1.136345175	-0.74576688	6
BRCC3	-0.276560738	1.109174606	-0.832613867	6
BRD1	-0.050095704	1.024106316	-0.974010613	6
BRD7	0.06164699	0.967750357	-1.029397346	6
BRINP1	-0.293878978	1.11401051	-0.820131532	6
BRIX1	-0.300583703	1.115816102	-0.815232399	6
BRWD3	-0.118006181	1.053767337	-0.935761156	6
BSN	-0.460960261	1.147342567	-0.686382307	6
BST2	-0.212857066	1.089291139	-0.876434073	6
BTK	-0.54352004	1.154052191	-0.61053215	6
BTN2A2	-0.436651132	1.144069506	-0.707418373	6
BTN3A2	-0.174028331	1.075591732	-0.901563401	6
BTRC	-0.251203258	1.101651163	-0.850447905	6
C10orf105	-0.590719391	1.154596538	-0.563877147	6
C11orf24	-0.573685551	1.154692801	-0.58100725	6
C11orf42	-0.521774085	1.152971037	-0.631196952	6
C11orf49	-0.613538247	1.153927972	-0.540389725	6
C11orf53	-0.359352041	1.130017845	-0.770665804	6
C11orf58	-0.382539774	1.134799161	-0.752259387	6
C12orf56	-0.01241657	1.006150469	-0.993733899	6
C14orf119	-0.150332388	1.066655039	-0.916322651	6
C17orf49	-0.35003932	1.127964756	-0.777925436	6
C17orf64	-0.539884593	1.153906928	-0.614022335	6
C17orf80	-0.296818118	1.114806624	-0.817988506	6
C17orf97	-0.139791453	1.062540558	-0.922749106	6
C19orf18	-0.525971702	1.15321915	-0.627247448	6
C19orf38	-0.48456839	1.149970874	-0.665402484	6
C1orf167	-0.11903762	1.054190883	-0.935153264	6
C1orf21	-0.619286616	1.153659342	-0.534372726	6
C1orf226	-0.176675472	1.076563082	-0.899887609	6
C1orf35	-0.211231163	1.088741233	-0.87751007	6
C1S	-0.01940882	1.009563137	-0.990154317	6
C2	-0.216863781	1.090637356	-0.873773574	6
C20orf204	-0.33010673	1.123318548	-0.793211818	6
C20orf27	-0.223357777	1.092792275	-0.869434499	6
C2orf42	-0.420829146	1.141638028	-0.720808882	6
C5orf24	-0.44648018	1.145461097	-0.698980918	6
C6orf47	-0.131997609	1.059443556	-0.927445946	6
C9orf153	-0.528772868	1.153374308	-0.62460144	6
C9orf64	-0.1721964	1.074916333	-0.902719933	6
C9orf72	-0.535468788	1.15371119	-0.618242402	6
CA2	0.046532468	0.97592146	-1.022453927	6
CA7	-0.184776598	1.079501872	-0.894725274	6
CABP1	-0.247398351	1.100477319	-0.853078968	6
CACNA1I	-0.433123698	1.143547769	-0.710424071	6
CACNA2D2	0.016738531	0.991525662	-1.008264193	6
CACNB3	-0.420931748	1.141654548	-0.720722801	6
CACNG1	-0.03145525	1.015356519	-0.983901269	6
CACTIN	0.042979342	0.97781738	-1.020796722	6
CAD	0.000864987	0.999567226	-1.000432213	6
CALB2	-0.455532158	1.14666098	-0.691128822	6
CALCOCO2	-0.548413201	1.154224908	-0.605811706	6
CALHM1	-0.340123368	1.125696065	-0.785572697	6

CALHM3	-0.431224397	1.143261992	-0.712037595	6
CALML3	-0.021503467	1.010578319	-0.989074852	6
CALN1	0.017541149	0.991114034	-1.008655184	6
CAMK1D	-0.410384073	1.139905193	-0.72952112	6
CAMK4	-0.21462492	1.089886685	-0.875261765	6
CAMKMT	-0.413504218	1.14043337	-0.726929152	6
CAPN12	-0.082027116	1.038487199	-0.956460083	6
CAPN7	-0.168802951	1.073658355	-0.904855403	6
CAPZA2	-0.185691727	1.079830634	-0.894138907	6
CARD6	-0.577092187	1.1547005	-0.577608313	6
CARM1	-0.010184798	1.005053499	-0.994868702	6
CASP2	-0.319291389	1.120655453	-0.801364065	6
CAST	-0.24967817	1.101182062	-0.851503892	6
CAT	-0.322286026	1.121402763	-0.799116737	6
CATSPER3	-0.565278395	1.154616979	-0.589338584	6
CBFA2T2	-0.611331876	1.154020229	-0.542688353	6
CBLB	-0.167601272	1.073210743	-0.905609471	6
CBLN2	-0.433190039	1.14355769	-0.710367651	6
CBR1	-0.544377946	1.154084369	-0.609706422	6
CBS	0.034896497	0.982094985	-1.016991482	6
CBWD2	-0.134783423	1.060555883	-0.92577246	6
CBWD3	-0.29357308	1.113927241	-0.820354161	6
CBWD5	-0.484861853	1.150000063	-0.66513821	6
CBWD6	-0.22236152	1.092463857	-0.870102337	6
CBX2	-0.488563491	1.150360818	-0.661797327	6
CBX7	0.032139303	0.983542923	-1.015682226	6
CC2D2A	-0.517409839	1.152693339	-0.6352835	6
CCDC107	-0.518407007	1.152758556	-0.634351549	6
CCDC120	-0.429665652	1.143024924	-0.713359272	6
CCDC142	-0.40289218	1.138600516	-0.735708336	6
CCDC146	0.005277741	0.997350684	-1.002628425	6
CCDC17	-0.147853017	1.065694936	-0.917841919	6
CCDC187	0.038582018	0.980150621	-1.018732638	6
CCDC25	-0.187236394	1.080384076	-0.893147682	6
CCDC28B	-0.479004009	1.149401103	-0.670397094	6
CCDC57	-0.211978423	1.088994227	-0.877015805	6
CCDC61	-0.229366371	1.094756296	-0.865389924	6
CCDC70	-0.307513902	1.117643044	-0.810129142	6
CCDC71	0.008358671	0.995794464	-1.004153135	6
CCDC8	-0.05255387	1.025240682	-0.972686812	6
CCDC90B	-0.558918107	1.154506431	-0.595588324	6
CCDC96	-0.459292704	1.147136221	-0.687843517	6
CCNDBP1	-0.279555113	1.110028402	-0.83047329	6
CCNE1	-0.572786534	1.154688545	-0.581902011	6
CCNE2	-0.300235236	1.11572318	-0.815487945	6
CCNG1	-0.615321001	1.153849043	-0.538528042	6
CCNT2	-0.626549585	1.153260931	-0.526711346	6
CCR4	-0.029654975	1.014497652	-0.984842676	6
CCSAP	-0.415750646	1.140808091	-0.725057445	6
CCT8L2	0.035458052	0.981799385	-1.017257437	6
CCZ1	-0.487895144	1.150296703	-0.662401558	6
CCZ1B	-0.442701721	1.144937018	-0.702235297	6
CD164	-0.505471392	1.151831911	-0.646360519	6
CD180	-0.436519049	1.144050181	-0.707531132	6
CD1A	-0.402063673	1.138453088	-0.736389414	6

CD2AP	-0.568168981	1.154652125	-0.586483144	6
CD300LG	-0.052642829	1.025281649	-0.97263882	6
CD40	-0.392803743	1.136762927	-0.743959184	6
CD40LG	-0.28157623	1.11060053	-0.8290243	6
CD48	-0.011052875	1.005480624	-0.994427749	6
CD99L2	-0.40540794	1.139044347	-0.733636407	6
CDC123	-0.032878381	1.016033738	-0.983155357	6
CDC16	-0.601851241	1.154348911	-0.55249767	6
CDC42EP1	-0.008465607	1.004205928	-0.995740321	6
CDC42EP2	-0.618960257	1.153675692	-0.534715435	6
CDC42SE1	0.025868595	0.986814727	-1.012683322	6
CDC7	-0.03910066	1.018976842	-0.979876183	6
CDCA3	0.066084488	0.965318728	-1.031403216	6
CDH2	0.067045025	0.964790426	-1.031835451	6
CDK3	-0.505443359	1.151829714	-0.646386355	6
CDK5	-0.039461326	1.019146544	-0.979685218	6
CDK5RAP3	-0.249585796	1.101153588	-0.851567792	6
CDKL1	-0.303368892	1.116555156	-0.813186264	6
CDKL4	-0.253396528	1.1023225	-0.848925972	6
CDKN2AIP	-0.579546911	1.154697749	-0.575150837	6
CDKN2C	-0.371353146	1.132551766	-0.76119862	6
CDT1	-0.609108591	1.154107145	-0.544998554	6
CEACAM3	-0.020714847	1.010196496	-0.989481649	6
CELF1	-0.610088032	1.154069602	-0.54398157	6
CELF2	-0.287501047	1.112258325	-0.824757277	6
CELF4	-0.501075767	1.151477563	-0.650401796	6
CELSR3	-0.557853629	1.154483493	-0.596629864	6
CENPP	-0.303806216	1.116670612	-0.812864396	6
CEP120	-0.380408037	1.134379476	-0.753971439	6
CEP152	-0.605404545	1.154238533	-0.548833988	6
CEP85L	-0.072529224	1.034289979	-0.961760755	6
CEPT1	-0.198812046	1.084472177	-0.885660131	6
CERS1	-0.173185595	1.075281354	-0.902095759	6
CFP	-0.040136654	1.019464038	-0.979327384	6
CHAC1	-0.511009568	1.152249963	-0.641240395	6
CHAD	-0.468997717	1.148299086	-0.679301369	6
CHCHD7	-0.135885255	1.060994186	-0.925108931	6
CHDH	-0.602904038	1.1543178	-0.551413762	6
CHMP5	-0.114127839	1.052167496	-0.938039657	6
CHRM1	-0.377259805	1.133752265	-0.75649246	6
CHRM4	0.006618805	0.996674169	-1.003292974	6
CHSY1	-0.594408362	1.154530854	-0.560122492	6
CIAO3	-0.234119655	1.096289622	-0.862169967	6
CIDEB	-0.442365048	1.14488966	-0.702524612	6
CIITA	-0.146826482	1.065296041	-0.918469558	6
CILK1	-0.602678736	1.154324571	-0.551645835	6
CKMT2	-0.407035353	1.139328376	-0.732293023	6
CKS1B	0.061395857	0.967887527	-1.029283384	6
CLEC10A	-0.475796628	1.14905866	-0.673262032	6
CLEC17A	-0.422657261	1.141930925	-0.719273664	6
CLEC2A	-0.580778057	1.154693741	-0.573915684	6
CLEC4E	-0.496090046	1.151051667	-0.654961621	6
CLEC4F	-0.516961849	1.152663699	-0.635701851	6
CLMN	-0.001020385	1.000509802	-0.999489417	6
CLTC	-0.119416609	1.054346307	-0.934929697	6

CNBP	-0.407012915	1.139324477	-0.732311562	6
CNGA2	0.003104306	0.998444233	-1.001548539	6
CNN1	-0.445773074	1.145364057	-0.699590983	6
CNNM2	-0.294554123	1.114194018	-0.819639895	6
CNNM3	-0.564768542	1.154609798	-0.589841256	6
CNOT1	-0.144281345	1.064303547	-0.920022202	6
CNOT10	0.041615839	0.978542415	-1.020158254	6
CNOT6L	-0.252427544	1.102026383	-0.849598839	6
CNPY4	-0.627274442	1.153217521	-0.525943079	6
CNTD1	-0.232103196	1.095641349	-0.863538152	6
CNTFR	-0.546028322	1.154144006	-0.608115684	6
CNTRL	-0.09892284	1.045785014	-0.946862174	6
COA8	-0.386333198	1.135535951	-0.749202753	6
COG4	-0.61179861	1.154001213	-0.542202603	6
COG8	-0.449650993	1.145890363	-0.69623937	6
COL10A1	-0.484520166	1.149966069	-0.665445903	6
COL15A1	-0.616376579	1.153800455	-0.537423876	6
COL25A1	-0.007983229	1.003967715	-0.995984486	6
COL9A3	-0.511920359	1.152315667	-0.640395308	6
COLQ	-0.389092767	1.136063836	-0.74697107	6
COMMD10	0.059354233	0.969000913	-1.028355146	6
COMMD3-BMI1	-0.615902577	1.153822444	-0.537919867	6
COPG1	-0.078704838	1.037026795	-0.958321958	6
COPG2	-0.258512832	1.103873437	-0.845360604	6
COPS7B	-0.072299811	1.034187757	-0.961887946	6
COQ2	-0.530664072	1.153474329	-0.622810257	6
COQ8A	-0.332579996	1.123913572	-0.791333576	6
COX11	-0.398765908	1.137860078	-0.73909417	6
COX14	-0.400631187	1.13819671	-0.737565523	6
COX15	-0.281608016	1.110609501	-0.829001485	6
COX20	-0.101870822	1.047036185	-0.945165363	6
COX5B	-0.305812228	1.117198166	-0.811385937	6
COX6B1	-0.541785358	1.153984673	-0.612199314	6
COX6C	-0.206664134	1.087185436	-0.880521302	6
COX7B	-0.457464889	1.146906941	-0.689442052	6
COX8A	-0.553326892	1.154371833	-0.601044941	6
CPAMD8	-0.603362394	1.154303837	-0.550941443	6
CPM	-0.317251533	1.120142068	-0.802890535	6
CPNE9	-0.361321318	1.130442307	-0.769120989	6
CPPED1	-0.354913236	1.129048677	-0.77413544	6
CPSF2	-0.346128543	1.127080138	-0.780951595	6
CPSF6	-0.232757654	1.095852107	-0.863094453	6
CPTP	-0.272203544	1.107919102	-0.835715558	6
CR1	-0.403432523	1.13869633	-0.735263807	6
CR1L	-0.156982009	1.069206648	-0.912224639	6
CRB3	-0.572320314	1.154685973	-0.58236566	6
CREB1	-0.151561901	1.067129397	-0.915567496	6
CREB5	-0.233329208	1.096035887	-0.862706679	6
CREG1	-0.303613591	1.116619778	-0.813006186	6
CRIM1	-0.538296481	1.153838963	-0.615542482	6
CRISPLD2	-0.547970864	1.154210374	-0.606239511	6
CRYBB2	-0.261066686	1.104639671	-0.843572986	6
CRYBG1	-0.352974468	1.128619981	-0.775645514	6
CRYBG3	-0.627662551	1.153194005	-0.525531454	6
CSNK1A1L	-0.195470698	1.083302904	-0.887832206	6

CSNK2A1	0.020853849	0.989409981	-1.01026383	6
CST11	-0.13185887	1.059388004	-0.927529134	6
CSTF1	-0.195623943	1.083356722	-0.887732779	6
CSTF2T	-0.257556775	1.10358523	-0.846028455	6
CT47A12	-0.135610165	1.060884844	-0.925274679	6
CT47A3	-0.152652425	1.067549158	-0.914896733	6
CT47A9	-0.202450561	1.085735477	-0.883284917	6
CT47B1	-0.500915175	1.151464241	-0.650549067	6
CTBP1	-0.558171289	1.15449047	-0.596319181	6
CTBP2	-0.218515668	1.091188675	-0.872673007	6
CTC1	-0.420409595	1.14157037	-0.721160775	6
CTDSPL2	-0.383453669	1.134977842	-0.751524173	6
CTNS	-0.06275748	1.02990071	-0.96714323	6
CUL1	-0.598285578	1.154444359	-0.55615878	6
CUL4A	-0.285183151	1.11161321	-0.826430059	6
CUL4B	0.064055536	0.96643238	-1.030487916	6
CWC22	-0.177202304	1.076755749	-0.899553445	6
CXADR	-0.567088122	1.154640093	-0.587551971	6
CXCR2	-0.056672425	1.027131075	-0.97045865	6
CXorf38	-0.61171077	1.154004812	-0.542294042	6
CYGB	-0.566157732	1.154628673	-0.588470941	6
CYP26A1	-0.0199762	1.009838445	-0.989862246	6
CYP3A7	-0.420265093	1.141547029	-0.721281936	6
CYP4F11	-0.424737641	1.142260455	-0.717522814	6
CYP4F12	-0.184710472	1.079478091	-0.894767619	6
CYTH4	0.054220639	0.971786618	-1.026007257	6
DAB2	-0.276274902	1.10909272	-0.832817817	6
DAD1	-0.16479094	1.072159563	-0.907368623	6
DAPP1	-0.147476144	1.065548583	-0.918072439	6
DBF4	-0.390891587	1.136404255	-0.745512667	6
DBH	-0.617784976	1.153733475	-0.535948498	6
DCAF12	-0.23875072	1.097766166	-0.859015447	6
DCAF12L1	-0.476815044	1.149168501	-0.672353457	6
DCAF17	-0.425392289	1.142363316	-0.716971027	6
DCAF6	-0.622101114	1.153512813	-0.531411699	6
DCAF8	-0.003142767	1.001567679	-0.998424913	6
DCAKD	-0.471574405	1.148592322	-0.677017916	6
DCLRE1A	-0.61743525	1.153750337	-0.536315087	6
DCP2	0.014038811	0.992906683	-1.006945495	6
DCTD	-0.425178041	1.142329697	-0.717151655	6
DCTN1	-0.565631186	1.154621776	-0.58899059	6
DCUN1D1	-0.488230802	1.150328959	-0.662098158	6
DDAH1	-0.600368486	1.15439046	-0.554021974	6
DDAH2	-0.224212469	1.093073398	-0.86886093	6
DDHD1	-0.536576138	1.153762256	-0.617186118	6
DDIAS	-0.040072365	1.019433828	-0.979361463	6
DDIT4	-0.540841992	1.153946578	-0.613104587	6
DDX17	-0.172411267	1.074995685	-0.902584418	6
DDX3X	-0.49028217	1.150523626	-0.660241456	6
DDX41	-0.115141342	1.052586678	-0.937445336	6
DDX47	-0.168771468	1.073646642	-0.904875174	6
DEGS2	-0.073615679	1.034773545	-0.961157866	6
DELE1	-0.613608378	1.153924941	-0.540316564	6
DENND10	-0.402530073	1.138536158	-0.736006085	6
DENND2D	-0.335298367	1.124561538	-0.789263171	6

DENND3	-0.45556641	1.146665371	-0.691098961	6
DEPP1	-0.425227996	1.14233754	-0.717109543	6
DERA	-0.199157058	1.084592412	-0.885435354	6
DERL2	-0.098075586	1.045424206	-0.94734862	6
DGCR2	-0.236512847	1.097054796	-0.86054195	6
DGKE	-0.049178358	1.023681826	-0.974503468	6
DHPS	-0.130568116	1.058870479	-0.928302363	6
DHRS2	-0.510885667	1.152240958	-0.641355291	6
DHRS7	-0.312005967	1.118805771	-0.806799804	6
DHX32	-0.586837058	1.15464829	-0.567811232	6
DHX57	-0.428776243	1.142888633	-0.71411239	6
DIP2A	-0.106389192	1.048941052	-0.94255186	6
DKK1	-0.342132592	1.126162609	-0.784030017	6
DLL1	-0.121513065	1.055204083	-0.933691018	6
DLST	-0.486801042	1.150190772	-0.66338973	6
DMTN	-0.129020619	1.058248333	-0.929227715	6
DNAAF10	-0.447085931	1.145543848	-0.698457917	6
DNAAF2	-0.093433077	1.04343751	-0.950004433	6
DNAAF6	-0.231870463	1.095566318	-0.863695855	6
DNAJA3	-0.551802691	1.154329113	-0.602526422	6
DNAJB13	-0.151612614	1.067148938	-0.915536324	6
DNAJC11	-0.499692658	1.151361961	-0.651669304	6
DNAJC16	-0.379936731	1.134286141	-0.75434941	6
DNAJC28	-0.166173666	1.072677524	-0.906503857	6
DNTTIP1	-0.487321676	1.15024133	-0.662919654	6
DOCK11	-0.349786129	1.127907885	-0.778121756	6
DOCK2	-0.188635734	1.08088384	-0.892248107	6
DOCK9	-0.005998219	1.002985617	-0.996987398	6
DOK1	-0.369196962	1.132105844	-0.762908882	6
DOT1L	-0.285197604	1.111617246	-0.826419642	6
DPCD	-0.519431465	1.152824469	-0.633393004	6
DPEP3	-0.511385127	1.15227716	-0.640892033	6
DPY30	-0.44929451	1.145842583	-0.696548073	6
DPYSL2	-0.197018576	1.083845657	-0.886827081	6
DRAP1	-0.462864882	1.147574939	-0.684710056	6
DRAXIN	-0.490846608	1.150576443	-0.659729835	6
DRC7	-0.475008971	1.148973003	-0.673964032	6
DUS4L	-0.27815353	1.10962968	-0.83147615	6
DUS4L-BCAP29	-0.10839053	1.049779827	-0.941389297	6
DUSP8	-0.097144377	1.045027018	-0.947882641	6
DVL1	-0.237484085	1.097364024	-0.859879939	6
DYM	-0.56973598	1.154667211	-0.584931231	6
DYNLL1	-0.522494551	1.153014949	-0.630520398	6
ECHDC3	0.053117898	0.972382424	-1.025500322	6
ECPAS	-0.101983526	1.047083888	-0.945100361	6
ECSIT	-0.075394081	1.035563164	-0.960169082	6
ECT2	0.040770003	0.978991481	-1.019761485	6
EDAR	-0.040364986	1.019571307	-0.979206321	6
EDEM2	-0.005301182	1.002640052	-0.997338871	6
EEF1A2	-0.150889055	1.066869951	-0.915980896	6
EEF1AKMT4	-0.563745708	1.154594506	-0.590848798	6
EEF1AKMT4-ECE2	-0.399233226	1.137944713	-0.738711487	6
EEF2K	-0.430219606	1.143109435	-0.71288983	6
EEF2KMT	-0.498311132	1.151244537	-0.652933406	6
EFNA2	-0.470718378	1.148495628	-0.67777725	6

EFNB2	-0.615274629	1.153851146	-0.538576517	6
EGR4	0.000329981	0.999834969	-1.00016495	6
EHBP1L1	0.060952762	0.968129432	-1.029082194	6
EHHADH	-0.612165075	1.153986094	-0.541821019	6
EIF2B1	-0.411746901	1.140136993	-0.728390092	6
EIF2B5	-0.037094203	1.018030976	-0.980936773	6
EIF2S1	-0.621873626	1.153525025	-0.531651399	6
EIF3M	-0.138220022	1.061919871	-0.92369985	6
EIF4G1	-0.182154513	1.078556269	-0.896401756	6
EIF6	-0.607843553	1.154153899	-0.546310346	6
ELF3	-0.28426235	1.111355704	-0.827093353	6
ELMOD2	-0.085232764	1.039888427	-0.954655663	6
ELMOD3	-0.102297462	1.047216712	-0.94491925	6
ELOA3DP	-0.590125537	1.154605605	-0.564480068	6
ELOC	0.034213664	0.982454106	-1.01666777	6
ELOVL1	-0.020679709	1.010179473	-0.989499764	6
ELOVL3	-0.548379187	1.154223798	-0.605844611	6
ELP1	-0.544223563	1.154078638	-0.609855074	6
ELP3	-0.518385123	1.152757136	-0.634372013	6
ELP6	-0.468303509	1.148218966	-0.679915457	6
EMB	-0.625648039	1.153313995	-0.527665956	6
EMC2	-0.287389618	1.112227413	-0.824837795	6
EMID1	-0.215681533	1.090241452	-0.874559919	6
EMILIN1	-0.308039375	1.117779931	-0.809740556	6
EMILIN3	-0.440568197	1.144635086	-0.704066889	6
EML2	-0.069357948	1.032873397	-0.963515449	6
ENTR1	-0.56216769	1.154568599	-0.592400909	6
EPB41L1	-0.381455255	1.134586153	-0.753130898	6
EPB42	-0.001053828	1.000526498	-0.999472669	6
EPOR	-0.587983376	1.154634856	-0.56665148	6
ERAP2	-0.052785923	1.025347532	-0.972561609	6
ERCC2	-0.203045585	1.085941082	-0.882895496	6
ERCC5	-0.223769347	1.09292772	-0.869158373	6
ERLEC1	-0.595882938	1.154500074	-0.558617136	6
ERMAP	-0.128005799	1.057839348	-0.929833549	6
ERN2	-0.295852447	1.114545847	-0.8186934	6
ERO1A	-0.623245456	1.153450398	-0.530204942	6
ERO1B	-0.620955869	1.153573633	-0.532617764	6
ERRFI1	-0.578371786	1.154699936	-0.576328149	6
ERVV-2	-0.546966984	1.154176593	-0.607209609	6
ESPNL	-0.23329868	1.096026078	-0.862727398	6
ESRRB	0.038299469	0.980300045	-1.018599515	6
ETFA	-0.401402813	1.138335043	-0.73693223	6
ETFDH	-0.401786723	1.138403666	-0.736616944	6
ETNK1	-0.085713114	1.040097725	-0.954384611	6
ETV7	-0.073226564	1.034600458	-0.961373894	6
EVA1A	-0.546459377	1.154159091	-0.607699714	6
EVI5	-0.073056934	1.034524967	-0.961468032	6
EXOC2	-0.238194132	1.097589614	-0.859395482	6
EXOC4	-0.048090836	1.023177768	-0.975086933	6
EXOSC4	-0.017502762	1.008636495	-0.991133732	6
EXOSC5	-0.626299292	1.153275766	-0.526976474	6
EXTL1	0.010445499	0.994736334	-1.005181833	6
FAAP20	-0.106718273	1.049079181	-0.942360908	6
FABP9	-0.45926959	1.147133341	-0.687863752	6

FAHD1	-0.482490283	1.149761704	-0.667271421	6
FAM120C	0.00836378	0.995791877	-1.004155657	6
FAM124A	-0.174139397	1.075632596	-0.901493199	6
FAM126B	0.009171732	0.995382588	-1.00455432	6
FAM13B	-0.43445721	1.143746387	-0.709289176	6
FAM156A	-0.039617474	1.019219985	-0.97960251	6
FAM156B	-0.285872674	1.11180558	-0.825932906	6
FAM162A	-0.197697752	1.084083213	-0.886385461	6
FAM167B	0.031337402	0.983962969	-1.015300371	6
FAM172A	-0.220609878	1.091884514	-0.871274636	6
FAM174C	-0.186222548	1.080021033	-0.893798485	6
FAM200B	-0.547065843	1.154179969	-0.607114126	6
FAM204A	-0.542087698	1.153996677	-0.611908978	6
FAM210A	-0.251724883	1.101811179	-0.850086296	6
FAM217B	-0.614391275	1.153890695	-0.53949942	6
FAM221A	-0.489542726	1.150453945	-0.660911219	6
FAM228B	-0.505306984	1.151819016	-0.646512032	6
FAM236A	-0.31838837	1.120428618	-0.802040248	6
FAM236B	-0.189900772	1.081334324	-0.891433551	6
FAM236C	-0.256747942	1.103340827	-0.846592885	6
FAM32A	-0.379441989	1.134187951	-0.754745963	6
FAM3A	-0.135425598	1.06081145	-0.925385852	6
FAM3B	-0.205817041	1.08689506	-0.881078019	6
FAM3D	-0.236205943	1.096956926	-0.860750983	6
FAM50A	-0.099441235	1.046005507	-0.946564272	6
FAM53A	-0.585897122	1.154658154	-0.568761031	6
FAM71F1	-0.525008447	1.153163866	-0.628155419	6
FAM89A	-0.39307538	1.136813612	-0.743738232	6
FANCB	-0.132309404	1.059568345	-0.927258941	6
FARP2	-0.4197784	1.141468274	-0.721689874	6
FARSB	0.025046419	0.987241517	-1.012287936	6
FASTK	-0.412368384	1.140242133	-0.727873749	6
FBLN1	-0.605939741	1.154220581	-0.54828084	6
FBXL13	-0.159045162	1.069991398	-0.910946236	6
FBXL17	-0.088736735	1.041411167	-0.952674432	6
FBXL3	-0.336850714	1.124928727	-0.788078013	6
FBXO22	-0.412281063	1.140227382	-0.727946319	6
FBXO3	-0.214672386	1.089902641	-0.875230255	6
FBXO5	-0.031878489	1.015558083	-0.983679593	6
FBXW9	-0.464295037	1.147747097	-0.68345206	6
FCER1G	-0.091192973	1.042473049	-0.951280076	6
FCGBP	-0.37829305	1.133959086	-0.755666036	6
FCGR1A	-0.210443947	1.088474235	-0.878030287	6
FCGR1B	-0.296709847	1.114777424	-0.818067577	6
FCHSD1	-0.398062113	1.137732242	-0.739670129	6
FCHSD2	-0.519061206	1.152800774	-0.633739568	6
FCN2	-0.257751102	1.103643871	-0.845892768	6
FDPS	-0.119126618	1.054227391	-0.935100773	6
FER	-0.59896752	1.154427286	-0.555459766	6
FES	-0.200209878	1.084958737	-0.884748859	6
FFAR1	-0.418110322	1.141196684	-0.723086362	6
FFAR3	-0.370798109	1.13243737	-0.761639262	6
FGF11	-0.073569497	1.034753008	-0.961183511	6
FGF12	-0.598348706	1.154442802	-0.556094096	6
FGL2	-0.499201147	1.151320409	-0.652119262	6

FGR	-0.542227012	1.154002174	-0.611775162	6
FHL1	-0.577363729	1.154700538	-0.577336809	6
FHL2	0.039732249	0.979541706	-1.019273955	6
FIBIN	-0.161759466	1.071018817	-0.909259351	6
FIGNL1	-0.441712693	1.144797591	-0.703084897	6
FIP1L1	-0.415889406	1.140831085	-0.724941679	6
FLG	0.017216601	0.991280539	-1.00849714	6
FNBP4	-0.15059676	1.066757134	-0.916160375	6
FNDC11	-0.200558112	1.085079712	-0.8845216	6
FNDC3B	-0.046719255	1.022540784	-0.975821529	6
FOXA3	0.029655853	0.984842218	-1.014498071	6
FOXN1	-0.235137152	1.096615505	-0.861478354	6
FOXN2	-0.102261807	1.04720163	-0.944939823	6
FRG2C	-0.139675296	1.062494743	-0.922819447	6
FRY	-0.001830388	1.000913937	-0.99908355	6
FSTL1	-0.223714496	1.092909677	-0.869195181	6
FUNDC1	-0.065930734	1.031333963	-0.965403229	6
FYB1	-0.006484535	1.003226499	-0.996741964	6
FYN	0.067083497	0.964769252	-1.031852749	6
FZD6	-0.493953053	1.150861357	-0.656908304	6
GAB3	-0.573265766	1.154690929	-0.581425163	6
GABRA1	-0.33841616	1.125296924	-0.786880764	6
GABRA2	-0.033581681	1.016367853	-0.982786171	6
GALE	-0.073230417	1.034602172	-0.961371755	6
GALNT10	0.04237619	0.978138275	-1.020514465	6
GALNT7	-0.098215541	1.045483844	-0.947268303	6
GANAB	-0.03542585	1.017242193	-0.981816342	6
GAS1	-0.221911761	1.092315335	-0.870403573	6
GATC	-0.371583997	1.132599265	-0.761015268	6
GBE1	-0.3735903	1.133010092	-0.759419792	6
GBP1	-0.512998559	1.152392331	-0.639393771	6
GCA	-0.28120172	1.110494769	-0.829293049	6
GCM2	-0.313100876	1.119086609	-0.805985733	6
GCOM1	-0.481148672	1.149624371	-0.668475699	6
GDF1	-0.182842407	1.078804863	-0.895962456	6
GFM1	-0.514947327	1.152527817	-0.63758049	6
GFRA4	-0.371225281	1.132525436	-0.761300156	6
GFY	-0.540483461	1.153931847	-0.613448386	6
GGPS1	-0.210313957	1.088430099	-0.878116142	6
GGT1	-0.057683826	1.02759335	-0.969909524	6
GH2	0.014198028	0.992825389	-1.007023417	6
GHRH	-0.492372726	1.150717636	-0.65834491	6
GIMAP4	-0.10093477	1.046639624	-0.945704853	6
GIMAP8	-0.160256979	1.070450798	-0.91019382	6
GINS4	-0.420214768	1.141538896	-0.721324128	6
GIT2	-0.019473426	1.009594497	-0.990121072	6
GLA	-0.558745326	1.154502794	-0.595757468	6
GLCCI1	-0.148352349	1.065888676	-0.917536327	6
GLCE	0.022700329	0.988456578	-1.011156906	6
GLE1	-0.539446755	1.153888463	-0.614441709	6
GLIPR1	-0.385729612	1.135419579	-0.749689967	6
GLOD4	-0.23389207	1.096216618	-0.862324548	6
GLT1D1	-0.345408345	1.126915788	-0.781507443	6
GLTP	-0.57461022	1.154696211	-0.580085991	6
GLUD1	-0.545135378	1.154112109	-0.608976732	6

GLUD2	-0.51234513	1.152346014	-0.640000884	6
GMDS	-0.55950104	1.154518455	-0.595017415	6
GML	-0.585677772	1.154660306	-0.568982535	6
GNA11	-0.420608066	1.141602396	-0.72099433	6
GNAI2	-0.181682998	1.078385658	-0.89670266	6
GNAQ	-0.398265839	1.137769293	-0.739503454	6
GNB1	-0.101256232	1.046775887	-0.945519655	6
GNG14	-0.623800404	1.153419537	-0.529619134	6
GNG5	-0.228825551	1.094580695	-0.865755144	6
GNG5P2	-0.602945571	1.154316545	-0.551370974	6
GOLGA3	-0.157774321	1.069508404	-0.911734083	6
GOLGA6L7	-0.328458411	1.122919095	-0.794460684	6
GOLGA8R	-0.177277906	1.07678338	-0.899505474	6
GON4L	-0.178473593	1.077219784	-0.89874619	6
GOPC	-0.088976195	1.041514891	-0.952538696	6
GP9	-0.535311718	1.153703839	-0.618392121	6
GPAT3	-0.014662972	1.007250857	-0.992587885	6
GPAT4	-0.541591324	1.153976917	-0.612385592	6
GPI	-0.29664653	1.114760344	-0.818113814	6
GPR107	-0.540370085	1.153927159	-0.613557074	6
GPR137C	-0.564404452	1.15460449	-0.590200038	6
GPR148	-0.568645606	1.15465701	-0.586011403	6
GPR150	-0.079936218	1.037569057	-0.957632839	6
GPR156	-0.086847292	1.040591215	-0.953743923	6
GPR17	-0.456093066	1.146732734	-0.690639668	6
GPR179	-0.568500184	1.154655547	-0.586155363	6
GPR37L1	-0.346038356	1.127059582	-0.781021226	6
GPR42	-0.458267805	1.147008057	-0.688740252	6
GPR50	-0.317877244	1.120299919	-0.802422675	6
GPR61	-0.235683298	1.096790083	-0.861106786	6
GPRASP1	-0.581056065	1.154692593	-0.573636528	6
GPS1	-0.50891945	1.152095924	-0.643176473	6
GPSM3	-0.491996172	1.150683017	-0.658686846	6
GPT2	-0.201716933	1.085481597	-0.883764664	6
GRAMD4	-0.448136647	1.145686552	-0.697549905	6
GRIA2	-0.489306681	1.150431585	-0.661124904	6
GRID2IP	-0.526354868	1.153240867	-0.626885999	6
GRIN3A	-0.400367556	1.138149324	-0.737781768	6
GRPEL1	-0.188024603	1.080665767	-0.892641164	6
GRPR	-0.131518261	1.059251561	-0.927733301	6
GSDMA	-0.080973357	1.038024892	-0.957051534	6
GSG1L2	-0.145086931	1.064618229	-0.919531298	6
GSK3A	-0.168736452	1.073633614	-0.904897162	6
GSTK1	-0.049331608	1.023752785	-0.974421176	6
GTF2I	-0.060373027	1.02881874	-0.968445713	6
GTF3A	-0.017335167	1.008554887	-0.99121972	6
GTPBP2	-0.401438206	1.138341375	-0.736903169	6
GTSE1	-0.382163375	1.134725352	-0.752561978	6
GYG1	-0.501405194	1.151504808	-0.650099614	6
GZF1	-0.177161867	1.076740968	-0.899579102	6
H2AZ1	-0.427021611	1.142617585	-0.715595974	6
H2BC11	-0.04486111	1.021675575	-0.976814465	6
H2BC18	-0.216383637	1.090476701	-0.874093065	6
H2BC4	-0.611419295	1.154016688	-0.542597393	6
H2BC6	-0.201001289	1.085233533	-0.884232244	6

H2BC7	0.038271907	0.980314618	-1.018586526	6
H3-3A	-0.496184412	1.151059964	-0.654875552	6
H3C1	-0.605504733	1.154235199	-0.548730466	6
H3C2	-0.42269968	1.141937684	-0.719238004	6
H4C11	-0.304160041	1.116763908	-0.812603866	6
H4C13	-0.3737528	1.13304321	-0.759290411	6
HACD2	-0.317697785	1.12025468	-0.802556895	6
HAGHL	-0.170864704	1.074423727	-0.903559023	6
HAO1	-0.549989725	1.154274953	-0.604285228	6
HAPLN3	-0.438082201	1.144277821	-0.70619562	6
HAPLN4	-0.058899647	1.028148038	-0.969248391	6
HAUS5	-0.332911269	1.123992874	-0.791081605	6
HBA1	-0.038306891	1.018603012	-0.980296121	6
HBA2	-0.128632021	1.058091816	-0.929459796	6
HBB	-0.619800972	1.153633303	-0.533832331	6
HBE1	-0.585820896	1.154658908	-0.568838012	6
HBG1	-0.523898552	1.153098944	-0.629200392	6
HBG2	-0.541397688	1.153969135	-0.612571447	6
HCAR1	-0.407066997	1.139333875	-0.732266878	6
HCLS1	-0.600860004	1.15437698	-0.553516976	6
HCN4	-0.349669035	1.127881565	-0.77821253	6
HCST	-0.238798699	1.097781374	-0.858982675	6
HDAC11	-0.434973633	1.143822854	-0.708849221	6
HDAC7	-0.470071856	1.148422121	-0.678350265	6
HDLBP	-0.57243149	1.154686609	-0.58225512	6
HECTD1	-0.421060687	1.141675296	-0.720614609	6
HELQ	-0.229872629	1.094920463	-0.865047834	6
HERC4	-0.191675404	1.081964167	-0.890288763	6
HIGD2A	-0.323949923	1.121814706	-0.797864784	6
HILPDA	-0.481372221	1.14964738	-0.668275159	6
HIPK2	-0.254732888	1.102729641	-0.847996754	6
HK2	-0.508290672	1.152048696	-0.643758025	6
HLA-DMB	-0.593072674	1.154556501	-0.561483828	6
HLA-DOB	-0.067128089	1.031872797	-0.964744708	6
HLA-DQA1	-0.111053196	1.050891049	-0.939837853	6
HMGA2	-0.138130727	1.061884545	-0.923753818	6
HMGB1	-0.102037194	1.047106599	-0.945069405	6
HMGB1P1	-0.018087302	1.008920962	-0.99083366	6
HMGB2	-0.620887284	1.153577223	-0.532689939	6
HMGN3	-0.621265747	1.153557339	-0.532291592	6
HNRNPA2B1	-0.091559103	1.042630944	-0.95107184	6
HNRNPA3	-0.477776772	1.149271282	-0.67149451	6
HNRNPAB	-0.411926012	1.140167331	-0.728241318	6
HNRNPF	-0.226012784	1.093663654	-0.867650871	6
HNRNPLL	-0.448464512	1.145730865	-0.697266353	6
HOXB2	-0.391073818	1.136438578	-0.745364761	6
HOXB9	0.011736644	0.994080021	-1.005816665	6
HPDL	-0.350093953	1.12797702	-0.777883067	6
HPS5	-0.567065335	1.154639825	-0.58757449	6
HS1BP3	-0.519681059	1.15284036	-0.633159301	6
HSBP1L1	0.014186167	0.992831446	-1.007017613	6
HSD11B1L	-0.031491348	1.015373715	-0.983882367	6
HSD17B8	-0.625740125	1.153308622	-0.527568496	6
HSF2BP	-0.225386176	1.093458505	-0.868072329	6
HSPA4L	-0.534167406	1.153649486	-0.619482079	6

HSPBAP1	-0.024273771	1.011915905	-0.987642134	6
HTATSF1	-0.377886001	1.133877722	-0.755991721	6
HTR1A	-0.096034478	1.044552755	-0.948518277	6
HTR1E	-0.496644856	1.151100316	-0.65445546	6
HTRA3	-0.128406094	1.058000766	-0.929594672	6
HTRA4	-0.365474501	1.131326356	-0.765851855	6
HUS1	0.004150044	0.99791852	-1.002068563	6
HUS1B	-0.575122188	1.154697676	-0.579575488	6
HYAL2	-0.610619742	1.154048728	-0.543428986	6
HYOU1	-0.386059466	1.135483215	-0.749423749	6
HYPK	-0.234172721	1.096306638	-0.862133917	6
IAH1	-0.36054511	1.130275406	-0.769730297	6
ICAM4	-0.607813022	1.154155003	-0.546341981	6
ICAM5	-0.507655142	1.152000546	-0.644345404	6
ICE2	-0.082468216	1.03868047	-0.956212254	6
IDH3A	-0.292751302	1.113703158	-0.820951857	6
IER3IP1	-0.301520327	1.116065358	-0.814545032	6
IER5L	-0.5750445	1.154697473	-0.579652973	6
IFI16	0.025247077	0.987137403	-1.012384479	6
IFNA10	-0.114563286	1.052347691	-0.937784405	6
IFNAR2	-0.466712821	1.148033598	-0.681320777	6
IFT122	-0.58816063	1.154632641	-0.566472011	6
IGF1R	-0.607246521	1.154175285	-0.546928764	6
IGF2	-0.422652328	1.141930138	-0.719277811	6
IGFBP3	-0.401733629	1.138394184	-0.736660555	6
IGFBP6	-0.16768093	1.07324045	-0.905559519	6
IGFN1	-0.196060564	1.083509958	-0.887449394	6
IGLL5	-0.238091471	1.097557022	-0.859465552	6
IGSF1	-0.275581258	1.108893727	-0.833312469	6
IGSF6	-0.099370691	1.045975514	-0.946604823	6
IHH	-0.147614187	1.065602203	-0.917988016	6
IKZF3	-0.558482792	1.154497203	-0.596014412	6
IL17RA	-0.107116526	1.049246235	-0.942129709	6
IL17RE	-0.244273393	1.09950453	-0.855231137	6
IL18RAP	-0.413974531	1.140512207	-0.726537675	6
IL2RA	-0.470730885	1.148497046	-0.677766161	6
IL2RB	-0.525902964	1.153215237	-0.627312274	6
IL34	-0.137071225	1.06146492	-0.924393695	6
IL5	-0.391587492	1.13653517	-0.744947679	6
IL6ST	-0.554036415	1.154390841	-0.600354426	6
ILK	-0.285425652	1.11168091	-0.826255258	6
IMP3	-0.064696141	1.03077724	-0.966081099	6
ING1	-0.385564032	1.135387598	-0.749823566	6
ING4	-0.384732512	1.135226622	-0.750494111	6
INO80B	-0.09450604	1.043898121	-0.94939208	6
INPP4A	-0.42124992	1.141705718	-0.720455798	6
INPP4B	-0.083697404	1.039218272	-0.955520868	6
INPP5A	-0.154903643	1.068412792	-0.91350915	6
INPP5K	-0.064215475	1.03056018	-0.966344705	6
INSIG2	-0.575327863	1.15469818	-0.579370316	6
INSL3	-0.360002151	1.130158346	-0.770156195	6
INSYN2A	-0.145360547	1.064724997	-0.91936445	6
INSYN2B	-0.248777564	1.100904165	-0.852126601	6
INTS1	-0.345607502	1.12696128	-0.781353778	6
IPO13	-0.049873963	1.024003766	-0.974129804	6

IQCK	-0.336817927	1.124920993	-0.788103066	6
IQGAP1	-0.347931028	1.127489504	-0.779558476	6
IRAK3	-0.201345722	1.085352974	-0.884007252	6
IRF2	-0.458910245	1.147088513	-0.688178269	6
IRF7	-0.404606415	1.138903569	-0.734297153	6
ISG20L2	-0.182543979	1.078697061	-0.896153082	6
ISM2	-0.545578478	1.154128046	-0.608549569	6
ISOC2	-0.471207087	1.148550919	-0.677343833	6
ISY1-RAB43	0.04022941	0.979278209	-1.019507619	6
ITGA11	-0.432624872	1.143473042	-0.71084817	6
ITGA4	-0.145836665	1.064910646	-0.919073981	6
ITGAE	-0.26156828	1.104789544	-0.843221263	6
ITGAX	-0.039395014	1.01911535	-0.979720336	6
ITLN1	-0.390869444	1.136400082	-0.745530637	6
ITPR1	0.006107415	0.996932305	-1.00303972	6
ITPRIP	-0.262901914	1.105187029	-0.842285115	6
ITPRIPL2	-0.041567611	1.020135646	-0.978568035	6
JADE3	-0.568543201	1.154655982	-0.586112781	6
JAK1	-0.198104323	1.084225247	-0.886120924	6
JMJD7-PLA2G4B	-0.627608114	1.153197315	-0.525589201	6
JPH4	-0.171573327	1.074686024	-0.903112697	6
KANK2	-0.230307211	1.095061224	-0.864754013	6
KANK3	-0.594958472	1.154519674	-0.559561202	6
KANSL1L	-0.165137485	1.072289516	-0.90715203	6
KASH5	-0.250192384	1.101340438	-0.851148054	6
KATNAL1	0.019965377	0.989867819	-1.009833196	6
KATNAL2	0.012131996	0.993878806	-1.006010802	6
KBTBD13	-0.512513965	1.152358024	-0.639844059	6
KBTBD3	-0.061485231	1.029323947	-0.967838716	6
KBTBD6	-0.517786934	1.152718124	-0.634931191	6
KBTBD7	-0.295415743	1.114427662	-0.819011919	6
KCNC3	-0.544659786	1.154094764	-0.609434978	6
KCNE3	-0.176064733	1.076339458	-0.900274725	6
KCNG2	0.026332631	0.986573623	-1.012906254	6
KCNH2	-0.613892638	1.153912594	-0.540019957	6
KCNH8	-0.593164049	1.154554814	-0.561390765	6
KCNK17	-0.191500546	1.081902217	-0.89040167	6
KCNK5	-0.530925246	1.153487841	-0.622562595	6
KCNQ2	-0.141074181	1.063045809	-0.921971628	6
KCTD20	0.059511418	0.968915305	-1.028426723	6
KCTD21	-0.326183357	1.122363962	-0.796180605	6
KDM3A	-0.506371104	1.151901986	-0.645530882	6
KDM4E	-0.016974238	1.008379067	-0.991404828	6
KHDC1	-0.269649916	1.107176074	-0.837526157	6
KHDC4	-0.111425879	1.051046153	-0.939620273	6
KHDRBS3	-0.515652811	1.152575887	-0.636923076	6
KIAA0100	-0.35623362	1.129338764	-0.773105144	6
KIAA0513	-0.015805611	1.007809119	-0.992003509	6
KIAA1143	-0.603271896	1.154306614	-0.551034718	6
KIAA1549L	-0.279440116	1.109995749	-0.830555633	6
KIDINS220	-0.122558053	1.055630394	-0.933072341	6
KIF22	-0.171359397	1.074606879	-0.903247482	6
KIF4B	-0.606329976	1.154207273	-0.547877297	6
KIF9	-0.346001955	1.127051283	-0.781049328	6
KLHDC10	-0.609471437	1.154093373	-0.544621937	6

KLHDC4	-0.289526201	1.112818334	-0.823292133	6
KLHL17	0.011473481	0.994213893	-1.005687374	6
KLHL28	-0.073912405	1.034905459	-0.960993053	6
KLHL29	0.044844913	0.976823109	-1.021668022	6
KLHL6	-0.024588049	1.012067284	-0.987479235	6
KLK14	-0.052584965	1.025255002	-0.972670037	6
KLK4	-0.6045948	1.154265032	-0.549670232	6
KMT2B	-0.389377078	1.136117834	-0.746740757	6
KPNA1	0.026656646	0.986405175	-1.013061821	6
KRBA1	-0.543935464	1.154067873	-0.610132409	6
KRBA2	0.054689292	0.97153313	-1.026222422	6
KRI1	-0.300701912	1.1158476	-0.815145688	6
KRR1	-0.100583433	1.046490607	-0.945907174	6
KRT12	-0.515339759	1.15255462	-0.637214861	6
KRT14	-0.189466398	1.081179783	-0.891713385	6
KRT17	-0.330861192	1.123500611	-0.792639419	6
KRT222	-0.549664663	1.154264858	-0.604600195	6
KRT71	-0.569655752	1.154666507	-0.585010754	6
KRT78	-0.109034388	1.050049025	-0.941014637	6
KRTAP10-4	-0.061258558	1.029221058	-0.9679625	6
KRTAP10-9	-0.569191166	1.154662283	-0.585471117	6
KRTAP13-4	-0.619905254	1.153627983	-0.533722729	6
KRTAP5-1	-0.159199612	1.070050013	-0.910850401	6
KRTAP5-5	-0.542358991	1.154007363	-0.611648372	6
KRTAP9-1	-0.140226525	1.062712069	-0.922485545	6
KRTCAP2	-0.043382283	1.020985134	-0.977602851	6
KTN1	-0.435408432	1.14388704	-0.708478607	6
KYAT1	-0.064935579	1.030885301	-0.965949723	6
L2HGDH	-0.526340634	1.153240063	-0.626899429	6
L3MBTL4	0.011675464	0.994111148	-1.005786612	6
LACRT	-0.091205933	1.04247864	-0.951272707	6
LAMB3	0.012264337	0.993811425	-1.006075762	6
LARGE1	-0.039445019	1.019138873	-0.979693854	6
LARP4B	-0.346201985	1.127096873	-0.780894887	6
LARS2	-0.104063471	1.047962504	-0.943899033	6
LASP1	-0.153467572	1.067862324	-0.914394752	6
LATS1	0.002057442	0.998969692	-1.001027133	6
LCE1B	-0.104446314	1.048123868	-0.943677554	6
LCE4A	-0.28879447	1.112616381	-0.823821912	6
LCN9	-0.244799535	1.099668866	-0.854869331	6
LCP1	0.000531408	0.99973419	-1.000265598	6
LDB3	-0.145345409	1.064719091	-0.919373683	6
LDHA	-0.627432863	1.153207945	-0.525775082	6
LDHD	0.019100174	0.990313097	-1.009413272	6
LDLRAD2	0.029052508	0.985157178	-1.014209686	6
LEP	-0.157929601	1.069567486	-0.911637885	6
LGALS2	-0.541327311	1.153966297	-0.612638986	6
LGALS4	-0.20838011	1.087771926	-0.879391816	6
LGALS8	-0.283650267	1.111184146	-0.827533878	6
LGMN	-0.334384502	1.124344409	-0.789959907	6
LILRA6	-0.27028842	1.107362358	-0.837073938	6
LILRB1	-0.528322841	1.153349945	-0.625027104	6
LIM2	-0.505517323	1.151835509	-0.646318185	6
LIMD2	-0.172902812	1.075177082	-0.902274271	6
LIME1	-0.32418886	1.121873669	-0.797684809	6

LIN37	-0.412721257	1.140301672	-0.727580415	6
LIN7A	-0.358617208	1.129858589	-0.771241381	6
LIPJ	-0.341945628	1.126119342	-0.784173714	6
LKAAEAR1	-0.038277837	1.01858932	-0.980311483	6
LMBR1	-0.066290306	1.031495891	-0.965205585	6
LNPEP	-0.289168826	1.112719756	-0.82355093	6
LONP1	-0.566037004	1.15462712	-0.588590116	6
LOXL4	-0.116625263	1.053199013	-0.936573749	6
LPCAT2	-0.419742895	1.14146252	-0.721719625	6
LPGAT1	-0.565827902	1.15462439	-0.588796488	6
LRIG2	-0.367200982	1.1316894	-0.764488418	6
LRIT3	-0.61081673	1.154040907	-0.543224177	6
LRRC24	-0.419307426	1.141391853	-0.722084427	6
LRRC27	-0.25334734	1.102307487	-0.848960146	6
LRRC37A	-0.212597992	1.089203656	-0.876605664	6
LRRC37A2	-0.01948221	1.009598761	-0.990116551	6
LRRC49	-0.238299829	1.097623161	-0.859323332	6
LRRC4B	-0.412730418	1.140303216	-0.727572799	6
LRRC58	-0.619219966	1.153662692	-0.534442726	6
LRRK2	-0.290247701	1.113017028	-0.822769328	6
LRTM2	-0.096327132	1.044677898	-0.948350765	6
LTB	-0.021170778	1.010417299	-0.989246521	6
LTF	-0.604223499	1.154276917	-0.550053417	6
LTN1	-0.457295198	1.146885491	-0.689590293	6
LTV1	-0.022161377	1.010896499	-0.988735122	6
LUC7L3	-0.382620161	1.134814908	-0.752194747	6
LY6G5C	-0.160985828	1.070726559	-0.909740731	6
LY75	-0.317455097	1.120193458	-0.802738362	6
LY75-CD302	-0.464840318	1.147812211	-0.682971893	6
LYPLAL1	-0.006179436	1.003075398	-0.996895962	6
LYSMD3	-0.246818209	1.100297317	-0.853479107	6
LZTS1	-0.470716342	1.148495397	-0.677779055	6
LZTS2	-0.559233475	1.154512983	-0.595279508	6
MACROH2A1	-0.58881936	1.154624084	-0.565804724	6
MAD1L1	-0.067280709	1.031941401	-0.964660692	6
MADCAM1	-0.003488791	1.001739831	-0.99825104	6
MAFIP	-0.319240433	1.120642672	-0.801402238	6
MAGEB17	-0.466328527	1.147988443	-0.681659915	6
MAGEF1	-0.110613728	1.050708015	-0.940094288	6
MALSU1	-0.503814059	1.151700642	-0.647886583	6
MAN1A1	-0.542208215	1.154001434	-0.611793219	6
MAN1B1	-0.409051989	1.139676973	-0.730624984	6
MAN2A1	-0.574605792	1.154696197	-0.580090405	6
MANF	-0.237890588	1.097493224	-0.859602636	6
MAP2K5	-0.470829432	1.148508213	-0.677678781	6
MAP2K7	-0.513693998	1.152441135	-0.638747137	6
MAP3K7	-0.205120976	1.086656032	-0.881535055	6
MAP4	-0.047546365	1.022925076	-0.975378712	6
MAP4K2	0.00718743	0.996386913	-1.003574343	6
MAPK11	-0.090803827	1.042305118	-0.951501291	6
MAPK14	-0.041553232	1.020128905	-0.978575672	6
MAPK3	-0.513430988	1.152422737	-0.638991749	6
MAPK8	-0.376725607	1.133644964	-0.756919357	6
MAPKAPK3	-0.337756228	1.125141963	-0.787385734	6
MARCHF1	-0.371248534	1.132530226	-0.761281692	6

MARS2	-0.474363005	1.148902296	-0.674539291	6
MARVELD1	-0.148057154	1.065774164	-0.91771701	6
MAST2	-0.533637754	1.15362385	-0.619986096	6
MAST3	-0.494629467	1.150922097	-0.65629263	6
MB21D2	-0.164840067	1.072177991	-0.907337924	6
MBD1	-0.503435072	1.151670227	-0.648235155	6
MBD3L3	-0.138490971	1.062027027	-0.923536055	6
MBD3L4	-0.158282381	1.069701646	-0.911419265	6
MBD3L5	-0.270952746	1.107555823	-0.836603077	6
MBNL2	-0.036831822	1.017907063	-0.981075241	6
MBTPS2	-0.253637081	1.102395894	-0.848758814	6
MCCC2	-0.536287996	1.153749096	-0.6174611	6
MCM3AP	-0.619939497	1.153626234	-0.533686737	6
MCM6	0.017953442	0.990902399	-1.008855842	6
MDK	0.068510474	0.963983079	-1.032493553	6
MDM1	-0.595331073	1.154511897	-0.559180824	6
MECR	-0.104519144	1.048154552	-0.943635408	6
MEFV	-0.430645392	1.143174198	-0.712528806	6
MEGF9	0.021211755	0.989225382	-1.010437136	6
MEI1	-0.509934001	1.152171262	-0.642237261	6
MEMO1	0.026921113	0.986267627	-1.01318874	6
MEN1	-0.520172814	1.152871478	-0.632698664	6
MEOX2	-0.618616503	1.153692771	-0.535076268	6
METAP2	-0.034608306	1.016854902	-0.982246596	6
METRN	-0.104289221	1.048057668	-0.943768448	6
METTL3	-0.60916874	1.154104873	-0.544936133	6
METTL4	-0.623868663	1.153415715	-0.529547052	6
MFSD14B	-0.393292368	1.136854052	-0.743561684	6
MFSD4B	-0.520965389	1.152921094	-0.631955705	6
MFSD6	-0.195930642	1.083464376	-0.887533734	6
MFSD8	0.013729788	0.993064413	-1.006794201	6
MGAT5	-0.288939839	1.112656537	-0.823716698	6
MGLL	-0.153362364	1.067821933	-0.914459569	6
MICOS10	-0.340729716	1.125837226	-0.78510751	6
MICOS10-NBL1	-0.474037995	1.148866564	-0.674828569	6
MICU1	-0.105991577	1.048774046	-0.94278247	6
MIEF1	-0.066632349	1.031649835	-0.965017486	6
MIEF2	-0.138865926	1.062175221	-0.923309295	6
MIGA2	-0.288377251	1.112501034	-0.824123783	6
MIS18BP1	-0.30243584	1.11630829	-0.81387245	6
MITD1	-0.077987352	1.036710309	-0.958722957	6
MKX	-0.186495349	1.080118797	-0.893623449	6
MLANA	-0.521792686	1.152972178	-0.631179491	6
MLKL	-0.547148637	1.154182788	-0.607034151	6
MMP2	-0.452829313	1.14631096	-0.693481647	6
MMP24	-0.06201867	1.029565925	-0.967547255	6
MNAT1	-0.39857206	1.137824913	-0.739252853	6
MNDA	-0.560085685	1.154530132	-0.594444446	6
MOK	-0.377146648	1.133729557	-0.756582909	6
MON1B	-0.538283002	1.153838374	-0.615555372	6
MPDU1	-0.17559781	1.076168298	-0.900570488	6
MPEG1	-0.100586115	1.046491745	-0.94590563	6
MPG	-0.607603456	1.154162552	-0.546559096	6
MPHOSPH8	-0.357794566	1.129679743	-0.771885177	6
MPP2	-0.38586189	1.13544511	-0.74958322	6

MPST	-0.575697006	1.154698962	-0.579001956	6
MPZ	-0.03362893	1.016390286	-0.982761356	6
MRO	-0.035736972	1.017389447	-0.981652475	6
MROH1	-0.151657946	1.067166403	-0.915508457	6
MRPL16	-0.233409286	1.096061615	-0.862652329	6
MRPL34	-0.475268071	1.149001248	-0.673733176	6
MRPL54	-0.211158437	1.088716587	-0.87755815	6
MRPS16	-0.109209268	1.050122088	-0.94091282	6
MRPS18B	-0.616883397	1.153776636	-0.536893238	6
MRPS34	-0.518656742	1.152774725	-0.634117984	6
MRPS5	-0.502807603	1.151619546	-0.648811942	6
MRS2	-0.44094801	1.144689153	-0.703741143	6
MRTO4	-0.232306705	1.095706922	-0.863400217	6
MS4A4E	-0.563614345	1.154592457	-0.590978112	6
MS4A6A	-0.267994743	1.106691626	-0.838696883	6
MS4A6E	-0.014042625	1.006947362	-0.992904736	6
MSL1	-0.443012666	1.14498066	-0.701967994	6
MSR1	-0.415682613	1.140796811	-0.725114198	6
MST1R	-0.063136444	1.030072274	-0.96693583	6
MT-CO1	-0.369678451	1.132205777	-0.762527325	6
MT-ND3	-0.612877865	1.153956214	-0.541078348	6
MT-ND6	-0.367063534	1.131660593	-0.76459706	6
MT1F	-0.08664666	1.040503989	-0.953857329	6
MTERF3	-0.49258065	1.15073669	-0.65815604	6
MTFMT	-0.382320926	1.134756262	-0.752435336	6
MTG1	-0.352217029	1.128451611	-0.776234582	6
MTHFD1	-0.111355575	1.051016902	-0.939661326	6
MTMR4	-0.246828265	1.100300439	-0.853472174	6
MTMR8	-0.611612136	1.154008842	-0.542396706	6
MTO1	-0.48515744	1.150029376	-0.664871936	6
MTOR	-0.270356599	1.10738223	-0.837025631	6
MTRNR2L1	-0.543311622	1.154044252	-0.61073263	6
MTRR	-0.129652262	1.058502496	-0.928850234	6
MUC2	0.056207698	0.970710709	-1.026918407	6
MYCBP	-0.53839014	1.153843047	-0.615452907	6
MYCBP2	-0.41824658	1.141218966	-0.722972386	6
MYL12A	-0.471411112	1.148573932	-0.677162821	6
MYO15A	-0.090687633	1.042254953	-0.95156732	6
MYO1H	-0.564409097	1.154604559	-0.590195462	6
MYO7A	0.00600106	0.996985965	-1.002987025	6
MYO9A	-0.215641755	1.090228112	-0.874586357	6
MYOM2	-0.601902045	1.15434744	-0.552445395	6
MZF1	-0.246725557	1.100268544	-0.853542987	6
NAA38	-0.154924725	1.068420862	-0.913496137	6
NAA60	-0.388556661	1.135961818	-0.747405157	6
NAALAD2	-0.620861359	1.153578579	-0.53271722	6
NAALADL1	-0.160289232	1.07046301	-0.910173778	6
NAGPA	-0.148057929	1.065774465	-0.917716536	6
NAGS	-0.441884586	1.14482189	-0.702937304	6
NAIF1	-0.045752346	1.022090886	-0.97633854	6
NAIP	-0.547993905	1.154211137	-0.606217231	6
NALF2	-0.376963578	1.133692795	-0.756729217	6
NANOS2	-0.517019567	1.15266753	-0.635647963	6
NAP1L3	-0.481692465	1.149680253	-0.667987788	6
NAP1L4	-0.583966452	1.154675168	-0.570708716	6

NARF	0.035084377	0.981996113	-1.017080489	6
NAT9	-0.120814255	1.054918534	-0.934104278	6
NAV1	-0.181689281	1.078387933	-0.896698652	6
NBPF12	0.046934393	0.975706398	-1.022640791	6
NCAPD2	-0.180614785	1.077998501	-0.897383715	6
NCBP1	-0.354680837	1.128997462	-0.774316625	6
NCF1	-0.527293007	1.153293379	-0.626000372	6
NCOA4	-0.331481441	1.123649923	-0.792168482	6
NCOA5	-0.074492704	1.035163246	-0.960670542	6
NCOR1	0.055255546	0.97122663	-1.026482176	6
NDN	-0.315514549	1.119702141	-0.804187593	6
NDRG1	-0.304522805	1.116859451	-0.812336647	6
NDRG3	-0.597703713	1.154458486	-0.556754773	6
NDUFA12	-0.278883453	1.10983753	-0.830954076	6
NDUFA5	-0.452713998	1.14629587	-0.693581873	6
NDUFA6	-0.506203076	1.151888962	-0.645685887	6
NDUFAF8	-0.075291216	1.035517556	-0.96022634	6
NDUFB10	0.029798806	0.984767553	-1.014566359	6
NDUFB5	-0.115012324	1.05253336	-0.937521035	6
NDUFC1	-0.395625471	1.137286186	-0.741660716	6
NDUFS7	-0.243049869	1.099121513	-0.856071644	6
NECAP2	-0.191240648	1.081810093	-0.890569445	6
NEDD4	-0.077242833	1.036381487	-0.959138654	6
NEIL1	-0.111655232	1.051141552	-0.939486321	6
NEK11	-0.316134493	1.119859447	-0.803724954	6
NES	-0.138120351	1.061880439	-0.923760089	6
NETO1	-0.163999887	1.071862574	-0.907862687	6
NEU2	-0.423420429	1.142052278	-0.718631849	6
NEU3	-0.442050254	1.144845282	-0.702795029	6
NEURL4	-0.377682743	1.133837038	-0.756154294	6
NF1	-0.07676019	1.036168101	-0.959407911	6
NFATC2	-0.259192913	1.104077998	-0.844885085	6
NFATC3	-0.447097257	1.145545392	-0.698448135	6
NFE2	-0.511167154	1.152261393	-0.641094239	6
NFS1	-0.062230633	1.029662016	-0.967431383	6
NHEJ1	-0.61665458	1.153787429	-0.537132849	6
NHLRC2	-0.163306011	1.071601671	-0.90829566	6
NHLRC4	-0.428663802	1.14287135	-0.714207548	6
NHS	-0.601058297	1.15437146	-0.553313163	6
NIF3L1	-0.252586278	1.102074943	-0.849488665	6
NIPA1	-0.241543818	1.098648403	-0.857104585	6
NISCH	-0.29953099	1.115535079	-0.816004089	6
NKG7	-0.403133877	1.138643407	-0.73550953	6
NKX1-1	-0.395334954	1.137232645	-0.741897692	6
NLN	-0.006573191	1.003270393	-0.996697202	6
NLRP8	-0.336526245	1.124852149	-0.788325903	6
NMNAT1	-0.497105328	1.151140455	-0.654035127	6
NOC2L	-0.486961567	1.150206389	-0.663244822	6
NOC3L	-0.494045311	1.150869669	-0.656824357	6
NOD2	-0.044226711	1.021379586	-0.977152874	6
NOL8	-0.600642331	1.154382986	-0.553740655	6
NOP14	-0.300001031	1.115660671	-0.81565964	6
NPB	-0.313513182	1.119192102	-0.80567892	6
NPBWR1	-0.576026683	1.154699528	-0.578672844	6
NPFFR1	-0.175676767	1.076197253	-0.900520486	6

NPL	-0.193338103	1.082552048	-0.889213945	6
NPR1	-0.541389962	1.153968824	-0.612578862	6
NPR2	-0.562131994	1.154567981	-0.592435987	6
NPTX1	-0.572106844	1.154684713	-0.582577869	6
NR1H3	-0.378372348	1.13397492	-0.755602572	6
NRBP2	-0.365536827	1.131339508	-0.765802681	6
NRIP2	-0.468471954	1.14823845	-0.679766496	6
NRK	-0.347541581	1.127401294	-0.779859713	6
NSD1	-0.369259812	1.1321189	-0.762859088	6
NUBP1	-0.30248591	1.116321556	-0.813835646	6
NUBPL	-0.35791976	1.129706999	-0.771787239	6
NUDCD3	-0.394282073	1.137037964	-0.742755891	6
NUDT17	-0.60484835	1.15425682	-0.54940847	6
NUDT3	-0.209761577	1.088242401	-0.878480824	6
NUFIP2	-0.037524876	1.018234255	-0.980709379	6
NUP205	-0.052258329	1.02510454	-0.972846211	6
NUP50	-0.228525888	1.094483296	-0.865957408	6
NUP58	-0.601533969	1.154358024	-0.552824055	6
NUSAP1	-0.386685153	1.135603657	-0.748918504	6
NUTM2E	-0.369373427	1.132142493	-0.762769066	6
NXPH3	0.055040274	0.97134318	-1.026383454	6
NYNRIN	-0.573220542	1.154690715	-0.581470174	6
OAS1	-0.547166497	1.154183395	-0.607016898	6
OCM2	0.028434751	0.985479378	-1.013914129	6
OGDH	-0.438442976	1.144330032	-0.705887055	6
OGFOD1	-0.244577326	1.099599488	-0.855022162	6
OGFR	-0.145647045	1.06483673	-0.919189685	6
OGT	0.026739177	0.986362257	-1.013101434	6
OLFML3	-0.604053636	1.154282298	-0.550228663	6
OMG	-0.28603688	1.111851335	-0.825814454	6
OMP	-0.337691045	1.125126636	-0.787435591	6
OOSP3	-0.616843122	1.15377854	-0.536935418	6
OPA1	-0.111236451	1.05096733	-0.939730878	6
OPN1SW	-0.43702534	1.144124165	-0.707098825	6
OPTN	-0.134052373	1.060264562	-0.926212189	6
OR10G4	0.054697302	0.971528796	-1.026226098	6
OR10G9	-0.554546757	1.154404167	-0.59985741	6
OR10H3	-0.020045081	1.009871852	-0.989826771	6
OR1D2	-0.049385132	1.023777563	-0.974392431	6
OR2C1	-0.263117836	1.105251248	-0.842133412	6
OR2C3	0.051349262	0.9733361	-1.024685362	6
OR2L8	-0.501675823	1.151527107	-0.649851284	6
OR2V2	-0.232755683	1.095851472	-0.86309579	6
OR4C46	-0.571609229	1.154681572	-0.583072342	6
OR56A4	-0.437617399	1.144210374	-0.706592975	6
OR56B4	-0.550337497	1.154285623	-0.603948126	6
OR6J1	-0.492617881	1.150740097	-0.658122217	6
OR6N2	0.056651883	0.970469795	-1.027121678	6
OR6Q1	-0.538754632	1.153858851	-0.615104218	6
OR8G5	-0.502980521	1.151633553	-0.648653032	6
OR8I2	-0.548980836	1.154243242	-0.605262406	6
ORC2	-0.611947421	1.153995093	-0.542047672	6
ORMDL1	-0.402295193	1.138494348	-0.736199156	6
OSBPL1A	-0.220452107	1.091832213	-0.871380105	6
OSBPL3	-0.394090444	1.137002424	-0.74291198	6

OSER1	-0.591279186	1.154587608	-0.563308423	6
OSGEPL1	-0.075573003	1.035642473	-0.960069471	6
OXNAD1	-0.512055423	1.152325337	-0.640269914	6
OXSM	-0.125735887	1.056921697	-0.93118581	6
OXSR1	-0.149357756	1.06627819	-0.916920434	6
P2RX5	-0.606264007	1.154209535	-0.547945528	6
P2RX7	-0.046316479	1.022353459	-0.976036981	6
P3R3URF-PIK3R3	-0.554620014	1.154406056	-0.599786043	6
P4HA3	-0.565378992	1.154618362	-0.589239369	6
PA2G4	-0.078573829	1.036969036	-0.958395207	6
PABIR2	-0.163583876	1.071706195	-0.908122319	6
PABPC1L	-0.22914707	1.094685118	-0.865538048	6
PAFAH2	-0.39094447	1.136414218	-0.745469748	6
PAGE5	-0.306642781	1.117415605	-0.810772824	6
PAK2	-0.28745168	1.112244631	-0.824792951	6
PALS1	-0.431790228	1.143347484	-0.711557257	6
PANK3	0.039429913	0.979701854	-1.019131767	6
PAPSS1	-0.466036816	1.147954069	-0.681917253	6
PARP1	0.055647658	0.971014248	-1.026661906	6
PARP11	-0.619578233	1.153644619	-0.534066387	6
PARP12	-0.18349839	1.079041581	-0.895543191	6
PARP4	-0.537901402	1.15382163	-0.615920228	6
PARP6	-0.156679182	1.069091187	-0.912412005	6
PARP8	-0.555940859	1.154439095	-0.598498236	6
PCBP1	-0.280074908	1.110175864	-0.830100956	6
PCDHB4	-0.142670965	1.063672999	-0.921002034	6
PCDHB5	-0.172661408	1.07508802	-0.902426611	6
PCNP	-0.479896935	1.149494617	-0.669597683	6
PCNX1	0.041033435	0.97885168	-1.019885114	6
PCNX4	-0.392660039	1.136736086	-0.744076047	6
PDCD11	0.047604653	0.975347486	-1.022952139	6
PDCD6	-0.524063794	1.153108692	-0.629044898	6
PDE3A	-0.509805415	1.152161772	-0.642356357	6
PDE6D	-0.594764802	1.154523651	-0.559758849	6
PDIA6	-0.593426495	1.154549914	-0.561123418	6
PDK1	-0.596407788	1.154488492	-0.558080704	6
PDK2	-0.578822819	1.154699285	-0.575876467	6
PECAM1	-0.525582446	1.153196928	-0.627614482	6
PEF1	-0.172855962	1.075159801	-0.90230384	6
PELP1	-0.582554084	1.154684857	-0.572130773	6
PES1	-0.052186414	1.025071402	-0.972884988	6
PEX10	-0.568280069	1.154653287	-0.586373217	6
PEX12	-0.443136742	1.144998049	-0.701861307	6
PEX3	-0.169047849	1.073749439	-0.90470159	6
PEX5	-0.407651389	1.13943526	-0.731783871	6
PFN1	-0.351708334	1.128338254	-0.77662992	6
PGAP2	-0.192178843	1.082142396	-0.889963554	6
PGBD1	-0.55452835	1.154403692	-0.599875342	6
PGP	-0.36012853	1.130185616	-0.770057086	6
PHACTR2	-0.122912281	1.055774715	-0.932862435	6
PHETA1	-0.310117079	1.118318913	-0.808201834	6
PHF1	-0.464343908	1.147752945	-0.683409037	6
PHF21B	-0.599754597	1.154406887	-0.55465229	6
PHF3	-0.605295569	1.154242145	-0.548946576	6
PHF6	-0.139567834	1.062452349	-0.922884514	6

PIAS3	-0.225569017	1.093518399	-0.867949382	6
PICK1	0.007343975	0.996307787	-1.003651762	6
PIGG	-0.350675719	1.128107457	-0.777431737	6
PIGH	-0.42141178	1.141731713	-0.720319933	6
PIGV	-0.247208279	1.100418375	-0.853210096	6
PIK3C2A	-0.416217835	1.140885437	-0.724667602	6
PIK3IP1	-0.307488664	1.117636464	-0.810147799	6
PIK3R3	-0.268070692	1.106713904	-0.838643213	6
PIP	-0.489739657	1.150472557	-0.660732899	6
PIP4K2C	-0.025287583	1.012403965	-0.987116381	6
PIP4P1	-0.560334444	1.154534984	-0.59420054	6
PKDREJ	-0.029613972	1.014478061	-0.98486409	6
PKN2	-0.502293957	1.151577756	-0.6492838	6
PLA2G12B	-0.4991007	1.151311886	-0.652211186	6
PLA2G4B	-0.524569498	1.153138346	-0.628568848	6
PLAAT4	-0.273715165	1.108356428	-0.834641263	6
PLCG2	0.0019447	0.999026232	-1.000970932	6
PLCXD1	-0.48605079	1.150117437	-0.664066647	6
PLD4	-0.336004195	1.124728749	-0.788724554	6
PLD5P1	-0.350464558	1.128060147	-0.777595589	6
PLEKHG3	-0.24907149	1.100994932	-0.851923442	6
PLEKHS1	-0.331196675	1.123581412	-0.792384738	6
PLIN2	-0.277389907	1.109411766	-0.832021859	6
PLK5	-0.033715277	1.016431278	-0.982716001	6
PLXNA2	0.009195335	0.995370624	-1.004565959	6
PLXNB3	-0.262147375	1.104962318	-0.842814944	6
PLXNC1	-0.39807006	1.137733688	-0.739663628	6
PM20D1	-0.251404168	1.101712821	-0.850308653	6
PM20D2	-0.273190395	1.108204819	-0.835014424	6
PMPCB	-0.611119148	1.154028808	-0.54290966	6
PMS1	-0.054132538	1.02596679	-0.971834252	6
PMS2	-0.211449208	1.088815101	-0.877365892	6
PNKP	-0.406536727	1.139241609	-0.732704883	6
PNMA6F	-0.586094877	1.154656164	-0.568561288	6
PNMA8C	-0.406305164	1.139201237	-0.732896073	6
PNPLA7	-0.01988593	1.00979466	-0.98990873	6
PNPO	-0.60434527	1.154273038	-0.549927767	6
POC5	-0.147199477	1.065441074	-0.918241597	6
POLB	-0.49914104	1.15131531	-0.65217427	6
POLD2	0.029383505	0.984984424	-1.014367929	6
POLG2	-0.566566858	1.154633816	-0.588066958	6
POLH	-0.568268858	1.15465317	-0.586384312	6
POLL	-0.53596138	1.15373407	-0.61777269	6
POLM	-0.488710524	1.150374863	-0.661664339	6
POLR3H	-0.367377128	1.131726292	-0.764349164	6
POM121L2	-0.481353909	1.149645497	-0.668291587	6
POPDC3	-0.045007773	1.02174396	-0.976736187	6
PORCN	-0.449914478	1.1459256	-0.696011122	6
POTEF	-0.235256326	1.09665362	-0.861397294	6
PP2D1	-0.544534507	1.154090154	-0.609555647	6
PPA2	-0.161724411	1.071005585	-0.909281173	6
PPAT	0.007468375	0.996244896	-1.003713271	6
PPBP	-0.56891699	1.154659675	-0.585742685	6
PPDPF	-0.192224485	1.082158545	-0.88993406	6
PPFIBP2	-0.573888474	1.154693633	-0.58080516	6

PPHLN1	-0.154368435	1.068207825	-0.913839389	6
PPIAL4H	-0.000293933	1.000146934	-0.999853001	6
PPIG	-0.077111466	1.036323424	-0.959211959	6
PPIL3	-0.255805462	1.103055372	-0.847249911	6
PPIP5K1	-0.031895968	1.015566404	-0.983670436	6
PPL	0.005918928	0.997027398	-1.002946326	6
PPME1	-0.604488008	1.154268467	-0.549780459	6
PPP1R12A	-0.452760621	1.146301973	-0.693541352	6
PPP1R14A	-0.252963105	1.102190141	-0.849227037	6
PPP1R14B	0.06417858	0.966364932	-1.030543511	6
PPP1R21	-0.168373367	1.073498468	-0.905125101	6
PPP1R3E	-0.055339911	1.026520855	-0.971180945	6
PPP1R7	-0.39230192	1.136669116	-0.744367197	6
PPP2R1A	-0.005493193	1.002735281	-0.997242088	6
PPP2R2C	-0.049832466	1.023984571	-0.974152105	6
PPP2R5B	-0.303054262	1.116471992	-0.813417731	6
PPP2R5C	-0.13627868	1.061150463	-0.924871784	6
PPP3CC	-0.19123956	1.081809707	-0.890570147	6
PPP5C	-0.441407109	1.144754323	-0.703347214	6
PPT1	-0.321978521	1.121326375	-0.799347854	6
PRAG1	-0.529665226	1.153421979	-0.623756753	6
PRCP	-0.356090817	1.129307464	-0.773216646	6
PRDM16	-0.036747909	1.017867423	-0.981119514	6
PRDX1	0.064616955	0.966124538	-1.030741493	6
PRELID3A	-0.609377028	1.154096972	-0.544719944	6
PREX1	-0.512764605	1.152375799	-0.639611193	6
PRF1	-0.146599034	1.065207548	-0.918608514	6
PRICKLE3	0.03252373	0.983341384	-1.015865114	6
PRIM2	-0.612146866	1.153986849	-0.541839983	6
PRKAB2	-0.502412806	1.15158745	-0.649174644	6
PRKACA	-0.314075661	1.119335788	-0.805260127	6
PRKAG3	-0.492612384	1.150739594	-0.65812721	6
PRKAR1A	-0.286873295	1.112084047	-0.825210752	6
PRKAR1B	-0.040707643	1.019732211	-0.979024568	6
PRKCD	-0.525833002	1.15321125	-0.627378249	6
PRKCE	0.008372127	0.995787651	-1.004159779	6
PRKX	-0.077580353	1.036530607	-0.958950254	6
PRM2	-0.060375485	1.028819857	-0.968444372	6
PROK1	-0.205518038	1.086792429	-0.881274391	6
PROK2	-0.200365856	1.085012935	-0.884647079	6
PROKR2	-0.534837587	1.15368149	-0.618843903	6
PRPF19	-0.24863356	1.100859669	-0.852226109	6
PRPF4	-0.494227229	1.150886033	-0.656658804	6
PRPSAP2	-0.431586795	1.143316782	-0.711729987	6
PRR13	-0.157361587	1.069351273	-0.911989685	6
PRR14L	-0.512456802	1.152353961	-0.639897159	6
PRR16	-0.322193001	1.121379663	-0.799186662	6
PRR19	-0.164479441	1.072042673	-0.907563232	6
PRR20A	-0.544386158	1.154084673	-0.609698515	6
PRR20B	-0.593546873	1.154547639	-0.561000766	6
PRR20C	-0.590932215	1.154593187	-0.563660972	6
PRR20D	-0.627880478	1.153180716	-0.525300239	6
PRR20E	-0.545387182	1.154121192	-0.608734011	6
PRRC1	-0.052332249	1.025138597	-0.972806349	6
PRRC2A	-0.620371235	1.153604047	-0.533232812	6

PRRT2	-0.131583226	1.059277592	-0.927694366	6
PRRT4	0.049179192	0.97450302	-1.023682212	6
PRSS35	-0.515759022	1.152583078	-0.636824057	6
PRXL2A	-0.556838079	1.154460429	-0.59762235	6
PSENEN	-0.244661249	1.099625695	-0.854964446	6
PSG1	-0.112836292	1.051632183	-0.93879589	6
PSMA6	-0.578344964	1.154699967	-0.576355003	6
PSMB1	-0.451178428	1.146093706	-0.694915278	6
PSMB7	-0.113731578	1.052003391	-0.938271813	6
PSMC6	-0.496424368	1.15108102	-0.654656652	6
PSMD3	-0.347891468	1.12748055	-0.779589081	6
PSME2	-0.241899994	1.098760457	-0.856860462	6
PSME3IP1	-0.426333475	1.142510498	-0.716177024	6
PSMG4	-0.53483789	1.153681504	-0.618843614	6
PTAFR	-0.063318964	1.030154866	-0.966835902	6
PTGDR2	-0.246358127	1.100154373	-0.853796246	6
PTGES3L	-0.02302393	1.011313157	-0.988289227	6
PTGFRN	-0.502644859	1.151606334	-0.648961475	6
PTPA	-0.017377351	1.008575429	-0.991198079	6
PTPRF	-0.392906087	1.136782031	-0.743875944	6
PTRH1	-0.270891118	1.107537891	-0.836646773	6
PUM3	-0.403471817	1.138703288	-0.735231471	6
PXK	-0.276446566	1.109141906	-0.832695339	6
PXMP2	-0.021822291	1.01073255	-0.988910259	6
PYGB	-0.623011604	1.153463287	-0.530451683	6
PYGO2	0.004038548	0.99797461	-1.002013158	6
QKI	-0.019678775	1.009694157	-0.990015381	6
QRSL1	-0.468746031	1.148270093	-0.679524062	6
RAB10	-0.184537062	1.079415711	-0.894878649	6
RAB25	-0.229288046	1.094730878	-0.865442832	6
RAB27A	-0.406099389	1.13916532	-0.733065931	6
RAB2B	-0.540797254	1.153944748	-0.613147494	6
RAB31	-0.310220335	1.118345605	-0.80812527	6
RAB3GAP1	-0.140130193	1.062674107	-0.922543913	6
RAB40B	-0.114867801	1.052473619	-0.937605818	6
RAB5A	-0.259718881	1.104235947	-0.844517066	6
RAB5IF	-0.452475072	1.146264564	-0.693789492	6
RAB8B	-0.254776719	1.102742971	-0.847966251	6
RABGAP1L	-0.461208996	1.147373115	-0.686164119	6
RABL6	-0.41381974	1.140486282	-0.726666542	6
RAD18	-0.202768322	1.085845311	-0.883076989	6
RAD23B	-0.272719184	1.108068491	-0.835349308	6
RAD52	-0.562594806	1.154575888	-0.591981083	6
RALA	-0.159632685	1.07021427	-0.910581585	6
RALGAPB	-0.606681471	1.154195126	-0.547513656	6
RALGDS	-0.436114165	1.143990842	-0.707876677	6
RALGPS2	-0.510143331	1.152186673	-0.642043342	6
RARB	-0.515595399	1.152571994	-0.636976595	6
RASAL2	-0.505396615	1.151826049	-0.646429434	6
RASD2	-0.038698914	1.018787697	-0.980088783	6
RASSF2	-0.373139731	1.13291814	-0.759778409	6
RASSF4	-0.308273313	1.117840799	-0.809567485	6
RAX2	-0.012909333	1.00639217	-0.993482838	6
RBM18	-0.378783214	1.134056869	-0.755273655	6
RBM22	-0.173591217	1.075430813	-0.901839596	6

RBM47	-0.103912798	1.047898967	-0.943986168	6
RBMX2	-0.513975163	1.152460724	-0.638485561	6
RBPJ	-0.074757268	1.03528069	-0.960523422	6
RCBTB1	-0.195832417	1.083429906	-0.887597489	6
RECQL	-0.074960662	1.035370944	-0.960410281	6
RECQL5	-0.502147907	1.151565824	-0.649417917	6
RENBP	-0.474079268	1.148871107	-0.674791839	6
REPIN1	-0.204859367	1.086566097	-0.88170673	6
RESF1	-0.171995649	1.074842161	-0.902846512	6
RETREG1	0.026214124	0.986635212	-1.012849336	6
RETSAT	-0.311795007	1.118751545	-0.806956538	6
REX1BD	-0.407318718	1.139377584	-0.732058866	6
RFC2	-0.326576583	1.122460227	-0.795883644	6
RFC3	-0.028890081	1.014132003	-0.985241922	6
RFFL	-0.349244865	1.127786121	-0.778541257	6
RFK	-0.330836613	1.123494687	-0.792658074	6
RFPL4A	-0.210705548	1.088563015	-0.877857468	6
RFT1	-0.587078926	1.154645584	-0.567566658	6
RFX5	-0.064230024	1.030566752	-0.966336729	6
RFXANK	-0.219690859	1.091579581	-0.871888722	6
RGR	-0.347548202	1.127402795	-0.779854593	6
RGS11	-0.211123865	1.08870487	-0.877581005	6
RGS14	-0.484122249	1.149926333	-0.665804084	6
RGS19	-0.118237359	1.053862337	-0.935624979	6
RGS3	-0.560210143	1.154532568	-0.594322425	6
RGS5	-0.258628605	1.103908286	-0.845279682	6
RHAG	-0.154161013	1.068128329	-0.913967316	6
RHBDD1	-0.40328718	1.138670584	-0.735383404	6
RHCG	-0.551837289	1.154330111	-0.602492823	6
RHOA	-0.624369319	1.153387498	-0.529018178	6
RHOV	-0.579707746	1.154697325	-0.57498958	6
RIC1	0.009323912	0.995305443	-1.004629355	6
RIC8A	-0.544481094	1.154088183	-0.609607089	6
RIDA	-0.114941647	1.052504146	-0.937562499	6
RIMBP2	-0.056614747	1.027104689	-0.970489942	6
RIMBP3	-0.249223915	1.101041975	-0.851818061	6
RIMBP3B	-0.085104581	1.039832546	-0.954727965	6
RIMBP3C	-0.562852066	1.15458018	-0.591728114	6
RIOK3	-0.440929638	1.144686541	-0.703756902	6
RIOX2	-0.241133291	1.098519125	-0.857385834	6
RIPK3	-0.128981834	1.058232717	-0.929250883	6
RIPOR2	-0.387783679	1.135814269	-0.74803059	6
RMND1	-0.251829891	1.101843365	-0.850013474	6
RMND5A	-0.288635648	1.112572489	-0.823936841	6
RMND5B	-0.181722023	1.078399785	-0.896677762	6
RNASEH1	-0.255202061	1.10287224	-0.847670178	6
RNASEH2B	-0.089060528	1.041551411	-0.952490882	6
RNASEL	-0.540987945	1.153952536	-0.61296459	6
RNF121	-0.133093127	1.059881685	-0.926788559	6
RNF14	-0.474814984	1.148951813	-0.674136828	6
RNF145	-0.111710811	1.051164664	-0.939453854	6
RNF166	-0.579002421	1.154698961	-0.57569654	6
RNF170	-0.513052146	1.152396109	-0.639343963	6
RNF181	-0.197594295	1.08404705	-0.886452755	6
RNF183	-0.494090663	1.150873751	-0.656783088	6

RNF212B	-0.314901938	1.119546378	-0.804644441	6
RNF213	-0.410165048	1.13986778	-0.729702732	6
RNF217	0.012354333	0.993765596	-1.006119929	6
RNF32	0.053634461	0.972103441	-1.025737902	6
RNF39	-0.519419965	1.152823735	-0.63340377	6
RNGTT	-0.623979387	1.153409502	-0.529430115	6
RNPC3	-0.421992179	1.141824726	-0.719832547	6
ROCK1	-0.531130815	1.153498425	-0.62236761	6
ROCK2	-0.488238627	1.15032971	-0.662091083	6
RORA	0.048524955	0.97485413	-1.023379086	6
RPF1	-0.162757483	1.071395156	-0.908637673	6
RPF2	-0.623839577	1.153417344	-0.529577768	6
RPGRIP1	-0.472813041	1.148730954	-0.675917912	6
RPH3A	-0.43528826	1.143869318	-0.708581058	6
RPL22L1	-0.555095242	1.154418167	-0.599322925	6
RPL31	-0.509724823	1.152155816	-0.642430993	6
RPL32	-0.620656639	1.153589253	-0.532932614	6
RPL4	-0.601933648	1.154346524	-0.552412876	6
RPS11	-0.483979792	1.149912068	-0.665932276	6
RPS6	-0.582259484	1.154686584	-0.572427101	6
RPS6KA5	-0.301061854	1.11594344	-0.814881586	6
RPS7	-0.517701996	1.152712555	-0.635010559	6
RPUSD2	-0.526405354	1.153243717	-0.626838363	6
RRN3	-0.555672561	1.154432541	-0.59875998	6
RRP1	0.026858	0.986300456	-1.013158456	6
RRP9	-0.426575527	1.142548217	-0.71597269	6
RSRP1	-0.542613057	1.154017298	-0.611404241	6
RTEL1	-0.141160051	1.063079587	-0.921919536	6
RTN4R	-0.515317374	1.152553096	-0.637235722	6
RTP2	-0.411525737	1.140099492	-0.728573755	6
RUFY3	-0.199905341	1.084852864	-0.884947523	6
RWDD2A	-0.179251248	1.077503017	-0.898251768	6
RWDD4	-0.543589583	1.154054829	-0.610465246	6
RXFP2	-0.608781908	1.154119406	-0.545337497	6
S100A2	-0.492006083	1.15068393	-0.658677847	6
S100A5	-0.37096387	1.132471563	-0.761507693	6
S100A9	-0.016682058	1.008236664	-0.991554606	6
S100Z	-0.399357988	1.137967275	-0.738609287	6
SAFB	-0.459895239	1.147211091	-0.687315852	6
SALL2	-0.581032794	1.154692692	-0.573659898	6
SAMD3	-0.10961471	1.050291389	-0.940676679	6
SAP30	-0.53941289	1.153887026	-0.614474136	6
SART3	-0.277352604	1.109401108	-0.832048505	6
SATB2	-0.419888797	1.141486157	-0.72159736	6
SAYSD1	-0.250937707	1.101569617	-0.85063191	6
SBDS	-0.5578833	1.154484149	-0.596600849	6
SCAF1	-0.136603276	1.061279311	-0.924676035	6
SCAF11	-0.569595196	1.15466597	-0.585070774	6
SCAMP2	-0.55929461	1.15451424	-0.595219631	6
SCARF1	0.00237208	0.99881185	-1.00118393	6
SCFD1	-0.055113321	1.026416957	-0.971303636	6
SCIMP	-0.277531715	1.109452269	-0.831920555	6
SCNN1A	-0.161860726	1.071057036	-0.90919631	6
SCNN1D	-0.465634433	1.147906519	-0.682272085	6
SCNN1G	-0.545853368	1.154137825	-0.608284457	6

SCRG1	-0.588424423	1.154629275	-0.566204852	6
SCRN3	-0.029238595	1.014298661	-0.985060065	6
SCUBE2	-0.067891106	1.032215606	-0.9643245	6
SDCCAG8	-0.566722914	1.154635727	-0.587912814	6
SDHD	-0.431633415	1.143323821	-0.711690406	6
SEC14L2	-0.464465342	1.147767465	-0.683302124	6
SEC22B	-0.35381426	1.128806075	-0.774991815	6
SEC22C	0.067415067	0.964586715	-1.032001782	6
SELENOH	-0.306913331	1.11748631	-0.810572979	6
SELENOK	-0.318203379	1.120382063	-0.802178684	6
SELENOM	-0.5986594	1.154435069	-0.555775669	6
SELPLG	-0.064573126	1.030721705	-0.96614858	6
SEMA6C	-0.568868272	1.154659203	-0.585790931	6
SENP7	-0.586898586	1.154647608	-0.567749022	6
SEPHS2	-0.609639481	1.154086941	-0.544447459	6
SERF2	-0.169531156	1.073929058	-0.904397902	6
SERINC3	-0.545179614	1.15411371	-0.608934096	6
SERP1	-0.381920885	1.134677735	-0.75275685	6
SERPINA1	-0.066898933	1.031769756	-0.964870822	6
SERPINH1	0.054842691	0.971450122	-1.026292814	6
SESN2	-0.122902121	1.055770577	-0.932868457	6
SETD6	-0.115890378	1.052895974	-0.937005596	6
SFI1	-0.190934193	1.0817014	-0.890767206	6
SFN	-0.255887044	1.10308011	-0.847193066	6
SGCB	-0.616256897	1.153806033	-0.537549137	6
SH3PXD2A	0.041644992	0.978526928	-1.02017192	6
SH3RF2	-0.052541743	1.025235097	-0.972693354	6
SHISA4	-0.263827103	1.10546193	-0.841634827	6
SHISA7	-0.373290513	1.132948931	-0.759658418	6
SHTN1	-0.290333336	1.113040583	-0.822707247	6
SIGLEC10	-0.25279047	1.10213738	-0.84934691	6
SIGLEC7	-0.217878219	1.090976182	-0.873097963	6
SIK2	-0.567490904	1.154644732	-0.587153828	6
SIRPB2	-0.231935259	1.095587212	-0.863651953	6
SIRT2	-0.049218039	1.023700201	-0.974482162	6
SIX4	-0.428706552	1.142877923	-0.71417137	6
SKP2	-0.519335067	1.152818314	-0.633483247	6
SLAMF6	-0.279184526	1.109923135	-0.830738609	6
SLAMF8	-0.158789549	1.06989435	-0.911104801	6
SLC10A1	-0.078768668	1.037054932	-0.958286264	6
SLC15A4	-0.270976099	1.107562617	-0.836586518	6
SLC17A9	-0.4646863	1.147793849	-0.683107549	6
SLC20A1	-0.345146105	1.126855833	-0.781709728	6
SLC22A1	-0.49191579	1.150675609	-0.658759819	6
SLC22A17	0.028236949	0.985582484	-1.013819433	6
SLC25A1	-0.424864012	1.142280342	-0.717416331	6
SLC25A12	-0.518154666	1.152742151	-0.634587485	6
SLC25A19	-0.238454919	1.097672368	-0.859217449	6
SLC25A2	-0.609169799	1.154104833	-0.544935034	6
SLC25A30	-0.098959753	1.045800721	-0.946840968	6
SLC25A35	-0.443400032	1.1450349	-0.701634869	6
SLC29A1	-0.356209018	1.129333373	-0.773124355	6
SLC29A3	-0.491411621	1.150628992	-0.659217371	6
SLC2A10	-0.400153983	1.138110889	-0.737956906	6
SLC2A12	-0.403507598	1.138709622	-0.735202024	6

SLC2A6	-0.217957885	1.091002756	-0.873044871	6
SLC2A8	0.004419695	0.997782827	-1.002202523	6
SLC30A7	-0.50577842	1.151855918	-0.646077498	6
SLC31A1	-0.258379587	1.103833314	-0.845453727	6
SLC33A1	-0.204445523	1.086423717	-0.881978194	6
SLC34A1	-0.446695171	1.145490507	-0.698795336	6
SLC35B4	-0.000753522	1.000376548	-0.999623026	6
SLC35C1	-0.238700709	1.097750313	-0.859049604	6
SLC35G2	-0.186306888	1.080051264	-0.893744376	6
SLC36A3	-0.063377983	1.030181567	-0.966803584	6
SLC36A4	-0.058518093	1.027974083	-0.96945599	6
SLC39A7	0.047747506	0.975270947	-1.023018453	6
SLC40A1	-0.247096372	1.100383657	-0.853287285	6
SLC45A3	-0.504482679	1.151753941	-0.647271262	6
SLC47A1	-0.282969383	1.110992941	-0.828023558	6
SLC7A6	-0.116434753	1.053120493	-0.93668574	6
SLC8A1	-0.15089588	1.066872584	-0.915976704	6
SLC8A3	-0.477522938	1.149244244	-0.671721306	6
SMAD5	0.061108922	0.968044195	-1.029153116	6
SMAP1	-0.578813213	1.154699302	-0.575886089	6
SMARCD2	-0.073365313	1.03466219	-0.961296876	6
SMCO4	0.002979729	0.998506806	-1.001486535	6
SMIM24	-0.125867637	1.056975067	-0.93110743	6
SMYD5	0.015263261	0.992281003	-1.007544264	6
SNAI3	0.070251424	0.963021849	-1.033273273	6
SNAPC3	0.022294548	0.988666316	-1.010960864	6
SNAPC5	-0.571572003	1.154681325	-0.583109323	6
SND1	-0.249828041	1.101228244	-0.851400203	6
SNRNP35	-0.245744942	1.099963597	-0.854218654	6
SNRPB	0.03751845	0.980712773	-1.018231223	6
SNTB2	-0.575746848	1.154699055	-0.578952207	6
SNURF	-0.151467726	1.067093105	-0.915625379	6
SNX1	-0.600791541	1.154378875	-0.553587335	6
SNX13	-0.495362834	1.150987427	-0.655624592	6
SNX14	-0.296888743	1.114825666	-0.817936923	6
SNX15	-0.525754214	1.153206754	-0.62745254	6
SNX19	-0.146761876	1.065270909	-0.918509033	6
SNX20	-0.129612563	1.058486531	-0.928873968	6
SNX27	-0.240451338	1.098304074	-0.857852736	6
SNX29	-0.183039699	1.078876093	-0.895836394	6
SNX5	-0.464803248	1.147807794	-0.683004546	6
SOAT1	-0.210407585	1.08846189	-0.878054305	6
SOCS1	-0.071107236	1.033655727	-0.962548492	6
SOGA1	-0.171873367	1.074796967	-0.902923599	6
SON	0.031637875	0.983805634	-1.015443509	6
SORT1	-0.303335103	1.116546228	-0.813211126	6
SOWAHB	-0.543802595	1.154062878	-0.610260283	6
SOX13	-0.35909808	1.129962859	-0.770864778	6
SOX15	-0.411643434	1.140119455	-0.72847602	6
SOX4	-0.557332136	1.154471793	-0.597139657	6
SP1	-0.209425552	1.088128102	-0.87870255	6
SP140	-0.35238217	1.128488362	-0.776106193	6
SPATA2L	-0.516225049	1.152614494	-0.636389445	6
SPATA3	-0.561418397	1.154555316	-0.59313692	6
SPATA46	-0.212930478	1.089315919	-0.876385441	6

SPATA7	-0.608713168	1.154121969	-0.545408801	6
SPDYC	0.008269766	0.995839471	-1.004109237	6
SPICE1	-0.293339243	1.113863535	-0.820524292	6
SPIN1	-0.130953903	1.059025293	-0.92807139	6
SPIN2A	-0.246759815	1.100279184	-0.853519368	6
SPP1	-0.299869371	1.115625511	-0.81575614	6
SPP2	-0.019233222	1.009477883	-0.99024466	6
SPTB	-0.370612525	1.13239906	-0.761786535	6
SPTBN4	-0.537004945	1.153781675	-0.616776731	6
SPTLC2	-0.544144961	1.15407571	-0.609930749	6
SPTSSB	-0.400770839	1.138221786	-0.737450947	6
SPX	-0.388862551	1.136020059	-0.747157508	6
SQOR	-0.450931972	1.146061048	-0.695129076	6
SRA1	-0.35518114	1.129107658	-0.773926518	6
SRGAP2	-0.145374999	1.064730635	-0.919355636	6
SRP54	-0.346018584	1.127055074	-0.78103649	6
SRP9	0.006681543	0.996642487	-1.00332403	6
SRPK2	-0.419247765	1.141382158	-0.722134393	6
SRPK3	-0.342443992	1.126234606	-0.783790613	6
SRRM2	-0.544585051	1.154092016	-0.609506965	6
SRRM5	-0.404656716	1.138912421	-0.734255704	6
SRSF10	-0.106114574	1.04882572	-0.942711147	6
SRSF8	-0.532759457	1.153580676	-0.620821219	6
SS18	-0.484037144	1.149917813	-0.665880669	6
SSB	-0.485064856	1.150020204	-0.664955349	6
SSC5D	-0.412075907	1.140192697	-0.72811679	6
SSH2	-0.453040648	1.146338582	-0.693297934	6
SSNA1	-0.003730775	1.001860168	-0.998129393	6
SSU72P4	0.060709894	0.968261962	-1.028971856	6
ST20	-0.536003726	1.153736024	-0.617732298	6
ST20-MTHFS	0.054150769	0.971824396	-1.025975165	6
ST6GAL2	-0.429674323	1.143026249	-0.713351926	6
ST6GALNAC2	-0.052067982	1.025016821	-0.972948839	6
ST8SIA4	-0.378166106	1.133933728	-0.755767622	6
STAP1	-0.466293295	1.147984296	-0.681691001	6
STAT2	-0.19998015	1.084878878	-0.884898728	6
STAT6	-0.551850645	1.154330496	-0.602479851	6
STIM1	-0.17821077	1.077123953	-0.898913183	6
STING1	-0.076415184	1.036015459	-0.959600275	6
STIP1	-0.481877766	1.149699228	-0.667821462	6
STK25	0.006595778	0.996685797	-1.003281575	6
STK36	-0.575623576	1.154698819	-0.579075242	6
STK38	-0.625025423	1.153350041	-0.528324618	6
STMP1	-0.427176242	1.142641588	-0.715465345	6
STN1	0.06254186	0.967261186	-1.029803046	6
STS	-0.24343714	1.099242876	-0.855805736	6
STT3A	-0.538801132	1.153860856	-0.615059724	6
STX16	-0.30484804	1.116945017	-0.812096978	6
STX17	-0.518881334	1.152789211	-0.633907877	6
STX3	-0.208726959	1.087890191	-0.879163232	6
STXBP3	-0.113803709	1.052033272	-0.938229562	6
STYXL1	-0.624635077	1.153372391	-0.528737315	6
SUGP2	-0.284376394	1.111387635	-0.827011241	6
SUGT1	-0.27549864	1.10887	-0.833371359	6
SULT1A1	-0.544272713	1.154080465	-0.609807752	6

SULT1A3	0.000383732	0.999808079	-1.000191811	6
SULT1A4	-0.096090604	1.04457676	-0.948486156	6
SUMF1	-0.055754779	1.026710986	-0.970956207	6
SUN1	-0.050136809	1.024125323	-0.973988513	6
SUSD3	-0.496106319	1.151053099	-0.654946779	6
SWAP70	-0.252148975	1.101941113	-0.849792138	6
SWT1	-0.001999321	1.000998162	-0.99899884	6
SYAP1	-0.250492362	1.101432732	-0.85094037	6
SYCP3	0.009696348	0.995116568	-1.004812916	6
SYDE1	-0.32281946	1.121535085	-0.798715625	6
SYNCRIP	-0.510710897	1.152228229	-0.641517332	6
SYT11	-0.199600687	1.084746877	-0.88514619	6
SYT12	-0.240456396	1.09830567	-0.857849274	6
TACSTD2	-0.549732496	1.154266974	-0.604534478	6
TAF1	-0.429898027	1.14306041	-0.713162383	6
TAF10	-0.228451213	1.094459014	-0.866007801	6
TAF11	-0.54619078	1.154149715	-0.607958935	6
TAF12	-0.112871138	1.051646642	-0.938775504	6
TAF5L	-0.446986445	1.145530282	-0.698543837	6
TAF7	-0.443967836	1.145114147	-0.701146312	6
TAOK1	-0.228764674	1.094560914	-0.86579624	6
TAP1	-0.091676503	1.042681551	-0.951005048	6
TARM1	-0.361005503	1.130374464	-0.769368961	6
TAS2R19	-0.104618607	1.048196451	-0.943577844	6
TAS2R42	0.053468892	0.972192883	-1.025661775	6
TAS2R60	-0.334544561	1.12438249	-0.789837928	6
TBC1D14	-0.553573374	1.1543785	-0.600805126	6
TBC1D21	-0.224606464	1.093202795	-0.868596331	6
TBC1D22A	-0.41783317	1.141151309	-0.723318139	6
TBC1D3C	0.021769253	0.988937645	-1.010706898	6
TBC1D3E	0.032432008	0.98338948	-1.015821488	6
TBCK	-0.047303474	1.022812278	-0.975508803	6
TBK1	-0.573467857	1.154691855	-0.581223998	6
TBL1XR1	-0.091065959	1.04241825	-0.951352291	6
TBL3	0.008704935	0.995619116	-1.004324051	6
TBRG4	-0.585799349	1.15465912	-0.568859771	6
TBX21	-0.612404255	1.153976137	-0.541571882	6
TCAF1	-0.627649675	1.153194788	-0.525545113	6
TCAF2	-0.381278372	1.134551312	-0.75327294	6
TCEAL3	0.051174659	0.973430121	-1.024604779	6
TCERG1L	-0.479986127	1.149503915	-0.669517788	6
TCHP	-0.164814058	1.072168235	-0.907354177	6
TCP11L1	-0.55018708	1.154281024	-0.604093945	6
TCP11L2	0.015620127	0.992098437	-1.007718564	6
TCTA	-0.123648329	1.056074293	-0.932425964	6
TDGF1P3	-0.2559501	1.103099227	-0.847149127	6
TDRD3	-0.591037126	1.154591515	-0.563554389	6
TEAD2	-0.206466491	1.087117736	-0.880651245	6
TEC	-0.09272307	1.043132233	-0.950409163	6
TECPR1	-0.577600335	1.154700502	-0.577100167	6
TECPR2	-0.291328726	1.113313928	-0.821985202	6
TEF	-0.06984229	1.033090239	-0.963247949	6
TEKT1	-0.276314372	1.109104031	-0.832789659	6
TEKT3	-0.501968305	1.151551121	-0.649582816	6
TEN1-CDK3	-0.164872649	1.072190212	-0.907317563	6

TENT5C	-0.519826825	1.152849611	-0.633022786	6
TESMIN	-0.346576246	1.127182079	-0.780605833	6
TESPA1	-0.392209166	1.136651752	-0.744442586	6
TEX49	0.04194716	0.978366366	-1.020313526	6
TEX52	-0.23877757	1.097774677	-0.858997107	6
TFEC	-0.396744505	1.137491706	-0.740747201	6
TFPT	-0.048467549	1.023352473	-0.974884923	6
TGDS	-0.49326812	1.150799378	-0.657531257	6
TGFB2	-0.482048921	1.149716723	-0.667667802	6
TGOLN2	-0.293219956	1.113831019	-0.820611063	6
THAP3	-0.553436384	1.154374803	-0.600938419	6
THAP4	-0.274220954	1.10850234	-0.834281386	6
THAP5	-0.32885614	1.123015692	-0.794159552	6
THAP9	-0.599238152	1.154420356	-0.555182204	6
THEM5	-0.209157803	1.088036964	-0.878879161	6
THOC6	-0.208628454	1.087856614	-0.879228159	6
THUMPD1	-0.574450413	1.154695691	-0.580245279	6
THUMPD3	-0.444404484	1.14517488	-0.700770397	6
TIGD1	-0.24605034	1.100058651	-0.85400831	6
TIGD4	-0.544874704	1.154102633	-0.609227929	6
TIGD7	-0.123555393	1.05603649	-0.932481097	6
TIMP2	-0.301292726	1.116004856	-0.81471213	6
TIPIN	-0.30329087	1.116534541	-0.813243671	6
TK1	-0.427386962	1.14267426	-0.715287298	6
TLE5	-0.609421722	1.15409527	-0.544673548	6
TLN1	-0.260799814	1.104559849	-0.843760035	6
TLR2	-0.145127453	1.064634045	-0.919506592	6
TLR8	-0.405619803	1.13908146	-0.733461657	6
TM2D1	-0.34269411	1.126292373	-0.783598263	6
TM2D2	-0.061425532	1.029296853	-0.967871321	6
TM6SF1	-0.21649183	1.090512918	-0.874021088	6
TMBIM4	-0.358918211	1.12992388	-0.771005669	6
TMCC1	0.033972108	0.982581063	-1.016553171	6
TMEM106A	-0.333992892	1.124251146	-0.790258255	6
TMEM116	-0.515564206	1.152569878	-0.637005672	6
TMEM117	-0.274103398	1.108468446	-0.834365048	6
TMEM121B	-0.16684462	1.072928325	-0.906083706	6
TMEM144	-0.465639376	1.147907104	-0.682267728	6
TMEM151B	-0.072707309	1.034369303	-0.961661994	6
TMEM154	0.017386503	0.991193383	-1.008579886	6
TMEM161A	-0.459808018	1.147200275	-0.687392258	6
TMEM161B	-0.222455171	1.092494762	-0.870039592	6
TMEM165	-0.450054872	1.145944348	-0.695889476	6
TMEM177	-0.227192452	1.094049026	-0.866856574	6
TMEM179	-0.13455557	1.060465128	-0.925909558	6
TMEM183A	-0.108775642	1.049940881	-0.941165239	6
TMEM19	-0.485095961	1.150023287	-0.664927326	6
TMEM203	-0.358570421	1.129848433	-0.771278012	6
TMEM212	0.052682592	0.972617366	-1.025299958	6
TMEM218	-0.375095563	1.133315982	-0.758220419	6
TMEM223	-0.556079739	1.154442456	-0.598362717	6
TMEM265	0.051965352	0.973004162	-1.024969513	6
TMEM30A	-0.595889126	1.154499939	-0.558610812	6
TMEM30B	-0.417357455	1.141073261	-0.723715806	6
TMEM33	-0.158483513	1.069778092	-0.911294579	6

TMEM39A	-0.224292348	1.093099642	-0.868807294	6
TMEM41A	-0.408548353	1.139590263	-0.73104191	6
TMEM45B	-0.420148453	1.141528175	-0.721379722	6
TMEM53	0.00112879	0.999435127	-1.000563917	6
TMEM63A	-0.030312489	1.014811617	-0.984499129	6
TMEM71	-0.042037687	1.020355936	-0.978318249	6
TMEM80	-0.374874012	1.133271086	-0.758397074	6
TMIGD2	-0.292483028	1.113629884	-0.821146856	6
TMLHE	-0.588773742	1.154624693	-0.565850951	6
TMOD2	-0.545528248	1.15412625	-0.608598003	6
TMOD3	-0.051709341	1.024851471	-0.97314213	6
TMSB10	-0.462176862	1.147491405	-0.685314543	6
TMSB15A	-0.272064588	1.107878807	-0.835814219	6
TMTC2	0.015981436	0.9919135	-1.007894936	6
TMX3	-0.427873409	1.142749527	-0.714876118	6
TNFAIP1	-0.246344419	1.100150111	-0.853805693	6
TNFAIP8L3	-0.146067283	1.065000507	-0.918933224	6
TNFRSF13C	-0.162741436	1.071389111	-0.908647675	6
TNFRSF19	-0.251210105	1.101653265	-0.850443159	6
TNFSF12-TNFSF13	0.027543592	0.98594367	-1.013487262	6
TNFSF13	-0.46471642	1.147797442	-0.683081021	6
TNFSF9	-0.352550608	1.128525823	-0.775975215	6
TNRC6A	-0.366053917	1.131448488	-0.765394571	6
TOB1	-0.306663222	1.117420949	-0.810757726	6
TOM1L1	-0.33422902	1.124307396	-0.790078377	6
TOMM20L	-0.241538532	1.098646739	-0.857108208	6
TOMM34	-0.15038593	1.06667572	-0.916289791	6
TOMM40	-0.278593168	1.109754922	-0.831161754	6
TOP3A	-0.26239818	1.105037062	-0.842638882	6
TOR1A	-0.503913204	1.151708575	-0.647795371	6
TOR2A	-0.226299174	1.093757313	-0.867458139	6
TOX2	-0.303460295	1.1165793	-0.813119005	6
TOX4	-0.067306675	1.031953071	-0.964646396	6
TP53	-0.26927839	1.107067527	-0.837789137	6
TP53I3	-0.468348898	1.148224219	-0.679875321	6
TP53TG3	-0.514801141	1.152517791	-0.63771665	6
TP53TG3C	-0.072503692	1.034278605	-0.961774913	6
TPD52	0.046406704	0.975988728	-1.022395433	6
TPMT	-0.401143651	1.138288642	-0.737144991	6
TPSG1	-0.49248224	1.150727677	-0.658245437	6
TRAF3IP1	-0.541156627	1.153959391	-0.612802764	6
TRAF3IP3	0.061590471	0.967781231	-1.029371703	6
TRAPPC14	-0.523430789	1.153071192	-0.629640402	6
TRAPPC6B	-0.162202246	1.071185879	-0.908983633	6
TRIM10	-0.18025811	1.077869031	-0.897610921	6
TRIM24	-0.217682853	1.090910992	-0.873228139	6
TRIM27	-0.413822422	1.140486731	-0.726664309	6
TRIM38	-0.548523154	1.154228487	-0.605705333	6
TRIM40	-0.468001794	1.148183997	-0.680182203	6
TRIM44	-0.345526261	1.126942727	-0.781416466	6
TRIM64	-0.558519566	1.154497991	-0.595978425	6
TRIM64B	-0.508514799	1.152065577	-0.643550778	6
TRIM66	-0.076274141	1.035953031	-0.95967889	6
TRIP6	-0.204612595	1.086481213	-0.881868618	6
TRMT13	-0.450202384	1.145964027	-0.695761642	6

TRNAU1AP	-0.521327122	1.152943519	-0.631616397	6
TRPM4	0.010448824	0.994734645	-1.005183469	6
TRPM7	-0.491758135	1.150661059	-0.658902924	6
TRPS1	-0.547998508	1.154211289	-0.606212781	6
TRPT1	-0.142151475	1.063469167	-0.921317692	6
TSC22D3	0.025239629	0.987141268	-1.012380896	6
TSHZ1	-0.007781937	1.003868259	-0.996086322	6
TSLP	-0.394165651	1.137016376	-0.742850725	6
TSNARE1	-0.586578741	1.154651104	-0.568072362	6
TSPAN18	-0.458442176	1.147029934	-0.688587758	6
TSPAN31	-0.539027688	1.153870595	-0.614842907	6
TSPAN32	-0.389915642	1.136219923	-0.746304281	6
TSPAN6	-0.103983113	1.04792862	-0.943945507	6
TTBK1	-0.615358335	1.153847348	-0.538489013	6
TTC1	-0.41108183	1.140024086	-0.728942256	6
TTC14	-0.612704231	1.15396355	-0.541259319	6
TTC22	-0.369074007	1.132080292	-0.763006285	6
TTC24	-0.558095845	1.154488823	-0.596392978	6
TTC28	-0.123317564	1.05593972	-0.932622156	6
TTC33	-0.244993965	1.099729538	-0.854735573	6
TTC37	-0.494463211	1.150907211	-0.656444	6
TTC39C	0.004590627	0.997696784	-1.002287411	6
TTI2	-0.113828047	1.052043353	-0.938215306	6
TTL	-0.278046505	1.109599167	-0.831552662	6
TTLL2	-0.335587659	1.124630123	-0.789042464	6
TTLL4	-0.149943387	1.066504716	-0.916561329	6
TUB	-0.163562926	1.071698317	-0.908135391	6
TUBB	-0.170466548	1.074276179	-0.90380963	6
TUBB4B	-0.617564077	1.153744143	-0.536180066	6
TUBB8B	-0.130715707	1.058929719	-0.928214013	6
TULP4	-0.059593129	1.028463924	-0.968870795	6
TXN2	-0.39312777	1.13682338	-0.74369561	6
TXNL1	0.06055346	0.968347303	-1.028900763	6
TXNL4A	-0.058814716	1.028109326	-0.969294611	6
TYW1B	-0.306576166	1.117398186	-0.81082202	6
U2AF1	-0.12469006	1.056497579	-0.93180752	6
U2SURP	-0.350986523	1.128177021	-0.777190498	6
UAP1L1	0.061810604	0.967660963	-1.029471568	6
UBALD2	-0.061819796	1.029475737	-0.967655941	6
UBB	-0.5861474	1.154655628	-0.568508229	6
UBE2F	-0.50779763	1.152011378	-0.644213748	6
UBE2Q2	-0.070553099	1.033408152	-0.962855052	6
UBE2S	-0.286470864	1.111972152	-0.825501288	6
UBE2T	-0.557946839	1.154485552	-0.596538713	6
UBE2V1	0.061722417	0.967709148	-1.029431565	6
UBE2W	-0.55752	1.154476043	-0.596956043	6
UBN2	-0.457837172	1.146953903	-0.68911673	6
UBP1	-0.314925667	1.119552418	-0.804626751	6
UBR1	-0.545030776	1.154108316	-0.609077539	6
UBR2	-0.389500387	1.136141231	-0.746640844	6
UBR7	-0.131903772	1.059405985	-0.927502213	6
UBTF	-0.044240517	1.02138603	-0.977145513	6
UBXN2B	-0.103989468	1.0479313	-0.943941832	6
UBXN7	-0.6153409	1.15384814	-0.53850724	6
UBXN8	-0.043515733	1.021047507	-0.977531774	6

UCN3	-0.608283358	1.154137866	-0.545854507	6
UFL1	-0.231280666	1.095375981	-0.864095315	6
UGT3A2	-0.130200292	1.058722767	-0.928522475	6
UMPS	-0.403257109	1.138665255	-0.735408146	6
UNK	-0.146079292	1.065005185	-0.918925893	6
UNKL	-0.288369	1.112498752	-0.824129751	6
UPB1	-0.163663379	1.071736091	-0.908072712	6
UPF3B	-0.544831572	1.154101058	-0.609269486	6
UPP2	-0.392033906	1.136618921	-0.744585015	6
UQCRC2	-0.627397747	1.153210071	-0.525812324	6
URB1	-0.624641916	1.153372001	-0.528730085	6
URM1	-0.624572859	1.153375936	-0.528803077	6
UROS	-0.088840858	1.041456274	-0.952615416	6
USP10	-0.387474967	1.135755192	-0.748280225	6
USP11	-0.056789592	1.027184667	-0.970395075	6
USP17L13	-0.626682525	1.15325302	-0.526570494	6
USP17L7	-0.482395803	1.149752092	-0.667356289	6
USP20	-0.034847254	1.016968149	-0.982120895	6
USP28	-0.606917173	1.154186897	-0.547269724	6
USP32	-0.500255386	1.151409231	-0.651153846	6
USP34	-0.399242087	1.137946316	-0.738704229	6
USP40	-0.140351781	1.06276142	-0.922409639	6
USP45	-0.566809354	1.154636774	-0.58782742	6
USP46	-0.063180128	1.030092044	-0.966911916	6
USP50	-0.238591416	1.09771566	-0.859124244	6
USP8	-0.067364126	1.031978891	-0.964614765	6
USP9X	0.047469567	0.975419849	-1.022889416	6
UTP15	-0.178180939	1.077113073	-0.898932134	6
UTP18	-0.456713063	1.146811693	-0.69009863	6
UVSSA	-0.069665346	1.033011041	-0.963345695	6
VAMP1	-0.503457127	1.151672001	-0.648214874	6
VAMP3	-0.268130827	1.10673154	-0.838600714	6
VAMP7	-0.615715772	1.153831033	-0.538115261	6
VAV3	-0.402120473	1.138463215	-0.736342742	6
VCP	-0.344099697	1.126616006	-0.782516309	6
VCPIP1	-0.023671346	1.011625526	-0.98795418	6
VDAC1	-0.386365422	1.135542154	-0.749176732	6
VPS13A	-0.477803665	1.149274143	-0.671470478	6
VPS13C	-0.576785877	1.154700355	-0.577914478	6
VPS29	-0.426567355	1.142546944	-0.715979589	6
VPS37A	-0.223926384	1.092979365	-0.869052981	6
VPS45	-0.295572879	1.114470206	-0.818897327	6
VPS4B	-0.023737696	1.011657521	-0.987919826	6
VPS72	-0.598774261	1.154432181	-0.55565792	6
VPS8	-0.270436369	1.107405475	-0.836969106	6
VRK1	-0.298240962	1.115189447	-0.816948485	6
VSIR	-0.613682274	1.153921741	-0.540239467	6
VSX2	-0.101942143	1.047066373	-0.94512423	6
VTI1A	-0.05363044	1.025736054	-0.972105613	6
VWA7	-0.537947902	1.153823679	-0.615875777	6
VWA8	-0.443038132	1.144984231	-0.701946098	6
WASHC4	-0.51467669	1.152509239	-0.637832548	6
WASHC5	0.043393985	0.977596619	-1.020990604	6
WBP1L	-0.202064662	1.085601985	-0.883537323	6
WBP2	-0.510463592	1.152210163	-0.641746571	6

WBP2NL	-0.070406718	1.033342713	-0.962935995	6
WDCP	-0.109434081	1.050215979	-0.940781898	6
WDFY3	-0.206858654	1.087252036	-0.880393382	6
WDR18	-0.149672074	1.066399803	-0.916727729	6
WDR27	-0.443174416	1.145003326	-0.70182891	6
WDR44	-0.078205419	1.03680654	-0.958601122	6
WDR45	-0.306611894	1.117407529	-0.810795635	6
WDR45B	-0.22921419	1.094706907	-0.865492717	6
WDR46	-0.13571064	1.060924787	-0.925214147	6
WDR59	0.05395542	0.971929998	-1.025885418	6
WDR5B	0.040247353	0.979268696	-1.019516048	6
WDR73	-0.428371167	1.142826315	-0.714455148	6
WDR77	-0.424364546	1.142201654	-0.717837108	6
WDR81	0.013168842	0.993350545	-1.006519387	6
WDR86	-0.039922074	1.019363194	-0.97944112	6
WDR88	-0.062068592	1.029588559	-0.967519968	6
WDR90	-0.387335094	1.135728396	-0.748393302	6
WDR91	-0.133554923	1.060066096	-0.926511173	6
WDR97	-0.336421081	1.124827309	-0.788406228	6
WDSUB1	-0.175299315	1.07605879	-0.900759475	6
WSB1	-0.536565247	1.15376176	-0.617196513	6
XAB2	-0.470970237	1.148524152	-0.677553916	6
XK	-0.399806774	1.138048316	-0.738241542	6
XPNPEP3	0.004926597	0.997527599	-1.002454197	6
XPO1	-0.257953766	1.103704994	-0.845751228	6
XPOT	-0.495607533	1.151009103	-0.65540157	6
XRRA1	-0.620309481	1.153607235	-0.533297754	6
YIPF1	-0.108456592	1.049807462	-0.94135087	6
YIPF4	-0.085893514	1.040176283	-0.954282769	6
YOD1	-0.035662876	1.017354384	-0.981691508	6
YTHDC2	-0.457177911	1.146870649	-0.689692738	6
YWHAZ	-0.311745956	1.118738931	-0.806992975	6
YY1	0.025244226	0.987138882	-1.012383108	6
ZBTB12	-0.591139331	1.154589874	-0.563450543	6
ZBTB18	-0.205060686	1.08663531	-0.881574624	6
ZBTB25	-0.309023775	1.118035748	-0.809011973	6
ZBTB3	-0.31688327	1.120049011	-0.80316574	6
ZBTB38	-0.494697529	1.150928183	-0.656230654	6
ZBTB44	-0.537300265	1.153794934	-0.616494668	6
ZC2HC1C	-0.46324854	1.147621318	-0.684372778	6
ZCCHC13	-0.483511114	1.149864995	-0.666353881	6
ZCCHC4	-0.287375412	1.112223472	-0.82484806	6
ZCCHC7	-0.046922509	1.022635268	-0.975712758	6
ZCRB1	-0.196986181	1.083834317	-0.886848137	6
ZDHHC1	0.060181135	0.968550346	-1.02873148	6
ZDHHC18	-0.083625112	1.039186674	-0.955561562	6
ZDHHC20	-0.433364353	1.143583737	-0.710219384	6
ZDHHC22	-0.165738605	1.072514712	-0.906776107	6
ZDHHC4	-0.015947765	1.007878504	-0.991930739	6
ZER1	-0.186818128	1.080234398	-0.89341627	6
ZFAND2A	-0.602313186	1.154335425	-0.552022239	6
ZFP14	-0.483459062	1.149859754	-0.666400691	6
ZFP82	-0.020929522	1.01030048	-0.989370959	6
ZFYVE27	-0.549383301	1.154256026	-0.604872725	6
ZGPAT	-0.184920166	1.079553492	-0.894633327	6

ZHX1	-0.569706028	1.154666949	-0.584960921	6
ZHX2	-0.373672002	1.133026746	-0.759354744	6
ZMYM2	-0.36032407	1.130227782	-0.769903712	6
ZMYM3	-0.483329393	1.149846684	-0.666517292	6
ZMYND8	-0.291807424	1.113445091	-0.821637667	6
ZNF121	-0.523971408	1.153103245	-0.629131838	6
ZNF138	-0.130685063	1.058917421	-0.928232358	6
ZNF185	-0.580877996	1.154693339	-0.573815343	6
ZNF205	-0.386596661	1.135586644	-0.748989983	6
ZNF215	-0.386693559	1.135605273	-0.748911714	6
ZNF23	-0.066372233	1.031532772	-0.965160539	6
ZNF250	-0.458448029	1.147030668	-0.688582639	6
ZNF275	-0.10348305	1.047717651	-0.944234601	6
ZNF28	0.012358438	0.993763505	-1.006121943	6
ZNF286A	-0.100016356	1.046249889	-0.946233533	6
ZNF316	-0.113990232	1.052110522	-0.938120289	6
ZNF334	-0.185693295	1.079831197	-0.894137902	6
ZNF354A	0.043565176	0.977505437	-1.021070613	6
ZNF354B	-0.406242805	1.139190357	-0.732947552	6
ZNF365	-0.289365337	1.112773974	-0.823408637	6
ZNF37A	-0.481743822	1.149685515	-0.667941693	6
ZNF446	-0.063282652	1.030138437	-0.966855784	6
ZNF480	-0.381856744	1.134665131	-0.752808387	6
ZNF491	-0.326788863	1.12251214	-0.795723277	6
ZNF493	-0.069027199	1.032725217	-0.963698019	6
ZNF500	-0.341819441	1.126090123	-0.784270683	6
ZNF503	-0.100392943	1.046409774	-0.94601683	6
ZNF507	-0.459496266	1.147161555	-0.687665288	6
ZNF516	-0.531424228	1.153513453	-0.622089225	6
ZNF530	-0.238508834	1.09768947	-0.859180635	6
ZNF540	-0.299901953	1.115634213	-0.81573226	6
ZNF546	-0.286657681	1.112024113	-0.825366432	6
ZNF560	-0.418794017	1.141308312	-0.722514296	6
ZNF562	-0.445779833	1.145364987	-0.699585154	6
ZNF566	-0.396708812	1.137485168	-0.740776356	6
ZNF587	-0.353435089	1.128722129	-0.77528704	6
ZNF587B	-0.409885078	1.139819893	-0.729934815	6
ZNF654	-0.022982601	1.011293206	-0.988310605	6
ZNF672	-0.405942156	1.13913785	-0.733195693	6
ZNF674	0.007474207	0.996241947	-1.003716154	6
ZNF675	-0.58361999	1.15467776	-0.57105777	6
ZNF692	-0.120491144	1.054786377	-0.934295233	6
ZNF7	-0.401555674	1.138362383	-0.736806709	6
ZNF705A	-0.452288934	1.146240136	-0.693951202	6
ZNF71	-0.145846361	1.064914425	-0.919068064	6
ZNF713	-0.184359596	1.079351847	-0.894992251	6
ZNF721	-0.141586593	1.063247289	-0.921660696	6
ZNF732	-0.606570383	1.154198981	-0.547628598	6
ZNF740	-0.508382011	1.152055582	-0.643673572	6
ZNF76	-0.252348919	1.102002322	-0.849653403	6
ZNF765	-0.416301293	1.140899233	-0.72459794	6
ZNF784	-0.507121327	1.151959778	-0.644838452	6
ZNF808	-0.247096321	1.100383641	-0.85328732	6
ZNF816	-0.188668352	1.080895472	-0.892227119	6
ZNF83	0.06021705	0.968530764	-1.028747814	6

ZNF830	-0.585616068	1.154660902	-0.569044833	6
ZNF862	-0.190114955	1.081410471	-0.891295516	6
ZNF875	-0.118355834	1.053911008	-0.935555174	6
ZNHIT2	-0.437916245	1.144253763	-0.706337517	6
ZP1	-0.215692717	1.090245203	-0.874552486	6
ZRANB2	-0.420025895	1.14150835	-0.721482456	6
ZRANB3	-0.463485671	1.147649913	-0.684164242	6
ZSCAN20	-0.440084997	1.144566104	-0.704481107	6
ZSCAN31	-0.400080039	1.138097572	-0.738017533	6
ZXDA	-0.340488651	1.125781143	-0.785292492	6
A3GALT2	0.743721393	-1.136817471	0.393096079	7
A4GNT	1.008905304	-0.99085019	-0.018055114	7
AADAT	0.899758274	-1.076637692	0.176879419	7
ABCA4	1.002925541	-0.997048555	-0.005876986	7
ABHD12B	1.063296273	-0.921585025	-0.141711248	7
ABHD14A	0.803629428	-1.119891873	0.316262445	7
ABHD17B	0.817658347	-1.114928402	0.297270056	7
ABHD2	1.075739101	-0.901310109	-0.174428992	7
ABLIM3	1.061005413	-0.925091904	-0.135913509	7
ABRAXAS2	0.816814706	-1.115238522	0.298423817	7
ABTB2	0.907721581	-1.071947474	0.164225893	7
ACAA2	1.075046279	-0.902497966	-0.172548313	7
ACADL	1.001966003	-0.998022332	-0.003943671	7
ACADS	1.087532807	-0.879853141	-0.207679666	7
ACOX3	0.856851497	-1.098764569	0.241913072	7
ACRV1	1.088649841	-0.877688301	-0.21096154	7
ACSM2A	1.029104667	-0.968102428	-0.061002239	7
ACTR1A	0.804043868	-1.119751057	0.315707189	7
ACY3	0.694617168	-1.146139131	0.451521963	7
ADAM17	0.849338241	-1.102141197	0.252802955	7
ADAM29	1.087573078	-0.879775528	-0.20779755	7
ADAM32	0.903921799	-1.074210075	0.170288276	7
ADAM33	1.080750415	-0.892488691	-0.188261724	7
ADAMTS16	0.888868642	-1.082740206	0.193871564	7
ADAMTS17	0.87464673	-1.090197643	0.215550913	7
ADAMTS4	0.755618678	-1.133970903	0.378352225	7
ADAMTS9	1.088793578	-0.877407913	-0.211385665	7
ADAP1	0.863750378	-1.095540363	0.231789986	7
ADAR	1.065062237	-0.918836468	-0.146225769	7
ADCK2	0.83965822	-1.106291458	0.266633238	7
ADCY9	1.045693971	-0.94698504	-0.098708932	7
ADD2	1.036647572	-0.958802342	-0.07784523	7
ADGRA3	0.870370854	-1.092331478	0.221960624	7
ADGRB3	1.0771092	-0.89893888	-0.17817032	7
ADGRE2	0.808881434	-1.118081466	0.309200032	7
ADGRF4	0.912840197	-1.068826892	0.155986695	7
ADGRF5	0.864684068	-1.0950947	0.230410632	7
ADH4	0.706549373	-1.144217787	0.437668413	7
ADH6	0.761753293	-1.132407711	0.370654418	7
ADIPOR1	0.872445732	-1.091302123	0.218856391	7
ADIPOR2	1.090085841	-0.874868093	-0.215217748	7
ADNP2	0.975351499	-1.022948661	0.047597162	7
ADO	0.906263918	-1.072820819	0.166556901	7
ADORA2B	1.014624512	-0.984703945	-0.029920566	7
ADORA3	1.029688888	-0.967398966	-0.062289921	7

AFP	0.742558881	-1.137082765	0.394523884	7
AGBL4	1.088276923	-0.878413804	-0.209863118	7
AGO3	0.944235155	-1.047717246	0.103482091	7
AHR	0.768934	-1.130493392	0.361559392	7
AIFM2	0.920939782	-1.063713158	0.142773376	7
AJUBA	0.73496404	-1.13876076	0.40379672	7
AK5	0.772381205	-1.129541432	0.357160227	7
AK6	0.864450471	-1.09520641	0.230755939	7
AKAP8	0.773219151	-1.12930676	0.35608761	7
AKR1A1	1.007575623	-0.992248171	-0.015327452	7
AKR1B1	1.034122939	-0.96196855	-0.07215439	7
AKR1B15	0.855855341	-1.09922025	0.243364909	7
ALCAM	1.042794067	-0.950856462	-0.091937605	7
ALDH16A1	0.916158485	-1.066758323	0.150599838	7
ALDH6A1	1.086344639	-0.882128798	-0.204215841	7
ALG11	0.960810679	-1.035051289	0.07424061	7
ALG3	0.756864977	-1.13365865	0.376793673	7
ALKBH3	0.970963664	-1.026704681	0.055741018	7
AMDHD1	0.679509457	-1.148271997	0.46876254	7
AMDHD2	0.88340212	-1.085673513	0.202271393	7
AMELY	0.738254909	-1.138045374	0.399790465	7
AMN	1.074316442	-0.903741277	-0.170575165	7
AMOT	1.069316521	-0.912046169	-0.157270352	7
AMPD2	1.068541899	-0.91330084	-0.155241058	7
AMPD3	0.719032904	-1.141976523	0.422943619	7
AMY1A	1.0100988	-0.989585581	-0.02051322	7
AMY1B	1.079439755	-0.89483586	-0.184603896	7
ANAPC15	0.987470975	-1.012074956	0.024603981	7
ANAPC16	0.890407153	-1.081899208	0.191492055	7
ANAPC2	1.001594887	-0.998397445	-0.003197442	7
ANGPTL1	0.886650206	-1.083940868	0.197290662	7
ANKEF1	0.838135372	-1.106924374	0.268789003	7
ANKRD1	0.853152582	-1.100444234	0.247291652	7
ANKRD17	1.045623945	-0.947079488	-0.098544457	7
ANKRD20A2P	0.768083835	-1.13072486	0.362641025	7
ANKRD28	1.003141956	-0.996828146	-0.00631381	7
ANKRD36C	0.984578123	-1.01473948	0.030161357	7
ANKRD40CL	1.060769915	-0.925448738	-0.135321178	7
ANKRD46	0.860878209	-1.096897304	0.236019095	7
ANKRD49	0.895160674	-1.079257095	0.184096421	7
ANKRD54	0.997304485	-1.002673892	0.005369406	7
ANKRD65	1.074838234	-0.902853212	-0.171985022	7
ANKRD66	1.04276482	-0.950895096	-0.091869724	7
ANKUB1	0.726095999	-1.140600859	0.41450486	7
ANO4	1.064927542	-0.919047523	-0.14588002	7
ANP32D	0.771664357	-1.129741172	0.358076815	7
ANTXR2	1.0284631	-0.96887178	-0.059591321	7
ANXA11	0.781411854	-1.126944092	0.345532238	7
ANXA2	1.023255976	-0.974996529	-0.048259447	7
ANXA3	1.076408484	-0.900155319	-0.176253165	7
ANXA5	0.957227005	-1.037887447	0.080660442	7
ANXA8	1.030281776	-0.966682244	-0.063599532	7
ANXA8L1	0.976172795	-1.022235258	0.046062463	7
AP1B1	1.011024156	-0.98859863	-0.022425527	7
AP1S1	0.780437942	-1.127231497	0.346793555	7

AP2A2	0.680953178	-1.148082357	0.467129179	7
AP4M1	1.05354848	-0.936074496	-0.117473984	7
AP5Z1	0.833061787	-1.108994643	0.275932856	7
APIP	1.077822804	-0.897691978	-0.180130826	7
APOBEC3F	1.056216841	-0.932217921	-0.12399892	7
APOBEC4	0.752883367	-1.134646785	0.381763418	7
APOC4-APOC2	0.932901686	-1.055747741	0.122846055	7
APOH	0.683021317	-1.147805524	0.464784207	7
APOO	0.990200065	-1.009519972	0.019319907	7
APPL2	0.874581611	-1.090230507	0.215648896	7
ARFGEF3	0.998495066	-1.00149817	0.003003105	7
ARHGAP23	1.039549617	-0.955093594	-0.084456023	7
ARHGAP27	1.02251722	-0.975848646	-0.046668574	7
ARHGAP28	1.077606142	-0.898071428	-0.179534714	7
ARHGAP45	1.04728821	-0.944821685	-0.102466524	7
ARHGAP5	1.014108512	-0.985267539	-0.028840973	7
ARHGEF19	0.895842963	-1.078872382	0.183029419	7
ARHGEF37	1.032115534	-0.964447266	-0.067668268	7
ARID4A	1.062907103	-0.922185388	-0.140721714	7
ARIH2	0.931488919	-1.056715098	0.12522618	7
ARL2	1.053079386	-0.936744343	-0.116335044	7
ARL3	0.719533361	-1.141881638	0.422348277	7
ARL4A	1.00912945	-0.990613401	-0.018516049	7
ARL4D	0.720493471	-1.141698505	0.421205035	7
ARMC2	0.695516462	-1.146001702	0.45048524	7
ARMC7	0.954040343	-1.040363112	0.086322769	7
ARMC9	1.043439469	-0.950001833	-0.093437636	7
ARMH3	1.009103024	-0.990641334	-0.018461689	7
ARNT2	1.040699387	-0.953603186	-0.087096201	7
ARNTL2	1.092901656	-0.869211542	-0.223690114	7
ARRDC4	0.844961269	-1.104045261	0.259083992	7
ARSJ	0.809932828	-1.117712239	0.307779411	7
ART3	0.945378167	-1.046879897	0.10150173	7
ART4	0.950440386	-1.043108655	0.092668269	7
ASAH1	0.777865235	-1.127982181	0.350116945	7
ASAP2	0.826062711	-1.111755405	0.285692694	7
ASB11	1.068559949	-0.913271699	-0.15528825	7
ASB6	0.941361598	-1.049799747	0.108438149	7
ASB7	1.006335871	-0.99354135	-0.012794521	7
ASCC1	0.982133783	-1.016956542	0.034822759	7
ASCL5	0.930171434	-1.057610676	0.127439242	7
ASIC2	0.720312695	-1.141733097	0.421420402	7
ASPM	0.925961547	-1.060430696	0.13446915	7
ASPSCR1	0.875631669	-1.089699171	0.214067502	7
ATAD3B	0.897055543	-1.078185229	0.181129686	7
ATAD5	0.979514294	-1.019298279	0.039783985	7
ATG9A	0.921246355	-1.063515262	0.142268907	7
ATL2	0.692427388	-1.1464688	0.454041411	7
ATP13A1	1.000520472	-0.999478714	-0.001041757	7
ATP13A3	0.712381127	-1.143200632	0.430819505	7
ATP1A1	0.982354323	-1.016757781	0.034403458	7
ATP1A2	1.062860277	-0.922257496	-0.140602781	7
ATP2A3	0.689777274	-1.14685839	0.457081115	7
ATP2C1	0.947861607	-1.045042684	0.097181077	7
ATP4B	1.083532449	-0.887407765	-0.196124684	7

ATP6V0D2	1.009306738	-0.990425879	-0.018880859	7
ATP6V1FNB	0.697697094	-1.145663503	0.447966408	7
ATP6V1G3	0.984549595	-1.014765536	0.030215941	7
ATRAID	0.831426848	-1.109649334	0.278222486	7
ATXN7L2	0.887607826	-1.083424316	0.19581649	7
AUP1	0.998805661	-1.001190075	0.002384415	7
AVPR1A	0.896794413	-1.078333581	0.181539168	7
AVPR2	0.856784569	-1.098795262	0.242010693	7
AZIN2	1.045067113	-0.947828803	-0.09723831	7
AZU1	0.991027399	-1.008737355	0.017709956	7
B3GALT5	1.014505315	-0.984834299	-0.029671015	7
B3GNT3	0.857786742	-1.098334503	0.240547761	7
B4GALT1	0.831100672	-1.109779228	0.278678556	7
B4GALT7	1.088664524	-0.877659677	-0.211004847	7
BACE2	0.899146959	-1.076989657	0.177842697	7
BAHD1	0.828126534	-1.110952665	0.282826131	7
BAIAP3	1.093343244	-0.868308706	-0.225034538	7
BARX1	0.81411474	-1.116220886	0.302106146	7
BBOF1	0.840547884	-1.105919215	0.265371331	7
BBS10	0.986651865	-1.012833943	0.026182078	7
BCAP31	0.976085255	-1.022311456	0.046226202	7
BCAR3	0.894430734	-1.079667141	0.185236407	7
BCL2L1	1.056283443	-0.932120639	-0.124162804	7
BDKRB2	1.08482969	-0.884990979	-0.199838712	7
BEND3	1.079785557	-0.894219411	-0.185566146	7
BFSP1	1.081186733	-0.891700809	-0.189485924	7
BHLHB9	1.069849818	-0.911177517	-0.158672302	7
BLID	0.792405621	-1.123574791	0.33116917	7
BLM	0.730863108	-1.139627484	0.408764376	7
BLOC1S2	1.005580288	-0.994324694	-0.011255594	7
BLOC1S4	1.034306419	-0.961740292	-0.072566126	7
BLOC1S5-TXNDC5	0.864595592	-1.095137027	0.230541435	7
BLOC1S6	0.774782204	-1.128865581	0.354083377	7
BMP1	0.923054551	-1.062341491	0.13928694	7
BMP10	0.691858171	-1.146553344	0.454695173	7
BMP4	1.073341085	-0.9053902	-0.167950885	7
BMPR1B	1.004319096	-0.995624202	-0.008694893	7
BOD1L1	0.681444092	-1.148017198	0.466573106	7
BORCS8-MEF2B	1.078861633	-0.895861991	-0.182999642	7
BPI	1.070433163	-0.910222759	-0.160210404	7
BPY2	1.067304256	-0.915288367	-0.152015888	7
BPY2B	0.937111078	-1.052821849	0.115710771	7
BPY2C	1.057336536	-0.930575704	-0.126760832	7
BRAT1	1.034301312	-0.961746649	-0.072554664	7
BRCA2	0.824556379	-1.11233539	0.287779011	7
BRD9	1.012450016	-0.987066691	-0.025383325	7
BRF1	0.744441037	-1.136652109	0.392211072	7
BRF2	1.006788107	-0.993070764	-0.013717343	7
BRINP3	0.865493708	-1.094706431	0.229212723	7
BRPF3	1.075424935	-0.901849676	-0.173575259	7
BSCL2	1.089111433	-0.876786382	-0.212325051	7
BSPRY	1.062741456	-0.922440348	-0.140301108	7
BTBD17	0.804274969	-1.119672383	0.315397414	7
BTBD19	0.904216103	-1.074036447	0.169820345	7
BTBD7	1.066229416	-0.916997665	-0.149231752	7

BUB1B-PAK6	0.789310999	-1.124546663	0.335235664	7
C10orf67	0.708769219	-1.143836727	0.435067507	7
C11orf91	0.856030719	-1.099140203	0.243109484	7
C11orf96	0.917655383	-1.065813234	0.148157851	7
C12orf40	1.047797033	-0.944125876	-0.103671157	7
C12orf42	1.041913908	-0.952015601	-0.089898307	7
C12orf60	1.044121014	-0.949095027	-0.095025987	7
C14orf93	0.702787674	-1.144846491	0.442058817	7
C16orf46	0.997828454	-1.002157491	0.004329037	7
C16orf54	0.954778817	-1.03979323	0.085014413	7
C16orf95	0.756904324	-1.133648748	0.376744424	7
C17orf78	0.788399673	-1.124829337	0.336429664	7
C18orf21	0.92428535	-1.061536072	0.137250721	7
C18orf32	0.990944801	-1.008815659	0.017870858	7
C19orf25	1.078522141	-0.896461985	-0.182060156	7
C19orf81	0.830267557	-1.11010992	0.279842363	7
C19orf85	0.944936659	-1.047203949	0.10226729	7
C1orf141	0.710647511	-1.143508433	0.432860922	7
C1orf185	0.72926031	-1.139958827	0.410698517	7
C1orf54	0.74597928	-1.136295729	0.390316448	7
C1orf87	1.075364592	-0.901953138	-0.173411454	7
C1orf94	1.007221977	-0.992618061	-0.014603915	7
C1QTNF2	1.091553448	-0.871941257	-0.219612192	7
C1QTNF8	0.993758084	-1.006127167	0.012369082	7
C1QTNF9B	0.873225254	-1.090912437	0.217687183	7
C20orf203	0.688826083	-1.146995729	0.458169646	7
C22orf31	0.723617408	-1.141092596	0.417475188	7
C2CD4C	1.079763312	-0.894259129	-0.185504183	7
C2orf66	0.984345189	-1.0149521	0.030606911	7
C2orf73	0.697958627	-1.145622468	0.447663841	7
C2orf74	0.948922123	-1.044250587	0.095328464	7
C2orf80	1.027027634	-0.970581295	-0.056446339	7
C2orf92	0.8350073	-1.108207716	0.273200416	7
C3	1.086080941	-0.882630138	-0.203450803	7
C3orf52	1.069134846	-0.912341179	-0.156793668	7
C3orf85	0.853727922	-1.100185189	0.246457268	7
C5AR1	0.706777498	-1.144178972	0.437401473	7
C5orf47	1.087132245	-0.880623404	-0.206508841	7
C5orf52	0.747492709	-1.135941203	0.388448494	7
C6orf58	0.915286206	-1.067305609	0.152019403	7
C7orf57	0.680220329	-1.148178991	0.467958662	7
C8orf88	0.984586536	-1.014731796	0.03014526	7
C9orf24	0.723101685	-1.141193687	0.418092002	7
C9orf43	1.002746308	-0.997230877	-0.005515431	7
C9orf50	1.081290357	-0.891513207	-0.18977715	7
C9orf57	1.04318119	-0.950344316	-0.092836874	7
C9orf85	0.864514158	-1.095175968	0.230661809	7
CA12	1.048299533	-0.943436145	-0.104863388	7
CAB39	1.027893402	-0.969552195	-0.058341207	7
CABIN1	1.024148927	-0.973961064	-0.050187863	7
CABP7	1.04510939	-0.947772019	-0.097337371	7
CABYR	0.794745203	-1.122827663	0.328082461	7
CACHD1	0.820074036	-1.114031998	0.293957962	7
CACNB2	1.015765661	-0.983451008	-0.032314652	7
CACNB4	1.076778347	-0.899514213	-0.177264134	7

CACNG4	0.828247713	-1.110905239	0.282657526	7
CADM1	0.887493125	-1.083486326	0.195993201	7
CADPS	0.751497786	-1.134984237	0.383486452	7
CAGE1	0.962248386	-1.033897684	0.071649298	7
CALCB	0.781221643	-1.127000364	0.345778721	7
CALHM4	0.749905502	-1.135367969	0.385462467	7
CALR3	0.694696465	-1.146127061	0.451430595	7
CAMKK1	0.936635077	-1.05315602	0.116520944	7
CAMLG	0.82635675	-1.111641611	0.285284861	7
CAMTA1	0.959579308	-1.036032105	0.076452797	7
CAND2	0.874690678	-1.090175457	0.21548478	7
CAP2	1.06468611	-0.919425237	-0.145260873	7
CAPN1	0.859046139	-1.097751921	0.238705782	7
CAPSL	0.699754184	-1.145338003	0.445583819	7
CAPZB	0.957760248	-1.037468954	0.079708706	7
CARD18	0.933363876	-1.055429682	0.122065806	7
CASP3	1.070786555	-0.909642013	-0.161144542	7
CASQ2	1.079721415	-0.894333907	-0.185387508	7
CASS4	0.68908818	-1.146958017	0.457869837	7
CATSPERD	0.830015241	-1.110209767	0.280194526	7
CATSPERG	0.979144483	-1.01962607	0.040481587	7
CAV2	0.846550986	-1.103358996	0.25680801	7
CAVIN2	1.076676531	-0.899690912	-0.176985619	7
CBLL1	0.986873256	-1.012629158	0.025755902	7
CBLN3	0.732198692	-1.139348211	0.407149519	7
CBLN4	0.955990049	-1.038853536	0.082863487	7
CBY1	1.063114388	-0.921865853	-0.141248535	7
CBY2	0.948969923	-1.044214778	0.095244854	7
CC2D2B	1.071121747	-0.909089523	-0.162032224	7
CCDC106	0.966460428	-1.03046479	0.064004362	7
CCDC126	0.798429558	-1.121629259	0.323199701	7
CCDC13	0.871867327	-1.091590222	0.219722896	7
CCDC14	0.89895281	-1.077101201	0.178148391	7
CCDC148	0.925743356	-1.060575141	0.134831785	7
CCDC154	0.88658576	-1.083975538	0.197389778	7
CCDC167	1.010487268	-0.989171945	-0.021315323	7
CCDC168	0.785751887	-1.125641581	0.339889694	7
CCDC169	0.89215837	-1.080933564	0.188775194	7
CCDC169-SOHLH2	0.696628751	-1.145830073	0.449201322	7
CCDC177	0.893779422	-1.080031685	0.186252263	7
CCDC186	0.830656538	-1.109955714	0.279299176	7
CCDC190	1.013024436	-0.98644567	-0.026578766	7
CCDC200	1.086547874	-0.881741495	-0.20480638	7
CCDC33	0.73146004	-1.139503021	0.408042981	7
CCDC43	0.799012702	-1.121437114	0.322424412	7
CCDC60	0.836560722	-1.107573201	0.271012479	7
CCDC63	0.914690458	-1.067677945	0.152987487	7
CCDC65	0.956045471	-1.038810389	0.082764918	7
CCDC71L	1.092828331	-0.869361034	-0.223467297	7
CCDC73	0.999540131	-1.000459235	0.000919104	7
CCDC80	1.063089683	-0.921903963	-0.14118572	7
CCDC85B	1.059072589	-0.92800077	-0.131071819	7
CCDC9B	0.699761682	-1.145336805	0.445575124	7
CCL1	0.721012395	-1.141598922	0.420586527	7
CCL13	0.957353085	-1.037788609	0.080435524	7

CCL15	0.810400727	-1.117547186	0.307146459	7
CCL3L1	0.965564574	-1.031201643	0.065637069	7
CCL7	1.092446159	-0.870138257	-0.222307903	7
CCNA1	0.985985846	-1.013448429	0.027462584	7
CCNB1	1.065469406	-0.918197034	-0.147272372	7
CCNH	1.027552458	-0.969958166	-0.057594291	7
CCT2	0.797633383	-1.121890505	0.324257122	7
CD1D	0.845901737	-1.103639996	0.25773826	7
CD1E	1.023114851	-0.975159627	-0.047955223	7
CD207	1.072522045	-0.906763853	-0.165758192	7
CD274	1.03972857	-0.954862418	-0.084866152	7
CD37	0.948443357	-1.044608739	0.096165383	7
CD44	1.079216331	-0.895233085	-0.183983246	7
CD59	0.72561161	-1.140697728	0.415086118	7
CD68	0.753866428	-1.134405363	0.380538935	7
CD74	0.738354	-1.138023557	0.399669557	7
CD82	0.749783488	-1.135397195	0.385613707	7
CDC20B	1.032301542	-0.964219008	-0.068082533	7
CDC37	0.93522612	-1.054140194	0.118914074	7
CDC42BPG	0.87261703	-1.09121663	0.2185996	7
CDC42EP4	1.045696517	-0.946981605	-0.098714912	7
CDH10	0.817676795	-1.114921604	0.297244809	7
CDH13	0.973559216	-1.024494067	0.050934852	7
CDH18	0.812730568	-1.116718554	0.303987985	7
CDH5	1.016801118	-0.982306261	-0.034494857	7
CDH8	1.01977061	-0.978981166	-0.040789444	7
CDK1	1.075460786	-0.901788181	-0.173672606	7
CDK11A	1.055846284	-0.932758255	-0.123088028	7
CDK12	0.959949495	-1.035737939	0.075788443	7
CDK15	0.924184578	-1.061602213	0.137417635	7
CDK17	1.053635734	-0.935949637	-0.117686096	7
CDK5RAP2	0.797347711	-1.121983935	0.324636224	7
CDNF	0.919294575	-1.064769683	0.145475107	7
CDY1B	1.014365874	-0.984986668	-0.029379206	7
CEACAM4	0.96793062	-1.029247563	0.061316943	7
CEACAM7	0.775850452	-1.128561478	0.352711026	7
CEBPA	1.040111968	-0.95436615	-0.085745818	7
CEBPB	0.69110747	-1.14666412	0.45555665	7
CEBPD	0.720624487	-1.141673403	0.421048917	7
CECR2	1.090341744	-0.874361032	-0.215980712	7
CELSR2	1.030486464	-0.96643414	-0.064052324	7
CENATAC	1.079374799	-0.894951431	-0.184423368	7
CEND1	0.861838342	-1.096446033	0.234607691	7
CENPF	0.790637903	-1.12413222	0.333494316	7
CENPJ	0.846362846	-1.103440528	0.257077682	7
CENPM	0.726978482	-1.140423418	0.413444936	7
CENPS	1.002070561	-0.997916496	-0.004154065	7
CENPS-CORT	0.742097126	-1.137187514	0.395090388	7
CENPX	1.093257017	-0.868485342	-0.224771675	7
CEP112	1.043911072	-0.949374832	-0.09453624	7
CEP126	0.982498492	-1.016627714	0.034129222	7
CEP290	0.750724475	-1.135171142	0.384446668	7
CEP44	1.063922063	-0.920615612	-0.143306452	7
CEP85	0.897484661	-1.077940997	0.180456336	7
CFAP161	1.000117241	-0.999882718	-0.000234523	7

CFAP300	0.9381004	-1.052124577	0.114024177	7
CFAP69	1.052244716	-0.937930314	-0.114314402	7
CFDP1	0.972538094	-1.025367593	0.052829499	7
CFHR5	0.803612377	-1.119897659	0.316285282	7
CFL1	0.80102881	-1.120767579	0.319738769	7
CGN	0.856369306	-1.098985449	0.242616143	7
CGREF1	0.693554112	-1.146300051	0.452745939	7
CHADL	1.003522219	-0.996440164	-0.007082055	7
CHAT	1.049464883	-0.941826669	-0.107638213	7
CHD3	1.056890832	-0.931231125	-0.125659707	7
CHID1	0.84339642	-1.104714944	0.261318524	7
CHKB-CPT1B	1.093595177	-0.867791657	-0.22580352	7
CHMP1A	1.043895364	-0.949395751	-0.094499613	7
CHMP2A	0.704465583	-1.144568694	0.440103111	7
CHMP2B	0.704866756	-1.144501647	0.43963489	7
CHMP4C	1.011574436	-0.988009024	-0.023565413	7
CHPF2	0.694143165	-1.146211088	0.452067923	7
CHRM3	0.772971459	-1.129376262	0.356404802	7
CHRNA5	0.819627943	-1.114198473	0.294570531	7
CHRNA6	1.081297864	-0.891499609	-0.189798254	7
CHRNB4	0.997653855	-1.002329747	0.004675891	7
CHTOP	0.813712754	-1.116365831	0.302653078	7
CIART	1.090732535	-0.873584034	-0.217148501	7
CIB2	1.04447343	-0.948624379	-0.095849051	7
CIB4	0.838427764	-1.106803268	0.268375504	7
CIC	0.990959939	-1.008801311	0.017841372	7
CIDEC	0.836542324	-1.107580748	0.271038424	7
CIPC	1.049589795	-0.941653323	-0.107936471	7
CISD3	0.968396076	-1.028860117	0.060464041	7
CIT	1.043307711	-0.950176626	-0.093131084	7
CITED1	1.06137124	-0.924536249	-0.136834991	7
CITED4	0.926266698	-1.060228403	0.133961706	7
CLASRP	0.919731747	-1.064489836	0.144758089	7
CLCA1	0.998094488	-1.001894681	0.003800192	7
CLCN5	0.755839462	-1.133915783	0.378076321	7
CLCNKA	1.040138741	-0.954331445	-0.085807296	7
CLDN10	0.923132144	-1.06229087	0.139158726	7
CLEC4M	1.090034177	-0.874970297	-0.215063879	7
CLN3	1.027534792	-0.969979176	-0.057555616	7
CLSPN	0.950445076	-1.043105112	0.092660037	7
CLSTN1	0.719239608	-1.14193738	0.422697772	7
CLTRN	0.720848236	-1.141630471	0.420782235	7
CMBL	0.78151486	-1.126913591	0.345398731	7
CMPK1	0.868400569	-1.09329841	0.224897841	7
CMPK2	0.81773089	-1.114901665	0.297170775	7
CMTM6	0.927123615	-1.059658572	0.132534957	7
CNBD2	1.020546819	-0.978101521	-0.042445298	7
CNEP1R1	0.930954699	-1.057078998	0.126124299	7
CNKSR3	0.850519607	-1.101619396	0.251099789	7
CNOT7	0.979188451	-1.019587134	0.040398682	7
CNPPD1	0.684806008	-1.147561714	0.462755706	7
CNTN3	1.07519924	-0.902236351	-0.172962889	7
CNTNAP4	0.85372136	-1.100188148	0.246466789	7
COASY	0.954045029	-1.040359502	0.086314473	7
COG5	0.983634626	-1.015598939	0.031964314	7

COL11A1	1.076621199	-0.899786872	-0.176834327	7
COL16A1	1.024647972	-0.973379731	-0.051268241	7
COL17A1	0.917690667	-1.065790866	0.148100199	7
COL1A2	1.078393346	-0.896689111	-0.181704236	7
COL4A1	1.019436419	-0.979358541	-0.040077878	7
COL4A4	0.987022032	-1.012491393	0.025469361	7
COL4A6	1.092570522	-0.869885696	-0.222684826	7
COL5A2	1.091559487	-0.871929118	-0.219630369	7
COMMD2	0.885463877	-1.084577163	0.199113286	7
COMP	0.841473157	-1.10553012	0.264056963	7
COMT	1.029929851	-0.967108018	-0.062821833	7
COPS5	0.933900563	-1.055059373	0.12115881	7
COQ10B	0.991609379	-1.008184558	0.016575179	7
COTL1	1.080806444	-0.8923877	-0.188418744	7
CPA3	0.712972944	-1.143094498	0.430121554	7
CPB2	0.948700716	-1.044416331	0.095715616	7
CPN1	0.970321127	-1.02724698	0.056925854	7
CPS1	0.952263135	-1.04172523	0.089462096	7
CPSF4L	0.907133937	-1.072300364	0.165166427	7
CPT1B	0.859823698	-1.097390243	0.237566545	7
CRABP1	0.928662296	-1.058628847	0.129966551	7
CREBBP	0.959374683	-1.036194451	0.076819769	7
CRHR2	0.960191551	-1.035545269	0.075353718	7
CRIP2	1.082770866	-0.888812315	-0.193958552	7
CRIP3	0.861690004	-1.096515906	0.234825902	7
CRISPLD1	0.950955666	-1.042718956	0.09176329	7
CRYBB1	1.093511923	-0.86796268	-0.225549243	7
CS	0.968427205	-1.028834169	0.060406964	7
CSAG2	0.835673109	-1.107936432	0.272263323	7
CSF3R	0.719585149	-1.141871797	0.422286648	7
CSHL1	1.08918525	-0.876641746	-0.212543504	7
CSKMT	0.987344224	-1.012192639	0.024848415	7
CSMD3	1.070786568	-0.909641991	-0.161144577	7
CSNK2B	1.023523784	-0.974686614	-0.04883717	7
CSRP3	1.071254286	-0.908870617	-0.162383669	7
CST2	1.051005148	-0.93967782	-0.111327329	7
CST9	1.076081444	-0.900720394	-0.175361049	7
CT45A10	0.74921942	-1.135531977	0.386312557	7
CTCF	0.993080617	-1.006778651	0.013698035	7
CTCFL	1.064866335	-0.919143351	-0.145722984	7
CTR9	1.066268072	-0.916936458	-0.149331614	7
CTSB	1.087973134	-0.879002752	-0.208970383	7
CTSG	1.093396138	-0.868200269	-0.225195869	7
CTSV	0.97678307	-1.021703007	0.044919937	7
CUEDC1	0.866735911	-1.094107424	0.227371513	7
CUEDC2	0.938341181	-1.051954316	0.113613135	7
CXCL10	1.088706057	-0.877578689	-0.211127369	7
CXCL16	1.072287156	-0.907155965	-0.165131191	7
CXCR6	0.999365601	-1.000633194	0.001267592	7
CYB5R2	0.889725008	-1.082272935	0.192547926	7
CYHR1	0.975279367	-1.023011159	0.047731792	7
CYP19A1	1.014179299	-0.985190331	-0.028988968	7
CYP24A1	0.954202745	-1.040237982	0.086035237	7
CYP26B1	1.06359077	-0.92112944	-0.14246133	7
CYP26C1	1.087073775	-0.880735576	-0.206338199	7

CYP27B1	0.988065371	-1.011521929	0.023456557	7
CYP4B1	0.821459786	-1.113512125	0.292052339	7
CYP4F8	0.95880784	-1.036643226	0.077835386	7
CYTH1	0.698159478	-1.145590886	0.447431408	7
CYTH3	1.031407988	-0.965312904	-0.066095084	7
DACH1	0.997998694	-1.001989362	0.003990668	7
DAGLB	1.042385419	-0.951395544	-0.090989876	7
DBF4B	0.814898184	-1.115937421	0.301039237	7
DCANP1	0.962779624	-1.033469105	0.070689481	7
DCBLD1	1.059646274	-0.927142064	-0.13250421	7
DCBLD2	1.045434818	-0.947334332	-0.098100486	7
DCDC1	1.0870974	-0.880690262	-0.206407138	7
DCDC2	0.706811089	-1.14417325	0.437362161	7
DCTN4	1.040890863	-0.953353804	-0.087537059	7
DCX	0.845932442	-1.10362673	0.257694288	7
DDHD2	1.035593018	-0.960131592	-0.075461426	7
DDX1	1.034370525	-0.961660473	-0.072710052	7
DDX10	0.983749636	-1.015494423	0.031744787	7
DDX28	0.830060588	-1.110191833	0.280131245	7
DDX39A	0.920776273	-1.063818573	0.1430423	7
DDX42	0.92580171	-1.060536526	0.134734817	7
DDX58	0.950920859	-1.042745314	0.091824455	7
DDX59	0.762954468	-1.132093887	0.369139419	7
DDX6	0.785438482	-1.125736835	0.340298354	7
DEAF1	1.046759199	-0.945542346	-0.101216853	7
DEFA1	1.036217965	-0.959345027	-0.076872939	7
DEFB106B	0.783865111	-1.126212213	0.342347103	7
DEFB107B	1.030255605	-0.966713942	-0.063541662	7
DEFB110	0.755178773	-1.134080474	0.378901701	7
DEFB118	1.072528933	-0.906752342	-0.16577659	7
DEFB132	0.8815347	-1.086656218	0.205121518	7
DENND2B	0.982207888	-1.016889783	0.034681895	7
DENND4A	0.866516123	-1.0942137	0.227697577	7
DENND5B	0.77605224	-1.128503797	0.352451557	7
DEPDC1B	0.984547297	-1.014767634	0.030220337	7
DEPDC4	0.954849797	-1.039738333	0.084888537	7
DES	1.075148915	-0.902322463	-0.172826452	7
DHFR	0.89774568	-1.077792167	0.180046487	7
DIAPH3	1.029687495	-0.967400647	-0.062286847	7
DIO1	0.996810746	-1.003159028	0.006348283	7
DIP2B	0.811501151	-1.117157218	0.305656068	7
DIPK1A	0.948531214	-1.044543085	0.096011871	7
DIPK1C	1.069176769	-0.912273144	-0.156903625	7
DIRAS2	1.021787739	-0.976686057	-0.045101683	7
DISP1	1.006381195	-0.993494246	-0.012886948	7
DLC1	0.848890875	-1.102337916	0.253447041	7
DLD	0.968541574	-1.028738798	0.060197224	7
DLG2	0.94049607	-1.050420731	0.109924661	7
DLG4	0.774166671	-1.129039853	0.354873182	7
DLGAP3	0.920762731	-1.063827299	0.143064568	7
DMC1	1.088838077	-0.877321023	-0.211517055	7
DMXL2	0.974347484	-1.0238163	0.049468816	7
DNAH3	0.835738215	-1.10790985	0.272171635	7
DNAH6	0.801941946	-1.120461652	0.318519706	7
DNAH8	0.744933718	-1.136538398	0.39160468	7

DNAJB14	1.084592438	-0.885435306	-0.199157132	7
DNAJB5	0.986089704	-1.013352762	0.027263058	7
DNAJC1	0.970471527	-1.027120218	0.056648691	7
DNAJC7	0.888261938	-1.08306997	0.194808033	7
DNASE1L2	0.733408005	-1.139092845	0.40568484	7
DNM3	0.869810404	-1.092607563	0.22279716	7
DNMBP	0.826000149	-1.111779593	0.285779444	7
DNMT3A	0.950373349	-1.043159273	0.092785925	7
DNTTIP2	0.886230771	-1.084166293	0.197935522	7
DOCK6	0.842984848	-1.104890119	0.261905271	7
DOK6	1.069520927	-0.911713697	-0.157807229	7
DOLPP1	1.017085587	-0.981990448	-0.035095139	7
DPF3	0.795549807	-1.122568238	0.327018431	7
DPM1	0.94023675	-1.050606222	0.110369471	7
DPRX	0.808542271	-1.118200086	0.309657815	7
DPYS	0.819965668	-1.114072479	0.294106811	7
DQX1	0.966235721	-1.030649959	0.064414238	7
DRD3	0.976626925	-1.021839364	0.045212439	7
DRP2	1.09353243	-0.867920568	-0.225611863	7
DSC1	0.685774716	-1.147427462	0.461652746	7
DSE	0.684249243	-1.147638265	0.463389022	7
DSEL	0.97507921	-1.023184451	0.048105241	7
DSP	0.955653236	-1.039115465	0.083462229	7
DTD1	1.054846748	-0.934208027	-0.120638721	7
DTHD1	1.07745235	-0.898340308	-0.179112042	7
DTWD2	0.981182637	-1.017810888	0.036628252	7
DUS1L	0.823666645	-1.112675632	0.289008987	7
DUSP12	0.77761516	-1.128054492	0.350439332	7
DUSP14	0.800311161	-1.121006837	0.320695677	7
DUSP15	1.083327212	-0.887787304	-0.195539908	7
DUSP16	1.019817469	-0.978928188	-0.040889281	7
DUSP6	0.757659914	-1.133458071	0.375798157	7
DVL3	0.737687132	-1.138170071	0.400482939	7
DYNC1H1	0.833440487	-1.108842137	0.275401651	7
DYNC2H1	0.964176499	-1.032336156	0.068159657	7
DYNLRB2	1.001658732	-0.998332972	-0.003325761	7
DYRK1A	0.954452911	-1.040045014	0.085592103	7
DYTN	1.04807485	-0.943744861	-0.10432999	7
DZANK1	1.085553133	-0.88362961	-0.201923523	7
E2F4	0.721193819	-1.141564007	0.420370188	7
E4F1	0.743428802	-1.136884455	0.393455654	7
EBF3	1.077560322	-0.898151576	-0.179408746	7
ECE1	1.01664603	-0.982478197	-0.034167833	7
ECI1	1.050022757	-0.941051229	-0.108971527	7
ECM2	0.871141018	-1.091950731	0.220809713	7
ECRG4	0.950866413	-1.042786534	0.091920121	7
EDA	0.973894479	-1.02420617	0.050311691	7
EDEM1	0.850515166	-1.101621364	0.251106199	7
EFCAB5	0.930804833	-1.057180899	0.126376066	7
EFEMP2	0.936652798	-1.053143594	0.116490796	7
EGFLAM	0.912915691	-1.068780233	0.155864542	7
EGFR	1.067370063	-0.915183211	-0.152186852	7
EHD4	1.090185663	-0.874670463	-0.2155152	7
EHMT1	0.701280439	-1.145092427	0.443811988	7
EID2B	0.93743127	-1.052596582	0.115165312	7

EIF1AD	1.060754287	-0.925472393	-0.135281894	7
EIF4E	1.074197688	-0.90394281	-0.170254878	7
EIF4G3	0.967453884	-1.029643361	0.062189478	7
EIF5AL1	0.982794359	-1.01636045	0.033566091	7
ELAC1	1.013921236	-0.985471637	-0.028449599	7
ELF5	0.831849208	-1.109480781	0.277631573	7
ELFN2	0.816558435	-1.115332427	0.298773992	7
ELL	0.815709452	-1.115642524	0.299933073	7
ELMO1	1.058605404	-0.92869718	-0.129908224	7
ELMOD1	1.026114607	-0.971660193	-0.054454414	7
ELSPBP1	0.770490692	-1.130066177	0.359575484	7
EMILIN2	1.062164147	-0.92332625	-0.138837896	7
ENAH	1.007076202	-0.992770298	-0.014305904	7
ENAM	0.78580509	-1.125625392	0.339820303	7
ENPEP	0.886985279	-1.083760424	0.196775146	7
ENPP4	0.689704399	-1.146868958	0.457164559	7
EOGT	1.031650598	-0.965016553	-0.066634044	7
EPB41L3	0.685530368	-1.147461453	0.461931085	7
EPHA2	1.054563201	-0.934617262	-0.11994594	7
EPHA3	0.82413569	-1.11249648	0.28836079	7
EPHA5	1.051269854	-0.939306017	-0.111963837	7
EPHX1	0.970322932	-1.02724546	0.056922528	7
EPHX3	0.950482183	-1.043077085	0.092594902	7
EPOP	1.044864101	-0.948101239	-0.096762862	7
EPS15L1	0.824268402	-1.112445704	0.288177302	7
EPSTI1	0.89447491	-1.07964237	0.185167459	7
ERC2	1.086217505	-0.882370669	-0.203846836	7
ERCC3	0.974266177	-1.023886349	0.049620172	7
ERCC4	0.933689825	-1.055204907	0.121515081	7
ERCC6	0.821568051	-1.113471334	0.291903283	7
ERI2	0.820771593	-1.113770822	0.292999229	7
ERICH3	0.993343467	-1.006526191	0.013182724	7
ERV3-1	0.976027075	-1.022362077	0.046335003	7
ERVK3-1	0.875864111	-1.089581152	0.213717041	7
ESRRA	1.092752914	-0.869514665	-0.223238249	7
ETHE1	0.687063739	-1.147246722	0.460182982	7
ETV3	0.768278549	-1.130671962	0.362393413	7
ETV4	0.756223645	-1.133819669	0.377596024	7
ETV6	0.892089536	-1.080971689	0.188882153	7
EXD1	0.97972745	-1.01910903	0.03938158	7
EXO1	0.790197183	-1.124270251	0.334073068	7
EYA1	0.964048925	-1.03243999	0.068391065	7
F11R	0.745104615	-1.13649886	0.391394245	7
F2	1.087886974	-0.879169453	-0.208717521	7
F2RL3	0.801079163	-1.120750753	0.319671589	7
F3	1.009042343	-0.99070546	-0.018336883	7
FAAH2	0.717527003	-1.142259673	0.42473267	7
FADS6	1.07192246	-0.907763166	-0.164159293	7
FAH	1.021188946	-0.977370492	-0.043818454	7
FAM102B	0.831710946	-1.109536002	0.277825056	7
FAM110B	0.729493261	-1.139910927	0.410417666	7
FAM110D	1.074661244	-0.903154908	-0.171506336	7
FAM136A	0.78750707	-1.125104655	0.337597585	7
FAM149A	0.853517012	-1.100280245	0.246763233	7
FAM163A	0.865159115	-1.094867093	0.229707978	7

FAM171A2	0.919962402	-1.064341924	0.144379522	7
FAM180A	0.993366487	-1.006504061	0.013137574	7
FAM184A	1.074925263	-0.902704687	-0.172220577	7
FAM189A2	1.014586534	-0.984745488	-0.029841046	7
FAM20C	0.769247277	-1.130407769	0.361160492	7
FAM214A	1.018514796	-0.980395072	-0.038119723	7
FAM222A	0.977455326	-1.021114566	0.04365924	7
FAM229B	0.956219976	-1.038674452	0.082454475	7
FAM234B	0.82694632	-1.111412869	0.284466549	7
FAM241B	0.93800334	-1.052193149	0.114189809	7
FAM25G	0.958676905	-1.036746691	0.078069786	7
FAM47B	0.991061263	-1.008705241	0.017643977	7
FAM71B	0.797557281	-1.12191541	0.32435813	7
FAM71C	0.981468514	-1.017554593	0.036086079	7
FAM78B	1.074429482	-0.903549241	-0.170880241	7
FAM83A	0.732271133	-1.13933298	0.407061847	7
FAM83B	0.841032685	-1.105715596	0.264682911	7
FAM98C	0.948273622	-1.044735491	0.096461869	7
FAN1	1.072696037	-0.906472874	-0.166223163	7
FANCM	1.045965159	-0.94661882	-0.09934634	7
FARP1	0.817795685	-1.114877774	0.297082088	7
FAT1	0.682201324	-1.147916016	0.465714692	7
FAXC	1.074440841	-0.903529934	-0.170910907	7
FAXDC2	0.772672471	-1.129460007	0.356787536	7
FBLN7	0.996374956	-1.003586044	0.007211088	7
FBN1	0.847062835	-1.103136755	0.25607392	7
FBRS	1.072177609	-0.907338561	-0.164839048	7
FBXL18	1.086506959	-0.881819531	-0.204687428	7
FBXO38	0.987297678	-1.012235834	0.024938156	7
FCGRT	1.011493193	-0.9880962	-0.023396993	7
FCRL3	1.085602671	-0.883536028	-0.202066643	7
FCRLA	1.028069646	-0.969341975	-0.05872767	7
FDX1	0.85426696	-1.099941752	0.245674792	7
FER1L6	1.084989894	-0.884690355	-0.200299539	7
FGB	0.847074789	-1.103131557	0.256056768	7
FGF13	1.086291034	-0.882230819	-0.204060215	7
FGF17	1.093671786	-0.867634144	-0.226037642	7
FGF18	0.907161524	-1.072283822	0.165122298	7
FGFR1	0.98637422	-1.013090393	0.026716172	7
FGFR4	1.055656871	-0.93303385	-0.122623021	7
FHIP1A	1.089586368	-0.875853843	-0.213732525	7
FKBP15	1.088633413	-0.877720319	-0.210913094	7
FKBP6	0.953540289	-1.040747704	0.087207415	7
FLG2	1.081744898	-0.890688089	-0.191056809	7
FLNA	1.086998419	-0.880880045	-0.206118373	7
FLOT2	1.049020865	-0.942441549	-0.106579317	7
FLRT2	1.011371221	-0.988226998	-0.023144223	7
FLRT3	0.939433113	-1.051179427	0.111746314	7
FLYWCH1	0.905616507	-1.073206558	0.167590052	7
FLYWCH2	1.039171474	-0.955581134	-0.083590339	7
FNIP2	0.932864098	-1.055773573	0.122909475	7
FNTA	1.080363087	-0.893185368	-0.187177719	7
FOLR1	0.917611935	-1.065840771	0.148228836	7
FOS	0.724592446	-1.14090032	0.416307875	7
FOXD4L6	1.074999182	-0.902578444	-0.172420738	7

FOXR1	1.062136499	-0.923368572	-0.138767927	7
FPGS	0.683405059	-1.147753486	0.464348427	7
FRAT2	0.901215935	-1.075793843	0.174577908	7
FREM1	0.819692295	-1.114174484	0.29448219	7
FRG1	0.979753163	-1.019086185	0.039333022	7
FRMD1	0.742483393	-1.137099913	0.39461652	7
FRRS1	0.789992954	-1.124334087	0.334341134	7
FSCN1	1.084933002	-0.884797169	-0.200135833	7
FSIP2	0.731875724	-1.139416009	0.407540285	7
FTHL17	1.05283383	-0.937094033	-0.115739797	7
FXYD6	0.818266864	-1.114703772	0.296436908	7
FXYD6-FXYD2	1.077501996	-0.898253552	-0.179248444	7
FZD3	0.946900696	-1.045756476	0.09885578	7
FZD4	1.08354836	-0.887378308	-0.196170052	7
FZD9	1.08665913	-0.881529139	-0.20512999	7
G0S2	0.912730609	-1.068894591	0.156163982	7
GABPB1	0.679894075	-1.148221765	0.46832769	7
GABRB2	1.059125172	-0.927922226	-0.131202946	7
GABRP	0.854205177	-1.09996969	0.245764514	7
GABRR1	0.875687117	-1.089671032	0.213983914	7
GABRR2	0.999411428	-1.000587534	0.001176106	7
GADD45GIP1	0.892761505	-1.08059891	0.187837405	7
GALNT1	1.052721819	-0.937253327	-0.115468493	7
GALNT12	0.984181348	-1.015101483	0.030920135	7
GALNT14	0.930739503	-1.057225294	0.126485791	7
GAMT	0.889313511	-1.082497731	0.19318422	7
GATA2	0.96789925	-1.02927364	0.06137439	7
GATAD2B	1.060179323	-0.926340658	-0.133838665	7
GATD3	0.915500955	-1.067171105	0.15167015	7
GBP2	1.087833415	-0.879273004	-0.208560412	7
GBP5	0.746264389	-1.136229237	0.389964848	7
GBX2	0.922904538	-1.062439299	0.139534761	7
GCSAML	1.07070743	-0.909772197	-0.160935233	7
GDAP1	0.884203645	-1.085248723	0.201045078	7
GDI1	0.702510702	-1.14489194	0.442381237	7
GEMIN6	0.763900326	-1.131844968	0.367944642	7
GFER	1.026352294	-0.971379951	-0.054972343	7
GFOD2	1.071425584	-0.90858732	-0.162838265	7
GHRL	0.874999269	-1.090019527	0.215020258	7
GJC3	0.744368134	-1.136668901	0.392300766	7
GLP2R	0.832702603	-1.10913899	0.276436387	7
GLRA1	1.057354111	-0.930549813	-0.126804298	7
GLYAT	1.022598801	-0.975754747	-0.046844054	7
GMCL2	1.045063674	-0.947833422	-0.097230252	7
GMEB1	0.930899424	-1.057116592	0.126217168	7
GMFB	0.865216456	-1.09483958	0.229623123	7
GMIP	0.689961422	-1.146831649	0.456870227	7
GMPR2	1.089667377	-0.875694318	-0.213973059	7
GNA12	0.746829248	-1.136097098	0.38926785	7
GNAT2	0.887057931	-1.083721257	0.196663326	7
GNAZ	0.959707511	-1.035930297	0.076222787	7
GNB3	0.742896323	-1.137005991	0.394109668	7
GNB5	0.895511711	-1.079059334	0.183547622	7
GNG12	0.941158481	-1.049945735	0.108787255	7
GNG2	0.810191026	-1.117621215	0.307430188	7

GNG3	0.972900588	-1.025058068	0.05215748	7
GNGT2	0.761871006	-1.132377069	0.370506063	7
GNL1	0.890440754	-1.081880763	0.191440009	7
GNPDA2	1.062259836	-0.923179699	-0.139080137	7
GOLGA6L1	0.76960669	-1.130309319	0.360702629	7
GOLGA6L2	1.077831004	-0.897677603	-0.180153401	7
GOLGA6L22	0.691580449	-1.146594422	0.455013973	7
GOLGA6L24P	0.95229552	-1.041700527	0.089405007	7
GOLGA6L25P	0.977365622	-1.021193216	0.043827594	7
GOLGA6L6	0.755938437	-1.133891046	0.377952609	7
GOLGA8K	0.968406841	-1.028851144	0.060444303	7
GOLGA8S	1.069768547	-0.911310151	-0.158458396	7
GOLGA8T	0.998644035	-1.001350471	0.002706436	7
GOLGB1	0.995535756	-1.004405242	0.008869486	7
GORASP1	0.944579517	-1.047465516	0.102886	7
GPC6	1.030473513	-0.966449848	-0.064023665	7
GPD1	1.022512053	-0.975854591	-0.046657462	7
GPD2	0.949957672	-1.043472738	0.093515066	7
GPHN	0.937192696	-1.052764464	0.115571768	7
GPIHBP1	0.697182056	-1.145744016	0.44856196	7
GPM6A	1.069643569	-0.911513936	-0.158129633	7
GPM6B	0.913917942	-1.068159017	0.154241075	7
GPR108	0.740786903	-1.137482803	0.396695901	7
GPR137	0.84314183	-1.104823351	0.261681521	7
GPR149	0.741360041	-1.137353985	0.395993944	7
GPR151	0.792177472	-1.123647077	0.331469605	7
GPR162	0.713906998	-1.142925889	0.429018891	7
GPR19	0.907030153	-1.072362575	0.165332422	7
GPR21	1.063291687	-0.921592111	-0.141699576	7
GPR39	0.961323836	-1.034640583	0.073316747	7
GPR55	1.052407863	-0.937699093	-0.11470877	7
GPR78	1.062112233	-0.92340571	-0.138706523	7
GPR85	0.683572132	-1.147730763	0.464158631	7
GPX7	1.064219441	-0.920153192	-0.144066249	7
GRAMD2A	0.714192623	-1.142874062	0.428681438	7
GREB1L	0.706775153	-1.144179371	0.437404218	7
GRHPR	0.792794462	-1.12345136	0.330656898	7
GRID2	0.968027305	-1.029167166	0.061139861	7
GRIN2D	1.020671507	-0.977959808	-0.0427117	7
GRM6	1.069953875	-0.911007561	-0.158946314	7
GRPEL2	0.956022925	-1.038827943	0.082805018	7
GRTP1	0.772431612	-1.129527351	0.357095739	7
GSAP	1.060929309	-0.925207294	-0.135722015	7
GSG1L	1.032333749	-0.964179456	-0.068154292	7
GSK3B	0.768937458	-1.130492448	0.36155499	7
GSTA1	0.836862987	-1.107449096	0.270586109	7
GSTA2	0.879023293	-1.087962522	0.208939228	7
GSTA3	0.769972025	-1.130209006	0.360236981	7
GTF2B	0.766528358	-1.13114499	0.364616631	7
GTF2IRD1	0.954434295	-1.040059383	0.085625088	7
GTF2IRD2	0.818261445	-1.114705776	0.296444331	7
GTF2IRD2B	0.75042123	-1.135244156	0.384822926	7
GTPBP8	0.866064392	-1.094431736	0.228367344	7
GUCY1A1	0.865737896	-1.094588996	0.228851099	7
GUCY2C	0.865697655	-1.094608359	0.228910703	7

GUK1	0.94104344	-1.050028349	0.108984909	7
GULP1	0.858648946	-1.097936087	0.239287142	7
GVQW3	0.757746489	-1.13343616	0.375689671	7
GYG2	1.009677269	-0.990033296	-0.019643974	7
H1-8	0.919045084	-1.0649291	0.145884016	7
H2AC12	0.694834413	-1.146106041	0.451271628	7
H2AC20	0.690875073	-1.146698246	0.455823173	7
H3C4	0.755430598	-1.134017791	0.378587192	7
H4C6	1.030196244	-0.966785817	-0.063410427	7
HAUS8	1.056922396	-0.931184783	-0.125737613	7
HDAC5	1.054955263	-0.934051173	-0.12090409	7
HDHD3	0.944408678	-1.047590459	0.103181781	7
HECTD2	0.952164661	-1.04180032	0.089635659	7
HECW1	1.092081881	-0.87087609	-0.22120579	7
HEG1	1.015030497	-0.984259224	-0.030771273	7
HELLS	1.06006305	-0.926515758	-0.133547292	7
HFE	0.72260874	-1.141289919	0.418681179	7
HHIP	1.090119532	-0.874801413	-0.215318119	7
HHIPL2	0.923015295	-1.062367093	0.139351798	7
HIC1	0.728281838	-1.140159072	0.411877234	7
HIPK1	0.941752643	-1.04951824	0.107765596	7
HIPK4	0.811850205	-1.117032994	0.305182789	7
HJURP	1.074279882	-0.903803344	-0.170476538	7
HLA-C	1.000574875	-0.999424132	-0.001150743	7
HLA-DOA	0.946976522	-1.045700285	0.098723763	7
HLA-E	0.930979374	-1.057062213	0.12608284	7
HLA-G	0.970767092	-1.026870794	0.056103702	7
HLCS	0.72193275	-1.141421261	0.419488511	7
HMCN1	0.811920836	-1.117007827	0.305086991	7
HMGB4	0.702188245	-1.144944708	0.442756463	7
HMGCS1	1.00714666	-0.992696734	-0.014449926	7
HMGCS2	0.908377264	-1.071552431	0.163175167	7
HMGXB4	1.01811546	-0.980842303	-0.037273157	7
HMHB1	0.682514617	-1.147873914	0.465359297	7
HMX2	0.902319061	-1.075150904	0.172831843	7
HNF4G	1.08332209	-0.887796766	-0.195525324	7
HNRNPA0	1.044392682	-0.948732324	-0.095660358	7
HNRNPL	0.761564322	-1.13245685	0.370892527	7
HOOK1	0.941832417	-1.049460739	0.107628322	7
HOOK2	1.015284866	-0.983980003	-0.031304863	7
HORMAD1	0.859038652	-1.097755397	0.238716745	7
HOXA3	0.904168106	-1.074064782	0.169896676	7
HOXA4	0.975097371	-1.023168735	0.048071364	7
HOXB3	1.015384607	-0.983870395	-0.031514212	7
HOXB7	1.076670216	-0.899701866	-0.17696835	7
HOXC11	1.064707204	-0.919392266	-0.145314937	7
HOXD11	1.048835007	-0.942698325	-0.106136682	7
HOXD4	1.046225505	-0.946266559	-0.099958946	7
HOXD9	1.069674612	-0.911463338	-0.158211275	7
HPCA	0.973993028	-1.02412144	0.050128413	7
HPS1	0.841133283	-1.105673276	0.264539993	7
HPS4	1.063371171	-0.921469262	-0.141901909	7
HRH4	0.691516293	-1.146603895	0.455087601	7
HRNR	0.797093542	-1.122066926	0.324973384	7
HS3ST3A1	1.049564518	-0.941688414	-0.107876103	7

HS3ST3B1	0.811492215	-1.117160395	0.305668181	7
HS6ST1	0.834526637	-1.108402935	0.273876298	7
HSF5	1.001475101	-0.998518342	-0.002956759	7
HSH2D	0.844875279	-1.104082211	0.259206933	7
HSPB8	0.767508955	-1.13088064	0.363371685	7
HTR1F	1.083093025	-0.888219448	-0.194873577	7
HTR3A	0.84751392	-1.102940379	0.25542646	7
HTR3B	1.030612414	-0.966281305	-0.064331109	7
HTR3E	0.728234785	-1.140168662	0.411933877	7
HTRA1	0.972237391	-1.025623878	0.053386487	7
HTRA2	0.887069606	-1.083714962	0.196645355	7
HYI	0.979997335	-1.018869084	0.038871749	7
IBA57	0.860728705	-1.096967362	0.236238656	7
ICA1	0.984523939	-1.014788964	0.030265025	7
ICAM2	1.083949427	-0.886634299	-0.197315127	7
ICAM3	0.715435629	-1.142647044	0.427211416	7
ICMT	1.086576004	-0.881687825	-0.204888179	7
ICOS	1.065590747	-0.918006055	-0.147584692	7
IDS	0.685180938	-1.147509913	0.462328975	7
IFFO2	1.012245295	-0.987287481	-0.024957814	7
IFI27L1	0.782480841	-1.126626578	0.344145736	7
IFI27L2	1.000134544	-0.999865402	-0.000269143	7
IFI30	1.076483409	-0.900025622	-0.176457786	7
IFI44	0.998123894	-1.001865606	0.003741712	7
IFIT1	0.939128295	-1.051396197	0.112267902	7
IFITM2	0.693254136	-1.14634516	0.453091024	7
IFNA6	0.862356592	-1.096201477	0.233844885	7
IFNA8	0.712290246	-1.143216882	0.430926637	7
IFNL2	0.711693896	-1.143323201	0.431629305	7
IFNL3	0.989036888	-1.010613906	0.021577018	7
IFT74	0.715826672	-1.142575131	0.426748459	7
IGDCC4	0.867841735	-1.093570807	0.225729071	7
IGFBP1	0.899968757	-1.076516243	0.176547485	7
IGFBP7	0.777124152	-1.128196134	0.351071982	7
IGFLR1	0.800402026	-1.1209766	0.320574574	7
IGSF5	0.690140172	-1.146805645	0.456665474	7
IGSF8	0.975567692	-1.02276119	0.047193497	7
IL12A	1.040887462	-0.953358236	-0.087529226	7
IL18R1	1.047742989	-0.944199904	-0.103543084	7
IL1R1	1.071807145	-0.907954756	-0.163852389	7
IL1RAP	0.693800608	-1.146262886	0.452462277	7
IL1RAPL2	1.01911745	-0.979717972	-0.039399478	7
IL20RA	0.739821504	-1.137698553	0.397877049	7
IL26	0.951901703	-1.042000638	0.090098935	7
IL31	0.923878973	-1.061802582	0.137923609	7
IL36G	0.706092063	-1.144295359	0.438203296	7
ILRUN	0.837402845	-1.107226917	0.269824072	7
IMP4	1.044022797	-0.949225981	-0.094796816	7
IMPA1	0.687130938	-1.147237234	0.460106296	7
INAFM1	0.728228716	-1.140169899	0.411941183	7
INMT-MINDY4	1.08999542	-0.87504693	-0.21494849	7
INO80	0.780923925	-1.127088304	0.346164379	7
INSRR	1.012420891	-0.987098119	-0.025322773	7
INTS11	0.983027313	-1.016149699	0.033122386	7
INTS14	0.934052404	-1.054954412	0.120902008	7

IP6K1	1.056775159	-0.931400852	-0.125374307	7
IQCA1	1.07706905	-0.89900879	-0.17806026	7
IQCH	1.087410591	-0.880088492	-0.207322098	7
IRAK2	0.72105523	-1.141590683	0.420535453	7
IRX4	0.972431997	-1.025458067	0.053026071	7
ISL1	0.714573334	-1.142804784	0.42823145	7
ISLR	1.079232924	-0.895203612	-0.184029312	7
ISY1	0.915544748	-1.067143657	0.15159891	7
ITGA2	0.725982303	-1.14062363	0.414641327	7
ITGA3	0.903723813	-1.074326727	0.170602914	7
ITIH6	1.00713352	-0.992710455	-0.014423065	7
IZUMO1R	0.782013974	-1.126765513	0.344751538	7
JADE2	0.682973497	-1.147811994	0.464838498	7
JAKMIP2	0.954258464	-1.040195026	0.085936562	7
JHY	1.04310543	-0.950444655	-0.092660775	7
JPH2	0.752582243	-1.134720403	0.382138161	7
JSRP1	0.928815086	-1.058526135	0.129711049	7
KANK1	1.025896443	-0.971917029	-0.053979415	7
KANK4	1.049456663	-0.94183807	-0.107618593	7
KAT5	1.070116547	-0.910741561	-0.159374986	7
KCNAB1	0.975437149	-1.022874417	0.047437268	7
KCNE5	1.031101791	-0.965686233	-0.065415559	7
KCNH1	0.900594733	-1.076154255	0.175559522	7
KCNH7	0.706160657	-1.144283744	0.438123087	7
KCNIP1	0.985454068	-1.013937366	0.028483298	7
KCNIP3	1.066605584	-0.916401208	-0.150204376	7
KCNJ14	0.747997709	-1.135822043	0.387824334	7
KCNK3	1.017422441	-0.981615738	-0.035806703	7
KCNN2	1.091227396	-0.872595468	-0.218631928	7
KCNQ1	1.043194533	-0.950326639	-0.092867893	7
KCNV1	1.093545267	-0.867894202	-0.225651066	7
KCTD16	0.865088418	-1.094901003	0.229812585	7
KCTD19	1.048325626	-0.94340026	-0.104925366	7
KDELR3	1.068711565	-0.913026738	-0.155684827	7
KDM5C	0.783256778	-1.126394756	0.343137978	7
KHDRBS1	0.856486152	-1.098931978	0.242445826	7
KIAA0319	1.079853698	-0.894097701	-0.185755997	7
KIAA0408	0.997409608	-1.002570417	0.005160809	7
KIAA0753	1.034853068	-0.961058528	-0.07379454	7
KIAA0895L	1.030424001	-0.966509888	-0.063914113	7
KIF18A	0.701797845	-1.145008385	0.44321054	7
KIF18B	0.792097504	-1.12367239	0.331574886	7
KIF24	0.731102673	-1.139577603	0.408474931	7
KIF26A	0.861242558	-1.096726333	0.235483775	7
KIF26B	1.080008749	-0.893820466	-0.186188283	7
KIF3B	1.046556852	-0.94581726	-0.100739592	7
KIR3DL2	1.080277394	-0.893339156	-0.186938238	7
KIRREL2	1.026625273	-0.971057556	-0.055567717	7
KLC1	1.059665241	-0.927113608	-0.132551633	7
KLC4	1.090565959	-0.873915627	-0.216650332	7
KLF5	0.995763399	-1.004183428	0.008420029	7
KLHDC7A	0.757209087	-1.133571959	0.376362873	7
KLHDC8A	0.97624624	-1.022171301	0.045925061	7
KLHDC8B	0.982023166	-1.01705614	0.035032974	7
KLHL1	0.970702669	-1.026925195	0.056222527	7

KLHL10	1.035749765	-0.959934627	-0.075815138	7
KLHL21	1.055685118	-0.932992776	-0.122692342	7
KLHL25	0.930030354	-1.057706206	0.127675852	7
KLHL26	0.813333369	-1.116502316	0.303168947	7
KLHL3	1.055108331	-0.933829697	-0.121278635	7
KLHL30	1.028065487	-0.969346938	-0.05871855	7
KLHL5	0.976486044	-1.021962288	0.045476244	7
KLHL7	0.950986399	-1.042695678	0.091709279	7
KLK8	0.875912595	-1.089556516	0.21364392	7
KMO	1.034003502	-0.962116982	-0.07188652	7
KMT2D	0.981056842	-1.017923533	0.036866691	7
KMT5C	1.06122245	-0.924762444	-0.136460007	7
KNG1	1.017188668	-0.981875868	-0.0353128	7
KNL1	1.054525487	-0.934671626	-0.119853861	7
KPNA5	0.715170182	-1.142695725	0.427525543	7
KRBOX4	0.826396883	-1.111626064	0.285229181	7
KRT25	1.052847425	-0.93707469	-0.115772735	7
KRT35	0.836870408	-1.107446046	0.270575638	7
KRT76	0.963434073	-1.032939397	0.069505324	7
KRT80	0.97206176	-1.025773366	0.053711606	7
KRT84	1.079344951	-0.895004514	-0.184340436	7
KRTAP12-4	1.070497297	-0.910117497	-0.1603798	7
KRTAP17-1	0.7486303	-1.135672166	0.387041865	7
KRTAP4-3	1.087088776	-0.880706804	-0.206381972	7
KRTAP5-10	0.682579308	-1.147865203	0.465285895	7
KRTAP5-6	1.046524495	-0.945861184	-0.10066331	7
KSR2	1.060502078	-0.925853753	-0.134648325	7
KXD1	0.97617649	-1.022232042	0.046055552	7
L1TD1	1.029951668	-0.967081653	-0.062870015	7
LAIR1	0.711810918	-1.143302381	0.431491463	7
LAMA2	1.018879714	-0.979985388	-0.038894326	7
LANCL2	0.751751084	-1.134922794	0.38317171	7
LANCL3	1.06911746	-0.912369387	-0.156748073	7
LARP1B	0.919222117	-1.064816003	0.145593886	7
LCE1C	1.074764261	-0.902979365	-0.171784896	7
LCE2A	0.861954499	-1.096391279	0.23443678	7
LCN15	0.872274774	-1.091387369	0.219112596	7
LCN2	0.705828876	-1.14433986	0.438510984	7
LCN8	1.086697424	-0.88145599	-0.205241434	7
LCP2	0.957386322	-1.037762542	0.08037622	7
LCT	0.899591199	-1.076733998	0.177142799	7
LDLRAD3	1.076604647	-0.899815568	-0.176789079	7
LEFTY2	1.050901409	-0.939823329	-0.11107808	7
LEXM	0.85385568	-1.100127556	0.246271876	7
LGALS9	0.769665298	-1.130293243	0.360627945	7
LGALS9B	0.855344986	-1.099452752	0.244107765	7
LGALS9C	0.714490494	-1.142819878	0.428329384	7
LGI1	1.03827809	-0.956727843	-0.081550246	7
LGI4	0.885562384	-1.08452448	0.198962096	7
LHFPL1	0.894188622	-1.079802799	0.185614178	7
LHFPL2	1.001931498	-0.998057245	-0.003874253	7
LIF	0.957226825	-1.037887589	0.080660765	7
LIG1	0.980980224	-1.017992103	0.037011879	7
LILRB4	1.075719204	-0.901344328	-0.174374875	7
LIMK2	1.055018492	-0.933959718	-0.121058774	7

LIMS1	1.084484821	-0.885636506	-0.198848315	7
LIN7B	0.704885306	-1.144498541	0.439613234	7
LINS1	0.828362008	-1.110860477	0.282498469	7
LIPE	0.713434428	-1.143011362	0.429576934	7
LIPH	0.77398908	-1.129090004	0.355100925	7
LIPT2	0.735987849	-1.138540104	0.402552255	7
LIX1	0.747973863	-1.135827679	0.387853816	7
LMF2	0.888631238	-1.082869369	0.194238131	7
LMNA	0.680816563	-1.148100429	0.467283866	7
LMO7	1.027065234	-0.970536724	-0.05652851	7
LMTK3	0.776176994	-1.128468099	0.352291106	7
LORICRIN	0.91211352	-1.069275069	0.157161549	7
LOXL2	0.988042701	-1.011543056	0.023500354	7
LPCAT4	1.067106166	-0.915604549	-0.151501617	7
LPIN1	0.740325617	-1.137586085	0.397260468	7
LPL	0.933506498	-1.055331378	0.12182488	7
LRCOL1	0.84848013	-1.102518107	0.254037977	7
LRFN4	0.744811994	-1.13656653	0.391754536	7
LRG1	0.767513251	-1.130879478	0.363366227	7
LRGUK	1.056069746	-0.932432597	-0.123637148	7
LRP1	0.824549963	-1.112337849	0.287787887	7
LRP10	0.993662649	-1.006219099	0.01255645	7
LRP11	0.784099317	-1.126141748	0.342042431	7
LRP3	0.788767905	-1.124715311	0.335947406	7
LRPAP1	0.821975583	-1.113317561	0.291341978	7
LRRC2	0.943130628	-1.04852152	0.105390892	7
LRRC3	0.882046514	-1.086387852	0.204341339	7
LRRC38	1.033466259	-0.962783147	-0.070683112	7
LRRC66	0.801595897	-1.120577785	0.318981889	7
LRRC8B	1.077528069	-0.898207974	-0.179320094	7
LRRIQ1	0.912722571	-1.068899555	0.156176983	7
LRRN2	1.009792753	-0.989910755	-0.019881997	7
LRSAM1	0.923431005	-1.062095702	0.138664697	7
LSM2	0.866962936	-1.093997517	0.22703458	7
LUC7L	0.87595776	-1.089533561	0.213575801	7
LY6G6F-LY6G6D	0.983155809	-1.016033328	0.032877518	7
LYPD8	0.882290559	-1.086259632	0.203969073	7
LYRM1	1.091881541	-0.871280632	-0.220600908	7
LYRM4	0.913685542	-1.068303357	0.154617815	7
LZIC	0.696641642	-1.145828073	0.449186431	7
LZTFL1	0.712440314	-1.143190042	0.430749728	7
MACIR	1.042218048	-0.951615883	-0.090602165	7
MACROH2A2	0.83017491	-1.110146599	0.27997169	7
MAD2L1	0.836782179	-1.107482294	0.270700115	7
MAF1	0.985191485	-1.014178242	0.028986758	7
MAGEA10	0.892472498	-1.080759401	0.188286903	7
MAGEC1	1.078229605	-0.89697747	-0.181252135	7
MAGI2	0.930188066	-1.05759941	0.127411343	7
MALRD1	1.080063369	-0.893722707	-0.186340661	7
MAMDC2	1.065306412	-0.918453267	-0.146853145	7
MAML3	1.013001282	-0.986470747	-0.026530535	7
MAN2B2	0.686750861	-1.147290812	0.460539952	7
MAOB	1.07901622	-0.895588154	-0.183428066	7
MAP1A	1.027823459	-0.969635553	-0.058187906	7
MAP3K10	1.046939098	-0.945297587	-0.101641511	7

MAP3K19	0.863092597	-1.095852987	0.23276039	7
MAP3K4	1.05991286	-0.926741695	-0.133171165	7
MAP3K8	1.004060662	-0.995889259	-0.008171403	7
MAP4K5	0.979198194	-1.019578505	0.040380311	7
MAP6	1.081343657	-0.891416638	-0.189927019	7
MAPK4	1.083431932	-0.887593744	-0.195838188	7
MARCHF4	1.058374201	-0.929040871	-0.12933333	7
MARCHF5	0.860341758	-1.097148423	0.236806666	7
MARCO	1.027688236	-0.969796602	-0.057891634	7
MASTL	1.001278888	-0.998716186	-0.002562702	7
MATN3	1.053003291	-0.936852778	-0.116150514	7
MAU2	0.935644811	-1.053848506	0.118203696	7
MAZ	0.868302348	-1.093346346	0.225043998	7
MBD3	1.045903165	-0.946702603	-0.099200562	7
MBOAT4	0.754310812	-1.134295683	0.379984872	7
MC2R	1.087912958	-0.879119196	-0.208793761	7
MCAM	0.798419286	-1.121632638	0.323213352	7
MCF2L2	0.958143891	-1.03716711	0.079023219	7
MCFD2	1.070843865	-0.909547663	-0.161296202	7
MCM3	0.987069503	-1.01244741	0.025377906	7
MCM7	0.98705342	-1.012462312	0.025408892	7
MCRS1	0.725119803	-1.140795697	0.415675894	7
MDH1B	0.744248991	-1.136696323	0.392447332	7
ME1	1.030301676	-0.966658139	-0.063643537	7
ME2	0.947972372	-1.044960167	0.096987795	7
MEA1	1.027315977	-0.970239211	-0.057076766	7
MECP2	0.734178851	-1.138928827	0.404749976	7
MED1	1.021329402	-0.977210186	-0.044119216	7
MED11	1.030099523	-0.966902867	-0.063196656	7
MED13L	1.080598604	-0.892762055	-0.187836549	7
MEI4	1.034333808	-0.961706194	-0.072627614	7
MEIS2	0.865075155	-1.094907363	0.229832208	7
MELK	1.083868674	-0.886784337	-0.197084337	7
MESP1	0.745716009	-1.136357006	0.390640997	7
MEST	0.933436753	-1.05537946	0.121942707	7
MET	0.737252664	-1.138265132	0.401012468	7
METTL21C	0.928491907	-1.058743291	0.130251384	7
METTL25	0.822426691	-1.113146927	0.290720237	7
METTL26	1.030191224	-0.966791894	-0.06339933	7
METTL8	0.717493492	-1.142265933	0.424772441	7
MFAP3	0.792857576	-1.123431297	0.330573721	7
MFSD13A	0.843171905	-1.104810553	0.261638648	7
MFSD4A	0.95274292	-1.041358802	0.088615882	7
MIA2	0.829767361	-1.11030772	0.280540359	7
MIB2	0.919471531	-1.064656486	0.145184955	7
MICALL2	1.055786603	-0.932845134	-0.122941469	7
MICB	1.090762081	-0.873525157	-0.217236924	7
MIER1	1.066622955	-0.916373619	-0.150249336	7
MINDY4	0.856618372	-1.09887143	0.242253058	7
MINK1	0.808195029	-1.118321286	0.310126257	7
MIPOL1	0.955485709	-1.039245566	0.083759856	7
MLEC	1.07976798	-0.894250795	-0.185517185	7
MLNR	1.063360687	-0.92148547	-0.141875218	7
MLXIPL	0.947519328	-1.045297363	0.097778035	7
MMACHC	0.956599355	-1.038378472	0.081779117	7

MMP13	1.022750057	-0.975580522	-0.047169535	7
MMP28	0.79594141	-1.122441515	0.326500105	7
MMP7	0.95252333	-1.041526629	0.089003299	7
MOB3A	0.862113584	-1.096316235	0.234202651	7
MOBP	0.988920442	-1.010723012	0.02180257	7
MOG	0.761031249	-1.132595127	0.371563878	7
MON1A	1.028520011	-0.968803667	-0.059716344	7
MOSPD3	0.928399165	-1.058805538	0.130406373	7
MOV10	0.835972947	-1.107813933	0.271840987	7
MOXD1	1.061031804	-0.925051874	-0.13597993	7
MPDZ	1.062648087	-0.92258391	-0.140064177	7
MPHOSPH9	0.795455061	-1.122598853	0.327143792	7
MPPED2	1.037434854	-0.957803628	-0.079631226	7
MPV17L	1.008479855	-0.991298739	-0.017181116	7
MRGPRX1	1.056451916	-0.931874335	-0.124577581	7
MRI1	0.927272684	-1.059559179	0.132286495	7
MRM2	0.968270487	-1.028964757	0.06069427	7
MRM3	0.809024474	-1.118031368	0.309006893	7
MRPL20	1.036731774	-0.958695788	-0.078035986	7
MRPL47	0.846128851	-1.103541813	0.257412962	7
MRPS10	1.058024031	-0.929560218	-0.128463813	7
MRPS12	0.723609969	-1.141094058	0.417484088	7
MRPS26	1.005917136	-0.993975916	-0.01194122	7
MS4A12	0.69341068	-1.146321637	0.452910957	7
MS4A15	0.991560418	-1.008231137	0.016670719	7
MS4A2	1.07325079	-0.905542129	-0.167708661	7
MSANTD3	1.093652556	-0.867673695	-0.225978862	7
MSC	0.710200786	-1.143587002	0.433386216	7
MSMB	1.073175753	-0.905668291	-0.167507463	7
MSMP	1.078872137	-0.895843397	-0.18302874	7
MSTN	0.801873471	-1.120484651	0.318611179	7
MT-ND2	1.000536016	-0.999463121	-0.001072895	7
MT1A	0.680691931	-1.148116893	0.467424962	7
MTA2	0.996255324	-1.003703074	0.00744775	7
MTHFD1L	1.057312809	-0.930610653	-0.126702156	7
MTHFD2L	1.009168891	-0.990571701	-0.01859719	7
MTMR3	0.772967799	-1.129377288	0.356409488	7
MTMR7	0.907550272	-1.072050461	0.164500189	7
MTRFR	0.814594754	-1.116047361	0.301452608	7
MTSS1	1.033565054	-0.962660828	-0.070904226	7
MTSS2	0.721437523	-1.141517023	0.4200795	7
MTURN	0.992399905	-1.007430646	0.015030741	7
MTX2	0.862088077	-1.096328271	0.234240194	7
MUC16	0.844334122	-1.104314346	0.259980224	7
MUC5AC	0.844873152	-1.104083125	0.259209973	7
MUC5B	1.093356848	-0.868280822	-0.225076025	7
MUCL1	0.961028971	-1.03487672	0.073847749	7
MVB12B	0.754978653	-1.134130209	0.379151556	7
MVK	0.995105082	-1.004824073	0.009718991	7
MYBBP1A	0.942269054	-1.049145576	0.106876522	7
MYH10	0.943044265	-1.048584205	0.10553994	7
MYH13	0.88984057	-1.082209717	0.192369147	7
MYH3	1.0111344	-0.98848067	-0.02265373	7
MYH8	0.889277284	-1.082517497	0.193240213	7
MYH9	1.063855826	-0.920718456	-0.143137369	7

MYMK	0.73749837	-1.13821141	0.40071304	7
MYMX	1.043170081	-0.950359032	-0.092811049	7
MYO1C	1.0845368	-0.885539352	-0.198997448	7
MYO1D	0.814693925	-1.11601145	0.301317526	7
MYO7B	0.870621165	-1.092207904	0.221586739	7
MYORG	1.04860238	-0.943019218	-0.105583162	7
MYOT	0.933748634	-1.05516431	0.121415676	7
N4BP1	0.868070947	-1.093459179	0.225388232	7
N4BP2	0.873025436	-1.091012482	0.217987046	7
NAA15	0.991416417	-1.008368053	0.016951637	7
NAA35	0.84458048	-1.104208754	0.259628273	7
NAA50	1.03788328	-0.957232323	-0.080650957	7
NAGLU	0.719944496	-1.141803395	0.421858899	7
NAP1L1	1.019011772	-0.979836893	-0.039174879	7
NAP1L5	1.073764713	-0.904675785	-0.169088929	7
NAPA	0.898837971	-1.077167125	0.178329154	7
NAT14	0.799080037	-1.121414884	0.322334847	7
NAT8L	1.010222392	-0.98945409	-0.020768302	7
NBPF1	1.016869214	-0.982230715	-0.034638499	7
NBPF10	0.942562965	-1.048933015	0.10637005	7
NBPF14	1.011497039	-0.988092074	-0.023404965	7
NBPF9	0.873897401	-1.090575122	0.216677721	7
NCAM2	1.080504334	-0.892931608	-0.187572725	7
NCAN	0.866508906	-1.094217188	0.227708282	7
NCF2	1.0908826	-0.873284806	-0.217597794	7
NCK1	0.833839799	-1.10868098	0.274841181	7
NCLN	0.700563818	-1.145208165	0.444644347	7
NCOA7	1.026741221	-0.970920443	-0.055820778	7
NCR2	1.019593228	-0.97918157	-0.040411657	7
NDC80	1.093039171	-0.868930858	-0.224108313	7
NDEL1	0.760966602	-1.132611862	0.37164526	7
NDFIP2	0.859732446	-1.097432767	0.237700322	7
NDST3	0.932942963	-1.055719368	0.122776405	7
NDUFA13	0.95502098	-1.039605851	0.084584871	7
NDUFS2	0.929389517	-1.058139233	0.128749716	7
NDUFS8	0.975868987	-1.022499541	0.046630554	7
NEB	1.047303683	-0.944800564	-0.102503119	7
NEDD4L	0.742040495	-1.137200336	0.395159841	7
NEK7	1.023780122	-0.974389463	-0.049390659	7
NELFCD	1.073457405	-0.905194304	-0.168263102	7
NEURL3	1.08641304	-0.881998536	-0.204414503	7
NEUROD1	0.689550189	-1.146891297	0.457341109	7
NEXN	0.877948006	-1.088516514	0.210568508	7
NF2	0.826940114	-1.111415281	0.284475167	7
NFASC	0.795369138	-1.122626602	0.327257464	7
NFATC4	0.813615356	-1.116400899	0.302785543	7
NFIC	0.79580455	-1.122485837	0.326681288	7
NFILZ	0.846387724	-1.103429752	0.257042028	7
NFKB1	0.931203791	-1.056909451	0.12570566	7
NFKBIE	1.085873312	-0.883023944	-0.202849368	7
NGDN	0.938996152	-1.051490061	0.112493909	7
NGRN	1.061420331	-0.92446156	-0.136958771	7
NHLH2	1.090146314	-0.874748392	-0.215397921	7
NHLRC1	1.0342041	-0.961867618	-0.072336482	7
NINL	1.053460739	-0.93619997	-0.117260768	7

NIPBL	0.845152102	-1.103963197	0.258811095	7
NKIRAS2	0.974329483	-1.023831811	0.049502328	7
NLRP14	1.080072789	-0.893705842	-0.186366947	7
NLRP3	1.043379935	-0.950080832	-0.093299103	7
NLRP7	1.088738948	-0.877514529	-0.211224419	7
NME4	0.791051952	-1.124002199	0.332950247	7
NME9	1.04053789	-0.953813261	-0.086724629	7
NMNAT3	0.811340075	-1.117214458	0.305874383	7
NMU	0.968818059	-1.028507988	0.059689929	7
NNMT	0.964863302	-1.031775902	0.0669126	7
NODAL	1.050306711	-0.940655293	-0.109651417	7
NOG	1.084309973	-0.885962944	-0.19834703	7
NOL9	1.091692647	-0.871661261	-0.220031386	7
NOSIP	0.835006803	-1.108207918	0.273201115	7
NOTCH2NLA	0.75178012	-1.134915744	0.383135623	7
NOTCH2NLB	0.842779106	-1.104977539	0.262198433	7
NOVA1	0.818277786	-1.114699733	0.296421947	7
NOX1	0.921878194	-1.063106388	0.141228195	7
NPC1	0.951937405	-1.041973457	0.090036052	7
NPFFR2	0.789605824	-1.124454871	0.334849047	7
NPHP4	0.79483284	-1.122799469	0.327966628	7
NPIPA2	1.075900815	-0.901031771	-0.174869044	7
NPIPA9	0.727178618	-1.140383003	0.413204385	7
NPIPB12	0.717216332	-1.142317646	0.425101314	7
NPIPB15	1.083372253	-0.887704076	-0.195668177	7
NPIPB3	0.978926294	-1.019819144	0.04089285	7
NPIPB4	1.08655056	-0.881736372	-0.204814188	7
NPIPB5	0.88679167	-1.083864726	0.197073057	7
NPIPB7	1.070081713	-0.910798552	-0.15928316	7
NPIPB8	1.044155581	-0.949048916	-0.095106665	7
NPIPB9	1.079206039	-0.895251362	-0.183954677	7
NPLOC4	0.926288307	-1.060214065	0.133925758	7
NPPB	0.99753145	-1.002450403	0.004918952	7
NPVF	1.09124795	-0.872554295	-0.218693654	7
NPY4R	0.896325197	-1.078599634	0.182274437	7
NR0B2	1.045895674	-0.946712724	-0.09918295	7
NR1D1	0.849837629	-1.101921033	0.252083404	7
NR1I3	0.710275618	-1.143573862	0.433298244	7
NR3C1	1.059193995	-0.927819374	-0.131374621	7
NR4A2	0.727876916	-1.140241489	0.412364573	7
NR4A3	0.815882718	-1.115579362	0.299696644	7
NRF1	1.005569411	-0.994335943	-0.011233468	7
NRIP3	0.684887688	-1.147550446	0.462662759	7
NRROS	0.73947382	-1.137775875	0.398302055	7
NRXN2	1.023267202	-0.97498355	-0.048283652	7
NSD2	0.995808239	-1.0041397	0.008331461	7
NT5C	1.045701348	-0.946975087	-0.098726261	7
NT5C3A	0.858752047	-1.09788832	0.239136273	7
NT5C3B	0.764000752	-1.131818446	0.367817694	7
NTAQ1	1.035477591	-0.960276499	-0.075201092	7
NTN1	0.930022889	-1.057711258	0.127688369	7
NTNG1	1.041931918	-0.951991955	-0.089939963	7
NUBP2	0.885809101	-1.084392413	0.198583311	7
NUDT10	0.813497075	-1.116443459	0.302946384	7
NUDT19	0.808736461	-1.118132199	0.309395738	7

NUP160	0.954510779	-1.04000034	0.085489561	7
NUP188	1.046782677	-0.945510422	-0.101272255	7
NXN	0.783285206	-1.126386242	0.343101036	7
NXPE1	1.010643404	-0.989005413	-0.021637991	7
NYAP1	1.035396989	-0.960377618	-0.07501937	7
NYAP2	0.711548229	-1.143349088	0.431800859	7
OAF	0.862295707	-1.096230243	0.233934536	7
OAZ1	0.940327596	-1.050541269	0.110213674	7
OBP2B	1.061935286	-0.923676297	-0.138258989	7
OC90	1.020096268	-0.978612647	-0.041483621	7
ODAD4	0.7501159	-1.135317512	0.385201612	7
OGFOD3	0.748235182	-1.135765859	0.387530677	7
OLAH	0.958642875	-1.036773569	0.078130693	7
OLFM4	1.035604077	-0.960117703	-0.075486374	7
OPALIN	1.088322451	-0.878325381	-0.20999707	7
OPHN1	0.84807903	-1.102693675	0.254614646	7
OPN3	0.904180924	-1.074057215	0.169876291	7
OR10AG1	0.680315179	-1.148166527	0.467851348	7
OR10G8	0.939070511	-1.05143725	0.112366739	7
OR10H4	0.974701643	-1.023510807	0.048809164	7
OR10H5	1.027439098	-0.970092944	-0.057346154	7
OR10W1	0.799745052	-1.121194846	0.321449793	7
OR11G2	0.791240901	-1.123942754	0.332701853	7
OR11L1	1.084873374	-0.884909054	-0.199964319	7
OR13A1	1.062097568	-0.92342815	-0.138669417	7
OR1R1P	0.953897299	-1.040473234	0.086575935	7
OR2A7	0.707635467	-1.144032283	0.436396816	7
OR2AG1	1.086769374	-0.881318477	-0.205450897	7
OR2AT4	1.041459214	-0.95261157	-0.088847644	7
OR2B11	0.789930523	-1.124353585	0.334423062	7
OR2L2	0.738394541	-1.138014626	0.399620086	7
OR2L3	0.98307894	-1.016102953	0.033024013	7
OR2M4	0.809553849	-1.117845592	0.308291743	7
OR2V1	0.860390172	-1.09712579	0.236735618	7
OR4B1	1.086221014	-0.882363999	-0.203857015	7
OR4F15	0.791806816	-1.123764298	0.331957482	7
OR4F16	0.961756705	-1.034293233	0.072536528	7
OR4F29	0.961458276	-1.034532792	0.073074516	7
OR4K2	1.092531724	-0.869964524	-0.222567201	7
OR4N2	0.978732411	-1.019990508	0.041258098	7
OR4N4	1.080554943	-0.892840603	-0.187714339	7
OR51A7	0.91244557	-1.069070491	0.156624921	7
OR51D1	1.085883073	-0.883005449	-0.202877624	7
OR51E2	0.706857357	-1.144165365	0.437308008	7
OR51S1	0.989767791	-1.009927379	0.020159588	7
OR52J3	0.768038615	-1.130737135	0.362698521	7
OR52N1	1.04848288	-0.943183844	-0.105299036	7
OR56B1	1.083637659	-0.887212905	-0.196424754	7
OR5AN1	0.771670269	-1.129739528	0.358069259	7
OR5D18	0.87114141	-1.091950536	0.220809126	7
OR5L2	1.074669681	-0.903140538	-0.171529143	7
OR5M11	1.073465089	-0.905181356	-0.168283733	7
OR5P2	0.966368526	-1.030540549	0.064172023	7
OR6A2	0.81835195	-1.1146723	0.296320349	7
OR6C6	0.894773982	-1.079474517	0.184700535	7

OR6C75	0.729395209	-1.139931099	0.41053589	7
OR6K2	0.987503504	-1.012044741	0.024541237	7
OR7C2	1.027371466	-0.970173305	-0.057198161	7
OR8H2	0.909465718	-1.070893618	0.161427899	7
ORAI1	0.99652186	-1.003442221	0.006920362	7
ORC4	0.732765014	-1.139228919	0.406463905	7
ORM1	1.08791501	-0.879115227	-0.208799783	7
OSTN	0.81053175	-1.117500887	0.306969136	7
OTC	0.935148093	-1.05419448	0.119046388	7
OTOA	0.793055305	-1.123368393	0.330313089	7
OTOP3	0.974423999	-1.023750351	0.049326351	7
OVCH2	0.710891456	-1.1434654	0.432573944	7
P2RX1	0.775232746	-1.128737579	0.353504834	7
P2RY6	0.966723081	-1.030248058	0.063524977	7
P3H3	0.950594142	-1.042992485	0.092398343	7
PABPC4L	0.749895604	-1.135370341	0.385474737	7
PACS2	1.084138486	-0.886282559	-0.197855927	7
PACSIN2	1.074331794	-0.903715208	-0.170616586	7
PADI1	0.978237201	-1.020427347	0.042190145	7
PAFAH1B2	1.05951678	-0.927336237	-0.132180543	7
PAGE3	0.699255714	-1.145417455	0.446161741	7
PAH	1.025931825	-0.9718754	-0.054056424	7
PAK3	0.810607617	-1.117474061	0.306866443	7
PAK4	1.091827896	-0.871388808	-0.220439088	7
PALLD	0.737887598	-1.138126105	0.400238507	7
PAMR1	0.823592675	-1.112703841	0.289111166	7
PAN2	0.851890126	-1.101009808	0.249119682	7
PAQR7	0.760041822	-1.132850454	0.372808632	7
PARD6B	0.698083277	-1.145602875	0.447519598	7
PARP10	1.07914538	-0.895359054	-0.183786326	7
PARPBP	1.049618842	-0.94161299	-0.108005852	7
PASD1	0.839826051	-1.106221377	0.266395326	7
PATE1	1.057685736	-0.930060592	-0.127625144	7
PAX6	0.770839728	-1.129969785	0.359130057	7
PAXBP1	1.067471026	-0.915021766	-0.15244926	7
PBOV1	0.868727967	-1.093138444	0.224410476	7
PBX1	0.777419285	-1.12811105	0.350691765	7
PCDH10	0.911130822	-1.069878417	0.158747595	7
PCDH17	0.865361814	-1.094769797	0.229407983	7
PCDH18	0.725547367	-1.140710547	0.41516318	7
PCDHB16	0.987416286	-1.012125743	0.024709457	7
PCGF6	0.866185501	-1.094373333	0.228187832	7
PCID2	1.071629101	-0.908250185	-0.163378916	7
PCIF1	0.986900394	-1.012604037	0.025703643	7
PCK1	1.062917194	-0.922169844	-0.140747351	7
PCLO	1.087047243	-0.880786456	-0.206260787	7
PCMTD1	0.848677214	-1.102431698	0.253754484	7
PCSK5	1.002110648	-0.997875902	-0.004234746	7
PDE1A	1.016658167	-0.982464748	-0.034193419	7
PDE2A	1.055266174	-0.933601037	-0.121665137	7
PDE4A	0.868913042	-1.093047892	0.22413485	7
PDE4B	1.020893957	-0.977706699	-0.043187257	7
PDE6B	1.090037197	-0.874964325	-0.215072872	7
PDE8B	0.868286261	-1.093354194	0.225067934	7
PDE9A	1.061500666	-0.924339271	-0.137161396	7

PDGFRA	1.007191871	-0.992649513	-0.014542358	7
PDILT	1.057018753	-0.931043247	-0.125975506	7
PDLIM3	0.742328384	-1.137135097	0.394806713	7
PDXDC1	0.994524734	-1.005386776	0.010862041	7
PDXK	0.853891845	-1.100111235	0.24621939	7
PDZD2	0.942840446	-1.048732028	0.105891583	7
PELO	1.05459337	-0.934573762	-0.120019608	7
PENK	1.088299777	-0.878369421	-0.209930356	7
PEX1	0.975862528	-1.022505155	0.046642627	7
PEX16	0.751235752	-1.135047684	0.383811932	7
PEX19	1.069162146	-0.912296878	-0.156865268	7
PF4V1	0.934130469	-1.054900415	0.120769946	7
PFDN6	0.991265904	-1.008511038	0.017245133	7
PFKFB2	0.811261711	-1.117242285	0.305980574	7
PGA3	1.079117007	-0.895409404	-0.183707603	7
PGAP6	1.06600759	-0.917348514	-0.148659076	7
PGD	0.993722423	-1.006161525	0.012439102	7
PGLYRP2	0.913887195	-1.068178124	0.154290929	7
PGLYRP4	0.9645027	-1.032070326	0.067567626	7
PHACTR1	0.858329444	-1.098083943	0.239754499	7
PHACTR3	0.955323537	-1.039371395	0.084047858	7
PHACTR4	0.897106927	-1.078156014	0.181049087	7
PHB2	0.750417101	-1.135245149	0.384828048	7
PHF8	1.045649871	-0.947044525	-0.098605347	7
PHLDA3	1.074766056	-0.902976305	-0.171789751	7
PHLPP1	1.011178892	-0.988433041	-0.022745851	7
PHOSPHO1	0.751858795	-1.134896633	0.383037838	7
PI4K2B	0.882953362	-1.085910557	0.202957195	7
PICALM	0.902481732	-1.075055777	0.172574045	7
PIGB	0.724235776	-1.14097083	0.416735054	7
PIGW	0.998941913	-1.001054739	0.002112826	7
PIGX	0.915006186	-1.067480764	0.152474578	7
PIK3R5	0.710511739	-1.143532345	0.433020605	7
PIM1	1.004643646	-0.995290746	-0.0093529	7
PIM3	0.889255459	-1.082529404	0.193273946	7
PIN1	1.012431158	-0.987087041	-0.025344117	7
PIP5KL1	0.909104891	-1.071112437	0.162007546	7
PIR	0.876371529	-1.089323006	0.212951477	7
PITPNM1	0.905854103	-1.073065148	0.167211045	7
PIWIL4	0.94258228	-1.048919034	0.106336754	7
PKD2L1	0.752553554	-1.134727409	0.382173855	7
PKHD1	0.896354144	-1.07858324	0.182229096	7
PKLR	0.758641505	-1.133208874	0.374567369	7
PKN3	0.716824438	-1.142390563	0.425566125	7
PLA2G4F	0.844250106	-1.104350323	0.260100217	7
PLAA	1.03275311	-0.963663667	-0.069089443	7
PLAAT1	1.023791935	-0.974375757	-0.049416178	7
PLAG1	1.021800708	-0.976671205	-0.045129503	7
PLB1	1.071215358	-0.908934939	-0.162280419	7
PLCD3	1.040534505	-0.953817662	-0.086716843	7
PLEC	0.800801814	-1.120843369	0.320041555	7
PLEKHG6	1.092656875	-0.869710124	-0.222946751	7
PLEKHG7	0.841615692	-1.105470004	0.263854312	7
PLG	1.015847802	-0.983360471	-0.032487331	7
PLGLB2	0.91600382	-1.066855547	0.150851727	7

PLIN4	0.99396905	-1.00592376	0.01195471	7
PLIN5	0.765542248	-1.131409087	0.36586684	7
PLPP3	0.726980757	-1.140422959	0.413442202	7
PLXNA3	0.936938778	-1.052942908	0.116004129	7
PLXND1	0.907014457	-1.072371981	0.165357524	7
PML	1.090915955	-0.873218233	-0.217697722	7
PMP2	0.999342908	-1.0006558	0.001312892	7
PNLIP	0.76665154	-1.131111877	0.364460337	7
PNOC	0.98321121	-1.015983129	0.032771919	7
PNPLA3	1.050113827	-0.940924336	-0.10918949	7
POLE2	1.018187002	-0.980762263	-0.037424739	7
POLE3	0.893002547	-1.080464868	0.187462321	7
POLR1A	0.714896459	-1.14274581	0.427849351	7
POLR2B	0.868459566	-1.093269605	0.224810038	7
POLR2I	0.890174341	-1.08202691	0.19185257	7
POLR2J3	0.968970172	-1.028380852	0.059410681	7
POM121	0.945207648	-1.047005144	0.101797496	7
POM121C	0.979023667	-1.019733009	0.040709342	7
POTEB2	0.847338386	-1.103016854	0.255678467	7
POU1F1	0.998396537	-1.001595787	0.00319925	7
POU5F1B	0.968244851	-1.028986107	0.060741256	7
PPA1	0.822264343	-1.113208387	0.290944044	7
PPAN-P2RY11	0.822969046	-1.112941195	0.289972149	7
PPIC	1.047542416	-0.944474387	-0.103068028	7
PPM1D	1.093595057	-0.867791903	-0.225803154	7
PPM1E	1.016946353	-0.982145095	-0.034801258	7
PPP1R17	1.051349976	-0.939193332	-0.112156643	7
PPP1R1A	0.890170758	-1.082028874	0.191858116	7
PPP1R1C	1.048809217	-0.942733928	-0.106075288	7
PPP1R8	0.692237162	-1.146497106	0.454259944	7
PPP2CB	0.977049529	-1.021470037	0.044420508	7
PPP2R2B	0.797849543	-1.121819702	0.323970158	7
PPP4R4	0.886309804	-1.084123855	0.19781405	7
PPP6R2	0.816513565	-1.115348855	0.29883529	7
PPWD1	1.081313651	-0.891471008	-0.189842643	7
PRAMEF27	0.875515219	-1.089758243	0.214243024	7
PRB3	0.886643438	-1.08394451	0.197301071	7
PRDM1	0.952952653	-1.041198321	0.088245668	7
PRDM13	0.69694609	-1.145780772	0.448834682	7
PRDM14	0.951430342	-1.042359	0.090928658	7
PRDM2	0.933641188	-1.055238472	0.121597285	7
PRDM8	1.050480911	-0.940411976	-0.110068936	7
PREB	0.861005765	-1.096837486	0.235831721	7
PRELID1	1.036340898	-0.959189901	-0.077150997	7
PRKCZ	1.006730953	-0.993130309	-0.013600645	7
PRKD1	1.069446581	-0.911834691	-0.15761189	7
PRKN	1.046345922	-0.946103403	-0.10024252	7
PROM2	1.024139864	-0.973971604	-0.05016826	7
PRPF40B	1.062614027	-0.922636251	-0.139977776	7
PRR15L	0.979872892	-1.018979768	0.039106875	7
PRR22	0.826159663	-1.111717906	0.285558243	7
PRR36	0.939991351	-1.050781518	0.110790167	7
PRRC2B	0.913605648	-1.068352936	0.154747288	7
PRSS8	0.753755927	-1.134432583	0.380676656	7
PRUNE1	0.703266122	-1.14476771	0.441501588	7

PRY2	0.917915931	-1.065647967	0.147732035	7
PSAP	0.750087994	-1.135324209	0.385236215	7
PSAT1	0.97429916	-1.023857937	0.049558776	7
PSG5	0.820086472	-1.114027351	0.293940879	7
PSG8	0.960909638	-1.034972177	0.07406254	7
PSMC4	1.062169774	-0.923317634	-0.13885214	7
PSMD13	0.855159609	-1.099537044	0.244377435	7
PSMD4	0.799688851	-1.121213476	0.321524625	7
PSMD5	0.859351179	-1.097610215	0.238259036	7
PSME4	0.933055457	-1.055642009	0.122586553	7
PSORS1C2	0.850281241	-1.101724952	0.251443711	7
PSTK	0.681098823	-1.148063062	0.466964239	7
PTCHD1	0.967844182	-1.029319405	0.061475223	7
PTDSS2	0.825418227	-1.112004161	0.286585934	7
PTGER3	0.958080977	-1.037216653	0.079135676	7
PTGIS	0.724799258	-1.140859343	0.416060085	7
PTGR1	0.693454066	-1.146315111	0.452861045	7
PTH2R	1.061497626	-0.9243439	-0.137153726	7
PTPN20	0.687098238	-1.147241852	0.460143613	7
PTPRB	1.040332036	-0.954080688	-0.086251348	7
PTPRC	1.092743222	-0.869534401	-0.223208821	7
PTPRD	1.058528225	-0.928811979	-0.129716247	7
PTPRG	1.012771675	-0.986719212	-0.026052463	7
PTPRR	0.995359172	-1.0045771	0.009217928	7
PTPRT	1.038438673	-0.956522255	-0.081916418	7
PTPRZ1	1.069739585	-0.911357395	-0.158382191	7
PTTG1IP2	0.960254321	-1.035495263	0.075240942	7
PTX3	0.878653232	-1.088153537	0.209500305	7
PURA	0.918396781	-1.065342362	0.146945581	7
PXYLP1	0.867980762	-1.093503116	0.225522355	7
PYCR1	1.043793035	-0.949531964	-0.094261071	7
PYCR3	0.851517392	-1.101176047	0.249658655	7
PYGO1	0.795799599	-1.12248744	0.326687841	7
QARS1	1.079737902	-0.894304485	-0.185433417	7
QTRT1	1.08526835	-0.884166687	-0.201101663	7
RAB11FIP1	0.806894726	-1.118772936	0.31187821	7
RAB11FIP2	1.037127381	-0.958194327	-0.078933053	7
RAB11FIP4	0.914145741	-1.068017363	0.153871623	7
RAB11FIP5	1.02501923	-0.97294602	-0.05207321	7
RAB18	0.779991447	-1.127362665	0.347371218	7
RAB26	1.021433126	-0.97709171	-0.044341416	7
RAB27B	0.954263106	-1.040191446	0.08592834	7
RAB37	1.05294322	-0.936938334	-0.116004886	7
RAB3IL1	1.092964133	-0.869084071	-0.223880062	7
RAB40AL	1.083947969	-0.88663701	-0.197310959	7
RAB4B-EGLN2	0.964359089	-1.032187418	0.067828329	7
RABEP2	0.909167668	-1.071074397	0.161906729	7
RABGEF1	0.867390044	-1.093790382	0.226400338	7
RACGAP1	0.736935355	-1.138334362	0.401399007	7
RACK1	1.052492341	-0.937579253	-0.114913088	7
RAD23A	1.037002062	-0.958353328	-0.078648734	7
RAD54L	1.022097439	-0.97633102	-0.045766418	7
RAD54L2	0.985544104	-1.013854689	0.028310584	7
RADX	0.709489297	-1.143711506	0.434222209	7
RAMP1	0.987123662	-1.012397216	0.025273554	7

RAMP3	0.879126891	-1.08790898	0.208782089	7
RANBP3L	1.088100841	-0.878755396	-0.209345445	7
RARG	0.877249727	-1.088874575	0.211624848	7
RARS2	0.96897187	-1.028379432	0.059407562	7
RASA4	0.963863401	-1.032590858	0.068727457	7
RASGRP2	1.08440307	-0.885789204	-0.198613866	7
RASL12	0.815984459	-1.115542243	0.299557784	7
RBBP5	0.7878005	-1.125014317	0.337213817	7
RBBP8	1.057831421	-0.929845272	-0.12798615	7
RBBP8NL	1.027915719	-0.969525589	-0.05839013	7
RBFOX3	1.028198606	-0.969187997	-0.059010609	7
RBM34	0.808638127	-1.118166586	0.309528459	7
RBP3	0.826114859	-1.111735238	0.285620379	7
RCC1L	1.013992561	-0.985393934	-0.028598627	7
RCC2	0.982403118	-1.016713772	0.034310654	7
RCN1	1.074815341	-0.902892262	-0.17192308	7
RCN2	0.893343444	-1.080275004	0.18693156	7
RCN3	0.774090555	-1.129061355	0.3549708	7
RCVRN	1.061732413	-0.923986051	-0.137746362	7
RDH16	0.876190188	-1.089415343	0.213225155	7
RDH5	1.05654189	-0.931742662	-0.124799228	7
REC114	1.007927555	-0.991879275	-0.01604828	7
REC8	0.792847765	-1.123434416	0.330586651	7
REEP6	1.020533552	-0.978116594	-0.042416957	7
REG1B	0.880531125	-1.08718032	0.206649196	7
RELT	0.708810475	-1.143829574	0.435019099	7
REM2	0.931973394	-1.056384186	0.124410792	7
RETREG2	0.994562457	-1.005350258	0.010787801	7
REV3L	1.018819507	-0.980053047	-0.03876646	7
RFX2	0.86484506	-1.09501763	0.23017257	7
RGL2	0.929239959	-1.05824008	0.129000122	7
RGN	0.956499655	-1.038456314	0.08195666	7
RGPD4	0.783364981	-1.126362339	0.342997358	7
RGS10	0.832579503	-1.109188394	0.27660889	7
RGS18	1.061605327	-0.924179832	-0.137425495	7
RGS20	1.078706727	-0.896135998	-0.182570728	7
RHBDL2	1.085540053	-0.883654311	-0.201885741	7
RHOBTB1	0.964452352	-1.032111388	0.067659036	7
RHOBTB2	0.741836908	-1.137246387	0.39540948	7
RHOF	0.809446006	-1.117883484	0.308437478	7
RHOJ	0.942954736	-1.048649157	0.105694422	7
RHPN1	1.093261816	-0.868475515	-0.224786302	7
RIBC2	1.023091079	-0.975187085	-0.047903994	7
RILPL1	1.007116851	-0.992727861	-0.014388989	7
RIMS4	1.065928951	-0.917472738	-0.148456213	7
RIN1	0.75608667	-1.133853966	0.377767297	7
RIN2	1.080364522	-0.893182792	-0.18718173	7
RING1	0.835799406	-1.107884858	0.272085452	7
RIOX1	1.040166902	-0.954294934	-0.085871968	7
RIPK1	1.021104928	-0.977466315	-0.043638613	7
RIPK2	0.949821625	-1.043575178	0.093753552	7
RIPK4	1.041472058	-0.952594761	-0.088877297	7
RLIM	1.02936856	-0.967785014	-0.061583546	7
RMI2	1.09324572	-0.868508471	-0.224737249	7
RNF128	1.070747486	-0.909706303	-0.161041183	7

RNF130	0.924220201	-1.061578836	0.137358635	7
RNF144B	0.795230323	-1.122671401	0.327441078	7
RNF222	0.742765018	-1.137035888	0.39427087	7
RNF25	0.905755168	-1.073124052	0.167368884	7
RNF4	1.077742196	-0.89783324	-0.179908956	7
ROPN1	0.966867882	-1.030128439	0.063260558	7
ROR2	0.985819336	-1.013601686	0.027782349	7
ROS1	0.746677856	-1.136132566	0.38945471	7
RP1	0.696296718	-1.145881498	0.44958478	7
RPA3	1.03021168	-0.96676713	-0.06344455	7
RPE65	0.744375088	-1.136667299	0.392292211	7
RPL19	0.807179711	-1.118674246	0.311494536	7
RPL26	0.683599025	-1.147727102	0.464128077	7
RPL27A	0.77589514	-1.12854871	0.35265357	7
RPS10-NUDT3	0.750587206	-1.135204213	0.384617006	7
RPS19BP1	0.911385208	-1.069722532	0.158337324	7
RPS6KA2	1.039239298	-0.955493784	-0.083745514	7
RPUSD3	0.994957539	-1.004967313	0.010009775	7
RREB1	1.042271372	-0.951545712	-0.09072566	7
RRM1	0.961086154	-1.034830957	0.073744803	7
RTKN2	0.704885325	-1.144498537	0.439613212	7
RTL6	1.076594504	-0.899833149	-0.176761355	7
RTL9	1.015603076	-0.983630073	-0.031973003	7
RTN2	0.882134621	-1.08634158	0.204206959	7
RTN3	1.076848806	-0.899391835	-0.177456971	7
RTN4RL1	0.695266276	-1.146040054	0.450773778	7
RUNDC3B	0.849530361	-1.102056568	0.252526207	7
RUNX1T1	1.076408228	-0.900155762	-0.176252466	7
RUNX2	1.021957286	-0.976491779	-0.045465507	7
RXFP3	1.081710619	-0.89075044	-0.190960179	7
S100B	0.680781256	-1.148105096	0.46732384	7
SAE1	0.79972604	-1.121201149	0.321475109	7
SAGE1	1.006416874	-0.993457156	-0.012959718	7
SAMD4B	1.06833662	-0.913631945	-0.154704675	7
SAMSN1	1.073304345	-0.905452033	-0.167852312	7
SAP30BP	1.09281067	-0.869397022	-0.223413648	7
SAXO1	1.037430467	-0.957809209	-0.079621258	7
SBK1	0.881490387	-1.086679419	0.205189032	7
SC5D	0.775109767	-1.128772555	0.353662788	7
SCAMP5	0.7789259	-1.127674188	0.348748287	7
SCART1	1.036052938	-0.959553062	-0.076499876	7
SCGB1D4	0.836649542	-1.107536754	0.270887213	7
SCGN	1.077643054	-0.898006836	-0.179636218	7
SCN2A	0.965857538	-1.030961078	0.06510354	7
SCN3A	1.079869767	-0.894068989	-0.185800778	7
SCRN2	1.049988527	-0.941098901	-0.108889626	7
SCYGR1	1.068771339	-0.912930078	-0.155841261	7
SCYGR5	0.721611556	-1.141483414	0.419871858	7
SCYGR8	0.892418727	-1.080789234	0.188370508	7
SCYL1	0.783934568	-1.126191327	0.342256758	7
SDC1	1.043742507	-0.949599186	-0.094143322	7
SDS	0.703166266	-1.14478418	0.441617914	7
SDSL	0.88346836	-1.085638476	0.202170116	7
SEC14L3	0.760885692	-1.132632797	0.371747105	7
SEC16B	1.086226953	-0.882352706	-0.203874247	7

SEC61A1	1.022890119	-0.975419039	-0.04747108	7
SEMA3E	0.69730504	-1.145724826	0.448419786	7
SEMA4C	0.90019522	-1.076385423	0.176190202	7
SENP1	1.046060324	-0.946490135	-0.09957019	7
SENP8	0.866172385	-1.094379659	0.228207274	7
SEPHS1	0.952829027	-1.041292937	0.08846391	7
SEPTIN5	1.000850649	-0.999147174	-0.001703475	7
SEPTIN9	1.084822661	-0.885004158	-0.199818504	7
SERPINB5	1.016433118	-0.982713965	-0.033719153	7
SERTAD2	0.847192047	-1.103080553	0.255888506	7
SERTM1	1.008404367	-0.991378203	-0.017026164	7
SETBP1	1.026650386	-0.971027868	-0.055622518	7
SETD1B	0.857463072	-1.098483587	0.241020515	7
SFRP4	1.035904933	-0.959739437	-0.076165496	7
SFXN3	0.902140361	-1.075255311	0.17311495	7
SGSM1	1.023335515	-0.974904541	-0.048430975	7
SGSM2	0.855580861	-1.099345374	0.243764513	7
SGTA	0.795276546	-1.122656488	0.327379942	7
SH2B3	1.030314349	-0.966642785	-0.063671564	7
SH3BGR	0.992360418	-1.007468386	0.015107968	7
SH3BGRL	0.860712243	-1.096975073	0.23626283	7
SH3BGRL3	0.864916679	-1.094983323	0.230066644	7
SH3BP2	0.941228948	-1.049895106	0.108666157	7
SHANK3	1.037413656	-0.957830591	-0.079583065	7
SHARPIN	1.057187474	-0.930795159	-0.126392315	7
SHE	0.882001041	-1.086411725	0.204410684	7
SHISAL2A	0.77528261	-1.12872339	0.35344078	7
SHISAL2B	0.748379408	-1.13573169	0.387352282	7
SHOC1	0.89509702	-1.079292915	0.184195896	7
SHROOM1	1.012016002	-0.987534437	-0.024481564	7
SHROOM3	0.727136007	-1.140391613	0.413255606	7
SIAH2	1.089085173	-0.876837809	-0.212247364	7
SIGLEC12	1.08984721	-0.875339697	-0.214507514	7
SINHCAF	0.780283049	-1.127277043	0.346993995	7
SIRPD	1.084747527	-0.885144973	-0.199602555	7
SIRT5	0.919528772	-1.064619847	0.145091075	7
SIVA1	0.816151883	-1.115481114	0.29932923	7
SKAP2	1.074066162	-0.904165769	-0.169900393	7
SKI	0.940081029	-1.050717485	0.110636457	7
SKOR1	0.912398131	-1.069099741	0.15670161	7
SKOR2	0.892863875	-1.080542003	0.187678128	7
SLC10A4	1.088669282	-0.877650401	-0.211018881	7
SLC12A6	1.04027417	-0.954155793	-0.086118377	7
SLC13A1	0.842308907	-1.105176952	0.262868045	7
SLC13A5	0.959537596	-1.036065213	0.076527617	7
SLC14A1	1.014509493	-0.984829731	-0.029679762	7
SLC15A3	0.907450129	-1.072110622	0.164660493	7
SLC16A10	1.032905103	-0.96347636	-0.069428743	7
SLC17A4	1.032612923	-0.963836252	-0.06877667	7
SLC18A1	0.915615635	-1.067099215	0.15148358	7
SLC1A2	0.848875232	-1.102344785	0.253469553	7
SLC22A11	0.955637049	-1.03912804	0.083490991	7
SLC22A24	0.945416432	-1.046851776	0.101435345	7
SLC25A10	1.05935724	-0.927575188	-0.131782052	7
SLC25A17	1.065735954	-0.917777261	-0.147958693	7

SLC25A44	0.917066806	-1.066185734	0.149118927	7
SLC26A11	0.882079543	-1.086370508	0.204290966	7
SLC26A4	0.768991258	-1.130477756	0.361486498	7
SLC27A3	1.011880963	-0.987679714	-0.024201249	7
SLC2A2	0.849723194	-1.101971537	0.252248343	7
SLC30A1	0.67958707	-1.148261877	0.468674808	7
SLC32A1	1.087197105	-0.880498895	-0.20669821	7
SLC35D2	0.974223229	-1.023923337	0.049700108	7
SLC35G1	0.937106775	-1.052824874	0.115718099	7
SLC38A11	0.917112324	-1.066156967	0.149044643	7
SLC38A7	0.971177695	-1.026523607	0.055345911	7
SLC38A8	0.706918051	-1.144155018	0.437236966	7
SLC39A13	0.991710569	-1.008088249	0.01637768	7
SLC3A1	0.828376417	-1.110854832	0.282478415	7
SLC43A1	0.983376428	-1.015833328	0.0324569	7
SLC44A4	1.031147043	-0.965631108	-0.065515935	7
SLC45A1	1.054516376	-0.934684758	-0.119831618	7
SLC47A2	0.991090322	-1.008677679	0.017587357	7
SLC4A8	0.979386169	-1.019411924	0.040025755	7
SLC50A1	0.941571072	-1.049649024	0.108077952	7
SLC6A5	0.852619823	-1.100683382	0.248063559	7
SLC6A8	1.060631121	-0.925658726	-0.134972396	7
SLC6A9	0.894103653	-1.079850367	0.185746715	7
SLC7A2	1.051490374	-0.938995711	-0.112494663	7
SLC8B1	1.031403164	-0.965318792	-0.066084372	7
SLC9A4	1.093677888	-0.867621594	-0.226056294	7
SLCO1A2	1.075121971	-0.902368551	-0.17275342	7
SLCO6A1	1.087680107	-0.879569093	-0.208111014	7
SLIT3	0.807597582	-1.118529236	0.310931654	7
SLITRK2	0.735196166	-1.138710882	0.403514716	7
SLITRK5	1.041712327	-0.952280051	-0.089432276	7
SLITRK6	0.753044829	-1.134607248	0.381562419	7
SLPI	0.68115317	-1.148055854	0.466902684	7
SMAD7	0.96660487	-1.030345641	0.063740771	7
SMARCD1	0.955459932	-1.039265574	0.083805641	7
SMARCD3	0.745008179	-1.136521177	0.391512998	7
SMARCE1	1.075021094	-0.902541006	-0.172480088	7
SMC1B	0.792383756	-1.123581724	0.331197967	7
SMC2	0.918850984	-1.065052978	0.146201995	7
SMC6	0.836208689	-1.107717476	0.271508787	7
SMCO3	0.758179698	-1.133326323	0.375146626	7
SMIM31	0.984164415	-1.015116913	0.030952498	7
SMIM8	1.072058595	-0.907536736	-0.16452186	7
SMOC2	0.891127318	-1.081503182	0.190375864	7
SMPDL3A	0.886980413	-1.083763047	0.196782635	7
SMTN	0.784466447	-1.126031081	0.341564633	7
SMURF2	0.714565639	-1.142806187	0.428240547	7
SMYD4	0.749794328	-1.135394599	0.385600271	7
SNAP47	1.050418234	-0.940499558	-0.109918676	7
SNF8	1.092970091	-0.869071909	-0.223898182	7
SNRPG	0.899745161	-1.076645254	0.176900093	7
SNTB1	1.070421102	-0.910242547	-0.160178555	7
SNTG1	1.091268357	-0.872513406	-0.218754951	7
SNTN	0.736561986	-1.138415611	0.401853625	7
SNX12	1.07751914	-0.898223584	-0.179295556	7

SNX24	1.091595587	-0.871856539	-0.219739048	7
SNX32	0.843176986	-1.10480839	0.261631404	7
SOCS3	0.727189627	-1.140380778	0.413191151	7
SOD2	1.063986762	-0.920515101	-0.143471661	7
SOGA3	0.873632841	-1.090708035	0.217075194	7
SOHLH1	1.052261657	-0.937906318	-0.114355339	7
SORCS1	1.080539584	-0.892868225	-0.18767136	7
SOST	0.765381451	-1.131451986	0.366070535	7
SOWAHC	1.066251792	-0.916962238	-0.149289554	7
SP110	0.950280402	-1.043229427	0.092949024	7
SPANXA1	0.914201584	-1.067982612	0.153781028	7
SPANXC	0.789647067	-1.124442017	0.33479495	7
SPATA17	1.006132361	-0.993752694	-0.012379667	7
SPATA31A1	0.888146731	-1.083132469	0.194985738	7
SPATA31A5	0.78868016	-1.124742506	0.336062346	7
SPATS1	1.063640134	-0.921052966	-0.142587168	7
SPATS2L	0.781225573	-1.126999202	0.345773629	7
SPC24	0.713768654	-1.142950947	0.429182293	7
SPDYE16	0.920147174	-1.064223306	0.144076132	7
SPDYE18	0.987737407	-1.011827304	0.024089897	7
SPDYE21	0.700012414	-1.145296698	0.445284284	7
SPDYE4	0.692409039	-1.146471532	0.454062494	7
SPESP1	1.085443827	-0.883835933	-0.201607894	7
SPG21	0.688997253	-1.146971111	0.457973859	7
SPINT2	0.867755353	-1.09361284	0.225857486	7
SPIRE1	0.929487898	-1.058072851	0.128584953	7
SPNS3	1.032680917	-0.963752564	-0.068928353	7
SPR	0.776492827	-1.128377596	0.351884769	7
SPRED1	1.072528741	-0.906752663	-0.165776078	7
SPRED2	1.043903014	-0.949385564	-0.09451745	7
SPRN	0.886465156	-1.084040385	0.197575229	7
SPRR2G	0.820414348	-1.113904711	0.293490363	7
SPRR3	0.899305795	-1.076898316	0.177592522	7
SPRY4	0.813027896	-1.116611991	0.303584095	7
SPTAN1	1.085910623	-0.882953237	-0.202957386	7
SPTBN2	0.820564953	-1.1138483	0.293283347	7
SQLE	1.038795265	-0.956064895	-0.082730371	7
SRGAP1	1.000435495	-0.999563935	-0.00087156	7
SRR	0.787308217	-1.125165782	0.337857565	7
SRRM4	0.786874324	-1.125298897	0.338424573	7
SRSF11	0.699191631	-1.145427643	0.446236012	7
SRSF12	0.807059994	-1.118715724	0.31165573	7
SSH1	0.685620469	-1.147448929	0.46182846	7
SSPN	0.687096467	-1.147242102	0.460145635	7
SSR2	0.778703176	-1.127739036	0.349035859	7
SSTR1	0.970774465	-1.026864567	0.056090102	7
SSUH2	1.039469667	-0.955196782	-0.084272886	7
SSX7	0.723801697	-1.141056371	0.417254674	7
ST18	1.08427813	-0.886022333	-0.198255797	7
ST6GALNAC4	0.960734458	-1.035112193	0.074377735	7
ST7	1.083384119	-0.887682143	-0.195701976	7
STARD3NL	0.856611495	-1.098874581	0.242263086	7
STIMATE-MUSTN1	1.057023563	-0.931036179	-0.125987384	7
STK32A	0.999818822	-1.00018108	0.000362259	7
STK32B	0.892392366	-1.080803856	0.18841149	7

STK40	0.776292391	-1.128435053	0.352142662	7
STMND1	0.768619867	-1.130579071	0.361959203	7
STOX1	0.787153409	-1.125213317	0.338059908	7
STX12	0.68030463	-1.148167914	0.467863283	7
STXBP2	0.701709947	-1.14502269	0.443312743	7
STXBP5	0.958811807	-1.03664009	0.077828283	7
STYXL2	0.819208752	-1.11435452	0.295145768	7
SUMO3	0.952152369	-1.04180969	0.089657321	7
SUN5	0.790683166	-1.124118022	0.333434856	7
SUPT7L	0.897169938	-1.078120176	0.180950238	7
SUSD2	0.784568799	-1.126000182	0.341431383	7
SUV39H1	1.030544534	-0.966363691	-0.064180843	7
SVEP1	1.05186347	-0.938469528	-0.113393943	7
SYS1	1.073889308	-0.90446515	-0.169424158	7
SYT2	1.0185632	-0.980340785	-0.038222416	7
SYT3	0.881762293	-1.086536971	0.204774678	7
SYT4	1.041078147	-0.953109551	-0.087968596	7
SYTL2	1.073597873	-0.904957469	-0.168640404	7
TAB2	1.048728431	-0.942845408	-0.105883023	7
TAB3	0.948372719	-1.044661503	0.096288784	7
TAF11L10	0.99983477	-1.000165148	0.000330377	7
TAF11L12	0.941587541	-1.049637166	0.108049625	7
TAF11L7	1.092003528	-0.87103441	-0.220969119	7
TAF2	0.904228028	-1.074029406	0.169801378	7
TAF3	0.829925693	-1.110245169	0.280319476	7
TAF5	0.871240323	-1.091901522	0.220661199	7
TAF6	0.90975094	-1.070720353	0.160969413	7
TAFA3	1.083930057	-0.886670299	-0.197259757	7
TAFAZZIN	0.964535193	-1.03204382	0.067508628	7
TAGLN3	1.066717928	-0.916222708	-0.15049522	7
TAS1R1	0.732627914	-1.139257845	0.406629932	7
TAS1R3	0.947859604	-1.045044176	0.097184573	7
TAS2R20	0.888222304	-1.083091476	0.194869172	7
TAS2R31	0.922197496	-1.062899242	0.140701746	7
TAS2R39	0.976256196	-1.022162629	0.045906433	7
TBC1D12	0.742070906	-1.137193451	0.395122546	7
TBC1D13	0.931689335	-1.056578311	0.124888976	7
TBC1D28	0.887225357	-1.083630939	0.196405582	7
TBC1D7	0.936291089	-1.053396984	0.117105895	7
TBCEL-TECTA	1.021437524	-0.977086684	-0.04435084	7
TBL1Y	1.010424959	-0.989238359	-0.0211866	7
TBX15	0.968174932	-1.029044323	0.060869391	7
TBX6	0.811756523	-1.117066359	0.305309837	7
TCEAL2	0.931654707	-1.056601955	0.124947248	7
TCEANC	0.888200827	-1.083103127	0.194902301	7
TCF23	0.853674145	-1.100209437	0.246535292	7
TCHH	1.013269099	-0.986180481	-0.027088618	7
TCHHL1	1.035295639	-0.960504687	-0.074790951	7
TCTN2	1.010592329	-0.989059907	-0.021532421	7
TDG	1.08046128	-0.893008993	-0.187452287	7
TDGF1	0.803558441	-1.119915958	0.316357518	7
TDP2	0.722835699	-1.14124566	0.418409961	7
TDRD12	1.00220144	-0.997783924	-0.004417516	7
TDRD15	1.074405166	-0.903590566	-0.1708146	7
TDRD7	0.974609528	-1.023590323	0.048980795	7

TEAD1	1.031367543	-0.96536226	-0.066005283	7
TECR	0.995859188	-1.004090002	0.008230814	7
TECRL	0.757762177	-1.133432188	0.375670011	7
TEKT2	0.996424256	-1.003537792	0.007113536	7
TEN1	0.793894351	-1.123100616	0.329206265	7
TENT4A	0.900681759	-1.076103835	0.175422076	7
TERF1	0.919114104	-1.064885019	0.145770915	7
TET3	1.075510398	-0.901703049	-0.173807349	7
TEX29	0.78024676	-1.127287707	0.347040947	7
TEX45	0.979956525	-1.01890539	0.038948865	7
TFB1M	0.899372614	-1.076859868	0.177487254	7
TFEB	1.045964404	-0.946619841	-0.099344563	7
TG	1.063185767	-0.921755694	-0.141430073	7
TGFB1	1.077739333	-0.897838255	-0.179901078	7
TGFBI	0.825531032	-1.111960686	0.286429654	7
TGM1	0.84709056	-1.103124699	0.256034139	7
TGM3	1.081917371	-0.890374058	-0.191543313	7
TGM4	1.044429775	-0.948682746	-0.095747029	7
TGS1	1.005662798	-0.994239328	-0.01142347	7
THAP12	1.014130394	-0.985243676	-0.028886718	7
THBS3	0.975151643	-1.023121762	0.047970119	7
THOC3	0.892317325	-1.08084547	0.188528146	7
THSD4	0.960354785	-1.035415193	0.075060408	7
THSD7A	1.053502318	-0.936140521	-0.117361797	7
THSD7B	1.07042948	-0.910228802	-0.160200677	7
TIE1	0.740003452	-1.137658009	0.397654556	7
TIMM17A	1.060777551	-0.925437178	-0.135340373	7
TIMM17B	0.74455231	-1.136626463	0.392074153	7
TIMM23	1.01600748	-0.983184337	-0.032823143	7
TIMM50	0.752451017	-1.134752436	0.382301419	7
TINAGL1	1.080826007	-0.892352425	-0.188473582	7
TINF2	0.723128028	-1.141188533	0.418060505	7
TIPRL	0.870189349	-1.092420981	0.222231632	7
TJAP1	0.962005914	-1.034092883	0.07208697	7
TKFC	0.988422238	-1.011188981	0.022766743	7
TLE3	0.758915502	-1.133139014	0.374223511	7
TLK2	1.024685176	-0.973336316	-0.05134886	7
TLR3	1.071466531	-0.908519538	-0.162946992	7
TLR9	0.796085744	-1.122394731	0.326308987	7
TLX2	1.077656762	-0.897982843	-0.179673919	7
TM4SF19	0.853944167	-1.100087616	0.246143448	7
TM4SF19-DYNLT2B	1.051198705	-0.939406025	-0.111792679	7
TM6SF2	1.082195333	-0.889866854	-0.192328479	7
TMC2	0.783659776	-1.126273907	0.342614131	7
TMC3	1.012620125	-0.986883014	-0.02573711	7
TMC5	0.863020544	-1.095887164	0.23286662	7
TMCO3	1.086569369	-0.881700487	-0.204868882	7
TMCO4	1.081249284	-0.891587588	-0.189661696	7
TMCO5A	0.850081528	-1.101813286	0.251731758	7
TMED4	1.022723892	-0.975610673	-0.047113219	7
TMED5	1.003704536	-0.996253829	-0.007450708	7
TMED7	1.042844017	-0.95079046	-0.092053556	7
TMEM115	0.950783776	-1.042849074	0.092065298	7
TMEM120B	0.709064808	-1.14378542	0.434720612	7
TMEM129	1.07656375	-0.89988645	-0.1766773	7

TMEM132B	0.972235237	-1.025625712	0.053390475	7
TMEM140	0.933734408	-1.055174132	0.121439725	7
TMEM14A	1.077674419	-0.897951934	-0.179722485	7
TMEM156	0.991878239	-1.007928542	0.016050303	7
TMEM163	0.859561838	-1.097512216	0.237950379	7
TMEM169	1.017797315	-0.981197789	-0.036599526	7
TMEM174	0.898733339	-1.077227155	0.178493816	7
TMEM181	1.028290112	-0.96907866	-0.059211453	7
TMEM184B	1.07649084	-0.900012753	-0.176478087	7
TMEM185B	0.981869894	-1.01719404	0.035324146	7
TMEM211	0.81875855	-1.114521692	0.295763142	7
TMEM216	0.9154512	-1.067202282	0.151751082	7
TMEM219	0.838779441	-1.106657344	0.267877903	7
TMEM225B	0.80769191	-1.118496453	0.310804542	7
TMEM253	0.780450026	-1.127227942	0.346777916	7
TMEM268	0.905501799	-1.073274766	0.167772967	7
TMEM31	1.051944756	-0.938354691	-0.113590065	7
TMEM42	1.076654301	-0.899729471	-0.176924829	7
TMEM61	1.053701331	-0.935855713	-0.117845617	7
TMEM63B	1.001815524	-0.998174533	-0.003640991	7
TMEM65	0.968859154	-1.028473652	0.059614498	7
TMEM82	1.035093208	-0.960758222	-0.074334986	7
TMEM86B	0.81372991	-1.116359652	0.302629742	7
TMEM87A	0.983949909	-1.015312257	0.031362347	7
TMOD1	0.944505787	-1.047519452	0.103013665	7
TMPRSS3	0.819335962	-1.114307205	0.294971243	7
TMTC3	0.758570185	-1.133227037	0.374656852	7
TNFRSF1B	0.870986934	-1.092027031	0.221040097	7
TNFRSF21	1.014023401	-0.985360326	-0.028663075	7
TNFSF11	0.880940749	-1.086966737	0.206025988	7
TNFSF14	1.065740299	-0.917770411	-0.147969888	7
TNFSF8	0.868261331	-1.093366356	0.225105026	7
TNIP2	0.68831544	-1.147068918	0.458753477	7
TNIP3	1.086220767	-0.882364469	-0.203856298	7
TNKS1BP1	0.997436172	-1.002544259	0.005108087	7
TNNI1	0.828767323	-1.110701508	0.281934185	7
TNNT1	1.089744853	-0.875541622	-0.21420323	7
TNP1	1.052889507	-0.937014802	-0.115874705	7
TNPO1	1.081795529	-0.890595956	-0.191199574	7
TOP1MT	0.936626813	-1.053161814	0.116535001	7
TOR4A	1.068362987	-0.913589448	-0.154773539	7
TP53AIP1	1.081527321	-0.891083501	-0.190443821	7
TPSB2	0.89137726	-1.081365384	0.189988124	7
TRABD	1.059668483	-0.927108743	-0.13255974	7
TRABD2B	1.08649647	-0.88183953	-0.20465694	7
TRAF3IP2	0.868565707	-1.093217759	0.224652052	7
TRAF7	0.818444965	-1.114637878	0.296192913	7
TRAIP	0.922579649	-1.062650858	0.140071209	7
TRANK1	0.784896414	-1.125901147	0.341004733	7
TRAPPC9	1.076470614	-0.900047777	-0.176422837	7
TRDMT1	1.076459618	-0.900066813	-0.176392805	7
TRDN	0.942968932	-1.04863886	0.105669928	7
TREM1	1.036559613	-0.958913585	-0.077646027	7
TREX1	0.846101983	-1.103553435	0.257451452	7
TRHR	0.937196669	-1.05276167	0.115565001	7

TRIB3	0.6834219	-1.147751197	0.464329297	7
TRIL	1.012525672	-0.986985025	-0.025540647	7
TRIM13	1.063573864	-0.921155622	-0.142418243	7
TRIM17	1.029443921	-0.967694267	-0.061749654	7
TRIM45	0.862860359	-1.095963099	0.23310274	7
TRIM51GP	1.064319519	-0.919997316	-0.144322203	7
TRIM60	0.980008055	-1.018859546	0.038851491	7
TRIM62	0.741428021	-1.137338669	0.395910648	7
TRIM63	0.695838096	-1.14595226	0.450114164	7
TRIM75P	1.015418895	-0.983832699	-0.031586196	7
TRIM9	1.044457741	-0.948645357	-0.095812384	7
TRIO	0.999004068	-1.000992965	0.001988897	7
TRIP13	0.931719807	-1.0565575	0.124837693	7
TRMT11	0.798743575	-1.121525875	0.3227823	7
TRMT12	0.950259821	-1.043244955	0.092985134	7
TRMT1L	0.698503842	-1.145536598	0.447032757	7
TRMT6	0.99040906	-1.00932263	0.01891357	7
TRMT61B	0.848120769	-1.102675423	0.254554654	7
TRMT9B	1.048722878	-0.942853069	-0.105869809	7
TRNP1	1.030259041	-0.96670978	-0.063549261	7
TRPC5	0.933068004	-1.055633378	0.122565374	7
TRPM3	1.027085252	-0.97051299	-0.056572261	7
TRPM8	0.790314004	-1.1242337	0.333919696	7
TRPV6	0.997995427	-1.00199259	0.003997164	7
TSFM	1.051271445	-0.939303779	-0.111967666	7
TSHR	1.051063606	-0.939595774	-0.111467833	7
TSHZ2	1.053622382	-0.935968748	-0.117653634	7
TSPAN14	1.082569313	-0.889182289	-0.193387024	7
TSPAN7	1.011406415	-0.988189267	-0.023217147	7
TSPO2	0.686350862	-1.147346974	0.460996112	7
TSTD2	1.085366544	-0.883981674	-0.201384869	7
TTC21A	0.982656516	-1.016485024	0.033828509	7
TTC27	0.8801245	-1.087391879	0.207267379	7
TTC30A	0.759729099	-1.132930796	0.373201697	7
TTC30B	0.999979383	-1.000020616	0.0000412	7
TTC36	0.774424311	-1.128966994	0.354542682	7
TTC9B	0.865822022	-1.094548502	0.22872648	7
TTF2	1.090658241	-0.873732	-0.21692624	7
TTLL13P	0.703592331	-1.144713799	0.441121468	7
TTN	0.92123569	-1.063522152	0.142286462	7
TTPAL	0.749985741	-1.135348735	0.385362994	7
TUBA3E	0.886837237	-1.083840188	0.197002951	7
TUBAL3	1.031688852	-0.964969781	-0.066719071	7
TUBE1	0.770017459	-1.130196514	0.360179055	7
TUFT1	0.864682364	-1.095095516	0.230413153	7
TUSC3	0.987724591	-1.011839224	0.024114633	7
TWSG1	0.833547571	-1.108798955	0.275251384	7
TXK	1.031114769	-0.965670425	-0.065444344	7
TXLNB	1.053921728	-0.935539795	-0.118381933	7
TXNDC8	0.799350493	-1.121325502	0.321975009	7
UBAP1	0.932170443	-1.05624935	0.124078907	7
UBAP2	0.913690685	-1.068300165	0.15460948	7
UBE2A	0.870546203	-1.092244928	0.221698725	7
UBE2D1	1.084309039	-0.885964687	-0.198344351	7
UBE2J2	0.759008021	-1.133115394	0.374107374	7

UBE2L5	1.043001602	-0.95058208	-0.092419522	7
UBQLNL	0.857921286	-1.098272454	0.240351168	7
UBR4	0.849520259	-1.102061021	0.252540762	7
UBTD2	0.83083203	-1.109886031	0.279054002	7
UCK1	1.092264221	-0.87050713	-0.221757091	7
UCP1	0.884937627	-1.08485814	0.199920513	7
UGT1A1	0.970362541	-1.027212085	0.056849544	7
UGT1A7	0.750537876	-1.135216089	0.384678214	7
UGT2B10	1.020903897	-0.977695381	-0.043208516	7
UGT2B28	1.067082599	-0.915642131	-0.151440468	7
ULK1	0.807611906	-1.118524259	0.310912353	7
ULK3	0.818807797	-1.114503426	0.295695629	7
ULK4	0.876674518	-1.089168529	0.21249401	7
UPK3BL1	0.701128943	-1.145116958	0.443988016	7
UPK3BL2	0.804718664	-1.119521032	0.314802367	7
UPP1	0.911481832	-1.069663267	0.158181435	7
UQCR11	1.069266774	-0.912126996	-0.157139777	7
URB2	1.047075765	-0.94511143	-0.101964335	7
USB1	1.001583516	-0.998408926	-0.00317459	7
USP18	0.792061483	-1.123683787	0.331622304	7
USP35	1.084815015	-0.885018494	-0.199796521	7
USP43	0.922163617	-1.062921237	0.140757621	7
USP44	1.050229721	-0.940762728	-0.109466994	7
USP49	1.073455048	-0.905198275	-0.168256773	7
USP5	0.735159976	-1.138718664	0.403558688	7
VANGL2	0.833992403	-1.108619295	0.274626892	7
VAT1L	0.976950759	-1.021556434	0.044605676	7
VIM	1.032735255	-0.963685658	-0.069049597	7
VOPP1	1.026847841	-0.970794267	-0.056053574	7
VPS13D	1.03140884	-0.965311863	-0.066096977	7
VPS16	0.740020398	-1.13765423	0.397633831	7
VPS33B	1.074839696	-0.902850718	-0.171988978	7
VPS53	0.921598109	-1.063287805	0.141689696	7
VSIG2	1.088687867	-0.877614164	-0.211073703	7
VSIG8	1.026554001	-0.971141786	-0.055412215	7
VSTM4	0.946930594	-1.045734323	0.098803729	7
VWA3A	1.067556429	-0.914885093	-0.152671336	7
VWC2	0.805506099	-1.11925144	0.313745341	7
WASH6P	0.913648782	-1.068326171	0.154677389	7
WASHC2A	0.955711748	-1.039069996	0.083358249	7
WDR1	1.025520569	-0.972358666	-0.053161903	7
WDR12	1.009382351	-0.990345839	-0.019036513	7
WDR19	1.09300232	-0.869006115	-0.223996205	7
WDR20	1.085103216	-0.884477415	-0.200625801	7
WDR31	1.076905124	-0.899293961	-0.177611163	7
WDR54	0.782221852	-1.126703701	0.344481849	7
WFDC10A	0.905806347	-1.073093585	0.167287238	7
WFDC5	0.986399096	-1.013067432	0.026668336	7
WIPF2	0.839821573	-1.106223247	0.266401674	7
WNT1	1.054485556	-0.93472917	-0.119756386	7
WNT5B	0.99398466	-1.0059087	0.011924039	7
WRAP53	0.828717368	-1.110721121	0.282003753	7
WSCD2	0.74240888	-1.137116831	0.394707951	7
WTIP	1.036953925	-0.958414365	-0.07853956	7
WWC1	1.020411998	-0.978254624	-0.042157374	7

WWOX	1.090321897	-0.874400407	-0.21592149	7
XAGE3	0.98430252	-1.014991017	0.030688498	7
XKR9	1.053386187	-0.936306515	-0.117079672	7
XPNPEP1	1.028098063	-0.969308057	-0.058790006	7
XPO7	0.856298189	-1.099017978	0.242719789	7
XRCC1	0.742843977	-1.137017913	0.394173936	7
YAF2	0.749269817	-1.135519956	0.386250139	7
YBX1	0.839249603	-1.106461811	0.267212209	7
YWHAQ	1.029459327	-0.967675709	-0.061783618	7
ZBTB17	0.963556022	-1.03284048	0.069284458	7
ZBTB47	0.762512996	-1.132209525	0.369696528	7
ZBTB6	0.810286722	-1.117587444	0.307300722	7
ZC3H11B	1.042120936	-0.951743607	-0.090377328	7
ZC3H8	0.913703782	-1.068292035	0.154588253	7
ZC3HAV1	1.017686801	-0.981321105	-0.036365696	7
ZCCHC3	1.046950342	-0.945282279	-0.101668062	7
ZCCHC9	1.009728163	-0.989979303	-0.01974886	7
ZCWPW1	1.039214457	-0.955525781	-0.083688675	7
ZDBF2	1.092340526	-0.870352513	-0.221988013	7
ZDHHC11	1.006666678	-0.993197248	-0.01346943	7
ZDHHC11B	0.860367089	-1.097136582	0.236769493	7
ZDHHC23	0.817113927	-1.115128703	0.298014776	7
ZFAND3	0.993594619	-1.006284601	0.012689982	7
ZFP28	0.958761236	-1.036680061	0.077918826	7
ZFP3	0.711995497	-1.143269499	0.431274002	7
ZFP30	1.079248372	-0.89517617	-0.184072202	7
ZFP36L2	0.952310117	-1.04168939	0.089379273	7
ZFP69B	0.843304951	-1.10475391	0.26144896	7
ZFP91	1.055447372	-0.9333382	-0.122109172	7
ZFP91-CNTF	0.933055318	-1.055642104	0.122586786	7
ZFP92	0.700457319	-1.1452253	0.444767981	7
ZFR2	0.95398913	-1.040402548	0.086413418	7
ZGRF1	1.030693165	-0.966183249	-0.064509916	7
ZKSCAN4	1.084892482	-0.884873208	-0.200019274	7
ZMAT1	1.05484508	-0.934210437	-0.120634643	7
ZMAT5	1.006474258	-0.993397486	-0.013076772	7
ZMYND10	0.937855986	-1.052297183	0.114441197	7
ZNF107	0.909431745	-1.070914238	0.161482493	7
ZNF117	1.021697255	-0.976789653	-0.044907602	7
ZNF132	0.703382013	-1.144748576	0.441366562	7
ZNF14	0.984899	-1.014446118	0.029547118	7
ZNF17	1.057530596	-0.930289617	-0.12724098	7
ZNF18	0.859864923	-1.097371025	0.237506102	7
ZNF267	1.039234986	-0.955499338	-0.083735647	7
ZNF268	0.768133883	-1.13071127	0.362577387	7
ZNF273	0.816764906	-1.115256782	0.298491876	7
ZNF276	0.908411177	-1.071531961	0.163120784	7
ZNF277	0.770805394	-1.129979277	0.359173883	7
ZNF280D	0.771161296	-1.129880783	0.358719487	7
ZNF285	1.026513823	-0.971189252	-0.055324571	7
ZNF330	0.738484418	-1.137994818	0.3995104	7
ZNF345	0.916453194	-1.066572845	0.150119652	7
ZNF346	1.038828588	-0.956022097	-0.082806491	7
ZNF358	1.062792612	-0.922361647	-0.140430965	7
ZNF415	0.797410331	-1.121963469	0.324553138	7

ZNF442	1.076536141	-0.899934289	-0.176601852	7
ZNF484	0.928296948	-1.05887411	0.130577161	7
ZNF488	1.088071307	-0.87881263	-0.209258677	7
ZNF492	0.842755591	-1.104987524	0.262231932	7
ZNF502	0.884752916	-1.084956576	0.20020366	7
ZNF525	1.065814553	-0.917653301	-0.148161252	7
ZNF529	0.842411298	-1.105133572	0.262722274	7
ZNF541	0.88112308	-1.086871519	0.205748439	7
ZNF550	0.812497782	-1.116801856	0.304304074	7
ZNF568	0.730567873	-1.139688829	0.409120956	7
ZNF570	0.74830081	-1.135750315	0.387449505	7
ZNF574	0.964084271	-1.032411229	0.068326958	7
ZNF575	0.683693732	-1.1477142	0.464020468	7
ZNF577	0.990926477	-1.008833025	0.017906548	7
ZNF582	1.020086737	-0.978623443	-0.041463294	7
ZNF594	0.719302727	-1.141925414	0.422622687	7
ZNF605	0.928892583	-1.058474007	0.129581424	7
ZNF624	0.982646595	-1.016493986	0.033847391	7
ZNF625-ZNF20	0.870866956	-1.092086399	0.221219442	7
ZNF629	0.940543934	-1.050386465	0.109842531	7
ZNF630	0.808083833	-1.118360045	0.310276212	7
ZNF644	0.960321378	-1.035441823	0.075120445	7
ZNF646	0.880286607	-1.087307593	0.207020986	7
ZNF670-ZNF695	0.882776385	-1.086003885	0.2032275	7
ZNF676	1.077598176	-0.898085365	-0.179512811	7
ZNF684	0.896813145	-1.078322946	0.181509801	7
ZNF687	0.863591217	-1.095616109	0.232024892	7
ZNF688	0.846299587	-1.103467923	0.257168336	7
ZNF69	1.061695572	-0.924042246	-0.137653326	7
ZNF696	0.825435387	-1.111997549	0.286562161	7
ZNF703	0.966151728	-1.030719114	0.064567386	7
ZNF746	1.081390362	-0.891331978	-0.190058384	7
ZNF747	0.716680228	-1.142417335	0.425737107	7
ZNF773	0.971986515	-1.025837365	0.05385085	7
ZNF775	1.072668169	-0.90651951	-0.16614866	7
ZNF8	0.825860552	-1.111833532	0.28597298	7
ZNF829	0.71910332	-1.141963196	0.422859876	7
ZNF85	1.024072599	-0.97404981	-0.050022788	7
ZNF880	0.813984972	-1.116267714	0.302282742	7
ZPBP2	1.078257853	-0.896927755	-0.181330098	7
ZRSR2	0.891440054	-1.081330736	0.189890682	7
ZSCAN32	1.03331791	-0.962966666	-0.070351244	7
ZUP1	0.802925468	-1.120130265	0.317204797	7
ABCA1	0.037571074	-1.018256052	0.980684978	8
ABCA3	0.552343069	-1.154344553	0.602001484	8
ABCB4	0.055426592	-1.026560592	0.971133999	8
ABCB9	0.36267022	-1.130731094	0.768060873	8
ABCC10	0.401430283	-1.138339958	0.736909675	8
ABCC4	0.11942577	-1.054350062	0.934924292	8
ABCD3	0.476278213	-1.149110729	0.672832517	8
ABCG4	-0.107579601	-0.941860727	1.049440327	8
ABCG5	0.326073091	-1.122336944	0.796263853	8
ABHD1	0.635543768	-1.152674926	0.517131158	8
ABHD12	0.410165165	-1.1398678	0.729702635	8
ABHD14A-ACY1	0.466836333	-1.14804808	0.681211747	8

ABHD14B	0.437528336	-1.144197427	0.706669091	8
ABHD15	0.064304647	-1.030600462	0.966295814	8
ABHD16B	0.643942723	-1.152033604	0.508090882	8
ABHD17C	0.25966273	-1.104219096	0.844556366	8
ABI3BP	0.095689986	-1.044405362	0.948715377	8
ABRAXAS1	0.612701782	-1.153963653	0.541261871	8
ABT1	0.676051198	-1.148714247	0.472663048	8
ACKR3	0.071858637	-1.033991066	0.962132429	8
ACP6	-0.052973583	-0.972460329	1.025433912	8
ACSM6	0.299374705	-1.11549328	0.816118575	8
ACTG1	-0.012919581	-0.993477614	1.006397195	8
ACTL6B	-0.03630987	-0.98135054	1.01766041	8
ACTL7A	0.137148914	-1.061495719	0.924346804	8
ACTL9	-0.031148388	-0.984061906	1.015210295	8
ACTR8	0.353252096	-1.12868157	0.775429474	8
ACVR1C	-0.019983323	-0.989858577	1.0098419	8
ACVR2B	0.247737576	-1.100582446	0.85284487	8
ACY1	0.388601408	-1.135970343	0.747368935	8
ADAM11	0.086139551	-1.040283385	0.954143834	8
ADAM12	0.642881077	-1.152119743	0.509238666	8
ADAMTS2	0.497034798	-1.151134321	0.654099523	8
ADAMTS20	0.159747659	-1.070257853	0.910510194	8
ADAMTS3	0.471661001	-1.148602063	0.676941062	8
ADAMTS6	0.274140184	-1.108479053	0.834338869	8
ADAP2	0.633873147	-1.1527916	0.518918453	8
ADARB2	0.473784734	-1.148838648	0.675053914	8
ADCY8	0.163230567	-1.071573281	0.908342714	8
ADGRG2	0.641543539	-1.152226167	0.510682629	8
ADGRG4	0.290566845	-1.113104781	0.822537936	8
ADGRG6	0.516033988	-1.152601642	0.636567654	8
ADH7	0.182799633	-1.078789416	0.895989782	8
ADIRF	0.108304485	-1.049743827	0.941439343	8
ADM2	0.422403327	-1.141890426	0.719487098	8
ADPGK	0.530716582	-1.153477051	0.622760469	8
ADRA2B	0.0070906	-1.003526446	0.996435846	8
ADSL	0.37577653	-1.133453704	0.757677174	8
AGMO	0.646603007	-1.151811258	0.505208251	8
AGPAT5	0.280545231	-1.110309099	0.829763869	8
AGR3	0.211306622	-1.088766801	0.877460179	8
AHCY	0.57045002	-1.154673157	0.584223137	8
AIFM3	0.388638294	-1.135977369	0.747339075	8
AIRE	-0.025629848	-0.986938712	1.01256856	8
AK8	0.613941677	-1.153910454	0.539968777	8
AKAP8L	0.044134751	-1.021336655	0.977201904	8
AKIRIN1	0.547071658	-1.154180167	0.607108509	8
AKIRIN2	0.672934159	-1.149098431	0.476164272	8
ALB	0.649062374	-1.151597403	0.502535029	8
ALDH1A2	0.348973235	-1.12772492	0.778751684	8
ALDH3B1	0.009707257	-1.004818291	0.995111034	8
ALPI	0.16970385	-1.073993195	0.904289345	8
AMBN	-0.026526721	-0.986472729	1.012999451	8
AMPH	0.661694633	-1.150371666	0.488677033	8
ANGPTL5	0.521877054	-1.152977346	0.631100293	8
ANGPTL7	0.021047207	-1.01035747	0.989310264	8
ANHX	0.59186119	-1.154577932	0.562716742	8

ANKDD1A	0.102228751	-1.047187647	0.944958896	8
ANKDD1B	0.209969452	-1.088313065	0.878343613	8
ANKRD16	0.132191704	-1.059521247	0.927329543	8
ANKRD18B	-0.122635856	-0.933026245	1.055662101	8
ANKRD26	0.403213665	-1.138657554	0.735443889	8
ANKRD29	0.627994589	-1.153173734	0.525179145	8
ANKRD33	0.390532212	-1.136336477	0.745804266	8
ANKRD53	-0.096442811	-0.948284534	1.044727345	8
ANO2	0.532187435	-1.153552111	0.621364676	8
ANO8	0.249069901	-1.100994442	0.851924541	8
ANXA10	-0.079932218	-0.957635079	1.037567297	8
ANXA2R	0.444094625	-1.145131801	0.701037176	8
AOPEP	0.235944462	-1.096873482	0.860929019	8
AP1S2	0.506276112	-1.151894627	0.645618515	8
AP2B1	0.643695248	-1.152053816	0.508358568	8
AP2S1	0.415131333	-1.14070525	0.725573917	8
AP3B1	0.621026132	-1.153569949	0.532543817	8
AP3S1	0.672783023	-1.149116713	0.47633369	8
AP5B1	0.112518035	-1.051500078	0.938982043	8
APCDD1	0.656489382	-1.150902742	0.49441336	8
APELA	-0.005593967	-0.997191282	1.002785249	8
APLN	-0.051408254	-0.973304328	1.024712582	8
APOA4	0.523904325	-1.153099285	0.62919496	8
APOBEC3C	0.665616484	-1.149947167	0.484330683	8
APOC1	0.417442342	-1.141087203	0.723644861	8
APOC2	-0.056614614	-0.970490014	1.027104629	8
APOL5	0.046416278	-1.022399886	0.975983608	8
APRT	-0.07574287	-0.959974877	1.035717747	8
APTX	0.131557691	-1.059267361	0.92770967	8
AQP8	0.367659396	-1.131785354	0.764125958	8
ARAF	0.652874615	-1.151250046	0.498375431	8
AREL1	0.677501744	-1.148530805	0.471029061	8
ARF6	0.194005635	-1.082787459	0.888781824	8
ARHGAP20	0.395819732	-1.137321945	0.741502213	8
ARHGAP21	0.521306765	-1.152942261	0.631635496	8
ARHGAP26	0.422271524	-1.141869381	0.719597857	8
ARHGAP31	-0.048951333	-0.974625342	1.023576675	8
ARHGAP36	-0.027972589	-0.985720238	1.013692827	8
ARHGAP4	0.341125995	-1.12592931	0.784803315	8
ARHGAP40	0.663932796	-1.150131987	0.486199191	8
ARHGAP6	0.195412833	-1.083282577	0.887869744	8
ARHGEF26	0.473861605	-1.148847128	0.674985523	8
ARHGEF38	0.477000405	-1.149188382	0.672187977	8
ARHGEF9	0.560538567	-1.154538914	0.594000347	8
ARID1A	0.308895469	-1.118002451	0.809106982	8
ARID3A	-0.123913068	-0.932268875	1.056181943	8
ARID5A	0.505265786	-1.15181578	0.646549994	8
ARL5B	0.013367754	-1.006616864	0.993249109	8
ARMCX6	0.565216876	-1.154616129	0.589399253	8
ARMH4	-0.080127725	-0.957525562	1.037653287	8
ARPIN	0.598703206	-1.154433969	0.555730764	8
ARRB2	0.245882357	-1.100006376	0.854124019	8
ARRDC5	0.46083114	-1.147326686	0.686495546	8
ARSB	0.645070104	-1.151940514	0.50687041	8
ARSI	-0.095703505	-0.948707643	1.044411148	8

ASAH2	0.02649733	-1.012985339	0.98648801	8
ASB16	0.101634293	-1.046936042	0.945301748	8
ASB5	-0.083558523	-0.955599042	1.039157565	8
ASCL4	0.007822995	-1.003888547	0.996065553	8
ASIP	0.126307541	-1.057153169	0.930845628	8
ASPHD1	0.110988302	-1.050864031	0.939875729	8
ASTE1	0.191982868	-1.08207304	0.890090172	8
ASTN1	0.622984979	-1.15346475	0.530479771	8
ATAD2	0.611411924	-1.154016987	0.542605063	8
ATAD2B	0.644302772	-1.152004056	0.507701284	8
ATP13A5	0.410482961	-1.13992207	0.729439109	8
ATP2A2	0.01720755	-1.008492731	0.991285182	8
ATP6AP1	0.119529261	-1.054392484	0.934863223	8
ATP6V1C1	0.344540388	-1.126717123	0.782176735	8
ATP8B2	0.009144863	-1.00454107	0.995396207	8
ATP8B3	0.268332846	-1.106790766	0.83845792	8
ATPAF1	0.414020134	-1.14051984	0.726499706	8
ATPSCKMT	0.110020326	-1.050460637	0.940440311	8
ATXN1L	0.530952739	-1.153489259	0.62253652	8
AXIN1	0.084189824	-1.039433398	0.955243574	8
AXIN2	0.165131973	-1.072287449	0.907155476	8
B3GALT1	0.584838449	-1.154668024	0.569829575	8
B3GNT5	0.21417246	-1.089734498	0.875562038	8
B3GNT7	0.193135171	-1.082480413	0.889345243	8
B3GNT9	-0.075934378	-0.959868207	1.035802585	8
B4GALT3	0.584585926	-1.154670184	0.570084258	8
B4GALT6	0.463588139	-1.147662252	0.684074113	8
BAALC	0.285000517	-1.111562191	0.826561674	8
BAG3	0.07517275	-1.035465021	0.960292272	8
BATF	-0.057904677	-0.969789513	1.02769419	8
BAX	-0.014878023	-0.992477977	1.007356	8
BBS12	0.200477715	-1.085051791	0.884574076	8
BBS7	0.324982876	-1.122069261	0.797086385	8
BBS9	0.450017391	-1.145939345	0.695921953	8
BCAM	0.443195744	-1.145006313	0.701810569	8
BCAS1	-0.080254955	-0.957454277	1.037709232	8
BCKDHB	-0.116631724	-0.936569951	1.053201675	8
BCKDK	0.451055032	-1.146077362	0.69502233	8
BCL2A1	0.09675632	-1.044861306	0.948104986	8
BCL7B	0.403466374	-1.138702324	0.73523595	8
BDP1	-0.107930443	-0.941656828	1.049587271	8
BEST4	0.114811395	-1.052450298	0.937638904	8
BEX5	0.093950911	-1.043659919	0.949709008	8
BIN3	0.239989517	-1.098158228	0.858168711	8
BIRC3	0.292736405	-1.113699091	0.820962686	8
BLMH	0.050065045	-1.02409214	0.974027095	8
BMP3	0.374149426	-1.133123948	0.758974522	8
BMP8B	0.200129187	-1.084930692	0.884801505	8
BOP1	0.124723076	-1.056510981	0.931787906	8
BORCS6	0.486733939	-1.150184236	0.663450297	8
BPIFB2	-0.006883576	-0.996540443	1.003424019	8
BPIFB4	-0.037883448	-0.980519948	1.018403396	8
BRD4	0.337709567	-1.125130992	0.787421424	8
BRIP1	-0.08795029	-0.953119916	1.041070206	8
BRMS1L	0.166452657	-1.072781852	0.906329195	8

BSPH1	0.327672047	-1.122727713	0.795055666	8
BTAF1	-0.084108857	-0.955289181	1.039398038	8
BTBD6	0.49836082	-1.151248794	0.652887974	8
BTBD9	0.05020566	-1.024157155	0.973951495	8
BTLA	0.078416996	-1.036899874	0.958482878	8
BUB3	0.588532907	-1.154627868	0.56609496	8
C11orf87	0.160614971	-1.070586296	0.909971325	8
C11orf97	0.040585765	-1.01967499	0.979089225	8
C15orf39	0.665348452	-1.149976849	0.484628398	8
C16orf92	-0.116182642	-0.9368339	1.053016542	8
C18orf63	0.211617408	-1.088872056	0.877254648	8
C19orf47	0.560449768	-1.15453721	0.594087442	8
C19orf73	0.330846785	-1.123497139	0.792650354	8
C1D	0.639352493	-1.152395462	0.513042969	8
C1orf122	0.238236968	-1.097603211	0.859366243	8
C1orf127	0.439516147	-1.14448461	0.704968463	8
C1orf146	0.539572595	-1.153893792	0.614321196	8
C1orf159	0.573772207	-1.154693162	0.580920955	8
C1orf210	0.351582426	-1.128310162	0.776727736	8
C1QTNF5	0.31080249	-1.118495923	0.807693433	8
C1QTNF9	0.049334662	-1.023754198	0.974419536	8
C1R	-0.066026693	-0.965350494	1.031377186	8
C21orf140	0.423189092	-1.14201555	0.718826458	8
C2CD3	-0.015565032	-0.992126628	1.007691661	8
C2orf16	0.282164059	-1.110766299	0.82860224	8
C3orf18	0.597428814	-1.154465019	0.557036205	8
C3orf20	0.087547564	-1.040895423	0.95334786	8
C4orf54	-0.106402749	-0.942543995	1.048946744	8
C5AR2	0.513229851	-1.152408618	0.639178768	8
C5orf58	0.347740838	-1.127446442	0.779705604	8
C6orf15	0.284818622	-1.111511351	0.826692729	8
C7	0.302332266	-1.116280842	0.813948576	8
C7orf50	0.143454485	-1.063980037	0.920525552	8
C8orf82	0.587495119	-1.154640767	0.567145648	8
CA10	0.105637269	-1.04862513	0.942987861	8
CA14	-0.012742426	-0.993567897	1.006310323	8
CA3	0.626397274	-1.153269968	0.526872695	8
CAAP1	0.495235177	-1.150976094	0.655740917	8
CABLES2	-0.044055613	-0.977244092	1.021299705	8
CACNA1G	0.126860983	-1.057377028	0.930516045	8
CACNA2D4	0.348036248	-1.127513314	0.779477066	8
CALCA	0.49431651	-1.150894052	0.656577542	8
CALY	0.242287856	-1.098882362	0.856594506	8
CAMSAP2	0.018433429	-1.009089285	0.990655855	8
CARD14	0.067889881	-1.032215056	0.964325175	8
CASC3	0.281266134	-1.110512968	0.829246833	8
CASP8	0.253095155	-1.102230483	0.849135328	8
CASP8AP2	0.509687326	-1.152153042	0.642465716	8
CASQ1	0.574750599	-1.154696642	0.579946043	8
CATSPER1	0.556622677	-1.154455389	0.597832712	8
CATSPERB	0.559804892	-1.154524571	0.59471968	8
CAVIN3	0.195935579	-1.083466108	0.887530529	8
CAVIN4	0.519813124	-1.152848742	0.633035618	8
CBFB	0.639244269	-1.152403663	0.513159393	8
CCAR2	0.163405496	-1.071639101	0.908233605	8

CCBE1	0.572677909	-1.154687968	0.582010059	8
CCDC115	0.625509731	-1.153322044	0.527812314	8
CCDC121	0.552308777	-1.154343583	0.602034806	8
CCDC127	0.286213274	-1.11190046	0.825687186	8
CCDC134	0.533652157	-1.153624551	0.619972394	8
CCDC137	0.267393031	-1.10651496	0.839121929	8
CCDC144A	0.515228179	-1.152547016	0.637318836	8
CCDC170	0.518706472	-1.152777937	0.634071465	8
CCDC174	0.299834561	-1.115616212	0.815781652	8
CCDC191	0.376865841	-1.133673156	0.756807316	8
CCDC196	0.566610595	-1.154634354	0.588023759	8
CCDC6	0.223131567	-1.092717774	0.869586207	8
CCDC68	0.243306699	-1.099202012	0.855895313	8
CCDC83	0.599282357	-1.154419215	0.555136858	8
CCDC88A	0.214216062	-1.089749171	0.875533108	8
CCDC88B	0.447058402	-1.145540095	0.698481693	8
CCL2	0.214646413	-1.08989391	0.875247497	8
CCL23	0.217242293	-1.090763875	0.873521582	8
CCL4L2	0.473991083	-1.148861398	0.674870315	8
CCN6	0.485421537	-1.150055493	0.664633956	8
CCNB3	0.278559042	-1.109745206	0.831186163	8
CCR10	0.313049697	-1.119073505	0.806023808	8
CCR6	0.196772771	-1.083759593	0.886986822	8
CCRL2	0.542396204	-1.154008823	0.611612618	8
CCSER1	0.367482159	-1.131748277	0.764266118	8
CD109	0.359132686	-1.129970355	0.770837669	8
CD163L1	0.628992822	-1.15311195	0.524119128	8
CD1B	0.291486507	-1.113357181	0.821870673	8
CD2BP2	0.583792207	-1.154676489	0.570884282	8
CD300C	0.37339505	-1.132970267	0.759575217	8
CD300E	0.352260435	-1.128461273	0.776200838	8
CD300LB	0.455630413	-1.146673572	0.691043159	8
CD69	0.5789466	-1.154699066	0.575752466	8
CD79B	0.018423889	-1.009084647	0.990660757	8
CD80	0.473238757	-1.148778251	0.675539495	8
CD8B	0.104498692	-1.048145936	0.943647244	8
CDC42EP3	0.556218347	-1.154445789	0.598227441	8
CDC42SE2	0.529594362	-1.153418224	0.623823862	8
CDCA2	0.41656058	-1.140942052	0.724381472	8
CDCA8	0.529791437	-1.153428653	0.623637216	8
CDH12	0.182353885	-1.078628357	0.896274472	8
CDH20	0.201356927	-1.085356858	0.883999931	8
CDH4	0.045141588	-1.021806341	0.976664752	8
CDHR4	0.399822433	-1.13805114	0.738228708	8
CDK14	0.039961221	-1.019381594	0.979420373	8
CDK5RAP1	0.268108594	-1.106725021	0.838616427	8
CDK6	0.530016819	-1.153440528	0.623423709	8
CDK7	-0.109187985	-0.940925213	1.050113198	8
CDKL2	0.454484459	-1.14652614	0.692041681	8
CDKN1A	0.301506761	-1.116061753	0.814554993	8
CDKN1C	0.460073796	-1.147233211	0.687159415	8
CDKN3	-0.065038931	-0.965893001	1.030931932	8
CDRT15	0.007471345	-1.003714739	0.996243394	8
CDRT15L2	0.579193006	-1.154698576	0.57550557	8
CDS1	0.293806829	-1.113990877	0.820184048	8

CDY1	0.527560972	-1.153308207	0.625747234	8
CEP70	0.351016106	-1.128183638	0.777167531	8
CEP76	0.110218347	-1.050543218	0.940324871	8
CEP78	0.25667303	-1.103318165	0.846645134	8
CEP97	-0.010187381	-0.99486739	1.005054771	8
CERCAM	0.338826844	-1.125393169	0.786566325	8
CES5A	0.19437755	-1.082918467	0.888540917	8
CFAP126	0.646009706	-1.151861653	0.505851947	8
CFAP206	0.613492584	-1.153929942	0.540437359	8
CFAP251	-0.012739976	-0.993569145	1.006309121	8
CFAP36	0.395149733	-1.13719847	0.742048737	8
CFAP44	0.045771698	-1.022099897	0.976328199	8
CFAP45	-0.030132244	-0.984593338	1.014725582	8
CFAP52	0.261623126	-1.104805919	0.843182793	8
CFAP61	0.20084	-1.085177569	0.88433757	8
CFAP73	0.547274899	-1.154187073	0.606912173	8
CFAP77	0.385552916	-1.13538545	0.749832534	8
CFAP91	0.40321631	-1.138658023	0.735441713	8
CFAP97D1	0.558174442	-1.154490539	0.596316097	8
CFD	0.43221518	-1.143411493	0.711196313	8
CGAS	0.375082501	-1.133313336	0.758230835	8
CGNL1	-0.117469542	-0.936077111	1.053546653	8
CHCHD4	0.396879681	-1.137516456	0.740636775	8
CHD1	0.378101004	-1.133920717	0.755819713	8
CHD7	0.516762814	-1.152650464	0.63588765	8
CHL1	0.369195047	-1.132105446	0.762910399	8
CHODL	0.205919586	-1.086930241	0.881010655	8
CHP1	-0.009678783	-0.995125478	1.004804262	8
CHRDL1	-0.01164313	-0.994127598	1.005770728	8
CHRNA1	0.307619436	-1.117670554	0.810051119	8
CHRNA4	0.263944092	-1.105496641	0.841552549	8
CHRNA9	-0.0983387	-0.947197613	1.045536313	8
CHST12	0.428462195	-1.142840332	0.714378137	8
CHST5	0.450856017	-1.146050971	0.695194954	8
CHSY3	0.382264997	-1.134745292	0.752480295	8
CHTF8	0.349797987	-1.12791055	0.778112562	8
CIR1	0.67412962	-1.148952697	0.474823077	8
CKAP2L	0.01421763	-1.007033009	0.992815379	8
CLCA4	0.365638753	-1.131361008	0.765722255	8
CLCN6	0.569086314	-1.154661296	0.585574982	8
CLDN1	-0.025715804	-0.986894079	1.012609883	8
CLDN11	0.522613401	-1.15302214	0.630408739	8
CLDN4	0.370747804	-1.132426989	0.761679185	8
CLDN8	0.501545871	-1.151516409	0.649970538	8
CLEC20A	0.237241444	-1.097286841	0.860045397	8
CLEC2L	0.628449716	-1.153145722	0.524696006	8
CLEC3A	0.440756083	-1.144661849	0.703905766	8
CLEC5A	0.588073664	-1.154633732	0.566560068	8
CLGN	0.376727625	-1.13364537	0.756917745	8
CLIC4	0.469245791	-1.148327602	0.679081811	8
CLIC6	0.017063836	-1.008422722	0.991358885	8
CLIP1	0.479953311	-1.149500495	0.669547184	8
CLIP2	0.295092584	-1.114340104	0.81924752	8
CLN8	0.169565701	-1.07394189	0.904376189	8
CLPS	0.579394498	-1.154698123	0.575303625	8

CLPSL2	0.509725048	-1.152155833	0.642430784	8
CLRN1	0.094754564	-1.044004684	0.949250121	8
CLTA	0.214048299	-1.089692707	0.875644408	8
CLTB	0.613261986	-1.153939852	0.540677866	8
CLYBL	0.53469984	-1.153674951	0.618975111	8
CMSS1	0.290416075	-1.113063335	0.822647261	8
CMTM3	0.320320523	-1.120913129	0.800592606	8
CMYA5	-0.117447457	-0.936090109	1.053537566	8
CNGB3	0.213199334	-1.089406633	0.8762073	8
CNTF	-0.061899481	-0.967612396	1.029511877	8
CNTNAP1	0.359291448	-1.130004731	0.770713282	8
COCH	-0.091457953	-0.951129379	1.042587333	8
COL21A1	0.613528037	-1.153928413	0.540400376	8
COL22A1	-0.007701804	-0.996126854	1.003828658	8
COL4A3	0.383901312	-1.135065091	0.751163778	8
COL7A1	0.678054344	-1.14846014	0.470405797	8
COL8A2	0.283051619	-1.111016055	0.827964435	8
COL9A2	-0.079304431	-0.95798655	1.03729098	8
COLCA2	0.385867975	-1.135446284	0.749578309	8
COMTD1	0.535395544	-1.153707766	0.618312222	8
COP1	0.352561136	-1.128528164	0.775967028	8
COPE	0.512385455	-1.152348885	0.63996343	8
COPS4	0.111227175	-1.050963469	0.939736294	8
COPZ2	0.256265929	-1.103194927	0.846928998	8
COQ3	-0.008592624	-0.995676	1.004268624	8
COQ4	0.046169388	-1.022285019	0.976115632	8
CORO7	0.355878746	-1.129260948	0.773382202	8
CORO7-PAM16	0.563651948	-1.154593046	0.590941098	8
CORT	0.28029048	-1.110236955	0.829946475	8
COX16	-0.069340242	-0.963525224	1.032865467	8
COX4I1	0.301212931	-1.115983634	0.814770703	8
COX7A2	0.636099085	-1.152635347	0.516536262	8
COX7B2	0.130326057	-1.058773284	0.928447227	8
CPA2	0.406329403	-1.139205466	0.732876062	8
CPN2	0.12379204	-1.056132736	0.932340696	8
CR2	0.320601165	-1.120983241	0.800382076	8
CRACR2A	0.51091719	-1.152243251	0.641326061	8
CRB1	0.240296036	-1.098255048	0.857959012	8
CRB2	0.453163416	-1.146354609	0.693191193	8
CRCT1	-0.076810338	-0.959379943	1.036190281	8
CREB3L1	0.040475962	-1.019623429	0.979147466	8
CREM	0.446775206	-1.145501444	0.698726239	8
CRPPA	0.327738134	-1.122743817	0.795005683	8
CRTAP	0.636599354	-1.152599351	0.515999997	8
CRTC1	-0.099380748	-0.946599042	1.04597979	8
CRTC2	-0.020836018	-0.989419175	1.010255194	8
CRYL1	0.66162985	-1.150378502	0.488748652	8
CSAG3	-0.03103658	-0.984120419	1.015156999	8
CSK	0.633435933	-1.152821542	0.519385609	8
CSMD1	0.436971383	-1.144116292	0.707144908	8
CSNK2A2	0.228572257	-1.094498372	0.865926115	8
CSNKA2IP	0.593762914	-1.154543513	0.560780599	8
CST1	0.655762732	-1.150973967	0.495211235	8
CST3	-0.001290388	-0.999354181	1.00064457	8
CST6	0.435492913	-1.14389949	0.708406577	8

CT47A1	0.087201417	-1.040745099	0.953543681	8
CTNNA2	0.6059557	-1.154220041	0.548264341	8
CTNNA3	0.066676155	-1.031669544	0.96499339	8
CTNNB1	0.060894401	-1.029055684	0.968161283	8
CTPS1	0.51040978	-1.152206224	0.641796443	8
CTSD	-0.077452354	-0.959021712	1.036474066	8
CTSL	0.411751751	-1.140137815	0.728386064	8
CTSS	-0.122735388	-0.932967269	1.055702657	8
CTXN2	0.306007888	-1.117249442	0.811241553	8
CXCL5	0.467595702	-1.14813679	0.680541087	8
CXCL9	-0.043575592	-0.977499888	1.02107548	8
CXCR4	-0.063329942	-0.966829891	1.030159833	8
CXorf66	0.051324555	-1.02467396	0.973349405	8
CXXC5	0.265771667	-1.106037448	0.840265782	8
CYB561A3	0.341452002	-1.126004964	0.784552962	8
CYB5D1	-0.010741569	-0.994585947	1.005327516	8
CYP17A1	0.571074791	-1.154677883	0.583603092	8
CYP1A1	-0.000724801	-0.999637403	1.000362203	8
CYP2A6	0.301711072	-1.11611603	0.814404958	8
CYP2C9	-0.005445582	-0.997266089	1.00271167	8
CYP2R1	0.631545653	-1.152948176	0.521402523	8
CYP2S1	0.32367238	-1.121746156	0.798073776	8
CYP8B1	0.632571264	-1.152880036	0.520308772	8
CYSTM1	0.508157266	-1.152038624	0.643881358	8
CYTIP	0.473690046	-1.148828195	0.675138149	8
DAAM2	0.287271606	-1.112194664	0.824923059	8
DACH2	0.081798765	-1.038387088	0.956588322	8
DAND5	0.432473877	-1.143450376	0.710976499	8
DAP3	0.397532272	-1.137635706	0.740103434	8
DAPL1	0.232404779	-1.095738511	0.863333733	8
DBI	0.173745682	-1.075487694	0.901742013	8
DCAF16	0.257148726	-1.103461997	0.846313271	8
DCAF4L2	0.18917849	-1.08107727	0.89189878	8
DCD	0.334155686	-1.124289932	0.790134246	8
DCHS1	0.641044527	-1.152265274	0.511220748	8
DCP1A	0.628918745	-1.153116578	0.524197833	8
DCPS	-0.03643954	-0.981282166	1.017721706	8
DCT	-0.076664038	-0.959461531	1.036125569	8
DDI1	-0.083307445	-0.955740333	1.039047778	8
DDX19A	0.578352089	-1.154699959	0.57634787	8
DDX19B	0.280461701	-1.11028545	0.829823749	8
DDX20	0.451359898	-1.146117715	0.694757817	8
DDX3Y	0.593983547	-1.154539241	0.560555694	8
DDX4	0.117485908	-1.053553386	0.936067478	8
DDX49	0.041272273	-1.019997157	0.978724884	8
DDX54	0.272682014	-1.10805773	0.835375716	8
DEDD	0.503804086	-1.151699844	0.647895758	8
DEFB103A	0.460773954	-1.147319648	0.686545694	8
DEFB103B	0.019867769	-1.009785851	0.989918081	8
DEFB104A	0.141345728	-1.063152606	0.921806878	8
DEFB104B	-0.091486974	-0.951112872	1.042599846	8
DEFB116	-0.090840846	-0.951480252	1.042321098	8
DEFB121	0.458692258	-1.147061259	0.688369	8
DEFB123	-0.044814631	-0.976839269	1.0216539	8
DEFB134	-0.094433571	-0.949433467	1.043867038	8

DEFB136	0.34926985	-1.127791748	0.778521898	8
DENND11	0.02911631	-1.014240195	0.985123884	8
DENND6A	0.520367514	-1.152883728	0.632516214	8
DEPDC7	0.621825206	-1.153527616	0.53170241	8
DERL1	0.471642434	-1.148599975	0.676957541	8
DERPC	0.372519177	-1.132791203	0.760272026	8
DESI2	0.381560859	-1.134606941	0.753046082	8
DEXI	0.175868554	-1.076267565	0.900399011	8
DGCR6L	-0.075876559	-0.959900415	1.035776974	8
DHCR24	0.560988744	-1.154547415	0.593558671	8
DHRS3	0.479969434	-1.149502175	0.669532741	8
DHX34	0.347355273	-1.127359049	0.780003776	8
DHX36	0.475844684	-1.149063866	0.673219183	8
DKK3	0.274492969	-1.108580725	0.834087756	8
DLG5	0.229112353	-1.094673847	0.865561494	8
DLGAP1	0.663113851	-1.15022048	0.48710663	8
DLGAP2	0.514655091	-1.152507753	0.637852661	8
DLL3	0.061410613	-1.029290082	0.967879468	8
DLX1	0.554761705	-1.154409694	0.599647988	8
DMRTC2	0.579676215	-1.154697411	0.575021196	8
DNAAF4	0.649112203	-1.151592988	0.502480785	8
DNAH14	0.651656153	-1.151363172	0.499707019	8
DNAH2	0.268755347	-1.106914523	0.838159175	8
DNAI4	0.098562413	-1.045631591	0.947069178	8
DNAI7	0.64106967	-1.152263311	0.511193641	8
DNAJA4	0.521305713	-1.152942196	0.631636483	8
DNAJB1	0.143239623	-1.063895886	0.920656263	8
DNAJB7	-0.015322152	-0.992250882	1.007573034	8
DNAJC18	0.04757645	-1.022939045	0.975362595	8
DNAL1	0.524745386	-1.153148596	0.62840321	8
DNASE1	0.360491421	-1.130263843	0.769772421	8
DNM1L	0.630944834	-1.152987469	0.522042635	8
DNM2	0.49900654	-1.151303888	0.652297348	8
DNPH1	0.416613578	-1.140950797	0.724337219	8
DOCK4	0.373218277	-1.132934182	0.759715905	8
DOK2	0.528422157	-1.15335534	0.624933183	8
DOK3	0.272617178	-1.108038956	0.835421778	8
DOK7	0.384525455	-1.135186442	0.750660986	8
DPEP2	0.319605818	-1.120734275	0.801128457	8
DPP10	0.201963497	-1.085566971	0.883603473	8
DPP7	0.27759941	-1.109471599	0.831872189	8
DPYSL5	0.33977521	-1.12561487	0.78583966	8
DRD5	0.511458107	-1.152282428	0.640824321	8
DRG2	0.268839349	-1.10693911	0.838099762	8
DSG4	0.154230785	-1.068155073	0.913924288	8
DSTN	-0.039794305	-0.979508826	1.019303131	8
DUOX2	-0.073015932	-0.961490784	1.034506716	8
DUOXA1	0.204408687	-1.086411037	0.882002351	8
DUSP1	0.552554616	-1.15435051	0.601795894	8
DUSP10	0.387545758	-1.135768746	0.748222989	8
DUSP2	0.454442689	-1.146520742	0.692078053	8
DUSP21	0.456414298	-1.146773691	0.690359393	8
DUSP23	0.60140491	-1.154361697	0.552956786	8
DUSP4	0.63178981	-1.152932077	0.521142267	8
DYDC2	-0.02841676	-0.985488757	1.013905517	8

DYNLT1	0.642184787	-1.152175437	0.50999065	8
DYNLT4	0.643290672	-1.152086685	0.508796013	8
DYRK3	0.424217201	-1.142178396	0.717961195	8
E2F2	0.640073262	-1.15234046	0.512267198	8
E2F6	0.617952443	-1.153725346	0.535772903	8
EDC4	0.129951304	-1.058622719	0.928671415	8
EDDM13	0.051925825	-1.024951291	0.973025467	8
EDDM3A	0.492850234	-1.150761329	0.657911096	8
EDDM3B	0.309736652	-1.118220497	0.808483845	8
EDIL3	0.497726945	-1.151194297	0.653467351	8
EDRF1	0.284796604	-1.111505195	0.826708591	8
EEF1AKMT3	0.612009215	-1.153992544	0.541983329	8
EEF1D	0.553608412	-1.154379442	0.60077103	8
EEF2	0.047560703	-1.022931734	0.975371031	8
EFCAB6	0.511807131	-1.152307546	0.640500415	8
EFCAB8	0.66982777	-1.149467793	0.479640022	8
EGR3	0.309092423	-1.118053557	0.808961135	8
EI24	0.427222639	-1.142648785	0.715426146	8
EID1	0.355274886	-1.129128282	0.773853396	8
EIF2AK4	0.603722096	-1.154292701	0.550570605	8
EIF2B2	0.372751574	-1.132838781	0.760087206	8
EIF2S2	0.087403645	-1.040832933	0.953429288	8
EIF3L	0.04168289	-1.020189684	0.978506794	8
ELAC2	0.249694661	-1.101187144	0.851492483	8
ELF1	0.252740732	-1.102122175	0.849381442	8
ELF4	0.496026341	-1.151046061	0.65501972	8
ELMO3	0.587230074	-1.154643858	0.567413784	8
ELOA	0.672149923	-1.149192948	0.477043025	8
ELOB	0.092209109	-1.04291101	0.950701901	8
ELOVL4	0.582128538	-1.15468732	0.572558781	8
ELOVL6	0.267276189	-1.10648062	0.839204431	8
EMC7	0.271446216	-1.107699298	0.836253082	8
EMCN	0.289137441	-1.112711094	0.823573653	8
EML6	0.586362824	-1.154653396	0.568290571	8
EMP1	0.368873529	-1.132038601	0.763165072	8
ENO2	0.017795815	-1.008779141	0.990983327	8
ENOX1	0.389642008	-1.136168086	0.746526078	8
ENPP3	0.587245005	-1.154643686	0.56739868	8
EP300	0.587219014	-1.154643985	0.56742497	8
EPB41L4A	0.228121298	-1.09435168	0.866230382	8
EPC1	0.169080944	-1.073761744	0.904680801	8
EPHB4	0.11167696	-1.051150588	0.939473628	8
EPM2AIP1	0.083664134	-1.03920373	0.955539596	8
EPN1	-0.076664368	-0.959461348	1.036125715	8
EPN2	0.320520709	-1.120963147	0.800442438	8
EPPIN	0.298841492	-1.115350517	0.816509025	8
EPS8L1	0.371670048	-1.132616959	0.76094691	8
ERC1	0.546689378	-1.154167056	0.607477679	8
ERFL	-0.023921451	-0.987824663	1.011746114	8
ERI3	0.627827534	-1.15318395	0.525356416	8
ESPL1	0.086997478	-1.040656489	0.953659011	8
ETF1	0.456278057	-1.146756332	0.690478275	8
EVA1C	0.116985518	-1.053347417	0.9363619	8
EVC	-0.054277994	-0.971755604	1.026033598	8
EXO5	0.56356444	-1.154591673	0.591027233	8

EXOC7	0.029449368	-1.014399407	0.984950039	8
EZH2	0.329344292	-1.123134066	0.793789774	8
EZHIP	-0.093666996	-0.949871007	1.043538003	8
FAAH	0.595464455	-1.154509072	0.559044617	8
FABP2	0.385846974	-1.135442232	0.749595258	8
FAIM2	-0.026712895	-0.986375925	1.01308882	8
FAM133B	0.555744919	-1.154434316	0.598689397	8
FAM13C	0.198284563	-1.084288171	0.886003608	8
FAM161A	0.640503801	-1.152307284	0.511803483	8
FAM162B	-0.077290985	-0.959111781	1.036402767	8
FAM168B	0.356680287	-1.129436553	0.772756266	8
FAM170B	0.572794492	-1.154688587	0.581894094	8
FAM174B	0.461509061	-1.147409886	0.685900825	8
FAM177B	0.138082877	-1.061865612	0.923782735	8
FAM187A	0.404765231	-1.138931509	0.734166279	8
FAM189A1	-0.024941385	-0.987296003	1.012237388	8
FAM189B	0.065157616	-1.030985471	0.965827855	8
FAM193B	0.552782684	-1.154356876	0.601574192	8
FAM200A	0.01846982	-1.009106976	0.990637157	8
FAM219A	0.454565628	-1.146536624	0.691970996	8
FAM227A	0.347309948	-1.127348767	0.780038818	8
FAM234A	0.19663362	-1.08371085	0.88707723	8
FAM240A	0.151922518	-1.067268306	0.915345788	8
FAM71A	0.383908334	-1.135066458	0.751158124	8
FAM71E2	0.248056485	-1.100681193	0.852624708	8
FAM81B	0.04357143	-1.021073535	0.977502105	8
FAM86B1	0.311240502	-1.118608835	0.807368333	8
FAM86B2	0.420500071	-1.141584974	0.721084903	8
FAM91A1	-0.095156777	-0.949020273	1.044177049	8
FAM98B	0.385087789	-1.135295477	0.750207688	8
FAR1	0.180632509	-1.078004932	0.897372422	8
FBLN2	0.090086701	-1.04199535	0.951908649	8
FBXL12	0.254993698	-1.102808933	0.847815234	8
FBXL7	0.512931956	-1.15238763	0.639455674	8
FBXO25	0.64600771	-1.151861822	0.505854112	8
FBXO39	0.176293246	-1.076423163	0.900129916	8
FBXO47	0.470808536	-1.148505846	0.67769731	8
FBXO48	0.608054223	-1.154146248	0.546092025	8
FBXW5	0.356506485	-1.129398523	0.772892038	8
FCAMR	0.06246714	-1.029769194	0.967302054	8
FCAR	0.612168853	-1.153985937	0.541817084	8
FCGR3A	0.663851643	-1.150140798	0.486289154	8
FCGR3B	0.464780816	-1.14780512	0.683024304	8
FCRL1	-0.099702173	-0.946414243	1.046116416	8
FCRL2	0.274647529	-1.108625237	0.833977708	8
FCRLB	-0.0508293	-0.973616024	1.024445323	8
FEM1A	0.356871935	-1.129478457	0.772606522	8
FHAD1	-0.053773588	-0.972028268	1.025801856	8
FHIP1B	0.518537377	-1.152767005	0.634229628	8
FHL3	0.545216829	-1.154115055	0.608898226	8
FILIP1	-0.025213032	-0.987155069	1.012368101	8
FKBP8	0.665912668	-1.149914251	0.484001583	8
FLACC1	0.243562588	-1.099282163	0.855719575	8
FLNB	-0.123020667	-0.932798189	1.055818855	8
FLVCR1	-0.069451768	-0.963463647	1.032915414	8

FMOD	0.538530129	-1.153849133	0.615319005	8
FMR1NB	0.153366977	-1.067823705	0.914456727	8
FN1	0.447333302	-1.14557754	0.698244238	8
FNIP1	0.15486683	-1.068398701	0.913531871	8
FOSB	0.458252419	-1.147006126	0.688753707	8
FOSL2	0.128942756	-1.058216981	0.929274225	8
FOXJ1	0.562957579	-1.154581918	0.591624339	8
FOXJ2	0.375985213	-1.133495827	0.757510614	8
FOXO3	0.148527828	-1.065956716	0.917428888	8
FOXO3B	0.003580091	-1.001785239	0.998205148	8
FOXO6	0.56291918	-1.154581287	0.591662107	8
FOXP3	-0.088701027	-0.952694669	1.041395696	8
FOXP4	0.461492011	-1.147407799	0.685915787	8
FOXRED1	0.527788432	-1.153320733	0.625532301	8
FOXRED2	0.414647676	-1.140624689	0.725977013	8
FPR2	0.346333255	-1.127126772	0.780793517	8
FRAS1	0.111845354	-1.051220604	0.939375249	8
FRMD5	0.220778008	-1.091940227	0.871162219	8
FSCB	0.108241806	-1.0497176	0.941475794	8
FSIP1	0.23874467	-1.097764249	0.859019579	8
FURIN	0.181673734	-1.078382304	0.89670857	8
FUT3	0.069160743	-1.032785057	0.963624314	8
FUT5	0.042340329	-1.020497675	0.978157345	8
FXR2	0.220646371	-1.091896608	0.871250237	8
FXYD1	0.06276072	-1.029902177	0.967141457	8
FYTTD1	0.311623498	-1.118707432	0.807083934	8
G2E3	-0.007845413	-0.996054212	1.003899625	8
G6PC2	0.216164348	-1.090403268	0.874238919	8
G6PC3	0.074848721	-1.035321275	0.960472554	8
GAB4	0.45540575	-1.146644768	0.691239018	8
GABARAPL1	0.479243008	-1.149426211	0.670183203	8
GABPB2	0.501566845	-1.151518137	0.649951291	8
GABRG1	0.165824559	-1.07254689	0.906722331	8
GABRR3	0.205873009	-1.086914262	0.881041254	8
GAD2	0.006770135	-1.003367879	0.996597744	8
GAGE1	0.551658293	-1.154324932	0.602666639	8
GAGE13	-0.071452186	-0.96235754	1.033809726	8
GAGE2A	-0.122531985	-0.933087785	1.05561977	8
GAGE2E	0.068810728	-1.032628191	0.963817463	8
GAL3ST2	0.175065876	-1.0759731	0.900907224	8
GALNT18	0.499457814	-1.151342138	0.651884325	8
GALNT3	0.18213065	-1.078547639	0.896416989	8
GALNT8	0.040105585	-1.019449439	0.979343854	8
GAP43	0.125195115	-1.0567025	0.931507385	8
GAS2L1	0.613814671	-1.153915991	0.540101319	8
GAS7	0.35142804	-1.128275697	0.776847658	8
GAST	0.186414825	-1.080089946	0.893675121	8
GATA4	0.151314552	-1.067034061	0.915719509	8
GC	-0.115850002	-0.937029311	1.052879313	8
GCNA	-0.080444159	-0.957348245	1.037792404	8
GCSAM	0.004963122	-1.002472324	0.997509202	8
GCSH	0.127871457	-1.057785147	0.929913691	8
GDA	0.585332584	-1.15466358	0.569330996	8
GDAP1L1	0.548337477	-1.154222435	0.605884958	8
GDF15	0.165536598	-1.072439065	0.906902468	8

GDNF	0.408361226	-1.139557987	0.731196761	8
GDPD5	0.271171435	-1.107619431	0.836447996	8
GEM	0.039105273	-1.018979013	0.979873741	8
GEMIN2	0.480052359	-1.149510814	0.669458454	8
GEMIN8	0.167571654	-1.073199697	0.905628043	8
GFOD1	0.406941656	-1.139312089	0.732370434	8
GFPT1	0.635597307	-1.152671127	0.517073821	8
GGH	0.019604842	-1.009658279	0.990053438	8
GGNBP2	0.241971823	-1.098783042	0.856811219	8
GHR	0.58966525	-1.154612347	0.564947097	8
GIT1	0.630854881	-1.152993312	0.522138431	8
GK5	0.097595227	-1.045219401	0.947624174	8
GLI1	0.511941513	-1.152317183	0.640375669	8
GLIPR1L2	0.093037125	-1.043267312	0.950230187	8
GLOD5	0.516479315	-1.152631539	0.636152224	8
GLRA3	0.65518175	-1.151030403	0.495848653	8
GLS	0.453875631	-1.146447295	0.692571665	8
GMCL1	-0.038728382	-0.980073193	1.018801575	8
GNB4	0.145007296	-1.064587144	0.919579848	8
GNG11	0.296991985	-1.114853494	0.817861509	8
GOLGA1	0.615281801	-1.153850821	0.538569019	8
GOLGA6L10	0.364074769	-1.131030099	0.76695533	8
GOLGA6L4	0.527103458	-1.153282845	0.626179387	8
GOLGA6L9	0.418987753	-1.141339865	0.722352112	8
GORASP2	0.545855096	-1.154137886	0.60828279	8
GOSR2	0.122596404	-1.055646024	0.93304962	8
GOT1	0.02625688	-1.012869873	0.986612993	8
GP6	0.583675644	-1.154677353	0.571001709	8
GPALPP1	0.538123432	-1.153831392	0.615707959	8
GPAM	0.409913617	-1.139824778	0.72991116	8
GPAT2	0.171324101	-1.074593817	0.903269716	8
GPATCH3	-0.047608487	-0.975345432	1.022953919	8
GPATCH8	0.655137624	-1.151034671	0.495897047	8
GPHA2	0.292577232	-1.113655621	0.821078389	8
GPLD1	-0.076950222	-0.959301917	1.036252139	8
GPR12	-0.098861276	-0.946897539	1.045758815	8
GPR132	0.53394449	-1.153638733	0.619694243	8
GPR135	0.556102251	-1.154442998	0.598340748	8
GPR157	0.449885716	-1.145921757	0.696036041	8
GPR183	0.343580148	-1.126496579	0.782916431	8
GPR35	0.232582227	-1.095795647	0.86321342	8
GPR6	0.315983173	-1.119821081	0.803837907	8
GPR84	0.557078717	-1.154465998	0.597387281	8
GPR88	0.423967776	-1.142138979	0.718171203	8
GPRC5C	0.010678942	-1.005296705	0.994617763	8
GPRIN2	0.646199391	-1.151845592	0.505646201	8
GPRIN3	-0.026193713	-0.986645819	1.012839532	8
GPX6	-0.0162615	-0.991770082	1.008031581	8
GREM1	0.108946616	-1.050012346	0.94106573	8
GRHL1	0.007000127	-1.003481688	0.996481561	8
GRIA4	0.215683684	-1.090242173	0.87455849	8
GRIK3	0.051060978	-1.024552302	0.973491324	8
GRIK5	0.523764618	-1.153091021	0.629326403	8
GRM3	0.204153307	-1.086323102	0.882169794	8
GRM4	-0.056895568	-0.970337564	1.027233132	8

GRM5	0.273765041	-1.108370825	0.834605785	8
GRM7	0.534307672	-1.153656224	0.619348552	8
GRSF1	0.040239934	-1.019512563	0.979272629	8
GSDME	0.615918367	-1.153821716	0.537903349	8
GSG1	0.381375138	-1.134570376	0.753195238	8
GTF2H3	0.052716242	-1.025315452	0.972599209	8
GTPBP10	0.10550252	-1.048568469	0.943065949	8
GTPBP3	0.133489813	-1.060040106	0.926550292	8
GUCA1ANB	0.349408794	-1.127823026	0.778414232	8
GUSB	0.404163972	-1.138825608	0.734661636	8
H1-5	0.105227069	-1.048452602	0.943225533	8
H2AC8	0.176166554	-1.07637676	0.900210206	8
H2AL3	0.591381985	-1.154585928	0.563203944	8
H2BU1	0.152774065	-1.067595922	0.914821858	8
H3C13	-0.120563701	-0.93425236	1.054816061	8
H3C3	0.576666719	-1.154700269	0.578033549	8
H3Y2	0.05419899	-1.025997314	0.971798324	8
H4C15	-0.105600763	-0.943009018	1.048609781	8
H4C4	-0.052208251	-0.972873214	1.025081464	8
HABP4	0.129737454	-1.058536753	0.928799298	8
HACD1	0.168003874	-1.073360832	0.905356958	8
HACD3	0.173522173	-1.075405381	0.901883209	8
HAS1	0.143357749	-1.063942155	0.920584405	8
HCAR2	0.407441112	-1.139398815	0.731957704	8
HCAR3	0.59266518	-1.154563904	0.561898724	8
HCFC1	0.302634811	-1.116360994	0.813726183	8
HCFC2	0.629997187	-1.153048507	0.523051321	8
HDAC10	0.323235171	-1.121638037	0.798402867	8
HDAC2	0.444942566	-1.145249471	0.700306905	8
HDAC6	0.64850742	-1.151646358	0.503138938	8
HEATR9	0.110733761	-1.050758022	0.940024262	8
HECW2	0.001613933	-1.00080599	0.999192056	8
HEMGN	0.481739633	-1.149685086	0.667945453	8
HEPACAM	-0.100917124	-0.945715017	1.046632142	8
HES3	0.154493162	-1.068255611	0.913762449	8
HEXB	0.019046816	-1.009387356	0.99034054	8
HEY1	0.377006092	-1.133701335	0.756695242	8
HFM1	0.483640308	-1.149877993	0.666237685	8
HGS	0.658651903	-1.150686562	0.492034659	8
HIF1A	-0.127820303	-0.929944203	1.057764506	8
HLA-DMA	0.396911043	-1.137522195	0.740611152	8
HMG20B	0.219155431	-1.091401617	0.872246185	8
HMGN5	0.594258948	-1.154533828	0.560274881	8
HMOX2	0.341787982	-1.126082837	0.784294855	8
HNF4A	0.066594801	-1.03163294	0.965038139	8
HNRNPCL1	0.611937094	-1.153995519	0.542058424	8
HNRNPCL3	0.030700637	-1.014996807	0.984296171	8
HNRNPCL4	0.584551447	-1.154670473	0.570119026	8
HNRNPU	-0.077934425	-0.958752522	1.036686947	8
HOMER1	0.52994398	-1.153436696	0.623492716	8
HOXC13	0.524341642	-1.153125018	0.628783377	8
HOXD12	0.057066227	-1.027311159	0.970244932	8
HOXD3	-0.114368366	-0.937898681	1.052267048	8
HR	0.440051415	-1.144561301	0.704509886	8
HS3ST2	0.449163678	-1.145825017	0.696661339	8

HS3ST6	0.679064432	-1.148329856	0.469265424	8
HSD17B11	0.459776661	-1.147196385	0.687419723	8
HSD17B2	0.53915099	-1.153875872	0.614724883	8
HSD3B1	0.323365023	-1.121670166	0.798305143	8
HSF1	0.663628185	-1.15016501	0.486536825	8
HSPBP1	0.292979324	-1.113765391	0.820786067	8
HTN3	0.667586045	-1.149726019	0.482139974	8
HTR2C	0.052273186	-1.025111385	0.972838199	8
HTR7	-0.129107959	-0.929175536	1.058283496	8
HYAL1	0.071733239	-1.033935133	0.962201893	8
HYDIN	0.194344583	-1.082906859	0.888562276	8
ICOSLG	0.194770655	-1.083056821	0.888286166	8
IDO1	0.649793128	-1.151532317	0.501739189	8
IDO2	0.028140681	-1.013773335	0.985632654	8
IER3	0.537331704	-1.15379634	0.616464635	8
IFI27	0.15215927	-1.067359448	0.915200178	8
IFI44L	0.337351642	-1.125046775	0.787695133	8
IFIT1B	0.138120932	-1.061880669	0.923759737	8
IFIT3	0.420923677	-1.141653249	0.720729573	8
IFIT5	-0.057668949	-0.969917606	1.027586555	8
IFITM1	0.33785647	-1.125165525	0.787309054	8
IFTAP	0.527436326	-1.153301319	0.625864993	8
IGBP1	0.048495077	-1.023365235	0.974870157	8
IGFALS	0.651276916	-1.151397977	0.500121061	8
IGFBP4	0.407691205	-1.139442156	0.731750951	8
IGFBPL1	0.221162722	-1.092067626	0.870904903	8
IGSF21	0.15253335	-1.067503369	0.914970019	8
IGSF22	0.112524976	-1.05150296	0.938977984	8
IK	0.346405179	-1.127143148	0.780737969	8
IKZF5	0.110129054	-1.050505984	0.940376929	8
IL10	0.462769307	-1.147563362	0.684794055	8
IL10RA	0.229770686	-1.094887421	0.865116736	8
IL12B	0.44335285	-1.145028302	0.701675451	8
IL16	0.534042952	-1.153643489	0.619600537	8
IL17RB	0.599722952	-1.154407721	0.55468477	8
IL19	0.504285329	-1.151738257	0.647452928	8
IL1RAPL1	0.21707707	-1.090708663	0.873631592	8
IL1RL1	0.585773579	-1.154659373	0.568885794	8
IL23R	0.029700613	-1.014519455	0.984818841	8
IL36RN	-0.072561673	-0.961742762	1.034304435	8
IL7	0.159358051	-1.070110123	0.910752072	8
IMMP2L	0.558229102	-1.154491728	0.596262626	8
IMPA2	0.161041274	-1.070747521	0.909706246	8
IMPACT	0.351181433	-1.128220603	0.77703917	8
INA	0.270815242	-1.107515809	0.836700566	8
INAFM2	0.22409921	-1.093036179	0.868936969	8
INHBB	-0.123228886	-0.932674741	1.055903627	8
INPP5J	0.090397787	-1.042129774	0.951731987	8
INSM2	0.185797783	-1.079868692	0.894070909	8
INTS5	0.099038999	-1.045834438	0.946795439	8
IP6K2	0.622925772	-1.153468	0.530542228	8
IPO11	0.330487745	-1.123410553	0.792922808	8
IQCA1L	0.02025136	-1.009971874	0.989720514	8
IQCJ	0.302266738	-1.116263471	0.813996733	8
IQGAP3	0.168338468	-1.073485473	0.905147005	8

IQSEC1	0.534747835	-1.153677232	0.618929396	8
IQSEC3	-0.050766167	-0.973649998	1.024416165	8
IRAG1	0.509903526	-1.152169014	0.642265488	8
IRF2BPL	0.429904022	-1.143061325	0.713157303	8
IRF9	0.23773546	-1.097443935	0.859708474	8
IST1	0.088973172	-1.041513582	0.95254041	8
ITGA5	-0.021967421	-0.98883531	1.010802731	8
ITGAV	0.578585037	-1.154699657	0.57611462	8
ITGBL1	0.487459188	-1.150254638	0.66279545	8
ITPA	0.228076123	-1.094336976	0.866260853	8
ITSN2	0.629833648	-1.153058925	0.523225277	8
IZUMO3	0.35740728	-1.129595341	0.772188061	8
IZUMO4	0.401473024	-1.138347603	0.736874579	8
JPT2	0.442935243	-1.144969802	0.702034559	8
KANSL2	0.127772438	-1.057745189	0.929972752	8
KANSL3	0.451776608	-1.146172729	0.69439612	8
KAT14	0.162534179	-1.071311018	0.908776839	8
KCNA4	-0.088607771	-0.952747516	1.041355287	8
KCNC1	0.244948613	-1.099715389	0.854766776	8
KCNC4	0.407216684	-1.139359874	0.73214319	8
KCNJ11	0.331811296	-1.123729195	0.791917899	8
KCNJ12	0.62499837	-1.153351597	0.528353226	8
KCNJ3	0.016666672	-1.008229164	0.991562492	8
KCNK16	0.123158268	-1.05587488	0.932716612	8
KCNK9	0.36195862	-1.130578946	0.768620326	8
KCTD1	0.004453326	-1.002219226	0.9977659	8
KCTD14	0.387076766	-1.135678862	0.748602096	8
KCTD2	-0.080090341	-0.957546506	1.037636847	8
KCTD3	0.114091179	-1.052152319	0.938061139	8
KCTD6	0.111175406	-1.050941922	0.939766516	8
KDELR2	0.196524136	-1.083672489	0.887148353	8
KDM2B	0.259581613	-1.104194747	0.844613134	8
KDM4A	0.591089824	-1.15459067	0.563500847	8
KDM4D	0.449406744	-1.145857639	0.696450895	8
KDM5A	0.5429101	-1.154028825	0.611118725	8
KDM6B	0.573942613	-1.154693847	0.580751235	8
KDR	0.070223284	-1.033260688	0.963037404	8
KEL	-0.127775538	-0.929970903	1.05774644	8
KHK	0.552518275	-1.15434949	0.601831215	8
KHSRP	0.359236829	-1.129992907	0.770756078	8
KIAA0232	0.303809202	-1.1166714	0.812862198	8
KIAA1109	0.596180848	-1.15449354	0.558312692	8
KIAA1755	-0.024140181	-0.987711355	1.011851536	8
KIF11	0.34472254	-1.126758869	0.78203633	8
KIF5B	-0.101946362	-0.945121797	1.047068159	8
KIR2DL3	0.194796269	-1.083065832	0.888269563	8
KIR2DL4	0.023768806	-1.011672522	0.987903716	8
KLF10	0.336516239	-1.124849786	0.788333546	8
KLF17	0.267618248	-1.106581119	0.838962871	8
KLF2	0.562432473	-1.154573142	0.592140669	8
KLF3	-0.029026519	-0.985170738	1.014197258	8
KLF4	0.222787307	-1.092604315	0.869817008	8
KLF6	0.417384307	-1.141077672	0.723693365	8
KLHDC7B	0.1357853	-1.060954463	0.925169163	8
KLHL18	-0.073634571	-0.961147375	1.034781945	8

KLHL24	0.105476175	-1.048557389	0.943081215	8
KLHL32	0.675069547	-1.148836709	0.473767162	8
KLHL38	0.511422235	-1.152279839	0.640857605	8
KLHL8	0.115338182	-1.052668	0.937329817	8
KLRC1	0.582608803	-1.154684525	0.572075721	8
KLRG2	0.035399255	-1.017229602	0.981830347	8
KPNA4	0.658464652	-1.150705531	0.492240879	8
KRT1	-0.092178662	-0.950719237	1.042897899	8
KRT15	0.074955281	-1.035368556	0.960413275	8
KRT20	-0.121548982	-0.933669767	1.05521875	8
KRT24	0.047776168	-1.023031757	0.975255588	8
KRT37	0.07642766	-1.03602098	0.959593321	8
KRT74	0.355880493	-1.129261331	0.773380838	8
KRTAP4-1	0.676345305	-1.148677292	0.472331988	8
KRTAP4-11	0.633181666	-1.152838843	0.519657177	8
KRTAP4-12	0.606594418	-1.154198149	0.547603731	8
KTI12	0.00597317	-1.002973206	0.997000035	8
L3HYPDH	0.328901162	-1.123026618	0.794125456	8
LACTB2	-0.077253516	-0.959132692	1.036386208	8
LAGE3	0.412033498	-1.140185523	0.728152024	8
LAIR2	0.479039471	-1.149404832	0.670365361	8
LAPTM5	0.208703437	-1.087882174	0.879178737	8
LBR	0.088965348	-1.041510194	0.952544846	8
LDAF1	0.197136955	-1.083887089	0.886750134	8
LDHAL6A	0.519841237	-1.152850524	0.633009287	8
LDLR	0.295092625	-1.114340115	0.81924749	8
LEAP2	0.596638154	-1.154483305	0.557845151	8
LENEP	0.217083513	-1.090710816	0.873627303	8
LGALS16	0.599471432	-1.154414312	0.55494288	8
LGI2	0.03058788	-1.014943022	0.984355141	8
LGR5	0.05341386	-1.025636467	0.972222607	8
LGSN	0.425945535	-1.142449933	0.716504398	8
LHFPL4	0.548645988	-1.154232469	0.605586481	8
LHFPL6	0.13168975	-1.059320268	0.927630518	8
LHX9	-0.064879517	-0.965980487	1.030860004	8
LIG4	0.046115413	-1.022259902	0.976144488	8
LILRA5	-0.052137918	-0.972911135	1.025049053	8
LIN28A	0.495778522	-1.151024214	0.655245692	8
LIN52	-0.019284776	-0.990218139	1.009502915	8
LIPF	0.14493104	-1.064557373	0.919626333	8
LIPK	0.041350322	-1.020033762	0.97868344	8
LLGL1	0.289815688	-1.112898108	0.823082419	8
LMO3	0.631423155	-1.152956225	0.52153307	8
LOX	0.441313352	-1.14474103	0.703427679	8
LPA	0.585683374	-1.154660252	0.568976878	8
LPAR3	0.110779466	-1.050777061	0.939997595	8
LRATD2	-0.027409959	-0.986013241	1.0134232	8
LRCH4	-0.055974674	-0.970837035	1.026811709	8
LRIT1	0.565831543	-1.154624438	0.588792895	8
LRP12	0.071425563	-1.033797844	0.962372281	8
LRP1B	0.612412056	-1.153975811	0.541563755	8
LRP5	0.232948939	-1.095913643	0.862964704	8
LRR1	0.38266336	-1.134823368	0.752160008	8
LRRC14B	0.455305715	-1.146631927	0.691326211	8
LRRC19	0.430364904	-1.143131555	0.712766651	8

LRRC20	0.597567674	-1.15446173	0.556894056	8
LRRC34	0.332705376	-1.123943597	0.791238221	8
LRRC42	0.475188161	-1.148992544	0.673804383	8
LRRC4C	0.629997121	-1.153048512	0.52305139	8
LRRC51	0.145682026	-1.064850368	0.919168342	8
LRRC59	0.279317413	-1.109960895	0.830643482	8
LRRC63	0.646818542	-1.151792836	0.504974294	8
LRRC8A	0.607357397	-1.154171346	0.54681395	8
LRRC8D	-0.122459138	-0.933130939	1.055590077	8
LRRIQ3	0.526733626	-1.153262181	0.626528554	8
LRRIQ4	0.542305596	-1.154005266	0.61169967	8
LRRN4	0.303636441	-1.116625809	0.812989369	8
LRRTM1	0.199958601	-1.084871385	0.884912784	8
LRRTM2	-0.089799598	-0.952071627	1.041871225	8
LRRTM4	0.215844137	-1.09029597	0.874451832	8
LSAMP	0.004235921	-1.002111232	0.997875311	8
LSMEM2	0.494597271	-1.150919217	0.656321946	8
LSP1	0.09621174	-1.044628562	0.948416822	8
LTBP3	0.197624523	-1.084057617	0.886433095	8
LTBR	0.665452813	-1.149965304	0.484512491	8
LY6E	0.073157096	-1.034569545	0.961412449	8
LY6G5B	0.585212332	-1.154664688	0.569452356	8
LY6G6D	0.520408964	-1.15288633	0.632477367	8
LY86	0.2988708	-1.11535837	0.81648757	8
LYRM2	-0.02792563	-0.985744702	1.013670332	8
LYZL6	0.345300665	-1.126891176	0.781590511	8
MAEL	0.576102417	-1.15469964	0.578597223	8
MAFB	-0.128504095	-0.929536171	1.058040266	8
MAFG	0.59132451	-1.154586869	0.563262359	8
MAGEA1	0.366787729	-1.13160274	0.764815011	8
MAGEB6B	0.634446967	-1.15275193	0.518304963	8
MAGED2	0.024405094	-1.011979169	0.987574075	8
MAGIX	0.129326595	-1.058371491	0.929044896	8
MAN1A2	0.444573145	-1.14519829	0.700625146	8
MANSC4	0.658781321	-1.150673424	0.491892104	8
MAP10	-0.056274157	-0.970674673	1.02694883	8
MAP1S	0.313257635	-1.119126735	0.8058691	8
MAP2K3	0.241660894	-1.098685247	0.857024352	8
MAP4K3	0.315504557	-1.119699604	0.804195046	8
MAPK12	0.27377455	-1.10837357	0.83459902	8
MAPK1IP1L	0.040049422	-1.019423047	0.979373624	8
MAPK8IP3	0.196596628	-1.08369789	0.887101262	8
MAPRE1	0.275248088	-1.108798008	0.833549919	8
MARCHF3	0.051653991	-1.024825944	0.973171953	8
MATR3	0.586096207	-1.154656151	0.568559943	8
MBD5	0.580551168	-1.154694612	0.574143444	8
MBD6	0.659510405	-1.150598987	0.491088582	8
MCCC1	0.191463534	-1.0818891	0.890425567	8
MCF2	-0.102777269	-0.944642301	1.04741957	8
MCHR2	0.138898195	-1.06218797	0.923289775	8
MCL1	0.556465301	-1.154451674	0.597986372	8
MEOX1	0.627589958	-1.153198418	0.52560846	8
MEP1A	0.171734749	-1.07474572	0.903010971	8
MEP1B	0.181220163	-1.07821802	0.896997857	8
MEPCE	0.162289948	-1.071218951	0.908929003	8

MEPE	-0.005787258	-0.997093811	1.002881069	8
METAP1D	0.155407201	-1.068605438	0.913198237	8
METTL13	0.27752552	-1.1094505	0.83192498	8
METTL15	0.161887265	-1.071067052	0.909179787	8
METTL27	0.61912419	-1.153667496	0.534543306	8
METTL2A	-0.076098908	-0.959776541	1.03587545	8
METTL7B	0.64058196	-1.152301235	0.511719275	8
MFGE8	0.379524476	-1.134204337	0.754679862	8
MFSD12	0.367844813	-1.131824113	0.7639793	8
MFSD9	0.462038429	-1.147474542	0.685436113	8
MGAT1	0.34960535	-1.127867245	0.778261895	8
MGAT3	0.368286469	-1.131916313	0.763629844	8
MGP	0.629874404	-1.153056332	0.523181928	8
MICA	0.397625096	-1.137652637	0.740027541	8
MICU2	0.626899323	-1.153240069	0.526340746	8
MICU3	-0.12930319	-0.929058883	1.058362073	8
MID1IP1	0.422918738	-1.141972564	0.719053826	8
MIDEAS	0.677703133	-1.148505102	0.470801969	8
MIDN	-0.055448415	-0.971122179	1.026570595	8
MIP	0.419896454	-1.141487397	0.721590944	8
MIS12	0.441577876	-1.144778513	0.703200637	8
MKRN2OS	-0.12297986	-0.932822378	1.055802238	8
MLF2	0.274279825	-1.10851931	0.834239485	8
MMAB	0.51193869	-1.15231698	0.64037829	8
MMADHC	0.274665897	-1.108630526	0.833964629	8
MMP17	0.613811215	-1.153916141	0.540104927	8
MMP27	0.215445912	-1.090162417	0.874716505	8
MN1	0.640384407	-1.152316508	0.511932101	8
MOCOS	0.258614211	-1.103903954	0.845289743	8
MOGAT1	0.196102011	-1.083524496	0.887422485	8
MORC1	-0.113213476	-0.938575171	1.051788647	8
MORN1	0.030405006	-1.014855769	0.984450763	8
MORN3	0.518671614	-1.152775686	0.634104072	8
MPLKIP	0.489291122	-1.15043011	0.661138988	8
MPV17L2	0.119289582	-1.054294225	0.935004643	8
MRAP	0.426351329	-1.142513282	0.716161954	8
MRGPRD	0.578659007	-1.154699549	0.576040542	8
MRM1	0.069786434	-1.033065242	0.963278807	8
MROH9	0.615048226	-1.153861374	0.538813148	8
MRPL24	0.537705271	-1.153812963	0.616107692	8
MRPL28	-0.129301448	-0.929059924	1.058361372	8
MRPL39	0.592561414	-1.154565758	0.562004343	8
MRPL40	-0.107467703	-0.941925739	1.049393442	8
MRPL45	0.527749759	-1.153318607	0.625568847	8
MRPS18A	0.654879605	-1.151059573	0.496179968	8
MSH2	0.566791881	-1.154636563	0.587844682	8
MSN	0.458734738	-1.147066574	0.688331836	8
MSTO1	0.62061163	-1.153591593	0.532979963	8
MT1G	0.653264386	-1.151213439	0.497949053	8
MT1HL1	-0.074135693	-0.960868988	1.03500468	8
MT1M	0.22484392	-1.093280721	0.868436801	8
MT1X	0.135141689	-1.060698501	0.925556813	8
MT2A	0.118877479	-1.054125177	0.935247698	8
MTA1	0.092787643	-1.043160013	0.950372369	8
MTA3	-0.011442274	-0.994229765	1.005672039	8

MTERF4	0.373499729	-1.132991622	0.759491893	8
MTHFSD	0.276188679	-1.109068006	0.832879327	8
MTMR1	0.491417255	-1.150629514	0.659212259	8
MTNR1B	0.284437553	-1.111404754	0.826967201	8
MTUS1	0.519705463	-1.15284191	0.633136447	8
MUC17	0.668101522	-1.149667256	0.481565734	8
MUTYH	0.61322839	-1.15394129	0.5407129	8
MYADM	0.445333448	-1.145303484	0.699970035	8
MYB	0.603207348	-1.154308589	0.551101241	8
MYC	-0.046454039	-0.975963411	1.02241745	8
MYG1	0.435132324	-1.143846301	0.708713977	8
MYL12B	0.627594204	-1.15319816	0.525603956	8
MYL4	0.188515236	-1.080840866	0.892325629	8
MYO9B	0.612879916	-1.153956127	0.541076211	8
MYOCD	0.428765663	-1.142887007	0.714121344	8
MYOCOS	0.655741887	-1.150976	0.495234113	8
MYOD1	0.195683322	-1.08337757	0.887694248	8
MYOZ1	0.084150819	-1.039416364	0.955265545	8
NAA10	0.163908754	-1.071828329	0.907919575	8
NAA16	0.664028824	-1.150121551	0.486092727	8
NAALADL2	0.14993333	-1.066500829	0.916567498	8
NABP1	0.030282461	-1.014797286	0.984514825	8
NACA	0.293938326	-1.114026656	0.82008833	8
NAF1	0.614774428	-1.153873659	0.539099231	8
NAGK	0.14107366	-1.063045604	0.921971944	8
NALF1	-0.02380731	-0.987883777	1.011691087	8
NAMPT	0.560039507	-1.154529223	0.594489716	8
NATD1	0.20215306	-1.085632575	0.883479515	8
NBN	-0.022777272	-0.988416794	1.011194066	8
NBPF20	-0.017292903	-0.9912414	1.008534304	8
NCBP3	0.304058722	-1.116737203	0.812678481	8
NCS1	0.488078033	-1.150314292	0.662236259	8
NDP	0.027097679	-1.013273445	0.986175766	8
NDRG4	0.483405618	-1.149854369	0.666448751	8
NDST4	-0.101534873	-0.945359063	1.046893935	8
NDUFA4L2	0.037671656	-1.018303504	0.980631848	8
NDUFAF5	0.674932116	-1.148853745	0.473921629	8
NDUFS3	0.640222322	-1.152329001	0.512106679	8
NDUFS5	-0.039261238	-0.979791172	1.01905241	8
NECAB3	0.239116703	-1.097882123	0.85876542	8
NECAP1	-0.041736563	-0.978478277	1.02021484	8
NEDD1	0.003326995	-1.001659347	0.998332352	8
NEDD8	0.263760282	-1.105442099	0.841681816	8
NEDD9	0.611432429	-1.154016155	0.542583726	8
NEFL	-0.062417658	-0.967329116	1.029746773	8
NELFB	0.414255809	-1.140559259	0.72630345	8
NELFE	0.029606498	-1.014474491	0.984867993	8
NELL1	0.67372043	-1.149002805	0.475282375	8
NEU1	0.359975353	-1.130152562	0.770177209	8
NEURL2	0.31901954	-1.120587238	0.801567698	8
NFATC1	0.496029335	-1.151046325	0.65501699	8
NFE2L2	0.253957722	-1.102493652	0.84853593	8
NFKBIZ	0.559529546	-1.154519033	0.594989487	8
NFU1	0.068569497	-1.032520025	0.963950529	8
NHP2	0.447102088	-1.145546051	0.698443962	8

NIM1K	0.454223273	-1.146492359	0.692269087	8
NINJ1	0.481062825	-1.149615522	0.668552697	8
NINJ2	0.647487024	-1.151735309	0.504248284	8
NIPAL3	0.420234416	-1.141542072	0.721307655	8
NIPSNAP3A	0.592596579	-1.154565131	0.561968551	8
NIT1	0.192575815	-1.082282794	0.889706979	8
NKD1	0.628433006	-1.153146755	0.524713749	8
NKPD1	0.250644903	-1.101479636	0.850834733	8
NKX1-2	0.662800849	-1.15025406	0.487453211	8
NKX2-4	0.439835467	-1.144530394	0.704694927	8
NKX6-2	0.0000714	-1.000035687	0.99996431	8
NLGN3	0.03282922	-1.016010369	0.983181149	8
NLRP13	0.495468643	-1.150996807	0.655528164	8
NLRP5	0.087171605	-1.040732148	0.953560543	8
NMBR	0.127075095	-1.057463571	0.930388476	8
NMRK1	0.114350896	-1.052259818	0.937908923	8
NMS	0.453920127	-1.14645307	0.692532943	8
NMUR2	-0.070884328	-0.962671837	1.033556165	8
NOC4L	0.304696674	-1.116905206	0.812208531	8
NOCT	0.4770288	-1.149191425	0.672162624	8
NOL6	0.184693727	-1.079472069	0.894778341	8
NOL7	0.606720736	-1.15419376	0.547473024	8
NOMO2	0.05108473	-1.024563267	0.973478537	8
NONO	0.472691724	-1.148717442	0.676025719	8
NOP2	0.024812207	-1.01217521	0.987363003	8
NOP58	0.612953563	-1.153953004	0.54099944	8
NOP9	0.585018835	-1.154666435	0.569647599	8
NOS2	0.651414967	-1.15138533	0.499970363	8
NOTCH1	0.51400323	-1.152462675	0.638459445	8
NOTCH2NLC	0.663096122	-1.150222386	0.487126263	8
NOX4	0.566624738	-1.154634528	0.58800979	8
NPHP1	0.309703019	-1.11821179	0.808508771	8
NPIPA7	0.371955379	-1.132675581	0.760720201	8
NPIPA8	0.60209805	-1.154341738	0.552243688	8
NPIPB13	0.670624509	-1.149374338	0.478749829	8
NR0B1	0.426512524	-1.142538404	0.716025881	8
NR3C2	0.446194485	-1.145421947	0.699227462	8
NR4A1	0.582003742	-1.154688002	0.57268426	8
NRAP	0.523607809	-1.153081721	0.629473912	8
NRBP1	0.529163696	-1.153395291	0.624231595	8
NRG2	0.481880489	-1.149699506	0.667819018	8
NRIP1	0.282389172	-1.110829706	0.828440534	8
NSG2	-0.01381271	-0.993022096	1.006834806	8
NSUN7	-0.087171205	-0.953560769	1.040731974	8
NT5C1B	-0.100712124	-0.945833077	1.046545201	8
NT5DC3	0.631643903	-1.152941707	0.521297804	8
NT5DC4	0.567910691	-1.154649371	0.58673868	8
NT5M	0.530207181	-1.153450515	0.623243334	8
NTHL1	0.158524743	-1.069793759	0.911269016	8
NTM	-0.025488706	-0.98701199	1.012500696	8
NTRK1	0.464978085	-1.147828616	0.682850531	8
NTSR1	0.365800145	-1.131395033	0.765594888	8
NUCKS1	0.474329651	-1.148898634	0.674568982	8
NUF2	0.338747296	-1.125374538	0.786627242	8
NUMBL	0.072639646	-1.034339167	0.961699521	8

NUP210	0.529418199	-1.153408868	0.623990668	8
NXPE2	-0.004373551	-0.997806051	1.002179603	8
NXPE4	0.47026779	-1.148444441	0.678176651	8
OBI1	0.312738982	-1.118993897	0.806254915	8
ODAD3	0.093773653	-1.04358381	0.949810157	8
ODF1	0.202332861	-1.085694774	0.883361914	8
OGN	0.169828083	-1.07403932	0.904211237	8
OLFML1	0.508875305	-1.152092621	0.643217316	8
ONECUT2	0.561168988	-1.154550755	0.593381767	8
OOSP2	0.399752048	-1.138038443	0.738286396	8
OPTC	0.284288123	-1.111362921	0.827074798	8
OR10A5	0.300676109	-1.115840726	0.815164617	8
OR10A7	0.336497263	-1.124845304	0.788348041	8
OR10J1	0.026898936	-1.013178099	0.986279163	8
OR10T2	0.442631632	-1.144927168	0.702295536	8
OR11H1	0.187548542	-1.08049569	0.892947148	8
OR12D3	-0.123874674	-0.93229166	1.056166334	8
OR13C8	0.219240667	-1.091429963	0.872189295	8
OR13D1	0.135663102	-1.06090589	0.925242787	8
OR13G1	0.365771393	-1.131388973	0.76561758	8
OR14A16	-0.082239298	-0.956340888	1.038580186	8
OR1B1	-0.099902814	-0.946298848	1.046201662	8
OR1N2	-0.062443448	-0.967315011	1.029758459	8
OR1S1	0.047129093	-1.022731267	0.975602175	8
OR2A14	0.603678737	-1.154294052	0.550615315	8
OR2A4	0.591865696	-1.154577855	0.562712159	8
OR2G3	0.357263377	-1.129563947	0.772300569	8
OR2G6	-0.0337963	-0.982673437	1.016469737	8
OR2H1	0.514136155	-1.152471903	0.638335748	8
OR2J1	0.547470505	-1.154193675	0.606723171	8
OR2T2	0.196255496	-1.083578323	0.887322827	8
OR2T33	0.540891051	-1.153948583	0.613057532	8
OR2T35	0.005168796	-1.002574379	0.997405583	8
OR2Y1	-0.052528707	-0.972700386	1.025229093	8
OR2Z1	0.061056784	-1.029129439	0.968072655	8
OR4C15	0.185238111	-1.079667753	0.894429642	8
OR4F21	0.397324869	-1.137597848	0.740272979	8
OR4F3	0.541961222	-1.153991667	0.612030445	8
OR4K13	0.561312841	-1.154553394	0.593240553	8
OR4X1	0.035717219	-1.0173801	0.981662881	8
OR51A2	-0.043997637	-0.977274996	1.021272633	8
OR51E1	0.426826337	-1.142587242	0.715760905	8
OR52B4	0.187800456	-1.08058571	0.892785255	8
OR5AK2	-0.075926808	-0.959872424	1.035799232	8
OR5B21	0.669794469	-1.14947168	0.479677211	8
OR5D3P	0.350199175	-1.128000633	0.777801458	8
OR5H1	0.024501275	-1.012025495	0.98752422	8
OR5K3	0.427279241	-1.142657563	0.715378322	8
OR5M8	0.283390903	-1.111111357	0.827720454	8
OR6C1	-0.078745884	-0.958299005	1.037044889	8
OR6C70	0.297900836	-1.115098087	0.817197251	8
OR6M1	0.009753638	-1.004841143	0.995087506	8
OR7C1	0.514393731	-1.152489732	0.638096001	8
OR7D4	0.323331244	-1.12166181	0.798330565	8
OR8B3	0.521492454	-1.152953723	0.631461269	8

OR8H1	0.657884919	-1.150763958	0.492879039	8
OR8H3	0.470452491	-1.148465448	0.678012957	8
ORAI2	0.324213806	-1.121879822	0.797666016	8
ORMDL3	0.3625261	-1.130700315	0.768174215	8
OSM	0.352103296	-1.128426286	0.77632299	8
OSMR	0.069498821	-1.032936485	0.963437664	8
OSTM1	0.334084863	-1.124273061	0.790188198	8
OTOG	0.530788749	-1.153480788	0.622692039	8
OTOL1	0.55546324	-1.154427372	0.598964132	8
OTUB1	0.486383459	-1.150150024	0.663766565	8
OTUD1	0.177247525	-1.076772277	0.899524752	8
OTUD4	0.265670682	-1.106007636	0.840336954	8
OVCH1	0.621492603	-1.153545333	0.532052731	8
OVGP1	-0.043955841	-0.977297273	1.021253114	8
OXCT1	0.675571713	-1.148774232	0.47320252	8
OXER1	0.201321257	-1.085344493	0.884023236	8
OXGR1	0.660658699	-1.150480288	0.489821589	8
OXTR	0.633986806	-1.152783776	0.51879697	8
PACS1	0.268283232	-1.106776224	0.838492992	8
PAGE2	-0.01410218	-0.992874331	1.00697651	8
PAK6	0.621277444	-1.153556721	0.532279277	8
PALB2	0.177825432	-1.076983355	0.899157923	8
PAOX	0.277684248	-1.109495818	0.83181157	8
PAPPA2	0.677353625	-1.148549673	0.471196048	8
PAQR3	0.426040795	-1.142464818	0.716424023	8
PAQR6	0.041383867	-1.020049493	0.978665626	8
PARP2	-0.017572393	-0.991098001	1.008670394	8
PAX8	0.45914357	-1.147117635	0.687974065	8
PAX9	0.675786378	-1.148747417	0.472961038	8
PAXIP1	-0.120470512	-0.934307424	1.054777936	8
PBX2	0.302011141	-1.116195683	0.814184542	8
PCBD1	-0.081415129	-0.95680368	1.038218809	8
PCDH19	0.573753047	-1.154693083	0.580940036	8
PCDH7	0.677462795	-1.14853577	0.471072975	8
PCDHB10	0.398753457	-1.137857821	0.739104364	8
PCDHB13	0.120699309	-1.054871528	0.934172219	8
PCDHB14	0.313698692	-1.119239521	0.805540828	8
PCDHB6	0.457484658	-1.146909438	0.68942478	8
PCSK2	0.537301198	-1.153794975	0.616493777	8
PCSK6	0.180490659	-1.077953455	0.897462796	8
PDCD10	0.59355166	-1.154547548	0.560995888	8
PDCL2	0.015325972	-1.0075749	0.992248928	8
PDCL3	-0.100232499	-0.946109169	1.046341668	8
PDE1C	0.48443595	-1.149957672	0.665521722	8
PDE5A	0.333622427	-1.124162798	0.790540371	8
PDGFA	0.357287047	-1.129569112	0.772282065	8
PDGFRL	0.433568319	-1.143614179	0.71004586	8
PDHA1	0.041038891	-1.019887674	0.978848784	8
PDIA2	0.274174445	-1.108488932	0.834314486	8
PDIA5	0.506196764	-1.151888473	0.645691709	8
PDLIM7	0.603382006	-1.154303234	0.550921228	8
PDX1	0.65550878	-1.150998691	0.495489911	8
PDZRN3	-0.004660928	-0.99766139	1.002322317	8
PELI1	0.514704629	-1.15251116	0.637806531	8
PER1	0.268137041	-1.106733363	0.838596322	8

PERP	0.455759525	-1.146690103	0.690930577	8
PEX13	0.355074606	-1.129084211	0.774009605	8
PFDN1	0.045434653	-1.021942911	0.976508258	8
PGLS	0.434462536	-1.143747176	0.709284641	8
PGM3	0.465281197	-1.147864645	0.682583448	8
PGM5	0.560048928	-1.154529409	0.594480481	8
PGS1	0.524624208	-1.153141538	0.62851733	8
PHAF1	0.596072061	-1.154495938	0.558423877	8
PHF12	0.436346776	-1.144024952	0.707678176	8
PHF24	0.567088865	-1.154640101	0.587551237	8
PHKA2	0.28808285	-1.112419555	0.824336706	8
PHLDA1	0.550032776	-1.154276281	0.604243505	8
PHTF2	0.344975986	-1.126816907	0.781840922	8
PHYHD1	0.64042776	-1.152313161	0.5118854	8
PI16	0.375798221	-1.133458084	0.757659864	8
PIGS	0.43572508	-1.143933672	0.708208592	8
PIGT	0.244948281	-1.099715285	0.854767004	8
PIK3C2G	-0.023687111	-0.987946018	1.011633128	8
PIK3CB	0.490847338	-1.150576511	0.659729174	8
PIK3CD	0.300602833	-1.1158212	0.815218367	8
PIK3R4	0.263273693	-1.105297579	0.842023886	8
PIN4	0.417859104	-1.141155558	0.723296454	8
PIP5K1C	0.377923136	-1.133885151	0.755962015	8
PITPNA	-0.013262129	-0.993302977	1.006565106	8
PKD1L3	0.395221014	-1.137211626	0.741990612	8
PKP4	-0.072383677	-0.961841454	1.034225131	8
PLA1A	0.289312237	-1.112759327	0.82344709	8
PLA2G2C	0.531513212	-1.153517992	0.62200478	8
PLA2G4D	0.662073519	-1.150331573	0.488258055	8
PLA2G5	0.146097469	-1.065012266	0.918914796	8
PLAAT3	0.567105241	-1.154640294	0.587535052	8
PLAU	0.459649327	-1.147180577	0.68753125	8
PLAUR	0.292124457	-1.113531854	0.821407397	8
PLBD2	0.427317814	-1.142663543	0.715345729	8
PLCE1	0.547764614	-1.154203524	0.60643891	8
PLCH1	0.312168182	-1.118847442	0.80667926	8
PLEKHA8	0.267077723	-1.106422265	0.839344543	8
PLEKHG2	0.070560827	-1.033411606	0.962850779	8
PLEKHM2	0.389778951	-1.136194037	0.746415086	8
PLEKHO2	0.474205394	-1.148884981	0.674679587	8
PLGLB1	0.382771339	-1.134844506	0.752073167	8
PLK3	0.312305331	-1.118882656	0.806577325	8
PLPP5	0.260262927	-1.104399089	0.844136162	8
PLPPR1	-0.111422394	-0.939622309	1.051044703	8
PLSCR2	-0.118750905	-0.935322324	1.054073229	8
PMEL	0.155517554	-1.068647629	0.913130076	8
PMP22	-0.072213636	-0.961935713	1.034149349	8
PMPCA	0.383735861	-1.135032864	0.751297003	8
PNKD	0.669791474	-1.149472029	0.479680556	8
PNP	0.137508819	-1.061638337	0.924129518	8
PNPLA2	0.174821303	-1.075883278	0.901061976	8
POFUT2	0.321461578	-1.12119778	0.799736202	8
POGK	-0.090829185	-0.951486879	1.042316064	8
POGLUT2	0.286088041	-1.111865585	0.825777545	8
POLR1F	-0.004625315	-0.99767932	1.002304635	8

POLR2A	0.41930424	-1.141391336	0.722087095	8
POLR2K	-0.083727076	-0.955504164	1.03923124	8
POMGNT2	0.198075369	-1.084215136	0.886139767	8
POP7	0.606034317	-1.154217373	0.548183056	8
POU5F1	0.60859009	-1.154126544	0.545536454	8
POU6F2	-0.104757455	-0.943497473	1.048254928	8
PPAN	-0.010038868	-0.994942773	1.004981641	8
PPARD	0.573044807	-1.154689863	0.581645056	8
PPEF2	-0.025025406	-0.987252418	1.012277824	8
PPIAL4C	0.651519272	-1.151375757	0.499856485	8
PPIF	0.384818563	-1.13524331	0.750424747	8
PPIH	0.587544283	-1.154640184	0.567095901	8
PPP1CB	-0.053025007	-0.972432571	1.025457578	8
PPP1R12B	0.123546476	-1.056032863	0.932486387	8
PPP1R15A	0.199396197	-1.084675696	0.885279499	8
PPP1R15B	0.61647281	-1.153795957	0.537323147	8
PPP4C	-0.050781839	-0.973641564	1.024423403	8
PRAMEF17	0.602359815	-1.154334049	0.551974235	8
PRB4	0.443594434	-1.145062067	0.701467633	8
PRG2	0.593238699	-1.154553429	0.56131473	8
PRG4	0.204527768	-1.086452024	0.881924256	8
PRKAB1	0.409110058	-1.139686956	0.730576898	8
PRKRIP1	-0.049030695	-0.974582742	1.023613437	8
PRLH	0.408937151	-1.139657222	0.730720071	8
PRMT5	0.261884198	-1.104883832	0.842999634	8
PRMT7	0.440760561	-1.144662486	0.703901925	8
PRMT9	0.344730018	-1.126760583	0.782030565	8
PROC	-0.013863399	-0.992996225	1.006859624	8
PROP1	0.48227632	-1.149739923	0.667463603	8
PRPF18	0.007486051	-1.00372201	0.996235959	8
PRPF40A	0.501732582	-1.151531774	0.649799192	8
PRPS1	0.055802497	-1.026732847	0.970930349	8
PRR23A	0.110560401	-1.050685795	0.940125395	8
PRR27	0.474812307	-1.14895152	0.674139213	8
PRR5L	0.496088657	-1.151051545	0.654962888	8
PRR9	0.577760985	-1.154700441	0.576939456	8
PRSS12	0.548689048	-1.154233862	0.605544814	8
PRSS2	0.423708538	-1.14209795	0.718389412	8
PRSS23	0.604595801	-1.154265	0.549669199	8
PRSS37	0.281713258	-1.110639197	0.82892594	8
PRSS54	0.545135753	-1.154112123	0.60897637	8
PRTFDC1	0.41442917	-1.140588223	0.726159052	8
PRTG	0.320366214	-1.120924548	0.800558334	8
PRXL2C	0.049475006	-1.023819165	0.974344159	8
PRY	0.477357968	-1.149226637	0.671868668	8
PRYP3	0.591333745	-1.154586718	0.563252973	8
PSG4	0.455367862	-1.146639905	0.691272043	8
PSMA1	0.469915057	-1.148404232	0.678489175	8
PSMC1	-0.006011546	-0.996980675	1.002992221	8
PSMD14	0.487210646	-1.150230571	0.663019925	8
PSMD2	0.506083812	-1.151879701	0.645795889	8
PSMD7	0.003468089	-1.001729534	0.998261445	8
PSMD8	-0.00604567	-0.996963459	1.003009129	8
PSMF1	0.37285552	-1.132860045	0.760004525	8
PSMG2	-0.077096703	-0.959220196	1.036316899	8

PSPH	-0.03312221	-0.983027405	1.016149615	8
PTAR1	0.142253404	-1.063509177	0.921255773	8
PTBP1	0.336075601	-1.124745641	0.78867004	8
PTBP3	0.031097422	-1.015186002	0.98408858	8
PTCH2	0.203209563	-1.085997694	0.88278813	8
PTGDR	-0.105638907	-0.942986911	1.048625818	8
PTGER2	0.544898408	-1.154103498	0.609205089	8
PTGES2	0.411866096	-1.140157186	0.72829109	8
PTK6	0.210300672	-1.088425588	0.878124916	8
PTPMT1	0.462366662	-1.147514495	0.685147834	8
PTPN12	0.124386756	-1.056374423	0.931987668	8
PTPN2	0.54856933	-1.154229986	0.605660656	8
PTPN5	-0.032494448	-0.983356739	1.015851187	8
PTPRK	-0.068365457	-0.964063044	1.032428501	8
PTTG1	0.61926842	-1.153660257	0.534391837	8
PURB	0.153206874	-1.067762224	0.91455535	8
PUS1	0.640183615	-1.15233198	0.512148364	8
PUS10	0.447190563	-1.145558106	0.698367543	8
PUSL1	0.278507361	-1.10973049	0.831223129	8
PWP2	0.492155246	-1.150697659	0.658542413	8
PWWP2B	0.677030473	-1.148590729	0.471560256	8
PWWP3B	0.558801801	-1.154503986	0.595702186	8
PXMP4	0.368139274	-1.131885603	0.76374633	8
PXT1	0.647123005	-1.151766708	0.504643703	8
PYCR2	0.439053911	-1.144418164	0.705364253	8
PYGM	0.046754849	-1.022557332	0.975802483	8
PZP	0.473739958	-1.148833706	0.675093748	8
QPRT	0.300865626	-1.115891205	0.815025579	8
R3HDM2	0.506037312	-1.151876086	0.645838774	8
RAB1B	0.068338754	-1.032416521	0.964077767	8
RAB23	0.152140448	-1.067352203	0.915211756	8
RAB33A	0.548159504	-1.154216599	0.606057095	8
RAB35	0.197824008	-1.084127334	0.886303326	8
RAB40A	0.12169418	-1.05527803	0.93358385	8
RAB5C	0.264063618	-1.105532094	0.841468475	8
RAD21L1	0.667675054	-1.149715898	0.482040845	8
RAET1E	0.559489202	-1.154518214	0.595029012	8
RAI14	0.155227629	-1.068536762	0.913309133	8
RANBP10	0.466018596	-1.14795192	0.681933324	8
RANBP2	0.033038372	-1.016109777	0.983071405	8
RAPGEF5	0.659657759	-1.150583856	0.490926096	8
RAPH1	0.008081246	-1.004016133	0.995934887	8
RARA	-0.002063866	-0.99896647	1.001030336	8
RARS1	0.306389398	-1.11734933	0.810959932	8
RASD1	0.004381705	-1.002183653	0.997801948	8
RASGEF1B	0.515351218	-1.152555401	0.637204182	8
RASGRF1	0.638808307	-1.152436542	0.513628235	8
RASSF10	0.184576074	-1.079429747	0.894853672	8
RASSF5	0.15043811	-1.066695873	0.916257763	8
RAX	0.278964637	-1.10986062	0.830895983	8
RBFOX1	0.317889957	-1.120303123	0.802413166	8
RBM15B	-0.121046703	-0.933966855	1.055013559	8
RBM17	0.531114492	-1.153497586	0.622383094	8
RBM46	0.4727749	-1.148726707	0.675951808	8
RBP2	0.584392897	-1.154671785	0.570278889	8

RBP7	0.034790746	-1.016941372	0.982150625	8
RCAN3	0.189973694	-1.081360253	0.891386559	8
RCE1	-0.011004012	-0.994452585	1.005456597	8
REEP4	0.351269714	-1.128240332	0.776970618	8
REL	0.610123349	-1.154068226	0.543944877	8
RERGL	0.49332058	-1.150804141	0.657483561	8
RET	0.448692768	-1.145761654	0.697068886	8
REXO4	0.554002928	-1.154389957	0.600387028	8
RFPL4B	-0.061379875	-0.967896255	1.02927613	8
RFX6	0.626576251	-1.153259346	0.526683095	8
RFX7	0.584841454	-1.154667997	0.569826543	8
RFX8	0.413107558	-1.14036672	0.727259163	8
RGCC	0.203442968	-1.086078238	0.88263527	8
RGL1	0.429939147	-1.143066685	0.713127537	8
RGPD3	0.351698279	-1.128336011	0.776637732	8
RGPD5	-0.029199446	-0.985080498	1.014279944	8
RGPD6	-0.023075001	-0.988262809	1.01133781	8
RGPD8	0.015253831	-1.007539657	0.992285826	8
RGS1	0.068666117	-1.032563354	0.963897237	8
RGS12	0.589150593	-1.154619588	0.565468996	8
RGS13	0.158899391	-1.06993606	0.911036669	8
RGS22	0.624115189	-1.15340186	0.529286671	8
RHBDF1	0.66195081	-1.150344579	0.488393769	8
RHOG	0.173215955	-1.075292545	0.902076591	8
RIC3	0.340868531	-1.125869498	0.785000967	8
RICTOR	0.604503233	-1.154267978	0.549764746	8
RINT1	0.493334126	-1.150805371	0.657471245	8
RIPOR1	0.277943487	-1.109569788	0.831626301	8
RIT2	0.130738895	-1.058939025	0.92820013	8
RLN2	0.314824232	-1.119526598	0.804702367	8
RLN3	0.225708077	-1.093563933	0.867855857	8
RNASEH2C	0.067125636	-1.031871694	0.964746058	8
RNF168	0.081994777	-1.038473023	0.956478247	8
RNF169	0.655980036	-1.150952742	0.494972706	8
RNF17	0.382406702	-1.134773081	0.752366379	8
RNF180	0.142902915	-1.063763942	0.920861027	8
RNF185	0.661748319	-1.150365997	0.488617678	8
RNF223	0.010934118	-1.005422225	0.994488107	8
RNF26	0.672553327	-1.149144437	0.47659111	8
RNF34	0.530383367	-1.153459725	0.623076358	8
RNF40	0.440356382	-1.144604874	0.704248492	8
RNF43	0.35710439	-1.129529241	0.77242485	8
RNF7	0.031175364	-1.015223152	0.984047788	8
RNFT1	0.437441116	-1.14418474	0.706743624	8
RNPEP	0.648320429	-1.151662762	0.503342333	8
RO60	0.425460356	-1.142373988	0.716913632	8
ROBO1	-0.033469854	-0.982844898	1.016314752	8
ROBO3	0.635356128	-1.152688209	0.517332081	8
ROBO4	0.367758755	-1.131806128	0.764047373	8
ROPN1B	0.537453729	-1.153801786	0.616348058	8
RPGR	0.044380278	-1.021451263	0.977070984	8
RPL37	0.516740154	-1.152648954	0.6359088	8
RPRD2	0.281646787	-1.110620442	0.828973655	8
RPS12	0.295044204	-1.114326988	0.819282785	8
RPS4Y1	-0.05444998	-0.971662591	1.026112571	8

RPS6KB2	0.332412246	-1.123873379	0.791461133	8
RPUSD1	0.020882473	-1.010277694	0.989395221	8
RRAGA	0.141342441	-1.063151314	0.921808873	8
RRBP1	0.423338991	-1.142039354	0.718700363	8
RSBN1L	0.24496577	-1.099720742	0.854754972	8
RSPH10B	0.576175014	-1.154699741	0.578524727	8
RSPH14	0.282623246	-1.110895593	0.828272348	8
RSPH4A	-0.050755331	-0.973655829	1.02441116	8
RSRC2	0.117819401	-1.053690552	0.93587115	8
RSU1	0.151495402	-1.067103771	0.915608369	8
RTL8C	0.566641646	-1.154634735	0.587993089	8
RTN4RL2	0.524411846	-1.153129131	0.628717285	8
RUNX3	-0.114841841	-0.937621045	1.052462886	8
RUSC2	0.121124058	-1.055045172	0.933921114	8
RXFP1	0.242884524	-1.099069661	0.856185137	8
RXRA	0.503436905	-1.151670375	0.648233469	8
RYK	0.13169058	-1.059320601	0.927630021	8
S1PR2	0.075268109	-1.03550731	0.960239201	8
S1PR3	0.25953312	-1.104180189	0.844647069	8
SAA1	0.275296731	-1.108811988	0.833515258	8
SAFB2	0.676024877	-1.148717548	0.47269267	8
SAMD10	-0.092840616	-0.950342184	1.0431828	8
SAMM50	0.533611102	-1.153622552	0.62001145	8
SAP130	0.485508557	-1.150064083	0.664555526	8
SAPCD2	-0.118892364	-0.935238921	1.054131285	8
SASH1	0.605209081	-1.154245002	0.549035921	8
SATL1	0.246545166	-1.100212505	0.853667338	8
SBSN	0.610995811	-1.154033756	0.543037945	8
SCIN	-0.107388723	-0.94197162	1.049360343	8
SCMH1	0.030528139	-1.014914521	0.984386382	8
SCML2	0.314745322	-1.119506506	0.804761185	8
SCN7A	0.120917518	-1.054960753	0.934043235	8
SCUBE3	0.496227864	-1.151063781	0.654835917	8
SDC4	-0.108284928	-0.941450716	1.049735644	8
SDF2	0.54558872	-1.154128412	0.608539692	8
SDHAF2	0.333154075	-1.124050939	0.790896864	8
SEC11C	0.454967011	-1.146588377	0.691621366	8
SEC16A	-0.057966525	-0.969755899	1.027722423	8
SEC24C	0.469288431	-1.148332497	0.679044066	8
SEC24D	0.604915994	-1.154254616	0.549338622	8
SEC31B	0.407058266	-1.139332358	0.732274092	8
SELENOT	0.370192998	-1.132312345	0.762119346	8
SELENOV	0.583600745	-1.1546779	0.571077155	8
SEM1	0.593744964	-1.154543857	0.560798894	8
SEMA3A	0.617663226	-1.153739362	0.536076136	8
SEMA6B	0.669859682	-1.149464067	0.479604385	8
SEMA7A	0.631727007	-1.152936225	0.521209219	8
SERBP1	-0.022360107	-0.988632438	1.010992546	8
SERP2	0.379548965	-1.134209201	0.754660236	8
SERPINA11	0.280978786	-1.110431758	0.829452972	8
SERPINB3	0.005807854	-1.002891278	0.997083424	8
SERPINB9	0.303587747	-1.116612955	0.813025208	8
SERTM2	0.211756935	-1.088919286	0.877162351	8
SET	0.609397819	-1.15409618	0.544698361	8
SETD1A	0.461084308	-1.147357809	0.686273501	8

SETD2	0.602282327	-1.154336334	0.552054007	8
SETD7	0.356843792	-1.129472305	0.772628513	8
SETD9	0.497985656	-1.151216589	0.653230933	8
SETMAR	0.050640781	-1.024358245	0.973717463	8
SF3A1	0.59865466	-1.154435188	0.555780528	8
SF3B4	0.624790492	-1.153363516	0.528573024	8
SFT2D3	-0.093250555	-0.950108514	1.043359069	8
SFTPD	0.313409412	-1.119165565	0.805756153	8
SGCZ	0.64051236	-1.152306622	0.511794262	8
SGO2	0.354576584	-1.128974472	0.774397887	8
SH2D1B	0.088256428	-1.041202987	0.952946559	8
SH2D3A	0.484576718	-1.149971703	0.665394986	8
SH3GL1	0.279671825	-1.110061532	0.830389707	8
SH3GL2	0.418106463	-1.141196053	0.72308959	8
SHISA9	0.206042653	-1.086972453	0.8809298	8
SHISAL1	0.241439891	-1.098615688	0.857175798	8
SHPK	0.620542776	-1.153595168	0.533052392	8
SIGLEC6	0.169882998	-1.074059705	0.904176707	8
SIK1	0.635208612	-1.152698621	0.517490008	8
SIKE1	0.296499942	-1.114720787	0.818220845	8
SIL1	0.263345979	-1.10531906	0.841973081	8
SIN3A	0.242416314	-1.098922711	0.856506396	8
SIN3B	0.261544878	-1.104782556	0.843237677	8
SIPA1L1	0.428766499	-1.142887136	0.714120637	8
SIRT7	0.230633298	-1.095166746	0.864533447	8
SIX1	0.089340203	-1.041672482	0.952332279	8
SKIL	0.340709002	-1.125832409	0.785123406	8
SLA2	0.617816296	-1.153731957	0.535915661	8
SLC13A2	0.497688186	-1.151190951	0.653502765	8
SLC13A4	-0.10414668	-0.943850905	1.047997585	8
SLC19A2	0.529280751	-1.153401544	0.624120793	8
SLC1A4	0.009149421	-1.004543318	0.995393897	8
SLC20A2	0.391828137	-1.13658034	0.744752202	8
SLC22A13	0.051575584	-1.024789779	0.973214195	8
SLC22A2	0.023890793	-1.011731335	0.987840542	8
SLC22A25	0.317877281	-1.120299928	0.802422647	8
SLC22A31	0.581031588	-1.154692697	0.573661109	8
SLC22A4	0.438592085	-1.144351575	0.705759489	8
SLC24A5	0.62544027	-1.153326078	0.527885808	8
SLC25A28	0.016568757	-1.008181427	0.99161267	8
SLC25A39	0.391380011	-1.136496184	0.745116173	8
SLC25A51	0.596206691	-1.154492968	0.558286277	8
SLC26A1	0.45801439	-1.146976211	0.688961821	8
SLC27A2	0.438540309	-1.144344096	0.705803787	8
SLC34A2	0.37485804	-1.133267847	0.758409808	8
SLC35C2	0.196886225	-1.083799323	0.886913097	8
SLC35D1	0.138132892	-1.061885401	0.923752509	8
SLC35F1	0.226582132	-1.093849786	0.867267654	8
SLC35F2	0.195411522	-1.083282117	0.887870595	8
SLC35F5	0.57939739	-1.154698116	0.575300726	8
SLC35G3	0.527433156	-1.153301144	0.625867988	8
SLC35G6	0.63476526	-1.152729743	0.517964483	8
SLC45A2	0.48295065	-1.149808415	0.666857765	8
SLC46A3	0.287120477	-1.112152709	0.825032232	8
SLC49A3	0.388815695	-1.136011143	0.747195449	8

SLC49A4	0.183397584	-1.079005226	0.895607642	8
SLC4A2	0.208424151	-1.087786948	0.879362797	8
SLC4A4	0.140568576	-1.062846808	0.922278232	8
SLC4A5	0.501731869	-1.151531715	0.649799847	8
SLC4A9	0.014388062	-1.007116397	0.992728335	8
SLC5A12	0.431368761	-1.143283833	0.711915072	8
SLC66A2	-0.05947124	-0.968937188	1.028408429	8
SLC6A11	0.043184056	-1.02089246	0.977708404	8
SLC6A17	0.624983201	-1.153352468	0.528369267	8
SLC6A2	0.270675222	-1.107475046	0.836799824	8
SLC7A8	0.181196336	-1.078209385	0.897013049	8
SLC9A2	0.218968109	-1.091339301	0.872371192	8
SLC9A3R1	-0.117003511	-0.936351316	1.053354827	8
SLC9A7	-0.013722738	-0.993068011	1.006790749	8
SLCO1B3	0.480669983	-1.149574935	0.668904953	8
SLCO1C1	0.070610099	-1.033433629	0.96282353	8
SLCO2B1	0.266673499	-1.106303313	0.839629814	8
SLITRK1	0.518455654	-1.152761711	0.634306057	8
SLTM	-0.025240538	-0.987140795	1.012381334	8
SMARCC2	-0.014440114	-0.992701746	1.00714186	8
SMG5	0.425153339	-1.142325818	0.717172479	8
SMG6	0.11567467	-1.052806947	0.937132277	8
SMIM14	-0.123371382	-0.93259024	1.055961622	8
SMIM19	0.12694202	-1.057409787	0.930467767	8
SMIM20	0.043773564	-1.021167977	0.977394413	8
SMIM26	-0.054490915	-0.97164045	1.026131364	8
SMIM28	0.068628485	-1.032546479	0.963917994	8
SMIM3	0.103256297	-1.047621925	0.944365628	8
SMKR1	-0.005301952	-0.997338482	1.002640435	8
SMOX	0.589322257	-1.154617208	0.565294951	8
SMPD4	0.60611558	-1.154214607	0.548099028	8
SMPDL3B	0.17309834	-1.075249187	0.902150847	8
SMYD2	0.20035491	-1.085009132	0.884654222	8
SMYD3	0.158207881	-1.069673322	0.911465441	8
SNAP23	0.457637162	-1.146928689	0.689291527	8
SNCAIP	0.41474293	-1.140640572	0.725897642	8
SNPH	0.065760037	-1.031257058	0.965497021	8
SNRPA1	0.611118114	-1.154028849	0.542910735	8
SNRPD2	-0.037822286	-0.980552266	1.018374552	8
SOCS4	0.39580163	-1.137318615	0.741516984	8
SOHLH2	0.607022297	-1.154183205	0.547160908	8
SORCS2	0.632049235	-1.152914887	0.520865652	8
SORCS3	0.17426346	-1.075678231	0.901414771	8
SOX2	0.418330052	-1.141232607	0.722902555	8
SP2	0.543133273	-1.154037421	0.610904148	8
SPA17	0.63588124	-1.152650921	0.516769681	8
SPAG1	0.434409977	-1.14373938	0.709329403	8
SPAG16	0.641426962	-1.152235332	0.510808371	8
SPARCL1	0.388107513	-1.135876149	0.747768636	8
SPATA19	0.507447056	-1.151984689	0.644537634	8
SPATA22	0.298968577	-1.115384564	0.816415987	8
SPATA25	0.281672284	-1.110627637	0.828955353	8
SPATA31A7	-0.054862913	-0.971439179	1.026302091	8
SPATA31D3	0.334424025	-1.124353814	0.78992979	8
SPATA5L1	0.531424316	-1.153513457	0.622089142	8

SPDYA	0.337608813	-1.125107296	0.787498483	8
SPDYE1	0.523235742	-1.153059551	0.629823809	8
SPDYE2	0.402236619	-1.138483914	0.736247295	8
SPDYE2B	0.336628136	-1.124876206	0.78824807	8
SPDYE5	0.265212176	-1.105872175	0.840659999	8
SPDYE6	0.633098668	-1.152844472	0.519745804	8
SPEF2	0.148017303	-1.0657587	0.917741397	8
SPHK1	0.336017575	-1.124731915	0.788714339	8
SPIN4	0.350179699	-1.127996264	0.777816564	8
SPINK13	0.235175035	-1.096627623	0.861452587	8
SPINK4	-0.071332765	-0.962423657	1.033756422	8
SPINT1	0.163100563	-1.071524349	0.908423786	8
SPNS1	-0.11323998	-0.938559657	1.051799637	8
SPPL2B	0.434401507	-1.143738124	0.709336617	8
SPRR2D	-0.101843278	-0.945181247	1.047024525	8
SPTLC3	0.478873042	-1.14938732	0.670514279	8
SRC	0.422107485	-1.141843167	0.719735682	8
SREK1	0.011212807	-1.005559255	0.994346448	8
SRI	-0.087002393	-0.953656232	1.040658625	8
SRL	0.300478899	-1.115788166	0.815309267	8
SRPRB	0.299666718	-1.115571364	0.815904646	8
SRPX	0.622098346	-1.153512962	0.531414616	8
SRSF6	-0.092085221	-0.950772435	1.042857655	8
SSR3	0.232454586	-1.095754551	0.863299965	8
SSTR2	0.384645396	-1.135209722	0.750564326	8
SSX1	-0.020947441	-0.989361718	1.010309159	8
SSX2IP	-0.013212372	-0.993328349	1.006540721	8
SSX5	0.653767074	-1.151165928	0.497398853	8
ST3GAL1	0.623048077	-1.153461281	0.530413204	8
ST6GAL1	0.126032151	-1.05704169	0.931009539	8
STAB1	-0.125924286	-0.931073724	1.05699801	8
STAM	0.596975804	-1.154475587	0.557499783	8
STARD13	0.285421601	-1.111679779	0.826258179	8
STARD5	0.547019378	-1.154178384	0.607159005	8
STAT4	0.668893466	-1.149576262	0.480682795	8
STATH	0.19184709	-1.08202497	0.89017788	8
STAU1	0.413908552	-1.140501159	0.726592607	8
STK24	0.362784573	-1.130755502	0.767970929	8
STK33	0.443787467	-1.145089007	0.70130154	8
STOML2	0.557921244	-1.154484987	0.596563743	8
STOX2	0.246785972	-1.100287306	0.853501334	8
STPG2	-0.121652564	-0.933608477	1.055261041	8
STRIP1	-0.042620999	-0.978008062	1.020629061	8
STX11	0.32730778	-1.122638881	0.795331101	8
STX1A	0.621869921	-1.153525223	0.531655302	8
STX4	0.195456514	-1.083297921	0.887841407	8
SUDS3	0.361309473	-1.130439764	0.769130291	8
SULT1A2	0.06268906	-1.029869723	0.967180663	8
SULT1C2	0.568657585	-1.154657129	0.585999544	8
SUN3	0.43275501	-1.14349256	0.71073755	8
SUPT20HL2	0.369708365	-1.132211979	0.762503613	8
SV2B	-0.038138804	-0.980384986	1.01852379	8
SVOPL	-0.117529213	-0.93604199	1.053571202	8
SYBU	-0.059305299	-0.96902756	1.028332859	8
SYF2	0.319446978	-1.120694467	0.801247489	8

SYN1	0.132551362	-1.059665132	0.927113771	8
SYN2	0.446950324	-1.145525354	0.69857503	8
SYNDIG1L	0.598649958	-1.154435306	0.555785347	8
SYNE4	-0.094249712	-0.949538449	1.043788161	8
SYNGAP1	0.528918113	-1.153382125	0.624464012	8
SYNPO2	-0.090196901	-0.951846076	1.042042977	8
SYT1	0.113707697	-1.051993497	0.9382858	8
SYT9	0.003172951	-1.0015827	0.998409749	8
SYVN1	0.671422742	-1.149279823	0.477857081	8
TAC4	0.179396796	-1.077555975	0.898159179	8
TAGAP	0.477013066	-1.149189739	0.672176673	8
TAGLN	0.247464066	-1.100497692	0.853033625	8
TAPBP	0.561426497	-1.154555463	0.593128966	8
TAS2R1	0.295091215	-1.114339733	0.819248518	8
TAS2R4	-0.011558009	-0.994170899	1.005728908	8
TAS2R7	0.633794119	-1.15279703	0.519002912	8
TBC1D10B	0.239357662	-1.097958409	0.858600747	8
TBC1D16	0.052086733	-1.025025463	0.97293873	8
TBC1D20	0.447432628	-1.145591052	0.698158424	8
TBC1D3H	0.114999049	-1.052527873	0.937528824	8
TBC1D3I	0.430269558	-1.143117042	0.712847484	8
TBC1D5	0.659270566	-1.150623554	0.491352988	8
TBCB	0.43889003	-1.144394557	0.705504528	8
TBCEL	0.569571727	-1.154665761	0.585094034	8
TBX19	0.090303765	-1.042089153	0.951785389	8
TCEA3	0.536669833	-1.153766516	0.617096683	8
TCEAL6	0.186901869	-1.080264376	0.893362507	8
TCEANC2	0.054085863	-1.025945349	0.971859486	8
TCERG1	0.165080283	-1.072268071	0.907187789	8
TCF7L1	-0.12729959	-0.930254682	1.057554273	8
TCL1A	0.282058649	-1.110736594	0.828677945	8
TCOF1	0.005591541	-1.002784046	0.997192505	8
TCP11	0.250508265	-1.101437623	0.850929358	8
TDRD1	0.031773601	-1.015508143	0.983734542	8
TDRD5	0.373765688	-1.133045836	0.759280148	8
TEDC2	0.16793205	-1.073334066	0.905402016	8
TENT2	0.596459829	-1.154487326	0.558027497	8
TENT5B	-0.00983586	-0.99504579	1.00488165	8
TEPP	0.063051138	-1.030033664	0.966982526	8
TEPSIN	0.5361797	-1.153744126	0.617564426	8
TESC	0.633720133	-1.152802107	0.519081974	8
TEX101	0.557090193	-1.154466262	0.597376069	8
TEX2	0.575360009	-1.154698254	0.579338245	8
TEX50	0.346090313	-1.127071425	0.780981112	8
TEX53	0.539269454	-1.153880927	0.614611473	8
TGFBR1	0.657458309	-1.150806662	0.493348353	8
TGFBR3	-0.099059265	-0.946783795	1.045843061	8
THAP8	0.596447606	-1.1544876	0.558039994	8
THBD	0.247942033	-1.100645763	0.85270373	8
THBS1	-0.023293944	-0.98814953	1.011443473	8
THEG	0.011429532	-1.005665777	0.994236245	8
THEGL	0.005890574	-1.002932275	0.997041701	8
THYN1	0.270617359	-1.107458197	0.836840838	8
TIAM1	0.457340233	-1.146891187	0.689550954	8
TICAM1	0.36702807	-1.131653158	0.764625088	8

TIMM13	0.17970446	-1.077667866	0.897963406	8
TIMP1	0.622245635	-1.153505021	0.531259387	8
TINAG	0.182606688	-1.078719719	0.896113031	8
TIPARP	0.043919722	-1.021236246	0.977316524	8
TJP2	0.269242627	-1.107057073	0.837814446	8
TLCD5	0.163967947	-1.071850573	0.907882626	8
TLL2	0.255335152	-1.102912658	0.847577506	8
TM2D3	0.633802222	-1.152796474	0.518994252	8
TM4SF20	0.127577544	-1.05766652	0.930088976	8
TM4SF5	0.50529825	-1.15181833	0.64652008	8
TM7SF3	-0.05987605	-0.968716642	1.028592692	8
TMC6	0.099218763	-1.045910906	0.946692143	8
TMC7	0.48116371	-1.149625921	0.66846221	8
TMCC2	0.655217798	-1.151026914	0.495809117	8
TMED2	0.510512778	-1.152213761	0.641700983	8
TMEFF2	-0.056127217	-0.970754344	1.026881561	8
TMEM108	0.190151871	-1.081423592	0.891271721	8
TMEM109	0.471479834	-1.148581675	0.677101841	8
TMEM127	0.314355589	-1.119407197	0.805051608	8
TMEM131L	0.036223865	-1.017619748	0.981395883	8
TMEM143	0.637743516	-1.15251581	0.514772294	8
TMEM14B	0.330065656	-1.123308622	0.793242966	8
TMEM158	0.353403525	-1.128715135	0.77531161	8
TMEM171	0.614532969	-1.153884416	0.539351447	8
TMEM178B	0.474291324	-1.148894424	0.6746031	8
TMEM202	0.436089963	-1.14398729	0.707897326	8
TMEM213	0.428062491	-1.142778723	0.714716233	8
TMEM215	-0.012198928	-0.993844729	1.006043657	8
TMEM236	-0.002895024	-0.998549345	1.001444369	8
TMEM250	0.485305663	-1.150044043	0.664738379	8
TMEM262	0.039725608	-1.019270832	0.979545224	8
TMEM263	-0.078358223	-0.958515728	1.03687395	8
TMEM272	0.313738506	-1.119249694	0.805511187	8
TMEM35B	0.588630866	-1.154626585	0.565995719	8
TMEM38A	0.422536765	-1.141911715	0.719374949	8
TMEM38B	0.604158035	-1.154278995	0.55012096	8
TMEM40	0.34716202	-1.127315197	0.780153177	8
TMEM64	0.482398514	-1.149752368	0.667353854	8
TMEM97	0.437246347	-1.144156384	0.706910037	8
TMPPE	0.435079313	-1.143838471	0.708759158	8
TMPRSS15	0.620413303	-1.153601873	0.53318857	8
TMSB15B	0.385426945	-1.135361101	0.749934157	8
TNF	0.356917042	-1.129488315	0.772571273	8
TNFAIP6	0.434727448	-1.143786432	0.709058984	8
TNFAIP8	0.139204188	-1.062308821	0.923104633	8
TNFRSF12A	0.299080924	-1.115414652	0.816333728	8
TNFRSF6B	0.407938021	-1.139484873	0.731546852	8
TNFSF13B	0.482679941	-1.149780973	0.667101032	8
TNFSF15	0.534212094	-1.153651635	0.619439541	8
TNMD	0.451112141	-1.146084928	0.694972787	8
TNNI3K	0.051434145	-1.024724528	0.973290383	8
TNRC18	0.292329309	-1.113587872	0.821258563	8
TNRC6C	0.588895082	-1.154623068	0.565727986	8
TOM1	0.37826997	-1.133954477	0.755684507	8
TOR3A	0.328253326	-1.122869233	0.794615907	8

TPD52L3	0.540118017	-1.153916687	0.61379867	8
TPO	0.245951181	-1.100027796	0.854076615	8
TPP1	-0.039584114	-0.979620182	1.019204296	8
TPR	0.062333356	-1.029708572	0.967375216	8
TPRA1	0.406608005	-1.139254027	0.732646022	8
TPSAB1	0.658651239	-1.15068663	0.49203539	8
TPTE2	0.549781279	-1.154268493	0.604487214	8
TRAF3	0.363689336	-1.130948219	0.767258883	8
TRAF5	0.199199577	-1.084607223	0.885407646	8
TRAPPC3	0.488083962	-1.150314862	0.6622309	8
TRAPPC4	0.060538168	-1.028893812	0.968355644	8
TRAPPC6A	0.190277048	-1.081468075	0.891191028	8
TREM2	0.425443233	-1.142371304	0.716928071	8
TRIB1	0.523320338	-1.153064605	0.629744267	8
TRIM28	0.240657512	-1.098369129	0.857711617	8
TRIM31	0.574425344	-1.154695607	0.580270264	8
TRIM4	0.166740539	-1.072889443	0.906148904	8
TRIM49D1	0.425440016	-1.1423708	0.716930784	8
TRIM49D2	-0.077152772	-0.95918891	1.036341683	8
TRIM65	0.0425159	-1.02057987	0.978063969	8
TRIM67	0.508268612	-1.152047032	0.64377842	8
TRIM7	-0.12385308	-0.932304475	1.056157555	8
TRIML1	-0.037833028	-0.98054659	1.018379618	8
TRIP4	0.037450852	-1.018199325	0.980748473	8
TRIR	0.481794029	-1.149690657	0.667896628	8
TRMU	0.622185167	-1.153508285	0.531323118	8
TRPC1	0.196437479	-1.083642119	0.88720464	8
TRPC7	-0.021735242	-0.988955206	1.010690447	8
TRRAP	0.606781464	-1.154191644	0.547410179	8
TSC2	-0.08451307	-0.955061446	1.039574516	8
TSPYL1	0.591921487	-1.154576906	0.562655419	8
TSSC4	-0.038451401	-0.980219704	1.018671105	8
TSSK1B	0.218470852	-1.091173747	0.872702894	8
TTC12	0.195515807	-1.083318747	0.887802941	8
TTC17	0.637210734	-1.152554922	0.515344188	8
TTC34	0.623899223	-1.153414002	0.529514779	8
TTC7A	0.654617052	-1.151084822	0.49646777	8
TTLL1	-0.00672379	-0.996621151	1.003344941	8
TTYH3	0.397699509	-1.137666204	0.739966694	8
TUBA1A	0.299273042	-1.115466079	0.816193037	8
TUBA1C	-0.086536709	-0.953919466	1.040456175	8
TUBA4B	-0.081478749	-0.956767974	1.038246723	8
TUBGCP2	-0.036531694	-0.981233566	1.01776526	8
TUBGCP4	0.46255095	-1.147536881	0.684985931	8
TUFM	0.194508759	-1.08296466	0.888455901	8
TULP1	-0.090563415	-0.951637899	1.042201313	8
TULP2	0.232479067	-1.095762434	0.863283367	8
TUNAR	0.591652695	-1.154581444	0.56292875	8
TWIST1	0.270824757	-1.107518578	0.836693821	8
TXNDC11	-0.078552358	-0.95840721	1.036959569	8
TXNRD3	0.087131601	-1.040714768	0.953583167	8
TYR	-0.013468509	-0.993197718	1.006666227	8
TYSND1	0.605303995	-1.154241867	0.548937872	8
UACA	0.066683342	-1.031672778	0.964989436	8
UAP1	-0.078263691	-0.958568558	1.03683225	8

UBA2	0.136573319	-1.061267423	0.924694104	8
UBAC1	0.598842543	-1.154430457	0.555587914	8
UBAC2	0.297932548	-1.115106609	0.817174061	8
UBE2D4	0.282020799	-1.110725926	0.828705126	8
UBE2K	0.570938425	-1.15467689	0.583738464	8
UBE2QL1	0.286992726	-1.112117229	0.825124503	8
UBE2V2	0.439320425	-1.1444565	0.705136075	8
UBE3C	0.342688624	-1.126291106	0.783602482	8
UBOX5	0.068350703	-1.032421882	0.964071179	8
UBQLN3	0.554939392	-1.154414223	0.599474831	8
UBXN1	0.001160648	-1.000579819	0.999419171	8
UBXN10	0.646930798	-1.151783217	0.504852419	8
UCK2	0.11690634	-1.053314809	0.936408469	8
UFD1	0.327007504	-1.122565569	0.795558065	8
UFM1	-0.041886122	-0.978398805	1.020284927	8
UGDH	0.285428567	-1.111681723	0.826253157	8
UGT1A5	-0.008437623	-0.995754491	1.004192114	8
UGT2B7	0.632848498	-1.152861386	0.520012888	8
UGT8	0.610591212	-1.154049857	0.543458646	8
UHRF1BP1	0.464652119	-1.147789771	0.683137651	8
ULBP1	0.001415106	-1.000706802	0.999291696	8
ULBP2	0.091955874	-1.042801937	0.950846064	8
ULK2	0.662690071	-1.150265912	0.487575842	8
UMAD1	0.37529478	-1.133356315	0.758061535	8
UNC119	0.416394383	-1.140914613	0.72452023	8
UNC13C	0.491188687	-1.150608297	0.65941961	8
UNC13D	0.212497698	-1.089169775	0.876672076	8
UNC45B	0.308376918	-1.11786774	0.809490822	8
UNC5B	0.157388209	-1.069361412	0.911973203	8
UNC5CL	0.552108139	-1.15433788	0.602229741	8
UNC79	0.64012001	-1.15233687	0.51221686	8
UPK1A	0.232183266	-1.095667152	0.863483887	8
UPK1B	0.335165861	-1.1245301	0.789364238	8
UROC1	0.146455583	-1.065151716	0.918696133	8
USE1	0.323173014	-1.121622653	0.798449639	8
USP14	0.382235454	-1.134739496	0.752504042	8
USP17L4	0.497826038	-1.151202843	0.653376805	8
USP2	0.455525133	-1.14666008	0.691134947	8
USP21	0.088107039	-1.041138201	0.953031162	8
USP24	0.660297477	-1.150517821	0.490220344	8
USP27X	0.231393884	-1.09541254	0.864018656	8
USP30	0.468546035	-1.14824701	0.679700975	8
USP31	0.380363086	-1.134370582	0.754007497	8
USP36	0.524840959	-1.153154152	0.628313193	8
USP4	0.166084332	-1.072644104	0.906559772	8
USP42	0.292579107	-1.113656133	0.821077026	8
USP6	0.327299623	-1.12263689	0.795337268	8
USP7	0.550050082	-1.154276814	0.604226732	8
UTS2B	-0.018096438	-0.990828968	1.008925406	8
UXS1	0.29506738	-1.114333271	0.819265892	8
VAMP8	0.67449248	-1.148908067	0.474415587	8
VAV2	0.350173729	-1.127994924	0.777821195	8
VCY	-0.126270508	-0.930867673	1.057138181	8
VDAC2	-0.063949389	-0.966490556	1.030439945	8
VGLL3	0.61599553	-1.153818154	0.537822624	8

VIP	0.271803126	-1.107802945	0.835999819	8
VPS26A	0.003701624	-1.001845674	0.99814405	8
VSIG10	0.632189716	-1.152905543	0.520715827	8
VSTM2L	0.628146897	-1.153164389	0.525017492	8
VTN	0.661296629	-1.150413571	0.489116942	8
VWDE	0.102900477	-1.047471633	0.944571157	8
WBP4	0.461066653	-1.147355641	0.686288988	8
WDHD1	0.416206484	-1.14088356	0.724677076	8
WDPCP	0.010158988	-1.005040791	0.994881804	8
WDR36	0.408521468	-1.139585628	0.73106416	8
WDR47	-0.019314072	-0.990203067	1.009517139	8
WDR89	0.384326361	-1.13514777	0.750821409	8
WFDC11	-0.00046804	-0.999765898	1.000233938	8
WFDC13	-0.01444741	-0.992698019	1.007145429	8
WHAMM	0.440587035	-1.144637771	0.704050736	8
WNK2	0.243718855	-1.099331084	0.855612229	8
WNK4	0.604341153	-1.154273169	0.549932016	8
WRNIP1	-0.123749792	-0.932365765	1.056115557	8
WSCD1	0.586952051	-1.154647012	0.567694961	8
WT1	0.079180032	-1.037236192	0.95805616	8
XAGE5	0.09092108	-1.042355729	0.951434649	8
XBP1	-0.126519692	-0.930719317	1.057239008	8
XCR1	0.227615855	-1.094187072	0.866571217	8
XDH	0.147396104	-1.065517487	0.918121383	8
XIAP	0.270729902	-1.107490966	0.836761065	8
XYLT2	-0.07873164	-0.958306971	1.03703861	8
YARS1	0.655885983	-1.150961936	0.495075953	8
YEATS2	0.423732559	-1.142101754	0.718369195	8
YEATS4	-0.02228336	-0.988672097	1.010955457	8
YTHDF1	0.1001324	-1.046299168	0.946166768	8
ZACN	0.63848152	-1.152461026	0.513979506	8
ZAR1L	-0.001885254	-0.99905604	1.000941294	8
ZBBX	0.053689073	-1.025763008	0.972073935	8
ZBED6	0.033125391	-1.016151126	0.983025736	8
ZBTB21	0.25646215	-1.103254343	0.846792194	8
ZBTB48	0.3293718	-1.123140731	0.793768931	8
ZC3H11A	-0.032615758	-0.983293121	1.015908879	8
ZCCHC18	0.084784095	-1.039692776	0.954908681	8
ZCCHC2	0.625962959	-1.153295576	0.527332617	8
ZCCHC24	0.3532326	-1.128677248	0.775444647	8
ZCWPW2	0.264026273	-1.105521018	0.841494745	8
ZDHHC19	0.507545193	-1.151992173	0.64444698	8
ZDHHC3	0.280302503	-1.110240361	0.829937858	8
ZDHHC6	0.539071156	-1.153872458	0.614801301	8
ZFP36	-0.078841804	-0.958245364	1.037087168	8
ZFPL1	0.017798204	-1.008780304	0.9909821	8
ZFPM2	0.131777384	-1.05935537	0.927577986	8
ZFY	0.254496544	-1.102657741	0.848161197	8
ZFYVE1	0.616988537	-1.153771654	0.536783118	8
ZKSCAN5	-0.077433578	-0.959032193	1.036465771	8
ZKSCAN8	0.66007885	-1.150540451	0.490461601	8
ZMYND11	0.240205879	-1.098226578	0.858020698	8
ZNF12	0.51599735	-1.152599173	0.636601823	8
ZNF155	0.397887541	-1.137700463	0.739812922	8
ZNF160	-0.043543611	-0.977516924	1.021060535	8

ZNF177	0.651919266	-1.151338911	0.499419645	8
ZNF207	0.594557817	-1.154527852	0.559970035	8
ZNF224	-0.052781184	-0.972564167	1.025345351	8
ZNF226	0.311337413	-1.118633795	0.807296382	8
ZNF253	0.276450004	-1.109142891	0.832692887	8
ZNF266	0.00045607	-1.000227957	0.999771887	8
ZNF280A	-0.076753369	-0.959411715	1.036165084	8
ZNF280B	0.604755379	-1.15425984	0.549504461	8
ZNF30	-0.048012116	-0.975129132	1.023141248	8
ZNF320	0.3429599	-1.126353701	0.783393801	8
ZNF391	0.006586683	-1.003277072	0.996690389	8
ZNF41	-0.095416677	-0.948871685	1.044288362	8
ZNF418	0.093771742	-1.043582989	0.949811247	8
ZNF423	0.448988719	-1.1458015	0.696812781	8
ZNF426	0.154412794	-1.068224821	0.913812027	8
ZNF44	0.242321723	-1.098893001	0.856571278	8
ZNF467	0.411698775	-1.140128837	0.728430062	8
ZNF483	-0.034856736	-0.982115906	1.016972642	8
ZNF501	0.395469536	-1.137257458	0.741787922	8
ZNF519	0.42244248	-1.141896674	0.719454194	8
ZNF551	0.244628416	-1.099615443	0.854987027	8
ZNF569	0.047138757	-1.022735757	0.975597001	8
ZNF571	0.35881552	-1.129901614	0.771086094	8
ZNF580	0.386989774	-1.135662168	0.748672395	8
ZNF585A	0.302585258	-1.116347872	0.813762613	8
ZNF608	0.04542934	-1.021940436	0.976511096	8
ZNF610	0.099296153	-1.045943818	0.946647665	8
ZNF619	0.316584242	-1.119973364	0.803389122	8
ZNF628	0.465698495	-1.1479141	0.682215605	8
ZNF639	0.471258523	-1.148556725	0.677298202	8
ZNF658	0.233728076	-1.096163987	0.862435911	8
ZNF66	-0.117255503	-0.936203068	1.053458572	8
ZNF668	0.296315248	-1.114670922	0.818355674	8
ZNF670	0.244968301	-1.099721531	0.85475323	8
ZNF671	0.445110479	-1.145272691	0.700162212	8
ZNF679	0.677396787	-1.148544178	0.471147391	8
ZNF681	0.413373529	-1.140411426	0.727037897	8
ZNF700	0.084460355	-1.039551508	0.955091153	8
ZNF705G	0.474611893	-1.148929588	0.674317695	8
ZNF709	-0.041258748	-0.978732065	1.019990814	8
ZNF766	0.333415531	-1.124113407	0.790697876	8
ZNF768	-0.077393133	-0.959054769	1.036447902	8
ZNF772	0.581792752	-1.154689115	0.572896362	8
ZNF783	0.104901858	-1.048315729	0.943413871	8
ZNF799	0.539296234	-1.153882067	0.614585833	8
ZNF835	0.111285363	-1.050987685	0.939702322	8
ZNF84	0.500689741	-1.151445496	0.650755755	8
ZNF845	0.004850515	-1.002416435	0.99756592	8
ZNF92	0.101167996	-1.046738493	0.945570497	8
ZNRF2	-0.049997549	-0.974063377	1.024060927	8
ZP4	-0.05849378	-0.969469215	1.027962995	8
ZPBP	0.318283005	-1.120402105	0.8021191	8
ZSCAN1	0.495908353	-1.151035667	0.655127314	8
ZSCAN12	-0.069633892	-0.963363068	1.03299696	8
ZSCAN21	0.508415917	-1.152058136	0.643642219	8

ZSCAN23	0.292959885	-1.113760088	0.820800203	8
ZSWIM1	0.144252745	-1.064292366	0.920039622	8
ZWILCH	0.482243193	-1.149736546	0.667493353	8
A2M	-1.010373521	0.989293165	0.021080356	9
AAK1	-0.854843698	1.099680496	-0.244836798	9
ABCE1	-1.079514166	0.89470338	0.184810786	9
ABCF2	-0.88696618	1.083770718	-0.196804539	9
ABCF2-H2BE1	-0.876097108	1.089462703	-0.213365595	9
ABCG2	-0.700536116	1.145212624	-0.444676508	9
ABHD10	-1.002830412	0.997145347	0.005685065	9
ABHD17A	-1.061079739	0.924979143	0.136100596	9
ACAD10	-0.8010472	1.120761434	-0.319714234	9
ACAP3	-0.966645904	1.030311774	-0.06366587	9
ACBD4	-0.879890223	1.087513561	-0.207623338	9
ACCS	-0.665777613	1.149929275	-0.484151662	9
ACKR4	-0.800920731	1.120803678	-0.319882947	9
ACOT2	-0.797737634	1.121856369	-0.324118735	9
ACP4	-0.816016624	1.115530504	-0.299513879	9
ACSBG1	-1.031855111	0.96476636	0.067088751	9
ACSL3	-0.819393566	1.114285768	-0.294892202	9
ACTA1	-0.866686046	1.094131547	-0.227445501	9
ACTA2	-1.03190908	0.964700279	0.067208801	9
ACTN2	-0.96326677	1.033074993	-0.069808223	9
ACYP1	-0.852359646	1.10079992	-0.248440274	9
ADCY3	-0.676305774	1.148682267	-0.472376493	9
ADCY4	-1.109179969	0.8326005	0.27657947	9
ADGRE1	-1.049362922	0.941968045	0.107394877	9
ADGRG1	-0.913081551	1.068677656	-0.155596106	9
ADH5	-0.872479213	1.091285419	-0.218806206	9
ADHFE1	-1.127059569	0.781021271	0.346038298	9
ADM	-0.760940876	1.13261852	-0.371677645	9
ADRB2	-0.829562223	1.110388679	-0.280826456	9
AFF4	-1.041515522	0.95253787	0.088977652	9
AGK	-0.823268019	1.112827511	-0.289559492	9
AGO4	-0.832490023	1.109224283	-0.276734261	9
AIF1	-1.089397043	0.876226141	0.213170902	9
AIG1	-1.083708802	0.887081029	0.196627773	9
AIM2	-0.897801552	1.077760283	-0.179958731	9
AK1	-1.09202636	0.870988289	0.221038071	9
AKAIN1	-1.040885393	0.953360932	0.087524461	9
AKAP1	-1.030457488	0.966469283	0.063988205	9
AKAP4	-1.100655236	0.852682605	0.247972631	9
ALDH1A1	-1.098612194	0.857183402	0.241428793	9
ALDH2	-0.959272737	1.036275266	-0.077002528	9
ALDH4A1	-1.114699522	0.818278356	0.296421166	9
ALDH8A1	-1.117596545	0.810260937	0.307335608	9
ALDOB	-0.661361849	1.150406719	-0.48904487	9
ALDOC	-0.914211958	1.067976155	-0.153764197	9
ALG1L2	-0.668173456	1.149659027	-0.481485571	9
ALKBH2	-0.988247471	1.011352121	-0.02310465	9
ALKBH4	-1.088675033	0.877639189	0.211035843	9
ALKBH5	-1.057723979	0.930004094	0.127719886	9
ALMS1	-0.726584774	1.140502735	-0.413917961	9
ALPG	-0.702123458	1.144955291	-0.442831833	9
AMACR	-1.108457118	0.834392999	0.274064118	9

AMIGO3	-0.72622529	1.140574941	-0.414349651	9
AMN1	-0.815480297	1.115725963	-0.300245666	9
AMOTL1	-0.889015972	1.082659967	-0.193643995	9
AMY2B	-0.766616187	1.131121383	-0.364505197	9
ANKIB1	-0.811090318	1.117303104	-0.306212786	9
ANKLE1	-0.765433942	1.131437987	-0.366004046	9
ANKRD13A	-0.749676877	1.135422711	-0.385745834	9
ANKRD13B	-0.731310362	1.139534284	-0.408223922	9
ANKRD13C	-0.933610774	1.055259456	-0.121648682	9
ANKRD40	-0.989296376	1.010370507	-0.02107413	9
ANKRD44	-0.663448032	1.15018448	-0.486736449	9
ANO6	-1.068035678	0.914116304	0.153919373	9
ANO9	-0.794309739	1.122967537	-0.328657798	9
ANP32B	-0.959688572	1.035945341	-0.076256769	9
ANTXR1	-0.883348198	1.085702026	-0.202353828	9
ANXA6	-0.824740932	1.112264598	-0.287523667	9
ANXA9	-0.805976465	1.119089799	-0.313113333	9
AP2A1	-0.885163579	1.084737595	-0.199574016	9
AP3M1	-0.906983753	1.072390377	-0.165406624	9
AP4E1	-1.020187134	0.978509684	0.04167745	9
APEH	-0.830313428	1.110091753	-0.279778325	9
APOA2	-0.94275672	1.048792704	-0.106035984	9
APOBR	-1.045054285	0.94784603	0.097208254	9
APOL2	-0.910911942	1.070012376	-0.159100435	9
APOOL	-1.08263563	0.889060635	0.193574995	9
APPL1	-1.112258267	0.824757428	0.287500839	9
AQP3	-0.637567776	1.152528752	-0.514960976	9
AQP4	-0.969618663	1.027837633	-0.058218969	9
ARF4	-0.870258119	1.092387079	-0.22212896	9
ARHGAP1	-0.959739935	1.035904538	-0.076164603	9
ARHGAP11A	-0.768091568	1.130722761	-0.362631192	9
ARHGAP11A-SCG5	-1.099675569	0.854854557	0.244821012	9
ARHGAP11B	-0.998782795	1.001212777	-0.002429982	9
ARHGAP12	-1.0424708	0.951283041	0.091187759	9
ARHGAP19	-0.750086479	1.135324573	-0.385238094	9
ARHGAP19-SLIT1	-0.822555407	1.113098159	-0.290542751	9
ARHGAP35	-0.784360002	1.126063194	-0.341703191	9
ARHGDIB	-0.904408505	1.073922792	-0.169514287	9
ARL1	-1.073531997	0.905068576	0.168463421	9
ARL13A	-0.767238337	1.130953766	-0.36371543	9
ARL15	-1.127933585	0.778033069	0.349900516	9
ARL5C	-0.787620035	1.125069896	-0.337449861	9
ARL6IP1	-0.728293944	1.140156604	-0.41186266	9
ARMC1	-1.090665989	0.873716574	0.216949415	9
ARMC6	-0.740399692	1.137569524	-0.397169832	9
ARMCX3	-0.789331122	1.124540403	-0.33520928	9
ARMT1	-1.100975716	0.851966471	0.249009245	9
ARNT	-0.653452925	1.151195659	-0.497742734	9
ARPP19	-0.88926085	1.082526463	-0.193265614	9
ARRDC1	-1.081932639	0.890346232	0.191586406	9
ARRDC2	-0.960576345	1.035238453	-0.074662108	9
ARSA	-1.115393543	0.816391443	0.2990021	9
ARSG	-0.907091484	1.072325816	-0.165234332	9
ARSK	-1.129206569	0.773575515	0.355631054	9
ASB2	-0.801231535	1.120699805	-0.31946827	9

ASCC2	-1.048289693	0.943449676	0.104840016	9
ASGR2	-0.800568597	1.120921129	-0.320352532	9
ASH2L	-0.719685789	1.14185266	-0.422166871	9
ASPRV1	-0.659051114	1.150645963	-0.49159485	9
ATF1	-0.993633152	1.006247503	-0.012614352	9
ATF6B	-0.87973107	1.087596138	-0.207865068	9
ATF7IP	-0.824584135	1.112324748	-0.287740613	9
ATF7IP2	-0.663526922	1.15017596	-0.486649037	9
ATG10	-1.101555871	0.85066291	0.250892961	9
ATG12	-0.629799598	1.15306109	-0.523261492	9
ATG3	-1.010169842	0.989510011	0.020659831	9
ATG7	-1.039197263	0.955547924	0.083649339	9
ATP11A	-0.715310239	1.142670054	-0.427359815	9
ATP2B3	-1.013745237	0.985663226	0.028082012	9
ATP5F1C	-0.698886106	1.145476129	-0.446590023	9
ATP5ME	-0.743960577	1.136762607	-0.392802031	9
ATP5MG	-0.746839419	1.136094713	-0.389255295	9
ATP5MJ	-0.97984387	1.01900557	-0.039161701	9
ATP5PB	-0.661287539	1.150414526	-0.489126987	9
ATP5PF	-0.864023434	1.095410262	-0.231386828	9
ATP6V0A1	-1.128619681	0.775646567	0.352973114	9
ATP6V1F	-0.930723254	1.057236333	-0.126513079	9
ATP6V1G2	-0.980614161	1.018319296	-0.037705135	9
ATP7A	-0.965158356	1.031534559	-0.066376203	9
ATP9A	-0.703491453	1.144730488	-0.441239034	9
ATR	-0.849105693	1.102243515	-0.253137821	9
ATXN3L	-0.62908051	1.153106462	-0.524025952	9
AURKA	-1.088551889	0.877879136	0.210672753	9
B2M	-0.999446977	1.000552107	-0.00110513	9
B3GALNT1	-1.069812906	0.911237769	0.158575137	9
B3GALT2	-0.955948183	1.03888612	-0.082937937	9
B3GAT2	-0.742503038	1.137095451	-0.394592413	9
B3GNT4	-1.000581243	0.999417742	0.001163502	9
B3GNT6	-0.756252101	1.13381254	-0.377560439	9
BACE1	-0.999307752	1.000690813	-0.00138306	9
BAG1	-1.127295672	0.780219652	0.347076019	9
BAIAP2L1	-0.635965946	1.152644873	-0.516678926	9
BANF1	-1.081424446	0.891270172	0.190154273	9
BAZ1B	-0.817669894	1.114924147	-0.297254253	9
BCAS4	-0.998102113	1.001887142	-0.003785029	9
BCL2L10	-1.079131116	0.895384368	0.183746748	9
BCL2L11	-1.118903013	0.80651837	0.312384643	9
BCL2L13	-1.112924137	0.823013937	0.2899102	9
BCL2L15	-1.129784066	0.771509973	0.358274093	9
BCL2L2	-1.023414783	0.974812819	0.048601964	9
BCL2L2-PABPN1	-1.126671236	0.782330924	0.344340312	9
BCL9	-1.023886485	0.974266018	0.049620467	9
BCO2	-1.044136061	0.949074957	0.095061104	9
BEST1	-1.099706069	0.854787324	0.244918746	9
BEX3	-0.953447905	1.040818643	-0.087370738	9
BEX4	-0.977037314	1.021480724	-0.044443411	9
BGLAP	-1.10061828	0.852765007	0.247853273	9
BHLHE41	-0.812941252	1.116643063	-0.303701811	9
BICD2	-1.107124741	0.837650571	0.26947417	9
BICRAL	-1.036725138	0.958704188	0.078020949	9

BLACAT1	-0.631091541	1.152977917	-0.521886376	9
BLVRA	-1.016879288	0.982219535	0.034659754	9
BLVRB	-0.68300502	1.147807729	-0.464802709	9
BMP15	-0.803799779	1.119834034	-0.316034255	9
BMP6	-0.955142288	1.039511895	-0.084369607	9
BMX	-1.069265469	0.912129116	0.157136353	9
BNC1	-0.86392481	1.095457274	-0.231532464	9
BNIP3	-1.09410435	0.866742265	0.227362084	9
BORCS7	-0.683917883	1.147683613	-0.463765731	9
BPIFA1	-1.106561678	0.839009626	0.267552052	9
BPIFA3	-1.043023915	0.950552555	0.09247136	9
BPIFB3	-0.981890882	1.017175165	-0.035284283	9
BRPF1	-0.667615414	1.149722681	-0.482107266	9
BSG	-0.753058232	1.134603964	-0.381545732	9
BTG1	-0.887631613	1.083411452	-0.19577984	9
BTG3	-0.871426859	1.091809018	-0.220382159	9
BTN2A1	-1.116773449	0.812577201	0.304196248	9
BTNL3	-1.096680255	0.861340628	0.235339627	9
BTNL9	-1.03386152	0.962293273	0.071568247	9
BUD23	-1.127987134	0.777848119	0.350139015	9
BYSL	-1.041689785	0.9523096	0.089380186	9
C11orf45	-0.752288008	1.134792186	-0.382504178	9
C11orf71	-0.944045247	1.047855871	-0.103810624	9
C12orf43	-1.121784373	0.797957294	0.32382708	9
C13orf42	-1.11291311	0.823042952	0.289870158	9
C15orf65	-1.110488419	0.829309172	0.281179247	9
C16orf72	-1.064650227	0.91948131	0.145168918	9
C16orf86	-0.866145367	1.094392691	-0.228247324	9
C16orf89	-0.694373512	1.146176161	-0.45180265	9
C16orf91	-0.818347617	1.114673903	-0.296326285	9
C17orf67	-0.967940611	1.029239258	-0.061298647	9
C17orf99	-0.883330835	1.085711205	-0.20238037	9
C19orf53	-0.733923274	1.138983315	-0.405060041	9
C19orf54	-0.857290792	1.098562834	-0.241272041	9
C1GALT1	-0.757099258	1.133599651	-0.376500392	9
C1orf115	-0.798820511	1.121500515	-0.322680004	9
C1orf131	-0.675556638	1.148776113	-0.473219475	9
C1orf56	-0.863562999	1.095629532	-0.232066532	9
C1QTNF3	-0.768568404	1.13059309	-0.362024686	9
C1QTNF3-AMACR	-0.977487898	1.021085998	-0.043598099	9
C22orf23	-0.919731426	1.064490041	-0.144758616	9
C22orf42	-0.910682392	1.0701527	-0.159470308	9
C3orf14	-0.654913356	1.151056321	-0.496142965	9
C3orf38	-0.929475059	1.058081516	-0.128606457	9
C3orf49	-1.076320001	0.90030837	0.176011631	9
C3orf56	-0.71804704	1.142162292	-0.424115252	9
C4orf19	-0.9751884	1.023089941	-0.047901542	9
C4orf3	-0.742923429	1.136999815	-0.394076387	9
C4orf36	-0.904177306	1.074059351	-0.169882045	9
C5	-0.79266264	1.123493238	-0.330830598	9
C5orf22	-0.903205123	1.074631758	-0.171426635	9
C5orf46	-0.95414683	1.040281076	-0.086134246	9
C6orf163	-1.032251747	0.964280141	0.067971606	9
C6orf226	-1.053572191	0.936040574	0.117531617	9
C7orf26	-0.978537398	1.020162681	-0.041625283	9

C8orf44-SGK3	-0.877673987	1.088657184	-0.210983196	9
C9	-1.1088946	0.833310302	0.275584298	9
C9orf78	-0.93186388	1.056459062	-0.124595182	9
CACNA1F	-0.753231979	1.134561363	-0.381329384	9
CACNA2D3	-1.000704379	0.999294129	0.00141025	9
CACNG2	-0.711692347	1.143323476	-0.431631129	9
CALML4	-0.961341597	1.034626348	-0.07328475	9
CALR	-1.067151724	0.915531878	0.151619846	9
CAMK2N1	-1.073074038	0.905839174	0.167234864	9
CAPN10	-1.130956561	0.767227984	0.363728577	9
CAPN14	-0.864113924	1.095367104	-0.23125318	9
CARD16	-1.107321964	0.837172095	0.270149868	9
CARHSP1	-0.977568983	1.021014858	-0.043445875	9
CARMIL1	-1.080564999	0.892822515	0.187742483	9
CARNMT1	-0.971375324	1.026356216	-0.054980892	9
CASD1	-0.884280366	1.085207968	-0.200927602	9
CASK	-0.748614529	1.13567591	-0.387061381	9
CASP1	-1.021609203	0.976890406	0.044718798	9
CASP6	-0.874383418	1.09033046	-0.215947042	9
CASTOR1	-1.111178707	0.827547826	0.283630881	9
CBX3	-0.892271264	1.080871005	-0.188599741	9
CBX4	-1.033409108	0.962853869	0.070555239	9
CBX5	-0.79631492	1.122320364	-0.326005444	9
CCDC110	-0.993333205	1.006536054	-0.013202849	9
CCDC116	-0.892675792	1.080646534	-0.187970742	9
CCDC117	-0.96695893	1.030053175	-0.063094244	9
CCDC149	-0.954105698	1.040312769	-0.086207071	9
CCDC163	-0.858510819	1.09800004	-0.239489221	9
CCDC22	-0.701897572	1.14499214	-0.443094568	9
CCDC34	-0.920910411	1.0637321	-0.142821689	9
CCDC39	-1.103615045	0.845959479	0.257655566	9
CCDC51	-0.864462936	1.095200453	-0.230737517	9
CCDC66	-0.680775532	1.148105852	-0.46733032	9
CCKBR	-0.630371667	1.153024524	-0.522652857	9
CCL17	-1.02504041	0.972921245	0.052119165	9
CCM2L	-1.115553236	0.815954336	0.2995989	9
CCNA2	-1.07161974	0.908265706	0.163354034	9
CCNC	-0.994880828	1.005041737	-0.010160909	9
CCNG2	-0.74769754	1.135892923	-0.388195383	9
CCPG1	-1.070761394	0.909683419	0.161077976	9
CCR1	-1.009615238	0.990099081	0.019516157	9
CCR8	-0.644282377	1.152005734	-0.507723357	9
CCT4	-0.868885007	1.093061614	-0.224176607	9
CCT5	-0.784500368	1.126020843	-0.341520475	9
CCT6B	-0.826653443	1.111526595	-0.284873152	9
CCT7	-0.824255053	1.112450813	-0.28819576	9
CCT8	-0.791640222	1.123816896	-0.332176675	9
CD14	-1.076356093	0.900245955	0.176110138	9
CD151	-0.85861928	1.097949827	-0.239330547	9
CD1C	-1.117295666	0.811111289	0.306184377	9
CD200R1	-0.667943357	1.149685325	-0.481741968	9
CD22	-0.760717992	1.132676151	-0.371958159	9
CD24	-1.062656747	0.922570599	0.140086148	9
CD244	-0.836666028	1.107529987	-0.270863959	9
CD28	-1.126019095	0.784506158	0.341512937	9

CD300LD	-1.050580888	0.940272188	0.1103087	9
CD302	-0.992278683	1.00754648	-0.015267797	9
CD33	-0.645535675	1.151901584	-0.506365909	9
CD3E	-0.994622033	1.00529257	-0.010670537	9
CD52	-0.720951974	1.141610539	-0.420658565	9
CD53	-1.122685729	0.795185901	0.327499828	9
CD84	-0.916774493	1.066370303	-0.149595809	9
CD8A	-0.789074708	1.124620109	-0.335545401	9
CD96	-1.122662683	0.795257345	0.327405338	9
CDC37L1	-0.971475798	1.026271043	-0.054795245	9
CDC40	-1.101157834	0.851558265	0.249599569	9
CDC42BPA	-0.956927756	1.038121766	-0.08119401	9
CDC45	-0.716673879	1.142418513	-0.425744634	9
CDH19	-0.729549163	1.139899419	-0.410350256	9
CDK10	-0.781909317	1.126796602	-0.344887285	9
CDK19	-0.83120316	1.10973844	-0.27853528	9
CDK4	-1.005026284	0.994896759	0.010129525	9
CDK5R1	-0.663147116	1.150216903	-0.487069787	9
CDK9	-0.859092452	1.097730422	-0.23863797	9
CDKN1B	-0.95043469	1.043112956	-0.092678266	9
CEACAM6	-1.124090113	0.790772107	0.333318006	9
CEBPG	-0.809530907	1.117853655	-0.308322748	9
CENPH	-0.742652895	1.137061394	-0.394408498	9
CENPL	-0.644084934	1.152021954	-0.507937019	9
CENPQ	-0.870780081	1.092129363	-0.221349282	9
CENPW	-1.00159421	0.998398129	0.003196081	9
CEP104	-1.066871032	0.915979175	0.150891857	9
CEP162	-0.904589596	1.073815713	-0.169226117	9
CEP20	-0.833557597	1.10879491	-0.275237313	9
CEP295NL	-1.102878791	0.847655161	0.255223629	9
CERK	-0.917154408	1.066130365	-0.148975957	9
CERKL	-0.902985773	1.074760502	-0.171774729	9
CETN2	-1.124366217	0.789890065	0.334476151	9
CFAP119	-0.744736716	1.136583915	-0.391847199	9
CFAP157	-0.812383055	1.116842869	-0.304459814	9
CFAP298-TCP10L	-1.123946471	0.791229093	0.332717377	9
CFAP299	-0.864280221	1.095287737	-0.231007516	9
CFHR3	-1.099707716	0.854783693	0.244924024	9
CHAC2	-0.808813993	1.118105072	-0.309291079	9
CHCHD2	-1.056214733	0.932220999	0.123993734	9
CHD1L	-0.694657163	1.146133044	-0.451475881	9
CHD5	-0.708529207	1.143878289	-0.435349081	9
CHML	-0.968567487	1.02871718	-0.060149693	9
CHMP6	-0.77184904	1.129689802	-0.357840762	9
CHPF	-0.927875631	1.059156355	-0.131280724	9
CHRNA10	-1.04154091	0.952504631	0.089036279	9
CHST11	-1.026662801	0.971013189	0.055649613	9
CHST13	-1.096107552	0.862555249	0.233552303	9
CIAO2B	-0.678069202	1.148458234	-0.470389032	9
CIP2A	-1.101780723	0.850155175	0.251625548	9
CIRBP	-0.866258193	1.09433826	-0.228080067	9
CISD1	-0.787146383	1.125215473	-0.33806909	9
CISD2	-0.772546908	1.129495128	-0.35694822	9
CITED2	-1.030190933	0.966792247	0.063398686	9
CKB	-0.687585257	1.147172916	-0.459587658	9

CLASP2	-0.645428797	1.151910546	-0.506481749	9
CLCN7	-0.997698165	1.002286048	-0.004587883	9
CLDN5	-0.805697013	1.119185887	-0.313488874	9
CLEC12A	-1.088529949	0.877921854	0.210608095	9
CLEC12B	-1.021728033	0.976754422	0.044973611	9
CLEC16A	-0.837504797	1.107184883	-0.269680086	9
CLEC1A	-1.002895509	0.997079118	0.005816391	9
CLEC2D	-0.761548971	1.132460839	-0.370911867	9
CLEC4D	-1.121199093	0.799732242	0.321466851	9
CLEC7A	-0.750816814	1.135148878	-0.384332064	9
CLIC2	-0.702957105	1.144818632	-0.441861527	9
CLINT1	-1.115045627	0.817339935	0.297705692	9
CLIP4	-1.115799283	0.815278682	0.3005206	9
CLK1	-0.705567991	1.144383868	-0.438815876	9
CLOCK	-1.097176812	0.860281014	0.236895798	9
CLPSL1	-0.831506322	1.109617649	-0.278111326	9
CLSTN2	-0.911912566	1.069398704	-0.157486138	9
CLUL1	-0.994021956	1.005872711	-0.011850756	9
CLVS1	-1.026441119	0.971275111	0.055166008	9
CMC1	-1.000370046	0.999629543	0.000740503	9
CMTR2	-0.932775459	1.055834468	-0.123059009	9
CNDP2	-1.083626713	0.887233188	0.196393525	9
CNMD	-0.637559478	1.152529362	-0.514969884	9
CNNM4	-1.090697065	0.873654691	0.217042374	9
CNOT4	-1.048573778	0.943058634	0.105515144	9
CNOT6	-0.883447794	1.085649356	-0.202201562	9
CNP	-1.050936295	0.939774408	0.111161887	9
CNTNAP5	-0.921800378	1.063156818	-0.14135644	9
CNTROB	-0.860671066	1.096994357	-0.23632329	9
COA5	-1.031621521	0.965052096	0.066569425	9
COG2	-0.791047297	1.124003662	-0.332956365	9
COL13A1	-0.661129422	1.150431112	-0.48930169	9
COL26A1	-1.120588378	0.801564298	0.31902408	9
COLEC10	-0.699430534	1.145389633	-0.445959099	9
COMMD1	-1.089014165	0.876976797	0.212037368	9
COMMD3	-0.726721322	1.140475254	-0.413753931	9
COMMD6	-0.691089005	1.146666834	-0.45557783	9
COMMD9	-0.935843923	1.053709563	-0.117865641	9
COPB2	-1.011274938	0.988330177	0.022944761	9
COPS2	-1.094954679	0.864976455	0.229978224	9
COQ10A	-0.854782424	1.099708292	-0.244925868	9
COQ5	-0.941767809	1.04950731	-0.107739502	9
COQ7	-1.023939731	0.97420419	0.049735541	9
COX5A	-1.039463878	0.955204252	0.084259626	9
COX7C	-1.124558585	0.789272668	0.335285917	9
CPNE2	-1.090617474	0.873813142	0.216804332	9
CPNE3	-1.045108466	0.94777326	0.097335207	9
CPSF1	-0.98849934	1.011116956	-0.022617616	9
CPT2	-0.945658206	1.046673959	-0.101015753	9
CPVL	-1.063762084	0.92086391	0.142898174	9
CRCP	-0.936400689	1.053320257	-0.116919569	9
CREB3	-0.916310557	1.066662651	-0.150352093	9
CREBZF	-1.02698207	0.970635291	0.05634678	9
CRKL	-1.119923492	0.803536229	0.316387263	9
CRLS1	-0.889579535	1.082352461	-0.192772927	9

CROT	-0.814175474	1.116198958	-0.302023483	9
CRYBA1	-1.053332632	0.936383017	0.116949615	9
CRYGB	-1.112132866	0.825083841	0.287049026	9
CRYGC	-1.115780652	0.815329935	0.300450717	9
CRYM	-1.082904406	0.888566788	0.194337618	9
CRYZL1	-0.943313417	1.04838875	-0.105075333	9
CSDC2	-0.675033141	1.148841224	-0.473808083	9
CSF1R	-1.024168536	0.973938257	0.050230279	9
CSF2RB	-0.896521734	1.078488274	-0.18196654	9
CSPG4	-0.985710024	1.013702218	-0.027992194	9
CSRP2	-0.676773282	1.1486233	-0.471850018	9
CST5	-1.068653477	0.913120627	0.15553285	9
CST9L	-0.93349296	1.055340712	-0.121847752	9
CSTF2	-0.982749696	1.016400825	-0.033651129	9
CT47A11	-0.89438788	1.079691165	-0.185303285	9
CT47A8	-0.809297861	1.117935499	-0.308637638	9
CTAGE4	-0.892034335	1.081002253	-0.188967917	9
CTAGE8	-1.021510328	0.977003475	0.044506853	9
CTH	-0.928751927	1.058568603	-0.129816676	9
CTRL	-1.055552495	0.933185545	0.12236695	9
CTSH	-0.702714001	1.144858591	-0.442144591	9
CTSO	-1.02651291	0.97119033	0.055322581	9
CTXND1	-0.763145379	1.132043774	-0.368898395	9
CUTC	-0.776547081	1.128362031	-0.35181495	9
CUZD1	-0.872165901	1.091441616	-0.219275715	9
CWC27	-1.095656667	0.863505941	0.232150726	9
CXCL6	-1.116897195	0.812230969	0.304666227	9
CXCR5	-0.990831619	1.008922895	-0.018091277	9
CYB5B	-0.8319471	1.109441658	-0.277494558	9
CYBB	-0.72962132	1.139884558	-0.410263238	9
CYCS	-0.924666291	1.061285721	-0.136619429	9
CYP20A1	-0.860826066	1.096921745	-0.236095679	9
CYP2A13	-0.986659753	1.012826651	-0.026166899	9
CYP2A7	-0.829562973	1.110388384	-0.28082541	9
CYP2U1	-0.771785428	1.129707503	-0.357922074	9
CYP4F2	-0.938430065	1.051891409	-0.113461344	9
CYSLTR1	-0.839224221	1.10647238	-0.267248159	9
DAB1	-0.780409224	1.127239945	-0.346830721	9
DALRD3	-0.635226612	1.152697352	-0.517470739	9
DAP	-1.126632553	0.78246079	0.344171763	9
DARS1	-0.692771728	1.146417426	-0.453645698	9
DARS2	-0.934661135	1.054532766	-0.11987163	9
DAXX	-0.786064102	1.125546502	-0.3394824	9
DBR1	-1.125463121	0.786337404	0.339125717	9
DCAF1	-0.652308934	1.151302812	-0.498993878	9
DCDC2C	-0.913917669	1.068159187	-0.154241518	9
DCTN3	-1.119063056	0.80605416	0.313008896	9
DCTPP1	-0.980968049	1.018002996	-0.037034947	9
DDB1	-0.835474768	1.108017352	-0.272542583	9
DDI2	-0.828269549	1.110896689	-0.282627141	9
DDO	-0.633664165	1.152805942	-0.519141777	9
DDR2	-0.721633171	1.141479237	-0.419846066	9
DDRGK1	-0.7242949	1.140959156	-0.416664256	9
DDX23	-0.659929537	1.150555869	-0.490626333	9
DDX24	-0.959863328	1.035806465	-0.075943137	9

DDX51	-0.96197166	1.034120437	-0.072148777	9
DDX56	-0.989126629	1.010529771	-0.021403142	9
DECR1	-0.706265788	1.144265928	-0.43800014	9
DECR2	-0.686180472	1.147370827	-0.461190355	9
DEFA4	-1.125013896	0.787801867	0.337212029	9
DEFB115	-1.09563475	0.863552027	0.232082723	9
DEK	-1.076781245	0.899509179	0.177272066	9
DENND1B	-0.727224725	1.140373683	-0.413148958	9
DENND6B	-0.870606996	1.092214903	-0.221607907	9
DFFB	-1.096979662	0.860702444	0.236277218	9
DGAT2L6	-0.758368508	1.133278349	-0.374909841	9
DHDDS	-1.009317279	0.990414723	0.018902555	9
DHRS1	-0.830119616	1.110168481	-0.280048866	9
DHRS12	-0.683582846	1.147729305	-0.464146458	9
DHRS7C	-1.067880343	0.914365827	0.153514516	9
DHX16	-0.944237734	1.047715363	-0.103477629	9
DHX58	-0.850851255	1.101472302	-0.250621047	9
DHX8	-0.837280144	1.107277473	-0.269997329	9
DIABLO	-0.730719688	1.139657302	-0.408937614	9
DIAPH2	-0.999787262	1.000212603	-0.000425341	9
DIPK1B	-1.078713971	0.896123193	0.182590778	9
DISP3	-0.801938993	1.120462643	-0.31852365	9
DKKL1	-0.65989369	1.150559566	-0.490665876	9
DLG1	-1.066357185	0.916795283	0.149561902	9
DLGAP4	-0.982031508	1.017048632	-0.035017124	9
DLX5	-0.957337373	1.03780093	-0.080463557	9
DMAC1	-0.805540649	1.119239582	-0.313698932	9
DMPK	-0.865068769	1.094910425	-0.229841656	9
DMXL1	-1.105050292	0.842607701	0.262442592	9
DNAAF11	-1.124846486	0.788344217	0.336502269	9
DNAJA1	-0.795153852	1.122696064	-0.327542211	9
DNAJB9	-0.728447523	1.140125272	-0.411677749	9
DNAJC13	-0.776558794	1.12835867	-0.351799876	9
DNAJC15	-1.128366427	0.776531759	0.351834668	9
DNAJC17	-0.789631308	1.124446929	-0.334815621	9
DNAJC4	-0.866491336	1.094225678	-0.227734342	9
DNAJC8	-1.051503131	0.938977743	0.112525388	9
DNASE2	-0.748141531	1.135788027	-0.387646496	9
DND1	-0.862641694	1.096066649	-0.233424955	9
DNPEP	-0.842372325	1.105150087	-0.262777762	9
DOP1A	-1.048449296	0.943230084	0.105219211	9
DOP1B	-0.967035974	1.029989458	-0.062953484	9
DPH3	-0.688768352	1.147004022	-0.45823567	9
DPH5	-0.990917107	1.008841904	-0.017924797	9
DPM2	-0.663490785	1.150179864	-0.486689079	9
DPY19L3	-0.777090515	1.128205821	-0.351115306	9
DRAM1	-0.967711442	1.029429661	-0.061718219	9
DROSHA	-0.807850178	1.118441406	-0.310591228	9
DSTYK	-0.819887526	1.114101653	-0.294214126	9
DTNBP1	-0.919263742	1.064789396	-0.145525654	9
DTX3	-1.010456195	0.989205068	0.021251127	9
DTX4	-1.029067397	0.968147211	0.060920186	9
DUSP28	-0.863845922	1.095494861	-0.231648939	9
DUSP5	-1.059211562	0.927793112	0.13141845	9
DYNC1I2	-0.838574505	1.106742414	-0.268167909	9

DYNLRB1	-1.001633576	0.998358379	0.003275198	9
DYRK2	-0.730875051	1.139624999	-0.408749948	9
DYRK4	-0.895296126	1.079180829	-0.183884704	9
EAF2	-0.948843543	1.044309434	-0.095465892	9
EBPL	-0.672711905	1.149125305	-0.4764134	9
ECHDC1	-0.761495984	1.132474604	-0.370978621	9
EDC3	-0.877103644	1.088949314	-0.21184567	9
EDEM3	-0.824338791	1.112418757	-0.288079966	9
EEF1AKMT1	-1.12899711	0.774317867	0.354679244	9
EEF1G	-1.000057142	0.999942849	0.000114293	9
EFCAB12	-1.047686881	0.944276729	0.103410152	9
EFCAB9	-0.808158636	1.118333974	-0.310175338	9
EFL1	-1.113374953	0.821823594	0.291551359	9
EFNA3	-1.074281685	0.903800284	0.170481401	9
EFNA4	-0.671141708	1.1493132	-0.478171492	9
EHD2	-0.973052667	1.024928024	-0.051875357	9
EIF1	-1.092010624	0.871020078	0.220990545	9
EIF3E	-0.79022876	1.124260374	-0.334031614	9
EIF3F	-0.955798656	1.039002436	-0.08320378	9
EIF3I	-0.846636512	1.103321905	-0.256685393	9
EIF4A2	-1.0449294	0.948013653	0.096915747	9
EIF4A3	-0.98964413	1.010043737	-0.020399607	9
EIF4E3	-1.098084855	0.858327471	0.239757384	9
EIF4EBP3	-0.960861315	1.035010814	-0.0741495	9
ELAVL2	-1.130043033	0.770574568	0.359468465	9
ELL3	-0.762092741	1.132319284	-0.370226543	9
ELOA3BP	-0.689592231	1.14688521	-0.457292979	9
ELOVL2	-0.765502872	1.131419597	-0.365916725	9
ELOVL5	-1.117562562	0.810357194	0.307205368	9
ELP2	-0.632937403	1.152855384	-0.519917982	9
EMC10	-1.040307542	0.954112483	0.086195059	9
EMC3	-0.706735107	1.14418619	-0.437451084	9
EMG1	-0.705089338	1.144464342	-0.439375004	9
ENDOG	-0.809396371	1.117900917	-0.308504546	9
ENGASE	-1.036361737	0.959163592	0.077198146	9
ENO1	-0.802788907	1.120176394	-0.317387487	9
ENO4	-0.665167689	1.149996812	-0.484829123	9
ENOPH1	-0.844869992	1.104084483	-0.259214491	9
ENTPD1	-1.088451357	0.878074796	0.210376561	9
ENTPD4	-1.089667895	0.875693297	0.213974598	9
ENTPD5	-1.094391539	0.866147755	0.228243784	9
EOLA2	-1.125834998	0.785114862	0.340720135	9
EPB41L2	-1.012041135	0.987507386	0.024533749	9
EPHB2	-1.112427782	0.824315218	0.288112564	9
EPRS1	-1.092721743	0.869578127	0.223143617	9
ERAL1	-0.894849106	1.079432312	-0.184583206	9
ERCC1	-1.053701217	0.935855876	0.117845341	9
ERICH6	-1.023807902	0.97435723	0.049450672	9
ERP44	-1.074766988	0.902974715	0.171792274	9
ERVFRD-1	-1.118893757	0.806545179	0.312348578	9
ESR1	-1.036520459	0.958963082	0.077557377	9
ETDB	-0.945081208	1.047097941	-0.102016733	9
ETFB	-1.014009491	0.985375485	0.028634006	9
EXOC1	-1.075613982	0.901525181	0.1740888	9
EXOC5	-0.897078843	1.078171982	-0.181093139	9

EXOC6B	-1.001653125	0.998338636	0.003314489	9
EXOC8	-1.050104309	0.940937602	0.109166706	9
EXOG	-0.793096015	1.123355433	-0.330259418	9
EXOSC1	-1.121975834	0.7973725	0.324603334	9
FABP5	-0.649353465	1.151571561	-0.502218097	9
FAHD2A	-1.012460091	0.987055818	0.025404273	9
FAM111A	-0.701815057	1.145005582	-0.443190525	9
FAM114A2	-0.650988512	1.151424318	-0.500435806	9
FAM118A	-0.662628689	1.150272472	-0.487643783	9
FAM166A	-0.758245382	1.133309641	-0.375064259	9
FAM166B	-1.076771928	0.899525358	0.17724657	9
FAM184B	-1.098317323	0.857824004	0.240493319	9
FAM205C	-1.100241763	0.853602424	0.246639339	9
FAM216A	-0.742689215	1.137053134	-0.394363919	9
FAM219B	-0.981890982	1.017175074	-0.035284092	9
FAM229A	-0.775905667	1.128545702	-0.352640036	9
FAM236D	-1.061469452	0.924386795	0.137082657	9
FAM53C	-1.115559377	0.815937503	0.299621873	9
FAM71F2	-0.773568475	1.129208551	-0.355640076	9
FAM78A	-0.86328526	1.095761535	-0.232476274	9
FAM83D	-1.020580208	0.978063584	0.042516624	9
FAS	-0.715337351	1.14266508	-0.427327729	9
FASTKD1	-0.787934978	1.12497286	-0.337037882	9
FAU	-0.943467781	1.048276526	-0.104808745	9
FBN2	-1.100184567	0.853729301	0.246455267	9
FBP2	-0.993471761	1.006402825	-0.012931064	9
FBXL4	-0.903419211	1.074505958	-0.171086747	9
FBXO16	-0.684884983	1.14755082	-0.462665837	9
FBXO30	-0.84102305	1.105719648	-0.264696599	9
FBXO4	-0.632510017	1.152884143	-0.520374127	9
FBXO42	-0.917778997	1.065734853	-0.147955856	9
FCER1A	-1.119806119	0.803881934	0.315924185	9
FCER2	-0.819752937	1.11415187	-0.294398933	9
FCHO1	-1.130628923	0.768436788	0.362192135	9
FCN1	-0.901106658	1.07585733	-0.174750671	9
FCSK	-1.085348032	0.884016567	0.201331466	9
FDCSP	-0.632282446	1.152899361	-0.520616915	9
FDXACB1	-0.715520872	1.142631388	-0.427110516	9
FER1L5	-0.694712755	1.14612458	-0.451411825	9
FERMT3	-0.946954934	1.045716285	-0.098761351	9
FGF20	-1.026711844	0.970955193	0.055756651	9
FGF23	-0.67714525	1.148576164	-0.471430913	9
FGF5	-0.889097309	1.082615642	-0.193518333	9
FGFBP2	-1.045237913	0.947599284	0.097638629	9
FGL1	-1.091034858	0.872980716	0.218054142	9
FHDC1	-1.106860165	0.838290453	0.268569712	9
FIG4	-0.710274436	1.14357407	-0.433299634	9
FKBP7	-0.734849209	1.138785402	-0.403936193	9
FKTN	-0.833761951	1.108712426	-0.274950475	9
FLI1	-0.854016975	1.100054738	-0.246037763	9
FMC1	-0.939455321	1.05116362	-0.111708299	9
FMN1	-0.998685866	1.001308973	-0.002623107	9
FMNL2	-1.010200903	0.989476959	0.020723944	9
FMO4	-1.101992684	0.849675256	0.252317428	9
FOCAD	-0.712255192	1.143223147	-0.430967954	9

FOXH1	-0.968089776	1.029115195	-0.061025419	9
FOXL1	-1.047431272	0.944626313	0.102804959	9
FRA10AC1	-0.841209923	1.105641018	-0.264431096	9
FRS2	-0.830283471	1.110103618	-0.279820148	9
FSCN3	-0.84676415	1.103266518	-0.256502369	9
FST	-0.799339373	1.12132918	-0.321989807	9
FTH1	-0.982423609	1.016695286	-0.034271678	9
FTL	-1.119476973	0.804847606	0.314629366	9
FUCA2	-1.034985542	0.960892924	0.074092617	9
FUT11	-0.970978559	1.026692087	-0.055713529	9
FUT6	-1.097270082	0.860081305	0.237188777	9
FXR1	-0.799669283	1.12121996	-0.321550677	9
G3BP2	-0.701897031	1.144992229	-0.443095197	9
GABARAP	-0.999331901	1.000666763	-0.001334862	9
GABBR2	-0.999198021	1.000800054	-0.001602033	9
GABPA	-1.127651708	0.779003034	0.348648674	9
GABRA3	-1.040391852	0.954003021	0.086388832	9
GADD45A	-0.82448979	1.112360914	-0.287871124	9
GADD45G	-0.995238399	1.004694536	-0.009456138	9
GALC	-0.790386786	1.124210914	-0.333824129	9
GALNT11	-1.065833192	0.917623894	0.148209298	9
GAPVD1	-0.824356512	1.112411971	-0.288055459	9
GAR1	-0.849115758	1.102239089	-0.253123331	9
GARNL3	-1.031173865	0.965598426	0.065575439	9
GAS2L3	-0.991076849	1.008690458	-0.017613609	9
GASK1B	-0.651184691	1.151406413	-0.500221722	9
GCC1	-1.020385446	0.978284761	0.042100685	9
GCDH	-1.066376522	0.916764635	0.149611887	9
GCM1	-0.764740754	1.13162246	-0.366881706	9
GCNT2	-1.083847743	0.886823208	0.197024535	9
GDF10	-0.873586962	1.090731066	-0.217144104	9
GDPD4	-1.130802163	0.767798838	0.363003325	9
GEMIN7	-1.06211414	0.923402791	0.138711349	9
GGA1	-0.954033114	1.040368679	-0.086335565	9
GGCT	-0.849057363	1.102264763	-0.2532074	9
GGCX	-1.085609651	0.883522838	0.202086813	9
GGT2	-1.049850101	0.941291565	0.108558536	9
GHITM	-1.09887617	0.856608024	0.242268146	9
GIGYF1	-0.655790982	1.150971211	-0.495180229	9
GIMAP1	-0.746118416	1.136263297	-0.390144882	9
GIMAP6	-0.679168187	1.148316392	-0.469148205	9
GIMAP7	-0.965799236	1.031008984	-0.065209748	9
GIN1	-0.910198633	1.070447865	-0.160249233	9
GINS1	-0.992933136	1.006920131	-0.013986995	9
GINS3	-0.846953334	1.103184352	-0.256231018	9
GIPC2	-0.893982687	1.079918051	-0.185935364	9
GJA1	-1.07064149	0.90988062	0.16076087	9
GJB5	-0.796529157	1.12225075	-0.325721593	9
GKAP1	-1.100313474	0.853443226	0.246870249	9
GLB1	-0.647671732	1.151719309	-0.504047577	9
GLIPR1L1	-1.060172879	0.926350367	0.133822512	9
GLT8D1	-0.785736543	1.125646249	-0.339909705	9
GLTPD2	-0.744987752	1.136525902	-0.39153815	9
GLYATL1B	-0.84734563	1.1030137	-0.25566807	9
GM2A	-0.932951839	1.055713265	-0.122761427	9

GMFG	-0.683610573	1.14772553	-0.464114957	9
GMNN	-0.906361325	1.072762668	-0.166401344	9
GNA13	-0.767973914	1.130754692	-0.362780779	9
GNL2	-0.825704353	1.111893835	-0.286189482	9
GNL3L	-0.695978501	1.14593063	-0.449952129	9
GNPAT	-0.81402577	1.116252995	-0.302227225	9
GNS	-0.723260803	1.141162542	-0.417901739	9
GOLGA2	-0.654136172	1.151130827	-0.496994655	9
GOLM1	-1.033962177	0.96216831	0.071793868	9
GOLT1B	-1.048692305	0.942895239	0.105797066	9
GP2	-0.818934283	1.114456489	-0.295522206	9
GPATCH2	-0.675697597	1.148758515	-0.473060918	9
GPN2	-0.881010627	1.086930256	-0.205919629	9
GPN3	-0.778331878	1.127846936	-0.349515058	9
GPR143	-0.904674626	1.073765399	-0.169090773	9
GPR146	-0.741829319	1.137248103	-0.395418783	9
GPR174	-0.810979992	1.117342221	-0.306362229	9
GPR31	-1.064689024	0.919420682	0.145268342	9
GPR34	-1.106464329	0.839243557	0.267220771	9
GPRC5A	-0.832320492	1.109292231	-0.276971739	9
GPRC5D	-1.061750731	0.923958104	0.137792627	9
GPSM2	-0.7055713	1.14438331	-0.43881201	9
GRAMD1B	-1.070999436	0.909291312	0.161708125	9
GRB2	-0.835020055	1.108202529	-0.273182474	9
GRHL2	-0.702857167	1.14483507	-0.441977903	9
GRHL3	-0.957026506	1.038044485	-0.081017979	9
GRIA3	-1.095542666	0.863745542	0.231797124	9
GRIK1	-1.035538194	0.960200432	0.075337762	9
GRIP2	-0.773418514	1.129250738	-0.355832225	9
GRK3	-0.828154743	1.110941628	-0.282786885	9
GSDMC	-0.920458447	1.064023216	-0.143564769	9
GSE1	-0.749247008	1.135525397	-0.386278389	9
GSKIP	-0.756781835	1.133679565	-0.376897729	9
GSPT1	-0.630875765	1.152991956	-0.522116192	9
GSTM3	-0.926425498	1.060123	-0.133697503	9
GTDC1	-0.77019835	1.130146741	-0.359948391	9
GTF2H1	-0.684828259	1.147558646	-0.462730386	9
GTF3C2	-0.998959806	1.001036958	-0.002077152	9
GTSF1	-0.630679391	1.153004682	-0.522325291	9
GUCA2A	-0.679308338	1.14829818	-0.468989842	9
GUCY2D	-0.828131343	1.110950783	-0.28281944	9
GUF1	-0.748004685	1.135820394	-0.387815709	9
GXYLT1	-1.124949898	0.788009414	0.336940484	9
GZMA	-1.107412483	0.836952062	0.270460421	9
GZMB	-0.644049113	1.152024891	-0.507975778	9
H1-1	-1.077455407	0.898334968	0.179120439	9
H2AC13	-1.059153718	0.927879572	0.131274147	9
H2AC15	-0.644609133	1.151978779	-0.507369646	9
H2AC17	-0.843311466	1.104751135	-0.261439669	9
H2AC4	-0.850849219	1.101473206	-0.250623987	9
H2AZ2	-0.910701184	1.070141219	-0.159440036	9
H2BC12	-0.78391439	1.126197395	-0.342283006	9
H2BS1	-0.976869303	1.02162765	-0.044758347	9
H3-2	-1.098579155	0.857255289	0.241323866	9
H3-4	-1.129951409	0.770906179	0.35904523	9

H3C11	-0.775603253	1.128632036	-0.353028783	9
H3C12	-0.907032989	1.072360875	-0.165327886	9
H3C8	-0.779469914	1.127515405	-0.348045491	9
H3Y1	-1.059008952	0.928095782	0.13091317	9
H4-16	-0.729423012	1.139925381	-0.410502369	9
H4C12	-0.692923212	1.14639477	-0.453471558	9
H4C2	-0.719750437	1.141840359	-0.422089922	9
HACD4	-0.820100938	1.114021945	-0.293921006	9
HADHA	-0.927177427	1.059622701	-0.132445274	9
HADHB	-0.648381329	1.151657425	-0.503276096	9
HAL	-0.9253105	1.0608612	-0.1355507	9
HAO2	-1.036088708	0.959507991	0.076580717	9
HARS1	-0.738708813	1.137945304	-0.39923649	9
HAUS2	-1.066311952	0.916866956	0.149444996	9
HAUS6	-0.974869248	1.023366021	-0.048496773	9
HAVCR1	-0.737752886	1.138155657	-0.400402771	9
HBD	-0.64852172	1.151645102	-0.503123382	9
HDAC8	-0.851956882	1.100979999	-0.249023117	9
HDAC9	-0.687047777	1.147248975	-0.460201198	9
HDC	-0.940234244	1.050608013	-0.110373768	9
HDGF	-0.804498677	1.119596123	-0.315097446	9
HECA	-0.775259484	1.128729971	-0.353470487	9
HERC3	-0.974336326	1.023825915	-0.049489589	9
HES7	-0.971150123	1.026546945	-0.055396822	9
HGF	-0.710285247	1.143572171	-0.433286923	9
HHEX	-1.091310625	0.872428689	0.218881937	9
HIBCH	-1.111656613	0.826318013	0.2853386	9
HIGD2B	-0.905421637	1.073322408	-0.167900771	9
HINT1	-1.129516558	0.77247024	0.357046318	9
HJV	-0.678656796	1.148382608	-0.469725812	9
HMSD	-1.097282555	0.860054582	0.237227972	9
HNRNPDL	-0.712208441	1.143231499	-0.431023058	9
HOXC10	-0.765517161	1.131415783	-0.365898623	9
HPSE	-1.094452134	0.866022074	0.22843006	9
HPX	-1.042994818	0.950591055	0.092403763	9
HRH1	-1.12239776	0.796076404	0.326321356	9
HS2ST1	-0.674067749	1.148960289	-0.47489254	9
HS3ST1	-0.913425204	1.068464837	-0.155039634	9
HS6ST3	-1.001991903	0.997996123	0.00399578	9
HSBP1	-0.916460393	1.066568311	-0.150107918	9
HSD17B10	-0.795327733	1.122639968	-0.327312235	9
HSD17B14	-0.853420966	1.100323496	-0.246902529	9
HSD17B4	-0.699698414	1.145346911	-0.445648497	9
HSDL2	-0.931844412	1.056472368	-0.124627956	9
HSP90B1	-0.927103291	1.059672117	-0.132568826	9
HSPA1L	-1.050025386	0.941047567	0.108977819	9
HSPA4	-0.943225699	1.048452481	-0.105226782	9
HSPA8	-1.018685868	0.980203132	0.038482736	9
HSPA9	-1.106665148	0.838760652	0.267904496	9
HSPB1	-0.864340427	1.095258985	-0.230918558	9
HSPB2	-0.706510425	1.144224406	-0.43771398	9
HSPD1	-0.753739121	1.134436721	-0.3806976	9
HSPE1	-0.984389211	1.014911939	-0.030522728	9
HSPE1-MOB4	-1.068929325	0.912674357	0.156254968	9
HTR5A	-1.066086011	0.917224555	0.148861456	9

IBSP	-1.118523495	0.807614104	0.310909392	9
ID4	-1.079368217	0.894963139	0.184405078	9
IDH1	-0.696785623	1.14580572	-0.449020097	9
IER5	-0.941597096	1.049630287	-0.108033191	9
IFFO1	-0.7358164	1.138577175	-0.402760775	9
IFI35	-0.63780602	1.152511198	-0.514705178	9
IFNA1	-0.792083363	1.123676864	-0.331593501	9
IFNA13	-0.757586321	1.133476687	-0.375890366	9
IFNA16	-1.072645946	0.906556692	0.166089254	9
IFNA7	-0.903074036	1.074708715	-0.171634678	9
IFNK	-1.128986049	0.774356969	0.35462908	9
IFT46	-1.127306118	0.780184089	0.347122029	9
IFT52	-0.680720506	1.14811312	-0.467392614	9
IGSF3	-0.834160078	1.108551458	-0.274391379	9
IL15	-1.081061159	0.891927901	0.189133259	9
IL17A	-0.680367923	1.14815959	-0.467791668	9
IL1RL2	-1.031971942	0.964623278	0.067348664	9
IL20	-0.664499449	1.150070219	-0.485570771	9
IL21	-0.88774894	1.083347976	-0.195599035	9
IL24	-0.879965584	1.087474435	-0.207508851	9
IL36A	-1.124382907	0.789836591	0.334546317	9
IL36B	-0.812695211	1.116731213	-0.304036002	9
IL7R	-1.110172783	0.830108743	0.28006404	9
IL9	-1.124821913	0.788423673	0.336398239	9
ILKAP	-0.784587354	1.125994578	-0.341407224	9
ING3	-1.086911272	0.881046979	0.205864293	9
INSL6	-1.128726623	0.775271247	0.353455376	9
INSR	-1.082303222	0.889669618	0.192633604	9
INTS13	-0.876648116	1.089182	-0.212533884	9
INTS3	-0.680245454	1.148175691	-0.467930237	9
INTS4	-1.102209314	0.849183458	0.253025856	9
INTS8	-0.656409013	1.150910654	-0.494501641	9
IPO8	-0.912551172	1.069005354	-0.156454181	9
IQCB1	-1.105073939	0.842551956	0.262521983	9
IRF3	-0.726992527	1.140420584	-0.413428056	9
ISCU	-0.703402195	1.144745241	-0.441343047	9
ITCH	-1.121841969	0.797781593	0.324060375	9
ITGB1BP2	-0.769352809	1.130378886	-0.361026077	9
ITGB5	-1.051008283	0.939673421	0.111334861	9
ITK	-0.920998671	1.063675169	-0.142676498	9
ITLN2	-0.855479738	1.099391425	-0.243911687	9
ITPKB	-1.063558781	0.92117898	0.142379801	9
ITPRID1	-0.967764448	1.029385642	-0.061621194	9
JAG1	-0.901426811	1.075671227	-0.174244416	9
JAG2	-0.969717939	1.0277543	-0.058036361	9
JAKMIP1	-0.676983579	1.148596675	-0.471613096	9
JAZF1	-0.97733163	1.021223008	-0.043891377	9
JCHAIN	-0.842810859	1.104964053	-0.262153193	9
JMJD7	-1.077196695	0.898786438	0.178410257	9
JMY	-0.872911082	1.091069689	-0.218158607	9
JOSD1	-0.694452707	1.146164135	-0.451711427	9
JOSD2	-1.045432535	0.947337406	0.098095128	9
JRK	-1.127593217	0.779203557	0.34838966	9
KAT7	-1.070051321	0.910848262	0.159203059	9
KBTBD2	-0.991131213	1.008638885	-0.017507673	9

KBTBD4	-0.967194292	1.02985844	-0.062664147	9
KCMF1	-1.047536368	0.944482657	0.103053711	9
KCNAB3	-0.731687272	1.139455491	-0.407768219	9
KCNE4	-0.63591995	1.152648158	-0.516728208	9
KCNJ1	-1.113737861	0.820859427	0.292878435	9
KCNJ15	-0.998266127	1.001724901	-0.003458774	9
KCNJ8	-0.661443506	1.150398132	-0.488954626	9
KCNK10	-0.780663318	1.127165146	-0.346501829	9
KCNK13	-0.819789084	1.114138387	-0.294349303	9
KCNK6	-1.119602462	0.804480091	0.315122371	9
KCNN4	-0.897790165	1.077766782	-0.179976617	9
KCP	-0.899100043	1.077016622	-0.177916578	9
KCTD15	-1.051365502	0.939171488	0.112194014	9
KDM1B	-0.69889687	1.145474423	-0.446577553	9
KDM4C	-0.712947749	1.143099027	-0.430151278	9
KDM5B	-0.773719568	1.129166003	-0.355446435	9
KDM7A	-0.784983263	1.125874858	-0.340891595	9
KHDC1L	-0.718319752	1.142111056	-0.423791305	9
KIAA0040	-0.663568914	1.150171421	-0.486602507	9
KIAA1328	-0.726540219	1.140511695	-0.413971476	9
KIAA1586	-1.126200953	0.78390256	0.342298393	9
KIAA1614	-0.875675758	1.089676797	-0.214001038	9
KIAA2026	-0.700907514	1.145152752	-0.444245238	9
KIF13A	-1.09705898	0.860533009	0.23652597	9
KIF16B	-1.053451014	0.936213871	0.117237143	9
KIF1B	-1.080369795	0.893173325	0.18719647	9
KIF6	-1.062517096	0.922785131	0.139731965	9
KIFBP	-0.734314488	1.138899866	-0.404585379	9
KLF11	-1.011407506	0.988188097	0.023219409	9
KLF13	-0.640462423	1.152310483	-0.511848059	9
KLHDC1	-1.092438336	0.870154134	0.222284202	9
KLHDC2	-1.110438806	0.829435092	0.281003713	9
KLHL11	-0.704261485	1.144602711	-0.440341225	9
KLRC3	-1.125014036	0.787801412	0.337212624	9
KLRD1	-1.116385182	0.813659016	0.302726166	9
KLRF1	-0.746409769	1.136195279	-0.38978551	9
KMT5B	-0.649958773	1.151517465	-0.501558692	9
KNSTRN	-1.080144439	0.893577513	0.186566926	9
KPNA3	-0.863537108	1.095641845	-0.232104737	9
KPNB1	-0.979140424	1.019629664	-0.04048924	9
KRBOX1	-1.079874256	0.894060966	0.18581329	9
KRT26	-0.911063478	1.069919649	-0.158856171	9
KRT6A	-1.106437774	0.839307316	0.267130458	9
KRT7	-0.670975223	1.149332921	-0.478357698	9
KRT85	-1.046780321	0.945513625	0.101266696	9
KRT86	-0.896434164	1.078537907	-0.182103743	9
KRTAP1-1	-1.048774569	0.942781749	0.10599282	9
KRTAP1-3	-0.894185108	1.079804767	-0.18561966	9
KRTAP10-10	-0.918077262	1.06554552	-0.147468258	9
KRTAP19-3	-1.005700826	0.994199969	0.011500857	9
KRTAP19-6	-1.085522848	0.883686797	0.201836051	9
KRTAP20-1	-1.118566343	0.807490751	0.311075592	9
KRTAP22-1	-0.707251737	1.144098028	-0.436846291	9
KRTAP23-1	-0.8621883	1.096280967	-0.234092667	9
KRTAP27-1	-1.110033983	0.830459213	0.279574771	9

KRTAP8-1	-0.895634165	1.078990261	-0.183356096	9
KRTCAP3	-0.744954338	1.13653363	-0.391579292	9
L3MBTL3	-0.927046668	1.059709845	-0.132663177	9
LACC1	-0.970063544	1.027463832	-0.057400288	9
LAMP1	-1.121126849	0.799950019	0.32117683	9
LAMTOR2	-0.904600881	1.073809036	-0.169208156	9
LANCL1	-0.796864141	1.122141719	-0.325277579	9
LAPTM4A	-0.972132902	1.025712831	-0.053579928	9
LARP7	-0.891407972	1.081348439	-0.189940466	9
LBHD1	-1.114308971	0.819331217	0.294977754	9
LCA5L	-0.786637084	1.125371528	-0.338734444	9
LCAT	-0.814936185	1.115923638	-0.300987453	9
LCE2C	-1.113572715	0.821298845	0.29227387	9
LCE5A	-0.951384772	1.042393597	-0.091008825	9
LDHAL6B	-0.672138338	1.149194338	-0.477056001	9
LDHB	-1.026997782	0.970616674	0.056381108	9
LDLRAP1	-0.794926056	1.122769463	-0.327843406	9
LEF1	-0.847178589	1.103086409	-0.255907821	9
LELP1	-1.034992997	0.960883601	0.074109396	9
LIAS	-0.671975026	1.14921391	-0.477238884	9
LILRB3	-1.013460477	0.985972761	0.027487716	9
LIN7C	-0.808750308	1.118127355	-0.309377047	9
LMAN1	-1.108944446	0.833186524	0.275757922	9
LMAN2	-1.099759422	0.854669655	0.245089767	9
LMAN2L	-0.888223229	1.083090974	-0.194867745	9
LMLN	-0.71741995	1.142279667	-0.424859717	9
LMOD2	-0.75691454	1.133646176	-0.376731637	9
LMTK2	-1.040457887	0.953917241	0.086540646	9
LOXHD1	-0.863481812	1.095668139	-0.232186327	9
LPAR1	-0.802883388	1.120144483	-0.317261095	9
LPAR6	-0.864770305	1.095053425	-0.230283121	9
LPCAT1	-0.681797637	1.147970059	-0.466172422	9
LPCAT3	-0.664878267	1.150028681	-0.485150414	9
LRCH1	-0.657417157	1.150810769	-0.493393612	9
LRPPRC	-1.125783387	0.785285095	0.340498292	9
LRRC23	-1.019037125	0.97980837	0.039228756	9
LRRC31	-1.067348746	0.915217282	0.152131464	9
LRRC43	-1.057312914	0.930610499	0.126702415	9
LRRC69	-1.104060968	0.844924722	0.259136245	9
LRRC75A	-1.069599988	0.911584944	0.158015044	9
LRRCC1	-0.678265908	1.148432972	-0.470167063	9
LRRN1	-0.794584546	1.122879311	-0.328294764	9
LRRN3	-0.99778016	1.002205155	-0.004424995	9
LSM11	-0.776802689	1.128288624	-0.351485936	9
LSM12	-1.130853888	0.767607836	0.363246052	9
LSM14B	-0.827998903	1.111002581	-0.283003677	9
LSM6	-1.089543768	0.875937679	0.213606089	9
LSM8	-1.042903552	0.950711763	0.092191789	9
LTA	-0.923914954	1.061779008	-0.137864054	9
LTB4R2	-1.054463991	0.934760238	0.119703753	9
LUM	-1.121034296	0.800228601	0.320805694	9
LURAP1	-0.956460469	1.038486897	-0.082026428	9
LURAP1L	-1.105154346	0.842362272	0.262792075	9
LUZP1	-1.060509894	0.925841946	0.134667947	9
LYG2	-0.699444062	1.145387478	-0.445943415	9

LYNX1-SLURP2	-0.842961231	1.104900159	-0.261938927	9
LYSMD4	-0.877880409	1.088551235	-0.210670826	9
LYVE1	-0.686960331	1.14726131	-0.460300978	9
M6PR	-0.796783021	1.122168143	-0.325385122	9
MAF	-0.663207068	1.150210453	-0.487003385	9
MAGEA2	-0.69569534	1.145974224	-0.450278884	9
MAGEA2B	-0.758630883	1.13321158	-0.374580697	9
MAGEA3	-1.09152881	0.871990772	0.219538037	9
MAGEA6	-0.909142178	1.071089844	-0.161947666	9
MAGEA8	-0.76360007	1.131924158	-0.368324088	9
MAGEB2	-0.799218772	1.121369052	-0.32215028	9
MAGOH	-1.093757234	0.867458302	0.226298932	9
MAGT1	-0.764909917	1.131577521	-0.366667604	9
MAK16	-0.737132798	1.138291303	-0.401158505	9
MAL	-0.942621481	1.048890655	-0.106269173	9
MAMDC4	-0.630347263	1.153026092	-0.52267883	9
MAML2	-0.645921617	1.151869096	-0.505947478	9
MANEAL	-0.704385766	1.144582004	-0.440196238	9
MAP2K4	-1.065962869	0.917419168	0.148543701	9
MAP2K6	-1.119673292	0.804272302	0.31540099	9
MAP7	-0.675337809	1.148803377	-0.473465568	9
MAPK13	-0.833914758	1.108650687	-0.274735929	9
MARCHF7	-0.680444245	1.148149546	-0.467705301	9
MARK1	-1.060499504	0.925857641	0.134641863	9
MATK	-0.92209356	1.062966708	-0.140873149	9
MB	-1.095272975	0.864311135	0.230961841	9
MBLAC1	-0.733238971	1.139128682	-0.405889711	9
MBLAC2	-0.848628306	1.10245315	-0.253824844	9
MBNL3	-1.052206765	0.93798406	0.114222705	9
MBOAT1	-1.009810607	0.989891802	0.019918806	9
MBP	-0.996382913	1.003578257	-0.007195344	9
MC3R	-0.813834429	1.116321994	-0.302487565	9
MCC	-0.635572925	1.152672858	-0.517099933	9
MCEE	-0.674784892	1.148871966	-0.474087074	9
MCM4	-0.737233994	1.13826921	-0.401035216	9
MCOLN2	-1.063707598	0.920948401	0.142759197	9
MCTS1	-1.035310842	0.960485632	0.07482521	9
MDH1	-0.685945346	1.147403675	-0.461458329	9
MEAF6	-0.965530652	1.031229473	-0.065698821	9
MED15	-0.686493443	1.147326982	-0.460833539	9
MED16	-1.093083742	0.868839791	0.224243951	9
MED19	-0.932209443	1.056222647	-0.124013204	9
MED20	-1.055294503	0.933559968	0.121734535	9
MED22	-0.961857023	1.034212616	-0.072355593	9
MED23	-1.106623831	0.838860112	0.267763719	9
MED24	-0.869085985	1.092963195	-0.22387721	9
MED4	-0.912737905	1.068890085	-0.156152179	9
MEF2A	-0.693444448	1.146316557	-0.452872109	9
MEGF8	-0.801337114	1.120664475	-0.319327362	9
METTL16	-0.847470894	1.102959131	-0.255488237	9
METTL23	-1.096305469	0.862136395	0.234169074	9
METTL5	-0.707874556	1.143991206	-0.43611665	9
METTL7A	-0.978098932	1.020549099	-0.042450167	9
MEX3B	-0.729015893	1.140008991	-0.410993098	9
MEX3C	-0.845387798	1.103861722	-0.258473924	9

MFF	-0.864735254	1.095070204	-0.23033495	9
MFRP	-0.844054545	1.104434001	-0.260379456	9
MFSD1	-0.838051973	1.106958881	-0.268906908	9
MFSD11	-0.856545411	1.098904847	-0.242359436	9
MFSD3	-0.87726781	1.088865319	-0.21159751	9
MGAM2	-1.063480922	0.921299502	0.142181419	9
MGAT4A	-1.001796709	0.998193554	0.003603156	9
MGAT4D	-1.12692628	0.781472015	0.345454265	9
MID2	-0.779761448	1.127430087	-0.34766864	9
MIGA1	-1.110011948	0.830514787	0.279497161	9
MILR1	-0.728460766	1.140122569	-0.411661803	9
MINDY2	-0.821604135	1.113457733	-0.291853597	9
MINDY4B	-0.915850022	1.066952147	-0.151102125	9
MIPEP	-0.88178264	1.086526304	-0.204743664	9
MISP3	-0.675716656	1.148756133	-0.473039477	9
MKLN1	-0.951640983	1.042198969	-0.090557987	9
MKNK2	-0.827214101	1.111308723	-0.284094622	9
MLLT10	-0.701900559	1.144991654	-0.443091095	9
MLLT3	-1.101235493	0.851383922	0.249851571	9
MLN	-0.738087486	1.138082199	-0.399994713	9
MME	-0.760118988	1.132830603	-0.372711615	9
MOB4	-0.883519154	1.085611601	-0.202092446	9
MORF4L2	-0.716985999	1.142360532	-0.425374533	9
MOSPD2	-0.734509007	1.138858281	-0.404349274	9
MPC1L	-1.045363011	0.947430998	0.097932014	9
MPP1	-0.741404318	1.13734401	-0.395939692	9
MPP3	-0.802515124	1.120268763	-0.317753639	9
MPZL2	-0.978166167	1.020489907	-0.04232374	9
MRC1	-1.089243212	0.876528098	0.212715114	9
MRGBP	-0.724716703	1.140875709	-0.416159005	9
MRGPRX4	-1.108952038	0.833167665	0.275784373	9
MROH7-TTC4	-1.041793221	0.952173974	0.089619247	9
MROH8	-1.061810302	0.923867187	0.137943115	9
MRPL10	-0.977436117	1.021131411	-0.043695295	9
MRPL14	-1.094911309	0.865066925	0.229844383	9
MRPL18	-1.097431573	0.859735009	0.237696565	9
MRPL19	-1.062340548	0.923055997	0.139284551	9
MRPL27	-0.796385989	1.122297281	-0.325911292	9
MRPL44	-1.052533456	0.937520898	0.115012558	9
MRPL53	-0.765500718	1.131420171	-0.365919453	9
MRPL55	-0.931607893	1.056633914	-0.12502602	9
MRPL57	-0.989799097	1.009897909	-0.020098812	9
MRPL58	-1.021716883	0.976767186	0.044949697	9
MRPS14	-1.003010817	0.996961739	0.006049078	9
MRPS24	-0.802136445	1.120396272	-0.318259827	9
MRPS25	-0.805624675	1.119210734	-0.313586059	9
MRPS28	-1.115239321	0.816812528	0.298426793	9
MRPS33	-0.894438221	1.079662943	-0.185224722	9
MRPS35	-0.828025616	1.110992136	-0.28296652	9
MRTFB	-1.070196062	0.910611402	0.15958466	9
MSANTD4	-0.689384672	1.146915236	-0.457530563	9
MSRB1	-0.70505458	1.144470173	-0.439415592	9
MTARC1	-0.977195768	1.021342028	-0.04414626	9
MTCH2	-1.066382287	0.916755498	0.149626789	9
MTFR1L	-1.122671513	0.795229974	0.32744154	9

MTFR2	-0.94053286	1.050394394	-0.109861534	9
MTHFD2	-1.038207538	0.956818095	0.081389443	9
MTIF3	-0.943883834	1.047973584	-0.10408975	9
MTM1	-0.821536467	1.113483236	-0.291946769	9
MTMR6	-1.076273449	0.900388842	0.175884607	9
MTR	-0.871492167	1.09177661	-0.220284444	9
MTRF1	-1.100449897	0.853139983	0.247309913	9
MTRF1L	-0.688566768	1.147032943	-0.458466175	9
MTRNR2L10	-1.123076417	0.793969964	0.329106453	9
MTRNR2L12	-0.839965803	1.106162971	-0.266197168	9
MTRNR2L5	-0.686305802	1.147353286	-0.461047484	9
MTRNR2L7	-0.745573555	1.136390114	-0.390816559	9
MUSK	-1.103155943	0.8470187	0.256137244	9
MXD3	-0.697405024	1.145709209	-0.448304184	9
MXI1	-0.992200313	1.007621324	-0.015421011	9
MXRA7	-0.963025225	1.033270542	-0.070245317	9
MYBL1	-0.857687456	1.098380263	-0.240692807	9
MYL2	-0.985553553	1.01384601	-0.028292457	9
MYSM1	-1.013899938	0.985494832	0.028405106	9
N4BP2L1	-0.732175227	1.139353142	-0.407177916	9
NAA40	-0.75944384	1.133003933	-0.373560093	9
NACC1	-0.733935713	1.138980665	-0.405044952	9
NADK2	-0.642223555	1.152172352	-0.509948797	9
NAPRT	-0.850236633	1.101744691	-0.251508058	9
NBDY	-0.670651991	1.149371099	-0.478719108	9
NBEAL1	-0.987086934	1.012431257	-0.025344324	9
NBL1	-0.816468665	1.115365289	-0.298896623	9
NCDN	-1.071190067	0.908976715	0.162213353	9
NCOA2	-0.874837543	1.090101278	-0.215263735	9
NDC1	-1.109137011	0.832707531	0.27642948	9
NDFIP1	-0.804071723	1.11974158	-0.315669857	9
NDST1	-0.995527942	1.004412851	-0.008884909	9
NDUFA1	-0.977632224	1.02095935	-0.043327125	9
NDUFA10	-1.050918712	0.939799067	0.111119645	9
NDUFAF2	-1.100906444	0.852121502	0.248784942	9
NDUFAF3	-0.659633819	1.150586316	-0.490952497	9
NDUFAF6	-0.86174771	1.096488731	-0.234741021	9
NDUFB1	-0.686384169	1.147342306	-0.460958138	9
NDUFB11	-0.778692125	1.127742251	-0.349050125	9
NDUFB2	-0.978199806	1.020460284	-0.042260478	9
NDUFB4	-1.072839141	0.906233218	0.166605923	9
NDUFC2-KCTD14	-0.831623068	1.109571078	-0.27794801	9
NDUFV2	-0.932725075	1.055869069	-0.123143995	9
NEK2	-0.665970214	1.149907842	-0.483937628	9
NEMP2	-1.050433723	0.940477919	0.109955804	9
NEUROG1	-1.108045312	0.835406184	0.272639128	9
NFAM1	-0.963170311	1.033153115	-0.069982803	9
NFXL1	-0.904338162	1.073964358	-0.169626196	9
NFYA	-0.72363704	1.14108874	-0.4174517	9
NFYC	-0.817567915	1.114961717	-0.297393802	9
NGLY1	-0.693169574	1.146357853	-0.453188279	9
NHLRC3	-0.72432569	1.140953074	-0.416627385	9
NHSL2	-1.130734748	0.768047411	0.362687337	9
NIPAL1	-0.789174022	1.124589252	-0.33541523	9
NIPSNAP1	-0.638575073	1.152454031	-0.513878957	9

NKAPD1	-0.758697065	1.133194719	-0.374497654	9
NLRP4	-1.089888077	0.875259017	0.21462906	9
NME3	-0.960155723	1.035573803	-0.075418079	9
NME7	-0.884102148	1.085302614	-0.201200467	9
NME8	-0.763669256	1.131905925	-0.368236669	9
NMI	-0.859981594	1.097316612	-0.237335018	9
NMUR1	-0.912715577	1.068903874	-0.156188296	9
NOB1	-1.050494007	0.940393672	0.110100335	9
NOMO3	-0.983297574	1.015904841	-0.032607267	9
NOVA2	-0.671836429	1.149230491	-0.477394062	9
NPAS2	-0.9734614	1.024577962	-0.051116562	9
NPEPL1	-1.021263975	0.977284878	0.043979097	9
NR2C2	-0.932139832	1.056270306	-0.124130474	9
NRDC	-1.021682505	0.976806535	0.044875969	9
NRG1	-0.917824777	1.065705812	-0.147881036	9
NRP1	-0.791588886	1.123833094	-0.332244208	9
NSA2	-0.757934905	1.133388429	-0.375453524	9
NSDHL	-1.065804671	0.917668892	0.148135779	9
NT5DC1	-0.996399648	1.003561879	-0.007162231	9
NT5E	-1.002078643	0.997908313	0.00417033	9
NUB1	-0.651627846	1.151365776	-0.499737931	9
NUDT12	-1.128474668	0.776154045	0.352320624	9
NUP155	-0.872797724	1.091126362	-0.218328638	9
NUP88	-0.873677837	1.090685443	-0.217007606	9
NUTF2	-0.980808502	1.018145676	-0.037337174	9
NWD2	-1.044287584	0.948872723	0.095414861	9
NXF1	-0.911924414	1.069391418	-0.157467005	9
NXT1	-0.985166423	1.014201213	-0.029034789	9
OAT	-1.102299198	0.84897901	0.253320188	9
OCM	-1.052446571	0.93764419	0.114802381	9
ODF3B	-0.872041729	1.091503448	-0.219461718	9
OGFOD2	-1.05184162	0.938500384	0.113341236	9
OGFRL1	-1.018161569	0.980790722	0.037370847	9
OIT3	-0.833393362	1.108861133	-0.27546777	9
OLFM2	-0.789951796	1.124346942	-0.334395146	9
OR10A4	-0.733971665	1.138973006	-0.405001341	9
OR10G7	-0.660526145	1.150494082	-0.489967937	9
OR1A2	-1.007071605	0.992775097	0.014296508	9
OR1J1	-1.064023016	0.920458758	0.143564259	9
OR1J2	-0.728113517	1.140193364	-0.412079846	9
OR1N1	-0.81469373	1.116011521	-0.301317792	9
OR2B2	-0.939560723	1.051088572	-0.111527849	9
OR2D3	-1.082220175	0.889821458	0.192398717	9
OR2F2	-1.084050908	0.886445578	0.19760533	9
OR2M5	-1.129432386	0.772771148	0.356661237	9
OR2T5	-1.07088206	0.909484757	0.161397303	9
OR2W1	-0.9600464	1.035660836	-0.075614436	9
OR4D6	-0.649836641	1.151528419	-0.501691778	9
OR4K15	-1.129231182	0.773488049	0.355743133	9
OR52B2	-1.033243066	0.963059182	0.070183884	9
OR52B6	-0.721331036	1.141537564	-0.420206528	9
OR52H1	-0.642993433	1.152110697	-0.509117264	9
OR52N4	-1.018983597	0.979868586	0.039115011	9
OR5B17	-1.082258548	0.889751314	0.192507233	9
OR5H6	-1.005368669	0.99454344	0.010825228	9

OR5K4	-1.052824254	0.937107656	0.115716598	9
OR5M10	-0.747930764	1.135837864	-0.387907101	9
OR6C3	-1.084493566	0.885620164	0.198873402	9
OR6K6	-0.904155386	1.07407229	-0.169916903	9
OR8B4	-1.108075311	0.835332571	0.27274274	9
ORAI3	-0.83711622	1.107344961	-0.270228741	9
ORC3	-0.922446759	1.062737288	-0.140290529	9
OSBPL8	-1.120465689	0.801929926	0.318535763	9
OSGEP	-0.778173311	1.127892938	-0.349719627	9
OST4	-1.07813733	0.89713978	0.180997549	9
OSTC	-1.045044119	0.94785968	0.097184439	9
OTULINL	-0.741097996	1.13741295	-0.396314954	9
OTX2	-1.010134007	0.989548134	0.020585872	9
P2RX4	-0.974787084	1.023437016	-0.048649933	9
P4HA1	-0.888663807	1.082851659	-0.194187852	9
PABIR3	-1.112636232	0.823769908	0.288866324	9
PABPN1	-1.114262348	0.819456479	0.294805869	9
PACRGL	-0.938271278	1.052003768	-0.11373249	9
PADI6	-1.12670964	0.782201889	0.344507751	9
PAIP2	-0.770088448	1.130176989	-0.360088541	9
PANX1	-0.63458094	1.152742607	-0.518161666	9
PARD6G	-0.652370474	1.151297092	-0.498926618	9
PARG	-0.881715854	1.086561315	-0.204845461	9
PARK7	-1.123668406	0.792110091	0.331558316	9
PARP16	-0.844305781	1.104326484	-0.260020703	9
PATE4	-1.104251269	0.844481327	0.259769943	9
PAX3	-0.631192415	1.152971333	-0.521778919	9
PBDC1	-1.083070694	0.888260605	0.194810089	9
PBK	-0.855574153	1.09934843	-0.243774277	9
PBRM1	-0.711475091	1.143362073	-0.431886982	9
PCBP4	-0.912472109	1.069054125	-0.156582017	9
PCDHA6	-0.877383996	1.088805828	-0.211421832	9
PCDHGA1	-1.124805921	0.78847536	0.336330562	9
PCDHGA10	-1.115985143	0.814766539	0.301218604	9
PCDHGA12	-1.029966048	0.967064272	0.062901776	9
PCDHGA4	-1.044910489	0.948039024	0.096871465	9
PCDHGA5	-1.033610028	0.962605117	0.071004911	9
PCDHGA6	-0.983517549	1.015705263	-0.032187714	9
PCDHGA8	-1.011733283	0.987838449	0.023894834	9
PCDHGA9	-1.043508064	0.949910768	0.093597295	9
PCDHGB1	-1.080039715	0.893765049	0.186274666	9
PCDHGB2	-1.002412246	0.99757017	0.004842076	9
PCDHGB3	-1.033539635	0.962692307	0.070847328	9
PCDHGB4	-1.022918754	0.975386006	0.047532748	9
PCDHGB5	-1.060710416	0.925538786	0.13517163	9
PCDHGB6	-1.113037961	0.82271416	0.2903238	9
PCDHGB7	-1.046005546	0.946564219	0.099441327	9
PCDHGC3	-0.964170951	1.032340673	-0.068169722	9
PCDHGC4	-1.093148943	0.868706496	0.224442447	9
PCDHGC5	-0.756692164	1.133702108	-0.377009945	9
PCED1B	-1.129294263	0.773263649	0.356030614	9
PCF11	-0.845304069	1.103897786	-0.258593717	9
PCK2	-1.075941556	0.900961585	0.174979972	9
PCM1	-0.959787848	1.035866464	-0.076078615	9
PCMT1	-0.711706895	1.143320889	-0.431613994	9

PCNX3	-0.701339156	1.145082909	-0.443743754	9
PCOLCE2	-1.084254563	0.886066276	0.198188286	9
PCP2	-0.980480181	1.01843888	-0.037958699	9
PCYT1A	-1.065467783	0.918199587	0.147268197	9
PDC	-1.014109131	0.985266864	0.028842267	9
PDCD1	-0.953696668	1.040627545	-0.086930877	9
PDCD4	-1.017761255	0.981238036	0.036523219	9
PDCD5	-0.870436471	1.0922991	-0.221862629	9
PDE3B	-1.115234379	0.816826004	0.298408375	9
PDE4D	-0.901361948	1.075708957	-0.174347009	9
PDE6A	-1.092195855	0.870645552	0.221550303	9
PDE7A	-0.628517078	1.153141554	-0.524624476	9
PDHA2	-1.050772869	0.940003466	0.110769403	9
PDIA3	-0.758643494	1.133208368	-0.374564874	9
PDIA4	-1.060806048	0.925394031	0.135412017	9
PDIK1L	-1.027490953	0.970031304	0.057459648	9
PDK4	-0.798175424	1.121712784	-0.32353736	9
PDS5A	-0.947883448	1.045026417	-0.097142969	9
PEAK3	-1.073754096	0.904693722	0.169060375	9
PER3	-0.880038487	1.087436571	-0.207398085	9
PET100	-1.021339683	0.977198446	0.044141237	9
PEX26	-1.055987625	0.93255234	0.123435285	9
PFDN5	-0.713923567	1.142922886	-0.42899932	9
PFKM	-0.890906019	1.081625038	-0.190719019	9
PGAM1	-0.692644163	1.146436478	-0.453792315	9
PGAP3	-0.714266865	1.14286057	-0.428593705	9
PGF	-0.927584431	1.059351064	-0.131766633	9
PGLYRP3	-0.830597991	1.109978946	-0.279380955	9
PGPEP1	-1.032171747	0.964378315	0.067793432	9
PHF13	-1.096773712	0.861141661	0.235632051	9
PHF20	-0.902398377	1.075104531	-0.172706154	9
PHF7	-0.780295275	1.12727345	-0.346978174	9
PHIP	-0.796623372	1.122220107	-0.325596735	9
PHKG1	-0.820624461	1.113825998	-0.293201537	9
PHLDB3	-1.055846957	0.932757274	0.123089684	9
PHYH	-0.742633087	1.137065898	-0.394432811	9
PID1	-0.94326024	1.048427389	-0.105167149	9
PIF1	-0.7478576	1.135855146	-0.387997546	9
PIGA	-1.012052854	0.98749477	0.024558084	9
PIGK	-1.104927759	0.842896291	0.262031468	9
PIGY	-1.037038344	0.958307309	0.078731035	9
PIH1D1	-1.096182614	0.862396505	0.233786109	9
PIP4K2A	-1.11801096	0.809082708	0.308928253	9
PISD	-0.915017541	1.067473667	-0.152456126	9
PITPNM3	-0.750583829	1.135205026	-0.384621197	9
PKP1	-1.008352635	0.991432639	0.016919996	9
PLAAT5	-0.695795831	1.145958766	-0.450162935	9
PLCB1	-1.076288759	0.90036238	0.175926379	9
PLEKHB1	-0.777411142	1.1281134	-0.350702258	9
PLEKHB2	-1.091657229	0.871732544	0.219924685	9
PLEKHH3	-1.028114916	0.969287937	0.058826979	9
PLEKHM3	-0.804844138	1.119478158	-0.31463402	9
PLGRKT	-1.010850804	0.988783953	0.022066851	9
PLIN3	-0.673779364	1.148995603	-0.475216239	9
PLK2	-0.920290859	1.064130985	-0.143840126	9

PLK4	-0.894949107	1.079376106	-0.184426999	9
PLPP7	-0.81640887	1.115387168	-0.298978298	9
PLRG1	-0.715109511	1.142706837	-0.427597326	9
PLXDC1	-0.682502473	1.147875549	-0.465373076	9
PLXDC2	-1.066409538	0.916712295	0.149697243	9
PMM1	-1.090973089	0.87310414	0.21786895	9
PNISR	-0.905014882	1.073563837	-0.168548956	9
PNRC1	-1.030942363	0.96588031	0.065062053	9
POC1A	-0.96149995	1.034499363	-0.072999414	9
PODXL	-0.649040204	1.151599367	-0.502559163	9
POLD1	-1.120132732	0.802918167	0.317214565	9
POLD4	-1.032467796	0.964014745	0.068453051	9
POLK	-0.648783555	1.15162205	-0.502838496	9
POLN	-1.058751718	0.928479354	0.130272365	9
POLR1E	-0.872805594	1.091122429	-0.218316834	9
POLR2F	-1.040665191	0.953647689	0.087017502	9
POLR2H	-0.785144037	1.125826156	-0.340682119	9
POLR2L	-0.659512267	1.150598796	-0.491086529	9
POLR2M	-0.699389598	1.145396152	-0.446006553	9
POLR3C	-0.853308639	1.10037405	-0.247065411	9
POLR3E	-0.630596994	1.153010007	-0.522413012	9
POLR3F	-0.644062118	1.152023825	-0.507961706	9
POLR3G	-0.739233174	1.137829276	-0.398596102	9
POLR3GL	-0.835972728	1.107814023	-0.271841295	9
POLR3K	-0.631577858	1.152946057	-0.521368199	9
POLRMT	-0.676554969	1.148650874	-0.472095905	9
POMC	-1.12226684	0.796479664	0.325787176	9
POMP	-0.862918708	1.095935446	-0.233016738	9
POMZP3	-0.896062339	1.078748391	-0.182686052	9
PPEF1	-1.057749011	0.929967103	0.127781908	9
PPIA	-0.754993319	1.134126566	-0.379133247	9
PPID	-0.805946458	1.119100124	-0.313153666	9
PPIL1	-0.888263674	1.083069028	-0.194805354	9
PPM1B	-0.741925868	1.137226273	-0.395300405	9
PPM1M	-0.923078036	1.062326172	-0.139248136	9
PPP1R10	-1.057231655	0.930730141	0.126501513	9
PPP1R3C	-1.07662373	0.899782484	0.176841246	9
PPP1R9B	-0.937814249	1.052326635	-0.114512386	9
PPP3CA	-0.696621899	1.145831136	-0.449209237	9
PPP3CB	-0.895205687	1.079231756	-0.18402607	9
PRAME	-0.670075558	1.149438823	-0.479363265	9
PRC1	-0.689949234	1.146833421	-0.456884186	9
PRDM11	-0.775160295	1.128758188	-0.353597893	9
PRDX2	-0.915442352	1.067207825	-0.151765472	9
PRDX3	-0.913967859	1.068127992	-0.154160133	9
PRH2	-0.920676826	1.063882642	-0.143205817	9
PRIMPOL	-1.028484538	0.968846125	0.059638413	9
PRKAR2A	-0.951252144	1.042494239	-0.091242095	9
PRKCQ	-0.71543776	1.142646653	-0.427208892	9
PRKRA	-1.079182723	0.895292765	0.183889958	9
PRMT2	-0.770683034	1.130013086	-0.359330052	9
PRODH	-0.79888439	1.121479451	-0.32259506	9
PRPF38A	-0.639835619	1.15235867	-0.51252305	9
PRPF4B	-0.840184522	1.106071471	-0.265886949	9
PRPH2	-0.872615769	1.09121726	-0.218601491	9

PRR14	-1.032273843	0.964253017	0.068020825	9
PRR20G	-0.788094581	1.124923613	-0.336829032	9
PRRG1	-0.689071258	1.146960455	-0.457889197	9
PRRG4	-0.658531085	1.150698807	-0.492167722	9
PRSS1	-0.843025922	1.104872655	-0.261846733	9
PRSS51	-0.985303598	1.014075442	-0.028771844	9
PSD2	-0.76368234	1.131902476	-0.368220136	9
PSKH1	-0.82550388	1.111971153	-0.286467273	9
PSMA7	-0.924885967	1.061141122	-0.136255155	9
PSMB3	-1.126382054	0.783299186	0.343082868	9
PSMD1	-1.096230381	0.862295415	0.233934966	9
PSMD10	-1.040810686	0.953458269	0.087352416	9
PSMD6	-1.095925512	0.862939667	0.232985845	9
PSMG3	-0.803574249	1.119910596	-0.316336347	9
PTCD3	-0.764089417	1.131795015	-0.367705598	9
PTCH1	-1.112032398	0.825344918	0.28668748	9
PTER	-1.027039564	0.970567154	0.05647241	9
PTGES	-0.724086487	1.141000283	-0.416913796	9
PTGES3	-1.082631339	0.88906851	0.193562829	9
PTMA	-0.770778368	1.129986747	-0.359208378	9
PTOV1	-1.09925729	0.855774129	0.243483161	9
PTP4A2	-0.948317267	1.04470291	-0.096385643	9
PTPN18	-0.847231975	1.103063178	-0.255831203	9
PTPN21	-0.902079349	1.075290935	-0.173211586	9
PTPRM	-0.638329311	1.152472383	-0.514143072	9
PTS	-0.902622959	1.074973123	-0.172350165	9
PTTG2	-1.080130706	0.893602117	0.186528589	9
PUM1	-0.952070012	1.041872456	-0.089802444	9
PVALB	-0.826900031	1.111430856	-0.284530825	9
PVRIG	-1.03527443	0.960531267	0.074743163	9
PYHIN1	-0.936133991	1.053506884	-0.117372893	9
PYROXD1	-0.973267001	1.024744556	-0.051477555	9
PYROXD2	-1.105452973	0.84165605	0.263796923	9
PYURF	-1.037038344	0.958307309	0.078731035	9
R3HDML	-1.078168045	0.897085769	0.181082276	9
RAB11A	-0.954884554	1.039711444	-0.08482689	9
RAB14	-1.046912049	0.945334408	0.101577641	9
RAB21	-1.130770176	0.76791683	0.362853346	9
RAB24	-1.085993489	0.882796106	0.203197384	9
RAB28	-0.714480807	1.142821642	-0.428340835	9
RAB29	-0.648447962	1.151651579	-0.503203617	9
RAB33B	-0.644949129	1.151950583	-0.507001454	9
RAB38	-1.06636052	0.916789998	0.149570523	9
RAB40C	-0.722794513	1.141253698	-0.418459185	9
RAB4A	-0.944540513	1.047494051	-0.102953538	9
RABGGTA	-1.011697279	0.987877126	0.023820152	9
RABIF	-0.870997075	1.092022011	-0.221024936	9
RAD1	-0.990781123	1.008970716	-0.018189593	9
RAD51B	-0.944564083	1.047476809	-0.102912726	9
RAE1	-1.117875547	0.809468603	0.308406944	9
RASGEF1A	-1.073988062	0.904298036	0.169690026	9
RASGRP1	-0.806308458	1.118975438	-0.312666979	9
RASL10A	-1.019263	0.97955405	0.03970895	9
RASL11B	-0.797934736	1.121791771	-0.323857036	9
RASSF1	-1.127627845	0.779084874	0.34854297	9

RASSF3	-0.824400555	1.112395103	-0.287994548	9
RB1	-0.939809639	1.050911172	-0.111101532	9
RB1CC1	-1.056993238	0.931080736	0.125912501	9
RBAK	-1.060857179	0.925316592	0.135540587	9
RBAK-RBAKDN	-1.087774458	0.879386926	0.208387532	9
RBL2	-0.981422922	1.017595496	-0.036172575	9
RBM12B	-0.815686222	1.115650988	-0.299964766	9
RBM19	-0.63753064	1.152531482	-0.515000842	9
RBM24	-0.639651129	1.152372755	-0.512721626	9
RBM38	-0.793481033	1.123232697	-0.329751664	9
RBM45	-0.699581421	1.145365582	-0.445784161	9
RC3H2	-1.016634117	0.982491398	0.034142719	9
RCBTB2	-0.807822352	1.118451088	-0.310628736	9
RCSD1	-0.718909859	1.141999792	-0.423089933	9
RDH11	-0.663237611	1.150207166	-0.486969555	9
RDH12	-0.734004955	1.138965912	-0.404960957	9
RDX	-0.789980107	1.1243381	-0.334357994	9
REEP5	-1.041978604	0.951930646	0.090047957	9
REG3A	-1.009412053	0.990314387	0.019097666	9
REG3G	-1.114359082	0.819196483	0.295162599	9
RER1	-0.678128901	1.148450573	-0.470321672	9
RETNLB	-1.108922292	0.833241548	0.275680745	9
RETREG3	-0.811290899	1.117231922	-0.305941023	9
REXO1	-0.653973744	1.151146296	-0.497172552	9
RFC5	-0.755088675	1.134102874	-0.379014199	9
RGMB	-0.950937501	1.042732712	-0.091795211	9
RHBDD3	-0.94126819	1.049866903	-0.108598713	9
RHBDL3	-1.053640234	0.935943195	0.117697039	9
RHOT2	-0.871212833	1.091915147	-0.220702314	9
RINL	-0.990096121	1.00961803	-0.019521909	9
RLF	-0.885096549	1.084773371	-0.199676822	9
RNASE1	-0.736766955	1.138371036	-0.401604081	9
RNASEH2A	-1.07224919	0.907219268	0.165029922	9
RNF11	-0.923080184	1.062324771	-0.139244587	9
RNF113B	-0.883056988	1.085855869	-0.202798881	9
RNF114	-0.802699634	1.12020653	-0.317506896	9
RNF187	-0.666893126	1.149804431	-0.482911305	9
RNF2	-0.668959437	1.149568642	-0.480609205	9
RNF20	-1.109317265	0.832257991	0.277059274	9
RNF214	-0.930066605	1.057681666	-0.127615061	9
RNPS1	-0.819647721	1.114191102	-0.294543381	9
RPA4	-0.95169956	1.042154434	-0.090454874	9
RPE	-0.675732285	1.14875418	-0.473021895	9
RPIA	-0.980283905	1.0186139	-0.038329994	9
RPL11	-1.115384122	0.816417194	0.298966928	9
RPL13	-0.890800395	1.081683147	-0.190882752	9
RPL21	-0.922430568	1.062747815	-0.140317247	9
RPL23A	-0.835947015	1.107824536	-0.271877521	9
RPL27	-1.067423614	0.915097598	0.152326016	9
RPL28	-0.700462566	1.145224456	-0.44476189	9
RPL35	-0.692267057	1.146492661	-0.454225604	9
RPL35A	-1.120122997	0.802946972	0.317176025	9
RPL36A	-1.085749255	0.883258846	0.20249041	9
RPL36A-HNRNPH2	-1.026886628	0.970748344	0.056138284	9
RPL37A	-1.130686497	0.768225072	0.362461424	9

RPL39	-1.10517591	0.842311367	0.262864543	9
RPL6	-0.872104558	1.091472167	-0.219367609	9
RPLP2	-0.72377466	1.141061689	-0.417287028	9
RPN1	-0.99651383	1.003450086	-0.006936256	9
RPP25L	-0.713035664	1.143083218	-0.430047553	9
RPS15	-1.104440552	0.844039228	0.260401324	9
RPS15A	-0.987720286	1.011843229	-0.024122943	9
RPS17	-1.10238252	0.848789286	0.253593233	9
RPS18	-1.117770952	0.809766074	0.308004877	9
RPS20	-0.843000634	1.104883407	-0.261882773	9
RPS21	-0.997394506	1.002585286	-0.00519078	9
RPS23	-0.860969667	1.096854419	-0.235884752	9
RPS25	-1.102431677	0.848677262	0.253754415	9
RPS27	-0.90301697	1.0747422	-0.17172523	9
RPS6KA1	-0.632850704	1.152861237	-0.520010533	9
RPS6KA3	-0.85244655	1.100761013	-0.248314462	9
RPS6KA4	-0.750824695	1.135146978	-0.384322283	9
RRAGD	-0.637630096	1.152524167	-0.514894071	9
RRP8	-0.789560204	1.124469086	-0.334908882	9
RSC1A1	-0.80881496	1.118104734	-0.309289774	9
RSL24D1	-0.711208155	1.143409396	-0.432201241	9
RSPH1	-0.927178016	1.059622308	-0.132444293	9
RSPH9	-0.650764409	1.15144471	-0.5006803	9
RSPO1	-0.694767491	1.146116241	-0.451348751	9
RSPO2	-1.053692657	0.935868135	0.117824522	9
RTF2	-1.11173235	0.826122325	0.285610026	9
RTN4IP1	-0.982947042	1.016222351	-0.033275309	9
RUFY1	-0.763807525	1.131869461	-0.368061935	9
RUFY2	-1.048326146	0.943399544	0.104926603	9
RWDD1	-0.884705794	1.084981673	-0.200275879	9
RWDD2B	-0.984317836	1.014977049	-0.030659213	9
RXRB	-0.676327519	1.148679531	-0.472352011	9
RYBP	-0.656896141	1.150862561	-0.49396642	9
S100A12	-0.919981179	1.064329875	-0.144348696	9
S100A3	-0.825723997	1.111886255	-0.286162258	9
S100G	-0.672703479	1.149126322	-0.476422843	9
S100PBP	-0.959324897	1.036233923	-0.076909026	9
SAAL1	-0.682835648	1.147830627	-0.464994979	9
SAMD8	-0.757518211	1.133493907	-0.375975696	9
SAMHD1	-0.743045562	1.136971976	-0.393926414	9
SAR1B	-1.013675977	0.985738563	0.027937414	9
SASS6	-1.109076389	0.832858465	0.276217924	9
SAT1	-1.101388065	0.851040911	0.250347154	9
SBNO1	-0.850407215	1.101669184	-0.251261969	9
SCCPDH	-1.127007294	0.781198202	0.345809092	9
SCFD2	-0.943414002	1.048315634	-0.104901633	9
SCML1	-1.040951108	0.953275269	0.087675839	9
SDCBP	-0.882879932	1.08594929	-0.203069358	9
SDE2	-1.10413512	0.844752079	0.25938304	9
SDHA	-0.816948281	1.115189522	-0.298241241	9
SDHAF1	-0.669545469	1.149500694	-0.479955226	9
SDHAF3	-0.666570159	1.149840753	-0.483270594	9
SDK2	-0.639980899	1.152347546	-0.512366648	9
SEC23A	-1.100580443	0.852849334	0.247731109	9
SEC24A	-0.977039486	1.021478824	-0.044439338	9

SEC24B	-1.125410174	0.786510705	0.33889947	9
SEC61A2	-0.95298175	1.041176042	-0.088194293	9
SEL1L3	-0.636422177	1.152612137	-0.51618996	9
SELENOI	-1.026024879	0.971765871	0.054259009	9
SELENOS	-0.931873938	1.056452187	-0.124578249	9
SELENOW	-1.069270546	0.912120868	0.157149679	9
SEMA3F	-0.982469039	1.016654295	-0.034185256	9
SEMA4B	-0.869163881	1.09292502	-0.223761139	9
SENP3-EIF4A1	-1.065380629	0.918336637	0.147043992	9
SEPTIN10	-1.012260207	0.987271408	0.024988799	9
SEPTIN7	-0.688175852	1.147088859	-0.458913007	9
SEPTIN8	-0.630313967	1.153028231	-0.522714264	9
SERINC4	-1.112345405	0.824530252	0.287815153	9
SERINC5	-0.781821026	1.126822812	-0.345001786	9
SERPINA5	-0.688362153	1.147062238	-0.458700085	9
SERPINB1	-0.741171184	1.137396492	-0.396225308	9
SERPINB2	-0.894277325	1.079753118	-0.185475792	9
SERPINB8	-0.902547994	1.075017004	-0.172469011	9
SERPIND1	-0.802432258	1.12029669	-0.317864433	9
SERPINF1	-0.741397198	1.137345614	-0.395948416	9
SERPINF2	-0.876541801	1.089236225	-0.212694424	9
SERPINI1	-0.666140759	1.149888821	-0.483748062	9
SESN3	-0.776707651	1.128315932	-0.351608281	9
SETD3	-0.635292897	1.152692675	-0.517399778	9
SETD5	-1.119223703	0.805586904	0.3136368	9
SETDB1	-0.72512282	1.140795097	-0.415672277	9
SETX	-0.890066147	1.082086203	-0.192020056	9
SEZ6L2	-0.729702168	1.139867896	-0.410165728	9
SF3B1	-0.790448994	1.124191431	-0.333742437	9
SF3B2	-0.953188433	1.041017691	-0.087829258	9
SF3B5	-0.988776397	1.010857876	-0.022081478	9
SF3B6	-1.099372783	0.855520682	0.2438521	9
SFRP2	-0.992434765	1.00739732	-0.014962555	9
SFT2D1	-1.009605095	0.990109836	0.019495259	9
SFTPA2	-0.79629837	1.122325738	-0.326027368	9
SGCG	-0.669410854	1.149516345	-0.48010549	9
SGK3	-0.920375273	1.064076714	-0.14370144	9
SGMS2	-0.78317001	1.126420736	-0.343250726	9
SH2D1A	-1.097952396	0.858613732	0.239338664	9
SH3D21	-0.938095059	1.052128352	-0.114033293	9
SH3GL3	-1.069422041	0.91187461	0.157547431	9
SH3RF1	-0.739975836	1.137664166	-0.397688331	9
SHLD3	-0.975695215	1.022650502	-0.046955287	9
SIGLEC11	-1.114370013	0.819167077	0.295202936	9
SIGLEC9	-0.775949755	1.128533102	-0.352583347	9
SIGMAR1	-1.114455772	0.818936214	0.295519558	9
SIRT4	-0.998852551	1.001143512	-0.002290961	9
SKA2	-1.045646042	0.947049689	0.098596353	9
SKIV2L	-0.857394793	1.098515004	-0.241120211	9
SLA	-0.80199383	1.120444218	-0.318450388	9
SLC10A3	-1.052240632	0.937936099	0.114304533	9
SLC11A1	-1.105425583	0.84172094	0.263704644	9
SLC16A1	-0.999072917	1.000924511	-0.001851594	9
SLC16A2	-0.871970436	1.09153893	-0.219568494	9
SLC16A6	-1.11154593	0.826603603	0.284942327	9

SLC25A14	-1.086221815	0.882362475	0.203859341	9
SLC25A16	-1.124977954	0.78791846	0.337059494	9
SLC25A23	-1.015373566	0.983882531	0.031491035	9
SLC25A24	-0.856847955	1.098766194	-0.241918239	9
SLC25A38	-1.057826137	0.929853087	0.12797305	9
SLC25A4	-0.642388325	1.152159222	-0.509770897	9
SLC25A42	-0.698210856	1.145582798	-0.447371942	9
SLC25A46	-0.936772874	1.053059369	-0.116286495	9
SLC25A5	-0.903799562	1.07428211	-0.170482548	9
SLC26A6	-0.843908391	1.10449648	-0.260588089	9
SLC26A8	-1.017964154	0.981011457	0.036952697	9
SLC28A3	-0.631630912	1.152942563	-0.521311651	9
SLC29A4	-1.078208423	0.897014741	0.181193683	9
SLC2A1	-0.750690977	1.135179216	-0.384488239	9
SLC2A11	-0.846640051	1.10332037	-0.256680318	9
SLC2A14	-0.657302347	1.150822213	-0.493519867	9
SLC2A3	-0.748750936	1.135643507	-0.386892571	9
SLC30A5	-0.659261633	1.150624467	-0.491362835	9
SLC35A3	-0.785725011	1.125649756	-0.339924745	9
SLC35E2B	-0.689019698	1.14696788	-0.457948182	9
SLC37A2	-0.64065063	1.152295914	-0.511645284	9
SLC38A6	-0.844135075	1.104399554	-0.260264479	9
SLC38A9	-0.74955762	1.13545123	-0.385893611	9
SLC43A2	-1.130760176	0.767953699	0.362806477	9
SLC43A3	-0.674295841	1.148932275	-0.474636434	9
SLC4A1	-1.067428084	0.915090449	0.152337635	9
SLC51A	-0.710491086	1.14353598	-0.433044894	9
SLC52A1	-0.92517417	1.06095116	-0.135776991	9
SLC5A7	-1.023216107	0.975042621	0.048173486	9
SLC9A6	-0.797617441	1.121895723	-0.324278282	9
SLC9A9	-0.892325549	1.08084091	-0.188515362	9
SLC9B1	-0.797938872	1.121790415	-0.323851543	9
SLCO4A1	-0.720924778	1.141615766	-0.420690988	9
SLF1	-0.794745034	1.122827718	-0.328082684	9
SLITRK3	-1.096792662	0.861101291	0.235691372	9
SLITRK4	-0.854771117	1.09971342	-0.244942303	9
SLX4IP	-1.091321898	0.872406089	0.218915809	9
SMAGP	-0.948711795	1.044408042	-0.095696247	9
SMARCA2	-0.917580474	1.065860707	-0.148280232	9
SMARCC1	-0.878181227	1.088396627	-0.2102154	9
SMC3	-1.018152119	0.980801294	0.037350824	9
SMCHD1	-0.684025355	1.147668923	-0.463643567	9
SMCP	-1.05029005	0.940678547	0.109611504	9
SMIM1	-0.939281088	1.051287583	-0.112006496	9
SMIM10	-1.016944007	0.9821477	0.034796307	9
SMIM12	-0.81078975	1.117409612	-0.306619862	9
SMIM15	-0.878081142	1.088448094	-0.210366952	9
SMIM18	-0.983835991	1.015415901	-0.03157991	9
SMIM30	-0.94751644	1.045299511	-0.097783071	9
SMIM35	-0.680761674	1.148107683	-0.467346009	9
SMIM39	-0.773025858	1.129361007	-0.356335149	9
SMIM4	-0.924368633	1.061481382	-0.137112748	9
SMIM40	-1.052313046	0.937833508	0.114479537	9
SMU1	-0.651993871	1.151332015	-0.499338145	9
SNAPIN	-1.054995425	0.933993088	0.121002337	9

SNRPA	-0.950781636	1.042850694	-0.092069058	9
SNU13	-0.991980197	1.00783135	-0.015851153	9
SNW1	-0.818267205	1.114703646	-0.296436441	9
SNX11	-0.846781552	1.103258963	-0.256477411	9
SNX17	-0.992112297	1.007705337	-0.01559304	9
SNX2	-1.086293935	0.8822253	0.204068634	9
SNX22	-1.069585565	0.911608439	0.157977126	9
SNX4	-0.849034829	1.102274668	-0.253239839	9
SNX6	-0.964334111	1.032207773	-0.067873662	9
SOCS6	-0.719649675	1.141859529	-0.422209854	9
SOX6	-0.795325999	1.122640528	-0.327314529	9
SPANXN3	-1.102931475	0.847534345	0.25539713	9
SPAST	-1.107180366	0.83751575	0.269664617	9
SPATA16	-0.962711584	1.033524068	-0.070812484	9
SPATC1L	-0.81233907	1.116858585	-0.304519515	9
SPCS1	-0.851612837	1.101133511	-0.249520674	9
SPIDR	-1.115596223	0.815836481	0.299759743	9
SPIN2B	-0.752881428	1.13464726	-0.381765832	9
SPINK8	-0.783075524	1.12644901	-0.343373486	9
SPNS2	-1.064653938	0.919475511	0.145178427	9
SPON1	-0.985499991	1.013895202	-0.028395211	9
SPON2	-1.083808968	0.886895194	0.196913774	9
SPOUT1	-0.745433667	1.136422592	-0.390988925	9
SPRR2A	-0.693785436	1.146265176	-0.45247974	9
SPRR2B	-0.891404916	1.081350125	-0.189945209	9
SPRR2E	-1.001161162	0.998834779	0.002326384	9
SPRR2F	-1.130353644	0.769444978	0.360908666	9
SPRR4	-1.088920217	0.877160531	0.211759685	9
SPRR5	-0.939751236	1.050952817	-0.111201581	9
SPRY2	-1.118616455	0.807346369	0.311270086	9
SRD5A2	-0.913595129	1.068359463	-0.154764333	9
SRGAP2B	-0.70399762	1.144646596	-0.440648976	9
SRGAP2C	-0.910787154	1.07008868	-0.159301526	9
SRP72	-0.945757447	1.046600902	-0.100843455	9
SRRD	-0.639671422	1.152371208	-0.512699786	9
SRSF2	-1.125773376	0.785318093	0.340455283	9
SRSF3	-0.696964816	1.145777858	-0.448813041	9
SRSF5	-0.876731163	1.089139621	-0.212408458	9
SRY	-0.797541805	1.121920474	-0.324378669	9
SS18L1	-0.891669363	1.081204109	-0.189534746	9
SSR1	-0.742551907	1.137084349	-0.394532442	9
ST6GALNAC5	-1.060193014	0.926320029	0.133872985	9
ST8SIA3	-1.022576439	0.97578049	0.046795949	9
STAC	-0.883677193	1.085527935	-0.201850742	9
STAG2	-0.98739278	1.012147568	-0.024754788	9
STAG3	-0.881246402	1.086807064	-0.205560662	9
STAU2	-0.903566507	1.074419324	-0.170852817	9
STEAP4	-1.087976864	0.878995533	0.20898133	9
STK17B	-0.918142634	1.065503983	-0.147361349	9
STK3	-0.691066841	1.146670092	-0.455603251	9
STK32C	-0.930840167	1.057156881	-0.126316714	9
STK38L	-0.647238924	1.151756728	-0.504517804	9
STKLD1	-1.0061855	0.993697535	0.012487966	9
STPG4	-1.130321713	0.76956149	0.360760223	9
STRBP	-0.744188146	1.136710318	-0.392522172	9

STRN3	-0.829523059	1.110404125	-0.280881066	9
STX10	-0.986093698	1.013349083	-0.027255385	9
STXBP1	-0.63110854	1.152976809	-0.521868269	9
STYX	-0.746701029	1.13612714	-0.389426111	9
SUCLG1	-0.808768734	1.118120909	-0.309352174	9
SUFU	-0.87146336	1.091790907	-0.220327547	9
SULT1B1	-0.6422226	1.152172428	-0.509949828	9
SULT2B1	-0.875594157	1.089718204	-0.214124047	9
SULT6B1	-1.018148534	0.980805305	0.037343229	9
SUPT4H1	-0.910726863	1.070125528	-0.159398666	9
SURF1	-0.713412039	1.143015403	-0.429603365	9
SURF2	-0.634843036	1.152724301	-0.517881265	9
SVIP	-0.732456893	1.139293887	-0.406836994	9
SYNC	-0.84542813	1.103844344	-0.258416214	9
SYNDIG1	-0.952527765	1.041523241	-0.088995476	9
SYNJ1	-0.786437703	1.125432485	-0.338994782	9
SYNJ2BP-COX16	-0.639407667	1.152391276	-0.512983608	9
SYNRG	-1.098882427	0.856594365	0.242288062	9
SYT7	-0.761228733	1.132543959	-0.371315226	9
SZT2	-0.850464515	1.101643805	-0.25117929	9
TACC1	-0.800385217	1.120982195	-0.320596978	9
TACC3	-0.971085011	1.026602045	-0.055517034	9
TACR2	-1.102731575	0.847992329	0.254739247	9
TAF15	-0.881719318	1.086559499	-0.204840182	9
TAF1D	-0.87788666	1.088548024	-0.210661364	9
TAFA2	-1.048778876	0.942775804	0.106003072	9
TALDO1	-0.790775034	1.124089194	-0.33331416	9
TAOK3	-0.734688165	1.138819925	-0.404131759	9
TAPT1	-0.709727231	1.143669956	-0.433942725	9
TARBP2	-0.798353394	1.121654305	-0.323300911	9
TARDBP	-0.785474859	1.125725788	-0.34025093	9
TARS1	-0.853154535	1.100443356	-0.247288821	9
TASOR2	-1.104339542	0.844275286	0.260064256	9
TATDN2	-0.757637692	1.133463693	-0.375826002	9
TATDN3	-0.927426082	1.059456817	-0.132030735	9
TBCE	-0.800260522	1.121023681	-0.320763159	9
TBPL2	-0.854672422	1.099758168	-0.245085747	9
TBX2	-0.790173638	1.124277614	-0.334103976	9
TBX20	-0.759766436	1.132921213	-0.373154777	9
TBXAS1	-1.016102833	0.983079073	0.03302376	9
TC2N	-1.119585625	0.804529448	0.315056177	9
TCAF2C	-0.645933702	1.151868075	-0.505934373	9
TCF7	-0.684042415	1.147666589	-0.463624174	9
TCIRG1	-0.922603418	1.062635393	-0.140031975	9
TCP10L2	-0.689518645	1.146895862	-0.457377217	9
TDRKH	-0.831788643	1.109504976	-0.277716333	9
TDRP	-1.054230749	0.935095945	0.119134803	9
TEP1	-0.757308647	1.133546839	-0.376238192	9
TESK1	-1.002519111	0.997461706	0.005057406	9
TEX13C	-1.069614817	0.911560785	0.158054032	9
TEX36	-1.036277378	0.959270072	0.077007306	9
TFAP4	-0.907546552	1.072052697	-0.164506145	9
TFCP2L1	-0.937367463	1.052641504	-0.115274041	9
TFR2	-0.651619058	1.151366585	-0.499747527	9
THAP1	-1.102581313	0.848335831	0.254245482	9

THG1L	-0.752104469	1.134836888	-0.38273242	9
THOC5	-1.081946634	0.890320725	0.191625909	9
THOC7	-1.131119405	0.766623543	0.364495862	9
THSD8	-1.075816984	0.90117611	0.174640874	9
TIA1	-0.816732074	1.115268816	-0.298536742	9
TICAM2	-0.726694197	1.140480715	-0.413786518	9
TIMM10B	-1.07850982	0.896483725	0.182026094	9
TIMM22	-1.071908686	0.907786061	0.164122626	9
TIMM8A	-0.956415561	1.038521939	-0.082106378	9
TIMP4	-1.068502134	0.913365024	0.15513711	9
TLDC2	-1.101831641	0.850040003	0.251791638	9
TLN2	-0.669542311	1.149501062	-0.47995875	9
TLR5	-0.854701394	1.099745035	-0.245043641	9
TLR6	-0.969360301	1.02805429	-0.058693989	9
TM9SF2	-0.87465269	1.090194635	-0.215541944	9
TM9SF3	-0.755019586	1.134120042	-0.379100456	9
TMA7	-0.997234245	1.002742995	-0.00550875	9
TMBIM6	-0.695903183	1.145942237	-0.450039053	9
TMC8	-0.812770581	1.116704224	-0.303933643	9
TMCC3	-0.660775089	1.150468157	-0.489693068	9
TMEM135	-0.860148967	1.097238493	-0.237089526	9
TMEM150B	-0.868288978	1.093352869	-0.225063891	9
TMEM167A	-0.935936693	1.053644776	-0.117708082	9
TMEM179B	-0.862541946	1.096113844	-0.233571898	9
TMEM18	-0.997618551	1.002364556	-0.004746005	9
TMEM185A	-0.751956531	1.134872877	-0.382916346	9
TMEM201	-1.027466978	0.970059805	0.057407173	9
TMEM207	-0.854484873	1.099843136	-0.245358264	9
TMEM221	-0.830518247	1.110010576	-0.27949233	9
TMEM222	-0.770478452	1.130069553	-0.359591101	9
TMEM230	-0.656440782	1.150907528	-0.494466746	9
TMEM237	-0.967845016	1.029318711	-0.061473695	9
TMEM238L	-1.030455786	0.966471347	0.063984439	9
TMEM243	-0.974696929	1.023514877	-0.048817948	9
TMEM255A	-1.117723751	0.809900144	0.307823607	9
TMEM259	-0.9709365	1.026727647	-0.055791148	9
TMEM260	-0.743775252	1.136805125	-0.393029873	9
TMEM269	-0.96704948	1.029978285	-0.062928805	9
TMEM50A	-1.118167656	0.808635064	0.309532592	9
TMEM52	-0.858578169	1.097968863	-0.239390693	9
TMEM59	-0.856590866	1.09888403	-0.242293164	9
TMEM63C	-0.687177358	1.147230676	-0.460053318	9
TMEM69	-1.028450033	0.968887416	0.059562616	9
TMEM72	-0.643586395	1.15206268	-0.508476285	9
TMEM8B	-1.058090722	0.929461417	0.128629305	9
TMPRSS11F	-0.70012341	1.145278913	-0.445155503	9
TMPRSS2	-0.635091035	1.152706899	-0.517615864	9
TMSB4X	-0.743492116	1.136869973	-0.393377856	9
TMX2	-0.967362172	1.029719382	-0.062357211	9
TMX4	-0.786798951	1.125321984	-0.338523033	9
TNFAIP8L1	-0.693330753	1.146333652	-0.453002898	9
TNFRSF10D	-1.048548022	0.94309412	0.105453902	9
TNNT3	-0.94892748	1.044246574	-0.095319094	9
TOMM20	-0.656563085	1.150895478	-0.494332393	9
TOMM5	-0.856632819	1.098864812	-0.242231993	9

TOMM6	-0.834621852	1.108364305	-0.273742453	9
TOMM7	-1.079072384	0.895488567	0.183583817	9
TOP2A	-0.738327176	1.138029464	-0.399702289	9
TOX3	-1.062443353	0.922898318	0.139545034	9
TP53RK	-1.068207313	0.913840213	0.1543671	9
TP53TG3D	-0.930443609	1.057426176	-0.126982567	9
TPGS2	-0.767809204	1.130799354	-0.36299015	9
TPI1	-0.690910326	1.146693075	-0.455782749	9
TPP2	-1.121286051	0.799469729	0.321816321	9
TRA2B	-1.091267571	0.872514982	0.218752589	9
TRABD2A	-0.817363264	1.115037046	-0.297673782	9
TRERF1	-0.913538561	1.068394553	-0.154855992	9
TRH	-1.012778079	0.986712287	0.026065792	9
TRIAP1	-0.930811931	1.057176074	-0.126364143	9
TRIM11	-1.124120221	0.790676158	0.333444062	9
TRIM29	-0.848655924	1.102441037	-0.253785112	9
TRIM43	-0.991006284	1.008757376	-0.017751092	9
TRIM56	-1.113398178	0.821762048	0.29163613	9
TRIM6-TRIM34	-0.94123512	1.04989067	-0.10865555	9
TRIM68	-0.921872451	1.06311011	-0.141237659	9
TRIM69	-0.693717738	1.146275391	-0.452557653	9
TRIQK	-1.122226811	0.796602764	0.325624047	9
TRMT10A	-0.720841512	1.141631762	-0.42079025	9
TRMT112	-0.690166907	1.146801752	-0.456634845	9
TRNT1	-1.128141658	0.777313178	0.350828481	9
TRPM2	-0.741376517	1.137350273	-0.395973756	9
TRPV1	-0.645445121	1.151909178	-0.506464057	9
TSKS	-0.990788916	1.008963337	-0.018174421	9
TSPYL2	-0.839817597	1.106224909	-0.266407312	9
TSR1	-0.967793662	1.029361377	-0.061567715	9
TSSK2	-0.884916465	1.084869423	-0.199952958	9
TSSK3	-0.73236592	1.139313039	-0.40694712	9
TTC16	-0.851484596	1.101190658	-0.249706062	9
TTC19	-0.745589395	1.136386434	-0.390797039	9
TTC23L	-0.995284876	1.004649353	-0.009364477	9
TTC3	-1.077795065	0.897740602	0.180054463	9
TTC32	-0.959757504	1.035890578	-0.076133074	9
TTC4	-0.708436429	1.143894331	-0.435457903	9
TTC9C	-0.887832803	1.08330258	-0.195469778	9
TTI1	-0.911927307	1.069389639	-0.157462332	9
TTYH2	-0.798399848	1.12163903	-0.323239182	9
TWF2	-0.988021318	1.011562981	-0.023541663	9
TXN	-0.962193584	1.033941824	-0.07174824	9
TXNDC16	-0.797795137	1.121837531	-0.324042394	9
TXNDC2	-1.09379951	0.867371242	0.226428268	9
TXNIP	-1.04008322	0.954403408	0.085679812	9
TYMP	-1.020998358	0.977587784	0.043410574	9
UBA3	-1.016608081	0.982520243	0.034087838	9
UBA52	-0.882138561	1.08633951	-0.204200949	9
UBALD1	-0.835882798	1.107850785	-0.271967988	9
UBE2D2	-0.906586208	1.072628301	-0.166042093	9
UBE2G2	-0.993535312	1.006341682	-0.01280637	9
UBE2I	-1.125957937	0.784708632	0.341249305	9
UBE2M	-0.833942976	1.10863928	-0.274696304	9
UBE3B	-1.012161283	0.987378006	0.024783277	9

UBFD1	-1.098275487	0.857914712	0.240360775	9
UBL5	-1.114711841	0.818245042	0.296466799	9
UBQLN1	-0.881031256	1.086919483	-0.205888227	9
UBQLN2	-0.727222588	1.140374115	-0.413151527	9
UBQLN4	-1.007839695	0.991971445	0.015868249	9
UBR3	-1.018467097	0.980448551	0.038018546	9
UBR5	-0.926882823	1.059818955	-0.132936132	9
UBXN4	-1.018615239	0.980282402	0.038332837	9
UCMA	-0.938881141	1.051571701	-0.11269056	9
UFC1	-1.032504478	0.963969646	0.068534832	9
UFSP2	-0.635198538	1.152699331	-0.517500793	9
UGGT1	-0.805041906	1.119410517	-0.314368612	9
UGP2	-0.794855158	1.122792286	-0.327937128	9
UMOD	-0.66082563	1.150462884	-0.489637253	9
UPF3A	-0.639410766	1.152391041	-0.512980274	9
UQCC2	-0.860771135	1.096947485	-0.23617635	9
UQCC3	-0.849767499	1.101951987	-0.252184487	9
UQCRH	-0.719215513	1.141941946	-0.422726434	9
UQCRQ	-0.932990652	1.055686579	-0.122695927	9
URGCP-MRPS24	-0.633439405	1.152821305	-0.5193819	9
URI1	-0.657682013	1.1507843	-0.493102286	9
USP16	-0.988743837	1.010888345	-0.022144508	9
USP17L11	-0.957124954	1.037967399	-0.080842444	9
USP17L15	-0.995846686	1.004102198	-0.008255512	9
USP17L18	-0.667009531	1.149791305	-0.482781773	9
USP17L19	-0.8549774	1.099619814	-0.244642414	9
USP17L23	-0.998144509	1.00184522	-0.003700711	9
USP17L24	-0.847250166	1.103055261	-0.255805096	9
USP17L25	-1.090112274	0.874815781	0.215296493	9
USP17L28	-1.07582194	0.901167581	0.174654359	9
USP17L29	-1.123633924	0.79221901	0.331414914	9
USP17L5	-1.090895351	0.873259358	0.217635993	9
USP22	-0.759799264	1.132912785	-0.373113521	9
USP33	-0.958103272	1.037199099	-0.079095827	9
USP48	-1.11783796	0.809575562	0.308262398	9
UTP4	-0.97031488	1.027252243	-0.056937362	9
UTP6	-0.990992633	1.008770318	-0.017777685	9
UTRN	-1.014827366	0.984481878	0.030345489	9
UXT	-0.870162598	1.092434165	-0.222271567	9
VAMP4	-0.85629654	1.099018732	-0.242722192	9
VARS1	-1.125873086	0.784989118	0.340883968	9
VAX1	-0.960567392	1.0352456	-0.074678208	9
VAX2	-0.727507727	1.140316405	-0.412808678	9
VBP1	-0.865463096	1.094721142	-0.229258046	9
VDAC3	-0.926142972	1.060310464	-0.134167492	9
VEGFB	-0.717840099	1.142201094	-0.424360995	9
VEGFC	-0.745301717	1.136453198	-0.391151481	9
VENTX	-0.803625968	1.119893048	-0.31626708	9
VKORC1	-0.764102836	1.131791468	-0.367688631	9
VNN1	-0.7213314	1.141537494	-0.420206094	9
VPS33A	-0.78460804	1.125988331	-0.34138029	9
VPS35	-0.909181997	1.071065712	-0.161883715	9
VPS50	-0.754691221	1.134201522	-0.379510301	9
VPS9D1	-0.989391714	1.010280988	-0.020889274	9
VRK2	-0.869371589	1.092823151	-0.223451562	9

VRTN	-0.947182561	1.045547483	-0.098364922	9
VSTM2B	-1.071124589	0.909084832	0.162039757	9
VWA5A	-0.708245162	1.143927362	-0.4356822	9
WAPL	-0.628839005	1.153121553	-0.524282548	9
WASF1	-0.785856826	1.125609644	-0.339752819	9
WDR25	-0.936006351	1.053596109	-0.117589758	9
WDR26	-0.820922012	1.113714366	-0.292792354	9
WDR3	-1.069513712	0.911725443	0.157788269	9
WDR33	-0.841447087	1.10554111	-0.264094023	9
WDR41	-1.003773461	0.996183331	0.00759013	9
WDR43	-0.924911905	1.061124038	-0.136212133	9
WDR49	-0.840488428	1.105944149	-0.265455721	9
WDR53	-0.81745864	1.115001951	-0.297543311	9
WDR55	-0.945142021	1.047053316	-0.101911295	9
WDR6	-1.126521101	0.782834357	0.343686745	9
WFDC2	-1.124870251	0.788267338	0.336602913	9
WFDC6	-1.034569667	0.961412296	0.073157371	9
WIF1	-0.760568795	1.132714681	-0.372145885	9
WLS	-1.060321243	0.926126714	0.134194529	9
WNT8B	-0.863049138	1.095873603	-0.232824465	9
WRAP73	-0.856114143	1.0991021	-0.242987957	9
WWP1	-1.07223571	0.907241737	0.164993973	9
XAGE1A	-0.659083387	1.150642672	-0.491559285	9
XAGE1B	-0.630996059	1.152984137	-0.521988078	9
XRCC6	-1.1311848	0.766380128	0.364804672	9
YAP1	-0.674273702	1.148934998	-0.474661295	9
YARS2	-0.713171126	1.143058836	-0.42988771	9
YIF1A	-0.696740819	1.145812679	-0.44907186	9
YIPF7	-1.079775598	0.894237193	0.185538405	9
YJEFN3	-1.004358721	0.995583525	0.008775196	9
YPEL3	-0.842048841	1.105287025	-0.263238185	9
YTHDC1	-0.729946146	1.139817553	-0.409871406	9
YWHAG	-0.934006727	1.054985995	-0.120979268	9
YWHAH	-1.039965146	0.954556354	0.085408792	9
ZAP70	-1.10019821	0.853699045	0.246499166	9
ZBTB11	-1.026439556	0.971276956	0.055162601	9
ZBTB24	-0.88746497	1.083501541	-0.196036571	9
ZBTB4	-0.720891624	1.141622136	-0.420730512	9
ZBTB43	-0.82642519	1.111615096	-0.285189906	9
ZBTB45	-0.695851529	1.145950192	-0.450098663	9
ZBTB49	-1.028300129	0.969066687	0.059233442	9
ZBTB9	-0.959081676	1.036426602	-0.077344926	9
ZC2HC1A	-0.751235124	1.135047836	-0.383812712	9
ZDHHC12	-0.754294381	1.134299744	-0.380005363	9
ZDHHC15	-0.725019917	1.140815548	-0.41579563	9
ZDHHC16	-0.924809317	1.061191595	-0.136382278	9
ZDHHC17	-0.950517234	1.043050604	-0.09253337	9
ZFAND5	-0.898370358	1.077435149	-0.179064791	9
ZFP36L1	-0.82172791	1.113411058	-0.291683148	9
ZFYVE21	-1.106024078	0.840297705	0.265726373	9
ZKSCAN3	-0.770109102	1.130171306	-0.360062204	9
ZMIZ2	-0.753232712	1.134561183	-0.381328471	9
ZNF131	-1.121772991	0.797991994	0.323780997	9
ZNF146	-0.753461332	1.134505049	-0.381043718	9
ZNF169	-0.817235801	1.115083918	-0.297848117	9

ZNF175	-1.065749605	0.917755738	0.147993867	9
ZNF180	-1.088800622	0.87739416	0.211406462	9
ZNF200	-0.870986265	1.092027362	-0.221041097	9
ZNF208	-0.965434287	1.031308502	-0.065874215	9
ZNF211	-0.818294831	1.114693429	-0.296398598	9
ZNF222	-0.638620764	1.15245061	-0.513829846	9
ZNF257	-0.667950733	1.149684484	-0.48173375	9
ZNF284	-0.949355871	1.043925309	-0.094569438	9
ZNF286A-TBC1D26	-1.020422612	0.978242576	0.042180036	9
ZNF292	-0.677557889	1.148523645	-0.470965756	9
ZNF300	-0.910017237	1.07055835	-0.160541113	9
ZNF317	-1.118668073	0.807197522	0.311470551	9
ZNF335	-0.649090223	1.151594936	-0.502504713	9
ZNF337	-1.020290104	0.978392933	0.04189717	9
ZNF354C	-0.832335148	1.10928636	-0.276951212	9
ZNF383	-0.936373434	1.053339342	-0.116965908	9
ZNF395	-0.903222582	1.074621504	-0.171398923	9
ZNF416	-0.824082181	1.112516943	-0.288434762	9
ZNF420	-0.864577887	1.095145495	-0.230567608	9
ZNF429	-0.85190106	1.101004926	-0.249103866	9
ZNF431	-0.65236188	1.151297891	-0.498936011	9
ZNF449	-0.939548675	1.051097152	-0.111548477	9
ZNF460	-0.837369497	1.10724066	-0.269871163	9
ZNF468	-1.013025785	0.98644421	0.026581576	9
ZNF474	-0.92597147	1.060424123	-0.134452654	9
ZNF487	-1.101851123	0.849995916	0.251855206	9
ZNF513	-0.812428815	1.116826514	-0.304397698	9
ZNF547	-0.761994199	1.132344975	-0.370350776	9
ZNF548	-0.755967494	1.133883781	-0.377916287	9
ZNF552	-0.73216545	1.139355197	-0.407189747	9
ZNF555	-0.906413082	1.072731757	-0.166318676	9
ZNF583	-1.00430341	0.995640302	0.008663107	9
ZNF595	-0.669501735	1.149505782	-0.480004047	9
ZNF609	-0.908153886	1.071687168	-0.163533282	9
ZNF649	-0.915963222	1.066881054	-0.150917832	9
ZNF682	-1.012869782	0.98661309	0.026256692	9
ZNF683	-0.716422972	1.142465013	-0.42604204	9
ZNF691	-0.718693306	1.142040686	-0.42334738	9
ZNF708	-0.723531253	1.141109514	-0.417578261	9
ZNF710	-1.055082534	0.933867041	0.121215494	9
ZNF717	-0.785586	1.125692023	-0.340106023	9
ZNF726	-0.888750811	1.082804334	-0.194053523	9
ZNF729	-0.735591243	1.138625786	-0.403034543	9
ZNF730	-0.935700175	1.053809887	-0.118109712	9
ZNF74	-0.984591635	1.014727138	-0.030135503	9
ZNF770	-1.040386697	0.954009716	0.086376981	9
ZNF804A	-0.664919068	1.150024195	-0.485105127	9
ZNF805	-1.119154597	0.805788065	0.313366531	9
ZNF81	-1.045789305	0.94685638	0.098932925	9
ZNF814	-0.774538113	1.128934772	-0.354396659	9
ZNF816-ZNF321P	-0.914230444	1.067964648	-0.153734204	9
ZNF827	-0.833562764	1.108792826	-0.275230062	9
ZNF836	-0.655610192	1.150988828	-0.495378636	9
ZNF878	-0.918002033	1.065593302	-0.147591269	9
ZNF888	-0.947415594	1.045374456	-0.097958862	9

ZNF99	-0.935679091	1.053824595	-0.118145504	9
ZNRF3	-1.122793996	0.794849844	0.327944153	9
ZSCAN2	-0.840553002	1.105917069	-0.265364066	9
ZSCAN26	-0.859064165	1.097743554	-0.238679388	9
ZSCAN29	-0.693823336	1.146259455	-0.452436119	9
ZSWIM6	-1.00853903	0.991236422	0.017302608	9
ZSWIM8	-0.841386821	1.10556651	-0.264179689	9
ZW10	-1.100409852	0.853229048	0.247180804	9
ZXDC	-0.858977038	1.097783989	-0.238806951	9
ZZZ3	-1.014776127	0.984537997	0.03023813	9
