# Supplementary material for: RBAD: The first database dedicated alterations of blood RNA in individuals with Alzheimer’s disease and their clinical relevance
Source: Neural Regen Res. 2025 Mar 25;21(6):2553–62. doi: 10.4103/NRR.NRR-D-24-01165 (PMC13211806; doi:10.4103/NRR.NRR-D-24-01165)
Supplement: Supplementary file 8 [file NRR-21-2553_Suppl5.pdf]

Additional Table 8. Pathway enrichment (GO and KEGG) of each cluster found in ROSMAP cohort dataset.	
Method	R package clusterProfiler
Description	Pathway enrichment of each cluster using over representation analysis. Corresponding to Figure 3D.

Cluster	ONTOLOGY	ID	Description	GeneRatio	BgRatio	P value	FDR	Q value	Symbol	Count
cluster1	BP	GO:0050907	detection of chemical stimulus involved in sensory perception	84/1639	474/18862	1.97E-10	0.00000152	0.00000152	/OR4N5/OR51A4/OR51	84
cluster1	BP	GO:0050911	etection of chemical stimulus involved in sensory perception of sme	76/1639	430/18862	1.65E-09	0.00000635	0.00000635	05/OR4F4/OR4F6/OR4K	76
cluster1	BP	GO:0007608	sensory perception of smell	78/1639	454/18862	3.78E-09	0.00000974	0.00000973	5/OR4F4/OR4F6/OR4K5	78
cluster1	BP	GO:0006413	translational initiation	40/1639	193/18862	0.000000178	0.000343133	0.000342758	0/PPP1CA/RPL12/RPL1	40
cluster1	BP	GO:0022613	ribonucleoprotein complex biogenesis	73/1639	473/18862	0.000000948	0.001463884	0.001462287	3/NOL3/NOM1/NOP10/	73
cluster1	BP	GO:0045047	protein targeting to ER	27/1639	120/18862	0.00000338	0.004345257	0.004340517	RPL7A/RPL9/RPS10/RI	27
cluster1	BP	GO:0002181	cytoplasmic translation	24/1639	102/18862	0.00000513	0.005577583	0.005571499	3J/EIF4A1/FTSJ1/RPL1	24
cluster1	BP	GO:0006613	cotranslational protein targeting to membrane	25/1639	110/18862	0.00000636	0.005577583	0.005571499	PL7A/RPL9/RPS10/RPS	25
cluster1	BP	GO:0072599	establishment of protein localization to endoplasmic reticulum	27/1639	124/18862	0.0000065	0.005577583	0.005571499	RPL7A/RPL9/RPS10/RI	27
cluster1	BP	GO:0006614	SRP-dependent cotranslational protein targeting to membrane	24/1639	105/18862	0.00000873	0.006738584	0.006731234	17/RPL7A/RPL9/RPS10/	24
cluster1	BP	GO:0042254	ribosome biogenesis	49/1639	307/18862	0.0000231	0.016193554	0.01617589	OP56/NPM1/NPM3/PRI	49
cluster1	CC	GO:0044391	ribosomal subunit	44/1724	187/19520	1.21E-09	0.00000118	0.00000115	S9/NDUFAB1/RPL10L/	44
cluster1	CC	GO:0005840	ribosome	48/1724	243/19520	8.77E-08	0.0000429	0.0000416	PS6/MRPS9/NDUFAB1/	48
cluster1	CC	GO:0022626	cytosolic ribosome	27/1724	107/19520	0.000000421	0.000137133	0.000133121	L1/RPL29/RPL34/RPL3	27
cluster1	CC	GO:0015934	large ribosomal subunit	26/1724	115/19520	0.00000624	0.001524962	0.001480354	UFAB1/RPL10L/RPL12	26
cluster1	CC	GO:0022625	cytosolic large ribosomal subunit	16/1724	57/19520	0.0000224	0.004375587	0.004247593	PL22/RPL23/RPL24/RP	16
cluster1	CC	GO:0015935	small ribosomal subunit	18/1724	74/19520	0.0000583	0.009500942	0.009223023	1/MRPS6/MRPS9/RPS1	18
cluster1	MF	GO:0004984	olfactory receptor activity	76/1670	430/18337	1.26E-08	0.0000236	0.0000236	05/OR4F4/OR4F6/OR4K	76
cluster1	MF	GO:0003735	structural constituent of ribosome	41/1670	180/18337	2.83E-08	0.0000264	0.0000264	/RPL12/RPL15/RPL17/	41
cluster1	KEGG	hsa04740	Olfactory transduction	76/844	439/8644	0.000000346	0.000115204	0.000115204	/OR4F6/OR4K5/OR4N	76
cluster1	KEGG	hsa03010	Ribosome	36/844	167/8644	0.00000357	0.000595031	0.000595031	7-C18orf32/RPL18/RPL	36
cluster2	BP	GO:0045165	cell fate commitment	50/1963	251/18862	4.87E-06	0.041531481	0.041056471	MITF/MNX1/NANOG/	50
cluster3	BP	GO:0030048	actin filament-based movement	44/2360	150/18862	3.24E-08	0.000290566	0.000279129	7/MYL3/MYL6/MYLK	44
cluster3	BP	GO:0006813	potassium ion transport	56/2360	232/18862	7.48E-07	0.003354542	0.003222505	KCNJ4/KCNJ6/KCNK15	56
cluster3	BP	GO:0035637	multicellular organismal signaling	50/2360	203/18862	0.00000153	0.003724088	0.003577505	A5/KCND1/KCND2/KC	50
cluster3	BP	GO:0071805	potassium ion transmembrane transport	51/2360	212/18862	2.55E-06	0.003724088	0.003577505	K15/KCNK2/KCNMB3	51
cluster3	BP	GO:0007411	axon guidance	62/2360	277/18862	3.02E-06	0.003724088	0.003577505	/MYPN/NEO1/NRP2/N	62
cluster3	BP	GO:0019226	transmission of nerve impulse	23/2360	67/18862	3.28E-06	0.003724088	0.003577505	CNMB3/KCNMB4/MA	23
cluster3	BP	GO:0097485	neuron projection guidance	62/2360	278/18862	0.00000342	0.003724088	0.003577505	/MYPN/NEO1/NRP2/N	62
cluster3	BP	GO:0042391	regulation of membrane potential	87/2360	431/18862	3.55E-06	0.003724088	0.003577505	NH4/KCNH6/KCNK15/	87
cluster3	BP	GO:0008544	epidermis development	92/2360	463/18862	3.74E-06	0.003724088	0.003577505	TAP5-4/KRTAP6-2/KR	92
cluster3	BP	GO:0050954	sensory perception of mechanical stimulus	42/2360	167/18862	5.84E-06	0.005238593	0.005032398	O3A/MYO6/PDZD7/PG	42
cluster3	BP	GO:0086070	SA node cell to atrial cardiac muscle cell communication	8/2360	11/18862	6.90E-06	0.005469237	0.005253964	GJA5/GJC1/KCNA5/RY	8
cluster3	BP	GO:0070252	actin-mediated cell contraction	33/2360	120/18862	7.64E-06	0.005469237	0.005253964	E1/MYBPC2/MYH6/M	33
cluster3	BP	GO:0098742	cell-cell adhesion via plasma-membrane adhesion molecules	60/2360	273/18862	7.93E-06	5.47E-03	5.25E-03	LRRC4/MAG/MDGA1/	60
cluster3	BP	GO:0007409	axonogenesis	90/2360	460/18862	9.04E-06	0.00574347	0.005517403	MX1A/LRP4/MAG/MA	90
cluster3	BP	GO:0009913	epidermal cell differentiation	74/2360	360/18862	9.60E-06	0.00574347	0.005517403	RTAP4-6/KRTAP5-4/K	74
cluster3	BP	GO:0021885	forebrain cell migration	19/2360	53/18862	1.13E-05	0.006342907	0.006093246	D1/DRD2/EFHC1/FBX	19
cluster3	BP	GO:0015849	organic acid transport	67/2360	324/18862	2.03E-05	0.010731386	0.01030899	1A7/SLC25A13/SLC25	67
cluster3	BP	GO:0007389	pattern specification process	83/2360	426/18862	2.33E-05	0.011315539	0.010870151	/IFT140/IFT57/IRX3/LE	83
cluster3	BP	GO:0003002	regionalization	67/2360	326/18862	2.50E-05	0.011315539	0.010870151	40/IFT57/IRX3/LRP2/L	67
cluster3	BP	GO:1905039	carboxylic acid transmembrane transport	38/2360	154/18862	2.52E-05	1.13E-02	1.09E-02	LC25A21/SLC25A29/SL	38
cluster3	BP	GO:0098657	import into cell	51/2360	230/18862	2.86E-05	1.20E-02	1.15E-02	A1/SLC15A2/SLC1A3/	51
cluster3	BP	GO:1903825	organic acid transmembrane transport	38/2360	155/18862	2.94E-05	1.20E-02	1.15E-02	LC25A21/SLC25A29/SL	38

cluster3	BP	GO:0001508	action potential	34/2360	135/18862	4.21E-05	1.64E-02	1.58E-02	CND2/KCNE1/KCNM	34
cluster3	BP	GO:0006865	amino acid transport	37/2360	152/18862	4.39E-05	1.64E-02	1.58E-02	SLC25A48/SLC36A1/S	37
cluster3	BP	GO:0007605	sensory perception of sound	36/2360	147/18862	4.83E-05	1.73E-02	1.66E-02	D3A/MYO6/PDZD7/PG	36
cluster3	BP	GO:0046942	carboxylic acid transport	59/2360	284/18862	5.38E-05	0.018571029	0.01784006	C1A7/SLC25A13/SLC2	59
cluster3	BP	GO:0022029	telencephalon cell migration	17/2360	50/18862	7.06E-05	0.022088014	0.021218613	RD1/DRD2/EFHC1/FB	17
cluster3	BP	GO:0045664	regulation of neuron differentiation	43/2360	190/18862	7.12E-05	0.022088014	0.021218613	ISL2/KCTD11/LBX1/L	43
cluster3	BP	GO:0098739	import across plasma membrane	38/2360	161/18862	7.14E-05	0.022088014	0.021218613	1/SLC15A2/SLC1A3/SI	38
cluster3	BP	GO:1901879	regulation of protein depolymerization	24/2360	85/18862	8.10E-05	0.023711516	0.022778213	1B/MTPN/NAV3/PDX	24
cluster3	BP	GO:1901880	negative regulation of protein depolymerization	21/2360	70/18862	8.54E-05	0.023711516	0.022778213	MAP1B/MTPN/NAV3/	21
cluster3	BP	GO:0086069	bundle of His cell to Purkinje myocyte communication	8/2360	14/18862	8.84E-05	0.023711516	0.022778213	GJA5/JUP/KCNA5/PK	8
cluster3	BP	GO:0086019	cell-cell signaling involved in cardiac conduction	13/2360	33/18862	8.93E-05	0.023711516	0.022778213	GJA5/GJC1/KCNA5/P	13
cluster3	BP	GO:0003333	amino acid transmembrane transport	26/2360	96/18862	8.99E-05	0.023711516	0.022778213	C36A1/SLC36A2/SLC3	26
cluster3	BP	GO:0031424	keratinization	48/2360	225/18862	0.000132094	0.033712188	0.032385251	/KRTAP16-1/KRTAP1	48
cluster3	BP	GO:0010975	regulation of neuron projection development	80/2360	428/18862	1.39E-04	0.033712188	0.032385251	MAG/MAP1B/MAP2/M	80
cluster3	BP	GO:0043588	skin development	78/2360	415/18862	1.39E-04	0.033712188	0.032385251	2-1/KRTAP12-2/KRTA	78
cluster3	BP	GO:0048638	regulation of developmental growth	63/2360	319/18862	0.000143861	0.033962567	0.032625775	MAP3K13/MAPK1/ME	63
cluster3	BP	GO:0006814	sodium ion transport	50/2360	239/18862	0.000160838	0.036996816	0.035540594	LC13A3/SLC17A6/SLC	50
cluster3	BP	GO:1903522	regulation of blood circulation	58/2360	290/18862	0.000182919	0.041024177	0.039409435	/GJC1/GRK2/HRC/HT	58
cluster3	BP	GO:0007611	learning or memory	49/2360	235/18862	2.02E-04	0.044172947	0.042434267	MAPK8IP2/NPAS4/NR	49
cluster3	BP	GO:0030834	regulation of actin filament depolymerization	17/2360	54/18862	2.07E-04	0.044172947	0.042434267	TPN/PDXP/PIK3CA/PL	17
cluster3	BP	GO:0086067	AV node cell to bundle of His cell communication	7/2360	12/18862	2.12E-04	4.43E-02	4.25E-02	5/GJC1/RYR2/SCN10A	7
cluster3	BP	GO:0086015	SA node cell action potential	6/2360	9/18862	2.28E-04	0.045537211	0.043744832	A1D/KCNA5/RYR2/S	6
cluster3	BP	GO:0086018	SA node cell to atrial cardiac muscle cell signaling	6/2360	9/18862	0.000228422	0.045537211	0.043744832	A1D/KCNA5/RYR2/S	6
cluster3	CC	GO:0098862	cluster of actin-based cell projections	43/2476	156/19520	0.00000047	0.000499261	0.000460217	LC11A2/SLC15A1/SL	43
cluster3	CC	GO:0097060	synaptic membrane	79/2476	368/19520	0.00000146	0.00054617	0.000503458	IL31RA/ITGA8/ITGB3/	79
cluster3	CC	GO:0005903	brush border	31/2476	101/19520	0.00000154	0.00054617	0.000503458	SLC15A1/SLC22A12/S	31
cluster3	CC	GO:0043679	axon terminus	33/2476	114/19520	0.00000306	0.000738705	0.000680935	OXT/PACSIN1/PDYN/	33
cluster3	CC	GO:0044306	neuron projection terminus	36/2476	130/19520	0.00000348	0.000738705	0.000680935	FF/NTRK2/OXT/PACS	36
cluster3	CC	GO:0045211	postsynaptic membrane	60/2476	265/19520	0.00000458	0.000810755	0.000747351	2/GLRA2/GRIN2B/GR	60
cluster3	CC	GO:0043025	neuronal cell body	93/2476	474/19520	0.0000101	0.001530965	0.001411237	A/KIF5C/KLHL14/KN	93
cluster3	CC	GO:0034703	cation channel complex	51/2476	222/19520	0.0000151	0.002007393	0.001850407	NG4/KCNH4/KCNJ4/K	51
cluster3	CC	GO:0031526	brush border membrane	20/2476	59/19520	0.0000215	0.002534302	0.00233611	12/SLC26A3/SLC27A4/	20
cluster3	CC	GO:0150034	distal axon	61/2476	287/19520	0.0000302	0.003045327	0.00280717	1B/MAP2/MYH14/ND	61
cluster3	CC	GO:0098936	intrinsic component of postsynaptic membrane	29/2476	105/19520	0.0000315	0.003045327	0.00280717	A4/GRM2/HTR2A/KC	29
cluster3	CC	GO:1902495	transmembrane transporter complex	66/2476	323/19520	0.000053	0.00469014	0.004323353	CN2/KCNA3/KCNA5/	66
cluster3	CC	GO:1990351	transporter complex	68/2476	338/19520	0.000068	0.005559069	0.005124327	/HCN1/HCN2/KCNA3/	68
cluster3	CC	GO:0008076	voltage-gated potassium channel complex	25/2476	89/19520	0.0000799	0.006064153	0.005589912	G4/KCNH4/KCNJ4/KC	25
cluster3	CC	GO:0099055	integral component of postsynaptic membrane	27/2476	100/19520	0.0000892	0.006315808	0.005821887	RA4/GRM2/HTR2A/KC	27
cluster3	CC	GO:0031253	cell projection membrane	67/2476	337/19520	0.00011095	0.007364283	0.006788367	G/PDPN/PDXP/PDZK1	67
cluster3	CC	GO:0099240	intrinsic component of synaptic membrane	34/2476	140/19520	0.000121137	0.007567471	0.006975664	TR1B/HTR2A/KCNA3/	34
cluster3	CC	GO:0034705	potassium channel complex	26/2476	99/19520	0.000194909	0.011499656	0.010600337	KCNG4/KCNH4/KCNJ4	26
cluster3	CC	GO:0034702	ion channel complex	60/2476	301/19520	0.000228941	0.011927806	0.010995004	NB1/KCND1/KCND2/	60
cluster3	CC	GO:0099568	cytoplasmic region	53/2476	258/19520	0.000239798	0.011927806	0.010995004	XW11/FMR1/GLI3/IFT	53
cluster3	CC	GO:0099699	integral component of synaptic membrane	31/2476	128/19520	0.000249003	0.011927806	0.010995004	M2/HTR1B/HTR2A/KC	31
cluster3	CC	GO:0043204	perikaryon	35/2476	151/19520	0.000257492	0.011927806	0.010995004	/KCNB1/KCND2/KIF5	35
cluster3	CC	GO:0036156	inner dynein arm	4/2476	4/19520	0.000258323	0.011927806	0.010995004	H1/DNAH7/DNAI3/DN	4
cluster3	CC	GO:0005911	cell-cell junction	88/2476	485/19520	0.000305887	0.013535494	0.012476965	LDR1/ITGB3/JAM2/JC	88
cluster3	CC	GO:0062023	collagen-containing extracellular matrix	78/2476	423/19520	0.000397151	0.016531118	0.015238319	5/L1CAM/LAD1/LAM	78
cluster3	CC	GO:0005938	cell cortex	59/2476	301/19520	0.000404717	0.016531118	0.015238319	1B1/PARD3/PARD3B/	59
cluster3	CC	GO:0097441	basal dendrite	5/2476	7/19520	0.000549991	0.020916755	0.019280981	MAP2/SLC17A8/SLC4A	5
cluster3	CC	GO:0098793	presynapse	87/2476	487/19520	0.000551478	0.020916755	0.019280981	PCDH8/PDYN/PDZD11	87
cluster3	CC	GO:0097014	ciliary plasm	30/2476	130/19520	0.000739434	0.02707857	0.024960918	DNAH9/DNAI3/DNHD	30
cluster3	CC	GO:0097440	apical dendrite	8/2476	18/19520	0.000889136	0.031475423	0.029013919	B/MAP2/SEZ6/SLC17A	8
cluster3	CC	GO:0016324	apical plasma membrane	65/2476	351/19520	0.001032765	0.035380531	0.032613632	M2/SHROOM4/SLC11	65
cluster3	CC	GO:0005930	axoneme	29/2476	128/19520	0.001221053	0.040011389	0.036882339	AH7/DNAH9/DNAI3/D	29

cluster3	CC	GO:0031674	I band	30/2476	134/19520	0.001243292	0.040011389	0.036882339	JP/KCNA5/KCNE1/KRT	30
cluster3	CC	GO:0045177	apical part of cell	74/2476	414/19520	0.001349997	0.042167561	0.03886989	SHROOM2/SHROOM4	74
cluster3	CC	GO:0016459	myosin complex	16/2476	57/19520	0.001483489	0.045013301	0.041493081	MYL9/MYO10/MYO16	16
cluster3	CC	GO:0098858	actin-based cell projection	43/2476	216/19520	0.001686138	0.049741082	0.045851131	/MYO1G/MYO3A/MYO	43
cluster3	MF	GO:0046873	metal ion transmembrane transporter activity	91/2389	425/18337	0.000000866	0.001808242	0.001737585	I3/NALCN/PKD1L1/PS	91
cluster3	MF	GO:0015267	channel activity	98/2389	477/18337	0.00000233	0.001808242	0.001737585	B1/KCND1/KCND2/KC	98
cluster3	MF	GO:0022803	passive transmembrane transporter activity	98/2389	478/18337	0.00000257	0.001808242	0.001737585	B1/KCND1/KCND2/KC	98
cluster3	MF	GO:0005261	cation channel activity	72/2389	333/18337	0.00000819	0.004323642	0.004154696	H6/KCNJ18/KCNJ4/KC	72
cluster3	MF	GO:0015081	sodium ion transmembrane transporter activity	37/2389	146/18337	0.0000414	0.017503964	0.016819997	/SLC24A1/SLC28A2/SL	37
cluster3	MF	GO:0005216	ion channel activity	84/2389	429/18337	0.0000735	0.019854145	0.019078345	CNG4/KCNH4/KCNH6	84
cluster3	MF	GO:0022836	gated channel activity	69/2389	337/18337	0.0000779	0.019854145	0.019078345	ND2/KCNE1/KCNG4/K	69
cluster3	MF	GO:0030898	actin-dependent ATPase activity	11/2389	24/18337	0.0000874	0.019854145	0.019078345	YO10/MYO1B/MYO1G/	11
cluster3	MF	GO:0005244	voltage-gated ion channel activity	45/2389	197/18337	0.000101689	0.019854145	0.019078345	KCNE1/KCNG4/KCNH	45
cluster3	MF	GO:0022832	voltage-gated channel activity	45/2389	197/18337	0.000101689	0.019854145	0.019078345	KCNE1/KCNG4/KCNH	45
cluster3	MF	GO:0015079	potassium ion transmembrane transporter activity	37/2389	152/18337	0.000103407	0.019854145	0.019078345	CNK15/KCNK2/KCNM	37
cluster3	MF	GO:0022843	voltage-gated cation channel activity	34/2389	139/18337	0.000175057	0.030809949	0.029606052	NA5/KCNB1/KCND1/K	34
cluster3	MF	GO:0017022	myosin binding	21/2389	71/18337	0.000190624	0.030969142	0.029759024	NKD2/NPC1L1/RAB39B	21
cluster3	MF	GO:0015291	secondary active transmembrane transporter activity	51/2389	239/18337	0.000226043	0.034100226	0.032767761	LC34A3/SLC35D3/SLC	51
cluster3	MF	GO:0015293	symporter activity	34/2389	143/18337	0.000310676	0.043743156	0.042033894	A1/SLC28A2/SLC34A3	34
cluster5	BP	GO:0050907	detection of chemical stimulus involved in sensory perception	99/1866	474/18862	3.81E-13	1.66E-09	1.65E-09	B/OR4S1/OR4X2/OR51	99
cluster5	BP	GO:0007608	sensory perception of smell	96/1866	454/18862	4.05E-13	1.66E-09	1.65E-09	/OR4K1/OR4K14/OR4L	96
cluster5	BP	GO:0050911	etection of chemical stimulus involved in sensory perception of sme	92/1866	430/18862	6.21E-13	1.7E-09	1.69E-09	14/OR4L1/OR4M1/OR4	92
cluster5	MF	GO:0004984	olfactory receptor activity	92/1919	430/18337	1.45E-11	2.67E-08	2.67E-08	14/OR4L1/OR4M1/OR4	92
cluster5	MF	GO:0030546	signaling receptor activator activity	80/1919	492/18337	0.0000419	0.029036039	0.029036039	B1/IFNE/IFNL1/IFNW1	80
cluster5	MF	GO:0048018	receptor ligand activity	79/1919	486/18337	0.0000472	0.029036039	0.029036039	IFNE/IFNL1/IFNW1/IL1	79
cluster5	KEGG	hsa04740	Olfactory transduction	94/896	439/8644	2.05E-12	6.79E-10	6.73E-10	4L1/OR4M1/OR4Q3/O	94
cluster6	BP	GO:0016236	macroautophagy	60/1878	311/18862	0.000000399	0.003326841	0.003207904	RKAB2/PRKACA/PRK	60
cluster6	BP	GO:0016241	regulation of macroautophagy	37/1878	160/18862	0.000000812	0.003384622	0.00326362	R/NEDD4/NOD2/NRB	37
cluster6	BP	GO:0010506	regulation of autophagy	60/1878	328/18862	0.00000249	0.006918913	0.006671558	MTMR8/MTOR/NEDD4	60
cluster6	BP	GO:0009411	response to UV	32/1878	146/18862	0.0000143	0.029849306	0.028782176	2/NEDD4/NOC2L/OPN	32
cluster6	BP	GO:0036293	response to decreased oxygen levels	61/1878	360/18862	0.0000245	0.040893813	0.039431835	LDHA/LEP/LONP1/MM	61
cluster6	BP	GO:0034976	response to endoplasmic reticulum stress	52/1878	296/18862	0.0000357	0.049568107	0.047796018	MAN1B1/MANF/MBT	52
cluster6	KEGG	hsa05131	Shigellosis	49/927	247/8644	0.0000128	0.004299401	0.003634058	MAPK11/MAPK14/MA	49
cluster6	KEGG	hsa04380	Osteoclast differentiation	30/927	135/8644	0.0000735	0.012315521	0.010409663	RB1/MAP2K7/MAP3K7	30
cluster6	KEGG	hsa04022	cGMP-PKG signaling pathway	33/927	167/8644	0.000351898	0.037878563	0.032016759	GNAQ/GTF2I/ITPR1/M	33
cluster6	KEGG	hsa03410	Base excision repair	13/927	44/8644	0.000495883	0.037878563	0.032016759	P1/PARP4/PNKP/POLB	13
cluster6	KEGG	hsa04611	Platelet activation	26/927	124/8644	0.000569662	0.037878563	0.032016759	PK11/MAPK14/MAPK	26
cluster6	KEGG	hsa04714	Thermogenesis	41/927	232/8644	0.000831892	0.037878563	0.032016759	1/MAPK14/MGLL/MTO	41
cluster6	KEGG	hsa04071	Sphingolipid signaling pathway	25/927	121/8644	0.000904563	0.037878563	0.032016759	K3/MAPK8/PIK3R3/PP	25
cluster6	KEGG	hsa04660	T cell receptor signaling pathway	25/927	121/8644	0.000904563	0.037878563	0.032016759	K3/MAPK8/NFATC2/N	25
cluster7	BP	GO:0010923	negative regulation of phosphatase activity	27/1940	104/18862	0.00000439	0.037887724	0.037138034	/PCIF1/PHACTR1/PHA	27
cluster9	BP	GO:0019080	viral gene expression	43/1777	198/18862	0.00000015	0.000554277	0.00055019	L35A/RPL36A/RPL37A	43
cluster9	BP	GO:1901998	toxin transport	16/1777	39/18862	0.000000166	0.000554277	0.00055019	T6B/CCT7/CCT8/COPB	16
cluster9	BP	GO:0019083	viral transcription	40/1777	180/18862	0.000000212	0.000554277	0.00055019	A/RPL36A/RPL37A/RP	40
cluster9	BP	GO:0000184	uclear-transcribed mRNA catabolic process, nonsense-mediated deca	30/1777	120/18862	0.000000492	0.000966525	0.000959399	PL36A/RPL37A/RPL39/	30
cluster9	BP	GO:0006613	cotranslational protein targeting to membrane	27/1777	110/18862	0.00000264	0.004150479	0.004119876	L39/RPL6/RPLP2/RPS1	27
cluster9	BP	GO:0006402	mRNA catabolic process	62/1777	375/18862	0.0000084	0.010997706	0.010916616	/PUM1/RBM24/RC3H2	62
cluster9	BP	GO:0006614	SRP-dependent cotranslational protein targeting to membrane	25/1777	105/18862	0.0000109	0.012210436	0.012120404	39/RPL6/RPLP2/RPS15/	25
cluster9	BP	GO:0006401	RNA catabolic process	65/1777	414/18862	0.0000279	0.023815528	0.023639927	MD6/PUM1/RBM24/RC	65
cluster9	BP	GO:0070972	protein localization to endoplasmic reticulum	31/1777	152/18862	0.0000292	0.023815528	0.023639927	L37A/RPL39/RPL6/RPL	31
cluster9	BP	GO:0006605	protein targeting	68/1777	441/18862	0.0000331	0.023815528	0.023639927	/RPL27/RPL28/RPL35/	68
cluster9	BP	GO:0006415	translational termination	24/1777	105/18862	0.0000333	0.023815528	0.023639927	MRPL55/MRPL57/MRF	24
cluster9	BP	GO:0045047	protein targeting to ER	26/1777	120/18862	0.0000428	0.028032607	0.027825912	RPL6/RPLP2/RPS15/RPS	26
cluster9	BP	GO:0000956	nuclear-transcribed mRNA catabolic process	38/1777	208/18862	0.0000527	0.031846389	0.031611573	L28/RPL35/RPL35A/RP	38
cluster9	BP	GO:0072599	establishment of protein localization to endoplasmic reticulum	26/1777	124/18862	0.0000768	0.043114705	0.042796804	RPL6/RPLP2/RPS15/RPS	26

cluster9	CC	GO:0005743	mitochondrial inner membrane	82/1855	493/19520	0.000000349	0.000361741	0.000339247	PS24/MRPS25/MRPS28	82
cluster9	CC	GO:0044391	ribosomal subunit	40/1855	187/19520	0.000000766	0.000396818	0.000372143	/RPL13/RPL21/RPL23A	40
cluster9	CC	GO:0005666	RNA polymerase III complex	10/1855	18/19520	0.00000126	0.000433447	0.000406494	/POLR3C/POLR3E/POL	10
cluster9	CC	GO:0098798	mitochondrial protein-containing complex	50/1855	265/19520	0.00000186	0.000482773	0.000452753	L58/MRPS14/MRPS24/I	50
cluster9	CC	GO:0022626	cytosolic ribosome	25/1855	107/19520	0.0000179	0.003716969	0.003485838	7A/RPL39/RPL6/RPLP2	25
cluster9	CC	GO:0005759	mitochondrial matrix	71/1855	476/19520	0.0000877	0.014915327	0.013987854	IRPL18/MRPL19/MRPI	71
cluster9	CC	GO:0005840	ribosome	42/1855	243/19520	0.000100779	0.014915327	0.013987854	3/RPL11/RPL13/RPL21	42
cluster9	CC	GO:0015934	large ribosomal subunit	24/1855	115/19520	0.000175893	0.022778188	0.021361784	L58/RPL11/RPL13/RPL2	24
cluster9	CC	GO:0016592	mediator complex	12/1855	40/19520	0.000232877	0.026806696	0.025139789	5/MED16/MED19/MEI	12
cluster9	CC	GO:0032993	protein-DNA complex	36/1855	208/19520	0.000296583	0.030726008	0.028815388	H3C12/H3C8/H3Y1/H4	36
cluster9	CC	GO:0098800	inner mitochondrial membrane protein complex	27/1855	142/19520	0.000359103	0.03382095	0.03171788	DUFA1/NDUFA10/NDU	27
cluster9	MF	GO:0034062	5'-3' RNA polymerase activity	15/1829	43/18337	0.00000919	0.007964082	0.007962988	R2M/POLR3C/POLR3E	15
cluster9	MF	GO:0097747	RNA polymerase activity	15/1829	43/18337	0.00000919	0.007964082	0.007962988	R2M/POLR3C/POLR3E	15
cluster9	MF	GO:0003899	DNA-directed 5'-3' RNA polymerase activity	14/1829	39/18337	0.0000125	0.007964082	0.007962988	POLR2M/POLR3C/POL	14
cluster9	MF	GO:0003735	structural constituent of ribosome	36/1829	180/18337	0.0000373	0.017848535	0.017846083	L23A/RPL27/RPL28/RI	36
cluster9	KEGG	hsa03020	RNA polymerase	12/876	34/8644	0.0000722	0.023746696	0.023476977	POLR2M/POLR3C/POI	12
cluster9	KEGG	hsa03010	Ribosome	32/876	167/8644	0.000277185	0.031785087	0.031424066	A/RPL36A/RPL36A-HN	32
cluster9	KEGG	hsa04623	Cytosolic DNA-sensing pathway	18/876	75/8644	0.000383286	0.031785087	0.031424066	2F/POLR2H/POLR2L/I	18
cluster9	KEGG	hsa04141	Protein processing in endoplasmic reticulum	32/876	170/8644	0.000386445	0.031785087	0.031424066	/PDIA4/RPN1/SAR1B/	32
