# Supplementary material for: RBAD: The first database dedicated alterations of blood RNA in individuals with Alzheimer’s disease and their clinical relevance
Source: Neural Regen Res. 2025 Mar 25;21(6):2553–62. doi: 10.4103/NRR.NRR-D-24-01165 (PMC13211806; doi:10.4103/NRR.NRR-D-24-01165)
Supplement: Supplementary file 9 [file NRR-21-2553_Suppl6.pdf]

Additional Table 9. Time-Series analysis of the expression of circulating mRNA using Homo dataset (integrated ACOM, Emory, SRP310421, and SRP223445 datasets).

Method	Time-Series Analysis by Mfuzz R package
Description	Data set: Homo dataset(integrated ACOM, Emory, SRP310421, and SRP223445 datasets) in RBAD.<br>Clusters of circulating mRNA expression trend from control to MCI to AD.<br>●Control/MCI/AD: Normalized average expression of each gene in control, MCI, and AD groups. ●Cluster: range from 1 to 9, corresponding to Figure 3C.

Symbol	**Control**	**MCI**	**AD**	**Cluster**
A1BG	-1.145014493	0.44325417	0.701760323	1
AADACL4	-1.036787176	0.078161531	0.958625645	1
AAR2	-0.846038051	-0.25754303	1.103581081	1
AATK	-1.084349907	0.19846147	0.885888437	1
ABAT	-1.130990855	0.363889963	0.767100893	1
ABCA6	-0.965262391	-0.066186978	1.031449369	1
ABCB9	-1.15293512	0.521191375	0.631743745	1
ABCG5	-0.89698488	-0.181240515	1.078225395	1

ABHD6	-1.139934446	0.410555508	0.729378937	1
ABI1	-1.049089768	0.106743502	0.942346265	1
ABO	-1.152663494	0.516958755	0.635704739	1
ABRA	-0.929033834	-0.129345106	1.05837894	1
ABTB1	-0.937956596	-0.114269564	1.05222616	1
ACHE	-1.147575141	0.684708587	0.462866554	1
ACMSD	-0.944629291	-0.102799802	1.047429093	1
ACP1	-0.981029728	-0.036918074	1.017947802	1
ACTA2	-1.149476186	0.669755847	0.479720339	1
ACTB	-1.152704115	0.517573514	0.635130602	1
ACTL7A	-1.154689254	0.572923698	0.581765557	1
ACTL9	-1.039203125	0.08366275	0.955540375	1
ACTR10	-1.152458076	0.513937079	0.638520997	1
ACTR3	-1.118348134	0.310230122	0.808118012	1
ACY3	-1.080532245	0.187650822	0.892881423	1
ADAD1	-1.003091402	0.006211744	0.996879658	1
ADAM30	-1.044994861	0.097069052	0.947925809	1
ADAM7	-0.90530681	-0.168083806	1.073390616	1
ADAM8	-0.920693184	-0.143178921	1.063872106	1
ADAMTS17	-1.009768136	0.019831253	0.989936883	1
ADCK1	-1.110823406	0.828456608	0.282366799	1
ADGRD1	-1.093538802	0.225631321	0.867907481	1
ADGRG3	-0.984002746	-0.031261416	1.015264162	1
ADIPOQ	-1.028907074	0.060567346	0.968339728	1

ADIPOR1	-1.102572606	0.25421689	0.848355716	1
ADM2	-0.994571114	-0.010770762	1.005341877	1
AGPAT3	-1.154685928	0.582373445	0.572312483	1
AIF1	-1.088811028	0.211437185	0.877373843	1
AJM1	-0.951295381	-0.091166057	1.042461438	1
AKAIN1	-1.142586618	0.426822327	0.715764291	1
AKR1B10	-0.918342711	-0.147034055	1.065376765	1
ALDH3B1	-0.907461728	-0.164641927	1.072103655	1
ALLC	-1.114415289	0.295370057	0.819045232	1
ALPL	-0.944794202	-0.102514141	1.047308343	1
ALPP	-0.855741715	-0.243530356	1.09927207	1
AMFR	-0.998871775	-0.002252644	1.001124419	1
ANGPTL1	-0.929232896	-0.129011945	1.058244841	1
ANGPTL5	-1.015869405	0.032532752	0.983336653	1
ANGPTL7	-1.13691968	0.393644992	0.743274688	1
ANHX	-1.136109533	0.746776189	0.389333344	1
ANKFN1	-1.10787467	0.272050325	0.835824345	1
ANKRD13A	-1.09451099	0.228611071	0.865899919	1
ANKRD13B	-0.927732182	-0.131520128	1.05925231	1
ANKRD18A	-0.916409916	-0.150190185	1.066600101	1
ANKRD30B	-0.943153104	-0.105352097	1.048505201	1
ANKRD55	-1.151682498	0.648094741	0.503587757	1
ANKRD62	-1.074367388	0.170712635	0.903654753	1
ANKRD63	-1.150997149	0.655524645	0.495472505	1

ANKS4B	-0.838814554	-0.267828205	1.106642759	1
ANO10	-1.129779171	0.35825157	0.771527601	1
ANO7	-0.854510966	-0.245320353	1.09983132	1
ANP32A	-1.108564107	0.834128824	0.274435283	1
ANXA8L1	-1.150853093	0.493861428	0.656991665	1
ANXA9	-1.154432633	0.598756308	0.555676326	1
AP2M1	-1.116704472	0.812769888	0.303934585	1
AP2S1	-1.137168789	0.394989006	0.742179783	1
AP4M1	-1.152597947	0.636618779	0.515979169	1
APBA2	-1.136098357	0.389274479	0.746823878	1
APLP2	-0.934442732	-0.120241471	1.054684203	1
APOBEC1	-1.105599262	0.264290179	0.841309083	1
APOBR	-1.154503616	0.558784258	0.595719358	1
APOL5	-1.081708519	0.190954259	0.89075426	1
APPL2	-1.154646368	0.58700944	0.567636928	1
AQP12B	-1.12273738	0.327711717	0.795025663	1
ARF1	-1.144495086	0.439589151	0.704905935	1
ARF5	-1.055574646	0.122421284	0.933153361	1
ARFGAP1	-1.148079583	0.467105453	0.68097413	1
ARHGAP15	-1.037451169	0.079668293	0.957782875	1
ARHGAP40	-1.037388701	0.079526373	0.957862328	1
ARHGDIB	-1.125496315	0.786228658	0.339267657	1
ARHGEF12	-0.963751263	-0.068930712	1.032681975	1
ARL6IP1	-1.064054096	0.143643654	0.920410442	1

ARL6IP6	-1.149591082	0.480826087	0.668764995	1
ARL8A	-0.854475177	-0.24537235	1.099847527	1
ARPC5	-0.980019292	-0.038830254	1.018849546	1
ARPIN-AP3S2	-0.974648245	-0.048908661	1.023556906	1
ARRB1	-0.887369879	-0.196183035	1.083552913	1
ARSA	-0.837528858	-0.269646103	1.10717496	1
ARSF	-0.952886463	-0.088362524	1.041248987	1
ARSH	-1.082632428	0.193565917	0.889066511	1
ART1	-1.149941565	0.665666967	0.484274598	1
ART3	-1.005239759	0.010563206	0.994676553	1
ART4	-1.15404938	0.610603263	0.543446117	1
ASAH1	-0.866722819	-0.227390939	1.094113758	1
ASB15	-1.144477303	0.439465244	0.705012058	1
ASCL4	-0.923668907	-0.138271214	1.061940121	1
ASGR2	-0.86455813	-0.230596812	1.095154943	1
ATAT1	-0.903703106	-0.170635814	1.07433892	1
ATF6	-1.144535463	0.439870864	0.704664599	1
ATF7	-1.154495713	0.558413411	0.596082302	1
ATG9A	-1.15467254	0.570372487	0.584300053	1
ATOH7	-1.135949355	0.747458095	0.388491261	1
ATP2A1	-1.10692959	0.268806822	0.838122768	1
ATP2A3	-0.893401915	-0.186840488	1.080242403	1
ATP5F1C	-0.948263031	-0.096480364	1.044743396	1
ATP5IF1	-1.112348236	0.824522868	0.287825368	1

ATP5MF-PTCD1	-1.127212416	0.780502789	0.346709627	1
ATP6AP1	-0.877298548	-0.211551036	1.088849584	1
ATP6V0C	-1.104404337	0.26028044	0.844123897	1
ATP6V0E1	-1.052666147	0.115333698	0.937332449	1
ATP6V1FNB	-1.13594738	0.388480899	0.747466481	1
ATXN7L2	-1.143779277	0.434679128	0.709100149	1
AWAT2	-0.885330436	-0.199318048	1.084648484	1
AXDND1	-0.98498399	-0.029384336	1.014368326	1
B2M	-0.910839695	-0.159216864	1.070056559	1
B3GNT5	-1.098468628	0.240973053	0.857495575	1
B3GNT7	-1.11203592	0.286700145	0.825335774	1
B4GALT5	-1.154281528	0.55020351	0.604078018	1
B9D2	-1.133197017	0.374508971	0.758688046	1
BAG3	-0.93122934	-0.125662708	1.056892048	1
BAGE5	-0.859460758	-0.238098494	1.097559252	1
BAIAP2	-0.993289513	-0.013288531	1.006578044	1
BAZ2B	-1.134967721	0.751565919	0.383401801	1
BCL2A1	-1.092774861	0.223304891	0.86946997	1
BEND7	-1.153633806	0.619791089	0.533842718	1
BEST1	-0.911793688	-0.157678093	1.069471781	1
BFAR	-1.130555657	0.768705775	0.361849883	1
BHLHE23	-1.132711258	0.760582055	0.372129203	1
BID	-0.984847965	-0.029644849	1.014492814	1
BLNK	-1.149576359	0.480683735	0.668892624	1

BLZF1	-1.072695256	0.166221075	0.906474181	1
BMPR1A	-1.138673331	0.403302679	0.735370652	1
BOD1L2	-1.149246657	0.671701083	0.477545575	1
BOK	-0.958148994	-0.079014097	1.037163091	1
BORCS8-MEF2B	-1.146349057	0.453120878	0.693228179	1
BPIFA1	-0.897362462	-0.180648141	1.078010603	1
BPIFA3	-1.105781494	0.264905562	0.840875931	1
BRDT	-1.131607797	0.366811826	0.764795971	1
BROX	-1.020897251	0.043194302	0.977702948	1
BST1	-1.111622596	0.826405833	0.285216763	1
BTBD11	-0.851693306	-0.249404326	1.101097632	1
C11orf49	-0.994392968	-0.011121299	1.005514267	1
C11orf87	-1.019141738	0.039451109	0.979690629	1
C12orf40	-1.031780956	0.066923839	0.964857118	1
C12orf50	-0.845737489	-0.257973435	1.103710925	1
C14orf180	-1.096316529	0.234203567	0.862112961	1
C15orf65	-0.989476383	-0.020725062	1.010201445	1
C16orf72	-1.096330733	0.234247872	0.862082861	1
C16orf89	-1.151132093	0.497009196	0.654122897	1
C17orf107	-0.849300373	-0.252857495	1.102157867	1
C17orf78	-0.863645246	-0.231945158	1.095590404	1
C17orf97	-0.932417649	-0.123662345	1.056079994	1
C19orf54	-1.134589968	0.753115336	0.381474632	1
C19orf85	-1.14566341	0.697697684	0.447965726	1

C1QL1	-0.932251982	-0.123941532	1.056193514	1
C1QL2	-0.892157865	-0.188775979	1.080933844	1
C1QTNF4	-1.003009592	0.006046606	0.996962986	1
C1QTNF7	-0.917094417	-0.149073868	1.066168285	1
C1QTNF8	-0.973944209	-0.05021921	1.024163419	1
C20orf202	-1.096792755	0.235691663	0.861101093	1
C2orf78	-0.892889492	-0.187638266	1.080527758	1
C3orf84	-0.927631725	-0.131687737	1.059319462	1
C4orf17	-1.100048974	0.246019237	0.854029736	1
C5AR1	-1.127055989	0.346022594	0.781033394	1
C5AR2	-1.153894093	0.539579736	0.614314358	1
C6orf141	-1.052378539	0.114637865	0.937740674	1
C6orf226	-0.925433931	-0.135345765	1.060779696	1
C6orf62	-1.150632554	0.491450044	0.65918251	1
C7orf50	-0.935993765	-0.117611139	1.053604903	1
C8A	-1.078278711	0.181387673	0.896891038	1
C8orf74	-1.076339509	0.176064873	0.900274637	1
CAB39	-0.959862276	-0.075945025	1.035807301	1
CABIN1	-0.912524258	-0.1564977	1.069021959	1
CABLES2	-1.101708379	0.251389692	0.850318687	1
CACFD1	-0.9519889	-0.089945344	1.041934244	1
CACNB4	-0.962535665	-0.071130412	1.033666077	1
CALHM3	-1.124025806	0.333048951	0.790976855	1
CALHM4	-0.884461296	-0.200650494	1.085111789	1

CALM2	-1.136293711	0.745987939	0.390305772	1
CALM3	-1.082366229	0.192811889	0.889554339	1
CALY	-1.109002869	0.275961531	0.833041337	1
CANT1	-1.146709396	0.690799027	0.455910369	1
CAP1	-1.152406193	0.639210834	0.513195359	1
CAPN13	-1.09826648	0.240332246	0.857934234	1
CAPN9	-1.152743084	0.634574095	0.518168989	1
CAPZA1	-0.965152352	-0.066387122	1.031539474	1
CAPZA2	-0.907151007	-0.165139122	1.072290129	1
CARD14	-1.147321906	0.4607923	0.686529606	1
CARD16	-1.036703329	0.077971538	0.958731791	1
CARNS1	-1.109664675	0.831388356	0.27827632	1
CASR	-1.124127513	0.333474602	0.79065291	1
CASS4	-0.859684899	-0.237770018	1.097454916	1
CATSPER1	-1.092779453	0.223318837	0.869460616	1
CATSPERD	-1.112054867	0.286768301	0.825286565	1
CBFA2T2	-0.911983268	-0.157371952	1.06935522	1
CBFB	-0.946485924	-0.099577512	1.046063437	1
CBS	-0.874200381	-0.216222296	1.090422676	1
CC2D2B	-1.136549751	0.744884609	0.391665142	1
CCDC124	-1.038311481	0.081626369	0.956685112	1
CCDC134	-1.152273137	0.51132945	0.640943688	1
CCDC14	-1.028796428	0.060323956	0.968472472	1
CCDC153	-1.011271516	0.022937674	0.988333842	1

CCDC166	-0.865703458	-0.228902109	1.094605567	1
CCDC171	-1.15283395	0.633253698	0.519580252	1
CCDC182	-1.130565295	0.768670419	0.361894875	1
CCDC185	-1.1334333	0.375675513	0.757757787	1
CCDC187	-1.14205251	0.718630618	0.423421893	1
CCDC190	-1.122979676	0.328707781	0.794271894	1
CCDC197	-1.057692506	0.127641914	0.930050592	1
CCDC27	-0.949510988	-0.09429781	1.043808798	1
CCDC54	-1.115998551	0.814729533	0.301269018	1
CCDC69	-1.15425508	0.604901755	0.549353325	1
CCDC87	-1.064317857	0.144317951	0.919999907	1
CCDC9B	-0.836221264	-0.271491063	1.107712327	1
CCIN	-1.016688199	0.034256735	0.982431464	1
CCL5	-1.086572987	0.204879404	0.881693583	1
CCNJL	-1.066526541	0.149999845	0.916526696	1
CCNK	-0.844208076	-0.260160239	1.104368315	1
CCPG1	-1.016048098	0.032908593	0.983139505	1
CCZ1B	-0.921566408	-0.141741913	1.063308321	1
CD151	-0.865834905	-0.228707394	1.094542299	1
CD207	-0.96304506	-0.070209433	1.033254493	1
CD226	-0.962075154	-0.071962014	1.034037168	1
CD300C	-1.10580798	0.264995094	0.840812887	1
CD300LF	-0.872639261	-0.218566268	1.091205529	1
CD33	-1.055978253	0.123412253	0.932566	1

CD46	-1.129385982	0.356449198	0.772936784	1
CD55	-1.149646689	0.668281183	0.481365507	1
CD58	-1.034442109	0.072870808	0.961571301	1
CD63	-0.923115385	-0.13918642	1.062301806	1
CD99	-1.147045796	0.68847705	0.458568745	1
CDA	-0.867773933	-0.225829867	1.0936038	1
CDC42	-1.141928116	0.719288479	0.422639637	1
CDH20	-1.053402513	0.117119324	0.936283189	1
CDHR2	-0.893989941	-0.185924052	1.079913993	1
CDK8	-1.044805066	0.096624679	0.948180387	1
CDKN2D	-1.096124598	0.233605387	0.862519211	1
CDRT1	-1.029664385	0.062235859	0.967428526	1
CEACAM20	-0.942943332	-0.105714096	1.048657428	1
CEACAM6	-0.871368043	-0.220470153	1.091838196	1
CELA2A	-0.872991285	-0.218038285	1.09102957	1
CENPT	-1.149377824	0.670594925	0.478782899	1
CENPVL1	-1.12023868	0.317634341	0.802604339	1
CENPVL2	-0.891802625	-0.189327827	1.081130451	1
CENPVL3	-0.865106042	-0.229786509	1.094892551	1
CENPW	-1.029259517	0.061343275	0.967916241	1
CEP295	-1.146677061	0.455657655	0.691019406	1
CER1	-1.126187031	0.342238184	0.783948848	1
CFAP100	-0.972302171	-0.053266532	1.025568703	1
CFHR3	-0.864957848	-0.230005748	1.094963596	1

CFHR4	-1.073406318	0.168125952	0.905280366	1
CGB3	-0.917611924	-0.148228853	1.065840778	1
CGB5	-1.043342035	0.093210927	0.950131108	1
CGB8	-1.152632969	0.516500687	0.636132282	1
CHAD	-1.128364717	0.776537718	0.351826999	1
CHAT	-1.148046328	0.681224942	0.466821386	1
CHMP4A	-0.987691698	-0.02417812	1.011869818	1
CHMP4B	-1.112579203	0.823919268	0.288659935	1
CHPF	-1.035080595	0.074306587	0.960774008	1
CHRFAM7A	-0.859410034	-0.238172813	1.097582847	1
CHRNA1	-0.853659974	-0.24655585	1.100215825	1
CHST12	-0.972627714	-0.052663412	1.025291127	1
CHST2	-1.14072391	0.725480364	0.415243546	1
CIB4	-1.037697391	0.080228023	0.957469368	1
CLDN10	-1.001682489	0.003373513	0.998308976	1
CLDN11	-0.925687051	-0.134925337	1.060612388	1
CLDN3	-1.111979449	0.286497094	0.825482356	1
CLDND1	-1.154303024	0.550914188	0.603388835	1
CLEC14A	-0.89672972	-0.181640583	1.078370302	1
CLEC4A	-0.916325578	-0.150327619	1.066653197	1
CLPP	-1.125028943	0.787753028	0.337275915	1
CMIP	-0.978843587	-0.041048681	1.019892268	1
CMTM1	-0.940338291	-0.110195329	1.05053362	1
CMTM2	-1.111776917	0.285769845	0.826007072	1

CMTM6	-1.091876301	0.220585101	0.8712912	1
CNFN	-1.144854628	0.44211649	0.702738138	1
CNTN1	-1.12498902	0.33710645	0.787882571	1
CNTNAP3	-1.154474351	0.597029335	0.557445016	1
COL4A2	-1.111385066	0.827017846	0.28436722	1
COQ10B	-1.090975083	0.217874924	0.873100158	1
COQ2	-1.149555366	0.480481101	0.669074265	1
CORO1C	-1.149992352	0.665208107	0.484784245	1
COX10	-1.113441794	0.291795383	0.821646411	1
COX6C	-0.868814476	-0.224281652	1.093096129	1
COX7B	-0.895380593	-0.18375265	1.079133243	1
COX7C	-1.132430148	0.37076311	0.761667037	1
CPE	-1.152662205	0.635722854	0.516939352	1
CPEB4	-1.119135767	0.313292934	0.805842833	1
CPSF4L	-0.892117504	-0.188838696	1.0809562	1
CPXCR1	-0.893235477	-0.187099696	1.080335173	1
CRADD	-1.039734482	0.084879706	0.954854776	1
CREB5	-1.153118387	0.628889768	0.524228619	1
CREBBP	-1.115096848	0.817200625	0.297896223	1
CRHR2	-1.022684121	0.047027628	0.975656493	1
CRYGA	-0.942203486	-0.106989463	1.049192949	1
CSF1	-1.152180554	0.642120405	0.510060149	1
CSF2	-0.890489414	-0.191364633	1.081854047	1
CST2	-0.940324288	-0.110219347	1.050543635	1

CST7	-1.152402434	0.513141938	0.639260496	1
CSTB	-1.148952315	0.674132737	0.474819578	1
CT45A2	-1.065721772	0.147922153	0.917799619	1
CT45A5	-1.149509589	0.480040604	0.669468985	1
CT45A7	-1.006288925	0.012698798	0.993590127	1
CT45A9	-1.138006182	0.399573319	0.738432863	1
CT47A1	-1.107692617	0.836269394	0.271423223	1
CT47A11	-1.14155948	0.721217318	0.420342162	1
CT47A2	-1.154185685	0.606951747	0.547233938	1
CT47A3	-1.120875769	0.80070468	0.320171089	1
CT47A4	-1.065690008	0.147840321	0.917849687	1
CT47A6	-1.127411661	0.347587319	0.779824341	1
CT47A7	-1.063593341	0.142467884	0.921125457	1
CT47A9	-1.148720728	0.675999517	0.47272121	1
CT47B1	-1.113894461	0.293452739	0.820441722	1
CTCFL	-1.072520174	0.165753196	0.906766979	1
CTF1	-0.927006331	-0.132730385	1.059736716	1
CTIF	-0.846117985	-0.257428528	1.103546514	1
CTSH	-1.12851991	0.775995897	0.352524013	1
CTSW	-1.154331132	0.551872733	0.6024584	1
CTSZ	-1.075168703	0.172880094	0.902288609	1
CXCL16	-0.889216022	-0.193334894	1.082550916	1
CXCR2	-1.13554068	0.749182917	0.386357763	1
CXorf65	-1.144147974	0.706959349	0.437188624	1

CYBB	-1.092788954	0.223347689	0.869441265	1
CYBC1	-0.907811959	-0.164081145	1.071893103	1
CYP1A2	-0.855157127	-0.244381045	1.099538172	1
CYP27A1	-1.114306538	0.819337754	0.294968784	1
CYP4F12	-1.147874982	0.465368302	0.68250668	1
CYREN	-0.838225456	-0.268661627	1.106887083	1
DAD1	-1.001573946	0.003155358	0.998418587	1
DAND5	-1.113936601	0.820329148	0.293607453	1
DAPK2	-0.940473139	-0.109964004	1.050437143	1
DAZ2	-0.977296744	-0.043956834	1.021253578	1
DAZ3	-0.943555375	-0.104657427	1.048212802	1
DAZAP2	-1.013404942	0.027371879	0.986033064	1
DCAF7	-1.038975449	0.08314209	0.955833359	1
DCTN3	-1.045710374	0.098747465	0.946962909	1
DDI1	-1.114344171	0.819236585	0.295107586	1
DDX46	-1.048837689	0.106143066	0.942694623	1
DEDD	-0.984556363	-0.030202991	1.014759354	1
DEFA5	-1.144247428	0.706374852	0.437872576	1
DEFB107A	-1.136771216	0.743923073	0.392848143	1
DEFB107B	-1.136771216	0.743923073	0.392848143	1
DEFB112	-0.989986391	-0.019735092	1.009721483	1
DEFB115	-1.028449515	0.059561479	0.968888036	1
DEFB123	-1.152351334	0.639931469	0.512419865	1
DEFB127	-1.151969694	0.644718882	0.507250813	1

DENND3	-1.025783099	0.05373278	0.972050319	1
DENND5A	-1.150054854	0.485415071	0.664639783	1
DGAT2	-0.939841631	-0.111046724	1.050888355	1
DGAT2L6	-0.837039402	-0.270337163	1.107376565	1
DGKG	-1.017642194	0.036271339	0.981370855	1
DGUOK	-1.09997937	0.245795603	0.854183767	1
DHH	-1.138242965	0.400888865	0.737354101	1
DHRS11	-1.057446616	0.12703314	0.930413476	1
DIO2	-1.154689323	0.572937224	0.581752099	1
DLGAP1	-1.112351734	0.82451374	0.287837994	1
DLK2	-0.887584666	-0.195852175	1.08343684	1
DLX4	-1.034379734	0.072730729	0.961649004	1
DMRTC1	-0.938571432	-0.113219864	1.051791296	1
DMRTC1B	-1.12537702	0.338757893	0.786619128	1
DNAAF3	-1.14303133	0.713323752	0.429707578	1
DNAJA2	-0.972241181	-0.053379468	1.02562065	1
DNAJB7	-0.855378185	-0.244059462	1.099437647	1
DNAJC25-GNG10	-1.138915325	0.734242101	0.404673225	1
DNAJC5G	-0.844207039	-0.26016172	1.104368759	1
DNASE2	-1.151248854	0.498361518	0.652887336	1
DNM3	-1.036825747	0.078248953	0.958576794	1
DOK2	-1.001392044	0.002789926	0.998602118	1
DPCD	-1.147118268	0.687969624	0.459148644	1
DPEP2	-1.153696241	0.618546295	0.535149946	1

DRC7	-0.835444226	-0.272585578	1.108029804	1
DRD5	-1.086234498	0.203896138	0.88233836	1
DRG1	-1.154685263	0.57219889	0.582486373	1
DSC2	-1.140884837	0.416214204	0.724670633	1
DSG3	-0.87847185	-0.209775175	1.088247024	1
DUOX1	-0.950096419	-0.093271768	1.043368187	1
DUSP1	-0.974188483	-0.049764771	1.023953254	1
DUSP10	-1.033972305	0.071816573	0.962155732	1
DUSP8	-1.145444343	0.446357845	0.699086498	1
DYNLL1	-1.041357623	0.088613161	0.952744462	1
DYNLL2	-1.07302113	0.167093131	0.905927999	1
DYNLT1	-1.135723198	0.748415229	0.387307969	1
DYNLT3	-0.853173044	-0.247261991	1.100435035	1
DYRK1A	-1.154408749	0.599683915	0.554724834	1
E2F3	-1.120344972	0.318056073	0.8022889	1
EBF1	-0.850264008	-0.251468571	1.101732579	1
EBI3	-1.039957731	0.085391776	0.954565955	1
EEPD1	-1.150989394	0.655604381	0.495385013	1
EFCAB3	-1.145857921	0.449408845	0.696449076	1
EFHD1	-1.111262103	0.283928267	0.827333836	1
EGFL6	-0.897423527	-0.180552299	1.077975826	1
EGLN1	-1.127840945	0.778352517	0.349488429	1
EGR1	-0.872833303	-0.218275274	1.091108578	1
EGR2	-1.122884744	0.794567636	0.328317109	1

EGR4	-1.137768649	0.398262296	0.739506352	1
EIF1B	-1.070190762	0.159570683	0.91062008	1
EIF2AK1	-0.975809058	-0.046742563	1.02255162	1
EIF2S2	-1.147592869	0.684579802	0.463013067	1
EIF3I	-1.057076202	0.126117391	0.930958811	1
EIF4EBP1	-1.126604419	0.782555172	0.344049247	1
ELAPOR1	-0.914161103	-0.153846701	1.068007804	1
ELF2	-1.099702766	0.244908159	0.854794607	1
ELL2	-1.016082728	0.032981455	0.983101273	1
EMC3	-1.045239212	0.097641674	0.947597538	1
ENDOD1	-1.148800572	0.675360346	0.473440227	1
ENG	-0.955857074	-0.08309993	1.038957004	1
EPHX3	-1.12148041	0.798881481	0.322598929	1
EPPIN-WFDC6	-0.940489553	-0.109935842	1.050425395	1
ERC2	-0.843780357	-0.260770815	1.104551172	1
ERFE	-1.153294069	0.625988636	0.527305433	1
ERI3	-1.063970683	0.143430597	0.920540086	1
ERP44	-1.153529678	0.621786631	0.531743047	1
ERVV-1	-1.154692589	0.58105688	0.57363571	1
ERVV-2	-0.914925919	-0.152605006	1.067530925	1
ESPNL	-1.126511726	0.343645987	0.78286574	1
ETF1	-0.910992034	-0.158971342	1.069963376	1
ETFA	-0.878912468	-0.209107298	1.088019766	1
EVA1B	-0.908495556	-0.162985459	1.071481015	1

EVA1C	-0.973996335	-0.05012226	1.024118596	1
EVI2B	-1.097021639	0.236408845	0.860612794	1
EVPLL	-1.152454182	0.513881132	0.63857305	1
EXD1	-1.143652728	0.433826984	0.709825743	1
EXOSC7	-0.93875613	-0.112904251	1.051660381	1
EYA2	-0.969254274	-0.058888838	1.028143111	1
F11R	-0.869782051	-0.222839457	1.092621508	1
F13A1	-1.152437939	0.638789704	0.513648235	1
FAAP20	-1.121221149	0.321555452	0.799665697	1
FADS1	-1.133727704	0.377137419	0.756590285	1
FAM126B	-1.144211792	0.706584633	0.437627159	1
FAM161B	-1.153684548	0.534902154	0.618782393	1
FAM166C	-1.071129775	0.162053503	0.909076272	1
FAM180A	-0.952668317	-0.088747525	1.041415842	1
FAM189A1	-0.97950736	-0.039797072	1.019304432	1
FAM200B	-1.069753764	0.158419495	0.911334269	1
FAM227B	-0.886215846	-0.197958459	1.084174305	1
FAM236C	-1.038581637	0.08224261	0.956339028	1
FAM237B	-1.056810796	0.125462219	0.931348577	1
FAM241B	-0.915850966	-0.151100588	1.066951555	1
FAM25C	-0.89712913	-0.181014256	1.078143387	1
FAM32A	-1.153619122	0.533540737	0.620078385	1
FAM71D	-0.989968555	-0.019769736	1.009738291	1
FAM83B	-0.90296188	-0.171812636	1.074774516	1

FAM83C	-1.060443561	0.134501436	0.925942125	1
FAM83E	-1.140221318	0.41224518	0.727976138	1
FAM90A1	-0.989982403	-0.019742839	1.009725241	1
FAM90A26	-1.035664985	0.075623799	0.960041186	1
FAM98B	-0.993549628	-0.012778278	1.006327905	1
FANCB	-0.995946389	-0.008058517	1.004004906	1
FAS	-1.126735592	0.782114635	0.344620957	1
FBRS	-1.06621231	0.149187566	0.917024744	1
FBXL13	-0.968628939	-0.060036964	1.028665903	1
FBXL7	-0.846838483	-0.256395761	1.103234244	1
FBXO2	-1.147126859	0.687909295	0.459217564	1
FBXO24	-0.900930846	-0.175028548	1.075959394	1
FCER1G	-1.138771796	0.403859169	0.734912627	1
FCGRT	-1.058793193	0.13037563	0.928417563	1
FCRLB	-1.055439651	0.122090244	0.933349407	1
FER1L6	-1.012264161	0.024997014	0.987267146	1
FFAR1	-1.146319482	0.452894475	0.693425007	1
FFAR2	-0.960653975	-0.074522502	1.035176477	1
FGD4	-1.142897675	0.4288351	0.714062575	1
FGF13	-1.11206703	0.825254968	0.286812062	1
FGF22	-1.100555699	0.24765124	0.852904459	1
FGF3	-0.88492883	-0.199934001	1.084862831	1
FGF6	-0.852678863	-0.247978052	1.100656914	1
FGFR1OP2	-1.043837269	0.094364174	0.949473095	1

FGFRL1	-0.874051956	-0.216445431	1.090497388	1
FJX1	-1.150508877	0.660383709	0.490125168	1
FKBP8	-1.134475201	0.380892455	0.753582746	1
FNDC10	-1.149975716	0.484617016	0.6653587	1
FNIP1	-1.147049933	0.458601784	0.688448149	1
FOSL1	-0.884596643	-0.200443138	1.085039781	1
FOXB1	-1.149137714	0.476528636	0.672609079	1
FOXC2	-1.060557051	0.134786355	0.925770696	1
FOXD1	-1.152389986	0.639424652	0.512965334	1
FOXD4	-1.114421413	0.295392666	0.819028747	1
FOXD4L1	-0.969248347	-0.058899728	1.028148075	1
FOXD4L3	-1.05865284	0.130026253	0.928626587	1
FOXD4L4	-1.139402702	0.407463525	0.731939177	1
FOXD4L5	-1.023203424	0.048146142	0.975057282	1
FOXD4L6	-1.118049203	0.309075636	0.808973567	1
FOXE1	-0.943861737	-0.104127953	1.04798969	1
FOXL2	-0.986228371	-0.026996573	1.013224943	1
FOXL2NB	-0.88316736	-0.202630229	1.085797589	1
FOXP2	-1.071748099	0.163695316	0.908052783	1
FPR2	-1.15452188	0.559670579	0.594851301	1
FRG2C	-0.917662964	-0.148145465	1.065808429	1
FRK	-0.889491314	-0.192909345	1.082400659	1
FRMD5	-1.154008498	0.542387932	0.611620566	1
FRMD7	-0.891030921	-0.190525358	1.081556279	1

FSTL1	-0.855973385	-0.243192996	1.09916638	1
FURIN	-0.930986168	-0.126071423	1.057057591	1
FXYD4	-1.141041399	0.723877779	0.41716362	1
FXYD7	-0.903515104	-0.17093446	1.074449564	1
G0S2	-1.150159887	0.66367552	0.486484367	1
G6PD	-1.133907528	0.7558725	0.378035028	1
GABARAPL2	-1.055501925	0.122242931	0.933258995	1
GABRB1	-0.931960573	-0.124432381	1.056392954	1
GADD45G	-1.1017853	0.251640475	0.850144825	1
GAGE12D	-1.097903573	0.239184441	0.858719132	1
GAGE12G	-0.843336745	-0.261403623	1.104740368	1
GAGE12H	-1.124094668	0.333337074	0.790757594	1
GAGE12J	-0.906640083	-0.165956003	1.072596086	1
GAGE13	-1.132591785	0.371547626	0.761044159	1
GAGE2A	-0.914172448	-0.153828297	1.068000745	1
GAL	-1.112682847	0.289035116	0.823647731	1
GAL3ST4	-1.117075687	0.305345363	0.811730324	1
GALK2	-1.140512034	0.726538532	0.413973502	1
GALNS	-0.847342136	-0.255673085	1.103015221	1
GALR1	-1.1200102	0.316729814	0.803280386	1
GALR3	-1.153159258	0.628230316	0.524928942	1
GAN	-1.09936794	0.243836624	0.855531316	1
GAS2L1	-0.933623046	-0.121627944	1.05525099	1
GCA	-1.15455675	0.593059137	0.561497614	1

GCLM	-0.93271473	-0.123161442	1.055876172	1
GDF3	-0.916005919	-0.150848308	1.066854228	1
GFI1B	-0.966364833	-0.064178759	1.030543592	1
GGT6	-1.056439304	0.124546519	0.931892785	1
GGTLC3	-1.034599183	0.0732237	0.961375483	1
GIT1	-1.137307663	0.74156554	0.395742123	1
GJA4	-0.843651394	-0.260954827	1.104606221	1
GJD4	-1.132492264	0.371064283	0.761427981	1
GLB1L	-1.143444437	0.432434337	0.7110101	1
GLIPR1	-0.951437977	-0.090915224	1.042353202	1
GLIS1	-1.140955179	0.724315037	0.416640141	1
GLT1D1	-1.129254007	0.773406891	0.355847115	1
GLTPD2	-1.041598782	0.08916994	0.952428842	1
GMFG	-1.021206812	0.043856703	0.977350109	1
GMPR	-1.146285408	0.452634105	0.693651303	1
GNG10	-0.866712223	-0.227406662	1.094118884	1
GNG14	-0.977505343	-0.043565352	1.021070695	1
GNG7	-1.12527671	0.338329986	0.786946724	1
GNPDA2	-1.154698399	0.575424342	0.579274057	1
GNRHR	-1.127671451	0.778935293	0.348736158	1
GOLGA6L10	-0.936659692	-0.116479068	1.05313876	1
GOLGA6L7	-1.133487708	0.375944975	0.757542733	1
GOLGA8G	-0.850171499	-0.251602004	1.101773504	1
GP1BA	-0.858283347	-0.239821906	1.098105254	1

GP2	-1.069109348	0.1567268	0.912382548	1
GPAT3	-1.141364592	0.419139726	0.722224866	1
GPATCH2L	-0.856842155	-0.241926699	1.098768854	1
GPR119	-1.133319155	0.758207926	0.37511123	1
GPR142	-0.891012824	-0.190553421	1.081566245	1
GPR143	-1.151615158	0.648861644	0.502753514	1
GPR148	-1.030418605	0.063902175	0.96651643	1
GPR151	-1.120474189	0.318569569	0.801904621	1
GPR20	-1.119173443	0.805733228	0.313440215	1
GPR21	-1.140203532	0.412139971	0.728063561	1
GPR27	-0.85048168	-0.25115452	1.101636201	1
GPR32	-0.898777513	-0.178424301	1.077201815	1
GPR33	-1.153132235	0.628667329	0.524464906	1
GPR45	-1.154660611	0.585646262	0.569014349	1
GPR62	-1.148149917	0.680441432	0.467708485	1
GPR78	-1.11664174	0.303696797	0.812944943	1
GPR88	-1.154123334	0.608676502	0.545446831	1
GPRC6A	-1.027802439	0.058141842	0.969660597	1
GPSM3	-1.110571056	0.281471819	0.829099237	1
GPX1	-1.012428224	0.025338018	0.987090206	1
GPX4	-1.1312031	0.364891164	0.766311936	1
GPX6	-1.137183956	0.395071119	0.742112836	1
GRIN2C	-1.044972977	0.097017797	0.94795518	1
GRIN3B	-0.854831551	-0.244854456	1.099686007	1

GRK1	-0.858359054	-0.239711197	1.098070251	1
GRK7	-1.121153239	0.321282741	0.799870499	1
GRN	-0.847075479	-0.256055779	1.103131257	1
GSG1	-0.910665087	-0.159498185	1.070163272	1
GSTM4	-0.860862502	-0.236042165	1.096904667	1
GTF2IRD1	-1.084735175	0.199567064	0.885168112	1
GUCA2A	-1.010855639	0.022076853	0.988778787	1
GYPA	-1.144845776	0.702792028	0.442053748	1
H1-1	-1.072943043	0.166884023	0.90605902	1
H1-5	-1.010102246	0.020520329	0.989581916	1
H1-6	-0.855330316	-0.244129109	1.099459425	1
H1-7	-1.123290071	0.793301164	0.329988907	1
H2AC12	-0.929857513	-0.12796563	1.057823143	1
H2AC14	-1.09398136	0.226985073	0.866996287	1
H2AC18	-1.034694222	0.073437324	0.961256899	1
H2AC19	-1.047764272	0.103593518	0.944170754	1
H2AC7	-0.967259471	-0.062544995	1.029804467	1
H2AZ1	-1.131408908	0.365865987	0.76554292	1
H2BC12	-1.047987061	0.104121717	0.943865344	1
H2BC4	-1.152791102	0.518910714	0.633880388	1
H2BC5	-1.079989435	0.186134413	0.893855022	1
H2BS1	-0.968141809	-0.060930085	1.029071894	1
H2BU1	-0.961716465	-0.072609094	1.034325559	1
H2BW2	-0.836961056	-0.27044773	1.107408785	1

H3-3A	-1.09485114	0.229658775	0.865192365	1
H3C11	-0.922070532	-0.140911119	1.062981651	1
H3C13	-0.933365677	-0.122062764	1.055428441	1
H3C14	-1.124292146	0.334164983	0.790127163	1
H3C15	-1.116395416	0.302764828	0.813630588	1
H3C2	-0.947119498	-0.098474771	1.045594269	1
H3Y1	-1.119117406	0.313221182	0.805896224	1
H4C3	-1.080762683	0.188296101	0.892466582	1
HACD1	-0.841388501	-0.264177301	1.105565802	1
HBA1	-0.842400879	-0.262737108	1.105137987	1
HBP1	-0.875411993	-0.214398582	1.089810575	1
HCAR1	-0.974412907	-0.049347006	1.023759913	1
HCRT	-0.88974288	-0.192520281	1.082263161	1
HDAC5	-1.15426358	0.604639797	0.549623783	1
HEATR9	-1.069477515	0.157693159	0.911784356	1
HELT	-0.898963107	-0.178132181	1.077095288	1
HEPACAM	-0.836613844	-0.270937561	1.107551405	1
HEPHL1	-1.122101348	0.325113344	0.796988004	1
HK1	-1.094955695	0.22998136	0.864974335	1
HLA-E	-1.154674202	0.570583167	0.584091035	1
HMGB1	-1.152900028	0.520627588	0.63227244	1
HMGB2	-1.118655028	0.807235149	0.311419879	1
HMGXB4	-1.141488754	0.41990483	0.721583924	1
HMX2	-0.990517728	-0.018702198	1.009219926	1

HOGA1	-1.153747465	0.536252426	0.617495039	1
HOXB7	-1.013058417	0.026649554	0.986408863	1
HOXB8	-0.963401337	-0.069564601	1.032965938	1
HOXD4	-0.867071502	-0.226873408	1.09394491	1
HPCAL1	-0.994024632	-0.011845497	1.005870128	1
HRURF	-1.15463039	0.588337764	0.566292626	1
HSD11B2	-0.862348589	-0.23385667	1.096205259	1
HSD17B11	-1.150514452	0.660329972	0.49018448	1
HSPA4L	-1.114514696	0.295737284	0.818777412	1
HSPB9	-1.128708416	0.353373203	0.775335213	1
HSPE1-MOB4	-0.900559875	-0.175614569	1.076174444	1
HTATIP2	-0.998310796	-0.003369891	1.001680687	1
HTR1A	-1.078120239	0.180950411	0.897169828	1
HTR1B	-1.117624462	0.810181823	0.307442638	1
HTR2B	-1.136266804	0.390163425	0.746103379	1
HTR5A	-0.906077403	-0.16685468	1.072932083	1
HTRA3	-0.991623012	-0.016548575	1.008171586	1
HYAL3	-1.092308279	0.221890409	0.870417871	1
HYAL4	-1.13542163	0.385740235	0.749681394	1
IFNA13	-1.151350414	0.499555779	0.651794635	1
IFNA14	-0.841449464	-0.264090644	1.105540108	1
IFNGR2	-0.909427018	-0.16149009	1.070917107	1
IFNL1	-1.129599679	0.772172509	0.35742717	1
IFT88	-0.875798065	-0.213816635	1.0896147	1

IGFALS	-1.133111484	0.759023333	0.37408815	1
IGFBP1	-0.884403801	-0.200738561	1.085142362	1
IGFL2	-1.123446709	0.330637616	0.792809093	1
IGFL3	-0.842332571	-0.262834358	1.105166929	1
IGSF22	-0.991914633	-0.015979223	1.007893856	1
IL17A	-1.144394113	0.705507165	0.438886948	1
IL17F	-0.901517926	-0.174100278	1.075618204	1
IL1R2	-1.1346651	0.752808515	0.381856584	1
IL6	-0.909947059	-0.160654005	1.070601065	1
IL6R	-0.843041779	-0.261824133	1.104865912	1
IMMT	-0.971085646	-0.055515862	1.026601508	1
INHBB	-0.83816873	-0.268741838	1.106910568	1
INO80C	-0.889721838	-0.19255283	1.082274669	1
INPP5E	-1.137263314	0.741762002	0.395501311	1
INSM1	-0.938824164	-0.112787962	1.051612126	1
INSM2	-0.986797737	-0.025901305	1.012699042	1
IQCF2	-1.026685369	0.055698866	0.970986503	1
IRAG2	-1.148270237	0.468747276	0.679522961	1
IRAK1	-1.130549233	0.768729338	0.361819896	1
IRS2	-1.153520607	0.621956063	0.531564544	1
ISOC2	-1.090320072	0.215916044	0.874404028	1
ITGAX	-0.872205157	-0.219216903	1.09142206	1
ITPK1	-1.148960155	0.474891319	0.674068836	1
JAK2	-0.938659634	-0.113069161	1.051728795	1

JAML	-1.154678879	0.5834644	0.571214478	1
JKAMP	-0.871652139	-0.220045039	1.091697178	1
JPH2	-0.956416277	-0.082105104	1.03852138	1
KAAG1	-1.075498855	0.173775995	0.90172286	1
KASH5	-0.882787934	-0.203209863	1.085997797	1
KBTBD12	-1.117994411	0.809129915	0.308864497	1
KBTBD13	-1.151144698	0.653990545	0.497154153	1
KCNE5	-1.137000845	0.394081937	0.742918908	1
KCNF1	-1.100395714	0.247135233	0.853260481	1
KCNG1	-1.129602049	0.772164011	0.357438038	1
KCNG4	-1.146603198	0.455082182	0.691521016	1
KCNIP4	-0.976952505	-0.044602403	1.021554907	1
KCNK12	-1.001256365	0.002517484	0.998738881	1
KCNK15	-1.032716142	0.069006949	0.963709194	1
KCNK7	-1.121686052	0.798256801	0.323429251	1
KCNU1	-0.840304498	-0.265716734	1.106021233	1
KCNV2	-1.130263468	0.769773788	0.36048968	1
KCP	-1.055802541	0.122980605	0.932821936	1
KDM4B	-1.097473487	0.237828463	0.859645024	1
KDM7A	-1.1546034	0.590272523	0.564330876	1
KIAA0319	-1.153171012	0.525132003	0.628039009	1
KIAA1109	-0.869485592	-0.223281599	1.092767191	1
KIAA1210	-1.064877176	0.145750795	0.919126381	1
KIAA1549	-0.955387648	-0.083934016	1.039321665	1

KIF4B	-0.936451278	-0.116833549	1.053284827	1
KIFC3	-0.8890006	-0.193667741	1.082668342	1
KLF5	-1.114670529	0.818356735	0.296313794	1
KLF6	-1.145030289	0.70166323	0.443367058	1
KLHL10	-1.134380816	0.753966004	0.380414812	1
KLHL13	-1.121534378	0.322816607	0.798717771	1
KMT5B	-0.871364684	-0.220475178	1.091839862	1
KNCN	-1.143686802	0.43405598	0.709630823	1
KPNA7	-1.154485776	0.557957007	0.596528768	1
KRT12	-0.850368096	-0.251318411	1.101686506	1
KRT25	-0.918950257	-0.146039378	1.064989636	1
KRT31	-1.059826712	0.132955544	0.926871169	1
KRT32	-0.941752695	-0.107765507	1.049518202	1
KRT33B	-1.068819198	0.15596655	0.912852648	1
KRTAP1-5	-0.90432324	-0.169649933	1.073973173	1
KRTAP10-1	-0.881819964	-0.204686767	1.086506732	1
KRTAP10-3	-1.152851357	0.519854379	0.632996978	1
KRTAP10-5	-1.070846965	0.161304407	0.909542558	1
KRTAP10-9	-1.051505148	0.112530245	0.938974903	1
KRTAP11-1	-0.934981599	-0.119328643	1.054310242	1
KRTAP16-1	-1.128008314	0.777774905	0.350233409	1
KRTAP17-1	-1.030989261	0.065166019	0.965823242	1
KRTAP19-2	-0.843641378	-0.260969116	1.104610495	1
KRTAP19-4	-1.118763913	0.311843109	0.806920803	1

KRTAP2-2	-1.149274835	0.477810175	0.671464661	1
KRTAP2-4	-1.154111339	0.608997212	0.545114128	1
KRTAP4-1	-1.149518881	0.480129864	0.669389017	1
KRTAP4-12	-1.00229619	0.004608307	0.997687883	1
KRTAP4-4	-0.865855058	-0.228677538	1.094532596	1
KRTAP5-2	-0.971708214	-0.054365626	1.02607384	1
KRTAP5-5	-0.879379639	-0.208398591	1.08777823	1
KRTAP5-9	-1.154170663	0.607376591	0.546794072	1
KRTAP9-8	-1.154228071	0.605717711	0.54851036	1
LACTB	-1.154005517	0.61169353	0.542311988	1
LALBA	-1.113738717	0.820857146	0.292881571	1
LAMB4	-1.011406876	0.023218102	0.988188773	1
LAMC3	-0.847182171	-0.25590268	1.103084851	1
LAMP2	-1.00399139	0.008031154	0.995960235	1
LAMTOR4	-0.977514183	-0.043548757	1.02106294	1
LAT2	-1.154576614	0.591938611	0.562638004	1
LBR	-1.05681991	0.125484703	0.931335207	1
LDLRAD1	-1.148063614	0.681094659	0.466968954	1
LDLRAD3	-0.994136261	-0.011626101	1.005762362	1
LEUTX	-1.151297222	0.498928145	0.652369077	1
LGALS2	-1.088861062	0.211584936	0.877276126	1
LGALS9C	-0.990752089	-0.018246115	1.008998205	1
LILRA1	-1.034005681	0.071891406	0.962114275	1
LILRB3	-1.154408619	0.599688882	0.554719737	1

LIMS1	-0.883993747	-0.201366392	1.085360139	1
LINGO4	-1.094682588	0.229139277	0.865543311	1
LKAAEAR1	-1.152981009	0.631044094	0.521936915	1
LMLN2	-0.879010606	-0.20895847	1.087969077	1
LMTK2	-0.868717133	-0.224426608	1.093143742	1
LNPK	-1.12027872	0.317793138	0.802485583	1
LOC100129307	-1.136291609	0.390294646	0.745996963	1
LOC100131107	-0.836908953	-0.270521252	1.107430204	1
LOC101059906	-1.112610168	0.823838186	0.288771982	1
LOC102723655	-0.893877709	-0.186099044	1.079976753	1
LOC102724334	-0.892150388	-0.188787598	1.080937986	1
LOC102724474	-1.089260927	0.212767578	0.876493349	1
LOC102725035	-1.154559211	0.561634428	0.592924783	1
LOC102725191	-0.978963353	-0.040823014	1.019786367	1
LOC105373102	-1.141792161	0.72000341	0.421788751	1
LOC105373989	-1.010739705	0.021837086	0.988902619	1
LOC105376341	-1.082098586	0.192055041	0.890043545	1
LOC107984025	-1.032718126	0.069011375	0.963706751	1
LOC107985678	-0.930046208	-0.127649267	1.057695474	1
LOC107985729	-1.036580966	0.077694381	0.958886586	1
LOC107985795	-1.056780478	0.125387427	0.931393051	1
LOC107987042	-1.039816313	0.085067351	0.954748962	1
LOC107987067	-0.955088452	-0.084465149	1.0395536	1
LOC107987125	-1.063574057	0.142418733	0.921155324	1

LOC107987235	-0.95152665	-0.090759204	1.042285854	1
LOC107987425	-0.855499421	-0.243883043	1.099382464	1
LOC107987432	-1.150338345	0.488328694	0.662009651	1
LOC107987462	-1.141047909	0.417203203	0.723844706	1
LOC112267859	-1.107590471	0.271071853	0.836518618	1
LOC112267897	-1.134853118	0.382815347	0.752037772	1
LOC112267904	-1.139099383	0.733377182	0.405722202	1
LOC112268186	-0.872581566	-0.21865277	1.091234336	1
LOC112268347	-1.152437848	0.513646931	0.638790917	1
LOC112268390	-0.951311426	-0.091137836	1.042449263	1
LOC112268395	-0.863711119	-0.231847934	1.095559053	1
LOC441239	-1.052456212	0.114825698	0.937630514	1
LOC645202	-1.078312206	0.181480146	0.89683206	1
LPAR2	-1.142427167	0.425799943	0.716627223	1
LPGAT1	-0.948145063	-0.096686354	1.044831416	1
LRFN5	-1.096593417	0.235068106	0.861525312	1
LRP10	-1.152646652	0.516705614	0.635941039	1
LRP3	-1.153340223	0.625195859	0.528144364	1
LRRC10	-0.905556628	-0.167685541	1.073242169	1
LRRC19	-1.098770016	0.241930394	0.856839622	1
LRRC49	-0.918535306	-0.146718872	1.065254178	1
LRRC69	-0.952214019	-0.089548669	1.041762688	1
LRRC70	-1.125915656	0.784848459	0.341067196	1
LRRC74B	-1.111490802	0.826745673	0.284745129	1

LRRC75B	-1.118829621	0.312098799	0.806730822	1
LRRC8E	-0.907294536	-0.164909495	1.072204031	1
LRRN4	-1.033651761	0.071098354	0.962553407	1
LRRTM1	-0.998390761	-0.003210747	1.001601508	1
LSAMP	-1.131066939	0.364248386	0.766818553	1
LSM1	-1.141954986	0.422808309	0.719146677	1
LSM14A	-0.922223663	-0.140658586	1.06288225	1
LSM2	-1.120176698	0.317388693	0.802788006	1
LTBR	-1.151567409	0.649400121	0.502167288	1
LUZP6	-0.902187186	-0.173040776	1.075227962	1
LYPD2	-0.969318726	-0.058770398	1.028089124	1
LYZL4	-1.151290283	0.498846621	0.652443662	1
LZTS2	-0.840505876	-0.265430958	1.105936834	1
M1AP	-1.096320958	0.234217383	0.862103575	1
MACROD1	-1.119752503	0.315712885	0.804039618	1
MACROH2A1	-1.073943737	0.169570674	0.904373063	1
MADCAM1	-1.028649007	0.059999822	0.968649184	1
MAFG	-0.881521525	-0.205141591	1.086663116	1
MAGEA10-MAGEA5	-1.122584164	0.795500528	0.327083636	1
MAGEA11	-0.915876297	-0.151059353	1.06693565	1
MAGEA12	-1.145633215	0.697890199	0.447743016	1
MAGEB16	-0.843656105	-0.260948105	1.104604211	1
MAGEB3	-1.152973656	0.631156847	0.52181681	1
MAML3	-1.056800772	0.125437488	0.931363283	1

MANBA	-1.126836784	0.345062841	0.781773943	1
MANEAL	-1.152497871	0.514511644	0.637986227	1
MANSC1	-1.150960597	0.655899684	0.495060913	1
MAP1S	-1.153394976	0.529157803	0.624237173	1
MAP3K15	-0.907424897	-0.164700878	1.072125775	1
MAP7D2	-1.13307682	0.373917827	0.759158994	1
MAPK14	-1.141936953	0.422695094	0.71924186	1
MAPK15	-1.016973984	0.034859568	0.982114416	1
MAPK3	-0.957257857	-0.080605412	1.037863268	1
MAPKAPK2	-0.9547649	-0.08503909	1.03980399	1
MAPT	-0.866874712	-0.227165532	1.094040244	1
MARCHF7	-0.86672311	-0.227390507	1.094113617	1
MARCOL	-1.128481403	0.35235089	0.776130513	1
MAS1	-1.074398039	0.170795361	0.903602678	1
MAT2B	-1.119411302	0.805039612	0.31437169	1
MBD2	-1.093588471	0.225783035	0.867805437	1
MBD3L1	-1.074564654	0.171245304	0.903319351	1
MBD6	-1.070776818	0.16111878	0.909658037	1
MCHR2	-1.108949298	0.275774826	0.833174472	1
MCM9	-1.153078708	0.523557091	0.629521617	1
MCOLN2	-1.116572656	0.303435139	0.813137516	1
MCRIP1	-1.15470045	0.576958053	0.577742396	1
MCTP1	-1.028779922	0.060287656	0.968492266	1
MCU	-1.022254296	0.046103368	0.976150928	1

MEA1	-1.09912456	0.243059586	0.856064974	1
MECP2	-1.026969053	0.056318338	0.970650715	1
MED26	-1.131763795	0.367556319	0.764207475	1
MEGF9	-1.126322638	0.783497395	0.342825243	1
MEIS2	-1.15417891	0.607144117	0.547034794	1
MESD	-0.919452255	-0.145216567	1.064668822	1
MESP1	-1.125876008	0.340896543	0.784979466	1
METRN	-0.976220787	-0.045972682	1.022193469	1
METRNL	-0.886720151	-0.197183076	1.083903228	1
METTL11B	-1.151540149	0.501834532	0.649705617	1
MGAM	-0.975397393	-0.047511491	1.022908884	1
MGLL	-1.152560827	0.63712986	0.515430967	1
MGST3	-1.014109499	0.028843035	0.985266464	1
MICAL2	-1.144180482	0.706768628	0.437411855	1
MINDY1	-0.957507671	-0.08015966	1.037667331	1
MITD1	-0.875595659	-0.214121784	1.089717442	1
MKNK1	-1.123730782	0.3318179	0.791912881	1
MLF1	-1.118877731	0.312286143	0.806591587	1
MLPH	-1.143498214	0.432792722	0.710705491	1
MMP25	-1.143828208	0.435009857	0.708818351	1
MMP26	-0.989588102	-0.020508328	1.01009643	1
MMP3	-1.038803936	0.082750177	0.956053759	1
MMP9	-1.153181052	0.627874982	0.52530607	1
MPHOSPH8	-0.912285616	-0.156883469	1.069169085	1

MPIG6B	-1.065276406	0.146776006	0.918500399	1
MPP1	-1.154612932	0.564988474	0.589624458	1
MPP7	-1.008484928	0.01719153	0.991293398	1
MRGPRF	-0.846060239	-0.257511248	1.103571488	1
MROH2B	-0.989035435	-0.021579833	1.010615268	1
MRPL27	-0.939974409	-0.110819203	1.050793612	1
MRPL32	-1.116839338	0.304446403	0.812392935	1
MRPL36	-1.084310557	0.198348703	0.885961855	1
MRPL49	-1.084083738	0.197699253	0.886384485	1
MRPS35	-1.15025488	0.662793185	0.487461695	1
MRPS6	-1.15381496	0.616064587	0.537750373	1
MSL1	-0.959676577	-0.076278292	1.035954869	1
MSMP	-1.151415957	0.651080189	0.500335768	1
MSRB1	-1.105510788	0.263991783	0.841519005	1
MSRB3	-1.141362021	0.722238106	0.419123915	1
MSX1	-1.060762578	0.135302733	0.925459845	1
MTF2	-1.086183556	0.20374835	0.882435206	1
MTG2	-0.890879578	-0.190760009	1.081639587	1
MTNR1B	-0.917508557	-0.148397709	1.065906266	1
MTO1	-1.151247063	0.498340611	0.652906452	1
MTRNR2L1	-1.069071584	0.156627785	0.912443799	1
MTRNR2L10	-1.149562149	0.480546534	0.669015615	1
MTRNR2L3	-1.148943485	0.474738847	0.674204638	1
MTRNR2L5	-1.113187105	0.822320579	0.290866526	1

MTRNR2L6	-1.1187879	0.311936424	0.806851475	1
MTRNR2L9	-0.939516053	-0.111604331	1.051120383	1
MTURN	-0.884303316	-0.200892456	1.085195773	1
MXD1	-0.899284016	-0.177626829	1.076910845	1
MYC	-1.097494153	0.237893514	0.859600639	1
MYEOV	-1.060767504	0.135315116	0.925452388	1
MYL12B	-0.963010607	-0.070271761	1.033282367	1
MYL9	-0.940915326	-0.109204964	1.050120291	1
MYLPF	-0.872821064	-0.218293633	1.091114696	1
MYMK	-0.849911235	-0.251977296	1.101888531	1
MYMX	-1.149736284	0.66749566	0.482240624	1
MYO15A	-0.855776887	-0.243479145	1.099256032	1
MYO16	-1.132242719	0.762386024	0.369856695	1
MYO5A	-1.100085014	0.246135083	0.85394993	1
MYOT	-1.154492058	0.596247783	0.558244275	1
N4BP2L2	-1.080926689	0.188755909	0.89217078	1
NAA10	-1.146749708	0.456226097	0.69052361	1
NAA11	-1.154598211	0.563986367	0.590611843	1
NAA50	-0.885006992	-0.199814157	1.084821149	1
NAIP	-0.912856564	-0.155960215	1.068816778	1
NAMPT	-1.154313313	0.551261064	0.603052249	1
NAP1L5	-1.059306919	0.131656426	0.927650493	1
NAPA	-1.147779277	0.464564214	0.683215063	1
NAPG	-0.873450481	-0.217349062	1.090799543	1

NBL1	-0.880098015	-0.207307628	1.087405643	1
NCKAP5L	-0.987305205	-0.024923644	1.01222885	1
NCMAP	-0.841624774	-0.263841398	1.105466172	1
NCOA4	-0.929528589	-0.128516796	1.058045385	1
NCOA5	-1.090077343	0.215192434	0.874884909	1
NCS1	-1.121191097	0.799756358	0.321434739	1
NDUFA4L2	-0.989873981	-0.019953412	1.009827393	1
NDUFC1	-0.976939433	-0.044626906	1.021566338	1
NECAB2	-1.144595928	0.704302211	0.440293717	1
NECTIN2	-1.001871474	0.003753515	0.998117959	1
NEDD8	-1.145400446	0.446037825	0.699362622	1
NEIL3	-1.040435615	0.086489439	0.953946176	1
NEK6	-0.846698817	-0.256596056	1.103294873	1
NENF	-0.855038435	-0.244553663	1.099592098	1
NEUROD4	-0.961148561	-0.073632434	1.034780995	1
NHLH1	-1.153657107	0.619331016	0.534326091	1
NINJ1	-1.116988054	0.811976306	0.305011748	1
NKAPL	-1.123050558	0.328999833	0.794050725	1
NKX2-4	-0.877383179	-0.211423068	1.088806247	1
NKX2-5	-0.917598087	-0.148251459	1.065849546	1
NKX3-2	-0.994089508	-0.011717998	1.005807506	1
NLRP6	-1.153793283	0.537263386	0.616529897	1
NLRX1	-0.865537288	-0.229148196	1.094685484	1
NME4	-1.102101322	0.252672532	0.84942879	1

NME9	-1.11685443	0.304503729	0.812350701	1
NMUR2	-0.990975886	-0.017810309	1.008786195	1
NNT	-0.982071516	-0.034941098	1.017012614	1
NODAL	-1.154697015	0.574878189	0.579818826	1
NOTCH1	-0.950092739	-0.093278221	1.04337096	1
NOTCH3	-1.072548713	0.165829431	0.906719283	1
NOTO	-1.144994201	0.701884928	0.443109273	1
NOX5	-0.889744041	-0.192518485	1.082262526	1
NPBWR2	-0.836350794	-0.271308477	1.107659271	1
NPFF	-1.106456903	0.267195514	0.839261389	1
NPFFR1	-1.145014239	0.443252354	0.701761885	1
NPIPB12	-0.872949902	-0.21810037	1.091050272	1
NPIPB2	-1.11467602	0.296334126	0.818341894	1
NPPA	-1.132328623	0.370271696	0.762056927	1
NPS	-1.099068177	0.242879792	0.856188385	1
NPY4R	-0.972288418	-0.053292	1.025580418	1
NPY4R2	-1.141009134	0.724041589	0.416967545	1
NR2F6	-1.154660332	0.568985244	0.585675088	1
NR4A1	-1.14039237	0.413260112	0.727132259	1
NRBF2	-1.154700332	0.576753007	0.577947326	1
NRDC	-1.151515527	0.649980352	0.501535176	1
NRIP3	-0.859179251	-0.238510861	1.097690113	1
NRTN	-0.854078791	-0.245948022	1.100026813	1
NT5C2	-0.841384063	-0.264183609	1.105567673	1

NTSR1	-0.907303134	-0.164895738	1.072198871	1
NTSR2	-1.0174877	0.035944648	0.981543052	1
NUDT5	-1.12383702	0.79157644	0.33226058	1
NUP188	-1.038149064	0.0812562	0.956892863	1
NUTF2	-1.121101963	0.321076994	0.800024969	1
NXNL1	-0.890738059	-0.190979367	1.081717427	1
NXPE1	-1.108814849	0.275306684	0.833508165	1
NXPE4	-1.085046616	0.200462816	0.884583801	1
OAZ1	-0.920674415	-0.143209781	1.063884195	1
OAZ2	-1.141829085	0.422019425	0.719809659	1
OBP2B	-1.109824686	0.278838306	0.830986381	1
OC90	-1.108465306	0.274092511	0.834372795	1
ODAD1	-0.838107426	-0.268828513	1.106935939	1
ODF3L2	-0.918030939	-0.147544004	1.065574944	1
OGFRL1	-1.049573106	0.107896615	0.941676492	1
OLFM2	-0.905718454	-0.16742745	1.073145904	1
OPN1MW	-1.130830467	0.76769435	0.363136117	1
OPN5	-0.888412584	-0.194575603	1.082988188	1
OPRM1	-0.885068063	-0.199720508	1.084788571	1
OR10A4	-0.902451363	-0.172622179	1.075073542	1
OR10D3	-0.887440384	-0.196074442	1.083514826	1
OR10H1	-1.116027164	0.301376627	0.814650537	1
OR10H5	-1.112547422	0.824002447	0.288544975	1
OR10J5	-0.986914822	-0.025675857	1.012590679	1

OR10T2	-1.149672459	0.481616448	0.668056011	1
OR10V1	-1.146310789	0.693482786	0.452828003	1
OR11A1	-0.940488542	-0.109937577	1.050426119	1
OR11G2	-1.12971094	0.357937868	0.771773072	1
OR11H6	-1.13665227	0.392211933	0.744440337	1
OR13C2	-0.950752888	-0.092119555	1.042872443	1
OR13C3	-1.000511191	0.001023168	0.999488023	1
OR13C4	-1.15146963	0.50098009	0.65048954	1
OR13C5	-0.946126194	-0.100202913	1.046329107	1
OR13G1	-0.885962742	-0.19834734	1.084310082	1
OR13J1	-0.895394013	-0.183731669	1.079125681	1
OR1D2	-1.034591704	0.073206892	0.961384812	1
OR1D5	-0.947513107	-0.09778888	1.045301988	1
OR1I1	-0.997766032	-0.004453064	1.002219096	1
OR1K1	-0.942430029	-0.106599169	1.049029198	1
OR1L8	-1.148822175	0.473635558	0.675186617	1
OR1N1	-1.141635292	0.420812165	0.720823128	1
OR2A25	-1.083665419	0.196503962	0.887161457	1
OR2B3	-1.116122118	0.301733996	0.814388122	1
OR2C3	-1.067504641	0.152536658	0.914967983	1
OR2D2	-1.011610917	0.023641052	0.987969865	1
OR2G6	-1.153371035	0.62465886	0.528712175	1
OR2M3	-0.950961987	-0.091752182	1.042714168	1
OR2T3	-1.023410976	0.04859375	0.974817225	1

OR2T4	-0.854999148	-0.244610791	1.099609939	1
OR4A47	-1.133988873	0.378442251	0.755546622	1
OR4C12	-1.117167149	0.811473217	0.305693932	1
OR4D10	-0.835858199	-0.272002639	1.107860838	1
OR4D9	-1.146614777	0.691442542	0.455172235	1
OR4F16	-0.867440423	-0.226325495	1.093765918	1
OR4F4	-1.154484451	0.557896942	0.596587509	1
OR4K17	-0.95825167	-0.078830528	1.037082198	1
OR4X2	-0.934634337	-0.11991702	1.054551357	1
OR51B2	-0.877010841	-0.211985924	1.088996765	1
OR51B4	-0.869509695	-0.22324566	1.092755355	1
OR51D1	-1.027069463	0.056537752	0.970531711	1
OR51F1	-1.036480118	0.077466054	0.959014064	1
OR51I2	-1.128363562	0.351821817	0.776541745	1
OR51M1	-1.154555935	0.561452545	0.593103391	1
OR52N1	-0.852649848	-0.248020074	1.100669923	1
OR56A4	-0.983975966	-0.031312575	1.015288541	1
OR56B4	-0.915550308	-0.151589864	1.067140172	1
OR5A1	-1.122102771	0.325119132	0.796983639	1
OR5AP2	-1.14838129	0.678667005	0.469714285	1
OR5AS1	-0.952644595	-0.088789379	1.041433974	1
OR5AU1	-1.113268077	0.291161551	0.822106526	1
OR5B21	-1.117352564	0.810950807	0.306401757	1
OR5C1	-1.065404478	0.147105333	0.918299145	1

OR5H6	-0.84824761	-0.254372321	1.102619932	1
OR5K3	-0.958927459	-0.07762118	1.036548639	1
OR5M8	-0.851131231	-0.250216687	1.101347918	1
OR6A2	-1.040105708	0.085731445	0.954374263	1
OR6C4	-1.07862972	0.182357657	0.896272063	1
OR6C65	-0.958757493	-0.077925526	1.036683019	1
OR6C74	-1.05572063	0.122779504	0.932941126	1
OR6K3	-1.119767293	0.803996135	0.315771158	1
OR6M1	-1.108356759	0.273716313	0.834640446	1
OR6V1	-1.14473015	0.703493493	0.441236657	1
OR7A17	-0.945953349	-0.100503226	1.046456575	1
OR7D4	-1.125332916	0.338569669	0.786763247	1
OR8B12	-0.886718331	-0.197185876	1.083904208	1
OR8B8	-1.112111214	0.286971075	0.82514014	1
OR8G1	-0.995365952	-0.009204552	1.004570504	1
OR8H2	-0.977254349	-0.044036371	1.02129072	1
OR8S1	-0.869232613	-0.223658711	1.092891324	1
ORAI2	-0.998153166	-0.003683492	1.001836658	1
ORC4	-1.138069129	0.738146935	0.399922193	1
OSBPL2	-0.985895456	-0.027636186	1.013531642	1
OSCAR	-0.986474153	-0.026523983	1.012998136	1
OSGIN1	-1.144572552	0.70444245	0.440130102	1
OSM	-1.12770888	0.778806787	0.348902093	1
OTUD6A	-1.131688171	0.367195118	0.764493053	1

OVCH2	-0.849702589	-0.252278038	1.101980627	1
OVOL3	-1.044684787	0.096343248	0.948341539	1
OXER1	-1.10779076	0.271761148	0.836029612	1
P2RX1	-1.141464297	0.41975386	0.721710437	1
P2RY1	-0.857039795	-0.241638362	1.098678157	1
P2RY13	-1.132303441	0.370149967	0.762153474	1
PAEP	-1.029692422	0.062297719	0.967394703	1
PAGE4	-0.863946681	-0.23150017	1.095446851	1
PAIP2	-1.036553229	0.077631573	0.958921656	1
PAK6	-0.839860048	-0.266347125	1.106207173	1
PAPLN	-0.862984105	-0.232920339	1.095904444	1
PAQR9	-1.153724934	0.535764021	0.617960913	1
PARD6A	-1.145716517	0.697358246	0.448358271	1
PARG	-1.085190499	0.200877258	0.884313241	1
PARK7	-0.986935254	-0.025636509	1.012571763	1
PARVB	-0.90331166	-0.171257513	1.074569174	1
PASD1	-1.082093072	0.192039462	0.89005361	1
PC	-0.97554379	-0.047238138	1.022781928	1
PCDH11X	-1.004844583	0.00976062	0.995083963	1
PCDH20	-0.934751177	-0.119719103	1.054470281	1
PCDH7	-0.922265501	-0.140589577	1.062855078	1
PCDHA1	-1.022053091	0.045671186	0.976381904	1
PCDHB1	-1.124081163	0.333280546	0.790800617	1
PCDHB10	-0.960502035	-0.07479572	1.035297755	1

PCDHB11	-1.005400914	0.010890787	0.994510127	1
PCDHB13	-0.963636482	-0.069138698	1.03277518	1
PCDHB14	-1.154694742	0.580515993	0.574178748	1
PCDHB2	-1.066945461	0.151084789	0.915860672	1
PCDHB3	-0.88134384	-0.205412267	1.086756107	1
PCDHB4	-1.088495433	0.210506395	0.877989038	1
PCDHB5	-1.035201357	0.074578543	0.960622814	1
PCDHB9	-1.138314438	0.737026751	0.401287687	1
PCDHGA11	-0.935340189	-0.118720601	1.05406079	1
PCDHGA3	-1.149315011	0.671126437	0.478188573	1
PCDHGA4	-1.109754936	0.831161717	0.278593219	1
PCDHGA8	-1.09882732	0.242112682	0.856714638	1
PCDHGA9	-1.112960247	0.290041357	0.82291889	1
PCDHGB2	-0.956793934	-0.081432495	1.038226429	1
PCDHGB6	-0.996283437	-0.007392144	1.00367558	1
PCDHGB7	-0.999456788	-0.00108554	1.000542328	1
PCDHGC3	-1.154637844	0.566898424	0.58773942	1
PCDHGC5	-0.851913927	-0.249085254	1.100999181	1
PCMT1	-1.117570292	0.307234988	0.810335304	1
PCOLCE2	-1.139135211	0.405927061	0.73320815	1
PCSK6	-1.125357159	0.338673113	0.786684046	1
PCTP	-1.117985305	0.30882942	0.809155885	1
PDCD4	-0.869305289	-0.223550391	1.09285568	1
PDE4DIP	-0.871844576	-0.219756961	1.091601536	1

PDE6A	-0.96359079	-0.069221477	1.032812266	1
PDLIM2	-1.106261645	0.26653201	0.839729635	1
PDLIM7	-1.059079852	0.131089929	0.927989923	1
PDSS1	-1.14951452	0.48008796	0.66942656	1
PDX1	-1.048651036	0.105698891	0.942952146	1
PELO	-0.985990258	-0.027454107	1.013444366	1
PERCC1	-1.154369148	0.601140838	0.55322831	1
PFN3	-1.149300891	0.671245455	0.478055437	1
PGK2	-1.148164139	0.680333343	0.467830795	1
PGLYRP3	-1.084456549	0.198767222	0.885689327	1
PHF20L1	-0.909173599	-0.161897203	1.071070802	1
PHLDA2	-0.970361668	-0.056851153	1.027212821	1
PHOSPHO2	-0.954419122	-0.085651972	1.040071094	1
PIEZO2	-1.142351471	0.717034697	0.425316774	1
PIGZ	-1.12465339	0.788967522	0.335685867	1
PIK3C2G	-0.883497142	-0.202126107	1.085623248	1
PILRA	-1.001140578	0.002285072	0.998855506	1
PIP4K2A	-1.140859098	0.416058604	0.724800494	1
PITX3	-1.107670239	0.836324024	0.271346215	1
PLA2G4D	-1.109931692	0.279214637	0.830717055	1
PLA2G4F	-1.118986825	0.80627543	0.312711395	1
PLAGL1	-1.032319013	0.06812146	0.964197553	1
PLAGL2	-1.15235154	0.639928785	0.512422754	1
PLAU	-1.133482169	0.757564642	0.375917527	1

PLAUR	-1.018893396	0.038923388	0.979970008	1
PLCH2	-1.090705364	0.217067201	0.873638163	1
PLCZ1	-1.147202131	0.45982298	0.687379151	1
PLD5	-1.143116469	0.430265794	0.712850675	1
PLEK	-1.068053072	0.153964729	0.914088343	1
PLEKHN1	-1.145283545	0.445189029	0.700094516	1
PLN	-0.880795597	-0.206246878	1.087042475	1
PLP2	-0.895738467	-0.183192925	1.078931393	1
PLPP7	-1.111627875	0.285235667	0.826392208	1
PLPPR2	-1.116758761	0.304140512	0.812618249	1
PLXNB2	-1.125722414	0.340236446	0.785485968	1
PNMA6F	-1.087075321	0.20634271	0.880732611	1
POC1B	-1.005660079	0.011417937	0.994242142	1
POLA2	-1.085843666	0.202763561	0.883080105	1
POLR3K	-1.151590931	0.649135398	0.502455533	1
POMC	-1.025068041	0.05217912	0.97288892	1
POTEA	-0.962889956	-0.070489981	1.033379937	1
POTEE	-1.128487412	0.352377897	0.776109515	1
POTEF	-1.136676931	0.392343674	0.744333256	1
POTEH	-0.886607994	-0.197355585	1.083963578	1
PPIF	-0.971916214	-0.053980922	1.025897136	1
PPP1R15A	-0.983464646	-0.032288639	1.015753284	1
PPP1R1B	-0.948800167	-0.09554174	1.044341907	1
PPP2CA	-1.083057003	0.194771171	0.888285832	1

PPP3R1	-1.149724399	0.482124102	0.667600297	1
PPRC1	-0.913376671	-0.155118246	1.068494917	1
PPWD1	-1.14603066	0.695327611	0.450703049	1
PRAMEF13	-0.967204257	-0.062645932	1.029850189	1
PRAMEF14	-1.118520868	0.310899205	0.807621663	1
PRAMEF19	-1.153785209	0.616701761	0.537083448	1
PRAMEF27	-0.960160245	-0.075409957	1.035570202	1
PRAMEF9	-1.058703979	0.130153525	0.928550454	1
PRCC	-1.133749755	0.377247296	0.756502459	1
PRDM16	-1.154294832	0.550641164	0.603653668	1
PRDX3	-1.149568435	0.668961229	0.480607206	1
PRDX6	-0.894597081	-0.184976754	1.079573834	1
PRELID1	-1.113588881	0.821255881	0.292333	1
PRKACG	-1.115059159	0.297756017	0.817303142	1
PRKCB	-0.942577954	-0.106344211	1.048922165	1
PRKCE	-1.014627666	0.029927171	0.984700495	1
PRMT8	-1.095627447	0.232060066	0.863567381	1
PROK2	-1.043572403	0.093747093	0.949825311	1
PRR15L	-1.003212018	0.006455288	0.996756729	1
PRR23C	-0.916422282	-0.150170032	1.066592313	1
PRR23D2	-0.876442342	-0.212844583	1.089286925	1
PRR27	-1.140467993	0.41371062	0.726757373	1
PRR32	-0.909540397	-0.161307881	1.070848278	1
PRR5-ARHGAP8	-1.028492947	0.059656885	0.968836062	1

PRRG1	-0.97246402	-0.052966744	1.025430765	1
PRRG2	-0.878801472	-0.209275593	1.088077065	1
PRSS48	-0.847964959	-0.254778577	1.102743536	1
PRSS54	-1.033572781	0.070921523	0.962651258	1
PRXL2B	-0.979524313	-0.039765076	1.019289389	1
PRY	-1.001567526	0.003142458	0.998425068	1
PSD3	-0.88776423	-0.195575471	1.083339701	1
PSG9	-1.154691466	0.5733816	0.581309866	1
PSMA6	-1.100279073	0.24675946	0.853519613	1
PSMB3	-1.147157737	0.687692166	0.459465571	1
PSMD8	-1.087332875	0.207094879	0.880237996	1
PTBP3	-0.966341353	-0.064221589	1.030562942	1
PTDSS2	-1.132160534	0.76270019	0.369460344	1
PTGDR	-1.154631037	0.588287115	0.566343923	1
PTGER1	-1.007247806	0.014656734	0.992591072	1
PTGER3	-0.878181166	-0.210215493	1.088396659	1
PTGIS	-1.012621357	0.025739675	0.986881683	1
PTP4A2	-0.939127075	-0.11226999	1.051397064	1
PTP4A3	-1.112180327	0.287219952	0.824960375	1
PTPN18	-0.948334744	-0.096355117	1.044689861	1
PTPRC	-1.153458021	0.623107296	0.530350725	1
PTPRJ	-1.079727289	0.185403864	0.894323425	1
PTPRN	-1.15465665	0.568609649	0.586047001	1
PXDC1	-0.953558268	-0.087175627	1.040733895	1

PXT1	-0.98321554	-0.032763665	1.015979205	1
PYGL	-1.053535545	0.117442546	0.936092999	1
QKI	-0.925229359	-0.135685391	1.06091475	1
QPCT	-1.123773174	0.331994462	0.791778713	1
R3HDM4	-1.063139332	0.141311968	0.921827364	1
R3HDML	-1.123879743	0.791440945	0.332438798	1
RAB1B	-1.111951674	0.286397266	0.825554408	1
RAB25	-1.016520977	0.033904263	0.982616713	1
RAB26	-1.119644119	0.315286204	0.804357915	1
RAB33A	-1.082967983	0.194518201	0.888449783	1
RAB36	-1.143114098	0.712863876	0.430250222	1
RAB3A	-1.037644117	0.080106872	0.957537245	1
RAB40AL	-0.984305528	-0.030682747	1.014988274	1
RAB7A	-0.869274979	-0.223595568	1.092870547	1
RAC1	-1.12418339	0.79047466	0.333708731	1
RAET1E	-1.025395622	0.05289039	0.972505232	1
RAF1	-1.154043963	0.610739901	0.543304062	1
RALGPS1	-1.143351067	0.431813986	0.711537081	1
RAMP1	-0.98912221	-0.021411704	1.010533914	1
RARG	-0.984361471	-0.030575776	1.014937247	1
RASD2	-0.850039355	-0.251792572	1.101831927	1
RASL12	-0.89427508	-0.185479296	1.079754376	1
RBBP8NL	-1.045598555	0.098484834	0.947113721	1
RBM47	-1.004908573	0.009890514	0.995018059	1

RBMXL2	-1.140403166	0.72707881	0.413324356	1
RBMXL3	-0.84286752	-0.262072463	1.104939984	1
RCCD1	-1.024794941	0.051586776	0.973208165	1
RCOR1	-1.109818742	0.831001331	0.27881741	1
RCOR2	-1.045799547	0.098956993	0.946842553	1
RD3	-1.091259236	0.218727555	0.872531682	1
RECK	-0.873452054	-0.2173467	1.090798754	1
REEP5	-0.853782669	-0.246377828	1.100160497	1
RELL1	-1.142033575	0.423302588	0.718730987	1
REM2	-0.937449662	-0.115133969	1.052583631	1
RETREG2	-0.985657775	-0.028092472	1.013750247	1
RETSAT	-1.154443569	0.598317626	0.556125943	1
RFC3	-1.113753868	0.292937091	0.820816777	1
RFTN1	-1.022450913	0.046525985	0.975924928	1
RGS19	-1.143117467	0.430272351	0.712845116	1
RGS22	-0.981918039	-0.035232699	1.017150737	1
RHBG	-0.916665389	-0.149773729	1.066439119	1
RHOA	-1.063549948	0.142357293	0.921192656	1
RILPL2	-1.149963296	0.484492351	0.665470945	1
RIMKLA	-1.033223442	0.070140008	0.963083433	1
RIT1	-0.914376246	-0.153497608	1.067873853	1
RLBP1	-0.913054871	-0.155639291	1.068694163	1
RMI1	-0.882455449	-0.203717456	1.086172905	1
RMND5A	-0.864610536	-0.230519344	1.09512988	1

RNASET2	-1.141822797	0.421980124	0.719842673	1
RNF13	-1.022576911	0.046796965	0.975779947	1
RNF150	-1.122145847	0.79685147	0.325294377	1
RNF151	-0.858643341	-0.239295343	1.097938683	1
RNF222	-1.093443004	0.225338868	0.868104136	1
RNF225	-1.133263585	0.374837019	0.758426566	1
RNF24	-1.0309427	0.065062799	0.9658799	1
RO60	-1.010560407	0.021466451	0.989093957	1
ROS1	-1.00941096	0.019095414	0.990315546	1
RPL17-C18orf32	-0.869979042	-0.222545534	1.092524577	1
RPRML	-1.109054938	0.276143099	0.83291184	1
RS1	-1.09194574	0.22079465	0.871151091	1
RSC1A1	-0.878586867	-0.209600886	1.088187753	1
RSPRY1	-0.966853835	-0.063286212	1.030140047	1
RTL8B	-1.11656916	0.303421905	0.813147255	1
RTN3	-1.073628929	0.168723863	0.904905067	1
RTP2	-0.923933808	-0.137832845	1.061766653	1
RTP5	-1.103934331	0.258715149	0.845219182	1
RUFY1	-0.879150748	-0.208745898	1.087896646	1
RUSC1	-0.913024725	-0.155688085	1.06871281	1
RXFP3	-1.070457068	0.160273538	0.91018353	1
S100A5	-1.12469405	0.335857578	0.788836473	1
S100A9	-1.072719941	0.166287078	0.906432863	1
S100G	-1.145202407	0.444602824	0.700599583	1

S100P	-1.13257801	0.761097345	0.371480665	1
S1PR2	-1.021537973	0.044566105	0.976971868	1
S1PR3	-0.85971706	-0.237722875	1.097439935	1
SAMSN1	-1.152160701	0.642369789	0.509790912	1
SANBR	-1.152515428	0.514766729	0.637748699	1
SAP25	-1.05755389	0.127298643	0.930255247	1
SAT1	-0.914658767	-0.153038952	1.067697719	1
SBK1	-0.936791826	-0.116254243	1.05304607	1
SCAMP4	-1.135071904	0.751135596	0.383936308	1
SCAPER	-0.955603461	-0.083550671	1.039154132	1
SCARB1	-1.128273468	0.351418055	0.776855413	1
SCARF2	-1.127587014	0.348362209	0.779224805	1
SCLT1	-1.151548927	0.501941536	0.649607391	1
SCTR	-1.151804994	0.505128621	0.646676373	1
SDCBP	-0.987511699	-0.024525429	1.012037128	1
SDCCAG8	-1.04907619	0.106711145	0.942365045	1
SECTM1	-0.851954321	-0.249026822	1.100981143	1
SEL1L2	-1.05366889	0.117766721	0.935902169	1
SEPTIN14	-1.138594819	0.402860096	0.735734722	1
SEPTIN7	-0.979877428	-0.039098307	1.018975735	1
SERINC2	-0.876860597	-0.212212937	1.089073534	1
SERINC4	-0.835952578	-0.271869684	1.107822262	1
SERPINA2	-0.952216705	-0.089543934	1.041760639	1
SERPINB11	-0.858537174	-0.239450667	1.097987841	1

SERPINB12	-1.143417796	0.711160711	0.432257084	1
SERPINB5	-0.871438981	-0.220364023	1.091803004	1
SEZ6	-0.884321005	-0.200865368	1.085186373	1
SF1	-0.948943631	-0.095290844	1.044234475	1
SFTPB	-0.896011683	-0.182765351	1.078777034	1
SGCD	-1.138190573	0.737593548	0.400597025	1
SH3BGRL	-0.973695682	-0.050681264	1.024376946	1
SH3BGRL3	-1.017162397	0.035257321	0.981905076	1
SHCBP1	-1.113833698	0.293229781	0.820603917	1
SIAH2	-1.131980787	0.763384981	0.368595806	1
SIGLEC5	-1.145030064	0.701664613	0.443365451	1
SIM2	-0.905208848	-0.168239926	1.073448773	1
SIRPA	-0.89770986	-0.180102744	1.077812604	1
SIRPB2	-0.91023874	-0.160184683	1.070423423	1
SIX1	-1.13305883	0.373829477	0.759229353	1
SKA2	-0.987132627	-0.02525628	1.012388906	1
SKAP2	-0.877549754	-0.211171137	1.088720891	1
SKP1	-1.007900482	0.015992801	0.991907681	1
SLBP	-1.141519641	0.420095683	0.721423958	1
SLC15A1	-1.068859077	0.156070973	0.912788105	1
SLC18A2	-1.097159213	0.236840539	0.860318674	1
SLC18A3	-0.995050743	-0.009826098	1.004876841	1
SLC19A1	-0.948690134	-0.095734114	1.044424248	1
SLC22A1	-0.849857578	-0.252054648	1.101912225	1

SLC22A13	-1.130316366	0.769580992	0.360735374	1
SLC22A14	-1.057062193	0.126082788	0.930979405	1
SLC24A5	-1.128688034	0.775406783	0.353281251	1
SLC25A10	-1.106069747	0.265881106	0.840188641	1
SLC25A44	-1.075968292	0.175052782	0.900915511	1
SLC25A48	-0.86087522	-0.236023486	1.096898706	1
SLC27A1	-0.959750242	-0.076146107	1.035896348	1
SLC28A2	-1.152994437	0.522156893	0.630837544	1
SLC29A2	-1.151990634	0.507525002	0.644465632	1
SLC2A14	-1.153265249	0.626476865	0.526788384	1
SLC2A3	-0.983651323	-0.031932447	1.01558377	1
SLC2A8	-0.966823308	-0.063341963	1.030165271	1
SLC30A10	-1.15425366	0.549308376	0.604945284	1
SLC30A2	-0.848628662	-0.253824332	1.102452994	1
SLC30A7	-0.932497658	-0.123527475	1.056025133	1
SLC34A3	-1.010100255	0.020516222	0.989584033	1
SLC35D3	-0.903877581	-0.170358557	1.074236138	1
SLC35F1	-1.043046308	0.092523388	0.95052292	1
SLC35F4	-1.145662249	0.447957155	0.697705094	1
SLC35G1	-0.842493612	-0.262605068	1.10509868	1
SLC35G2	-0.879119504	-0.208793295	1.087912799	1
SLC35G4	-1.152572739	0.636966357	0.515606382	1
SLC36A2	-1.111181619	0.28364126	0.827540359	1
SLC41A1	-0.926877144	-0.13294559	1.059822735	1

SLC43A2	-1.150370712	0.66170367	0.488667042	1
SLC46A2	-0.921604283	-0.141679526	1.063283809	1
SLC47A2	-0.835157941	-0.272988484	1.108146426	1
SLC5A11	-1.131734259	0.367415181	0.764319077	1
SLC5A8	-0.876088883	-0.213378004	1.089466887	1
SLC6A4	-0.849223187	-0.252968648	1.102191835	1
SLC6A6	-1.089776717	0.214297931	0.875478786	1
SLC7A9	-1.132307911	0.762136344	0.370171566	1
SLC9C1	-0.89890507	-0.178223541	1.077128611	1
SLCO4A1	-0.884793589	-0.200141321	1.084934909	1
SLCO6A1	-1.145989163	0.695598124	0.450391039	1
SLITRK4	-1.153820346	0.615948071	0.537872275	1
SLPI	-0.928531656	-0.130184946	1.058716602	1
SMAD1	-1.094772781	0.229417179	0.865355601	1
SMCHD1	-1.140418134	0.413413466	0.727004668	1
SMCO1	-1.15249128	0.514416133	0.638075147	1
SMCO4	-1.135624762	0.748829784	0.386794978	1
SMG8	-1.15445019	0.598047432	0.556402758	1
SMIM18	-1.149386754	0.670519089	0.478867665	1
SMIM28	-1.030630105	0.064370278	0.966259827	1
SMIM32	-1.037823048	0.080513883	0.957309164	1
SMIM4	-0.880526096	-0.206656843	1.087182939	1
SMIM5	-0.932216767	-0.124000865	1.056217631	1
SMPD3	-1.139806105	0.730001561	0.409804544	1

SNAPC4	-0.98392386	-0.031412103	1.015335963	1
SNRNP27	-0.915577952	-0.151544889	1.067122842	1
SNRPA	-1.011102082	0.022586825	0.988515257	1
SNUPN	-1.14659268	0.69159224	0.45500044	1
SNURF	-0.913048364	-0.155649824	1.068698188	1
SNW1	-0.917876553	-0.147796406	1.065672959	1
SNX14	-1.007262366	0.014686509	0.992575857	1
SNX21	-1.111903684	0.286224856	0.825678829	1
SNX3	-1.06460288	0.145047608	0.919555272	1
SNX32	-1.011748838	0.023927101	0.987821736	1
SOD2	-1.130817237	0.767743199	0.363074038	1
SORL1	-1.058727175	0.130211264	0.92851591	1
SOS2	-0.976586364	-0.045288401	1.021874765	1
SOWAHB	-1.150454293	0.489546403	0.66090789	1
SOX11	-1.140531588	0.414090338	0.72644125	1
SOX12	-0.920880018	-0.14287168	1.063751698	1
SOX15	-0.870147172	-0.222294594	1.092441766	1
SOX2	-1.143022676	0.429650941	0.713371735	1
SOX21	-0.960172751	-0.07538749	1.035560242	1
SOX30	-0.851888187	-0.249122486	1.101010674	1
SOX7	-1.146041595	0.450785381	0.695256214	1
SPA17	-1.150176384	0.486653392	0.663522993	1
SPACA5	-1.118203152	0.808533498	0.309669654	1
SPACA5B	-0.942357905	-0.106723447	1.049081353	1

SPANXB1	-1.12851695	0.776006249	0.352510701	1
SPATA19	-1.147376939	0.461240169	0.68613677	1
SPATA2	-1.137377307	0.741256456	0.396120851	1
SPATA21	-0.96907567	-0.059216945	1.028292614	1
SPATA48	-1.113525975	0.821423009	0.292102966	1
SPATC1	-1.077538012	0.179347422	0.89819059	1
SPATC1L	-1.069796044	0.158530758	0.911265287	1
SPATS1	-1.017883121	0.03678114	0.981101982	1
SPINK14	-1.14435322	0.705749738	0.438603482	1
SPINT1	-0.8491253	-0.253109592	1.102234893	1
SPINT3	-1.07672168	0.177109103	0.899612577	1
SPIRE2	-1.017625083	0.036235149	0.981389935	1
SPTLC2	-1.082060955	0.191948728	0.890112227	1
SRGN	-1.147337788	0.686416402	0.460921386	1
SRSF9	-1.137116497	0.742410353	0.394706144	1
SSTR4	-0.884041364	-0.20129351	1.085334874	1
SSX5	-1.114868074	0.817821984	0.297046091	1
STEAP4	-1.099869544	0.245443	0.854426545	1
STIMATE-MUSTN1	-1.154267728	0.549756694	0.604511034	1
STK35	-1.142317325	0.717218059	0.425099265	1
STMP1	-0.901353876	-0.174359774	1.075713651	1
STRAP	-0.979327218	-0.040136967	1.019464185	1
STRIT1	-0.900660595	-0.175455505	1.076116099	1
STX1B	-1.151943514	0.645034091	0.506909423	1

STX3	-1.037079836	0.07882517	0.958254666	1
STX6	-1.128686286	0.353273366	0.77541292	1
STXBP2	-0.906283571	-0.166525518	1.072809089	1
SULT1B1	-1.117827335	0.30822155	0.809605784	1
SUPT20HL1	-1.123041351	0.79407947	0.328961881	1
SUPT20HL2	-1.081732223	0.191021077	0.890711146	1
SUPT4H1	-1.139789948	0.730079728	0.40971022	1
SUSD3	-1.141898835	0.71944281	0.422456025	1
SUSD6	-0.932983707	-0.122707647	1.055691354	1
SV2C	-1.154121077	0.608737106	0.545383971	1
SVIL	-1.12846084	0.352258488	0.776202352	1
SYNC	-1.041145101	0.088122948	0.953022153	1
SYNDIG1L	-0.858803554	-0.239060893	1.097864447	1
TAAR5	-1.08766025	0.20805284	0.87960741	1
TAAR6	-1.154678366	0.571142196	0.58353617	1
TAAR9	-1.153744052	0.536178092	0.617565961	1
TAB2	-1.009532538	0.019345791	0.990186748	1
TAB3	-0.891156717	-0.190330266	1.081486983	1
TACR2	-1.140863268	0.416083805	0.724779463	1
TAF6	-1.105787858	0.264927074	0.840860784	1
TAGLN2	-1.153129631	0.628709244	0.524420387	1
TAL1	-1.072184193	0.164856602	0.907327591	1
TALDO1	-1.088539407	0.210635967	0.87790344	1
TANGO2	-1.154410581	0.599614175	0.554796406	1

TAS2R30	-0.845513435	-0.258294142	1.103807577	1
TAS2R39	-1.13474623	0.752476451	0.382269779	1
TAS2R41	-1.02243143	0.046484096	0.975947334	1
TAS2R46	-0.83729931	-0.269970268	1.107269578	1
TAS2R9	-1.028247802	0.059118578	0.969129224	1
TAX1BP1	-1.110357614	0.280716646	0.829640967	1
TAX1BP3	-0.865291755	-0.229511683	1.094803438	1
TBATA	-0.96498783	-0.066686262	1.031674091	1
TBC1D2	-1.153691465	0.6186429	0.535048565	1
TBC1D20	-1.098869785	0.242247822	0.856621963	1
TBC1D3B	-1.117545925	0.307141627	0.810404298	1
TBC1D3I	-0.947179214	-0.098370751	1.045549966	1
TBCA	-1.148172592	0.680269038	0.467903554	1
TBXAS1	-1.110563678	0.281445688	0.82911799	1
TCEAL8	-1.123051553	0.794047619	0.329003934	1
TCTN1	-1.079856053	0.185762558	0.894093494	1
TDP2	-1.107751955	0.836124457	0.271627499	1
TENM1	-1.136297434	0.390325471	0.745971963	1
TERF2IP	-1.083169218	0.195090261	0.888078957	1
TERT	-1.02402411	0.049917945	0.974106165	1
TEX101	-1.153360484	0.624843464	0.52851702	1
TEX13C	-1.119455136	0.31454366	0.804911476	1
TEX38	-1.027520389	0.057524084	0.969996305	1
TEX47	-1.154687053	0.572510667	0.582176386	1

TEX49	-0.960550299	-0.074708943	1.035259242	1
TEX51	-0.875899784	-0.213663242	1.089563026	1
TFCP2L1	-1.03373522	0.071285271	0.962449949	1
TFE3	-1.110140829	0.830189488	0.279951341	1
TFF2	-1.005894408	0.011894936	0.993999472	1
TGFB1	-0.999145939	-0.00170594	1.000851878	1
TGFB2	-0.982470927	-0.034181665	1.016652592	1
TGFBR3L	-1.026875401	0.056113764	0.970761637	1
TGFBRAP1	-1.151932022	0.506760073	0.645171949	1
THAP4	-0.930484368	-0.126914156	1.057398524	1
THEG	-0.96632989	-0.064242498	1.030572388	1
THG1L	-1.110218688	0.82999268	0.280226008	1
THOP1	-1.129269357	0.773352285	0.355917072	1
TICAM1	-1.019378941	0.039955577	0.979423364	1
TICAM2	-0.891086833	-0.190438653	1.081525486	1
TIGD6	-1.128939584	0.354418459	0.774521124	1
TIMP2	-0.952493668	-0.089055616	1.041549284	1
TK1	-1.066019115	0.148688812	0.917330302	1
TLCD1	-1.132427863	0.370752039	0.761675824	1
TLCD4	-1.144426965	0.439115058	0.705311907	1
TLE3	-0.92214069	-0.14079543	1.06293612	1
TLR4	-0.965662879	-0.065458085	1.031120964	1
TLR5	-0.84772047	-0.255129826	1.102850296	1
TM2D3	-1.134195978	0.754713587	0.379482391	1

TM4SF20	-1.122738784	0.327717476	0.795021307	1
TM6SF1	-1.088301461	0.209935309	0.878366152	1
TMBIM4	-1.06665388	0.150329387	0.916324493	1
TMC4	-1.044512378	0.095940087	0.948572291	1
TMCO1	-1.133869361	0.377844213	0.756025147	1
TMCO3	-1.065006595	0.146082912	0.918923683	1
TMCO5A	-1.153668436	0.619105424	0.534563012	1
TMEM114	-0.85327599	-0.247112748	1.100388738	1
TMEM127	-1.13226629	0.369970494	0.762295797	1
TMEM131	-0.920587739	-0.143352269	1.063940008	1
TMEM150C	-1.111657702	0.826315201	0.285342501	1
TMEM190	-1.088743453	0.211237716	0.877505738	1
TMEM209	-0.992250362	-0.015323168	1.00757353	1
TMEM211	-1.090112954	0.215298518	0.874814435	1
TMEM217	-1.09052484	0.216527449	0.87399739	1
TMEM232	-0.985710619	-0.027991052	1.013701671	1
TMEM255A	-0.861724365	-0.234775361	1.096499726	1
TMEM271	-0.84482789	-0.259274677	1.104102567	1
TMEM45A	-0.902285121	-0.17288562	1.075170741	1
TMEM50A	-0.969386472	-0.058645886	1.028032358	1
TMEM51	-1.078471195	0.181919336	0.896551859	1
TMEM52	-1.110881052	0.82830948	0.282571572	1
TMEM61	-0.949144733	-0.094939009	1.044083742	1
TMEM71	-1.118352417	0.310246695	0.808105723	1

TMEM91	-0.993927632	-0.012036082	1.005963714	1
TMEM95	-1.023052846	0.047821607	0.975231239	1
TMF1	-1.154111406	0.608995452	0.545115953	1
TMPRSS13	-1.040938256	0.087646231	0.953292025	1
TMPRSS6	-0.973874927	-0.050348048	1.024222974	1
TMSB4X	-0.915922156	-0.150984694	1.06690685	1
TNFAIP8L2-SCNM1	-0.85346672	-0.246836175	1.100302895	1
TNFRSF10C	-0.917202944	-0.148896733	1.066099677	1
TNFRSF1B	-0.896543356	-0.18193266	1.078476016	1
TNFRSF8	-1.148995182	0.673782803	0.475212379	1
TNFSF13B	-1.090414763	0.216198668	0.874216095	1
TNNC2	-1.091465139	0.219346467	0.872118672	1
TNS4	-0.947940275	-0.097043808	1.044984083	1
TOM1L2	-1.130146111	0.359945471	0.77020064	1
TOP1	-1.11273319	0.289217506	0.823515684	1
TOPORS	-1.100372914	0.24706175	0.853311164	1
TP53AIP1	-1.135161403	0.750764876	0.384396527	1
TPD52L2	-0.855377962	-0.244059787	1.099437749	1
TPM4	-1.150080349	0.485673553	0.664406796	1
TPPP2	-1.08685343	0.205695733	0.881157697	1
TPRG1L	-1.105822739	0.265044989	0.84077775	1
TPSD1	-1.116701194	0.303922155	0.812779039	1
TPST1	-1.056568308	0.124864326	0.931703982	1
TPTE2	-1.039056772	0.083328009	0.955728763	1

TRAIP	-1.07447026	0.170990341	0.903479919	1
TRAPPC4	-1.151487333	0.501193737	0.650293596	1
TREM1	-1.151332596	0.651987593	0.499345003	1
TRIM3	-1.151608106	0.648941438	0.502666668	1
TRIM43	-0.845620023	-0.258141589	1.103761612	1
TRIM43B	-1.093932315	0.22683483	0.867097485	1
TRIM46	-1.042382659	0.090983479	0.95139918	1
TRIM47	-0.991264586	-0.017247704	1.00851229	1
TRIM49	-1.141894847	0.422431029	0.719463818	1
TRIM49B	-0.911961146	-0.157407681	1.069368828	1
TRIM49C	-1.154048281	0.610631048	0.543417232	1
TRIM62	-1.125926392	0.784812966	0.341113426	1
TRIM64C	-1.036807798	0.078208269	0.958599529	1
TRIM8	-0.954924638	-0.08475579	1.039680428	1
TRNT1	-1.154604068	0.564375893	0.590228175	1
TRPC5	-1.024373908	0.050674688	0.973699221	1
TRPC5OS	-1.113010817	0.29022512	0.822785696	1
TRPV6	-1.089293888	0.21286521	0.876428678	1
TSC22D3	-0.919414805	-0.14527798	1.064692785	1
TSNAX	-1.107703008	0.836244023	0.271458984	1
TSPAN10	-1.113066428	0.290427323	0.822639105	1
TSPAN32	-0.901920125	-0.173463724	1.07538385	1
TSPYL6	-1.137078944	0.39450325	0.742575693	1
TTC7B	-0.860312473	-0.236849637	1.097162111	1

TTLL12	-1.135560964	0.749097807	0.386463157	1
TTLL4	-1.108149869	0.835149481	0.273000388	1
TUBA4B	-1.133296441	0.374999106	0.758297334	1
TUBB4B	-1.152692757	0.517401009	0.635291747	1
TVP23B	-0.999348677	-0.001301375	1.000650052	1
TVP23C	-0.914793975	-0.152819358	1.067613333	1
TWIST1	-0.835929439	-0.271902282	1.107831721	1
TYMP	-1.144368087	0.70566161	0.438706477	1
UBA2	-0.866068956	-0.228360581	1.094429536	1
UBE2D3	-1.060603791	0.134903744	0.925700047	1
UBE2H	-0.973410628	-0.051210861	1.024621489	1
UBE2J1	-1.150687121	0.492040725	0.658646396	1
UBE2L3	-1.066084416	0.14885734	0.917227076	1
UBE2M	-0.939689036	-0.111308119	1.050997154	1
UBE2Q1	-0.979782982	-0.039276706	1.019059688	1
UBE2R2	-1.121862299	0.32414277	0.797719529	1
UBE2V1	-1.151100287	0.654455764	0.496644523	1
UBL4B	-1.154573963	0.562480831	0.592093133	1
UBOX5	-1.151286573	0.498803057	0.652483516	1
UBQLN3	-1.061100696	0.136153358	0.924947337	1
UBTD1	-1.150424088	0.489227662	0.661196425	1
UCMA	-0.939481645	-0.111663236	1.051144881	1
UCP2	-0.90571093	-0.167439451	1.073150381	1
UGP2	-0.884596757	-0.200442965	1.085039721	1

UIMC1	-0.971696702	-0.054386913	1.026083614	1
ULK4	-1.154700066	0.576445899	0.578254167	1
UMOD	-1.003468486	0.006973444	0.996495042	1
UMODL1	-1.079086755	0.183623679	0.895463076	1
USP11	-1.019320091	0.03983038	0.979489711	1
USP17L1	-0.889953061	-0.192195081	1.082148142	1
USP17L11	-1.152807511	0.633641261	0.51916625	1
USP17L12	-1.116235245	0.302160285	0.81407496	1
USP17L13	-0.994225525	-0.01145061	1.005676135	1
USP17L15	-0.996286767	-0.007385556	1.003672323	1
USP17L17	-1.00027384	0.000547905	0.999725935	1
USP17L18	-1.153112371	0.628986088	0.524126283	1
USP17L19	-1.120368906	0.318151118	0.802217788	1
USP17L20	-1.108649595	0.83391746	0.274732134	1
USP17L21	-1.13498712	0.383501228	0.751485892	1
USP17L22	-0.904680051	-0.169082137	1.073762188	1
USP17L24	-1.153869198	0.538995092	0.614874106	1
USP17L25	-0.908150768	-0.163538279	1.071689047	1
USP17L26	-1.091646786	0.219893229	0.871753557	1
USP17L27	-1.083290911	0.195436555	0.887854355	1
USP17L28	-0.841380478	-0.264188705	1.105569183	1
USP17L29	-1.120174473	0.317379877	0.802794596	1
USP17L30	-0.919220167	-0.145597082	1.064817249	1
USP17L5	-1.05589247	0.123201478	0.932690992	1

USP17L7	-1.15430635	0.60328051	0.55102584	1
UTS2B	-1.106927181	0.268798591	0.83812859	1
VCAN	-0.945896512	-0.100601952	1.046498464	1
VCX	-1.108632704	0.274673463	0.833959241	1
VGF	-0.887558059	-0.195893168	1.083451226	1
VMP1	-1.087212575	0.206743389	0.880469186	1
VPS4B	-1.151061776	0.496205037	0.65485674	1
VPS52	-0.860188177	-0.237032005	1.097220182	1
VSIG10L2	-0.876029892	-0.213466997	1.089496889	1
VSIR	-1.124866889	0.788278218	0.33658867	1
WAPL	-1.128306	0.776742223	0.351563777	1
WASHC5	-0.898483401	-0.178887015	1.077370416	1
WBP1L	-0.928501139	-0.130235954	1.058737093	1
WBP2	-1.067002579	0.1512329	0.91576968	1
WDR1	-1.118734155	0.311727384	0.807006771	1
WDR43	-1.128870632	0.774764395	0.354106237	1
WDR93	-0.884312601	-0.200878239	1.085190839	1
WEE1	-0.907654499	-0.164333315	1.071987814	1
WFDC11	-0.904335498	-0.169630434	1.073965932	1
WFDC5	-0.882420089	-0.20377142	1.086191509	1
WFDC8	-1.111896466	0.825697536	0.28619893	1
WIPI1	-1.137588902	0.740313013	0.39727589	1
WLS	-0.862538667	-0.233576728	1.096115395	1
WTAP	-1.049567344	0.107882854	0.94168449	1

XPNPEP2	-1.122100007	0.796992117	0.32510789	1
XPO6	-1.144022739	0.436331678	0.707691061	1
XRCC6	-1.140173842	0.411964478	0.728209364	1
YIPF3	-1.148818983	0.473606673	0.67521231	1
YIPF6	-1.046158941	0.099802254	0.946356687	1
YIPF7	-0.900831383	-0.17518571	1.076017094	1
YME1L1	-0.962868761	-0.070528311	1.033397071	1
YPEL5	-0.946932913	-0.098799691	1.045732604	1
YTHDF1	-1.150552355	0.490588763	0.659963592	1
YWHAH	-1.03013732	0.063280185	0.966857135	1
ZBED3	-0.919902281	-0.144478214	1.064380495	1
ZBTB2	-1.152482067	0.638199191	0.514282876	1
ZC2HC1C	-1.07859053	0.182249257	0.896341273	1
ZCCHC7	-1.008582883	0.017392656	0.991190226	1
ZDHHC1	-1.144601445	0.44033236	0.704269085	1
ZDHHC2	-1.151011273	0.655379208	0.495632065	1
ZDHHC8	-1.131379455	0.365726238	0.765653216	1
ZFAND6	-0.93156641	-0.125095816	1.056662226	1
ZFP91	-1.130228667	0.360328177	0.76990049	1
ZFYVE1	-1.105919398	0.26537195	0.840547448	1
ZG16	-1.079080035	0.183605038	0.895474996	1
ZMAT2	-1.044283913	0.095406288	0.948877626	1
ZNF143	-0.964361642	-0.067823695	1.032185337	1
ZNF226	-1.153113894	0.524152174	0.62896172	1

ZNF227	-1.054384069	0.119508731	0.934875338	1
ZNF280B	-1.081322588	0.189867771	0.891454817	1
ZNF394	-1.038972219	0.083134707	0.955837512	1
ZNF418	-0.885927303	-0.198401775	1.084329078	1
ZNF423	-1.12308103	0.329125477	0.793955553	1
ZNF429	-1.150135256	0.486232559	0.663902697	1
ZNF444	-1.141245932	0.722834307	0.418411625	1
ZNF503	-1.097007827	0.23636553	0.860642297	1
ZNF516	-0.927202567	-0.132403372	1.059605939	1
ZNF552	-1.154692847	0.573696528	0.58099632	1
ZNF580	-1.132479639	0.761476599	0.37100304	1
ZNF586	-0.966853831	-0.063286219	1.03014005	1
ZNF648	-1.134782178	0.75232907	0.382453108	1
ZNF750	-0.977403318	-0.043756852	1.02116017	1
ZNF777	-1.108330664	0.273625933	0.834704731	1
ZNF782	-1.147756544	0.464373991	0.683382552	1
ZNF804A	-1.122962381	0.328636567	0.794325813	1
ZNF806	-1.010496455	0.021334303	0.989162152	1
ZNF816-ZNF321P	-1.149156695	0.476705121	0.672451573	1
ZNF821	-1.125293446	0.786892115	0.33840133	1
ZNF90	-1.150894971	0.494326748	0.656568223	1
ZNRF2	-0.844012022	-0.260440163	1.104452185	1
ZNRF4	-1.017527803	0.036029432	0.98149837	1
ZSCAN18	-1.057664411	0.127572321	0.93009209	1

ZSCAN31	-0.93199051	-0.124381969	1.056372479	1
AACS	0.944479087	0.103059893	-1.047538979	2
AAK1	1.073007965	-0.167057868	-0.905950096	2
AARS1	0.984590145	0.030138354	-1.014728499	2
AARS2	1.056028461	-0.123535657	-0.932492805	2
AARSD1	0.877267752	0.211597598	-1.088865349	2
AASDH	1.072783392	-0.166456775	-0.906326617	2
ABCA5	1.028300899	-0.059235132	-0.969065767	2
ABCB1	1.001420444	-0.002846967	-0.998573477	2
ABCB6	1.010979034	-0.022332147	-0.988646887	2
ABCB8	1.005845182	-0.011794703	-0.994050479	2
ABCC10	0.968393988	0.060467869	-1.028861857	2
ABCC2	1.068698125	-0.15564966	-0.913048466	2
ABCF3	0.912414914	0.15667448	-1.069089394	2
ABCG1	0.875328543	0.214524318	-1.089852861	2
ABHD12	0.892354051	0.188471055	-1.080825106	2
ABHD17A	1.051385516	-0.112242191	-0.939143326	2
ABHD5	0.914314386	0.153597997	-1.067912383	2
ACAD9	0.985509237	0.028377475	-1.013886711	2
ACADVL	0.887034496	0.196699396	-1.083733892	2
ACAT1	0.953949484	0.086483584	-1.040433069	2
ACAT2	1.052998087	-0.116137895	-0.936860191	2
ACKR3	1.022076732	-0.045721953	-0.97635478	2
ACO2	1.033043688	-0.069738278	-0.963305409	2

ACOT1	1.065054486	-0.146205865	-0.91884862	2
ACOT13	1.029219409	-0.061254925	-0.967964484	2
ACOT2	1.091557377	-0.219624019	-0.871933359	2
ACP2	0.979753454	0.039332473	-1.019085927	2
ACSS2	1.051964935	-0.113638762	-0.938326173	2
ACTL6A	0.889171563	0.193403598	-1.082575161	2
ACTR5	0.978859589	0.041018534	-1.019878123	2
ACVRL1	0.959300385	0.076952969	-1.036253353	2
ADAMDEC1	1.08553618	-0.201874557	-0.883661623	2
ADAMTS10	1.093034486	-0.224094059	-0.868940427	2
ADAMTSL5	1.031374658	-0.06602108	-0.965353578	2
ADARB1	0.983257762	0.032683173	-1.015940935	2
ADCK5	1.01556069	-0.031883965	-0.983676725	2
ADCY7	0.915621849	0.151473468	-1.067095318	2
ADCY9	1.093963542	-0.226930482	-0.867033059	2
ADGRB2	1.090749151	-0.217198225	-0.873550926	2
ADH5	0.962902621	0.070467077	-1.033369698	2
ADPRH	1.0746708	-0.171532168	-0.903138632	2
ADSS2	1.058884754	-0.130603677	-0.928281077	2
ADTRP	0.984337465	0.03062168	-1.014959146	2
AEBP1	0.991672792	0.016451419	-1.008124211	2
AEN	0.950979891	0.091720717	-1.042700608	2
AFF3	1.09884135	-0.242157327	-0.856684023	2
AFG3L2	1.027411383	-0.057285504	-0.970125879	2

AGAP5	1.082132624	-0.192151226	-0.889981398	2
AGK	1.106664859	-0.267903511	-0.838761348	2
AHCYL1	1.079734059	-0.185422717	-0.894311342	2
AIFM1	0.944369504	0.103249588	-1.047619092	2
AIFM3	0.931453853	0.125285163	-1.056739016	2
AIM2	0.96474719	0.067123579	-1.031870769	2
AIP	0.894620633	0.184939984	-1.079560617	2
AK2	1.023078231	-0.047876307	-0.975201924	2
AK5	1.053028169	-0.116210834	-0.936817334	2
AKR7A2	1.035691643	-0.075683956	-0.960007687	2
AKT2	0.975450892	0.04741161	-1.022862501	2
ALAD	0.889064514	0.193569003	-1.082633517	2
ALDH1B1	0.892379492	0.188431505	-1.080810997	2
ALDH8A1	1.025712673	-0.053579585	-0.972133088	2
ALDOC	0.895995435	0.182790785	-1.07878622	2
ALG13	0.881572693	0.205063629	-1.086636322	2
ALG14	0.889717517	0.192559515	-1.082277032	2
ALG2	1.086672263	-0.205168208	-0.881504055	2
ALG8	0.885869731	0.198490201	-1.084359931	2
ALG9	1.118997133	-0.312751606	-0.806245527	2
ALKBH2	1.067148152	-0.151610576	-0.915537577	2
ALKBH3	0.868285099	0.225069662	-1.093354761	2
ALKBH4	1.036084239	-0.076570617	-0.959513622	2
ALKBH5	1.095560915	-0.231853709	-0.863707206	2

ALS2CL	1.078655582	-0.182429203	-0.896226379	2
ALYREF	1.016928766	-0.034764148	-0.982164619	2
AMACR	0.951179722	0.091369442	-1.042549165	2
AMBRA1	0.875518288	0.214238399	-1.089756687	2
AMDHD1	0.89640671	0.182146753	-1.078553463	2
AMDHD2	1.021663824	-0.04483591	-0.976827913	2
AMER1	1.093589612	-0.225786517	-0.867803094	2
AMMECR1	0.920809243	0.14298808	-1.063797324	2
AMMECR1L	0.956422176	0.082094602	-1.038516778	2
AMN1	0.925398699	0.135404266	-1.060802966	2
AMY2B	1.078961873	-0.183277402	-0.895684471	2
AMZ2	0.875371693	0.214459306	-1.089830999	2
ANAPC2	0.944844071	0.102427738	-1.047271808	2
ANAPC4	0.940942181	0.109158842	-1.050101023	2
ANAPC7	1.006935338	-0.01401806	-0.992917278	2
ANK1	0.96948648	0.058462038	-1.027948518	2
ANKAR	0.925253725	0.135644947	-1.060898672	2
ANKHD1	1.016822596	-0.034540158	-0.982282437	2
ANKRA2	0.98791566	0.023745741	-1.011661401	2
ANKRD11	0.951224589	0.091290551	-1.04251514	2
ANKRD16	1.025517565	-0.053155373	-0.972362191	2
ANKRD54	1.042600992	-0.091489632	-0.95111136	2
ANKRD6	0.873989031	0.216540014	-1.090529044	2
ANKS6	1.084928011	-0.200121476	-0.884806535	2

ANKZF1	1.018080861	-0.037199863	-0.980880998	2
ANTKMT	1.073455732	-0.168258609	-0.905197123	2
AOPEP	0.950302662	0.092909966	-1.043212629	2
AP1G2	0.926854833	0.132982752	-1.059837585	2
AP4B1	1.014305751	-0.029253428	-0.985052323	2
AP4E1	0.993812959	0.012261323	-1.006074283	2
AP5Z1	0.961006744	0.073887762	-1.034894506	2
APBB1	0.961716733	0.072608609	-1.034325343	2
APC	0.911153587	0.158710888	-1.069864475	2
APEX1	0.926724024	0.13320059	-1.059924614	2
APH1A	1.060381646	-0.134346063	-0.926035583	2
APOBEC3A	0.926111746	0.13421942	-1.060331166	2
APOBEC3A_B	1.031486203	-0.066268786	-0.965217417	2
APOBEC3B	1.07188511	-0.164059868	-0.907825242	2
APOBEC3H	0.907926054	0.163898373	-1.071824428	2
AQP3	1.10370394	-0.25795027	-0.84575367	2
ARAF	0.878510535	0.209716558	-1.088227093	2
ARFIP1	1.072505498	-0.165713996	-0.906791502	2
ARFRP1	1.025717233	-0.053589503	-0.97212773	2
ARHGAP11B	1.013087842	-0.026710858	-0.986376984	2
ARHGAP30	1.012026414	-0.024503184	-0.98752323	2
ARHGAP35	1.111152397	-0.283537115	-0.827615282	2
ARHGEF18	1.075156332	-0.172846559	-0.902309774	2
ARHGEF3	1.112674266	-0.289004038	-0.823670228	2

ARHGEF39	1.000035758	-0.0000715	-0.999964238	2
ARHGEF5	0.914940659	0.152581056	-1.067521715	2
ARHGEF6	1.115437888	-0.299167715	-0.816270173	2
ARIH1	0.936580208	0.116614277	-1.053194485	2
ARIH2	1.09839674	-0.240745049	-0.857651691	2
ARL10	0.986799964	0.025897017	-1.012696981	2
ARL16	1.059937227	-0.133232169	-0.926705058	2
ARL6IP4	1.060239075	-0.133988463	-0.926250612	2
ARMC5	0.999451295	0.001096507	-1.000547803	2
ARMCX5	0.980711437	0.03752098	-1.018232416	2
ARMCX5-GPRASP2	1.104941798	-0.262078548	-0.84286325	2
ARMT1	1.002775303	-0.005573908	-0.997201395	2
ARPC4	1.027617755	-0.057737268	-0.969880486	2
ARPIN	1.101560296	-0.250907367	-0.85065293	2
ARRDC2	1.049946237	-0.108788455	-0.941157782	2
ASH2L	1.091135177	-0.218355089	-0.872780088	2
ASMT	0.903552646	0.170874833	-1.074427479	2
ASMTL	0.888148489	0.194983027	-1.083131516	2
ASNS	1.100794025	-0.248421209	-0.852372816	2
ATF7-NPFF	1.028864762	-0.06047426	-0.968390502	2
ATG101	1.030973436	-0.065130935	-0.965842501	2
ATG16L1	1.07037243	-0.160050046	-0.910322384	2
ATG4A	1.105051079	-0.262445234	-0.842605846	2
ATG4B	1.074305936	-0.170546821	-0.903759115	2

ATL3	1.068441294	-0.154978112	-0.913463183	2
ATM	1.068390005	-0.154844112	-0.913545893	2
ATP1B2	1.085807714	-0.202659524	-0.88314819	2
ATP5MC1	0.916815381	0.149529121	-1.066344503	2
ATP5PO	0.954620034	0.085295922	-1.039915956	2
ATP6V0A2	0.975153494	0.047966666	-1.02312016	2
ATP6V1E2	0.966510256	0.063913441	-1.030423698	2
ATP8A2	0.949502634	0.094312441	-1.043815075	2
ATP8B2	0.997635344	0.004712655	-1.002347999	2
ATXN7L3B	0.993582179	0.012714396	-1.006296575	2
AUP1	0.975993903	0.046397028	-1.022390931	2
AURKA	0.88182151	0.204684411	-1.086505921	2
AVEN	1.012517745	-0.025524162	-0.986993583	2
B3GALT6	0.876101608	0.213358806	-1.089460414	2
B3GAT3	1.042703174	-0.09172667	-0.950976503	2
B4GALNT3	0.985456731	0.02847819	-1.013934921	2
B4GALT3	1.112153387	-0.287122921	-0.825030467	2
B4GALT4	0.915251426	0.152075953	-1.067327378	2
B4GAT1	1.046869567	-0.101477342	-0.945392225	2
BAALC	1.079533885	-0.184865628	-0.894668257	2
BACH1	1.068955567	-0.156323718	-0.912631849	2
BACH2	1.102095402	-0.252653174	-0.849442228	2
BAHCC1	0.865927308	0.22857049	-1.094497798	2
BAHD1	0.889288891	0.193222274	-1.082511165	2

BAIAP2L1	1.067742799	-0.1531563	-0.914586499	2
BAIAP3	1.01232656	-0.025126688	-0.987199872	2
BANP	1.098858609	-0.242212251	-0.856646358	2
BBS4	1.099803843	-0.245232214	-0.854571628	2
BCAS1	1.109270452	-0.276895602	-0.83237485	2
BCL2L12	0.991583754	0.016625183	-1.008208938	2
BCL9	1.114348556	-0.295123765	-0.819224791	2
BCO2	0.961035344	0.073836276	-1.034871621	2
BCR	1.073614872	-0.168686083	-0.904928789	2
BEND2	1.082686116	-0.193718144	-0.888967972	2
BFSP1	1.021419247	-0.044311679	-0.977107567	2
BICDL1	1.103285776	-0.256565994	-0.846719781	2
BIRC3	1.057075932	-0.126116723	-0.930959208	2
BLCAP	1.03011103	-0.063222084	-0.966888946	2
BMF	0.917696618	0.148090475	-1.065787093	2
BMP8A	0.945562198	0.101182398	-1.046744597	2
BMS1	1.045660133	-0.098629449	-0.947030685	2
BMX	0.911186453	0.158657891	-1.069844344	2
BOP1	0.892590355	0.188103627	-1.080693982	2
BPHL	0.876136179	0.213306646	-1.089442825	2
BPNT1	1.050190578	-0.109373252	-0.940817326	2
BPTF	0.995341853	0.009252092	-1.004593945	2
BRAT1	0.865824812	0.228722347	-1.094547159	2
BRD9	1.077512305	-0.179276773	-0.898235532	2

BRF2	0.966067376	0.064721156	-1.030788532	2
BRPF1	1.031053272	-0.065307954	-0.965745319	2
BTBD19	1.067796185	-0.153295308	-0.914500878	2
BTBD6	1.101664228	-0.251245821	-0.850418407	2
BTN3A1	1.048511626	-0.105367369	-0.943144257	2
BTN3A2	1.104404718	-0.260281713	-0.844123005	2
BTN3A3	0.922081807	0.140892528	-1.062974335	2
BUD13	0.902384661	0.172727891	-1.075112552	2
BYSL	1.01824783	-0.037553648	-0.980694183	2
BZW2	1.102704405	-0.254649916	-0.848054489	2
C10orf95	1.074990767	-0.172397946	-0.902592821	2
C11orf42	0.984567658	0.030181381	-1.014749039	2
C12orf73	1.0542727	-0.119237091	-0.935035609	2
C14orf28	1.03681011	-0.07821351	-0.958596601	2
C15orf48	0.999529156	0.000941023	-1.000470179	2
C15orf62	0.946528382	0.099503665	-1.046032047	2
C18orf21	1.11813956	-0.309424143	-0.808715417	2
C18orf25	0.905754989	0.167369169	-1.073124159	2
C19orf71	0.96446051	0.067644225	-1.032104735	2
C1orf100	0.883888908	0.201526834	-1.085415742	2
C1orf131	1.060036984	-0.133481993	-0.926554991	2
C1orf146	0.987111307	0.02529736	-1.012408668	2
C1orf216	1.032832974	-0.069267703	-0.963565272	2
C1orf35	1.034081254	-0.072060885	-0.962020369	2

C1orf50	0.912096799	0.157188563	-1.069285362	2
C1QBP	0.876616993	0.212580885	-1.089197877	2
C22orf15	0.865903457	0.228605829	-1.094509286	2
C22orf39	1.00081856	-0.001639135	-0.999179425	2
C2orf42	0.867193618	0.226692083	-1.093885702	2
C2orf92	1.061986572	-0.138388662	-0.92359791	2
C3orf18	0.965338628	0.066048281	-1.031386909	2
C4BPB	1.048222756	-0.104681061	-0.943541695	2
C4orf51	0.940706759	0.109563077	-1.050269835	2
C5orf15	0.956354064	0.082215847	-1.038569911	2
C5orf24	0.998875133	0.00224595	-1.001121083	2
C5orf34	0.88076671	0.206290831	-1.087057541	2
C6orf163	1.056512925	-0.124727865	-0.93178506	2
C6orf89	1.105059637	-0.262473965	-0.842585672	2
C8orf33	1.006391915	-0.012908811	-0.993483104	2
C8orf76	0.971242611	0.055226033	-1.026468643	2
C9orf163	0.946898362	0.098859844	-1.045758205	2
C9orf72	0.939373262	0.111848756	-1.051222018	2
C9orf85	1.059019768	-0.130940131	-0.928079637	2
CA11	0.975759842	0.046834533	-1.022594375	2
CA5B	1.082539405	-0.193302279	-0.889237126	2
CACNA1F	0.95232719	0.089349174	-1.041676364	2
CACNA1I	0.956046181	0.082763657	-1.038809837	2
CACNA2D2	1.057306105	-0.12668558	-0.930620526	2

CACNA2D4	0.921582029	0.141716183	-1.063298212	2
CACNB2	0.936010623	0.1175825	-1.053593123	2
CACNB3	0.882862939	0.203095312	-1.085958252	2
CACNG6	0.990909978	0.017938683	-1.00884866	2
CAD	0.905895331	0.16714526	-1.073040591	2
CALHM6	1.107424802	-0.270502705	-0.836922096	2
CALML4	1.069477647	-0.157693507	-0.911784141	2
CALML6	1.017169764	-0.035272878	-0.981896886	2
CAPN14	0.986812056	0.025873737	-1.012685793	2
CAPN3	1.033516068	-0.070794579	-0.962721489	2
CAPRIN2	0.963805073	0.068833186	-1.032638259	2
CAPS	1.10852233	-0.274290304	-0.834232026	2
CARMIL2	0.989278584	0.021108624	-1.010387208	2
CASK	1.037614096	-0.080038611	-0.957575485	2
CASP1	0.970190359	0.057166749	-1.027357109	2
CASP10	1.036373896	-0.077225657	-0.95914824	2
CASP2	0.965261395	0.06618879	-1.031450185	2
CASP7	1.086310574	-0.204116937	-0.882193637	2
CATSPER2	0.976855379	0.044784442	-1.02163982	2
CBLIF	0.896088757	0.182644692	-1.07873345	2
CBX5	1.039877556	-0.085207826	-0.95466973	2
CBY1	1.030092353	-0.063180812	-0.966911542	2
CCDC102B	1.004000228	-0.008049046	-0.995951181	2
CCDC115	1.118410073	-0.310469872	-0.807940201	2

CCDC142	1.102939935	-0.255424997	-0.847514938	2
CCDC163	1.112961972	-0.290047623	-0.822914349	2
CCDC167	1.031978682	-0.067363662	-0.96461502	2
CCDC17	1.064114972	-0.143799203	-0.920315769	2
CCDC186	1.078852331	-0.182973873	-0.895878457	2
CCDC24	0.949507939	0.09430315	-1.043811089	2
CCDC28B	0.902761227	0.172130915	-1.074892141	2
CCDC57	1.108205549	-0.273192922	-0.835012627	2
CCDC65	1.030377667	-0.063811609	-0.966566057	2
CCDC70	1.086742108	-0.205371507	-0.881370601	2
CCDC71	0.987100638	0.025317918	-1.012418556	2
CCDC86	1.050804766	-0.110845983	-0.939958782	2
CCDC88C	1.077988564	-0.180587402	-0.897401162	2
CCDC9	0.930117105	0.127530368	-1.057647473	2
CCDC90B	0.936760741	0.11630714	-1.053067881	2
CCDC97	1.052889517	-0.115874729	-0.937014788	2
CCL17	1.072082434	-0.164585376	-0.907497058	2
CCL7	1.028375258	-0.059398396	-0.968976862	2
CCN6	1.057857457	-0.128050692	-0.929806765	2
CCNB1	1.093688325	-0.226088201	-0.867600123	2
CCR5	1.059261938	-0.131544158	-0.927717781	2
CCR9	0.969486036	0.058462854	-1.02794889	2
CCSER2	1.09917645	-0.243225126	-0.855951324	2
CCT3	1.01794101	-0.036903693	-0.981037317	2

CCT4	0.998593843	0.002806407	-1.00140025	2
CCT8	0.954616935	0.085301415	-1.03991835	2
CD163	1.101278479	-0.249991135	-0.851287345	2
CD2	1.10877997	-0.275185339	-0.833594631	2
CD22	1.043686624	-0.09401312	-0.949673504	2
CD248	0.903482562	0.170986143	-1.074468705	2
CD27	1.027101243	-0.056607213	-0.970494029	2
CD300E	0.998592697	0.002808689	-1.001401386	2
CD6	1.077147246	-0.178274639	-0.898872607	2
CD69	0.992684483	0.014473907	-1.00715839	2
CD7	1.023258969	-0.0482659	-0.974993069	2
CD72	1.118379509	-0.310351543	-0.808027966	2
CD79A	0.881443322	0.205260732	-1.086704054	2
CD79B	1.00309317	-0.006215314	-0.996877857	2
CD83	1.018618566	-0.038339896	-0.98027867	2
CD8A	0.91638347	0.150233283	-1.066616753	2
CD99L2	1.090905681	-0.217666939	-0.873238741	2
CDC37	0.927619529	0.131708083	-1.059327612	2
CDC42SE1	1.089360247	-0.213061835	-0.876298412	2
CDCA3	0.963177245	0.069970256	-1.033147501	2
CDH23	0.903109357	0.171578628	-1.074687985	2
CDH26	1.006119808	-0.012354086	-0.993765721	2
CDIPT	0.969656409	0.058149545	-1.027805954	2
CDK15	1.102008398	-0.252368771	-0.849639626	2

CDK16	0.949256576	0.094743265	-1.043999841	2
CDK5RAP3	0.997897612	0.0041916	-1.002089211	2
CDK7	0.933016849	0.122651715	-1.055668564	2
CDKN2AIPNL	0.884531047	0.200543639	-1.085074686	2
CDKN3	1.11492879	-0.297271494	-0.817657295	2
CDRT4	1.048230371	-0.104699144	-0.943531228	2
CEACAM1	1.068750729	-0.155787317	-0.912963412	2
CEBPE	1.103136819	-0.25607413	-0.847062689	2
CELA1	0.947060568	0.098577407	-1.045637975	2
CELF1	1.067465565	-0.152435062	-0.915030502	2
CELSR3	0.894042325	0.185842363	-1.079884687	2
CENPP	0.989562332	0.020558327	-1.010120659	2
CEP104	1.076605372	-0.176791062	-0.89981431	2
CEP250	0.929901805	0.127891382	-1.057793187	2
CEP63	0.948686364	0.095740703	-1.044427068	2
CEP68	0.900124927	0.176301118	-1.076426045	2
CEPT1	1.108511393	-0.274252358	-0.834259035	2
CERS3	1.041403603	-0.088719277	-0.952684326	2
CES2	1.01417193	-0.02897356	-0.98519837	2
CFAP58	0.927754939	0.131482153	-1.059237092	2
CHAF1B	1.035730915	-0.075772589	-0.959958326	2
CHD7	0.990220812	0.019279579	-1.009500391	2
CHFR	1.005522653	-0.011138355	-0.994384298	2
CHI3L2	1.045877207	-0.099139534	-0.946737673	2

CHKA	0.999787441	0.000424982	-1.000212423	2
CHM	0.873129829	0.217830399	-1.090960228	2
CHML	0.882075274	0.204297476	-1.08637275	2
CHN2	0.907147653	0.165144487	-1.07229214	2
CHRAC1	1.090996259	-0.217938406	-0.873057853	2
CHRNA10	0.868058159	0.225407251	-1.09346541	2
CHTOP	0.954778815	0.085014417	-1.039793231	2
CHUK	1.005242382	-0.010568536	-0.994673846	2
CHURC1	0.981722955	0.035603175	-1.01732613	2
CIB3	0.99663303	0.006700269	-1.003333299	2
CIC	1.02522683	-0.052523794	-0.972703036	2
CIITA	0.978853492	0.04103002	-1.019883512	2
CILK1	0.871858961	0.219735421	-1.091594383	2
CIPC	1.056357842	-0.124345933	-0.932011909	2
CIR1	1.114590322	-0.296016935	-0.818573386	2
CIT	1.047806466	-0.103693515	-0.944112951	2
CITED2	0.901760843	0.173715877	-1.07547672	2
CIZ1	0.999147912	0.001702004	-1.000849915	2
CKAP5	0.974553331	0.049085483	-1.023638814	2
CKMT2	1.016553336	-0.033972456	-0.98258088	2
CLC	0.944399009	0.103198518	-1.047597527	2
CLCC1	0.868521152	0.224718374	-1.093239526	2
CLDN15	1.000606934	-0.001214975	-0.999391959	2
CLDN7	1.045680793	-0.098677976	-0.947002817	2

CLEC10A	0.985811907	0.027796613	-1.01360852	2
CLEC12B	0.960303709	0.075152197	-1.035455906	2
CLEC16A	0.892765063	0.18783187	-1.080596933	2
CLEC18A	1.009023393	-0.018297912	-0.990725481	2
CLEC1A	1.046822893	-0.101367167	-0.945455725	2
CLEC4D	0.87510265	0.21486458	-1.08996723	2
CLEC9A	1.019054906	-0.039266542	-0.979788364	2
CLIP4	1.100467817	-0.247367703	-0.853100114	2
CLK4	1.077863893	-0.180243961	-0.897619932	2
CLN6	0.876903616	0.212147943	-1.089051559	2
CLPB	1.054702021	-0.120285003	-0.934417018	2
CLPTM1	0.936354377	0.116998306	-1.053352684	2
CLPTM1L	1.071961806	-0.164264054	-0.907697751	2
CLTA	1.069141683	-0.156811597	-0.912330086	2
CLU	0.959413737	0.076749743	-1.03616348	2
CMKLR1	1.109041003	-0.276094498	-0.832946506	2
CMTM8	1.020969019	-0.04334781	-0.977621209	2
CMTR1	1.007253956	-0.01466931	-0.992584646	2
CMTR2	1.036056727	-0.076508438	-0.959548289	2
CNBD2	0.941587697	0.108049358	-1.049637054	2
CNDP2	1.049195355	-0.106995198	-0.942200157	2
CNN3	1.003141596	-0.006313083	-0.996828513	2
CNOT2	1.114934405	-0.29729235	-0.817642055	2
CNOT4	1.032370417	-0.068236001	-0.964134416	2

CNPY2	1.035607446	-0.075493974	-0.960113471	2
COA4	0.93252045	0.12348905	-1.0560095	2
COA6	0.870560609	0.221677205	-1.092237814	2
COA8	1.039189236	-0.083630974	-0.955558262	2
COBLL1	0.865597789	0.229058606	-1.094656395	2
COG1	1.01731693	-0.035583737	-0.981733193	2
COG2	1.09428605	-0.227919701	-0.866366349	2
COG8	0.944816383	0.102475712	-1.047292095	2
COMMD1	0.954138584	0.086148846	-1.040287431	2
COMMD3	0.98429442	0.030703984	-1.014998404	2
COPB1	0.895981047	0.182813306	-1.078794354	2
COPE	0.983271815	0.032656381	-1.015928196	2
COPRS	1.072998064	-0.167031352	-0.905966712	2
COPS6	0.967211569	0.062632567	-1.029844135	2
COQ10A	0.935817281	0.117910881	-1.053728162	2
COQ3	1.028362692	-0.059370803	-0.968991889	2
COQ8A	0.994584306	0.010744797	-1.005329104	2
CORO2A	1.035678338	-0.075653931	-0.960024407	2
COX20	0.947308966	0.098144689	-1.045453654	2
COX7A2	0.944465826	0.103082851	-1.047548677	2
CPA3	0.933047954	0.122599216	-1.05564717	2
CPAMD8	0.873262246	0.217631658	-1.090893904	2
CPD	0.98261237	0.033912529	-1.016524899	2
CPOX	0.977794541	0.04302225	-1.020816791	2

CPSF1	0.981870831	0.035322368	-1.017193198	2
CPSF3	1.113461646	-0.291867892	-0.821593754	2
CPT1B	0.949729327	0.093915307	-1.043644634	2
CR2	1.110449774	-0.281042512	-0.829407262	2
CRACR2A	0.948495145	0.096074898	-1.044570043	2
CREBL2	1.014278773	-0.029196996	-0.985081777	2
CRLF3	0.917564799	0.14830584	-1.065870639	2
CRNKL1	1.084179672	-0.197973826	-0.886205847	2
CRTC3	0.911973601	0.157387566	-1.069361167	2
CRY2	0.941336442	0.108481395	-1.049817837	2
CRYGS	1.047126102	-0.102083282	-0.94504282	2
CSN1S1	0.92590833	0.134557611	-1.060465942	2
CSNK2A2	0.99012824	0.019459496	-1.009587736	2
CST5	0.982222888	0.034653379	-1.016876267	2
CSTA	0.944304902	0.103361398	-1.047666299	2
CTC1	0.899884165	0.176680904	-1.076565069	2
CTNNBL1	0.89000489	0.192114869	-1.082119759	2
CTSK	0.98189314	0.035279993	-1.017173133	2
CTTNBP2NL	1.085896044	-0.202915176	-0.882980868	2
CTU1	1.02914076	-0.061081712	-0.968059048	2
CTU2	0.957617893	0.079962902	-1.037580795	2
CUL1	1.084971137	-0.20024556	-0.884725577	2
CUTC	0.871108422	0.220858455	-1.091966877	2
CUZD1	1.064989792	-0.14603978	-0.918950012	2

CWC15	1.009415355	-0.019104464	-0.990310891	2
CWC25	1.110233995	-0.280280034	-0.829953962	2
CXCR4	0.895371023	0.183767613	-1.079138636	2
CXCR5	0.903751208	0.170559384	-1.074310593	2
CXXC1	1.093264798	-0.224795389	-0.868469409	2
CYB561	1.038492618	-0.082039479	-0.956453139	2
CYB561D1	1.08624076	-0.203914308	-0.882326452	2
CYB5A	1.112054141	-0.286765689	-0.825288452	2
CYB5R2	0.999664238	0.000671187	-1.000335424	2
CYB5RL	1.03394081	-0.071745967	-0.962194844	2
CYC1	0.917293265	0.148749284	-1.066042549	2
CYP2J2	0.98771226	0.024138434	-1.011850694	2
CYP2U1	0.995015124	0.009896298	-1.004911422	2
CYSLTR2	0.915310886	0.151979273	-1.067290159	2
CZIB	0.881023512	0.205900015	-1.086923527	2
DAGLB	1.020834914	-0.043060999	-0.977773915	2
DAZ1	0.883754606	0.201732321	-1.085486927	2
DAZAP1	0.98949236	0.020694071	-1.010186431	2
DBP	1.075176915	-0.172902359	-0.902274557	2
DBT	1.081229785	-0.189606895	-0.89162289	2
DCAF4	1.019818339	-0.040891135	-0.978927204	2
DCAF5	1.046816904	-0.101353033	-0.945463871	2
DCAF8	1.031374913	-0.066021646	-0.965353267	2
DCHS1	1.01669644	-0.03427411	-0.98242233	2

DCPS	1.083007494	-0.194630461	-0.888377033	2
DCTPP1	0.9128306	0.156002222	-1.068832822	2
DDIAS	0.966635587	0.063684704	-1.030320291	2
DDIT4	0.900001797	0.176495369	-1.076497167	2
DDOST	0.972664068	0.052596031	-1.025260098	2
DDX10	1.051545897	-0.112628397	-0.938917499	2
DDX19B	1.060784298	-0.135357335	-0.925426963	2
DDX20	0.897992126	0.179659332	-1.077651458	2
DDX23	0.936469732	0.116802168	-1.0532719	2
DDX28	1.087213305	-0.206745521	-0.880467784	2
DDX3Y	1.014093369	-0.028809317	-0.985284052	2
DDX41	0.866531145	0.227675296	-1.094206441	2
DDX42	1.080623366	-0.187905873	-0.892717493	2
DDX49	1.040115769	-0.085754545	-0.954361224	2
DDX51	0.893244403	0.187085797	-1.0803302	2
DDX52	0.984967733	0.029415476	-1.014383209	2
DDX56	0.920082305	0.144182659	-1.064264964	2
DELE1	1.102933788	-0.255404748	-0.84752904	2
DENND11	0.913483024	0.154945969	-1.068428993	2
DENND6A	0.934360905	0.12037999	-1.054740895	2
DEPDC7	1.080112677	-0.186478265	-0.893634412	2
DEXI	0.885141177	0.199608376	-1.084749553	2
DFFA	0.970575	0.056457946	-1.027032945	2
DGCR8	1.033737649	-0.07129071	-0.962446938	2

DGKA	0.9378195	0.114503431	-1.052322931	2
DGKQ	0.979049115	0.040661374	-1.01971049	2
DHPS	0.962106637	0.071905191	-1.034011829	2
DHX35	1.091559039	-0.219629021	-0.871930018	2
DHX37	0.920671887	0.143213937	-1.063885823	2
DHX38	1.011258084	-0.022909853	-0.988348231	2
DHX57	1.021212805	-0.043869534	-0.977343272	2
DHX58	0.960765012	0.074322771	-1.035087783	2
DHX8	1.112571164	-0.288630852	-0.823940312	2
DHX9	1.061132291	-0.136232915	-0.924899375	2
DIABLO	0.887728092	0.195631166	-1.083359258	2
DIDO1	0.877323187	0.211513782	-1.088836969	2
DIP2A	0.908424458	0.163099486	-1.071523944	2
DIP2C	0.889664772	0.1926411	-1.082305872	2
DIS3L2	0.929383388	0.128759979	-1.058143367	2
DISC1	0.986893327	0.025717252	-1.012610579	2
DKK4	1.117731808	-0.307854542	-0.809877266	2
DMAC1	0.921714767	0.141497509	-1.063212276	2
DMAC2	0.964427519	0.067704116	-1.032131635	2
DMAP1	1.100624021	-0.247871813	-0.852752208	2
DNAAF9	1.009625734	-0.019537783	-0.990087951	2
DNAH1	0.960206864	0.075326206	-1.035533071	2
DNAJB12	0.982352667	0.034406607	-1.016759274	2
DNAJB9	1.057156406	-0.12631554	-0.930840866	2

DNAJC11	0.959648972	0.07632782	-1.035976792	2
DNAJC14	0.95909715	0.077317202	-1.036414351	2
DNAJC18	1.115120045	-0.297982549	-0.817137496	2
DNAJC9	0.947028668	0.09863296	-1.045661628	2
DNASE1	1.053878936	-0.118277761	-0.935601175	2
DND1	1.070191728	-0.15957323	-0.910618498	2
DNHD1	1.038946332	-0.083075538	-0.955870794	2
DNPH1	1.045596901	-0.098480951	-0.94711595	2
DPF2	0.879750953	0.207834872	-1.087585825	2
DPH2	1.090153731	-0.215420025	-0.874733706	2
DPP4	0.944323696	0.103328871	-1.047652567	2
DPPA3	1.047454518	-0.102859971	-0.944594547	2
DPYD	1.039877626	-0.085207986	-0.95466964	2
DRAM2	1.081777014	-0.191147361	-0.890629653	2
DRC3	0.998711228	0.00257258	-1.001283808	2
DRICH1	1.012168893	-0.024799086	-0.987369807	2
DUS2	1.030145907	-0.063299164	-0.966846744	2
DUSP12	1.082075904	-0.191990958	-0.890084946	2
DUSP18	1.078182528	-0.181122233	-0.897060295	2
DUSP3	1.001872694	-0.003755969	-0.998116725	2
DUT	0.998348646	0.003294567	-1.001643213	2
DVL1	1.050428439	-0.109943138	-0.940485301	2
DVL2	1.05094101	-0.111173215	-0.939767795	2
DYNC1LI1	1.050242156	-0.109496777	-0.94074538	2

DYNC1LI2	1.058272956	-0.129081778	-0.929191178	2
DYNLT2	0.896469111	0.182048992	-1.078518103	2
DZIP3	1.03117282	-0.065573119	-0.965599701	2
E4F1	0.871148452	0.220798596	-1.091947048	2
EAPP	0.924174647	0.137434082	-1.061608729	2
EARS2	0.923142939	0.139140887	-1.062283826	2
ECHDC2	1.091699971	-0.220053454	-0.871646517	2
ECI2	0.912244449	0.156949997	-1.069194446	2
EDA	0.990886892	0.017983642	-1.008870535	2
EDARADD	0.922944876	0.13946813	-1.062413006	2
EDC3	0.875686345	0.213985079	-1.089671424	2
EDRF1	1.021707765	-0.044930141	-0.976777624	2
EEF1A1	0.87653028	0.212711819	-1.089242099	2
EEF1AKNMT	0.917373795	0.148617795	-1.06599159	2
EEF1D	1.032527552	-0.068586279	-0.963941273	2
EEF1G	0.872363987	0.218978907	-1.091342894	2
EEF2K	0.948414415	0.096215945	-1.04463036	2
EEF2KMT	1.074619981	-0.171394805	-0.903225176	2
EEFSEC	1.117981066	-0.308813093	-0.809167973	2
EFCAB14	0.95819317	0.078935122	-1.037128292	2
EHMT1	1.093872614	-0.226652016	-0.867220599	2
EI24	0.97504064	0.048177182	-1.023217821	2
EID2B	0.999584814	0.000829856	-1.00041467	2
EID3	1.030541992	-0.064175218	-0.966366775	2

EIF1AD	0.996835122	0.006299987	-1.00313511	2
EIF1AX	1.015424963	-0.031598937	-0.983826027	2
EIF2AK3	0.996051831	0.007850119	-1.00390195	2
EIF2B1	0.966300004	0.064297006	-1.03059701	2
EIF2D	0.93121604	0.125685068	-1.056901107	2
EIF3A	0.90901132	0.162157794	-1.071169114	2
EIF3F	1.024317086	-0.050551698	-0.973765389	2
EIF3K	0.974908638	0.048423336	-1.023331974	2
EIF4A1	0.976366367	0.045700265	-1.022066633	2
EIF4E2	1.018799175	-0.038723286	-0.980075889	2
EIF4ENIF1	1.081930855	-0.191581371	-0.890349484	2
EIF4G2	0.907687773	0.164280034	-1.071967806	2
EIF4H	0.967276197	0.062514416	-1.029790613	2
ELAC1	0.896177427	0.182505858	-1.078683286	2
ELAVL1	0.970503762	0.056589273	-1.027093035	2
ELL3	1.084465436	-0.198792712	-0.885672725	2
ELMOD2	1.059357702	-0.131783206	-0.927574496	2
ELMOD3	1.077693518	-0.179775024	-0.897918494	2
ELOA	1.055318407	-0.121793099	-0.933525308	2
ELP1	1.110749732	-0.282105267	-0.828644465	2
ELP2	0.932415438	0.123666071	-1.056081509	2
ELP5	0.947104868	0.098500253	-1.045605121	2
EMG1	0.969867375	0.057761399	-1.027628774	2
EML2	0.900976069	0.174957081	-1.07593315	2

ENKD1	0.915946379	0.150945256	-1.066891635	2
ENPP3	1.012395695	-0.025270392	-0.987125303	2
ENPP4	0.907154852	0.165132971	-1.072287823	2
EOGT	0.893172757	0.187197354	-1.080370111	2
EOLA1	1.028685698	-0.06008048	-0.968605219	2
EOLA2	0.962009823	0.072079914	-1.034089738	2
EPB41L2	0.959206491	0.077121265	-1.036327756	2
EPG5	0.944164166	0.103604915	-1.047769082	2
EPHA1	1.110708084	-0.281957508	-0.828750575	2
EPHA4	1.049623691	-0.108017435	-0.941606256	2
EPM2AIP1	0.973020585	0.051934882	-1.024955467	2
EPPK1	0.924290111	0.137242834	-1.061532946	2
ERAL1	0.993266001	0.013334634	-1.006600635	2
ERCC1	0.87302234	0.217991691	-1.091014031	2
ERCC2	1.009704954	-0.019701027	-0.990003927	2
ERCC3	0.97682675	0.044838091	-1.021664841	2
ERCC5	1.031174851	-0.065577625	-0.965597226	2
ERCC8	1.046869231	-0.101476549	-0.945392682	2
ERLEC1	1.079335802	-0.184315021	-0.895020781	2
ERLIN1	0.903218229	0.171405832	-1.074624061	2
ERMN	1.042474516	-0.091196374	-0.951278143	2
ERMP1	1.077040921	-0.177983165	-0.899057756	2
ESD	1.043234254	-0.09296025	-0.950274004	2
ESYT1	1.012299237	-0.025069904	-0.987229332	2

ESYT2	1.08076245	-0.188295446	-0.892467004	2
ETFDH	0.906888357	0.165559158	-1.072447515	2
ETS1	0.961848213	0.072371484	-1.034219698	2
EVI5L	0.922015919	0.141001163	-1.063017082	2
EWSR1	1.106865967	-0.26858952	-0.838276447	2
EXO5	0.972457618	0.052978606	-1.025436224	2
EXOC1	1.011809176	-0.024052284	-0.987756892	2
EXOC2	0.970627123	0.05636184	-1.026988963	2
EXOSC3	0.940891128	0.109246521	-1.050137649	2
EXOSC6	1.10845685	-0.27406319	-0.83439366	2
EXT2	1.000248699	-0.000497583	-0.999751116	2
EXTL3	1.060662383	-0.135050934	-0.925611449	2
FAAP100	0.955971423	0.08289661	-1.038868033	2
FADS3	1.048073709	-0.104327283	-0.943746427	2
FAF2	0.923381005	0.138747371	-1.062128376	2
FAHD2B	0.971960644	0.053898721	-1.025859364	2
FAM118A	1.040335039	-0.086258249	-0.95407679	2
FAM136A	0.923969397	0.137773932	-1.061743329	2
FAM13A	1.105642852	-0.264437286	-0.841205566	2
FAM149B1	0.962330441	0.071501126	-1.033831568	2
FAM207A	1.080769757	-0.188315924	-0.892453833	2
FAM20A	0.870592891	0.22162898	-1.09222187	2
FAM3B	1.049395441	-0.107472473	-0.941922967	2
FAM3D	0.974972252	0.048304721	-1.023276973	2

FAM50B	1.029272555	-0.061372	-0.967900555	2
FAM76A	1.035956506	-0.07628199	-0.959674516	2
FAM8A1	0.919198867	0.145631995	-1.064830862	2
FAM91A1	1.036283112	-0.077020276	-0.959262836	2
FANCC	1.073730825	-0.168997792	-0.904733033	2
FANCE	0.987185643	0.025154111	-1.012339754	2
FANCF	1.047593068	-0.103187961	-0.944405108	2
FANCG	1.116454441	-0.3029879	-0.813466541	2
FARP1	0.975023558	0.04820904	-1.023232598	2
FARSB	1.020710766	-0.042795601	-0.977915165	2
FASN	0.898588163	0.178722228	-1.077310391	2
FASTKD1	1.100370784	-0.247054887	-0.853315897	2
FASTKD2	1.013874903	-0.028352809	-0.985522095	2
FASTKD3	0.976130771	0.046141072	-1.022271843	2
FASTKD5	1.105901475	-0.265311301	-0.840590174	2
FBXL12	1.109310974	-0.277037276	-0.832273699	2
FBXL6	0.916623195	0.149842526	-1.066465722	2
FBXO31	1.110277837	-0.280434815	-0.829843021	2
FBXO33	0.998468363	0.003056268	-1.001524631	2
FBXO44	0.896485647	0.182023084	-1.078508731	2
FCER1A	1.026257345	-0.054765392	-0.971491953	2
FCGBP	1.070977392	-0.161649735	-0.909327657	2
FCRL1	0.960433525	0.074918881	-1.035352406	2
FCRL3	1.022902985	-0.047498787	-0.975404198	2

FCRL5	0.893342993	0.186932262	-1.080275255	2
FCSK	0.87983277	0.207710608	-1.087543378	2
FGD6	0.966003754	0.064837115	-1.030840869	2
FH	1.102601382	-0.254311392	-0.84828999	2
FIG4	1.030396075	-0.063852331	-0.966543744	2
FITM2	1.093261302	-0.224784733	-0.868476569	2
FKBP11	0.955487192	0.083757222	-1.039244415	2
FKBP4	0.877541146	0.211184159	-1.088725305	2
FLT3	0.879722796	0.207877632	-1.087600428	2
FLT3LG	1.074308196	-0.170552918	-0.903755278	2
FLYWCH2	1.000650768	-0.00130281	-0.999347959	2
FMNL3	1.058562228	-0.129800819	-0.92876141	2
FOXJ2	0.994152998	0.0115932	-1.005746198	2
FOXK1	1.046979229	-0.101736286	-0.945242943	2
FOXO1	1.103823316	-0.258346392	-0.845476924	2
FOXP3	1.102854617	-0.25514405	-0.847710568	2
FOXRED1	1.117554548	-0.307174664	-0.810379884	2
FPGS	0.97770116	0.043197662	-1.020898822	2
FRG1	1.063163731	-0.141374021	-0.921789709	2
FRS2	1.103815647	-0.258320931	-0.845494716	2
FRYL	0.997807567	0.00437054	-1.002178107	2
FSTL3	0.948326196	0.096370047	-1.044696243	2
FTO	1.03021072	-0.063442429	-0.966768292	2
FUT11	0.942957415	0.105689799	-1.048647214	2

FYCO1	1.114186487	-0.294526386	-0.819660101	2
FZD1	0.916839259	0.149490174	-1.066329433	2
G3BP1	0.952003811	0.089919076	-1.041922887	2
GAB3	0.869449249	0.223335785	-1.092785034	2
GABPB2	1.041265973	-0.088401705	-0.952864268	2
GAK	0.975017491	0.048220355	-1.023237846	2
GALC	0.885326996	0.199323326	-1.084650322	2
GALNT11	0.929283222	0.128927695	-1.058210916	2
GALNT4	0.96165355	0.072722534	-1.034376084	2
GAMT	0.882014408	0.2043903	-1.086404708	2
GART	1.00446094	-0.008982393	-0.995478547	2
GATB	0.932995038	0.122688525	-1.055683563	2
GATC	0.935003376	0.119291729	-1.054295106	2
GATD3A	1.101449055	-0.250545441	-0.850903614	2
GBP4	1.082917051	-0.194373529	-0.888543523	2
GBP5	0.966220975	0.064441128	-1.030662102	2
GCC1	1.053939476	-0.118425144	-0.935514332	2
GCDH	0.871047519	0.220949518	-1.091997037	2
GCH1	1.092941286	-0.223810591	-0.869130695	2
GCLC	0.919731345	0.144758749	-1.064490093	2
GCNT4	1.100804471	-0.248454994	-0.852349477	2
GCSAM	0.953020909	0.088125145	-1.041146054	2
GDF9	1.019256641	-0.039695427	-0.979561214	2
GEMIN4	0.923831075	0.13800288	-1.061833956	2

GFER	0.965882624	0.065057838	-1.030940461	2
GFM1	0.928968182	0.129454952	-1.058423135	2
GGA1	0.914650653	0.153052127	-1.067702781	2
GGA3	0.964900402	0.066845176	-1.031745578	2
GGPS1	0.90524282	0.168185788	-1.073428608	2
GGT7	0.97739475	0.043772931	-1.021167681	2
GIGYF1	0.926823356	0.133035177	-1.059858533	2
GIMAP1	0.877445592	0.211328683	-1.088774275	2
GIMAP7	0.883006651	0.202875787	-1.085882438	2
GIMAP8	1.039345575	-0.083988748	-0.955356827	2
GK5	1.081651141	-0.190792563	-0.890858578	2
GKN2	1.095696047	-0.232272947	-0.8634231	2
GLB1L2	0.994652215	0.01061112	-1.005263336	2
GLB1L3	1.04038598	-0.086375334	-0.954010646	2
GLI1	1.054991519	-0.12099278	-0.933998738	2
GLIPR1L1	1.012929694	-0.026381442	-0.986548252	2
GLS2	1.085516742	-0.201818418	-0.883698324	2
GLT8D1	0.894516159	0.185103076	-1.079619235	2
GLUD1	0.870275661	0.222102768	-1.09237843	2
GML	1.05266691	-0.115335545	-0.937331365	2
GMPPB	1.042245937	-0.090666751	-0.951579186	2
GNAL	1.04672033	-0.101125143	-0.945595187	2
GNB4	0.9390004	0.112486644	-1.051487044	2
GNL3L	1.10532584	-0.263368796	-0.841957044	2

GNPAT	1.067601172	-0.152787721	-0.914813451	2
GNPDA1	0.965520094	0.065718039	-1.031238133	2
GOLGA5	0.938071067	0.114074238	-1.052145305	2
GOLGA7B	1.044797776	-0.096607617	-0.948190158	2
GOLGA8A	1.0734809	-0.168326189	-0.905154711	2
GOLGA8B	0.93795367	0.114274556	-1.052228226	2
GOLGA8M	0.904834863	0.16883566	-1.073670523	2
GOLGA8O	0.971945478	0.05392678	-1.025872258	2
GOLGA8Q	0.880017386	0.207430146	-1.087447532	2
GOLGA8S	1.105067093	-0.262498997	-0.842568096	2
GOSR2	0.888826152	0.193937184	-1.082763335	2
GOT1	0.884826976	0.200090143	-1.08491712	2
GPAA1	0.885266431	0.199416245	-1.084682676	2
GPATCH3	1.116159748	-0.301875731	-0.814284017	2
GPBP1L1	0.944908929	0.102315347	-1.047224277	2
GPCPD1	0.958028164	0.079230064	-1.037258229	2
GPN1	0.96864612	0.060005445	-1.028651565	2
GPN2	0.98508971	0.029181794	-1.014271505	2
GPR132	0.943371443	0.104975132	-1.048346575	2
GPR153	1.045587321	-0.098458456	-0.947128865	2
GPR174	0.881073308	0.205824212	-1.08689752	2
GPR18	1.010090606	-0.02049631	-0.989594295	2
GPR35	0.900387795	0.17588626	-1.076274055	2
GPRASP1	1.089458433	-0.213352931	-0.876105502	2

GPX7	0.888308043	0.194736904	-1.083044947	2
GRAMD1A	1.038982117	-0.083157333	-0.955824784	2
GRAP	1.09231923	-0.221923551	-0.870395679	2
GSTP1	0.898587962	0.178722544	-1.077310506	2
GSTZ1	1.010225272	-0.020774247	-0.989451025	2
GTF2H1	1.022841457	-0.047366298	-0.97547516	2
GTF3C1	1.090316758	-0.215906157	-0.874410601	2
GTF3C2	1.065970931	-0.148564498	-0.917406433	2
GUF1	1.118146593	-0.309451286	-0.808695306	2
GZMK	0.946071916	0.100297232	-1.046369148	2
H2BE1	1.091772267	-0.220271349	-0.871500918	2
H4C14	0.927574156	0.131783773	-1.059357929	2
H4C15	1.07134308	-0.162619259	-0.908723821	2
H4C8	0.933791573	0.121343089	-1.055134661	2
HADHA	1.019193425	-0.039560999	-0.979632426	2
HARS1	1.055779174	-0.122923228	-0.932855946	2
HAUS2	1.07874066	-0.182664651	-0.896076009	2
HAUS4	0.889992662	0.192133794	-1.082126456	2
HAUS7	1.054792483	-0.120506068	-0.934286415	2
HAX1	1.015347052	-0.031435378	-0.983911674	2
HCCS	0.907242188	0.164993253	-1.07223544	2
HCFC1	0.871518521	0.220245009	-1.091763529	2
HDAC1	0.940627255	0.10969954	-1.050326795	2
HDAC10	1.102463935	-0.253860223	-0.848603711	2

HDAC11	0.882078338	0.204292802	-1.086371141	2
HDC	1.034611144	-0.073250581	-0.961360563	2
HDHD3	0.977962984	0.042705729	-1.020668713	2
HDLBP	0.989287059	0.021092194	-1.010379253	2
HEATR6	0.875964752	0.213565255	-1.089530007	2
HELZ2	0.982796167	0.033562649	-1.016358816	2
HEMK1	0.918190795	0.147282576	-1.065473372	2
HENMT1	0.952914327	0.088313333	-1.04122766	2
HERC2	0.992169591	0.015481062	-1.007650653	2
HERC5	0.95346855	0.087334244	-1.040802793	2
HERC6	1.072418399	-0.165481426	-0.906936974	2
HGH1	1.053288688	-0.116842924	-0.936445765	2
HIBCH	0.916608034	0.149867246	-1.066475279	2
HIGD1C	0.904145131	0.169933212	-1.074078343	2
HIRA	1.065848826	-0.148249603	-0.917599224	2
HIRIP3	0.881777859	0.204750951	-1.08652881	2
HIVEP2	0.979802934	0.039239022	-1.019041956	2
HLA-DMB	0.882178081	0.204140666	-1.086318748	2
HLA-DOA	0.928201044	0.130737369	-1.058938413	2
HLA-DOB	0.922869751	0.139592218	-1.062461969	2
HLA-DPA1	1.055966658	-0.123383757	-0.932582901	2
HLA-DPB1	1.068647412	-0.155516984	-0.913130427	2
HLA-DQB1	1.105494536	-0.263936995	-0.841557541	2
HLA-DRB1	1.101625335	-0.251119132	-0.850506204	2

HLF	1.112391925	-0.287983074	-0.824408851	2
HMCES	1.02587982	-0.053943238	-0.971936583	2
HMGCR	1.075547808	-0.173908977	-0.901638831	2
HMGN4	0.965790346	0.06522594	-1.031016287	2
HNRNPA1L2	1.098703561	-0.241719103	-0.856984457	2
HNRNPF	0.999573918	0.000851619	-1.000425538	2
HNRNPH1	0.946363727	0.099790013	-1.046153741	2
HNRNPM	1.012056038	-0.024564696	-0.987491342	2
HNRNPUL2	1.063131304	-0.141291552	-0.921839752	2
HOMEZ	1.079938258	-0.1859917	-0.893946557	2
HPF1	1.116364334	-0.302647424	-0.81371691	2
HPS3	0.9063232	0.166462232	-1.072785432	2
HPS6	0.967498496	0.062107872	-1.029606368	2
HPSE	1.106157301	-0.266177938	-0.839979363	2
HS3ST1	0.879653562	0.207982765	-1.087636327	2
HSD17B13	1.093336077	-0.225012683	-0.868323393	2
HSDL2	0.883825016	0.201624598	-1.085449613	2
HSFX4	1.082195356	-0.192328545	-0.889866811	2
HSH2D	0.973587376	0.05088253	-1.024469906	2
HSPA1L	1.110604339	-0.281589728	-0.829014612	2
HSPB11	1.106087195	-0.265940241	-0.840146954	2
HSPBP1	1.023146757	-0.048023991	-0.975122767	2
HTRA4	0.983152236	0.032884329	-1.016036565	2
HYLS1	0.884027339	0.201314978	-1.085342317	2

HYPK	1.054541339	-0.11989256	-0.934648778	2
IARS1	1.079213417	-0.183975157	-0.89523826	2
IARS2	1.039797121	-0.085023336	-0.954773785	2
IBA57	0.874192201	0.216234595	-1.090426795	2
ICA1L	0.918796055	0.146291957	-1.065088012	2
ICAM4	0.896084794	0.182650898	-1.078735691	2
ICMT	1.111427904	-0.284520276	-0.826907628	2
ICOS	0.941564263	0.108089662	-1.049653925	2
ID2	1.063813302	-0.14302885	-0.920784452	2
ID3	1.052016803	-0.113763953	-0.93825285	2
IDH1	0.971944523	0.053928547	-1.02587307	2
IDH2	0.957488886	0.080193187	-1.037682073	2
IER5	1.022856391	-0.047398452	-0.975457939	2
IFI16	1.005100792	-0.010280857	-0.994819935	2
IFI35	1.015236075	-0.031202479	-0.984033596	2
IFI44	1.068935216	-0.1562704	-0.912664816	2
IFIH1	1.065926113	-0.148448893	-0.91747722	2
IFIT1	1.074702359	-0.171617493	-0.903084866	2
IFIT2	1.078337862	-0.18155099	-0.896786872	2
IFIT3	1.03018303	-0.063381217	-0.966801813	2
IFIT5	0.885915741	0.198419534	-1.084335275	2
IFNG	0.954289184	0.085882153	-1.040171337	2
IGHMBP2	0.97789273	0.04283776	-1.02073049	2
IGIP	1.047912554	-0.103945016	-0.943967538	2

IGSF23	0.912873922	0.155932129	-1.068806051	2
IKBKE	1.046564758	-0.100758231	-0.945806527	2
IKZF1	0.99838739	0.003217455	-1.001604846	2
IKZF3	1.051916484	-0.113521843	-0.938394641	2
IL12RB2	1.042561349	-0.091397697	-0.951163652	2
IL18	1.072685734	-0.166195616	-0.906490118	2
IL18R1	0.895932468	0.182889344	-1.078821812	2
IL1B	0.877775368	0.210829795	-1.088605163	2
IL1RL1	0.934546305	0.120066104	-1.05461241	2
IL20RB	1.105998443	-0.265639547	-0.840358896	2
IL22	1.093671184	-0.226035802	-0.867635383	2
IL23A	1.067927362	-0.15363703	-0.914290332	2
IL24	1.049376446	-0.107427146	-0.9419493	2
IL26	0.889717918	0.192558894	-1.082276812	2
IL32	0.876151744	0.213283162	-1.089434906	2
IL4	1.040381945	-0.086366057	-0.954015887	2
IL4I1	0.98232012	0.034468504	-1.016788623	2
IL7R	0.959942404	0.075801176	-1.03574358	2
ILF2	0.904411264	0.169509897	-1.073921161	2
ILVBL	0.876142752	0.213296729	-1.089439481	2
IMP3	0.866539137	0.227663441	-1.094202578	2
IMP4	0.991031659	0.017701656	-1.008733315	2
ING4	1.011953027	-0.024350825	-0.987602202	2
INO80E	1.002661159	-0.005343734	-0.997317424	2

INPP5F	1.073665432	-0.168821976	-0.904843456	2
INTS10	0.996542594	0.006879319	-1.003421912	2
INTS14	1.002288543	-0.004592907	-0.997695636	2
INTS6	1.061200419	-0.136404506	-0.924795913	2
INTS7	1.116537452	-0.303301888	-0.813235564	2
INVS	1.011134758	-0.022654472	-0.988480286	2
IPMK	0.922385669	0.140391331	-1.062777	2
IPO4	0.994526133	0.010859288	-1.005385422	2
IPO8	0.88614792	0.198062842	-1.084210761	2
IPO9	0.924270653	0.137275067	-1.06154572	2
IPP	1.105344303	-0.263430941	-0.841913363	2
IPPK	1.044122685	-0.095029887	-0.949092798	2
IQCC	1.107790542	-0.271760399	-0.836030143	2
IRF2BP1	0.984385568	0.030529694	-1.014915263	2
IRGQ	0.940466242	0.109975838	-1.05044208	2
ISG20L2	0.968284813	0.060668011	-1.028952824	2
ISOC1	0.985379753	0.028625822	-1.014005575	2
ISYNA1	0.883707657	0.201804142	-1.085511799	2
ITGAD	1.015360201	-0.031462979	-0.983897222	2
ITGAE	1.040781165	-0.087284446	-0.953496719	2
ITGB1BP1	0.95178684	0.090301209	-1.042088049	2
ITGB7	0.880522473	0.206662353	-1.087184826	2
ITIH1	1.107501376	-0.27076566	-0.836735716	2
ITIH4	0.978486342	0.041721385	-1.020207726	2

ITPA	1.069509222	-0.15777647	-0.911732751	2
ITPKB	1.077533439	-0.179334855	-0.898198584	2
ITPKC	0.940127667	0.110556504	-1.050684172	2
ITPR3	0.977856151	0.042906496	-1.020762647	2
IVD	1.102586971	-0.254264063	-0.848322908	2
JAGN1	0.870510814	0.221751588	-1.092262402	2
JCHAIN	0.993020758	0.013815332	-1.00683609	2
JHY	1.047044877	-0.101891357	-0.94515352	2
JMJD4	1.100976833	-0.249012862	-0.851963971	2
JMJD8	0.89540441	0.183715412	-1.079119822	2
JRK	0.922146187	0.140786365	-1.062932552	2
KANK1	0.941472119	0.108248125	-1.049720245	2
KAT14	1.025052057	-0.052144436	-0.972907621	2
KAT2A	1.118653299	-0.311413163	-0.807240136	2
KAT5	0.962889407	0.070490975	-1.033380382	2
KATNBL1	0.871434082	0.220371354	-1.091805435	2
KATNIP	0.952541541	0.088971177	-1.041512718	2
KBTBD2	1.039224845	-0.083712445	-0.955512401	2
KBTBD6	1.000021747	-0.0000435	-0.999978251	2
KCNA6	0.979421134	0.039959786	-1.019380919	2
KCNC4	0.944426358	0.103151175	-1.047577534	2
KCNJ10	1.033540045	-0.070848246	-0.962691799	2
KCNK17	0.963113998	0.070084706	-1.033198704	2
KCNQ5	1.10082862	-0.248533104	-0.852295516	2

KCTD11	1.014189581	-0.029010468	-0.985179113	2
KCTD6	0.945384929	0.101489999	-1.046874928	2
KCTD9	1.065496856	-0.147343009	-0.918153847	2
KDM1B	0.880511719	0.206678708	-1.087190427	2
KDM2B	0.888173464	0.194944507	-1.08311797	2
KDM5B	1.108940575	-0.275744434	-0.833196141	2
KIAA0040	1.08243999	-0.193020698	-0.889419292	2
KIAA0100	1.025153304	-0.052364172	-0.972789132	2
KIAA0232	1.070348795	-0.159987653	-0.910361142	2
KIAA0586	1.075249211	-0.173098407	-0.902150805	2
KIF18B	1.113641448	-0.292525351	-0.821116097	2
KIF3B	0.919356253	0.145373986	-1.06473024	2
KIFBP	0.883939508	0.201449402	-1.08538891	2
KIN	0.88537942	0.199242889	-1.084622309	2
KLC1	0.996230815	0.007496224	-1.003727039	2
KLF12	0.99786496	0.004256492	-1.002121452	2
KLF7	0.870584889	0.221640933	-1.092225822	2
KLF8	0.8847491	0.200209508	-1.084958608	2
KLHDC7B	0.905856753	0.167206816	-1.073063569	2
KLHDC9	1.049857882	-0.108577141	-0.941280741	2
KLHL11	0.90986761	0.160781795	-1.070649404	2
KLHL12	0.907183153	0.165087698	-1.072270851	2
KLHL26	0.969006643	0.059343711	-1.028350354	2
KLHL28	0.966476289	0.063975422	-1.030451711	2

KLHL3	0.892227853	0.188667212	-1.080895065	2
KLHL9	0.895321167	0.183845557	-1.079166724	2
KLKB1	1.047396328	-0.102722274	-0.944674053	2
KLRC3	1.04868864	-0.105788347	-0.942900293	2
KLRK1	0.970832505	0.055983032	-1.026815537	2
KMT2D	0.958951943	0.077577327	-1.03652927	2
KMT2E	1.093503241	-0.225522736	-0.867980505	2
KRBA1	0.879359572	0.208429046	-1.087788618	2
KRBOX1	1.115497275	-0.299389639	-0.816107636	2
KRBOX4	1.089331957	-0.212977998	-0.876353958	2
KRI1	0.996372188	0.007216564	-1.003588752	2
KRT2	0.95951547	0.076567302	-1.036082772	2
KRT77	0.879546606	0.208145153	-1.087691759	2
L3HYPDH	0.969656027	0.058150248	-1.027806275	2
L3MBTL2	0.992871141	0.014108427	-1.006979568	2
LAIR1	0.916805995	0.149544431	-1.066350426	2
LAMP3	1.108864603	-0.275479851	-0.833384752	2
LAMTOR3	1.03271901	-0.069013347	-0.963705663	2
LANCL1	1.114798926	-0.296789569	-0.818009356	2
LANCL2	1.058276235	-0.129089924	-0.929186311	2
LARS2	0.963950208	0.068570077	-1.032520286	2
LCE1D	0.876594494	0.212614859	-1.089209353	2
LCK	1.03667033	-0.077896782	-0.958773549	2
LCMT2	1.054923394	-0.120826142	-0.934097252	2

LDB1	0.949565965	0.094201515	-1.04376748	2
LDHB	1.028194271	-0.059001096	-0.969193175	2
LETMD1	1.011785017	-0.024002158	-0.987782859	2
LIAS	0.9917272	0.016345216	-1.008072416	2
LILRA4	1.10645649	-0.267194109	-0.839262381	2
LMAN2	0.96007235	0.075567831	-1.035640181	2
LMBRD2	0.898284719	0.179199445	-1.077484164	2
LMF2	1.103870855	-0.258504258	-0.845366597	2
LMNTD2	0.962117867	0.071884921	-1.034002789	2
LMOD3	0.997218619	0.005539745	-1.002758364	2
LOC101927375	0.873072847	0.217915907	-1.090988754	2
LOC101930420	1.011266962	-0.022928241	-0.988338721	2
LOC102724159	0.997446373	0.005087839	-1.002534212	2
LOC105370706	1.064046414	-0.143624028	-0.920422386	2
LOC107984124	1.069076643	-0.156641049	-0.912435594	2
LOC107984153	1.086666483	-0.205151388	-0.881515095	2
LOC107987373	1.016336667	-0.033516004	-0.982820663	2
LOC112268342	1.107822603	-0.27187086	-0.835951743	2
LOC112268384	0.932150993	0.124111672	-1.056262666	2
LOC441155	0.899726859	0.176928948	-1.076655807	2
LOC728392	1.006479979	-0.013088442	-0.993391537	2
LONP1	0.89718987	0.180918967	-1.078108837	2
LRATD2	0.929491902	0.128578246	-1.058070148	2
LRRC36	1.077762932	-0.179966022	-0.897796911	2

LRRC46	0.871941914	0.219611207	-1.091553121	2
LRRC57	1.080961763	-0.188854303	-0.89210746	2
LRRC59	1.034750351	-0.073563522	-0.961186829	2
LRRCC1	1.012912038	-0.026344676	-0.986567362	2
LRRK2	0.953461021	0.087347552	-1.040808573	2
LSG1	0.944201731	0.103539924	-1.047741655	2
LSS	0.969071383	0.059224817	-1.0282962	2
LTK	1.098245809	-0.240266778	-0.857979031	2
LY96	1.100547156	-0.247623667	-0.852923489	2
LYAR	1.055364457	-0.121905939	-0.933458518	2
LYG1	1.020152373	-0.041603293	-0.97854908	2
LYRM4	1.012747352	-0.026001839	-0.986745512	2
LYRM7	1.069661414	-0.158176564	-0.911484851	2
LYZL6	1.116857434	-0.304515143	-0.812342292	2
MACF1	0.967035165	0.062954963	-1.029990128	2
MAD2L1	1.058071346	-0.128581218	-0.929490128	2
MAGEE2	0.885034124	0.199772553	-1.084806677	2
MAN2C1	0.960441178	0.074905124	-1.035346302	2
MANF	1.082990122	-0.194581101	-0.888409022	2
MANSC4	1.068469312	-0.155051327	-0.913417985	2
MAP2K1	0.876628811	0.212563037	-1.089191848	2
MAP2K5	1.088824483	-0.21147691	-0.877347572	2
MAP2K6	1.102380825	-0.253587677	-0.848793147	2
MAP3K1	1.114373333	-0.295215187	-0.819158146	2

MAP4	0.908122455	0.163583659	-1.071706114	2
MAPK7	0.926547802	0.133493959	-1.060041761	2
MAPRE1	1.057862489	-0.128063166	-0.929799322	2
MARCHF9	0.979836485	0.03917565	-1.019012135	2
MARCO	1.013329995	-0.027215584	-0.986114411	2
MARS2	1.118715299	-0.311654078	-0.807061221	2
MARVELD3	1.057700867	-0.127662625	-0.930038241	2
MASTL	0.974452759	0.049272796	-1.023725555	2
MAU2	0.870463168	0.221822756	-1.092285924	2
MB21D2	1.116290392	-0.302368301	-0.813922091	2
MBD4	1.008959721	-0.018166988	-0.990792734	2
MBOAT1	0.911320839	0.158441157	-1.069761996	2
MBOAT4	0.978120666	0.042409302	-1.020529967	2
MBTPS1	0.904538847	0.169306884	-1.073845731	2
MCEE	0.965679294	0.065428194	-1.031107488	2
MCM3	0.924180279	0.137424755	-1.061605034	2
MCM3AP	0.976819911	0.044850905	-1.021670816	2
MCM7	0.920848122	0.14292414	-1.063772262	2
MCM8	0.868553735	0.224669873	-1.093223608	2
MCMBP	0.882918688	0.203010161	-1.085928849	2
MCRS1	0.873468617	0.217321829	-1.090790446	2
MDC1	1.045224132	-0.097606319	-0.947617813	2
ME3	1.039451525	-0.084231336	-0.955220189	2
MEAF6	1.006879235	-0.013903454	-0.992975781	2

MEAK7	0.980715298	0.037513668	-1.018228967	2
MECOM	0.944097299	0.10372059	-1.047817889	2
MED19	0.925735801	0.134844337	-1.060580139	2
MED22	0.917086787	0.149086321	-1.066173107	2
MED23	1.037060492	-0.078781281	-0.958279211	2
MED24	0.903477149	0.17099474	-1.074471889	2
MED25	0.997394324	0.005191141	-1.002585465	2
MED31	0.913908425	0.154256507	-1.068164932	2
MED6	0.911218444	0.158606303	-1.069824747	2
MED9	1.0762465	-0.175811088	-0.900435412	2
MELK	1.022191685	-0.04596885	-0.976222835	2
MEMO1	1.05102047	-0.111364152	-0.939656318	2
MEN1	1.041165688	-0.088170417	-0.952995271	2
METAP1	0.915531173	0.151620993	-1.067152166	2
METTL4	1.032452571	-0.068419111	-0.964033461	2
METTL9	1.020812531	-0.043013141	-0.97779939	2
MEX3C	0.914753271	0.152885471	-1.067638743	2
MFAP3	1.115380966	-0.298955145	-0.816425821	2
MFF	0.909484846	0.16139716	-1.070882006	2
MFGE8	0.97929763	0.040192779	-1.019490408	2
MFSD10	0.941491366	0.108215028	-1.049706395	2
MGAT4A	0.913991759	0.154121374	-1.068113133	2
MIA	1.070734183	-0.161005993	-0.90972819	2
MIB2	0.868151441	0.225268504	-1.093419945	2

MICA	1.060567325	-0.134812156	-0.925755168	2
MICB	0.918283906	0.147130264	-1.06541417	2
MICOS10	0.888712552	0.194112596	-1.082825148	2
MICOS13	0.906254463	0.166571998	-1.072826462	2
MIEF2	1.074057762	-0.169877765	-0.904179997	2
MIGA1	0.937886312	0.114389468	-1.052275779	2
MINDY3	1.074266903	-0.170441529	-0.903825374	2
MIPEP	0.915117136	0.15229426	-1.067411395	2
MIX23	1.085967772	-0.203122887	-0.882844885	2
MKRN2	1.034810533	-0.073698865	-0.961111668	2
MLYCD	0.884762005	0.20018973	-1.084951735	2
MMACHC	0.930643583	0.126646866	-1.057290449	2
MMP11	1.060276202	-0.134081562	-0.92619464	2
MMP14	1.077251736	-0.178561259	-0.898690477	2
MMP19	1.012871253	-0.026259753	-0.9866115	2
MMP23B	1.043831042	-0.094349658	-0.949481384	2
MMUT	0.909535117	0.161316367	-1.070851484	2
MNDA	0.883119881	0.202702782	-1.085822664	2
MOAP1	1.009813804	-0.019925396	-0.989888408	2
MOCS3	1.019749543	-0.040744563	-0.97900498	2
MORN1	0.945526668	0.101244062	-1.04677073	2
MORN3	0.997589947	0.004802807	-1.002392753	2
MPC2	0.940294979	0.110269613	-1.050564593	2
MPDU1	1.077337582	-0.178796868	-0.898540714	2

MPHOSPH10	1.073540291	-0.1684857	-0.905054591	2
MPRIP	1.084324823	-0.198389581	-0.885935242	2
MR1	0.956946153	0.08116122	-1.038107373	2
MRI1	1.07358476	-0.16860517	-0.904979591	2
MRM1	1.036709609	-0.077985766	-0.958723843	2
MRPL10	0.982848672	0.033462667	-1.016311339	2
MRPL11	0.963299968	0.06974813	-1.033048097	2
MRPL12	0.908309868	0.163283231	-1.071593099	2
MRPL15	0.937491726	0.11506228	-1.052554006	2
MRPL17	1.065295673	-0.146825536	-0.918470137	2
MRPL21	0.960847781	0.074173852	-1.035021633	2
MRPL30	0.923484773	0.138575784	-1.062060557	2
MRPL33	0.91484112	0.152742773	-1.067583893	2
MRPL34	1.069592049	-0.15799417	-0.911597878	2
MRPL38	0.896557592	0.181910353	-1.078467945	2
MRPL39	0.98547031	0.028452144	-1.013922454	2
MRPL44	0.982750136	0.03365029	-1.016400427	2
MRPL54	1.077160343	-0.178310555	-0.898849788	2
MRPS10	0.887321448	0.196257619	-1.083579067	2
MRPS11	1.031342102	-0.065948801	-0.965393301	2
MRPS15	1.100711295	-0.248153754	-0.85255754	2
MRPS17	1.018785345	-0.038693919	-0.980091425	2
MRPS18B	0.954159895	0.086111114	-1.040271009	2
MRPS18C	1.09125984	-0.218729366	-0.872530473	2

MRPS31	1.061322961	-0.136713286	-0.924609674	2
MRPS5	0.895223368	0.183998434	-1.079221802	2
MRPS7	0.87178316	0.219848911	-1.091632071	2
MRPS9	1.006184728	-0.012486392	-0.993698336	2
MSANTD2	0.995837481	0.008273697	-1.004111178	2
MSI2	0.866219279	0.228137758	-1.094357037	2
MST1	1.036943731	-0.078516443	-0.958427288	2
MSTO1	1.013088745	-0.026712739	-0.986376006	2
MTA1	1.111289509	-0.284026051	-0.827263458	2
MTERF1	1.047658816	-0.103343671	-0.944315144	2
MTERF3	1.028463903	-0.059593084	-0.968870819	2
MTG1	1.090300521	-0.215857715	-0.874442806	2
MTHFD1	0.955678511	0.083417315	-1.039095826	2
MTMR10	1.118114473	-0.309327345	-0.808787128	2
MTMR11	0.958622236	0.078167632	-1.036789868	2
MTMR4	1.062859041	-0.140599642	-0.922259399	2
MTMR6	0.892180948	0.188740108	-1.080921055	2
MTRFR	1.012153054	-0.024766184	-0.98738687	2
MUS81	1.086260012	-0.203970177	-0.882289835	2
MVB12B	1.052209936	-0.114230366	-0.93797957	2
MYBBP1A	1.039120616	-0.08347401	-0.955646606	2
MYBPC3	0.926013687	0.134382467	-1.060396154	2
MYCBP	0.932206397	0.124018336	-1.056224733	2
MYL6	0.974531339	0.049126447	-1.023657786	2

MYO19	1.016388628	-0.033625439	-0.982763189	2
MYO1A	1.067339152	-0.152106539	-0.915232613	2
MYO1D	1.118435627	-0.310568842	-0.807866785	2
MYO1G	1.00750331	-0.015179439	-0.992323871	2
MYO7A	1.077126644	-0.178218148	-0.898908496	2
MYPOP	0.887954476	0.195282206	-1.083236682	2
N6AMT1	0.941300484	0.108543204	-1.049843689	2
NAA40	1.05252054	-0.114981308	-0.937539232	2
NADSYN1	0.972057325	0.053719814	-1.025777139	2
NANS	0.905225631	0.168213181	-1.073438812	2
NAPEPLD	1.047871734	-0.103848232	-0.944023502	2
NAPRT	0.920672726	0.143212557	-1.063885283	2
NARS2	1.048723093	-0.10587032	-0.942852772	2
NAT10	1.020124408	-0.04154364	-0.978580768	2
NAT9	1.024434168	-0.050805146	-0.973629022	2
NAXD	1.069415825	-0.157531104	-0.911884721	2
NBPF12	0.980106387	0.038665638	-1.018772025	2
NBPF8	1.05026559	-0.109552907	-0.940712683	2
NCBP1	1.095510838	-0.231698458	-0.86381238	2
NCBP2AS2	1.046448958	-0.100485276	-0.945963682	2
NDUFA10	0.96746839	0.062162943	-1.029631333	2
NDUFA13	1.074486057	-0.171033001	-0.903453056	2
NDUFA4	1.094457332	-0.228446041	-0.866011291	2
NDUFB4	1.097975808	-0.23941264	-0.858563168	2

NDUFB7	0.96962232	0.058212245	-1.027834565	2
NDUFV1	0.957629772	0.079941693	-1.037571465	2
NEDD8-MDP1	0.9350247	0.119255583	-1.054280283	2
NEIL1	1.111187466	-0.283662102	-0.827525364	2
NEMP2	0.909540816	0.161307207	-1.070848023	2
NEU3	1.028282908	-0.059195637	-0.969087271	2
NEURL4	1.072732379	-0.166320337	-0.906412042	2
NF2	0.980551029	0.037824627	-1.018375656	2
NFATC2	0.923894868	0.137897299	-1.061792168	2
NFKBID	1.056602431	-0.124948421	-0.93165401	2
NGDN	0.958935222	0.077607277	-1.036542498	2
NGLY1	0.981485123	0.036054568	-1.017539691	2
NHLRC3	0.949200939	0.094840644	-1.044041584	2
NHLRC4	1.031194682	-0.065621625	-0.965573057	2
NIFK	0.953938759	0.086502567	-1.040441325	2
NIPSNAP2	0.920452246	0.143574959	-1.064027205	2
NKTR	0.932571687	0.123402664	-1.055974351	2
NKX3-1	0.95502299	0.084581305	-1.039604295	2
NLN	1.069170846	-0.156888088	-0.912282758	2
NLRP1	1.026101944	-0.054426834	-0.97167511	2
NLRP3	0.929718722	0.128198245	-1.057916967	2
NME8	0.968944159	0.059458443	-1.028402601	2
NMI	1.018262366	-0.037584456	-0.98067791	2
NMNAT1	0.884954637	0.199894433	-1.08484907	2

NMRK1	0.917793782	0.147931693	-1.065725475	2
NOB1	0.982578958	0.033976114	-1.016555072	2
NOC2L	0.956056299	0.08274566	-1.038801959	2
NOD1	0.920045795	0.144242608	-1.064288403	2
NOL11	0.91024691	0.160171534	-1.070418443	2
NOL3	0.935990058	0.117617435	-1.053607493	2
NOL4L	0.967642733	0.061843968	-1.0294867	2
NOL6	0.890227826	0.19176976	-1.081997586	2
NOL8	1.029151647	-0.061105685	-0.968045961	2
NOM1	0.890630753	0.191145656	-1.081776409	2
NOP14	0.975483218	0.04735125	-1.022834468	2
NOP2	0.958737005	0.077962204	-1.036699209	2
NOP53	0.902378837	0.17273712	-1.075115957	2
NOP56	0.900610887	0.175534012	-1.076144898	2
NOP9	0.890174544	0.191852255	-1.082026799	2
NOXRED1	1.082735417	-0.193857978	-0.888877439	2
NPIPA1	0.998469142	0.003054718	-1.00152386	2
NPIPA5	1.083132476	-0.194985758	-0.888146718	2
NPIPA7	0.873700072	0.216974205	-1.090674277	2
NPIPB3	0.874257587	0.216136278	-1.090393865	2
NPIPB6	1.091670893	-0.219965847	-0.871705046	2
NPRL2	0.867260248	0.226593131	-1.093853379	2
NRBP2	1.065706509	-0.14788283	-0.917823679	2
NRDE2	0.98918532	0.021289402	-1.010474722	2

NRF1	1.111664429	-0.285366601	-0.826297828	2
NRIP2	0.907719779	0.164228778	-1.071948558	2
NSD2	1.061755076	-0.137803601	-0.923951474	2
NSDHL	1.077969148	-0.1805339	-0.897435249	2
NSMAF	0.957519704	0.080138181	-1.037657886	2
NSUN2	0.915291482	0.152010823	-1.067302306	2
NSUN7	1.09808513	-0.239758254	-0.858326876	2
NT5DC2	0.91633909	0.150305602	-1.066644692	2
NUBP1	0.877659679	0.211004844	-1.088664523	2
NUBP2	0.956896895	0.081249015	-1.03814591	2
NUDT1	1.052505286	-0.114944404	-0.937560882	2
NUDT13	1.02756206	-0.057615315	-0.969946745	2
NUDT16L1	1.05664139	-0.12504445	-0.93159694	2
NUDT19	1.016302118	-0.03344325	-0.982858868	2
NUDT2	1.061393245	-0.136890472	-0.924502773	2
NUDT4	1.116537072	-0.30330045	-0.813236622	2
NUGGC	0.91091824	0.159090285	-1.070008524	2
NUP160	1.090796667	-0.217340452	-0.873456215	2
NUP35	1.100012208	-0.245901094	-0.854111114	2
NUP37	0.980993036	0.036987604	-1.017980639	2
NUP62	0.926593957	0.133417132	-1.060011089	2
NUP93	1.117790814	-0.308081189	-0.809709625	2
NUP98	1.035274295	-0.074742859	-0.960531436	2
NUTM2D	1.116304921	-0.302423126	-0.813881795	2

NVL	0.915870073	0.151069485	-1.066939558	2
NXF1	1.107217268	-0.269791013	-0.837426254	2
NXT1	0.934838014	0.119571977	-1.054409991	2
OCIAD2	0.951617403	0.090599489	-1.042216892	2
ODF2L	0.97504725	0.048164853	-1.023212103	2
OMA1	0.879859157	0.207670529	-1.087529685	2
OMG	0.943544825	0.104675654	-1.048220478	2
OPA3	0.969987872	0.057539608	-1.02752748	2
OPLAH	0.986623233	0.026237176	-1.012860409	2
OR10AD1	1.009152961	-0.018564416	-0.990588544	2
OR10G2	1.007286712	-0.0147363	-0.992550412	2
OR13A1	0.867690058	0.225954541	-1.093644599	2
OR1J2	1.047956692	-0.104049686	-0.943907005	2
OR4C11	1.015379049	-0.031502544	-0.983876505	2
OR4D1	0.952206948	0.089561131	-1.041768079	2
ORMDL1	0.881495156	0.205181766	-1.086676922	2
OSBPL5	0.989738968	0.020215539	-1.009954507	2
OSGIN2	0.969360373	0.058693856	-1.028054229	2
OSTM1	0.885983805	0.198314985	-1.084298789	2
OTUD1	0.941092062	0.108901376	-1.049993438	2
OVGP1	0.990256484	0.019210238	-1.009466722	2
OXLD1	0.922932081	0.139489267	-1.062421347	2
P2RX4	1.118915032	-0.312431477	-0.806483555	2
P2RX5	1.003317318	-0.006667983	-0.996649335	2

P2RY10	1.098292645	-0.24041513	-0.857877515	2
P2RY8	1.106054892	-0.265830768	-0.840224124	2
P3H1	1.08870473	-0.211123451	-0.877581278	2
PABIR1	0.999480041	0.001039108	-1.000519149	2
PABPC4	0.945699809	0.100943528	-1.046643337	2
PABPN1L	0.901734953	0.173756855	-1.075491808	2
PACC1	1.041930696	-0.089937137	-0.951993559	2
PADI6	0.876530953	0.212710803	-1.089241756	2
PAFAH1B3	0.866686254	0.227445193	-1.094131446	2
PAGE2	0.871609787	0.220108428	-1.091718214	2
PAK1IP1	0.96680624	0.063373133	-1.030179373	2
PAM	1.006874471	-0.013893725	-0.992980747	2
PAM16	1.109438267	-0.277482686	-0.831955582	2
PAN2	0.954085738	0.086242408	-1.040328146	2
PAPOLG	1.080468592	-0.187472738	-0.892995854	2
PAQR6	1.080390234	-0.187253611	-0.893136623	2
PAQR7	0.952037161	0.089860322	-1.041897483	2
PARP10	0.926631695	0.133354309	-1.059986004	2
PARP14	1.037595279	-0.079995831	-0.957599448	2
PARP3	0.971260784	0.055192469	-1.026453253	2
PARVG	1.021679659	-0.044869867	-0.976809792	2
PATZ1	1.037715585	-0.080269405	-0.95744618	2
PAX4	1.085426783	-0.201558698	-0.883868085	2
PAX5	1.071898325	-0.164095043	-0.907803282	2

PCBD2	0.986258864	0.026937959	-1.013196822	2
PCDHGA5	1.072915096	-0.166809206	-0.906105891	2
PCED1B	1.115677684	-0.300064757	-0.815612927	2
PCID2	0.879793634	0.207770051	-1.087563685	2
PCNA	1.042626585	-0.091548993	-0.951077592	2
PCNX1	1.088556885	-0.21068748	-0.877869405	2
PCNX4	0.889278395	0.193238496	-1.082516891	2
PCSK7	1.095247029	-0.23088157	-0.864365459	2
PCYOX1	0.997091135	0.005792565	-1.0028837	2
PCYT2	1.02669005	-0.055709083	-0.970980967	2
PDCD2	0.966320843	0.064258998	-1.030579841	2
PDCD6IP	1.113188315	-0.290870935	-0.822317381	2
PDCD7	1.037799768	-0.080460913	-0.957338855	2
PDCL3	1.093174649	-0.224520731	-0.868653918	2
PDE12	1.085342705	-0.201316099	-0.884026607	2
PDE6B	1.10094969	-0.248924958	-0.852024732	2
PDE8A	0.98678023	0.025935008	-1.012715238	2
PDP2	0.986039777	0.027358982	-1.013398759	2
PDPK1	0.878803327	0.20927278	-1.088076107	2
PDRG1	0.995474876	0.008989636	-1.004464512	2
PDXP	0.875685005	0.213987099	-1.089672104	2
PDZD2	0.867710597	0.225924013	-1.09363461	2
PEG10	0.96552122	0.065715989	-1.031237209	2
PES1	1.110382227	-0.280803647	-0.829578581	2

PEX12	0.918080987	0.147462166	-1.065543153	2
PEX14	1.010223157	-0.02076988	-0.989453276	2
PEX2	1.108920633	-0.275674966	-0.833245668	2
PEX26	0.942815801	0.10593409	-1.048749891	2
PEX7	1.106872762	-0.268612723	-0.838260039	2
PFDN5	0.903031288	0.171702512	-1.0747338	2
PFDN6	1.112658054	-0.28894533	-0.823712724	2
PFN1	0.925784425	0.13476354	-1.060547965	2
PGAP3	1.099903043	-0.245550517	-0.854352526	2
PGBD2	1.047352161	-0.102617785	-0.944734376	2
PGGT1B	0.975140439	0.047991022	-1.023131461	2
PHAF1	1.00197119	-0.003954106	-0.998017084	2
PHB	0.984837168	0.029665523	-1.014502691	2
PHF10	1.023233738	-0.048211499	-0.97502224	2
PHF11	0.893152204	0.187229354	-1.080381557	2
PHF12	0.930947138	0.126137004	-1.057084142	2
PHF19	1.066140209	-0.149001373	-0.917138836	2
PHF8	1.100846513	-0.248590991	-0.852255522	2
PHOSPHO1	0.905065052	0.168469035	-1.073534087	2
PHPT1	0.969614813	0.058226051	-1.027840864	2
PI4K2B	1.067358698	-0.152157322	-0.915201376	2
PI4KB	1.056697047	-0.125181668	-0.931515379	2
PIAS3	0.948228972	0.096539842	-1.044768814	2
PIAS4	1.029401887	-0.061656999	-0.967744888	2

PICK1	1.076610176	-0.176804193	-0.899805983	2
PIGL	1.033099854	-0.069863775	-0.963236079	2
PIGM	1.045654318	-0.098615791	-0.947038528	2
PIGT	1.052534315	-0.115014635	-0.93751968	2
PIGV	1.118691066	-0.311559892	-0.807131174	2
PIGW	0.957223439	0.080666803	-1.037890242	2
PIH1D1	1.091448324	-0.219295889	-0.872152435	2
PIK3C3	0.913353854	0.155155201	-1.068509055	2
PIK3CG	1.078981796	-0.18333263	-0.895649167	2
PIK3R4	0.996249202	0.007459858	-1.00370906	2
PIM2	0.938717108	0.112970943	-1.051688051	2
PIN4	1.095997213	-0.233208864	-0.862788349	2
PINX1	1.062100087	-0.138675792	-0.923424295	2
PIP4K2C	0.918803882	0.146279138	-1.06508302	2
PITPNA	0.872599498	0.218625885	-1.091225384	2
PITRM1	1.045877269	-0.099139681	-0.946737588	2
PJA1	0.966944769	0.063120114	-1.030064884	2
PKD1	0.906421347	0.166305473	-1.07272682	2
PKD1L1	1.020297739	-0.041913467	-0.978384272	2
PKN3	0.965247876	0.066213382	-1.031461258	2
PLA2G10	1.07332165	-0.167898738	-0.905422912	2
PLAC8	1.03444967	-0.072887789	-0.961561881	2
PLCB2	0.947088107	0.098529445	-1.045617552	2
PLCD1	0.958374288	0.078611245	-1.036985533	2

PLCG1	1.113792661	-0.29307929	-0.820713371	2
PLCXD1	1.031685739	-0.066712152	-0.964973587	2
PLD2	1.017587882	-0.036156472	-0.98143141	2
PLD3	0.92859583	0.130077673	-1.058673502	2
PLEKHA1	1.027848079	-0.058241864	-0.969606215	2
PLEKHB2	0.946654255	0.099284688	-1.045938943	2
PLEKHD1	0.888910054	0.193807604	-1.082717658	2
PLEKHF2	0.926255806	0.133979823	-1.060235629	2
PLEKHM2	0.909657313	0.161119945	-1.070777258	2
PLGRKT	0.971626001	0.054517625	-1.026143626	2
PLIN2	1.021432626	-0.044340345	-0.977092281	2
PLIN4	0.94014055	0.110534418	-1.050674968	2
PLLP	0.91796432	0.147652928	-1.065617248	2
PLPBP	0.882535693	0.203594978	-1.086130671	2
PLPP6	0.892838473	0.187717654	-1.080556127	2
PMEL	0.928544714	0.13016312	-1.058707834	2
PMM2	0.901100426	0.174760523	-1.075860949	2
PMPCA	1.074802483	-0.171888292	-0.902914192	2
PNMA3	1.112564573	-0.288607013	-0.823957561	2
PNMA6A	1.064882207	-0.1457637	-0.919118507	2
PNO1	1.051030519	-0.111388302	-0.939642216	2
PNPLA8	1.073289543	-0.167812605	-0.905476939	2
PNPT1	0.966232788	0.064419587	-1.030652374	2
POGK	1.030614636	-0.064336029	-0.966278607	2

POLB	1.058549924	-0.129770215	-0.92877971	2
POLD2	0.928607509	0.130058147	-1.058665657	2
POLD4	0.960273058	0.075207274	-1.035480333	2
POLDIP3	0.93702493	0.115857461	-1.052882391	2
POLE	0.910302718	0.160081703	-1.070384421	2
POLR1A	1.038473031	-0.081994794	-0.956478237	2
POLR1B	1.054269833	-0.1192301	-0.935039733	2
POLR1F	0.910235785	0.160189439	-1.070425224	2
POLR1G	1.016396767	-0.033642582	-0.982754185	2
POLR1H	0.880475497	0.206733792	-1.087209289	2
POLR2I	0.921000336	0.142673759	-1.063674095	2
POLR3B	0.952270917	0.089448377	-1.041719294	2
POLR3D	1.04886623	-0.106211017	-0.942655213	2
POLR3H	1.104908331	-0.261966325	-0.842942006	2
POMK	1.025769937	-0.053704147	-0.97206579	2
POP1	1.035733413	-0.075778227	-0.959955186	2
POP5	1.060847577	-0.135516441	-0.925331136	2
POPDC2	0.954102083	0.086213472	-1.040315555	2
POU2AF1	1.003841628	-0.007728048	-0.99611358	2
POU2F1	0.941862273	0.107576939	-1.049439212	2
POU6F1	0.893429287	0.186797853	-1.080227139	2
PPA1	1.001391131	-0.002788092	-0.998603039	2
PPAN	1.076819329	-0.177376287	-0.899443042	2
PPAN-P2RY11	0.941016691	0.109030859	-1.05004755	2

PPARD	1.042029147	-0.090164899	-0.951864248	2
PPFIA4	0.985314159	0.028751594	-1.014065754	2
PPID	1.078444577	-0.181845776	-0.8965988	2
PPIE	0.961488023	0.073020908	-1.034508931	2
PPIL2	1.081266962	-0.189711385	-0.891555577	2
PPIP5K1	1.025110357	-0.052270955	-0.972839402	2
PPM1G	1.071977049	-0.164304646	-0.907672403	2
PPM1N	1.004029154	-0.008107609	-0.995921545	2
PPOX	0.969355588	0.058702651	-1.028058239	2
PPP1R13L	1.037455083	-0.079677188	-0.957777895	2
PPP1R21	1.077730511	-0.179876803	-0.897853708	2
PPP1R26	0.994184805	0.01153067	-1.005715475	2
PPP2R1A	0.904691205	0.169064381	-1.073755586	2
PPP2R5A	1.093398097	-0.225201847	-0.86819625	2
PPP5C	0.892763327	0.18783457	-1.080597897	2
PPP6R3	0.898242866	0.179265244	-1.07750811	2
PRDM15	1.101294003	-0.250041548	-0.851252455	2
PREB	1.006446538	-0.013020224	-0.993426314	2
PREPL	1.014842393	-0.030376976	-0.984465417	2
PRG2	1.052310174	-0.114472596	-0.937837578	2
PRH1-TAS2R14	1.103608402	-0.257633551	-0.845974851	2
PRIM2	1.025714969	-0.053584579	-0.97213039	2
PRKAB1	0.905244363	0.168183329	-1.073427692	2
PRKCQ	1.023917737	-0.049688004	-0.974229732	2

PRKDC	0.891680202	0.189517919	-1.081198121	2
PRKX	0.943034381	0.105556996	-1.048591377	2
PRMT5	1.077886406	-0.18030596	-0.897580446	2
PRMT6	0.946261149	0.09996835	-1.0462295	2
PRMT7	0.882279118	0.203986528	-1.086265646	2
PRMT9	1.066460614	-0.149829316	-0.916631297	2
PRORP	0.927906855	0.131228605	-1.05913546	2
PROSER1	0.973994104	0.05012641	-1.024120514	2
PRPF3	1.060207974	-0.133910487	-0.926297487	2
PRPF38B	0.922743868	0.1398001	-1.062543969	2
PRPF4	0.932080621	0.124230209	-1.056310831	2
PRPF8	1.01956002	-0.040340958	-0.979219063	2
PRR12	0.969013365	0.059331367	-1.028344732	2
PRR14L	1.013771193	-0.028136209	-0.985634984	2
PRRC1	0.980393064	0.038123523	-1.018516587	2
PRRC2C	1.110185339	-0.280108334	-0.830077005	2
PRSS1	0.92934136	0.128830354	-1.058171714	2
PRSS41	0.936393898	0.116931115	-1.053325013	2
PRX	1.047176122	-0.102201509	-0.944974613	2
PSMA2	0.88043421	0.206796575	-1.087230785	2
PSMA3	0.949011121	0.095172787	-1.044183908	2
PSMA5	0.917804738	0.147913787	-1.065718525	2
PSMC3IP	1.064218746	-0.144064471	-0.920154275	2
PSMD1	0.957494213	0.080183679	-1.037677893	2

PSMD3	0.991112644	0.017543859	-1.008656503	2
PSMD5	0.934797835	0.119640055	-1.05443789	2
PSME3IP1	0.956376531	0.082175855	-1.038552386	2
PTCD2	0.999020657	0.001955818	-1.000976475	2
PTCD3	1.013488437	-0.027546042	-0.985942394	2
PTDSS1	1.053119571	-0.116432517	-0.936687054	2
PTPDC1	0.946081742	0.100280158	-1.0463619	2
PTPMT1	0.902943991	0.171841017	-1.074785008	2
PTPN4	0.901428654	0.174241501	-1.075670155	2
PTPN9	0.896949343	0.181296244	-1.078245588	2
PUM3	1.111395979	-0.284406201	-0.826989777	2
PUS1	0.938495297	0.113349925	-1.051845222	2
PUS3	1.03683451	-0.078268816	-0.958565695	2
PVRIG	0.902057328	0.173246462	-1.07530379	2
PWP1	1.030269144	-0.0635716	-0.966697544	2
PYDC1	0.965727558	0.0653403	-1.031067858	2
PYM1	1.05907458	-0.131076783	-0.927997797	2
QDPR	0.914270541	0.153669144	-1.067939685	2
QRICH1	1.069303666	-0.157236607	-0.912067059	2
QTRT1	0.94728468	0.098187007	-1.045471687	2
R3HCC1L	0.951185902	0.091358576	-1.042544479	2
R3HDM2	1.001994947	-0.004001905	-0.997993042	2
RAB11FIP3	0.918582515	0.146641595	-1.06522411	2
RAB11FIP5	1.101709258	-0.251392556	-0.850316702	2

RAB20	1.097223494	-0.237042409	-0.860181085	2
RAB33B	0.961384673	0.073207143	-1.034591816	2
RAB44	0.975973675	0.04643485	-1.022408525	2
RAD51	0.962439717	0.071303755	-1.033743472	2
RAD51D	1.066454474	-0.149813439	-0.916641035	2
RAD9A	0.973501748	0.051041613	-1.024543362	2
RAE1	0.952610422	0.088849668	-1.04146009	2
RAG1	0.998187955	0.003614293	-1.001802248	2
RALBP1	0.991130821	0.017508435	-1.008639256	2
RALGDS	1.065503965	-0.147361303	-0.918142662	2
RANBP6	1.115878953	-0.300819618	-0.815059335	2
RANGAP1	0.892231915	0.188660899	-1.080892814	2
RASA4	0.93901882	0.112455144	-1.051473964	2
RASGRP2	1.015508561	-0.031774479	-0.983734082	2
RASGRP3	0.892895926	0.187628255	-1.08052418	2
RBBP4	0.90319563	0.171441702	-1.074637333	2
RBBP5	0.952863127	0.088403718	-1.041266845	2
RBBP6	0.913310562	0.155225314	-1.068535876	2
RBBP9	1.061429729	-0.13698247	-0.924447259	2
RBFA	1.115756822	-0.300361351	-0.815395471	2
RBL2	1.029485136	-0.061840519	-0.967644617	2
RBM10	0.896103404	0.182621761	-1.078725165	2
RBM12	1.066589114	-0.150161752	-0.916427362	2
RBM14	0.940651816	0.109657386	-1.050309202	2

RBM15B	0.908076246	0.163657715	-1.071733961	2
RBM17	0.884394462	0.200752866	-1.085147328	2
RBM19	0.932986566	0.122702822	-1.055689388	2
RBM7	0.901081523	0.174790403	-1.075871927	2
RBMS3	0.894325661	0.185400375	-1.079726036	2
RBMX2	1.025618245	-0.053374239	-0.972244005	2
RBPJ	0.869682811	0.22298749	-1.092670301	2
RCC1	1.019569929	-0.040362054	-0.979207876	2
RDH14	0.963230202	0.069874412	-1.033104614	2
REELD1	0.993551795	0.012774025	-1.00632582	2
REG1A	1.053723314	-0.117899088	-0.935824226	2
REPIN1	1.063089751	-0.141185893	-0.921903859	2
REST	1.04220611	-0.090574522	-0.951631588	2
RFC2	1.010264446	-0.020855121	-0.989409325	2
RFX5	1.081750252	-0.191071904	-0.890678348	2
RGP1	1.038971677	-0.083133469	-0.955838208	2
RGPD5	0.949207055	0.09482994	-1.044036996	2
RGPD8	1.066852418	-0.150843618	-0.9160088	2
RHBDD3	0.956169468	0.082544343	-1.038713811	2
RHOF	0.991867053	0.016072149	-1.007939202	2
RHOH	1.111204303	-0.283722127	-0.827482176	2
RIF1	1.112086679	-0.286882766	-0.825203913	2
RIMBP3	1.100914355	-0.24881055	-0.852103805	2
RIMBP3C	1.09970764	-0.244923779	-0.854783861	2

RINT1	1.072796371	-0.166491496	-0.906304875	2
RIOK2	1.083345334	-0.195591513	-0.887753821	2
RIOX2	1.022796184	-0.047268826	-0.975527357	2
RIPK2	0.954269926	0.085916261	-1.040186187	2
RITA1	0.877336941	0.211492986	-1.088829927	2
RMND5B	1.07634244	-0.176072871	-0.900269569	2
RNASEH2A	1.054557217	-0.119931328	-0.934625889	2
RNF114	0.86741837	0.226358258	-1.093776627	2
RNF14	1.063521003	-0.142283536	-0.921237467	2
RNF144B	1.089958434	-0.2148384	-0.875120034	2
RNF145	1.007451388	-0.015073184	-0.992378204	2
RNF157	1.116809485	-0.304333038	-0.812476447	2
RNF220	0.972419548	0.053049131	-1.025468679	2
RNF25	1.108105558	-0.272847241	-0.835258317	2
RNF38	1.057397226	-0.126910946	-0.93048628	2
RNF43	0.983037284	0.033103387	-1.016140671	2
RNH1	1.016825102	-0.034545444	-0.982279657	2
RNPS1	1.071928712	-0.164175938	-0.907752774	2
RPF2	1.027825098	-0.058191499	-0.9696336	2
RPGRIP1	0.956741807	0.081525369	-1.038267176	2
RPL10	0.913142596	0.155497284	-1.06863988	2
RPL12	0.907799462	0.164101161	-1.071900623	2
RPL13	0.906647928	0.165943467	-1.072591395	2
RPL13A	1.026188398	-0.054615161	-0.971573237	2

RPL17	0.973187055	0.051625961	-1.024813015	2
RPL18	0.879467627	0.208265044	-1.087732671	2
RPL18A	0.895580067	0.183440715	-1.079020782	2
RPL19	1.112177255	-0.287208887	-0.824968368	2
RPL21	0.990061118	0.01958992	-1.009651038	2
RPL23A	0.890563042	0.191250569	-1.081813611	2
RPL27	1.09959751	-0.244570992	-0.855026518	2
RPL27A	1.095300469	-0.231046915	-0.864253554	2
RPL29	1.057923127	-0.128213521	-0.929709605	2
RPL3	0.912037824	0.157283832	-1.069321656	2
RPL30	0.99268398	0.014474892	-1.007158872	2
RPL36	0.919583009	0.145002111	-1.06458512	2
RPL5	0.976260076	0.045899172	-1.022159249	2
RPL7A	0.983250554	0.032696915	-1.015947469	2
RPL8	0.978580855	0.041543475	-1.02012433	2
RPLP0	1.009906091	-0.020115686	-0.989790405	2
RPLP1	0.956526719	0.081908469	-1.038435188	2
RPP14	0.975320091	0.047655787	-1.022975878	2
RPP40	1.046420403	-0.100417988	-0.946002415	2
RPRD1A	1.050664738	-0.11050987	-0.940154868	2
RPS15	0.940450698	0.110002506	-1.050453204	2
RPS2	1.026292418	-0.054841829	-0.971450589	2
RPS21	1.095562524	-0.231858696	-0.863703828	2
RPS25	1.070314299	-0.159896606	-0.910417693	2

RPS28	1.118331258	-0.310164832	-0.808166427	2
RPS3	1.051839464	-0.113336035	-0.938503428	2
RPS5	0.989663674	0.020361679	-1.010025353	2
RPS6	1.057033167	-0.126011101	-0.931022066	2
RPS8	1.022454176	-0.046533002	-0.975921174	2
RPSAP58	0.889583193	0.192767268	-1.082350462	2
RRBP1	1.010429894	-0.021196795	-0.989233099	2
RRP7A	0.904765584	0.168945969	-1.073711553	2
RRS1	0.957997134	0.079285518	-1.037282651	2
RSAD1	0.918840296	0.146219499	-1.065059795	2
RSKR	0.870281275	0.222094387	-1.092375662	2
RSL24D1	0.969954352	0.057601312	-1.027555664	2
RTL10	1.066602563	-0.150196558	-0.916406005	2
RTL6	0.908566741	0.162871277	-1.071438017	2
RTN4IP1	0.936332337	0.117035776	-1.053368113	2
RWDD2A	1.074156316	-0.170143346	-0.90401297	2
RWDD2B	1.062539456	-0.139788659	-0.922750797	2
RXRB	0.872263249	0.219129864	-1.091393113	2
S100A12	0.878259294	0.21009717	-1.088356464	2
S100A2	1.040913841	-0.087589988	-0.953323853	2
S100A8	1.046131643	-0.099738007	-0.946393636	2
S1PR1	0.942629609	0.106255162	-1.04888477	2
SAA4	1.087026877	-0.206201378	-0.880825499	2
SAAL1	1.091933166	-0.220756694	-0.871176471	2

SAFB2	0.942905588	0.105779212	-1.0486848	2
SAMD10	0.870397313	0.221921111	-1.092318424	2
SAP30BP	0.942031282	0.107286009	-1.049317291	2
SAP30L	1.023825879	-0.049489511	-0.974336368	2
SAR1B	0.891460695	0.189858648	-1.081319343	2
SARM1	1.111908763	-0.286243097	-0.825665666	2
SARNP	1.116698614	-0.303912373	-0.812786242	2
SARS1	0.995084607	0.00975935	-1.004843958	2
SARS2	0.88024092	0.207090435	-1.087331355	2
SAYSD1	1.045498559	-0.098250079	-0.947248481	2
SBSPON	1.019170841	-0.039512981	-0.97965786	2
SCAI	1.097361695	-0.237476763	-0.859884932	2
SCGB1D2	1.05796806	-0.128324963	-0.929643097	2
SCGB3A2	0.956768709	0.08147744	-1.038246148	2
SCML4	0.931383929	0.125402768	-1.056786697	2
SCO2	1.050099758	-0.109155812	-0.940943945	2
SCRIB	0.984773723	0.029786995	-1.014560718	2
SCRN2	1.045988357	-0.099400898	-0.946587459	2
SCYL1	0.980902586	0.037158968	-1.018061554	2
SDHA	0.975782444	0.046792297	-1.022574742	2
SDHC	0.989153968	0.021350163	-1.010504131	2
SDR39U1	0.965057379	0.066559821	-1.031617199	2
SDS	0.973380711	0.051266421	-1.024647132	2
SEC11C	0.978292373	0.042086366	-1.020378739	2

SEC24B	0.874840044	0.215259971	-1.090100015	2
SEC24C	1.070422415	-0.160182022	-0.910240393	2
SEC61A2	0.985982148	0.027469686	-1.013451834	2
SEL1L	1.018369014	-0.037810543	-0.980558471	2
SELENOF	0.933265244	0.122232377	-1.055497622	2
SEMA4C	0.996759357	0.006450083	-1.00320944	2
SEMA4F	0.911578849	0.158024881	-1.06960373	2
SEMA4G	0.915109918	0.152305991	-1.067415909	2
SEMG1	0.968213362	0.060798966	-1.029012328	2
SEPTIN1	0.908760678	0.162560114	-1.071320792	2
SEPTIN9	0.981128863	0.036730189	-1.017859051	2
SERBP1	0.941394012	0.108382423	-1.049776435	2
SERGEF	1.041449561	-0.088825361	-0.9526242	2
SERHL2	1.107183247	-0.269674482	-0.837508765	2
SERPINB9	1.09878444	-0.241976272	-0.856808168	2
SERPINH1	0.992774765	0.014297157	-1.007071922	2
SESN1	1.057877351	-0.128100013	-0.929777338	2
SETD4	0.935172515	0.119004976	-1.054177491	2
SETDB1	1.07770239	-0.179799431	-0.897902959	2
SFMBT1	1.068404855	-0.154882906	-0.913521949	2
SFMBT2	0.951304958	0.091149213	-1.042454171	2
SFR1	1.035333702	-0.074876726	-0.960456975	2
SFT2D2	1.037807501	-0.080478507	-0.957328993	2
SFXN1	1.023632256	-0.049071324	-0.974560932	2

SFXN4	0.972425249	0.053038571	-1.02546382	2
SGF29	1.092550097	-0.222622899	-0.869927198	2
SGK3	1.011290238	-0.022976454	-0.988313784	2
SGMS2	0.889384927	0.193073826	-1.082458752	2
SGPL1	0.998242625	0.003505534	-1.001748159	2
SGSM2	0.894003884	0.18590231	-1.079906194	2
SGSM3	0.888599562	0.194287028	-1.082886591	2
SH2B3	0.869049277	0.223931901	-1.092981179	2
SH3BP5L	0.930364143	0.127115928	-1.057480072	2
SHFL	0.988088758	0.023411373	-1.011500131	2
SHISA2	0.971163702	0.055371749	-1.026535451	2
SHMT1	0.927748623	0.131492693	-1.059241315	2
SHMT2	0.995748726	0.008449007	-1.004197734	2
SHQ1	1.068837554	-0.156014611	-0.912822943	2
SIAH1	1.018321004	-0.037708755	-0.980612249	2
SIGLEC16	0.938794546	0.11283859	-1.051633136	2
SIGLEC6	0.867719522	0.225910747	-1.093630269	2
SIGLECL1	0.95496291	0.084687897	-1.039650807	2
SIKE1	1.022745556	-0.047159846	-0.97558571	2
SIN3B	0.934955317	0.119373188	-1.054328505	2
SIPA1L3	1.080117807	-0.186492584	-0.893625223	2
SKAP1	1.080163214	-0.186619342	-0.893543872	2
SKIL	1.046902101	-0.101554153	-0.945347949	2
SKP2	0.955870192	0.083076608	-1.0389468	2

SLC12A1	0.883340876	0.202365021	-1.085705897	2
SLC12A3	0.87922857	0.208627832	-1.087856401	2
SLC12A4	1.07887195	-0.183028222	-0.895843728	2
SLC20A1	0.959845672	0.07597483	-1.035820502	2
SLC22A3	1.115642952	-0.299934674	-0.815708278	2
SLC23A1	0.933629869	0.121616413	-1.055246282	2
SLC23A2	1.030820489	-0.064791958	-0.966028531	2
SLC23A3	0.972924557	0.052113022	-1.025037579	2
SLC24A1	0.883387765	0.202293339	-1.085681105	2
SLC25A12	1.028477115	-0.059622105	-0.968855009	2
SLC25A13	0.910033655	0.160514699	-1.070548354	2
SLC25A20	0.877660446	0.211003683	-1.08866413	2
SLC25A22	1.003594258	-0.007227695	-0.996366562	2
SLC25A3	1.021673787	-0.044857274	-0.976816512	2
SLC25A32	1.066605137	-0.150203218	-0.916401918	2
SLC25A35	1.041631585	-0.089245716	-0.952385869	2
SLC25A51	0.938696557	0.113006065	-1.051702621	2
SLC25A6	1.014430176	-0.029513754	-0.984916421	2
SLC26A1	1.033429229	-0.070600256	-0.962828974	2
SLC26A6	1.072429922	-0.165512185	-0.906917736	2
SLC27A2	0.939347554	0.111892754	-1.051240308	2
SLC35A4	0.921484416	0.141876953	-1.063361369	2
SLC35B2	1.111219484	-0.283776257	-0.827443227	2
SLC35C2	1.117075535	-0.305344783	-0.811730752	2

SLC35E2A	1.112036327	-0.28670161	-0.825334717	2
SLC35E2B	1.114175435	-0.294485689	-0.819689746	2
SLC35F2	0.930915459	0.126190228	-1.057105687	2
SLC38A5	1.079368511	-0.184405896	-0.894962615	2
SLC38A7	0.989368838	0.020933634	-1.010302472	2
SLC41A3	1.115759042	-0.300369678	-0.815389365	2
SLC45A3	1.109614705	-0.278101002	-0.831513703	2
SLC48A1	1.118910395	-0.312413409	-0.806496987	2
SLC4A2	0.948615636	0.095864333	-1.044479969	2
SLC4A9	0.921111265	0.142491238	-1.063602503	2
SLC51A	0.88934663	0.193133026	-1.082479656	2
SLC6A16	1.090745376	-0.217186928	-0.873558448	2
SLC7A1	1.098278638	-0.240370757	-0.857907881	2
SLC7A5	0.992720897	0.014402623	-1.00712352	2
SLC7A6OS	0.897388784	0.18060683	-1.077995614	2
SLC9A3	0.922243331	0.140626146	-1.062869477	2
SLFN12L	0.886876191	0.196943014	-1.083819206	2
SMAD4	1.106371016	-0.266903511	-0.839467505	2
SMAD7	0.942276572	0.106863571	-1.049140143	2
SMARCA4	0.898697782	0.178549766	-1.077247547	2
SMCR8	0.907681505	0.164290071	-1.071971576	2
SMIM14	1.066456895	-0.1498197	-0.916637195	2
SMIM19	1.042466964	-0.091178866	-0.951288097	2
SMIM24	0.977530674	0.043517799	-1.021048472	2

SMIM38	1.082362951	-0.192802613	-0.889560338	2
SMPD4	0.88612335	0.198100595	-1.084223945	2
SMYD4	0.9659444	0.064945277	-1.030889677	2
SNAPC3	1.014999399	-0.03070607	-0.984293329	2
SNAPIN	0.993423977	0.013024808	-1.006448785	2
SND1	0.871439259	0.220363607	-1.091802866	2
SNED1	1.070956212	-0.16159364	-0.909362572	2
SNIP1	1.091325102	-0.218925438	-0.872399664	2
SNORC	1.041594963	-0.089161119	-0.952433844	2
SNRNP200	1.071655366	-0.163448733	-0.908206633	2
SNRNP70	1.114300089	-0.294945002	-0.819355087	2
SNRPD3	1.117372293	-0.306477168	-0.810895125	2
SNX1	1.008368163	-0.016951861	-0.991416302	2
SNX17	0.925314819	0.13554353	-1.060858349	2
SNX20	0.865962619	0.228518166	-1.094480785	2
SNX29	0.91455779	0.153202911	-1.067760702	2
SNX8	0.921944933	0.141118188	-1.063063121	2
SOCS5	0.958458871	0.078459944	-1.036918815	2
SP140	1.006970831	-0.014090576	-0.992880255	2
SP2	1.060598101	-0.134889451	-0.92570865	2
SP3	1.104177159	-0.259523029	-0.84465413	2
SP4	0.875509065	0.214252299	-1.089761364	2
SPAG16	1.049027229	-0.106594477	-0.942432751	2
SPATA20	1.046143574	-0.099766085	-0.946377489	2

SPATA5L1	0.9110712	0.158843722	-1.069914922	2
SPHK2	1.066230003	-0.149233267	-0.916996735	2
SPIN2A	0.969584785	0.058281275	-1.02786606	2
SPN	0.928422363	0.130367607	-1.058789971	2
SPPL3	1.015746491	-0.03227436	-0.983472131	2
SPRING1	1.116059738	-0.301499175	-0.814560562	2
SPRY3	0.942082992	0.107196974	-1.049279965	2
SPSB3	1.085377583	-0.201416721	-0.883960862	2
SPTAN1	0.964892042	0.066860369	-1.031752411	2
SPTBN1	0.881291681	0.205491706	-1.086783387	2
SREBF1	1.007052154	-0.014256755	-0.992795399	2
SRP14	0.978428424	0.041830385	-1.020258809	2
SRP19	0.967447686	0.062200814	-1.0296485	2
SRP54	1.059872333	-0.133069718	-0.926802615	2
SRP68	0.948971409	0.095242255	-1.044213665	2
SRPRB	0.879219601	0.208641439	-1.08786104	2
SRR	1.035255499	-0.07470051	-0.960554989	2
SRRM1	1.06292338	-0.140763063	-0.922160316	2
SRSF10	1.061490362	-0.137135402	-0.92435496	2
SRSF11	1.023659932	-0.049131082	-0.97452885	2
SRSF2	0.912948198	0.155811937	-1.068760136	2
SS18L1	1.04760443	-0.103214866	-0.944389564	2
SS18L2	1.07098013	-0.161656987	-0.909323143	2
SSR2	1.06401787	-0.143551112	-0.920466757	2

SSR3	1.105186217	-0.262899187	-0.842287031	2
SSRP1	0.939684469	0.111315939	-1.051000409	2
STAB1	0.938656427	0.11307464	-1.051731067	2
STAG3	0.988054618	0.023477331	-1.01153195	2
STAMBP	1.037458567	-0.079685105	-0.957773462	2
STARD7	1.029348237	-0.061538757	-0.96780948	2
STAT2	0.967420422	0.06225068	-1.029671102	2
STBD1	0.908888658	0.162354711	-1.071243369	2
STIM2	1.060512508	-0.134674511	-0.925837997	2
STK11IP	1.087732725	-0.208265201	-0.879467524	2
STK19	0.975451461	0.047410548	-1.022862008	2
STK25	1.100144865	-0.246327544	-0.853817321	2
STMN1	1.040044871	-0.085591774	-0.954453097	2
STUB1	0.9086957	0.162664381	-1.071360081	2
STX8	0.918138992	0.147367305	-1.065506297	2
STYK1	0.898073906	0.17953082	-1.077604726	2
STYX	1.037466567	-0.079703283	-0.957763284	2
SUCLG2	0.965076305	0.066525408	-1.031601714	2
SUN1	1.056338275	-0.124297764	-0.932040511	2
SUN2	0.972889389	0.052178251	-1.02506764	2
SUN3	1.069482579	-0.157706464	-0.911776115	2
SUPT20H	1.048931409	-0.106366225	-0.942565184	2
SUPT6H	1.077110189	-0.178173032	-0.898937157	2
SUPV3L1	1.072604803	-0.165979297	-0.906625506	2

SUV39H1	1.001232974	-0.002470526	-0.998762448	2
SUZ12	1.017442736	-0.0358496	-0.981593136	2
SYCE1	0.940838675	0.109336594	-1.050175269	2
SYNJ2	1.080253938	-0.18687271	-0.893381228	2
SYTL1	0.917094703	0.149073401	-1.066168104	2
SYTL3	1.051973183	-0.113658669	-0.938314515	2
TAB1	0.890799042	0.19088485	-1.081683892	2
TACC1	0.94521492	0.101784885	-1.046999805	2
TACO1	1.09110185	-0.218255088	-0.872846762	2
TACSTD2	1.052946999	-0.116014046	-0.936932953	2
TAF1B	1.09510857	-0.230453487	-0.864655082	2
TAF3	0.958535472	0.078322894	-1.036858367	2
TAF6L	1.082507529	-0.193211974	-0.889295555	2
TAF9	0.965868081	0.065084332	-1.030952414	2
TAFA2	0.968720235	0.059869456	-1.028589692	2
TANGO6	1.040317381	-0.086217669	-0.954099712	2
TARDBP	1.060601231	-0.134897314	-0.925703917	2
TARS2	1.054947283	-0.120884571	-0.934062712	2
TAS1R3	1.073996561	-0.169712914	-0.904283647	2
TAS2R4	0.910002934	0.160564123	-1.070567057	2
TATDN2	1.105749604	-0.264797799	-0.840951805	2
TBC1D10C	1.010164157	-0.020648097	-0.98951606	2
TBC1D21	0.905023891	0.168534604	-1.073558495	2
TBC1D22A	0.905612579	0.167596316	-1.073208895	2

TBC1D3D	1.055944469	-0.123329233	-0.932615236	2
TBC1D4	1.070450592	-0.160256433	-0.910194159	2
TBC1D8	0.975974472	0.04643336	-1.022407831	2
TBK1	1.108309493	-0.273552624	-0.834756869	2
TBL3	0.978913976	0.040916061	-1.019830037	2
TBRG4	0.932292927	0.12387254	-1.056165466	2
TCEA1	0.945618471	0.101084727	-1.046703198	2
TCEANC2	0.904029911	0.170116413	-1.074146324	2
TCFL5	1.040873986	-0.087498187	-0.953375799	2
TCHP	1.057732206	-0.127740267	-0.929991938	2
TCIRG1	0.908304229	0.163292273	-1.071596501	2
TCL1A	1.117448489	-0.306768581	-0.810679908	2
TCL1B	0.982238357	0.034623969	-1.016862326	2
TCP11L1	1.067166046	-0.151657019	-0.915509027	2
TDG	1.011717712	-0.023862535	-0.987855177	2
TDP1	0.906862286	0.165600839	-1.072463125	2
TECR	0.898456825	0.178928813	-1.077385638	2
TELO2	1.014380091	-0.029408953	-0.984971138	2
TENT4A	0.921324192	0.142140773	-1.063464966	2
TERF1	0.868880038	0.224184008	-1.093064046	2
TERF2	1.028200382	-0.059014506	-0.969185876	2
TESK2	0.963091355	0.070125675	-1.03321703	2
TEX30	0.957357298	0.080428008	-1.037785305	2
TEX43	1.095972943	-0.233133361	-0.862839582	2

TEX53	1.109954042	-0.279293291	-0.83066075	2
TFAP2E	0.871389197	0.220438506	-1.091827703	2
TFAP4	1.056702989	-0.125196321	-0.931506668	2
TFIP11	0.890715072	0.191014992	-1.081730065	2
TFRC	0.952003144	0.089920252	-1.041923396	2
TGDS	1.032686054	-0.068939814	-0.96374624	2
TGFA	0.876167451	0.213259462	-1.089426913	2
TGFBI	0.934069449	0.120873175	-1.054942624	2
TGIF1	1.050717921	-0.110637501	-0.940080419	2
THAP2	1.048033554	-0.104232007	-0.943801547	2
THBS3	1.098813241	-0.242067888	-0.856745353	2
THEM4	0.959011501	0.077470646	-1.036482147	2
THOC1	1.06848568	-0.155094105	-0.913391575	2
THOC6	0.968014135	0.061163984	-1.029178119	2
THRA	1.026467455	-0.055223441	-0.971244014	2
THRAP3	0.915915759	0.150995109	-1.066910868	2
THTPA	0.952272189	0.089446135	-1.041718324	2
THUMPD3	1.080781527	-0.188348908	-0.892432619	2
THYN1	0.868898358	0.224156721	-1.09305508	2
TIAL1	1.035525058	-0.075308135	-0.960216923	2
TIGD1	0.961167554	0.073598233	-1.034765787	2
TIGD7	0.883186828	0.202600478	-1.085787306	2
TIMM10	0.873405567	0.2174165	-1.090822067	2
TIMM13	0.915418968	0.151803507	-1.067222474	2

TIMM17A	1.006952529	-0.014053182	-0.992899347	2
TIMM50	0.881897864	0.204568006	-1.08646587	2
TIMM8A	1.000615177	-0.001231491	-0.999383686	2
TIPIN	0.912788016	0.156071116	-1.068859132	2
TK2	0.872464113	0.21882884	-1.091292953	2
TKTL1	0.877217165	0.211674075	-1.08889124	2
TLE5	1.095947924	-0.233055542	-0.862892382	2
TLNRD1	0.985543873	0.028311028	-1.013854901	2
TLR3	1.067265902	-0.151916276	-0.915349627	2
TLR7	1.074685061	-0.171570724	-0.903114337	2
TLR8	0.985628684	0.028148298	-1.013776982	2
TM4SF1	1.115934333	-0.301027634	-0.814906699	2
TMC2	0.953459231	0.087350717	-1.040809948	2
TMED6	1.069889175	-0.158775921	-0.911113254	2
TMEM101	0.874468359	0.215819278	-1.090287636	2
TMEM102	0.936752848	0.116320571	-1.053073419	2
TMEM106A	0.922117007	0.140834484	-1.062951491	2
TMEM117	0.939599494	0.111461462	-1.051060956	2
TMEM132C	0.99715105	0.005673756	-1.002824806	2
TMEM144	1.050142182	-0.109257372	-0.940884809	2
TMEM147	0.918767631	0.146338506	-1.065106137	2
TMEM150A	0.983429646	0.032355401	-1.015785046	2
TMEM156	0.97198632	0.053851211	-1.025837531	2
TMEM161B	0.905022698	0.168536505	-1.073559203	2

TMEM170A	1.006743174	-0.013625595	-0.993117579	2
TMEM18	1.011694587	-0.023814569	-0.987880018	2
TMEM183A	1.099759005	-0.245088431	-0.854670575	2
TMEM184C	0.929348595	0.128818239	-1.058166835	2
TMEM186	0.965768709	0.065265352	-1.031034061	2
TMEM19	0.886114021	0.19811493	-1.08422895	2
TMEM191B	1.115750491	-0.300337615	-0.815412876	2
TMEM191C	0.876640837	0.212544876	-1.089185713	2
TMEM201	0.904845745	0.168818332	-1.073664077	2
TMEM204	0.994289922	0.01132398	-1.005613901	2
TMEM208	1.069135416	-0.156795162	-0.912340254	2
TMEM220	1.094381309	-0.228212342	-0.866168967	2
TMEM223	1.021366489	-0.044198655	-0.977167834	2
TMEM237	0.887648526	0.195753779	-1.083402304	2
TMEM245	1.011745876	-0.023920958	-0.987824919	2
TMEM259	0.953249313	0.087721701	-1.040971013	2
TMEM260	0.959986213	0.075722516	-1.035708729	2
TMEM265	1.038566018	-0.082206963	-0.956359055	2
TMEM267	0.919653447	0.144886558	-1.064540005	2
TMEM30B	1.10583845	-0.265098113	-0.840740337	2
TMEM38A	0.870819468	0.221290418	-1.092109887	2
TMEM50B	0.972324131	0.053225864	-1.025549995	2
TMEM62	1.096428385	-0.234552596	-0.861875789	2
TMEM81	0.884915147	0.199954978	-1.084870125	2

TMEM94	0.964900063	0.066845792	-1.031745855	2
TMX2	1.049723937	-0.10825695	-0.941466987	2
TNFAIP8L1	0.893495689	0.18669441	-1.080190099	2
TNFRSF10B	0.980178006	0.038530243	-1.018708249	2
TNFRSF10D	0.998891923	0.002212483	-1.001104406	2
TNFRSF4	0.876785124	0.212326951	-1.089112075	2
TNFSF11	0.996776612	0.006415903	-1.003192515	2
TNIP2	0.927913473	0.131217557	-1.05913103	2
TNKS	0.940894761	0.109240282	-1.050135043	2
TNRC6B	0.884752155	0.200204827	-1.084956982	2
TOMM22	1.052286447	-0.114415249	-0.937871198	2
TOMM34	1.071903213	-0.164108056	-0.907795157	2
TOMM40	1.061691857	-0.137643943	-0.924047914	2
TOMM40L	1.102398553	-0.253645798	-0.848752755	2
TOR3A	0.970260846	0.057036911	-1.027297757	2
TPGS2	0.980199604	0.038489407	-1.018689011	2
TPMT	1.063624702	-0.142547827	-0.921076875	2
TRA2A	1.117997358	-0.308875849	-0.809121509	2
TRAF1	0.928008646	0.131058669	-1.059067315	2
TRAF3IP3	1.042690496	-0.091697256	-0.95099324	2
TRAF6	1.006956176	-0.014060634	-0.992895542	2
TRAP1	1.01745066	-0.035866349	-0.981584311	2
TRAPPC12	1.076917599	-0.177645324	-0.899272275	2
TRAPPC2L	1.079040898	-0.183496495	-0.895544403	2

TRAPPC3	1.006971023	-0.014090968	-0.992880055	2
TRAPPC6A	0.960982645	0.07393114	-1.034913785	2
TREML4	1.013045645	-0.026622947	-0.986422698	2
TRIB2	0.923204414	0.139039289	-1.062243703	2
TRIM16	1.116009574	-0.301310469	-0.814699105	2
TRIM22	1.08746202	-0.207472528	-0.879989492	2
TRIM26	1.09328784	-0.224865622	-0.868422219	2
TRIM37	0.971754615	0.054279824	-1.026034439	2
TRIM39	0.959541231	0.076521097	-1.036062328	2
TRIM56	0.904536685	0.169310325	-1.07384701	2
TRIM6	1.040634814	-0.086947602	-0.953687212	2
TRIM64	0.881571785	0.205065013	-1.086636797	2
TRIM64B	0.979520923	0.039771475	-1.019292397	2
TRIM65	1.087911207	-0.208788624	-0.879122583	2
TRIM66	1.117688351	-0.307687724	-0.810000627	2
TRIM69	1.034257593	-0.07245653	-0.961801062	2
TRIT1	1.088909189	-0.211727103	-0.877182086	2
TRMT10B	1.089523865	-0.213547031	-0.875976834	2
TRMT2A	0.904886715	0.168753089	-1.073639804	2
TRMT2B	1.088687052	-0.2110713	-0.877615752	2
TRMT5	0.951585597	0.090655467	-1.042241065	2
TRNAU1AP	0.963157826	0.070005398	-1.033163224	2
TRPM6	0.948823656	0.095500667	-1.044324323	2
TRPV5	0.866284667	0.228040815	-1.094325483	2

TSEN15	1.003500188	-0.007037521	-0.996462667	2
TSHZ1	0.931942978	0.124462009	-1.056404986	2
TSNARE1	0.916129751	0.150646641	-1.066776391	2
TSPAN17	0.934592086	0.119988577	-1.054580663	2
TSPAN4	0.883761569	0.201721668	-1.085483237	2
TSPYL2	0.966968325	0.063077082	-1.030045407	2
TSPYL4	0.982946227	0.033276861	-1.016223088	2
TSSK4	0.908749744	0.16257766	-1.071327404	2
TTC12	0.976973495	0.044563055	-1.02153655	2
TTC17	0.949160702	0.094911064	-1.044071766	2
TTC21A	0.95071062	0.092193796	-1.042904416	2
TTC30B	1.085125106	-0.20068885	-0.884436256	2
TTC31	1.003374704	-0.006783925	-0.996590779	2
TTF2	1.079370955	-0.184412687	-0.894958268	2
TTLL5	1.10017551	-0.246426127	-0.853749384	2
TUBB	0.975693197	0.046959056	-1.022652254	2
TUBB8B	1.096267124	-0.234049506	-0.862217618	2
TUBE1	1.015026712	-0.030763337	-0.984263375	2
TUBG1	0.951561634	0.09069764	-1.042259274	2
TUBG2	0.922661886	0.139935456	-1.062597342	2
TUBGCP2	1.031794018	-0.066952883	-0.964841135	2
TWNK	1.048449228	-0.10521905	-0.943230178	2
TXK	0.95959595	0.076422944	-1.036018893	2
TXLNA	1.091968244	-0.220862582	-0.871105662	2

TXN	0.90487071	0.168778577	-1.073649287	2
TXNDC15	1.104431382	-0.260370711	-0.844060671	2
TXNDC9	0.990034222	0.019642173	-1.009676395	2
TYSND1	1.028790861	-0.060311713	-0.968479149	2
TYW1B	0.953270346	0.087684538	-1.040954884	2
U2AF1	1.008118505	-0.016439718	-0.991678787	2
UAP1L1	0.987474874	0.024596462	-1.012071335	2
UBA3	1.038283067	-0.081561592	-0.956721475	2
UBA6	0.889928618	0.192232906	-1.082161524	2
UBAC1	1.112776351	-0.289373953	-0.823402398	2
UBE2D1	0.965176525	0.06634316	-1.031519685	2
UBE2G2	1.090115418	-0.21530586	-0.874809558	2
UBE2I	0.998166195	0.003657576	-1.001823771	2
UBE4A	0.932970728	0.122729551	-1.055700279	2
UBIAD1	1.045734759	-0.098804755	-0.946930004	2
UBN2	0.935597134	0.11828462	-1.053881754	2
UBP1	0.952200303	0.089572844	-1.041773147	2
UBQLN2	0.952694841	0.088700724	-1.041395565	2
UBQLNL	1.005413166	-0.010915698	-0.994497468	2
UBR4	0.908183406	0.163485964	-1.071669371	2
UBXN8	1.068198318	-0.154343626	-0.913854692	2
UCK1	0.953486278	0.087302904	-1.040789182	2
UCP1	0.935451866	0.11853114	-1.053983006	2
UCP3	1.064768475	-0.145472011	-0.919296464	2

UFL1	0.96023847	0.075269423	-1.035507892	2
UGT2B11	1.103552479	-0.257448284	-0.846104194	2
UGT2B17	1.078654693	-0.182426744	-0.896227949	2
UHRF1	0.869474375	0.223298323	-1.092772698	2
ULK3	0.937320109	0.115354723	-1.052674832	2
UNC119B	1.002546052	-0.0051117	-0.997434351	2
UNC45A	0.933848104	0.121247512	-1.055095616	2
UNC93B1	1.044558549	-0.096048025	-0.948510524	2
UNG	0.96881962	0.059687063	-1.028506683	2
UNK	0.986224227	0.027004538	-1.013228765	2
UPRT	0.955206519	0.084255601	-1.03946212	2
UQCC3	1.10276709	-0.254856043	-0.847911046	2
URGCP	0.904247731	0.169770041	-1.074017772	2
URGCP-MRPS24	1.04353213	-0.093653323	-0.949878807	2
USP14	1.010457338	-0.021253487	-0.989203851	2
USP19	1.068589163	-0.15536464	-0.913224523	2
USP21	1.046291996	-0.100115508	-0.946176487	2
USP25	0.891233674	0.190210895	-1.081444568	2
USP3	0.939553797	0.111539707	-1.051093505	2
USP36	1.035469555	-0.075182972	-0.960286583	2
USP41	0.903332244	0.171224834	-1.074557078	2
USP46	0.890566468	0.191245261	-1.081811729	2
USP5	0.952304152	0.08938979	-1.041693942	2
USP7	1.04574966	-0.098839766	-0.946909895	2

UST	0.939542854	0.111558444	-1.051101298	2
UTP18	0.992272546	0.015279797	-1.007552343	2
UTP25	1.031120383	-0.065456795	-0.965663588	2
UXT	1.039359708	-0.084021101	-0.955338606	2
VAC14	1.00579335	-0.011689181	-0.994104169	2
VAV1	1.029942094	-0.062848871	-0.967093223	2
VNN3	0.905145065	0.168341559	-1.073486624	2
VPS11	0.933842681	0.121256682	-1.055099363	2
VPS13D	1.078288659	-0.181415136	-0.896873523	2
VPS18	0.947579559	0.097673023	-1.045252582	2
VPS33A	0.876037799	0.213455069	-1.089492868	2
VPS4A	1.077525446	-0.179312886	-0.89821256	2
VPS53	1.065787393	-0.148091247	-0.917696145	2
WASHC1	1.043211519	-0.092907387	-0.950304132	2
WASHC3	1.036920573	-0.07846393	-0.958456643	2
WDCP	1.077614633	-0.179558062	-0.898056571	2
WDFY2	1.107655909	-0.27129691	-0.836358999	2
WDFY4	0.97783145	0.042952907	-1.020784357	2
WDR12	1.101510498	-0.250745304	-0.850765194	2
WDR20	0.957930891	0.079403883	-1.037334773	2
WDR3	1.066134711	-0.148987176	-0.917147535	2
WDR54	1.052612698	-0.115204316	-0.937408382	2
WDR55	0.973210995	0.051581524	-1.024792519	2
WDR6	1.075739941	-0.174431276	-0.901308665	2

WDR61	0.868537486	0.22469406	-1.093231546	2
WDR81	0.971624589	0.054520236	-1.026144825	2
WNT10B	1.035284511	-0.074765878	-0.960518633	2
WRAP53	1.091457329	-0.219322976	-0.872134354	2
WRAP73	0.902523168	0.172508364	-1.075031532	2
XAB2	1.04671005	-0.101100891	-0.945609159	2
XPA	1.026433235	-0.055148816	-0.971284419	2
XPNPEP3	1.088517804	-0.210572308	-0.877945496	2
XPO5	0.961121308	0.073681508	-1.034802816	2
XXYLT1	1.107185858	-0.269683424	-0.837502434	2
YARS1	0.965406899	0.065924056	-1.031330955	2
YBEY	0.917078271	0.149100218	-1.066178489	2
YDJC	1.03931096	-0.083909516	-0.955401445	2
YIF1A	0.875065888	0.214919942	-1.08998583	2
YIPF2	1.001674172	-0.003356795	-0.998317377	2
YIPF5	1.07598964	-0.175110925	-0.900878715	2
YJU2B	0.948622789	0.095851831	-1.04447462	2
YKT6	1.114653291	-0.29624997	-0.818403321	2
YPEL1	0.875646255	0.214045516	-1.08969177	2
ZADH2	0.947937948	0.097047869	-1.044985817	2
ZBTB21	1.02029437	-0.041906276	-0.978388094	2
ZBTB22	1.002192577	-0.004399673	-0.997792905	2
ZBTB33	1.072419472	-0.165484288	-0.906935184	2
ZBTB4	1.094947756	-0.229956856	-0.864990899	2

ZBTB43	0.929784223	0.128088474	-1.057872697	2
ZBTB48	1.109378159	-0.277272291	-0.832105869	2
ZBTB49	1.101645618	-0.251185197	-0.850460421	2
ZBTB5	0.984126473	0.03102501	-1.015151483	2
ZBTB9	1.118185571	-0.309601761	-0.80858381	2
ZC3H12D	0.99367826	0.012525805	-1.006204065	2
ZC3H3	0.959899758	0.07587774	-1.035777497	2
ZC3H4	1.097867425	-0.239070294	-0.858797131	2
ZC3H6	1.114004442	-0.293856676	-0.820147766	2
ZC3H7B	1.027397072	-0.057254189	-0.970142883	2
ZCCHC3	1.02444669	-0.050832259	-0.973614431	2
ZCCHC9	1.106775904	-0.268282141	-0.838493763	2
ZDBF2	1.11579463	-0.300503146	-0.815291484	2
ZDHHC12	1.025141256	-0.052338019	-0.972803237	2
ZDHHC16	1.03014419	-0.063295369	-0.966848822	2
ZDHHC23	0.986563461	0.026352182	-1.012915643	2
ZFP2	0.889539804	0.192834366	-1.08237417	2
ZFP30	0.886582269	0.197395146	-1.083977415	2
ZFP57	0.893849611	0.186142849	-1.07999246	2
ZFP69	0.913185931	0.155427126	-1.068613056	2
ZFP90	0.890048258	0.192047746	-1.082096004	2
ZFYVE21	0.999349676	0.001299381	-1.000649057	2
ZGPAT	1.018547853	-0.038189854	-0.980357999	2
ZGRF1	0.942471574	0.106527572	-1.048999146	2

ZHX2	1.055771642	-0.122904735	-0.932866907	2
ZIK1	1.03241946	-0.068345305	-0.964074155	2
ZKSCAN4	0.926934759	0.132849621	-1.05978438	2
ZKSCAN5	0.99550878	0.008922729	-1.004431508	2
ZKSCAN8	0.997406378	0.005167219	-1.002573597	2
ZMYM3	1.021860094	-0.045256919	-0.976603175	2
ZNF101	1.060207618	-0.133909594	-0.926298023	2
ZNF12	0.90094443	0.175007082	-1.075951512	2
ZNF124	0.922539718	0.140137118	-1.062676836	2
ZNF133	1.031986865	-0.06738187	-0.964604994	2
ZNF134	1.045492705	-0.098236336	-0.947256368	2
ZNF138	1.103731326	-0.258041106	-0.84569022	2
ZNF142	1.016443835	-0.03374173	-0.982702105	2
ZNF155	1.115780093	-0.300448619	-0.815331474	2
ZNF180	1.116363686	-0.302644974	-0.813718711	2
ZNF182	0.971842259	0.054117727	-1.025959987	2
ZNF2	0.927555762	0.131814456	-1.059370218	2
ZNF20	1.070007092	-0.15908651	-0.910920581	2
ZNF200	1.09639051	-0.234434379	-0.861956131	2
ZNF202	1.06458064	-0.144990634	-0.919590005	2
ZNF208	1.039128661	-0.08349241	-0.955636251	2
ZNF211	0.935342938	0.118715938	-1.054058876	2
ZNF212	0.965740447	0.065316826	-1.031057273	2
ZNF221	0.94421495	0.103517052	-1.047732002	2

ZNF224	0.993395252	0.013081154	-1.006476406	2
ZNF232	1.048803493	-0.106061663	-0.942741829	2
ZNF234	1.021421482	-0.044316468	-0.977105014	2
ZNF239	0.937814228	0.114512423	-1.05232665	2
ZNF248	0.916377237	0.150243441	-1.066620677	2
ZNF250	1.099765843	-0.245110354	-0.854655489	2
ZNF251	0.999988203	0.0000236	-1.000011797	2
ZNF26	1.052149212	-0.114083676	-0.938065536	2
ZNF263	0.97849437	0.041706275	-1.020200644	2
ZNF264	1.098271978	-0.240349662	-0.857922317	2
ZNF266	1.102639203	-0.254435631	-0.848203571	2
ZNF273	0.997921678	0.004143766	-1.002065444	2
ZNF275	1.011041768	-0.022461977	-0.988579791	2
ZNF28	0.935876266	0.117810713	-1.053686979	2
ZNF286A	1.051962413	-0.113632675	-0.938329738	2
ZNF30	0.953925365	0.086526269	-1.040451634	2
ZNF300	1.100805999	-0.248459934	-0.852346065	2
ZNF329	1.051488624	-0.112490448	-0.938998175	2
ZNF330	1.057747293	-0.127777651	-0.929969642	2
ZNF333	0.978194945	0.042269619	-1.020464565	2
ZNF34	1.11802319	-0.308975379	-0.809047811	2
ZNF343	1.083027676	-0.194687816	-0.88833986	2
ZNF347	1.07512133	-0.172751682	-0.902369648	2
ZNF383	1.01621774	-0.033265602	-0.982952138	2

ZNF384	1.040835847	-0.087410354	-0.953425493	2
ZNF410	0.903097321	0.171597729	-1.074695049	2
ZNF416	1.116594637	-0.303518372	-0.813076266	2
ZNF426	1.099358823	-0.243807488	-0.855551335	2
ZNF433	0.964334457	0.067873034	-1.032207492	2
ZNF438	1.101491076	-0.250682115	-0.850808961	2
ZNF439	0.960784581	0.074287565	-1.035072146	2
ZNF44	1.116120337	-0.301727287	-0.814393049	2
ZNF443	0.8794622	0.208273282	-1.087735482	2
ZNF445	1.015911073	-0.032620372	-0.983290701	2
ZNF486	1.010777525	-0.021915293	-0.988862232	2
ZNF496	1.078780154	-0.182773988	-0.896006166	2
ZNF500	1.080008079	-0.186186415	-0.893821664	2
ZNF506	1.038975624	-0.083142491	-0.955833133	2
ZNF511	0.896769671	0.181577956	-1.078347627	2
ZNF514	0.890655256	0.191107688	-1.081762944	2
ZNF518B	0.891831988	0.189282226	-1.081114214	2
ZNF526	0.890886338	0.190749529	-1.081635867	2
ZNF527	0.908490085	0.162994234	-1.071484319	2
ZNF540	0.954944222	0.084721049	-1.039665271	2
ZNF543	1.080891092	-0.188656071	-0.892235021	2
ZNF546	1.105533294	-0.264067666	-0.841465628	2
ZNF547	0.974278424	0.049597376	-1.0238758	2
ZNF548	0.963091069	0.070126192	-1.033217261	2

ZNF549	1.033201447	-0.070090838	-0.963110609	2
ZNF554	0.892541585	0.188179473	-1.080721058	2
ZNF557	0.937929734	0.114315393	-1.052245126	2
ZNF558	1.111267966	-0.283949181	-0.827318785	2
ZNF561	0.938608565	0.113156422	-1.051764986	2
ZNF57	0.955611451	0.083536475	-1.039147926	2
ZNF576	1.010708569	-0.021772707	-0.988935862	2
ZNF585A	1.050438113	-0.109966329	-0.940471784	2
ZNF585B	1.052314125	-0.114482145	-0.937831979	2
ZNF587	1.001161973	-0.002328012	-0.998833962	2
ZNF587B	1.058596781	-0.129886771	-0.92871001	2
ZNF589	1.069850474	-0.158674027	-0.911176447	2
ZNF594	1.097480883	-0.237851742	-0.859629141	2
ZNF597	1.035892118	-0.076136554	-0.959755565	2
ZNF611	1.061665118	-0.13757643	-0.924088688	2
ZNF613	0.965795752	0.065216094	-1.031011846	2
ZNF615	0.946501231	0.09955089	-1.046052121	2
ZNF619	1.033204755	-0.070098233	-0.963106522	2
ZNF620	1.100520878	-0.247538871	-0.852982007	2
ZNF622	0.955981251	0.082879133	-1.038860384	2
ZNF623	0.986374727	0.026715198	-1.013089925	2
ZNF629	1.057396356	-0.126908792	-0.930487563	2
ZNF644	0.894664813	0.184871006	-1.079535819	2
ZNF646	1.044161977	-0.095121594	-0.949040383	2

ZNF652	0.970511499	0.05657501	-1.027086509	2
ZNF660-ZNF197	0.984971632	0.029408008	-1.01437964	2
ZNF665	1.068649238	-0.155521761	-0.913127477	2
ZNF675	0.981070643	0.036840535	-1.017911179	2
ZNF681	0.910499446	0.159764967	-1.070264413	2
ZNF688	0.911481382	0.158182161	-1.069663543	2
ZNF691	1.114804602	-0.29681062	-0.817993982	2
ZNF706	1.078330029	-0.181529361	-0.896800668	2
ZNF707	1.044437962	-0.095766162	-0.9486718	2
ZNF720	1.077403957	-0.178979119	-0.898424837	2
ZNF737	1.045020845	-0.097129916	-0.947890929	2
ZNF738	1.018939845	-0.039022057	-0.979917788	2
ZNF74	1.070850941	-0.16131493	-0.909536011	2
ZNF740	0.876420125	0.212878122	-1.089298247	2
ZNF747	1.036666319	-0.077887694	-0.958778625	2
ZNF75D	0.984664878	0.029995341	-1.014660218	2
ZNF772	1.010253212	-0.020831927	-0.989421285	2
ZNF784	0.902855753	0.171980992	-1.074836745	2
ZNF79	1.019306958	-0.039802444	-0.979504513	2
ZNF805	0.999760678	0.000478472	-1.00023915	2
ZNF813	1.069595356	-0.158002865	-0.911592491	2
ZNF831	0.901692491	0.173824059	-1.07551655	2
ZNF837	0.990634717	0.018474567	-1.009109284	2
ZNF841	1.048018424	-0.104196114	-0.94382231	2

ZNF846	1.002673888	-0.005369398	-0.997304489	2
ZNF852	1.056413886	-0.124483925	-0.931929961	2
ZNF862	1.114509826	-0.295719284	-0.818790543	2
ZNF891	1.005145021	-0.010370708	-0.994774313	2
ZNF91	0.911807261	0.157656179	-1.069463439	2
ZNF92	1.101707862	-0.251388007	-0.850319855	2
ZNF93	0.928938714	0.129504253	-1.058442967	2
ZNFX1	0.982571965	0.033989421	-1.016561386	2
ZPR1	0.885671751	0.198794207	-1.084465958	2
ZRANB2	0.980603065	0.037726139	-1.018329204	2
ZSCAN2	1.035920449	-0.076200542	-0.959719907	2
ZSCAN22	0.893866536	0.186116463	-1.079982999	2
ZSCAN30	0.869095139	0.223863571	-1.09295871	2
ZSCAN9	1.006623326	-0.013380944	-0.993242382	2
ZSWIM1	1.086618564	-0.205011968	-0.881606596	2
ZW10	0.90932866	0.161648123	-1.070976784	2
ZWINT	1.101547285	-0.250865017	-0.850682268	2
ZZEF1	0.941279347	0.108579536	-1.049858883	2
AAMDC	1.059628989	-0.927167995	-0.132460995	3
AAMP	1.148146628	-0.467680219	-0.680466409	3
ABCD2	1.115581687	-0.815876342	-0.299705346	3
ABCD4	1.152778733	-0.518718799	-0.634059934	3
ABHD10	1.153490344	-0.622516561	-0.530973783	3
ABHD18	1.140312818	-0.727525426	-0.412787392	3

ABLIM1	1.122379039	-0.796134128	-0.326244911	3
ABRAXAS2	1.152821675	-0.633433983	-0.519387692	3
ACAD10	1.108463441	-0.834377397	-0.274086045	3
ACAD11	0.973302992	-1.024713727	0.051410735	3
ACAD8	0.990677968	-1.009068361	0.018390393	3
ACIN1	1.146284063	-0.452623835	-0.693660228	3
ACLY	1.113618561	-0.821176971	-0.29244159	3
ACOD1	1.152638115	-0.516577687	-0.636060429	3
ACOT8	1.127313339	-0.347153835	-0.780159504	3
ACSF2	1.143328932	-0.431667268	-0.711661664	3
ACSF3	1.154109269	-0.545057025	-0.609052244	3
ACSL6	1.061162482	-0.924853533	-0.136308949	3
ACSM3	1.152063354	-0.643578116	-0.508485238	3
ACTL7B	1.127141633	-0.346398527	-0.780743107	3
ACYP1	1.153232493	-0.627025702	-0.52620679	3
ADAT1	1.131956249	-0.368478027	-0.763478222	3
ADAT2	1.154005156	-0.611702368	-0.542302788	3
ADCY6	1.074890319	-0.902764337	-0.172125981	3
ADGRE1	1.153564169	-0.621136121	-0.532428048	3
ADGRG7	1.151445452	-0.500689217	-0.650756235	3
AEBP2	1.153207838	-0.525773194	-0.627434643	3
AGAP6	1.123197088	-0.329604521	-0.793592567	3
AGAP9	1.037546352	-0.957661745	-0.079884607	3
AGL	0.962560293	-1.033646204	0.071085911	3

AGMAT	0.901055034	-1.075887309	0.174832275	3
AGO3	0.957021598	-1.038048328	0.08102673	3
AGRN	1.130983031	-0.363853134	-0.767129898	3
AHCTF1	1.14747703	-0.46205884	-0.68541819	3
AIMP1	1.122089192	-0.797025281	-0.325063911	3
AIRE	1.145198695	-0.700622635	-0.44457606	3
AK6	1.152970304	-0.521762142	-0.631208163	3
AKAP5	1.141858607	-0.422204081	-0.719654526	3
AKAP8	0.898235692	-1.077512214	0.179276522	3
AKR1C3	1.15194195	-0.506889084	-0.645052866	3
ALCAM	1.154631241	-0.566360119	-0.588271122	3
ALDH16A1	1.151901982	-0.645530935	-0.506371046	3
ALDH18A1	1.068051178	-0.914091387	-0.153959792	3
ALDH1A2	1.016096069	-0.983086543	-0.033009526	3
ALDH6A1	1.023391996	-0.97483919	-0.048552806	3
ALG1	1.129943825	-0.770933594	-0.359010232	3
ALX3	1.019914185	-0.97881879	-0.041095396	3
AMELY	1.094146443	-0.866655246	-0.227491197	3
AMIGO1	1.152814259	-0.633542613	-0.519271646	3
AMN	1.15321805	-0.627265683	-0.525952367	3
AMY2A	1.129314171	-0.356121411	-0.77319276	3
ANAPC10	0.993846345	-1.006042099	0.012195754	3
ANAPC13	1.12437289	-0.789868686	-0.334504205	3
ANAPC15	1.131947079	-0.368434031	-0.763513049	3

ANKEF1	1.137572202	-0.397184486	-0.740387715	3
ANKFY1	1.112898991	-0.823080095	-0.289818896	3
ANKRD2	1.149365739	-0.670697442	-0.478668298	3
ANKRD40	1.139760935	-0.730219976	-0.409540959	3
ANKS1B	1.125799604	-0.340567983	-0.785231622	3
ANO9	1.135798464	-0.74809742	-0.387701043	3
ANTXR1	1.151073933	-0.654730404	-0.496343529	3
ANXA2R	1.031993141	-0.964597304	-0.067395837	3
AP1S1	1.153984119	-0.541771481	-0.612212638	3
AP3B1	1.145898109	-0.696189235	-0.449708874	3
AP3M2	1.154503332	-0.558770781	-0.595732551	3
AP3S2	1.051765486	-0.93860786	-0.113157627	3
AP4S1	1.121175286	-0.799804037	-0.321371249	3
AP5M1	1.139027711	-0.733714661	-0.40531305	3
AP5S1	1.088251742	-0.878462692	-0.20978905	3
APAF1	1.071237156	-0.908898924	-0.162338232	3
APBA3	1.137594252	-0.39730518	-0.740289073	3
APBB3	1.154209718	-0.547951038	-0.606258679	3
API5	1.113444653	-0.821638828	-0.291805825	3
APOBEC3F	1.154651439	-0.568103972	-0.586547467	3
APOL3	1.123557572	-0.33109765	-0.792459922	3
AQP11	1.14168265	-0.720576242	-0.421106407	3
AQR	1.146801788	-0.690166663	-0.456635125	3
ARGLU1	1.048836079	-0.942696846	-0.106139233	3

ARHGAP17	1.130409118	-0.361166767	-0.76924235	3
ARHGAP19	1.147874249	-0.682512129	-0.46536212	3
ARHGEF17	1.152269408	-0.640991536	-0.511277872	3
ARHGEF19	1.055352608	-0.933475706	-0.121876902	3
ARID1B	1.146695955	-0.455805261	-0.690890694	3
ARID2	1.125740791	-0.340315336	-0.785425454	3
ARL13A	1.151663062	-0.648317004	-0.503346058	3
ARL14EP	1.134404605	-0.753869503	-0.380535103	3
ARMC12	1.146467154	-0.454028717	-0.692438437	3
ASB1	1.101675668	-0.850392573	-0.251283095	3
ASB7	1.097424695	-0.859749772	-0.237674922	3
ASXL1	1.133453404	-0.375775044	-0.75767836	3
ASXL2	1.074356116	-0.9036739	-0.170682216	3
ATAD1	0.889872321	-1.082192341	0.19232002	3
ATAD3A	0.988127727	-1.011463802	0.023336074	3
ATAD3B	1.15183396	-0.50549755	-0.64633641	3
ATAD5	0.946858644	-1.045787629	0.098928984	3
ATG14	1.124324892	-0.790022389	-0.334302503	3
ATMIN	1.145075825	-0.443692983	-0.701382842	3
ATP10A	1.070227668	-0.910559641	-0.159668027	3
ATP10D	1.125287559	-0.338376235	-0.786911324	3
ATP1A1	1.123305226	-0.79325362	-0.330051606	3
ATP2B1	1.120732625	-0.319599231	-0.801133394	3
ATP2B4	1.105006438	-0.842711039	-0.262295399	3

ATP5MC2	0.954588953	-1.039939967	0.085351014	3
ATP6V1D	1.119619585	-0.315189703	-0.804429882	3
ATP6V1G1	1.08471749	-0.885201238	-0.199516253	3
ATPSCKMT	0.928027819	-1.059054475	0.131026656	3
ATXN7	0.947206298	-1.045529868	0.098323571	3
ATXN7L3	1.154245132	-0.60520516	-0.549039972	3
AUH	1.14639481	-0.45347187	-0.692922941	3
AXIN1	1.137876327	-0.739020787	-0.39885554	3
B3GLCT	0.919608017	-1.064569104	0.144961087	3
B3GNT4	1.004960038	-0.994965035	-0.009995003	3
BACE2	1.153101759	-0.523946229	-0.62915553	3
BAG2	1.142672454	-0.427375307	-0.715297147	3
BAG4	1.14817957	-0.467963651	-0.680215919	3
BANF1	0.9064373	-1.072717291	0.166279991	3
BAZ1A	1.032813	-0.963589886	-0.069223114	3
BBS10	1.154685105	-0.5825127	-0.572172406	3
BCDIN3D	1.148979774	-0.47507103	-0.673908744	3
BCL2	1.116587034	-0.813097454	-0.30348958	3
BCL2L11	1.129452335	-0.772699887	-0.356752448	3
BCL2L14	1.153125247	-0.628779701	-0.524345546	3
BCL2L15	1.151369084	-0.651591882	-0.499777202	3
BCL7B	1.148719787	-0.676007016	-0.472712772	3
BCS1L	1.153711123	-0.53546736	-0.618243763	3
BDH1	1.152849323	-0.633027034	-0.51982229	3

BDH2	1.140777315	-0.725212228	-0.415565087	3
BHLHB9	1.083499437	-0.887468864	-0.196030574	3
BICD1	1.126356559	-0.342972292	-0.783384267	3
BICRAL	1.052804892	-0.937135199	-0.115669694	3
BLM	1.133934787	-0.755763378	-0.378171409	3
BLOC1S5	1.097832983	-0.858871417	-0.238961567	3
BNIP1	1.122585246	-0.327088065	-0.795497181	3
BNIPL	1.154628253	-0.566124943	-0.58850331	3
BOD1	1.153968645	-0.612583136	-0.541385509	3
BOLA1	0.915414022	-1.067225572	0.15181155	3
BOLA2	1.142751541	-0.427886443	-0.714865097	3
BOLA2-SMG1P6	1.082079943	-0.890077574	-0.19200237	3
BOLA2B	0.942826083	-1.048742439	0.105916356	3
BORA	1.150375394	-0.488716081	-0.661659312	3
BORCS5	1.074282652	-0.903798642	-0.17048401	3
BPY2	1.154700538	-0.577350269	-0.577350269	3
BPY2B	1.154700538	-0.577350269	-0.577350269	3
BPY2C	1.154700538	-0.577350269	-0.577350269	3
BRD1	1.135049451	-0.383821003	-0.751228448	3
BRD7	1.113610516	-0.821198362	-0.292412154	3
BRIX1	1.068998621	-0.912562085	-0.156436536	3
BSPH1	1.154700538	-0.577350269	-0.577350269	3
BTBD10	1.150437789	-0.489372106	-0.661065682	3
BTBD2	1.019372771	-0.979430321	-0.03994245	3

BTD	0.959763239	-1.03588602	0.076122782	3
BTF3L4	1.146371014	-0.693081812	-0.453289202	3
BTLA	1.148024985	-0.466639425	-0.68138556	3
BTN2A2	1.149767154	-0.482543887	-0.667223267	3
C10orf88	1.014280626	-0.985079754	-0.029200872	3
C11orf1	1.153505446	-0.53126767	-0.622237776	3
C11orf24	1.112212608	-0.824876346	-0.287336262	3
C11orf94	1.135831677	-0.38787473	-0.747956947	3
C12orf57	1.147430028	-0.461673737	-0.685756292	3
C17orf100	1.141140239	-0.723374625	-0.417765614	3
C18orf54	1.141414546	-0.419447145	-0.721967401	3
C19orf18	0.993231101	-1.006634163	0.013403063	3
C1orf109	1.145024823	-0.443327985	-0.701696838	3
C1orf162	1.128959606	-0.77445041	-0.354509196	3
C1orf174	1.135354253	-0.749962727	-0.385391527	3
C1orf52	1.153805568	-0.616266891	-0.537538677	3
C1orf54	0.985794241	-1.013624771	0.02783053	3
C1orf74	1.102229968	-0.849136499	-0.253093468	3
C2orf49	0.994998676	-1.004927388	0.009928712	3
C2orf69	1.151376544	-0.499865846	-0.651510698	3
C4orf33	1.151318424	-0.499177739	-0.652140685	3
C4orf46	1.133433294	-0.757757811	-0.375675483	3
CACHD1	1.12505301	-0.337378123	-0.787674886	3
CACTIN	1.119558836	-0.314950886	-0.80460795	3

CAMSAP3	1.091560605	-0.871926872	-0.219633732	3
CAPN10	1.1508166	-0.657358676	-0.493457924	3
CAPNS1	1.145650184	-0.697782052	-0.447868132	3
CARD11	1.141850717	-0.422154712	-0.719696005	3
CASP6	0.916235233	-1.066710048	0.150474815	3
CBR1	1.129938949	-0.770951217	-0.358987732	3
CBX3	1.150161819	-0.486504148	-0.663657671	3
CCDC120	1.140623663	-0.414641526	-0.725982137	3
CCDC141	1.147031455	-0.458454304	-0.688577151	3
CCDC146	1.149956552	-0.484424724	-0.665531828	3
CCDC39	0.914136214	-1.068023291	0.153887077	3
CCDC77	1.063857207	-0.920716312	-0.143140896	3
CCDC78	0.914840579	-1.067584232	0.152743653	3
CCDC93	1.127346148	-0.347298405	-0.780047743	3
CCL3	0.913006828	-1.06872388	0.155717052	3
CCL8	1.126586159	-0.782616403	-0.343969756	3
CCND2	1.092436054	-0.870158764	-0.22227729	3
CCNE1	1.146045893	-0.450817754	-0.695228138	3
CCNL1	1.127903667	-0.778136306	-0.349767362	3
CCNL2	1.153990938	-0.541942838	-0.612048099	3
CCNT1	1.02972388	-0.967356745	-0.062367134	3
CCR6	1.154652784	-0.568231863	-0.586420922	3
CCT6B	1.101493661	-0.850803134	-0.250690527	3
CCZ1	1.152626391	-0.636223981	-0.51640241	3

CD1D	1.067614205	-0.914792578	-0.152821627	3
CD274	1.148442242	-0.470248467	-0.678193775	3
CD38	1.134999995	-0.383567242	-0.751432753	3
CD3D	1.062051745	-0.923498252	-0.138553493	3
CD4	1.15463787	-0.566900566	-0.587737304	3
CD48	1.131947376	-0.368435455	-0.763511921	3
CD52	1.153377152	-0.624551486	-0.528825667	3
CDC20	1.119860222	-0.316137552	-0.803722671	3
CDC25B	1.122825221	-0.794752796	-0.328072425	3
CDC40	1.135488464	-0.386086695	-0.749401769	3
CDCA4	1.151507338	-0.501435857	-0.650071481	3
CDCA7L	0.961008363	-1.03489321	0.073884847	3
CDH24	1.154650659	-0.586620082	-0.568030576	3
CDK10	1.102041228	-0.849565165	-0.252476063	3
CDK13	1.016393837	-0.982757427	-0.033636409	3
CDKN2AIP	1.148219558	-0.679910939	-0.468308619	3
CDKN2C	1.137476443	-0.396661185	-0.740815258	3
CDR1	1.153952478	-0.61296595	-0.540986528	3
CEBPZ	0.896889941	-1.078279334	0.181389393	3
CELA2B	1.125796586	-0.34055501	-0.785241576	3
CELA3B	1.129587469	-0.772216281	-0.357371187	3
CENATAC	1.149137873	-0.476530112	-0.672607761	3
CENPL	0.968200131	-1.029023344	0.060823213	3
CENPS	1.125035128	-0.787732952	-0.337302176	3

CEP164	1.14770282	-0.463925628	-0.683777192	3
CEP41	1.151152642	-0.497245637	-0.653907005	3
CEP78	0.893492767	-1.080191729	0.186698962	3
CEP95	1.153556761	-0.532280066	-0.621276695	3
CERK	1.154346788	-0.55242224	-0.601924548	3
CERKL	1.146541617	-0.454604298	-0.691937319	3
CERS6	1.152447165	-0.638666742	-0.513780424	3
CERT1	0.89313625	-1.080390442	0.187254192	3
CFAP157	1.145442201	-0.446342216	-0.699099985	3
CFH	0.930231297	-1.05757012	0.127338822	3
CFL2	1.153381133	-0.624481473	-0.52889966	3
CHAC2	0.933002923	-1.05567814	0.122675217	3
CHAF1A	1.134026563	-0.378631179	-0.755395383	3
CHCHD1	1.153624581	-0.533652776	-0.619971806	3
CHCHD4	1.127027726	-0.345898654	-0.781129072	3
CHMP5	1.097832473	-0.858872516	-0.238959957	3
CHMP7	1.147296741	-0.460588024	-0.686708716	3
CHORDC1	1.114259873	-0.819463124	-0.294796749	3
CHRM5	1.149920357	-0.665857806	-0.484062552	3
CHTF8	1.077998274	-0.897384114	-0.18061416	3
CIAO2B	1.126216322	-0.342364877	-0.783851445	3
CILP	1.142884896	-0.714132973	-0.428751923	3
CLCF1	1.093112753	-0.868780495	-0.224332258	3
CLEC17A	0.965592221	-1.031178958	0.065586736	3

CLEC18C	1.090535666	-0.873975866	-0.2165598	3
CLEC2A	1.153826203	-0.615820934	-0.538005269	3
CLEC4C	1.154688128	-0.572707714	-0.581980414	3
CLEC7A	1.018558752	-0.980345774	-0.038212978	3
CLK2	1.151773183	-0.504725494	-0.647047689	3
CLN8	1.149054388	-0.475757207	-0.673297181	3
CLUAP1	1.144538453	-0.439891745	-0.704646708	3
CLYBL	1.140363884	-0.413090695	-0.727273189	3
CMPK2	0.958891698	-1.036576923	0.077685226	3
CMSS1	1.130189189	-0.360145093	-0.770044096	3
CNNM4	0.961757749	-1.034292395	0.072534646	3
CNOT10	1.154638364	-0.566941971	-0.587696393	3
CNTF	1.082046485	-0.890138629	-0.191907856	3
CNTROB	1.152300227	-0.640594971	-0.511705256	3
COA1	1.055670159	-0.93301453	-0.122655628	3
COA5	0.924114029	-1.061648497	0.137534468	3
COG5	1.152650111	-0.516757516	-0.635892595	3
COL13A1	0.996438413	-1.003523933	0.00708552	3
COL3A1	1.154697168	-0.574932464	-0.579764704	3
COLQ	1.022400247	-0.975983192	-0.046417055	3
COMMD10	1.093571793	-0.867839708	-0.225732085	3
COPS4	1.141285744	-0.722630169	-0.418655575	3
COPS7B	1.154630798	-0.588305858	-0.56632494	3
COPS8	1.004522068	-0.995415737	-0.009106331	3

COQ5	1.148479539	-0.677902983	-0.470576555	3
COQ6	1.153368699	-0.624699793	-0.528668906	3
COQ7	1.143765847	-0.709177369	-0.434588479	3
CORT	1.147598893	-0.463062889	-0.684536004	3
COX11	1.153971852	-0.541465171	-0.61250668	3
COX18	1.107842353	-0.83590343	-0.271938923	3
COX4I1	1.141482265	-0.419864762	-0.721617503	3
CPSF6	1.119189103	-0.31350145	-0.805687653	3
CPSF7	1.151239849	-0.652983396	-0.498256453	3
CPT2	1.153621007	-0.620041621	-0.533579386	3
CREB3L2	1.052290442	-0.937865537	-0.114424905	3
CRIPT	1.151636794	-0.648616209	-0.503020585	3
CRISP2	1.14461511	-0.440428112	-0.704186998	3
CROT	1.019577783	-0.979199009	-0.040378774	3
CRTAP	0.987734193	-1.011830293	0.0240961	3
CRTC2	1.153019446	-0.630450597	-0.522568849	3
CRY1	1.129188902	-0.773638268	-0.355550634	3
CRYBA1	1.151941153	-0.645062442	-0.506878711	3
CRYBB1	1.051157504	-0.939463912	-0.111693592	3
CRYZ	1.153990883	-0.541941452	-0.612049431	3
CSE1L	0.905011876	-1.07356562	0.168553744	3
CSF1R	1.143918344	-0.435620927	-0.708297416	3
CSKMT	1.154679593	-0.571316689	-0.583362904	3
CTDP1	1.154688301	-0.581947963	-0.572740339	3

CTPS1	1.138776341	-0.403884898	-0.734891443	3
CTSF	1.139106091	-0.40576054	-0.733345551	3
CUBN	1.034696998	-0.961253434	-0.073443564	3
CUL2	1.078668712	-0.89620318	-0.182465531	3
CYB561A3	1.154442863	-0.598346246	-0.556096616	3
CYB5R1	0.977391673	-1.021170379	0.043778707	3
CYP17A1	1.151585275	-0.502386124	-0.649199151	3
CYP20A1	1.034242468	-0.961819883	-0.072422585	3
CYP4V2	1.033762394	-0.962416252	-0.071346142	3
CYTH1	1.14731441	-0.460731415	-0.686582995	3
CYTH3	1.132399348	-0.37061392	-0.761785429	3
DAP3	1.027720104	-0.969758661	-0.057961443	3
DAPK1	0.930112854	-1.057650351	0.127537497	3
DARS1	1.070876945	-0.909493183	-0.161383762	3
DARS2	1.139287317	-0.732488085	-0.406799232	3
DAZL	1.153655862	-0.619355733	-0.534300129	3
DBF4	1.135424314	-0.385754138	-0.749670176	3
DBNDD1	1.15267978	-0.635475276	-0.517204504	3
DCAF1	1.149815438	-0.48302005	-0.666795388	3
DCANP1	0.969077477	-1.028291101	0.059213624	3
DCBLD1	1.10466723	-0.843508363	-0.261158866	3
DCLRE1B	1.143042055	-0.429777792	-0.713264264	3
DCUN1D3	1.137974782	-0.399399519	-0.738575263	3
DDO	1.106759587	-0.838533107	-0.268226481	3

DDRGK1	1.150275305	-0.662602175	-0.487673129	3
DDTL	1.146561422	-0.454757805	-0.691803617	3
DDX31	0.948320321	-1.044700629	0.096380308	3
DDX47	1.148437394	-0.470205888	-0.678231506	3
DDX6	1.066128145	-0.91715792	-0.148970225	3
DDX60	1.146905934	-0.457456917	-0.689449018	3
DEFB1	1.11359993	-0.821226508	-0.292373422	3
DEFB105B	1.154700538	-0.577350269	-0.577350269	3
DEFB116	1.154700538	-0.577350269	-0.577350269	3
DEFB121	1.154700538	-0.577350269	-0.577350269	3
DEFB124	1.154700538	-0.577350269	-0.577350269	3
DEFB126	1.154700538	-0.577350269	-0.577350269	3
DENND2D	1.122229643	-0.325635582	-0.79659406	3
DENR	0.989690555	-1.010000064	0.020309508	3
DFFB	0.983511341	-1.015710899	0.032199558	3
DGKE	1.030120273	-0.966877763	-0.06324251	3
DHODH	1.123379409	-0.793020696	-0.330358713	3
DHRS13	1.131895485	-0.368186626	-0.763708858	3
DHRS4L2	1.108284213	-0.834819106	-0.273465107	3
DHX15	1.125242527	-0.787058208	-0.338184319	3
DHX16	1.131453995	-0.366080076	-0.765373919	3
DHX32	1.149033072	-0.475560737	-0.673472335	3
DHX40	0.923986495	-1.061732122	0.137745627	3
DIS3	0.960183689	-1.03555153	0.075367841	3

DLD	1.10217853	-0.849253425	-0.252925104	3
DLEC1	1.128571024	-0.352753994	-0.77581703	3
DMRTB1	1.008413742	-0.991368337	-0.017045405	3
DNAAF5	1.150459862	-0.489605285	-0.660854576	3
DNAI7	1.129268313	-0.355912312	-0.773356001	3
DNAJB13	1.095900435	-0.86299256	-0.232907875	3
DNAJB14	1.140509415	-0.726551557	-0.413957859	3
DNAJC1	0.934930401	-1.054345818	0.119415417	3
DNAJC24	1.145091024	-0.701289096	-0.443801927	3
DNAJC25	1.151629174	-0.50292642	-0.648702753	3
DNAJC3	1.101414109	-0.850982294	-0.250431815	3
DNAL4	1.120651119	-0.319274108	-0.801377011	3
DNASE1L2	0.958998213	-1.036492662	0.077494449	3
DNMT3A	1.090810162	-0.873429307	-0.217380856	3
DNTT	1.14019951	-0.728083325	-0.412116185	3
DOLK	1.151045074	-0.655029945	-0.496015129	3
DSEL	1.047901792	-0.943982294	-0.103919498	3
DSTN	0.965767161	-1.031035332	0.065268171	3
DTD2	1.153782541	-0.616758379	-0.537024161	3
DTNB	1.010601017	-0.989050638	-0.021550379	3
DTX2	1.14823776	-0.468465985	-0.679771775	3
DTX3L	1.154635548	-0.587927375	-0.566708173	3
DUS4L	1.108930803	-0.833220411	-0.275710392	3
DUSP14	1.033627209	-0.962583831	-0.071043378	3

DUSP2	1.07878131	-0.896004121	-0.182777188	3
DXO	1.146162185	-0.694465541	-0.451696644	3
DYNC1I2	0.996460356	-1.003502451	0.007042095	3
DYNC2I1	1.122608887	-0.327184889	-0.795423998	3
DYRK2	0.953266638	-1.040957727	0.087691089	3
DYRK4	1.149538732	-0.480320824	-0.669217908	3
E2F6	1.154627553	-0.566070544	-0.588557009	3
EBNA1BP2	1.150466444	-0.489674933	-0.660791511	3
EBPL	1.129750347	-0.358119001	-0.771631346	3
ECHDC1	1.133224569	-0.758579878	-0.374644691	3
ECPAS	1.10263756	-0.848207326	-0.254430234	3
EDEM3	1.154223522	-0.605852779	-0.548370743	3
EEF1B2	0.910183465	-1.070457108	0.160273643	3
EEF1E1	1.13258382	-0.761074911	-0.37150891	3
EGFL8	1.138275134	-0.737206867	-0.401068267	3
EGLN3	1.137728715	-0.739685987	-0.398042728	3
EIF2A	1.078498138	-0.896504335	-0.181993803	3
EIF2AK2	1.151265057	-0.652714147	-0.498550911	3
EIF2B4	1.147086697	-0.458895704	-0.688190993	3
EIF2S1	1.067138741	-0.915552591	-0.15158615	3
EIF3E	1.119329799	-0.314052195	-0.805277604	3
EIF3J	0.953310116	-1.040924379	0.087614263	3
EIF3L	1.152687477	-0.635366489	-0.517320988	3
EIF4E	1.081400155	-0.891314222	-0.190085934	3

EIF4EBP2	1.153339065	-0.625215914	-0.528123151	3
ELMO3	1.129492458	-0.356936001	-0.772556457	3
ELOC	1.136700068	-0.392467357	-0.744232711	3
EMC6	1.152410981	-0.513263462	-0.639147519	3
EMC9	0.964402647	-1.032151913	0.067749265	3
EME1	1.124399943	-0.789781992	-0.334617951	3
EML3	1.154571819	-0.562354885	-0.592216934	3
EML4	1.123773676	-0.791777126	-0.33199655	3
ENGASE	1.152640958	-0.516620262	-0.636020697	3
ENOSF1	1.145538989	-0.698488702	-0.447050287	3
ENPP2	1.151974052	-0.507307793	-0.64466626	3
ENSA	1.153830735	-0.615722283	-0.538108452	3
ENTPD7	1.145935006	-0.695950109	-0.449984897	3
ENTR1	1.121098014	-0.321061154	-0.80003686	3
EOMES	1.139239082	-0.40652222	-0.732716862	3
EP400	1.147653338	-0.684139232	-0.463514106	3
EPC1	1.151325598	-0.499262375	-0.652063223	3
EPHX1	1.125705009	-0.340161746	-0.785543264	3
EPHX2	1.123265375	-0.329886765	-0.79337861	3
EPM2A	1.135451209	-0.3858935	-0.749557709	3
EPOP	0.95369788	-1.040626614	0.086928734	3
EPRS1	1.150602776	-0.659473466	-0.49112931	3
EPS8L2	1.142569421	-0.715857664	-0.426711757	3
ERAP1	1.141005568	-0.416945887	-0.724059681	3

ERCC6	1.149683387	-0.667960336	-0.481723051	3
ERCC6L2	1.145610081	-0.698037451	-0.44757263	3
ERH	1.154055917	-0.543618289	-0.610437628	3
ERO1B	1.139239966	-0.406527295	-0.732712671	3
ERV3-1	1.152153096	-0.642465049	-0.509688046	3
ETS2	1.078966091	-0.895676997	-0.183289094	3
ETV2	1.101819321	-0.850067875	-0.251751446	3
EVL	1.153149515	-0.52476117	-0.628388345	3
EXD2	1.123251592	-0.329829774	-0.793421818	3
EXOSC9	1.120971478	-0.320554063	-0.800417415	3
EXPH5	1.126437803	-0.343324822	-0.783112982	3
EXTL2	1.024517394	-0.973532024	-0.050985369	3
EYA3	0.977574889	-1.021009675	0.043434786	3
FABP9	1.154700538	-0.577350269	-0.577350269	3
FAHD2A	1.085111785	-0.884461305	-0.20065048	3
FAM102A	1.136523477	-0.744998236	-0.391525241	3
FAM120AOS	1.127166792	-0.346509062	-0.78065773	3
FAM153A	1.147533368	-0.462522009	-0.685011358	3
FAM156A	1.148395597	-0.678556148	-0.469839448	3
FAM156B	1.151032791	-0.495875729	-0.655157061	3
FAM167B	1.118349364	-0.808114483	-0.310234881	3
FAM184B	0.993345889	-1.006523862	0.013177973	3
FAM236A	1.079916297	-0.893985823	-0.185930474	3
FAM24B	1.15444918	-0.556360332	-0.598088849	3

FAM25A	1.154700538	-0.577350269	-0.577350269	3
FAM25G	1.154700538	-0.577350269	-0.577350269	3
FAM3C	1.150176955	-0.486659242	-0.663517712	3
FAM43A	1.122243005	-0.32569003	-0.796552975	3
FAM47E-STBD1	1.153077626	-0.523538889	-0.629538736	3
FAM83H	1.150591903	-0.491012471	-0.659579432	3
FAN1	1.083894848	-0.88673572	-0.197159128	3
FANCD2	1.142720571	-0.715034475	-0.427686096	3
FAR1	1.140782504	-0.415596358	-0.725186146	3
FARS2	1.13967534	-0.730632847	-0.409042494	3
FARSA	1.148066417	-0.466992903	-0.681073514	3
FAU	1.152916936	-0.520898556	-0.632018381	3
FBLN2	1.135153374	-0.384355199	-0.750798174	3
FBXL16	1.030266243	-0.966701058	-0.063565185	3
FBXL3	1.152553551	-0.515324058	-0.637229493	3
FBXO21	1.136747444	-0.392720836	-0.744026608	3
FBXO22	1.116876337	-0.812289376	-0.304586962	3
FBXO25	1.112828394	-0.823265699	-0.289562695	3
FBXO28	1.115860973	-0.815108863	-0.30075211	3
FBXO3	1.147777705	-0.464551054	-0.683226651	3
FBXO32	1.145763066	-0.69705982	-0.448703247	3
FBXO39	1.086754065	-0.881347744	-0.20540632	3
FBXO42	1.013759341	-0.985647881	-0.02811146	3
FBXO6	1.053248191	-0.936503573	-0.116744618	3

FBXW8	1.144599417	-0.704281261	-0.440318157	3
FBXW9	0.965184368	-1.031513264	0.066328896	3
FCHSD2	1.132860181	-0.372856184	-0.760003997	3
FEM1C	1.131161461	-0.364694417	-0.766467045	3
FHIT	1.148334415	-0.679029278	-0.469305137	3
FIGNL1	1.15410494	-0.609166969	-0.54493797	3
FITM1	1.1030389	-0.847287754	-0.255751146	3
FLAD1	1.126349692	-0.783407176	-0.342942516	3
FLNB	1.152769074	-0.51856935	-0.634199725	3
FMC1-LUC7L2	0.973975485	-1.024136526	0.050161041	3
FMO4	1.154040939	-0.543225019	-0.61081592	3
FMR1NB	1.057085786	-0.93094472	-0.126141066	3
FNDC7	1.051114047	-0.939524951	-0.111589096	3
FOXJ3	1.150067325	-0.664525902	-0.485541423	3
FTCDNL1	1.146768705	-0.69039355	-0.456375155	3
FTSJ1	1.144134101	-0.43709346	-0.70704064	3
FUBP3	1.061274972	-0.92468263	-0.136592341	3
FUCA1	0.90228091	-1.075173202	0.172892292	3
FUS	1.013012961	-0.986458099	-0.026554862	3
FXN	1.146013229	-0.695441331	-0.450571897	3
FXR2	1.154461811	-0.597564272	-0.556897538	3
FYN	1.132427101	-0.370748348	-0.761678753	3
FZD2	1.134611869	-0.381585905	-0.753025964	3
FZD6	1.12347001	-0.330734243	-0.792735767	3

GALNT6	1.04190032	-0.952033438	-0.089866882	3
GALT	1.152070434	-0.643491029	-0.508579405	3
GAR1	1.137021954	-0.394195726	-0.742826228	3
GATAD1	0.956145321	-1.038732623	0.082587303	3
GBE1	1.152073282	-0.508617318	-0.643455964	3
GCC2	1.149569943	-0.668948181	-0.480621762	3
GCNA	1.153634521	-0.533857459	-0.619777062	3
GDAP2	1.024802461	-0.973199383	-0.051603078	3
GDF7	1.154656655	-0.586046496	-0.568610159	3
GDPGP1	1.153513027	-0.531415896	-0.622097131	3
GEMIN5	1.122504017	-0.326755637	-0.795748381	3
GEMIN6	1.141502234	-0.721514133	-0.419988102	3
GEMIN7	1.027290397	-0.970269585	-0.057020812	3
GEN1	0.979408059	-1.019392514	0.039984455	3
GFM2	1.135241738	-0.384810454	-0.750431283	3
GGACT	1.154698297	-0.579319328	-0.57537897	3
GGCX	1.0341044	-0.961991598	-0.072112801	3
GH1	1.132188047	-0.369592957	-0.76259509	3
GID8	1.152216974	-0.510556725	-0.641660249	3
GIMAP6	1.136742227	-0.392692908	-0.744049319	3
GKAP1	1.027987094	-0.969440471	-0.058546623	3
GLB1	1.151838978	-0.505561649	-0.64627733	3
GLIS2	1.116838455	-0.812395407	-0.304443048	3
GLMN	1.150574575	-0.659747993	-0.490826582	3

GLRX2	1.060524633	-0.925819679	-0.134704953	3
GLYCTK	1.154682972	-0.582857372	-0.571825601	3
GLYR1	1.122534898	-0.795652926	-0.326881973	3
GM2A	1.077896363	-0.89756298	-0.180333383	3
GMEB1	1.142852246	-0.428539602	-0.714312644	3
GMEB2	1.135188864	-0.384537933	-0.750650931	3
GNA11	1.143896163	-0.435470335	-0.708425829	3
GNA12	1.119289999	-0.3138963	-0.8053937	3
GNE	0.951356975	-1.042414696	0.091057721	3
GNGT2	1.153068435	-0.62968389	-0.523384545	3
GNMT	1.104094042	-0.844847738	-0.259246304	3
GOLGA3	1.127152913	-0.346448077	-0.780704836	3
GOLGA8H	1.137409244	-0.396294765	-0.741114479	3
GOLGA8J	1.144333417	-0.438466401	-0.705867017	3
GOLGA8K	1.153024735	-0.52265636	-0.630368375	3
GOLGA8N	1.127690571	-0.778869661	-0.34882091	3
GOLGA8R	1.04771895	-0.944232823	-0.103486127	3
GOLGA8T	1.14965328	-0.481429625	-0.668223654	3
GOLPH3L	1.154652637	-0.586434925	-0.568217712	3
GORASP1	0.961443281	-1.034544819	0.073101538	3
GOT2	1.138339068	-0.401425312	-0.736913756	3
GP5	1.030307252	-0.966651384	-0.063655868	3
GPATCH11	1.117136038	-0.811560717	-0.305575321	3
GPATCH8	1.144531799	-0.43984528	-0.70468652	3

GPKOW	1.146774592	-0.456421373	-0.690353218	3
GPR141	1.153248033	-0.626766126	-0.526481907	3
GPR15	1.114844706	-0.817885329	-0.296959377	3
GPR183	1.15367449	-0.534690136	-0.618984353	3
GPR34	1.122507094	-0.795738873	-0.326768221	3
GPR75	1.01878618	-0.980090487	-0.038695694	3
GRAMD4	1.132061175	-0.36898206	-0.763079115	3
GRK2	0.944483451	-1.047535788	0.103052337	3
GRSF1	1.134525395	-0.753378512	-0.381146883	3
GRXCR2	1.100063545	-0.853997474	-0.246066072	3
GSKIP	1.010544265	-0.989111173	-0.021433092	3
GSPT2	1.119568057	-0.314987123	-0.804580934	3
GSR	1.114430632	-0.819003923	-0.295426709	3
GSTM3	1.154267313	-0.604523928	-0.549743385	3
GTF2A1L	1.154700538	-0.577350269	-0.577350269	3
GTF2B	1.153599791	-0.620453547	-0.533146245	3
GTF2E1	0.888557746	-1.082909321	0.194351575	3
GTF2IRD2	1.154311651	-0.603106907	-0.551204744	3
GTF2IRD2B	1.057691562	-0.930051987	-0.127639575	3
GTF3C3	1.138538552	-0.735995022	-0.40254353	3
GTF3C6	1.149958318	-0.665515895	-0.484442423	3
GTPBP1	1.141002811	-0.724073666	-0.416929145	3
GTPBP3	1.141454123	-0.721763031	-0.419691092	3
GTSF1L	1.154699942	-0.5783664	-0.576333542	3

GUCA1B	1.126640935	-0.344208277	-0.782432659	3
GZMA	1.099616277	-0.854985189	-0.244631089	3
H3C6	1.151386468	-0.499983913	-0.651402555	3
H6PD	0.941859866	-1.049440948	0.107581081	3
HAAO	1.154354653	-0.601651841	-0.552702812	3
HAGHL	0.975958817	-1.022421446	0.046462629	3
HARBI1	1.154493767	-0.596170585	-0.558323182	3
HARS2	1.096072926	-0.86262843	-0.233444496	3
HAUS1	1.132083646	-0.762993503	-0.369090143	3
HAUS5	1.139303747	-0.406893679	-0.732410068	3
HBQ1	1.146002006	-0.450487524	-0.695514481	3
HCST	1.119153907	-0.80579007	-0.313363837	3
HDAC8	1.138767115	-0.734934437	-0.403832678	3
HDDC3	1.153660416	-0.619265254	-0.534395162	3
HDGFL2	0.980751824	-1.018196331	0.037444507	3
HEATR1	0.98051071	-1.01841164	0.03790093	3
HEATR3	1.124810012	-0.336347871	-0.788462141	3
HELZ	1.126191898	-0.342259228	-0.78393267	3
HGS	1.154359937	-0.552893093	-0.601466844	3
HIF1AN	1.144423587	-0.439091582	-0.705332004	3
HILPDA	1.137459919	-0.740888894	-0.396571025	3
HINFP	1.153437823	-0.529965392	-0.623472431	3
HINT1	0.894218235	-1.079786216	0.185567982	3
HIP1R	1.154590164	-0.591121333	-0.563468831	3

HIVEP3	1.147926931	-0.465807005	-0.682119926	3
HK2	1.094769984	-0.865361424	-0.22940856	3
HKDC1	1.107012736	-0.837921735	-0.269091001	3
HLA-DQB2	1.120977143	-0.320576748	-0.800400395	3
HLCS	1.104145721	-0.844727383	-0.259418338	3
HMGN1	1.025837615	-0.971986222	-0.053851393	3
HMGXB3	1.133528977	-0.376149579	-0.757379398	3
HNMT	1.127651098	-0.348645969	-0.779005128	3
HNRNPA1	1.14260886	-0.715643423	-0.426965436	3
HNRNPAB	1.060387904	-0.926026138	-0.134361766	3
HNRNPR	0.895802606	-1.078895176	0.18309257	3
HOXB2	1.129786099	-0.358283446	-0.771502653	3
HPCAL4	1.153588558	-0.620669998	-0.53291856	3
HPGDS	0.972410626	-1.025476284	0.053065658	3
HSD17B4	1.121477114	-0.798891473	-0.322585642	3
HSF2	1.147842265	-0.465092819	-0.682749445	3
HSF4	1.152604457	-0.516075789	-0.636528669	3
HSPA14	1.026241091	-0.971511119	-0.054729972	3
HSPB1	1.146709752	-0.45591315	-0.690796601	3
HTATSF1	0.886288067	-1.084135529	0.197847462	3
HTD2	0.95955469	-1.036051646	0.076496956	3
HTR3A	1.09875738	-0.856867168	-0.241890212	3
HVCN1	1.123122161	-0.32929516	-0.793827001	3
ICAM5	1.134647267	-0.381765868	-0.752881399	3

IDO2	1.150638184	-0.491510816	-0.659127368	3
IDUA	1.108702071	-0.83378759	-0.274914481	3
IFFO2	1.099718802	-0.854759249	-0.244959554	3
IFI44L	1.145318555	-0.699875848	-0.445442707	3
IFNA5	1.105113812	-0.842457918	-0.262655894	3
IFT46	0.97331586	-1.024702703	0.051386843	3
IGBP1	1.129866293	-0.358652708	-0.771213585	3
IGSF9B	1.154000001	-0.542171866	-0.611828135	3
IKBKB	1.152661738	-0.516932307	-0.63572943	3
IL10	1.062741779	-0.922439852	-0.140301928	3
IL10RA	1.15304496	-0.522992201	-0.630052759	3
IL11RA	0.994557922	-1.005354649	0.010796727	3
IL12A	1.15444978	-0.556385509	-0.598064271	3
IL15RA	1.02994113	-0.967094388	-0.062846742	3
IL17RB	1.153634091	-0.619785495	-0.533848596	3
IL17RE	1.034807654	-0.961115264	-0.07369239	3
IL27	1.154115526	-0.545229876	-0.60888565	3
IL27RA	1.052344567	-0.937788834	-0.114555732	3
IL2RB	1.139880633	-0.410240266	-0.729640368	3
IL2RG	1.088371581	-0.878229914	-0.210141667	3
ILF3	1.154614366	-0.565090435	-0.589523931	3
IMPA1	1.010736378	-0.988906171	-0.021830207	3
ING3	0.994677825	-1.005238527	0.010560702	3
ING5	0.945515306	-1.046779085	0.101263779	3

INPP5B	0.97241646	-1.025471312	0.053054852	3
INSL6	1.154269444	-0.60445756	-0.549811884	3
INTS12	1.028351362	-0.969005437	-0.059345925	3
INTS13	0.965997405	-1.03084609	0.064848686	3
INTS4	1.08185258	-0.890492087	-0.191360492	3
INTS6L	1.153674225	-0.534684577	-0.618989649	3
IP6K2	1.146564783	-0.691780912	-0.45478387	3
IRAK1BP1	1.145406963	-0.699321675	-0.446085288	3
IRF2	1.133060903	-0.373839659	-0.759221245	3
IRF4	1.1540247	-0.611221189	-0.54280351	3
IRF7	0.975901354	-1.022471406	0.046570052	3
IRGM	1.032071986	-0.964500665	-0.06757132	3
ISG15	1.150066341	-0.664534892	-0.485531449	3
IST1	1.059327752	-0.92761932	-0.131708432	3
ITFG2	1.145679021	-0.69759802	-0.448081001	3
ITGA10	0.892052995	-1.080991922	0.188938927	3
ITGA3	0.903703149	-1.074338895	0.170635746	3
ITGA4	1.136009085	-0.388804878	-0.747204207	3
ITGA6	1.154360637	-0.552918423	-0.601442215	3
ITIH2	1.133221493	-0.758591958	-0.374629535	3
ITK	1.145544939	-0.447093933	-0.698451006	3
ITLN2	1.154474362	-0.597028847	-0.557445516	3
ITM2C	1.153371699	-0.528724481	-0.624647218	3
IWS1	1.12913512	-0.773829144	-0.355305976	3

JADE1	1.047575631	-0.944428961	-0.10314667	3
JAK1	1.143468001	-0.432591272	-0.710876728	3
JUN	0.969640704	-1.027819135	0.058178431	3
KAT7	1.145692334	-0.448179386	-0.697512948	3
KATNA1	1.143124175	-0.712807759	-0.430316417	3
KBTBD11	1.124167351	-0.790525845	-0.333641506	3
KCNH7	1.148458007	-0.678070977	-0.47038703	3
KCNIP2	1.027366664	-0.97017901	-0.057187654	3
KCNJ14	1.129355385	-0.356309481	-0.773045903	3
KCNMB1	1.148641457	-0.472011873	-0.676629585	3
KCTD10	1.154700091	-0.576469892	-0.578230199	3
KCTD13	1.068293649	-0.913701181	-0.154592468	3
KCTD5	1.120219439	-0.802661378	-0.317558061	3
KDM3A	1.038952188	-0.955863266	-0.083088922	3
KDM4A	1.154032172	-0.542996821	-0.61103535	3
KDM4E	0.95231604	-1.041684872	0.089368832	3
KDM6A	1.029650584	-0.967445172	-0.062205412	3
KIAA0895L	1.141526251	-0.721389697	-0.420136554	3
KIAA1143	1.141243588	-0.418397275	-0.722846313	3
KICS2	1.152123944	-0.509295113	-0.642828831	3
KIF27	1.134625644	-0.381655918	-0.752969726	3
KIF4A	1.144932288	-0.702264226	-0.442668062	3
KLC2	1.153493174	-0.622464452	-0.531028722	3
KLHL17	1.154700445	-0.57775206	-0.576948386	3

KLHL36	1.030959454	-0.965859514	-0.06509994	3
KLHL42	1.13553073	-0.386306085	-0.749224646	3
KLHL6	1.05248463	-0.937590194	-0.114894437	3
KLK2	1.092530169	-0.869967683	-0.222562486	3
KLRB1	1.15353687	-0.531885056	-0.621651813	3
KLRC4-KLRK1	1.122642061	-0.327320812	-0.795321249	3
KLRF1	1.151728937	-0.647560644	-0.504168293	3
KMO	1.091166568	-0.872717264	-0.218449303	3
KNOP1	1.136200015	-0.389810511	-0.746389504	3
KPNA1	1.143320935	-0.711706637	-0.431614299	3
KRAS	1.018014331	-0.980955379	-0.037058952	3
KRTAP10-11	1.069792109	-0.911271707	-0.158520402	3
KRTAP10-6	0.901253522	-1.075771998	0.174518476	3
KRTAP5-8	1.154344437	-0.552338968	-0.602005469	3
KYAT1	1.139996782	-0.410921355	-0.729075426	3
LAG3	1.012928113	-0.986549964	-0.02637815	3
LARP4	1.149096117	-0.672953272	-0.476142844	3
LARP7	0.929899748	-1.057794579	0.127894831	3
LARS1	1.154286664	-0.550371643	-0.603915021	3
LAS1L	1.151129736	-0.654147619	-0.496982116	3
LBH	1.132329551	-0.370276181	-0.76205337	3
LCE3C	1.154700538	-0.577350269	-0.577350269	3
LCE5A	0.962969259	-1.033315813	0.070346554	3
LDAH	1.093692352	-0.867591838	-0.226100515	3

LEAP2	1.129249486	-0.355826517	-0.773422969	3
LELP1	0.968788056	-1.028533052	0.059744996	3
LEMD3	0.970543766	-1.027059294	0.056515528	3
LEO1	1.052391871	-0.93772177	-0.114670101	3
LETM1	0.896745502	-1.078361345	0.181615843	3
LGALS14	1.150923798	-0.656275315	-0.494648483	3
LGALS8	1.116548573	-0.813204596	-0.303343978	3
LGMN	1.135315378	-0.750124793	-0.385190585	3
LIMD1	1.082473916	-0.889357148	-0.193116768	3
LIPA	1.141075229	-0.723705796	-0.417369433	3
LIPT2	0.988188005	-1.011407592	0.023219587	3
LMAN2L	1.149627149	-0.668451518	-0.481175631	3
LOC100421372	1.105031541	-0.842651894	-0.262379647	3
LOC100996709	1.064606468	-0.919549669	-0.145056799	3
LOC102724200	1.039114175	-0.955654896	-0.08345928	3
LOC105375817	1.154700538	-0.577350269	-0.577350269	3
LOC107984126	1.128288585	-0.35148576	-0.776802825	3
LOC107984156	0.986003549	-1.013432127	0.027428578	3
LOC107984189	1.154700538	-0.577350269	-0.577350269	3
LONP2	1.124701404	-0.335888645	-0.78881276	3
LONRF1	0.940436074	-1.050463668	0.110027594	3
LRFN4	1.146327527	-0.452956021	-0.693371506	3
LRIG1	1.08568907	-0.883372702	-0.202316369	3
LRIT3	1.1516802	-0.648121059	-0.503559142	3

LRRC27	1.011076226	-0.988542926	-0.0225333	3
LRRC37A	1.154641067	-0.587469673	-0.567171394	3
LRRC37A2	1.047319296	-0.94477925	-0.102540046	3
LRRC37B	1.154556542	-0.593070481	-0.56148606	3
LRRC47	1.128157413	-0.350898878	-0.777258535	3
LRRC72	1.051038631	-0.93963083	-0.111407801	3
LRRC8C	1.142987274	-0.713567802	-0.429419472	3
LRRK1	1.139887429	-0.410280044	-0.729607385	3
LSM3	1.008185169	-0.991608737	-0.016576432	3
LSMEM2	1.010463837	-0.989196923	-0.021266915	3
LTB	1.152495567	-0.514478241	-0.638017326	3
LTB4R2	1.15007827	-0.66442582	-0.485652451	3
LTBP3	1.131048868	-0.766885659	-0.364163209	3
LTO1	1.030551665	-0.966355038	-0.064196627	3
LYPD6B	1.152911609	-0.52081304	-0.632098568	3
LYRM9	1.136933471	-0.743214303	-0.393719167	3
LZTR1	1.154700537	-0.577300005	-0.577400532	3
MACROH2A2	1.094104896	-0.866741136	-0.22736376	3
MAD2L1BP	1.144974078	-0.442965728	-0.702008351	3
MAGEH1	1.1339184	-0.755828988	-0.378089412	3
MAGOH	1.148589546	-0.471549741	-0.677039805	3
MAJIN	1.135184982	-0.384517937	-0.750667045	3
MAK16	0.940546924	-1.050384324	0.1098374	3
MALT1	1.145983332	-0.695636077	-0.450347256	3

MAML2	1.154582246	-0.591604692	-0.562977554	3
MAN1B1	1.067525821	-0.914934087	-0.152591734	3
MAN1C1	1.109004677	-0.833036843	-0.275967834	3
MAP3K2	1.132848719	-0.37280015	-0.760048569	3
MAP3K7	1.139362163	-0.732132295	-0.407229867	3
MAPKAPK3	1.037548922	-0.957658473	-0.079890448	3
MAPKAPK5	1.152148976	-0.509632393	-0.642516583	3
MARCHF1	1.15387596	-0.614722919	-0.539153041	3
MASP2	1.154700494	-0.577626884	-0.57707361	3
MAT2A	1.154333993	-0.551972262	-0.602361731	3
MAVS	1.153471621	-0.530611912	-0.62285971	3
MBD1	1.154034721	-0.610971701	-0.54306302	3
MBD3	1.154228698	-0.548529654	-0.605699044	3
MCCC1	1.064377721	-0.919906607	-0.144471114	3
MCEMP1	1.120025927	-0.803233937	-0.31679199	3
MCM2	1.149160511	-0.476740646	-0.672419865	3
MCM4	1.019558322	-0.97922098	-0.040337342	3
MCMDC2	1.142493189	-0.42622251	-0.716270679	3
MCTP2	1.138611852	-0.735655824	-0.402956028	3
MCUB	1.114400236	-0.819085753	-0.295314483	3
MDFIC	1.154579343	-0.591777785	-0.562801558	3
MDM4	1.031535894	-0.965156726	-0.066379167	3
MED10	1.151788661	-0.504921381	-0.64686728	3
MED11	1.126969161	-0.781327143	-0.345642019	3

MED13L	1.147042098	-0.688502871	-0.458539227	3
MED16	1.132902327	-0.759839991	-0.373062336	3
MED28	1.14058416	-0.414404841	-0.726179318	3
MED29	1.148908428	-0.674489545	-0.474418883	3
MED8	1.153060627	-0.523253749	-0.629806878	3
METAP1D	1.141629983	-0.720850778	-0.420779205	3
METTL1	1.124413502	-0.334674977	-0.789738525	3
METTL14	1.141678027	-0.720600365	-0.421077661	3
METTL2A	1.153753531	-0.617368641	-0.53638489	3
METTL2B	1.154663252	-0.585367888	-0.569295364	3
METTL6	1.010427598	-0.989235547	-0.021192051	3
MFAP1	1.091546016	-0.871956195	-0.21958982	3
MFN1	0.959417592	-1.036160423	0.076742831	3
MFNG	0.939846864	-1.050884622	0.111037758	3
MGAT1	1.125717765	-0.34021649	-0.785501275	3
MGAT5	1.153780215	-0.536972543	-0.616807671	3
MIDEAS	1.023152799	-0.975115786	-0.048037013	3
MIEN1	1.135970543	-0.388602457	-0.747368086	3
MIER3	1.123581239	-0.792385284	-0.331195955	3
MIIP	1.060689939	-0.925569767	-0.135120172	3
MIOS	1.147750539	-0.464323794	-0.683426744	3
MLLT3	1.153691132	-0.535041512	-0.61864962	3
MMAB	1.084588196	-0.885443241	-0.199144955	3
MMGT1	0.941194099	-1.049920147	0.108726047	3

MMS19	1.149094684	-0.672965102	-0.476129582	3
MN1	1.139267135	-0.732583854	-0.406683282	3
MOG	1.014963298	-0.984332913	-0.030630385	3
MOGAT1	1.154542726	-0.593803771	-0.560738955	3
MON2	1.094815018	-0.865267632	-0.229547386	3
MORC2	1.131646225	-0.366995005	-0.76465122	3
MORC4	0.971761253	-1.026028801	0.054267549	3
MOSMO	0.913151225	-1.068634539	0.155483314	3
MPHOSPH6	1.127680249	-0.778905097	-0.348775151	3
MPV17	1.150839119	-0.657132421	-0.493706698	3
MRGBP	1.132794655	-0.760258625	-0.372536029	3
MRPL22	1.021645189	-0.976849236	-0.044795953	3
MRPL28	1.094257427	-0.866425617	-0.22783181	3
MRPL35	1.127311051	-0.347143757	-0.780167294	3
MRPL37	1.133295528	-0.374994603	-0.758300926	3
MRPL46	1.154700394	-0.577849877	-0.576850517	3
MRPL47	0.964695585	-1.031912913	0.067217328	3
MRPL57	1.121831643	-0.324018538	-0.797813106	3
MRPS14	1.1464565	-0.453946566	-0.692509934	3
MRPS25	1.153564224	-0.532429153	-0.621135072	3
MRPS27	1.115878036	-0.815061861	-0.300816175	3
MRPS28	1.004729262	-0.99520267	-0.009526592	3
MRPS33	0.948851036	-1.044303824	0.095452789	3
MRRF	1.140732483	-0.725437358	-0.415295125	3

MRTO4	1.147927238	-0.465809596	-0.682117641	3
MS4A14	1.147821844	-0.682900646	-0.464921198	3
MS4A5	1.154700538	-0.577350269	-0.577350269	3
MSH3	1.154700037	-0.576417941	-0.578282096	3
MTA2	1.117303334	-0.81108967	-0.306213663	3
MTAP	0.979527558	-1.01928651	0.039758953	3
MTFMT	1.152464254	-0.514025964	-0.63843829	3
MTMR1	1.154678395	-0.583532234	-0.57114616	3
MTRR	1.099546119	-0.855139641	-0.244406477	3
MUTYH	1.152251138	-0.511025746	-0.641225391	3
MVK	0.903711366	-1.074334056	0.17062269	3
MYH3	1.096672884	-0.861356312	-0.235316572	3
MYORG	1.119067787	-0.313027372	-0.806040416	3
MZB1	1.150098091	-0.664244254	-0.485853837	3
NAA20	1.097509098	-0.859568537	-0.237940561	3
NABP1	1.075015646	-0.902550315	-0.172465331	3
NAGK	1.131666991	-0.764572936	-0.367094055	3
NANOS2	1.141819082	-0.719862179	-0.421956903	3
NANP	1.153317294	-0.625591401	-0.527725893	3
NBEAL1	1.057856667	-0.929807934	-0.128048733	3
NBPF15	1.150444395	-0.48944184	-0.661002555	3
NBPF4	1.075387368	-0.901914093	-0.173473275	3
NBPF9	1.129532295	-0.357118379	-0.772413916	3
NCK1	1.15393907	-0.613280242	-0.540658828	3

NCOA3	1.151352411	-0.651772982	-0.499579429	3
NCOA6	1.042308297	-0.951497105	-0.090811192	3
NDC1	1.057454958	-0.930401175	-0.127053783	3
NDST2	0.957576022	-1.037613674	0.080037653	3
NDUFA12	1.147798735	-0.683071471	-0.464727264	3
NDUFAF4	1.147764914	-0.683320918	-0.464443996	3
NDUFB2	1.147431059	-0.46168217	-0.685748889	3
NDUFB8	0.909599111	-1.070812618	0.161213507	3
NECAB3	1.129577053	-0.772253611	-0.357323442	3
NEK3	1.118138182	-0.808719356	-0.309418825	3
NEK9	1.118034679	-0.809015024	-0.309019656	3
NELFB	1.107279171	-0.837276021	-0.27000315	3
NELL2	1.154083427	-0.609730888	-0.54435254	3
NEMP1	1.130448822	-0.769097155	-0.361351667	3
NEPRO	1.154029649	-0.611098197	-0.542931452	3
NFATC1	0.956019348	-1.038830728	0.08281138	3
NFATC3	1.023075348	-0.975205254	-0.047870094	3
NFE2L3	0.889169161	-1.082576471	0.19340731	3
NFIA	1.154452762	-0.556511292	-0.59794147	3
NFKB1	1.154675868	-0.583874805	-0.570801063	3
NFRKB	1.144430954	-0.439142779	-0.705288175	3
NFU1	1.097743881	-0.859063459	-0.238680422	3
NFX1	0.957392987	-1.037757314	0.080364328	3
NHEJ1	1.127113106	-0.346273248	-0.780839858	3

NICN1	1.150644656	-0.491580722	-0.659063934	3
NKX6-3	0.92276749	-1.062528586	0.139761095	3
NLE1	1.127929104	-0.349880567	-0.778048537	3
NLRC3	1.131695089	-0.367228135	-0.764466953	3
NLRP4	1.135626657	-0.386804844	-0.748821813	3
NME1	1.152142982	-0.642591503	-0.509551479	3
NME2	1.087637656	-0.879651	-0.207986656	3
NME3	0.985035497	-1.014321162	0.029285665	3
NMT2	1.124240409	-0.333947842	-0.790292567	3
NOC3L	0.963640584	-1.03277185	0.069131266	3
NOG	0.915630974	-1.067089596	0.151458622	3
NOL10	1.012896542	-0.986584134	-0.026312408	3
NOL9	1.041840035	-0.952112557	-0.089727478	3
NONO	0.930864841	-1.057140107	0.126275266	3
NOPCHAP1	1.134068064	-0.755228664	-0.378839399	3
NPAT	1.049526477	-0.941741212	-0.107785265	3
NPBWR1	0.991504967	-1.008283873	0.016778906	3
NPIPA2	1.13560514	-0.386692867	-0.748912273	3
NR2C1	1.15378693	-0.537121733	-0.616665197	3
NRBP1	0.907839061	-1.071876794	0.164037733	3
NRROS	1.151280534	-0.652548321	-0.498732214	3
NSA2	1.046801045	-0.945485442	-0.101315602	3
NSD3	1.080496113	-0.892946387	-0.187549726	3
NSG1	1.080772045	-0.892449711	-0.188322334	3

NSMCE1	1.120128146	-0.802931737	-0.317196409	3
NT5E	1.137387509	-0.741211117	-0.396176392	3
NTMT1	1.126526952	-0.782814768	-0.343712184	3
NTPCR	1.139264283	-0.406666902	-0.732597382	3
NUDCD2	1.133427316	-0.375645895	-0.757781421	3
NUDT9	0.98669354	-1.012795414	0.026101875	3
NUFIP2	1.134690851	-0.381987652	-0.7527032	3
NUMA1	1.139572398	-0.731127647	-0.40844475	3
NUP133	1.145929453	-0.44994332	-0.695986133	3
NUP153	1.149885095	-0.483710968	-0.666174128	3
NUP155	1.124263471	-0.334044613	-0.790218858	3
NUP210L	1.056686841	-0.931530338	-0.125156503	3
NUP43	1.118123838	-0.808760362	-0.309363476	3
NUS1	1.122446398	-0.326520063	-0.795926336	3
NUSAP1	0.892041906	-1.080998061	0.188956155	3
NXN	1.141706915	-0.720449548	-0.421257367	3
NXPE3	1.152866229	-0.632776643	-0.520089586	3
OAS1	1.144189813	-0.437475986	-0.706713827	3
OAS2	1.09058917	-0.873869458	-0.216719712	3
OASL	1.055984707	-0.932556593	-0.123428114	3
OBSCN	1.132360238	-0.370424614	-0.761935624	3
OCIAD1	0.891417632	-1.081343109	0.189925477	3
OGT	1.103215699	-0.846881182	-0.256334517	3
OR52N4	1.082547509	-0.889222267	-0.193325242	3

OR5K2	1.146294368	-0.45270252	-0.693591848	3
OR5M1	1.154651113	-0.586577916	-0.568073197	3
ORC2	1.052212932	-0.937975328	-0.114237604	3
ORC3	1.152457521	-0.638528418	-0.513929103	3
OSBPL3	1.153085555	-0.629413163	-0.523672392	3
OSGEPL1	1.135408145	-0.749737746	-0.385670398	3
OTUD3	1.136191725	-0.746424976	-0.389766749	3
OTULIN	0.903222545	-1.074621526	0.171398981	3
OXCT2	1.148587334	-0.471530095	-0.67705724	3
OXSR1	1.117211079	-0.811349587	-0.305861492	3
P2RY11	1.153798277	-0.616423214	-0.537375063	3
PAAF1	1.004812564	-0.995116931	-0.009695633	3
PACRGL	1.150932935	-0.494750705	-0.65618223	3
PAGE1	1.153752219	-0.617396007	-0.536356212	3
PAGE5	1.134528775	-0.381164028	-0.753364747	3
PAICS	1.014539028	-0.98479744	-0.029741587	3
PALB2	0.919606774	-1.0645699	0.144963126	3
PANK4	1.129751439	-0.35812402	-0.771627418	3
PAPSS1	1.151080538	-0.49641887	-0.654661668	3
PARD6B	1.147924073	-0.682141247	-0.465782826	3
PARP1	1.129378372	-0.35641444	-0.772963932	3
PARP12	1.103884262	-0.845335472	-0.25854879	3
PARP15	0.964717732	-1.031894827	0.067177095	3
PASK	1.154672939	-0.570422543	-0.584250396	3

PATJ	1.123337484	-0.330185107	-0.793152377	3
PBX2	1.128105586	-0.77743822	-0.350667366	3
PCBD1	1.083782677	-0.88694399	-0.196838687	3
PCCB	1.125328303	-0.338549989	-0.786778314	3
PCDHB15	1.077727785	-0.897858483	-0.179869301	3
PCGF1	1.058807605	-0.928396085	-0.13041152	3
PCYT1A	1.154318765	-0.551446723	-0.602872042	3
PDCD11	1.066623635	-0.916372539	-0.150251096	3
PDE6C	1.151407612	-0.500236046	-0.651171566	3
PDGFD	1.137776576	-0.398305913	-0.739470663	3
PDIA3	1.151653656	-0.503229359	-0.648424297	3
PDIA4	1.072584612	-0.906659269	-0.165925343	3
PDIA6	1.146828157	-0.689985447	-0.45684271	3
PDXDC1	1.138527891	-0.736044281	-0.40248361	3
PDZD9	1.095873998	-0.863048305	-0.232825694	3
PECR	1.071635085	-0.908240265	-0.16339482	3
PEX10	1.024297722	-0.973787933	-0.050509789	3
PEX19	1.009902233	-0.989794504	-0.020107729	3
PEX5	1.130799816	-0.362992317	-0.767807499	3
PEX6	1.119901308	-0.316299684	-0.803601624	3
PFAS	1.154693859	-0.573945542	-0.580748316	3
PFKFB1	1.154110842	-0.609010428	-0.545100415	3
PFKFB3	0.902137225	-1.075257142	0.173119918	3
PGAP2	1.143767392	-0.434598901	-0.709168491	3

PGAP4	1.151851236	-0.646132788	-0.505718448	3
PGBD4	0.919477975	-1.064652361	0.145174386	3
PGM1	1.129950421	-0.359040673	-0.770909749	3
PHACTR4	0.944833716	-1.047279395	0.102445679	3
PHC1	1.152132347	-0.642724194	-0.509408153	3
PHF5A	1.154674374	-0.584068973	-0.570605401	3
PHLDB3	1.154204746	-0.547801283	-0.606403462	3
PHRF1	1.143498212	-0.432792715	-0.710705497	3
PI16	1.145185069	-0.444477869	-0.700707201	3
PIDD1	1.138610591	-0.402948926	-0.735661665	3
PIGB	1.147787513	-0.464633206	-0.683154307	3
PIGBOS1	1.152086533	-0.508793986	-0.643292547	3
PIGG	1.005594493	-0.99431	-0.011284494	3
PIGK	1.127270931	-0.780303843	-0.346967088	3
PIK3AP1	1.147211355	-0.459897369	-0.687313987	3
PIP4K2B	1.132557457	-0.371380794	-0.761176663	3
PITPNB	1.111093027	-0.827767405	-0.283325623	3
PKD2L1	1.154699889	-0.57628904	-0.578410849	3
PKN1	1.129497047	-0.356957005	-0.772540042	3
PLA2G6	1.154625969	-0.588677563	-0.565948406	3
PLAAT2	1.072048171	-0.907554084	-0.164494087	3
PLAC9	1.132473895	-0.37097518	-0.761498715	3
PLCB3	1.148653428	-0.676534721	-0.472118707	3
PLD6	1.141620301	-0.420719124	-0.720901177	3

PLEKHG4	1.139460461	-0.731663532	-0.407796929	3
PLSCR1	1.136352372	-0.390616437	-0.745735936	3
PLXNA3	0.905262885	-1.073416697	0.168153812	3
PMF1-BGLAP	1.154693533	-0.580830197	-0.573863336	3
PNOC	1.143034907	-0.713303916	-0.429730992	3
PNPLA4	1.131722744	-0.367360178	-0.764362565	3
PNPO	1.132258471	-0.369932738	-0.762325733	3
POC1A	1.153915183	-0.613833238	-0.540081944	3
POFUT1	1.140306955	-0.412752599	-0.727554356	3
POGZ	1.154167184	-0.546693077	-0.607474107	3
POLD1	1.151847513	-0.646176735	-0.505670778	3
POLE3	0.909209382	-1.071049112	0.16183973	3
POLE4	1.021284612	-0.977261322	-0.044023291	3
POLH	0.989335308	-1.010333957	0.020998649	3
POLQ	1.086312802	-0.882189397	-0.204123405	3
POLR1E	1.154700399	-0.576858862	-0.577841537	3
POLR2D	1.148609333	-0.471725676	-0.676883658	3
POLR2E	1.00090342	-0.999094125	-0.001809295	3
POLR2M	1.137114546	-0.742418946	-0.394695601	3
POLR3A	1.026073322	-0.971708825	-0.054364497	3
POLR3E	1.150091248	-0.664306983	-0.485784265	3
POLR3GL	1.153696267	-0.53515051	-0.618545758	3
POMGNT2	0.965615186	-1.031160112	0.065544926	3
POU1F1	1.120120938	-0.317167874	-0.802953064	3

PPDPF	1.138909254	-0.734270536	-0.404638717	3
PPEF1	1.14881852	-0.675216037	-0.473602483	3
PPM1D	1.12894085	-0.774516654	-0.354424196	3
PPM1K	1.069566026	-0.911640262	-0.157925764	3
PPP1R35	1.151140134	-0.497101637	-0.654038496	3
PPP1R3E	1.096328613	-0.862087353	-0.23424126	3
PPP1R3F	1.153705454	-0.535346177	-0.618359277	3
PPP2R5B	1.131984654	-0.368614369	-0.763370284	3
PPP2R5D	1.125964433	-0.341277298	-0.784687136	3
PPP3CC	1.151484391	-0.650326193	-0.501158198	3
PPP4R3A	1.148914575	-0.674439656	-0.47447492	3
PQBP1	1.137263039	-0.395499823	-0.741763216	3
PRDM2	0.924524298	-1.061379097	0.136854799	3
PRKAR2A	1.097624863	-0.85931967	-0.238305194	3
PRKCH	1.154602668	-0.590320916	-0.564281752	3
PRKCI	1.128696478	-0.353319342	-0.775377136	3
PRKD3	1.153731356	-0.535902664	-0.617828692	3
PRKRA	1.148794161	-0.473382324	-0.675411837	3
PRL	1.082082386	-0.890073115	-0.192009271	3
PRLH	1.154700538	-0.577350269	-0.577350269	3
PROX2	1.100711187	-0.85255778	-0.248153407	3
PRPF39	1.15469726	-0.574965634	-0.579731626	3
PRPF4B	1.150706708	-0.658453017	-0.492253691	3
PRPS1	1.111989502	-0.825456272	-0.28653323	3

PRPSAP2	1.050630499	-0.940202783	-0.110427717	3
PRR3	0.958247342	-1.037085608	0.078838266	3
PRR4	1.069801244	-0.911256801	-0.158544443	3
PRSS16	1.131454848	-0.366084131	-0.765370718	3
PRSS35	1.015921101	-0.98327964	-0.032641461	3
PSG8	1.153701282	-0.535257199	-0.618444083	3
PSMA8	1.153288111	-0.62608999	-0.52719812	3
PSMB11	1.125382528	-0.338781405	-0.786601122	3
PSMB2	1.1352714	-0.384963491	-0.750307909	3
PSMB4	1.15210315	-0.509016142	-0.643087008	3
PSMD12	1.154031983	-0.611040046	-0.542991937	3
PSMG1	1.153997837	-0.611880789	-0.542117047	3
PSMG4	1.134923588	-0.383175775	-0.751747813	3
PTGES3	1.125253613	-0.787022062	-0.338231551	3
PTPN1	0.899941018	-1.076532256	0.176591239	3
PTX3	1.14480222	-0.703056793	-0.441745427	3
PUS10	1.154248184	-0.605112441	-0.549135742	3
PWP2	1.152141856	-0.509536287	-0.642605569	3
PYGM	1.14134695	-0.419031281	-0.722315669	3
QTRT2	1.143920503	-0.435635594	-0.708284909	3
RAB18	1.138081389	-0.73809117	-0.399990219	3
RAB28	1.049324652	-0.942021082	-0.107303569	3
RAB35	1.132437855	-0.761637398	-0.370800457	3
RAB39B	1.12043783	-0.31842499	-0.80201284	3

RAB40B	1.150005636	-0.665087651	-0.484917986	3
RAB43	1.153941251	-0.613229305	-0.540711946	3
RABEP2	1.153139811	-0.628545215	-0.524594595	3
RABGAP1	0.902124313	-1.075264682	0.173140369	3
RABGEF1	1.138284509	-0.40112058	-0.737163929	3
RABIF	0.904819047	-1.073679891	0.168860844	3
RABL2B	1.153057313	-0.523198318	-0.629858995	3
RAC3	1.153381269	-0.528902173	-0.624479096	3
RACK1	1.138925412	-0.404730563	-0.734194849	3
RAD17	1.136828555	-0.393155534	-0.743673021	3
RAD52	1.085243893	-0.88421274	-0.201031153	3
RAD54L2	1.151387226	-0.499992945	-0.651394281	3
RADX	1.141734694	-0.421430346	-0.720304347	3
RALGAPA1	0.927075464	-1.05969066	0.132615195	3
RALGAPA2	1.14806712	-0.466998914	-0.681068206	3
RAMP3	1.139650867	-0.408900213	-0.730750654	3
RANGRF	1.154663067	-0.569275367	-0.5853877	3
RAPGEF3	1.144135775	-0.707030831	-0.437104944	3
RAPGEF6	1.145587568	-0.698180558	-0.44740701	3
RAPGEFL1	1.154393272	-0.554128671	-0.600264601	3
RASAL1	1.142259066	-0.424728819	-0.717530247	3
RASGRF2	1.102147597	-0.849323704	-0.252823893	3
RASGRP1	0.942507287	-1.048973308	0.106466021	3
RASSF7	1.146444717	-0.453855765	-0.692588952	3

RBM14-RBM4	1.144066938	-0.436633576	-0.707433362	3
RBM22	1.120828514	-0.80084633	-0.319982184	3
RBM28	1.1522859	-0.640779652	-0.511506248	3
RBM34	1.143962145	-0.435918742	-0.708043403	3
RBM4	1.13822966	-0.400814708	-0.737414951	3
RBM6	1.100100949	-0.853914633	-0.246186316	3
RBMX	1.132802275	-0.372573239	-0.760229036	3
RBSN	1.129670375	-0.771918826	-0.357751549	3
RCBTB1	1.073552426	-0.905034128	-0.168518298	3
RCC2	0.889061325	-1.082635254	0.193573929	3
RCE1	1.153302769	-0.62584023	-0.527462539	3
RCL1	1.153578424	-0.532714117	-0.620864307	3
RDH16	1.122814285	-0.328027492	-0.794786793	3
RELCH	0.988532534	-1.011085937	0.022553403	3
RELL2	1.100624968	-0.852750097	-0.247874871	3
RETNLB	1.154700538	-0.577350269	-0.577350269	3
RFC5	1.147357528	-0.686275511	-0.461082017	3
RFNG	1.131702396	-0.764439379	-0.367263017	3
RFPL2	1.154691639	-0.57341984	-0.581271799	3
RFPL4A	1.153690755	-0.535033523	-0.618657232	3
RFPL4AL1	1.154376487	-0.553498694	-0.600877793	3
RFT1	1.141404464	-0.722019406	-0.419385058	3
RFWD3	0.924163109	-1.0616163	0.137453191	3
RHOBTB2	1.129123095	-0.773871789	-0.355251305	3

RHOXF1	1.116695801	-0.812794094	-0.303901707	3
RIMBP3B	1.147383033	-0.461289863	-0.68609317	3
RIMKLB	1.131254711	-0.76611946	-0.365135251	3
RINL	1.153423258	-0.623733861	-0.529689398	3
RIPK1	0.975072527	-1.023190233	0.048117706	3
RMDN1	1.108268728	-0.834857215	-0.273411513	3
RMI2	1.143787734	-0.434736238	-0.709051496	3
RMND1	1.150907887	-0.494470753	-0.656437134	3
RNF103	0.902516611	-1.075035369	0.172518757	3
RNF121	1.131358255	-0.765732555	-0.3656257	3
RNF125	1.079194823	-0.895271279	-0.183923544	3
RNF141	1.128608967	-0.352924848	-0.775684119	3
RNF144A	1.14540066	-0.44603938	-0.69936128	3
RNF169	1.038714972	-0.956167977	-0.082546996	3
RNF182	1.133294728	-0.374990655	-0.758304073	3
RNF185	1.078374886	-0.896721643	-0.181653242	3
RNF207	1.153778973	-0.616833958	-0.536945015	3
RNF213	1.120377799	-0.31818644	-0.802191359	3
RNF214	1.133542326	-0.757326527	-0.376215799	3
RNF227	1.153553484	-0.532214775	-0.621338709	3
RNF4	1.078624262	-0.896281704	-0.182342558	3
RNF41	1.118031308	-0.809024644	-0.309006664	3
RNF7	0.967254587	-1.029808512	0.062553925	3
RNF8	1.007430588	-0.992399965	-0.015030623	3

ROGDI	0.980538484	-1.018386853	0.037848368	3
RP2	1.020569419	-0.978075845	-0.042493574	3
RPAP3	1.145929196	-0.449941396	-0.6959878	3
RPF1	1.15188368	-0.64574865	-0.50613503	3
RPL10A	1.133319997	-0.375115388	-0.75820461	3
RPL11	1.120835848	-0.800824354	-0.320011494	3
RPL14	1.090044333	-0.874950211	-0.215094121	3
RPL15	1.116604337	-0.813049232	-0.303555104	3
RPL22	1.131894823	-0.368183458	-0.763711366	3
RPL26L1	1.140994769	-0.416880322	-0.724114448	3
RPL31	0.999388652	-1.000610229	0.001221577	3
RPL34	1.124937088	-0.788050925	-0.336886164	3
RPL37A	1.154599601	-0.59052182	-0.564077781	3
RPL41	1.149915309	-0.484012144	-0.665903165	3
RPL6	0.980867759	-1.018092698	0.037224939	3
RPL9	0.91469893	-1.067672659	0.152973729	3
RPP21	1.153875066	-0.539132112	-0.614742954	3
RPRD2	1.154392658	-0.554105334	-0.600287323	3
RPRM	1.128447188	-0.35219716	-0.776250028	3
RPS13	1.154124017	-0.608658127	-0.54546589	3
RPS14	1.066061027	-0.917264056	-0.148796972	3
RPS16	1.119157467	-0.313377754	-0.805779713	3
RPS17	1.032509306	-0.96396371	-0.068545595	3
RPS19	1.154305707	-0.603301503	-0.551004203	3

RPS20	0.89917777	-1.076971944	0.177794174	3
RPS27A	1.150469493	-0.660762283	-0.48970721	3
RPS27L	1.127080385	-0.346129628	-0.780950758	3
RPS3A	1.145373727	-0.699530346	-0.445843381	3
RPS4X	0.984310508	-1.014983733	0.030673225	3
RPS4Y1	0.892178307	-1.080922518	0.188744211	3
RPS6KB2	1.150598041	-0.491078407	-0.659519634	3
RPS7	1.133496733	-0.757507029	-0.375989705	3
RPS9	1.117361652	-0.810925161	-0.306436491	3
RPSA	1.121000309	-0.320669527	-0.800330782	3
RPUSD1	1.090683442	-0.873681822	-0.21700162	3
RPUSD4	1.144328974	-0.438435658	-0.705893316	3
RRM1	0.965142786	-1.031547305	0.06640452	3
RRP1	1.154184856	-0.547209511	-0.606975345	3
RRP1B	1.151290925	-0.652436772	-0.498854153	3
RRP8	1.154573562	-0.592116415	-0.562457146	3
RSBN1L	0.931593957	-1.056643426	0.125049468	3
RSL1D1	1.031285272	-0.965462618	-0.065822654	3
RTL5	1.13644645	-0.74533082	-0.391115629	3
RTP4	1.024682755	-0.973339142	-0.051343612	3
RTRAF	1.140026453	-0.41109575	-0.728930703	3
RUNX1	1.152496346	-0.514489538	-0.638006808	3
RXYLT1	1.088143138	-0.878673397	-0.209469741	3
RYK	1.154561311	-0.561752173	-0.592809138	3

S100A1	1.152473598	-0.638312997	-0.514160601	3
S100A7	1.15408651	-0.609650708	-0.544435802	3
S100PBP	1.144904689	-0.442471805	-0.702432884	3
SAA2-SAA4	0.942949462	-1.048652983	0.105703521	3
SAG	1.144464419	-0.439375537	-0.705088882	3
SAPCD1	1.154700538	-0.577350269	-0.577350269	3
SATB1	1.147841373	-0.46508532	-0.682756053	3
SAV1	1.083485115	-0.887495365	-0.19598975	3
SCAF4	1.153383849	-0.528950189	-0.624433659	3
SCN3A	1.150856467	-0.493898828	-0.65695764	3
SCN5A	1.043953209	-0.949318707	-0.094634502	3
SCUBE3	1.138742578	-0.403693874	-0.735048704	3
SCYL3	0.973479739	-1.024562236	0.051082497	3
SDC3	1.126560056	-0.782703887	-0.343856169	3
SDE2	1.112296618	-0.824657481	-0.287639136	3
SDR42E1	1.125937599	-0.784775906	-0.341161693	3
SEC31B	0.945711464	-1.046634757	0.100923293	3
SENP2	1.146410408	-0.692818675	-0.453591733	3
SENP3	1.146978819	-0.68894369	-0.45803513	3
SEPSECS	1.150395357	-0.661469879	-0.488925477	3
SERINC5	1.124781916	-0.788552916	-0.336229001	3
SERPINB6	0.996114365	-1.003840861	0.007726496	3
SERPINF2	1.153182786	-0.525336181	-0.627846605	3
SETD5	1.154699429	-0.578735996	-0.575963433	3

SETD6	1.020265338	-0.97842102	-0.041844318	3
SF3A3	0.924376632	-1.061476128	0.137099497	3
SFN	0.943906163	-1.047957306	0.104051143	3
SFSWAP	1.151934179	-0.506788085	-0.645146094	3
SFXN2	1.131716436	-0.764386379	-0.367330057	3
SFXN3	1.118821868	-0.806753249	-0.31206862	3
SGPP1	1.024631916	-0.973398464	-0.051233452	3
SGTB	1.154510072	-0.559092702	-0.59541737	3
SH2D3A	1.147908701	-0.465652869	-0.682255832	3
SH3YL1	1.084785463	-0.885073886	-0.199711577	3
SIGLEC11	1.031726206	-0.964924098	-0.066802108	3
SIL1	1.150708907	-0.658431276	-0.492277631	3
SIN3A	1.119582888	-0.315045418	-0.80453747	3
SINHCAF	1.129144428	-0.355348301	-0.773796127	3
SIRT3	0.905962425	-1.073000618	0.167038193	3
SIT1	1.141455219	-0.419697851	-0.721757368	3
SLC11A2	1.150745499	-0.658068562	-0.492676937	3
SLC12A7	1.153653241	-0.619407722	-0.534245519	3
SLC15A2	1.114973304	-0.817536453	-0.297436851	3
SLC15A3	1.062680417	-0.922534212	-0.140146206	3
SLC16A10	1.064563124	-0.919617354	-0.14494577	3
SLC17A3	1.041870459	-0.952072632	-0.089797828	3
SLC18A1	0.996700071	-1.00326758	0.006567509	3
SLC1A4	1.131511741	-0.366354555	-0.765157186	3

SLC24A4	0.945803947	-1.046566658	0.100762711	3
SLC25A15	1.14152781	-0.420146197	-0.721381614	3
SLC25A16	1.153944457	-0.613154304	-0.540790153	3
SLC25A25	1.138603482	-0.402908885	-0.735694597	3
SLC25A26	1.147607534	-0.684473147	-0.463134387	3
SLC25A33	1.133172125	-0.374386423	-0.758785702	3
SLC25A5	1.14567359	-0.448040885	-0.697632705	3
SLC26A11	1.153842474	-0.61546548	-0.538376994	3
SLC27A4	1.152215414	-0.510535383	-0.641680031	3
SLC2A6	1.020740615	-0.977881214	-0.042859402	3
SLC30A6	1.102377748	-0.848800156	-0.253577592	3
SLC35B3	1.130331494	-0.360805682	-0.769525812	3
SLC35F5	1.123194805	-0.793599714	-0.329595092	3
SLC36A3	0.921870653	-1.063111276	0.141240623	3
SLC38A1	1.148618387	-0.471806263	-0.676812124	3
SLC38A11	0.938686025	-1.051710087	0.113024062	3
SLC38A9	1.142777691	-0.714721892	-0.428055799	3
SLC39A10	1.123781659	-0.332029813	-0.791751846	3
SLC39A6	1.146765783	-0.690413568	-0.456352215	3
SLC39A9	0.939890025	-1.050853832	0.110963807	3
SLC47A1	1.150757169	-0.492804657	-0.657952512	3
SLC52A2	1.153622541	-0.533610872	-0.620011669	3
SLC5A10	1.128868318	-0.354095765	-0.774772553	3
SLC5A3	1.153917245	-0.613785833	-0.540131412	3

SLC66A3	1.121973948	-0.324595678	-0.79737827	3
SLC6A13	1.137690883	-0.397834943	-0.73985594	3
SLC7A6	1.12348196	-0.330783812	-0.792698148	3
SMARCC1	1.152763943	-0.518490098	-0.634273845	3
SMG6	1.145971294	-0.450256901	-0.695714393	3
SMIM10	1.007843423	-0.991967536	-0.015875887	3
SMIM13	1.133421701	-0.375618107	-0.757803593	3
SMIM17	1.154700538	-0.577350269	-0.577350269	3
SMIM29	1.116075856	-0.814516021	-0.301559836	3
SMUG1	1.084677662	-0.885275818	-0.199401845	3
SMURF2	0.899347897	-1.076874092	0.177526195	3
SMYD5	0.908762655	-1.071319596	0.162556941	3
SNAP29	1.143119473	-0.712833947	-0.430285526	3
SNAP47	1.131189368	-0.364826261	-0.766363107	3
SNRPB2	1.144371965	-0.438733355	-0.705638611	3
SNX11	1.154670925	-0.584497202	-0.570173724	3
SNX19	1.107570712	-0.836566788	-0.271003924	3
SNX2	0.999764779	-1.000235055	0.000470276	3
SNX30	1.00049045	-0.999508827	-0.000981623	3
SOD1	1.151347985	-0.65182098	-0.499527005	3
SOS1	0.978811214	-1.01992088	0.041109666	3
SP100	1.143117599	-0.430273213	-0.712844385	3
SP140L	1.151467387	-0.500953068	-0.65051432	3
SPAG1	1.114524292	-0.818751538	-0.295772754	3

SPAG8	1.152901071	-0.63225681	-0.520644261	3
SPANXN1	1.154700538	-0.577350269	-0.577350269	3
SPATA25	1.147313968	-0.686586142	-0.460727825	3
SPATA33	1.139738731	-0.730327208	-0.409411522	3
SPATA6L	1.148028373	-0.466668288	-0.681360084	3
SPATS2L	1.133303362	-0.375033267	-0.758270095	3
SPCS3	1.146016458	-0.69542027	-0.450596188	3
SPDYA	1.154700538	-0.577333696	-0.577366842	3
SPEF2	1.140090936	-0.728615619	-0.411475317	3
SPG7	0.946802375	-1.045829303	0.099026928	3
SPIN3	1.135408664	-0.385673085	-0.749735579	3
SPIN4	0.932809552	-1.055811049	0.123001498	3
SPINK4	1.128296842	-0.351522747	-0.776774095	3
SPOUT1	1.152644236	-0.635974848	-0.516669389	3
SPPL2B	1.093025587	-0.868958602	-0.224066985	3
SPRYD4	1.127219887	-0.780477402	-0.346742485	3
SPTSSB	0.964019818	-1.03246367	0.068443852	3
SPTY2D1OS	1.114493742	-0.8188339	-0.295659842	3
SRBD1	1.022835923	-0.975481541	-0.047354383	3
SRD5A3	1.129868001	-0.358660575	-0.771207425	3
SREK1IP1	1.152095929	-0.643176406	-0.508919523	3
SRSF8	1.126139915	-0.34203451	-0.784105405	3
SSTR3	1.142654004	-0.715397716	-0.427256288	3
ST20-MTHFS	1.148102199	-0.467299024	-0.680803175	3

ST3GAL1	1.149008738	-0.67367185	-0.475336889	3
STAC3	1.126287022	-0.34267093	-0.783616091	3
STARD5	1.14482822	-0.4419294	-0.702898821	3
STAT1	1.143865995	-0.708600257	-0.435265738	3
STATH	1.154700538	-0.577350269	-0.577350269	3
STEEP1	1.154203826	-0.547773659	-0.606430167	3
STK36	0.936209144	-1.053454321	0.117245177	3
STK38	1.083564722	-0.887348013	-0.196216709	3
STK38L	1.086763709	-0.881329306	-0.205434403	3
STS	1.078499649	-0.896501668	-0.181997981	3
STX12	1.125468794	-0.786318822	-0.339149972	3
SUGP2	1.119708054	-0.315537828	-0.804170226	3
SUN5	1.100517457	-0.852989623	-0.247527834	3
SUPT3H	1.151891864	-0.645651384	-0.50624048	3
SUPT7L	1.129954236	-0.359058279	-0.770895957	3
SURF2	1.12385401	-0.79152257	-0.332331439	3
SYCE2	1.142192442	-0.717886271	-0.424306171	3
SYCP2	1.125741324	-0.340317626	-0.785423698	3
SYF2	0.942370492	-1.049072252	0.106701759	3
SYNJ2BP	0.943606834	-1.048175352	0.104568518	3
SYT11	1.153849852	-0.538546692	-0.615303161	3
SZRD1	1.153856305	-0.615160616	-0.538695689	3
TAC4	1.153892253	-0.614356037	-0.539536216	3
TAF11	1.061955624	-0.923645215	-0.138310409	3

TAF15	1.152539159	-0.5151131	-0.63742606	3
TAF1C	1.142861568	-0.428600194	-0.714261374	3
TAFAZZIN	1.092662387	-0.86969891	-0.222963477	3
TARBP1	1.106711494	-0.838649019	-0.268062475	3
TAS2R14	1.14068757	-0.415025081	-0.72566249	3
TASOR2	1.057911021	-0.929727521	-0.1281835	3
TBC1D10A	1.074021581	-0.904241281	-0.169780299	3
TBC1D2B	0.949663357	-1.043694255	0.094030898	3
TBC1D3E	1.101274448	-0.851296404	-0.249978044	3
TBC1D3F	1.142970545	-0.71366034	-0.429310205	3
TBCC	1.154634064	-0.588047051	-0.566587013	3
TBCD	1.135626918	-0.386806202	-0.748820716	3
TBL2	1.020474844	-0.978183274	-0.04229157	3
TCAP	0.89529685	-1.079180422	0.183883572	3
TCEA3	0.89462549	-1.079557891	0.1849324	3
TCEAL3	1.149619091	-0.668521656	-0.481097435	3
TCF20	1.152360386	-0.512547211	-0.639813174	3
TDRD3	1.072813056	-0.906276924	-0.166536132	3
TDRD9	1.149157849	-0.476715866	-0.672441984	3
TEF	1.056818303	-0.931337564	-0.12548074	3
TEX10	0.92407919	-1.061671347	0.137592157	3
TEX12	1.148273646	-0.679496804	-0.468776842	3
TEX29	1.154227274	-0.605741411	-0.548485864	3
TEX33	1.085150533	-0.884388433	-0.2007621	3

TFAM	1.154538776	-0.59400738	-0.560531396	3
TFB1M	1.123493148	-0.330830227	-0.792662921	3
TFB2M	1.154629139	-0.56619416	-0.588434979	3
TGFBR2	1.130791964	-0.767836468	-0.362955497	3
TGIF2	1.141212326	-0.418205968	-0.723006358	3
TGM1	1.138305268	-0.737068799	-0.401236469	3
TGS1	1.139716846	-0.409284038	-0.730432808	3
THADA	1.07579145	-0.901220053	-0.174571397	3
THAP12	1.152189232	-0.510178156	-0.642011076	3
THAP6	1.059694166	-0.927070202	-0.132623964	3
THNSL1	1.15451297	-0.559232869	-0.595280101	3
THNSL2	0.938380591	-1.051926428	0.113545837	3
TIGD2	1.137423169	-0.741052528	-0.396370641	3
TIMELESS	1.144917626	-0.702353856	-0.44256377	3
TIMM10B	1.141967205	-0.422885067	-0.719082138	3
TIMM44	1.083718943	-0.887062223	-0.196656719	3
TJAP1	1.147858564	-0.465229979	-0.682628584	3
TLDC2	1.08003335	-0.893776442	-0.186256908	3
TLE2	1.150954676	-0.655960259	-0.494994417	3
TLK2	1.089658309	-0.875712182	-0.213946127	3
TLN1	0.975298701	-1.02299441	0.047695709	3
TM7SF2	1.09379583	-0.867378822	-0.226417008	3
TM9SF3	1.128159341	-0.777251845	-0.350907496	3
TMC8	1.153852455	-0.538606732	-0.615245723	3

TMCC1	1.153471749	-0.622857377	-0.530614372	3
TMCO6	1.147605511	-0.463117644	-0.684487867	3
TMED5	1.13334181	-0.758118693	-0.375223117	3
TMED7	1.145266258	-0.445063934	-0.700202323	3
TMEM109	0.955916904	-1.038910459	0.082993555	3
TMEM119	1.154684823	-0.572125123	-0.582559699	3
TMEM131L	0.949461921	-1.043845664	0.094383744	3
TMEM134	1.149506659	-0.480012468	-0.669494191	3
TMEM14A	1.071810128	-0.907949801	-0.163860327	3
TMEM161A	1.122006226	-0.324726744	-0.797279481	3
TMEM165	1.150809061	-0.657434277	-0.493374784	3
TMEM167A	0.901296004	-1.075747302	0.174451299	3
TMEM187	1.149351226	-0.478530829	-0.670820397	3
TMEM218	1.148020551	-0.681418894	-0.466601657	3
TMEM225B	1.146614044	-0.69144751	-0.455166534	3
TMEM263	1.128731371	-0.775254563	-0.353476808	3
TMEM268	1.132417438	-0.761715905	-0.370701533	3
TMEM39A	1.152886918	-0.520418316	-0.632468601	3
TMEM41A	1.145056987	-0.701498928	-0.443558059	3
TMEM41B	1.045311815	-0.947499887	-0.097811927	3
TMEM42	0.89566712	-1.078971665	0.183304545	3
TMEM8B	1.141195034	-0.7230948	-0.418100233	3
TMIGD2	1.062606578	-0.922647696	-0.139958882	3
TMOD2	1.116859091	-0.812337656	-0.304521435	3

TMPO	1.141022101	-0.417046317	-0.723975784	3
TMSB15B	1.079497928	-0.894732298	-0.18476563	3
TMX3	1.154200531	-0.547674922	-0.606525609	3
TNFRSF10A	1.154652459	-0.568200733	-0.586451725	3
TNFRSF17	1.141960951	-0.422845773	-0.719115178	3
TNFSF10	1.142363731	-0.425394936	-0.716968795	3
TNNC1	1.146808626	-0.6901197	-0.456688926	3
TNRC6C	1.127365232	-0.779982699	-0.347382532	3
TOMM20L	1.131764319	-0.764205493	-0.367558826	3
TOMM7	1.065482559	-0.918176343	-0.147306216	3
TOMT	1.140962033	-0.72428033	-0.416681703	3
TOP3B	1.033314513	-0.962970866	-0.070343647	3
TOPBP1	0.930464229	-1.057412187	0.126947958	3
TOR1B	0.91936299	-1.064725931	0.14536294	3
TP53BP2	1.145042398	-0.443453659	-0.701588739	3
TP53I13	1.15391408	-0.613858566	-0.540055514	3
TP53RK	1.139451873	-0.407747322	-0.731704551	3
TPH1	1.15284901	-0.519817343	-0.633031666	3
TPR	1.080946688	-0.892134677	-0.188812012	3
TRABD2A	1.04519748	-0.947653643	-0.097543837	3
TRAF4	1.128354591	-0.351781587	-0.776573004	3
TRAF5	1.017443751	-0.981592006	-0.035851746	3
TRAM2	1.148805436	-0.675321263	-0.473484173	3
TRAPPC11	1.136635194	-0.74451444	-0.392120753	3

TRAPPC13	1.110553392	-0.829144132	-0.28140926	3
TRERF1	1.154359178	-0.601493485	-0.552865693	3
TRIM59	0.956291059	-1.038619042	0.082327983	3
TRIM68	1.125854565	-0.34080429	-0.785050275	3
TRIM74	1.148911132	-0.474443525	-0.674467607	3
TRIP13	1.10852847	-0.834216863	-0.274311607	3
TRMT1	1.154665915	-0.585076909	-0.569589006	3
TRMT10A	1.085581715	-0.883575622	-0.202006093	3
TRMT13	1.150308703	-0.488019877	-0.662288826	3
TRMT1L	1.15440094	-0.554422146	-0.599978794	3
TRMT6	1.141471336	-0.4197973	-0.721674037	3
TRMU	1.145510429	-0.446840986	-0.698669443	3
TRPC4AP	1.151195487	-0.497740745	-0.653454743	3
TRPC6	1.029391292	-0.967757646	-0.061633646	3
TRPT1	1.154416086	-0.599403198	-0.555012888	3
TRPV2	1.152720189	-0.517818443	-0.634901745	3
TSC22D2	1.154586161	-0.563218371	-0.59136779	3
TSFM	1.146362737	-0.693137009	-0.453225729	3
TSPAN14	0.902583658	-1.07499613	0.172412472	3
TSPAN18	0.960098189	-1.035619612	0.075521423	3
TSPYL5	1.140108768	-0.411580419	-0.728528349	3
TSTD2	0.997155529	-1.002820402	0.005664873	3
TTC19	1.1183564	-0.808094293	-0.310262107	3
TTC21B	1.140958953	-0.724295927	-0.416663026	3

TTC38	0.943156281	-1.048502895	0.105346615	3
TTC39B	0.892658039	-1.080656395	0.187998355	3
TTLL1	1.125035171	-0.787732812	-0.337302359	3
TTYH2	0.917926005	-1.065641572	0.147715567	3
TUBB1	1.154155795	-0.607791098	-0.546364697	3
TUBB6	1.122063313	-0.324958701	-0.797104612	3
TUBB8	1.142791293	-0.428143964	-0.71464733	3
TUBD1	1.152813195	-0.519255011	-0.633558184	3
TUSC2	0.957760644	-1.037468643	0.079707999	3
TVP23A	1.123389105	-0.330398879	-0.792990226	3
TWF1	1.134436388	-0.753740474	-0.380695915	3
TXLNG	0.90031564	-1.076315796	0.176000156	3
TYW3	0.925019844	-1.061052916	0.136033072	3
UAP1	1.095994247	-0.862794611	-0.233199636	3
UBA7	1.119643454	-0.315283588	-0.804359866	3
UBAP1L	1.147993949	-0.681618625	-0.466375325	3
UBE2A	0.946430697	-1.046104257	0.09967356	3
UBE2T	0.928760865	-1.058562595	0.12980173	3
UBE2W	1.138636623	-0.735540993	-0.40309563	3
UBR3	1.154385196	-0.553823517	-0.600561679	3
UEVLD	0.919563079	-1.064597882	0.145034803	3
UGCG	1.124369181	-0.334488614	-0.789880568	3
UGGT1	1.014578055	-0.984754762	-0.029823294	3
UGT2A2	1.154700538	-0.577350269	-0.577350269	3

UGT2B15	1.150807352	-0.493355947	-0.657451404	3
UHRF1BP1	1.127548522	-0.348191916	-0.779356606	3
UHRF1BP1L	1.149916841	-0.484027435	-0.665889406	3
UMAD1	1.14577743	-0.448809863	-0.696967567	3
UNC45B	0.986245505	-1.013209142	0.026963637	3
UNC5CL	1.13731732	-0.395794592	-0.741522727	3
UQCC2	1.148649039	-0.676569516	-0.472079523	3
UQCRQ	1.153009088	-0.522397866	-0.630611222	3
URB2	0.955782345	-1.039015117	0.083232772	3
UROS	1.134882424	-0.751917262	-0.382965162	3
USF2	0.956643397	-1.038344071	0.081700674	3
USP16	1.128341735	-0.351723942	-0.776617794	3
USP20	1.148043525	-0.681246056	-0.466797469	3
USP27X	1.146230028	-0.452211984	-0.694018044	3
USP35	1.144737444	-0.441288063	-0.70344938	3
USP38	1.121951225	-0.324503444	-0.797447781	3
USP42	1.030450226	-0.966478089	-0.063972137	3
USP44	1.087992746	-0.878964787	-0.209027959	3
USPL1	0.981673131	-1.017370893	0.035697762	3
UTP11	1.068015332	-0.914149006	-0.153866326	3
UTP14A	1.146495239	-0.454245517	-0.692249722	3
UVRAG	1.137564748	-0.740421043	-0.397143705	3
UVSSA	1.144324148	-0.438402275	-0.705921873	3
VAMP1	1.001686128	-0.9983053	-0.003380828	3

VAMP3	1.142509439	-0.716182759	-0.426326679	3
VBP1	1.130204459	-0.769988565	-0.360215894	3
VDAC2	1.081657053	-0.890847832	-0.190809221	3
VEZF1	1.14431239	-0.438320978	-0.705991413	3
VHL	1.085804146	-0.883154946	-0.2026492	3
VMAC	0.951231841	-1.042509639	0.091277798	3
VPS35L	1.136909675	-0.743318477	-0.393591198	3
VPS51	1.140093515	-0.411490515	-0.728603	3
VPS72	1.072506125	-0.906790454	-0.165715671	3
VRK3	1.134264107	-0.379825614	-0.754438493	3
VSIG1	1.014014974	-0.985369509	-0.028645465	3
VWCE	1.154626059	-0.565955341	-0.588670718	3
VXN	1.035007593	-0.960865344	-0.074142248	3
WBP1	1.136122902	-0.389403779	-0.746719123	3
WDHD1	1.13534044	-0.385320107	-0.750020333	3
WDR11	0.906167713	-1.072878223	0.166710511	3
WDR13	1.04142584	-0.952655236	-0.088770604	3
WDR19	1.153716156	-0.535575226	-0.61814093	3
WDR27	1.142372358	-0.425449954	-0.716922404	3
WDR33	1.145013165	-0.443244678	-0.701768486	3
WDR36	1.125588085	-0.785927628	-0.339660457	3
WDR37	0.998617416	-1.001376873	0.002759457	3
WDR45B	0.923699053	-1.061920393	0.138221339	3
WDR59	1.116085558	-0.814489208	-0.30159635	3

WDR5B	1.079470424	-0.894781269	-0.184689156	3
WDR89	0.961207131	-1.034734091	0.07352696	3
WDR97	1.13122142	-0.364977777	-0.766243643	3
WFDC10A	1.153258393	-0.526666126	-0.626592268	3
XAF1	1.148480198	-0.470582362	-0.677897836	3
XBP1	1.154017821	-0.542626491	-0.61139133	3
XKRX	1.108438995	-0.834437707	-0.274001288	3
XPO7	1.154558334	-0.561585544	-0.59297279	3
XPOT	1.151089135	-0.654572109	-0.496517026	3
XRCC2	1.144656873	-0.44072113	-0.703935743	3
XYLT1	0.944325993	-1.047650889	0.103324896	3
XYLT2	1.085351992	-0.884009104	-0.201342887	3
YEATS4	1.150645199	-0.659058608	-0.491586592	3
ZBED4	1.132568794	-0.37143588	-0.761132914	3
ZBED5	1.12729953	-0.347093012	-0.780206518	3
ZBP1	1.145300939	-0.445315012	-0.699985927	3
ZBTB11	1.132238334	-0.369835529	-0.762402805	3
ZBTB24	1.150583917	-0.659657162	-0.490926755	3
ZBTB40	1.0324567	-0.964028386	-0.068428315	3
ZBTB6	1.150477394	-0.489790904	-0.660686489	3
ZC3H11B	1.140685307	-0.415011481	-0.725673826	3
ZC3H15	0.945975272	-1.046440414	0.100465142	3
ZCCHC13	1.07255906	-0.906701988	-0.165857072	3
ZCCHC18	1.126418699	-0.343241884	-0.783176815	3

ZDHHC5	1.112958259	-0.822924124	-0.290034135	3
ZFHX3	0.919527625	-1.064620581	0.145092956	3
ZFP3	1.15197316	-0.644677034	-0.507296126	3
ZFP64	1.065229913	-0.918573405	-0.146656508	3
ZFYVE28	1.149806267	-0.66687683	-0.482929437	3
ZHX1-C8orf76	1.152366322	-0.639735461	-0.512630861	3
ZKSCAN3	1.143253388	-0.431167558	-0.71208583	3
ZMPSTE24	1.119791584	-0.803924696	-0.315866889	3
ZMYM1	1.152858299	-0.63289424	-0.51996406	3
ZMYM4	1.140758851	-0.725304997	-0.415453854	3
ZMYND10	1.15339251	-0.624280787	-0.529111724	3
ZNF10	0.932941385	-1.055720452	0.122779067	3
ZNF106	1.150600531	-0.659495361	-0.491105169	3
ZNF107	1.087291452	-0.880317635	-0.206973816	3
ZNF121	1.121953503	-0.324512689	-0.797440814	3
ZNF131	1.009772677	-0.989932064	-0.019840613	3
ZNF132	1.122128496	-0.79690472	-0.325223776	3
ZNF141	1.113426064	-0.821688126	-0.291737937	3
ZNF154	1.152877917	-0.520275084	-0.632602834	3
ZNF16	1.154330554	-0.60247792	-0.551852634	3
ZNF17	0.984799321	-1.014537308	0.029737987	3
ZNF177	1.152926542	-0.521053067	-0.631873475	3
ZNF18	1.124607415	-0.789115572	-0.335491843	3
ZNF181	1.151424014	-0.500432168	-0.650991847	3

ZNF222	1.132405642	-0.3706444	-0.761761242	3
ZNF25	1.153830665	-0.538106873	-0.615723793	3
ZNF283	1.148030701	-0.46668813	-0.681342571	3
ZNF316	1.154607906	-0.56463756	-0.589970346	3
ZNF317	1.12486263	-0.788291998	-0.336570631	3
ZNF318	1.135807339	-0.38774744	-0.748059899	3
ZNF32	1.152282704	-0.511461935	-0.640820769	3
ZNF320	1.154682533	-0.571756802	-0.582925731	3
ZNF322	1.148544955	-0.677390685	-0.47115427	3
ZNF324B	1.148362696	-0.678810883	-0.469551812	3
ZNF337	0.979212825	-1.019565546	0.040352721	3
ZNF33B	1.101938447	-0.849798178	-0.25214027	3
ZNF35	1.133193775	-0.758700769	-0.374493005	3
ZNF354B	1.152187212	-0.642036548	-0.510150664	3
ZNF365	1.084728708	-0.885180226	-0.199548482	3
ZNF382	1.119612976	-0.804449266	-0.31516371	3
ZNF397	1.104335159	-0.844285522	-0.260049637	3
ZNF41	1.111062658	-0.827845169	-0.28321749	3
ZNF415	1.131069225	-0.766810061	-0.364259163	3
ZNF419	1.153438699	-0.529982052	-0.623456647	3
ZNF431	0.988586863	-1.011035157	0.022448294	3
ZNF432	1.032879503	-0.963507922	-0.06937158	3
ZNF449	1.084940375	-0.884783329	-0.200157046	3
ZNF45	1.146419275	-0.692759351	-0.453659924	3

ZNF473	1.151893426	-0.506260621	-0.645632805	3
ZNF483	1.123524499	-0.330960332	-0.792564167	3
ZNF485	1.120793218	-0.800952054	-0.319841164	3
ZNF490	1.153916016	-0.6138141	-0.540101915	3
ZNF493	1.151645279	-0.648519704	-0.503125576	3
ZNF502	1.154607121	-0.564583613	-0.590023508	3
ZNF507	1.154582442	-0.591592907	-0.562989535	3
ZNF510	0.906791616	-1.07250543	0.165713813	3
ZNF512	1.154572404	-0.562389095	-0.592183308	3
ZNF513	1.135249456	-0.384850263	-0.750399193	3
ZNF517	1.140297858	-0.41269863	-0.727599228	3
ZNF518A	1.122769793	-0.794925031	-0.327844762	3
ZNF519	1.147088868	-0.458913081	-0.688175787	3
ZNF528	1.075033149	-0.902520406	-0.172512743	3
ZNF529	1.153905452	-0.614056032	-0.53984942	3
ZNF544	1.003102417	-0.996868436	-0.006233981	3
ZNF550	1.134466193	-0.380846823	-0.75361937	3
ZNF562	0.952494873	-1.041548363	0.089053491	3
ZNF564	1.098200113	-0.858078024	-0.240122089	3
ZNF570	0.887831078	-1.083303514	0.195472436	3
ZNF573	1.149573201	-0.668919974	-0.480653226	3
ZNF578	1.105073666	-0.842552598	-0.262521068	3
ZNF593	1.133611292	-0.376558233	-0.757053059	3
ZNF610	1.138407961	-0.736597188	-0.401810772	3

ZNF630	1.128315658	-0.776708603	-0.351607055	3
ZNF639	1.114825926	-0.817936217	-0.296889709	3
ZNF655	1.080261446	-0.893367764	-0.186893682	3
ZNF66	1.154178031	-0.607168998	-0.547009032	3
ZNF660	0.977489239	-1.021084821	0.043595582	3
ZNF662	1.114768967	-0.818090473	-0.296678493	3
ZNF669	1.129461305	-0.77266783	-0.356793475	3
ZNF674	1.152678243	-0.635496972	-0.517181271	3
ZNF682	1.154677558	-0.571029773	-0.583647785	3
ZNF684	1.062750689	-0.922426147	-0.140324542	3
ZNF689	1.140421905	-0.413435924	-0.726985981	3
ZNF69	1.15451526	-0.55934433	-0.595170929	3
ZNF708	1.154270352	-0.604429237	-0.549841115	3
ZNF713	1.153035924	-0.630194013	-0.522841911	3
ZNF714	1.148497756	-0.470737144	-0.677760611	3
ZNF717	1.000382661	-0.999616899	-0.000765762	3
ZNF749	1.148977893	-0.673924105	-0.475053789	3
ZNF764	1.141899125	-0.422457839	-0.719441286	3
ZNF765-ZNF761	1.099829869	-0.854514169	-0.2453157	3
ZNF77	1.020372244	-0.978299744	-0.0420725	3
ZNF780B	1.134619823	-0.381626328	-0.752993495	3
ZNF783	1.138555334	-0.402637898	-0.735917437	3
ZNF785	1.135201989	-0.384605547	-0.750596442	3
ZNF789	0.953147554	-1.041049025	0.087901471	3

ZNF791	1.011595005	-0.987986946	-0.02360806	3
ZNF8	1.154573288	-0.592132242	-0.562441046	3
ZNF808	1.146507805	-0.454342636	-0.692165169	3
ZNF814	1.095396351	-0.864052605	-0.231343746	3
ZNF835	1.15405253	-0.610523564	-0.543528965	3
ZNF836	1.154618483	-0.565387847	-0.589230636	3
ZNF839	1.131132488	-0.766574878	-0.364557609	3
ZNF844	1.01171534	-0.987857725	-0.023857615	3
ZNF85	1.154186383	-0.547254545	-0.606931838	3
ZNF853	1.144831284	-0.441951091	-0.702880193	3
ZNF860	1.153216197	-0.525919812	-0.627296385	3
ZNF875	1.148109368	-0.467360452	-0.680748916	3
ZXDA	1.141828249	-0.719814048	-0.422014201	3
ZXDB	1.129572822	-0.772268771	-0.357304051	3
AAAS	0.861123166	0.235659228	-1.096782394	4
AATF	0.502146616	0.649419102	-1.151565719	4
ABCA2	0.810088221	0.307569253	-1.117657474	4
ABCC6	0.764376239	0.367342883	-1.131719122	4
ABCE1	0.65032099	0.501163871	-1.151484861	4
ABCF2-H2BE1	0.601221193	0.553145697	-1.154366889	4
ABHD14A-ACY1	0.570632922	0.584041665	-1.154674587	4
ABHD14B	0.692009712	0.45452117	-1.146530882	4
ABHD15	0.809665924	0.308140262	-1.117806186	4
ABHD17C	0.459718513	0.687470655	-1.147189168	4

ABL2	0.479005513	0.670395748	-1.149401261	4
ABR	0.622373202	0.53112492	-1.153498122	4
ABT1	0.527898485	0.625428288	-1.153326773	4
ACAA2	0.630473519	0.522544451	-1.15301797	4
ACADS	0.864929562	0.230047589	-1.094977151	4
ACAP1	0.816718464	0.298555341	-1.115273805	4
ACAP3	0.70803871	0.435924243	-1.143962954	4
ACBD3	0.710315117	0.433251806	-1.143566923	4
ACOT4	0.583734497	0.570942422	-1.154676919	4
ACOT9	0.640359527	0.511958901	-1.152318428	4
ACSS1	0.628329419	0.524823732	-1.153153152	4
ACTG1	0.542416349	0.611593263	-1.154009612	4
ACTN4	0.733975498	0.404996692	-1.138972189	4
ACTR6	0.777763196	0.350248505	-1.1280117	4
ACVR1C	0.538740024	0.615118196	-1.15385822	4
ADA	0.757138938	0.376450711	-1.133589649	4
ADAM28	0.832455467	0.276782671	-1.109238138	4
ADAMTS4	0.665036651	0.484974604	-1.150011255	4
ADAR	0.755972882	0.377909551	-1.133882433	4
ADAT3	0.554154716	0.60023924	-1.154393956	4
ADCY10	0.83602021	0.271774395	-1.107794605	4
ADGRA2	0.671930001	0.477289298	-1.1492193	4
ADGRG1	0.698973891	0.446488321	-1.145462212	4
ADGRG5	0.46754163	0.680588862	-1.148130492	4

ADGRL1	0.526411811	0.62683227	-1.153244081	4
ADO	0.750657371	0.384529942	-1.135187313	4
ADPRHL1	0.478256616	0.671065604	-1.14932222	4
ADPRM	0.754887351	0.379265526	-1.134152877	4
ADPRS	0.776345322	0.352074566	-1.128419887	4
ADRM1	0.76002364	0.37283149	-1.13285513	4
ADSL	0.674758315	0.474116938	-1.148875253	4
AFF1	0.50676268	0.645169543	-1.151932223	4
AGA	0.770463454	0.359610236	-1.13007369	4
AGAP3	0.781741004	0.345105552	-1.126846556	4
AGBL2	0.833851334	0.274824984	-1.108676319	4
AGFG1	0.470369558	0.678086462	-1.14845602	4
AGO2	0.68872708	0.458282869	-1.147009948	4
AGPAT4	0.512862334	0.639520377	-1.152382711	4
AHCY	0.819818927	0.294308326	-1.114127253	4
AHNAK	0.831000734	0.278818245	-1.109818979	4
AIMP2	0.822586448	0.290499945	-1.113086393	4
AK1	0.786003363	0.33956165	-1.125565013	4
AKAP1	0.842769059	0.262212746	-1.104981805	4
AKAP13	0.638355341	0.514115102	-1.152470443	4
AKAP17A	0.556768216	0.597690584	-1.1544588	4
AKAP8L	0.747001468	0.389055234	-1.136056702	4
AKAP9	0.476481372	0.672651254	-1.149132626	4
AKR1A1	0.730285409	0.409461979	-1.139747388	4

AKTIP	0.859798705	0.237603187	-1.097401892	4
ALDH2	0.773205969	0.356104492	-1.129310462	4
ALDH9A1	0.79817199	0.323541921	-1.121713912	4
ALG11	0.786167838	0.339347032	-1.12551487	4
ALG1L2	0.701306474	0.443781733	-1.145088207	4
ALG5	0.779335127	0.348219671	-1.127554797	4
ALG6	0.777706834	0.350321163	-1.128027997	4
ALKBH1	0.853131954	0.247321552	-1.100453506	4
ALKBH7	0.744074727	0.392661663	-1.13673639	4
ALMS1	0.777499792	0.350588021	-1.128087813	4
ALOX5	0.552205033	0.602135607	-1.15434064	4
AMIGO3	0.738508954	0.399480454	-1.137989408	4
AMOT	0.844834746	0.259264877	-1.104099622	4
AMPD3	0.692714383	0.45371161	-1.146425993	4
AMY1B	0.856771453	0.242029822	-1.098801275	4
ANAPC1	0.801819058	0.318683863	-1.12050292	4
ANG	0.632169595	0.520737288	-1.152906883	4
ANGEL2	0.55855683	0.595941957	-1.154498788	4
ANGPTL6	0.78822799	0.336654421	-1.12488241	4
ANKHD1-EIF4EBP3	0.861806695	0.234654249	-1.096460945	4
ANKIB1	0.826123259	0.28560873	-1.111731989	4
ANKRD22	0.714973924	0.427757724	-1.142731647	4
ANKRD24	0.677970553	0.47050033	-1.148470883	4
ANKRD27	0.594560879	0.559966912	-1.154527791	4

ANKRD34B	0.486422237	0.663731579	-1.150153815	4
ANKRD35	0.588608391	0.566018489	-1.15462688	4
ANKRD44	0.593197381	0.561356816	-1.154554197	4
ANKRD52	0.848380074	0.254181866	-1.102561939	4
ANKS1A	0.84020109	0.265863445	-1.106064535	4
ANKS3	0.499202659	0.652117878	-1.151320537	4
ANKUB1	0.487766285	0.662518004	-1.150284289	4
ANXA3	0.775428051	0.353253924	-1.128681976	4
ANXA4	0.648788719	0.502832876	-1.151621595	4
ANXA5	0.551499096	0.602821201	-1.154320297	4
ANXA6	0.827878331	0.283171372	-1.111049703	4
AP1M1	0.820711097	0.293082418	-1.113793514	4
AP2A1	0.532684991	0.620891986	-1.153576977	4
AP3M1	0.533307344	0.620300362	-1.153607706	4
AP3S1	0.706646574	0.437554684	-1.144201258	4
AP5B1	0.624554424	0.528822561	-1.153376985	4
APBB1IP	0.85252721	0.248197675	-1.100724884	4
APEH	0.735476227	0.403174358	-1.138650586	4
APH1B	0.811598403	0.305524231	-1.117122633	4
APOBEC2	0.511019987	0.641230733	-1.152250719	4
APOBEC3D	0.828561723	0.282220469	-1.110782192	4
APOBEC3G	0.810342998	0.307224577	-1.117567575	4
APOL1	0.631544343	0.521403919	-1.152948262	4
APOL2	0.467317609	0.680786759	-1.148104368	4

APOL6	0.85909285	0.238637387	-1.097730237	4
APRT	0.846402253	0.257021204	-1.103423457	4
APTX	0.588031582	0.566602675	-1.154634257	4
AQP9	0.817675903	0.29724603	-1.114921933	4
ARCN1	0.754226937	0.380089474	-1.134316411	4
AREL1	0.854314306	0.24560603	-1.099920336	4
ARF6	0.501699754	0.649829321	-1.151529075	4
ARFGAP3	0.587508334	0.567132276	-1.15464061	4
ARG1	0.814955916	0.300960566	-1.115916481	4
ARHGAP1	0.572362732	0.582323486	-1.154686218	4
ARHGAP22	0.812900913	0.303756611	-1.116657524	4
ARHGAP25	0.753842681	0.380568533	-1.134411214	4
ARHGAP27	0.622251913	0.531252769	-1.153504682	4
ARHGAP45	0.73600404	0.402532561	-1.138536601	4
ARHGEF1	0.820924134	0.292789435	-1.113713569	4
ARHGEF2	0.524280314	0.628841108	-1.153121422	4
ARHGEF40	0.527135073	0.626149532	-1.153284604	4
ARID1A	0.756412565	0.377359747	-1.133772311	4
ARID3A	0.499246392	0.652077852	-1.151324244	4
ARL17A	0.683957983	0.463720151	-1.147678134	4
ARL2	0.587462494	0.567178657	-1.154641151	4
ARL2BP	0.787507847	0.337596569	-1.125104416	4
ARL4C	0.794451374	0.32847071	-1.122922084	4
ARL4D	0.816344672	0.299065978	-1.11541065	4

ARMC10	0.607666518	0.546493767	-1.154160286	4
ARMC6	0.803476004	0.316467911	-1.119943915	4
ARMC9	0.573544857	0.581147339	-1.154692196	4
ARMH2	0.850157723	0.251621874	-1.101779596	4
ARNT	0.844127496	0.260275301	-1.104402797	4
ARNTL	0.729527337	0.410376575	-1.139903913	4
ARPC1A	0.82250534	0.290611792	-1.113117133	4
ARPC5L	0.839167171	0.267328959	-1.10649613	4
ARRDC1	0.59487334	0.559648088	-1.154521428	4
ARSB	0.650499329	0.500969415	-1.151468744	4
ARSD	0.861914197	0.234496084	-1.096410281	4
ARSG	0.710677471	0.432825683	-1.143503153	4
ASB13	0.710202994	0.433383621	-1.143586614	4
ASB14	0.720127917	0.421640486	-1.141768402	4
ASB17	0.737259094	0.401004633	-1.138263727	4
ASGR1	0.4564665	0.690313837	-1.146780337	4
ASH1L	0.728563282	0.41153835	-1.140101632	4
ASL	0.632431569	0.520457827	-1.152889396	4
ASNSD1	0.623105815	0.530352287	-1.153458103	4
ASTE1	0.754149971	0.380185449	-1.13433542	4
ASTN2	0.861558393	0.23501946	-1.096577853	4
ATAD3C	0.653071061	0.49816056	-1.151231621	4
ATF6B	0.572577897	0.582109528	-1.154687425	4
ATG16L2	0.752357145	0.382418187	-1.134775333	4

ATG2B	0.751794961	0.383117178	-1.134912139	4
ATG5	0.647361388	0.504384777	-1.151746165	4
ATIC	0.771750523	0.35796669	-1.129717212	4
ATN1	0.757438893	0.376075057	-1.13351395	4
ATP5F1A	0.767033531	0.363975489	-1.131009021	4
ATP5PB	0.738458841	0.399541615	-1.138000456	4
ATP6V1F	0.683816764	0.463880656	-1.147697421	4
ATP6V1H	0.809787092	0.307976463	-1.117763555	4
ATP8B3	0.732157054	0.407199907	-1.139356961	4
ATPAF2	0.856131295	0.242962969	-1.099094264	4
ATR	0.774562499	0.354365365	-1.128927864	4
ATXN2L	0.616323121	0.537479828	-1.153802949	4
AURKAIP1	0.685775225	0.461652167	-1.147427391	4
AVIL	0.607713928	0.546444651	-1.154158579	4
AVL9	0.545461214	0.608662636	-1.15412385	4
B3GALT9	0.818607673	0.295969947	-1.114577619	4
B3GNT9	0.467876409	0.68029303	-1.148169439	4
B4GALT7	0.482925171	0.666880664	-1.149805835	4
B9D1	0.757441841	0.376071364	-1.133513205	4
BABAM2	0.835664616	0.272275283	-1.107939899	4
BAG5	0.687348129	0.459858395	-1.147206523	4
BAG6	0.687979556	0.459137297	-1.147116853	4
BAK1	0.678150266	0.470297564	-1.14844783	4
BAP1	0.486625434	0.663548224	-1.150173657	4

BAX	0.653249841	0.497964968	-1.151214809	4
BAZ2A	0.825066927	0.287072444	-1.11213937	4
BBC3	0.729868475	0.409965116	-1.13983359	4
BBS12	0.762301211	0.369963665	-1.132264876	4
BBS2	0.607826079	0.546328452	-1.154154531	4
BCKDHA	0.841955272	0.263371317	-1.105326589	4
BCL7C	0.783621447	0.342663967	-1.126285414	4
BCL9L	0.764245344	0.367508432	-1.131753775	4
BET1L	0.762376246	0.369869028	-1.132245274	4
BICDL2	0.849930239	0.251949899	-1.101880138	4
BIN1	0.81431952	0.301827397	-1.116146917	4
BIN3	0.689223586	0.457714909	-1.146938495	4
BLK	0.800870933	0.319949369	-1.120820302	4
BLOC1S3	0.472990759	0.675759961	-1.14875072	4
BLOC1S4	0.852356371	0.248445015	-1.100801386	4
BLVRA	0.683207997	0.464572238	-1.147780235	4
BOLA3	0.618110559	0.535607081	-1.15371764	4
BORCS6	0.665958858	0.483950249	-1.149909107	4
BRCC3	0.796483148	0.32578256	-1.122265708	4
BRD2	0.490674358	0.659886001	-1.150560359	4
BRD3OS	0.8558804	0.243328416	-1.099208817	4
BRD8	0.607305486	0.546867707	-1.154173193	4
BRF1	0.788245842	0.336631052	-1.124876894	4
BRI3BP	0.855829272	0.24340287	-1.099232141	4

BRICD5	0.722586895	0.41870728	-1.141294175	4
BRMS1	0.857214483	0.241383428	-1.098597911	4
BRWD1	0.668754005	0.480838343	-1.149592349	4
BSDC1	0.768084406	0.362640299	-1.130724705	4
BST2	0.612524285	0.541446829	-1.153971114	4
BTBD9	0.847222925	0.255844192	-1.103067117	4
BTG1	0.589110595	0.565509544	-1.154620138	4
BTN2A1	0.604856486	0.54940007	-1.154256555	4
BTNL3	0.636286677	0.51633521	-1.152621887	4
BTNL8	0.688141311	0.458952477	-1.147093788	4
BUB3	0.718245408	0.423879627	-1.142125035	4
BUD23	0.850382686	0.25129736	-1.101680046	4
C10orf105	0.775548733	0.35309885	-1.128647582	4
C10orf143	0.626179113	0.527103748	-1.153282861	4
C11orf80	0.61049664	0.543556951	-1.154053592	4
C11orf98	0.816256047	0.299187005	-1.115443052	4
C12orf42	0.454789937	0.691775628	-1.146565565	4
C14orf119	0.665422709	0.484545927	-1.149968636	4
C14orf93	0.785639706	0.340035992	-1.125675698	4
C16orf74	0.573741753	0.580951283	-1.154693036	4
C16orf86	0.772070747	0.357557304	-1.129628052	4
C17orf49	0.624162385	0.529236814	-1.153399199	4
C17orf58	0.622633403	0.530850583	-1.153483985	4
C19orf12	0.496702917	0.654402472	-1.151105389	4

C19orf47	0.661172149	0.489254484	-1.150426633	4
C1orf43	0.837227704	0.270071365	-1.107299069	4
C1QL3	0.608604494	0.545521515	-1.15412601	4
C1QTNF12	0.536627581	0.617137015	-1.153764596	4
C1QTNF6	0.763471405	0.368486638	-1.131958043	4
C2orf15	0.814606842	0.301436144	-1.116042985	4
C2orf50	0.546426509	0.607731438	-1.154157948	4
C2orf81	0.722432333	0.41889193	-1.141324264	4
C4BPA	0.771904394	0.357769999	-1.129674393	4
C5orf63	0.46021817	0.687032903	-1.147251074	4
C6orf120	0.813412319	0.30306162	-1.116473938	4
C6orf201	0.782575583	0.344022751	-1.126598333	4
C6orf47	0.548667452	0.605565712	-1.154233164	4
C7orf26	0.674155927	0.474793541	-1.148949468	4
C8orf58	0.800135959	0.320929133	-1.121065091	4
C8orf82	0.561720674	0.592840077	-1.154560751	4
CA2	0.539470845	0.61441864	-1.153889485	4
CA6	0.851135475	0.250210557	-1.101346031	4
CACNA1H	0.76537157	0.366083051	-1.131454621	4
CACNA2D3	0.798996976	0.322445329	-1.121442305	4
CACNG8	0.475756656	0.673297672	-1.149054328	4
CACYBP	0.820743922	0.293037281	-1.113781202	4
CALCOCO2	0.715948024	0.426604742	-1.142552765	4
CALHM1	0.627765873	0.525421839	-1.153187712	4

CALR	0.621100472	0.532465572	-1.153566044	4
CAMKK2	0.847505367	0.25543874	-1.102944107	4
CAMP	0.544520563	0.609569077	-1.15408964	4
CAPN15	0.622925425	0.530542594	-1.153468019	4
CAPN5	0.751615308	0.383340436	-1.134955743	4
CAPRIN1	0.747680618	0.388216296	-1.135896915	4
CAPS2	0.704490285	0.440074287	-1.144564572	4
CARD17	0.811339347	0.30587537	-1.117214717	4
CARD9	0.863256776	0.232518285	-1.095775061	4
CARM1	0.695170067	0.450884712	-1.146054778	4
CARS1	0.812462038	0.304352599	-1.116814637	4
CARS2	0.768485936	0.362129609	-1.130615546	4
CASP5	0.689418212	0.457492176	-1.146910388	4
CAST	0.825808042	0.286045768	-1.11185381	4
CASTOR2	0.686367449	0.460977201	-1.14734465	4
CAVIN1	0.547749308	0.606453706	-1.154203014	4
CBL	0.779235259	0.348348704	-1.127583962	4
CBR3	0.747781822	0.388091215	-1.135873037	4
CBX4	0.635690002	0.51697454	-1.152664542	4
CBX7	0.802350986	0.317973081	-1.120324067	4
CC2D1B	0.774199983	0.354830456	-1.129030439	4
CCAR2	0.752248149	0.382553751	-1.134801899	4
CCDC12	0.466050265	0.681905391	-1.147955656	4
CCDC126	0.683927323	0.463755	-1.147682324	4

CCDC137	0.523769459	0.629321849	-1.153091308	4
CCDC144A	0.475943402	0.673131152	-1.149074554	4
CCDC170	0.635299094	0.517393145	-1.152692238	4
CCDC22	0.851003859	0.250400669	-1.101404529	4
CCDC28A	0.816918364	0.298282136	-1.1152005	4
CCDC32	0.817781008	0.297102178	-1.114883186	4
CCDC51	0.72718238	0.413199862	-1.140382243	4
CCDC71L	0.486632236	0.663542085	-1.150174321	4
CCDC85B	0.619343385	0.5343131	-1.153656484	4
CCDC85C	0.690184907	0.456614223	-1.14679913	4
CCL28	0.852012446	0.248942733	-1.100955179	4
CCM2	0.631995236	0.520923236	-1.152918472	4
CCNB1IP1	0.748258988	0.387501233	-1.135760222	4
CCNF	0.816628243	0.298678619	-1.115306862	4
CCNI2	0.777496024	0.350592877	-1.128088901	4
CCNQ	0.718206542	0.423925798	-1.14213234	4
CCR3	0.684376025	0.463244848	-1.147620873	4
CCRL2	0.82049599	0.293378148	-1.113874137	4
CCT5	0.720615893	0.421059158	-1.14167505	4
CCT6A	0.453500581	0.692897967	-1.146398548	4
CD101	0.750144698	0.385165902	-1.1353106	4
CD160	0.861891665	0.234529238	-1.096420902	4
CD177	0.491343289	0.659279365	-1.150622654	4
CD180	0.781700863	0.345157599	-1.126858462	4

CD19	0.762533013	0.369671276	-1.132204289	4
CD1A	0.814319506	0.301827416	-1.116146922	4
CD1B	0.756888657	0.376764034	-1.133652691	4
CD1C	0.852653427	0.248014891	-1.100668318	4
CD200R1	0.844195242	0.260178566	-1.104373808	4
CD200R1L	0.847878161	0.254903294	-1.102781454	4
CD209	0.702318714	0.442604662	-1.144923376	4
CD247	0.802376102	0.317939507	-1.120315608	4
CD2AP	0.498085837	0.653139365	-1.151225203	4
CD2BP2	0.58794956	0.566685714	-1.154635274	4
CD300H	0.48244728	0.66731005	-1.14975733	4
CD302	0.64566384	0.506226977	-1.151890817	4
CD37	0.595378708	0.559132183	-1.154510891	4
CD44	0.571997682	0.582686366	-1.154684048	4
CD8B	0.716295136	0.42619353	-1.142488666	4
CD96	0.720231062	0.42151764	-1.141748701	4
CDC23	0.809861979	0.307875212	-1.117737191	4
CDC42EP1	0.836402637	0.271235388	-1.107638025	4
CDC42EP2	0.543435188	0.610613776	-1.154048964	4
CDC7	0.838786889	0.267867362	-1.106654251	4
CDH1	0.453428805	0.692960398	-1.146389203	4
CDH13	0.73869294	0.399255869	-1.137948809	4
CDK11A	0.571559042	0.583122197	-1.154681239	4
CDK11B	0.746879426	0.389205907	-1.136085333	4

CDK2AP2	0.56011129	0.594419344	-1.154530634	4
CDK4	0.829861194	0.280409463	-1.110270657	4
CDK5	0.822467723	0.290663663	-1.113131385	4
CDK5RAP1	0.859578055	0.237926612	-1.097504667	4
CDK9	0.771957326	0.357702327	-1.129659653	4
CDKN1C	0.502953293	0.648678056	-1.151631349	4
CDRT15	0.748553628	0.387136739	-1.135690367	4
CDT1	0.531772528	0.621758644	-1.153531173	4
CDYL	0.694709687	0.45141536	-1.146125047	4
CEACAM21	0.833126803	0.27584168	-1.108968483	4
CEBPA	0.476109692	0.672982843	-1.149092535	4
CEBPG	0.602898048	0.551419933	-1.154317981	4
CEMIP2	0.836807154	0.270664881	-1.107472035	4
CENPB	0.671592518	0.477667088	-1.149259606	4
CENPC	0.710927169	0.432531923	-1.143459092	4
CENPH	0.655582998	0.495408477	-1.150991474	4
CENPX	0.594876232	0.559645137	-1.154521368	4
CEP170	0.451508567	0.694628794	-1.146137362	4
CEP192	0.555991994	0.598448341	-1.154440335	4
CEP85L	0.469186013	0.679134724	-1.148320736	4
CEP89	0.838663804	0.268041554	-1.106705358	4
CES1	0.799554542	0.32170343	-1.121257971	4
CES4A	0.710932771	0.432525331	-1.143458103	4
CFAP20	0.830432511	0.279612056	-1.110044568	4

CFAP298	0.757270403	0.376286088	-1.13355649	4
CFAP45	0.655832992	0.495134119	-1.150967111	4
CFAP97D2	0.771384632	0.358434227	-1.129818859	4
CHD2	0.687034764	0.460216047	-1.147250811	4
CHD3	0.760815138	0.371835905	-1.132651043	4
CHD4	0.732542711	0.406733096	-1.139275807	4
CHD6	0.843688033	0.260902552	-1.104590585	4
CHD8	0.711264089	0.432135401	-1.143399489	4
CHERP	0.784364278	0.341697626	-1.126061904	4
CHID1	0.476192105	0.672909331	-1.149101436	4
CHKB	0.614610005	0.539270987	-1.153880992	4
CHMP2B	0.555641977	0.598789812	-1.154431789	4
CHST14	0.758786825	0.374385013	-1.133171839	4
CIAO1	0.756219192	0.377601593	-1.133820784	4
CIAO2A	0.659586722	0.491004432	-1.150591154	4
CIAPIN1	0.762916155	0.369187782	-1.132103936	4
CIB1	0.858500172	0.239504795	-1.098004967	4
CIRBP	0.848350745	0.254224038	-1.102574783	4
CISD1	0.863660733	0.2319223	-1.095583034	4
CISH	0.828142346	0.282804133	-1.110946478	4
CKB	0.677398852	0.471145063	-1.148543915	4
CKLF-CMTM1	0.689324911	0.457598957	-1.146923869	4
CKS1B	0.797126335	0.32492989	-1.122056225	4
CLASP1	0.448598814	0.697150172	-1.145748987	4

CLASRP	0.680710319	0.467404147	-1.148114465	4
CLCN5	0.80636471	0.312591329	-1.118956038	4
CLDN12	0.803766095	0.316079381	-1.119845476	4
CLDN20	0.813160879	0.303403391	-1.116564269	4
CLDN23	0.659289062	0.4913326	-1.150621662	4
CLDN24	0.648955574	0.502651283	-1.151606856	4
CLEC4E	0.831664564	0.277889953	-1.109554517	4
CLEC4F	0.7415702	0.395736412	-1.137306612	4
CLEC5A	0.71220429	0.43102795	-1.14323224	4
CLEC6A	0.772499113	0.357009376	-1.129508489	4
CLECL1	0.846812494	0.256433036	-1.10324553	4
CLP1	0.756448646	0.377314613	-1.133763259	4
CLTB	0.570283794	0.584388031	-1.154671825	4
CLTC	0.520267956	0.632609513	-1.152877469	4
CLTCL1	0.827194736	0.284121524	-1.11131626	4
CLUH	0.617101663	0.536664617	-1.153766279	4
CNEP1R1	0.716116374	0.426405326	-1.142521701	4
CNIH3	0.775130892	0.353635657	-1.128766549	4
CNIH4	0.544664933	0.60943002	-1.154094953	4
CNN2	0.522848716	0.630187617	-1.153036334	4
CNOT1	0.729108776	0.410881164	-1.13998994	4
CNOT6	0.479778727	0.669703557	-1.149482283	4
CNOT8	0.448905364	0.696884922	-1.145790286	4
CNPY3-GNMT	0.664293878	0.4857988	-1.150092678	4

CNR2	0.786277258	0.339204224	-1.125481482	4
CNTD1	0.622540798	0.530948228	-1.153489026	4
COASY	0.490849732	0.659727002	-1.150576735	4
COCH	0.670689976	0.478676644	-1.14936662	4
COIL	0.693881906	0.452368702	-1.146250608	4
COL19A1	0.725030971	0.415782381	-1.140813352	4
COL4A4	0.573182373	0.58150816	-1.154690533	4
COL6A2	0.559485705	0.595032438	-1.154518143	4
COLGALT1	0.681973012	0.465973598	-1.147946609	4
COMMD2	0.557907981	0.596576714	-1.154484694	4
COMMD4	0.586513481	0.568138321	-1.154651802	4
COMMD5	0.853922275	0.246175224	-1.100097499	4
COMMD9	0.816374833	0.299024786	-1.115399619	4
COPG1	0.769144327	0.3612916	-1.130435927	4
COPZ1	0.610279165	0.543782973	-1.154062138	4
COQ4	0.668348618	0.481290339	-1.149638958	4
COQ9	0.752953857	0.381675673	-1.13462953	4
CORO1A	0.732048131	0.407331707	-1.139379838	4
CORO1B	0.642787814	0.509339425	-1.152127239	4
CORO7	0.694628472	0.451508939	-1.146137411	4
COX14	0.573594846	0.581097567	-1.154692413	4
COX19	0.66424227	0.485856037	-1.150098308	4
COX8A	0.481226875	0.66840555	-1.149632426	4
CPED1	0.858599358	0.239359693	-1.097959052	4

CPNE1	0.852092073	0.248827525	-1.100919598	4
CPNE8	0.797846829	0.323973762	-1.121820591	4
CPO	0.767853951	0.362933274	-1.130787225	4
CPQ	0.714435238	0.428394702	-1.142829939	4
CPTP	0.637598098	0.514928424	-1.152526522	4
CRACR2B	0.452967775	0.693361288	-1.146329063	4
CREB1	0.487120945	0.663100924	-1.150221869	4
CREB3	0.710286676	0.433285244	-1.14357192	4
CREBZF	0.864467706	0.230730467	-1.095198173	4
CRIM1	0.55779968	0.596682617	-1.154482296	4
CRIP1	0.667913531	0.481775198	-1.149688729	4
CRIP3	0.74397973	0.39277848	-1.13675821	4
CRISP3	0.746129784	0.390130862	-1.136260646	4
CRK	0.481473524	0.668184265	-1.14965779	4
CROCC	0.799289759	0.322055827	-1.121345587	4
CRTAM	0.800169835	0.320883997	-1.121053832	4
CRYBG1	0.488575769	0.661786223	-1.150361992	4
CSK	0.514099515	0.638369846	-1.152469361	4
CSNK1D	0.719863965	0.421954776	-1.141818741	4
CSNK1G2	0.760013843	0.372843807	-1.13285765	4
CSNK2A1	0.647758717	0.503953042	-1.151711759	4
CSNK2A3	0.775323062	0.353388812	-1.128711875	4
CSTF1	0.759937615	0.372939631	-1.132877246	4
CSTF2T	0.797006048	0.325089416	-1.122095464	4

CTAG2	0.776153265	0.352321626	-1.128474891	4
CTBP2	0.690649203	0.456082136	-1.146731339	4
CTDNEP1	0.813823326	0.302502669	-1.116325996	4
CTDSP2	0.66365319	0.486509113	-1.150162304	4
CTNNA1	0.556449943	0.598001367	-1.15445131	4
CTNNB1	0.494870532	0.6560731	-1.150943632	4
CTR9	0.833704416	0.275031243	-1.108735659	4
CTSO	0.755888683	0.3780148	-1.133903483	4
CUL9	0.848079119	0.254614518	-1.102693636	4
CUTA	0.796412659	0.325875957	-1.122288616	4
CUX2	0.548351262	0.605871624	-1.154222885	4
CWF19L1	0.571680197	0.58300184	-1.154682037	4
CX3CR1	0.455556123	0.691107929	-1.146664052	4
CXCR1	0.689070564	0.457889991	-1.146960555	4
CXCR6	0.788607834	0.336157076	-1.124764911	4
CXorf38	0.849196356	0.253007284	-1.10220364	4
CYB561D2	0.452126709	0.69409211	-1.146218819	4
CYB5R4	0.808338245	0.309933084	-1.118271329	4
CYHR1	0.648781931	0.502840263	-1.151622193	4
CYLD	0.680484465	0.467659784	-1.14814425	4
CYP27B1	0.632918104	0.519938584	-1.152856688	4
CYP2R1	0.791610995	0.332215124	-1.123826118	4
CYP3A5	0.814237062	0.301939651	-1.116176713	4
CYP3A7-CYP3A51P	0.752222604	0.382585518	-1.134808122	4

CYP4F2	0.81935502	0.294945094	-1.114300114	4
CYSLTR1	0.626891473	0.526349067	-1.153240539	4
CYTH2	0.856418205	0.242544872	-1.098963077	4
CYTIP	0.517428747	0.635265838	-1.152694585	4
CYTL1	0.621590736	0.531949385	-1.15354012	4
DALRD3	0.854993464	0.244619055	-1.09961252	4
DAPK3	0.552159993	0.602179365	-1.154339358	4
DBF4B	0.666472186	0.483379556	-1.149851742	4
DBNDD2	0.771883605	0.357796576	-1.129680181	4
DBNL	0.543032714	0.61100084	-1.154033554	4
DBR1	0.805484525	0.313774317	-1.119258843	4
DCAF15	0.854740456	0.244986868	-1.099727324	4
DCAF16	0.836425975	0.271202483	-1.107628458	4
DCDC2B	0.712410562	0.430784804	-1.143195366	4
DCLRE1C	0.532415298	0.621148234	-1.153563532	4
DCTN1	0.827161499	0.284167695	-1.111329194	4
DCTN5	0.854147336	0.245848502	-1.099995838	4
DCUN1D2	0.847664995	0.255209506	-1.102874501	4
DCXR	0.691674775	0.454905707	-1.146580482	4
DDB2	0.659058017	0.491587242	-1.150645259	4
DDI2	0.783272953	0.343116959	-1.126389912	4
DDX27	0.719052966	0.42291976	-1.141972727	4
DDX39A	0.829534395	0.28086526	-1.110399655	4
DDX43	0.740405936	0.397162191	-1.137568127	4

DDX5	0.701606729	0.443432745	-1.145039474	4
DDX54	0.827973437	0.283039099	-1.111012536	4
DDX55	0.824227516	0.288233836	-1.112461351	4
DDX60L	0.778264997	0.349601348	-1.127866345	4
DEF6	0.671971593	0.477242728	-1.149214321	4
DEF8	0.727919391	0.412313465	-1.140232856	4
DEFA1	0.529523311	0.623891144	-1.153414455	4
DEFA1B	0.51041685	0.641789891	-1.152206741	4
DEFA3	0.676836352	0.471778969	-1.148615321	4
DEFA4	0.725877813	0.414766726	-1.140644539	4
DEGS1	0.611824054	0.542176115	-1.154000168	4
DENND1C	0.691313461	0.455320344	-1.146633805	4
DENND4B	0.749995616	0.385350751	-1.135346367	4
DERL3	0.595850424	0.558650357	-1.15450078	4
DERPC	0.485919633	0.664184925	-1.150104558	4
DESI1	0.818449355	0.296186898	-1.114636252	4
DET1	0.457167809	0.689701561	-1.14686937	4
DGAT1	0.706891793	0.437267702	-1.144159495	4
DGCR6L	0.862464117	0.233686534	-1.096150651	4
DGLUCY	0.864078541	0.23130544	-1.095383982	4
DHFR2	0.837752996	0.269329458	-1.107082454	4
DHRS12	0.450403601	0.695587235	-1.145990836	4
DHRS4	0.645294703	0.506627066	-1.151921769	4
DHRS7	0.691011284	0.45566697	-1.146678254	4

DHRS7B	0.674695667	0.474187328	-1.148882995	4
DHRS9	0.710050888	0.433562409	-1.143613297	4
DHTKD1	0.835390791	0.272660796	-1.108051586	4
DICER1	0.476963281	0.672221122	-1.149184403	4
DIP2B	0.448445066	0.697283173	-1.145728239	4
DIPK1A	0.770100268	0.360073468	-1.130173736	4
DIS3L	0.81281293	0.303876124	-1.116689054	4
DISP2	0.680801832	0.467300545	-1.148102376	4
DKC1	0.767819056	0.362977627	-1.130796683	4
DMPK	0.476700692	0.672455527	-1.149156219	4
DMRTA1	0.457806019	0.689143959	-1.146949978	4
DMWD	0.684517973	0.463083399	-1.147601372	4
DMXL1	0.643201517	0.508892382	-1.152093899	4
DNAAF1	0.743642728	0.393192766	-1.136835494	4
DNAAF2	0.700066723	0.445221275	-1.145287998	4
DNAI2	0.745122543	0.391372166	-1.136494709	4
DNAJA3	0.659946893	0.490607186	-1.150554079	4
DNAJA4	0.6976129	0.448063791	-1.145676691	4
DNAJB11	0.852194546	0.24867924	-1.100873786	4
DNAJB4	0.81011519	0.307532774	-1.117647964	4
DNAJC10	0.698583407	0.446940623	-1.14552403	4
DNAJC17	0.531925692	0.621613232	-1.153538924	4
DNAJC19	0.565063962	0.589550033	-1.154613995	4
DNAJC21	0.76837375	0.362272324	-1.130646074	4

DNAJC30	0.805780926	0.313376124	-1.11915705	4
DNAJC8	0.77034312	0.359763745	-1.130106865	4
DNASE1L1	0.736538359	0.401882386	-1.138420745	4
DNM2	0.808638396	0.309528095	-1.118166491	4
DNMBP	0.51161557	0.640678206	-1.152293776	4
DNPEP	0.848584965	0.25388719	-1.102472155	4
DNTTIP1	0.638569935	0.51388448	-1.152454415	4
DOCK2	0.642650638	0.509487607	-1.152138245	4
DOCK8	0.689029267	0.457937235	-1.146966502	4
DOK1	0.758647629	0.374559686	-1.133207314	4
DOLPP1	0.717338329	0.424956569	-1.142294899	4
DPEP3	0.713751295	0.429202794	-1.142954089	4
DPH5	0.683558982	0.464173571	-1.147732553	4
DPM3	0.829341934	0.28113358	-1.110475514	4
DPP7	0.855056903	0.244526806	-1.099583709	4
DSC1	0.615791322	0.538036243	-1.153827565	4
DSTYK	0.838240136	0.268640868	-1.106881004	4
DTD1	0.852717403	0.247922229	-1.100639632	4
DTNBP1	0.540676488	0.613263308	-1.153939795	4
DUSP11	0.746345439	0.389864871	-1.13621031	4
DUSP13	0.707494047	0.436562492	-1.144056539	4
DUSP7	0.852071435	0.248857387	-1.100928822	4
DVL3	0.627412583	0.52579659	-1.153209173	4
DYM	0.621615243	0.531923574	-1.153538817	4

DYNC1H1	0.802230004	0.318134791	-1.120364796	4
ECD	0.81006769	0.307597022	-1.117664712	4
ECH1	0.751761913	0.383158252	-1.134920165	4
ECHDC3	0.679726713	0.468516936	-1.148243649	4
ECHS1	0.656269776	0.494654567	-1.150924342	4
ECT2L	0.736685769	0.401702931	-1.1383887	4
EDAR	0.683674811	0.464041968	-1.147716779	4
EDC4	0.718341304	0.423765698	-1.142107002	4
EDEM2	0.689703028	0.45716613	-1.146869157	4
EEF1AKMT3	0.610672858	0.543373766	-1.154046624	4
EEF2	0.7327505	0.406481483	-1.139231982	4
EFCAB7	0.756493711	0.37725824	-1.133751951	4
EFCAB8	0.584601472	0.570068581	-1.154670053	4
EFEMP2	0.834593952	0.273781675	-1.108375627	4
EFNA4	0.838984767	0.267587249	-1.106572015	4
EHBP1L1	0.519144705	0.633661425	-1.15280613	4
EID2	0.759879384	0.373012824	-1.132892208	4
EIF2B3	0.455702355	0.69098043	-1.146682785	4
EIF2B5	0.800355102	0.320637116	-1.120992217	4
EIF2S3	0.553701181	0.600680749	-1.15438193	4
EIF3C	0.676953649	0.471646819	-1.148600468	4
EIF3CL	0.843553438	0.261094571	-1.104648009	4
EIF3G	0.725082254	0.415720907	-1.140803161	4
EIF4A2	0.783512797	0.342805221	-1.126318018	4

EIF4B	0.619797901	0.533835558	-1.153633459	4
EIF4G1	0.68414701	0.463505263	-1.147652273	4
EIF5	0.566054462	0.588572884	-1.154627345	4
EIF5A	0.678623272	0.469763664	-1.148386936	4
EIF6	0.535525022	0.618188793	-1.153713815	4
ELAC2	0.664378179	0.485705297	-1.150083475	4
ELMO1	0.814240521	0.301934943	-1.116175463	4
ELOVL1	0.640981005	0.511289224	-1.152270229	4
ELOVL3	0.509972372	0.642201717	-1.15217409	4
EME2	0.658265098	0.492460596	-1.150725694	4
EMP3	0.61546303	0.538379556	-1.153842586	4
ENDOG	0.812378618	0.304465837	-1.116844455	4
ENHO	0.527621814	0.625689749	-1.153311562	4
ENO1	0.617230119	0.536530037	-1.153760157	4
ENPP5	0.505161123	0.646646429	-1.151807552	4
ENTPD5	0.726065999	0.414540871	-1.14060687	4
EP300	0.678571244	0.469822406	-1.14839365	4
EPB41	0.470376219	0.678080558	-1.148456777	4
EPC2	0.700126218	0.445152245	-1.145278463	4
EPDR1	0.653419013	0.497779847	-1.151198861	4
ERAP2	0.823921061	0.288657456	-1.112578518	4
ERG28	0.797126601	0.324929537	-1.122056138	4
ERGIC3	0.645745914	0.506137997	-1.15188391	4
ERI1	0.849355431	0.252778197	-1.102133628	4

ERICH4	0.470368013	0.678087831	-1.148455844	4
ERP27	0.623974372	0.529435411	-1.153409783	4
ERP29	0.617915253	0.535811901	-1.153727154	4
ESAM	0.627887119	0.525293192	-1.153180311	4
ESRP2	0.451483205	0.694650807	-1.146134012	4
ESRRA	0.769078019	0.361376033	-1.130454052	4
ESS2	0.800260495	0.320763195	-1.12102369	4
ETV7	0.617066573	0.536701375	-1.153767948	4
EXOC3	0.538202931	0.615631943	-1.153834874	4
EXOC7	0.734637786	0.40419293	-1.138830716	4
EXOSC10	0.720520223	0.421173158	-1.141693382	4
EXOSC4	0.532013173	0.621530166	-1.153543339	4
EXOSC8	0.541730892	0.612251608	-1.1539825	4
EZH1	0.854760392	0.244957891	-1.099718284	4
EZR	0.55602478	0.598416348	-1.154441128	4
F2RL1	0.728583492	0.41151401	-1.140097502	4
F8A1	0.643156668	0.508940856	-1.152097524	4
F8A2	0.449290738	0.696551338	-1.145842077	4
FAAH2	0.764450366	0.367249118	-1.131699484	4
FAHD1	0.48466323	0.665317087	-1.149980316	4
FAM107B	0.826865168	0.284579232	-1.1114444	4
FAM111A	0.746872051	0.389215011	-1.136087063	4
FAM117B	0.780517261	0.346690896	-1.127208157	4
FAM118B	0.689776272	0.457082263	-1.146858535	4

FAM120B	0.774733204	0.354146276	-1.128879479	4
FAM124B	0.612135301	0.541852028	-1.153987328	4
FAM151B	0.797625559	0.324267507	-1.121893066	4
FAM162A	0.84633207	0.257121787	-1.103453857	4
FAM168A	0.771762051	0.357951955	-1.129714006	4
FAM174A	0.466279075	0.681703546	-1.147982621	4
FAM174B	0.817721668	0.297183396	-1.114905064	4
FAM174C	0.638059143	0.514433324	-1.152492467	4
FAM177B	0.57960238	0.575095226	-1.154697606	4
FAM210A	0.853895912	0.246213487	-1.100109399	4
FAM220A	0.74790447	0.387939606	-1.135844076	4
FAM236D	0.603380179	0.550923111	-1.15430329	4
FAM241A	0.761188585	0.371365782	-1.132554367	4
FAM50A	0.788553153	0.33622869	-1.124781843	4
FAM78A	0.774085998	0.354976644	-1.129062642	4
FAM86B1	0.779187524	0.348410373	-1.127597896	4
FAM86B2	0.768100187	0.362620234	-1.13072042	4
FAM89A	0.817416062	0.297601558	-1.11501762	4
FAM89B	0.596374655	0.558114578	-1.154489233	4
FAM9B	0.491574801	0.659069307	-1.150644108	4
FANCA	0.787504261	0.337601258	-1.125105519	4
FANCD2OS	0.84009186	0.266018389	-1.106110249	4
FASTK	0.850949387	0.25047934	-1.101428727	4
FBH1	0.481616446	0.668056013	-1.149672459	4

FBLL1	0.473188902	0.675583819	-1.148772722	4
FBLN5	0.744821075	0.391743358	-1.136564432	4
FBXL14	0.856210751	0.242847203	-1.099057954	4
FBXL18	0.495965851	0.655074884	-1.151040734	4
FBXO11	0.638277793	0.514198426	-1.15247622	4
FBXO34	0.717741307	0.424478286	-1.142219594	4
FBXO41	0.755507793	0.37849076	-1.133998553	4
FBXO46	0.614842548	0.539028064	-1.153870611	4
FBXW2	0.759843514	0.373057908	-1.132901422	4
FBXW5	0.451212163	0.694886009	-1.146098172	4
FCRL6	0.793903331	0.329194411	-1.123097743	4
FCRLA	0.64951864	0.502038208	-1.151556848	4
FDFT1	0.575539098	0.579159548	-1.154698646	4
FDPS	0.554930986	0.599483024	-1.154414009	4
FDX1	0.814911564	0.301021004	-1.115932568	4
FDXACB1	0.795436873	0.327167856	-1.122604728	4
FEM1A	0.629103403	0.524001625	-1.153105028	4
FEM1B	0.736141316	0.402365564	-1.13850688	4
FEN1	0.738169059	0.399895203	-1.138064263	4
FES	0.80676764	0.312049253	-1.118816893	4
FGD2	0.770422316	0.359662718	-1.130085034	4
FGD3	0.571296194	0.583383257	-1.154679451	4
FHIP1B	0.767226686	0.363730225	-1.130956911	4
FHIP2B	0.852605554	0.248084224	-1.100689778	4

FIBP	0.691950484	0.454589181	-1.146539665	4
FIP1L1	0.675860356	0.472877804	-1.14873816	4
FIS1	0.838836897	0.26779658	-1.106633477	4
FIZ1	0.546334975	0.607819783	-1.154154758	4
FKBP15	0.865322602	0.229466025	-1.094788627	4
FKBP2	0.577740323	0.576960127	-1.154700451	4
FKBPL	0.618176707	0.5355377	-1.153714406	4
FLII	0.846966997	0.256211418	-1.103178415	4
FLNA	0.755862547	0.378047468	-1.133910015	4
FLVCR2	0.724304216	0.4166531	-1.140957316	4
FMNL1	0.859703238	0.237743137	-1.097446374	4
FMNL2	0.781478974	0.345445245	-1.126924219	4
FMR1	0.653967004	0.497179933	-1.151146937	4
FNBP4	0.790505058	0.333668808	-1.124173866	4
FNDC3A	0.765429953	0.366009098	-1.131439051	4
FOLR2	0.646403937	0.505424282	-1.151828219	4
FOLR3	0.557139346	0.597328044	-1.154467391	4
FOXL3	0.653079544	0.498151281	-1.151230824	4
FOXN3	0.461733051	0.685704226	-1.147437277	4
FOXO3B	0.451111806	0.694973077	-1.146084883	4
FOXP4	0.770903599	0.359048524	-1.129952122	4
FSD2	0.825846248	0.285992809	-1.111839057	4
FTCD	0.711397041	0.43197888	-1.143375921	4
FTL	0.574650174	0.580046162	-1.154696336	4

FTSJ3	0.717266008	0.425042379	-1.142308387	4
FUCA2	0.69301856	0.453361932	-1.146380492	4
FUT1	0.693625986	0.452663238	-1.146289224	4
GAB2	0.546649799	0.607515891	-1.15416569	4
GABRR2	0.701200219	0.443905202	-1.145105421	4
GADD45GIP1	0.864742252	0.230324602	-1.095066854	4
GALE	0.784659962	0.341312682	-1.125972645	4
GALM	0.802491292	0.317785504	-1.120276796	4
GALNT14	0.644178553	0.507835716	-1.152014269	4
GALNT3	0.779853868	0.347549139	-1.127403007	4
GALNT7	0.792164949	0.331486092	-1.123651042	4
GAPDHS	0.673483161	0.475548591	-1.149031753	4
GATAD2B	0.694181474	0.452023811	-1.146205285	4
GATD1	0.853829677	0.246309613	-1.10013929	4
GBA2	0.80338893	0.316584499	-1.119973429	4
GBF1	0.770108855	0.360062518	-1.130171374	4
GBGT1	0.646384757	0.505445093	-1.15182985	4
GBP1	0.712902611	0.430204528	-1.143107139	4
GBP6	0.78557829	0.340116076	-1.125694366	4
GCNT1	0.819755792	0.294395014	-1.114150806	4
GDE1	0.676138696	0.472564569	-1.148703265	4
GDI2	0.771779957	0.357929067	-1.129709025	4
GDPD3	0.48454533	0.665423246	-1.149968576	4
GDPD5	0.855835798	0.243393367	-1.099229165	4

GFI1	0.68708601	0.460157568	-1.147243578	4
GFOD2	0.553053928	0.601310444	-1.154364372	4
GGH	0.497255292	0.653898188	-1.15115348	4
GGT2	0.529543635	0.623871898	-1.153415533	4
GHDC	0.62853483	0.524605624	-1.153140454	4
GIGYF2	0.716034361	0.426502479	-1.14253684	4
GIMAP1-GIMAP5	0.688235996	0.458844274	-1.14708027	4
GIMAP2	0.781043557	0.346009431	-1.127052987	4
GIMAP4	0.53801695	0.615809766	-1.153826717	4
GJB6	0.680998289	0.467078095	-1.148076384	4
GJD3	0.519249075	0.63356374	-1.152812815	4
GK	0.602116345	0.552224858	-1.154341203	4
GLMP	0.832320868	0.276971213	-1.109292081	4
GLO1	0.544022981	0.610048172	-1.154071152	4
GLOD4	0.860172421	0.237055119	-1.097227541	4
GLUD2	0.784044495	0.342113757	-1.126158252	4
GMCL2	0.553427392	0.600947167	-1.15437456	4
GNB1L	0.752080506	0.382762214	-1.13484272	4
GNL2	0.703285973	0.441478461	-1.144764434	4
GNL3	0.578153017	0.576547149	-1.154700166	4
GNLY	0.522451666	0.630560684	-1.15301235	4
GNRH1	0.684276277	0.463358281	-1.147634558	4
GNS	0.824652162	0.287646496	-1.112298658	4
GOLGA2	0.599689799	0.554718795	-1.154408594	4

GOLGA6L4	0.842439624	0.262681942	-1.105121566	4
GOLGB1	0.863614773	0.23199013	-1.095604903	4
GON7	0.667374507	0.48237552	-1.149750027	4
GPALPP1	0.803474146	0.316470398	-1.119944545	4
GPAT2	0.658590576	0.492102204	-1.15069278	4
GPBAR1	0.847945492	0.254806549	-1.102752041	4
GPHA2	0.791220018	0.332729309	-1.123949327	4
GPHN	0.851639706	0.249481827	-1.101121533	4
GPN3	0.690207043	0.456588863	-1.146795905	4
GPR157	0.816920728	0.298278904	-1.115199632	4
GPR19	0.798851031	0.322639422	-1.121490452	4
GPR65	0.560409025	0.5941274	-1.154536425	4
GPR68	0.779112118	0.34850778	-1.127619898	4
GPRIN3	0.652046381	0.499280777	-1.151327157	4
GPS1	0.816254587	0.299188999	-1.115443586	4
GPX3	0.588041632	0.5665925	-1.154634132	4
GRAMD1C	0.564628741	0.589979037	-1.154607778	4
GRAPL	0.79511066	0.327599328	-1.122709988	4
GRK6	0.62904251	0.524066332	-1.153108842	4
GSDMD	0.845654476	0.258092272	-1.103746749	4
GSS	0.478956684	0.670439441	-1.149396125	4
GSTK1	0.677817652	0.470672808	-1.14849046	4
GTF2A2	0.759258544	0.373792819	-1.133051363	4
GTF2H2C	0.471097435	0.677441099	-1.148538534	4

GTF2H2C_2	0.6042835	0.549991508	-1.154275008	4
GTF2I	0.75410604	0.380240226	-1.134346266	4
GTF3A	0.76412929	0.367655183	-1.131784473	4
GTF3C5	0.806690528	0.31215302	-1.118843548	4
GTPBP10	0.609633173	0.54445401	-1.154087183	4
GTPBP4	0.744360241	0.392310477	-1.136670718	4
GTPBP6	0.749361934	0.386136041	-1.135497975	4
GUCD1	0.452399624	0.693855042	-1.146254666	4
GUCY2C	0.773172521	0.356147332	-1.129319853	4
GVQW3	0.774293368	0.35471067	-1.129004039	4
GXYLT1	0.766586501	0.364542862	-1.131129364	4
GYG1	0.55773193	0.59674886	-1.154480789	4
GYPC	0.564167821	0.59043314	-1.15460096	4
GYS1	0.668408456	0.481223637	-1.149632092	4
GZMB	0.627710468	0.52548062	-1.153191088	4
GZMH	0.706009799	0.438299482	-1.14430928	4
GZMM	0.601067241	0.553303969	-1.15437121	4
H1-0	0.582110861	0.572576556	-1.154687418	4
H1-10	0.60897664	0.545135473	-1.154112113	4
H2AX	0.559589436	0.594930809	-1.154520245	4
H3-5	0.849374684	0.252750467	-1.102125151	4
HACD2	0.75975183	0.373173132	-1.132924962	4
HACL1	0.823444254	0.289316154	-1.112760407	4
HAPLN3	0.480633084	0.668938031	-1.149571115	4

HAUS3	0.712865673	0.430248102	-1.143113775	4
HAVCR1	0.48969545	0.660772933	-1.150468382	4
HAVCR2	0.830863437	0.279010116	-1.109873553	4
HCAR2	0.494108159	0.656767167	-1.150875326	4
HDAC7	0.63172083	0.521215803	-1.152936633	4
HDHD5	0.54827791	0.605942576	-1.154220485	4
HEATR4	0.497946946	0.653266312	-1.151213258	4
HEBP1	0.769654409	0.360641821	-1.13029623	4
HEBP2	0.859627682	0.23785388	-1.097481562	4
HECA	0.586699223	0.567950578	-1.154649801	4
HECTD4	0.813907449	0.302388223	-1.116295672	4
HEXB	0.528617769	0.624748167	-1.153365936	4
HEXD	0.790206536	0.334060789	-1.124267325	4
HEXIM1	0.60301662	0.551297774	-1.154314394	4
HGF	0.720195222	0.421560327	-1.141755549	4
HHAT	0.674283455	0.474650344	-1.148933799	4
HIGD2A	0.700238214	0.445022284	-1.145260499	4
HINT2	0.736796161	0.401568518	-1.138364679	4
HIP1	0.719726055	0.422118945	-1.141844999	4
HLA-DMA	0.750301512	0.384971426	-1.135272938	4
HLA-DQA1	0.861582031	0.2349847	-1.096566731	4
HLA-DQA2	0.840586628	0.265316334	-1.105902963	4
HLA-DRA	0.527802599	0.625518911	-1.153321511	4
HLA-DRB3	0.679543125	0.468724483	-1.148267608	4

HLA-DRB5	0.818899659	0.295569682	-1.114469341	4
HMBOX1	0.742492395	0.394605473	-1.137097869	4
HMG20A	0.489181554	0.661238156	-1.15041971	4
HMGA1	0.560303602	0.594230784	-1.154534386	4
HMGCL	0.804879784	0.314586188	-1.119465972	4
HMOX1	0.70871076	0.435136098	-1.143846858	4
HMOX2	0.742840011	0.394178805	-1.137018816	4
HNRNPA0	0.849228038	0.252961663	-1.102189701	4
HNRNPA2B1	0.624111417	0.529290655	-1.153402073	4
HNRNPA3	0.581370413	0.573320773	-1.154691186	4
HNRNPL	0.716486174	0.425967135	-1.142453309	4
HNRNPU	0.56651497	0.588118204	-1.154633174	4
HOOK2	0.760916422	0.371708426	-1.132624848	4
HOPX	0.545369503	0.608751054	-1.154120557	4
HORMAD1	0.601267183	0.55309841	-1.154365593	4
HOXA1	0.463110698	0.684493974	-1.147604671	4
HPS5	0.655665875	0.495317532	-1.150983407	4
HS3ST3B1	0.815602239	0.300079338	-1.115681576	4
HSBP1L1	0.713298563	0.42973731	-1.143035872	4
HSCB	0.494678067	0.656248377	-1.150926443	4
HSD17B10	0.851120411	0.250232318	-1.101352729	4
HSD17B7	0.786200535	0.33930436	-1.125504895	4
HSFX3	0.828412947	0.28242757	-1.110840518	4
HSP90AB1	0.794931042	0.327836815	-1.122767857	4

HSPA1B	0.609276298	0.544824501	-1.154100799	4
HSPA8	0.722390549	0.418941842	-1.141332391	4
HSPA9	0.743838991	0.392951518	-1.136790509	4
HSPD1	0.586546984	0.56810446	-1.154651444	4
HSPG2	0.759975138	0.372892462	-1.132867601	4
HTR7	0.778295527	0.349561959	-1.127857486	4
HTRA1	0.57166461	0.583017325	-1.154681935	4
HTRA2	0.778543652	0.349241773	-1.127785425	4
HUWE1	0.674285329	0.47464824	-1.148933568	4
HYKK	0.713535145	0.42945803	-1.142993175	4
HYOU1	0.702506598	0.442386015	-1.144892612	4
IAH1	0.813697508	0.302673813	-1.116371322	4
ICE2	0.762368993	0.369878176	-1.13224717	4
IDH3B	0.765825735	0.365507608	-1.131333343	4
IDH3G	0.581954274	0.572733994	-1.154688268	4
IDNK	0.632291339	0.520607429	-1.152898767	4
IFFO1	0.788635259	0.336121157	-1.124756416	4
IFI30	0.627575416	0.525623886	-1.153199301	4
IFITM3	0.657309785	0.493511687	-1.150821472	4
IFNGR1	0.742026442	0.395177075	-1.137203517	4
IFNLR1	0.724372113	0.416571789	-1.140943902	4
IFRD2	0.647021634	0.504753786	-1.151775421	4
IFT20	0.55344815	0.600926971	-1.154375121	4
IGF2	0.480886956	0.668710415	-1.149597371	4

IGF2R	0.484340947	0.665607245	-1.149948191	4
IGFBP3	0.776623138	0.351717063	-1.128340201	4
IK	0.497586967	0.65359524	-1.151182207	4
IL13RA1	0.644286046	0.507719386	-1.152005432	4
IL15	0.84004253	0.266088356	-1.106130885	4
IL16	0.787217875	0.337975654	-1.125193528	4
IL17B	0.650732188	0.500715448	-1.151447636	4
IL17REL	0.645119357	0.506817052	-1.151936409	4
IL18BP	0.861024947	0.235803541	-1.096828488	4
IL2RA	0.715673561	0.426929755	-1.142603316	4
IL9R	0.711472227	0.431890354	-1.143362581	4
ILRUN	0.826091041	0.285653408	-1.11174445	4
IMPDH1	0.463186493	0.684427334	-1.147613827	4
IMPDH2	0.863111697	0.232732228	-1.095843925	4
INAFM2	0.775154547	0.353605276	-1.128759823	4
INCENP	0.614096433	0.539807249	-1.153903681	4
INO80B	0.629172789	0.523927887	-1.153100677	4
INPPL1	0.680938731	0.467145538	-1.14808427	4
INTS1	0.846199661	0.257311516	-1.103511177	4
INTS11	0.66517599	0.484819906	-1.149995896	4
INTS3	0.829781766	0.280520266	-1.110302032	4
IPO13	0.776166102	0.352305115	-1.128471217	4
IPO7	0.601504389	0.552854479	-1.154358868	4
IQCG	0.626938303	0.526299432	-1.153237735	4

IRAG1	0.823477438	0.289270326	-1.112747764	4
IRF1	0.618822222	0.534860346	-1.153682568	4
IRF2BP2	0.68725222	0.459967873	-1.147220093	4
IRF2BPL	0.566775327	0.587861036	-1.154636363	4
IRF3	0.50958153	0.642563679	-1.152145209	4
IRF5	0.840096446	0.266011884	-1.10610833	4
IRF9	0.63867256	0.513774169	-1.152446729	4
ISCA2	0.481930936	0.66777373	-1.149704666	4
ISG20	0.67590329	0.472829495	-1.148732785	4
ISL2	0.690043658	0.456776034	-1.146819692	4
ISM1	0.802607439	0.317630195	-1.120237634	4
ISY1	0.573121769	0.581568471	-1.15469024	4
ISY1-RAB43	0.495737149	0.655283412	-1.15102056	4
ITGA5	0.688222246	0.458859988	-1.147082234	4
ITLN1	0.649828811	0.50170031	-1.151529121	4
ITM2A	0.514132459	0.638339188	-1.152471647	4
ITPRIPL2	0.54583658	0.60830065	-1.15413723	4
ITSN2	0.684440373	0.463171663	-1.147612036	4
JAG1	0.79827702	0.323402388	-1.121679408	4
JAM2	0.556942126	0.597520718	-1.154462844	4
JMJD7	0.518873283	0.633915409	-1.152788693	4
JOSD1	0.449075428	0.69673773	-1.145813159	4
JRKL	0.658915622	0.491744144	-1.150659767	4
JUND	0.47113492	0.67740785	-1.148542769	4

JUP	0.496700381	0.654404787	-1.151105168	4
KALRN	0.85514082	0.244404764	-1.099545583	4
KANK3	0.721699839	0.419766508	-1.141466347	4
KANSL2	0.848524289	0.253974465	-1.102498754	4
KAT6A	0.856359725	0.242630108	-1.098989833	4
KAT8	0.843370065	0.261356109	-1.104726174	4
KBTBD4	0.842753349	0.262235126	-1.104988475	4
KCNE1B	0.500957467	0.650510285	-1.151467752	4
KCNK13	0.664987078	0.485029634	-1.150016713	4
KCTD12	0.457894752	0.689066403	-1.146961154	4
KCTD2	0.565831621	0.588792818	-1.154624439	4
KCTD3	0.463310007	0.684318729	-1.147628736	4
KDM1A	0.781228879	0.345769346	-1.126998225	4
KDM2A	0.704834974	0.439671993	-1.144506967	4
KDM5A	0.796341756	0.325969893	-1.122311649	4
KDM5C	0.854333155	0.245578653	-1.099911808	4
KHNYN	0.723233785	0.417934048	-1.141167833	4
KHSRP	0.779933923	0.347445614	-1.127379537	4
KIAA1328	0.543975117	0.610094243	-1.15406936	4
KIAA1614	0.483994607	0.665918946	-1.149913553	4
KIAA1671	0.582966815	0.571715451	-1.154682266	4
KIF19	0.571358534	0.583321349	-1.154679882	4
KIZ	0.749968871	0.385383909	-1.13535278	4
KLB	0.682464493	0.465416166	-1.147880659	4

KLF1	0.464554362	0.683223738	-1.1477781	4
KLF13	0.667528997	0.482203502	-1.149732499	4
KLF2	0.648247864	0.503421251	-1.151669115	4
KLF4	0.865446478	0.229282649	-1.094729127	4
KLF9	0.64379037	0.508255686	-1.152046057	4
KLHDC2	0.567292188	0.587350278	-1.154642466	4
KLHL20	0.612028839	0.541962895	-1.153991734	4
KLHL22	0.7352836	0.403408472	-1.138692071	4
KLHL29	0.573601013	0.581091427	-1.15469244	4
KLHL34	0.74450356	0.392134141	-1.136637701	4
KLRC1	0.757709543	0.375735969	-1.133445512	4
KLRC4	0.804688918	0.314842273	-1.119531191	4
KLRD1	0.646550283	0.505265473	-1.151815755	4
KLRG1	0.485385645	0.664666302	-1.150051948	4
KMT2A	0.749853817	0.385526535	-1.135380352	4
KMT2B	0.525104621	0.628064809	-1.15316943	4
KMT5C	0.818965001	0.295480083	-1.114445085	4
KPNA2	0.776158165	0.352315324	-1.128473489	4
KPNB1	0.72862312	0.411466283	-1.140089403	4
KPTN	0.464312216	0.683436937	-1.147749153	4
KRBA2	0.764744316	0.366877199	-1.131621515	4
KREMEN2	0.591149822	0.563439882	-1.154589705	4
KRT10	0.805382388	0.31391149	-1.119293878	4
KRT72	0.697036682	0.448729988	-1.14576667	4

KRT73	0.682477463	0.465401451	-1.147878914	4
KRTAP20-4	0.750526392	0.384692462	-1.135218853	4
KRTAP4-6	0.562525917	0.59204881	-1.154574727	4
KRTCAP2	0.758772987	0.37440238	-1.133175367	4
KSR1	0.519552569	0.633279618	-1.152832188	4
KTI12	0.509581689	0.642563532	-1.152145221	4
L2HGDH	0.448200243	0.697494912	-1.145695156	4
LAGE3	0.616458793	0.53733782	-1.153796613	4
LAIR2	0.608138978	0.546004177	-1.154143155	4
LAP3	0.473220472	0.675555752	-1.148776223	4
LARP1	0.834214875	0.2743144	-1.108529275	4
LARP4B	0.820006261	0.294051057	-1.114057318	4
LASP1	0.524608322	0.628532289	-1.153140611	4
LATS2	0.759306423	0.37373269	-1.133039113	4
LAX1	0.788760496	0.335957113	-1.124717608	4
LCMT1	0.765627334	0.365759035	-1.131386368	4
LCN10	0.601377743	0.552984724	-1.154362467	4
LDHA	0.558994714	0.595513318	-1.154508033	4
LDLR	0.740452793	0.397104853	-1.137557646	4
LDLRAD4	0.596495671	0.557990849	-1.154486521	4
LEMD2	0.85919982	0.238480737	-1.097680558	4
LEP	0.851460748	0.249740533	-1.101201281	4
LEPROTL1	0.632329659	0.520566551	-1.152896209	4
LETM2	0.565167533	0.58944791	-1.154615443	4

LGALS4	0.596134712	0.558359847	-1.154494559	4
LGALS9	0.555288087	0.599134923	-1.15442301	4
LGSN	0.59861261	0.555823631	-1.154436241	4
LHX4	0.605619343	0.548612027	-1.15423137	4
LIG1	0.795371781	0.327253967	-1.122625748	4
LIG4	0.625930872	0.527366587	-1.153297458	4
LIM2	0.832450482	0.276789655	-1.109240137	4
LIMD2	0.726394429	0.414146564	-1.140540993	4
LIME1	0.769757349	0.360510631	-1.130267981	4
LIN37	0.636684876	0.515908289	-1.152593166	4
LIN52	0.818234502	0.296481237	-1.114715738	4
LINS1	0.718144874	0.423999051	-1.142143925	4
LIPE	0.829288378	0.281208231	-1.110496609	4
LIPH	0.782999995	0.343471605	-1.1264716	4
LIPN	0.806447307	0.312480235	-1.118927542	4
LIPT1	0.576103273	0.578596368	-1.154699641	4
LIX1L	0.801626267	0.318941336	-1.120567603	4
LLCFC1	0.650154858	0.501344978	-1.151499836	4
LLGL2	0.781441575	0.345493719	-1.126935293	4
LOC100133315	0.463653396	0.684016709	-1.147670105	4
LOC105371063	0.75277115	0.381903088	-1.134674238	4
LOC105378979	0.668474215	0.481150326	-1.149624542	4
LOC107984203	0.787648236	0.337412979	-1.125061215	4
LOC107984832	0.745801684	0.390535394	-1.136337078	4

LOC107986217	0.489872651	0.660612453	-1.150485104	4
LOC107986860	0.697513231	0.448179059	-1.14569229	4
LOC107987285	0.819694178	0.294479604	-1.114173782	4
LOC107987423	0.820261594	0.293700282	-1.113961876	4
LOC107987464	0.698158539	0.447432495	-1.145591034	4
LOC107987477	0.720124111	0.421645017	-1.141769129	4
LOC112267855	0.621961567	0.531558745	-1.153520312	4
LOC112267992	0.562553921	0.592021279	-1.1545752	4
LOC112268260	0.828182968	0.282747614	-1.110930582	4
LOC390877	0.49860183	0.652667578	-1.151269407	4
LOC391322	0.559294672	0.59521957	-1.154514242	4
LONRF3	0.607186105	0.54699132	-1.154177425	4
LPAR3	0.79438614	0.328556884	-1.122943024	4
LPAR6	0.579992665	0.574703836	-1.154696501	4
LPCAT1	0.577273501	0.577427034	-1.154700535	4
LPIN1	0.682316273	0.465584312	-1.147900585	4
LPXN	0.456639288	0.690163029	-1.146802317	4
LRCH4	0.554105034	0.600287615	-1.15439265	4
LRIF1	0.565399125	0.589219512	-1.154618637	4
LRRC14	0.853043175	0.247450226	-1.100493402	4
LRRC3	0.626607957	0.526649503	-1.15325746	4
LRRC45	0.810880374	0.306497145	-1.117377519	4
LRRC51	0.625922093	0.52737588	-1.153297973	4
LRRC63	0.727904581	0.412331285	-1.140235867	4

LRRC75A	0.510736594	0.641493509	-1.152230103	4
LRRC8A	0.716383462	0.426088864	-1.142472326	4
LRWD1	0.542450838	0.611560125	-1.154010963	4
LSM10	0.848282649	0.254321947	-1.102604596	4
LSM14B	0.753666759	0.380787774	-1.134454533	4
LSP1	0.755317319	0.378728682	-1.134046001	4
LTA4H	0.500252942	0.651156085	-1.151409027	4
LTBP2	0.557455417	0.597019169	-1.154474586	4
LTBP4	0.846311948	0.257150622	-1.10346257	4
LTV1	0.653816408	0.497344839	-1.151161247	4
LUZP1	0.576667686	0.578032584	-1.154700269	4
LY75	0.828511004	0.282291077	-1.110802081	4
LYL1	0.847883678	0.254895366	-1.102779044	4
LYPLA2	0.570073567	0.584596528	-1.154670095	4
LYSMD4	0.767640719	0.363204268	-1.130844987	4
LZTFL1	0.810217545	0.307394313	-1.117611858	4
MAB21L3	0.758783841	0.374388759	-1.133172599	4
MAD1L1	0.686817416	0.460464029	-1.147281445	4
MAF	0.851761119	0.249306264	-1.101067383	4
MAF1	0.504465918	0.647286692	-1.15175261	4
MAFB	0.754243475	0.38006885	-1.134312325	4
MAFF	0.825622928	0.286302322	-1.11192525	4
MAFK	0.664777782	0.485261936	-1.150039718	4
MAGED2	0.838791083	0.267861426	-1.106652509	4

MAGED4B	0.584026944	0.570647757	-1.154674701	4
MAGEF1	0.764067415	0.367733416	-1.131800831	4
MAL	0.697845051	0.44779525	-1.145640301	4
MAML1	0.681628415	0.466364229	-1.147992644	4
MAMLD1	0.702916867	0.441908385	-1.144825252	4
MAN2A1	0.502962251	0.648669823	-1.151632074	4
MAN2B1	0.77536795	0.353331144	-1.128699094	4
MAP2K2	0.610070876	0.543999394	-1.154070269	4
MAP2K7	0.604596636	0.549668337	-1.154264973	4
MAP3K12	0.855292446	0.244184206	-1.099476651	4
MAP3K14	0.549895179	0.604376849	-1.154272029	4
MAP3K3	0.58700147	0.567644988	-1.154646458	4
MAP3K9	0.789060909	0.335563485	-1.124624395	4
MAP4K1	0.695320528	0.450711218	-1.146031746	4
MAP7D1	0.467677579	0.680468742	-1.148146321	4
MAP7D3	0.515234709	0.637312752	-1.152547461	4
MAPK13	0.667263588	0.482499003	-1.149762591	4
MAPKBP1	0.804612234	0.314945139	-1.119557373	4
MARCKSL1	0.461288746	0.68609415	-1.147382896	4
MARK2	0.634951355	0.517765355	-1.15271671	4
MARK3	0.499186306	0.652132844	-1.15131915	4
MARS1	0.850801283	0.2506932	-1.101494483	4
MAST1	0.833573987	0.275214312	-1.108788299	4
MAST2	0.512527369	0.639831607	-1.152358976	4

MATK	0.760835367	0.371810446	-1.132645813	4
MAZ	0.76294467	0.369151787	-1.132096457	4
MBLAC1	0.656382687	0.494530558	-1.150913244	4
MBLAC2	0.851036419	0.250353642	-1.101390061	4
MCRIP2	0.787562322	0.337525335	-1.125087657	4
MCTS1	0.534538531	0.619128737	-1.153667268	4
MDH1	0.851593471	0.249548672	-1.101142143	4
MDH2	0.776051505	0.352452503	-1.128504008	4
MDM2	0.680837286	0.467260404	-1.14809769	4
ME2	0.544276312	0.609804287	-1.154080599	4
MED1	0.628886026	0.524232594	-1.153118621	4
MED12	0.863107993	0.23273769	-1.095845683	4
MED15	0.756412946	0.377359269	-1.133772216	4
MED17	0.858006726	0.240226302	-1.098233027	4
MED18	0.564309726	0.590293359	-1.154603085	4
MED30	0.731244481	0.408303551	-1.139548033	4
MEF2D	0.642290272	0.509876767	-1.15216704	4
MEGF6	0.599951589	0.554450076	-1.154401665	4
MEGF8	0.496785556	0.654327048	-1.151112604	4
MEIKIN	0.646219517	0.505624368	-1.151843885	4
METTL23	0.54719638	0.60698803	-1.15418441	4
METTL26	0.864933903	0.230041167	-1.09497507	4
METTL3	0.843927936	0.260560192	-1.104488128	4
METTL7B	0.512948418	0.639440374	-1.152388792	4

MFHAS1	0.492629675	0.658111501	-1.150741176	4
MFSD13A	0.660962462	0.489486127	-1.150448589	4
MFSD14B	0.61846037	0.53524011	-1.153700479	4
MFSD3	0.736284333	0.40219155	-1.138475884	4
MFSD5	0.838537771	0.268219881	-1.106757653	4
MGA	0.632289793	0.520609078	-1.152898871	4
MGAT2	0.765705729	0.365659696	-1.131365424	4
MGAT3	0.703699233	0.440996864	-1.144696097	4
MGMT	0.53389876	0.619737761	-1.153636521	4
MICAL1	0.657168335	0.493667214	-1.150835549	4
MID1IP1	0.474593183	0.674334355	-1.148927538	4
MILR1	0.66456528	0.485497735	-1.150063015	4
MIS12	0.796936437	0.325181722	-1.122118159	4
MKS1	0.735391664	0.403277141	-1.138668805	4
MLH1	0.861268102	0.235446232	-1.096714333	4
MLKL	0.692432938	0.454035035	-1.146467973	4
MLLT6	0.804744398	0.314767843	-1.119512241	4
MLST8	0.68767896	0.459480653	-1.147159613	4
MLX	0.794448928	0.328473942	-1.12292287	4
MNT	0.826227364	0.285464343	-1.111691707	4
MOGS	0.795442855	0.327159941	-1.122602796	4
MON1A	0.578388589	0.576311326	-1.154699916	4
MON1B	0.535252171	0.618448874	-1.153701046	4
MOSPD3	0.847122811	0.255987863	-1.103110673	4

MPC1	0.592550193	0.562015765	-1.154565957	4
MPI	0.83443567	0.274004151	-1.108439821	4
MPLKIP	0.531974441	0.621566944	-1.153541385	4
MPPE1	0.493637679	0.657195198	-1.150832878	4
MPZL1	0.670602937	0.478773943	-1.14937688	4
MPZL2	0.760997042	0.371606941	-1.132603983	4
MRFAP1L1	0.588136765	0.566496176	-1.154632941	4
MRM2	0.856036903	0.243100477	-1.09913738	4
MROH6	0.859870455	0.23749799	-1.097368445	4
MRPL16	0.679283503	0.469017906	-1.148301409	4
MRPL23	0.544882181	0.609220724	-1.154102906	4
MRPL24	0.794620374	0.328247424	-1.122867797	4
MRPL3	0.556304	0.598143838	-1.154447837	4
MRPL4	0.823803	0.288820601	-1.112623601	4
MRPL40	0.862316079	0.233904541	-1.096220619	4
MRPL43	0.739984722	0.397677462	-1.137662185	4
MRPL48	0.806492043	0.312420059	-1.118912102	4
MRPL50	0.692833706	0.453574454	-1.14640816	4
MRPL52	0.608544699	0.545583527	-1.154128227	4
MRPL53	0.808019551	0.310362889	-1.11838244	4
MRPL55	0.808176993	0.310150581	-1.118327575	4
MRPL9	0.861829726	0.234620367	-1.096450093	4
MRPS16	0.571460427	0.583220151	-1.154680578	4
MRPS18A	0.738974078	0.398912586	-1.137886664	4

MRPS2	0.82729881	0.283976934	-1.111275744	4
MRPS22	0.857186332	0.241424516	-1.098610848	4
MRPS24	0.617976273	0.535747913	-1.153724187	4
MRTFA	0.654630821	0.49645268	-1.151083501	4
MS4A1	0.780560795	0.346634547	-1.127195341	4
MS4A4A	0.660960096	0.48948874	-1.150448836	4
MS4A4E	0.722955684	0.418266545	-1.141222229	4
MS4A7	0.463847525	0.683845917	-1.147693442	4
MSH6	0.602065458	0.552277231	-1.154342689	4
MSL2	0.631301239	0.521662977	-1.152964216	4
MTARC1	0.684345419	0.463279655	-1.147625073	4
MTCP1	0.84298127	0.261910371	-1.10489164	4
MTFP1	0.554457766	0.599944098	-1.154401864	4
MTHFD1L	0.634393405	0.518362246	-1.152755651	4
MTIF2	0.678357738	0.470063422	-1.148421159	4
MTLN	0.452355314	0.693893537	-1.146248851	4
MTR	0.787919341	0.337058341	-1.124977683	4
MTX3	0.817743686	0.297153261	-1.114896947	4
MVP	0.763831862	0.368031178	-1.131863039	4
MXD4	0.694780028	0.451334303	-1.146114331	4
MXRA7	0.825578145	0.286364377	-1.111942521	4
MYBPH	0.465711469	0.682204165	-1.147915635	4
MYCL	0.815312663	0.300474268	-1.115786931	4
MYD88	0.817801151	0.297074607	-1.114875758	4

MYDGF	0.547348715	0.606840854	-1.154189569	4
MYH7B	0.788906533	0.335765784	-1.124672317	4
MYLIP	0.577236362	0.577464169	-1.154700531	4
MYO15B	0.514916557	0.637609152	-1.152525709	4
MYO18A	0.54029089	0.613632986	-1.153923876	4
MYO1F	0.498823339	0.652464961	-1.1512883	4
MYO9B	0.490964545	0.659622894	-1.150587439	4
MYOM2	0.668935776	0.4806356	-1.149571376	4
MZF1	0.673176683	0.475892345	-1.149069028	4
MZT2A	0.776620724	0.35172017	-1.128340894	4
MZT2B	0.78182533	0.344996205	-1.126821535	4
NAA25	0.666166547	0.483719395	-1.149885942	4
NAA35	0.509064385	0.643042366	-1.152106751	4
NAA38	0.718305188	0.423808607	-1.142113795	4
NAA80	0.666791939	0.483023887	-1.149815826	4
NAAA	0.630812192	0.52218389	-1.152996082	4
NAB2	0.765074012	0.366459868	-1.13153388	4
NACA	0.543826876	0.610236916	-1.154063792	4
NACA2	0.807355995	0.31125712	-1.118613115	4
NACC1	0.668180898	0.481477277	-1.149658175	4
NADK	0.513798568	0.638649863	-1.15244843	4
NAGS	0.53645799	0.617298881	-1.153756871	4
NAP1L2	0.689712971	0.457154745	-1.146867716	4
NAPSA	0.822937631	0.290015497	-1.112953129	4

NASP	0.651681515	0.499679322	-1.151360837	4
NAT1	0.510329003	0.641871301	-1.152200304	4
NATD1	0.451374132	0.694745465	-1.146119597	4
NAV1	0.581967348	0.57272085	-1.154688198	4
NBPF1	0.577087701	0.577612797	-1.154700499	4
NBR1	0.50353517	0.648143104	-1.151678275	4
NCALD	0.831924089	0.277526768	-1.109450857	4
NCAPD2	0.736634307	0.401765585	-1.138399892	4
NCAPD3	0.852984215	0.247535672	-1.100519887	4
NCAPH2	0.759943865	0.372931774	-1.132875639	4
NCF1	0.566703853	0.587931643	-1.154635495	4
NCKAP1L	0.58961327	0.564999822	-1.154613092	4
NCL	0.626848519	0.52639459	-1.153243109	4
NCLN	0.668039861	0.481634444	-1.149674305	4
NCOR1	0.775417997	0.353266843	-1.12868484	4
NCOR2	0.492158413	0.658539538	-1.150697951	4
NCR3	0.520596025	0.632302029	-1.152898054	4
NDE1	0.449387056	0.696467943	-1.145854999	4
NDRG1	0.605193013	0.549052519	-1.154245532	4
NDRG3	0.758313389	0.374978971	-1.133292361	4
NDUFA11	0.795295433	0.327354959	-1.122650393	4
NDUFA2	0.452958049	0.693369743	-1.146327792	4
NDUFA3	0.831510839	0.278105009	-1.109615848	4
NDUFA7	0.771947678	0.357714662	-1.12966234	4

NDUFAB1	0.493730739	0.657110553	-1.150841292	4
NDUFB5	0.464284364	0.683461456	-1.14774582	4
NDUFB9	0.627176454	0.526046975	-1.153223429	4
NDUFC2-KCTD14	0.478581821	0.67077479	-1.149356612	4
NDUFS1	0.514752899	0.637761579	-1.152514478	4
NDUFS2	0.830270166	0.279838721	-1.110108887	4
NDUFS7	0.690070641	0.456745125	-1.146815766	4
NDUFS8	0.615005911	0.538857368	-1.153863279	4
NECAP2	0.81568314	0.29996897	-1.11565211	4
NEDD9	0.812876122	0.303790288	-1.11666641	4
NEFL	0.492043541	0.658643839	-1.15068738	4
NELFA	0.774275929	0.354733042	-1.12900897	4
NELFCD	0.694107444	0.452109054	-1.146216498	4
NELFE	0.564481074	0.590124546	-1.15460562	4
NEU1	0.494266814	0.656622775	-1.15088959	4
NFATC2IP	0.768929361	0.361565298	-1.130494659	4
NFE2L1	0.719398306	0.422508976	-1.141907283	4
NFIC	0.575576597	0.579122127	-1.154698724	4
NFIX	0.765188887	0.366314414	-1.1315033	4
NFKB2	0.61484832	0.539022033	-1.153870353	4
NFKBIE	0.68559008	0.461863075	-1.147453155	4
NGRN	0.596942473	0.557533882	-1.154476355	4
NHLRC2	0.605336885	0.548903893	-1.154240778	4
NIPSNAP1	0.810360746	0.307200562	-1.117561308	4

NIT1	0.772664913	0.356797209	-1.129462122	4
NIT2	0.767452105	0.363443908	-1.130896013	4
NKAPD1	0.566452982	0.588179421	-1.154632404	4
NLRC5	0.753978681	0.380399009	-1.13437769	4
NLRP12	0.644198721	0.507813891	-1.152012613	4
NLRP2	0.621188123	0.532373309	-1.153561432	4
NMB	0.561728484	0.592832406	-1.15456089	4
NME6	0.725233727	0.415539311	-1.140773037	4
NMRAL1	0.516572195	0.636065553	-1.152637748	4
NMT1	0.843869279	0.260643912	-1.104513192	4
NMUR1	0.592521142	0.562045332	-1.154566473	4
NOCT	0.512401839	0.639948212	-1.152350051	4
NOL7	0.626314534	0.526960331	-1.153274865	4
NOLC1	0.828864954	0.281798208	-1.110663162	4
NPDC1	0.621365521	0.532186546	-1.153552067	4
NPIPA3	0.597684567	0.556774376	-1.154458943	4
NPIPB13	0.862014597	0.23434834	-1.096362937	4
NPIPB4	0.654304092	0.496810706	-1.151114798	4
NPIPB5	0.79083417	0.333236459	-1.124070629	4
NR1H2	0.500949039	0.650518014	-1.151467053	4
NR1H3	0.797922381	0.323873442	-1.121795823	4
NR3C1	0.513860269	0.638592461	-1.152452729	4
NRG4	0.773989979	0.355099772	-1.129089751	4
NRXN2	0.592131761	0.562441535	-1.154573296	4

NSF	0.512892258	0.639492568	-1.152384826	4
NSL1	0.85617375	0.242901114	-1.099074865	4
NSMF	0.837210523	0.270095621	-1.107306144	4
NSUN4	0.594789716	0.559733426	-1.154523142	4
NSUN5	0.861785113	0.234686	-1.096471113	4
NT5C	0.8291632	0.281382689	-1.110545889	4
NT5C1A	0.524240065	0.628878994	-1.153119059	4
NT5C1B	0.528071216	0.625265013	-1.153336228	4
NTHL1	0.687769908	0.459376781	-1.147146689	4
NUCKS1	0.564669342	0.589939025	-1.154608367	4
NUDC	0.75798665	0.37538866	-1.13337531	4
NUDT15	0.499766672	0.651601525	-1.151368197	4
NUDT17	0.831698099	0.277843032	-1.109541131	4
NUDT18	0.58316014	0.571520844	-1.154680984	4
NUDT22	0.851285452	0.249993869	-1.101279322	4
NUDT3	0.526227222	0.627006427	-1.15323365	4
NUDT7	0.673540054	0.475484764	-1.149024819	4
NUP107	0.517042714	0.635626351	-1.152669065	4
NUTM2A	0.791864236	0.33188192	-1.123746156	4
NUTM2E	0.570629138	0.58404542	-1.154674558	4
NUTM2G	0.642446233	0.509708366	-1.152154599	4
OARD1	0.503203507	0.648448063	-1.15165157	4
OCM2	0.710442958	0.43310149	-1.143544448	4
ODAD4	0.453461796	0.692931703	-1.146393499	4

OFD1	0.784160845	0.341962374	-1.126123219	4
OGA	0.649103245	0.502490536	-1.151593782	4
OGDH	0.775951724	0.352580814	-1.128532539	4
OGFR	0.564360277	0.590243559	-1.154603836	4
OGG1	0.802169921	0.318215091	-1.120385012	4
OLA1	0.797305966	0.324691608	-1.121997574	4
OLAH	0.558632263	0.595868133	-1.154500396	4
OLFM4	0.536691601	0.617075904	-1.153767505	4
OLR1	0.657930061	0.492829363	-1.150759425	4
OOSP4B	0.631652782	0.52128834	-1.152941122	4
OR1F1	0.678430195	0.469981636	-1.148411831	4
OR2A1	0.661603353	0.488777943	-1.150381296	4
OR2A42	0.655027978	0.496017286	-1.151045264	4
OR2T10	0.730819727	0.408816779	-1.139636507	4
OR4E1	0.632843597	0.520018119	-1.152861716	4
OR52K1	0.466000973	0.681948867	-1.14794984	4
OR52R1	0.463741739	0.68393899	-1.14768073	4
OR56B1	0.588340599	0.566289754	-1.154630354	4
ORAI3	0.545854325	0.608283534	-1.154137859	4
OSBP	0.847370612	0.255632207	-1.10300282	4
OSBPL9	0.471551761	0.677038012	-1.148589773	4
OSER1	0.781666381	0.345202306	-1.126868687	4
OSTC	0.806953995	0.31179843	-1.118752425	4
OTUB1	0.590650254	0.563947361	-1.154597615	4

OTUD4	0.592562718	0.562003016	-1.154565734	4
OTUD5	0.476337667	0.672779475	-1.149117142	4
OTULINL	0.761069428	0.371515812	-1.13258524	4
OVCH1	0.464750096	0.683051362	-1.147801457	4
OXA1L	0.822838509	0.290152261	-1.11299077	4
OXNAD1	0.841932516	0.263403692	-1.105336208	4
P2RX6	0.536821694	0.616951706	-1.153773401	4
P2RX7	0.774204002	0.354825302	-1.129029304	4
P2RY2	0.854412786	0.245462985	-1.099875772	4
P2RY6	0.642021706	0.510166683	-1.152188389	4
P4HB	0.762754867	0.369391346	-1.132146213	4
PA2G4	0.608700446	0.545421997	-1.154122443	4
PABPC1	0.714584825	0.428217865	-1.14280269	4
PABPC3	0.486597345	0.663573572	-1.150170917	4
PABPN1	0.752134935	0.382694538	-1.134829472	4
PAF1	0.499434737	0.65190545	-1.151340187	4
PAFAH2	0.774049197	0.355023837	-1.129073034	4
PAG1	0.65605563	0.494889713	-1.150945343	4
PAGR1	0.623338293	0.530106969	-1.153445262	4
PANK1	0.833590364	0.275191328	-1.108781691	4
PANK3	0.799625617	0.321608811	-1.121234429	4
PAOX	0.51028688	0.641910335	-1.152197215	4
PAQR4	0.579402314	0.57529579	-1.154698104	4
PARL	0.827330302	0.283933178	-1.11126348	4

PARP11	0.86396903	0.231467169	-1.095436199	4
PARP4	0.540316592	0.61360835	-1.153924942	4
PARP8	0.500483005	0.650945255	-1.15142826	4
PARP9	0.570501265	0.584172297	-1.154673562	4
PARS2	0.842132599	0.263118993	-1.105251592	4
PAX8	0.816656521	0.298639982	-1.115296502	4
PAXX	0.710843391	0.432630495	-1.143473886	4
PBLD	0.772079802	0.357545726	-1.129625528	4
PCBP1	0.612705915	0.541257564	-1.153963479	4
PCDH1	0.480754817	0.668828896	-1.149583713	4
PCF11	0.648323275	0.503339237	-1.151662513	4
PCIF1	0.807076495	0.311633514	-1.118710009	4
PCM1	0.688656167	0.458363958	-1.147020125	4
PCOLCE	0.534986915	0.61870164	-1.153688555	4
PDCD2L	0.765877728	0.365441707	-1.131319435	4
PDCD5	0.703473707	0.441259715	-1.144733422	4
PDCL	0.461514084	0.685896417	-1.1474105	4
PDE3B	0.838785816	0.267868881	-1.106654697	4
PDE4B	0.833768854	0.274940785	-1.108709639	4
PDE6D	0.826308007	0.285352481	-1.111660488	4
PDF	0.70801574	0.435951169	-1.143966909	4
PDGFB	0.549335179	0.604919328	-1.154254507	4
PDHX	0.559928069	0.594598953	-1.154527021	4
PDIK1L	0.484169646	0.665761428	-1.149931074	4

PDK3	0.714235795	0.428630422	-1.142866217	4
PDPR	0.702627094	0.442245761	-1.144872855	4
PDSS2	0.67397039	0.475001834	-1.148972224	4
PDZK1	0.73155973	0.407922449	-1.139482179	4
PEAK1	0.851186498	0.250136844	-1.101323342	4
PEBP1	0.620022149	0.533599856	-1.153622004	4
PEBP4	0.672241127	0.476940873	-1.149182	4
PELI3	0.685865869	0.461548891	-1.14741476	4
PELP1	0.750173655	0.385129994	-1.135303649	4
PEX11B	0.82749893	0.283698842	-1.111197772	4
PEX13	0.803868074	0.315942756	-1.11981083	4
PEX16	0.719877754	0.42193836	-1.141816114	4
PFKL	0.844879808	0.259200457	-1.104080265	4
PGAM1	0.468881862	0.679403886	-1.148285748	4
PGAM5	0.643489123	0.508581465	-1.152070589	4
PGBD3	0.616296527	0.537507661	-1.153804188	4
PGLS	0.685963224	0.461437956	-1.14740118	4
PGM3	0.788616168	0.336146161	-1.124762329	4
PGP	0.833307775	0.275587844	-1.108895619	4
PGPEP1	0.502821352	0.648799308	-1.15162066	4
PHACTR1	0.629275978	0.523818215	-1.153094194	4
PHAX	0.713006357	0.430082132	-1.143088489	4
PHB2	0.792065774	0.331616656	-1.12368243	4
PHETA1	0.59087864	0.563715395	-1.154594035	4

PHETA2	0.763014537	0.369063589	-1.132078126	4
PHF1	0.851742921	0.249332581	-1.101075502	4
PHF14	0.74046816	0.397086047	-1.137554207	4
PHF2	0.733357005	0.405746657	-1.139103662	4
PHF3	0.565028502	0.589584994	-1.154613496	4
PHKG2	0.769302871	0.361089685	-1.130392556	4
PI4K2A	0.863090997	0.232762749	-1.095853746	4
PI4KA	0.843319059	0.261428843	-1.104747901	4
PID1	0.719780935	0.422053619	-1.141834554	4
PIEZO1	0.794669351	0.328182703	-1.122852054	4
PIGC	0.73835767	0.399665079	-1.138022749	4
PIGH	0.633653792	0.519152861	-1.152806653	4
PIGR	0.734032339	0.404927737	-1.138960076	4
PIGU	0.465817448	0.682110718	-1.147928166	4
PIGY	0.845881488	0.257767255	-1.103648744	4
PIK3C2B	0.844750468	0.259385344	-1.104135812	4
PIK3CD	0.657078252	0.493766248	-1.1508445	4
PIK3R2	0.684471188	0.463136614	-1.147607803	4
PILRB	0.521190137	0.631744906	-1.152935043	4
PIN1	0.645547135	0.506353487	-1.151900622	4
PINK1	0.475165646	0.673824444	-1.14899009	4
PIP5K1B	0.572064191	0.582620264	-1.154684455	4
PISD	0.456097528	0.690635776	-1.146733304	4
PITPNC1	0.755966502	0.377917527	-1.133884029	4

PITPNM1	0.674165696	0.474782572	-1.148948268	4
PJVK	0.630943469	0.522044089	-1.152987558	4
PKD2L2	0.806893386	0.311880015	-1.1187734	4
PKP4	0.724301863	0.416655917	-1.140957781	4
PLA2G1B	0.770876707	0.359082853	-1.12995956	4
PLA2G2C	0.855475839	0.243917362	-1.099393201	4
PLA2G4B	0.595140798	0.559375091	-1.154515889	4
PLA2G7	0.850437415	0.251218395	-1.101655809	4
PLAAT4	0.7779876	0.349959155	-1.127946756	4
PLAAT5	0.848274227	0.254334056	-1.102608282	4
PLAC8L1	0.816474141	0.298889144	-1.115363285	4
PLBD1	0.632752786	0.520115049	-1.152867836	4
PLBD2	0.56289091	0.591689911	-1.154580821	4
PLCE1	0.78183465	0.344984119	-1.126818769	4
PLCG2	0.787569466	0.337515993	-1.125085459	4
PLCXD2	0.520506205	0.632386224	-1.152892429	4
PLD4	0.596452253	0.558035243	-1.154487496	4
PLEC	0.696332021	0.449544017	-1.145876038	4
PLEKHA2	0.806962063	0.31178757	-1.118749633	4
PLEKHJ1	0.694084656	0.452135292	-1.146219948	4
PLEKHM1	0.517130779	0.635544122	-1.152674901	4
PLGLB1	0.568443449	0.586211521	-1.154654969	4
PLOD1	0.669830532	0.479636938	-1.14946747	4
PLPP5	0.743299988	0.393613913	-1.1369139	4

PLXND1	0.684316428	0.463312624	-1.147629051	4
PMAIP1	0.836458091	0.2711572	-1.107615292	4
PMF1	0.59957613	0.554835447	-1.154411578	4
PMM1	0.473701678	0.675127801	-1.148829479	4
PNKP	0.749246305	0.386279259	-1.135525565	4
PNMA5	0.647133003	0.504632845	-1.151765848	4
PNMA8B	0.568930435	0.58572937	-1.154659805	4
PNN	0.452735782	0.69356294	-1.146298722	4
PNPLA1	0.519973833	0.632885084	-1.152858917	4
PNPLA2	0.574208969	0.580485883	-1.154694852	4
POC5	0.733523589	0.405544724	-1.139068313	4
PODXL	0.771590947	0.358170627	-1.129761574	4
POGLUT1	0.661363976	0.489042519	-1.150406496	4
POLA1	0.652184543	0.499129815	-1.151314358	4
POLDIP2	0.687589375	0.459582956	-1.147172331	4
POLG	0.844019612	0.260429327	-1.104448939	4
POLI	0.487588263	0.662678849	-1.150267112	4
POLR2B	0.505406929	0.64641993	-1.151826858	4
POLR2C	0.533123187	0.620475469	-1.153598656	4
POLR2F	0.709849928	0.433798567	-1.143648496	4
POLR3F	0.63898467	0.513438601	-1.152423271	4
POM121	0.800594196	0.320318403	-1.120912599	4
POM121C	0.720379773	0.421340494	-1.141720268	4
POMGNT1	0.651643216	0.499721147	-1.151364362	4

PON2	0.483244363	0.666593742	-1.149838105	4
POP7	0.645519106	0.506383868	-1.151902974	4
POR	0.805024507	0.314391964	-1.119416471	4
POTEI	0.62132294	0.532231378	-1.153554318	4
POU2F2	0.855445487	0.243961531	-1.099407018	4
POU5F1B	0.638927038	0.513500574	-1.152427612	4
PPARGC1A	0.739653851	0.398082011	-1.137735862	4
PPARGC1B	0.502612734	0.64899099	-1.151603723	4
PPFIA1	0.586475758	0.568176446	-1.154652204	4
PPHLN1	0.727852798	0.412393592	-1.14024639	4
PPIAL4H	0.497658542	0.653529849	-1.151188391	4
PPIH	0.839627874	0.266676249	-1.106304123	4
PPIL6	0.568542398	0.586113576	-1.154655974	4
PPIP5K2	0.610117054	0.543951417	-1.154068471	4
PPL	0.583324842	0.571355016	-1.154679858	4
PPM1F	0.591209539	0.5633792	-1.15458874	4
PPM1M	0.709785082	0.433874759	-1.143659841	4
PPME1	0.794734911	0.328096062	-1.122830973	4
PPP1CA	0.67737621	0.471170588	-1.148546798	4
PPP1CC	0.636005555	0.516636487	-1.152642041	4
PPP1R10	0.751219065	0.383832655	-1.135051721	4
PPP1R12B	0.511258659	0.641009359	-1.152268018	4
PPP1R12C	0.623699816	0.529725344	-1.15342516	4
PPP1R37	0.687660376	0.459501876	-1.147162252	4

PPP1R8	0.667875982	0.48181703	-1.149693012	4
PPP2R2B	0.643193867	0.508900651	-1.152094518	4
PPP2R3B	0.598408106	0.556033226	-1.154441332	4
PPP2R3C	0.526462161	0.626784759	-1.15324692	4
PPP2R5C	0.51662776	0.636013699	-1.152641459	4
PPP4C	0.473233733	0.675543961	-1.148777694	4
PPP6R2	0.707355513	0.436724757	-1.14408027	4
PRAM1	0.556411406	0.598038989	-1.154450395	4
PRCD	0.545904912	0.608234738	-1.15413965	4
PRCP	0.776431471	0.351963721	-1.128395192	4
PRDM1	0.730547624	0.409145407	-1.139693031	4
PRDX1	0.687398288	0.459801133	-1.147199421	4
PRDX4	0.850463408	0.251180888	-1.101644296	4
PREP	0.792960513	0.330438046	-1.123398559	4
PREX1	0.60633295	0.547874221	-1.15420717	4
PRF1	0.857671938	0.240715474	-1.098387412	4
PRICKLE3	0.619458762	0.534191902	-1.153650664	4
PRIM1	0.759498134	0.373491889	-1.132990023	4
PRKAG1	0.673845752	0.475141732	-1.148987484	4
PRKAG3	0.776988299	0.351246946	-1.128235244	4
PRKCSH	0.644972647	0.50697598	-1.151948627	4
PRKD2	0.626943903	0.526293496	-1.153237399	4
PRMT1	0.642341591	0.509821359	-1.15216295	4
PRMT2	0.716950965	0.425416082	-1.142367047	4

PRNP	0.722592939	0.418700059	-1.141292997	4
PROB1	0.853390205	0.246947138	-1.100337343	4
PROC	0.768344208	0.362309901	-1.130654109	4
PROCA1	0.516838828	0.635816695	-1.152655523	4
PRPF38A	0.859722012	0.237715617	-1.097437628	4
PRPF40A	0.510737333	0.641492824	-1.152230157	4
PRPF6	0.835016219	0.273187869	-1.108204088	4
PRRC2B	0.829709022	0.280621732	-1.110330754	4
PRRT3	0.683996895	0.46367592	-1.147672815	4
PRSS36	0.705897144	0.438431183	-1.144328327	4
PRXL2C	0.706811698	0.437361448	-1.144173146	4
PSD4	0.749974849	0.385376498	-1.135351347	4
PSEN2	0.487514631	0.662745367	-1.150259998	4
PSENEN	0.690733091	0.455985966	-1.146719057	4
PSMA1	0.848878971	0.253464173	-1.102343144	4
PSMA7	0.568591781	0.58606469	-1.154656471	4
PSMB10	0.632132565	0.520776783	-1.152909347	4
PSMB5	0.830542947	0.279457833	-1.11000078	4
PSMC1	0.527667787	0.625646309	-1.153314096	4
PSMC4	0.734143848	0.404792447	-1.138936295	4
PSMC5	0.560953622	0.593593138	-1.15454676	4
PSMC6	0.807818972	0.310633292	-1.118452264	4
PSMD2	0.582797381	0.571885973	-1.154683354	4
PSMD7	0.707091171	0.437034301	-1.144125472	4

PSMD9	0.640504101	0.51180316	-1.152307261	4
PSMG2	0.788094846	0.336828685	-1.124923531	4
PSMG3	0.864269319	0.231023623	-1.095292942	4
PSTK	0.810096337	0.307558276	-1.117654612	4
PTBP1	0.801382969	0.319266155	-1.120649124	4
PTCD1	0.853691764	0.24650973	-1.100201493	4
PTER	0.679641741	0.468613003	-1.148254744	4
PTPN23	0.4982574	0.652982531	-1.151239931	4
PTPN6	0.513707733	0.638734361	-1.152442094	4
PTPN7	0.640774539	0.511511758	-1.152286297	4
PTPRE	0.579434275	0.575263753	-1.154698028	4
PTPRN2	0.797025831	0.325063182	-1.122089013	4
PUM1	0.632259626	0.520641258	-1.152900883	4
PURA	0.628371663	0.524778882	-1.153150545	4
PURB	0.533006697	0.620586216	-1.153592913	4
PVR	0.777751308	0.35026383	-1.128015138	4
PWWP2B	0.776665526	0.351662504	-1.12832803	4
PYHIN1	0.476864827	0.672309017	-1.149173844	4
PYROXD2	0.504362983	0.64738145	-1.151744433	4
QARS1	0.840683076	0.265179413	-1.105862489	4
QRSL1	0.642798841	0.509327512	-1.152126354	4
R3HCC1	0.72935594	0.410583234	-1.139939174	4
RAB11FIP4	0.517926763	0.634800515	-1.152727277	4
RAB15	0.649465645	0.502095928	-1.151561573	4

RAB29	0.569209828	0.585452629	-1.154662457	4
RAB2A	0.705346464	0.439074691	-1.144421155	4
RAB32	0.838111042	0.268823401	-1.106934443	4
RAB3GAP1	0.855183721	0.244342364	-1.099526085	4
RAB40C	0.50852433	0.643541964	-1.152066294	4
RAB41	0.688181225	0.458906866	-1.147088091	4
RAB9A	0.638990277	0.513432571	-1.152422848	4
RABGGTA	0.59258642	0.561978892	-1.154565312	4
RABGGTB	0.83658888	0.270972769	-1.107561648	4
RABL6	0.670015682	0.479430149	-1.149445831	4
RAD51C	0.797416557	0.324544876	-1.121961433	4
RALGAPB	0.657369812	0.493445678	-1.15081549	4
RAMAC	0.863414053	0.232286294	-1.095700347	4
RANBP1	0.784337947	0.341731897	-1.126069845	4
RAP1GAP2	0.635315466	0.517375616	-1.152691082	4
RAP2B	0.574795395	0.57990138	-1.154696775	4
RAPGEF1	0.624826	0.528535483	-1.153361484	4
RAPGEF2	0.449222101	0.696610762	-1.145832863	4
RAPSN	0.647409223	0.504332811	-1.151742034	4
RASA4B	0.862114399	0.234201451	-1.09631585	4
RASAL2	0.533829854	0.619803329	-1.153633183	4
RASAL3	0.765183018	0.366321846	-1.131504863	4
RASGEF1A	0.567547134	0.587098231	-1.154645365	4
RASSF1	0.738219559	0.399833594	-1.138053153	4

RASSF5	0.635183034	0.517517389	-1.152700423	4
RB1	0.780938514	0.346145484	-1.127083999	4
RBCK1	0.544643951	0.609450231	-1.154094182	4
RBKS	0.845873669	0.257778453	-1.103652122	4
RBM23	0.674315328	0.47461455	-1.148929879	4
RBM25	0.747364008	0.388607495	-1.135971503	4
RBM38	0.457543221	0.689373613	-1.146916834	4
RBM39	0.811952675	0.305043803	-1.116996478	4
RBM4B	0.692923754	0.453470935	-1.146394689	4
RBMS1	0.54178196	0.612202577	-1.153984537	4
RBMS2	0.571613214	0.583068384	-1.154681598	4
RBMY1D	0.849963676	0.251901691	-1.101865367	4
RBP5	0.647244517	0.504511729	-1.151756246	4
RCN2	0.783798475	0.342433767	-1.126232243	4
RDH13	0.798919751	0.322548036	-1.121467787	4
RDM1	0.657463717	0.493342406	-1.150806123	4
RECQL5	0.550413975	0.603873977	-1.154287952	4
REEP4	0.613581119	0.540345001	-1.15392612	4
RENBP	0.457056271	0.689798971	-1.146855242	4
RER1	0.756039334	0.377826478	-1.133865812	4
RETN	0.560621548	0.59391895	-1.154540498	4
RETREG1	0.692994716	0.453389348	-1.146384064	4
RETREG3	0.690696145	0.456028323	-1.146724468	4
REV1	0.777200193	0.350974035	-1.128174227	4

REXO1	0.477958593	0.671332017	-1.14929061	4
REXO2	0.85347349	0.246826357	-1.100299846	4
RFC1	0.822600841	0.290480095	-1.113080936	4
RFX8	0.607121258	0.547058459	-1.154179717	4
RGL2	0.584402434	0.570269274	-1.154671707	4
RGL4	0.806861535	0.311922884	-1.118784419	4
RGMA	0.535877222	0.617852957	-1.153730179	4
RGPD4	0.831742227	0.277781285	-1.109523513	4
RGPD6	0.823664534	0.289011903	-1.112676438	4
RGS14	0.648688154	0.502942306	-1.15163046	4
RHAG	0.491250699	0.659363359	-1.150614058	4
RHBDF2	0.742165979	0.395005938	-1.137171917	4
RHEB	0.642235587	0.509935807	-1.152171395	4
RHEBL1	0.811364491	0.305841294	-1.117205785	4
RHEX	0.64966202	0.501882027	-1.151544047	4
RHNO1	0.821246147	0.292346396	-1.113592543	4
RHOB	0.61302279	0.540927272	-1.153950062	4
RHOC	0.771135519	0.358752405	-1.129887925	4
RHOT2	0.840181299	0.265891521	-1.10607282	4
RHOU	0.486898533	0.663301727	-1.15020026	4
RIBC1	0.625173222	0.528168307	-1.153341529	4
RIC1	0.795375313	0.327249295	-1.122624608	4
RIC3	0.812590905	0.30417764	-1.116768545	4
RIC8A	0.656317596	0.494602048	-1.150919644	4

RIN1	0.540512612	0.613420438	-1.15393305	4
RIN2	0.624058781	0.529346256	-1.153405037	4
RING1	0.861725389	0.234773854	-1.096499244	4
RIOK1	0.791820586	0.331939361	-1.123759948	4
RIOX1	0.858660878	0.239269682	-1.09793056	4
RIPK4	0.777967987	0.349984449	-1.127952436	4
RMC1	0.513523986	0.638905265	-1.152429251	4
RMDN3	0.704587151	0.439961251	-1.144548402	4
RNASE4	0.718093595	0.424059958	-1.142153554	4
RNASEH2C	0.758167485	0.375161939	-1.133329424	4
RNF10	0.670002615	0.479444745	-1.14944736	4
RNF113A	0.817007267	0.298160604	-1.115167871	4
RNF126	0.615246831	0.538605574	-1.153852405	4
RNF135	0.555321398	0.599102444	-1.154423842	4
RNF139	0.723867219	0.417176259	-1.141043478	4
RNF165	0.66976926	0.479705361	-1.149474621	4
RNF170	0.602769981	0.551551855	-1.154321836	4
RNF181	0.580008752	0.5746877	-1.154696452	4
RNF187	0.503231915	0.648421947	-1.151653862	4
RNF2	0.597148771	0.557322809	-1.154471581	4
RNF26	0.652920669	0.498325062	-1.151245731	4
RNF31	0.76731818	0.363614024	-1.130932205	4
RNF34	0.8010805	0.319669806	-1.120750306	4
RNF40	0.654913108	0.496143237	-1.151056345	4

RNFT1	0.574264478	0.580430573	-1.154695051	4
RNPC3	0.494230797	0.656655557	-1.150886354	4
RNPEPL1	0.604182958	0.550095247	-1.154278204	4
RORC	0.850115212	0.251683183	-1.101798394	4
RPA2	0.752064691	0.382781878	-1.134846569	4
RPA3	0.681691472	0.46629276	-1.147984233	4
RPAIN	0.470318127	0.678132042	-1.14845017	4
RPL24	0.800443778	0.320518924	-1.120962702	4
RPL28	0.690860738	0.455839611	-1.146700349	4
RPL32	0.654584224	0.496503749	-1.151087973	4
RPL37	0.724340507	0.41660964	-1.140950147	4
RPL4	0.843383389	0.261337108	-1.104720497	4
RPL7	0.758952334	0.374177279	-1.133129613	4
RPL7L1	0.828496353	0.282311473	-1.110807826	4
RPLP2	0.614796062	0.53907663	-1.153872692	4
RPN2	0.715520504	0.427110952	-1.142631456	4
RPP25	0.729640703	0.410239861	-1.139880564	4
RPP25L	0.756541414	0.377198562	-1.133739976	4
RPP30	0.614324191	0.539569469	-1.153893659	4
RPP38	0.715846838	0.426724577	-1.142571415	4
RPS10	0.484180795	0.665751394	-1.149932189	4
RPS11	0.841452991	0.26408563	-1.105538621	4
RPS12	0.770988943	0.358939567	-1.12992851	4
RPS18	0.834320861	0.274165488	-1.108486349	4

RPS19BP1	0.790970868	0.333056819	-1.124027687	4
RPS27	0.6118172	0.542183249	-1.15400045	4
RPTOR	0.788997408	0.335646704	-1.124644112	4
RRAGA	0.537994274	0.615831446	-1.15382572	4
RRAS	0.48076162	0.668822797	-1.149584417	4
RRM2B	0.73216847	0.407186092	-1.139354562	4
RRN3	0.734542816	0.404308231	-1.138851047	4
RRP9	0.702818333	0.442023119	-1.144841453	4
RSPH3	0.523920911	0.629179354	-1.153100265	4
RSRP1	0.781529914	0.345379217	-1.126909131	4
RTCB	0.824450057	0.287926082	-1.112376139	4
RTF1	0.551818766	0.602510811	-1.154329577	4
RTL8A	0.731230918	0.408319944	-1.139550862	4
RTTN	0.78607286	0.339470972	-1.125543832	4
RUBCN	0.522953228	0.630089391	-1.153042619	4
RUBCNL	0.789698641	0.334727298	-1.124425939	4
RUFY3	0.750982783	0.384126042	-1.135108825	4
RUNDC1	0.608109162	0.546035082	-1.154144244	4
RUSC2	0.7184898	0.42358925	-1.142079049	4
RUVBL1	0.798640187	0.322919748	-1.121559935	4
RUVBL2	0.827154192	0.284177844	-1.111332036	4
RXFP2	0.648278782	0.503387627	-1.151666409	4
RXRA	0.677386441	0.471159054	-1.148545495	4
S1PR5	0.461489444	0.68591804	-1.147407484	4

SAFB	0.648070278	0.503614355	-1.151684634	4
SAMD3	0.738358473	0.399664098	-1.138022572	4
SAMD4B	0.620761716	0.532822064	-1.153583781	4
SAMD7	0.511121607	0.641136485	-1.152258092	4
SAMD9	0.726672352	0.41381276	-1.140485113	4
SAMD9L	0.845230046	0.258699608	-1.103929654	4
SAMHD1	0.717739249	0.42448073	-1.142219979	4
SAMM50	0.777083472	0.351124377	-1.128207849	4
SAP30	0.827485806	0.283717082	-1.111202888	4
SARDH	0.479089378	0.6703207	-1.149410078	4
SART1	0.730258909	0.409493966	-1.139752875	4
SASH3	0.844728778	0.259416344	-1.104145123	4
SAT2	0.849801125	0.252136022	-1.101937147	4
SBF1	0.741988031	0.395224179	-1.13721221	4
SBF2	0.456068906	0.690660744	-1.14672965	4
SBNO1	0.554703958	0.599704256	-1.154408214	4
SBNO2	0.791994486	0.331710495	-1.12370498	4
SCAF1	0.554167149	0.600227133	-1.154394282	4
SCAF11	0.707640248	0.436391214	-1.144031462	4
SCAMP3	0.841271216	0.264343994	-1.10561521	4
SCARA5	0.717602943	0.424642536	-1.142245479	4
SCD	0.58423279	0.57044029	-1.15467308	4
SCGB2B2	0.788954862	0.335702457	-1.124657319	4
SCO1	0.781213473	0.345789306	-1.12700278	4

SDHAF1	0.820831681	0.292916594	-1.113748275	4
SDHD	0.754993209	0.379133385	-1.134126594	4
SEC13	0.753058071	0.381545932	-1.134604003	4
SEC14L4	0.458299402	0.688712622	-1.147012024	4
SEC16A	0.707230103	0.436871626	-1.144101728	4
SEC22B	0.818157297	0.296586979	-1.114744276	4
SEC23IP	0.797476988	0.324464686	-1.121941674	4
SEC61B	0.707878441	0.436112098	-1.143990538	4
SELENOH	0.798319673	0.323345716	-1.12166539	4
SELENOK	0.648240691	0.503429051	-1.151669743	4
SELENOS	0.822508105	0.29060798	-1.113116085	4
SELENOT	0.606694744	0.54749992	-1.154194665	4
SEMA6C	0.620690124	0.532897387	-1.153587511	4
SEMA7A	0.484065453	0.665855195	-1.149920648	4
SERPINB2	0.722023647	0.419379995	-1.141403642	4
SERPINE3	0.545469522	0.608654626	-1.154124147	4
SERTAD2	0.568765177	0.585893017	-1.154658194	4
SET	0.804792652	0.314703102	-1.119495755	4
SETD1B	0.461195369	0.686176073	-1.147371443	4
SETMAR	0.726232966	0.414340435	-1.140573401	4
SF3A1	0.582878153	0.571804687	-1.154682839	4
SF3B2	0.574280077	0.580415028	-1.154695106	4
SF3B3	0.767692681	0.363138238	-1.130830919	4
SF3B5	0.700188156	0.445080374	-1.14526853	4

SFPQ	0.734635678	0.404195489	-1.138831167	4
SFT2D1	0.584980415	0.569686361	-1.154666776	4
SFTPD	0.767671136	0.363165617	-1.130836753	4
SGCG	0.780474255	0.346746558	-1.127220813	4
SGK1	0.811352726	0.305857237	-1.117209964	4
SH2D1A	0.719879506	0.421936275	-1.141815781	4
SH3GL1	0.563086705	0.591497324	-1.154584029	4
SH3PXD2A	0.834816561	0.273468686	-1.108285247	4
SHARPIN	0.812537682	0.304249904	-1.116787586	4
SHC1	0.812835181	0.303845901	-1.116681081	4
SHISA4	0.547022209	0.607156271	-1.154178481	4
SHLD2	0.825735555	0.286146238	-1.111881794	4
SHPK	0.765638556	0.365744814	-1.131383371	4
SHROOM1	0.722702513	0.418569129	-1.141271642	4
SIDT2	0.700828112	0.444337458	-1.145165569	4
SIGIRR	0.772219469	0.35736711	-1.129586579	4
SIGLEC1	0.72864652	0.411438099	-1.140084619	4
SIK3	0.596680872	0.557801464	-1.154482336	4
SIPA1L1	0.477498106	0.671743489	-1.149241595	4
SIRT1	0.515124158	0.637415756	-1.152539915	4
SIRT4	0.863437439	0.232251793	-1.095689232	4
SKI	0.840382724	0.265605735	-1.105988459	4
SKIV2L	0.713804236	0.429140269	-1.142944505	4
SLAMF1	0.737899807	0.400223619	-1.138123425	4

SLAMF7	0.59341372	0.561136434	-1.154550154	4
SLAMF8	0.566942411	0.587695958	-1.154638369	4
SLAMF9	0.806058577	0.313002958	-1.119061535	4
SLC15A4	0.737527766	0.40067721	-1.138204976	4
SLC16A13	0.728873909	0.411164179	-1.140038088	4
SLC16A6	0.755944384	0.377945175	-1.133889559	4
SLC16A7	0.658690569	0.491992071	-1.15068264	4
SLC22A15	0.775901957	0.352644805	-1.128546762	4
SLC22A16	0.682808282	0.46502604	-1.147834322	4
SLC22A31	0.518121242	0.634618731	-1.152739973	4
SLC22A5	0.488980892	0.66141974	-1.150400632	4
SLC25A1	0.597025628	0.557448808	-1.154474437	4
SLC25A14	0.854795088	0.24490746	-1.099702548	4
SLC25A18	0.680476128	0.46766922	-1.148145348	4
SLC25A29	0.775105822	0.353667854	-1.128773677	4
SLC25A38	0.550766903	0.603531711	-1.154298615	4
SLC25A45	0.850512302	0.251110331	-1.101622633	4
SLC26A2	0.676285867	0.472398904	-1.148684771	4
SLC26A8	0.806611878	0.312258843	-1.118870722	4
SLC29A1	0.517284309	0.635400745	-1.152685055	4
SLC2A1	0.534871666	0.618811439	-1.153683104	4
SLC2A4RG	0.722761892	0.41849817	-1.141260062	4
SLC2A5	0.567561798	0.587083732	-1.154645529	4
SLC30A9	0.642540034	0.509607067	-1.152147101	4

SLC31A1	0.449406778	0.696450866	-1.145857644	4
SLC35A1	0.767316799	0.363615779	-1.130932578	4
SLC35B1	0.833612057	0.275160881	-1.108772938	4
SLC35F3	0.634314422	0.518446708	-1.152761131	4
SLC36A1	0.765405158	0.366040506	-1.131445664	4
SLC36A4	0.691709409	0.454865953	-1.146575361	4
SLC37A2	0.539687984	0.614210678	-1.153898662	4
SLC37A4	0.800295937	0.320715964	-1.121011901	4
SLC39A11	0.775228815	0.353509882	-1.128738697	4
SLC3A2	0.604043948	0.550238657	-1.154282604	4
SLC46A3	0.70100073	0.444136963	-1.145137693	4
SLC5A5	0.466272578	0.681709278	-1.147981856	4
SLC5A9	0.609733927	0.544349383	-1.15408331	4
SLC66A2	0.649802576	0.501728895	-1.151531471	4
SLC9A3R1	0.593289691	0.561262788	-1.154552479	4
SLF2	0.73264394	0.406610526	-1.139254466	4
SLFN11	0.696524981	0.449321182	-1.145846162	4
SLFN13	0.592093501	0.562480456	-1.154573957	4
SLFN5	0.804368269	0.315272321	-1.11964059	4
SMAGP	0.636268886	0.51635428	-1.152623166	4
SMARCA2	0.812057303	0.304901868	-1.11695917	4
SMARCAD1	0.78099847	0.34606783	-1.127066301	4
SMARCC2	0.524325346	0.628798717	-1.153124063	4
SMARCD2	0.800259012	0.320765171	-1.121024183	4

SMC1A	0.766130161	0.365121682	-1.131251843	4
SMC4	0.773567931	0.355640772	-1.129208704	4
SMC5	0.521314803	0.631627955	-1.152942758	4
SMCO3	0.669512417	0.479992123	-1.149504539	4
SMG1	0.60028183	0.554110976	-1.154392806	4
SMIM10L1	0.474252029	0.674638078	-1.148890107	4
SMIM12	0.753180602	0.381393363	-1.134573965	4
SMIM35	0.840580497	0.265325038	-1.105905535	4
SNAPC5	0.721710014	0.419754366	-1.141464379	4
SNRNP35	0.598183651	0.556263211	-1.154446863	4
SNRNP40	0.738953306	0.398937955	-1.13789126	4
SNRNP48	0.618216059	0.535496421	-1.15371248	4
SNRPB	0.565475882	0.5891438	-1.154619682	4
SNRPC	0.681838779	0.466125782	-1.147964561	4
SNRPD2	0.848022151	0.25469639	-1.102718541	4
SNRPG	0.815309308	0.300478843	-1.115788151	4
SNTA1	0.764800242	0.366806421	-1.131606663	4
SNTB2	0.789088001	0.335527979	-1.12461598	4
SNU13	0.792231935	0.331397896	-1.123629831	4
SNX13	0.587775829	0.566861574	-1.154637403	4
SNX18	0.835527327	0.27246859	-1.107995917	4
SNX27	0.606743704	0.547449256	-1.15419296	4
SNX33	0.732043369	0.407337468	-1.139380837	4
SOCS4	0.700139741	0.445136554	-1.145276295	4

SOCS7	0.599394671	0.555021636	-1.154416308	4
SOGA1	0.707832054	0.43616646	-1.143998515	4
SON	0.650700288	0.500750244	-1.151450532	4
SORD	0.469597589	0.678770348	-1.148367937	4
SOWAHD	0.571081482	0.583596449	-1.154677931	4
SOX4	0.514742036	0.637771695	-1.152513731	4
SP1	0.644101951	0.507918606	-1.152020558	4
SPAG7	0.667877943	0.481814845	-1.149692788	4
SPATA13	0.68302924	0.464775212	-1.147804452	4
SPATA9	0.662851614	0.487397009	-1.150248623	4
SPC24	0.467702428	0.680446784	-1.148149212	4
SPCS2	0.858025676	0.240198604	-1.09822428	4
SPDYE1	0.57593424	0.578765141	-1.154699382	4
SPDYE10P	0.494683088	0.656243805	-1.150926892	4
SPDYE13	0.459585925	0.687586775	-1.1471727	4
SPDYE14	0.605175717	0.549070385	-1.154246102	4
SPDYE16	0.634083957	0.518693118	-1.152777075	4
SPDYE18	0.796816751	0.325340406	-1.122157157	4
SPDYE2	0.771432593	0.358372956	-1.129805549	4
SPDYE21	0.667614682	0.482108081	-1.149722764	4
SPDYE2B	0.67181282	0.477420492	-1.149233313	4
SPDYE5	0.723085998	0.418110758	-1.141196755	4
SPESP1	0.521153033	0.631779711	-1.152932744	4
SPI1	0.679716964	0.468527958	-1.148244922	4

SPIB	0.493849157	0.657002829	-1.150851986	4
SPIDR	0.680553284	0.467581898	-1.148135182	4
SPINDOC	0.572345849	0.582340272	-1.154686121	4
SPINK2	0.582749254	0.571934402	-1.154683656	4
SPP1	0.648718519	0.502909265	-1.151627784	4
SPR	0.754412165	0.379858455	-1.13427062	4
SPRED1	0.766876728	0.364174546	-1.131051274	4
SPRED3	0.632356336	0.520538091	-1.152894427	4
SPRTN	0.769725547	0.360551163	-1.13027671	4
SPRY2	0.690356542	0.456417564	-1.146774107	4
SPTB	0.574645374	0.580050947	-1.154696321	4
SPTBN5	0.755956947	0.377929471	-1.133886418	4
SRCAP	0.740393013	0.397178004	-1.137571017	4
SREK1	0.535212226	0.618486943	-1.15369917	4
SRGAP2	0.746941952	0.389128716	-1.136070668	4
SRGAP2B	0.641562163	0.510662539	-1.152224702	4
SRGAP2C	0.680035828	0.468167371	-1.148203199	4
SRP72	0.639455724	0.512931903	-1.152387626	4
SRRM2	0.599488758	0.554925102	-1.15441386	4
SRSF1	0.626311744	0.526963285	-1.15327503	4
SRSF4	0.65924372	0.491382579	-1.150626299	4
SRSF7	0.530205976	0.623244476	-1.153450452	4
SSB	0.650176414	0.501321481	-1.151497895	4
SSBP4	0.448269805	0.697434757	-1.145704562	4

SSH3	0.842038293	0.263253193	-1.105291486	4
ST6GALNAC1	0.480875259	0.668720904	-1.149596163	4
ST6GALNAC2	0.557204112	0.597264762	-1.154468874	4
ST6GALNAC3	0.589823988	0.564786062	-1.15461005	4
ST6GALNAC4	0.658443102	0.492264609	-1.150707711	4
STAP2	0.724021013	0.416992177	-1.141013189	4
STARD10	0.695005103	0.45107489	-1.146079993	4
STAT4	0.850741357	0.250779717	-1.101521074	4
STAT5A	0.671667121	0.477583588	-1.14925071	4
STAT5B	0.625556393	0.527762939	-1.153319331	4
STAT6	0.791621309	0.332201556	-1.123822864	4
STIMATE	0.582507597	0.572177539	-1.154685136	4
STIP1	0.774242744	0.354775609	-1.129018353	4
STK10	0.580786953	0.573906752	-1.154693706	4
STK17A	0.799577287	0.321673151	-1.121250438	4
STK32C	0.725056256	0.415752072	-1.140808328	4
STK4	0.519943837	0.632913183	-1.15285702	4
STMN3	0.725856161	0.414792708	-1.140648869	4
STOML1	0.673472336	0.475560735	-1.149033071	4
STOML2	0.76151307	0.370957096	-1.132470166	4
STOX1	0.476879411	0.672295998	-1.149175408	4
STRA8	0.576445516	0.57825455	-1.154700066	4
STRADA	0.819157081	0.295216648	-1.114373729	4
STRBP	0.845222654	0.258710182	-1.103932836	4

STRN3	0.451093244	0.69498918	-1.146082425	4
STT3B	0.732381811	0.406927884	-1.139309695	4
STX4	0.504174594	0.647554845	-1.151729439	4
SUCLA2	0.728347142	0.411798613	-1.140145755	4
SUFU	0.850453065	0.251195812	-1.101648877	4
SUGT1	0.829511152	0.280897668	-1.11040882	4
SULT1A3	0.779440249	0.348083829	-1.127524078	4
SULT1A4	0.806942984	0.311813252	-1.118756236	4
SUMF1	0.732655728	0.406596251	-1.139251979	4
SUOX	0.557369808	0.59710284	-1.154472648	4
SUPT5H	0.78050463	0.346707245	-1.127211874	4
SURF4	0.596754307	0.557726358	-1.154480665	4
SUSD4	0.846232713	0.257264159	-1.103496872	4
SYNCRIP	0.762189779	0.370104189	-1.132293968	4
SYNE3	0.814091154	0.302138246	-1.1162294	4
SYTL2	0.816156112	0.299323457	-1.115479569	4
SZT2	0.862879397	0.23307468	-1.095954077	4
TADA2B	0.723005735	0.418206713	-1.141212448	4
TAF12	0.635783331	0.516874569	-1.1526579	4
TAF1L	0.712332747	0.430876538	-1.143209284	4
TAF5	0.690492506	0.456261747	-1.146754253	4
TAF8	0.688345748	0.458718837	-1.147064584	4
TAFA1	0.78511284	0.34072277	-1.12583561	4
TAGLN	0.857521231	0.240935587	-1.098456818	4

TAL2	0.62624602	0.527032893	-1.153278913	4
TAOK2	0.600640459	0.553742578	-1.154383037	4
TARP	0.57259439	0.582093125	-1.154687515	4
TAS2R20	0.646169379	0.505678757	-1.151848136	4
TAS2R5	0.636975339	0.515596746	-1.152572086	4
TASL	0.71982607	0.421999889	-1.14182596	4
TATDN3	0.708685671	0.435165534	-1.143851205	4
TBC1D1	0.642818153	0.509306649	-1.152124802	4
TBC1D3L	0.603646454	0.550648602	-1.154295056	4
TBC1D5	0.57384317	0.580850282	-1.154693451	4
TBC1D9	0.771218726	0.358646142	-1.129864868	4
TBCEL-TECTA	0.76410484	0.367686097	-1.131790938	4
TBX6	0.659740804	0.490834511	-1.150575315	4
TC2N	0.860436828	0.236667145	-1.097103973	4
TCEA2	0.680829304	0.467269441	-1.148098745	4
TCEANC	0.764917584	0.366657899	-1.131575483	4
TCERG1	0.667612168	0.482110882	-1.14972305	4
TCF12	0.534351302	0.619307013	-1.153658316	4
TCF3	0.515528193	0.637039241	-1.152567433	4
TCN1	0.723909186	0.417126031	-1.141035216	4
TCOF1	0.840921062	0.264841465	-1.105762527	4
TCP1	0.59610234	0.558392932	-1.154495272	4
TDRD12	0.545800907	0.608335059	-1.154135965	4
TDRD7	0.637027192	0.515541119	-1.152568311	4

TECPR1	0.548034356	0.606178118	-1.154212474	4
TEFM	0.718491573	0.423587143	-1.142078715	4
TENT5A	0.553184141	0.601183801	-1.154367941	4
TEP1	0.622573747	0.530913486	-1.153487234	4
TERB2	0.851980954	0.248988294	-1.100969247	4
TESK1	0.628456564	0.524688734	-1.153145298	4
TESMIN	0.50209473	0.649466744	-1.151561475	4
TET3	0.636277058	0.516345521	-1.152622579	4
TEX264	0.787359052	0.33779111	-1.125150163	4
TF	0.546323032	0.607831309	-1.154154341	4
TFDP1	0.698722936	0.446779031	-1.145501967	4
TFG	0.789062789	0.335561022	-1.124623811	4
TGM2	0.467471005	0.680651256	-1.148122261	4
TGM3	0.596479093	0.5580078	-1.154486893	4
THAP11	0.829519999	0.280885333	-1.110405332	4
THAP8	0.737708998	0.40045628	-1.138165279	4
THBD	0.635038718	0.517671858	-1.152710577	4
THOC3	0.738975557	0.39891078	-1.137886337	4
TIAM2	0.680022811	0.468182094	-1.148204905	4
TIE1	0.70075213	0.444425695	-1.145177826	4
TIFA	0.621959204	0.531561234	-1.153520439	4
TIFAB	0.689560282	0.457329554	-1.146889836	4
TIGD5	0.756683501	0.377020784	-1.133704286	4
TIGIT	0.800287931	0.320726633	-1.121014564	4

TIMM22	0.719754448	0.422085148	-1.141839596	4
TIMM29	0.620252007	0.53335819	-1.153610198	4
TINF2	0.748626016	0.387047167	-1.135673183	4
TKFC	0.851361965	0.249883303	-1.101245268	4
TLE4	0.73448239	0.404381584	-1.138863975	4
TLN2	0.697776545	0.447874503	-1.145651048	4
TLR1	0.593910532	0.560630129	-1.154540661	4
TLR10	0.636131347	0.516501688	-1.152633036	4
TM2D1	0.721472553	0.420037708	-1.141510262	4
TM9SF1	0.515116946	0.637422476	-1.152539422	4
TMC5	0.729534389	0.410368072	-1.139902461	4
TMC6	0.853193456	0.247232401	-1.100425857	4
TMCO4	0.834214549	0.274314857	-1.108529406	4
TMED1	0.783158333	0.343265898	-1.126424231	4
TMED7-TICAM2	0.854184894	0.245793967	-1.09997886	4
TMEM115	0.716379727	0.42609329	-1.142473017	4
TMEM126B	0.847852354	0.254940371	-1.102792725	4
TMEM128	0.785380302	0.340374196	-1.125754497	4
TMEM141	0.449291474	0.696550701	-1.145842176	4
TMEM14B	0.484044907	0.665873684	-1.149918591	4
TMEM168	0.858746994	0.239143669	-1.097890662	4
TMEM181	0.586759905	0.567889233	-1.154649138	4
TMEM199	0.794814552	0.327990802	-1.122805354	4
TMEM200A	0.79290692	0.330508686	-1.123415606	4

TMEM203	0.832599885	0.276580331	-1.109180216	4
TMEM230	0.584523894	0.570146809	-1.154670703	4
TMEM236	0.794639111	0.328222664	-1.122861775	4
TMEM241	0.827300596	0.283974453	-1.111275049	4
TMEM243	0.528039242	0.625295239	-1.15333448	4
TMEM244	0.546047682	0.608097006	-1.154144688	4
TMEM251	0.687418664	0.459777871	-1.147196535	4
TMEM252	0.840191299	0.265877335	-1.106068634	4
TMEM255B	0.791543671	0.332303684	-1.123847355	4
TMEM272	0.597992955	0.556458559	-1.154451514	4
TMEM35B	0.557084823	0.597381315	-1.154466138	4
TMEM40	0.815234828	0.300580391	-1.115815219	4
TMEM45B	0.567674582	0.586972204	-1.154646786	4
TMEM53	0.770855919	0.35910939	-1.129965309	4
TMEM59	0.724312971	0.416642616	-1.140955587	4
TMEM65	0.796424049	0.325860866	-1.122284915	4
TMEM69	0.747744284	0.388137611	-1.135881895	4
TMEM87A	0.623907372	0.529506172	-1.153413544	4
TMIE	0.530943165	0.6225456	-1.153488765	4
TMIGD3	0.841079187	0.264616849	-1.105696036	4
TMPRSS11E	0.718846193	0.423165629	-1.142011822	4
TMPRSS3	0.579336027	0.575362232	-1.154698259	4
TMTC4	0.861028202	0.235798758	-1.09682696	4
TMUB1	0.614724627	0.539151256	-1.153875884	4

TMX1	0.849313149	0.252839094	-1.102152243	4
TNFAIP2	0.673012582	0.47607635	-1.149088932	4
TNFAIP6	0.663114336	0.487106092	-1.150220428	4
TNFAIP8	0.76777421	0.363034626	-1.130808836	4
TNFRSF12A	0.65358293	0.497600441	-1.151183371	4
TNFRSF13C	0.470644208	0.677843008	-1.148487216	4
TNFRSF14	0.67653505	0.472118337	-1.148653387	4
TNFRSF9	0.819916325	0.294174578	-1.114090902	4
TNFSF12	0.456846096	0.68998249	-1.146828587	4
TNFSF13	0.516659643	0.635983943	-1.152643586	4
TNFSF15	0.595830064	0.558671158	-1.154501222	4
TNFSF8	0.779084708	0.348543185	-1.127627893	4
TNK2	0.50481227	0.646967774	-1.151780044	4
TNNI3K	0.828032658	0.282956724	-1.110989383	4
TNPO2	0.794469891	0.328446247	-1.122916139	4
TNPO3	0.856725661	0.242096607	-1.098822267	4
TNS1	0.494634077	0.656288433	-1.15092251	4
TNS3	0.69391997	0.452324887	-1.146244857	4
TOB2	0.607360992	0.546810227	-1.154171218	4
TOGARAM2	0.845254168	0.258665103	-1.103919271	4
TOM1	0.586611931	0.568038816	-1.154650747	4
TOR1AIP1	0.779883724	0.347510531	-1.127394255	4
TOX4	0.521929271	0.63105127	-1.152980542	4
TP53	0.854939465	0.24469757	-1.099637036	4

TP53I3	0.674419853	0.474497161	-1.148917014	4
TP53TG5	0.7528292	0.381830839	-1.13466004	4
TPM2	0.785499886	0.3402183	-1.125718187	4
TPM3	0.451212565	0.69488566	-1.146098225	4
TPPP3	0.650259628	0.501230768	-1.151490396	4
TPRA1	0.811237188	0.306013802	-1.117250991	4
TPRG1	0.763935563	0.367900101	-1.131835664	4
TPT1	0.676427896	0.472238997	-1.148666893	4
TPTEP2-CSNK1E	0.607225718	0.546950305	-1.154176023	4
TRADD	0.62127565	0.532281165	-1.153556816	4
TRAF2	0.765968795	0.36532627	-1.131295064	4
TRAF3	0.602937675	0.551379109	-1.154316784	4
TRAF3IP2	0.83611512	0.271640656	-1.107755776	4
TRAFD1	0.689318905	0.457605831	-1.146924736	4
TRANK1	0.854233245	0.245723754	-1.099956999	4
TRAPPC10	0.459283342	0.687851713	-1.147135055	4
TRAPPC14	0.673339363	0.475709896	-1.149049258	4
TRAT1	0.688684774	0.458331246	-1.14701602	4
TRDMT1	0.535046314	0.618645044	-1.153691359	4
TREM2	0.578769836	0.575929538	-1.154699374	4
TRIM11	0.815850632	0.299740432	-1.115591063	4
TRIM13	0.541965696	0.612026149	-1.153991845	4
TRIM17	0.767497052	0.363386807	-1.130883859	4
TRIM21	0.830177098	0.279968636	-1.110145733	4

TRIM25	0.532678101	0.620898533	-1.153576635	4
TRIM28	0.854855628	0.244819456	-1.099675084	4
TRIM32	0.609373983	0.544723104	-1.154097088	4
TRIM33	0.79185233	0.331897589	-1.123749918	4
TRIM34	0.858004714	0.240229242	-1.098233956	4
TRIM38	0.846499983	0.256881124	-1.103381107	4
TRIM4	0.847695795	0.255165269	-1.102861063	4
TRIM44	0.84455871	0.259659381	-1.104218091	4
TRIM49D1	0.786204686	0.339298944	-1.125503629	4
TRIM49D2	0.797719794	0.324142419	-1.121862212	4
TRIM52	0.854208455	0.245759754	-1.099968208	4
TRIM61	0.771915427	0.357755894	-1.129671321	4
TRIM7	0.461103344	0.686256803	-1.147360147	4
TRIM72	0.534963687	0.61872377	-1.153687457	4
TRIM73	0.492101149	0.658591534	-1.150692683	4
TRIO	0.524235345	0.628883437	-1.153118782	4
TRIP10	0.861453346	0.23517392	-1.096627266	4
TRIP4	0.514749056	0.637765158	-1.152514214	4
TRIP6	0.699685413	0.445663574	-1.145348987	4
TRMT10C	0.490402445	0.660132463	-1.150534908	4
TRMT11	0.482552685	0.667215363	-1.149768048	4
TRMT112	0.663478371	0.486702833	-1.150181204	4
TRMT44	0.55965683	0.594864774	-1.154521604	4
TRMT61A	0.761716527	0.370700749	-1.132417276	4

TRNP1	0.817143718	0.297974041	-1.115117759	4
TRPM2	0.731910271	0.407498494	-1.139408765	4
TRRAP	0.854526531	0.24529774	-1.099824271	4
TSC1	0.517796202	0.634922529	-1.152718732	4
TSC2	0.45623587	0.690515084	-1.146750954	4
TSGA10	0.77596622	0.352562175	-1.128528395	4
TSHZ2	0.655067931	0.495973475	-1.151041406	4
TSN	0.82366675	0.289008842	-1.112675592	4
TSPOAP1	0.660450025	0.490051967	-1.150501992	4
TSPYL1	0.638145914	0.514340113	-1.152486026	4
TSR1	0.81588494	0.299693611	-1.115578551	4
TSSC4	0.589472945	0.565142144	-1.154615089	4
TSSK6	0.792114276	0.331552806	-1.123667082	4
TTBK2	0.818716572	0.295820685	-1.114537257	4
TTC16	0.516222404	0.636391913	-1.152614317	4
TTC3	0.795552283	0.327015155	-1.122567438	4
TTC32	0.760223623	0.372580046	-1.132803669	4
TTC39A	0.71741597	0.424864439	-1.142280409	4
TTC4	0.722782707	0.418473294	-1.141256001	4
TTC5	0.523492869	0.629582019	-1.153074888	4
TTC9C	0.53417411	0.619475698	-1.153649808	4
TTI2	0.79974634	0.321448079	-1.121194419	4
TTLL3	0.459398373	0.687751003	-1.147149377	4
TTN	0.492936961	0.657832279	-1.15076924	4

TTPAL	0.675272899	0.473538553	-1.148811452	4
TUBA1B	0.704134016	0.440489909	-1.144623924	4
TUBGCP6	0.845216424	0.258719094	-1.103935518	4
TUFM	0.658784224	0.491888906	-1.15067313	4
TUFT1	0.613028325	0.540921501	-1.153949826	4
TULP4	0.853677054	0.246531071	-1.100208125	4
TUT1	0.785607892	0.340077477	-1.125685369	4
TWF2	0.469084952	0.679224168	-1.148309121	4
TXNDC12	0.668518485	0.48110097	-1.149619455	4
TXNDC17	0.715003997	0.427722149	-1.142726147	4
TXNDC5	0.606277294	0.547931786	-1.15420908	4
TXNIP	0.730664651	0.409004085	-1.139668736	4
TXNL4A	0.610984666	0.543049536	-1.154034202	4
U2AF1L5	0.490851819	0.65972511	-1.150576929	4
UBA1	0.702490651	0.442404575	-1.144895226	4
UBA5	0.630583646	0.522427222	-1.153010868	4
UBA52	0.612418653	0.541556882	-1.153975535	4
UBAP2L	0.798602127	0.322970341	-1.121572468	4
UBB	0.700728532	0.444453099	-1.145181631	4
UBE2J2	0.561093643	0.59345572	-1.154549363	4
UBE2N	0.790032838	0.334288789	-1.124321627	4
UBE2S	0.840640135	0.265240377	-1.105880512	4
UBL7	0.449988549	0.695946945	-1.145935494	4
UBQLN4	0.789175128	0.33541378	-1.124588909	4

UBTF	0.804679413	0.314855024	-1.119534437	4
UBXN1	0.711653419	0.431676979	-1.143330397	4
UBXN10	0.580066589	0.574629682	-1.154696272	4
UBXN4	0.742682333	0.394372366	-1.137054699	4
UBXN6	0.454531351	0.692000847	-1.146532197	4
UCK2	0.661572243	0.488812332	-1.150384575	4
UCKL1	0.723449673	0.417675849	-1.141125522	4
UFD1	0.705841481	0.43849625	-1.144337731	4
UFSP1	0.82325738	0.28957418	-1.11283156	4
UMPS	0.48407459	0.665846973	-1.149921563	4
UNC50	0.793551111	0.329659214	-1.123210326	4
UPB1	0.851696563	0.249399616	-1.101096179	4
UPF1	0.77587675	0.352677215	-1.128553965	4
UPF3A	0.772522007	0.356980082	-1.129502089	4
UPF3B	0.677436968	0.471102092	-1.14853906	4
UQCRC1	0.550438109	0.603850576	-1.154288685	4
UQCRFS1	0.562073264	0.592493696	-1.15456696	4
URM1	0.851148749	0.25019138	-1.101340129	4
USF1	0.673384938	0.475658775	-1.149043713	4
USF3	0.679982767	0.468227385	-1.148210152	4
USP10	0.58017624	0.57451968	-1.15469592	4
USP28	0.727603751	0.41269319	-1.140296941	4
USP49	0.652521647	0.498761374	-1.15128302	4
USP8	0.733966613	0.40500747	-1.138974083	4

UTP15	0.689478565	0.457423096	-1.146901661	4
UTP23	0.819577911	0.294639207	-1.114217118	4
UTP3	0.67988141	0.468342013	-1.148223422	4
UTRN	0.640003317	0.51234251	-1.152345827	4
UTS2	0.804389905	0.315243309	-1.119633215	4
VAMP5	0.834463451	0.273965107	-1.108428558	4
VAPB	0.539561173	0.614332135	-1.153893309	4
VASH1	0.731424024	0.408086523	-1.139510547	4
VAT1	0.702259408	0.442673667	-1.144933076	4
VCP	0.805571623	0.313657327	-1.11922895	4
VCPKMT	0.715444334	0.427201112	-1.142645446	4
VDAC1	0.731933575	0.407470301	-1.139403877	4
VEGFB	0.629074808	0.524032012	-1.153106819	4
VILL	0.536427052	0.617328406	-1.153755458	4
VNN1	0.632289766	0.520609106	-1.152898872	4
VPS13B	0.565553703	0.589067032	-1.154620735	4
VPS16	0.82084181	0.292902663	-1.113744474	4
VPS26B	0.585500079	0.56916193	-1.154662009	4
VPS26C	0.809030887	0.308998233	-1.118029121	4
VPS35	0.84630074	0.257166683	-1.103467423	4
VPS39	0.747950106	0.387883188	-1.135833294	4
VSIG4	0.528826839	0.624550377	-1.153377216	4
VSTM1	0.73876251	0.399170933	-1.137933443	4
VTI1A	0.63855514	0.513900382	-1.152455522	4

VWDE	0.624715196	0.528652624	-1.15336782	4
WASHC2A	0.662293252	0.48801498	-1.150308232	4
WBP11	0.686011471	0.461382974	-1.147394445	4
WDPCP	0.719613916	0.422252412	-1.141866328	4
WDR24	0.831833976	0.277652891	-1.109486867	4
WDR25	0.764603022	0.367055989	-1.131659012	4
WDR35	0.860560751	0.236485247	-1.097045998	4
WDR46	0.773365482	0.355900166	-1.129265648	4
WDR53	0.626478758	0.526786379	-1.153265137	4
WDR74	0.667356054	0.482396064	-1.149752118	4
WDR75	0.452786998	0.693518427	-1.146305425	4
WDR82	0.751659752	0.38328521	-1.134944961	4
WDR83	0.854916525	0.244730923	-1.099647448	4
WDR83OS	0.791955016	0.331762445	-1.123717461	4
WIPF2	0.50887239	0.643220013	-1.152092403	4
WNT16	0.556944185	0.597518707	-1.154462892	4
WNT3	0.789862252	0.334512647	-1.124374899	4
WSB1	0.657813945	0.492957135	-1.15077108	4
WTIP	0.701311485	0.44377591	-1.145087395	4
WWP1	0.848004065	0.254722381	-1.102726446	4
WWP2	0.700172957	0.445098011	-1.145270968	4
XCL1	0.757707548	0.375738469	-1.133446017	4
XCL2	0.835073759	0.273106924	-1.108180683	4
XIAP	0.842536756	0.262543629	-1.105080385	4

XKR3	0.848250215	0.254368576	-1.102618792	4
XRCC1	0.644195746	0.507817111	-1.152012857	4
YARS2	0.822756483	0.29026542	-1.113021903	4
YBX1	0.475386964	0.673627222	-1.149014186	4
YES1	0.799944668	0.321183957	-1.121128625	4
YIPF1	0.789516845	0.334965747	-1.124482592	4
YIPF4	0.465119072	0.682726313	-1.147845386	4
YTHDF2	0.590370718	0.564231194	-1.154601912	4
YWHAB	0.79662913	0.325589104	-1.122218234	4
YY1	0.518148629	0.634593129	-1.152741758	4
ZAP70	0.836457161	0.271158512	-1.107615673	4
ZBTB1	0.863886646	0.231588814	-1.09547546	4
ZBTB17	0.52330812	0.629755755	-1.153063875	4
ZBTB18	0.849626328	0.252387933	-1.102014262	4
ZBTB20	0.68646014	0.460871513	-1.147331654	4
ZBTB3	0.795805699	0.326679767	-1.122485465	4
ZBTB37	0.532305912	0.621252144	-1.153558056	4
ZBTB38	0.7776752	0.350361942	-1.128037142	4
ZBTB45	0.748140335	0.387647976	-1.135788311	4
ZC3H11A	0.772444673	0.35707903	-1.129523702	4
ZC3HAV1	0.830822354	0.279067521	-1.109889875	4
ZCCHC2	0.534490973	0.619174025	-1.153664998	4
ZCRB1	0.849062352	0.253200218	-1.10226257	4
ZCWPW1	0.847994338	0.25473636	-1.102730697	4

ZDHHC13	0.771296415	0.358546915	-1.12984333	4
ZDHHC14	0.616850337	0.536927862	-1.153778199	4
ZDHHC20	0.717903382	0.424285854	-1.142189235	4
ZDHHC6	0.82969945	0.280635082	-1.110334532	4
ZEB1	0.557816477	0.596666192	-1.154482669	4
ZFP14	0.761830066	0.370557663	-1.132387729	4
ZFP62	0.830512672	0.279500115	-1.110012787	4
ZFP82	0.835710719	0.272210359	-1.107921078	4
ZFP92	0.779872675	0.347524819	-1.127397494	4
ZFPL1	0.528116719	0.625221995	-1.153338714	4
ZFR	0.607957403	0.546192368	-1.154149771	4
ZFX	0.783203271	0.343207508	-1.126410779	4
ZFYVE26	0.840009029	0.266135867	-1.106144896	4
ZFYVE9	0.850739327	0.250782648	-1.101521975	4
ZG16B	0.809469196	0.308406142	-1.117875338	4
ZHX1	0.569633942	0.585032372	-1.154666314	4
ZKSCAN1	0.626612757	0.526644417	-1.153257174	4
ZKSCAN7	0.455980392	0.690737953	-1.146718345	4
ZMAT5	0.764561017	0.367109136	-1.131670153	4
ZMIZ1	0.802759425	0.317426923	-1.120186348	4
ZMYND19	0.760060798	0.372784776	-1.132845574	4
ZNF114	0.572780356	0.581908156	-1.154688513	4
ZNF136	0.773151304	0.356174505	-1.129325809	4
ZNF14	0.780248761	0.347038358	-1.127287119	4

ZNF140	0.81790794	0.296928422	-1.114836362	4
ZNF146	0.484072386	0.665848956	-1.149921342	4
ZNF157	0.853339647	0.247020451	-1.100360098	4
ZNF160	0.548209813	0.606008439	-1.154218252	4
ZNF165	0.806833595	0.31196049	-1.118794084	4
ZNF174	0.855517184	0.243857191	-1.099374376	4
ZNF184	0.511259494	0.641008585	-1.152268078	4
ZNF189	0.58385689	0.570819113	-1.154676003	4
ZNF195	0.690712519	0.456009551	-1.14672207	4
ZNF213	0.525683059	0.627519628	-1.153202687	4
ZNF225	0.720842866	0.420788636	-1.141631502	4
ZNF233	0.575569341	0.579129368	-1.154698709	4
ZNF253	0.840829359	0.264971702	-1.105801061	4
ZNF254	0.839251145	0.267210025	-1.106461169	4
ZNF257	0.709070655	0.434713749	-1.143784404	4
ZNF268	0.505679048	0.646169111	-1.151848159	4
ZNF274	0.519861515	0.632990294	-1.152851809	4
ZNF276	0.817148129	0.297968008	-1.115116138	4
ZNF277	0.68979147	0.45706486	-1.14685633	4
ZNF287	0.819865236	0.294244737	-1.114109972	4
ZNF302	0.617493734	0.536253793	-1.153747528	4
ZNF304	0.760332514	0.372443104	-1.132775619	4
ZNF319	0.505671962	0.646175643	-1.151847605	4
ZNF335	0.704164186	0.44045472	-1.144618906	4

ZNF345	0.717230634	0.425084347	-1.142314981	4
ZNF362	0.727919866	0.412312894	-1.14023276	4
ZNF37A	0.847711017	0.255143404	-1.102854421	4
ZNF385A	0.696177136	0.449722843	-1.145899978	4
ZNF395	0.692734311	0.453688706	-1.146423017	4
ZNF396	0.775045532	0.353745281	-1.128790813	4
ZNF408	0.789712892	0.334708603	-1.124421495	4
ZNF414	0.50489151	0.646894793	-1.151786304	4
ZNF428	0.716658132	0.425763302	-1.142421434	4
ZNF43	0.615748058	0.538081494	-1.153829552	4
ZNF440	0.744096951	0.392634332	-1.136731283	4
ZNF461	0.76320598	0.368821872	-1.132027852	4
ZNF468	0.851529066	0.24964178	-1.101170846	4
ZNF484	0.478237261	0.671082908	-1.14932017	4
ZNF525	0.721670541	0.419801471	-1.141472012	4
ZNF530	0.515766298	0.636817273	-1.152583571	4
ZNF565	0.448399214	0.697322834	-1.145722048	4
ZNF567	0.59430752	0.560225344	-1.154532864	4
ZNF568	0.811312187	0.305912176	-1.117224363	4
ZNF569	0.622399309	0.531097398	-1.153496707	4
ZNF574	0.683887375	0.463800406	-1.147687781	4
ZNF581	0.607445321	0.546722891	-1.154168212	4
ZNF592	0.78774559	0.337285645	-1.125031235	4
ZNF595	0.775920377	0.352621121	-1.128541498	4

ZNF598	0.625387098	0.527942063	-1.153329162	4
ZNF600	0.673304037	0.475749518	-1.149053554	4
ZNF605	0.741699801	0.395577561	-1.137277362	4
ZNF609	0.821314841	0.292251854	-1.113566695	4
ZNF627	0.845673993	0.258064335	-1.103738328	4
ZNF641	0.586619934	0.568030726	-1.15465066	4
ZNF653	0.51567572	0.636901719	-1.152577439	4
ZNF654	0.700562298	0.444646111	-1.14520841	4
ZNF658	0.532002808	0.621540008	-1.153542817	4
ZNF668	0.783540748	0.342768884	-1.126309632	4
ZNF677	0.826509326	0.285073162	-1.111582488	4
ZNF678	0.514118726	0.638351968	-1.152470694	4
ZNF687	0.45458996	0.691949806	-1.146539766	4
ZNF696	0.542749116	0.611273474	-1.15402259	4
ZNF699	0.692040874	0.454485385	-1.146526259	4
ZNF7	0.793591353	0.329606122	-1.123197475	4
ZNF700	0.638481181	0.513979871	-1.152461051	4
ZNF71	0.854375075	0.245517765	-1.09989284	4
ZNF710	0.56595101	0.588674993	-1.154626003	4
ZNF721	0.79796563	0.323816009	-1.121781639	4
ZNF736	0.72854409	0.411561462	-1.140105553	4
ZNF75A	0.792995531	0.330391886	-1.123387417	4
ZNF76	0.60326603	0.551040764	-1.154306794	4
ZNF768	0.594992271	0.559526704	-1.154518975	4

ZNF770	0.691890193	0.454658408	-1.146548601	4
ZNF776	0.771346689	0.358482697	-1.129829386	4
ZNF780A	0.718868299	0.423139346	-1.142007645	4
ZNF787	0.592212532	0.562359364	-1.154571896	4
ZNF799	0.620123829	0.533492961	-1.15361679	4
ZNF816	0.625649087	0.527664847	-1.153313934	4
ZNF83	0.673525808	0.475500747	-1.149026555	4
ZNF830	0.765941901	0.365360361	-1.131302263	4
ZNF84	0.515072829	0.637463578	-1.152536406	4
ZNF845	0.554626639	0.599779588	-1.154406227	4
ZNF878	0.556069373	0.598372833	-1.154442205	4
ZNF879	0.639508465	0.512875152	-1.152383617	4
ZNF880	0.4751689	0.673821545	-1.148990445	4
ZNHIT1	0.758634691	0.374575919	-1.13321061	4
ZNHIT2	0.807832898	0.310614521	-1.118447419	4
ZNHIT3	0.827014021	0.284372532	-1.111386554	4
ZP3	0.837338136	0.269915447	-1.107253583	4
ZSCAN16	0.803596085	0.316307103	-1.119903187	4
ZSCAN32	0.842517551	0.262570978	-1.105088529	4
ZSWIM3	0.863466664	0.232208676	-1.09567534	4
ZSWIM8	0.839418486	0.266972964	-1.10639145	4
A1CF	-0.593061815	-0.561494886	1.154556701	5
A2ML1	-0.818389772	-0.296268533	1.114658305	5
A4GALT	-0.517658302	-0.635051384	1.152709687	5

A4GNT	-0.784766609	-0.341173801	1.12594041	5
AADACL3	-0.651570113	-0.499800972	1.151371085	5
AADAT	-0.539569939	-0.61432374	1.153893679	5
ABCA12	-0.682062496	-0.465872132	1.147934627	5
ABCA4	-0.540864128	-0.613083356	1.153947483	5
ABCA8	-0.554940595	-0.599473659	1.154414254	5
ABCB11	-0.800410841	-0.320562826	1.120973666	5
ABCC12	-0.584431339	-0.570240131	1.15467147	5
ABCC3	-0.510562153	-0.641655218	1.152217371	5
ABCC8	-0.561844947	-0.592718009	1.154562955	5
ABCC9	-0.586651676	-0.567998642	1.154650317	5
ABCG2	-0.702245451	-0.442689906	1.144935358	5
ABCG4	-0.509373216	-0.642756535	1.152129751	5
ABHD13	-0.772705254	-0.356745578	1.129450832	5
ABHD2	-0.584763604	-0.569905068	1.154668672	5
ABHD4	-0.612782719	-0.541177519	1.153960238	5
ABL1	-0.604810206	-0.549447855	1.15425806	5
ABLIM2	-0.515506958	-0.637059034	1.152565991	5
ABLIM3	-0.502657946	-0.648949451	1.151607398	5
ACADL	-0.539318027	-0.614564968	1.153882995	5
ACAN	-0.502277377	-0.649299026	1.151576403	5
ACBD7	-0.548315541	-0.605906177	1.154221717	5
ACCSL	-0.514866896	-0.637655408	1.152522304	5
ACE	-0.587344235	-0.567298301	1.154642536	5

ACE2	-0.572167804	-0.582517274	1.154685078	5
ACKR1	-0.535109435	-0.618584898	1.153694334	5
ACKR2	-0.619716355	-0.533921254	1.153637609	5
ACOT6	-0.648846455	-0.502770044	1.151616499	5
ACOT7	-0.67819173	-0.470250774	1.148442505	5
ACOX2	-0.522782937	-0.630249435	1.153032372	5
ACP4	-0.719438678	-0.422460942	1.14189962	5
ACP7	-0.547008922	-0.607169105	1.154178027	5
ACRBP	-0.549398625	-0.604857885	1.15425651	5
ACRV1	-0.555603818	-0.59882703	1.154430849	5
ACSBG1	-0.514099299	-0.638370047	1.152469346	5
ACSL1	-0.75682429	-0.376844596	1.133668886	5
ACSM6	-0.671082303	-0.478237938	1.149320241	5
ACSS3	-0.54583063	-0.60830639	1.15413702	5
ACTA1	-0.630978296	-0.522006997	1.152985293	5
ACTBL2	-0.599720552	-0.554687233	1.154407785	5
ACTC1	-0.562836515	-0.591743408	1.154579922	5
ACTL6B	-0.554569797	-0.599834965	1.154404762	5
ACTL8	-0.563178032	-0.591407477	1.15458551	5
ACTN1	-0.57950682	-0.57519103	1.15469785	5
ACTN2	-0.556571625	-0.597882561	1.154454187	5
ACTR3B	-0.537063764	-0.61672056	1.153784323	5
ACTRT2	-0.574274587	-0.5804205	1.154695086	5
ADAM10	-0.804808501	-0.314681838	1.119490339	5

ADAM11	-0.521860038	-0.631116266	1.152976305	5
ADAM12	-0.552217015	-0.602123965	1.15434098	5
ADAM2	-0.587277907	-0.567365399	1.154643306	5
ADAM32	-0.505770949	-0.646084386	1.151855335	5
ADAM33	-0.653779014	-0.497385781	1.151164795	5
ADAMTS13	-0.517293436	-0.635392222	1.152685658	5
ADAMTS14	-0.656181675	-0.494751314	1.150932989	5
ADAMTS15	-0.526015102	-0.627206516	1.153221618	5
ADAMTS18	-0.566801746	-0.587834936	1.154636682	5
ADAMTS19	-0.535321792	-0.61838252	1.153704312	5
ADAMTS2	-0.656629165	-0.494259794	1.150888959	5
ADAMTS20	-0.538984446	-0.614884295	1.153868741	5
ADAMTS3	-0.526964856	-0.626310262	1.153275118	5
ADAMTS6	-0.508613979	-0.643459052	1.152073031	5
ADAMTS7	-0.548666355	-0.605566773	1.154233128	5
ADAMTS8	-0.537192435	-0.616597669	1.153790104	5
ADAMTSL1	-0.580634574	-0.574059725	1.154694299	5
ADAMTSL2	-0.526733843	-0.62652835	1.153262193	5
ADAMTSL3	-0.559553677	-0.594965845	1.154519522	5
ADARB2	-0.534250472	-0.619403006	1.153653479	5
ADCY1	-0.558986081	-0.595521771	1.154507852	5
ADCY2	-0.555875189	-0.598562309	1.154437498	5
ADCY3	-0.576258616	-0.578441235	1.154699851	5
ADCY5	-0.540645988	-0.613292554	1.153938542	5

ADCY8	-0.554802722	-0.59960802	1.154410742	5
ADCYAP1	-0.550634954	-0.60365969	1.154294644	5
ADCYAP1R1	-0.533651402	-0.619973112	1.153624515	5
ADGB	-0.534414419	-0.619246919	1.153661338	5
ADGRA1	-0.570950306	-0.583726671	1.154676977	5
ADGRA3	-0.511206808	-0.641057457	1.152264265	5
ADGRB1	-0.567726068	-0.586921287	1.154647355	5
ADGRB3	-0.553819983	-0.600565119	1.154385102	5
ADGRF1	-0.724294677	-0.416664523	1.1409592	5
ADGRF2	-0.624925986	-0.528429767	1.153355753	5
ADGRF3	-0.680621561	-0.467504618	1.148126179	5
ADGRF4	-0.607807068	-0.54634815	1.154155218	5
ADGRG2	-0.775883657	-0.352668335	1.128551991	5
ADGRG4	-0.582590028	-0.572094611	1.154684639	5
ADGRL3	-0.543180692	-0.610858549	1.154039241	5
ADGRV1	-0.551874365	-0.602456815	1.154331179	5
ADH1A	-0.605406797	-0.548831661	1.154238458	5
ADH1C	-0.504464073	-0.64728839	1.151752464	5
ADH7	-0.535608543	-0.618109165	1.153717708	5
ADIG	-0.69345831	-0.452856162	1.146314472	5
ADIPOR2	-0.570258698	-0.584412923	1.154671621	5
ADM5	-0.763385213	-0.368595513	1.131980726	5
ADORA1	-0.534493809	-0.619171324	1.153665133	5
ADORA2B	-0.730679949	-0.408985609	1.139665558	5

ADPGK	-0.635087722	-0.51761941	1.152707132	5
ADRA1A	-0.700607113	-0.444594081	1.145201194	5
ADRA1B	-0.776089094	-0.35240416	1.128493255	5
ADRA1D	-0.687524237	-0.459657334	1.147181572	5
ADRA2A	-0.818310308	-0.296377396	1.114687704	5
ADRB3	-0.655103209	-0.495934789	1.151037997	5
AFAP1	-0.513271835	-0.639139734	1.152411569	5
AFF2	-0.505627615	-0.646216524	1.151844139	5
AGAP1	-0.523196943	-0.629860288	1.153057231	5
AGAP4	-0.690134526	-0.456671941	1.146806467	5
AGBL3	-0.538429217	-0.615415531	1.153844748	5
AGBL4	-0.679336561	-0.468957949	1.148294509	5
AGBL5	-0.593360318	-0.561190839	1.154551157	5
AGFG2	-0.655274904	-0.49574648	1.151021384	5
AGPAT1	-0.734749666	-0.40405708	1.138806746	5
AGR2	-0.562700818	-0.591876847	1.154577666	5
AGR3	-0.623177849	-0.530276282	1.153454131	5
AGT	-0.645889092	-0.505982749	1.151871841	5
AGTR2	-0.585332741	-0.569330837	1.154663579	5
AGTRAP	-0.760548197	-0.3721718	1.132719997	5
AGXT2	-0.628542264	-0.524597729	1.153139993	5
AHDC1	-0.502836003	-0.648785846	1.151621848	5
AHI1	-0.659422417	-0.491185592	1.150608009	5
AIPL1	-0.814453024	-0.301645623	1.116098647	5

AJAP1	-0.55605359	-0.598388235	1.154441824	5
AJUBA	-0.502565019	-0.649034824	1.151599843	5
AK4	-0.506915321	-0.645028646	1.151943967	5
AK7	-0.567324546	-0.587318292	1.154642838	5
AK8	-0.706765128	-0.437415951	1.144181078	5
AK9	-0.544174828	-0.609901995	1.154076823	5
AKAP14	-0.534049472	-0.619594332	1.153643804	5
AKAP4	-0.819610953	-0.294593852	1.114204805	5
AKAP6	-0.55077053	-0.603528193	1.154298723	5
AKIRIN2	-0.627520791	-0.525681826	1.153202617	5
AKNAD1	-0.714047235	-0.428853223	1.142900459	5
AKT1	-0.660454763	-0.490046737	1.1505015	5
ALDH1L2	-0.58349757	-0.571181072	1.154678643	5
ALDH3B2	-0.59497354	-0.559545822	1.154519363	5
ALDH7A1	-0.53485878	-0.618823714	1.153682494	5
ALK	-0.578542738	-0.576156979	1.154699717	5
ALKAL2	-0.644621851	-0.507355876	1.151977727	5
ALOX12	-0.793262553	-0.330039826	1.123302379	5
ALOX12B	-0.741015454	-0.396416046	1.137431499	5
ALPG	-0.63626372	-0.516359817	1.152623537	5
ALX1	-0.529791063	-0.62363757	1.153428633	5
ALX4	-0.556792863	-0.597666512	1.154459375	5
AMBN	-0.568874911	-0.585784356	1.154659267	5
AMD1	-0.596611111	-0.557872806	1.154483917	5

AMELX	-0.50625898	-0.645634319	1.151893299	5
AMER3	-0.604072347	-0.55020936	1.154281707	5
AMOTL2	-0.516532355	-0.636102731	1.152635086	5
AMPD2	-0.532231379	-0.621322939	1.153554318	5
AMPH	-0.688515629	-0.458524641	1.14704027	5
AMZ1	-0.731689089	-0.407766022	1.139455111	5
ANGPT1	-0.50196142	-0.649589137	1.151550557	5
ANGPT2	-0.751171154	-0.383892153	1.135063307	5
ANGPTL2	-0.527194114	-0.626093774	1.153287888	5
ANGPTL4	-0.544182945	-0.60989418	1.154077125	5
ANGPTL8	-0.526924669	-0.626348205	1.153272873	5
ANK3	-0.590455608	-0.564145009	1.154600617	5
ANKLE1	-0.542945912	-0.611084295	1.154030208	5
ANKLE2	-0.542220088	-0.611781813	1.154001902	5
ANKRD18B	-0.509916949	-0.642253055	1.152170004	5
ANKRD20A1	-0.605350999	-0.548889311	1.15424031	5
ANKRD31	-0.584915294	-0.569752057	1.154667351	5
ANKRD33	-0.760225206	-0.372578055	1.132803261	5
ANKRD33B	-0.599486643	-0.554927272	1.154413915	5
ANKRD34A	-0.651765653	-0.499587433	1.151353086	5
ANKRD34C	-0.649814125	-0.501716311	1.151530436	5
ANKRD36C	-0.512229478	-0.640108292	1.15233777	5
ANKRD45	-0.55813226	-0.596357359	1.154489619	5
ANKRD53	-0.78500763	-0.34085985	1.12586748	5

ANKRD66	-0.610275277	-0.543787014	1.15406229	5
ANKRD7	-0.684515067	-0.463086705	1.147601772	5
ANO3	-0.603284644	-0.551021579	1.154306223	5
ANO4	-0.597525736	-0.556936989	1.154462725	5
ANO6	-0.503257842	-0.648398111	1.151655953	5
ANOS1	-0.553401957	-0.600971913	1.154373871	5
ANP32C	-0.659692379	-0.490887917	1.150580296	5
ANPEP	-0.700693211	-0.444494113	1.145187324	5
ANTXR2	-0.525074374	-0.628093306	1.153167681	5
ANXA13	-0.518283735	-0.634466815	1.15275055	5
ANXA7	-0.540990615	-0.612962029	1.153952644	5
AOC2	-0.70466028	-0.439875905	1.144536185	5
AOC3	-0.602939958	-0.551376758	1.154316715	5
AP1M2	-0.54360611	-0.610449346	1.154055455	5
AP1S2	-0.67735163	-0.471198297	1.148549927	5
AP1S3	-0.515298499	-0.637253311	1.15255181	5
AP3B2	-0.687710627	-0.459444487	1.147155114	5
AP3D1	-0.729612944	-0.410273339	1.139886283	5
APBA1	-0.546365859	-0.607789976	1.154155835	5
APC2	-0.572251462	-0.582434111	1.154685572	5
APCDD1	-0.571186999	-0.583491686	1.154678685	5
APELA	-0.53085577	-0.622628484	1.153484253	5
APMAP	-0.692727974	-0.453695989	1.146423963	5
APOA5	-0.521629892	-0.631332291	1.152962183	5

APOBEC4	-0.539601281	-0.614293723	1.153895004	5
APOD	-0.727004331	-0.413413871	1.140418202	5
APOF	-0.822596523	-0.29048605	1.113082573	5
APOO	-0.624553615	-0.528823417	1.153377031	5
APP	-0.521268896	-0.631671023	1.152939919	5
AQP12A	-0.69626093	-0.4496261	1.145887031	5
AQP6	-0.615095314	-0.538763938	1.153859252	5
ARC	-0.626039521	-0.527251558	1.153291079	5
ARG2	-0.539553343	-0.614339635	1.153892977	5
ARHGAP11A-SCG5	-0.524505398	-0.628629204	1.153134602	5
ARHGAP18	-0.508346511	-0.643706396	1.152052907	5
ARHGAP32	-0.515818044	-0.636769026	1.15258707	5
ARHGAP33	-0.559090233	-0.595419788	1.154510021	5
ARHGAP36	-0.654498871	-0.496597286	1.151096157	5
ARHGAP39	-0.547525767	-0.606669766	1.154195533	5
ARHGAP44	-0.601272195	-0.553093257	1.154365452	5
ARHGAP6	-0.51891816	-0.633873421	1.152791581	5
ARHGAP8	-0.668165596	-0.481494331	1.149659926	5
ARHGDIA	-0.71118196	-0.432232074	1.143414034	5
ARHGDIG	-0.546630151	-0.60753486	1.154165011	5
ARHGEF16	-0.556521852	-0.59793116	1.154453012	5
ARHGEF26	-0.593828673	-0.560713572	1.154542246	5
ARHGEF37	-0.523240827	-0.629819028	1.153059855	5
ARHGEF38	-0.591458386	-0.563126286	1.154584672	5

ARHGEF4	-0.575790822	-0.578908313	1.154699136	5
ARID3C	-0.536913075	-0.616864457	1.153777531	5
ARID5B	-0.663573133	-0.486597831	1.150170964	5
ARL14	-0.509933791	-0.642237455	1.152171246	5
ARL9	-0.541867004	-0.61212092	1.153987924	5
ARMC3	-0.508817117	-0.643271148	1.152088265	5
ARMC7	-0.82591406	-0.285898803	1.111812862	5
ARMCX3	-0.697207709	-0.448532306	1.145740015	5
ARMH4	-0.540368099	-0.613558977	1.153927077	5
ARNT2	-0.530366089	-0.623092734	1.153458823	5
ARPC1B	-0.677038204	-0.471551545	1.148589749	5
ARRDC4	-0.510085758	-0.64209668	1.152182439	5
ARSI	-0.585182836	-0.569482121	1.154664957	5
ARSJ	-0.608596719	-0.545529579	1.154126298	5
ARSL	-0.559933038	-0.594594082	1.15452712	5
ART5	-0.506488345	-0.64542271	1.151911056	5
ARTN	-0.59676747	-0.557712895	1.154480365	5
ARVCF	-0.558864127	-0.595641171	1.154505298	5
ARX	-0.562211656	-0.592357703	1.154569359	5
AS3MT	-0.529428241	-0.623981161	1.153409402	5
ASAP3	-0.513448003	-0.638975926	1.15242393	5
ASB10	-0.55720494	-0.597263954	1.154468893	5
ASB4	-0.679876539	-0.46834752	1.14822406	5
ASB5	-0.650476527	-0.50099428	1.151470807	5

ASCL1	-0.530556869	-0.622911892	1.153468761	5
ASCL3	-0.679795294	-0.468439392	1.148234686	5
ASCL5	-0.680359464	-0.467801239	1.148160703	5
ASDURF	-0.677820666	-0.470669409	1.148490075	5
ASIC1	-0.551417192	-0.602900709	1.154317901	5
ASIC2	-0.603223177	-0.551084928	1.154308105	5
ASIC3	-0.569689326	-0.584977476	1.154666802	5
ASIC4	-0.504299422	-0.647439956	1.151739378	5
ASPA	-0.634776983	-0.51795194	1.152728923	5
ASPM	-0.511453885	-0.640828239	1.152282124	5
ASPN	-0.72172631	-0.419734917	1.141461227	5
ASTN1	-0.549234708	-0.605016619	1.154251327	5
ASXL3	-0.552510793	-0.601838487	1.15434928	5
ATAD2B	-0.511866953	-0.640444885	1.152311838	5
ATCAY	-0.555343688	-0.59908071	1.154424398	5
ATE1	-0.510330326	-0.641870075	1.152200401	5
ATF4	-0.701003572	-0.444133662	1.145137233	5
ATL1	-0.502858413	-0.648765252	1.151623665	5
ATOH1	-0.535821637	-0.617905968	1.153727605	5
ATOX1	-0.777023848	-0.351201165	1.128225013	5
ATP10B	-0.565450536	-0.589168801	1.154619338	5
ATP11A	-0.595662398	-0.558842444	1.154504842	5
ATP11B	-0.563931195	-0.590666173	1.154597367	5
ATP13A5	-0.537114285	-0.61667231	1.153786595	5

ATP1A2	-0.55586249	-0.598574698	1.154437189	5
ATP1A3	-0.605501704	-0.548733595	1.1542353	5
ATP1B1	-0.520432819	-0.632455008	1.152887828	5
ATP1B4	-0.564085576	-0.590514143	1.154599719	5
ATP2B2	-0.621219424	-0.532340358	1.153559782	5
ATP2B3	-0.597191813	-0.557278765	1.154470578	5
ATP5F1E	-0.730620199	-0.409057768	1.139677967	5
ATP6V0A1	-0.786028403	-0.33952898	1.125557383	5
ATP6V0A4	-0.789787166	-0.334611163	1.124398329	5
ATP6V0B	-0.735154579	-0.403565245	1.138719824	5
ATP6V1B1	-0.555900709	-0.59853741	1.154438119	5
ATP9A	-0.51890589	-0.633884901	1.152790792	5
ATRNL1	-0.533013519	-0.620579731	1.15359325	5
ATRX	-0.504571677	-0.647189323	1.151761001	5
ATXN2	-0.558308808	-0.596184648	1.154493456	5
ATXN3L	-0.83422303	-0.274302942	1.108525973	5
ATXN7L1	-0.728538183	-0.411568576	1.140106759	5
AURKB	-0.568908725	-0.58575087	1.154659595	5
AUTS2	-0.5379971	-0.615828744	1.153825844	5
AVP	-0.652415137	-0.498877801	1.151292938	5
AVPR1B	-0.63431116	-0.518450197	1.152761357	5
AZU1	-0.53240463	-0.621158368	1.153562998	5
B3GALT1	-0.623519083	-0.529916147	1.15343523	5
B3GALT4	-0.823655448	-0.289024455	1.112679903	5

B3GALT5	-0.646657851	-0.505148726	1.151806576	5
B4GALNT1	-0.553098431	-0.601267163	1.154365594	5
B4GALNT4	-0.531474024	-0.62204197	1.153515994	5
B4GALT1	-0.643593641	-0.50846845	1.152062091	5
B4GALT2	-0.506137481	-0.64574639	1.15188387	5
BARHL1	-0.524423593	-0.628706225	1.153129818	5
BARHL2	-0.538588326	-0.615263332	1.153851658	5
BARX1	-0.665988478	-0.483917329	1.149905807	5
BARX2	-0.52839268	-0.62496106	1.15335374	5
BASP1	-0.677527281	-0.471000268	1.148527549	5
BBLN	-0.614852174	-0.539018006	1.15387018	5
BBOF1	-0.509363614	-0.642765423	1.152129038	5
BBS1	-0.667745106	-0.48196282	1.149707926	5
BCAN	-0.536308232	-0.617441791	1.153750023	5
BCAP31	-0.539414308	-0.614472779	1.153887087	5
BCAR3	-0.571153406	-0.58352504	1.154678446	5
BCHE	-0.546453317	-0.607705563	1.15415888	5
BCL10	-0.683494154	-0.46424722	1.147741374	5
BCL11A	-0.617828102	-0.535903282	1.153731385	5
BCL2L10	-0.551814899	-0.602514566	1.154329465	5
BCL3	-0.773516634	-0.355706506	1.12922314	5
BCO1	-0.593207993	-0.561346006	1.154554	5
BDNF	-0.550463405	-0.603826049	1.154289454	5
BEAN1	-0.672657737	-0.476474107	1.149131844	5

BECN2	-0.508377943	-0.643677333	1.152055276	5
BEGAIN	-0.60934758	-0.544750513	1.154098092	5
BEST2	-0.540563293	-0.613371846	1.153935139	5
BEST3	-0.524602036	-0.628538209	1.153140245	5
BET1	-0.536257423	-0.617490271	1.153747694	5
BEX1	-0.515780845	-0.63680371	1.152584555	5
BHLHA9	-0.818017705	-0.296778138	1.114795843	5
BHLHE22	-0.551230901	-0.603081523	1.154312423	5
BHLHE41	-0.504976215	-0.646816772	1.151792987	5
BICD2	-0.603024789	-0.551289357	1.154314146	5
BIN2	-0.699293362	-0.446118106	1.145411467	5
BIRC5	-0.53088171	-0.622603883	1.153485593	5
BIRC6	-0.685691622	-0.461747409	1.147439031	5
BIVM-ERCC5	-0.603172469	-0.551137185	1.154309654	5
BLID	-0.799333285	-0.321997909	1.121331194	5
BLOC1S6	-0.583828367	-0.570847851	1.154676218	5
BMP2	-0.56395932	-0.590638477	1.154597798	5
BMP4	-0.508285665	-0.643762654	1.152048319	5
BMP5	-0.540698612	-0.613242092	1.153940704	5
BMP6	-0.545543353	-0.608583438	1.154126791	5
BMP7	-0.549684072	-0.604581392	1.154265464	5
BMPER	-0.524198841	-0.628917796	1.153116638	5
BMPR1B	-0.553057619	-0.601306854	1.154364473	5
BOC	-0.533610435	-0.620012085	1.15362252	5

BPI	-0.708837682	-0.434987174	1.143824855	5
BPIFA2	-0.584698779	-0.569970449	1.154669228	5
BPIFB3	-0.539383201	-0.614502564	1.153885766	5
BPIFB4	-0.666273551	-0.483600433	1.149873983	5
BRCA2	-0.518233918	-0.634513393	1.15274731	5
BRD3	-0.667605666	-0.482118123	1.149723789	5
BRINP1	-0.553178749	-0.601189045	1.154367794	5
BRINP2	-0.563891935	-0.59070483	1.154596765	5
BRINP3	-0.5727849	-0.581903637	1.154688536	5
BRK1	-0.533798982	-0.619832703	1.153631686	5
BRME1	-0.626454503	-0.526812074	1.153266576	5
BRSK1	-0.538123963	-0.615707452	1.153831415	5
BRSK2	-0.585992335	-0.568664866	1.154657201	5
BRWD3	-0.773876321	-0.355245496	1.129121817	5
BSCL2	-0.650238201	-0.501254127	1.151492328	5
BSN	-0.507479935	-0.644507263	1.151987198	5
BSX	-0.549886815	-0.604384954	1.154271769	5
BTBD17	-0.539448103	-0.614440418	1.15388852	5
BTC	-0.536789952	-0.616982011	1.153771964	5
BTNL2	-0.593881358	-0.560659869	1.154541227	5
BUB1B-PAK6	-0.817113253	-0.298015697	1.11512895	5
BVES	-0.538853132	-0.615009965	1.153863097	5
C10orf120	-0.681747159	-0.46622964	1.1479768	5
C10orf53	-0.640737766	-0.511551388	1.152289153	5

C10orf62	-0.504545629	-0.647213307	1.151758935	5
C10orf71	-0.568565747	-0.586090463	1.154656209	5
C10orf90	-0.686700387	-0.460597525	1.147297912	5
C11orf16	-0.565070691	-0.589543399	1.154614089	5
C11orf86	-0.561826271	-0.592736354	1.154562625	5
C11orf96	-0.682172271	-0.465747642	1.147919913	5
C11orf97	-0.534742011	-0.618934944	1.153676955	5
C12orf54	-0.7115255	-0.431827624	1.143353124	5
C14orf132	-0.543053902	-0.610980468	1.15403437	5
C14orf39	-0.551252874	-0.603060198	1.154313072	5
C16orf46	-0.618655475	-0.535035367	1.153690842	5
C16orf82	-0.771751406	-0.35796556	1.129716967	5
C16orf87	-0.789269639	-0.335289887	1.124559526	5
C16orf90	-0.680772951	-0.467333242	1.148106193	5
C16orf92	-0.614124837	-0.539777597	1.153902435	5
C16orf95	-0.735612452	-0.403008758	1.138621211	5
C16orf96	-0.718572305	-0.423491198	1.142063504	5
C18orf63	-0.505142661	-0.646663438	1.151806099	5
C19orf33	-0.523827813	-0.629266948	1.153094762	5
C19orf44	-0.706526958	-0.437694638	1.144221596	5
C1orf116	-0.557676438	-0.596803113	1.154479551	5
C1orf141	-0.612343122	-0.541635567	1.153978689	5
C1orf158	-0.786642445	-0.338727443	1.125369888	5
C1orf167	-0.696807224	-0.44899514	1.145802364	5

C1orf194	-0.600949805	-0.553424681	1.154374486	5
C1orf198	-0.538539001	-0.615310517	1.153849519	5
C1orf210	-0.504769888	-0.647006806	1.151776694	5
C1orf53	-0.527542395	-0.625764786	1.153307181	5
C1orf87	-0.528802496	-0.624573409	1.153375905	5
C1QL4	-0.530884973	-0.622600789	1.153485762	5
C1R	-0.519000101	-0.633796749	1.15279685	5
C20orf144	-0.582148872	-0.572538335	1.154687207	5
C20orf173	-0.619077836	-0.534591982	1.153669817	5
C20orf203	-0.64660306	-0.505208194	1.151811254	5
C20orf96	-0.573116568	-0.581573647	1.154690215	5
C21orf58	-0.647309427	-0.504441223	1.15175065	5
C21orf62	-0.673061947	-0.476021002	1.149082948	5
C22orf31	-0.689324214	-0.457599755	1.146923969	5
C22orf42	-0.648851573	-0.502764474	1.151616047	5
C2CD4A	-0.82725981	-0.284031119	1.111290929	5
C2CD4B	-0.599755732	-0.554651125	1.154406857	5
C2CD4C	-0.524589044	-0.628550443	1.153139487	5
C2CD6	-0.554310122	-0.600087902	1.154398024	5
C2orf66	-0.506408845	-0.645496062	1.151904907	5
C2orf68	-0.522428526	-0.630582421	1.153010947	5
C2orf72	-0.52529195	-0.627888289	1.153180239	5
C2orf73	-0.541711497	-0.612270228	1.153981725	5
C2orf76	-0.601987534	-0.552357424	1.154344959	5

C2orf80	-0.559437278	-0.59507988	1.154517158	5
C2orf83	-0.773596236	-0.3556045	1.129200736	5
C2orf88	-0.544324864	-0.609757537	1.154082401	5
C3orf52	-0.631118733	-0.521857411	1.152976144	5
C3orf70	-0.770304969	-0.359812408	1.130117377	5
C3orf80	-0.770387478	-0.35970716	1.130094639	5
C4orf36	-0.53479361	-0.618885794	1.153679404	5
C4orf45	-0.811193874	-0.30607249	1.117266364	5
C4orf47	-0.581112791	-0.573579556	1.154692347	5
C5orf49	-0.544200764	-0.609877025	1.154077789	5
C6orf118	-0.545876338	-0.6082623	1.154138639	5
C6orf15	-0.566116247	-0.588511894	1.154628141	5
C7	-0.518807007	-0.633977416	1.152784423	5
C7orf31	-0.54259909	-0.611417664	1.154016754	5
C7orf33	-0.560455168	-0.594082145	1.154537314	5
C8B	-0.638672142	-0.513774619	1.152446761	5
C8orf34	-0.628809362	-0.524314038	1.1531234	5
C9orf116	-0.535583464	-0.618133076	1.15371654	5
C9orf153	-0.686627198	-0.460681002	1.1473082	5
C9orf24	-0.827825403	-0.283244976	1.111070379	5
C9orf40	-0.613481177	-0.540449257	1.153930434	5
C9orf50	-0.708656992	-0.435199179	1.143856172	5
C9orf57	-0.511978469	-0.64034136	1.152319829	5
C9orf64	-0.698562955	-0.446964306	1.145527261	5

CA10	-0.537974238	-0.6158506	1.153824838	5
CA12	-0.562115021	-0.592452665	1.154567686	5
CA13	-0.50277815	-0.648839007	1.151617157	5
CA14	-0.532585729	-0.620986308	1.153572038	5
CA9	-0.529303199	-0.624099543	1.153402742	5
CABCOCO1	-0.597378978	-0.557087215	1.154466193	5
CABP1	-0.70036485	-0.444875314	1.145240164	5
CABP7	-0.657810054	-0.492961416	1.15077147	5
CABS1	-0.572845653	-0.5818432	1.154688853	5
CABYR	-0.543928336	-0.610139269	1.154067605	5
CACNA1A	-0.639468392	-0.512918271	1.152386664	5
CACNA1B	-0.539178646	-0.614698408	1.153877054	5
CACNA1C	-0.552693354	-0.601661035	1.154354389	5
CACNA1G	-0.537643881	-0.616166361	1.153810241	5
CACNA1S	-0.566782106	-0.58785434	1.154636445	5
CACNA2D1	-0.547655101	-0.606544767	1.154199868	5
CACNB1	-0.743656813	-0.393175455	1.136832268	5
CACNG2	-0.58467208	-0.569997375	1.154669455	5
CACNG3	-0.531894426	-0.621642917	1.153537343	5
CACNG4	-0.54859029	-0.605640376	1.154230666	5
CACNG5	-0.57913679	-0.575561904	1.154698694	5
CACNG7	-0.546220622	-0.607930139	1.154150761	5
CADM1	-0.565965448	-0.588660743	1.154626191	5
CADM3	-0.548742708	-0.605492886	1.154235594	5

CADPS	-0.550926794	-0.603376606	1.1543034	5
CALB1	-0.558795776	-0.595708083	1.154503859	5
CALB2	-0.533345628	-0.620263954	1.153609582	5
CALCA	-0.722908156	-0.418323357	1.141231513	5
CALCB	-0.526608478	-0.626646677	1.153255155	5
CALCOCO1	-0.520390672	-0.63249451	1.152885182	5
CALCR	-0.639630855	-0.512743445	1.1523743	5
CALHM5	-0.636308355	-0.516311974	1.152620329	5
CALML3	-0.572677331	-0.582010634	1.154687965	5
CALN1	-0.601202356	-0.553165064	1.15436742	5
CAMK1G	-0.557698776	-0.596781274	1.15448005	5
CAMK2A	-0.5700534	-0.584616526	1.154669926	5
CAMK2B	-0.567626908	-0.587019348	1.154646257	5
CAMK2N2	-0.551776232	-0.602552117	1.154328349	5
CAMKV	-0.549871978	-0.604399331	1.154271309	5
CAND2	-0.653110386	-0.498117541	1.151227927	5
CAP2	-0.549847261	-0.604423282	1.154270543	5
CAPN6	-0.549380253	-0.604875677	1.15425593	5
CAPSL	-0.54684568	-0.607326757	1.154172436	5
CARD19	-0.519123689	-0.633681094	1.152804783	5
CARD6	-0.543019555	-0.611013492	1.154033048	5
CARMIL1	-0.506493424	-0.645418025	1.151911448	5
CARMIL3	-0.568639904	-0.586017049	1.154656953	5
CASKIN1	-0.557913984	-0.596570843	1.154484827	5

CASQ2	-0.702122475	-0.442832976	1.144955451	5
CASZ1	-0.63086256	-0.522130254	1.152992814	5
CATSPER4	-0.790224784	-0.334036834	1.124261618	5
CATSPERG	-0.677922168	-0.470554914	1.148477082	5
CAVIN3	-0.593947545	-0.560592398	1.154539942	5
CAVIN4	-0.553899097	-0.600488109	1.154387206	5
CBARP	-0.546993137	-0.607184351	1.154177487	5
CBLB	-0.766892513	-0.36415451	1.131047022	5
CBLC	-0.513041896	-0.639353491	1.152395387	5
CBLL2	-0.6192714	-0.534388707	1.153660107	5
CBLN1	-0.539372883	-0.614512445	1.153885327	5
CBLN2	-0.552670443	-0.601683307	1.15435375	5
CBLN3	-0.666522399	-0.483323713	1.149846112	5
CBX2	-0.527903915	-0.625423155	1.153327071	5
CBX8	-0.761791306	-0.370606513	1.132397818	5
CBY2	-0.745164885	-0.391320019	1.136484904	5
CBY3	-0.748524762	-0.387172456	1.135697218	5
CC2D2A	-0.738045993	-0.400045326	1.138091319	5
CCBE1	-0.590385571	-0.564216115	1.154601686	5
CCDC103	-0.559051931	-0.595457294	1.154509225	5
CCDC105	-0.695824952	-0.450129332	1.145954284	5
CCDC110	-0.535136651	-0.618558965	1.153695615	5
CCDC136	-0.519324367	-0.633493263	1.15281763	5
CCDC160	-0.528668491	-0.624700186	1.153368677	5

CCDC168	-0.587220848	-0.567423115	1.154643964	5
CCDC169	-0.55179406	-0.602534804	1.154328864	5
CCDC169-SOHLH2	-0.541782458	-0.612202099	1.153984557	5
CCDC173	-0.541567198	-0.612408751	1.153975949	5
CCDC175	-0.511409771	-0.640869168	1.15227894	5
CCDC177	-0.561103507	-0.593446039	1.154549546	5
CCDC18	-0.773365145	-0.355900598	1.129265743	5
CCDC181	-0.521175563	-0.631758577	1.152934141	5
CCDC183	-0.581989864	-0.572698213	1.154688077	5
CCDC198	-0.599799863	-0.554605828	1.154405691	5
CCDC3	-0.551396715	-0.602920585	1.154317301	5
CCDC33	-0.544456304	-0.609630964	1.154087268	5
CCDC38	-0.583869219	-0.570806691	1.15467591	5
CCDC40	-0.568249405	-0.586403562	1.154652968	5
CCDC47	-0.761676965	-0.370750601	1.132427566	5
CCDC60	-0.653443027	-0.497753567	1.151196594	5
CCDC74A	-0.556262101	-0.598184735	1.154446836	5
CCDC74B	-0.561686473	-0.592873668	1.154560141	5
CCDC8	-0.643461744	-0.508611068	1.152072812	5
CCDC83	-0.616198997	-0.537609728	1.153808726	5
CCDC91	-0.503392094	-0.648274675	1.151666769	5
CCDC92	-0.710744317	-0.432747051	1.143491367	5
CCDC92B	-0.657662936	-0.493123273	1.150786209	5
CCER1	-0.531996199	-0.621546284	1.153542483	5

CCK	-0.562932279	-0.591649223	1.154581502	5
CCKAR	-0.509713959	-0.642441053	1.152155013	5
CCKBR	-0.572779522	-0.581908986	1.154688508	5
CCL22	-0.624884327	-0.528473816	1.153358142	5
CCL25	-0.511354094	-0.640920824	1.152274918	5
CCN2	-0.521808086	-0.631165036	1.152973122	5
CCNB2	-0.505850864	-0.646010705	1.151861568	5
CCND3	-0.712758141	-0.430374941	1.143133082	5
CCNE2	-0.5070751	-0.644881135	1.151956234	5
CCNO	-0.553836735	-0.600548813	1.154385548	5
CCSER1	-0.621607266	-0.531931975	1.153539241	5
CD164	-0.578494212	-0.57620557	1.154699782	5
CD276	-0.539544053	-0.614348532	1.153892585	5
CD300LB	-0.509877973	-0.642289156	1.152167129	5
CD68	-0.677274737	-0.471284972	1.148559709	5
CD82	-0.653163013	-0.498059966	1.151222979	5
CD84	-0.531486212	-0.622030404	1.153516616	5
CDC14C	-0.574502268	-0.580193595	1.154695863	5
CDC20B	-0.55870889	-0.595793133	1.154502022	5
CDC42SE2	-0.505205839	-0.64660523	1.151811068	5
CDC45	-0.525136605	-0.628034673	1.153171278	5
CDCA5	-0.562309032	-0.592262003	1.154571034	5
CDCA7	-0.536920652	-0.616857222	1.153777874	5
CDH10	-0.515531952	-0.637035737	1.152567689	5

CDH11	-0.509735396	-0.642421202	1.152156598	5
CDH12	-0.602719741	-0.551603602	1.154323343	5
CDH16	-0.530879337	-0.622606134	1.153485471	5
CDH17	-0.578663871	-0.576035671	1.154699541	5
CDH18	-0.599581499	-0.554829938	1.154411437	5
CDH19	-0.544840277	-0.609261099	1.154101376	5
CDH2	-0.542918553	-0.611110598	1.154029151	5
CDH22	-0.545839542	-0.608297793	1.154137335	5
CDH4	-0.556854944	-0.597605877	1.154460821	5
CDH6	-0.544908046	-0.609195804	1.154103849	5
CDH7	-0.571755244	-0.582927279	1.154682523	5
CDH8	-0.528877587	-0.62450236	1.153379946	5
CDH9	-0.503181794	-0.648468024	1.151649818	5
CDHR4	-0.565076181	-0.589537986	1.154614166	5
CDK1	-0.513346411	-0.639070394	1.152416805	5
CDK14	-0.569176202	-0.585485941	1.154662143	5
CDK18	-0.574642181	-0.58005413	1.154696311	5
CDK19	-0.657561973	-0.493234335	1.150796308	5
CDK2AP1	-0.524919733	-0.628238991	1.153158724	5
CDK5R1	-0.559067195	-0.595442347	1.154509542	5
CDK5R2	-0.7413233	-0.396038959	1.137362259	5
CDKL2	-0.545231393	-0.608884187	1.154115581	5
CDKL3	-0.733170033	-0.405973252	1.139143284	5
CDKL4	-0.525204778	-0.627970436	1.153175214	5

CDKN1A	-0.683958734	-0.463719298	1.147678031	5
CDKN2B	-0.70614536	-0.438140975	1.144286335	5
CDO1	-0.541923827	-0.612066356	1.153990183	5
CDON	-0.556149223	-0.598294906	1.154444129	5
CDR2L	-0.516702853	-0.635943615	1.152646468	5
CDRT15L2	-0.519524884	-0.63330554	1.152830424	5
CDS1	-0.508038728	-0.64399093	1.152029658	5
CDX1	-0.757142921	-0.376445724	1.133588644	5
CDX2	-0.592532343	-0.562033932	1.154566275	5
CDX4	-0.640248439	-0.512078551	1.152326991	5
CDY2A	-0.521955577	-0.631026574	1.15298215	5
CDY2B	-0.525087487	-0.628080952	1.153168439	5
CEACAM16	-0.676070484	-0.472641344	1.148711827	5
CEACAM18	-0.535637804	-0.618081266	1.15371907	5
CEACAM19	-0.674107611	-0.474847787	1.148955398	5
CEACAM5	-0.684735789	-0.462835604	1.147571393	5
CECR2	-0.543765033	-0.610296429	1.154061462	5
CEL	-0.524292939	-0.628829223	1.153122163	5
CELA3A	-0.680148717	-0.468039675	1.148188393	5
CELF2	-0.710093913	-0.43351184	1.143605753	5
CELF3	-0.545584735	-0.608543535	1.15412827	5
CELF4	-0.55707169	-0.597394146	1.154465836	5
CELF5	-0.549420866	-0.604836345	1.154257211	5
CELSR1	-0.583194782	-0.571485968	1.15468075	5

CELSR2	-0.544191821	-0.609885635	1.154077456	5
CEMIP	-0.585401023	-0.569261919	1.154662942	5
CENPA	-0.504319943	-0.647421068	1.151741011	5
CEP128	-0.50667234	-0.645252921	1.151925261	5
CEP131	-0.539718687	-0.614181269	1.153899956	5
CERS1	-0.555614278	-0.598816829	1.154431107	5
CERS5	-0.815817586	-0.299785526	1.115603112	5
CES3	-0.620895016	-0.532681802	1.153576819	5
CETP	-0.529565567	-0.62385113	1.153416697	5
CFAP126	-0.554176811	-0.600217725	1.154394536	5
CFAP206	-0.538360675	-0.615481088	1.153841763	5
CFAP20DC	-0.56833464	-0.586319212	1.154653852	5
CFAP221	-0.57006551	-0.584604518	1.154670028	5
CFAP299	-0.700319146	-0.44492836	1.145247505	5
CFAP300	-0.548011352	-0.606200362	1.154211714	5
CFAP43	-0.554338456	-0.600060307	1.154398763	5
CFAP46	-0.568279481	-0.5863738	1.154653281	5
CFAP47	-0.554544563	-0.599859548	1.154404111	5
CFAP52	-0.548750941	-0.605484919	1.154235859	5
CFAP54	-0.573459259	-0.581232558	1.154691817	5
CFAP57	-0.533078922	-0.620517554	1.153596476	5
CFAP61	-0.553007257	-0.601355831	1.154363088	5
CFAP65	-0.54207876	-0.611917564	1.153996323	5
CFAP69	-0.564345247	-0.590258366	1.154603613	5

CFAP74	-0.576473723	-0.578226372	1.154700095	5
CFAP77	-0.591544918	-0.563038322	1.15458324	5
CFAP91	-0.510019552	-0.642158014	1.152177565	5
CFAP97D1	-0.566733457	-0.587902399	1.154635855	5
CFAP99	-0.539095705	-0.614777803	1.153873508	5
CFC1	-0.612992247	-0.540959114	1.153951361	5
CFC1B	-0.663135182	-0.487083004	1.150218187	5
CFHR2	-0.712344151	-0.430863093	1.143207245	5
CFHR5	-0.511453972	-0.640828158	1.15228213	5
CFTR	-0.549807922	-0.604461399	1.154269321	5
CGA	-0.50779046	-0.644220373	1.152010833	5
CGB2	-0.737240152	-0.401027713	1.138267865	5
CGB7	-0.621469665	-0.532076885	1.15354655	5
CGN	-0.520265018	-0.632612266	1.152877284	5
CH25H	-0.768322282	-0.36233779	1.130660072	5
CHAC1	-0.644107663	-0.507912426	1.152020089	5
CHADL	-0.785485824	-0.340236635	1.125722458	5
CHAMP1	-0.509592484	-0.642553536	1.152146021	5
CHD5	-0.551658254	-0.602666677	1.154324931	5
CHDH	-0.698228765	-0.447351213	1.145579977	5
CHGA	-0.700742588	-0.444436777	1.145179365	5
CHGB	-0.532278373	-0.621278302	1.153556676	5
CHIC2	-0.671408446	-0.477873078	1.149281524	5
CHL1	-0.521027096	-0.631897834	1.15292493	5

CHODL	-0.597602437	-0.556858466	1.154460903	5
CHP2	-0.644314896	-0.507688162	1.152003058	5
CHRD	-0.527617279	-0.625694034	1.153311312	5
CHRDL1	-0.545739459	-0.608394324	1.154133783	5
CHRDL2	-0.649642895	-0.501902861	1.151545756	5
CHRM1	-0.637173069	-0.515384604	1.152557673	5
CHRM2	-0.517874061	-0.634849769	1.15272383	5
CHRM3	-0.511411909	-0.640867185	1.152279094	5
CHRNA2	-0.525221159	-0.627955	1.153176159	5
CHRNA3	-0.504437145	-0.647313181	1.151750326	5
CHRNA4	-0.532221467	-0.621332353	1.15355382	5
CHRNA6	-0.567760683	-0.586887053	1.154647736	5
CHRNA7	-0.666851726	-0.48295737	1.149809095	5
CHRNB4	-0.537659903	-0.616151049	1.153810952	5
CHRNG	-0.626390653	-0.526879707	1.15327036	5
CHST1	-0.790476771	-0.333705958	1.124182729	5
CHST15	-0.62960704	-0.523466264	1.153073304	5
CHST3	-0.545706208	-0.608426392	1.1541326	5
CHST4	-0.631639226	-0.521302789	1.152942015	5
CHST5	-0.642264856	-0.509904209	1.152169064	5
CHST6	-0.581754622	-0.572934688	1.15468931	5
CHST8	-0.560023716	-0.594505196	1.154528912	5
CHST9	-0.617636259	-0.536104405	1.153740664	5
CHURC1-FNTB	-0.514508332	-0.637989311	1.152497642	5

CIB2	-0.568789855	-0.585868582	1.154658437	5
CIBAR1	-0.506838994	-0.645099103	1.151938098	5
CIBAR2	-0.572674049	-0.582013899	1.154687947	5
CIDEC	-0.594982684	-0.55953649	1.154519174	5
CILP2	-0.553830634	-0.600554752	1.154385386	5
CITED1	-0.538856113	-0.615007112	1.153863225	5
CKMT1A	-0.542655879	-0.611363086	1.154018966	5
CKMT1B	-0.545416872	-0.608705386	1.154122259	5
CLCA2	-0.687179067	-0.460051368	1.147230434	5
CLCN2	-0.758848076	-0.374308141	1.133156217	5
CLCN4	-0.734917641	-0.403853079	1.13877072	5
CLCNKA	-0.541764393	-0.612219444	1.153983837	5
CLCNKB	-0.531423735	-0.622089692	1.153513428	5
CLDN1	-0.527241555	-0.626048969	1.153290524	5
CLDN14	-0.683283563	-0.464486422	1.147769984	5
CLDN16	-0.758307887	-0.374985872	1.133293759	5
CLDN18	-0.507848624	-0.644166625	1.152015249	5
CLDN19	-0.568973267	-0.585686951	1.154660217	5
CLDN2	-0.587026911	-0.567619261	1.154646171	5
CLDN22	-0.564787913	-0.589822163	1.154610077	5
CLDN4	-0.583098777	-0.571582619	1.154681396	5
CLDN5	-0.768364227	-0.362284438	1.130648664	5
CLDN6	-0.519219406	-0.63359151	1.152810916	5
CLDN8	-0.587433872	-0.567207616	1.154641488	5

CLEC11A	-0.510614329	-0.641606853	1.152221182	5
CLEC19A	-0.53805609	-0.615772347	1.153828437	5
CLEC2L	-0.642229439	-0.509942445	1.152171884	5
CLEC3A	-0.509798319	-0.642362929	1.152161248	5
CLEC4G	-0.537586984	-0.616220732	1.153807716	5
CLGN	-0.538851242	-0.615011773	1.153863015	5
CLHC1	-0.704654995	-0.439882072	1.144537068	5
CLIC6	-0.551096925	-0.603211535	1.154308461	5
CLIP1	-0.734005983	-0.40495971	1.138965693	5
CLIP2	-0.678511862	-0.469889446	1.148401307	5
CLIP3	-0.556235467	-0.598210732	1.154446199	5
CLMP	-0.536082701	-0.617656963	1.153739664	5
CLPS	-0.682023497	-0.465916354	1.147939851	5
CLPSL2	-0.523310269	-0.629753735	1.153064004	5
CLRN1	-0.780452607	-0.346774576	1.127227183	5
CLRN2	-0.582255171	-0.572431438	1.154686609	5
CLRN3	-0.540767415	-0.613176111	1.153943526	5
CLSTN2	-0.557586084	-0.596891444	1.154477528	5
CLTRN	-0.523107532	-0.629944345	1.153051877	5
CLUL1	-0.561992966	-0.592572592	1.154565558	5
CLVS1	-0.67306641	-0.476015997	1.149082407	5
CLVS2	-0.549409006	-0.604847831	1.154256837	5
CMA1	-0.508061103	-0.643970249	1.152031351	5
CMBL	-0.645847508	-0.506027842	1.151875349	5

CMC2	-0.557194779	-0.597273882	1.154468661	5
CMYA5	-0.585814439	-0.568844532	1.154658972	5
CNBD1	-0.709936162	-0.433697238	1.143633399	5
CNGA3	-0.573154253	-0.581536145	1.154690398	5
CNGB1	-0.555231218	-0.599190368	1.154421586	5
CNKSR3	-0.513855795	-0.638596623	1.152452418	5
CNN1	-0.535770511	-0.617954725	1.153725235	5
CNNM1	-0.82837654	-0.282478244	1.110854784	5
CNNM2	-0.541513026	-0.612460749	1.153973775	5
CNOT3	-0.571209293	-0.583469549	1.154678842	5
CNPY1	-0.581134269	-0.573557984	1.154692253	5
CNPY3	-0.629159724	-0.523941772	1.153101496	5
CNTFR	-0.54236238	-0.611645116	1.154007496	5
CNTN3	-0.545257748	-0.608858784	1.154116532	5
CNTN4	-0.553333948	-0.601038077	1.154372025	5
CNTN5	-0.510845178	-0.641392834	1.152238012	5
CNTN6	-0.670479159	-0.478912294	1.149391453	5
CNTNAP1	-0.624432422	-0.528951497	1.153383919	5
CNTNAP2	-0.539488892	-0.614401357	1.153890249	5
CNTNAP4	-0.545888747	-0.60825033	1.154139078	5
CNTNAP5	-0.561843153	-0.592719771	1.154562924	5
COL11A1	-0.55565313	-0.598778933	1.154432063	5
COL12A1	-0.832765541	-0.276348177	1.109113718	5
COL14A1	-0.504336362	-0.647405955	1.151742317	5

COL16A1	-0.531436485	-0.622077593	1.153514079	5
COL20A1	-0.669608891	-0.479884421	1.149493312	5
COL21A1	-0.523714034	-0.62937399	1.153088024	5
COL22A1	-0.578468041	-0.576231776	1.154699817	5
COL23A1	-0.532977253	-0.620614206	1.153591459	5
COL24A1	-0.507488726	-0.644499143	1.151987868	5
COL25A1	-0.557460533	-0.597014168	1.154474702	5
COL26A1	-0.527299465	-0.625994272	1.153293738	5
COL27A1	-0.533946897	-0.619691952	1.15363885	5
COL28A1	-0.72053854	-0.421151334	1.141689873	5
COL2A1	-0.539747247	-0.614153911	1.153901158	5
COL4A1	-0.514928569	-0.637597963	1.152526532	5
COL4A5	-0.548958453	-0.605284072	1.154242526	5
COL4A6	-0.550038876	-0.604237593	1.154276469	5
COL5A3	-0.534440727	-0.619221869	1.153662596	5
COL6A1	-0.549895955	-0.604376098	1.154272053	5
COL6A5	-0.784869454	-0.341039851	1.125909304	5
COL6A6	-0.746302504	-0.389917834	1.136220338	5
COL8A1	-0.617160157	-0.536603337	1.153763494	5
COL9A1	-0.554914732	-0.599498865	1.154413596	5
COLCA2	-0.736520819	-0.401903737	1.138424556	5
COLEC10	-0.560537377	-0.594001514	1.154538891	5
COLEC12	-0.544404086	-0.609681251	1.154085336	5
COMT	-0.59283335	-0.561727523	1.154560873	5

COPS3	-0.597159002	-0.557312341	1.154471343	5
CORO2B	-0.590230721	-0.564373308	1.154604029	5
COX4I2	-0.654675093	-0.496404156	1.151079249	5
COX5B	-0.530656881	-0.622817074	1.153473956	5
COX6A2	-0.535701498	-0.618020533	1.153722031	5
CP	-0.579523106	-0.575174703	1.154697809	5
CPA2	-0.551865723	-0.602465208	1.154330931	5
CPA4	-0.598999758	-0.555426707	1.154426465	5
CPA6	-0.520776253	-0.632133061	1.152909314	5
CPB1	-0.619050924	-0.534620239	1.153671163	5
CPB2	-0.68807976	-0.459022809	1.147102569	5
CPEB1	-0.818510178	-0.296103554	1.114613733	5
CPEB3	-0.513742615	-0.638701914	1.152444528	5
CPLX2	-0.575376035	-0.579322256	1.154698291	5
CPLX4	-0.657645156	-0.493142833	1.150787989	5
CPN2	-0.557192266	-0.597276337	1.154468603	5
CPNE2	-0.60278874	-0.551532533	1.154321273	5
CPNE4	-0.53125757	-0.622247358	1.153504928	5
CPNE5	-0.609215467	-0.544887638	1.154103105	5
CPNE6	-0.627439099	-0.525768469	1.153207568	5
CPNE9	-0.554518382	-0.599885052	1.154403434	5
CPS1	-0.538706283	-0.615150479	1.153856762	5
CPT1C	-0.545994234	-0.608148571	1.154142804	5
CPVL	-0.511909567	-0.640405327	1.152314893	5

CPXM1	-0.554089717	-0.60030253	1.154392246	5
CR1L	-0.592082029	-0.562492126	1.154574155	5
CRABP1	-0.517060056	-0.635610159	1.152670215	5
CRABP2	-0.538740593	-0.615117652	1.153858244	5
CRAMP1	-0.600328646	-0.554062893	1.15439154	5
CRB2	-0.540972156	-0.612979736	1.153951892	5
CRBN	-0.745294156	-0.391160794	1.13645495	5
CRCT1	-0.620951893	-0.532621948	1.153573841	5
CREB3L3	-0.681154851	-0.46690078	1.148055631	5
CREG2	-0.617580269	-0.536163094	1.153743363	5
CRH	-0.56256553	-0.592009866	1.154575395	5
CRHR1	-0.564137999	-0.590462512	1.154600511	5
CRIP2	-0.502568729	-0.649031416	1.151600145	5
CRISP1	-0.688894845	-0.458091	1.146985845	5
CRISPLD1	-0.532085415	-0.621461565	1.15354698	5
CRISPLD2	-0.540799826	-0.613145027	1.153944853	5
CRLF1	-0.517983816	-0.63474719	1.152731006	5
CRMP1	-0.545095784	-0.609014891	1.154110675	5
CRP	-0.576914663	-0.577785766	1.154700429	5
CRPPA	-0.502037906	-0.649518917	1.151556823	5
CRTAC1	-0.584211856	-0.570461391	1.154673247	5
CRX	-0.593825314	-0.560716997	1.15454231	5
CRYAA	-0.633074215	-0.519771914	1.152846129	5
CRYAB	-0.592232845	-0.562338697	1.154571542	5

CRYGB	-0.649661921	-0.501882134	1.151544055	5
CRYGD	-0.59224097	-0.562330431	1.154571401	5
CSAG2	-0.555955848	-0.59848361	1.154439458	5
CSAG3	-0.551732807	-0.602594286	1.154327093	5
CSDC2	-0.681647937	-0.466342104	1.14799004	5
CSGALNACT2	-0.741599654	-0.395700313	1.137299967	5
CSH1	-0.528716838	-0.624654449	1.153371287	5
CSH2	-0.539066844	-0.614805429	1.153872273	5
CSMD2	-0.590313757	-0.564289019	1.154602776	5
CSMD3	-0.567766042	-0.586881752	1.154647795	5
CSN3	-0.559027621	-0.595481098	1.154508719	5
CSNK1E	-0.543772451	-0.61028929	1.154061742	5
CSNKA2IP	-0.628001234	-0.525172093	1.153173327	5
CSPG4	-0.508249301	-0.643796274	1.152045575	5
CSPG5	-0.533582463	-0.620038694	1.153621157	5
CSRNP3	-0.53678657	-0.616985241	1.153771811	5
CSRP2	-0.539915471	-0.613992751	1.153908222	5
CSRP3	-0.588260215	-0.566371164	1.15463138	5
CST1	-0.57625943	-0.578440422	1.154699852	5
CST3	-0.521697216	-0.631269103	1.152966319	5
CST4	-0.533585145	-0.620036143	1.153621288	5
CST6	-0.666090874	-0.483803515	1.149894389	5
CST9L	-0.776043906	-0.352462275	1.128506181	5
CT45A1	-0.767831098	-0.362962322	1.13079342	5

CT45A10	-0.57943674	-0.575261282	1.154698022	5
CT45A3	-0.794377272	-0.328568598	1.12294587	5
CT45A6	-0.831394677	-0.278267479	1.109662156	5
CT55	-0.502802624	-0.648816518	1.151619142	5
CT83	-0.577350269	-0.577350269	1.154700538	5
CTBP1	-0.741876935	-0.395360403	1.137237339	5
CTCF	-0.655235005	-0.495790244	1.151025249	5
CTDSPL	-0.513845981	-0.638605753	1.152451734	5
CTNNA2	-0.53994344	-0.613965954	1.153909394	5
CTNNAL1	-0.554498146	-0.599904764	1.15440291	5
CTNND1	-0.540526839	-0.613406798	1.153933636	5
CTNND2	-0.542519325	-0.611494316	1.154013641	5
CTSG	-0.586258486	-0.568395997	1.154654484	5
CTTNBP2	-0.507717923	-0.644287398	1.152005321	5
CTXN1	-0.53780932	-0.616008246	1.153817566	5
CTXN3	-0.509461798	-0.642674532	1.15213633	5
CUL7	-0.697849445	-0.447790166	1.145639611	5
CUX1	-0.701902109	-0.443089292	1.144991401	5
CWH43	-0.773380722	-0.355880641	1.129261364	5
CXCL13	-0.505390706	-0.64643488	1.151825586	5
CXCL14	-0.522669099	-0.630356406	1.153025505	5
CXCL17	-0.787665849	-0.337389944	1.125055793	5
CXCL3	-0.511930294	-0.640386084	1.152316379	5
CXCL5	-0.744901131	-0.391644801	1.136545932	5

CXorf49	-0.589424517	-0.565191256	1.154615773	5
CXorf49B	-0.505033438	-0.64676406	1.151797498	5
CXorf66	-0.810954242	-0.306397104	1.117351346	5
CXXC4	-0.516966762	-0.635697263	1.152664026	5
CXXC5	-0.757216436	-0.376353669	1.133570106	5
CYB5R3	-0.697294112	-0.44843242	1.145726532	5
CYCS	-0.640389984	-0.511926094	1.152316078	5
CYFIP1	-0.607274445	-0.54689985	1.154174295	5
CYLC2	-0.82101552	-0.292663725	1.113679245	5
CYP11B1	-0.542673873	-0.611345792	1.154019666	5
CYP11B2	-0.521780395	-0.631191029	1.152971424	5
CYP21A2	-0.596888903	-0.557588684	1.154477586	5
CYP26A1	-0.524310616	-0.628812584	1.153123199	5
CYP26C1	-0.729161201	-0.41081798	1.13997918	5
CYP27C1	-0.562605958	-0.591970118	1.154576076	5
CYP2A13	-0.572350363	-0.582335784	1.154686147	5
CYP2A7	-0.752844277	-0.381812075	1.134656351	5
CYP2B6	-0.809858818	-0.307879486	1.117738304	5
CYP2C19	-0.68833682	-0.458729041	1.147065861	5
CYP2F1	-0.606694508	-0.547500165	1.154194673	5
CYP2W1	-0.518984589	-0.633811264	1.152795853	5
CYP39A1	-0.563499852	-0.591090803	1.154590655	5
CYP3A43	-0.782174073	-0.344543842	1.126717915	5
CYP46A1	-0.559327041	-0.595187865	1.154514906	5

CYP4F11	-0.7524458	-0.382307909	1.134753709	5
CYP4F3	-0.590175925	-0.564428927	1.154604852	5
CYP8B1	-0.784118123	-0.342017962	1.126136085	5
CYSRT1	-0.55313464	-0.601231946	1.154366587	5
DAAM1	-0.520370252	-0.632513648	1.1528839	5
DAB1	-0.538362154	-0.615479674	1.153841827	5
DAB2	-0.52603867	-0.627184287	1.153222957	5
DACH1	-0.550712137	-0.603584832	1.154296969	5
DACT2	-0.594810723	-0.559711989	1.154522712	5
DACT3	-0.555051932	-0.599365141	1.154417074	5
DAG1	-0.527231966	-0.626058025	1.153289991	5
DAGLA	-0.768144996	-0.362563255	1.130708252	5
DAO	-0.757749492	-0.375685908	1.1334354	5
DAOA	-0.50524437	-0.646569727	1.151814097	5
DAP	-0.668109158	-0.481557225	1.149666383	5
DAPL1	-0.559050854	-0.595458348	1.154509202	5
DAPP1	-0.718240214	-0.423885798	1.142126011	5
DAW1	-0.560361419	-0.594174087	1.154535506	5
DAZ4	-0.742663856	-0.394395046	1.137058901	5
DBH	-0.725860178	-0.414787888	1.140648066	5
DBI	-0.760807875	-0.371845046	1.132652921	5
DBN1	-0.677857518	-0.470627841	1.148485359	5
DBX2	-0.510815706	-0.641420161	1.152235867	5
DCAF8L1	-0.640599336	-0.511700553	1.152299889	5

DCAF8L2	-0.594646562	-0.559879495	1.154526057	5
DCBLD2	-0.523262149	-0.62979898	1.153061129	5
DCDC2C	-0.832838346	-0.276246127	1.109084472	5
DCHS2	-0.543754357	-0.610306702	1.154061059	5
DCLK1	-0.537383216	-0.616415425	1.153798641	5
DCLK2	-0.539370321	-0.614514898	1.153885219	5
DCST2	-0.671797835	-0.477437268	1.149235104	5
DCSTAMP	-0.553031763	-0.601331999	1.154363762	5
DCT	-0.561066733	-0.593482132	1.154548865	5
DCTD	-0.562187375	-0.592381565	1.15456894	5
DCTN4	-0.529774357	-0.623653394	1.15342775	5
DCX	-0.553730011	-0.60065269	1.154382701	5
DDAH1	-0.535715469	-0.618007211	1.15372268	5
DDIT3	-0.585313366	-0.569350393	1.154663758	5
DDIT4L	-0.553110768	-0.601255164	1.154365932	5
DDN	-0.527421239	-0.625879245	1.153300484	5
DDX11	-0.790502578	-0.333672065	1.124174643	5
DDX19A	-0.575523991	-0.579174623	1.154698615	5
DDX25	-0.604930508	-0.549323635	1.154254142	5
DDX39B	-0.505989035	-0.645883295	1.15187233	5
DDX3X	-0.520553425	-0.632341963	1.152895387	5
DDX4	-0.551879152	-0.602452165	1.154331317	5
DEFB104A	-0.65362906	-0.497549946	1.151179006	5
DEFB104B	-0.644774937	-0.507190111	1.151965048	5

DEFB110	-0.614860373	-0.53900944	1.153869813	5
DEFB118	-0.562699117	-0.59187852	1.154577637	5
DEFB119	-0.582614336	-0.572070154	1.154684491	5
DEFB128	-0.55001938	-0.604256487	1.154275868	5
DEFB129	-0.565802161	-0.588821889	1.15462405	5
DEFB132	-0.61570836	-0.538123013	1.153831373	5
DEFB134	-0.673926735	-0.475050836	1.148977571	5
DEFB135	-0.688682504	-0.458333842	1.147016346	5
DENND2A	-0.511180509	-0.641081852	1.15226236	5
DENND2B	-0.552228679	-0.602112633	1.154341312	5
DENND2C	-0.508180242	-0.643860118	1.15204036	5
DENND4C	-0.60425767	-0.55001816	1.15427583	5
DENND6B	-0.724594876	-0.416304963	1.140899839	5
DEPDC1	-0.515353233	-0.637202305	1.152555538	5
DEPDC1B	-0.507488553	-0.644499302	1.151987855	5
DEPDC4	-0.653566748	-0.497618155	1.151184902	5
DES	-0.624293559	-0.529098229	1.153391788	5
DGCR2	-0.619673822	-0.533965949	1.153639771	5
DGCR6	-0.613115243	-0.540830881	1.153946124	5
DGKB	-0.542657189	-0.611361828	1.154019017	5
DGKI	-0.533634544	-0.61998915	1.153623694	5
DHCR24	-0.515855636	-0.636733974	1.15258961	5
DHCR7	-0.72280939	-0.418441405	1.141250795	5
DHRS7C	-0.592920508	-0.561638781	1.154559289	5

DIAPH3	-0.514524822	-0.637973958	1.15249878	5
DIPK1B	-0.615367727	-0.538479194	1.153846921	5
DIPK1C	-0.580472718	-0.574222181	1.154694899	5
DIPK2A	-0.526429561	-0.626815521	1.153245082	5
DIRAS1	-0.519967356	-0.632891152	1.152858508	5
DIRAS2	-0.558516668	-0.595981261	1.154497929	5
DISP3	-0.580947861	-0.573745189	1.15469305	5
DKK3	-0.55396837	-0.600420673	1.154389042	5
DKKL1	-0.541458064	-0.612513502	1.153971566	5
DLAT	-0.555023607	-0.599392751	1.154416357	5
DLG5	-0.537059131	-0.616724984	1.153784115	5
DLGAP2	-0.580917935	-0.57377524	1.154693175	5
DLGAP3	-0.626713772	-0.526537385	1.153251157	5
DLK1	-0.526728494	-0.626533399	1.153261893	5
DLL1	-0.539174173	-0.61470269	1.153876863	5
DLL3	-0.54618801	-0.607961608	1.154149618	5
DLX1	-0.541916915	-0.612072993	1.153989908	5
DLX2	-0.541976901	-0.612015388	1.153992289	5
DLX3	-0.60183268	-0.552516768	1.154349448	5
DLX5	-0.626995099	-0.52623923	1.153234329	5
DLX6	-0.568787787	-0.58587063	1.154658417	5
DMBX1	-0.523327697	-0.629737347	1.153065044	5
DMC1	-0.503236378	-0.648417844	1.151654222	5
DMD	-0.548163827	-0.606052913	1.154216741	5

DMGDH	-0.514520843	-0.637977662	1.152498505	5
DMRT1	-0.616534491	-0.537258577	1.153793068	5
DMRT3	-0.548159811	-0.606056798	1.154216609	5
DMRTA2	-0.563232053	-0.591354329	1.154586382	5
DMRTC2	-0.710919047	-0.43254148	1.143460527	5
DMTN	-0.607249523	-0.546925656	1.154175179	5
DNAAF6	-0.602567435	-0.551760457	1.154327893	5
DNAAF8	-0.659902683	-0.490655957	1.150558639	5
DNAH10	-0.568998699	-0.585661762	1.154660461	5
DNAH11	-0.571758597	-0.582923947	1.154682544	5
DNAH12	-0.525076563	-0.628091244	1.153167807	5
DNAH14	-0.510565316	-0.641652286	1.152217602	5
DNAH2	-0.542867405	-0.611159768	1.154027174	5
DNAH3	-0.719004223	-0.422977725	1.141981949	5
DNAH5	-0.622067073	-0.531447572	1.153514645	5
DNAH7	-0.57265554	-0.582032308	1.154687848	5
DNAH9	-0.567094158	-0.587546006	1.154640164	5
DNAI1	-0.552689333	-0.601664944	1.154354277	5
DNAI3	-0.5567822	-0.597676926	1.154459126	5
DNAJB3	-0.754313474	-0.379981551	1.134295025	5
DNAJB5	-0.561500087	-0.593056708	1.154556795	5
DNAJC12	-0.510539732	-0.641676	1.152215732	5
DNAJC22	-0.520466938	-0.63242303	1.152889968	5
DNAJC28	-0.718657185	-0.423390314	1.1420475	5

DNALI1	-0.544310445	-0.609771421	1.154081866	5
DNER	-0.554907893	-0.599505529	1.154413423	5
DNM1	-0.541233164	-0.612729328	1.153962492	5
DNMT3B	-0.502437653	-0.649151821	1.151589474	5
DOC2A	-0.531342923	-0.622166375	1.153509298	5
DOC2B	-0.515106115	-0.637432567	1.152538682	5
DOCK4	-0.543314589	-0.610729776	1.154044365	5
DOK4	-0.631191916	-0.52177945	1.152971366	5
DOK5	-0.537848805	-0.615970505	1.15381931	5
DOK6	-0.52794982	-0.625379767	1.153329587	5
DOP1B	-0.636647161	-0.515948734	1.152595895	5
DOT1L	-0.524066169	-0.629042663	1.153108832	5
DPF1	-0.555386373	-0.599039088	1.154425462	5
DPF3	-0.588365895	-0.566264134	1.154630029	5
DPH7	-0.567989713	-0.586660508	1.154650221	5
DPP10	-0.548379781	-0.605844036	1.154223817	5
DPP6	-0.589798454	-0.564811968	1.154610421	5
DPPA2	-0.732274368	-0.407057932	1.1393323	5
DPPA5	-0.587003068	-0.567643371	1.15464644	5
DPY19L1	-0.699937052	-0.445371711	1.145308763	5
DPY19L2	-0.508166345	-0.643872964	1.15203931	5
DPYSL3	-0.513457424	-0.638967165	1.15242459	5
DPYSL4	-0.599024589	-0.555401243	1.154425832	5
DPYSL5	-0.550185785	-0.6040952	1.154280985	5

DRAXIN	-0.517086627	-0.635585349	1.152671976	5
DRC1	-0.520616795	-0.632282558	1.152899353	5
DRD1	-0.567970744	-0.586679274	1.154650018	5
DRD2	-0.576408593	-0.578291434	1.154700027	5
DRD4	-0.614691406	-0.53918596	1.153877366	5
DRGX	-0.55567314	-0.598759415	1.154432555	5
DSCAM	-0.833063741	-0.275930116	1.108993857	5
DSCAML1	-0.569196918	-0.585465419	1.154662337	5
DSG2	-0.541115043	-0.612842661	1.153957704	5
DSG4	-0.527217986	-0.626071228	1.153289214	5
DSPP	-0.800689506	-0.320191323	1.120880829	5
DTHD1	-0.599414515	-0.555001277	1.154415792	5
DTNA	-0.544307365	-0.609774386	1.154081752	5
DTX1	-0.771896581	-0.357779988	1.129676568	5
DUOX2	-0.540262168	-0.613660516	1.153922684	5
DUOXA1	-0.656106689	-0.494833653	1.150940341	5
DUOXA2	-0.596516103	-0.557969958	1.154486061	5
DUSP15	-0.506116198	-0.645766019	1.151882217	5
DUSP26	-0.6294893	-0.52359145	1.15308075	5
DUSP4	-0.52542955	-0.627758605	1.153188155	5
DUSP9	-0.507471553	-0.644515005	1.151986558	5
DYDC2	-0.571332454	-0.583347249	1.154679702	5
DYNC1I1	-0.513327787	-0.639087711	1.152415498	5
DYNC2I2	-0.808172998	-0.31015597	1.118328968	5

DYNLRB2	-0.539636843	-0.614259663	1.153896505	5
DYTN	-0.523917856	-0.629182228	1.153100084	5
DZIP1	-0.541622163	-0.612355989	1.153978152	5
E2F7	-0.527818704	-0.625503691	1.153322395	5
EBF2	-0.559491143	-0.595027111	1.154518254	5
EBF4	-0.537165815	-0.616623095	1.153788909	5
EBLN1	-0.620302908	-0.533304666	1.153607574	5
ECE1	-0.557485416	-0.596989848	1.154475263	5
ECEL1	-0.541283324	-0.612681197	1.153964521	5
ECM1	-0.520944805	-0.63197501	1.152919814	5
ECM2	-0.823794586	-0.288832227	1.112626813	5
EDA2R	-0.538298538	-0.615540514	1.153839052	5
EDDM3B	-0.779423542	-0.34810542	1.127528962	5
EDIL3	-0.541609757	-0.612367898	1.153977655	5
EDN2	-0.743834503	-0.392957035	1.136791538	5
EDNRA	-0.539816483	-0.614087586	1.153904069	5
EDNRB	-0.53300486	-0.620587963	1.153592822	5
EEA1	-0.53995174	-0.613958001	1.153909741	5
EEF1A2	-0.545073089	-0.609036763	1.154109852	5
EEF1AKMT4-ECE2	-0.52292056	-0.630120096	1.153040655	5
EFCAB1	-0.570626802	-0.584047738	1.15467454	5
EFCAB12	-0.626090294	-0.527197798	1.153288093	5
EFCAB13	-0.516201622	-0.636411299	1.15261292	5
EFCAB5	-0.554407288	-0.599993267	1.154400554	5

EFCAB6	-0.603627556	-0.550668087	1.154295643	5
EFHC2	-0.509362102	-0.642766823	1.152128925	5
EFNA2	-0.573293453	-0.581397605	1.154691059	5
EFNA3	-0.67025448	-0.479163371	1.149417851	5
EFNA5	-0.555506165	-0.598922271	1.154428436	5
EFNB2	-0.510421965	-0.641785151	1.152207116	5
EFNB3	-0.550071475	-0.604205998	1.154277473	5
EFR3A	-0.58559378	-0.569067335	1.154661116	5
EFR3B	-0.554923248	-0.599490565	1.154413813	5
EFS	-0.541054952	-0.61290031	1.153955263	5
EGF	-0.50992272	-0.64224771	1.15217043	5
EGFR	-0.513967392	-0.638492791	1.152460184	5
EHD3	-0.544555351	-0.609535572	1.154090922	5
EHMT2	-0.67073181	-0.478629874	1.149361685	5
EIF1	-0.669719086	-0.479761387	1.149480473	5
EIF1AY	-0.550749195	-0.603548888	1.154298083	5
EIF2B2	-0.651102879	-0.500311007	1.151413886	5
EIF3D	-0.739470349	-0.398306297	1.137776646	5
EIF4A3	-0.596736794	-0.55774427	1.154481064	5
EIF4E1B	-0.574118914	-0.580575607	1.154694521	5
EIF4E3	-0.655040384	-0.496003682	1.151044066	5
EIF4G3	-0.502484741	-0.649108568	1.15159331	5
ELANE	-0.756794228	-0.376882221	1.133676448	5
ELAVL2	-0.540743017	-0.613199508	1.153942526	5

ELAVL3	-0.548813209	-0.605424655	1.154237865	5
ELAVL4	-0.544076957	-0.609996214	1.154073171	5
ELF1	-0.610952473	-0.543083017	1.15403549	5
ELFN1	-0.53513263	-0.618562796	1.153695426	5
ELFN2	-0.533059702	-0.620535827	1.153595528	5
ELMOD1	-0.550858495	-0.603442864	1.154301359	5
ELOA2	-0.636704697	-0.515887033	1.152591731	5
ELOVL2	-0.5410541	-0.612901128	1.153955228	5
ELOVL4	-0.539668381	-0.614229454	1.153897836	5
ELOVL7	-0.517544193	-0.635157994	1.152702187	5
EMC7	-0.532111523	-0.621436771	1.153548294	5
EMILIN1	-0.692932626	-0.453460734	1.146393361	5
EMILIN2	-0.540734298	-0.61320787	1.153942168	5
EMILIN3	-0.543421373	-0.610627065	1.154048438	5
EML1	-0.504383138	-0.647362897	1.151746035	5
EML5	-0.502581545	-0.649019643	1.151601187	5
EMP2	-0.817216225	-0.297874889	1.115091114	5
EMX1	-0.610991479	-0.543042451	1.154033929	5
EMX2	-0.558696426	-0.595805332	1.154501758	5
EN1	-0.575990186	-0.578709285	1.154699471	5
EN2	-0.558459639	-0.596037068	1.154496707	5
ENKUR	-0.51260313	-0.639761225	1.152364355	5
ENO4	-0.752950472	-0.381679887	1.134630359	5
ENOX1	-0.635289473	-0.517403444	1.152692917	5

ENPP1	-0.566289791	-0.588340563	1.154630354	5
ENPP6	-0.578144515	-0.576555659	1.154700174	5
ENPP7	-0.612068748	-0.541921336	1.153990084	5
ENTPD3	-0.534712342	-0.618963204	1.153675545	5
ENTPD8	-0.667279641	-0.482481133	1.149760774	5
EPB41L1	-0.537236686	-0.616555401	1.153792087	5
EPB41L4B	-0.528107668	-0.625230552	1.153338219	5
EPCAM	-0.522217603	-0.630780531	1.152998134	5
EPHA10	-0.576052495	-0.578647072	1.154699567	5
EPHA3	-0.536738356	-0.61703127	1.153769626	5
EPHA5	-0.562055994	-0.592510665	1.154566659	5
EPHA6	-0.596027782	-0.558469129	1.15449691	5
EPHA7	-0.51562559	-0.636948452	1.152574041	5
EPHA8	-0.504999253	-0.64679555	1.151794804	5
EPHB3	-0.602035277	-0.552308292	1.154343569	5
EPO	-0.536033854	-0.617703559	1.153737414	5
EPS15L1	-0.759862791	-0.373033679	1.13289647	5
EPS8	-0.570771535	-0.58390411	1.154675645	5
EPS8L3	-0.532283961	-0.621272995	1.153556956	5
EPYC	-0.682488604	-0.465388811	1.147877415	5
EQTN	-0.52024587	-0.632630209	1.152876079	5
ERBB2	-0.626277641	-0.526999404	1.153277046	5
ERBB3	-0.660548246	-0.489943538	1.150491784	5
ERBB4	-0.530057382	-0.623385277	1.153442659	5

ERC1	-0.531840904	-0.621693732	1.153534636	5
ERFL	-0.573174883	-0.581515614	1.154690497	5
ERICH2	-0.580254011	-0.574441652	1.154695662	5
ERICH5	-0.609775883	-0.544305811	1.154081694	5
ERICH6	-0.623792327	-0.529627662	1.153419989	5
ERICH6B	-0.620141264	-0.53347463	1.153615894	5
ERN2	-0.597587368	-0.556873894	1.154461262	5
ERO1A	-0.511918514	-0.640397021	1.152315535	5
ERVMER34-1	-0.564006936	-0.590591588	1.154598524	5
ERVW-1	-0.60715057	-0.547028112	1.154178682	5
ESCO2	-0.504099802	-0.647623674	1.151723476	5
ESPL1	-0.548799478	-0.605437944	1.154237423	5
ESRP1	-0.524273708	-0.628847326	1.153121034	5
ESRRB	-0.608804615	-0.545313942	1.154118558	5
ESRRG	-0.564848488	-0.589762455	1.154610944	5
ESX1	-0.642016992	-0.510171771	1.152188763	5
ESYT3	-0.820744511	-0.29303647	1.113780981	5
ETNK2	-0.604512782	-0.549754889	1.154267672	5
ETNPPL	-0.581591335	-0.573098793	1.154690128	5
ETV1	-0.543782592	-0.610279532	1.154062124	5
ETV3L	-0.531956259	-0.621584209	1.153540468	5
ETV4	-0.58526725	-0.569396934	1.154664184	5
ETV5	-0.531447227	-0.6220674	1.153514627	5
EVA1A	-0.555537546	-0.598891666	1.154429213	5

EVC	-0.556526208	-0.597926906	1.154453115	5
EVC2	-0.80614589	-0.312885576	1.119031466	5
EVPL	-0.504632096	-0.647133693	1.151765789	5
EVX1	-0.549054637	-0.605190963	1.1542456	5
EXOC1L	-0.808884712	-0.309195607	1.118080319	5
EXOC3L1	-0.579286266	-0.575412106	1.154698372	5
EXOC3L2	-0.508460841	-0.643600677	1.152061518	5
EXOC3L4	-0.623502451	-0.529933704	1.153436155	5
EXTL1	-0.572385061	-0.582301284	1.154686345	5
EYA4	-0.555354376	-0.599070289	1.154424665	5
EZH2	-0.509337128	-0.642789941	1.152127069	5
EZHIP	-0.713196347	-0.429857946	1.143054293	5
F13B	-0.528935891	-0.62444719	1.153383081	5
F2	-0.642015848	-0.510173006	1.152188854	5
F2R	-0.74967796	-0.385744491	1.135422451	5
F2RL3	-0.624508786	-0.528870795	1.153379581	5
F3	-0.547608563	-0.606589747	1.154198311	5
F5	-0.632966845	-0.519886549	1.152853394	5
FABP3	-0.517070878	-0.635600055	1.152670932	5
FABP6	-0.584071308	-0.570603048	1.154674356	5
FABP7	-0.571195666	-0.58348308	1.154678746	5
FADS6	-0.707076292	-0.437051721	1.144128013	5
FAIM	-0.77940009	-0.348135726	1.127535816	5
FAIM2	-0.552826335	-0.601531752	1.154358087	5

FAM104B	-0.51865547	-0.634119173	1.152774643	5
FAM110B	-0.547061756	-0.607118074	1.15417983	5
FAM110D	-0.772197159	-0.357395644	1.129592803	5
FAM131A	-0.608582935	-0.545543875	1.154126809	5
FAM131B	-0.513754636	-0.638690731	1.152445367	5
FAM131C	-0.585818234	-0.568840701	1.154658934	5
FAM133A	-0.521976209	-0.631007203	1.152983412	5
FAM135B	-0.577936215	-0.576764125	1.15470034	5
FAM13C	-0.507767694	-0.64424141	1.152009104	5
FAM161A	-0.614283046	-0.539612429	1.153895475	5
FAM162B	-0.503324139	-0.648337157	1.151661296	5
FAM163A	-0.565407403	-0.589211346	1.15461875	5
FAM163B	-0.7487789	-0.38685796	1.13563686	5
FAM166A	-0.819493893	-0.294754523	1.114248415	5
FAM169A	-0.516564155	-0.636073057	1.152637211	5
FAM171A1	-0.591530481	-0.563052999	1.154583479	5
FAM171A2	-0.558791761	-0.595712013	1.154503774	5
FAM171B	-0.538465067	-0.61538124	1.153846307	5
FAM178B	-0.610073258	-0.543996919	1.154070177	5
FAM181A	-0.514071607	-0.638395817	1.152467424	5
FAM181B	-0.524187479	-0.628928491	1.15311597	5
FAM183A	-0.625885776	-0.527414325	1.153300102	5
FAM185A	-0.514276154	-0.638205448	1.152481602	5
FAM186A	-0.545000508	-0.609106708	1.154107216	5

FAM186B	-0.814880534	-0.301063287	1.115943821	5
FAM187A	-0.654728805	-0.496345281	1.151074087	5
FAM187B	-0.7507696	-0.384390664	1.135160264	5
FAM189A2	-0.551397805	-0.602919527	1.154317332	5
FAM189B	-0.68238129	-0.465510559	1.147891848	5
FAM20B	-0.777408136	-0.350706131	1.128114267	5
FAM216B	-0.551705089	-0.602621201	1.15432629	5
FAM219B	-0.674130372	-0.474822233	1.148952605	5
FAM221B	-0.790198155	-0.334071792	1.124269947	5
FAM222A	-0.647001517	-0.504775631	1.151777148	5
FAM228B	-0.612010007	-0.541982505	1.153992511	5
FAM229B	-0.511109225	-0.64114797	1.152257194	5
FAM3A	-0.743998943	-0.392754855	1.136753798	5
FAM43B	-0.558694379	-0.595807336	1.154501715	5
FAM47A	-0.581838438	-0.57285044	1.154688878	5
FAM47C	-0.520074248	-0.632791013	1.152865261	5
FAM47E	-0.550646403	-0.603648587	1.15429499	5
FAM71B	-0.607905907	-0.546245732	1.15415164	5
FAM71C	-0.650765292	-0.500679337	1.15144463	5
FAM71F1	-0.540408804	-0.613519957	1.153928761	5
FAM72A	-0.595926057	-0.558573077	1.154499135	5
FAM72C	-0.565048576	-0.589565203	1.154613779	5
FAM72D	-0.649752745	-0.501783187	1.151535932	5
FAM81B	-0.517985229	-0.634745869	1.152731098	5

FAM83D	-0.502487268	-0.649106248	1.151593515	5
FAM9A	-0.545974882	-0.60816724	1.154142122	5
FANK1	-0.515992368	-0.636606469	1.152598837	5
FAP	-0.56097668	-0.59357051	1.15454719	5
FAT1	-0.548198019	-0.606019845	1.154217865	5
FAT2	-0.516420667	-0.636206946	1.152627614	5
FAT3	-0.524522673	-0.628612939	1.153135612	5
FAT4	-0.536141165	-0.61760119	1.153742355	5
FAXC	-0.576975913	-0.577724545	1.154700457	5
FBLIM1	-0.514645606	-0.637861494	1.1525071	5
FBLN1	-0.522579373	-0.63044071	1.153020083	5
FBN3	-0.543198811	-0.610841124	1.154039935	5
FBP2	-0.511174966	-0.641086993	1.152261959	5
FBRSL1	-0.654484564	-0.496612964	1.151097528	5
FBXL2	-0.578161285	-0.576538874	1.154700158	5
FBXL4	-0.765116206	-0.366406445	1.131522651	5
FBXO10	-0.666837558	-0.482973133	1.149810691	5
FBXO16	-0.540616717	-0.613320621	1.153937338	5
FBXO17	-0.53623732	-0.617509452	1.153746772	5
FBXO27	-0.648796451	-0.502824461	1.151620912	5
FBXO36	-0.562832596	-0.591747261	1.154579858	5
FBXO43	-0.537861782	-0.6159581	1.153819883	5
FBXO47	-0.622591661	-0.530894598	1.153486259	5
FCAMR	-0.807202816	-0.311463422	1.118666238	5

FCMR	-0.507332973	-0.644643004	1.151975977	5
FER	-0.506162652	-0.645723173	1.151885825	5
FER1L5	-0.716588888	-0.425845387	1.142434275	5
FERD3L	-0.520936792	-0.631982524	1.152919316	5
FERMT1	-0.56494179	-0.589670481	1.154612271	5
FERMT3	-0.781521543	-0.345390068	1.126911611	5
FEV	-0.627248858	-0.525970206	1.153219065	5
FEZ1	-0.507756446	-0.644251803	1.152008249	5
FEZF1	-0.574774191	-0.579922522	1.154696713	5
FEZF2	-0.558021564	-0.596465632	1.154487195	5
FGD1	-0.525776878	-0.62743117	1.153208048	5
FGF10	-0.599712576	-0.554695419	1.154407995	5
FGF14	-0.63273429	-0.520134791	1.152869081	5
FGF16	-0.66207877	-0.488252246	1.150331016	5
FGF17	-0.832295604	-0.277006597	1.109302201	5
FGF19	-0.546694277	-0.607472948	1.154167225	5
FGF2	-0.505053883	-0.646745226	1.151799109	5
FGF23	-0.625532399	-0.527788328	1.153320727	5
FGF4	-0.658335562	-0.492383018	1.15071858	5
FGF5	-0.718575476	-0.42348743	1.142062906	5
FGF7	-0.715060978	-0.427654743	1.142715721	5
FGF9	-0.623198537	-0.530254453	1.15345299	5
FGFBP3	-0.561197205	-0.593354069	1.154551274	5
FGFR1	-0.634346931	-0.518411945	1.152758876	5

FGFR2	-0.536396702	-0.61735737	1.153754071	5
FGFR3	-0.531431795	-0.622082045	1.153513839	5
FGFR4	-0.6465505	-0.505265236	1.151815737	5
FHAD1	-0.624043748	-0.529362134	1.153405883	5
FHL1	-0.532961425	-0.620629252	1.153590677	5
FHOD1	-0.77575527	-0.352833389	1.128588659	5
FHOD3	-0.548899644	-0.605340998	1.154240641	5
FIBCD1	-0.656882913	-0.493980958	1.150863872	5
FIGN	-0.532657796	-0.620917828	1.153575625	5
FIGNL2	-0.542881256	-0.611146453	1.15402771	5
FKBP10	-0.527612479	-0.625698569	1.153311048	5
FKBP1A	-0.530486043	-0.622979033	1.153465076	5
FKBP1B	-0.658118579	-0.492621884	1.150740464	5
FKBP6	-0.551377599	-0.602939141	1.15431674	5
FKBP7	-0.543161544	-0.610876962	1.154038506	5
FLRT1	-0.575433228	-0.579265191	1.154698419	5
FLRT2	-0.564162231	-0.590438645	1.154600876	5
FLRT3	-0.53284215	-0.620742626	1.153584776	5
FLT4	-0.622135055	-0.531375931	1.153510986	5
FMN2	-0.550703543	-0.603593167	1.154296711	5
FMO3	-0.505059963	-0.646739625	1.151799588	5
FN3K	-0.507114229	-0.644845006	1.151959234	5
FNBP1L	-0.515463946	-0.637099123	1.152563069	5
FNDC11	-0.680691312	-0.467425663	1.148116975	5

FNDC4	-0.533198523	-0.62040384	1.153602363	5
FNDC5	-0.548888462	-0.605351821	1.154240282	5
FNIP2	-0.555362728	-0.599062145	1.154424873	5
FOLH1	-0.530058819	-0.623383916	1.153442735	5
FOLH1B	-0.566595653	-0.588038518	1.154634171	5
FOLR1	-0.549219002	-0.605031827	1.154250829	5
FOS	-0.627679549	-0.525513422	1.153192971	5
FOSB	-0.576137735	-0.578561955	1.15469969	5
FOXA1	-0.570797686	-0.583878157	1.154675842	5
FOXA2	-0.584615975	-0.570053956	1.154669931	5
FOXB2	-0.599798541	-0.554607185	1.154405726	5
FOXC1	-0.765893392	-0.365421852	1.131315244	5
FOXD3	-0.606167594	-0.548045239	1.154212833	5
FOXE3	-0.619814587	-0.533818022	1.153632609	5
FOXF2	-0.555233738	-0.599187911	1.154421649	5
FOXG1	-0.576859691	-0.577840708	1.154700399	5
FOXI3	-0.554445085	-0.59995645	1.154401536	5
FOXJ1	-0.550195079	-0.604086191	1.15428127	5
FOXL1	-0.553231634	-0.601137605	1.154369239	5
FOXN1	-0.713461139	-0.429545401	1.14300654	5
FOXN4	-0.564506714	-0.590099282	1.154605996	5
FOXO6	-0.530924829	-0.622562991	1.153487819	5
FOXR1	-0.647061829	-0.504710138	1.151771968	5
FOXR2	-0.511799617	-0.640507389	1.152307006	5

FOXRED2	-0.798030105	-0.323730383	1.121760487	5
FOXS1	-0.52442853	-0.628701577	1.153130107	5
FREM1	-0.534619227	-0.619051888	1.153671115	5
FREM2	-0.543328797	-0.610716111	1.154044908	5
FREM3	-0.586823903	-0.567824532	1.154648435	5
FRG2	-0.755720101	-0.378225492	1.133945592	5
FRMD3	-0.526369259	-0.626872421	1.15324168	5
FRMD4B	-0.643750685	-0.508298611	1.152049295	5
FRMPD2	-0.65623802	-0.49468944	1.15092746	5
FRMPD3	-0.515939193	-0.636656058	1.152595251	5
FRMPD4	-0.599292505	-0.555126449	1.154418953	5
FRRS1L	-0.581460787	-0.573229973	1.15469076	5
FRS3	-0.784672369	-0.341296526	1.125968896	5
FRZB	-0.518310244	-0.634442029	1.152752273	5
FSCB	-0.664074634	-0.486041933	1.150116568	5
FSD1	-0.640569312	-0.511732902	1.152302214	5
FSIP2	-0.558904066	-0.59560207	1.154506136	5
FSTL5	-0.548277187	-0.605943275	1.154220462	5
FTMT	-0.605013896	-0.54923752	1.154251416	5
FUT2	-0.77444745	-0.354512994	1.128960444	5
FUT5	-0.517238926	-0.63544313	1.152682056	5
FUT9	-0.560922501	-0.593623677	1.154546178	5
FXYD3	-0.596880066	-0.557597723	1.154477789	5
FXYD6	-0.608628177	-0.545496954	1.15412513	5

FYB1	-0.642205933	-0.509967821	1.152173754	5
FYB2	-0.560107081	-0.594423471	1.154530552	5
FZD10	-0.504683968	-0.647085928	1.151769896	5
FZD3	-0.541858657	-0.612128935	1.153987592	5
G3BP2	-0.559763739	-0.59476001	1.154523749	5
G6PC1	-0.622789375	-0.530686096	1.153475471	5
GAB1	-0.522516291	-0.630499975	1.153016265	5
GAB4	-0.71493967	-0.427798241	1.142737911	5
GABARAPL1	-0.773530113	-0.355689234	1.129219347	5
GABBR2	-0.589473791	-0.565141286	1.154615077	5
GABRA1	-0.58212963	-0.572557684	1.154687314	5
GABRA2	-0.542904678	-0.611123937	1.154028615	5
GABRA3	-0.545205357	-0.608909283	1.15411464	5
GABRA4	-0.535309499	-0.618394236	1.153703735	5
GABRA5	-0.62877086	-0.524354937	1.153125798	5
GABRA6	-0.599896116	-0.554507024	1.15440314	5
GABRB2	-0.547722552	-0.60647957	1.154202122	5
GABRB3	-0.544499925	-0.609588954	1.154088878	5
GABRD	-0.579172919	-0.575525699	1.154698618	5
GABRE	-0.561342016	-0.593211911	1.154553927	5
GABRG1	-0.568827358	-0.585831446	1.154658804	5
GABRG2	-0.575736317	-0.578962719	1.154699036	5
GABRG3	-0.737385985	-0.40085001	1.138235995	5
GABRQ	-0.547560561	-0.60663614	1.154196701	5

GAD1	-0.54819478	-0.606022978	1.154217758	5
GAD2	-0.550344725	-0.603941118	1.154285844	5
GADD45A	-0.692856065	-0.453548751	1.146404816	5
GADD45B	-0.770696363	-0.359313041	1.130009404	5
GADL1	-0.760020512	-0.372835422	1.132855935	5
GAGE1	-0.621840622	-0.531686169	1.153526791	5
GAGE12F	-0.756065775	-0.377793421	1.133859196	5
GAL3ST1	-0.562337329	-0.59223419	1.154571519	5
GAL3ST2	-0.650521856	-0.500944849	1.151466705	5
GAL3ST3	-0.536253486	-0.617494028	1.153747513	5
GALNT10	-0.654146278	-0.496983585	1.151129863	5
GALNT13	-0.555852073	-0.598584862	1.154436935	5
GALNT15	-0.504847519	-0.646935311	1.15178283	5
GALNT16	-0.540934998	-0.613015379	1.153950377	5
GALNT17	-0.570798308	-0.583877539	1.154675847	5
GALNT5	-0.527182887	-0.626104377	1.153287264	5
GALNT8	-0.542020112	-0.61197389	1.153994002	5
GALNT9	-0.576837552	-0.577862835	1.154700387	5
GALNTL6	-0.528662479	-0.624705873	1.153368352	5
GAP43	-0.550076307	-0.604201315	1.154277622	5
GAREM1	-0.556384017	-0.598065727	1.154449744	5
GAREM2	-0.698297923	-0.447271158	1.145569082	5
GARNL3	-0.508312335	-0.643737995	1.15205033	5
GAS2L3	-0.507029265	-0.644923453	1.151952718	5

GAS6	-0.699275197	-0.446139159	1.145414357	5
GASK1A	-0.546154664	-0.607993784	1.154148448	5
GAST	-0.507479931	-0.644507266	1.151987197	5
GATA4	-0.749001701	-0.386582153	1.135583854	5
GBA	-0.69595952	-0.449974035	1.145933556	5
GBX2	-0.527262544	-0.626029145	1.153291689	5
GC	-0.504260566	-0.64747572	1.151736286	5
GCG	-0.561406048	-0.593149044	1.154555092	5
GCK	-0.586816086	-0.567832435	1.154648521	5
GCM2	-0.528927375	-0.624455248	1.153382623	5
GCSH	-0.733036512	-0.406135032	1.139171544	5
GDA	-0.57003023	-0.584639502	1.154669732	5
GDAP1L1	-0.550322481	-0.603962685	1.154285165	5
GDF1	-0.698702818	-0.446802332	1.14550515	5
GDF10	-0.653010335	-0.498226987	1.151237322	5
GDF5	-0.552277199	-0.602065489	1.154342688	5
GDF6	-0.514280163	-0.638201717	1.15248188	5
GDI1	-0.506046736	-0.645830082	1.151876819	5
GDNF	-0.615097333	-0.538761828	1.153859161	5
GDPD2	-0.542019176	-0.611974789	1.153993965	5
GDPD4	-0.764842354	-0.366753122	1.131595476	5
GEM	-0.538761107	-0.615098023	1.15385913	5
GET1-SH3BGR	-0.554653512	-0.599753406	1.154406918	5
GFAP	-0.619437595	-0.534214138	1.153651733	5

GFPT2	-0.520866038	-0.632048873	1.152914911	5
GFRA1	-0.568724183	-0.585933607	1.15465779	5
GFRA2	-0.520238	-0.632637583	1.152875584	5
GFRA3	-0.530819094	-0.622663264	1.153482358	5
GGN	-0.592668362	-0.561895485	1.154563847	5
GGTA1	-0.653820929	-0.497339889	1.151160818	5
GH2	-0.54609053	-0.608055665	1.154146196	5
GHR	-0.510265942	-0.641929736	1.152195679	5
GHSR	-0.70243468	-0.442469715	1.144904394	5
GIMD1	-0.570500674	-0.584172883	1.154673557	5
GIPC1	-0.736200738	-0.402293267	1.138494005	5
GIPC3	-0.537609239	-0.616199465	1.153808704	5
GJA10	-0.641444278	-0.510789694	1.152233972	5
GJB3	-0.598247678	-0.556197613	1.154445291	5
GJC1	-0.540313101	-0.613611697	1.153924798	5
GJD2	-0.524708047	-0.628438376	1.153146423	5
GJE1	-0.554115672	-0.600277257	1.15439293	5
GK2	-0.585022747	-0.569643653	1.1546664	5
GKN1	-0.504961157	-0.646830642	1.1517918	5
GLDC	-0.531491741	-0.622025157	1.153516898	5
GLDN	-0.679628596	-0.468627863	1.14825646	5
GLI2	-0.542898851	-0.611129539	1.15402839	5
GLI3	-0.543349393	-0.610696301	1.154045694	5
GLIS3	-0.522913546	-0.630126688	1.153040234	5

GLP1R	-0.602973152	-0.551342559	1.154315711	5
GLP2R	-0.556304207	-0.598143635	1.154447842	5
GLRA1	-0.542700308	-0.611320386	1.154020694	5
GLRA2	-0.564291026	-0.59031178	1.154602806	5
GLRB	-0.563401925	-0.591187176	1.154589101	5
GLT6D1	-0.754013803	-0.380355223	1.134369027	5
GLYATL1	-0.514185023	-0.638290268	1.152475291	5
GLYATL3	-0.628287963	-0.524867744	1.153155708	5
GMNC	-0.551649571	-0.602675108	1.154324679	5
GNAI1	-0.525243433	-0.62793401	1.153177443	5
GNAO1	-0.715601329	-0.427015272	1.142616601	5
GNAS	-0.657420531	-0.493389901	1.150810432	5
GNAT1	-0.7872313	-0.337958105	1.125189406	5
GNAZ	-0.815800158	-0.299809309	1.115609466	5
GNG11	-0.634760456	-0.517969622	1.152730078	5
GNG3	-0.562844477	-0.591735577	1.154580054	5
GNG8	-0.522352259	-0.630654061	1.15300632	5
GNGT1	-0.55523591	-0.599185793	1.154421703	5
GOLGA6D	-0.69939286	-0.446002772	1.145395632	5
GOLGA6L2	-0.549224742	-0.605026269	1.154251011	5
GOLGA6L22	-0.521102522	-0.63182709	1.152929612	5
GOLGA6L6	-0.509005637	-0.643096728	1.152102365	5
GOLGA7	-0.713216818	-0.429833787	1.143050605	5
GOLGA8F	-0.809230815	-0.308728209	1.117959024	5

GOLM2	-0.792457202	-0.331101233	1.123558435	5
GOSR1	-0.744019805	-0.392729201	1.136749007	5
GP1BB	-0.709181164	-0.434584023	1.143765187	5
GP6	-0.56936934	-0.585294592	1.154663932	5
GP9	-0.658582971	-0.492110579	1.150693551	5
GPAT4	-0.68190518	-0.466050505	1.147955684	5
GPATCH1	-0.536949941	-0.616829254	1.153779195	5
GPC2	-0.526116718	-0.627110669	1.153227387	5
GPC3	-0.526473588	-0.626773976	1.153247564	5
GPC6	-0.557155842	-0.597311927	1.154467769	5
GPLD1	-0.644438866	-0.507553977	1.151992842	5
GPM6A	-0.553741093	-0.600641904	1.154382997	5
GPM6B	-0.592781971	-0.561779832	1.154561803	5
GPR101	-0.678532372	-0.46986629	1.148398663	5
GPR12	-0.56860282	-0.586053762	1.154656582	5
GPR137	-0.816178128	-0.299293398	1.115471526	5
GPR137C	-0.546332327	-0.607822339	1.154154666	5
GPR139	-0.602182245	-0.552157029	1.154339274	5
GPR149	-0.618339481	-0.535366945	1.153706427	5
GPR156	-0.614159629	-0.539741278	1.153900907	5
GPR158	-0.555462904	-0.59896446	1.154427364	5
GPR161	-0.533986065	-0.619654678	1.153640743	5
GPR173	-0.551908745	-0.602423423	1.154332169	5
GPR176	-0.564960003	-0.589652526	1.154612529	5

GPR26	-0.666658351	-0.483172497	1.149830849	5
GPR31	-0.662054362	-0.488279243	1.150333605	5
GPR37	-0.574862429	-0.579834541	1.15469697	5
GPR39	-0.508481543	-0.643581533	1.152063076	5
GPR42	-0.526742262	-0.626520403	1.153262665	5
GPR50	-0.575764712	-0.578934376	1.154699088	5
GPR52	-0.805068934	-0.314332332	1.119401267	5
GPR6	-0.685391344	-0.462089411	1.147480755	5
GPR87	-0.516110347	-0.636496436	1.152606783	5
GPRC5A	-0.559607411	-0.594913197	1.154520608	5
GPRC5B	-0.543606486	-0.610448984	1.15405547	5
GPRC5C	-0.529929993	-0.623505966	1.153435959	5
GPRIN1	-0.626680715	-0.526572413	1.153253127	5
GPRIN2	-0.592648747	-0.561915452	1.154564198	5
GPSM1	-0.586812052	-0.567836513	1.154648565	5
GPT2	-0.572855809	-0.581833097	1.154688906	5
GPX8	-0.531680025	-0.621846454	1.153526479	5
GRAMD1B	-0.530679725	-0.622795416	1.153475141	5
GRAMD2A	-0.777234822	-0.350929425	1.128164248	5
GRB10	-0.514717728	-0.637794332	1.152512061	5
GRB14	-0.516424525	-0.636203347	1.152627872	5
GRB7	-0.614406144	-0.539483894	1.153890038	5
GREB1L	-0.695451144	-0.45056058	1.146011724	5
GREM1	-0.823515948	-0.289217141	1.112733089	5

GREM2	-0.560282541	-0.594251437	1.154533977	5
GRHL1	-0.518443424	-0.634317493	1.152760918	5
GRHL2	-0.592323681	-0.562246275	1.154569956	5
GRIA1	-0.556912827	-0.597549339	1.154462165	5
GRIA2	-0.559174117	-0.595337641	1.154511758	5
GRIA3	-0.564938586	-0.58967364	1.154612226	5
GRIA4	-0.581570661	-0.573119569	1.15469023	5
GRID1	-0.583947862	-0.570727449	1.154675311	5
GRID2	-0.573856842	-0.580836665	1.154693507	5
GRID2IP	-0.506171363	-0.645715138	1.151886501	5
GRIK1	-0.649760045	-0.501775234	1.151535279	5
GRIK2	-0.581341245	-0.573350076	1.154691321	5
GRIK3	-0.545362744	-0.60875757	1.154120314	5
GRIK5	-0.503904386	-0.647803484	1.151707869	5
GRIN1	-0.539595311	-0.614299441	1.153894752	5
GRIN2A	-0.574233548	-0.580461392	1.15469494	5
GRIN2B	-0.600678501	-0.553703491	1.154381992	5
GRIN2D	-0.614905623	-0.538962161	1.153867784	5
GRIP1	-0.509502119	-0.642637203	1.152139322	5
GRIP2	-0.583027422	-0.571654447	1.154681869	5
GRIPAP1	-0.620950059	-0.532623878	1.153573937	5
GRK4	-0.514456948	-0.638037149	1.152494097	5
GRM2	-0.531265861	-0.622239492	1.153505353	5
GRM3	-0.546402579	-0.607754535	1.154157115	5

GRM4	-0.60946389	-0.544629772	1.154093661	5
GRM5	-0.5750358	-0.57966165	1.15469745	5
GRM6	-0.707112428	-0.437009413	1.144121841	5
GRM7	-0.779850911	-0.347552962	1.127403874	5
GRM8	-0.54160297	-0.612374413	1.153977383	5
GRP	-0.550387897	-0.603899262	1.154287159	5
GRPR	-0.535112506	-0.618581972	1.153694478	5
GRXCR1	-0.617907725	-0.535819795	1.15372752	5
GSC2	-0.70354578	-0.441175722	1.144721502	5
GSDMA	-0.651029035	-0.500391589	1.151420624	5
GSG1L	-0.566633666	-0.588000971	1.154634637	5
GSK3B	-0.667955544	-0.481728391	1.149683934	5
GSTA4	-0.525428862	-0.627759253	1.153188116	5
GSTA5	-0.52844967	-0.624907163	1.153356833	5
GSTM5	-0.539099802	-0.614773881	1.153873684	5
GSTO1	-0.693809916	-0.452451565	1.146261481	5
GSTT2	-0.562784932	-0.591794135	1.154579067	5
GSTT2B	-0.569494704	-0.585170366	1.15466507	5
GSX1	-0.629462835	-0.523619585	1.153082421	5
GSX2	-0.516078095	-0.636526517	1.152604612	5
GUCA1C	-0.747679942	-0.388217132	1.135897074	5
GUCY1A1	-0.509955277	-0.642217553	1.15217283	5
GUCY1A2	-0.526707626	-0.626553096	1.153260722	5
GUCY2F	-0.672294956	-0.476880577	1.149175533	5

GULP1	-0.734053394	-0.404902193	1.138955587	5
GXYLT2	-0.539828269	-0.614076296	1.153904564	5
GYG2	-0.552030378	-0.60230528	1.154335658	5
GYPE	-0.833549109	-0.275249226	1.108798335	5
GZF1	-0.795068733	-0.327654769	1.122723502	5
H1-2	-0.698487134	-0.447052102	1.145539236	5
H1-3	-0.587600397	-0.567039119	1.154639516	5
H1-4	-0.582061166	-0.572626525	1.15468769	5
H2AC1	-0.551047625	-0.603259372	1.154306997	5
H2AC13	-0.596506015	-0.557980273	1.154486288	5
H2AC15	-0.576838408	-0.577861979	1.154700387	5
H2AC16	-0.737902943	-0.400219793	1.138122736	5
H2AC20	-0.597233933	-0.557235662	1.154469595	5
H2AC21	-0.810397443	-0.307150903	1.117548346	5
H2AC4	-0.594743174	-0.559780919	1.154524093	5
H2AC8	-0.713015033	-0.430071895	1.143086929	5
H2AJ	-0.824018155	-0.288523263	1.112541418	5
H2BC13	-0.614913715	-0.538953706	1.153867421	5
H2BC14	-0.619328254	-0.534328992	1.153657246	5
H2BC17	-0.570421509	-0.584251422	1.154672931	5
H2BC3	-0.565135735	-0.589479265	1.154615	5
H2BC6	-0.636176094	-0.516453733	1.152629827	5
H2BC8	-0.555491729	-0.59893635	1.154428078	5
H2BW1	-0.807843576	-0.310600127	1.118443703	5

H3-4	-0.702859125	-0.441975622	1.144834748	5
H3C1	-0.580235558	-0.574460166	1.154695724	5
H3C12	-0.719471177	-0.422422272	1.141893449	5
H3C4	-0.589015645	-0.56560579	1.154621436	5
H3C7	-0.625950729	-0.527345564	1.153296293	5
H4-16	-0.649229645	-0.502352922	1.151582567	5
H4C1	-0.638815663	-0.513620326	1.152435989	5
H4C13	-0.51895903	-0.633835179	1.15279421	5
H4C2	-0.580747506	-0.573946356	1.154693862	5
H4C6	-0.674178515	-0.47476818	1.148946694	5
H4C7	-0.651443277	-0.499939456	1.151382733	5
H4C9	-0.762233207	-0.370049426	1.132282633	5
HABP2	-0.584245733	-0.570427243	1.154672977	5
HACD4	-0.710865169	-0.432604872	1.143470041	5
HADH	-0.818992302	-0.295442646	1.114434948	5
HAL	-0.519248307	-0.633564459	1.152812766	5
HAO2	-0.581791582	-0.572897539	1.154689121	5
HAP1	-0.552173787	-0.602165964	1.154339751	5
HAPLN1	-0.544819195	-0.609281411	1.154100605	5
HAPLN2	-0.548341511	-0.605881056	1.154222567	5
HAPLN4	-0.571175431	-0.583503172	1.154678603	5
HAS2	-0.515288744	-0.637262401	1.152551145	5
HAS3	-0.581933852	-0.572754524	1.154688376	5
HBA2	-0.774608067	-0.354306887	1.128914954	5

HCN1	-0.562277717	-0.59229278	1.154570497	5
HCN3	-0.574014735	-0.580679392	1.154694127	5
HCN4	-0.554394956	-0.600005278	1.154400234	5
HCRTR1	-0.532383381	-0.621178555	1.153561936	5
HCRTR2	-0.535554948	-0.618160263	1.153715211	5
HDAC2	-0.683584131	-0.464144999	1.14772913	5
HDAC6	-0.518538777	-0.634228318	1.152767096	5
HDGFL1	-0.822065752	-0.291217738	1.11328349	5
HEATR5A	-0.701809657	-0.443196805	1.145006462	5
HECTD2	-0.508680435	-0.643397584	1.15207802	5
HEPACAM2	-0.612193091	-0.54179184	1.153984931	5
HEPH	-0.550216931	-0.604065008	1.154281939	5
HERPUD1	-0.530872052	-0.622613043	1.153485095	5
HES1	-0.504550352	-0.647208957	1.15175931	5
HES2	-0.744014385	-0.392735867	1.136750251	5
HES5	-0.508550263	-0.643517981	1.152068244	5
HES6	-0.610843262	-0.543196589	1.15403985	5
HES7	-0.555569725	-0.598860283	1.154430008	5
HEYL	-0.568156878	-0.58649512	1.154651998	5
HFE	-0.611153152	-0.542874288	1.15402744	5
HFM1	-0.525322348	-0.627859642	1.15318199	5
HGFAC	-0.553652711	-0.60072792	1.154380631	5
HHIP	-0.565214871	-0.58940123	1.154616101	5
HHIPL1	-0.553245152	-0.601124456	1.154369608	5

HHLA1	-0.712231288	-0.43099613	1.143227418	5
HID1	-0.574173041	-0.58052168	1.154694721	5
HIF1A	-0.734997131	-0.403756524	1.138753655	5
HIF3A	-0.506484173	-0.645426561	1.151910733	5
HIGD1A	-0.691399303	-0.455221851	1.146621154	5
HIGD2B	-0.604979155	-0.549273398	1.154252553	5
HIPK3	-0.644241826	-0.507767243	1.152009069	5
HIVEP1	-0.555378129	-0.599047128	1.154425256	5
HJURP	-0.50530199	-0.646516633	1.151818624	5
HLA-B	-0.791177554	-0.332785137	1.123962691	5
HLA-C	-0.518277446	-0.634472696	1.152750141	5
HLTF	-0.519978739	-0.632880488	1.152859227	5
HMCN2	-0.537366654	-0.616431247	1.153797901	5
HMG20B	-0.818172719	-0.296565858	1.114738577	5
HMGA2	-0.556404338	-0.598045889	1.154450227	5
HMGB4	-0.824723899	-0.287547236	1.112271135	5
HMGCLL1	-0.524989299	-0.628173458	1.153162757	5
HMGCS2	-0.504153125	-0.647574603	1.151727728	5
HMSD	-0.70915408	-0.434615818	1.143769899	5
HMX1	-0.617214988	-0.536545891	1.153760879	5
HMX3	-0.69920154	-0.446224528	1.145426068	5
HNF1A	-0.549828498	-0.604441462	1.15426996	5
HNF1B	-0.557632691	-0.596845882	1.154478573	5
HNF4G	-0.550143647	-0.604136045	1.154279692	5

HNRNPCL4	-0.679264072	-0.469039864	1.148303935	5
HNRNPUL1	-0.700644295	-0.444550911	1.145195206	5
HOATZ	-0.528676164	-0.624692927	1.153369091	5
HOMER1	-0.526993759	-0.626282972	1.153276731	5
HORMAD2	-0.547230912	-0.60695467	1.154185582	5
HOXA11	-0.617846697	-0.535883786	1.153730483	5
HOXA3	-0.519342907	-0.633475908	1.152818815	5
HOXA4	-0.724203707	-0.416773454	1.14097716	5
HOXB1	-0.519923845	-0.63293191	1.152855755	5
HOXB13	-0.694715649	-0.45140849	1.146124139	5
HOXB6	-0.737872535	-0.400256875	1.138129411	5
HOXB9	-0.583026813	-0.57165506	1.154681873	5
HOXC10	-0.548994629	-0.605249054	1.154243683	5
HOXC13	-0.551341162	-0.602974508	1.15431567	5
HOXC4	-0.502441702	-0.649148103	1.151589804	5
HOXC6	-0.744681205	-0.391915524	1.136596729	5
HOXC8	-0.540879461	-0.613068649	1.15394811	5
HOXC9	-0.518390132	-0.634367329	1.152757461	5
HOXD10	-0.537648069	-0.616162359	1.153810427	5
HOXD11	-0.502196908	-0.649372922	1.15156983	5
HOXD12	-0.732595511	-0.406669167	1.139264678	5
HOXD13	-0.661106471	-0.489327046	1.150433517	5
HOXD3	-0.572515957	-0.582171125	1.154687083	5
HPCA	-0.532390093	-0.621172178	1.153562271	5

HPN	-0.514314785	-0.63816949	1.152484275	5
HPRT1	-0.759103025	-0.3739881	1.133091125	5
HPSE2	-0.555833898	-0.598602593	1.154436492	5
HR	-0.515805325	-0.636780885	1.15258621	5
HRC	-0.508499009	-0.64356538	1.15206439	5
HRCT1	-0.627201076	-0.526020869	1.153221945	5
HRH3	-0.550341853	-0.603943903	1.154285756	5
HROB	-0.535627954	-0.618090657	1.153718611	5
HS3ST3A1	-0.82857115	-0.282207345	1.110778495	5
HS3ST4	-0.559835999	-0.594689193	1.154525192	5
HS3ST5	-0.60988317	-0.544194382	1.154077551	5
HS6ST1	-0.714415469	-0.428418069	1.142833538	5
HS6ST2	-0.559385545	-0.595130558	1.154516103	5
HS6ST3	-0.595085267	-0.559431779	1.154517046	5
HSBP1	-0.525875541	-0.627338135	1.153213675	5
HSD11B1L	-0.813096312	-0.303491133	1.116587444	5
HSD17B12	-0.612774482	-0.541186105	1.153960586	5
HSD17B2	-0.54225583	-0.611747479	1.154003309	5
HSD3B2	-0.70390206	-0.440760404	1.144662464	5
HSF1	-0.720232571	-0.421515842	1.141748413	5
HSF2BP	-0.6496554	-0.501889238	1.151544638	5
HSFX1	-0.6360725	-0.516564751	1.152637251	5
HSFX2	-0.56554834	-0.589072322	1.154620662	5
HSPA2	-0.534088534	-0.619557153	1.153645687	5

HSPB6	-0.761160963	-0.371400564	1.132561526	5
HTN3	-0.559803072	-0.594721463	1.154524535	5
HTR1D	-0.579890327	-0.574806481	1.154696808	5
HTR1E	-0.664785985	-0.485252833	1.150038818	5
HTR1F	-0.532357832	-0.621202825	1.153560657	5
HTR2A	-0.520683174	-0.632220329	1.152903503	5
HTR2C	-0.541361647	-0.612606036	1.153967683	5
HTR3C	-0.504621435	-0.647143509	1.151764944	5
HTR4	-0.652056235	-0.49927001	1.151326245	5
HUNK	-0.544716794	-0.609380062	1.154096856	5
HUS1B	-0.534200039	-0.619451016	1.153651055	5
HYDIN	-0.56229422	-0.59227656	1.15457078	5
IAPP	-0.568390994	-0.586263439	1.154654433	5
IBSP	-0.575918072	-0.578781283	1.154699355	5
ICAM1	-0.674591048	-0.474304863	1.148895911	5
ID1	-0.660995601	-0.489449522	1.150445123	5
ID4	-0.55958745	-0.594932754	1.154520205	5
IDS	-0.823475504	-0.289272997	1.112748501	5
IER3	-0.615118136	-0.538740087	1.153858223	5
IFNA10	-0.738083198	-0.399999943	1.138083142	5
IFNA16	-0.622170494	-0.531338582	1.153509076	5
IFNA17	-0.66369937	-0.486457935	1.150157304	5
IFNA4	-0.581931494	-0.572756895	1.154688389	5
IFNA6	-0.505708208	-0.646142229	1.151850437	5

IFNA8	-0.632418656	-0.520471604	1.15289026	5
IFNB1	-0.608705185	-0.545417081	1.154122266	5
IFT140	-0.686063463	-0.461323721	1.147387184	5
IFT81	-0.52695584	-0.626318774	1.153274614	5
IGDCC4	-0.539078593	-0.614794183	1.153872776	5
IGF2BP1	-0.538147123	-0.615685307	1.15383243	5
IGF2BP3	-0.545685476	-0.608446387	1.154131862	5
IGFBP2	-0.565987998	-0.588638486	1.154626484	5
IGFBP4	-0.50691681	-0.645027271	1.151944081	5
IGFBPL1	-0.536337369	-0.617413988	1.153751357	5
IGLON5	-0.573520488	-0.581171601	1.154692089	5
IGSF1	-0.526145269	-0.627083738	1.153229007	5
IGSF11	-0.510202639	-0.641988392	1.152191031	5
IGSF21	-0.544959422	-0.609146299	1.154105721	5
IGSF3	-0.540042233	-0.613871292	1.153913525	5
IGSF5	-0.571316943	-0.583362652	1.154679595	5
IGSF9	-0.558325937	-0.596167889	1.154493827	5
IKBKG	-0.555741884	-0.598692358	1.154434242	5
IKZF4	-0.64404463	-0.507980629	1.152025258	5
IL10RB	-0.713613137	-0.429365943	1.14297908	5
IL13	-0.818467973	-0.296161387	1.11462936	5
IL17C	-0.709903879	-0.433735174	1.143639052	5
IL17D	-0.523522024	-0.629554599	1.153076623	5
IL17RD	-0.55420081	-0.600194356	1.154395165	5

IL1RAPL1	-0.561111871	-0.59343783	1.1545497	5
IL1RAPL2	-0.684745276	-0.46282481	1.147570086	5
IL1RL2	-0.651467472	-0.49991304	1.151380513	5
IL20	-0.618643586	-0.535047845	1.153691431	5
IL20RA	-0.534003222	-0.619638349	1.153641571	5
IL22RA1	-0.606700662	-0.547493797	1.154194459	5
IL31	-0.620137879	-0.53347819	1.153616068	5
IL36B	-0.592937817	-0.561621156	1.154558973	5
IL36RN	-0.721924098	-0.419498839	1.141422937	5
IL5	-0.800647862	-0.32024685	1.120894712	5
IL7	-0.721224222	-0.420333928	1.14155815	5
IL9	-0.609055318	-0.545053835	1.154109153	5
ILDR2	-0.544992811	-0.609114125	1.154106936	5
ILK	-0.808527436	-0.309677834	1.11820527	5
ILKAP	-0.55813919	-0.59635058	1.15448977	5
IMPG1	-0.588374846	-0.566255069	1.154629914	5
INA	-0.548758061	-0.605478028	1.154236089	5
INAVA	-0.551712217	-0.602614279	1.154326496	5
INF2	-0.595617915	-0.558887882	1.154505797	5
INHA	-0.511824094	-0.640484669	1.152308763	5
INHBC	-0.515726758	-0.636854137	1.152580895	5
INPP4B	-0.506465816	-0.645443498	1.151909314	5
INS	-0.739946294	-0.397724457	1.137670751	5
INS-IGF2	-0.524751347	-0.628397596	1.153148943	5

INSL4	-0.672362086	-0.476805376	1.149167463	5
INSYN1	-0.566559466	-0.588074259	1.154633725	5
INSYN2A	-0.553972089	-0.600417052	1.154389141	5
IP6K3	-0.733394286	-0.405701469	1.139095755	5
IQANK1	-0.64321622	-0.50887649	1.15209271	5
IQCA1	-0.527693188	-0.625622306	1.153315494	5
IQCF3	-0.531650889	-0.62187411	1.153524999	5
IQCF6	-0.548151582	-0.606064756	1.154216338	5
IQCH	-0.548284379	-0.605936318	1.154220697	5
IQCJ	-0.568736621	-0.585921292	1.154657913	5
IQCJ-SCHIP1	-0.503139567	-0.648506843	1.151646409	5
IQSEC2	-0.512560533	-0.639800799	1.152361331	5
IQUB	-0.569292637	-0.58537059	1.154663226	5
IRAK2	-0.502358706	-0.649224333	1.151583039	5
IRGC	-0.502993264	-0.64864132	1.151634584	5
IRS4	-0.555175565	-0.599244624	1.154420189	5
IRX2	-0.549271321	-0.604981166	1.154252487	5
IRX4	-0.684716291	-0.462857788	1.14757408	5
IRX5	-0.51300535	-0.639387459	1.15239281	5
ISL1	-0.549254472	-0.604997481	1.154251954	5
ISLR	-0.680711611	-0.467402684	1.148114295	5
ISLR2	-0.547767928	-0.606435707	1.154203635	5
ISM2	-0.541750709	-0.612232582	1.153983291	5
ISX	-0.530455703	-0.623007793	1.153463496	5

ITFG1	-0.681253718	-0.46678879	1.148042507	5
ITGA2	-0.527544134	-0.625763143	1.153307277	5
ITGA2B	-0.610433228	-0.543622862	1.15405609	5
ITGA7	-0.523042787	-0.630005209	1.153047996	5
ITGAM	-0.68947899	-0.457422609	1.146901599	5
ITGB1	-0.673670905	-0.475337949	1.149008854	5
ITGB3	-0.782102498	-0.344636703	1.126739201	5
ITGB5	-0.524912705	-0.628245612	1.153158317	5
ITGB8	-0.557363704	-0.597108806	1.15447251	5
ITIH6	-0.767889423	-0.362888186	1.130777608	5
ITM2B	-0.766482181	-0.364675215	1.131157396	5
ITPKA	-0.507848354	-0.644166875	1.152015229	5
ITPRID1	-0.800528362	-0.320406171	1.120934533	5
IVL	-0.549243639	-0.605007971	1.15425161	5
IYD	-0.541098666	-0.612858373	1.153957039	5
IZUMO1	-0.537084867	-0.616700406	1.153785273	5
IZUMO3	-0.574548694	-0.58014732	1.154696014	5
JMJD6	-0.603240551	-0.551067022	1.154307574	5
JPH1	-0.620673788	-0.532914573	1.153588361	5
JPH3	-0.555651347	-0.598780672	1.154432019	5
JPH4	-0.589043887	-0.565577164	1.154621051	5
JSRP1	-0.663687285	-0.486471328	1.150158613	5
JUNB	-0.570312885	-0.584359176	1.15467206	5
KANK4	-0.551925009	-0.602407627	1.154332636	5

KANSL1	-0.689437475	-0.457470129	1.146907603	5
KAT6B	-0.569595756	-0.585070219	1.154665975	5
KAZALD1	-0.549532386	-0.604728331	1.154260717	5
KCMF1	-0.769050765	-0.361410735	1.1304615	5
KCNA10	-0.733580358	-0.405475898	1.139056256	5
KCNA4	-0.539561928	-0.614331413	1.153893341	5
KCNA7	-0.737426243	-0.400800947	1.13822719	5
KCNB1	-0.532247587	-0.621307545	1.153555131	5
KCNB2	-0.556226258	-0.59821972	1.154445978	5
KCNC1	-0.526025105	-0.627197081	1.153222186	5
KCNC2	-0.574099	-0.580595447	1.154694447	5
KCNC3	-0.551330683	-0.602984679	1.154315362	5
KCND2	-0.547325093	-0.606863678	1.154188771	5
KCND3	-0.51373321	-0.638710663	1.152443872	5
KCNE4	-0.528155902	-0.62518495	1.153340852	5
KCNG3	-0.64896215	-0.502644124	1.151606275	5
KCNH1	-0.50377151	-0.647925725	1.151697235	5
KCNH2	-0.705344897	-0.439076522	1.144421419	5
KCNH4	-0.598698063	-0.555736036	1.154434099	5
KCNH5	-0.59280905	-0.561752263	1.154561313	5
KCNH6	-0.616823719	-0.536955738	1.153779457	5
KCNIP1	-0.559821648	-0.594703258	1.154524906	5
KCNIP3	-0.54260852	-0.611408601	1.154017121	5
KCNJ13	-0.639573814	-0.512804831	1.152378645	5

KCNJ16	-0.652712136	-0.498553109	1.151265245	5
KCNJ18	-0.708935226	-0.434872703	1.143807929	5
KCNJ3	-0.528464473	-0.624893163	1.153357636	5
KCNJ4	-0.50951935	-0.64262125	1.1521406	5
KCNJ5	-0.518034965	-0.634699381	1.152734346	5
KCNJ6	-0.743384368	-0.393510247	1.136894615	5
KCNJ9	-0.558702738	-0.595799154	1.154501892	5
KCNK10	-0.510168673	-0.642019863	1.152188535	5
KCNK3	-0.578935565	-0.575763521	1.154699086	5
KCNK5	-0.54533394	-0.608785337	1.154119278	5
KCNK6	-0.6151369	-0.538720475	1.153857376	5
KCNMA1	-0.547927579	-0.606281361	1.154208941	5
KCNMB2	-0.561678761	-0.592881243	1.154560004	5
KCNN1	-0.556619283	-0.597836025	1.154455309	5
KCNN2	-0.508713838	-0.643366687	1.152080525	5
KCNN3	-0.545852949	-0.608284861	1.15413781	5
KCNQ2	-0.581585366	-0.573104791	1.154690158	5
KCNQ3	-0.569486136	-0.585178857	1.154664993	5
KCNQ4	-0.54837478	-0.605848874	1.154223654	5
KCNRG	-0.680034952	-0.468168362	1.148203314	5
KCNS2	-0.666039225	-0.483860925	1.14990015	5
KCNT1	-0.823957969	-0.288606448	1.112564417	5
KCNT2	-0.519428746	-0.63339555	1.152824295	5
KCNV1	-0.696302124	-0.449578538	1.145880662	5

KCTD19	-0.528501665	-0.624857987	1.153359652	5
KCTD20	-0.654627912	-0.496455868	1.15108378	5
KCTD4	-0.716483205	-0.425970653	1.142453859	5
KCTD8	-0.554949222	-0.599465251	1.154414473	5
KDELR1	-0.732413078	-0.406890034	1.139303113	5
KDM8	-0.735331093	-0.403350756	1.138681848	5
KERA	-0.527649907	-0.625663204	1.153313111	5
KHDC1L	-0.507697648	-0.644306131	1.152003779	5
KHDC3L	-0.509135325	-0.642976718	1.152112043	5
KHDRBS1	-0.597947743	-0.556504867	1.15445261	5
KHDRBS2	-0.544940369	-0.609164658	1.154105027	5
KHDRBS3	-0.504097522	-0.647625773	1.151723294	5
KIAA0319L	-0.709098123	-0.434681507	1.143779629	5
KIAA0513	-0.722185075	-0.419187244	1.141372319	5
KIAA0895	-0.541578999	-0.612397423	1.153976422	5
KIAA1191	-0.815260478	-0.30054542	1.115805899	5
KIAA1549L	-0.550233327	-0.604049114	1.154282441	5
KIAA1586	-0.615711216	-0.538120026	1.153831242	5
KIAA1755	-0.628305262	-0.524849379	1.153154641	5
KIAA2012	-0.729846748	-0.409991327	1.139838075	5
KIAA2013	-0.619339214	-0.534317481	1.153656694	5
KIF12	-0.562386825	-0.59218554	1.154572365	5
KIF18A	-0.509289267	-0.642834242	1.152123509	5
KIF1A	-0.551042815	-0.603264039	1.154306855	5

KIF20A	-0.515383256	-0.637174326	1.152557581	5
KIF20B	-0.534934102	-0.618751957	1.153686059	5
KIF21A	-0.513692358	-0.638748663	1.152441021	5
KIF21B	-0.750698576	-0.384478808	1.135177384	5
KIF24	-0.626403036	-0.526866591	1.153269627	5
KIF25	-0.572253023	-0.582432559	1.154685582	5
KIF26B	-0.524407638	-0.628721246	1.153128884	5
KIF2B	-0.629617693	-0.523454937	1.15307263	5
KIF3A	-0.512788115	-0.639589348	1.152377462	5
KIF3C	-0.558497	-0.596000508	1.154497508	5
KIF5A	-0.549903176	-0.6043691	1.154272276	5
KIF5C	-0.524457369	-0.628674426	1.153131794	5
KIF6	-0.647292314	-0.504459811	1.151752126	5
KIF7	-0.51249009	-0.639866237	1.152356328	5
KIFC1	-0.517069606	-0.635601242	1.152670848	5
KIR3DL3	-0.517863566	-0.634859577	1.152723143	5
KIRREL1	-0.533277727	-0.620328526	1.153606253	5
KIRREL2	-0.540006582	-0.613905454	1.153912035	5
KIRREL3	-0.57497243	-0.579724848	1.154697278	5
KISS1	-0.597782278	-0.556674324	1.154456602	5
KITLG	-0.505261966	-0.646553514	1.15181548	5
KLC4	-0.679274241	-0.469028372	1.148302613	5
KLF10	-0.618853836	-0.53482716	1.153680995	5
KLF15	-0.525836935	-0.62737454	1.153211475	5

KLF17	-0.631274103	-0.521691889	1.152965992	5
KLHDC1	-0.504992557	-0.646801719	1.151794276	5
KLHDC8A	-0.71803412	-0.424130596	1.142164716	5
KLHDC8B	-0.584400246	-0.570271479	1.154671725	5
KLHL1	-0.519323589	-0.633493991	1.15281758	5
KLHL21	-0.804711283	-0.31481227	1.119523553	5
KLHL23	-0.549868258	-0.604402936	1.154271194	5
KLHL25	-0.730292624	-0.40945327	1.139745894	5
KLHL30	-0.649914692	-0.501606729	1.151521421	5
KLHL31	-0.528890382	-0.624490252	1.153380635	5
KLHL35	-0.78633265	-0.339131922	1.125464572	5
KLHL4	-0.518455437	-0.634306259	1.152761697	5
KLHL40	-0.666641747	-0.483190967	1.149832714	5
KLK10	-0.524173791	-0.628941374	1.153115165	5
KLK11	-0.502709827	-0.648901785	1.151611612	5
KLK12	-0.520155187	-0.632715179	1.152870367	5
KLK13	-0.826989165	-0.284407052	1.111396217	5
KLK3	-0.758974558	-0.374149381	1.133123939	5
KLK5	-0.808197765	-0.310122567	1.118320332	5
KLK6	-0.546284614	-0.607868386	1.154153	5
KLK9	-0.729122476	-0.410864652	1.139987128	5
KMT2C	-0.521379265	-0.631567475	1.15294674	5
KNDC1	-0.528140757	-0.625199269	1.153340026	5
KNG1	-0.511489234	-0.640795439	1.152284673	5

KRT15	-0.557693965	-0.596785978	1.154479943	5
KRT20	-0.530456972	-0.62300659	1.153463562	5
KRT222	-0.519438554	-0.633386367	1.152824921	5
KRT27	-0.572539246	-0.582147966	1.154687212	5
KRT28	-0.512039104	-0.640285065	1.152324169	5
KRT39	-0.634104087	-0.518671598	1.152775685	5
KRT6C	-0.51591914	-0.636674758	1.152593898	5
KRT75	-0.602510882	-0.551818693	1.154329575	5
KRT76	-0.507977157	-0.644047838	1.152024995	5
KRT8	-0.53187838	-0.621658152	1.153536532	5
KRT80	-0.755902528	-0.377997495	1.133900023	5
KRT83	-0.528570087	-0.62479327	1.153363357	5
KRT84	-0.64070509	-0.5115866	1.15229169	5
KRTAP1-1	-0.549113095	-0.605134368	1.154247463	5
KRTAP1-4	-0.716948752	-0.425418706	1.142367459	5
KRTAP10-10	-0.659482896	-0.491118913	1.150601809	5
KRTAP10-12	-0.659508625	-0.491090545	1.15059917	5
KRTAP10-2	-0.799257411	-0.32209887	1.121356281	5
KRTAP10-4	-0.612229871	-0.541753532	1.153983403	5
KRTAP10-7	-0.828114632	-0.282842689	1.110957321	5
KRTAP10-8	-0.833103619	-0.275874194	1.108977813	5
KRTAP12-4	-0.807141339	-0.311546205	1.118687544	5
KRTAP13-2	-0.512589094	-0.639774265	1.152363359	5
KRTAP13-3	-0.736144165	-0.402362098	1.138506263	5

KRTAP15-1	-0.816364888	-0.299038368	1.115403256	5
KRTAP19-1	-0.740527599	-0.397013306	1.137540905	5
KRTAP19-7	-0.580501187	-0.574193609	1.154694796	5
KRTAP2-1	-0.810671043	-0.306780582	1.117451625	5
KRTAP21-1	-0.603792742	-0.550497754	1.154290496	5
KRTAP21-2	-0.522532256	-0.630484976	1.153017232	5
KRTAP21-3	-0.573428568	-0.58126311	1.154691679	5
KRTAP22-2	-0.698916819	-0.446554443	1.145471261	5
KRTAP24-1	-0.707012534	-0.437126364	1.144138898	5
KRTAP25-1	-0.59117327	-0.563416056	1.154589326	5
KRTAP27-1	-0.51551475	-0.63705177	1.152566521	5
KRTAP29-1	-0.566585547	-0.588048499	1.154634046	5
KRTAP3-1	-0.600741324	-0.553638938	1.154380262	5
KRTAP3-2	-0.56477576	-0.589834142	1.154609902	5
KRTAP4-8	-0.787978151	-0.336981393	1.124959544	5
KRTAP5-1	-0.610427971	-0.543628325	1.154056297	5
KRTAP5-11	-0.754474146	-0.379781138	1.134255284	5
KRTAP5-3	-0.679796076	-0.468438508	1.148234584	5
KRTAP5-4	-0.59323145	-0.561322114	1.154553564	5
KRTAP5-7	-0.748668559	-0.38699452	1.135663079	5
KRTAP9-2	-0.715412574	-0.427238702	1.142651277	5
KRTAP9-4	-0.785145066	-0.340680778	1.125825845	5
KRTAP9-6	-0.588389357	-0.566240371	1.154629727	5
KRTAP9-7	-0.51585876	-0.636731062	1.152589821	5

KRTAP9-9	-0.632695314	-0.520176389	1.152871703	5
KSR2	-0.623891919	-0.529522492	1.153414411	5
L1CAM	-0.548045989	-0.606166869	1.154212858	5
LACRT	-0.548171947	-0.60604506	1.154217008	5
LAD1	-0.573069213	-0.58162077	1.154689983	5
LAMA1	-0.546454843	-0.60770409	1.154158933	5
LAMA3	-0.512556969	-0.63980411	1.152361078	5
LAMA5	-0.578413782	-0.576286103	1.154699885	5
LAMB1	-0.539715766	-0.614184067	1.153899833	5
LAMB2	-0.53882777	-0.615034235	1.153862004	5
LAMC2	-0.628829106	-0.524293064	1.15312217	5
LAMP1	-0.603639333	-0.550655944	1.154295277	5
LAMP5	-0.624253803	-0.529140233	1.153394036	5
LAMTOR1	-0.607903403	-0.546248327	1.154151731	5
LAPTM4B	-0.507645392	-0.644354411	1.151999804	5
LARP6	-0.524571207	-0.628567239	1.153138446	5
LBP	-0.645200761	-0.506728856	1.151929618	5
LBX1	-0.540026353	-0.613886509	1.153912862	5
LBX2	-0.651864837	-0.499479101	1.151343938	5
LCA5	-0.514718523	-0.637793592	1.152512115	5
LCE1A	-0.50807139	-0.64396074	1.15203213	5
LCE1C	-0.55770029	-0.596779794	1.154480084	5
LCE2C	-0.555293432	-0.599129711	1.154423143	5
LCE2D	-0.539308499	-0.61457409	1.153882589	5

LCE3A	-0.525402334	-0.627784257	1.153186591	5
LCE3D	-0.632732107	-0.520137121	1.152869228	5
LCE3E	-0.588845784	-0.565777946	1.15462373	5
LCE4A	-0.531839036	-0.621695506	1.153534541	5
LCE6A	-0.727624005	-0.412668828	1.140292833	5
LCN1	-0.593438027	-0.56111167	1.154549697	5
LCN12	-0.733054288	-0.406113496	1.139167784	5
LCT	-0.559295253	-0.595219	1.154514253	5
LDB3	-0.622839122	-0.530633627	1.153472749	5
LDHAL6B	-0.703441039	-0.441297783	1.144738822	5
LDHC	-0.60650383	-0.547697454	1.154201284	5
LECT2	-0.78154415	-0.345360763	1.126904914	5
LEFTY1	-0.587213089	-0.567430964	1.154644053	5
LEFTY2	-0.560976209	-0.593570972	1.154547181	5
LEKR1	-0.663707748	-0.486448648	1.150156397	5
LEMD1	-0.553358172	-0.601014511	1.154372683	5
LEPR	-0.554974014	-0.599441088	1.154415101	5
LGALS12	-0.670267444	-0.479148886	1.14941633	5
LGALS16	-0.569215744	-0.585446769	1.154662513	5
LGALS7	-0.581757553	-0.572931742	1.154689295	5
LGALS7B	-0.534978134	-0.618710006	1.15368814	5
LGALS9B	-0.665438243	-0.484528674	1.149966917	5
LGI1	-0.541220809	-0.612741183	1.153961992	5
LGI3	-0.562753163	-0.591825375	1.154578539	5

LGR4	-0.521311686	-0.631630879	1.152942565	5
LGR5	-0.541193718	-0.612767177	1.153960895	5
LHCGR	-0.608838794	-0.545278486	1.15411728	5
LHFPL2	-0.665585252	-0.484365378	1.14995063	5
LHFPL3	-0.596855999	-0.557622342	1.154478341	5
LHFPL4	-0.666837557	-0.482973134	1.149810691	5
LHFPL5	-0.55852465	-0.59597345	1.1544981	5
LHFPL6	-0.53063846	-0.62283454	1.153473	5
LHX1	-0.558898785	-0.59560724	1.154506026	5
LHX2	-0.572789532	-0.581899028	1.154688561	5
LHX3	-0.614819404	-0.539052243	1.153871648	5
LHX5	-0.547372298	-0.606818067	1.154190366	5
LHX9	-0.520969056	-0.631952267	1.152921322	5
LILRA6	-0.630813816	-0.52218216	1.152995976	5
LILRB2	-0.777382314	-0.350739404	1.128121717	5
LIMS3	-0.532315401	-0.621243131	1.153558532	5
LIN28B	-0.516435821	-0.636192808	1.152628628	5
LINC02210-CRHR1	-0.627585168	-0.525613541	1.153198709	5
LINGO1	-0.535480399	-0.618231333	1.153711732	5
LINGO2	-0.663074374	-0.487150348	1.150224723	5
LIPC	-0.514217882	-0.638259686	1.152477568	5
LIPF	-0.510700545	-0.641526929	1.152227474	5
LIPG	-0.556588602	-0.597865984	1.154454587	5
LIPI	-0.550238349	-0.604044246	1.154282595	5

LIX1	-0.581537246	-0.573153147	1.154690392	5
LMBRD1	-0.677704265	-0.470800693	1.148504957	5
LMLN	-0.535776467	-0.617949045	1.153725511	5
LMNA	-0.583776816	-0.570899789	1.154676604	5
LMNTD1	-0.626310436	-0.526964671	1.153275107	5
LMO1	-0.59028071	-0.564322566	1.154603276	5
LMO3	-0.545786212	-0.608349232	1.154135444	5
LMO7	-0.668427955	-0.481201899	1.149629854	5
LMOD2	-0.561700116	-0.592860269	1.154560385	5
LMTK3	-0.543044772	-0.610989247	1.154034019	5
LMX1A	-0.546814122	-0.607357231	1.154171352	5
LMX1B	-0.556295912	-0.598151732	1.154447644	5
LOC100132202	-0.523058689	-0.62999026	1.153048949	5
LOC101930434	-0.677463035	-0.471072704	1.148535739	5
LOC102723532	-0.589217418	-0.565401248	1.154618666	5
LOC102723713	-0.588032987	-0.566601253	1.154634239	5
LOC102723728	-0.574952919	-0.579744306	1.154697225	5
LOC102724770	-0.610878977	-0.543159449	1.154038426	5
LOC102724788	-0.518516934	-0.634248747	1.152765681	5
LOC102724971	-0.671055851	-0.478267524	1.149323375	5
LOC102725023	-0.506613828	-0.64530692	1.151920748	5
LOC105369591	-0.510787373	-0.64144643	1.152233803	5
LOC105371419	-0.787504969	-0.337600332	1.125105301	5
LOC105372704	-0.665011668	-0.485002338	1.150014006	5

LOC105373057	-0.662945939	-0.487292571	1.150238511	5
LOC105374301	-0.562435816	-0.592137383	1.154573199	5
LOC105375816	-0.624043025	-0.529362898	1.153405924	5
LOC105376722	-0.549288847	-0.604964195	1.154253042	5
LOC105379554	-0.540525858	-0.613407738	1.153933596	5
LOC107983983	-0.600636574	-0.55374657	1.154383144	5
LOC107984512	-0.702352613	-0.442565217	1.144917829	5
LOC107985555	-0.622596583	-0.530889408	1.153485991	5
LOC107985805	-0.672489794	-0.476662298	1.149152092	5
LOC107987243	-0.772281195	-0.35728816	1.129569355	5
LOC107987248	-0.661326532	-0.489083899	1.15041043	5
LOC112268052	-0.654921045	-0.496134535	1.15105558	5
LOC112268092	-0.615443761	-0.538399702	1.153843463	5
LOC112268320	-0.565352469	-0.589265529	1.154617998	5
LOC112268387	-0.723563686	-0.41753946	1.141103147	5
LOC118142757	-0.510578972	-0.641639627	1.1522186	5
LOC389831	-0.549184946	-0.605064802	1.154249748	5
LOC647264	-0.638671337	-0.513775484	1.152446821	5
LOC730098	-0.603842295	-0.55044665	1.154288945	5
LONRF2	-0.563018944	-0.591563979	1.154582923	5
LORICRIN	-0.717962467	-0.424215691	1.142178158	5
LOX	-0.632223656	-0.520679625	1.152903281	5
LOXL4	-0.651267804	-0.500131007	1.151398811	5
LPIN3	-0.60486253	-0.549393829	1.154256358	5

LRAT	-0.56362995	-0.590962751	1.154592701	5
LRATD1	-0.523116705	-0.629935722	1.153052427	5
LRCH2	-0.53767124	-0.616140215	1.153811455	5
LRFN3	-0.677201142	-0.471367922	1.148569064	5
LRIG3	-0.527638087	-0.625674373	1.153312459	5
LRIT1	-0.532868844	-0.620717254	1.153586098	5
LRP11	-0.547860102	-0.6063466	1.154206702	5
LRP2	-0.554513182	-0.599890117	1.1544033	5
LRP2BP	-0.560615552	-0.593924832	1.154540384	5
LRP4	-0.545902112	-0.608237438	1.154139551	5
LRP5	-0.55263211	-0.601720569	1.154352679	5
LRP6	-0.506517956	-0.645395388	1.151913344	5
LRRC10B	-0.554007352	-0.600382722	1.154390073	5
LRRC17	-0.59153324	-0.563050194	1.154583434	5
LRRC2	-0.642568176	-0.509576673	1.152144849	5
LRRC31	-0.752994953	-0.381624513	1.134619466	5
LRRC32	-0.507383936	-0.644595935	1.151979871	5
LRRC3B	-0.598421022	-0.55601999	1.154441012	5
LRRC4B	-0.542950223	-0.611080151	1.154030374	5
LRRC4C	-0.551963363	-0.602370375	1.154333738	5
LRRC53	-0.530841708	-0.622641818	1.153483527	5
LRRC55	-0.53543621	-0.618273457	1.153709667	5
LRRC71	-0.570459463	-0.584213769	1.154673232	5
LRRC73	-0.538923339	-0.614942778	1.153866117	5

LRRC74A	-0.754788563	-0.379388824	1.134177387	5
LRRC8B	-0.533514904	-0.620102957	1.153617861	5
LRRC9	-0.545324345	-0.608794587	1.154118932	5
LRRD1	-0.673056683	-0.476026903	1.149083586	5
LRRIQ1	-0.550619463	-0.603674714	1.154294177	5
LRRN1	-0.548166334	-0.606050489	1.154216823	5
LRRN2	-0.525852028	-0.627360307	1.153212335	5
LRRN4CL	-0.615062478	-0.538798254	1.153860732	5
LRRTM2	-0.605725565	-0.548502242	1.154227807	5
LRRTM3	-0.549097714	-0.605149259	1.154246973	5
LRRTM4	-0.548465782	-0.605760839	1.154226621	5
LRTM1	-0.541287711	-0.612676987	1.153964698	5
LRTM2	-0.559377837	-0.595138108	1.154515945	5
LRTOMT	-0.577710614	-0.576989849	1.154700463	5
LSM12	-0.721720704	-0.419741607	1.141462311	5
LTBP1	-0.515553637	-0.637015524	1.152569161	5
LUC7L	-0.65250384	-0.498780839	1.151284679	5
LURAP1L	-0.737665313	-0.400509539	1.138174852	5
LUZP2	-0.573546664	-0.58114554	1.154692204	5
LUZP4	-0.682241732	-0.465668861	1.147910593	5
LVRN	-0.535345321	-0.618360093	1.153705414	5
LY6G5C	-0.672775326	-0.476342317	1.149117643	5
LY6G6C	-0.525284934	-0.627894901	1.153179835	5
LY6G6D	-0.52219914	-0.63079787	1.15299701	5

LY6G6F-LY6G6D	-0.642795688	-0.509330919	1.152126607	5
LY6H	-0.547621115	-0.606577616	1.154198731	5
LY6K	-0.833974054	-0.27465266	1.108626715	5
LYN	-0.793095648	-0.330259902	1.12335555	5
LYNX1-SLURP2	-0.533895446	-0.619740915	1.15363636	5
LYPD1	-0.550142176	-0.60413747	1.154279647	5
LYPD5	-0.565295028	-0.589322181	1.154617209	5
LYPD6	-0.534708015	-0.618967325	1.15367534	5
LZTS1	-0.573586171	-0.581106205	1.154692376	5
LZTS3	-0.614980995	-0.538883405	1.153864399	5
MAB21L1	-0.753705644	-0.380739319	1.134444963	5
MAB21L4	-0.642770157	-0.5093585	1.152128657	5
MAEL	-0.532252676	-0.621302711	1.153555387	5
MAGEA3	-0.722876181	-0.418361576	1.141237757	5
MAGEA4	-0.697366062	-0.448349234	1.145715296	5
MAGEA8	-0.549861658	-0.604409332	1.154270989	5
MAGEA9	-0.720880328	-0.420743979	1.141624307	5
MAGEA9B	-0.519500413	-0.633328452	1.152828865	5
MAGEB1	-0.553728726	-0.60065394	1.154382667	5
MAGEB10	-0.689401918	-0.457510825	1.146912743	5
MAGEB2	-0.528180954	-0.625161264	1.153342219	5
MAGEB5	-0.773258088	-0.356037737	1.129295825	5
MAGEB6	-0.558002075	-0.596484692	1.154486767	5
MAGED1	-0.644372902	-0.507625378	1.15199828	5

MAGED4	-0.571697163	-0.582984985	1.154682147	5
MAGEL2	-0.553902649	-0.600484651	1.1543873	5
MAGI2	-0.534186996	-0.619463431	1.153650428	5
MAGI3	-0.532476425	-0.621090161	1.153566586	5
MAIP1	-0.512420907	-0.639930502	1.152351408	5
MAL2	-0.563280984	-0.591306184	1.154587168	5
MALL	-0.818860073	-0.295623959	1.114484031	5
MAMDC4	-0.615124453	-0.538733485	1.153857938	5
MAN1A1	-0.703476286	-0.44125671	1.144732996	5
MAN2A2	-0.538982044	-0.614886594	1.153868638	5
MANBAL	-0.68248276	-0.465395442	1.147878201	5
MAOB	-0.510460133	-0.641749777	1.15220991	5
MAP1A	-0.534550861	-0.619116996	1.153667856	5
MAP1LC3B	-0.671172244	-0.478137335	1.149309579	5
MAP1LC3B2	-0.75695614	-0.376679563	1.133635704	5
MAP1LC3C	-0.573097816	-0.581592307	1.154690123	5
MAP2	-0.541594432	-0.612382609	1.153977041	5
MAP3K19	-0.567276901	-0.587365389	1.15464229	5
MAP3K21	-0.702031747	-0.442938515	1.144970261	5
MAP3K5	-0.751702829	-0.383231679	1.134934508	5
MAP4K5	-0.746192414	-0.390053622	1.136246036	5
MAP6	-0.542138524	-0.611860161	1.153998685	5
MAP9	-0.547183397	-0.607000573	1.154183969	5
MAPK10	-0.535657611	-0.618062381	1.153719991	5

MAPK12	-0.516020528	-0.636580207	1.152600735	5
MAPK4	-0.591327313	-0.563259511	1.154586823	5
MAPK8IP1	-0.51619381	-0.636418586	1.152612396	5
MAPK8IP2	-0.540668984	-0.613270503	1.153939487	5
MAPRE2	-0.521515031	-0.631440083	1.152955114	5
MAPRE3	-0.626565571	-0.52669441	1.153259981	5
MARCHF11	-0.569572076	-0.585093688	1.154665764	5
MARCHF2	-0.522345195	-0.630660696	1.153005891	5
MARCHF4	-0.558586112	-0.5959133	1.154499413	5
MARCHF6	-0.612197362	-0.541787391	1.153984754	5
MARVELD2	-0.505282046	-0.646535011	1.151817057	5
MAS1L	-0.700102344	-0.445179947	1.14528229	5
MASP1	-0.554946052	-0.59946834	1.154414392	5
MAST4	-0.552626712	-0.601725816	1.154352528	5
MATN2	-0.523579888	-0.629500175	1.153080063	5
MATN3	-0.543984157	-0.610085542	1.154069698	5
MATN4	-0.707763441	-0.436246866	1.144010307	5
MBL2	-0.529868339	-0.623564371	1.153432711	5
MBOAT7	-0.808417786	-0.309825778	1.118243565	5
MBTPS2	-0.53474706	-0.618930135	1.153677195	5
MC2R	-0.53236714	-0.621193983	1.153561123	5
MC4R	-0.591580861	-0.563001782	1.154582643	5
MCC	-0.514497968	-0.63799896	1.152496928	5
MCHR1	-0.528150545	-0.625190015	1.15334056	5

MCIDAS	-0.596969915	-0.557505808	1.154475723	5
MCM10	-0.510797267	-0.641437257	1.152234524	5
MDFI	-0.534129451	-0.619518208	1.153647659	5
MDGA2	-0.585323997	-0.569339663	1.15466366	5
MDH1B	-0.542226201	-0.611775942	1.154002142	5
MDK	-0.700883338	-0.444273317	1.145156656	5
MDM1	-0.736263046	-0.402217454	1.138480499	5
ME1	-0.509269282	-0.64285274	1.152122022	5
MED12L	-0.635935784	-0.516711243	1.152647027	5
MED7	-0.732986171	-0.406196021	1.139182192	5
MEF2B	-0.559682166	-0.594839947	1.154522113	5
MEGF10	-0.55643895	-0.598012099	1.154451049	5
MEGF11	-0.630865839	-0.522126762	1.152992601	5
MEIOB	-0.525805174	-0.627404489	1.153209663	5
MEIS1	-0.518837764	-0.633948641	1.152786405	5
MEIS3	-0.552573358	-0.601777677	1.154351035	5
MEP1B	-0.667487641	-0.482249553	1.149737195	5
METTL24	-0.558352135	-0.596142258	1.154494392	5
MEX3A	-0.562354533	-0.592217281	1.154571813	5
MEX3B	-0.523095158	-0.629955978	1.153051136	5
MFAP2	-0.542633542	-0.611384554	1.154018096	5
MFAP4	-0.540220404	-0.613700545	1.153920949	5
MFRP	-0.507732433	-0.644273991	1.152006424	5
MFSD1	-0.827696931	-0.283423607	1.111120538	5

MFSD2A	-0.631483695	-0.521468554	1.152952249	5
MGAT4B	-0.780681236	-0.346478631	1.127159867	5
MGAT4C	-0.59993968	-0.554462302	1.154401982	5
MGAT4D	-0.502671007	-0.648937452	1.151608459	5
MGAT5B	-0.556939897	-0.597522895	1.154462793	5
MIA2	-0.608337769	-0.545798096	1.154135866	5
MICU3	-0.715652827	-0.426954303	1.14260713	5
MID1	-0.528039495	-0.625294999	1.153334494	5
MIER2	-0.683694053	-0.464020103	1.147714157	5
MINAR2	-0.752116305	-0.382717703	1.134834007	5
MINDY2	-0.629361402	-0.523727415	1.153088817	5
MINDY4	-0.580808826	-0.573884793	1.154693618	5
MINK1	-0.56371227	-0.590881717	1.154593987	5
MIOX	-0.565550536	-0.589070156	1.154620692	5
MIP	-0.83422503	-0.274300133	1.108525163	5
MIPOL1	-0.538127179	-0.615704377	1.153831556	5
MIS18A	-0.680551945	-0.467583413	1.148135359	5
MISP	-0.539593242	-0.614301422	1.153894664	5
MISP3	-0.643435125	-0.508639848	1.152074973	5
MITF	-0.783713331	-0.342544493	1.126257824	5
MLH3	-0.531312187	-0.622195539	1.153507725	5
MLIP	-0.572105186	-0.582579517	1.154684703	5
MLN	-0.503382969	-0.648283065	1.151666034	5
MLXIP	-0.608612606	-0.545513103	1.154125708	5

MLXIPL	-0.538747676	-0.615110874	1.15385855	5
MMD	-0.523775989	-0.629315706	1.153091695	5
MMD2	-0.549956879	-0.604317059	1.154273938	5
MMP10	-0.561150696	-0.593399722	1.154550418	5
MMP13	-0.514181726	-0.638293337	1.152475062	5
MMP15	-0.553064952	-0.601299723	1.154364675	5
MMP16	-0.552751812	-0.601604206	1.154356017	5
MMP21	-0.79381925	-0.329305391	1.123124641	5
MMP24	-0.576591007	-0.578109199	1.154700206	5
MMP24-AS1-EDEM2	-0.580112782	-0.574583343	1.154696125	5
MMP8	-0.677350218	-0.471199888	1.148550106	5
MMRN1	-0.505777545	-0.646078305	1.15185585	5
MND1	-0.514717878	-0.637794193	1.152512071	5
MNS1	-0.529835606	-0.623595378	1.153430984	5
MNX1	-0.724240281	-0.41672966	1.140969941	5
MOB1B	-0.818388678	-0.296270032	1.11465871	5
MOBP	-0.588965206	-0.565656915	1.154622121	5
MOCS1	-0.574972866	-0.579724414	1.15469728	5
MOGAT2	-0.706223333	-0.438049792	1.144273125	5
MOGAT3	-0.798873476	-0.322609574	1.12148305	5
MOK	-0.644926195	-0.507026295	1.15195249	5
MORF4L1	-0.782567245	-0.344033575	1.12660082	5
MORN5	-0.55038595	-0.60390115	1.154287099	5
MOV10L1	-0.790894284	-0.333157465	1.124051749	5

MPDZ	-0.506463597	-0.645445546	1.151909143	5
MPP2	-0.571049467	-0.583628233	1.1546777	5
MPP3	-0.519615353	-0.63322083	1.152836183	5
MPPED1	-0.562133949	-0.592434066	1.154568015	5
MPPED2	-0.554314319	-0.600083814	1.154398134	5
MPZL3	-0.779807784	-0.347608728	1.127416512	5
MRAP2	-0.593697043	-0.560847734	1.154544777	5
MRC2	-0.832870075	-0.276201648	1.109071723	5
MRE11	-0.827468228	-0.283741512	1.11120974	5
MRGPRD	-0.802864656	-0.317286156	1.120150811	5
MRGPRG	-0.710313572	-0.433253622	1.143567194	5
MRGPRX2	-0.66111107	-0.489321965	1.150433035	5
MRGPRX3	-0.510684287	-0.641542002	1.152226288	5
MRGPRX4	-0.552010029	-0.602325046	1.154335075	5
MROH8	-0.591251509	-0.563336549	1.154588059	5
MRPL41	-0.70946714	-0.434248231	1.143715371	5
MRPS26	-0.629566103	-0.523509792	1.153075895	5
MRPS30	-0.542829914	-0.611195809	1.154025722	5
MS4A10	-0.679732521	-0.468510369	1.14824289	5
MS4A12	-0.69815759	-0.447433593	1.145591183	5
MS4A15	-0.527139476	-0.626145373	1.15328485	5
MS4A18	-0.644430055	-0.507563514	1.151993569	5
MS4A2	-0.556543994	-0.597909541	1.154453535	5
MS4A6E	-0.510524159	-0.641690434	1.152214593	5

MS4A8	-0.556728014	-0.597729846	1.15445786	5
MSH5	-0.726495283	-0.414025447	1.140520729	5
MSI1	-0.549143104	-0.605105314	1.154248418	5
MSRA	-0.811577501	-0.305552567	1.117130068	5
MSX2	-0.778537068	-0.349250272	1.127787339	5
MT1A	-0.684311084	-0.4633187	1.147629784	5
MT1B	-0.529438835	-0.62397113	1.153409965	5
MT1HL1	-0.728014401	-0.412199135	1.140213535	5
MT1X	-0.529312284	-0.624090942	1.153403226	5
MT2A	-0.520239504	-0.632636175	1.152875678	5
MT3	-0.605161341	-0.549085234	1.154246575	5
MTHFD2	-0.668168068	-0.481491575	1.149659643	5
MTMR8	-0.814473689	-0.301617484	1.116091172	5
MTNR1A	-0.801763635	-0.318757887	1.120521523	5
MTRF1L	-0.821946778	-0.291381664	1.113328442	5
MTRNR2L2	-0.763703968	-0.368192806	1.131896774	5
MUC13	-0.538878802	-0.614985399	1.153864201	5
MUC16	-0.545492173	-0.608632786	1.154124959	5
MUC22	-0.555112333	-0.599306265	1.154418598	5
MUC5AC	-0.544383441	-0.609701131	1.154084572	5
MUC5B	-0.508320102	-0.643730814	1.152050916	5
MUSK	-0.595621477	-0.558884243	1.15450572	5
MXRA5	-0.55627396	-0.598173159	1.15444712	5
MYADM	-0.728818864	-0.411230496	1.14004936	5

MYADML2	-0.541669089	-0.612310941	1.15398003	5
MYBPC1	-0.547501285	-0.606693425	1.154194711	5
MYBPHL	-0.753375803	-0.381150257	1.13452606	5
MYCN	-0.516676522	-0.63596819	1.152644712	5
MYCT1	-0.580724551	-0.573969401	1.154693952	5
MYH1	-0.552120534	-0.6022177	1.154338234	5
MYH10	-0.529618508	-0.623800997	1.153419504	5
MYH13	-0.593747679	-0.560796126	1.154543805	5
MYH2	-0.646835251	-0.504956154	1.151791405	5
MYH4	-0.610431944	-0.543624196	1.15405614	5
MYH6	-0.565905588	-0.588719822	1.15462541	5
MYH7	-0.576109253	-0.578590397	1.15469965	5
MYH8	-0.577859424	-0.576840965	1.154700389	5
MYH9	-0.591443903	-0.563141008	1.15458491	5
MYL1	-0.634862828	-0.517860088	1.152722915	5
MYL12A	-0.822994982	-0.289936358	1.11293134	5
MYL7	-0.562032126	-0.592534117	1.154566243	5
MYLK	-0.526927569	-0.626345466	1.153273035	5
MYO18B	-0.634493497	-0.518255197	1.152748694	5
MYO1C	-0.579692242	-0.575005125	1.154697367	5
MYO3A	-0.672827309	-0.476284051	1.149111359	5
MYO3B	-0.517592225	-0.635113121	1.152705345	5
MYO5B	-0.642404354	-0.509753588	1.152157943	5
MYO6	-0.504104841	-0.647619038	1.151723878	5

MYOC	-0.555986385	-0.598453814	1.154440199	5
MYOCD	-0.594347444	-0.560184626	1.15453207	5
MYOG	-0.547025105	-0.607153475	1.154178579	5
MYOZ1	-0.568998635	-0.585661826	1.154660461	5
MYOZ2	-0.631177796	-0.521794492	1.152972288	5
MYOZ3	-0.542210806	-0.61179073	1.154001536	5
MYPN	-0.737950218	-0.400162139	1.138112357	5
MYRF	-0.625194452	-0.528145852	1.153340304	5
MYRFL	-0.750728035	-0.384442249	1.135170284	5
MYT1	-0.537907912	-0.615914005	1.153821917	5
MYT1L	-0.566126063	-0.588502205	1.154628267	5
N4BP1	-0.640807264	-0.51147649	1.152283754	5
NAALADL2	-0.653192435	-0.498027776	1.151220212	5
NACAD	-0.54624539	-0.607906238	1.154151628	5
NAE1	-0.670939486	-0.478397663	1.149337149	5
NALCN	-0.559965064	-0.59456269	1.154527754	5
NALF1	-0.533067928	-0.620528006	1.153595934	5
NALF2	-0.576888121	-0.577812294	1.154700415	5
NANOGNB	-0.801180691	-0.319536119	1.12071681	5
NAT14	-0.618873162	-0.534806872	1.153680033	5
NAT16	-0.610509317	-0.543543775	1.154053092	5
NAT8L	-0.53523857	-0.618461837	1.153700407	5
NAV2	-0.523754102	-0.629336297	1.153090398	5
NAV3	-0.532271899	-0.621284452	1.153556351	5

NCAM1	-0.505205085	-0.646605924	1.151811009	5
NCAM2	-0.567096466	-0.587543725	1.154640191	5
NCAN	-0.555597185	-0.5988335	1.154430685	5
NCBP2L	-0.517891456	-0.634833512	1.152724968	5
NCCRP1	-0.51912548	-0.633679417	1.152804898	5
NCK2	-0.526533831	-0.626717126	1.153250957	5
NCKAP1	-0.504237859	-0.647496619	1.151734479	5
NCKAP5	-0.526552667	-0.62669935	1.153252017	5
NCOA1	-0.579576311	-0.575121362	1.154697674	5
NCR2	-0.583253608	-0.571426741	1.154680349	5
NCR3LG1	-0.719888701	-0.421925328	1.141814029	5
NDEL1	-0.584536859	-0.570133736	1.154670595	5
NDNF	-0.536815619	-0.616957507	1.153773126	5
NDP	-0.595038303	-0.559479719	1.154518022	5
NDRG4	-0.51421082	-0.638266259	1.152477078	5
NDST3	-0.513352558	-0.639064679	1.152417237	5
NDST4	-0.563327152	-0.591260756	1.154587908	5
NDUFS6	-0.575853862	-0.578845385	1.154699247	5
NECAB1	-0.549636362	-0.604627611	1.154263974	5
NECAP1	-0.555886463	-0.598551309	1.154437772	5
NECTIN1	-0.581118021	-0.573574303	1.154692324	5
NECTIN3	-0.510485189	-0.641726554	1.152211743	5
NECTIN4	-0.696393013	-0.449473587	1.1458666	5
NEDD4L	-0.567857818	-0.586790979	1.154648797	5

NEFH	-0.639024854	-0.513395388	1.152420241	5
NEFM	-0.538755891	-0.615103014	1.153858905	5
NEK1	-0.526058918	-0.627165189	1.153224107	5
NEK10	-0.639662295	-0.512709609	1.152371904	5
NEK2	-0.509369802	-0.642759695	1.152129497	5
NEK4	-0.733320939	-0.405790371	1.13911131	5
NEK5	-0.671653274	-0.477599088	1.149252362	5
NELL1	-0.603960228	-0.550325014	1.154285243	5
NEMF	-0.543987617	-0.610082211	1.154069828	5
NEU4	-0.608144932	-0.545998005	1.154142937	5
NEURL1	-0.58127685	-0.573414766	1.154691616	5
NEURL2	-0.757535644	-0.375953856	1.1334895	5
NEUROD1	-0.578672948	-0.57602658	1.154699528	5
NEUROD2	-0.596957375	-0.557518637	1.154476012	5
NEUROD6	-0.577593489	-0.577107015	1.154700504	5
NEUROG1	-0.577395379	-0.577305158	1.154700537	5
NEUROG2	-0.543748717	-0.61031213	1.154060846	5
NEUROG3	-0.831816307	-0.277677618	1.109493926	5
NEXMIF	-0.521427597	-0.631522126	1.152949723	5
NEXN	-0.521353994	-0.631591186	1.15294518	5
NFASC	-0.534598278	-0.619071839	1.153670117	5
NFATC4	-0.553115629	-0.601250437	1.154366066	5
NFKBIA	-0.734221377	-0.404698373	1.13891975	5
NFKBIL1	-0.610704911	-0.543340441	1.154045353	5

NFS1	-0.523044021	-0.630004049	1.15304807	5
NGEF	-0.545109212	-0.60900195	1.154111161	5
NGF	-0.624008126	-0.529399761	1.153407886	5
NGFR	-0.588981741	-0.565640155	1.154621896	5
NHLH2	-0.605221664	-0.549022924	1.154244587	5
NID1	-0.515194778	-0.637349959	1.152544737	5
NIM1K	-0.581738068	-0.572951326	1.154689394	5
NINL	-0.545968995	-0.608172919	1.154141914	5
NIPAL2	-0.52795174	-0.625377952	1.153329692	5
NKAIN1	-0.552036979	-0.602298867	1.154335847	5
NKAIN4	-0.531191849	-0.622309709	1.153501558	5
NKD2	-0.548691499	-0.605542441	1.154233941	5
NKX1-2	-0.622055242	-0.531460039	1.153515281	5
NKX2-1	-0.550487707	-0.603802484	1.154290191	5
NKX2-2	-0.751879756	-0.383011784	1.13489154	5
NKX2-6	-0.772933302	-0.356453657	1.129386958	5
NKX2-8	-0.664936191	-0.485086121	1.150022312	5
NKX6-1	-0.505162832	-0.646644854	1.151807686	5
NLGN1	-0.540301152	-0.61362315	1.153924302	5
NLGN2	-0.544570064	-0.609521401	1.154091464	5
NLGN3	-0.508738515	-0.64334386	1.152082376	5
NLGN4X	-0.540803175	-0.613141815	1.15394499	5
NLRP10	-0.784302936	-0.341777464	1.126080401	5
NLRP11	-0.668061556	-0.481610269	1.149671825	5

NLRP13	-0.801695533	-0.318848839	1.120544372	5
NLRP14	-0.514953346	-0.637574883	1.152528229	5
NLRP8	-0.596706889	-0.557774855	1.154481745	5
NMBR	-0.528624145	-0.624742136	1.153366281	5
NME5	-0.56982001	-0.584847931	1.154667941	5
NMNAT2	-0.53445846	-0.619204984	1.153663444	5
NMNAT3	-0.743756765	-0.393052599	1.136809364	5
NMRK2	-0.53734537	-0.616451581	1.15379695	5
NMS	-0.718985559	-0.42299992	1.141985479	5
NMU	-0.51754053	-0.635161416	1.152701946	5
NNAT	-0.527501855	-0.625803087	1.153304942	5
NOBOX	-0.577215838	-0.57748469	1.154700528	5
NOL4	-0.565872805	-0.588752175	1.15462498	5
NOS2	-0.516031182	-0.636570271	1.152601453	5
NOS3	-0.506317832	-0.645580028	1.15189786	5
NOTCH2	-0.759785161	-0.373131245	1.132916406	5
NOTCH4	-0.611214834	-0.542810122	1.154024956	5
NOVA1	-0.542420892	-0.611588898	1.15400979	5
NPAS1	-0.69341867	-0.452901765	1.146320435	5
NPAS2	-0.562964334	-0.591617695	1.154582029	5
NPAS3	-0.544984869	-0.609121778	1.154106647	5
NPAS4	-0.541964881	-0.612026931	1.153991812	5
NPB	-0.5648798	-0.58973159	1.15461139	5
NPC1L1	-0.507889395	-0.644128947	1.152018343	5

NPEPPS	-0.617206061	-0.536555244	1.153761305	5
NPFFR2	-0.580136427	-0.574559623	1.154696049	5
NPHP4	-0.808578707	-0.309608647	1.118187354	5
NPHS1	-0.698503947	-0.447032635	1.145536582	5
NPL	-0.520613371	-0.632285768	1.152899139	5
NPM2	-0.742935739	-0.394061272	1.136997011	5
NPPC	-0.587171915	-0.56747261	1.154644525	5
NPSR1	-0.50415159	-0.647576015	1.151727606	5
NPTN	-0.591336281	-0.563250395	1.154586677	5
NPTX1	-0.604662595	-0.549600249	1.154262844	5
NPTX2	-0.566384447	-0.5882471	1.154631546	5
NPTXR	-0.685660411	-0.461782963	1.147443374	5
NPY	-0.570817017	-0.58385897	1.154675987	5
NPY5R	-0.562869504	-0.591710964	1.154580468	5
NR0B1	-0.58204586	-0.572641914	1.154687774	5
NR0B2	-0.511546183	-0.640742595	1.152288779	5
NR1H4	-0.636192214	-0.516436456	1.152628671	5
NR2E1	-0.551907526	-0.602424608	1.154332134	5
NR2E3	-0.620512371	-0.533084373	1.153596744	5
NR2F1	-0.502704048	-0.648907094	1.151611142	5
NR2F2	-0.82710865	-0.284241104	1.111349754	5
NR4A2	-0.616922884	-0.536851882	1.153774766	5
NR6A1	-0.777106223	-0.351095074	1.128201297	5
NRAP	-0.545179718	-0.608933996	1.154113714	5

NRCAM	-0.543072923	-0.610962179	1.154035102	5
NREP	-0.538442858	-0.615402484	1.153845341	5
NRG2	-0.542551907	-0.611463006	1.154014913	5
NRG3	-0.548875602	-0.605364268	1.15423987	5
NRP2	-0.512619694	-0.639745836	1.15236553	5
NRSN1	-0.555145351	-0.599274078	1.154419429	5
NRSN2	-0.677573248	-0.470948438	1.148521686	5
NRXN1	-0.574864528	-0.579832448	1.154696976	5
NRXN3	-0.55904541	-0.595463679	1.154509089	5
NSG2	-0.559142254	-0.595368845	1.154511099	5
NT5DC3	-0.806496205	-0.312414461	1.118910665	5
NT5M	-0.532540556	-0.62102923	1.153569786	5
NTF3	-0.558931094	-0.595575609	1.154506703	5
NTM	-0.558632057	-0.595868334	1.154500391	5
NTN1	-0.541104302	-0.612852966	1.153957268	5
NTN3	-0.599961224	-0.554440185	1.154401408	5
NTNG1	-0.549471451	-0.604787352	1.154258803	5
NTNG2	-0.625576176	-0.527742004	1.15331818	5
NTRK2	-0.55201951	-0.602315837	1.154335347	5
NTS	-0.531363372	-0.622146972	1.153510344	5
NUMBL	-0.723472965	-0.417647987	1.141120952	5
NUP62CL	-0.505593503	-0.646247968	1.151841471	5
NWD1	-0.65695053	-0.493906642	1.150857172	5
NWD2	-0.522601163	-0.630420237	1.1530214	5

NXF2	-0.517751436	-0.634964362	1.152715798	5
NXF2B	-0.541184895	-0.612775642	1.153960537	5
NXF5	-0.714551683	-0.428257047	1.14280873	5
NXPE2	-0.726666516	-0.413819772	1.140486287	5
NXPH2	-0.575798147	-0.578901002	1.154699149	5
NXPH3	-0.539625553	-0.614270476	1.153896029	5
NXPH4	-0.740671662	-0.396836976	1.137508639	5
NYAP1	-0.524251798	-0.62886795	1.153119748	5
NYAP2	-0.611445453	-0.542570173	1.154015626	5
NYNRIN	-0.667180547	-0.48259144	1.149771986	5
OCA2	-0.544715105	-0.609381689	1.154096794	5
OCSTAMP	-0.716587448	-0.425847094	1.142434542	5
ODAD2	-0.530754348	-0.622724659	1.153479007	5
ODAD3	-0.687742147	-0.459408489	1.147150635	5
ODAM	-0.5630552	-0.591528315	1.154583515	5
ODF1	-0.556460606	-0.597990957	1.154451562	5
ODF3	-0.542309226	-0.611696183	1.154005409	5
ODF3L1	-0.636824366	-0.51575869	1.152583056	5
ODF4	-0.808051782	-0.31031943	1.118371212	5
OGDHL	-0.540540199	-0.613393988	1.153934187	5
OGN	-0.586964326	-0.567682548	1.154646874	5
OLFM1	-0.582414407	-0.572271281	1.154685688	5
OLFM3	-0.547232056	-0.606953565	1.154185621	5
OLFML2B	-0.681242546	-0.466801444	1.148043991	5

OMD	-0.687529162	-0.459651711	1.147180873	5
ONECUT3	-0.527058923	-0.626221441	1.153280364	5
OOEP	-0.564680776	-0.589927757	1.154608532	5
OOSP2	-0.576838795	-0.577861592	1.154700387	5
OPALIN	-0.652561819	-0.498717457	1.151279276	5
OPCML	-0.568268564	-0.586384603	1.154653167	5
OPHN1	-0.735723652	-0.402873557	1.138597209	5
OPN1LW	-0.605231277	-0.549012993	1.15424427	5
OPN1MW2	-0.580277985	-0.574417596	1.154695581	5
OPN1MW3	-0.573859712	-0.580833806	1.154693518	5
OPN4	-0.615016295	-0.538846517	1.153862812	5
OPRK1	-0.580516543	-0.574178197	1.15469474	5
OPTC	-0.505281354	-0.646535649	1.151817003	5
OR10A5	-0.521694842	-0.631271332	1.152966174	5
OR10A7	-0.624676406	-0.528693628	1.153370034	5
OR10G7	-0.617486774	-0.536261088	1.153747862	5
OR10H3	-0.520990943	-0.63193174	1.152922683	5
OR10H4	-0.509051618	-0.643054181	1.152105799	5
OR10J1	-0.733232042	-0.405898108	1.13913015	5
OR10J3	-0.74042883	-0.397134176	1.137563006	5
OR10K1	-0.810344249	-0.307222884	1.117567133	5
OR10Q1	-0.651771425	-0.499581129	1.151352554	5
OR10W1	-0.753743443	-0.380692214	1.134435657	5
OR10Z1	-0.607252669	-0.546922399	1.154175067	5

OR11H2	-0.517956183	-0.634773017	1.1527292	5
OR11H4	-0.590200282	-0.564404205	1.154604487	5
OR11L1	-0.552765327	-0.601591066	1.154356393	5
OR12D1	-0.635656665	-0.517010247	1.152666912	5
OR12D2	-0.613994741	-0.539913395	1.153908135	5
OR12D3	-0.508045016	-0.643985118	1.152030134	5
OR13C8	-0.571854343	-0.582828811	1.154683154	5
OR13C9	-0.621813572	-0.531714666	1.153528238	5
OR13D1	-0.564469256	-0.59013619	1.154605446	5
OR13F1	-0.677482228	-0.471051065	1.148533293	5
OR13H1	-0.626111529	-0.527175314	1.153286843	5
OR14A16	-0.802043888	-0.318383505	1.120427394	5
OR14I1	-0.66395653	-0.486172879	1.150129409	5
OR14J1	-0.825818873	-0.286030756	1.111849628	5
OR1A1	-0.779428073	-0.348099565	1.127527637	5
OR1A2	-0.581919442	-0.572769011	1.154688453	5
OR1B1	-0.673688806	-0.475317862	1.149006668	5
OR1E1	-0.618761381	-0.534924211	1.153685591	5
OR1E2	-0.57632727	-0.578372664	1.154699935	5
OR1G1	-0.740884078	-0.396576922	1.137461	5
OR1J4	-0.597212647	-0.557257445	1.154470092	5
OR1L1	-0.592024628	-0.562550514	1.154575142	5
OR1L3	-0.546693787	-0.607473421	1.154167209	5
OR1L4	-0.590545003	-0.564054241	1.154599244	5

OR1L6	-0.514894844	-0.637629376	1.15252422	5
OR1M1	-0.608330447	-0.545805688	1.154136135	5
OR1S2	-0.568763809	-0.585894372	1.154658181	5
OR2A2	-0.507258764	-0.644711539	1.151970303	5
OR2AG1	-0.654366888	-0.496741906	1.151108794	5
OR2AG2	-0.520547729	-0.632347302	1.152895031	5
OR2AJ1	-0.552145574	-0.602193373	1.154338948	5
OR2AK2	-0.784481937	-0.341544469	1.126026406	5
OR2AT4	-0.681756729	-0.466218793	1.147975522	5
OR2B6	-0.553665731	-0.600715249	1.15438098	5
OR2D3	-0.780309462	-0.346959817	1.12726928	5
OR2F1	-0.619731966	-0.53390485	1.153636815	5
OR2G2	-0.632605411	-0.520272333	1.152877744	5
OR2G3	-0.532935432	-0.620653961	1.153589393	5
OR2H1	-0.599628812	-0.554781384	1.154410197	5
OR2J1	-0.560153472	-0.594377989	1.154531461	5
OR2J2	-0.512456966	-0.639897007	1.152353973	5
OR2L13	-0.815116361	-0.30074189	1.11585825	5
OR2L2	-0.691493855	-0.455113351	1.146607206	5
OR2L8	-0.787744171	-0.3372875	1.125031672	5
OR2M4	-0.629701166	-0.523366174	1.15306734	5
OR2S2	-0.550601951	-0.603691697	1.154293648	5
OR2T11	-0.754105216	-0.380241253	1.134346469	5
OR2T27	-0.600988264	-0.553385151	1.154373415	5

OR2T29	-0.582059062	-0.57262864	1.154687702	5
OR2T34	-0.711597131	-0.43174327	1.143340401	5
OR2T5	-0.58439553	-0.570276234	1.154671764	5
OR2T6	-0.834846687	-0.273426319	1.108273006	5
OR2T7	-0.834854153	-0.27341582	1.108269973	5
OR2W1	-0.730338961	-0.409397335	1.139736296	5
OR2W3	-0.516284073	-0.636334384	1.152618457	5
OR2Y1	-0.612376813	-0.54160047	1.153977283	5
OR2Z1	-0.555368227	-0.599056783	1.15442501	5
OR3A2	-0.647225613	-0.504532262	1.151757875	5
OR3A3	-0.609597617	-0.544490929	1.154088546	5
OR4A15	-0.687590826	-0.4595813	1.147172125	5
OR4A16	-0.640170455	-0.512162537	1.152332992	5
OR4A5	-0.656397242	-0.494514571	1.150911813	5
OR4C13	-0.519635877	-0.633201612	1.152837488	5
OR4C16	-0.606700677	-0.547493781	1.154194458	5
OR4C3	-0.540650251	-0.613288466	1.153938717	5
OR4C45	-0.609575711	-0.544513674	1.154089386	5
OR4C46	-0.692708809	-0.453718017	1.146426826	5
OR4C6	-0.648686122	-0.502944517	1.151630639	5
OR4D11	-0.733143152	-0.406005823	1.139148976	5
OR4D5	-0.648133879	-0.503545202	1.151679081	5
OR4D6	-0.523306427	-0.629757348	1.153063774	5
OR4F15	-0.599974614	-0.554426437	1.154401052	5

OR4F17	-0.59821811	-0.556227907	1.154446018	5
OR4F21	-0.605325222	-0.548915942	1.154241164	5
OR4F29	-0.520393713	-0.63249166	1.152885373	5
OR4F3	-0.745395716	-0.391035681	1.136431398	5
OR4F5	-0.723450019	-0.417675435	1.141125454	5
OR4K1	-0.625681617	-0.52763042	1.153312037	5
OR4K13	-0.70912023	-0.434655555	1.143775785	5
OR4K15	-0.55871806	-0.595784157	1.154502217	5
OR4K5	-0.581553042	-0.573137274	1.154690316	5
OR4L1	-0.562168013	-0.592400592	1.154568605	5
OR4M1	-0.512195981	-0.640139399	1.15233538	5
OR4M2	-0.515922772	-0.636671371	1.152594143	5
OR4N2	-0.509147406	-0.642965538	1.152112944	5
OR4N4	-0.629046871	-0.524061698	1.153108569	5
OR4N5	-0.574720348	-0.579976203	1.154696551	5
OR4X1	-0.625673197	-0.52763933	1.153312528	5
OR51A2	-0.517313041	-0.635373912	1.152686952	5
OR51B5	-0.703076004	-0.441723051	1.144799056	5
OR51B6	-0.775832631	-0.352733936	1.128566568	5
OR51E2	-0.604518623	-0.54974886	1.154267484	5
OR51G1	-0.537960081	-0.615864134	1.153824215	5
OR51I1	-0.69908264	-0.446362315	1.145444955	5
OR51L1	-0.576200901	-0.578498875	1.154699776	5
OR51Q1	-0.777656323	-0.350386275	1.128042598	5

OR51V1	-0.702546826	-0.442339193	1.144886019	5
OR52A5	-0.711624152	-0.431711448	1.143335599	5
OR52B2	-0.686391869	-0.460949358	1.147341227	5
OR52B6	-0.746432963	-0.389756896	1.136189858	5
OR52E4	-0.611255786	-0.542767518	1.154023304	5
OR52E5	-0.694746114	-0.451373384	1.146119498	5
OR52E6	-0.673952	-0.475022477	1.148974477	5
OR52E8	-0.640224531	-0.5121043	1.152328831	5
OR52I2	-0.564858245	-0.589752838	1.154611083	5
OR52J3	-0.691875943	-0.454674769	1.146550712	5
OR52L1	-0.686122313	-0.461256647	1.14737896	5
OR52N5	-0.814265816	-0.301900509	1.116166325	5
OR56A5	-0.523333508	-0.629731882	1.153065391	5
OR5AC2	-0.52086992	-0.632045233	1.152915153	5
OR5AK2	-0.734605369	-0.404232288	1.138837657	5
OR5B17	-0.543581305	-0.61047321	1.154054515	5
OR5B3	-0.692877203	-0.453524452	1.146401655	5
OR5D14	-0.515737751	-0.636843888	1.152581639	5
OR5D16	-0.572975359	-0.581714157	1.154689516	5
OR5H2	-0.627433926	-0.525773955	1.153207881	5
OR5K1	-0.556775233	-0.597683731	1.154458963	5
OR5K4	-0.673724964	-0.475277287	1.149002251	5
OR5M10	-0.619232415	-0.534429651	1.153662067	5
OR5M11	-0.7406521	-0.396860922	1.137513022	5

OR5P2	-0.673058676	-0.476024669	1.149083345	5
OR5T3	-0.54362631	-0.61042991	1.15405622	5
OR5V1	-0.728311606	-0.411841396	1.140153002	5
OR5W2	-0.656342086	-0.494575151	1.150917237	5
OR6B1	-0.69088844	-0.455807846	1.146696286	5
OR6B2	-0.833169148	-0.275782292	1.108951441	5
OR6C3	-0.61158955	-0.542420213	1.154009764	5
OR6C6	-0.597987968	-0.556463667	1.154451635	5
OR6C70	-0.814020744	-0.302234065	1.116254809	5
OR6C76	-0.684458543	-0.463150997	1.14760954	5
OR6J1	-0.729729684	-0.410132539	1.139862223	5
OR6P1	-0.70957881	-0.434117075	1.143695885	5
OR6T1	-0.555794019	-0.598641498	1.154435518	5
OR7A10	-0.809005792	-0.309032122	1.118037914	5
OR7C1	-0.8165558	-0.298777592	1.115333392	5
OR7C2	-0.568927655	-0.585732123	1.154659778	5
OR7E24	-0.711487716	-0.431872116	1.143359832	5
OR7G1	-0.655655928	-0.495328448	1.150984376	5
OR7G2	-0.738670751	-0.399282957	1.137953708	5
OR7G3	-0.575399719	-0.579298625	1.154698344	5
OR8A1	-0.58273655	-0.571947186	1.154683736	5
OR8B2	-0.597232575	-0.557237051	1.154469627	5
OR8B3	-0.719440389	-0.422458906	1.141899295	5
OR8B4	-0.673821187	-0.475169302	1.148990489	5

OR8D2	-0.649679904	-0.501862544	1.151542448	5
OR8H1	-0.557317533	-0.597153928	1.154471461	5
OR8H3	-0.511022598	-0.641228311	1.152250909	5
OR8I2	-0.513455625	-0.638968839	1.152424464	5
OR8J1	-0.642952893	-0.509161069	1.152113963	5
OR8J3	-0.751862345	-0.383033425	1.13489577	5
OR8K1	-0.692301694	-0.454185816	1.14648751	5
OR9A4	-0.562344623	-0.59222702	1.154571644	5
OR9G1	-0.631114875	-0.52186152	1.152976395	5
OR9I1	-0.689185981	-0.457757938	1.146943919	5
OR9K2	-0.572305813	-0.582380076	1.154685889	5
OR9Q1	-0.562863534	-0.591716836	1.154580369	5
OSBPL11	-0.766507922	-0.364642559	1.131150481	5
OSBPL6	-0.540140008	-0.613777595	1.153917603	5
OST4	-0.554127386	-0.600265852	1.154393238	5
OSTF1	-0.803651222	-0.316233256	1.119884477	5
OTOF	-0.729566663	-0.410329152	1.139895816	5
OTOGL	-0.550040614	-0.604235909	1.154276522	5
OTOP1	-0.771648208	-0.358097452	1.12974566	5
OTOS	-0.783573572	-0.342726212	1.126299783	5
OTP	-0.523305883	-0.629757859	1.153063742	5
OTX1	-0.812157199	-0.30476633	1.116923529	5
OTX2	-0.542635683	-0.611382496	1.154018179	5
OVOL1	-0.617605483	-0.536136664	1.153742148	5

P2RX2	-0.553918336	-0.60046938	1.154387717	5
P2RX3	-0.544476625	-0.609611394	1.154088018	5
P3H3	-0.605144934	-0.549102181	1.154247115	5
P3H4	-0.632694247	-0.520177528	1.152871775	5
P3R3URF-PIK3R3	-0.683962347	-0.46371519	1.147677537	5
PABIR3	-0.61609056	-0.537723197	1.153813757	5
PABPC1L2A	-0.818529475	-0.296077112	1.114606587	5
PABPC1L2B	-0.552575893	-0.601775213	1.154351106	5
PABPC4L	-0.51226514	-0.640075173	1.152340314	5
PABPC5	-0.54526584	-0.608850983	1.154116824	5
PACRG	-0.806064516	-0.312994975	1.11905949	5
PACS2	-0.71144617	-0.431921035	1.143367205	5
PACSIN1	-0.734649931	-0.404178184	1.138828115	5
PACSIN3	-0.544659723	-0.609435039	1.154094762	5
PADI1	-0.734174868	-0.404754809	1.138929677	5
PADI2	-0.803461996	-0.316486668	1.119948664	5
PADI3	-0.686021754	-0.461371255	1.147393009	5
PAFAH1B2	-0.539350408	-0.614533964	1.153884372	5
PAGE3	-0.701512073	-0.44354278	1.145054852	5
PAH	-0.520837631	-0.63207551	1.152913141	5
PAK3	-0.555125547	-0.599293384	1.154418931	5
PAK5	-0.580378716	-0.574316518	1.154695234	5
PALS2	-0.5473321	-0.606856908	1.154189008	5
PAMR1	-0.533275985	-0.620330182	1.153606167	5

PANX2	-0.634472177	-0.518278	1.152750177	5
PAPOLB	-0.545339599	-0.608779882	1.154119481	5
PAPPA	-0.568938403	-0.585721479	1.154659882	5
PAPPA2	-0.558469904	-0.596027022	1.154496927	5
PARD3	-0.510719384	-0.641509464	1.152228848	5
PARM1	-0.557432536	-0.597041533	1.154474069	5
PATE1	-0.625510803	-0.527811179	1.153321982	5
PATE2	-0.623281704	-0.53016669	1.153448394	5
PATE4	-0.764658484	-0.366985813	1.131644297	5
PAX1	-0.770915713	-0.359033058	1.129948771	5
PAX2	-0.585714924	-0.568945021	1.154659946	5
PAX3	-0.527728339	-0.62558909	1.153317429	5
PAX6	-0.555291808	-0.599131294	1.154423103	5
PAX7	-0.541557827	-0.612417747	1.153975573	5
PBK	-0.531206484	-0.622295826	1.153502309	5
PBOV1	-0.76423802	-0.367517693	1.131755713	5
PBX1	-0.510085408	-0.642097005	1.152182413	5
PCARE	-0.584235919	-0.570437136	1.154673055	5
PCBP3	-0.787752527	-0.33727657	1.125029098	5
PCDH10	-0.549620169	-0.604643298	1.154263467	5
PCDH11Y	-0.800634141	-0.320265145	1.120899286	5
PCDH15	-0.563905526	-0.590691447	1.154596974	5
PCDH18	-0.518184711	-0.634559397	1.152744108	5
PCDH19	-0.539645766	-0.614251115	1.153896882	5

PCDH9	-0.764098105	-0.367694613	1.131792718	5
PCDHA11	-0.544217206	-0.609861195	1.154078401	5
PCDHA12	-0.55632856	-0.598119863	1.154448423	5
PCDHA13	-0.539804604	-0.614098966	1.15390357	5
PCDHA2	-0.515837503	-0.636750882	1.152588385	5
PCDHA3	-0.555835016	-0.598601502	1.154436519	5
PCDHA5	-0.556600684	-0.597854188	1.154454871	5
PCDHA6	-0.550470861	-0.603818819	1.15428968	5
PCDHA7	-0.556879482	-0.59758191	1.154461391	5
PCDHA8	-0.562451318	-0.592122145	1.154573463	5
PCDHA9	-0.753699484	-0.380746995	1.134446479	5
PCDHAC1	-0.527737692	-0.625580251	1.153317943	5
PCDHAC2	-0.551375966	-0.602940726	1.154316692	5
PCDHB16	-0.533348975	-0.620260771	1.153609746	5
PCDHB6	-0.787237055	-0.337950584	1.125187639	5
PCDHB7	-0.576474695	-0.578225401	1.154700096	5
PCDHB8	-0.545891774	-0.608247411	1.154139185	5
PCDHGA1	-0.584607012	-0.570062995	1.154670006	5
PCDHGA2	-0.600796049	-0.553582702	1.154378751	5
PCDHGA6	-0.789771664	-0.334631501	1.124403165	5
PCDHGB1	-0.52578063	-0.627427632	1.153208262	5
PCDHGB3	-0.541493214	-0.612479765	1.153972979	5
PCDHGB4	-0.556464009	-0.597987634	1.154451643	5
PCGF5	-0.674397167	-0.474522641	1.148919808	5

PCLO	-0.541032218	-0.61292212	1.153954338	5
PCMTD1	-0.773741833	-0.355417896	1.129159729	5
PCP4	-0.545316488	-0.608802161	1.154118649	5
PCP4L1	-0.530840515	-0.62264295	1.153483465	5
PCSK1	-0.539507609	-0.614383433	1.153891042	5
PCSK2	-0.556440179	-0.598010899	1.154451078	5
PCSK4	-0.653815529	-0.497345801	1.15116133	5
PCYT1B	-0.719131877	-0.422825912	1.141957789	5
PDC	-0.615973055	-0.537846137	1.153819192	5
PDE10A	-0.516301218	-0.636318389	1.152619607	5
PDE11A	-0.597450189	-0.557014325	1.154464514	5
PDE1A	-0.538594602	-0.615257328	1.15385193	5
PDE1B	-0.821327964	-0.292233792	1.113561757	5
PDE1C	-0.5369627	-0.616817071	1.153779771	5
PDE3A	-0.505992843	-0.645879784	1.151872627	5
PDE5A	-0.644895176	-0.507059892	1.151955068	5
PDGFA	-0.514272493	-0.638208856	1.152481349	5
PDGFC	-0.510827264	-0.641409444	1.152236708	5
PDGFRA	-0.510556945	-0.641660046	1.15221699	5
PDGFRB	-0.582352493	-0.572333557	1.15468605	5
PDGFRL	-0.544215683	-0.609862661	1.154078344	5
PDHA1	-0.792746343	-0.330720307	1.12346665	5
PDHA2	-0.522486796	-0.630527683	1.153014479	5
PDIA2	-0.546953723	-0.607222417	1.15417614	5

PDPN	-0.524826366	-0.628326939	1.153153305	5
PDYN	-0.587989453	-0.566645327	1.15463478	5
PDZD7	-0.626421298	-0.526847247	1.153268545	5
PDZK1IP1	-0.544809606	-0.609290649	1.154100255	5
PDZRN3	-0.502134577	-0.649430158	1.151564734	5
PDZRN4	-0.528720343	-0.624651133	1.153371476	5
PEAR1	-0.525118687	-0.628051556	1.153170243	5
PECAM1	-0.715624101	-0.426988313	1.142612413	5
PELI1	-0.647066363	-0.504705215	1.151771578	5
PELI2	-0.749199916	-0.386336711	1.135536627	5
PENK	-0.507001487	-0.644949099	1.151950586	5
PER1	-0.580406129	-0.574289008	1.154695137	5
PERM1	-0.611390055	-0.542627818	1.154017873	5
PEX5L	-0.5871424	-0.567502462	1.154644862	5
PF4	-0.526813511	-0.626453146	1.153266657	5
PF4V1	-0.532065015	-0.621480937	1.153545952	5
PFN2	-0.533619095	-0.620003847	1.153622942	5
PGAP1	-0.535965198	-0.617769047	1.153734246	5
PGBD5	-0.56276017	-0.591818485	1.154578655	5
PGC	-0.701702153	-0.443321805	1.145023958	5
PGD	-0.749340511	-0.386162577	1.135503088	5
PGK1	-0.764846965	-0.366747286	1.131594251	5
PGLYRP4	-0.590243722	-0.564360111	1.154603834	5
PHF21B	-0.540934709	-0.613015656	1.153950365	5

PHGDH	-0.565944909	-0.588681014	1.154625923	5
PHIP	-0.75410976	-0.380235587	1.134345348	5
PHKB	-0.57199151	-0.5826925	1.15468401	5
PHLPP1	-0.522200066	-0.630797	1.152997066	5
PHOSPHO2-KLHL23	-0.524533409	-0.62860283	1.153136239	5
PHOX2B	-0.553155829	-0.601211338	1.154367167	5
PHYHIP	-0.574446896	-0.580248784	1.15469568	5
PHYHIPL	-0.551303911	-0.603010663	1.154314575	5
PI15	-0.561699463	-0.59286091	1.154560373	5
PIANP	-0.758725243	-0.374462295	1.133187538	5
PIAS1	-0.790016726	-0.334309934	1.124326661	5
PIBF1	-0.584823993	-0.569844156	1.154668149	5
PIFO	-0.561610487	-0.592948295	1.154558782	5
PIGS	-0.551133236	-0.603176301	1.154309537	5
PIH1D2	-0.574372853	-0.580322576	1.154695429	5
PIMREG	-0.542388515	-0.611620006	1.154008521	5
PIP4P2	-0.624727169	-0.528639966	1.153367136	5
PIRT	-0.605308832	-0.548932875	1.154241707	5
PITX1	-0.531449122	-0.622065602	1.153514724	5
PIWIL1	-0.626100169	-0.527187342	1.153287512	5
PKD1L2	-0.618594814	-0.53509903	1.153693844	5
PKD1L3	-0.515172727	-0.637370505	1.152543232	5
PKDCC	-0.521900346	-0.631078426	1.152978772	5
PKDREJ	-0.830486182	-0.279537109	1.110023291	5

PKHD1	-0.734355822	-0.404535213	1.138891035	5
PKM	-0.666263819	-0.483611252	1.149875071	5
PKMYT1	-0.830184896	-0.27995775	1.110142647	5
PKN2	-0.573253989	-0.581436884	1.154690874	5
PKNOX2	-0.544713592	-0.609383147	1.154096739	5
PKP2	-0.588335136	-0.566295288	1.154630424	5
PLA2G12A	-0.725120111	-0.415675525	1.140795636	5
PLA2G12B	-0.570376608	-0.584295965	1.154672573	5
PLA2G2E	-0.803248642	-0.316772306	1.120020948	5
PLA2G2F	-0.760681936	-0.372003531	1.132685466	5
PLA2G3	-0.534859265	-0.618823252	1.153682517	5
PLA2G4C	-0.741487207	-0.395838121	1.137325329	5
PLA2G4E	-0.637919165	-0.51458367	1.152502835	5
PLA2G5	-0.506905408	-0.645037797	1.151943205	5
PLA2R1	-0.597872217	-0.556582219	1.154454436	5
PLAAT1	-0.588854791	-0.565768818	1.154623609	5
PLCB1	-0.535302072	-0.618401315	1.153703387	5
PLCD4	-0.507041195	-0.644912438	1.151953633	5
PLCH1	-0.524340476	-0.628784474	1.15312495	5
PLEKHA5	-0.506377382	-0.64552509	1.151902472	5
PLEKHA6	-0.510872192	-0.641367786	1.152239978	5
PLEKHG4B	-0.557774288	-0.596707444	1.154481732	5
PLEKHG5	-0.553276685	-0.601093782	1.154370467	5
PLEKHH1	-0.544485244	-0.609603093	1.154088337	5

PLEKHH3	-0.521611632	-0.631349428	1.15296106	5
PLEKHO1	-0.755338972	-0.378701638	1.13404061	5
PLEKHS1	-0.694777867	-0.451336794	1.14611466	5
PLIN1	-0.610852019	-0.543187483	1.154039501	5
PLK1	-0.573024859	-0.581664904	1.154689763	5
PLK5	-0.649928021	-0.501592204	1.151520225	5
PLOD2	-0.513054125	-0.639342123	1.152396249	5
PLP1	-0.557508902	-0.596966891	1.154475793	5
PLPP3	-0.524605289	-0.628535146	1.153140434	5
PLPP4	-0.783056509	-0.34339819	1.126454698	5
PLPPR1	-0.539564692	-0.614328766	1.153893457	5
PLPPR3	-0.541033791	-0.612920611	1.153954402	5
PLPPR4	-0.55406676	-0.600324881	1.154391642	5
PLPPR5	-0.529814897	-0.623614994	1.153429891	5
PLSCR5	-0.607994655	-0.546153762	1.154148417	5
PLTP	-0.625449827	-0.527875697	1.153325523	5
PLVAP	-0.820304219	-0.293641711	1.11394593	5
PLXDC2	-0.595551217	-0.558956007	1.154507224	5
PLXNA1	-0.646830571	-0.504961234	1.151791806	5
PLXNA2	-0.584822648	-0.569845513	1.154668161	5
PLXNA4	-0.513606697	-0.638828339	1.152435037	5
PLXNB1	-0.552053827	-0.602282502	1.154336329	5
PLXNB3	-0.51828091	-0.634469456	1.152750366	5
PMEPA1	-0.507581173	-0.644413741	1.151994914	5

PMP2	-0.576550085	-0.578150084	1.154700169	5
PNCK	-0.566791102	-0.587845452	1.154636554	5
PNLIPRP1	-0.503941183	-0.647769628	1.151710811	5
PNMA2	-0.555220045	-0.59920126	1.154421306	5
PNMA8A	-0.553369528	-0.601003463	1.154372991	5
PNMA8C	-0.576434005	-0.578266049	1.154700054	5
PNPLA3	-0.560600966	-0.59393914	1.154540106	5
PNPLA5	-0.529361759	-0.624044104	1.153405863	5
PNRC1	-0.785058243	-0.340793909	1.125852152	5
POC1B-GALNT4	-0.797872861	-0.323939198	1.121812058	5
PODN	-0.648201219	-0.503471976	1.151673195	5
PODNL1	-0.684250634	-0.46338744	1.147638074	5
PODXL2	-0.557481932	-0.596993252	1.154475185	5
POPDC3	-0.589987067	-0.564620592	1.154607659	5
POTEB	-0.562001494	-0.592564214	1.154565708	5
POTEB2	-0.551351184	-0.60296478	1.154315964	5
POTEB3	-0.816113899	-0.299381089	1.115494988	5
POTEG	-0.779100546	-0.348522727	1.127623274	5
POTEJ	-0.676779475	-0.471843042	1.148622517	5
POU3F2	-0.590425699	-0.564175375	1.154601074	5
POU3F3	-0.688058288	-0.459047343	1.147105631	5
POU3F4	-0.523491785	-0.629583038	1.153074824	5
POU4F3	-0.558364126	-0.596130525	1.154494651	5
POU6F2	-0.54467154	-0.609423656	1.154095196	5

PPBP	-0.509613471	-0.642534104	1.152147575	5
PPFIA2	-0.554237371	-0.600158752	1.154396123	5
PPIAL4A	-0.622156733	-0.531353085	1.153509818	5
PPIAL4F	-0.75943357	-0.373572993	1.133006563	5
PPIB	-0.749175074	-0.386367476	1.13554255	5
PPIC	-0.509890577	-0.642277482	1.152168059	5
PPM1E	-0.541181694	-0.612778714	1.153960408	5
PPP1R13B	-0.559575346	-0.594944614	1.15451996	5
PPP1R14A	-0.523175924	-0.629880049	1.153055973	5
PPP1R14C	-0.542890027	-0.611138022	1.154028049	5
PPP1R14D	-0.622675297	-0.530806405	1.153481701	5
PPP1R15B	-0.678959862	-0.469383549	1.148343411	5
PPP1R1A	-0.53123464	-0.622269113	1.153503753	5
PPP1R2C	-0.634299001	-0.518463199	1.1527622	5
PPP1R42	-0.586331552	-0.568322171	1.154653723	5
PPP1R9A	-0.524580209	-0.628558762	1.153138971	5
PPP2R2C	-0.538738799	-0.615119368	1.153858167	5
PPP4R3C	-0.562369086	-0.592202976	1.154572062	5
PPT2	-0.789432663	-0.335076141	1.124508804	5
PRAC1	-0.541252968	-0.612710325	1.153963293	5
PRAG1	-0.62865658	-0.524476323	1.153132903	5
PRAMEF1	-0.818035803	-0.296753356	1.11478916	5
PRAMEF12	-0.585638768	-0.569021915	1.154660683	5
PRAMEF15	-0.618803784	-0.534879701	1.153683485	5

PRAMEF17	-0.618215755	-0.53549674	1.153712495	5
PRAMEF18	-0.794223658	-0.328771485	1.122995143	5
PRAMEF2	-0.676280749	-0.472404665	1.148685414	5
PRAMEF22	-0.790713786	-0.33339463	1.124108415	5
PRAMEF25	-0.636140354	-0.516492036	1.15263239	5
PRAMEF26	-0.561498019	-0.593058739	1.154556758	5
PRAMEF33	-0.613551878	-0.540375506	1.153927383	5
PRAMEF5	-0.64425836	-0.507749349	1.15200771	5
PRAMEF6	-0.59718719	-0.557283496	1.154470686	5
PRAMEF8	-0.53923992	-0.614639748	1.153879668	5
PRAP1	-0.626770207	-0.526477582	1.153247789	5
PRB2	-0.52657654	-0.626676819	1.15325336	5
PRB3	-0.550022626	-0.604253342	1.154275968	5
PRDM11	-0.576936553	-0.577763887	1.15470044	5
PRDM12	-0.546632592	-0.607532503	1.154165095	5
PRDM13	-0.512940077	-0.639448126	1.152388203	5
PRDM14	-0.766351444	-0.364841055	1.131192499	5
PRDM6	-0.747808848	-0.388057809	1.135866657	5
PRDX5	-0.833915781	-0.274734492	1.108650273	5
PRELID3A	-0.512505656	-0.639851777	1.152357434	5
PRG3	-0.785906398	-0.339688152	1.125594551	5
PRICKLE1	-0.502293523	-0.649284197	1.151577721	5
PRICKLE4	-0.747389443	-0.388576073	1.135965517	5
PRIMA1	-0.561701785	-0.592858629	1.154560414	5

PRKAA2	-0.531362666	-0.622147641	1.153510307	5
PRKAR1A	-0.670076261	-0.47936248	1.14943874	5
PRKAR1B	-0.525291665	-0.627888558	1.153180223	5
PRKCD	-0.678056421	-0.470403453	1.148459874	5
PRKCG	-0.522462744	-0.630550277	1.153013022	5
PRKG1	-0.575448712	-0.579249741	1.154698453	5
PRKN	-0.743430809	-0.393453188	1.136883997	5
PRM1	-0.804731471	-0.314785186	1.119516657	5
PRM3	-0.712232084	-0.430995191	1.143227275	5
PRND	-0.567341611	-0.587301422	1.154643034	5
PRODH	-0.534705758	-0.618969474	1.153675232	5
PROM1	-0.549700727	-0.604565257	1.154265984	5
PROM2	-0.544440669	-0.609646021	1.15408669	5
PROS1	-0.523002082	-0.630043471	1.153045553	5
PROSER2	-0.564414667	-0.590189974	1.154604641	5
PRPF40B	-0.74856704	-0.387120144	1.135687184	5
PRPH	-0.521180223	-0.631754206	1.152934429	5
PRR15	-0.503270534	-0.648386442	1.151656976	5
PRR16	-0.526553662	-0.626698411	1.153252073	5
PRR18	-0.625681291	-0.527630765	1.153312056	5
PRR19	-0.774627894	-0.354281441	1.128909335	5
PRR23D1	-0.61128863	-0.542733347	1.154021977	5
PRR25	-0.791260138	-0.332676559	1.123936698	5
PRR30	-0.617597955	-0.536144556	1.153742511	5

PRR35	-0.797050597	-0.325030338	1.122080935	5
PRR36	-0.551024221	-0.603282081	1.154306302	5
PRR7	-0.736171787	-0.402328491	1.138500279	5
PRRG3	-0.56785657	-0.586792214	1.154648784	5
PRRT1	-0.652015307	-0.499314725	1.151330033	5
PRRT2	-0.573580277	-0.581112073	1.15469235	5
PRRT4	-0.6501301	-0.501371965	1.151502065	5
PRSS12	-0.527698479	-0.625617307	1.153315785	5
PRSS27	-0.613873638	-0.540039785	1.153913423	5
PRSS37	-0.832042353	-0.277361216	1.109403569	5
PRSS47	-0.805027571	-0.314387852	1.119415423	5
PRSS50	-0.520204665	-0.63266882	1.152873485	5
PRSS53	-0.551254567	-0.603058555	1.154313121	5
PRSS55	-0.625649446	-0.527664467	1.153313913	5
PRSS56	-0.530281842	-0.62317258	1.153454422	5
PRSS8	-0.55543739	-0.59898934	1.15442673	5
PRTFDC1	-0.509235962	-0.64288358	1.152119542	5
PRTG	-0.515621045	-0.636952689	1.152573733	5
PRTN3	-0.554364323	-0.600035114	1.154399437	5
PSAP	-0.817157339	-0.297955415	1.115112754	5
PSAPL1	-0.60268463	-0.551639764	1.154324394	5
PSAT1	-0.626756829	-0.526491759	1.153248588	5
PSCA	-0.600872702	-0.553503926	1.154376628	5
PSD	-0.527556053	-0.625751883	1.153307935	5

PSD2	-0.557564533	-0.596912511	1.154477044	5
PSG1	-0.527097801	-0.626184729	1.15328253	5
PSG11	-0.814894319	-0.301044504	1.115938823	5
PSG2	-0.552849003	-0.601509713	1.154358716	5
PSG5	-0.589825698	-0.564784327	1.154610025	5
PSMB6	-0.573704589	-0.580988292	1.154692881	5
PSORS1C2	-0.541195567	-0.612765403	1.15396097	5
PSPC1	-0.54413555	-0.609939808	1.154075359	5
PSPH	-0.792294371	-0.331315681	1.123610052	5
PSTPIP1	-0.761955618	-0.370399411	1.132355029	5
PTCH1	-0.673225687	-0.47583739	1.149063076	5
PTCH2	-0.735492163	-0.403154988	1.138647151	5
PTCHD1	-0.555083111	-0.599334749	1.154417861	5
PTCHD4	-0.539349161	-0.614535158	1.153884319	5
PTF1A	-0.649874793	-0.501650206	1.151524999	5
PTGES3L	-0.518721499	-0.634057408	1.152778907	5
PTGES3L-AARSD1	-0.511890827	-0.640422723	1.15231355	5
PTGFR	-0.629147632	-0.523954623	1.153102255	5
PTGFRN	-0.543735302	-0.610325038	1.15406034	5
PTGR1	-0.542061752	-0.611933899	1.15399565	5
PTGS2	-0.511592502	-0.640699613	1.152292115	5
PTH	-0.545986355	-0.608156171	1.154142526	5
PTH1R	-0.578917917	-0.575781201	1.154699118	5
PTH2	-0.627899859	-0.525279673	1.153179532	5

PTK7	-0.52950795	-0.623905689	1.153413639	5
PTN	-0.563816377	-0.590779224	1.154595601	5
PTP4A1	-0.507865638	-0.644150902	1.15201654	5
PTPN3	-0.506830329	-0.645107102	1.151937431	5
PTPN5	-0.549281022	-0.604971772	1.154252794	5
PTPRD	-0.553733018	-0.600649764	1.154382782	5
PTPRF	-0.523224214	-0.629834648	1.153058862	5
PTPRG	-0.520648735	-0.632252616	1.152901351	5
PTPRQ	-0.643875257	-0.508163865	1.152039122	5
PTPRS	-0.531292914	-0.622213825	1.153506739	5
PTPRT	-0.591056739	-0.563534462	1.154591201	5
PTPRU	-0.534569987	-0.619098781	1.153668769	5
PTPRZ1	-0.551308393	-0.603006314	1.154314707	5
PTX4	-0.511933529	-0.640383082	1.15231661	5
PURG	-0.68876948	-0.45823438	1.14700386	5
PVALB	-0.525215234	-0.627960583	1.153175817	5
PVALEF	-0.759632065	-0.373323626	1.132955691	5
PWWP3B	-0.539581792	-0.614312388	1.15389418	5
PXDN	-0.543097432	-0.610938613	1.154036044	5
PXMP2	-0.60065256	-0.553730144	1.154382705	5
PYCR1	-0.543541621	-0.61051139	1.15405301	5
PYDC2	-0.582680669	-0.572003414	1.154684083	5
PYGO1	-0.547276914	-0.606910227	1.154187141	5
PYGO2	-0.566431167	-0.588200964	1.154632131	5

PYY	-0.744857347	-0.391698704	1.136556051	5
QRFPR	-0.508368487	-0.643686076	1.152054563	5
QSOX1	-0.804616679	-0.314939177	1.119555856	5
RAB11FIP2	-0.583777886	-0.570898711	1.154676596	5
RAB27B	-0.51106444	-0.641189506	1.152253946	5
RAB31	-0.786222812	-0.339275287	1.125498099	5
RAB37	-0.54985122	-0.604419446	1.154270666	5
RAB3B	-0.554144576	-0.600249114	1.15439369	5
RAB5A	-0.578918029	-0.575781089	1.154699118	5
RAB5B	-0.619195477	-0.534468445	1.153663921	5
RAB6C	-0.612320777	-0.541658844	1.15397962	5
RAB6D	-0.682554485	-0.465314061	1.147868546	5
RAB8A	-0.684736698	-0.46283457	1.147571268	5
RABAC1	-0.74500099	-0.39152185	1.13652284	5
RABGAP1L	-0.742835759	-0.394184025	1.137019784	5
RAD50	-0.62092907	-0.532645966	1.153575036	5
RAD51AP2	-0.561090947	-0.593458366	1.154549313	5
RADIL	-0.514623129	-0.637882423	1.152505553	5
RAET1G	-0.504582206	-0.647179629	1.151761835	5
RAET1L	-0.548037095	-0.606175469	1.154212564	5
RAI1	-0.592540675	-0.562025451	1.154566126	5
RALYL	-0.57655793	-0.578142246	1.154700176	5
RAP1B	-0.811064739	-0.306247436	1.117312175	5
RARB	-0.533181729	-0.620419808	1.153601537	5

RARRES1	-0.525157607	-0.628014883	1.153172491	5
RASA1	-0.605648691	-0.548581695	1.154230387	5
RASEF	-0.627679068	-0.525513932	1.153193	5
RASGEF1C	-0.557686509	-0.596793267	1.154479776	5
RASGRF1	-0.556103385	-0.598339641	1.154443026	5
RASL10B	-0.57660342	-0.578096797	1.154700216	5
RASL11B	-0.572128928	-0.582555917	1.154684846	5
RASSF6	-0.528648504	-0.624719093	1.153367597	5
RAX	-0.538515594	-0.615332908	1.153848503	5
RAX2	-0.720721335	-0.420933495	1.14165483	5
RBFOX1	-0.620949993	-0.532623947	1.153573941	5
RBFOX2	-0.53019675	-0.623253219	1.153449969	5
RBFOX3	-0.694027893	-0.452200644	1.146228538	5
RBM20	-0.661220023	-0.48920159	1.150421612	5
RBM24	-0.54107682	-0.612879332	1.153956152	5
RBM27	-0.701119836	-0.443998596	1.145118432	5
RBM46	-0.733450325	-0.40563354	1.139083865	5
RBP1	-0.527672962	-0.625641419	1.153314381	5
RBP2	-0.554920377	-0.599493363	1.15441374	5
RBP3	-0.589211907	-0.565406835	1.154618742	5
RBPJL	-0.82186952	-0.291488097	1.113357616	5
RBPMS2	-0.668699801	-0.480898793	1.149598594	5
RBX1	-0.708558598	-0.435314607	1.143873204	5
RCAN2	-0.526080945	-0.627144412	1.153225358	5

RCOR3	-0.799348304	-0.321977922	1.121326226	5
RD3L	-0.517488796	-0.635209745	1.152698541	5
RDH8	-0.621080324	-0.53248678	1.153567103	5
RECQL4	-0.705209473	-0.439234703	1.144444177	5
REEP1	-0.551931515	-0.602401309	1.154332823	5
REEP2	-0.526263083	-0.626972596	1.153235679	5
REEP6	-0.789168643	-0.335422281	1.124590924	5
REG3A	-0.53995709	-0.613952875	1.153909965	5
REG3G	-0.506671195	-0.645253978	1.151925173	5
RELN	-0.545213291	-0.608901636	1.154114927	5
RELT	-0.651749471	-0.499605107	1.151354578	5
REM1	-0.514027876	-0.638436511	1.152464387	5
RESP18	-0.503864637	-0.647840053	1.15170469	5
RET	-0.547495171	-0.606699334	1.154194505	5
RFLNA	-0.555734521	-0.598699541	1.154434062	5
RFPL1	-0.799702623	-0.321506288	1.121208911	5
RFX3	-0.612131092	-0.541856411	1.153987503	5
RFX4	-0.54312548	-0.610911642	1.154037122	5
RFX6	-0.565121078	-0.589493717	1.154614795	5
RGR	-0.655259923	-0.495762913	1.151022836	5
RGS10	-0.674329303	-0.474598857	1.14892816	5
RGS11	-0.536876636	-0.61689925	1.153775885	5
RGS12	-0.5412538	-0.612709527	1.153963327	5
RGS16	-0.600996219	-0.553376974	1.154373193	5

RGS17	-0.505787856	-0.646068798	1.151856654	5
RGS20	-0.528521945	-0.624838805	1.153360751	5
RGS21	-0.660940621	-0.489510251	1.150450872	5
RGS3	-0.621299255	-0.532256315	1.153555569	5
RGS4	-0.5142512	-0.638228675	1.152479875	5
RGS6	-0.56765758	-0.586989017	1.154646598	5
RGS7	-0.640009996	-0.512335319	1.152345315	5
RGS7BP	-0.706523332	-0.437698881	1.144222212	5
RGS8	-0.520135935	-0.632733218	1.152869153	5
RGS9	-0.714742207	-0.428031775	1.142773983	5
RGSL1	-0.679762722	-0.468476222	1.148238944	5
RHBDL1	-0.74227145	-0.39487656	1.13714801	5
RHBDL2	-0.574333112	-0.58036218	1.154695292	5
RHBDL3	-0.531309247	-0.622198327	1.153507575	5
RHCE	-0.580790227	-0.573903466	1.154693693	5
RHCG	-0.53632352	-0.617427203	1.153750723	5
RHD	-0.60888132	-0.545234368	1.154115688	5
RHO	-0.591161223	-0.563428298	1.154589521	5
RHOBTB1	-0.509255282	-0.642865698	1.15212098	5
RHOD	-0.535691286	-0.618030271	1.153721557	5
RHOV	-0.633295117	-0.519536017	1.152831134	5
RICTOR	-0.522762104	-0.630269012	1.153031116	5
RIIAD1	-0.541745793	-0.612237302	1.153983094	5
RIMBP2	-0.543057229	-0.610977269	1.154034498	5

RIMS3	-0.539416075	-0.614471087	1.153887162	5
RIMS4	-0.544740354	-0.609357366	1.15409772	5
RIPOR1	-0.736740568	-0.40163621	1.138376778	5
RIPOR3	-0.607796808	-0.546358781	1.154155589	5
RIPPLY1	-0.548622157	-0.605609541	1.154231698	5
RIPPLY3	-0.578040326	-0.576659938	1.154700263	5
RIT2	-0.548377251	-0.605846484	1.154223734	5
RNASE11	-0.514147839	-0.638324875	1.152472713	5
RNASE12	-0.66822388	-0.481429374	1.149653254	5
RNASE8	-0.673620921	-0.475394035	1.149014955	5
RND2	-0.547391457	-0.606799556	1.154191012	5
RND3	-0.539663665	-0.614233972	1.153897637	5
RNF11	-0.529616105	-0.623803272	1.153419377	5
RNF111	-0.73195323	-0.407446524	1.139399754	5
RNF113B	-0.54924623	-0.605005463	1.154251692	5
RNF133	-0.665239967	-0.484748869	1.149988835	5
RNF148	-0.52482806	-0.628325343	1.153153403	5
RNF180	-0.516614081	-0.636026464	1.152640546	5
RNF183	-0.740965316	-0.396477446	1.137442762	5
RNF186	-0.717176079	-0.425149068	1.142325147	5
RNF208	-0.519687702	-0.63315308	1.152840782	5
RNF215	-0.710024596	-0.43359331	1.143617906	5
RNF223	-0.813721457	-0.30264124	1.116362697	5
RNF224	-0.623285177	-0.530163025	1.153448202	5

RNF39	-0.576425289	-0.578274755	1.154700045	5
RNF44	-0.556013108	-0.598427738	1.154440846	5
RNFT2	-0.565039678	-0.589573976	1.154613654	5
ROBO1	-0.53311792	-0.620480477	1.153598397	5
ROBO2	-0.54019467	-0.613725209	1.153919879	5
ROBO3	-0.682243668	-0.465666666	1.147910334	5
ROCK1	-0.580454235	-0.574240731	1.154694966	5
ROCK2	-0.51435785	-0.638129403	1.152487253	5
ROPN1	-0.775350495	-0.35335357	1.128704064	5
ROR1	-0.73133645	-0.408192388	1.139528838	5
ROR2	-0.568926673	-0.585733095	1.154659769	5
RORB	-0.560407804	-0.594128598	1.154536402	5
RP1	-0.620669978	-0.532918581	1.153588559	5
RPA1	-0.569676172	-0.584990514	1.154666687	5
RPE65	-0.527903605	-0.625423449	1.153327054	5
RPH3AL	-0.661944517	-0.488400728	1.150345246	5
RPL10L	-0.635556556	-0.517117463	1.152674019	5
RPL36A-HNRNPH2	-0.641952136	-0.510241769	1.152193904	5
RPS6KA2	-0.540490678	-0.613441467	1.153932145	5
RPS6KA6	-0.549698	-0.604567899	1.154265899	5
RRAGB	-0.524988046	-0.628174638	1.153162684	5
RRP36	-0.668632838	-0.480973465	1.149606304	5
RSPH1	-0.541236934	-0.61272571	1.153962645	5
RSPH6A	-0.515991094	-0.636607658	1.152598751	5

RSPH9	-0.710185052	-0.433404711	1.143589764	5
RSPO1	-0.547604589	-0.606593589	1.154198177	5
RSPO2	-0.549930527	-0.604342596	1.154273123	5
RSPO3	-0.532805415	-0.62077754	1.153582955	5
RSPO4	-0.560230397	-0.594302566	1.154532963	5
RTKN	-0.697446888	-0.448255777	1.145702665	5
RTL1	-0.512068314	-0.640257944	1.152326259	5
RTL3	-0.612540761	-0.541429662	1.153970423	5
RTL9	-0.595028618	-0.559489605	1.154518223	5
RTN2	-0.569545359	-0.585120166	1.154665525	5
RTN4RL1	-0.642819024	-0.509305708	1.152124732	5
RTN4RL2	-0.551489165	-0.602830841	1.154320007	5
RTP1	-0.811609951	-0.305508574	1.117118525	5
RUNDC3B	-0.545084164	-0.609026089	1.154110253	5
RXRG	-0.529939918	-0.623496564	1.153436482	5
RYBP	-0.543728727	-0.610331365	1.154060092	5
RYR2	-0.552333671	-0.602010617	1.154344287	5
RYR3	-0.540694931	-0.613245622	1.153940553	5
S100A14	-0.52661084	-0.626644448	1.153255288	5
S100A7A	-0.652492984	-0.498792707	1.151285691	5
SAA1	-0.508089849	-0.643943677	1.152033526	5
SAE1	-0.671278827	-0.478018102	1.149296929	5
SALL1	-0.555287195	-0.599135792	1.154422987	5
SALL2	-0.545692173	-0.608439928	1.154132101	5

SALL3	-0.529054492	-0.624334952	1.153389445	5
SALL4	-0.532842198	-0.62074258	1.153584778	5
SAMD11	-0.560861657	-0.593683381	1.154545038	5
SAMD14	-0.538201587	-0.615633228	1.153834815	5
SAMD5	-0.516863797	-0.635793387	1.152657184	5
SAXO1	-0.724367571	-0.416577229	1.140944799	5
SBDS	-0.508249927	-0.643795695	1.152045622	5
SBK2	-0.577680845	-0.57701963	1.154700475	5
SCAMP1	-0.726419765	-0.414116139	1.140535904	5
SCAMP5	-0.565987248	-0.588639226	1.154626475	5
SCEL	-0.572304386	-0.582381495	1.154685881	5
SCFD2	-0.70545799	-0.438944402	1.144402392	5
SCG2	-0.556944899	-0.59751801	1.154462909	5
SCG3	-0.540026215	-0.613886641	1.153912856	5
SCG5	-0.542345122	-0.611661696	1.154006819	5
SCGB1A1	-0.65812854	-0.492610921	1.15073946	5
SCGB1C2	-0.513435916	-0.638987166	1.152423083	5
SCGB1D4	-0.523016747	-0.630029686	1.153046433	5
SCGB2A1	-0.577350269	-0.577350269	1.154700538	5
SCHIP1	-0.526473792	-0.626773784	1.153247576	5
SCML1	-0.611559524	-0.542451463	1.154010987	5
SCN10A	-0.568896903	-0.585762577	1.154659481	5
SCN1A	-0.578834685	-0.57586458	1.154699265	5
SCN2A	-0.547757046	-0.606446226	1.154203272	5

SCN2B	-0.630762711	-0.522236577	1.152999289	5
SCN3B	-0.560602859	-0.593937283	1.154540142	5
SCN4A	-0.533334251	-0.620274774	1.153609025	5
SCN8A	-0.526268896	-0.626967111	1.153236008	5
SCN9A	-0.520152811	-0.632717406	1.152870217	5
SCNM1	-0.697646031	-0.448025472	1.145671503	5
SCNN1G	-0.568368849	-0.586285356	1.154654205	5
SCPEP1	-0.570616242	-0.584058216	1.154674458	5
SCRT1	-0.55965177	-0.594869731	1.154521502	5
SCRT2	-0.801691661	-0.31885401	1.120545671	5
SCUBE1	-0.560880513	-0.593664879	1.154545392	5
SDC1	-0.519257477	-0.633555876	1.152813352	5
SDC2	-0.524404277	-0.628724411	1.153128687	5
SDC4	-0.517779125	-0.634938488	1.152717613	5
SDK1	-0.543669208	-0.610388636	1.154057843	5
SEC14L2	-0.832467515	-0.276765794	1.109233308	5
SEC14L5	-0.526160156	-0.627069694	1.153229851	5
SEC14L6	-0.571941695	-0.582742006	1.154683702	5
SEC61A1	-0.553280495	-0.601090076	1.154370571	5
SECISBP2	-0.774253366	-0.354761983	1.12901535	5
SEH1L	-0.509850323	-0.642314765	1.152165088	5
SEL1L3	-0.533158501	-0.620441893	1.153600394	5
SELENOW	-0.804738547	-0.314775693	1.11951424	5
SELP	-0.542270487	-0.611733399	1.154003886	5

SEMA3A	-0.532281985	-0.621274872	1.153556857	5
SEMA3E	-0.594200314	-0.560334674	1.154534988	5
SEMA4D	-0.741078366	-0.396338996	1.137417362	5
SEMA5A	-0.572666559	-0.582021348	1.154687907	5
SEMA5B	-0.538045901	-0.615782088	1.153827989	5
SEMA6A	-0.538014658	-0.615811958	1.153826616	5
SEMA6D	-0.54190973	-0.612079893	1.153989623	5
SENP6	-0.754624049	-0.379594118	1.134218167	5
SEPTIN3	-0.557951195	-0.596534453	1.154485648	5
SEPTIN4	-0.565312472	-0.589304977	1.154617449	5
SEPTIN5	-0.5297802	-0.623647859	1.153428059	5
SERF2	-0.745859864	-0.390463674	1.136323538	5
SERPINA11	-0.575356196	-0.57934205	1.154698245	5
SERPINA12	-0.602562634	-0.551765402	1.154328036	5
SERPINA4	-0.582544519	-0.572140395	1.154684914	5
SERPINA7	-0.536464669	-0.617292507	1.153757176	5
SERPINA9	-0.567889918	-0.586759227	1.154649146	5
SERPINB1	-0.657182811	-0.493651299	1.15083411	5
SERPINB13	-0.668010574	-0.481667077	1.149677651	5
SERPINB3	-0.638905199	-0.513524056	1.152429256	5
SERPINB4	-0.554417709	-0.599983116	1.154400825	5
SERPINB7	-0.627462343	-0.525743817	1.15320616	5
SERPINE1	-0.564295122	-0.590307745	1.154602867	5
SERPINE2	-0.529398646	-0.624009181	1.153407827	5

SERTAD4	-0.53156412	-0.621956466	1.153520586	5
SERTM1	-0.522277844	-0.630723955	1.153001798	5
SERTM2	-0.685913715	-0.461494373	1.147408088	5
SEZ6L	-0.559854916	-0.594670653	1.154525568	5
SEZ6L2	-0.542983879	-0.611047793	1.154031673	5
SF3B1	-0.703393826	-0.441352798	1.144746624	5
SFRP2	-0.630080082	-0.522963132	1.153043214	5
SFTPA1	-0.629740586	-0.523324252	1.153064838	5
SFTPA2	-0.617073768	-0.536693838	1.153767606	5
SGCA	-0.567668778	-0.586977944	1.154646722	5
SGCZ	-0.787761802	-0.337264438	1.12502624	5
SGO1	-0.515769711	-0.63681409	1.152583801	5
SH2D4A	-0.600335908	-0.554055435	1.154391343	5
SH2D4B	-0.5478021	-0.606402673	1.154204773	5
SH2D5	-0.559316411	-0.595198277	1.154514688	5
SH2D7	-0.62332995	-0.530115774	1.153445724	5
SH3BGRL2	-0.710732511	-0.432760938	1.143493449	5
SH3BP4	-0.511426027	-0.640854086	1.152280113	5
SH3GL2	-0.598432262	-0.556008471	1.154440734	5
SH3GL3	-0.588616781	-0.566009989	1.15462677	5
SH3PXD2B	-0.517227199	-0.635454082	1.152681281	5
SH3RF1	-0.520003893	-0.632856924	1.152860817	5
SHANK1	-0.548404978	-0.605819661	1.154224639	5
SHANK2	-0.531517566	-0.622000648	1.153518214	5

SHC2	-0.509200769	-0.642916152	1.152116921	5
SHC3	-0.56162618	-0.592932883	1.154559063	5
SHC4	-0.561303172	-0.593250045	1.154553218	5
SHD	-0.563261208	-0.591325643	1.154586851	5
SHH	-0.648705894	-0.502923003	1.151628897	5
SHISA6	-0.531276133	-0.622229746	1.153505879	5
SHISA7	-0.682440865	-0.465442972	1.147883838	5
SHISA9	-0.576331052	-0.578368887	1.154699939	5
SHISAL1	-0.549581643	-0.604680619	1.154262261	5
SHOC2	-0.830321902	-0.279766494	1.110088396	5
SHOX	-0.732991048	-0.406190112	1.13918116	5
SHOX2	-0.538149151	-0.615683368	1.153832519	5
SHPRH	-0.539546157	-0.614346517	1.153892674	5
SHROOM2	-0.508372387	-0.64368247	1.152054857	5
SI	-0.573634999	-0.581057587	1.154692586	5
SIDT1	-0.622948187	-0.530518583	1.15346677	5
SIK1	-0.524204307	-0.628912652	1.153116959	5
SIK1B	-0.510184933	-0.642004797	1.15218973	5
SIRT2	-0.68472352	-0.462849564	1.147573084	5
SIX2	-0.507895529	-0.644123279	1.152018808	5
SIX3	-0.546379421	-0.607776887	1.154156308	5
SIX4	-0.525641501	-0.627558809	1.15320031	5
SIX6	-0.509930757	-0.642240266	1.152171022	5
SKA3	-0.506175634	-0.645711198	1.151886833	5

SKIDA1	-0.543356758	-0.610689218	1.154045975	5
SKOR2	-0.508978739	-0.643121617	1.152100356	5
SLAIN1	-0.513120557	-0.639280372	1.152400929	5
SLC10A3	-0.628246571	-0.524911686	1.153158258	5
SLC10A5	-0.749630309	-0.385803542	1.13543385	5
SLC11A1	-0.6622907	-0.488017804	1.150308503	5
SLC12A5	-0.570501153	-0.584172408	1.154673561	5
SLC12A9	-0.818540311	-0.296062262	1.114602573	5
SLC13A1	-0.638428931	-0.514036022	1.152464953	5
SLC13A4	-0.580174875	-0.574521049	1.154695925	5
SLC13A5	-0.624491685	-0.528888868	1.153380553	5
SLC15A5	-0.831431646	-0.278215775	1.109647421	5
SLC16A12	-0.631530232	-0.521418959	1.15294919	5
SLC16A2	-0.549842192	-0.604428193	1.154270385	5
SLC16A8	-0.697535785	-0.448152976	1.145688761	5
SLC16A9	-0.535418327	-0.618290505	1.153708831	5
SLC17A2	-0.506328074	-0.645570579	1.151898654	5
SLC17A4	-0.657337668	-0.493481026	1.150818694	5
SLC17A6	-0.541401725	-0.612567573	1.153969298	5
SLC17A7	-0.544845542	-0.609256026	1.154101568	5
SLC17A8	-0.541851656	-0.612135657	1.153987314	5
SLC19A3	-0.795175923	-0.327513024	1.122688947	5
SLC1A1	-0.546492183	-0.607668047	1.154160231	5
SLC1A2	-0.548481115	-0.605746004	1.15422712	5

SLC1A6	-0.566152796	-0.588475815	1.15462861	5
SLC22A10	-0.785288136	-0.340494328	1.125782464	5
SLC22A11	-0.539711981	-0.614187692	1.153899673	5
SLC22A12	-0.645642408	-0.506250211	1.151892619	5
SLC22A24	-0.591641771	-0.562939856	1.154581627	5
SLC22A7	-0.756359231	-0.377426456	1.133785687	5
SLC22A9	-0.833792487	-0.274907607	1.108700093	5
SLC24A2	-0.795277437	-0.327378764	1.1226562	5
SLC24A3	-0.532568053	-0.621003104	1.153571157	5
SLC25A2	-0.668381275	-0.481253937	1.149635212	5
SLC25A21	-0.510677369	-0.641548415	1.152225784	5
SLC25A23	-0.825604714	-0.286327561	1.111932275	5
SLC25A27	-0.524476853	-0.628656081	1.153132934	5
SLC25A31	-0.668753114	-0.480839337	1.149592451	5
SLC26A9	-0.686042229	-0.461347921	1.14739015	5
SLC27A6	-0.560840605	-0.593704038	1.154544643	5
SLC28A3	-0.644237584	-0.507771834	1.152009418	5
SLC29A4	-0.564669847	-0.589938527	1.154608374	5
SLC2A10	-0.537540125	-0.616265507	1.153805633	5
SLC2A12	-0.547452817	-0.606740263	1.15419308	5
SLC2A13	-0.670982218	-0.478349875	1.149332093	5
SLC30A1	-0.64547707	-0.50642943	1.1519065	5
SLC30A3	-0.63682841	-0.515754353	1.152582762	5
SLC30A8	-0.629886426	-0.523169141	1.153055567	5

SLC34A1	-0.659366822	-0.491246882	1.150613704	5
SLC34A2	-0.5153863	-0.637171489	1.152557789	5
SLC35G6	-0.696820868	-0.448979376	1.145800244	5
SLC37A1	-0.519680519	-0.633159807	1.152840326	5
SLC37A3	-0.827846066	-0.283216242	1.111062308	5
SLC38A3	-0.522029871	-0.630956819	1.15298669	5
SLC38A4	-0.583892463	-0.570783271	1.154675734	5
SLC38A8	-0.550738802	-0.603558969	1.154297771	5
SLC39A12	-0.572118095	-0.582566685	1.154684781	5
SLC39A5	-0.511939964	-0.640377107	1.152317072	5
SLC41A2	-0.622045657	-0.531470139	1.153515796	5
SLC44A2	-0.834576384	-0.27380637	1.108382754	5
SLC44A5	-0.510852829	-0.64138574	1.152238569	5
SLC45A1	-0.737483061	-0.4007317	1.13821476	5
SLC45A4	-0.832709789	-0.276426316	1.109136105	5
SLC4A11	-0.679961174	-0.468251807	1.14821298	5
SLC4A3	-0.585032437	-0.569633876	1.154666313	5
SLC4A4	-0.641001275	-0.511267373	1.152268648	5
SLC4A5	-0.545771292	-0.608363622	1.154134914	5
SLC5A1	-0.531277393	-0.62222855	1.153505944	5
SLC5A7	-0.511554427	-0.640734945	1.152289372	5
SLC66A1	-0.646401239	-0.50542721	1.151828448	5
SLC6A1	-0.551107873	-0.603200912	1.154308785	5
SLC6A11	-0.75859627	-0.374624126	1.133220395	5

SLC6A15	-0.551082583	-0.603225452	1.154308035	5
SLC6A17	-0.557294159	-0.59717677	1.154470929	5
SLC6A2	-0.649047718	-0.502550983	1.151598701	5
SLC6A20	-0.629620562	-0.523451886	1.153072448	5
SLC6A3	-0.592042471	-0.562532365	1.154574836	5
SLC6A5	-0.520353214	-0.632529615	1.152882829	5
SLC6A7	-0.749220607	-0.386311086	1.135531693	5
SLC7A10	-0.535973587	-0.617761046	1.153734633	5
SLC7A14	-0.575430878	-0.579267536	1.154698414	5
SLC7A3	-0.545616002	-0.608513385	1.154129386	5
SLC7A4	-0.55405638	-0.600334988	1.154391368	5
SLC7A8	-0.567231469	-0.587410296	1.154641765	5
SLC8A2	-0.51415971	-0.638313826	1.152473536	5
SLC8A3	-0.522359887	-0.630646896	1.153006783	5
SLC9A2	-0.562775893	-0.591803024	1.154578917	5
SLC9A4	-0.671994543	-0.477217031	1.149211573	5
SLC9A5	-0.695355513	-0.450670872	1.146026385	5
SLC9A6	-0.5756922	-0.579006753	1.154698953	5
SLC9A7	-0.531625417	-0.621898288	1.153523704	5
SLC9B2	-0.605416525	-0.54882161	1.154238135	5
SLC9C2	-0.555615085	-0.598816042	1.154431126	5
SLCO1B1	-0.597932096	-0.556520893	1.154452989	5
SLCO1B3	-0.514410506	-0.638080385	1.152490891	5
SLCO1B3-SLCO1B7	-0.541109908	-0.612847588	1.153957496	5

SLCO1C1	-0.72089339	-0.420728407	1.141621797	5
SLF1	-0.733470821	-0.405608694	1.139079515	5
SLIT1	-0.550679341	-0.603616641	1.154295982	5
SLIT3	-0.518232513	-0.634514706	1.152747219	5
SLITRK1	-0.557508767	-0.596967023	1.15447579	5
SLITRK2	-0.555176166	-0.599244038	1.154420204	5
SLITRK3	-0.551531278	-0.602789958	1.154321236	5
SLITRK6	-0.547194865	-0.606989494	1.154184359	5
SLN	-0.560301459	-0.594232885	1.154534345	5
SLURP1	-0.540106033	-0.613810154	1.153916187	5
SLURP2	-0.577350269	-0.577350269	1.154700538	5
SLX4	-0.594743837	-0.559780242	1.154524079	5
SMAD5	-0.76807456	-0.362652818	1.130727378	5
SMARCA1	-0.526768716	-0.626495431	1.153264148	5
SMARCD1	-0.74239469	-0.394725362	1.137120052	5
SMARCE1	-0.572294505	-0.582391319	1.154685824	5
SMCP	-0.572644104	-0.582043682	1.154687786	5
SMIM10L2B	-0.585026088	-0.569640282	1.15466637	5
SMIM2	-0.620081162	-0.533537817	1.153618979	5
SMIM21	-0.719227541	-0.422712126	1.141939667	5
SMIM27	-0.698030218	-0.447581	1.145611218	5
SMIM43	-0.535002351	-0.618686933	1.153689284	5
SMO	-0.535095341	-0.618598329	1.15369367	5
SMPX	-0.515433299	-0.637127687	1.152560986	5

SMR3A	-0.599424974	-0.554990547	1.15441552	5
SMTN	-0.514041607	-0.638423734	1.152465341	5
SMYD1	-0.543155587	-0.610882691	1.154038278	5
SNAP23	-0.621705839	-0.531828152	1.15353399	5
SNAP25	-0.55296224	-0.601399607	1.154361847	5
SNAP91	-0.554570863	-0.599833927	1.15440479	5
SNCAIP	-0.506354013	-0.64554665	1.151900663	5
SNCB	-0.801583113	-0.318998958	1.120582071	5
SNRK	-0.719793611	-0.422038529	1.141832141	5
SNTG1	-0.586401082	-0.568251912	1.154652994	5
SNTG2	-0.504905659	-0.646881761	1.151787421	5
SNTN	-0.609458874	-0.544634979	1.154093853	5
SNX16	-0.621536808	-0.532006179	1.153542987	5
SNX22	-0.7219425	-0.419476871	1.141419371	5
SNX31	-0.74117003	-0.396226722	1.137396752	5
SNX9	-0.67578793	-0.472959292	1.148747222	5
SOBP	-0.517170403	-0.635507121	1.152677524	5
SOGA3	-0.523825149	-0.629269455	1.153094604	5
SOHLH1	-0.573751986	-0.580941092	1.154693079	5
SOHLH2	-0.699001529	-0.446456299	1.145457828	5
SORBS3	-0.691608403	-0.454981889	1.146590292	5
SORCS1	-0.544705035	-0.60939139	1.154096425	5
SORT1	-0.576277902	-0.578421973	1.154699875	5
SOSTDC1	-0.726502434	-0.414016858	1.140519292	5

SOX1	-0.744068566	-0.392669239	1.136737805	5
SOX10	-0.524174747	-0.628940474	1.153115222	5
SOX14	-0.599975745	-0.554425276	1.154401021	5
SOX18	-0.711009165	-0.432435438	1.143444603	5
SOX3	-0.55084311	-0.603457789	1.154300899	5
SOX5	-0.52763342	-0.625678782	1.153312202	5
SOX9	-0.558502053	-0.595995564	1.154497616	5
SP110	-0.773774969	-0.355375422	1.129150391	5
SP5	-0.526165278	-0.627064862	1.153230141	5
SP6	-0.54654015	-0.607621745	1.154161895	5
SP7	-0.578450519	-0.57624932	1.154699839	5
SP8	-0.557921317	-0.596563672	1.154484989	5
SP9	-0.55717505	-0.597293159	1.154468209	5
SPACA1	-0.700083642	-0.445201646	1.145285287	5
SPACA4	-0.802896146	-0.317244026	1.120140173	5
SPACA9	-0.682708733	-0.465139024	1.147847757	5
SPAG17	-0.54132652	-0.612639746	1.153966265	5
SPAG6	-0.53835591	-0.615485646	1.153841555	5
SPANXA1	-0.787158346	-0.338053457	1.125211802	5
SPANXA2	-0.794475133	-0.328439323	1.122914456	5
SPANXN4	-0.571837575	-0.582845473	1.154683048	5
SPATA16	-0.594232633	-0.560301717	1.15453435	5
SPATA17	-0.558757967	-0.595745094	1.154503061	5
SPATA18	-0.502904635	-0.648722774	1.151627409	5

SPATA31A1	-0.567485834	-0.58715884	1.154644674	5
SPATA31A3	-0.598348086	-0.556094731	1.154442817	5
SPATA31A5	-0.612129789	-0.541857768	1.153987557	5
SPATA31A6	-0.70724119	-0.436858642	1.144099832	5
SPATA31A7	-0.594923927	-0.55959646	1.154520387	5
SPATA31C2	-0.557876393	-0.596607604	1.154483996	5
SPATA31D3	-0.576218257	-0.578481542	1.154699799	5
SPATA31D4	-0.611605238	-0.542403885	1.154009124	5
SPATA31E1	-0.516302771	-0.636316941	1.152619712	5
SPATA32	-0.810029482	-0.307648699	1.117678181	5
SPATS2	-0.5608726	-0.593672643	1.154545243	5
SPC25	-0.660193393	-0.49033521	1.150528603	5
SPDEF	-0.630298981	-0.522730212	1.153029193	5
SPDYE4	-0.592383262	-0.562185648	1.15456891	5
SPEF1	-0.644334651	-0.50766678	1.152001431	5
SPEG	-0.567106933	-0.587533381	1.154640313	5
SPEM1	-0.575037434	-0.57966002	1.154697454	5
SPEM2	-0.64419645	-0.507816349	1.152012799	5
SPEN	-0.574759679	-0.57993699	1.154696669	5
SPHKAP	-0.526713948	-0.626547129	1.153261077	5
SPINK1	-0.548988572	-0.605254918	1.154243489	5
SPO11	-0.830749483	-0.279169334	1.109918817	5
SPOCD1	-0.59965564	-0.554753852	1.154409493	5
SPOCK1	-0.564671366	-0.58993703	1.154608396	5

SPOCK3	-0.535543629	-0.618171054	1.153714683	5
SPPL2A	-0.690498287	-0.456255121	1.146753408	5
SPPL2C	-0.602203204	-0.552135455	1.154338659	5
SPRR2F	-0.505337862	-0.646483577	1.15182144	5
SPRR2G	-0.692517137	-0.453938289	1.146455426	5
SPRR4	-0.569649881	-0.585016574	1.154666455	5
SPRY1	-0.577753792	-0.576946653	1.154700444	5
SPSB1	-0.612976391	-0.540975643	1.153952034	5
SPSB4	-0.549564961	-0.604696777	1.154261738	5
SPTBN4	-0.517584726	-0.635120126	1.152704852	5
SPTLC3	-0.507806396	-0.644205647	1.152012043	5
SPTSSA	-0.75345414	-0.381052676	1.134506816	5
SPX	-0.527036858	-0.626242277	1.153279134	5
SPZ1	-0.626958469	-0.526278057	1.153236526	5
SQSTM1	-0.739251647	-0.398573533	1.13782518	5
SRARP	-0.524259354	-0.628860838	1.153120192	5
SRC	-0.617453319	-0.53629615	1.153749469	5
SRCIN1	-0.5550216	-0.599394706	1.154416307	5
SRD5A2	-0.633581986	-0.519229581	1.152811567	5
SREBF2	-0.516465976	-0.63616467	1.152630647	5
SRGAP1	-0.578217704	-0.576482399	1.154700104	5
SRGAP3	-0.551891516	-0.602440157	1.154331673	5
SRPX2	-0.705227146	-0.439214063	1.144441209	5
SRRM3	-0.527950971	-0.625378679	1.15332965	5

SRRM4	-0.550543264	-0.60374861	1.154291874	5
SRSF12	-0.63758534	-0.514942121	1.15252746	5
SSBP2	-0.543302687	-0.610741224	1.154043911	5
SSC4D	-0.751410715	-0.383594618	1.135005333	5
SSPN	-0.587450897	-0.567190391	1.154641288	5
SSR1	-0.719529389	-0.422353003	1.141882393	5
SST	-0.531975531	-0.621565909	1.15354144	5
SSTR1	-0.515973661	-0.636623915	1.152597576	5
SSTR2	-0.597709273	-0.55674908	1.154458352	5
SSTR5	-0.583268951	-0.571411293	1.154680244	5
ST18	-0.551470979	-0.602848496	1.154319475	5
ST3GAL5	-0.641832756	-0.510370597	1.152203353	5
ST6GAL2	-0.514865563	-0.637656649	1.152522212	5
ST6GALNAC5	-0.636095352	-0.516540262	1.152635615	5
ST7	-0.694557075	-0.451591196	1.146148272	5
ST8SIA2	-0.556829598	-0.597630633	1.154460231	5
ST8SIA3	-0.512940903	-0.639447359	1.152388262	5
ST8SIA5	-0.622723168	-0.530755921	1.153479089	5
ST8SIA6	-0.513176457	-0.639228406	1.152404863	5
STAC2	-0.576111667	-0.578587986	1.154699653	5
STARD6	-0.570801725	-0.583874148	1.154675873	5
STC1	-0.524035114	-0.629071888	1.153107002	5
STC2	-0.559059792	-0.595449596	1.154509388	5
STEAP1	-0.513388812	-0.639030968	1.15241978	5

STEAP2	-0.552023937	-0.602311536	1.154335474	5
STH	-0.750362369	-0.384895941	1.13525831	5
STK24	-0.509248751	-0.642871742	1.152120494	5
STK32A	-0.553133614	-0.601232944	1.154366559	5
STK33	-0.50278305	-0.648834504	1.151617554	5
STK40	-0.736419869	-0.402026609	1.138446478	5
STMN2	-0.544221162	-0.609857387	1.154078548	5
STMN4	-0.559650977	-0.594870509	1.154521486	5
STMND1	-0.694367582	-0.45180948	1.146177061	5
STOM	-0.792252604	-0.33137068	1.123623284	5
STOML3	-0.539304634	-0.614577791	1.153882425	5
STON1	-0.510662717	-0.641561998	1.152224715	5
STON1-GTF2A1L	-0.526832996	-0.626434752	1.153267747	5
STOX2	-0.522767811	-0.630263649	1.15303146	5
STPG1	-0.736495241	-0.401934871	1.138430112	5
STPG2	-0.536493029	-0.61726544	1.153758469	5
STPG3	-0.568834336	-0.585824536	1.154658872	5
STPG4	-0.643116558	-0.508984206	1.152100764	5
STRA6	-0.536055103	-0.61768329	1.153738393	5
STRADB	-0.648556735	-0.50308529	1.151642025	5
STRIP2	-0.531676574	-0.62184973	1.153526304	5
STRN	-0.570644619	-0.584030058	1.154674677	5
STRN4	-0.691622132	-0.454966132	1.146588263	5
STUM	-0.530486547	-0.622978555	1.153465103	5

STX11	-0.645972449	-0.505892353	1.151864802	5
STX1A	-0.612277795	-0.541703615	1.15398141	5
STX7	-0.650461247	-0.501010942	1.151472189	5
STXBP1	-0.553716528	-0.600665813	1.154382341	5
STXBP5L	-0.564302868	-0.590300114	1.154602983	5
STYXL2	-0.604197548	-0.550080194	1.154277741	5
SUCNR1	-0.53140599	-0.622106531	1.153512522	5
SULF1	-0.531965633	-0.621575307	1.153540941	5
SULF2	-0.684841611	-0.462715193	1.147556804	5
SULT1C2	-0.627763665	-0.525424182	1.153187847	5
SULT1C3	-0.599079192	-0.555345245	1.154424437	5
SULT1C4	-0.554882004	-0.59953076	1.154412764	5
SULT1E1	-0.586753843	-0.567895362	1.154649205	5
SULT4A1	-0.559773911	-0.594750042	1.154523952	5
SV2A	-0.579648022	-0.575049464	1.154697486	5
SVEP1	-0.544205667	-0.609872304	1.154077971	5
SYBU	-0.53957945	-0.614314632	1.153894081	5
SYCP1	-0.766633436	-0.364483309	1.131116745	5
SYDE2	-0.771977784	-0.357676171	1.129653955	5
SYN1	-0.579616657	-0.575080912	1.154697569	5
SYN2	-0.739906956	-0.397772562	1.137679518	5
SYN3	-0.57904375	-0.575655131	1.154698881	5
SYNDIG1	-0.656922671	-0.493937262	1.150859933	5
SYNE2	-0.621942589	-0.53157874	1.15352133	5

SYNGR3	-0.764889481	-0.366693472	1.131582953	5
SYNGR4	-0.511658429	-0.64063843	1.15229686	5
SYNPO2L	-0.536182283	-0.617561962	1.153744245	5
SYNPR	-0.574580048	-0.580116066	1.154696115	5
SYP	-0.516141157	-0.636467699	1.152608856	5
SYT1	-0.545428975	-0.608693718	1.154122693	5
SYT10	-0.534565974	-0.619102603	1.153668577	5
SYT12	-0.556178133	-0.598266691	1.154444824	5
SYT13	-0.552485999	-0.601862584	1.154348583	5
SYT14	-0.555354845	-0.599069831	1.154424676	5
SYT16	-0.588616985	-0.566009782	1.154626767	5
SYT2	-0.807916391	-0.31050197	1.118418361	5
SYT3	-0.558176273	-0.596314306	1.154490579	5
SYT4	-0.555670449	-0.59876204	1.154432489	5
SYT5	-0.58240316	-0.572282594	1.154685754	5
SYT6	-0.550367438	-0.603919097	1.154286536	5
SYT7	-0.534023352	-0.619619191	1.153642543	5
SYT8	-0.794321969	-0.328641644	1.122963614	5
SYT9	-0.564275692	-0.590326885	1.154602577	5
TAAR2	-0.562646971	-0.591929794	1.154576765	5
TAC1	-0.536480254	-0.617277632	1.153757887	5
TAC3	-0.753401486	-0.381118266	1.134519752	5
TACC2	-0.519323037	-0.633494508	1.152817545	5
TACC3	-0.785452946	-0.340279497	1.125732443	5

TACR1	-0.79354221	-0.329670958	1.123213168	5
TACR3	-0.542217884	-0.61178393	1.154001815	5
TADA1	-0.581840287	-0.572848582	1.154688869	5
TAF10	-0.585474343	-0.56918791	1.154662253	5
TAF7L	-0.509303479	-0.642821087	1.152124566	5
TAFA3	-0.528412639	-0.624942184	1.153354824	5
TAFA4	-0.509221175	-0.642897265	1.152118441	5
TAFA5	-0.534863934	-0.618818804	1.153682738	5
TAGLN3	-0.546327346	-0.607827146	1.154154492	5
TANC2	-0.541788671	-0.612196134	1.153984805	5
TAP2	-0.571520115	-0.583160864	1.154680979	5
TAPBP	-0.798085809	-0.323656398	1.121742207	5
TAS1R1	-0.732804193	-0.406416454	1.139220646	5
TAS2R1	-0.800191016	-0.320855776	1.121046792	5
TAS2R13	-0.533199737	-0.620402685	1.153602422	5
TAS2R16	-0.524754189	-0.62839492	1.153149108	5
TAS2R7	-0.570129796	-0.584540766	1.154670563	5
TAS2R8	-0.777518117	-0.350564405	1.128082522	5
TAT	-0.637474953	-0.515060618	1.152535571	5
TBC1D13	-0.50269847	-0.648912219	1.151610689	5
TBC1D16	-0.514996967	-0.637534249	1.152531216	5
TBC1D26	-0.579309175	-0.575389146	1.15469832	5
TBC1D31	-0.74372544	-0.393091104	1.136816544	5
TBC1D32	-0.565750139	-0.588873223	1.154623362	5

TBC1D3K	-0.62043945	-0.53316107	1.153600521	5
TBL1Y	-0.50621442	-0.645675423	1.151889843	5
TBPL1	-0.717647671	-0.424589443	1.142237114	5
TBR1	-0.570048468	-0.584621417	1.154669885	5
TBX10	-0.536401861	-0.617352446	1.153754307	5
TBX15	-0.517387176	-0.635304669	1.152691844	5
TBX20	-0.585090928	-0.569574861	1.154665789	5
TBX22	-0.781672217	-0.345194739	1.126866956	5
TBX4	-0.553011469	-0.601351735	1.154363204	5
TBX5	-0.556381127	-0.598068549	1.154449675	5
TBXA2R	-0.724358355	-0.416588265	1.14094662	5
TBXT	-0.637781858	-0.514731124	1.152512981	5
TCAF1	-0.513845326	-0.638606363	1.152451689	5
TCEAL2	-0.513462457	-0.638962485	1.152424942	5
TCEAL5	-0.539476789	-0.614412948	1.153889737	5
TCEAL7	-0.55522907	-0.599192462	1.154421532	5
TCEAL9	-0.506505307	-0.645407059	1.151912367	5
TCERG1L	-0.549655327	-0.604609239	1.154264566	5
TCF24	-0.585852751	-0.568805843	1.154658594	5
TCF4	-0.538181811	-0.615652138	1.153833949	5
TCP11	-0.548209077	-0.606009151	1.154218228	5
TCP11X1	-0.617238202	-0.536521568	1.153759771	5
TCP11X2	-0.580681279	-0.574012841	1.15469412	5
TCTA	-0.521039532	-0.63188617	1.152925702	5

TCTE1	-0.530426667	-0.623035316	1.153461983	5
TDGF1	-0.591082215	-0.563508577	1.154590793	5
TDRD5	-0.508379537	-0.643675858	1.152055396	5
TDRP	-0.507383994	-0.644595881	1.151979875	5
TEAD1	-0.531551563	-0.621968383	1.153519946	5
TEAD2	-0.568764416	-0.585893771	1.154658187	5
TECRL	-0.652586699	-0.498690257	1.151276956	5
TECTB	-0.560875527	-0.593669771	1.154545298	5
TEDC2	-0.614702694	-0.539174169	1.153876863	5
TEKT1	-0.54234565	-0.611661189	1.154006839	5
TEKT2	-0.518074133	-0.634662768	1.152736901	5
TEKT5	-0.57820765	-0.576492463	1.154700114	5
TENM2	-0.55280793	-0.601549647	1.154357577	5
TENM3	-0.535417239	-0.618291541	1.15370878	5
TENM4	-0.558518728	-0.595979245	1.154497973	5
TENT5B	-0.66362162	-0.4865441	1.15016572	5
TENT5D	-0.5850886	-0.56957721	1.15466581	5
TERB1	-0.576344498	-0.578355456	1.154699955	5
TET2	-0.583264172	-0.571416105	1.154680277	5
TEX15	-0.53209458	-0.621452861	1.153547441	5
TEX19	-0.503317189	-0.648343547	1.151660736	5
TEX26	-0.641123241	-0.511135886	1.152259127	5
TEX44	-0.712043938	-0.431216923	1.143260861	5
TEX45	-0.759823245	-0.373083382	1.132906627	5

TEX50	-0.566241328	-0.588388412	1.15462974	5
TEX55	-0.621590459	-0.531949676	1.153540135	5
TEX9	-0.505691441	-0.646157686	1.151849127	5
TFAP2A	-0.558663574	-0.595837487	1.154501061	5
TFAP2B	-0.530013124	-0.623427209	1.153440333	5
TFAP2C	-0.578162396	-0.576537761	1.154700157	5
TFPI	-0.516899755	-0.635759819	1.152659575	5
TGFB1I1	-0.549745822	-0.604521567	1.154267389	5
TH	-0.587291835	-0.56735131	1.154643145	5
THAP10	-0.524109684	-0.62900171	1.153111394	5
THAP9	-0.671719295	-0.477525188	1.149244484	5
THEGL	-0.638219522	-0.514261033	1.152480556	5
THPO	-0.568648937	-0.586008106	1.154657043	5
THRB	-0.519729997	-0.633113472	1.152843468	5
THSD4	-0.535768481	-0.61795666	1.153725141	5
THSD7A	-0.510741967	-0.641488527	1.152230494	5
THSD7B	-0.572967367	-0.581722109	1.154689476	5
THUMPD2	-0.701191102	-0.443915795	1.145106897	5
THY1	-0.543951507	-0.610116967	1.154068475	5
TICRR	-0.513257229	-0.639153313	1.152410543	5
TIMM21	-0.591795532	-0.562783511	1.154579043	5
TIMM23B	-0.562863163	-0.5917172	1.154580363	5
TIMM8B	-0.54477183	-0.609327042	1.154098873	5
TIMM9	-0.566561936	-0.588071819	1.154633755	5

TIMP1	-0.600920822	-0.55345447	1.154375292	5
TIPARP	-0.59412362	-0.56041288	1.154536499	5
TJP2	-0.560794106	-0.593749661	1.154543767	5
TLCD3B	-0.549844709	-0.604425755	1.154270464	5
TLCD4-RWDD3	-0.541808906	-0.612176706	1.153985611	5
TLCD5	-0.659594089	-0.490996309	1.150590398	5
TLE1	-0.705626508	-0.438747497	1.144374006	5
TLK1	-0.506002468	-0.645870908	1.151873375	5
TLL2	-0.523425327	-0.629645539	1.153070866	5
TLX1	-0.506981733	-0.644967336	1.151949069	5
TLX2	-0.605218201	-0.549026501	1.154244702	5
TLX3	-0.557173623	-0.597294553	1.154468176	5
TMA7	-0.665240621	-0.484748142	1.149988763	5
TMBIM6	-0.584635136	-0.570034633	1.154669769	5
TMC1	-0.704723363	-0.439802277	1.144525639	5
TMC3	-0.576955286	-0.577745162	1.154700448	5
TMC7	-0.543564796	-0.610489093	1.154053889	5
TMED10	-0.714031751	-0.428871518	1.142903268	5
TMEFF2	-0.568534326	-0.586121567	1.154655892	5
TMEM104	-0.533333298	-0.62027568	1.153608978	5
TMEM108	-0.555176952	-0.599243271	1.154420224	5
TMEM126A	-0.576125525	-0.578574148	1.154699673	5
TMEM130	-0.558833237	-0.595671411	1.154504648	5
TMEM132A	-0.551483948	-0.602835906	1.154319854	5

TMEM132D	-0.581373784	-0.573317386	1.15469117	5
TMEM132E	-0.620892835	-0.532684098	1.153576933	5
TMEM145	-0.545777096	-0.608358025	1.15413512	5
TMEM151A	-0.589535223	-0.565078983	1.154614206	5
TMEM151B	-0.558959796	-0.595547507	1.154507303	5
TMEM163	-0.520380743	-0.632503816	1.152884559	5
TMEM164	-0.67551009	-0.473271828	1.148781918	5
TMEM17	-0.528298588	-0.625050037	1.153348626	5
TMEM170B	-0.508334092	-0.643717878	1.152051971	5
TMEM174	-0.541014211	-0.612939395	1.153953605	5
TMEM178B	-0.553400501	-0.600973331	1.154373831	5
TMEM179	-0.586893698	-0.567753965	1.154647662	5
TMEM196	-0.562113036	-0.592454616	1.154567652	5
TMEM198	-0.520674704	-0.63222827	1.152902974	5
TMEM200B	-0.523808269	-0.629285336	1.153093605	5
TMEM200C	-0.550594208	-0.603699207	1.154293414	5
TMEM202	-0.556867646	-0.597593471	1.154461116	5
TMEM215	-0.624968799	-0.528384497	1.153353295	5
TMEM221	-0.57372741	-0.580965567	1.154692976	5
TMEM229A	-0.686677929	-0.460623141	1.14730107	5
TMEM231	-0.622508779	-0.530981988	1.153490767	5
TMEM233	-0.643137809	-0.508961239	1.152099048	5
TMEM239	-0.753085448	-0.381511847	1.134597294	5
TMEM249	-0.695843362	-0.450108088	1.14595145	5

TMEM253	-0.579752289	-0.574944913	1.154697203	5
TMEM270	-0.574522314	-0.580173614	1.154695929	5
TMEM30A	-0.707836299	-0.436161487	1.143997785	5
TMEM35A	-0.550607528	-0.603686288	1.154293817	5
TMEM37	-0.524390583	-0.628737302	1.153127886	5
TMEM47	-0.546931723	-0.607243664	1.154175387	5
TMEM52B	-0.687359693	-0.459845193	1.147204886	5
TMEM59L	-0.552590324	-0.601761187	1.15435151	5
TMEM63C	-0.641287315	-0.510958973	1.152246288	5
TMEM72	-0.52717941	-0.62610766	1.153287071	5
TMEM74	-0.542974234	-0.611057066	1.154031301	5
TMEM82	-0.58941412	-0.565201799	1.154615919	5
TMEM88B	-0.821965339	-0.291356092	1.113321431	5
TMEM98	-0.554813154	-0.599597855	1.154411009	5
TMEM9B	-0.749795137	-0.385599268	1.135394406	5
TMPRSS11A	-0.651924838	-0.499413558	1.151338396	5
TMPRSS11D	-0.790730603	-0.333372536	1.124103139	5
TMPRSS11F	-0.777248532	-0.350911764	1.128160296	5
TMPRSS12	-0.50261015	-0.648993363	1.151603513	5
TMPRSS4	-0.785483004	-0.340240311	1.125723315	5
TMPRSS5	-0.666922566	-0.482878547	1.149801113	5
TMPRSS7	-0.522953306	-0.630089317	1.153042624	5
TMSB15A	-0.542496306	-0.611516436	1.154012741	5
TMSB4Y	-0.615455716	-0.538387203	1.153842919	5

TMUB2	-0.756082869	-0.377772048	1.133854918	5
TMX4	-0.72215747	-0.419220208	1.141377679	5
TNC	-0.547627954	-0.606571006	1.15419896	5
TNFRSF11A	-0.546426501	-0.607731447	1.154157948	5
TNFRSF11B	-0.547498732	-0.606695893	1.154194625	5
TNFRSF13B	-0.523601393	-0.629479947	1.15308134	5
TNFRSF19	-0.544907182	-0.609196636	1.154103818	5
TNFRSF21	-0.522925333	-0.630115609	1.153040942	5
TNN	-0.657986573	-0.492767173	1.150753746	5
TNNI1	-0.59871202	-0.555721728	1.154433748	5
TNNI3	-0.54020393	-0.613716334	1.153920264	5
TNNT2	-0.560689787	-0.593852007	1.154541795	5
TNP2	-0.507825335	-0.644188146	1.152013481	5
TNR	-0.57681952	-0.577880856	1.154700376	5
TNRC18	-0.644821968	-0.507139178	1.151961147	5
TNXB	-0.602047423	-0.552295792	1.154343215	5
TOB1	-0.738308036	-0.399725643	1.138033679	5
TOGARAM1	-0.585320809	-0.56934288	1.154663689	5
TOP2A	-0.522287608	-0.630714784	1.153002392	5
TOX	-0.765920624	-0.365387333	1.131307957	5
TOX2	-0.523151805	-0.629902724	1.153054529	5
TOX3	-0.55181856	-0.602511011	1.154329571	5
TP53INP1	-0.691668444	-0.454912974	1.146581418	5
TP53TG3	-0.698461269	-0.44708205	1.145543319	5

TP73	-0.689705193	-0.457163651	1.146868843	5
TPBG	-0.561759107	-0.592802328	1.154561435	5
TPD52L3	-0.606005824	-0.548212516	1.154218341	5
TPH2	-0.581778697	-0.57291049	1.154689187	5
TPM1	-0.799844833	-0.321316921	1.121161754	5
TPPP	-0.601986546	-0.552358442	1.154344988	5
TPSG1	-0.643496665	-0.508573311	1.152069976	5
TPST2	-0.588171896	-0.566460603	1.154632498	5
TPX2	-0.517798395	-0.63492048	1.152718875	5
TRABD2B	-0.559637419	-0.594883794	1.154521213	5
TRAF7	-0.505862558	-0.645999922	1.15186248	5
TRAK1	-0.707736115	-0.436278887	1.144015002	5
TRAM1L1	-0.555652249	-0.598779792	1.154432041	5
TRAPPC3L	-0.52573795	-0.627467875	1.153205825	5
TRARG1	-0.536052643	-0.617685637	1.15373828	5
TRDN	-0.658444072	-0.492263541	1.150707613	5
TREH	-0.731872242	-0.407544497	1.139416739	5
TREML1	-0.636903097	-0.515674241	1.152577339	5
TRH	-0.544132009	-0.609943218	1.154075226	5
TRIB1	-0.783876311	-0.342332535	1.126208846	5
TRIM10	-0.688892729	-0.45809342	1.146986149	5
TRIM2	-0.520131305	-0.632737556	1.152868861	5
TRIM24	-0.763433937	-0.368533968	1.131967905	5
TRIM36	-0.525318538	-0.627863233	1.15318177	5

TRIM40	-0.529884256	-0.623549294	1.15343355	5
TRIM45	-0.577421949	-0.577278586	1.154700535	5
TRIM51	-0.52833658	-0.625014111	1.153350692	5
TRIM54	-0.585360461	-0.56930286	1.154663321	5
TRIM55	-0.692897351	-0.453501289	1.14639864	5
TRIM6-TRIM34	-0.61788896	-0.535839471	1.153728432	5
TRIM63	-0.609461198	-0.544632566	1.154093764	5
TRIM67	-0.537191868	-0.61659821	1.153790078	5
TRIM77	-0.502961961	-0.648670089	1.151632051	5
TRIM9	-0.587011703	-0.567634639	1.154646343	5
TRIML2	-0.669417322	-0.480098271	1.149515593	5
TRIQK	-0.765755836	-0.365596196	1.131352032	5
TRIR	-0.523348987	-0.629717328	1.153066314	5
TRMT9B	-0.543898443	-0.61016804	1.154066483	5
TRO	-0.544576591	-0.609515114	1.154091705	5
TROAP	-0.51051549	-0.64169847	1.15221396	5
TRPC3	-0.60673554	-0.547457704	1.154193245	5
TRPC4	-0.537453747	-0.61634804	1.153801787	5
TRPC7	-0.649914874	-0.50160653	1.151521404	5
TRPM1	-0.623363121	-0.530080766	1.153443887	5
TRPM3	-0.558006155	-0.596480702	1.154486857	5
TRPM4	-0.546959245	-0.607217084	1.154176329	5
TRPM8	-0.581292139	-0.573399407	1.154691547	5
TRPV4	-0.776177523	-0.352290425	1.128467948	5

TSBP1	-0.619192007	-0.534472088	1.153664095	5
TSGA13	-0.692164524	-0.454343377	1.146507901	5
TSPAN11	-0.542506712	-0.611506436	1.154013148	5
TSPAN19	-0.544302131	-0.609779426	1.154081557	5
TSPAN2	-0.736116925	-0.402395238	1.138512163	5
TSPAN33	-0.757187886	-0.37638942	1.133577306	5
TSPAN6	-0.533355421	-0.620254641	1.153610062	5
TSPAN8	-0.544625738	-0.609467775	1.154093513	5
TSPAN9	-0.514032567	-0.638432147	1.152464713	5
TSPY2	-0.680825182	-0.467274108	1.14809929	5
TSSK1B	-0.564188533	-0.590412738	1.154601272	5
TTBK1	-0.555651347	-0.598780672	1.154432019	5
TTC28	-0.527483814	-0.625820131	1.153303945	5
TTC29	-0.521759302	-0.631210828	1.15297013	5
TTC36	-0.62326577	-0.530183506	1.153449275	5
TTC6	-0.562424638	-0.592148371	1.154573009	5
TTC9B	-0.55692651	-0.597535972	1.154462483	5
TTLL10	-0.515755244	-0.636827579	1.152582823	5
TTLL10-AS1	-0.669504726	-0.480000707	1.149505434	5
TTLL11	-0.517129168	-0.635545626	1.152674794	5
TTLL6	-0.651747243	-0.499607541	1.151354783	5
TTLL7	-0.530827853	-0.622654957	1.153482811	5
TTLL9	-0.519621355	-0.63321521	1.152836565	5
TTPA	-0.541741318	-0.612241598	1.153982916	5

TTR	-0.542454188	-0.611556906	1.154011094	5
TTYH1	-0.544348433	-0.609734842	1.154083275	5
TUB	-0.553293949	-0.601076988	1.154370937	5
TUBA3E	-0.700363271	-0.444877146	1.145240417	5
TUBA4A	-0.827122074	-0.284222458	1.111344532	5
TUBB3	-0.574626232	-0.580070029	1.154696261	5
TUBB4A	-0.60584726	-0.548376449	1.154223708	5
TULP1	-0.523194272	-0.629862799	1.153057071	5
TULP3	-0.644813136	-0.507148744	1.151961879	5
TUSC3	-0.547845278	-0.606360931	1.154206209	5
TUT4	-0.54070172	-0.613239111	1.153940831	5
TXNDC11	-0.592620968	-0.561943727	1.154564695	5
TXNDC2	-0.688826462	-0.458169213	1.146995675	5
TYMS	-0.513030365	-0.639364208	1.152394574	5
TYR	-0.53818392	-0.615650122	1.153834042	5
TYRO3	-0.56986641	-0.58480193	1.154668341	5
TYRP1	-0.538125913	-0.615705587	1.1538315	5
UBALD2	-0.749385895	-0.386106359	1.135492254	5
UBE2B	-0.754293529	-0.380006426	1.134299955	5
UBE2C	-0.517885015	-0.634839532	1.152724547	5
UBE2D4	-0.777089206	-0.351116991	1.128206197	5
UBE2G1	-0.512737063	-0.639636785	1.152373849	5
UBL4A	-0.516629403	-0.636012166	1.152641568	5
UBR7	-0.795667447	-0.326862754	1.122530201	5

UBTFL1	-0.571484635	-0.583196107	1.154680741	5
UBXN2B	-0.761496051	-0.370978536	1.132474587	5
UCHL1	-0.552875181	-0.60148426	1.154359441	5
UCN2	-0.720396859	-0.42132014	1.141716999	5
UCN3	-0.64461772	-0.507360348	1.151978069	5
UGGT2	-0.824621715	-0.287688622	1.112310336	5
UGT1A3	-0.566527844	-0.58810549	1.154633334	5
UGT1A4	-0.638577817	-0.513876008	1.152453825	5
UGT1A6	-0.675866569	-0.472870813	1.148737383	5
UGT1A8	-0.624410597	-0.52897456	1.153385157	5
UGT2B7	-0.534010127	-0.619631778	1.153641905	5
UGT3A1	-0.566295683	-0.588334746	1.154630429	5
ULBP1	-0.613490357	-0.540439682	1.153930038	5
ULBP2	-0.705350728	-0.439069711	1.144420438	5
UNC13A	-0.50888684	-0.643206644	1.152093484	5
UNC13C	-0.708787813	-0.435045691	1.143833503	5
UNC5B	-0.545570194	-0.608557557	1.15412775	5
UNC5C	-0.538472355	-0.615374269	1.153846624	5
UNC5D	-0.540258077	-0.613664437	1.153922514	5
UNC79	-0.591275136	-0.563312539	1.154587674	5
UNC80	-0.548372078	-0.605851488	1.154223565	5
UNC93A	-0.725175357	-0.415609292	1.14078465	5
UNCX	-0.57335146	-0.581339867	1.154691327	5
UPK1A	-0.565167244	-0.589448195	1.154615439	5

UPK2	-0.743636634	-0.393200256	1.13683689	5
UPP2	-0.629426656	-0.523658047	1.153084704	5
UQCC1	-0.608166132	-0.54597603	1.154142162	5
UQCR10	-0.570389622	-0.584283055	1.154672677	5
UQCRH	-0.503409482	-0.648258686	1.151668168	5
UQCRHL	-0.681512448	-0.466495649	1.148008098	5
URAD	-0.749840219	-0.38554339	1.135383609	5
USH1C	-0.652784403	-0.498474086	1.151258489	5
USH2A	-0.585732755	-0.568927017	1.154659772	5
USP17L10	-0.802469311	-0.317814894	1.120284204	5
USP17L2	-0.831947798	-0.277493581	1.109441379	5
USP17L3	-0.773073539	-0.356274093	1.129347632	5
USP17L4	-0.82365408	-0.289026346	1.112680425	5
USP17L8	-0.733417978	-0.405672751	1.139090729	5
USP2	-0.535726334	-0.617996851	1.153723185	5
USP22	-0.602281051	-0.55205532	1.154336372	5
USP29	-0.744276544	-0.39241344	1.136689984	5
USP4	-0.717273381	-0.425033631	1.142307012	5
USP43	-0.821026667	-0.29264839	1.113675057	5
USP50	-0.508840023	-0.643249958	1.15208998	5
USP54	-0.529649481	-0.623771664	1.153421145	5
VANGL2	-0.545689643	-0.608442368	1.154132011	5
VAPA	-0.597068831	-0.557404606	1.154473437	5
VARS1	-0.668465621	-0.481159908	1.149625529	5

VARS2	-0.514288843	-0.638193637	1.15248248	5
VASH2	-0.691499643	-0.45510671	1.146606352	5
VASN	-0.635533946	-0.517141676	1.152675622	5
VAT1L	-0.539026024	-0.6148445	1.153870524	5
VAV2	-0.570935134	-0.583741731	1.154676865	5
VAX1	-0.585979718	-0.56867761	1.154657328	5
VAX2	-0.720827303	-0.420807188	1.141634491	5
VCX2	-0.53703159	-0.616751286	1.153782875	5
VCX3A	-0.68498004	-0.462557654	1.147537695	5
VCX3B	-0.596179425	-0.558314146	1.154493572	5
VEGFA	-0.577208367	-0.577492159	1.154700527	5
VEGFD	-0.529884194	-0.623549353	1.153433546	5
VEPH1	-0.526407773	-0.62683608	1.153243853	5
VGLL1	-0.807049995	-0.311669193	1.118719188	5
VGLL2	-0.580851903	-0.573841542	1.154693445	5
VGLL4	-0.548376204	-0.605847496	1.1542237	5
VIPR2	-0.599148397	-0.555274267	1.154422664	5
VIRMA	-0.509759567	-0.642398818	1.152158385	5
VIT	-0.689345794	-0.457575058	1.146920853	5
VKORC1	-0.63807476	-0.514416548	1.152491308	5
VN1R2	-0.585908589	-0.56874945	1.154658039	5
VPS37B	-0.810566687	-0.306921848	1.117488535	5
VPS37D	-0.538370804	-0.6154714	1.153842204	5
VRTN	-0.565083555	-0.589530714	1.15461427	5

VSIG10L	-0.673273843	-0.475783382	1.149057225	5
VSIG2	-0.546312269	-0.607841697	1.154153966	5
VSNL1	-0.543316514	-0.610727925	1.154044439	5
VSTM2A	-0.583999533	-0.57067538	1.154674913	5
VSTM2B	-0.593155818	-0.561399149	1.154554967	5
VSTM2L	-0.537170406	-0.616618709	1.153789115	5
VSTM4	-0.733704526	-0.405325341	1.139029867	5
VSX1	-0.814521816	-0.301551943	1.116073759	5
VSX2	-0.602723086	-0.551600157	1.154323243	5
VTA1	-0.76781786	-0.362979147	1.130797007	5
VTCN1	-0.628827911	-0.524294334	1.153122244	5
VWA1	-0.509619439	-0.642528578	1.152148017	5
VWA2	-0.548710416	-0.605524136	1.154234552	5
VWA3A	-0.54768683	-0.606514099	1.154200929	5
VWA3B	-0.619952457	-0.533673114	1.153625571	5
VWA5B1	-0.731221919	-0.408330821	1.13955274	5
VWA5B2	-0.579797735	-0.57489934	1.154697075	5
VWC2	-0.589812689	-0.564797525	1.154610214	5
VWC2L	-0.551715405	-0.602611183	1.154326589	5
VWF	-0.502310724	-0.649268401	1.151579125	5
WASF1	-0.546538552	-0.607623287	1.154161839	5
WASF3	-0.513845621	-0.638606088	1.152451709	5
WDR17	-0.526855916	-0.626413113	1.15326903	5
WDR31	-0.50614945	-0.64573535	1.1518848	5

WDR38	-0.548327818	-0.605894301	1.154222119	5
WDR44	-0.718085985	-0.424068997	1.142154982	5
WDR49	-0.512228063	-0.640109606	1.152337669	5
WDR5	-0.562931095	-0.591650388	1.154581483	5
WDR7	-0.545649034	-0.60848153	1.154130564	5
WDR72	-0.520499101	-0.632392883	1.152891984	5
WDR86	-0.556606162	-0.597848838	1.154455	5
WDR87	-0.558468691	-0.59602821	1.154496901	5
WDR90	-0.774713787	-0.354171199	1.128884986	5
WDTC1	-0.589411281	-0.565204678	1.154615959	5
WEE2	-0.610156616	-0.543910313	1.154066929	5
WFDC1	-0.52511388	-0.628056084	1.153169965	5
WFDC10B	-0.557764837	-0.596716685	1.154481522	5
WFDC2	-0.535259687	-0.618441712	1.153701398	5
WFDC9	-0.733424268	-0.405665126	1.139089394	5
WFIKKN2	-0.561013977	-0.593533908	1.154547885	5
WFS1	-0.675347609	-0.473454548	1.148802158	5
WHRN	-0.639433387	-0.512955936	1.152389323	5
WIF1	-0.535184781	-0.618513099	1.153697879	5
WIPF1	-0.684501518	-0.463102116	1.147603634	5
WIPF3	-0.524621518	-0.628519863	1.153141381	5
WNK2	-0.551730055	-0.602596958	1.154327013	5
WNK3	-0.547476565	-0.606717315	1.154193879	5
WNT11	-0.58888008	-0.56574319	1.15462327	5

WNT2B	-0.541073535	-0.612882483	1.153956018	5
WNT3A	-0.53329721	-0.620309998	1.153607209	5
WNT4	-0.507255577	-0.644714481	1.151970059	5
WNT5A	-0.563634385	-0.590958386	1.154592771	5
WNT7A	-0.642465296	-0.50968778	1.152153076	5
WNT7B	-0.55143267	-0.602885684	1.154318354	5
WNT8A	-0.744624448	-0.391985378	1.136609825	5
WNT8B	-0.569539079	-0.585126389	1.154665469	5
WNT9A	-0.521832915	-0.631141728	1.152974643	5
WNT9B	-0.585086962	-0.569578862	1.154665825	5
WSCD1	-0.533098947	-0.620498515	1.153597462	5
WSCD2	-0.548967462	-0.605275352	1.154242814	5
WT1	-0.564376765	-0.590227316	1.154604081	5
WWC1	-0.555602221	-0.598828588	1.154430809	5
XDH	-0.548592483	-0.605638254	1.154230737	5
XG	-0.824516978	-0.287833515	1.112350493	5
XIRP1	-0.515886465	-0.636705227	1.152591692	5
XKR4	-0.596092622	-0.558402864	1.154495486	5
XKR5	-0.566058819	-0.588568582	1.154627402	5
XKR7	-0.560861712	-0.593683327	1.154545039	5
XYLB	-0.663710925	-0.486445127	1.150156053	5
YAP1	-0.524056735	-0.629051542	1.153108276	5
YBX2	-0.52474035	-0.628407953	1.153148303	5
YIF1B	-0.746899051	-0.389181681	1.136080731	5

YJEFN3	-0.828319864	-0.282557122	1.110876985	5
YPEL4	-0.541140227	-0.612818499	1.153958726	5
YWHAZ	-0.532256448	-0.621299128	1.153555576	5
ZACN	-0.55710572	-0.597360899	1.154466619	5
ZAR1L	-0.535442298	-0.618267654	1.153709952	5
ZASP	-0.645205964	-0.50672322	1.151929183	5
ZBBX	-0.531997243	-0.621545293	1.153542536	5
ZBED9	-0.531761891	-0.621768742	1.153530633	5
ZBTB16	-0.818956927	-0.295491156	1.114448083	5
ZBTB39	-0.640136315	-0.512199301	1.152335617	5
ZBTB7C	-0.520099144	-0.632767688	1.152866832	5
ZBTB8B	-0.566566624	-0.588067189	1.154633813	5
ZC2HC1A	-0.777682639	-0.350352352	1.128034991	5
ZC3H12A	-0.600059821	-0.554338955	1.154398776	5
ZC3H12B	-0.660509363	-0.489986463	1.150495827	5
ZCCHC12	-0.54220161	-0.611799563	1.154001173	5
ZCCHC17	-0.683900672	-0.463785293	1.147685965	5
ZDHHC11B	-0.536216902	-0.617528933	1.153745835	5
ZDHHC15	-0.509279876	-0.642842934	1.15212281	5
ZDHHC22	-0.566299392	-0.588331084	1.154630475	5
ZDHHC4	-0.50474165	-0.64703281	1.151774461	5
ZDHHC7	-0.572860245	-0.581828684	1.154688929	5
ZDHHC9	-0.608988863	-0.54512279	1.154111653	5
ZFAND3	-0.800140913	-0.320922531	1.121063445	5

ZFAT	-0.736473727	-0.401961057	1.138434784	5
ZFHX2	-0.560838105	-0.59370649	1.154544596	5
ZFHX4	-0.537399423	-0.616399941	1.153799364	5
ZFP36	-0.595679778	-0.558824691	1.154504468	5
ZFP42	-0.586549292	-0.568102127	1.15465142	5
ZFR2	-0.634851304	-0.517872419	1.152723722	5
ZIC1	-0.546945286	-0.607230565	1.154175851	5
ZIC2	-0.552006898	-0.602328088	1.154334986	5
ZIC3	-0.538236696	-0.615599655	1.153836351	5
ZIC4	-0.553729474	-0.600653213	1.154382687	5
ZIC5	-0.544477038	-0.609610995	1.154088034	5
ZIM3	-0.536760197	-0.617010419	1.153770616	5
ZMAT4	-0.575835315	-0.578863899	1.154699214	5
ZMYND12	-0.509045538	-0.643059807	1.152105345	5
ZNF117	-0.627005825	-0.526227861	1.153233686	5
ZNF135	-0.635593338	-0.517078071	1.152671409	5
ZNF185	-0.537027339	-0.616755345	1.153782684	5
ZNF19	-0.580091159	-0.574605035	1.154696194	5
ZNF205	-0.515780893	-0.636803665	1.152584558	5
ZNF214	-0.560831181	-0.593713284	1.154544465	5
ZNF219	-0.650288809	-0.501198956	1.151487765	5
ZNF236	-0.525066087	-0.628101114	1.153167201	5
ZNF280A	-0.691080752	-0.455587295	1.146668047	5
ZNF292	-0.5929005	-0.561659154	1.154559653	5

ZNF311	-0.511842924	-0.64046719	1.152310114	5
ZNF334	-0.547722241	-0.606479871	1.154202111	5
ZNF354A	-0.799302282	-0.322039165	1.121341446	5
ZNF385B	-0.558453484	-0.59604309	1.154496574	5
ZNF385C	-0.570760994	-0.583914571	1.154675565	5
ZNF385D	-0.522812389	-0.630221757	1.153034146	5
ZNF391	-0.619085993	-0.534583415	1.153669409	5
ZNF474	-0.574252191	-0.580442816	1.154695007	5
ZNF48	-0.555576443	-0.598853731	1.154430173	5
ZNF488	-0.574001576	-0.5806925	1.154694077	5
ZNF512B	-0.614771385	-0.53910241	1.153873795	5
ZNF521	-0.510114061	-0.64207046	1.152184521	5
ZNF536	-0.576200194	-0.578499582	1.154699775	5
ZNF541	-0.692070973	-0.454450819	1.146521793	5
ZNF551	-0.69841766	-0.447132541	1.145550201	5
ZNF560	-0.737271478	-0.400989544	1.138261022	5
ZNF575	-0.675036065	-0.473804797	1.148840862	5
ZNF579	-0.608630024	-0.545495037	1.154125062	5
ZNF583	-0.509104404	-0.643005334	1.152109737	5
ZNF599	-0.712600707	-0.430560609	1.143161316	5
ZNF618	-0.569583653	-0.585082214	1.154665867	5
ZNF664-RFLNA	-0.548343902	-0.605878743	1.154222645	5
ZNF679	-0.55209512	-0.602242389	1.154337509	5
ZNF705B	-0.77831407	-0.349538035	1.127852105	5

ZNF711	-0.534015171	-0.619626978	1.153642148	5
ZNF716	-0.681674559	-0.46631193	1.147986489	5
ZNF723	-0.551286823	-0.603027248	1.154314072	5
ZNF724	-0.528969572	-0.624415317	1.153384889	5
ZNF730	-0.509032791	-0.643071602	1.152104393	5
ZNF735	-0.578669281	-0.576030252	1.154699533	5
ZNF775	-0.6830845	-0.464712471	1.147796971	5
ZNF827	-0.603796619	-0.550493756	1.154290374	5
ZNF843	-0.569794202	-0.584873515	1.154667718	5
ZNRF1	-0.795257903	-0.3274046	1.122662503	5
ZNRF3	-0.555177654	-0.599242588	1.154420241	5
ZP4	-0.536811279	-0.61696165	1.153772929	5
ZPBP	-0.806970212	-0.311776599	1.118746812	5
ZSCAN1	-0.639027553	-0.513392485	1.152420038	5
ZSCAN23	-0.647094431	-0.504674734	1.151769165	5
ZSCAN4	-0.610893771	-0.543144064	1.154037835	5
ZSWIM2	-0.583871563	-0.570804329	1.154675892	5
ZSWIM4	-0.621814204	-0.531714	1.153528204	5
ZSWIM5	-0.517918769	-0.634807986	1.152726754	5
ZYG11A	-0.715756349	-0.426831732	1.142588081	5
A3GALT2	-0.391893185	1.13659254	-0.744699355	6
AANAT	-0.371661682	1.132615239	-0.760953557	6
ABCC11	-0.456866951	1.146831234	-0.689964282	6
ABCF2	-0.572305059	1.154685885	-0.582380826	6

ABHD8	-0.770992116	1.129927631	-0.358935515	6
ABTB2	-0.762321413	1.1322596	-0.369938187	6
ACBD4	-0.42567777	1.142408047	-0.716730277	6
ACO1	-0.556316156	1.154448128	-0.598131972	6
ACOT11	-0.581090573	1.154692444	-0.573601871	6
ACOXL	-0.424610168	1.14224038	-0.717630211	6
ACP3	-0.549652753	1.154264486	-0.604611733	6
ACSL5	-1.092600584	0.869824592	0.222775992	6
ACSM4	-0.418744503	1.141300243	-0.72255574	6
ACTR8	-0.365406697	1.131312045	-0.765905348	6
ACTRT1	-1.051527949	0.938942784	0.112585165	6
ACY1	-0.29174346	1.113427576	-0.821684116	6
ADAM19	-1.015274705	0.983991166	0.031283539	6
ADAM20	-0.358160229	1.129759313	-0.771599083	6
ADAMTSL4	-0.328318728	1.122885138	-0.79456641	6
ADCY4	-0.626295682	1.153275979	-0.526980297	6
ADGRE5	-0.359933123	1.130143445	-0.770210322	6
ADGRL2	-1.10441506	0.844098831	0.260316228	6
ADK	-0.726603676	1.140498932	-0.413895256	6
ADORA2A	-0.763521494	1.131944855	-0.368423361	6
ADRA2B	-0.930537445	1.057362505	-0.12682506	6
AGAP2	-1.052841429	0.937083221	0.115758207	6
AGBL1	-1.00302646	0.996945809	0.00608065	6
AHRR	-0.280188781	1.110208139	-0.830019358	6

AICDA	-1.057261691	0.930685926	0.126575764	6
AIFM2	-0.366235222	1.131486643	-0.765251421	6
AKT3	-0.990222356	1.009498935	-0.019276578	6
ALAS1	-0.683552445	1.147733443	-0.464180998	6
ALDH4A1	-1.073008556	0.905949104	0.167059451	6
ALG3	-0.373901377	1.133073471	-0.759172094	6
ALOX15	-1.052594882	0.937433685	0.115161197	6
ALOX5AP	-1.069895725	0.911102555	0.15879317	6
AMER2	-1.000277944	0.999721824	0.00055612	6
AMH	-0.587900208	1.154635882	-0.566735675	6
ANAPC11	-0.439432556	1.144472609	-0.705040053	6
ANGPT4	-0.936603393	1.053178233	-0.116574841	6
ANK2	-0.472457674	1.148691335	-0.676233661	6
ANKK1	-0.360591573	1.130285412	-0.769693838	6
ANKMY2	-0.884388863	1.085150304	-0.200761442	6
ANKRD26	-0.323838123	1.121787101	-0.797948978	6
ANKRD30A	-0.780707495	1.127152129	-0.346444634	6
ANKRD30BL	-0.586023155	1.154656891	-0.568633736	6
ANKRD36	-1.031807249	0.964824944	0.066982305	6
ANKRD39	-0.697253106	1.145732932	-0.448479826	6
ANKRD40CL	-0.969950452	1.027558943	-0.057608491	6
ANKRD60	-1.004590669	0.995345221	0.009245448	6
ANKRD61	-0.978633292	1.020078042	-0.041444749	6
ANP32E	-0.278843042	1.109826034	-0.830982992	6

ANXA10	-0.766417646	1.131174727	-0.364757082	6
AOC1	-0.491198588	1.150609217	-0.659410628	6
AP2A2	-0.488116607	1.150317998	-0.662201391	6
AP2B1	-0.589999896	1.15460747	-0.564607574	6
APCDD1L	-0.830139056	1.110160789	-0.280021732	6
APEX2	-0.624018072	1.153407327	-0.529389255	6
APOBEC3C	-0.416467807	1.140926738	-0.724458932	6
ARF4	-1.055790007	0.93284018	0.122949826	6
ARGFX	-0.834880337	1.108259332	-0.273378995	6
ARHGEF10L	-0.245735444	1.099960639	-0.854225195	6
ARID3B	-0.973397611	1.024632647	-0.051235036	6
ARL17B	-0.343826839	1.126553314	-0.782726475	6
ARL4A	-0.344534185	1.126715701	-0.782181516	6
ARL5A	-1.059710317	0.927045961	0.132664356	6
ARL6IP5	-0.800441506	1.120963458	-0.320521952	6
ARPC2	-1.039233899	0.955500739	0.08373316	6
ARPC3	-1.074501209	0.903427288	0.171073921	6
ARPP21	-0.995725804	1.004220081	-0.008494277	6
ARR3	-0.790860498	1.124062361	-0.333201863	6
ARRDC3	-0.780170088	1.12731023	-0.347140142	6
ASAP1	-1.050854397	0.939889232	0.110965166	6
ASB11	-0.989417716	1.010256565	-0.020838849	6
ASB18	-0.309658196	1.118200185	-0.808541989	6
ASCC1	-0.781307697	1.126974914	-0.345667217	6

ASF1B	-0.662037072	1.150335438	-0.488298367	6
ASPG	-0.938900045	1.051558285	-0.11265824	6
ASPRV1	-0.752206038	1.134812158	-0.38260612	6
ASPSCR1	-0.723800166	1.141056672	-0.417256505	6
ASRGL1	-0.357677494	1.129654243	-0.771976749	6
ATG13	-0.459385239	1.147147742	-0.687762503	6
ATG3	-0.724999642	1.140819575	-0.415819933	6
ATG4D	-1.049318606	0.942029459	0.107289147	6
ATP13A1	-0.482899558	1.149803241	-0.666903684	6
ATP13A2	-0.540189944	1.153919682	-0.613729738	6
ATP5MC3	-0.581868919	1.154688719	-0.5728198	6
ATP6V1B2	-1.068153876	0.913926214	0.154227662	6
ATP6V1C1	-0.62545119	1.153325444	-0.527874254	6
AWAT1	-0.837863487	1.10703681	-0.269173324	6
B3GAT1	-0.53116287	1.153500071	-0.622337201	6
B3GNT6	-0.491020897	1.150592687	-0.65957179	6
B4GALNT2	-0.862042034	1.096349995	-0.234307961	6
BAD	-1.035593027	0.960131581	0.075461445	6
BCL2L1	-0.441528786	1.144771562	-0.703242776	6
BCL6	-0.832053245	1.109399212	-0.277345967	6
BCORL1	-0.366885983	1.131623358	-0.764737375	6
BECN1	-0.465685273	1.147912535	-0.682227263	6
BEND6	-0.938531113	1.051819857	-0.113288744	6
BGLAP	-1.086139327	0.882519249	0.203620078	6

BHLHA15	-0.315900612	1.11980014	-0.803899528	6
BICC1	-0.531201394	1.153502048	-0.622300654	6
BIRC2	-0.957421932	1.037734609	-0.080312677	6
BLOC1S1	-0.781475839	1.126925148	-0.345449308	6
BNIP2	-0.234800945	1.096507917	-0.861706972	6
BNIP3L	-0.606949976	1.154185747	-0.547235771	6
BPGM	-0.450743088	1.146035979	-0.695292891	6
BPIFC	-0.842560125	1.105070474	-0.262510349	6
BRPF3	-0.74512327	1.136494541	-0.391371271	6
BRS3	-0.821877267	1.113354691	-0.291477424	6
BSND	-1.062901453	0.922194089	0.140707365	6
BSPRY	-1.076946533	0.899221966	0.177724567	6
BTBD16	-0.439548203	1.144489211	-0.704941008	6
C10orf67	-0.401238782	1.138305682	-0.7370669	6
C11orf68	-0.666340675	1.149866474	-0.483525798	6
C12orf60	-0.609582651	1.15408912	-0.544506469	6
C12orf71	-1.000210986	0.99978888	0.000422106	6
C13orf46	-0.340761106	1.125844525	-0.785083419	6
C15orf39	-0.565438964	1.15461918	-0.589180216	6
C15orf61	-0.418169759	1.141206406	-0.723036647	6
C16orf78	-1.089312734	0.876391693	0.212921041	6
C17orf50	-0.65566958	1.150983046	-0.495313466	6
C17orf75	-0.565597894	1.15462133	-0.589023435	6
C17orf80	-1.040854638	0.953401009	0.087453629	6

C18orf12	-0.558616932	1.154500069	-0.595883137	6
C19orf53	-0.438589196	1.144351157	-0.705761961	6
C19orf67	-0.264792089	1.105747914	-0.840955825	6
C1GALT1C1L	-0.726318083	1.140556322	-0.414238239	6
C1orf127	-0.695250197	1.146042516	-0.450792319	6
C1orf226	-1.060480587	0.925886212	0.134594375	6
C1RL	-1.090169045	0.874703378	0.215465667	6
C20orf204	-0.863369684	1.095721431	-0.232351748	6
C3AR1	-0.347500001	1.127391868	-0.779891867	6
C3orf22	-0.591218445	1.154588595	-0.56337015	6
C3orf62	-0.96784532	1.029318459	-0.061473138	6
C3orf85	-0.584851957	1.154667906	-0.569815949	6
C4A	-0.309702486	1.118211652	-0.808509166	6
C4orf19	-0.492328901	1.150713614	-0.658384714	6
C4orf48	-0.396880938	1.137516686	-0.740635748	6
C5orf46	-0.308358904	1.117863057	-0.809504153	6
C5orf52	-0.555288701	1.154423025	-0.599134324	6
C9orf131	-0.24770324	1.100571809	-0.85286857	6
CA4	-0.613925158	1.153911176	-0.539986017	6
CACUL1	-1.017788224	0.981207936	0.036580288	6
CAGE1	-1.096382401	0.861973327	0.234409074	6
CALR3	-0.26957152	1.107153179	-0.837581658	6
CAMK2G	-0.293144746	1.113810513	-0.820665766	6
CAMKK1	-0.990018125	1.00969157	-0.019673445	6

CAMKMT	-0.84182439	1.105381897	-0.263557507	6
CAPN11	-0.379357122	1.134171086	-0.754813964	6
CAPNS2	-0.253924168	1.102483426	-0.848559257	6
CARD8	-0.731406644	1.139514178	-0.408107534	6
CASP4	-0.778884046	1.127686381	-0.348802335	6
CAT	-0.23058264	1.095150358	-0.864567718	6
CAV2	-0.857788039	1.098333905	-0.240545866	6
CBFA2T3	-0.712718237	1.143140242	-0.430422004	6
CBWD1	-0.431984809	1.143376814	-0.711392005	6
CBWD2	-0.303559718	1.116605555	-0.813045837	6
CBWD6	-0.623039265	1.153461766	-0.5304225	6
CCDC102A	-0.745932357	1.136306659	-0.390374301	6
CCDC113	-0.324651266	1.12198764	-0.797336373	6
CCDC125	-0.568238116	1.15465285	-0.586414733	6
CCDC13	-0.686508404	1.147324882	-0.460816478	6
CCDC157	-0.3803831	1.134374542	-0.753991442	6
CCDC184	-0.83852343	1.106763601	-0.268240171	6
CCDC196	-0.671109662	1.149316999	-0.478207337	6
CCDC30	-1.074879182	0.902783344	0.172095838	6
CCDC59	-0.712838777	1.143118606	-0.430279829	6
CCDC96	-1.031299567	0.965445184	0.065854382	6
CCL16	-1.0269383	0.970687147	0.056251153	6
CCL19	-1.085373111	0.883969293	0.201403818	6
CCL26	-0.616805628	1.153780311	-0.536974684	6

CCN3	-0.905685147	1.073165724	-0.167480577	6
CCNDBP1	-0.613592436	1.153925631	-0.540333195	6
CCNP	-0.920912634	1.063730666	-0.142818032	6
CCR10	-0.256336651	1.103216345	-0.846879695	6
CD14	-1.020511457	0.978141691	0.042369766	6
CD320	-0.367830937	1.131821214	-0.763990276	6
CD36	-0.870854995	1.092092315	-0.22123732	6
CD3E	-0.520498831	1.152891967	-0.632393137	6
CD74	-0.394132632	1.137010251	-0.742877619	6
CDADC1	-0.678897717	1.148351459	-0.469453742	6
CDC26	-0.699305903	1.145409472	-0.44610357	6
CDC42EP3	-1.081563249	0.891018265	0.190544984	6
CDC5L	-0.882325292	1.086241369	-0.203916078	6
CDCP1	-1.086672742	0.881503141	0.205169601	6
CDHR5	-1.103608938	0.845973611	0.257635328	6
CDK5RAP2	-0.789493861	1.12448975	-0.334995889	6
CDKL1	-0.912636579	1.068952647	-0.156316068	6
CDKN2A	-0.446292016	1.145435321	-0.699143305	6
CEBPB	-0.381166331	1.134529229	-0.753362898	6
CEBPD	-0.305574047	1.117135703	-0.811561656	6
CEND1	-1.004247946	0.995697217	0.00855073	6
CENPE	-1.071351624	0.908709689	0.162641934	6
CENPM	-1.07157124	0.908346096	0.163225144	6
CENPS-CORT	-0.383536495	1.134993999	-0.751457504	6

CEP112	-0.718241218	1.142125823	-0.423884604	6
CEP162	-0.374974004	1.133291354	-0.75831735	6
CEP170B	-0.624758419	1.15336535	-0.528606931	6
CEP19	-0.405565058	1.139071874	-0.733506816	6
CEP295NL	-0.598652992	1.15443523	-0.555782238	6
CEP43	-0.694739218	1.146120549	-0.451381331	6
CEP72	-0.428933345	1.142912761	-0.713979416	6
CEP83	-0.707292218	1.144091103	-0.436798885	6
CERCAM	-0.39204986	1.136621911	-0.744572051	6
CFAP298-TCP10L	-0.83174931	1.109520684	-0.277771374	6
CFP	-0.588730227	1.154625272	-0.565895045	6
CGB1	-0.611968488	1.153994225	-0.542025737	6
CGNL1	-0.86329436	1.095757213	-0.232462853	6
CHCHD5	-0.407922488	1.139482186	-0.731559698	6
CHCHD6	-0.729658466	1.139876904	-0.410218438	6
CHD1	-0.445557497	1.145334377	-0.69977688	6
CHMP1A	-0.449342671	1.145849045	-0.696506375	6
CHMP2A	-1.068492303	0.913380889	0.155111414	6
CHRND	-1.073022738	0.905925301	0.167097437	6
CHRNE	-1.01842072	0.980500533	0.037920187	6
CHST11	-0.527496122	1.153304625	-0.625808503	6
CHSY3	-0.593231938	1.154553555	-0.561321617	6
CHTF18	-0.824743375	1.11226366	-0.287520285	6
CIAO3	-0.786716077	1.125347356	-0.338631278	6

CISD3	-0.930955973	1.057078132	-0.126122158	6
CITED4	-0.428644298	1.142868351	-0.714224053	6
CKAP4	-1.105899162	0.840595686	0.265303476	6
CKLF	-0.960751785	1.035098351	-0.074346567	6
CLCN7	-0.471908417	1.148629854	-0.676721437	6
CLDN17	-0.286691827	1.112033607	-0.82534178	6
CLDN34	-0.884643314	1.085014939	-0.200371625	6
CLDN9	-0.359977253	1.130152972	-0.770175719	6
CLDND2	-0.818645313	1.114563671	-0.295918358	6
CLEC1B	-0.402286641	1.138492825	-0.736206184	6
CLEC2D	-1.105828574	0.840763854	0.265064721	6
CLIC1	-0.836288479	1.1076848	-0.271396321	6
CLK3	-0.237570279	1.09739143	-0.859821151	6
CLPSL1	-0.245975893	1.100035486	-0.854059593	6
CNBP	-0.764301336	1.131738956	-0.36743762	6
CNGA1	-0.563928662	1.154597328	-0.590668667	6
CNKSR1	-1.061060347	0.92500857	0.136051776	6
CNOT7	-0.684420922	1.147614708	-0.463193786	6
CNP	-0.364715565	1.131165939	-0.766450374	6
CNTNAP3C	-1.10650939	0.839135313	0.267374077	6
COA7	-0.656607212	1.150891125	-0.494283913	6
COG7	-0.829551513	1.110392904	-0.28084139	6
COL10A1	-1.100313109	0.853444036	0.246869073	6
COL9A2	-0.653237611	1.15121596	-0.497978349	6

COL9A3	-0.307114323	1.117538798	-0.810424474	6
COMTD1	-0.382672932	1.134825242	-0.75215231	6
COP1	-0.97231962	1.025553838	-0.053234219	6
COPS5	-0.332142585	1.123808719	-0.791666134	6
COPS9	-0.259058544	1.104037611	-0.844979067	6
CORO6	-0.765478542	1.131426089	-0.365947547	6
CORO7-PAM16	-0.312436478	1.118916315	-0.806479837	6
COTL1	-0.460226263	1.147252074	-0.687025811	6
COX17	-1.040992691	0.953221042	0.087771649	6
COX5A	-0.410153494	1.139865805	-0.729712311	6
COX6A1	-0.682475514	1.147879177	-0.465403663	6
COX6B1	-0.840224519	1.106054727	-0.265830208	6
COX6B2	-0.904046343	1.074136632	-0.170090289	6
CPHXL	-0.522279266	1.153001885	-0.630722619	6
CPNE7	-0.307592752	1.117663599	-0.810070848	6
CPPED1	-0.571851965	1.154683139	-0.582831174	6
CPSF2	-0.245168799	1.099784069	-0.854615271	6
CPSF4	-1.060408325	0.925995317	0.134413007	6
CRAT	-0.889561016	1.08236258	-0.192801564	6
CREG1	-0.826847403	1.111451301	-0.284603898	6
CRLF2	-0.229227224	1.094711138	-0.865483914	6
CROCC2	-0.620477462	1.153598553	-0.533121091	6
CRYBB3	-0.438140596	1.14428628	-0.706145684	6
CRYGN	-0.729183839	1.139974533	-0.410790694	6

CS	-0.953671992	1.040646512	-0.08697452	6
CSDE1	-1.037936795	0.957164024	0.08077277	6
CSF2RA	-0.946796903	1.045833355	-0.099036452	6
CSF3R	-1.007004455	0.992845176	0.014159279	6
CSNK1A1L	-0.365730265	1.131380304	-0.765650039	6
CST8	-1.107025777	0.837890185	0.269135592	6
CT45A8	-1.087779456	0.87937727	0.208402186	6
CT47A10	-1.078113889	0.89718099	0.180932899	6
CT47A12	-1.043261457	0.950237948	0.093023509	6
CT47A5	-1.022482886	0.975888148	0.046594738	6
CT47A8	-0.792173102	1.123648461	-0.331475358	6
CTDSPL2	-0.544325389	1.154082421	-0.609757031	6
CTSB	-0.939916182	1.050835167	-0.110918985	6
CTSS	-0.93157881	1.056653764	-0.125074953	6
CTXND2	-0.27206348	1.107878486	-0.835815006	6
CXCL1	-1.081774089	0.890634975	0.191139114	6
CXCR3	-0.397849378	1.137693513	-0.739844135	6
CYP1B1	-0.817177021	1.115105522	-0.297928501	6
CYP2D6	-0.367305538	1.131711302	-0.764405764	6
CYP2S1	-0.394042654	1.136993555	-0.742950902	6
CYP4F22	-0.932786047	1.055827196	-0.123041149	6
CYRIB	-0.708524416	1.143879118	-0.435354702	6
CYSTM1	-0.947274363	1.045479346	-0.098204983	6
D2HGDH	-0.614827761	1.153871274	-0.539043512	6

DACH2	-0.277155293	1.109344718	-0.832189425	6
DCAF10	-0.585976472	1.154657361	-0.568680889	6
DCAF12	-0.61466246	1.153878656	-0.539216196	6
DCAF13	-0.467444472	1.148119168	-0.680674696	6
DCAF4L1	-0.779236507	1.127583598	-0.348347091	6
DCAF6	-0.679373382	1.148289718	-0.468916337	6
DCC	-1.05408508	0.935305302	0.118779778	6
DCD	-1.018464693	0.980451246	0.038013447	6
DCP1B	-0.977598681	1.020988794	-0.043390112	6
DCUN1D1	-1.025140612	0.972803991	0.052336621	6
DDR1	-0.336508311	1.124847913	-0.788339603	6
DDX17	-0.470173367	1.14843369	-0.678260322	6
DDX53	-0.996396088	1.003565363	-0.007169274	6
DECR1	-0.328563419	1.122944611	-0.794381193	6
DECR2	-0.907100013	1.072320703	-0.16522069	6
DEFB103A	-0.361077964	1.130390038	-0.769312074	6
DEFB103B	-0.237114769	1.097246528	-0.860131759	6
DEFB130A	-0.577350269	1.154700538	-0.577350269	6
DEFB130B	-0.577350269	1.154700538	-0.577350269	6
DEFB131A	-0.488857748	1.150388904	-0.661531157	6
DEFB136	-0.577350269	1.154700538	-0.577350269	6
DEGS2	-0.410429296	1.139912912	-0.729483616	6
DEK	-0.613889503	1.153912731	-0.540023228	6
DGKH	-1.000867328	0.999130409	0.001736919	6

DHDDS	-1.066536036	0.916511626	0.15002441	6
DLG2	-1.027796884	0.969667214	0.05812967	6
DMP1	-0.610383224	1.154058056	-0.543674832	6
DMRT2	-0.654814761	1.151065818	-0.496251057	6
DNAJB2	-1.044859403	0.948107539	0.096751864	6
DNAJC6	-0.771051795	1.129911112	-0.358859317	6
DOHH	-0.617509548	1.153746767	-0.536237219	6
DOK3	-0.479262555	1.149428262	-0.670165707	6
DPH1	-0.710498966	1.143534593	-0.433035627	6
DPP9	-1.092404033	0.870223731	0.222180302	6
DUSP29	-0.434144194	1.143699916	-0.709555721	6
DUX4	-0.893974901	1.079922406	-0.185947505	6
DUXA	-0.366792405	1.131603721	-0.764811316	6
DYDC1	-0.398669765	1.137842642	-0.739172876	6
E2F4	-0.321632477	1.121240318	-0.799607841	6
EBP	-1.101871317	0.849950208	0.251921109	6
ECRG4	-0.463674754	1.147672674	-0.68399792	6
EDDM3A	-0.869895652	1.092565622	-0.222669971	6
EDF1	-0.340189694	1.125711522	-0.785521828	6
EED	-0.45465352	1.14654797	-0.69189445	6
EFNA1	-1.097837341	0.858862019	0.238975323	6
EIF4EBP3	-0.426746939	1.142574894	-0.715827955	6
EIF5A2	-0.294123554	1.114077031	-0.819953477	6
ELL	-0.317525382	1.120211194	-0.802685812	6

ELOVL5	-1.006998237	0.992851663	0.014146573	6
ELSPBP1	-0.98274078	1.016408883	-0.033668103	6
EMC1	-0.992218068	1.00760437	-0.015386302	6
EMC4	-0.558145713	1.154489913	-0.596344199	6
EMC8	-0.464106005	1.147724457	-0.683618452	6
ENAM	-1.025589051	0.972278284	0.053310767	6
ENTPD2	-0.827011277	1.11138762	-0.284376343	6
EPHB1	-1.083767777	0.886971638	0.196796139	6
EPHX4	-0.552126154	1.154338394	-0.60221224	6
EPN3	-0.371718802	1.13262698	-0.760908179	6
EPOR	-1.064499486	0.919716688	0.144782797	6
EPPIN	-0.34084314	1.125863596	-0.785020456	6
ERI2	-0.744049061	1.136742286	-0.392693225	6
ERVFRD-1	-0.611732728	1.154003913	-0.542271185	6
ETDA	-1.010836663	0.988799061	0.022037602	6
ETDB	-0.670389215	1.149402029	-0.479012814	6
ETNK1	-1.076955976	0.899205544	0.177750432	6
EXD3	-0.62422558	1.153395631	-0.529170052	6
EXOSC5	-0.324144699	1.121862775	-0.797718076	6
F12	-0.381001309	1.134496683	-0.753495374	6
F2RL2	-0.82340751	1.112774403	-0.289366893	6
FA2H	-0.579115933	1.154698737	-0.575582804	6
FAAP24	-0.330668571	1.123454175	-0.792785604	6
FABP12	-0.699377623	1.145398058	-0.446020435	6

FAM120A	-0.56528669	1.154617094	-0.589330404	6
FAM133B	-0.898468454	1.077378978	-0.178910524	6
FAM166B	-0.448537892	1.145740768	-0.697202877	6
FAM168B	-1.019965955	0.978760204	0.041205751	6
FAM170B	-0.507453393	1.151985173	-0.64453178	6
FAM172A	-1.080398477	0.89312182	0.187276656	6
FAM177A1	-1.060992639	0.925111278	0.135881361	6
FAM205C	-0.335588128	1.124630234	-0.789042106	6
FAM209A	-0.485119201	1.150025589	-0.664906388	6
FAM214B	-0.340797152	1.125852905	-0.785055754	6
FAM217A	-0.992390646	1.007439495	-0.015048849	6
FAM227A	-0.855553376	1.099357894	-0.243804518	6
FAM229A	-0.729404287	1.139929233	-0.410524946	6
FAM240A	-0.388082086	1.135871294	-0.747789208	6
FAM240B	-0.373106129	1.132911275	-0.759805146	6
FAM53A	-0.310132174	1.118322816	-0.808190642	6
FAM71A	-0.465471016	1.147887162	-0.682416146	6
FAM71E1	-0.958015154	1.037268469	-0.079253315	6
FAM83A	-0.596894157	1.154477466	-0.557583309	6
FAM83G	-1.065789844	0.91769228	0.148097564	6
FAR2	-0.760562407	1.13271633	-0.372153923	6
FAXDC2	-0.559528897	1.15451902	-0.594990123	6
FBF1	-1.081739421	0.890698052	0.19104137	6
FBXL15	-0.344536828	1.126716307	-0.782179479	6

FBXL5	-0.757505458	1.13349713	-0.375991673	6
FBXL8	-0.451151443	1.146090133	-0.69493869	6
FBXO48	-0.602648857	1.154325464	-0.551676607	6
FBXO8	-0.351699176	1.128336211	-0.776637035	6
FBXW12	-0.77270379	1.129451242	-0.356747451	6
FCAR	-0.578174067	1.154700146	-0.57652608	6
FCER2	-0.660315387	1.150515964	-0.490200578	6
FCGR1A	-0.884262554	1.085217431	-0.200954877	6
FCGR2A	-1.082395533	0.889500699	0.192894834	6
FCN1	-0.960451879	1.035337767	-0.074885888	6
FETUB	-0.94074172	1.050244779	-0.10950306	6
FGL2	-0.430110686	1.143092841	-0.712982156	6
FHL3	-0.425616912	1.142398518	-0.716781606	6
FKBP5	-0.762731777	1.132152261	-0.369420484	6
FKBP9	-0.558230125	1.15449175	-0.596261625	6
FLCN	-0.311543887	1.118686947	-0.80714306	6
FLOT2	-0.42304391	1.141992475	-0.718948564	6
FN3KRP	-1.095021732	0.864836495	0.230185237	6
FNDC8	-0.433506992	1.14360503	-0.710098038	6
FNDC9	-0.620250595	1.15361027	-0.533359675	6
FOXA3	-1.013200193	0.986255209	0.026944983	6
FOXQ1	-0.910168848	1.070466014	-0.160297166	6
FPR1	-0.89628396	1.078622985	-0.182339025	6
FRAS1	-0.25507846	1.10283469	-0.84775623	6

FRG2B	-0.371885415	1.132661213	-0.760775798	6
FRMD1	-1.09152018	0.872008112	0.219512068	6
FRRS1	-0.436421496	1.144035898	-0.707614402	6
FRY	-0.985690866	1.01371983	-0.028028964	6
FSCN2	-0.429700464	1.143030243	-0.713329779	6
FSHR	-0.967344394	1.029734114	-0.06238972	6
FTH1	-1.001077349	0.998919157	0.002158192	6
FUNDC2	-0.362009234	1.130589782	-0.768580548	6
FUT6	-1.006057446	0.993830426	0.01222702	6
FUT7	-0.926716135	1.05992986	-0.133213725	6
FUZ	-0.29232156	1.113585753	-0.821264193	6
FXR1	-0.606460936	1.154202765	-0.547741829	6
FXYD6-FXYD2	-0.983683375	1.015554647	-0.031871272	6
FZD7	-0.835398203	1.108048565	-0.272650362	6
FZD9	-0.289171088	1.11272038	-0.823549293	6
GABRR3	-0.304830421	1.116940384	-0.812109963	6
GAGE12B	-0.476546306	1.149139616	-0.67259331	6
GAGE12C	-0.391905684	1.136594884	-0.7446892	6
GAGE12E	-1.028059351	0.969354261	0.058705089	6
GALNT18	-0.340228682	1.125720606	-0.785491923	6
GALNTL5	-0.459482081	1.147159791	-0.68767771	6
GANC	-0.363902503	1.130993519	-0.767091017	6
GAPDH	-0.745050568	1.136511369	-0.391460801	6
GAS1	-0.375653878	1.133428929	-0.757775051	6

GATA1	-0.650323916	1.151484597	-0.501160681	6
GCAT	-0.237721253	1.09743942	-0.859718167	6
GCM1	-0.291538745	1.113371496	-0.821832751	6
GCNT3	-1.050023834	0.941049729	0.108974105	6
GDF15	-0.285056525	1.11157784	-0.826521315	6
GFRA4	-0.861192965	1.096749624	-0.235556658	6
GGA2	-0.474870869	1.148957921	-0.674087052	6
GGT1	-0.234913588	1.096543973	-0.861630386	6
GGTLC1	-0.451685053	1.146160656	-0.694475603	6
GHITM	-0.399350637	1.137965946	-0.738615309	6
GID4	-0.528089321	1.153337217	-0.625247896	6
GINM1	-1.010545421	0.989109939	0.021435481	6
GIP	-0.646653514	1.151806947	-0.505153432	6
GIT2	-0.270032805	1.107287822	-0.837255017	6
GJA3	-0.591641789	1.154581627	-0.562939837	6
GJA8	-0.87002196	1.092503445	-0.222481485	6
GJB7	-0.729928667	1.139821163	-0.409892496	6
GLI4	-0.571360813	1.154679898	-0.583319085	6
GLUL	-0.272788161	1.108088459	-0.835300298	6
GLYATL1B	-0.257394012	1.103536091	-0.846142079	6
GMDS	-0.274638234	1.108622561	-0.833984327	6
GMPPA	-0.917758387	1.065747925	-0.147989538	6
GNAI3	-0.335149862	1.124526303	-0.78937644	6
GNAQ	-0.493364458	1.150808124	-0.657443666	6

GNG4	-0.919339471	1.064740973	-0.145401502	6
GNG5	-0.281471697	1.110571021	-0.829099325	6
GNRH2	-1.085948234	0.882881935	0.203066299	6
GOLGA6A	-0.826517744	1.111579224	-0.285061481	6
GOPC	-0.460920629	1.147337695	-0.686417066	6
GORASP2	-1.091418969	0.872211362	0.219207607	6
GOT1L1	-0.655436863	1.151005677	-0.495568814	6
GPBP1	-1.099629447	0.854956181	0.244673267	6
GPC1	-0.245856053	1.099998188	-0.854142135	6
GPR152	-0.62373259	1.153423329	-0.529690739	6
GPR171	-0.923918135	1.061776923	-0.137858788	6
GPR22	-0.680866186	1.148093868	-0.467227682	6
GPR84	-0.95127419	1.042477515	-0.091203324	6
GRB2	-1.10661952	0.838870486	0.267749034	6
GREB1	-0.486218062	1.150133836	-0.663915774	6
GSG1L2	-0.620040117	1.153621084	-0.533580967	6
GSTO2	-0.414349171	1.14057486	-0.72622569	6
GSTT4	-0.616891298	1.153776262	-0.536884964	6
GTF2F2	-1.015210698	0.984061463	0.031149235	6
GTF2H2	-1.050133792	0.940896506	0.109237286	6
GYPB	-0.559895713	1.15452638	-0.594630667	6
H1-8	-1.083206537	0.888010107	0.19519643	6
H2AB1	-0.37802245	1.133905013	-0.755882563	6
H2AP	-0.915315878	1.067287033	-0.151971155	6

HAS1	-0.435719982	1.143932922	-0.70821294	6
HBB	-0.416903238	1.140998544	-0.724095306	6
HBD	-0.538333032	1.153840558	-0.615507525	6
HBE1	-0.645050637	1.151942136	-0.506891498	6
HBG1	-0.631201629	1.152970731	-0.521769103	6
HBG2	-0.625228275	1.153338351	-0.528110076	6
HBM	-0.309798079	1.118236396	-0.808438317	6
HBS1L	-0.952158236	1.041805218	-0.089646981	6
HBZ	-0.723497079	1.141116221	-0.417619141	6
HCG22	-0.860531184	1.097059834	-0.23652865	6
HDAC3	-0.498743234	1.151281474	-0.65253824	6
HDGFL3	-0.263387145	1.105331292	-0.841944147	6
HECW1	-0.791153903	1.123970133	-0.33281623	6
HEMGN	-0.334661094	1.124410201	-0.789749107	6
HIGD1B	-0.324086291	1.121848364	-0.797762073	6
HLA-DRB4	-0.268296942	1.106780243	-0.8384833	6
HLX	-0.332859703	1.123980536	-0.791120833	6
HMBS	-0.760266337	1.132792668	-0.372526331	6
HMGN3	-0.524627612	1.153141736	-0.628514124	6
HNF4A	-0.936666978	1.053133651	-0.116466672	6
HNRNPH2	-0.620216717	1.153612015	-0.533395297	6
HOMER2	-0.693862318	1.146253568	-0.45239125	6
HOXA10	-1.065217116	0.918593495	0.146623621	6
HOXC11	-0.243258796	1.099187002	-0.855928206	6

HOXC5	-0.804750719	1.119510082	-0.314759363	6
HP	-0.480548857	1.14956239	-0.669013533	6
HPD	-0.930607945	1.057314648	-0.126706703	6
HRH2	-0.656555584	1.150896217	-0.494340634	6
HS3ST6	-1.066548002	0.916492633	0.150055369	6
HSD17B1	-0.787657285	1.125058429	-0.337401144	6
HSPA12A	-0.521278687	1.152940525	-0.631661837	6
HSPA6	-0.543849385	1.154064639	-0.610215254	6
HYAL1	-0.895571337	1.079025706	-0.18345437	6
HYAL2	-0.809024787	1.118031258	-0.309006471	6
IDI1	-1.002096393	0.997890339	0.004206053	6
IFITM10	-0.642776638	1.152128137	-0.5093515	6
IFITM2	-0.291077792	1.113245096	-0.822167304	6
IFT27	-0.927038958	1.059714982	-0.132676024	6
IGF2BP2	-0.7928657	1.123428714	-0.330563014	6
IGFL1	-0.265793048	1.10604376	-0.840250711	6
IGLL5	-0.279574967	1.110034039	-0.830459072	6
IGSF6	-0.645753728	1.151883252	-0.506129525	6
IHO1	-0.455333379	1.146635479	-0.6913021	6
IL17RA	-0.373677344	1.133027835	-0.759350491	6
IL17RC	-0.831514879	1.109614236	-0.278099357	6
IL18RAP	-0.255068788	1.102831751	-0.847762963	6
IL1RAP	-0.712110376	1.143249007	-0.431138632	6
IL3RA	-0.416161714	1.140876157	-0.724714443	6

IL5RA	-0.357130017	1.129534836	-0.772404819	6
ILDR1	-0.681862802	1.14796135	-0.466098548	6
INCA1	-0.785343608	1.125765633	-0.340422025	6
ING2	-1.089977203	0.87508294	0.214894263	6
INKA2	-0.270585719	1.107448982	-0.836863263	6
INPP1	-0.71148223	1.143360806	-0.431878576	6
INPP5J	-1.102099252	0.849433488	0.252665764	6
INSYN2B	-0.540220969	1.153920972	-0.613700004	6
INTS5	-0.482097499	1.149721684	-0.667624184	6
IP6K1	-0.35445128	1.128946827	-0.774495547	6
IQCA1L	-0.398498435	1.137811548	-0.739313113	6
IQCM	-0.577350269	1.154700538	-0.577350269	6
IQGAP2	-0.945963087	1.046449397	-0.10048631	6
ITGA11	-0.847213303	1.103071304	-0.255858002	6
IZUMO2	-0.769494039	1.130340201	-0.360846162	6
JPT1	-0.871481979	1.091781667	-0.220299688	6
JTB	-0.539905732	1.153907814	-0.614002082	6
KATNAL1	-0.335329215	1.124568855	-0.78923964	6
KAZN	-0.610351287	1.15405931	-0.543708023	6
KCND1	-0.621254558	1.153557929	-0.532303371	6
KCNJ1	-0.813732157	1.116358843	-0.302626686	6
KCNJ15	-0.539106673	1.153873978	-0.614767305	6
KCNJ2	-0.285786076	1.111781442	-0.825995366	6
KCNQ1	-0.329577616	1.123190575	-0.793612959	6

KCTD16	-0.921499404	1.063351674	-0.14185227	6
KCTD21	-0.42221485	1.141860327	-0.719645478	6
KDELR2	-0.403844487	1.138769202	-0.734924714	6
KDM6B	-0.352911194	1.128605935	-0.775694742	6
KIAA0754	-0.548829327	1.154238383	-0.605409056	6
KIR2DL1	-0.817051793	1.115151523	-0.29809973	6
KIR2DL2	-0.269549926	1.107146871	-0.837596945	6
KIR2DL3	-0.645121201	1.151936255	-0.506815054	6
KIR2DL4	-0.688736306	1.147008624	-0.458272317	6
KIR2DL5A	-0.46854537	1.148246934	-0.679701563	6
KIR2DL5B	-0.284468654	1.111413458	-0.826944804	6
KIR2DS1	-0.751309566	1.135029823	-0.383720257	6
KIR2DS2	-0.752246569	1.134802284	-0.382555715	6
KIR2DS4	-0.845544009	1.103794395	-0.258250386	6
KIR3DL1	-0.601661524	1.154354375	-0.552692851	6
KIR3DL2	-0.593694538	1.154544824	-0.560850286	6
KIR3DS1	-0.601552264	1.154357502	-0.552805238	6
KISS1R	-0.527458241	1.153302531	-0.62584429	6
KLF18	-0.24998349	1.101276125	-0.851292635	6
KLF3	-0.339959193	1.12565779	-0.785698598	6
KLHL2	-0.959535624	1.036066778	-0.076531155	6
KLK15	-0.560991528	1.154547467	-0.593555939	6
KLLN	-0.705537664	1.144388977	-0.438851313	6
KLRC2	-0.727720941	1.140273166	-0.412552225	6

KLRG2	-0.796325468	1.122316938	-0.32599147	6
KNSTRN	-0.245041402	1.099744336	-0.854702934	6
KRT14	-0.864598266	1.095135748	-0.230537482	6
KRT36	-1.070041472	0.910864369	0.159177102	6
KRT40	-1.096322481	0.862100349	0.234222132	6
KRTAP12-3	-1.019683425	0.979079695	0.04060373	6
KRTAP2-3	-0.834688943	1.108337074	-0.273648131	6
KRTAP7-1	-0.91565474	1.067074691	-0.151419951	6
KRTAP8-1	-1.047639679	0.944341334	0.103298345	6
KRTDAP	-0.322302331	1.121406811	-0.79910448	6
KYAT3	-0.852720375	1.100638299	-0.247917924	6
LACTBL1	-1.06208395	0.923448986	0.138634965	6
LAPTM5	-0.300848287	1.115886588	-0.815038301	6
LARGE2	-0.327490168	1.122683373	-0.795193205	6
LBHD1	-0.766338014	1.131196103	-0.364858088	6
LCAT	-0.33897606	1.125428103	-0.786452043	6
LCE3B	-0.741105673	1.137411224	-0.396305551	6
LCN8	-0.438453305	1.144331525	-0.70587822	6
LCORL	-0.269575674	1.107154392	-0.837578718	6
LCP1	-0.711272378	1.14339802	-0.432125642	6
LDHD	-0.487488882	1.150257509	-0.662768627	6
LENG9	-0.335249163	1.124549866	-0.789300702	6
LGALS1	-0.710588556	1.14351882	-0.432930264	6
LGALS13	-0.615699599	1.153831775	-0.538132176	6

LGALS3	-0.439501413	1.144482495	-0.704981082	6
LGR6	-0.576582141	1.154700198	-0.578118057	6
LHB	-1.087863419	0.879215002	0.208648417	6
LHPP	-0.978248243	1.02041762	-0.042169376	6
LILRA3	-0.438962284	1.144404969	-0.705442685	6
LILRA5	-0.341553613	1.126028526	-0.784474913	6
LILRB4	-0.642526392	1.152148192	-0.5096218	6
LIMK1	-0.25089213	1.101555615	-0.850663486	6
LIMS4	-0.757356866	1.133534666	-0.3761778	6
LIN7A	-1.082323338	0.889632822	0.192690515	6
LINGO3	-0.401680489	1.138384691	-0.736704202	6
LIPK	-0.812119646	1.116936929	-0.304817283	6
LITAFD	-0.657533863	1.150799117	-0.493265254	6
LMF1	-0.493435457	1.150814564	-0.657379107	6
LOC100286986	-0.844306382	1.104326226	-0.260019845	6
LOC100505841	-0.97729355	1.021256376	-0.043962826	6
LOC100509620	-0.986302065	1.013156972	-0.026854907	6
LOC100652777	-0.460049411	1.147230192	-0.687180781	6
LOC100996598	-0.641552034	1.152225499	-0.510673464	6
LOC100996701	-0.441300719	1.144739239	-0.70343852	6
LOC101060341	-0.793353505	1.123273382	-0.329919877	6
LOC101060588	-1.039659038	0.954952276	0.084706761	6
LOC101929601	-0.75832553	1.133289275	-0.374963745	6
LOC101929627	-0.759559755	1.132974231	-0.373414476	6

LOC102723502	-0.61837472	1.153704695	-0.535329975	6
LOC102723971	-0.503379782	1.151665777	-0.648285996	6
LOC102723996	-0.597185817	1.154470718	-0.557284901	6
LOC102724265	-1.067413887	0.915113151	0.152300737	6
LOC102724485	-0.413110799	1.140367265	-0.727256466	6
LOC105370980	-0.553045105	1.154364129	-0.601319024	6
LOC105371095	-0.325711498	1.122248273	-0.796536775	6
LOC105371253	-1.103842504	0.845432402	0.258410102	6
LOC105372204	-0.331558314	1.123668406	-0.792110092	6
LOC105374103	-0.563929362	1.154597339	-0.590667977	6
LOC105375107	-0.577350269	1.154700538	-0.577350269	6
LOC105375116	-0.531094758	1.153496572	-0.622401814	6
LOC105375809	-0.28481805	1.111511191	-0.826693141	6
LOC105376335	-0.915670185	1.067065004	-0.151394819	6
LOC105376714	-1.053734984	0.935807509	0.117927475	6
LOC105376747	-0.330133361	1.123324982	-0.793191621	6
LOC105377805	-0.575202872	1.154697879	-0.579495007	6
LOC105378193	-0.361082483	1.130391009	-0.769308526	6
LOC105379417	-0.509082064	1.152108071	-0.643026007	6
LOC105379473	-0.880592328	1.087148438	-0.20655611	6
LOC105379545	-0.396202344	1.137392275	-0.741189931	6
LOC105379561	-0.407305527	1.139375295	-0.732069768	6
LOC105379589	-0.571705124	1.154682199	-0.582977075	6
LOC107983989	-0.475876216	1.149067281	-0.673191066	6

LOC107984014	-0.843971506	1.104469506	-0.260498	6
LOC107984264	-0.75164994	1.134947342	-0.383297402	6
LOC107984351	-0.577350269	1.154700538	-0.577350269	6
LOC107984590	-0.743723491	1.13681699	-0.393093499	6
LOC107984638	-0.584949312	1.154667051	-0.56971774	6
LOC107984817	-0.853663772	1.100214113	-0.246550341	6
LOC107985021	-0.312679846	1.118978737	-0.806298891	6
LOC107985022	-0.37339954	1.132971183	-0.759571643	6
LOC107985149	-0.351299263	1.128246934	-0.776947671	6
LOC107985773	-0.297979356	1.115119187	-0.817139831	6
LOC107986755	-0.467295031	1.148101733	-0.680806702	6
LOC107986762	-0.365491725	1.131329991	-0.765838266	6
LOC107986800	-0.643976534	1.152030837	-0.508054303	6
LOC107987004	-0.988825073	1.010812315	-0.021987242	6
LOC107987158	-0.315612353	1.11972698	-0.804114627	6
LOC107987238	-0.341221036	1.125951375	-0.784730339	6
LOC107987269	-1.025475693	0.97241132	0.053064373	6
LOC107987372	-0.417662643	1.141123356	-0.723460713	6
LOC107987457	-0.801492265	1.120612517	-0.319120253	6
LOC107987545	-0.620128262	1.153616562	-0.5334883	6
LOC112267881	-0.434530798	1.143757298	-0.7092265	6
LOC112268080	-0.310815906	1.118499384	-0.807683478	6
LOC112268131	-0.31248444	1.118928621	-0.80644418	6
LOC112268145	-0.766615014	1.131121699	-0.364506685	6

LOC112268350	-1.02409574	0.974022909	0.05007283	6
LOC112268355	-0.839084585	1.106530498	-0.267445913	6
LOC112268444	-0.239090387	1.097873789	-0.858783402	6
LOC389199	-0.762215543	1.132287244	-0.3700717	6
LOXL1	-1.070823859	0.909580604	0.161243255	6
LPAR1	-0.458463584	1.147032618	-0.688569034	6
LPCAT2	-0.381767155	1.13464752	-0.752880365	6
LPIN2	-0.979037192	1.01972104	-0.040683848	6
LPO	-0.298472864	1.115251681	-0.816778818	6
LRFN1	-0.696701124	1.145818842	-0.449117718	6
LRFN2	-0.954472898	1.040029586	-0.085556689	6
LRG1	-0.746247856	1.136233097	-0.389985241	6
LRP1B	-0.741427896	1.137338697	-0.395910802	6
LRRC18	-0.275421017	1.108847701	-0.833426685	6
LRRC26	-0.643591569	1.152062259	-0.50847069	6
LRRC29	-0.533097517	1.153597392	-0.620499874	6
LRRC38	-1.078763794	0.8960351	0.182728694	6
LRRC4	-0.757902991	1.133396518	-0.375493527	6
LRRC40	-0.323328969	1.121661247	-0.798332277	6
LRRC42	-0.434586041	1.143765486	-0.709179445	6
LRRC56	-0.891554004	1.081267831	-0.189713826	6
LRRFIP2	-1.010986328	0.988639088	0.02234724	6
LRSAM1	-0.248462291	1.100806727	-0.852344437	6
LSR	-0.980946714	1.018022083	-0.037075369	6

LST1	-0.813067874	1.116597648	-0.303529774	6
LTA	-1.002183719	0.99780188	0.004381839	6
LTB4R	-0.490064833	1.150503203	-0.66043837	6
LTC4S	-0.834696805	1.108333882	-0.273637077	6
LTF	-0.370356404	1.132346139	-0.761989735	6
LY6L	-0.925151048	1.060966411	-0.135815363	6
LYG2	-0.317835621	1.120289429	-0.802453808	6
LYPD3	-0.350384636	1.12804223	-0.777657594	6
LYPD4	-0.310189426	1.118337616	-0.80814819	6
LYPD8	-0.294957008	1.114303345	-0.819346337	6
LYZL1	-0.97875981	1.019966303	-0.041206493	6
MAB21L2	-0.420590053	1.141599491	-0.721009438	6
MAFA	-0.894359763	1.079706925	-0.185347162	6
MAGEA10	-0.916448952	1.066575517	-0.150126564	6
MAGEC2	-0.82125082	1.113590785	-0.292339965	6
MAP1B	-0.509685812	1.15215293	-0.642467119	6
MAP2K4	-0.641419864	1.15223589	-0.510816026	6
MAP6D1	-0.759424292	1.133008939	-0.373584648	6
MAPK1	-0.256096501	1.103143598	-0.847047097	6
MARCHF3	-0.855163036	1.099535487	-0.244372451	6
MARCKS	-0.378488011	1.133998005	-0.755509994	6
MARVELD1	-0.62527003	1.153335938	-0.528065909	6
MBD3L2	-0.245881873	1.100006225	-0.854124352	6
MBD3L3	-0.651631902	1.151365403	-0.499733502	6

MBD3L4	-1.051470832	0.93902323	0.112447602	6
MBD3L5	-0.452941611	1.146325643	-0.693384032	6
MBP	-1.081721183	0.890731227	0.190989956	6
MCAM	-0.39128904	1.136479078	-0.745190038	6
MCCC2	-1.055833659	0.932776637	0.123057022	6
MCM5	-1.056963922	0.9311238	0.125840122	6
MCOLN1	-0.912851954	1.068819627	-0.155967674	6
MDFIC2	-0.478450489	1.149342735	-0.670892246	6
MED21	-1.034381672	0.96164659	0.072735082	6
MEIOSIN	-0.652995964	1.15123867	-0.498242706	6
METTL27	-1.088020979	0.878910118	0.209110861	6
MFN2	-0.344300632	1.126662132	-0.7823615	6
MFSD2B	-0.935711168	1.053802218	-0.11809105	6
MICU2	-0.356263171	1.129345239	-0.773082068	6
MIDN	-0.629828933	1.153059225	-0.523230292	6
MIF	-0.554294045	1.154397605	-0.600103559	6
MIF4GD	-0.499594592	1.151353691	-0.651759099	6
MKRN1	-0.260515354	1.104474702	-0.843959349	6
MLC1	-0.720199397	1.141754751	-0.421555354	6
MLF2	-0.268741625	1.106910505	-0.83816888	6
MME	-0.266872967	1.106362028	-0.839489061	6
MMP12	-0.420777284	1.141629673	-0.720852389	6
MMP17	-0.698874126	1.145478027	-0.446603902	6
MOCOS	-0.748657024	1.135665819	-0.387008795	6

MOSPD2	-1.018389066	0.980536004	0.037853062	6
MOV10	-0.423793601	1.14211142	-0.718317819	6
MPND	-0.337116834	1.124991468	-0.787874634	6
MPST	-1.10055669	0.852902253	0.247654437	6
MROH1	-0.843928993	1.104487676	-0.260558683	6
MROH2A	-0.562144496	1.154568198	-0.592423702	6
MROH9	-0.762215014	1.132287382	-0.370072368	6
MRPL2	-0.242286828	1.09888204	-0.856595211	6
MRPS34	-0.488082904	1.15031476	-0.662231857	6
MS4A13	-0.794804504	1.122808587	-0.328004082	6
MS4A6A	-0.682736943	1.147843952	-0.465107009	6
MSANTD5	-0.403023164	1.138623767	-0.735600603	6
MSC	-0.330439474	1.123398904	-0.79295943	6
MSGN1	-0.432231062	1.143413882	-0.71118282	6
MSLN	-0.615488512	1.153841425	-0.538352912	6
MSMO1	-0.563363026	1.154588482	-0.591225456	6
MSS51	-0.307894508	1.117742216	-0.809847708	6
MT1E	-0.274931936	1.108707093	-0.833775157	6
MT1F	-0.243655696	1.099311314	-0.855655618	6
MT4	-0.577350269	1.154700538	-0.577350269	6
MTF1	-0.7729742	1.129375493	-0.356401294	6
MTHFSD	-0.236260883	1.096974452	-0.860713568	6
MTRNR2L4	-0.4574078	1.146899728	-0.689491928	6
MTRNR2L7	-0.782249466	1.126695484	-0.344446018	6

MTRNR2L8	-0.427656962	1.142716064	-0.715059102	6
MUC1	-0.811995918	1.116981061	-0.304985144	6
MUC12	-1.080532406	0.892881135	0.187651271	6
MVB12A	-0.948487519	1.044575741	-0.096088222	6
MX2	-0.657082795	1.150844049	-0.493761254	6
MXD3	-0.582505128	1.154685151	-0.572180023	6
MYG1	-0.329904766	1.123269728	-0.793364962	6
MYO1H	-0.546955	1.154176183	-0.607221183	6
MYO7B	-1.040477986	0.953891124	0.086586861	6
MYOCOS	-0.294234647	1.11410723	-0.819872583	6
NADK2	-0.233698644	1.096154539	-0.862455895	6
NANOG	-0.870533067	1.092251415	-0.221718348	6
NANOGP8	-1.061677844	0.924069282	0.137608562	6
NARF	-0.530585082	1.153470228	-0.622885145	6
NBPF19	-0.461886389	1.147456	-0.685569611	6
NBPF20	-0.268244822	1.106764964	-0.838520143	6
NCAPG2	-0.704811355	1.14451092	-0.439699565	6
NCF2	-0.831454057	1.109638488	-0.27818443	6
NDN	-0.867627736	1.093674902	-0.226047166	6
NDUFA9	-0.562395644	1.154572515	-0.592176871	6
NDUFB3	-0.412580963	1.140278014	-0.727697052	6
NDUFV3	-0.812239605	1.116894111	-0.304654506	6
NEK8	-0.526289671	1.153237183	-0.626947512	6
NEURL3	-0.899331424	1.076883571	-0.177552147	6

NFAM1	-0.923434646	1.062093323	-0.138658677	6
NFE2	-1.027332105	0.970220057	0.057112048	6
NFE2L2	-1.004211802	0.995734296	0.008477506	6
NIBAN1	-0.759636254	1.132954616	-0.373318363	6
NIP7	-0.937207085	1.052754345	-0.115547259	6
NKAIN3	-0.93060752	1.057314936	-0.126707416	6
NKG7	-0.334283351	1.124320332	-0.790036981	6
NKIRAS2	-0.687293722	1.147214223	-0.459920501	6
NKPD1	-0.53623863	1.153746832	-0.617508202	6
NKX6-2	-0.761474433	1.132480202	-0.371005769	6
NLRP2B	-0.678174273	1.148444747	-0.470270474	6
NLRP7	-0.228955687	1.094622971	-0.865667283	6
NOC4L	-1.089976537	0.875084257	0.214892281	6
NOL12	-1.044349957	0.948789413	0.095560543	6
NOP10	-0.677931916	1.148475833	-0.470543917	6
NOS1AP	-1.089408287	0.87620405	0.213204237	6
NOTCH2NLA	-0.759250571	1.133053403	-0.373802832	6
NOTCH2NLB	-1.015427808	0.983822899	0.031604909	6
NOX3	-0.397058393	1.137549151	-0.740490758	6
NPIPB11	-0.476146395	1.1490965	-0.672950105	6
NPIPB15	-0.684929411	1.147544687	-0.462615276	6
NPLOC4	-0.580319871	1.154695438	-0.574375568	6
NQO2	-0.716714046	1.14241106	-0.425697014	6
NRARP	-1.101432613	0.850940638	0.250491975	6

NRM	-1.104425184	0.844075161	0.260350024	6
NRN1L	-0.399939195	1.138072193	-0.738132998	6
NSFL1C	-0.358549124	1.129843809	-0.771294686	6
NSUN3	-0.404742607	1.138927531	-0.734184923	6
NT5DC1	-0.995615977	1.004327109	-0.008711133	6
NTRK1	-0.511796216	1.152306762	-0.640510546	6
NUDT14	-0.66723215	1.149766149	-0.482533999	6
NUDT4B	-0.270488684	1.107420717	-0.836932033	6
NUFIP1	-1.057471404	0.930376925	0.12709448	6
NUMB	-1.100527307	0.852967691	0.247559617	6
NUP58	-1.048997816	0.942473412	0.106524404	6
NUP88	-0.519595464	1.152834918	-0.633239454	6
NUTM2B	-0.539226909	1.153879113	-0.614652204	6
NXNL2	-0.754081523	1.134352317	-0.380270794	6
NYX	-0.892694522	1.080636129	-0.187941607	6
OAF	-0.915260614	1.067321628	-0.152061014	6
OAT	-0.48882375	1.150385664	-0.661561914	6
OCEL1	-0.523916405	1.153099998	-0.629183594	6
OCLN	-0.9272547	1.059571174	-0.132316473	6
ODF3B	-0.840889702	1.105775707	-0.264886005	6
OLFML1	-0.756150931	1.13383788	-0.377686949	6
OLIG1	-0.584707841	1.15466915	-0.569961309	6
OMP	-0.245930784	1.100021448	-0.854090664	6
OPN3	-0.706905043	1.144157236	-0.437252193	6

OPRD1	-0.923663835	1.06194344	-0.138279605	6
OPRL1	-0.708456743	1.14389082	-0.435434077	6
OR10A2	-0.837893205	1.107024529	-0.269131324	6
OR10A3	-0.896321238	1.078601876	-0.182280639	6
OR10AC1	-0.297763174	1.115061083	-0.817297909	6
OR10G3	-1.022646549	0.975699768	0.046946781	6
OR10G6	-0.272208643	1.10792058	-0.835711937	6
OR10K2	-0.993189243	1.006674365	-0.013485122	6
OR14A2	-1.035463256	0.960294487	0.075168768	6
OR1J1	-0.724341868	1.140949878	-0.41660801	6
OR2A4	-0.942002182	1.049338291	-0.10733611	6
OR2A5	-0.807700906	1.118493325	-0.310792419	6
OR2B11	-0.691273838	1.146639641	-0.455365803	6
OR2L5	-0.661882346	1.150351827	-0.488469481	6
OR2T12	-0.730973599	1.13960449	-0.408630891	6
OR2T8	-1.031378076	0.965349408	0.066028668	6
OR3A1	-0.694554009	1.146148738	-0.451594729	6
OR4K2	-0.964407052	1.032148322	-0.067741271	6
OR4K3	-0.22912046	1.094676479	-0.865556019	6
OR51A4	-0.99551601	1.004424469	-0.008908459	6
OR51G2	-1.090485319	0.87407594	0.216409379	6
OR52A1	-0.440919781	1.144685139	-0.703765358	6
OR52H1	-1.092489039	0.870051214	0.222437825	6
OR52W1	-0.788546084	1.124784031	-0.336237948	6

OR5F1	-0.810460629	1.117526023	-0.307065394	6
OR5H1	-0.446630216	1.145481626	-0.69885141	6
OR5H14	-0.864883956	1.094999	-0.230115044	6
OR6C68	-0.883475082	1.08563492	-0.202159838	6
OR6C75	-1.027528649	0.969986481	0.057542168	6
OR6N2	-1.092413911	0.870203692	0.22221022	6
OR6X1	-1.05708398	0.930947375	0.126136605	6
OR6Y1	-0.420004351	1.141504864	-0.721500513	6
OR7A5	-1.07320042	0.905626827	0.167573592	6
OR8D1	-0.561805913	1.154562265	-0.592756352	6
OR8D4	-0.295949355	1.114572052	-0.818622697	6
ORAI1	-0.819920467	1.114089356	-0.294168888	6
ORM1	-0.54167863	1.153980412	-0.612301781	6
ORM2	-0.436043603	1.143980484	-0.707936881	6
OSBPL7	-0.761897367	1.132370204	-0.370472837	6
OSGEP	-0.349284362	1.127795015	-0.778510654	6
OXT	-0.324086543	1.121848426	-0.797761883	6
P2RY14	-0.462264948	1.147502126	-0.685237178	6
P2RY4	-1.088251252	0.878463643	0.209787609	6
P4HA1	-0.643851056	1.1520411	-0.508190044	6
P4HA2	-0.688220651	1.147082462	-0.458861811	6
PACS1	-0.698627759	1.14551702	-0.44688926	6
PACSIN2	-0.76100199	1.132602702	-0.371600712	6
PAK1	-0.324381306	1.121921124	-0.797539818	6

PAN3	-0.259366833	1.104130252	-0.844763419	6
PARP16	-0.27110469	1.107600021	-0.836495332	6
PARP6	-1.101997773	0.849663718	0.252334054	6
PATE3	-0.838536964	1.106757987	-0.268221023	6
PBDC1	-0.232656524	1.095819562	-0.863163038	6
PCDHA10	-0.948625514	1.044472582	-0.095847067	6
PCDHA4	-1.057526979	0.930294954	0.127232025	6
PCDHGA10	-0.54377629	1.154061886	-0.610285596	6
PCDHGA12	-0.751794996	1.134912131	-0.383117135	6
PCDHGA7	-0.933569225	1.055288119	-0.121718894	6
PCDHGB5	-1.077887404	0.897578694	0.18030871	6
PCDHGC4	-0.825452157	1.111991087	-0.28653893	6
PCED1A	-0.46964978	1.148373911	-0.67872413	6
PCK2	-0.278757641	1.109801735	-0.831044094	6
PCSK1N	-0.42160636	1.141762931	-0.720156571	6
PDE4C	-0.7812646	1.126987662	-0.345723062	6
PDE6G	-0.9834995	1.015721648	-0.032222148	6
PDK2	-0.861227887	1.096733223	-0.235505336	6
PDZD11	-1.075628402	0.901500407	0.174127995	6
PDZD3	-1.043542351	0.949865232	0.093677119	6
PDZD8	-1.092869662	0.869276785	0.223592877	6
PEA15	-0.278754125	1.109800735	-0.83104661	6
PEAK3	-0.99620047	1.003756707	-0.007556236	6
PER3	-0.641359426	1.152240634	-0.510881208	6

PET117	-0.676107732	1.148707153	-0.472599421	6
PFDN1	-0.602497922	1.15432996	-0.551832038	6
PFDN2	-0.23289681	1.095896876	-0.863000066	6
PGAM2	-0.353969901	1.128840497	-0.774870596	6
PGAP6	-0.536632763	1.153764832	-0.617132069	6
PGGHG	-0.576650839	1.154700256	-0.578049417	6
PGLYRP1	-0.539578098	1.153894024	-0.614315926	6
PGLYRP2	-0.543309988	1.15404419	-0.610734202	6
PGM2L1	-1.009043178	0.990704578	0.0183386	6
PHF20	-0.768201439	1.130692919	-0.36249148	6
PHF21A	-0.666258048	1.149875717	-0.483617669	6
PHF24	-0.692377586	1.146476216	-0.454098629	6
PHLDA1	-0.261737898	1.104840178	-0.84310228	6
PI3	-0.500339778	1.151416293	-0.651076515	6
PICALM	-0.721951135	1.141417698	-0.419466563	6
PIGX	-0.807567551	1.118539669	-0.310972118	6
PIK3R6	-0.590105135	1.154605909	-0.564500774	6
PITHD1	-0.388933524	1.13603356	-0.747100036	6
PLA2G2D	-0.54025362	1.153922329	-0.613668709	6
PLB1	-0.362888681	1.130777714	-0.767889033	6
PLEKHB1	-0.377405428	1.133781471	-0.756376043	6
PLEKHF1	-0.580916682	1.15469318	-0.573776498	6
PLEKHG3	-1.050555291	0.940307988	0.110247303	6
PLEKHO2	-1.078012109	0.897359818	0.180652291	6

PLET1	-0.249970582	1.10127215	-0.851301568	6
PLIN5	-0.463140429	1.147608264	-0.684467834	6
PLSCR3	-0.509718682	1.152155362	-0.64243668	6
PLXNC1	-0.415307734	1.140734579	-0.725426845	6
PML	-1.08157151	0.89100326	0.19056825	6
PMVK	-0.740760084	1.137488818	-0.396728734	6
PNLIP	-0.306871329	1.117475337	-0.810604008	6
PNMA6E	-0.710462735	1.143540968	-0.433078233	6
PNP	-1.064015051	0.920471138	0.143543913	6
POLR2J2	-0.445111659	1.145272854	-0.700161196	6
POLR3C	-0.53170869	1.153527935	-0.621819245	6
POP4	-0.885950063	1.084316878	-0.198366815	6
PORCN	-0.611828734	1.153999976	-0.542171242	6
POTEC	-1.059041665	0.928046946	0.130994719	6
POTED	-0.403870716	1.138773836	-0.73490312	6
POTEM	-0.599845383	1.154404486	-0.554559103	6
POU2F3	-0.70134427	1.14508208	-0.44373781	6
POU3F1	-0.669246914	1.14953537	-0.480288456	6
PPFIBP2	-0.85401988	1.100053425	-0.246033545	6
PPP1CB	-0.835773633	1.107895386	-0.272121752	6
PPP1R11	-1.019396307	0.979403781	0.039992526	6
PPP1R27	-0.420789463	1.141631635	-0.720842172	6
PPP1R3G	-0.315533002	1.119706828	-0.804173826	6
PPP4R2	-0.499129689	1.151314347	-0.652184658	6

PPT1	-0.61250117	1.153972083	-0.541470913	6
PPTC7	-0.318044811	1.120342136	-0.802297324	6
PRAF2	-0.852589012	1.100697192	-0.24810818	6
PRELID2	-0.706039043	1.144304333	-0.43826529	6
PRELID3B	-0.98464671	1.01467682	-0.030030111	6
PRH1	-0.253794422	1.102443875	-0.848649453	6
PRLHR	-0.397584537	1.13764524	-0.740060703	6
PRPF18	-0.539622139	1.153895885	-0.614273746	6
PRPSAP1	-0.317436735	1.120188825	-0.802752089	6
PRR20A	-0.662885822	1.150244957	-0.487359135	6
PRR20B	-0.826370234	1.111636388	-0.285266154	6
PRR20C	-0.964917573	1.03173154	-0.066813967	6
PRR20D	-0.917678733	1.065798432	-0.1481197	6
PRR20E	-0.864045685	1.095399651	-0.231353966	6
PRR20G	-0.389592585	1.136158717	-0.746566131	6
PRR5	-0.443865395	1.145099873	-0.701234478	6
PRSS38	-0.794164837	1.123013998	-0.328849162	6
PRY2	-0.88691746	1.083796972	-0.196879512	6
PSMA4	-0.524535404	1.153136355	-0.628600951	6
PSMB7	-1.095423519	0.863995629	0.23142789	6
PSMB8	-0.996104342	1.003850653	-0.007746311	6
PSMB9	-0.929184344	1.058277561	-0.129093217	6
PSMC2	-1.0709584	0.909358965	0.161599436	6
PSMD13	-1.086331523	0.882153765	0.204177758	6

PSMD6	-0.685426377	1.147475893	-0.462049516	6
PTEN	-0.651484923	1.151378911	-0.499893988	6
PTGES2	-0.347846222	1.127470307	-0.779624085	6
PTPRCAP	-0.403575537	1.138721645	-0.735146109	6
PYCARD	-0.326814797	1.12251848	-0.795703683	6
PYCR3	-0.285915031	1.111817385	-0.825902354	6
RAB24	-0.396802677	1.137502359	-0.740699682	6
RAB2B	-1.076563943	0.899886116	0.176677827	6
RAB3C	-0.662814253	1.150252624	-0.487438372	6
RAB3D	-0.957682324	1.037530186	-0.079847862	6
RAB42	-0.454599109	1.146540947	-0.691941838	6
RAB4B	-0.27685094	1.109257674	-0.832406733	6
RAB5IF	-0.597108998	1.154472505	-0.557363507	6
RAB8B	-1.064313411	0.920006835	0.144306576	6
RAI2	-1.027168457	0.970414308	0.056754149	6
RALB	-0.481009149	1.149609986	-0.668600837	6
RARA	-0.354594467	1.128978416	-0.774383949	6
RASSF2	-0.47188418	1.148627134	-0.676742955	6
RASSF4	-0.389301842	1.136103552	-0.746801711	6
RBBP8	-0.353985996	1.128844056	-0.774858059	6
RBM18	-0.995511824	1.004428544	-0.008916721	6
RBM42	-0.915320507	1.067284135	-0.151963629	6
RBP7	-0.952261046	1.041726824	-0.089465778	6
RCN3	-0.487746406	1.150282372	-0.662535967	6

RDH5	-0.271744015	1.107785786	-0.836041771	6
REC8	-0.462291419	1.147505346	-0.685213927	6
RECQL	-0.877254282	1.088872243	-0.211617961	6
REG4	-0.28489023	1.111531369	-0.826641139	6
RERGL	-1.088910903	0.877178735	0.211732168	6
RESF1	-0.395591556	1.13727994	-0.741688384	6
RFPL3	-0.295609988	1.11448025	-0.818870262	6
RFX1	-0.69308887	1.146369956	-0.453281085	6
RFX2	-0.390338156	1.13629983	-0.745961674	6
RGCC	-0.702722323	1.144857225	-0.442134902	6
RGPD1	-0.522050925	1.152987975	-0.63093705	6
RGPD2	-0.5115548	1.152289399	-0.6407346	6
RGS2	-0.784443372	1.126038044	-0.341594672	6
RHBDD2	-0.967285199	1.029783156	-0.062497957	6
RHOG	-0.732566428	1.139270809	-0.40670438	6
RHPN1	-1.028935162	0.968306015	0.060629147	6
RHPN2	-0.635594206	1.152671347	-0.517077141	6
RIMS2	-0.954921617	1.039682766	-0.084761149	6
RIN3	-0.6317989	1.152931476	-0.521132576	6
RIOK3	-0.979132862	1.01963636	-0.040503498	6
RLN3	-0.231676749	1.095503834	-0.863827085	6
RNASE10	-0.677061311	1.148586818	-0.471525507	6
RNASE2	-0.575618637	1.154698809	-0.579080172	6
RNASE3	-0.473948405	1.148856696	-0.674908291	6

RNASE7	-1.064881162	0.919120142	0.14576102	6
RNASEK	-0.993856943	1.006031882	-0.012174939	6
RNF123	-0.890564136	1.08181301	-0.191248874	6
RNF149	-0.877267852	1.088865298	-0.211597446	6
RNF17	-0.629523704	1.153078576	-0.523554871	6
RNLS	-0.944399079	1.047597476	-0.103198397	6
ROPN1L	-1.070419974	0.910244398	0.160175576	6
RPAP1	-1.092758807	0.869502666	0.22325614	6
RPE	-0.56738433	1.154643522	-0.587259193	6
RPH3A	-1.062114821	0.923401749	0.138713072	6
RPL36A	-0.942264701	1.049148721	-0.106884021	6
RPL3L	-0.269770821	1.107211373	-0.837440553	6
RPS6KA4	-0.415295978	1.140732625	-0.725436647	6
RPS6KA5	-1.034805974	0.961117363	0.073688611	6
RPS6KB1	-0.864162303	1.095344022	-0.231181719	6
RPUSD3	-0.351619189	1.128318366	-0.776699177	6
RRAGD	-0.302156708	1.116234296	-0.814077588	6
RSPH14	-1.02481512	0.973184596	0.051630524	6
RSRC1	-0.814761683	1.115986903	-0.30122522	6
RTEL1	-0.33822975	1.12525319	-0.78702344	6
RTF2	-0.244127827	1.099459024	-0.855331198	6
RXFP4	-0.766565579	1.131134987	-0.364569408	6
S100A10	-0.40033724	1.138143871	-0.737806631	6
S100A11	-0.242466093	1.098938342	-0.856472249	6

S100A4	-0.44506527	1.145266442	-0.700201172	6
S100A6	-1.059097467	0.927963614	0.131133852	6
SAMD15	-0.857609191	1.098416316	-0.240807125	6
SAPCD2	-1.072099688	0.907468334	0.164631354	6
SCAND1	-0.589173708	1.15461927	-0.565445562	6
SCARF1	-0.768763396	1.130539946	-0.36177655	6
SCGB2A2	-0.361362365	1.130451118	-0.769088753	6
SCGB3A1	-0.373566833	1.133005307	-0.759438474	6
SCLY	-0.247689824	1.100567653	-0.85287783	6
SCNN1A	-1.067831238	0.91444464	0.153386598	6
SCP2D1	-0.801281042	1.120683241	-0.319402199	6
SCRG1	-0.645581779	1.151897713	-0.506315934	6
SDF2	-0.30631655	1.117330266	-0.811013716	6
SDHAF3	-0.299610844	1.115556428	-0.815945585	6
SDHB	-0.634199035	1.152769122	-0.518570087	6
SEBOX	-0.95008429	1.043377329	-0.093293039	6
SEC14L1	-0.304788322	1.116929313	-0.812140991	6
SEC22C	-0.670557349	1.14938225	-0.478824901	6
SEC62	-0.292813372	1.113720103	-0.820906731	6
SELENOM	-0.687892207	1.147129292	-0.459237084	6
SELL	-0.697787714	1.145649296	-0.447861582	6
SEMA4A	-0.922177348	1.062912322	-0.140734974	6
SEMA4B	-0.373519254	1.132995605	-0.75947635	6
SERINC1	-1.005760936	0.994137738	0.011623198	6

SERPINA1	-0.54900766	1.1542441	-0.605236439	6
SERPIND1	-0.408471986	1.139577095	-0.731105109	6
SERPING1	-0.876768515	1.089120554	-0.212352039	6
SERTAD3	-0.417462728	1.141090551	-0.723627823	6
SETBP1	-0.408955886	1.139660445	-0.730704559	6
SETD1A	-0.263774889	1.105446434	-0.841671545	6
SF3A2	-0.360957549	1.130364155	-0.769406606	6
SF3B4	-0.776762334	1.128300222	-0.351537887	6
SFRP5	-1.001152081	0.998843923	0.002308158	6
SFXN5	-1.045754914	0.946902805	0.098852109	6
SGSH	-0.343035939	1.126371235	-0.783335296	6
SH2D1B	-0.289399024	1.112783265	-0.823384242	6
SH2D6	-0.770358972	1.130102496	-0.359743524	6
SH3GLB1	-0.830268285	1.110109632	-0.279841347	6
SH3KBP1	-0.243674915	1.09931733	-0.855642416	6
SHCBP1L	-1.051759723	0.938615992	0.113143732	6
SHISA8	-0.289147031	1.112713741	-0.82356671	6
SHROOM3	-0.447732834	1.145631833	-0.697898999	6
SIAE	-0.883707482	1.085511892	-0.20180441	6
SIGLEC12	-0.704961739	1.144485737	-0.439523998	6
SIGLEC9	-1.091553677	0.871940797	0.21961288	6
SIRPB1	-0.778924689	1.127674541	-0.348749851	6
SLC14A2	-0.403836336	1.138767761	-0.734931425	6
SLC16A14	-0.713469306	1.143005066	-0.42953576	6

SLC16A3	-0.638882435	1.152430969	-0.513548534	6
SLC16A4	-0.429035471	1.142928433	-0.713892962	6
SLC16A5	-0.46247027	1.147527085	-0.685056814	6
SLC1A3	-0.895835001	1.07887688	-0.183041878	6
SLC22A18	-0.277159706	1.10934598	-0.832186274	6
SLC22A25	-1.08045058	0.89302822	0.18742236	6
SLC25A42	-0.316989744	1.120075928	-0.803086184	6
SLC29A3	-0.398206279	1.137758465	-0.739552186	6
SLC2A4	-0.498450816	1.151256498	-0.652805682	6
SLC35C1	-0.498563988	1.151266175	-0.652702187	6
SLC35G3	-0.433358515	1.143582865	-0.71022435	6
SLC39A13	-0.445754957	1.145361564	-0.699606607	6
SLC39A3	-1.004496575	0.995441935	0.00905464	6
SLC43A3	-0.341941232	1.126118325	-0.784177093	6
SLC49A4	-0.575308041	1.154698133	-0.579390093	6
SLC4A1AP	-0.80619178	1.119015655	-0.312823876	6
SLC5A2	-0.792692632	1.123483713	-0.330791081	6
SLC6A14	-1.058416483	0.928978065	0.129438418	6
SLC6A19	-0.733934764	1.138980867	-0.405046104	6
SLC6A9	-1.096295181	0.862158191	0.23413699	6
SLC9A8	-1.085985337	0.882811571	0.203173766	6
SLCO4C1	-0.326886556	1.122536018	-0.795649463	6
SLFN14	-1.019101085	0.979736393	0.039364692	6
SLIT2	-0.511050959	1.152252968	-0.641202009	6

SLITRK5	-0.357994875	1.129723345	-0.771728471	6
SLK	-0.964761413	1.031859152	-0.067097739	6
SLU7	-1.071817243	0.907937986	0.163879257	6
SLX4IP	-1.041063398	0.953128799	0.087934599	6
SMAP2	-0.456561852	1.14679247	-0.690230618	6
SMARCAL1	-0.706077193	1.144297877	-0.438220684	6
SMARCD3	-0.390689348	1.136366127	-0.745676779	6
SMC3	-0.278759377	1.109802229	-0.831042852	6
SMIM1	-0.397477469	1.137625706	-0.740148238	6
SMIM26	-0.485301991	1.15004368	-0.664741689	6
SMIM31	-0.289147678	1.112713919	-0.823566241	6
SMIM40	-0.263520726	1.105370973	-0.841850247	6
SMOX	-1.087906299	0.879132076	0.208774223	6
SMTNL1	-0.40285078	1.138593164	-0.735742384	6
SNX15	-0.39100432	1.136425492	-0.745421172	6
SNX24	-0.99815749	1.001832381	-0.003674892	6
SNX5	-0.258598004	1.103899076	-0.845301072	6
SNX6	-0.798920795	1.121467442	-0.322546647	6
SOCS1	-0.7635693	1.131932264	-0.368362964	6
SORCS2	-0.566919292	1.154638093	-0.587718801	6
SPAG11A	-0.499336068	1.15133184	-0.651995772	6
SPAG11B	-0.768470582	1.130619725	-0.362149143	6
SPAG9	-0.698005777	1.14561506	-0.447609283	6
SPANXC	-0.577350269	1.154700538	-0.577350269	6

SPANXN3	-0.515180437	1.152543758	-0.637363321	6
SPATA2L	-0.543907421	1.15406682	-0.610159399	6
SPATA3	-0.300040082	1.115671097	-0.815631015	6
SPATA6	-0.857268067	1.098573281	-0.241305214	6
SPDYE12P	-0.594434483	1.154530331	-0.560095848	6
SPDYE15	-0.461190257	1.147370815	-0.686180558	6
SPDYE17	-0.298571262	1.115278075	-0.816706812	6
SPDYE3	-0.817281327	1.115067181	-0.297785854	6
SPDYE7P	-0.449579189	1.145880749	-0.69630156	6
SPECC1	-0.283664829	1.111188231	-0.827523401	6
SPEM3	-0.87600003	1.089512072	-0.213512042	6
SPG11	-0.61155221	1.154011285	-0.542459075	6
SPG21	-1.066450711	0.916647005	0.149803706	6
SPINK9	-0.50916468	1.152114232	-0.642949551	6
SPOP	-0.231705424	1.095513086	-0.863807661	6
SPP2	-0.554362127	1.15439938	-0.600037252	6
SPRYD3	-0.974200912	1.023942553	-0.049741641	6
SRI	-0.589169503	1.154619328	-0.565449825	6
SRM	-1.105093047	0.842506898	0.262586149	6
SRPK1	-0.641914764	1.152196864	-0.5102821	6
SRPK2	-0.723853174	1.141046242	-0.417193068	6
SRXN1	-0.573460012	1.15469182	-0.581231809	6
SSX2	-0.516491642	1.152632364	-0.636140722	6
SSX2B	-0.259771616	1.104251771	-0.844480155	6

SSX3	-0.533947361	1.153638872	-0.619691511	6
SSX4	-0.853030795	1.100498964	-0.247468169	6
SSX4B	-0.377803242	1.133861161	-0.75605792	6
ST3GAL6	-1.035379396	0.960399682	0.074979715	6
STAR	-0.464506472	1.14777238	-0.683265908	6
STARD3NL	-0.717246308	1.142312059	-0.425065752	6
STAU1	-0.949321233	1.043951313	-0.09463008	6
STEAP3	-0.523584312	1.153080326	-0.629496013	6
STING1	-0.247240069	1.100428235	-0.853188166	6
STK3	-1.068585725	0.913230075	0.15535565	6
STX10	-0.746908546	1.136078504	-0.389169958	6
STXBP3	-0.738621387	1.137964604	-0.399343217	6
SUDS3	-0.938935019	1.051533462	-0.112598443	6
SULT1A1	-0.975996404	1.022388757	-0.046392353	6
SULT2B1	-0.458349088	1.147018259	-0.688669171	6
SULT6B1	-0.488115633	1.150317904	-0.662202271	6
SUMO2	-0.248891266	1.100939285	-0.85204802	6
SUPT16H	-0.867357356	1.093806251	-0.226448895	6
SVOP	-0.820581956	1.113841929	-0.293259972	6
SWSAP1	-0.34887539	1.127702858	-0.778827468	6
SYNGAP1	-0.811103561	1.117298407	-0.306194846	6
SYNGR1	-0.601643097	1.154354903	-0.552711806	6
TAF7	-0.485990731	1.150111542	-0.664120811	6
TARS1	-0.988088653	1.011500228	-0.023411575	6

TAS2R10	-0.574605052	1.154696194	-0.580091142	6
TAS2R19	-0.930042328	1.0576981	-0.127655772	6
TAS2R31	-1.103308646	0.846667075	0.256641571	6
TAS2R38	-0.330222815	1.123346592	-0.793123777	6
TAS2R60	-1.003591184	0.996369703	0.007221482	6
TBC1D22B	-0.448722413	1.145765649	-0.697043236	6
TBC1D23	-0.910203663	1.0704448	-0.160241138	6
TBC1D28	-0.932382376	1.056104172	-0.123721796	6
TBC1D3G	-0.920994121	1.063678105	-0.142683984	6
TBCB	-1.096651943	0.861400862	0.235251081	6
TBCE	-1.056543156	0.931740808	0.124802348	6
TBKBP1	-0.707480592	1.144058845	-0.436578253	6
TBX21	-0.528957994	1.153384268	-0.624426274	6
TCAF2	-0.866678033	1.094135422	-0.227457389	6
TCF23	-0.914750627	1.067640393	-0.152889766	6
TCN2	-0.429724564	1.143033925	-0.713309361	6
TDRKH	-0.98519589	1.014174204	-0.028978315	6
TEAD3	-0.322208062	1.121383404	-0.799175342	6
TENT5C	-0.410783368	1.139973285	-0.729189917	6
TEX11	-0.901159063	1.075826888	-0.174667825	6
TEX22	-0.489783563	1.150476701	-0.660693138	6
TEX261	-0.32354004	1.121713446	-0.798173407	6
TEX28	-0.349133784	1.127761101	-0.778627317	6
TFDP2	-0.575010871	1.154697383	-0.579686512	6

TFDP3	-0.859685066	1.097454839	-0.237769772	6
TFEB	-0.356794056	1.129461432	-0.772667376	6
TGFBR1	-0.540554957	1.153934795	-0.613379839	6
TGIF2LX	-0.921282563	1.063491868	-0.142209305	6
THAP5	-0.410331023	1.139896135	-0.729565112	6
THEG5	-1.078065223	0.897266518	0.180798705	6
THEM5	-0.801875694	1.120483904	-0.31860821	6
THSD8	-0.407059045	1.139332493	-0.732273448	6
TIMM23	-0.461610603	1.147422309	-0.685811706	6
TJP3	-0.523802798	1.153093282	-0.629290484	6
TLR2	-0.507472345	1.151986619	-0.644514274	6
TMCC3	-0.472486603	1.148694565	-0.676207961	6
TMDD1	-0.329123586	1.123080572	-0.793956986	6
TMED2	-0.684414819	1.147615546	-0.463200727	6
TMEM106C	-0.890208379	1.082008249	-0.19179987	6
TMEM121	-0.797332763	1.121988819	-0.324656056	6
TMEM121B	-0.417008265	1.141015838	-0.724007572	6
TMEM143	-0.560218247	1.154532726	-0.594314479	6
TMEM150B	-0.58426051	1.154672858	-0.570412348	6
TMEM154	-0.491903114	1.15067444	-0.658771326	6
TMEM158	-0.940634519	1.050321592	-0.109687073	6
TMEM160	-0.327683927	1.122730608	-0.795046681	6
TMEM167B	-0.49612441	1.15105469	-0.654930279	6
TMEM175	-0.619774117	1.153634671	-0.533860554	6

TMEM176B	-0.321277905	1.121152034	-0.79987413	6
TMEM214	-0.752645855	1.134704865	-0.382059009	6
TMEM222	-0.245254125	1.099810674	-0.854556549	6
TMEM225	-1.041873502	0.952068639	0.089804863	6
TMEM229B	-0.360029504	1.130164249	-0.770134746	6
TMEM238	-0.580326201	1.154695416	-0.574369215	6
TMEM238L	-0.273722327	1.108358495	-0.834636168	6
TMEM242	-0.256624333	1.10330343	-0.846679097	6
TMEM25	-0.633349437	1.152827437	-0.519478	6
TMEM262	-0.617253963	1.153759018	-0.536505055	6
TMEM275	-0.461205325	1.147372664	-0.686167339	6
TMEM33	-0.896977004	1.07822987	-0.181252866	6
TMEM60	-0.515612431	1.152573149	-0.636960718	6
TMEM63B	-0.693394176	1.146324118	-0.452929942	6
TMEM80	-0.954149796	1.040278791	-0.086128994	6
TMEM86A	-1.095033609	0.864811692	0.230221917	6
TMEM88	-0.250524647	1.101442661	-0.850918014	6
TMEM89	-0.403727763	1.13874857	-0.735020807	6
TMIGD1	-0.395889645	1.137334806	-0.741445162	6
TMPRSS11B	-1.068400681	0.913528679	0.154872002	6
TMPRSS9	-0.329345189	1.123134284	-0.793789095	6
TMTC2	-0.673235068	1.149061937	-0.475826869	6
TNFRSF1A	-0.255684302	1.103018624	-0.847334322	6
TNFSF9	-0.61039548	1.154057574	-0.543662095	6

TNIP3	-0.552412318	1.154346508	-0.60193419	6
TNNI2	-0.296156552	1.114628053	-0.818471502	6
TOLLIP	-1.093286485	0.868424996	0.224861489	6
TOMM6	-1.103248864	0.846804814	0.25644405	6
TONSL	-0.292698997	1.113688877	-0.82098988	6
TOP2B	-0.279731129	1.110078362	-0.830347232	6
TOR2A	-0.487110305	1.150220837	-0.663110532	6
TOR4A	-0.78718031	1.12520506	-0.338024751	6
TP53I11	-0.72090132	1.141620274	-0.420718954	6
TPBGL	-0.691365778	1.146626096	-0.455260318	6
TPGS1	-0.539022865	1.153870389	-0.614847523	6
TPSAB1	-0.564510721	1.154606055	-0.590095335	6
TPSB2	-0.738971107	1.137887322	-0.398916215	6
TPTE	-0.230481519	1.095117641	-0.864636121	6
TRAPPC2B	-1.105160573	0.842347574	0.262812998	6
TRAPPC8	-0.920151981	1.064220219	-0.144068238	6
TREML2	-1.060055594	0.926526982	0.133528612	6
TREX1	-0.476356458	1.149119168	-0.672762709	6
TREX2	-0.40994751	1.139830578	-0.729883068	6
TRIB3	-0.48089931	1.149598647	-0.668699337	6
TRIL	-0.740374797	1.137575091	-0.397200294	6
TRIM39-RPP21	-0.9050078	1.073568036	-0.168560236	6
TRIM48	-0.413221312	1.140385849	-0.727164537	6
TRIM58	-1.092798345	0.869422132	0.223376214	6

TRIP11	-0.71759376	1.142247195	-0.424653435	6
TRIP12	-0.29939118	1.115497687	-0.816106508	6
TRPA1	-0.861753922	1.096485805	-0.234731883	6
TRPS1	-0.572351895	1.154686155	-0.58233426	6
TSACC	-0.35668606	1.129437815	-0.772751756	6
TSC22D4	-1.099527764	0.855180028	0.244347737	6
TSHR	-1.073154058	0.905704753	0.167449305	6
TSHZ3	-0.656833222	1.150868791	-0.494035569	6
TSPEAR	-0.364296824	1.131077212	-0.766780388	6
TTC33	-0.56913387	1.154661745	-0.585527875	6
TTC34	-0.668501489	1.149621408	-0.481119919	6
TTI1	-0.516847958	1.152656131	-0.635808173	6
TTLL8	-0.807857645	1.118438808	-0.310581163	6
TUBA8	-0.865027124	1.094930392	-0.229903268	6
TUBB2A	-1.070437389	0.910215824	0.160221565	6
TUBB2B	-0.901085959	1.075869351	-0.174783392	6
TUT7	-0.625017542	1.153350495	-0.528332952	6
TVP23C-CDRT4	-0.62111496	1.153565282	-0.532450323	6
TXN2	-0.23273231	1.095843952	-0.863111641	6
TXNRD1	-0.755378729	1.134030711	-0.378651982	6
U2AF1L4	-1.098492834	0.857442977	0.241049857	6
U2AF2	-0.408047756	1.139503847	-0.731456091	6
UACA	-0.408831175	1.139638985	-0.730807809	6
UBAP1	-1.02998754	0.967038292	0.062949248	6

UBE2E2	-1.100490169	0.853050371	0.247439797	6
UBE2O	-0.310280371	1.11836112	-0.808080749	6
UBE2QL1	-0.930310731	1.057516284	-0.127205552	6
UBE2U	-1.09595565	0.862876078	0.233079572	6
UBE2V2	-0.986327964	1.013133078	-0.026805114	6
UBL5	-1.096486369	0.861752725	0.234733644	6
UBN1	-0.771987889	1.12965114	-0.357663252	6
ULBP3	-0.637568121	1.152528727	-0.514960606	6
UNC5A	-0.725788659	1.140662365	-0.414873706	6
UPK3BL1	-0.357709471	1.129661209	-0.771951738	6
USB1	-0.873346525	1.090851667	-0.217505142	6
USO1	-0.582665688	1.154684176	-0.572018487	6
USP1	-0.570593734	1.154674284	-0.58408055	6
USP12	-0.44005785	1.144562222	-0.704504372	6
USP15	-0.915491531	1.067177011	-0.151685479	6
USP26	-0.674707097	1.148881582	-0.474174485	6
UTP6	-0.664693458	1.15004897	-0.485355512	6
UTS2R	-0.971711533	1.026071022	-0.054359489	6
VAMP2	-0.531478234	1.153516209	-0.622037975	6
VDR	-0.521816203	1.152973619	-0.631157416	6
VHLL	-0.955276668	1.03940774	-0.084131073	6
VMO1	-0.362813096	1.130761588	-0.767948493	6
VNN2	-1.09558994	0.863646221	0.231943719	6
VPS28	-0.412531412	1.140269654	-0.727738242	6

VPS37C	-0.312755847	1.11899822	-0.806242373	6
VPS9D1	-0.77118173	1.129875121	-0.358693391	6
VSIG8	-0.391907231	1.136595174	-0.744687943	6
VTI1B	-0.813279617	1.116521629	-0.303242012	6
VWA7	-0.7193981	1.141907322	-0.422509222	6
WASF2	-0.413042787	1.140355823	-0.727313037	6
WASHC4	-0.330045709	1.123303801	-0.793258092	6
WDFY3	-0.614115531	1.153902843	-0.539787312	6
WDR18	-0.539445679	1.153888418	-0.614442739	6
WDR26	-0.955144068	1.039510516	-0.084366448	6
WDR4	-0.831407579	1.109657014	-0.278249435	6
WDR62	-0.956650081	1.03833885	-0.081688769	6
WDR64	-0.321876847	1.121301101	-0.799424254	6
WDR88	-0.481005844	1.149609645	-0.668603801	6
WFIKKN1	-0.542885737	1.154027883	-0.611142146	6
WIPI2	-0.451571355	1.146145652	-0.694574298	6
WNT1	-1.09987127	0.854422733	0.245448537	6
WNT6	-0.439867174	1.144534935	-0.704667761	6
WWOX	-0.965427581	1.031314	-0.065886419	6
XAGE1A	-0.293808317	1.113991282	-0.820182965	6
XAGE1B	-0.372059914	1.13269704	-0.760637126	6
XAGE2	-0.400191435	1.138117632	-0.737926197	6
XAGE5	-0.380097036	1.134317909	-0.754220873	6
XCR1	-0.239584353	1.098030135	-0.858445782	6

XK	-0.631633118	1.152942418	-0.5213093	6
XKR8	-1.089649412	0.875729707	0.213919705	6
YJU2	-0.5258207	1.153210549	-0.627389848	6
YPEL3	-0.623393543	1.153442201	-0.530048657	6
YTHDC1	-0.318650677	1.120494579	-0.801843902	6
YY1AP1	-1.106794607	0.838448656	0.268345951	6
ZAR1	-0.290135618	1.11298619	-0.822850572	6
ZBTB32	-0.453971404	1.146459722	-0.692488318	6
ZBTB42	-0.41409324	1.140532073	-0.726438834	6
ZBTB44	-0.274779643	1.10866327	-0.833883627	6
ZDHHC19	-0.280728492	1.110360965	-0.829632474	6
ZDHHC3	-0.321469606	1.121199778	-0.799730173	6
ZER1	-0.494939022	1.15094974	-0.656010718	6
ZFP36L1	-0.872566053	1.09124208	-0.218676028	6
ZFP41	-0.878154407	1.088410422	-0.210256015	6
ZIM2	-1.009887098	0.989810579	0.020076519	6
ZNF197	-0.574954798	1.15469723	-0.579742432	6
ZNF33A	-0.609303605	1.154099763	-0.544796158	6
ZNF341	-0.480328361	1.149539515	-0.669211154	6
ZNF346	-0.740071026	1.137642937	-0.397571911	6
ZNF350	-0.339085004	1.125453596	-0.786368592	6
ZNF446	-1.084981418	0.884706272	0.200275147	6
ZNF454	-0.863207585	1.095798417	-0.232590831	6
ZNF467	-0.737050771	1.1383092	-0.401258429	6

ZNF497	-0.766551637	1.131138734	-0.364587097	6
ZNF534	-1.067647937	0.914738541	0.152909397	6
ZNF667	-0.447460255	1.145594809	-0.698134554	6
ZNF683	-0.464583768	1.147781612	-0.683197844	6
ZNF695	-0.386783628	1.135622581	-0.748838953	6
ZNF701	-0.89492967	1.079387033	-0.184457363	6
ZNF703	-0.411735269	1.140135022	-0.728399753	6
ZNF705D	-0.869360777	1.092828457	-0.22346768	6
ZNF705G	-1.054862096	0.934185849	0.120676247	6
ZNF709	-0.628875313	1.153119289	-0.524243976	6
ZNF771	-0.310476536	1.118411794	-0.807935258	6
ZNF773	-0.58639596	1.154653048	-0.568257088	6
ZNF786	-0.374499818	1.133195158	-0.758695341	6
ZNF800	-0.710927947	1.143458955	-0.432531008	6
ZNRD2	-1.085552244	0.883631289	0.201920955	6
ZP2	-1.080368494	0.893175661	0.187192833	6
ZRANB1	-0.407421917	1.139395487	-0.73197357	6
ZSCAN10	-0.658971143	1.150654114	-0.491682971	6
ZSCAN26	-0.649269976	1.151578985	-0.502309008	6
ZSCAN5A	-0.301616211	1.116090834	-0.814474623	6
ZSCAN5C	-1.061540409	0.924278744	0.137261665	6
AASDHPPT	0.095576308	-1.044356705	0.948780397	7
ABCA10	0.653993042	-1.15114446	0.497151419	7
ABCA3	0.717152899	-1.142329465	0.425176567	7

ABCA7	0.209527607	-1.088162825	0.878635219	7
ABCA9	0.010452848	-1.00518545	0.994732602	7
ABCB4	0.863816081	-1.095509076	0.231692995	7
ABCC1	0.58042999	-1.154695053	0.574265062	7
ABCC5	0.803438123	-1.119956757	0.316518634	7
ABCD3	0.656970452	-1.150855197	0.493884745	7
ABCF1	0.243124339	-1.09914486	0.856020521	7
ABHD1	0.691302501	-1.14663542	0.455332918	7
ABHD17B	0.134200834	-1.060323757	0.926122922	7
ABRACL	0.223648746	-1.092888045	0.869239299	7
ABRAXAS1	0.371289939	-1.132538752	0.761248814	7
ACADSB	0.505026982	-1.151796989	0.646770007	7
ACBD6	0.319290425	-1.120655212	0.801364787	7
ACCS	0.545764694	-1.15413468	0.608369986	7
ACER3	0.267993103	-1.106691145	0.838698042	7
ACKR4	0.800484333	-1.120949197	0.320464864	7
ACP6	0.045472648	-1.021960613	0.976487964	7
ACTN3	0.416106142	-1.140866964	0.724760822	7
ACTR3C	0.122623563	-1.055657092	0.933033529	7
ACTRT3	0.061207007	-1.029197654	0.967990646	7
ACVR1	-0.096133323	-0.948461707	1.044595029	7
ACVR1B	-0.029634991	-0.984853113	1.014488104	7
ACYP2	0.77260436	-1.129479061	0.356874702	7
ADAP2	-0.088344456	-0.952896698	1.041241154	7

ADI1	0.816308844	-1.115423751	0.299114907	7
ADNP	0.285273387	-1.111638407	0.82636502	7
AFMID	0.114068534	-1.052142943	0.938074409	7
AGER	0.648171231	-1.151675817	0.503504586	7
AGPAT5	0.588893738	-1.154623086	0.565729347	7
AGPS	0.014199452	-1.007024114	0.992824662	7
AGTPBP1	0.153895449	-1.068026502	0.914131053	7
AHSG	0.324490924	-1.12194814	0.797457216	7
AIG1	-0.073800662	-0.961055127	1.034855789	7
AKAP10	0.832332368	-1.109287474	0.276955106	7
AKAP3	0.12604191	-1.057045642	0.931003732	7
AKAP7	0.241483799	-1.098629511	0.857145712	7
AKR1C4	0.031826245	-1.015533209	0.983706964	7
AKR7A3	0.465763047	-1.147921735	0.682158687	7
ALG10	0.128357627	-1.057981228	0.929623601	7
ALG10B	0.330745778	-1.123472791	0.792727013	7
ALKAL1	0.788703935	-1.124735139	0.336031204	7
ALKBH8	0.61805864	-1.153720174	0.535661534	7
ALS2	0.012912253	-1.006393602	0.993481349	7
AMIGO2	0.225552481	-1.093512983	0.867960503	7
AMT	0.729803327	-1.139847034	0.410043707	7
AMY1A	0.615809375	-1.153826735	0.53801736	7
AMY1C	0.312895118	-1.11903391	0.806138792	7
ANAPC16	0.471753177	-1.148612424	0.676859246	7

ANKDD1A	0.029290266	-1.014323361	0.985033095	7
ANKMY1	0.304760052	-1.116921877	0.812161825	7
ANKRD36B	0.243307448	-1.099202247	0.855894799	7
ANKRD37	0.335461558	-1.124600236	0.789138678	7
ANO8	0.627056998	-1.153230613	0.526173616	7
APLP1	-0.058293787	-0.969577981	1.027871768	7
APOA4	0.293670043	-1.113953644	0.820283601	7
APOC3	0.106635111	-1.049044282	0.942409172	7
APOC4	0.297321839	-1.114942345	0.817620506	7
APPBP2	0.382183891	-1.134729379	0.752545487	7
ARFGEF1	0.657805963	-1.15077188	0.492965917	7
ARFIP2	0.288333018	-1.112488797	0.824155779	7
ARL1	-0.054458422	-0.971658025	1.026116447	7
ARL13B	0.152766731	-1.067593104	0.914826372	7
ARL5B	0.670735612	-1.149361236	0.478625624	7
ARL5C	0.143717409	-1.064082963	0.920365554	7
ARL8B	0.318443414	-1.120442464	0.80199905	7
ARMCX6	0.143136694	-1.063855561	0.920718867	7
ARMH3	0.631927762	-1.152922947	0.520995185	7
ARPC4-TTLL3	0.044916609	-1.021701455	0.976784846	7
ARPP19	0.021446465	-1.010550736	0.989104271	7
ARV1	0.577676512	-1.154700477	0.577023965	7
ASAH2B	0.01190812	-1.005900882	0.993992762	7
ASB6	0.270709136	-1.107484921	0.836775785	7

ASB8	0.059043011	-1.028213371	0.96917036	7
ASB9	0.292388253	-1.113603984	0.821215731	7
ASCC3	0.165914895	-1.072580702	0.906665807	7
ASF1A	0.359979697	-1.130153499	0.770173803	7
ASIP	0.37154774	-1.132591808	0.761044068	7
ASPDH	0.790263545	-1.124249491	0.333985946	7
ATF1	0.665682851	-1.149939802	0.484256951	7
ATP13A3	0.475532238	-1.149029977	0.673497738	7
ATP13A4	-0.065778835	-0.965486694	1.031265528	7
ATP23	0.153084798	-1.067715332	0.914630534	7
ATP2A2	-0.096741544	-0.948113451	1.044854994	7
ATP5ME	0.676254882	-1.148688667	0.472433785	7
ATP5MF	-0.067453693	-0.964565445	1.032019138	7
ATP7A	0.646335265	-1.151834058	0.505498793	7
ATP8	0.057289914	-1.027413398	0.970123484	7
ATP8B4	0.670099606	-1.149436006	0.4793364	7
ATP9B	0.288348095	-1.112492968	0.824144873	7
ATRAID	0.868536619	-1.09323197	0.224695352	7
ATRN	0.712266033	-1.14322121	0.430955177	7
ATXN10	0.223886665	-1.092966304	0.869079639	7
AURKC	0.70071444	-1.145183902	0.444469463	7
AXL	0.864011753	-1.095415831	0.231404079	7
B3GALNT2	0.771311659	-1.129839102	0.358527444	7
B3GALT2	0.60291715	-1.154317404	0.551400254	7

B4GALT6	0.095467358	-1.044310062	0.948842705	7
BAG1	0.080837635	-1.037965286	0.957127651	7
BARD1	0.466152883	-1.147967756	0.681814873	7
BATF	0.55712743	-1.154467117	0.597339687	7
BATF3	0.613909458	-1.153911861	0.540002403	7
BBIP1	-0.102308356	-0.944912963	1.047221319	7
BBS5	0.136197346	-1.061118165	0.92492082	7
BCAP29	0.118388467	-1.053924412	0.935535945	7
BCCIP	0.706216003	-1.144274367	0.438058364	7
BCL2L2-PABPN1	0.236366267	-1.097008062	0.860641795	7
BCL7A	0.680703121	-1.148115416	0.467412294	7
BCLAF3	0.007157045	-1.003559313	0.996402269	7
BCOR	0.076549731	-1.036074998	0.959525267	7
BEX2	0.089901777	-1.041915408	0.952013631	7
BEX5	0.011484555	-1.005692816	0.994208261	7
BHLHE40	0.64040804	-1.152314684	0.511906643	7
BLMH	0.599273599	-1.154419442	0.555145842	7
BMP10	0.500482	-1.151428176	0.650946176	7
BMP8B	0.677477806	-1.148533857	0.471056051	7
BMT2	0.389777039	-1.136193674	0.746416636	7
BNIP3	0.669585677	-1.149496015	0.479910338	7
BORCS7	-0.027666667	-0.985879584	1.013546251	7
BPIFB1	0.301549105	-1.116073005	0.8145239	7
BPIFB2	0.023158361	-1.011378044	0.988219683	7

BPNT2	-0.091127681	-0.9513172	1.042444881	7
BTAF1	0.42158712	-1.141759846	0.720172726	7
C10orf99	0.853099324	-1.100468172	0.247368848	7
C11orf21	0.527818658	-1.153322393	0.625503735	7
C12orf29	-0.039335499	-0.979751852	1.01908735	7
C12orf4	0.531358296	-1.153510084	0.622151788	7
C12orf43	0.531170294	-1.153500452	0.622330158	7
C15orf40	0.661196013	-1.150424131	0.489228118	7
C17orf98	0.35788682	-1.129699829	0.771813009	7
C19orf73	0.251767544	-1.101824256	0.850056712	7
C19orf84	0.093401802	-1.043424071	0.950022269	7
C1orf189	0.705699568	-1.144361685	0.438662118	7
C1QA	0.227259789	-1.09407099	0.8668112	7
C20orf141	0.421106702	-1.141682697	0.720575995	7
C21orf91	-0.016547066	-0.991623785	1.008170851	7
C2CD2	0.739414858	-1.137788968	0.39837411	7
C2CD2L	0.396880958	-1.137516689	0.740635732	7
C2CD3	0.502819747	-1.15162053	0.648800783	7
C2orf74	0.576292401	-1.154699893	0.578407492	7
C3orf20	0.108003299	-1.049617773	0.941614474	7
C3orf38	0.120360468	-1.054732906	0.934372439	7
C4orf50	0.309020489	-1.118034895	0.809014406	7
C5	0.269712134	-1.107194241	0.837482106	7
C6orf52	0.711942913	-1.143278872	0.431335959	7

C8orf89	0.362113942	-1.130612193	0.768498251	7
C9orf135	0.124754991	-1.056523936	0.931768944	7
C9orf43	0.657149468	-1.150837425	0.493687957	7
CALM1	0.180609773	-1.077996682	0.897386909	7
CAMK4	0.095132452	-1.044166629	0.949034176	7
CAMLG	0.123149143	-1.055871165	0.932722022	7
CAND1	0.881227858	-1.086816758	0.2055889	7
CANX	0.057064419	-1.027310333	0.970245913	7
CAPG	0.801459622	-1.120623453	0.319163831	7
CAPZB	0.467206599	-1.148091405	0.680884806	7
CARF	0.001803228	-1.000900395	0.999097167	7
CATSPER3	0.704365275	-1.14458542	0.440220145	7
CAV3	-0.031721822	-0.983761665	1.015483487	7
CBWD5	0.374880085	-1.133272317	0.758392232	7
CCDC107	-0.03819995	-0.980352661	1.018552612	7
CCDC117	0.146562214	-1.065193219	0.918631005	7
CCDC127	-0.100350081	-0.9460415	1.046391581	7
CCDC149	0.343863349	-1.126561707	0.782698357	7
CCDC154	0.016375478	-1.008087175	0.991711697	7
CCDC172	-0.03459555	-0.982253305	1.016848854	7
CCDC174	0.659457809	-1.150604381	0.491146572	7
CCDC180	0.670249135	-1.149418478	0.479169343	7
CCDC191	0.087008013	-1.040661067	0.953653055	7
CCDC42	-0.057185176	-0.970180355	1.027365531	7

CCDC43	0.094852051	-1.044046473	0.949194422	7
CCDC6	-0.093953779	-0.949707371	1.04366115	7
CCDC7	0.88573585	-1.084431642	0.198695792	7
CCDC73	-0.073028135	-0.961484013	1.034512148	7
CCDC88A	0.278405182	-1.109701389	0.831296206	7
CCHCR1	0.446506913	-1.145464757	0.698957844	7
CCL24	-0.030395741	-0.984455607	1.014851348	7
CCNB3	0.243385286	-1.099226633	0.855841347	7
CCNY	0.073040022	-1.034517439	0.961477417	7
CCNYL1	0.153841616	-1.068005854	0.914164238	7
CCR1	0.418195609	-1.141210633	0.723015024	7
CCR4	0.479805719	-1.149485101	0.669679382	7
CCR7	0.096688582	-1.044832368	0.948143787	7
CCSAP	-0.006077005	-0.996947649	1.003024654	7
CCT2	0.584016107	-1.154674785	0.570658679	7
CD28	0.034797039	-1.016944354	0.982147315	7
CD3G	0.093307173	-1.043383404	0.950076231	7
CD40	0.727905518	-1.140235676	0.412330159	7
CD40LG	0.319945977	-1.120819453	0.800873476	7
CD5	0.615180106	-1.153855424	0.538675317	7
CD53	0.783884931	-1.126206254	0.342321323	7
CDC14A	0.293296532	-1.113851894	0.820555362	7
CDC14B	0.484484918	-1.149962555	0.665477637	7
CDC37L1	0.43283175	-1.143504062	0.710672312	7

CDHR3	0.754772066	-1.134181478	0.379409412	7
CDK20	0.74478187	-1.136573488	0.391791618	7
CDKAL1	0.839828313	-1.106220432	0.266392118	7
CDR2	0.515077403	-1.152536719	0.637459316	7
CEBPZOS	0.374012899	-1.133096172	0.759083273	7
CELF6	0.806358418	-1.118958209	0.312599791	7
CENPQ	-0.027574948	-0.985927344	1.013502292	7
CEP120	0.379946756	-1.134288128	0.754341372	7
CEP76	0.830178059	-1.110145353	0.279967294	7
CEP85	0.665233287	-1.149989573	0.484756285	7
CFAP36	0.496715281	-1.151106469	0.654391188	7
CFAP418	0.87145775	-1.091793691	0.220335941	7
CFAP97	0.808773096	-1.118119383	0.309346287	7
CFDP1	0.259162741	-1.104068931	0.84490619	7
CFHR1	-0.057251034	-0.970144596	1.02739563	7
CGAS	0.580565595	-1.154694558	0.574128964	7
CGRRF1	-0.0976099	-0.947615759	1.04522566	7
CHEK2	0.272629021	-1.108042385	0.835413364	7
CHIA	-0.038497046	-0.980195564	1.01869261	7
CHIC1	0.033331279	-1.016248938	0.982917658	7
CHMP1B	-0.021497202	-0.989078086	1.010575287	7
CHMP3	0.323860155	-1.121792542	0.797932387	7
CHRNB1	0.620445975	-1.153600183	0.533154208	7
CHSY1	0.475503377	-1.149026841	0.673523464	7

CKS2	0.709502669	-1.143709174	0.434206505	7
CLCN6	0.695229044	-1.146045754	0.45081671	7
CLEC20A	0.280509574	-1.110299005	0.829789431	7
CLNS1A	0.070191115	-1.0332463	0.963055186	7
CLSTN3	0.133526643	-1.060054808	0.926528165	7
CNGA4	0.205688994	-1.086851117	0.881162123	7
CNNM3	0.312578063	-1.118952636	0.806374573	7
CNOT11	0.753155278	-1.134580175	0.381424897	7
CNOT6L	0.466319132	-1.147987337	0.681668205	7
CNOT9	0.847558235	-1.102921061	0.255362826	7
CNPY4	0.239269509	-1.097930506	0.858660996	7
COG3	0.773236907	-1.129301774	0.356064867	7
COMMD3-BMI1	-0.072675491	-0.961679642	1.034355132	7
COMMD8	0.693428161	-1.146319007	0.452890846	7
COPA	0.874403231	-1.090320473	0.215917243	7
COPG2	0.668903587	-1.149575093	0.480671506	7
COPZ2	0.815232006	-1.115816245	0.300584239	7
COX1	0.355691878	-1.129219927	0.77352805	7
COX16	0.416458601	-1.140925218	0.724466617	7
COX3	0.327058917	-1.122578127	0.79551921	7
COX7A2L	0.215572774	-1.090204976	0.874632202	7
COX7B2	-0.087882427	-0.953158335	1.041040762	7
CPEB2	-0.041054529	-0.978840483	1.019895012	7
CPNE3	0.482719326	-1.14978497	0.667065644	7

CREB3L4	0.286356767	-1.111940404	0.825583637	7
CRELD1	0.522259281	-1.15300067	0.630741389	7
CRLS1	-0.003443017	-0.998274046	1.001717063	7
CRYAA2	0.708953006	-1.143804842	0.434851836	7
CRYL1	-0.059359357	-0.968998122	1.02835748	7
CRYZL1	0.368311669	-1.131921568	0.763609899	7
CSAD	0.17095119	-1.074455761	0.90350457	7
CSF3	-0.098145016	-0.947308778	1.045453794	7
CSN2	0.506591554	-1.151919028	0.645327475	7
CSNK2B	0.050133504	-1.024123794	0.973990291	7
CSRNP2	0.427791372	-1.142736849	0.714945477	7
CSTF3	0.083054355	-1.038937063	0.955882708	7
CTAG1A	-0.092220572	-0.950695374	1.042915947	7
CTAGE4	-0.056477389	-0.970564453	1.027041843	7
CTAGE6	0.132114604	-1.059490389	0.927375785	7
CTAGE8	0.275018896	-1.108732108	0.833713212	7
CTAGE9	-0.10098295	-0.945677101	1.046660052	7
CTLA4	0.867896345	-1.093544224	0.225647878	7
CTNS	0.013027393	-1.006450052	0.993422659	7
CTXN2	0.231147774	-1.095333056	0.864185283	7
CTXND1	0.216841132	-1.090629781	0.873788649	7
CUEDC1	-0.055744714	-0.970961661	1.026706375	7
CXCL6	-0.016813303	-0.991487335	1.008300638	7
CYB5D1	0.684847573	-1.147555981	0.462708409	7

CYBRD1	0.40159085	-1.138368671	0.736777821	7
CYP4B1	0.130699406	-1.058923177	0.928223772	7
CYP4F8	0.074922458	-1.035353993	0.960431535	7
DAAM2	0.713660466	-1.142970522	0.429310057	7
DCAF4L2	0.545492616	-1.154124975	0.608632358	7
DCK	0.481775233	-1.149688733	0.6679135	7
DCP1A	0.227120607	-1.094025587	0.86690498	7
DCUN1D5	-0.029131143	-0.985116144	1.014247287	7
DDX1	0.593930402	-1.154540275	0.560609874	7
DDX21	0.813489796	-1.116446077	0.302956281	7
DDX58	0.728610241	-1.140092036	0.411481795	7
DDX59	0.700882459	-1.145156797	0.444274338	7
DEFB109B	0.619330068	-1.153657155	0.534327086	7
DEFB125	0.026661934	-1.013064359	0.986402426	7
DENND1B	0.881132027	-1.086866844	0.205734816	7
DEPDC5	-0.047149359	-0.975591324	1.022740684	7
DERL2	0.514053943	-1.152466198	0.638412254	7
DGKD	0.153236115	-1.067773454	0.914537339	7
DGKK	-0.094234057	-0.949547387	1.043781444	7
DHFR	0.674783112	-1.148872187	0.474089074	7
DHRS3	-0.008750072	-0.995596252	1.004346324	7
DHX29	0.353232593	-1.128677246	0.775444653	7
DHX33	0.382612348	-1.134813378	0.752201029	7
DHX34	-0.102003554	-0.94508881	1.047092363	7

DIAPH1	0.059306641	-1.02833347	0.96902683	7
DISP1	0.072284882	-1.034181103	0.961896222	7
DLG3	0.073862928	-1.034883467	0.961020539	7
DLST	0.058249247	-1.027851447	0.969602201	7
DNAAF11	0.855087988	-1.099569588	0.2444816	7
DNAH8	0.692350259	-1.146480283	0.454130024	7
DNAJC13	-0.016188348	-0.991807548	1.007995896	7
DNAJC16	0.440885695	-1.144680291	0.703794597	7
DNAJC4	-0.048879069	-0.974664127	1.023543197	7
DNAL1	0.671463618	-1.149274959	0.477811341	7
DNMT3L	-0.010012664	-0.994956072	1.004968736	7
DOCK10	0.713644444	-1.14297342	0.429328975	7
DOCK9	-0.014531675	-0.992654971	1.007186646	7
DONSON	0.125294815	-1.05674293	0.931448114	7
DPH3	0.704467924	-1.144568303	0.440100379	7
DR1	0.644465688	-1.15199063	0.507524942	7
DRAM1	0.544692152	-1.154095952	0.6094038	7
DRG2	0.783027838	-1.126463274	0.343435436	7
DTWD1	0.795161686	-1.122693538	0.327531851	7
DTWD2	0.041446914	-1.020079057	0.978632143	7
DUS4L-BCAP29	0.132577879	-1.059675737	0.927097858	7
DUSP28	0.227970752	-1.094302673	0.866331921	7
DUSP5	0.783606586	-1.126289875	0.342683289	7
DYNLT4	0.258675867	-1.10392251	0.845246643	7

E2F1	-0.055419632	-0.971137769	1.026557401	7
EBAG9	0.825348497	-1.11203102	0.286682523	7
ECE2	0.287554555	-1.112273165	0.824718609	7
EDEM1	0.546953875	-1.154176145	0.60722227	7
EEF1AKMT2	0.236212748	-1.096959097	0.860746349	7
EFCAB10	0.200969618	-1.085222546	0.884252927	7
EHD1	-0.077539107	-0.958973282	1.036512389	7
EHD4	-0.077893581	-0.958775336	1.036668917	7
EHF	0.163285627	-1.071594001	0.908308374	7
EIF3H	0.664268829	-1.150095411	0.485826582	7
ELF4	0.516251611	-1.152616278	0.636364667	7
ELP3	0.175704153	-1.076207295	0.900503142	7
ELP6	0.286031338	-1.111849791	0.825818452	7
EMC10	0.029067977	-1.014217083	0.985149106	7
EMID1	-0.074857365	-0.960467745	1.035325111	7
EMSY	0.814392194	-1.116120646	0.301728452	7
ENDOV	0.491387466	-1.150626752	0.659239286	7
ENO3	0.789517972	-1.124482241	0.334964269	7
ENOPH1	0.097248086	-1.045071286	0.947823199	7
ENTPD4	0.866169322	-1.094381137	0.228211815	7
ENTPD6	0.730997935	-1.139599422	0.408601487	7
ENY2	-0.034622375	-0.982239195	1.016861571	7
EPB41L3	0.866661742	-1.094143302	0.22748156	7
EPSTI1	0.039151302	-1.019000677	0.979849374	7

EREG	0.450174944	-1.145960368	0.695785424	7
ERMAP	0.804691862	-1.119530186	0.314838325	7
ERMARD	0.294836117	-1.114270554	0.819434437	7
ESCO1	-0.062979269	-0.967021862	1.030001131	7
ETAA1	0.604535935	-1.154266927	0.549730993	7
ETFBKMT	0.805136813	-1.119378029	0.314241216	7
ETV3	-0.019544046	-0.990084728	1.009628774	7
EVI2A	0.20797231	-1.087632757	0.879660448	7
EXOC5	0.106300988	-1.048904015	0.942603027	7
EXOC6	-0.022034895	-0.98880046	1.010835355	7
EXOC6B	0.327999318	-1.122807427	0.794808109	7
EXT1	0.029176544	-1.014268995	0.985092451	7
FAAH	-0.096979242	-0.947977273	1.044956515	7
FAM111B	0.05776005	-1.027628158	0.969868107	7
FAM13B	0.184972857	-1.079572434	0.894599577	7
FAM153B	0.745189674	-1.136479162	0.391289488	7
FAM170A	-0.069930761	-0.963199068	1.033129829	7
FAM193A	0.472020343	-1.148642407	0.676622064	7
FAM199X	-0.034387268	-0.982362836	1.016750104	7
FAM214A	0.624927562	-1.153355663	0.528428101	7
FAM217B	0.27238658	-1.107972155	0.835585575	7
FAM221A	0.491947006	-1.150678487	0.658731481	7
FAM222B	0.303510362	-1.116592522	0.813082161	7
FAM234A	-0.09255118	-0.950507089	1.043058269	7

FAM236B	0.649334769	-1.151573225	0.502238455	7
FANCI	-0.032344144	-0.983435547	1.015779691	7
FBL	-0.029763459	-0.984786017	1.014549475	7
FBXL17	-0.044296164	-0.977115841	1.021412005	7
FBXO15	0.473294636	-1.148784446	0.67548981	7
FBXO4	0.350091504	-1.12797647	0.777884967	7
FBXO45	0.135463265	-1.060826431	0.925363165	7
FBXW4	-0.08743988	-0.953408788	1.040848668	7
FCGR2B	-0.062944338	-0.96704098	1.029985317	7
FCHO1	0.77647858	-1.128381682	0.351903103	7
FCRL2	0.019104732	-1.009415485	0.990310753	7
FERMT2	-0.009605092	-0.995162857	1.004767949	7
FEZ2	0.712193058	-1.143234246	0.431041189	7
FFAR3	0.397485176	-1.137627113	0.740141937	7
FGF21	0.635353921	-1.152688365	0.517334444	7
FHL2	0.178325846	-1.077165919	0.898840073	7
FKTN	0.742529363	-1.137089472	0.394560109	7
FLACC1	0.410323012	-1.139894767	0.729571755	7
FMC1	0.799746857	-1.121194247	0.32144739	7
FOSL2	0.726495781	-1.140520629	0.414024849	7
FOXP1	0.216022804	-1.090355848	0.874333044	7
FRMD8	-0.072696979	-0.961667724	1.034364703	7
FSCN3	0.188026354	-1.080666392	0.892640039	7
FTHL17	0.116344238	-1.053083177	0.936738939	7

FUBP1	0.656428166	-1.150908769	0.494480603	7
FUT8	0.737303221	-1.138254086	0.400950864	7
FXYD5	0.238293657	-1.097621202	0.859327545	7
FYTTD1	-0.013603457	-0.993128874	1.006732331	7
FZD8	0.027121468	-1.013284856	0.986163388	7
G6PC3	0.43008547	-1.143088998	0.713003528	7
GABBR1	-0.01027135	-0.994824762	1.005096111	7
GABPA	0.447164722	-1.145554586	0.698389864	7
GABPB1	-0.021045408	-0.989311191	1.010356599	7
GAGE10	0.558861485	-1.154505242	0.595643758	7
GALNT12	0.365215497	-1.131271667	0.76605617	7
GALNT2	0.233135691	-1.095973692	0.862838001	7
GANAB	0.231691774	-1.095508682	0.863816908	7
GARS1	-0.081798674	-0.956588374	1.038387048	7
GASK1B	0.173444789	-1.075376874	0.901932084	7
GATA3	0.434986957	-1.143824823	0.708837866	7
GBP3	0.307867468	-1.117735174	0.809867707	7
GCFC2	0.648590074	-1.151639093	0.503049019	7
GCN1	0.597029318	-1.154474351	0.557445033	7
GCNT2	0.627974157	-1.153174986	0.525200828	7
GDF2	-0.061635975	-0.967756374	1.029392349	7
GEMIN2	0.535017939	-1.15369002	0.61867208	7
GET1	0.244394407	-1.099542348	0.85514794	7
GET4	0.824631716	-1.112306501	0.287674785	7

GFPT1	0.635720583	-1.152662367	0.516941784	7
GGCT	0.06831047	-1.032403831	0.964093361	7
GHRHR	0.014661724	-1.007250246	0.992588523	7
GIMAP5	0.880453698	-1.087220639	0.206766941	7
GINS1	0.081677032	-1.038333702	0.95665667	7
GINS3	0.750402685	-1.135248616	0.384845931	7
GINS4	0.545037506	-1.15410856	0.609071054	7
GJC2	0.252503589	-1.102049649	0.84954606	7
GLA	0.386801928	-1.135626097	0.748824169	7
GLE1	0.398530817	-1.137817427	0.739286609	7
GLRX	-0.073978234	-0.96095648	1.034934715	7
GLS	-0.043699773	-0.977433731	1.021133504	7
GMCL1	0.705426075	-1.144407764	0.438981689	7
GMFB	0.297937781	-1.115108015	0.817170235	7
GNB1	0.257119786	-1.103453252	0.846333466	7
GNL1	0.014270199	-1.007058732	0.992788533	7
GNPNAT1	0.736113319	-1.138512944	0.402399625	7
GNPTG	0.004973834	-1.00247764	0.997503806	7
GOLGA1	0.573579415	-1.154692347	0.581112931	7
GON4L	0.734537619	-1.138852159	0.40431454	7
GORAB	0.854231453	-1.09995781	0.245726357	7
GPAM	0.276801825	-1.10924362	0.832441795	7
GPANK1	0.086638085	-1.040500261	0.953862176	7
GPATCH4	-0.020124119	-0.989786061	1.009910181	7

GPD1	0.053033099	-1.025461302	0.972428203	7
GPD1L	0.488783928	-1.150381867	0.661597938	7
GPER1	0.151041132	-1.066928621	0.91588749	7
GPI	0.494076429	-1.15087247	0.656796041	7
GPR107	0.328199653	-1.122856178	0.794656524	7
GPR146	0.138162413	-1.061897081	0.923734668	7
GPR155	0.463405966	-1.147640308	0.684234342	7
GPR160	0.289739052	-1.112876996	0.823137944	7
GPR162	0.567437785	-1.15464413	0.587206346	7
GPR17	0.09075482	-1.042283961	0.951529141	7
GPR180	0.006125591	-1.003048724	0.996923133	7
GPR55	0.229668966	-1.094854444	0.865185478	7
GPR82	0.580906604	-1.154693221	0.573786617	7
GPSM2	0.124455692	-1.056402421	0.931946729	7
GRHPR	0.732407824	-1.139304219	0.406896395	7
GRIFIN	-0.101198643	-0.945552838	1.046751482	7
GRIN3A	0.794384576	-1.122943526	0.328558949	7
GRK3	0.486520491	-1.150163415	0.663642924	7
GRM1	0.41344985	-1.140424243	0.726974393	7
GRTP1	0.765666193	-1.131375988	0.365709795	7
GSC	-0.075894773	-0.959890269	1.035785043	7
GSDMB	0.435374809	-1.143882083	0.708507273	7
GSE1	0.180486037	-1.077951778	0.897465741	7
GSK3A	0.516558564	-1.152636838	0.636078274	7

GSTM1	0.23511816	-1.09660943	0.86149127	7
GSTM2	-0.065574022	-0.965599205	1.031173227	7
GTF2A1	0.566975286	-1.15463876	0.587663474	7
GTF2H3	0.141936326	-1.063384688	0.921448362	7
GTF2H5	0.219975743	-1.091674178	0.871698435	7
GTPBP2	-0.044990303	-0.976745513	1.021735815	7
GTPBP8	-0.034231276	-0.982444848	1.016676124	7
GUCY1B1	0.245934906	-1.100022731	0.854087825	7
H2AW	0.752668023	-1.134699448	0.382031426	7
H2BC1	0.59109031	-1.154590663	0.563500353	7
H2BC11	0.228810077	-1.094575667	0.86576559	7
H2BC9	0.016004367	-1.007906126	0.99190176	7
H3C10	0.139172724	-1.062296398	0.923123673	7
H4C12	0.083748169	-1.039240458	0.955492289	7
HACD3	0.025566245	-1.01253798	0.986971735	7
HADHB	0.414125903	-1.140537537	0.726411634	7
HASPIN	0.027964436	-1.013688921	0.985724486	7
HAT1	-0.052542917	-0.972692721	1.025235638	7
HAUS6	0.376433126	-1.133586108	0.757152982	7
HAUS8	0.300899565	-1.115900241	0.815000677	7
HDDC2	-0.032742744	-0.983226515	1.015969258	7
HEATR5B	0.609676426	-1.154085522	0.544409096	7
HELQ	-0.000473889	-0.999762971	1.00023686	7
HERC1	-0.016439113	-0.991679097	1.00811821	7

HERPUD2	0.317037353	-1.12008796	0.803050607	7
HEXIM2	0.047808999	-1.023046994	0.975237995	7
HGSNAT	0.488801707	-1.150383562	0.661581855	7
HHLA2	0.815130564	-1.115853093	0.300722528	7
HIKESHI	0.245202	-1.099794422	0.854592422	7
HINT3	0.298355809	-1.115220273	0.816864464	7
HIPK2	0.336501462	-1.124846296	0.788344834	7
HLA-F	0.689331846	-1.146922867	0.457591021	7
HMGN2	0.55087846	-1.154301956	0.603423497	7
HMHB1	0.416768934	-1.140976415	0.724207481	7
HNRNPD	0.681129508	-1.148058992	0.466929484	7
HOOK3	0.210325103	-1.088433884	0.878108781	7
HOXA5	0.481764055	-1.149687588	0.667923533	7
HOXA9	0.762895153	-1.132109444	0.369214291	7
HOXB4	0.579073457	-1.154698822	0.575625365	7
HOXB5	0.673329948	-1.149050403	0.475720455	7
HP1BP3	0.266726429	-1.106318896	0.839592468	7
HPS4	0.38163217	-1.134620972	0.752988802	7
HPX	0.561853797	-1.154563112	0.592709315	7
HSD11B1	0.511976469	-1.152319686	0.640343218	7
HSD17B14	0.343884272	-1.126566515	0.782682244	7
HSPE1	0.549298394	-1.154253344	0.604954951	7
HSPH1	0.00091744	-1.000458404	0.999540964	7
HUS1	0.607825171	-1.154154563	0.546329392	7

ICAM2	0.168937362	-1.073708352	0.904770989	7
IDE	0.197280776	-1.08393741	0.886656634	7
IDH3A	0.573986515	-1.154694018	0.580707503	7
IDI2	0.061785906	-1.029460365	0.967674459	7
IER3IP1	0.293145673	-1.113810765	0.820665093	7
IFI27L1	0.075599257	-1.035654109	0.960054852	7
IFI6	0.468186376	-1.148205401	0.680019025	7
IFNA21	0.262049655	-1.104933182	0.842883527	7
IFNE	0.513971211	-1.152460449	0.638489238	7
IFT172	0.489991147	-1.150496268	0.660505121	7
IFT43	0.703133587	-1.144789568	0.441655981	7
IFT57	0.496021705	-1.151045653	0.655023948	7
IFT80	0.402929962	-1.138607224	0.735677262	7
IGSF8	0.769408215	-1.130363714	0.360955499	7
IKZF2	0.260618596	-1.104505613	0.843887017	7
IKZF5	0.80040793	-1.120974635	0.320566705	7
IL19	-0.068225302	-0.964140313	1.032365616	7
IL21R	0.646006254	-1.151861945	0.505855691	7
IL6ST	-0.067605622	-0.964481773	1.032087395	7
IMMP1L	0.29858297	-1.115281214	0.816698245	7
IMPA2	0.166316384	-1.0727309	0.906414517	7
IMPACT	0.04245799	-1.020552761	0.978094771	7
INKA1	0.729253995	-1.139960125	0.41070613	7
INO80	0.074428893	-1.035134912	0.960706019	7

INSL5	-0.041394716	-0.978659865	1.02005458	7
INTS8	0.478797646	-1.149379378	0.670581732	7
INTU	0.701800595	-1.145007937	0.443207342	7
IPO5	-0.018403586	-0.990671189	1.009074775	7
IQSEC3	0.285845553	-1.111798021	0.825952468	7
IRAK3	0.057557854	-1.027535814	0.96997796	7
ITIH3	-0.081882487	-0.956541309	1.038423797	7
ITPR1	0.316583159	-1.11997309	0.803389931	7
IZUMO1R	-0.053665297	-0.972086781	1.025752078	7
IZUMO4	0.589505282	-1.154614631	0.565109349	7
JAM3	0.356739746	-1.129449557	0.772709811	7
JMJD7-PLA2G4B	0.018440845	-1.00909289	0.990652045	7
KANSL3	-0.006001945	-0.996985519	1.002987464	7
KATNAL2	0.793797752	-1.123131515	0.329333763	7
KATNB1	0.192079618	-1.082107284	0.890027666	7
KBTBD3	0.34674936	-1.12722145	0.78047209	7
KBTBD8	0.06143353	-1.029300483	0.967866952	7
KCNA3	0.861392504	-1.096655872	0.235263367	7
KCNE2	0.790414475	-1.124202243	0.333787768	7
KCNMB3	0.67023795	-1.14941979	0.47918184	7
KCTD14	0.50466075	-1.151768058	0.647107308	7
KCTD18	0.545964125	-1.154141742	0.608177617	7
KCTD7	0.488264794	-1.150332219	0.662067425	7
KDM3B	0.380617488	-1.134420891	0.753803403	7

KDM4C	0.217168286	-1.090739147	0.873570861	7
KHDC4	0.767453687	-1.130895585	0.363441898	7
KIAA0408	-0.055498199	-0.971095214	1.026593413	7
KIAA0753	0.824651813	-1.112298792	0.287646979	7
KIDINS220	0.356321174	-1.129357946	0.773036772	7
KIF14	0.396437589	-1.137435451	0.740997863	7
KIF9	0.784970592	-1.125878695	0.340908103	7
KLHDC10	0.004642364	-1.0023131	0.997670736	7
KLHL14	0.794234313	-1.122991727	0.328757414	7
KLHL18	0.036867963	-1.017924134	0.981056171	7
KLHL32	0.033123781	-1.016150361	0.983026581	7
KLHL7	0.050224417	-1.024165826	0.973941409	7
KLRF2	-0.093214918	-0.950128832	1.04334375	7
KNTC1	0.013274527	-1.006571182	0.993296654	7
KPNA5	0.667999445	-1.149678922	0.481679476	7
KRCC1	0.443207894	-1.145008015	0.70180012	7
KRR1	0.402624784	-1.138553003	0.735928219	7
KRT13	0.701543046	-1.145049822	0.443506776	7
KRT17	0.028488509	-1.01393986	0.985451351	7
KRT18	0.212249944	-1.089086045	0.876836101	7
KRT24	-0.022310333	-0.988658159	1.010968493	7
KRT26	0.846252814	-1.103488172	0.257235358	7
KRT33A	-0.066954613	-0.964840183	1.031794796	7
KRT7	0.129539862	-1.058457291	0.928917428	7

KRT74	0.149161858	-1.066202356	0.917040498	7
KRTAP12-1	0.693300384	-1.146338214	0.453037831	7
KRTAP19-5	0.109365331	-1.05018727	0.940821939	7
KRTAP22-1	0.666892549	-1.149804496	0.482911948	7
KRTAP9-1	-0.010802974	-0.994554748	1.005357722	7
KRTCAP3	0.775478382	-1.128667635	0.353189253	7
L3MBTL1	0.378905866	-1.134081304	0.755175438	7
L3MBTL3	0.315127976	-1.119603888	0.804475912	7
LANCL3	0.554401488	-1.154400403	0.599998915	7
LATS1	0.287722677	-1.112319776	0.824597099	7
LCLAT1	-0.053307095	-0.972280267	1.025587362	7
LCOR	-0.043920691	-0.977316008	1.021236698	7
LEF1	0.793299288	-1.123290669	0.329991382	7
LENG1	0.602345069	-1.154334485	0.551989416	7
LGALSL	0.614818289	-1.153871697	0.539053408	7
LIG3	0.640200863	-1.152330653	0.51212979	7
LIMK2	0.211063411	-1.088684378	0.877620967	7
LIPJ	-0.079789507	-0.957715003	1.03750451	7
LMAN1	0.172075052	-1.074871502	0.90279645	7
LMO4	0.497313214	-1.151158505	0.653845291	7
LNPEP	0.354411916	-1.128938139	0.774526224	7
LOC102724813	0.196197974	-1.083558152	0.887360178	7
LOC107987236	0.506223078	-1.151890514	0.645667436	7
LOC388813	-0.016187985	-0.991807734	1.007995719	7

LOXL3	0.081072826	-1.038068567	0.956995741	7
LPCAT3	0.296486476	-1.114717152	0.818230676	7
LPCAT4	0.41875642	-1.141302185	0.722545765	7
LPP	0.536166264	-1.153743509	0.617577245	7
LRBA	0.167567907	-1.073198299	0.905630392	7
LRCH3	0.428360776	-1.142824714	0.714463938	7
LRIG2	0.854654461	-1.099766309	0.245111848	7
LRP1	-0.068163695	-0.964174273	1.032337968	7
LRPPRC	0.529658075	-1.1534216	0.623763526	7
LRRC20	0.015927151	-1.007868443	0.991941292	7
LRRC23	0.542022538	-1.153994098	0.61197156	7
LRRC34	0.450594009	-1.146016169	0.695422159	7
LRRC39	0.743035854	-1.136974189	0.393938336	7
LRRC61	0.34359404	-1.126499776	0.782905736	7
LRRC66	0.817245863	-1.11508022	0.297834357	7
LRRN3	0.316674843	-1.119996292	0.803321449	7
LSM5	0.600679433	-1.154381966	0.553702533	7
LSMEM1	0.147202419	-1.065442218	0.918239799	7
LUC7L2	0.08954496	-1.041761083	0.952216123	7
LUC7L3	0.653525935	-1.151188761	0.497662827	7
LXN	0.084457278	-1.039550165	0.955092887	7
LY75-CD302	0.852710876	-1.100642559	0.247931684	7
LYZL2	0.011321291	-1.00561258	0.994291289	7
MADD	-0.02420393	-0.987678325	1.011882254	7

MAGEB17	0.310635196	-1.118452755	0.80781756	7
MAGEC1	-0.081305792	-0.956865037	1.038170829	7
MAGEE1	0.54553605	-1.15412653	0.60859048	7
MAGIX	0.386656736	-1.135598195	0.748941459	7
MANEA	0.380228383	-1.134343921	0.754115538	7
MAP3K20	0.345999062	-1.127050623	0.781051561	7
MAP3K7CL	0.397879544	-1.137699007	0.739819463	7
MAP4K2	0.305812844	-1.117198327	0.811385483	7
MAPK11	-0.005479567	-0.997248957	1.002728524	7
MAPK1IP1L	0.780104427	-1.12732951	0.347225083	7
MAPK8IP3	0.863400684	-1.0957067	0.232306016	7
MAPK9	0.303579881	-1.116610878	0.813030997	7
MAPKAP1	0.314111516	-1.119344938	0.805233422	7
MARCHF10	0.108137683	-1.049674025	0.941536342	7
MARK1	-0.058953035	-0.969219335	1.028172369	7
MBD5	0.367977147	-1.131851757	0.76387461	7
MBIP	0.343506881	-1.126479719	0.782972838	7
MCF2	0.270309299	-1.107368444	0.837059145	7
MCF2L2	0.419107957	-1.141359425	0.722251468	7
MCM6	-0.026055243	-0.986717768	1.012773011	7
MCUR1	-0.096386526	-0.948316761	1.044703287	7
MDP1	0.254317065	-1.10260311	0.848286044	7
MECR	0.842809312	-1.10496471	0.262155399	7
MED4	0.753869544	-1.134404595	0.380535052	7

MEIOC	-0.006455568	-0.996756588	1.003212156	7
MESP2	0.179243384	-1.077500155	0.898256771	7
MEST	0.531235866	-1.153503816	0.62226795	7
METAP2	0.352377468	-1.128487316	0.776109849	7
METTL15	0.558672558	-1.154501252	0.595828694	7
METTL17	0.804400592	-1.119629572	0.31522898	7
METTL25B	0.614180596	-1.153899985	0.53971939	7
METTL5	0.789728131	-1.124416743	0.334688612	7
METTL8	0.814053794	-1.116242883	0.302189089	7
MFSD11	0.211007198	-1.088665321	0.877658123	7
MFSD14A	0.284147069	-1.111323416	0.827176347	7
MFSD14C	-0.096751079	-0.948107989	1.044859067	7
MFSD4B	0.667303254	-1.1497581	0.482454846	7
MFSD8	0.606897661	-1.154187581	0.54728992	7
MGME1	0.219276054	-1.091441729	0.872165675	7
MIA3	0.28266139	-1.110906326	0.828244936	7
MICOS10-NBL1	0.164906085	-1.072202752	0.907296667	7
MIER1	0.059194862	-1.028282555	0.969087693	7
MINPP1	0.1356464	-1.06089925	0.92525285	7
MKKS	0.645062672	-1.151941133	0.506878462	7
MLANA	-0.091122371	-0.951320219	1.04244259	7
MLLT11	0.755067599	-1.134108112	0.379040513	7
MMAA	0.352347369	-1.12848062	0.776133251	7
MMEL1	0.14757831	-1.065588269	0.918009959	7

MMP27	0.393439815	-1.136881507	0.743441693	7
MMP28	0.702623862	-1.144873385	0.442249523	7
MNAT1	0.275088924	-1.108752248	0.833663324	7
MOB3C	-0.001359317	-0.999319649	1.000678965	7
MOB4	0.229054467	-1.094655051	0.865600584	7
MORF4L2	0.131741628	-1.059341049	0.927599421	7
MORN4	0.057879455	-1.027682676	0.96980322	7
MOS	0.091655445	-1.042672474	0.95101703	7
MPHOSPH9	-0.004928358	-0.997526713	1.002455071	7
MPZ	0.388728063	-1.135994463	0.7472664	7
MRLN	-0.054429894	-0.971673455	1.026103349	7
MRM3	0.712468144	-1.14318506	0.430716916	7
MRNIP	0.882552939	-1.086121592	0.203568653	7
MROH7	0.212689679	-1.089234622	0.876544943	7
MRPL13	0.85080054	-1.101494813	0.250694273	7
MRPL18	0.885897986	-1.08434479	0.198446804	7
MRPL20	0.243684574	-1.099320354	0.85563578	7
MRPL45	0.389039358	-1.136053684	0.747014327	7
MRPS21	0.586476099	-1.1546522	0.568176101	7
MRPS23	0.783857043	-1.126214639	0.342357596	7
MRPS36	0.222815173	-1.092613502	0.869798329	7
MSANTD3	0.32363086	-1.121735895	0.798105035	7
MSANTD4	-0.026982935	-0.986235466	1.013218401	7
MSH2	0.095929758	-1.04450796	0.948578201	7

MTBP	0.091941682	-1.042795823	0.950854141	7
MTCH2	0.505318619	-1.151819929	0.64650131	7
MTDH	0.013923984	-1.006889286	0.992965301	7
MTERF2	0.548285574	-1.154220736	0.605935162	7
MTERF4	0.663764406	-1.150150258	0.486385852	7
MTMR12	-0.040068764	-0.979363372	1.019432136	7
MTMR14	0.159232084	-1.070062334	0.91083025	7
MTMR9	0.356765508	-1.12945519	0.772689682	7
MTOR	0.658004938	-1.150751899	0.492746962	7
MTPAP	0.299816758	-1.115611456	0.815794699	7
MTREX	0.738723229	-1.13794212	0.399218891	7
MTRF1	0.429840169	-1.143051579	0.713211411	7
MX1	0.697415138	-1.145707628	0.44829249	7
MYBL1	0.335435906	-1.124594154	0.789158248	7
MYCBPAP	0.875154544	-1.089940969	0.214786425	7
MYL5	0.3965881	-1.137463049	0.740874949	7
MYLK4	-0.095824171	-0.948638614	1.044462785	7
MYOD1	0.293425699	-1.113887094	0.820461395	7
MYOF	-0.059446295	-0.968950775	1.02839707	7
NAA15	0.143528468	-1.064009004	0.920480536	7
NAB1	0.121821831	-1.055330134	0.933508302	7
NAF1	0.09079924	-1.042303138	0.951503898	7
NAP1L1	-0.071101491	-0.962551671	1.033653162	7
NAP1L3	0.312646293	-1.118970134	0.80632384	7

NBEA	0.231101649	-1.095318154	0.864216506	7
NBPF11	0.828746696	-1.110709607	0.281962911	7
NBPF3	-0.100939847	-0.94570193	1.046641776	7
NCBP2	0.615092783	-1.153859366	0.538766583	7
NCDN	0.290750083	-1.113155126	0.822405043	7
ND1	0.293145317	-1.113810668	0.820665351	7
ND2	0.155835093	-1.068768982	0.912933889	7
ND3	0.38509247	-1.135296383	0.750203913	7
ND4	0.254744895	-1.102733293	0.847988398	7
NDC80	0.083340359	-1.039062173	0.955721814	7
NDFIP1	0.286196122	-1.111895684	0.825699562	7
NDFIP2	-0.050702755	-0.973684119	1.024386874	7
NDUFA6	0.213907995	-1.089645469	0.875737473	7
NDUFA8	0.05604861	-1.026845568	0.970796958	7
NDUFAF5	0.472002565	-1.148640414	0.676637849	7
NDUFAF6	0.407145207	-1.139347462	0.732202254	7
NDUFAF7	0.845491221	-1.103817153	0.258325932	7
NDUFB11	0.601492257	-1.154359213	0.552866956	7
NDUFC2	0.200545723	-1.08507541	0.884529687	7
NDUFS5	0.254330213	-1.102607113	0.848276899	7
NFKBIB	0.604576755	-1.154265613	0.549688859	7
NFKBIZ	0.78212036	-1.12673389	0.34461353	7
NFXL1	-0.068568829	-0.963950897	1.032519726	7
NFYA	-0.081002683	-0.957035086	1.038037769	7

NHLRC1	0.044420743	-1.021470146	0.977049404	7
NIPA2	0.797037415	-1.122085235	0.32504782	7
NIPAL3	0.594025382	-1.154538425	0.560513042	7
NIPBL	0.299536962	-1.115536676	0.815999714	7
NIPSNAP3B	0.047063693	-1.022700879	0.975637187	7
NKIRAS1	0.051520771	-1.024764493	0.973243722	7
NKRF	0.424017585	-1.142146855	0.71812927	7
NLRC4	0.138462214	-1.062015657	0.923553442	7
NLRP5	-0.049238514	-0.974471168	1.023709682	7
NMD3	0.37994324	-1.134287431	0.754344191	7
NOA1	0.38611879	-1.13549465	0.74937586	7
NOMO1	0.391443918	-1.136508196	0.745064279	7
NOMO2	0.634603229	-1.152741054	0.518137824	7
NOMO3	0.544240545	-1.154079269	0.609838725	7
NOSIP	0.220002167	-1.091682949	0.871680782	7
NPC1	0.557338548	-1.154471939	0.597133391	7
NPHP3	0.873057006	-1.090996683	0.217939677	7
NPHS2	0.080253672	-1.037708668	0.957454996	7
NPIPB8	0.658604799	-1.150691338	0.492086539	7
NPM3	0.88368347	-1.08552461	0.201841139	7
NPR2	0.6467901	-1.15179527	0.50500517	7
NPRL3	0.283603185	-1.111170937	0.827567751	7
NR1D2	0.383004373	-1.134890091	0.751885718	7
NR1I3	0.417461933	-1.14109042	0.723628487	7

NR2C2	0.050000046	-1.024062081	0.974062036	7
NR2C2AP	0.662182528	-1.150320002	0.488137473	7
NRIP1	0.570577329	-1.154674157	0.584096828	7
NSMCE2	-0.075198892	-0.960277723	1.035476616	7
NSMCE3	0.403548902	-1.138716932	0.73516803	7
NSMCE4A	0.477974078	-1.149292255	0.671318176	7
NSUN6	0.687759287	-1.147148199	0.459388912	7
NT5C3B	0.095732509	-1.044423561	0.948691052	7
NT5DC4	0.880671735	-1.087107057	0.206435322	7
NUCB2	0.654518	-1.151094324	0.496576324	7
NUDCD1	0.448703021	-1.145763036	0.697060015	7
NUDCD3	-0.081674974	-0.956657826	1.0383328	7
NUDT10	-0.040312764	-0.979234012	1.019546777	7
NUDT11	0.024626764	-1.012085927	0.987459163	7
NUP205	0.839740667	-1.106257039	0.266516372	7
NUP210	0.739066394	-1.13786623	0.398799835	7
OAS3	0.812244951	-1.116892203	0.304647252	7
OAZ3	-0.098335021	-0.947199725	1.045534746	7
OBI1	-0.100281929	-0.946080723	1.046362652	7
OCM	0.185803897	-1.079870886	0.894066989	7
ODAPH	0.277463473	-1.10943278	0.831969307	7
ODF2	0.175323818	-1.076067782	0.900743964	7
OGFOD1	0.143814391	-1.064120915	0.920306524	7
OGFOD2	0.619946897	-1.153625855	0.533678958	7

OR14K1	-0.055594646	-0.971042968	1.026637614	7
OR1S1	0.181451849	-1.078301957	0.896850108	7
OR4E2	0.039474955	-1.019152954	0.979678	7
OR4F6	0.337922778	-1.125181106	0.787258328	7
OR4K14	0.780373304	-1.127250509	0.346877206	7
OR52B4	-0.008335494	-0.995806197	1.004141692	7
OR5M3	-0.094389849	-0.949458434	1.043848283	7
OR5T1	0.754233801	-1.134314715	0.380080913	7
OR8K3	0.347760744	-1.127450951	0.779690207	7
OR8U1	0.039090725	-1.018972166	0.979881441	7
ORC5	-0.021620343	-0.989014524	1.010634866	7
OSBPL10	0.070781664	-1.033510297	0.962728633	7
OTOR	0.146388279	-1.065125515	0.918737236	7
OTUD6B	-0.078173967	-0.958618696	1.036792663	7
OXCT1	0.456963763	-1.146843515	0.689879752	7
PAIP2B	0.029486513	-1.014417158	0.984930645	7
PARN	0.021112341	-1.010389008	0.989276666	7
PATL1	0.449342271	-1.145848992	0.69650672	7
PAXBP1	0.245538758	-1.09989938	0.854360622	7
PAXIP1	0.743885902	-1.136779746	0.392893845	7
PBX3	-0.041725218	-0.978484305	1.020209523	7
PCDH12	0.159012888	-1.069979147	0.910966259	7
PCGF3	0.704404485	-1.144578883	0.440174399	7
PCGF6	0.020676452	-1.010177895	0.989501443	7

PCNP	0.115580789	-1.052768189	0.937187399	7
PCNX2	0.300189282	-1.115710919	0.815521637	7
PCP2	-0.101133203	-0.945590543	1.046723746	7
PCSK5	0.805225466	-1.119347664	0.314122199	7
PCYOX1L	0.318523908	-1.120462708	0.8019388	7
PDAP1	0.048140097	-1.02320062	0.975060523	7
PDCD1LG2	0.078958388	-1.037138544	0.958180157	7
PDE7A	0.509637125	-1.152149327	0.642512202	7
PDE8B	0.698714666	-1.145503275	0.446788609	7
PDIA5	0.074703767	-1.035256944	0.960553177	7
PDILT	0.769534177	-1.1303292	0.360795023	7
PDK1	0.019452221	-1.009584205	0.990131984	7
PDLIM1	0.321914543	-1.121310472	0.799395929	7
PDZD4	-0.094652716	-0.949308303	1.043961019	7
PEDS1-UBE2V1	0.858369542	-1.098065401	0.239695858	7
PEG3	0.408294832	-1.139546527	0.731251695	7
PEPD	-0.016268636	-0.991766426	1.008035062	7
PEX1	0.451165158	-1.146091949	0.694926791	7
PEX11A	0.463441657	-1.14764461	0.684202953	7
PFKFB2	0.858873039	-1.097832231	0.238959191	7
PFKP	0.216588898	-1.090545403	0.873956505	7
PGBD1	0.035882419	-1.017458263	0.981575843	7
PGS1	0.337671838	-1.12512212	0.787450282	7
PHC3	0.444771406	-1.145225774	0.700454368	7

PHF13	0.138474192	-1.062020392	0.923546201	7
PHKA2	0.254705714	-1.102721377	0.848015663	7
PHYH	0.753323668	-1.134538861	0.381215193	7
PHYKPL	0.832906342	-1.109057148	0.276150806	7
PIGA	0.436913917	-1.144107903	0.707193987	7
PIGO	0.231836208	-1.095555271	0.863719063	7
PIGQ	0.595757063	-1.154502802	0.558745739	7
PIK3R1	0.676989308	-1.148595949	0.47160664	7
PIP5K1A	0.382749278	-1.134840188	0.75209091	7
PIWIL4	0.728857056	-1.14004154	0.411184483	7
PKHD1L1	-0.095463813	-0.948844731	1.044308544	7
PKP3	0.28811367	-1.112428089	0.824314419	7
PLAA	-0.073207629	-0.961384403	1.034592032	7
PLCL1	0.132130219	-1.059496639	0.92736642	7
PLD1	0.55573843	-1.154434157	0.598695727	7
PLEKHM3	0.121564043	-1.0552249	0.933660856	7
PLG	0.145305584	-1.064703555	0.91939797	7
PLS1	0.291694065	-1.113414048	0.821719983	7
PMS2	0.166253368	-1.072707334	0.906453966	7
PNLIPRP3	-0.052311285	-0.972817654	1.025128939	7
PNMA1	0.198208439	-1.084261598	0.88605316	7
POLG2	0.808832366	-1.118098642	0.309266276	7
POLR1C	0.4838326	-1.149897308	0.666064708	7
POLR2H	0.880311127	-1.087294838	0.206983711	7

POM121L12	-0.058758629	-0.96932513	1.028083759	7
POMP	0.504115711	-1.151724745	0.647609034	7
POMT1	0.268449562	-1.106824969	0.838375406	7
POT1	0.846149835	-1.103532736	0.257382901	7
PP2D1	0.06100796	-1.029107265	0.968099306	7
PPA2	0.024572237	-1.01205967	0.987487433	7
PPAT	-0.019629802	-0.99004059	1.009670392	7
PPDPFL	0.637500249	-1.152533714	0.515033465	7
PPEF2	0.653060368	-1.151232625	0.498172258	7
PPIAL4E	0.114057633	-1.05213843	0.938080797	7
PPIG	-0.036910935	-0.981033496	1.01794443	7
PPIL1	0.77136211	-1.129825108	0.358462998	7
PPIL3	0.056369822	-1.026992617	0.970622794	7
PPIL4	0.299237122	-1.115456466	0.816219344	7
PPP1R32	0.323570218	-1.121720906	0.798150689	7
PPP2R1B	0.681764486	-1.147974486	0.46621	7
PPP2R2A	0.869052512	-1.092979594	0.223927082	7
PPP2R2D	-0.012124547	-0.993882598	1.006007145	7
PPP2R5E	-0.012536403	-0.993672861	1.006209264	7
PPP3CA	0.253547604	-1.1023686	0.848820996	7
PPP3CB	0.111858715	-1.051226158	0.939367443	7
PPP4R3B	0.706754776	-1.144182841	0.437428066	7
PPP6C	0.205759035	-1.086875155	0.88111612	7
PRAME	-0.094943469	-0.949142184	1.044085653	7

PRDM10	0.209109731	-1.088020595	0.878910864	7
PRDM4	0.443309072	-1.145022177	0.701713105	7
PRELP	0.032903157	-1.016045514	0.983142357	7
PRKAA1	0.091849377	-1.042756053	0.950906676	7
PRKAG2	0.488510177	-1.15035572	0.661845543	7
PRKAR2B	0.555410548	-1.154426063	0.599015515	7
PRKCA	0.290570095	-1.113105674	0.822535579	7
PRMT3	0.021790449	-1.01071715	0.988926701	7
PRPF19	-0.092487243	-0.950543508	1.043030752	7
PRPF31	0.688702199	-1.14701352	0.458311321	7
PRPS1L1	0.865194318	-1.094850203	0.229655885	7
PRR11	0.19660309	-1.083700154	0.887097064	7
PRR23A	0.406043279	-1.13915552	0.733112241	7
PRR29	0.136741756	-1.061334255	0.9245925	7
PRR5L	0.823864244	-1.112600218	0.288735974	7
PRSS2	0.290301911	-1.11303194	0.822730029	7
PSG7	0.208569689	-1.087836579	0.87926689	7
PSMD11	0.841925657	-1.105339108	0.263413451	7
PTAR1	0.105665119	-1.048636839	0.942971719	7
PTCHD3	0.065949686	-1.0313425	0.965392814	7
PTK2	0.778923049	-1.127675018	0.348751969	7
PTPN11	0.371190637	-1.1325183	0.761327663	7
PTRHD1	0.834449208	-1.108434333	0.273985125	7
PUS7L	0.657346778	-1.150817786	0.493471008	7

PWWP3A	0.481471998	-1.149657633	0.668185635	7
PXK	0.635777314	-1.152658329	0.516881015	7
PYCR2	0.641095609	-1.152261286	0.511165677	7
QRFP	0.333982348	-1.124248633	0.790266285	7
R3HDM1	0.767626384	-1.130848868	0.363222484	7
RAB1A	0.511762632	-1.15230435	0.640541719	7
RAB3IL1	0.854165016	-1.099987846	0.24582283	7
RABEP1	0.403745103	-1.138751636	0.735006533	7
RABEPK	0.82308979	-1.112895306	0.289805516	7
RABL2A	0.186765799	-1.080215663	0.893449863	7
RABL3	0.549992691	-1.154275044	0.604282353	7
RACGAP1	0.374847362	-1.133265682	0.75841832	7
RAD23B	-0.041466321	-0.978621836	1.020088156	7
RALGPS2	-0.007733854	-0.996110643	1.003844497	7
RASA2	0.425044482	-1.142308717	0.717264235	7
RASGEF1B	0.702695534	-1.144861623	0.442166089	7
RB1CC1	0.139618867	-1.062472483	0.922853616	7
RBAK	0.781122281	-1.127029732	0.345907452	7
RBAK-RBAKDN	0.687445733	-1.1471927	0.459746967	7
RBIS	0.144227718	-1.064282581	0.920054864	7
RBL1	0.520185945	-1.152872305	0.63268636	7
RBM11	0.120453803	-1.054771099	0.934317296	7
RBM26	0.873009709	-1.091020352	0.218010643	7
RBM3	0.082895124	-1.038867383	0.955972259	7

RBM33	0.198648934	-1.084415301	0.885766367	7
RBM43	0.198810892	-1.084471775	0.885660882	7
RBM44	0.6528089	-1.151256197	0.498447297	7
RBM48	-0.057854012	-0.969817048	1.02767106	7
RBM5	0.112364596	-1.05143636	0.939071764	7
RBMY1A1	0.330809646	-1.123488188	0.792678541	7
RBMY1E	0.643203749	-1.152093718	0.508889969	7
RBP4	-0.06908424	-0.963666538	1.032750778	7
RC3H1	0.444503238	-1.145188591	0.700685353	7
RCSD1	0.074005978	-1.034947044	0.960941066	7
RDH12	0.205502005	-1.086786924	0.881284919	7
REG1B	-0.090623737	-0.951603626	1.042227363	7
REP15	-0.000161094	-0.999919443	1.000080537	7
REPS1	0.017421117	-1.008596741	0.991175624	7
RFFL	0.625570454	-1.153318513	0.527748059	7
RFK	-0.080623666	-0.957247623	1.037871289	7
RGS1	0.45875422	-1.14706901	0.688314791	7
RGS13	0.215114587	-1.090051205	0.874936618	7
RHBDD1	0.225856133	-1.093612397	0.867756264	7
RHBDF1	-0.037572301	-0.98068433	1.018256631	7
RHOT1	0.227237262	-1.094063642	0.86682638	7
RILPL1	0.673091654	-1.149079346	0.475987692	7
RLF	0.042701085	-1.02066654	0.977965455	7
RLN1	0.100465424	-1.046440534	0.94597511	7

RNASEH1	0.81297518	-1.116630898	0.303655718	7
RNF122	0.619640773	-1.153641448	0.534000676	7
RNF19A	-0.043917686	-0.977317609	1.021235295	7
RNF19B	-0.016476121	-0.991660136	1.008136257	7
RNF216	0.507985667	-1.15202564	0.644039973	7
RNF32	0.037658189	-1.018297151	0.980638962	7
RNGTT	0.749363691	-1.135497555	0.386133864	7
RNMT	0.213263188	-1.08942817	0.876164982	7
ROPN1B	0.854687334	-1.099751408	0.245064074	7
RORA	0.449921729	-1.145926569	0.696004839	7
RP9	-0.087622293	-0.953305572	1.040927865	7
RPGR	0.638061775	-1.152492271	0.514430496	7
RPL22L1	0.361659173	-1.130514788	0.768855615	7
RPL23	0.832835888	-1.10908546	0.276249572	7
RPL26	0.33398782	-1.124249937	0.790262117	7
RPL35	0.564336283	-1.15460348	0.590267196	7
RPL35A	0.588082591	-1.15463362	0.566551029	7
RPL38	0.710521091	-1.143530699	0.433009607	7
RPL39	0.604669331	-1.154262626	0.549593295	7
RPL39L	0.310238979	-1.118350423	0.808111444	7
RPS10-NUDT3	-0.010027722	-0.99494843	1.004976152	7
RPS15A	0.649003887	-1.151602582	0.502598694	7
RPS24	0.844278012	-1.104338375	0.260060363	7
RPS29	0.162860408	-1.071433924	0.908573516	7

RPS6KA3	-0.0848641	-0.954863575	1.039727675	7
RPS6KC1	-0.036871112	-0.981054509	1.017925622	7
RPUSD2	0.192924123	-1.082405879	0.889481756	7
RRAGC	0.32754632	-1.122697065	0.795150745	7
RRAS2	0.19583772	-1.083431767	0.887594047	7
RRH	0.717735018	-1.142220771	0.424485752	7
RRM2	0.002338672	-1.001167285	0.998828613	7
RRP15	0.195012148	-1.083141756	0.888129607	7
RSAD2	0.753574549	-1.134477217	0.380902668	7
RSF1	-0.002638525	-0.998678127	1.001316652	7
RSU1	-0.051680132	-0.973157869	1.024838	7
RTN4	-0.10107426	-0.945624501	1.046698761	7
RUNX2	0.066080955	-1.031401625	0.96532067	7
S100Z	0.276145991	-1.109055768	0.832909776	7
SACM1L	0.208224799	-1.087718939	0.87949414	7
SAP130	0.458725964	-1.147065476	0.688339512	7
SAP18	0.484065055	-1.149920608	0.665855553	7
SART3	0.068384313	-1.032436961	0.964052648	7
SC5D	0.035488988	-1.017272082	0.981783094	7
SCAF8	0.564212471	-1.154601631	0.59038916	7
SCCPDH	0.096619115	-1.044802688	0.948183573	7
SCIMP	0.140901212	-1.062977753	0.92207654	7
SCN1B	-0.091427499	-0.951146702	1.042574201	7
SCNN1D	0.473956649	-1.148857604	0.674900955	7

SCP2	0.789941607	-1.124350124	0.334408517	7
SCT	-0.067282575	-0.964659665	1.03194224	7
SDCBP2	0.677836351	-1.148488068	0.470651717	7
SDF4	0.673237455	-1.149061647	0.475824192	7
SDHAF2	0.190538189	-1.081560836	0.891022647	7
SDHAF4	0.815545845	-1.115702107	0.300156262	7
SEC24D	0.65200734	-1.15133077	0.499323429	7
SEC31A	0.142873956	-1.06375259	0.920878634	7
SEC61G	0.499650328	-1.151358393	0.651708065	7
SELENON	-0.001857585	-0.999069913	1.000927499	7
SEM1	-0.063307504	-0.966842177	1.030149681	7
SENP5	0.542277433	-1.154004159	0.611726725	7
SEPHS1	0.793784098	-1.123135882	0.329351784	7
SERF1A	0.656656563	-1.150886254	0.494229691	7
SERF1B	0.787079271	-1.125236066	0.338156795	7
SERPINC1	0.133919101	-1.06021141	0.926292309	7
SF3B6	-0.050899551	-0.973578216	1.024477766	7
SFI1	0.633448658	-1.152820674	0.519372016	7
SFRP1	0.094815764	-1.044030919	0.949215155	7
SGPP2	0.747943104	-1.135834948	0.387891845	7
SH3D21	0.532803555	-1.153582863	0.620779308	7
SHISAL2A	-0.093420566	-0.950011568	1.043432134	7
SHLD1	0.319699764	-1.12075781	0.801058046	7
SHLD3	0.869296828	-1.09285983	0.223563003	7

SIMC1	-0.099276779	-0.9466588	1.04593558	7
SIRT5	0.046506801	-1.02244199	0.975935189	7
SLC12A6	0.252432957	-1.102028039	0.849595082	7
SLC17A5	0.198691109	-1.084430009	0.8857389	7
SLC20A2	0.601775106	-1.154351109	0.552576003	7
SLC22A17	0.27806644	-1.109604851	0.831538411	7
SLC22A23	0.737748323	-1.138156658	0.400408335	7
SLC22A6	0.488508755	-1.150355584	0.66184683	7
SLC25A17	-0.034035209	-0.982547903	1.016583112	7
SLC25A28	0.656082213	-1.150942739	0.494860526	7
SLC25A4	-0.084651135	-0.954983632	1.039634767	7
SLC25A40	0.203239402	-1.086007993	0.882768591	7
SLC25A43	0.096689861	-1.044832915	0.948143054	7
SLC25A46	0.148074434	-1.065780869	0.917706435	7
SLC25A53	0.747519424	-1.13593491	0.388415486	7
SLC27A5	0.463104864	-1.147603966	0.684499103	7
SLC2A11	-0.001208741	-0.999395082	1.000603823	7
SLC33A1	-0.048302394	-0.974973499	1.023275893	7
SLC35A5	0.697578039	-1.145682149	0.448104109	7
SLC35B4	0.512850581	-1.152381881	0.639531299	7
SLC35D1	0.525450645	-1.153189367	0.627738722	7
SLC38A2	0.088574209	-1.041340743	0.952766533	7
SLC39A2	0.12329469	-1.055930411	0.932635721	7
SLC39A4	-0.045673587	-0.976380622	1.022054209	7

SLC39A8	0.284307155	-1.11136825	0.827061095	7
SLC46A1	0.594562406	-1.15452776	0.559965354	7
SLC49A3	0.24728622	-1.100442549	0.853156329	7
SLC4A10	0.317912174	-1.120308721	0.802396547	7
SLC52A3	0.508143126	-1.152037555	0.643894429	7
SMAD3	0.270306072	-1.107367503	0.837061431	7
SMC1B	0.652957614	-1.151242267	0.498284653	7
SMC6	0.111887795	-1.051238246	0.939350452	7
SMDT1	0.536950786	-1.153779234	0.616828448	7
SMIM11A	0.601139018	-1.154369199	0.553230182	7
SMIM11B	0.781641263	-1.126876133	0.345234871	7
SMIM20	0.077030661	-1.036287703	0.959257042	7
SMIM34A	0.699967708	-1.145303856	0.445336148	7
SMIM34B	-0.033738796	-0.982703646	1.016442442	7
SMKR1	0.779670258	-1.127456791	0.347786534	7
SMN1	-0.099767475	-0.946376689	1.046144164	7
SMN2	0.812099162	-1.116944238	0.304845076	7
SMNDC1	-0.060128578	-0.968578999	1.028707576	7
SMPD2	0.885082452	-1.084780893	0.199698442	7
SMS	-0.068858388	-0.963791168	1.032649556	7
SMYD3	0.457894518	-1.146961125	0.689066607	7
SNAPC1	0.647406933	-1.151742232	0.504335299	7
SNRPD1	0.012456648	-1.006170134	0.993713486	7
SNRPE	0.123912474	-1.056181701	0.932269227	7

SNRPF	0.724857944	-1.140847703	0.415989759	7
SNX12	0.456750998	-1.146816512	0.690065514	7
SOAT1	-0.000293167	-0.999853384	1.000146551	7
SOCS3	0.538554691	-1.153850199	0.615295508	7
SORCS3	0.007984871	-1.003968526	0.995983655	7
SPACA3	-0.023477886	-0.988054331	1.011532218	7
SPANXN5	-0.09653004	-0.948234585	1.044764625	7
SPART	0.186690062	-1.080188542	0.89349848	7
SPAST	-0.075370141	-0.960182409	1.03555255	7
SPATA24	0.648557192	-1.151641984	0.503084792	7
SPATA45	-0.066746548	-0.964954665	1.031701213	7
SPIN2B	0.789617638	-1.12445119	0.334833552	7
SPINK6	-0.046964669	-0.975690193	1.022654862	7
SPRR2A	0.191724	-1.08198138	0.890257379	7
SPTY2D1	0.157265153	-1.06931454	0.912049388	7
SRPRA	0.408163109	-1.13952378	0.731360671	7
SRRM5	0.728524528	-1.140109549	0.411585021	7
SS18	0.087135237	-1.040716348	0.95358111	7
SSBP1	0.50862754	-1.152074049	0.643446509	7
SSH1	0.796377057	-1.122300183	0.325923126	7
SSMEM1	-0.034630472	-0.982234937	1.016865409	7
ST7L	0.781206324	-1.127004893	0.345798568	7
STAM	0.083316498	-1.039051737	0.95573524	7
STAMBPL1	0.701931602	-1.144986594	0.443054992	7

STAP1	0.681942446	-1.147950699	0.466008253	7
STARD3	0.63192445	-1.152923166	0.520998716	7
STIL	0.182188479	-1.078568552	0.896380073	7
STIM1	0.020145867	-1.009920726	0.989774859	7
STK26	0.172899773	-1.075175962	0.902276188	7
STK31	0.167975758	-1.073350355	0.905374597	7
STON2	0.06971412	-1.033032874	0.963318754	7
STRIP1	0.816875589	-1.115216193	0.298340604	7
STT3A	0.203450264	-1.086080755	0.882630491	7
STX16	0.257498975	-1.103567783	0.846068808	7
STX17	0.511543083	-1.152288555	0.640745472	7
STX18	0.104677882	-1.048221417	0.943543535	7
STX2	0.659706242	-1.15057887	0.490872628	7
STXBP5	-0.088415096	-0.952856682	1.041271778	7
SUGCT	0.495432995	-1.150993648	0.655560653	7
SURF6	0.600971647	-1.154373878	0.553402231	7
SVBP	0.201653296	-1.085459555	0.883806259	7
SVOPL	0.384024399	-1.13508905	0.751064651	7
SYAP1	0.5924757	-1.154567279	0.562091579	7
SYCE3	0.291712543	-1.113419109	0.821706566	7
SYCP2L	0.336095687	-1.124750392	0.788654705	7
SYNGR2	0.332754441	-1.123955343	0.791200902	7
SYNJ2BP-COX16	0.185149273	-1.079635835	0.894486562	7
SYNRG	0.842908678	-1.104922495	0.262013817	7

SYS1	0.603198012	-1.154308874	0.551110862	7
TAF1A	0.607889508	-1.154152234	0.546262726	7
TAF4	0.290255195	-1.11301909	0.822763895	7
TAF5L	0.823618154	-1.112694126	0.289075973	7
TAGAP	0.242537499	-1.098960762	0.856423263	7
TAPBPL	0.180398476	-1.077919994	0.897521518	7
TAPT1	0.055692265	-1.026682345	0.970990079	7
TAS2R42	0.177549561	-1.076882626	0.899333066	7
TATDN1	0.486015633	-1.150113986	0.664098354	7
TBC1D19	0.341363535	-1.125984444	0.784620908	7
TBC1D25	-0.051551354	-0.973227247	1.024778602	7
TBC1D3	0.841826165	-1.105381147	0.263554982	7
TBC1D30	0.615449765	-1.15384319	0.538393425	7
TBC1D3H	-0.06583256	-0.965457175	1.031289735	7
TBC1D9B	0.451773969	-1.146172381	0.694398412	7
TBP	0.583086673	-1.154681477	0.571594803	7
TBRG1	0.327369186	-1.122653864	0.795284678	7
TCAIM	0.058312503	-1.027880307	0.969567804	7
TCF7	0.62826976	-1.153156829	0.524887069	7
TCP10L	-0.051534021	-0.973236585	1.024770606	7
TENT2	0.227518456	-1.094155329	0.866636872	7
TESPA1	0.577171347	-1.15470052	0.577529172	7
TEX13A	0.676024525	-1.148717592	0.472693067	7
TEX46	0.788455184	-1.124812164	0.33635698	7

TFAP2D	-0.023196036	-0.988200191	1.011396227	7
TFCP2	0.277227167	-1.109365263	0.832138096	7
TFPT	0.089251113	-1.041633921	0.952382808	7
THAP1	0.137639932	-1.061690268	0.924050336	7
THAP3	0.114958024	-1.052510916	0.937552892	7
THOC7	-0.063034312	-0.966991736	1.030026047	7
THUMPD1	0.151715136	-1.067188435	0.915473299	7
TIMD4	0.151116973	-1.066957874	0.915840901	7
TIMMDC1	0.288161407	-1.112441304	0.824279897	7
TLCD2	0.347823518	-1.127465166	0.779641648	7
TM7SF3	0.110488776	-1.050655947	0.940167172	7
TMA16	0.644748698	-1.151967223	0.507218525	7
TMED3	0.050516262	-1.024300713	0.973784451	7
TMED8	-0.08615689	-0.954134041	1.040290931	7
TMEM106B	-0.019833633	-0.989935658	1.009769291	7
TMEM116	0.847336065	-1.103017865	0.255681799	7
TMEM123	0.555486875	-1.154427958	0.598941083	7
TMEM129	0.584480812	-1.154671061	0.570190249	7
TMEM138	0.061691424	-1.029417505	0.967726081	7
TMEM139	0.16373156	-1.071761725	0.908030165	7
TMEM140	-0.047578915	-0.975361274	1.02294019	7
TMEM169	-0.012144593	-0.993872393	1.006016986	7
TMEM179B	0.409208133	-1.139703809	0.730495676	7
TMEM185B	0.775840274	-1.128564385	0.35272411	7

TMEM234	0.493524439	-1.150822627	0.657298189	7
TMEM254	0.754470105	-1.134256284	0.37978618	7
TMEM256	0.831467167	-1.109633261	0.278166094	7
TMEM258	0.653688452	-1.151173381	0.497484929	7
TMEM43	0.720335719	-1.141728695	0.421392976	7
TMEM63A	0.103555513	-1.047748234	0.944192721	7
TMEM74B	0.45929829	-1.147136916	0.687838627	7
TMEM87B	0.188994154	-1.081011602	0.892017448	7
TMOD3	-0.086469252	-0.953957583	1.040426835	7
TMOD4	0.201282935	-1.085331208	0.884048273	7
TNFRSF6B	0.667871861	-1.149693482	0.481821621	7
TNFSF12-TNFSF13	0.12216956	-1.055472003	0.933302443	7
TNFSF4	0.150523063	-1.066728679	0.916205617	7
TNKS2	-0.089485291	-0.952249976	1.041735267	7
TNNT1	0.291379716	-1.113327908	0.821948192	7
TNNT3	0.353932516	-1.128832231	0.774899715	7
TNP1	0.075291916	-1.035517866	0.96022595	7
TOE1	0.018529424	-1.009135952	0.990606527	7
TOMM20	0.225340546	-1.093443554	0.868103008	7
TOMM5	-0.070827352	-0.962703358	1.033530711	7
TOMM70	0.834140998	-1.108559181	0.274418183	7
TOP3A	0.86853601	-1.093232267	0.224696257	7
TOR1A	0.227306186	-1.094086121	0.866779936	7
TP53BP1	0.000434369	-1.000217114	0.999782745	7

TPCN1	0.328959196	-1.1230407	0.794081504	7
TPD52	0.257425537	-1.10354561	0.846120073	7
TPP2	0.208627844	-1.087856406	0.879228562	7
TPRKB	0.322797603	-1.121529668	0.798732065	7
TRA2B	0.712451054	-1.143188119	0.430737065	7
TRIAP1	0.73000603	-1.139805181	0.409799152	7
TRIM31	0.01126456	-1.005584695	0.994320135	7
TRIM35	0.009109537	-1.004523649	0.995414112	7
TRIM41	0.866527008	-1.09420844	0.227681432	7
TRMT61B	0.588244001	-1.154631586	0.566387584	7
TRPC1	0.032586563	-1.015894996	0.983308433	7
TRPM7	-0.039632134	-0.979594745	1.019226879	7
TRUB2	0.575922751	-1.154699363	0.578776611	7
TSC22D1	0.354967073	-1.129060534	0.774093461	7
TSR2	0.727545236	-1.140308803	0.412763567	7
TST	0.685011333	-1.147533371	0.462522038	7
TSTD3	0.039207132	-1.01902695	0.979819818	7
TTC14	0.353808862	-1.128804881	0.774996019	7
TTC26	0.510941676	-1.152245031	0.641303354	7
TTC27	0.148105066	-1.065792755	0.917687689	7
TTC37	0.295507699	-1.114452562	0.818944862	7
TTC9	0.526174374	-1.153230656	0.627056282	7
TTLL2	0.408770621	-1.139628559	0.730857938	7
TUBA1C	0.018839107	-1.009286452	0.990447346	7

TUBA3C	0.313435226	-1.119172168	0.805736941	7
TUBGCP5	0.037412777	-1.018181357	0.98076858	7
TWIST2	0.869295983	-1.092860245	0.223564262	7
TXNDC8	-0.09669152	-0.948142104	1.044833624	7
TYW1	-0.030743565	-0.984273717	1.015017282	7
TYW5	0.836109859	-1.107757929	0.27164807	7
UBAP2	0.572212275	-1.154685342	0.582473067	7
UBASH3A	0.513718676	-1.152442858	0.638724182	7
UBASH3B	0.093523098	-1.043476188	0.949953091	7
UBE2E1	0.811377706	-1.11720109	0.305823384	7
UBE2E3	0.195301229	-1.083243366	0.887942137	7
UBE3A	0.826259003	-1.11167946	0.285420457	7
UBE3C	0.533469236	-1.153615631	0.620146395	7
UBL3	0.018323058	-1.009035621	0.990712563	7
UBLCP1	-0.028141484	-0.985632235	1.013773719	7
UBR5	0.204264216	-1.086361297	0.882097082	7
UBXN2A	0.101649997	-1.046942692	0.945292695	7
UBXN7	0.555893255	-1.154437938	0.598544683	7
UFM1	0.313821581	-1.119270916	0.805449335	7
UGT1A5	-0.022650799	-0.988482185	1.011132984	7
UGT8	0.113428029	-1.0518776	0.938449571	7
UNKL	0.821653191	-1.113439238	0.291786047	7
UPF2	-0.01424947	-0.992799119	1.007048589	7
UQCRB	-0.053290865	-0.972289031	1.025579896	7

URB1	0.315579019	-1.119718515	0.804139497	7
URI1	0.081959603	-1.038457604	0.956498002	7
USP18	0.807704327	-1.118492136	0.310787809	7
USP24	0.187598479	-1.080513539	0.892915059	7
USP30	0.741332832	-1.137360113	0.396027281	7
USP40	0.148464219	-1.065932055	0.917467836	7
USP48	0.231846658	-1.095558641	0.863711983	7
USP51	0.065908767	-1.031324068	0.9654153	7
USP53	0.311739821	-1.118737354	0.806997533	7
USP9X	-0.069913967	-0.963208347	1.033122314	7
UTP14C	0.50105567	-1.151475898	0.650420228	7
UTP20	0.640287428	-1.152323987	0.51203656	7
UTP4	-0.011577113	-0.994161181	1.005738294	7
VAMP4	0.286144647	-1.111881351	0.825736703	7
VAMP7	-0.032498159	-0.983354793	1.015852952	7
VAMP8	0.255845756	-1.103067591	0.847221835	7
VCPIP1	0.267083331	-1.106423915	0.839340584	7
VIPAS39	0.172041728	-1.074859189	0.902817461	7
VIPR1	0.392834762	-1.136768718	0.743933956	7
VMA21	0.70126251	-1.145095332	0.443832821	7
VPS13C	0.711031047	-1.143440734	0.432409687	7
VPS26A	0.486432189	-1.150154788	0.663722599	7
VPS36	0.100061079	-1.046268882	0.946207803	7
VPS50	0.27435407	-1.108540707	0.834186637	7

VPS54	0.053810663	-1.025818896	0.972008233	7
VSIG10	0.59966081	-1.154409357	0.554748547	7
VWA5A	0.721807648	-1.141445488	0.41963784	7
VWA8	0.090895541	-1.042344706	0.951449165	7
WARS2	0.552216684	-1.154340971	0.602124287	7
WBP4	0.88201786	-1.086402896	0.204385036	7
WDR73	0.741208672	-1.137388059	0.396179388	7
WDR76	0.789777852	-1.124401235	0.334623383	7
WDR91	0.808320957	-1.118277362	0.309956404	7
WFDC13	0.296153854	-1.114627325	0.818473471	7
WHAMM	0.472685665	-1.148716767	0.676031102	7
XPC	0.070350265	-1.033317472	0.962967207	7
XPNPEP1	0.123424176	-1.055983105	0.932558929	7
XRCC4	0.186320259	-1.080056057	0.893735797	7
XRN1	0.335967938	-1.12472017	0.788752232	7
YAF2	0.380708831	-1.13443894	0.753730109	7
YPEL2	0.836757416	-1.107492465	0.270735049	7
YRDC	0.858999013	-1.097773793	0.23877478	7
YTHDF3	0.476380503	-1.14912176	0.672741256	7
ZBED6CL	0.821606174	-1.113456964	0.291850791	7
ZBTB14	0.732484368	-1.1392881	0.406803732	7
ZBTB25	0.880948394	-1.086962747	0.206014353	7
ZBTB26	0.013984305	-1.006918815	0.992934509	7
ZBTB34	0.058052173	-1.027761516	0.969709343	7

ZBTB47	-0.064732608	-0.966061093	1.030793701	7
ZBTB8OS	0.199887006	-1.084846487	0.884959482	7
ZC2HC1B	0.591934483	-1.154576685	0.562642201	7
ZC3H10	0.72884964	-1.140043058	0.411193419	7
ZC3H8	0.872004311	-1.091522072	0.219517761	7
ZC3HC1	0.88329766	-1.085728742	0.202431081	7
ZCCHC10	0.462521207	-1.14753327	0.685012063	7
ZCCHC4	0.226081505	-1.093686134	0.867604629	7
ZCCHC8	0.119254904	-1.054280005	0.935025101	7
ZDHHC17	0.44846563	-1.145731016	0.697265386	7
ZFAND2B	0.215699307	-1.090247412	0.874548105	7
ZFAND5	0.532720257	-1.153578729	0.620858473	7
ZFP28	0.013241419	-1.006554957	0.993313537	7
ZFP69B	0.309708054	-1.118213094	0.808505039	7
ZFY	0.826330975	-1.111651593	0.285320619	7
ZFYVE16	0.201133273	-1.085279313	0.88414604	7
ZFYVE19	0.668574656	-1.149612997	0.481038341	7
ZGLP1	-0.070892923	-0.962667082	1.033560005	7
ZMAT1	0.832613894	-1.109174595	0.276560701	7
ZMAT3	0.009252843	-1.004594315	0.995341472	7
ZMYM6	0.06223467	-1.029663846	0.967429176	7
ZMYND8	0.070966924	-1.033593061	0.962626137	7
ZNF169	0.773712279	-1.129168056	0.355455777	7
ZNF175	0.288495041	-1.112533614	0.824038573	7

ZNF22	0.137157414	-1.061499088	0.924341674	7
ZNF223	0.340979538	-1.125895293	0.784915755	7
ZNF23	0.784610707	-1.125987525	0.341376818	7
ZNF24	0.384122595	-1.135108154	0.750985559	7
ZNF256	0.123978895	-1.056208702	0.932229807	7
ZNF285	-0.04687748	-0.975736858	1.022614339	7
ZNF331	0.49922667	-1.151322573	0.652095902	7
ZNF354C	0.766016433	-1.131282309	0.365265876	7
ZNF367	-0.042494318	-0.978075449	1.020569767	7
ZNF404	0.590296527	-1.154603037	0.56430651	7
ZNF417	0.865199096	-1.09484791	0.229648814	7
ZNF425	0.051638786	-1.024818931	0.973180145	7
ZNF430	0.558917304	-1.154506414	0.595589109	7
ZNF436	0.15183288	-1.067233787	0.915400907	7
ZNF441	0.565276333	-1.154616951	0.589340618	7
ZNF451	-0.035551117	-0.981750374	1.01730149	7
ZNF480	0.759948706	-1.132874395	0.372925689	7
ZNF555	0.863819568	-1.095507415	0.231687847	7
ZNF559	0.037465679	-1.018206322	0.980740643	7
ZNF559-ZNF177	-0.060447351	-0.968405179	1.02885253	7
ZNF566	0.834974485	-1.10822106	0.273246576	7
ZNF572	0.742004949	-1.137208381	0.395203432	7
ZNF577	0.341410302	-1.125995292	0.784584991	7
ZNF582	0.455503548	-1.146657312	0.691153765	7

ZNF584	0.70914017	-1.143772318	0.434632148	7
ZNF607	0.61241177	-1.153975823	0.541564054	7
ZNF614	0.214408253	-1.089813828	0.875405575	7
ZNF616	0.028900767	-1.014137114	0.985236347	7
ZNF621	0.633874116	-1.152791533	0.518917417	7
ZNF624	0.100568843	-1.046484417	0.945915574	7
ZNF626	0.55643921	-1.154451055	0.598011845	7
ZNF638	0.20574737	-1.086871152	0.881123782	7
ZNF649	0.485644766	-1.150077513	0.664432747	7
ZNF664	0.427677094	-1.142719178	0.715042084	7
ZNF671	0.729799999	-1.139847721	0.410047722	7
ZNF680	0.051883998	-1.024932008	0.97304801	7
ZNF692	0.607780463	-1.154156179	0.546375716	7
ZNF705A	0.151858247	-1.067243556	0.915385309	7
ZNF718	0.343685834	-1.126520892	0.782835058	7
ZNF778	0.227327964	-1.094093224	0.866765259	7
ZNF781	0.549062186	-1.15424584	0.605183655	7
ZNF792	0.341815383	-1.126089184	0.7842738	7
ZNF81	0.242179941	-1.098848457	0.856668515	7
ZNF829	0.827529601	-1.111185814	0.283656212	7
ZNF850	0.137465979	-1.061621366	0.924155387	7
ZPBP2	0.825903221	-1.11181705	0.285913829	7
ZRSR2	0.710847799	-1.143473108	0.432625309	7
ZSCAN12	0.082524834	-1.038705267	0.956180433	7

ZSCAN21	-0.038812856	-0.980028498	1.018841354	7
ZSCAN29	-0.083721085	-0.955507536	1.039228622	7
ZUP1	0.011166206	-1.005536345	0.994370139	7
ZXDC	0.05002199	-1.02407223	0.97405024	7
ZYX	0.172773593	-1.075129414	0.902355821	7
AADAC	-0.21273259	-0.876516523	1.089249113	8
AARD	-0.306388683	-0.81096046	1.117349143	8
AASS	-0.487840393	-0.662451037	1.15029143	8
ABCA1	-0.471127625	-0.677414321	1.148541945	8
ABCA13	-0.497142801	-0.65400091	1.151143711	8
ABCB5	-0.456779625	-0.690040523	1.146820148	8
ABCC4	-0.500959199	-0.650508697	1.151467896	8
ABHD11	-0.395480639	-0.741778865	1.137259504	8
ABHD14A	-0.155978072	-0.912845527	1.068823599	8
ABHD16A	-0.454448694	-0.692072824	1.146521518	8
ABHD16B	-0.404145704	-0.734676681	1.138822385	8
ABI2	-0.188076509	-0.892607791	1.080684301	8
ABI3BP	-0.492045188	-0.658642344	1.150687532	8
ABITRAM	-0.445873115	-0.699504701	1.145377815	8
ACACB	-0.303634858	-0.812990533	1.116625392	8
ACADM	-0.346559324	-0.780618905	1.127178229	8
ACAP2	-0.169102771	-0.904667088	1.07376986	8
ACER1	-0.238174292	-0.859409024	1.097583316	8
ACER2	-0.438211241	-0.706085268	1.14429651	8

ACR	-0.492355411	-0.658360636	1.150716047	8
ACSBG2	-0.223507057	-0.869334361	1.092841418	8
ACSL4	-0.501820782	-0.649718238	1.15153902	8
ACSM2A	-0.483613967	-0.666261377	1.149875344	8
ACSM2B	-0.457686211	-0.689248665	1.146934876	8
ACSM5	-0.387893167	-0.747942035	1.135835201	8
ACTG2	-0.350276041	-0.777741836	1.128017877	8
ACVR2A	-0.489730489	-0.660741202	1.150471691	8
ACVR2B	-0.491181078	-0.659426512	1.150607589	8
ADA2	-0.175194499	-0.900825821	1.07602032	8
ADAD2	-0.199333616	-0.88532029	1.084653905	8
ADAL	-0.323680346	-0.798067779	1.121748124	8
ADAM17	-0.288549968	-0.823998834	1.112548802	8
ADAM18	-0.474981545	-0.673988464	1.148970009	8
ADAM21	-0.452881558	-0.693436235	1.146317793	8
ADAM22	-0.406275067	-0.73292092	1.139195986	8
ADAM23	-0.443797163	-0.701293196	1.145090359	8
ADAM9	-0.485084453	-0.664937694	1.150022146	8
ADAMTS1	-0.399439887	-0.738542191	1.137982078	8
ADAMTS12	-0.497070497	-0.654066929	1.151137426	8
ADAMTS16	-0.491165098	-0.659441006	1.150606104	8
ADAMTS5	-0.449006399	-0.696797479	1.145803878	8
ADAMTS9	-0.463096575	-0.68450639	1.147602965	8
ADD3	-0.440175551	-0.704403497	1.144579048	8

ADGRD2	-0.491896933	-0.658776937	1.15067387	8
ADGRF5	-0.428468997	-0.714372382	1.142841379	8
ADGRG6	-0.420637179	-0.720969911	1.141607091	8
ADGRL4	-0.458003566	-0.688971283	1.146974849	8
ADH1B	-0.412440305	-0.727813972	1.140254277	8
ADH4	-0.410928296	-0.729069667	1.139997963	8
ADH6	-0.4653099	-0.682558153	1.147868052	8
ADHFE1	-0.233297054	-0.862728501	1.096025555	8
ADIRF	-0.411525935	-0.728573591	1.140099525	8
ADNP2	-0.414414694	-0.726171111	1.140585805	8
ADRA2C	-0.380771005	-0.753680216	1.134451221	8
ADSS1	-0.251286233	-0.850390398	1.101676631	8
AFAP1L1	-0.427618978	-0.715091209	1.142710187	8
AFAP1L2	-0.469426216	-0.678922087	1.148348304	8
AFDN	-0.480314754	-0.669223348	1.149538102	8
AFF4	-0.417936379	-0.723231836	1.141168215	8
AFG1L	-0.239408488	-0.858566006	1.097974494	8
AFM	-0.480770769	-0.668814593	1.149585363	8
AFP	-0.438258018	-0.706045263	1.14430328	8
AGGF1	-0.116473254	-0.93666311	1.053136363	8
AGO1	-0.245998038	-0.854044339	1.100042377	8
AGTR1	-0.348459087	-0.779149814	1.1276089	8
AGXT	-0.424774342	-0.71749189	1.142266232	8
AHCYL2	-0.384532536	-0.75065528	1.135187817	8

AHNAK2	-0.464740387	-0.683059913	1.1478003	8
AHR	-0.291538845	-0.821832679	1.113371524	8
AIF1L	-0.418553591	-0.722715516	1.141269107	8
AKAP11	-0.366624307	-0.764944121	1.131568429	8
AKAP12	-0.437440762	-0.706743927	1.144184689	8
AKIP1	-0.398323191	-0.739456526	1.137779716	8
AKIRIN1	-0.184766528	-0.894731723	1.079498251	8
AKR1B15	-0.472759854	-0.675965178	1.148725032	8
AKR1C2	-0.373850165	-0.759212878	1.133063043	8
AKR1D1	-0.488695662	-0.661677782	1.150373445	8
AKR1E2	-0.486744429	-0.663440829	1.150185258	8
ALB	-0.452017024	-0.694187368	1.146204392	8
ALDH1A3	-0.475921276	-0.673150883	1.149072159	8
ALDH1L1	-0.466103649	-0.681858303	1.147961952	8
ALDH3A1	-0.421981397	-0.719841604	1.141823001	8
ALDOB	-0.417200137	-0.723847268	1.141047404	8
ALOXE3	-0.213480752	-0.876020773	1.089501525	8
ALPI	-0.39274536	-0.744006665	1.136752024	8
ALPK2	-0.18841661	-0.892389073	1.080805683	8
ALPK3	-0.438314026	-0.705997358	1.144311384	8
AMBP	-0.444474682	-0.700709945	1.145184627	8
AMHR2	-0.45880485	-0.688270492	1.147075342	8
AMOTL1	-0.468059613	-0.680131093	1.148190705	8
AMPD1	-0.268415069	-0.838399793	1.106814862	8

AMTN	-0.271199284	-0.836428244	1.107627528	8
ANAPC5	-0.499952047	-0.651431744	1.151383791	8
ANGPTL3	-0.441176737	-0.703544909	1.144721646	8
ANKDD1B	-0.482751987	-0.667036297	1.149788284	8
ANKRD1	-0.307909707	-0.809836467	1.117746174	8
ANKRD10	-0.301955844	-0.814225166	1.11618101	8
ANKRD13C	-0.460929668	-0.686409138	1.147338806	8
ANKRD13D	-0.372267672	-0.760471988	1.13273966	8
ANKRD17	-0.435837434	-0.708112761	1.143950195	8
ANKRD28	-0.492561186	-0.658173723	1.150734908	8
ANKRD29	-0.460803654	-0.686519649	1.147323304	8
ANKRD46	-0.300940022	-0.814970991	1.115911012	8
ANKRD50	-0.406208017	-0.732976269	1.139184286	8
ANKRD65	-0.452394613	-0.693859396	1.146254009	8
ANLN	-0.487127567	-0.663094945	1.150222512	8
ANO1	-0.447870013	-0.697780426	1.145650439	8
ANO2	-0.472467811	-0.676224655	1.148692467	8
ANO5	-0.433280076	-0.710291072	1.143571147	8
ANP32D	-0.297667303	-0.817368	1.115035304	8
ANTXRL	-0.400647843	-0.737551859	1.138199702	8
AOAH	-0.404750927	-0.734178067	1.138928994	8
AOX1	-0.42049373	-0.721090221	1.141583951	8
AP1AR	-0.103084689	-0.944464764	1.047549453	8
AP1G1	-0.23388094	-0.862332107	1.096213047	8

APBB2	-0.455156181	-0.691456533	1.146612713	8
APCS	-0.441334123	-0.703409853	1.144743976	8
APLF	-0.50036057	-0.651057461	1.151418031	8
APLN	-0.492212839	-0.658490116	1.150702954	8
APLNR	-0.429415601	-0.713571081	1.142986682	8
APOA1	-0.28262842	-0.828268629	1.110897049	8
APOA2	-0.30382312	-0.812851952	1.116675072	8
APOB	-0.363251039	-0.767603911	1.13085495	8
APOC1	-0.375285539	-0.758068906	1.133354445	8
APOC2	-0.281574691	-0.829025404	1.110600096	8
APOE	-0.429304872	-0.713664856	1.142969728	8
APOH	-0.424503341	-0.717720203	1.142223544	8
APOL4	-0.209396812	-0.87872151	1.088118322	8
APOLD1	-0.439363531	-0.705099163	1.144462694	8
APOM	-0.488959066	-0.661439489	1.150398555	8
APPL1	-0.436632446	-0.707434327	1.144066773	8
AQP1	-0.110133793	-0.940374167	1.05050796	8
AQP10	-0.298892495	-0.816471687	1.115364183	8
AQP2	-0.497420161	-0.653747612	1.151167773	8
AQP4	-0.489416051	-0.661025902	1.150441953	8
AQP5	-0.49318642	-0.657605533	1.150791953	8
AQP7	-0.317007726	-0.803072746	1.120080473	8
AQP8	-0.449635678	-0.696252635	1.145888313	8
AR	-0.497909342	-0.653300678	1.15121002	8

ARAP2	-0.328055441	-0.794765647	1.122821087	8
AREG	-0.25541627	-0.847521016	1.102937286	8
ARF3	-0.465827819	-0.682101572	1.147929392	8
ARFGEF2	-0.218418337	-0.872737914	1.091156251	8
ARFGEF3	-0.494946935	-0.65600351	1.150950445	8
ARHGAP10	-0.351455927	-0.776825997	1.128281924	8
ARHGAP11A	-0.453580698	-0.692828275	1.146408973	8
ARHGAP20	-0.460285911	-0.686973536	1.147259448	8
ARHGAP21	-0.483753347	-0.666136005	1.149889352	8
ARHGAP23	-0.406258202	-0.732934842	1.139193044	8
ARHGAP28	-0.455901654	-0.690806628	1.146708282	8
ARHGAP29	-0.441763403	-0.703041359	1.144804762	8
ARHGAP31	-0.383752129	-0.751283905	1.135036034	8
ARHGAP5	-0.448495751	-0.697239331	1.145735082	8
ARHGEF10	-0.459659724	-0.687522144	1.147181868	8
ARHGEF15	-0.391517473	-0.745004545	1.136522018	8
ARHGEF25	-0.49920689	-0.652114006	1.151320896	8
ARHGEF28	-0.462721211	-0.684836323	1.147557533	8
ARHGEF33	-0.470002477	-0.678411731	1.148414209	8
ARHGEF35	-0.301022277	-0.81491063	1.115932907	8
ARHGEF7	-0.207694176	-0.879843589	1.087537764	8
ARHGEF9	-0.182622387	-0.896103004	1.078725391	8
ARID5A	-0.477855626	-0.671424042	1.149279668	8
ARL14EPL	-0.448509441	-0.697227488	1.145736929	8

ARL15	-0.477825306	-0.671451139	1.149276444	8
ARL3	-0.425464213	-0.71691038	1.142374593	8
ARL6	-0.213983115	-0.875687647	1.089670763	8
ARMC2	-0.299369241	-0.816122577	1.115491818	8
ARMC8	-0.473555013	-0.675258259	1.148813272	8
ARMCX1	-0.474143936	-0.674734287	1.148878223	8
ARMCX2	-0.302670491	-0.813699951	1.116370442	8
ARMCX4	-0.407432213	-0.731965059	1.139397272	8
ARMS2	-0.265043414	-0.840778859	1.105822273	8
ARNTL2	-0.447951877	-0.697709657	1.145661534	8
ARSK	-0.429922474	-0.713141667	1.143064141	8
ASAH2	-0.353425242	-0.775294705	1.128719947	8
ASAP2	-0.103907988	-0.94398895	1.047896938	8
ASB12	-0.420771314	-0.720857397	1.141628711	8
ASIC5	-0.409001645	-0.730666671	1.139668316	8
ASPH	-0.47994734	-0.669552532	1.149499873	8
ASPHD1	-0.424655116	-0.717592344	1.14224746	8
ASS1	-0.495724128	-0.655295282	1.15101941	8
ATF2	-0.440088848	-0.704477806	1.144566654	8
ATF3	-0.498568573	-0.652697993	1.151266567	8
ATF5	-0.474472834	-0.674441513	1.148914347	8
ATF7IP	-0.489440157	-0.661004079	1.150444236	8
ATF7IP2	-0.252324354	-0.84967045	1.101994804	8
ATG10	-0.39601033	-0.741346667	1.137356997	8

ATG4C	-0.279888248	-0.830234687	1.110122936	8
ATOH8	-0.348093286	-0.779432931	1.127526217	8
ATP11C	-0.239335415	-0.858615952	1.097951367	8
ATP12A	-0.319679773	-0.801073029	1.120752803	8
ATP1A4	-0.345461967	-0.781466073	1.12692804	8
ATP2C1	-0.348526798	-0.779097395	1.127624193	8
ATP4A	-0.397099686	-0.740457015	1.137556701	8
ATP4B	-0.397234992	-0.740346439	1.137581431	8
ATP5MG	-0.339450659	-0.786088428	1.125539086	8
ATP5MJ	-0.411423315	-0.728658794	1.14008211	8
ATP5MK	-0.130928793	-0.928086427	1.05901522	8
ATP5PF	-0.184145745	-0.895129112	1.079274857	8
ATP6	-0.480514887	-0.669043982	1.149558869	8
ATP6AP2	-0.405458131	-0.733595012	1.139053143	8
ATP6V0D2	-0.396062785	-0.741303853	1.137366638	8
ATP6V1C2	-0.288033885	-0.824372112	1.112405997	8
ATP6V1E1	-0.425696649	-0.716714353	1.142411003	8
ATP6V1G2	-0.487665889	-0.662608717	1.150274606	8
ATP6V1G3	-0.106953036	-0.942224635	1.049177671	8
ATP7B	-0.42736981	-0.715301793	1.142671602	8
ATP8A1	-0.451006266	-0.695064633	1.146070899	8
ATP8B1	-0.477202601	-0.672007429	1.14921003	8
ATRIP	-0.23638956	-0.86062593	1.09701549	8
AUNIP	-0.116101755	-0.936881425	1.05298318	8

AVPR1A	-0.14309806	-0.920742363	1.063840423	8
AVPR2	-0.243085444	-0.856047223	1.099132667	8
AXIN2	-0.45977884	-0.687417815	1.147196655	8
AZGP1	-0.409249593	-0.730461337	1.139710931	8
AZI2	-0.397356178	-0.740247388	1.137603566	8
AZIN1	-0.346262788	-0.780847935	1.127110724	8
B3GALNT1	-0.448474945	-0.697257329	1.145732273	8
B3GAT2	-0.278336279	-0.831345481	1.10968176	8
B3GNT2	-0.38285115	-0.752008974	1.134860124	8
B3GNT3	-0.47964126	-0.669826662	1.149467922	8
BAAT	-0.478416535	-0.67092261	1.149339145	8
BACE1	-0.41683684	-0.724150766	1.140987606	8
BAIAP2L2	-0.314685451	-0.804805808	1.119491259	8
BAMBI	-0.498110937	-0.653116423	1.151227359	8
BANF2	-0.483448151	-0.666410503	1.149858655	8
BANK1	-0.492572524	-0.658163423	1.150735946	8
BATF2	-0.171693092	-0.903037225	1.074730317	8
BBOX1	-0.357576858	-0.772055455	1.129632314	8
BBS7	-0.295871229	-0.818679698	1.114550927	8
BBS9	-0.47158155	-0.677011575	1.148593126	8
BBX	-0.499751151	-0.651615739	1.15136689	8
BCAM	-0.495707434	-0.655310501	1.151017935	8
BCAR1	-0.42382793	-0.718288924	1.142116854	8
BCAT1	-0.418140522	-0.723061102	1.141201624	8

BCKDHB	-0.262301464	-0.842706781	1.105008246	8
BCL11B	-0.170010811	-0.90409633	1.074107141	8
BCL2L2	-0.385762904	-0.749663102	1.135426006	8
BCL6B	-0.397351104	-0.740251535	1.137602639	8
BDKRB2	-0.386465558	-0.749095868	1.135561426	8
BDP1	-0.466674733	-0.681354396	1.148029129	8
BEND3	-0.467579124	-0.680555735	1.148134859	8
BEND4	-0.423234893	-0.718787933	1.142022825	8
BEND5	-0.468036655	-0.680151387	1.148188042	8
BEX3	-0.44087963	-0.703799799	1.144679429	8
BEX4	-0.302068317	-0.814142534	1.116210852	8
BGN	-0.379900548	-0.754378419	1.134278967	8
BHMT	-0.442867741	-0.70209259	1.144960331	8
BHMT2	-0.461897918	-0.685559489	1.147457407	8
BIRC7	-0.237371607	-0.859956644	1.097328251	8
BIVM	-0.361258714	-0.769170151	1.130428865	8
BMERB1	-0.474195985	-0.674687961	1.148883946	8
BMI1	-0.160402176	-0.910103591	1.070505767	8
BMP1	-0.336452715	-0.788382067	1.124834782	8
BMP15	-0.469920827	-0.678484063	1.14840489	8
BMP2K	-0.455460344	-0.691191427	1.146651772	8
BMPR2	-0.431131687	-0.712116269	1.143247956	8
BNC1	-0.496400191	-0.65467871	1.151078901	8
BNC2	-0.438556344	-0.705790068	1.144346413	8

BNIP5	-0.43662772	-0.707438362	1.144066081	8
BOLL	-0.273085441	-0.835089029	1.10817447	8
BPIFB6	-0.242479585	-0.856462994	1.098942579	8
BRAF	-0.128867833	-0.929318976	1.058186809	8
BRAP	-0.329565266	-0.793622319	1.123187585	8
BRCA1	-0.260308466	-0.844104268	1.104412734	8
BRIP1	-0.49975604	-0.651611262	1.151367302	8
BRMS1L	-0.405619807	-0.733461654	1.139081461	8
BTBD3	-0.49198135	-0.658700302	1.150681652	8
BTBD8	-0.433391973	-0.710195889	1.143587862	8
BTG3	-0.22112041	-0.87093321	1.09205362	8
BTG4	-0.430858614	-0.712347951	1.143206565	8
BTK	-0.453614078	-0.692799236	1.146413314	8
BTNL10	-0.403121065	-0.735520069	1.138641135	8
BTNL9	-0.411989617	-0.72818848	1.140178097	8
BTRC	-0.395959769	-0.741387932	1.137347702	8
BUB1	-0.15448082	-0.913770063	1.068250883	8
BUB1B	-0.500313606	-0.651100497	1.151414104	8
C10orf82	-0.279175987	-0.830744721	1.109920708	8
C11orf52	-0.499598353	-0.651755655	1.151354008	8
C11orf53	-0.119780082	-0.934715182	1.054495264	8
C11orf91	-0.303294109	-0.813241288	1.116535396	8
C12orf56	-0.256142011	-0.847015377	1.103157388	8
C12orf75	-0.491236582	-0.659376165	1.150612747	8

C12orf76	-0.455743043	-0.690944951	1.146687993	8
C17orf113	-0.400742323	-0.737474344	1.138216667	8
C17orf67	-0.475917058	-0.673154645	1.149071703	8
C18orf32	-0.188965271	-0.892036039	1.08100131	8
C19orf81	-0.490925721	-0.6596581	1.150583821	8
C1GALT1	-0.424551976	-0.717679234	1.14223121	8
C1orf112	-0.31865936	-0.801837402	1.120496762	8
C1orf115	-0.380617665	-0.753803261	1.134420926	8
C1orf185	-0.458167978	-0.688827542	1.14699552	8
C1orf21	-0.306192372	-0.811105387	1.117297759	8
C1orf232	-0.491774906	-0.658887702	1.150662608	8
C1orf68	-0.48004685	-0.669463389	1.14951024	8
C1orf94	-0.413117418	-0.727250961	1.140368379	8
C1QB	-0.402565831	-0.735976687	1.138542519	8
C1QC	-0.45716602	-0.689703123	1.146869143	8
C1QTNF1	-0.422297543	-0.719575994	1.141873537	8
C1QTNF2	-0.238612816	-0.85910963	1.097722446	8
C1QTNF3	-0.323743949	-0.79801989	1.121763839	8
C1QTNF5	-0.410613567	-0.729330779	1.139944346	8
C1QTNF9	-0.432109494	-0.711286096	1.14339559	8
C1QTNF9B	-0.366961971	-0.764677326	1.131639297	8
C1S	-0.269231945	-0.837822005	1.10705395	8
C2	-0.445718247	-0.699638266	1.145356513	8
C20orf85	-0.496543599	-0.654547862	1.151091461	8

C22orf23	-0.108813455	-0.941143233	1.049956688	8
C2CD5	-0.25435993	-0.84825623	1.102616159	8
C2orf16	-0.321412623	-0.799772967	1.12118559	8
C3	-0.499023456	-0.65228187	1.151305326	8
C3orf14	-0.492764508	-0.657988995	1.150753502	8
C3orf33	-0.190890422	-0.890795447	1.081685869	8
C3orf56	-0.224607516	-0.868595624	1.09320314	8
C4orf3	-0.17422421	-0.901439585	1.075663795	8
C4orf54	-0.478188796	-0.671126239	1.149315034	8
C5orf22	-0.301574862	-0.814504987	1.116079849	8
C5orf47	-0.299210399	-0.816238914	1.115449314	8
C5orf51	-0.267077594	-0.839344633	1.106422227	8
C6	-0.484560134	-0.665409918	1.149970051	8
C6orf132	-0.479728617	-0.669748434	1.149477051	8
C6orf136	-0.222094023	-0.870281518	1.092375541	8
C6orf58	-0.235785707	-0.861037085	1.096822793	8
C7orf57	-0.48975832	-0.660715998	1.150474318	8
C8orf44-SGK3	-0.468175113	-0.680028983	1.148204096	8
C8orf48	-0.495429603	-0.655563744	1.150993347	8
C8orf88	-0.405381489	-0.733658222	1.139039711	8
C9	-0.299693436	-0.815885069	1.115578504	8
C9orf152	-0.42631196	-0.716195183	1.142507143	8
CA3	-0.320887691	-0.800167063	1.121054754	8
CA5A	-0.475055544	-0.673922541	1.148978085	8

CA7	-0.371342847	-0.761206799	1.132549646	8
CA8	-0.420715987	-0.720903808	1.141619795	8
CAAP1	-0.434183913	-0.709521905	1.143705817	8
CAB39L	-0.310410309	-0.80798438	1.118394689	8
CABLES1	-0.493126761	-0.657659766	1.150786527	8
CABP2	-0.461343642	-0.686045984	1.147389625	8
CABP5	-0.493493743	-0.657326103	1.150819847	8
CACNA1D	-0.447620413	-0.697996159	1.145616571	8
CACNA1E	-0.153379286	-0.914449145	1.06782843	8
CACNG1	-0.422977401	-0.719004496	1.141981897	8
CADM2	-0.456131945	-0.690605752	1.146737697	8
CADM4	-0.406521914	-0.732717115	1.139239028	8
CADPS2	-0.479372685	-0.670067125	1.14943981	8
CALCRL	-0.424086017	-0.718071655	1.142157672	8
CALD1	-0.474410755	-0.674496782	1.148907536	8
CALML5	-0.4439752	-0.701139974	1.145115173	8
CALU	-0.298642218	-0.816654884	1.115297102	8
CAMK2D	-0.322317648	-0.799092966	1.121410614	8
CAMSAP1	-0.422041137	-0.719791421	1.141832558	8
CAMSAP2	-0.469431953	-0.678917008	1.148348961	8
CAMTA1	-0.134581286	-0.925894087	1.060475373	8
CAPN1	-0.442300167	-0.702580354	1.144880521	8
CAPN2	-0.309211066	-0.808873261	1.118084328	8
CAPN7	-0.190030328	-0.89135006	1.081380389	8

CAPN8	-0.369260659	-0.762858417	1.132119076	8
CAPZA3	-0.467429961	-0.680687515	1.148117476	8
CARD10	-0.420221197	-0.721318738	1.141539935	8
CARD18	-0.383251552	-0.751686837	1.134938389	8
CARNMT1	-0.413614439	-0.726837425	1.140451864	8
CARTPT	-0.466361318	-0.681630983	1.147992301	8
CASD1	-0.12656775	-0.930690699	1.057258449	8
CASKIN2	-0.416178494	-0.724700438	1.140878932	8
CASP14	-0.314460466	-0.804973468	1.119433934	8
CASP8AP2	-0.147078201	-0.918315728	1.06539393	8
CASP9	-0.358163269	-0.771596704	1.129759974	8
CASQ1	-0.221713844	-0.870536082	1.092249926	8
CATSPERB	-0.352430688	-0.776068467	1.128499156	8
CATSPERE	-0.47664139	-0.672508454	1.149149844	8
CAV1	-0.427805331	-0.714933675	1.142739007	8
CAVIN2	-0.383418281	-0.751552656	1.134970937	8
CBLN4	-0.487621184	-0.662649107	1.150270291	8
CBX6	-0.494930185	-0.656018766	1.150948952	8
CCDC112	-0.355670747	-0.77354454	1.129215287	8
CCDC116	-0.479294311	-0.670137282	1.149431593	8
CCDC122	-0.435754292	-0.708183677	1.143937969	8
CCDC138	-0.500436695	-0.650987698	1.151424393	8
CCDC148	-0.500478685	-0.650949214	1.151427899	8
CCDC15	-0.475432996	-0.673586196	1.149019192	8

CCDC150	-0.485778448	-0.664312228	1.150090676	8
CCDC152	-0.371312394	-0.761230982	1.132543376	8
CCDC158	-0.494829346	-0.656110611	1.150939957	8
CCDC178	-0.375971902	-0.757521239	1.133493141	8
CCDC188	-0.448895677	-0.696893305	1.145788982	8
CCDC189	-0.180035704	-0.897752546	1.07778825	8
CCDC34	-0.395114072	-0.742077815	1.137191887	8
CCDC50	-0.214723437	-0.875196363	1.0899198	8
CCDC62	-0.363018615	-0.767786808	1.130805422	8
CCDC63	-0.397534475	-0.740101633	1.137636108	8
CCDC66	-0.237589682	-0.859807917	1.097397599	8
CCDC68	-0.438135734	-0.706149842	1.144285576	8
CCDC80	-0.399517509	-0.738478593	1.137996102	8
CCDC81	-0.404606988	-0.734296681	1.13890367	8
CCDC82	-0.381442465	-0.75314117	1.134583635	8
CCDC85A	-0.405918389	-0.733215306	1.139133695	8
CCDC89	-0.272342013	-0.835617227	1.10795924	8
CCER2	-0.185354136	-0.894355294	1.07970943	8
CCL1	-0.118838857	-0.93527047	1.054109327	8
CCL11	-0.493406293	-0.657405626	1.150811919	8
CCL13	-0.266971433	-0.839419567	1.106391	8
CCL14	-0.385391526	-0.749962727	1.135354253	8
CCL15	-0.482234435	-0.667501218	1.149735654	8
CCL18	-0.397790602	-0.739892203	1.137682805	8

CCL2	-0.416570934	-0.724372827	1.140943761	8
CCL20	-0.133256886	-0.926690213	1.059947099	8
CCL21	-0.415229378	-0.725492177	1.140721555	8
CCL27	-0.176411816	-0.900054762	1.076466579	8
CCM2L	-0.414077351	-0.726452064	1.140529415	8
CCN1	-0.482680987	-0.667100093	1.14978108	8
CCN4	-0.467885308	-0.680285164	1.148170472	8
CCN5	-0.199228289	-0.885388935	1.084617224	8
CCNA1	-0.464091182	-0.683631498	1.14772268	8
CCNA2	-0.478738881	-0.670634303	1.149373184	8
CCND1	-0.416159771	-0.724716064	1.140875835	8
CCNG1	-0.119556833	-0.934846951	1.054403785	8
CCNG2	-0.148674266	-0.917339211	1.066013477	8
CCNJ	-0.13580379	-0.925158022	1.060961812	8
CCP110	-0.312126061	-0.806710563	1.118836624	8
CCT7	-0.480099256	-0.66941644	1.149515696	8
CCT8L2	-0.256093494	-0.847049193	1.103142687	8
CD109	-0.490624896	-0.659930839	1.150555735	8
CD163L1	-0.450101735	-0.695848867	1.145950602	8
CD164L2	-0.47148117	-0.677100655	1.148581825	8
CD200	-0.466352941	-0.681638375	1.147991316	8
CD24	-0.169004604	-0.904728754	1.073733358	8
CD300LG	-0.383674761	-0.751346195	1.135020957	8
CD34	-0.434534256	-0.709223555	1.143757811	8

CD47	-0.330635862	-0.792810424	1.123446286	8
CD5L	-0.468315765	-0.67990462	1.148220385	8
CDC25A	-0.455908473	-0.690800681	1.146709154	8
CDC25C	-0.471360331	-0.677207878	1.148568208	8
CDC42BPA	-0.49730201	-0.653855523	1.151157533	8
CDC42BPB	-0.480913153	-0.668686924	1.149600077	8
CDC42BPG	-0.321936528	-0.79937941	1.121315937	8
CDC42EP5	-0.395080025	-0.742105575	1.1371856	8
CDC6	-0.360545792	-0.769729761	1.130275553	8
CDCA2	-0.492919192	-0.657848428	1.15076762	8
CDCP2	-0.356425923	-0.772954964	1.129380886	8
CDH15	-0.366265063	-0.765227858	1.131492921	8
CDH5	-0.429338473	-0.713636401	1.142974874	8
CDHR1	-0.300954495	-0.81496037	1.115914865	8
CDIN1	-0.469256173	-0.679072621	1.148328794	8
CDIP1	-0.474330195	-0.674568498	1.148898693	8
CDK12	-0.222543874	-0.869980155	1.092524029	8
CDK17	-0.44056907	-0.70406614	1.14463521	8
CDK2	-0.356539467	-0.772866275	1.129405742	8
CDK6	-0.319838994	-0.80095368	1.120792675	8
CDKN1B	-0.234640771	-0.861815857	1.096456628	8
CDNF	-0.175330387	-0.900739806	1.076070192	8
CDS2	-0.491488397	-0.65914771	1.150636107	8
CDSN	-0.415749194	-0.725058657	1.140807851	8

CDY1	-0.205857492	-0.881051447	1.086908939	8
CDY1B	-0.140921675	-0.92206413	1.062985805	8
CDYL2	-0.443727325	-0.701353293	1.145080617	8
CEACAM7	-0.356589771	-0.772826979	1.12941675	8
CEMP1	-0.413327389	-0.727076287	1.140403675	8
CENPF	-0.45182413	-0.694354862	1.146178992	8
CENPI	-0.483319454	-0.666526228	1.149845682	8
CENPJ	-0.312141525	-0.80669907	1.118840596	8
CENPK	-0.484787702	-0.665204994	1.149992696	8
CENPN	-0.286237478	-0.825669721	1.111907199	8
CENPO	-0.351807153	-0.776553139	1.128360292	8
CENPU	-0.399462739	-0.738523468	1.137986207	8
CENPV	-0.499185563	-0.652133524	1.151319087	8
CEP126	-0.44360686	-0.701456943	1.145063802	8
CEP135	-0.484363879	-0.665586601	1.149950481	8
CEP152	-0.152299939	-0.915113642	1.06741358	8
CEP20	-0.241105018	-0.857405198	1.098510217	8
CEP290	-0.443846944	-0.701250356	1.145097301	8
CEP44	-0.269507206	-0.837627186	1.107134392	8
CEP55	-0.381622556	-0.752996525	1.134619081	8
CEP57	-0.110249366	-0.940306785	1.050556151	8
CEP57L1	-0.437150184	-0.706992187	1.144142371	8
CEP70	-0.211938686	-0.877042099	1.088980785	8
CEP97	-0.315649635	-0.804086811	1.119736446	8

CERS2	-0.152035943	-0.915276033	1.067311976	8
CES5A	-0.461337888	-0.686051032	1.14738892	8
CETN1	-0.410988168	-0.729019985	1.140008152	8
CETN2	-0.231125027	-0.864200681	1.095325707	8
CETN3	-0.218742689	-0.872521586	1.091264275	8
CFAP161	-0.499605198	-0.651749387	1.151354586	8
CFAP251	-0.414449347	-0.726142244	1.140591592	8
CFAP44	-0.194202514	-0.88865431	1.082856824	8
CFAP53	-0.424946627	-0.717346708	1.142293335	8
CFAP70	-0.213545705	-0.875977713	1.089523418	8
CFAP73	-0.162428951	-0.908842405	1.071271356	8
CFB	-0.433124122	-0.710423711	1.143547833	8
CFI	-0.481580187	-0.668088552	1.149668739	8
CGGBP1	-0.452051525	-0.694157406	1.146208931	8
CGREF1	-0.460938103	-0.68640174	1.147339843	8
CHCHD2	-0.159604774	-0.910598914	1.070203688	8
CHD1L	-0.151591244	-0.91554946	1.067140704	8
CHD9	-0.501411895	-0.650093466	1.151505361	8
CHEK1	-0.40756468	-0.731855556	1.139420237	8
CHI3L1	-0.441963383	-0.702869636	1.144833019	8
CHMP4C	-0.416079661	-0.724782922	1.140862583	8
CHMP6	-0.110356788	-0.940244147	1.050600935	8
CHN1	-0.412525986	-0.727742753	1.140268739	8
CHPF2	-0.486689526	-0.663490381	1.150179907	8

CHRM4	-0.47695561	-0.672227971	1.149183581	8
CHRNA5	-0.49044968	-0.660089654	1.150539334	8
CHRNA9	-0.350820022	-0.777319743	1.128139765	8
CHRNB2	-0.49606133	-0.65498781	1.151049141	8
CHRNB3	-0.446446015	-0.699010405	1.14545642	8
CHST10	-0.497211246	-0.653938411	1.151149657	8
CIART	-0.45142225	-0.694703708	1.146125958	8
CIDEA	-0.482520942	-0.66724388	1.149764822	8
CIP2A	-0.491092624	-0.659506739	1.150599363	8
CKAP2	-0.370510388	-0.761867575	1.132377963	8
CKAP2L	-0.494681597	-0.656245162	1.150926759	8
CKM	-0.465893877	-0.682043319	1.147937196	8
CLASP2	-0.386874867	-0.74876524	1.135640107	8
CLBA1	-0.426756685	-0.715819725	1.14257641	8
CLCA1	-0.299057261	-0.816351054	1.115408316	8
CLCA4	-0.409571952	-0.730194298	1.13976625	8
CLCN3	-0.469838305	-0.678557161	1.148395466	8
CLDN25	-0.445324272	-0.699977945	1.145302217	8
CLEC3B	-0.41633921	-0.724566289	1.140905498	8
CLEC4M	-0.391500954	-0.74501796	1.136518914	8
CLIC4	-0.478071484	-0.67123111	1.149302594	8
CLIC5	-0.29757604	-0.817434716	1.115010756	8
CLINT1	-0.186845195	-0.893398894	1.080244088	8
CLK1	-0.392455058	-0.74424271	1.136697768	8

CLMN	-0.437970406	-0.706291211	1.144261617	8
CLN5	-0.382064037	-0.752641815	1.134705852	8
CLOCK	-0.393064397	-0.743747167	1.136811564	8
CLSPN	-0.455921023	-0.690789735	1.146710758	8
CMAS	-0.49322345	-0.657571869	1.150795319	8
CMC1	-0.232899631	-0.862998152	1.095897783	8
CMC4	-0.109902655	-0.940508895	1.05041155	8
CMKLR2	-0.408855271	-0.730787862	1.139643132	8
CMPK1	-0.236721745	-0.860399625	1.09712137	8
CMTM4	-0.359195628	-0.770788358	1.129983986	8
CMTM5	-0.461719148	-0.685716431	1.147435578	8
CMTM7	-0.326154995	-0.796202018	1.122357013	8
CNDP1	-0.168787287	-0.90486524	1.073652527	8
CNGA2	-0.242368795	-0.856538992	1.098907786	8
CNGB3	-0.394347878	-0.742702283	1.137050161	8
CNIH1	-0.210481581	-0.878005429	1.08848701	8
CNIH2	-0.446053824	-0.699348819	1.145402643	8
CNMD	-0.461683387	-0.685747821	1.147431208	8
CNRIP1	-0.474439852	-0.674470877	1.148910729	8
CNST	-0.298519244	-0.816744879	1.115264123	8
CNTLN	-0.496649782	-0.654450965	1.151100747	8
CNTN2	-0.488895649	-0.661496867	1.150392516	8
CNTRL	-0.207864316	-0.879731565	1.087595881	8
COBL	-0.495016711	-0.655939951	1.150956662	8

COG4	-0.325573044	-0.796641247	1.122214291	8
COG6	-0.356060527	-0.773240295	1.129300822	8
COL11A2	-0.453910518	-0.692541305	1.146451823	8
COL15A1	-0.419291196	-0.72209802	1.141389216	8
COL17A1	-0.494258087	-0.656630719	1.150888806	8
COL1A1	-0.390642676	-0.745714646	1.136357323	8
COL1A2	-0.474500362	-0.674417003	1.148917365	8
COL5A1	-0.462628964	-0.684917383	1.147546347	8
COL5A2	-0.495662325	-0.655351624	1.151013948	8
COL6A3	-0.399638257	-0.73837965	1.138017907	8
COL7A1	-0.422635075	-0.719292314	1.141927389	8
COLEC11	-0.485331157	-0.664715406	1.150046563	8
COLGALT2	-0.403818082	-0.734946453	1.138764535	8
COMMD6	-0.250697364	-0.850798399	1.101495763	8
COMMD7	-0.327146519	-0.795453	1.122599519	8
COMP	-0.274754159	-0.833901775	1.108655935	8
CORIN	-0.422857292	-0.719105492	1.141962785	8
COX15	-0.105350347	-0.943154118	1.048504465	8
COX2	-0.130465801	-0.9283636	1.058829401	8
COX7A1	-0.436567539	-0.707489739	1.144057277	8
COX8C	-0.432757247	-0.710735649	1.143492896	8
CPA1	-0.362732035	-0.768012254	1.130744289	8
CPLANE1	-0.472685814	-0.67603097	1.148716784	8
CPLANE2	-0.387454961	-0.748296399	1.13575136	8

CPLX1	-0.490514051	-0.660031312	1.150545363	8
CPLX3	-0.481453236	-0.668202469	1.149655706	8
CPN1	-0.211988728	-0.877008985	1.088997713	8
CPT1A	-0.274196277	-0.834298949	1.108495226	8
CPXM2	-0.494448628	-0.656457276	1.150905904	8
CPZ	-0.419426847	-0.721984403	1.14141125	8
CRACD	-0.124783474	-0.931752022	1.056535496	8
CRCP	-0.347564656	-0.779841868	1.127406524	8
CREB3L1	-0.498647924	-0.652625419	1.151273343	8
CREBRF	-0.435352897	-0.708525954	1.143878852	8
CREM	-0.345008674	-0.781815715	1.126824389	8
CRHBP	-0.354924218	-0.774126878	1.129051096	8
CRKL	-0.326227088	-0.796147586	1.122374674	8
CRNN	-0.42875155	-0.714133288	1.142884839	8
CRTC1	-0.319500914	-0.801207073	1.120707987	8
CRYBA2	-0.296291828	-0.818372769	1.114664597	8
CRYBA4	-0.259291045	-0.84481644	1.104107484	8
CRYBB2	-0.409855762	-0.729959112	1.139814875	8
CRYBG3	-0.293685783	-0.820272146	1.113957929	8
CRYGC	-0.42705685	-0.715566207	1.142623057	8
CRYM	-0.291031355	-0.822200997	1.113232352	8
CRYZL2P-SEC16B	-0.375160463	-0.758168662	1.133329125	8
CSF2RB	-0.433621981	-0.7100002	1.143622181	8
CSHL1	-0.401113567	-0.737169685	1.138283252	8

CSMD1	-0.389989644	-0.746244286	1.13623393	8
CSNK1G1	-0.488645879	-0.661722812	1.150368691	8
CSNK1G3	-0.495669375	-0.655345196	1.151014572	8
CSPP1	-0.458120309	-0.68886922	1.146989529	8
CSRNP1	-0.253485078	-0.848864445	1.102349523	8
CST11	-0.431525259	-0.711782229	1.143307487	8
CST9	-0.290281854	-0.822744569	1.113026423	8
CTAG1B	-0.453931141	-0.692523358	1.146454499	8
CTAGE15	-0.1524924	-0.914995219	1.067487619	8
CTH	-0.465397053	-0.682481339	1.147878393	8
CTHRC1	-0.473303959	-0.67548152	1.148785479	8
CTNNA3	-0.28076033	-0.829609643	1.110369973	8
CTNNBIP1	-0.474332705	-0.674566264	1.148898969	8
CTPS2	-0.265385099	-0.840538185	1.105923284	8
CTRB1	-0.368374511	-0.76356016	1.131934672	8
CTRB2	-0.398620978	-0.739212813	1.13783379	8
CTSA	-0.447023265	-0.698512039	1.145535304	8
CTSD	-0.406924896	-0.732384279	1.139309175	8
CTSE	-0.439725446	-0.704789184	1.14451463	8
CTSV	-0.494737216	-0.656194514	1.15093173	8
CTTN	-0.323151742	-0.798465646	1.121617388	8
CUL4B	-0.238437709	-0.859229199	1.097666909	8
CUL5	-0.448289733	-0.697417523	1.145707255	8
CWC27	-0.405115701	-0.733877386	1.138993087	8

CX3CL1	-0.476938472	-0.672243271	1.149181743	8
CXCL11	-0.449567863	-0.696311369	1.145879232	8
CXCL12	-0.442023904	-0.702817659	1.144841564	8
CXCL2	-0.47230943	-0.67636534	1.148674771	8
CXCL8	-0.448049266	-0.697625459	1.145674725	8
CXCL9	-0.39109587	-0.74534686	1.13644273	8
CXorf51A	-0.464570091	-0.683209888	1.147779979	8
CXorf51B	-0.464570091	-0.683209888	1.147779979	8
CYB5D2	-0.110119554	-0.940382468	1.050502022	8
CYGB	-0.470616683	-0.67786741	1.148484093	8
CYLC1	-0.498547115	-0.652717618	1.151264733	8
CYP11A1	-0.226664772	-0.867212009	1.093876781	8
CYP19A1	-0.466113481	-0.68184963	1.147963111	8
CYP1A1	-0.375842605	-0.757624441	1.133467046	8
CYP24A1	-0.223208501	-0.869534615	1.092743116	8
CYP26B1	-0.49215761	-0.658540267	1.150697877	8
CYP2A6	-0.484783936	-0.665208385	1.149992321	8
CYP2C18	-0.473256984	-0.675523288	1.148780272	8
CYP2C8	-0.242683	-0.856323433	1.099006433	8
CYP2C9	-0.394097184	-0.742906491	1.137003675	8
CYP2E1	-0.39919854	-0.738739898	1.137938438	8
CYP3A4	-0.35303557	-0.775597972	1.128633542	8
CYP3A7	-0.369274571	-0.762847394	1.132121965	8
CYP4A11	-0.4698989	-0.678503486	1.148402387	8

CYP4A22	-0.165355608	-0.907015654	1.072371263	8
CYP4X1	-0.470776056	-0.67772611	1.148502166	8
CYP4Z1	-0.414454299	-0.72613812	1.140592419	8
CYP51A1	-0.137886428	-0.923901436	1.061787865	8
CYP7A1	-0.320198707	-0.800683968	1.120882675	8
CYRIA	-0.44367505	-0.701398272	1.145073322	8
CYS1	-0.482143706	-0.667582694	1.1497264	8
CYTB	-0.36602017	-0.765421213	1.131441383	8
CYYR1	-0.450141699	-0.695814235	1.145955934	8
DAB2IP	-0.484672054	-0.665309141	1.149981194	8
DACT1	-0.420326448	-0.721230494	1.141556942	8
DBX1	-0.410989675	-0.729018734	1.140008409	8
DCAF12L1	-0.105803281	-0.942891636	1.048694917	8
DCAF12L2	-0.459285583	-0.687849751	1.147135334	8
DCAF17	-0.407923815	-0.731558601	1.139482415	8
DCAKD	-0.41481733	-0.725835642	1.140652972	8
DCDC2	-0.496341805	-0.654731977	1.151073782	8
DCLK3	-0.366761138	-0.76483602	1.131597159	8
DCLRE1A	-0.451381522	-0.694739052	1.146120574	8
DCN	-0.36813739	-0.76374782	1.13188521	8
DCST1	-0.259486385	-0.844679771	1.104166156	8
DCTN6	-0.389563277	-0.746589882	1.136153159	8
DCUN1D4	-0.440034787	-0.704524136	1.144558923	8
DDAH2	-0.351573677	-0.776734533	1.128308209	8

DDC	-0.485227492	-0.664808818	1.150036311	8
DDR2	-0.453556789	-0.692849073	1.146405862	8
DEAF1	-0.21924169	-0.872188613	1.091430303	8
DEFA6	-0.448986969	-0.696814296	1.145801265	8
DEFB106A	-0.376834899	-0.756832038	1.133666937	8
DEFB106B	-0.237223749	-0.860057462	1.097281211	8
DEFB4A	-0.377328792	-0.756437311	1.133766103	8
DEFB4B	-0.285409486	-0.826266912	1.111676399	8
DENND4A	-0.426936206	-0.715668113	1.142604318	8
DENND5B	-0.491918205	-0.658757627	1.150675831	8
DEPTOR	-0.393141682	-0.743684291	1.136825973	8
DERA	-0.285128991	-0.826469092	1.111598083	8
DERL1	-0.329477498	-0.793688835	1.123166333	8
DEUP1	-0.157757342	-0.9117446	1.069501942	8
DHRS2	-0.445186697	-0.700096525	1.145283223	8
DHX36	-0.155711958	-0.913009975	1.068721933	8
DIO1	-0.153593852	-0.91431694	1.067910793	8
DIO3	-0.447179841	-0.698376805	1.145556646	8
DIPK2B	-0.42617747	-0.71630869	1.14248616	8
DIRAS3	-0.493786244	-0.657060062	1.150846306	8
DIXDC1	-0.481889621	-0.667810819	1.14970044	8
DKK1	-0.497972936	-0.653242558	1.151215495	8
DKK2	-0.441009923	-0.70368803	1.144697953	8
DLC1	-0.416662541	-0.724296332	1.140958873	8

DLG1	-0.379530591	-0.754674961	1.134205552	8
DLG4	-0.31599614	-0.803828229	1.119824369	8
DLGAP5	-0.495542919	-0.655460466	1.151003385	8
DLL4	-0.378364352	-0.755608972	1.133973324	8
DMBT1	-0.481303303	-0.668336988	1.149640292	8
DMKN	-0.171887864	-0.902914461	1.074802325	8
DNA2	-0.47804848	-0.671251673	1.149300153	8
DNAAF10	-0.216110875	-0.87427448	1.090385355	8
DNAAF4	-0.450609838	-0.695408435	1.146018273	8
DNAH6	-0.295384358	-0.819034804	1.114419163	8
DNAI4	-0.501487537	-0.650024064	1.151511601	8
DNAJB1	-0.461073273	-0.686283181	1.147356454	8
DNAJB6	-0.458008424	-0.688967036	1.14697546	8
DNAJB8	-0.33960786	-0.785967945	1.125575804	8
DNAJC15	-0.271804668	-0.835998724	1.107803392	8
DNAJC2	-0.331688874	-0.792010911	1.123699785	8
DNAJC27	-0.356831901	-0.772637805	1.129469706	8
DNAJC5B	-0.319026139	-0.801562756	1.120588895	8
DNASE1L3	-0.380074184	-0.754239198	1.134313381	8
DNASE2B	-0.494192767	-0.656690169	1.150882936	8
DNMT1	-0.356020358	-0.773271655	1.129292013	8
DOCK1	-0.473753338	-0.675081845	1.148835183	8
DOCK3	-0.473950397	-0.674906519	1.148856916	8
DOCK6	-0.443240358	-0.701772201	1.14501256	8

DOCK7	-0.453011493	-0.693323281	1.146334774	8
DPEP1	-0.422826991	-0.71913097	1.141957961	8
DPPA4	-0.341205349	-0.784742385	1.125947734	8
DPRX	-0.361186517	-0.769226843	1.13041336	8
DPT	-0.29825623	-0.816937315	1.115193546	8
DPY19L3	-0.413589125	-0.726858492	1.140447617	8
DPY19L4	-0.157848345	-0.911688227	1.069536571	8
DPY30	-0.111211066	-0.939745698	1.050956765	8
DPYS	-0.498917795	-0.652378546	1.151296341	8
DPYSL2	-0.412789083	-0.72752402	1.140313103	8
DQX1	-0.145384489	-0.919349848	1.064734337	8
DROSHA	-0.292611106	-0.821053767	1.113664874	8
DRP2	-0.478735552	-0.67063728	1.149372833	8
DSC3	-0.436512144	-0.707537026	1.14404917	8
DSCC1	-0.378241649	-0.755707171	1.13394882	8
DSE	-0.312807328	-0.806204086	1.119011415	8
DSG1	-0.448765794	-0.6970057	1.145771494	8
DSN1	-0.22764375	-0.866552412	1.094196162	8
DSP	-0.489028741	-0.661376444	1.150405185	8
DST	-0.478247611	-0.671073655	1.149321266	8
DTL	-0.492959604	-0.6578117	1.150771305	8
DTX4	-0.266922273	-0.839454263	1.106376536	8
DUSP16	-0.134897278	-0.925703939	1.060601217	8
DUSP19	-0.491052154	-0.659543443	1.150595597	8

DUSP21	-0.475546193	-0.673485299	1.149031492	8
DUSP22	-0.358337319	-0.771460487	1.129797806	8
DUSP6	-0.312543004	-0.80640064	1.118943644	8
DYNAP	-0.420376586	-0.721188454	1.14156504	8
DYNC2H1	-0.499331832	-0.651999649	1.151331481	8
DYNC2LI1	-0.405632303	-0.733451346	1.139083649	8
DYNLRB1	-0.162223623	-0.908970318	1.07119394	8
DYNLT2B	-0.457413702	-0.689486771	1.146900474	8
DYNLT5	-0.491766174	-0.658895628	1.150661802	8
DZANK1	-0.366133461	-0.765331771	1.131465231	8
E2F5	-0.211605631	-0.877262438	1.088868069	8
E2F8	-0.488264988	-0.66206725	1.150332238	8
EAF1	-0.362846341	-0.767922341	1.130768682	8
EAF2	-0.487975134	-0.662329266	1.1503044	8
EBF3	-0.4936912	-0.657146518	1.150837718	8
ECSCR	-0.405570871	-0.733502021	1.139072892	8
ECT2	-0.495556533	-0.655448058	1.15100459	8
EDN1	-0.459596107	-0.687577858	1.147173966	8
EDN3	-0.308495245	-0.809403254	1.1178985	8
EEF1AKMT1	-0.371585895	-0.76101376	1.132599655	8
EFCAB2	-0.469495775	-0.678860501	1.148356276	8
EFCAB9	-0.404906675	-0.7340497	1.138956374	8
EFCC1	-0.434058349	-0.709628806	1.143687155	8
EFEMP1	-0.475055889	-0.673922234	1.148978122	8

EFHB	-0.468910855	-0.679378232	1.148289087	8
EFHC1	-0.401195501	-0.737102429	1.138297931	8
EFNB1	-0.149447784	-0.916865246	1.066313031	8
EGFLAM	-0.462173849	-0.685317189	1.147491038	8
EGR3	-0.46409228	-0.683630532	1.147722811	8
EHBP1	-0.46367652	-0.683996367	1.147672887	8
EHD2	-0.433228777	-0.710334704	1.143563481	8
EIF2AK4	-0.132180184	-0.927336453	1.059516636	8
EIF5B	-0.229567825	-0.865253822	1.094821647	8
ELAPOR2	-0.417771518	-0.723369689	1.141141206	8
ELF3	-0.269752342	-0.837453637	1.107205979	8
ELF5	-0.202221268	-0.883434905	1.085656173	8
ELK1	-0.493143011	-0.657644994	1.150788005	8
ELK3	-0.209883423	-0.878400402	1.088283825	8
ELMO2	-0.389789633	-0.746406427	1.13619606	8
ELN	-0.479787645	-0.669695569	1.149483214	8
ELOVL6	-0.354992777	-0.774073418	1.129066195	8
ELP4	-0.441669418	-0.703122051	1.144791469	8
EMC2	-0.379017512	-0.755086022	1.134103534	8
EMCN	-0.467676601	-0.680469606	1.148146207	8
EMD	-0.400323234	-0.737818117	1.138141351	8
EML6	-0.26608576	-0.84004436	1.10613012	8
ENAH	-0.451223746	-0.694875959	1.146099705	8
ENO2	-0.199518241	-0.885199941	1.084718182	8

ENPEP	-0.241789361	-0.856936301	1.098725662	8
ENTHD1	-0.425790671	-0.716635045	1.142425716	8
EPAS1	-0.416015165	-0.724836744	1.140851909	8
EPB41L4A	-0.442052602	-0.702793012	1.144845614	8
EPB41L5	-0.460276152	-0.686982089	1.147258242	8
EPHA2	-0.497934584	-0.65327761	1.151212194	8
EPS15	-0.483312124	-0.666532818	1.149844943	8
EPX	-0.306218994	-0.811085735	1.117304729	8
ERAS	-0.44553379	-0.69979732	1.14533111	8
ERBIN	-0.38352377	-0.751467747	1.134991517	8
ERCC4	-0.308165335	-0.809647375	1.11781271	8
ERG	-0.415166745	-0.725544395	1.14071114	8
ERGIC2	-0.159451152	-0.910694284	1.070145435	8
ERICH1	-0.442038884	-0.702804794	1.144843678	8
ERICH3	-0.416407465	-0.724509309	1.140916774	8
ERN1	-0.249105177	-0.851900154	1.101005331	8
ERRFI1	-0.475133566	-0.673853027	1.148986593	8
ERV3-1-ZNF117	-0.232892093	-0.863003265	1.095895359	8
ERVH48-1	-0.481186205	-0.668442033	1.149628238	8
ESF1	-0.440166618	-0.704411153	1.144577772	8
ESM1	-0.398237174	-0.739526907	1.137764082	8
ESR1	-0.203897133	-0.882337708	1.086234841	8
ETFRF1	-0.238706033	-0.859045968	1.097752001	8
ETV6	-0.441903495	-0.702921066	1.144824561	8

EVX2	-0.485619657	-0.664455382	1.150075039	8
EXO1	-0.41225233	-0.727970197	1.140222526	8
EXOG	-0.482054517	-0.667662778	1.149717295	8
EYA1	-0.488328073	-0.662010213	1.150338286	8
F10	-0.445455778	-0.699864579	1.145320357	8
F7	-0.43740084	-0.706778039	1.144178879	8
F9	-0.419121895	-0.722239797	1.141361692	8
FABP1	-0.354578623	-0.774396298	1.128974921	8
FABP2	-0.45671598	-0.690096083	1.146812064	8
FABP4	-0.402090323	-0.736367516	1.13845784	8
FABP5	-0.440019535	-0.704537206	1.144556741	8
FAF1	-0.126900484	-0.930492513	1.057392997	8
FAH	-0.415638715	-0.725150816	1.140789531	8
FAM102B	-0.111316619	-0.939684073	1.051000692	8
FAM107A	-0.392717525	-0.744029301	1.136746826	8
FAM110A	-0.187415147	-0.893032854	1.080448001	8
FAM114A2	-0.236553403	-0.860514321	1.097067724	8
FAM120C	-0.180070207	-0.897730578	1.077800784	8
FAM124A	-0.491933074	-0.658744128	1.150677202	8
FAM126A	-0.413305293	-0.72709467	1.140399963	8
FAM135A	-0.398323673	-0.739456131	1.137779804	8
FAM149A	-0.472933026	-0.675811276	1.148744302	8
FAM151A	-0.16166831	-0.909316095	1.070984405	8
FAM167A	-0.501280471	-0.650214035	1.151494506	8

FAM180B	-0.262328626	-0.842687713	1.105016339	8
FAM184A	-0.482994155	-0.666818663	1.149812818	8
FAM193B	-0.45781924	-0.689132404	1.146951644	8
FAM205A	-0.465557565	-0.682339852	1.147897417	8
FAM20C	-0.429477861	-0.713518348	1.142996209	8
FAM216A	-0.439483134	-0.704996737	1.144479871	8
FAM219A	-0.447453831	-0.698140104	1.145593935	8
FAM228A	-0.318715828	-0.801795125	1.120510954	8
FAM234B	-0.309255303	-0.808840495	1.118095797	8
FAM237A	-0.156592407	-0.912465682	1.069058089	8
FAM240C	-0.46879144	-0.679483889	1.148275329	8
FAM243A	-0.272261166	-0.835674641	1.107935807	8
FAM243B	-0.277571622	-0.831892043	1.109463665	8
FAM24A	-0.417344796	-0.723726385	1.141071181	8
FAM47B	-0.463193935	-0.684420791	1.147614726	8
FAM71E2	-0.384670809	-0.750543844	1.135214653	8
FAM72B	-0.475318875	-0.673687903	1.149006778	8
FAM81A	-0.407456726	-0.731944797	1.139401523	8
FAM9C	-0.409685724	-0.730100027	1.139785751	8
FANCL	-0.483927672	-0.665979172	1.149906844	8
FANCM	-0.483672927	-0.666208345	1.149881272	8
FATE1	-0.219145451	-0.872252846	1.091398297	8
FBN1	-0.487346631	-0.662897115	1.150243746	8
FBN2	-0.419596406	-0.721842362	1.141438768	8

FBXL19	-0.271705096	-0.836069391	1.107774487	8
FBXL20	-0.171935078	-0.902884698	1.074819776	8
FBXO30	-0.123100104	-0.932751096	1.0558512	8
FBXO40	-0.319478574	-0.801223814	1.120702387	8
FBXO5	-0.227845073	-0.866416674	1.094261747	8
FBXW10	-0.28657429	-0.825426632	1.112000922	8
FBXW11	-0.123036499	-0.932788803	1.055825302	8
FBXW7	-0.460295574	-0.686965068	1.147260642	8
FCF1	-0.338371467	-0.786914974	1.125286441	8
FCN2	-0.286618389	-0.825394798	1.112013187	8
FCN3	-0.398187945	-0.739567186	1.137755131	8
FCRL4	-0.441919756	-0.702907102	1.144826858	8
FDCSP	-0.215960593	-0.874374409	1.090335002	8
FGA	-0.214506553	-0.875340334	1.089846887	8
FGB	-0.460324878	-0.686939385	1.147264263	8
FGD5	-0.399512878	-0.738482388	1.137995265	8
FGF1	-0.442189525	-0.702675403	1.144864928	8
FGF11	-0.500192474	-0.651211489	1.151403963	8
FGF12	-0.487916044	-0.66238267	1.150298714	8
FGF18	-0.482105697	-0.667616823	1.14972252	8
FGF20	-0.315588417	-0.804132485	1.119720902	8
FGF8	-0.455529683	-0.69113098	1.146660663	8
FGFBP1	-0.205320042	-0.881404388	1.086724429	8
FGG	-0.417501848	-0.723595125	1.141096973	8

FGGY	-0.478745718	-0.670628187	1.149373905	8
FGL1	-0.475615468	-0.673423546	1.149039014	8
FHDC1	-0.404086711	-0.734725265	1.138811976	8
FHIP1A	-0.433235379	-0.710329089	1.143564468	8
FHL5	-0.372273753	-0.760467154	1.132740906	8
FIBIN	-0.479752903	-0.669726684	1.149479587	8
FIGLA	-0.37173516	-0.760895182	1.132630342	8
FILIP1	-0.425741582	-0.716676453	1.142418035	8
FILIP1L	-0.41224755	-0.727974169	1.140221719	8
FKBP14	-0.124072004	-0.932174541	1.056246545	8
FKBP3	-0.217735154	-0.873193293	1.090928447	8
FKRP	-0.354627641	-0.774358091	1.128985732	8
FLG	-0.327011335	-0.79555517	1.122566505	8
FLG2	-0.486756363	-0.663430058	1.15018642	8
FLI1	-0.247384251	-0.853088697	1.100472948	8
FLNC	-0.464657767	-0.683132678	1.147790444	8
FLT1	-0.379235243	-0.754911612	1.134146855	8
FMO1	-0.473174891	-0.675596276	1.148771167	8
FMO2	-0.425158073	-0.717168488	1.142326561	8
FMOD	-0.446222207	-0.699203542	1.14542575	8
FN1	-0.448413804	-0.697310214	1.145724018	8
FNDC1	-0.406653912	-0.73260811	1.139262022	8
FOCAD	-0.31247237	-0.806453154	1.118925524	8
FOXF1	-0.463782293	-0.683903311	1.147685604	8

FOXH1	-0.384317544	-0.750828513	1.135146057	8
FOXI1	-0.353284389	-0.775404341	1.12868873	8
FOXI2	-0.439012204	-0.705399955	1.144412159	8
FOXK2	-0.401818565	-0.736590787	1.138409352	8
FOXM1	-0.297206081	-0.817705093	1.114911174	8
FPGT-TNNI3K	-0.468764856	-0.679507408	1.148272264	8
FRA10AC1	-0.343905585	-0.782665829	1.126571413	8
FRMD6	-0.461774686	-0.685667677	1.147442363	8
FRMPD1	-0.408073742	-0.731434596	1.139508338	8
FSBP	-0.433007619	-0.710522782	1.143530401	8
FSD1L	-0.471997184	-0.676642626	1.148639811	8
FSHB	-0.489966655	-0.660527306	1.150493961	8
FSIP1	-0.457486991	-0.689422742	1.146909733	8
FST	-0.45916618	-0.687954274	1.147120454	8
FSTL4	-0.103520002	-0.944213245	1.047733247	8
FUNDC1	-0.253275446	-0.849010093	1.102285539	8
FUT3	-0.484390171	-0.665562934	1.149953105	8
FXYD1	-0.479861582	-0.669629348	1.14949093	8
G2E3	-0.179627016	-0.898012692	1.077639708	8
G6PC2	-0.441654922	-0.703134496	1.144789418	8
GAA	-0.399384132	-0.738587869	1.137972001	8
GABRP	-0.492441313	-0.658282613	1.150723926	8
GABRR1	-0.458080891	-0.688903683	1.146984574	8
GALP	-0.496427832	-0.654653491	1.151081324	8

GAPVD1	-0.429985864	-0.713087947	1.143073811	8
GARRE1	-0.371761575	-0.760874196	1.132635771	8
GAS2	-0.208763867	-0.879138903	1.08790277	8
GAS2L2	-0.417259371	-0.723797772	1.141057143	8
GAS8	-0.381066542	-0.753443009	1.134509551	8
GATA2	-0.392775552	-0.743982111	1.136757663	8
GATA5	-0.486893701	-0.663306089	1.15019979	8
GATA6	-0.445240804	-0.700049891	1.145290695	8
GATM	-0.404152609	-0.734670994	1.138823603	8
GBP7	-0.242752177	-0.856275964	1.099028141	8
GBX1	-0.50037968	-0.651039948	1.151419629	8
GCGR	-0.501276247	-0.65021791	1.151494157	8
GCKR	-0.388633313	-0.747343108	1.13597642	8
GCOM1	-0.282433364	-0.828408785	1.110842149	8
GCSAML	-0.464906618	-0.68291349	1.147820108	8
GDAP1	-0.476549678	-0.672590301	1.149139979	8
GDF11	-0.332506334	-0.791389592	1.123895926	8
GDPD1	-0.435651861	-0.708271037	1.143922897	8
GEMIN8	-0.132865822	-0.926925033	1.059790855	8
GFRAL	-0.492039138	-0.658647837	1.150686975	8
GFY	-0.408890165	-0.730758973	1.139649138	8
GHRH	-0.18351156	-0.89553477	1.07904633	8
GIN1	-0.160978024	-0.909745585	1.070723609	8
GINS2	-0.104940305	-0.943391609	1.048331915	8

GIPC2	-0.440144963	-0.704429714	1.144574676	8
GJA1	-0.370487231	-0.761885947	1.132373178	8
GJA5	-0.429606245	-0.713409599	1.143015844	8
GJB1	-0.448394328	-0.69732706	1.145721388	8
GJB2	-0.45163576	-0.694518393	1.146154153	8
GJB4	-0.500861426	-0.650598351	1.151459777	8
GJB5	-0.498992676	-0.652310034	1.15130271	8
GJC3	-0.169528552	-0.904399539	1.073928091	8
GLCE	-0.161476368	-0.909435557	1.070911925	8
GLG1	-0.343110596	-0.783277849	1.126388445	8
GLOD5	-0.383844687	-0.751209377	1.135054064	8
GLRA3	-0.445568853	-0.699767089	1.145335942	8
GLRX3	-0.465381399	-0.682495137	1.147876536	8
GLT8D2	-0.436408291	-0.707625673	1.144033964	8
GLYAT	-0.49341855	-0.657394481	1.150813031	8
GLYATL2	-0.455077177	-0.691525377	1.146602554	8
GMNN	-0.342690978	-0.783600672	1.12629165	8
GMPS	-0.453550911	-0.692854187	1.146405097	8
GNA13	-0.42398054	-0.718160457	1.142140998	8
GNA14	-0.462731107	-0.684827626	1.147558733	8
GNA15	-0.429027609	-0.713899618	1.142927227	8
GNAT3	-0.392086675	-0.744542134	1.136628809	8
GNB3	-0.46980258	-0.678588804	1.148391384	8
GNB5	-0.172016431	-0.90283341	1.074849841	8

GNG12	-0.468967228	-0.679328349	1.148295577	8
GNG13	-0.384995403	-0.75028218	1.135277583	8
GOLGA6L1	-0.481606389	-0.668065038	1.149671427	8
GOLGA6L9	-0.323289821	-0.79836174	1.121651561	8
GOLIM4	-0.391787372	-0.74478532	1.136572691	8
GOLM1	-0.420685755	-0.720929167	1.141614922	8
GOLPH3	-0.471753194	-0.676859232	1.148612426	8
GOLT1A	-0.176811749	-0.899801191	1.07661294	8
GOLT1B	-0.238990211	-0.858851847	1.097842058	8
GPATCH2	-0.293267519	-0.820576467	1.113843986	8
GPC5	-0.501051741	-0.650423831	1.151475572	8
GPD2	-0.386760645	-0.74885752	1.135618165	8
GPHB5	-0.47599399	-0.673086037	1.149080027	8
GPIHBP1	-0.422379579	-0.719507056	1.141886635	8
GPNMB	-0.49904947	-0.652258065	1.151307536	8
GPR108	-0.19250656	-0.88975175	1.082258309	8
GPR137B	-0.204539655	-0.881916459	1.086456114	8
GPR179	-0.350024547	-0.777936892	1.127961439	8
GPR182	-0.456260177	-0.690493876	1.146754053	8
GPR3	-0.487916664	-0.66238211	1.150298774	8
GPR4	-0.352332904	-0.776144497	1.128477401	8
GPR61	-0.321771334	-0.799503528	1.121274862	8
GPR75-ASB3	-0.489391708	-0.661047938	1.150439646	8
GPR83	-0.455523785	-0.691136122	1.146659907	8

GPR89A	-0.398530603	-0.739286785	1.137817388	8
GPR89B	-0.301797343	-0.814341595	1.116138938	8
GPRASP2	-0.441340684	-0.703404223	1.144744906	8
GPT	-0.452858495	-0.693456282	1.146314777	8
GPX5	-0.385074007	-0.750218801	1.135292808	8
GRAMD2B	-0.313801314	-0.805464425	1.119265739	8
GRAP2	-0.365471164	-0.765854488	1.131325652	8
GRHL3	-0.320952912	-0.800118111	1.121071022	8
GRIK4	-0.478140708	-0.671169229	1.149309936	8
GRK5	-0.313501643	-0.805687509	1.119189152	8
GRPEL1	-0.348847793	-0.778848841	1.127696635	8
GRPEL2	-0.117206079	-0.936232148	1.053438228	8
GSAP	-0.141601174	-0.921651846	1.063253019	8
GSDMC	-0.408085076	-0.731425221	1.139510297	8
GSDME	-0.381745045	-0.752898128	1.134643173	8
GSTA1	-0.482421516	-0.667333193	1.149754709	8
GSTA2	-0.361824252	-0.768725915	1.130550166	8
GSTA3	-0.482916817	-0.666888173	1.149804989	8
GSTCD	-0.396154696	-0.741228829	1.137383524	8
GTDC1	-0.417440039	-0.723646786	1.141086825	8
GTF2H4	-0.409460214	-0.730286872	1.139747085	8
GTF3C4	-0.258488913	-0.845377322	1.103866235	8
GTSE1	-0.49433124	-0.656564134	1.150895374	8
GYS2	-0.398999168	-0.73890318	1.137902348	8

H2AC11	-0.151882442	-0.915370432	1.067252874	8
H2AC17	-0.413141828	-0.727230657	1.140372484	8
H2AC6	-0.467479105	-0.6806441	1.148123205	8
H2AZ2	-0.434601666	-0.709166135	1.143767802	8
H2BC10	-0.475609895	-0.673428514	1.149038409	8
H2BC15	-0.439688073	-0.7048212	1.144509273	8
H2BC18	-0.47052882	-0.677945299	1.148474119	8
H2BC21	-0.157035173	-0.912191738	1.069226911	8
H2BC7	-0.315147451	-0.80446139	1.119608841	8
H3C3	-0.418116931	-0.723080834	1.141197765	8
H3C8	-0.375315526	-0.758044987	1.133360513	8
H4C11	-0.310541296	-0.80788722	1.118428515	8
H4C4	-0.470479932	-0.677988634	1.148468566	8
H4C5	-0.126524036	-0.93071673	1.057240766	8
HABP4	-0.203572327	-0.882550532	1.086122859	8
HACE1	-0.4135468	-0.726893716	1.140440516	8
HAMP	-0.449750148	-0.696153484	1.145903631	8
HAND1	-0.445103986	-0.700167808	1.145271794	8
HAND2	-0.415410541	-0.725341117	1.140751658	8
HAO1	-0.399951874	-0.738122605	1.138074479	8
HBEGF	-0.188588205	-0.892278686	1.080866891	8
HCN2	-0.479544725	-0.669913101	1.149457826	8
HDAC9	-0.407433997	-0.731963584	1.139397582	8
HDX	-0.491429498	-0.659201151	1.150630649	8

HECW2	-0.372512899	-0.760277018	1.132789917	8
HEG1	-0.484041315	-0.665876916	1.149918231	8
HERC3	-0.499812478	-0.651559575	1.151372054	8
HES3	-0.392875278	-0.743901003	1.136776281	8
HESX1	-0.292384153	-0.82121871	1.113602863	8
HEXA	-0.316593261	-0.803382386	1.119975647	8
HEY1	-0.495252991	-0.655724685	1.150977677	8
HEY2	-0.433722129	-0.709914979	1.143637109	8
HGD	-0.489902464	-0.66058545	1.150487914	8
HHATL	-0.109102553	-0.940974954	1.050077507	8
HIBADH	-0.117177809	-0.936248781	1.05342659	8
HIC2	-0.389807771	-0.746391725	1.136199496	8
HIPK4	-0.392530722	-0.744181195	1.136711917	8
HJV	-0.498141751	-0.653088255	1.151230006	8
HLA-A	-0.400860984	-0.737376979	1.138237964	8
HMCN1	-0.481335401	-0.668308192	1.149643593	8
HMGCS1	-0.487606624	-0.662662261	1.150268885	8
HMGN5	-0.494649025	-0.656274821	1.150923847	8
HMMR	-0.483816041	-0.666079606	1.149895646	8
HNRNPCL1	-0.478890897	-0.670498303	1.1493892	8
HNRNPCL2	-0.445938701	-0.699448129	1.14538683	8
HNRNPCL3	-0.493785865	-0.657060407	1.150846272	8
HNRNPDL	-0.112509346	-0.938987124	1.05149647	8
HNRNPH3	-0.482217973	-0.667516002	1.149733975	8

HNRNPLL	-0.403970296	-0.734821129	1.138791425	8
HOOK1	-0.353745435	-0.775045412	1.128790847	8
HOXA13	-0.430994951	-0.712232288	1.143227239	8
HOXA2	-0.388441813	-0.747498116	1.13593993	8
HOXA6	-0.483452229	-0.666406837	1.149859066	8
HOXA7	-0.422140894	-0.719707614	1.141848508	8
HOXB3	-0.347287173	-0.780056426	1.127343599	8
HOXC12	-0.48390222	-0.666002073	1.149904292	8
HOXD1	-0.42094345	-0.720712982	1.141656432	8
HOXD8	-0.441768734	-0.703036782	1.144805516	8
HOXD9	-0.466971493	-0.681092417	1.148063911	8
HPGD	-0.459038041	-0.688066429	1.14710447	8
HRG	-0.364008667	-0.767007399	1.131016066	8
HRH1	-0.410273494	-0.729612816	1.13988631	8
HRNR	-0.33644766	-0.788385928	1.124833588	8
HS2ST1	-0.16294479	-0.908520911	1.071465702	8
HS3ST2	-0.487223287	-0.66300851	1.150231796	8
HSD17B3	-0.464766676	-0.683036758	1.147803434	8
HSD17B6	-0.359255175	-0.770741704	1.129996879	8
HSD17B8	-0.466560011	-0.681455648	1.14801566	8
HSD3B1	-0.480211789	-0.669315613	1.149527402	8
HSDL1	-0.299023768	-0.816375578	1.115399346	8
HSF5	-0.207546999	-0.879940474	1.087487473	8
HSFY1	-0.394929837	-0.742228019	1.137157857	8

HSFY2	-0.395895388	-0.741440475	1.137335863	8
HSPA12B	-0.391563147	-0.744967451	1.136530598	8
HSPA13	-0.198824452	-0.88565205	1.084476502	8
HSPA5	-0.106969268	-0.942215211	1.049184479	8
HSPB2	-0.130524116	-0.928328699	1.058852814	8
HSPB3	-0.392348921	-0.744328991	1.136677912	8
HSPB7	-0.460734104	-0.686580637	1.147314741	8
HSPB8	-0.430673866	-0.712504657	1.143178523	8
HTR3D	-0.311660455	-0.807056485	1.11871694	8
HTR3E	-0.238734725	-0.859026371	1.097761096	8
HTR6	-0.16704638	-0.905957295	1.073003675	8
HTT	-0.371147852	-0.761361634	1.132509486	8
HYI	-0.427363491	-0.715307132	1.142670623	8
ICA1	-0.48101896	-0.668592038	1.149610998	8
ICE1	-0.185885645	-0.89401457	1.079900215	8
IFI27	-0.289807246	-0.823088536	1.112895782	8
IFI27L2	-0.380372539	-0.753999914	1.134372453	8
IFITM5	-0.384530648	-0.750656802	1.13518745	8
IFNA1	-0.289409669	-0.823376532	1.112786201	8
IFNA2	-0.114497422	-0.937823023	1.052320445	8
IFNA7	-0.419533863	-0.721894758	1.141428621	8
IFNK	-0.329757109	-0.793476905	1.123234014	8
IFNL2	-0.435935705	-0.708028932	1.143964638	8
IFNL3	-0.393982469	-0.742999915	1.136982384	8

IFNW1	-0.343496949	-0.782980484	1.126477433	8
IFRD1	-0.296929416	-0.817907214	1.11483663	8
IFT122	-0.480127922	-0.669390757	1.149518679	8
IFT22	-0.475210937	-0.673784088	1.148995025	8
IFT74	-0.46976705	-0.678620273	1.148387323	8
IFTAP	-0.412585931	-0.727692921	1.140278853	8
IGDCC3	-0.492345093	-0.658370007	1.150715101	8
IGF1	-0.497068643	-0.654068622	1.151137265	8
IGFBP5	-0.464714018	-0.683083137	1.147797155	8
IGFBP6	-0.193382471	-0.889185235	1.082567706	8
IGFN1	-0.40795717	-0.731531015	1.139488184	8
IGLL1	-0.471066556	-0.677468488	1.148535044	8
IGSF10	-0.325473214	-0.796716566	1.122189779	8
IHH	-0.458381263	-0.688641033	1.147022295	8
IL11	-0.453826301	-0.692614591	1.146440891	8
IL12B	-0.372908761	-0.759962173	1.132870934	8
IL13RA2	-0.456993795	-0.689853528	1.146847323	8
IL1A	-0.262719485	-0.842413257	1.105132742	8
IL1F10	-0.46714451	-0.680939639	1.148084149	8
IL1R1	-0.229859214	-0.865056901	1.094916115	8
IL2	-0.321208871	-0.799925963	1.121134834	8
IL22RA2	-0.500870953	-0.650589616	1.151460568	8
IL25	-0.438437092	-0.705892089	1.144329181	8
IL3	-0.32942504	-0.793728588	1.123153628	8

IL33	-0.424498113	-0.717724607	1.14222272	8
IL34	-0.301556827	-0.81451823	1.116075057	8
IL36A	-0.178103588	-0.898981269	1.077084857	8
IL36G	-0.433834134	-0.709819658	1.143653792	8
IL37	-0.257316114	-0.846196452	1.103512566	8
IL4R	-0.257180096	-0.84629138	1.103471476	8
IMPG2	-0.248661214	-0.852207002	1.100868215	8
INHBA	-0.449224798	-0.696608427	1.145833225	8
INHBE	-0.314252192	-0.805128636	1.119380828	8
INMT	-0.392197322	-0.744452213	1.136649534	8
INO80D	-0.128442514	-0.929572932	1.058015446	8
INPP5A	-0.204018159	-0.882258386	1.086276545	8
INSIG1	-0.447210061	-0.698350701	1.145560762	8
INSIG2	-0.375107496	-0.758210903	1.133318399	8
INSRR	-0.398182891	-0.739571321	1.137754212	8
INTS2	-0.319184601	-0.801444064	1.120628664	8
IPCEF1	-0.44435777	-0.700810622	1.145168392	8
IPO11	-0.336189473	-0.788583098	1.124772571	8
IQCD	-0.419683345	-0.721769522	1.141452867	8
IQCF1	-0.50182515	-0.649714229	1.151539378	8
IQCF5	-0.423803181	-0.718309755	1.142112936	8
IQCK	-0.484254134	-0.665685386	1.14993952	8
IQGAP3	-0.472202213	-0.676460564	1.148662777	8
IREB2	-0.201002229	-0.88423163	1.085233859	8

IRF6	-0.305851253	-0.811357143	1.117208395	8
IRF8	-0.2945581	-0.819636998	1.114195098	8
IRS1	-0.205377197	-0.881366865	1.086744062	8
IRX1	-0.477342873	-0.671882152	1.149225024	8
IRX3	-0.462004769	-0.68546567	1.147470439	8
IRX6	-0.462862297	-0.684712328	1.147574626	8
ISCA1	-0.254141063	-0.848408448	1.102549511	8
ITGA1	-0.267252606	-0.839221082	1.106473687	8
ITGA8	-0.482242828	-0.667493681	1.149736509	8
ITGA9	-0.343094018	-0.783290606	1.126384624	8
ITGAV	-0.378473839	-0.755521338	1.133995177	8
ITGB3BP	-0.452093537	-0.69412092	1.146214457	8
ITGB4	-0.451843917	-0.694337682	1.146181599	8
ITGB6	-0.420257256	-0.721288507	1.141545763	8
ITGBL1	-0.419470872	-0.721947525	1.141418398	8
ITIH5	-0.465844286	-0.682087052	1.147931337	8
ITPR2	-0.346799329	-0.780433481	1.12723281	8
ITPRID2	-0.299963518	-0.815687137	1.115650655	8
ITSN1	-0.458214786	-0.688786613	1.1470014	8
IVNS1ABP	-0.444362165	-0.700806837	1.145169002	8
JADE2	-0.236725744	-0.8603969	1.097122644	8
JADE3	-0.414429775	-0.726158549	1.140588324	8
JAG2	-0.487145714	-0.663078559	1.150224273	8
JAKMIP2	-0.341288326	-0.784678667	1.125966993	8

JAKMIP3	-0.362686148	-0.768048346	1.130734494	8
JARID2	-0.290890046	-0.822303517	1.113193562	8
JCAD	-0.398808839	-0.739059023	1.137867862	8
JMY	-0.384228876	-0.750899946	1.135128822	8
JPT2	-0.150169986	-0.91642231	1.066592296	8
KANK2	-0.486646882	-0.663528868	1.150175749	8
KANSL1L	-0.372523294	-0.760268752	1.132792046	8
KCNA1	-0.425110075	-0.717208948	1.142319023	8
KCNA2	-0.109816183	-0.940559289	1.050375471	8
KCNAB1	-0.3525463	-0.775978565	1.128524866	8
KCNH8	-0.311853268	-0.806913257	1.118766524	8
KCNJ11	-0.403081363	-0.735552729	1.138634093	8
KCNJ12	-0.500110423	-0.651286662	1.151397085	8
KCNJ8	-0.433898988	-0.709764459	1.143663447	8
KCNK1	-0.319277394	-0.801374549	1.120651943	8
KCNK16	-0.224556398	-0.868629961	1.093186359	8
KCNK18	-0.492880693	-0.657883415	1.150764109	8
KCNK2	-0.480643111	-0.668929042	1.149572153	8
KCNK4	-0.382390853	-0.752379121	1.134769974	8
KCNK9	-0.479871598	-0.669620377	1.149491975	8
KCNMB4	-0.43539156	-0.708492993	1.143884552	8
KCNS3	-0.481662645	-0.668014551	1.149677196	8
KCTD1	-0.424920807	-0.717368468	1.142289275	8
KCTD15	-0.317766318	-0.802505641	1.120271959	8

KCTD17	-0.123228376	-0.932675044	1.055903419	8
KDELR3	-0.494286682	-0.656604692	1.150891374	8
KDM4D	-0.477652422	-0.671605622	1.149258044	8
KDM5D	-0.372428835	-0.760343859	1.132772695	8
KDR	-0.429406968	-0.713578392	1.14298536	8
KIAA1217	-0.470721539	-0.677774448	1.148495986	8
KIAA1522	-0.472451811	-0.676238869	1.14869068	8
KIF11	-0.472839706	-0.675894215	1.148733921	8
KIF13B	-0.414206243	-0.72634473	1.140550973	8
KIF15	-0.458642087	-0.688412892	1.147054979	8
KIF16B	-0.446362429	-0.699082542	1.145444971	8
KIF17	-0.453254184	-0.693112264	1.146366448	8
KIF1C	-0.245865496	-0.854135632	1.100001128	8
KIF23	-0.478117681	-0.671189814	1.149307495	8
KIF26A	-0.402987252	-0.735630142	1.138617394	8
KIF2A	-0.374068112	-0.759039295	1.133107407	8
KIF2C	-0.49635176	-0.654722895	1.151074655	8
KIFAP3	-0.499199229	-0.652121017	1.151320246	8
KIT	-0.392147689	-0.74449255	1.136640239	8
KL	-0.309735072	-0.808485016	1.118220088	8
KLHDC3	-0.428555632	-0.714299081	1.142854712	8
KLHL15	-0.247458849	-0.853037226	1.100496074	8
KLHL38	-0.454758154	-0.691803313	1.146561467	8
KLHL41	-0.339237063	-0.786252099	1.125489161	8

KLHL5	-0.389212419	-0.746874151	1.13608657	8
KLHL8	-0.465139286	-0.682708503	1.147847788	8
KLK1	-0.45798356	-0.688988772	1.146972332	8
KLK14	-0.385306106	-0.750031626	1.135337732	8
KLK4	-0.483662597	-0.666217637	1.149880234	8
KLK7	-0.236981214	-0.860222798	1.097204012	8
KLK8	-0.341446143	-0.784557462	1.126003605	8
KMT5A	-0.47302412	-0.675730307	1.148754427	8
KNL1	-0.483037817	-0.666779418	1.149817235	8
KPNA3	-0.376551596	-0.757058361	1.133609957	8
KPRP	-0.424471487	-0.717747034	1.142218522	8
KRT16	-0.317887278	-0.802415169	1.120302448	8
KRT19	-0.445466663	-0.699855195	1.145321858	8
KRT3	-0.494807121	-0.656130852	1.150937973	8
KRT34	-0.465638169	-0.682268792	1.147906961	8
KRT35	-0.442214951	-0.702653561	1.144868512	8
KRT37	-0.434280973	-0.70943926	1.143720233	8
KRT38	-0.299504614	-0.816023412	1.115528026	8
KRT4	-0.287767645	-0.824564594	1.11233224	8
KRT5	-0.351715554	-0.776624311	1.128339864	8
KRT6A	-0.319719596	-0.801043181	1.120762777	8
KRT6B	-0.351992301	-0.77640926	1.12840156	8
KRT71	-0.475610666	-0.673427826	1.149038492	8
KRT78	-0.467932325	-0.680243608	1.148175933	8

KRT82	-0.397438521	-0.740180078	1.137618598	8
KRT85	-0.412527193	-0.72774175	1.140268942	8
KRT9	-0.462780065	-0.6847846	1.147564666	8
KRTAP1-3	-0.474104968	-0.674768968	1.148873936	8
KRTAP12-2	-0.234163184	-0.862140396	1.09630358	8
KRTAP13-1	-0.402055074	-0.73639648	1.138451554	8
KRTAP13-4	-0.483444174	-0.66641408	1.149858254	8
KRTAP19-3	-0.230689198	-0.864495628	1.095184826	8
KRTAP19-6	-0.405250605	-0.733766155	1.139016759	8
KRTAP19-8	-0.265244289	-0.840637379	1.105881668	8
KRTAP20-1	-0.434898242	-0.708913464	1.143811706	8
KRTAP20-2	-0.351044776	-0.777145274	1.12819005	8
KRTAP20-3	-0.443989808	-0.7011274	1.145117208	8
KRTAP23-1	-0.229989282	-0.86496898	1.094958262	8
KRTAP26-1	-0.340974971	-0.784919262	1.125894232	8
KRTAP3-3	-0.321909966	-0.799399369	1.121309334	8
KRTAP4-11	-0.42914097	-0.713803643	1.142944613	8
KRTAP4-3	-0.246830177	-0.853470856	1.100301032	8
KRTAP4-5	-0.271853391	-0.835964142	1.107817533	8
KRTAP4-7	-0.477682649	-0.671578614	1.149261263	8
KRTAP4-9	-0.487245721	-0.66298825	1.150233971	8
KRTAP5-10	-0.302792864	-0.813609973	1.116402837	8
KRTAP5-6	-0.467225193	-0.680868385	1.148093577	8
KRTAP6-1	-0.481025089	-0.668586541	1.14961163	8

KRTAP6-2	-0.428826063	-0.714070224	1.142896287	8
KRTAP6-3	-0.424706701	-0.717548883	1.142255584	8
KRTAP9-3	-0.484145029	-0.665783582	1.149928612	8
KTN1	-0.130434654	-0.92838224	1.058816895	8
L1TD1	-0.36013177	-0.770054545	1.130186315	8
L3MBTL4	-0.44872137	-0.697044139	1.145765509	8
LACC1	-0.297683566	-0.817356111	1.115039677	8
LAMA2	-0.445707567	-0.699647476	1.145355043	8
LAMA4	-0.422355737	-0.719527092	1.141882829	8
LAMC1	-0.471271388	-0.677286789	1.148558177	8
LARGE1	-0.469532378	-0.678828092	1.14836047	8
LARP1B	-0.47415089	-0.674728097	1.148878988	8
LAT	-0.249005514	-0.85196905	1.100974564	8
LAYN	-0.335010984	-0.789482351	1.124493334	8
LCA5L	-0.356546953	-0.772860427	1.12940738	8
LCE1B	-0.38434338	-0.750807697	1.135151077	8
LCE1F	-0.460065756	-0.687166459	1.147232216	8
LCE2A	-0.401795745	-0.736609532	1.138405278	8
LCE2B	-0.346863427	-0.780383951	1.127247378	8
LCN15	-0.197396069	-0.886581669	1.083977738	8
LCN6	-0.378224471	-0.755720918	1.133945389	8
LCN9	-0.347592862	-0.779820055	1.127412917	8
LCNL1	-0.417899305	-0.723262838	1.141162143	8
LCP2	-0.149290529	-0.91696164	1.066252169	8

LDB2	-0.435599184	-0.708315959	1.143915143	8
LDLRAD2	-0.303453634	-0.813123906	1.116577541	8
LDLRAP1	-0.155219706	-0.913314025	1.068533731	8
LENEP	-0.444871865	-0.700367821	1.145239686	8
LEPROT	-0.17789383	-0.899114489	1.07700832	8
LGI2	-0.501277797	-0.650216488	1.151494285	8
LGI4	-0.486027978	-0.66408722	1.150115198	8
LHFPL1	-0.311694057	-0.807031526	1.118725583	8
LHX6	-0.346399955	-0.780742004	1.127141959	8
LHX8	-0.421412104	-0.720319661	1.141731765	8
LIF	-0.462730956	-0.684827759	1.147558715	8
LIFR	-0.488811514	-0.661572984	1.150384497	8
LIMA1	-0.309594435	-0.808589239	1.118183674	8
LIMCH1	-0.439246537	-0.705199342	1.144445878	8
LIMS2	-0.469068382	-0.679238833	1.148307215	8
LIN28A	-0.484214023	-0.665721489	1.149935511	8
LIN54	-0.485990436	-0.664121076	1.150111513	8
LIN7C	-0.434433457	-0.709309407	1.143742863	8
LIN9	-0.466268819	-0.681712595	1.147981414	8
LMAN1L	-0.218798756	-0.872484183	1.09128294	8
LMBR1	-0.330521628	-0.792897101	1.123418729	8
LMBR1L	-0.471903022	-0.676726227	1.148629249	8
LMCD1	-0.454435467	-0.692084341	1.146519808	8
LMNB2	-0.469081496	-0.679227227	1.148308723	8

LMO2	-0.112229018	-0.939151026	1.051380044	8
LMOD1	-0.468355073	-0.679869861	1.148224934	8
LNP1	-0.333979656	-0.790268336	1.124247992	8
LNX1	-0.379633981	-0.7545921	1.134226081	8
LNX2	-0.350933865	-0.777231376	1.128165241	8
LOC100653049	-0.462040172	-0.685434583	1.147474755	8
LOC100653133	-0.275066366	-0.833679395	1.10874576	8
LOC100996750	-0.104935485	-0.943394401	1.048329885	8
LOC101929773	-0.436340576	-0.707683467	1.144024043	8
LOC101929937	-0.422409325	-0.719482058	1.141891383	8
LOC102723623	-0.461471261	-0.685933998	1.147405258	8
LOC105372440	-0.204334307	-0.882051124	1.086385431	8
LOC107985556	-0.471194162	-0.677355298	1.14854946	8
LOC112268119	-0.329871611	-0.7933901	1.12326171	8
LOC112268437	-0.492542853	-0.658190376	1.15073323	8
LOC112577516	-0.227252184	-0.866816325	1.094068509	8
LOC112694756	-0.114321851	-0.937925948	1.052247799	8
LOC389895	-0.461242409	-0.686134805	1.147377214	8
LOC401040	-0.336945212	-0.788005801	1.124951013	8
LOXHD1	-0.396174633	-0.741212553	1.137387186	8
LOXL2	-0.355659152	-0.773553589	1.12921274	8
LPA	-0.381726783	-0.752912798	1.134639582	8
LPAR4	-0.499134835	-0.652179949	1.151314784	8
LPAR5	-0.360702993	-0.769606404	1.130309397	8

LPL	-0.430087654	-0.713001677	1.143089331	8
LRCH1	-0.341780298	-0.784300759	1.126081057	8
LRGUK	-0.490054	-0.660448184	1.150502183	8
LRIT2	-0.491923438	-0.658752876	1.150676314	8
LRP12	-0.15546593	-0.913161963	1.068627893	8
LRP8	-0.322858073	-0.79868658	1.121544654	8
LRPAP1	-0.37450582	-0.758690557	1.133196377	8
LRRC1	-0.448823274	-0.696955962	1.145779235	8
LRRC15	-0.458647105	-0.688408502	1.147055608	8
LRRC28	-0.467327726	-0.680777823	1.148105549	8
LRRC30	-0.403772329	-0.73498412	1.138756449	8
LRRC3C	-0.392275033	-0.744389051	1.136664084	8
LRRC43	-0.482374766	-0.667375184	1.14974995	8
LRRC52	-0.401852747	-0.736562708	1.138415455	8
LRRC58	-0.429713242	-0.713318954	1.143032195	8
LRRC8D	-0.145622813	-0.919204469	1.064827282	8
LRRIQ3	-0.387448261	-0.748301816	1.135750077	8
LRRIQ4	-0.493319597	-0.657484455	1.150804052	8
LSM8	-0.346629445	-0.780564736	1.127194181	8
LUM	-0.352563228	-0.775965401	1.128528629	8
LURAP1	-0.210259023	-0.87815242	1.088411443	8
LY6D	-0.143968634	-0.920212626	1.06418126	8
LY6E	-0.292356528	-0.821238784	1.113595312	8
LY6G5B	-0.208334156	-0.879422094	1.08775625	8

LY6G6F	-0.500599359	-0.650838607	1.151437966	8
LYNX1	-0.314682249	-0.804808194	1.119490443	8
LYRM1	-0.499481936	-0.651862241	1.151344177	8
LYSMD3	-0.406000753	-0.733147337	1.13914809	8
LYVE1	-0.246283967	-0.853847349	1.100131316	8
LZIC	-0.336950511	-0.788001751	1.124952263	8
M6PR	-0.452157084	-0.694065729	1.146222812	8
MACC1	-0.432281106	-0.711140302	1.143421408	8
MACIR	-0.477045816	-0.672147431	1.149193247	8
MACROD2	-0.246130329	-0.853953205	1.100083535	8
MAG	-0.422944286	-0.719032343	1.141976629	8
MAGEA1	-0.216509166	-0.874009554	1.090518721	8
MAGEA2	-0.4755029	-0.67352389	1.149026789	8
MAGEA2B	-0.479955983	-0.66954479	1.149500773	8
MAGEA6	-0.325979306	-0.796334651	1.122313956	8
MAGEB18	-0.457353284	-0.689539553	1.146892837	8
MAGEB4	-0.279218098	-0.830714578	1.109932676	8
MAGEC3	-0.436524511	-0.707526469	1.144050981	8
MAGI1	-0.501748446	-0.649784632	1.151533078	8
MAGOHB	-0.234690021	-0.86178238	1.0964724	8
MALRD1	-0.47242258	-0.676264835	1.148687415	8
MALSU1	-0.247777624	-0.852817227	1.100594851	8
MAN1A2	-0.463056869	-0.684541297	1.147598165	8
MAOA	-0.420064096	-0.721450436	1.141514531	8

MAP3K13	-0.234285692	-0.862057165	1.096342857	8
MAP3K4	-0.450369632	-0.69561668	1.145986313	8
MAP3K8	-0.252572814	-0.849498011	1.102070825	8
MAP4K3	-0.456355255	-0.690410915	1.14676617	8
MAP7	-0.441465317	-0.703297255	1.144762572	8
MAPK6	-0.311268926	-0.807347231	1.118616156	8
MAPK8	-0.246707662	-0.853555324	1.100262986	8
MAT1A	-0.399594127	-0.738415813	1.138009939	8
MATN1	-0.262349037	-0.842673384	1.105022421	8
MAX	-0.188999046	-0.892014299	1.081013345	8
MB	-0.448937758	-0.696856887	1.145794645	8
MBNL1	-0.158003103	-0.911592344	1.069595446	8
MBNL2	-0.490910411	-0.659671982	1.150582393	8
MBOAT2	-0.449571536	-0.696308188	1.145879724	8
MBTD1	-0.413908644	-0.726592531	1.140501174	8
MC1R	-0.459392579	-0.687756076	1.147148655	8
MC3R	-0.498605629	-0.652664103	1.151269732	8
MC5R	-0.431753998	-0.711588022	1.143342019	8
MCCD1	-0.415645252	-0.725145363	1.140790615	8
MCF2L	-0.479302836	-0.670129651	1.149432487	8
MCPH1	-0.330001764	-0.793291415	1.123293179	8
MDGA1	-0.487482016	-0.662774829	1.150256845	8
MDN1	-0.103277026	-0.944353652	1.047630677	8
MED14	-0.289286814	-0.823465499	1.112752313	8

MED20	-0.501003371	-0.65046819	1.151471561	8
MED27	-0.352162155	-0.776277239	1.128439394	8
MEDAG	-0.448155035	-0.697534005	1.14568904	8
MEF2A	-0.318960639	-0.801611811	1.12057245	8
MEF2C	-0.399417049	-0.738560902	1.13797795	8
MEFV	-0.18937184	-0.891774282	1.081146122	8
MEI4	-0.324433533	-0.797500464	1.121933997	8
MEIG1	-0.399498157	-0.738494449	1.137992606	8
MELTF	-0.457085946	-0.689773056	1.146859002	8
MEOX1	-0.460370426	-0.686899463	1.147269889	8
MEOX2	-0.43609933	-0.707889334	1.143988665	8
MEP1A	-0.479377686	-0.670062648	1.149440334	8
MEPCE	-0.289425578	-0.82336501	1.112790589	8
MEPE	-0.2223305	-0.870123118	1.092453618	8
MERTK	-0.293886021	-0.820126406	1.114012426	8
MET	-0.446712671	-0.698780228	1.145492899	8
METTL22	-0.147245094	-0.918213711	1.065458804	8
METTL25	-0.497980658	-0.653235501	1.151216159	8
MEX3D	-0.380917687	-0.753562494	1.134480182	8
MFAP3L	-0.41438274	-0.726197728	1.140580468	8
MFAP5	-0.276729155	-0.832493667	1.109222822	8
MFSD4A	-0.381506312	-0.753089893	1.134596205	8
MFSD6	-0.404351577	-0.73450711	1.138858687	8
MGARP	-0.435059392	-0.708776136	1.143835528	8

MGP	-0.412561095	-0.727713567	1.140274663	8
MGST2	-0.37077717	-0.76165588	1.132433049	8
MIB1	-0.436010016	-0.707965537	1.143975553	8
MICAL3	-0.463698787	-0.683976778	1.147675565	8
MICU1	-0.234999767	-0.861571785	1.096571552	8
MID2	-0.299297485	-0.816175135	1.11547262	8
MIGA2	-0.486184422	-0.663946118	1.15013054	8
MINAR1	-0.412462432	-0.727795581	1.140258012	8
MKI67	-0.464892039	-0.682926333	1.147818372	8
MKX	-0.44605825	-0.699345001	1.145403251	8
MLEC	-0.486300214	-0.663841666	1.15014188	8
MLLT10	-0.360563672	-0.769715732	1.130279404	8
MLNR	-0.47767403	-0.671586316	1.149260346	8
MMP1	-0.459863596	-0.687343572	1.147207168	8
MMP2	-0.497694033	-0.653497423	1.151191456	8
MMP20	-0.474435188	-0.674475029	1.148910217	8
MMP7	-0.482161882	-0.667566372	1.149728254	8
MMRN2	-0.437772012	-0.70646082	1.144232832	8
MMS22L	-0.391732692	-0.744829738	1.136562431	8
MOB1A	-0.37166346	-0.760952144	1.132615604	8
MOB2	-0.39468743	-0.742425605	1.137113035	8
MOB3B	-0.455797992	-0.690897033	1.146695025	8
MOCS2	-0.370319954	-0.762018649	1.132338603	8
MORC1	-0.500221291	-0.651185085	1.151406377	8

MORC3	-0.419589945	-0.721847774	1.14143772	8
MORN2	-0.475371282	-0.673641199	1.14901248	8
MOSPD1	-0.42020712	-0.72133054	1.14153766	8
MOXD1	-0.468041294	-0.680147286	1.14818858	8
MPC1L	-0.411634663	-0.728483304	1.140117967	8
MPEG1	-0.385696401	-0.749716766	1.135413166	8
MPL	-0.241578529	-0.857080798	1.098659328	8
MPP4	-0.496769747	-0.654341477	1.151111224	8
MRAP	-0.486709539	-0.663472319	1.150181858	8
MRAS	-0.439044992	-0.705371888	1.14441688	8
MRC1	-0.312122066	-0.806713532	1.118835597	8
MRGPRX1	-0.425194963	-0.717137391	1.142332354	8
MRO	-0.382405156	-0.752367622	1.134772778	8
MRPL1	-0.153987414	-0.914074357	1.068061771	8
MRPL42	-0.158175679	-0.911485399	1.069661078	8
MRS2	-0.180769644	-0.897285038	1.078054682	8
MRTFB	-0.412772062	-0.727538173	1.140310235	8
MS4A3	-0.3713456	-0.761204612	1.132550212	8
MSANTD1	-0.483738882	-0.666149017	1.149887899	8
MSH4	-0.464721135	-0.683076869	1.147798004	8
MSMB	-0.366573229	-0.76498447	1.1315577	8
MST1R	-0.385205924	-0.750112423	1.135318347	8
MSTN	-0.347646814	-0.779778328	1.127425142	8
MT1G	-0.437521131	-0.706675248	1.144196379	8

MT1H	-0.41997751	-0.72152301	1.14150052	8
MT1M	-0.34474531	-0.782018776	1.126764086	8
MTA3	-0.42585884	-0.716577539	1.142436379	8
MTARC2	-0.445576274	-0.69976069	1.145336964	8
MTCL1	-0.494874514	-0.656069473	1.150943987	8
MTFR1	-0.494194114	-0.656688942	1.150883057	8
MTFR1L	-0.142643742	-0.921018581	1.063662323	8
MTFR2	-0.184722971	-0.894759615	1.079482586	8
MTHFD2L	-0.380647264	-0.753779512	1.134426775	8
MTM1	-0.282564328	-0.828314685	1.110879013	8
MTMR2	-0.431269868	-0.711999006	1.143268873	8
MTMR7	-0.499458681	-0.651883531	1.151342212	8
MTPN	-0.500599758	-0.650838241	1.151437999	8
MTSS1	-0.113437823	-0.938443837	1.05188166	8
MTSS2	-0.478971236	-0.67042642	1.149397656	8
MTTP	-0.469656474	-0.678718203	1.148374677	8
MTUS1	-0.43241147	-0.711029532	1.143441002	8
MTX2	-0.292065183	-0.821450455	1.113515639	8
MUC15	-0.468742486	-0.679527199	1.148269684	8
MUC17	-0.350793749	-0.777340135	1.128133884	8
MUC19	-0.328511805	-0.794420265	1.122932071	8
MUC2	-0.436154708	-0.707842082	1.143996791	8
MUC20	-0.366086502	-0.765368845	1.131455348	8
MUC21	-0.368705365	-0.763298237	1.132003602	8

MUC3A	-0.394065717	-0.742932118	1.136997836	8
MUC4	-0.43613494	-0.70785895	1.14399389	8
MUC6	-0.47559569	-0.673441177	1.149036867	8
MUC7	-0.439684483	-0.704824274	1.144508758	8
MUCL1	-0.493185015	-0.65760681	1.150791825	8
MUCL3	-0.479849047	-0.669640575	1.149489622	8
MUSTN1	-0.478066723	-0.671235366	1.149302089	8
MXRA8	-0.474079241	-0.674791864	1.148871104	8
MYB	-0.301185358	-0.814790941	1.1159763	8
MYBPC2	-0.29104515	-0.822190989	1.113236138	8
MYCBP2	-0.136173926	-0.924934938	1.061108864	8
MYEF2	-0.501430418	-0.650076472	1.15150689	8
MYF5	-0.445750102	-0.699610795	1.145360896	8
MYF6	-0.306832476	-0.81063271	1.117465186	8
MYH14	-0.415772466	-0.725039243	1.140811708	8
MYH15	-0.3930865	-0.743729185	1.136815685	8
MYL10	-0.48334963	-0.666499095	1.149848725	8
MYL2	-0.467544982	-0.6805859	1.148130882	8
MYL3	-0.404251293	-0.734589715	1.138841008	8
MYL6B	-0.350952145	-0.777217185	1.128169331	8
MYLK2	-0.39170381	-0.7448532	1.13655701	8
MYLK3	-0.321025461	-0.800063654	1.121089115	8
MYO10	-0.476997	-0.672191017	1.149188017	8
MYO1B	-0.493052733	-0.657727056	1.150779789	8

MYO5C	-0.44537321	-0.69993576	1.14530897	8
MYO9A	-0.347735125	-0.779710023	1.127445148	8
MYOM1	-0.121592881	-0.933643793	1.055236674	8
MYOM3	-0.489643469	-0.660820002	1.150463471	8
MYRIP	-0.413986676	-0.726527564	1.14051424	8
MYSM1	-0.233112945	-0.862853435	1.09596638	8
MYZAP	-0.497689459	-0.653501602	1.151191061	8
MZT1	-0.368302618	-0.763617063	1.131919681	8
N4BP2	-0.494814062	-0.656124531	1.150938593	8
N4BP2L1	-0.266573486	-0.839700376	1.106273861	8
N4BP3	-0.498892214	-0.652401951	1.151294165	8
NAA16	-0.195962703	-0.887512923	1.083475625	8
NAA30	-0.261213155	-0.843470301	1.104683456	8
NAA60	-0.493575291	-0.657251941	1.150827232	8
NAALAD2	-0.432651585	-0.710825465	1.14347705	8
NABP2	-0.256241408	-0.846946092	1.103187499	8
NAGA	-0.479153875	-0.670262978	1.149416854	8
NANOS1	-0.16978181	-0.904240331	1.074022141	8
NANOS3	-0.379466203	-0.754726559	1.134192762	8
NAPB	-0.104436627	-0.943683159	1.048119786	8
NAT2	-0.408445862	-0.731126728	1.139572589	8
NAT8	-0.469455823	-0.678895875	1.148351698	8
NBAS	-0.120093278	-0.934530258	1.054623535	8
NCAPG	-0.462390982	-0.68512647	1.147517451	8

NCAPH	-0.499347915	-0.651984928	1.151332842	8
NCEH1	-0.30717509	-0.81037957	1.11755466	8
NCOA7	-0.481169375	-0.668457129	1.149626504	8
NCR1	-0.431681444	-0.711649628	1.143331071	8
NCSTN	-0.185022663	-0.894567673	1.079590336	8
ND4L	-0.131439151	-0.927780707	1.059219858	8
ND5	-0.172480728	-0.902540602	1.07502133	8
ND6	-0.374533603	-0.758668416	1.133202019	8
NDRG2	-0.157664389	-0.911802176	1.069466564	8
NDST1	-0.239781229	-0.858311165	1.098092394	8
NDUFA1	-0.397499354	-0.740130346	1.1376297	8
NDUFA5	-0.499817034	-0.651555403	1.151372437	8
NDUFAF2	-0.441256137	-0.703476777	1.144732914	8
NDUFAF3	-0.318335713	-0.802079656	1.120415369	8
NDUFB1	-0.444690424	-0.700524129	1.145214553	8
NDUFS4	-0.138180542	-0.923723711	1.061904253	8
NDUFV2	-0.377296378	-0.756463223	1.133759602	8
NEBL	-0.485017296	-0.664998193	1.150015489	8
NEGR1	-0.485420519	-0.664634874	1.150055392	8
NEK11	-0.398523876	-0.73929229	1.137816167	8
NEK7	-0.310032899	-0.808264247	1.118297147	8
NEO1	-0.501243227	-0.6502482	1.151491427	8
NES	-0.454868586	-0.691707115	1.146575701	8
NET1	-0.440666929	-0.703982225	1.144649154	8

NETO1	-0.389694042	-0.746483906	1.136177949	8
NETO2	-0.382585692	-0.752222465	1.134808156	8
NEU2	-0.464647222	-0.683141964	1.147789186	8
NF1	-0.350747393	-0.777376113	1.128123506	8
NFAT5	-0.414023023	-0.726497301	1.140520324	8
NFIB	-0.477382245	-0.671846984	1.149229229	8
NFYB	-0.211393994	-0.877402405	1.088796399	8
NGB	-0.474409286	-0.674498089	1.148907375	8
NIBAN2	-0.220012457	-0.871673908	1.091686364	8
NIPA1	-0.368625547	-0.763361435	1.131986982	8
NIPAL1	-0.253334988	-0.848968728	1.102303716	8
NIPSNAP3A	-0.185782122	-0.894080951	1.079863073	8
NKAIN2	-0.498068461	-0.653155248	1.15122371	8
NKD1	-0.452480735	-0.693784572	1.146265306	8
NKX2-3	-0.489612869	-0.66084771	1.150460579	8
NLGN4Y	-0.489501152	-0.660948859	1.150450011	8
NLRP9	-0.4330862	-0.71045596	1.14354216	8
NME7	-0.43599647	-0.707977093	1.143973563	8
NNMT	-0.406734095	-0.732541886	1.139275981	8
NOS1	-0.379920335	-0.754362555	1.13428289	8
NOSTRIN	-0.429936409	-0.713129857	1.143066267	8
NOTUM	-0.501245047	-0.650246531	1.151491577	8
NOVA2	-0.49840288	-0.652849516	1.151252396	8
NOX1	-0.357859668	-0.77183425	1.129693918	8

NOX4	-0.478369654	-0.670964532	1.149334186	8
NPAP1	-0.458196828	-0.688802316	1.146999144	8
NPHP1	-0.352567345	-0.775962199	1.128529544	8
NPIPA8	-0.148617508	-0.917373971	1.065991479	8
NPNT	-0.490146274	-0.660364587	1.150510861	8
NPPB	-0.400377794	-0.737773371	1.138151165	8
NPR1	-0.432476778	-0.710974034	1.143450812	8
NPR3	-0.442232082	-0.702638845	1.144870927	8
NPVF	-0.463908816	-0.683791985	1.147700802	8
NPW	-0.393735405	-0.743201084	1.136936489	8
NPY1R	-0.400518706	-0.737657794	1.1381765	8
NPY2R	-0.426981245	-0.715630071	1.142611316	8
NQO1	-0.138428696	-0.923573707	1.062002403	8
NR1I2	-0.485185891	-0.664846302	1.150032193	8
NR3C2	-0.230120699	-0.864880132	1.095000831	8
NR4A3	-0.501852215	-0.649689385	1.1515416	8
NR5A1	-0.414602448	-0.726014697	1.140617145	8
NR5A2	-0.437766041	-0.706465924	1.144231966	8
NRAS	-0.211238597	-0.877505155	1.088743752	8
NRK	-0.495937443	-0.655100789	1.151038231	8
NRN1	-0.462874588	-0.684701525	1.147576114	8
NRP1	-0.463258218	-0.684364268	1.147622487	8
NSD1	-0.259822889	-0.844444266	1.104267154	8
NT5C1B-RDH14	-0.501014338	-0.650458133	1.151472471	8

NT5C3A	-0.469548353	-0.678813946	1.148362299	8
NTF4	-0.490834247	-0.659741043	1.15057529	8
NTN4	-0.481661938	-0.668015186	1.149677124	8
NTN5	-0.420155516	-0.721373801	1.141529317	8
NUAK1	-0.436396226	-0.707635971	1.144032197	8
NUAK2	-0.501423977	-0.650082382	1.151506358	8
NUBPL	-0.152340154	-0.915088899	1.067429053	8
NUCB1	-0.242867705	-0.85619668	1.099064385	8
NUDT12	-0.454175404	-0.692310757	1.146486161	8
NUDT21	-0.342585348	-0.783681912	1.12626726	8
NUDT6	-0.238656248	-0.859079969	1.097736217	8
NUF2	-0.462257576	-0.685243652	1.147501229	8
NUP54	-0.164642111	-0.907461613	1.072103724	8
NUPR1	-0.41451102	-0.726090868	1.140601888	8
NUTM1	-0.474571514	-0.67435365	1.148925164	8
NXF3	-0.483670884	-0.666210183	1.149881067	8
NXPH1	-0.496090664	-0.654961058	1.151051722	8
NXT2	-0.401448417	-0.736894785	1.138343202	8
OCRL	-0.39235672	-0.744322652	1.136679372	8
ODC1	-0.390423162	-0.745892726	1.136315888	8
ODR4	-0.18593389	-0.893983632	1.079917522	8
OIP5	-0.454590146	-0.691949644	1.14653979	8
OIT3	-0.420659678	-0.720951041	1.141610718	8
OLFML2A	-0.443018488	-0.701962989	1.144981477	8

OLFML3	-0.471605697	-0.676990146	1.148595842	8
OLIG3	-0.501466722	-0.650043162	1.151509884	8
ONECUT1	-0.500591605	-0.650845715	1.15143732	8
ONECUT2	-0.500784312	-0.650669055	1.151453366	8
OPA1	-0.392828635	-0.743938939	1.136767574	8
OPN1SW	-0.450877069	-0.695176695	1.146053764	8
OPRPN	-0.345694482	-0.781286656	1.126981138	8
OR10AG1	-0.330530597	-0.792890296	1.123420893	8
OR10C1	-0.426951415	-0.715655266	1.142606682	8
OR10G4	-0.184233365	-0.895073041	1.079306406	8
OR10G8	-0.381718909	-0.752919124	1.134638033	8
OR10G9	-0.492033354	-0.658653088	1.150686442	8
OR10H2	-0.113446143	-0.938438965	1.051885108	8
OR10P1	-0.436098604	-0.707889954	1.143988558	8
OR10R2	-0.470624273	-0.677860681	1.148484954	8
OR10S1	-0.413222985	-0.727163145	1.14038613	8
OR10X1	-0.306488697	-0.810886612	1.117375309	8
OR11H1	-0.403096102	-0.735540605	1.138636707	8
OR11H12	-0.47117418	-0.677373024	1.148547204	8
OR14C36	-0.42329828	-0.718734612	1.142032891	8
OR1C1	-0.272097023	-0.835791191	1.107888214	8
OR1N2	-0.408980144	-0.730684474	1.139664618	8
OR1Q1	-0.425949978	-0.71650065	1.142450627	8
OR2A12	-0.463468528	-0.68417932	1.147647848	8

OR2A14	-0.497043959	-0.654091159	1.151135118	8
OR2AE1	-0.149164114	-0.917039116	1.06620323	8
OR2AP1	-0.250148983	-0.851178096	1.101327079	8
OR2B2	-0.15634408	-0.912619258	1.068963338	8
OR2F2	-0.262551532	-0.842531206	1.105082739	8
OR2H2	-0.111210828	-0.939745838	1.050956665	8
OR2J3	-0.468743027	-0.67952672	1.148269747	8
OR2K2	-0.362717403	-0.768023763	1.130741166	8
OR2L3	-0.274315694	-0.834213954	1.108529648	8
OR2M2	-0.377699639	-0.756140782	1.133840421	8
OR2M5	-0.451162053	-0.694929485	1.146091538	8
OR2M7	-0.488127271	-0.662191751	1.150319022	8
OR2T1	-0.3778775	-0.755998521	1.133876021	8
OR2T2	-0.386763825	-0.748854952	1.135618776	8
OR2T35	-0.456317441	-0.690443911	1.146761352	8
OR2V2	-0.481406124	-0.66824474	1.149650865	8
OR4B1	-0.36314741	-0.767685463	1.130832874	8
OR4C15	-0.494667227	-0.656258247	1.150925474	8
OR4C5	-0.44933029	-0.696517094	1.145847384	8
OR4D2	-0.458520948	-0.688518859	1.147039808	8
OR4N4C	-0.470730645	-0.677766374	1.148497019	8
OR4P4	-0.323411093	-0.798270468	1.121681561	8
OR4Q3	-0.278384839	-0.831310755	1.109695594	8
OR4S1	-0.498021909	-0.653197798	1.151219707	8

OR4S2	-0.443138706	-0.701859619	1.144998325	8
OR51A7	-0.247748674	-0.85283721	1.100585884	8
OR51E1	-0.500859363	-0.650600243	1.151459605	8
OR51F2	-0.484221609	-0.66571466	1.14993627	8
OR51J1	-0.287529256	-0.824736892	1.112266149	8
OR51S1	-0.460930668	-0.686408261	1.147338929	8
OR51T1	-0.157944415	-0.911628706	1.069573122	8
OR52D1	-0.461645289	-0.685781261	1.14742655	8
OR52E2	-0.362463511	-0.768223431	1.130686943	8
OR52I1	-0.393735497	-0.743201009	1.136936506	8
OR52M1	-0.452724426	-0.69357281	1.146297235	8
OR52N2	-0.202254275	-0.883413317	1.085667592	8
OR56A3	-0.312994109	-0.806065159	1.119059269	8
OR5A2	-0.208801195	-0.879114296	1.087915491	8
OR5AR1	-0.405863613	-0.733260506	1.139124119	8
OR5B12	-0.298586576	-0.816695606	1.115282181	8
OR5B2	-0.252773999	-0.849358346	1.102132345	8
OR5D13	-0.478338868	-0.67099206	1.149330928	8
OR5D18	-0.254697295	-0.848021521	1.102718816	8
OR5H15	-0.38028342	-0.754071397	1.134354816	8
OR5I1	-0.488688468	-0.661684289	1.150372758	8
OR5J2	-0.372094029	-0.760610012	1.132704041	8
OR5L1	-0.491061904	-0.659534601	1.150596505	8
OR5L2	-0.40790581	-0.731573491	1.139479301	8

OR5M9	-0.435749135	-0.708188076	1.14393721	8
OR5P3	-0.443835386	-0.701260304	1.145095689	8
OR5T2	-0.446749901	-0.698748086	1.145497987	8
OR6B3	-0.464897223	-0.682921766	1.14781899	8
OR6C1	-0.159158166	-0.91087612	1.070034286	8
OR6C2	-0.317518708	-0.802690802	1.12020951	8
OR6F1	-0.482137587	-0.667588188	1.149725775	8
OR6K2	-0.302207923	-0.814039955	1.116247877	8
OR6K6	-0.450353112	-0.695631	1.145984112	8
OR6Q1	-0.329871631	-0.793390085	1.123261715	8
OR6S1	-0.358885322	-0.771031428	1.12991675	8
OR8G5	-0.466766854	-0.681273082	1.148039935	8
OR8K5	-0.465592382	-0.682309158	1.14790154	8
OR8U3	-0.442750487	-0.702193381	1.144943868	8
OR8U8	-0.501119765	-0.650361443	1.151481209	8
OR8U9	-0.441074482	-0.703632644	1.144707126	8
OR9A2	-0.451489856	-0.694645034	1.14613489	8
OR9G4	-0.476163577	-0.672934779	1.149098356	8
OR9G9	-0.494743151	-0.656189109	1.15093226	8
OR9Q2	-0.498490754	-0.652769161	1.151259915	8
ORC1	-0.403007371	-0.735613593	1.138620964	8
ORC6	-0.458438022	-0.688591391	1.147029414	8
ORMDL3	-0.495511356	-0.655489235	1.151000591	8
OSBPL1A	-0.473929288	-0.674925301	1.148854589	8

OSBPL8	-0.175122766	-0.900871221	1.075993987	8
OSMR	-0.418758138	-0.722544327	1.141302465	8
OSR1	-0.399755351	-0.738283689	1.138039039	8
OSR2	-0.492965906	-0.657805973	1.150771879	8
OSTN	-0.487774678	-0.66251042	1.150285098	8
OTC	-0.459133963	-0.687982474	1.147116437	8
OTOA	-0.424796827	-0.717472944	1.142269771	8
OTOG	-0.42339248	-0.718655364	1.142047843	8
OTOL1	-0.463972482	-0.683735961	1.147708443	8
OTOP2	-0.487213389	-0.663017448	1.150230837	8
OTOP3	-0.323058677	-0.798535669	1.121594346	8
OTUD7B	-0.362261917	-0.768381931	1.130643848	8
OVCA2	-0.24277704	-0.856258902	1.099035942	8
OVOL2	-0.427222117	-0.715426587	1.142648704	8
OXGR1	-0.439960596	-0.704587713	1.144548308	8
OXR1	-0.248113206	-0.852585541	1.100698747	8
OXTR	-0.500217673	-0.651188401	1.151406074	8
P2RY12	-0.347601574	-0.779813317	1.127414891	8
P4HA3	-0.433664275	-0.709964211	1.143628486	8
PABIR2	-0.389536604	-0.746611497	1.136148101	8
PAFAH1B1	-0.487029422	-0.663183561	1.150212983	8
PALD1	-0.467659887	-0.680484375	1.148144262	8
PALLD	-0.49024116	-0.660278616	1.150519776	8
PALM	-0.48248092	-0.667279832	1.149760752	8

PALM2AKAP2	-0.400203131	-0.737916606	1.138119737	8
PALM3	-0.490952771	-0.65963357	1.150586342	8
PALMD	-0.420545007	-0.721047217	1.141592225	8
PALS1	-0.38409145	-0.751010646	1.135102096	8
PANX1	-0.289446868	-0.823349591	1.11279646	8
PANX3	-0.479566104	-0.669893959	1.149460063	8
PAPSS2	-0.383583745	-0.751419468	1.135003213	8
PAQR3	-0.278959805	-0.830899441	1.109859246	8
PAQR5	-0.440376233	-0.704231474	1.144607707	8
PARD3B	-0.490526722	-0.660019828	1.150546549	8
PARD6G	-0.490427105	-0.660110114	1.150537219	8
PARP2	-0.490823015	-0.659751227	1.150574242	8
PARPBP	-0.460782392	-0.686538294	1.147320686	8
PARVA	-0.438909316	-0.705488021	1.144397337	8
PAWR	-0.491153153	-0.65945184	1.150604993	8
PAX9	-0.419335271	-0.722061106	1.141396377	8
PBX4	-0.150187689	-0.916411447	1.066599136	8
PBXIP1	-0.490210335	-0.660306545	1.150516881	8
PCCA	-0.43159131	-0.711726154	1.143317464	8
PCDH17	-0.474991278	-0.673979794	1.148971072	8
PCDH8	-0.46949004	-0.678865579	1.148355619	8
PCDHB12	-0.493805811	-0.657042262	1.150848073	8
PCGF2	-0.440270948	-0.704321728	1.144592677	8
PCK1	-0.478638391	-0.670724193	1.149362583	8

PCLAF	-0.473139099	-0.675628096	1.148767195	8
PCMTD2	-0.475625375	-0.673414713	1.149040089	8
PCNT	-0.402697428	-0.735868489	1.138565917	8
PCSK9	-0.499681366	-0.651679644	1.15136101	8
PDCD10	-0.427932159	-0.714826443	1.142758602	8
PDCL2	-0.425263017	-0.717080019	1.142343036	8
PDE2A	-0.454605808	-0.691936003	1.146541812	8
PDE4D	-0.452626086	-0.693658271	1.146284358	8
PDE6H	-0.242001759	-0.856790694	1.098792453	8
PDE7B	-0.39028378	-0.746005776	1.136289555	8
PDE9A	-0.4103296	-0.729566292	1.139895892	8
PDLIM3	-0.487222235	-0.663009459	1.150231694	8
PDLIM4	-0.45700542	-0.689843376	1.146848797	8
PDLIM5	-0.159153865	-0.910878789	1.070032653	8
PDP1	-0.30809464	-0.809699675	1.117794315	8
PDS5A	-0.183329438	-0.895651207	1.078980645	8
PDS5B	-0.379633333	-0.754592619	1.134225952	8
PEDS1	-0.274391213	-0.834160197	1.10855141	8
PEF1	-0.131428467	-0.92778711	1.059215576	8
PER2	-0.443229002	-0.701781968	1.14501097	8
PERP	-0.470826393	-0.677681476	1.148507869	8
PET100	-0.436843769	-0.707253891	1.14409766	8
PEX3	-0.154390711	-0.913825649	1.06821636	8
PFDN4	-0.433515666	-0.710090658	1.143606324	8

PFKM	-0.171229122	-0.903329543	1.074558665	8
PGM5	-0.306904532	-0.81057948	1.117484011	8
PGR	-0.495190559	-0.65578157	1.150972129	8
PGRMC1	-0.277346748	-0.832052687	1.109399435	8
PGRMC2	-0.201702612	-0.883774025	1.085476637	8
PHACTR2	-0.468016333	-0.680169352	1.148185684	8
PHACTR3	-0.496207175	-0.654854789	1.151061964	8
PHEX	-0.308084797	-0.809706956	1.117791753	8
PHF6	-0.463317413	-0.684312216	1.147629629	8
PHF7	-0.496367866	-0.654708201	1.151076067	8
PHGR1	-0.377833097	-0.756034039	1.133867136	8
PHKA1	-0.500778111	-0.650674739	1.151452851	8
PHKG1	-0.315395083	-0.804276707	1.119671791	8
PHLDB1	-0.470932241	-0.677587612	1.148519853	8
PHLDB2	-0.483555865	-0.666313635	1.149869499	8
PHLPP2	-0.361548446	-0.768942599	1.130491044	8
PHOX2A	-0.453333021	-0.693043704	1.146376725	8
PHTF1	-0.321378428	-0.799798646	1.121177074	8
PHTF2	-0.391668735	-0.74488169	1.136550425	8
PIAS2	-0.425118732	-0.71720165	1.142320383	8
PIF1	-0.3747368	-0.758506458	1.133243259	8
PIGN	-0.11713979	-0.936271148	1.053410939	8
PIK3C2A	-0.408517728	-0.731067255	1.139584983	8
PIK3CB	-0.419065655	-0.722286888	1.141352543	8

PIK3IP1	-0.346658699	-0.780542136	1.127200834	8
PIK3R3	-0.434789838	-0.70900583	1.143795668	8
PIKFYVE	-0.183028614	-0.895843478	1.078872091	8
PIM3	-0.316281384	-0.803615288	1.119896672	8
PINLYP	-0.223075987	-0.869623476	1.092699462	8
PIP4P1	-0.418312507	-0.722917233	1.14122974	8
PIPOX	-0.469456766	-0.67889504	1.148351806	8
PIR	-0.470321788	-0.678128798	1.148450586	8
PITPNM2	-0.32842774	-0.7944839	1.12291164	8
PITPNM3	-0.41017396	-0.729695343	1.139869303	8
PITX2	-0.496816684	-0.654298636	1.151115319	8
PIWIL2	-0.230682002	-0.864500497	1.095182499	8
PIWIL3	-0.280925224	-0.829491389	1.110416613	8
PJA2	-0.322199666	-0.799181653	1.121381318	8
PKD2	-0.424840506	-0.717436138	1.142276644	8
PKIA	-0.467946468	-0.680231107	1.148177575	8
PKIB	-0.45027502	-0.695698688	1.145973709	8
PKIG	-0.31983981	-0.800953069	1.120792879	8
PKLR	-0.434002838	-0.709676061	1.143678899	8
PKP1	-0.392492308	-0.744212427	1.136704734	8
PLA1A	-0.458988677	-0.688109632	1.147098309	8
PLA2G2A	-0.388672836	-0.747311112	1.135983948	8
PLA2G4A	-0.384274277	-0.750863371	1.135137647	8
PLAAT3	-0.265222256	-0.840652899	1.105875155	8

PLAC1	-0.388348648	-0.747573517	1.135922165	8
PLAG1	-0.46381524	-0.683874323	1.147689563	8
PLAT	-0.446306264	-0.69913101	1.145437274	8
PLCB4	-0.494792807	-0.656143888	1.150936695	8
PLCXD3	-0.406013197	-0.733137067	1.139150264	8
PLEKHA4	-0.423072065	-0.718924886	1.141996951	8
PLEKHA8	-0.297825751	-0.817252155	1.115077906	8
PLEKHG1	-0.477472253	-0.671766584	1.149238837	8
PLEKHG2	-0.500004244	-0.651383931	1.151388175	8
PLEKHH2	-0.49951694	-0.651830194	1.151347135	8
PLK2	-0.440572843	-0.704062905	1.144635748	8
PLK3	-0.340221432	-0.785497485	1.125718916	8
PLK4	-0.423523008	-0.71854554	1.142068548	8
PLPP1	-0.472334246	-0.676343299	1.148677545	8
PLPP2	-0.406882945	-0.732418934	1.13930188	8
PLS3	-0.4848231	-0.665173113	1.149996213	8
PLSCR4	-0.475996637	-0.673083677	1.149080313	8
PM20D2	-0.118602165	-0.935410004	1.054012169	8
PMFBP1	-0.497841338	-0.653362824	1.151204162	8
PMS1	-0.426696056	-0.715870922	1.142566978	8
PNKD	-0.222516335	-0.869998608	1.092514944	8
PNMT	-0.399337021	-0.738626463	1.137963484	8
POF1B	-0.459593036	-0.687580548	1.147173584	8
POFUT2	-0.489596193	-0.660862809	1.150459002	8

POGLUT2	-0.475553688	-0.673478618	1.149032306	8
POGLUT3	-0.189737193	-0.89153895	1.081276143	8
POLE2	-0.49479687	-0.656140188	1.150937058	8
POLK	-0.454938382	-0.691646308	1.146584691	8
POLN	-0.485873519	-0.664226507	1.150100026	8
POLR2G	-0.492006602	-0.658677376	1.150683978	8
POLR2K	-0.191268235	-0.890551639	1.081819874	8
POLR3G	-0.48488156	-0.66512046	1.15000202	8
POM121L2	-0.469207953	-0.679115304	1.148323256	8
POMT2	-0.343368806	-0.783079126	1.126447933	8
POMZP3	-0.223081377	-0.869619861	1.092701238	8
PON1	-0.430994768	-0.712232443	1.143227211	8
PON3	-0.409446096	-0.730298567	1.139744663	8
POSTN	-0.487958591	-0.662344218	1.150302808	8
POU4F1	-0.460437255	-0.686840885	1.147278141	8
POU4F2	-0.491350074	-0.659273209	1.150623283	8
PPFIA3	-0.487893038	-0.662403462	1.1502965	8
PPFIBP1	-0.439287102	-0.705164609	1.14445171	8
PPIAL4D	-0.339337974	-0.786174779	1.125512753	8
PPIAL4G	-0.24328496	-0.85591024	1.0991952	8
PPM1A	-0.425335824	-0.717018636	1.14235446	8
PPM1B	-0.148029566	-0.917733892	1.065763459	8
PPM1H	-0.410922133	-0.729074781	1.139996914	8
PPM1L	-0.481404714	-0.668246005	1.14965072	8

PPP1R17	-0.491249487	-0.659364459	1.150613946	8
PPP1R1C	-0.470108492	-0.678317805	1.148426297	8
PPP1R2	-0.108741002	-0.941185397	1.049926399	8
PPP1R36	-0.261122567	-0.843533811	1.104656378	8
PPP1R3A	-0.279846861	-0.830264335	1.110111196	8
PPP1R3C	-0.444027772	-0.701094722	1.145122495	8
PPP2CB	-0.147059923	-0.9183269	1.065386823	8
PPP4R4	-0.480018155	-0.669489096	1.149507251	8
PPY	-0.310940109	-0.807591307	1.118531416	8
PRAC2	-0.442498279	-0.702410134	1.144908414	8
PRAMEF10	-0.289431867	-0.823360456	1.112792323	8
PRAMEF11	-0.466494841	-0.681513161	1.148008003	8
PRAMEF20	-0.414520299	-0.726083138	1.140603436	8
PRAMEF4	-0.144034969	-0.920172238	1.064207207	8
PRAMEF7	-0.34964369	-0.778232176	1.127875866	8
PRB4	-0.320482551	-0.800471065	1.120953616	8
PRC1	-0.459566041	-0.687604188	1.147170229	8
PRDM5	-0.413879236	-0.726617013	1.140496249	8
PRDM7	-0.489260144	-0.661167027	1.15042717	8
PRDM9	-0.234800991	-0.861706941	1.096507931	8
PREX2	-0.446576136	-0.698898094	1.145474229	8
PRG4	-0.145361733	-0.919363727	1.06472546	8
PRICKLE2	-0.477755148	-0.671513833	1.149268981	8
PRKAB2	-0.492669613	-0.658075217	1.150744829	8

PRKACB	-0.129525641	-0.928925929	1.05845157	8
PRKD1	-0.495663251	-0.65535078	1.15101403	8
PRKG2	-0.430557555	-0.712603297	1.143160852	8
PRKRIP1	-0.46657978	-0.681438202	1.148017982	8
PRLR	-0.444565856	-0.700631424	1.145197279	8
PRM2	-0.468576257	-0.679674244	1.148250501	8
PROCR	-0.155778863	-0.912968636	1.068747499	8
PRODH2	-0.420838723	-0.720800847	1.14163957	8
PROK1	-0.407795325	-0.731664858	1.139460183	8
PROKR1	-0.450452587	-0.695544769	1.145997356	8
PROP1	-0.331659919	-0.792032908	1.123692827	8
PROX1	-0.435615065	-0.708302416	1.143917481	8
PRPS2	-0.479660969	-0.669809013	1.149469982	8
PRR23B	-0.168132375	-0.905276336	1.073408711	8
PRR9	-0.465073024	-0.682766887	1.147839911	8
PRRX1	-0.432424554	-0.711018414	1.143442968	8
PRRX2	-0.396305012	-0.741106113	1.137411125	8
PRSS22	-0.133179465	-0.92673671	1.059916176	8
PRSS3	-0.44041727	-0.704196293	1.144613563	8
PRSS58	-0.184591996	-0.894843479	1.079435475	8
PRUNE1	-0.436930954	-0.707179436	1.144110391	8
PRUNE2	-0.501603086	-0.649918035	1.151521121	8
PSEN1	-0.391545698	-0.744981622	1.13652732	8
PSG3	-0.207233756	-0.880146624	1.08738038	8

PSG4	-0.357613452	-0.772026837	1.129640289	8
PSG6	-0.454845481	-0.691727243	1.146572723	8
PSIP1	-0.229066072	-0.865592747	1.094658819	8
PSKH2	-0.450386217	-0.695602304	1.145988521	8
PSORS1C1	-0.258098898	-0.845649848	1.103748746	8
PSPN	-0.498939861	-0.652358358	1.151298219	8
PSRC1	-0.443668153	-0.701404206	1.14507236	8
PSTPIP2	-0.498475492	-0.652783117	1.151258609	8
PTBP2	-0.437844045	-0.706399243	1.144243288	8
PTCRA	-0.263188535	-0.842083732	1.105272267	8
PTGIR	-0.464022662	-0.683691802	1.147714463	8
PTGR2	-0.367759941	-0.764046435	1.131806375	8
PTGS1	-0.297734331	-0.817318997	1.115053328	8
PTH2R	-0.420851172	-0.720790403	1.141641575	8
PTHLH	-0.475470792	-0.673552508	1.1490233	8
PTMA	-0.398967144	-0.738929404	1.137896548	8
PTMS	-0.500000635	-0.651387237	1.151387872	8
PTPN12	-0.192867299	-0.889518507	1.082385805	8
PTPN13	-0.498371614	-0.652878105	1.151249719	8
PTPN14	-0.450623962	-0.695396189	1.146020151	8
PTPN21	-0.466771755	-0.681268755	1.14804051	8
PTPN22	-0.250658055	-0.850825624	1.101483679	8
PTPRA	-0.233884525	-0.862329672	1.096214197	8
PTPRB	-0.42053322	-0.721057103	1.141590323	8

PTPRH	-0.350122395	-0.777861009	1.127983404	8
PTPRK	-0.489129552	-0.661285217	1.15041477	8
PTPRM	-0.440552982	-0.704079935	1.144632917	8
PTPRR	-0.435733955	-0.708201022	1.143934977	8
PTS	-0.410766213	-0.729204149	1.139970362	8
PTTG1IP	-0.378650246	-0.755380119	1.134030364	8
PUS7	-0.488791909	-0.661590719	1.150382628	8
PWWP2A	-0.362759224	-0.767990868	1.130750092	8
PXDNL	-0.418916323	-0.722411913	1.141328236	8
PXMP4	-0.40508006	-0.73390677	1.13898683	8
PYGB	-0.290299508	-0.822731771	1.113031279	8
QRICH2	-0.385467435	-0.749901495	1.135368929	8
QSER1	-0.391441997	-0.745065839	1.136507835	8
QSOX2	-0.262585129	-0.842507614	1.105092743	8
RAB11A	-0.238521095	-0.859172264	1.097693358	8
RAB11B	-0.485472009	-0.664588467	1.150060476	8
RAB12	-0.121685421	-0.933589034	1.055274455	8
RAB13	-0.448623856	-0.697128508	1.145752364	8
RAB14	-0.392383972	-0.744300499	1.136684471	8
RAB17	-0.483043129	-0.666774644	1.149817773	8
RAB22A	-0.440581824	-0.704055204	1.144637028	8
RAB23	-0.481091543	-0.66852694	1.149618483	8
RAB30	-0.482379692	-0.66737076	1.149750452	8
RAB38	-0.426574342	-0.71597369	1.142548032	8

RAB39A	-0.321516222	-0.799695162	1.121211384	8
RAB3GAP2	-0.221159475	-0.870907076	1.092066551	8
RAB3IP	-0.462376141	-0.685139507	1.147515647	8
RAB40A	-0.445276255	-0.700019334	1.14529559	8
RAB4A	-0.355883014	-0.77337887	1.129261884	8
RAB6B	-0.49320975	-0.657584324	1.150794074	8
RAB9B	-0.478638785	-0.67072384	1.149362625	8
RAD18	-0.223586476	-0.869281079	1.092867555	8
RAD21	-0.379381182	-0.754794686	1.134175868	8
RAD21L1	-0.28336695	-0.827737682	1.111104631	8
RAD51AP1	-0.466683451	-0.681346701	1.148030152	8
RAD54B	-0.383734182	-0.751298355	1.135032537	8
RAD54L	-0.48076755	-0.66881748	1.14958503	8
RAG2	-0.366963506	-0.764676113	1.131639619	8
RAI14	-0.45330245	-0.693070291	1.146372741	8
RALA	-0.300612821	-0.815211041	1.115823862	8
RAMP2	-0.428167527	-0.714627401	1.142794928	8
RANBP17	-0.489499647	-0.660950222	1.150449869	8
RANBP3	-0.350798319	-0.777336588	1.128134907	8
RANBP3L	-0.469336534	-0.679001484	1.148338018	8
RANBP9	-0.467299488	-0.680802765	1.148102253	8
RAP1GDS1	-0.3724688	-0.760312083	1.132780883	8
RAP2C	-0.320100083	-0.800757927	1.12085801	8
RAPGEF4	-0.452248603	-0.693986236	1.146234839	8

RAPGEF5	-0.438069305	-0.706206648	1.144275952	8
RAPH1	-0.437046613	-0.707080655	1.144127268	8
RARRES2	-0.471399842	-0.67717282	1.148572662	8
RARS1	-0.207527514	-0.8799533	1.087480814	8
RARS2	-0.122914666	-0.932861021	1.055775687	8
RASA3	-0.406870199	-0.732429464	1.139299663	8
RASIP1	-0.405168012	-0.733834256	1.139002268	8
RASSF10	-0.496780399	-0.654331755	1.151112154	8
RASSF8	-0.482128591	-0.667596267	1.149724857	8
RASSF9	-0.449395662	-0.696460491	1.145856153	8
RAVER2	-0.40295482	-0.735656817	1.138611637	8
RBM12B	-0.206976293	-0.880316006	1.087292299	8
RBM15	-0.131397129	-0.927805887	1.059203016	8
RBM41	-0.44232429	-0.702559629	1.14488392	8
RBM45	-0.339929335	-0.785721492	1.125650827	8
RBMY1B	-0.430986887	-0.71223913	1.143226017	8
RBMY1F	-0.126742882	-0.930586396	1.057329278	8
RBMY1J	-0.268861418	-0.838084151	1.106945569	8
RBPMS	-0.350745049	-0.777377932	1.128122981	8
RCAN3	-0.170691576	-0.903668009	1.074359584	8
RCHY1	-0.354451215	-0.774495598	1.128946813	8
RCN1	-0.458662056	-0.688395423	1.147057479	8
RCVRN	-0.333879805	-0.790344385	1.12422419	8
RDH10	-0.472148124	-0.676508598	1.148656722	8

RDH11	-0.353243986	-0.775435786	1.128679772	8
RDX	-0.45431544	-0.692188848	1.146504287	8
REEP3	-0.493468841	-0.657348749	1.15081759	8
REN	-0.456106042	-0.690628349	1.146734391	8
REPS2	-0.290335321	-0.822705808	1.113041129	8
RERG	-0.46299906	-0.684592116	1.147591175	8
REV3L	-0.477491771	-0.671749148	1.149240919	8
REXO4	-0.197977957	-0.886203158	1.084181115	8
REXO5	-0.424924389	-0.717365449	1.142289839	8
RFESD	-0.274215763	-0.83428508	1.108500843	8
RFLNB	-0.457326241	-0.689563177	1.146889417	8
RFPL4B	-0.296966531	-0.817880103	1.114846634	8
RFTN2	-0.451155606	-0.694935078	1.146090684	8
RFX7	-0.35774898	-0.771920835	1.129669815	8
RFXAP	-0.283330413	-0.82776396	1.111094372	8
RGL1	-0.481239747	-0.668394004	1.149633751	8
RGMB	-0.449556598	-0.696321125	1.145877723	8
RGN	-0.458591976	-0.688456729	1.147048705	8
RGPD3	-0.325381942	-0.796785419	1.122167362	8
RGS18	-0.463493595	-0.684157273	1.147650868	8
RGS5	-0.482919984	-0.666885326	1.14980531	8
RHOBTB3	-0.499852606	-0.651522824	1.15137543	8
RHOJ	-0.437810137	-0.70642823	1.144238367	8
RHOXF2B	-0.316650225	-0.803339838	1.119990062	8

RIBC2	-0.455260795	-0.691365362	1.146626157	8
RIC8B	-0.181584906	-0.896765237	1.078350143	8
RIDA	-0.300985029	-0.814937964	1.115922993	8
RIMS1	-0.48142157	-0.668230882	1.149652452	8
RIPOR2	-0.369339106	-0.762796261	1.132135367	8
RIPPLY2	-0.490073571	-0.660430453	1.150504025	8
RLN2	-0.442403703	-0.7024914	1.144895103	8
RMDN2	-0.47059585	-0.677885878	1.148481729	8
RNASE13	-0.493205024	-0.65758862	1.150793644	8
RNASE9	-0.361428491	-0.769036819	1.13046531	8
RNASEH2B	-0.106480558	-0.942498853	1.048979411	8
RND1	-0.452841651	-0.693470923	1.146312574	8
RNF112	-0.337841901	-0.7873202	1.125162101	8
RNF115	-0.311400614	-0.807249455	1.118650069	8
RNF128	-0.49730713	-0.653850847	1.151157977	8
RNF138	-0.116662226	-0.936552018	1.053214244	8
RNF166	-0.398598317	-0.739231361	1.137829678	8
RNF168	-0.417742893	-0.723393621	1.141136514	8
RNF212B	-0.501146314	-0.650337093	1.151483407	8
RNF217	-0.466688188	-0.68134252	1.148030708	8
RNF6	-0.187642461	-0.892886796	1.080529257	8
ROBO4	-0.403721619	-0.735025865	1.138747484	8
ROMO1	-0.469671948	-0.678704499	1.148376447	8
RP1L1	-0.439394406	-0.705072723	1.14446713	8

RPAP2	-0.428369792	-0.714456311	1.142826103	8
RPGRIP1L	-0.431014271	-0.712215897	1.143230167	8
RPN1	-0.450621921	-0.695397958	1.146019879	8
RPRD1B	-0.4580951	-0.68889126	1.14698636	8
RPS26	-0.492076276	-0.658614118	1.150690394	8
RPS4Y2	-0.157883699	-0.911666324	1.069550023	8
RPS6KL1	-0.471255469	-0.677300911	1.14855638	8
RPTN	-0.321900248	-0.799406671	1.121306918	8
RRAD	-0.442462688	-0.702440718	1.144903406	8
RREB1	-0.434655197	-0.709120535	1.143775732	8
RSBN1	-0.173398267	-0.901961466	1.075359733	8
RSPH10B	-0.409670095	-0.730112978	1.139783073	8
RSPH10B2	-0.292174221	-0.821371244	1.113545465	8
RSPH4A	-0.482037261	-0.667678271	1.149715532	8
RTBDN	-0.469103344	-0.679207891	1.148311235	8
RTKN2	-0.334319999	-0.790009058	1.124329056	8
RTL4	-0.492038193	-0.658648695	1.150686888	8
RTN1	-0.451217128	-0.694881701	1.146098829	8
RTP3	-0.26505586	-0.840770094	1.105825954	8
RUFY2	-0.467522695	-0.68060559	1.148128285	8
RUFY4	-0.346598345	-0.780588762	1.127187106	8
RUNDC3A	-0.193143825	-0.889339644	1.082483469	8
RUNX1T1	-0.463739248	-0.683941182	1.14768043	8
RXFP1	-0.499375737	-0.65195946	1.151335197	8

RYR1	-0.475349463	-0.673660644	1.149010107	8
S100A13	-0.260568376	-0.843922202	1.104490578	8
S100A16	-0.425104554	-0.717213602	1.142318155	8
S100A3	-0.426576356	-0.715971989	1.142548346	8
S1PR4	-0.496318994	-0.654752787	1.151071781	8
SAA2	-0.372160462	-0.760557209	1.132717671	8
SACS	-0.413642275	-0.726814258	1.140456533	8
SAGE1	-0.170463845	-0.903811332	1.074275176	8
SAMD1	-0.499585464	-0.651767456	1.15135292	8
SAMD12	-0.194051584	-0.888752067	1.082803651	8
SAMD13	-0.463041948	-0.684554414	1.147596362	8
SARAF	-0.493145707	-0.657642543	1.15078825	8
SASH1	-0.469324488	-0.679012148	1.148336635	8
SASS6	-0.424557247	-0.717674794	1.142232041	8
SATB2	-0.49369454	-0.65714348	1.15083802	8
SATL1	-0.472031806	-0.676611886	1.148643692	8
SAXO2	-0.152079038	-0.915249528	1.067328566	8
SBK3	-0.425012767	-0.717290966	1.142303733	8
SBSN	-0.35258997	-0.775944604	1.128534574	8
SCAMP2	-0.488512413	-0.661843521	1.150355934	8
SCARA3	-0.432006166	-0.711373866	1.143380031	8
SCARB2	-0.105370311	-0.943142552	1.048512863	8
SCFD1	-0.156862221	-0.912298763	1.069160984	8
SCGB1C1	-0.500387146	-0.651033107	1.151420253	8

SCGN	-0.415162438	-0.725547986	1.140710424	8
SCIN	-0.314011403	-0.805307984	1.119319387	8
SCMH1	-0.300794639	-0.815077662	1.1158723	8
SCML2	-0.488977891	-0.661422456	1.150400347	8
SCN11A	-0.44544556	-0.699873389	1.145318948	8
SCN4B	-0.498923106	-0.652373687	1.151296793	8
SCN7A	-0.492330282	-0.658383459	1.150713741	8
SCNN1B	-0.307056123	-0.810467479	1.117523602	8
SCOC	-0.43151601	-0.71179008	1.14330609	8
SCRN1	-0.195262064	-0.88796754	1.083229604	8
SCRN3	-0.319849821	-0.800945564	1.120795385	8
SCUBE2	-0.44052474	-0.704104151	1.144628891	8
SCX	-0.257639678	-0.845970573	1.103610251	8
SCYL2	-0.298269353	-0.816927715	1.115197068	8
SDR9C7	-0.443150086	-0.701849832	1.144999919	8
SEC16B	-0.142851706	-0.920892162	1.063743868	8
SEC22A	-0.186609579	-0.893550138	1.080159718	8
SEC23A	-0.257696177	-0.845931123	1.1036273	8
SEC23B	-0.286597643	-0.825409774	1.112007417	8
SEC24A	-0.43738281	-0.706793445	1.144176255	8
SEC63	-0.243172776	-0.855987267	1.099160043	8
SECISBP2L	-0.426726688	-0.715845056	1.142571744	8
SELE	-0.435963453	-0.708005261	1.143968714	8
SELENOI	-0.482731166	-0.667055006	1.149786172	8

SELENOP	-0.443730262	-0.701350765	1.145081027	8
SELENOV	-0.467374124	-0.680736839	1.148110963	8
SEMA3C	-0.382223899	-0.75251333	1.134737229	8
SEMA3D	-0.450773075	-0.695266886	1.146039961	8
SEMA3F	-0.487728761	-0.66255191	1.150280671	8
SEMA3G	-0.391724835	-0.744836121	1.136560956	8
SEMA6B	-0.494231339	-0.656655063	1.150886402	8
SEMG2	-0.404840968	-0.734103858	1.138944826	8
SENP1	-0.123426508	-0.932557546	1.055984054	8
SENP7	-0.150004813	-0.916523648	1.066528461	8
SENP8	-0.393650847	-0.743269922	1.136920769	8
SEPTIN10	-0.439821114	-0.704707224	1.144528338	8
SEPTIN11	-0.491373278	-0.659252158	1.150625436	8
SEPTIN12	-0.305644492	-0.811509691	1.117154182	8
SEPTIN2	-0.493180181	-0.657611205	1.150791385	8
SEPTIN6	-0.299319915	-0.816158706	1.115478621	8
SEPTIN8	-0.371967954	-0.760710208	1.132678163	8
SERAC1	-0.157727097	-0.911763335	1.069490432	8
SERINC3	-0.267867485	-0.838786802	1.106654287	8
SERP1	-0.125484083	-0.931335576	1.056819658	8
SERP2	-0.212431064	-0.876716196	1.08914726	8
SERPINA10	-0.369933859	-0.762324845	1.132258703	8
SERPINA3	-0.434762064	-0.709029493	1.143791557	8
SERPINA5	-0.409386864	-0.730347635	1.139734499	8

SERPINA6	-0.461781067	-0.685662075	1.147443142	8
SERPINB10	-0.164158846	-0.907763446	1.071922292	8
SERPINB8	-0.349867394	-0.77805875	1.127926144	8
SERPINF1	-0.131471259	-0.927761467	1.059232726	8
SERPINI1	-0.178804718	-0.898535724	1.077340441	8
SERPINI2	-0.192435322	-0.889797798	1.08223312	8
SESN2	-0.393554505	-0.743348345	1.13690285	8
SESTD1	-0.47186189	-0.676762743	1.148624633	8
SETD2	-0.469921624	-0.678483357	1.148404981	8
SETD3	-0.29689622	-0.817931462	1.114827681	8
SETD7	-0.214097416	-0.875611824	1.089709241	8
SETD9	-0.216198695	-0.874216077	1.090414772	8
SETDB2	-0.129943764	-0.928675924	1.058619689	8
SETDB2-PHF11	-0.460043003	-0.687186396	1.147229399	8
SFRP4	-0.417202482	-0.723845308	1.14104779	8
SFT2D3	-0.432532027	-0.71092708	1.143459108	8
SFTPC	-0.418460425	-0.722793475	1.1412539	8
SGCB	-0.449170485	-0.696655446	1.145825931	8
SGCE	-0.426650419	-0.715909457	1.142559876	8
SGK2	-0.152770803	-0.914823866	1.067594669	8
SGMS1	-0.475253445	-0.67374621	1.148999655	8
SGO2	-0.381917509	-0.752759563	1.134677072	8
SGSM1	-0.447015667	-0.698518601	1.145534268	8
SH2B1	-0.458990958	-0.688107635	1.147098593	8

SH3BGR	-0.327968206	-0.794831647	1.122799853	8
SH3D19	-0.427881477	-0.714869297	1.142750773	8
SH3TC2	-0.476116637	-0.672976648	1.149093285	8
SHANK3	-0.371111726	-0.761390315	1.132502042	8
SHB	-0.491231574	-0.659380707	1.150612282	8
SHBG	-0.496374829	-0.654701849	1.151076678	8
SHE	-0.431364275	-0.711918879	1.143283155	8
SHISA3	-0.438065591	-0.706209823	1.144275414	8
SHISAL2B	-0.434211085	-0.709498769	1.143709854	8
SHROOM4	-0.469857108	-0.678540506	1.148397614	8
SIGLEC14	-0.187546308	-0.892948583	1.080494891	8
SIK2	-0.207797267	-0.879775714	1.087572981	8
SIM1	-0.50132017	-0.650177617	1.151497787	8
SIPA1L2	-0.235308438	-0.861361845	1.096670283	8
SIRT7	-0.278833256	-0.830989993	1.10982325	8
SIX5	-0.500394954	-0.651025951	1.151420905	8
SKA1	-0.426042534	-0.716422556	1.14246509	8
SKOR1	-0.475994815	-0.673085301	1.149080116	8
SLA2	-0.324706997	-0.797294366	1.122001363	8
SLAIN2	-0.406208941	-0.732975506	1.139184447	8
SLC10A2	-0.459760655	-0.687433744	1.147194399	8
SLC10A4	-0.329590251	-0.793603382	1.123193633	8
SLC10A6	-0.408585312	-0.731011323	1.139596634	8
SLC10A7	-0.378830311	-0.755235943	1.134066253	8

SLC12A2	-0.490676287	-0.659884252	1.150560539	8
SLC13A2	-0.395555922	-0.741717454	1.137273376	8
SLC13A3	-0.467685196	-0.680462011	1.148147207	8
SLC16A1	-0.310536869	-0.807890503	1.118427372	8
SLC17A1	-0.243716725	-0.855613693	1.099330417	8
SLC19A2	-0.24925233	-0.851798413	1.101050743	8
SLC22A2	-0.423053868	-0.71894019	1.141994058	8
SLC22A8	-0.470411082	-0.678049659	1.148460741	8
SLC25A24	-0.193521072	-0.889095537	1.082616609	8
SLC25A30	-0.263421945	-0.841919686	1.105341631	8
SLC25A36	-0.481146468	-0.668477676	1.149624144	8
SLC25A41	-0.325240761	-0.79689191	1.122132671	8
SLC25A47	-0.498308084	-0.652936192	1.151244276	8
SLC26A3	-0.442777688	-0.70217	1.144947689	8
SLC26A4	-0.348961111	-0.778761075	1.127722187	8
SLC26A7	-0.465106997	-0.682736953	1.14784395	8
SLC28A1	-0.147355977	-0.918145918	1.065501895	8
SLC2A2	-0.361253222	-0.769174464	1.130427686	8
SLC30A5	-0.193663882	-0.889003099	1.082666981	8
SLC32A1	-0.495713359	-0.655305099	1.151018459	8
SLC35D2	-0.173879631	-0.901657375	1.075537006	8
SLC35E1	-0.269461921	-0.837659242	1.107121162	8
SLC35E3	-0.412402088	-0.727845736	1.140247825	8
SLC38A10	-0.424960402	-0.717335099	1.142295501	8

SLC38A6	-0.257391719	-0.84614368	1.103535399	8
SLC39A1	-0.466057624	-0.6818989	1.147956524	8
SLC39A14	-0.454569168	-0.691967913	1.146537081	8
SLC3A1	-0.314008514	-0.805310136	1.119318649	8
SLC40A1	-0.401174946	-0.737119302	1.138294249	8
SLC44A1	-0.42230225	-0.719572039	1.141874289	8
SLC44A3	-0.417606979	-0.723507246	1.141114225	8
SLC44A4	-0.460811609	-0.686512673	1.147324283	8
SLC45A2	-0.370404202	-0.761951818	1.13235602	8
SLC4A7	-0.422547414	-0.719365999	1.141913413	8
SLC4A8	-0.414992173	-0.72568992	1.140682093	8
SLC50A1	-0.443943315	-0.701167417	1.145110731	8
SLC51B	-0.44314983	-0.701850052	1.144999883	8
SLC5A12	-0.467128956	-0.680953375	1.148082331	8
SLC5A4	-0.477636994	-0.671619407	1.149256401	8
SLC5A6	-0.138206581	-0.923707974	1.061914554	8
SLC6A18	-0.465634917	-0.682271659	1.147906576	8
SLC7A11	-0.448373576	-0.697345009	1.145718585	8
SLC7A13	-0.473652454	-0.675171588	1.148824042	8
SLC7A2	-0.491608128	-0.659039064	1.150647192	8
SLC9A1	-0.432558413	-0.710904656	1.143463069	8
SLC9A3R2	-0.406122004	-0.733047266	1.139169269	8
SLC9A9	-0.42605216	-0.716414433	1.142466593	8
SLC9B1	-0.444200121	-0.700946358	1.145146478	8

SLCO1A2	-0.496146893	-0.654909774	1.151056666	8
SLCO2A1	-0.451700099	-0.694462541	1.146162641	8
SLCO2B1	-0.426169072	-0.716315777	1.142484849	8
SLIRP	-0.255288214	-0.847610191	1.102898405	8
SLMAP	-0.45893957	-0.688152607	1.147092176	8
SMAD2	-0.361118792	-0.769280019	1.130398811	8
SMAD6	-0.411715111	-0.728416495	1.140131605	8
SMAD9	-0.408671889	-0.730939665	1.139611554	8
SMARCA5	-0.451033071	-0.69504138	1.146074452	8
SMC2	-0.259595296	-0.844603559	1.104198855	8
SMCO2	-0.427329167	-0.715336137	1.142665303	8
SMIM10L2A	-0.498475069	-0.652783504	1.151258573	8
SMIM15	-0.177882429	-0.899121729	1.077004158	8
SMIM22	-0.436739208	-0.707343175	1.144082382	8
SMIM23	-0.179532327	-0.898072947	1.077605274	8
SMIM3	-0.434509823	-0.709244366	1.143754188	8
SMIM30	-0.115580783	-0.937187403	1.052768186	8
SMIM7	-0.352086602	-0.776335966	1.128422568	8
SMIM8	-0.122069326	-0.933361792	1.055431118	8
SMIM9	-0.393761554	-0.743179795	1.136941349	8
SMLR1	-0.38122945	-0.753312221	1.134541671	8
SMOC1	-0.492400265	-0.658319897	1.150720162	8
SMOC2	-0.483629401	-0.666247496	1.149876897	8
SMPD1	-0.388066756	-0.74780161	1.135868366	8

SMR3B	-0.475644871	-0.673397334	1.149042205	8
SMTNL2	-0.490339289	-0.660189696	1.150528985	8
SMURF1	-0.413825835	-0.726661469	1.140487303	8
SMYD2	-0.157905756	-0.911652659	1.069558414	8
SNAI1	-0.50045564	-0.650970334	1.151425975	8
SNAI2	-0.432364184	-0.711069713	1.143433897	8
SNCG	-0.457919664	-0.689044626	1.146964291	8
SNN	-0.458787263	-0.68828588	1.147073143	8
SNPH	-0.466736115	-0.681300215	1.14803633	8
SNRPN	-0.129902924	-0.928700349	1.058603274	8
SNTB1	-0.498555311	-0.652710122	1.151265433	8
SNX25	-0.168596746	-0.904984879	1.073581625	8
SNX4	-0.18359656	-0.895480418	1.079076978	8
SNX7	-0.475539584	-0.67349119	1.149030774	8
SOCS2	-0.302642794	-0.813720314	1.116363108	8
SOCS6	-0.393600476	-0.743310925	1.136911401	8
SOD3	-0.441489864	-0.703276185	1.144766049	8
SORBS1	-0.474558826	-0.674364947	1.148923774	8
SORBS2	-0.497889821	-0.653318518	1.151208339	8
SOST	-0.466634497	-0.681389909	1.148024406	8
SOWAHA	-0.436907149	-0.707199766	1.144106915	8
SOX17	-0.413760628	-0.726715748	1.140476376	8
SOX8	-0.440246905	-0.704342338	1.144589243	8
SPAAR	-0.334533784	-0.789846142	1.124379926	8

SPACA6	-0.474121141	-0.674754574	1.148875715	8
SPAG4	-0.276365335	-0.832753299	1.109118634	8
SPAG5	-0.360305183	-0.769918528	1.130223711	8
SPAM1	-0.328851755	-0.794162873	1.123014628	8
SPANXN2	-0.476959424	-0.672224566	1.149183989	8
SPARC	-0.394228663	-0.742799399	1.137028062	8
SPARCL1	-0.458967326	-0.688128316	1.147095643	8
SPATA12	-0.259465305	-0.844694521	1.104159826	8
SPATA22	-0.435111352	-0.708731851	1.143843203	8
SPATA31C1	-0.32839795	-0.794506449	1.122904399	8
SPATA31D1	-0.46903679	-0.679266792	1.148303582	8
SPATA4	-0.486613124	-0.663559333	1.150172456	8
SPATA46	-0.460573492	-0.686721456	1.147294949	8
SPATA7	-0.448160516	-0.697529266	1.145689782	8
SPCS1	-0.378071055	-0.755843675	1.13391473	8
SPDL1	-0.225990649	-0.867665764	1.093656413	8
SPDYC	-0.202617149	-0.883175919	1.085793068	8
SPECC1L	-0.21599779	-0.874349676	1.090347467	8
SPEGNB	-0.471339028	-0.677226778	1.148565806	8
SPHK1	-0.475810059	-0.673250056	1.149060116	8
SPIC	-0.477143183	-0.672060489	1.149203673	8
SPICE1	-0.388103318	-0.74777203	1.135875348	8
SPIN1	-0.425727638	-0.716688215	1.142415853	8
SPINK13	-0.44847215	-0.697259746	1.145731896	8

SPINK5	-0.490419577	-0.660116936	1.150536513	8
SPINK7	-0.237770059	-0.859684871	1.097454929	8
SPINT4	-0.342775925	-0.783535332	1.126311257	8
SPIRE1	-0.466174337	-0.681795947	1.147970284	8
SPNS1	-0.254087923	-0.848445401	1.102533324	8
SPNS2	-0.402004954	-0.736437661	1.138442615	8
SPON1	-0.395953381	-0.741393146	1.137346527	8
SPRED2	-0.484823664	-0.665172605	1.149996269	8
SPRN	-0.358050688	-0.7716848	1.129735488	8
SPRR1A	-0.381683541	-0.752947536	1.134631077	8
SPRR1B	-0.459738108	-0.687453492	1.1471916	8
SPRR2B	-0.26227199	-0.842727472	1.104999462	8
SPRR2D	-0.422303648	-0.719570864	1.141874512	8
SPRR2E	-0.436444244	-0.707594985	1.144039229	8
SPRR3	-0.452920907	-0.693402031	1.146322937	8
SPRY4	-0.48052359	-0.669036181	1.149559771	8
SPRYD7	-0.392387512	-0.744297621	1.136685133	8
SPTA1	-0.414859599	-0.725800416	1.140660015	8
SRFBP1	-0.480912991	-0.668687069	1.14960006	8
SRL	-0.405944639	-0.733193644	1.139138284	8
SRMS	-0.412734785	-0.727569167	1.140303952	8
SRP9	-0.12492605	-0.931667304	1.056593354	8
SRPK3	-0.415378039	-0.725368221	1.14074626	8
SRPX	-0.466179329	-0.681791543	1.147970873	8

SRSF5	-0.32007506	-0.800776691	1.120851751	8
SRSF6	-0.130311911	-0.928455691	1.058767602	8
SRY	-0.417526325	-0.723574666	1.141100991	8
SSC5D	-0.473275076	-0.675507202	1.148782278	8
SSR4	-0.465387714	-0.682489571	1.147877285	8
SSUH2	-0.48787614	-0.662418733	1.150294873	8
SSX2IP	-0.459856496	-0.687349792	1.147206288	8
ST3GAL3	-0.276967121	-0.832323789	1.10929091	8
ST3GAL4	-0.335297409	-0.789263902	1.12456131	8
ST6GAL1	-0.420317934	-0.721237633	1.141555567	8
ST8SIA1	-0.229455004	-0.865330048	1.094785052	8
STAB2	-0.416000399	-0.724849065	1.140849464	8
STAC	-0.488971781	-0.661427984	1.150399765	8
STARD13	-0.459071598	-0.68803706	1.147108658	8
STARD4	-0.492998399	-0.657776441	1.15077484	8
STARD8	-0.420479171	-0.72110243	1.141581601	8
STARD9	-0.366112905	-0.765348	1.131460905	8
STAT3	-0.187158005	-0.893198029	1.080356035	8
STAU2	-0.485138677	-0.664888841	1.150027518	8
STEAP1B	-0.485281802	-0.664759881	1.150041683	8
STK32B	-0.476972325	-0.672213048	1.149185372	8
STK39	-0.274100371	-0.834367202	1.108467573	8
STKLD1	-0.44521915	-0.700068555	1.145287705	8
STRC	-0.316148774	-0.803714293	1.119863067	8

STX19	-0.398192297	-0.739563625	1.137755922	8
STXBP4	-0.494834521	-0.656105898	1.150940419	8
STXBP6	-0.489510358	-0.660940524	1.150450882	8
SUB1	-0.385181006	-0.750132518	1.135313524	8
SUCO	-0.458217408	-0.688784321	1.147001729	8
SULT2A1	-0.442389674	-0.702503454	1.144893128	8
SUMF2	-0.112275175	-0.939124043	1.051399218	8
SUMO1	-0.467719712	-0.680431511	1.148151223	8
SUMO3	-0.40855662	-0.731035068	1.139591689	8
SUSD1	-0.441441996	-0.703317271	1.144759267	8
SUSD5	-0.434133882	-0.709564501	1.143698383	8
SUV39H2	-0.378872609	-0.755202071	1.134074679	8
SV2B	-0.448683878	-0.697076578	1.145760456	8
SVIP	-0.475521183	-0.673507593	1.149028776	8
SWI5	-0.386121784	-0.749373443	1.135495227	8
SWT1	-0.439110218	-0.705316051	1.144426269	8
SYDE1	-0.437509009	-0.706685607	1.144194616	8
SYNJ1	-0.456530974	-0.690257568	1.146788542	8
SYNM	-0.449771417	-0.696135059	1.145906476	8
SYNPO	-0.387344048	-0.748386064	1.135730112	8
SYNPO2	-0.464842529	-0.682969946	1.147812475	8
SYPL2	-0.485724539	-0.664360831	1.15008537	8
SYT17	-0.447143757	-0.698407973	1.145551729	8
SYTL4	-0.499710083	-0.651653347	1.15136343	8

SYTL5	-0.287582672	-0.82469829	1.112280962	8
TAAR1	-0.470389678	-0.67806863	1.148458308	8
TAAR8	-0.493741499	-0.657100765	1.150842264	8
TADA2A	-0.284116409	-0.827198418	1.111314827	8
TADA3	-0.250932738	-0.850635352	1.101568091	8
TAF13	-0.274729498	-0.833919338	1.108648836	8
TAF2	-0.336516519	-0.788333333	1.124849852	8
TAF4B	-0.257534465	-0.846044031	1.103578496	8
TAF9B	-0.362309201	-0.768344758	1.130653959	8
TAMALIN	-0.24327605	-0.855916359	1.099192408	8
TAMM41	-0.383133526	-0.751781807	1.134915334	8
TANC1	-0.497077685	-0.654060366	1.151138051	8
TAOK3	-0.432865366	-0.710643733	1.143509099	8
TAP1	-0.132311686	-0.927257572	1.059569258	8
TARS3	-0.474370805	-0.674532346	1.148903152	8
TAS1R2	-0.429071807	-0.7138622	1.142934007	8
TAS2R3	-0.238608713	-0.859112432	1.097721145	8
TAS2R50	-0.44517047	-0.700110511	1.145280981	8
TASP1	-0.336659631	-0.788224009	1.12488364	8
TBC1D12	-0.263491703	-0.84187065	1.105362353	8
TBC1D14	-0.344528246	-0.782186094	1.126714339	8
TBC1D15	-0.397650932	-0.740006415	1.137657348	8
TBC1D7-LOC100130357	-0.28485469	-0.826666744	1.111521434	8
TBC1D8B	-0.484007857	-0.665907023	1.14991488	8

TBCEL	-0.433071908	-0.710468114	1.143540022	8
TBCK	-0.451785554	-0.694388354	1.146173908	8
TBL1XR1	-0.338788254	-0.786595877	1.125384132	8
TBPL2	-0.490067143	-0.660436277	1.15050342	8
TBX1	-0.491062311	-0.659534232	1.150596543	8
TBX18	-0.485307314	-0.664736892	1.150044206	8
TBX2	-0.462277703	-0.685225974	1.147503677	8
TBX3	-0.465440615	-0.682442943	1.147883558	8
TCEAL1	-0.362105476	-0.768504905	1.130610382	8
TCEAL4	-0.31153428	-0.807150195	1.118684475	8
TCEAL6	-0.488013376	-0.662294702	1.150308077	8
TCF15	-0.151145031	-0.915823664	1.066968695	8
TCF19	-0.207650772	-0.879872163	1.087522935	8
TCF21	-0.486990048	-0.66321911	1.150209157	8
TCF7L1	-0.398259073	-0.73950899	1.137768063	8
TCHH	-0.313881339	-0.80540484	1.119286179	8
TCHHL1	-0.487586726	-0.662680238	1.150266964	8
TCIM	-0.355463052	-0.773706603	1.129169655	8
TCTN2	-0.411325339	-0.728740134	1.140065473	8
TDO2	-0.314161549	-0.805196156	1.119357705	8
TDRD10	-0.379233336	-0.75491314	1.134146476	8
TDRD15	-0.330283095	-0.793078055	1.123361151	8
TDRD6	-0.415037674	-0.725651992	1.140689666	8
TEAD4	-0.393662549	-0.743260395	1.136922945	8

TEC	-0.427804469	-0.714934405	1.142738874	8
TECPR2	-0.416107871	-0.724759379	1.14086725	8
TECTA	-0.497983595	-0.653232816	1.151216412	8
TEK	-0.381068393	-0.753441523	1.134509917	8
TEKT3	-0.500949321	-0.650517755	1.151467076	8
TEKT4	-0.500562439	-0.650872449	1.151434888	8
TET1	-0.242160363	-0.856681942	1.098842304	8
TEX13B	-0.461436149	-0.68596481	1.147400959	8
TEX14	-0.434388249	-0.709347907	1.143736156	8
TEX2	-0.218013684	-0.873007682	1.091021366	8
TEX35	-0.495031343	-0.655926622	1.150957965	8
TEX36	-0.205360487	-0.881377836	1.086738322	8
TEX37	-0.481241355	-0.668392562	1.149633916	8
TFEC	-0.300665053	-0.815172727	1.11583778	8
TFF1	-0.398989507	-0.738911092	1.137900598	8
TFF3	-0.262718637	-0.842413852	1.10513249	8
TFPI2	-0.479144768	-0.67027113	1.149415897	8
TGFB3	-0.486856305	-0.663339846	1.150196151	8
TGIF2LY	-0.48415451	-0.66577505	1.14992956	8
TGM5	-0.467180532	-0.680907827	1.148088359	8
TGM6	-0.381287474	-0.753265632	1.134553106	8
TGM7	-0.412357913	-0.727882451	1.140240364	8
TGOLN2	-0.365781875	-0.765609308	1.131391183	8
THBS1	-0.493809638	-0.65703878	1.150848419	8

THBS2	-0.483003134	-0.666810592	1.149813727	8
THBS4	-0.396081602	-0.741288493	1.137370096	8
THOC2	-0.494382207	-0.65651774	1.150899948	8
THRSP	-0.436567807	-0.707489509	1.144057317	8
THSD1	-0.3828765	-0.751988583	1.134865083	8
TIA1	-0.244112779	-0.85534154	1.099454319	8
TIAM1	-0.23464792	-0.861810998	1.096458917	8
TIGD4	-0.483239974	-0.666597688	1.149837662	8
TIMP3	-0.475445945	-0.673574654	1.1490206	8
TIMP4	-0.475745237	-0.673307853	1.14905309	8
TINAG	-0.488089937	-0.662225499	1.150315436	8
TINAGL1	-0.363904848	-0.767089169	1.130994018	8
TJP1	-0.439734446	-0.704781474	1.14451592	8
TKTL2	-0.237160241	-0.86010076	1.097261	8
TLCD3A	-0.485056378	-0.664962986	1.150019364	8
TLE6	-0.451077346	-0.695002972	1.146080318	8
TLL1	-0.425918252	-0.716527417	1.142445668	8
TM2D2	-0.270099748	-0.8372076	1.107307347	8
TM4SF18	-0.405827777	-0.733290076	1.139117852	8
TM4SF4	-0.498054115	-0.653168361	1.151222476	8
TM4SF5	-0.437781936	-0.706452337	1.144234273	8
TM6SF2	-0.49195549	-0.658723779	1.150679269	8
TM9SF2	-0.440283226	-0.704311204	1.14459443	8
TMBIM1	-0.367218546	-0.764474534	1.13169308	8

TMCO2	-0.481840976	-0.667854487	1.149695463	8
TMED4	-0.318964637	-0.801608817	1.120573454	8
TMEFF1	-0.498430949	-0.652823849	1.151254798	8
TMEM100	-0.451790829	-0.694383775	1.146174603	8
TMEM107	-0.273914396	-0.834499532	1.108413928	8
TMEM120B	-0.276345158	-0.832767695	1.109112853	8
TMEM125	-0.299676197	-0.8158977	1.115573897	8
TMEM135	-0.403823732	-0.734941802	1.138765534	8
TMEM178A	-0.489986787	-0.66050907	1.150495857	8
TMEM182	-0.360003404	-0.770155212	1.130158616	8
TMEM185A	-0.484679944	-0.665302036	1.14998198	8
TMEM192	-0.250097246	-0.851213906	1.101311152	8
TMEM207	-0.20111948	-0.884155049	1.085274529	8
TMEM210	-0.161336253	-0.909522744	1.070858997	8
TMEM212	-0.486181719	-0.663948556	1.150130275	8
TMEM219	-0.33480901	-0.789636349	1.124445358	8
TMEM235	-0.478597025	-0.670761192	1.149358217	8
TMEM240	-0.462336291	-0.685174511	1.147510803	8
TMEM247	-0.483038292	-0.666778991	1.149817283	8
TMEM248	-0.429358657	-0.713619307	1.142977965	8
TMEM26	-0.457673997	-0.689259339	1.146933336	8
TMEM266	-0.460822877	-0.686502792	1.14732567	8
TMEM269	-0.455569623	-0.69109616	1.146665783	8
TMEM31	-0.477954329	-0.671335828	1.149290157	8

TMEM38B	-0.434265463	-0.709452468	1.14371793	8
TMEM39B	-0.321653068	-0.799592373	1.121245442	8
TMEM44	-0.291679949	-0.821730233	1.113410181	8
TMEM54	-0.457519978	-0.689393921	1.146913899	8
TMEM64	-0.488503363	-0.661851706	1.150355069	8
TMEM67	-0.496269098	-0.654798304	1.151067402	8
TMEM68	-0.246879997	-0.853436503	1.100316501	8
TMEM70	-0.449550628	-0.696326295	1.145876923	8
TMEM9	-0.411197362	-0.728846366	1.140043729	8
TMEM97	-0.312493337	-0.806437566	1.118930903	8
TMLHE	-0.288802906	-0.823815806	1.112618712	8
TMPPE	-0.494197544	-0.656685821	1.150883365	8
TMPRSS15	-0.471579325	-0.677013551	1.148592875	8
TMPRSS2	-0.497096945	-0.654042781	1.151139726	8
TMTC1	-0.39106401	-0.745372722	1.136436732	8
TMTC3	-0.31696158	-0.803107228	1.120068809	8
TNFAIP1	-0.123863459	-0.932298315	1.056161775	8
TNFAIP3	-0.260444638	-0.844008887	1.104453525	8
TNFAIP8L3	-0.337165992	-0.787837058	1.12500305	8
TNFRSF25	-0.219824855	-0.871799228	1.091624083	8
TNFSF18	-0.421908461	-0.719902868	1.141811329	8
TNIK	-0.277534543	-0.831918534	1.109453077	8
TNKS1BP1	-0.485086317	-0.664936014	1.150022331	8
TNMD	-0.466118066	-0.681845586	1.147963652	8

TNPO1	-0.193358467	-0.889200768	1.082559235	8
TNRC6A	-0.43336342	-0.710220178	1.143583598	8
TNS2	-0.204679685	-0.88182461	1.086504295	8
TOM1L1	-0.491784697	-0.658878815	1.150663512	8
TOP1MT	-0.327226051	-0.795392884	1.122618934	8
TOPAZ1	-0.174603357	-0.901199839	1.075803197	8
TP53INP2	-0.449301491	-0.696542029	1.14584352	8
TP53TG3B	-0.463926577	-0.683776357	1.147702934	8
TP53TG3C	-0.205567319	-0.88124203	1.086809349	8
TP53TG3D	-0.458576818	-0.688469988	1.147046807	8
TP53TG3E	-0.217587471	-0.873291683	1.090879154	8
TP53TG3F	-0.167647071	-0.905580753	1.073227823	8
TP63	-0.45381871	-0.692621196	1.146439906	8
TPCN2	-0.344118147	-0.782502096	1.126620243	8
TPD52L1	-0.453723984	-0.692703617	1.146427601	8
TPI1	-0.303629214	-0.812994688	1.116623902	8
TPO	-0.409636324	-0.730140961	1.139777285	8
TPP1	-0.49667756	-0.654425614	1.151103174	8
TPRN	-0.307093672	-0.810439734	1.117533406	8
TPRX1	-0.495469553	-0.655527335	1.150996888	8
TRAF3IP1	-0.421006188	-0.720660341	1.141666529	8
TRAM1	-0.138187571	-0.923719463	1.061907034	8
TRAPPC1	-0.161030524	-0.909712933	1.070743457	8
TRAPPC2	-0.315234621	-0.804396385	1.119631006	8

TRAPPC9	-0.126286995	-0.930857859	1.057144854	8
TRHDE	-0.500971976	-0.65049698	1.151468956	8
TRHR	-0.471707106	-0.676900141	1.148607246	8
TRIM15	-0.438880658	-0.705512549	1.144393207	8
TRIM16L	-0.266076537	-0.840050863	1.1061274	8
TRIM29	-0.210772971	-0.877812917	1.088585889	8
TRIM42	-0.354876814	-0.77416384	1.129040653	8
TRIM50	-0.341401608	-0.784591668	1.125993276	8
TRIM60	-0.248073739	-0.852612794	1.100686533	8
TRIM71	-0.495341457	-0.655644073	1.15098553	8
TRIML1	-0.425696339	-0.716714615	1.142410954	8
TRPM5	-0.306270386	-0.811047797	1.117318183	8
TRPV1	-0.290587572	-0.822522905	1.113110477	8
TRPV3	-0.429966035	-0.713104752	1.143070786	8
TRUB1	-0.381845062	-0.752817773	1.134662835	8
TSEN34	-0.393289626	-0.743563915	1.136853541	8
TSKU	-0.491952874	-0.658726153	1.150679028	8
TSLP	-0.444180961	-0.700962852	1.145143813	8
TSNAXIP1	-0.471318254	-0.67724521	1.148563463	8
TSPAN1	-0.418820559	-0.722492078	1.141312637	8
TSPAN12	-0.467982196	-0.680199527	1.148181723	8
TSPAN13	-0.482931133	-0.666875306	1.149806439	8
TSPAN15	-0.462902109	-0.684677336	1.147579445	8
TSPAN3	-0.359035209	-0.770914029	1.129949237	8

TSPO	-0.380671298	-0.753760227	1.134431525	8
TSPY1	-0.452839127	-0.693473117	1.146312244	8
TSPY10	-0.327137877	-0.795459532	1.122597409	8
TSPY3	-0.493819176	-0.657030103	1.15084928	8
TSPY4	-0.417954443	-0.72321673	1.141171173	8
TSPY8	-0.494692471	-0.65623526	1.150927731	8
TSSK2	-0.353576139	-0.775177232	1.128753371	8
TSSK3	-0.427472678	-0.715214861	1.142687539	8
TTC1	-0.339241014	-0.786249071	1.125490085	8
TTC23	-0.471092766	-0.677445241	1.148538006	8
TTC23L	-0.498737787	-0.652543222	1.151281009	8
TTC39C	-0.485955796	-0.664152315	1.150108111	8
TTC8	-0.402707889	-0.735859888	1.138567777	8
TTK	-0.48060299	-0.668965009	1.149567999	8
TTL	-0.293520024	-0.820392766	1.11391279	8
TTYH3	-0.469249968	-0.679078114	1.148328082	8
TUBAL3	-0.461404688	-0.685992418	1.147397105	8
TUBGCP4	-0.276389188	-0.83273628	1.109125468	8
TULP2	-0.381995296	-0.752697056	1.134692353	8
TWSG1	-0.491455707	-0.659177372	1.150633078	8
TXLNB	-0.149279346	-0.916968494	1.06624784	8
TXNDC16	-0.408999553	-0.730668403	1.139667956	8
TXNL1	-0.411075946	-0.728947139	1.140023085	8
TXNL4B	-0.197190062	-0.88671561	1.083905672	8

TXNRD3	-0.153402875	-0.914434612	1.067837487	8
TYK2	-0.348513312	-0.779107835	1.127621147	8
UBAC2	-0.295270821	-0.819117586	1.114388407	8
UBD	-0.454386539	-0.692126944	1.146513483	8
UBE2D2	-0.294190002	-0.819905093	1.114095095	8
UBE2F	-0.48810532	-0.662211594	1.150316914	8
UBE2K	-0.194941175	-0.888175624	1.083116799	8
UBE2L5	-0.385272946	-0.75005837	1.135331316	8
UBE2L6	-0.458604077	-0.688446143	1.14705022	8
UBE2Q2	-0.421661386	-0.720110367	1.141771753	8
UBE3B	-0.259188496	-0.844888175	1.104076671	8
UBE3D	-0.236613815	-0.860473163	1.097086978	8
UBFD1	-0.159045869	-0.910945798	1.069991666	8
UBR1	-0.343580977	-0.782915793	1.12649677	8
UBTD2	-0.249253849	-0.851797363	1.101051212	8
UBXN11	-0.498226962	-0.653010358	1.15123732	8
UCHL3	-0.462199917	-0.685294295	1.147494212	8
UFSP2	-0.405675827	-0.73341544	1.139091267	8
UGDH	-0.455764839	-0.690925944	1.146690783	8
UGT1A1	-0.38401367	-0.751073292	1.135086962	8
UGT1A10	-0.441267929	-0.703466658	1.144734587	8
UGT1A7	-0.475202005	-0.673792047	1.148994052	8
UGT1A9	-0.443269101	-0.701747482	1.145016583	8
UGT2A1	-0.379970537	-0.754322305	1.134292842	8

UGT2A3	-0.419717779	-0.72174067	1.141458449	8
UGT2B10	-0.478091616	-0.671213114	1.14930473	8
UGT2B28	-0.495270996	-0.65570828	1.150979275	8
UGT2B4	-0.46153092	-0.685881641	1.147412561	8
UGT3A2	-0.431354701	-0.711927006	1.143281707	8
ULK2	-0.212420411	-0.876723249	1.08914366	8
UNC13B	-0.499203858	-0.65211678	1.151320639	8
UNC13D	-0.424769549	-0.717495929	1.142265478	8
UPK1B	-0.189152682	-0.891915396	1.081068078	8
UPK3BL2	-0.481872754	-0.667825961	1.149698715	8
UQCR11	-0.439078606	-0.705343113	1.144421719	8
UROC1	-0.395884402	-0.74144944	1.137333842	8
USH1G	-0.403391261	-0.735297762	1.138689023	8
USHBP1	-0.427805077	-0.714933891	1.142738968	8
USP13	-0.465199015	-0.682655871	1.147854886	8
USP31	-0.475826948	-0.673234997	1.149061945	8
USP33	-0.328364225	-0.794531975	1.1228962	8
USP34	-0.287334244	-0.824877805	1.112212048	8
USP37	-0.471845666	-0.676777146	1.148622811	8
USP39	-0.396191972	-0.741198399	1.137390371	8
USP45	-0.423646024	-0.718442022	1.142088047	8
USP47	-0.110944387	-0.939901358	1.050845745	8
USP6NL	-0.434824407	-0.708976377	1.143800784	8
USP9Y	-0.485276839	-0.664764353	1.150041192	8

UTF1	-0.43929257	-0.705159927	1.144452496	8
UTY	-0.477202272	-0.672007723	1.149209995	8
UXS1	-0.495450159	-0.65554501	1.150995169	8
VANGL1	-0.45814574	-0.688846985	1.146992725	8
VAV3	-0.177704193	-0.899234901	1.076939094	8
VCAM1	-0.489562076	-0.6608937	1.150455776	8
VCL	-0.352094805	-0.77632959	1.128424395	8
VCY	-0.471725301	-0.676883991	1.148609291	8
VCY1B	-0.46958328	-0.678783018	1.148366299	8
VDAC3	-0.218321439	-0.872802524	1.091123963	8
VEGFC	-0.480422828	-0.669126493	1.149549321	8
VEZT	-0.24639852	-0.853768409	1.100166929	8
VGLL3	-0.482433763	-0.667322192	1.149755955	8
VIL1	-0.355047312	-0.77403089	1.129078202	8
VIP	-0.49866244	-0.652612142	1.151274582	8
VKORC1L1	-0.495509313	-0.655491096	1.15100041	8
VLDLR	-0.247433139	-0.853054965	1.100488104	8
VN1R1	-0.272621319	-0.835418836	1.108040155	8
VN1R4	-0.492043115	-0.658644226	1.150687341	8
VOPP1	-0.217078153	-0.873630872	1.090709024	8
VPS13A	-0.467572997	-0.680561149	1.148134145	8
VPS37A	-0.376125155	-0.757398898	1.133524053	8
VPS41	-0.155434774	-0.913181207	1.068615981	8
VPS8	-0.37124639	-0.761283394	1.132529784	8

VRK2	-0.228815503	-0.865761927	1.09457743	8
VSTM5	-0.462298922	-0.685207337	1.147506258	8
VTN	-0.380053068	-0.75425613	1.134309198	8
WASL	-0.43145102	-0.711845249	1.143296269	8
WDFY1	-0.241410836	-0.857195705	1.098606541	8
WDR41	-0.239448653	-0.85853855	1.097987204	8
WFDC12	-0.470856477	-0.677654799	1.148511276	8
WFDC3	-0.237882212	-0.859608351	1.097490563	8
WFDC6	-0.22044673	-0.8713837	1.09183043	8
WNK4	-0.383679008	-0.751342776	1.135021785	8
WNT2	-0.375902028	-0.757577013	1.13347904	8
WNT5B	-0.390902404	-0.745503889	1.136406293	8
WRN	-0.393623139	-0.743292477	1.136915616	8
WRNIP1	-0.207678344	-0.879854011	1.087532356	8
WWC2	-0.476112677	-0.672980181	1.149092858	8
WWTR1	-0.408413704	-0.731153338	1.139567042	8
XAGE3	-0.471679071	-0.676925024	1.148604095	8
XIRP2	-0.462512422	-0.685019781	1.147532204	8
XKR9	-0.239249995	-0.858674333	1.097924328	8
XPO1	-0.193475634	-0.889124944	1.082600578	8
XPO4	-0.117624336	-0.935985996	1.053610332	8
XPR1	-0.451931431	-0.694261695	1.146193126	8
YAE1	-0.144653257	-0.91979563	1.064448887	8
YEATS2	-0.235537581	-0.861205946	1.096743528	8

YLPM1	-0.399751865	-0.738286546	1.13803841	8
YTHDC2	-0.431394936	-0.711892855	1.143287791	8
YWHAE	-0.476837579	-0.67233334	1.14917092	8
YWHAQ	-0.479143714	-0.670272073	1.149415786	8
ZAN	-0.423037164	-0.718954238	1.141991402	8
ZBED8	-0.448321976	-0.697389637	1.145711613	8
ZBTB10	-0.427733597	-0.71499432	1.142727917	8
ZBTB12	-0.487118779	-0.66310288	1.150221659	8
ZBTB41	-0.306423429	-0.810934805	1.117358234	8
ZBTB46	-0.46589121	-0.682045671	1.147936881	8
ZBTB7B	-0.195411566	-0.887870566	1.083282132	8
ZC3H12C	-0.497582496	-0.653599324	1.15118182	8
ZC3H13	-0.432447984	-0.710998503	1.143446487	8
ZC3H7A	-0.321920929	-0.799391131	1.12131206	8
ZC3HAV1L	-0.227713203	-0.866505588	1.094218791	8
ZC4H2	-0.312823107	-0.806192352	1.119015458	8
ZCCHC14	-0.308643873	-0.809293246	1.117937119	8
ZCCHC24	-0.449536632	-0.696338416	1.145875048	8
ZCWPW2	-0.451141843	-0.694947019	1.146088862	8
ZDHHC11	-0.338412408	-0.786883636	1.125296044	8
ZDHHC21	-0.35487434	-0.774165768	1.129040108	8
ZFAND1	-0.141098863	-0.921956656	1.063055519	8
ZFAND2A	-0.427989778	-0.714777721	1.1427675	8
ZFAND4	-0.355531702	-0.773653041	1.129184742	8

ZFC3H1	-0.26129687	-0.843411604	1.104708474	8
ZFP1	-0.272456948	-0.835535597	1.107992544	8
ZFP37	-0.400947643	-0.737305865	1.138253508	8
ZFPM2	-0.501457629	-0.650051506	1.151509134	8
ZFTA	-0.468980949	-0.679316207	1.148297157	8
ZFYVE27	-0.383082593	-0.751822788	1.134905381	8
ZHX3	-0.41164376	-0.72847575	1.14011951	8
ZKSCAN2	-0.411762707	-0.728376965	1.140139671	8
ZMIZ2	-0.431856309	-0.71150114	1.143357449	8
ZMYM2	-0.267348122	-0.83915364	1.106501762	8
ZMYM5	-0.497684389	-0.653506234	1.151190623	8
ZMYND11	-0.316868325	-0.803176906	1.120045232	8
ZNF100	-0.240586332	-0.857760341	1.098346673	8
ZNF112	-0.453045406	-0.693293798	1.146339204	8
ZNF148	-0.421542239	-0.720210408	1.141752648	8
ZNF215	-0.46246125	-0.685064739	1.147525989	8
ZNF229	-0.497242612	-0.653909767	1.15115238	8
ZNF260	-0.466861458	-0.681189566	1.148051024	8
ZNF280C	-0.492547981	-0.658185718	1.150733699	8
ZNF280D	-0.343015907	-0.783350709	1.126366616	8
ZNF284	-0.427717193	-0.715008188	1.14272538	8
ZNF3	-0.472243092	-0.676424259	1.148667351	8
ZNF326	-0.37511157	-0.758207654	1.133319224	8
ZNF358	-0.483089615	-0.666732859	1.149822473	8

ZNF366	-0.370240218	-0.762081895	1.132322113	8
ZNF442	-0.329379069	-0.793763423	1.123142492	8
ZNF462	-0.488459716	-0.661891177	1.150350893	8
ZNF469	-0.40934276	-0.730384168	1.139726928	8
ZNF470	-0.205707364	-0.881150058	1.086857422	8
ZNF471	-0.499695059	-0.651667105	1.151362164	8
ZNF479	-0.453079511	-0.693264147	1.146343657	8
ZNF501	-0.156413036	-0.912576618	1.068989653	8
ZNF532	-0.466676318	-0.681352997	1.148029315	8
ZNF563	-0.441157079	-0.703561777	1.144718856	8
ZNF608	-0.494258812	-0.656630059	1.150888871	8
ZNF625	-0.432283561	-0.711138216	1.143421777	8
ZNF672	-0.143153135	-0.920708868	1.063862003	8
ZNF676	-0.402714606	-0.735854365	1.13856897	8
ZNF697	-0.344364907	-0.782311972	1.126676879	8
ZNF705E	-0.114160317	-0.938020624	1.052180941	8
ZNF726	-0.434042289	-0.709642477	1.143684767	8
ZNF727	-0.305558911	-0.811572821	1.117131732	8
ZNF728	-0.495881858	-0.655151473	1.151033331	8
ZNF729	-0.424934619	-0.717356829	1.142291447	8
ZNF732	-0.46972558	-0.678657002	1.148382582	8
ZNF765	-0.147254724	-0.918207823	1.065462547	8
ZNF790	-0.231481733	-0.863959167	1.0954409	8
ZNF793	-0.167897491	-0.905423694	1.073321185	8

ZNF804B	-0.492242012	-0.658463623	1.150705635	8
ZNF823	-0.406510517	-0.732726525	1.139237042	8
ZNF865	-0.457621372	-0.689305325	1.146926697	8
ZNF883	-0.339082719	-0.786370343	1.125453062	8
ZNF98	-0.459724885	-0.687465074	1.147189959	8
ZNF99	-0.491981296	-0.658700351	1.150681647	8
ZNHIT6	-0.241957255	-0.856821207	1.098778461	8
ZPLD1	-0.485310574	-0.664733954	1.150044528	8
ZRANB3	-0.415323802	-0.725413447	1.140737249	8
ZSCAN20	-0.12022773	-0.934450849	1.054678578	8
ZSCAN5B	-0.487252174	-0.662982422	1.150234596	8
ZSWIM7	-0.302251411	-0.814007997	1.116259408	8
ZSWIM9	-0.436017348	-0.707959281	1.143976629	8
ZWILCH	-0.453512707	-0.692887419	1.146400126	8
ZZZ3	-0.294814988	-0.819449834	1.114264822	8
A2M	0.425549158	0.716838747	-1.142387905	9
AADACL2	-0.000848418	1.000423939	-0.999575521	9
AAGAB	0.369669222	0.762534641	-1.132203863	9
ABCB10	0.234875142	0.861656526	-1.096531668	9
ABCB7	0.371837156	0.760814144	-1.1326513	9
ABCD1	0.293803851	0.820186216	-1.113990067	9
ABCG8	0.39572968	0.741575693	-1.137305373	9
ABHD12B	0.076254067	0.959690078	-1.035944145	9
ABHD3	0.25774331	0.84589821	-1.10364152	9

ABI3	0.433487804	0.710114362	-1.143602167	9
ACAA1	0.174797851	0.901076812	-1.075874663	9
ACACA	0.373341532	0.759617813	-1.132959345	9
ACBD5	0.356972173	0.772528188	-1.129500361	9
ACD	0.379236336	0.754910736	-1.134147072	9
ACOT12	0.274312265	0.834216395	-1.108528659	9
ACOX1	-0.204929028	1.08659005	-0.881661022	9
ACOX3	0.437607083	0.706601792	-1.144208875	9
ACP5	0.385050501	0.750237754	-1.135288256	9
ACSL3	-0.127854559	1.057778329	-0.92992377	9
ACSM1	-0.058735214	1.028073084	-0.969337871	9
ACTL10	-0.130269233	1.05875046	-0.928481227	9
ACTR1A	0.429314558	0.713656653	-1.142971212	9
ACTR1B	-0.027487163	1.013460213	-0.985973049	9
ACTR2	0.316402144	0.803525117	-1.119927261	9
ADAM15	0.432756351	0.710736411	-1.143492761	9
ADAM29	0.088099954	0.953035174	-1.041135128	9
ADAP1	0.109650528	0.940655811	-1.050306339	9
ADCK2	0.390488948	0.745839362	-1.13632831	9
ADD1	0.204369631	0.882027961	-1.086397593	9
ADD2	0.124187281	0.932106108	-1.056293389	9
ADGRE2	-0.046469769	1.022424767	-0.975954998	9
ADGRE3	-0.014713394	1.007275512	-0.992562118	9
ADM	0.407324769	0.732053865	-1.139378634	9

ADORA3	0.224130066	0.868916254	-1.09304632	9
ADRB1	-0.086706067	1.04052982	-0.953823753	9
ADRB2	0.360159048	0.770033151	-1.130192199	9
AFTPH	0.113315509	0.938515445	-1.051830954	9
AGMO	0.393033159	0.743772579	-1.136805738	9
AGO4	0.074334977	0.960758227	-1.035093204	9
AGPAT2	0.007417759	0.996270486	-1.003688246	9
AGRP	-0.122821388	1.055737693	-0.932916305	9
AHSA1	0.00393846	0.998024953	-1.001963413	9
AHSP	0.099130724	0.946742735	-1.045873459	9
AIDA	0.220686512	0.871223399	-1.091909911	9
AK3	0.402772092	0.735807094	-1.138579186	9
AKNA	0.386707176	0.748900714	-1.13560789	9
AKR1B1	0.198780058	0.885680966	-1.084461025	9
AKR1C1	0.390086071	0.746166103	-1.136252174	9
AKT1S1	0.444797426	0.700431953	-1.145229379	9
ALAS2	0.015564402	0.992126951	-1.007691353	9
ALDH1A1	0.024735086	0.987402997	-1.012138082	9
ALDH3A2	-0.008342467	1.004145134	-0.995802668	9
ALDH5A1	-0.098414201	1.045568473	-0.947154272	9
ALDOA	0.055544523	0.971070121	-1.026614644	9
ALG12	0.014239658	0.99280413	-1.007043788	9
ALKBH6	0.061219807	0.967983658	-1.029203465	9
ALOX15B	0.136480343	0.924750179	-1.061230523	9

ALPK1	0.238801607	0.858980689	-1.097782296	9
ANGEL1	0.075764994	0.959962556	-1.03572755	9
ANKH	-0.206532642	1.087140399	-0.880607756	9
ANKRD12	0.283355572	0.827745865	-1.111101437	9
ANKRD23	-0.116461761	1.053131626	-0.936669865	9
ANKRD42	0.344266677	0.782387662	-1.12665434	9
ANKRD49	0.062694523	0.967177675	-1.029872197	9
ANKRD9	-0.03237247	1.015793167	-0.983420696	9
ANP32B	-0.170989556	1.074469969	-0.903480413	9
ANXA1	0.128099392	0.929777708	-1.0578771	9
ANXA11	-0.087305202	1.04079018	-0.953484978	9
ANXA2	0.072243046	0.961919411	-1.034162457	9
ANXA8	-0.179499347	1.077593279	-0.898093931	9
AP1B1	0.440957936	0.703732628	-1.144690564	9
APIP	0.253656191	0.848745532	-1.102401723	9
APOOL	0.274122061	0.834351766	-1.108473827	9
ARAP1	0.08939629	0.952300465	-1.041696755	9
ARAP3	0.15711635	0.912141497	-1.069257847	9
ARFGAP2	-0.107038173	1.049213378	-0.942175205	9
ARHGAP12	-0.217418702	1.090822802	-0.8734041	9
ARHGAP24	0.432694346	0.710789118	-1.143483464	9
ARHGAP26	0.263506146	0.841860497	-1.105366643	9
ARHGAP4	-0.147167768	1.065428749	-0.918260981	9
ARHGAP42	0.12145203	0.933727128	-1.055179158	9

ARHGAP9	0.101537524	0.945357535	-1.046895058	9
ARHGEF11	0.307669117	0.810014385	-1.117683502	9
ARID4A	0.26123112	0.843457705	-1.104688826	9
ARID4B	0.325894204	0.796398886	-1.122293091	9
ARL11	0.263977501	0.84152905	-1.105506552	9
ARMC1	0.382373667	0.752392938	-1.134766604	9
ARMH1	0.089966641	0.95197681	-1.041943452	9
ARRB2	-0.031300691	1.015282878	-0.983982187	9
ARRDC5	0.248560847	0.852276349	-1.100837196	9
ASB16	-0.188575506	1.080862362	-0.892286856	9
ASB2	0.411937786	0.728231538	-1.140169324	9
ASB3	0.149028885	0.91712198	-1.066150865	9
ASCC2	-0.039109147	1.018980837	-0.97987169	9
ASCL2	0.26193863	0.84296144	-1.10490007	9
ASPHD2	0.140899191	0.922077766	-1.062976957	9
ASTL	0.072595172	0.961724185	-1.034319357	9
ASZ1	-0.106327698	1.048915231	-0.942587534	9
ATAD2	0.147834344	0.917853343	-1.065687687	9
ATG12	0.391902556	0.744691742	-1.136594297	9
ATG2A	0.358315995	0.771477177	-1.129793172	9
ATG7	0.16359834	0.908113295	-1.071711634	9
ATG9B	-0.12219794	1.055483577	-0.933285638	9
ATL2	0.000527886	0.999735952	-1.000263839	9
ATP1B3	0.005041649	0.997469644	-1.002511292	9

ATP2C2	-0.156174945	1.068898776	-0.912723832	9
ATP5F1B	0.34398865	0.78260185	-1.1265905	9
ATP5F1D	0.425370551	0.716989357	-1.142359907	9
ATP5MGL	0.149396409	0.916896741	-1.066293149	9
ATP5PD	-0.029742836	1.014539624	-0.984796788	9
ATP6V0D1	0.12055516	0.934257407	-1.054812567	9
ATP6V0E2	0.089507739	0.95223724	-1.04174498	9
ATP6V1A	0.144325125	0.919995537	-1.064320662	9
ATPAF1	0.427067724	0.715557022	-1.142624745	9
ATXN1	0.159130153	0.910893502	-1.070023655	9
ATXN1L	0.27823327	0.831419137	-1.109652407	9
ATXN3	0.234827502	0.861688916	-1.096516418	9
AVPI1	0.007908426	0.996022333	-1.003930759	9
AZIN2	0.30539299	0.8116952	-1.11708819	9
B3GNT8	-0.086604973	1.040485862	-0.953880889	9
B3GNTL1	0.430545248	0.712613733	-1.143158981	9
BABAM1	0.318232235	0.802157092	-1.120389327	9
BAZ1B	0.228169856	0.866197627	-1.094367483	9
BCAS2	0.28851509	0.824024068	-1.112539158	9
BCAS3	0.439945452	0.704600689	-1.144546141	9
BCAS4	0.191715579	0.890262818	-1.081978397	9
BCAT2	0.063608917	0.966677103	-1.03028602	9
BCKDK	0.402505775	0.73602606	-1.138531835	9
BCL2L13	-0.12484881	1.056562011	-0.931713202	9

BCLAF1	0.097830837	0.94748904	-1.045319877	9
BDKRB1	0.331108668	0.792451557	-1.123560225	9
BEST4	0.375975736	0.757518179	-1.133493915	9
BFSP2	0.349067711	0.778678503	-1.127746214	9
BICRA	0.385996522	0.749474558	-1.13547108	9
BIK	0.133863751	0.92632558	-1.060189331	9
BLOC1S2	0.414199513	0.726350335	-1.140549848	9
BLVRB	0.071930216	0.962092773	-1.034022988	9
BMP3	0.187228539	0.893152727	-1.080381266	9
BOD1L1	0.43931124	0.70514394	-1.14445518	9
BORCS8	0.312983231	0.806073251	-1.119056482	9
BRD4	0.420209234	0.721328768	-1.141538001	9
BRI3	-0.12039696	1.05474784	-0.934350879	9
BSG	-0.16774844	1.073265622	-0.905517181	9
BTBD1	0.05559857	0.971040842	-1.026639412	9
BTBD18	0.378725206	0.755320102	-1.134045308	9
BTBD7	0.000693403	0.999653118	-1.000346521	9
BTF3	-0.167266038	1.073085673	-0.905819635	9
BTG2	0.415638093	0.725151334	-1.140789428	9
BTN1A1	0.407125876	0.732218228	-1.139344104	9
BUD31	0.079844219	0.957684364	-1.037528583	9
BZW1	0.415504504	0.725262756	-1.14076726	9
C11orf40	0.383825027	0.751225208	-1.135050235	9
C11orf54	-0.011741094	1.005818851	-0.994077756	9

C11orf58	0.408421102	0.731147216	-1.139568318	9
C11orf65	0.065006792	0.96591064	-1.030917432	9
C11orf71	0.439012341	0.705399838	-1.144412178	9
C13orf42	-0.049632344	1.023891982	-0.974259637	9
C16orf54	0.047489701	0.975409065	-1.022898766	9
C16orf91	0.272978056	0.835165353	-1.108143409	9
C17orf64	0.318635061	0.801855593	-1.120490654	9
C17orf99	-0.088186701	1.04117275	-0.952986049	9
C19orf25	0.050248427	0.973928498	-1.024176926	9
C19orf38	0.100039904	0.946219985	-1.04625989	9
C1D	-0.06400478	1.030464979	-0.966460199	9
C1GALT1C1	-0.172637822	1.075079315	-0.902441493	9
C1orf105	0.412359317	0.727881285	-1.140240601	9
C1orf122	0.359571051	0.770494167	-1.130065218	9
C1orf159	-0.217653689	1.090901258	-0.873247569	9
C1orf56	-0.160920074	1.070701698	-0.909781624	9
C20orf27	-0.207445922	1.087452925	-0.880007003	9
C2CD4D	0.215843225	0.874452439	-1.090295664	9
C3orf49	0.325578667	0.796637005	-1.122215672	9
C3orf86	0.267228322	0.839238226	-1.106466549	9
C4B	-0.157515128	1.069409742	-0.911894614	9
C4B_2	0.41376493	0.726712167	-1.140477097	9
C5orf58	0.447939641	0.697720235	-1.145659876	9
C7orf25	-0.138806261	1.062151647	-0.923345385	9

C7orf61	0.422350913	0.719531145	-1.141882059	9
C8G	-0.000290188	1.000145062	-0.999854875	9
C9orf47	0.251675143	0.850120786	-1.10179593	9
C9orf78	0.33872395	0.786645119	-1.125369069	9
CA1	0.099320628	0.946633598	-1.045954226	9
CABP4	-0.157605711	1.069444228	-0.911838518	9
CALHM2	0.133409303	0.92659866	-1.060007963	9
CAMK1	0.166002431	0.906611029	-1.07261346	9
CAMK1D	0.217676941	0.873232078	-1.090909019	9
CAMK2N1	0.404730284	0.73419508	-1.138925363	9
CAMTA2	-0.08513404	1.03984539	-0.954711349	9
CAPN12	0.395681613	0.741614911	-1.137296524	9
CARHSP1	-0.12722724	1.057525046	-0.930297806	9
CASC3	0.419129336	0.722233567	-1.141362902	9
CASP3	-0.000670668	1.000335165	-0.999664498	9
CASP8	0.249277724	0.851780854	-1.101058578	9
CASTOR1	0.131571392	0.927701459	-1.059272851	9
CATIP	0.343880016	0.782685521	-1.126565537	9
CATSPERZ	0.180979913	0.897151022	-1.078130936	9
CBLL1	0.37869769	0.755342133	-1.134039823	9
CBR4	0.036480018	0.981260819	-1.017740838	9
CBSL	0.349133915	0.778627216	-1.127761131	9
CBWD3	0.27517261	0.8336037	-1.10877631	9
CBX1	0.117776546	0.935896384	-1.05367293	9

CC2D1A	0.237977029	0.859543652	-1.097520681	9
CCAR1	-0.213549439	1.089524677	-0.875975237	9
CCDC106	0.375268931	0.758082152	-1.133351084	9
CCDC121	0.286658384	0.825365924	-1.112024308	9
CCDC159	0.214777722	0.875160322	-1.089938044	9
CCDC179	0.068056899	0.964233136	-1.032290036	9
CCDC192	0.172201945	0.902716436	-1.074918381	9
CCDC194	0.042221725	0.978220411	-1.020442136	9
CCDC200	-0.018169045	1.008960722	-0.990791677	9
CCDC25	0.365179356	0.766084675	-1.131264031	9
CCDC61	-0.168282286	1.07346455	-0.905182264	9
CCDC88B	-0.164295539	1.071973629	-0.90767809	9
CCL23	0.264324404	0.841285001	-1.105609405	9
CCL3L1	-0.071857923	1.033990748	-0.962132825	9
CCL3L3	-0.106853219	1.0491358	-0.942282581	9
CCL4	-0.204824727	1.086554184	-0.881729457	9
CCL4L1	0.32991938	0.793353882	-1.123273262	9
CCL4L2	-0.131854307	1.059386177	-0.92753187	9
CCNC	0.38643036	0.749124293	-1.135554653	9
CCNH	0.010589912	0.994662988	-1.0052529	9
CCNI	0.218467058	0.872705424	-1.091172483	9
CCNT2	0.432369924	0.711064835	-1.143434759	9
CCR2	-0.133707804	1.060127111	-0.926419307	9
CCR8	0.29577264	0.818751621	-1.114524261	9

CCS	-0.187204854	1.080372794	-0.89316794	9
CD1E	0.428042749	0.714732928	-1.142775677	9
CD244	-0.047630644	1.022964205	-0.975333562	9
CD300A	-0.08971658	1.041835322	-0.952118741	9
CD300LD	0.31007055	0.808236333	-1.118306883	9
CD59	0.148729256	0.917305532	-1.066034788	9
CD70	0.282053116	0.828681919	-1.110735035	9
CD80	0.398369967	0.739418248	-1.137788215	9
CD81	0.063407633	0.966787347	-1.03019498	9
CD86	0.105291824	0.943188022	-1.048479846	9
CD8B2	0.437367072	0.706806892	-1.144173965	9
CD9	-0.124915471	1.056589062	-0.931673591	9
CD93	0.181333337	0.89692569	-1.078259026	9
CDAN1	0.408531985	0.731055456	-1.139587441	9
CDC123	0.17669139	0.899877516	-1.076568906	9
CDC16	0.243624822	0.855676826	-1.099301649	9
CDC27	0.076893948	0.959333308	-1.036227256	9
CDC34	0.251838359	0.850007601	-1.10184596	9
CDC42EP4	0.392918441	0.743865895	-1.136784337	9
CDC73	0.091743496	0.950966929	-1.042710425	9
CDCA8	0.323474819	0.798222501	-1.121697321	9
CDH3	0.069911072	0.963209947	-1.033121019	9
CDKL5	0.088476021	0.952822166	-1.041298187	9
CDPF1	0.059387876	0.968982591	-1.028370467	9

CDV3	-0.011361817	1.005632498	-0.994270681	9
CEACAM3	-0.187496136	1.080476956	-0.892980821	9
CEACAM4	0.146793281	0.918489845	-1.065283126	9
CEACAM8	-0.175614357	1.076174366	-0.900560009	9
CENPBD1	0.042505305	0.978069605	-1.02057491	9
CEP350	0.383806663	0.751239995	-1.135046658	9
CERS4	0.185889201	0.89401229	-1.079901491	9
CFAP410	0.284986452	0.826571809	-1.111558261	9
CFAP92	0.390511147	0.745821354	-1.136332501	9
CFD	-0.134587253	1.06047775	-0.925890497	9
CFL1	0.333658792	0.790512684	-1.124171476	9
CFLAR	0.076134223	0.959756863	-1.035891087	9
CHCHD10	0.299233481	0.81622201	-1.115455492	9
CHCHD3	-0.149419672	1.066302152	-0.91688248	9
CHCHD7	0.403525046	0.735187664	-1.13871271	9
CHIT1	0.4041756	0.734652059	-1.138827659	9
CHP1	0.13342084	0.926591729	-1.060012569	9
CHPT1	0.04954036	0.974309053	-1.023849413	9
CHST13	0.247988761	0.852671469	-1.100660229	9
CHST7	0.261191904	0.843485201	-1.104677105	9
CIDEB	0.33909832	0.786358391	-1.125456712	9
CINP	0.40568258	0.733409869	-1.139092449	9
CISD2	0.212139199	0.876909403	-1.089048603	9
CLCN1	0.323124455	0.798486177	-1.121610633	9

CLEC12A	-0.042455724	1.0205517	-0.978095976	9
CLEC18B	0.16995788	0.904129618	-1.074087498	9
CLEC2B	0.160578375	0.909994074	-1.07057245	9
CLIC2	0.422308911	0.719566441	-1.141875352	9
CLIC3	0.278371617	0.83132021	-1.109691827	9
CLN3	0.433352406	0.710229547	-1.143581953	9
CLNK	0.216834449	0.873793097	-1.090627546	9
CLPX	0.010053363	0.994935416	-1.00498878	9
CLSTN1	0.275150566	0.833619406	-1.108769972	9
CMTM3	0.407256083	0.73211063	-1.139366714	9
CNKSR2	0.284445248	0.82696166	-1.111406908	9
CNPPD1	0.143613392	0.920428858	-1.06404225	9
CNR1	-0.03383231	1.016486829	-0.982654518	9
CNTNAP3B	0.360961879	0.769403207	-1.130365086	9
COA3	0.235899323	0.860959748	-1.096859071	9
COL18A1	0.083584746	0.955584283	-1.039169028	9
COL4A3	0.394922333	0.742234137	-1.13715647	9
COL8A2	0.339195018	0.786284311	-1.12547933	9
COPB2	0.222173569	0.870228241	-1.09240181	9
COPS2	-0.165331239	1.072362132	-0.907030892	9
COPS7A	-0.154993112	1.068447035	-0.913453923	9
COQ8B	0.101469009	0.945397028	-1.046866037	9
CPA5	0.163490791	0.908180395	-1.071671186	9
CPM	0.443636546	0.701431402	-1.145067947	9

CR1	-0.138639774	1.062085851	-0.923446077	9
CRACDL	0.147348427	0.918150535	-1.065498961	9
CRB1	0.143899942	0.920254446	-1.064154388	9
CRB3	0.351454946	0.776826759	-1.128281706	9
CRELD2	0.410244479	0.729636874	-1.139881353	9
CRYBG2	0.339097756	0.786358823	-1.12545658	9
CSGALNACT1	0.374704155	0.75853248	-1.133236636	9
CSNK1A1	0.233882841	0.862330815	-1.096213657	9
CSRP1	0.203168867	0.882814779	-1.085983645	9
CSTF2	0.246663352	0.853585871	-1.100249223	9
CSTL1	-0.002556205	1.001275652	-0.998719447	9
CTAGE1	-0.057283783	1.027410596	-0.970126814	9
CTBS	-0.095721468	1.044418836	-0.948697368	9
CTDSP1	0.406675565	0.732590227	-1.139265792	9
CTRC	0.424636399	0.717608113	-1.142244512	9
CTRL	-0.134179384	1.060315205	-0.926135821	9
CTSC	0.398790831	0.739073766	-1.137864597	9
CTSL	0.278052935	0.831548066	-1.109601	9
CUEDC2	0.318081838	0.802269623	-1.120351461	9
CUL3	-0.149955402	1.066509361	-0.916553959	9
CUL4A	-0.029225085	1.014292201	-0.985067117	9
CWC22	0.118418486	0.935518256	-1.053936742	9
CWF19L2	-0.147481314	1.065550592	-0.918069278	9
CXADR	-0.076727967	1.036153848	-0.959425881	9

CXCL10	0.039630191	0.979595774	-1.019225965	9
CXorf58	-0.08121221	1.038129755	-0.956917545	9
CYB5B	0.050108887	0.974003525	-1.024112412	9
CYBA	0.133612899	0.926476338	-1.060089236	9
CYFIP2	-0.165199644	1.072312815	-0.907113171	9
CYP7B1	0.260057118	0.844280284	-1.104337402	9
CYTH4	-0.013238178	1.006553368	-0.99331519	9
DAXX	0.410797018	0.729178592	-1.13997561	9
DCAF11	0.241115622	0.857397936	-1.098513558	9
DCDC1	-0.153322024	1.067806444	-0.91448442	9
DCP2	0.251942125	0.849935631	-1.101877756	9
DCTN2	-0.098606669	1.045650435	-0.947043765	9
DDA1	-0.044712331	1.021606187	-0.976893856	9
DDB1	0.157057865	0.912177695	-1.069235559	9
DDHD1	0.40932023	0.73040283	-1.13972306	9
DDHD2	-0.031188369	1.01522935	-0.984040981	9
DDT	-0.0139338	1.006894091	-0.992960291	9
DDX18	0.093771243	0.949811532	-1.043582775	9
DDX24	0.256245097	0.84694352	-1.103188617	9
DDX50	0.099123345	0.946746976	-1.04587032	9
DEDD2	0.078192093	0.958608568	-1.036800661	9
DEFB105A	-0.210861353	1.088615866	-0.877754513	9
DEFB108B	-0.110197447	1.050534503	-0.940337057	9
DEFB131B	0.315525994	0.804179055	-1.119705048	9

DENND10	0.439256678	0.705190658	-1.144447337	9
DENND1A	0.083719993	0.955508151	-1.039228144	9
DEPP1	0.014759957	0.992538322	-1.007298279	9
DESI2	0.32264542	0.79884652	-1.12149194	9
DGKZ	0.367610432	0.764164682	-1.131775114	9
DHDH	0.028047171	0.98568138	-1.01372855	9
DHRS1	-0.179502824	1.077594543	-0.89809172	9
DHRSX	-0.146557826	1.065191511	-0.918633686	9
DHX30	-0.108829358	1.049963336	-0.941133978	9
DIAPH2	0.19057879	0.890996463	-1.081575253	9
DIMT1	0.442198587	0.702667618	-1.144866205	9
DLEU7	-0.000612836	1.000306277	-0.999693441	9
DLGAP4	0.196895993	0.88690675	-1.083802743	9
DMAC2L	0.307573651	0.81008497	-1.11765862	9
DMTF1	0.316140245	0.80372066	-1.119860905	9
DMXL2	-0.222189172	1.092406962	-0.87021779	9
DNAH17	-0.051198267	1.024615676	-0.97341741	9
DNAJA1	0.089028986	0.952508766	-1.041537753	9
DNAJC5	0.445401592	0.699911292	-1.145312885	9
DNAJC7	0.052728548	0.97259257	-1.025321117	9
DNLZ	0.269933414	0.837325411	-1.107258826	9
DNM1L	0.428176375	0.714619917	-1.142796292	9
DNTTIP2	-0.206806184	1.087234074	-0.88042789	9
DOCK11	-0.11390992	1.052077263	-0.938167343	9

DOCK5	0.323733004	0.798028131	-1.121761135	9
DOK7	-0.182220661	1.07858019	-0.896359529	9
DOP1A	0.37722605	0.756519442	-1.133745492	9
DPAGT1	0.257320955	0.846193072	-1.103514028	9
DPEP2NB	0.054350687	0.971716293	-1.026066981	9
DPH6	0.423218083	0.718802072	-1.142020155	9
DPM1	-0.131664835	1.059310288	-0.927645453	9
DPM2	0.146204555	0.918849421	-1.065053975	9
DPP3	0.277839859	0.831700367	-1.109540226	9
DPP8	0.364620418	0.766525374	-1.131145792	9
DRAP1	0.402207175	0.736271493	-1.138478668	9
DRD3	0.251190434	0.850456792	-1.101647226	9
DTX3	0.434321819	0.709404478	-1.143726298	9
DTYMK	0.072375935	0.961845746	-1.034221681	9
DUS1L	-0.026292576	1.012887017	-0.986594441	9
DUS3L	0.376854459	0.75681641	-1.133670869	9
DUSP23	0.356898019	0.772586139	-1.129484158	9
DUXB	0.34662646	0.780567042	-1.127193502	9
DYRK1B	-0.144598476	1.064427486	-0.91982901	9
DYRK3	-0.170815902	1.074405649	-0.903589746	9
DYSF	0.253173417	0.849080969	-1.102254385	9
DZIP1L	0.24039473	0.857891475	-1.098286206	9
E2F2	0.008907798	0.995516345	-1.004424143	9
EBLN2	0.419338923	0.722058048	-1.14139697	9

ECI1	-0.149896579	1.06648662	-0.916590041	9
ECSIT	0.087592621	0.953322363	-1.040914984	9
EDDM13	0.357961098	0.771754898	-1.129715995	9
EEF1AKMT4	0.357931588	0.771777985	-1.129709573	9
EFCAB11	-0.142774762	1.063713701	-0.920938939	9
EFHD2	-0.054377776	1.026079419	-0.971701643	9
EFL1	0.364053142	0.766972366	-1.131025508	9
EFTUD2	0.39438823	0.742669408	-1.137057639	9
EGFL7	0.27113687	0.83647251	-1.10760938	9
EGLN2	0.123731798	0.932376442	-1.056108239	9
EHHADH	0.411172091	0.728867343	-1.140039433	9
EID1	0.012361375	0.99376201	-1.006123384	9
EIF2S3B	0.213533494	0.875985809	-1.089519302	9
EIF3B	0.0030657	0.998463626	-1.001529325	9
EIF3M	0.14824233	0.917603675	-1.065846005	9
EIF5AL1	0.375975286	0.757518538	-1.133493824	9
EIPR1	-0.004584899	1.002284567	-0.997699667	9
ELK4	0.187975279	0.892672875	-1.080648154	9
ELOB	-0.124592238	1.056457867	-0.931865628	9
ELOF1	0.029360008	0.98499669	-1.014356698	9
EMB	-0.105800253	1.048693644	-0.942893391	9
EMP1	-0.078018258	1.03672395	-0.958705692	9
ENC1	-0.079916633	1.037560441	-0.957643808	9
ENDOU	-0.145869781	1.064923552	-0.919053771	9

ENOX2	0.158401882	0.911345188	-1.069747069	9
ENTPD1	-0.217370817	1.090806809	-0.873435993	9
EPB42	0.08248007	0.956205592	-1.038685662	9
EPGN	-0.076942718	1.036248821	-0.959306103	9
EPHB2	-0.064303514	1.03059995	-0.966296436	9
EPHB4	0.12045352	0.934317463	-1.054770983	9
EPHB6	0.447597209	0.698016211	-1.14561342	9
EPN1	0.346673937	0.780530363	-1.1272043	9
EPN2	0.243479376	0.855776729	-1.099256105	9
EPS8L1	0.43352759	0.710080513	-1.143608103	9
ERCC6L	0.115875339	0.93701443	-1.052889768	9
ERF	0.319949922	0.800870518	-1.120820441	9
ERGIC1	0.302116766	0.814106937	-1.116223703	9
ERLIN2	0.336008942	0.78872093	-1.124729872	9
ERVFC1	-0.005866982	1.002920583	-0.997053601	9
ESPN	0.345933909	0.781101858	-1.127035766	9
ESR2	0.09955369	0.946499621	-1.046053311	9
ETDC	-0.047337525	1.022828094	-0.975490568	9
ETFB	-0.227734078	1.094225592	-0.866491514	9
ETHE1	0.240929309	0.85752553	-1.098454839	9
EVI5	0.004335177	0.997825364	-1.002160541	9
EXOC4	0.342663172	0.783622059	-1.12628523	9
EXOC8	0.386062583	0.749421234	-1.135483816	9
EXOSC1	-0.010076843	1.005000342	-0.994923499	9

EXOSC2	-0.093571254	1.043496877	-0.949925623	9
EYS	0.419139455	0.722225093	-1.141364548	9
F11	-0.161393114	1.070880478	-0.909487364	9
F8	0.110042678	0.940427282	-1.05046996	9
F8A3	0.386902866	0.748742618	-1.135645484	9
FADD	0.433204688	0.710355192	-1.14355988	9
FADS2	0.234026886	0.862232983	-1.096259869	9
FAM104A	-0.158359519	1.069730968	-0.911371449	9
FAM110C	0.229285205	0.865444751	-1.094729956	9
FAM114A1	0.302615128	0.813740654	-1.116355782	9
FAM117A	0.406332319	0.732873656	-1.139205974	9
FAM200A	0.35381867	0.774988381	-1.128807051	9
FAM204A	0.207991134	0.879648051	-1.087639184	9
FAM209B	0.207344022	0.880074066	-1.087418087	9
FAM210B	0.034054149	0.982537949	-1.016592098	9
FAM53B	0.152176563	0.915189541	-1.067366103	9
FAM53C	0.24937359	0.851714562	-1.101088152	9
FAM71F2	0.30053442	0.815268547	-1.115802966	9
FAM76B	0.428727613	0.714153547	-1.14288116	9
FAM78B	-0.222180191	1.092403996	-0.870223806	9
FAM83F	0.180645141	0.897364374	-1.078009515	9
FAM98A	0.340217032	0.785500859	-1.125717891	9
FAM98C	0.387218972	0.748487166	-1.135706138	9
FARP2	0.293505386	0.820403417	-1.113908803	9

FASLG	0.164272237	0.907692641	-1.071964879	9
FBLN7	0.236037557	0.86086564	-1.096903196	9
FBP1	0.282573516	0.828308083	-1.110881599	9
FBXL22	-0.019197196	1.009460389	-0.990263193	9
FBXO38	0.011989608	0.993951288	-1.005940896	9
FBXO7	0.422518784	0.719390063	-1.141908847	9
FBXO9	0.337838878	0.787322512	-1.12516139	9
FCGR3A	0.19424835	0.888624618	-1.082872969	9
FCGR3B	-0.195312803	1.083247433	-0.88793463	9
FCHO2	0.385169904	0.750141471	-1.135311375	9
FCHSD1	-0.04314563	1.020874492	-0.977728862	9
FDX2	0.321968874	0.799355104	-1.121323977	9
FDXR	0.260877194	0.843705806	-1.104583	9
FECH	-0.086106541	1.040269018	-0.954162477	9
FFAR4	0.151836842	0.915398471	-1.067235313	9
FGFBP2	0.283633232	0.827546134	-1.111179367	9
FGR	0.288815564	0.823806645	-1.11262221	9
FHIP2A	-0.039403949	1.019119553	-0.979715604	9
FICD	0.352854681	0.775738706	-1.128593387	9
FLJ44635	0.340870994	0.784999076	-1.125870071	9
FLOT1	0.103008446	0.944508802	-1.047517247	9
FLVCR1	0.370282333	0.76204849	-1.132330823	9
FLYWCH1	-0.05023077	1.024168763	-0.973937993	9
FMN1	0.298679683	0.816627464	-1.115307147	9

FMO5	0.276997573	0.832302047	-1.10929962	9
FNBP1	0.277943028	0.831626629	-1.109569657	9
FNDC3B	0.367102757	0.764566058	-1.131668816	9
FNTA	0.194573708	0.888413813	-1.08298752	9
FNTB	0.094673442	0.949296463	-1.043969906	9
FOXD2	-0.044415735	1.02146781	-0.977052075	9
FOXN2	-0.165865629	1.072562263	-0.906696634	9
FOXO3	0.094481526	0.949406081	-1.043887607	9
FOXO4	-0.015480991	1.007650618	-0.992169628	9
FPGT	0.216153073	0.874246418	-1.090399491	9
FPR3	-0.086977114	1.04064764	-0.953670526	9
FRAT1	0.295192716	0.819174528	-1.114367244	9
FRAT2	0.2761792	0.832886088	-1.109065288	9
FRMD4A	-0.046423749	1.022403361	-0.975979612	9
FSCN1	0.153650817	0.914281836	-1.067932653	9
FUOM	0.363174728	0.767663966	-1.130838694	9
FUT10	0.214066295	0.87563247	-1.089698765	9
FUT4	0.359703237	0.770390553	-1.130093791	9
FXYD2	0.35309536	0.775551448	-1.128646808	9
FZD4	0.102786505	0.944636968	-1.047423473	9
FZD5	0.267567622	0.838998629	-1.106566251	9
FZR1	0.119724761	0.934747838	-1.054472599	9
GABARAP	-0.204948657	1.086596798	-0.881648142	9
GALK1	0.314148189	0.805206107	-1.119354296	9

GALNT1	0.198562132	0.885822893	-1.084385025	9
GALR2	0.055239482	0.971235328	-1.026474811	9
GAPT	-0.136703962	1.061319261	-0.9246153	9
GAS7	-0.03757178	1.018256385	-0.980684606	9
GATAD2A	0.193977579	0.888799993	-1.082777572	9
GATD3B	0.160937282	0.909770923	-1.070708205	9
GBP2	0.418832733	0.722481888	-1.141314621	9
GCHFR	-0.050893594	1.024475016	-0.973581421	9
GET3	0.396435464	0.740999598	-1.137435062	9
GFOD1	0.182713349	0.896044903	-1.078758251	9
GFUS	0.439318667	0.70513758	-1.144456247	9
GGNBP2	-0.079027052	1.037168799	-0.958141747	9
GGT5	0.198344498	0.885964592	-1.08430909	9
GGTLC2	0.195336604	0.887919192	-1.083255796	9
GHRL	0.19464673	0.888366489	-1.083013219	9
GIPR	0.286979492	0.825134061	-1.112113552	9
GLCCI1	-0.061829433	1.029480108	-0.967650675	9
GLIPR1L2	0.056463146	0.970572179	-1.027035325	9
GLIPR2	-0.146289312	1.065086982	-0.91879767	9
GLRX5	0.21231217	0.876794909	-1.089107079	9
GLTP	-0.061034622	1.029119374	-0.968084753	9
GMIP	0.216156662	0.874244031	-1.090400693	9
GMPR2	-0.055886158	1.026771169	-0.970885011	9
GNAI2	0.30423302	0.812550117	-1.116783138	9

GNAT2	0.412513567	0.727753076	-1.140266643	9
GNB2	-0.092137263	1.04288007	-0.950742807	9
GNG2	0.426096017	0.716377426	-1.142473443	9
GNPTAB	-0.064166646	1.03053812	-0.966371474	9
GOLGA4	0.321190401	0.79993983	-1.121130231	9
GOLGA6B	0.222081629	0.870289819	-1.092371448	9
GOLGA6C	0.084816839	0.954890221	-1.03970706	9
GPA33	0.095181167	0.94900633	-1.044187498	9
GPC4	0.256568619	0.846717951	-1.10328657	9
GPR135	-0.035615494	1.01733196	-0.981716466	9
GPR150	0.406281298	0.732915776	-1.139197074	9
GPR25	-0.162963166	1.071472621	-0.908509455	9
GPR37L1	-0.205293227	1.086715218	-0.881421991	9
GPR63	-0.169360126	1.073865516	-0.90450539	9
GPR85	0.23762165	0.859786111	-1.097407761	9
GPRC5D	-0.140850568	1.062957822	-0.922107253	9
GPS2	0.012414411	0.993734999	-1.00614941	9
GPX2	-0.010592296	1.005254074	-0.994661777	9
GRINA	0.218786099	0.872492627	-1.091278726	9
GRWD1	-0.020160081	1.009927618	-0.989767537	9
GSN	-0.033089345	1.016133999	-0.983044654	9
GSPT1	0.122084188	0.933352993	-1.055437181	9
GSTT1	-0.064373519	1.030631569	-0.96625805	9
GTF2E2	0.167408621	0.905730258	-1.073138879	9

GTF2F1	0.147424801	0.918103835	-1.065528637	9
GTSF1	-0.216465889	1.090504236	-0.874038346	9
GUCA2B	0.263134574	0.842121651	-1.105256224	9
GUCY2D	0.240757563	0.857643124	-1.098400687	9
GUK1	0.444783301	0.700444121	-1.145227422	9
GUSB	0.390438625	0.745880183	-1.136318808	9
H2AB2	-0.003820475	1.001904764	-0.998084289	9
H2AB3	-0.010803854	1.005358155	-0.994554301	9
H3-2	0.43509175	0.708748558	-1.143840308	9
H3-3B	0.071142321	0.962529074	-1.033671395	9
H3Y2	0.13323634	0.926702553	-1.059938893	9
HAGH	-0.08400539	1.039352845	-0.955347455	9
HCAR3	0.265920906	0.840160585	-1.106081491	9
HCFC1R1	-0.083620396	1.039184612	-0.955564216	9
HCFC2	0.364243025	0.766822777	-1.131065802	9
HCK	0.221660605	0.870571721	-1.092232326	9
HCLS1	0.08723929	0.95352226	-1.040761551	9
HDAC4	-0.135245801	1.060739928	-0.925494127	9
HDGF	0.105342132	0.943158878	-1.048501009	9
HDHD2	0.288975739	0.823690712	-1.112666452	9
HECTD1	0.015763251	0.99202519	-1.007788441	9
HECTD3	0.088323012	0.952908845	-1.041231857	9
HELB	0.243081102	0.856050203	-1.099131306	9
HELLS	-0.020321737	1.010005992	-0.989684255	9

HERC4	-0.190233068	1.081452448	-0.89121938	9
HES4	-0.020565109	1.010123946	-0.989558836	9
HHEX	0.181535749	0.896796594	-1.078332343	9
HHIPL2	-0.105921924	1.048744779	-0.942822855	9
HIC1	0.444190102	0.700954983	-1.145145085	9
HIPK1	0.071811518	0.962158532	-1.03397005	9
HK3	0.379734085	0.754511864	-1.134245949	9
HLA-G	0.366769877	0.764829116	-1.131598993	9
HM13	-0.079016352	1.037164085	-0.958147733	9
HMGB3	0.362706389	0.768032426	-1.130738815	9
HNRNPC	-0.075086299	1.035426678	-0.960340379	9
HNRNPK	0.041523093	0.978591682	-1.020114775	9
HOMER3	0.38083962	0.753625151	-1.134464771	9
HPDL	-0.19447006	1.082951037	-0.888480977	9
HPR	0.122456031	0.933132779	-1.05558881	9
HPS1	0.284764454	0.826731752	-1.111496206	9
HRAS	-0.182064081	1.078523561	-0.89645948	9
HRH4	0.030540346	0.984379998	-1.014920345	9
HRK	0.066184303	0.965263861	-1.031448164	9
HS1BP3	-0.086472964	1.040428449	-0.953955485	9
HSD3B7	0.209373725	0.87873674	-1.088110465	9
HSP90AA1	0.381699813	0.752934465	-1.134634278	9
HSP90B1	0.445585826	0.699752454	-1.14533828	9
HSPA1A	0.162685509	0.908682533	-1.071368041	9

HSPA4	0.375275063	0.758077262	-1.133352325	9
HSPBAP1	0.27389333	0.834514519	-1.108407849	9
HTR3B	0.412863613	0.727462046	-1.140325659	9
IBTK	-0.127824219	1.057766086	-0.929941867	9
ICAM3	0.282628448	0.828268609	-1.110897057	9
ICOSLG	-0.062752423	1.02989842	-0.967145997	9
IDO1	-0.02038216	1.010035281	-0.989653121	9
IER2	-0.142304036	1.063529049	-0.921225012	9
IER5L	0.127556766	0.930101365	-1.057658131	9
IFIT1B	-0.188744905	1.080922766	-0.892177861	9
IFITM1	0.365178465	0.766085378	-1.131263843	9
IFNAR1	0.384569584	0.750625425	-1.135195008	9
IFNAR2	-0.010496734	1.005207048	-0.994710314	9
IFT52	-0.078689264	1.03701993	-0.958330666	9
IGF1R	-0.004883512	1.002432813	-0.997549301	9
IGFBP7	0.119576015	0.934835631	-1.054411646	9
IGFL4	0.034150105	0.982487516	-1.016637621	9
IGFLR1	-0.121121624	1.055044177	-0.933922554	9
IKBIP	0.185392765	0.894330538	-1.079723303	9
IL12RB1	0.174447082	0.90129867	-1.075745752	9
IL1RN	-0.170995269	1.074472085	-0.903476816	9
IL21	0.258729393	0.845209224	-1.103938617	9
IL23R	-0.043687576	1.021127805	-0.977440229	9
IL31RA	0.367689054	0.764102502	-1.131791556	9

IMMP2L	-0.073115239	1.034550917	-0.961435678	9
INAFM1	0.064908955	0.965964332	-1.030873288	9
ING1	0.445961569	0.699428403	-1.145389972	9
INIP	0.102637351	0.944723081	-1.047360432	9
INPP4A	0.236409057	0.86061265	-1.097021707	9
INPP5D	0.402936884	0.735671569	-1.138608453	9
INPP5K	0.222203415	0.870208249	-1.092411665	9
INSC	0.363633656	0.767302724	-1.13093638	9
INSL3	0.032715021	0.983241057	-1.015956078	9
INSR	0.153886539	0.914136546	-1.068023085	9
INTS9	0.272472399	0.835524621	-1.107997021	9
IQCB1	-0.141228256	1.063106412	-0.921878157	9
IQCE	-0.214689895	1.089908527	-0.875218631	9
IQCN	0.32460689	0.79736982	-1.12197671	9
IQGAP1	0.242374158	0.856535313	-1.098909471	9
IQSEC1	0.102253004	0.944944902	-1.047197906	9
IRAK4	0.177787278	0.899182149	-1.076969427	9
ISCU	0.181421949	0.896869178	-1.078291127	9
ITCH	-0.00828546	1.004116987	-0.995831526	9
ITGAL	0.222705418	0.869871894	-1.092577313	9
ITGB1BP2	0.090275962	0.951801179	-1.042077141	9
ITGB2	0.240556956	0.857780448	-1.098337404	9
ITPRIP	0.049106917	0.974541824	-1.023648741	9
ITPRIPL1	0.431061782	0.712175585	-1.143237367	9

JAK3	0.435001635	0.708825358	-1.143826993	9
JAKMIP1	0.391958789	0.744646052	-1.136604841	9
JAZF1	-0.192471966	1.082246078	-0.889774112	9
JDP2	-0.21244071	1.08915052	-0.876709809	9
JMJD1C	0.082021939	0.956462991	-1.03848493	9
JOSD2	0.248191447	0.85253151	-1.100722958	9
KARS1	0.012827311	0.99352464	-1.006351951	9
KAT2B	0.23252433	0.863252678	-1.095777008	9
KBTBD7	0.409535946	0.730224129	-1.139760076	9
KCNA5	0.421034512	0.720636574	-1.141671086	9
KCNAB2	0.433799477	0.709849154	-1.143648631	9
KCNAB3	0.03737544	0.980788296	-1.018163736	9
KCNE1	0.43075052	0.712439642	-1.143190162	9
KCNE3	0.408494603	0.731086393	-1.139580996	9
KCNG2	-0.22244561	1.092491608	-0.870045997	9
KCNH3	-0.001005056	1.000502149	-0.999497093	9
KCNN4	0.277767964	0.831751747	-1.109519711	9
KCNS1	0.208927695	0.879030897	-1.087958593	9
KDF1	-0.175192104	1.07601944	-0.900827337	9
KDSR	0.324525144	0.797431428	-1.121956572	9
KEAP1	-0.17263312	1.07507758	-0.90244446	9
KEL	0.199784498	0.885026334	-1.084810832	9
KHDC1	-0.132500564	1.059644816	-0.927144252	9
KHK	0.277592484	0.831877137	-1.109469622	9

KIAA0825	0.055426693	0.971133945	-1.026560638	9
KIAA0930	0.356185861	0.773142437	-1.129328298	9
KIAA1958	0.388392559	0.74753798	-1.135930539	9
KIAA2026	0.106899854	0.942255509	-1.049155363	9
KIF13A	-0.128480415	1.058030722	-0.929550307	9
KIF1B	-0.067962593	1.032247701	-0.964285108	9
KIF22	0.191612077	0.890329657	-1.081941733	9
KIF5B	0.345409593	0.78150648	-1.126916073	9
KIFC2	0.003028176	0.998482473	-1.001510649	9
KIR2DS3	-0.023101919	1.011350803	-0.988248883	9
KIR2DS5	0.044915076	0.976785664	-1.02170074	9
KLC3	-0.075189934	1.035472642	-0.960282709	9
KLF11	0.160213197	0.910221024	-1.07043422	9
KLF14	0.269100662	0.8379149	-1.107015562	9
KLF16	-0.154229528	1.068154591	-0.913925063	9
KLHDC4	0.37864538	0.755384014	-1.134029394	9
KLHDC7A	0.236464643	0.860574786	-1.09703943	9
KLHL24	0.275909591	0.833078377	-1.108987969	9
KLHL33	0.302038599	0.814164369	-1.116202968	9
KPNA4	0.024301405	0.987627814	-1.011929219	9
KPNA6	0.426392611	0.716127107	-1.142519719	9
KREMEN1	0.276831511	0.832420604	-1.109252114	9
KRIT1	0.338486613	0.786826832	-1.125313445	9
KRT1	0.119473384	0.934896197	-1.05436958	9

KRT23	0.294312908	0.81981559	-1.114128498	9
KRT79	0.156339327	0.912622197	-1.068961524	9
KRT81	0.128359691	0.929622369	-1.057982061	9
KRT86	0.214834984	0.875122302	-1.089957286	9
KRTAP4-2	-0.067017757	1.03182319	-0.964805433	9
KXD1	0.303761673	0.812897187	-1.11665886	9
KY	0.423790352	0.718320553	-1.142110905	9
KYNU	-0.065571909	1.031172274	-0.965600365	9
LACTB2	0.259352295	0.84477359	-1.104125885	9
LAMB3	0.399979617	0.738099862	-1.138079479	9
LAMTOR2	0.336172387	0.788596144	-1.124768531	9
LAMTOR5	0.2131836	0.876217726	-1.089401326	9
LAPTM4A	0.396922215	0.740602024	-1.13752424	9
LBHD2	-0.019926627	1.009814401	-0.989887774	9
LCE1E	-0.122350168	1.055545652	-0.933195485	9
LCN2	0.403817713	0.734946757	-1.13876447	9
LCTL	-0.098125501	1.045445478	-0.947319977	9
LDAF1	0.245225307	0.854576383	-1.099801689	9
LDHAL6A	-0.099227892	1.045914789	-0.946686896	9
LDOC1	0.141669666	0.921610269	-1.063279934	9
LENG8	0.170068774	0.904059875	-1.074128649	9
LEXM	0.395474065	0.741784227	-1.137258292	9
LFNG	0.127897241	0.92989831	-1.057795551	9
LGALS3BP	-0.18599021	1.079937723	-0.893947513	9

LILRA2	0.365193276	0.766073696	-1.131266972	9
LILRB1	0.407441896	0.731957056	-1.139398951	9
LILRB5	-0.034805717	1.016948466	-0.982142749	9
LIN7B	-0.059026361	1.028205784	-0.969179423	9
LIPM	-0.134780458	1.060554702	-0.925774244	9
LITAF	0.085035426	0.954766967	-1.039802393	9
LLGL1	0.174324016	0.901376486	-1.075700501	9
LLPH	0.005351052	0.997313736	-1.002664788	9
LMNB1	0.097929327	0.947432539	-1.045361866	9
LOC100129098	-0.227914069	1.094284216	-0.866370147	9
LOC100288966	-0.18948316	1.081185749	-0.891702589	9
LOC100505502	0.160570915	0.909998712	-1.070569627	9
LOC100507221	-0.124993082	1.056620551	-0.931627469	9
LOC101059915	0.306380856	0.810966239	-1.117347095	9
LOC101927345	-0.141839972	1.063346843	-0.921506871	9
LOC101928268	-0.218540137	1.091196826	-0.872656689	9
LOC101929805	0.041713484	0.978490539	-1.020204024	9
LOC102723382	-0.190042722	1.081384795	-0.891342072	9
LOC102723407	-0.044214815	1.021374032	-0.977159217	9
LOC102723750	0.248333432	0.852433448	-1.10076688	9
LOC102723930	-0.012009646	1.005950735	-0.993941089	9
LOC102724014	0.102804107	0.944626805	-1.047430912	9
LOC102724052	-0.085037494	1.039803294	-0.954765801	9
LOC102724197	-0.161253641	1.070827784	-0.909574143	9

LOC102724250	0.188824986	0.892126327	-1.080951313	9
LOC102724488	-0.184116782	1.079264427	-0.895147645	9
LOC102724877	0.029163099	0.985099467	-1.014262566	9
LOC105369669	0.005480469	0.997248502	-1.002728971	9
LOC105369869	-0.216671957	1.090573194	-0.873901237	9
LOC105369914	0.321952206	0.799367628	-1.121319835	9
LOC105371932	0.331021776	0.792517524	-1.1235393	9
LOC105372412	0.267430549	0.839095434	-1.106525984	9
LOC105373091	-0.224832396	1.09327694	-0.868444544	9
LOC105375112	0.185516673	0.894251123	-1.079767796	9
LOC105376875	-0.209633832	1.088198959	-0.878565127	9
LOC105377310	0.270524887	0.836906377	-1.107431263	9
LOC105378148	-0.067108997	1.031864213	-0.964755217	9
LOC105378947	0.086639841	0.953861183	-1.040501024	9
LOC105379198	0.261424263	0.84332227	-1.104746534	9
LOC105379752	0.381426111	0.753154303	-1.134580415	9
LOC107983958	-0.067362218	1.031978033	-0.964615815	9
LOC107984449	0.031277937	0.983994098	-1.015272035	9
LOC107984833	-0.215572346	1.090204833	-0.874632487	9
LOC107984876	-0.170354246	1.074234539	-0.903880294	9
LOC107985876	-0.139852488	1.062564628	-0.92271214	9
LOC107986982	0.008602416	0.995671041	-1.004273457	9
LOC107987044	-0.215389343	1.090143435	-0.874754092	9
LOC107987211	0.107227319	0.942065368	-1.049292687	9

LOC107987254	0.315904787	0.803896412	-1.119801199	9
LOC107987288	-0.177657798	1.076922154	-0.899264356	9
LOC107987289	-0.224794829	1.093264614	-0.868469785	9
LOC107987433	-0.162430388	1.071271898	-0.90884151	9
LOC107987441	-0.130781855	1.058956265	-0.92817441	9
LOC107987478	0.383697056	0.751328246	-1.135025302	9
LOC107987479	0.260839248	0.843732399	-1.104571648	9
LOC110384692	0.281262962	0.829249109	-1.110512071	9
LOC112267876	-0.219733884	1.091593872	-0.871859988	9
LOC112267968	0.070127573	0.963090306	-1.033217879	9
LOC112268076	-0.199457941	1.084697192	-0.885239251	9
LOC112268168	-0.159129233	1.070023306	-0.910894073	9
LOC112268334	-0.143800079	1.064115315	-0.920315236	9
LOC112268336	-0.13881216	1.062153978	-0.923341817	9
LOC112268337	-0.149724038	1.066419901	-0.916695864	9
LOC112268340	-0.119938724	1.054560246	-0.934621522	9
LOC112268354	-0.064017713	1.030470823	-0.96645311	9
LOC112577592	-0.15086246	1.066859689	-0.915997229	9
LOC114841035	0.380821866	0.753639399	-1.134461265	9
LOC400499	0.421619303	0.720145703	-1.141765006	9
LRCOL1	-0.113415348	1.051872343	-0.938456995	9
LRMDA	-0.222810648	1.09261201	-0.869801362	9
LRR1	-0.110235475	1.05055036	-0.940314884	9
LRRC14B	-0.217726844	1.090925674	-0.87319883	9

LRRC24	-0.165341988	1.072366159	-0.907024171	9
LRRC25	0.146142554	0.918887273	-1.065029827	9
LRRC37A3	0.388085184	0.747786701	-1.135871885	9
LRRC41	-0.179682255	1.077659793	-0.897977538	9
LRRC7	-0.084560628	1.039595272	-0.955034644	9
LRRFIP1	-0.043400358	1.020993583	-0.977593225	9
LSM11	0.253127214	0.849113061	-1.102240275	9
LSM4	0.342983073	0.783375972	-1.126359045	9
LSM6	0.438500755	0.705837627	-1.144338382	9
LSM7	0.277944552	0.831625539	-1.109570092	9
LTN1	0.050349869	0.973873947	-1.024223816	9
LY86	0.201062232	0.884192441	-1.085254673	9
LY9	0.209661197	0.878547069	-1.088208266	9
LYPLA1	0.429550652	0.713456691	-1.143007343	9
LYPLAL1	0.056478086	0.970564076	-1.027042161	9
LYRM2	0.343302542	0.78313013	-1.126432672	9
LYSMD1	0.105283442	0.943192877	-1.04847632	9
LYSMD2	-0.052661863	1.025290413	-0.97262855	9
LYST	0.245835572	0.854156241	-1.099991813	9
LYZ	0.446907382	0.698612111	-1.145519493	9
MACO1	0.431552793	0.711758853	-1.143311647	9
MAD2L2	0.437293016	0.706870165	-1.144163182	9
MAEA	0.051642842	0.973177959	-1.024820802	9
MAGT1	-0.166742311	1.072890105	-0.906147794	9

MAK	-0.172556911	1.075049452	-0.902492541	9
MAMDC2	0.041671097	0.978513059	-1.020184156	9
MAMSTR	0.307518616	0.810125657	-1.117644273	9
MAN2B2	0.356071895	0.77323142	-1.129303315	9
MAP10	0.439644399	0.704858611	-1.14450301	9
MAP1LC3A	-0.18614731	1.079994059	-0.893846749	9
MAP2K3	-0.017755863	1.008759698	-0.991003835	9
MAP3K10	0.247514147	0.852999068	-1.100513215	9
MAP3K11	0.329866408	0.793394044	-1.123260452	9
MAP3K6	0.414685739	0.725945298	-1.140631037	9
MAP4K4	-0.174499937	1.075765183	-0.901265246	9
MARCHF5	-0.145911679	1.06493988	-0.919028201	9
MARCHF8	-0.165616476	1.072468981	-0.906852506	9
MARF1	0.327510767	0.795177629	-1.122688396	9
MARK4	0.090896845	0.951448424	-1.042345269	9
MAST3	-0.035794242	1.017416545	-0.981622303	9
MATR3	0.414404016	0.726180006	-1.140584022	9
MBD3L2B	-0.107689435	1.04948634	-0.941796904	9
MBNL3	0.379487877	0.75470919	-1.134197068	9
MCAT	0.410576894	0.729361199	-1.139938093	9
MCFD2	0.289798955	0.823094543	-1.112893498	9
MCL1	0.063335044	0.966827097	-1.030162141	9
MCOLN3	0.257909287	0.845782295	-1.103691582	9
MED13	0.065969325	0.965382022	-1.031351347	9

MEI1	0.227842608	0.866418336	-1.094260944	9
METTL16	-0.123522045	1.056022924	-0.932500879	9
METTL18	-0.007037424	1.00350014	-0.996462716	9
METTL21A	0.129650038	0.928851563	-1.058501602	9
METTL21C	0.022623157	0.988496476	-1.011119632	9
METTL7A	0.400178212	0.737937039	-1.138115251	9
MFSD12	0.17968138	0.897978095	-1.077659475	9
MFSD6L	0.431246405	0.712018918	-1.143265323	9
MFSD9	0.037492915	0.980726259	-1.018219174	9
MGAM2	0.23474251	0.861746698	-1.096489208	9
MGRN1	0.031742519	0.983750824	-1.015493343	9
MGST1	0.320352366	0.800568722	-1.120921087	9
MICALL1	0.161301509	0.909544361	-1.07084587	9
MICALL2	-0.061556573	1.029356321	-0.967799748	9
MIEF1	0.066017565	0.96535551	-1.031373075	9
MINDY4B	-0.143312111	1.06392428	-0.920612169	9
MIS18BP1	-0.040152614	1.019471537	-0.979318923	9
MIXL1	0.317802098	0.802478881	-1.120280979	9
MKLN1	-0.041631704	1.020165691	-0.978533987	9
MKNK2	0.329880432	0.793383412	-1.123263844	9
MKRN2OS	0.019832954	0.989936007	-1.009768961	9
MKRN3	0.206515355	0.880619122	-1.087134477	9
MLLT1	0.191390869	0.890472478	-1.081863347	9
MMADHC	0.196632644	0.887077864	-1.083710508	9

MMP24OS	-0.107377466	1.049355625	-0.941978159	9
MOB3A	0.41604177	0.724814542	-1.140856312	9
MPG	-0.186737062	1.080205373	-0.893468311	9
MPO	-0.169674255	1.073982206	-0.90430795	9
MPV17L	0.066832038	0.964907631	-1.031739668	9
MPV17L2	0.230642895	0.864526955	-1.09516985	9
MREG	0.433495007	0.710108234	-1.143603242	9
MRFAP1	0.135164935	0.925542817	-1.060707752	9
MRGPRE	0.187450218	0.893010323	-1.08046054	9
MRPL14	0.443977703	0.701137819	-1.145115522	9
MRPL19	0.387613909	0.748167882	-1.135781791	9
MRPL51	0.220155401	0.8715784	-1.091733801	9
MRPL58	0.415824597	0.724995751	-1.140820348	9
MRPS12	-0.014347994	1.007096795	-0.992748801	9
MSANTD3-TMEFF1	-0.119556692	1.054403727	-0.934847035	9
MSL3	-0.170995605	1.074472209	-0.903476604	9
MSN	0.398995775	0.738905959	-1.137901734	9
MSR1	0.202098607	0.883515125	-1.085613733	9
MSRB2	0.144758011	0.919731794	-1.064489805	9
MTCH1	0.007299983	0.996330025	-1.003630008	9
MTHFR	-0.132377932	1.059595762	-0.92721783	9
MTHFS	0.334462844	0.789900207	-1.124363051	9
MTIF3	-0.172339458	1.07496917	-0.902629711	9
MTMR3	0.316180624	0.803690516	-1.119871139	9

MTRES1	0.189862416	0.891458268	-1.081320683	9
MTUS2	0.095747458	0.9486825	-1.044429958	9
MTX1	0.211202769	0.877528842	-1.088731611	9
MUC8	0.261469328	0.843290666	-1.104759994	9
MUL1	0.388916031	0.747114202	-1.136030233	9
MVD	-0.03902289	1.018940237	-0.979917347	9
MXI1	0.278647128	0.831123154	-1.109770283	9
MYBL2	0.107430981	0.941947072	-1.049378053	9
MYH11	0.37820332	0.755737843	-1.133941163	9
MYL4	0.247198574	0.85321679	-1.100415364	9
MYNN	-0.132279273	1.059556289	-0.927277016	9
MYO1E	0.445980226	0.69941231	-1.145392535	9
NAALADL1	0.145607417	0.919213862	-1.064821279	9
NACC2	-0.20209186	1.085611398	-0.883519537	9
NAGLU	0.248458795	0.852346852	-1.100805647	9
NAGPA	0.304623432	0.812262503	-1.116885935	9
NAIF1	0.228186456	0.866186429	-1.094372885	9
NAP1L4	0.443161738	0.701839813	-1.145001551	9
NARS1	-0.169368425	1.0738686	-0.904500175	9
NAXE	0.397426567	0.740189849	-1.137616416	9
NBDY	-0.184329638	1.079341064	-0.895011426	9
NBEAL2	0.18069547	0.897332304	-1.078027775	9
NBN	-0.150116719	1.066571712	-0.916454993	9
NBPF10	-0.115744862	1.052835921	-0.937091058	9

NBPF14	0.081072194	0.956996095	-1.038068289	9
NBPF26	-0.031094305	1.015184516	-0.984090211	9
NBPF6	0.370873522	0.761579407	-1.132452929	9
NCBP3	0.116323496	0.936751129	-1.053074625	9
NCF4	-0.046576466	1.022474389	-0.975897923	9
NCKIPSD	0.395091231	0.742096439	-1.137187669	9
NCOA2	0.288552773	0.823996805	-1.112549578	9
NDOR1	0.33957514	0.785993024	-1.125568164	9
NDUFAF1	0.292564887	0.821087362	-1.113652249	9
NDUFAF8	-0.025217642	1.012370319	-0.987152677	9
NDUFB10	0.064224908	0.966339533	-1.030564441	9
NDUFB6	0.149103408	0.917076316	-1.066179724	9
NDUFS3	0.365858121	0.765549129	-1.13140725	9
NEB	-0.033261552	1.016215816	-0.982954264	9
NEDD1	0.104240983	0.943796354	-1.048037337	9
NEDD4	0.320512433	0.800448647	-1.12096108	9
NEIL2	0.237551262	0.859834122	-1.097385384	9
NEURL1B	-0.122601384	1.055648054	-0.933046669	9
NFIL3	0.227049404	0.86695295	-1.094002354	9
NFILZ	0.134398839	0.92600384	-1.060402679	9
NFYC	0.078155338	0.958629105	-1.036784443	9
NHP2	0.431015465	0.712214883	-1.143230348	9
NHS	0.227948846	0.866346694	-1.09429554	9
NHSL1	0.329161711	0.793928105	-1.123089815	9

NHSL2	0.427354166	0.715315012	-1.142669178	9
NIBAN3	0.143139432	0.920717202	-1.063856634	9
NID2	0.362905632	0.767875698	-1.13078133	9
NIF3L1	0.406090871	0.733072962	-1.139163833	9
NIN	0.167580401	0.905622558	-1.073202959	9
NINJ2	-0.216866747	1.090638347	-0.873771601	9
NIPAL4	0.030369085	0.984469542	-1.014838627	9
NISCH	0.41634718	0.724559635	-1.140906815	9
NKAP	-0.197149354	1.083891428	-0.886742074	9
NKX1-1	-0.049518859	1.023839462	-0.974320603	9
NLK	0.04539896	0.976527321	-1.021926282	9
NME1-NME2	0.076649916	0.959469406	-1.036119322	9
NOD2	0.073666342	0.96112973	-1.034796072	9
NOP16	0.400010736	0.73807435	-1.138085087	9
NOP58	0.431646308	0.71167946	-1.143325768	9
NOTCH2NLC	0.011863759	0.994015338	-1.005879098	9
NOXA1	0.072158132	0.961966475	-1.034124608	9
NOXO1	-0.072778167	1.034400859	-0.961622692	9
NPC2	0.086513143	0.953932782	-1.040445925	9
NPEPL1	-0.149049728	1.066158937	-0.917109209	9
NPIPB9	0.12539001	0.931391515	-1.056781525	9
NPM1	0.196293122	0.887298393	-1.083591515	9
NR1D1	0.332898579	0.791091259	-1.123989838	9
NRG1	-0.061448173	1.029307129	-0.967858955	9

NRGN	-0.129808119	1.058565163	-0.928757044	9
NRL	0.010784689	0.994564038	-1.005348728	9
NSRP1	0.303320279	0.813222033	-1.116542312	9
NTAN1	0.135644587	0.925253942	-1.060898529	9
NTAQ1	0.193726735	0.88896241	-1.082689145	9
NTRK3	-0.11491577	1.052493449	-0.937577679	9
NUB1	0.262797164	0.842358697	-1.105155861	9
NUDT16	0.289869828	0.823043191	-1.112913019	9
NUDT8	-0.098853928	1.045755688	-0.94690176	9
NUP214	-0.000614257	1.000306987	-0.99969273	9
NUP42	0.392820062	0.743945912	-1.136765974	9
NUP50	0.446328531	0.699111795	-1.145440326	9
NUP85	0.439291963	0.705160446	-1.144452409	9
NUPR2	-0.153987418	1.068061772	-0.914074355	9
NUTM2F	0.145701042	0.91915674	-1.064857781	9
OBP2A	-0.028927955	1.014150118	-0.985222163	9
OBSL1	-0.002265433	1.001130792	-0.998865359	9
OGFOD3	0.397722671	0.739947755	-1.137670426	9
OLIG2	0.182347926	0.896278276	-1.078626203	9
OOSP3	0.378944542	0.755144464	-1.134089006	9
OPTN	0.295218132	0.819155999	-1.114374131	9
OR10A6	-0.191742291	1.081987858	-0.890245567	9
OR1F12	0.219499313	0.872016628	-1.091515942	9
OR2A7	0.399598224	0.738412455	-1.138010679	9

OR2C1	0.225753517	0.867825292	-1.093578809	9
OR2T33	0.318734858	0.801780878	-1.120515736	9
OR2V1	-0.225181648	1.093391476	-0.868209828	9
OR52K2	0.158605407	0.911218999	-1.069824406	9
OR56A1	-0.109035496	1.050049488	-0.941013992	9
OR5AN1	-0.050105219	1.024110716	-0.974005497	9
OR6N1	0.081865988	0.956550575	-1.038416563	9
OR7D2	0.157658183	0.91180602	-1.069464202	9
ORMDL2	0.198016921	0.886177803	-1.084194724	9
OS9	0.243917127	0.855476	-1.099393127	9
OSBP2	0.187969694	0.892676466	-1.080646159	9
OSCP1	0.318062873	0.802283812	-1.120346685	9
OTUB2	0.109991125	0.940457331	-1.050448457	9
OTUD7A	0.438013118	0.706254692	-1.144267809	9
OXSM	-0.045499163	1.021972965	-0.976473802	9
P3H2	0.069889964	0.96322161	-1.033111574	9
P4HTM	0.301206581	0.814775364	-1.115981945	9
PABPC1L	0.090620246	0.95160561	-1.042225856	9
PADI4	-0.082157928	1.038544531	-0.956386602	9
PAGE2B	0.111017172	0.939858879	-1.050876051	9
PAIP1	0.297861977	0.817225666	-1.115087643	9
PAK2	-0.040146072	1.019468463	-0.979322391	9
PAK4	0.26053117	0.843948268	-1.104479438	9
PANK2	0.298162332	0.817006003	-1.115168335	9

PANO1	0.38363313	0.751379712	-1.135012842	9
PAPOLA	0.198531418	0.885842892	-1.084374311	9
PAQR8	0.420601519	0.720999821	-1.14160134	9
PATL2	-0.126624406	1.057281365	-0.930656959	9
PBRM1	0.408710926	0.730907352	-1.139618279	9
PCBP2	0.409913402	0.729911339	-1.139824741	9
PCBP4	0.042566417	0.978037098	-1.020603515	9
PCNX3	0.326917833	0.795625828	-1.122543661	9
PCOTH	0.108769242	0.941168963	-1.049938205	9
PDCD1	-0.068589819	1.032529139	-0.96393932	9
PDCD6	0.201110905	0.88416065	-1.085271555	9
PDE4A	0.274555182	0.834043463	-1.108598644	9
PDHB	0.404605317	0.734298058	-1.138903376	9
PDK4	0.088270331	0.952938684	-1.041209015	9
PDXK	0.386275686	0.74924919	-1.135524876	9
PEMT	-0.151555112	1.067126781	-0.915571669	9
PEX11G	0.191388896	0.890473752	-1.081862647	9
PFKFB4	0.027012166	0.986220258	-1.013232424	9
PFN4	0.399102647	0.738818437	-1.137921084	9
PGA3	0.205076652	0.881564146	-1.086640798	9
PGA4	0.194024489	0.888769615	-1.082794103	9
PGA5	0.246338825	0.853809547	-1.100148372	9
PGAM4	0.055411363	0.971142248	-1.026553611	9
PGF	0.178881269	0.898487055	-1.077368323	9

PGM2	-0.172584486	1.07505963	-0.902475145	9
PGPEP1L	-0.150080941	1.066557885	-0.916476944	9
PHC2	-0.018159519	1.008956089	-0.99079657	9
PHF23	0.414292135	0.726273195	-1.14056533	9
PHLDA3	0.130783886	0.928173194	-1.05895708	9
PHYHD1	-0.169508601	1.073920679	-0.904412079	9
PIGF	0.43288504	0.710627007	-1.143512046	9
PIGP	0.313124423	0.805968215	-1.119092638	9
PIK3CA	0.4092772	0.730438472	-1.139715672	9
PIK3R5	0.302007052	0.814187546	-1.116194598	9
PIM1	0.152275844	0.915128465	-1.067404309	9
PIP	0.161636446	0.909335929	-1.070972375	9
PIP5K1C	-0.14770464	1.065637329	-0.917932689	9
PIP5KL1	-0.207818045	1.087580078	-0.879762033	9
PKNOX1	0.207376158	0.880052917	-1.087429075	9
PLA2G15	-0.058654729	1.02803639	-0.969381661	9
PLCD3	0.213202303	0.876205332	-1.089407635	9
PLCL2	0.351968325	0.776427893	-1.128396218	9
PLEK2	0.044378681	0.977071836	-1.021450517	9
PLEKHA3	0.069088745	0.963664052	-1.032752797	9
PLEKHA7	0.213351807	0.876106247	-1.089458054	9
PLEKHG6	0.199654693	0.885110978	-1.084765671	9
PLEKHG7	-0.018376123	1.009061423	-0.9906853	9
PLGLB2	0.271961664	0.835887286	-1.107848951	9

PLIN3	0.307694531	0.809995593	-1.117690124	9
PLOD3	0.096665383	0.948157074	-1.044822457	9
PLRG1	-0.193246118	1.082519582	-0.889273464	9
PLSCR2	0.362749768	0.767998306	-1.130748074	9
PLXDC1	0.274909215	0.833791341	-1.108700556	9
PM20D1	-0.197326864	1.083953532	-0.886626668	9
PMCH	-0.186029834	1.079951934	-0.8939221	9
PMP22	0.028487251	0.985452007	-1.013939258	9
PMPCB	0.128283894	0.929667608	-1.057951502	9
PNISR	0.408114127	0.731401191	-1.139515317	9
PNLDC1	0.324370288	0.79754812	-1.121918408	9
PNPLA6	0.219383501	0.872093949	-1.09147745	9
PNPLA7	-0.196123218	1.083531934	-0.887408717	9
PNRC2	0.131559034	0.927708865	-1.059267899	9
POLD3	0.416450733	0.724473187	-1.140923919	9
POLL	-0.211016034	1.088668317	-0.877652283	9
POLM	0.393472187	0.743415346	-1.136887533	9
POLR1D	-0.188087941	1.080688382	-0.892600441	9
POLR2A	0.145937147	0.919012657	-1.064949804	9
POLR2J	0.30207761	0.814135707	-1.116213317	9
POLR2J3	-0.162323615	1.071231645	-0.90890803	9
POLR2L	0.17249296	0.902532886	-1.075025846	9
POLRMT	-0.132211819	1.059529297	-0.927317478	9
POU5F1	-0.145781923	1.06488931	-0.919107387	9

POU5F2	0.015999825	0.991904085	-1.00790391	9
PPARA	-0.133161311	1.059908924	-0.926747613	9
PPARG	-0.160877085	1.070685442	-0.909808357	9
PPCDC	0.159648435	0.910571806	-1.070220241	9
PPCS	0.296098978	0.818513518	-1.114612496	9
PPIA	0.343638382	0.782871595	-1.126509977	9
PPIAL4C	-0.101796405	1.047004682	-0.945208277	9
PPM1J	0.262253885	0.842740182	-1.104994066	9
PPP1R12A	0.166520781	0.906286538	-1.072807318	9
PPP1R14B	0.190673406	0.89093544	-1.081608845	9
PPP1R16A	0.415182287	0.725531437	-1.140713725	9
PPP1R16B	0.214014204	0.875667025	-1.089681229	9
PPP1R18	0.377494196	0.756305069	-1.133799265	9
PPP1R2B	0.190488634	0.891054604	-1.081543237	9
PPP1R3B	0.115643107	0.93715081	-1.052793917	9
PPP1R3D	0.23101163	0.864277437	-1.095289066	9
PPP1R7	0.288645684	0.823929579	-1.112575264	9
PPP1R9B	0.386125691	0.749370289	-1.13549598	9
PPP2R3A	0.178648999	0.898634711	-1.07728371	9
PPP3R2	0.029483407	0.984932267	-1.014415674	9
PPP4R1	0.021549427	0.98905113	-1.010600557	9
PPP6R1	0.42127304	0.720436393	-1.141709433	9
PRADC1	0.180258548	0.897610642	-1.07786919	9
PRDM8	0.435997896	0.707975877	-1.143973773	9

PRDX2	0.14018807	0.922508846	-1.062696916	9
PRH2	0.227155727	0.866881318	-1.094037045	9
PRIMPOL	0.115555085	0.937202491	-1.052757576	9
PRKACA	0.219346065	0.87211894	-1.091465005	9
PRKCZ	0.206681463	0.880509908	-1.08719137	9
PROKR2	0.344389292	0.782293181	-1.126682473	9
PROSER3	0.193366383	0.889195646	-1.082562029	9
PROZ	0.131138163	0.927961032	-1.059099195	9
PRPH2	-0.033757091	1.016451126	-0.982694035	9
PRR13	0.115691577	0.937122349	-1.052813926	9
PRR14	0.111816328	0.939392209	-1.051208537	9
PRR22	-0.019000207	1.009364716	-0.990364509	9
PRRC2A	0.410282539	0.729605316	-1.139887855	9
PRRG4	-0.126534051	1.057244817	-0.930710766	9
PRRT1B	-0.139910493	1.0625875	-0.922677007	9
PRSS21	-0.029919005	1.014623766	-0.984704761	9
PRSS23	0.141833683	0.92151069	-1.063344373	9
PRSS33	-0.062234018	1.02966355	-0.967429533	9
PRSS57	0.340336647	0.785409107	-1.125745754	9
PRXL2A	-0.138745336	1.062127572	-0.923382235	9
PSKH1	0.091161173	0.951298158	-1.04245933	9
PSMB1	0.140589882	0.922265316	-1.062855198	9
PSMC3	0.098824166	0.946918855	-1.045743021	9
PSMD10	0.252744369	0.849378918	-1.102123286	9

PSMD14	0.116177751	0.936836774	-1.053014525	9
PSMD4	-0.199584245	1.084741155	-0.88515691	9
PSME1	0.241688041	0.857005747	-1.098693788	9
PSME2	0.129848738	0.928732754	-1.058581492	9
PSME3	-0.143659637	1.064060352	-0.920400715	9
PSME4	-0.021661227	1.010654645	-0.988993418	9
PSMF1	0.002398382	0.998798652	-1.001197034	9
PTAFR	0.199722534	0.885066742	-1.084789275	9
PTGDR2	-0.002651092	1.001322911	-0.998671818	9
PTGDS	-0.129226109	1.058331052	-0.929104944	9
PTGER2	0.393931014	0.743041816	-1.13697283	9
PTGER4	0.097860022	0.947472299	-1.04533232	9
PTGES	-0.010318558	1.005119351	-0.994800793	9
PTK2B	0.138170828	0.923729582	-1.06190041	9
PTK6	0.387122468	0.748565162	-1.13568763	9
PTOV1	0.400583326	0.737604786	-1.138188112	9
PTPA	0.33884484	0.786552544	-1.125397383	9
PTPN2	-0.192272631	1.082175578	-0.889902946	9
PTPN20	0.231383883	0.864025428	-1.095409311	9
PTPRO	0.404044235	0.734760244	-1.138804479	9
PTRH1	-0.119604419	1.054423287	-0.934818868	9
PTRH2	-0.048167711	1.023213429	-0.975045717	9
PTTG1	0.035275212	0.981895657	-1.017170869	9
PTTG1IP2	-0.173986144	1.075576208	-0.901590064	9

PTTG2	-0.196038542	1.083502233	-0.887463691	9
PUDP	0.105032423	0.943338267	-1.04837069	9
PUF60	0.334158155	0.790132365	-1.12429052	9
PUM2	-0.02481759	1.012177801	-0.987360211	9
PUSL1	0.357742055	0.771926252	-1.129668307	9
PXN	0.186267683	0.893769529	-1.080037212	9
PXYLP1	-0.198338063	1.084306844	-0.885968781	9
PYDC5	-0.115817198	1.052865775	-0.937048577	9
PYROXD1	0.041922974	0.97837922	-1.020302194	9
PYURF	0.251690468	0.85011016	-1.101800628	9
PZP	-0.034781284	1.016936887	-0.982155604	9
QPCTL	0.098833233	0.946913647	-1.04574688	9
QPRT	0.243329855	0.855879413	-1.099209267	9
RAB10	-0.208388945	1.08777494	-0.879385995	9
RAB11FIP1	0.349884345	0.778045607	-1.127929953	9
RAB19	0.252267982	0.849709567	-1.101977548	9
RAB21	0.395005471	0.742166359	-1.137171831	9
RAB27A	-0.117398387	1.053517375	-0.936118988	9
RAB34	-0.122349993	1.055545581	-0.933195588	9
RAB5C	0.013858362	0.992998796	-1.006857158	9
RAB6A	-0.103051384	1.047535385	-0.944484002	9
RAB7B	0.277551607	0.831906342	-1.10945795	9
RAC2	0.295624019	0.818860029	-1.114484048	9
RAD1	-0.131094278	1.059081596	-0.927987318	9

RAD23A	-0.067245741	1.031925684	-0.964679943	9
RAD51B	0.291555848	0.821820335	-1.113376183	9
RAD9B	-0.015788162	1.007800602	-0.99201244	9
RALY	0.414364236	0.726213142	-1.140577377	9
RAN	-0.008941148	1.004440594	-0.995499447	9
RANBP10	0.389901489	0.746315754	-1.136217243	9
RANBP2	0.28059738	0.829726481	-1.110323861	9
RAP1A	0.308959141	0.809059836	-1.118018976	9
RAP1GAP	0.137727831	0.923997245	-1.061725076	9
RAP2A	0.07154884	0.96230402	-1.03385286	9
RASD1	0.006481532	0.99674348	-1.003225012	9
RASGRP4	0.074127463	0.960873561	-1.035001024	9
RASL10A	-0.107129439	1.04925165	-0.94212221	9
RASL11A	0.245960915	0.85406991	-1.100030825	9
RASSF3	0.172552894	0.902495076	-1.07504797	9
RAVER1	0.441341657	0.703403387	-1.144745044	9
RBBP7	-0.180592376	1.077990369	-0.897397993	9
RBM8A	0.368694068	0.763307182	-1.13200125	9
RBMXL1	0.428652905	0.71421677	-1.142869675	9
RC3H2	0.364695518	0.766466177	-1.131161695	9
RCAN1	0.059820855	0.96874672	-1.028567576	9
RCBTB2	0.327438966	0.79523192	-1.122670886	9
RCC1L	-0.018806977	1.009270841	-0.990463865	9
REC114	0.445140574	0.700136276	-1.14527685	9

REL	0.271160849	0.836455503	-1.107616353	9
RELA	0.342710403	0.783585731	-1.126296134	9
RELB	0.147555674	0.918023802	-1.065579477	9
RERE	0.372336196	0.760417512	-1.132753708	9
REX1BD	-0.040410655	1.019592757	-0.979182102	9
RFC4	-0.103936451	1.047908942	-0.943972491	9
RFXANK	0.031623242	0.983813298	-1.01543654	9
RGL3	-0.093283043	1.043373033	-0.950089989	9
RGS9BP	0.370452751	0.761913302	-1.132366053	9
RHOQ	0.016446911	0.991675101	-1.008122013	9
RHOXF2	0.141202335	0.921893883	-1.063096218	9
RILP	0.296126159	0.818493682	-1.114619841	9
RIPK3	-0.095135272	1.044167837	-0.949032565	9
RLIM	0.398877274	0.739002991	-1.137880266	9
RNASE1	-0.095244996	1.044214839	-0.948969842	9
RNASE6	0.227903367	0.866377364	-1.094280731	9
RNASEL	0.348895139	0.778812173	-1.127707312	9
RNF103-CHMP3	0.446465189	0.698993856	-1.145459045	9
RNF130	-0.226246291	1.093740024	-0.867493733	9
RNF146	0.242898693	0.856175412	-1.099074105	9
RNF152	-0.163456841	1.071658416	-0.908201575	9
RNF167	0.23653386	0.860527635	-1.097061495	9
RNF175	0.283233996	0.827833299	-1.111067295	9
RNF20	0.17244438	0.902563531	-1.075007911	9

RNF212	0.204190885	0.882145159	-1.086336044	9
RNF5	0.302442866	0.813867285	-1.116310152	9
RNPEP	0.035561186	0.98174507	-1.017306256	9
ROM1	-0.169838244	1.074043092	-0.904204848	9
RPA4	0.062593095	0.967233161	-1.029826256	9
RPEL1	0.062975443	0.967023956	-1.029999399	9
RPIA	0.044374825	0.977073893	-1.021448718	9
RPL36AL	0.052757359	0.972577023	-1.025334382	9
RPS23	0.407809667	0.731652999	-1.139462665	9
RPS6KA1	0.36405046	0.766974478	-1.131024939	9
RRP12	0.122627921	0.933030947	-1.055658868	9
RSRC2	-0.054325954	1.026055623	-0.971729669	9
RTCA	0.107847037	0.941705309	-1.049552346	9
RTL8C	0.289348683	0.823420697	-1.112769381	9
RTN4R	0.392485793	0.744217723	-1.136703516	9
RUNX3	0.414379722	0.726200242	-1.140579964	9
RUSF1	0.397995952	0.73972425	-1.137720201	9
RWDD1	0.312410304	0.806499295	-1.118909598	9
RWDD3	0.288023224	0.824379821	-1.112403045	9
RWDD4	0.343985314	0.782604419	-1.126589733	9
S100B	0.26448386	0.84117279	-1.105656649	9
SAC3D1	0.30648119	0.810892156	-1.117373345	9
SAMD4A	0.332360182	0.791500718	-1.1238609	9
SAMD8	0.345386389	0.781524381	-1.12691077	9

SAR1A	0.197974259	0.886205564	-1.084179824	9
SCAP	0.261401019	0.843338571	-1.10473959	9
SCD5	0.309126995	0.80893553	-1.118062525	9
SCGB1D1	-0.078350654	1.036870612	-0.958519958	9
SDAD1	0.169329025	0.904524934	-1.073853959	9
SDF2L1	0.107838578	0.941710226	-1.049548804	9
SDK2	-0.110013668	1.05045786	-0.940444192	9
SDR16C5	0.443041045	0.701943594	-1.144984639	9
SDR42E2	-0.025656956	1.012581593	-0.986924637	9
SDSL	0.326103555	0.796240854	-1.122344409	9
SEC11A	0.356769189	0.772686806	-1.129455995	9
SEC14L3	0.046132063	0.976135587	-1.02226765	9
SELENBP1	-0.049790369	1.023965096	-0.974174728	9
SELENOO	0.220667436	0.871236153	-1.091903589	9
SELPLG	0.271424167	0.836268724	-1.107692892	9
SEMA3B	0.031609627	0.983820428	-1.015430055	9
SEPHS2	0.370278374	0.762051631	-1.132330004	9
SERTAD1	0.396099449	0.741273926	-1.137373375	9
SESN3	-0.030708358	1.01500049	-0.984292132	9
SETSIP	0.378765041	0.755288207	-1.134053248	9
SETX	0.130136602	0.928560578	-1.05869718	9
SFTA2	0.33215324	0.791658035	-1.123811275	9
SGIP1	-0.133634753	1.060097959	-0.926463205	9
SGTA	0.316494278	0.803456313	-1.119950591	9

SH2B2	-0.017790312	1.008776463	-0.990986151	9
SH2D2A	0.283435995	0.82768802	-1.111124015	9
SH2D3C	0.362204168	0.768427329	-1.130631497	9
SH3BP1	0.155507749	0.913136132	-1.068643881	9
SH3BP2	0.38716677	0.748529357	-1.135696127	9
SH3BP5	0.006591013	0.996688203	-1.003279216	9
SH3GLB2	0.301422964	0.814616518	-1.116039482	9
SH3RF2	0.04170327	0.978495966	-1.020199236	9
SH3RF3	-0.028663731	1.014023715	-0.985359983	9
SH3TC1	0.38060482	0.753813567	-1.134418387	9
SHF	0.311881875	0.806892003	-1.118773878	9
SHISA5	0.234612196	0.86183528	-1.096447476	9
SHKBP1	0.078585945	0.958388433	-1.036974378	9
SHOC1	-0.097451095	1.045157915	-0.94770682	9
SHTN1	-0.115643313	1.052794002	-0.937150689	9
SIAH3	0.139597212	0.922866727	-1.062463939	9
SIGLEC10	-0.026216115	1.012850292	-0.986634178	9
SIGLEC15	0.151830538	0.915402347	-1.067232885	9
SIGLEC7	0.156025533	0.912816192	-1.068841725	9
SIGLEC8	0.351556672	0.776747742	-1.128304414	9
SIGMAR1	0.039740597	0.979537283	-1.01927788	9
SIPA1	0.322543592	0.798923092	-1.121466684	9
SIRPD	0.126211475	0.930902813	-1.057114287	9
SIRPG	-0.167104126	1.073025235	-0.905921109	9

SIRT6	0.3317454	0.791967967	-1.123713366	9
SIVA1	-0.055143276	1.026430694	-0.971287418	9
SLA	-0.18148868	1.078315297	-0.896826617	9
SLAMF6	0.263855237	0.841615041	-1.105470278	9
SLC10A1	0.166394709	0.906365479	-1.072760187	9
SLC12A8	0.002754625	0.998619842	-1.001374467	9
SLC14A1	-0.091499391	1.0426052	-0.951105808	9
SLC16A11	0.197012429	0.886831076	-1.083843506	9
SLC17A9	-0.172207961	1.074920603	-0.902712642	9
SLC18B1	0.360309425	0.7699152	-1.130224625	9
SLC1A5	-0.044444807	1.021481376	-0.977036569	9
SLC1A7	0.319530619	0.801184813	-1.120715432	9
SLC22A4	0.41738842	0.723689928	-1.141078348	9
SLC25A11	0.219281602	0.872161971	-1.091443574	9
SLC25A19	0.371294573	0.761245134	-1.132539706	9
SLC25A34	0.252986589	0.849210728	-1.102197317	9
SLC25A37	0.28950221	0.82330951	-1.112811719	9
SLC25A39	0.378658923	0.755373171	-1.134032095	9
SLC25A52	-0.135714557	1.060926344	-0.925211787	9
SLC26A5	0.094850813	0.949195129	-1.044045942	9
SLC27A3	0.334529234	0.78984961	-1.124378844	9
SLC2A7	-0.138544155	1.062048053	-0.923503898	9
SLC2A9	0.282926849	0.828054135	-1.110980984	9
SLC30A4	-0.128866743	1.05818637	-0.929319627	9

SLC31A2	0.303331827	0.813213536	-1.116545363	9
SLC35A2	0.146808518	0.918480535	-1.065289053	9
SLC35A3	0.246021116	0.854028442	-1.100049558	9
SLC35E4	0.234067324	0.862205515	-1.096272839	9
SLC35F6	0.00931243	0.995311264	-1.004623694	9
SLC35G5	-0.02103695	1.010352504	-0.989315554	9
SLC39A7	0.327127832	0.795467124	-1.122594956	9
SLC43A1	0.184777789	0.894724512	-1.079502301	9
SLC4A1	0.118659589	0.935376155	-1.054035744	9
SLC52A1	-0.095560104	1.044349768	-0.948789665	9
SLC6A12	0.437475638	0.706714125	-1.144189762	9
SLC6A8	0.291709117	0.821709054	-1.11341817	9
SLC7A7	0.036161728	0.981428639	-1.017590367	9
SLC8A1	0.348076257	0.779446108	-1.127522365	9
SLC8B1	0.317483026	0.802717481	-1.120200506	9
SLCO3A1	-0.069243771	1.032822254	-0.963578483	9
SLCO5A1	0.278431702	0.83127724	-1.109708942	9
SLFN12	0.004671825	0.997655903	-1.002327728	9
SLFNL1	-0.185474649	1.079752707	-0.894278058	9
SLTM	0.309595983	0.808588092	-1.118184075	9
SLX1A	0.006752781	0.996606509	-1.00335929	9
SLX1B	-0.083354829	1.039068501	-0.955713672	9
SMAP1	0.24467777	0.854953083	-1.099630854	9
SMARCB1	0.382825952	0.752029242	-1.134855194	9

SMG5	0.254485768	0.848168694	-1.102654461	9
SMG7	-0.093827565	1.04360696	-0.949779395	9
SMG9	-0.187004125	1.080300975	-0.893296849	9
SMIM33	0.023725497	0.987926142	-1.011651639	9
SMIM41	0.297183428	0.817721645	-1.114905073	9
SMIM6	-0.005511758	1.002744486	-0.997232729	9
SMPDL3A	0.431220723	0.712040713	-1.143261436	9
SMPDL3B	0.167005552	0.905982878	-1.07298843	9
SMU1	0.333840924	0.790373995	-1.124214919	9
SNAI3	0.011648485	0.994124874	-1.005773358	9
SNAPC2	0.419467168	0.721950628	-1.141417796	9
SNCA	0.153319242	0.914486134	-1.067805376	9
SNF8	0.046852832	0.97575005	-1.022602881	9
SNRNP25	0.033414915	0.982873746	-1.016288661	9
SNRPA1	-0.081832798	1.03840201	-0.956569212	9
SNX10	0.030195799	0.984560122	-1.014755921	9
SOAT2	-0.049552553	1.023855056	-0.974302504	9
SOWAHC	0.321314278	0.799846817	-1.121161096	9
SOX13	0.273460741	0.834822211	-1.108282951	9
SOX6	0.433113152	0.71043304	-1.143546192	9
SPACA7	-0.103158355	1.047580566	-0.944422211	9
SPANXD	-0.22529104	1.093427331	-0.868136291	9
SPATA1	-0.204238569	1.086352466	-0.882113897	9
SPATA5	0.405078865	0.733907755	-1.13898662	9

SPDYE11	0.253159657	0.849090526	-1.102250183	9
SPDYE6	0.411283778	0.728774635	-1.140058413	9
SPDYE8	0.358709591	0.771169045	-1.129878636	9
SPDYE9	0.419298717	0.722091721	-1.141390438	9
SPINK8	0.249228346	0.851814997	-1.101043343	9
SPINT2	0.400159152	0.737952667	-1.138111819	9
SPNS3	0.089424492	0.952284467	-1.041708959	9
SPOCK2	0.172708484	0.902396907	-1.075105391	9
SPON2	0.419421829	0.721988606	-1.141410435	9
SPOPL	0.217332586	0.873461453	-1.090794039	9
SPSB2	0.222506057	0.870005495	-1.092511553	9
SPTBN2	0.209942829	0.878361187	-1.088304017	9
SPTLC1	-0.04176622	1.020228739	-0.97846252	9
SQLE	-0.220345287	1.09179679	-0.871451504	9
SQOR	-0.162546169	1.071315537	-0.908769368	9
SRA1	0.360262482	0.769952022	-1.130214505	9
SRD5A1	0.352227018	0.776226817	-1.128453835	9
SRF	0.277879476	0.831672053	-1.109551528	9
SRRD	0.354678468	0.774318471	-1.12899694	9
SRRT	0.061755715	0.967690955	-1.02944667	9
SRSF3	-0.030541013	1.014920663	-0.98437965	9
SSBP3	-0.008757657	1.004350067	-0.99559241	9
SSH2	0.230409905	0.86468456	-1.095094465	9
SSNA1	0.051695289	0.973149702	-1.024844991	9

SSU72	0.420039448	0.721471096	-1.141510543	9
SSX1	-0.100389124	1.046408152	-0.946019028	9
SSX7	-0.167887164	1.073317336	-0.905430172	9
ST13	0.031984379	0.983624112	-1.015608491	9
ST14	-0.053067976	1.025477351	-0.972409375	9
ST3GAL2	0.210623489	0.877911684	-1.088535172	9
ST6GALNAC6	0.226988809	0.86699377	-1.093982579	9
ST8SIA4	0.034171651	0.98247619	-1.016647842	9
STAG1	-0.130285367	1.058756941	-0.928471574	9
STAG2	-0.196974798	1.083830333	-0.886855534	9
STAM2	0.03656966	0.981213542	-1.017783202	9
STK11	0.212287518	0.876811228	-1.089098747	9
STK16	0.374844371	0.758420705	-1.133265076	9
STK17B	-0.105435496	1.048540281	-0.943104785	9
STN1	0.114013736	0.938106518	-1.052120254	9
STX5	0.350021302	0.777939409	-1.127960711	9
STYXL1	0.027234274	0.986104685	-1.013338958	9
SUCLG1	0.153634602	0.914291829	-1.067926431	9
SUGP1	0.07868353	0.958333872	-1.037017402	9
SULT1A2	0.267206216	0.839253834	-1.106460049	9
SUMO4	0.354762529	0.774252941	-1.12901547	9
SURF1	0.437768848	0.706463525	-1.144232373	9
SUSD2	0.177861948	0.899134735	-1.076996683	9
SWAP70	0.376325305	0.757239087	-1.133564392	9

SYCE1L	0.114419783	0.93786854	-1.052288323	9
SYCN	-0.215981178	1.0903419	-0.874360722	9
SYCP3	0.082386157	0.95625837	-1.038644526	9
SYK	-0.166857835	1.072933262	-0.906075427	9
SYMPK	0.317751282	0.802516887	-1.120268168	9
SYNE1	-0.09336277	1.043407298	-0.950044528	9
SYNE4	0.283058983	0.827959141	-1.111018124	9
SYPL1	0.290612861	0.822504565	-1.113117426	9
SYT15	0.090555275	0.951642523	-1.042197798	9
SYVN1	-0.089543244	1.041760341	-0.952217097	9
TAF1	0.394691645	0.742422169	-1.137113815	9
TAF1D	0.425206864	0.717127359	-1.142334222	9
TANK	-0.018541824	1.009141979	-0.990600155	9
TAOK1	-0.190284738	1.081470808	-0.891186069	9
TARBP2	0.215014191	0.875003298	-1.090017489	9
TARM1	-0.044928619	1.021707055	-0.976778436	9
TAS2R40	0.149616575	0.916761761	-1.066378335	9
TAS2R43	0.09651385	0.948243857	-1.044757706	9
TAS2R45	0.340525518	0.785264205	-1.125789723	9
TASOR	0.389941061	0.746283674	-1.136224735	9
TBC1D10B	0.438340867	0.7059744	-1.144315267	9
TBC1D17	0.444492183	0.700694873	-1.145187056	9
TBC1D24	0.410625074	0.729321234	-1.139946308	9
TBC1D29P	0.069812373	0.963264477	-1.033076851	9

TBC1D3C	0.338462251	0.786845482	-1.125307732	9
TBC1D7	0.186163628	0.893836282	-1.07999991	9
TBCCD1	0.274213825	0.834286459	-1.108500285	9
TBL1X	0.276556338	0.832617008	-1.109173345	9
TBX19	-0.046950081	1.022648082	-0.975698001	9
TCF25	0.263069244	0.842167555	-1.105236799	9
TCF7L2	0.144711002	0.919760442	-1.064471444	9
TCP11L2	0.028354481	0.985521223	-1.013875704	9
TCTN3	0.050305392	0.973897866	-1.024203258	9
TDRD1	0.270215838	0.837125361	-1.107341199	9
TEDC1	0.13547141	0.925358259	-1.06082967	9
TEDDM1	0.365389806	0.765918673	-1.131308479	9
TEN1	-0.148313756	1.065873709	-0.917559953	9
TENT4B	0.21023197	0.878170285	-1.088402255	9
TEPP	-0.09781332	1.045312408	-0.947499089	9
TEPSIN	-0.061012872	1.029109496	-0.968096625	9
TES	0.377711302	0.756131454	-1.133842756	9
TESC	0.208754915	0.879144804	-1.087899719	9
TEX13D	0.305202767	0.811835474	-1.117038242	9
TEX48	-0.18669267	1.080189476	-0.893496806	9
TEX52	-0.02215815	1.010894939	-0.988736789	9
TFR2	0.292589	0.821069836	-1.113658836	9
TG	-0.023374173	1.011482183	-0.988108011	9
TGFBR3	-0.061137913	1.029166281	-0.968028368	9

TGIF2-RAB5IF	0.352773655	0.775801736	-1.128575391	9
TGM4	0.132458686	0.92716938	-1.059628066	9
THAP7	0.302874751	0.813549756	-1.116424507	9
THEM6	0.119530009	0.934862781	-1.054392791	9
THEMIS	0.314944051	0.804613046	-1.119557096	9
THEMIS2	0.360275528	0.769941789	-1.130217317	9
THOC5	0.117302106	0.936175646	-1.053477752	9
TIAF1	0.319463088	0.801235418	-1.120698506	9
TIGAR	-0.089277897	1.041645515	-0.952367617	9
TIGD3	0.01943357	0.990141581	-1.009575151	9
TIMM17B	-0.207109615	1.087337916	-0.880228302	9
TIPRL	-0.169279991	1.073835736	-0.904555745	9
TIRAP	-0.031969473	1.015601395	-0.983631922	9
TKT	0.179273083	0.89823788	-1.077510962	9
TLE7	-0.228024706	1.094320238	-0.866295532	9
TLR6	0.205280778	0.881430163	-1.086710941	9
TLR9	-0.200062074	1.084907362	-0.884845288	9
TM4SF19	-0.195619092	1.083355018	-0.887735927	9
TM9SF4	0.421220236	0.720480712	-1.141700948	9
TMCC2	0.227178306	0.866866105	-1.094044411	9
TMED9	0.038634842	0.980122678	-1.01875752	9
TMEM11	0.175257585	0.90078589	-1.076043475	9
TMEM120A	0.335824224	0.78886193	-1.124686154	9
TMEM132B	0.364576335	0.766560119	-1.131136455	9

TMEM14C	0.188775342	0.892158275	-1.080933617	9
TMEM171	-0.153197679	1.067758692	-0.914561013	9
TMEM176A	0.255253438	0.847634406	-1.102887844	9
TMEM177	0.254548104	0.848125326	-1.10267343	9
TMEM184A	-0.036781194	1.017883147	-0.981101953	9
TMEM184B	-0.085701412	1.040092628	-0.954391216	9
TMEM205	0.133055375	0.926811228	-1.059866603	9
TMEM213	-0.021382512	1.010519787	-0.989137275	9
TMEM216	0.3988538	0.739022211	-1.137876011	9
TMEM250	0.316537151	0.803424293	-1.119961444	9
TMEM273	-0.188884706	1.080972599	-0.892087893	9
TMEM79	-0.158585446	1.069816823	-0.911231377	9
TMEM86B	0.201479966	0.883919536	-1.085399502	9
TMEM92	0.086664522	0.953847234	-1.040511756	9
TMOD1	0.055002206	0.971363787	-1.026365993	9
TMSB10	0.182772839	0.8960069	-1.078779739	9
TNF	0.264110857	0.841435245	-1.105546102	9
TNFAIP8L2	0.363024074	0.767782512	-1.130806586	9
TNFRSF18	0.260066473	0.844273734	-1.104340207	9
TNFSF14	0.285709487	0.826050601	-1.111760088	9
TNIP1	-0.00794039	1.003946551	-0.996006161	9
TNK1	-0.074026328	1.034956087	-0.960929759	9
TOR1AIP2	0.087242864	0.953520239	-1.040763103	9
TPK1	0.146956067	0.918390373	-1.06534644	9

TRABD	0.352903549	0.775700689	-1.128604238	9
TRAK2	0.345255329	0.781625482	-1.126880811	9
TRAPPC5	0.413126349	0.727243532	-1.140369881	9
TRAPPC6B	0.272577065	0.835450274	-1.108027339	9
TRIM14	-0.035079645	1.017078248	-0.981998603	9
TRIM23	-0.055457841	1.026574915	-0.971117074	9
TRIM27	-0.035644911	1.017345882	-0.981700971	9
TRIM5	0.290851834	0.822331236	-1.11318307	9
TRIOBP	0.392080015	0.744547547	-1.136627561	9
TRMO	0.415960624	0.724882255	-1.140842879	9
TRMT12	0.442162923	0.702698254	-1.144861177	9
TSEN2	0.248237598	0.852499638	-1.100737236	9
TSEN54	0.345546425	0.781400908	-1.126947332	9
TSG101	0.150895575	0.915976891	-1.066872467	9
TSGA10IP	0.250774516	0.85074496	-1.101519476	9
TSHB	-0.062278516	1.029683719	-0.967405202	9
TSKS	0.209508782	0.878647639	-1.088156421	9
TSPAN16	0.446057218	0.699345891	-1.145403109	9
TSPAN31	0.419181118	0.722190205	-1.141371323	9
TSPAN5	0.199524631	0.885195776	-1.084720407	9
TSPAN7	0.032281669	0.9834683	-1.015749968	9
TSPO2	-0.153787957	1.06798527	-0.914197313	9
TSR3	0.144293977	0.920014508	-1.064308486	9
TSTD1	0.434665236	0.709111983	-1.143777219	9

TTC13	0.285081133	0.826503581	-1.111584715	9
TTC22	0.305904522	0.811317834	-1.117222357	9
TTC24	0.316657208	0.803334622	-1.11999183	9
TTC30A	-0.184301981	1.079331108	-0.895029127	9
TTC7A	0.245486764	0.854396417	-1.099883181	9
TTF1	-0.217423788	1.090824501	-0.873400713	9
TUBA1A	0.353053258	0.775584209	-1.128637467	9
TUBA3D	-0.103425504	1.047693361	-0.944267857	9
TUBGCP3	-0.2139304	1.089653013	-0.875722613	9
TUSC1	0.070639574	0.962807228	-1.033446802	9
TXNRD2	0.232824691	0.863048985	-1.095873676	9
TYROBP	0.03904589	0.979905173	-1.018951064	9
U2SURP	0.444894991	0.700347897	-1.145242887	9
UBALD1	0.389380508	0.746737978	-1.136118485	9
UBC	0.206929121	0.880347034	-1.087276155	9
UBE2Z	-0.139756467	1.06252676	-0.922770293	9
UBE4B	0.045685534	0.976374238	-1.022059773	9
UBQLN1	-0.130548077	1.058862434	-0.928314357	9
UBR2	0.00679483	0.996585271	-1.003380101	9
UCHL5	-0.135468501	1.060828513	-0.925360012	9
UCN	0.442324911	0.702559096	-1.144884007	9
UFC1	0.246912371	0.85341418	-1.100326551	9
UHMK1	0.121365322	0.933778421	-1.055143743	9
UHRF2	0.241473709	0.857152626	-1.098626335	9

ULK1	0.200048992	0.884853822	-1.084902814	9
UNC119	0.368996036	0.763068045	-1.132064081	9
UPK3A	0.429664393	0.713360338	-1.143024732	9
UPK3B	-0.206384897	1.087089778	-0.880704881	9
UPP1	0.307182631	0.810373997	-1.117556628	9
UQCRC2	0.301063898	0.814880086	-1.115943984	9
UROD	0.232025041	0.863591116	-1.095616157	9
USE1	0.179773544	0.897919436	-1.07769298	9
USP32	0.081043565	0.957012154	-1.03805572	9
USP6	0.016366616	0.991716237	-1.008082853	9
VASP	0.008662548	0.995640586	-1.004303134	9
VENTX	-0.158058938	1.069616683	-0.911557745	9
VIM	0.325292979	0.796852524	-1.122145504	9
VPREB1	0.40831905	0.731231658	-1.139550708	9
VPREB3	0.01051846	0.99469928	-1.00521774	9
VPS25	0.250206529	0.851138263	-1.101344792	9
VPS29	0.260370763	0.844060634	-1.104431397	9
VPS33B	-0.008350368	1.004149036	-0.995798667	9
VPS45	0.364608117	0.76653507	-1.131143186	9
VRK1	0.202912153	0.882982846	-1.085895	9
WAC	0.374918651	0.758361484	-1.133280135	9
WARS1	0.376083443	0.757432198	-1.133515641	9
WAS	0.295122417	0.819225774	-1.114348191	9
WASHC2C	0.237373132	0.859955604	-1.097328736	9

WBP2NL	0.194588089	0.888404493	-1.082992582	9
WDR45	0.023955437	0.98780706	-1.011762497	9
WDR47	-0.00635355	1.003161637	-0.996808087	9
WDR48	0.434003112	0.709675828	-1.14367894	9
WDR70	0.444737198	0.700483837	-1.145221035	9
WDR77	0.27556069	0.833327131	-1.108887821	9
WDSUB1	-0.064655278	1.030758794	-0.966103516	9
WIZ	0.146209333	0.918846503	-1.065055836	9
WNK1	0.23621501	0.860744809	-1.096959819	9
WNT10A	0.084113149	0.955286763	-1.039399912	9
WSB2	-0.197673525	1.084074746	-0.88640122	9
WWC3	0.13910245	0.923166198	-1.062268648	9
XKR6	0.292766437	0.820940854	-1.11370729	9
XRCC3	0.070438769	0.962918274	-1.033357043	9
XRCC5	-0.057095465	1.027324525	-0.97022906	9
XRN2	0.405066221	0.733918179	-1.1389844	9
XRRA1	-0.170971186	1.074463166	-0.90349198	9
YBX3	0.381178479	0.753353145	-1.134531624	9
YOD1	0.042380355	0.97813606	-1.020516415	9
YWHAG	0.136248605	0.924889916	-1.061138521	9
YY2	0.342115457	0.784043188	-1.126158645	9
ZBED1	0.389799937	0.746398075	-1.136198012	9
ZBED2	-0.228447833	1.094457914	-0.866010081	9
ZBED6	0.255979646	0.847128537	-1.103108183	9

ZBTB7A	0.361562905	0.76893124	-1.130494146	9
ZBTB8A	-0.028071141	1.013740031	-0.98566889	9
ZC3H14	-0.13056018	1.058867293	-0.928307113	9
ZC3H18	0.375609784	0.757810235	-1.133420019	9
ZDHHC18	-0.195943534	1.0834689	-0.887525366	9
ZDHHC24	0.111456091	0.939602631	-1.051058721	9
ZEB2	0.148564076	0.917406692	-1.065970768	9
ZFP36L2	0.35938736	0.770638127	-1.130025487	9
ZFPM1	-0.133885561	1.060198031	-0.92631247	9
ZMYND15	0.316954174	0.803112762	-1.120066937	9
ZNF207	0.405132964	0.733863153	-1.138996117	9
ZNF217	0.385121241	0.750180713	-1.135301954	9
ZNF230	0.066316763	0.965191039	-1.031507802	9
ZNF235	0.427348659	0.715319665	-1.142668325	9
ZNF267	0.049118301	0.974535712	-1.023654013	9
ZNF281	0.381111645	0.753406801	-1.134518447	9
ZNF282	-0.156989975	1.069209684	-0.912219709	9
ZNF296	-0.059271778	1.028317591	-0.969045813	9
ZNF324	-0.202692734	1.085819191	-0.883126457	9
ZNF398	0.040337129	0.979221093	-1.019558222	9
ZNF407	0.082731651	0.956064175	-1.038795826	9
ZNF420	0.19255984	0.889717307	-1.082277147	9
ZNF460	0.373046652	0.75985247	-1.132899122	9
ZNF487	-0.065741457	1.031248686	-0.965507229	9

ZNF491	0.127898014	0.929897849	-1.057795863	9
ZNF492	-0.018364859	1.009055946	-0.990691087	9
ZNF524	0.183958745	0.895248759	-1.079207505	9
ZNF556	0.170173998	0.903993689	-1.074167687	9
ZNF571	0.150652819	0.916125958	-1.066778777	9
ZNF596	0.397796099	0.739887708	-1.137683807	9
ZNF606	-0.025013683	1.012272182	-0.987258499	9
ZNF628	0.079206081	0.958041584	-1.037247665	9
ZNF670	0.376318779	0.757244298	-1.133563077	9
ZNF70	0.447456169	0.698138084	-1.145594253	9
ZNF704	-0.078378701	1.036882983	-0.958504282	9
ZNF746	0.123075671	0.932765581	-1.055841252	9
ZNF761	0.352342033	0.776137399	-1.128479432	9
ZNF763	0.204836284	0.881721874	-1.086558159	9
ZNF766	0.314231659	0.805143932	-1.119375591	9
ZNF774	0.182841902	0.895962778	-1.07880468	9
ZNF80	0.212940532	0.87637878	-1.089319312	9
ZNF888	-0.095414943	1.044287619	-0.948872677	9
ZP1	0.021511062	0.989070932	-1.010581994	9
ZSCAN25	0.444604622	0.700598034	-1.145202656	9
ZSWIM6	0.107124028	0.942125353	-1.04924938	9
ZYG11B	0.149483631	0.91684327	-1.066326902	9
