# Supplementary material for: RBAD: The first database dedicated alterations of blood RNA in individuals with Alzheimer’s disease and their clinical relevance
Source: Neural Regen Res. 2025 Mar 25;21(6):2553–62. doi: 10.4103/NRR.NRR-D-24-01165 (PMC13211806; doi:10.4103/NRR.NRR-D-24-01165)
Supplement: Supplementary file 10 [file NRR-21-2553_Suppl7.pdf]

**Additional Table 10. The overlapped genes between clusters identified from ROSMAP and Homo datasets.**	
Method	scRNA-seq
Description	Genes that identified with similar trend from control to MCI to AD in two data sets (ROSMAP and Homo datasets in RBAD).<br>Clusters of circulating mRNA expression trend from control to MCI to AD.

Symbol	Cluster	Project
ACMSD	1	ROSMAP & Homo validated
ALLC	1	ROSMAP & Homo validated
ATF6	1	ROSMAP & Homo validated
ATP5MF-PTCD	1	ROSMAP & Homo validated
ATP6V0C	1	ROSMAP & Homo validated
AWAT2	1	ROSMAP & Homo validated
C6orf62	1	ROSMAP & Homo validated
CCDC153	1	ROSMAP & Homo validated
CCDC182	1	ROSMAP & Homo validated
CCDC197	1	ROSMAP & Homo validated
CD58	1	ROSMAP & Homo validated
CD63	1	ROSMAP & Homo validated
CDK8	1	ROSMAP & Homo validated
CEP295	1	ROSMAP & Homo validated
CLEC4A	1	ROSMAP & Homo validated
CMIP	1	ROSMAP & Homo validated
CMTM2	1	ROSMAP & Homo validated
CPEB4	1	ROSMAP & Homo validated
CSF2	1	ROSMAP & Homo validated
CSTB	1	ROSMAP & Homo validated
CT45A5	1	ROSMAP & Homo validated
CTIF	1	ROSMAP & Homo validated
CTSW	1	ROSMAP & Homo validated
CYP27A1	1	ROSMAP & Homo validated
DDX46	1	ROSMAP & Homo validated
DEFA5	1	ROSMAP & Homo validated
DEFB112	1	ROSMAP & Homo validated
DEFB127	1	ROSMAP & Homo validated
DHRS11	1	ROSMAP & Homo validated
DSC2	1	ROSMAP & Homo validated
EIF4EBP1	1	ROSMAP & Homo validated
EVI2B	1	ROSMAP & Homo validated
FAM161B	1	ROSMAP & Homo validated
FGFR1OP2	1	ROSMAP & Homo validated
FNDC10	1	ROSMAP & Homo validated
FOXE1	1	ROSMAP & Homo validated
FXYD7	1	ROSMAP & Homo validated
GABRB1	1	ROSMAP & Homo validated
GAL3ST4	1	ROSMAP & Homo validated
GGTLC3	1	ROSMAP & Homo validated

GPX1	1	ROSMAP & Homo validated
GPX4	1	ROSMAP & Homo validated
GRK1	1	ROSMAP & Homo validated
H2AC18	1	ROSMAP & Homo validated
H2AC19	1	ROSMAP & Homo validated
H4C3	1	ROSMAP & Homo validated
HPCAL1	1	ROSMAP & Homo validated
HTATIP2	1	ROSMAP & Homo validated
IFNGR2	1	ROSMAP & Homo validated
IFT88	1	ROSMAP & Homo validated
IL17F	1	ROSMAP & Homo validated
IRAK1	1	ROSMAP & Homo validated
JAK2	1	ROSMAP & Homo validated
KCNIP4	1	ROSMAP & Homo validated
KDM4B	1	ROSMAP & Homo validated
KRTAP11-1	1	ROSMAP & Homo validated
KRTAP5-2	1	ROSMAP & Homo validated
LALBA	1	ROSMAP & Homo validated
LAMTOR4	1	ROSMAP & Homo validated
LEUTX	1	ROSMAP & Homo validated
LILRA1	1	ROSMAP & Homo validated
LRRC10	1	ROSMAP & Homo validated
LSM1	1	ROSMAP & Homo validated
MAGEB16	1	ROSMAP & Homo validated
MAGEB3	1	ROSMAP & Homo validated
MARCOL	1	ROSMAP & Homo validated
METRNL	1	ROSMAP & Homo validated
MICAL2	1	ROSMAP & Homo validated
MLPH	1	ROSMAP & Homo validated
MMP3	1	ROSMAP & Homo validated
MRPL36	1	ROSMAP & Homo validated
MRPS6	1	ROSMAP & Homo validated
MSX1	1	ROSMAP & Homo validated
MTF2	1	ROSMAP & Homo validated
MXD1	1	ROSMAP & Homo validated
NECAB2	1	ROSMAP & Homo validated
NECTIN2	1	ROSMAP & Homo validated
NEIL3	1	ROSMAP & Homo validated
NEK6	1	ROSMAP & Homo validated
NLRX1	1	ROSMAP & Homo validated
NOTCH3	1	ROSMAP & Homo validated
NUDT5	1	ROSMAP & Homo validated
OR10J5	1	ROSMAP & Homo validated
OR13C3	1	ROSMAP & Homo validated
OR13C4	1	ROSMAP & Homo validated
OR4C12	1	ROSMAP & Homo validated
OR4F4	1	ROSMAP & Homo validated
OR51I2	1	ROSMAP & Homo validated
OR5C1	1	ROSMAP & Homo validated
OR6C65	1	ROSMAP & Homo validated
OR6V1	1	ROSMAP & Homo validated
OR8B12	1	ROSMAP & Homo validated
OR8G1	1	ROSMAP & Homo validated
OR8S1	1	ROSMAP & Homo validated
P2RY1	1	ROSMAP & Homo validated

PAPLN	1	ROSMAP & Homo validated
PCDHGA11	1	ROSMAP & Homo validated
PCDHGA3	1	ROSMAP & Homo validated
PERCC1	1	ROSMAP & Homo validated
PLN	1	ROSMAP & Homo validated
PPP3R1	1	ROSMAP & Homo validated
PRR32	1	ROSMAP & Homo validated
PYGL	1	ROSMAP & Homo validated
RAB7A	1	ROSMAP & Homo validated
RCOR1	1	ROSMAP & Homo validated
RIT1	1	ROSMAP & Homo validated
RMI1	1	ROSMAP & Homo validated
RNF150	1	ROSMAP & Homo validated
RNF151	1	ROSMAP & Homo validated
RPL17-C18orf3[illegible]	1	ROSMAP & Homo validated
RTP5	1	ROSMAP & Homo validated
RUSC1	1	ROSMAP & Homo validated
SCAMP4	1	ROSMAP & Homo validated
SEL1L2	1	ROSMAP & Homo validated
SF1	1	ROSMAP & Homo validated
SKP1	1	ROSMAP & Homo validated
SLBP	1	ROSMAP & Homo validated
SLC27A1	1	ROSMAP & Homo validated
SMAD1	1	ROSMAP & Homo validated
SMG8	1	ROSMAP & Homo validated
SNUPN	1	ROSMAP & Homo validated
SPATA48	1	ROSMAP & Homo validated
SPINT3	1	ROSMAP & Homo validated
SRGN	1	ROSMAP & Homo validated
SSTR4	1	ROSMAP & Homo validated
STRIT1	1	ROSMAP & Homo validated
STX6	1	ROSMAP & Homo validated
TAAR9	1	ROSMAP & Homo validated
TAS2R9	1	ROSMAP & Homo validated
TBCA	1	ROSMAP & Homo validated
TCEAL8	1	ROSMAP & Homo validated
TEX38	1	ROSMAP & Homo validated
TGFBRAP1	1	ROSMAP & Homo validated
TMEM51	1	ROSMAP & Homo validated
TNNC2	1	ROSMAP & Homo validated
TRPC5OS	1	ROSMAP & Homo validated
TTLL12	1	ROSMAP & Homo validated
TVP23B	1	ROSMAP & Homo validated
TVP23C	1	ROSMAP & Homo validated
UBE2D3	1	ROSMAP & Homo validated
UMODL1	1	ROSMAP & Homo validated
USP17L12	1	ROSMAP & Homo validated
USP17L17	1	ROSMAP & Homo validated
USP17L20	1	ROSMAP & Homo validated
USP17L21	1	ROSMAP & Homo validated
USP17L26	1	ROSMAP & Homo validated
USP17L27	1	ROSMAP & Homo validated
USP17L30	1	ROSMAP & Homo validated
VCAN	1	ROSMAP & Homo validated
VCX	1	ROSMAP & Homo validated

ZBTB2	1	ROSMAP & Homo validated
ZFAND6	1	ROSMAP & Homo validated
ZG16	1	ROSMAP & Homo validated
ZMAT2	1	ROSMAP & Homo validated
ZNF143	1	ROSMAP & Homo validated
ZNF750	1	ROSMAP & Homo validated
ZNF90	1	ROSMAP & Homo validated
AACS	2	ROSMAP & Homo validated
AARS2	2	ROSMAP & Homo validated
ADAMTS10	2	ROSMAP & Homo validated
ADCK5	2	ROSMAP & Homo validated
ADCY7	2	ROSMAP & Homo validated
AFG3L2	2	ROSMAP & Homo validated
AMMECR1	2	ROSMAP & Homo validated
ANAPC7	2	ROSMAP & Homo validated
ANK1	2	ROSMAP & Homo validated
APBB1	2	ROSMAP & Homo validated
APC	2	ROSMAP & Homo validated
APH1A	2	ROSMAP & Homo validated
APOBEC3H	2	ROSMAP & Homo validated
ARHGEF3	2	ROSMAP & Homo validated
ASMTL	2	ROSMAP & Homo validated
ATP6V0A2	2	ROSMAP & Homo validated
ATXN7L3B	2	ROSMAP & Homo validated
AVEN	2	ROSMAP & Homo validated
B3GALT6	2	ROSMAP & Homo validated
BACH1	2	ROSMAP & Homo validated
BLCAP	2	ROSMAP & Homo validated
BZW2	2	ROSMAP & Homo validated
C15orf48	2	ROSMAP & Homo validated
C15orf62	2	ROSMAP & Homo validated
C22orf39	2	ROSMAP & Homo validated
C6orf89	2	ROSMAP & Homo validated
C8orf33	2	ROSMAP & Homo validated
CA11	2	ROSMAP & Homo validated
CARMIL2	2	ROSMAP & Homo validated
CATSPER2	2	ROSMAP & Homo validated
CBLIF	2	ROSMAP & Homo validated
CCDC88C	2	ROSMAP & Homo validated
CCDC9	2	ROSMAP & Homo validated
CCR5	2	ROSMAP & Homo validated
CD7	2	ROSMAP & Homo validated
CD72	2	ROSMAP & Homo validated
CDH23	2	ROSMAP & Homo validated
CEACAM1	2	ROSMAP & Homo validated
CEP63	2	ROSMAP & Homo validated
CFAP58	2	ROSMAP & Homo validated
CLEC18A	2	ROSMAP & Homo validated
CMKLR1	2	ROSMAP & Homo validated
CNN3	2	ROSMAP & Homo validated
CNPY2	2	ROSMAP & Homo validated
CTU1	2	ROSMAP & Homo validated
CYB5RL	2	ROSMAP & Homo validated
CZIB	2	ROSMAP & Homo validated
DDOST	2	ROSMAP & Homo validated

DISC1	2	ROSMAP & Homo validated
DNAJB12	2	ROSMAP & Homo validated
DPF2	2	ROSMAP & Homo validated
DUS2	2	ROSMAP & Homo validated
DUSP18	2	ROSMAP & Homo validated
ECI2	2	ROSMAP & Homo validated
EFCAB14	2	ROSMAP & Homo validated
EIF2AK3	2	ROSMAP & Homo validated
EIF4E2	2	ROSMAP & Homo validated
EOLA1	2	ROSMAP & Homo validated
EWSR1	2	ROSMAP & Homo validated
EXT2	2	ROSMAP & Homo validated
FAAP100	2	ROSMAP & Homo validated
FBXL6	2	ROSMAP & Homo validated
FKBP11	2	ROSMAP & Homo validated
FMNL3	2	ROSMAP & Homo validated
FOXK1	2	ROSMAP & Homo validated
GALNT4	2	ROSMAP & Homo validated
GATB	2	ROSMAP & Homo validated
GBP4	2	ROSMAP & Homo validated
GEMIN4	2	ROSMAP & Homo validated
GGA3	2	ROSMAP & Homo validated
GNAL	2	ROSMAP & Homo validated
GOLGA8B	2	ROSMAP & Homo validated
GOLGA8O	2	ROSMAP & Homo validated
GPBP1L1	2	ROSMAP & Homo validated
HDAC1	2	ROSMAP & Homo validated
HERC2	2	ROSMAP & Homo validated
HIRA	2	ROSMAP & Homo validated
HMGN4	2	ROSMAP & Homo validated
HNRNPUL2	2	ROSMAP & Homo validated
HOMEZ	2	ROSMAP & Homo validated
HPF1	2	ROSMAP & Homo validated
HPS3	2	ROSMAP & Homo validated
HPS6	2	ROSMAP & Homo validated
HSPB11	2	ROSMAP & Homo validated
IARS1	2	ROSMAP & Homo validated
IDH2	2	ROSMAP & Homo validated
IKZF1	2	ROSMAP & Homo validated
IL20RB	2	ROSMAP & Homo validated
INVS	2	ROSMAP & Homo validated
IPPK	2	ROSMAP & Homo validated
ITGB7	2	ROSMAP & Homo validated
ITIH1	2	ROSMAP & Homo validated
ITPKC	2	ROSMAP & Homo validated
IVD	2	ROSMAP & Homo validated
KCNJ10	2	ROSMAP & Homo validated
KLF8	2	ROSMAP & Homo validated
LAMP3	2	ROSMAP & Homo validated
LILRA4	2	ROSMAP & Homo validated
LSS	2	ROSMAP & Homo validated
LTK	2	ROSMAP & Homo validated
MACF1	2	ROSMAP & Homo validated
MAP3K1	2	ROSMAP & Homo validated
MAPK7	2	ROSMAP & Homo validated

MEAK7	2	ROSMAP & Homo validated
MLYCD	2	ROSMAP & Homo validated
MMP14	2	ROSMAP & Homo validated
MOCS3	2	ROSMAP & Homo validated
MPRIP	2	ROSMAP & Homo validated
MRPL33	2	ROSMAP & Homo validated
MRPS11	2	ROSMAP & Homo validated
MST1	2	ROSMAP & Homo validated
MTMR11	2	ROSMAP & Homo validated
NLRP1	2	ROSMAP & Homo validated
NPIPA1	2	ROSMAP & Homo validated
NPIPA5	2	ROSMAP & Homo validated
NRDE2	2	ROSMAP & Homo validated
NUDT16L1	2	ROSMAP & Homo validated
NUDT4	2	ROSMAP & Homo validated
ODF2L	2	ROSMAP & Homo validated
OR4D1	2	ROSMAP & Homo validated
OSBPL5	2	ROSMAP & Homo validated
PAM16	2	ROSMAP & Homo validated
PAPOLG	2	ROSMAP & Homo validated
PDCD2	2	ROSMAP & Homo validated
PDCD6IP	2	ROSMAP & Homo validated
PEX7	2	ROSMAP & Homo validated
PHF19	2	ROSMAP & Homo validated
PHPT1	2	ROSMAP & Homo validated
PIK3CG	2	ROSMAP & Homo validated
PIM2	2	ROSMAP & Homo validated
PINX1	2	ROSMAP & Homo validated
PKD1	2	ROSMAP & Homo validated
PLCD1	2	ROSMAP & Homo validated
PLPBP	2	ROSMAP & Homo validated
PLPP6	2	ROSMAP & Homo validated
PNMA3	2	ROSMAP & Homo validated
POLR1B	2	ROSMAP & Homo validated
POLR3B	2	ROSMAP & Homo validated
POMK	2	ROSMAP & Homo validated
POU2F1	2	ROSMAP & Homo validated
PPIE	2	ROSMAP & Homo validated
PPIL2	2	ROSMAP & Homo validated
PPP1R13L	2	ROSMAP & Homo validated
PPP6R3	2	ROSMAP & Homo validated
PSMA3	2	ROSMAP & Homo validated
PTCD2	2	ROSMAP & Homo validated
PYDC1	2	ROSMAP & Homo validated
PYM1	2	ROSMAP & Homo validated
RALBP1	2	ROSMAP & Homo validated
RASGRP3	2	ROSMAP & Homo validated
RBM10	2	ROSMAP & Homo validated
RITA1	2	ROSMAP & Homo validated
RNF157	2	ROSMAP & Homo validated
RNF220	2	ROSMAP & Homo validated
RPL3	2	ROSMAP & Homo validated
RPL8	2	ROSMAP & Homo validated
RPLP1	2	ROSMAP & Homo validated
RPS5	2	ROSMAP & Homo validated

SCML4	2	ROSMAP & Homo validated
SCO2	2	ROSMAP & Homo validated
SDR39U1	2	ROSMAP & Homo validated
SERGEF	2	ROSMAP & Homo validated
SFT2D2	2	ROSMAP & Homo validated
SGPL1	2	ROSMAP & Homo validated
SIPA1L3	2	ROSMAP & Homo validated
SLC25A20	2	ROSMAP & Homo validated
SLC35A4	2	ROSMAP & Homo validated
SLC6A16	2	ROSMAP & Homo validated
SLFN12L	2	ROSMAP & Homo validated
SMCR8	2	ROSMAP & Homo validated
SMIM38	2	ROSMAP & Homo validated
SPHK2	2	ROSMAP & Homo validated
SSRP1	2	ROSMAP & Homo validated
STIM2	2	ROSMAP & Homo validated
STK19	2	ROSMAP & Homo validated
STMN1	2	ROSMAP & Homo validated
SUPT20H	2	ROSMAP & Homo validated
SYCE1	2	ROSMAP & Homo validated
TAF1B	2	ROSMAP & Homo validated
TAF9	2	ROSMAP & Homo validated
TBC1D3D	2	ROSMAP & Homo validated
TBC1D4	2	ROSMAP & Homo validated
TELO2	2	ROSMAP & Homo validated
THAP2	2	ROSMAP & Homo validated
THRAP3	2	ROSMAP & Homo validated
TK2	2	ROSMAP & Homo validated
TLR7	2	ROSMAP & Homo validated
TM4SF1	2	ROSMAP & Homo validated
TMED6	2	ROSMAP & Homo validated
TMEM191C	2	ROSMAP & Homo validated
TMEM208	2	ROSMAP & Homo validated
TMEM220	2	ROSMAP & Homo validated
TRAPPC12	2	ROSMAP & Homo validated
TREML4	2	ROSMAP & Homo validated
TRIM22	2	ROSMAP & Homo validated
TRIM39	2	ROSMAP & Homo validated
TRIM6	2	ROSMAP & Homo validated
TSPAN17	2	ROSMAP & Homo validated
TSPAN4	2	ROSMAP & Homo validated
TUBG1	2	ROSMAP & Homo validated
UBE4A	2	ROSMAP & Homo validated
UBIAD1	2	ROSMAP & Homo validated
UCP3	2	ROSMAP & Homo validated
UNC119B	2	ROSMAP & Homo validated
UPRT	2	ROSMAP & Homo validated
UTP25	2	ROSMAP & Homo validated
VAV1	2	ROSMAP & Homo validated
XPO5	2	ROSMAP & Homo validated
YIPF2	2	ROSMAP & Homo validated
YIPF5	2	ROSMAP & Homo validated
ZBTB22	2	ROSMAP & Homo validated
ZC3H12D	2	ROSMAP & Homo validated
ZC3H6	2	ROSMAP & Homo validated

ZC3H7B	2	ROSMAP & Homo validated
ZFP90	2	ROSMAP & Homo validated
ZNF124	2	ROSMAP & Homo validated
ZNF232	2	ROSMAP & Homo validated
ZNF333	2	ROSMAP & Homo validated
ZNF34	2	ROSMAP & Homo validated
ZNF384	2	ROSMAP & Homo validated
ZNF410	2	ROSMAP & Homo validated
ZNF506	2	ROSMAP & Homo validated
ZNF526	2	ROSMAP & Homo validated
ZNF549	2	ROSMAP & Homo validated
ZNF554	2	ROSMAP & Homo validated
ZNF557	2	ROSMAP & Homo validated
ZNF597	2	ROSMAP & Homo validated
ZNF813	2	ROSMAP & Homo validated
ZNF93	2	ROSMAP & Homo validated
ZSCAN22	2	ROSMAP & Homo validated
ZSCAN30	2	ROSMAP & Homo validated
ZSCAN9	2	ROSMAP & Homo validated
ZZEF1	2	ROSMAP & Homo validated
ACOD1	3	ROSMAP & Homo validated
ACTL7B	3	ROSMAP & Homo validated
AMY2A	3	ROSMAP & Homo validated
ANKRD2	3	ROSMAP & Homo validated
ANKS1B	3	ROSMAP & Homo validated
ARHGAP17	3	ROSMAP & Homo validated
ARHGEF17	3	ROSMAP & Homo validated
ARID2	3	ROSMAP & Homo validated
ATAD3A	3	ROSMAP & Homo validated
ATP10A	3	ROSMAP & Homo validated
ATXN7	3	ROSMAP & Homo validated
BAG4	3	ROSMAP & Homo validated
BCL2	3	ROSMAP & Homo validated
BCS1L	3	ROSMAP & Homo validated
BDH2	3	ROSMAP & Homo validated
BNIPL	3	ROSMAP & Homo validated
BORA	3	ROSMAP & Homo validated
BORCS5	3	ROSMAP & Homo validated
C11orf94	3	ROSMAP & Homo validated
C1orf109	3	ROSMAP & Homo validated
C2orf69	3	ROSMAP & Homo validated
CAPNS1	3	ROSMAP & Homo validated
CCDC141	3	ROSMAP & Homo validated
CCL3	3	ROSMAP & Homo validated
CCL8	3	ROSMAP & Homo validated
CD4	3	ROSMAP & Homo validated
CELA3B	3	ROSMAP & Homo validated
CERS6	3	ROSMAP & Homo validated
CHRM5	3	ROSMAP & Homo validated
CREB3L2	3	ROSMAP & Homo validated
CRY1	3	ROSMAP & Homo validated
CTSF	3	ROSMAP & Homo validated
CUL2	3	ROSMAP & Homo validated
DEFB1	3	ROSMAP & Homo validated
DEFB124	3	ROSMAP & Homo validated

DIS3	3	ROSMAP & Homo validated
DTNB	3	ROSMAP & Homo validated
DXO	3	ROSMAP & Homo validated
DYNC2I1	3	ROSMAP & Homo validated
EBNA1BP2	3	ROSMAP & Homo validated
EGFL8	3	ROSMAP & Homo validated
EIF2AK2	3	ROSMAP & Homo validated
EML3	3	ROSMAP & Homo validated
EP400	3	ROSMAP & Homo validated
EPHX2	3	ROSMAP & Homo validated
EPM2A	3	ROSMAP & Homo validated
ERAP1	3	ROSMAP & Homo validated
ETS2	3	ROSMAP & Homo validated
EXD2	3	ROSMAP & Homo validated
FAM153A	3	ROSMAP & Homo validated
FAM25A	3	ROSMAP & Homo validated
FANCD2	3	ROSMAP & Homo validated
FBXL16	3	ROSMAP & Homo validated
FHIT	3	ROSMAP & Homo validated
FTCDNL1	3	ROSMAP & Homo validated
FXN	3	ROSMAP & Homo validated
GATAD1	3	ROSMAP & Homo validated
GH1	3	ROSMAP & Homo validated
GMEB2	3	ROSMAP & Homo validated
GNE	3	ROSMAP & Homo validated
GOLGA8J	3	ROSMAP & Homo validated
GP5	3	ROSMAP & Homo validated
GRK2	3	ROSMAP & Homo validated
GSPT2	3	ROSMAP & Homo validated
GTF2E1	3	ROSMAP & Homo validated
GTPBP1	3	ROSMAP & Homo validated
HBQ1	3	ROSMAP & Homo validated
HELZ	3	ROSMAP & Homo validated
HIF1AN	3	ROSMAP & Homo validated
HTD2	3	ROSMAP & Homo validated
ISG15	3	ROSMAP & Homo validated
KLHL42	3	ROSMAP & Homo validated
KRTAP10-6	3	ROSMAP & Homo validated
LARP4	3	ROSMAP & Homo validated
LEMD3	3	ROSMAP & Homo validated
LZTR1	3	ROSMAP & Homo validated
MAJIN	3	ROSMAP & Homo validated
MCM2	3	ROSMAP & Homo validated
MED8	3	ROSMAP & Homo validated
MFAP1	3	ROSMAP & Homo validated
MFN1	3	ROSMAP & Homo validated
MFNG	3	ROSMAP & Homo validated
MIEN1	3	ROSMAP & Homo validated
MS4A5	3	ROSMAP & Homo validated
NDUFB8	3	ROSMAP & Homo validated
NFRKB	3	ROSMAP & Homo validated
NICN1	3	ROSMAP & Homo validated
NOL10	3	ROSMAP & Homo validated
NR2C1	3	ROSMAP & Homo validated
NSG1	3	ROSMAP & Homo validated

NUP210L	3	ROSMAP & Homo validated
NUP43	3	ROSMAP & Homo validated
OCIAD1	3	ROSMAP & Homo validated
OXCT2	3	ROSMAP & Homo validated
PATJ	3	ROSMAP & Homo validated
PDZD9	3	ROSMAP & Homo validated
PEX6	3	ROSMAP & Homo validated
PFAS	3	ROSMAP & Homo validated
PKN1	3	ROSMAP & Homo validated
PLD6	3	ROSMAP & Homo validated
PNPLA4	3	ROSMAP & Homo validated
POLQ	3	ROSMAP & Homo validated
PQBP1	3	ROSMAP & Homo validated
PRL	3	ROSMAP & Homo validated
PRSS16	3	ROSMAP & Homo validated
PSMB11	3	ROSMAP & Homo validated
PTPN1	3	ROSMAP & Homo validated
QTRT2	3	ROSMAP & Homo validated
RAB39B	3	ROSMAP & Homo validated
RAD17	3	ROSMAP & Homo validated
RALGAPA2	3	ROSMAP & Homo validated
RAPGEFL1	3	ROSMAP & Homo validated
RBM14-RBM4	3	ROSMAP & Homo validated
RBM4	3	ROSMAP & Homo validated
RELCH	3	ROSMAP & Homo validated
RFPL2	3	ROSMAP & Homo validated
RPS16	3	ROSMAP & Homo validated
RPS27L	3	ROSMAP & Homo validated
RSL1D1	3	ROSMAP & Homo validated
SAG	3	ROSMAP & Homo validated
SCN5A	3	ROSMAP & Homo validated
SDC3	3	ROSMAP & Homo validated
SERPINB6	3	ROSMAP & Homo validated
SGPP1	3	ROSMAP & Homo validated
SLC11A2	3	ROSMAP & Homo validated
SLC15A2	3	ROSMAP & Homo validated
SLC25A26	3	ROSMAP & Homo validated
SLC27A4	3	ROSMAP & Homo validated
SLC6A13	3	ROSMAP & Homo validated
SPATA33	3	ROSMAP & Homo validated
SRD5A3	3	ROSMAP & Homo validated
SUPT3H	3	ROSMAP & Homo validated
TCF20	3	ROSMAP & Homo validated
TEX33	3	ROSMAP & Homo validated
TIMELESS	3	ROSMAP & Homo validated
TIMM44	3	ROSMAP & Homo validated
TLE2	3	ROSMAP & Homo validated
TMEM187	3	ROSMAP & Homo validated
TNNC1	3	ROSMAP & Homo validated
TOPBP1	3	ROSMAP & Homo validated
TPH1	3	ROSMAP & Homo validated
TUBB1	3	ROSMAP & Homo validated
TUBB6	3	ROSMAP & Homo validated
UGCG	3	ROSMAP & Homo validated
VEZF1	3	ROSMAP & Homo validated

VPS35L	3	ROSMAP & Homo validated
VWCE	3	ROSMAP & Homo validated
WDR13	3	ROSMAP & Homo validated
WDR37	3	ROSMAP & Homo validated
XKRX	3	ROSMAP & Homo validated
XRCC2	3	ROSMAP & Homo validated
ZDHHC5	3	ROSMAP & Homo validated
ZMYM1	3	ROSMAP & Homo validated
ZNF106	3	ROSMAP & Homo validated
ZNF528	3	ROSMAP & Homo validated
ZNF544	3	ROSMAP & Homo validated
ZNF564	3	ROSMAP & Homo validated
ZNF573	3	ROSMAP & Homo validated
ZNF655	3	ROSMAP & Homo validated
ZNF714	3	ROSMAP & Homo validated
ZNF749	3	ROSMAP & Homo validated
ZNF764	3	ROSMAP & Homo validated
ZNF785	3	ROSMAP & Homo validated
ZNF791	3	ROSMAP & Homo validated
ZXDB	3	ROSMAP & Homo validated
ADPRHL1	4	ROSMAP & Homo validated
AIMP2	4	ROSMAP & Homo validated
AKAP13	4	ROSMAP & Homo validated
ALG5	4	ROSMAP & Homo validated
ALOX5	4	ROSMAP & Homo validated
ANKRD22	4	ROSMAP & Homo validated
ANKRD35	4	ROSMAP & Homo validated
ANXA4	4	ROSMAP & Homo validated
APOL1	4	ROSMAP & Homo validated
ARCN1	4	ROSMAP & Homo validated
ARFGAP3	4	ROSMAP & Homo validated
ARHGAP25	4	ROSMAP & Homo validated
ARHGEF40	4	ROSMAP & Homo validated
ARMC10	4	ROSMAP & Homo validated
ASGR1	4	ROSMAP & Homo validated
ATG16L2	4	ROSMAP & Homo validated
BBS2	4	ROSMAP & Homo validated
BCL7C	4	ROSMAP & Homo validated
BLOC1S3	4	ROSMAP & Homo validated
BRI3BP	4	ROSMAP & Homo validated
BRMS1	4	ROSMAP & Homo validated
C1orf43	4	ROSMAP & Homo validated
C1QTNF12	4	ROSMAP & Homo validated
CACNA1H	4	ROSMAP & Homo validated
CACYBP	4	ROSMAP & Homo validated
CAMKK2	4	ROSMAP & Homo validated
CAPN5	4	ROSMAP & Homo validated
CAPRIN1	4	ROSMAP & Homo validated
CARD9	4	ROSMAP & Homo validated
CARS2	4	ROSMAP & Homo validated
CBL	4	ROSMAP & Homo validated
CC2D1B	4	ROSMAP & Homo validated
CCDC12	4	ROSMAP & Homo validated
CCDC32	4	ROSMAP & Homo validated
CCNQ	4	ROSMAP & Homo validated

CEACAM21	4	ROSMAP & Homo validated
CENPC	4	ROSMAP & Homo validated
CFAP97D2	4	ROSMAP & Homo validated
CHST14	4	ROSMAP & Homo validated
CIAPIN1	4	ROSMAP & Homo validated
CIB1	4	ROSMAP & Homo validated
CKLF-CMTM1	4	ROSMAP & Homo validated
CPNE1	4	ROSMAP & Homo validated
CPNE8	4	ROSMAP & Homo validated
CRIP1	4	ROSMAP & Homo validated
CSNK1D	4	ROSMAP & Homo validated
CSNK2A3	4	ROSMAP & Homo validated
CTAG2	4	ROSMAP & Homo validated
CTNNA1	4	ROSMAP & Homo validated
CUTA	4	ROSMAP & Homo validated
CX3CR1	4	ROSMAP & Homo validated
CYB561D2	4	ROSMAP & Homo validated
YP3A7-CYP3A5	4	ROSMAP & Homo validated
DCDC2B	4	ROSMAP & Homo validated
DCLRE1C	4	ROSMAP & Homo validated
DDX55	4	ROSMAP & Homo validated
DEGS1	4	ROSMAP & Homo validated
DHFR2	4	ROSMAP & Homo validated
DICER1	4	ROSMAP & Homo validated
DNAI2	4	ROSMAP & Homo validated
DNAJC10	4	ROSMAP & Homo validated
DNAJC21	4	ROSMAP & Homo validated
DNASE1L1	4	ROSMAP & Homo validated
DOCK8	4	ROSMAP & Homo validated
DPM3	4	ROSMAP & Homo validated
DUSP7	4	ROSMAP & Homo validated
ECH1	4	ROSMAP & Homo validated
ECT2L	4	ROSMAP & Homo validated
EPB41	4	ROSMAP & Homo validated
ERG28	4	ROSMAP & Homo validated
ESRP2	4	ROSMAP & Homo validated
EXOSC10	4	ROSMAP & Homo validated
EXOSC8	4	ROSMAP & Homo validated
EZR	4	ROSMAP & Homo validated
FAM117B	4	ROSMAP & Homo validated
FAM118B	4	ROSMAP & Homo validated
FAM241A	4	ROSMAP & Homo validated
FAM89B	4	ROSMAP & Homo validated
FBH1	4	ROSMAP & Homo validated
FBXW2	4	ROSMAP & Homo validated
FEM1B	4	ROSMAP & Homo validated
FGD2	4	ROSMAP & Homo validated
FGD3	4	ROSMAP & Homo validated
FHIP2B	4	ROSMAP & Homo validated
FIBP	4	ROSMAP & Homo validated
FKBP2	4	ROSMAP & Homo validated
FMNL1	4	ROSMAP & Homo validated
FOXN3	4	ROSMAP & Homo validated
GAPDHS	4	ROSMAP & Homo validated
GBA2	4	ROSMAP & Homo validated

GDPD3	4	ROSMAP & Homo validated
GIGYF2	4	ROSMAP & Homo validated
GJD3	4	ROSMAP & Homo validated
GNB1L	4	ROSMAP & Homo validated
GNL3	4	ROSMAP & Homo validated
GPX3	4	ROSMAP & Homo validated
GRAMD1C	4	ROSMAP & Homo validated
GZMM	4	ROSMAP & Homo validated
H1-0	4	ROSMAP & Homo validated
H2AX	4	ROSMAP & Homo validated
HACL1	4	ROSMAP & Homo validated
HMBOX1	4	ROSMAP & Homo validated
HUWE1	4	ROSMAP & Homo validated
HYKK	4	ROSMAP & Homo validated
IDH3G	4	ROSMAP & Homo validated
IDNK	4	ROSMAP & Homo validated
IFNGR1	4	ROSMAP & Homo validated
IGF2R	4	ROSMAP & Homo validated
IL18BP	4	ROSMAP & Homo validated
IMPDH1	4	ROSMAP & Homo validated
IRF2BP2	4	ROSMAP & Homo validated
KLHL22	4	ROSMAP & Homo validated
KSR1	4	ROSMAP & Homo validated
LARP1	4	ROSMAP & Homo validated
LATS2	4	ROSMAP & Homo validated
LCN10	4	ROSMAP & Homo validated
LDLRAD4	4	ROSMAP & Homo validated
LHX4	4	ROSMAP & Homo validated
LIX1L	4	ROSMAP & Homo validated
LYPLA2	4	ROSMAP & Homo validated
MAML1	4	ROSMAP & Homo validated
MAP4K1	4	ROSMAP & Homo validated
MAP7D3	4	ROSMAP & Homo validated
MAST1	4	ROSMAP & Homo validated
MDM2	4	ROSMAP & Homo validated
MED18	4	ROSMAP & Homo validated
MICAL1	4	ROSMAP & Homo validated
MKS1	4	ROSMAP & Homo validated
MLH1	4	ROSMAP & Homo validated
MLLT6	4	ROSMAP & Homo validated
MPZL1	4	ROSMAP & Homo validated
MROH6	4	ROSMAP & Homo validated
MRPL23	4	ROSMAP & Homo validated
MRPL48	4	ROSMAP & Homo validated
MS4A1	4	ROSMAP & Homo validated
MS4A4A	4	ROSMAP & Homo validated
MS4A7	4	ROSMAP & Homo validated
MTFP1	4	ROSMAP & Homo validated
MYCL	4	ROSMAP & Homo validated
MYH7B	4	ROSMAP & Homo validated
MYO18A	4	ROSMAP & Homo validated
MZT2A	4	ROSMAP & Homo validated
NAA25	4	ROSMAP & Homo validated
NAA80	4	ROSMAP & Homo validated
NBR1	4	ROSMAP & Homo validated

NCAPD3	4	ROSMAP & Homo validated
NDUFA3	4	ROSMAP & Homo validated
NDUFB9	4	ROSMAP & Homo validated
NDUFS1	4	ROSMAP & Homo validated
NSF	4	ROSMAP & Homo validated
NSL1	4	ROSMAP & Homo validated
NSMF	4	ROSMAP & Homo validated
NUDT22	4	ROSMAP & Homo validated
NUTM2A	4	ROSMAP & Homo validated
OR2T10	4	ROSMAP & Homo validated
OSBPL9	4	ROSMAP & Homo validated
OTUD5	4	ROSMAP & Homo validated
P2RY2	4	ROSMAP & Homo validated
P4HB	4	ROSMAP & Homo validated
PAG1	4	ROSMAP & Homo validated
PAGR1	4	ROSMAP & Homo validated
PAQR4	4	ROSMAP & Homo validated
PARL	4	ROSMAP & Homo validated
PARP9	4	ROSMAP & Homo validated
PCOLCE	4	ROSMAP & Homo validated
PDCL	4	ROSMAP & Homo validated
PDK3	4	ROSMAP & Homo validated
PEBP1	4	ROSMAP & Homo validated
PEX11B	4	ROSMAP & Homo validated
PHETA2	4	ROSMAP & Homo validated
PHF14	4	ROSMAP & Homo validated
PIK3C2B	4	ROSMAP & Homo validated
PILRB	4	ROSMAP & Homo validated
PINK1	4	ROSMAP & Homo validated
PKD2L2	4	ROSMAP & Homo validated
PLA2G7	4	ROSMAP & Homo validated
PNPLA1	4	ROSMAP & Homo validated
POLR2C	4	ROSMAP & Homo validated
POU2F2	4	ROSMAP & Homo validated
PPIP5K2	4	ROSMAP & Homo validated
PPP1R37	4	ROSMAP & Homo validated
PRAM1	4	ROSMAP & Homo validated
PREP	4	ROSMAP & Homo validated
PSD4	4	ROSMAP & Homo validated
RAB41	4	ROSMAP & Homo validated
RAP1GAP2	4	ROSMAP & Homo validated
RBM39	4	ROSMAP & Homo validated
RBMS1	4	ROSMAP & Homo validated
RGL4	4	ROSMAP & Homo validated
RHBDF2	4	ROSMAP & Homo validated
RHNO1	4	ROSMAP & Homo validated
RMC1	4	ROSMAP & Homo validated
RNF135	4	ROSMAP & Homo validated
RNF165	4	ROSMAP & Homo validated
RNPEPL1	4	ROSMAP & Homo validated
RPA2	4	ROSMAP & Homo validated
RPN2	4	ROSMAP & Homo validated
RPP25	4	ROSMAP & Homo validated
RUBCN	4	ROSMAP & Homo validated
RUNDC1	4	ROSMAP & Homo validated

RUVBL1	4	ROSMAP & Homo validated
SAMD9L	4	ROSMAP & Homo validated
SASH3	4	ROSMAP & Homo validated
SBF2	4	ROSMAP & Homo validated
SCAMP3	4	ROSMAP & Homo validated
SEC13	4	ROSMAP & Homo validated
SEC23IP	4	ROSMAP & Homo validated
SEC61B	4	ROSMAP & Homo validated
SHLD2	4	ROSMAP & Homo validated
SIGIRR	4	ROSMAP & Homo validated
SLAMF1	4	ROSMAP & Homo validated
SLAMF9	4	ROSMAP & Homo validated
SLC22A15	4	ROSMAP & Homo validated
SLC25A18	4	ROSMAP & Homo validated
SLC30A9	4	ROSMAP & Homo validated
SLC35A1	4	ROSMAP & Homo validated
SLC5A9	4	ROSMAP & Homo validated
SMARCAD1	4	ROSMAP & Homo validated
SMG1	4	ROSMAP & Homo validated
SPATA9	4	ROSMAP & Homo validated
SPCS2	4	ROSMAP & Homo validated
STAP2	4	ROSMAP & Homo validated
STARD10	4	ROSMAP & Homo validated
STAT5B	4	ROSMAP & Homo validated
STK10	4	ROSMAP & Homo validated
STMN3	4	ROSMAP & Homo validated
STT3B	4	ROSMAP & Homo validated
TAF8	4	ROSMAP & Homo validated
TBC1D3L	4	ROSMAP & Homo validated
TBC1D9	4	ROSMAP & Homo validated
TGM2	4	ROSMAP & Homo validated
TIGIT	4	ROSMAP & Homo validated
TIMM29	4	ROSMAP & Homo validated
TLR1	4	ROSMAP & Homo validated
TMEM141	4	ROSMAP & Homo validated
TMEM168	4	ROSMAP & Homo validated
TNFRSF14	4	ROSMAP & Homo validated
TNFSF12	4	ROSMAP & Homo validated
TOGARAM2	4	ROSMAP & Homo validated
TPM2	4	ROSMAP & Homo validated
TPPP3	4	ROSMAP & Homo validated
TRAFD1	4	ROSMAP & Homo validated
TRIM52	4	ROSMAP & Homo validated
TRMT44	4	ROSMAP & Homo validated
TRMT61A	4	ROSMAP & Homo validated
TSPOAP1	4	ROSMAP & Homo validated
TTBK2	4	ROSMAP & Homo validated
TTC5	4	ROSMAP & Homo validated
TUT1	4	ROSMAP & Homo validated
UBA1	4	ROSMAP & Homo validated
UBAP2L	4	ROSMAP & Homo validated
UBL7	4	ROSMAP & Homo validated
UBXN6	4	ROSMAP & Homo validated
UNC50	4	ROSMAP & Homo validated
VAMP5	4	ROSMAP & Homo validated

VAT1	4	ROSMAP & Homo validated
VPS39	4	ROSMAP & Homo validated
WDR24	4	ROSMAP & Homo validated
WDR82	4	ROSMAP & Homo validated
WNT3	4	ROSMAP & Homo validated
YWHAB	4	ROSMAP & Homo validated
ZBTB20	4	ROSMAP & Homo validated
ZBTB37	4	ROSMAP & Homo validated
ZDHHC14	4	ROSMAP & Homo validated
ZFR	4	ROSMAP & Homo validated
ZFX	4	ROSMAP & Homo validated
ZFYVE26	4	ROSMAP & Homo validated
ZKSCAN1	4	ROSMAP & Homo validated
ZKSCAN7	4	ROSMAP & Homo validated
ZNF174	4	ROSMAP & Homo validated
ZNF189	4	ROSMAP & Homo validated
ZNF195	4	ROSMAP & Homo validated
ZNF274	4	ROSMAP & Homo validated
ZNF319	4	ROSMAP & Homo validated
ZNF362	4	ROSMAP & Homo validated
ZNF414	4	ROSMAP & Homo validated
ZNF428	4	ROSMAP & Homo validated
ZNF43	4	ROSMAP & Homo validated
ZNF440	4	ROSMAP & Homo validated
ZNF567	4	ROSMAP & Homo validated
ZNF581	4	ROSMAP & Homo validated
ZNF641	4	ROSMAP & Homo validated
A1CF	5	ROSMAP & Homo validated
A4GALT	5	ROSMAP & Homo validated
AADACL3	5	ROSMAP & Homo validated
ACAN	5	ROSMAP & Homo validated
ACCSL	5	ROSMAP & Homo validated
ACKR2	5	ROSMAP & Homo validated
ACTBL2	5	ROSMAP & Homo validated
ACTN1	5	ROSMAP & Homo validated
ADAM2	5	ROSMAP & Homo validated
ADAMTS19	5	ROSMAP & Homo validated
ADCYAP1	5	ROSMAP & Homo validated
ADCYAP1R1	5	ROSMAP & Homo validated
ADGB	5	ROSMAP & Homo validated
ADH1A	5	ROSMAP & Homo validated
ADM5	5	ROSMAP & Homo validated
AGBL3	5	ROSMAP & Homo validated
AKAP14	5	ROSMAP & Homo validated
ALK	5	ROSMAP & Homo validated
AMELX	5	ROSMAP & Homo validated
ANKRD34A	5	ROSMAP & Homo validated
APOA5	5	ROSMAP & Homo validated
ARHGAP39	5	ROSMAP & Homo validated
ARHGEF16	5	ROSMAP & Homo validated
ASB4	5	ROSMAP & Homo validated
ASCL1	5	ROSMAP & Homo validated
ASCL3	5	ROSMAP & Homo validated
ATOH1	5	ROSMAP & Homo validated
ATP6V1B1	5	ROSMAP & Homo validated

ATRNL1	5	ROSMAP & Homo validated
ATRX	5	ROSMAP & Homo validated
BCHE	5	ROSMAP & Homo validated
BCL3	5	ROSMAP & Homo validated
BEST2	5	ROSMAP & Homo validated
BEX1	5	ROSMAP & Homo validated
BMP2	5	ROSMAP & Homo validated
BPIFA2	5	ROSMAP & Homo validated
BRSK1	5	ROSMAP & Homo validated
C10orf120	5	ROSMAP & Homo validated
C10orf71	5	ROSMAP & Homo validated
C11orf16	5	ROSMAP & Homo validated
C1orf116	5	ROSMAP & Homo validated
C1orf198	5	ROSMAP & Homo validated
C1orf53	5	ROSMAP & Homo validated
C2CD6	5	ROSMAP & Homo validated
C4orf45	5	ROSMAP & Homo validated
C7orf33	5	ROSMAP & Homo validated
C9orf40	5	ROSMAP & Homo validated
CA9	5	ROSMAP & Homo validated
CABCOCO1	5	ROSMAP & Homo validated
CABS1	5	ROSMAP & Homo validated
CACNG5	5	ROSMAP & Homo validated
CADM3	5	ROSMAP & Homo validated
CALCR	5	ROSMAP & Homo validated
CAMK2A	5	ROSMAP & Homo validated
CBY3	5	ROSMAP & Homo validated
CCDC105	5	ROSMAP & Homo validated
CCDC160	5	ROSMAP & Homo validated
CCDC175	5	ROSMAP & Homo validated
CCDC183	5	ROSMAP & Homo validated
CDKL3	5	ROSMAP & Homo validated
CDKN2B	5	ROSMAP & Homo validated
CDX1	5	ROSMAP & Homo validated
CDX4	5	ROSMAP & Homo validated
CEACAM16	5	ROSMAP & Homo validated
CELF3	5	ROSMAP & Homo validated
CENPA	5	ROSMAP & Homo validated
CEP131	5	ROSMAP & Homo validated
CERS5	5	ROSMAP & Homo validated
CFTR	5	ROSMAP & Homo validated
CHRM2	5	ROSMAP & Homo validated
CHRNG	5	ROSMAP & Homo validated
CHST15	5	ROSMAP & Homo validated
CHST9	5	ROSMAP & Homo validated
CHURC1-FNTB	5	ROSMAP & Homo validated
CLCN2	5	ROSMAP & Homo validated
CLDN14	5	ROSMAP & Homo validated
CLDN2	5	ROSMAP & Homo validated
CLHC1	5	ROSMAP & Homo validated
CLRN2	5	ROSMAP & Homo validated
CMC2	5	ROSMAP & Homo validated
CNBD1	5	ROSMAP & Homo validated
CNTN6	5	ROSMAP & Homo validated
COL6A5	5	ROSMAP & Homo validated

COL6A6	5	ROSMAP & Homo validated
CPA6	5	ROSMAP & Homo validated
CPB1	5	ROSMAP & Homo validated
CPLX4	5	ROSMAP & Homo validated
CPNE4	5	ROSMAP & Homo validated
CRH	5	ROSMAP & Homo validated
CRISP1	5	ROSMAP & Homo validated
CRP	5	ROSMAP & Homo validated
CSRNP3	5	ROSMAP & Homo validated
CT45A1	5	ROSMAP & Homo validated
CT45A3	5	ROSMAP & Homo validated
CT45A6	5	ROSMAP & Homo validated
CT55	5	ROSMAP & Homo validated
CT83	5	ROSMAP & Homo validated
CTTNBP2	5	ROSMAP & Homo validated
CTXN3	5	ROSMAP & Homo validated
CXXC4	5	ROSMAP & Homo validated
CYB5R3	5	ROSMAP & Homo validated
CYP2C19	5	ROSMAP & Homo validated
CYP2F1	5	ROSMAP & Homo validated
CYP39A1	5	ROSMAP & Homo validated
CYP46A1	5	ROSMAP & Homo validated
DAGLA	5	ROSMAP & Homo validated
DAW1	5	ROSMAP & Homo validated
DBN1	5	ROSMAP & Homo validated
DBX2	5	ROSMAP & Homo validated
DCAF8L1	5	ROSMAP & Homo validated
DCSTAMP	5	ROSMAP & Homo validated
DDIT4L	5	ROSMAP & Homo validated
DEFB128	5	ROSMAP & Homo validated
DEFB135	5	ROSMAP & Homo validated
DMGDH	5	ROSMAP & Homo validated
DNAH12	5	ROSMAP & Homo validated
DOC2A	5	ROSMAP & Homo validated
DOK4	5	ROSMAP & Homo validated
DPH7	5	ROSMAP & Homo validated
DRC1	5	ROSMAP & Homo validated
DUSP26	5	ROSMAP & Homo validated
DYNC1I1	5	ROSMAP & Homo validated
E2F7	5	ROSMAP & Homo validated
EDA2R	5	ROSMAP & Homo validated
EDN2	5	ROSMAP & Homo validated
EDNRA	5	ROSMAP & Homo validated
EFCAB13	5	ROSMAP & Homo validated
EPO	5	ROSMAP & Homo validated
EQTN	5	ROSMAP & Homo validated
ERBB4	5	ROSMAP & Homo validated
ERICH6B	5	ROSMAP & Homo validated
ERVW-1	5	ROSMAP & Homo validated
ESYT3	5	ROSMAP & Homo validated
F13B	5	ROSMAP & Homo validated
FAM104B	5	ROSMAP & Homo validated
FAM131A	5	ROSMAP & Homo validated
FAM171A1	5	ROSMAP & Homo validated
FAM171B	5	ROSMAP & Homo validated

FAM185A	5	ROSMAP & Homo validated
FAM47C	5	ROSMAP & Homo validated
FAT3	5	ROSMAP & Homo validated
FBRSL1	5	ROSMAP & Homo validated
FGF7	5	ROSMAP & Homo validated
FHOD1	5	ROSMAP & Homo validated
FKBP1B	5	ROSMAP & Homo validated
FMN2	5	ROSMAP & Homo validated
FMO3	5	ROSMAP & Homo validated
FRMPD4	5	ROSMAP & Homo validated
FRZB	5	ROSMAP & Homo validated
FSTL5	5	ROSMAP & Homo validated
FXYD3	5	ROSMAP & Homo validated
FYB2	5	ROSMAP & Homo validated
GABRA6	5	ROSMAP & Homo validated
GABRQ	5	ROSMAP & Homo validated
GADD45B	5	ROSMAP & Homo validated
GAGE12F	5	ROSMAP & Homo validated
GALNT16	5	ROSMAP & Homo validated
GAS6	5	ROSMAP & Homo validated
GET1-SH3BGR	5	ROSMAP & Homo validated
GIMD1	5	ROSMAP & Homo validated
GJE1	5	ROSMAP & Homo validated
GK2	5	ROSMAP & Homo validated
GKN1	5	ROSMAP & Homo validated
GLDC	5	ROSMAP & Homo validated
GLDN	5	ROSMAP & Homo validated
GLYATL3	5	ROSMAP & Homo validated
GPR158	5	ROSMAP & Homo validated
GPR176	5	ROSMAP & Homo validated
GPR26	5	ROSMAP & Homo validated
GPR87	5	ROSMAP & Homo validated
GRB14	5	ROSMAP & Homo validated
GREM2	5	ROSMAP & Homo validated
GRID1	5	ROSMAP & Homo validated
GRIK2	5	ROSMAP & Homo validated
GRK4	5	ROSMAP & Homo validated
GRP	5	ROSMAP & Homo validated
GRXCR1	5	ROSMAP & Homo validated
GSTA4	5	ROSMAP & Homo validated
GSTM5	5	ROSMAP & Homo validated
GSTO1	5	ROSMAP & Homo validated
GYPE	5	ROSMAP & Homo validated
H2AC16	5	ROSMAP & Homo validated
H2AC21	5	ROSMAP & Homo validated
H2BC14	5	ROSMAP & Homo validated
H2BC3	5	ROSMAP & Homo validated
H2BC8	5	ROSMAP & Homo validated
H4C1	5	ROSMAP & Homo validated
HAPLN1	5	ROSMAP & Homo validated
HCRTR2	5	ROSMAP & Homo validated
HDGFL1	5	ROSMAP & Homo validated
HEPACAM2	5	ROSMAP & Homo validated
HES6	5	ROSMAP & Homo validated
HIGD1A	5	ROSMAP & Homo validated

HORMAD2	5	ROSMAP & Homo validated
HPN	5	ROSMAP & Homo validated
HSFX1	5	ROSMAP & Homo validated
HSFX2	5	ROSMAP & Homo validated
IAPP	5	ROSMAP & Homo validated
IFNB1	5	ROSMAP & Homo validated
IGLON5	5	ROSMAP & Homo validated
IL13	5	ROSMAP & Homo validated
IL17C	5	ROSMAP & Homo validated
IL17RD	5	ROSMAP & Homo validated
IMPG1	5	ROSMAP & Homo validated
INSL4	5	ROSMAP & Homo validated
IQCF3	5	ROSMAP & Homo validated
IQUB	5	ROSMAP & Homo validated
JMJD6	5	ROSMAP & Homo validated
JPH3	5	ROSMAP & Homo validated
JUNB	5	ROSMAP & Homo validated
KAT6B	5	ROSMAP & Homo validated
KAZALD1	5	ROSMAP & Homo validated
KCNC2	5	ROSMAP & Homo validated
KCNJ16	5	ROSMAP & Homo validated
KCTD8	5	ROSMAP & Homo validated
KHDC3L	5	ROSMAP & Homo validated
KIF20B	5	ROSMAP & Homo validated
KIR3DL3	5	ROSMAP & Homo validated
KIRREL1	5	ROSMAP & Homo validated
KLK10	5	ROSMAP & Homo validated
KLK3	5	ROSMAP & Homo validated
KRT6C	5	ROSMAP & Homo validated
KRTAP13-2	5	ROSMAP & Homo validated
KRTAP19-1	5	ROSMAP & Homo validated
KRTAP19-7	5	ROSMAP & Homo validated
KRTAP21-2	5	ROSMAP & Homo validated
KRTAP24-1	5	ROSMAP & Homo validated
KRTAP5-3	5	ROSMAP & Homo validated
KRTAP5-7	5	ROSMAP & Homo validated
LBP	5	ROSMAP & Homo validated
LCA5	5	ROSMAP & Homo validated
LCE3D	5	ROSMAP & Homo validated
LEPR	5	ROSMAP & Homo validated
LGALS12	5	ROSMAP & Homo validated
LHX1	5	ROSMAP & Homo validated
LINGO2	5	ROSMAP & Homo validated
LIPC	5	ROSMAP & Homo validated
LMBRD1	5	ROSMAP & Homo validated
LMO1	5	ROSMAP & Homo validated
LMX1B	5	ROSMAP & Homo validated
LPIN3	5	ROSMAP & Homo validated
LRAT	5	ROSMAP & Homo validated
LRIG3	5	ROSMAP & Homo validated
LRRC3B	5	ROSMAP & Homo validated
LRRC53	5	ROSMAP & Homo validated
LRRD1	5	ROSMAP & Homo validated
LRRTM3	5	ROSMAP & Homo validated
LRTM1	5	ROSMAP & Homo validated

LRTOMT	5	ROSMAP & Homo validated
LUZP4	5	ROSMAP & Homo validated
LY6K	5	ROSMAP & Homo validated
MAGEB1	5	ROSMAP & Homo validated
MAGED1	5	ROSMAP & Homo validated
MAGED4	5	ROSMAP & Homo validated
MAIP1	5	ROSMAP & Homo validated
MARCHF11	5	ROSMAP & Homo validated
MARCHF2	5	ROSMAP & Homo validated
MAST4	5	ROSMAP & Homo validated
MBL2	5	ROSMAP & Homo validated
MCHR1	5	ROSMAP & Homo validated
MCM10	5	ROSMAP & Homo validated
MEX3A	5	ROSMAP & Homo validated
MID1	5	ROSMAP & Homo validated
MLH3	5	ROSMAP & Homo validated
MMP16	5	ROSMAP & Homo validated
MMP21	5	ROSMAP & Homo validated
MMP8	5	ROSMAP & Homo validated
MRE11	5	ROSMAP & Homo validated
MS4A8	5	ROSMAP & Homo validated
MSRA	5	ROSMAP & Homo validated
MT3	5	ROSMAP & Homo validated
MTNR1A	5	ROSMAP & Homo validated
MUC13	5	ROSMAP & Homo validated
MYH2	5	ROSMAP & Homo validated
MYL1	5	ROSMAP & Homo validated
MYOZ2	5	ROSMAP & Homo validated
NACAD	5	ROSMAP & Homo validated
NCAM1	5	ROSMAP & Homo validated
NCBP2L	5	ROSMAP & Homo validated
NCK2	5	ROSMAP & Homo validated
NCR3LG1	5	ROSMAP & Homo validated
NECAB1	5	ROSMAP & Homo validated
NECTIN3	5	ROSMAP & Homo validated
NEFM	5	ROSMAP & Homo validated
NEK4	5	ROSMAP & Homo validated
NEMF	5	ROSMAP & Homo validated
NEXMIF	5	ROSMAP & Homo validated
NFKBIL1	5	ROSMAP & Homo validated
NME5	5	ROSMAP & Homo validated
NPY	5	ROSMAP & Homo validated
NPY5R	5	ROSMAP & Homo validated
NRCAM	5	ROSMAP & Homo validated
NRG3	5	ROSMAP & Homo validated
NRSN1	5	ROSMAP & Homo validated
NTN3	5	ROSMAP & Homo validated
NTS	5	ROSMAP & Homo validated
NUP62CL	5	ROSMAP & Homo validated
NXPH2	5	ROSMAP & Homo validated
OCA2	5	ROSMAP & Homo validated
OCSTAMP	5	ROSMAP & Homo validated
ODAD2	5	ROSMAP & Homo validated
ODAM	5	ROSMAP & Homo validated
ODF3	5	ROSMAP & Homo validated

OLFM3	5	ROSMAP & Homo validated
OMD	5	ROSMAP & Homo validated
OOEP	5	ROSMAP & Homo validated
OPN4	5	ROSMAP & Homo validated
OR10Z1	5	ROSMAP & Homo validated
OR11H2	5	ROSMAP & Homo validated
OR11H4	5	ROSMAP & Homo validated
OR13F1	5	ROSMAP & Homo validated
OR14J1	5	ROSMAP & Homo validated
OR1E2	5	ROSMAP & Homo validated
OR2A2	5	ROSMAP & Homo validated
OR2AG2	5	ROSMAP & Homo validated
OR2T11	5	ROSMAP & Homo validated
OR2T27	5	ROSMAP & Homo validated
OR2W3	5	ROSMAP & Homo validated
OR4A16	5	ROSMAP & Homo validated
OR4C16	5	ROSMAP & Homo validated
OR4D11	5	ROSMAP & Homo validated
OR4F5	5	ROSMAP & Homo validated
OR4K1	5	ROSMAP & Homo validated
OR4L1	5	ROSMAP & Homo validated
OR4M1	5	ROSMAP & Homo validated
OR51B6	5	ROSMAP & Homo validated
OR52E4	5	ROSMAP & Homo validated
OR52E5	5	ROSMAP & Homo validated
OR52E6	5	ROSMAP & Homo validated
OR52N5	5	ROSMAP & Homo validated
OR5D16	5	ROSMAP & Homo validated
OR5H2	5	ROSMAP & Homo validated
OR5K1	5	ROSMAP & Homo validated
OR5V1	5	ROSMAP & Homo validated
OR6B2	5	ROSMAP & Homo validated
OR6C76	5	ROSMAP & Homo validated
OR6P1	5	ROSMAP & Homo validated
OR6T1	5	ROSMAP & Homo validated
OR7E24	5	ROSMAP & Homo validated
OR7G2	5	ROSMAP & Homo validated
OR8D2	5	ROSMAP & Homo validated
OR8J3	5	ROSMAP & Homo validated
OR9G1	5	ROSMAP & Homo validated
OR9K2	5	ROSMAP & Homo validated
OR9Q1	5	ROSMAP & Homo validated
OTOF	5	ROSMAP & Homo validated
OTOP1	5	ROSMAP & Homo validated
PABPC5	5	ROSMAP & Homo validated
PACSIN3	5	ROSMAP & Homo validated
PAK5	5	ROSMAP & Homo validated
PALS2	5	ROSMAP & Homo validated
PAPOLB	5	ROSMAP & Homo validated
PAX2	5	ROSMAP & Homo validated
PCDH15	5	ROSMAP & Homo validated
PCP4	5	ROSMAP & Homo validated
PDE1B	5	ROSMAP & Homo validated
PDGFC	5	ROSMAP & Homo validated
PELI2	5	ROSMAP & Homo validated

PERM1	5	ROSMAP & Homo validated
PFN2	5	ROSMAP & Homo validated
PHYHIP	5	ROSMAP & Homo validated
PHYHIPL	5	ROSMAP & Homo validated
PIAS1	5	ROSMAP & Homo validated
PIH1D2	5	ROSMAP & Homo validated
PLEKHG4B	5	ROSMAP & Homo validated
PLK1	5	ROSMAP & Homo validated
PLPPR4	5	ROSMAP & Homo validated
PLPPR5	5	ROSMAP & Homo validated
PNMA2	5	ROSMAP & Homo validated
POTEJ	5	ROSMAP & Homo validated
POU3F4	5	ROSMAP & Homo validated
PPFIA2	5	ROSMAP & Homo validated
PPP1R42	5	ROSMAP & Homo validated
PPP4R3C	5	ROSMAP & Homo validated
PRAMEF33	5	ROSMAP & Homo validated
PRICKLE4	5	ROSMAP & Homo validated
PRKAA2	5	ROSMAP & Homo validated
PRM3	5	ROSMAP & Homo validated
PROSER2	5	ROSMAP & Homo validated
PRR23D1	5	ROSMAP & Homo validated
PSG11	5	ROSMAP & Homo validated
PSG2	5	ROSMAP & Homo validated
PSPC1	5	ROSMAP & Homo validated
PTF1A	5	ROSMAP & Homo validated
PTGS2	5	ROSMAP & Homo validated
PTH1R	5	ROSMAP & Homo validated
PTK7	5	ROSMAP & Homo validated
PURG	5	ROSMAP & Homo validated
QSOX1	5	ROSMAP & Homo validated
RABAC1	5	ROSMAP & Homo validated
RAD51AP2	5	ROSMAP & Homo validated
RALYL	5	ROSMAP & Homo validated
RARRES1	5	ROSMAP & Homo validated
RASA1	5	ROSMAP & Homo validated
RASSF6	5	ROSMAP & Homo validated
RBM20	5	ROSMAP & Homo validated
RESP18	5	ROSMAP & Homo validated
RFX3	5	ROSMAP & Homo validated
RGS17	5	ROSMAP & Homo validated
RGS21	5	ROSMAP & Homo validated
RGS9	5	ROSMAP & Homo validated
RIPOR3	5	ROSMAP & Homo validated
RPA1	5	ROSMAP & Homo validated
RXRG	5	ROSMAP & Homo validated
S100A7A	5	ROSMAP & Homo validated
SAMD11	5	ROSMAP & Homo validated
SCG3	5	ROSMAP & Homo validated
SCRT2	5	ROSMAP & Homo validated
SDC2	5	ROSMAP & Homo validated
SEMA6A	5	ROSMAP & Homo validated
SERPINA7	5	ROSMAP & Homo validated
SERPINE1	5	ROSMAP & Homo validated
SI	5	ROSMAP & Homo validated

SLC12A5	5	ROSMAP & Homo validated
SLC15A5	5	ROSMAP & Homo validated
SLC22A9	5	ROSMAP & Homo validated
SLC24A2	5	ROSMAP & Homo validated
SLC25A31	5	ROSMAP & Homo validated
SLC27A6	5	ROSMAP & Homo validated
SLC2A13	5	ROSMAP & Homo validated
SLC30A3	5	ROSMAP & Homo validated
SLC4A3	5	ROSMAP & Homo validated
SLC7A3	5	ROSMAP & Homo validated
SLC9A5	5	ROSMAP & Homo validated
SLCO1B1	5	ROSMAP & Homo validated
CO1B3-SLCO1	5	ROSMAP & Homo validated
SLN	5	ROSMAP & Homo validated
SMIM21	5	ROSMAP & Homo validated
SMIM27	5	ROSMAP & Homo validated
SMIM43	5	ROSMAP & Homo validated
SOX10	5	ROSMAP & Homo validated
SOX14	5	ROSMAP & Homo validated
SPACA9	5	ROSMAP & Homo validated
SPAG6	5	ROSMAP & Homo validated
SPATA31A3	5	ROSMAP & Homo validated
SPATA31D4	5	ROSMAP & Homo validated
SPATA32	5	ROSMAP & Homo validated
SPEN	5	ROSMAP & Homo validated
SPOCK1	5	ROSMAP & Homo validated
SPOCK3	5	ROSMAP & Homo validated
SPRY1	5	ROSMAP & Homo validated
SREBF2	5	ROSMAP & Homo validated
SSC4D	5	ROSMAP & Homo validated
STC1	5	ROSMAP & Homo validated
STPG1	5	ROSMAP & Homo validated
STUM	5	ROSMAP & Homo validated
SUCNR1	5	ROSMAP & Homo validated
SYT6	5	ROSMAP & Homo validated
TAAR2	5	ROSMAP & Homo validated
TAC1	5	ROSMAP & Homo validated
TAC3	5	ROSMAP & Homo validated
TAFA4	5	ROSMAP & Homo validated
TAS2R13	5	ROSMAP & Homo validated
TAS2R16	5	ROSMAP & Homo validated
TAT	5	ROSMAP & Homo validated
TBX22	5	ROSMAP & Homo validated
TCF4	5	ROSMAP & Homo validated
TCP11X1	5	ROSMAP & Homo validated
TCP11X2	5	ROSMAP & Homo validated
TENT5D	5	ROSMAP & Homo validated
TEX26	5	ROSMAP & Homo validated
TEX55	5	ROSMAP & Homo validated
TFAP2B	5	ROSMAP & Homo validated
THUMPD2	5	ROSMAP & Homo validated
TMEM104	5	ROSMAP & Homo validated
TMEM130	5	ROSMAP & Homo validated
TMEM132D	5	ROSMAP & Homo validated
TMEM196	5	ROSMAP & Homo validated

TMEM198	5	ROSMAP & Homo validated
TMEM37	5	ROSMAP & Homo validated
TMPRSS11A	5	ROSMAP & Homo validated
TMPRSS5	5	ROSMAP & Homo validated
TMSB4Y	5	ROSMAP & Homo validated
TNC	5	ROSMAP & Homo validated
TNFRSF13B	5	ROSMAP & Homo validated
TNNT2	5	ROSMAP & Homo validated
TPBG	5	ROSMAP & Homo validated
TRAPPC3L	5	ROSMAP & Homo validated
TRPC3	5	ROSMAP & Homo validated
TRPC4	5	ROSMAP & Homo validated
TRPM1	5	ROSMAP & Homo validated
TSPAN19	5	ROSMAP & Homo validated
TSPAN2	5	ROSMAP & Homo validated
TTC29	5	ROSMAP & Homo validated
TTLL6	5	ROSMAP & Homo validated
TTR	5	ROSMAP & Homo validated
TUBB4A	5	ROSMAP & Homo validated
TULP3	5	ROSMAP & Homo validated
TYRP1	5	ROSMAP & Homo validated
UBL4A	5	ROSMAP & Homo validated
UGT1A4	5	ROSMAP & Homo validated
UGT1A8	5	ROSMAP & Homo validated
UGT3A1	5	ROSMAP & Homo validated
UNC5C	5	ROSMAP & Homo validated
UQCR10	5	ROSMAP & Homo validated
URAD	5	ROSMAP & Homo validated
USP17L3	5	ROSMAP & Homo validated
VASN	5	ROSMAP & Homo validated
VCX3A	5	ROSMAP & Homo validated
VCX3B	5	ROSMAP & Homo validated
VEGFD	5	ROSMAP & Homo validated
VGLL1	5	ROSMAP & Homo validated
VPS37D	5	ROSMAP & Homo validated
VTCN1	5	ROSMAP & Homo validated
VWF	5	ROSMAP & Homo validated
WASF3	5	ROSMAP & Homo validated
WFS1	5	ROSMAP & Homo validated
WHRN	5	ROSMAP & Homo validated
WNT9A	5	ROSMAP & Homo validated
ZBTB39	5	ROSMAP & Homo validated
ZC3H12A	5	ROSMAP & Homo validated
ZCCHC12	5	ROSMAP & Homo validated
ZDHHC7	5	ROSMAP & Homo validated
ZNF214	5	ROSMAP & Homo validated
ZNF385D	5	ROSMAP & Homo validated
ZSWIM2	5	ROSMAP & Homo validated
ABHD8	6	ROSMAP & Homo validated
ACP3	6	ROSMAP & Homo validated
ACTRT1	6	ROSMAP & Homo validated
AHRR	6	ROSMAP & Homo validated
AKT3	6	ROSMAP & Homo validated
ALOX15	6	ROSMAP & Homo validated
ARHGEF10L	6	ROSMAP & Homo validated

ARL17B	6	ROSMAP & Homo validated
ARPC3	6	ROSMAP & Homo validated
ASAP1	6	ROSMAP & Homo validated
ASF1B	6	ROSMAP & Homo validated
ATG13	6	ROSMAP & Homo validated
BNIP2	6	ROSMAP & Homo validated
BPIFC	6	ROSMAP & Homo validated
C17orf80	6	ROSMAP & Homo validated
C1orf226	6	ROSMAP & Homo validated
C20orf204	6	ROSMAP & Homo validated
CAMKMT	6	ROSMAP & Homo validated
CAT	6	ROSMAP & Homo validated
CBWD2	6	ROSMAP & Homo validated
CBWD6	6	ROSMAP & Homo validated
CCDC96	6	ROSMAP & Homo validated
CCNDBP1	6	ROSMAP & Homo validated
CDKL1	6	ROSMAP & Homo validated
CFP	6	ROSMAP & Homo validated
CIAO3	6	ROSMAP & Homo validated
CNBP	6	ROSMAP & Homo validated
COL10A1	6	ROSMAP & Homo validated
COL9A3	6	ROSMAP & Homo validated
COX6B1	6	ROSMAP & Homo validated
CPPED1	6	ROSMAP & Homo validated
CPSF2	6	ROSMAP & Homo validated
CREG1	6	ROSMAP & Homo validated
CSNK1A1L	6	ROSMAP & Homo validated
CT47A12	6	ROSMAP & Homo validated
CTDSPL2	6	ROSMAP & Homo validated
DCAF12	6	ROSMAP & Homo validated
DCAF6	6	ROSMAP & Homo validated
DCUN1D1	6	ROSMAP & Homo validated
DDX17	6	ROSMAP & Homo validated
DEGS2	6	ROSMAP & Homo validated
EPOR	6	ROSMAP & Homo validated
ETNK1	6	ROSMAP & Homo validated
EXOSC5	6	ROSMAP & Homo validated
FAM172A	6	ROSMAP & Homo validated
FAM53A	6	ROSMAP & Homo validated
FCGR1A	6	ROSMAP & Homo validated
FGL2	6	ROSMAP & Homo validated
FOXA3	6	ROSMAP & Homo validated
FRY	6	ROSMAP & Homo validated
GAS1	6	ROSMAP & Homo validated
GFRA4	6	ROSMAP & Homo validated
GGT1	6	ROSMAP & Homo validated
GIT2	6	ROSMAP & Homo validated
GMDS	6	ROSMAP & Homo validated
GNAQ	6	ROSMAP & Homo validated
GNG5	6	ROSMAP & Homo validated
GOPC	6	ROSMAP & Homo validated
GSG1L2	6	ROSMAP & Homo validated
HBB	6	ROSMAP & Homo validated
HBE1	6	ROSMAP & Homo validated
HBG1	6	ROSMAP & Homo validated

HBG2	6	ROSMAP & Homo validated
HMGN3	6	ROSMAP & Homo validated
HYAL2	6	ROSMAP & Homo validated
IGLL5	6	ROSMAP & Homo validated
IGSF6	6	ROSMAP & Homo validated
IL17RA	6	ROSMAP & Homo validated
IL18RAP	6	ROSMAP & Homo validated
INSYN2B	6	ROSMAP & Homo validated
ITGA11	6	ROSMAP & Homo validated
KATNAL1	6	ROSMAP & Homo validated
KCTD21	6	ROSMAP & Homo validated
KRT14	6	ROSMAP & Homo validated
LCP1	6	ROSMAP & Homo validated
LDHD	6	ROSMAP & Homo validated
LIN7A	6	ROSMAP & Homo validated
LPCAT2	6	ROSMAP & Homo validated
LTF	6	ROSMAP & Homo validated
MARVELD1	6	ROSMAP & Homo validated
MBD3L3	6	ROSMAP & Homo validated
MBD3L4	6	ROSMAP & Homo validated
MBD3L5	6	ROSMAP & Homo validated
MCCC2	6	ROSMAP & Homo validated
MPST	6	ROSMAP & Homo validated
MROH1	6	ROSMAP & Homo validated
MRPS34	6	ROSMAP & Homo validated
MS4A6A	6	ROSMAP & Homo validated
MT1F	6	ROSMAP & Homo validated
MYO1H	6	ROSMAP & Homo validated
NARF	6	ROSMAP & Homo validated
NDN	6	ROSMAP & Homo validated
NFE2	6	ROSMAP & Homo validated
NKG7	6	ROSMAP & Homo validated
NUP58	6	ROSMAP & Homo validated
OMP	6	ROSMAP & Homo validated
OR6N2	6	ROSMAP & Homo validated
PARP6	6	ROSMAP & Homo validated
PDK2	6	ROSMAP & Homo validated
PLEKHG3	6	ROSMAP & Homo validated
PLXNC1	6	ROSMAP & Homo validated
PORCN	6	ROSMAP & Homo validated
PPFIBP2	6	ROSMAP & Homo validated
PPT1	6	ROSMAP & Homo validated
PRR20A	6	ROSMAP & Homo validated
PRR20B	6	ROSMAP & Homo validated
PRR20C	6	ROSMAP & Homo validated
PRR20D	6	ROSMAP & Homo validated
PRR20E	6	ROSMAP & Homo validated
PSMB7	6	ROSMAP & Homo validated
RAB2B	6	ROSMAP & Homo validated
RAB5IF	6	ROSMAP & Homo validated
RAB8B	6	ROSMAP & Homo validated
RASSF2	6	ROSMAP & Homo validated
RASSF4	6	ROSMAP & Homo validated
RBM18	6	ROSMAP & Homo validated
RECQL	6	ROSMAP & Homo validated

RESF1	6	ROSMAP & Homo validated
RIOK3	6	ROSMAP & Homo validated
RPH3A	6	ROSMAP & Homo validated
RPS6KA5	6	ROSMAP & Homo validated
RTEL1	6	ROSMAP & Homo validated
SCARF1	6	ROSMAP & Homo validated
SCNN1A	6	ROSMAP & Homo validated
SCRG1	6	ROSMAP & Homo validated
SEC22C	6	ROSMAP & Homo validated
SELENOM	6	ROSMAP & Homo validated
SERPINA1	6	ROSMAP & Homo validated
SLC29A3	6	ROSMAP & Homo validated
SLC35C1	6	ROSMAP & Homo validated
SNX15	6	ROSMAP & Homo validated
SNX5	6	ROSMAP & Homo validated
SOCS1	6	ROSMAP & Homo validated
SPATA2L	6	ROSMAP & Homo validated
SPATA3	6	ROSMAP & Homo validated
SPP2	6	ROSMAP & Homo validated
SRPK2	6	ROSMAP & Homo validated
STING1	6	ROSMAP & Homo validated
STXBP3	6	ROSMAP & Homo validated
SULT1A1	6	ROSMAP & Homo validated
TAF7	6	ROSMAP & Homo validated
TAS2R19	6	ROSMAP & Homo validated
TAS2R60	6	ROSMAP & Homo validated
TBX21	6	ROSMAP & Homo validated
TCAF2	6	ROSMAP & Homo validated
TENT5C	6	ROSMAP & Homo validated
THAP5	6	ROSMAP & Homo validated
THEM5	6	ROSMAP & Homo validated
TLR2	6	ROSMAP & Homo validated
TMEM121B	6	ROSMAP & Homo validated
TMEM154	6	ROSMAP & Homo validated
TMEM33	6	ROSMAP & Homo validated
TMEM80	6	ROSMAP & Homo validated
TMTC2	6	ROSMAP & Homo validated
TNFSF9	6	ROSMAP & Homo validated
TOR2A	6	ROSMAP & Homo validated
TRPS1	6	ROSMAP & Homo validated
TTC33	6	ROSMAP & Homo validated
TXN2	6	ROSMAP & Homo validated
VWA7	6	ROSMAP & Homo validated
WASHC4	6	ROSMAP & Homo validated
WDFY3	6	ROSMAP & Homo validated
WDR18	6	ROSMAP & Homo validated
WDR88	6	ROSMAP & Homo validated
XK	6	ROSMAP & Homo validated
ZBTB44	6	ROSMAP & Homo validated
ZER1	6	ROSMAP & Homo validated
ZNF446	6	ROSMAP & Homo validated
ABHD17B	7	ROSMAP & Homo validated
AMY1A	7	ROSMAP & Homo validated
ANAPC16	7	ROSMAP & Homo validated
ARMH3	7	ROSMAP & Homo validated

ASB6	7	ROSMAP & Homo validated
ATP13A3	7	ROSMAP & Homo validated
ATRAID	7	ROSMAP & Homo validated
BMP10	7	ROSMAP & Homo validated
C2orf74	7	ROSMAP & Homo validated
C9orf43	7	ROSMAP & Homo validated
CAMLG	7	ROSMAP & Homo validated
CAPZB	7	ROSMAP & Homo validated
CCDC154	7	ROSMAP & Homo validated
CCDC43	7	ROSMAP & Homo validated
CCDC73	7	ROSMAP & Homo validated
CCT2	7	ROSMAP & Homo validated
CEP85	7	ROSMAP & Homo validated
CFDP1	7	ROSMAP & Homo validated
CSNK2B	7	ROSMAP & Homo validated
CUEDC1	7	ROSMAP & Homo validated
CYP4B1	7	ROSMAP & Homo validated
CYP4F8	7	ROSMAP & Homo validated
DDX1	7	ROSMAP & Homo validated
DDX58	7	ROSMAP & Homo validated
DDX59	7	ROSMAP & Homo validated
DHFR	7	ROSMAP & Homo validated
DISP1	7	ROSMAP & Homo validated
DNAH8	7	ROSMAP & Homo validated
DTWD2	7	ROSMAP & Homo validated
EDEM1	7	ROSMAP & Homo validated
EHD4	7	ROSMAP & Homo validated
EPB41L3	7	ROSMAP & Homo validated
EPSTI1	7	ROSMAP & Homo validated
ETV3	7	ROSMAP & Homo validated
FAM214A	7	ROSMAP & Homo validated
FTHL17	7	ROSMAP & Homo validated
GABPB1	7	ROSMAP & Homo validated
GALNT12	7	ROSMAP & Homo validated
GMFB	7	ROSMAP & Homo validated
GNL1	7	ROSMAP & Homo validated
GPD1	7	ROSMAP & Homo validated
GPR162	7	ROSMAP & Homo validated
GPR55	7	ROSMAP & Homo validated
GRHPR	7	ROSMAP & Homo validated
GRTP1	7	ROSMAP & Homo validated
GTPBP8	7	ROSMAP & Homo validated
HAUS8	7	ROSMAP & Homo validated
HMHB1	7	ROSMAP & Homo validated
HPS4	7	ROSMAP & Homo validated
ICAM2	7	ROSMAP & Homo validated
IFI27L1	7	ROSMAP & Homo validated
IGSF8	7	ROSMAP & Homo validated
INO80	7	ROSMAP & Homo validated
IZUMO1R	7	ROSMAP & Homo validated
KIAA0408	7	ROSMAP & Homo validated
KIAA0753	7	ROSMAP & Homo validated
KLHL7	7	ROSMAP & Homo validated
KPNA5	7	ROSMAP & Homo validated
LANCL3	7	ROSMAP & Homo validated

LIMK2	7	ROSMAP & Homo validated
LPCAT4	7	ROSMAP & Homo validated
LRP1	7	ROSMAP & Homo validated
LRRC66	7	ROSMAP & Homo validated
MAGEC1	7	ROSMAP & Homo validated
MCF2L2	7	ROSMAP & Homo validated
MEST	7	ROSMAP & Homo validated
METTL8	7	ROSMAP & Homo validated
MIER1	7	ROSMAP & Homo validated
MMP28	7	ROSMAP & Homo validated
MPHOSPH9	7	ROSMAP & Homo validated
MRM3	7	ROSMAP & Homo validated
MRPL20	7	ROSMAP & Homo validated
MSANTD3	7	ROSMAP & Homo validated
NAA15	7	ROSMAP & Homo validated
NAP1L1	7	ROSMAP & Homo validated
NDC80	7	ROSMAP & Homo validated
NDFIP2	7	ROSMAP & Homo validated
NHLRC1	7	ROSMAP & Homo validated
NIPBL	7	ROSMAP & Homo validated
NOSIP	7	ROSMAP & Homo validated
NPC1	7	ROSMAP & Homo validated
NPIPB8	7	ROSMAP & Homo validated
NR1I3	7	ROSMAP & Homo validated
NT5C3B	7	ROSMAP & Homo validated
NUDT10	7	ROSMAP & Homo validated
PAXBP1	7	ROSMAP & Homo validated
PCGF6	7	ROSMAP & Homo validated
PCSK5	7	ROSMAP & Homo validated
PDE8B	7	ROSMAP & Homo validated
PDILT	7	ROSMAP & Homo validated
PEX1	7	ROSMAP & Homo validated
PFKFB2	7	ROSMAP & Homo validated
PIWIL4	7	ROSMAP & Homo validated
PLAA	7	ROSMAP & Homo validated
PLG	7	ROSMAP & Homo validated
RAB3IL1	7	ROSMAP & Homo validated
RACGAP1	7	ROSMAP & Homo validated
REG1B	7	ROSMAP & Homo validated
RILPL1	7	ROSMAP & Homo validated
RPL26	7	ROSMAP & Homo validated
RPS10-NUDT3	7	ROSMAP & Homo validated
RUNX2	7	ROSMAP & Homo validated
SC5D	7	ROSMAP & Homo validated
SEPHS1	7	ROSMAP & Homo validated
SHISAL2A	7	ROSMAP & Homo validated
SIRT5	7	ROSMAP & Homo validated
SLC12A6	7	ROSMAP & Homo validated
SLC25A17	7	ROSMAP & Homo validated
SMC1B	7	ROSMAP & Homo validated
SMC6	7	ROSMAP & Homo validated
SNX12	7	ROSMAP & Homo validated
SOCS3	7	ROSMAP & Homo validated
SSH1	7	ROSMAP & Homo validated
STXBP5	7	ROSMAP & Homo validated

SYS1	7	ROSMAP & Homo validated
TMEM129	7	ROSMAP & Homo validated
TMEM140	7	ROSMAP & Homo validated
TMEM169	7	ROSMAP & Homo validated
TMEM185B	7	ROSMAP & Homo validated
TNNT1	7	ROSMAP & Homo validated
TNP1	7	ROSMAP & Homo validated
TRMT61B	7	ROSMAP & Homo validated
TTC27	7	ROSMAP & Homo validated
TXNDC8	7	ROSMAP & Homo validated
UBAP2	7	ROSMAP & Homo validated
USP18	7	ROSMAP & Homo validated
XPNPEP1	7	ROSMAP & Homo validated
YAF2	7	ROSMAP & Homo validated
ZBTB47	7	ROSMAP & Homo validated
ZC3H8	7	ROSMAP & Homo validated
ZFP28	7	ROSMAP & Homo validated
ZFP69B	7	ROSMAP & Homo validated
ZMAT1	7	ROSMAP & Homo validated
ZNF285	7	ROSMAP & Homo validated
ZNF577	7	ROSMAP & Homo validated
ZNF582	7	ROSMAP & Homo validated
ZNF624	7	ROSMAP & Homo validated
ZNF829	7	ROSMAP & Homo validated
ZPBP2	7	ROSMAP & Homo validated
ZRSR2	7	ROSMAP & Homo validated
ZUP1	7	ROSMAP & Homo validated
ABCA1	8	ROSMAP & Homo validated
ABCC4	8	ROSMAP & Homo validated
ABHD16B	8	ROSMAP & Homo validated
ABI3BP	8	ROSMAP & Homo validated
ACVR2B	8	ROSMAP & Homo validated
ADGRG6	8	ROSMAP & Homo validated
ADIRF	8	ROSMAP & Homo validated
AKIRIN1	8	ROSMAP & Homo validated
ALB	8	ROSMAP & Homo validated
ALPI	8	ROSMAP & Homo validated
ANKDD1B	8	ROSMAP & Homo validated
ANKRD29	8	ROSMAP & Homo validated
ANO2	8	ROSMAP & Homo validated
APLN	8	ROSMAP & Homo validated
APOC1	8	ROSMAP & Homo validated
APOC2	8	ROSMAP & Homo validated
AQP8	8	ROSMAP & Homo validated
ARHGAP20	8	ROSMAP & Homo validated
ARHGAP21	8	ROSMAP & Homo validated
ARHGAP31	8	ROSMAP & Homo validated
ARHGEF9	8	ROSMAP & Homo validated
ARID5A	8	ROSMAP & Homo validated
ASAH2	8	ROSMAP & Homo validated
ASPHD1	8	ROSMAP & Homo validated
AXIN2	8	ROSMAP & Homo validated
BBS7	8	ROSMAP & Homo validated
BBS9	8	ROSMAP & Homo validated
BCAM	8	ROSMAP & Homo validated

BCKDHB	8	ROSMAP & Homo validated
BDP1	8	ROSMAP & Homo validated
BRIP1	8	ROSMAP & Homo validated
BRMS1L	8	ROSMAP & Homo validated
C1QTNF5	8	ROSMAP & Homo validated
C1QTNF9	8	ROSMAP & Homo validated
C2orf16	8	ROSMAP & Homo validated
C4orf54	8	ROSMAP & Homo validated
CA3	8	ROSMAP & Homo validated
CAAP1	8	ROSMAP & Homo validated
CAMSAP2	8	ROSMAP & Homo validated
CASP8AP2	8	ROSMAP & Homo validated
CASQ1	8	ROSMAP & Homo validated
CATSPERB	8	ROSMAP & Homo validated
CCDC68	8	ROSMAP & Homo validated
CCL2	8	ROSMAP & Homo validated
CD109	8	ROSMAP & Homo validated
CD163L1	8	ROSMAP & Homo validated
CDCA2	8	ROSMAP & Homo validated
CDK6	8	ROSMAP & Homo validated
CDY1	8	ROSMAP & Homo validated
CEP70	8	ROSMAP & Homo validated
CEP97	8	ROSMAP & Homo validated
CES5A	8	ROSMAP & Homo validated
CFAP251	8	ROSMAP & Homo validated
CFAP44	8	ROSMAP & Homo validated
CFAP73	8	ROSMAP & Homo validated
CHRNA9	8	ROSMAP & Homo validated
CKAP2L	8	ROSMAP & Homo validated
CLCA4	8	ROSMAP & Homo validated
CLIC4	8	ROSMAP & Homo validated
CNGB3	8	ROSMAP & Homo validated
COL7A1	8	ROSMAP & Homo validated
CREB3L1	8	ROSMAP & Homo validated
CREM	8	ROSMAP & Homo validated
CRTC1	8	ROSMAP & Homo validated
CSMD1	8	ROSMAP & Homo validated
CTNNA3	8	ROSMAP & Homo validated
CTSD	8	ROSMAP & Homo validated
CXCL9	8	ROSMAP & Homo validated
CYP1A1	8	ROSMAP & Homo validated
CYP2A6	8	ROSMAP & Homo validated
CYP2C9	8	ROSMAP & Homo validated
DERL1	8	ROSMAP & Homo validated
DHX36	8	ROSMAP & Homo validated
DNAAF4	8	ROSMAP & Homo validated
DNAI4	8	ROSMAP & Homo validated
DNAJB1	8	ROSMAP & Homo validated
DUSP21	8	ROSMAP & Homo validated
EGR3	8	ROSMAP & Homo validated
EIF2AK4	8	ROSMAP & Homo validated
ELOVL6	8	ROSMAP & Homo validated
EMCN	8	ROSMAP & Homo validated
EML6	8	ROSMAP & Homo validated
ENO2	8	ROSMAP & Homo validated

EPB41L4A	8	ROSMAP & Homo validated
FABP2	8	ROSMAP & Homo validated
FAM193B	8	ROSMAP & Homo validated
FAM219A	8	ROSMAP & Homo validated
FAM71E2	8	ROSMAP & Homo validated
FILIP1	8	ROSMAP & Homo validated
FMOD	8	ROSMAP & Homo validated
FN1	8	ROSMAP & Homo validated
FSIP1	8	ROSMAP & Homo validated
FUT3	8	ROSMAP & Homo validated
FXYD1	8	ROSMAP & Homo validated
G2E3	8	ROSMAP & Homo validated
G6PC2	8	ROSMAP & Homo validated
GEMIN8	8	ROSMAP & Homo validated
GLOD5	8	ROSMAP & Homo validated
GLRA3	8	ROSMAP & Homo validated
GOLGA6L9	8	ROSMAP & Homo validated
GSDME	8	ROSMAP & Homo validated
H3C3	8	ROSMAP & Homo validated
H4C4	8	ROSMAP & Homo validated
HABP4	8	ROSMAP & Homo validated
HECW2	8	ROSMAP & Homo validated
HES3	8	ROSMAP & Homo validated
HEY1	8	ROSMAP & Homo validated
HMGN5	8	ROSMAP & Homo validated
HNRNPCL1	8	ROSMAP & Homo validated
HNRNPCL3	8	ROSMAP & Homo validated
HS3ST2	8	ROSMAP & Homo validated
HSD3B1	8	ROSMAP & Homo validated
IFI27	8	ROSMAP & Homo validated
IFTAP	8	ROSMAP & Homo validated
IL12B	8	ROSMAP & Homo validated
IPO11	8	ROSMAP & Homo validated
IQGAP3	8	ROSMAP & Homo validated
ITGAV	8	ROSMAP & Homo validated
ITGBL1	8	ROSMAP & Homo validated
JPT2	8	ROSMAP & Homo validated
KCNJ11	8	ROSMAP & Homo validated
KCNJ12	8	ROSMAP & Homo validated
KCNK16	8	ROSMAP & Homo validated
KCNK9	8	ROSMAP & Homo validated
KCTD1	8	ROSMAP & Homo validated
KDM4D	8	ROSMAP & Homo validated
KDR	8	ROSMAP & Homo validated
KIF11	8	ROSMAP & Homo validated
KLHL38	8	ROSMAP & Homo validated
KLHL8	8	ROSMAP & Homo validated
KRT37	8	ROSMAP & Homo validated
KRTAP4-11	8	ROSMAP & Homo validated
LENEP	8	ROSMAP & Homo validated
LGI2	8	ROSMAP & Homo validated
LIN28A	8	ROSMAP & Homo validated
LPA	8	ROSMAP & Homo validated
LRP12	8	ROSMAP & Homo validated
LRRC8D	8	ROSMAP & Homo validated

LRRIQ3	8	ROSMAP & Homo validated
LRRIQ4	8	ROSMAP & Homo validated
LY6E	8	ROSMAP & Homo validated
LY6G5B	8	ROSMAP & Homo validated
MAGEA1	8	ROSMAP & Homo validated
MAN1A2	8	ROSMAP & Homo validated
MAP4K3	8	ROSMAP & Homo validated
MEOX1	8	ROSMAP & Homo validated
MEP1A	8	ROSMAP & Homo validated
MEPCE	8	ROSMAP & Homo validated
MEPE	8	ROSMAP & Homo validated
MGP	8	ROSMAP & Homo validated
MORC1	8	ROSMAP & Homo validated
MRAP	8	ROSMAP & Homo validated
MT1G	8	ROSMAP & Homo validated
MT1M	8	ROSMAP & Homo validated
MTA3	8	ROSMAP & Homo validated
MTUS1	8	ROSMAP & Homo validated
MUC17	8	ROSMAP & Homo validated
MYB	8	ROSMAP & Homo validated
NAA16	8	ROSMAP & Homo validated
NIPSNAP3A	8	ROSMAP & Homo validated
NKD1	8	ROSMAP & Homo validated
NOX4	8	ROSMAP & Homo validated
NPHP1	8	ROSMAP & Homo validated
NPIPA8	8	ROSMAP & Homo validated
NR3C2	8	ROSMAP & Homo validated
NUF2	8	ROSMAP & Homo validated
ONECUT2	8	ROSMAP & Homo validated
OR11H1	8	ROSMAP & Homo validated
OR1N2	8	ROSMAP & Homo validated
OR2A14	8	ROSMAP & Homo validated
OR2T2	8	ROSMAP & Homo validated
OR2T35	8	ROSMAP & Homo validated
OR4C15	8	ROSMAP & Homo validated
OR51E1	8	ROSMAP & Homo validated
OR6C1	8	ROSMAP & Homo validated
ORMDL3	8	ROSMAP & Homo validated
OSMR	8	ROSMAP & Homo validated
OTOG	8	ROSMAP & Homo validated
OTOL1	8	ROSMAP & Homo validated
OXGR1	8	ROSMAP & Homo validated
OXTR	8	ROSMAP & Homo validated
PAQR3	8	ROSMAP & Homo validated
PARP2	8	ROSMAP & Homo validated
PAX9	8	ROSMAP & Homo validated
PDCD10	8	ROSMAP & Homo validated
PDCL2	8	ROSMAP & Homo validated
PERP	8	ROSMAP & Homo validated
PGM5	8	ROSMAP & Homo validated
PHTF2	8	ROSMAP & Homo validated
PIK3CB	8	ROSMAP & Homo validated
PLA1A	8	ROSMAP & Homo validated
PLAAT3	8	ROSMAP & Homo validated
PLEKHA8	8	ROSMAP & Homo validated

PLEKHG2	8	ROSMAP & Homo validated
PLK3	8	ROSMAP & Homo validated
PNKD	8	ROSMAP & Homo validated
POFUT2	8	ROSMAP & Homo validated
POGLUT2	8	ROSMAP & Homo validated
POLR2K	8	ROSMAP & Homo validated
PRB4	8	ROSMAP & Homo validated
PRG4	8	ROSMAP & Homo validated
PRKRIP1	8	ROSMAP & Homo validated
PROP1	8	ROSMAP & Homo validated
PRR9	8	ROSMAP & Homo validated
PSG4	8	ROSMAP & Homo validated
PTPN12	8	ROSMAP & Homo validated
PTPRK	8	ROSMAP & Homo validated
PXMP4	8	ROSMAP & Homo validated
RAB23	8	ROSMAP & Homo validated
RAB40A	8	ROSMAP & Homo validated
RAD21L1	8	ROSMAP & Homo validated
RAI14	8	ROSMAP & Homo validated
RAPGEF5	8	ROSMAP & Homo validated
RAPH1	8	ROSMAP & Homo validated
RARS1	8	ROSMAP & Homo validated
RASSF10	8	ROSMAP & Homo validated
RCAN3	8	ROSMAP & Homo validated
REXO4	8	ROSMAP & Homo validated
RFPL4B	8	ROSMAP & Homo validated
RFX7	8	ROSMAP & Homo validated
RGL1	8	ROSMAP & Homo validated
RGPD3	8	ROSMAP & Homo validated
RLN2	8	ROSMAP & Homo validated
RNF168	8	ROSMAP & Homo validated
ROBO4	8	ROSMAP & Homo validated
RSPH10B	8	ROSMAP & Homo validated
RSPH4A	8	ROSMAP & Homo validated
RXFP1	8	ROSMAP & Homo validated
SASH1	8	ROSMAP & Homo validated
SATL1	8	ROSMAP & Homo validated
SBSN	8	ROSMAP & Homo validated
SCIN	8	ROSMAP & Homo validated
SCMH1	8	ROSMAP & Homo validated
SCML2	8	ROSMAP & Homo validated
SCN7A	8	ROSMAP & Homo validated
SELENOV	8	ROSMAP & Homo validated
SEMA6B	8	ROSMAP & Homo validated
SERP2	8	ROSMAP & Homo validated
SETD2	8	ROSMAP & Homo validated
SETD7	8	ROSMAP & Homo validated
SETD9	8	ROSMAP & Homo validated
SFT2D3	8	ROSMAP & Homo validated
SGO2	8	ROSMAP & Homo validated
SIRT7	8	ROSMAP & Homo validated
SLA2	8	ROSMAP & Homo validated
SLC13A2	8	ROSMAP & Homo validated
SLC19A2	8	ROSMAP & Homo validated
SLC22A2	8	ROSMAP & Homo validated

SLC45A2	8	ROSMAP & Homo validated
SLC5A12	8	ROSMAP & Homo validated
SLCO2B1	8	ROSMAP & Homo validated
SMIM3	8	ROSMAP & Homo validated
SMYD2	8	ROSMAP & Homo validated
SNPH	8	ROSMAP & Homo validated
SPARCL1	8	ROSMAP & Homo validated
SPATA22	8	ROSMAP & Homo validated
SPHK1	8	ROSMAP & Homo validated
SPINK13	8	ROSMAP & Homo validated
SPNS1	8	ROSMAP & Homo validated
SPRR2D	8	ROSMAP & Homo validated
SRL	8	ROSMAP & Homo validated
SRPX	8	ROSMAP & Homo validated
SRSF6	8	ROSMAP & Homo validated
SSX2IP	8	ROSMAP & Homo validated
ST6GAL1	8	ROSMAP & Homo validated
STARD13	8	ROSMAP & Homo validated
SV2B	8	ROSMAP & Homo validated
SYNPO2	8	ROSMAP & Homo validated
TBCEL	8	ROSMAP & Homo validated
TCEAL6	8	ROSMAP & Homo validated
TCF7L1	8	ROSMAP & Homo validated
TEX2	8	ROSMAP & Homo validated
THBS1	8	ROSMAP & Homo validated
TIAM1	8	ROSMAP & Homo validated
TINAG	8	ROSMAP & Homo validated
TM4SF5	8	ROSMAP & Homo validated
TMEM38B	8	ROSMAP & Homo validated
TMEM64	8	ROSMAP & Homo validated
TMEM97	8	ROSMAP & Homo validated
TMPPE	8	ROSMAP & Homo validated
TMPRSS15	8	ROSMAP & Homo validated
TNMD	8	ROSMAP & Homo validated
TPO	8	ROSMAP & Homo validated
TPP1	8	ROSMAP & Homo validated
TRIML1	8	ROSMAP & Homo validated
TTYH3	8	ROSMAP & Homo validated
TUBGCP4	8	ROSMAP & Homo validated
TULP2	8	ROSMAP & Homo validated
TXNRD3	8	ROSMAP & Homo validated
UBAC2	8	ROSMAP & Homo validated
UBE2K	8	ROSMAP & Homo validated
UGDH	8	ROSMAP & Homo validated
ULK2	8	ROSMAP & Homo validated
UNC13D	8	ROSMAP & Homo validated
UPK1B	8	ROSMAP & Homo validated
UROC1	8	ROSMAP & Homo validated
USP31	8	ROSMAP & Homo validated
UXS1	8	ROSMAP & Homo validated
VCY	8	ROSMAP & Homo validated
VGLL3	8	ROSMAP & Homo validated
VIP	8	ROSMAP & Homo validated
VTN	8	ROSMAP & Homo validated
WNK4	8	ROSMAP & Homo validated

WRNIP1	8	ROSMAP & Homo validated
YEATS2	8	ROSMAP & Homo validated
ZCCHC24	8	ROSMAP & Homo validated
ZCWPW2	8	ROSMAP & Homo validated
ZFPM2	8	ROSMAP & Homo validated
ZMYND11	8	ROSMAP & Homo validated
ZNF501	8	ROSMAP & Homo validated
ZNF608	8	ROSMAP & Homo validated
ZWILCH	8	ROSMAP & Homo validated
A2M	9	ROSMAP & Homo validated
ACSL3	9	ROSMAP & Homo validated
ADM	9	ROSMAP & Homo validated
ADRB2	9	ROSMAP & Homo validated
AGO4	9	ROSMAP & Homo validated
ALDH1A1	9	ROSMAP & Homo validated
ANP32B	9	ROSMAP & Homo validated
APOOL	9	ROSMAP & Homo validated
ARHGAP12	9	ROSMAP & Homo validated
ARMC1	9	ROSMAP & Homo validated
ASB2	9	ROSMAP & Homo validated
ASCC2	9	ROSMAP & Homo validated
ATG12	9	ROSMAP & Homo validated
ATG7	9	ROSMAP & Homo validated
BAZ1B	9	ROSMAP & Homo validated
BCAS4	9	ROSMAP & Homo validated
BCL2L13	9	ROSMAP & Homo validated
BLVRB	9	ROSMAP & Homo validated
BSG	9	ROSMAP & Homo validated
C11orf71	9	ROSMAP & Homo validated
C13orf42	9	ROSMAP & Homo validated
C16orf91	9	ROSMAP & Homo validated
C17orf99	9	ROSMAP & Homo validated
C1orf56	9	ROSMAP & Homo validated
C3orf49	9	ROSMAP & Homo validated
C9orf78	9	ROSMAP & Homo validated
CAMK2N1	9	ROSMAP & Homo validated
CARHSP1	9	ROSMAP & Homo validated
CASTOR1	9	ROSMAP & Homo validated
CCNC	9	ROSMAP & Homo validated
CCR8	9	ROSMAP & Homo validated
CD244	9	ROSMAP & Homo validated
CD300LD	9	ROSMAP & Homo validated
CHST13	9	ROSMAP & Homo validated
CISD2	9	ROSMAP & Homo validated
CLEC12A	9	ROSMAP & Homo validated
CLIC2	9	ROSMAP & Homo validated
COPB2	9	ROSMAP & Homo validated
COPS2	9	ROSMAP & Homo validated
CSTF2	9	ROSMAP & Homo validated
CTRL	9	ROSMAP & Homo validated
CYB5B	9	ROSMAP & Homo validated
DAXX	9	ROSMAP & Homo validated
DDB1	9	ROSMAP & Homo validated
DDX24	9	ROSMAP & Homo validated
DHRS1	9	ROSMAP & Homo validated

DIAPH2	9	ROSMAP & Homo validated
DLGAP4	9	ROSMAP & Homo validated
DNAJA1	9	ROSMAP & Homo validated
DOP1A	9	ROSMAP & Homo validated
DPM2	9	ROSMAP & Homo validated
DTX3	9	ROSMAP & Homo validated
EFL1	9	ROSMAP & Homo validated
ENTPD1	9	ROSMAP & Homo validated
EPHB2	9	ROSMAP & Homo validated
ETFB	9	ROSMAP & Homo validated
EXOC8	9	ROSMAP & Homo validated
EXOSC1	9	ROSMAP & Homo validated
FAM53C	9	ROSMAP & Homo validated
FAM71F2	9	ROSMAP & Homo validated
FGFBP2	9	ROSMAP & Homo validated
FMN1	9	ROSMAP & Homo validated
GABARAP	9	ROSMAP & Homo validated
GPRC5D	9	ROSMAP & Homo validated
GSPT1	9	ROSMAP & Homo validated
GTSF1	9	ROSMAP & Homo validated
GUCY2D	9	ROSMAP & Homo validated
H3-2	9	ROSMAP & Homo validated
HDGF	9	ROSMAP & Homo validated
HHEX	9	ROSMAP & Homo validated
HSP90B1	9	ROSMAP & Homo validated
HSPA4	9	ROSMAP & Homo validated
IFT52	9	ROSMAP & Homo validated
IL21	9	ROSMAP & Homo validated
INSR	9	ROSMAP & Homo validated
IQCB1	9	ROSMAP & Homo validated
ISCU	9	ROSMAP & Homo validated
ITCH	9	ROSMAP & Homo validated
ITGB1BP2	9	ROSMAP & Homo validated
JAKMIP1	9	ROSMAP & Homo validated
JAZF1	9	ROSMAP & Homo validated
JOSD2	9	ROSMAP & Homo validated
KCNAB3	9	ROSMAP & Homo validated
KCNN4	9	ROSMAP & Homo validated
KIAA2026	9	ROSMAP & Homo validated
KIF13A	9	ROSMAP & Homo validated
KIF1B	9	ROSMAP & Homo validated
KLF11	9	ROSMAP & Homo validated
KRT86	9	ROSMAP & Homo validated
LAMTOR2	9	ROSMAP & Homo validated
LAPTM4A	9	ROSMAP & Homo validated
LSM11	9	ROSMAP & Homo validated
LSM6	9	ROSMAP & Homo validated
MAGT1	9	ROSMAP & Homo validated
MBNL3	9	ROSMAP & Homo validated
METTL16	9	ROSMAP & Homo validated
METTL7A	9	ROSMAP & Homo validated
MGAM2	9	ROSMAP & Homo validated
MINDY4B	9	ROSMAP & Homo validated
MKLN1	9	ROSMAP & Homo validated
MKNK2	9	ROSMAP & Homo validated

MRPL14	9	ROSMAP & Homo validated
MRPL19	9	ROSMAP & Homo validated
MRPL58	9	ROSMAP & Homo validated
MTIF3	9	ROSMAP & Homo validated
MXI1	9	ROSMAP & Homo validated
NBDY	9	ROSMAP & Homo validated
NCOA2	9	ROSMAP & Homo validated
NFYC	9	ROSMAP & Homo validated
NHSL2	9	ROSMAP & Homo validated
NPEPL1	9	ROSMAP & Homo validated
NRG1	9	ROSMAP & Homo validated
NUB1	9	ROSMAP & Homo validated
PBRM1	9	ROSMAP & Homo validated
PCBP4	9	ROSMAP & Homo validated
PCNX3	9	ROSMAP & Homo validated
PDCD1	9	ROSMAP & Homo validated
PDK4	9	ROSMAP & Homo validated
PGF	9	ROSMAP & Homo validated
PLIN3	9	ROSMAP & Homo validated
PLRG1	9	ROSMAP & Homo validated
PLXDC1	9	ROSMAP & Homo validated
PNISR	9	ROSMAP & Homo validated
POLR2L	9	ROSMAP & Homo validated
POLRMT	9	ROSMAP & Homo validated
PPIA	9	ROSMAP & Homo validated
PPP1R9B	9	ROSMAP & Homo validated
PRDX2	9	ROSMAP & Homo validated
PRH2	9	ROSMAP & Homo validated
PRIMPOL	9	ROSMAP & Homo validated
PRPH2	9	ROSMAP & Homo validated
PRR14	9	ROSMAP & Homo validated
PRRG4	9	ROSMAP & Homo validated
PSKH1	9	ROSMAP & Homo validated
PSMD10	9	ROSMAP & Homo validated
PTGES	9	ROSMAP & Homo validated
PTOV1	9	ROSMAP & Homo validated
PTTG2	9	ROSMAP & Homo validated
PYROXD1	9	ROSMAP & Homo validated
PYURF	9	ROSMAP & Homo validated
RAB21	9	ROSMAP & Homo validated
RAD1	9	ROSMAP & Homo validated
RAD51B	9	ROSMAP & Homo validated
RASL10A	9	ROSMAP & Homo validated
RASSF3	9	ROSMAP & Homo validated
RC3H2	9	ROSMAP & Homo validated
RCBTB2	9	ROSMAP & Homo validated
RNASE1	9	ROSMAP & Homo validated
RNF20	9	ROSMAP & Homo validated
RPA4	9	ROSMAP & Homo validated
RPIA	9	ROSMAP & Homo validated
RPS23	9	ROSMAP & Homo validated
RPS6KA1	9	ROSMAP & Homo validated
RWDD1	9	ROSMAP & Homo validated
SAMD8	9	ROSMAP & Homo validated
SDK2	9	ROSMAP & Homo validated

SESN3	9	ROSMAP & Homo validated
SETX	9	ROSMAP & Homo validated
SIGMAR1	9	ROSMAP & Homo validated
SLA	9	ROSMAP & Homo validated
SLC35A3	9	ROSMAP & Homo validated
SLC4A1	9	ROSMAP & Homo validated
SLC52A1	9	ROSMAP & Homo validated
SMU1	9	ROSMAP & Homo validated
SOX6	9	ROSMAP & Homo validated
SPINK8	9	ROSMAP & Homo validated
SPON2	9	ROSMAP & Homo validated
SRRD	9	ROSMAP & Homo validated
SRSF3	9	ROSMAP & Homo validated
STAG2	9	ROSMAP & Homo validated
STK17B	9	ROSMAP & Homo validated
SUCLG1	9	ROSMAP & Homo validated
SURF1	9	ROSMAP & Homo validated
TAF1D	9	ROSMAP & Homo validated
TARBP2	9	ROSMAP & Homo validated
TFR2	9	ROSMAP & Homo validated
THOC5	9	ROSMAP & Homo validated
TLR6	9	ROSMAP & Homo validated
TSKS	9	ROSMAP & Homo validated
UBALD1	9	ROSMAP & Homo validated
UBQLN1	9	ROSMAP & Homo validated
UFC1	9	ROSMAP & Homo validated
VENTX	9	ROSMAP & Homo validated
YWHAG	9	ROSMAP & Homo validated
ZNF420	9	ROSMAP & Homo validated
ZNF460	9	ROSMAP & Homo validated
ZNF487	9	ROSMAP & Homo validated
ZNF888	9	ROSMAP & Homo validated
ZSWIM6	9	ROSMAP & Homo validated
