# Supplementary material for: RBAD: The first database dedicated alterations of blood RNA in individuals with Alzheimer’s disease and their clinical relevance
Source: Neural Regen Res. 2025 Mar 25;21(6):2553–62. doi: 10.4103/NRR.NRR-D-24-01165 (PMC13211806; doi:10.4103/NRR.NRR-D-24-01165)
Supplement: Supplementary file 11 [file NRR-21-2553_Suppl8.pdf]

Additional Table 11. Pathway enrichment of each cluster (GO and KEGG) found in Homo dataset.	
Method	R package clusterProfiler
Description	Pathway enrichment of each cluster using over representation analysis. Corresponding to Figure 3E.

Cluster	ONTOLOGY	ID	Description	GeneRatio	BgRatio	P value	FDR	Q value	Symbol	Count
cluster1	BP	GO:0050907	detection of chemical stimulus involved in sensory perception	78/1407	477/18903	3.04E-11	0.000000238	0.000000232	CST2/GPR148/OR10A4/OR10D3/OR10H1/OR10H5/OR10J5/OR10T2/OR10V1/OR11A1/OR11G2/OR11H6/OR13C2/OR13C3/OR13C4/OR13C5/OR13G1/OR13J1/OR1D2/OR1D5/OR1I1/OR1K1/OR1L8/OR1N1/OR2A25/OR2B3/OR2C3/OR2D2/OR2G6/OR2M3/OR2T3/OR2T4/OR4A47/OR4C12/OR4D10/OR4D9/OR4F16/OR4F4/OR4K17/OR4X2/OR51B2/OR51B4/OR51D1/OR51F1/OR51I2/OR51M1/OR52N1/OR56A4/OR56B4/OR5A1/OR5AP2/OR5AS1/OR5AU1/OR5B21/OR5C1/OR5H6/OR5K3/OR5M8/OR6A2/OR6C4/OR6C65/OR6C74/OR6K3/OR6M1/OR6V1/OR7A17/OR7D4/OR8B12/OR8B8/OR8G1/OR8H2/OR8S1/RTP2/RTP5/TAS2R30/TAS2R39/TAS2R46/TAS2R9	78
cluster1	BP	GO:0050911	detection of chemical stimulus involved in sensory perception of smell	71/1407	431/18903	1.69E-10	0.000000662	0.000000646	GPR148/OR10A4/OR10D3/OR10H1/OR10H5/OR10J5/OR10T2/OR10V1/OR11A1/OR11G2/OR11H6/OR13C2/OR13C3/OR13C4/OR13C5/OR13G1/OR13J1/OR1D2/OR1D5/OR1I1/OR1K1/OR1L8/OR1N1/OR2A25/OR2B3/OR2C3/OR2D2/OR2G6/OR2M3/OR2T3/OR2T4/OR4A47/OR4C12/OR4D10/OR4D9/OR4F16/OR4F4/OR4K17/OR4X2/OR51B2/OR51B4/OR51D1/OR51F1/OR51I2/OR51M1/OR52N1/OR56A4/OR56B4/OR5A1/OR5AP2/OR5AS1/OR5AU1/OR5B21/OR5C1/OR5H6/OR5K3/OR5M8/OR6A2/OR6C4/OR6C65/OR6C74/OR6K3/OR6M1/OR6V1/OR7A17/OR7D4/OR8B12/OR8B8/OR8G1/OR8H2/OR8S1	71
cluster1	BP	GO:0007608	sensory perception of smell	73/1407	457/18903	3.92E-10	0.00000102	0.000000995	B2M/GPR148/OBP2B/OR10A4/OR10D3/OR10H1/OR10H5/OR10J5/OR10T2/OR10V1/OR11A1/OR11G2/OR11H6/OR13C2/OR13C3/OR13C4/OR13C5/OR13G1/OR13J1/OR1D2/OR1D5/OR1I1/OR1K1/OR1L8/OR1N1/OR2A25/OR2B3/OR2C3/OR2D2/OR2G6/OR2M3/OR2T3/OR2T4/OR4A47/OR4C12/OR4D10/OR4D9/OR4F16/OR4F4/OR4K17/OR4X2/OR51B2/OR51B4/OR51D1/OR51F1/OR51I2/OR51M1/OR52N1/OR56A4/OR56B4/OR5A1/OR5AP2/OR5AS1/OR5AU1/OR5B21/OR5C1/OR5H6/OR5K3/OR5M8/OR6A2/OR6C4/OR6C65/OR6C74/OR6K3/OR6M1/OR6V1/OR7A17/OR7D4/OR8B12/OR8B8/OR8G1/OR8H2/OR8S1	73

cluster1	BP	GO:0007156	homophilic cell adhesion via plasma membrane adhesion molecules	32/1407	170/18903	0.000000942	0.001840016	0.001794848	CDH20/CDHR2/CEACAM6/DSC2/DSG3/MYOT/NECTIN2/PCDH11X/PCDH20/PCDH7/PCDHA1/PCDHB1/PCDHB10/PCDHB11/PCDHB13/PCDHB14/PCDHB2/PCDHB3/PCDHB4/PCDHB5/PCDHB9/PCDHGA11/PCDHGA3/PCDHGA4/PCDHGA8/PCDHGA9/PCDHGB2/PCDHGB6/PCDHGB7/PCDHGC3/PCDHGC5/PLXNB2	32
cluster1	BP	GO:0098742	cell-cell adhesion via plasma-membrane adhesion molecules	44/1407	282/18903	0.00000227	0.003554541	0.003467284	ADIPOQ/CDH20/CDHR2/CEACAM6/CLDN10/CLDN11/CLDN3/DSC2/DSG3/FGFRL1/JAML/LRFN5/MAPK14/MYOT/NECTIN2/PCDH11X/PCDH20/PCDH7/PCDHA1/PCDHB1/PCDHB10/PCDHB11/PCDHB13/PCDHB14/PCDHB2/PCDHB3/PCDHB4/PCDHB5/PCDHB9/PCDHGA11/PCDHGA3/PCDHGA4/PCDHGA8/PCDHGA9/PCDHGB2/PCDHGB6/PCDHGB7/PCDHGC3/PCDHGC5/PLXNB2/SCARF2/TENM1/TGFB2/UMOD	44
cluster1	BP	GO:0007218	neuropeptide signaling pathway	23/1407	110/18903	0.00000505	0.006578754	0.006417259	CPE/GAL/GALR1/GALR3/GPR143/HCRT/MCHR2/NMUR2/NPBWR2/NPFF/NPFFR1/NPPA/NPS/NPY4R2/NTSR1/NTSR2/OPRM1/POMC/PROK2/RXFP3/SORL1/SSTR4/TENM1	23
cluster1	BP	GO:0052547	regulation of peptidase activity	60/1407	459/18903	0.0000138	0.0153911	0.01501328	ANXA8L1/APLP2/ARL6IP1/ATP2A3/BID/BOK/CARD16/CDKN2D/CLDN3/CRADD/CST2/CST7/CSTB/CTSH/FAS/FNIP1/FOXL2/FURIN/GPX1/GRN/HMGB1/JAK2/MAPT/MMP9/MYC/NAIP/NLRP6/NODAL/NR4A1/NRDC/P2RX1/PAPLN/PARK7/PCOLCE2/PLAUR/PRDX3/PRELID1/R3HDML/RAF1/RECK/RHOA/S100A9/SERPINA2/SERPINB11/SERPINB12/SERPINB5/SIAH2/SLPI/SORL1/SOX2/SOX7/SPINK14/SPINT1/SPINT3/TIMP2/UMODL1/VSIR/WFDC11/WFDC5/WFDC8	60

cluster1	BP	GO:0030509	BMP signaling pathway	28/1407	167/18903	0.0000413	0.033963157	0.033129431	BMPR1A/CER1/DAND5/EGR1/ENG/FKBP8/FOXD1/FSTL1/GDF3/HTRA3/KCP/MAPK3/MSX1/NBL1/NODAL/NOTCH1/PCSK6/PDCD4/PELO/SMAD1/SMPD3/SORL1/SOX11/TGFB1/TGFB2/TMPRSS6/UBE2D3/ZNF423	28
cluster1	BP	GO:0052548	regulation of endopeptidase activity	55/1407	428/18903	0.0000499	0.033963157	0.033129431	ANXA8L1/APLP2/ARL6IP1/ATP2A3/BID/BOK/CARD16/CDKN2D/CRADD/CST2/CST7/CSTB/CTSH/FAS/FNIP1/FOXL2/FURIN/GPX1/HMGB1/JAK2/MAPT/MMP9/MYC/NAIP/NLRP6/NODAL/NR4A1/NRDC/P2RX1/PAPLN/PARK7/PLAUR/PRDX3/PRELID1/RAF1/RECK/RHOA/S100A9/SERPINA2/SERPINB11/SERPINB12/SERPINB5/SIAH2/SLPI/SORL1/SOX2/SOX7/SPINK14/SPINT1/SPINT3/TIMP2/VSIR/WFDC11/WFDC5/WFDC8	55
cluster1	BP	GO:0001659	temperature homeostasis	29/1407	178/18903	0.0000522	0.033963157	0.033129431	ABAT/ABHD6/ACHE/ADIPOQ/ADIPOR1/ALPL/APPL2/DIO2/EGR1/FOXC2/G0S2/GADD45G/GPX1/HCRT/JAK2/NOTCH1/NRDC/NTSR1/PCTP/PRDM16/PTGER3/SLC25A44/SLC27A1/TFE3/TLE3/TLR4/UCP2/ZNF423/ZNF516	29
cluster1	BP	GO:0071772	response to BMP	29/1407	178/18903	0.0000522	0.033963157	0.033129431	BMPR1A/CER1/DAND5/EGR1/ENG/FKBP8/FOXD1/FSTL1/GDF3/HTRA3/KCP/MAPK3/MSX1/NBL1/NODAL/NOTCH1/PCSK6/PDCD4/PELO/SMAD1/SMPD3/SORL1/SOX11/SPINT1/TGFB1/TGFB2/TMPRSS6/UBE2D3/ZNF423	29

cluster1	BP	GO:0071773	cellular response to BMP stimulus	29/1407	178/18903	0.0000522	0.033963157	0.033129431	BMPR1A/CER1/DAND5/EGR1/ENG/FKBP8/FOXD1/FSTL1/GDF3/HTRA3/KCP/MAPK3/MSX1/NBL1/NODAL/NOTCH1/PCSK6/PDCD4/PELO/SMAD1/SMPD3/SORL1/SOX11/SPINT1/TGFB1/TGFB2/TMPRSS6/UBE2D3/ZNF423	29
cluster1	CC	GO:0030667	secretory granule membrane	54/1494	313/19869	7.35E-09	0.00000652	0.00000642	ADAM8/ADGRG3/ALDH3B1/APLP2/ARL8A/ATP6V0C/BST1/C5AR1/CD33/CD46/CD55/CD58/CD63/CEACAM6/CMTM6/CPE/CXCR2/CYBB/DYNLL1/FCER1G/FPR2/GLIPR1/GP2/HYAL3/IRAG2/ITGAX/LAMP2/LILRB3/MANBA/MGAM/MMP25/P2RX1/PCDH7/PLAU/PLAUR/PTPRC/PTPRJ/RAB26/RAB3A/RAB7A/RAC1/RHOA/SERPINB12/SIGLEC5/SIRPA/SLC2A3/STX3/TEX101/TICAM2/TMEM190/TMEM95/TNFRSF1B/VGF/ZG16	54
cluster1	CC	GO:0101002	ficolin-1-rich granule	37/1494	185/19869	3.49E-08	0.0000155	0.0000152	A1BG/ACTR10/ADAM8/ARL8A/ARPC5/ASAH1/ATP6V0C/CAB39/CANT1/CD55/CD58/CDA/CSTB/CTSH/CTSZ/DYNLL1/DYNLT1/FCER1G/FPR2/GMFG/HMGB1/ITGAX/LAMP2/MAPK14/MGAM/MMP9/PYGL/QPCT/RAC1/RHOA/SERPINB12/SIGLEC5/SIRPA/SLC2A3/TIMP2/XRCC6/YPEL5	37
cluster1	CC	GO:0070820	tertiary granule	32/1494	164/19869	0.000000515	0.000152291	0.000150004	ADAM8/ARL8A/ASAH1/ATP6V0C/B2M/CANT1/CD33/CD55/CD58/CDA/CSTB/CTSH/CYBB/DYNLL1/FCER1G/FPR2/ITGAX/LAMP2/MGAM/MMP9/OSCAR/PLAU/QPCT/RAC1/RHOA/SERPINB12/SIGLEC5/SIRPA/SLC2A3/STXBP2/TIMP2/YPEL5	32

cluster1	CC	GO:0101003	ficolin-1-rich granule membrane	17/1494	61/19869	0.00000159	0.000353456	0.00034815	ADAM8/ARL8A/ATP6V0C/CD55/CD58/DYNLL1/FCER1G/FPR2/ITGAX/LAMP2/MGAM/RAC1/RHOA/SERPINB12/SIGLEC5/SIRPA/SLC2A3	17
cluster1	CC	GO:0031225	anchored component of membrane	28/1494	169/19869	0.0000616	0.010926233	0.010762206	ACHE/ALPL/ALPP/ART1/ART3/ART4/BST1/CD55/CEACAM6/CNTN1/DPEP2/EEPD1/GP1BA/GP2/LSAMP/LYPD2/MMP25/PLAUR/PRMT8/RAB26/RAB3A/RAB7A/RECK/RHBG/TEX101/TNFRSF10C/UMOD/XPNPEP2	28
cluster1	CC	GO:0000786	nucleosome	23/1494	133/19869	0.000140149	0.019465255	0.01917304	ACTB/H1-1/H1-5/H1-6/H2AC12/H2AC18/H2AC19/H2AC7/H2AZ1/H2BC12/H2BC4/H2BC5/H2BW2/H3-3A/H3C11/H3C13/H3C14/H3C15/H3C2/H3Y1/H4C3/MACROH2A1/MPHOSPH8	23
cluster1	CC	GO:0042581	specific granule	26/1494	160/19869	0.000153615	0.019465255	0.01917304	ADAM8/ADGRG3/ALDH3B1/B2M/BST1/CANT1/CD33/CMTM6/CTSZ/CYBB/ERP44/FPR2/FRK/MMP25/OSCAR/P2RX1/PLAU/PLAUR/PTPRJ/QPCT/SLC2A3/SLPI/STX3/STXBP2/TIMP2/TNFRSF1B	26

cluster1	MF	GO:0004984	olfactory receptor activity	71/1449	429/18432	1.46E-09	0.00000244	0.00000237	GPR148/OR10A4/OR10D3/OR10H1/OR10H5/OR10J5/OR10T2/OR10V1/OR11A1/OR11G2/OR11H6/OR13C2/OR13C3/OR13C4/OR13C5/OR13G1/OR13J1/OR1D2/OR1D5/OR1I1/OR1K1/OR1L8/OR1N1/OR2A25/OR2B3/OR2C3/OR2D2/OR2G6/OR2M3/OR2T3/OR2T4/OR4A47/OR4C12/OR4D10/OR4D9/OR4F16/OR4F4/OR4K17/OR4X2/OR51B2/OR51B4/OR51D1/OR51F1/OR51I2/OR51M1/OR52N1/OR56A4/OR56B4/OR5A1/OR5AP2/OR5AS1/OR5AU1/OR5B21/OR5C1/OR5H6/OR5K3/OR5M8/OR6A2/OR6C4/OR6C65/OR6C74/OR6K3/OR6M1/OR6V1/OR7A17/OR7D4/OR8B12/OR8B8/OR8G1/OR8H2/OR8S1	71
cluster1	MF	GO:0030527	structural constituent of chromatin	21/1449	99/18432	0.0000231	0.01934627	0.01885421	H1-1/H1-5/H1-6/H2AC12/H2AC18/H2AC19/H2AC7/H2AZ1/H2BC12/H2BC4/H2BC5/H2BW2/H3-3A/H3C11/H3C13/H3C14/H3C15/H3C2/H3Y1/H4C3/MACROH2A1	21
cluster1	MF	GO:0008188	neuropeptide receptor activity	13/1449	46/18432	0.0000361	0.020116729	0.019605072	GAL/GALR1/GALR3/GPR143/NMUR2/NPBWR2/NPFFR1/NPY4R2/NTSR1/NTSR2/OPRM1/SSTR4/TACR2	13
cluster1	MF	GO:0005536	glucose binding	6/1449	11/18432	0.0000764	0.031931665	0.031119502	G6PD/HK1/PYGL/SLC2A3/SLC2A8/UGP2	6

cluster1	MF	GO:0046982	protein heterodimerization activity	47/1449	341/18432	0.000114678	0.038348234	0.03737287	ABCG5/ATF6/BCL2A1/BOK/CEACAM6/CENPT/CENPW/CYBB/GCA/H2AC12/H2AC18/H2AC19/H2AC7/H2AZ1/H2BC12/H2BC4/H2BC5/H2BW2/H3-3A/H3C11/H3C13/H3C14/H3C15/H3C2/H3Y1/H4C3/IL17A/IL17F/IRAK1/KRT25/MACROH2A1/MAFG/NR4A1/P2RY1/PDSS1/PGLYRP3/PPP2CA/SDCBP/SIM2/SOS2/SUPT4H1/TAF6/TENM1/TLR4/TPM4/UBA2/YWHAH	47
cluster1	MF	GO:0001653	peptide receptor activity	26/1449	153/18432	0.000147545	0.041115774	0.040070019	CRHR2/CXCR2/FPR2/GAL/GALR1/GALR3/GP1BA/GPR143/GPR32/MAS1/MCHR2/NLRP6/NMUR2/NPBWR2/NPFFR1/NPY4R2/NTSR1/NTSR2/OGFRL1/OPRM1/RAMP1/RXFP3/S1PR2/SCTR/SSTR4/TACR2	26
cluster1	MF	GO:0008528	G protein-coupled peptide receptor activity	25/1449	147/18432	0.000193656	0.046256227	0.045079728	CRHR2/CXCR2/FPR2/GAL/GALR1/GALR3/GP1BA/GPR143/GPR32/MAS1/MCHR2/NLRP6/NMUR2/NPBWR2/NPFFR1/NPY4R2/NTSR1/NTSR2/OPRM1/RAMP1/RXFP3/S1PR2/SCTR/SSTR4/TACR2	25
cluster1	KEGG	hsa04740	Olfactory transduction	74/663	441/8779	2.53E-11	8.16E-09	7.42E-09	ARRB1/CALM2/CALM3/GNG7/OR10A4/OR10D3/OR10H1/OR10H5/OR10J5/OR10T2/OR10V1/OR11A1/OR11G2/OR11H6/OR13C2/OR13C3/OR13C4/OR13C5/OR13G1/OR13J1/OR1D2/OR1D5/OR1I1/OR1K1/OR1L8/OR1N1/OR2A25/OR2B3/OR2C3/OR2D2/OR2G6/OR2M3/OR2T3/OR2T4/OR4A47/OR4C12/OR4D10/OR4D9/OR4F16/OR4K17/OR4X2/OR51B2/OR51B4/OR51D1/OR51F1/OR51I2/OR51M1/OR52N1/OR56A4/OR56B4/OR5A1/OR5AP2/OR5AS1/OR5AU1/OR5B21/OR5C1/OR5H6/OR5K3/OR5M8/OR6A2/OR6C4/OR6C65/OR6C74/OR6K3/OR6M1/OR6V1/OR7A17/OR7D4/OR8B12/OR8B8/OR8G1/OR8H2/OR8S1/PRKACG	74

cluster1	KEGG	hsa05034	Alcoholism	34/663	188/8779	0.00000132	0.000213896	0.000194483	CALM2/CALM3/CREB5/GNG10/GNG7/GRIN2C/GRIN3B/H2AC12/H2AC14/H2AC18/H2AC19/H2AC7/H2AZ1/H2BC12/H2BC4/H2BC5/H2BW2/H3-3A/H3C11/H3C13/H3C14/H3C15/H3C2/H4C3/HDAC5/LOC102724334/MACROH2A1/MAPK3/PPP1R1B/PRKACG/RAF1/SLC18A2/SLC29A2/SOS2	34
cluster1	KEGG	hsa04613	Neutrophil extracellular trap formation	33/663	191/8779	0.00000533	0.000574337	0.000522209	ACTB/C5AR1/CYBB/FPR2/GP1BA/H2AC12/H2AC14/H2AC18/H2AC19/H2AC7/H2AZ1/H2BC12/H2BC4/H2BC5/H2BW2/H3-3A/H3C11/H3C13/H3C14/H3C15/H3C2/H4C3/HDAC5/HMGB1/LOC102724334/MACROH2A1/MAPK14/MAPK3/PPIF/PRKCB/RAC1/RAF1/TLR4	33
cluster1	KEGG	hsa04080	Neuroactive ligand-receptor interaction	51/663	366/8779	0.0000121	0.000978243	0.000889456	ADM2/C5AR1/CHRNA1/CRHR2/DRD5/FPR2/GABRB1/GAL/GALR1/GALR3/GNRHR/GRIN2C/GRIN3B/HCRT/HTR1A/HTR1B/HTR2B/HTR5A/LPAR2/MAS1/MCHR2/MTNR1B/NMUR2/NPBWR2/NPFF/NPFFR1/NPS/NPY4R/NPY4R2/NTSR1/NTSR2/OPRM1/P2RX1/P2RY1/P2RY13/PAQR9/POMC/PTGDR/PTGER1/PTGER3/RXFP3/S1PR2/S1PR3/SCTR/SSTR4/TAAR5/TAAR6/TAAR9/TACR2/UTS2B/VGF	51
cluster1	KEGG	hsa04217	Necroptosis	25/663	159/8779	0.000338751	0.020894974	0.018998526	BID/CHMP4A/CHMP4B/CYBB/FAS/H2AC12/H2AC14/H2AC18/H2AC19/H2AC7/H2AZ1/HMGB1/IFNA13/IFNA14/IFNGR2/JAK2/MACROH2A1/PLA2G4D/PLA2G4F/PYGL/SPATA2/TICAM1/TICAM2/TLR4/VPS4B	25

cluster1	KEGG	hsa05132	Salmonella infection	34/663	247/8779	0.000435119	0.020894974	0.018998526	ABI1/ACTB/ACTR10/ACTR3/ARF1/ARL8A/ARPC5/CDC42/DCTN3/DYNLL1/DYNLL2/DYNLT1/DYNLT3/IL6/IRAK1/MAPK14/MAPK3/MYC/MYL12B/MYL9/NAIP/PFN3/PIK3C2G/PTPRC/RAB7A/RAC1/RAF1/RHOA/SKP1/TAB2/TAB3/TLR4/TLR5/TUBB4B	34
cluster1	KEGG	hsa05161	Hepatitis B	25/663	162/8779	0.000452832	0.020894974	0.018998526	ATP6AP1/BID/CREB5/CREBBP/E2F3/EGR2/FAS/IFNA13/IFNA14/IL6/IRAK1/JAK2/MAPK14/MAPK3/MMP9/MYC/PRKCB/RAF1/SOS2/TAB2/TGFB1/TGFB2/TICAM1/TICAM2/TLR4	25
cluster1	KEGG	hsa05131	Shigellosis	33/663	247/8779	0.000893975	0.036094226	0.032818279	ACTB/ACTR3/ARF1/ARPC5/CCL5/CDC42/CSF2/H3-3A/H3C11/H3C13/H3C14/H3C15/H3C2/HK1/MAPK14/MAPK3/MYL12B/MYL9/NAIP/PFN3/PLCZ1/PRKCE/RAC1/RHOA/SEPTIN7/SKP1/TAB2/TAB3/TLR4/TLR5/UBE2D3/UBE2V1/WIPI1	33
cluster1	KEGG	hsa05322	Systemic lupus erythematosus	21/663	137/8779	0.00137609	0.049386336	0.044903985	C8A/H2AC12/H2AC14/H2AC18/H2AC19/H2AC7/H2AZ1/H2BC12/H2BC4/H2BC5/H2BW2/H3-3A/H3C11/H3C13/H3C14/H3C15/H3C2/H4C3/LOC102724334/MACROH2A1/RO60	21

cluster2	BP	GO:0042254	ribosome biogenesis	93/2030	310/18903	6.52E-21	5.13E-17	4.94E-17	BMS1/BOP1/BYSL/C1QBP/CHD7/DDX10/DDX28/DDX49/DDX51/DDX52/DDX56/DHX37/EMG1/ERAL1/ERCC2/EXOSC3/EXOSC6/FASTKD2/FRG1/GEMIN4/GNL3L/IMP3/IMP4/ISG20L2/KRI1/LOC102724159/LSG1/LYAR/MPHOSPH10/MRM1/MRPL44/MRPS11/MRPS7/MTERF3/MYBBP1A/NAT10/NGDN/NIFK/NOB1/NOC2L/NOL11/NOL6/NOL8/NOM1/NOP14/NOP2/NOP53/NOP56/NOP9/NVL/PAK1IP1/PES1/PIH1D1/PIN4/PINX1/POP5/PPAN/PRKDC/PWP1/RBFA/RIOK2/RIOX2/RPF2/RPL10/RPL23A/RPL27/RPL5/RPL7A/RPLP0/RPP40/RPS15/RPS21/RPS25/RPS28/RPS5/RPS6/RPS8/RRP7A/RRS1/RSL24D1/SHQ1/SPATA5L1/SUV39H1/TBL3/TRMT2B/USP36/UTP18/UTP25/WDR12/WDR3/WDR55/YBEY/ZNF622	93
cluster2	BP	GO:0022613	ribonucleoprotein complex biogenesis	119/2030	489/18903	3.58E-18	1.41E-14	1.36E-14	ATM/BMS1/BOP1/BUD13/BYSL/C1QBP/CELF1/CHD7/CRNKL1/DDX10/DDX20/DDX23/DDX28/DDX49/DDX51/DDX52/DDX56/DHX37/DHX9/EIF2D/EIF3A/EIF3F/EIF3K/EIF4H/EMG1/ERAL1/ERCC2/EXOSC3/EXOSC6/FASTKD2/FRG1/GEMIN4/GNL3L/IMP3/IMP4/ISG20L2/KRI1/LOC102724159/LSG1/LYAR/MPHOSPH10/MRM1/MRPL44/MRPS11/MRPS7/MTERF3/MYBBP1A/NAT10/NCBP1/NGDN/NIFK/NOB1/NOC2L/NOL11/NOL3/NOL6/NOL8/NOM1/NOP14/NOP2/NOP53/NOP56/NOP9/NVL/PAK1IP1/PES1/PIH1D1/PIN4/PINX1/POP5/PPAN/PRKDC/PRMT5/PRMT7/PRPF3/PRPF8/PWP1/RBFA/RBMX2/RIOK2/RIOX2/RPF2/RPL10/RPL13A/RPL23A/RPL27/RPL5/RPL7A/RPLP0/RPP40/RPS15/RPS21/RPS25/RPS28/RPS5/RPS6/RPS8/RRP7A/RRS1/RSL24D1/SHQ1/SNIP1/SNRNP200/SNRPD3/SPATA5L1/SRSF10/SUV39H1/TAF9/TBL3/TRMT2B/USP36/UTP18/UTP25/WDR12/WDR3/WDR55/XAB2/YBEY/ZNF622	119
cluster2	BP	GO:0034470	ncRNA processing	109/2030	439/18903	2.05E-17	5.38E-14	5.18E-14	AARS1/AARS2/ANKRD16/BMS1/BOP1/BYSL/CHD7/CTU1/CTU2/DDX10/DDX49/DDX51/DDX52/DDX56/DGCR8/DHX37/DUS2/ELAC1/ELP1/ELP2/ELP5/EMG1/ERCC2/EXOSC3/EXOSC6/FRG1/GEMIN4/HENMT1/IMP3/IMP4/INTS10/INTS14/INTS6/INTS7/KRI1/LCMT2/LOC102724159/LYAR/MAP2K1/MOCS3/MPHOSPH10/MRM1/MRPL44/MRPS11/NAT10/NCBP1/NGDN/NIFK/NOB1/NOL11/NOL6/NOL8/NOP14/NOP2/NOP53/NOP56/NOP9/NSUN2/NVL/PES1/PIH1D1/PIN4/POP1/POP5/PPAN/PRKDC/PRORP/PUS1/PUS3/PWP1/QTRT1/RBFA/RIOK2/RPF2/RPL27/RPL5/RPL7A/RPP14/RPP40/RPS15/RPS21/RPS25/RPS28/RPS6/RPS8/RRP7A/RRS1/SARS1/SHQ1/SNIP1/SUV39H1/TBL3/THUMPD3/TRIT1/TRMT10B/TRMT2A/TRMT2B/TRMT5/TSEN15/TYW1B/USP36/UTP18/UTP25/WDR12/WDR3/WDR55/WDR6/YBEY/ZC3H7B	109
cluster2	BP	GO:0006364	rRNA processing	71/2030	229/18903	4.03E-17	7.94E-14	7.64E-14	BMS1/BOP1/BYSL/CHD7/DDX10/DDX49/DDX51/DDX52/DDX56/DHX37/EMG1/ERCC2/EXOSC3/EXOSC6/FRG1/GEMIN4/IMP3/IMP4/KRI1/LOC102724159/LYAR/MPHOSPH10/MRM1/MRPL44/MRPS11/NAT10/NGDN/NIFK/NOB1/NOL11/NOL6/NOL8/NOP14/NOP2/NOP53/NOP56/NOP9/NVL/PES1/PIH1D1/PIN4/POP5/PPAN/PRKDC/PWP1/RBFA/RIOK2/RPF2/RPL27/RPL5/RPL7A/RPP40/RPS15/RPS21/RPS25/RPS28/RPS6/RPS8/RRP7A/RRS1/SHQ1/SUV39H1/TBL3/TRMT2B/USP36/UTP18/UTP25/WDR12/WDR3/WDR55/YBEY	71

cluster2	BP	GO:0016072	rRNA metabolic process	78/2030	268/18903	6.56E-17	1.03E-13	9.95E-14	BMS1/BOP1/BYSL/CHD7/DDX10/DDX49/DDX51/DDX52/DDX56/DHX37/EMG1/ERCC2/EXOSC3/EXOSC6/FRG1/GEMIN4/GTF3C1/GTF3C2/IMP3/IMP4/IPPK/KRI1/LOC102724159/LYAR/MPHOSPH10/MRM1/MRPL44/MRPS11/NAT10/NGDN/NIFK/NOB1/NOL11/NOL6/NOL8/NOP14/NOP2/NOP53/NOP56/NOP9/NVL/PES1/PIH1D1/PIN4/POLR1B/POLR1G/POP5/PPAN/PRKDC/PWP1/RBFA/RIOK2/RPF2/RPL27/RPL5/RPL7A/RPP40/RPS15/RPS21/RPS25/RPS28/RPS6/RPS8/RRP7A/RRS1/SHQ1/SMARCA4/SUV39H1/TAF1B/TBL3/TRMT2B/USP36/UTP18/UTP25/WDR12/WDR3/WDR55/YBEY	78
cluster2	BP	GO:0042274	ribosomal small subunit biogenesis	36/2030	78/18903	2.25E-15	2.95E-12	2.84E-12	BMS1/BYSL/DDX52/DHX37/EMG1/ERAL1/ERCC2/IMP3/IMP4/KRI1/LOC102724159/MPHOSPH10/MRPS11/MRPS7/NAT10/NGDN/NOB1/NOL11/NOM1/NOP14/NOP9/PRKDC/RIOK2/RPP40/RPS15/RPS21/RPS25/RPS28/RPS5/RPS6/RPS8/RRP7A/RRS1/TBL3/UTP25/WDR3	36
cluster2	BP	GO:0140053	mitochondrial gene expression	52/2030	163/18903	1.84E-13	2.07E-10	1.99E-10	AARS2/C1QBP/EARS2/FASTKD1/FASTKD2/FASTKD3/FASTKD5/GATB/GATC/GFM1/HARS1/IARS2/LARS2/METTL4/MRPL10/MRPL11/MRPL12/MRPL15/MRPL17/MRPL21/MRPL30/MRPL33/MRPL34/MRPL38/MRPL39/MRPL44/MRPL54/MRPS10/MRPS11/MRPS15/MRPS17/MRPS18B/MRPS18C/MRPS31/MRPS5/MRPS7/MRPS9/MTERF1/MTG1/PNPT1/PRORP/PTCD3/PUS1/SARS2/SHMT2/SUPV3L1/TACO1/TARS2/TBRG4/TRIT1/TRMT5/TWNK	52
cluster2	BP	GO:0030490	maturation of SSU-rRNA	26/2030	55/18903	8.68E-12	8.54E-09	8.22E-09	BMS1/BYSL/DDX52/DHX37/ERCC2/IMP3/IMP4/KRI1/LOC102724159/MPHOSPH10/MRPS11/NAT10/NGDN/NOB1/NOL11/NOP14/NOP9/RIOK2/RPP40/RPS21/RPS28/RPS8/RRS1/TBL3/UTP25/WDR3	26

cluster2	BP	GO:0032543	mitochondrial translation	40/2030	131/18903	4.87E-10	0.000000426	0.00000041	AARS2/C1QBP/EARS2/FASTKD2/FASTKD3/GATB/GATC/GFM1/HARS1/IARS2/LARS2/MRPL10/MRPL11/MRPL12/MRPL15/MRPL17/MRPL21/MRPL30/MRPL33/MRPL34/MRPL38/MRPL39/MRPL44/MRPL54/MRPS10/MRPS11/MRPS15/MRPS17/MRPS18B/MRPS18C/MRPS31/MRPS5/MRPS7/MRPS9/MTG1/PTCD3/SARS2/SHMT2/TACO1/TARS2	40
cluster2	BP	GO:0042255	ribosome assembly	24/2030	61/18903	5.5E-09	0.00000433	0.00000417	BOP1/C1QBP/DDX28/DHX37/ERAL1/FASTKD2/LOC102724159/MRPS11/MRPS7/MTERF3/NOP2/NOP53/PPAN/PRKDC/RPF2/RPL10/RPL23A/RPL5/RPLP0/RPS15/RPS28/RPS5/RRP7A/RRS1	24
cluster2	BP	GO:0006399	tRNA metabolic process	49/2030	195/18903	9.2E-09	0.00000658	0.00000634	AARS1/AARS2/AARSD1/ANKRD16/CTU1/CTU2/DUS2/EARS2/ELAC1/ELP1/ELP2/ELP5/EXOSC3/FARSB/GATB/GATC/GTF3C1/GTF3C2/HARS1/IARS1/IARS2/LARS2/LCMT2/MARS2/MOCS3/NARS2/NAT10/NSUN2/POP1/POP5/PRORP/PUS1/PUS3/QTRT1/RPP14/RPP40/SARS1/SARS2/TARS2/THUMPD3/TRIT1/TRMT10B/TRMT2A/TRMT2B/TRMT5/TSEN15/TYW1B/WDR6/YARS1	49
cluster2	BP	GO:0000959	mitochondrial RNA metabolic process	20/2030	50/18903	7.39E-08	0.0000485	0.0000466	AARS2/EARS2/FASTKD1/FASTKD2/FASTKD3/FASTKD5/METTL4/MRPL12/MTERF1/PDE12/PNPT1/PRORP/PUS1/SARS2/SUPV3L1/TARS2/TBRG4/TRIT1/TRMT5/TWNK	20

cluster2	BP	GO:0002181	cytoplasmic translation	41/2030	161/18903	9.81E-08	0.0000594	0.0000572	AARS1/DHX9/DPH2/EIF2D/EIF3A/EIF3F/EIF3K/EIF4A1/EIF4H/NCBP1/RPL10/RPL12/RPL13/RPL13A/RPL17/RPL18/RPL18A/RPL19/RPL21/RPL23A/RPL27/RPL27A/RPL29/RPL3/RPL30/RPL36/RPL5/RPL7A/RPL8/RPLP0/RPLP1/RPS15/RPS2/RPS21/RPS25/RPS28/RPS3/RPS5/RPS6/RPS8/UNK	41
cluster2	BP	GO:0071826	ribonucleoprotein complex subunit organization	53/2030	242/18903	0.000000315	0.000162947	0.000156857	ATM/BOP1/BUD13/CELF1/CRNKL1/DDX20/DDX23/DDX28/DHX8/DHX9/EIF2D/EIF3A/EIF3F/EIF3K/EIF4H/ERAL1/FASTKD2/GEMIN4/KLC1/LOC102724159/MRPS11/MRPS7/NCBP1/NOL3/NOP2/NOP53/PIH1D1/PPAN/PRKDC/PRMT5/PRMT7/PRPF3/PRPF8/RBMX2/RPF2/RPL10/RPL13A/RPL23A/RPL5/RPLP0/RPS15/RPS28/RPS5/RRP7A/RRS1/SHQ1/SNIP1/SNRNP200/SNRPD3/SRSF10/TAF9/TFIP11/XAB2	53
cluster2	BP	GO:0043038	amino acid activation	18/2030	45/18903	0.000000331	0.000162947	0.000156857	AARS1/AARS2/AARSD1/AASDH/EARS2/FARSB/GATB/GATC/HARS1/IARS1/IARS2/LARS2/MARS2/NARS2/SARS1/SARS2/TARS2/YARS1	18
cluster2	BP	GO:1902570	protein localization to nucleolus	11/2030	18/18903	0.000000331	0.000162947	0.000156857	BYSL/MCRS1/NOL8/NOP53/NVL/PINX1/POLR1A/RPF2/RRP7A/RRS1/UTP25	11

cluster2	BP	GO:0034655	nucleobase-containing compound catabolic process	80/2030	428/18903	0.000000511	0.000236629	0.000227786	ACAT1/AIFM1/ALKBH5/APEX1/APOBEC3A/APOBEC3B/APOBEC3H/ATM/CELF1/CNOT2/CNOT4/DAZ1/DCPS/DCTPP1/DDX49/DFFA/DHX9/DIS3L2/DNASE1/DND1/DNPH1/DPYD/DUT/EDC3/EIF4ENIF1/ELAVL1/ENPP3/ENPP4/EXOSC3/EXOSC6/FASTKD1/FASTKD2/FASTKD3/FASTKD5/FITM2/FTO/HELZ2/HNRNPM/IKBKE/ITPA/MBD4/MLYCD/MUS81/NCBP1/NEIL1/NRDE2/NSUN2/NUDT1/NUDT13/NUDT19/NUDT4/PABPC4/PAN2/PCID2/PDE12/PDE8A/PNPT1/POP1/PYM1/RBM10/RBM7/RNASEH2A/RNH1/RNPS1/SARM1/SERBP1/SND1/SUCLG2/SUPV3L1/TARDBP/TBRG4/TDG/TENT4A/THRAP3/TNRC6B/UNG/ZC3H12D/ZC3H4/ZHX2/ZPR1	80
cluster2	BP	GO:0000462	maturation of SSU-rRNA from tricistronic rRNA transcript (SSU-rRNA, 5.8S rRNA, LSU-rRNA)	16/2030	39/18903	0.000000993	0.000434306	0.000418075	BMS1/BYSL/DHX37/ERCC2/KRI1/LOC102724159/MRPS11/NGDN/NOP14/NOP9/RPP40/RPS21/RPS8/RRS1/TBL3/UTP25	16
cluster2	BP	GO:0043039	tRNA aminoacylation	17/2030	44/18903	0.00000125	0.000518539	0.00049916	AARS1/AARS2/AARSD1/EARS2/FARSB/GATB/GATC/HARS1/IARS1/IARS2/LARS2/MARS2/NARS2/SARS1/SARS2/TARS2/YARS1	17
cluster2	BP	GO:0090305	nucleic acid phosphodiester bond hydrolysis	53/2030	253/18903	0.00000135	0.000518539	0.00049916	AEN/APEX1/BMS1/BOP1/CNOT2/CPSF1/CPSF3/DCPS/DIS3L2/DNASE1/EDC3/ELAC1/ENPP3/ERCC1/ERCC2/ERCC3/ERCC5/EXO5/EXOSC3/G3BP1/HELZ2/ISG20L2/KRI1/MRPL44/MUS81/NCBP1/NOB1/NOP14/NOP9/PAN2/PDE12/PLD3/PNPT1/POLE/POLR1H/POLR2I/POP1/POP5/PRORP/RAD9A/RAG1/RNASEH2A/RPP14/RPP40/RPS21/RRS1/SND1/TATDN2/TBL3/TDP1/XPA/YBEY/ZC3H12D	53

cluster2	BP	GO:0000963	mitochondrial RNA processing	11/2030	20/18903	0.00000142	0.000518539	0.00049916	FASTKD1/FASTKD2/FASTKD3/FASTKD5/PNPT1/PRORP/PUS1/SUPV3L1/TBRG4/TRIT1/TRMT5	11
cluster2	BP	GO:0022618	ribonucleoprotein complex assembly	50/2030	234/18903	0.00000145	0.000518539	0.00049916	ATM/BOP1/BUD13/CELF1/CRNKL1/DDX20/DDX23/DDX28/DHX9/EIF2D/EIF3A/EIF3F/EIF3K/EIF4H/ERAL1/FASTKD2/GEMIN4/LOC102724159/MRPS11/MRPS7/NCBP1/NOL3/NOP2/NOP53/PIH1D1/PPAN/PRKDC/PRMT5/PRMT7/PRPF3/PRPF8/RBMX2/RPF2/RPL10/RPL13A/RPL23A/RPL5/RPLP0/RPS15/RPS28/RPS5/RRP7A/RRS1/SHQ1/SNIP1/SNRNP200/SNRPD3/SRSF10/TAF9/XAB2	50
cluster2	BP	GO:0019439	aromatic compound catabolic process	86/2030	489/18903	0.00000268	0.000916469	0.000882219	ABCC2/ACAT1/AIFM1/ALDH8A1/ALKBH5/APEX1/APOBEC3A/APOBEC3B/APOBEC3H/ATM/CELF1/CNOT2/CNOT4/DAZ1/DCPS/DCTPP1/DDX49/DFFA/DHX9/DIS3L2/DNASE1/DND1/DNPH1/DPYD/DUT/EDC3/EIF4ENIF1/ELAVL1/ENPP3/ENPP4/EXOSC3/EXOSC6/FASTKD1/FASTKD2/FASTKD3/FASTKD5/FITM2/FTO/GSTZ1/HELZ2/HNRNPM/IKBKE/IL4I1/ITPA/MBD4/MLYCD/MUS81/NCBP1/NEIL1/NRDE2/NSUN2/NUDT1/NUDT13/NUDT19/NUDT4/PABPC4/PAN2/PCID2/PDE12/PDE8A/PDXP/PNPT1/POP1/PYM1/QDPR/RBM10/RBM7/RNASEH2A/RNH1/RNPS1/SARM1/SERBP1/SND1/SUCLG2/SUPV3L1/TARDBP/TBRG4/TDG/TENT4A/THRAP3/TNRC6B/UNG/ZC3H12D/ZC3H4/ZHX2/ZPR1	86
cluster2	BP	GO:0046700	heterocycle catabolic process	83/2030	468/18903	0.00000284	0.000932483	0.000897634	ABCC2/ACAT1/AIFM1/ALKBH5/APEX1/APOBEC3A/APOBEC3B/APOBEC3H/ATM/CELF1/CNOT2/CNOT4/DAZ1/DCPS/DCTPP1/DDX49/DFFA/DHX9/DIS3L2/DNASE1/DND1/DNPH1/DPYD/DUT/EDC3/EIF4ENIF1/ELAVL1/ENPP3/ENPP4/EXOSC3/EXOSC6/FASTKD1/FASTKD2/FASTKD3/FASTKD5/FITM2/FTO/HELZ2/HNRNPM/IKBKE/IL4I1/ITPA/MBD4/MLYCD/MUS81/NCBP1/NEIL1/NRDE2/NSUN2/NUDT1/NUDT13/NUDT19/NUDT4/PABPC4/PAN2/PCID2/PDE12/PDE8A/PDXP/PNPT1/POP1/PYM1/RBM10/RBM7/RNASEH2A/RNH1/RNPS1/SARM1/SERBP1/SND1/SUCLG2/SUPV3L1/TARDBP/TBRG4/TDG/TENT4A/THRAP3/TNRC6B/UNG/ZC3H12D/ZC3H4/ZHX2/ZPR1	83

cluster2	BP	GO:0009451	RNA modification	40/2030	176/18903	0.00000326	0.001024812	0.000986512	AARS1/AARS2/ADARB1/ALKBH3/ALKBH5/ANKRD16/APOBEC3A/APOBEC3B/APOBEC3H/CMTR1/CMTR2/CTU1/CTU2/DUS2/ELP1/ELP2/ELP5/EMG1/FTO/HENMT1/LCMT2/METTL4/MOCS3/MRM1/NAT10/NOP2/NSUN2/PUS1/PUS3/QTRT1/RBM15B/SNRPD3/THUMPD3/TRIT1/TRMT10B/TRMT2A/TRMT2B/TRMT5/TYW1B/WDR6	40
cluster2	BP	GO:0006839	mitochondrial transport	42/2030	189/18903	0.00000345	0.00104449	0.001005455	AGK/AIFM1/AIP/ARHGAP11B/ARIH2/CPT1B/CSNK2A2/GCLC/HAX1/HSPA1L/KIFBP/LRRK2/MFF/MIPEP/MOAP1/NDUFA13/NOL3/PAM16/PITRM1/PMPCA/RTL10/SLC25A13/SLC25A20/SLC25A32/SLC25A6/SREBF1/THEM4/TIMM10/TIMM13/TIMM17A/TIMM50/TIMM8A/TMEM102/TOMM22/TOMM34/TOMM40/TOMM40L/TRMT10B/UCP1/UCP3/USP36/VPS11	42
cluster2	BP	GO:0051168	nuclear export	38/2030	165/18903	0.00000406	0.001184962	0.001140677	AHCYL1/ALKBH5/ALYREF/CHTOP/DDX19B/DHX9/EIF4ENIF1/IL1B/LSG1/MCM3AP/NCBP1/NOL6/NOP9/NRDE2/NSUN2/NUP160/NUP62/NUP93/NXF1/NXT1/PCID2/PKD1/POLDIP3/RAE1/RANGAP1/RBM15B/RIOK2/RITA1/RPS15/RRS1/SARNP/STYX/SUPT6H/THOC1/THOC6/TXN/UBE2I/XPO5	38
cluster2	BP	GO:0044270	cellular nitrogen compound catabolic process	83/2030	474/18903	0.00000473	0.001330694	0.001280963	ABCC2/ACAT1/AIFM1/ALKBH5/APEX1/APOBEC3A/APOBEC3B/APOBEC3H/ATM/CELF1/CNOT2/CNOT4/DAZ1/DCPS/DCTPP1/DDX49/DFFA/DHX9/DIS3L2/DNASE1/DND1/DNPH1/DPYD/DUT/EDC3/EIF4ENIF1/ELAVL1/ENPP3/ENPP4/EXOSC3/EXOSC6/FASTKD1/FASTKD2/FASTKD3/FASTKD5/FITM2/FTO/HELZ2/HNRNPM/IKBKE/IL4I1/ITPA/MBD4/MLYCD/MUS81/NCBP1/NEIL1/NRDE2/NSUN2/NUDT1/NUDT13/NUDT19/NUDT4/PABPC4/PAN2/PCID2/PDE12/PDE8A/PDXP/PNPT1/POP1/PYM1/RBM10/RBM7/RNASEH2A/RNH1/RNPS1/SARM1/SERBP1/SND1/SUCLG2/SUPV3L1/TARDBP/TBRG4/TDG/TENT4A/THRAP3/TNRC6B/UNG/ZC3H12D/ZC3H4/ZHX2/ZPR1	83

cluster2	BP	GO:0000027	ribosomal large subunit assembly	12/2030	26/18903	0.00000519	0.001409374	0.001356703	BOP1/DDX28/FASTKD2/NOP2/NOP53/PPAN/RPF2/RPL10/RPL23A/RPL5/RPLP0/RRS1	12
cluster2	BP	GO:0044528	regulation of mitochondrial mRNA stability	6/2030	7/18903	0.00000969	0.002374065	0.002285341	FASTKD1/FASTKD2/FASTKD3/FASTKD5/PDE12/TBRG4	6
cluster2	BP	GO:0006626	protein targeting to mitochondrion	27/2030	104/18903	0.0000098	0.002374065	0.002285341	AGK/AIFM1/AIP/ARIH2/CSNK2A2/HAX1/HSPA1L/LRRK2/MFF/MIPEP/NDUFA13/PAM16/PITRM1/PMPCA/SREBF1/TIMM10/TIMM13/TIMM17A/TIMM50/TIMM8A/TOMM22/TOMM34/TOMM40/TOMM40L/TRMT10B/USP36/VPS11	27
cluster2	BP	GO:0042273	ribosomal large subunit biogenesis	22/2030	76/18903	0.00000992	0.002374065	0.002285341	BOP1/DDX28/FASTKD2/NIFK/NOC2L/NOP2/NOP53/NVL/PAK1IP1/PES1/PPAN/RPF2/RPL10/RPL23A/RPL5/RPL7A/RPLP0/RRS1/RSL24D1/SPATA5L1/WDR12/ZNF622	22

cluster2	BP	GO:0008033	tRNA processing	32/2030	134/18903	0.0000103	0.002374065	0.002285341	AARS1/AARS2/ANKRD16/CTU1/CTU2/DUS2/ELAC1/ELP1/ELP2/ELP5/LCMT2/MOCS3/NAT10/NSUN2/POP1/POP5/PRORP/PUS1/PUS3/QTRT1/RPP14/RPP40/SARS1/THUMPD3/TRIT1/TRMT10B/TRMT2A/TRMT2B/TRMT5/TSEN15/TYW1B/WDR6	32
cluster2	BP	GO:0000478	endonucleolytic cleavage involved in rRNA processing	9/2030	16/18903	0.0000106	0.002374065	0.002285341	BMS1/BOP1/KRI1/NOP14/NOP9/RPP40/RPS21/RRS1/TBL3	9
cluster2	BP	GO:0000479	endonucleolytic cleavage of tricistronic rRNA transcript (SSU-rRNA, 5.8S rRNA, LSU-rRNA)	9/2030	16/18903	0.0000106	0.002374065	0.002285341	BMS1/BOP1/KRI1/NOP14/NOP9/RPP40/RPS21/RRS1/TBL3	9
cluster2	BP	GO:0006418	tRNA aminoacylation for protein translation	15/2030	41/18903	0.0000116	0.002531051	0.00243646	AARS1/AARS2/AARSD1/EARS2/FARSB/HARS1/IARS1/IARS2/LARS2/MARS2/NARS2/SARS1/SARS2/TARS2/YARS1	15

cluster2	BP	GO:0006283	transcription-coupled nucleotide-excision repair	7/2030	10/18903	0.0000146	0.003103246	0.00298727	ERCC2/ERCC3/ERCC5/ERCC8/POLR2I/USP7/XAB2	7
cluster2	BP	GO:0009615	response to virus	72/2030	409/18903	0.0000165	0.003408348	0.00328097	ABCF3/ADARB1/AIM2/APOBEC3A/APOBEC3B/APOBEC3H/ARMC5/ATG16L1/AUP1/BIRC3/C1QBP/CASP1/CHUK/CLPB/CLU/CXCR4/DDIT4/DDX56/DHX58/DHX9/EEF1G/ELMOD2/EXOC1/FOXP3/G3BP1/GPATCH3/HERC5/IFI16/IFI44/IFIH1/IFIT1/IFIT2/IFIT3/IFIT5/IFNG/IKBKE/IL1B/IL23A/IL4/MICA/MICB/NCBP1/NLRP1/NLRP3/NMI/NOP53/PDE12/PIM2/PLA2G10/POLR3B/POLR3D/POLR3H/POU2AF1/PSMA2/SAP30BP/SHFL/SKP2/STAT2/STMN1/TBK1/TLR3/TLR7/TLR8/TRIM22/TRIM26/TRIM56/TRIM6/UNC93B1/WDFY4/YJU2B/ZCCHC3/ZNFX1	72
cluster2	BP	GO:0006405	RNA export from nucleus	24/2030	90/18903	0.0000185	0.003666593	0.003529564	ALKBH5/ALYREF/CHTOP/DDX19B/DHX9/MCM3AP/NCBP1/NOL6/NRDE2/NSUN2/NUP160/NUP62/NUP93/NXF1/NXT1/PCID2/POLDIP3/RAE1/RBM15B/SARNP/SUPT6H/THOC1/THOC6/XPO5	24
cluster2	BP	GO:0008380	RNA splicing	80/2030	469/18903	0.0000187	0.003666593	0.003529564	ALYREF/ARL6IP4/BUD13/C1QBP/CELF1/CIR1/CLK4/CRNKL1/CTNNBL1/CWC15/CWC25/DAZAP1/DCPS/DDX20/DDX23/DDX41/DHX35/DHX38/DHX8/DHX9/FRG1/GEMIN4/HNRNPA1L2/HNRNPF/HNRNPH1/HNRNPM/KAT2A/METTL4/MPHOSPH10/NCBP1/NOL3/NRDE2/NUP98/PDCD7/PPIE/PPP2R1A/PRMT5/PRMT7/PRPF3/PRPF38B/PRPF4/PRPF8/PRX/PUS1/RBM10/RBM12/RBM14/RBM15B/RBM17/RBM19/RBM7/RBMX2/REST/RNPS1/SGF29/SNIP1/SNRNP200/SNRNP70/SNRPD3/SRRM1/SRSF10/SRSF11/SRSF2/SUPT20H/SUPT6H/TAF6L/TAF9/TARDBP/TFIP11/THOC1/THOC6/THRAP3/TRA2A/TSEN15/TTF2/U2AF1/XAB2/YJU2B/ZPR1/ZRANB2	80

cluster2	BP	GO:0051607	defense response to virus	57/2030	304/18903	0.0000194	0.003666593	0.003529564	ABCF3/ADARB1/AIM2/APOBEC3A/APOBEC3B/APOBEC3H/ARMC5/ATG16L1/BIRC3/C1QBP/CASP1/CLPB/DDIT4/DDX56/DHX58/DHX9/ELMOD2/EXOC1/FOXP3/G3BP1/GPATCH3/HERC5/IFI16/IFIH1/IFIT1/IFIT2/IFIT3/IFIT5/IFNG/IKBKE/IL1B/IL23A/IL4/MICA/MICB/NCBP1/NLRP1/NOP53/PDE12/PLA2G10/POLR3B/POLR3D/POLR3H/SHFL/SKP2/STAT2/TBK1/TLR3/TLR7/TLR8/TRIM22/TRIM26/TRIM56/TRIM6/UNC93B1/ZCCHC3/ZNFX1	57
cluster2	BP	GO:0006403	RNA localization	42/2030	202/18903	0.0000196	0.003666593	0.003529564	ALKBH5/ALYREF/ATM/CCT3/CCT4/CCT8/CHTOP/CKAP5/DDX19B/DHX9/HNRNPA1L2/MCM3AP/NCBP1/NOL6/NPIPA1/NRDE2/NSUN2/NUP160/NUP35/NUP37/NUP62/NUP93/NUP98/NXF1/NXT1/PCID2/PEG10/PIH1D1/PNPT1/POLDIP3/RAE1/RBM15B/SARNP/SHQ1/SUPT6H/TERF1/THOC1/THOC6/TNKS/WRAP53/XPO5/ZC3H3	42
cluster2	BP	GO:0140546	defense response to symbiont	57/2030	305/18903	0.0000215	0.003928015	0.003781215	ABCF3/ADARB1/AIM2/APOBEC3A/APOBEC3B/APOBEC3H/ARMC5/ATG16L1/BIRC3/C1QBP/CASP1/CLPB/DDIT4/DDX56/DHX58/DHX9/ELMOD2/EXOC1/FOXP3/G3BP1/GPATCH3/HERC5/IFI16/IFIH1/IFIT1/IFIT2/IFIT3/IFIT5/IFNG/IKBKE/IL1B/IL23A/IL4/MICA/MICB/NCBP1/NLRP1/NOP53/PDE12/PLA2G10/POLR3B/POLR3D/POLR3H/SHFL/SKP2/STAT2/TBK1/TLR3/TLR7/TLR8/TRIM22/TRIM26/TRIM56/TRIM6/UNC93B1/ZCCHC3/ZNFX1	57
cluster2	BP	GO:1990542	mitochondrial transmembrane transport	24/2030	91/18903	0.0000226	0.004047018	0.003895771	ABCB8/AFG3L2/AIFM1/CPT1B/MPC2/PAM16/PNPT1/SFXN1/SFXN4/SLC25A12/SLC25A13/SLC25A20/SLC25A22/SLC25A3/SLC25A32/SLC25A51/SLC25A6/SLC41A3/TIMM17A/TIMM50/TOMM40/TOMM40L/UCP1/UCP3	24

cluster2	BP	GO:0051236	establishment of RNA localization	36/2030	166/18903	0.0000292	0.005115236	0.004924068	ALKBH5/ALYREF/ATM/CHTOP/CKAP5/DDX19B/DHX9/HNRNPA1L2/MCM3AP/NCBP1/NOL6/NPIPA1/NRDE2/NSUN2/NUP160/NUP35/NUP37/NUP62/NUP93/NUP98/NXF1/NXT1/PCID2/PEG10/PNPT1/POLDIP3/RAE1/RBM15B/SARNP/SUPT6H/TERF1/THOC1/THOC6/TNKS/XPO5/ZC3H3	36
cluster2	BP	GO:0006400	tRNA modification	24/2030	93/18903	0.0000334	0.005709559	0.005496179	AARS1/AARS2/ANKRD16/CTU1/CTU2/DUS2/ELP1/ELP2/ELP5/LCMT2/MOCS3/NAT10/NSUN2/PUS1/PUS3/QTRT1/THUMPD3/TRIT1/TRMT10B/TRMT2A/TRMT2B/TRMT5/TYW1B/WDR6	24
cluster2	BP	GO:0006520	cellular amino acid metabolic process	54/2030	289/18903	0.0000351	0.005877364	0.005657713	AARS1/AARS2/AARSD1/AASDH/ACAT1/ADSS2/ALDH8A1/AMDHD1/ARHGAP11B/ASNS/BPHL/CAD/DBT/DPYD/EARS2/FARSB/FPGS/GART/GATB/GATC/GCDH/GCLC/GLS2/GLUD1/GOT1/GSTZ1/HARS1/HDC/HIBCH/IARS1/IARS2/ICMT/IL4I1/ILVBL/IVD/LARS2/MARS2/MMUT/MRI1/MTHFD1/NARS2/NOXRED1/PCBD2/QDPR/SARS1/SARS2/SDS/SHMT1/SHMT2/SLC25A12/SLC25A13/SRR/TARS2/YARS1	54
cluster2	BP	GO:0006354	DNA-templated transcription elongation	47/2030	241/18903	0.0000367	0.006025332	0.005800151	AIP/ALYREF/CNOT2/DHX9/DPF2/EAPP/ELL3/ELOA/ELP2/ERCC2/ERCC3/EWSR1/INTS10/INTS14/INTS6/INTS7/KAT5/KDM2B/LDB1/MED19/MED22/MED23/MED24/MED25/MED31/MED6/MED9/NCBP1/NMI/PCID2/PHF10/PHF8/PIAS3/PIAS4/POLR2I/POU2AF1/SAP30L/SND1/SUPT20H/SUPT6H/TCEA1/TDG/THOC1/THRAP3/UXT/ZC3H4/ZNF410	47

cluster2	BP	GO:0006406	mRNA export from nucleus	19/2030	67/18903	0.0000538	0.00863932	0.008316448	ALKBH5/ALYREF/CHTOP/DDX19B/MCM3AP/NCBP1/NSUN2/NUP160/NUP93/NXF1/NXT1/PCID2/POLDIP3/RAE1/RBM15B/SARNP/SUPT6H/THOC1/THOC6	19
cluster2	BP	GO:0090501	RNA phosphodiester bond hydrolysis	34/2030	158/18903	0.0000567	0.008930914	0.008597145	APEX1/BMS1/BOP1/CNOT2/CPSF1/CPSF3/DCPS/DIS3L2/ELAC1/EXOSC3/HELZ2/ISG20L2/KRI1/NCBP1/NOB1/NOP14/NOP9/PAN2/PDE12/PNPT1/POLR1H/POLR2I/POP1/POP5/PRORP/RNASEH2A/RPP14/RPP40/RPS21/RRS1/SND1/TBL3/YBEY/ZC3H12D	34
cluster2	BP	GO:0000028	ribosomal small subunit assembly	9/2030	19/18903	0.0000631	0.009737824	0.009373899	ERAL1/LOC102724159/MRPS11/MRPS7/PRKDC/RPS15/RPS28/RPS5/RRP7A	9
cluster2	BP	GO:0031647	regulation of protein stability	58/2030	325/18903	0.0000713	0.010566217	0.010171332	AAK1/ATP1B2/AURKA/CAPN3/CCDC88C/CCT3/CCT4/CCT8/CDC37/CDK7/CHFR/CLU/COA8/CREBL2/DDOST/DVL1/EPHA4/GGA3/GNL3L/HCFC1/HYPK/KAT2A/LRRK2/LSS/MCM8/NF2/NOP53/P3H1/PDCL3/PDRG1/PEX2/PFDN5/PFDN6/PFN1/PIH1D1/PIM2/PINX1/PRKDC/RPL5/SEL1L/SMAD7/SREBF1/STUB1/TAF9/TARDBP/TELO2/TMEM183A/TNIP2/TRIM39/USP19/USP3/USP36/USP7/UTP25/UXT/VPS11/WDR81/WNT10B	58

cluster2	BP	GO:0009303	rRNA transcription	13/2030	37/18903	0.0000722	0.010566217	0.010171332	ERCC2/GTF3C1/GTF3C2/IPPK/NIFK/NOL11/NOP53/PIH1D1/POLR1B/POLR1G/PWP1/SMARCA4/TAF1B	13
cluster2	BP	GO:0002381	immunoglobulin production involved in immunoglobulin-mediated immune response	20/2030	74/18903	0.0000731	0.010566217	0.010171332	ERCC1/EXOSC3/EXOSC6/FOXP3/HLA-DMB/HLA-DOA/HLA-DOB/HLA-DPA1/HLA-DPB1/HLA-DQB1/HLA-DRB1/HMCES/IL4/NSD2/PARP3/RIF1/SUPT6H/TFRC/THOC1/UNG	20
cluster2	BP	GO:0006304	DNA modification	29/2030	128/18903	0.0000738	0.010566217	0.010171332	ALKBH2/ALKBH3/ALKBH4/APEX1/APOBEC3A/APOBEC3B/APOBEC3H/DMAP1/DPPA3/EHMT1/EXOSC3/EXOSC6/FTO/HEMK1/KDM1B/KMT2E/MBD4/METTL4/N6AMT1/NEIL1/PARP3/PICK1/PRMT5/PRMT7/TDG/UNG/USP7/ZDBF2/ZFP57	29
cluster2	BP	GO:0098781	ncRNA transcription	18/2030	63/18903	0.0000753	0.010589282	0.010193536	CDK7/ELL3/ERCC2/GTF3C1/GTF3C2/IPPK/NFATC2/NIFK/NOL11/NOP53/PIH1D1/POLR1B/POLR1G/PWP1/SMARCA4/SNAPC3/TAF1B/ZC3H4	18

cluster2	BP	GO:0000469	cleavage involved in rRNA processing	11/2030	28/18903	0.0000805	0.011114595	0.010699216	BMS1/BOP1/EXOSC3/KRI1/NOB1/NOP14/NOP9/RPP40/RPS21/RRS1/TBL3	11
cluster2	BP	GO:0072655	establishment of protein localization to mitochondrion	28/2030	123/18903	0.0000893	0.012116336	0.01166352	AGK/AIFM1/AIP/ARIH2/CSNK2A2/HAX1/HSPA1L/LRRK2/MFF/MIPEP/MOAP1/NDUFA13/PAM16/PITRM1/PMPCA/SREBF1/TIMM10/TIMM13/TIMM17A/TIMM50/TIMM8A/TOMM22/TOMM34/TOMM40/TOMM40L/TRMT10B/USP36/VPS11	28
cluster2	BP	GO:0051028	mRNA transport	29/2030	130/18903	0.0000991	0.013216259	0.012722336	ALKBH5/ALYREF/CHTOP/DDX19B/DHX9/HNRNPA1L2/MCM3AP/NCBP1/NPIPA1/NSUN2/NUP160/NUP35/NUP37/NUP62/NUP93/NUP98/NXF1/NXT1/PCID2/PEG10/POLDIP3/RAE1/RBM15B/SARNP/SUPT6H/THOC1/THOC6/TNKS/ZC3H3	29
cluster2	BP	GO:0050657	nucleic acid transport	34/2030	163/18903	0.000108796	0.014038205	0.013513564	ALKBH5/ALYREF/CHTOP/CKAP5/DDX19B/DHX9/HNRNPA1L2/MCM3AP/NCBP1/NOL6/NPIPA1/NRDE2/NSUN2/NUP160/NUP35/NUP37/NUP62/NUP93/NUP98/NXF1/NXT1/PCID2/PEG10/PNPT1/POLDIP3/RAE1/RBM15B/SARNP/SUPT6H/THOC1/THOC6/TNKS/XPO5/ZC3H3	34

cluster2	BP	GO:0050658	RNA transport	34/2030	163/18903	0.000108796	0.014038205	0.013513564	ALKBH5/ALYREF/CHTOP/CKAP5/DDX19B/DHX9/HNRNPA1L2/MCM3AP/NCBP1/NOL6/NPIPA1/NRDE2/NSUN2/NUP160/NUP35/NUP37/NUP62/NUP93/NUP98/NXF1/NXT1/PCID2/PEG10/PNPT1/POLDIP3/RAE1/RBM15B/SARNP/SUPT6H/THOC1/THOC6/TNKS/XPO5/ZC3H3	34
cluster2	BP	GO:0072594	establishment of protein localization to organelle	74/2030	449/18903	0.000117782	0.014952628	0.014393813	AGK/AIFM1/AIP/ARIH2/CCT3/CCT4/CCT8/CLU/CRY2/CSNK2A2/DMAP1/EIF4ENIF1/ELAVL1/GGA3/HAX1/HSPA1L/IFNG/IPO4/IPO8/IPO9/LAMP3/LRRK2/MFF/MIPEP/MOAP1/NDUFA13/NUP35/NUP62/NUP93/NUP98/NXT1/PAM16/PEX12/PEX14/PEX2/PEX26/PEX7/PIH1D1/PIK3C3/PIK3R4/PITRM1/PMPCA/RANBP6/RGPD5/RGPD8/SCRIB/SEC61A2/SGF29/SREBF1/SRP14/SRP19/SRP54/SRP68/SRPRB/SSR3/TARDBP/TERF1/TIMM10/TIMM13/TIMM17A/TIMM50/TIMM8A/TOMM22/TOMM34/TOMM40/TOMM40L/TRMT10B/TSPAN17/USP36/VPS11/VPS13D/VPS4A/WRAP53/ZPR1	74
cluster2	BP	GO:0032259	methylation	66/2030	390/18903	0.000123307	0.015405545	0.014829804	ANTKMT/ARMT1/ASH2L/ASMT/ASMTL/CHTOP/CMTR1/CMTR2/COPRS/COQ3/CXXC1/DMAP1/DPPA3/EEF2KMT/EHMT1/EMG1/GAMT/HCFC1/HEMK1/HENMT1/ICMT/KDM1B/KMT2D/KMT2E/LCMT2/MCRS1/MECOM/MEN1/METTL4/METTL9/MRM1/N6AMT1/NOP2/NSD2/NSUN2/NSUN7/PAX5/PHF19/PICK1/PIH1D1/PRDM15/PRMT5/PRMT6/PRMT7/PRMT9/PWP1/RBBP5/RBM15B/RIF1/SETD4/SETDB1/SMAD4/SMYD4/SNRPD3/SUPT6H/SUV39H1/SUZ12/THUMPD3/TPMT/TRMT10B/TRMT2A/TRMT2B/TRMT5/WDR6/ZDBF2/ZFP57	66
cluster2	BP	GO:0006605	protein targeting	57/2030	325/18903	0.000132973	0.016353634	0.01574246	AGK/AIFM1/AIP/AKT2/AP4B1/AP4E1/ARIH2/CDC37/CHM/CLU/CSNK2A2/GGA3/GOLGA7B/HAX1/HSPA1L/ICMT/ITGB1BP1/LRRK2/MFF/MIEF2/MIPEP/NDUFA13/PAM16/PEX12/PEX14/PEX2/PEX26/PEX7/PIK3C3/PIK3R4/PITRM1/PMPCA/SEC61A2/SREBF1/SRP14/SRP19/SRP54/SRP68/SRPRB/SSR2/SSR3/TIMM10/TIMM13/TIMM17A/TIMM50/TIMM8A/TOMM22/TOMM34/TOMM40/TOMM40L/TRMT10B/USP36/VPS11/VPS13D/VPS4A/ZDHHC12/ZDHHC23	57

cluster2	BP	GO:0016570	histone modification	77/2030	474/18903	0.000139271	0.016864624	0.016234353	ACTL6A/APBB1/ASH2L/BRPF1/C6orf89/CHTOP/COPRS/CXXC1/DMAP1/EHMT1/FOXP3/HCFC1/HDAC1/HDAC10/HDAC11/HPF1/ING4/KAT14/KAT2A/KAT5/KDM1B/KDM2B/KDM5B/KMT2D/KMT2E/LDB1/LRRK2/MCM3AP/MCRS1/MEAF6/MECOM/MED24/MEN1/MTA1/MYBBP1A/N6AMT1/NAA40/NOC2L/NSD2/PAX5/PHF19/PHF8/PIH1D1/PRMT5/PRMT6/PRMT7/PWP1/RAD51/RAG1/RBBP4/RBBP5/RBM14/REST/RIF1/RIOX2/SAP30BP/SETD4/SETDB1/SGF29/SIN3B/SMAD4/SPHK2/SREBF1/SUPT20H/SUPT6H/SUV39H1/SUZ12/TAF6L/TAF9/TRIM16/TRIM37/UFL1/UHRF1/USP21/USP3/USP36/USP7	77
cluster2	BP	GO:0000466	maturation of 5.8S rRNA from tricistronic rRNA transcript (SSU-rRNA, 5.8S rRNA, LSU-rRNA)	10/2030	25/18903	0.00014249	0.01699298	0.016357912	BOP1/EXOSC3/KRI1/NOP14/NOP9/PES1/RPP40/RPS21/RRS1/WDR12	10
cluster2	BP	GO:0106074	aminoacyl-tRNA metabolism involved in translational fidelity	7/2030	13/18903	0.000155617	0.01828148	0.017598258	AARS1/AARS2/AARSD1/IARS1/IARS2/LARS2/TARS2	7
cluster2	BP	GO:0006450	regulation of translational fidelity	9/2030	21/18903	0.000164376	0.019026488	0.018315423	AARS1/AARS2/AARSD1/GATC/IARS1/IARS2/LARS2/RPS5/TARS2	9

cluster2	BP	GO:0070585	protein localization to mitochondrion	28/2030	128/18903	0.00018436	0.021030392	0.020244437	AGK/AIFM1/AIP/ARIH2/CSNK2A2/HAX1/HSPA1L/LRRK2/MFF/MIPEP/MOAP1/NDUFA13/PAM16/PITRM1/PMPCA/SREBF1/TIMM10/TIMM13/TIMM17A/TIMM50/TIMM8A/TOMM22/TOMM34/TOMM40/TOMM40L/TRMT10B/USP36/VPS11	28
cluster2	BP	GO:0046434	organophosphate catabolic process	33/2030	161/18903	0.000192762	0.021674738	0.020864702	ABHD12/ACAT1/BPNT1/DCTPP1/DNPH1/DPYD/DUT/ENPP3/FITM2/GPCPD1/IDH1/INPP5F/ITPA/MBD4/MLYCD/NAPEPLD/NEIL1/NUDT13/NUDT19/NUDT4/PDE8A/PDXP/PLA2G10/PLCG1/PLD2/PLPP6/PNPLA8/SARM1/SMPD4/SUCLG2/SYNJ2/TDG/UNG	33
cluster2	BP	GO:0046488	phosphatidylinositol metabolic process	33/2030	162/18903	0.00021756	0.023828972	0.022938427	ATM/BMX/BPNT1/CDIPT/FIG4/GPAA1/INPP5F/IPMK/ITPKB/ITPKC/MTMR10/MTMR11/MTMR4/MTMR6/PGAP3/PI4K2B/PI4KB/PIGL/PIGM/PIGT/PIGV/PIGW/PIK3C3/PIK3CG/PIK3R4/PIP4K2C/PLCB2/PLCD1/PLCG1/SOCS5/SYNJ2/TMEM150A/VAC14	33
cluster2	BP	GO:0000447	endonucleolytic cleavage in ITS1 to separate SSU-rRNA from 5.8S rRNA and LSU-rRNA from tricistronic rRNA transcript (SSU-rRNA, 5.8S rRNA, LSU-rRNA)	6/2030	10/18903	0.000217976	0.023828972	0.022938427	KRI1/NOP14/NOP9/RPP40/RPS21/RRS1	6

cluster2	BP	GO:0008654	phospholipid biosynthetic process	47/2030	259/18903	0.000222903	0.024033843	0.023135641	ABHD5/ATM/BMX/BPNT1/CDIPT/CEPT1/CHKA/DGKA/DGKQ/FIG4/FITM2/GGPS1/GNPAT/GPAA1/IDH1/INPP5F/IPMK/ISYNA1/ITPKB/ITPKC/MBOAT1/MTMR4/MTMR6/ORMDL1/PCYT2/PGAP3/PI4K2B/PI4KB/PIGL/PIGM/PIGT/PIGV/PIGW/PIK3C3/PIK3CG/PIK3R4/PIP4K2C/PLD2/PPARD/PTDSS1/PTPMT1/SGMS2/SOCS5/SPHK2/SYNJ2/TMEM150A/VAC14	47
cluster2	BP	GO:0090502	RNA phosphodiester bond hydrolysis, endonucleolytic	21/2030	86/18903	0.000234862	0.024981058	0.024047457	APEX1/BMS1/BOP1/CPSF3/ELAC1/KRI1/NOB1/NOP14/NOP9/POP1/POP5/PRORP/RNASEH2A/RPP14/RPP40/RPS21/RRS1/SND1/TBL3/YBEY/ZC3H12D	21
cluster2	BP	GO:0071806	protein transmembrane transport	17/2030	64/18903	0.000311442	0.032684806	0.031463298	AIFM1/AKT2/BCR/HPSE/PAM16/PEX12/PEX14/PEX2/PEX26/PEX7/SEC61A2/SRP54/TIMM17A/TIMM50/TOMM22/TOMM40/TOMM40L	17
cluster2	BP	GO:0006401	RNA catabolic process	52/2030	299/18903	0.000315879	0.032714273	0.031491663	ALKBH5/APEX1/ATM/CELF1/CNOT2/CNOT4/DAZ1/DCPS/DDX49/DHX9/DIS3L2/DND1/EDC3/EIF4ENIF1/ELAVL1/EXOSC3/EXOSC6/FASTKD1/FASTKD2/FASTKD3/FASTKD5/FTO/HELZ2/HNRNPM/IKBKE/NCBP1/NRDE2/NSUN2/PABPC4/PAN2/PCID2/PDE12/PNPT1/POP1/PYM1/RBM10/RBM7/RNASEH2A/RNH1/RNPS1/SERBP1/SND1/SUPV3L1/TARDBP/TBRG4/TENT4A/THRAP3/TNRC6B/ZC3H12D/ZC3H4/ZHX2/ZPR1	52

cluster2	BP	GO:0080111	DNA demethylation	11/2030	32/18903	0.000322279	0.032846817	0.031619253	ALKBH2/ALKBH3/ALKBH4/APEX1/APOBEC3A/APOBEC3B/APOBEC3H/DPPA3/FTO/TDG/USP7	11
cluster2	BP	GO:0065002	intracellular protein transmembrane transport	15/2030	53/18903	0.000328641	0.032846817	0.031619253	AIFM1/AKT2/BCR/PAM16/PEX12/PEX14/PEX2/PEX26/PEX7/SEC61A2/SRP54/TIMM17A/TIMM50/TOMM40/TOMM40L	15
cluster2	BP	GO:0006913	nucleocytoplasmic transport	56/2030	329/18903	0.000333852	0.032846817	0.031619253	AHCYL1/ALKBH5/ALYREF/ANKRD54/BACH2/CHTOP/CRY2/DDX19B/DHX9/DMAP1/EIF4ENIF1/ELAVL1/IFNG/IL1B/IPO4/IPO8/IPO9/LRRK2/LSG1/MCM3AP/NCBP1/NOL6/NOP9/NRDE2/NSUN2/NUP160/NUP35/NUP37/NUP62/NUP93/NUP98/NXF1/NXT1/PCID2/PKD1/POLDIP3/RAE1/RANBP6/RANGAP1/RBM15B/RGPD5/RGPD8/RIOK2/RITA1/RPS15/RRS1/SARNP/STYX/SUPT6H/TARDBP/THOC1/THOC6/TXN/UBE2I/XPO5/ZPR1	56
cluster2	BP	GO:0051169	nuclear transport	56/2030	329/18903	0.000333852	0.032846817	0.031619253	AHCYL1/ALKBH5/ALYREF/ANKRD54/BACH2/CHTOP/CRY2/DDX19B/DHX9/DMAP1/EIF4ENIF1/ELAVL1/IFNG/IL1B/IPO4/IPO8/IPO9/LRRK2/LSG1/MCM3AP/NCBP1/NOL6/NOP9/NRDE2/NSUN2/NUP160/NUP35/NUP37/NUP62/NUP93/NUP98/NXF1/NXT1/PCID2/PKD1/POLDIP3/RAE1/RANBP6/RANGAP1/RBM15B/RGPD5/RGPD8/RIOK2/RITA1/RPS15/RRS1/SARNP/STYX/SUPT6H/TARDBP/THOC1/THOC6/TXN/UBE2I/XPO5/ZPR1	56

cluster2	BP	GO:0000966	RNA 5'-end processing	9/2030	23/18903	0.000374373	0.036378909	0.035019343	NOP14/NOP9/PNPT1/POP1/POP5/PRORP/RPP14/RPP40/TBL3	9
cluster2	BP	GO:0006661	phosphatidylinositol biosynthetic process	28/2030	134/18903	0.000410244	0.039039661	0.037580657	ATM/BMX/BPNT1/CDIPT/FIG4/GPAA1/INPP5F/IPMK/ITPKB/ITPKC/MTMR4/MTMR6/PGAP3/PI4K2B/PI4KB/PIGL/PIGM/PIGT/PIGV/PIGW/PIK3C3/PIK3CG/PIK3R4/PIP4K2C/SOCS5/SYNJ2/TMEM150A/VAC14	28
cluster2	BP	GO:0015931	nucleobase-containing compound transport	41/2030	223/18903	0.000411675	0.039039661	0.037580657	ALKBH5/ALYREF/CHTOP/CKAP5/DDX19B/DHX9/EPG5/HNRNPA1L2/MCM3AP/NCBP1/NOL6/NPIPA1/NRDE2/NSUN2/NUP160/NUP35/NUP37/NUP62/NUP93/NUP98/NXF1/NXT1/PCID2/PEG10/PNPT1/POLDIP3/RAE1/RBM15B/SARNP/SLC25A32/SLC25A51/SLC25A6/SLC35A4/SLC35B2/SLC35C2/SUPT6H/THOC1/THOC6/TNKS/XPO5/ZC3H3	41
cluster2	BP	GO:0032434	regulation of proteasomal ubiquitin-dependent protein catabolic process	29/2030	141/18903	0.000430885	0.040374939	0.038866032	ARAF/ARIH1/ARIH2/AURKA/CHFR/CLU/COMMD1/DVL1/GCLC/HSPBP1/LRRK2/MKRN2/NOP53/PABIR1/RNF14/RNF144B/SMAD7/SOCS5/STUB1/STYX/TAF9/TRIB2/TRIM39/UBQLN2/UFL1/USP14/USP5/USP7/WNT10B	29

cluster2	BP	GO:0043414	macromolecule methylation	58/2030	348/18903	0.000458812	0.042486018	0.040898215	ANTKMT/ARMT1/ASH2L/CHTOP/CMTR1/CMTR2/COPRS/CXXC1/DMAP1/DPPA3/EEF2KMT/EHMT1/EMG1/HCFC1/HEMK1/HENMT1/ICMT/KDM1B/KMT2D/KMT2E/LCMT2/MCRS1/MECOM/MEN1/METTL4/METTL9/MRM1/N6AMT1/NOP2/NSD2/NSUN2/PAX5/PHF19/PICK1/PIH1D1/PRMT5/PRMT6/PRMT7/PRMT9/PWP1/RBBP5/RBM15B/RIF1/SETD4/SETDB1/SMAD4/SNRPD3/SUPT6H/SUV39H1/SUZ12/THUMPD3/TRMT10B/TRMT2A/TRMT2B/TRMT5/WDR6/ZDBF2/ZFP57	58
cluster2	BP	GO:2000058	regulation of ubiquitin-dependent protein catabolic process	33/2030	170/18903	0.000542838	0.049682338	0.047825591	ARAF/ARIH1/ARIH2/AURKA/CHFR/CLU/COMMD1/CSNK2A2/DISC1/DVL1/GCLC/HSPBP1/LRRK2/MKRN2/NOP53/PABIR1/PDCL3/RNF14/RNF144B/RPL5/SMAD7/SOCS5/STUB1/STYX/TAF9/TRIB2/TRIM39/UBQLN2/UFL1/USP14/USP5/USP7/WNT10B	33
cluster2	CC	GO:0005759	mitochondrial matrix	108/2104	483/19869	2.58E-14	2.79E-11	2.56E-11	ACADVL/ACAT1/ACO2/ACOT13/ACOT2/ACSS2/ALDH1B1/ARHGAP11B/BCO2/C1QBP/CA5B/CCNB1/COQ3/DBT/DDX28/EARS2/ERAL1/ETFDH/FASTKD1/FASTKD2/FASTKD3/FASTKD5/FH/FPGS/GCDH/GFM1/GLS2/GLUD1/GSTZ1/GUF1/HADHA/HIBCH/HSPA1L/IARS2/IBA57/IDH2/IVD/LARS2/LIAS/LONP1/LRRC59/LRRK2/LYRM4/LYRM7/MARS2/MCEE/ME3/METTL4/MIPEP/MLYCD/MMUT/MRM1/MRPL10/MRPL11/MRPL12/MRPL15/MRPL17/MRPL21/MRPL30/MRPL33/MRPL34/MRPL38/MRPL39/MRPL44/MRPL54/MRPS10/MRPS11/MRPS15/MRPS17/MRPS18B/MRPS18C/MRPS31/MRPS5/MRPS7/MRPS9/MTERF1/MTG1/NARS2/NAXD/NDUFA10/NUDT1/NUDT13/NUDT2/PAM16/PDE12/PDP2/PIN4/PITRM1/PMPCA/PNPT1/PRORP/PTCD3/PUS1/RAD51/RPS3/SARS2/SCO2/SHMT2/SUCLG2/SUPV3L1/TARS2/TBRG4/THEM4/TK2/TRAP1/TRIT1/TRMT5/TWNK	108
cluster2	CC	GO:0005743	mitochondrial inner membrane	107/2104	498/19869	5.22E-13	2.82E-10	2.59E-10	ABCB8/ACAD9/ACADVL/AFG3L2/AGK/AIFM1/AIFM3/ATP5MC1/ATP5PO/C12orf73/C15orf48/CKMT2/CLU/COA8/COQ10A/COQ3/COQ8A/COX20/COX7A2/CPOX/CYC1/CYP2U1/DELE1/DMAC1/DNAJC11/DUSP18/ERAL1/ETFDH/FOXRED1/FPGS/GUF1/HADHA/HCCS/LDHB/LETMD1/LRRK2/MICOS10/MICOS13/MPC2/MRPL10/MRPL11/MRPL12/MRPL15/MRPL17/MRPL21/MRPL30/MRPL33/MRPL34/MRPL38/MRPL39/MRPL44/MRPL54/MRPS10/MRPS11/MRPS15/MRPS17/MRPS18B/MRPS18C/MRPS31/MRPS5/MRPS7/MRPS9/MTG1/NDUFA10/NDUFA13/NDUFA4/NDUFB4/NDUFB7/NDUFV1/OMA1/PAM16/PMPCA/PPOX/PTCD3/PTPMT1/RPS3/SCO2/SDHA/SDHC/SFXN1/SFXN4/SHMT2/SLC25A12/SLC25A13/SLC25A20/SLC25A22/SLC25A3/SLC25A32/SLC25A35/SLC25A51/SLC25A6/SLC41A3/SPHK2/THEM4/TIMM10/TIMM13/TIMM17A/TIMM50/TIMM8A/TMEM186/TMEM223/TOMM40/TRAP1/TRMT10B/UCP1/UCP3/UQCC3	107

cluster2	CC	GO:0044391	ribosomal subunit	54/2104	188/19869	4.07E-12	1.46E-09	1.35E-09	MRPL10/MRPL11/MRPL12/MRPL15/MRPL17/MRPL21/MRPL30/MRPL33/MRPL34/MRPL38/MRPL39/MRPL44/MRPL54/MRPS10/MRPS11/MRPS15/MRPS17/MRPS18B/MRPS18C/MRPS31/MRPS5/MRPS7/MRPS9/PTCD3/RPL10/RPL12/RPL13/RPL13A/RPL17/RPL18/RPL18A/RPL19/RPL21/RPL23A/RPL27/RPL27A/RPL29/RPL3/RPL30/RPL36/RPL5/RPL7A/RPL8/RPLP0/RPLP1/RPS15/RPS2/RPS21/RPS25/RPS28/RPS3/RPS5/RPS6/RPS8	54
cluster2	CC	GO:0005840	ribosome	61/2104	232/19869	1.1E-11	2.96E-09	2.72E-09	APEX1/DHX9/MRPL10/MRPL11/MRPL12/MRPL15/MRPL17/MRPL21/MRPL30/MRPL33/MRPL34/MRPL38/MRPL39/MRPL44/MRPL54/MRPS10/MRPS11/MRPS15/MRPS17/MRPS18B/MRPS18C/MRPS31/MRPS5/MRPS7/MRPS9/MTG1/PNPT1/PTCD3/RPL10/RPL12/RPL13/RPL13A/RPL17/RPL18/RPL18A/RPL19/RPL21/RPL23A/RPL27/RPL27A/RPL29/RPL3/RPL30/RPL36/RPL5/RPL7A/RPL8/RPLP0/RPLP1/RPS15/RPS2/RPS21/RPS25/RPS28/RPS3/RPS5/RPS6/RPS8/RRBP1/RSL24D1/SRP68	61
cluster2	CC	GO:0030686	90S preribosome	17/2104	29/19869	3.7E-10	0.00000008	7.35E-08	BMS1/BOP1/IMP3/IMP4/KRI1/LOC102724159/MPHOSPH10/NOC2L/NOL6/NOP14/NOP9/PES1/RRP7A/TBL3/UTP18/WDR12/WDR3	17
cluster2	CC	GO:0030684	preribosome	28/2104	76/19869	1.29E-09	0.000000231	0.000000213	BMS1/BOP1/BYSL/EMG1/IMP3/IMP4/KRI1/LOC102724159/MPHOSPH10/NGDN/NOB1/NOC2L/NOL6/NOP14/NOP56/NOP9/PES1/PPAN/PRKDC/RIOK2/RRP7A/RRS1/TBL3/UTP18/UTP25/WDR12/WDR3/ZNF622	28

cluster2	CC	GO:0015934	large ribosomal subunit	34/2104	115/19869	1.67E-08	0.00000258	0.00000237	MRPL10/MRPL11/MRPL12/MRPL15/MRPL17/MRPL21/MRPL30/MRPL33/MRPL34/MRPL38/MRPL39/MRPL44/MRPL54/RPL10/RPL12/RPL13/RPL13A/RPL17/RPL18/RPL18A/RPL19/RPL21/RPL23A/RPL27/RPL27A/RPL29/RPL3/RPL30/RPL36/RPL5/RPL7A/RPL8/RPLP0/RPLP1	34
cluster2	CC	GO:0022626	cytosolic ribosome	31/2104	105/19869	7.33E-08	0.00000989	0.00000909	MRPS11/RPL10/RPL12/RPL13/RPL13A/RPL17/RPL18/RPL18A/RPL19/RPL21/RPL23A/RPL27/RPL27A/RPL29/RPL3/RPL30/RPL36/RPL5/RPL7A/RPL8/RPLP0/RPLP1/RPS15/RPS2/RPS21/RPS25/RPS28/RPS3/RPS5/RPS6/RPS8	31
cluster2	CC	GO:0098798	mitochondrial protein-containing complex	62/2104	295/19869	9.57E-08	0.0000115	0.0000106	AFG3L2/AGK/ATP5MC1/ATP5PO/C15orf48/COX7A2/CYC1/DBT/DNAJC11/FOXRED1/HADHA/MICOS10/MICOS13/MPC2/MRPL10/MRPL11/MRPL12/MRPL15/MRPL17/MRPL21/MRPL30/MRPL33/MRPL34/MRPL38/MRPL39/MRPL44/MRPL54/MRPS10/MRPS11/MRPS15/MRPS17/MRPS18B/MRPS18C/MRPS31/MRPS5/MRPS7/MRPS9/NDUFA10/NDUFA13/NDUFA4/NDUFB4/NDUFB7/NDUFV1/PAM16/PMPCA/PNPT1/PRORP/PTCD3/SDHA/SDHC/SLC25A6/SUCLG2/SUPV3L1/TIMM10/TIMM13/TIMM17A/TIMM50/TIMM8A/TOMM22/TOMM40/TOMM40L/TRMT10B	62
cluster2	CC	GO:0022625	cytosolic large ribosomal subunit	21/2104	59/19869	0.000000291	0.0000314	0.0000289	RPL10/RPL12/RPL13/RPL13A/RPL17/RPL18/RPL18A/RPL19/RPL21/RPL23A/RPL27/RPL27A/RPL29/RPL3/RPL30/RPL36/RPL5/RPL7A/RPL8/RPLP0/RPLP1	21

cluster2	CC	GO:0000313	organellar ribosome	25/2104	89/19869	0.00000357	0.000321699	0.000295675	MRPL10/MRPL11/MRPL12/MRPL15/MRPL17/MRPL21/MRPL30/MRPL33/MRPL34/MRPL38/MRPL39/MRPL44/MRPL54/MRPS10/MRPS11/MRPS15/MRPS17/MRPS18B/MRPS18C/MRPS31/MRPS5/MRPS7/MRPS9/MTG1/PTCD3	25
cluster2	CC	GO:0005761	mitochondrial ribosome	25/2104	89/19869	0.00000357	0.000321699	0.000295675	MRPL10/MRPL11/MRPL12/MRPL15/MRPL17/MRPL21/MRPL30/MRPL33/MRPL34/MRPL38/MRPL39/MRPL44/MRPL54/MRPS10/MRPS11/MRPS15/MRPS17/MRPS18B/MRPS18C/MRPS31/MRPS5/MRPS7/MRPS9/MTG1/PTCD3	25
cluster2	CC	GO:0032040	small-subunit processome	14/2104	39/19869	0.0000248	0.002058221	0.001891718	EMG1/IMP3/IMP4/LOC102724159/MPHOSPH10/NGDN/NOL6/NOP14/NOP56/PRKDC/TBL3/UTP18/UTP25/WDR3	14
cluster2	CC	GO:0015935	small ribosomal subunit	20/2104	77/19869	0.000110163	0.008498275	0.007810793	MRPS10/MRPS11/MRPS15/MRPS17/MRPS18B/MRPS18C/MRPS31/MRPS5/MRPS7/MRPS9/PTCD3/RPS15/RPS2/RPS21/RPS25/RPS28/RPS3/RPS5/RPS6/RPS8	20

cluster2	CC	GO:0005844	polysome	18/2104	66/19869	0.000121912	0.008612662	0.007915926	DHX9/DIS3L2/HDLBP/MCRS1/MSI2/PNPT1/RPL18/RPL18A/RPL19/RPL30/RPL36/RPL7A/RPL8/RPS21/RPS28/RPS3/RPS6/UNK	18
cluster2	CC	GO:0071013	catalytic step 2 spliceosome	22/2104	90/19869	0.00013547	0.008612662	0.007915926	ALYREF/CRNKL1/CWC15/DDX23/DDX41/DHX35/DHX38/DHX8/FRG1/HNRNPA1L2/HNRNPF/HNRNPH1/HNRNPM/PPIE/PPIL2/PRPF8/SNRNP200/SNRPD3/SRRM1/TFIP11/U2AF1/XAB2	22
cluster2	CC	GO:0005758	mitochondrial intermembrane space	21/2104	84/19869	0.00013557	0.008612662	0.007915926	AGK/AIFM1/AK2/CLPB/COA4/COA6/CPOX/DIABLO/GFER/HAX1/MIX23/NDUFB7/NLN/PNPT1/PPOX/SHMT2/THEM4/TIMM10/TIMM13/TIMM8A/TRAP1	21
cluster2	CC	GO:0042788	polysomal ribosome	11/2104	31/19869	0.000206719	0.01240317	0.011399794	DHX9/PNPT1/RPL18/RPL18A/RPL19/RPL30/RPL36/RPL7A/RPL8/RPS21/RPS28	11

cluster2	CC	GO:0031970	organelle envelope lumen	22/2104	94/19869	0.000265088	0.014660307	0.013474337	AGK/AIFM1/AK2/CLPB/COA4/COA6/CPOX/DIABLO/GFER/HAX1/MIX23/NDUFB7/NLN/PNPT1/PPOX/SHMT2/THEM4/TIMM10/TIMM13/TIMM8A/TRAP1/TUBB	22
cluster2	CC	GO:0000314	organellar small ribosomal subunit	11/2104	32/19869	0.000285062	0.014660307	0.013474337	MRPS10/MRPS11/MRPS15/MRPS17/MRPS18B/MRPS18C/MRPS31/MRPS5/MRPS7/MRPS9/PTCD3	11
cluster2	CC	GO:0005763	mitochondrial small ribosomal subunit	11/2104	32/19869	0.000285062	0.014660307	0.013474337	MRPS10/MRPS11/MRPS15/MRPS17/MRPS18B/MRPS18C/MRPS31/MRPS5/MRPS7/MRPS9/PTCD3	11
cluster2	CC	GO:0031304	intrinsic component of mitochondrial inner membrane	16/2104	61/19869	0.000462171	0.022688382	0.020852967	AFG3L2/AGK/C12orf73/DNAJC11/ETFDH/MICOS10/MICOS13/MPC2/PPOX/SCO2/SFXN1/SFXN4/SLC25A3/TIMM17A/TMEM223/UQCC3	16

cluster2	CC	GO:0009295	nucleoid	13/2104	45/19869	0.000569993	0.025649689	0.023574714	ACADVL/DBT/DDX28/FASTKD2/FASTKD5/HADHA/LONP1/LRRC59/MTERF1/PRORP/SHMT2/SUPV3L1/TWNK	13
cluster2	CC	GO:0042645	mitochondrial nucleoid	13/2104	45/19869	0.000569993	0.025649689	0.023574714	ACADVL/DBT/DDX28/FASTKD2/FASTKD5/HADHA/LONP1/LRRC59/MTERF1/PRORP/SHMT2/SUPV3L1/TWNK	13
cluster2	CC	GO:0005681	spliceosomal complex	36/2104	195/19869	0.000643464	0.027797666	0.025548927	ALYREF/BUD13/CRNKL1/CTNNBL1/CWC15/CWC25/DDX23/DDX41/DHX35/DHX38/DHX8/FRG1/HNRNPA1L2/HNRNPF/HNRNPH1/HNRNPM/PDCD7/PPIE/PPIL2/PRPF3/PRPF38B/PRPF4/PRPF8/RBM17/RBMX2/SNIP1/SNRNP200/SNRNP70/SNRPD3/SRRM1/TFIP11/TRA2A/TTF2/U2AF1/XAB2/YJU2B	36
cluster2	CC	GO:0042613	MHC class II protein complex	7/2104	17/19869	0.001100008	0.044165284	0.040592459	HLA-DMB/HLA-DOA/HLA-DOB/HLA-DPA1/HLA-DPB1/HLA-DQB1/HLA-DRB1	7

cluster2	CC	GO:0031305	integral component of mitochondrial inner membrane	15/2104	60/19869	0.001179644	0.044165284	0.040592459	AFG3L2/AGK/C12orf73/DNAJC11/ETFDH/MICOS10/MICOS13/MPC2/SCO2/SFXN1/SFXN4/SLC25A3/TIMM17A/TMEM223/UQCC3	15
cluster2	CC	GO:0034457	Mpp10 complex	3/2104	3/19869	0.00118592	0.044165284	0.040592459	IMP3/IMP4/MPHOSPH10	3
cluster2	CC	GO:0070545	PeBoW complex	3/2104	3/19869	0.00118592	0.044165284	0.040592459	BOP1/PES1/WDR12	3
cluster2	MF	GO:0140098	catalytic activity, acting on RNA	93/2076	384/18432	4.13E-13	8.25E-10	7.89E-10	AARS1/AARS2/AARSD1/ALKBH3/ALKBH5/APEX1/CMTR1/CMTR2/CNOT2/CPSF3/DCPS/DDX10/DDX19B/DDX20/DDX23/DDX28/DDX3Y/DDX41/DDX42/DDX49/DDX51/DDX52/DDX56/DHX35/DHX37/DHX38/DHX57/DHX58/DHX8/DHX9/DIS3L2/DUS2/EARS2/EIF4A1/ELAC1/EMG1/EXOSC3/FARSB/FTO/G3BP1/GATB/GATC/HARS1/HELZ2/HENMT1/IARS1/IARS2/IFIH1/IGHMBP2/ISG20L2/LARS2/LCMT2/MARS2/METTL4/MRM1/NARS2/NOB1/NOP2/NSUN2/PAN2/PDE12/PNPT1/POLR1A/POLR1B/POLR1H/POLR2I/POLR3B/POLR3H/POP1/POP5/PRORP/PUS1/PUS3/QTRT1/RNASEH2A/RPP14/RPP40/SARS1/SARS2/SND1/SNRNP200/SUPV3L1/TARS2/THUMPD3/TRIT1/TRMT10B/TRMT2A/TRMT2B/TRMT5/TYW1B/YARS1/YBEY/ZC3H12D	93

cluster2	MF	GO:0003735	structural constituent of ribosome	51/2076	176/18432	1.05E-10	0.000000105	0.0000001	MRPL10/MRPL11/MRPL12/MRPL15/MRPL17/MRPL21/MRPL30/MRPL33/MRPL34/MRPL44/MRPL54/MRPS11/MRPS15/MRPS17/MRPS18B/MRPS18C/MRPS31/MRPS5/MRPS7/MRPS9/RPL10/RPL12/RPL13/RPL13A/RPL17/RPL18/RPL18A/RPL19/RPL21/RPL23A/RPL27/RPL27A/RPL29/RPL3/RPL30/RPL36/RPL5/RPL7A/RPL8/RPLP0/RPLP1/RPS15/RPS2/RPS21/RPS25/RPS28/RPS3/RPS5/RPS6/RPS8/RSL24D1	51
cluster2	MF	GO:0019843	rRNA binding	28/2076	67/18432	1.67E-10	0.000000111	0.000000106	DDX28/EMG1/ERAL1/FASTKD2/FASTKD5/IMP3/IMP4/KDM2B/MRPL11/MRPS11/MRPS17/MRPS18C/MRPS7/NOP53/PPAN/PTCD3/RPF2/RPL12/RPL23A/RPL3/RPL5/RPL8/RPLP0/RPS3/RPS5/RRS1/TACO1/UTP25	28
cluster2	MF	GO:0140101	catalytic activity, acting on a tRNA	37/2076	127/18432	3.17E-08	0.0000158	0.0000151	AARS1/AARS2/AARSD1/DUS2/EARS2/ELAC1/FARSB/FTO/GATB/GATC/HARS1/IARS1/IARS2/LARS2/LCMT2/MARS2/NARS2/NSUN2/POP1/POP5/PRORP/PUS1/PUS3/QTRT1/RPP14/RPP40/SARS1/SARS2/TARS2/THUMPD3/TRIT1/TRMT10B/TRMT2A/TRMT2B/TRMT5/TYW1B/YARS1	37
cluster2	MF	GO:0003724	RNA helicase activity	26/2076	77/18432	0.000000139	0.0000557	0.0000533	DDX10/DDX19B/DDX20/DDX23/DDX28/DDX3Y/DDX41/DDX42/DDX49/DDX51/DDX52/DDX56/DHX35/DHX37/DHX38/DHX57/DHX58/DHX8/DHX9/EIF4A1/G3BP1/HELZ2/IFIH1/IGHMBP2/SNRNP200/SUPV3L1	26

cluster2	MF	GO:0000049	tRNA binding	25/2076	73/18432	0.000000177	0.0000589	0.0000563	AARS1/AARS2/CTU1/CTU2/DUS2/EARS2/EEF1A1/EEFSEC/ELP1/ELP5/IARS1/IARS2/IFIT5/IGHMBP2/MTRFR/NAT10/NSUN2/PUS1/SARS1/SARS2/THUMPD3/TRMT10B/TRNAU1AP/XPO5/YARS1	25
cluster2	MF	GO:0008186	ATP-dependent activity, acting on RNA	26/2076	79/18432	0.000000249	0.000071	0.0000679	DDX10/DDX19B/DDX20/DDX23/DDX28/DDX3Y/DDX41/DDX42/DDX49/DDX51/DDX52/DDX56/DHX35/DHX37/DHX38/DHX57/DHX58/DHX8/DHX9/EIF4A1/G3BP1/HELZ2/IFIH1/IGHMBP2/SNRNP200/SUPV3L1	26
cluster2	MF	GO:0004386	helicase activity	39/2076	155/18432	0.000000945	0.000235678	0.000225462	CHD7/DDX10/DDX19B/DDX20/DDX23/DDX28/DDX3Y/DDX41/DDX42/DDX49/DDX51/DDX52/DDX56/DHX35/DHX37/DHX38/DHX57/DHX58/DHX8/DHX9/EIF4A1/ERCC2/ERCC3/G3BP1/HELZ2/IFIH1/IGHMBP2/MCM3/MCM7/MCM8/RAD51/RFC2/SMARCA4/SNRNP200/SUPV3L1/TTF2/TWNK/ZGRF1/ZNFX1	39
cluster2	MF	GO:0043021	ribonucleoprotein complex binding	39/2076	159/18432	0.00000187	0.000415368	0.000397362	BOP1/C1QBP/CBX5/CKAP5/DHX9/EEFSEC/EIF3K/EIF4H/ERAL1/GEMIN4/GUF1/IFIH1/IGHMBP2/LETMD1/MTRFR/NVL/PES1/PRMT5/PRMT7/PTCD3/PYM1/RPLP1/SEC61A2/SERBP1/SHFL/SND1/SPATA5L1/SRP19/SRP54/SRP68/TACO1/TIMM50/TMEM147/TMEM223/UNG/UNK/WDR12/XPO5/ZNF622	39

cluster2	MF	GO:0030515	snoRNA binding	14/2076	32/18432	0.00000332	0.000662232	0.000633525	BMS1/BYSL/DHX37/IMP3/IMP4/NOP14/NOP56/NUDT1/NUDT16L1/NUDT4/PRKDC/TBL3/UTP25/WDR3	14
cluster2	MF	GO:0016741	transferase activity, transferring one-carbon groups	48/2076	225/18432	0.00000899	0.001631888	0.001561146	ANTKMT/ARMT1/ASH2L/ASMT/ASMTL/CAD/CMTR1/CMTR2/COQ3/CXXC1/EEF2KMT/EHMT1/EMG1/GAMT/GART/HEMK1/HENMT1/ICMT/KMT2D/LCMT2/MECOM/METTL4/METTL9/MRM1/N6AMT1/NOP2/NSD2/NSUN2/NSUN7/PRDM15/PRMT5/PRMT6/PRMT7/PRMT9/RBBP5/SETD4/SETDB1/SHMT1/SHMT2/SMYD4/SUV39H1/SUZ12/THUMPD3/TPMT/TRMT10B/TRMT2A/TRMT2B/TRMT5	48
cluster2	MF	GO:0008757	S-adenosylmethionine-dependent methyltransferase activity	37/2076	162/18432	0.0000194	0.002776524	0.002656162	ANTKMT/ARMT1/ASH2L/ASMT/CMTR1/CMTR2/COQ3/CXXC1/EEF2KMT/EHMT1/EMG1/GAMT/HEMK1/ICMT/KMT2D/MECOM/METTL4/METTL9/MRM1/N6AMT1/NOP2/NSD2/NSUN2/PRMT5/PRMT6/PRMT7/PRMT9/RBBP5/SETD4/SETDB1/SUV39H1/THUMPD3/TPMT/TRMT10B/TRMT2A/TRMT2B/TRMT5	37
cluster2	MF	GO:0004812	aminoacyl-tRNA ligase activity	15/2076	41/18432	0.0000206	0.002776524	0.002656162	AARS1/AARS2/AARSD1/EARS2/FARSB/HARS1/IARS1/IARS2/LARS2/MARS2/NARS2/SARS1/SARS2/TARS2/YARS1	15

cluster2	MF	GO:0016875	ligase activity, forming carbon-oxygen bonds	15/2076	41/18432	0.0000206	0.002776524	0.002656162	AARS1/AARS2/AARSD1/EARS2/FARSB/HARS1/IARS1/IARS2/LARS2/MARS2/NARS2/SARS1/SARS2/TARS2/YARS1	15
cluster2	MF	GO:0042393	histone binding	52/2076	258/18432	0.0000209	0.002776524	0.002656162	APBB1/BPTF/BRD9/CBX5/CHAF1B/CHD7/COPRS/CXXC1/DNAJC9/DPF2/DPPA3/HIRA/HPF1/ING4/IPO9/KAT5/KDM1B/KDM5B/KMT2D/KMT2E/L3MBTL2/MCM3AP/NOC2L/PHF10/PHF19/PHF8/PIH1D1/PRMT6/PRMT7/PWP1/RAG1/RBBP4/RBBP5/RCC1/SAP30L/SCML4/SFMBT1/SFMBT2/SGF29/SMARCA4/SPHK2/SPIN2A/SSRP1/SUPT6H/SUZ12/TNKS/TSPYL2/TSPYL4/UHRF1/USP3/WRAP53/ZZEF1	52
cluster2	MF	GO:0016874	ligase activity	37/2076	165/18432	0.0000297	0.003708686	0.003547915	AACS/AARS1/AARS2/AARSD1/AASDH/ACSS2/ADSS2/ASNS/ATP5PO/CAD/DIP2A/EARS2/FARSB/FPGS/GART/GATB/GATC/GCLC/HARS1/IARS1/IARS2/LARS2/MARS2/MOCS3/MTHFD1/NADSYN1/NAPRT/NARS2/SARS1/SARS2/SLC27A2/SUCLG2/TARS2/TTLL5/UBA3/UBA6/YARS1	37
cluster2	MF	GO:0008168	methyltransferase activity	44/2076	212/18432	0.0000421	0.004947508	0.004733034	ANTKMT/ARMT1/ASH2L/ASMT/ASMTL/CMTR1/CMTR2/COQ3/CXXC1/EEF2KMT/EHMT1/EMG1/GAMT/HEMK1/HENMT1/ICMT/KMT2D/LCMT2/MECOM/METTL4/METTL9/MRM1/N6AMT1/NOP2/NSD2/NSUN2/NSUN7/PRDM15/PRMT5/PRMT6/PRMT7/PRMT9/RBBP5/SETD4/SETDB1/SMYD4/SUV39H1/SUZ12/THUMPD3/TPMT/TRMT10B/TRMT2A/TRMT2B/TRMT5	44

cluster2	MF	GO:0047429	nucleoside triphosphate diphosphatase activity	7/2076	11/18432	0.0000498	0.005519492	0.005280223	ASMTL/DCTPP1/DHX9/DUT/ENPP3/ITPA/NUDT1	7
cluster2	MF	GO:0140097	catalytic activity, acting on DNA	48/2076	241/18432	0.0000582	0.006110354	0.005845471	ALKBH2/ALKBH3/ALKBH4/APEX1/BPTF/CDK7/CHD7/CHRAC1/DHX9/DNASE1/ERCC1/ERCC2/ERCC3/ERCC5/EXO5/FTO/G3BP1/IGHMBP2/MBD4/MCM3/MCM7/MCM8/METTL4/MUS81/N6AMT1/NEIL1/PCNA/PLD3/POLB/POLD4/POLE/RAD51/RAD51D/RAD9A/RAG1/RBBP4/RFC2/RPS3/SMARCA4/SUPV3L1/TDG/TDP1/TERF1/TERF2/TTF2/TWNK/UNG/ZGRF1	48
cluster2	MF	GO:0051539	4 iron, 4 sulfur cluster binding	14/2076	43/18432	0.000166537	0.016620429	0.015899935	ACO2/DPH2/DPYD/ERCC2/ETFDH/EXO5/LIAS/NDUFV1/NUBP1/NUBP2/POLE/PRIM2/RSAD1/TYW1B	14
cluster2	MF	GO:0002161	aminoacyl-tRNA editing activity	7/2076	13/18432	0.000210825	0.020038376	0.019169716	AARS1/AARS2/AARSD1/IARS1/IARS2/LARS2/TARS2	7

cluster2	MF	GO:0008276	protein methyltransferase activity	22/2076	92/18432	0.000454511	0.041236572	0.039448973	ANTKMT/ARMT1/ASH2L/CXXC1/EEF2KMT/EHMT1/HEMK1/ICMT/KMT2D/MECOM/METTL9/N6AMT1/NSD2/PRMT5/PRMT6/PRMT7/PRMT9/RBBP5/SETD4/SETDB1/SUV39H1/SUZ12	22
cluster2	MF	GO:0008171	O-methyltransferase activity	9/2076	23/18432	0.000535162	0.04644276	0.044429473	ARMT1/ASMT/ASMTL/CMTR1/CMTR2/COQ3/HENMT1/ICMT/MRM1	9
cluster2	KEGG	hsa03010	Ribosome	49/1017	170/8779	6.05E-10	0.000000202	0.000000194	MRPL10/MRPL11/MRPL12/MRPL15/MRPL17/MRPL21/MRPL30/MRPL33/MRPL34/MRPS10/MRPS11/MRPS15/MRPS17/MRPS18C/MRPS5/MRPS7/MRPS9/RPL10/RPL12/RPL13/RPL13A/RPL17/RPL18/RPL18A/RPL19/RPL21/RPL23A/RPL27/RPL27A/RPL29/RPL3/RPL30/RPL36/RPL5/RPL7A/RPL8/RPLP0/RPLP1/RPS15/RPS2/RPS21/RPS25/RPS28/RPS3/RPS5/RPS6/RPS8/RPSAP58/RSL24D1	49
cluster3	BP	GO:0034470	ncRNA processing	78/1496	439/18903	9.03E-12	6.48E-08	6.42E-08	ADAT1/ADAT2/AGO3/BCDIN3D/BRIX1/C2orf49/DDX47/DIS3/DUS4L/EBNA1BP2/EXOSC9/FARS2/FTSJ1/GAR1/GRSF1/GTPBP3/HEATR1/INTS12/INTS13/INTS4/INTS6L/LARP7/LAS1L/MAK16/METTL1/METTL2A/METTL2B/METTL6/MPHOSPH6/MRTO4/MTFMT/NOL10/NOL9/NSA2/NUP155/OSGEPL1/PDCD11/PRKRA/PUS10/PWP2/QTRT2/RCL1/RIPK1/RPF1/RPL10A/RPL11/RPL14/RPP21/RPS14/RPS16/RPS17/RPS19/RPS7/RPUSD1/RPUSD4/RRP1/RRP1B/RRP8/RSL1D1/RTRAF/SPOUT1/TARBP1/TFB1M/TFB2M/THADA/TP53RK/TRMT1/TRMT10A/TRMT13/TRMT1L/TRMT6/TRMU/TRPT1/TYW3/UTP11/UTP14A/WDR36/ZMPSTE24	78

cluster3	BP	GO:0006399	tRNA metabolic process	43/1496	195/18903	5.43E-10	0.00000195	0.00000193	ADAT1/ADAT2/BCDIN3D/C2orf49/DARS1/DARS2/DTD2/DUS4L/EPRS1/EXOSC9/FARS2/FARSA/FTSJ1/GRSF1/GTF3C3/GTF3C6/GTPBP3/HARS2/LARS1/LRRC47/METTL1/METTL2A/METTL2B/METTL6/MTFMT/OSGEPL1/PUS10/QTRT2/RPP21/RPUSD4/RTRAF/SEPSECS/TARBP1/THADA/TP53RK/TRMT1/TRMT10A/TRMT13/TRMT1L/TRMT6/TRMU/TRPT1/TYW3	43
cluster3	BP	GO:0002181	cytoplasmic translation	36/1496	161/18903	9.2E-09	0.000022	0.0000218	DENR/DNAJC24/EIF3E/EIF3J/EIF3L/FAU/FTSJ1/NCK1/RACK1/RBM4/RPL10A/RPL11/RPL14/RPL15/RPL22/RPL26L1/RPL31/RPL34/RPL37A/RPL41/RPL6/RPL9/RPS13/RPS14/RPS16/RPS17/RPS19/RPS20/RPS27A/RPS3A/RPS4X/RPS7/RPS9/RPSA/ZC3H15/ZCCHC13	36
cluster3	BP	GO:0008033	tRNA processing	31/1496	134/18903	4.22E-08	0.0000755	0.0000749	ADAT1/ADAT2/BCDIN3D/C2orf49/DUS4L/FARS2/FTSJ1/GRSF1/GTPBP3/METTL1/METTL2A/METTL2B/METTL6/MTFMT/OSGEPL1/PUS10/QTRT2/RPP21/RPUSD4/RTRAF/TARBP1/THADA/TP53RK/TRMT1/TRMT10A/TRMT13/TRMT1L/TRMT6/TRMU/TRPT1/TYW3	31
cluster3	BP	GO:0030488	tRNA methylation	15/1496	42/18903	0.000000355	0.000508895	0.000504814	BCDIN3D/FTSJ1/GTPBP3/METTL1/METTL2A/METTL2B/METTL6/TARBP1/THADA/TRMT1/TRMT10A/TRMT13/TRMT1L/TRMT6/TYW3	15

cluster3	BP	GO:0006400	tRNA modification	23/1496	93/18903	0.000000653	0.000779657	0.000773405	ADAT2/BCDIN3D/DUS4L/FTSJ1/GTPBP3/METTL1/METTL2A/METTL2B/METTL6/MTFMT/OSGEPL1/PUS10/QTRT2/RPUSD4/TARBP1/THADA/TRMT1/TRMT10A/TRMT13/TRMT1L/TRMT6/TRMU/TYW3	23
cluster3	BP	GO:1905168	positive regulation of double-strand break repair via homologous recombination	14/1496	39/18903	0.000000812	0.000789087	0.00078276	ARID2/EP400/EPC1/ERCC6/FUS/HDGFL2/ING3/MMS19/MRGBP/PARP1/POGZ/TIMELESS/VPS72/YEATS4	14
cluster3	BP	GO:0022613	ribonucleoprotein complex biogenesis	70/1496	489/18903	0.000000881	0.000789087	0.00078276	AGO3/BRIX1/C1orf109/CPSF6/CPSF7/DDX31/DDX47/DENR/DIS3/EBNA1BP2/EIF2A/EIF3E/EIF3J/EIF3L/EXOSC9/GAR1/GEMIN5/GEMIN6/GEMIN7/HEATR1/HEATR3/HTATSF1/LAS1L/LTO1/MAK16/MPHOSPH6/MRPL22/MRTO4/NLE1/NOL10/NOL9/NOPCHAP1/NSA2/PDCD11/PHF5A/POLR2D/PRPF39/PTGES3/PWP2/RBMX/RCL1/RPF1/RPL10A/RPL11/RPL14/RPL26L1/RPL6/RPS14/RPS16/RPS17/RPS19/RPS27L/RPS7/RPSA/RPUSD1/RPUSD4/RRP1/RRP1B/RRP8/RSL1D1/SF3A3/SFSWAP/SNRPB2/TFB1M/TFB2M/TGS1/URB2/UTP11/UTP14A/WDR36	70
cluster3	BP	GO:0000724	double-strand break repair via homologous recombination	33/1496	169/18903	0.00000106	0.000843083	0.000836324	AP5S1/ARID2/BLM/EP400/EPC1/ERCC6/EXD2/FAN1/FIGNL1/FUS/GEN1/HDGFL2/ING3/MCM2/MCM4/MCMDC2/MMS19/MRGBP/NABP1/NSMCE1/PALB2/PARP1/POGZ/POLQ/RAD52/RADX/RFWD3/RMI2/TIMELESS/VPS72/XRCC2/YEATS4/ZNF365	33

cluster3	BP	GO:0000725	recombinational repair	33/1496	173/18903	0.00000182	0.001290986	0.001280635	AP5S1/ARID2/BLM/EP400/EPC1/ERCC6/EXD2/FAN1/FIGNL1/FUS/GEN1/HDGFL2/ING3/MCM2/MCM4/MCMDC2/MMS19/MRGBP/NABP1/NSMCE1/PALB2/PARP1/POGZ/POLQ/RAD52/RADX/RFWD3/RMI2/TIMELESS/VPS72/XRCC2/YEATS4/ZNF365	33
cluster3	BP	GO:0010569	regulation of double-strand break repair via homologous recombination	19/1496	72/18903	0.00000212	0.001290986	0.001280635	ARID2/EP400/EPC1/ERCC6/FIGNL1/FUS/HDGFL2/ING3/MMS19/MRGBP/PARP1/POGZ/POLQ/RADX/RMI2/TIMELESS/VPS72/YEATS4/ZNF365	19
cluster3	BP	GO:0006302	double-strand break repair	48/1496	300/18903	0.00000216	0.001290986	0.001280635	AP5S1/ARID1B/ARID2/BCL7B/BLM/BRD7/DCLRE1B/DNTT/DTX3L/EME1/EP400/EPC1/ERCC6/EXD2/EYA3/FAN1/FANCD2/FIGNL1/FUS/GEN1/HDGFL2/ING3/MCM2/MCM4/MCMDC2/MMS19/MRGBP/NABP1/NHEJ1/NSMCE1/PALB2/PARP1/POGZ/POLQ/RAD52/RADX/RFWD3/RMI2/RNF169/RNF8/SMARCC1/TIMELESS/TRIP13/UVRAG/VPS72/XRCC2/YEATS4/ZNF365	48
cluster3	BP	GO:0042254	ribosome biogenesis	49/1496	310/18903	0.00000242	0.001336821	0.001326102	BRIX1/C1orf109/DDX31/DDX47/DIS3/EBNA1BP2/EIF2A/EXOSC9/GAR1/HEATR1/HEATR3/LAS1L/LTO1/MAK16/MPHOSPH6/MRPL22/MRTO4/NLE1/NOL10/NOL9/NSA2/PDCD11/PWP2/RCL1/RPF1/RPL10A/RPL11/RPL14/RPL26L1/RPL6/RPS14/RPS16/RPS17/RPS19/RPS27L/RPS7/RPSA/RPUSD1/RPUSD4/RRP1/RRP1B/RRP8/RSL1D1/TFB1M/TFB2M/URB2/UTP11/UTP14A/WDR36	49

cluster3	BP	GO:0006310	DNA recombination	51/1496	332/18903	0.00000356	0.001821681	0.001807075	AP5S1/ARID2/ATAD5/BLM/CCR6/CENPS/CLCF1/CORT/DCAF1/EME1/EP400/EPC1/ERCC6/EXD2/FAN1/FANCD2/FIGNL1/FUS/GEN1/HDGFL2/IL10/IL27RA/ING3/KPNA1/MCM2/MCM4/MCMDC2/MMS19/MRGBP/MSH3/NABP1/NFRKB/NHEJ1/NONO/NSMCE1/PALB2/PARP1/POGZ/POLQ/RAD52/RADX/RFWD3/RMI2/RNF8/TIMELESS/TOPBP1/TRIP13/VPS72/XRCC2/YEATS4/ZNF365	51
cluster3	BP	GO:0032784	regulation of DNA-templated transcription elongation	23/1496	104/18903	0.00000515	0.002461638	0.0024419	AXIN1/CCNT1/CDK13/ERCC6/HMGN1/HTATSF1/INTS12/INTS13/INTS4/KAT7/LARP7/LEO1/MED10/MED11/MED16/MED28/MED29/MED8/NELFB/RNF8/SETD5/TSFM/VHL	23
cluster3	BP	GO:0009451	RNA modification	32/1496	176/18903	0.00000746	0.003340937	0.003314149	ADAT2/APOBEC3F/BCDIN3D/DUS4L/FTSJ1/GAR1/GTPBP3/HNRNPAB/LARP7/METTL1/METTL14/METTL2A/METTL2B/METTL6/MTFMT/OSGEPL1/PUS10/QTRT2/RPUSD1/RPUSD4/TARBP1/TFB1M/TFB2M/TGS1/THADA/TRMT1/TRMT10A/TRMT13/TRMT1L/TRMT6/TRMU/TYW3	32
cluster3	BP	GO:0006282	regulation of DNA repair	36/1496	212/18903	0.0000103	0.004342287	0.00430747	ARID1B/ARID2/ATXN7/ATXN7L3/BCL7B/BRD7/DTX3L/EP400/EPC1/ERCC6/EYA3/FIGNL1/FUS/HDGFL2/ING3/KAT7/MMS19/MRGBP/NFRKB/PARP1/POGZ/POLH/POLQ/RAD52/RADX/RMI2/RNF169/RNF8/SMARCC1/SUPT3H/SUPT7L/TIMELESS/TMEM161A/VPS72/YEATS4/ZNF365	36

cluster3	BP	GO:0000018	regulation of DNA recombination	26/1496	133/18903	0.0000139	0.005552387	0.005507867	ARID2/ATAD5/BLM/CLCF1/EP400/EPC1/ERCC6/FIGNL1/FUS/HDGFL2/IL10/IL27RA/ING3/KPNA1/MMS19/MRGBP/MSH3/PARP1/POGZ/POLQ/RADX/RMI2/TIMELESS/VPS72/YEATS4/ZNF365	26
cluster3	BP	GO:0016072	rRNA metabolic process	42/1496	268/18903	0.0000154	0.005751309	0.005705195	BRIX1/DDX47/DIS3/EBNA1BP2/ERCC6/EXOSC9/GAR1/GTF3C3/GTF3C6/HEATR1/LAS1L/MACROH2A2/MAK16/MPHOSPH6/MRTO4/NOL10/NOL9/NSA2/PDCD11/POLR1E/PWP2/RCL1/RPF1/RPL10A/RPL11/RPL14/RPS14/RPS16/RPS17/RPS19/RPS7/RPUSD1/RPUSD4/RRP1/RRP1B/RRP8/RSL1D1/TFB1M/TFB2M/UTP11/UTP14A/WDR36	42
cluster3	BP	GO:0006261	DNA-templated DNA replication	30/1496	166/18903	0.000016	0.005751309	0.005705195	ATAD5/BAZ1A/BLM/CCNE1/CENPS/CHTF8/CORT/DBF4/EME1/EXD2/GEN1/KAT7/MCM2/MCM4/NOC3L/ORC2/ORC3/PARP1/POLD1/POLE3/POLE4/POLQ/RFC5/RFWD3/RRM1/TIMELESS/TOPBP1/WDHD1/ZMPSTE24/ZNF365	30
cluster3	BP	GO:0001510	RNA methylation	20/1496	90/18903	0.0000193	0.006493819	0.006441751	BCDIN3D/FTSJ1/GTPBP3/LARP7/METTL1/METTL14/METTL2A/METTL2B/METTL6/TARBP1/TFB1M/TFB2M/TGS1/THADA/TRMT1/TRMT10A/TRMT13/TRMT1L/TRMT6/TYW3	20

cluster3	BP	GO:0042273	ribosomal large subunit biogenesis	18/1496	76/18903	0.0000199	0.006493819	0.006441751	BRIX1/C1orf109/EBNA1BP2/HEATR3/LAS1L/LTO1/MAK16/MRTO4/NLE1/NOL9/NSA2/RPF1/RPL10A/RPL11/RPL14/RPL26L1/RPL6/RSL1D1	18
cluster3	BP	GO:0006364	rRNA processing	37/1496	229/18903	0.0000245	0.007633218	0.007572015	BRIX1/DDX47/DIS3/EBNA1BP2/EXOSC9/GAR1/HEATR1/LAS1L/MAK16/MPHOSPH6/MRTO4/NOL10/NOL9/NSA2/PDCD11/PWP2/RCL1/RPF1/RPL10A/RPL11/RPL14/RPS14/RPS16/RPS17/RPS19/RPS7/RPUSD1/RPUSD4/RRP1/RRP1B/RRP8/RSL1D1/TFB1M/TFB2M/UTP11/UTP14A/WDR36	37
cluster3	BP	GO:0016573	histone acetylation	31/1496	180/18903	0.0000312	0.009316062	0.009241365	ATXN7/ATXN7L3/BRD1/BRD7/CRTC2/EOMES/EP400/EPC1/ERCC6/GLYR1/GTF2B/ING3/ING5/IRF4/IWS1/JADE1/KAT7/MAP3K7/MBD3/MLLT3/MRGBP/NCOA3/OGT/POLE3/SETD5/SIN3A/SUPT3H/SUPT7L/VPS72/YEATS4/ZMPSTE24	31
cluster3	BP	GO:0006354	DNA-templated transcription elongation	38/1496	241/18903	0.0000334	0.009573541	0.009496779	AEBP2/AXIN1/CCNT1/CDK13/CTDP1/ERCC6/FUS/HDGFL2/HMGN1/HTATSF1/INTS12/INTS13/INTS4/KAT7/KDM3A/LARP7/LEO1/MED10/MED11/MED13L/MED16/MED28/MED29/MED8/MTA2/NELFB/NPAT/POLR1E/RNF8/SETD5/TAF15/TCEA3/TFB2M/TRIP13/TSFM/VHL/ZXDA/ZXDB	38

cluster3	BP	GO:0016032	viral process	57/1496	421/18903	0.0000458	0.012572136	0.012471331	APOBEC3F/BANF1/BCL2/BICD1/CCL3/CCL8/CCNT1/CD4/CHMP5/CHMP7/CSF1R/CTDP1/DDX6/DENR/EIF2AK2/EIF3L/GPR15/GRK2/GTF2B/HTATSF1/ILF3/IRF7/ISG15/IST1/JUN/LARP7/MAN1B1/MAVS/MDFIC/MGAT1/MGAT5/MOG/MORC2/NFIA/NMT2/NUP153/OAS1/OAS2/OASL/PLSCR1/PTX3/RAB43/RPSA/RRP1B/SLC52A2/SPCS3/ST3GAL1/STAT1/TAF11/TMEM39A/TMEM41B/TRIM59/TRIM68/USF2/UVRAG/ZNF502/ZNF639	57
cluster3	BP	GO:2000781	positive regulation of double-strand break repair	19/1496	88/18903	0.0000474	0.012572136	0.012471331	ARID1B/ARID2/BCL7B/BRD7/DTX3L/EP400/EPC1/ERCC6/FUS/HDGFL2/ING3/MMS19/MRGBP/PARP1/POGZ/SMARCC1/TIMELESS/VPS72/YEATS4	19
cluster3	BP	GO:2000779	regulation of double-strand break repair	25/1496	135/18903	0.0000523	0.013383997	0.013276683	ARID1B/ARID2/BCL7B/BRD7/DTX3L/EP400/EPC1/ERCC6/FIGNL1/FUS/HDGFL2/ING3/MMS19/MRGBP/PARP1/POGZ/POLQ/RADX/RMI2/RNF169/SMARCC1/TIMELESS/VPS72/YEATS4/ZNF365	25
cluster3	BP	GO:0070106	interleukin-27-mediated signaling pathway	5/1496	7/18903	0.0000566	0.013980923	0.013868823	IL27RA/OAS1/OAS2/OASL/STAT1	5

cluster3	BP	GO:0018393	internal peptidyl-lysine acetylation	31/1496	186/18903	0.0000595	0.014227709	0.01411363	ATXN7/ATXN7L3/BRD1/BRD7/CRTC2/EOMES/EP400/EPC1/ERCC6/GLYR1/GTF2B/ING3/ING5/IRF4/IWS1/JADE1/KAT7/MAP3K7/MBD3/MLLT3/MRGBP/NCOA3/OGT/POLE3/SETD5/SIN3A/SUPT3H/SUPT7L/VPS72/YEATS4/ZMPSTE24	31
cluster3	BP	GO:0006475	internal protein amino acid acetylation	31/1496	188/18903	0.0000732	0.016934136	0.016798357	ATXN7/ATXN7L3/BRD1/BRD7/CRTC2/EOMES/EP400/EPC1/ERCC6/GLYR1/GTF2B/ING3/ING5/IRF4/IWS1/JADE1/KAT7/MAP3K7/MBD3/MLLT3/MRGBP/NCOA3/OGT/POLE3/SETD5/SIN3A/SUPT3H/SUPT7L/VPS72/YEATS4/ZMPSTE24	31
cluster3	BP	GO:0034243	regulation of transcription elongation by RNA polymerase II	18/1496	86/18903	0.000113674	0.025463082	0.025258917	AXIN1/CCNT1/CDK13/ERCC6/INTS12/INTS13/INTS4/LARP7/LEO1/MED10/MED11/MED16/MED28/MED29/MED8/NELFB/RNF8/VHL	18
cluster3	BP	GO:0045911	positive regulation of DNA recombination	16/1496	72/18903	0.000128238	0.027154607	0.026936879	ARID2/ATAD5/CLCF1/EP400/EPC1/ERCC6/FUS/HDGFL2/ING3/MMS19/MRGBP/PARP1/POGZ/TIMELESS/VPS72/YEATS4	16

cluster3	BP	GO:2001020	regulation of response to DNA damage stimulus	44/1496	312/18903	0.000128803	0.027154607	0.026936879	ARID1B/ARID2/ATAD5/ATXN7/ATXN7L3/BCL2/BCL7B/BRD7/CRY1/DTX3L/EEF1E1/EP400/EPC1/ERCC6/EYA3/FIGNL1/FUS/FXR2/HDGFL2/ING3/KAT7/MMS19/MRGBP/NFRKB/NSMCE1/PARP1/POGZ/POLH/POLQ/RAD52/RADX/RFWD3/RMI2/RNF169/RNF8/SMARCC1/SUPT3H/SUPT7L/TIMELESS/TMEM161A/VPS72/YEATS4/ZMPSTE24/ZNF365	44
cluster3	BP	GO:0032259	methylation	52/1496	390/18903	0.000143577	0.029404615	0.029168846	ATPSCKMT/BCDIN3D/BOD1/COQ5/CSKMT/DNMT3A/EOMES/FTSJ1/GNMT/GTPBP3/HNMT/IWS1/KDM3A/KDM4A/KDM6A/LARP7/MBD3/METTL1/METTL14/METTL2A/METTL2B/METTL6/MTA2/MTAP/MTRR/NCOA6/NSD3/NTMT1/OGT/PARP1/PLD6/PPM1D/PRDM2/RRP8/SETD5/SETD6/SMYD5/SPOUT1/TARBP1/TDRD9/TFB1M/TFB2M/TGS1/THADA/TRMT1/TRMT10A/TRMT13/TRMT1L/TRMT6/TYW3/WDR5B/ZMPSTE24	52
cluster3	BP	GO:0006260	DNA replication	41/1496	286/18903	0.000150531	0.029972397	0.029732076	ATAD5/BAZ1A/BLM/CCNE1/CENPS/CHAF1A/CHTF8/CORT/DBF4/EME1/EXD2/GEN1/ING5/JADE1/KAT7/KCTD13/MCM2/MCM4/NFIA/NFRKB/NOC3L/ORC2/ORC3/PARP1/POLD1/POLE3/POLE4/POLH/POLQ/RAD17/RFC5/RFWD3/RMI2/RRM1/SDE2/SIN3A/TIMELESS/TOPBP1/WDHD1/ZMPSTE24/ZNF365	41
cluster3	BP	GO:0045739	positive regulation of DNA repair	23/1496	128/18903	0.000164305	0.031830824	0.031575601	ARID1B/ARID2/BCL7B/BRD7/DTX3L/EP400/EPC1/ERCC6/EYA3/FUS/HDGFL2/ING3/MMS19/MRGBP/NFRKB/PARP1/POGZ/RNF8/SMARCC1/TIMELESS/TMEM161A/VPS72/YEATS4	23

cluster3	BP	GO:0018394	peptidyl-lysine acetylation	31/1496	197/18903	0.000176817	0.033353252	0.033085823	ATXN7/ATXN7L3/BRD1/BRD7/CRTC2/EOMES/EP400/EPC1/ERCC6/GLYR1/GTF2B/ING3/ING5/IRF4/IWS1/JADE1/KAT7/MAP3K7/MBD3/MLLT3/MRGBP/NCOA3/OGT/POLE3/SETD5/SIN3A/SUPT3H/SUPT7L/VPS72/YEATS4/ZMPSTE24	31
cluster3	BP	GO:0043414	macromolecule methylation	47/1496	348/18903	0.000217723	0.040016405	0.03969555	ATPSCKMT/BCDIN3D/BOD1/CSKMT/DNMT3A/EOMES/FTSJ1/GTPBP3/IWS1/KDM3A/KDM4A/KDM6A/LARP7/MBD3/METTL1/METTL14/METTL2A/METTL2B/METTL6/MTA2/MTRR/NCOA6/NSD3/NTMT1/OGT/PARP1/PLD6/PPM1D/PRDM2/RRP8/SETD5/SETD6/SMYD5/TARBP1/TDRD9/TFB1M/TFB2M/TGS1/THADA/TRMT1/TRMT10A/TRMT13/TRMT1L/TRMT6/TYW3/WDR5B/ZMPSTE24	47
cluster3	BP	GO:0006413	translational initiation	22/1496	123/18903	0.00024165	0.043303642	0.042956429	DAZL/DENR/DNAJC3/EIF2A/EIF2AK2/EIF2B4/EIF2S1/EIF3E/EIF3J/EIF3L/EIF4E/EIF4EBP2/HSPB1/LTO1/MTFMT/NCK1/POLR2D/RBM4/RPS17/RPS3A/RPS6KB2/TPR	22
cluster3	CC	GO:0044391	ribosomal subunit	45/1558	188/19869	8.65E-12	8.39E-09	7.98E-09	CHCHD1/DAP3/EIF2A/FAU/FXR2/ISG15/MRPL22/MRPL28/MRPL35/MRPL37/MRPL46/MRPL47/MRPL57/MRPS14/MRPS25/MRPS27/MRPS28/MRPS33/RACK1/RPL10A/RPL11/RPL14/RPL15/RPL22/RPL26L1/RPL31/RPL34/RPL37A/RPL41/RPL6/RPL9/RPS13/RPS14/RPS16/RPS17/RPS19/RPS20/RPS27A/RPS27L/RPS3A/RPS4X/RPS4Y1/RPS7/RPS9/RPSA	45

cluster3	CC	GO:0005840	ribosome	50/1558	232/19869	3.82E-11	1.69E-08	1.61E-08	CHCHD1/DAP3/EIF2A/EIF2AK2/FAU/FUBP3/FXR2/HSPA14/ISG15/MRPL22/MRPL28/MRPL35/MRPL37/MRPL46/MRPL47/MRPL57/MRPS14/MRPS25/MRPS27/MRPS28/MRPS33/NCK1/NUFIP2/RACK1/RPL10A/RPL11/RPL14/RPL15/RPL22/RPL26L1/RPL31/RPL34/RPL37A/RPL41/RPL6/RPL9/RPS13/RPS14/RPS16/RPS17/RPS19/RPS20/RPS27A/RPS27L/RPS3A/RPS4X/RPS4Y1/RPS7/RPS9/RPSA	50
cluster3	CC	GO:0022626	cytosolic ribosome	31/1558	105/19869	5.23E-11	1.69E-08	1.61E-08	EIF2A/FAU/FXR2/ISG15/RACK1/RPL10A/RPL11/RPL14/RPL15/RPL22/RPL26L1/RPL31/RPL34/RPL37A/RPL41/RPL6/RPL9/RPS13/RPS14/RPS16/RPS17/RPS19/RPS20/RPS27A/RPS27L/RPS3A/RPS4X/RPS4Y1/RPS7/RPS9/RPSA	31
cluster3	CC	GO:0015935	small ribosomal subunit	25/1558	77/19869	4.05E-10	9.82E-08	9.34E-08	CHCHD1/DAP3/EIF2A/FAU/ISG15/MRPS14/MRPS25/MRPS27/MRPS28/MRPS33/RACK1/RPS13/RPS14/RPS16/RPS17/RPS19/RPS20/RPS27A/RPS27L/RPS3A/RPS4X/RPS4Y1/RPS7/RPS9/RPSA	25
cluster3	CC	GO:0022627	cytosolic small ribosomal subunit	18/1558	45/19869	2.52E-09	0.000000488	0.000000465	EIF2A/FAU/ISG15/RACK1/RPS13/RPS14/RPS16/RPS17/RPS19/RPS20/RPS27A/RPS27L/RPS3A/RPS4X/RPS4Y1/RPS7/RPS9/RPSA	18

cluster3	CC	GO:0030684	preribosome	17/1558	76/19869	0.0000653	0.009416539	0.008960825	EBNA1BP2/HEATR1/LAS1L/MAK16/MRTO4/NOL10/NSA2/PDCD11/PWP2/RPF1/RPS7/RRP1/RRP1B/RSL1D1/UTP11/UTP14A/WDR36	17
cluster3	CC	GO:0000123	histone acetyltransferase complex	19/1558	91/19869	0.000068	0.009416539	0.008960825	ATXN7/ATXN7L3/BRD1/EP400/EPC1/ING3/ING5/JADE1/KAT7/MAP3K7/MLLT3/MRGBP/OGT/POLE3/POLE4/SUPT3H/SUPT7L/VPS72/YEATS4	19
cluster3	CC	GO:0031248	protein acetyltransferase complex	20/1558	101/19869	0.0000969	0.010434334	0.009929365	ATXN7/ATXN7L3/BRD1/EP400/EPC1/ING3/ING5/JADE1/KAT7/MAP3K7/MLLT3/MRGBP/NAA20/OGT/POLE3/POLE4/SUPT3H/SUPT7L/VPS72/YEATS4	20
cluster3	CC	GO:1902493	acetyltransferase complex	20/1558	101/19869	0.0000969	0.010434334	0.009929365	ATXN7/ATXN7L3/BRD1/EP400/EPC1/ING3/ING5/JADE1/KAT7/MAP3K7/MLLT3/MRGBP/NAA20/OGT/POLE3/POLE4/SUPT3H/SUPT7L/VPS72/YEATS4	20

cluster3	CC	GO:0030687	preribosome, large subunit precursor	8/1558	21/19869	0.000112956	0.010945457	0.010415751	EBNA1BP2/LAS1L/MAK16/MRTO4/NSA2/RPF1/RRP1/RRP1B	8
cluster3	CC	GO:0005759	mitochondrial matrix	61/1558	483/19869	0.000146985	0.012948086	0.012321463	ABHD10/ACAD10/ACAD8/ACSF2/ACSF3/ACSM3/ALDH6A1/ATAD3A/AUH/BDH1/BTD/C2orf69/CHCHD1/COQ5/DAP3/DARS2/DLD/EXD2/FARS2/FLAD1/FXN/GFM2/GLRX2/GOT2/GRSF1/GSR/HARS2/HTD2/LIPT2/MCCC1/MMAB/MRPL22/MRPL28/MRPL35/MRPL37/MRPL46/MRPL47/MRPL57/MRPS14/MRPS25/MRPS27/MRPS28/MRPS33/MRRF/NDUFB8/NUDT9/OXCT2/PCCB/PPM1K/PTPN1/RPUSD4/SIRT3/SLC25A5/SOD1/TFAM/TFB1M/TFB2M/TIMM44/TSFM/UQCC2/VDAC2	61
cluster3	CC	GO:0001650	fibrillar center	24/1558	145/19869	0.0003778	0.030507319	0.029030918	BTBD10/CDCA7L/CHCHD1/DCAF1/EIF3L/FHIT/GAR1/HEATR1/IP6K2/KDM4A/LEO1/LIPA/MALT1/NHEJ1/NONO/POLR1E/SMUG1/SNRPB2/TAF1C/TIMM44/TRAF4/TRERF1/WDR33/ZNF415	24
cluster3	CC	GO:0022625	cytosolic large ribosomal subunit	13/1558	59/19869	0.000537551	0.040068196	0.038129096	FXR2/RPL10A/RPL11/RPL14/RPL15/RPL22/RPL26L1/RPL31/RPL34/RPL37A/RPL41/RPL6/RPL9	13

cluster3	CC	GO:0015934	large ribosomal subunit	20/1558	115/19869	0.000588658	0.040743514	0.038771732	FXR2/MRPL22/MRPL28/MRPL35/MRPL37/MRPL46/MRPL47/MRPL57/RPL10A/RPL11/RPL14/RPL15/RPL22/RPL26L1/RPL31/RPL34/RPL37A/RPL41/RPL6/RPL9	20
cluster3	MF	GO:0003735	structural constituent of ribosome	40/1538	176/18432	3.86E-09	0.00000698	0.00000686	DAP3/FAU/ISG15/MRPL22/MRPL28/MRPL35/MRPL37/MRPL46/MRPL47/MRPL57/MRPS14/MRPS25/MRPS33/RPL10A/RPL11/RPL14/RPL15/RPL22/RPL26L1/RPL31/RPL34/RPL37A/RPL41/RPL6/RPL9/RPS13/RPS14/RPS16/RPS17/RPS19/RPS20/RPS27A/RPS27L/RPS3A/RPS4X/RPS4Y1/RPS7/RPS9/RPSA/SRBD1	40
cluster3	MF	GO:0140101	catalytic activity, acting on a tRNA	29/1538	127/18432	0.000000467	0.000422242	0.000415243	BCDIN3D/DARS1/DARS2/DTD2/DUS4L/EPRS1/FARS2/FARSA/FTSJ1/HARS2/LARS1/LRRC47/METTL1/METTL2A/METTL2B/METTL6/MTFMT/PUS10/QTRT2/RPP21/SEPSECS/TARBP1/TRMT1/TRMT10A/TRMT13/TRMT1L/TRMU/TRPT1/TYW3	29
cluster3	MF	GO:0008175	tRNA methyltransferase activity	12/1538	34/18432	0.0000105	0.006352938	0.006247641	BCDIN3D/FTSJ1/METTL1/METTL2A/METTL2B/METTL6/TARBP1/TRMT1/TRMT10A/TRMT13/TRMT1L/TYW3	12

cluster3	MF	GO:0008173	RNA methyltransferase activity	16/1538	65/18432	0.0000648	0.02933193	0.028845765	BCDIN3D/FTSJ1/METTL1/METTL14/METTL2A/METTL2B/METTL6/TARBP1/TFB1M/TFB2M/TGS1/TRMT1/TRMT10A/TRMT13/TRMT1L/TYW3	16
cluster3	KEGG	hsa05171	Coronavirus disease - COVID-19	41/754	233/8779	0.00000646	0.002156361	0.002120342	EIF2AK2/FAU/IFNA5/IKBKB/IL12A/ISG15/JAK1/JUN/MAP3K7/MASP2/MAVS/NFKB1/OAS1/OAS2/RPL10A/RPL11/RPL14/RPL15/RPL22/RPL26L1/RPL31/RPL34/RPL37A/RPL41/RPL6/RPL9/RPS13/RPS14/RPS16/RPS17/RPS19/RPS20/RPS27A/RPS27L/RPS3A/RPS4X/RPS4Y1/RPS7/RPS9/RPSA/STAT1	41
cluster3	KEGG	hsa03010	Ribosome	31/754	170/8779	0.0000426	0.007109726	0.006990969	FAU/MRPL22/MRPL28/MRPL35/MRPS14/RPL10A/RPL11/RPL14/RPL15/RPL22/RPL26L1/RPL31/RPL34/RPL37A/RPL41/RPL6/RPL9/RPS13/RPS14/RPS16/RPS17/RPS19/RPS20/RPS27A/RPS27L/RPS3A/RPS4X/RPS4Y1/RPS7/RPS9/RPSA	31
cluster4	BP	GO:0044270	cellular nitrogen compound catabolic process	90/2130	474/18903	0.000000416	0.002053062	0.001922131	ADA/AGO2/AHCY/AMPD3/ANG/ANGEL2/APOBEC2/APOBEC3D/APOBEC3G/BAX/BLVRA/CIRBP/CNOT1/CNOT6/CNOT8/CYP3A5/DDX5/DICER1/DIS3L/DKC1/DNASE1L1/EDC4/ELAC2/ENDOG/ENTPD5/EXOSC10/EXOSC4/EXOSC8/FASTK/FBH1/FEN1/FMR1/GIGYF2/HMOX1/HMOX2/HNRNPA0/HNRNPU/HSPA1B/ISG20/KHSRP/LARP1/LARP4B/METTL3/MLH1/MYD88/NIT1/NOCT/NT5C/NT5C1A/NT5C1B/NTHL1/NUDT15/NUDT17/NUDT18/NUDT3/NUDT7/OGG1/PABPC1/PDE4B/PHAX/POR/PPP1R8/PUM1/RBM38/RNASEH2C/SAMD4B/SAMHD1/SETMAR/SIDT2/SLFN13/SMG1/SSB/SUCLA2/SYNCRIP/TENT5A/TET3/TRAF2/TRAF3IP2/TRDMT1/TTC5/UPB1/UPF1/UPF3A/UPF3B/VCP/WDR82/YBX1/YTHDF2/ZC3HAV1/ZSWIM8	90

cluster4	BP	GO:0034655	nucleobase-containing compound catabolic process	83/2130	428/18903	0.000000474	0.002053062	0.001922131	ADA/AGO2/AHCY/AMPD3/ANG/ANGEL2/APOBEC2/APOBEC3D/APOBEC3G/BAX/CIRBP/CNOT1/CNOT6/CNOT8/DDX5/DICER1/DIS3L/DKC1/DNASE1L1/EDC4/ELAC2/ENDOG/ENTPD5/EXOSC10/EXOSC4/EXOSC8/FASTK/FBH1/FEN1/FMR1/GIGYF2/HNRNPA0/HNRNPU/HSPA1B/ISG20/KHSRP/LARP1/LARP4B/METTL3/MLH1/MYD88/NOCT/NT5C/NT5C1A/NT5C1B/NTHL1/NUDT15/NUDT17/NUDT18/NUDT3/NUDT7/OGG1/PABPC1/PDE4B/PHAX/PPP1R8/PUM1/RBM38/RNASEH2C/SAMD4B/SAMHD1/SETMAR/SIDT2/SLFN13/SMG1/SSB/SUCLA2/SYNCRIP/TENT5A/TRAF2/TRAF3IP2/TRDMT1/TTC5/UPB1/UPF1/UPF3A/UPF3B/VCP/WDR82/YBX1/YTHDF2/ZC3HAV1/ZSWIM8	83
cluster4	BP	GO:2001022	positive regulation of response to DNA damage stimulus	41/2130	166/18903	0.000000862	0.002053062	0.001922131	ARID1A/ATR/BABAM2/BCL7C/BRCC3/BRD8/CCAR2/CEBPG/DDX5/ENDOG/EPC2/FAM168A/FBH1/FMR1/H2AX/KMT5C/MGMT/PARP9/PMAIP1/PNKP/PYHIN1/RUVBL1/RUVBL2/SETMAR/SHLD2/SIRT1/SLF2/SMARCA2/SMARCC2/SMARCD2/SPIDR/SPRED1/TRIM28/TRRAP/TTI2/UBE2N/XRCC1/YY1/ZCWPW1/ZNF385A/ZNHIT1	41
cluster4	BP	GO:2001020	regulation of response to DNA damage stimulus	64/2130	312/18903	0.00000135	0.002053062	0.001922131	ARID1A/ATR/BABAM2/BCL7C/BRCC3/BRD8/CCAR2/CD44/CDK9/CEBPG/DDX5/ENDOG/EPC2/FAM168A/FBH1/FEM1B/FMR1/H2AX/KDM1A/KMT5C/MAGEF1/MDM2/MGMT/OGG1/OTUB1/PARP9/PMAIP1/PNKP/PPP1R10/PPP4C/PYHIN1/RECQL5/RPA2/RUVBL1/RUVBL2/SETMAR/SF3B3/SF3B5/SHLD2/SIRT1/SLF2/SMARCA2/SMARCC2/SMARCD2/SMG1/SOX4/SPIDR/SPRED1/TADA2B/TAF12/TAF5/TP53/TPT1/TRIM28/TRIM32/TRRAP/TTI2/UBE2N/UBQLN4/XRCC1/YY1/ZCWPW1/ZNF385A/ZNHIT1	64
cluster4	BP	GO:0046700	heterocycle catabolic process	87/2130	468/18903	0.00000161	0.002053062	0.001922131	ADA/AGO2/AHCY/AMPD3/ANG/ANGEL2/APOBEC2/APOBEC3D/APOBEC3G/BAX/BLVRA/CIRBP/CNOT1/CNOT6/CNOT8/DDX5/DICER1/DIS3L/DKC1/DNASE1L1/EDC4/ELAC2/ENDOG/ENTPD5/EXOSC10/EXOSC4/EXOSC8/FASTK/FBH1/FEN1/FMR1/GIGYF2/HMOX1/HMOX2/HNRNPA0/HNRNPU/HSPA1B/ISG20/KHSRP/LARP1/LARP4B/METTL3/MLH1/MYD88/NOCT/NT5C/NT5C1A/NT5C1B/NTHL1/NUDT15/NUDT17/NUDT18/NUDT3/NUDT7/OGG1/PABPC1/PDE4B/PHAX/PPP1R8/PUM1/RBM38/RNASEH2C/SAMD4B/SAMHD1/SETMAR/SIDT2/SLFN13/SMG1/SSB/SUCLA2/SYNCRIP/TENT5A/TET3/TRAF2/TRAF3IP2/TRDMT1/TTC5/UPB1/UPF1/UPF3A/UPF3B/VCP/WDR82/YBX1/YTHDF2/ZC3HAV1/ZSWIM8	87

cluster4	BP	GO:0019439	aromatic compound catabolic process	90/2130	489/18903	0.00000166	0.002053062	0.001922131	ADA/AGO2/AHCY/AMPD3/ANG/ANGEL2/APOBEC2/APOBEC3D/APOBEC3G/BAX/BLVRA/CIRBP/CNOT1/CNOT6/CNOT8/DDX5/DICER1/DIS3L/DKC1/DNASE1L1/EDC4/ELAC2/ENDOG/ENTPD5/EXOSC10/EXOSC4/EXOSC8/FASTK/FBH1/FEN1/FMR1/GIGYF2/HMOX1/HMOX2/HNRNPA0/HNRNPU/HSPA1B/ISG20/KHSRP/LARP1/LARP4B/METTL3/MLH1/MYD88/NOCT/NT5C/NT5C1A/NT5C1B/NTHL1/NUDT15/NUDT17/NUDT18/NUDT3/NUDT7/OGG1/PABPC1/PDE4B/PHAX/PON2/PPP1R8/PUM1/RBM38/RNASEH2C/SAMD4B/SAMHD1/SETMAR/SIDT2/SLFN13/SMG1/SSB/SUCLA2/SULT1A3/SULT1A4/SYNCRIP/TENT5A/TET3/TRAF2/TRAF3IP2/TRDMT1/TTC5/UPB1/UPF1/UPF3A/UPF3B/VCP/WDR82/YBX1/YTHDF2/ZC3HAV1/ZSWIM8	90
cluster4	BP	GO:0008380	RNA splicing	87/2130	469/18903	0.00000177	0.002053062	0.001922131	AHNAK/AKAP17A/AKAP8L/CCAR2/CD2BP2/CIRBP/CLASRP/CLP1/COIL/CWF19L1/DBR1/DDX39A/DDX5/DNAJC17/ECD/ESRP2/ESS2/FAM50A/FASTK/FMR1/HNRNPA2B1/HNRNPA3/HNRNPL/HNRNPU/HSPA8/IK/ISY1/KDM1A/KHSRP/LSM10/METTL3/NCL/PABPC1/PNN/PPARGC1A/PPIH/PPP1R8/PRMT1/PRPF38A/PRPF40A/PRPF6/PTBP1/RBM23/RBM25/RBM38/RBM39/RBM4B/RBMY1D/RNF113A/RNPC3/RTCB/SART1/SCAF1/SCAF11/SF3A1/SF3B2/SF3B3/SF3B5/SFPQ/SNRNP35/SNRNP40/SNRNP48/SNRPB/SNRPC/SNRPD2/SNRPG/SNU13/SON/SREK1/SRRM2/SRSF1/SRSF4/SRSF7/SYNCRIP/TADA2B/TAF12/TCERG1/THOC3/TRRAP/TXNL4A/USP49/WBP11/WDR83/YBX1/ZCRB1/ZMAT5/ZNF830	87
cluster4	BP	GO:0002831	regulation of response to biotic stimulus	71/2130	361/18903	0.00000185	0.002053062	0.001922131	ADAR/APOBEC3G/ARG1/ATG5/CARD9/CD160/CD180/CD96/CLEC6A/COCH/CRK/CRTAM/EP300/F2RL1/HAVCR2/HEXIM1/HLA-DRB3/HSPD1/HTRA1/IFNLR1/IL15/ILRUN/IRF1/IRF3/KLRC1/KLRC4/KLRD1/LEP/LGALS9/MAPKBP1/MED1/METTL3/NCF1/NCR3/NLRC5/NR1H2/NR1H3/PARP9/PLCG2/POLR3F/PPP2R3C/PTPN6/PUM1/PVR/PYHIN1/RNF135/RNF26/RNF31/SAMHD1/SELENOK/SFPQ/SH2D1A/SIGIRR/SLAMF8/SLC15A4/SPI1/STAT5B/SUSD4/TASL/TKFC/TRAF3/TRAF3IP2/TRAFD1/TREM2/TRIM21/TRIM38/TRIM44/UFD1/VSIG4/XIAP/YTHDF2	71
cluster4	BP	GO:0009259	ribonucleotide metabolic process	86/2130	466/18903	0.00000251	0.002153339	0.002016012	ABCC6/ABHD14B/ACOT4/ACOT9/ACSS1/ADA/ADCY10/ADSL/AK1/AMPD3/APRT/ARL2/ARNT/ATIC/ATP5F1A/ATP5PB/COASY/DGAT1/DHTKD1/DNAJC30/EIF6/ELOVL1/ELOVL3/ENO1/ENTPD5/EP300/FIS1/GAPDHS/GUCY2C/HACD2/HSPA1B/HSPA8/IMPDH1/IMPDH2/LDHA/MLST8/NADK/NCOR1/NDUFA11/NDUFA2/NDUFA3/NDUFA7/NDUFAB1/NDUFB5/NDUFB9/NDUFS1/NDUFS2/NDUFS7/NDUFS8/NME6/NT5C/NT5C1A/NUDT18/NUDT3/NUDT7/OGDH/OLA1/P2RX7/PANK1/PANK3/PDE4B/PDHX/PDK3/PFKL/PGAM1/PID1/PINK1/PPARGC1A/PRKAG1/PRKAG3/PRXL2C/RPTOR/SDHD/SLC25A1/SLC26A2/STOML2/SUCLA2/SULT1A3/SULT1A4/TREM2/UCK2/UCKL1/UMPS/UPB1/VCP/ZBTB20	86

cluster4	BP	GO:0090305	nucleic acid phosphodiester bond hydrolysis	54/2130	253/18903	0.00000256	0.002153339	0.002016012	ABT1/AGO2/ANG/ANGEL2/APTX/ASTE1/C11orf80/CNOT1/CNOT6/CNOT8/CSTF2T/DBR1/DCLRE1C/DICER1/DIS3L/DNASE1L1/EDC4/ELAC2/EME2/ENDOG/ERI1/EXOSC10/EXOSC4/EXOSC8/FEN1/FIP1L1/HORMAD1/INTS11/ISG20/KHNYN/MBLAC1/NOCT/NTHL1/OGG1/PCF11/PLD4/PNKP/POLG/POP7/PPP1R8/REXO1/REXO2/RNASE4/RPP25/RPP30/RPP38/SAMHD1/SETMAR/SLFN13/TATDN3/TSN/TSR1/TUT1/UTP23	54
cluster4	BP	GO:0009144	purine nucleoside triphosphate metabolic process	53/2130	247/18903	0.00000267	0.002153339	0.002016012	ABCC6/ADA/AK1/ARL2/ARNT/ATP5F1A/ATP5PB/DHTKD1/DNAJC30/EIF6/ENO1/EP300/FIS1/GAPDHS/HSPA1B/HSPA8/IMPDH1/IMPDH2/LDHA/MLST8/NADK/NCOR1/NDUFA11/NDUFA2/NDUFA3/NDUFA7/NDUFAB1/NDUFB5/NDUFB9/NDUFS1/NDUFS2/NDUFS7/NDUFS8/NME6/NUDT15/OGDH/OLA1/P2RX7/PFKL/PGAM1/PID1/PINK1/PPARGC1A/PRKAG1/PRKAG3/PRXL2C/RPTOR/SAMHD1/SDHD/STOML2/TREM2/VCP/ZBTB20	53
cluster4	BP	GO:0009141	nucleoside triphosphate metabolic process	56/2130	267/18903	0.00000297	0.002195785	0.002055752	ABCC6/ADA/AK1/ARL2/ARNT/ATP5F1A/ATP5PB/DHTKD1/DNAJC30/EIF6/ENO1/EP300/FIS1/GAPDHS/HSPA1B/HSPA8/IMPDH1/IMPDH2/LDHA/MLST8/NADK/NCOR1/NDUFA11/NDUFA2/NDUFA3/NDUFA7/NDUFAB1/NDUFB5/NDUFB9/NDUFS1/NDUFS2/NDUFS7/NDUFS8/NME6/NUDT15/OGDH/OLA1/P2RX7/PFKL/PGAM1/PID1/PINK1/PPARGC1A/PRKAG1/PRKAG3/PRXL2C/RPTOR/RRM2B/SAMHD1/SDHD/STOML2/TREM2/UCK2/UCKL1/VCP/ZBTB20	56
cluster4	BP	GO:0016570	histone modification	86/2130	474/18903	0.00000505	0.003443123	0.003223542	AKAP8L/ASH1L/ATG5/BABAM2/BAP1/BAZ2A/BRCC3/BRD8/BRMS1/CARM1/CDK9/CHD3/CHD4/CTNNB1/CTR9/DDB2/EP300/EPC2/EZH1/FBLL1/GATAD2B/GFI1/HDAC7/HOPX/HUWE1/IGF2/KANSL2/KAT6A/KAT8/KDM1A/KDM2A/KDM5A/KDM5C/KMT2A/KMT2B/KMT5C/METTL23/MLLT6/MSL2/NAP1L2/NELFA/NELFE/OTUB1/PAF1/PAGR1/PHF1/PHF2/PINK1/PPARGC1A/PRDM1/PRKD2/PRMT1/PRMT2/PWWP2B/RING1/RIOX1/RNF2/RNF40/RTF1/RUVBL1/RUVBL2/SET/SETD1B/SETMAR/SF3B3/SF3B5/SFPQ/SIRT1/SKI/SMARCAD1/SPI1/SRCAP/TADA2B/TAF12/TAF1L/TAF5/TET3/TP53/TRMT112/TRRAP/UBE2N/USP49/WDR82/ZNF274/ZNF304/ZNF335	86

cluster4	BP	GO:0019693	ribose phosphate metabolic process	86/2130	475/18903	0.00000549	0.003481774	0.003259728	ABCC6/ABHD14B/ACOT4/ACOT9/ACSS1/ADA/ADCY10/ADSL/AK1/AMPD3/APRT/ARL2/ARNT/ATIC/ATP5F1A/ATP5PB/COASY/DGAT1/DHTKD1/DNAJC30/EIF6/ELOVL1/ELOVL3/ENO1/ENTPD5/EP300/FIS1/GAPDHS/GUCY2C/HACD2/HSPA1B/HSPA8/IMPDH1/IMPDH2/LDHA/MLST8/NADK/NCOR1/NDUFA11/NDUFA2/NDUFA3/NDUFA7/NDUFAB1/NDUFB5/NDUFB9/NDUFS1/NDUFS2/NDUFS7/NDUFS8/NME6/NT5C/NT5C1A/NUDT18/NUDT3/NUDT7/OGDH/OLA1/P2RX7/PANK1/PANK3/PDE4B/PDHX/PDK3/PFKL/PGAM1/PID1/PINK1/PPARGC1A/PRKAG1/PRKAG3/PRXL2C/RPTOR/SDHD/SLC25A1/SLC26A2/STOML2/SUCLA2/SULT1A3/SULT1A4/TREM2/UCK2/UCKL1/UMPS/UPB1/VCP/ZBTB20	86
cluster4	BP	GO:0002703	regulation of leukocyte mediated immunity	51/2130	241/18903	0.0000062	0.003663986	0.00343032	ARG1/BLK/BST2/C4BPA/CD160/CD177/CD1A/CD1B/CD1C/CD96/CRK/CRTAM/CX3CR1/F2RL1/FES/GAB2/HAVCR2/HLA-DRA/HLA-DRB3/HMOX1/HSPD1/KLRC1/KLRC4/KLRD1/KMT5C/LEP/LGALS9/MLH1/NCKAP1L/NCR3/P2RX7/PLCG2/PRAM1/PTPN6/PVR/SASH3/SH2D1A/SHLD2/SLAMF1/SLC15A4/SPI1/STAT5B/STAT6/STX4/SUSD4/TNFSF13/TRAF2/TREM2/XCL1/ZBTB1/ZP3	51
cluster4	BP	GO:0046034	ATP metabolic process	47/2130	217/18903	0.00000738	0.003921252	0.00367118	ABCC6/AK1/ARL2/ARNT/ATP5F1A/ATP5PB/DHTKD1/DNAJC30/EIF6/ENO1/EP300/FIS1/GAPDHS/HSPA1B/HSPA8/LDHA/MLST8/NADK/NCOR1/NDUFA11/NDUFA2/NDUFA3/NDUFA7/NDUFAB1/NDUFB5/NDUFB9/NDUFS1/NDUFS2/NDUFS7/NDUFS8/OGDH/OLA1/P2RX7/PFKL/PGAM1/PID1/PINK1/PPARGC1A/PRKAG1/PRKAG3/PRXL2C/RPTOR/SDHD/STOML2/TREM2/VCP/ZBTB20	47
cluster4	BP	GO:0009199	ribonucleoside triphosphate metabolic process	52/2130	249/18903	0.00000751	0.003921252	0.00367118	ABCC6/AK1/ARL2/ARNT/ATP5F1A/ATP5PB/DHTKD1/DNAJC30/EIF6/ENO1/EP300/FIS1/GAPDHS/HSPA1B/HSPA8/IMPDH1/IMPDH2/LDHA/MLST8/NADK/NCOR1/NDUFA11/NDUFA2/NDUFA3/NDUFA7/NDUFAB1/NDUFB5/NDUFB9/NDUFS1/NDUFS2/NDUFS7/NDUFS8/NME6/OGDH/OLA1/P2RX7/PFKL/PGAM1/PID1/PINK1/PPARGC1A/PRKAG1/PRKAG3/PRXL2C/RPTOR/SDHD/STOML2/TREM2/UCK2/UCKL1/VCP/ZBTB20	52

cluster4	BP	GO:0034470	ncRNA processing	80/2130	439/18903	0.00000903	0.004450806	0.004166961	ABT1/ADAR/ADAT3/AGO2/ALKBH1/BUD23/CDK5RAP1/CLP1/DALRD3/DDX27/DDX5/DDX54/DICER1/DKC1/EIF6/ELAC2/ERI1/EXOSC10/EXOSC4/EXOSC8/FBLL1/FDXACB1/FTSJ3/GON7/GTPBP4/HNRNPA2B1/HSD17B10/INTS1/INTS11/INTS3/ISG20/KTI12/LAGE3/MAP2K2/METTL3/MRM2/NCOR1/NCOR2/NOLC1/NSUN4/NSUN5/PA2G4/PELP1/POP7/PTCD1/PUM1/RIOK1/RNF113A/RPL7/RPL7L1/RPP25/RPP25L/RPP30/RPP38/RPS27/RRP9/RTCB/SART1/SNU13/SSB/TP53/TRDMT1/TRMT10C/TRMT11/TRMT112/TRMT44/TRMT61A/TSN/TSR1/TUT1/URM1/UTP15/UTP23/UTP3/WBP11/WDR46/WDR74/WDR75/YTHDF2/ZNHIT3	80
cluster4	BP	GO:0009150	purine ribonucleotide metabolic process	81/2130	447/18903	0.00000987	0.004567012	0.004275757	ABCC6/ABHD14B/ACOT4/ACOT9/ACSS1/ADA/ADCY10/ADSL/AK1/AMPD3/APRT/ARL2/ARNT/ATIC/ATP5F1A/ATP5PB/COASY/DGAT1/DHTKD1/DNAJC30/EIF6/ELOVL1/ELOVL3/ENO1/EP300/FIS1/GAPDHS/GUCY2C/HACD2/HSPA1B/HSPA8/IMPDH1/IMPDH2/LDHA/MLST8/NADK/NCOR1/NDUFA11/NDUFA2/NDUFA3/NDUFA7/NDUFAB1/NDUFB5/NDUFB9/NDUFS1/NDUFS2/NDUFS7/NDUFS8/NME6/NT5C/NT5C1A/NUDT18/NUDT3/NUDT7/OGDH/OLA1/P2RX7/PANK1/PANK3/PDE4B/PDHX/PDK3/PFKL/PGAM1/PID1/PINK1/PPARGC1A/PRKAG1/PRKAG3/PRXL2C/RPTOR/SDHD/SLC25A1/SLC26A2/STOML2/SUCLA2/SULT1A3/SULT1A4/TREM2/VCP/ZBTB20	81
cluster4	BP	GO:0001909	leukocyte mediated cytotoxicity	33/2130	134/18903	0.0000104	0.004567012	0.004275757	ARG1/CD160/CD1A/CD1B/CD1C/CEBPG/CORO1A/CRK/CRTAM/CX3CR1/F2RL1/GZMB/GZMM/HAVCR2/HLA-DRA/KLRC1/KLRC4/KLRD1/LEP/LGALS9/MYD88/NCKAP1L/NCR3/P2RX7/PRDX1/PRF1/PTPN6/PVR/SH2D1A/SLAMF7/SPI1/STAT5B/XCL1	33
cluster4	BP	GO:0090501	RNA phosphodiester bond hydrolysis	37/2130	158/18903	0.0000108	0.004567012	0.004275757	ABT1/AGO2/ANG/ANGEL2/CNOT1/CNOT6/CNOT8/CSTF2T/DBR1/DICER1/DIS3L/ELAC2/ENDOG/ERI1/EXOSC10/EXOSC4/EXOSC8/FEN1/FIP1L1/INTS11/ISG20/KHNYN/MBLAC1/NOCT/PCF11/POP7/PPP1R8/REXO2/RNASE4/RPP25/RPP30/RPP38/SAMHD1/SLFN13/TSR1/TUT1/UTP23	37

cluster4	BP	GO:0045088	regulation of innate immune response	50/2130	240/18903	0.000012	0.004818846	0.00451153	ADAR/ARG1/ATG5/CARD9/CD160/CD96/CLEC6A/COCH/CRK/CRTAM/EP300/HAVCR2/HEXIM1/IRF1/IRF3/KLRC1/KLRC4/KLRD1/LEP/LGALS9/MED1/METTL3/NCF1/NCR3/NLRC5/NR1H2/NR1H3/PARP9/PLCG2/POLR3F/PTPN6/PVR/PYHIN1/RNF135/SAMHD1/SFPQ/SH2D1A/SLAMF8/SLC15A4/SPI1/STAT5B/SUSD4/TASL/TKFC/TRAFD1/TREM2/TRIM21/VSIG4/XIAP/YTHDF2	50
cluster4	BP	GO:0051054	positive regulation of DNA metabolic process	59/2130	301/18903	0.0000148	0.005371439	0.005028882	ANXA3/ARID1A/ATR/BABAM2/BAX/BCL7C/BRCC3/BRD8/CCT5/CCT6A/CDC7/CDT1/CEBPG/CTNNB1/DBF4B/DKC1/ENDOG/EPC2/FAM168A/GNL3/H2AX/HGF/HMBOX1/HNRNPA2B1/HSP90AB1/KLF4/KMT5C/LPIN1/MAP2K7/MGMT/MLH1/OTUD4/PARP9/PDGFB/PNKP/PPARGC1A/PPP1R10/PRKD2/RUVBL1/RUVBL2/SETMAR/SHLD2/SIRT1/SLF2/SMARCA2/SMARCC2/SMARCD2/SPIDR/STAT6/STOML2/TCP1/TINF2/TNFSF13/TRIM28/TRRAP/UBE2N/XRCC1/YY1/ZCWPW1	59
cluster4	BP	GO:0016072	rRNA metabolic process	54/2130	268/18903	0.000015	0.005371439	0.005028882	ABT1/ANG/BRF1/BUD23/CAVIN1/DDX27/DDX54/DIS3L/DKC1/EIF6/ERI1/EXOSC10/EXOSC4/EXOSC8/FBLL1/FDXACB1/FTSJ3/GTF3A/GTF3C5/GTPBP4/ISG20/MARS1/MRM2/NCL/NOLC1/NSUN4/NSUN5/PA2G4/PELP1/POP7/RIOK1/RPL7/RPL7L1/RPP25/RPP30/RPP38/RPS27/RRP9/SART1/SLFN13/SNU13/TCOF1/TP53/TRMT112/TSR1/UTP15/UTP23/UTP3/WBP11/WDR46/WDR74/WDR75/YTHDF2/ZNHIT3	54
cluster4	BP	GO:0009205	purine ribonucleoside triphosphate metabolic process	50/2130	242/18903	0.0000151	0.005371439	0.005028882	ABCC6/AK1/ARL2/ARNT/ATP5F1A/ATP5PB/DHTKD1/DNAJC30/EIF6/ENO1/EP300/FIS1/GAPDHS/HSPA1B/HSPA8/IMPDH1/IMPDH2/LDHA/MLST8/NADK/NCOR1/NDUFA11/NDUFA2/NDUFA3/NDUFA7/NDUFAB1/NDUFB5/NDUFB9/NDUFS1/NDUFS2/NDUFS7/NDUFS8/NME6/OGDH/OLA1/P2RX7/PFKL/PGAM1/PID1/PINK1/PPARGC1A/PRKAG1/PRKAG3/PRXL2C/RPTOR/SDHD/STOML2/TREM2/VCP/ZBTB20	50

cluster4	BP	GO:0022613	ribonucleoprotein complex biogenesis	86/2130	489/18903	0.0000173	0.00589883	0.005522639	AATF/ABCE1/ABT1/ADAR/AGO2/ATR/BUD23/CD2BP2/CLP1/COIL/DDX27/DDX54/DICER1/DKC1/EIF2S3/EIF3C/EIF3CL/EIF3G/EIF4B/EIF5/EIF6/ERI1/EXOSC10/EXOSC4/EXOSC8/FBLL1/FDXACB1/FTSJ3/GNL2/GTF3A/GTPBP10/GTPBP4/HSP90AB1/ISG20/ISY1/LTV1/MCTS1/MRM2/MRPS2/NGRN/NOLC1/NSUN4/NSUN5/PA2G4/PELP1/POP7/PRPF6/RAMAC/RIOK1/RPL24/RPL7/RPL7L1/RPP25/RPP30/RPP38/RPS27/RRN3/RRP9/RUVBL1/RUVBL2/SART1/SCAF11/SF3A1/SF3B2/SF3B3/SF3B5/SNRPB/SNRPC/SNRPD2/SNRPG/SNU13/SRSF1/TRMT112/TSC1/TSR1/TXNL4A/UTP15/UTP23/UTP3/WBP11/WDR46/WDR74/WDR75/YTHDF2/ZNF658/ZNHIT3	86
cluster4	BP	GO:0006163	purine nucleotide metabolic process	84/2130	476/18903	0.000019	0.006205137	0.005809412	ABCC6/ABHD14B/ACOT4/ACOT9/ACSS1/ADA/ADCY10/ADSL/AK1/AMPD3/APRT/ARL2/ARNT/ATIC/ATP5F1A/ATP5PB/COASY/DGAT1/DHTKD1/DNAJC30/EIF6/ELOVL1/ELOVL3/ENO1/EP300/FIS1/GAPDHS/GUCY2C/HACD2/HSPA1B/HSPA8/IMPDH1/IMPDH2/LDHA/MLST8/NADK/NCOR1/NDUFA11/NDUFA2/NDUFA3/NDUFA7/NDUFAB1/NDUFB5/NDUFB9/NDUFS1/NDUFS2/NDUFS7/NDUFS8/NME6/NT5C/NT5C1A/NT5C1B/NUDT15/NUDT18/NUDT3/NUDT7/OGDH/OLA1/P2RX7/PANK1/PANK3/PDE4B/PDHX/PDK3/PFKL/PGAM1/PID1/PINK1/PPARGC1A/PRKAG1/PRKAG3/PRXL2C/RPTOR/SAMHD1/SDHD/SLC25A1/SLC26A2/STOML2/SUCLA2/SULT1A3/SULT1A4/TREM2/VCP/ZBTB20	84
cluster4	BP	GO:0006282	regulation of DNA repair	45/2130	212/18903	0.0000196	0.006205137	0.005809412	ARID1A/ATR/BABAM2/BCL7C/BRCC3/BRD8/CDK9/CEBPG/EPC2/FAM168A/FBH1/H2AX/KDM1A/KMT5C/MAGEF1/MGMT/OGG1/OTUB1/PARP9/PNKP/PPP4C/RECQL5/RPA2/RUVBL1/RUVBL2/SETMAR/SF3B3/SF3B5/SHLD2/SIRT1/SLF2/SMARCA2/SMARCC2/SMARCD2/SPIDR/TADA2B/TAF12/TAF5/TRIM28/TRRAP/UBE2N/UBQLN4/XRCC1/YY1/ZCWPW1	45
cluster4	BP	GO:0001910	regulation of leukocyte mediated cytotoxicity	24/2130	87/18903	0.0000223	0.006810267	0.006375951	ARG1/CD160/CD1A/CD1B/CD1C/CRK/CRTAM/CX3CR1/F2RL1/HAVCR2/HLA-DRA/KLRC1/KLRC4/KLRD1/LEP/LGALS9/NCKAP1L/NCR3/P2RX7/PVR/SH2D1A/SPI1/STAT5B/XCL1	24

cluster4	BP	GO:0006302	double-strand break repair	58/2130	300/18903	0.0000265	0.007838515	0.007338623	APTX/ARID1A/ATR/BABAM2/BCL7C/BRCC3/BRD8/CDC7/CIB1/DCLRE1C/EME2/EPC2/FBH1/FEN1/H2AX/IFFO1/INTS3/KDM1A/KDM2A/KMT5C/LIG4/MAGEF1/MGMT/MLH1/NUCKS1/OGG1/OTUB1/PARP9/PAXX/PNKP/POLA1/PPP4C/RAD51C/RECQL5/RPA2/RPA3/RUVBL1/RUVBL2/SAMHD1/SETMAR/SFPQ/SHLD2/SLF2/SMARCA2/SMARCAD1/SMARCC2/SMARCD2/SMC5/SPIDR/TP53/TRRAP/UBE2N/UBQLN4/VCP/XRCC1/YY1/ZCWPW1/ZFYVE26	58
cluster4	BP	GO:0002705	positive regulation of leukocyte mediated immunity	33/2130	140/18903	0.0000275	0.007856431	0.007355397	ARG1/CD160/CD177/CD1A/CD1B/CD1C/CRTAM/F2RL1/HLA-DRA/HLA-DRB3/HSPD1/KLRC4/KLRD1/KMT5C/MLH1/NCR3/P2RX7/PLCG2/PVR/SASH3/SH2D1A/SHLD2/SLAMF1/SPI1/STAT5B/STAT6/STX4/TNFSF13/TRAF2/TREM2/XCL1/ZBTB1/ZP3	33
cluster4	BP	GO:0019080	viral gene expression	26/2130	100/18903	0.0000321	0.008904501	0.008336627	ATG5/CDK9/EDEM2/EIF3G/EP300/HEXIM1/IFITM3/MAN2A1/MCTS1/MGAT2/MOGS/MON1B/NUCKS1/PARP9/PRMT1/PTBP1/SP1/SSB/ST6GALNAC2/ST6GALNAC3/ST6GALNAC4/TRIM11/TRIM13/TRIM21/TRIM32/USF1	26
cluster4	BP	GO:2001252	positive regulation of chromosome organization	27/2130	106/18903	0.0000339	0.009116166	0.008534794	ATR/CCT5/CCT6A/CDT1/CTNNB1/DKC1/FEN1/GNL3/HMBOX1/HNRNPA2B1/LIG4/MAP2K7/NCAPD2/PNKP/PPHLN1/PPP1R10/RUVBL1/RUVBL2/SFPQ/SLF2/SMC4/SMC5/TCP1/TINF2/TRIM28/YY1/ZNF304	27

cluster4	BP	GO:1903578	regulation of ATP metabolic process	21/2130	73/18903	0.000036	0.009395834	0.008796626	ARL2/ARNT/DNAJC30/EIF6/ENO1/EP300/FIS1/GAPDHS/MLST8/NCOR1/P2RX7/PGAM1/PID1/PINK1/PPARGC1A/PRKAG1/PRXL2C/RPTOR/TREM2/VCP/ZBTB20	21
cluster4	BP	GO:0002706	regulation of lymphocyte mediated immunity	39/2130	179/18903	0.0000371	0.009395834	0.008796626	ARG1/C4BPA/CD160/CD1A/CD1B/CD1C/CD96/CRK/CRTAM/HAVCR2/HLA-DRA/HLA-DRB3/HSPD1/KLRC1/KLRC4/KLRD1/KMT5C/LEP/LGALS9/MLH1/NCKAP1L/NCR3/P2RX7/PTPN6/PVR/SASH3/SH2D1A/SHLD2/SLAMF1/SLC15A4/STAT5B/STAT6/SUSD4/TNFSF13/TRAF2/TREM2/XCL1/ZBTB1/ZP3	39
cluster4	BP	GO:0033044	regulation of chromosome organization	50/2130	252/18903	0.0000464	0.011127537	0.010417892	ANAPC1/ARID1A/ATR/BCL7C/BUB3/CCT5/CCT6A/CDC23/CDT1/CTNNB1/DKC1/EXOSC10/FEN1/GNL3/HMBOX1/HNRNPA2B1/HNRNPU/IK/INCENP/INO80B/KLHL22/LCMT1/LIG4/MAD1L1/MAP2K7/NCAPD2/PNKP/PPHLN1/PPP1R10/PSMG2/RB1/RUVBL1/RUVBL2/SETMAR/SFPQ/SLF2/SMARCA2/SMARCC2/SMARCD2/SMC4/SMC5/SMG1/SPC24/TCP1/TINF2/TRIM28/UPF1/XRCC1/YY1/ZNF304	50
cluster4	BP	GO:0031341	regulation of cell killing	26/2130	102/18903	0.0000464	0.011127537	0.010417892	ARG1/CD160/CD1A/CD1B/CD1C/CRK/CRTAM/CX3CR1/F2RL1/HAVCR2/HLA-DRA/HSP90AB1/KLRC1/KLRC4/KLRD1/LEP/LGALS9/NCKAP1L/NCR3/P2RX7/PRF1/PVR/SH2D1A/SPI1/STAT5B/XCL1	26

cluster4	BP	GO:0140053	mitochondrial gene expression	36/2130	163/18903	0.0000537	0.012518052	0.011719729	ALKBH1/AURKAIP1/CDK5RAP1/ELAC2/FASTK/GADD45GIP1/HSD17B10/MRPL16/MRPL23/MRPL24/MRPL3/MRPL4/MRPL40/MRPL43/MRPL48/MRPL50/MRPL52/MRPL53/MRPL55/MRPL9/MRPS16/MRPS18A/MRPS2/MRPS22/MRPS24/MTIF2/NDUFA7/NGRN/PPARGC1B/PTCD1/QRSL1/TEFM/THAP11/TRMT10C/TUFM/YARS2	36
cluster4	BP	GO:0006399	tRNA metabolic process	41/2130	195/18903	0.0000564	0.012518052	0.011719729	ADAT3/ALKBH1/ANG/BRF1/CARS1/CARS2/CDK5RAP1/CLP1/DALRD3/DICER1/DTD1/ELAC2/EXOSC10/EXOSC8/GON7/GTF3C5/HSD17B10/KTI12/LAGE3/MARS1/PARS2/POP7/PSTK/PTCD1/QARS1/QRSL1/RPP25/RPP25L/RPP30/RPP38/RTCB/SLFN13/SSB/TRDMT1/TRMT10C/TRMT11/TRMT112/TRMT44/TRMT61A/URM1/YARS2	41
cluster4	BP	GO:0009260	ribonucleotide biosynthetic process	47/2130	234/18903	0.0000564	0.012518052	0.011719729	ACSS1/ADA/ADCY10/ADSL/AK1/AMPD3/APRT/ATIC/ATP5F1A/ATP5PB/COASY/DNAJC30/ELOVL1/ELOVL3/ENO1/GUCY2C/HACD2/IMPDH1/IMPDH2/NDUFA11/NDUFA2/NDUFA3/NDUFA7/NDUFAB1/NDUFB5/NDUFB9/NDUFS1/NDUFS2/NDUFS7/NDUFS8/NME6/PANK1/PANK3/PDHX/PDK3/PID1/PINK1/PPARGC1A/SDHD/SLC25A1/SLC26A2/STOML2/TREM2/UCK2/UCKL1/UMPS/VCP	47
cluster4	BP	GO:0001906	cell killing	40/2130	189/18903	0.0000592	0.012812347	0.011995255	ARG1/ATN1/CD160/CD1A/CD1B/CD1C/CEBPG/CORO1A/CRK/CRTAM/CX3CR1/DEFA1/DEFA1B/DEFA3/DEFA4/F2RL1/GNLY/GZMB/GZMM/HAVCR2/HLA-DRA/HSP90AB1/KLRC1/KLRC4/KLRD1/LEP/LGALS9/MYD88/NCKAP1L/NCR3/P2RX7/PRDX1/PRF1/PTPN6/PVR/SH2D1A/SLAMF7/SPI1/STAT5B/XCL1	40

cluster4	BP	GO:0010822	positive regulation of mitochondrion organization	21/2130	76/18903	0.0000692	0.014285613	0.013374566	BAK1/BAX/BBC3/CAMKK2/ENDOG/FAM162A/FIS1/GZMB/HTRA2/NMT1/PDCD5/PGAM5/PINK1/PMAIP1/PPARGC1A/STOML2/TP53/TREM2/VDAC1/VPS35/ZDHHC6	21
cluster4	BP	GO:0002832	negative regulation of response to biotic stimulus	28/2130	116/18903	0.0000692	0.014285613	0.013374566	ADAR/ARG1/ATG5/CD96/CRK/HAVCR2/HTRA1/ILRUN/KLRC1/KLRD1/LGALS9/MAPKBP1/METTL3/NLRC5/NR1H2/NR1H3/RNF26/SAMHD1/SIGIRR/SLAMF8/SUSD4/TKFC/TRAFD1/TRIM21/TRIM38/UFD1/VSIG4/YTHDF2	28
cluster4	BP	GO:0051607	defense response to virus	57/2130	304/18903	0.0000754	0.015193101	0.01422418	ADAR/AKAP1/APOBEC3D/APOBEC3G/ATG5/BST2/CARD9/DDX60L/DEFA1/DEFA1B/DEFA3/EXOSC4/F2RL1/GBP1/HTRA1/IFITM3/IFNLR1/IL15/ILRUN/IRF1/IRF3/IRF5/IRF9/ISG20/MAP3K14/MLKL/MYD88/NLRC5/NMB/PARP9/PHB2/PMAIP1/POLR3F/PRF1/PUM1/RNF135/RNF26/SAMHD1/SELENOK/SLFN11/SLFN13/TKFC/TRAF3/TRAF3IP2/TRIM11/TRIM13/TRIM21/TRIM25/TRIM28/TRIM32/TRIM34/TRIM38/TRIM44/TRIM52/TTC4/UFD1/ZC3HAV1	57
cluster4	BP	GO:0140546	defense response to symbiont	57/2130	305/18903	0.0000827	0.016250425	0.015214074	ADAR/AKAP1/APOBEC3D/APOBEC3G/ATG5/BST2/CARD9/DDX60L/DEFA1/DEFA1B/DEFA3/EXOSC4/F2RL1/GBP1/HTRA1/IFITM3/IFNLR1/IL15/ILRUN/IRF1/IRF3/IRF5/IRF9/ISG20/MAP3K14/MLKL/MYD88/NLRC5/NMB/PARP9/PHB2/PMAIP1/POLR3F/PRF1/PUM1/RNF135/RNF26/SAMHD1/SELENOK/SLFN11/SLFN13/TKFC/TRAF3/TRAF3IP2/TRIM11/TRIM13/TRIM21/TRIM25/TRIM28/TRIM32/TRIM34/TRIM38/TRIM44/TRIM52/TTC4/UFD1/ZC3HAV1	57

cluster4	BP	GO:1903580	positive regulation of ATP metabolic process	13/2130	36/18903	0.0000859	0.016250425	0.015214074	ARNT/ENO1/GAPDHS/MLST8/P2RX7/PID1/PINK1/PPARGC1A/PRXL2C/RPTOR/TREM2/VCP/ZBTB20	13
cluster4	BP	GO:0000375	RNA splicing, via transesterification reactions	61/2130	333/18903	0.0000861	0.016250425	0.015214074	CD2BP2/CIRBP/COIL/CWF19L1/DBR1/DDX39A/DDX5/DNAJC17/ESRP2/ESS2/FMR1/HNRNPA2B1/HNRNPA3/HNRNPL/HNRNPU/HSPA8/IK/ISY1/KDM1A/KHSRP/METTL3/NCL/PABPC1/PNN/PPIH/PRPF38A/PRPF40A/PRPF6/PTBP1/RBM23/RBM25/RBM38/RBM39/RBM4B/RNF113A/RNPC3/SART1/SCAF11/SF3A1/SF3B2/SF3B3/SF3B5/SFPQ/SNRNP35/SNRNP40/SNRPB/SNRPC/SNRPD2/SNRPG/SNU13/SON/SRRM2/SRSF1/SRSF4/SRSF7/SYNCRIP/TXNL4A/USP49/WBP11/WDR83/ZCRB1	61
cluster4	BP	GO:0006401	RNA catabolic process	56/2130	299/18903	0.0000895	0.016534946	0.015480451	AGO2/ANG/ANGEL2/CIRBP/CNOT1/CNOT6/CNOT8/DDX5/DICER1/DIS3L/DKC1/EDC4/ELAC2/EXOSC10/EXOSC4/EXOSC8/FASTK/FEN1/FMR1/GIGYF2/HNRNPA0/HNRNPU/HSPA1B/ISG20/KHSRP/LARP1/LARP4B/METTL3/MLH1/MYD88/NOCT/PABPC1/PHAX/PPP1R8/PUM1/RBM38/RNASEH2C/SAMD4B/SIDT2/SLFN13/SMG1/SSB/SYNCRIP/TENT5A/TRAF2/TRAF3IP2/TRDMT1/TTC5/UPF1/UPF3A/UPF3B/WDR82/YBX1/YTHDF2/ZC3HAV1/ZSWIM8	56
cluster4	BP	GO:0009152	purine ribonucleotide biosynthetic process	44/2130	220/18903	0.000106215	0.018290053	0.017123628	ACSS1/ADA/ADCY10/ADSL/AK1/AMPD3/APRT/ATIC/ATP5F1A/ATP5PB/COASY/DNAJC30/ELOVL1/ELOVL3/ENO1/GUCY2C/HACD2/IMPDH1/IMPDH2/NDUFA11/NDUFA2/NDUFA3/NDUFA7/NDUFAB1/NDUFB5/NDUFB9/NDUFS1/NDUFS2/NDUFS7/NDUFS8/NME6/PANK1/PANK3/PDHX/PDK3/PID1/PINK1/PPARGC1A/SDHD/SLC25A1/SLC26A2/STOML2/TREM2/VCP	44

cluster4	BP	GO:0032543	mitochondrial translation	30/2130	131/18903	0.000109454	0.018290053	0.017123628	ALKBH1/AURKAIP1/CDK5RAP1/GADD45GIP1/MRPL16/MRPL23/MRPL24/MRPL3/MRPL4/MRPL40/MRPL43/MRPL48/MRPL50/MRPL52/MRPL53/MRPL55/MRPL9/MRPS16/MRPS18A/MRPS2/MRPS22/MRPS24/MTIF2/NDUFA7/NGRN/PTCD1/QRSL1/TRMT10C/TUFM/YARS2	30
cluster4	BP	GO:0000377	RNA splicing, via transesterification reactions with bulged adenosine as nucleophile	60/2130	329/18903	0.000111336	0.018290053	0.017123628	CD2BP2/CIRBP/COIL/CWF19L1/DBR1/DDX39A/DDX5/DNAJC17/ESRP2/ESS2/FMR1/HNRNPA2B1/HNRNPA3/HNRNPL/HNRNPU/HSPA8/IK/ISY1/KDM1A/METTL3/NCL/PABPC1/PNN/PPIH/PRPF38A/PRPF40A/PRPF6/PTBP1/RBM23/RBM25/RBM38/RBM39/RBM4B/RNF113A/RNPC3/SART1/SCAF11/SF3A1/SF3B2/SF3B3/SF3B5/SFPQ/SNRNP35/SNRNP40/SNRPB/SNRPC/SNRPD2/SNRPG/SNU13/SON/SRRM2/SRSF1/SRSF4/SRSF7/SYNCRIP/TXNL4A/USP49/WBP11/WDR83/ZCRB1	60
cluster4	BP	GO:0000398	mRNA splicing, via spliceosome	60/2130	329/18903	0.000111336	0.018290053	0.017123628	CD2BP2/CIRBP/COIL/CWF19L1/DBR1/DDX39A/DDX5/DNAJC17/ESRP2/ESS2/FMR1/HNRNPA2B1/HNRNPA3/HNRNPL/HNRNPU/HSPA8/IK/ISY1/KDM1A/METTL3/NCL/PABPC1/PNN/PPIH/PRPF38A/PRPF40A/PRPF6/PTBP1/RBM23/RBM25/RBM38/RBM39/RBM4B/RNF113A/RNPC3/SART1/SCAF11/SF3A1/SF3B2/SF3B3/SF3B5/SFPQ/SNRNP35/SNRNP40/SNRPB/SNRPC/SNRPD2/SNRPG/SNU13/SON/SRRM2/SRSF1/SRSF4/SRSF7/SYNCRIP/TXNL4A/USP49/WBP11/WDR83/ZCRB1	60
cluster4	BP	GO:0006913	nucleocytoplasmic transport	60/2130	329/18903	0.000111336	0.018290053	0.017123628	AAAS/ABCE1/AGFG1/AKAP13/AKAP8L/CALR/CDH1/CDK5/DDX39A/DESI1/EFCAB7/EIF6/FLNA/HNRNPA2B1/HSP90AB1/HSPA9/IPO13/IPO7/JUP/KPNA2/KPNB1/LEP/LTV1/MED1/NOLC1/NUP107/PABPN1/PHAX/PHB2/PIK3R2/POM121/POM121C/PPP1CC/PPP1R10/PRKAG1/RANBP1/RGPD4/RGPD6/RGS14/RPAIN/SEC13/SMG1/SSB/STK4/STRADA/SUFU/THOC3/TMEM53/TNPO2/TNPO3/TP53/TRAF3IP2/TRIM28/TSC1/TSC2/TXNIP/UPF1/UPF3A/UPF3B/ZC3H11A	60

cluster4	BP	GO:0051169	nuclear transport	60/2130	329/18903	0.000111336	0.018290053	0.017123628	AAAS/ABCE1/AGFG1/AKAP13/AKAP8L/CALR/CDH1/CDK5/DDX39A/DESI1/EFCAB7/EIF6/FLNA/HNRNPA2B1/HSP90AB1/HSPA9/IPO13/IPO7/JUP/KPNA2/KPNB1/LEP/LTV1/MED1/NOLC1/NUP107/PABPN1/PHAX/PHB2/PIK3R2/POM121/POM121C/PPP1CC/PPP1R10/PRKAG1/RANBP1/RGPD4/RGPD6/RGS14/RPAIN/SEC13/SMG1/SSB/STK4/STRADA/SUFU/THOC3/TMEM53/TNPO2/TNPO3/TP53/TRAF3IP2/TRIM28/TSC1/TSC2/TXNIP/UPF1/UPF3A/UPF3B/ZC3H11A	60
cluster4	BP	GO:0018205	peptidyl-lysine modification	72/2130	414/18903	0.000116218	0.018744968	0.017549532	ARNT/ASH1L/ATG5/BAG6/BRD8/CBX4/CTNNB1/CTR9/DESI1/DIP2B/EEF1AKMT3/EP300/EPC2/EZH1/GFI1/GNL3/HDAC7/HINT2/HMG20A/KANSL2/KAT6A/KAT8/KDM1A/KMT2A/KMT2B/KMT5C/LIPT1/MDM2/MLLT6/MSL2/NAP1L2/NDUFAB1/NELFA/NELFE/NFATC2IP/PAGR1/PHF1/PLOD1/POR/PPARGC1A/PWWP2B/RNF113A/RTF1/RUVBL1/RUVBL2/SET/SETD1B/SETMAR/SF3B3/SF3B5/SIRT1/SIRT4/SLF2/SMC5/SOX4/SPI1/SRCAP/TADA2B/TAF12/TAF1L/TAF5/TET3/TRIM28/TRIM38/TRMT112/TRRAP/VCPKMT/WDR82/ZMIZ1/ZNF274/ZNF304/ZNF335	72
cluster4	BP	GO:0046390	ribose phosphate biosynthetic process	47/2130	241/18903	0.000118999	0.01885076	0.017648576	ACSS1/ADA/ADCY10/ADSL/AK1/AMPD3/APRT/ATIC/ATP5F1A/ATP5PB/COASY/DNAJC30/ELOVL1/ELOVL3/ENO1/GUCY2C/HACD2/IMPDH1/IMPDH2/NDUFA11/NDUFA2/NDUFA3/NDUFA7/NDUFAB1/NDUFB5/NDUFB9/NDUFS1/NDUFS2/NDUFS7/NDUFS8/NME6/PANK1/PANK3/PDHX/PDK3/PID1/PINK1/PPARGC1A/SDHD/SLC25A1/SLC26A2/STOML2/TREM2/UCK2/UCKL1/UMPS/VCP	47
cluster4	BP	GO:0006403	RNA localization	41/2130	202/18903	0.000128396	0.019982541	0.01870818	AAAS/AGFG1/AKAP8L/ATR/CCT5/CCT6A/DDX39A/DKC1/EXOSC10/FMR1/HNRNPA2B1/HNRNPU/KHSRP/KPNB1/MVP/NUP107/PABPN1/PARP11/PHAX/POM121/POM121C/PRPF6/RUVBL1/RUVBL2/SEC13/SIDT2/SMG1/SRSF1/SRSF7/SSB/TCP1/THOC3/TSC1/UPF1/UPF3A/UPF3B/YBX1/YY1/ZC3H11A/ZNF385A/ZNHIT3	41

cluster4	BP	GO:0023035	CD40 signaling pathway	8/2130	16/18903	0.000143146	0.021522865	0.020150271	FANCA/ITGA5/PHB2/RNF31/SHARPIN/SLAMF1/TRAF3IP2/TREM2	8
cluster4	BP	GO:0099116	tRNA 5'-end processing	8/2130	16/18903	0.000143146	0.021522865	0.020150271	HSD17B10/POP7/RPP25/RPP25L/RPP30/RPP38/SSB/TRMT10C	8
cluster4	BP	GO:0043516	regulation of DNA damage response, signal transduction by p53 class mediator	13/2130	38/18903	0.000162069	0.02352932	0.022028768	ATR/CD44/DDX5/KDM1A/MDM2/PMAIP1/PYHIN1/SIRT1/SOX4/SPRED1/TP53/ZNF385A/ZNHIT1	13
cluster4	BP	GO:0006739	NADP metabolic process	14/2130	43/18903	0.000167424	0.02352932	0.022028768	DCXR/MDH1/ME2/MLST8/NADK/NOCT/NUDT17/PGAM1/PGLS/RBKS/RPTOR/SHPK/TP53/TP53I3	14

cluster4	BP	GO:0034504	protein localization to nucleus	56/2130	306/18903	0.000168697	0.02352932	0.022028768	ARL2/ARL2BP/CALR/CCT5/CCT6A/CD2AP/CDH1/CDK5/CNEP1R1/CTDNEP1/CTNNA1/DKC1/EFCAB7/FLNA/HNRNPU/HSP90AB1/ILRUN/IPO13/IPO7/JUP/KPNA2/KPNB1/LATS2/LEP/MDM2/MED1/MFHAS1/NOLC1/NUP107/PAF1/PARP9/PHB2/PIK3R2/PIN1/POM121/POM121C/PPP1R10/PYHIN1/RASSF5/RGPD4/RGPD6/RPAIN/SEC13/STK4/SUFU/TAF8/TCP1/TESK1/TNPO2/TNPO3/TOR1AIP1/TP53/TRAF3IP2/TRIM28/TSC2/TXNIP	56
cluster4	BP	GO:0045785	positive regulation of cell adhesion	81/2130	484/18903	0.000168816	0.02352932	0.022028768	ABL2/ADA/ADGRG1/ALOX5/APBB1IP/ARID1A/ARL2/CALR/CCL28/CD160/CD209/CD44/CDH13/CIB1/CORO1A/CRK/CSK/DNAJA3/DNM2/DOCK8/EFEMP2/FLNA/FUT1/GCNT1/HAVCR2/HLA-DMA/HLA-DQA1/HLA-DQA2/HLA-DRA/HLA-DRB3/HLA-DRB5/HSPD1/IGF2/IL15/IL2RA/ITGA5/JUP/LEP/LGALS9/NCKAP1L/NEDD9/OLFM4/P4HB/PDGFB/PIEZO1/PIK3R2/PLEKHA2/PODXL/POLDIP2/PPM1F/PREX1/PRKD2/PTPN23/PTPN6/RASAL3/RIN2/RRAS/SART1/SASH3/SELENOK/SLAMF1/SMARCA2/SMARCC2/SMARCD2/SOX4/STAT5B/STK4/STX4/TESK1/TGM2/TNFRSF13C/TNFRSF14/TSC1/UTRN/VNN1/XCL1/YES1/ZAP70/ZBTB1/ZMIZ1/ZP3	81
cluster4	BP	GO:2001242	regulation of intrinsic apoptotic signaling pathway	36/2130	172/18903	0.000170769	0.02352932	0.022028768	ARHGEF2/ARMC10/BAG5/BAX/BBC3/CCAR2/CD44/CREB3/CYLD/EIF5A/ENO1/ERP29/FBH1/FIS1/HTRA2/HYOU1/KDM1A/MDM2/P4HB/PARL/PINK1/PMAIP1/RRM2B/RRN3/SELENOS/SFPQ/SIRT1/SLC9A3R1/TP53/TPT1/TREM2/TRIM32/TXNDC12/UBB/VNN1/ZNF385A	36
cluster4	BP	GO:0045739	positive regulation of DNA repair	29/2130	128/18903	0.000172405	0.02352932	0.022028768	ARID1A/BABAM2/BCL7C/BRCC3/BRD8/CEBPG/EPC2/FAM168A/H2AX/KMT5C/MGMT/PARP9/PNKP/RUVBL1/RUVBL2/SETMAR/SHLD2/SIRT1/SLF2/SMARCA2/SMARCC2/SMARCD2/SPIDR/TRIM28/TRRAP/UBE2N/XRCC1/YY1/ZCWPW1	29

cluster4	BP	GO:0031348	negative regulation of defense response	51/2130	272/18903	0.000175256	0.023556006	0.022053751	ADA/ADAR/ALOX5/ARG1/ASH1L/ATG5/C1QTNF12/CD200R1/CD96/CNR2/CRK/CYLD/FEM1A/HAVCR2/HGF/HTRA1/IL2RA/ILRUN/KLF4/KLRC1/KLRD1/LDLR/LGALS9/MAPKBP1/METTL3/MFHAS1/NLRC5/NLRP12/NR1H2/NR1H3/PROC/PSMA1/RB1/RHBDF2/RNF26/SAMHD1/SELENOS/SHARPIN/SLAMF8/SUSD4/TKFC/TNFAIP6/TRAFD1/TREM2/TRIM21/TRIM38/UFD1/VPS35/VSIG4/YES1/YTHDF2	51
cluster4	BP	GO:0030522	intracellular receptor signaling pathway	50/2130	266/18903	0.000189513	0.024846736	0.023262167	ACTN4/ARID1A/ARNT/CALR/CARM1/CNOT1/CTBP2/CYLD/CYP27B1/DDX5/DDX54/DEFA1/DEFA1B/DEFA3/EP300/ESRRA/FAM120B/HSPA1B/IRF3/KDM1A/KLF2/LEP/MED1/NCOR1/NCOR2/NR1H2/NR1H3/NR3C1/PAGR1/PDK3/PHB2/PPARGC1B/PRCP/PRMT2/PUM1/RNF135/RNF34/RORC/RXRA/SAFB/SIRT1/SLC15A4/STRN3/TIFA/TKFC/TRIP4/UBA5/UFD1/XIAP/ZMIZ1	50
cluster4	BP	GO:0001912	positive regulation of leukocyte mediated cytotoxicity	17/2130	59/18903	0.000190461	0.024846736	0.023262167	ARG1/CD160/CD1A/CD1B/CD1C/CRTAM/F2RL1/HLA-DRA/KLRC4/KLRD1/NCR3/P2RX7/PVR/SH2D1A/SPI1/STAT5B/XCL1	17
cluster4	BP	GO:2000779	regulation of double-strand break repair	30/2130	135/18903	0.000193463	0.024872659	0.023286437	ARID1A/ATR/BCL7C/BRD8/EPC2/FBH1/KDM1A/KMT5C/MAGEF1/MGMT/OGG1/OTUB1/PARP9/PNKP/PPP4C/RECQL5/RPA2/RUVBL1/RUVBL2/SETMAR/SHLD2/SLF2/SMARCA2/SMARCC2/SMARCD2/SPIDR/TRRAP/UBE2N/UBQLN4/ZCWPW1	30

cluster4	BP	GO:0016032	viral process	72/2130	421/18903	0.000196368	0.024885476	0.023298436	ADAR/APOBEC3D/APOBEC3G/ATG16L2/ATG5/BST2/CALCOCO2/CD209/CDK9/CHMP2B/CLEC5A/CTBP2/CXCR6/EDEM2/EIF3G/EP300/FAM111A/FMR1/GFI1/HAVCR1/HEXIM1/HSP90AB1/HSPA1B/HSPA8/IFITM3/IGF2R/ISG20/ITGA5/KPNA2/LARP1/LDLR/LGALS9/LIG4/MAN2A1/MCTS1/MGAT2/MOGS/MON1B/NUCKS1/P4HB/PABPC1/PARP9/PCBP1/PPIH/PRMT1/PTBP1/PVR/SIGLEC1/SLAMF1/SLC3A2/SP1/SSB/ST6GALNAC2/ST6GALNAC3/ST6GALNAC4/TNFRSF14/TP53/TRIM11/TRIM13/TRIM21/TRIM25/TRIM28/TRIM32/TRIM34/TRIM38/USF1/VAPB/VCP/WWP1/WWP2/ZC3HAV1/ZDHHC20	72
cluster4	BP	GO:0030520	intracellular estrogen receptor signaling pathway	16/2130	54/18903	0.000204271	0.025470919	0.023846544	ARID1A/CARM1/CNOT1/DDX5/DDX54/DEFA1/DEFA1B/DEFA3/MED1/PAGR1/PHB2/PPARGC1B/SAFB/STRN3/TRIP4/UBA5	16
cluster4	BP	GO:0006140	regulation of nucleotide metabolic process	24/2130	99/18903	0.00020673	0.025470919	0.023846544	ARL2/ARNT/DNAJC30/EIF6/ENO1/EP300/FIS1/GAPDHS/ME2/MLST8/NCOR1/P2RX7/PDK3/PGAM1/PID1/PINK1/PPARGC1A/PRKAG1/PRXL2C/RPTOR/TP53/TREM2/VCP/ZBTB20	24
cluster4	BP	GO:0034976	response to endoplasmic reticulum stress	49/2130	261/18903	0.000225156	0.027361055	0.025616139	ALOX5/ATF6B/BAG6/BAK1/BAX/BBC3/CALR/CREB3/CREBZF/DERL3/DNAJC10/EDEM2/EIF2B5/EIF4G1/EP300/ERP27/ERP29/HYOU1/NR1H2/NR1H3/P4HB/PIK3R2/PMAIP1/PSMC6/RNF139/RNFT1/SEC16A/SEC61B/SELENOK/SELENOS/SIRT1/STT3B/TMTC4/TMUB1/TMX1/TP53/TRAF2/TRIM13/TRIM25/TXNDC12/UBA5/UBE2J2/UBXN1/UBXN10/UBXN4/UBXN6/UFD1/VAPB/VCP	49

cluster4	BP	GO:0009615	response to virus	70/2130	409/18903	0.000233836	0.028031847	0.026244151	ADAR/AKAP1/APOBEC3D/APOBEC3G/ATG5/BAX/BST2/CALR/CARD9/CCT5/CREBZF/DDX60L/DEFA1/DEFA1B/DEFA3/EIF5A/ENO1/EXOSC4/F2RL1/FMR1/GBF1/GBP1/HTRA1/IFITM3/IFNGR1/IFNLR1/IL15/ILRUN/IRF1/IRF3/IRF5/IRF9/ISG20/LGALS9/MAP3K14/MLKL/MYD88/NLRC5/NMB/PARP9/PHB2/PMAIP1/POLR3F/POU2F2/PRF1/PUM1/RNF135/RNF26/SAMHD1/SELENOK/SLFN11/SLFN13/TKFC/TPT1/TRAF3/TRAF3IP2/TRIM11/TRIM13/TRIM21/TRIM25/TRIM28/TRIM32/TRIM34/TRIM38/TRIM44/TRIM52/TTC4/UFD1/XCL1/ZC3HAV1	70
cluster4	BP	GO:1904951	positive regulation of establishment of protein localization	56/2130	310/18903	0.000238553	0.028216047	0.026416605	ANG/ARF6/BAP1/BBC3/BLK/C1QTNF12/CCT5/CCT6A/CD2AP/CDH1/CDK5/CIB1/DKC1/EDEM2/EFCAB7/ERGIC3/EZR/F2RL1/FIS1/FLNA/GPR68/GZMB/HCAR2/HSP90AB1/HTRA2/HUWE1/JUP/LEP/LRRC8A/MYO18A/NMT1/NR1H2/OSBP/P2RX7/PCM1/PDCD5/PDZK1/PIK3R2/PINK1/PLA2G1B/PRNP/PSMD9/PTPN23/RAB29/RHOU/RNF31/RUFY3/SOX4/STX4/TCP1/TREM2/TRIM28/TRPM2/TTN/UBE2J2/VPS35	56
cluster4	BP	GO:0002228	natural killer cell mediated immunity	20/2130	77/18903	0.000256055	0.029202818	0.027340445	CD160/CD96/CEBPG/CORO1A/CRK/CRTAM/GZMB/HAVCR2/KLRC1/KLRC4/KLRD1/LEP/LGALS9/NCR3/PRDX1/PTPN6/PVR/SH2D1A/SLAMF7/STAT5B	20
cluster4	BP	GO:0045824	negative regulation of innate immune response	20/2130	77/18903	0.000256055	0.029202818	0.027340445	ADAR/ARG1/ATG5/CD96/CRK/HAVCR2/KLRC1/KLRD1/LGALS9/METTL3/NLRC5/NR1H2/NR1H3/SAMHD1/SLAMF8/SUSD4/TRAFD1/TRIM21/VSIG4/YTHDF2	20

cluster4	BP	GO:1901798	positive regulation of signal transduction by p53 class mediator	11/2130	30/18903	0.000256771	0.029202818	0.027340445	ATR/DDX5/EIF5A/HEXIM1/PMAIP1/PYHIN1/RPL37/SPRED1/UBB/ZNF385A/ZNHIT1	11
cluster4	BP	GO:0010821	regulation of mitochondrion organization	32/2130	150/18903	0.000266448	0.02960674	0.027718607	ACAA2/BAK1/BAX/BBC3/CAMKK2/ENDOG/EP300/FAM162A/FIS1/GZMB/HGF/HTRA2/HUWE1/NMT1/PARL/PDCD5/PGAM5/PID1/PINK1/PISD/PMAIP1/PPARGC1A/RHOT2/STOML2/STOX1/TP53/TREM2/TSC2/VAT1/VDAC1/VPS35/ZDHHC6	32
cluster4	BP	GO:0031343	positive regulation of cell killing	18/2130	66/18903	0.000266998	0.02960674	0.027718607	ARG1/CD160/CD1A/CD1B/CD1C/CRTAM/F2RL1/HLA-DRA/KLRC4/KLRD1/NCR3/P2RX7/PRF1/PVR/SH2D1A/SPI1/STAT5B/XCL1	18
cluster4	BP	GO:0002708	positive regulation of lymphocyte mediated immunity	27/2130	119/18903	0.000277082	0.030345653	0.028410397	CD160/CD1A/CD1B/CD1C/CRTAM/HLA-DRA/HLA-DRB3/HSPD1/KLRC4/KLRD1/KMT5C/MLH1/NCR3/P2RX7/PVR/SASH3/SH2D1A/SHLD2/SLAMF1/STAT5B/STAT6/TNFSF13/TRAF2/TREM2/XCL1/ZBTB1/ZP3	27

cluster4	BP	GO:0009155	purine deoxyribonucleotide catabolic process	6/2130	10/18903	0.00028525	0.030859145	0.028891142	ADA/NT5C/NT5C1A/NUDT15/NUDT18/SAMHD1	6
cluster4	BP	GO:0043414	macromolecule methylation	61/2130	348/18903	0.000300211	0.032086357	0.03004009	ASH1L/BAZ2A/BUD23/CARM1/CTNNB1/CTR9/DALRD3/EEF1AKMT3/EZH1/FBLL1/FBXO11/FDXACB1/FTSJ3/GFI1/HSD17B10/KANSL2/KAT8/KDM1A/KMT2A/KMT2B/KMT5C/LCMT1/METTL23/METTL3/MGMT/MLLT6/MRM2/NELFA/NELFE/NSUN4/NSUN5/PAF1/PAGR1/PCIF1/PHF1/PRDM1/PRMT1/PRMT2/RAMAC/RTF1/SETD1B/SETMAR/SIRT1/SNRPB/SNRPD2/SNRPG/SPI1/TDRD12/TET3/TRDMT1/TRIM28/TRMT10C/TRMT11/TRMT112/TRMT44/TRMT61A/VCPKMT/WDR82/ZNF274/ZNF304/ZNF335	61
cluster4	BP	GO:0043470	regulation of carbohydrate catabolic process	16/2130	56/18903	0.000324183	0.034236093	0.032052729	ARL2/ARNT/EIF6/EP300/GAPDHS/MLST8/NCOR1/P2RX7/PGAM1/PHKG2/PPP1CA/PRKAG1/PRXL2C/RPTOR/TP53/ZBTB20	16
cluster4	BP	GO:0006260	DNA replication	52/2130	286/18903	0.000332624	0.03441853	0.032223531	ATR/CARM1/CDC7/CDK9/CDT1/CENPX/DBF4B/DNAJA3/DTD1/EME2/ENDOG/FAM111A/FBH1/FEN1/GTPBP4/HMGA1/INO80B/LIG1/LIG4/LPIN1/LRWD1/NASP/NFIC/NFIX/NUCKS1/PNKP/POLA1/POLG/POLI/PRIM1/PURA/RBMS1/RECQL5/REV1/RFC1/RPA2/RPA3/RPAIN/RRM2B/RUVBL1/RUVBL2/SAMHD1/SET/SETMAR/SLFN11/STOML2/STRA8/TP53/UPF1/YY1/ZBTB38/ZNF830	52

cluster4	BP	GO:0002275	myeloid cell activation involved in immune response	23/2130	96/18903	0.000333671	0.03441853	0.032223531	ANXA3/BLK/CBL/CD177/CX3CR1/DOCK2/F2RL1/FES/GAB2/HAVCR2/HMOX1/LGALS9/MILR1/MYD88/PIK3CD/PLCG2/PRAM1/PTPN6/SBNO2/SLAMF1/SPI1/STX4/TREM2	23
cluster4	BP	GO:0032785	negative regulation of DNA-templated transcription, elongation	9/2130	22/18903	0.00036302	0.037015509	0.034654892	HEXIM1/HNRNPU/NELFA/NELFCD/NELFE/RECQL5/SUPT5H/TCERG1/WDR82	9
cluster4	BP	GO:0016078	tRNA catabolic process	7/2130	14/18903	0.000381788	0.03796808	0.035546713	ANG/DICER1/ELAC2/EXOSC10/EXOSC8/SLFN13/TRDMT1	7
cluster4	BP	GO:1905214	regulation of RNA binding	7/2130	14/18903	0.000381788	0.03796808	0.035546713	CARM1/CDK9/EIF3C/EIF4G1/FMR1/HNRNPL/NUCKS1	7

cluster4	BP	GO:0006368	transcription elongation by RNA polymerase II promoter	25/2130	109/18903	0.000385202	0.03796808	0.035546713	ADRM1/CBX7/CDK9/CTNNB1/CTR9/HEXIM1/HNRNPU/INTS1/INTS11/INTS3/MED1/MED15/MED17/MED18/MED30/NELFA/NELFCD/NELFE/PAF1/PWWP2B/RECQL5/RTF1/SUPT5H/TCEA2/TCERG1	25
cluster4	BP	GO:0031330	negative regulation of cellular catabolic process	46/2130	247/18903	0.000414433	0.040400412	0.037823927	ANGEL2/BAG5/BAG6/CCAR2/CIRBP/CPTP/CSNK2A1/DKC1/EIF4G1/FMR1/GOLGA2/HGF/HMOX1/HNRNPA0/HNRNPU/HSP90AB1/KLHL22/LARP1/LARP4B/LEP/MLST8/MYD88/NOCT/PABPC1/PHAX/PINK1/POLDIP2/RBM38/RPTOR/RRAGA/RUBCN/SEC22B/SMG1/SUFU/SYNCRIP/TENT5A/TP53/TRAF2/TRAF3IP2/TRDMT1/TREM2/TSC1/TSC2/UBQLN4/UBXN1/YBX1	46
cluster4	BP	GO:0042267	natural killer cell mediated cytotoxicity	19/2130	74/18903	0.000423838	0.040814386	0.0382115	CD160/CEBPG/CORO1A/CRK/CRTAM/GZMB/HAVCR2/KLRC1/KLRC4/KLRD1/LEP/LGALS9/NCR3/PRDX1/PTPN6/PVR/SH2D1A/SLAMF7/STAT5B	19
cluster4	BP	GO:0042254	ribosome biogenesis	55/2130	310/18903	0.000427882	0.040814386	0.0382115	AATF/ABCE1/ABT1/BUD23/DDX27/DDX54/DKC1/EIF6/ERI1/EXOSC10/EXOSC4/EXOSC8/FBLL1/FDXACB1/FTSJ3/GNL2/GTF3A/GTPBP10/GTPBP4/ISG20/LTV1/MRM2/MRPS2/NGRN/NOLC1/NSUN4/NSUN5/PA2G4/PELP1/POP7/RIOK1/RPL24/RPL7/RPL7L1/RPP25/RPP30/RPP38/RPS27/RRN3/RRP9/SART1/SNU13/TRMT112/TSC1/TSR1/UTP15/UTP23/UTP3/WBP11/WDR46/WDR74/WDR75/YTHDF2/ZNF658/ZNHIT3	55

cluster4	BP	GO:0000723	telomere maintenance	32/2130	154/18903	0.000436405	0.041184564	0.03855807	ATR/CCT5/CCT6A/CTNNB1/DCLRE1C/DKC1/EXOSC10/FEN1/GNL3/HMBOX1/HNRNPA2B1/HNRNPU/HSP90AB1/MAP2K7/PNKP/PPP1R10/RAD51C/RFC1/RPA2/RPA3/RUVBL1/RUVBL2/SHLD2/SLF2/SMC5/SMG1/TCP1/TEP1/TINF2/UPF1/XRCC1/YY1	32
cluster4	BP	GO:0006354	DNA-templated transcription elongation	45/2130	241/18903	0.000448757	0.041904491	0.039232085	ADRM1/ARID3A/BTG1/CBX7/CCAR2/CDK9/CTNNB1/CTR9/EP300/HEXIM1/HMGA1/HNRNPU/INTS1/INTS11/INTS3/KAT6A/KAT8/KDM2A/LPXN/MED1/MED12/MED15/MED17/MED18/MED30/NAB2/NELFA/NELFCD/NELFE/PAF1/PHF2/PPARGC1A/PPARGC1B/PWWP2B/RECQL5/RTF1/SAP30/SMARCD2/SUPT5H/TCEA2/TCERG1/TEFM/TRRAP/WDR82/ZNF653	45
cluster4	BP	GO:0032259	methylation	66/2130	390/18903	0.000476618	0.043752199	0.040961957	ASH1L/BAZ2A/BUD23/CARM1/CTNNB1/CTR9/DALRD3/DPH5/EEF1AKMT3/EZH1/FAM86B1/FAM86B2/FBLL1/FBXO11/FDXACB1/FTSJ3/GFI1/HSD17B10/KANSL2/KAT8/KDM1A/KMT2A/KMT2B/KMT5C/LCMT1/METTL23/METTL3/METTL7B/MGMT/MLLT6/MRM2/MTR/NELFA/NELFE/NSUN4/NSUN5/PAF1/PAGR1/PCIF1/PHF1/PRDM1/PRMT1/PRMT2/RAMAC/RTF1/SETD1B/SETMAR/SIRT1/SNRPB/SNRPD2/SNRPG/SPI1/TDRD12/TET3/TRDMT1/TRIM28/TRMT10C/TRMT11/TRMT112/TRMT44/TRMT61A/VCPKMT/WDR82/ZNF274/ZNF304/ZNF335	66
cluster4	BP	GO:0002443	leukocyte mediated immunity	76/2130	463/18903	0.000478409	0.043752199	0.040961957	ANXA3/ARG1/BLK/BST2/C4BPA/CARD9/CBL/CD160/CD177/CD19/CD1A/CD1B/CD1C/CD96/CEBPG/CORO1A/CRK/CRTAM/CX3CR1/F2RL1/FES/GAB2/GZMB/GZMM/HAVCR2/HLA-DMA/HLA-DQA1/HLA-DQA2/HLA-DRA/HLA-DRB3/HLA-DRB5/HMOX1/HSPD1/IL9R/JAG1/KLRC1/KLRC4/KLRD1/KMT5C/LEP/LGALS9/LIG4/MILR1/MLH1/MSH6/MYD88/NCKAP1L/NCR3/P2RX7/PIK3CD/PLA2G1B/PLCG2/PRAM1/PRDX1/PRF1/PTPN6/PVR/SASH3/SH2D1A/SHLD2/SLAMF1/SLAMF7/SLAMF9/SLC15A4/SPI1/STAT5B/STAT6/STX4/SUSD4/TNFSF13/TRAF2/TRAF3IP2/TREM2/XCL1/ZBTB1/ZP3	76

cluster4	BP	GO:0009201	ribonucleoside triphosphate biosynthetic process	27/2130	123/18903	0.00048465	0.043870693	0.041072895	ATP5F1A/ATP5PB/DNAJC30/ENO1/IMPDH1/IMPDH2/NDUFA11/NDUFA2/NDUFA3/NDUFA7/NDUFAB1/NDUFB5/NDUFB9/NDUFS1/NDUFS2/NDUFS7/NDUFS8/NME6/PID1/PINK1/PPARGC1A/SDHD/STOML2/TREM2/UCK2/UCKL1/VCP	27
cluster4	BP	GO:0016052	carbohydrate catabolic process	32/2130	155/18903	0.000491618	0.044051955	0.041242597	ARL2/ARNT/DHTKD1/EIF6/ENO1/EP300/FUT1/GALE/GALM/GAPDHS/GK/HEXB/IDNK/LDHA/MLST8/NCOR1/NEU1/OGDH/P2RX7/PFKL/PGAM1/PHKG2/PPP1CA/PRKAG1/PRKAG3/PRXL2C/RBKS/RPTOR/SORD/TKFC/TP53/ZBTB20	32
cluster4	BP	GO:0031056	regulation of histone modification	35/2130	175/18903	0.000513159	0.045522294	0.042619167	AKAP8L/ATG5/CDK9/CTNNB1/CTR9/GFI1/IGF2/KANSL2/KAT8/KDM1A/KDM5A/KMT2A/MLLT6/NAP1L2/NELFA/NELFE/OTUB1/PAF1/PHF1/PINK1/PPARGC1A/PRKD2/PWWP2B/RTF1/RUVBL2/SET/SIRT1/SKI/SPI1/TADA2B/TP53/UBE2N/ZNF274/ZNF304/ZNF335	35
cluster4	BP	GO:0006364	rRNA processing	43/2130	229/18903	0.000525584	0.045772478	0.042853396	ABT1/BUD23/DDX27/DDX54/DKC1/EIF6/ERI1/EXOSC10/EXOSC4/EXOSC8/FBLL1/FDXACB1/FTSJ3/GTPBP4/ISG20/MRM2/NOLC1/NSUN4/NSUN5/PA2G4/PELP1/POP7/RIOK1/RPL7/RPL7L1/RPP25/RPP30/RPP38/RPS27/RRP9/SART1/SNU13/TRMT112/TSR1/UTP15/UTP23/UTP3/WBP11/WDR46/WDR74/WDR75/YTHDF2/ZNHIT3	43

cluster4	BP	GO:1901796	regulation of signal transduction by p53 class mediator	24/2130	105/18903	0.000526298	0.045772478	0.042853396	ARMC10/ATR/CD44/DDX5/EIF5A/EP300/HEXIM1/KAT6A/KDM1A/MDM2/PMAIP1/PYHIN1/RNF34/RPL37/RRM2B/RRN3/SGK1/SIRT1/SOX4/SPRED1/TP53/UBB/ZNF385A/ZNHIT1	24
cluster4	BP	GO:0000966	RNA 5'-end processing	9/2130	23/18903	0.000537083	0.046256893	0.043306918	ABT1/HSD17B10/POP7/RPP25/RPP25L/RPP30/RPP38/SSB/TRMT10C	9
cluster4	BP	GO:1902415	regulation of mRNA binding	6/2130	11/18903	0.000567632	0.048417927	0.045330135	CARM1/CDK9/EIF3C/EIF4G1/FMR1/HNRNPL	6
cluster4	BP	GO:0043489	RNA stabilization	18/2130	70/18903	0.000583066	0.049260768	0.046119224	ANGEL2/CIRBP/DKC1/HNRNPA0/HNRNPU/LARP1/LARP4B/MYD88/NOCT/PABPC1/PHAX/RBM38/SYNCRIP/TENT5A/TRAF2/TRAF3IP2/TRDMT1/YBX1	18

cluster4	CC	GO:0005759	mitochondrial matrix	99/2222	483/19869	1.35E-09	0.00000153	0.00000137	ABCE1/ACAA2/ACADS/ACOT9/ACSS1/ADPRS/ALDH2/ALKBH7/ARL2/ARL2BP/ATP5F1A/ATP5PB/AURKAIP1/BCKDHA/BOLA3/CARS2/CCAR2/COASY/DGLUCY/DHFR2/DHTKD1/DNAJA3/DNAJC19/ECHS1/ELAC2/FAHD1/FASTK/FDX1/GADD45GIP1/GLUD2/GSTK1/HMGCL/HSD17B10/HSPA9/HSPD1/HYKK/IDH3B/IDH3G/ISCA2/LIPT1/MDH2/ME2/MRM2/MRPL16/MRPL23/MRPL24/MRPL3/MRPL4/MRPL40/MRPL43/MRPL48/MRPL50/MRPL52/MRPL53/MRPL55/MRPL9/MRPS16/MRPS18A/MRPS2/MRPS22/MRPS24/MTHFD1L/NAGS/NDUFA7/NDUFAB1/NDUFS1/NDUFS2/NDUFS7/NDUFS8/NR3C1/NSUN4/OGDH/OGG1/OXA1L/PARS2/PDHX/PDK3/PDPR/PDSS2/POLDIP2/POLG/PTCD1/PYROXD2/QARS1/REXO2/SARDH/SDHAF1/SHC1/SIRT4/SUCLA2/SUOX/TDRD7/TEFM/TP53/TRMT10C/TTC5/TUFM/VDAC1/YARS2	99
cluster4	CC	GO:0005681	spliceosomal complex	50/2222	195/19869	1.22E-08	0.00000692	0.00000619	ADAR/AKAP17A/CCDC12/CIRBP/CWF19L1/DDX5/DNAJC17/ESS2/HNRNPA2B1/HNRNPA3/HNRNPU/HSPA8/IK/ISY1/NCL/PABPC1/PNN/PPIH/PPP1R8/PRPF38A/PRPF40A/PRPF6/RHEB/RNF113A/RNPC3/SART1/SF3A1/SF3B2/SF3B3/SF3B5/SNRNP35/SNRNP40/SNRNP48/SNRPB/SNRPC/SNRPD2/SNRPG/SNU13/SREK1/SRRM2/SRSF1/SYNCRIP/TXNL4A/UPF1/WBP11/WDR83/YBX1/ZCRB1/ZMAT5/ZNF830	50
cluster4	CC	GO:0060205	cytoplasmic vesicle lumen	68/2222	325/19869	0.000000227	0.000078	0.0000698	ACTN4/ADA/AGA/ALOX5/AMPD3/APRT/ARG1/ARHGAP45/ARSB/C6orf120/CAMP/CNN2/COMMD9/CRISP3/DBNL/DEFA1/DEFA1B/DEFA3/DEFA4/DNASE1L1/DOCK2/DPP7/DYNC1H1/EEF2/FOLR3/FTL/FUCA2/GDI2/GGH/GHDC/GNS/GSDMD/GYG1/HEBP2/HEXB/HGF/HSP90AB1/HSPA8/HUWE1/IGF2/IMPDH1/IMPDH2/JUP/KPNB1/MAGED2/MAN2B1/MVP/NEU1/NHLRC2/NIT2/OLA1/OLFM4/PA2G4/PDGFB/PFKL/PGAM1/PRDX4/PSMD2/PSMD7/PTPN6/RETN/TCN1/TEX264/TF/TXNDC5/VAT1/VCP/VEGFB	68
cluster4	CC	GO:0031983	vesicle lumen	68/2222	327/19869	0.000000289	0.000078	0.0000698	ACTN4/ADA/AGA/ALOX5/AMPD3/APRT/ARG1/ARHGAP45/ARSB/C6orf120/CAMP/CNN2/COMMD9/CRISP3/DBNL/DEFA1/DEFA1B/DEFA3/DEFA4/DNASE1L1/DOCK2/DPP7/DYNC1H1/EEF2/FOLR3/FTL/FUCA2/GDI2/GGH/GHDC/GNS/GSDMD/GYG1/HEBP2/HEXB/HGF/HSP90AB1/HSPA8/HUWE1/IGF2/IMPDH1/IMPDH2/JUP/KPNB1/MAGED2/MAN2B1/MVP/NEU1/NHLRC2/NIT2/OLA1/OLFM4/PA2G4/PDGFB/PFKL/PGAM1/PRDX4/PSMD2/PSMD7/PTPN6/RETN/TCN1/TEX264/TF/TXNDC5/VAT1/VCP/VEGFB	68

cluster4	CC	GO:0034774	secretory granule lumen	67/2222	322/19869	0.000000344	0.000078	0.0000698	ACTN4/AGA/ALOX5/AMPD3/APRT/ARG1/ARHGAP45/ARSB/C6orf120/CAMP/CNN2/COMMD9/CRISP3/DBNL/DEFA1/DEFA1B/DEFA3/DEFA4/DNASE1L1/DOCK2/DPP7/DYNC1H1/EEF2/FOLR3/FTL/FUCA2/GDI2/GGH/GHDC/GNS/GSDMD/GYG1/HEBP2/HEXB/HGF/HSP90AB1/HSPA8/HUWE1/IGF2/IMPDH1/IMPDH2/JUP/KPNB1/MAGED2/MAN2B1/MVP/NEU1/NHLRC2/NIT2/OLA1/OLFM4/PA2G4/PDGFB/PFKL/PGAM1/PRDX4/PSMD2/PSMD7/PTPN6/RETN/TCN1/TEX264/TF/TXNDC5/VAT1/VCP/VEGFB	67
cluster4	CC	GO:0016607	nuclear speck	77/2222	419/19869	0.00000747	0.00141151	0.001262865	ADAMTS4/AKAP17A/AKAP8L/ATPAF2/ATXN2L/BAZ2A/BRD2/CBX4/CD2BP2/CDYL/CTR9/DDX39A/DDX5/DNAAF1/DUSP11/FIBP/FNBP4/GATAD2B/GTF2H2C/GTF2H2C_2/H2AX/HNRNPU/HSPA1B/IK/IL15/IL16/ILRUN/INPPL1/KAT6A/LPXN/MAML1/METTL3/NR3C1/NSL1/OGG1/PABPN1/PCBP1/PIN1/PNN/POLI/PPIH/PPP1CC/PPP1R8/PRPF40A/PRPF6/PYHIN1/RBM25/RBM39/RBM4B/RING1/RNF113A/RNF34/SART1/SETD1B/SF3A1/SF3B2/SFPQ/SGK1/SLC2A4RG/SMC4/SMC5/SNRNP40/SON/SPRTN/SREK1/SRRM2/SRSF1/SRSF4/SRSF7/STK17A/TCERG1/TCF12/THOC3/TUT1/ZBTB18/ZNF395/ZNF830	77
cluster4	CC	GO:0000791	euchromatin	19/2222	60/19869	0.0000171	0.002762023	0.002471155	ALKBH1/CREB1/CTNNB1/CTR9/EXOSC10/EXOSC4/H1-0/H3-5/ICE2/KLF4/NSMF/PELP1/RNF2/RUVBL2/SIRT1/SP1/TCF3/TRIM28/TRNP1	19
cluster4	CC	GO:0035580	specific granule lumen	19/2222	62/19869	0.0000287	0.003897207	0.003486794	ARG1/CAMP/CNN2/CRISP3/DEFA4/DNASE1L1/DOCK2/FOLR3/GGH/GHDC/GSDMD/JUP/KPNB1/NEU1/NIT2/OLFM4/PTPN6/RETN/TCN1	19

cluster4	CC	GO:0005689	U12-type spliceosomal complex	12/2222	29/19869	0.000031	0.003897207	0.003486794	RNPC3/SF3B2/SF3B3/SF3B5/SNRNP35/SNRNP48/SNRPB/SNRPD2/SNRPG/YBX1/ZCRB1/ZMAT5	12
cluster4	CC	GO:0035578	azurophil granule lumen	24/2222	91/19869	0.0000441	0.004992365	0.004466621	AGA/ARG1/ARHGAP45/ARSB/C6orf120/DEFA1/DEFA1B/DEFA3/DPP7/DYNC1H1/FTL/FUCA2/GDI2/GGH/GNS/HEBP2/HEXB/IMPDH1/MAN2B1/PA2G4/RETN/TXNDC5/VAT1/VCP	24
cluster4	CC	GO:0098798	mitochondrial protein-containing complex	56/2222	295/19869	0.0000501	0.005038774	0.004508142	ATP5F1A/ATP5PB/AURKAIP1/BAX/BCKDHA/COX8A/DNAJC19/GADD45GIP1/HSD17B10/HSPA9/IDH3B/IDH3G/MPC1/MRPL16/MRPL23/MRPL24/MRPL3/MRPL4/MRPL40/MRPL43/MRPL48/MRPL50/MRPL52/MRPL53/MRPL55/MRPL9/MRPS16/MRPS18A/MRPS2/MRPS22/MRPS24/MTX3/NDUFA11/NDUFA2/NDUFA3/NDUFA7/NDUFAB1/NDUFB5/NDUFB9/NDUFC2-KCTD14/NDUFS1/NDUFS2/NDUFS7/NDUFS8/NSUN4/PDHX/PHB2/POLG/SAMM50/SDHD/TIMM22/TIMM29/TRMT10C/UQCRC1/UQCRFS1/VDAC1	56
cluster4	CC	GO:0034708	methyltransferase complex	24/2222	92/19869	0.0000534	0.005038774	0.004508142	C17orf49/CHD8/EZH1/HSD17B10/KAT8/KMT2A/KMT2B/METTL3/MGA/PAGR1/PELP1/PRMT1/RAMAC/RIOK1/RNF2/RUVBL1/RUVBL2/SETD1B/SNRPB/SNRPD2/SNRPG/TRMT10C/TRMT61A/WDR82	24

cluster4	CC	GO:0042581	specific granule	35/2222	160/19869	0.0000717	0.006250894	0.005592615	ANXA3/AP1M1/ARG1/CAMP/CD177/CLEC5A/CNN2/CRISP3/DEFA4/DEGS1/DGAT1/DNASE1L1/DOCK2/FOLR3/GGH/GHDC/GSDMD/HMOX2/JUP/KPNB1/NEU1/NIT2/OLFM4/OLR1/PTPN6/RAP2B/RETN/SLC15A4/SLC2A5/STK10/STX4/TCN1/TMC6/TOM1/TRPM2	35
cluster4	CC	GO:0090734	site of DNA damage	26/2222	106/19869	0.0000824	0.006669312	0.005966969	ADPRS/ARPC1A/CNTD1/DDB2/H2AX/IFFO1/INTS3/OARD1/PARP9/PAXX/PHF1/PNKP/RPA2/RPA3/SAMHD1/SETMAR/SHLD2/SLF2/SLFN11/SMARCAD1/SMC5/SPINDOC/TP53/UBQLN4/VCP/XRCC1	26
cluster4	CC	GO:0005925	focal adhesion	73/2222	422/19869	0.000099	0.007474456	0.006687324	ACTG1/ACTN4/AHNAK/ANXA5/ANXA6/APBB1IP/ARF6/ARHGAP22/ARHGEF2/ARL2/ARPC5L/AVIL/CALR/CAPN5/CBL/CD44/CDC42EP1/CDH13/CLASP1/CLTC/CNN2/CORO1B/CTNNA1/CTNNB1/DNM2/EZR/FES/FLII/FLNA/GDI2/HMGA1/HSPA1B/HSPA8/HSPA9/HSPG2/HYOU1/IGF2R/IL16/ITGA5/JUP/LAP3/LASP1/LPXN/MAP2K2/MPZL1/NEDD9/P4HB/PABPC1/PEAK1/PI4KA/PIK3R2/PLEC/PPFIA1/PPP1CC/PVR/REXO2/RHOB/RHOU/RPL4/RPL7/RPLP2/RPS10/RPS11/RPS18/RRAS/SNTB2/TGM2/TLN2/TNS1/TNS3/TRIP6/YES1/YWHAB	73
cluster4	CC	GO:0005766	primary lysosome	33/2222	155/19869	0.000195386	0.013021929	0.011650595	AGA/ARG1/ARHGAP45/ARSB/BST2/C6orf120/CPNE1/DEFA1/DEFA1B/DEFA3/DEFA4/DPP7/DYNC1H1/FTL/FUCA2/GDI2/GGH/GNS/HEBP2/HEXB/IMPDH1/LPCAT1/MAN2B1/PA2G4/PIGR/PRCP/RETN/SURF4/TOM1/TXNDC5/VAT1/VCP/VNN1	33

cluster4	CC	GO:0042582	azurophil granule	33/2222	155/19869	0.000195386	0.013021929	0.011650595	AGA/ARG1/ARHGAP45/ARSB/BST2/C6orf120/CPNE1/DEFA1/DEFA1B/DEFA3/DEFA4/DPP7/DYNC1H1/FTL/FUCA2/GDI2/GGH/GNS/HEBP2/HEXB/IMPDH1/LPCAT1/MAN2B1/PA2G4/PIGR/PRCP/RETN/SURF4/TOM1/TXNDC5/VAT1/VCP/VNN1	33
cluster4	CC	GO:0030055	cell-substrate junction	73/2222	432/19869	0.000207335	0.013050578	0.011676227	ACTG1/ACTN4/AHNAK/ANXA5/ANXA6/APBB1IP/ARF6/ARHGAP22/ARHGEF2/ARL2/ARPC5L/AVIL/CALR/CAPN5/CBL/CD44/CDC42EP1/CDH13/CLASP1/CLTC/CNN2/CORO1B/CTNNA1/CTNNB1/DNM2/EZR/FES/FLII/FLNA/GDI2/HMGA1/HSPA1B/HSPA8/HSPA9/HSPG2/HYOU1/IGF2R/IL16/ITGA5/JUP/LAP3/LASP1/LPXN/MAP2K2/MPZL1/NEDD9/P4HB/PABPC1/PEAK1/PI4KA/PIK3R2/PLEC/PPFIA1/PPP1CC/PVR/REXO2/RHOB/RHOU/RPL4/RPL7/RPLP2/RPS10/RPS11/RPS18/RRAS/SNTB2/TGM2/TLN2/TNS1/TNS3/TRIP6/YES1/YWHAB	73
cluster4	CC	GO:0005732	sno(s)RNA-containing ribonucleoprotein complex	11/2222	30/19869	0.000240269	0.013060044	0.011684696	DKC1/FBLL1/NOLC1/POP7/RPP25/RPP25L/RPP30/RPP38/RRP9/SNRNP40/SNU13	11
cluster4	CC	GO:0000313	organellar ribosome	22/2222	89/19869	0.000250266	0.013060044	0.011684696	AURKAIP1/GADD45GIP1/MRPL16/MRPL23/MRPL24/MRPL3/MRPL4/MRPL40/MRPL43/MRPL48/MRPL50/MRPL52/MRPL53/MRPL55/MRPL9/MRPS16/MRPS18A/MRPS2/MRPS22/MRPS24/NDUFA7/NSUN4	22

cluster4	CC	GO:0005761	mitochondrial ribosome	22/2222	89/19869	0.000250266	0.013060044	0.011684696	AURKAIP1/GADD45GIP1/MRPL16/MRPL23/MRPL24/MRPL3/MRPL4/MRPL40/MRPL43/MRPL48/MRPL50/MRPL52/MRPL53/MRPL55/MRPL9/MRPS16/MRPS18A/MRPS2/MRPS22/MRPS24/NDUFA7/NSUN4	22
cluster4	CC	GO:0071005	U2-type precatalytic spliceosome	15/2222	50/19869	0.000253593	0.013060044	0.011684696	IK/PRPF38A/PRPF6/RNF113A/SART1/SF3A1/SF3B2/SF3B3/SF3B5/SNRPB/SNRPD2/SNRPG/SNU13/SRRM2/TXNL4A	15
cluster4	CC	GO:0000315	organellar large ribosomal subunit	16/2222	56/19869	0.000297708	0.013644204	0.012207338	GADD45GIP1/MRPL16/MRPL23/MRPL24/MRPL3/MRPL4/MRPL40/MRPL43/MRPL48/MRPL50/MRPL52/MRPL53/MRPL55/MRPL9/MRPS18A/NSUN4	16
cluster4	CC	GO:0005762	mitochondrial large ribosomal subunit	16/2222	56/19869	0.000297708	0.013644204	0.012207338	GADD45GIP1/MRPL16/MRPL23/MRPL24/MRPL3/MRPL4/MRPL40/MRPL43/MRPL48/MRPL50/MRPL52/MRPL53/MRPL55/MRPL9/MRPS18A/NSUN4	16

cluster4	CC	GO:0072357	PTW/PP1 phosphatase complex	5/2222	7/19869	0.000301064	0.013644204	0.012207338	PPP1CA/PPP1CC/PPP1R10/TOX4/WDR82	5
cluster4	CC	GO:0000812	Swr1 complex	7/2222	14/19869	0.000364282	0.015874302	0.014202585	ACTR6/BRD8/RUVBL1/RUVBL2/SRCAP/TRRAP/ZNHIT1	7
cluster4	CC	GO:0101031	chaperone complex	13/2222	42/19869	0.000461369	0.019360399	0.017321563	CCT5/CCT6A/DNAAF2/DNAJB11/HSP90AB1/HSPA8/PSMG2/RUVBL1/RUVBL2/STIP1/TCP1/TSC1/WDR83OS	13
cluster4	CC	GO:1904813	ficolin-1-rich granule lumen	27/2222	124/19869	0.000493467	0.019907465	0.017811018	ALOX5/AMPD3/APEH/ARSB/COMMD9/DBNL/EEF2/GNS/GSDMD/GYG1/HSP90AB1/HSPA1B/HSPA8/HUWE1/IMPDH1/IMPDH2/JUP/KPNB1/LTA4H/MVP/PFKL/PGAM1/PRDX4/PSMD2/PSMD7/TNFAIP6/VCP	27

cluster4	CC	GO:0071011	precatalytic spliceosome	15/2222	53/19869	0.000509547	0.019907465	0.017811018	IK/PRPF38A/PRPF6/RNF113A/SART1/SF3A1/SF3B2/SF3B3/SF3B5/SNRPB/SNRPD2/SNRPG/SNU13/SRRM2/TXNL4A	15
cluster4	CC	GO:0098687	chromosomal region	65/2222	388/19869	0.000568517	0.021387286	0.019134999	ATR/BUB3/CDT1/CENPB/CENPC/CENPH/CENPX/CHD4/CHMP2B/CLASP1/DCTN1/DCTN5/EZH1/FEN1/FMR1/GATAD2B/H2AX/HMBOX1/HNRNPA2B1/HNRNPU/INCENP/KAT8/KDM1A/KMT5C/LIG4/LRIF1/LRWD1/MAD1L1/MEIKIN/MIS12/NCAPD2/NCAPD3/NDE1/NLRP2/NSL1/NUP107/PHF2/PMF1/POLR2B/PPP1CA/PPP1CC/PPP1R10/PPP2R5C/PSEN2/PURA/RPA2/SEC13/SLF2/SMARCC2/SMARCD2/SMC1A/SMC4/SMC5/SPC24/SSB/SUGT1/TEP1/TERB2/THOC3/TINF2/TOX4/UPF1/WDR82/XRCC1/ZNF276	65
cluster4	CC	GO:0005838	proteasome regulatory particle	8/2222	19/19869	0.000585177	0.021387286	0.019134999	ADRM1/PSMC1/PSMC4/PSMC5/PSMC6/PSMD2/PSMD7/PSMD9	8
cluster4	CC	GO:1905348	endonuclease complex	12/2222	38/19869	0.0006187	0.021905842	0.019598946	AGO2/CLP1/DICER1/EME2/HSD17B10/POP7/RPP25/RPP25L/RPP30/RPP38/TRMT10C/TSN	12

cluster4	CC	GO:0071013	catalytic step 2 spliceosome	21/2222	90/19869	0.000780978	0.025289682	0.022626435	DDX5/ESS2/HNRNPA2B1/HNRNPA3/HNRNPU/ISY1/PABPC1/PNN/PRPF6/SART1/SF3A1/SF3B2/SF3B3/SNRNP40/SNRPB/SNRPD2/SNRPG/SRRM2/SRSF1/SYNCRIP/WDR83	21
cluster4	CC	GO:0010494	cytoplasmic stress granule	20/2222	84/19869	0.000785915	0.025289682	0.022626435	ATXN2L/CAPRIN1/CIRBP/EIF4G1/FMR1/GIGYF2/KPNB1/LARP1/LARP4B/MCRIP2/PABPC1/PABPC3/PUM1/RNF135/RPTOR/TRIM25/UBAP2L/VCP/YBX1/YTHDF2	20
cluster4	CC	GO:0015934	large ribosomal subunit	25/2222	115/19869	0.000802374	0.025289682	0.022626435	GADD45GIP1/MRPL16/MRPL23/MRPL24/MRPL3/MRPL4/MRPL40/MRPL43/MRPL48/MRPL50/MRPL52/MRPL53/MRPL55/MRPL9/MRPS18A/NSUN4/RPL24/RPL28/RPL32/RPL37/RPL4/RPL7/RPL7L1/RPLP2/UBA52	25
cluster4	CC	GO:1902555	endoribonuclease complex	11/2222	34/19869	0.000824779	0.025289682	0.022626435	AGO2/CLP1/DICER1/HSD17B10/POP7/RPP25/RPP25L/RPP30/RPP38/TRMT10C/TSN	11

cluster4	CC	GO:0000779	condensed chromosome, centromeric region	34/2222	174/19869	0.000825877	0.025289682	0.022626435	BUB3/CDT1/CENPB/CENPC/CENPH/CENPX/CHMP2B/CLASP1/DCTN1/DCTN5/HNRNPU/INCENP/KAT8/KMT5C/LRWD1/MAD1L1/MEIKIN/MIS12/NCAPD2/NCAPD3/NDE1/NSL1/NUP107/PHF2/PMF1/PPP1CC/PSEN2/SEC13/SMARCC2/SMARCD2/SMC1A/SPC24/SUGT1/ZNF276	34
cluster4	CC	GO:0005774	vacuolar membrane	74/2222	461/19869	0.000874477	0.026073226	0.023327465	ABCA2/AHNAK/ANXA6/AP1M1/AP2A1/AP3M1/AP5B1/ATG16L2/ATP6V1F/ATP6V1H/BORCS6/BST2/CALCOCO2/CD1B/CHMP2B/CLCN5/CLTC/CPNE1/GLMP/HLA-DMA/HLA-DQA1/HLA-DQA2/HLA-DRA/HLA-DRB3/HLA-DRB5/HSP90AB1/HSPA8/IFITM3/KPTN/LDLR/LPCAT1/LRRC8A/NEU1/NSF/PI4K2A/PIGR/PLEKHM1/PRCP/PSEN2/RAB2A/RHEB/RMC1/RPN2/RPTOR/RRAGA/RUBCNL/SBF2/SEC13/SIDT2/SLC15A4/SLC36A1/SLC3A2/SLC46A3/SURF4/SZT2/TASL/TECPR1/TEX264/THBD/TM9SF1/TMEM199/TMEM59/TOM1/TRPM2/UBA1/UBA52/UBXN6/VNN1/VPS16/VPS35/VPS39/WDR24/YWHAB/ZFYVE26	74
cluster4	CC	GO:1904949	ATPase complex	21/2222	94/19869	0.001413196	0.039617509	0.035445405	ACTR6/ARID1A/BCL7C/BRD8/C17orf49/CHD3/CHD4/CSNK2A1/GATAD2B/INO80B/RB1/RUVBL1/RUVBL2/SMARCA2/SMARCC2/SMARCD2/SRCAP/TRRAP/VCP/YY1/ZNHIT1	21
cluster4	CC	GO:0090575	RNA polymerase II transcription regulator complex	44/2222	249/19869	0.001427785	0.039617509	0.035445405	ARNT/ATF6B/BCL9L/CEBPA/CEBPG/CHD4/CREB1/CREB3/CREBZF/CTNNB1/GTF2A2/GTF2H2C/GTF2H2C_2/HMGA1/IRF9/JUND/MAF/MAFB/MAFF/MED1/MED15/MED17/MED18/MED30/NR1H2/NR1H3/RB1/RXRA/SFPQ/STAT4/STAT5A/STAT5B/STAT6/TAF12/TAF1L/TAF5/TAF8/TCF12/TCF3/TFDP1/TLE4/TRIM28/TRIP4/TRRAP	44

cluster4	CC	GO:0016363	nuclear matrix	26/2222	126/19869	0.001433643	0.039617509	0.035445405	AKAP8L/ALOX5/ATN1/GFI1/GMCL2/HNRNPA2B1/HNRNPU/IFFO1/KAT8/LRIF1/NCOR2/NSMF/OGG1/PHB2/POLA1/PRPF40A/RUVBL1/RUVBL2/SFPQ/SMARCC2/SMARCD2/SMC1A/TEP1/TINF2/TP53/YY1	26
cluster4	CC	GO:0008540	proteasome regulatory particle, base subcomplex	5/2222	9/19869	0.001488231	0.040146795	0.035918952	PSMC1/PSMC4/PSMC5/PSMC6/PSMD2	5
cluster4	CC	GO:0044391	ribosomal subunit	35/2222	188/19869	0.001692967	0.044607705	0.039910085	AURKAIP1/GADD45GIP1/MRPL16/MRPL23/MRPL24/MRPL3/MRPL4/MRPL40/MRPL43/MRPL48/MRPL50/MRPL52/MRPL53/MRPL55/MRPL9/MRPS16/MRPS18A/MRPS2/MRPS22/MRPS24/NSUN4/RPL24/RPL28/RPL32/RPL37/RPL4/RPL7/RPL7L1/RPLP2/RPS10/RPS11/RPS12/RPS18/RPS27/UBA52	35
cluster4	CC	GO:0035861	site of double-strand break	18/2222	77/19869	0.001757958	0.04490102	0.040172511	ARPC1A/CNTD1/H2AX/IFFO1/INTS3/PAXX/PHF1/PNKP/RPA2/RPA3/SAMHD1/SETMAR/SHLD2/SLF2/SMARCAD1/SMC5/TP53/VCP	18

cluster4	CC	GO:0030027	lamellipodium	37/2222	203/19869	0.001879555	0.04490102	0.040172511	AMOT/APBB1IP/AVIL/CAPRIN1/CD177/CD44/CDH1/CDK5/CIB1/CORO1A/CORO1B/CTNNA1/CTNNB1/DBNL/DNM2/DOCK8/FAM89B/FGD2/FGD3/INPPL1/KPTN/MYO9B/NEDD9/P4HB/PABPC1/PIEZO1/PLCE1/PLXND1/PODXL/RUFY3/SPATA13/SRGAP2/STX4/TESK1/TIAM2/TSC1/TWF2	37
cluster4	CC	GO:0044665	MLL1/2 complex	10/2222	32/19869	0.001899993	0.04490102	0.040172511	C17orf49/CHD8/KAT8/KMT2A/KMT2B/MGA/PELP1/RNF2/RUVBL1/RUVBL2	10
cluster4	CC	GO:0097346	INO80-type complex	9/2222	27/19869	0.001924823	0.04490102	0.040172511	ACTR6/BRD8/INO80B/RUVBL1/RUVBL2/SRCAP/TRRAP/YY1/ZNHIT1	9
cluster4	CC	GO:0070603	SWI/SNF superfamily-type complex	20/2222	90/19869	0.001940621	0.04490102	0.040172511	ACTR6/ARID1A/BCL7C/BRD8/C17orf49/CHD3/CHD4/CSNK2A1/GATAD2B/INO80B/RB1/RUVBL1/RUVBL2/SMARCA2/SMARCC2/SMARCD2/SRCAP/TRRAP/YY1/ZNHIT1	20

cluster4	CC	GO:0071204	histone pre-mRNA 3'end processing complex	4/2222	6/19869	0.00194188	0.04490102	0.040172511	ERI1/SNRPB/SYNCRIP/YBX1	4
cluster4	CC	GO:0005775	vacuolar lumen	33/2222	176/19869	0.002000282	0.04532639	0.040553085	AGA/ARG1/ARHGAP45/ARSB/C6orf120/CHID1/DEFA1/DEFA1B/DEFA3/DPP7/DYNC1H1/EPDR1/FTL/FUCA2/GDI2/GGH/GNS/GYG1/HEBP2/HEXB/HSPA8/HSPG2/IFI30/IMPDH1/MAN2B1/NAAA/NEU1/PA2G4/PLBD2/RETN/TXNDC5/VAT1/VCP	33
cluster4	MF	GO:0016922	nuclear receptor binding	36/2205	139/18432	0.00000477	0.010469039	0.009949695	ACTN4/ARID1A/BAZ2A/BRD8/CALR/CNOT1/CTBP2/CTNNB1/DDX5/DDX54/EP300/HMGA1/JUND/KDM1A/LRIF1/MED1/MED12/MED17/MED30/NCOR1/NCOR2/NR1H2/PAGR1/PHB2/PPARGC1A/PPARGC1B/PRMT2/PRPF6/RXRA/SIRT1/SLC30A9/STAT5B/TOB2/TRIP4/TRIP6/ZNHIT3	36
cluster4	MF	GO:0140297	DNA-binding transcription factor binding	88/2205	476/18432	0.000019	0.020811471	0.019779064	ACTN4/ANXA4/ARID1A/ARNT/BAZ2A/BBS2/BRD8/BRMS1/CALR/CEBPA/CEBPG/CHD4/CNOT1/CPNE1/CREB1/CTBP2/CTNNB1/DAPK3/DDX5/DDX54/DHRS7B/EP300/FAM220A/FIZ1/FLNA/GTF2A2/GTF2I/HDAC7/HMGA1/JUND/JUP/KAT6A/KAT8/KDM1A/KLF4/LRIF1/MED1/MED12/MED17/MED30/MEF2D/METTL23/MLX/NAAA/NCL/NCOR1/NCOR2/NR1H2/NUCKS1/PAGR1/PARP9/PHB2/PPARGC1A/PPARGC1B/PRMT2/PRPF6/PSMC5/PSMD9/PURA/PURB/RB1/RXRA/SIRT1/SKI/SLC30A9/SP1/SPI1/STAT5B/STK4/TAF12/TBX6/TCERG1/TCF12/TCF3/TFDP1/TLE4/TOB2/TP53/TPT1/TRIM32/TRIP4/TRIP6/USF1/WWP2/YY1/ZBTB17/ZNF653/ZNHIT3	88

cluster4	MF	GO:0004518	nuclease activity	45/2205	208/18432	0.000052	0.038017172	0.036131232	AGO2/ANG/ANGEL2/APTX/ASTE1/CNOT1/CNOT6/CNOT8/DBR1/DCLRE1C/DICER1/DIS3L/DNASE1L1/ELAC2/EME2/ENDOG/ERI1/EXOSC10/EXOSC4/FEN1/INTS11/ISG20/KHNYN/MBLAC1/NOCT/NTHL1/OGG1/PLD4/PNKP/POLG/POP7/PPP1R8/RAD51C/REXO1/REXO2/RNASE4/RPP25/RPP30/RPP38/SAMHD1/SETMAR/SLFN13/TATDN3/TSN/XRCC1	45
cluster5	BP	GO:0061564	axon development	217/3710	490/18903	2.37E-36	2.48E-32	2.13E-32	ABL1/ACTBL2/ADCY1/ADGRB1/ANK3/ANOS1/APOD/APP/ARTN/ARX/ATL1/ATOH1/AUTS2/BARHL2/BDNF/BMP7/BMPR1B/BOC/BRSK1/BRSK2/CCK/CCKAR/CDH11/CDH2/CDH4/CDK5R1/CDK5R2/CDKL3/CHL1/CHODL/CNTN4/CNTN5/CNTN6/CNTNAP1/CRABP2/CRMP1/CRPPA/CRTAC1/CSPG5/CTNNA2/CYFIP1/DAB1/DAG1/DBN1/DCLK1/DLX5/DPYSL5/DRAXIN/DRD2/DRGX/DSCAM/DSCAML1/ECE1/EDN2/EDNRA/EFNA2/EFNA3/EFNA5/EFNB2/EFNB3/EPHA10/EPHA3/EPHA5/EPHA6/EPHA7/EPHA8/EPHB3/ERBB2/ETV1/EVX1/FEZ1/FEZF1/FEZF2/FGFR2/FKBP1B/FLRT2/FLRT3/FOLR1/FOXG1/FZD3/GAP43/GBX2/GDI1/GDNF/GFRA3/GLI2/GLI3/GRM7/GSK3B/HDAC6/IGSF9/ISL1/ISLR2/ITGB1/KIF5A/KIF5C/L1CAM/LAMA1/LAMA3/LAMA5/LAMB2/LAMC2/LGI1/LHX1/LHX2/LHX3/LHX9/LMX1A/LRP4/LRRC4C/LRTM1/LRTM2/MAP1A/MAP2/MAP6/MT3/MYPN/NCAM1/NCAM2/NDEL1/NECTIN1/NEFH/NEXN/NFASC/NGF/NGFR/NKX2-1/NKX2-8/NKX6-1/NLGN3/NOTCH2/NPTN/NPTX1/NR2E1/NR4A2/NRCAM/NREP/NRP2/NRXN1/NRXN3/NTN1/NTN3/NTNG1/NTNG2/NTRK2/NUMBL/OLFM1/OPHN1/OTX2/PAK3/PARD3/PAX2/PAX6/PHOX2B/PLP1/PLPPR4/PLXNA1/PLXNA2/PLXNA4/PLXNB1/PLXNB3/POU3F2/POU4F3/PTCH1/PTN/PTPRF/PTPRS/PTPRZ1/RAB8A/RELN/RET/RND2/ROBO1/ROBO2/ROBO3/RTN4RL1/RTN4RL2/SEMA3A/SEMA3E/SEMA4D/SEMA5A/SEMA5B/SEMA6A/SEMA6D/SHH/SHOX2/SLC9A6/SLIT1/SLIT3/SLITRK1/SLITRK2/SLITRK3/SLITRK6/SMO/SPTBN4/STK24/STXBP1/TBR1/THY1/TNC/TNFRSF21/TNN/TNR/TSPAN2/TUBB3/UCHL1/UNC5B/UNC5C/UNC5D/VASH2/VAX1/VAX2/VEGFA/VSTM2L/WNT3A/WNT5A/WNT7A	217
cluster5	BP	GO:0007409	axonogenesis	199/3710	438/18903	2.69E-35	1.41E-31	1.21E-31	ABL1/ACTBL2/ADCY1/ADGRB1/ANK3/ANOS1/APP/ARTN/ARX/ATL1/ATOH1/AUTS2/BARHL2/BDNF/BMP7/BMPR1B/BOC/BRSK1/BRSK2/CCK/CCKAR/CDH11/CDH2/CDH4/CDK5R1/CDK5R2/CDKL3/CHL1/CHODL/CNTN4/CNTN5/CNTN6/CNTNAP1/CRABP2/CRMP1/CRPPA/CTNNA2/CYFIP1/DAB1/DAG1/DBN1/DCLK1/DLX5/DPYSL5/DRAXIN/DRD2/DRGX/DSCAM/DSCAML1/ECE1/EDN2/EDNRA/EFNA2/EFNA3/EFNA5/EFNB2/EFNB3/EPHA10/EPHA3/EPHA5/EPHA6/EPHA7/EPHA8/EPHB3/ERBB2/ETV1/EVX1/FEZ1/FEZF1/FEZF2/FGFR2/FLRT2/FLRT3/FOXG1/FZD3/GAP43/GBX2/GDI1/GDNF/GFRA3/GLI2/GLI3/GSK3B/HDAC6/IGSF9/ISL1/ISLR2/ITGB1/KIF5A/KIF5C/L1CAM/LAMA1/LAMA3/LAMA5/LAMB2/LAMC2/LGI1/LHX1/LHX2/LHX3/LHX9/LMX1A/LRP4/LRRC4C/LRTM1/LRTM2/MAP1A/MAP2/MAP6/MT3/MYPN/NCAM1/NDEL1/NECTIN1/NEFH/NEXN/NFASC/NGF/NGFR/NKX2-1/NKX2-8/NKX6-1/NLGN3/NOTCH2/NPTN/NPTX1/NR2E1/NR4A2/NRCAM/NRP2/NRXN1/NRXN3/NTN1/NTN3/NTNG1/NTNG2/NTRK2/NUMBL/OLFM1/OPHN1/OTX2/PAK3/PARD3/PAX2/PAX6/PHOX2B/PLPPR4/PLXNA1/PLXNA2/PLXNA4/PLXNB1/PLXNB3/POU3F2/POU4F3/PTCH1/PTPRS/PTPRZ1/RAB8A/RELN/RET/RND2/ROBO1/ROBO2/ROBO3/SEMA3A/SEMA3E/SEMA4D/SEMA5A/SEMA5B/SEMA6A/SEMA6D/SHH/SHOX2/SLC9A6/SLIT1/SLIT3/SLITRK1/SLITRK2/SLITRK3/SLITRK6/SMO/SPTBN4/STXBP1/TBR1/THY1/TNN/TNR/TUBB3/UCHL1/UNC5B/UNC5C/UNC5D/VAX1/VAX2/VEGFA/VSTM2L/WNT3A/WNT5A/WNT7A	199
cluster5	BP	GO:0097485	neuron projection guidance	127/3710	237/18903	1.49E-31	5.2E-28	4.46E-28	ANOS1/APP/ARTN/ARX/ATOH1/BDNF/BMP7/BMPR1B/BOC/CDH4/CDK5R1/CDK5R2/CHL1/CNTN4/CNTN5/CNTN6/CRMP1/CRPPA/CYFIP1/DAG1/DLX5/DPYSL4/DPYSL5/DRAXIN/DRGX/DSCAM/DSCAML1/ECE1/EDNRA/EFNA2/EFNA3/EFNA5/EFNB2/EFNB3/EPHA10/EPHA3/EPHA5/EPHA6/EPHA7/EPHA8/EPHB3/ERBB2/ETV1/EVX1/FEZ1/FEZF1/FEZF2/FLRT2/FLRT3/FOXG1/FZD3/GAP43/GBX2/GDNF/GFRA3/GLI2/GLI3/IGSF9/ISL1/KIF5A/KIF5C/L1CAM/LAMA1/LAMA3/LAMA5/LAMB2/LAMC2/LGI1/LHX1/LHX2/LHX3/LHX9/LMX1A/LRTM1/LRTM2/MYPN/NCAM1/NECTIN1/NEXN/NFASC/NGFR/NKX2-1/NOTCH2/NPTN/NRCAM/NRP2/NRXN1/NRXN3/NTN1/NTN3/OPHN1/OTX2/PAX6/PLXNA1/PLXNA2/PLXNA4/PLXNB1/PLXNB3/POU4F3/PTCH1/RELN/RET/ROBO1/ROBO2/ROBO3/SEMA3A/SEMA3E/SEMA4D/SEMA5A/SEMA5B/SEMA6A/SEMA6D/SHH/SLIT1/SLIT3/SMO/TBR1/TNR/TUBB3/UNC5B/UNC5C/UNC5D/VAX1/VEGFA/VSTM2L/WNT3A/WNT5A	127

cluster5	BP	GO:0007411	axon guidance	126/3710	236/18903	4.16E-31	1.09E-27	9.36E-28	ANOS1/APP/ARTN/ARX/ATOH1/BDNF/BMP7/BMPR1B/BOC/CDH4/CDK5R1/CDK5R2/CHL1/CNTN4/CNTN5/CNTN6/CRMP1/CRPPA/CYFIP1/DAG1/DLX5/DPYSL5/DRAXIN/DRGX/DSCAM/DSCAML1/ECE1/EDNRA/EFNA2/EFNA3/EFNA5/EFNB2/EFNB3/EPHA10/EPHA3/EPHA5/EPHA6/EPHA7/EPHA8/EPHB3/ERBB2/ETV1/EVX1/FEZ1/FEZF1/FEZF2/FLRT2/FLRT3/FOXG1/FZD3/GAP43/GBX2/GDNF/GFRA3/GLI2/GLI3/IGSF9/ISL1/KIF5A/KIF5C/L1CAM/LAMA1/LAMA3/LAMA5/LAMB2/LAMC2/LGI1/LHX1/LHX2/LHX3/LHX9/LMX1A/LRTM1/LRTM2/MYPN/NCAM1/NECTIN1/NEXN/NFASC/NGFR/NKX2-1/NOTCH2/NPTN/NRCAM/NRP2/NRXN1/NRXN3/NTN1/NTN3/OPHN1/OTX2/PAX6/PLXNA1/PLXNA2/PLXNA4/PLXNB1/PLXNB3/POU4F3/PTCH1/RELN/RET/ROBO1/ROBO2/ROBO3/SEMA3A/SEMA3E/SEMA4D/SEMA5A/SEMA5B/SEMA6A/SEMA6D/SHH/SLIT1/SLIT3/SMO/TBR1/TNR/TUBB3/UNC5B/UNC5C/UNC5D/VAX1/VEGFA/VSTM2L/WNT3A/WNT5A	126
cluster5	BP	GO:0050808	synapse organization	182/3710	432/18903	2.21E-27	4.63E-24	3.98E-24	ABL1/ACTBL2/ACTN1/ADAM10/ADGRB1/ADGRB3/ADGRF1/ADGRL3/ANK3/APP/ARC/ARHGAP39/ARHGAP44/ASIC2/BCAN/BDNF/BSN/CACNA1S/CACNB1/CACNG2/CAMK2B/CAMKV/CBLN1/CBLN2/CBLN3/CDH2/CDH8/CDK5R1/CHRDL1/CHRNA7/CLSTN2/CNTN5/CNTNAP1/COL4A1/COL4A5/CTNNA2/CTNND2/CTTNBP2/CYFIP1/DAG1/DBN1/DLG5/DLGAP3/DNER/DRD1/DRD2/DSCAM/EFNA5/EFNB2/ELFN1/EPHA7/EPHB3/ERBB2/ERBB4/ERC1/F2R/FLRT2/FLRT3/FRMPD4/FRRS1L/GABRA1/GABRA2/GABRB2/GABRB3/GABRG2/GAP43/GDNF/GHSR/GJA10/GLRB/GPC6/GPM6A/GRID2/GRIN2B/GRIPAP1/GRM5/HAPLN4/HDAC6/HOMER1/IGSF21/IGSF9/IL1RAPL1/IL1RAPL2/INA/INS/ITGAM/ITPKA/KIF1A/KIRREL3/L1CAM/LAMB2/LHFPL4/LILRB2/LMX1A/LRFN3/LRP4/LRRC4B/LRRC4C/LRRN1/LRRTM2/LRRTM3/LRTM2/LZTS3/MUSK/NECTIN1/NEFH/NEURL1/NEUROD2/NFASC/NFATC4/NGEF/NLGN1/NLGN2/NLGN3/NLGN4X/NPAS4/NPTN/NPTX1/NRCAM/NRP2/NRXN1/NTN1/NTNG1/NTNG2/NTRK2/OPHN1/P2RX2/PAK3/PCDHB16/PCDHB6/PCLO/PDZRN3/PFN2/PLXNB1/PPFIA2/PRRT1/PTPRD/PTPRF/PTPRS/RELN/ROBO2/SDK1/SEMA3E/SEMA4D/SEZ6L/SEZ6L2/SHANK1/SHANK2/SHISA6/SHISA7/SIX4/SLC1A1/SLC6A1/SLC8A2/SLC8A3/SLIT1/SLITRK1/SLITRK2/SLITRK3/SLITRK6/SNCB/SRCIN1/SRPX2/ST8SIA2/SYBU/SYN1/SYNDIG1/TANC2/TMEM108/TNC/TNR/TPBG/UNC13A/UNC13C/WASF1/WASF3/WNT3A/WNT5A/WNT7A/WNT7B/YWHAZ/ZDHHC15	182
cluster5	BP	GO:0050804	modulation of chemical synaptic transmission	185/3710	448/18903	1.51E-26	2.63E-23	2.26E-23	ABL1/ACE/ACP4/ADCY1/ADCY8/ADGRB1/ADORA1/ADORA2B/ADRA1A/ADRA2A/AGT/APBA1/APP/ARC/ASIC1/ATF4/ATP1A2/BCHE/BDNF/BEGAIN/BRSK1/CACNA1A/CACNA1B/CACNG2/CACNG3/CACNG4/CACNG5/CACNG7/CALB1/CALB2/CAMK2A/CAMK2B/CBLN1/CDH11/CDH2/CELF4/CHRDL1/CHRM2/CHRNA3/CHRNA6/CHRNA7/CHRNB4/CLSTN2/CNTN4/CNTNAP4/CPEB3/CPLX2/CPLX4/CSPG5/CYFIP1/CYP46A1/DBN1/DGKI/DLGAP2/DLGAP3/DNM1/DRD1/DRD2/EIF4A3/ELAVL4/ERC1/FRRS1L/GFAP/GHSR/GIPC1/GRIA1/GRID1/GRID2/GRID2IP/GRIK1/GRIK2/GRIK3/GRIK5/GRIN1/GRIN2A/GRIN2B/GRIN2D/GRM2/GRM3/GRM4/GRM5/GRM6/GRM7/GRM8/GSK3B/HAP1/HCN1/HOMER1/HTR2A/IGSF11/INS/IQSEC2/ITGB1/ITPKA/JPH3/JPH4/KCNB1/KISS1/LGI1/LILRB2/LRRC4C/LRRTM2/LZTS1/MAP1A/MAPK8IP2/MPP2/NALCN/NEURL1/NEUROD2/NFATC4/NGF/NLGN1/NLGN2/NLGN3/NLGN4X/NMU/NPAS4/NPTN/NPTX1/NPTX2/NPTXR/NPY5R/NR2E1/NRG3/NRXN1/NTF3/NTNG1/NTNG2/NTRK2/OPHN1/P2RX3/PFN2/PLCB1/PLPPR4/PPP1R9A/PRKAR1B/PRKCG/PRKN/PRRT1/PRRT2/PTGS2/PTN/PTPRD/RAB5A/RAB8A/RAP1B/RASGRF1/RELN/RGS4/RIMS3/RIMS4/ROR2/SEPTIN5/SERPINE2/SHANK1/SHANK2/SHISA6/SHISA7/SHISA9/SLC1A1/SLC24A2/SLC30A1/SLC6A1/SLC8A2/SLC8A3/SNAP25/SNCAIP/SQSTM1/SRC/STX1A/STXBP1/SYN1/SYN3/SYP/SYT1/SYT12/SYT4/SYT7/TACR1/TMEM108/TNR/UNC13A/UNC13C/WNT3A/WNT7A	185
cluster5	BP	GO:0098742	cell-cell adhesion via plasma-membrane adhesion molecules	134/3710	282/18903	1.96E-26	2.72E-23	2.34E-23	ADGRL3/AJUBA/ARVCF/BMP2/CADM1/CADM3/CBLN1/CD164/CD84/CDH10/CDH11/CDH12/CDH16/CDH17/CDH18/CDH19/CDH2/CDH22/CDH4/CDH6/CDH7/CDH8/CDH9/CDHR4/CEACAM19/CEACAM5/CELSR1/CELSR2/CLDN1/CLDN14/CLDN16/CLDN18/CLDN19/CLDN2/CLDN22/CLDN4/CLDN5/CLDN6/CLDN8/CLSTN2/CNTN4/CNTN6/CRB2/DAB1/DCHS2/DSCAM/DSCAML1/DSG2/DSG4/EFNA5/FAT1/FAT2/FAT3/FAT4/FLRT3/GPC6/GRID2/ICAM1/IGSF11/IGSF21/IGSF9/IL1RAPL1/ITGAM/ITGB1/KIRREL3/L1CAM/LGALS7B/LRFN3/LRRC4B/LRRC4C/MMP24/MYADM/MYPN/NECTIN1/NECTIN3/NECTIN4/NEXN/NLGN1/NPTN/NTNG1/NTNG2/PCDH10/PCDH11Y/PCDH15/PCDH18/PCDH19/PCDH9/PCDHA11/PCDHA12/PCDHA13/PCDHA2/PCDHA3/PCDHA5/PCDHA6/PCDHA7/PCDHA8/PCDHA9/PCDHAC1/PCDHAC2/PCDHB16/PCDHB6/PCDHB7/PCDHB8/PCDHGA1/PCDHGA2/PCDHGA6/PCDHGB1/PCDHGB3/PCDHGB4/PECAM1/PLXNB3/PSG11/PSG2/PSG5/PTPRD/PTPRF/PTPRS/PTPRT/REG3A/RET/ROBO1/ROBO2/ROBO3/SDK1/SELP/SLITRK1/SLITRK2/SLITRK3/TENM2/TENM3/TENM4/TRO/UNC5D/VSTM2L	134

cluster5	BP	GO:0099177	regulation of trans-synaptic signaling	185/3710	449/18903	2.08E-26	2.72E-23	2.34E-23	ABL1/ACE/ACP4/ADCY1/ADCY8/ADGRB1/ADORA1/ADORA2B/ADRA1A/ADRA2A/AGT/APBA1/APP/ARC/ASIC1/ATF4/ATP1A2/BCHE/BDNF/BEGAIN/BRSK1/CACNA1A/CACNA1B/CACNG2/CACNG3/CACNG4/CACNG5/CACNG7/CALB1/CALB2/CAMK2A/CAMK2B/CBLN1/CDH11/CDH2/CELF4/CHRDL1/CHRM2/CHRNA3/CHRNA6/CHRNA7/CHRNB4/CLSTN2/CNTN4/CNTNAP4/CPEB3/CPLX2/CPLX4/CSPG5/CYFIP1/CYP46A1/DBN1/DGKI/DLGAP2/DLGAP3/DNM1/DRD1/DRD2/EIF4A3/ELAVL4/ERC1/FRRS1L/GFAP/GHSR/GIPC1/GRIA1/GRID1/GRID2/GRID2IP/GRIK1/GRIK2/GRIK3/GRIK5/GRIN1/GRIN2A/GRIN2B/GRIN2D/GRM2/GRM3/GRM4/GRM5/GRM6/GRM7/GRM8/GSK3B/HAP1/HCN1/HOMER1/HTR2A/IGSF11/INS/IQSEC2/ITGB1/ITPKA/JPH3/JPH4/KCNB1/KISS1/LGI1/LILRB2/LRRC4C/LRRTM2/LZTS1/MAP1A/MAPK8IP2/MPP2/NALCN/NEURL1/NEUROD2/NFATC4/NGF/NLGN1/NLGN2/NLGN3/NLGN4X/NMU/NPAS4/NPTN/NPTX1/NPTX2/NPTXR/NPY5R/NR2E1/NRG3/NRXN1/NTF3/NTNG1/NTNG2/NTRK2/OPHN1/P2RX3/PFN2/PLCB1/PLPPR4/PPP1R9A/PRKAR1B/PRKCG/PRKN/PRRT1/PRRT2/PTGS2/PTN/PTPRD/RAB5A/RAB8A/RAP1B/RASGRF1/RELN/RGS4/RIMS3/RIMS4/ROR2/SEPTIN5/SERPINE2/SHANK1/SHANK2/SHISA6/SHISA7/SHISA9/SLC1A1/SLC24A2/SLC30A1/SLC6A1/SLC8A2/SLC8A3/SNAP25/SNCAIP/SQSTM1/SRC/STX1A/STXBP1/SYN1/SYN3/SYP/SYT1/SYT12/SYT4/SYT7/TACR1/TMEM108/TNR/UNC13A/UNC13C/WNT3A/WNT7A	185
cluster5	BP	GO:0007389	pattern specification process	187/3710	472/18903	2.89E-24	3.36E-21	2.89E-21	AHI1/ALX4/APC2/ARC/ASCL1/BARX1/BASP1/BHLHE41/BMP2/BMP4/BMP5/BMP7/BMPR1B/CC2D2A/CCDC103/CCDC40/CDON/CDX1/CDX2/CDX4/CELSR2/CFAP52/CFC1/CFC1B/CHRD/CHRDL1/CRAMP1/CRB2/CXXC4/CYP26C1/DAW1/DDIT3/DLL1/DLL3/DLX1/DLX2/DMRT3/DMRTA2/DNAH11/DNAH5/DNAI1/DOP1B/DRC1/DSCAML1/EMX1/EMX2/EN1/ENKUR/ERBB4/FEZF1/FEZF2/FGF10/FGF2/FGFR2/FOLR1/FOXA1/FOXA2/FOXC1/FOXG1/FOXJ1/FOXN4/GATA4/GBX2/GDNF/GLI2/GLI3/GPC3/GPR161/GREM1/GREM2/GSX2/HES1/HES2/HES5/HES6/HES7/HEYL/HHIP/HIF1A/HNF1B/HOXA11/HOXA3/HOXA4/HOXB1/HOXB6/HOXB9/HOXC10/HOXC13/HOXC4/HOXC6/HOXC8/HOXC9/HOXD10/HOXD11/HOXD12/HOXD13/HOXD3/IFT140/IRX2/IRX4/ISL1/ITGAM/LAMA5/LBX1/LEFTY1/LHX1/LHX2/LHX3/LMX1B/LRP2/LRP4/LRP5/LRP6/MDFI/MEIS1/MEIS3/MID1/MMP21/MNS1/MSX2/NDRG4/NEUROD1/NEUROG1/NKX2-1/NKX2-2/NOTCH2/NR2F2/NRG3/NRP2/ODAD2/ODAD3/OOEP/OTX1/OTX2/PAX2/PAX6/PBX1/PGAP1/PIFO/PLXNA2/PTCH1/RAX/RELN/RFX3/RFX4/RIPPLY1/RIPPLY3/RNF111/ROBO1/ROBO2/SFRP2/SHH/SIX2/SIX3/SMAD5/SMO/SOSTDC1/SOX1/SOX18/SP8/SPRY1/STC1/TAF10/TBC1D32/TBR1/TBX20/TBX5/TBXT/TDGF1/TDRD5/TLL2/TULP3/UNCX/VANGL2/VAX2/WNT11/WNT2B/WNT3A/WNT5A/WNT7A/WNT7B/WNT8A/WNT8B/WT1/ZBTB16/ZIC1/ZIC3	187
cluster5	BP	GO:0007608	sensory perception of smell	177/3710	457/18903	9.38E-22	9.83E-19	8.44E-19	ADCY3/BBS1/BEST2/CFAP69/CNGB1/DRD2/NAV2/NCAM2/OR10A5/OR10A7/OR10G7/OR10H3/OR10H4/OR10J1/OR10J3/OR10K1/OR10Q1/OR10W1/OR10Z1/OR11H2/OR11H4/OR11L1/OR12D1/OR12D2/OR12D3/OR13C8/OR13C9/OR13D1/OR13F1/OR13H1/OR14A16/OR14I1/OR14J1/OR1A1/OR1A2/OR1B1/OR1E1/OR1E2/OR1G1/OR1J4/OR1L1/OR1L3/OR1L4/OR1L6/OR1M1/OR1S2/OR2A2/OR2AG1/OR2AG2/OR2AJ1/OR2AK2/OR2AT4/OR2B6/OR2D3/OR2F1/OR2G2/OR2G3/OR2H1/OR2J1/OR2J2/OR2L13/OR2L2/OR2L8/OR2M4/OR2S2/OR2T11/OR2T27/OR2T29/OR2T34/OR2T5/OR2T6/OR2T7/OR2W1/OR2W3/OR2Y1/OR2Z1/OR3A2/OR3A3/OR4A15/OR4A16/OR4A5/OR4C13/OR4C16/OR4C3/OR4C45/OR4C46/OR4C6/OR4D11/OR4D5/OR4D6/OR4F15/OR4F17/OR4F21/OR4F29/OR4F3/OR4F5/OR4K1/OR4K13/OR4K15/OR4K5/OR4L1/OR4M1/OR4M2/OR4N2/OR4N4/OR4N5/OR4X1/OR51A2/OR51B5/OR51B6/OR51E2/OR51G1/OR51I1/OR51L1/OR51Q1/OR51V1/OR52A5/OR52B2/OR52B6/OR52E4/OR52E5/OR52E6/OR52E8/OR52I2/OR52J3/OR52L1/OR52N5/OR56A5/OR5AC2/OR5AK2/OR5B17/OR5B3/OR5D14/OR5D16/OR5H2/OR5K1/OR5K4/OR5M10/OR5M11/OR5P2/OR5T3/OR5V1/OR5W2/OR6B1/OR6B2/OR6C3/OR6C6/OR6C70/OR6C76/OR6J1/OR6P1/OR6T1/OR7A10/OR7C1/OR7C2/OR7E24/OR7G1/OR7G2/OR7G3/OR8A1/OR8B2/OR8B3/OR8B4/OR8D2/OR8H1/OR8H3/OR8I2/OR8J1/OR8J3/OR8K1/OR9A4/OR9G1/OR9I1/OR9K2/OR9Q1/SLC6A3/SYT10	177
cluster5	BP	GO:0050911	detection of chemical stimulus involved in sensory perception of smell	168/3710	431/18903	4.82E-21	4.59E-18	3.94E-18	CNGB1/OR10A5/OR10A7/OR10G7/OR10H3/OR10H4/OR10J1/OR10J3/OR10K1/OR10Q1/OR10W1/OR10Z1/OR11H2/OR11H4/OR11L1/OR12D1/OR12D2/OR12D3/OR13C8/OR13C9/OR13D1/OR13F1/OR13H1/OR14A16/OR14I1/OR14J1/OR1A1/OR1A2/OR1B1/OR1E1/OR1E2/OR1G1/OR1J4/OR1L1/OR1L3/OR1L4/OR1L6/OR1M1/OR1S2/OR2A2/OR2AG1/OR2AG2/OR2AJ1/OR2AK2/OR2AT4/OR2B6/OR2D3/OR2F1/OR2G2/OR2G3/OR2H1/OR2J1/OR2J2/OR2L13/OR2L2/OR2L8/OR2M4/OR2S2/OR2T11/OR2T27/OR2T29/OR2T34/OR2T5/OR2T6/OR2T7/OR2W1/OR2W3/OR2Y1/OR2Z1/OR3A2/OR3A3/OR4A15/OR4A16/OR4A5/OR4C13/OR4C16/OR4C3/OR4C45/OR4C46/OR4C6/OR4D11/OR4D5/OR4D6/OR4F15/OR4F17/OR4F21/OR4F29/OR4F3/OR4F5/OR4K1/OR4K13/OR4K15/OR4K5/OR4L1/OR4M1/OR4M2/OR4N2/OR4N4/OR4N5/OR4X1/OR51A2/OR51B5/OR51B6/OR51E2/OR51G1/OR51I1/OR51L1/OR51Q1/OR51V1/OR52A5/OR52B2/OR52B6/OR52E4/OR52E5/OR52E6/OR52E8/OR52I2/OR52J3/OR52L1/OR52N5/OR56A5/OR5AC2/OR5AK2/OR5B17/OR5B3/OR5D14/OR5D16/OR5H2/OR5K1/OR5K4/OR5M10/OR5M11/OR5P2/OR5T3/OR5V1/OR5W2/OR6B1/OR6B2/OR6C3/OR6C6/OR6C70/OR6C76/OR6J1/OR6P1/OR6T1/OR7A10/OR7C1/OR7C2/OR7E24/OR7G1/OR7G2/OR7G3/OR8A1/OR8B2/OR8B3/OR8B4/OR8D2/OR8H1/OR8H3/OR8I2/OR8J1/OR8J3/OR8K1/OR9A4/OR9G1/OR9I1/OR9K2/OR9Q1	168

cluster5	BP	GO:0030900	forebrain development	154/3710	383/18903	6.34E-21	5.54E-18	4.75E-18	AKIRIN2/ALK/APP/ARX/ASCL1/ASPM/ATOH1/ATP1A2/ATP1A3/ATRX/BBS1/BCAN/BMP2/BMP4/CCKAR/CDH2/CDK5R1/CDK5R2/CDON/CHD5/CNTNAP2/DAB1/DCLK1/DCLK2/DCT/DLX1/DLX2/DLX5/DMRTA2/DNAH5/DRAXIN/DRD1/DRD2/EFNA2/EGFR/ELAVL4/EMX1/EMX2/EPHA5/EPHB3/ERBB4/EZH2/FAT4/FEZ1/FEZF1/FEZF2/FGF10/FGF2/FGFR2/FOXG1/GBX2/GLI2/GLI3/GSK3B/GSX1/GSX2/HAP1/HES1/HES5/HIF1A/HPRT1/HSD3B2/ID4/IGF2BP1/ISL1/ITGAM/KCNC1/KCNC2/KIRREL3/LAMB1/LHX1/LHX2/LHX3/LHX5/LMX1A/LRP2/MBOAT7/MDK/MFSD2A/NDEL1/NDNF/NEUROD1/NEUROD6/NEUROG3/NHLH2/NKX2-1/NKX2-6/NPY/NR0B1/NR2E1/NR2F2/NR4A2/NRG3/NRP2/NTRK2/NUMBL/OPHN1/OTP/OTX1/OTX2/PAX6/PCSK1/PGAP1/PITX1/PLCB1/PLXNA1/PLXNA4/POU3F2/POU3F3/PRKG1/PTCHD1/RARB/RAX/RELN/RFX4/ROBO1/ROBO2/RTN4RL1/SALL1/SECISBP2/SEMA3A/SEMA3E/SEMA5A/SHH/SIX3/SLC1A2/SLC6A3/SLIT1/SMO/SOX1/SOX3/SRC/SRD5A2/SSTR1/SSTR2/SYNE2/TACC2/TACC3/TBR1/TH/TMEM108/TNR/TOX/TYRO3/UNCX/VAX2/WNT2B/WNT3A/WNT4/WNT5A/WNT7A/WNT7B/ZIC1/ZIC3	154
cluster5	BP	GO:0050907	detection of chemical stimulus involved in sensory perception	180/3710	477/18903	1.16E-20	9.31E-18	8E-18	ASIC3/CNGB1/CST1/CST4/GNAT1/OR10A5/OR10A7/OR10G7/OR10H3/OR10H4/OR10J1/OR10J3/OR10K1/OR10Q1/OR10W1/OR10Z1/OR11H2/OR11H4/OR11L1/OR12D1/OR12D2/OR12D3/OR13C8/OR13C9/OR13D1/OR13F1/OR13H1/OR14A16/OR14I1/OR14J1/OR1A1/OR1A2/OR1B1/OR1E1/OR1E2/OR1G1/OR1J4/OR1L1/OR1L3/OR1L4/OR1L6/OR1M1/OR1S2/OR2A2/OR2AG1/OR2AG2/OR2AJ1/OR2AK2/OR2AT4/OR2B6/OR2D3/OR2F1/OR2G2/OR2G3/OR2H1/OR2J1/OR2J2/OR2L13/OR2L2/OR2L8/OR2M4/OR2S2/OR2T11/OR2T27/OR2T29/OR2T34/OR2T5/OR2T6/OR2T7/OR2W1/OR2W3/OR2Y1/OR2Z1/OR3A2/OR3A3/OR4A15/OR4A16/OR4A5/OR4C13/OR4C16/OR4C3/OR4C45/OR4C46/OR4C6/OR4D11/OR4D5/OR4D6/OR4F15/OR4F17/OR4F21/OR4F29/OR4F3/OR4F5/OR4K1/OR4K13/OR4K15/OR4K5/OR4L1/OR4M1/OR4M2/OR4N2/OR4N4/OR4N5/OR4X1/OR51A2/OR51B5/OR51B6/OR51E2/OR51G1/OR51I1/OR51L1/OR51Q1/OR51V1/OR52A5/OR52B2/OR52B6/OR52E4/OR52E5/OR52E6/OR52E8/OR52I2/OR52J3/OR52L1/OR52N5/OR56A5/OR5AC2/OR5AK2/OR5B17/OR5B3/OR5D14/OR5D16/OR5H2/OR5K1/OR5K4/OR5M10/OR5M11/OR5P2/OR5T3/OR5V1/OR5W2/OR6B1/OR6B2/OR6C3/OR6C6/OR6C70/OR6C76/OR6J1/OR6P1/OR6T1/OR7A10/OR7C1/OR7C2/OR7E24/OR7G1/OR7G2/OR7G3/OR8A1/OR8B2/OR8B3/OR8B4/OR8D2/OR8H1/OR8H3/OR8I2/OR8J1/OR8J3/OR8K1/OR9A4/OR9G1/OR9I1/OR9K2/OR9Q1/PKD1L3/RTP1/TAS1R1/TAS2R1/TAS2R13/TAS2R16/TAS2R7/TAS2R8	180
cluster5	BP	GO:0003002	regionalization	144/3710	360/18903	2.11E-19	1.58E-16	1.35E-16	AHI1/ALX4/ARC/ASCL1/BARX1/BASP1/BHLHE41/BMP2/BMP4/BMPR1B/CDON/CDX1/CDX2/CDX4/CELSR2/CFC1/CFC1B/CHRD/CRB2/CXXC4/CYP26C1/DDIT3/DLL1/DLL3/DLX1/DLX2/DMRT3/DMRTA2/DSCAML1/EMX1/EMX2/EN1/FEZF1/FEZF2/FGF10/FGF2/FGFR2/FOXA1/FOXA2/FOXC1/FOXG1/FOXJ1/FOXN4/GATA4/GBX2/GDNF/GLI2/GLI3/GPC3/GPR161/GREM1/GREM2/GSX2/HES1/HES2/HES5/HES6/HES7/HEYL/HHIP/HNF1B/HOXA11/HOXA3/HOXA4/HOXB1/HOXB6/HOXB9/HOXC10/HOXC13/HOXC4/HOXC6/HOXC8/HOXC9/HOXD10/HOXD11/HOXD13/HOXD3/IFT140/IRX2/ISL1/ITGAM/LAMA5/LHX1/LHX2/LHX3/LMX1B/LRP2/LRP4/LRP5/LRP6/MDFI/MNS1/MSX2/NEUROD1/NEUROG1/NKX2-1/NKX2-2/NOTCH2/NR2F2/NRP2/OTX1/OTX2/PAX2/PAX6/PBX1/PGAP1/PIFO/PLXNA2/PTCH1/RELN/RFX4/RIPPLY1/ROBO1/ROBO2/SFRP2/SHH/SIX2/SIX3/SMO/SOSTDC1/SOX1/SP8/SPRY1/TAF10/TBC1D32/TBR1/TBX20/TBXT/TDGF1/TDRD5/TLL2/TULP3/VAX2/WNT11/WNT2B/WNT3A/WNT5A/WNT7A/WNT7B/WNT8A/WNT8B/WT1/ZBTB16/ZIC3	144
cluster5	BP	GO:0051960	regulation of nervous system development	170/3710	456/18903	5.81E-19	4.06E-16	3.48E-16	ABCC8/ACE/ADGRB1/ADGRB3/AKT1/ASCL1/ASIC2/ASPA/ASPM/ATOH1/BDNF/BHLHE41/BMP2/BMP7/BRINP1/CAMK2B/CBLN1/CBLN2/CDH4/CDKL3/CDKN2B/CHODL/CLSTN2/CRABP2/CUL7/CUX1/CYFIP1/DAB1/DAG1/DBN1/DCT/DLG5/DLL1/DLL3/DLX1/DLX2/DMRTA2/DPYSL5/DRAXIN/DRD2/DSCAM/EFNA5/EFNB3/EPHA7/EPHB3/EZH2/F2/FAIM/FERD3L/FEZF1/FEZF2/FGF2/FLRT2/FLRT3/FOXG1/FZD3/GBX2/GDI1/GFAP/GLI3/GRID2/GRM5/GSX2/HAP1/HDAC2/HES1/HES2/HES5/HES6/HES7/HEYL/HIF1A/HLTF/ID1/ID4/IL1RAPL1/ISLR2/ITGB1/ITPKA/KHDC3L/L1CAM/LHX2/LRP2/LRP4/LRRC4B/LRRN1/LRRTM2/LRRTM3/LRTM2/LYN/MAP2/MAP6/MDK/MT3/MYRF/NDEL1/NEURL1/NFATC4/NGF/NKX2-2/NKX6-1/NLGN1/NLGN2/NLGN3/NPTN/NR2E1/NRXN1/NTN1/NTRK2/NUMBL/OTP/PAK3/PARD3/PAX6/PLXNA1/PLXNA2/PLXNA4/PLXNB1/PLXNB3/PRTG/PTN/PTPRD/PTPRS/PTPRZ1/RELN/RND2/ROBO1/ROBO2/RXRG/SEMA3A/SEMA3E/SEMA4D/SEMA5A/SEMA5B/SEMA6A/SEMA6D/SERPINE2/SHH/SHOX2/SIRT2/SLIT1/SLITRK1/SLITRK2/SLITRK3/SLITRK6/SMO/SOX10/SPEN/SRPX2/ST8SIA2/SYNDIG1/SYT4/TENM4/THY1/TLX2/TMEM98/TNFRSF21/TNR/TP73/TPBG/TPPP/TTBK1/VAX1/VEGFA/WASF3/WNT3A/WNT5A/WNT7A/YAP1/ZNF488	170

cluster5	BP	GO:0072073	kidney epithelium development	76/3710	146/18903	1.88E-18	1.23E-15	1.06E-15	AGT/AGTR2/AHI1/ARG2/BASP1/BMP2/BMP4/BMP7/BMPER/CALB1/CITED1/CRLF1/DLL1/EDNRA/EDNRB/EFNB2/EPCAM/EPHA7/FGF2/FGFR2/FOXC1/FOXJ1/GDNF/GLI3/GPC3/GREB1L/GREM1/GZF1/HES1/HES5/HEYL/HNF1B/HOXA11/HOXD11/HS3ST3A1/ILK/IRX2/KIF26B/KLF15/LAMA5/LAMB2/LGR4/LHX1/MAGED1/MAGI2/NOTCH2/NPHS1/PAX2/PBX1/PECAM1/POU3F3/PROM1/PTCH1/RARB/RET/ROBO2/SALL1/SDC1/SDC4/SHH/SIX2/SIX4/SMAD5/SMO/SOX9/SPRY1/TFAP2B/TMEM59L/VEGFA/WNT11/WNT2B/WNT4/WNT7B/WNT9B/WT1/YAP1	76
cluster5	BP	GO:0007156	homophilic cell adhesion via plasma membrane adhesion molecules	84/3710	170/18903	2.14E-18	1.32E-15	1.13E-15	CADM1/CADM3/CD84/CDH10/CDH11/CDH12/CDH16/CDH17/CDH18/CDH19/CDH2/CDH22/CDH4/CDH6/CDH7/CDH8/CDH9/CDHR4/CEACAM5/CELSR1/CELSR2/CLSTN2/CNTN4/CNTN6/DCHS2/DSCAM/DSCAML1/DSG2/DSG4/FAT1/FAT2/FAT3/FAT4/IGSF11/IGSF21/IGSF9/ITGB1/KIRREL3/L1CAM/MYPN/NECTIN1/NECTIN3/NECTIN4/NEXN/NPTN/PCDH10/PCDH11Y/PCDH15/PCDH18/PCDH19/PCDH9/PCDHA11/PCDHA12/PCDHA13/PCDHA2/PCDHA3/PCDHA5/PCDHA6/PCDHA7/PCDHA8/PCDHA9/PCDHAC1/PCDHAC2/PCDHB16/PCDHB6/PCDHB7/PCDHB8/PCDHGA1/PCDHGA2/PCDHGA6/PCDHGB1/PCDHGB3/PCDHGB4/PECAM1/PLXNB3/PTPRT/RET/ROBO1/ROBO2/ROBO3/SDK1/TENM3/TRO/VSTM2L	84
cluster5	BP	GO:0072001	renal system development	128/3710	318/18903	1.18E-17	6.89E-15	5.91E-15	ACE/ADAMTS6/AGT/AGTR2/AHI1/ANGPT1/ANGPT2/ARG2/ARID5B/BASP1/BMP2/BMP4/BMP6/BMP7/BMPER/CALB1/CC2D2A/CITED1/COL4A1/CRLF1/CYP26A1/DACT2/DCHS2/DLG5/DLL1/EDNRA/EDNRB/EFNB2/EMX2/EPCAM/EPHA7/ERBB4/FGF10/FGF2/FGFR2/FOXC1/FOXJ1/FREM2/GDF6/GDNF/GFRA1/GLI2/GLI3/GPC3/GREB1L/GREM1/GZF1/HAS2/HES1/HES5/HEYL/HMGCS2/HNF1B/HOXA11/HOXD11/HS3ST3A1/ILK/IRX2/ITGB3/JMJD6/KIF26B/KIRREL3/KLF15/LAMA5/LAMB2/LGR4/LGR5/LHX1/LRP2/LRP4/MAGED1/MAGI2/MYOCD/NID1/NOTCH2/NPHS1/OVOL1/PAX2/PBX1/PDGFA/PDGFRA/PDGFRB/PECAM1/PKD1L3/PKHD1/POU3F3/PROM1/PTCH1/PTK7/PYGO1/PYGO2/RARB/RET/ROBO2/SALL1/SDC1/SDC4/SEC61A1/SERPINB7/SHH/SIX2/SIX4/SLC34A1/SMAD5/SMO/SOX9/SPRY1/STRA6/SULF1/SULF2/TBC1D32/TFAP2A/TFAP2B/TIPARP/TMEM59L/TP73/VANGL2/VEGFA/WFS1/WNT11/WNT2B/WNT4/WNT5A/WNT7B/WNT9B/WT1/YAP1/ZBTB16	128
cluster5	BP	GO:0048880	sensory system development	150/3710	398/18903	2.29E-17	1.27E-14	1.09E-14	ADAMTS18/AHI1/ATF4/ATP2B2/BARHL2/BCAR3/BMP4/BMP6/BMP7/BMPR1B/CACNA1C/CACNA1S/CALB1/CC2D2A/CDON/CELF4/CHRDL1/CLCN2/COL4A1/COL8A1/CRB2/CRYAA/CRYAB/CRYGB/CRYGD/DCX/DLL1/DLX1/DLX2/DRD2/DSCAM/EGFR/FAT1/FAT3/FGF10/FGF2/FGF9/FOXC1/FOXE3/FOXF2/FOXN4/FREM2/GJE1/GLI3/GNAT1/GNGT1/GPM6A/GRHL2/GRM6/HCN1/HDAC2/HES1/HES5/HIF1A/HPCA/IFT140/ISL1/ITGAM/JMJD6/KERA/LAMA1/LAMB2/LHX1/LHX2/LRP5/MAB21L1/MDM1/MEGF11/MEIS1/MEIS3/MFAP2/MFRP/MFSD2A/MIP/MITF/NDP/NECTIN1/NECTIN3/NEUROD1/NOTCH2/NPHP4/NR2E1/NR2E3/NTRK2/OLFM3/OPN4/PAX2/PAX6/PBX1/PDGFRA/PDGFRB/PHOX2B/PLAAT1/PROM1/PRSS56/PTF1A/PXDN/PYGO2/RARB/RAX/RET/RHO/RORB/RP1/RPE65/SALL2/SDK1/SEMA3A/SH3PXD2B/SHH/SHROOM2/SIX3/SIX6/SLC17A6/SLC17A7/SLC17A8/SLC1A1/SLC39A5/SLC4A5/SLC6A3/SLITRK6/SOX1/SOX9/SPRY1/STRA6/TBC1D32/TENM3/TFAP2A/TFAP2B/TH/THRB/THY1/TMEM132E/TMEM231/TUB/TULP1/TULP3/USH1C/VAX1/VAX2/VEGFA/VSTM4/VSX1/WNT2B/WNT5A/WNT7A/WNT7B/WNT9A/WNT9B/WT1	150

cluster5	BP	GO:0042391	regulation of membrane potential	159/3710	431/18903	2.54E-17	1.33E-14	1.14E-14	ABL1/ACTN2/ADORA1/ADRA1A/AKAP6/AKT1/ANK3/APP/ASIC1/ASIC2/ATP1A2/ATP1A3/ATP1B1/BEST2/BVES/CACNA1C/CACNA1G/CACNA2D1/CACNG2/CASQ2/CBLN1/CELF4/CFTR/CHRM1/CHRNA2/CHRNA3/CHRNA4/CHRNA6/CHRNA7/CHRNB4/CHRNG/CLDN19/CNGB1/CNTNAP1/DMD/DRD1/DRD2/DRD4/DSG2/EHD3/EIF4A3/FHL1/FKBP1B/GABRA1/GABRA2/GABRA3/GABRA4/GABRA5/GABRA6/GABRB2/GABRB3/GABRD/GABRE/GABRG1/GABRG2/GABRG3/GABRQ/GJC1/GJD2/GLRA1/GLRA2/GLRB/GRIA1/GRIA3/GRID1/GRID2/GRIK1/GRIK2/GRIK3/GRIK5/GRIN1/GRIN2A/GRIN2B/GRIN2D/GRM5/GSK3B/HCN1/HCN3/HCN4/HTR3C/IGSF11/INSYN1/INSYN2A/KCNB1/KCNC1/KCNC2/KCND2/KCND3/KCNE4/KCNH1/KCNH2/KCNH4/KCNH5/KCNH6/KCNJ3/KCNJ5/KCNK10/KCNK3/KCNK5/KCNK6/KCNMA1/KCNMB2/KCNN2/KCNQ3/MAPK8IP2/MPP2/MYOC/NALCN/NEDD4L/NLGN1/NLGN2/NLGN3/NLGN4X/NPAS4/NRCAM/NRXN1/NTRK2/P2RX2/P2RX3/PHOX2B/PKP2/POPDC3/PRKAR1B/PRKN/PTPN3/PYCR1/RELN/RGS4/RGS7BP/RIMS3/RIMS4/RYR2/SCN10A/SCN1A/SCN2A/SCN2B/SCN3B/SCN4A/SCN8A/SCN9A/SHANK1/SLC1A6/SLC34A1/SLC4A11/SLC4A3/SLC4A4/SLC8A2/SLC8A3/SRC/STX1A/TACR1/TAFA4/TBX5/TMEM108/TRDN/TRPM4/UCN3/WNT7A/ZACN	159
cluster5	BP	GO:0072006	nephron development	76/3710	152/18903	3.76E-17	1.88E-14	1.61E-14	AGT/AGTR2/AHI1/ANGPT1/ANGPT2/BASP1/BMP2/BMP4/BMP7/CALB1/CITED1/DCHS2/DLL1/EDNRA/EDNRB/ERBB4/FGF2/FOXC1/FOXJ1/GDNF/GLI3/GPC3/GREB1L/GREM1/GZF1/HES1/HES5/HEYL/HNF1B/HOXA11/HOXD11/HS3ST3A1/ILK/IRX2/ITGB3/KIF26B/KIRREL3/KLF15/LAMA5/LAMB2/LGR4/LHX1/MAGED1/MAGI2/NID1/NOTCH2/NPHS1/PAX2/PBX1/PDGFRA/PDGFRB/PECAM1/POU3F3/PROM1/PTCH1/RET/SALL1/SEC61A1/SERPINB7/SHH/SIX2/SIX4/SMO/SOX9/SULF1/SULF2/TFAP2B/TMEM59L/VEGFA/WNT11/WNT2B/WNT4/WNT7B/WNT9B/WT1/YAP1	76
cluster5	BP	GO:0034329	cell junction assembly	158/3710	430/18903	4.89E-17	2.3E-14	1.98E-14	ABL1/ACE/ACE2/ACTN1/ACTN2/ADGRB1/ADGRB3/ADGRF1/ADGRL3/AGT/AJUBA/APOD/APP/ARHGAP6/ARVCF/ASIC2/BDNF/BSN/CBLN1/CBLN2/CDH10/CDH11/CDH12/CDH18/CDH19/CDH2/CDH22/CDH6/CDH7/CDH8/CDH9/CLDN1/CLDN14/CLDN16/CLDN18/CLDN19/CLDN2/CLDN22/CLDN4/CLDN5/CLDN6/CLDN8/CLSTN2/CNTN5/CNTNAP1/CNTNAP2/COL16A1/CORO2B/CTNND1/CTNND2/DLG5/DMTN/DNER/DRD1/DRD2/DSCAM/EFNA5/EFNB2/EPHA3/EPHA7/EPHB3/ERBB4/FER/FLRT2/FLRT3/FRMPD2/GABRA1/GABRA2/GABRB2/GABRB3/GABRG2/GAP43/GHSR/GJA10/GJC1/GPC6/GPM6A/GPM6B/GREM1/GRHL2/GRID2/HAPLN4/IL1RAPL1/IL1RAPL2/ITGA2/ITGB3/KIRREL3/LAMA3/LHFPL4/LRFN3/LRP4/LRRC4B/LRRN1/LRRTM2/LRRTM3/LRTM2/MARVELD2/MPDZ/MUSK/MYO1C/MYOC/NECTIN1/NLGN1/NLGN2/NLGN3/NLGN4X/NPAS4/NPHP4/NPHS1/NPTN/NPTX1/NR1H4/NRCAM/NRXN1/NTN1/NTNG2/NTRK2/OPHN1/PARD3/PCDHB16/PCDHB6/PCLO/PECAM1/PKN2/PKP2/PLXNB1/PTPRD/RAP1B/RHOD/ROBO2/ROCK1/ROCK2/SDC4/SDK1/SEMA4D/SHANK2/SIX4/SLIT1/SLITRK1/SLITRK2/SLITRK3/SLITRK6/SRC/SRPX2/ST8SIA2/STON1/STRN/SYNDIG1/TBX5/THY1/TPBG/TRPV4/VEGFA/WNT11/WNT3A/WNT4/WNT5A/WNT7A	158
cluster5	BP	GO:0001822	kidney development	124/3710	309/18903	5.08E-17	2.3E-14	1.98E-14	ACE/ADAMTS6/AGT/AGTR2/AHI1/ANGPT1/ANGPT2/ARG2/ARID5B/BASP1/BMP2/BMP4/BMP6/BMP7/BMPER/CALB1/CC2D2A/CITED1/CRLF1/CYP26A1/DACT2/DCHS2/DLG5/DLL1/EDNRA/EDNRB/EFNB2/EPCAM/EPHA7/ERBB4/FGF10/FGF2/FGFR2/FOXC1/FOXJ1/FREM2/GDF6/GDNF/GFRA1/GLI2/GLI3/GPC3/GREB1L/GREM1/GZF1/HAS2/HES1/HES5/HEYL/HMGCS2/HNF1B/HOXA11/HOXD11/HS3ST3A1/ILK/IRX2/ITGB3/JMJD6/KIF26B/KIRREL3/KLF15/LAMA5/LAMB2/LGR4/LHX1/LRP2/LRP4/MAGED1/MAGI2/NID1/NOTCH2/NPHS1/OVOL1/PAX2/PBX1/PDGFA/PDGFRA/PDGFRB/PECAM1/PKD1L3/PKHD1/POU3F3/PROM1/PTCH1/PTK7/PYGO1/PYGO2/RARB/RET/ROBO2/SALL1/SDC1/SDC4/SEC61A1/SERPINB7/SHH/SIX2/SIX4/SLC34A1/SMAD5/SMO/SOX9/SPRY1/STRA6/SULF1/SULF2/TBC1D32/TFAP2A/TFAP2B/TIPARP/TMEM59L/TP73/VANGL2/VEGFA/WFS1/WNT11/WNT2B/WNT4/WNT5A/WNT7B/WNT9B/WT1/YAP1/ZBTB16	124

cluster5	BP	GO:0045664	regulation of neuron differentiation	90/3710	196/18903	5.27E-17	2.3E-14	1.98E-14	ALK/APP/ASCL1/ASPM/ATOH1/BCL11A/BDNF/BMP2/BMP4/BMP6/BMP7/BRINP1/BRINP2/BRINP3/CASZ1/CDK5R1/CDON/CNTN4/CSNK1E/DAB1/DISP3/DLL1/DLX1/DLX2/DTX1/DUOXA1/EDNRB/EFNA3/EPO/ESRP1/FERD3L/FEZ1/FEZF1/FEZF2/FGF2/FGFR1/FOXA1/FOXG1/GDF5/GDF6/GLI3/GPRC5B/GSK3B/HES1/HES5/HEYL/HMG20B/HOXD3/ID4/ISL1/ITGB1/LBX1/LMX1A/MEIS1/MMD/MMD2/NCOA1/NEUROD1/NEUROD2/NEUROG1/NEUROG3/NGF/NKX2-2/NKX6-1/NLGN1/NR2E1/NRCAM/NREP/NTF3/PAX6/PBX1/PCP4/PHOX2B/RELN/RET/ROCK1/SFRP2/SH3GL3/SHH/SIX3/SOX3/SOX9/TCF4/TLX3/TP73/VWC2/VWC2L/WNT3A/ZFHX2/ZNF536	90
cluster5	BP	GO:0001823	mesonephros development	58/3710	102/18903	9.53E-17	3.91E-14	3.35E-14	AGT/AGTR2/ARG2/BASP1/BMP2/BMP4/BMP7/BMPER/CALB1/CITED1/CRLF1/EPCAM/FGF10/FGF2/FGFR2/FOXC1/FOXJ1/GDNF/GLI3/GPC3/GREB1L/GREM1/GZF1/HES1/HNF1B/HOXA11/HOXD11/HS3ST3A1/ILK/KIF26B/LAMA5/LGR4/LHX1/MAGED1/PAX2/PBX1/PTCH1/RARB/RET/ROBO2/SALL1/SDC1/SDC4/SHH/SIX2/SIX4/SMAD5/SMO/SOX9/SPRY1/TMEM59L/VEGFA/WNT11/WNT2B/WNT4/WNT9B/WT1/ZBTB16	58
cluster5	BP	GO:0060078	regulation of postsynaptic membrane potential	73/3710	145/18903	9.69E-17	3.91E-14	3.35E-14	ADORA1/AKT1/APP/CBLN1/CELF4/CHRM1/CHRNA2/CHRNA3/CHRNA4/CHRNA6/CHRNA7/CHRNB4/CHRNG/DRD2/DRD4/EIF4A3/GABRA1/GABRA2/GABRA3/GABRA4/GABRA5/GABRA6/GABRB2/GABRB3/GABRD/GABRE/GABRG1/GABRG2/GABRG3/GABRQ/GLRA1/GLRA2/GLRB/GRIA1/GRIA3/GRID1/GRID2/GRIK1/GRIK2/GRIK3/GRIK5/GRIN1/GRIN2A/GRIN2B/GRIN2D/GRM5/GSK3B/HTR3C/IGSF11/INSYN1/INSYN2A/KCND2/MAPK8IP2/MPP2/NLGN1/NLGN2/NLGN3/NLGN4X/NPAS4/NRXN1/P2RX2/P2RX3/PRKAR1B/RELN/RGS4/RGS7BP/SHANK1/SLC8A2/SLC8A3/STX1A/TMEM108/WNT7A/ZACN	73
cluster5	BP	GO:0001657	ureteric bud development	56/3710	97/18903	1.26E-16	4.89E-14	4.2E-14	AGT/AGTR2/ARG2/BASP1/BMP2/BMP4/BMP7/BMPER/CALB1/CITED1/CRLF1/EPCAM/FGF2/FGFR2/FOXC1/FOXJ1/GDNF/GLI3/GPC3/GREB1L/GREM1/GZF1/HES1/HNF1B/HOXA11/HOXD11/HS3ST3A1/ILK/KIF26B/LAMA5/LGR4/LHX1/MAGED1/PAX2/PBX1/PTCH1/RARB/RET/ROBO2/SALL1/SDC1/SDC4/SHH/SIX2/SIX4/SMAD5/SMO/SOX9/SPRY1/TMEM59L/VEGFA/WNT11/WNT2B/WNT4/WNT9B/WT1	56

cluster5	BP	GO:0050803	regulation of synapse structure or activity	95/3710	215/18903	1.63E-16	6.11E-14	5.24E-14	ABL1/ADGRB1/ADGRB3/APP/ARC/ARHGAP44/ASIC2/BDNF/CAMK2B/CAMKV/CBLN1/CBLN2/CDH2/CDH8/CDK5R1/CLSTN2/CTNNA2/CTTNBP2/CYFIP1/DAG1/DBN1/DLG5/DRD2/EFNA5/EPHA7/EPHB3/FLRT2/FLRT3/FRMPD4/GHSR/GPC6/GPM6A/GRID2/GRIN2B/GRIPAP1/HOMER1/IGSF9/IL1RAPL1/IL1RAPL2/INS/ITPKA/KIF1A/LHFPL4/LILRB2/LRFN3/LRP4/LRRC4B/LRRN1/LRRTM2/LRRTM3/LRTM2/LZTS3/MUSK/NECTIN1/NEURL1/NEUROD2/NFATC4/NGEF/NLGN1/NLGN2/NLGN3/NPTX1/NRCAM/NRP2/NRXN1/NTN1/NTNG2/NTRK2/PAK3/PPFIA2/PTPRD/RELN/ROBO2/SEMA4D/SHANK2/SIX4/SLC17A6/SLC17A7/SLC17A8/SLIT1/SLITRK1/SLITRK2/SLITRK3/SLITRK6/SRCIN1/SRPX2/ST8SIA2/SYNDIG1/TANC2/TPBG/WNT3A/WNT5A/WNT7A/YWHAZ/ZDHHC15	95
cluster5	BP	GO:0001654	eye development	145/3710	388/18903	1.83E-16	6.63E-14	5.69E-14	ADAMTS18/AHI1/ATF4/ATP2B2/BARHL2/BCAR3/BMP4/BMP6/BMP7/BMPR1B/CACNA1C/CACNA1S/CALB1/CC2D2A/CDON/CELF4/CHRDL1/CLCN2/COL4A1/COL8A1/CRB2/CRYAA/CRYAB/CRYGB/CRYGD/DCX/DLL1/DLX1/DLX2/DRD2/DSCAM/EGFR/FAT1/FAT3/FGF10/FGF2/FGF9/FOXC1/FOXE3/FOXF2/FOXN4/FREM2/GJE1/GLI3/GNAT1/GNGT1/GPM6A/GRHL2/GRM6/HCN1/HDAC2/HES1/HES5/HIF1A/HPCA/IFT140/JMJD6/KERA/LAMA1/LAMB2/LHX1/LHX2/LRP5/MAB21L1/MDM1/MEGF11/MEIS1/MEIS3/MFAP2/MFRP/MFSD2A/MIP/MITF/NDP/NECTIN1/NECTIN3/NEUROD1/NOTCH2/NPHP4/NR2E1/NR2E3/NTRK2/OLFM3/OPN4/PAX2/PAX6/PBX1/PDGFRA/PDGFRB/PLAAT1/PROM1/PRSS56/PTF1A/PXDN/PYGO2/RARB/RAX/RET/RHO/RORB/RP1/RPE65/SALL2/SDK1/SH3PXD2B/SHH/SHROOM2/SIX3/SIX6/SLC17A6/SLC17A7/SLC17A8/SLC1A1/SLC39A5/SLC4A5/SLC6A3/SLITRK6/SOX1/SOX9/SPRY1/STRA6/TBC1D32/TENM3/TFAP2A/TFAP2B/TH/THRB/THY1/TMEM231/TUB/TULP1/TULP3/USH1C/VAX1/VAX2/VEGFA/VSTM4/VSX1/WNT2B/WNT5A/WNT7A/WNT7B/WNT9A/WNT9B/WT1	145
cluster5	BP	GO:0150063	visual system development	146/3710	392/18903	2.02E-16	7.06E-14	6.06E-14	ADAMTS18/AHI1/ATF4/ATP2B2/BARHL2/BCAR3/BMP4/BMP6/BMP7/BMPR1B/CACNA1C/CACNA1S/CALB1/CC2D2A/CDON/CELF4/CHRDL1/CLCN2/COL4A1/COL8A1/CRB2/CRYAA/CRYAB/CRYGB/CRYGD/DCX/DLL1/DLX1/DLX2/DRD2/DSCAM/EGFR/FAT1/FAT3/FGF10/FGF2/FGF9/FOXC1/FOXE3/FOXF2/FOXN4/FREM2/GJE1/GLI3/GNAT1/GNGT1/GPM6A/GRHL2/GRM6/HCN1/HDAC2/HES1/HES5/HIF1A/HPCA/IFT140/ITGAM/JMJD6/KERA/LAMA1/LAMB2/LHX1/LHX2/LRP5/MAB21L1/MDM1/MEGF11/MEIS1/MEIS3/MFAP2/MFRP/MFSD2A/MIP/MITF/NDP/NECTIN1/NECTIN3/NEUROD1/NOTCH2/NPHP4/NR2E1/NR2E3/NTRK2/OLFM3/OPN4/PAX2/PAX6/PBX1/PDGFRA/PDGFRB/PLAAT1/PROM1/PRSS56/PTF1A/PXDN/PYGO2/RARB/RAX/RET/RHO/RORB/RP1/RPE65/SALL2/SDK1/SH3PXD2B/SHH/SHROOM2/SIX3/SIX6/SLC17A6/SLC17A7/SLC17A8/SLC1A1/SLC39A5/SLC4A5/SLC6A3/SLITRK6/SOX1/SOX9/SPRY1/STRA6/TBC1D32/TENM3/TFAP2A/TFAP2B/TH/THRB/THY1/TMEM231/TUB/TULP1/TULP3/USH1C/VAX1/VAX2/VEGFA/VSTM4/VSX1/WNT2B/WNT5A/WNT7A/WNT7B/WNT9A/WNT9B/WT1	146
cluster5	BP	GO:0001505	regulation of neurotransmitter levels	96/3710	219/18903	2.14E-16	7.23E-14	6.21E-14	ADCY1/ADORA2B/ADRA1A/ADRA2A/APBA1/ASIC1/ATP1A2/BCHE/BLOC1S6/BRSK1/CADPS/CAMK2A/CHRM2/CHRNA3/CHRNA6/CHRNB4/COMT/CPLX2/CPLX4/CSPG5/DAGLA/DBH/DOC2A/DOC2B/DRD1/DRD2/DRD4/GABRA2/GAD1/GAD2/GDNF/GFAP/GIPC1/GPM6B/GRIK5/GRM4/GSK3B/HRH3/HTR2A/ITGB1/ITGB3/MAOB/NLGN1/NRXN1/OTOF/PAH/PCLO/PDE1B/PFN2/PRIMA1/PRKCG/PRKN/PRRT2/RAB3B/RAB5A/RAP1B/RIMS3/RIMS4/RPH3AL/SEPTIN5/SLC17A8/SLC1A2/SLC1A6/SLC29A4/SLC30A1/SLC5A7/SLC6A1/SLC6A11/SLC6A2/SLC6A3/SLC6A5/SNAP23/SNAP25/SNCAIP/STX11/STX1A/STXBP1/SV2A/SYN1/SYN2/SYN3/SYNGR3/SYP/SYT1/SYT12/SYT2/SYT4/SYT5/SYT7/SYT8/SYT9/TH/UNC13A/UNC13C/WNT7A/ZNF219	96

cluster5	BP	GO:0072163	mesonephric epithelium development	56/3710	98/18903	2.38E-16	7.56E-14	6.49E-14	AGT/AGTR2/ARG2/BASP1/BMP2/BMP4/BMP7/BMPER/CALB1/CITED1/CRLF1/EPCAM/FGF2/FGFR2/FOXC1/FOXJ1/GDNF/GLI3/GPC3/GREB1L/GREM1/GZF1/HES1/HNF1B/HOXA11/HOXD11/HS3ST3A1/ILK/KIF26B/LAMA5/LGR4/LHX1/MAGED1/PAX2/PBX1/PTCH1/RARB/RET/ROBO2/SALL1/SDC1/SDC4/SHH/SIX2/SIX4/SMAD5/SMO/SOX9/SPRY1/TMEM59L/VEGFA/WNT11/WNT2B/WNT4/WNT9B/WT1	56
cluster5	BP	GO:0072164	mesonephric tubule development	56/3710	98/18903	2.38E-16	7.56E-14	6.49E-14	AGT/AGTR2/ARG2/BASP1/BMP2/BMP4/BMP7/BMPER/CALB1/CITED1/CRLF1/EPCAM/FGF2/FGFR2/FOXC1/FOXJ1/GDNF/GLI3/GPC3/GREB1L/GREM1/GZF1/HES1/HNF1B/HOXA11/HOXD11/HS3ST3A1/ILK/KIF26B/LAMA5/LGR4/LHX1/MAGED1/PAX2/PBX1/PTCH1/RARB/RET/ROBO2/SALL1/SDC1/SDC4/SHH/SIX2/SIX4/SMAD5/SMO/SOX9/SPRY1/TMEM59L/VEGFA/WNT11/WNT2B/WNT4/WNT9B/WT1	56
cluster5	BP	GO:0051962	positive regulation of nervous system development	113/3710	279/18903	5.66E-16	1.73E-13	1.49E-13	ACE/ADGRB1/ADGRB3/ASCL1/ASIC2/ASPA/ASPM/BDNF/BMP2/CAMK2B/CBLN1/CBLN2/CDH4/CDKL3/CHODL/CLSTN2/CRABP2/CUL7/CUX1/CYFIP1/DAG1/DBN1/DCT/DLG5/DMRTA2/DRD2/DSCAM/EFNA5/EPHB3/FAIM/FGF2/FLRT2/FLRT3/FOXG1/FZD3/GDI1/GFAP/GLI3/GRID2/GRM5/GSX2/HAP1/HDAC2/HES1/HIF1A/ID4/IL1RAPL1/ISLR2/ITGB1/ITPKA/KHDC3L/L1CAM/LRP2/LRRC4B/LRRN1/LRRTM2/LRRTM3/LRTM2/LYN/MAP6/MDK/NDEL1/NEURL1/NGF/NKX2-2/NKX6-1/NLGN1/NLGN2/NLGN3/NPTN/NR2E1/NRXN1/NTN1/NTRK2/NUMBL/OTP/PAK3/PAX6/PLXNA1/PLXNA2/PLXNA4/PLXNB1/PLXNB3/PTN/PTPRD/PTPRZ1/RELN/RND2/ROBO1/ROBO2/SEMA4D/SEMA5A/SERPINE2/SHH/SHOX2/SLITRK1/SLITRK2/SLITRK3/SLITRK6/SMO/SOX10/SPEN/SRPX2/ST8SIA2/SYNDIG1/TENM4/TP73/TPBG/TTBK1/VEGFA/WNT3A/WNT7A/ZNF488	113
cluster5	BP	GO:0001655	urogenital system development	136/3710	360/18903	5.79E-16	1.73E-13	1.49E-13	ACE/ADAMTS6/AGT/AGTR2/AHI1/ANGPT1/ANGPT2/ARG2/ARID5B/BASP1/BMP2/BMP4/BMP6/BMP7/BMPER/CALB1/CC2D2A/CITED1/COL4A1/CRLF1/CYP26A1/DACT2/DCHS2/DLG5/DLL1/EDNRA/EDNRB/EFNB2/EMX2/EPCAM/EPHA7/EPHB3/ERBB4/FGF10/FGF2/FGFR2/FOXA1/FOXC1/FOXJ1/FREM2/GDF6/GDNF/GFRA1/GLI2/GLI3/GPC3/GREB1L/GREM1/GZF1/HAS2/HES1/HES5/HEYL/HMGCS2/HNF1B/HOXA11/HOXB13/HOXD11/HOXD13/HS3ST3A1/ID4/ILK/IRX2/ITGB3/JMJD6/KIF26B/KIRREL3/KLF15/LAMA5/LAMB2/LGR4/LGR5/LHX1/LRP2/LRP4/MAGED1/MAGI2/MYOCD/NID1/NOTCH2/NPHS1/OVOL1/PAX2/PBX1/PDGFA/PDGFRA/PDGFRB/PECAM1/PKD1L3/PKHD1/POU3F3/PROM1/PSAP/PSAPL1/PTCH1/PTK7/PYGO1/PYGO2/RARB/RET/ROBO2/SALL1/SDC1/SDC4/SEC61A1/SERPINB7/SHH/SIX2/SIX4/SLC34A1/SMAD5/SMO/SOX9/SPRY1/STRA6/SULF1/SULF2/TBC1D32/TFAP2A/TFAP2B/TIPARP/TMEM59L/TNC/TP73/VANGL2/VEGFA/WFS1/WNT11/WNT2B/WNT4/WNT5A/WNT7B/WNT9B/WT1/YAP1/ZBTB16	136

cluster5	BP	GO:0050807	regulation of synapse organization	92/3710	209/18903	6.54E-16	1.91E-13	1.64E-13	ABL1/ADGRB1/ADGRB3/APP/ARC/ARHGAP44/ASIC2/BDNF/CAMK2B/CAMKV/CBLN1/CBLN2/CDH2/CDH8/CDK5R1/CLSTN2/CTNNA2/CTTNBP2/CYFIP1/DAG1/DBN1/DLG5/DRD2/EFNA5/EPHA7/EPHB3/FLRT2/FLRT3/FRMPD4/GHSR/GPC6/GPM6A/GRID2/GRIN2B/GRIPAP1/HOMER1/IGSF9/IL1RAPL1/IL1RAPL2/INS/ITPKA/KIF1A/LHFPL4/LILRB2/LRFN3/LRP4/LRRC4B/LRRN1/LRRTM2/LRRTM3/LRTM2/LZTS3/MUSK/NECTIN1/NEURL1/NEUROD2/NFATC4/NGEF/NLGN1/NLGN2/NLGN3/NPTX1/NRCAM/NRP2/NRXN1/NTN1/NTNG2/NTRK2/PAK3/PPFIA2/PTPRD/RELN/ROBO2/SEMA4D/SHANK2/SIX4/SLIT1/SLITRK1/SLITRK2/SLITRK3/SLITRK6/SRCIN1/SRPX2/ST8SIA2/SYNDIG1/TANC2/TPBG/WNT3A/WNT5A/WNT7A/YWHAZ/ZDHHC15	92
cluster5	BP	GO:0001764	neuron migration	82/3710	177/18903	6.76E-16	1.91E-13	1.64E-13	ADGRL3/ARX/ASCL1/ASPM/ASTN1/ATOH1/AUTS2/BARHL1/BARHL2/BBS1/CAMK2A/CAMK2B/CCK/CCKAR/CDK5R1/CDK5R2/CELSR1/CELSR2/CHL1/CTNNA2/DAB1/DCLK1/DCX/DNER/DRD1/DRD2/DRGX/EMX2/EVX1/FAT3/FEZF1/FEZF2/FGFR1/FLRT2/FOXG1/FZD3/GAS6/GFRA3/GPM6A/GPR173/KIAA0319L/KIF20B/KIRREL3/LHX1/MDK/NDEL1/NDNF/NEXMIF/NKX2-1/NKX6-1/NR2F2/NR4A2/NRCAM/NRG3/NRP2/NTN1/NTNG1/NTNG2/NTRK2/PAX6/PHOX2B/PLXNA1/PRKG1/RELN/SCRT1/SCRT2/SEMA3A/SEMA3E/SEMA6A/SEPTIN4/SH3RF1/SOX1/SOX14/SPOCK1/TBX20/TLX3/TNN/TYRO3/UNC5C/UNC5D/VAX1/VEGFA	82
cluster5	BP	GO:0007416	synapse assembly	84/3710	184/18903	8.82E-16	2.43E-13	2.09E-13	ADGRB1/ADGRB3/ADGRF1/ADGRL3/APP/ASIC2/BDNF/BSN/CBLN1/CBLN2/CDH2/CLSTN2/CNTN5/DLG5/DNER/DRD1/DRD2/DSCAM/EFNA5/EFNB2/EPHA7/EPHB3/ERBB4/FLRT2/FLRT3/GABRA1/GABRA2/GABRB2/GABRB3/GABRG2/GAP43/GHSR/GJA10/GPC6/GPM6A/GRID2/HAPLN4/IL1RAPL1/IL1RAPL2/KIRREL3/LHFPL4/LRFN3/LRP4/LRRC4B/LRRN1/LRRTM2/LRRTM3/LRTM2/MUSK/NECTIN1/NLGN1/NLGN2/NLGN3/NLGN4X/NPAS4/NPTN/NPTX1/NRCAM/NRXN1/NTN1/NTNG2/NTRK2/PCDHB16/PCDHB6/PCLO/PLXNB1/PTPRD/ROBO2/SDK1/SEMA4D/SHANK2/SIX4/SLIT1/SLITRK1/SLITRK2/SLITRK3/SLITRK6/SRPX2/ST8SIA2/SYNDIG1/TPBG/WNT3A/WNT5A/WNT7A	84
cluster5	BP	GO:0010975	regulation of neuron projection development	159/3710	446/18903	9.11E-16	2.45E-13	2.1E-13	ABL1/ACP4/ADGRB3/AGT/AKT1/ALK/ALKAL2/ARC/ARHGAP44/BCL11A/BDNF/BMP5/BMP7/BRSK1/BRSK2/CAMK2B/CDH2/CDH4/CDK5R1/CDKL3/CHODL/CHRNA3/CRABP2/CRMP1/CSMD3/CTNNA2/CUL7/CUX1/CYFIP1/DAB1/DAB2/DBN1/DPYSL3/DPYSL5/DRAXIN/DSCAM/EFHC2/EFNA5/EFNB2/EFNB3/ELAVL4/EPHA3/EPHA7/EPHB3/EPO/EZH2/FAT3/FEZ1/FEZF2/FKBP1B/FUT9/GDI1/GFAP/GPC2/GRID2/GSK3B/HDAC2/HES1/ID1/IL1RAPL1/ISLR2/ITPKA/KHDC3L/KIF1A/KLK6/KNDC1/L1CAM/LRP4/LRRC4C/LYN/LZTS1/LZTS3/MAGI2/MAP2/MAP6/MDK/MFSD2A/MT3/NDEL1/NDNF/NDRG4/NEDD4L/NEU4/NEUROG3/NFATC4/NGEF/NGF/NLGN1/NPTN/NR2E1/NR2F1/NRCAM/NTN1/NTNG1/NTNG2/NTRK2/PACSIN1/PAK3/PLA2G3/PLK5/PLPPR5/PLXNA1/PLXNA2/PLXNA4/PLXNB1/PLXNB3/POU3F2/PPFIA2/PRAG1/PTK7/PTN/PTPRD/PTPRF/PTPRG/PTPRS/RELN/RET/RIT2/RND2/ROBO1/ROBO2/ROR1/ROR2/RTN4RL1/RTN4RL2/SDC2/SEMA3A/SEMA3E/SEMA4D/SEMA5A/SEMA5B/SEMA6A/SEMA6D/SFRP2/SHOX2/SLC39A12/SLIT1/SLITRK1/SNAP25/SPOCK1/SRCIN1/STK24/STMN2/TANC2/TBR1/TENM3/THY1/TLX2/TMEM30A/TNN/TNR/TOX/TRIM67/TRPV4/VEGFA/WNT3A/WNT5A/WNT7A/ZDHHC15	159

cluster5	BP	GO:0030198	extracellular matrix organization	123/3710	318/18903	1.87E-15	4.9E-13	4.2E-13	ABL1/ADAM10/ADAMTS13/ADAMTS14/ADAMTS15/ADAMTS18/ADAMTS19/ADAMTS2/ADAMTS20/ADAMTS3/ADAMTS6/ADAMTS7/ADAMTS8/ADAMTSL1/ADAMTSL2/ADAMTSL3/AGT/APP/B4GALT1/BCL3/BMP2/C6orf15/CCN2/CHADL/CMA1/COL11A1/COL12A1/COL14A1/COL16A1/COL22A1/COL23A1/COL24A1/COL27A1/COL28A1/COL2A1/COL4A1/COL4A5/COL4A6/COL5A3/COL6A5/COL6A6/COL8A1/COL9A1/CPB2/CRISPLD2/CST3/CTSG/DAG1/ECM2/ELANE/EMILIN1/ERO1A/FAP/FBLN1/FERMT1/FGFR4/FKBP10/FLRT2/FOXC1/FOXF2/GAS6/GFAP/GPM6B/GREM1/HAPLN2/HAS2/HAS3/HPN/HPSE2/HSD17B12/IBSP/IMPG1/ITGB1/ITGB3/KAZALD1/KLK5/LAMA1/LAMB1/LAMB2/LOX/LOXL4/MATN2/MATN3/MATN4/MFAP4/MMP10/MMP13/MMP15/MMP16/MMP21/MMP24/MMP8/MPZL3/NDNF/NID1/NPHS1/NR2E1/NTNG1/NTNG2/OLFML2B/OPTC/P3H4/PDGFRA/PDPN/PXDN/QSOX1/SFRP2/SH3PXD2B/SLC2A10/SOX9/ST7/SULF1/SULF2/THSD4/TLL2/TNFRSF11B/TNR/TNXB/VIT/VWA1/WDR72/WNT3A/WT1	123
cluster5	BP	GO:0006836	neurotransmitter transport	92/3710	212/18903	1.93E-15	4.95E-13	4.24E-13	ADCY1/ADORA2B/ADRA1A/ADRA2A/APBA1/ASIC1/ATP1A2/BLOC1S6/BRSK1/CADPS/CAMK2A/CHRM2/CHRNA3/CHRNA6/CHRNB4/CPLX2/CPLX4/CSPG5/DOC2A/DOC2B/DRD1/DRD2/DRD4/GABRA2/GABRQ/GDNF/GFAP/GIPC1/GPM6B/GRIK5/GRM4/GSK3B/HRH3/HTR2A/ITGB1/ITGB3/NLGN1/NRXN1/OTOF/PCLO/PFN2/PRKCG/PRKN/PRRT2/RAB3B/RAB5A/RAP1B/RIMS3/RIMS4/RPH3AL/SEPTIN5/SLC17A6/SLC17A7/SLC17A8/SLC1A1/SLC1A2/SLC1A6/SLC29A4/SLC30A1/SLC5A7/SLC6A1/SLC6A11/SLC6A15/SLC6A17/SLC6A2/SLC6A3/SLC6A5/SLC6A7/SNAP23/SNAP25/SNCAIP/STX11/STX1A/STXBP1/SV2A/SYN1/SYN2/SYN3/SYNGR3/SYP/SYT1/SYT12/SYT2/SYT4/SYT5/SYT7/SYT8/SYT9/TH/UNC13A/UNC13C/WNT7A	92
cluster5	BP	GO:0043010	camera-type eye development	129/3710	340/18903	2.22E-15	5.54E-13	4.76E-13	AHI1/ATF4/ATP2B2/BARHL2/BCAR3/BMP4/BMP7/BMPR1B/CACNA1C/CACNA1S/CALB1/CC2D2A/CDON/CELF4/CLCN2/COL4A1/COL8A1/CRB2/CRYAA/CRYAB/CRYGB/CRYGD/DCX/DLL1/DLX1/DLX2/DRD2/DSCAM/EGFR/FAT1/FAT3/FGF10/FGF2/FOXC1/FOXE3/FOXF2/FOXN4/GJE1/GLI3/GNAT1/GPM6A/GRHL2/GRM6/HCN1/HDAC2/HES1/HES5/HIF1A/HPCA/IFT140/JMJD6/KERA/LAMA1/LAMB2/LHX1/LHX2/LRP5/MAB21L1/MDM1/MEGF11/MEIS1/MFRP/MFSD2A/MIP/MITF/NDP/NECTIN1/NECTIN3/NEUROD1/NOTCH2/NPHP4/NR2E1/NR2E3/NTRK2/OPN4/PAX2/PAX6/PDGFRA/PDGFRB/PLAAT1/PROM1/PRSS56/PTF1A/PYGO2/RAX/RET/RHO/RORB/RP1/RPE65/SDK1/SHH/SHROOM2/SIX3/SLC17A6/SLC17A7/SLC17A8/SLC1A1/SLC4A5/SLC6A3/SLITRK6/SOX1/SOX9/SPRY1/STRA6/TBC1D32/TENM3/TFAP2A/TFAP2B/TH/THRB/THY1/TMEM231/TUB/TULP1/TULP3/USH1C/VAX1/VAX2/VEGFA/VSTM4/VSX1/WNT2B/WNT5A/WNT7A/WNT7B/WNT9A/WNT9B/WT1	129
cluster5	BP	GO:0072009	nephron epithelium development	61/3710	116/18903	2.32E-15	5.65E-13	4.85E-13	AGT/AGTR2/AHI1/BASP1/BMP2/BMP4/CALB1/CITED1/DLL1/EDNRA/EDNRB/FGF2/FOXC1/FOXJ1/GDNF/GLI3/GPC3/GREB1L/GREM1/GZF1/HES1/HES5/HEYL/HNF1B/HOXA11/HOXD11/HS3ST3A1/ILK/IRX2/KIF26B/KLF15/LAMA5/LAMB2/LGR4/LHX1/MAGED1/MAGI2/NOTCH2/NPHS1/PAX2/PBX1/PECAM1/POU3F3/PROM1/PTCH1/SALL1/SHH/SIX2/SIX4/SMO/SOX9/TFAP2B/TMEM59L/VEGFA/WNT11/WNT2B/WNT4/WNT7B/WNT9B/WT1/YAP1	61

cluster5	BP	GO:0043062	extracellular structure organization	123/3710	319/18903	2.46E-15	5.86E-13	5.03E-13	ABL1/ADAM10/ADAMTS13/ADAMTS14/ADAMTS15/ADAMTS18/ADAMTS19/ADAMTS2/ADAMTS20/ADAMTS3/ADAMTS6/ADAMTS7/ADAMTS8/ADAMTSL1/ADAMTSL2/ADAMTSL3/AGT/APP/B4GALT1/BCL3/BMP2/C6orf15/CCN2/CHADL/CMA1/COL11A1/COL12A1/COL14A1/COL16A1/COL22A1/COL23A1/COL24A1/COL27A1/COL28A1/COL2A1/COL4A1/COL4A5/COL4A6/COL5A3/COL6A5/COL6A6/COL8A1/COL9A1/CPB2/CRISPLD2/CST3/CTSG/DAG1/ECM2/ELANE/EMILIN1/ERO1A/FAP/FBLN1/FERMT1/FGFR4/FKBP10/FLRT2/FOXC1/FOXF2/GAS6/GFAP/GPM6B/GREM1/HAPLN2/HAS2/HAS3/HPN/HPSE2/HSD17B12/IBSP/IMPG1/ITGB1/ITGB3/KAZALD1/KLK5/LAMA1/LAMB1/LAMB2/LOX/LOXL4/MATN2/MATN3/MATN4/MFAP4/MMP10/MMP13/MMP15/MMP16/MMP21/MMP24/MMP8/MPZL3/NDNF/NID1/NPHS1/NR2E1/NTNG1/NTNG2/OLFML2B/OPTC/P3H4/PDGFRA/PDPN/PXDN/QSOX1/SFRP2/SH3PXD2B/SLC2A10/SOX9/ST7/SULF1/SULF2/THSD4/TLL2/TNFRSF11B/TNR/TNXB/VIT/VWA1/WDR72/WNT3A/WT1	123
cluster5	BP	GO:0045229	external encapsulating structure organization	123/3710	321/18903	4.25E-15	9.89E-13	8.49E-13	ABL1/ADAM10/ADAMTS13/ADAMTS14/ADAMTS15/ADAMTS18/ADAMTS19/ADAMTS2/ADAMTS20/ADAMTS3/ADAMTS6/ADAMTS7/ADAMTS8/ADAMTSL1/ADAMTSL2/ADAMTSL3/AGT/APP/B4GALT1/BCL3/BMP2/C6orf15/CCN2/CHADL/CMA1/COL11A1/COL12A1/COL14A1/COL16A1/COL22A1/COL23A1/COL24A1/COL27A1/COL28A1/COL2A1/COL4A1/COL4A5/COL4A6/COL5A3/COL6A5/COL6A6/COL8A1/COL9A1/CPB2/CRISPLD2/CST3/CTSG/DAG1/ECM2/ELANE/EMILIN1/ERO1A/FAP/FBLN1/FERMT1/FGFR4/FKBP10/FLRT2/FOXC1/FOXF2/GAS6/GFAP/GPM6B/GREM1/HAPLN2/HAS2/HAS3/HPN/HPSE2/HSD17B12/IBSP/IMPG1/ITGB1/ITGB3/KAZALD1/KLK5/LAMA1/LAMB1/LAMB2/LOX/LOXL4/MATN2/MATN3/MATN4/MFAP4/MMP10/MMP13/MMP15/MMP16/MMP21/MMP24/MMP8/MPZL3/NDNF/NID1/NPHS1/NR2E1/NTNG1/NTNG2/OLFML2B/OPTC/P3H4/PDGFRA/PDPN/PXDN/QSOX1/SFRP2/SH3PXD2B/SLC2A10/SOX9/ST7/SULF1/SULF2/THSD4/TLL2/TNFRSF11B/TNR/TNXB/VIT/VWA1/WDR72/WNT3A/WT1	123
cluster5	BP	GO:0099003	vesicle-mediated transport in synapse	91/3710	212/18903	6.18E-15	1.41E-12	1.21E-12	ADCY1/ADORA2B/ADRA1A/ADRA2A/AMPH/AP3B2/AP3D1/APBA1/ARC/BLOC1S6/BRSK1/BRSK2/BSN/CADPS/CDH2/CDK5R1/CHRM2/CHRNA6/CPLX2/CPLX4/CSPG5/CYFIP1/DNM1/DOC2A/DOC2B/DRD1/DRD2/DRD4/EFNB2/GIPC1/GRIK5/GRIP2/GRIPAP1/GSG1L/GSK3B/HPCA/HTR2A/ITGB3/NLGN1/NLGN2/NLGN3/NLGN4X/OPHN1/OTOF/PACSIN1/PCLO/PFN2/PRKAR1B/PRKCG/PRRT2/RAB27B/RAB3B/RAB5A/RAB8A/RAP1B/RIMS3/RIMS4/ROCK1/SEPTIN5/SH3GL2/SH3GL3/SLC17A6/SLC17A7/SLC17A8/SNAP23/SNAP25/SNAP91/SNCB/STON1/STX11/STX1A/STXBP1/SV2A/SYN1/SYN2/SYN3/SYNDIG1/SYP/SYT1/SYT12/SYT2/SYT4/SYT5/SYT7/SYT8/SYT9/TH/UNC13A/UNC13C/WNT3A/WNT7A	91
cluster5	BP	GO:0016358	dendrite development	98/3710	237/18903	1.03E-14	2.29E-12	1.97E-12	ABL1/ACTL6B/ADGRB3/ALK/APP/ARC/ARHGAP44/BBS1/BCL11A/BMP5/BMP7/CAMK2A/CAMK2B/CDK5R1/CDKL3/CELSR2/CHRNA3/CHRNA7/CPEB3/CSMD3/CTNNA2/CTNND2/CUL7/CUX1/CYFIP1/DAB1/DBN1/DCLK1/DLG5/DPYSL5/DSCAM/ELAVL4/EPHB3/EZH2/FAT3/FEZF2/FLRT1/FOXO6/GPR37/GRIP1/GSK3B/HDAC2/HDAC6/HPRT1/ID1/IGF2BP1/IGSF9/IL1RAPL1/ITGB1/ITPKA/KHDC3L/KIF1A/KLHL1/KNDC1/LAMC2/LRP4/LZTS1/LZTS3/MAP1A/MAP2/MAP6/MAPK8IP2/MFSD2A/MINK1/NCK2/NEDD4L/NEURL1/NEUROG3/NFATC4/NGEF/NLGN1/NLGN2/NR2E1/NTN1/NTN3/PACSIN1/PAK3/PPFIA2/PRKG1/PTN/PTPRD/PTPRS/RELN/SDC2/SDK1/SEMA3A/SEMA4D/SHANK1/SLC12A5/SRCIN1/STRN/SULT4A1/TANC2/TLX2/TPBG/TULP1/WNT7A/ZDHHC15	98

cluster5	BP	GO:0099565	chemical synaptic transmission, postsynaptic	64/3710	130/18903	2.92E-14	6.38E-12	5.48E-12	ADORA1/AKT1/APP/CBLN1/CELF4/CHRNA2/CHRNA3/CHRNA4/CHRNA6/CHRNA7/CHRNB4/CHRNG/DRD2/DRD4/EIF4A3/GABRA1/GABRA2/GABRA3/GABRA4/GABRA5/GABRA6/GABRB2/GABRB3/GABRD/GABRE/GABRG1/GABRG2/GABRG3/GABRQ/GLRA1/GLRA2/GLRB/GRID2/GRIK2/GRIK5/GRIN1/GRIN2A/GRIN2B/GRIN2D/GSK3B/HTR3C/IGSF11/INSYN1/INSYN2A/MAPK8IP2/MPP2/NLGN1/NLGN2/NLGN3/NLGN4X/NPAS4/NRXN1/P2RX2/P2RX3/PRKAR1B/RELN/RGS4/SHANK1/SLC8A2/SLC8A3/STX1A/TMEM108/WNT7A/ZACN	64
cluster5	BP	GO:0090596	sensory organ morphogenesis	107/3710	272/18903	3.42E-14	7.32E-12	6.28E-12	AHI1/ATF4/ATOH1/ATP6V1B1/BCAR3/BMP4/BMP7/CALB1/CDON/CLRN1/CLRN2/COL11A1/COL2A1/COL8A1/CRB2/CRYGB/DLL1/DLX5/DLX6/DSCAM/EDNRA/FAT1/FAT3/FGF10/FGF2/FGF9/FGFR2/FOXE3/FOXF2/FOXG1/FOXN4/FRZB/FZD3/GBX2/GLI3/GNAT1/GNGT1/HCN1/HDAC2/HIF1A/HMX3/HOXC13/HPN/KCNQ4/LHFPL5/LHX1/LRIG3/LRP5/MEGF11/MEIS1/MFAP2/MFRP/MFSD2A/MYO3A/MYO3B/MYO6/NECTIN1/NECTIN3/NEUROG1/NOTCH2/NR2E3/NTN1/NTRK2/OLFM3/OTOP1/OTX1/PAX2/PAX6/PDZD7/POU3F4/POU4F3/PROM1/PTF1A/PTK7/RARB/RORB/RP1/RPE65/SDK1/SHROOM2/SIX2/SIX3/SIX4/SLC1A1/SLITRK6/SOBP/SOX1/SOX9/STRA6/TENM3/TFAP2A/TFAP2B/TH/THRB/THY1/TULP1/USH1C/VAX2/VEGFA/VSX1/WHRN/WNT2B/WNT3A/WNT5A/WNT9A/ZIC1/ZIC3	107
cluster5	BP	GO:0099504	synaptic vesicle cycle	83/3710	192/18903	5.9E-14	1.22E-11	1.05E-11	ADCY1/ADORA2B/ADRA1A/ADRA2A/AMPH/AP3B2/AP3D1/APBA1/BLOC1S6/BRSK1/BRSK2/BSN/CADPS/CDH2/CDK5R1/CHRM2/CHRNA6/CPLX2/CPLX4/CSPG5/CYFIP1/DNM1/DOC2A/DOC2B/DRD1/DRD2/GIPC1/GRIK5/GRIPAP1/GSK3B/HTR2A/NLGN1/NLGN2/NLGN3/NLGN4X/OPHN1/OTOF/PACSIN1/PCLO/PFN2/PRKAR1B/PRKCG/PRRT2/RAB27B/RAB3B/RAB5A/RAP1B/RIMS3/RIMS4/ROCK1/SEPTIN5/SH3GL2/SH3GL3/SLC17A6/SLC17A7/SLC17A8/SNAP23/SNAP25/SNAP91/SNCB/STON1/STX11/STX1A/STXBP1/SV2A/SYN1/SYN2/SYN3/SYNDIG1/SYP/SYT1/SYT12/SYT2/SYT4/SYT5/SYT7/SYT8/SYT9/TH/UNC13A/UNC13C/WNT3A/WNT7A	83
cluster5	BP	GO:0061326	renal tubule development	53/3710	99/18903	5.93E-14	1.22E-11	1.05E-11	AGT/AGTR2/AHI1/BMP2/BMP4/CALB1/CITED1/COL4A1/DLL1/FGF2/GDNF/GLI3/GPC3/GREB1L/GREM1/GZF1/HES1/HES5/HEYL/HNF1B/HOXA11/HOXD11/HS3ST3A1/ILK/IRX2/KIF26B/LAMA5/LGR4/LGR5/LHX1/MAGED1/NOTCH2/PAX2/PBX1/POU3F3/PROM1/PTCH1/SALL1/SHH/SIX2/SIX4/SMO/SOX9/TFAP2B/TMEM59L/VEGFA/WNT11/WNT2B/WNT4/WNT7B/WNT9B/WT1/YAP1	53

cluster5	BP	GO:0035249	synaptic transmission, glutamatergic	54/3710	102/18903	6.37E-14	1.28E-11	1.1E-11	ADORA1/ATP1A2/CACNG2/CACNG3/CACNG4/CACNG5/CACNG7/CDH2/CDH8/DGKI/DRD1/DRD2/FRRS1L/GRIA2/GRID1/GRID2/GRIK1/GRIK2/GRIK3/GRIK5/GRIN1/GRIN2A/GRIN2B/GRIN2D/GRM2/GRM3/GRM4/GRM5/GRM6/GRM7/GRM8/HCN1/HOMER1/HTR2A/MAPK8IP2/NLGN1/NLGN2/NLGN3/NRXN1/OPHN1/PLPPR4/PRKN/PTGS2/RELN/ROR2/SERPINE2/SLC17A6/SLC17A7/SLC17A8/STXBP1/SYT1/TNR/UNC13A/UNC13C	54
cluster5	BP	GO:0050890	cognition	118/3710	314/18903	7.61E-14	1.51E-11	1.29E-11	ABCC8/ABL1/ADAM2/ADCY1/ADCY3/ADCY8/ADGRB3/ADGRF1/ADORA1/AFF2/AGT/APP/ARC/ASIC1/ATP1A2/B4GALT2/BCHE/BDNF/BRINP1/BRSK1/CALB1/CHL1/CHRM1/CHRNA4/CHRNA7/CLDN5/CNTNAP2/CPEB3/CRH/CYFIP1/DBH/DGCR2/DNAH11/DOP1B/DRD1/DRD2/EGFR/EHMT2/EIF4A3/ELAVL4/EN1/FOS/FOXO6/GABRA5/GHSR/GLP1R/GRIA1/GRIN1/GRIN2A/GRIN2B/GRM5/GRPR/HIF1A/HRH3/HTR2A/INS/ITGB1/JPH3/JPH4/KCNK10/LHCGR/LILRB2/LMX1A/MAP1A/MAPK8IP2/MDK/MFSD2A/MUSK/NDRG4/NEUROD2/NEUROG1/NFATC4/NGF/NLGN3/NLGN4X/NPAS4/NPTN/NPTX2/NRXN1/NRXN3/NTF3/NTRK2/OPRK1/PAK5/PDE1B/PLCB1/PRKAR1B/PRKCG/PRKN/PRRT1/PTCHD1/PTGS2/PTN/PTPRZ1/RASGRF1/RELN/SCN2A/SHANK1/SHANK2/SHISA7/SLC12A5/SLC1A1/SLC6A1/SLC8A2/SLC8A3/SNAP25/SOBP/SRC/STRA6/SYT4/TACR1/TBR1/TH/TNR/TPBG/TTBK1/TTC36/TUSC3	118
cluster5	BP	GO:0050767	regulation of neurogenesis	135/3710	377/18903	9.39E-14	1.82E-11	1.56E-11	ABCC8/ACE/ASCL1/ASPA/ASPM/ATOH1/BDNF/BHLHE41/BMP2/BMP7/BRINP1/CAMK2B/CDH4/CDKL3/CDKN2B/CHODL/CRABP2/CUL7/CUX1/CYFIP1/DAB1/DAG1/DBN1/DCT/DLL1/DLL3/DLX1/DLX2/DMRTA2/DPYSL5/DRAXIN/DRD2/DSCAM/EFNA5/EFNB3/EPHA7/EZH2/F2/FAIM/FERD3L/FEZF1/FEZF2/FGF2/FOXG1/FZD3/GDI1/GFAP/GLI3/GRM5/GSX2/HAP1/HDAC2/HES1/HES2/HES5/HES6/HES7/HEYL/HIF1A/HLTF/ID1/ID4/IL1RAPL1/ISLR2/ITGB1/ITPKA/KHDC3L/L1CAM/LHX2/LRP2/LRP4/LYN/MAP2/MAP6/MDK/MT3/NDEL1/NEURL1/NFATC4/NGF/NKX2-2/NKX6-1/NPTN/NR2E1/NTN1/NTRK2/NUMBL/OTP/PAK3/PAX6/PLXNA1/PLXNA2/PLXNA4/PLXNB1/PLXNB3/PRTG/PTN/PTPRD/PTPRS/PTPRZ1/RELN/RND2/ROBO1/ROBO2/SEMA3A/SEMA3E/SEMA4D/SEMA5A/SEMA5B/SEMA6A/SEMA6D/SERPINE2/SHH/SHOX2/SIRT2/SLIT1/SLITRK1/SMO/SOX10/SPEN/SYT4/TENM4/THY1/TLX2/TMEM98/TNR/TP73/TTBK1/VAX1/VEGFA/WNT3A/WNT5A/WNT7A/YAP1/ZNF488	135
cluster5	BP	GO:0048167	regulation of synaptic plasticity	87/3710	207/18903	1.05E-13	2E-11	1.72E-11	ABL1/ACE/ACP4/ADCY1/ADCY8/ADGRB1/ADORA1/AGT/APP/ARC/ATF4/BRSK1/CALB1/CALB2/CAMK2A/CAMK2B/CBLN1/CHRDL1/CHRNA7/CNTN4/CPEB3/CPLX2/CYP46A1/DBN1/DRD1/DRD2/ERC1/GFAP/GIPC1/GRIA1/GRID2/GRID2IP/GRIK2/GRIN1/GRIN2A/GRIN2B/GRIN2D/GRM5/GSK3B/IGSF11/INS/ITPKA/JPH3/JPH4/KCNB1/LILRB2/LRRTM2/LZTS1/MAP1A/MPP2/NEURL1/NEUROD2/NFATC4/NPAS4/NPTN/NR2E1/NTRK2/P2RX3/PRKAR1B/PRKCG/PRRT1/PRRT2/PTGS2/PTN/RAB5A/RAB8A/RASGRF1/RELN/RIMS3/SERPINE2/SHANK2/SHISA6/SHISA7/SHISA9/SLC1A1/SLC24A2/SLC8A2/SLC8A3/SNAP25/SQSTM1/STXBP1/SYP/SYT12/SYT4/SYT7/TNR/UNC13C	87

cluster5	BP	GO:0021953	central nervous system neuron differentiation	77/3710	174/18903	1.07E-13	2E-11	1.72E-11	AGBL4/ARX/ASCL1/BMPR1B/CBLN1/CDH11/CHD5/DCLK1/DCLK2/DLX1/DLX2/DMRT3/DRAXIN/DRD1/DRD2/ELAVL4/EMX1/EPHB3/FAIM2/FEZF2/FGFR2/FOXG1/FOXN4/GBX2/GLI2/GLI3/GRID2/GSX1/GSX2/HES1/HES5/HOXC10/HOXD10/HPRT1/ID4/ISL1/KNDC1/LBX1/LHX1/LHX3/LHX5/LMX1A/MAP2/MDGA2/MNX1/NDEL1/NDNF/NHLH2/NKX2-1/NKX2-2/NKX6-1/NPY/NR2E1/NR4A2/NRP2/NTRK2/OPHN1/OTP/PAX6/PHOX2B/PLXNA1/PLXNA4/PTCH1/SECISBP2/SEMA3E/SHH/SMO/SOX1/SPOCK1/SPTBN4/TBR1/TBX20/TOX/TTC36/TULP3/WNT3A/WNT7A	77
cluster5	BP	GO:0060079	excitatory postsynaptic potential	61/3710	124/18903	1.22E-13	2.24E-11	1.92E-11	ADORA1/AKT1/APP/CBLN1/CELF4/CHRNA2/CHRNA3/CHRNA4/CHRNA6/CHRNA7/CHRNB4/CHRNG/DRD2/EIF4A3/GABRA1/GABRA2/GABRA3/GABRA4/GABRA5/GABRA6/GABRB2/GABRB3/GABRD/GABRE/GABRG1/GABRG2/GABRG3/GABRQ/GLRA1/GLRA2/GLRB/GRID2/GRIK2/GRIK5/GRIN1/GRIN2A/GRIN2B/GRIN2D/GSK3B/HTR3C/IGSF11/MAPK8IP2/MPP2/NLGN1/NLGN2/NLGN3/NLGN4X/NPAS4/NRXN1/P2RX2/P2RX3/PRKAR1B/RELN/RGS4/SHANK1/SLC8A2/SLC8A3/STX1A/TMEM108/WNT7A/ZACN	61
cluster5	BP	GO:0072080	nephron tubule development	51/3710	96/18903	2.63E-13	4.76E-11	4.08E-11	AGT/AGTR2/AHI1/BMP2/BMP4/CALB1/CITED1/DLL1/FGF2/GDNF/GLI3/GPC3/GREB1L/GREM1/GZF1/HES1/HES5/HEYL/HNF1B/HOXA11/HOXD11/HS3ST3A1/ILK/IRX2/KIF26B/LAMA5/LGR4/LHX1/MAGED1/NOTCH2/PAX2/PBX1/POU3F3/PROM1/PTCH1/SALL1/SHH/SIX2/SIX4/SMO/SOX9/TFAP2B/TMEM59L/VEGFA/WNT11/WNT2B/WNT4/WNT7B/WNT9B/WT1/YAP1	51
cluster5	BP	GO:0007611	learning or memory	105/3710	273/18903	3.26E-13	5.79E-11	4.97E-11	ABCC8/ABL1/ADAM2/ADCY1/ADCY3/ADCY8/ADGRB3/ADGRF1/AFF2/AGT/APP/ARC/ASIC1/ATP1A2/B4GALT2/BCHE/BDNF/BRINP1/BRSK1/CALB1/CHRNA7/CLDN5/CNTNAP2/CPEB3/CRH/DBH/DNAH11/DRD1/DRD2/EGFR/EHMT2/EIF4A3/ELAVL4/EN1/FOS/FOXO6/GABRA5/GHSR/GLP1R/GRIA1/GRIN1/GRIN2A/GRIN2B/GRM5/GRPR/HIF1A/HTR2A/ITGB1/JPH3/JPH4/KCNK10/LILRB2/LMX1A/MAP1A/MAPK8IP2/MDK/MUSK/NDRG4/NEUROD2/NEUROG1/NFATC4/NGF/NLGN3/NLGN4X/NPAS4/NPTN/NPTX2/NRXN1/NRXN3/NTF3/NTRK2/OPRK1/PAK5/PDE1B/PLCB1/PRKAR1B/PRKCG/PRKN/PRRT1/PTCHD1/PTGS2/PTN/PTPRZ1/RASGRF1/RELN/SCN2A/SHANK1/SHANK2/SHISA7/SLC12A5/SLC1A1/SLC6A1/SLC8A2/SLC8A3/SNAP25/SRC/STRA6/SYT4/TACR1/TBR1/TH/TNR/TPBG/TTBK1/TTC36	105

cluster5	BP	GO:0072028	nephron morphogenesis	45/3710	80/18903	4.43E-13	7.73E-11	6.64E-11	AGT/AGTR2/AHI1/BMP2/BMP4/BMP7/CITED1/ERBB4/FGF2/GDNF/GLI3/GPC3/GREB1L/GREM1/GZF1/HES1/HES5/HNF1B/HOXA11/HOXD11/HS3ST3A1/ILK/IRX2/KIF26B/LAMA5/LGR4/LHX1/MAGED1/PAX2/PBX1/PDGFRB/PTCH1/SALL1/SHH/SIX2/SIX4/SMO/SOX9/TMEM59L/VEGFA/WNT11/WNT2B/WNT4/WNT9B/WT1	45
cluster5	BP	GO:0060041	retina development in camera-type eye	71/3710	159/18903	5.62E-13	9.66E-11	8.29E-11	AHI1/ATP2B2/BARHL2/BMPR1B/CALB1/CDON/CELF4/CLCN2/COL4A1/CRB2/DCX/DLL1/DLX1/DLX2/DSCAM/FAT3/FOXN4/GNAT1/GPM6A/GRM6/HCN1/HES1/HIF1A/HPCA/JMJD6/LAMA1/LAMB2/LHX1/LHX2/LRP5/MDM1/MEGF11/MFRP/MFSD2A/NDP/NECTIN1/NECTIN3/NEUROD1/NPHP4/NR2E1/NR2E3/NTRK2/PAX2/PAX6/PDGFRA/PDGFRB/PROM1/PTF1A/RET/RHO/RORB/RP1/RPE65/SDK1/SIX3/SLC17A7/SLC17A8/SLC1A1/SLC4A5/SOX9/TBC1D32/TFAP2A/TFAP2B/THRB/THY1/TUB/TULP1/USH1C/VAX2/VSTM4/VSX1	71
cluster5	BP	GO:0060560	developmental growth involved in morphogenesis	93/3710	233/18903	5.94E-13	1E-10	8.62E-11	ABL1/APP/AUTS2/BARHL2/BCL11A/BDNF/BMP4/CACNG7/CDH4/CDKL3/CPNE5/CPNE6/CPNE9/CRABP2/CYFIP1/DBN1/DCLK1/DRAXIN/DSCAM/EDN2/EDNRA/EFNA5/EMX1/EPHA7/FGF10/FGFR2/FLRT1/FLRT3/GDI1/GSK3B/HDAC6/HNF1B/HOXD13/ISLR2/ITGB1/KIF26B/L1CAM/LAMB2/LHX1/LHX2/MAGI2/MAP2/MT3/NDEL1/NEDD4L/NGF/NKX6-1/NLGN3/NRCAM/NRP2/NTN1/OLFM1/PLXNA1/PLXNA4/POU4F3/PRKN/PTK7/PTPRS/RND2/SALL1/SEMA3A/SEMA3E/SEMA4D/SEMA5A/SEMA5B/SEMA6A/SEMA6D/SFRP2/SH3GL2/SHH/SIX4/SLC9A6/SLIT1/SLIT3/SOX9/SPAG6/SPRY1/ST8SIA2/SYT1/SYT2/SYT3/SYT4/TMEM108/TNN/TNR/UNC13A/VEGFA/WASF1/WNT11/WNT3A/WNT5A/WNT7B/YAP1	93
cluster5	BP	GO:0051966	regulation of synaptic transmission, glutamatergic	44/3710	78/18903	7.02E-13	1.17E-10	1E-10	ADORA1/ATP1A2/CACNG2/CACNG3/CACNG4/CACNG5/CACNG7/CDH2/DGKI/DRD1/DRD2/FRRS1L/GRIK1/GRIK2/GRIK3/GRIN1/GRIN2A/GRIN2B/GRIN2D/GRM2/GRM3/GRM4/GRM5/GRM6/GRM7/GRM8/HCN1/HOMER1/HTR2A/MAPK8IP2/NLGN1/NLGN2/NLGN3/NRXN1/OPHN1/PLPPR4/PTGS2/RELN/ROR2/SERPINE2/STXBP1/SYT1/TNR/UNC13A	44

cluster5	BP	GO:0021537	telencephalon development	99/3710	255/18903	7.94E-13	1.3E-10	1.12E-10	AKIRIN2/ALK/ARX/ASCL1/ASPM/ATOH1/ATP1A2/ATP1A3/BBS1/BCAN/BMP2/BMP4/CDH2/CDK5R1/CDK5R2/CDON/CNTNAP2/DAB1/DCLK2/DLX1/DLX2/DLX5/DMRTA2/DNAH5/DRAXIN/DRD1/DRD2/EFNA2/EGFR/EMX1/EMX2/EPHA5/EPHB3/ERBB4/EZH2/FAT4/FEZ1/FEZF1/FEZF2/FOXG1/GLI3/GSK3B/GSX2/HES1/HES5/HIF1A/HPRT1/HSD3B2/ID4/IGF2BP1/KIRREL3/LAMB1/LHX1/LHX2/LHX5/LMX1A/MBOAT7/MDK/MFSD2A/NDEL1/NEUROD1/NEUROD6/NKX2-1/NPY/NR2E1/NRG3/NTRK2/NUMBL/PAX6/PLCB1/PLXNA4/POU3F2/POU3F3/RARB/RELN/RFX4/ROBO1/ROBO2/RTN4RL1/SALL1/SECISBP2/SEMA3A/SHH/SIX3/SLC1A2/SMO/SRD5A2/SYNE2/TACC2/TACC3/TBR1/TH/TMEM108/TNR/UNCX/WNT3A/WNT5A/ZIC1/ZIC3	99
cluster5	BP	GO:0021536	diencephalon development	43/3710	76/18903	1.11E-12	1.78E-10	1.53E-10	ARX/BMP2/BMP4/CNTNAP2/DRD2/FGF10/FGF2/GBX2/GLI2/GSX1/HAP1/HES1/ISL1/KCNC1/KCNC2/LHX3/NDNF/NHLH2/NKX2-1/NKX2-6/NR0B1/NR4A2/NRP2/OTP/OTX1/PAX6/PCSK1/PITX1/PLXNA1/POU3F2/PTCHD1/RAX/SALL1/SEMA3E/SEMA5A/SHH/SIX3/SLC6A3/SMO/SOX3/SRD5A2/WNT4/WNT5A	43
cluster5	BP	GO:2000027	regulation of animal organ morphogenesis	61/3710	129/18903	1.12E-12	1.78E-10	1.53E-10	ABL1/AGT/AGTR2/AHI1/AJAP1/APCDD1/BMP2/BMP4/BMP7/CELSR1/CELSR2/DAAM1/DAB2/DMRT3/FGF10/FGF2/FGF7/FZD3/GDNF/GPC3/GPC6/GREM1/HOXA11/LGR4/LHX1/MAGED1/MAGI2/NGFR/PAX2/PDGFA/PKHD1/PRICKLE1/PTK7/ROBO1/ROBO2/ROR1/ROR2/RSPO2/RSPO3/SFRP2/SHH/SIX2/SIX4/SMO/SOX9/SP6/SPEF1/SPRY1/SULF1/TNFRSF11B/VANGL2/VEGFA/WNT11/WNT2B/WNT3A/WNT4/WNT5A/WNT7A/WNT9B/WT1/ZNRF3	61
cluster5	BP	GO:0031644	regulation of nervous system process	66/3710	145/18903	1.24E-12	1.94E-10	1.66E-10	ADORA1/AGT/APP/AVP/BEGAIN/CACNG4/CACNG5/CBLN1/CELF4/CHRNA7/CHRNB4/DAG1/DLGAP2/DLGAP3/EDNRB/EIF4A3/F2R/GHSR/GLRA1/GRIN1/GRIN2A/GRIN2B/GRIN2D/HOMER1/HTR2C/IGSF11/ITGA2/MYRF/NLGN1/NLGN2/NLGN3/NLGN4X/NOS3/NPTX1/NPTX2/NPTXR/NRXN1/OPRK1/P2RX3/PARD3/PRKAR1B/RELN/RGS4/ROCK2/SHANK1/SHISA6/SHISA7/SHISA9/SLC8A2/SLC8A3/SMR3A/SOX10/SPX/SRC/STX1A/TAFA4/TENM4/TMEM108/TMEM98/TNFRSF21/TNR/TPPP/WASF3/WNT7A/ZFHX2/ZNF488	66

cluster5	BP	GO:0061333	renal tubule morphogenesis	44/3710	79/18903	1.29E-12	1.98E-10	1.7E-10	AGT/AGTR2/AHI1/BMP2/BMP4/CITED1/COL4A1/FGF2/GDNF/GLI3/GPC3/GREB1L/GREM1/GZF1/HES1/HES5/HNF1B/HOXA11/HOXD11/HS3ST3A1/ILK/IRX2/KIF26B/LAMA5/LGR4/LGR5/LHX1/MAGED1/PAX2/PBX1/PTCH1/SALL1/SHH/SIX2/SIX4/SMO/SOX9/TMEM59L/VEGFA/WNT11/WNT2B/WNT4/WNT9B/WT1	44
cluster5	BP	GO:0045165	cell fate commitment	106/3710	282/18903	1.38E-12	2.1E-10	1.8E-10	APC2/ARX/ASCL1/ATOH1/BARHL2/BMP2/BMP4/CDON/DLL1/DLX1/DLX2/DMRT3/DMRTA2/DSCAML1/EBF2/EDNRA/EHMT2/ERBB4/ESRP1/FEV/FEZF2/FGF10/FGF2/FGFR2/FOXA1/FOXA2/FOXG1/FOXN1/FOXN4/GAP43/GATA4/GLI2/GLI3/GSX1/GSX2/HDAC2/HES1/HES5/HNF1B/HOXA11/HOXC10/HOXD10/IL7/ISL1/ITGB1/LBX1/LHX3/MITF/MNX1/MYOG/MYT1L/NEUROD1/NEUROG1/NKX2-1/NKX2-2/NOTCH2/NOTCH4/NR2E1/NR2F2/PAX2/PAX6/PDPN/PITX1/POU3F2/POU6F2/PRDM14/PTCH1/PTCH2/PTF1A/SFRP2/SHH/SIX2/SMAD5/SMO/SOSTDC1/SOX1/SOX18/SOX5/SOX9/SPDEF/TBR1/TBX10/TBX15/TBX20/TBX22/TBX4/TBX5/TBXT/TENM4/TGFB1I1/TLX3/TNXB/TOX/WNT11/WNT2B/WNT3A/WNT4/WNT5A/WNT7A/WNT7B/WNT8A/WNT8B/WNT9A/WNT9B/WT1/ZNF521	106
cluster5	BP	GO:0060675	ureteric bud morphogenesis	39/3710	66/18903	1.89E-12	2.83E-10	2.43E-10	AGT/AGTR2/BMP2/BMP4/CITED1/FGF2/GDNF/GLI3/GPC3/GREB1L/GREM1/GZF1/HES1/HNF1B/HOXA11/HOXD11/HS3ST3A1/ILK/KIF26B/LAMA5/LGR4/LHX1/MAGED1/PAX2/PBX1/PTCH1/SALL1/SHH/SIX2/SIX4/SMO/SOX9/TMEM59L/VEGFA/WNT11/WNT2B/WNT4/WNT9B/WT1	39
cluster5	BP	GO:0060993	kidney morphogenesis	50/3710	97/18903	2.04E-12	3.01E-10	2.59E-10	AGT/AGTR2/AHI1/BASP1/BMP2/BMP4/BMP7/CALB1/CITED1/ERBB4/FGF10/FGF2/FOXJ1/GDNF/GLI3/GPC3/GREB1L/GREM1/GZF1/HES1/HES5/HNF1B/HOXA11/HOXD11/HS3ST3A1/ILK/IRX2/KIF26B/LAMA5/LGR4/LHX1/MAGED1/PAX2/PBX1/PDGFRB/PTCH1/SALL1/SHH/SIX2/SIX4/SMO/SOX9/TMEM59L/VEGFA/WNT11/WNT2B/WNT4/WNT7B/WNT9B/WT1	50

cluster5	BP	GO:0072078	nephron tubule morphogenesis	42/3710	75/18903	3.25E-12	4.73E-10	4.06E-10	AGT/AGTR2/AHI1/BMP2/BMP4/CITED1/FGF2/GDNF/GLI3/GPC3/GREB1L/GREM1/GZF1/HES1/HES5/HNF1B/HOXA11/HOXD11/HS3ST3A1/ILK/IRX2/KIF26B/LAMA5/LGR4/LHX1/MAGED1/PAX2/PBX1/PTCH1/SALL1/SHH/SIX2/SIX4/SMO/SOX9/TMEM59L/VEGFA/WNT11/WNT2B/WNT4/WNT9B/WT1	42
cluster5	BP	GO:0051963	regulation of synapse assembly	52/3710	104/18903	3.46E-12	4.96E-10	4.26E-10	ADGRB1/ADGRB3/APP/ASIC2/BDNF/CBLN1/CBLN2/CLSTN2/DLG5/EFNA5/EPHA7/EPHB3/FLRT2/FLRT3/GHSR/GPC6/GRID2/IL1RAPL1/IL1RAPL2/LHFPL4/LRFN3/LRRC4B/LRRN1/LRRTM2/LRRTM3/LRTM2/MUSK/NECTIN1/NLGN1/NLGN2/NLGN3/NPTX1/NRXN1/NTN1/NTNG2/NTRK2/PTPRD/ROBO2/SEMA4D/SIX4/SLIT1/SLITRK1/SLITRK2/SLITRK3/SLITRK6/SRPX2/ST8SIA2/SYNDIG1/TPBG/WNT3A/WNT5A/WNT7A	52
cluster5	BP	GO:0072171	mesonephric tubule morphogenesis	39/3710	67/18903	3.67E-12	5.2E-10	4.46E-10	AGT/AGTR2/BMP2/BMP4/CITED1/FGF2/GDNF/GLI3/GPC3/GREB1L/GREM1/GZF1/HES1/HNF1B/HOXA11/HOXD11/HS3ST3A1/ILK/KIF26B/LAMA5/LGR4/LHX1/MAGED1/PAX2/PBX1/PTCH1/SALL1/SHH/SIX2/SIX4/SMO/SOX9/TMEM59L/VEGFA/WNT11/WNT2B/WNT4/WNT9B/WT1	39
cluster5	BP	GO:0006813	potassium ion transport	95/3710	247/18903	4.33E-12	6.05E-10	5.19E-10	ABCC8/ABCC9/ACTN2/ADORA1/ADRA2A/AKAP6/ANK3/ANO6/ATF4/ATP1A2/ATP1A3/ATP1B1/ATP1B4/CASQ2/CRBN/DPP10/DPP6/DRD1/DRD2/FHL1/FXYD3/GCK/GRP/HCN1/HCN3/HCN4/HPN/HTR2A/ITGB1/KCNA10/KCNA4/KCNA7/KCNB1/KCNB2/KCNC1/KCNC2/KCNC3/KCND2/KCND3/KCNE4/KCNG3/KCNH1/KCNH2/KCNH4/KCNH5/KCNH6/KCNIP1/KCNIP3/KCNJ13/KCNJ16/KCNJ18/KCNJ3/KCNJ4/KCNJ5/KCNJ6/KCNJ9/KCNK10/KCNK3/KCNK5/KCNK6/KCNMA1/KCNMB2/KCNN1/KCNN2/KCNN3/KCNQ2/KCNQ3/KCNQ4/KCNRG/KCNS2/KCNT1/KCNT2/KCNV1/LRRC55/NALCN/NEDD4L/NOS3/OPRK1/RGS4/RGS7/SLC12A5/SLC12A9/SLC17A6/SLC17A7/SLC24A2/SLC24A3/SLC9A2/SLC9A4/SLC9A5/SLC9A6/SLC9A7/SLC9C2/SNAP25/WNK2/WNK3	95

cluster5	BP	GO:0001658	branching involved in ureteric bud morphogenesis	36/3710	60/18903	7.15E-12	9.76E-10	8.38E-10	AGT/AGTR2/BMP2/BMP4/CITED1/FGF2/GDNF/GLI3/GPC3/GREB1L/GREM1/GZF1/HNF1B/HOXA11/HOXD11/HS3ST3A1/ILK/LAMA5/LGR4/LHX1/MAGED1/PAX2/PBX1/PTCH1/SALL1/SHH/SIX2/SIX4/SMO/SOX9/TMEM59L/VEGFA/WNT2B/WNT4/WNT9B/WT1	36
cluster5	BP	GO:0048839	inner ear development	79/3710	193/18903	7.17E-12	9.76E-10	8.38E-10	ADAM10/ADGRV1/AHI1/ATOH1/ATP6V1B1/BMP2/BMP4/BMPER/CALB1/CECR2/CLRN1/CLRN2/COL11A1/COL2A1/DLL1/DLX5/DLX6/ESRP1/ESRRB/EYA4/FGF10/FGF2/FGF9/FGFR2/FOXG1/FREM2/FRZB/FZD3/GABRA5/GABRB2/GBX2/GLI3/GRXCR1/HES1/HES5/HMX3/HPCA/HPN/KCNK3/KCNQ4/LGR5/LHFPL5/LHX3/LRIG3/MYO3A/MYO3B/MYO6/NEUROD1/NEUROG1/NTN1/OTOP1/OTX1/PAX2/PCDH15/PDGFA/PDGFRB/PDZD7/PHOX2B/PLPPR4/POU3F4/POU4F3/PTK7/PVALB/ROR1/SDC4/SHH/SIX4/SLC17A8/SLITRK6/SOBP/SOX9/TFAP2A/TMC1/USH1C/USH2A/WHRN/WNT3A/WNT5A/ZIC1	79
cluster5	BP	GO:0001708	cell fate specification	53/3710	109/18903	8.64E-12	1.16E-09	9.97E-10	APC2/ASCL1/ATOH1/CDON/DLL1/DMRT3/DMRTA2/EHMT2/ESRP1/FEV/FGF2/FOXA1/FOXA2/GLI2/GLI3/GSX2/HDAC2/HNF1B/HOXA11/HOXC10/HOXD10/ISL1/ITGB1/LHX3/MNX1/MYT1L/NKX2-2/PAX2/PAX6/POU3F2/PRDM14/PTCH1/PTCH2/SFRP2/SHH/SIX2/SMO/SOX1/SOX18/SOX9/TBR1/TBX10/TBX15/TBX20/TBX22/TBX4/TBX5/TBXT/TENM4/TLX3/WNT11/WNT3A/WNT5A	53
cluster5	BP	GO:0072088	nephron epithelium morphogenesis	42/3710	77/18903	1.05E-11	1.39E-09	1.2E-09	AGT/AGTR2/AHI1/BMP2/BMP4/CITED1/FGF2/GDNF/GLI3/GPC3/GREB1L/GREM1/GZF1/HES1/HES5/HNF1B/HOXA11/HOXD11/HS3ST3A1/ILK/IRX2/KIF26B/LAMA5/LGR4/LHX1/MAGED1/PAX2/PBX1/PTCH1/SALL1/SHH/SIX2/SIX4/SMO/SOX9/TMEM59L/VEGFA/WNT11/WNT2B/WNT4/WNT9B/WT1	42

cluster5	BP	GO:0048562	embryonic organ morphogenesis	107/3710	294/18903	1.11E-11	1.46E-09	1.25E-09	AHI1/ALX1/ALX4/ATOH1/ATP6V1B1/BMP4/BMP7/CCDC103/CCDC40/CLRN1/CLRN2/COL11A1/COL2A1/CRB2/DLL1/DLX2/DLX5/DLX6/DSCAML1/EDNRA/EIF4A3/FGF10/FGF9/FGFR2/FOLR1/FOXF2/FOXG1/FOXN4/FRZB/FZD3/GATA4/GBX2/GLI3/GRHL2/HES1/HIF1A/HMX3/HNF1B/HOXA11/HOXA3/HOXA4/HOXB1/HOXB6/HOXB9/HOXC4/HOXC9/HOXD10/HOXD3/HPN/IFT140/IRX5/KCNQ4/LBX1/LHFPL5/LHX1/LRIG3/MDFI/MFAP2/MMP16/MYO3A/MYO3B/MYO6/NDRG4/NEUROD1/NEUROG1/NOTCH2/NTN1/OTOP1/OTX1/PAX2/PAX6/PDGFRA/PDZD7/POU3F4/POU4F3/PTK7/RARB/RBPMS2/RYR2/SHH/SHOX2/SIX2/SIX3/SIX4/SLITRK6/SMO/SOBP/SOX18/SOX9/STRA6/TBX15/TBX20/TEAD2/TFAP2A/TH/TULP3/USH1C/VANGL2/VAX2/WHRN/WNT11/WNT3A/WNT5A/WNT9B/YAP1/ZIC1/ZIC3	107
cluster5	BP	GO:0001763	morphogenesis of a branching structure	81/3710	203/18903	1.86E-11	2.4E-09	2.06E-09	ABL1/AGT/AGTR2/BCL11A/BMP2/BMP4/BMP7/CELSR1/CITED1/COL4A1/DAG1/DLG5/DLX2/DRD2/EDNRA/EGF/EPHA7/FGF10/FGF2/FGF7/FGFR2/FOXA1/GBX2/GDNF/GLI2/GLI3/GPC3/GREB1L/GREM1/GRHL2/GZF1/HHIP/HNF1B/HOXA11/HOXB13/HOXD11/HOXD13/HS3ST3A1/ILK/LAMA1/LAMA5/LGR4/LHX1/LRP5/MAGED1/MDK/MSX2/NFATC4/NKX2-1/NOTCH4/PAX2/PBX1/PDGFA/PKHD1/PLXNA1/PTCH1/RSPO2/RSPO3/SALL1/SEMA3E/SFRP2/SHH/SHOX2/SIX2/SIX4/SMO/SOX10/SOX9/SPRY1/SRC/SULF1/TBX20/TDGF1/TMEM59L/VEGFA/WNT2B/WNT4/WNT5A/WNT9B/WT1/YAP1	81
cluster5	BP	GO:0021761	limbic system development	53/3710	111/18903	2.1E-11	2.68E-09	2.3E-09	ALK/ATP1A2/BBS1/BCAN/CDK5R1/CDK5R2/CNTNAP2/DAB1/DCLK2/DLX1/DLX2/DRD1/DRD2/EMX2/EPHA5/EZH2/FEZ1/FEZF2/FGFR2/GLI3/GSK3B/GSX1/HAP1/HSD3B2/ID4/KIRREL3/LHX5/LMX1A/MDK/MFSD2A/NDNF/NEUROD1/NEUROD6/NHLH2/NKX2-1/NKX2-6/NR0B1/NR2E1/NRP2/OTP/PLXNA1/POU3F2/RAX/RELN/SEMA3E/SMO/SOX3/SRD5A2/TBR1/TMEM108/WNT3A/ZIC1/ZIC3	53
cluster5	BP	GO:0043583	ear development	86/3710	221/18903	2.16E-11	2.73E-09	2.34E-09	ADAM10/ADGRV1/AHI1/ATOH1/ATP6V1B1/BMP2/BMP4/BMP5/BMPER/CALB1/CECR2/CLRN1/CLRN2/COL11A1/COL2A1/DLL1/DLX5/DLX6/ECE1/EDNRA/ESRP1/ESRRB/EYA4/FGF10/FGF2/FGF9/FGFR2/FOXG1/FREM2/FRZB/FZD3/GABRA5/GABRB2/GBX2/GLI3/GRXCR1/HES1/HES5/HMX3/HPCA/HPN/KCNK3/KCNQ4/LGR5/LHFPL5/LHX3/LRIG3/MYO3A/MYO3B/MYO6/NEUROD1/NEUROG1/NTN1/OTOP1/OTX1/PAX2/PCDH15/PDGFA/PDGFRB/PDZD7/PHOX2B/PLPPR4/POU3F4/POU4F3/PTK7/PVALB/ROR1/SDC4/SHH/SHROOM2/SIX2/SIX4/SLC17A8/SLITRK6/SOBP/SOX9/STRA6/TFAP2A/TMC1/USH1C/USH2A/WHRN/WNT3A/WNT5A/ZIC1/ZIC3	86

cluster5	BP	GO:0007612	learning	65/3710	150/18903	2.69E-11	3.33E-09	2.85E-09	ABCC8/ABL1/ADAM2/ADCY3/ADGRB3/AGT/APP/ARC/ASIC1/ATP1A2/B4GALT2/BCHE/BRSK1/CLDN5/CNTNAP2/DBH/DRD1/DRD2/EIF4A3/ELAVL4/EN1/FOS/GABRA5/GRIN1/GRIN2A/GRM5/HIF1A/ITGB1/JPH3/JPH4/MAP1A/MAPK8IP2/NDRG4/NEUROD2/NLGN3/NLGN4X/NPAS4/NPTN/NPTX2/NRXN1/NRXN3/NTRK2/OPRK1/PAK5/PDE1B/PLCB1/PRKN/PTGS2/PTN/RELN/SHANK1/SHANK2/SLC12A5/SLC1A1/SLC6A1/SLC8A2/SLC8A3/SNAP25/STRA6/TACR1/TBR1/TH/TNR/TPBG/TTC36	65
cluster5	BP	GO:0035637	multicellular organismal signaling	71/3710	170/18903	2.7E-11	3.33E-09	2.85E-09	ABCC9/ACE2/AGT/ANK3/ATP1A2/ATP1A3/ATP1B1/ATP2B2/ATP2B3/AVP/CACNA1C/CACNA1G/CACNA2D1/CACNG2/CACNG3/CACNG4/CACNG5/CACNG7/CASQ2/CHRNB4/CLDN19/CNTNAP1/CNTNAP2/DMRT3/DRD1/DSG2/EHD3/FKBP1B/GHSR/GJC1/GJD2/GLRA1/GRIK2/HCN1/HCN3/HCN4/HRC/ISL1/ITGA2/KCND2/KCND3/KCNE4/KCNH2/KCNH6/KCNJ3/KCNJ5/KCNMB2/KCNN2/NFASC/NRCAM/NTRK2/P2RX3/PKP2/RGS21/RYR2/SCN10A/SCN1A/SCN2A/SCN2B/SCN3B/SCN4A/SCN8A/SCN9A/SLC4A3/SLC8A2/SLC8A3/SPTBN4/TBX5/TNR/TRDN/TRPM4	71
cluster5	BP	GO:0006936	muscle contraction	121/3710	351/18903	3.25E-11	3.96E-09	3.4E-09	ABCC9/ACE2/ACTA1/ADORA1/ADORA2B/ADRA1A/ADRA1B/ADRA2A/AGT/ARG2/ATP1A2/ATP1B1/CACNA1C/CACNA1G/CACNA1S/CACNA2D1/CACNB1/CALCA/CASQ2/CCN2/CHGA/CHRM2/CHRM3/CHRNA3/CHRNB4/CHRNG/CNN1/CRYAB/CSRP3/DES/DMD/DOCK4/DRD1/DRD2/DSG2/DTNA/EDN2/EDNRA/EDNRB/EHD3/F2R/FKBP1B/GATA4/GDNF/GHSR/GJC1/GLRA1/GNAO1/GRIP2/GSTO1/GUCY1A1/HCN4/HOMER1/HRC/HSBP1/HTR1D/HTR2A/ITGA2/JSRP1/KCNB2/KCND3/KCNE4/KCNH2/KCNJ3/KCNJ5/KCNMA1/KCNN2/LMOD2/MYH1/MYH13/MYH2/MYH4/MYH6/MYH7/MYH8/MYL1/MYLK/MYOCD/NEDD4L/NEUROG1/NMU/P2RX2/P2RX3/PDE5A/PKP2/PRKG1/PTGS2/PVALEF/ROCK1/ROCK2/RYR2/SCN10A/SCN1A/SCN2B/SCN3B/SCN4A/SGCA/SLC8A3/SMAD5/SMPX/SMTN/SPX/SSPN/SSTR2/STAC2/STC1/SULF1/SULF2/TACR1/TACR3/TBX20/TBXA2R/TNNI1/TNNI3/TNNT2/TPM1/TRDN/TRIM63/TRPM4/TRPV4/ZC3H12A	121
cluster5	BP	GO:1901890	positive regulation of cell junction assembly	51/3710	106/18903	3.49E-11	4.21E-09	3.61E-09	ABL1/ACE2/ADGRB1/ADGRB3/AGT/ASIC2/BDNF/CBLN1/CBLN2/CLDN1/CLDN19/CLDN5/CLSTN2/CNTNAP2/COL16A1/DLG5/EFNA5/EPHB3/FLRT2/FLRT3/GRID2/IL1RAPL1/LRRC4B/LRRN1/LRRTM2/LRRTM3/LRTM2/MYOC/NLGN1/NLGN2/NLGN3/NPHP4/NRXN1/NTRK2/PTPRD/ROCK1/SDC4/SEMA4D/SLITRK1/SLITRK2/SLITRK3/SLITRK6/SRPX2/ST8SIA2/SYNDIG1/TBX5/THY1/TPBG/VEGFA/WNT4/WNT7A	51

cluster5	BP	GO:0006814	sodium ion transport	95/3710	255/18903	3.55E-11	4.23E-09	3.63E-09	AKT1/ANK3/ANO6/ASIC1/ASIC2/ASIC3/ASIC4/ATP1A2/ATP1A3/ATP1B1/ATP1B4/ATP6V1B1/CACNA1G/CATSPER4/CHP2/CLCNKB/CNKSR3/DMD/DRD2/DRD4/EDNRA/EDNRB/FGF14/FXYD3/FXYD6/GRP/HCN1/HCN3/HCN4/NALCN/NEDD4L/NKAIN1/NKAIN4/NOS3/PER1/PKP2/PRSS8/PTPN3/SCN10A/SCN1A/SCN2A/SCN2B/SCN3B/SCN4A/SCN8A/SCN9A/SCNN1G/SERPINE2/SHROOM2/SIK1/SLC10A5/SLC13A1/SLC13A4/SLC13A5/SLC17A2/SLC17A4/SLC17A6/SLC17A7/SLC17A8/SLC24A2/SLC24A3/SLC28A3/SLC34A1/SLC34A2/SLC38A3/SLC38A4/SLC38A8/SLC4A11/SLC4A4/SLC4A5/SLC5A1/SLC5A7/SLC6A1/SLC6A11/SLC6A15/SLC6A17/SLC6A2/SLC6A20/SLC6A3/SLC6A5/SLC6A7/SLC8A2/SLC8A3/SLC9A2/SLC9A4/SLC9A5/SLC9A6/SLC9A7/SLC9B2/SLC9C2/SPTBN4/STOM/TRPM4/WNK2/WNK3	95
cluster5	BP	GO:0007215	glutamate receptor signaling pathway	32/3710	52/18903	4.06E-11	4.78E-09	4.1E-09	APP/CDK5R1/DAGLA/FRRS1L/GRIA1/GRIA2/GRIA3/GRIA4/GRID1/GRID2/GRIK1/GRIK2/GRIK3/GRIK5/GRIN1/GRIN2A/GRIN2B/GRIN2D/GRM2/GRM3/GRM4/GRM5/GRM6/GRM7/GRM8/HOMER1/KCNB1/PLCB1/PLP1/SLC1A1/SSTR1/TRPM1	32
cluster5	BP	GO:0048568	embryonic organ development	146/3710	449/18903	4.39E-11	5.11E-09	4.39E-09	AHI1/AKT1/ALX1/ALX4/APELA/ATF4/ATOH1/ATP6V1B1/BIRC6/BMP4/BMP5/BMP7/CC2D2A/CCDC103/CCDC40/CDX2/CDX4/CITED1/CLRN1/CLRN2/COL11A1/COL2A1/CRB2/DLL1/DLX2/DLX5/DLX6/DSCAML1/E2F7/ECE1/EDNRA/EGFR/EIF4A3/EN1/EN2/FGF10/FGF9/FGFR2/FOLR1/FOXC1/FOXF2/FOXG1/FOXN4/FRZB/FZD3/GATA4/GBX2/GDNF/GLI2/GLI3/GRHL2/HES1/HIF1A/HMX3/HNF1B/HOXA11/HOXA3/HOXA4/HOXB1/HOXB6/HOXB9/HOXC4/HOXC9/HOXD10/HOXD3/HPN/HS6ST1/IFT140/IRX5/JUNB/KCNQ4/KITLG/LBX1/LHFPL5/LHX1/LRIG3/MDFI/MFAP2/MMP16/MYO3A/MYO3B/MYO6/NCOA1/NDRG4/NEUROD1/NEUROG1/NKX2-6/NOTCH2/NR2F2/NTN1/OTOP1/OTX1/PAX2/PAX6/PBX1/PDGFA/PDGFC/PDGFRA/PDZD7/PIFO/PKDCC/PLXNA4/POU3F4/POU4F3/PTCH1/PTK7/RARB/RBPMS2/RSPO3/RYR2/SALL1/SHH/SHOX2/SIX2/SIX3/SIX4/SLITRK6/SMO/SOBP/SOX18/SOX9/STRA6/TAF10/TBX15/TBX20/TBX4/TEAD1/TEAD2/TFAP2A/TH/TTPA/TULP3/USH1C/VANGL2/VASH2/VAX2/VEGFA/WHRN/WNT11/WNT3A/WNT5A/WNT7B/WNT9B/YAP1/ZIC1/ZIC3	146
cluster5	BP	GO:0051952	regulation of amine transport	48/3710	98/18903	5.92E-11	6.82E-09	5.86E-09	ACE2/ADORA1/ADRA2A/AGT/ATP1A2/AVP/AVPR1B/CHGA/CHRNA3/CHRNA4/CHRNA6/CLTRN/CRH/DRD1/DRD2/DRD4/GDNF/GHSR/GRM2/GRM7/HRH3/HTR2A/ITGB1/KCNB1/NPY5R/OPRK1/PRKN/RAB3B/RGS4/SLC17A8/SLC38A3/SLC6A1/STX1A/STXBP1/SV2A/SYT1/SYT10/SYT12/SYT13/SYT2/SYT3/SYT4/SYT5/SYT6/SYT7/SYT8/SYT9/TRH	48

cluster5	BP	GO:0008038	neuron recognition	30/3710	48/18903	9.48E-11	1.07E-08	9.19E-09	APP/CDK5R1/CNTN4/CNTN6/CNTNAP2/CRTAC1/DSCAM/DSCAML1/EFNB3/EPHA3/EPHB3/FEZF2/FOXG1/GAP43/IGSF9/MYPN/NCAM2/NEXN/NPTN/NRCAM/NTM/OPCML/ROBO1/ROBO2/ROBO3/SEMA5A/TNFRSF21/TNN/VSTM2L/YWHAZ	30
cluster5	BP	GO:0048754	branching morphogenesis of an epithelial tube	66/3710	157/18903	9.5E-11	1.07E-08	9.19E-09	ABL1/AGT/AGTR2/BMP2/BMP4/BMP7/CELSR1/CITED1/COL4A1/DAG1/DLG5/EDNRA/EGF/FGF10/FGF2/FGFR2/FOXA1/GBX2/GDNF/GLI2/GLI3/GPC3/GREB1L/GREM1/GZF1/HHIP/HNF1B/HOXA11/HOXD11/HS3ST3A1/ILK/LAMA1/LAMA5/LGR4/LHX1/LRP5/MAGED1/MDK/MSX2/NFATC4/NKX2-1/NOTCH4/PAX2/PBX1/PKHD1/PTCH1/RSPO2/SALL1/SEMA3E/SFRP2/SHH/SIX2/SIX4/SMO/SOX9/SPRY1/SRC/TBX20/TMEM59L/VEGFA/WNT2B/WNT4/WNT5A/WNT9B/WT1/YAP1	66
cluster5	BP	GO:0045666	positive regulation of neuron differentiation	46/3710	93/18903	9.67E-11	1.08E-08	9.26E-09	ASCL1/ATOH1/BMP2/BMP4/BMP6/BMP7/BRINP1/BRINP2/BRINP3/CDON/CSNK1E/DAB1/DLX1/DLX2/DUOXA1/EPO/FEZ1/FEZF1/FEZF2/FGF2/FGFR1/FOXA1/FOXG1/GDF5/GDF6/GPRC5B/HEYL/HMG20B/HOXD3/MMD/MMD2/NCOA1/NEUROD1/NEUROD2/NEUROG1/NEUROG3/NGF/NKX2-2/NKX6-1/NRCAM/PCP4/PHOX2B/SH3GL3/TCF4/VWC2/VWC2L	46
cluster5	BP	GO:0071805	potassium ion transmembrane transport	85/3710	223/18903	9.84E-11	1.09E-08	9.32E-09	ABCC8/ABCC9/ACTN2/AKAP6/ANK3/ANO6/ATP1A2/ATP1A3/ATP1B1/CASQ2/CRBN/DPP10/DPP6/FHL1/GRP/HCN1/HCN3/HCN4/HPN/ITGB1/KCNA10/KCNA4/KCNA7/KCNB1/KCNB2/KCNC1/KCNC2/KCNC3/KCND2/KCND3/KCNE4/KCNG3/KCNH1/KCNH2/KCNH4/KCNH5/KCNH6/KCNIP1/KCNIP3/KCNJ13/KCNJ16/KCNJ18/KCNJ3/KCNJ4/KCNJ5/KCNJ6/KCNJ9/KCNK10/KCNK3/KCNK5/KCNK6/KCNMA1/KCNMB2/KCNN1/KCNN2/KCNN3/KCNQ2/KCNQ3/KCNQ4/KCNRG/KCNS2/KCNT1/KCNT2/KCNV1/LRRC55/NALCN/NEDD4L/OPRK1/RGS4/RGS7/SLC12A5/SLC12A9/SLC17A6/SLC17A7/SLC24A2/SLC24A3/SLC9A2/SLC9A4/SLC9A5/SLC9A6/SLC9A7/SLC9C2/SNAP25/WNK2/WNK3	85

cluster5	BP	GO:0061138	morphogenesis of a branching epithelium	75/3710	188/18903	1.04E-10	1.12E-08	9.63E-09	ABL1/AGT/AGTR2/BMP2/BMP4/BMP7/CELSR1/CITED1/COL4A1/DAG1/DLG5/EDNRA/EGF/FGF10/FGF2/FGF7/FGFR2/FOXA1/GBX2/GDNF/GLI2/GLI3/GPC3/GREB1L/GREM1/GRHL2/GZF1/HHIP/HNF1B/HOXA11/HOXB13/HOXD11/HOXD13/HS3ST3A1/ILK/LAMA1/LAMA5/LGR4/LHX1/LRP5/MAGED1/MDK/MSX2/NFATC4/NKX2-1/NOTCH4/PAX2/PBX1/PDGFA/PKHD1/PLXNA1/PTCH1/RSPO2/RSPO3/SALL1/SEMA3E/SFRP2/SHH/SIX2/SIX4/SMO/SOX10/SOX9/SPRY1/SRC/SULF1/TBX20/TMEM59L/VEGFA/WNT2B/WNT4/WNT5A/WNT9B/WT1/YAP1	75
cluster5	BP	GO:1903522	regulation of blood circulation	96/3710	263/18903	1.05E-10	1.12E-08	9.63E-09	ABCC9/ABL1/ACE/ACE2/ADM5/ADORA1/ADRA1A/ADRA1B/ADRA1D/ADRA2A/AGT/AGTR2/APELA/ASIC2/ATP1A2/ATP1A3/ATP1B1/ATP2B2/ATP2B3/AVP/AVPR1B/BVES/CACNA1C/CACNA1G/CACNA2D1/CALCA/CASQ2/CCN2/CELF2/CHGA/CHRM2/CHRM3/CSRP3/DBH/DES/DMD/DOCK4/DRD2/DSG2/ECE1/EDN2/EDNRA/EDNRB/EHD3/F2R/FKBP1B/FOXN4/GATA4/GJC1/GLP1R/GSTO1/HCN1/HCN3/HCN4/HRC/HTR2A/ISL1/KCND3/KCNE4/KCNH2/KCNH6/KCNJ3/KCNJ5/KCNMB2/KCNN2/MYH6/MYH7/NOS3/PDE5A/PKP2/PTGS2/RGS4/RYR2/SCN10A/SCN2B/SCN3B/SHOX2/SLC1A1/SLC4A3/SLC8A2/SLC8A3/SPTBN4/SPX/STC1/TACR1/TACR3/TBX5/TBXA2R/TH/THRB/TNNI3/TNNT2/TPM1/TRDN/TRPM4/ZC3H12A	96
cluster5	BP	GO:0003341	cilium movement	77/3710	195/18903	1.05E-10	1.12E-08	9.63E-09	ADCY3/AKAP4/BBS1/C2CD6/CABS1/CABYR/CATSPER4/CCDC103/CCDC40/CELF3/CELSR2/CEP131/CFAP206/CFAP221/CFAP43/CFAP46/CFAP47/CFAP52/CFAP54/CFAP61/CFAP65/CFAP69/CFAP91/CFAP97D1/DAW1/DDX4/DNAAF6/DNAH11/DNAH5/DNAH7/DNAH9/DNAI1/DNAI3/DRC1/DZIP1/ENKUR/ENO4/FSIP2/GK2/HOATZ/HYDIN/IFT81/LDHC/MNS1/NEK10/NEURL1/NME5/NPHP4/ODAD2/ODAD3/PLA2G3/PLTP/PRDM14/PRM3/PRSS55/RFX3/ROPN1/RSPH6A/RSPH9/SEPTIN4/SLC9B2/SMCP/SPAG17/SPAG6/SPEF1/SPEM1/TACR1/TACR3/TCTE1/TEKT1/TEKT2/TEKT5/TTC29/TTLL6/TTLL9/UBE2B/ZBBX	77
cluster5	BP	GO:0007269	neurotransmitter secretion	64/3710	151/18903	1.2E-10	1.26E-08	1.08E-08	ADCY1/ADORA2B/ADRA1A/ADRA2A/APBA1/ASIC1/BLOC1S6/BRSK1/CADPS/CAMK2A/CHRM2/CHRNA3/CHRNA6/CHRNB4/CPLX2/CPLX4/CSPG5/DOC2A/DOC2B/DRD1/DRD2/GIPC1/GRIK5/GRM4/GSK3B/HRH3/HTR2A/NLGN1/NRXN1/OTOF/PCLO/PFN2/PRKCG/PRKN/PRRT2/RAB5A/RAP1B/RIMS3/RIMS4/RPH3AL/SEPTIN5/SLC30A1/SNAP23/SNAP25/SNCAIP/STX11/STX1A/STXBP1/SV2A/SYN1/SYN2/SYN3/SYP/SYT1/SYT12/SYT2/SYT4/SYT5/SYT7/SYT8/SYT9/UNC13A/UNC13C/WNT7A	64

cluster5	BP	GO:0099643	signal release from synapse	64/3710	151/18903	1.2E-10	1.26E-08	1.08E-08	ADCY1/ADORA2B/ADRA1A/ADRA2A/APBA1/ASIC1/BLOC1S6/BRSK1/CADPS/CAMK2A/CHRM2/CHRNA3/CHRNA6/CHRNB4/CPLX2/CPLX4/CSPG5/DOC2A/DOC2B/DRD1/DRD2/GIPC1/GRIK5/GRM4/GSK3B/HRH3/HTR2A/NLGN1/NRXN1/OTOF/PCLO/PFN2/PRKCG/PRKN/PRRT2/RAB5A/RAP1B/RIMS3/RIMS4/RPH3AL/SEPTIN5/SLC30A1/SNAP23/SNAP25/SNCAIP/STX11/STX1A/STXBP1/SV2A/SYN1/SYN2/SYN3/SYP/SYT1/SYT12/SYT2/SYT4/SYT5/SYT7/SYT8/SYT9/UNC13A/UNC13C/WNT7A	64
cluster5	BP	GO:0015837	amine transport	50/3710	106/18903	1.33E-10	1.38E-08	1.19E-08	ACE2/ADORA1/ADRA2A/AGT/ATP1A2/AVP/AVPR1B/CHGA/CHRNA3/CHRNA4/CHRNA6/CLTRN/CRH/DRD1/DRD2/DRD4/GDNF/GHSR/GRM2/GRM7/HRH3/HTR2A/ITGB1/KCNB1/NPY5R/OPRK1/PRKN/RAB3B/RGS4/RHCG/SLC17A8/SLC38A3/SLC6A1/STX1A/STXBP1/SV2A/SYT1/SYT10/SYT12/SYT13/SYT2/SYT3/SYT4/SYT5/SYT6/SYT7/SYT8/SYT9/TH/TRH	50
cluster5	BP	GO:0009953	dorsal/ventral pattern formation	46/3710	94/18903	1.54E-10	1.58E-08	1.35E-08	ASCL1/BMP4/BMPR1B/CHRD/CXXC4/DDIT3/DMRT3/DSCAML1/EN1/FOXA1/FOXG1/FOXN4/GLI2/GLI3/GPR161/GREM1/GREM2/GSX2/HHIP/HOXA11/HOXD11/LHX1/LHX2/LHX3/LMX1B/LRP4/MDFI/NKX2-1/NKX2-2/PAX6/PTCH1/RFX4/SHH/SIX3/SMO/SOSTDC1/SOX1/SP8/TBC1D32/TBX20/TLL2/TULP3/VAX2/WNT3A/WNT7A/WNT8B	46
cluster5	BP	GO:0001539	cilium or flagellum-dependent cell motility	66/3710	159/18903	1.83E-10	1.83E-08	1.57E-08	ADCY3/AKAP4/BBS1/C2CD6/CABS1/CATSPER4/CCDC40/CELF3/CEP131/CFAP206/CFAP221/CFAP43/CFAP46/CFAP47/CFAP52/CFAP54/CFAP65/CFAP69/CFAP97D1/DDX4/DNAAF6/DNAH11/DNAH14/DNAH2/DNAH3/DNAH5/DNAH7/DNAI1/DNAI3/DRC1/DZIP1/EFHC2/ENKUR/ENO4/FSIP2/GK2/HOATZ/IFT81/LDHC/MNS1/NEURL1/NPHP4/ODAD3/PLA2G3/PLTP/PRDM14/PRM3/PRSS55/RFX3/ROPN1/RSPH6A/RSPH9/SEPTIN4/SLC9B2/SMCP/SPAG6/SPEM1/TACR1/TACR3/TCTE1/TEKT1/TEKT2/TEKT5/TTLL6/TTLL9/UBE2B	66

cluster5	BP	GO:0060285	cilium-dependent cell motility	66/3710	159/18903	1.83E-10	1.83E-08	1.57E-08	ADCY3/AKAP4/BBS1/C2CD6/CABS1/CATSPER4/CCDC40/CELF3/CEP131/CFAP206/CFAP221/CFAP43/CFAP46/CFAP47/CFAP52/CFAP54/CFAP65/CFAP69/CFAP97D1/DDX4/DNAAF6/DNAH11/DNAH14/DNAH2/DNAH3/DNAH5/DNAH7/DNAI1/DNAI3/DRC1/DZIP1/EFHC2/ENKUR/ENO4/FSIP2/GK2/HOATZ/IFT81/LDHC/MNS1/NEURL1/NPHP4/ODAD3/PLA2G3/PLTP/PRDM14/PRM3/PRSS55/RFX3/ROPN1/RSPH6A/RSPH9/SEPTIN4/SLC9B2/SMCP/SPAG6/SPEM1/TACR1/TACR3/TCTE1/TEKT1/TEKT2/TEKT5/TTLL6/TTLL9/UBE2B	66
cluster5	BP	GO:1901888	regulation of cell junction assembly	79/3710	204/18903	1.84E-10	1.83E-08	1.57E-08	ABL1/ACE/ACE2/ADGRB1/ADGRB3/AGT/APOD/APP/ARHGAP6/ASIC2/BDNF/CBLN1/CBLN2/CLDN1/CLDN19/CLDN5/CLSTN2/CNTNAP2/COL16A1/DLG5/DMTN/EFNA5/EPHA3/EPHA7/EPHB3/FLRT2/FLRT3/GHSR/GPC6/GPM6B/GREM1/GRID2/IL1RAPL1/IL1RAPL2/LHFPL4/LRFN3/LRRC4B/LRRN1/LRRTM2/LRRTM3/LRTM2/MUSK/MYO1C/MYOC/NECTIN1/NLGN1/NLGN2/NLGN3/NPHP4/NPTX1/NRXN1/NTN1/NTNG2/NTRK2/PTPRD/RAP1B/ROBO2/ROCK1/ROCK2/SDC4/SEMA4D/SIX4/SLIT1/SLITRK1/SLITRK2/SLITRK3/SLITRK6/SRC/SRPX2/ST8SIA2/SYNDIG1/TBX5/THY1/TPBG/VEGFA/WNT3A/WNT4/WNT5A/WNT7A	79
cluster5	BP	GO:0048663	neuron fate commitment	37/3710	68/18903	1.95E-10	1.93E-08	1.65E-08	ASCL1/ATOH1/BMP4/DLL1/DLX1/DLX2/DMRT3/DMRTA2/EHMT2/ESRP1/FEV/FEZF2/FOXA1/FOXG1/FOXN4/GLI2/GLI3/GSX1/GSX2/HOXC10/HOXD10/ISL1/LBX1/LHX3/MNX1/MYT1L/NKX2-1/NKX2-2/PAX6/POU3F2/PTF1A/SHH/SOX1/SOX9/TBR1/TLX3/ZNF521	37
cluster5	BP	GO:0010720	positive regulation of cell development	107/3710	307/18903	2.15E-10	2.11E-08	1.81E-08	ABL1/ACE/ASCL1/ASPA/ASPM/BDNF/BMP2/BMP4/CAMK2B/CARMIL1/CDH4/CDKL3/CHODL/CLDN5/CRABP2/CSPG5/CUL7/CUX1/CYFIP1/DAB2/DAG1/DBN1/DCT/DMRTA2/DMTN/DRD2/DSCAM/EFNA5/FAIM/FGF2/FOXG1/FZD3/GDI1/GFAP/GLI3/GRM5/GSX2/HAP1/HAS2/HDAC2/HES1/HIF1A/HOXA11/ID4/IL1RAPL1/IL5/ILK/ISLR2/ITGB1/ITGB3/ITPKA/KHDC3L/L1CAM/LRP2/LYN/MAP6/MDK/MYADM/MYOC/MYOG/NDEL1/NEURL1/NGF/NKX2-2/NKX6-1/NPTN/NR2E1/NTN1/NTRK2/NUMBL/OTP/PAK3/PAX6/PDE3A/PDE5A/PLXNA1/PLXNA2/PLXNA4/PLXNB1/PLXNB3/PTN/PTPRD/PTPRZ1/RELN/RET/RFX3/RND2/ROBO1/ROBO2/SEMA4D/SEMA5A/SERPINE2/SHH/SHOX2/SIRT2/SLC9B2/SLITRK1/SMO/SOX10/SPEN/TENM4/TP73/TRIB1/TTBK1/VEGFA/WNT3A/ZNF488	107

cluster5	BP	GO:0003338	metanephros morphogenesis	23/3710	32/18903	2.24E-10	2.16E-08	1.85E-08	AGTR2/BASP1/BMP4/CALB1/FGF10/FOXJ1/GDNF/GREM1/HES1/HES5/KIF26B/LGR4/LHX1/PAX2/PDGFRB/SALL1/SIX2/SMO/SOX9/WNT4/WNT7B/WNT9B/WT1	23
cluster5	BP	GO:0048665	neuron fate specification	23/3710	32/18903	2.24E-10	2.16E-08	1.85E-08	ASCL1/ATOH1/DLL1/DMRT3/DMRTA2/EHMT2/ESRP1/FEV/FOXA1/GLI2/GLI3/GSX2/HOXC10/HOXD10/ISL1/LHX3/MNX1/MYT1L/NKX2-2/POU3F2/SOX1/SOX9/TLX3	23
cluster5	BP	GO:0001656	metanephros development	45/3710	92/18903	2.47E-10	2.36E-08	2.02E-08	AGTR2/BASP1/BMP4/BMP7/CALB1/CITED1/DLG5/FGF10/FOXJ1/GDF6/GDNF/GLI3/GPC3/GREB1L/GREM1/HES1/HES5/HNF1B/HOXA11/IRX2/KIF26B/LAMB2/LGR4/LHX1/PAX2/PDGFA/PDGFRA/PDGFRB/POU3F3/PTCH1/RET/ROBO2/SALL1/SHH/SIX2/SIX4/SMO/SOX9/SPRY1/TFAP2B/WNT4/WNT7B/WNT9B/WT1/YAP1	45
cluster5	BP	GO:0051965	positive regulation of synapse assembly	35/3710	63/18903	2.79E-10	2.63E-08	2.26E-08	ADGRB1/ADGRB3/ASIC2/BDNF/CBLN1/CBLN2/CLSTN2/DLG5/EFNA5/EPHB3/FLRT2/FLRT3/GRID2/IL1RAPL1/LRRC4B/LRRN1/LRRTM2/LRRTM3/LRTM2/NLGN1/NLGN2/NLGN3/NRXN1/NTRK2/PTPRD/SEMA4D/SLITRK1/SLITRK2/SLITRK3/SLITRK6/SRPX2/ST8SIA2/SYNDIG1/TPBG/WNT7A	35

cluster5	BP	GO:0050953	sensory perception of light stimulus	84/3710	224/18903	3.27E-10	3.06E-08	2.63E-08	ABCA4/ADGRV1/AIPL1/AOC2/BBS1/CABP1/CLDN19/CLRN1/CNGA3/CNGB1/COL11A1/COL2A1/CPLX4/CRB2/CRX/CRYAA/CRYGB/CRYGD/EYA4/FAM161A/GJA10/GJC1/GJD2/GLRA1/GLRB/GNAT1/GRM6/GRM8/GUCA1C/GUCY2F/IMPG1/IRX5/KERA/LAMB2/LOC118142757/LRAT/MFRP/MIP/MYO3A/MYO3B/NDP/NR2E1/NR2E3/OPN1LW/OPN1MW2/OPN1MW3/OPN4/PAX2/PAX6/PCARE/PCDH15/PDC/POU4F3/POU6F2/RAX/RAX2/RBP3/RDH8/REEP6/RGR/RGS16/RGS9/RHO/RORB/RP1/RPE65/SEMA5B/SIX3/SIX6/SLC24A2/SLITRK6/SOX14/TH/TRPM1/TULP1/TYR/USH1C/USH2A/VAX2/VSX1/VSX2/WFS1/WHRN/ZIC2	84
cluster5	BP	GO:0033555	multicellular organismal response to stress	41/3710	81/18903	4.04E-10	3.75E-08	3.21E-08	ADCYAP1R1/ADRA2A/AKT1/ASIC1/ASIC4/ATP1A2/BRINP1/CALCA/CRHR1/DBH/DRD1/DRD4/EDNRB/GABRA5/GRIK2/GRP/GRPR/HMGCS2/HTR2C/LRP11/LYPD1/MAPK8IP2/MDK/NEUROD2/NPAS2/NR2E1/NR4A2/P2RX2/P2RX3/PENK/PIRT/PRKAR1B/PRKCG/RELN/RET/SCN9A/SLC1A1/SLC6A2/TAC1/TACR1/VWA1	41
cluster5	BP	GO:0023061	signal release	148/3710	470/18903	4.26E-10	3.92E-08	3.36E-08	ABCA12/ABCC8/ADCY1/ADCY5/ADCY8/ADCYAP1/ADORA1/ADORA2B/ADRA1A/ADRA2A/AGT/APBA1/ASIC1/BLOC1S6/BMP6/BRSK1/BRSK2/CADPS/CAMK2A/CCKAR/CFTR/CGA/CHGA/CHRM2/CHRNA3/CHRNA4/CHRNA6/CHRNB4/CLTRN/CPLX2/CPLX4/CRH/CRHR1/CSPG5/DAB2/DOC2A/DOC2B/DRD1/DRD2/EDNRB/EFNA5/EPHA5/EXOC3L1/F2R/FGF23/FKBP1B/FOXA2/GCG/GCK/GDNF/GHSR/GIPC1/GLP1R/GPLD1/GRIK5/GRM2/GRM4/GRP/GSK3B/HADH/HFE/HIF1A/HNF1A/HNF1B/HRH3/HTR2A/HTR2C/ILDR2/INHA/INS/ISL1/KCNB1/KISS1/LRP5/LYN/MAOB/MC4R/NEUROD1/NKX6-1/NLGN1/NLGN2/NNAT/NOS2/NR0B2/NR1H4/NRXN1/OPRK1/OR51E2/OTOF/PCLO/PFN2/PLA2G3/PRKCG/PRKN/PRRT2/PTGS2/RAB11FIP2/RAB5A/RAP1B/RASL10B/RFX3/RFX6/RIMS3/RIMS4/RPH3AL/SCG5/SEPTIN5/SLC16A2/SLC30A1/SLC30A8/SLC9B2/SNAP23/SNAP25/SNCAIP/SPINK1/SSTR5/STX11/STX1A/STXBP1/SUCNR1/SV2A/SYN1/SYN2/SYN3/SYP/SYT1/SYT10/SYT12/SYT13/SYT2/SYT3/SYT4/SYT5/SYT6/SYT7/SYT8/SYT9/TACR1/TFAP2B/TNFRSF11A/TRH/TRPM4/TRPV4/UCN3/UNC13A/UNC13C/VSNL1/WNT7A	148
cluster5	BP	GO:0035107	appendage morphogenesis	60/3710	142/18903	5.23E-10	4.72E-08	4.05E-08	ALX4/ATRX/BMP4/BMP7/CACNA1C/CIBAR1/COL2A1/CRABP2/DLX5/DLX6/ECE1/EN1/FGF10/FGF4/FGF9/FGFR2/FREM2/GDF5/GLI3/GPC3/GREM1/GRHL2/HDAC2/HOXA11/HOXC10/HOXD10/HOXD12/HOXD13/IFT140/LRP4/LRP5/MSX2/NOTCH2/PBX1/PITX1/PKDCC/PLXNA2/PTCH1/RARB/RSPO2/SALL1/SALL4/SFRP2/SHH/SHOX2/SOX9/SP8/SP9/TBC1D32/TBX4/TBX5/TFAP2A/TFAP2B/TMEM231/TULP3/WNT5A/WNT7A/ZBTB16/ZIC3/ZNF219	60

cluster5	BP	GO:0035108	limb morphogenesis	60/3710	142/18903	5.23E-10	4.72E-08	4.05E-08	ALX4/ATRX/BMP4/BMP7/CACNA1C/CIBAR1/COL2A1/CRABP2/DLX5/DLX6/ECE1/EN1/FGF10/FGF4/FGF9/FGFR2/FREM2/GDF5/GLI3/GPC3/GREM1/GRHL2/HDAC2/HOXA11/HOXC10/HOXD10/HOXD12/HOXD13/IFT140/LRP4/LRP5/MSX2/NOTCH2/PBX1/PITX1/PKDCC/PLXNA2/PTCH1/RARB/RSPO2/SALL1/SALL4/SFRP2/SHH/SHOX2/SOX9/SP8/SP9/TBC1D32/TBX4/TBX5/TFAP2A/TFAP2B/TMEM231/TULP3/WNT5A/WNT7A/ZBTB16/ZIC3/ZNF219	60
cluster5	BP	GO:0048592	eye morphogenesis	65/3710	159/18903	5.39E-10	4.83E-08	4.14E-08	AHI1/ATF4/BCAR3/BMP4/BMP7/CALB1/CDON/COL8A1/CRB2/CRYGB/DLL1/DSCAM/FAT1/FAT3/FGF2/FOXE3/FOXF2/FOXN4/GLI3/GNAT1/GNGT1/HCN1/HIF1A/LHX1/LRP5/MEGF11/MEIS1/MFAP2/MFRP/MFSD2A/NECTIN1/NECTIN3/NOTCH2/NR2E3/NTRK2/OLFM3/PAX2/PAX6/PROM1/PTF1A/RARB/RORB/RP1/RPE65/SDK1/SHROOM2/SIX3/SLC1A1/SOX1/SOX9/STRA6/TENM3/TFAP2A/TFAP2B/TH/THRB/THY1/TULP1/USH1C/VAX2/VEGFA/VSX1/WNT2B/WNT5A/WNT9A	65
cluster5	BP	GO:0030534	adult behavior	60/3710	143/18903	7.29E-10	6.48E-08	5.56E-08	ADAM2/ALK/APP/ATP1A2/ATP6V1B1/BBS1/CHL1/CHRNA3/CHRNA4/CHRNB4/CNTNAP2/CRHR1/DAB1/DBH/DMBX1/DMRT3/DRD1/DRD2/DRD4/EFNB3/EHMT2/EN1/EPS8/FOXA2/GABRG2/GDNF/GHSR/GLRA1/GLRB/GRIN2D/GRM2/HDAC2/HOMER1/HOXD10/HTR2A/KLHL1/NLGN2/NLGN3/NLGN4X/NPY/NR4A2/NRXN1/NRXN3/OPRK1/PRKN/RNF180/SCN1A/SDK1/SEPTIN5/SHANK1/SHANK2/SLC1A1/SLC1A2/SLITRK1/SLITRK6/SPTBN4/TRH/UCHL1/ZFHX2/ZIC1	60
cluster5	BP	GO:1904062	regulation of cation transmembrane transport	128/3710	394/18903	7.37E-10	6.49E-08	5.57E-08	ABCC8/ABCC9/ABL1/ACE2/ACTN2/ADCYAP1R1/ADRA2A/AGT/AKAP6/ANK3/ANO6/APP/ARC/ATP1A2/ATP1B1/CABP1/CACNA1C/CACNA2D1/CACNB1/CACNG2/CACNG3/CACNG4/CACNG5/CACNG7/CALCR/CASQ2/CBARP/CEMIP/CHP2/CLTRN/CNKSR3/CRBN/CRH/CRHR1/DHRS7C/DMD/DPP10/DPP6/DRD1/DRD2/DRD4/EDNRA/EHD3/EPO/F2/F2R/F2RL3/FGF14/FHL1/FKBP1A/FKBP1B/FXYD3/FXYD6/GEM/GRIN1/GRIN2A/GRIN2B/GRIN2D/GRM6/GRP/GSG1L/GSTO1/HAP1/HCN1/HCN3/HCN4/HOMER1/HPCA/HRC/HSPA2/IL13/ITGB1/ITGB3/JPH1/JPH3/JPH4/JSRP1/KCNC1/KCNC2/KCNE4/KCNH2/KCNIP1/KCNIP3/KCNK3/KCNN2/KCNRG/KCNS2/LACRT/LHCGR/LRRC55/LYN/MAPK8IP2/MINK1/NEDD4L/NLGN1/NLGN2/NLGN3/NPSR1/OPRK1/P2RX2/P2RX3/PIRT/PLP1/PRRT1/PTPN3/RASGRF1/RELN/REM1/RGS4/RGS7/RYR2/SCN2B/SCN3B/SHANK1/SHISA6/SHISA7/SHISA9/SLC30A1/SLN/STAC2/STOM/THY1/TMBIM6/TMC1/TRDN/TRPC3/WNK2/WNK3	128

cluster5	BP	GO:0050769	positive regulation of neurogenesis	85/3710	231/18903	7.67E-10	0.000000067	5.75E-08	ACE/ASCL1/ASPA/ASPM/BDNF/BMP2/CAMK2B/CDH4/CDKL3/CHODL/CRABP2/CUL7/CUX1/CYFIP1/DAG1/DBN1/DCT/DMRTA2/DRD2/DSCAM/EFNA5/FAIM/FGF2/FOXG1/FZD3/GDI1/GFAP/GLI3/GRM5/GSX2/HAP1/HDAC2/HES1/HIF1A/ID4/IL1RAPL1/ISLR2/ITGB1/ITPKA/KHDC3L/L1CAM/LRP2/LYN/MAP6/MDK/NDEL1/NEURL1/NGF/NKX2-2/NKX6-1/NPTN/NR2E1/NTN1/NTRK2/NUMBL/OTP/PAK3/PAX6/PLXNA1/PLXNA2/PLXNA4/PLXNB1/PLXNB3/PTN/PTPRD/PTPRZ1/RELN/RND2/ROBO1/ROBO2/SEMA4D/SEMA5A/SERPINE2/SHH/SHOX2/SLITRK1/SMO/SOX10/SPEN/TENM4/TP73/TTBK1/VEGFA/WNT3A/ZNF488	85
cluster5	BP	GO:0009582	detection of abiotic stimulus	59/3710	140/18903	8.21E-10	7.11E-08	0.000000061	ABCA4/ADGRV1/ADORA1/AIPL1/ANO3/ASIC2/ASIC3/CALCA/CDH2/CDS1/CNGB1/COL11A1/CSRP3/DRGX/ELOVL4/GJA10/GNAT1/GNGT1/GPR52/GRIK2/GRK4/GRM6/GUCY2F/HPN/HTR2A/ITGA2/LHFPL5/LOC118142757/MMP24/NGFR/NPFFR2/NR2E3/OPN1LW/OPN1MW2/OPN1MW3/OPN4/OTOP1/PDC/PDZD7/PKD1L2/PKD1L3/PKDREJ/PRDM12/REEP6/RGR/RHO/RP1/RPE65/SCN1A/SEMA5B/SERPINE2/TAC1/TACR1/TMC1/TRPC3/TRPM3/TRPM8/TULP1/WHRN	59
cluster5	BP	GO:0007018	microtubule-based movement	133/3710	415/18903	9.31E-10	0.00000008	6.87E-08	ADCY3/AGBL4/AKAP4/AP3B2/AP3D1/APBA1/APP/ARMCX3/BBS1/BICD2/BLOC1S6/C2CD6/CABS1/CABYR/CATSPER4/CCDC103/CCDC40/CELF3/CELSR2/CEP131/CFAP206/CFAP221/CFAP43/CFAP46/CFAP47/CFAP52/CFAP54/CFAP61/CFAP65/CFAP69/CFAP91/CFAP97D1/CLIP3/DAW1/DDX4/DNAAF6/DNAH10/DNAH11/DNAH12/DNAH14/DNAH2/DNAH3/DNAH5/DNAH7/DNAH9/DNAI1/DNAI3/DRC1/DYNC1I1/DYNC2I2/DYNLRB2/DZIP1/ENKUR/ENO4/FEZ1/FMN2/FSIP2/GK2/HAP1/HDAC6/HIF1A/HOATZ/HSBP1/HYDIN/IFT140/IFT81/KIF12/KIF18A/KIF1A/KIF20A/KIF20B/KIF21A/KIF21B/KIF24/KIF25/KIF2B/KIF3A/KIF3C/KIF5A/KIF5C/KIF6/KIF7/KIFC1/KLC4/LAMP1/LCA5/LDHC/MAP1A/MAP2/MNS1/NDEL1/NEFH/NEK10/NEURL1/NME5/NPHP4/ODAD2/ODAD3/PLA2G3/PLTP/PRDM14/PRM3/PRSS55/RAB27B/RFX3/ROPN1/RSPH6A/RSPH9/SEPTIN4/SLC9B2/SMCP/SPAG17/SPAG6/SPEF1/SPEM1/SYBU/SYNE2/TACR1/TACR3/TCTE1/TEKT1/TEKT2/TEKT5/TMEM108/TRAK1/TTC29/TTLL6/TTLL9/TUB/UBE2B/UCHL1/WASF1/ZBBX	133
cluster5	BP	GO:0016079	synaptic vesicle exocytosis	50/3710	111/18903	0.000000001	8.56E-08	7.34E-08	ADCY1/ADORA2B/ADRA1A/ADRA2A/APBA1/BLOC1S6/CADPS/CHRM2/CHRNA6/CPLX2/CPLX4/CSPG5/DOC2A/DOC2B/DRD1/DRD2/GIPC1/GRIK5/GSK3B/HTR2A/NLGN1/OTOF/PCLO/PFN2/PRKCG/PRRT2/RAB5A/RAP1B/RIMS3/RIMS4/SEPTIN5/SNAP23/SNAP25/STX11/STX1A/STXBP1/SV2A/SYN1/SYP/SYT1/SYT12/SYT2/SYT4/SYT5/SYT7/SYT8/SYT9/UNC13A/UNC13C/WNT7A	50

cluster5	BP	GO:0008016	regulation of heart contraction	80/3710	214/18903	1.02E-09	0.000000086	7.38E-08	ABCC9/ACE2/ADM5/ADORA1/ADRA1A/ADRA1B/ADRA1D/AGT/AGTR2/APELA/ATP1A2/ATP1A3/ATP1B1/ATP2B2/ATP2B3/BVES/CACNA1C/CACNA1G/CACNA2D1/CALCA/CASQ2/CCN2/CELF2/CHGA/CHRM2/CSRP3/DES/DMD/DRD2/DSG2/EDN2/EDNRA/EDNRB/EHD3/FKBP1B/FOXN4/GATA4/GJC1/GLP1R/GSTO1/HCN1/HCN3/HCN4/HRC/ISL1/KCND3/KCNE4/KCNH2/KCNH6/KCNJ3/KCNJ5/KCNN2/MYH6/MYH7/NOS3/PDE5A/PKP2/RGS4/RYR2/SCN10A/SCN2B/SCN3B/SHOX2/SLC1A1/SLC4A3/SLC8A2/SLC8A3/SPTBN4/SPX/STC1/TACR3/TBX5/TH/THRB/TNNI3/TNNT2/TPM1/TRDN/TRPM4/ZC3H12A	80
cluster5	BP	GO:0009952	anterior/posterior pattern specification	81/3710	218/18903	1.12E-09	0.000000094	8.07E-08	ALX4/ARC/BARX1/BASP1/BHLHE41/BMP2/BMP4/CDON/CDX1/CDX2/CDX4/CELSR2/CFC1/CFC1B/CRB2/CYP26C1/DDIT3/DLL1/DLL3/EMX2/EN1/FEZF1/FEZF2/FOXA2/FOXC1/GATA4/GBX2/GLI3/GPC3/HES1/HES2/HES5/HES6/HES7/HEYL/HNF1B/HOXA11/HOXA3/HOXA4/HOXB1/HOXB6/HOXB9/HOXC10/HOXC13/HOXC4/HOXC6/HOXC8/HOXC9/HOXD10/HOXD13/HOXD3/LHX1/LRP5/LRP6/MSX2/NEUROD1/NEUROG1/NR2F2/OTX1/OTX2/PAX6/PBX1/PGAP1/PLXNA2/RIPPLY1/SFRP2/SHH/SIX2/SMO/TAF10/TBXT/TDGF1/TDRD5/TULP3/WNT2B/WNT3A/WNT5A/WNT8A/WT1/ZBTB16/ZIC3	81
cluster5	BP	GO:0006816	calcium ion transport	139/3710	440/18903	1.14E-09	9.49E-08	8.15E-08	ABL1/ACE/ADCYAP1R1/ADRA1A/ADRA2A/AGT/AKAP6/ANO6/ASIC1/ATP1A2/ATP1B1/ATP2B2/ATP2B3/CABP1/CACNA1A/CACNA1B/CACNA1C/CACNA1G/CACNA1S/CACNA2D1/CACNB1/CACNG2/CACNG3/CACNG4/CACNG5/CACNG7/CALCR/CAMK2A/CAMK2B/CASQ2/CATSPER4/CBARP/CCN2/CD84/CEMIP/CHRNA4/CHRNA7/CRHR1/DDIT3/DHRS7C/DMD/DRD1/DRD2/DRD4/EDNRA/EDNRB/EGF/EHD3/EPO/ERO1A/F2/F2R/F2RL3/FGF2/FKBP1A/FKBP1B/GAS6/GCG/GCK/GEM/GP1BB/GP9/GPM6A/GRAMD2A/GRIN1/GRIN2A/GRIN2B/GRIN2D/GRM6/GSTO1/HAP1/HES1/HOMER1/HPCA/HRC/HSPA2/HTR2A/HTR2C/IL13/ITGB3/JPH1/JPH3/JPH4/JSRP1/LACRT/LHCGR/LILRB2/LYN/MAIP1/MCHR1/MICU3/MYLK/NALCN/NALF1/NALF2/NOS3/NPSR1/P2RX2/P2RX3/PACSIN3/PDGFRB/PKD1L2/PKD1L3/PKDREJ/PLP1/PLPP4/PTGS2/REM1/RGS4/RYR2/RYR3/SEC61A1/SLC24A2/SLC24A3/SLC25A23/SLC30A1/SLC8A2/SLC8A3/SLN/SPINK1/STAC2/STC1/STC2/THY1/TMBIM6/TMC1/TMEM37/TRDN/TRPC3/TRPC4/TRPC7/TRPM1/TRPM3/TRPM4/TRPM8/TRPV4/WFS1/WNK3/WNT3A	139
cluster5	BP	GO:0048565	digestive tract development	56/3710	132/18903	1.63E-09	0.000000134	0.000000115	AGR2/AHI1/ALX4/ASCL1/BMP4/CCDC103/CCDC40/CCKBR/CDX2/CLDN18/CLMP/CPS1/EDNRB/EGFR/EPHB3/FGF10/FGF9/FGFR2/FOXF2/FOXL1/GATA4/GLI2/GLI3/HES1/HIF1A/HMGCS2/HNF1B/HOXD13/LGR4/MYOCD/NKX2-2/NKX2-6/NPY/PDGFC/PDGFRA/PKDCC/PYY/RARB/RBPMS2/RET/SALL1/SFRP2/SHH/SHOX2/SIX2/SMO/SOX10/SOX9/SPDEF/SRC/STRA6/TYMS/WNT11/WNT5A/YAP1/ZIC3	56

cluster5	BP	GO:0048593	camera-type eye morphogenesis	55/3710	129/18903	1.82E-09	0.000000149	0.000000128	AHI1/ATF4/BCAR3/BMP4/BMP7/CALB1/CDON/COL8A1/CRB2/CRYGB/DLL1/DSCAM/FAT1/FAT3/FOXE3/FOXF2/FOXN4/GLI3/HCN1/HIF1A/LHX1/LRP5/MEGF11/MEIS1/MFSD2A/NECTIN1/NECTIN3/NOTCH2/NTRK2/PAX2/PAX6/PROM1/PTF1A/RORB/RP1/RPE65/SDK1/SHROOM2/SIX3/SLC1A1/SOX1/SOX9/STRA6/TENM3/TFAP2A/TFAP2B/TH/THRB/THY1/USH1C/VEGFA/VSX1/WNT2B/WNT5A/WNT9A	55
cluster5	BP	GO:0050806	positive regulation of synaptic transmission	67/3710	170/18903	1.84E-09	0.000000149	0.000000128	ABL1/ADCY1/ADCY8/ADORA1/ADRA1A/APP/ARC/CACNG2/CACNG3/CACNG4/CACNG5/CACNG7/CALB1/CALB2/CHRNA7/CLSTN2/CYP46A1/DRD1/DRD2/GFAP/GRIK2/GRIN1/GRIN2A/GRIN2B/GRIN2D/GSK3B/HAP1/IGSF11/INS/KISS1/LGI1/LILRB2/LRRTM2/MPP2/NALCN/NFATC4/NLGN1/NLGN2/NLGN3/NMU/NPTN/NR2E1/NRXN1/NTRK2/PRKAR1B/PRKCG/PRRT1/PTGS2/PTN/RELN/RIMS3/ROR2/SERPINE2/SHANK2/SHISA7/SLC1A1/SLC24A2/SLC8A2/SLC8A3/SNAP25/SQSTM1/STX1A/STXBP1/SYT1/SYT12/TACR1/TNR	67
cluster5	BP	GO:0007601	visual perception	81/3710	220/18903	1.85E-09	0.000000149	0.000000128	ABCA4/ADGRV1/AIPL1/AOC2/BBS1/CABP1/CLDN19/CLRN1/CNGA3/CNGB1/COL11A1/COL2A1/CPLX4/CRB2/CRX/CRYAA/CRYGB/CRYGD/EYA4/FAM161A/GJA10/GJC1/GJD2/GLRA1/GLRB/GNAT1/GRM6/GRM8/GUCA1C/GUCY2F/IMPG1/IRX5/KERA/LAMB2/LOC118142757/LRAT/MFRP/MIP/MYO3A/MYO3B/NDP/NR2E1/NR2E3/OPN1LW/OPN1MW2/OPN1MW3/OPN4/PAX2/PAX6/PCARE/PDC/POU4F3/POU6F2/RAX/RAX2/RBP3/RDH8/REEP6/RGR/RGS16/RGS9/RHO/RORB/RP1/RPE65/SEMA5B/SIX3/SIX6/SLC24A2/SLITRK6/SOX14/TH/TRPM1/TULP1/TYR/USH2A/VAX2/VSX1/VSX2/WFS1/ZIC2	81
cluster5	BP	GO:0002065	columnar/cuboidal epithelial cell differentiation	51/3710	116/18903	1.94E-09	0.000000156	0.000000134	ABL1/AGR2/AKT1/ASCL1/BMP2/BMP4/BMP5/BMP6/BMP7/CDH2/CDX2/CLCN2/DLG5/DLL1/DLX3/DSPP/EMX1/FGF2/FGFR2/FOXA1/GATA4/GPAT4/GSK3B/HES1/HIF1A/IL13/LHX3/NEUROD1/NKX2-2/NKX6-1/NPY/OTP/PAX6/POU3F2/PYY/RARB/RFX3/RFX6/SERPINE1/SLC9A4/SMO/SOX9/SPDEF/SRC/TMEM231/TYMS/VAX1/WNT11/WNT4/WNT5A/YAP1	51

cluster5	BP	GO:0060047	heart contraction	89/3710	250/18903	2.11E-09	0.000000168	0.000000144	ABCC9/ACE/ACE2/ACTC1/ADM5/ADORA1/ADRA1A/ADRA1B/ADRA1D/AGT/AGTR2/APELA/ATP1A2/ATP1A3/ATP1B1/ATP2B2/ATP2B3/BVES/CACNA1C/CACNA1G/CACNA2D1/CALCA/CASQ2/CCN2/CELF2/CHGA/CHRM2/CSRP3/DES/DMD/DRD2/DSG2/EDN2/EDNRA/EDNRB/EHD3/FKBP1B/FOXN4/GATA4/GJC1/GLP1R/GSTO1/HCN1/HCN3/HCN4/HRC/ISL1/KCND3/KCNE4/KCNH2/KCNH6/KCNJ3/KCNJ5/KCNN2/MYH6/MYH7/MYL7/NEDD4L/NOS3/PDE5A/PKP2/RGS4/RPS6KA2/RYR2/SCN10A/SCN1A/SCN2B/SCN3B/SGCZ/SHOX2/SLC1A1/SLC4A3/SLC8A2/SLC8A3/SMAD5/SPTBN4/SPX/STC1/TACR3/TBX5/TH/THRB/TNNI1/TNNI3/TNNT2/TPM1/TRDN/TRPM4/ZC3H12A	89
cluster5	BP	GO:0071542	dopaminergic neuron differentiation	26/3710	42/18903	2.3E-09	0.000000181	0.000000156	CSNK1E/DMRTA2/EN1/EN2/FERD3L/FOXA1/FOXA2/GSK3B/HIF1A/HPRT1/LMX1A/LMX1B/LRP6/NR4A2/OTP/OTX2/PHOX2B/RSPO2/SFRP2/SHH/SMO/VEGFA/VEGFD/WNT3A/WNT5A/WNT9B	26
cluster5	BP	GO:0048813	dendrite morphogenesis	58/3710	140/18903	2.48E-09	0.000000194	0.000000166	ADGRB3/ARC/ARHGAP44/CAMK2B/CDK5R1/CDKL3/CELSR2/CHRNA3/CHRNA7/CTNNA2/CTNND2/CUL7/CUX1/DBN1/DCLK1/DPYSL5/DSCAM/ELAVL4/EPHB3/GSK3B/HDAC6/HPRT1/ID1/IGF2BP1/IL1RAPL1/ITGB1/ITPKA/KIF1A/KNDC1/LRP4/LZTS1/LZTS3/MAP2/MAP6/MAPK8IP2/MINK1/NEDD4L/NEUROG3/NFATC4/NGEF/NLGN1/NR2E1/PAK3/PPFIA2/PTN/PTPRD/RELN/SDC2/SEMA3A/SEMA4D/SHANK1/SRCIN1/SULT4A1/TANC2/TLX2/TPBG/WNT7A/ZDHHC15	58
cluster5	BP	GO:0007188	adenylate cyclase-modulating G protein-coupled receptor signaling pathway	88/3710	248/18903	3.12E-09	0.000000242	0.000000208	ADCY1/ADCY2/ADCY3/ADCY5/ADCY8/ADCYAP1/ADCYAP1R1/ADGRB1/ADGRB3/ADGRF1/ADGRF2/ADGRF3/ADGRF4/ADGRG2/ADGRG4/ADGRL3/ADM5/ADORA1/ADORA2B/ADRA1A/ADRA1B/ADRA1D/ADRA2A/ADRB3/AVPR1B/CALCA/CALCB/CALCR/CHGA/CHRM1/CHRM2/CHRM3/CRHR1/DRD1/DRD2/DRD4/EDNRA/GABBR2/GCG/GLP1R/GLP2R/GNAI1/GNAO1/GNAS/GNAT1/GNAZ/GPR101/GPR12/GPR161/GPR176/GPR26/GPR37/GPR6/GRIK3/GRM2/GRM3/GRM4/GRM5/GRM6/GRM7/GRM8/HRH3/HTR1D/HTR1E/HTR1F/HTR4/IAPP/ITGB3/LHCGR/MC2R/MC4R/MCHR1/MRAP2/MTNR1A/OPRK1/PF4/PSAP/PSAPL1/PTGFR/PTH/PTH1R/RIT2/SSTR2/TBXA2R/TCP11/UCN2/UCN3/VIPR2	88

cluster5	BP	GO:1990138	neuron projection extension	67/3710	172/18903	3.25E-09	0.00000025	0.000000215	ABL1/AUTS2/BARHL2/BCL11A/CACNG7/CDH4/CDKL3/CPNE5/CPNE6/CPNE9/CYFIP1/DBN1/DCLK1/DRAXIN/DSCAM/EDN2/EDNRA/EMX1/FLRT1/FLRT3/GDI1/GSK3B/ISLR2/ITGB1/L1CAM/LAMB2/LHX2/MAP2/MT3/NDEL1/NEDD4L/NKX6-1/NLGN3/NRCAM/NRP2/NTN1/OLFM1/PLXNA1/PLXNA4/POU4F3/PRKN/PTPRS/SEMA3A/SEMA3E/SEMA4D/SEMA5A/SEMA5B/SEMA6A/SEMA6D/SH3GL2/SLC9A6/SLIT1/SLIT3/SPAG6/ST8SIA2/SYT1/SYT2/SYT3/SYT4/TMEM108/TNN/TNR/UNC13A/VEGFA/WASF1/WNT3A/WNT5A	67
cluster5	BP	GO:0098657	import into cell	89/3710	252/18903	3.32E-09	0.000000253	0.000000217	ABCC8/ABCC9/ACE2/ACSL1/AGT/AKT1/ATP1A2/ATP1A3/ATP1B1/CACNA1A/CACNA1B/CACNA1C/CACNA1S/CACNA2D1/CALCR/CLTRN/CNGA3/DRD1/DRD2/DRD4/FABP3/FOLR1/GDNF/GFAP/GPM6B/GRM6/HCN4/HFE/ITGB1/ITGB3/KCNH2/KCNJ13/KCNJ16/KCNJ18/KCNJ3/KCNJ4/KCNJ5/KCNJ6/KCNJ9/KCNK5/LRP2/NALF1/NALF2/PLPPR4/PRKN/RAB3B/RGS4/SCNN1G/SLC12A5/SLC16A2/SLC17A8/SLC1A1/SLC1A2/SLC1A6/SLC24A2/SLC29A4/SLC2A10/SLC30A1/SLC34A1/SLC38A3/SLC39A12/SLC39A5/SLC5A1/SLC6A1/SLC6A11/SLC6A2/SLC6A20/SLC6A3/SLC6A5/SLC7A3/SLC7A8/SLC8A2/SLC8A3/SLC9A2/SLC9A4/SLC9A5/SLC9A6/SLC9A7/SLC9C2/SNAP25/SPX/STEAP2/STRA6/SYNGR3/TRPM1/TRPM4/TRPV4/WNK2/WNK3	89
cluster5	BP	GO:0007626	locomotory behavior	74/3710	197/18903	3.33E-09	0.000000253	0.000000217	ADCY5/ADCY8/ALK/APBA1/APP/ASTN1/ATP1A2/AVP/B4GALT2/BSX/CALB1/CHL1/CHRNA3/CHRNB4/CRBN/CRH/DAB1/DBH/DMBX1/DMRT3/DRD1/DRD2/DRD4/DSCAM/EFNB3/ELAVL4/EN1/EPS8/FEZF2/FOXA2/GAD1/GDNF/GHSR/GLRA1/GLRB/GPR37/GPR52/GRIN2D/GRM5/GRM6/HOXD10/HPRT1/HTR2C/KCND2/KLHL1/LMX1A/MEIS1/NAV2/NKX2-1/NLGN2/NMS/NR4A2/OPRK1/PAK5/PDE1B/PENK/PRKN/RELN/SCN1A/SHANK2/SLC1A1/SLC6A3/SLITRK6/SLURP1/SNAP25/SOBP/SPTBN4/STRN/TH/TNR/TRH/UCHL1/USP2/ZIC1	74
cluster5	BP	GO:0072507	divalent inorganic cation homeostasis	116/3710	356/18903	3.78E-09	0.000000285	0.000000244	ABL1/ADCY8/ADORA1/AKAP6/ANK3/AP3D1/APP/ATF4/ATP13A5/ATP1A2/ATP1B1/ATP2B2/ATP2B3/ATP6V1B1/BCAP31/CACNA1C/CALB1/CALB2/CALCA/CALCB/CASQ2/CCDC47/CEMIP/CHRNA7/CIB2/CNNM1/CNNM2/CSRP3/DDIT3/DHRS7C/DMD/DRD1/DRD2/DRD4/EDN2/EDNRA/ELANE/ERO1A/F2/F2R/F2RL3/FGF2/FGF23/FKBP1A/FKBP1B/GCM2/GP1BB/GP9/GPR12/GRIA1/GRIK2/GRIN1/GRM5/GSTO1/HAP1/HCRTR1/HCRTR2/HERPUD1/HRC/HTR2A/HTR2C/IL13/ITGB3/JPH1/JPH3/JPH4/JSRP1/LACRT/LHCGR/LYN/MAIP1/MICU3/MT1A/MT1B/MT1HL1/MT1X/MT2A/MT3/NPSR1/NPTN/PACS2/PKHD1/PRKN/PTH/PTH1R/RYR2/RYR3/S100A14/SLC11A1/SLC1A1/SLC24A2/SLC24A3/SLC25A23/SLC30A1/SLC30A3/SLC30A8/SLC39A12/SLC39A5/SLC8A2/SLC8A3/STC1/STC2/SV2A/TFAP2B/THY1/TMBIM6/TNNI3/TRDN/TRIM24/TRPC3/TRPC4/TRPC7/TRPM8/TRPV4/WFS1/WNT5A	116

cluster5	BP	GO:0099601	regulation of neurotransmitter receptor activity	33/3710	62/18903	3.92E-09	0.000000294	0.000000252	APP/ARC/BEGAIN/CACNG2/CACNG3/CACNG4/CACNG5/CACNG7/CRH/DLGAP2/DLGAP3/GSG1L/HOMER1/MAPK8IP2/MINK1/NLGN1/NLGN2/NLGN3/NPTX1/NPTX2/NPTXR/PATE1/PATE4/PRRT1/PSCA/RASGRF1/RELN/SHANK1/SHISA6/SHISA7/SHISA9/SLURP2/SRC	33
cluster5	BP	GO:0051588	regulation of neurotransmitter transport	47/3710	105/18903	4.07E-09	0.000000302	0.000000259	ADCY1/ADORA2B/ADRA1A/ADRA2A/APBA1/ASIC1/ATP1A2/CAMK2A/CHRM2/CHRNA3/CHRNA6/CHRNB4/CPLX2/CPLX4/CSPG5/DRD1/DRD2/DRD4/GDNF/GFAP/GIPC1/GPM6B/GSK3B/HTR2A/ITGB1/ITGB3/NLGN1/PFN2/PRKCG/PRKN/RAB3B/RAB5A/RAP1B/RIMS3/RIMS4/SEPTIN5/SLC17A8/SLC30A1/SNCAIP/STX1A/STXBP1/SYN1/SYP/SYT1/SYT12/SYT4/WNT7A	47
cluster5	BP	GO:0099173	postsynapse organization	65/3710	166/18903	4.34E-09	0.000000321	0.000000275	ACTBL2/ACTN1/ADAM10/ARC/ARHGAP39/ARHGAP44/CAMK2B/CBLN1/CDH2/CDK5R1/CHRDL1/CHRNA7/CNTNAP1/CTNND2/DBN1/EPHA7/EPHB3/FRRS1L/GAP43/GDNF/GHSR/GLRB/GRID2/GRIN2B/HDAC6/HOMER1/IL1RAPL1/INA/INS/ITPKA/KIF1A/LHFPL4/LILRB2/LRP4/LRRC4B/LRRTM2/LZTS3/MUSK/NEFH/NGEF/NLGN1/NLGN2/NLGN3/NLGN4X/NPTX1/NRCAM/NRP2/NRXN1/NTNG2/OPHN1/PAK3/PPFIA2/PTPRD/RELN/SHANK1/SHANK2/SHISA6/SHISA7/SLITRK3/SRCIN1/TANC2/TMEM108/WNT5A/WNT7A/ZDHHC15	65
cluster5	BP	GO:0035270	endocrine system development	56/3710	135/18903	4.39E-09	0.000000322	0.000000276	AKT1/ARID5B/ASCL1/BMP2/BMP4/BMP5/BMP6/CDH2/CGA/CRH/CRHR1/DKK3/DLL1/DRD2/EDNRA/FGF10/FGF2/FOXA2/GCM2/GLI2/GSK3B/GSX1/HES1/HOXA3/HOXD3/ISL1/LHX3/MDK/MNX1/NEUROD1/NEUROG3/NKX2-1/NKX2-2/NKX6-1/NR0B1/OTP/PAX6/PBX1/PCSK1/PDGFRA/PITX1/POU3F2/RFX3/RFX6/SALL1/SHH/SIX3/SLC6A3/SMO/SOX3/SOX9/STRA6/WNT11/WNT4/WNT5A/WT1	56

cluster5	BP	GO:0003015	heart process	91/3710	261/18903	4.62E-09	0.000000337	0.000000289	ABCC9/ACE/ACE2/ACTC1/ADM5/ADORA1/ADRA1A/ADRA1B/ADRA1D/AGT/AGTR2/APELA/ATP1A2/ATP1A3/ATP1B1/ATP2B2/ATP2B3/BVES/CACNA1C/CACNA1G/CACNA2D1/CALCA/CASQ2/CCN2/CELF2/CHGA/CHRM2/CSRP3/DES/DMD/DRD2/DSG2/EDN2/EDNRA/EDNRB/EHD3/FKBP1B/FOXN4/GATA4/GJC1/GLP1R/GSTO1/HCN1/HCN3/HCN4/HRC/ISL1/KCND3/KCNE4/KCNH2/KCNH6/KCNJ3/KCNJ5/KCNN2/MYH6/MYH7/MYL7/NEDD4L/NOS3/PDE5A/PKP2/RGS4/RPS6KA2/RYR2/SCN10A/SCN1A/SCN2B/SCN3B/SGCZ/SHOX2/SLC1A1/SLC4A3/SLC8A2/SLC8A3/SMAD5/SPTBN4/SPX/SRC/STC1/TACR3/TBX5/TH/THRB/TNNI1/TNNI3/TNNT2/TPM1/TRDN/TRPM4/YAP1/ZC3H12A	91
cluster5	BP	GO:0035725	sodium ion transmembrane transport	70/3710	184/18903	4.72E-09	0.000000341	0.000000293	ANK3/ANO6/ASIC1/ASIC2/ASIC3/ASIC4/ATP1A2/ATP1A3/ATP1B1/CACNA1G/CHP2/CNKSR3/DMD/DRD4/FGF14/FXYD3/FXYD6/GRP/HCN1/HCN3/HCN4/NALCN/NEDD4L/PTPN3/SCN10A/SCN1A/SCN2A/SCN2B/SCN3B/SCN4A/SCN8A/SCN9A/SCNN1G/SHROOM2/SLC17A2/SLC17A4/SLC17A6/SLC17A7/SLC17A8/SLC24A2/SLC24A3/SLC28A3/SLC34A1/SLC34A2/SLC4A11/SLC4A4/SLC4A5/SLC5A1/SLC6A1/SLC6A11/SLC6A15/SLC6A17/SLC6A2/SLC6A20/SLC6A3/SLC6A5/SLC6A7/SLC8A2/SLC8A3/SLC9A2/SLC9A4/SLC9A5/SLC9A6/SLC9A7/SLC9B2/SLC9C2/STOM/TRPM4/WNK2/WNK3	70
cluster5	BP	GO:0060294	cilium movement involved in cell motility	59/3710	146/18903	5.57E-09	0.0000004	0.000000343	ADCY3/AKAP4/BBS1/C2CD6/CABS1/CATSPER4/CCDC40/CELF3/CEP131/CFAP206/CFAP221/CFAP43/CFAP46/CFAP47/CFAP52/CFAP54/CFAP65/CFAP69/CFAP97D1/DDX4/DNAAF6/DNAH11/DNAH5/DNAI1/DNAI3/DZIP1/ENKUR/ENO4/FSIP2/GK2/HOATZ/IFT81/LDHC/MNS1/NEURL1/NPHP4/ODAD3/PLA2G3/PLTP/PRDM14/PRM3/PRSS55/ROPN1/RSPH6A/RSPH9/SEPTIN4/SLC9B2/SMCP/SPAG6/SPEM1/TACR1/TACR3/TCTE1/TEKT1/TEKT2/TEKT5/TTLL6/TTLL9/UBE2B	59
cluster5	BP	GO:0021543	pallium development	67/3710	174/18903	5.66E-09	0.000000404	0.000000346	AKIRIN2/ALK/ARX/ASCL1/ASPM/ATOH1/ATP1A3/BBS1/BCAN/CDH2/CDK5R1/CDK5R2/CDON/CNTNAP2/DAB1/DCLK2/DLX1/DLX2/DMRTA2/DRD1/EGFR/EMX1/EMX2/EPHA5/EZH2/FAT4/FEZ1/FEZF2/FOXG1/GLI3/GSK3B/HIF1A/HSD3B2/ID4/IGF2BP1/KIRREL3/LAMB1/LHX2/LHX5/LMX1A/MBOAT7/MDK/MFSD2A/NDEL1/NEUROD1/NEUROD6/NKX2-1/NPY/NR2E1/NTRK2/PAX6/PLCB1/POU3F2/POU3F3/RELN/ROBO1/SMO/SRD5A2/SYNE2/TACC2/TACC3/TBR1/TH/TMEM108/WNT3A/ZIC1/ZIC3	67

cluster5	BP	GO:0051932	synaptic transmission, GABAergic	31/3710	57/18903	5.82E-09	0.000000412	0.000000354	ADORA1/ADRA1A/CNTNAP4/DRD2/EZH2/GABBR2/GABRA1/GABRA2/GABRA3/GABRA4/GABRA5/GABRA6/GABRB2/GABRB3/GABRE/GABRG1/GABRG2/GABRG3/HAP1/HAPLN4/NALCN/NLGN1/NLGN2/NPAS4/NPY5R/SLC6A1/SLITRK3/STXBP1/SYN3/TACR1/TPBG	31
cluster5	BP	GO:0055123	digestive system development	58/3710	143/18903	6.37E-09	0.000000448	0.000000385	AGR2/AHI1/ALX4/ASCL1/BARX1/BMP4/CCDC103/CCDC40/CCKBR/CDX2/CLDN18/CLMP/CPS1/EDNRB/EGFR/EPHB3/FGF10/FGF9/FGFR2/FOXF2/FOXL1/GATA4/GLI2/GLI3/HES1/HIF1A/HMGCS2/HNF1B/HOXD13/LGR4/MYOCD/NKX2-2/NKX2-6/NPY/PDGFC/PDGFRA/PKDCC/PTF1A/PYY/RARB/RBPMS2/RET/SALL1/SFRP2/SHH/SHOX2/SIX2/SMO/SOX10/SOX9/SPDEF/SRC/STRA6/TYMS/WNT11/WNT5A/YAP1/ZIC3	58
cluster5	BP	GO:0021675	nerve development	40/3710	84/18903	6.5E-09	0.000000454	0.00000039	BDNF/CNGB1/DAG1/DRGX/ECE1/EDNRA/ERBB3/GABRA5/GABRB2/GLI3/HES1/HOXA3/HOXB1/HOXD3/ILK/ISL1/NAV2/NEUROG1/NGF/NGFR/NKX2-2/NPTX1/NRP2/NTF3/PAX2/PHOX2B/PLXNA1/PLXNA4/POU4F3/PRKCG/RET/SALL1/SEMA3A/SERPINE2/SIX4/SLITRK6/SULF1/SULF2/TFAP2A/TMEM126A	40
cluster5	BP	GO:0035418	protein localization to synapse	35/3710	69/18903	7.1E-09	0.000000492	0.000000422	ADAM10/ARHGAP44/ASIC2/BSN/CACNG2/CACNG3/CACNG7/DAG1/GHSR/GPC6/GRIN2A/GRIP1/GRIP2/GRIPAP1/HOMER1/IQSEC2/KIF5A/KIF5C/LGI1/MAP1A/NLGN1/NLGN2/NPHS1/NPTX1/NRXN1/PCLO/RAB27B/RAB8A/RELN/SHANK1/SLITRK3/SNAP25/WNT5A/WNT7A/ZDHHC15	35

cluster5	BP	GO:0021871	forebrain regionalization	18/3710	24/18903	7.13E-09	0.000000492	0.000000422	BMP2/BMP4/DMRTA2/EMX1/EMX2/FEZF1/FEZF2/GLI3/GSX2/LHX1/LHX2/NKX2-1/PAX6/PGAP1/SHH/SIX3/WNT2B/WNT7B	18
cluster5	BP	GO:0060485	mesenchyme development	104/3710	313/18903	7.52E-09	0.000000515	0.000000442	ACTA1/ACTC1/ALX1/AMELX/BASP1/BMP2/BMP4/BMP5/BMP7/CDH2/CRB2/CUL7/CYP26C1/DAB2/DACT3/DAG1/DLG5/DLL3/EDNRA/EDNRB/EMP2/EPHA3/ERBB3/ERBB4/EZH2/FAM83D/FGF10/FGF19/FGF9/FGFR1/FGFR2/FOLR1/FOXA1/FOXA2/FOXC1/FOXF2/FRZB/GATA4/GBX2/GDNF/GREM1/GSK3B/HAS2/HDAC2/HES1/HEYL/HIF1A/HMGA2/HPN/IL17RD/ISL1/KITLG/LAMA5/LRP6/MDK/MSX2/NKX2-1/NOS3/NOTCH4/NRP2/OLFM1/PAX2/PDGFRB/PDPN/PHOX2B/PTK7/RADIL/RBM24/RET/ROBO1/ROBO2/ROCK1/ROCK2/SEMA3A/SEMA3E/SEMA4D/SEMA5A/SEMA5B/SEMA6A/SEMA6D/SERPINB3/SFRP2/SHH/SIX2/SIX4/SMO/SOX10/SOX9/SPRY1/TAF10/TBX20/TBX5/TEAD2/TGFB1I1/TSPY2/VASN/WNT11/WNT3A/WNT4/WNT5A/WNT8A/WT1/YAP1/ZIC3	104
cluster5	BP	GO:0009581	detection of external stimulus	56/3710	137/18903	8.29E-09	0.000000564	0.000000484	ABCA4/ADGRV1/ADORA1/AIPL1/ANO3/ASIC2/ASIC3/CALCA/CDH2/CDS1/CNGB1/COL11A1/CSRP3/DRGX/ELOVL4/GJA10/GNAT1/GNGT1/GPR52/GRIK2/GRK4/GRM6/GUCY2F/HPN/HTR2A/ITGA2/LHFPL5/LOC118142757/MMP24/NGFR/NR2E3/OPN1LW/OPN1MW2/OPN1MW3/OPN4/OTOP1/PDC/PDZD7/PKD1L2/PKD1L3/PKDREJ/PRDM12/REEP6/RGR/RHO/RP1/RPE65/SCN1A/SEMA5B/SERPINE2/TMC1/TRPC3/TRPM3/TRPM8/TULP1/WHRN	56
cluster5	BP	GO:0070588	calcium ion transmembrane transport	112/3710	345/18903	8.8E-09	0.000000595	0.000000511	ABL1/ADCYAP1R1/ADRA1A/ADRA2A/AKAP6/ANO6/ASIC1/ATP1A2/ATP1B1/ATP2B2/ATP2B3/CABP1/CACNA1A/CACNA1B/CACNA1C/CACNA1G/CACNA1S/CACNA2D1/CACNB1/CACNG2/CACNG3/CACNG4/CACNG5/CACNG7/CALCR/CASQ2/CATSPER4/CBARP/CCN2/CEMIP/CHRNA7/CRHR1/DDIT3/DHRS7C/DMD/DRD1/DRD2/DRD4/EDNRA/EDNRB/EHD3/EPO/ERO1A/F2/F2R/F2RL3/FGF2/FKBP1A/FKBP1B/GAS6/GEM/GP1BB/GP9/GPM6A/GRIN1/GRIN2A/GRIN2B/GRIN2D/GRM6/GSTO1/HAP1/HPCA/HRC/HSPA2/HTR2A/HTR2C/IL13/ITGB3/JPH1/JPH3/JPH4/JSRP1/LACRT/LHCGR/LYN/MAIP1/MICU3/NALCN/NALF1/NALF2/NPSR1/P2RX2/P2RX3/PKD1L2/PKD1L3/PKDREJ/PLP1/REM1/RYR2/RYR3/SEC61A1/SLC24A2/SLC24A3/SLC25A23/SLC8A2/SLC8A3/SLN/STAC2/THY1/TMBIM6/TMC1/TMEM37/TRDN/TRPC3/TRPC4/TRPC7/TRPM1/TRPM3/TRPM4/TRPM8/TRPV4/WNT3A	112

cluster5	BP	GO:0051216	cartilage development	74/3710	201/18903	9.14E-09	0.000000614	0.000000527	ADAMTS7/AMELX/BARX2/BBS1/BMP2/BMP4/BMP5/BMP6/BMP7/BMPR1B/CCN2/CHADL/CHRDL2/COL11A1/COL27A1/COL2A1/DLX2/ECM1/EPYC/EVC/FGF2/FGF4/FGF9/FGFR3/FRZB/GDF5/GDF6/GHR/GLI3/GPLD1/GREM1/HES5/HIF1A/HMGA2/HOXA11/HOXA3/HOXC4/HOXD3/ITGB8/MATN3/MDK/MMP13/MSX2/NPPC/OGN/PITX1/PKDCC/PTH/PTH1R/RARB/RFLNA/RSPO2/SFRP2/SHOX2/SIX2/SMAD5/SOX5/SOX9/STC1/SULF1/SULF2/TIMP1/TRPV4/TYMS/UNCX/WNT11/WNT2B/WNT5A/WNT7A/WNT7B/WNT9A/ZBTB16/ZNF219/ZNF664-RFLNA	74
cluster5	BP	GO:2000179	positive regulation of neural precursor cell proliferation	30/3710	55/18903	9.33E-09	0.000000623	0.000000535	ASCL1/ASPM/CDON/DCT/DISP3/DMRTA2/DRD2/EGF/FGF2/FOXG1/FZD3/GLI3/HIF1A/ID4/ITGB1/LHX1/LHX2/LHX5/LRP2/LYN/MDK/NR2E1/OTP/PAX6/SHH/SMO/SOX10/TOX/VEGFA/WNT3A	30
cluster5	BP	GO:0010001	glial cell differentiation	81/3710	227/18903	9.75E-09	0.000000647	0.000000555	ABL1/AKT1/APP/ASCL1/ASPA/BMP2/CDH2/CDK1/CNTNAP1/CSPG5/DAB1/DAG1/DLL1/DLX1/DLX2/DNER/DRD1/DUSP15/EIF2B2/EMX1/ERBB2/ERBB3/F2/FGF10/FGF2/FGF5/GAP43/GFAP/GLI3/GSX2/HDAC2/HES1/HES5/ID4/ILK/KLF15/LAMB2/LYN/MDK/MMP24/MT3/MYOC/MYRF/NKX2-1/NKX2-2/NKX6-1/NR2E1/NTRK2/OPALIN/PARD3/PAX2/PAX6/PHGDH/PHOX2B/PLP1/PLPP3/POU3F2/PTN/PTPRZ1/RELN/ROR1/ROR2/SERPINE2/SHH/SIRT2/SLC8A3/SMO/SOX1/SOX10/SOX9/TENM4/TMEM98/TNFRSF21/TP73/TPPP/TRPC4/TSPAN2/TTBK1/VAX1/WASF3/ZNF488	81
cluster5	BP	GO:0050954	sensory perception of mechanical stimulus	68/3710	180/18903	1.09E-08	0.000000716	0.000000615	ADGRV1/ALDH7A1/ASIC2/ASIC3/ATP2B2/ATP6V0A4/ATP6V1B1/BARHL1/BIRC5/CEACAM16/CEMIP/CLRN1/CLRN2/CLRN3/CNTN5/COL11A1/COL2A1/DDIT3/DRGX/EPYC/EYA4/GABRA5/GABRB2/GRM7/GRXCR1/HPN/HTR2A/ITGA2/KCNQ4/LHFPL3/LHFPL4/LHFPL5/LRP2/MARVELD2/MYO3A/MYO3B/MYO6/NAV2/OTOF/OTOGL/OTOS/P2RX2/PAX3/PCDH15/PDZD7/PGAP1/POU3F4/POU4F3/ROR1/SCN1A/SERPINE2/SLC17A8/SLITRK6/SOBP/SPTBN4/SRRM4/TFAP2A/TH/THRB/TIMM8B/TIMM9/TMC1/TUB/USH1C/USH2A/WFS1/WHRN/ZNF354A	68

cluster5	BP	GO:0007605	sensory perception of sound	62/3710	159/18903	1.18E-08	0.000000774	0.000000664	ADGRV1/ALDH7A1/ASIC2/ATP2B2/ATP6V0A4/ATP6V1B1/BARHL1/BIRC5/CEACAM16/CEMIP/CLRN1/CLRN2/CLRN3/CNTN5/COL11A1/COL2A1/DDIT3/EPYC/EYA4/GABRA5/GABRB2/GRM7/GRXCR1/HPN/KCNQ4/LHFPL3/LHFPL4/LHFPL5/LRP2/MARVELD2/MYO3A/MYO3B/MYO6/NAV2/OTOF/OTOGL/OTOS/P2RX2/PAX3/PCDH15/PDZD7/PGAP1/POU3F4/POU4F3/ROR1/SLC17A8/SLITRK6/SOBP/SPTBN4/SRRM4/TFAP2A/TH/THRB/TIMM8B/TIMM9/TMC1/TUB/USH1C/USH2A/WFS1/WHRN/ZNF354A	62
cluster5	BP	GO:0010959	regulation of metal ion transport	131/3710	423/18903	1.33E-08	0.000000866	0.000000743	ABCC8/ABCC9/ABL1/ACE/ACTN2/ADCYAP1R1/ADORA1/ADRA2A/AGT/AKAP6/AKT1/ANK3/ANO6/ATF4/ATP1A2/ATP1B1/CABP1/CACNA1C/CACNA2D1/CACNB1/CALCR/CAMK2A/CAMK2B/CASQ2/CBARP/CD84/CEMIP/CHP2/CNKSR3/CRBN/CRHR1/DHRS7C/DMD/DPP10/DPP6/DRD1/DRD2/DRD4/EGF/EHD3/EPO/F2/F2R/F2RL3/FGF14/FHL1/FKBP1A/FKBP1B/FXYD3/FXYD6/GCG/GCK/GEM/GRAMD2A/GRIN1/GRM6/GRP/GSTO1/HAP1/HES1/HFE/HOMER1/HPCA/HRC/HSPA2/HTR2A/IL13/ITGB1/ITGB3/JPH1/JPH3/JPH4/JSRP1/KCNC1/KCNC2/KCNE4/KCNH2/KCNIP1/KCNIP3/KCNK3/KCNN2/KCNRG/KCNS2/LACRT/LHCGR/LILRB2/LRRC55/LYN/MCHR1/MYLK/NEDD4L/NKAIN1/NKAIN4/NOS3/NPSR1/OPRK1/P2RX2/P2RX3/PACSIN3/PDGFRB/PER1/PKP2/PLP1/PLPP4/PRSS8/PTGS2/PTPN3/REM1/RGS4/RGS7/RYR2/SCN2B/SCN3B/SERPINE2/SIK1/SLC30A1/SLN/SPINK1/SPTBN4/STAC2/STC1/STC2/STOM/THY1/TMBIM6/TMC1/TRDN/TRPC3/WFS1/WNK2/WNK3	131
cluster5	BP	GO:0048588	developmental cell growth	81/3710	229/18903	1.53E-08	0.000000993	0.000000852	ABL1/ADRA1A/AGT/AKAP6/APP/AUTS2/BARHL2/BCL11A/BDNF/CACNG7/CDH4/CDKL3/CPNE5/CPNE6/CPNE9/CRABP2/CYFIP1/DBN1/DCLK1/DRAXIN/DSCAM/EDN2/EDNRA/EFNA5/EMX1/EPHA7/FLRT1/FLRT3/GATA4/GDI1/GSK3B/HDAC6/ISLR2/ITGB1/L1CAM/LAMB2/LHX2/MAP2/MT3/NDEL1/NEDD4L/NGF/NKX6-1/NLGN3/NRCAM/NRP2/NTN1/OLFM1/PLXNA1/PLXNA4/POU4F3/PRKN/PTPRS/RGS4/RND2/SEMA3A/SEMA3E/SEMA4D/SEMA5A/SEMA5B/SEMA6A/SEMA6D/SH3GL2/SLC9A6/SLIT1/SLIT3/SOX9/SPAG6/ST8SIA2/SYT1/SYT2/SYT3/SYT4/TMEM108/TNN/TNR/UNC13A/VEGFA/WASF1/WNT3A/WNT5A	81
cluster5	BP	GO:0048736	appendage development	67/3710	178/18903	1.64E-08	0.00000105	0.000000902	ALX4/ATRX/BMP4/BMP7/CACNA1C/CIBAR1/COL2A1/CRABP2/DLX5/DLX6/ECE1/EN1/FGF10/FGF4/FGF9/FGFR2/FOXN1/FREM2/GDF5/GLI3/GPC3/GREM1/GRHL2/HDAC2/HOXA11/HOXC10/HOXC13/HOXD10/HOXD12/HOXD13/IFT140/KRT84/LRP4/LRP5/MSX2/NOTCH2/PBX1/PITX1/PKDCC/PLXNA2/PTCH1/RARB/RAX/RSPO2/RSPO4/SALL1/SALL4/SFRP2/SHH/SHOX2/SOX9/SP8/SP9/TAF10/TBC1D32/TBX4/TBX5/TFAP2A/TFAP2B/TMEM231/TULP3/WNT5A/WNT7A/ZBTB16/ZIC3/ZNF219/ZNRF3	67

cluster5	BP	GO:0060173	limb development	67/3710	178/18903	1.64E-08	0.00000105	0.000000902	ALX4/ATRX/BMP4/BMP7/CACNA1C/CIBAR1/COL2A1/CRABP2/DLX5/DLX6/ECE1/EN1/FGF10/FGF4/FGF9/FGFR2/FOXN1/FREM2/GDF5/GLI3/GPC3/GREM1/GRHL2/HDAC2/HOXA11/HOXC10/HOXC13/HOXD10/HOXD12/HOXD13/IFT140/KRT84/LRP4/LRP5/MSX2/NOTCH2/PBX1/PITX1/PKDCC/PLXNA2/PTCH1/RARB/RAX/RSPO2/RSPO4/SALL1/SALL4/SFRP2/SHH/SHOX2/SOX9/SP8/SP9/TAF10/TBC1D32/TBX4/TBX5/TFAP2A/TFAP2B/TMEM231/TULP3/WNT5A/WNT7A/ZBTB16/ZIC3/ZNF219/ZNRF3	67
cluster5	BP	GO:0035082	axoneme assembly	42/3710	93/18903	1.95E-08	0.00000124	0.00000106	CC2D2A/CCDC103/CCDC40/CEP131/CFAP206/CFAP43/CFAP46/CFAP47/CFAP65/CFAP69/CFAP74/CFAP91/CFAP97D1/DAW1/DCX/DNAAF6/DNAAF8/DNAH2/DNAH5/DNAH7/DNAI1/DNAI3/DRC1/FOXJ1/FSIP2/HOATZ/HYDIN/MNS1/NEURL1/ODAD2/ODAD3/PLA2G3/RP1/RSPH1/RSPH6A/RSPH9/SPAG17/SPAG6/SPEF1/TEKT2/TOGARAM1/UBE2B	42
cluster5	BP	GO:2000311	regulation of AMPA receptor activity	18/3710	25/18903	2.08E-08	0.00000131	0.00000113	ARC/CACNG2/CACNG3/CACNG4/CACNG5/CACNG7/GSG1L/MAPK8IP2/MINK1/NLGN1/NLGN2/NLGN3/PRRT1/RELN/SHANK1/SHISA6/SHISA7/SHISA9	18
cluster5	BP	GO:2000177	regulation of neural precursor cell proliferation	41/3710	90/18903	2.11E-08	0.00000132	0.00000114	ASCL1/ASPM/CDH2/CDON/DCT/DISP3/DMRTA2/DRD2/EGF/EMX1/FGF2/FOXG1/FZD3/GLI3/HIF1A/ID4/ILK/ITGB1/LHX1/LHX2/LHX5/LRP2/LYN/MDK/NR2E1/OTP/PAX6/PTN/PTPRZ1/SHH/SIRT2/SIX3/SLC16A2/SMO/SOX10/TAFA3/TOX/VAX1/VEGFA/WNT3A/WNT5A	41

cluster5	BP	GO:0006939	smooth muscle contraction	48/3710	113/18903	2.22E-08	0.00000138	0.00000119	ADORA1/ADORA2B/ADRA1A/ADRA2A/AGT/ATP1A2/CALCA/CHRM2/CHRM3/CHRNA3/CHRNB4/CNN1/DOCK4/DRD1/DRD2/EDN2/EDNRA/EDNRB/F2R/FKBP1B/GDNF/GHSR/GRIP2/GUCY1A1/HTR1D/HTR2A/ITGA2/KCNB2/KCNMA1/MYLK/MYOCD/NEUROG1/NMU/P2RX2/P2RX3/PRKG1/PTGS2/ROCK1/ROCK2/SMTN/SPX/SSTR2/SULF1/SULF2/TACR1/TACR3/TBXA2R/TNNI3	48
cluster5	BP	GO:0003012	muscle system process	138/3710	455/18903	2.33E-08	0.00000144	0.00000124	ABCC8/ABCC9/ACE2/ACTA1/ADORA1/ADORA2B/ADRA1A/ADRA1B/ADRA2A/AGT/AKAP6/ARG2/ATP1A2/ATP1B1/CACNA1C/CACNA1G/CACNA1S/CACNA2D1/CACNB1/CALCA/CAMK2B/CASQ2/CCN2/CHGA/CHRM2/CHRM3/CHRNA3/CHRNB4/CHRNG/CNN1/CRYAB/CSRP3/DAG1/DES/DMD/DOCK4/DRD1/DRD2/DSG2/DTNA/EDN2/EDNRA/EDNRB/EHD3/EZH2/F2R/FKBP1B/GATA4/GDNF/GHSR/GJC1/GLRA1/GNAO1/GRIP2/GSTO1/GUCY1A1/HCN4/HDAC2/HOMER1/HRC/HSBP1/HTR1D/HTR2A/ITGA2/JSRP1/KCNB2/KCND3/KCNE4/KCNH2/KCNJ3/KCNJ5/KCNMA1/KCNN2/KLF15/LMNA/LMOD2/MLIP/MYH1/MYH13/MYH2/MYH4/MYH6/MYH7/MYH8/MYL1/MYLK/MYOC/MYOCD/MYOG/MYOZ1/MYOZ2/NEDD4L/NEUROG1/NMU/NOS3/P2RX2/P2RX3/PDE5A/PKP2/PRKG1/PTGS2/PVALEF/RGS4/ROCK1/ROCK2/RYR2/SCN10A/SCN1A/SCN2B/SCN3B/SCN4A/SGCA/SLC8A3/SLN/SMAD5/SMPX/SMTN/SPX/SSPN/SSTR2/STAC2/STC1/SULF1/SULF2/TACR1/TACR3/TBX20/TBXA2R/TNNI1/TNNI3/TNNT2/TPM1/TRDN/TRIM63/TRPC3/TRPM4/TRPV4/ZC3H12A	138
cluster5	BP	GO:0072503	cellular divalent inorganic cation homeostasis	105/3710	323/18903	2.35E-08	0.00000145	0.00000124	ABL1/ADCY8/ADORA1/AKAP6/AP3D1/APP/ATF4/ATP13A5/ATP1A2/ATP1B1/ATP2B2/ATP2B3/BCAP31/CACNA1C/CALB1/CALB2/CALCA/CALCB/CASQ2/CCDC47/CEMIP/CHRNA7/CSRP3/DDIT3/DHRS7C/DMD/DRD1/DRD2/DRD4/EDN2/EDNRA/ELANE/ERO1A/F2/F2R/F2RL3/FGF2/FKBP1A/FKBP1B/GCM2/GP1BB/GP9/GPR12/GRIA1/GRIK2/GRM5/GSTO1/HAP1/HCRTR1/HCRTR2/HERPUD1/HRC/HTR2A/HTR2C/IL13/ITGB3/JPH1/JPH3/JPH4/JSRP1/LACRT/LHCGR/LYN/MAIP1/MICU3/MT1A/MT1B/MT1HL1/MT1X/MT2A/MT3/NPSR1/NPTN/PACS2/PKHD1/PTH/PTH1R/RYR2/RYR3/SLC11A1/SLC1A1/SLC24A2/SLC24A3/SLC25A23/SLC30A1/SLC30A3/SLC30A8/SLC39A12/SLC39A5/SLC8A2/SLC8A3/STC1/STC2/SV2A/THY1/TMBIM6/TNNI3/TRDN/TRPC3/TRPC4/TRPC7/TRPM8/TRPV4/WFS1/WNT5A	105
cluster5	BP	GO:0007157	heterophilic cell-cell adhesion via plasma membrane cell adhesion molecules	28/3710	51/18903	0.000000024	0.00000146	0.00000125	CADM1/CADM3/CBLN1/CD164/CDH2/CDH4/CEACAM19/CEACAM5/CRB2/FAT4/GRID2/ICAM1/IGSF21/IL1RAPL1/LGALS7B/NECTIN1/NECTIN3/NECTIN4/NLGN1/PSG11/PSG2/PSG5/PTPRD/REG3A/SELP/TENM2/TENM3/TENM4	28

cluster5	BP	GO:0060688	regulation of morphogenesis of a branching structure	28/3710	51/18903	0.000000024	0.00000146	0.00000125	ABL1/AGT/AGTR2/BCL11A/BMP4/BMP7/FGF10/FGF7/FGFR2/GDNF/GREM1/HOXD13/LGR4/LHX1/MAGED1/MDK/PAX2/PDGFA/SHH/SHOX2/SIX2/SIX4/SMO/SOX9/SULF1/VEGFA/WNT2B/WNT5A	28
cluster5	BP	GO:0060562	epithelial tube morphogenesis	108/3710	335/18903	2.44E-08	0.00000148	0.00000127	ABL1/AGT/AGTR2/AHI1/BCL10/BMP2/BMP4/BMP5/BMP7/CC2D2A/CCDC103/CCDC40/CECR2/CELSR1/CITED1/COL4A1/DAG1/DLG5/DLL1/EDNRA/EFNB2/EGF/EPHA7/FGF10/FGF2/FGFR2/FOLR1/FOXA1/FOXN4/FZD3/GATA4/GBX2/GDNF/GLI2/GLI3/GPC3/GREB1L/GREM1/GRHL2/GZF1/HES1/HES5/HHIP/HIF1A/HNF1B/HOXA11/HOXD11/HS3ST3A1/ILK/IRX2/KIF20B/KIF26B/LAMA1/LAMA5/LBX1/LGR4/LGR5/LHX1/LHX2/LRP2/LRP5/MAGED1/MDK/MSX2/NDRG4/NFATC4/NKX2-1/NOTCH2/NOTCH4/NTN1/PAX2/PBX1/PKHD1/PRICKLE1/PTCH1/PTK7/RET/RSPO2/RYR2/SALL1/SALL4/SDC4/SEMA3E/SFRP2/SHH/SIX2/SIX4/SMO/SOSTDC1/SOX18/SOX9/SPRY1/SRC/TBX20/TEAD2/TMEM59L/TULP3/VANGL2/VEGFA/WNT11/WNT2B/WNT3A/WNT4/WNT5A/WNT9B/WT1/YAP1/ZIC3	108
cluster5	BP	GO:0043270	positive regulation of ion transport	96/3710	289/18903	2.85E-08	0.00000172	0.00000147	ABCB11/ABCC8/ABL1/ACE2/ACTN2/ADCYAP1R1/ADORA1/ADRA2A/AGT/AKAP6/AKT1/ANK3/ANO6/ARC/ATF4/ATP1B1/AVP/AVPR1B/CACNA2D1/CACNG2/CACNG3/CACNG4/CALCR/CAMK2A/CEMIP/CFTR/CHP2/CHRM1/CLTRN/CNKSR3/DMD/DRD1/DRD2/DRD4/EDNRA/EHD3/F2/F2R/F2RL3/FHL1/GCG/GDNF/GRIN1/GRM6/GSTO1/HAP1/HOMER1/HSPA2/IL13/ITGB1/KCNB1/KCNC1/KCNC2/KCNH2/KCNK3/LACRT/LHCGR/LRRC55/MCHR1/MYLK/NLGN3/NPSR1/OPRK1/P2RX2/P2RX3/PDGFRB/PIRT/PKP2/PLA2G3/PLA2R1/PLP1/PRSS8/RAB3B/RELN/RGS7/RYR2/SCN3B/SLC17A8/SLC34A1/SLC38A3/SLC6A1/STAC2/STC1/STX1A/STXBP1/SYT1/SYT4/TCAF1/THY1/TNFRSF11A/TRDN/TRH/TRPC3/WFS1/WNK2/WNK3	96
cluster5	BP	GO:0017156	calcium-ion regulated exocytosis	33/3710	66/18903	0.00000003	0.0000018	0.00000154	ADRA2A/CACNA1G/CADPS/CBARP/CDK5R2/CLTRN/DOC2A/DOC2B/EQTN/KCNB1/MYH9/RAP1B/RIMS3/RPH3AL/SCAMP5/STX1A/STXBP1/SYN2/SYT1/SYT10/SYT12/SYT13/SYT2/SYT3/SYT4/SYT5/SYT6/SYT7/SYT8/SYT9/UNC13A/UNC13C/ZP4	33

cluster5	BP	GO:0008306	associative learning	39/3710	85/18903	3.63E-08	0.00000216	0.00000186	ABCC8/ABL1/ADAM2/ADCY3/AGT/APP/ASIC1/ATP1A2/B4GALT2/BRSK1/DBH/DRD1/DRD2/EIF4A3/ELAVL4/FOS/GABRA5/GRIN1/GRIN2A/HIF1A/ITGB1/MAP1A/NDRG4/NEUROD2/NPTN/NPTX2/OPRK1/PDE1B/RELN/SHANK1/SHANK2/SLC1A1/SLC6A1/SNAP25/TACR1/TBR1/TNR/TPBG/TTC36	39
cluster5	BP	GO:0061351	neural precursor cell proliferation	58/3710	149/18903	3.71E-08	0.0000022	0.00000189	ARTN/ARX/ASCL1/ASPM/BBS1/CDH2/CDON/DAGLA/DBN1/DCT/DISP3/DMRTA2/DRD2/EGF/EML1/EMX1/EMX2/FGF2/FGFR2/FOXG1/FZD3/GBX2/GLI3/HHIP/HIF1A/ID4/IGF2BP1/ILK/ITGB1/LHX1/LHX2/LHX5/LRP2/LYN/MDK/NR2E1/NUMBL/OTP/PAX6/POU3F2/POU3F3/PTN/PTPRZ1/SHH/SIRT2/SIX3/SLC16A2/SLC39A5/SMO/SOX10/SOX5/TAFA3/TOX/VAX1/VEGFA/WNT3A/WNT5A/WNT7A	58
cluster5	BP	GO:0030326	embryonic limb morphogenesis	49/3710	118/18903	3.82E-08	0.00000224	0.00000192	ALX4/BMP4/BMP7/CACNA1C/CRABP2/DLX5/DLX6/ECE1/EN1/FGF4/FGF9/FREM2/GDF5/GLI3/GPC3/GREM1/GRHL2/HDAC2/HOXA11/HOXC10/HOXD10/HOXD12/HOXD13/IFT140/LRP4/LRP5/MSX2/NOTCH2/PBX1/PITX1/PTCH1/RARB/RSPO2/SALL1/SALL4/SFRP2/SHH/SHOX2/SP8/SP9/TBC1D32/TBX4/TBX5/TFAP2A/TMEM231/TULP3/WNT5A/WNT7A/ZBTB16	49
cluster5	BP	GO:0035113	embryonic appendage morphogenesis	49/3710	118/18903	3.82E-08	0.00000224	0.00000192	ALX4/BMP4/BMP7/CACNA1C/CRABP2/DLX5/DLX6/ECE1/EN1/FGF4/FGF9/FREM2/GDF5/GLI3/GPC3/GREM1/GRHL2/HDAC2/HOXA11/HOXC10/HOXD10/HOXD12/HOXD13/IFT140/LRP4/LRP5/MSX2/NOTCH2/PBX1/PITX1/PTCH1/RARB/RSPO2/SALL1/SALL4/SFRP2/SHH/SHOX2/SP8/SP9/TBC1D32/TBX4/TBX5/TFAP2A/TMEM231/TULP3/WNT5A/WNT7A/ZBTB16	49

cluster5	BP	GO:0099054	presynapse assembly	27/3710	49/18903	3.85E-08	0.00000224	0.00000192	APP/BSN/CBLN1/CBLN2/CNTN5/EFNB2/GPC6/IL1RAPL1/IL1RAPL2/LRFN3/LRP4/LRRC4B/LRRTM3/NLGN1/NLGN2/NLGN3/NLGN4X/NTNG2/PCLO/PTPRD/SLITRK1/SLITRK2/SLITRK3/SLITRK6/WNT3A/WNT5A/WNT7A	27
cluster5	BP	GO:0060541	respiratory system development	75/3710	211/18903	4.08E-08	0.00000236	0.00000203	ABCA12/ACE/ADAMTS2/ADAMTSL2/AGR2/ASCL1/BASP1/BMP4/CCBE1/CCDC40/CCN2/CELSR1/CLCN2/CRH/CRISPLD2/DAG1/DLG5/DLX5/EDN2/FGF10/FGF2/FGF7/FGF9/FGFR2/FLT4/FOXA1/FOXJ1/GLI2/GLI3/GPC3/GRHL2/HES1/HHIP/HMGCS2/HS6ST1/HYDIN/IL13/JMJD6/LAMA1/LAMA5/LHX3/LOX/MYOCD/NKX2-1/NKX2-8/NOS3/PDGFA/PDGFRA/PDGFRB/PDPN/PHOX2B/PKDCC/PTK7/RSPO2/SHH/SIX4/SOX9/SPDEF/SPRY1/STK40/STRA6/TBX4/TBX5/THRB/TP73/TULP3/VEGFA/WNT11/WNT2B/WNT5A/WNT7B/WT1/YAP1/YWHAZ/ZIC3	75
cluster5	BP	GO:0019226	transmission of nerve impulse	36/3710	76/18903	4.35E-08	0.00000251	0.00000215	AGT/ANK3/AVP/CACNA1G/CACNG2/CACNG3/CACNG4/CACNG5/CACNG7/CHRNB4/CLDN19/CNTNAP1/CNTNAP2/DMRT3/DRD1/FKBP1B/GHSR/GJD2/GLRA1/GRIK2/ITGA2/KCND2/KCNMB2/NFASC/NRCAM/NTRK2/P2RX3/RGS21/SCN10A/SCN1A/SCN2A/SCN4A/SCN8A/SCN9A/SPTBN4/TNR	36
cluster5	BP	GO:0017158	regulation of calcium ion-dependent exocytosis	24/3710	41/18903	4.52E-08	0.00000259	0.00000222	ADRA2A/CACNA1G/CBARP/CDK5R2/DOC2A/DOC2B/KCNB1/RAP1B/RPH3AL/SCAMP5/STX1A/STXBP1/SYT1/SYT10/SYT12/SYT13/SYT2/SYT3/SYT4/SYT5/SYT6/SYT7/SYT8/SYT9	24

cluster5	BP	GO:0045665	negative regulation of neuron differentiation	35/3710	73/18903	4.55E-08	0.00000259	0.00000222	APP/ASCL1/ASPM/BMP7/CNTN4/DISP3/DLL1/DLX1/DLX2/DTX1/FEZF2/FOXG1/GLI3/GSK3B/HES1/HES5/ID4/ISL1/ITGB1/LBX1/LMX1A/MEIS1/NKX2-2/NR2E1/PAX6/PBX1/PHOX2B/SHH/SIX3/SOX3/SOX9/TLX3/TP73/WNT3A/ZNF536	35
cluster5	BP	GO:0010469	regulation of signaling receptor activity	65/3710	175/18903	4.84E-08	0.00000274	0.00000235	ACE2/ADH7/ADORA1/ADRA2A/AGTR2/APP/ARC/BEGAIN/BTC/CACNG2/CACNG3/CACNG4/CACNG5/CACNG7/CALCR/CBLB/CBLC/CGA/CRH/DKK3/DKKL1/DLGAP2/DLGAP3/EGF/GPRC5A/GREM1/GREM2/GSG1L/HDAC2/HDAC6/HFE/HIF1A/HOMER1/ITGB1/LY6G6D/LY6H/LYPD1/LYPD6/MAPK8IP2/MINK1/MTRNR2L2/NCK2/NEURL1/NLGN1/NLGN2/NLGN3/NPTX1/NPTX2/NPTXR/PATE1/PATE4/PRKCD/PRRT1/PSCA/RASGRF1/RELN/SERPINE1/SHANK1/SHISA6/SHISA7/SHISA9/SLURP2/SRC/TAFA4/WFIKKN2	65
cluster5	BP	GO:0030324	lung development	68/3710	187/18903	6.29E-08	0.00000354	0.00000304	ABCA12/ACE/ADAMTS2/ADAMTSL2/AGR2/ASCL1/BMP4/CCBE1/CCDC40/CCN2/CELSR1/CLCN2/CRH/CRISPLD2/DAG1/DLG5/EDN2/FGF10/FGF2/FGF7/FGF9/FGFR2/FLT4/FOXA1/FOXJ1/GLI2/GLI3/GPC3/GRHL2/HES1/HHIP/HMGCS2/HS6ST1/IL13/JMJD6/LAMA1/LAMA5/LHX3/LOX/MYOCD/NKX2-1/NKX2-8/NOS3/PDGFA/PDGFRA/PDGFRB/PDPN/PKDCC/PTK7/RSPO2/SHH/SOX9/SPDEF/SPRY1/STK40/STRA6/TBX4/TBX5/THRB/TP73/VEGFA/WNT11/WNT2B/WNT5A/WNT7B/YAP1/YWHAZ/ZIC3	68
cluster5	BP	GO:0003018	vascular process in circulatory system	90/3710	270/18903	6.37E-08	0.00000357	0.00000306	ABCC3/ABCC8/ABCC9/ABCG2/ABL1/ACE/ACE2/ADORA1/ADORA2B/ADRA1A/ADRA1B/ADRA1D/ADRA2A/ADRB3/AGT/AGTR2/ANGPT1/ASIC2/ATP1A2/AVP/AVPR1B/AZU1/BMP6/C2CD4A/C2CD4B/CALCA/CHRM3/CLDN5/CPS1/CRP/DBH/DDAH1/DOCK4/DRD1/ECE1/EDN2/EDNRA/EDNRB/F2R/FGFBP3/FOXC1/GRIP2/GUCY1A1/HTR1D/HTR2A/INS/KCNMA1/KCNMB2/KNG1/LEPR/LRP2/MFSD2A/NOS3/NPPC/NTS/PDE3A/PLVAP/PRKG1/PTGS2/ROCK1/ROCK2/SCPEP1/SH3GL2/SLC16A12/SLC16A2/SLC1A1/SLC1A2/SLC24A3/SLC29A4/SLC2A10/SLC2A13/SLC38A3/SLC4A3/SLC4A4/SLC5A1/SLC6A1/SLC6A17/SLC6A20/SLC7A3/SLC7A8/SLC8A2/SLCO1C1/SRC/TACR1/TBXA2R/TJP2/TRPM4/TRPV4/VEGFA/VSTM4	90

cluster5	BP	GO:0030323	respiratory tube development	69/3710	191/18903	6.79E-08	0.00000379	0.00000325	ABCA12/ACE/ADAMTS2/ADAMTSL2/AGR2/ASCL1/BMP4/CCBE1/CCDC40/CCN2/CELSR1/CLCN2/CRH/CRISPLD2/DAG1/DLG5/EDN2/FGF10/FGF2/FGF7/FGF9/FGFR2/FLT4/FOXA1/FOXJ1/GLI2/GLI3/GPC3/GRHL2/HES1/HHIP/HMGCS2/HS6ST1/IL13/JMJD6/LAMA1/LAMA5/LHX3/LOX/MYOCD/NKX2-1/NKX2-8/NOS3/PDGFA/PDGFRA/PDGFRB/PDPN/PKDCC/PTK7/RSPO2/SHH/SOX9/SPDEF/SPRY1/STK40/STRA6/TBX4/TBX5/THRB/TP73/TULP3/VEGFA/WNT11/WNT2B/WNT5A/WNT7B/YAP1/YWHAZ/ZIC3	69
cluster5	BP	GO:0042063	gliogenesis	100/3710	310/18903	0.000000077	0.00000427	0.00000367	ABCC8/ABL1/AKT1/APCDD1/APP/ASCL1/ASPA/ATOH1/AZU1/BMP2/CDH2/CDK1/CDK5R1/CDK5R2/CDKN2B/CHRM1/CNTNAP1/CSPG4/CSPG5/DAB1/DAG1/DLL1/DLX1/DLX2/DNER/DRD1/DUSP15/EIF2B2/EMX1/ERBB2/ERBB3/EZH2/F2/FGF10/FGF2/FGF5/FOXG1/GAP43/GCM2/GFAP/GLI3/GSX2/HDAC2/HES1/HES5/ID4/ILK/KLF15/LAMB1/LAMB2/LEPR/LRP2/LYN/MBOAT7/MDK/MMP24/MT3/MYOC/MYRF/NKX2-1/NKX2-2/NKX6-1/NR2E1/NTN1/NTRK2/OPALIN/PARD3/PAX2/PAX6/PENK/PHGDH/PHOX2B/PLP1/PLPP3/POU3F2/PTN/PTPRZ1/RELN/ROR1/ROR2/SERPINE2/SHH/SIRT2/SLC8A3/SMO/SOX1/SOX10/SOX9/SYNE2/TENM4/TMEM98/TNFRSF21/TP73/TPPP/TRPC4/TSPAN2/TTBK1/VAX1/WASF3/ZNF488	100
cluster5	BP	GO:0001578	microtubule bundle formation	50/3710	124/18903	8.59E-08	0.00000474	0.00000407	CAPN6/CC2D2A/CCDC103/CCDC40/CEP131/CFAP206/CFAP43/CFAP46/CFAP47/CFAP65/CFAP69/CFAP74/CFAP91/CFAP97D1/CLIP1/DAW1/DCX/DNAAF6/DNAAF8/DNAH2/DNAH5/DNAH7/DNAI1/DNAI3/DRC1/FOXJ1/FSIP2/HOATZ/HYDIN/KIF20A/MAP2/MNS1/NCKAP5/NEURL1/ODAD2/ODAD3/PLA2G3/PLK1/RP1/RSPH1/RSPH6A/RSPH9/SPAG17/SPAG6/SPEF1/TEKT2/TOGARAM1/TPPP/TTLL6/UBE2B	50
cluster5	BP	GO:0045055	regulated exocytosis	82/3710	242/18903	0.000000109	0.000006	0.00000515	ABCA12/ADCY1/ADORA2B/ADRA1A/ADRA2A/APBA1/BLOC1S6/CACNA1G/CADPS/CBARP/CD84/CDK5R2/CHGA/CHRM2/CHRNA6/CLTRN/CPLX2/CPLX4/CSPG5/DMTN/DOC2A/DOC2B/DRD1/DRD2/EQTN/FER/GIPC1/GRIK5/GRP/GSK3B/HTR2A/IL13/ITGAM/KCNB1/LAMP1/LYN/MRGPRX2/MYH9/NLGN1/OTOF/PCLO/PFN2/PLA2G3/PRKCG/PRRT2/RAB11FIP2/RAB31/RAB5A/RAP1B/RIMS3/RIMS4/RPH3AL/SCAMP5/SEPTIN5/SNAP23/SNAP25/STEAP2/STX11/STX1A/STXBP1/SV2A/SYN1/SYN2/SYNGR3/SYP/SYT1/SYT10/SYT12/SYT13/SYT2/SYT3/SYT4/SYT5/SYT6/SYT7/SYT8/SYT9/TMED10/UNC13A/UNC13C/WNT7A/ZP4	82

cluster5	BP	GO:1902414	protein localization to cell junction	42/3710	98/18903	0.000000122	0.00000665	0.00000571	ADAM10/ARHGAP44/ASIC2/BSN/CACNG2/CACNG3/CACNG7/DAG1/DLG5/GHSR/GPC6/GRIN2A/GRIP1/GRIP2/GRIPAP1/HOMER1/IQSEC2/KIF3A/KIF5A/KIF5C/LGI1/MAP1A/NECTIN1/NECTIN3/NLGN1/NLGN2/NPHS1/NPTX1/NRXN1/PCLO/PECAM1/RAB27B/RAB8A/RELN/SHANK1/SLITRK3/SNAP25/TJP2/WNT5A/WNT7A/ZDHHC15/ZDHHC7	42
cluster5	BP	GO:0055074	calcium ion homeostasis	99/3710	309/18903	0.000000129	0.00000701	0.00000602	ABL1/ADCY8/ADORA1/AKAP6/APP/ATF4/ATP13A5/ATP1A2/ATP1B1/ATP2B2/ATP2B3/ATP6V1B1/BCAP31/CACNA1C/CALB1/CALB2/CALCA/CALCB/CASQ2/CCDC47/CEMIP/CHRNA7/CIB2/CSRP3/DDIT3/DHRS7C/DMD/DRD1/DRD2/DRD4/EDN2/EDNRA/ELANE/ERO1A/F2/F2R/F2RL3/FGF2/FGF23/FKBP1A/FKBP1B/GCM2/GP1BB/GP9/GPR12/GRIA1/GRIK2/GRIN1/GRM5/GSTO1/HAP1/HCRTR1/HCRTR2/HERPUD1/HRC/HTR2A/HTR2C/IL13/ITGB3/JPH1/JPH3/JPH4/JSRP1/LACRT/LHCGR/LYN/MAIP1/MICU3/NPSR1/NPTN/PACS2/PKHD1/PTH/PTH1R/RYR2/RYR3/S100A14/SLC24A2/SLC24A3/SLC25A23/SLC30A1/SLC8A2/SLC8A3/STC1/STC2/SV2A/TFAP2B/THY1/TMBIM6/TNNI3/TRDN/TRIM24/TRPC3/TRPC4/TRPC7/TRPM8/TRPV4/WFS1/WNT5A	99
cluster5	BP	GO:0098739	import across plasma membrane	71/3710	202/18903	0.000000154	0.00000831	0.00000713	ABCC8/ABCC9/ACE2/ACSL1/AGT/AKT1/ATP1A2/ATP1A3/ATP1B1/CACNA1A/CACNA1B/CACNA1C/CACNA1S/CACNA2D1/CALCR/CLTRN/CNGA3/FOLR1/GFAP/GRM6/HCN4/HFE/ITGB1/KCNH2/KCNJ13/KCNJ16/KCNJ18/KCNJ3/KCNJ4/KCNJ5/KCNJ6/KCNJ9/KCNK5/LRP2/NALF1/NALF2/RGS4/SCNN1G/SLC12A5/SLC16A2/SLC17A8/SLC1A1/SLC1A2/SLC1A6/SLC24A2/SLC2A10/SLC30A1/SLC34A1/SLC38A3/SLC39A12/SLC39A5/SLC5A1/SLC6A1/SLC6A20/SLC6A5/SLC7A3/SLC7A8/SLC8A2/SLC8A3/SLC9A2/SLC9A4/SLC9A5/SLC9A6/SLC9A7/SLC9C2/STEAP2/TRPM1/TRPM4/TRPV4/WNK2/WNK3	71
cluster5	BP	GO:0098659	inorganic cation import across plasma membrane	50/3710	126/18903	0.000000156	0.00000835	0.00000716	ABCC8/ABCC9/ATP1A2/ATP1A3/ATP1B1/CACNA1A/CACNA1B/CACNA1C/CACNA1S/CACNA2D1/CALCR/CNGA3/GRM6/HCN4/HFE/KCNH2/KCNJ13/KCNJ16/KCNJ18/KCNJ3/KCNJ4/KCNJ5/KCNJ6/KCNJ9/KCNK5/NALF1/NALF2/SCNN1G/SLC12A5/SLC24A2/SLC30A1/SLC34A1/SLC39A12/SLC39A5/SLC5A1/SLC6A1/SLC8A2/SLC8A3/SLC9A2/SLC9A4/SLC9A5/SLC9A6/SLC9A7/SLC9C2/STEAP2/TRPM1/TRPM4/TRPV4/WNK2/WNK3	50

cluster5	BP	GO:0099587	inorganic ion import across plasma membrane	50/3710	126/18903	0.000000156	0.00000835	0.00000716	ABCC8/ABCC9/ATP1A2/ATP1A3/ATP1B1/CACNA1A/CACNA1B/CACNA1C/CACNA1S/CACNA2D1/CALCR/CNGA3/GRM6/HCN4/HFE/KCNH2/KCNJ13/KCNJ16/KCNJ18/KCNJ3/KCNJ4/KCNJ5/KCNJ6/KCNJ9/KCNK5/NALF1/NALF2/SCNN1G/SLC12A5/SLC24A2/SLC30A1/SLC34A1/SLC39A12/SLC39A5/SLC5A1/SLC6A1/SLC8A2/SLC8A3/SLC9A2/SLC9A4/SLC9A5/SLC9A6/SLC9A7/SLC9C2/STEAP2/TRPM1/TRPM4/TRPV4/WNK2/WNK3	50
cluster5	BP	GO:0016055	Wnt signaling pathway	135/3710	456/18903	0.000000159	0.00000844	0.00000724	ABL1/AMER3/AMOTL2/APC2/APCDD1/APP/ASPM/BARX1/BMP2/CALCOCO1/CDH2/CDK14/CELSR1/CELSR2/CITED1/CRBN/CSNK1E/CTNND1/CTNND2/CXXC4/DAAM1/DAB2/DACT3/DDIT3/DDX3X/DEPDC1B/DKK3/DKKL1/DLX3/DLX5/DRAXIN/DRD2/EDNRA/EDNRB/EGF/EGFR/FERMT1/FGF10/FGF2/FGF9/FGFR2/FOLR1/FOXL1/FRZB/FZD10/FZD3/GLI3/GPC3/GPC6/GPRC5B/GRB10/GREM1/GSK3B/HNF1B/IGFBP2/IGFBP4/ILK/ISL1/KLF15/LBX2/LGR4/LGR5/LRP4/LRP5/LRP6/LYPD6/MAGI2/MCC/MDFI/MDK/MITF/MYOC/NDP/NFATC4/NKD2/NPHP4/NR4A2/PLPP3/PRICKLE1/PRKAA2/PRKN/PTK7/PTPRU/PYGO1/PYGO2/RAB5A/RBX1/ROR1/ROR2/RSPO1/RSPO2/RSPO3/RSPO4/SALL1/SCEL/SDC1/SEMA5A/SFRP2/SHH/SHISA6/SOSTDC1/SOX10/SOX9/SPEF1/SRC/STRN/SULF1/SULF2/TGFB1I1/TLE1/TMEM170B/TMEM198/TNN/TPBG/TRABD2B/TRPM4/UBE2B/VANGL2/VAX2/VGLL4/WIF1/WNK2/WNT11/WNT2B/WNT3A/WNT4/WNT5A/WNT7A/WNT7B/WNT8A/WNT8B/WNT9A/WNT9B/YAP1/ZNRF3	135
cluster5	BP	GO:0051937	catecholamine transport	34/3710	73/18903	0.000000171	0.00000907	0.00000779	ADRA2A/AGT/CHGA/CHRNA4/CHRNA6/CRH/DRD1/DRD2/DRD4/GDNF/GHSR/GRM2/HRH3/HTR2A/KCNB1/OPRK1/PRKN/RAB3B/SLC29A4/SLC6A2/SLC6A3/STX1A/SYT1/SYT10/SYT12/SYT13/SYT2/SYT3/SYT4/SYT5/SYT6/SYT7/SYT8/SYT9	34
cluster5	BP	GO:0030317	flagellated sperm motility	51/3710	130/18903	0.000000181	0.00000947	0.00000813	ADCY3/AKAP4/C2CD6/CABS1/CATSPER4/CCDC40/CELF3/CEP131/CFAP206/CFAP221/CFAP43/CFAP47/CFAP52/CFAP54/CFAP65/CFAP69/CFAP97D1/DDX4/DNAAF6/DNAH11/DNAH5/DNAI1/DZIP1/ENKUR/ENO4/FSIP2/GK2/HOATZ/IFT81/LDHC/MNS1/NEURL1/NPHP4/ODAD3/PLA2G3/PLTP/PRDM14/PRM3/PRSS55/ROPN1/SEPTIN4/SLC9B2/SMCP/SPAG6/SPEM1/TACR1/TACR3/TCTE1/TEKT2/TTLL9/UBE2B	51

cluster5	BP	GO:0097722	sperm motility	51/3710	130/18903	0.000000181	0.00000947	0.00000813	ADCY3/AKAP4/C2CD6/CABS1/CATSPER4/CCDC40/CELF3/CEP131/CFAP206/CFAP221/CFAP43/CFAP47/CFAP52/CFAP54/CFAP65/CFAP69/CFAP97D1/DDX4/DNAAF6/DNAH11/DNAH5/DNAI1/DZIP1/ENKUR/ENO4/FSIP2/GK2/HOATZ/IFT81/LDHC/MNS1/NEURL1/NPHP4/ODAD3/PLA2G3/PLTP/PRDM14/PRM3/PRSS55/ROPN1/SEPTIN4/SLC9B2/SMCP/SPAG6/SPEM1/TACR1/TACR3/TCTE1/TEKT2/TTLL9/UBE2B	51
cluster5	BP	GO:0003073	regulation of systemic arterial blood pressure	41/3710	96/18903	0.000000192	0.00001	0.00000861	ACE/ACE2/ADM5/ADORA1/ADRA1A/ADRA1B/ADRA1D/ADRB3/AGT/AGTR2/ASIC2/AVP/AVPR1B/CALCA/CMA1/CORO2B/CTSG/CYP11B2/DDAH1/DRD2/ECE1/EDN2/EDNRB/EMP2/F2R/GAS6/KCNK6/KLK3/NAV2/NOS3/NR2F2/OR51E2/P2RX2/RASL10B/RPS6KA2/SLC4A5/SPX/SUCNR1/TACR1/TNNI3/TPM1	41
cluster5	BP	GO:0072273	metanephric nephron morphogenesis	17/3710	25/18903	0.000000196	0.0000102	0.00000872	BMP4/GDNF/GREM1/HES1/HES5/KIF26B/LGR4/LHX1/PAX2/PDGFRB/SALL1/SIX2/SMO/SOX9/WNT4/WNT9B/WT1	17
cluster5	BP	GO:0001976	nervous system process involved in regulation of systemic arterial blood pressure	12/3710	14/18903	0.000000197	0.0000102	0.00000872	ACE2/ADRA1A/AGT/AGTR2/ASIC2/CALCA/DRD2/NAV2/P2RX2/RPS6KA2/TACR1/TNNI3	12

cluster5	BP	GO:0021872	forebrain generation of neurons	27/3710	52/18903	0.000000199	0.0000102	0.00000872	ASCL1/ASPM/DCLK2/DCT/DLX1/DLX2/FEZF2/FGFR2/FOXG1/GBX2/GLI3/HES1/HES5/LHX5/NDNF/NHLH2/NKX2-1/NR2E1/NRP2/OTP/PAX6/PLXNA1/SECISBP2/SEMA3E/SOX1/TBR1/WNT3A	27
cluster5	BP	GO:0099172	presynapse organization	27/3710	52/18903	0.000000199	0.0000102	0.00000872	APP/BSN/CBLN1/CBLN2/CNTN5/EFNB2/GPC6/IL1RAPL1/IL1RAPL2/LRFN3/LRP4/LRRC4B/LRRTM3/NLGN1/NLGN2/NLGN3/NLGN4X/NTNG2/PCLO/PTPRD/SLITRK1/SLITRK2/SLITRK3/SLITRK6/WNT3A/WNT5A/WNT7A	27
cluster5	BP	GO:0090497	mesenchymal cell migration	30/3710	61/18903	0.000000203	0.0000103	0.00000882	ALX1/BMP4/BMP7/CDH2/EDNRA/EDNRB/ERBB4/FGF19/FOLR1/GBX2/GDNF/HIF1A/ISL1/KITLG/LAMA5/NRP2/PHOX2B/RADIL/RET/SEMA3A/SEMA3E/SEMA4D/SEMA5A/SEMA5B/SEMA6A/SEMA6D/SHH/SMO/SOX10/TPBG	30
cluster5	BP	GO:0006874	cellular calcium ion homeostasis	92/3710	284/18903	0.000000203	0.0000103	0.00000882	ABL1/ADCY8/ADORA1/AKAP6/APP/ATF4/ATP13A5/ATP1A2/ATP1B1/ATP2B2/ATP2B3/BCAP31/CACNA1C/CALB1/CALB2/CALCA/CALCB/CASQ2/CCDC47/CEMIP/CHRNA7/CSRP3/DDIT3/DHRS7C/DMD/DRD1/DRD2/DRD4/EDN2/EDNRA/ELANE/ERO1A/F2/F2R/F2RL3/FGF2/FKBP1A/FKBP1B/GCM2/GP1BB/GP9/GPR12/GRIA1/GRIK2/GRM5/GSTO1/HAP1/HCRTR1/HCRTR2/HERPUD1/HRC/HTR2A/HTR2C/IL13/ITGB3/JPH1/JPH3/JPH4/JSRP1/LACRT/LHCGR/LYN/MAIP1/MICU3/NPSR1/NPTN/PACS2/PKHD1/PTH/PTH1R/RYR2/RYR3/SLC24A2/SLC24A3/SLC25A23/SLC30A1/SLC8A2/SLC8A3/STC1/STC2/SV2A/THY1/TMBIM6/TNNI3/TRDN/TRPC3/TRPC4/TRPC7/TRPM8/TRPV4/WFS1/WNT5A	92

cluster5	BP	GO:0007613	memory	48/3710	120/18903	0.000000206	0.0000104	0.00000892	ABCC8/ADCY1/ADCY8/ADGRF1/ARC/ASIC1/B4GALT2/BDNF/BRINP1/CALB1/CHRNA7/CPEB3/DBH/DRD1/DRD2/EHMT2/FOXO6/GRIA1/GRIN2A/HTR2A/JPH3/KCNK10/LMX1A/MAP1A/MDK/MUSK/NFATC4/NGF/NPAS4/NTF3/PAK5/PLCB1/PTCHD1/PTGS2/PTN/RASGRF1/RELN/SCN2A/SHANK1/SHISA7/SLC1A1/SLC6A1/SLC8A2/SLC8A3/SYT4/TACR1/TH/TTC36	48
cluster5	BP	GO:0198738	cell-cell signaling by wnt	135/3710	458/18903	0.000000209	0.0000105	0.000009	ABL1/AMER3/AMOTL2/APC2/APCDD1/APP/ASPM/BARX1/BMP2/CALCOCO1/CDH2/CDK14/CELSR1/CELSR2/CITED1/CRBN/CSNK1E/CTNND1/CTNND2/CXXC4/DAAM1/DAB2/DACT3/DDIT3/DDX3X/DEPDC1B/DKK3/DKKL1/DLX3/DLX5/DRAXIN/DRD2/EDNRA/EDNRB/EGF/EGFR/FERMT1/FGF10/FGF2/FGF9/FGFR2/FOLR1/FOXL1/FRZB/FZD10/FZD3/GLI3/GPC3/GPC6/GPRC5B/GRB10/GREM1/GSK3B/HNF1B/IGFBP2/IGFBP4/ILK/ISL1/KLF15/LBX2/LGR4/LGR5/LRP4/LRP5/LRP6/LYPD6/MAGI2/MCC/MDFI/MDK/MITF/MYOC/NDP/NFATC4/NKD2/NPHP4/NR4A2/PLPP3/PRICKLE1/PRKAA2/PRKN/PTK7/PTPRU/PYGO1/PYGO2/RAB5A/RBX1/ROR1/ROR2/RSPO1/RSPO2/RSPO3/RSPO4/SALL1/SCEL/SDC1/SEMA5A/SFRP2/SHH/SHISA6/SOSTDC1/SOX10/SOX9/SPEF1/SRC/STRN/SULF1/SULF2/TGFB1I1/TLE1/TMEM170B/TMEM198/TNN/TPBG/TRABD2B/TRPM4/UBE2B/VANGL2/VAX2/VGLL4/WIF1/WNK2/WNT11/WNT2B/WNT3A/WNT4/WNT5A/WNT7A/WNT7B/WNT8A/WNT8B/WNT9A/WNT9B/YAP1/ZNRF3	135
cluster5	BP	GO:0021700	developmental maturation	97/3710	304/18903	0.000000214	0.0000107	0.00000916	ABHD2/ACTL6B/ADAMTS7/ADGRB3/ANGPTL8/AP3D1/APP/ASCL1/BCAN/BCL11A/BLOC1S6/BMP2/BRCA2/C2CD6/CABYR/CAMK2B/CATSPER4/CDK5R1/CDK5R2/CFTR/CHRDL1/CNGB1/CNTNAP2/DAG1/DDIT3/DMC1/EDNRA/EDNRB/EPHA8/EPO/FERMT1/FEV/FGFR3/FOXA1/GLDN/GPAT4/GREM1/HES1/HES5/HIF1A/HOXB13/IGSF21/KCNB1/MBTPS2/MSX2/MYOC/NEURL1/NEUROD2/NFATC4/NKX6-1/NLGN1/NPPC/NR4A2/NRCAM/NRXN1/PAX2/PCSK4/PDE3A/PLA2G3/PLXNB1/PTH/PTH1R/RELN/RET/RFLNA/RFX3/ROPN1/RPS6KA2/SEMA4D/SEZ6L/SEZ6L2/SHANK1/SIRT2/SIX3/SOX10/SOX18/SPINK1/SPTBN4/SRRM4/STXBP1/SYBU/SYP/TCP11/TCP11X1/TDRD5/TUT4/TYMS/UNC13A/UNC13C/VEGFA/VSX1/WEE2/WNT5A/YWHAZ/ZBTB16/ZDHHC15/ZNF664-RFLNA	97
cluster5	BP	GO:0050770	regulation of axonogenesis	58/3710	156/18903	0.00000024	0.0000119	0.0000102	BDNF/BRSK1/BRSK2/CDH2/CDH4/CDKL3/CHODL/CRABP2/CYFIP1/DAB1/DBN1/DRAXIN/DSCAM/EFNA5/EFNB3/EPHA7/EPHB3/GDI1/GSK3B/ISLR2/L1CAM/LRP4/LRRC4C/MAP2/MAP6/MT3/NDEL1/NGF/NTN1/NTRK2/PAK3/PLXNA1/PLXNA2/PLXNA4/PLXNB1/PLXNB3/POU3F2/PTPRS/RET/RND2/ROBO1/ROBO2/SEMA3A/SEMA3E/SEMA4D/SEMA5A/SEMA5B/SEMA6A/SEMA6D/SHOX2/SLIT1/SLITRK1/THY1/TNR/VEGFA/WNT3A/WNT5A/WNT7A	58

cluster5	BP	GO:0072210	metanephric nephron development	23/3710	41/18903	0.000000253	0.0000125	0.0000107	AGTR2/BMP4/GDNF/GREM1/HES1/HES5/IRX2/KIF26B/LAMB2/LGR4/LHX1/PAX2/PDGFRA/PDGFRB/RET/SALL1/SIX2/SMO/SOX9/TFAP2B/WNT4/WNT9B/WT1	23
cluster5	BP	GO:0002027	regulation of heart rate	44/3710	107/18903	0.000000259	0.0000128	0.0000109	ADM5/ADRA1A/ADRA1B/ADRA1D/AGT/AGTR2/BVES/CACNA1C/CACNA1G/CACNA2D1/CASQ2/DMD/DRD2/DSG2/EDN2/EDNRA/EDNRB/FKBP1B/HCN1/HCN3/HCN4/HRC/ISL1/KCND3/KCNE4/KCNH2/KCNH6/KCNJ3/KCNJ5/MYH6/MYH7/PKP2/RGS4/RYR2/SCN10A/SCN2B/SCN3B/SHOX2/SLC1A1/SPTBN4/SPX/TACR3/TPM1/TRPM4	44
cluster5	BP	GO:2001257	regulation of cation channel activity	69/3710	197/18903	0.000000266	0.000013	0.0000112	ABCC8/ABCC9/ACTN2/AKAP6/ANK3/APP/ARC/CABP1/CACNA2D1/CACNB1/CACNG2/CACNG3/CACNG4/CACNG5/CACNG7/CASQ2/CBARP/CRBN/CRH/CRHR1/DMD/DRD2/DRD4/EDNRA/EHD3/EPO/FGF14/FKBP1A/FKBP1B/GEM/GRP/GSG1L/GSTO1/HAP1/HCN1/HCN3/HCN4/HOMER1/HPCA/HRC/ITGB1/JPH1/JPH3/JPH4/JSRP1/KCNC1/KCNC2/KCNE4/KCNK3/KCNRG/KCNS2/LRRC55/MAPK8IP2/MINK1/NLGN1/NLGN2/NLGN3/PIRT/PRRT1/RASGRF1/RELN/REM1/SHANK1/SHISA6/SHISA7/SHISA9/STAC2/STOM/TRDN	69
cluster5	BP	GO:0090183	regulation of kidney development	20/3710	33/18903	0.000000274	0.0000134	0.0000115	AGT/AGTR2/BASP1/BMP4/GDNF/GREM1/HNF1B/LGR4/LHX1/MAGED1/PAX2/RET/SIX2/SIX4/SMO/SOX9/VEGFA/WNT2B/WNT4/WT1	20

cluster5	BP	GO:0061448	connective tissue development	89/3710	274/18903	0.000000279	0.0000135	0.0000116	ADAMTS7/AMELX/ARID5B/BARX2/BBS1/BMP2/BMP4/BMP5/BMP6/BMP7/BMPR1B/CCN2/CHADL/CHRDL2/COL11A1/COL27A1/COL2A1/DLX2/EBF2/ECM1/EPYC/EVC/FGF2/FGF4/FGF9/FGFR3/FOXA1/FRZB/GDF5/GDF6/GHR/GLI3/GPLD1/GREM1/HES5/HIF1A/HMGA2/HMGCS2/HOXA11/HOXA3/HOXC4/HOXD3/ID4/ITGB3/ITGB8/LOX/LRP5/MATN3/MDK/MMP13/MSX2/NCOA1/NPPC/NR1H4/OGN/PDGFRB/PITX1/PKDCC/PTH/PTH1R/RARB/RFLNA/RSPO2/SERPINB7/SFRP2/SH3PXD2B/SHOX2/SIX2/SMAD5/SOX5/SOX9/STC1/SULF1/SULF2/TIMP1/TRPM4/TRPV4/TYMS/UNCX/WNT11/WNT2B/WNT5A/WNT7A/WNT7B/WNT9A/WT1/ZBTB16/ZNF219/ZNF664-RFLNA	89
cluster5	BP	GO:0098815	modulation of excitatory postsynaptic potential	24/3710	44/18903	0.000000283	0.0000137	0.0000117	APP/CBLN1/CELF4/CHRNA7/EIF4A3/GRIN1/GRIN2A/GRIN2B/GRIN2D/IGSF11/NLGN1/NLGN2/NLGN3/NLGN4X/NRXN1/PRKAR1B/RELN/RGS4/SHANK1/SLC8A2/SLC8A3/STX1A/TMEM108/WNT7A	24
cluster5	BP	GO:0015844	monoamine transport	37/3710	84/18903	0.000000293	0.0000141	0.0000121	ADRA2A/AGT/CHGA/CHRNA4/CHRNA6/CRH/DRD1/DRD2/DRD4/GDNF/GHSR/GPM6B/GRM2/HRH3/HTR2A/ITGB3/KCNB1/MAOB/OPRK1/PRKN/RAB3B/SLC29A4/SLC6A2/SLC6A3/STX1A/SYT1/SYT10/SYT12/SYT13/SYT2/SYT3/SYT4/SYT5/SYT6/SYT7/SYT8/SYT9	37
cluster5	BP	GO:0050919	negative chemotaxis	25/3710	47/18903	0.000000306	0.0000146	0.0000126	EFNA5/EPHA7/FLRT2/FLRT3/ITGB3/LRTM1/LRTM2/NRG3/NRP2/NTN1/PDGFA/PLXNA4/ROBO1/ROBO2/SEMA3A/SEMA3E/SEMA4D/SEMA5A/SEMA5B/SEMA6A/SEMA6D/SLIT1/SLIT3/UNC5C/WNT5A	25

cluster5	BP	GO:0002052	positive regulation of neuroblast proliferation	18/3710	28/18903	0.000000308	0.0000147	0.0000126	ASPM/DCT/DMRTA2/DRD2/FGF2/FOXG1/FZD3/GLI3/HIF1A/ID4/ITGB1/NR2E1/OTP/PAX6/SHH/SMO/SOX10/VEGFA	18
cluster5	BP	GO:0007422	peripheral nervous system development	36/3710	81/18903	0.000000322	0.0000153	0.0000131	ADGRB1/AKT1/ARTN/ASCL1/ASIC2/BDNF/CDK1/CLDN1/CNTNAP1/DAG1/EDNRB/ERBB2/ERBB3/ETV1/GDNF/GFRA3/HAPLN2/HOXD10/ILK/ISL1/LAMB2/MYOC/NEFH/NEUROG3/NFASC/NGF/NHLH2/NTF3/NTRK2/PARD3/PLXNA4/POU3F2/RXRG/SCN8A/SIRT2/SOX10	36
cluster5	BP	GO:0071248	cellular response to metal ion	69/3710	198/18903	0.000000331	0.0000156	0.0000134	ADCY1/ADCY8/ADGRV1/AKT1/ANK3/APP/ATF4/BMP6/CHP2/CLDN1/CPNE2/CPNE4/CPNE5/CPNE6/CPNE9/CYP11B1/CYP11B2/DMTN/EGFR/FOS/FOSB/GLRA1/GLRA2/GPLD1/HFE/HPCA/HSF1/ITPKA/JUNB/KCNB1/KCNH1/KCNK3/LOC118142757/MT1A/MT1B/MT1HL1/MT1X/MT2A/MT3/MYOG/NEUROD2/NFATC4/NLGN1/NPTX1/PRKAA2/PRKN/PTGS2/RYR3/SHH/SLC13A5/SLC1A1/SLC25A23/SLC34A1/SYT1/SYT10/SYT12/SYT13/SYT2/SYT3/SYT4/SYT5/SYT6/SYT7/SYT8/SYT9/TFAP2A/TH/TPH2/WNT5A	69
cluster5	BP	GO:0022898	regulation of transmembrane transporter activity	93/3710	291/18903	0.000000348	0.0000164	0.000014	ABCC8/ABCC9/ACE2/ACTN2/ADRA2A/AKAP6/ANK3/APP/ARC/ATP1A2/ATP1B1/CABP1/CACNA2D1/CACNB1/CACNG2/CACNG3/CACNG4/CACNG5/CACNG7/CASQ2/CBARP/CFTR/CHP2/CHRM3/CLTRN/CNKSR3/CRBN/CRH/CRHR1/DMD/DRD2/DRD4/EDNRA/EHD3/EPO/FGF14/FHL1/FKBP1A/FKBP1B/FXYD3/FXYD6/GEM/GRM5/GRP/GSG1L/GSTO1/HAP1/HCN1/HCN3/HCN4/HOMER1/HPCA/HRC/HSPA2/INS/ITGB1/JPH1/JPH3/JPH4/JSRP1/KCNC1/KCNC2/KCNE4/KCNK3/KCNRG/KCNS2/LRRC55/MAPK8IP2/MINK1/NEDD4L/NLGN1/NLGN2/NLGN3/PIRT/PRRT1/PTPN3/RASGRF1/RELN/REM1/RYR2/SCN2B/SCN3B/SHANK1/SHISA6/SHISA7/SHISA9/SLN/STAC2/STOM/TCAF1/TRDN/WNK2/WNK3	93

cluster5	BP	GO:0048762	mesenchymal cell differentiation	83/3710	252/18903	0.000000361	0.0000169	0.0000145	ALX1/AMELX/BMP2/BMP4/BMP5/BMP7/CDH2/CRB2/CUL7/CYP26C1/DAB2/DACT3/DAG1/DLG5/EDNRA/EDNRB/EMP2/EPHA3/ERBB4/EZH2/FAM83D/FGF10/FGF19/FGFR1/FGFR2/FOLR1/FOXA1/FOXA2/FOXC1/FOXF2/FRZB/GBX2/GDNF/GREM1/GSK3B/HAS2/HDAC2/HES1/HEYL/HIF1A/HMGA2/HPN/IL17RD/ISL1/KITLG/LAMA5/LRP6/MDK/MSX2/NKX2-1/NOTCH4/NRP2/OLFM1/PAX2/PDPN/PHOX2B/RADIL/RET/ROCK1/ROCK2/SEMA3A/SEMA3E/SEMA4D/SEMA5A/SEMA5B/SEMA6A/SEMA6D/SERPINB3/SFRP2/SHH/SIX2/SMO/SOX10/SOX9/SPRY1/TBX20/TBX5/TGFB1I1/VASN/WNT11/WNT4/WNT5A/WNT8A	83
cluster5	BP	GO:0090189	regulation of branching involved in ureteric bud morphogenesis	15/3710	21/18903	0.00000039	0.0000181	0.0000156	AGT/AGTR2/BMP4/GDNF/GREM1/LGR4/LHX1/MAGED1/PAX2/SIX2/SIX4/SMO/SOX9/VEGFA/WNT2B	15
cluster5	BP	GO:0048638	regulation of developmental growth	103/3710	332/18903	0.000000419	0.0000194	0.0000167	ABL1/ADRB3/AGR2/AKAP6/AKT1/APP/ARX/BARHL2/BASP1/BCL11A/BDNF/BMP4/CACNG7/CDH4/CDK1/CDKL3/CDKN1A/CGA/CPNE5/CPNE6/CPNE9/CRABP2/CYFIP1/DBN1/DLL1/DRAXIN/DRD2/DSCAM/DUSP9/EFNA5/EPHA7/ERBB4/FGF2/FGF9/FGFR2/FGFR3/FOXC1/FOXS1/GDF5/GDI1/GHR/GHSR/GPAT4/GSK3B/HNF1B/IL7/ISLR2/L1CAM/MAEL/MAP2/MFSD2A/MT3/MUSK/MYH6/MYOZ1/NDEL1/NEDD4L/NGF/NKX6-1/NPPC/NRCAM/NTN1/OLFM1/PLCB1/PLXNA4/POU3F2/PPIB/PRKN/PTCH1/PTPRS/RAI1/RGS4/RND2/SEMA3A/SEMA3E/SEMA4D/SEMA5A/SEMA5B/SEMA6A/SEMA6D/SIX4/SLC6A3/SLIT1/SMO/SPTBN4/STC2/SYT1/SYT2/SYT3/SYT4/TBX20/TBX5/TLL2/TNR/TP73/UNC13A/VEGFA/VGLL4/WNT3A/WNT5A/WT1/WWC1/YAP1	103
cluster5	BP	GO:0032835	glomerulus development	32/3710	69/18903	0.000000443	0.0000204	0.0000175	AGTR2/ANGPT1/ANGPT2/BASP1/BMP4/BMP7/EDNRA/EDNRB/FOXC1/FOXJ1/HES1/HEYL/ITGB3/KIRREL3/KLF15/LAMB2/LGR4/LHX1/MAGI2/NID1/NOTCH2/NPHS1/PAX2/PDGFRA/PDGFRB/PECAM1/PROM1/RET/SERPINB7/SULF1/SULF2/WT1	32

cluster5	BP	GO:0021983	pituitary gland development	23/3710	42/18903	0.000000456	0.0000209	0.000018	BMP2/BMP4/DRD2/FGF10/FGF2/GLI2/GSX1/HES1/ISL1/LHX3/NKX2-1/NR0B1/OTP/PAX6/PCSK1/PITX1/POU3F2/SALL1/SIX3/SLC6A3/SOX3/WNT4/WNT5A	23
cluster5	BP	GO:0035235	ionotropic glutamate receptor signaling pathway	17/3710	26/18903	0.000000462	0.0000211	0.0000181	APP/CDK5R1/GRIA1/GRIA2/GRIA3/GRIA4/GRID1/GRID2/GRIK1/GRIK2/GRIK3/GRIK5/GRIN1/GRIN2A/GRIN2B/GRIN2D/PLP1	17
cluster5	BP	GO:1990806	ligand-gated ion channel signaling pathway	17/3710	26/18903	0.000000462	0.0000211	0.0000181	APP/CDK5R1/GRIA1/GRIA2/GRIA3/GRIA4/GRID1/GRID2/GRIK1/GRIK2/GRIK3/GRIK5/GRIN1/GRIN2A/GRIN2B/GRIN2D/PLP1	17
cluster5	BP	GO:0032412	regulation of ion transmembrane transporter activity	90/3710	281/18903	0.00000048	0.0000218	0.0000187	ABCC8/ABCC9/ACTN2/ADRA2A/AKAP6/ANK3/APP/ARC/ATP1A2/ATP1B1/CABP1/CACNA2D1/CACNB1/CACNG2/CACNG3/CACNG4/CACNG5/CACNG7/CASQ2/CBARP/CFTR/CHP2/CHRM3/CNKSR3/CRBN/CRH/CRHR1/DMD/DRD2/DRD4/EDNRA/EHD3/EPO/FGF14/FHL1/FKBP1A/FKBP1B/FXYD3/FXYD6/GEM/GRM5/GRP/GSG1L/GSTO1/HAP1/HCN1/HCN3/HCN4/HOMER1/HPCA/HRC/HSPA2/ITGB1/JPH1/JPH3/JPH4/JSRP1/KCNC1/KCNC2/KCNE4/KCNK3/KCNRG/KCNS2/LRRC55/MAPK8IP2/MINK1/NEDD4L/NLGN1/NLGN2/NLGN3/PIRT/PRRT1/PTPN3/RASGRF1/RELN/REM1/RYR2/SCN2B/SCN3B/SHANK1/SHISA6/SHISA7/SHISA9/SLN/STAC2/STOM/TCAF1/TRDN/WNK2/WNK3	90

cluster5	BP	GO:0099072	regulation of postsynaptic membrane neurotransmitter receptor levels	30/3710	63/18903	0.000000495	0.0000224	0.0000192	ADAM10/ARC/ARHGAP44/CACNG2/CACNG3/CACNG4/CACNG5/CACNG7/CPT1C/CTNND1/DAG1/DRD4/EFNB2/EPS8/FRRS1L/GHSR/GPC6/GRIP1/GRIP2/GRIPAP1/GSG1L/HPCA/IQSEC2/ITGB3/LGI1/NPTX1/OPHN1/RAB8A/SHISA6/SNAP25	30
cluster5	BP	GO:0007193	adenylate cyclase-inhibiting G protein-coupled receptor signaling pathway	35/3710	79/18903	0.000000513	0.0000231	0.0000198	ADCY5/ADORA1/ADRA2A/CHRM1/CHRM2/CHRM3/DRD2/DRD4/EDNRA/GABBR2/GNAI1/GNAT1/GNAZ/GPR176/GPR37/GRIK3/GRM2/GRM3/GRM4/GRM5/GRM6/GRM7/GRM8/HRH3/HTR1D/HTR1E/HTR1F/HTR4/ITGB3/MCHR1/MTNR1A/OPRK1/PSAP/PSAPL1/SSTR2	35
cluster5	BP	GO:0021511	spinal cord patterning	14/3710	19/18903	0.000000525	0.0000235	0.0000202	ASCL1/CHRD/DMRT3/FOXN4/GLI2/GLI3/LHX3/NKX2-2/RELN/RFX4/SHH/SMO/SOX1/TULP3	14
cluster5	BP	GO:0050433	regulation of catecholamine secretion	28/3710	57/18903	0.000000529	0.0000236	0.0000203	ADRA2A/AGT/CHGA/CHRNA4/CHRNA6/CRH/DRD2/GDNF/GHSR/GRM2/HRH3/HTR2A/KCNB1/OPRK1/PRKN/STX1A/SYT1/SYT10/SYT12/SYT13/SYT2/SYT3/SYT4/SYT5/SYT6/SYT7/SYT8/SYT9	28

cluster5	BP	GO:0015872	dopamine transport	26/3710	51/18903	0.000000533	0.0000237	0.0000203	CHRNA4/CHRNA6/DRD1/DRD2/DRD4/GDNF/GRM2/HTR2A/OPRK1/PRKN/RAB3B/SLC29A4/SLC6A2/SLC6A3/SYT1/SYT10/SYT12/SYT13/SYT2/SYT3/SYT4/SYT5/SYT6/SYT7/SYT8/SYT9	26
cluster5	BP	GO:0060563	neuroepithelial cell differentiation	20/3710	34/18903	0.000000543	0.000024	0.0000206	ABL1/ASCL1/BMP2/CDH2/DLG5/DLL1/DLX3/DSPP/EMX1/FGF2/HES1/LHX3/NKX2-2/OTP/POU3F2/SERPINE1/TMEM231/VAX1/WNT11/WNT4	20
cluster5	BP	GO:0045216	cell-cell junction organization	71/3710	208/18903	0.000000553	0.0000244	0.0000209	ABCC8/ACE/ACE2/ADAM10/AGT/ARVCF/BMP6/CDH10/CDH11/CDH12/CDH18/CDH19/CDH2/CDH22/CDH6/CDH7/CDH8/CDH9/CLDN1/CLDN14/CLDN16/CLDN18/CLDN19/CLDN2/CLDN22/CLDN4/CLDN5/CLDN6/CLDN8/CNTNAP1/CNTNAP2/CTNND1/CTNND2/DLG5/DSG2/EFNB2/F2R/FER/FRMPD2/GJC1/GRHL1/GRHL2/INAVA/KIRREL1/MARVELD2/MPDZ/MYO1C/NECTIN1/NLGN2/NLGN4X/NPHP4/NPHS1/NR1H4/NUMBL/PARD3/PECAM1/PKHD1/PKN2/PKP2/PRTN3/ROCK1/ROCK2/SRC/STRN/SVEP1/TBX5/TJP2/TRPV4/VEGFA/WHRN/WNT11	71
cluster5	BP	GO:0097553	calcium ion transmembrane import into cytosol	64/3710	182/18903	0.000000605	0.0000265	0.0000228	ABL1/ADCYAP1R1/ADRA1A/AKAP6/ATP1A2/CACNA1A/CACNA1B/CACNA1C/CACNA1S/CACNA2D1/CALCR/CASQ2/CCN2/CEMIP/DDIT3/DHRS7C/DMD/DRD1/DRD2/EPO/ERO1A/F2/F2R/F2RL3/FGF2/FKBP1A/FKBP1B/GP1BB/GP9/GRIN1/GRIN2A/GRIN2B/GRIN2D/GRM6/GSTO1/HAP1/HRC/HTR2A/HTR2C/IL13/ITGB3/JPH1/JPH3/JPH4/JSRP1/LACRT/LHCGR/LYN/NALF1/NALF2/NPSR1/P2RX2/P2RX3/RYR2/RYR3/SLC24A2/SLC8A2/SLC8A3/THY1/TMBIM6/TRDN/TRPC3/TRPM1/TRPV4	64

cluster5	BP	GO:0002067	glandular epithelial cell differentiation	33/3710	73/18903	0.000000612	0.0000267	0.000023	AGR2/AKT1/ASCL1/BMP2/BMP4/BMP5/BMP6/CDH2/CLCN2/DLL1/FGF2/FGFR2/FOXA1/GPAT4/GSK3B/HES1/HIF1A/IL13/LHX3/NEUROD1/NKX2-2/NKX6-1/PAX6/RARB/RFX3/RFX6/SLC9A4/SMO/SOX9/SPDEF/WNT4/WNT5A/YAP1	33
cluster5	BP	GO:0042472	inner ear morphogenesis	42/3710	103/18903	0.000000631	0.0000274	0.0000236	ATOH1/ATP6V1B1/CLRN1/CLRN2/COL11A1/COL2A1/DLX5/DLX6/FGF10/FGF9/FGFR2/FOXG1/FRZB/FZD3/GBX2/HMX3/HPN/KCNQ4/LHFPL5/LRIG3/MYO3A/MYO3B/MYO6/NEUROG1/NTN1/OTOP1/OTX1/PAX2/PDZD7/POU3F4/POU4F3/PTK7/SIX4/SLITRK6/SOBP/SOX9/TFAP2A/USH1C/WHRN/WNT3A/WNT5A/ZIC1	42
cluster5	BP	GO:0099068	postsynapse assembly	18/3710	29/18903	0.000000662	0.0000287	0.0000246	CBLN1/CDH2/GAP43/GRID2/LRP4/LRRC4B/LRRTM2/NLGN1/NLGN2/NLGN3/NLGN4X/NPTX1/NRXN1/NTNG2/PTPRD/SLITRK3/WNT5A/WNT7A	18
cluster5	BP	GO:0021513	spinal cord dorsal/ventral patterning	13/3710	17/18903	0.000000674	0.0000291	0.0000249	ASCL1/CHRD/DMRT3/FOXN4/GLI2/GLI3/LHX3/NKX2-2/RFX4/SHH/SMO/SOX1/TULP3	13

cluster5	BP	GO:0030856	regulation of epithelial cell differentiation	59/3710	164/18903	0.00000069	0.0000297	0.0000255	ABCA12/AHI1/AJAP1/ASCL1/ATOH1/BMP2/BMP4/BMP6/BMP7/CDKN2B/CLDN5/DLL1/DSPP/ESRP1/ETV4/EZH2/FGF10/FGF2/FOXC1/FOXE3/FOXJ1/FOXN1/FRZB/GDNF/GRHL1/GRHL2/HES1/HES5/ID1/IL13/IL20/KRT84/LHX1/MSX2/NKX2-2/NKX6-1/NOTCH4/PAX2/PAX6/PLCB1/PROM1/PTCH1/PTCH2/REG3A/REG3G/RFX3/ROCK1/ROCK2/SERPINE1/SIX2/SMO/SOX9/SPRY1/TP73/VEGFA/WNT9B/XDH/YAP1/ZFP36	59
cluster5	BP	GO:0007405	neuroblast proliferation	31/3710	67/18903	0.000000711	0.0000304	0.0000261	ARTN/ASCL1/ASPM/DAGLA/DCT/DMRTA2/DRD2/EML1/FGF2/FGFR2/FOXG1/FZD3/GLI3/HHIP/HIF1A/ID4/ITGB1/NR2E1/NUMBL/OTP/PAX6/PTN/SHH/SIX3/SMO/SOX10/SOX5/TAFA3/VAX1/VEGFA/WNT3A	31
cluster5	BP	GO:0048709	oligodendrocyte differentiation	41/3710	100/18903	0.000000719	0.0000306	0.0000263	ASCL1/ASPA/CNTNAP1/DAG1/DLX1/DLX2/DUSP15/EIF2B2/ERBB2/GLI3/GSX2/HDAC2/HES1/HES5/ID4/LYN/MDK/MYRF/NKX2-1/NKX2-2/NKX6-1/NTRK2/OPALIN/PAX6/PLP1/PTN/PTPRZ1/SHH/SLC8A3/SOX1/SOX10/SOX9/TENM4/TMEM98/TNFRSF21/TP73/TPPP/TRPC4/TSPAN2/WASF3/ZNF488	41
cluster5	BP	GO:1902692	regulation of neuroblast proliferation	22/3710	40/18903	0.000000733	0.0000311	0.0000267	ASPM/DCT/DMRTA2/DRD2/FGF2/FOXG1/FZD3/GLI3/HIF1A/ID4/ITGB1/NR2E1/OTP/PAX6/PTN/SHH/SIX3/SMO/SOX10/TAFA3/VAX1/VEGFA	22

cluster5	BP	GO:0046530	photoreceptor cell differentiation	30/3710	64/18903	0.000000757	0.000032	0.0000275	AHI1/BBS1/CNGB1/CRB2/DCX/DLX1/DLX2/DSCAM/GNAT1/GNGT1/HCN1/IFT140/MFRP/MFSD2A/NPHP4/NR2E3/NTRK2/OLFM3/PAX6/PCARE/PROM1/RORB/RP1/SOX9/TH/THRB/THY1/TULP1/USH1C/VEGFA	30
cluster5	BP	GO:0042596	fear response	23/3710	43/18903	0.000000798	0.0000334	0.0000287	ADRA2A/ASIC1/ASIC4/ATP1A2/BRINP1/CRHR1/DBH/DRD1/DRD4/GABRA5/GRIK2/GRP/GRPR/HTR2C/LYPD1/MAPK8IP2/MDK/NEUROD2/NPAS2/NR2E1/PENK/PRKAR1B/SLC1A1	23
cluster5	BP	GO:0048713	regulation of oligodendrocyte differentiation	23/3710	43/18903	0.000000798	0.0000334	0.0000287	ASPA/DAG1/DLX1/DLX2/DUSP15/GSX2/HDAC2/HES1/HES5/ID4/MDK/NKX2-2/NKX6-1/OPALIN/PTN/PTPRZ1/SHH/SOX1/TENM4/TMEM98/TNFRSF21/TP73/ZNF488	23
cluster5	BP	GO:0050432	catecholamine secretion	28/3710	58/18903	0.000000832	0.0000346	0.0000297	ADRA2A/AGT/CHGA/CHRNA4/CHRNA6/CRH/DRD2/GDNF/GHSR/GRM2/HRH3/HTR2A/KCNB1/OPRK1/PRKN/STX1A/SYT1/SYT10/SYT12/SYT13/SYT2/SYT3/SYT4/SYT5/SYT6/SYT7/SYT8/SYT9	28

cluster5	BP	GO:0061005	cell differentiation involved in kidney development	28/3710	58/18903	0.000000832	0.0000346	0.0000297	BASP1/BMP4/EDNRA/EDNRB/FOXJ1/GDNF/GLI3/GREM1/HES1/KLF15/LAMB2/LGR4/LHX1/MAGI2/NOTCH2/NPHS1/PAX2/POU3F3/PROM1/PTCH1/SALL1/SHH/SIX2/SMO/WNT4/WNT9B/WT1/YAP1	28
cluster5	BP	GO:0071277	cellular response to calcium ion	37/3710	87/18903	0.000000848	0.0000351	0.0000302	ADCY1/ADCY8/ADGRV1/CHP2/CPNE2/CPNE4/CPNE5/CPNE6/CPNE9/DMTN/FOS/FOSB/GPLD1/HPCA/ITPKA/JUNB/KCNB1/KCNH1/LOC118142757/NEUROD2/NLGN1/PRKAA2/RYR3/SLC25A23/SYT1/SYT10/SYT12/SYT13/SYT2/SYT3/SYT4/SYT5/SYT6/SYT7/SYT8/SYT9/WNT5A	37
cluster5	BP	GO:0060291	long-term synaptic potentiation	42/3710	104/18903	0.000000859	0.0000354	0.0000304	ABL1/ADCY1/ADCY8/ADORA1/APP/ARC/CALB1/CALB2/CHRNA7/CYP46A1/DRD1/DRD2/GFAP/GRIN2A/GRIN2B/GRIN2D/GSK3B/IGSF11/INS/LILRB2/LRRTM2/MPP2/NFATC4/NPTN/NR2E1/NTRK2/PRKAR1B/PRKCG/PRRT1/PTN/RELN/SERPINE2/SHANK2/SHISA7/SLC1A1/SLC24A2/SLC8A2/SLC8A3/SNAP25/SQSTM1/SYT12/TNR	42
cluster5	BP	GO:0010721	negative regulation of cell development	65/3710	188/18903	0.000000984	0.0000404	0.0000347	ABCC8/ATOH1/BCL11A/BMP7/BRINP1/CDKL3/CDKN2B/CLDN18/DAB1/DLL3/DLX1/DLX2/DMTN/DPYSL5/DRAXIN/EDNRB/EFNA5/EFNB3/EPHA7/F2/FBLN1/FRZB/GDI1/GSK3B/HES1/HES5/ID1/ID4/LHX2/LRP4/MAP2/MT3/NFATC4/NKX6-1/NPPC/NR2E1/NTN1/PAX6/PRTG/PTN/PTPRS/RFLNA/SEMA3A/SEMA3E/SEMA4D/SEMA5A/SEMA5B/SEMA6A/SEMA6D/SIRT2/SLIT1/SOX10/SYT4/THY1/TLX2/TMEM98/TNR/TTPA/VAX1/VEGFA/WEE2/WNT3A/WNT5A/WNT7A/ZNF664-RFLNA	65

cluster5	BP	GO:0021895	cerebral cortex neuron differentiation	17/3710	27/18903	0.00000102	0.0000417	0.0000358	ARX/ASCL1/CHD5/DLX1/DLX2/DRD1/DRD2/ELAVL4/EMX1/FEZF2/HES1/HPRT1/ID4/NKX2-1/NR2E1/OPHN1/TOX	17
cluster5	BP	GO:0046928	regulation of neurotransmitter secretion	38/3710	91/18903	0.00000105	0.0000429	0.0000368	ADCY1/ADORA2B/ADRA1A/ADRA2A/APBA1/ASIC1/CAMK2A/CHRM2/CHRNA3/CHRNA6/CHRNB4/CPLX2/CPLX4/CSPG5/DRD1/DRD2/GIPC1/GSK3B/HTR2A/NLGN1/PFN2/PRKCG/PRKN/RAB5A/RAP1B/RIMS3/RIMS4/SEPTIN5/SLC30A1/SNCAIP/STX1A/STXBP1/SYN1/SYP/SYT1/SYT12/SYT4/WNT7A	38
cluster5	BP	GO:0021510	spinal cord development	40/3710	98/18903	0.00000112	0.0000456	0.0000391	ACTL6B/ASCL1/CHRD/DAB1/DLL1/DMRT3/DRAXIN/DRGX/FOXN4/GDNF/GLI2/GLI3/GSX1/GSX2/HOXC10/HOXD10/ISL1/LBX1/LHX1/LHX3/LHX5/MDGA2/MNX1/NEUROG3/NKX2-2/PAX6/PHGDH/PTCH1/RELN/RFX4/SHH/SLIT1/SMO/SOX1/TBX20/TULP3/UNCX/VIT/WNT3A/ZIC1	40
cluster5	BP	GO:0098661	inorganic anion transmembrane transport	48/3710	126/18903	0.00000113	0.0000456	0.0000391	ADAMTS8/ANO3/ANO4/ANO6/AQP6/BEST2/BEST3/CFTR/CLCA2/CLCN2/CLCN4/CLCNKA/CLCNKB/CLDN4/CLIC6/FXYD3/GABRA1/GABRA2/GABRA3/GABRA4/GABRA5/GABRA6/GABRB2/GABRB3/GABRD/GABRE/GABRG1/GABRG2/GABRG3/GABRQ/GLRA1/GLRA2/GLRB/GRM5/SLC12A5/SLC12A9/SLC13A1/SLC13A4/SLC17A6/SLC17A7/SLC17A8/SLC1A1/SLC26A9/SLC34A1/SLC37A1/SLC4A11/SLC6A1/TTYH1	48

cluster5	BP	GO:0006937	regulation of muscle contraction	61/3710	174/18903	0.00000123	0.0000498	0.0000427	ACE2/ADORA1/ADORA2B/ADRA1A/ADRA1B/ADRA2A/ATP1A2/ATP1B1/CACNA1C/CACNA1S/CACNB1/CALCA/CASQ2/CCN2/CHGA/CHRM2/CHRM3/CHRNA3/CHRNB4/CNN1/DMD/DOCK4/DSG2/EDN2/EHD3/F2R/FKBP1B/GATA4/GHSR/GSTO1/GUCY1A1/HCN4/HRC/ITGA2/KCNB2/KCNMA1/MYH7/MYOCD/NMU/PDE5A/PKP2/PRKG1/PTGS2/PVALEF/RYR2/SCN10A/SCN4A/SLC8A3/SPX/SSTR2/STC1/TACR1/TACR3/TBXA2R/TNNI1/TNNI3/TNNT2/TPM1/TRPM4/TRPV4/ZC3H12A	61
cluster5	BP	GO:0001755	neural crest cell migration	28/3710	59/18903	0.00000129	0.0000517	0.0000444	ALX1/BMP4/BMP7/EDNRA/EDNRB/ERBB4/FGF19/FOLR1/GBX2/GDNF/HIF1A/ISL1/KITLG/LAMA5/NRP2/PHOX2B/RADIL/RET/SEMA3A/SEMA3E/SEMA4D/SEMA5A/SEMA5B/SEMA6A/SEMA6D/SHH/SMO/SOX10	28
cluster5	BP	GO:0010038	response to metal ion	108/3710	360/18903	0.00000135	0.000054	0.0000464	ABCC8/ACTA1/ADAMTS13/ADCY1/ADCY8/ADGRV1/AKT1/AMELX/ANK3/APP/ASCL1/ATF4/BMP6/CACNA1G/CACNG2/CASQ2/CDK1/CHP2/CLDN1/CNGA3/CPNE2/CPNE4/CPNE5/CPNE6/CPNE9/CPS1/CYP11B1/CYP11B2/DMTN/DRD2/EGFR/FGF23/FOS/FOSB/GDI1/GLRA1/GLRA2/GPLD1/HFE/HIF1A/HMGCS2/HOMER1/HPCA/HSF1/IGFBP2/ITPKA/JUNB/KCNB1/KCNC1/KCNC2/KCNH1/KCNK3/KCNMA1/KCNMB2/LOC118142757/MAOB/MT1A/MT1B/MT1HL1/MT1X/MT2A/MT3/MYOG/NEUROD2/NFATC4/NLGN1/NPTX1/PCSK1/PENK/PRKAA2/PRKN/PTGS2/PTH/RYR2/RYR3/SDC1/SHH/SLC13A5/SLC1A1/SLC25A23/SLC30A1/SLC30A3/SLC30A8/SLC34A1/SLC6A1/SLC6A3/SUCNR1/SYT1/SYT10/SYT12/SYT13/SYT2/SYT3/SYT4/SYT5/SYT6/SYT7/SYT8/SYT9/TAT/TFAP2A/TH/TNFRSF11B/TNNT2/TPH2/TRPC3/WNT5A/ZACN	108
cluster5	BP	GO:0042461	photoreceptor cell development	25/3710	50/18903	0.00000141	0.000056	0.0000481	AHI1/BBS1/CNGB1/CRB2/DCX/GNAT1/GNGT1/HCN1/IFT140/MFRP/MFSD2A/NPHP4/NR2E3/NTRK2/OLFM3/PAX6/PCARE/RORB/RP1/TH/THRB/THY1/TULP1/USH1C/VEGFA	25

cluster5	BP	GO:1903305	regulation of regulated secretory pathway	53/3710	145/18903	0.00000143	0.0000567	0.0000487	ADCY1/ADORA2B/ADRA1A/ADRA2A/APBA1/CACNA1G/CBARP/CD84/CDK5R2/CHRM2/CHRNA6/CSPG5/DOC2A/DOC2B/DRD1/DRD2/FER/GIPC1/GSK3B/HTR2A/IL13/ITGAM/KCNB1/LAMP1/LYN/NLGN1/PFN2/PLA2G3/PRKCG/RAB5A/RAP1B/RIMS3/RIMS4/RPH3AL/SCAMP5/SEPTIN5/STX1A/STXBP1/SYN1/SYP/SYT1/SYT10/SYT12/SYT13/SYT2/SYT3/SYT4/SYT5/SYT6/SYT7/SYT8/SYT9/WNT7A	53
cluster5	BP	GO:0021766	hippocampus development	35/3710	82/18903	0.0000015	0.0000594	0.000051	ALK/BBS1/BCAN/CDK5R1/CDK5R2/DAB1/DCLK2/DLX1/DLX2/DRD1/EMX2/EPHA5/EZH2/FEZ1/FEZF2/GLI3/GSK3B/HSD3B2/ID4/KIRREL3/LHX5/LMX1A/MDK/MFSD2A/NEUROD1/NEUROD6/NKX2-1/NR2E1/RELN/SMO/SRD5A2/TMEM108/WNT3A/ZIC1/ZIC3	35
cluster5	BP	GO:0051968	positive regulation of synaptic transmission, glutamatergic	19/3710	33/18903	0.00000165	0.000065	0.0000558	CACNG2/CACNG3/CACNG4/CACNG5/CACNG7/DRD1/GRIN1/GRIN2A/GRIN2B/GRIN2D/NLGN1/NLGN2/NLGN3/NRXN1/PTGS2/RELN/ROR2/STXBP1/TNR	19
cluster5	BP	GO:0003407	neural retina development	34/3710	79/18903	0.00000168	0.000066	0.0000566	AHI1/ATP2B2/BARHL2/CALB1/CRB2/DLX1/DLX2/DSCAM/FAT3/FOXN4/GPM6A/HCN1/HES1/LHX1/MEGF11/NEUROD1/NTRK2/PROM1/PTF1A/RORB/RP1/RPE65/SDK1/SIX3/SLC17A7/SLC17A8/SLC1A1/SOX9/TFAP2A/TFAP2B/THRB/THY1/USH1C/VSX1	34

cluster5	BP	GO:0001508	action potential	53/3710	146/18903	0.00000182	0.0000711	0.0000611	ADRA1A/AKAP6/ANK3/ATP1A2/ATP1B1/CACNA1C/CACNA1G/CACNA2D1/CHRNA4/CHRNB4/CLDN19/CNTNAP1/DMD/DRD1/DSG2/FKBP1B/GJC1/GJD2/GLRA1/GRIK2/HCN1/HCN3/HCN4/KCNB1/KCNC2/KCND2/KCND3/KCNE4/KCNH2/KCNH6/KCNJ3/KCNJ5/KCNMB2/KCNN2/NEDD4L/NRCAM/NTRK2/P2RX3/PKP2/PTPN3/RYR2/SCN10A/SCN1A/SCN2A/SCN2B/SCN3B/SCN4A/SCN8A/SCN9A/SLC4A3/SLC8A2/TACR1/TRPM4	53
cluster5	BP	GO:0060042	retina morphogenesis in camera-type eye	29/3710	63/18903	0.00000184	0.0000714	0.0000613	AHI1/CALB1/CDON/CRB2/DLL1/DSCAM/FAT3/FOXN4/HCN1/LHX1/LRP5/MEGF11/MFSD2A/NECTIN3/NTRK2/PROM1/PTF1A/RORB/RP1/RPE65/SDK1/SLC1A1/SOX9/TFAP2A/TFAP2B/THRB/THY1/USH1C/VSX1	29
cluster5	BP	GO:0008217	regulation of blood pressure	65/3710	191/18903	0.00000184	0.0000714	0.0000613	ABCC9/ACE/ACE2/ADM5/ADORA1/ADRA1A/ADRA1B/ADRA1D/ADRB3/AGT/AGTR2/AGTRAP/ANPEP/ASIC2/ATP1A2/AVP/AVPR1B/CALCA/CHGA/CMA1/CORO2B/CTSG/CYP11B1/CYP11B2/DDAH1/DLL1/DRD2/ECE1/EDN2/EDNRA/EDNRB/EMILIN1/EMILIN2/EMP2/F2R/GAS6/GLP1R/GRIP2/GUCY1A1/KCNK6/KLK3/LRP5/LVRN/MYH6/NAV2/NOS2/NOS3/NPY/NR2F2/OR51E2/P2RX2/PTGS2/RASL10B/RPS6KA2/SCNN1G/SCPEP1/SLC4A5/SPX/SUCNR1/TAC3/TACR1/TACR3/TBXA2R/TNNI3/TPM1	65
cluster5	BP	GO:1905332	positive regulation of morphogenesis of an epithelium	20/3710	36/18903	0.0000019	0.0000734	0.000063	ABL1/AGT/AGTR2/BMP4/EGF/FGF2/GDNF/GREM1/LBX2/LGR4/LHX1/MAGED1/MDK/PAX2/SIX4/SMO/SOX9/VEGFA/WNT2B/WNT4	20

cluster5	BP	GO:0090190	positive regulation of branching involved in ureteric bud morphogenesis	13/3710	18/18903	0.00000199	0.0000763	0.0000655	AGT/AGTR2/GDNF/GREM1/LGR4/LHX1/MAGED1/PAX2/SIX4/SMO/SOX9/VEGFA/WNT2B	13
cluster5	BP	GO:0098962	regulation of postsynaptic neurotransmitter receptor activity	13/3710	18/18903	0.00000199	0.0000763	0.0000655	BEGAIN/CACNG4/CACNG5/DLGAP2/DLGAP3/HOMER1/NPTX1/NPTX2/NPTXR/SHISA6/SHISA7/SHISA9/SRC	13
cluster5	BP	GO:0050805	negative regulation of synaptic transmission	32/3710	73/18903	0.00000207	0.0000794	0.0000681	ADCY8/ADORA1/ARC/ASIC1/BCHE/CBLN1/CELF4/DRD1/DRD2/EIF4A3/GRIA1/GRID2/GRID2IP/GRIK2/GRIK3/HCN1/HTR2A/KCNB1/LILRB2/NLGN4X/NPY5R/PRKN/PRRT1/PTGS2/RAP1B/SHANK2/SLC24A2/SLC30A1/SLC6A1/STXBP1/SYT4/TNR	32
cluster5	BP	GO:0051592	response to calcium ion	53/3710	147/18903	0.00000231	0.0000879	0.0000755	ADCY1/ADCY8/ADGRV1/AMELX/CACNG2/CASQ2/CHP2/CPNE2/CPNE4/CPNE5/CPNE6/CPNE9/DMTN/FOS/FOSB/GDI1/GPLD1/HOMER1/HPCA/ITPKA/JUNB/KCNB1/KCNH1/KCNMA1/KCNMB2/LOC118142757/NEUROD2/NLGN1/PCSK1/PENK/PRKAA2/RYR2/RYR3/SDC1/SLC25A23/SLC6A1/SUCNR1/SYT1/SYT10/SYT12/SYT13/SYT2/SYT3/SYT4/SYT5/SYT6/SYT7/SYT8/SYT9/TNNT2/TPH2/TRPC3/WNT5A	53

cluster5	BP	GO:0031346	positive regulation of cell projection organization	105/3710	352/18903	0.00000251	0.0000953	0.0000818	ABL1/AGT/ALK/ALKAL2/AUTS2/BDNF/BMP5/BMP7/BRK1/CAMK2B/CARMIL1/CDH4/CDKL3/CHODL/CLRN1/CRABP2/CUL7/CUX1/CYFIP1/DBN1/DPYSL3/DSCAM/DZIP1/EFNA5/ELAVL4/EPHA3/EPO/EPS8/EPS8L3/EZH2/FEZ1/FKBP1B/FNBP1L/FUT9/GDI1/GPC2/GPM6A/GRIP1/GSK3B/HAP1/IL1RAPL1/INS/ISLR2/ITGA2/ITPKA/KHDC3L/L1CAM/LYN/MAGI2/MAP6/MDK/MNS1/MYO3A/MYO3B/NCKAP1/NDEL1/NDNF/NDRG4/NEURL1/NGF/NLGN1/NPTN/NTN1/NTRK2/PACSIN1/PAK3/PLA2G3/PLK5/PLPPR5/PLXNA1/PLXNA2/PLXNA4/PLXNB1/PLXNB3/PROM2/PTK7/PTN/PTPRD/RELN/RET/RIT2/RND2/ROBO1/ROBO2/ROR1/ROR2/RP1/SAXO1/SEMA4D/SEMA5A/SHOX2/SLITRK1/SRC/STK24/STMN2/TENM2/TENM3/TMEM30A/TNN/TOX/TRIM67/VEGFA/WNT3A/WNT5A/ZDHHC15	105
cluster5	BP	GO:0007158	neuron cell-cell adhesion	12/3710	16/18903	0.00000265	0.000100105	0.0000859	ASTN1/CDK5R1/CNTN4/NCAM2/NLGN1/NLGN2/NLGN3/NLGN4X/NRXN1/NRXN3/RET/TNR	12
cluster5	BP	GO:0006887	exocytosis	109/3710	369/18903	0.0000027	0.000101712	0.0000873	ABCA12/ADCY1/ADORA2B/ADRA1A/ADRA2A/APBA1/ARHGAP44/ATP9A/BLOC1S6/BRSK2/CACNA1G/CADPS/CBARP/CD84/CDK5R2/CFTR/CHGA/CHRM2/CHRNA6/CLTRN/CPLX2/CPLX4/CSPG5/DMTN/DOC2A/DOC2B/DRD1/DRD2/EQTN/EXOC1L/EXOC3L1/EXOC3L2/EXOC3L4/FER/GIPC1/GRIK5/GRP/GSK3B/HAP1/HTR2A/IL13/IL1RAPL1/ITGAM/KCNB1/LAMP1/LGI3/LYN/MRGPRX2/MYH9/NKD2/NLGN1/OTOF/PCLO/PFN2/PLA2G3/PRKCG/PRKN/PRRT2/PRSS12/RAB11FIP2/RAB27B/RAB31/RAB3B/RAB5A/RAB8A/RAP1B/RIMS3/RIMS4/RPH3AL/SCAMP1/SCAMP5/SCFD2/SDC1/SDC4/SEPTIN4/SEPTIN5/SNAP23/SNAP25/SRCIN1/STEAP2/STX11/STX1A/STXBP1/STXBP5L/SV2A/SYN1/SYN2/SYNGR3/SYP/SYT1/SYT10/SYT12/SYT13/SYT16/SYT2/SYT3/SYT4/SYT5/SYT6/SYT7/SYT8/SYT9/TMED10/TRARG1/UNC13A/UNC13C/VSNL1/WNT7A/ZP4	109
cluster5	BP	GO:0061337	cardiac conduction	40/3710	101/18903	0.00000278	0.000104134	0.0000894	ABCC9/ACE2/AGT/ATP1A2/ATP1A3/ATP1B1/ATP2B2/ATP2B3/CACNA1C/CACNA1G/CACNA2D1/CASQ2/DSG2/EHD3/FKBP1B/GJC1/HCN1/HCN3/HCN4/HRC/ISL1/KCND3/KCNE4/KCNH2/KCNH6/KCNJ3/KCNJ5/KCNN2/PKP2/RYR2/SCN10A/SCN2B/SCN3B/SLC4A3/SLC8A2/SLC8A3/SPTBN4/TBX5/TRDN/TRPM4	40

cluster5	BP	GO:1902476	chloride transmembrane transport	40/3710	101/18903	0.00000278	0.000104134	0.0000894	ANO3/ANO4/ANO6/BEST2/BEST3/CFTR/CLCA2/CLCN2/CLCN4/CLCNKA/CLCNKB/CLDN4/CLIC6/FXYD3/GABRA1/GABRA2/GABRA3/GABRA4/GABRA5/GABRA6/GABRB2/GABRB3/GABRD/GABRE/GABRG1/GABRG2/GABRG3/GABRQ/GLRA1/GLRA2/GLRB/SLC12A5/SLC12A9/SLC17A6/SLC17A7/SLC17A8/SLC1A1/SLC26A9/SLC6A1/TTYH1	40
cluster5	BP	GO:0044458	motile cilium assembly	28/3710	61/18903	0.00000295	0.000110201	0.0000946	AHI1/AKAP4/BBOF1/CC2D2A/CCDC40/CEP131/CFAP206/CFAP221/CFAP43/CFAP47/CFAP54/CFAP65/CFAP69/CFAP97D1/DZIP1/FOXJ1/FSIP2/GK2/IFT81/MCIDAS/MNS1/NEURL1/PLA2G3/RSPH6A/RSPH9/SPAG17/SPAG6/UBE2B	28
cluster5	BP	GO:0009954	proximal/distal pattern formation	19/3710	34/18903	0.00000305	0.000112978	0.000097	DLL1/DLX1/DLX2/EN1/FGF10/GLI2/GLI3/GREM1/HES5/HOXA11/HOXB9/HOXC10/HOXC9/HOXD10/IRX2/LRP4/PBX1/SIX3/SP8	19
cluster5	BP	GO:0099084	postsynaptic specialization organization	19/3710	34/18903	0.00000305	0.000112978	0.000097	CBLN1/CDH2/CNTNAP1/GAP43/GRID2/LILRB2/LRRC4B/LRRTM2/NLGN1/NLGN2/NPTX1/NRXN1/NTNG2/OPHN1/PTPRD/RELN/SHANK2/SLITRK3/TMEM108	19

cluster5	BP	GO:0017157	regulation of exocytosis	70/3710	213/18903	0.00000314	0.000115888	0.0000995	ADCY1/ADORA2B/ADRA1A/ADRA2A/APBA1/ATP9A/CACNA1G/CADPS/CBARP/CD84/CDK5R2/CFTR/CHRM2/CHRNA6/CPLX2/CSPG5/DOC2A/DOC2B/DRD1/DRD2/FER/GIPC1/GSK3B/HAP1/HTR2A/IL13/IL1RAPL1/ITGAM/KCNB1/LAMP1/LGI3/LYN/NLGN1/PCLO/PFN2/PLA2G3/PRKCG/PRKN/RAB27B/RAB3B/RAB5A/RAB8A/RAP1B/RIMS3/RIMS4/RPH3AL/SCAMP5/SDC1/SDC4/SEPTIN4/SEPTIN5/STX1A/STXBP1/STXBP5L/SYN1/SYP/SYT1/SYT10/SYT12/SYT13/SYT2/SYT3/SYT4/SYT5/SYT6/SYT7/SYT8/SYT9/VSNL1/WNT7A	70
cluster5	BP	GO:0007216	G protein-coupled glutamate receptor signaling pathway	11/3710	14/18903	0.00000331	0.00012133	0.000104139	DAGLA/GRIK3/GRM2/GRM3/GRM4/GRM5/GRM6/GRM7/GRM8/HOMER1/TRPM1	11
cluster5	BP	GO:0008212	mineralocorticoid metabolic process	11/3710	14/18903	0.00000331	0.00012133	0.000104139	BMP2/BMP5/BMP6/CLCN2/CYP11B1/CYP11B2/CYP21A2/DAB2/DKK3/EDNRB/WNT4	11
cluster5	BP	GO:0071241	cellular response to inorganic substance	74/3710	229/18903	0.00000335	0.000122107	0.000104806	ADCY1/ADCY8/ADGRV1/AKT1/ANK3/APP/ATF4/ATRX/BMP6/CHP2/CLDN1/CPNE2/CPNE4/CPNE5/CPNE6/CPNE9/CYP11B1/CYP11B2/DDX11/DMTN/EGFR/EIF4A3/FOS/FOSB/GLRA1/GLRA2/GPLD1/HFE/HPCA/HSF1/ITPKA/JUNB/KCNB1/KCNC2/KCNH1/KCNK3/LOC118142757/MT1A/MT1B/MT1HL1/MT1X/MT2A/MT3/MYOG/NEUROD2/NFATC4/NLGN1/NPTX1/PHOX2B/PRKAA2/PRKN/PTGS2/RYR3/SHH/SLC13A5/SLC1A1/SLC25A23/SLC34A1/SYT1/SYT10/SYT12/SYT13/SYT2/SYT3/SYT4/SYT5/SYT6/SYT7/SYT8/SYT9/TFAP2A/TH/TPH2/WNT5A	74

cluster5	BP	GO:0014046	dopamine secretion	20/3710	37/18903	0.00000337	0.000122107	0.000104806	CHRNA4/CHRNA6/DRD2/GDNF/GRM2/HTR2A/OPRK1/PRKN/SYT1/SYT10/SYT12/SYT13/SYT2/SYT3/SYT4/SYT5/SYT6/SYT7/SYT8/SYT9	20
cluster5	BP	GO:0014059	regulation of dopamine secretion	20/3710	37/18903	0.00000337	0.000122107	0.000104806	CHRNA4/CHRNA6/DRD2/GDNF/GRM2/HTR2A/OPRK1/PRKN/SYT1/SYT10/SYT12/SYT13/SYT2/SYT3/SYT4/SYT5/SYT6/SYT7/SYT8/SYT9	20
cluster5	BP	GO:0021879	forebrain neuron differentiation	22/3710	43/18903	0.00000368	0.00013298	0.000114139	ASCL1/DCLK2/DLX1/DLX2/FEZF2/FGFR2/FOXG1/GBX2/HES1/HES5/LHX5/NDNF/NHLH2/NKX2-1/NRP2/OTP/PAX6/PLXNA1/SECISBP2/SEMA3E/SOX1/TBR1	22
cluster5	BP	GO:0006705	mineralocorticoid biosynthetic process	10/3710	12/18903	0.00000375	0.000134946	0.000115826	BMP2/BMP5/BMP6/CLCN2/CYP11B1/CYP11B2/CYP21A2/DAB2/DKK3/WNT4	10

cluster5	BP	GO:0022412	cellular process involved in reproduction in multicellular organism	123/3710	430/18903	0.0000038	0.000136311	0.000116997	ABHD2/ACRBP/ADAM2/AKAP4/AKT1/ANGPT2/ASPM/BMP4/BMPR1B/BRCA2/BRME1/C14orf39/C16orf92/C2CD6/CABYR/CATSPER4/CCDC136/CCNB2/CELF4/CEP131/CFAP206/CFAP221/CFAP43/CFAP47/CFAP54/CFAP65/CFAP69/CFAP97D1/CFTR/CGA/CRISP1/DDX25/DDX4/DMC1/DMRT1/DMRTC2/DPY19L2/DZIP1/EDNRA/EHMT2/EQTN/FAM9A/FER/FKBP6/FMN2/FOLR1/FOXC1/FSIP2/GK2/GPR149/HSF2BP/HSPA2/IFT81/ITGB1/IZUMO1/KIF18A/LGR5/MAEL/MDK/MEIOB/MLH3/MNS1/MOV10L1/NECTIN3/NEURL1/NME5/NOBOX/NPM2/NPPC/OCA2/PACRG/PCSK4/PDE3A/PDE5A/PIWIL1/PLA2G3/PLK1/PRDM14/PRM1/PRSS37/PTCH1/PTN/PYGO1/PYGO2/ROBO2/ROPN1/RPL10L/RPS6KA2/RSPH1/RSPH6A/SEPTIN4/SIRT2/SLC25A31/SLIT3/SMAD5/SOHLH1/SOHLH2/SPACA1/SPAG17/SPAG6/SPATA16/SPEM1/SPINK1/SPO11/SRC/SYCP1/TBPL1/TCP11/TCP11X1/TDRD5/TEX15/TEX19/TOP2A/TSSK1B/TUT4/UBE2B/UCHL1/WEE2/WNT4/WT1/YBX2/ZBTB16/ZPBP	123
cluster5	BP	GO:0030902	hindbrain development	54/3710	153/18903	0.0000039	0.000139644	0.000119858	ABL1/AHI1/ASCL1/B4GALT2/BMP5/BMP7/CBLN1/CDK5R1/CDK5R2/CTNNA2/DAB1/DLL1/EGF/EN1/EN2/EZH2/FAIM2/FGF2/FOXC1/GBX2/GDF10/GLI2/GRID2/GSX2/HAP1/HES1/HNF1B/HOXB1/ITGB1/KCNC1/KLHL1/KNDC1/LHX1/LHX5/LMX1A/MDK/NEUROD1/NEUROD2/NEUROG3/NHLH2/NLGN4X/OPHN1/OTX1/PHOX2B/PLXNA2/PTF1A/SERPINE2/SHH/SMO/SSTR1/SSTR2/TBR1/WHRN/WNT7A	54
cluster5	BP	GO:0060537	muscle tissue development	121/3710	422/18903	0.000004	0.000142537	0.000122341	ABL1/ACTA1/ACTC1/ACTN2/ADRA1A/AGT/AKAP6/ANKRD33/BARX2/BMP2/BMP4/BMP5/BMP7/BVES/CACNA1G/CACNA1S/CDK1/CDON/CNTNAP1/COL11A1/CSRP2/CSRP3/DIPK2A/DLL1/DMD/DMRTA2/DNER/DSG2/EDNRA/EFNB2/ERBB3/ERBB4/FGF2/FGF9/FGFR2/FHOD3/FKBP1A/FOS/FOXC1/GATA4/GJC1/GREM1/HCN4/HEYL/HMG20B/HOMER1/HOXD10/ISL1/ITGA7/ITGB1/KLHL40/LMNA/LOX/LRP2/LUC7L/MEGF10/MSX2/MYH6/MYH7/MYL7/MYLK/MYO18B/MYOCD/MYOG/MYOZ1/MYOZ2/NDRG4/NEURL1/NKX2-6/NOTCH2/NPHS1/NR2F2/NRAP/P2RX2/PDGFRA/PDGFRB/PHOX2B/PITX1/PKP2/POPDC3/PRKAR1A/PTCH1/RARB/RBFOX1/RBM24/RGS4/RXRG/RYR2/SGCZ/SHH/SHOX2/SIK1/SIRT2/SIX4/SMAD5/SMO/SMYD1/SOX9/STRA6/TBX20/TBX5/TENM4/TFAP2B/TIPARP/TLL2/TNN/TNNI1/TNNI3/TNNT2/TP73/TPM1/VAX1/VEGFA/VGLL2/VGLL4/WNT3A/WNT5A/WNT8A/WT1/YAP1/ZIC3	121
cluster5	BP	GO:1905330	regulation of morphogenesis of an epithelium	29/3710	65/18903	0.00000401	0.000142616	0.000122409	ABL1/AGT/AGTR2/BMP4/BMP7/EGF/FGF10/FGF2/FGF7/GDNF/GREM1/HOXD13/LBX2/LGR4/LHX1/MAGED1/MDK/PAX2/PDGFA/SHH/SIX2/SIX4/SMO/SOX9/SULF1/VEGFA/WNT2B/WNT4/WNT5A	29

cluster5	BP	GO:0003156	regulation of animal organ formation	17/3710	29/18903	0.00000418	0.000148021	0.000127048	BMP2/BMP4/BMP7/FGF10/FGF2/GDNF/HOXA11/ROBO1/ROBO2/SHH/SPRY1/SULF1/WNT11/WNT2B/WNT3A/WNT5A/WT1	17
cluster5	BP	GO:0048706	embryonic skeletal system development	47/3710	128/18903	0.00000476	0.000167985	0.000144183	ALX1/ALX4/BMP4/BMP7/COL11A1/COL2A1/DLX1/DLX2/DSCAML1/EDNRA/EIF4A3/FGF9/FGFR2/GLI3/GRHL2/HOXA11/HOXA3/HOXA4/HOXB1/HOXB6/HOXB9/HOXC4/HOXC6/HOXC9/HOXD10/HOXD3/IFT140/IRX5/LHX1/MDFI/MMP16/PBX1/PDGFRA/SHH/SHOX2/SIX2/SIX4/SLC2A10/SULF1/SULF2/TBX15/TFAP2A/TULP3/WNT11/WNT5A/WNT9A/WNT9B	47
cluster5	BP	GO:0007218	neuropeptide signaling pathway	42/3710	110/18903	0.00000481	0.000169068	0.000145113	ADCYAP1/CALCA/ECEL1/GLRA1/GLRA2/GLRB/GPR139/GPR149/GPR37/GRP/GRPR/HCRTR1/HCRTR2/MC2R/MCHR1/NMBR/NMS/NMU/NPB/NPFFR2/NPSR1/NPY/NPY5R/NTS/NXPH2/NXPH3/NXPH4/OPRK1/PDYN/PENK/PTH2/PYY/QRFPR/SCG5/SORCS1/SORT1/SSTR1/SSTR2/SSTR5/TAC1/TAC3/TYRO3	42
cluster5	BP	GO:0050905	neuromuscular process	54/3710	154/18903	0.00000488	0.000170909	0.000146693	ABL1/ADCY5/APP/ASCL1/CLRN1/CNTNAP1/CTNNA2/DMD/DRD1/DRD2/EDNRA/FOXS1/GLRA1/GLRB/GRID2/GRIN2A/GRIN2D/GSTO1/HMX3/HOMER1/HOXC10/HOXD10/JPH3/JPH4/JSRP1/KCNH1/MAP1A/MYH7/MYH8/NEUROG1/NLGN2/NPAS1/NRXN1/PCDH15/PENK/POU4F3/PRKN/PRRT2/PVALEF/RBFOX1/SCN1A/SCN4A/SHANK1/SLC6A3/SLC8A3/SLITRK6/SLURP1/STAC2/STRA6/TNNI1/TNNI3/TNR/UCHL1/USH1C	54

cluster5	BP	GO:0009798	axis specification	38/3710	96/18903	0.00000496	0.000173261	0.000148712	AHI1/BASP1/BMP4/CDX1/CDX2/CDX4/CHRDL1/CXXC4/DDIT3/DLL1/FGF10/FOXA2/GPC3/IRX4/LHX1/MDFI/MNS1/NEUROG1/NOTCH2/OTX2/PAX6/PGAP1/PTCH1/RIPPLY1/SHH/SIX2/SIX3/SMO/STC1/TBXT/TDGF1/TDRD5/VAX2/WNT5A/WNT7A/WNT8A/WT1/ZIC3	38
cluster5	BP	GO:0097120	receptor localization to synapse	26/3710	56/18903	0.0000051	0.000177642	0.000152473	ADAM10/ARHGAP44/CACNG2/CACNG3/CACNG4/CACNG5/CACNG7/DAG1/DBN1/GHSR/GPC6/GRIP1/GRIP2/GRIPAP1/IQSEC2/KIF5A/KIF5C/LGI1/NLGN1/NPTN/NPTX1/RAB8A/RELN/SHISA6/SNAP25/STX7	26
cluster5	BP	GO:1904862	inhibitory synapse assembly	13/3710	19/18903	0.00000515	0.00017887	0.000153526	CBLN1/GABRA1/GABRA2/GABRB2/GABRB3/GABRG2/HAPLN4/LHFPL4/NLGN2/NPAS4/PLXNB1/SEMA4D/WNT5A	13
cluster5	BP	GO:0048732	gland development	125/3710	441/18903	0.00000521	0.000180216	0.000154682	ABL1/AKT1/ARID5B/ASCL1/BMP2/BMP4/BMP7/BRCA2/BSX/CADM1/CCDC40/CCKBR/CCNB2/CDKN2B/CDO1/CGA/CLCN2/CLDN1/CPB2/CPS1/CRH/CRHR1/CSN3/DAG1/DKK3/DRD2/E2F7/EDNRA/EGF/EGFR/EPHB3/ERBB4/EZH2/FGF10/FGF2/FGF7/FGFR2/FOXA1/FOXC1/FOXN1/FRZB/GCM2/GLI2/GLI3/GPAT4/GSX1/HES1/HIF1A/HMGCS2/HNF1A/HNF1B/HOXA11/HOXA3/HOXB13/HOXB9/HOXD13/HOXD3/HPN/ID4/IGSF3/ISL1/ITGA2/KRT76/LAMA1/LAMA5/LHX3/LRP5/MDK/MSX2/NEURL1/NKX2-1/NKX2-8/NOTCH2/NOTCH4/NR0B1/NRG3/NTN1/OTP/PAX6/PBX1/PCSK1/PDGFA/PDGFRA/PITX1/PLXNA1/POU3F2/PSAP/PSAPL1/PTCH1/PTF1A/PTN/PTPN3/PYGO2/ROBO1/SALL1/SERPINE2/SHH/SIX3/SIX4/SLC6A3/SMO/SOSTDC1/SOX10/SOX3/SOX9/SRC/STRA6/SULF1/TAF10/TDGF1/TNC/TNFRSF11A/TYMS/TYR/UGT1A6/UGT1A8/VEGFA/WNT11/WNT3A/WNT4/WNT5A/WNT7B/WT1/XDH/ZIC3	125

cluster5	BP	GO:0002062	chondrocyte differentiation	43/3710	114/18903	0.00000538	0.000185421	0.00015915	ADAMTS7/AMELX/BMP2/BMP4/BMP6/BMPR1B/CCN2/CHADL/COL11A1/COL27A1/COL2A1/ECM1/FGF9/FGFR3/GDF5/GDF6/GLI3/GPLD1/GREM1/HIF1A/HMGA2/HOXA11/MDK/MSX2/NPPC/PKDCC/PTH/PTH1R/RARB/RFLNA/SFRP2/SHOX2/SIX2/SOX5/SOX9/SULF1/SULF2/WNT2B/WNT7A/WNT9A/ZBTB16/ZNF219/ZNF664-RFLNA	43
cluster5	BP	GO:0051961	negative regulation of nervous system development	53/3710	151/18903	0.00000575	0.000197588	0.000169592	ABCC8/ATOH1/BMP7/BRINP1/CBLN1/CDKL3/CDKN2B/DAB1/DLL3/DLX1/DLX2/DPYSL5/DRAXIN/EFNB3/EPHA7/F2/GDI1/HES1/HES5/ID1/ID4/LHX2/LRP4/MAP2/MT3/NFATC4/NKX6-1/NR2E1/NTN1/PAX6/PRTG/PTN/PTPRS/ROBO2/SEMA3A/SEMA3E/SEMA4D/SEMA5A/SEMA5B/SEMA6A/SEMA6D/SIRT2/SLIT1/SOX10/SYT4/THY1/TLX2/TMEM98/TNR/VAX1/WNT3A/WNT5A/WNT7A	53
cluster5	BP	GO:2001222	regulation of neuron migration	23/3710	47/18903	0.00000589	0.000201932	0.000173321	CAMK2A/CAMK2B/CTNNA2/DRD1/DRD2/FLRT2/GPR173/KIF20B/MDK/NEXMIF/NKX6-1/NRG3/NTNG1/NTNG2/RELN/SCRT1/SCRT2/SEMA3A/SEMA6A/SOX14/TNN/UNC5C/UNC5D	23
cluster5	BP	GO:0042417	dopamine metabolic process	21/3710	41/18903	0.00000597	0.00020278	0.000174049	COMT/DAO/DBH/DRD1/DRD2/DRD4/GPR37/GRIN2A/HPRT1/ITGAM/MAOB/NPY/NR4A2/PDE1B/PRKN/SLC1A1/SLC6A3/SNCAIP/SNCB/TACR3/TH	21

cluster5	BP	GO:0001504	neurotransmitter uptake	22/3710	44/18903	0.000006	0.00020278	0.000174049	ATP1A2/DRD1/DRD2/DRD4/GDNF/GFAP/GPM6B/ITGB1/ITGB3/PRKN/RAB3B/SLC17A8/SLC1A2/SLC1A6/SLC29A4/SLC6A1/SLC6A11/SLC6A2/SLC6A3/SLC6A5/SNAP25/SYNGR3	22
cluster5	BP	GO:0001709	cell fate determination	22/3710	44/18903	0.000006	0.00020278	0.000174049	ASCL1/ATOH1/BARHL2/BMP4/DLL1/DSCAML1/EBF2/FEZF2/FOXG1/HES1/ISL1/LBX1/NKX2-2/NOTCH2/NOTCH4/PAX2/PAX6/POU6F2/PTCH1/PTCH2/TNXB/WNT7A	22
cluster5	BP	GO:0014075	response to amine	22/3710	44/18903	0.000006	0.00020278	0.000174049	ADAMTS13/ASIC1/CDK1/CPS1/DBH/DRD1/DRD2/DRD4/EDNRA/GLDC/GRIN2A/HDAC2/HPRT1/ITGA2/KCNC1/KCNC2/NR4A2/PDE1B/RGS4/RGS7/SLC1A1/TH	22
cluster5	BP	GO:0048675	axon extension	45/3710	122/18903	0.00000655	0.000220663	0.000189398	ABL1/AUTS2/BARHL2/CDH4/CDKL3/CYFIP1/DBN1/DCLK1/DRAXIN/DSCAM/EDN2/EDNRA/GDI1/GSK3B/ISLR2/ITGB1/L1CAM/LAMB2/LHX2/MAP2/MT3/NDEL1/NKX6-1/NLGN3/NRCAM/NRP2/NTN1/OLFM1/PLXNA4/POU4F3/PTPRS/SEMA3A/SEMA3E/SEMA4D/SEMA5A/SEMA5B/SEMA6A/SEMA6D/SLC9A6/SLIT1/SLIT3/TNR/VEGFA/WNT3A/WNT5A	45

cluster5	BP	GO:0003401	axis elongation	16/3710	27/18903	0.00000663	0.000222873	0.000191295	BMP4/FGF10/FGFR2/HNF1B/HOXD13/MAGI2/PTK7/SFRP2/SHH/SIX4/SOX9/SPRY1/WNT11/WNT3A/WNT5A/YAP1	16
cluster5	BP	GO:0034767	positive regulation of ion transmembrane transport	62/3710	186/18903	0.00000673	0.000225316	0.000193392	ABCC8/ABL1/ACE2/ACTN2/ADCYAP1R1/AGT/AKAP6/ANK3/ANO6/ARC/ATP1B1/CACNA2D1/CACNG2/CACNG3/CACNG4/CALCR/CEMIP/CFTR/CHP2/CLTRN/CNKSR3/DMD/DRD1/DRD4/EDNRA/EHD3/F2/F2R/F2RL3/GRIN1/GRM6/GSTO1/HAP1/HSPA2/IL13/ITGB1/KCNC1/KCNC2/KCNH2/KCNK3/LACRT/LHCGR/LRRC55/NLGN3/NPSR1/OPRK1/P2RX2/P2RX3/PIRT/PLP1/RELN/RGS7/RYR2/SLC17A8/SLC34A1/STAC2/TCAF1/THY1/TRDN/TRPC3/WNK2/WNK3	62
cluster5	BP	GO:0030003	cellular cation homeostasis	138/3710	499/18903	0.00000709	0.000236568	0.00020305	ABL1/ADCY8/ADORA1/AGT/AKAP6/AP3D1/APP/ATF4/ATOX1/ATP13A5/ATP1A2/ATP1A3/ATP1B1/ATP2B2/ATP2B3/ATP6V0A1/ATP6V0A4/ATP6V0B/ATP6V1B1/AVP/AVPR1B/BCAP31/BMP6/CACNA1C/CALB1/CALB2/CALCA/CALCB/CASQ2/CCDC47/CEMIP/CFTR/CHRNA7/CLDN16/CP/CSRP3/DDIT3/DHRS7C/DMD/DRD1/DRD2/DRD4/EDN2/EDNRA/ELANE/ERO1A/F2/F2R/F2RL3/FGF2/FKBP1A/FKBP1B/FTMT/GCM2/GP1BB/GP9/GPR12/GRIA1/GRIK2/GRM5/GSTO1/HAP1/HCRTR1/HCRTR2/HEPH/HERPUD1/HFE/HIF1A/HRC/HTR2A/HTR2C/IL13/ITGB3/JPH1/JPH3/JPH4/JSRP1/KCNMA1/LACRT/LHCGR/LYN/MAIP1/MICU3/MT1A/MT1B/MT1HL1/MT1X/MT2A/MT3/NPSR1/NPTN/PACS2/PKHD1/PRND/PTH/PTH1R/RYR2/RYR3/SCNN1G/SLC11A1/SLC12A5/SLC1A1/SLC24A2/SLC24A3/SLC25A23/SLC30A1/SLC30A3/SLC30A8/SLC39A12/SLC39A5/SLC4A11/SLC4A3/SLC4A4/SLC4A5/SLC8A2/SLC8A3/SLC9A2/SLC9A4/SLC9A5/SLC9A6/SLC9A7/SLC9C2/STC1/STC2/STEAP2/SV2A/TFAP2B/THY1/TMBIM6/TNNI3/TRDN/TRPC3/TRPC4/TRPC7/TRPM8/TRPV4/WFS1/WNT5A	138
cluster5	BP	GO:0060070	canonical Wnt signaling pathway	93/3710	310/18903	0.00000711	0.000236612	0.000203087	AMER3/APC2/ASPM/BMP2/CDH2/CDK14/CSNK1E/CTNND1/CTNND2/DAB2/DACT3/DDIT3/DDX3X/DKK3/DKKL1/DLX5/DRAXIN/EDNRA/EDNRB/EGF/EGFR/FERMT1/FGF10/FGF2/FGF9/FGFR2/FOLR1/FRZB/FZD10/FZD3/GLI3/GPC3/GPRC5B/GREM1/GSK3B/IGFBP2/IGFBP4/ILK/ISL1/LGR4/LGR5/LRP4/LRP5/LRP6/LYPD6/MCC/MDK/NKD2/NPHP4/NR4A2/PLPP3/PRICKLE1/PRKN/PTK7/PTPRU/PYGO1/PYGO2/RAB5A/RBX1/RSPO1/RSPO2/RSPO3/SCEL/SDC1/SEMA5A/SFRP2/SHH/SHISA6/SOSTDC1/SOX10/SOX9/SRC/TLE1/TMEM170B/TMEM198/TNN/TPBG/TRPM4/UBE2B/WNK2/WNT11/WNT2B/WNT3A/WNT4/WNT5A/WNT7A/WNT7B/WNT8A/WNT8B/WNT9A/WNT9B/YAP1/ZNRF3	93

cluster5	BP	GO:0007631	feeding behavior	41/3710	108/18903	0.00000732	0.000242931	0.000208511	ACE/ACE2/ADRB3/AGT/APP/BSX/CALCA/CCK/CNTFR/DACH1/DMBX1/DRD1/DRD2/EN1/FOS/GCG/GHSR/HCRTR1/HCRTR2/HTR2C/IAPP/INS/LEPR/MC4R/MCHR1/MRAP2/NMU/NPB/NPSR1/NPY/NPY5R/NTRK2/OPRK1/PYY/STRA6/TACR1/TACR3/TBR1/TH/TRH/UCHL1	41
cluster5	BP	GO:0045956	positive regulation of calcium ion-dependent exocytosis	12/3710	17/18903	0.00000738	0.000243952	0.000209387	CACNA1G/CDK5R2/DOC2B/KCNB1/SCAMP5/STX1A/STXBP1/SYT1/SYT10/SYT4/SYT7/SYT9	12
cluster5	BP	GO:0048168	regulation of neuronal synaptic plasticity	26/3710	57/18903	0.00000764	0.000251689	0.000216028	ACP4/AGT/APP/ARC/CAMK2A/CAMK2B/DBN1/DRD2/GRIK2/GRIN1/GRIN2A/GRIN2B/GRIN2D/GRM5/JPH3/NEURL1/NPTN/RAB5A/RAB8A/RASGRF1/SHISA6/SHISA7/SHISA9/SLC8A2/SYP/SYT4	26
cluster5	BP	GO:0042475	odontogenesis of dentin-containing tooth	36/3710	91/18903	0.00000885	0.000290337	0.0002492	AMBN/AMELX/APCDD1/BMP2/BMP4/BMP7/CFTR/DLX1/DLX2/DLX3/DMRT3/DSPP/FGF10/FGF4/FOXC1/GLI2/GLI3/HDAC2/KLK5/LAMA5/LRP4/MSX2/NECTIN1/NGFR/ODAM/PDGFRA/RELT/RSPO2/SCN10A/SERPINE1/SHH/SLC34A1/SMO/SOSTDC1/TNFRSF11B/WDR72	36

cluster5	BP	GO:0006875	cellular metal ion homeostasis	119/3710	420/18903	0.00000886	0.000290337	0.0002492	ABL1/ADCY8/ADORA1/AGT/AKAP6/AP3D1/APP/ATF4/ATOX1/ATP13A5/ATP1A2/ATP1A3/ATP1B1/ATP2B2/ATP2B3/BCAP31/BMP6/CACNA1C/CALB1/CALB2/CALCA/CALCB/CASQ2/CCDC47/CEMIP/CHRNA7/CLDN16/CP/CSRP3/DDIT3/DHRS7C/DMD/DRD1/DRD2/DRD4/EDN2/EDNRA/ELANE/ERO1A/F2/F2R/F2RL3/FGF2/FKBP1A/FKBP1B/FTMT/GCM2/GP1BB/GP9/GPR12/GRIA1/GRIK2/GRM5/GSTO1/HAP1/HCRTR1/HCRTR2/HEPH/HERPUD1/HFE/HIF1A/HRC/HTR2A/HTR2C/IL13/ITGB3/JPH1/JPH3/JPH4/JSRP1/KCNMA1/LACRT/LHCGR/LYN/MAIP1/MICU3/MT1A/MT1B/MT1HL1/MT1X/MT2A/MT3/NPSR1/NPTN/PACS2/PKHD1/PRND/PTH/PTH1R/RYR2/RYR3/SCNN1G/SLC11A1/SLC1A1/SLC24A2/SLC24A3/SLC25A23/SLC30A1/SLC30A3/SLC30A8/SLC39A12/SLC39A5/SLC8A2/SLC8A3/STC1/STC2/STEAP2/SV2A/THY1/TMBIM6/TNNI3/TRDN/TRPC3/TRPC4/TRPC7/TRPM8/TRPV4/WFS1/WNT5A	119
cluster5	BP	GO:0007281	germ cell development	94/3710	316/18903	0.00000933	0.000304609	0.00026145	ABHD2/ACRBP/AKAP4/AKT1/ANGPT2/ASPM/BMP4/BMPR1B/BRCA2/C14orf39/C2CD6/CABYR/CATSPER4/CCDC136/CELF4/CEP131/CFAP206/CFAP221/CFAP43/CFAP47/CFAP54/CFAP65/CFAP69/CFAP97D1/CFTR/DDX25/DMC1/DMRT1/DMRTC2/DPY19L2/DZIP1/EDNRA/EHMT2/FAM9A/FER/FMN2/FSIP2/GK2/GPR149/HSPA2/IFT81/LGR5/MDK/MNS1/MOV10L1/NECTIN3/NEURL1/NME5/NOBOX/NPM2/NPPC/OCA2/PACRG/PCSK4/PDE3A/PDE5A/PIWIL1/PLA2G3/PRDM14/PRM1/PTCH1/PTN/PYGO1/PYGO2/ROPN1/RPS6KA2/RSPH1/RSPH6A/SIRT2/SMAD5/SOHLH1/SOHLH2/SPACA1/SPAG17/SPAG6/SPATA16/SPEM1/SPINK1/SPO11/SRC/SYCP1/TBPL1/TCP11/TCP11X1/TDRD5/TSSK1B/TUT4/UBE2B/WEE2/WNT4/WT1/YBX2/ZBTB16/ZPBP	94
cluster5	BP	GO:0097484	dendrite extension	19/3710	36/18903	0.00000941	0.000305386	0.000262117	AUTS2/BCL11A/CACNG7/CDKL3/CPNE5/CPNE6/CPNE9/CYFIP1/NEDD4L/PRKN/SH3GL2/SLC9A6/SYT1/SYT2/SYT3/SYT4/TMEM108/UNC13A/WASF1	19
cluster5	BP	GO:0120316	sperm flagellum assembly	19/3710	36/18903	0.00000941	0.000305386	0.000262117	AKAP4/CEP131/CFAP206/CFAP221/CFAP43/CFAP47/CFAP54/CFAP65/CFAP69/CFAP97D1/DZIP1/FSIP2/GK2/IFT81/MNS1/NEURL1/PLA2G3/SPAG6/UBE2B	19

cluster5	BP	GO:0050768	negative regulation of neurogenesis	51/3710	146/18903	0.00000997	0.000322515	0.000276819	ABCC8/ATOH1/BMP7/BRINP1/CDKL3/CDKN2B/DAB1/DLL3/DLX1/DLX2/DPYSL5/DRAXIN/EFNB3/EPHA7/F2/GDI1/HES1/HES5/ID1/ID4/LHX2/LRP4/MAP2/MT3/NFATC4/NKX6-1/NR2E1/NTN1/PAX6/PRTG/PTN/PTPRS/SEMA3A/SEMA3E/SEMA4D/SEMA5A/SEMA5B/SEMA6A/SEMA6D/SIRT2/SLIT1/SOX10/SYT4/THY1/TLX2/TMEM98/TNR/VAX1/WNT3A/WNT5A/WNT7A	51
cluster5	BP	GO:1904936	interneuron migration	11/3710	15/18903	0.0000102	0.000328854	0.00028226	ARX/DRD1/DRD2/EVX1/FAT3/FEZF1/FEZF2/NKX2-1/NR2F2/RELN/SOX1	11
cluster5	BP	GO:0021854	hypothalamus development	15/3710	25/18903	0.0000105	0.000337563	0.000289735	GSX1/HAP1/NDNF/NHLH2/NKX2-1/NKX2-6/NR0B1/NRP2/OTP/PLXNA1/POU3F2/RAX/SEMA3E/SOX3/SRD5A2	15
cluster5	BP	GO:0042471	ear morphogenesis	45/3710	124/18903	0.0000107	0.000342228	0.000293739	ATOH1/ATP6V1B1/CLRN1/CLRN2/COL11A1/COL2A1/DLX5/DLX6/EDNRA/FGF10/FGF9/FGFR2/FOXG1/FRZB/FZD3/GBX2/HMX3/HPN/KCNQ4/LHFPL5/LRIG3/MYO3A/MYO3B/MYO6/NEUROG1/NTN1/OTOP1/OTX1/PAX2/PDZD7/POU3F4/POU4F3/PTK7/SIX2/SIX4/SLITRK6/SOBP/SOX9/TFAP2A/USH1C/WHRN/WNT3A/WNT5A/ZIC1/ZIC3	45

cluster5	BP	GO:0051924	regulation of calcium ion transport	82/3710	268/18903	0.0000108	0.000346168	0.000297121	ABL1/ACE/ADCYAP1R1/ADRA2A/AGT/AKAP6/ATP1A2/ATP1B1/CABP1/CACNA1C/CACNA2D1/CACNB1/CALCR/CAMK2A/CAMK2B/CASQ2/CBARP/CD84/CEMIP/CRHR1/DHRS7C/DMD/DRD1/DRD2/DRD4/EGF/EHD3/EPO/F2/F2R/F2RL3/FKBP1A/FKBP1B/GCG/GEM/GRAMD2A/GRIN1/GRM6/GSTO1/HAP1/HES1/HOMER1/HPCA/HRC/HSPA2/IL13/ITGB3/JPH1/JPH3/JPH4/JSRP1/LACRT/LHCGR/LILRB2/LYN/MCHR1/MYLK/NOS3/NPSR1/P2RX2/P2RX3/PACSIN3/PDGFRB/PLP1/PLPP4/PTGS2/REM1/RGS4/RYR2/SLC30A1/SLN/SPINK1/STAC2/STC1/STC2/THY1/TMBIM6/TMC1/TRDN/TRPC3/WFS1/WNK3	82
cluster5	BP	GO:0048863	stem cell differentiation	77/3710	248/18903	0.0000111	0.000353624	0.00030352	ABL1/ACE/ALX1/BMP4/BMP7/CDH2/CDX2/CYP26C1/DMRTA2/EDNRA/EDNRB/EEF1AKMT4-ECE2/EPCAM/ERBB4/ESRRB/EZH2/FGF19/FGF2/FGFR2/FOLR1/FOXA1/FOXC1/FRZB/GATA4/GBX2/GDNF/GPM6A/GREM1/GSK3B/HDAC2/HES1/HES5/HIF1A/HMGA2/HNF1B/ISL1/ITGB1/KITLG/LAMA5/LRP6/MSX2/MYOCD/NRP2/PAX2/PDGFRA/PHOX2B/PRICKLE1/PTN/RADIL/RBM24/RET/SEMA3A/SEMA3E/SEMA4D/SEMA5A/SEMA5B/SEMA6A/SEMA6D/SFRP2/SHC4/SHH/SLC4A11/SMO/SOX10/SOX18/SOX5/SOX9/SP7/TBX5/TBXT/TEAD2/WNT3A/WNT7A/WNT8A/YAP1/ZFP36/ZIC3	77
cluster5	BP	GO:0016198	axon choice point recognition	7/3710	7/18903	0.0000112	0.000353624	0.00030352	APP/EFNB3/FOXG1/GAP43/ROBO1/ROBO2/ROBO3	7
cluster5	BP	GO:0032808	lacrimal gland development	7/3710	7/18903	0.0000112	0.000353624	0.00030352	FGF10/FGFR2/FOXC1/IGSF3/PAX6/SOX10/SOX9	7

cluster5	BP	GO:0021987	cerebral cortex development	43/3710	117/18903	0.0000115	0.000364538	0.000312888	AKIRIN2/ARX/ASCL1/ASPM/ATOH1/ATP1A3/BBS1/CDH2/CDK5R1/CDK5R2/CDON/CNTNAP2/DAB1/DMRTA2/EGFR/EMX1/EMX2/FAT4/FOXG1/GLI3/GSK3B/HIF1A/LAMB1/LHX2/MBOAT7/MDK/NDEL1/NKX2-1/NPY/NR2E1/NTRK2/PAX6/PLCB1/POU3F2/POU3F3/RELN/ROBO1/SMO/SYNE2/TACC2/TACC3/TBR1/TH	43
cluster5	BP	GO:0006584	catecholamine metabolic process	25/3710	55/18903	0.0000123	0.000385291	0.0003307	AOC2/COMT/DAO/DBH/DRD1/DRD2/DRD4/EDNRA/GPR37/GRIN2A/HPRT1/ITGAM/MAOB/NPY/NR4A2/PAH/PDE1B/PRKN/RNF180/SLC1A1/SLC6A3/SNCAIP/SNCB/TACR3/TH	25
cluster5	BP	GO:0009712	catechol-containing compound metabolic process	25/3710	55/18903	0.0000123	0.000385291	0.0003307	AOC2/COMT/DAO/DBH/DRD1/DRD2/DRD4/EDNRA/GPR37/GRIN2A/HPRT1/ITGAM/MAOB/NPY/NR4A2/PAH/PDE1B/PRKN/RNF180/SLC1A1/SLC6A3/SNCAIP/SNCB/TACR3/TH	25
cluster5	BP	GO:0007214	gamma-aminobutyric acid signaling pathway	16/3710	28/18903	0.0000127	0.00039599	0.000339884	ATF4/GABBR2/GABRA1/GABRA2/GABRA3/GABRA4/GABRA5/GABRA6/GABRB2/GABRB3/GABRE/GABRG1/GABRG2/GABRG3/GPR156/SHISA7	16

cluster5	BP	GO:0050773	regulation of dendrite development	39/3710	103/18903	0.000013	0.000405723	0.000348237	ABL1/ADGRB3/ALK/BCL11A/BMP5/BMP7/CAMK2B/CDKL3/CHRNA3/CSMD3/CUL7/CUX1/CYFIP1/DBN1/DPYSL5/ELAVL4/EZH2/FAT3/GSK3B/ID1/IL1RAPL1/ITPKA/KHDC3L/KNDC1/LZTS1/MFSD2A/NEDD4L/NEUROG3/NFATC4/NR2E1/PACSIN1/PAK3/PTN/PTPRD/RELN/SDC2/SEMA4D/TLX2/ZDHHC15	39
cluster5	BP	GO:0043410	positive regulation of MAPK cascade	136/3710	496/18903	0.0000131	0.000408632	0.000350734	ABL1/ADCYAP1/ADORA1/ADRA1A/ADRA1B/ADRA1D/ADRA2A/ADRB3/AJUBA/ALK/ALKAL2/ALOX12B/ANGPT1/APELA/APP/ARHGAP8/AVPR1B/BCAR3/BMP2/BMP4/BMPER/CALCR/CAVIN3/CCL22/CCL25/CCN2/CDH2/CDON/CHRNA7/CSPG4/CXCL17/DENND2B/DOK4/DOK5/DOK6/DRD2/DRD4/EDA2R/EGF/EGFR/ELANE/EPHA8/EPO/ERBB2/ERBB4/ERN2/EZH2/F2R/FGF10/FGF19/FGF2/FGF23/FGF4/FGFR1/FGFR2/FGFR3/FGFR4/FLT4/FZD10/GADD45A/GADD45B/GAREM1/GAS6/GATA4/GCG/GDF6/GHR/GPR37/GRM4/GRM5/HCRTR1/HTR2A/HTR2C/IAPP/ICAM1/IGFBP4/INAVA/INS/ITGB3/KISS1/KITLG/LAMTOR1/MAGED1/MAP3K5/MAPK8IP1/MAPK8IP2/MID1/MINK1/MMP8/MT3/NDRG4/NEK10/NOTCH2/NPSR1/NPTN/NPY/NPY5R/NTF3/NTRK2/OPRK1/OR2AT4/PDE5A/PDGFA/PDGFC/PDGFRA/PDGFRB/PELI2/PLA2G5/PLCB1/RAP1B/RET/RIT2/ROBO1/ROCK1/ROCK2/ROR2/SEMA3A/SH3RF1/SHC2/SORBS3/SRC/TDGF1/THPO/TNFRSF11A/TNFRSF19/TP73/TPBG/TRAF7/TRPV4/VEGFA/WNT5A/WNT7A/WNT7B/WWC1/XDH/ZC3H12A	136
cluster5	BP	GO:0006940	regulation of smooth muscle contraction	28/3710	65/18903	0.0000133	0.000410496	0.000352334	ADORA1/ADORA2B/ADRA1A/ADRA2A/ATP1A2/CALCA/CHRM2/CHRM3/CHRNA3/CHRNB4/CNN1/DOCK4/EDN2/F2R/GHSR/GUCY1A1/ITGA2/KCNB2/KCNMA1/MYOCD/NMU/PRKG1/PTGS2/SPX/TACR1/TACR3/TBXA2R/TNNI3	28
cluster5	BP	GO:0021978	telencephalon regionalization	10/3710	13/18903	0.0000134	0.000410496	0.000352334	BMP2/BMP4/DMRTA2/EMX1/EMX2/GSX2/LHX2/PAX6/SHH/SIX3	10

cluster5	BP	GO:0032341	aldosterone metabolic process	10/3710	13/18903	0.0000134	0.000410496	0.000352334	BMP2/BMP5/BMP6/CLCN2/CYP11B1/CYP11B2/DAB2/DKK3/EDNRB/WNT4	10
cluster5	BP	GO:0097475	motor neuron migration	10/3710	13/18903	0.0000134	0.000410496	0.000352334	ASCL1/CELSR2/DAB1/FZD3/LHX1/NTN1/PHOX2B/RELN/TBX20/VEGFA	10
cluster5	BP	GO:0048864	stem cell development	35/3710	89/18903	0.0000137	0.000419025	0.000359655	ALX1/BMP4/BMP7/CDH2/CYP26C1/EDNRA/EDNRB/ERBB4/FGF19/FGF2/FOLR1/FOXC1/GBX2/GDNF/HES1/HIF1A/ISL1/KITLG/LAMA5/NRP2/PHOX2B/RADIL/RET/SEMA3A/SEMA3E/SEMA4D/SEMA5A/SEMA5B/SEMA6A/SEMA6D/SHH/SMO/SOX10/SOX9/WNT7A	35
cluster5	BP	GO:0003044	regulation of systemic arterial blood pressure mediated by a chemical signal	23/3710	49/18903	0.0000142	0.000431618	0.000370464	ACE/ACE2/ADRA1A/ADRA1B/ADRA1D/ADRB3/AGT/AGTR2/AVPR1B/CMA1/CTSG/CYP11B2/ECE1/EDN2/EDNRB/F2R/NOS3/OR51E2/RASL10B/RPS6KA2/SUCNR1/TACR1/TPM1	23

cluster5	BP	GO:0048546	digestive tract morphogenesis	23/3710	49/18903	0.0000142	0.000431618	0.000370464	AGR2/AHI1/BMP4/EGFR/EPHB3/FGF10/FGFR2/GATA4/GLI2/GLI3/HIF1A/HNF1B/HOXD13/PDGFRA/RBPMS2/SFRP2/SHH/SHOX2/SIX2/SOX10/STRA6/WNT11/WNT5A	23
cluster5	BP	GO:0097106	postsynaptic density organization	17/3710	31/18903	0.0000143	0.000433212	0.000371831	CBLN1/CDH2/CNTNAP1/GRID2/LILRB2/LRRC4B/LRRTM2/NLGN1/NLGN2/NPTX1/NRXN1/OPHN1/PTPRD/RELN/SHANK2/SLITRK3/TMEM108	17
cluster5	BP	GO:1904064	positive regulation of cation transmembrane transport	57/3710	171/18903	0.0000155	0.000469832	0.000403263	ABCC8/ABL1/ACE2/ACTN2/ADCYAP1R1/AGT/AKAP6/ANK3/ANO6/ARC/ATP1B1/CACNA2D1/CACNG2/CACNG3/CACNG4/CALCR/CEMIP/CHP2/CLTRN/CNKSR3/DMD/DRD1/DRD4/EDNRA/EHD3/F2/F2R/F2RL3/GRIN1/GRM6/GSTO1/HAP1/HSPA2/IL13/KCNC1/KCNC2/KCNH2/KCNK3/LACRT/LHCGR/LRRC55/NLGN3/NPSR1/OPRK1/P2RX2/P2RX3/PIRT/PLP1/RELN/RGS7/RYR2/STAC2/THY1/TRDN/TRPC3/WNK2/WNK3	57
cluster5	BP	GO:0001662	behavioral fear response	19/3710	37/18903	0.0000158	0.00047424	0.000407047	ASIC1/ASIC4/ATP1A2/BRINP1/DRD1/DRD4/GABRA5/GRIK2/GRP/GRPR/HTR2C/LYPD1/MAPK8IP2/MDK/NEUROD2/NPAS2/NR2E1/PENK/SLC1A1	19

cluster5	BP	GO:0007588	excretion	19/3710	37/18903	0.0000158	0.00047424	0.000407047	ABCG2/ACE/ADORA1/ADRA1A/AGT/ATP6V0A4/ATP6V1B1/AVP/CHRNA3/CHRNB4/DRD2/EDNRB/KCNMA1/MDK/NPSR1/SLC22A12/SPX/STC1/TACR1	19
cluster5	BP	GO:0010092	specification of animal organ identity	19/3710	37/18903	0.0000158	0.00047424	0.000407047	BMP2/BMP4/FGF10/FGF2/FGFR2/GDNF/GLI3/HOXA11/ISL1/LRP2/PAX2/ROBO1/ROBO2/SPRY1/TBR1/WNT11/WNT2B/WNT3A/WNT5A	19
cluster5	BP	GO:0018958	phenol-containing compound metabolic process	41/3710	111/18903	0.0000159	0.000475943	0.000408508	AOC2/CGA/CITED1/COMT/DAO/DBH/DCT/DRD1/DRD2/DRD4/DUOX2/DUOXA1/DUOXA2/EDNRA/GIPC1/GPR37/GRIN2A/HPN/HPRT1/ITGAM/IYD/MAOB/NPY/NR4A2/OCA2/PAH/PDE1B/PRKN/RNF180/SLC16A2/SLC1A1/SLC6A3/SLCO1C1/SNCAIP/SNCB/TACR3/TH/TPH2/TYR/TYRP1/WNT5A	41
cluster5	BP	GO:0021889	olfactory bulb interneuron differentiation	9/3710	11/18903	0.000016	0.000476654	0.000409119	ARX/DLX5/ERBB4/GSX2/ROBO1/ROBO2/SALL1/UNCX/WNT5A	9

cluster5	BP	GO:0032342	aldosterone biosynthetic process	9/3710	11/18903	0.000016	0.000476654	0.000409119	BMP2/BMP5/BMP6/CLCN2/CYP11B1/CYP11B2/DAB2/DKK3/WNT4	9
cluster5	BP	GO:0007196	adenylate cyclase-inhibiting G protein-coupled glutamate receptor signaling pathway	8/3710	9/18903	0.0000163	0.000482969	0.000414539	GRIK3/GRM2/GRM3/GRM4/GRM5/GRM6/GRM7/GRM8	8
cluster5	BP	GO:0048645	animal organ formation	29/3710	69/18903	0.0000166	0.000492481	0.000422703	BMP2/BMP4/BMP7/EMP2/FGF10/FGF2/FGFR2/FOLR1/GDNF/GLI3/HES1/HOXA11/HOXA3/ISL1/LRP2/PAX2/RBM20/ROBO1/ROBO2/SHH/SPRY1/SULF1/TBR1/TBX5/WNT11/WNT2B/WNT3A/WNT5A/WT1	29
cluster5	BP	GO:0008544	epidermis development	104/3710	362/18903	0.0000169	0.000498989	0.000428289	ABCA12/ALX4/APCDD1/ATOH1/BMP4/CCN2/CLRN1/CLRN2/CRABP2/CST6/DCT/DLL1/DLX3/DSG4/EDA2R/EGFR/ESRP1/ETV4/EVPL/EZH2/FERMT1/FGF10/FGF2/FGF7/FGFR2/FOXC1/FOXN1/FZD3/GLI2/GRHL1/GRHL2/GRXCR1/HDAC2/HES1/HES5/HOXB13/HOXC13/IL20/IVL/KLK5/KRT15/KRT27/KRT6C/KRT75/KRT76/KRT8/KRT80/KRT83/KRT84/KRTAP21-1/LAMA3/LAMA5/LAMC2/LCE1A/LCE1C/LCE2C/LCE2D/LCE3A/LCE3D/LCE3E/LCE4A/LCE6A/LGR4/LGR5/LHFPL5/LHX2/LORICRIN/LRP4/MSX2/MYO6/NGFR/OVOL1/PAX6/PDGFA/PDZD7/POU3F2/POU4F3/PTCH1/PTCH2/RBP2/REG3A/REG3G/ROCK1/ROCK2/SCEL/SHH/SLITRK6/SMO/SOSTDC1/SOX18/SOX9/SPRR2F/SPRR2G/SPRR4/SVEP1/TMC1/TMEM132E/TNFRSF19/USH1C/USH2A/WHRN/WNT5A/YAP1/ZFP36	104

cluster5	BP	GO:0051580	regulation of neurotransmitter uptake	12/3710	18/18903	0.0000182	0.000533214	0.000457665	ATP1A2/DRD1/DRD2/DRD4/GDNF/GFAP/GPM6B/ITGB1/ITGB3/PRKN/RAB3B/SLC17A8	12
cluster5	BP	GO:0072077	renal vesicle morphogenesis	12/3710	18/18903	0.0000182	0.000533214	0.000457665	BMP4/GDNF/GREM1/KIF26B/LHX1/PAX2/SALL1/SIX2/SMO/SOX9/WNT4/WNT9B	12
cluster5	BP	GO:0014032	neural crest cell development	33/3710	83/18903	0.0000182	0.000533742	0.000458118	ALX1/BMP4/BMP7/CDH2/CYP26C1/EDNRA/EDNRB/ERBB4/FGF19/FOLR1/FOXC1/GBX2/GDNF/HES1/HIF1A/ISL1/KITLG/LAMA5/NRP2/PHOX2B/RADIL/RET/SEMA3A/SEMA3E/SEMA4D/SEMA5A/SEMA5B/SEMA6A/SEMA6D/SHH/SMO/SOX10/SOX9	33
cluster5	BP	GO:0048705	skeletal system morphogenesis	71/3710	227/18903	0.0000183	0.000533742	0.000458118	ALX1/ALX4/ARID5B/BARX2/BMP4/BMP6/BMP7/BMPR1B/CCN2/CDX1/COL11A1/COL27A1/COL2A1/DLX2/DLX5/DSCAML1/EIF4A3/FGF4/FGFR1/FGFR2/FGFR3/FOXC1/FREM1/GHR/GLI3/GREM1/GRHL2/HAS2/HHIP/HOXA11/HOXA3/HOXA4/HOXB1/HOXB6/HOXB9/HOXC4/HOXC8/HOXC9/HOXD10/HOXD3/IFT140/IRX5/LHX1/LRP5/MDFI/MMP13/MMP16/MSX2/NEUROG1/NPPC/PAPPA2/PDGFRA/RARB/RFLNA/SHOX2/SIX2/SIX4/SOX5/SOX9/SP5/STC1/TBX15/TBX4/TFAP2A/TIPARP/TRPV4/TULP3/UNCX/WNT7A/WNT9B/ZNF664-RFLNA	71

cluster5	BP	GO:0010976	positive regulation of neuron projection development	53/3710	157/18903	0.0000204	0.000593605	0.000509499	ABL1/AGT/ALK/ALKAL2/BDNF/BMP5/BMP7/CAMK2B/CYFIP1/DBN1/DPYSL3/ELAVL4/EPHA3/EPO/EZH2/FEZ1/FKBP1B/FUT9/GPC2/ITPKA/KHDC3L/LYN/MAGI2/MDK/NDEL1/NDNF/NDRG4/NLGN1/NPTN/NTRK2/PACSIN1/PAK3/PLA2G3/PLK5/PLPPR5/PLXNB3/PTK7/PTN/RELN/RET/RIT2/ROR1/ROR2/STK24/STMN2/TENM3/TMEM30A/TNN/TOX/TRIM67/VEGFA/WNT5A/ZDHHC15	53
cluster5	BP	GO:0090257	regulation of muscle system process	78/3710	256/18903	0.0000207	0.000600291	0.000515238	ABCC8/ACE2/ADORA1/ADORA2B/ADRA1A/ADRA1B/ADRA2A/AGT/AKAP6/ATP1A2/ATP1B1/CACNA1C/CACNA1S/CACNB1/CALCA/CAMK2B/CASQ2/CCN2/CHGA/CHRM2/CHRM3/CHRNA3/CHRNB4/CNN1/DAG1/DMD/DOCK4/DSG2/EDN2/EHD3/F2R/FKBP1B/GATA4/GHSR/GSTO1/GUCY1A1/HCN4/HRC/ITGA2/KCNB2/KCNMA1/LMNA/MLIP/MYH7/MYOCD/MYOG/NEUROG1/NMU/NOS3/PDE5A/PKP2/PRKG1/PTGS2/PVALEF/RGS4/ROCK1/ROCK2/RYR2/SCN10A/SCN4A/SGCA/SLC8A3/SLN/SPX/SSTR2/STC1/TACR1/TACR3/TBXA2R/TNNI1/TNNI3/TNNT2/TPM1/TRIM63/TRPC3/TRPM4/TRPV4/ZC3H12A	78
cluster5	BP	GO:0032409	regulation of transporter activity	95/3710	326/18903	0.0000209	0.000603873	0.000518312	ABCC8/ABCC9/ACE2/ACTN2/ADRA2A/AKAP6/ANK3/APP/ARC/ATP1A2/ATP1B1/CABP1/CACNA2D1/CACNB1/CACNG2/CACNG3/CACNG4/CACNG5/CACNG7/CASQ2/CBARP/CFTR/CHP2/CHRM3/CLTRN/CNKSR3/CRBN/CRH/CRHR1/DMD/DRD2/DRD4/EDNRA/EHD3/EPO/FGF14/FHL1/FKBP1A/FKBP1B/FXYD3/FXYD6/GEM/GRM5/GRP/GSG1L/GSTO1/HAP1/HCN1/HCN3/HCN4/HOMER1/HPCA/HRC/HSPA2/INS/ITGB1/JPH1/JPH3/JPH4/JSRP1/KCNC1/KCNC2/KCNE4/KCNK3/KCNRG/KCNS2/LRRC55/MAPK8IP2/MINK1/NEDD4L/NLGN1/NLGN2/NLGN3/PIRT/PRKCD/PRRT1/PTPN3/RASGRF1/RELN/REM1/RYR2/SCN2B/SCN3B/SHANK1/SHISA6/SHISA7/SHISA9/SLN/STAC2/STOM/SYNGR3/TCAF1/TRDN/WNK2/WNK3	95
cluster5	BP	GO:0099175	regulation of postsynapse organization	34/3710	87/18903	0.0000211	0.000608987	0.000522702	ARC/ARHGAP44/CAMK2B/CBLN1/CDH2/CDK5R1/DBN1/EPHA7/GHSR/GRID2/GRIN2B/HOMER1/IL1RAPL1/INS/ITPKA/KIF1A/LILRB2/LRRC4B/LRRTM2/LZTS3/NGEF/NLGN1/NPTX1/NRCAM/NRP2/PAK3/PPFIA2/PTPRD/RELN/SRCIN1/TANC2/WNT5A/WNT7A/ZDHHC15	34

cluster5	BP	GO:0021515	cell differentiation in spinal cord	23/3710	50/18903	0.0000214	0.000615676	0.000528443	ASCL1/DMRT3/DRAXIN/FOXN4/GLI2/GLI3/GSX1/GSX2/HOXC10/HOXD10/ISL1/LBX1/LHX1/LHX3/LHX5/MDGA2/MNX1/NKX2-2/PTCH1/SHH/SOX1/TBX20/WNT3A	23
cluster5	BP	GO:0060402	calcium ion transport into cytosol	16/3710	29/18903	0.0000231	0.000661206	0.000567522	ADCYAP1R1/ADRA1A/CACNA1C/CACNA2D1/EPO/GRIN1/JPH1/JPH3/JPH4/LHCGR/P2RX2/P2RX3/RYR2/TMBIM6/TRPC3/TRPM1	16
cluster5	BP	GO:0060441	epithelial tube branching involved in lung morphogenesis	16/3710	29/18903	0.0000231	0.000661206	0.000567522	BMP4/CELSR1/DAG1/DLG5/FGF10/FGFR2/FOXA1/HHIP/LAMA1/NKX2-1/RSPO2/SHH/SOX9/SPRY1/WNT2B/YAP1	16
cluster5	BP	GO:2000300	regulation of synaptic vesicle exocytosis	26/3710	60/18903	0.0000236	0.000673279	0.000577884	ADCY1/ADORA2B/ADRA1A/ADRA2A/APBA1/CHRM2/CHRNA6/CSPG5/DRD1/DRD2/GIPC1/GSK3B/HTR2A/NLGN1/PFN2/PRKCG/RAB5A/RAP1B/RIMS3/RIMS4/SEPTIN5/SYN1/SYP/SYT1/SYT4/WNT7A	26

cluster5	BP	GO:0006941	striated muscle contraction	59/3710	181/18903	0.0000236	0.000673504	0.000578078	ABCC9/ACE2/ADORA1/ADRA1A/ADRA1B/ARG2/ATP1A2/ATP1B1/CACNA1C/CACNA1G/CACNA1S/CACNA2D1/CASQ2/CCN2/CHGA/CSRP3/DMD/DSG2/DTNA/EHD3/FKBP1B/GATA4/GJC1/GSTO1/HCN4/HOMER1/HRC/JSRP1/KCND3/KCNE4/KCNH2/KCNJ3/KCNJ5/KCNN2/MYH6/MYH7/MYH8/NEDD4L/PDE5A/PKP2/PVALEF/RYR2/SCN10A/SCN1A/SCN2B/SCN3B/SCN4A/SLC8A3/SMAD5/SMPX/STAC2/STC1/TNNI1/TNNI3/TNNT2/TPM1/TRPM4/TRPV4/ZC3H12A	59
cluster5	BP	GO:1903351	cellular response to dopamine	35/3710	91/18903	0.000024	0.000682275	0.000585606	ABL1/ADCY5/ALK/ATF4/CHRM1/CHRM2/CHRM3/DRD1/DRD2/DRD4/GNAO1/GNAS/GNAZ/GSK3B/HCN3/HDAC2/HRH3/HTR1D/HTR1E/HTR1F/HTR2A/HTR2C/HTR3C/HTR4/ID1/NSG2/OR10H3/OR10H4/OR11H4/OR5T3/OR6T1/PRKN/RGS4/RGS8/SLC1A1	35
cluster5	BP	GO:0002064	epithelial cell development	67/3710	213/18903	0.0000254	0.000718433	0.000616641	ABCA12/AKT1/ARHGEF26/ATF4/ATRX/B4GALT1/BMP4/BMP5/BMP6/CDH2/CLDN1/CLDN5/COL22A1/CRYGB/DACT2/DLL1/DMRT1/EDNRA/EDNRB/FAT1/FER/FOXA1/FOXJ1/FRZB/GPAT4/GRHL2/GSK3B/HAPLN2/HIF1A/HOXB13/HYDIN/ICAM1/LAMB2/MAGI2/MARVELD2/MYADM/NKX2-2/NKX6-1/NOTCH2/NOTCH4/NPHS1/PAX6/PECAM1/PKHD1/PLCB1/RAP1B/RARB/RFX3/ROCK1/ROCK2/SDC1/SIX3/SLC4A5/SLC9A4/SMO/SOX18/SOX9/SPDEF/SRC/STC1/TJP2/TYMS/VEGFA/WNT5A/WNT7A/WNT7B/YAP1	67
cluster5	BP	GO:0021988	olfactory lobe development	18/3710	35/18903	0.0000257	0.00072633	0.000623419	ARX/ATP1A2/DLX2/DLX5/EFNA2/ERBB4/FEZF1/GSX2/LHX2/NR2E1/ROBO1/ROBO2/SALL1/SEMA3A/UNCX/WNT5A/ZIC1/ZIC3	18

cluster5	BP	GO:0002209	behavioral defense response	19/3710	38/18903	0.0000258	0.00072633	0.000623419	ASIC1/ASIC4/ATP1A2/BRINP1/DRD1/DRD4/GABRA5/GRIK2/GRP/GRPR/HTR2C/LYPD1/MAPK8IP2/MDK/NEUROD2/NPAS2/NR2E1/PENK/SLC1A1	19
cluster5	BP	GO:0030111	regulation of Wnt signaling pathway	97/3710	336/18903	0.0000259	0.00072633	0.000623419	ABL1/AMER3/APC2/APCDD1/APP/ASPM/BARX1/BMP2/CDH2/CDK14/CITED1/CRBN/CSNK1E/CTNND1/CTNND2/CXXC4/DAB2/DACT3/DDIT3/DDX3X/DEPDC1B/DKK3/DKKL1/DLX5/DRAXIN/EGF/EGFR/FERMT1/FGF10/FGF2/FGF9/FGFR2/FOLR1/FOXL1/FRZB/GLI3/GPC3/GPRC5B/GRB10/GREM1/GSK3B/HNF1B/IGFBP2/IGFBP4/ILK/ISL1/KLF15/LBX2/LGR4/LGR5/LRP4/LYPD6/MCC/MDFI/MDK/NFATC4/NKD2/NPHP4/PLPP3/PRICKLE1/PRKN/PTK7/PTPRU/RBX1/RSPO1/RSPO2/RSPO3/RSPO4/SALL1/SCEL/SEMA5A/SFRP2/SHH/SHISA6/SOSTDC1/SOX10/SOX9/SPEF1/SRC/SULF1/SULF2/TLE1/TMEM170B/TMEM198/TNN/TPBG/TRABD2B/TRPM4/UBE2B/VGLL4/WIF1/WNK2/WNT11/WNT3A/WNT5A/YAP1/ZNRF3	97
cluster5	BP	GO:0042060	wound healing	122/3710	442/18903	0.0000259	0.00072633	0.000623419	ABCC8/ADAMTS13/ADAMTS18/ADIPOR2/ADRA2A/AJAP1/AJUBA/ALOX12/ANO6/B4GALT1/BLOC1S6/CDKN1A/CLDN1/CLDN19/CLDN4/CPB2/CTSG/CYP4F11/DAG1/DCBLD2/DGKB/DGKI/DMTN/DUOX2/EMILIN1/EMILIN2/EPB41L4B/ERBB2/ERBB3/EVPL/F13B/F2/F2R/F2RL3/F3/F5/FAP/FBLN1/FER1L5/FERMT1/FERMT3/FGF10/FGF2/FGFR2/FKBP10/FOXA2/GAS6/GATA4/GLI3/GNAS/GP1BB/GP6/GP9/HGFAC/HIF1A/ILK/INS/ITGA2/ITGB1/ITGB3/KNG1/LACRT/LOX/LYN/MAP3K5/MMRN1/MSX2/MYH9/MYL12A/MYLK/MYOZ1/NDNF/NOS3/NOTCH2/NOTCH4/ODAM/PDGFA/PDGFRA/PDGFRB/PDPN/PEAR1/PF4/PF4V1/PLPP3/PRKCD/PRKG1/PROS1/PRSS56/PTK7/REG3A/REG3G/SAA1/SCUBE1/SDC1/SDC4/SELP/SERPINE1/SERPINE2/SHH/SLC11A1/SRC/STXBP1/SYT7/TAFA5/TBXA2R/TFPI/TIMP1/TMEFF2/TPM1/TREML1/TSPAN8/TYRO3/VAV2/VEGFA/VKORC1/VWF/WFDC1/WNT3A/WNT4/WNT5A/WNT7A/YAP1	122
cluster5	BP	GO:0007187	G protein-coupled receptor signaling pathway, coupled to cyclic nucleotide second messenger	28/3710	67/18903	0.0000262	0.000733465	0.000629543	ADRB3/AGT/AGTR2/CHRM1/CHRM2/CHRM3/DRD1/DRD4/HRH3/HTR1D/HTR1E/HTR1F/HTR2A/HTR2C/HTR4/LHCGR/MC2R/MTNR1A/NPY/OR10H3/OR10H4/OR11H4/OR5T3/OR6T1/PTH1R/SSTR1/SSTR2/SSTR5	28

cluster5	BP	GO:0072176	nephric duct development	11/3710	16/18903	0.0000268	0.000747353	0.000641463	AHI1/BMP4/EFNB2/EPHA7/GPC3/GREB1L/HNF1B/LHX1/PAX2/WNT11/WNT9B	11
cluster5	BP	GO:0015698	inorganic anion transport	60/3710	186/18903	0.0000288	0.000799406	0.000686141	ABCC3/ABCC9/ADAMTS8/ANO3/ANO4/ANO6/AQP6/ATF4/BEST2/BEST3/CFTR/CLCA2/CLCN2/CLCN4/CLCNKA/CLCNKB/CLDN4/CLIC6/ENPP1/FGF23/FXYD3/GABRA1/GABRA2/GABRA3/GABRA4/GABRA5/GABRA6/GABRB2/GABRB3/GABRD/GABRE/GABRG1/GABRG2/GABRG3/GABRQ/GLRA1/GLRA2/GLRB/GRM5/LRRC8B/SLC11A1/SLC12A5/SLC12A9/SLC13A1/SLC13A4/SLC17A6/SLC17A7/SLC17A8/SLC1A1/SLC22A11/SLC26A9/SLC34A1/SLC34A2/SLC37A1/SLC4A11/SLC4A3/SLC4A4/SLC4A5/SLC6A1/TTYH1	60
cluster5	BP	GO:0032330	regulation of chondrocyte differentiation	24/3710	54/18903	0.000029	0.00080294	0.000689175	ADAMTS7/BMP4/BMP6/BMPR1B/CCN2/CHADL/GDF5/GDF6/GLI3/GREM1/HOXA11/MDK/PKDCC/PTH/RARB/RFLNA/SHOX2/SIX2/SOX5/SOX9/WNT9A/ZBTB16/ZNF219/ZNF664-RFLNA	24
cluster5	BP	GO:1903350	response to dopamine	35/3710	92/18903	0.0000315	0.000871572	0.000748082	ABL1/ADCY5/ALK/ATF4/CHRM1/CHRM2/CHRM3/DRD1/DRD2/DRD4/GNAO1/GNAS/GNAZ/GSK3B/HCN3/HDAC2/HRH3/HTR1D/HTR1E/HTR1F/HTR2A/HTR2C/HTR3C/HTR4/ID1/NSG2/OR10H3/OR10H4/OR11H4/OR5T3/OR6T1/PRKN/RGS4/RGS8/SLC1A1	35

cluster5	BP	GO:0021954	central nervous system neuron development	33/3710	85/18903	0.0000325	0.000888236	0.000762385	AGBL4/ARX/ASCL1/CDH11/DCLK1/DCLK2/DRAXIN/DRD1/DRD2/EPHB3/FEZF2/FGFR2/FOXG1/GBX2/GLI2/HPRT1/MAP2/NDEL1/NDNF/NHLH2/NPY/NR2E1/NR4A2/NRP2/NTRK2/PHOX2B/PLXNA1/PLXNA4/SECISBP2/SEMA3E/SOX1/SPTBN4/TTC36	33
cluster5	BP	GO:0016048	detection of temperature stimulus	14/3710	24/18903	0.0000325	0.000888236	0.000762385	ADORA1/ANO3/ASIC3/CALCA/DRGX/GRIK2/HTR2A/MMP24/NGFR/OPN4/PRDM12/RHO/TRPM3/TRPM8	14
cluster5	BP	GO:0030539	male genitalia development	14/3710	24/18903	0.0000325	0.000888236	0.000762385	BMP5/BMP6/DHCR24/FGF10/GREB1L/HOXD13/LGR4/LHCGR/PDGFRA/ROR2/SHH/SRD5A2/WNT9B/WT1	14
cluster5	BP	GO:0060571	morphogenesis of an epithelial fold	14/3710	24/18903	0.0000325	0.000888236	0.000762385	BMP4/BMP5/BMP7/CECR2/EGFR/FGF10/FGFR2/HIF1A/HOXD13/SHH/SOSTDC1/SULF1/WNT2B/WNT5A	14

cluster5	BP	GO:0099560	synaptic membrane adhesion	14/3710	24/18903	0.0000325	0.000888236	0.000762385	EFNA5/FLRT3/GPC6/LRFN3/LRRC4B/LRRC4C/NTNG1/NTNG2/PTPRD/PTPRF/PTPRS/SLITRK1/SLITRK2/SLITRK3	14
cluster5	BP	GO:0035150	regulation of tube size	50/3710	148/18903	0.0000337	0.000917155	0.000787207	ABL1/ACE/ACE2/ADORA1/ADORA2B/ADRA1A/ADRA1B/ADRA1D/ADRA2A/ADRB3/AGT/AGTR2/ASIC2/ATP1A2/AVP/AVPR1B/CALCA/CHRM3/CPS1/CRP/DBH/DOCK4/DRD1/ECE1/EDN2/EDNRA/EDNRB/F2R/FOXC1/GRIP2/GUCY1A1/HTR1D/HTR2A/INS/KCNMA1/KCNMB2/KNG1/NOS3/NPPC/NTS/PRKG1/PTGS2/ROCK1/ROCK2/SCPEP1/TACR1/TBXA2R/TRPM4/VSTM4/WNT9B	50
cluster5	BP	GO:0007043	cell-cell junction assembly	51/3710	152/18903	0.0000348	0.000944894	0.000811015	ACE/ACE2/AGT/ARVCF/CDH10/CDH11/CDH12/CDH18/CDH19/CDH2/CDH22/CDH6/CDH7/CDH8/CDH9/CLDN1/CLDN14/CLDN16/CLDN18/CLDN19/CLDN2/CLDN22/CLDN4/CLDN5/CLDN6/CLDN8/CNTNAP1/CNTNAP2/CTNND1/CTNND2/DLG5/FER/FRMPD2/GJC1/GRHL2/MARVELD2/MPDZ/MYO1C/NPHP4/NPHS1/NR1H4/PARD3/PECAM1/PKN2/PKP2/ROCK1/ROCK2/STRN/TBX5/TRPV4/WNT11	51
cluster5	BP	GO:0001738	morphogenesis of a polarized epithelium	36/3710	96/18903	0.0000351	0.000948392	0.000814018	ABL1/AHI1/AJAP1/BRSK1/BRSK2/CELSR1/CELSR2/DAAM1/DAB2/DLG5/ERBB4/FAT1/FOXF2/FZD3/GPC3/GPC6/LAMA1/LAMA3/LAMA5/MAGI2/OPHN1/PKHD1/PRICKLE1/PTK7/ROR1/ROR2/RSPO3/SFRP2/SPEF1/VANGL2/WNT11/WNT5A/WNT7A/WNT9B/ZDHHC7/ZNRF3	36

cluster5	BP	GO:0014033	neural crest cell differentiation	36/3710	96/18903	0.0000351	0.000948392	0.000814018	ALX1/BMP4/BMP7/CDH2/CYP26C1/EDNRA/EDNRB/ERBB4/FGF19/FOLR1/FOXC1/FRZB/GBX2/GDNF/HES1/HIF1A/ISL1/KITLG/LAMA5/LRP6/NRP2/PHOX2B/RADIL/RET/SEMA3A/SEMA3E/SEMA4D/SEMA5A/SEMA5B/SEMA6A/SEMA6D/SHH/SMO/SOX10/SOX9/WNT8A	36
cluster5	BP	GO:0009583	detection of light stimulus	28/3710	68/18903	0.0000363	0.0009785	0.000839859	ABCA4/AIPL1/ASIC2/CDS1/CNGB1/ELOVL4/GJA10/GNAT1/GNGT1/GPR52/GRK4/GRM6/GUCY2F/LOC118142757/NR2E3/OPN1LW/OPN1MW2/OPN1MW3/OPN4/PDC/REEP6/RGR/RHO/RP1/RPE65/SEMA5B/TRPC3/TULP1	28
cluster5	BP	GO:0048639	positive regulation of developmental growth	55/3710	168/18903	0.0000384	0.001030909	0.000884843	AGR2/AKAP6/AKT1/ARX/BASP1/BDNF/CACNG7/CDH4/CDK1/CPNE5/CPNE6/CPNE9/CRABP2/CYFIP1/DBN1/DLL1/DRD2/DSCAM/EFNA5/ERBB4/FGF2/FGF9/FGFR2/FOXS1/GDI1/GHR/GHSR/IL7/ISLR2/L1CAM/NDEL1/NEDD4L/NGF/NTN1/PLCB1/POU3F2/PPIB/PRKN/RND2/SEMA4D/SEMA5A/SLC6A3/SMO/SPTBN4/SYT1/SYT2/SYT3/SYT4/TBX20/TBX5/UNC13A/VEGFA/WNT3A/WT1/YAP1	55
cluster5	BP	GO:0097091	synaptic vesicle clustering	10/3710	14/18903	0.0000385	0.00103114	0.000885041	BRSK1/BRSK2/CDH2/NLGN1/NLGN2/PCLO/SYN1/SYN2/SYN3/SYNDIG1	10

cluster5	BP	GO:0050878	regulation of body fluid levels	109/3710	390/18903	0.0000389	0.00104085	0.000893375	ABCA12/ADAMTS13/ADAMTS18/ADORA1/ADRA2A/AGR2/ALOX12/ALOX12B/ANO6/AQP6/ATP6V1B1/AVP/BLOC1S6/CDO1/CEL/CELSR2/CFTR/CHRM1/CHRM3/CLDN1/CLDN4/CORO2B/CPB2/CSN3/CTSG/CYP11B2/CYP4F11/DGKB/DGKI/DMTN/DRD2/EDNRB/EMILIN1/EMILIN2/EMP2/ERBB4/F13B/F2/F2R/F2RL3/F3/F5/FAP/FBLN1/FERMT3/FGF10/FOXA2/GAS6/GNAS/GP1BB/GP6/GP9/GPAT4/GRHL1/HAS2/HGFAC/HIF1A/ILK/ITGA2/ITGB3/KNG1/LACRT/LYN/MMRN1/MYH9/MYL12A/MYO5B/NEURL1/NEUROG1/NOS3/OPRK1/PDGFA/PDGFRA/PDPN/PEAR1/PF4/PF4V1/PRKCD/PRKG1/PROS1/PRSS56/SAA1/SCNN1G/SCUBE1/SELP/SERPINE1/SERPINE2/SHH/SLC4A5/SLC6A3/SPINK1/SRC/STXBP1/TACR1/TBXA2R/TFAP2B/TFPI/TMPRSS11F/TREML1/TRPV4/TSPAN8/TYRO3/VAV2/VEGFA/VKORC1/VWF/WFS1/WNT3A/XDH	109
cluster5	BP	GO:0010463	mesenchymal cell proliferation	20/3710	42/18903	0.0000393	0.001048874	0.000900263	BMP2/BMP4/BMP7/CHRD/DCHS2/FGF4/FGF7/FGF9/FGFR2/GPC3/LMNA/LRP5/PDGFA/SHH/SHOX2/SIX2/SMO/SOX9/WNT11/WNT5A	20
cluster5	BP	GO:0072087	renal vesicle development	12/3710	19/18903	0.0000405	0.00107385	0.0009217	BMP4/GDNF/GREM1/KIF26B/LHX1/PAX2/SALL1/SIX2/SMO/SOX9/WNT4/WNT9B	12
cluster5	BP	GO:2000463	positive regulation of excitatory postsynaptic potential	16/3710	30/18903	0.0000405	0.00107385	0.0009217	CHRNA7/GRIN1/GRIN2A/GRIN2B/GRIN2D/IGSF11/NLGN1/NLGN2/NLGN3/NRXN1/PRKAR1B/RELN/RGS4/SHANK1/STX1A/WNT7A	16

cluster5	BP	GO:0007340	acrosome reaction	19/3710	39/18903	0.0000411	0.001086693	0.000932723	ABHD2/ADCY3/B4GALT1/CRISP1/EQTN/GLRA1/GLRB/PCSK4/PKDREJ/PLCB1/PLCD4/PRND/PRSS37/SPINK1/STXBP1/SYT6/TNP2/TRIM36/ZP4	19
cluster5	BP	GO:0021772	olfactory bulb development	17/3710	33/18903	0.0000419	0.001103666	0.000947292	ARX/DLX2/DLX5/EFNA2/ERBB4/FEZF1/GSX2/LHX2/NR2E1/ROBO1/ROBO2/SALL1/SEMA3A/UNCX/WNT5A/ZIC1/ZIC3	17
cluster5	BP	GO:0045777	positive regulation of blood pressure	18/3710	36/18903	0.000042	0.001103666	0.000947292	ACE/ADORA1/ADRA1A/ADRA1B/ADRA1D/AGT/AVP/CYP11B2/GLP1R/GRIP2/NR2F2/OR51E2/SPX/TAC3/TACR1/TACR3/TBXA2R/TPM1	18
cluster5	BP	GO:0062237	protein localization to postsynapse	18/3710	36/18903	0.000042	0.001103666	0.000947292	ADAM10/ARHGAP44/CACNG2/CACNG3/CACNG7/DAG1/GHSR/GPC6/GRIN2A/GRIP1/GRIP2/GRIPAP1/IQSEC2/LGI1/NPTX1/RAB8A/SNAP25/ZDHHC15	18

cluster5	BP	GO:0014706	striated muscle tissue development	77/3710	257/18903	0.000044	0.001152761	0.000989431	ABL1/ACTC1/ACTN2/ADRA1A/AGT/AKAP6/BMP2/BMP4/BMP5/BMP7/BVES/CACNA1G/CDK1/COL11A1/CSRP3/DIPK2A/DLL1/DSG2/EDNRA/EFNB2/ERBB3/ERBB4/FGF2/FGF9/FGFR2/FHOD3/FKBP1A/FOXC1/GATA4/GJC1/GREM1/HCN4/ISL1/ITGB1/LMNA/LOX/LRP2/LUC7L/MSX2/MYH6/MYH7/MYL7/MYO18B/MYOCD/NDRG4/NKX2-6/NOTCH2/NRAP/PDGFRA/PDGFRB/PKP2/PRKAR1A/RARB/RGS4/RYR2/SGCZ/SHH/SHOX2/SIK1/SIRT2/SMAD5/TBX20/TBX5/TENM4/TNNI1/TNNI3/TNNT2/TP73/TPM1/VEGFA/VGLL4/WNT3A/WNT5A/WNT8A/WT1/YAP1/ZIC3	77
cluster5	BP	GO:0061387	regulation of extent of cell growth	39/3710	108/18903	0.0000457	0.00118748	0.00101923	ABL1/BARHL2/BDNF/CDH4/CDKL3/CRABP2/CYFIP1/DBN1/DRAXIN/DSCAM/EFNA5/EPHA7/GDI1/GSK3B/ISLR2/L1CAM/MAP2/MT3/NDEL1/NGF/NKX6-1/NRCAM/NTN1/OLFM1/PLXNA4/PTPRS/RND2/SEMA3A/SEMA3E/SEMA4D/SEMA5A/SEMA5B/SEMA6A/SEMA6D/SLIT1/TNR/VEGFA/WNT3A/WNT5A	39
cluster5	BP	GO:0071867	response to monoamine	39/3710	108/18903	0.0000457	0.00118748	0.00101923	ABL1/ADCY5/ALK/APP/ATF4/CHRM1/CHRM2/CHRM3/DRD1/DRD2/DRD4/GNAO1/GNAS/GNAZ/GSK3B/HCN3/HDAC2/HRH3/HTR1D/HTR1E/HTR1F/HTR2A/HTR2C/HTR3C/HTR4/ID1/NSG2/OR10H3/OR10H4/OR11H4/OR5T3/OR6T1/PENK/PRKN/RGS4/RGS8/RYR2/SIRT2/SLC1A1	39
cluster5	BP	GO:0071869	response to catecholamine	39/3710	108/18903	0.0000457	0.00118748	0.00101923	ABL1/ADCY5/ALK/APP/ATF4/CHRM1/CHRM2/CHRM3/DRD1/DRD2/DRD4/GNAO1/GNAS/GNAZ/GSK3B/HCN3/HDAC2/HRH3/HTR1D/HTR1E/HTR1F/HTR2A/HTR2C/HTR3C/HTR4/ID1/NSG2/OR10H3/OR10H4/OR11H4/OR5T3/OR6T1/PENK/PRKN/RGS4/RGS8/RYR2/SIRT2/SLC1A1	39

cluster5	BP	GO:0048738	cardiac muscle tissue development	73/3710	241/18903	0.0000463	0.00120099	0.001030826	ABL1/ACTC1/ACTN2/ADRA1A/AGT/AKAP6/BMP2/BMP4/BMP5/BMP7/BVES/CACNA1G/CDK1/COL11A1/CSRP3/DIPK2A/DLL1/DSG2/EDNRA/EFNB2/ERBB3/ERBB4/FGF2/FGF9/FGFR2/FHOD3/FKBP1A/FOXC1/GATA4/GJC1/GREM1/HCN4/ISL1/ITGB1/LMNA/LRP2/MSX2/MYH6/MYH7/MYL7/MYO18B/MYOCD/NDRG4/NKX2-6/NOTCH2/NRAP/PDGFRA/PDGFRB/PKP2/PRKAR1A/RARB/RGS4/RYR2/SGCZ/SHOX2/SIK1/SMAD5/TBX20/TBX5/TENM4/TNNI1/TNNI3/TNNT2/TP73/TPM1/VEGFA/VGLL4/WNT3A/WNT5A/WNT8A/WT1/YAP1/ZIC3	73
cluster5	BP	GO:0009880	embryonic pattern specification	30/3710	76/18903	0.0000506	0.001306687	0.001121547	BASP1/BMP7/CHRDL1/CXXC4/DLL1/DOP1B/ERBB4/FGF10/FGFR2/LAMA5/LHX1/MEIS1/MEIS3/NEUROG1/NRP2/OOEP/PGAP1/PTCH1/RIPPLY1/RIPPLY3/SHH/SMAD5/TBXT/TDGF1/TDRD5/WNT5A/WNT7A/WT1/ZBTB16/ZIC3	30
cluster5	BP	GO:0045685	regulation of glial cell differentiation	30/3710	76/18903	0.0000506	0.001306687	0.001121547	ASPA/BMP2/CDK1/DAB1/DAG1/DLX1/DLX2/DUSP15/F2/GSX2/HDAC2/HES1/HES5/ID4/MDK/NKX2-2/NKX6-1/NR2E1/OPALIN/PTN/PTPRZ1/SERPINE2/SHH/SOX1/TENM4/TMEM98/TNFRSF21/TP73/TTBK1/ZNF488	30
cluster5	BP	GO:0071695	anatomical structure maturation	75/3710	250/18903	0.0000523	0.001347841	0.00115687	ABHD2/ACTL6B/ADAMTS7/ADGRB3/ANGPTL8/APP/ASCL1/BCL11A/BMP2/BRCA2/C2CD6/CABYR/CATSPER4/CDK5R1/CDK5R2/CFTR/CNGB1/CNTNAP2/DAG1/DDIT3/DMC1/EDNRA/EDNRB/EPHA8/EPO/FERMT1/FEV/FGFR3/FOXA1/GLDN/GPAT4/GREM1/HES1/HES5/HIF1A/HOXB13/KCNB1/MBTPS2/MSX2/MYOC/NKX6-1/NPPC/NR4A2/NRCAM/PAX2/PCSK4/PDE3A/PLA2G3/PLXNB1/PTH/PTH1R/RET/RFLNA/RFX3/ROPN1/RPS6KA2/SEMA4D/SIRT2/SIX3/SOX10/SOX18/SPINK1/SPTBN4/SRRM4/TCP11/TCP11X1/TDRD5/TUT4/TYMS/VEGFA/VSX1/WEE2/WNT5A/ZBTB16/ZNF664-RFLNA	75

cluster5	BP	GO:0072178	nephric duct morphogenesis	9/3710	12/18903	0.0000528	0.00135618	0.001164028	AHI1/BMP4/EFNB2/EPHA7/GPC3/HNF1B/LHX1/PAX2/WNT9B	9
cluster5	BP	GO:0048704	embryonic skeletal system morphogenesis	35/3710	94/18903	0.0000532	0.001363769	0.001170542	ALX1/ALX4/BMP4/BMP7/COL11A1/COL2A1/DLX2/DSCAML1/EIF4A3/FGFR2/GLI3/GRHL2/HOXA11/HOXA3/HOXA4/HOXB1/HOXB6/HOXB9/HOXC4/HOXC9/HOXD10/HOXD3/IFT140/IRX5/LHX1/MDFI/MMP16/PDGFRA/SHOX2/SIX2/SIX4/TBX15/TFAP2A/TULP3/WNT9B	35
cluster5	BP	GO:0042476	odontogenesis	46/3710	135/18903	0.0000537	0.001367635	0.001173859	ACP4/AMBN/AMELX/APCDD1/AQP6/ASPN/BMP2/BMP4/BMP7/CFTR/DLX1/DLX2/DLX3/DMRT3/DSPP/FGF10/FGF4/FGFR2/FOXC1/GLI2/GLI3/HDAC2/KLK5/LAMA5/LAMB1/LRP4/MSX2/NECTIN1/NGFR/ODAM/PDGFRA/RELT/RSPO2/SCN10A/SDC1/SERPINE1/SHH/SLC34A1/SMO/SOSTDC1/SP6/SP7/SRC/TFAP2A/TNFRSF11B/WDR72	46
cluster5	BP	GO:0071868	cellular response to monoamine stimulus	38/3710	105/18903	0.0000538	0.001367635	0.001173859	ABL1/ADCY5/ALK/APP/ATF4/CHRM1/CHRM2/CHRM3/DRD1/DRD2/DRD4/GNAO1/GNAS/GNAZ/GSK3B/HCN3/HDAC2/HRH3/HTR1D/HTR1E/HTR1F/HTR2A/HTR2C/HTR3C/HTR4/ID1/NSG2/OR10H3/OR10H4/OR11H4/OR5T3/OR6T1/PRKN/RGS4/RGS8/RYR2/SIRT2/SLC1A1	38

cluster5	BP	GO:0071870	cellular response to catecholamine stimulus	38/3710	105/18903	0.0000538	0.001367635	0.001173859	ABL1/ADCY5/ALK/APP/ATF4/CHRM1/CHRM2/CHRM3/DRD1/DRD2/DRD4/GNAO1/GNAS/GNAZ/GSK3B/HCN3/HDAC2/HRH3/HTR1D/HTR1E/HTR1F/HTR2A/HTR2C/HTR3C/HTR4/ID1/NSG2/OR10H3/OR10H4/OR11H4/OR5T3/OR6T1/PRKN/RGS4/RGS8/RYR2/SIRT2/SLC1A1	38
cluster5	BP	GO:0009914	hormone transport	87/3710	300/18903	0.0000551	0.001397779	0.001199733	ABCA12/ABCC8/ADCY5/ADCY8/ADCYAP1/ADORA1/ADRA2A/AGT/BMP6/BRSK2/CCKAR/CFTR/CGA/CHGA/CLTRN/CRH/CRHR1/DAB2/DOC2B/DRD2/EFNA5/EPHA5/EXOC3L1/FGF23/FKBP1B/FOXA2/GCG/GCK/GHSR/GLP1R/GPLD1/GRP/HADH/HFE/HIF1A/HNF1A/HNF1B/HTR2C/ILDR2/INHA/INS/ISL1/KCNB1/KISS1/LRP5/LYN/MC4R/NEUROD1/NKX6-1/NLGN2/NNAT/NOS2/NR0B2/NR1H4/OPRK1/PCLO/PLA2G3/PRKN/RAB11FIP2/RASL10B/RFX3/RFX6/RPH3AL/SCG5/SERPINA7/SLC16A2/SLC22A9/SLC30A8/SLC7A8/SLC9B2/SLCO1B1/SLCO1C1/SNAP23/SNAP25/SPINK1/SSTR5/STX1A/SYT7/SYT9/TACR1/TFAP2B/TRH/TRPM4/TRPV4/TTR/UCN3/VSNL1	87
cluster5	BP	GO:0021517	ventral spinal cord development	21/3710	46/18903	0.0000557	0.001402336	0.001203644	ASCL1/DAB1/DMRT3/FOXN4/GLI2/GLI3/HOXC10/HOXD10/ISL1/LBX1/LHX1/LHX3/MDGA2/MNX1/NKX2-2/PAX6/PTCH1/RELN/SHH/SOX1/TBX20	21
cluster5	BP	GO:0048806	genitalia development	21/3710	46/18903	0.0000557	0.001402336	0.001203644	BMP5/BMP6/DHCR24/FGF10/FOXF2/GREB1L/HOXD13/LGR4/LHCGR/LHX1/LRP2/NEUROG1/PDGFRA/ROR2/SHH/SRD5A2/STRA6/TYRO3/WNT5A/WNT9B/WT1	21

cluster5	BP	GO:1900271	regulation of long-term synaptic potentiation	21/3710	46/18903	0.0000557	0.001402336	0.001203644	ABL1/ADCY1/ADCY8/ADORA1/APP/ARC/CALB1/CALB2/CHRNA7/CYP46A1/DRD2/GSK3B/IGSF11/INS/LILRB2/NPTN/PRKAR1B/PTN/RELN/SHISA7/SQSTM1	21
cluster5	BP	GO:0031214	biomineral tissue development	56/3710	174/18903	0.000056	0.001407899	0.001208419	ADGRV1/AMBN/AMELX/ANO6/ASPN/ATF4/BMP2/BMP4/BMP6/BMP7/BMPR1B/CFTR/DSPP/ECM1/ENPP1/FGF23/FGFR2/FGFR3/GAS6/GPC3/GPM6B/GREM1/HIF1A/HTN3/IBSP/KLF10/LGR4/LOX/MMP13/MSX2/NECTIN1/NELL1/NOS3/ODAM/OMD/OTOP1/PKDCC/PTGS2/PTH/PTH1R/PTN/RFLNA/ROCK1/ROCK2/ROR2/RSPO2/SBDS/SLC24A3/SOX9/SP7/TFAP2A/TRPM4/WDR72/WNT11/WNT4/ZNF664-RFLNA	56
cluster5	BP	GO:0051582	positive regulation of neurotransmitter uptake	6/3710	6/18903	0.000057	0.001428614	0.001226198	DRD2/DRD4/ITGB1/PRKN/RAB3B/SLC17A8	6
cluster5	BP	GO:0035296	regulation of tube diameter	49/3710	147/18903	0.0000593	0.001478839	0.001269308	ABL1/ACE/ACE2/ADORA1/ADORA2B/ADRA1A/ADRA1B/ADRA1D/ADRA2A/ADRB3/AGT/AGTR2/ASIC2/ATP1A2/AVP/AVPR1B/CALCA/CHRM3/CPS1/CRP/DBH/DOCK4/DRD1/ECE1/EDN2/EDNRA/EDNRB/F2R/FOXC1/GRIP2/GUCY1A1/HTR1D/HTR2A/INS/KCNMA1/KCNMB2/KNG1/NOS3/NPPC/NTS/PRKG1/PTGS2/ROCK1/ROCK2/SCPEP1/TACR1/TBXA2R/TRPM4/VSTM4	49

cluster5	BP	GO:0097746	blood vessel diameter maintenance	49/3710	147/18903	0.0000593	0.001478839	0.001269308	ABL1/ACE/ACE2/ADORA1/ADORA2B/ADRA1A/ADRA1B/ADRA1D/ADRA2A/ADRB3/AGT/AGTR2/ASIC2/ATP1A2/AVP/AVPR1B/CALCA/CHRM3/CPS1/CRP/DBH/DOCK4/DRD1/ECE1/EDN2/EDNRA/EDNRB/F2R/FOXC1/GRIP2/GUCY1A1/HTR1D/HTR2A/INS/KCNMA1/KCNMB2/KNG1/NOS3/NPPC/NTS/PRKG1/PTGS2/ROCK1/ROCK2/SCPEP1/TACR1/TBXA2R/TRPM4/VSTM4	49
cluster5	BP	GO:0016339	calcium-dependent cell-cell adhesion via plasma membrane cell adhesion molecules	20/3710	43/18903	0.00006	0.001487226	0.001276506	AJUBA/ARVCF/CDH10/CDH11/CDH12/CDH16/CDH17/CDH18/CDH19/CDH2/CDH22/CDH6/CDH7/CDH8/CDH9/NLGN1/PCDHB16/PCDHB6/PCDHGB4/SELP	20
cluster5	BP	GO:0051954	positive regulation of amine transport	20/3710	43/18903	0.00006	0.001487226	0.001276506	ACE2/AGT/AVP/AVPR1B/CLTRN/DRD2/DRD4/GDNF/ITGB1/KCNB1/OPRK1/RAB3B/SLC17A8/SLC38A3/SLC6A1/STX1A/STXBP1/SYT1/SYT4/TRH	20
cluster5	BP	GO:0060428	lung epithelium development	20/3710	43/18903	0.00006	0.001487226	0.001276506	ADAMTSL2/AGR2/ASCL1/BMP4/FGF10/FGF7/FGFR2/FOXA1/FOXJ1/GRHL2/IL13/NKX2-1/SHH/SOX9/SPDEF/STRA6/THRB/TP73/WNT7B/YAP1	20

cluster5	BP	GO:0001964	startle response	14/3710	25/18903	0.0000606	0.001497947	0.001285708	CTNNA2/DRD1/DRD2/GLRA1/GLRB/GRID2/GRIN2A/GRIN2D/KCNH1/NPAS1/PENK/PRKN/SLC6A3/SLITRK6	14
cluster5	BP	GO:0048791	calcium ion-regulated exocytosis of neurotransmitter	11/3710	17/18903	0.0000624	0.001532559	0.001315416	DOC2B/RIMS3/STXBP1/SYT1/SYT2/SYT4/SYT5/SYT7/SYT8/UNC13A/UNC13C	11
cluster5	BP	GO:0070593	dendrite self-avoidance	11/3710	17/18903	0.0000624	0.001532559	0.001315416	CNTN4/CNTN6/DSCAM/DSCAML1/IGSF9/MYPN/NEXN/NPTN/ROBO3/TNN/VSTM2L	11
cluster5	BP	GO:1900006	positive regulation of dendrite development	11/3710	17/18903	0.0000624	0.001532559	0.001315416	ABL1/ALK/BMP5/BMP7/CYFIP1/ELAVL4/EZH2/KHDC3L/PACSIN1/PTN/ZDHHC15	11

cluster5	BP	GO:0002068	glandular epithelial cell development	18/3710	37/18903	0.0000669	0.001634263	0.00140271	AKT1/BMP4/BMP5/BMP6/CDH2/DLL1/GPAT4/GSK3B/HIF1A/NKX2-2/NKX6-1/PAX6/RARB/RFX3/SLC9A4/SMO/SPDEF/WNT5A	18
cluster5	BP	GO:0042048	olfactory behavior	8/3710	10/18903	0.0000672	0.001634263	0.00140271	ADCY3/ATP6V1B1/BBS1/CFAP69/LMX1A/SHANK1/TPBG/WFS1	8
cluster5	BP	GO:0098976	excitatory chemical synaptic transmission	8/3710	10/18903	0.0000672	0.001634263	0.00140271	CHRDL1/GRIN1/GRIN2A/GRIN2B/GRIN2D/PTCHD1/PVALB/SHISA6	8
cluster5	BP	GO:2001054	negative regulation of mesenchymal cell apoptotic process	8/3710	10/18903	0.0000672	0.001634263	0.00140271	BMP7/GDF5/HIF1A/HNF1B/PAX2/POU3F4/SHH/SOX9	8

cluster5	BP	GO:0060048	cardiac muscle contraction	47/3710	140/18903	0.0000681	0.001644047	0.001411108	ABCC9/ACE2/ADORA1/ADRA1A/ADRA1B/ATP1A2/ATP1B1/CACNA1C/CACNA1G/CACNA2D1/CASQ2/CCN2/CHGA/CSRP3/DMD/DSG2/EHD3/FKBP1B/GATA4/GJC1/GSTO1/HCN4/HRC/KCND3/KCNE4/KCNH2/KCNJ3/KCNJ5/KCNN2/MYH6/MYH7/NEDD4L/PDE5A/PKP2/RYR2/SCN10A/SCN1A/SCN2B/SCN3B/SMAD5/STC1/TNNI1/TNNI3/TNNT2/TPM1/TRPM4/ZC3H12A	47
cluster5	BP	GO:0007517	muscle organ development	98/3710	348/18903	0.0000682	0.001644047	0.001411108	ACTA1/ACTC1/ADGRB1/ALX4/ANKRD33/ARID5B/BARX2/BASP1/BMP2/BMP4/BVES/CACNA1S/CAVIN4/CD164/CDON/CHODL/CNTFR/CNTNAP1/COL11A1/CRYAB/CSRP3/DAG1/DES/DLL1/DMD/DMRTA2/DNER/DPF3/EDNRA/ERBB3/ETV1/EVC/FGFR2/FHL1/FKBP1A/FOS/FOXC1/HEYL/HMG20B/HOMER1/HOXD10/ISL1/ITGA7/ITGB1/JPH1/KLHL40/LAMA5/LBX1/LOX/LRP2/LUC7L/MAPK12/MEF2B/MEGF10/MYH6/MYH7/MYLK/MYOCD/MYOG/MYOZ1/MYOZ2/NEURL1/NEUROG1/NPHS1/NR2F2/P2RX2/PAX3/PAX7/PHOX2B/PITX1/PKP2/POPDC3/RBFOX1/RBM24/RXRG/RYR2/SGCA/SHH/SHOX2/SIRT2/SIX4/SMO/SMTN/SMYD1/SPEG/STRA6/TBX20/TLL2/TNNI1/TNNI3/TNNT2/TPM1/USP2/VAX1/VGLL2/WNT3A/WNT5A/WT1	98
cluster5	BP	GO:0099174	regulation of presynapse organization	17/3710	34/18903	0.0000685	0.001644047	0.001411108	APP/CBLN1/CBLN2/GPC6/IL1RAPL1/IL1RAPL2/LRFN3/LRRC4B/NLGN2/NTNG2/PTPRD/SLITRK1/SLITRK2/SLITRK3/SLITRK6/WNT3A/WNT7A	17
cluster5	BP	GO:0110110	positive regulation of animal organ morphogenesis	17/3710	34/18903	0.0000685	0.001644047	0.001411108	BMP2/BMP4/FGF10/FGF2/GDNF/HOXA11/NGFR/ROBO1/ROBO2/SIX4/SOX9/SPRY1/WNT11/WNT2B/WNT3A/WNT4/WNT5A	17

cluster5	BP	GO:1905144	response to acetylcholine	17/3710	34/18903	0.0000685	0.001644047	0.001411108	CDK5R1/CHRM1/CHRM2/CHRM3/CHRNA2/CHRNA3/CHRNA7/EDNRA/HRH3/LY6G6D/LY6H/LYPD1/PLCB1/RGS10/RGS8/ROCK2/SLURP2	17
cluster5	BP	GO:1905606	regulation of presynapse assembly	17/3710	34/18903	0.0000685	0.001644047	0.001411108	APP/CBLN1/CBLN2/GPC6/IL1RAPL1/IL1RAPL2/LRFN3/LRRC4B/NLGN2/NTNG2/PTPRD/SLITRK1/SLITRK2/SLITRK3/SLITRK6/WNT3A/WNT7A	17
cluster5	BP	GO:0003014	renal system process	43/3710	125/18903	0.0000721	0.001724763	0.001480388	ABCG2/ACE/ADORA1/ADRA1A/AGT/AGTR2/AQP6/ATP6V0A4/ATP6V1B1/AVP/BMP4/CHRNA3/CHRNB4/CLCNKA/CLCNKB/CLDN4/CORO2B/CYP11B2/DRD2/EDNRA/EDNRB/EMP2/F2R/FOLR1/GAS6/HAS2/HNF1A/KCNMA1/KIRREL1/MYO5B/OR51E2/SLC22A12/SLC4A5/SLC5A1/SPX/STC1/SUCNR1/SULF1/SULF2/TACR1/TFAP2B/TMEM63C/WFS1	43
cluster5	BP	GO:0032344	regulation of aldosterone metabolic process	7/3710	8/18903	0.000074	0.00176325	0.001513422	BMP2/BMP5/BMP6/CLCN2/DAB2/DKK3/WNT4	7

cluster5	BP	GO:0032347	regulation of aldosterone biosynthetic process	7/3710	8/18903	0.000074	0.00176325	0.001513422	BMP2/BMP5/BMP6/CLCN2/DAB2/DKK3/WNT4	7
cluster5	BP	GO:0006821	chloride transport	41/3710	118/18903	0.0000813	0.001932702	0.001658864	ANO3/ANO4/ANO6/BEST2/BEST3/CFTR/CLCA2/CLCN2/CLCN4/CLCNKA/CLCNKB/CLDN4/CLIC6/FXYD3/GABRA1/GABRA2/GABRA3/GABRA4/GABRA5/GABRA6/GABRB2/GABRB3/GABRD/GABRE/GABRG1/GABRG2/GABRG3/GABRQ/GLRA1/GLRA2/GLRB/GRM5/SLC12A5/SLC12A9/SLC17A6/SLC17A7/SLC17A8/SLC1A1/SLC26A9/SLC6A1/TTYH1	41
cluster5	BP	GO:0042053	regulation of dopamine metabolic process	12/3710	20/18903	0.000083	0.001956116	0.001678961	DRD1/DRD4/GPR37/HPRT1/ITGAM/MAOB/NPY/NR4A2/PDE1B/PRKN/SLC6A3/TACR3	12
cluster5	BP	GO:0042069	regulation of catecholamine metabolic process	12/3710	20/18903	0.000083	0.001956116	0.001678961	DRD1/DRD4/GPR37/HPRT1/ITGAM/MAOB/NPY/NR4A2/PDE1B/PRKN/SLC6A3/TACR3	12

cluster5	BP	GO:0060004	reflex	12/3710	20/18903	0.000083	0.001956116	0.001678961	ADRA1A/ASCL1/CACNG2/GLRA1/GLRB/HPN/NMBR/NPSR1/SHANK1/SLC1A1/SLITRK6/TMC1	12
cluster5	BP	GO:0060231	mesenchymal to epithelial transition	12/3710	20/18903	0.000083	0.001956116	0.001678961	BASP1/BMP4/CITED1/GDNF/GREM1/PAX2/SALL1/SIX2/SMO/WNT4/WNT9B/WT1	12
cluster5	BP	GO:0042445	hormone metabolic process	72/3710	241/18903	0.0000845	0.00198482	0.001703598	ACE/ACE2/ADAM10/ADH1A/ADH1C/ADH7/BBS1/BCHE/BCO1/BMP2/BMP5/BMP6/CEL/CGA/CHST8/CHST9/CLCN2/CMA1/CRABP1/CRABP2/CTSG/CYP11B1/CYP11B2/CYP21A2/CYP26A1/CYP26C1/CYP27C1/CYP2W1/CYP46A1/DAB2/DHCR7/DKK3/DUOX2/DUOXA1/DUOXA2/ECE1/EDNRB/ERO1A/FOXA1/GHR/HFE/HIF1A/HPN/HSD17B12/HSD17B2/HSD3B2/IYD/KLK6/LHCGR/LRAT/PCSK1/PCSK2/PCSK4/PDGFRA/RBP1/RDH8/RPE65/SCG5/SCPEP1/SHH/SLC16A2/SLC30A8/SLCO1C1/SRD5A2/STC2/SULT1E1/TIPARP/TTR/UGT1A3/UGT1A8/UGT2B7/WNT4	72
cluster5	BP	GO:0034764	positive regulation of transmembrane transport	71/3710	237/18903	0.0000857	0.002009837	0.00172507	ABCC8/ABL1/ACE2/ACSL1/ACTN2/ADCYAP1R1/AGT/AKAP6/AKT1/ANK3/ANO6/ARC/ATP1B1/CACNA2D1/CACNG2/CACNG3/CACNG4/CALCR/CEMIP/CFTR/CHP2/CLIP3/CLTRN/CNKSR3/DMD/DRD1/DRD4/EDNRA/EHD3/F2/F2R/F2RL3/FGF19/GPC3/GRIN1/GRM6/GSTO1/HAP1/HSPA2/IL13/INS/ITGB1/KCNC1/KCNC2/KCNH2/KCNK3/KLF15/LACRT/LHCGR/LRRC55/NLGN3/NPSR1/OPRK1/P2RX2/P2RX3/PIRT/PLP1/PTH/RELN/RGS7/RYR2/SLC17A8/SLC1A2/SLC34A1/STAC2/TCAF1/THY1/TRDN/TRPC3/WNK2/WNK3	71

cluster5	BP	GO:0098656	anion transmembrane transport	78/3710	266/18903	0.0000873	0.002043239	0.00175374	ACE2/ADAMTS8/AGT/ANO3/ANO4/ANO6/AQP6/ATP1A2/BEST2/BEST3/CFTR/CLCA2/CLCN2/CLCN4/CLCNKA/CLCNKB/CLDN4/CLIC6/CLTRN/FOLR1/FXYD3/GABRA1/GABRA2/GABRA3/GABRA4/GABRA5/GABRA6/GABRB2/GABRB3/GABRD/GABRE/GABRG1/GABRG2/GABRG3/GABRQ/GFAP/GLRA1/GLRA2/GLRB/GRM5/ITGB1/LRP2/LRRC8B/RGS4/SLC11A1/SLC12A5/SLC12A9/SLC13A1/SLC13A4/SLC13A5/SLC16A12/SLC16A8/SLC16A9/SLC17A6/SLC17A7/SLC17A8/SLC1A1/SLC1A2/SLC1A6/SLC25A2/SLC25A21/SLC25A31/SLC26A9/SLC34A1/SLC37A1/SLC38A3/SLC4A11/SLC4A3/SLC4A4/SLC4A5/SLC66A1/SLC6A1/SLC6A20/SLC6A5/SLC7A3/SLC7A8/TCAF1/TTYH1	78
cluster5	BP	GO:0050772	positive regulation of axonogenesis	30/3710	78/18903	0.0000893	0.002083718	0.001788483	BDNF/CDH4/CHODL/CRABP2/CYFIP1/DBN1/DSCAM/EFNA5/GDI1/ISLR2/L1CAM/MAP6/NDEL1/NGF/NTN1/NTRK2/PLXNA1/PLXNA2/PLXNA4/PLXNB1/PLXNB3/RND2/ROBO1/ROBO2/SEMA4D/SEMA5A/SHOX2/SLITRK1/VEGFA/WNT3A	30
cluster5	BP	GO:0007189	adenylate cyclase-activating G protein-coupled receptor signaling pathway	51/3710	157/18903	0.0000901	0.002096446	0.001799408	ADCY1/ADCY2/ADCY3/ADCY5/ADCY8/ADCYAP1/ADGRB1/ADGRB3/ADGRF1/ADGRF2/ADGRF3/ADGRF4/ADGRG2/ADGRG4/ADGRL3/ADM5/ADORA2B/ADRA1A/ADRA1B/ADRA1D/ADRA2A/ADRB3/CALCA/CALCB/CALCR/CHGA/CRHR1/DRD1/DRD2/GCG/GLP1R/GNAS/GPR101/GPR12/GPR161/GPR26/GPR6/IAPP/LHCGR/MC2R/MC4R/MRAP2/PF4/PTGFR/PTH/PTH1R/RIT2/TBXA2R/TCP11/UCN2/UCN3	51
cluster5	BP	GO:0009410	response to xenobiotic stimulus	117/3710	432/18903	0.0000902	0.002096446	0.001799408	ABCB11/ABCC3/ABCC8/ABL1/ACE/ACSL1/ADCY1/ADCYAP1R1/ADRA1A/AMELX/AOC2/AS3MT/BCAR3/BCHE/CA9/CCNO/CDK1/CERS1/CES3/CMBL/CPB2/CPS1/CSAG2/CSAG3/CYP26A1/CYP2A13/CYP2A7/CYP2B6/CYP2C19/CYP2F1/CYP2W1/CYP46A1/DRD1/DRD2/EHMT2/EMX1/EMX2/FOS/FOSB/FZD3/GABRG3/GAD2/GAS6/GATA4/GPLD1/GPR52/GRIN1/GRIN2A/GRM2/GSTA4/GSTA5/GSTO1/HADH/HDAC2/HMGCS2/HNF1B/HSF1/HTR2A/IGFBP2/ITGA2/ITGB3/KCNC1/KCNH2/KCNK3/LHCGR/LOX/LYN/MAOB/MDK/NEUROD1/NKX6-1/NOS2/NPAS2/NPPC/PCSK1/PDE3A/PDGFA/PPM1E/PRKAA2/PTCH1/PTGS2/PTH/RAB6C/RET/SCGB1A1/SFRP2/SHANK2/SLC12A5/SLC1A1/SLC1A2/SLC22A12/SLC22A7/SLC34A1/SLC6A11/SLC6A2/SLC6A3/SLCO1B1/SLCO1B3/SOX10/SRC/SRD5A2/SST/SULT1C3/SULT1C4/TBXA2R/TFAP2B/TH/TNFRSF11B/TP73/TYMS/UBE2B/UCHL1/UGT1A3/UGT1A6/UGT1A8/VAV2/VKORC1	117

cluster5	BP	GO:0001837	epithelial to mesenchymal transition	54/3710	169/18903	0.0000933	0.002162879	0.001856428	ALX1/AMELX/BMP2/BMP4/BMP5/BMP7/CRB2/CUL7/DAB2/DACT3/DAG1/DLG5/EDNRA/EMP2/EPHA3/EZH2/FAM83D/FGFR1/FGFR2/FOXA1/FOXA2/FOXC1/FOXF2/GREM1/GSK3B/HAS2/HDAC2/HEYL/HIF1A/HMGA2/HPN/IL17RD/ISL1/LRP6/MDK/MSX2/NKX2-1/NOTCH4/OLFM1/PDPN/ROCK1/ROCK2/SERPINB3/SFRP2/SOX9/SPRY1/TBX20/TBX5/TGFB1I1/VASN/WNT11/WNT4/WNT5A/WNT8A	54
cluster5	BP	GO:0110148	biomineralization	57/3710	181/18903	0.0000947	0.002183711	0.001874308	ADGRV1/ADORA1/AMBN/AMELX/ANO6/ASPN/ATF4/BMP2/BMP4/BMP6/BMP7/BMPR1B/CFTR/DSPP/ECM1/ENPP1/FGF23/FGFR2/FGFR3/GAS6/GPC3/GPM6B/GREM1/HIF1A/HTN3/IBSP/KLF10/LGR4/LOX/MMP13/MSX2/NECTIN1/NELL1/NOS3/ODAM/OMD/OTOP1/PKDCC/PTGS2/PTH/PTH1R/PTN/RFLNA/ROCK1/ROCK2/ROR2/RSPO2/SBDS/SLC24A3/SOX9/SP7/TFAP2A/TRPM4/WDR72/WNT11/WNT4/ZNF664-RFLNA	57
cluster5	BP	GO:0007286	spermatid development	59/3710	189/18903	0.0000948	0.002183711	0.001874308	ABHD2/ACRBP/AKAP4/C2CD6/CABYR/CATSPER4/CCDC136/CEP131/CFAP206/CFAP221/CFAP43/CFAP47/CFAP54/CFAP65/CFAP69/CFAP97D1/CFTR/DDX25/DMC1/DMRTC2/DPY19L2/DZIP1/EHMT2/FAM9A/FSIP2/GK2/HSPA2/IFT81/MNS1/NECTIN3/NEURL1/NME5/OCA2/PACRG/PCSK4/PIWIL1/PLA2G3/PRM1/PTCH1/PYGO1/PYGO2/ROPN1/RSPH1/RSPH6A/SPACA1/SPAG17/SPAG6/SPATA16/SPEM1/SPINK1/SPO11/SYCP1/TBPL1/TCP11/TCP11X1/TDRD5/TSSK1B/UBE2B/ZPBP	59
cluster5	BP	GO:0072283	metanephric renal vesicle morphogenesis	10/3710	15/18903	0.000095	0.002183711	0.001874308	BMP4/GDNF/GREM1/KIF26B/LHX1/PAX2/SALL1/SIX2/SMO/WNT9B	10

cluster5	BP	GO:0097152	mesenchymal cell apoptotic process	10/3710	15/18903	0.000095	0.002183711	0.001874308	BMP7/EDNRA/GDF5/HIF1A/HNF1B/MSX2/PAX2/POU3F4/SHH/SOX9	10
cluster5	BP	GO:0030048	actin filament-based movement	45/3710	134/18903	0.0000953	0.002186576	0.001876768	ABCC9/ACTC1/ADORA1/ATP1A2/CACNA1C/CACNA1G/CACNA2D1/DSG2/EMP2/FNBP1L/GATA4/GJC1/HCN4/KCND3/KCNE4/KCNH2/KCNJ3/KCNJ5/KCNN2/MYH10/MYH2/MYH4/MYH6/MYH7/MYH8/MYH9/MYL1/MYO1C/MYO5B/MYO6/NEDD4L/PDPN/PKP2/ROCK1/RYR2/SCN1A/SCN2B/SCN3B/STC1/SYNE2/TNNT2/TPM1/TRPM4/WIPF1/WIPF3	45
cluster5	BP	GO:0021846	cell proliferation in forebrain	13/3710	23/18903	0.0000982	0.002247386	0.001928962	ARX/DCT/EMX2/FGFR2/GLI3/IGF2BP1/LHX5/NUMBL/POU3F2/POU3F3/SIX3/WNT3A/WNT7A	13
cluster5	BP	GO:0031016	pancreas development	31/3710	82/18903	0.000100158	0.002287276	0.001963199	AKT1/BMP4/BMP5/BMP6/CCDC40/CDH2/DLL1/FGF10/FOXA2/GSK3B/HES1/HNF1A/HNF1B/ILDR2/ISL1/MNX1/NEUROD1/NEUROG3/NKX2-2/NKX6-1/PAX6/PCSK1/PTF1A/RFX3/RFX6/SHH/SMO/SOX9/WFS1/WNT5A/ZIC3	31

cluster5	BP	GO:0072010	glomerular epithelium development	14/3710	26/18903	0.000107593	0.002447833	0.002101009	BASP1/BMP4/EDNRA/EDNRB/FOXC1/FOXJ1/KLF15/LAMB2/MAGI2/NOTCH2/NPHS1/PECAM1/PROM1/WT1	14
cluster5	BP	GO:0032526	response to retinoic acid	38/3710	108/18903	0.000107656	0.002447833	0.002101009	ASCL1/ATP1A3/BMP6/BRINP1/BRINP2/BRINP3/CYP26A1/EPHA3/FGFR2/FZD10/GJB3/GSK3B/HDAC2/HSD17B2/IGFBP2/LRAT/LYN/PAX2/PDGFA/PDGFRB/PTCH1/PTK7/RET/RORB/RXRG/SLC10A3/SOX9/STRA6/TEAD2/WNT11/WNT3A/WNT5A/WNT7B/WNT8A/WNT8B/WNT9A/WNT9B/YAP1	38
cluster5	BP	GO:0031128	developmental induction	17/3710	35/18903	0.000108992	0.002456891	0.002108783	BMP2/BMP4/FGF10/FGF2/GDNF/HOXA11/NKX2-1/ROBO1/ROBO2/SALL1/SOX9/SPRY1/WNT11/WNT2B/WNT3A/WNT4/WNT5A	17
cluster5	BP	GO:0042462	eye photoreceptor cell development	17/3710	35/18903	0.000108992	0.002456891	0.002108783	CRB2/GNAT1/GNGT1/HCN1/MFRP/NR2E3/NTRK2/OLFM3/PAX6/RORB/RP1/TH/THRB/THY1/TULP1/USH1C/VEGFA	17

cluster5	BP	GO:0098664	G protein-coupled serotonin receptor signaling pathway	17/3710	35/18903	0.000108992	0.002456891	0.002108783	CHRM1/CHRM2/CHRM3/DRD4/GNAZ/HRH3/HTR1D/HTR1E/HTR1F/HTR2A/HTR2C/HTR4/OR10H3/OR10H4/OR11H4/OR5T3/OR6T1	17
cluster5	BP	GO:1903539	protein localization to postsynaptic membrane	17/3710	35/18903	0.000108992	0.002456891	0.002108783	ADAM10/ARHGAP44/CACNG2/CACNG3/CACNG7/DAG1/GHSR/GPC6/GRIN2A/GRIP1/GRIP2/GRIPAP1/IQSEC2/LGI1/NPTX1/RAB8A/SNAP25	17
cluster5	BP	GO:0007263	nitric oxide mediated signal transduction	15/3710	29/18903	0.00011186	0.002505384	0.002150405	AGT/AGTR2/DDAH1/EGFR/GUCY1A1/GUCY1A2/INS/KCNC2/NDNF/NEUROD1/NOS2/NOS3/PDE5A/SPINK1/VEGFA	15
cluster5	BP	GO:0048265	response to pain	15/3710	29/18903	0.00011186	0.002505384	0.002150405	AKT1/CALCA/DBH/EDNRB/P2RX2/P2RX3/PIRT/PRKCG/RELN/RET/SCN9A/SLC6A2/TAC1/TACR1/VWA1	15

cluster5	BP	GO:0060074	synapse maturation	15/3710	29/18903	0.00011186	0.002505384	0.002150405	BCAN/CAMK2B/CHRDL1/IGSF21/NEURL1/NEUROD2/NFATC4/NLGN1/NRXN1/RELN/SEZ6L/SEZ6L2/SHANK1/SYBU/YWHAZ	15
cluster5	BP	GO:0036465	synaptic vesicle recycling	30/3710	79/18903	0.000117141	0.002618072	0.002247127	AMPH/AP3B2/AP3D1/CYFIP1/DNM1/GRIPAP1/NLGN1/NLGN2/NLGN3/NLGN4X/OPHN1/PACSIN1/RAB27B/RAB5A/ROCK1/SH3GL2/SH3GL3/SLC17A7/SNAP91/SNCB/STON1/STX1A/SYP/SYT1/SYT2/SYT5/SYT7/SYT8/WNT3A/WNT7A	30
cluster5	BP	GO:0035850	epithelial cell differentiation involved in kidney development	21/3710	48/18903	0.000119135	0.002645715	0.002270853	BASP1/BMP4/EDNRA/EDNRB/FOXJ1/GDNF/GREM1/KLF15/LAMB2/LHX1/MAGI2/NOTCH2/NPHS1/PAX2/PROM1/SALL1/SIX2/SMO/WNT9B/WT1/YAP1	21
cluster5	BP	GO:0048483	autonomic nervous system development	21/3710	48/18903	0.000119135	0.002645715	0.002270853	ARX/ASCL1/EDNRA/EDNRB/FZD3/GBX2/GDNF/GFRA3/HES1/HOXB1/ISX/NAV2/NRP2/PHOX2B/PLXNA4/RET/SEMA3A/SOX10/TFAP2A/TFAP2B/TLX2	21

cluster5	BP	GO:1990089	response to nerve growth factor	21/3710	48/18903	0.000119135	0.002645715	0.002270853	AKT1/APP/BDNF/CALCA/CSNK1E/EIF4A3/ELAVL4/HES1/ID1/KCNC1/KCNC2/MAGI2/NGF/NTF3/NTRK2/SH3GL2/SORT1/STMN2/TAC1/TMEM108/WASF1	21
cluster5	BP	GO:0035567	non-canonical Wnt signaling pathway	28/3710	72/18903	0.000120985	0.002681108	0.002301231	ABL1/CELSR1/CELSR2/CSNK1E/DAAM1/DAB2/FRZB/FZD10/FZD3/GPC3/GPC6/LBX2/MAGI2/MYOC/PRICKLE1/PTK7/ROR1/ROR2/RSPO3/SFRP2/SPEF1/VANGL2/WNT11/WNT4/WNT5A/WNT7A/WNT9B/ZNRF3	28
cluster5	BP	GO:0006865	amino acid transport	48/3710	147/18903	0.000124072	0.002743713	0.002354965	ACE2/ADORA1/AGT/APBA1/ATP1A2/AVP/AVPR1B/CLTRN/GFAP/GIPC1/GRM2/GRM7/HRH3/ITGB1/NPY5R/NTRK2/OCA2/RGS4/SLC11A1/SLC16A2/SLC17A6/SLC17A7/SLC17A8/SLC1A1/SLC1A2/SLC1A6/SLC25A2/SLC38A3/SLC38A4/SLC38A8/SLC66A1/SLC6A1/SLC6A11/SLC6A15/SLC6A17/SLC6A20/SLC6A5/SLC6A7/SLC7A10/SLC7A14/SLC7A3/SLC7A4/SLC7A8/STXBP1/SV2A/SYT4/TRH/TRPC4	48
cluster5	BP	GO:0007635	chemosensory behavior	11/3710	18/18903	0.000131999	0.00289568	0.002485401	ADCY3/ATP6V1B1/BBS1/CFAP69/LMX1A/P2RX3/PRKCG/SCN9A/SHANK1/TPBG/WFS1	11

cluster5	BP	GO:0060602	branch elongation of an epithelium	11/3710	18/18903	0.000131999	0.00289568	0.002485401	BMP4/FGF10/FGFR2/HNF1B/HOXD13/SHH/SIX4/SOX9/SPRY1/WNT5A/YAP1	11
cluster5	BP	GO:0006775	fat-soluble vitamin metabolic process	20/3710	45/18903	0.000132049	0.00289568	0.002485401	BCO1/CRABP1/CYP26A1/CYP26C1/CYP2W1/CYP4F11/CYP4F3/FGF23/GC/LRAT/LRP2/NPC1L1/PLTP/RBP1/RBP2/RPE65/TTPA/UGT1A3/UGT1A4/VKORC1	20
cluster5	BP	GO:0051955	regulation of amino acid transport	20/3710	45/18903	0.000132049	0.00289568	0.002485401	ACE2/ADORA1/AGT/ATP1A2/AVP/AVPR1B/CLTRN/GRM2/GRM7/HRH3/ITGB1/NPY5R/RGS4/SLC17A8/SLC38A3/SLC6A1/STXBP1/SV2A/SYT4/TRH	20
cluster5	BP	GO:0045471	response to ethanol	43/3710	128/18903	0.00013354	0.002922277	0.00250823	ABCB11/ADCYAP1R1/ADH7/AVP/CDK1/CDO1/CLDN1/CLDN18/CLDN5/CRHR1/DBH/DRD2/DRD4/EHMT2/EPS8/FGF19/FGFR2/FOS/GLRA1/GLRA2/GRIN1/GRIN2A/GRIN2B/HDAC2/HMGCS2/IL13/KCNC2/MAOB/NPPC/NR0B2/OPRK1/PENK/PTH/RGS4/RGS7/SLC6A3/SPINK1/TACR1/TBXA2R/TH/TNFRSF11A/TP53INP1/TYMS	43

cluster5	BP	GO:0015849	organic acid transport	91/3710	324/18903	0.000134634	0.002940074	0.002523505	ABCB11/ABCC3/ABCG2/ACE/ACE2/ACSL1/ADORA1/AGT/AKT1/APBA1/ATP1A2/AVP/AVPR1B/CLTRN/CRABP1/CRABP2/DRD2/DRD4/FABP3/FABP6/FABP7/FGF19/FOLR1/GFAP/GIPC1/GRM2/GRM7/HRH3/ITGB1/LHCGR/LRP2/MFSD2A/NPY5R/NR0B2/NTRK2/OCA2/PLA2G12A/PLA2G12B/PLA2G2E/PLA2G2F/PLA2G3/PLA2G5/PLA2R1/PMP2/RBP1/RBP2/RGS4/SLC10A3/SLC10A5/SLC11A1/SLC13A5/SLC16A2/SLC16A8/SLC17A6/SLC17A7/SLC17A8/SLC1A1/SLC1A2/SLC1A6/SLC22A9/SLC25A2/SLC25A21/SLC26A9/SLC27A6/SLC29A4/SLC38A3/SLC38A4/SLC38A8/SLC66A1/SLC6A1/SLC6A11/SLC6A15/SLC6A17/SLC6A20/SLC6A5/SLC6A7/SLC7A10/SLC7A14/SLC7A3/SLC7A4/SLC7A8/SLCO1B1/SLCO1B3/SLCO1C1/SPX/STXBP1/SV2A/SYT4/TNFRSF11A/TRH/TRPC4	91
cluster5	BP	GO:0030858	positive regulation of epithelial cell differentiation	27/3710	69/18903	0.000140597	0.003063914	0.002629799	AHI1/ATOH1/BMP2/BMP4/BMP6/BMP7/CDKN2B/ETV4/FGF2/FOXC1/FOXJ1/FOXN1/GDNF/IL13/IL20/LHX1/NKX2-2/NKX6-1/PAX2/PAX6/PROM1/PTCH1/PTCH2/RFX3/SERPINE1/SOX9/TP73	27
cluster5	BP	GO:2001053	regulation of mesenchymal cell apoptotic process	9/3710	13/18903	0.000141513	0.003077474	0.002641437	BMP7/GDF5/HIF1A/HNF1B/MSX2/PAX2/POU3F4/SHH/SOX9	9
cluster5	BP	GO:0035176	social behavior	22/3710	52/18903	0.000150906	0.003274944	0.002810928	AVP/BRINP1/CNTNAP2/DRD4/EN1/GAD1/GRID1/GRP/GRPR/MAPK8IP2/NLGN2/NLGN3/NLGN4X/NPAS4/NR2E1/NRXN1/NRXN3/PTCHD1/SEPTIN5/SHANK1/SHANK2/TH	22

cluster5	BP	GO:0048515	spermatid differentiation	60/3710	196/18903	0.000152286	0.003298052	0.002830762	ABHD2/ACRBP/AKAP4/C2CD6/CABYR/CATSPER4/CCDC136/CEP131/CFAP206/CFAP221/CFAP43/CFAP47/CFAP54/CFAP65/CFAP69/CFAP97D1/CFTR/DDX25/DMC1/DMRTC2/DPY19L2/DZIP1/EHMT2/FAM9A/FSIP2/GK2/HSPA2/IFT81/MNS1/NECTIN3/NEURL1/NME5/OCA2/PACRG/PCSK4/PIWIL1/PLA2G3/PRM1/PTCH1/PYGO1/PYGO2/ROPN1/RSPH1/RSPH6A/SEPTIN4/SPACA1/SPAG17/SPAG6/SPATA16/SPEM1/SPINK1/SPO11/SYCP1/TBPL1/TCP11/TCP11X1/TDRD5/TSSK1B/UBE2B/ZPBP	60
cluster5	BP	GO:0060348	bone development	69/3710	233/18903	0.000156822	0.003389306	0.002909087	ADAMTS7/BMP2/BMP4/BMP6/BMPR1B/CDX1/CLDN18/COL27A1/COL2A1/DLX5/ECM1/EPYC/EVC/FGF4/FGFR2/FGFR3/FOXC1/FREM1/GHR/GLI3/GNAS/GP1BB/GP9/GREM1/HAS2/HOXA11/LEPR/LOX/LRP5/LRRC17/MBTPS2/MEIS1/MMP13/MMP16/MSX2/MYOC/NEUROG1/NOTCH2/NPPC/OGN/PAPPA2/PDGFC/PLXNB1/PPIB/PTH/RARB/RFLNA/SBDS/SEMA4D/SH3PXD2B/SHOX2/SLC9B2/SMAD5/SOX9/SP5/SRC/SRD5A2/STC1/SULF1/SULF2/TFAP2A/THPO/TNN/TRIM45/TRPV4/TULP3/VKORC1/ZBTB16/ZNF664-RFLNA	69
cluster5	BP	GO:0001990	regulation of systemic arterial blood pressure by hormone	18/3710	39/18903	0.000157611	0.003392362	0.00291171	ACE/ACE2/AGT/AGTR2/AVPR1B/CMA1/CTSG/CYP11B2/ECE1/EDN2/EDNRB/F2R/NOS3/OR51E2/RASL10B/RPS6KA2/SUCNR1/TACR1	18
cluster5	BP	GO:0003016	respiratory system process	18/3710	39/18903	0.000157611	0.003392362	0.00291171	ADORA1/ATP1A2/CCBE1/CFAP221/CFAP43/CFAP54/DNAH9/ECEL1/FLT4/GLRA1/GSX2/NEK10/NLGN2/NLGN3/PHOX2B/STK40/TLX3/YWHAZ	18

cluster5	BP	GO:0061035	regulation of cartilage development	28/3710	73/18903	0.000159859	0.003433685	0.002947178	ADAMTS7/BMP2/BMP4/BMP6/BMPR1B/CCN2/CHADL/FRZB/GDF5/GDF6/GLI3/GREM1/HOXA11/MDK/PKDCC/PTH/RARB/RFLNA/SHOX2/SIX2/SOX5/SOX9/WNT11/WNT5A/WNT9A/ZBTB16/ZNF219/ZNF664-RFLNA	28
cluster5	BP	GO:0042490	mechanoreceptor differentiation	26/3710	66/18903	0.00016315	0.003497226	0.003001716	ADGRV1/ATOH1/BMP4/CECR2/CLRN1/CLRN2/DLL1/ESRP1/FGF2/GABRA5/GABRB2/GRXCR1/HES1/HES5/LHFPL5/MYO6/NTRK2/PDZD7/POU4F3/SDC4/SLITRK6/TMC1/TMEM132E/USH1C/USH2A/WHRN	26
cluster5	BP	GO:0051899	membrane depolarization	31/3710	84/18903	0.000167857	0.003590769	0.003082005	ABL1/ANK3/ATP1A2/BEST2/CACNA1C/CACNA1G/CACNA2D1/CACNG2/CHRNA4/CHRNA6/CNGB1/FHL1/HCN1/HCN3/HCN4/KCNH2/MYOC/NEDD4L/PHOX2B/PTPN3/SCN10A/SCN1A/SCN2A/SCN2B/SCN3B/SCN4A/SCN8A/SCN9A/SRC/TBX5/TRPM4	31
cluster5	BP	GO:0086010	membrane depolarization during action potential	17/3710	36/18903	0.000168922	0.003598865	0.003088955	ANK3/ATP1A2/CACNA1C/CACNA1G/CACNA2D1/HCN4/KCNH2/PTPN3/SCN10A/SCN1A/SCN2A/SCN2B/SCN3B/SCN4A/SCN8A/SCN9A/TRPM4	17

cluster5	BP	GO:0098810	neurotransmitter reuptake	17/3710	36/18903	0.000168922	0.003598865	0.003088955	ATP1A2/DRD1/DRD2/DRD4/GDNF/GPM6B/ITGB1/ITGB3/PRKN/RAB3B/SLC17A8/SLC1A2/SLC29A4/SLC6A1/SLC6A11/SLC6A2/SLC6A3	17
cluster5	BP	GO:0007602	phototransduction	21/3710	49/18903	0.000170199	0.003618711	0.003105989	ABCA4/AIPL1/ASIC2/CDS1/CNGB1/GNAT1/GNGT1/GPR52/GRK4/GUCY2F/LOC118142757/NR2E3/OPN1LW/OPN1MW2/OPN1MW3/OPN4/PDC/RGR/RHO/RP1/TRPC3	21
cluster5	BP	GO:0007548	sex differentiation	82/3710	288/18903	0.000173413	0.003679588	0.00315824	ACE/ACSBG1/ADCYAP1R1/ARID5B/ASPM/ATRX/BASP1/BMP5/BMP6/BMPR1B/BRCA2/CBX2/CDKL2/CGA/CNTFR/DACH1/DHCR24/DMC1/DMRT1/DMRT3/DMRTA2/DMRTC2/EIF2B2/FER/FGF10/FGF9/FOXC1/FOXF2/GATA4/GFRA1/GPR149/GREB1L/HOXA11/HOXD13/INHA/IRX5/KIF18A/KITLG/LGR4/LHCGR/LHFPL2/LHX1/LHX9/LRP2/NEUROG1/NHLH2/NKX2-1/NOS3/NR0B1/NR2F2/PBX1/PCYT1B/PDGFA/PDGFRA/PDGFRB/ROBO2/ROR2/SALL1/SDC1/SFRP2/SHH/SIX4/SLIT3/SMAD5/SOX9/SPO11/SRC/SRD5A2/STRA6/TEX19/TFAP2C/TIPARP/TSPY2/TYRO3/VEGFA/WNT2B/WNT4/WNT5A/WNT7A/WNT9B/WT1/ZFP42	82
cluster5	BP	GO:0043266	regulation of potassium ion transport	39/3710	114/18903	0.000174276	0.003690427	0.003167543	ABCC8/ABCC9/ACTN2/ADORA1/ADRA2A/AKAP6/ANK3/ANO6/ATF4/ATP1B1/CASQ2/CRBN/DPP10/DPP6/DRD1/DRD2/FHL1/GCK/GRP/HTR2A/ITGB1/KCNC1/KCNC2/KCNE4/KCNH2/KCNIP1/KCNIP3/KCNK3/KCNN2/KCNRG/KCNS2/LRRC55/NEDD4L/NOS3/OPRK1/RGS4/RGS7/WNK2/WNK3	39

cluster5	BP	GO:0001759	organ induction	13/3710	24/18903	0.000175792	0.003700109	0.003175853	BMP2/BMP4/FGF10/FGF2/GDNF/HOXA11/ROBO1/ROBO2/SPRY1/WNT11/WNT2B/WNT3A/WNT5A	13
cluster5	BP	GO:0072202	cell differentiation involved in metanephros development	13/3710	24/18903	0.000175792	0.003700109	0.003175853	BMP4/GDNF/GREM1/LAMB2/LGR4/PAX2/POU3F3/SALL1/SIX2/SMO/WNT4/WNT9B/YAP1	13
cluster5	BP	GO:0099637	neurotransmitter receptor transport	13/3710	24/18903	0.000175792	0.003700109	0.003175853	AP3D1/ARHGAP44/CACNG2/CACNG3/CACNG4/CACNG5/CACNG7/GRIP1/GRIP2/GRIPAP1/RAB8A/SLC1A1/SNAP25	13
cluster5	BP	GO:0043279	response to alkaloid	35/3710	99/18903	0.000177569	0.003730017	0.003201524	ADCY8/BCHE/CACNA1S/CACNG4/CASQ2/DRD1/DRD2/DRD4/EHMT2/ELAVL4/EN1/FOSB/GHR/GRM2/HDAC2/HES1/HOMER1/HTR2A/OPRK1/PCSK1/PENK/PRKCG/RGS4/RYR2/RYR3/SDK1/SLC1A1/SLC1A2/SLC34A1/SLC6A1/SLC6A3/ST8SIA2/TACR1/TACR3/TH	35

cluster5	BP	GO:0048566	embryonic digestive tract development	16/3710	33/18903	0.00017793	0.003730126	0.003201618	FGF10/FGF9/FGFR2/FOXF2/GLI2/GLI3/HNF1B/PDGFRA/PKDCC/RARB/RBPMS2/SALL1/SHH/SHOX2/SIX2/STRA6	16
cluster5	BP	GO:0030325	adrenal gland development	14/3710	27/18903	0.00018319	0.003818825	0.003277749	ARID5B/ASCL1/CRH/CRHR1/DKK3/MDK/NR0B1/PBX1/PDGFRA/SALL1/STRA6/WNT11/WNT4/WT1	14
cluster5	BP	GO:0001975	response to amphetamine	15/3710	30/18903	0.000183254	0.003818825	0.003277749	ASIC1/DBH/DRD1/DRD2/DRD4/EDNRA/GRIN2A/HDAC2/HPRT1/NR4A2/PDE1B/RGS4/RGS7/SLC1A1/TH	15
cluster5	BP	GO:0070168	negative regulation of biomineral tissue development	15/3710	30/18903	0.000183254	0.003818825	0.003277749	ASPN/ECM1/ENPP1/FGF23/GAS6/GREM1/HIF1A/NOS3/PTH/RFLNA/ROCK1/ROCK2/SOX9/TRPM4/ZNF664-RFLNA	15

cluster5	BP	GO:0021545	cranial nerve development	23/3710	56/18903	0.000184299	0.003825381	0.003283376	CNGB1/DRGX/ERBB3/GLI3/HES1/HOXA3/HOXB1/HOXD3/ISL1/NAV2/NEUROG1/NKX2-2/NRP2/PAX2/PHOX2B/PLXNA1/PLXNA4/POU4F3/SALL1/SIX4/SLITRK6/TFAP2A/TMEM126A	23
cluster5	BP	GO:0042733	embryonic digit morphogenesis	23/3710	56/18903	0.000184299	0.003825381	0.003283376	ALX4/BMP4/ECE1/FREM2/GLI3/GRHL2/HDAC2/HOXA11/HOXD12/HOXD13/IFT140/LRP4/LRP5/MSX2/SALL1/SFRP2/SHH/TBC1D32/TMEM231/TULP3/WNT5A/WNT7A/ZBTB16	23
cluster5	BP	GO:0085029	extracellular matrix assembly	20/3710	46/18903	0.000190814	0.003952781	0.003392725	AGT/DAG1/EMILIN1/FKBP10/GAS6/GPM6B/HAPLN2/HAS2/HAS3/LAMB1/LAMB2/LOX/MFAP4/NTNG1/NTNG2/PXDN/QSOX1/SOX9/THSD4/TNXB	20
cluster5	BP	GO:0043588	skin development	85/3710	302/18903	0.000203558	0.004162303	0.003572561	ABCA12/ADAMTS2/AHDC1/ALOX12/ALOX12B/ALX4/APCDD1/CLDN1/CLDN4/COL5A3/DACT2/DHCR24/DLL1/DLX3/DSG4/EGFR/ETV4/EVPL/EZH2/FERMT1/FGF10/FGF7/FGFR2/FOXC1/FOXN1/FZD3/GJB3/GLI2/GRHL1/GRHL2/HDAC2/HOXC13/IL20/ITGA2/IVL/KLK5/KRT27/KRT6C/KRT75/KRT76/KRT8/KRT80/KRT83/KRT84/KRTAP21-1/LAMA5/LCE1A/LCE1C/LCE2C/LCE2D/LCE3A/LCE3D/LCE3E/LCE4A/LCE6A/LGR4/LGR5/LHX2/LORICRIN/LRP4/MSX2/NGFR/OVOL1/PAX6/PDGFA/PTCH2/REG3A/REG3G/ROCK1/ROCK2/SCEL/SHH/SLC2A10/SMO/SOSTDC1/SOX18/SOX9/SPRR2F/SPRR2G/SPRR4/TFAP2B/TNFRSF19/WNT5A/YAP1/ZFP36	85

cluster5	BP	GO:0021892	cerebral cortex GABAergic interneuron differentiation	8/3710	11/18903	0.000203707	0.004162303	0.003572561	ARX/ASCL1/DLX1/DLX2/DRD1/DRD2/FEZF2/NKX2-1	8
cluster5	BP	GO:0060433	bronchus development	8/3710	11/18903	0.000203707	0.004162303	0.003572561	ADAMTSL2/AGR2/BMP4/IL13/SOX9/SPDEF/TULP3/WNT7B	8
cluster5	BP	GO:0060579	ventral spinal cord interneuron fate commitment	8/3710	11/18903	0.000203707	0.004162303	0.003572561	ASCL1/DMRT3/FOXN4/GLI2/GLI3/LHX3/NKX2-2/SOX1	8
cluster5	BP	GO:0060581	cell fate commitment involved in pattern specification	8/3710	11/18903	0.000203707	0.004162303	0.003572561	ASCL1/DMRT3/FOXN4/GLI2/GLI3/LHX3/NKX2-2/SOX1	8

cluster5	BP	GO:0060601	lateral sprouting from an epithelium	8/3710	11/18903	0.000203707	0.004162303	0.003572561	BMP4/BMP7/CELSR1/FGF10/FGFR2/SHH/SULF1/WNT5A	8
cluster5	BP	GO:2000826	regulation of heart morphogenesis	8/3710	11/18903	0.000203707	0.004162303	0.003572561	BMP2/BMP4/ROBO1/ROBO2/SMO/WNT11/WNT3A/WNT5A	8
cluster5	BP	GO:0021542	dentate gyrus development	10/3710	16/18903	0.000208648	0.004205867	0.003609953	DRD1/EMX2/FEZF2/LMX1A/MDK/NEUROD1/NEUROD6/NR2E1/SMO/TMEM108	10
cluster5	BP	GO:0061101	neuroendocrine cell differentiation	10/3710	16/18903	0.000208648	0.004205867	0.003609953	ASCL1/BMP2/FGF2/HES1/LHX3/NKX2-2/OTP/POU3F2/WNT11/WNT4	10

cluster5	BP	GO:0072044	collecting duct development	10/3710	16/18903	0.000208648	0.004205867	0.003609953	BMP4/CALB1/DACT2/DLG5/PAX2/PTCH1/SHH/TFAP2B/WNT7B/WNT9B	10
cluster5	BP	GO:0072578	neurotransmitter-gated ion channel clustering	10/3710	16/18903	0.000208648	0.004205867	0.003609953	CHRDL1/FRRS1L/GLRB/LHFPL4/NLGN1/NLGN2/RELN/SHISA6/SHISA7/SLITRK3	10
cluster5	BP	GO:0097154	GABAergic neuron differentiation	10/3710	16/18903	0.000208648	0.004205867	0.003609953	ARX/ASCL1/DLX1/DLX2/DRD1/DRD2/FEZF2/GSX2/NKX2-1/TLX3	10
cluster5	BP	GO:0099633	protein localization to postsynaptic specialization membrane	10/3710	16/18903	0.000208648	0.004205867	0.003609953	ADAM10/CACNG2/CACNG3/CACNG7/DAG1/GHSR/GPC6/IQSEC2/LGI1/NPTX1	10

cluster5	BP	GO:0099645	neurotransmitter receptor localization to postsynaptic specialization membrane	10/3710	16/18903	0.000208648	0.004205867	0.003609953	ADAM10/CACNG2/CACNG3/CACNG7/DAG1/GHSR/GPC6/IQSEC2/LGI1/NPTX1	10
cluster5	BP	GO:0007585	respiratory gaseous exchange by respiratory system	28/3710	74/18903	0.000209487	0.004214661	0.0036175	ADORA1/ATP1A2/CCBE1/CFAP221/CFAP43/CFAP54/COX5B/DACH1/DNAH9/ECEL1/EDNRA/FLT4/GLRA1/GRP/GRPR/GSX2/NEK10/NLGN2/NLGN3/NMBR/NR4A2/PHOX2B/SFTPA1/SFTPA2/STK40/TLX3/TMPRSS11D/YWHAZ	28
cluster5	BP	GO:0060071	Wnt signaling pathway, planar cell polarity pathway	22/3710	53/18903	0.000210558	0.004228101	0.003629036	ABL1/CELSR1/CELSR2/DAAM1/DAB2/FZD3/GPC3/GPC6/MAGI2/PRICKLE1/PTK7/ROR1/ROR2/RSPO3/SFRP2/SPEF1/VANGL2/WNT11/WNT5A/WNT7A/WNT9B/ZNRF3	22
cluster5	BP	GO:0042491	inner ear auditory receptor cell differentiation	19/3710	43/18903	0.000212384	0.004256615	0.00365351	ATOH1/BMP4/CLRN1/CLRN2/DLL1/ESRP1/FGF2/GRXCR1/HES1/HES5/LHFPL5/MYO6/PDZD7/POU4F3/SLITRK6/TMC1/USH1C/USH2A/WHRN	19

cluster5	BP	GO:0002791	regulation of peptide secretion	56/3710	182/18903	0.00021416	0.004272158	0.003666851	ABCA12/ABCC8/ADCY5/ADCY8/ADCYAP1/ADORA1/ADRA2A/BRSK2/CFTR/CHGA/CRH/DOC2B/DRD2/EFNA5/EPHA5/FKBP1B/FOXA2/GCG/GCK/GHSR/GPLD1/GRP/HADH/HFE/HIF1A/HTR2C/INS/ISL1/KCNB1/KISS1/LRP5/NEUROD1/NKX6-1/NLGN2/NNAT/NOS2/NR0B2/NR1H4/PRKN/RASL10B/RFX3/RFX6/RPH3AL/SLC30A8/SLC9B2/SNAP25/SPINK1/SSTR5/STX1A/SYT7/SYT9/TFAP2B/TRH/TRPM4/UCN3/VSNL1	56
cluster5	BP	GO:0042303	molting cycle	39/3710	115/18903	0.000214382	0.004272158	0.003666851	ALX4/APCDD1/DLX3/DSG4/EGFR/EPS8L3/FERMT1/FGF10/FGF7/FGFR2/FOXN1/FZD3/GLI2/HDAC2/HOXC13/KRT27/KRT83/KRT84/KRTAP21-1/KRTAP4-8/LAMA5/LGR4/LGR5/LHX2/LRP4/MPZL3/MSX2/NGFR/PDGFA/PER1/PTCH2/PTGS2/SHH/SMO/SOSTDC1/SOX18/SOX9/TNFRSF19/WNT5A	39
cluster5	BP	GO:0042633	hair cycle	39/3710	115/18903	0.000214382	0.004272158	0.003666851	ALX4/APCDD1/DLX3/DSG4/EGFR/EPS8L3/FERMT1/FGF10/FGF7/FGFR2/FOXN1/FZD3/GLI2/HDAC2/HOXC13/KRT27/KRT83/KRT84/KRTAP21-1/KRTAP4-8/LAMA5/LGR4/LGR5/LHX2/LRP4/MPZL3/MSX2/NGFR/PDGFA/PER1/PTCH2/PTGS2/SHH/SMO/SOSTDC1/SOX18/SOX9/TNFRSF19/WNT5A	39
cluster5	BP	GO:0046942	carboxylic acid transport	82/3710	290/18903	0.00022177	0.004410992	0.003786014	ABCB11/ABCC3/ABCG2/ACE/ACE2/ADORA1/AGT/APBA1/ATP1A2/AVP/AVPR1B/CLTRN/DRD2/DRD4/FGF19/FOLR1/GFAP/GIPC1/GRM2/GRM7/HRH3/ITGB1/LHCGR/LRP2/NOS2/NPY5R/NR0B2/NTRK2/PLA2G12A/PLA2G12B/PLA2G2E/PLA2G2F/PLA2G3/PLA2G5/PLA2R1/PSAP/PTGS2/RGS4/SLC10A3/SLC10A5/SLC11A1/SLC13A5/SLC16A12/SLC16A2/SLC16A8/SLC16A9/SLC17A2/SLC17A4/SLC17A6/SLC17A7/SLC17A8/SLC1A1/SLC1A2/SLC1A6/SLC22A9/SLC25A2/SLC25A21/SLC26A9/SLC38A3/SLC38A4/SLC38A8/SLC66A1/SLC6A1/SLC6A11/SLC6A15/SLC6A17/SLC6A20/SLC6A5/SLC6A7/SLC7A10/SLC7A3/SLC7A8/SLCO1B1/SLCO1B3/SLCO1C1/STXBP1/SV2A/SYT4/TNFRSF11A/TRH/TRPC4/UGT1A3	82

cluster5	BP	GO:0030282	bone mineralization	41/3710	123/18903	0.000228207	0.004530424	0.003888524	ADGRV1/ANO6/ASPN/ATF4/BMP2/BMP4/BMP6/BMP7/BMPR1B/ECM1/ENPP1/FGF23/FGFR2/FGFR3/GPC3/GPM6B/GREM1/HIF1A/IBSP/KLF10/LGR4/LOX/MMP13/NELL1/OMD/PKDCC/PTGS2/PTH/PTH1R/PTN/RFLNA/ROR2/RSPO2/SBDS/SLC24A3/SOX9/TFAP2A/TRPM4/WNT11/WNT4/ZNF664-RFLNA	41
cluster5	BP	GO:0070252	actin-mediated cell contraction	36/3710	104/18903	0.000233719	0.004625334	0.003969987	ABCC9/ACTC1/ADORA1/ATP1A2/CACNA1C/CACNA1G/CACNA2D1/DSG2/EMP2/GATA4/GJC1/HCN4/KCND3/KCNE4/KCNH2/KCNJ3/KCNJ5/KCNN2/MYH2/MYH4/MYH6/MYH7/MYH8/MYL1/NEDD4L/PDPN/PKP2/ROCK1/RYR2/SCN1A/SCN2B/SCN3B/STC1/TNNT2/TPM1/TRPM4	36
cluster5	BP	GO:0007210	serotonin receptor signaling pathway	18/3710	40/18903	0.000234311	0.004625334	0.003969987	CHRM1/CHRM2/CHRM3/DRD4/GNAZ/HRH3/HTR1D/HTR1E/HTR1F/HTR2A/HTR2C/HTR3C/HTR4/OR10H3/OR10H4/OR11H4/OR5T3/OR6T1	18
cluster5	BP	GO:0045823	positive regulation of heart contraction	18/3710	40/18903	0.000234311	0.004625334	0.003969987	ACE2/ADM5/ADRA1A/ADRA1B/ADRA1D/APELA/ATP1A2/CCN2/CHGA/EDN2/HRC/RGS4/RYR2/SCN3B/SLC1A1/TACR3/TPM1/TRPM4	18

cluster5	BP	GO:0002066	columnar/cuboidal epithelial cell development	21/3710	50/18903	0.000239597	0.004711923	0.004044307	AKT1/BMP4/BMP5/BMP6/CDH2/DLL1/GPAT4/GSK3B/HIF1A/NKX2-2/NKX6-1/PAX6/RARB/RFX3/SLC9A4/SMO/SPDEF/SRC/TYMS/WNT5A/YAP1	21
cluster5	BP	GO:0090102	cochlea development	21/3710	50/18903	0.000239597	0.004711923	0.004044307	ADAM10/CALB1/CECR2/FRZB/GABRA5/GABRB2/HES1/HPN/KCNK3/MYO3A/MYO3B/NEUROG1/PAX2/POU3F4/PTK7/PVALB/SLC17A8/SLITRK6/SOBP/SOX9/WNT5A	21
cluster5	BP	GO:0030516	regulation of axon extension	33/3710	93/18903	0.000247968	0.004867418	0.004177771	ABL1/BARHL2/CDH4/CDKL3/CYFIP1/DBN1/DRAXIN/DSCAM/GDI1/GSK3B/ISLR2/L1CAM/MAP2/MT3/NDEL1/NKX6-1/NRCAM/NTN1/OLFM1/PLXNA4/PTPRS/SEMA3A/SEMA3E/SEMA4D/SEMA5A/SEMA5B/SEMA6A/SEMA6D/SLIT1/TNR/VEGFA/WNT3A/WNT5A	33
cluster5	BP	GO:0090175	regulation of establishment of planar polarity	23/3710	57/18903	0.000252085	0.004934114	0.004235017	ABL1/CELSR1/CELSR2/DAAM1/DAB2/FZD3/GPC3/GPC6/MAGI2/PKHD1/PRICKLE1/PTK7/ROR1/ROR2/RSPO3/SFRP2/SPEF1/VANGL2/WNT11/WNT5A/WNT7A/WNT9B/ZNRF3	23

cluster5	BP	GO:0090276	regulation of peptide hormone secretion	55/3710	179/18903	0.000252307	0.004934114	0.004235017	ABCA12/ABCC8/ADCY5/ADCY8/ADCYAP1/ADRA2A/BRSK2/CFTR/CHGA/CRH/DOC2B/DRD2/EFNA5/EPHA5/FKBP1B/FOXA2/GCG/GCK/GHSR/GPLD1/GRP/HADH/HFE/HIF1A/HTR2C/INS/ISL1/KCNB1/KISS1/LRP5/NEUROD1/NKX6-1/NLGN2/NNAT/NOS2/NR0B2/NR1H4/PRKN/RASL10B/RFX3/RFX6/RPH3AL/SLC30A8/SLC9B2/SNAP25/SPINK1/SSTR5/STX1A/SYT7/SYT9/TFAP2B/TRH/TRPM4/UCN3/VSNL1	55
cluster5	BP	GO:0051279	regulation of release of sequestered calcium ion into cytosol	30/3710	82/18903	0.000253368	0.004945622	0.004244894	ABL1/AKAP6/ATP1A2/CACNA1C/CASQ2/CEMIP/DHRS7C/DMD/DRD1/F2/F2R/F2RL3/FKBP1A/FKBP1B/GSTO1/HAP1/HRC/IL13/ITGB3/JPH1/JPH3/JPH4/JSRP1/LACRT/LHCGR/LYN/NPSR1/RYR2/THY1/TRDN	30
cluster5	BP	GO:0086019	cell-cell signaling involved in cardiac conduction	17/3710	37/18903	0.000255681	0.004981506	0.004275694	CACNA1C/CACNA1G/CACNA2D1/CASQ2/GJC1/HCN1/HCN3/HCN4/KCNJ3/KCNJ5/KCNN2/PKP2/RYR2/SCN10A/SCN3B/TBX5/TRPM4	17
cluster5	BP	GO:0021516	dorsal spinal cord development	11/3710	19/18903	0.000257869	0.005005517	0.004296303	ASCL1/DRAXIN/DRGX/GDNF/GSX1/GSX2/LHX1/LHX3/LHX5/UNCX/WNT3A	11

cluster5	BP	GO:0071625	vocalization behavior	11/3710	19/18903	0.000257869	0.005005517	0.004296303	BRINP1/CNTNAP2/GLI3/NEUROG1/NLGN3/NLGN4X/NRXN1/NRXN3/SHANK1/SHANK2/SRPX2	11
cluster5	BP	GO:0071774	response to fibroblast growth factor	40/3710	120/18903	0.00027031	0.005237319	0.004495261	CCN2/CPS1/CXCL13/FGF10/FGF16/FGF17/FGF19/FGF2/FGF23/FGF4/FGF5/FGF7/FGF9/FGFBP3/FGFR1/FGFR2/FGFR3/FGFR4/FLRT1/FLRT2/FLRT3/FRS3/HHIP/KCNC1/LHX1/NDNF/NGFR/NPTN/OTX2/PRDM14/PTH/SCGB1A1/SHOC2/SPRY1/SULF1/SULF2/TDGF1/WNT4/WNT5A/ZFP36	40
cluster5	BP	GO:0031018	endocrine pancreas development	20/3710	47/18903	0.00027134	0.005247572	0.004504062	AKT1/BMP4/BMP5/BMP6/CDH2/DLL1/FOXA2/GSK3B/HES1/MNX1/NEUROD1/NEUROG3/NKX2-2/NKX6-1/PAX6/RFX3/RFX6/SMO/SOX9/WNT5A	20
cluster5	BP	GO:0031589	cell-substrate adhesion	100/3710	369/18903	0.00027489	0.005306448	0.004554596	ABL1/ACTN1/ACTN2/ADAMTS13/AGR2/AJAP1/AJUBA/ANGPT1/ANGPT2/APOD/ARHGAP6/ATRNL1/BVES/CARMIL1/CCL25/CCN2/COL16A1/COL26A1/COL5A3/COL8A1/CORO2B/CSPG5/DAB2/DAG1/DEFB118/DMTN/ECM2/EDIL3/EFNA5/EMILIN1/EMP2/EPHA3/EPHB3/FAT2/FBLN1/FER/FERMT1/FERMT3/FREM1/GAS6/GPM6B/GREM1/GSK3B/HAS2/HOXD3/HSD17B12/ID1/ILK/ITGA2/ITGA2B/ITGA7/ITGAM/ITGB1/ITGB3/ITGB5/ITGB8/L1CAM/LAMA5/LAMB1/LAMB2/MDK/MINK1/MYADM/MYOC/NDNF/NEXMIF/NID1/NPY/NTNG1/NTNG2/PDPN/PKHD1/PKP2/PPFIA2/RADIL/RASA1/RHOD/ROCK1/ROCK2/SDC4/SEMA3E/SERPINE1/SORBS3/SPOCK1/SRC/SRCIN1/STON1/THY1/TMEFF2/TNN/TNXB/TTYH1/TYRO3/USH2A/VEGFA/VIT/VWA2/VWC2/VWF/WNT4	100

cluster5	BP	GO:0097105	presynaptic membrane assembly	7/3710	9/18903	0.000276143	0.005320823	0.004566934	IL1RAPL1/LRP4/NLGN1/NLGN2/NLGN3/NLGN4X/PTPRD	7
cluster5	BP	GO:1903169	regulation of calcium ion transmembrane transport	57/3710	188/18903	0.000289219	0.005514124	0.004732847	ABL1/ADCYAP1R1/ADRA2A/AKAP6/ATP1A2/ATP1B1/CABP1/CACNA1C/CACNA2D1/CACNB1/CALCR/CASQ2/CBARP/CEMIP/CRHR1/DHRS7C/DMD/DRD1/DRD2/DRD4/EHD3/EPO/F2/F2R/F2RL3/FKBP1A/FKBP1B/GEM/GRIN1/GRM6/GSTO1/HAP1/HPCA/HRC/HSPA2/IL13/ITGB3/JPH1/JPH3/JPH4/JSRP1/LACRT/LHCGR/LYN/NPSR1/P2RX2/P2RX3/PLP1/REM1/RYR2/SLN/STAC2/THY1/TMBIM6/TMC1/TRDN/TRPC3	57
cluster5	BP	GO:0007596	blood coagulation	66/3710	225/18903	0.000289236	0.005514124	0.004732847	ADAMTS13/ADAMTS18/ADRA2A/ALOX12/ANO6/BLOC1S6/CPB2/CTSG/CYP4F11/DGKB/DGKI/DMTN/EMILIN1/EMILIN2/F13B/F2/F2R/F2RL3/F3/F5/FAP/FBLN1/FERMT3/FOXA2/GAS6/GNAS/GP1BB/GP6/GP9/HGFAC/ILK/ITGA2/ITGB3/KNG1/LYN/MMRN1/MYH9/MYL12A/NOS3/PDGFA/PDGFRA/PDPN/PEAR1/PF4/PF4V1/PRKCD/PRKG1/PROS1/PRSS56/SAA1/SCUBE1/SELP/SERPINE1/SERPINE2/SHH/SRC/STXBP1/TBXA2R/TFPI/TREML1/TSPAN8/TYRO3/VAV2/VKORC1/VWF/WNT3A	66
cluster5	BP	GO:0030072	peptide hormone secretion	66/3710	225/18903	0.000289236	0.005514124	0.004732847	ABCA12/ABCC8/ADCY5/ADCY8/ADCYAP1/ADRA2A/BRSK2/CFTR/CHGA/CLTRN/CRH/CRHR1/DOC2B/DRD2/EFNA5/EPHA5/EXOC3L1/FKBP1B/FOXA2/GCG/GCK/GHSR/GPLD1/GRP/HADH/HFE/HIF1A/HNF1A/HNF1B/HTR2C/ILDR2/INS/ISL1/KCNB1/KISS1/LRP5/MC4R/NEUROD1/NKX6-1/NLGN2/NNAT/NOS2/NR0B2/NR1H4/PCLO/PRKN/RAB11FIP2/RASL10B/RFX3/RFX6/RPH3AL/SLC16A2/SLC30A8/SLC9B2/SNAP25/SPINK1/SSTR5/STX1A/SYT7/SYT9/TFAP2B/TRH/TRPM4/TRPV4/UCN3/VSNL1	66

cluster5	BP	GO:0051703	biological process involved in intraspecies interaction between organisms	22/3710	54/18903	0.000290026	0.005514124	0.004732847	AVP/BRINP1/CNTNAP2/DRD4/EN1/GAD1/GRID1/GRP/GRPR/MAPK8IP2/NLGN2/NLGN3/NLGN4X/NPAS4/NR2E1/NRXN1/NRXN3/PTCHD1/SEPTIN5/SHANK1/SHANK2/TH	22
cluster5	BP	GO:0002035	brain renin-angiotensin system	5/3710	5/18903	0.000290586	0.005514124	0.004732847	ACE2/AGT/AGTR2/RPS6KA2/TACR1	5
cluster5	BP	GO:0014050	negative regulation of glutamate secretion	5/3710	5/18903	0.000290586	0.005514124	0.004732847	ADORA1/GRM7/HRH3/NPY5R/TRH	5
cluster5	BP	GO:0016199	axon midline choice point recognition	5/3710	5/18903	0.000290586	0.005514124	0.004732847	APP/FOXG1/ROBO1/ROBO2/ROBO3	5

cluster5	BP	GO:0110150	negative regulation of biomineralization	15/3710	31/18903	0.000290909	0.005514124	0.004732847	ASPN/ECM1/ENPP1/FGF23/GAS6/GREM1/HIF1A/NOS3/PTH/RFLNA/ROCK1/ROCK2/SOX9/TRPM4/ZNF664-RFLNA	15
cluster5	BP	GO:1905145	cellular response to acetylcholine	15/3710	31/18903	0.000290909	0.005514124	0.004732847	CDK5R1/CHRM1/CHRM2/CHRM3/CHRNA3/CHRNA7/HRH3/LY6G6D/LY6H/LYPD1/PLCB1/RGS10/RGS8/ROCK2/SLURP2	15
cluster5	BP	GO:0090087	regulation of peptide transport	56/3710	184/18903	0.000292234	0.005529241	0.004745823	ABCA12/ABCC8/ADCY5/ADCY8/ADCYAP1/ADORA1/ADRA2A/BRSK2/CFTR/CHGA/CRH/DOC2B/DRD2/EFNA5/EPHA5/FKBP1B/FOXA2/GCG/GCK/GHSR/GPLD1/GRP/HADH/HFE/HIF1A/HTR2C/INS/ISL1/KCNB1/KISS1/LRP5/NEUROD1/NKX6-1/NLGN2/NNAT/NOS2/NR0B2/NR1H4/PRKN/RASL10B/RFX3/RFX6/RPH3AL/SLC30A8/SLC9B2/SNAP25/SPINK1/SSTR5/STX1A/SYT7/SYT9/TFAP2B/TRH/TRPM4/UCN3/VSNL1	56
cluster5	BP	GO:0060113	inner ear receptor cell differentiation	24/3710	61/18903	0.000293863	0.005540051	0.0047551	ADGRV1/ATOH1/BMP4/CECR2/CLRN1/CLRN2/DLL1/ESRP1/FGF2/GABRA5/GABRB2/GRXCR1/HES1/HES5/LHFPL5/MYO6/PDZD7/POU4F3/SDC4/SLITRK6/TMC1/USH1C/USH2A/WHRN	24

cluster5	BP	GO:0086065	cell communication involved in cardiac conduction	24/3710	61/18903	0.000293863	0.005540051	0.0047551	ATP1A2/ATP1A3/ATP1B1/CACNA1C/CACNA1G/CACNA2D1/CASQ2/DSG2/FKBP1B/GJC1/HCN1/HCN3/HCN4/HRC/KCNJ3/KCNJ5/KCNN2/PKP2/RYR2/SCN10A/SCN3B/TBX5/TRDN/TRPM4	24
cluster5	BP	GO:0007224	smoothened signaling pathway	46/3710	144/18903	0.000298345	0.005614454	0.004818962	BOC/CC2D2A/CDON/CIBAR1/DISP3/DLG5/DZIP1/ENPP1/EVC/EVC2/FGF10/FGF9/FGFR2/FOXA1/GLI2/GLI3/GPC2/GPC3/GPR161/HES1/HES5/HHIP/IFT140/IFT81/IQUB/KIF7/NKX2-2/NKX6-1/OTX2/PAX6/PTCH1/PTCH2/PTCHD1/RFX4/SCUBE1/SERPINE2/SHH/SHOX2/SMO/TBC1D32/TEDC2/TMEM17/TMEM231/TULP3/ZIC1/ZIC3	46
cluster5	BP	GO:0044331	cell-cell adhesion mediated by cadherin	14/3710	28/18903	0.000300436	0.005636505	0.004837888	BMP6/CDH2/CTNND1/EPCAM/FER/FOXA1/FOXA2/MMP24/NEXMIF/NOTCH4/PTPRU/VEGFA/WNT3A/WNT5A	14
cluster5	BP	GO:0048714	positive regulation of oligodendrocyte differentiation	13/3710	25/18903	0.000300592	0.005636505	0.004837888	ASPA/DAG1/GSX2/HDAC2/MDK/NKX2-2/NKX6-1/PTN/PTPRZ1/SHH/TENM4/TP73/ZNF488	13

cluster5	BP	GO:0016049	cell growth	128/3710	493/18903	0.000309945	0.005801508	0.004979512	ABL1/ADAM10/ADRA1A/AGT/AGTR2/AKAP6/AKT1/APP/AUTS2/AVP/BARHL2/BCL11A/BDNF/CACNG7/CDH4/CDKL3/CDKN1A/CPNE5/CPNE6/CPNE9/CRABP2/CRYAB/CYFIP1/DAB2/DACT3/DBN1/DCBLD2/DCLK1/DCSTAMP/DDX3X/DRAXIN/DSCAM/EDN2/EDNRA/EFNA5/EGFR/EMX1/ENPP1/EPHA7/ERBB2/F2/FHL1/FLRT1/FLRT3/FRZB/GATA4/GDI1/GREM1/GSK3B/HDAC6/HPN/IGFBP4/IGFBPL1/IL9/INS/ISLR2/ITGB1/KAZALD1/L1CAM/LAMB2/LAMTOR1/LGI1/LHX2/LMX1A/MAP2/MFSD2A/MT3/MYOCD/NDEL1/NEDD4L/NGF/NKX6-1/NLGN3/NRCAM/NRG3/NRP2/NTN1/OLFM1/PAK5/PAPPA2/PLXNA1/PLXNA4/POU4F3/PRDM11/PRKN/PTCH2/PTPRS/RGS4/RICTOR/RND2/SEMA3A/SEMA3E/SEMA4D/SEMA5A/SEMA5B/SEMA6A/SEMA6D/SERPINE2/SFRP2/SH3BP4/SH3GL2/SLC9A6/SLIT1/SLIT3/SOX9/SPAG6/SPOCK1/ST8SIA2/SYT1/SYT2/SYT3/SYT4/TEAD1/TMEM108/TNC/TNN/TNR/TRIM40/UNC13A/VEGFA/VGLL4/WASF1/WFDC1/WNT11/WNT3A/WNT5A/WT1/YAP1	128
cluster5	BP	GO:0061458	reproductive system development	86/3710	310/18903	0.000315313	0.00589146	0.00505672	ACE/ACSBG1/ARID5B/ASPM/ATRX/BASP1/BMP4/BMP5/BMP6/BMP7/BMPR1B/BRCA2/CGA/DHCR24/DMC1/DMRT1/EIF2B2/FER/FGF10/FGF9/FGFR2/FOXA1/FOXC1/FOXF2/GATA4/GFRA1/GLI3/GPR149/GREB1L/HOXA11/HOXB13/HOXD13/ID4/INHA/IRX5/KIF18A/KITLG/LGR4/LHCGR/LHX1/LHX9/LRP2/MYOCD/NEUROG1/NHLH2/NKX2-1/NOS3/NR0B1/NR2F2/PCYT1B/PDGFA/PDGFRA/PDGFRB/PLEKHA5/PSAP/PSAPL1/PTCH1/ROBO2/ROR2/SALL1/SDC1/SERPINE2/SFRP2/SHH/SIX4/SLIT3/SOX9/SPO11/SRC/SRD5A2/STRA6/SULF1/TEX19/TFAP2C/TIPARP/TNC/TSPY2/TYRO3/VEGFA/WNT2B/WNT4/WNT5A/WNT7A/WNT9B/WT1/ZFP42	86
cluster5	BP	GO:0007204	positive regulation of cytosolic calcium ion concentration	61/3710	205/18903	0.000317064	0.005913647	0.005075763	ABL1/ACKR2/ADCY5/ADCY8/ADCYAP1/ADCYAP1R1/ADRA1A/ADRA1B/ADRA1D/AGT/AVP/AVPR1B/BCAP31/CACNA1A/CACNA1C/CACNA2D1/CALCA/CALCR/CCKBR/CIB2/CXCL13/DRD1/DRD2/EDN2/EDNRA/EDNRB/EPO/F2R/F2RL3/FKBP1B/GLP1R/GNG3/GPR6/GRIN1/HTR2A/HTR2C/IAPP/JPH1/JPH3/JPH4/KISS1/KNG1/LHCGR/LRP6/MCHR1/NPTN/P2RX2/P2RX3/PDGFRA/PTGFR/RYR2/SAA1/SPINK1/TAC1/TACR1/TBXA2R/TMBIM6/TRPC3/TRPM1/TRPM4/TRPV4	61
cluster5	BP	GO:0002790	peptide secretion	67/3710	230/18903	0.000324343	0.006027947	0.005173868	ABCA12/ABCC8/ADCY5/ADCY8/ADCYAP1/ADORA1/ADRA2A/BRSK2/CFTR/CHGA/CLTRN/CRH/CRHR1/DOC2B/DRD2/EFNA5/EPHA5/EXOC3L1/FKBP1B/FOXA2/GCG/GCK/GHSR/GPLD1/GRP/HADH/HFE/HIF1A/HNF1A/HNF1B/HTR2C/ILDR2/INS/ISL1/KCNB1/KISS1/LRP5/MC4R/NEUROD1/NKX6-1/NLGN2/NNAT/NOS2/NR0B2/NR1H4/PCLO/PRKN/RAB11FIP2/RASL10B/RFX3/RFX6/RPH3AL/SLC16A2/SLC30A8/SLC9B2/SNAP25/SPINK1/SSTR5/STX1A/SYT7/SYT9/TFAP2B/TRH/TRPM4/TRPV4/UCN3/VSNL1	67

cluster5	BP	GO:0050817	coagulation	67/3710	230/18903	0.000324343	0.006027947	0.005173868	ADAMTS13/ADAMTS18/ADRA2A/ALOX12/ANO6/BLOC1S6/CPB2/CTSG/CYP4F11/DGKB/DGKI/DMTN/EMILIN1/EMILIN2/F13B/F2/F2R/F2RL3/F3/F5/FAP/FBLN1/FERMT3/FOXA2/GAS6/GNAS/GP1BB/GP6/GP9/HGFAC/HS3ST5/ILK/ITGA2/ITGB3/KNG1/LYN/MMRN1/MYH9/MYL12A/NOS3/PDGFA/PDGFRA/PDPN/PEAR1/PF4/PF4V1/PRKCD/PRKG1/PROS1/PRSS56/SAA1/SCUBE1/SELP/SERPINE1/SERPINE2/SHH/SRC/STXBP1/TBXA2R/TFPI/TREML1/TSPAN8/TYRO3/VAV2/VKORC1/VWF/WNT3A	67
cluster5	BP	GO:0048608	reproductive structure development	85/3710	306/18903	0.000325358	0.006036109	0.005180874	ACE/ACSBG1/ARID5B/ASPM/ATRX/BASP1/BMP4/BMP5/BMP6/BMP7/BMPR1B/BRCA2/CGA/DHCR24/DMC1/DMRT1/EIF2B2/FER/FGF10/FGF9/FGFR2/FOXA1/FOXC1/FOXF2/GATA4/GFRA1/GLI3/GPR149/GREB1L/HOXA11/HOXB13/HOXD13/ID4/INHA/IRX5/KIF18A/KITLG/LGR4/LHCGR/LHX1/LHX9/LRP2/MYOCD/NEUROG1/NHLH2/NKX2-1/NOS3/NR0B1/NR2F2/PCYT1B/PDGFA/PDGFRA/PDGFRB/PSAP/PSAPL1/PTCH1/ROBO2/ROR2/SALL1/SDC1/SERPINE2/SFRP2/SHH/SIX4/SLIT3/SOX9/SPO11/SRC/SRD5A2/STRA6/SULF1/TEX19/TFAP2C/TIPARP/TNC/TSPY2/TYRO3/VEGFA/WNT2B/WNT4/WNT5A/WNT7A/WNT9B/WT1/ZFP42	85
cluster5	BP	GO:0034309	primary alcohol biosynthetic process	9/3710	14/18903	0.000327003	0.006055905	0.005197865	BMP2/BMP5/BMP6/CLCN2/CYP11B1/CYP11B2/DAB2/DKK3/WNT4	9
cluster5	BP	GO:0050731	positive regulation of peptidyl-tyrosine phosphorylation	58/3710	193/18903	0.00033173	0.006101451	0.005236958	ABL1/ACE/ADORA1/ADRA1A/ADRA2A/AGT/ALK/ALKAL2/ANGPT1/BMP6/BTC/CRLF1/CSH1/CSH2/CSPG4/EFNA5/EGF/EGFR/EPO/ERBB3/ERBB4/FGF10/FGF7/FGFR3/GAS6/GFRA1/GH2/GHR/GPRC5B/GREM1/GRM5/HDAC2/HES1/HES5/HSF1/HTR2A/IL13/IL20/IL5/ISL1/ITGB3/KITLG/LACRT/LRP4/LYN/NEURL1/NTF3/PECAM1/PIBF1/PLPP3/RELN/RICTOR/SEMA4D/SRC/SRCIN1/TDGF1/VEGFA/WNT3A	58

cluster5	BP	GO:0070444	oligodendrocyte progenitor proliferation	6/3710	7/18903	0.00033179	0.006101451	0.005236958	CDH2/EMX1/LRP2/LYN/PTPRZ1/SIRT2	6
cluster5	BP	GO:0070445	regulation of oligodendrocyte progenitor proliferation	6/3710	7/18903	0.00033179	0.006101451	0.005236958	CDH2/EMX1/LRP2/LYN/PTPRZ1/SIRT2	6
cluster5	BP	GO:0098943	neurotransmitter receptor transport, postsynaptic endosome to lysosome	6/3710	7/18903	0.00033179	0.006101451	0.005236958	AP3D1/CACNG2/CACNG3/CACNG4/CACNG5/CACNG7	6
cluster5	BP	GO:0035315	hair cell differentiation	21/3710	51/18903	0.000332628	0.006106147	0.005240988	ATOH1/BMP4/CLRN1/CLRN2/DLL1/DLX3/ESRP1/FGF2/GRXCR1/HES1/HES5/LHFPL5/MYO6/PDZD7/POU4F3/SLITRK6/TMC1/TMEM132E/USH1C/USH2A/WHRN	21

cluster5	BP	GO:0034331	cell junction maintenance	18/3710	41/18903	0.000341595	0.006248861	0.005363481	ADGRB3/BSN/CBLN1/CBLN2/CBLN3/CLDN1/CNTNAP1/ERC1/F2R/INAVA/KIRREL1/NLGN1/NLGN2/OPHN1/PCLO/PRTN3/SHANK2/WHRN	18
cluster5	BP	GO:1903524	positive regulation of blood circulation	18/3710	41/18903	0.000341595	0.006248861	0.005363481	ACE2/ADM5/ADRA1A/ADRA1B/ADRA1D/APELA/ATP1A2/CCN2/CHGA/EDN2/HRC/RGS4/RYR2/SCN3B/SLC1A1/TACR3/TPM1/TRPM4	18
cluster5	BP	GO:0008543	fibroblast growth factor receptor signaling pathway	31/3710	87/18903	0.000346367	0.006314114	0.005419489	CCN2/FGF10/FGF16/FGF17/FGF19/FGF2/FGF23/FGF4/FGF5/FGF7/FGF9/FGFBP3/FGFR1/FGFR2/FGFR3/FGFR4/FLRT1/FLRT2/FLRT3/FRS3/HHIP/NGFR/NPTN/OTX2/PRDM14/SHOC2/SPRY1/SULF1/SULF2/WNT4/WNT5A	31
cluster5	BP	GO:2000243	positive regulation of reproductive process	31/3710	87/18903	0.000346367	0.006314114	0.005419489	ANKRD31/APELA/CFAP69/DMRT1/EDNRB/FGF9/GLRA1/HDAC2/LHFPL2/MSX2/NPM2/OOEP/OVOL1/PDE3A/PDE5A/PLCB1/PRDM14/PRSS37/SHH/SIRT2/SOX9/SRC/TACR1/TACR3/UBE2B/UNC5C/VEGFA/WNT4/WNT5A/WT1/ZP4	31

cluster5	BP	GO:0001942	hair follicle development	32/3710	91/18903	0.000367971	0.006696302	0.005747527	ALX4/APCDD1/DLX3/DSG4/EGFR/FERMT1/FGF10/FGF7/FGFR2/FOXN1/FZD3/GLI2/HDAC2/HOXC13/KRT27/KRT84/KRTAP21-1/LAMA5/LGR4/LGR5/LHX2/LRP4/MSX2/NGFR/PDGFA/SHH/SMO/SOSTDC1/SOX18/SOX9/TNFRSF19/WNT5A	32
cluster5	BP	GO:0001754	eye photoreceptor cell differentiation	20/3710	48/18903	0.000380057	0.00688516	0.005909625	CRB2/DSCAM/GNAT1/GNGT1/HCN1/MFRP/NR2E3/NTRK2/OLFM3/PAX6/PROM1/RORB/RP1/SOX9/TH/THRB/THY1/TULP1/USH1C/VEGFA	20
cluster5	BP	GO:0042220	response to cocaine	20/3710	48/18903	0.000380057	0.00688516	0.005909625	CACNG4/DRD1/DRD2/DRD4/EHMT2/ELAVL4/EN1/GRM2/HDAC2/HOMER1/HTR2A/OPRK1/RGS4/SDK1/SLC1A1/SLC1A2/SLC6A1/SLC6A3/ST8SIA2/TACR3	20
cluster5	BP	GO:0001503	ossification	113/3710	429/18903	0.000380319	0.00688516	0.005909625	ADAMTS7/ADGRV1/AKT1/AMELX/ANO6/ASPN/ATF4/ATP6V0A4/ATP6V1B1/BMP2/BMP4/BMP5/BMP6/BMP7/BMPR1B/CALCA/CCDC47/CCN2/CDH11/CHRD/CHRDL1/CHRDL2/CITED1/CLEC11A/CLEC3A/COL11A1/COL2A1/COL6A1/DLX5/DNAI3/ECM1/EGFR/ENPP1/FGF2/FGF23/FGF9/FGFR2/FGFR3/FOXC1/GDF10/GDF5/GDPD2/GLI2/GLI3/GPC3/GPLD1/GPM6B/GREM1/GSK3B/HIF1A/IBSP/ID4/ILK/JUNB/KAZALD1/KLF10/LGR4/LOX/LRP4/LRP5/MDK/MMP13/MMP16/MRC2/MSX2/MYOC/MYOG/NELL1/NPPC/OMD/OSTF1/PBX1/PENK/PKDCC/PLXNB1/PTCH1/PTGS2/PTH/PTH1R/PTN/RFLNA/ROR2/RORB/RSPO2/SBDS/SEMA4D/SFRP2/SHH/SHOX2/SIX2/SLC24A3/SLC34A1/SMAD5/SMO/SORT1/SOX9/SP7/STC1/TACR1/TFAP2A/TNC/TNFRSF11A/TNN/TOB1/TRPM4/WNT11/WNT3A/WNT4/WNT5A/WNT7B/YAP1/ZBTB16/ZNF664-RFLNA	113

cluster5	BP	GO:0008344	adult locomotory behavior	29/3710	80/18903	0.000381312	0.006891235	0.00591484	APP/ATP1A2/CHL1/DAB1/DMBX1/DMRT3/DRD1/DRD2/DRD4/EFNB3/EN1/EPS8/FOXA2/GDNF/GLRA1/GLRB/GRIN2D/HOXD10/KLHL1/NLGN2/NR4A2/PRKN/SCN1A/SHANK2/SLITRK6/SPTBN4/TRH/UCHL1/ZIC1	29
cluster5	BP	GO:0034109	homotypic cell-cell adhesion	33/3710	95/18903	0.000387862	0.006985514	0.005995761	ADAMTS18/ALOX12/ANK3/CEACAM5/CTSG/DMTN/DSG2/EMILIN2/FERMT3/GNAS/GP6/ILK/ITGB3/LYN/MEGF10/MEGF11/MMRN1/MYH9/MYL12A/PDGFRA/PDPN/PEAR1/PKP2/PLPP3/PRKCD/PRKG1/PTPRU/SERPINE2/STXBP1/TJP2/TYRO3/WNT3A/XG	33
cluster5	BP	GO:0098773	skin epidermis development	33/3710	95/18903	0.000387862	0.006985514	0.005995761	ALX4/APCDD1/DLL1/DLX3/DSG4/EGFR/FERMT1/FGF10/FGF7/FGFR2/FOXN1/FZD3/GLI2/HDAC2/HOXC13/KRT27/KRT84/KRTAP21-1/LAMA5/LGR4/LGR5/LHX2/LRP4/MSX2/NGFR/PDGFA/SHH/SMO/SOSTDC1/SOX18/SOX9/TNFRSF19/WNT5A	33
cluster5	BP	GO:0030178	negative regulation of Wnt signaling pathway	53/3710	174/18903	0.000408872	0.007348133	0.006307002	APC2/APCDD1/BARX1/BMP2/CDH2/CITED1/CSNK1E/CTNND1/CXXC4/DAB2/DACT3/DDIT3/DKK3/DKKL1/DRAXIN/FERMT1/FGF9/FRZB/GLI3/GPC3/GRB10/GREM1/GSK3B/IGFBP2/IGFBP4/ISL1/LRP4/MCC/MDFI/MDK/NFATC4/NKD2/NPHP4/PRICKLE1/PRKN/PTPRU/RBX1/SFRP2/SHH/SHISA6/SOSTDC1/SOX10/SOX9/TLE1/TMEM170B/TNN/TPBG/TRABD2B/VGLL4/WIF1/WNT11/WNT5A/ZNRF3	53

cluster5	BP	GO:0035050	embryonic heart tube development	30/3710	84/18903	0.000409398	0.007348133	0.006307002	AHI1/APELA/CCDC103/CCDC40/DLL1/ECE1/EDNRA/FOLR1/FOXC1/FOXN4/GATA4/HES1/HIF1A/LBX1/NDRG4/NKX2-6/NOTCH2/PIFO/PLXNA4/RYR2/SHH/SMO/SOX18/TBX20/TEAD2/VANGL2/WNT3A/WNT5A/YAP1/ZIC3	30
cluster5	BP	GO:0046879	hormone secretion	81/3710	291/18903	0.000413482	0.007408743	0.006359024	ABCA12/ABCC8/ADCY5/ADCY8/ADCYAP1/ADORA1/ADRA2A/AGT/BMP6/BRSK2/CCKAR/CFTR/CGA/CHGA/CLTRN/CRH/CRHR1/DAB2/DOC2B/DRD2/EFNA5/EPHA5/EXOC3L1/FGF23/FKBP1B/FOXA2/GCG/GCK/GHSR/GLP1R/GPLD1/GRP/HADH/HFE/HIF1A/HNF1A/HNF1B/HTR2C/ILDR2/INHA/INS/ISL1/KCNB1/KISS1/LRP5/LYN/MC4R/NEUROD1/NKX6-1/NLGN2/NNAT/NOS2/NR0B2/NR1H4/OPRK1/PCLO/PLA2G3/PRKN/RAB11FIP2/RASL10B/RFX3/RFX6/RPH3AL/SCG5/SLC16A2/SLC30A8/SLC9B2/SNAP23/SNAP25/SPINK1/SSTR5/STX1A/SYT7/SYT9/TACR1/TFAP2B/TRH/TRPM4/TRPV4/UCN3/VSNL1	81
cluster5	BP	GO:0035137	hindlimb morphogenesis	16/3710	35/18903	0.0004151	0.00742504	0.006373012	ALX4/BMP4/FGF4/GDF5/GPC3/HOXD10/MSX2/PITX1/PTCH1/RARB/RSPO2/SHH/TBX4/TFAP2B/WNT7A/ZBTB16	16
cluster5	BP	GO:0061548	ganglion development	10/3710	17/18903	0.00041753	0.00745579	0.006399405	ASCL1/FZD3/NRP2/PHOX2B/RGS4/SEMA3A/SIX4/TUBB3/TULP3/UNC5C	10

cluster5	BP	GO:0009799	specification of symmetry	45/3710	142/18903	0.000419758	0.007470117	0.006411702	AHI1/BMP4/CC2D2A/CCDC103/CCDC40/CFAP52/CFC1/CFC1B/DAW1/DDIT3/DLL1/DNAH11/DNAH5/DNAI1/DRC1/ENKUR/FGF10/FOLR1/FOXJ1/FOXN4/GATA4/GREM1/GREM2/HIF1A/IFT140/LBX1/LEFTY1/MMP21/NDRG4/NOTCH2/ODAD2/ODAD3/RFX3/SHH/SMO/SOSTDC1/SOX18/TBC1D32/TBX20/TDGF1/VANGL2/WNT3A/WNT5A/WNT8B/ZIC3	45
cluster5	BP	GO:0010977	negative regulation of neuron projection development	45/3710	142/18903	0.000419758	0.007470117	0.006411702	ACP4/BCL11A/CDKL3/CRMP1/DAB1/DAB2/DPYSL3/DRAXIN/EFNB2/EFNB3/EPHA7/FAT3/GDI1/GFAP/HDAC2/HES1/LRP4/MAP2/MT3/NEU4/NGEF/NLGN1/NR2F1/NTN1/PRAG1/PTPRG/PTPRS/RIT2/RTN4RL1/RTN4RL2/SEMA3A/SEMA3E/SEMA4D/SEMA5A/SEMA5B/SEMA6A/SEMA6D/SLIT1/SPOCK1/STMN2/THY1/TNR/TRPV4/WNT3A/WNT5A	45
cluster5	BP	GO:0071300	cellular response to retinoic acid	25/3710	66/18903	0.000439963	0.007816427	0.006708945	ATP1A3/BRINP1/BRINP2/BRINP3/EPHA3/FGFR2/FZD10/GJB3/GSK3B/HDAC2/LYN/PAX2/PTK7/RET/RORB/SOX9/TEAD2/WNT11/WNT3A/WNT5A/WNT7B/WNT8B/WNT9A/WNT9B/YAP1	25
cluster5	BP	GO:0061036	positive regulation of cartilage development	15/3710	32/18903	0.000448785	0.00795967	0.006831892	BMP2/BMP4/BMP6/BMPR1B/GDF5/GDF6/GLI3/HOXA11/MDK/PKDCC/SOX5/SOX9/WNT5A/ZBTB16/ZNF219	15

cluster5	BP	GO:0022037	metencephalon development	37/3710	111/18903	0.000449804	0.007964266	0.006835837	ABL1/ASCL1/B4GALT2/CBLN1/CDK5R1/CDK5R2/DAB1/DLL1/EN1/EZH2/FAIM2/FOXC1/GBX2/GDF10/GLI2/GRID2/HAP1/HOXB1/KCNC1/KLHL1/KNDC1/LHX1/LHX5/LMX1A/MDK/NEUROD1/NEUROD2/NLGN4X/OPHN1/OTX1/PTF1A/SERPINE2/SMO/SSTR1/SSTR2/WHRN/WNT7A	37
cluster5	BP	GO:0055001	muscle cell development	57/3710	191/18903	0.000450939	0.007970896	0.006841527	ACTA1/ACTC1/ACTN1/ACTN2/ADRA1A/AGT/AKAP6/BMP4/BVES/CACNA1S/CDK1/CNTNAP1/CSRP2/CSRP3/DMD/DNER/EDNRA/FER1L5/FHOD3/GATA4/HES1/HOMER1/ISL1/ITGB1/KLHL40/LDB3/LMNA/LMOD2/LOX/MEGF10/MYH6/MYH7/MYO18B/MYOG/MYOZ1/MYOZ2/MYPN/NFATC4/NKX2-6/NRAP/P2RX2/PDGFRA/PDGFRB/PRKAR1A/RGS4/SDC1/SGCZ/SHOX2/SIX4/SMO/SYNPO2L/TBX5/TNNT2/TPM1/UCHL1/VEGFA/WFIKKN2	57
cluster5	BP	GO:0051480	regulation of cytosolic calcium ion concentration	21/3710	52/18903	0.00045573	0.00803251	0.006894412	ADCY8/ADORA1/ATP2B2/ATP2B3/CALB1/CALB2/CALCA/CALCB/F2/FKBP1B/GRIA1/GRM5/HCRTR1/HCRTR2/HRC/RYR2/SLC8A2/TRPC3/TRPC4/TRPC7/WNT5A	21
cluster5	BP	GO:0045604	regulation of epidermal cell differentiation	23/3710	59/18903	0.000455957	0.00803251	0.006894412	ABCA12/ATOH1/BMP4/DLL1/ESRP1/ETV4/EZH2/FGF2/FOXC1/GRHL1/GRHL2/HES1/HES5/IL20/KRT84/MSX2/PTCH1/PTCH2/REG3A/REG3G/ROCK1/ROCK2/ZFP36	23

cluster5	BP	GO:0060996	dendritic spine development	32/3710	92/18903	0.000459376	0.008079156	0.006934449	ARC/ARHGAP44/CAMK2A/CAMK2B/CDK5R1/CPEB3/CTNND2/DBN1/DLG5/EPHB3/FOXO6/HDAC2/HDAC6/ITPKA/KIF1A/LZTS3/NCK2/NEURL1/NGEF/NLGN1/NLGN2/PAK3/PPFIA2/PTPRS/RELN/SDK1/SHANK1/SLC12A5/SRCIN1/TANC2/WNT7A/ZDHHC15	32
cluster5	BP	GO:0015850	organic hydroxy compound transport	81/3710	292/18903	0.000463913	0.008145288	0.00699121	ABCA12/ABCA4/ABCA8/ABCB11/ABCC3/ABCG4/ADRA2A/AGT/APOA5/ASIC3/BMP6/CEL/CETP/CFTR/CHGA/CHRNA4/CHRNA6/CRH/CRHR1/CYP8B1/DAB2/DRD1/DRD2/DRD4/EGF/ENPP7/FGF19/GDNF/GHSR/GPM6B/GRAMD1B/GRM2/HRH3/HTR2A/ITGB3/KCNB1/LAMTOR1/LIPC/LIPG/LRP6/MAOB/NFKBIA/NPC1L1/NR0B2/OPRK1/OSBPL11/OSBPL6/PLTP/PRKN/PTCH1/RAB3B/SHH/SLC10A3/SLC10A5/SLC16A8/SLC22A24/SLC29A4/SLC2A13/SLC5A1/SLC6A2/SLC6A3/SLCO1B1/SLCO1B3/SLCO1C1/SREBF2/STRA6/STX1A/SYT1/SYT10/SYT12/SYT13/SYT2/SYT3/SYT4/SYT5/SYT6/SYT7/SYT8/SYT9/VAPA/YJEFN3	81
cluster5	BP	GO:0032332	positive regulation of chondrocyte differentiation	11/3710	20/18903	0.000471543	0.008224144	0.007058894	BMP6/BMPR1B/GDF5/GDF6/GLI3/HOXA11/PKDCC/SOX5/SOX9/ZBTB16/ZNF219	11
cluster5	BP	GO:0055062	phosphate ion homeostasis	11/3710	20/18903	0.000471543	0.008224144	0.007058894	ENPP1/FGF23/FGFR4/GCM2/PTH/SLC17A6/SLC17A7/SLC17A8/SLC34A1/SLC34A2/TFAP2B	11

cluster5	BP	GO:0072506	trivalent inorganic anion homeostasis	11/3710	20/18903	0.000471543	0.008224144	0.007058894	ENPP1/FGF23/FGFR4/GCM2/PTH/SLC17A6/SLC17A7/SLC17A8/SLC34A1/SLC34A2/TFAP2B	11
cluster5	BP	GO:0098698	postsynaptic specialization assembly	11/3710	20/18903	0.000471543	0.008224144	0.007058894	CBLN1/GAP43/GRID2/LRRC4B/LRRTM2/NLGN2/NPTX1/NRXN1/NTNG2/PTPRD/SLITRK3	11
cluster5	BP	GO:0095500	acetylcholine receptor signaling pathway	14/3710	29/18903	0.00047644	0.008295748	0.007120352	CDK5R1/CHRM1/CHRM2/CHRM3/CHRNA3/CHRNA7/HRH3/LY6G6D/LY6H/LYPD1/PLCB1/RGS10/RGS8/SLURP2	14
cluster5	BP	GO:0086091	regulation of heart rate by cardiac conduction	18/3710	42/18903	0.000488997	0.008453242	0.007255532	CACNA1C/CACNA1G/CACNA2D1/DSG2/HCN1/HCN3/HCN4/ISL1/KCND3/KCNE4/KCNH2/KCNH6/KCNJ3/KCNJ5/PKP2/SCN2B/SCN3B/TRPM4	18

cluster5	BP	GO:0010842	retina layer formation	13/3710	26/18903	0.000493591	0.008453242	0.007255532	AHI1/CALB1/DSCAM/FAT3/FOXN4/LHX1/MEGF11/PROM1/PTF1A/SDK1/SLC1A1/TFAP2A/TFAP2B	13
cluster5	BP	GO:0014047	glutamate secretion	13/3710	26/18903	0.000493591	0.008453242	0.007255532	ADORA1/APBA1/AVP/AVPR1B/GIPC1/GRM2/GRM7/HRH3/NPY5R/NTRK2/STXBP1/SYT4/TRH	13
cluster5	BP	GO:0050951	sensory perception of temperature stimulus	13/3710	26/18903	0.000493591	0.008453242	0.007255532	ADORA1/ADRA2A/ASIC3/CALCA/GRIK2/HTR2A/MMP24/OPN4/OPRK1/PRDM12/RHO/TRPM3/TRPM8	13
cluster5	BP	GO:0060384	innervation	13/3710	26/18903	0.000493591	0.008453242	0.007255532	ECE1/EDNRA/GABRA5/GABRB2/ISL1/NPTX1/PRKCG/RET/SEMA3A/SERPINE2/SLITRK6/SULF1/SULF2	13

cluster5	BP	GO:0060740	prostate gland epithelium morphogenesis	13/3710	26/18903	0.000493591	0.008453242	0.007255532	BMP4/BMP7/FGF10/FGFR2/FOXA1/HOXB13/HOXD13/ID4/SHH/SOX9/SULF1/TNC/WNT5A	13
cluster5	BP	GO:0099590	neurotransmitter receptor internalization	13/3710	26/18903	0.000493591	0.008453242	0.007255532	ARC/CACNG2/CACNG3/CACNG4/CACNG5/CACNG7/DRD4/EFNB2/GSG1L/HPCA/ITGB3/OPHN1/SNAP25	13
cluster5	BP	GO:0016338	calcium-independent cell-cell adhesion via plasma membrane cell-adhesion molecules	12/3710	23/18903	0.000494356	0.008453242	0.007255532	BMP2/CLDN1/CLDN14/CLDN16/CLDN18/CLDN19/CLDN2/CLDN22/CLDN4/CLDN5/CLDN6/CLDN8	12
cluster5	BP	GO:0072311	glomerular epithelial cell differentiation	12/3710	23/18903	0.000494356	0.008453242	0.007255532	BASP1/BMP4/EDNRA/EDNRB/FOXJ1/KLF15/LAMB2/MAGI2/NOTCH2/NPHS1/PROM1/WT1	12

cluster5	BP	GO:0097164	ammonium ion metabolic process	12/3710	23/18903	0.000494356	0.008453242	0.007255532	ALDH7A1/BCHE/CHDH/DMGDH/ENPP6/GRIN2A/PDE1B/PRG3/RNF180/SLC29A4/TPH2/TRH	12
cluster5	BP	GO:1900273	positive regulation of long-term synaptic potentiation	12/3710	23/18903	0.000494356	0.008453242	0.007255532	ADCY1/ADCY8/APP/CHRNA7/DRD2/IGSF11/INS/NPTN/PRKAR1B/RELN/SHISA7/SQSTM1	12
cluster5	BP	GO:0002087	regulation of respiratory gaseous exchange by nervous system process	8/3710	12/18903	0.000505547	0.008560809	0.007347858	ADORA1/ATP1A2/GLRA1/GSX2/NLGN2/NLGN3/PHOX2B/TLX3	8
cluster5	BP	GO:0003337	mesenchymal to epithelial transition involved in metanephros morphogenesis	8/3710	12/18903	0.000505547	0.008560809	0.007347858	BMP4/GDNF/GREM1/PAX2/SALL1/SIX2/SMO/WNT9B	8

cluster5	BP	GO:0021979	hypothalamus cell differentiation	8/3710	12/18903	0.000505547	0.008560809	0.007347858	HAP1/NDNF/NHLH2/NRP2/OTP/PLXNA1/POU3F2/SEMA3E	8
cluster5	BP	GO:0035881	amacrine cell differentiation	8/3710	12/18903	0.000505547	0.008560809	0.007347858	BARHL2/DLX1/DLX2/FOXN4/HES1/NEUROD1/PTF1A/RORB	8
cluster5	BP	GO:0051583	dopamine uptake involved in synaptic transmission	8/3710	12/18903	0.000505547	0.008560809	0.007347858	DRD1/DRD2/DRD4/GDNF/PRKN/RAB3B/SLC6A2/SLC6A3	8
cluster5	BP	GO:0051934	catecholamine uptake involved in synaptic transmission	8/3710	12/18903	0.000505547	0.008560809	0.007347858	DRD1/DRD2/DRD4/GDNF/PRKN/RAB3B/SLC6A2/SLC6A3	8

cluster5	BP	GO:0021885	forebrain cell migration	24/3710	63/18903	0.000515534	0.008715847	0.007480929	ARX/CDK5R1/CDK5R2/DAB1/DRD1/DRD2/EGFR/EMX2/FEZF2/FOXG1/GLI3/LAMB1/MBOAT7/NDEL1/NKX2-1/NR2E1/NRG3/POU3F2/POU3F3/RELN/ROBO1/SYNE2/TNR/TYRO3	24
cluster5	BP	GO:0051146	striated muscle cell differentiation	81/3710	293/18903	0.000519831	0.008774339	0.007531134	ACTA1/ACTC1/ACTN2/ADAM12/ADAMTS15/ADGRB1/ADGRB3/ADRA1A/AGT/AKAP6/AKT1/ATP11A/BARX2/BDNF/BHLHE41/BMP2/BMP4/BVES/CACNA1S/CDH2/CDK1/CDON/CEACAM5/CNTNAP1/CSRP2/CSRP3/DLL1/DNER/EFNB2/ERVW-1/EZH2/FER1L5/FHOD3/GATA4/GREM1/HOMER1/ISL1/ITGB1/KCNH1/KLHL40/LDB3/LMNA/LMOD2/MYH6/MYH7/MYH9/MYO18B/MYOCD/MYOG/MYOZ1/MYOZ2/MYPN/NKX2-6/NPHS1/NRAP/P2RX2/PDGFRA/PDGFRB/PLEKHO1/POPDC3/PRKAR1A/PTGFRN/RARB/RBM24/RGS4/SDC1/SHH/SHOX2/SIK1/SIX4/SMO/SMYD1/SORT1/SYNPO2L/TBX5/TNNT2/TPM1/VEGFA/WNT3A/WNT8A/WT1	81
cluster5	BP	GO:0045933	positive regulation of muscle contraction	20/3710	49/18903	0.000524787	0.008843761	0.00759072	ACE2/ADRA1A/CACNA1S/CACNB1/CCN2/CHGA/CHRM3/EDN2/F2R/GHSR/GSTO1/ITGA2/MYOCD/NMU/PTGS2/SPX/TACR1/TACR3/TBXA2R/TRPV4	20
cluster5	BP	GO:0001736	establishment of planar polarity	27/3710	74/18903	0.000530841	0.008917115	0.00765368	ABL1/BRSK1/BRSK2/CELSR1/CELSR2/DAAM1/DAB2/ERBB4/FOXF2/FZD3/GPC3/GPC6/MAGI2/PKHD1/PRICKLE1/PTK7/ROR1/ROR2/RSPO3/SFRP2/SPEF1/VANGL2/WNT11/WNT5A/WNT7A/WNT9B/ZNRF3	27

cluster5	BP	GO:0007164	establishment of tissue polarity	27/3710	74/18903	0.000530841	0.008917115	0.00765368	ABL1/BRSK1/BRSK2/CELSR1/CELSR2/DAAM1/DAB2/ERBB4/FOXF2/FZD3/GPC3/GPC6/MAGI2/PKHD1/PRICKLE1/PTK7/ROR1/ROR2/RSPO3/SFRP2/SPEF1/VANGL2/WNT11/WNT5A/WNT7A/WNT9B/ZNRF3	27
cluster5	BP	GO:0035265	organ growth	54/3710	180/18903	0.00054582	0.009154056	0.00785705	ABL1/ADRA1A/AGT/AKAP6/AKT1/ARX/BASP1/CDK1/CGA/COL27A1/DIPK2A/DLL1/DUSP9/ECM1/EHMT2/ERBB4/EVC/FGF10/FGF2/FGF7/FGF9/FGFR2/FGFR3/FOXC1/GATA4/GJE1/IL7/LEPR/MAEL/MMP13/MSX2/MYH6/NDRG4/NLGN4X/NPPC/PDGFRB/PRKAR1A/PSAP/PSAPL1/RARB/RGS4/RSPO2/SHH/SMO/SOX9/STC1/TBX20/TBX5/TENM4/TP73/VGLL4/WT1/WWC1/YAP1	54
cluster5	BP	GO:0046883	regulation of hormone secretion	67/3710	234/18903	0.000547149	0.009161689	0.007863601	ABCA12/ABCC8/ADCY5/ADCY8/ADCYAP1/ADORA1/ADRA2A/AGT/BMP6/BRSK2/CCKAR/CFTR/CHGA/CRH/CRHR1/DAB2/DOC2B/DRD2/EFNA5/EPHA5/FGF23/FKBP1B/FOXA2/GCG/GCK/GHSR/GPLD1/GRP/HADH/HFE/HIF1A/HTR2C/INHA/INS/ISL1/KCNB1/KISS1/LRP5/NEUROD1/NKX6-1/NLGN2/NNAT/NOS2/NR0B2/NR1H4/OPRK1/PLA2G3/PRKN/RASL10B/RFX3/RFX6/RPH3AL/SCG5/SLC30A8/SLC9B2/SNAP25/SPINK1/SSTR5/STX1A/SYT7/SYT9/TACR1/TFAP2B/TRH/TRPM4/UCN3/VSNL1	67
cluster5	BP	GO:0035136	forelimb morphogenesis	17/3710	39/18903	0.000549399	0.009184697	0.007883349	ALX4/ATRX/CACNA1C/CRABP2/EN1/GDF5/HOXA11/HOXD10/MSX2/RSPO2/SHH/SHOX2/TBX5/TFAP2A/TFAP2B/WNT7A/ZBTB16	17

cluster5	BP	GO:1903034	regulation of response to wounding	52/3710	172/18903	0.000560366	0.00933824	0.008015138	ABCC8/ADAMTS18/ADRA2A/AJAP1/ALOX12/ANO6/CLDN1/CLDN19/CLDN4/CPB2/DMTN/DUOX2/EMILIN1/EMILIN2/F2/F2R/F3/FAP/FERMT1/FGF2/FKBP1B/FOXA2/ITGB1/KNG1/MDK/MMRN1/MYLK/MYOZ1/NDEL1/NOS3/PDGFA/PDGFRA/PRKCD/PRKG1/PROS1/PTN/PTPRF/PTPRS/REG3A/REG3G/RTN4RL1/SERPINE1/SERPINE2/STK24/TAFA5/TBXA2R/TFPI/TNR/TSPAN8/VKORC1/WFDC1/WNT4	52
cluster5	BP	GO:1905475	regulation of protein localization to membrane	52/3710	172/18903	0.000560366	0.00933824	0.008015138	ADAM10/AGR2/AKT1/ANK3/ARHGAP44/ARHGEF16/CACNG2/CDH2/CDK5R1/CEMIP/CLIP3/CSRP3/DAB2/DAG1/DMTN/DPP10/EGFR/EPHA3/ERBB2/FRRS1L/GDI1/GHSR/GPC2/GPC3/GPC6/GRIPAP1/HPCA/INS/IQSEC2/ITGAM/ITGB1/KCNB1/LRP4/LYPD1/MRAP2/MYO1C/NKD2/NLGN2/PKDCC/PRKN/SHISA6/SHISA7/SLC1A1/SQSTM1/STAC2/STOM/STX7/TCAF1/USP17L2/WNK3/WNT3A/ZDHHC7	52
cluster5	BP	GO:0044070	regulation of anion transport	32/3710	93/18903	0.000570278	0.009488343	0.008143973	ABCB11/ACE2/ADORA1/AGT/ATF4/ATP1A2/AVP/AVPR1B/CFTR/CLTRN/FGF23/GABRE/GRM2/GRM5/GRM7/HRH3/ITGB1/NPY5R/PLA2G3/PLA2R1/RGS4/SLC17A8/SLC34A1/SLC38A3/SLC6A1/STC1/STXBP1/SV2A/SYT4/TCAF1/TNFRSF11A/TRH	32
cluster5	BP	GO:0060828	regulation of canonical Wnt signaling pathway	73/3710	260/18903	0.000592577	0.009843732	0.008449008	AMER3/APC2/ASPM/BMP2/CDH2/CDK14/CSNK1E/CTNND1/CTNND2/DAB2/DACT3/DDIT3/DDX3X/DKK3/DKKL1/DLX5/DRAXIN/EGF/EGFR/FERMT1/FGF10/FGF2/FGF9/FGFR2/FOLR1/FRZB/GLI3/GPC3/GPRC5B/GREM1/GSK3B/IGFBP2/IGFBP4/ILK/ISL1/LGR4/LGR5/LRP4/LYPD6/MCC/MDK/NKD2/NPHP4/PRICKLE1/PRKN/PTK7/PTPRU/RBX1/RSPO1/RSPO2/RSPO3/SCEL/SEMA5A/SFRP2/SHH/SHISA6/SOSTDC1/SOX10/SOX9/SRC/TLE1/TMEM170B/TMEM198/TNN/TPBG/TRPM4/UBE2B/WNK2/WNT11/WNT3A/WNT5A/YAP1/ZNRF3	73

cluster5	BP	GO:1990090	cellular response to nerve growth factor stimulus	19/3710	46/18903	0.000602216	0.009988012	0.008572846	AKT1/APP/BDNF/CALCA/CSNK1E/EIF4A3/ELAVL4/HES1/ID1/MAGI2/NGF/NTF3/NTRK2/SH3GL2/SORT1/STMN2/TAC1/TMEM108/WASF1	19
cluster5	BP	GO:0048708	astrocyte differentiation	29/3710	82/18903	0.000610081	0.010102475	0.008671091	ABL1/APP/BMP2/DAB1/DLL1/DRD1/F2/GAP43/GFAP/HES1/HES5/ID4/LAMB2/MT3/NKX2-2/NR2E1/PAX6/PLP1/PLPP3/POU3F2/ROR1/ROR2/SERPINE2/SHH/SMO/SOX9/TSPAN2/TTBK1/VAX1	29
cluster5	BP	GO:0021549	cerebellum development	34/3710	101/18903	0.000614337	0.010156911	0.008717813	ABL1/B4GALT2/CBLN1/CDK5R1/CDK5R2/DAB1/DLL1/EN1/EZH2/FAIM2/FOXC1/GBX2/GDF10/GLI2/GRID2/HAP1/KCNC1/KLHL1/KNDC1/LHX1/LHX5/LMX1A/MDK/NEUROD1/NEUROD2/NLGN4X/OPHN1/PTF1A/SERPINE2/SMO/SSTR1/SSTR2/WHRN/WNT7A	34
cluster5	BP	GO:0009584	detection of visible light	21/3710	53/18903	0.000616625	0.01017868	0.008736498	ABCA4/AIPL1/CNGB1/ELOVL4/GJA10/GNAT1/GPR52/GRK4/GRM6/GUCY2F/OPN1LW/OPN1MW2/OPN1MW3/OPN4/REEP6/RGR/RHO/RP1/RPE65/SEMA5B/TULP1	21

cluster5	BP	GO:0009913	epidermal cell differentiation	67/3710	235/18903	0.000620947	0.010233913	0.008783906	ABCA12/ATOH1/BMP4/CLRN1/CLRN2/DLL1/DLX3/DSG4/ESRP1/ETV4/EVPL/EZH2/FGF2/FOXC1/FOXN1/GRHL1/GRHL2/GRXCR1/HDAC2/HES1/HES5/IL20/IVL/KLK5/KRT6C/KRT75/KRT76/KRT8/KRT80/KRT83/KRT84/LCE1A/LCE1C/LCE2C/LCE2D/LCE3A/LCE3D/LCE3E/LCE4A/LCE6A/LHFPL5/LORICRIN/MSX2/MYO6/OVOL1/PAX6/PDZD7/POU4F3/PTCH1/PTCH2/REG3A/REG3G/ROCK1/ROCK2/SCEL/SLITRK6/SPRR2F/SPRR2G/SPRR4/TMC1/TMEM132E/USH1C/USH2A/WHRN/WNT5A/YAP1/ZFP36	67
cluster5	BP	GO:0007599	hemostasis	66/3710	231/18903	0.000637611	0.010492048	0.009005466	ADAMTS13/ADAMTS18/ADRA2A/ALOX12/ANO6/BLOC1S6/CPB2/CTSG/CYP4F11/DGKB/DGKI/DMTN/EMILIN1/EMILIN2/F13B/F2/F2R/F2RL3/F3/F5/FAP/FBLN1/FERMT3/FOXA2/GAS6/GNAS/GP1BB/GP6/GP9/HGFAC/ILK/ITGA2/ITGB3/KNG1/LYN/MMRN1/MYH9/MYL12A/NOS3/PDGFA/PDGFRA/PDPN/PEAR1/PF4/PF4V1/PRKCD/PRKG1/PROS1/PRSS56/SAA1/SCUBE1/SELP/SERPINE1/SERPINE2/SHH/SRC/STXBP1/TBXA2R/TFPI/TREML1/TSPAN8/TYRO3/VAV2/VKORC1/VWF/WNT3A	66
cluster5	BP	GO:0018212	peptidyl-tyrosine modification	101/3710	382/18903	0.000655877	0.01077571	0.009248937	ABL1/ACE/ADORA1/ADRA1A/ADRA2A/AGT/ALK/ALKAL2/ANGPT1/APP/BMP6/BTC/CBLB/CBLC/CRLF1/CSH1/CSH2/CSPG4/DMTN/EFNA5/EGF/EGFR/EPHA10/EPHA3/EPHA5/EPHA6/EPHA7/EPHA8/EPHB3/EPO/ERBB2/ERBB3/ERBB4/FER/FGF10/FGF7/FGFR1/FGFR2/FGFR3/FGFR4/FLT4/GAS6/GFRA1/GH2/GHR/GPRC5A/GPRC5B/GREM1/GRM5/HDAC2/HES1/HES5/HIPK3/HSF1/HTR2A/IL13/IL20/IL5/IL7/IL9/ISL1/ITGB3/KITLG/LACRT/LRP4/LYN/MUSK/NCK2/NEK1/NEURL1/NTF3/NTRK2/PBK/PDGFA/PDGFC/PDGFRA/PDGFRB/PECAM1/PIBF1/PKDCC/PKM/PLPP3/PRKCD/RELN/RET/RICTOR/ROR1/ROR2/SEMA4D/SFRP2/SPINK1/SRC/SRCIN1/TDGF1/THY1/TPST2/TTBK1/TYRO3/VEGFA/WEE2/WNT3A	101
cluster5	BP	GO:0045927	positive regulation of growth	73/3710	261/18903	0.000667264	0.01094564	0.00939479	ADAM10/AGR2/AKAP6/AKT1/ARX/AVP/BASP1/BDNF/CACNG7/CDH4/CDK1/CPNE5/CPNE6/CPNE9/CRABP2/CSH1/CSH2/CYFIP1/DBN1/DDX3X/DLL1/DRD2/DSCAM/EFNA5/EGFR/ERBB2/ERBB4/F2/FGF2/FGF9/FGFR2/FOXS1/GDI1/GH2/GHR/GHSR/HPN/IL7/IL9/INS/ISLR2/L1CAM/LGI1/MFSD2A/NDEL1/NEDD4L/NGF/NTN1/PLCB1/POU3F2/PPIB/PRKN/RICTOR/RND2/SEMA4D/SEMA5A/SFRP2/SLC6A3/SMO/SPTBN4/SYT1/SYT2/SYT3/SYT4/TBX20/TBX5/TEAD1/UNC13A/VEGFA/WFS1/WNT3A/WT1/YAP1	73

cluster5	BP	GO:0021782	glial cell development	38/3710	117/18903	0.000671817	0.010986719	0.00943005	AKT1/APP/ASCL1/ASPA/CNTNAP1/CSPG5/DAG1/DLL1/DRD1/EIF2B2/GFAP/HES5/ID4/ILK/LAMB2/LYN/MDK/MT3/MYOC/MYRF/NKX2-2/NTRK2/PARD3/PHGDH/PLP1/POU3F2/ROR1/ROR2/SHH/SIRT2/SMO/SOX10/TENM4/TPPP/TSPAN2/TTBK1/WASF3/ZNF488	38
cluster5	BP	GO:0019229	regulation of vasoconstriction	24/3710	64/18903	0.000673121	0.010986719	0.00943005	ABL1/ACE/ACE2/ADRA1A/ADRA1B/ADRA1D/ADRA2A/AGT/ASIC2/ATP1A2/AVP/AVPR1B/CHRM3/DBH/DOCK4/ECE1/EDN2/F2R/HTR2A/KCNMB2/PTGS2/TACR1/TBXA2R/TRPM4	24
cluster5	BP	GO:0048484	enteric nervous system development	9/3710	15/18903	0.000675009	0.010986719	0.00943005	ARX/EDNRA/EDNRB/GDNF/ISX/PHOX2B/RET/SOX10/TLX2	9
cluster5	BP	GO:0060080	inhibitory postsynaptic potential	9/3710	15/18903	0.000675009	0.010986719	0.00943005	CHRNA4/DRD4/GLRA1/GRIK2/INSYN1/INSYN2A/NLGN2/NLGN3/NPAS4	9

cluster5	BP	GO:0060572	morphogenesis of an epithelial bud	9/3710	15/18903	0.000675009	0.010986719	0.00943005	BMP4/BMP7/FGF10/FGFR2/SHH/SOSTDC1/SULF1/WNT2B/WNT5A	9
cluster5	BP	GO:0045494	photoreceptor cell maintenance	18/3710	43/18903	0.000688158	0.011183365	0.009598833	ABCA4/ADGRV1/BBS1/CIB2/CLRN1/CNGB1/CRB2/ESRRB/LCA5/NPHP4/PCDH15/PROM1/RHO/RP1/TUB/TULP1/USH1C/USH2A	18
cluster5	BP	GO:0050796	regulation of insulin secretion	46/3710	149/18903	0.000690305	0.011200896	0.00961388	ABCA12/ABCC8/ADCY5/ADCY8/ADRA2A/BRSK2/CFTR/CHGA/DOC2B/DRD2/EFNA5/EPHA5/FKBP1B/FOXA2/GCG/GCK/GHSR/GPLD1/HADH/HIF1A/ISL1/KCNB1/LRP5/NEUROD1/NKX6-1/NLGN2/NNAT/NOS2/NR0B2/NR1H4/PRKN/RFX3/RFX6/RPH3AL/SLC30A8/SLC9B2/SNAP25/SSTR5/STX1A/SYT7/SYT9/TFAP2B/TRH/TRPM4/UCN3/VSNL1	46
cluster5	BP	GO:0042692	muscle cell differentiation	107/3710	409/18903	0.000693897	0.011216538	0.009627306	ACTA1/ACTC1/ACTN1/ACTN2/ADAM12/ADAMTS15/ADGRB1/ADGRB3/ADRA1A/AGT/AKAP6/AKIRIN2/AKT1/ATP11A/BARX2/BDNF/BHLHE41/BMP2/BMP4/BVES/CACNA1S/CDH2/CDK1/CDON/CEACAM5/CNTNAP1/CSRP2/CSRP3/DLL1/DMD/DNER/EDNRA/EDNRB/EFNB2/ERVW-1/EZH2/FER1L5/FGF10/FGF9/FGFR2/FHOD3/GATA4/GREM1/HES1/HOMER1/ISL1/ITGB1/KCNH1/KLHL40/LAMA1/LAMB1/LAMB2/LBX2/LDB3/LMNA/LMOD2/LOX/MAPK12/MEGF10/MYH6/MYH7/MYH9/MYO18B/MYOCD/MYOG/MYOZ1/MYOZ2/MYPN/NFATC4/NID1/NKX2-6/NPHS1/NRAP/P2RX2/PDGFRA/PDGFRB/PLEKHO1/POPDC3/PRDM6/PRKAR1A/PTGFRN/RARB/RBM24/RBPMS2/RGS4/SDC1/SGCZ/SHH/SHOX2/SIK1/SIX4/SMO/SMYD1/SORT1/SOX9/SPEG/SYNPO2L/TBX5/TNNT2/TPM1/UCHL1/VEGFA/WFIKKN2/WNT3A/WNT4/WNT8A/WT1	107

cluster5	BP	GO:0030168	platelet activation	42/3710	133/18903	0.000694391	0.011216538	0.009627306	ADAMTS13/ADAMTS18/ADRA2A/ALOX12/CTSG/DGKB/DGKI/DMTN/EMILIN2/F2/F2R/F2RL3/FERMT3/GNAS/GP1BB/GP6/GP9/ILK/ITGB3/LYN/MMRN1/MYH9/MYL12A/NOS3/PDGFA/PDGFRA/PDPN/PEAR1/PF4/PF4V1/PRKCD/PRKG1/SAA1/SELP/SERPINE2/SRC/STXBP1/TREML1/TYRO3/VAV2/VWF/WNT3A	42
cluster5	BP	GO:0009855	determination of bilateral symmetry	44/3710	141/18903	0.000695173	0.011216538	0.009627306	AHI1/CC2D2A/CCDC103/CCDC40/CFAP52/CFC1/CFC1B/DAW1/DDIT3/DLL1/DNAH11/DNAH5/DNAI1/DRC1/ENKUR/FGF10/FOLR1/FOXJ1/FOXN4/GATA4/GREM1/GREM2/HIF1A/IFT140/LBX1/LEFTY1/MMP21/NDRG4/NOTCH2/ODAD2/ODAD3/RFX3/SHH/SMO/SOSTDC1/SOX18/TBC1D32/TBX20/TDGF1/VANGL2/WNT3A/WNT5A/WNT8B/ZIC3	44
cluster5	BP	GO:0061041	regulation of wound healing	43/3710	137/18903	0.00069555	0.011216538	0.009627306	ABCC8/ADAMTS18/ADRA2A/AJAP1/ALOX12/ANO6/CLDN1/CLDN19/CLDN4/CPB2/DMTN/DUOX2/EMILIN1/EMILIN2/F2/F2R/F3/FAP/FERMT1/FGF2/FOXA2/ITGB1/KNG1/MMRN1/MYLK/MYOZ1/NOS3/PDGFA/PDGFRA/PRKCD/PRKG1/PROS1/REG3A/REG3G/SERPINE1/SERPINE2/TAFA5/TBXA2R/TFPI/TSPAN8/VKORC1/WFDC1/WNT4	43
cluster5	BP	GO:0022404	molting cycle process	32/3710	94/18903	0.000704104	0.01131966	0.009715817	ALX4/APCDD1/DLX3/DSG4/EGFR/FERMT1/FGF10/FGF7/FGFR2/FOXN1/FZD3/GLI2/HDAC2/HOXC13/KRT27/KRT84/KRTAP21-1/LAMA5/LGR4/LGR5/LHX2/LRP4/MSX2/NGFR/PDGFA/SHH/SMO/SOSTDC1/SOX18/SOX9/TNFRSF19/WNT5A	32

cluster5	BP	GO:0022405	hair cycle process	32/3710	94/18903	0.000704104	0.01131966	0.009715817	ALX4/APCDD1/DLX3/DSG4/EGFR/FERMT1/FGF10/FGF7/FGFR2/FOXN1/FZD3/GLI2/HDAC2/HOXC13/KRT27/KRT84/KRTAP21-1/LAMA5/LGR4/LGR5/LHX2/LRP4/MSX2/NGFR/PDGFA/SHH/SMO/SOSTDC1/SOX18/SOX9/TNFRSF19/WNT5A	32
cluster5	BP	GO:0060292	long-term synaptic depression	14/3710	30/18903	0.000732977	0.011765796	0.010098742	ADCY8/ADORA1/ARC/CBLN1/DRD1/GRIA1/GRID2/GRID2IP/KCNB1/LILRB2/PRRT1/SHANK2/SLC24A2/STXBP1	14
cluster5	BP	GO:0003129	heart induction	7/3710	10/18903	0.000763681	0.012155019	0.010432817	BMP2/BMP4/ROBO1/ROBO2/WNT11/WNT3A/WNT5A	7
cluster5	BP	GO:0048672	positive regulation of collateral sprouting	7/3710	10/18903	0.000763681	0.012155019	0.010432817	BDNF/CRABP2/EFNA5/NGF/RND2/SEMA4D/WNT3A	7

cluster5	BP	GO:0097090	presynaptic membrane organization	7/3710	10/18903	0.000763681	0.012155019	0.010432817	IL1RAPL1/LRP4/NLGN1/NLGN2/NLGN3/NLGN4X/PTPRD	7
cluster5	BP	GO:0097104	postsynaptic membrane assembly	7/3710	10/18903	0.000763681	0.012155019	0.010432817	CDH2/LRP4/NLGN1/NLGN2/NLGN3/NLGN4X/NRXN1	7
cluster5	BP	GO:1905809	negative regulation of synapse organization	7/3710	10/18903	0.000763681	0.012155019	0.010432817	CBLN1/EPHA7/NEUROD2/NFATC4/ROBO2/SLIT1/WNT5A	7
cluster5	BP	GO:0042310	vasoconstriction	29/3710	83/18903	0.000764182	0.012155019	0.010432817	ABL1/ACE/ACE2/ADRA1A/ADRA1B/ADRA1D/ADRA2A/AGT/ASIC2/ATP1A2/AVP/AVPR1B/CHRM3/CRP/DBH/DOCK4/ECE1/EDN2/EDNRA/EDNRB/F2R/GRIP2/HTR1D/HTR2A/KCNMB2/PTGS2/TACR1/TBXA2R/TRPM4	29

cluster5	BP	GO:0003007	heart morphogenesis	72/3710	258/18903	0.000773739	0.012262406	0.010524989	ACTC1/AHI1/BMP2/BMP4/BMP5/BMP7/CCDC103/CCDC40/CLDN5/COL11A1/COL2A1/DLL1/DNAH11/EDNRA/EMP2/FGFR2/FKBP1A/FLRT2/FOLR1/FOXC1/FOXN4/GATA4/GRHL2/HAS2/HES1/HEYL/HIF1A/ILK/ISL1/LBX1/LEFTY1/LRP2/MSX2/MYH6/MYH7/NDRG4/NOS3/NOTCH2/NPY5R/NRP2/OLFM1/PKP2/PTCH1/RBM20/ROBO1/ROBO2/RYR2/SFRP2/SHH/SHOX2/SLIT3/SMO/SOX18/SOX9/SPRY1/SYNPO2L/TBX20/TBX5/TBXT/TEAD2/TH/TNNI1/TNNI3/TNNT2/TPM1/VANGL2/VEGFA/WNT11/WNT3A/WNT5A/YAP1/ZIC3	72
cluster5	BP	GO:0019896	axonal transport of mitochondrion	10/3710	18/18903	0.000774443	0.012262406	0.010524989	AGBL4/ARMCX3/FEZ1/HAP1/HDAC6/HIF1A/HSBP1/SYBU/TRAK1/UCHL1	10
cluster5	BP	GO:1902683	regulation of receptor localization to synapse	10/3710	18/18903	0.000774443	0.012262406	0.010524989	ADAM10/ARHGAP44/DAG1/DBN1/GHSR/GPC6/GRIPAP1/IQSEC2/NPTN/STX7	10
cluster5	BP	GO:0010460	positive regulation of heart rate	13/3710	27/18903	0.000781821	0.012323381	0.010577325	ADM5/ADRA1A/ADRA1B/ADRA1D/EDN2/HRC/RGS4/RYR2/SCN3B/SLC1A1/TACR3/TPM1/TRPM4	13

cluster5	BP	GO:0072207	metanephric epithelium development	13/3710	27/18903	0.000781821	0.012323381	0.010577325	CALB1/HES1/HES5/LAMB2/LGR4/PAX2/POU3F3/SOX9/WNT4/WNT7B/WNT9B/WT1/YAP1	13
cluster5	BP	GO:1904861	excitatory synapse assembly	13/3710	27/18903	0.000781821	0.012323381	0.010577325	CBLN1/GRID2/LRRC4B/LRRTM2/NLGN1/NLGN2/NPTN/NPTX1/NRXN1/PTPRD/SLITRK3/WNT5A/WNT7A	13
cluster5	BP	GO:0044344	cellular response to fibroblast growth factor stimulus	37/3710	114/18903	0.000796315	0.012532991	0.010757236	CCN2/CPS1/CXCL13/FGF10/FGF16/FGF17/FGF19/FGF2/FGF23/FGF4/FGF5/FGF7/FGF9/FGFBP3/FGFR1/FGFR2/FGFR3/FGFR4/FLRT1/FLRT2/FLRT3/FRS3/HHIP/LHX1/NDNF/NGFR/NPTN/OTX2/PRDM14/SHOC2/SPRY1/SULF1/SULF2/TDGF1/WNT4/WNT5A/ZFP36	37
cluster5	BP	GO:0015833	peptide transport	71/3710	254/18903	0.000797723	0.012536331	0.010760102	ABCA12/ABCC8/ADCY5/ADCY8/ADCYAP1/ADORA1/ADRA2A/BRSK2/CDH17/CFTR/CHGA/CLTRN/CRH/CRHR1/DOC2B/DRD2/EFNA5/EPHA5/EXOC3L1/FKBP1B/FOXA2/GCG/GCK/GHSR/GPLD1/GRP/HADH/HFE/HIF1A/HNF1A/HNF1B/HTR2C/ILDR2/INS/ISL1/KCNB1/KISS1/LRP5/MC4R/NEUROD1/NKX6-1/NLGN2/NNAT/NOS2/NR0B2/NR1H4/PCLO/PRKN/RAB11FIP2/RASL10B/RFX3/RFX6/RPH3AL/SLC15A5/SLC16A2/SLC30A8/SLC9B2/SNAP25/SPINK1/SSTR5/STX1A/SYT7/SYT9/TAP2/TAPBP/TFAP2B/TRH/TRPM4/TRPV4/UCN3/VSNL1	71

cluster5	BP	GO:0043576	regulation of respiratory gaseous exchange	12/3710	24/18903	0.000812919	0.012756019	0.010948663	ADORA1/ATP1A2/GLRA1/GRP/GRPR/GSX2/NLGN2/NLGN3/NMBR/NR4A2/PHOX2B/TLX3	12
cluster5	BP	GO:0003206	cardiac chamber morphogenesis	40/3710	126/18903	0.00081858	0.012825647	0.011008426	BMP2/BMP4/BMP5/BMP7/COL11A1/DNAH11/EDNRA/FGFR2/FKBP1A/FOXC1/GATA4/GRHL2/HES1/HEYL/HIF1A/ISL1/LRP2/MSX2/MYH6/MYH7/NOS3/NOTCH2/NPY5R/NRP2/PKP2/ROBO1/ROBO2/RYR2/SFRP2/SHOX2/SLIT3/SMO/TBX20/TBX5/TNNI1/TNNI3/TNNT2/TPM1/WNT11/WNT5A	40
cluster5	BP	GO:0048469	cell maturation	55/3710	187/18903	0.000824192	0.012860107	0.011038004	ABHD2/ACTL6B/ADGRB3/ANGPTL8/APP/ASCL1/BCL11A/BRCA2/C2CD6/CABYR/CATSPER4/CFTR/CNTNAP2/DMC1/EDNRA/EDNRB/EPHA8/EPO/FEV/FOXA1/GLDN/GPAT4/HES1/HES5/HIF1A/HOXB13/KCNB1/MYOC/NKX6-1/NPPC/NR4A2/NRCAM/PCSK4/PDE3A/PLA2G3/PTH1R/RET/RFX3/ROPN1/RPS6KA2/SIRT2/SIX3/SOX10/SOX18/SPINK1/SPTBN4/SRRM4/TCP11/TCP11X1/TDRD5/TUT4/TYMS/VEGFA/VSX1/WEE2	55
cluster5	BP	GO:0018108	peptidyl-tyrosine phosphorylation	100/3710	380/18903	0.000824254	0.012860107	0.011038004	ABL1/ACE/ADORA1/ADRA1A/ADRA2A/AGT/ALK/ALKAL2/ANGPT1/APP/BMP6/BTC/CBLB/CBLC/CRLF1/CSH1/CSH2/CSPG4/DMTN/EFNA5/EGF/EGFR/EPHA10/EPHA3/EPHA5/EPHA6/EPHA7/EPHA8/EPHB3/EPO/ERBB2/ERBB3/ERBB4/FER/FGF10/FGF7/FGFR1/FGFR2/FGFR3/FGFR4/FLT4/GAS6/GFRA1/GH2/GHR/GPRC5A/GPRC5B/GREM1/GRM5/HDAC2/HES1/HES5/HIPK3/HSF1/HTR2A/IL13/IL20/IL5/IL7/IL9/ISL1/ITGB3/KITLG/LACRT/LRP4/LYN/MUSK/NCK2/NEK1/NEURL1/NTF3/NTRK2/PBK/PDGFA/PDGFC/PDGFRA/PDGFRB/PECAM1/PIBF1/PKDCC/PKM/PLPP3/PRKCD/RELN/RET/RICTOR/ROR1/ROR2/SEMA4D/SFRP2/SPINK1/SRC/SRCIN1/TDGF1/THY1/TTBK1/TYRO3/VEGFA/WEE2/WNT3A	100

cluster5	BP	GO:0030857	negative regulation of epithelial cell differentiation	21/3710	54/18903	0.00082446	0.012860107	0.011038004	DLL1/EZH2/FGF10/FOXE3/FRZB/GRHL2/HES1/HES5/ID1/IL13/MSX2/NOTCH4/REG3A/REG3G/SIX2/SMO/SOX9/SPRY1/VEGFA/XDH/YAP1	21
cluster5	BP	GO:0030073	insulin secretion	54/3710	183/18903	0.000840273	0.013087278	0.011232987	ABCA12/ABCC8/ADCY5/ADCY8/ADCYAP1/ADRA2A/BRSK2/CFTR/CHGA/CLTRN/DOC2B/DRD2/EFNA5/EPHA5/FKBP1B/FOXA2/GCG/GCK/GHSR/GPLD1/HADH/HIF1A/HNF1A/HNF1B/ILDR2/ISL1/KCNB1/LRP5/MC4R/NEUROD1/NKX6-1/NLGN2/NNAT/NOS2/NR0B2/NR1H4/PCLO/PRKN/RAB11FIP2/RFX3/RFX6/RPH3AL/SLC30A8/SLC9B2/SNAP25/SSTR5/STX1A/SYT7/SYT9/TFAP2B/TRH/TRPM4/UCN3/VSNL1	54
cluster5	BP	GO:0001941	postsynaptic membrane organization	16/3710	37/18903	0.000883554	0.013700316	0.011759166	CDH2/CHRDL1/FRRS1L/GDNF/GLRB/LHFPL4/LRP4/MUSK/NLGN1/NLGN2/NLGN3/NLGN4X/NRXN1/RELN/SHISA6/SHISA7	16
cluster5	BP	GO:0035883	enteroendocrine cell differentiation	16/3710	37/18903	0.000883554	0.013700316	0.011759166	AKT1/BMP4/BMP5/BMP6/CDH2/DLL1/GSK3B/HES1/NEUROD1/NKX2-2/NKX6-1/PAX6/RFX3/RFX6/SMO/WNT5A	16

cluster5	BP	GO:0038179	neurotrophin signaling pathway	16/3710	37/18903	0.000883554	0.013700316	0.011759166	AGT/AGTR2/BDNF/CYFIP1/DOK5/GFRA1/HAP1/MAGI2/NGF/NTF3/NTRK2/SORT1/SPRY1/SRC/TMEM108/WASF1	16
cluster5	BP	GO:0015711	organic anion transport	98/3710	372/18903	0.000888821	0.013761628	0.011811791	ABCB11/ABCC3/ABCG2/ACE/ACE2/ADORA1/AGT/APBA1/ASIC3/ATP1A2/AVP/AVPR1B/CFTR/CLTRN/DRD2/DRD4/FGF19/FOLR1/GFAP/GIPC1/GRM2/GRM7/HRH3/ITGB1/LHCGR/LRP2/MFSD2A/NOS2/NPY5R/NR0B2/NTRK2/PLA2G12A/PLA2G12B/PLA2G2E/PLA2G2F/PLA2G3/PLA2G5/PLA2R1/PSAP/PTGS2/RGS4/SLC10A3/SLC10A5/SLC11A1/SLC13A5/SLC16A12/SLC16A2/SLC16A8/SLC16A9/SLC17A2/SLC17A4/SLC17A6/SLC17A7/SLC17A8/SLC1A1/SLC1A2/SLC1A6/SLC22A10/SLC22A11/SLC22A12/SLC22A24/SLC22A7/SLC22A9/SLC25A2/SLC25A21/SLC25A23/SLC25A31/SLC26A9/SLC27A6/SLC2A10/SLC38A3/SLC38A4/SLC38A8/SLC4A11/SLC4A3/SLC4A4/SLC4A5/SLC66A1/SLC6A1/SLC6A11/SLC6A15/SLC6A17/SLC6A20/SLC6A5/SLC6A7/SLC7A10/SLC7A3/SLC7A8/SLCO1B1/SLCO1B3/SLCO1C1/STXBP1/SV2A/SYT4/TNFRSF11A/TRH/TRPC4/UGT1A3	98
cluster5	BP	GO:0009948	anterior/posterior axis specification	22/3710	58/18903	0.000928888	0.01435106	0.012317708	BASP1/BMP4/CDX1/CDX2/CDX4/DDIT3/FOXA2/GPC3/LHX1/NEUROG1/OTX2/PGAP1/RIPPLY1/SHH/SIX2/TBXT/TDGF1/TDRD5/WNT5A/WNT8A/WT1/ZIC3	22
cluster5	BP	GO:0014013	regulation of gliogenesis	35/3710	107/18903	0.000929629	0.01435106	0.012317708	ABCC8/ASPA/ATOH1/BMP2/CDKN2B/DAB1/DAG1/DLX1/DLX2/EZH2/F2/GFAP/GSX2/HDAC2/HES1/HES5/ID4/LRP2/LYN/MDK/NKX2-2/NKX6-1/NR2E1/NTN1/PTN/PTPRZ1/SERPINE2/SHH/SIRT2/SOX10/TENM4/TMEM98/TP73/TTBK1/ZNF488	35

cluster5	BP	GO:0071526	semaphorin-plexin signaling pathway	18/3710	44/18903	0.00095306	0.014691146	0.012609609	ARHGDIA/ECE1/EDNRA/GDNF/NCAM1/NRP2/PLXNA1/PLXNA2/PLXNA4/PLXNB1/PLXNB3/SEMA3A/SEMA3E/SEMA4D/SEMA5A/SEMA5B/SEMA6A/SEMA6D	18
cluster5	BP	GO:0060425	lung morphogenesis	20/3710	51/18903	0.00096155	0.014778545	0.012684625	BMP4/CELSR1/DAG1/DLG5/FGF10/FGF7/FGFR2/FOXA1/GRHL2/HHIP/LAMA1/NKX2-1/RSPO2/SHH/SOX9/SPRY1/STK40/WNT2B/WNT7B/YAP1	20
cluster5	BP	GO:1903793	positive regulation of anion transport	20/3710	51/18903	0.00096155	0.014778545	0.012684625	ABCB11/ACE2/AGT/ATF4/AVP/AVPR1B/CFTR/CLTRN/ITGB1/PLA2G3/PLA2R1/SLC17A8/SLC34A1/SLC38A3/SLC6A1/STXBP1/SYT4/TCAF1/TNFRSF11A/TRH	20
cluster5	BP	GO:0051282	regulation of sequestering of calcium ion	40/3710	127/18903	0.000971951	0.014916525	0.012803054	ABL1/AKAP6/ATP1A2/CACNA1C/CASQ2/CEMIP/DDIT3/DHRS7C/DMD/DRD1/DRD2/ERO1A/F2/F2R/F2RL3/FGF2/FKBP1A/FKBP1B/GP1BB/GP9/GSTO1/HAP1/HRC/HTR2A/HTR2C/IL13/ITGB3/JPH1/JPH3/JPH4/JSRP1/LACRT/LHCGR/LYN/NPSR1/RYR2/RYR3/SLC25A23/THY1/TRDN	40

cluster5	BP	GO:0090287	regulation of cellular response to growth factor stimulus	90/3710	338/18903	0.00098607	0.015111089	0.012970052	ABL1/ADAMTS3/ADAMTSL2/AGT/AGTR2/ASPN/BMP2/BMP4/BMPER/CCBE1/CDKN2B/CHRD/CHRDL1/CHRDL2/CITED1/CRB2/CXCL13/CYFIP1/DAB2/DKK3/DLL1/DLX1/DOK5/EMILIN1/FERMT1/FGF10/FGF16/FGF2/FGF4/FGF9/FGFBP3/FGFR1/FKBP1A/FOLR1/GATA4/GDF5/GIPC1/GPC3/GRB10/GREM1/GREM2/HDAC2/HES1/HES5/HHIP/HIF1A/IL17RD/ILK/ITGB3/LOX/LRP2/LTBP1/MSX2/MT3/MYO1C/MYOCD/NGFR/NKX2-1/NOTCH2/NPTN/NREP/OTX2/PMEPA1/PRDM14/RASL11B/RBPMS2/RNF111/ROBO1/SEMA6A/SFRP2/SKOR2/SLC2A10/SOSTDC1/SPRY1/SULF1/SULF2/TBX20/TFAP2B/TGFB1I1/TMEM108/TOB1/VASN/VEPH1/VWC2/VWC2L/WASF1/WFIKKN2/WNT4/WNT5A/XDH	90
cluster5	BP	GO:0033238	regulation of cellular amine metabolic process	15/3710	34/18903	0.00098976	0.0151455	0.012999587	ATCAY/DRD1/DRD4/GPR37/HPRT1/INS/ITGAM/MAOB/NPY/NR1H4/NR4A2/PDE1B/PRKN/SLC6A3/TACR3	15
cluster5	BP	GO:0031279	regulation of cyclase activity	27/3710	77/18903	0.001074151	0.016412909	0.014087421	ADCYAP1/ADGRV1/ADORA2B/ADRB3/CACNA1C/CALCA/CAP2/CRHR1/DRD1/DRD2/EDNRA/EDNRB/GABBR2/GLP1R/GNAS/GPR87/GRM2/GRM3/GRM7/GUCA1C/HPCA/LHCGR/LOC118142757/NOS2/NOS3/NPFFR2/VIPR2	27
cluster5	BP	GO:0021514	ventral spinal cord interneuron differentiation	8/3710	13/18903	0.001088	0.016456592	0.014124915	ASCL1/DMRT3/FOXN4/GLI2/GLI3/LHX3/NKX2-2/SOX1	8

cluster5	BP	GO:0021681	cerebellar granular layer development	8/3710	13/18903	0.001088	0.016456592	0.014124915	CBLN1/FAIM2/GRID2/KNDC1/MDK/OPHN1/SERPINE2/WNT7A	8
cluster5	BP	GO:0045605	negative regulation of epidermal cell differentiation	8/3710	13/18903	0.001088	0.016456592	0.014124915	DLL1/EZH2/GRHL2/HES1/HES5/MSX2/REG3A/REG3G	8
cluster5	BP	GO:0045683	negative regulation of epidermis development	8/3710	13/18903	0.001088	0.016456592	0.014124915	DLL1/EZH2/GRHL2/HES1/HES5/MSX2/REG3A/REG3G	8
cluster5	BP	GO:0099550	trans-synaptic signaling, modulating synaptic transmission	8/3710	13/18903	0.001088	0.016456592	0.014124915	CSPG5/EFNB3/F2R/GRM5/GUCY1A1/NPTN/NTRK2/SYT4	8

cluster5	BP	GO:1903961	positive regulation of anion transmembrane transport	8/3710	13/18903	0.001088	0.016456592	0.014124915	ACE2/AGT/CFTR/CLTRN/ITGB1/SLC17A8/SLC34A1/TCAF1	8
cluster5	BP	GO:1905874	regulation of postsynaptic density organization	8/3710	13/18903	0.001088	0.016456592	0.014124915	CBLN1/CDH2/GRID2/LILRB2/LRRC4B/LRRTM2/NPTX1/PTPRD	8
cluster5	BP	GO:0003309	type B pancreatic cell differentiation	14/3710	31/18903	0.001097033	0.016545465	0.014201196	AKT1/BMP4/BMP5/BMP6/CDH2/DLL1/GSK3B/NKX2-2/NKX6-1/PAX6/RFX3/RFX6/SMO/WNT5A	14
cluster5	BP	GO:0140058	neuron projection arborization	14/3710	31/18903	0.001097033	0.016545465	0.014201196	CHRNA7/GRIP1/IGF2BP1/LRP2/MFSD2A/NLGN1/NTNG1/NTNG2/PTN/ROCK1/SEMA3A/SULT4A1/TPBG/WNT5A	14

cluster5	BP	GO:0014016	neuroblast differentiation	6/3710	8/18903	0.001105115	0.016571986	0.01422396	ASCL1/BCHE/DLX1/DLX2/SIX3/TAFA3	6
cluster5	BP	GO:0021940	positive regulation of cerebellar granule cell precursor proliferation	6/3710	8/18903	0.001105115	0.016571986	0.01422396	EGF/FGF2/LHX1/LHX5/SHH/SMO	6
cluster5	BP	GO:0072050	S-shaped body morphogenesis	6/3710	8/18903	0.001105115	0.016571986	0.01422396	BMP4/HES1/HES5/LHX1/PDGFRB/WT1	6
cluster5	BP	GO:2000969	positive regulation of AMPA receptor activity	6/3710	8/18903	0.001105115	0.016571986	0.01422396	ARC/CACNG2/CACNG3/CACNG4/NLGN3/RELN	6

cluster5	BP	GO:0048488	synaptic vesicle endocytosis	24/3710	66/18903	0.001117278	0.016682782	0.014319057	AMPH/AP3B2/AP3D1/DNM1/NLGN1/NLGN2/NLGN3/NLGN4X/OPHN1/PACSIN1/RAB27B/ROCK1/SH3GL2/SH3GL3/SLC17A7/SNAP91/SNCB/STON1/STX1A/SYP/SYT1/SYT2/SYT5/SYT8	24
cluster5	BP	GO:0048814	regulation of dendrite morphogenesis	24/3710	66/18903	0.001117278	0.016682782	0.014319057	ADGRB3/CAMK2B/CDKL3/CHRNA3/CUL7/CUX1/DBN1/DPYSL5/GSK3B/ID1/IL1RAPL1/ITPKA/KNDC1/LZTS1/NEDD4L/NEUROG3/NFATC4/NR2E1/PAK3/PTPRD/RELN/SDC2/SEMA4D/TLX2	24
cluster5	BP	GO:0140238	presynaptic endocytosis	24/3710	66/18903	0.001117278	0.016682782	0.014319057	AMPH/AP3B2/AP3D1/DNM1/NLGN1/NLGN2/NLGN3/NLGN4X/OPHN1/PACSIN1/RAB27B/ROCK1/SH3GL2/SH3GL3/SLC17A7/SNAP91/SNCB/STON1/STX1A/SYP/SYT1/SYT2/SYT5/SYT8	24
cluster5	BP	GO:0007528	neuromuscular junction development	19/3710	48/18903	0.001119392	0.016690558	0.014325732	ANK3/APP/CACNA1S/CACNB1/CACNG2/CNTNAP1/COL4A1/COL4A5/ERBB2/F2R/LAMB2/LRP4/MUSK/P2RX2/PDZRN3/SIX4/TNC/UNC13A/UNC13C	19

cluster5	BP	GO:0001570	vasculogenesis	28/3710	81/18903	0.001128816	0.016807166	0.014425817	APELA/ASB4/CITED1/CUL7/EMP2/GJC1/HAS2/ITGB8/JUNB/MYO18B/MYOCD/NTRK2/PDGFRB/RASA1/SHH/SMO/SOX18/TBX20/TBX5/TEAD2/TIPARP/TNNI3/VEGFA/WNT7A/WNT7B/WT1/XDH/YAP1	28
cluster5	BP	GO:0051209	release of sequestered calcium ion into cytosol	39/3710	124/18903	0.00115018	0.017100972	0.014677996	ABL1/AKAP6/ATP1A2/CACNA1C/CASQ2/CEMIP/DDIT3/DHRS7C/DMD/DRD1/DRD2/ERO1A/F2/F2R/F2RL3/FGF2/FKBP1A/FKBP1B/GP1BB/GP9/GSTO1/HAP1/HRC/HTR2A/HTR2C/IL13/ITGB3/JPH1/JPH3/JPH4/JSRP1/LACRT/LHCGR/LYN/NPSR1/RYR2/RYR3/THY1/TRDN	39
cluster5	BP	GO:0097305	response to alcohol	70/3710	253/18903	0.001165698	0.017307145	0.014854956	ABCB11/ACE/ADCY1/ADCY2/ADCY3/ADCY5/ADCY8/ADCYAP1R1/ADH7/AKT1/AVP/CDK1/CDO1/CFTR/CLDN1/CLDN18/CLDN5/CRHR1/CYP8B1/DAG1/DBH/DEFB104A/DEFB104B/DRD2/DRD4/EFNA5/EHMT2/EPS8/FGF19/FGFR2/FOS/FOSB/GLRA1/GLRA2/GNAI1/GPLD1/GRAMD1B/GRIN1/GRIN2A/GRIN2B/HDAC2/HMGCS2/HSD3B2/IL13/KCNC2/LRP6/MAOB/NPAS4/NPPC/NR0B2/OPRK1/PENK/PRKAA2/PTCH1/PTGFR/PTH/RGS4/RGS7/SCNN1G/SLC6A3/SLIT3/SMO/SPINK1/TACR1/TBXA2R/TH/TNFRSF11A/TP53INP1/TYMS/UCN3	70
cluster5	BP	GO:0050730	regulation of peptidyl-tyrosine phosphorylation	73/3710	266/18903	0.001182452	0.017531058	0.015047144	ABL1/ACE/ADORA1/ADRA1A/ADRA2A/AGT/ALK/ALKAL2/ANGPT1/APP/BMP6/BTC/CBLB/CBLC/CRLF1/CSH1/CSH2/CSPG4/DMTN/EFNA5/EGF/EGFR/EPHA7/EPO/ERBB3/ERBB4/FGF10/FGF7/FGFR3/GAS6/GFRA1/GH2/GHR/GPRC5A/GPRC5B/GREM1/GRM5/HDAC2/HES1/HES5/HSF1/HTR2A/IL13/IL20/IL5/IL7/IL9/ISL1/ITGB3/KITLG/LACRT/LRP4/LYN/NCK2/NEURL1/NTF3/PDGFA/PDGFC/PECAM1/PIBF1/PLPP3/PRKCD/RELN/RICTOR/SEMA4D/SFRP2/SPINK1/SRC/SRCIN1/TDGF1/THY1/VEGFA/WNT3A	73

cluster5	BP	GO:0007288	sperm axoneme assembly	13/3710	28/18903	0.001199022	0.017640744	0.015141289	CEP131/CFAP206/CFAP43/CFAP47/CFAP65/CFAP69/CFAP97D1/FSIP2/MNS1/NEURL1/PLA2G3/SPAG6/UBE2B	13
cluster5	BP	GO:0060479	lung cell differentiation	13/3710	28/18903	0.001199022	0.017640744	0.015141289	AGR2/ASCL1/FGF10/FOXA1/FOXJ1/GRHL2/IL13/NKX2-1/SOX9/SPDEF/THRB/TP73/YAP1	13
cluster5	BP	GO:0060487	lung epithelial cell differentiation	13/3710	28/18903	0.001199022	0.017640744	0.015141289	AGR2/ASCL1/FGF10/FOXA1/FOXJ1/GRHL2/IL13/NKX2-1/SOX9/SPDEF/THRB/TP73/YAP1	13
cluster5	BP	GO:0060512	prostate gland morphogenesis	13/3710	28/18903	0.001199022	0.017640744	0.015141289	BMP4/BMP7/FGF10/FGFR2/FOXA1/HOXB13/HOXD13/ID4/SHH/SOX9/SULF1/TNC/WNT5A	13

cluster5	BP	GO:0030193	regulation of blood coagulation	25/3710	70/18903	0.001199766	0.017640744	0.015141289	ADAMTS18/ALOX12/ANO6/CPB2/DMTN/EMILIN1/EMILIN2/F2/F2R/F3/FAP/FOXA2/KNG1/NOS3/PDGFA/PDGFRA/PRKCD/PRKG1/PROS1/SERPINE1/SERPINE2/TBXA2R/TFPI/TSPAN8/VKORC1	25
cluster5	BP	GO:0070371	ERK1 and ERK2 cascade	90/3710	340/18903	0.001199948	0.017640744	0.015141289	ABL1/ACE2/ADCYAP1/ADRA1A/AGT/ALKAL2/ANGPT1/APELA/APP/ARHGAP8/AVP/BMP2/BMP4/BMPER/C1QL4/CALCR/CAVIN3/CCL22/CCL25/CCN2/CDK1/CHRNA7/CNKSR3/CXCL17/DAB2/DENND2B/DRD2/DUSP26/DUSP4/DUSP9/EGF/EGFR/EMILIN1/EPHA7/EPO/ERBB2/ERBB4/F2R/FAM83D/FBLN1/FGF10/FGF19/FGF2/FGF23/FGF4/FGFR2/FGFR3/FGFR4/FLT4/GAREM1/GAS6/GATA4/GCG/GRB10/HCRTR1/HTR2A/HTR2C/ICAM1/ITGB3/LMO3/LYN/MT3/NDRG4/NEK10/NOTCH2/NPSR1/NPTN/NPY/NPY5R/OR2AT4/PDGFA/PDGFC/PDGFRA/PDGFRB/PKHD1/PLA2G5/PSCA/RAP1B/RPS6KA6/SEMA6A/SOX9/SPRY1/SRC/THPO/TNFRSF11A/TPBG/TRAF7/TRPV4/WNK2/YWHAZ	90
cluster5	BP	GO:0031345	negative regulation of cell projection organization	56/3710	194/18903	0.001209422	0.017755132	0.01523947	ACP4/ARHGAP44/BCL11A/CDKL3/CRMP1/DAB1/DAB2/DPYSL3/DPYSL5/DRAXIN/EFNB2/EFNB3/EPHA7/FAT3/GDI1/GFAP/GRIN2B/HDAC2/HES1/ID1/KIF24/LRP4/MAP2/MT3/NEU4/NFATC4/NGEF/NLGN1/NR2F1/NTN1/PFN2/PLXNB3/PRAG1/PRKCD/PTPRG/PTPRS/RIT2/RTN4RL1/RTN4RL2/SEMA3A/SEMA3E/SEMA4D/SEMA5A/SEMA5B/SEMA6A/SEMA6D/SLIT1/SPOCK1/STMN2/THY1/TLX2/TNR/TRPV4/WNT3A/WNT5A/YAP1	56
cluster5	BP	GO:0009612	response to mechanical stimulus	60/3710	211/18903	0.0012395	0.018171246	0.015596626	ACTA1/ADGRV1/AGT/ANGPT2/ANO3/ASIC2/ASIC3/ATP1A2/BCL10/BMP6/CDH2/CNTNAP2/COL11A1/CSRP3/DAG1/DMD/DRD2/ETV1/FGF2/FOS/FOSB/GADD45A/GATA4/GDF5/HPN/HTR2A/IGFBP2/IL13/ITGA2/ITGB3/KCNC1/LHFPL5/LRP11/MDK/NEUROG1/NFKBIA/NRXN1/P2RX3/PDZD7/PKD1L2/PKD1L3/PKDREJ/PSPH/PTCH1/PTGS2/PTN/RYR2/SCEL/SCN1A/SERPINE2/SLITRK6/SOX9/SRC/STRA6/TACR1/TMC1/TNFRSF11A/TRPV4/WHRN/WNT11	60

cluster5	BP	GO:0019228	neuronal action potential	16/3710	38/18903	0.001250779	0.018285445	0.015694645	ANK3/CACNA1G/CHRNB4/DRD1/GJD2/GLRA1/GRIK2/KCND2/KCNMB2/P2RX3/SCN10A/SCN1A/SCN2A/SCN4A/SCN8A/SCN9A	16
cluster5	BP	GO:0098926	postsynaptic signal transduction	16/3710	38/18903	0.001250779	0.018285445	0.015694645	CDK5R1/CHRM1/CHRM2/CHRM3/CHRNA3/CHRNA7/HRH3/LY6G6D/LY6H/LYPD1/PLCB1/PRR7/RGS10/RGS8/SLURP2/WNT3A	16
cluster5	BP	GO:0051781	positive regulation of cell division	31/3710	93/18903	0.001254503	0.018314347	0.015719451	AURKB/BIRC5/BTC/CDC14C/DRD2/FGF2/FGF4/FGF5/FGF7/FGF9/FGFR2/GAREM1/GIPC1/GKN1/KIF20B/MDK/MRGPRX2/OPN1LW/OPN1MW2/OR1A2/PDGFA/PDGFC/PKN2/PPBP/PTN/SHH/SIRT2/SSTR5/TAS2R13/VEGFA/VEGFD	31
cluster5	BP	GO:0033275	actin-myosin filament sliding	9/3710	16/18903	0.001274616	0.018479292	0.015861026	ACTC1/MYH2/MYH4/MYH6/MYH7/MYH8/MYL1/TNNT2/TPM1	9

cluster5	BP	GO:0045836	positive regulation of meiotic nuclear division	9/3710	16/18903	0.001274616	0.018479292	0.015861026	DMRT1/MSX2/NPM2/OOEP/PLCB1/SIRT2/UBE2B/WNT4/WNT5A	9
cluster5	BP	GO:0045986	negative regulation of smooth muscle contraction	9/3710	16/18903	0.001274616	0.018479292	0.015861026	ADORA1/ADORA2B/ADRA2A/CALCA/DOCK4/GUCY1A1/KCNMA1/PRKG1/PTGS2	9
cluster5	BP	GO:0046184	aldehyde biosynthetic process	9/3710	16/18903	0.001274616	0.018479292	0.015861026	BMP2/BMP5/BMP6/CLCN2/CYP11B1/CYP11B2/DAB2/DKK3/WNT4	9
cluster5	BP	GO:1902285	semaphorin-plexin signaling pathway involved in neuron projection guidance	9/3710	16/18903	0.001274616	0.018479292	0.015861026	ECE1/EDNRA/NRP2/PLXNA1/PLXNA2/PLXNA4/PLXNB1/PLXNB3/SEMA3A	9

cluster5	BP	GO:0003081	regulation of systemic arterial blood pressure by renin-angiotensin	12/3710	25/18903	0.001285837	0.018513931	0.015890757	ACE/ACE2/AGT/AGTR2/CMA1/CTSG/EDNRB/F2R/OR51E2/RPS6KA2/SUCNR1/TACR1	12
cluster5	BP	GO:0003323	type B pancreatic cell development	12/3710	25/18903	0.001285837	0.018513931	0.015890757	AKT1/BMP4/BMP5/BMP6/CDH2/DLL1/GSK3B/NKX2-2/NKX6-1/RFX3/SMO/WNT5A	12
cluster5	BP	GO:0032331	negative regulation of chondrocyte differentiation	12/3710	25/18903	0.001285837	0.018513931	0.015890757	ADAMTS7/BMP4/CHADL/GDF5/GLI3/GREM1/PTH/RARB/RFLNA/SOX9/WNT9A/ZNF664-RFLNA	12
cluster5	BP	GO:0045932	negative regulation of muscle contraction	12/3710	25/18903	0.001285837	0.018513931	0.015890757	ADORA1/ADORA2B/ADRA2A/ATP1A2/CALCA/DOCK4/GUCY1A1/KCNMA1/PDE5A/PRKG1/PTGS2/ZC3H12A	12

cluster5	BP	GO:0098719	sodium ion import across plasma membrane	12/3710	25/18903	0.001285837	0.018513931	0.015890757	HCN4/SCNN1G/SLC34A1/SLC5A1/SLC6A1/SLC9A2/SLC9A4/SLC9A5/SLC9A6/SLC9A7/SLC9C2/TRPM4	12
cluster5	BP	GO:0042551	neuron maturation	18/3710	45/18903	0.001300242	0.018695655	0.016046733	ACTL6B/ADGRB3/APP/BCL11A/CNTNAP2/EDNRA/EDNRB/EPHA8/FEV/GLDN/KCNB1/MYOC/NR4A2/NRCAM/RET/SPTBN4/SRRM4/VSX1	18
cluster5	BP	GO:0071772	response to BMP	52/3710	178/18903	0.001325937	0.019012957	0.016319078	ABL1/ADAMTS7/BMP2/BMP4/BMP5/BMP6/BMP7/BMPER/BMPR1B/CHRD/CHRDL1/CHRDL2/COL2A1/CRB2/DLX1/DLX3/DLX5/DSG4/GATA4/GDF1/GDF5/GDF6/GPC3/GREM1/GREM2/HES1/HES5/HEYL/HFE/HIVEP1/ILK/LEFTY1/LEFTY2/LRP2/MSX2/NOTCH2/PHOX2B/RBPMS2/SFRP2/SKOR2/SLC39A5/SMAD5/SOSTDC1/SOX9/SULF1/TBX20/TFAP2B/TOB1/VSTM2A/VWC2/VWC2L/WNT5A	52
cluster5	BP	GO:0071773	cellular response to BMP stimulus	52/3710	178/18903	0.001325937	0.019012957	0.016319078	ABL1/ADAMTS7/BMP2/BMP4/BMP5/BMP6/BMP7/BMPER/BMPR1B/CHRD/CHRDL1/CHRDL2/COL2A1/CRB2/DLX1/DLX3/DLX5/DSG4/GATA4/GDF1/GDF5/GDF6/GPC3/GREM1/GREM2/HES1/HES5/HEYL/HFE/HIVEP1/ILK/LEFTY1/LEFTY2/LRP2/MSX2/NOTCH2/PHOX2B/RBPMS2/SFRP2/SKOR2/SLC39A5/SMAD5/SOSTDC1/SOX9/SULF1/TBX20/TFAP2B/TOB1/VSTM2A/VWC2/VWC2L/WNT5A	52

cluster5	BP	GO:0061318	renal filtration cell differentiation	11/3710	22/18903	0.00134269	0.019200656	0.016480182	BASP1/BMP4/EDNRA/EDNRB/KLF15/LAMB2/MAGI2/NOTCH2/NPHS1/PROM1/WT1	11
cluster5	BP	GO:0072112	podocyte differentiation	11/3710	22/18903	0.00134269	0.019200656	0.016480182	BASP1/BMP4/EDNRA/EDNRB/KLF15/LAMB2/MAGI2/NOTCH2/NPHS1/PROM1/WT1	11
cluster5	BP	GO:0001502	cartilage condensation	10/3710	19/18903	0.001348415	0.019203918	0.016482982	BARX2/BMPR1B/CCN2/COL11A1/COL2A1/FGF4/SOX5/SOX9/UNCX/WNT7A	10
cluster5	BP	GO:0072074	kidney mesenchyme development	10/3710	19/18903	0.001348415	0.019203918	0.016482982	BASP1/BMP4/BMP7/PAX2/PDGFRB/SHH/SIX2/SIX4/WNT4/WT1	10

cluster5	BP	GO:0099563	modification of synaptic structure	10/3710	19/18903	0.001348415	0.019203918	0.016482982	ABL1/ARHGAP44/CAMKV/CTTNBP2/CYFIP1/DLGAP3/GRIPAP1/PFN2/WASF1/WASF3	10
cluster5	BP	GO:0070372	regulation of ERK1 and ERK2 cascade	84/3710	315/18903	0.001358031	0.019293786	0.016560117	ABL1/ACE2/ADCYAP1/ADRA1A/ALKAL2/ANGPT1/APELA/APP/ARHGAP8/BMP2/BMP4/BMPER/C1QL4/CALCR/CAVIN3/CCL22/CCL25/CCN2/CHRNA7/CNKSR3/CXCL17/DAB2/DENND2B/DRD2/DUSP26/DUSP4/DUSP9/EGFR/EMILIN1/EPHA7/EPO/ERBB2/ERBB4/F2R/FAM83D/FBLN1/FGF10/FGF19/FGF2/FGF23/FGF4/FGFR2/FGFR3/FGFR4/FLT4/GAREM1/GAS6/GATA4/GCG/HCRTR1/HTR2A/HTR2C/ICAM1/ITGB3/LMO3/LYN/MT3/NDRG4/NEK10/NOTCH2/NPSR1/NPTN/NPY/NPY5R/OR2AT4/PDGFA/PDGFC/PDGFRA/PDGFRB/PKHD1/PLA2G5/PSCA/RAP1B/RPS6KA6/SEMA6A/SPRY1/SRC/THPO/TNFRSF11A/TPBG/TRAF7/TRPV4/WNK2/YWHAZ	84
cluster5	BP	GO:0009308	amine metabolic process	37/3710	117/18903	0.001358985	0.019293786	0.016560117	ALDH7A1/AMD1/AOC2/AOC3/ATCAY/CHDH/COMT/DAO/DBH/DMGDH/DRD1/DRD2/DRD4/EDNRA/GPR37/GRIN2A/HPRT1/INS/ITGAM/MAOB/NPY/NR1H4/NR4A2/PAH/PDE1B/PRG3/PRKN/RNF180/SLC1A1/SLC29A4/SLC6A3/SNCAIP/SNCB/SULT1C2/TACR3/TH/TRH	37
cluster5	BP	GO:0051283	negative regulation of sequestering of calcium ion	39/3710	125/18903	0.001360247	0.019293786	0.016560117	ABL1/AKAP6/ATP1A2/CACNA1C/CASQ2/CEMIP/DDIT3/DHRS7C/DMD/DRD1/DRD2/ERO1A/F2/F2R/F2RL3/FGF2/FKBP1A/FKBP1B/GP1BB/GP9/GSTO1/HAP1/HRC/HTR2A/HTR2C/IL13/ITGB3/JPH1/JPH3/JPH4/JSRP1/LACRT/LHCGR/LYN/NPSR1/RYR2/RYR3/THY1/TRDN	39

cluster5	BP	GO:0043954	cellular component maintenance	24/3710	67/18903	0.001421341	0.020133108	0.017280519	ADGRB3/BSN/CBLN1/CBLN2/CBLN3/CLDN1/CLRN2/CNTNAP1/ERC1/F2R/GRIN2B/HOMER1/INAVA/INS/ITPKA/KIRREL1/MYOCD/NLGN1/NLGN2/OPHN1/PCLO/PRTN3/SHANK2/WHRN	24
cluster5	BP	GO:0045761	regulation of adenylate cyclase activity	21/3710	56/18903	0.001425489	0.02016461	0.017307557	ADCYAP1/ADGRV1/ADORA2B/ADRB3/CACNA1C/CALCA/CAP2/CRHR1/DRD1/DRD2/EDNRA/EDNRB/GABBR2/GLP1R/GPR87/GRM2/GRM3/HPCA/LHCGR/NPFFR2/VIPR2	21
cluster5	BP	GO:0002088	lens development in camera-type eye	29/3710	86/18903	0.001447719	0.020251294	0.017381959	ATF4/BCAR3/BMP4/CDON/CRYAA/CRYAB/CRYGB/CRYGD/FAT1/FGF2/FOXE3/GJE1/MEIS1/MIP/NECTIN1/NECTIN3/PAX6/PLAAT1/PYGO2/SHROOM2/SIX3/SLITRK6/SOX1/SPRY1/TBC1D32/WNT2B/WNT5A/WNT7A/WNT7B	29
cluster5	BP	GO:0019695	choline metabolic process	5/3710	6/18903	0.001458665	0.020251294	0.017381959	ALDH7A1/BCHE/CHDH/DMGDH/ENPP6	5

cluster5	BP	GO:0021910	smoothened signaling pathway involved in ventral spinal cord patterning	5/3710	6/18903	0.001458665	0.020251294	0.017381959	GLI2/GLI3/RFX4/SMO/TULP3	5
cluster5	BP	GO:0035502	metanephric part of ureteric bud development	5/3710	6/18903	0.001458665	0.020251294	0.017381959	BASP1/CALB1/FOXJ1/LHX1/WT1	5
cluster5	BP	GO:0060482	lobar bronchus development	5/3710	6/18903	0.001458665	0.020251294	0.017381959	ADAMTSL2/AGR2/IL13/SPDEF/WNT7B	5
cluster5	BP	GO:0060684	epithelial-mesenchymal cell signaling	5/3710	6/18903	0.001458665	0.020251294	0.017381959	BMP4/FOXA1/PDGFA/SHH/SMO	5

cluster5	BP	GO:0060685	regulation of prostatic bud formation	5/3710	6/18903	0.001458665	0.020251294	0.017381959	BMP4/BMP7/SHH/SULF1/WNT5A	5
cluster5	BP	GO:0072038	mesenchymal stem cell maintenance involved in nephron morphogenesis	5/3710	6/18903	0.001458665	0.020251294	0.017381959	BMP7/HNF1B/PAX2/SIX2/WNT9B	5
cluster5	BP	GO:0072049	comma-shaped body morphogenesis	5/3710	6/18903	0.001458665	0.020251294	0.017381959	BMP4/HES1/HES5/LHX1/PDGFRB	5
cluster5	BP	GO:0072215	regulation of metanephros development	5/3710	6/18903	0.001458665	0.020251294	0.017381959	AGTR2/BASP1/PAX2/RET/WT1	5

cluster5	BP	GO:1900019	regulation of protein kinase C activity	5/3710	6/18903	0.001458665	0.020251294	0.017381959	CEMIP/DIPK2A/EGFR/ROR2/WNT5A	5
cluster5	BP	GO:1900020	positive regulation of protein kinase C activity	5/3710	6/18903	0.001458665	0.020251294	0.017381959	CEMIP/DIPK2A/EGFR/ROR2/WNT5A	5
cluster5	BP	GO:1900454	positive regulation of long-term synaptic depression	5/3710	6/18903	0.001458665	0.020251294	0.017381959	ADCY8/CBLN1/GRID2/KCNB1/LILRB2	5
cluster5	BP	GO:1904158	axonemal central apparatus assembly	5/3710	6/18903	0.001458665	0.020251294	0.017381959	CFAP91/HYDIN/RSPH9/SPAG17/SPEF1	5

cluster5	BP	GO:0003025	regulation of systemic arterial blood pressure by baroreceptor feedback	4/3710	4/18903	0.001481863	0.02041115	0.017519166	ADRA1A/ASIC2/CALCA/NAV2	4
cluster5	BP	GO:0021759	globus pallidus development	4/3710	4/18903	0.001481863	0.02041115	0.017519166	ARX/KCNC1/KCNC2/NKX2-1	4
cluster5	BP	GO:0060686	negative regulation of prostatic bud formation	4/3710	4/18903	0.001481863	0.02041115	0.017519166	BMP4/BMP7/SULF1/WNT5A	4
cluster5	BP	GO:0061205	paramesonephric duct development	4/3710	4/18903	0.001481863	0.02041115	0.017519166	GREB1L/LHX1/STRA6/WNT4	4

cluster5	BP	GO:0097376	interneuron axon guidance	4/3710	4/18903	0.001481863	0.02041115	0.017519166	DLX5/EVX1/LHX1/LHX9	4
cluster5	BP	GO:0097402	neuroblast migration	4/3710	4/18903	0.001481863	0.02041115	0.017519166	ABCC8/ATOH1/EDNRB/SIX3	4
cluster5	BP	GO:0043524	negative regulation of neuron apoptotic process	46/3710	154/18903	0.001485375	0.020432683	0.017537648	ANGPT1/BARHL1/BDNF/CHL1/CITED1/CNTFR/CRLF1/DLX1/DRAXIN/EN1/EN2/ERBB3/F2R/FAIM2/GABRA5/GABRB2/GDF5/GDNF/GRIK2/HIF1A/ISL1/MDK/MT3/NDNF/NFATC4/NGF/NR4A2/NTF3/NTRK2/PLA2G3/PRKCG/PRKN/PTPRZ1/RASA1/ROCK1/SEMA3E/SIX4/SLC1A1/SNCB/STXBP1/TFAP2B/TOX3/TYRO3/UNC5B/VSTM2L/WFS1	46
cluster5	BP	GO:0061178	regulation of insulin secretion involved in cellular response to glucose stimulus	19/3710	49/18903	0.001495974	0.020551511	0.01763964	ABCA12/ADCY5/ADCY8/ADRA2A/BRSK2/CFTR/EFNA5/EPHA5/FKBP1B/FOXA2/GCG/GPLD1/HIF1A/LRP5/NR1H4/RFX6/SLC9B2/TRPM4/VSNL1	19

cluster5	BP	GO:0003351	epithelial cilium movement involved in extracellular fluid movement	17/3710	42/18903	0.001506158	0.020664333	0.017736476	CABYR/CCDC103/CCDC40/CFAP221/CFAP43/CFAP54/DAW1/DNAH11/DNAH5/DNAH9/DNAI1/NEK10/NME5/ODAD3/RFX3/SPAG17/SPAG6	17
cluster5	BP	GO:0022029	telencephalon cell migration	22/3710	60/18903	0.001561245	0.02139212	0.018361145	ARX/CDK5R1/CDK5R2/DAB1/DRD1/DRD2/EGFR/FEZF2/FOXG1/GLI3/LAMB1/MBOAT7/NDEL1/NKX2-1/NR2E1/NRG3/POU3F2/POU3F3/RELN/ROBO1/SYNE2/TNR	22
cluster5	BP	GO:0032890	regulation of organic acid transport	26/3710	75/18903	0.001591187	0.021745525	0.018664478	ACE2/ACSL1/ADORA1/AGT/AKT1/ATP1A2/AVP/AVPR1B/CLTRN/FABP3/GRM2/GRM7/HRH3/ITGB1/NPY5R/PLA2G3/PLA2R1/RGS4/SLC17A8/SLC38A3/SLC6A1/STXBP1/SV2A/SYT4/TNFRSF11A/TRH	26
cluster5	BP	GO:0050818	regulation of coagulation	26/3710	75/18903	0.001591187	0.021745525	0.018664478	ADAMTS18/ALOX12/ANO6/CPB2/DMTN/EMILIN1/EMILIN2/F2/F2R/F3/FAP/FOXA2/HS3ST5/KNG1/NOS3/PDGFA/PDGFRA/PRKCD/PRKG1/PROS1/SERPINE1/SERPINE2/TBXA2R/TFPI/TSPAN8/VKORC1	26

cluster5	BP	GO:0010644	cell communication by electrical coupling	14/3710	32/18903	0.001601226	0.021825808	0.018733386	ANK3/ATP1A2/ATP1A3/ATP1B1/CACNA1C/CASQ2/DBN1/FKBP1B/GJC1/HRC/PKP2/RYR2/TBX5/TRDN	14
cluster5	BP	GO:0061037	negative regulation of cartilage development	14/3710	32/18903	0.001601226	0.021825808	0.018733386	ADAMTS7/BMP4/CHADL/FRZB/GDF5/GLI3/GREM1/PTH/RARB/RFLNA/SOX9/WNT11/WNT9A/ZNF664-RFLNA	14
cluster5	BP	GO:0043271	negative regulation of ion transport	49/3710	167/18903	0.001621109	0.02206814	0.018941383	ACE/ACTN2/ADORA1/ADRA2A/ANK3/ATF4/ATP1A2/BEST3/CASQ2/CBARP/CRBN/CRHR1/DRD2/DRD4/EPO/FKBP1B/GABRE/GEM/GHSR/GPM6B/GRM7/GRP/GSTO1/HES1/HRH3/HTR2A/KCNE4/KCNH2/KCNRG/LILRB2/MAOB/NEDD4L/NOS3/NPY5R/PACSIN3/PLA2R1/PTGS2/REM1/RGS4/SERPINE2/SLC30A1/SLN/SPINK1/STC1/TMBIM6/TRDN/TRH/WNK2/WNK3	49
cluster5	BP	GO:0086001	cardiac muscle cell action potential	27/3710	79/18903	0.00166069	0.022577627	0.019378682	ANK3/ATP1A2/ATP1B1/CACNA1C/CACNA1G/CACNA2D1/DMD/DSG2/GJC1/HCN1/HCN3/HCN4/KCND3/KCNE4/KCNH2/KCNJ3/KCNJ5/KCNN2/NEDD4L/PKP2/RYR2/SCN10A/SCN1A/SCN2B/SCN3B/SLC4A3/TRPM4	27

cluster5	BP	GO:0043523	regulation of neuron apoptotic process	61/3710	218/18903	0.001736872	0.023401756	0.020086043	ABL1/ANGPT1/ASCL1/ATF4/BARHL1/BDNF/CDK5R1/CHL1/CITED1/CNTFR/CRLF1/DDIT3/DLX1/DRAXIN/EN1/EN2/EPHA7/ERBB3/F2R/FAIM2/GABRA5/GABRB2/GDF5/GDNF/GRID2/GRIK2/GRIK5/GRM4/HIF1A/ISL1/ITGAM/KCNB1/MDK/MT3/NAE1/NDNF/NFATC4/NGF/NR4A2/NTF3/NTRK2/PAK3/PLA2G3/PRKCG/PRKN/PTPRZ1/RASA1/ROCK1/SEMA3E/SIX4/SLC1A1/SNCB/STXBP1/TFAP2A/TFAP2B/TOX3/TRIM2/TYRO3/UNC5B/VSTM2L/WFS1	61
cluster5	BP	GO:0007494	midgut development	7/3710	11/18903	0.001743634	0.023401756	0.020086043	CPS1/EDNRB/FOXL1/HMGCS2/RET/SMO/WNT5A	7
cluster5	BP	GO:0021527	spinal cord association neuron differentiation	7/3710	11/18903	0.001743634	0.023401756	0.020086043	ASCL1/GSX1/GSX2/LHX1/LHX3/LHX5/WNT3A	7
cluster5	BP	GO:0042487	regulation of odontogenesis of dentin-containing tooth	7/3710	11/18903	0.001743634	0.023401756	0.020086043	APCDD1/BMP2/BMP4/DMRT3/NGFR/RSPO2/TNFRSF11B	7

cluster5	BP	GO:0048505	regulation of timing of cell differentiation	7/3710	11/18903	0.001743634	0.023401756	0.020086043	ASCL1/DLL1/FGF9/HES1/NR2E1/PAX6/SERPINE2	7
cluster5	BP	GO:0051386	regulation of neurotrophin TRK receptor signaling pathway	7/3710	11/18903	0.001743634	0.023401756	0.020086043	AGT/AGTR2/CYFIP1/DOK5/SPRY1/TMEM108/WASF1	7
cluster5	BP	GO:0060174	limb bud formation	7/3710	11/18903	0.001743634	0.023401756	0.020086043	COL2A1/FGF10/FGFR2/PLXNA2/SHH/SOX9/ZNF219	7
cluster5	BP	GO:0071679	commissural neuron axon guidance	7/3710	11/18903	0.001743634	0.023401756	0.020086043	DAG1/FZD3/GDNF/NCAM1/PTCH1/SMO/VEGFA	7

cluster5	BP	GO:0072173	metanephric tubule morphogenesis	7/3710	11/18903	0.001743634	0.023401756	0.020086043	HES1/HES5/LGR4/PAX2/SOX9/WNT4/WNT9B	7
cluster5	BP	GO:0072205	metanephric collecting duct development	7/3710	11/18903	0.001743634	0.023401756	0.020086043	BMP4/CALB1/DLG5/PAX2/PTCH1/SHH/WNT7B	7
cluster5	BP	GO:0035272	exocrine system development	18/3710	46/18903	0.001748973	0.02344339	0.020121778	BMP7/CLCN2/DAG1/EGFR/FGF10/FGF7/FGFR2/FOXC1/IGSF3/LAMA1/LAMA5/PAX6/PDGFA/PLXNA1/PTF1A/SHH/SOX10/SOX9	18
cluster5	BP	GO:0060037	pharyngeal system development	13/3710	29/18903	0.00178612	0.023849815	0.020470618	BMP4/BMP5/BMP7/ECE1/EDNRA/FOLR1/HES1/ISL1/NKX2-6/PLXNA2/PTCH1/RIPPLY3/SIX4	13

cluster5	BP	GO:0072012	glomerulus vasculature development	13/3710	29/18903	0.00178612	0.023849815	0.020470618	ANGPT1/ANGPT2/BMP4/BMP7/EDNRA/HES1/ITGB3/NOTCH2/PDGFRA/PDGFRB/PECAM1/SERPINB7/WT1	13
cluster5	BP	GO:0097254	renal tubular secretion	13/3710	29/18903	0.00178612	0.023849815	0.020470618	ABCG2/ACE/ADORA1/AGT/ATP6V0A4/ATP6V1B1/AVP/DRD2/EDNRB/SLC22A12/SPX/STC1/TACR1	13
cluster5	BP	GO:0042886	amide transport	79/3710	296/18903	0.00180386	0.024056051	0.020647634	ABCA12/ABCC8/ABCG2/ADCY5/ADCY8/ADCYAP1/ADORA1/ADRA2A/BRSK2/CDH17/CFTR/CHGA/CLTRN/CRH/CRHR1/DOC2B/DRD2/EFNA5/EPHA5/EXOC3L1/FKBP1B/FOLR1/FOXA2/GCG/GCK/GHSR/GPLD1/GRP/HADH/HFE/HIF1A/HNF1A/HNF1B/HTR2C/ILDR2/INS/ISL1/KCNB1/KISS1/LRP2/LRP5/MC4R/NEUROD1/NKX6-1/NLGN2/NNAT/NOS2/NR0B2/NR1H4/PCLO/PLTP/POU3F3/PRKN/PSAP/RAB11FIP2/RASL10B/RFX3/RFX6/RPH3AL/SLC15A5/SLC16A2/SLC30A8/SLC38A3/SLC9B2/SNAP25/SPINK1/SSTR5/STX1A/SYT7/SYT9/TAP2/TAPBP/TFAP2B/TRH/TRPM4/TRPV4/UCN3/VAPA/VSNL1	79
cluster5	BP	GO:0051208	sequestering of calcium ion	40/3710	131/18903	0.001866756	0.024863197	0.021340418	ABL1/AKAP6/ATP1A2/CACNA1C/CASQ2/CEMIP/DDIT3/DHRS7C/DMD/DRD1/DRD2/ERO1A/F2/F2R/F2RL3/FGF2/FKBP1A/FKBP1B/GP1BB/GP9/GSTO1/HAP1/HRC/HTR2A/HTR2C/IL13/ITGB3/JPH1/JPH3/JPH4/JSRP1/LACRT/LHCGR/LYN/NPSR1/RYR2/RYR3/SLC25A23/THY1/TRDN	40

cluster5	BP	GO:0019233	sensory perception of pain	33/3710	103/18903	0.00188932	0.025111841	0.021553832	ACE/ADORA1/ASIC3/CALCA/CHRNA4/EDNRB/F2R/GRIN2A/GRIN2D/HTR2A/IAPP/ITGA2/KCND2/MMP24/MRGPRX2/NLGN2/OPRK1/P2RX3/PENK/PRDM12/PRKAR1B/PTGS2/SCN10A/SCN1A/SCN3B/SCN9A/SMR3A/SPX/TAC1/TACR1/TAFA4/UCHL1/ZFHX2	33
cluster5	BP	GO:1900046	regulation of hemostasis	25/3710	72/18903	0.001890216	0.025111841	0.021553832	ADAMTS18/ALOX12/ANO6/CPB2/DMTN/EMILIN1/EMILIN2/F2/F2R/F3/FAP/FOXA2/KNG1/NOS3/PDGFA/PDGFRA/PRKCD/PRKG1/PROS1/SERPINE1/SERPINE2/TBXA2R/TFPI/TSPAN8/VKORC1	25
cluster5	BP	GO:0019932	second-messenger-mediated signaling	83/3710	314/18903	0.001893967	0.025129826	0.021569269	ACKR2/ADCY1/ADCY2/ADCYAP1/ADCYAP1R1/ADORA2B/AGT/AGTR2/AKAP6/ATP1A2/ATP1B1/AZU1/BCAP31/C10orf71/CACNA1C/CAP2/CASQ2/CHP2/CHRM3/DDAH1/DMD/DMTN/EDN2/EDNRB/EGFR/EPHA5/ERBB3/FKBP1B/GLP1R/GNAI1/GRIN1/GRIN2A/GRIN2B/GRIN2D/GRM5/GSK3B/GSTO1/GUCY1A1/GUCY1A2/GUCY2F/HPCA/HRC/HTR2C/INS/KCNC2/KSR2/LACRT/LHCGR/LOC118142757/MYOZ1/MYOZ2/NDNF/NEUROD1/NEUROD2/NFATC4/NOS2/NOS3/NPPC/OR51E2/P2RX2/P2RX3/PDE10A/PDE11A/PDE3A/PDE5A/PEX5L/PPP1R9A/PRKG1/PTGFR/RCAN2/RIT2/RYR2/SAMD14/SELP/SLC8A2/SOX9/SPINK1/TBXA2R/TNNI3/TRPM4/TRPM8/UBE2B/VEGFA	83
cluster5	BP	GO:0032963	collagen metabolic process	34/3710	107/18903	0.001901299	0.025195217	0.021625395	ADAMTS14/ADAMTS2/ADAMTS3/AMELX/BMP4/CCN2/CST3/EMILIN1/F2/F2R/FAP/HDAC2/HIF1A/ITGA2/ITGB1/KLK6/LARP6/MFAP4/MMP10/MMP13/MMP15/MMP16/MMP21/MMP24/MMP8/MRC2/NPPC/P3H3/P3H4/PDGFRB/PRTN3/SERPINB7/TNXB/WNT4	34

cluster5	BP	GO:0030318	melanocyte differentiation	12/3710	26/18903	0.001964905	0.025907083	0.022236399	ADAMTS20/BLOC1S6/CITED1/EDNRB/ENPP1/GLI3/KITLG/MITF/OCA2/OR51E2/SOX10/TYRP1	12
cluster5	BP	GO:0051957	positive regulation of amino acid transport	12/3710	26/18903	0.001964905	0.025907083	0.022236399	ACE2/AGT/AVP/AVPR1B/CLTRN/ITGB1/SLC17A8/SLC38A3/SLC6A1/STXBP1/SYT4/TRH	12
cluster5	BP	GO:0060314	regulation of ryanodine-sensitive calcium-release channel activity	12/3710	26/18903	0.001964905	0.025907083	0.022236399	AKAP6/CASQ2/DMD/FKBP1A/FKBP1B/GSTO1/HRC/JPH1/JPH3/JPH4/JSRP1/TRDN	12
cluster5	BP	GO:1903859	regulation of dendrite extension	12/3710	26/18903	0.001964905	0.025907083	0.022236399	BCL11A/CACNG7/CPNE5/CPNE6/CPNE9/NEDD4L/PRKN/SYT1/SYT2/SYT3/SYT4/UNC13A	12

cluster5	BP	GO:0051339	regulation of lyase activity	26/3710	76/18903	0.001973864	0.025970749	0.022291045	ADCYAP1/ADGRV1/ADORA2B/ADRB3/CACNA1C/CALCA/CAP2/CRHR1/DRD1/DRD2/EDNRA/EDNRB/FTMT/GABBR2/GLP1R/GPR87/GRM2/GRM3/GUCA1C/HPCA/LHCGR/LOC118142757/NOS2/NOS3/NPFFR2/VIPR2	26
cluster5	BP	GO:1990573	potassium ion import across plasma membrane	19/3710	50/18903	0.001974689	0.025970749	0.022291045	ABCC8/ABCC9/ATP1A2/ATP1A3/ATP1B1/HCN4/KCNH2/KCNJ13/KCNJ16/KCNJ18/KCNJ3/KCNJ4/KCNJ5/KCNJ6/KCNJ9/KCNK5/SLC12A5/WNK2/WNK3	19
cluster5	BP	GO:0007616	long-term memory	15/3710	36/18903	0.001998409	0.026249779	0.02253054	ADCY1/ADCY8/ARC/CALB1/CPEB3/DRD2/EHMT2/GRIA1/NFATC4/NPAS4/PTCHD1/RASGRF1/RELN/SHANK1/TACR1	15
cluster5	BP	GO:0045687	positive regulation of glial cell differentiation	17/3710	43/18903	0.002041005	0.026775733	0.022981973	ASPA/BMP2/DAG1/GSX2/HDAC2/HES1/MDK/NKX2-2/NKX6-1/PTN/PTPRZ1/SERPINE2/SHH/TENM4/TP73/TTBK1/ZNF488	17

cluster5	BP	GO:0014068	positive regulation of phosphatidylinositol 3-kinase signaling	27/3710	80/18903	0.00204521	0.026797365	0.02300054	AGT/ANGPT1/EGF/ERBB3/ERBB4/F2/F2R/FGF2/FGFR1/INS/MYOC/NTRK2/PDGFA/PDGFC/PDGFRA/PDGFRB/PLXNB1/RELN/ROR1/ROR2/SELP/SEMA3E/SEMA4D/SERPINA12/SOX9/SRC/UNC5B	27
cluster5	BP	GO:0030049	muscle filament sliding	8/3710	14/18903	0.002102664	0.027379031	0.023499792	MYH2/MYH4/MYH6/MYH7/MYH8/MYL1/TNNT2/TPM1	8
cluster5	BP	GO:0048148	behavioral response to cocaine	8/3710	14/18903	0.002102664	0.027379031	0.023499792	DRD1/DRD2/DRD4/EHMT2/HOMER1/HTR2A/OPRK1/SDK1	8
cluster5	BP	GO:0098814	spontaneous synaptic transmission	8/3710	14/18903	0.002102664	0.027379031	0.023499792	APP/CBLN2/DOC2A/DOC2B/ITGB1/PRKN/RPH3AL/SYT1	8

cluster5	BP	GO:1904321	response to forskolin	8/3710	14/18903	0.002102664	0.027379031	0.023499792	ADCY1/ADCY2/ADCY3/ADCY5/ADCY8/CFTR/EFNA5/GNAI1	8
cluster5	BP	GO:1904322	cellular response to forskolin	8/3710	14/18903	0.002102664	0.027379031	0.023499792	ADCY1/ADCY2/ADCY3/ADCY5/ADCY8/CFTR/EFNA5/GNAI1	8
cluster5	BP	GO:0014821	phasic smooth muscle contraction	11/3710	23/18903	0.00212033	0.027472563	0.023580072	DRD1/DRD2/EDN2/EDNRB/GDNF/GHSR/HTR1D/NEUROG1/P2RX2/P2RX3/SSTR2	11
cluster5	BP	GO:0048169	regulation of long-term neuronal synaptic plasticity	11/3710	23/18903	0.00212033	0.027472563	0.023580072	AGT/APP/CAMK2B/DRD2/GRIK2/GRM5/NEURL1/NPTN/RAB5A/RAB8A/SYP	11

cluster5	BP	GO:0072170	metanephric tubule development	11/3710	23/18903	0.00212033	0.027472563	0.023580072	CALB1/HES1/HES5/LGR4/PAX2/POU3F3/SOX9/WNT4/WNT7B/WNT9B/YAP1	11
cluster5	BP	GO:1903861	positive regulation of dendrite extension	11/3710	23/18903	0.00212033	0.027472563	0.023580072	CACNG7/CPNE5/CPNE6/CPNE9/NEDD4L/PRKN/SYT1/SYT2/SYT3/SYT4/UNC13A	11
cluster5	BP	GO:0050771	negative regulation of axonogenesis	23/3710	65/18903	0.002129386	0.027555828	0.023651539	CDKL3/DAB1/DRAXIN/EFNB3/EPHA7/GDI1/LRP4/MAP2/MT3/NTN1/PTPRS/SEMA3A/SEMA3E/SEMA4D/SEMA5A/SEMA5B/SEMA6A/SEMA6D/SLIT1/THY1/TNR/WNT3A/WNT5A	23
cluster5	BP	GO:0014048	regulation of glutamate secretion	10/3710	20/18903	0.002225287	0.028688882	0.024624055	ADORA1/AVP/AVPR1B/GRM2/GRM7/HRH3/NPY5R/STXBP1/SYT4/TRH	10

cluster5	BP	GO:1902473	regulation of protein localization to synapse	10/3710	20/18903	0.002225287	0.028688882	0.024624055	ADAM10/ARHGAP44/DAG1/GHSR/GPC6/GRIPAP1/IQSEC2/NLGN2/WNT5A/WNT7A	10
cluster5	BP	GO:0001991	regulation of systemic arterial blood pressure by circulatory renin-angiotensin	9/3710	17/18903	0.002238839	0.028688882	0.024624055	ACE/ACE2/AGTR2/CMA1/CTSG/EDNRB/F2R/OR51E2/SUCNR1	9
cluster5	BP	GO:0042481	regulation of odontogenesis	9/3710	17/18903	0.002238839	0.028688882	0.024624055	APCDD1/BMP2/BMP4/DMRT3/NGFR/RSPO2/SHH/SP6/TNFRSF11B	9
cluster5	BP	GO:0048557	embryonic digestive tract morphogenesis	9/3710	17/18903	0.002238839	0.028688882	0.024624055	FGF10/FGFR2/GLI3/HNF1B/PDGFRA/RBPMS2/SHH/SHOX2/SIX2	9

cluster5	BP	GO:0072224	metanephric glomerulus development	9/3710	17/18903	0.002238839	0.028688882	0.024624055	AGTR2/LAMB2/LGR4/LHX1/PAX2/PDGFRA/PDGFRB/RET/WT1	9
cluster5	BP	GO:0090494	dopamine uptake	9/3710	17/18903	0.002238839	0.028688882	0.024624055	DRD1/DRD2/DRD4/GDNF/PRKN/RAB3B/SLC29A4/SLC6A2/SLC6A3	9
cluster5	BP	GO:0097107	postsynaptic density assembly	9/3710	17/18903	0.002238839	0.028688882	0.024624055	CBLN1/GRID2/LRRC4B/LRRTM2/NLGN2/NPTX1/NRXN1/PTPRD/SLITRK3	9
cluster5	BP	GO:0045907	positive regulation of vasoconstriction	14/3710	33/18903	0.002284078	0.029197199	0.02506035	ABL1/ACE/ADRA1A/ADRA1B/ADRA1D/AVP/AVPR1B/DBH/F2R/HTR2A/PTGS2/TACR1/TBXA2R/TRPM4	14

cluster5	BP	GO:0098801	regulation of renal system process	14/3710	33/18903	0.002284078	0.029197199	0.02506035	ACE/ADORA1/AGT/AVP/CORO2B/DRD2/EDNRB/EMP2/F2R/GAS6/OR51E2/SPX/STC1/TACR1	14
cluster5	BP	GO:0031503	protein-containing complex localization	51/3710	178/18903	0.002310762	0.029502323	0.025322243	ADAM10/AKIRIN2/AP3D1/ARC/ARHGAP44/BIRC5/CACNG2/CACNG3/CACNG4/CACNG5/CACNG7/CEP131/DAG1/DBN1/DRD4/DYNC2I2/DZIP1/EFNB2/EXOC3L1/EXOC3L2/EXOC3L4/GHSR/GPC6/GRIP1/GRIP2/GRIPAP1/GSG1L/HPCA/IFT140/IFT81/IQSEC2/ITGB3/KIF5A/KIF5C/KLHL21/LCA5/LGI1/LMNA/NLGN1/NPTN/NPTX1/OPHN1/RAB8A/RELN/SEH1L/SHISA6/SLC1A1/SNAP25/SPAG17/STX7/TUB	51
cluster5	BP	GO:0016331	morphogenesis of embryonic epithelium	45/3710	153/18903	0.002325079	0.029649001	0.025448138	ABL1/BCL10/BMP4/BMP5/BMP7/CC2D2A/CECR2/CELSR1/FGF10/FGFR2/FOLR1/FZD3/GDNF/GREM1/GRHL2/HES5/HIF1A/HNF1B/IRX2/KIF20B/LAMA5/LHX2/LRP2/PAX2/PRICKLE1/PTCH1/PTK7/RET/SALL4/SDC4/SFRP2/SHH/SIX4/SOX9/SULF1/TEAD2/TFAP2A/TGFB1I1/TULP3/VANGL2/WNT2B/WNT4/WNT5A/WNT7B/WNT9B	45
cluster5	BP	GO:0051402	neuron apoptotic process	69/3710	255/18903	0.002334526	0.029733293	0.025520487	ABL1/AGTR2/ANGPT1/APP/ASCL1/ATF4/BARHL1/BDNF/CDK5R1/CHL1/CITED1/CNTFR/CRLF1/DDIT3/DLX1/DRAXIN/EN1/EN2/EPHA7/ERBB3/F2R/FAIM2/GABRA5/GABRB2/GDF5/GDNF/GRID2/GRIK2/GRIK5/GRM4/HIF1A/ISL1/ITGAM/KCNB1/MDK/MT3/NAE1/NDNF/NFATC4/NGF/NGFR/NR4A2/NTF3/NTRK2/PAK3/PLA2G3/POU4F3/PRKCG/PRKN/PTPRZ1/RASA1/ROCK1/SCN2A/SEMA3E/SIX4/SLC1A1/SNCB/STXBP1/TFAP2A/TFAP2B/THRB/TMBIM6/TNFRSF21/TOX3/TRIM2/TYRO3/UNC5B/VSTM2L/WFS1	69

cluster5	BP	GO:0050795	regulation of behavior	25/3710	73/18903	0.002347154	0.029857849	0.025627395	ADORA1/AHI1/CNTNAP4/CRH/DRD2/GHSR/GRP/GRPR/HCRTR2/HDAC2/HTR1D/INS/LEPR/MC4R/MDK/NLGN1/NMU/NPAS2/NPSR1/NPY/OPRK1/PENK/RELN/STRA6/TACR3	25
cluster5	BP	GO:0060997	dendritic spine morphogenesis	21/3710	58/18903	0.00236539	0.030053355	0.0257952	ARC/ARHGAP44/CAMK2B/CDK5R1/CTNND2/DBN1/EPHB3/HDAC6/ITPKA/KIF1A/LZTS3/NGEF/NLGN1/PAK3/PPFIA2/RELN/SHANK1/SRCIN1/TANC2/WNT7A/ZDHHC15	21
cluster5	BP	GO:0034332	adherens junction organization	19/3710	51/18903	0.00257624	0.03264876	0.028022871	ADAM10/BMP6/CDH10/CDH11/CDH12/CDH18/CDH19/CDH22/CDH6/CDH7/CDH8/CDH9/DLG5/EFNB2/FER/INAVA/NUMBL/SRC/VEGFA	19
cluster5	BP	GO:0050982	detection of mechanical stimulus	19/3710	51/18903	0.00257624	0.03264876	0.028022871	ADGRV1/ANO3/ASIC2/ASIC3/CDH2/COL11A1/CSRP3/HPN/HTR2A/ITGA2/LHFPL5/PDZD7/PKD1L2/PKD1L3/PKDREJ/SCN1A/SERPINE2/TMC1/WHRN	19

cluster5	BP	GO:0008037	cell recognition	63/3710	230/18903	0.002579009	0.03264876	0.028022871	ADAM2/ADAM32/ADGRB1/APP/B4GALT1/CADM1/CDK5R1/CLGN/CNTN4/CNTN6/CNTNAP2/COLEC10/COLEC12/CRISP1/CRP/CRTAC1/DSCAM/DSCAML1/EFNB3/EPHA3/EPHB3/FEZF2/FOLR1/FOXG1/GAP43/IGSF9/IZUMO1/JMJD6/LBP/LY6K/MBL2/MEGF10/MYPN/NCAM2/NCK2/NEXN/NPTN/NRCAM/NTM/OPCML/PCDHA7/PCDHB6/PCSK4/PEAR1/PECAM1/PLA2G5/PRSS37/PRSS55/ROBO1/ROBO2/ROBO3/SEMA5A/SFTPA1/SPACA4/TMPRSS12/TNFRSF21/TNN/TUB/TULP1/VSTM2L/YWHAZ/ZP4/ZPBP	63
cluster5	BP	GO:0010464	regulation of mesenchymal cell proliferation	13/3710	30/18903	0.002591464	0.032766856	0.028124235	BMP4/CHRD/FGF9/FGFR2/LMNA/LRP5/PDGFA/SHH/SHOX2/SMO/SOX9/WNT11/WNT5A	13
cluster5	BP	GO:0048678	response to axon injury	29/3710	89/18903	0.002605535	0.032905081	0.028242875	APOD/CDK1/CSPG5/DAG1/DPYSL3/DRD2/EPO/FGF2/FKBP1B/FLRT3/FOLR1/GAP43/ISL1/KCNB1/LAMB2/LYN/NDEL1/NREP/PCSK1/PTN/PTPRF/PTPRS/RTN4RL1/RTN4RL2/SLC1A1/STK24/THY1/TNC/TNR	29
cluster5	BP	GO:0006942	regulation of striated muscle contraction	31/3710	97/18903	0.00266163	0.033573053	0.028816205	ACE2/ADORA1/ADRA1A/ADRA1B/ATP1A2/ATP1B1/CACNA1C/CASQ2/CCN2/CHGA/DMD/DSG2/EHD3/FKBP1B/GATA4/GSTO1/HCN4/HRC/MYH7/PDE5A/PKP2/RYR2/SCN10A/SCN4A/SLC8A3/STC1/TNNI1/TNNI3/TRPM4/TRPV4/ZC3H12A	31

cluster5	BP	GO:0007229	integrin-mediated signaling pathway	35/3710	113/18903	0.002666395	0.033592726	0.02883309	ABL1/ADAM10/ADAM11/ADAMTS13/CCN2/CDH17/COL16A1/DMTN/EMP2/FERMT1/FERMT3/FYB1/FYB2/ILK/ITGA2/ITGA2B/ITGA7/ITGAM/ITGB1/ITGB3/ITGB5/ITGB8/LAMA1/LAMA3/LAMA5/LAMB1/LAMB2/MYH9/NID1/PLPP3/PTN/SLC2A10/SRC/THY1/TIMP1	35
cluster5	BP	GO:0045682	regulation of epidermis development	23/3710	66/18903	0.002670745	0.033607145	0.028845467	ABCA12/ATOH1/BMP4/DLL1/ESRP1/ETV4/EZH2/FGF2/FOXC1/GRHL1/GRHL2/HES1/HES5/IL20/KRT84/MSX2/PTCH1/PTCH2/REG3A/REG3G/ROCK1/ROCK2/ZFP36	23
cluster5	BP	GO:0045214	sarcomere organization	17/3710	44/18903	0.002726007	0.033953576	0.029142812	ACTN2/CSRP2/CSRP3/FHOD3/ITGB1/LDB3/LMOD2/MYH6/MYH7/MYOZ1/MYOZ2/MYPN/PRKAR1A/SIX4/SYNPO2L/TNNT2/TPM1	17
cluster5	BP	GO:0048846	axon extension involved in axon guidance	15/3710	37/18903	0.002758537	0.033953576	0.029142812	DSCAM/NRP2/PLXNA4/SEMA3A/SEMA3E/SEMA4D/SEMA5A/SEMA5B/SEMA6A/SEMA6D/SLIT1/SLIT3/VEGFA/WNT3A/WNT5A	15

cluster5	BP	GO:1902284	neuron projection extension involved in neuron projection guidance	15/3710	37/18903	0.002758537	0.033953576	0.029142812	DSCAM/NRP2/PLXNA4/SEMA3A/SEMA3E/SEMA4D/SEMA5A/SEMA5B/SEMA6A/SEMA6D/SLIT1/SLIT3/VEGFA/WNT3A/WNT5A	15
cluster5	BP	GO:0002016	regulation of blood volume by renin-angiotensin	6/3710	9/18903	0.00276306	0.033953576	0.029142812	ACE/ACE2/AGT/AGTR2/RPS6KA2/TACR1	6
cluster5	BP	GO:0019227	neuronal action potential propagation	6/3710	9/18903	0.00276306	0.033953576	0.029142812	CLDN19/CNTNAP1/FKBP1B/NRCAM/NTRK2/SCN1A	6
cluster5	BP	GO:0021520	spinal cord motor neuron cell fate specification	6/3710	9/18903	0.00276306	0.033953576	0.029142812	GLI3/HOXC10/HOXD10/ISL1/LHX3/MNX1	6

cluster5	BP	GO:0021683	cerebellar granular layer morphogenesis	6/3710	9/18903	0.00276306	0.033953576	0.029142812	CBLN1/GRID2/KNDC1/OPHN1/SERPINE2/WNT7A	6
cluster5	BP	GO:0032253	dense core granule localization	6/3710	9/18903	0.00276306	0.033953576	0.029142812	KIF1A/KIF5A/MAP2/PPFIA2/SYT4/TANC2	6
cluster5	BP	GO:0045607	regulation of inner ear auditory receptor cell differentiation	6/3710	9/18903	0.00276306	0.033953576	0.029142812	ATOH1/DLL1/ESRP1/FGF2/HES1/HES5	6
cluster5	BP	GO:0045631	regulation of mechanoreceptor differentiation	6/3710	9/18903	0.00276306	0.033953576	0.029142812	ATOH1/DLL1/ESRP1/FGF2/HES1/HES5	6

cluster5	BP	GO:0048149	behavioral response to ethanol	6/3710	9/18903	0.00276306	0.033953576	0.029142812	CRHR1/DBH/DRD2/DRD4/EPS8/HDAC2	6
cluster5	BP	GO:0060513	prostatic bud formation	6/3710	9/18903	0.00276306	0.033953576	0.029142812	BMP4/BMP7/FGF10/SHH/SULF1/WNT5A	6
cluster5	BP	GO:0072177	mesonephric duct development	6/3710	9/18903	0.00276306	0.033953576	0.029142812	GPC3/GREB1L/HNF1B/LHX1/WNT11/WNT9B	6
cluster5	BP	GO:0098870	action potential propagation	6/3710	9/18903	0.00276306	0.033953576	0.029142812	CLDN19/CNTNAP1/FKBP1B/NRCAM/NTRK2/SCN1A	6

cluster5	BP	GO:0098953	receptor diffusion trapping	6/3710	9/18903	0.00276306	0.033953576	0.029142812	CACNG2/CACNG3/CACNG4/CACNG5/CACNG7/SHISA6	6
cluster5	BP	GO:0098970	postsynaptic neurotransmitter receptor diffusion trapping	6/3710	9/18903	0.00276306	0.033953576	0.029142812	CACNG2/CACNG3/CACNG4/CACNG5/CACNG7/SHISA6	6
cluster5	BP	GO:0099519	dense core granule cytoskeletal transport	6/3710	9/18903	0.00276306	0.033953576	0.029142812	KIF1A/KIF5A/MAP2/PPFIA2/SYT4/TANC2	6
cluster5	BP	GO:0099628	neurotransmitter receptor diffusion trapping	6/3710	9/18903	0.00276306	0.033953576	0.029142812	CACNG2/CACNG3/CACNG4/CACNG5/CACNG7/SHISA6	6

cluster5	BP	GO:1901950	dense core granule transport	6/3710	9/18903	0.00276306	0.033953576	0.029142812	KIF1A/KIF5A/MAP2/PPFIA2/SYT4/TANC2	6
cluster5	BP	GO:2000980	regulation of inner ear receptor cell differentiation	6/3710	9/18903	0.00276306	0.033953576	0.029142812	ATOH1/DLL1/ESRP1/FGF2/HES1/HES5	6
cluster5	BP	GO:0044089	positive regulation of cellular component biogenesis	123/3710	498/18903	0.002865201	0.035167486	0.030184728	ABL1/ACE2/ADGRB1/ADGRB3/AGT/AJUBA/ANKRD53/ASIC2/AUTS2/BDNF/BMP7/BRK1/CARMIL1/CBLN1/CBLN2/CCN2/CDH17/CDK5R1/CHGA/CLDN1/CLDN19/CLDN5/CLIP1/CLRN1/CLSTN2/CNTNAP2/COL16A1/CRBN/CXCL13/CYFIP1/DAG1/DDX3X/DLG5/DPYSL3/DZIP1/EFNA5/EMILIN1/EPHB3/EPS8/EPS8L3/FER/FERMT1/FHOD1/FLRT2/FLRT3/FNBP1L/FNIP2/G3BP2/GPM6A/GRID2/GSK3B/HAP1/HAS3/HSF1/ID1/IL1RAPL1/IL5/KIRREL1/LMOD2/LRRC4B/LRRN1/LRRTM2/LRRTM3/LRTM2/MNS1/MYO3A/MYO3B/MYOC/NAV3/NCK2/NCKAP1/NDEL1/NEURL1/NLGN1/NLGN2/NLGN3/NPHP4/NPHS1/NRXN1/NTRK2/PFN2/PLPPR5/PPM1E/PTPRD/RAP1B/RICTOR/ROCK1/ROCK2/RP1/SAXO1/SDC1/SDC4/SEMA4D/SLAIN1/SLF1/SLITRK1/SLITRK2/SLITRK3/SLITRK6/SLX4/SNX9/SORBS3/SOX9/SRC/SRPX2/ST8SIA2/SYNDIG1/SYNPO2L/TACR1/TBX5/TENM2/THY1/TOGARAM1/TPBG/TPM1/TPPP/TRABD2B/TTBK1/USP50/VEGFA/WNT11/WNT4/WNT7A	123
cluster5	BP	GO:0001963	synaptic transmission, dopaminergic	12/3710	27/18903	0.002911372	0.035609098	0.03056377	CNTNAP4/CRH/DRD1/DRD2/DRD4/GDNF/PRKN/PTGS2/RAB3B/SLC6A2/SLC6A3/TH	12

cluster5	BP	GO:0021544	subpallium development	12/3710	27/18903	0.002911372	0.035609098	0.03056377	ASCL1/BBS1/CNTNAP2/DLX1/DLX2/DRD1/DRD2/GLI3/GSX2/HPRT1/RARB/SECISBP2	12
cluster5	BP	GO:0051953	negative regulation of amine transport	12/3710	27/18903	0.002911372	0.035609098	0.03056377	ADORA1/ADRA2A/CHGA/CRH/DRD2/GHSR/GRM7/HRH3/NPY5R/RGS4/SYT4/TRH	12
cluster5	BP	GO:0055117	regulation of cardiac muscle contraction	26/3710	78/18903	0.002978826	0.036391671	0.031235462	ACE2/ADORA1/ADRA1A/ADRA1B/ATP1A2/ATP1B1/CACNA1C/CASQ2/CCN2/CHGA/DMD/DSG2/EHD3/FKBP1B/GATA4/GSTO1/HCN4/HRC/PDE5A/PKP2/RYR2/SCN10A/STC1/TNNI3/TRPM4/ZC3H12A	26
cluster5	BP	GO:0035773	insulin secretion involved in cellular response to glucose stimulus	21/3710	59/18903	0.003003845	0.036611981	0.031424558	ABCA12/ADCY5/ADCY8/ADRA2A/BRSK2/CFTR/CLTRN/EFNA5/EPHA5/FKBP1B/FOXA2/GCG/GPLD1/HIF1A/LRP5/NR1H4/RAB11FIP2/RFX6/SLC9B2/TRPM4/VSNL1	21

cluster5	BP	GO:0061098	positive regulation of protein tyrosine kinase activity	21/3710	59/18903	0.003003845	0.036611981	0.031424558	ACE/ADORA1/ADRA1A/ADRA2A/AGT/ALK/ALKAL2/BTC/EGF/EGFR/ERBB3/ERBB4/GAS6/GPRC5B/GREM1/GRM5/NEURL1/RELN/SRC/SRCIN1/WNT3A	21
cluster5	BP	GO:0070374	positive regulation of ERK1 and ERK2 cascade	61/3710	223/18903	0.003084379	0.037549894	0.032229581	ABL1/ADCYAP1/ADRA1A/ALKAL2/ANGPT1/APELA/APP/ARHGAP8/BMP2/BMP4/BMPER/CALCR/CAVIN3/CCL22/CCL25/CCN2/CHRNA7/CXCL17/DENND2B/DRD2/EGFR/EPO/ERBB4/F2R/FGF10/FGF19/FGF2/FGF23/FGF4/FGFR2/FGFR3/FGFR4/FLT4/GAREM1/GAS6/GATA4/GCG/HCRTR1/HTR2A/HTR2C/ICAM1/ITGB3/MT3/NDRG4/NOTCH2/NPSR1/NPTN/NPY/NPY5R/OR2AT4/PDGFA/PDGFC/PDGFRA/PDGFRB/PLA2G5/RAP1B/SRC/THPO/TNFRSF11A/TPBG/TRPV4	61
cluster5	BP	GO:0002793	positive regulation of peptide secretion	30/3710	94/18903	0.003160786	0.038435455	0.03298967	ADCY8/ADCYAP1/ADORA1/CFTR/CRH/DOC2B/DRD2/GCG/GCK/GPLD1/GRP/HFE/HIF1A/INS/ISL1/KISS1/NKX6-1/NLGN2/NNAT/NR0B2/NR1H4/RASL10B/RFX6/RPH3AL/SLC30A8/SPINK1/TRH/TRPM4/UCN3/VSNL1	30
cluster5	BP	GO:0090313	regulation of protein targeting to membrane	14/3710	34/18903	0.0031901	0.038746962	0.033257041	ANK3/CDK5R1/CEMIP/DMTN/ERBB2/GDI1/HPCA/ITGAM/KCNB1/MYO1C/SLC1A1/STOM/TCAF1/USP17L2	14

cluster5	BP	GO:0120178	steroid hormone biosynthetic process	16/3710	41/18903	0.003196681	0.038781954	0.033287075	BMP2/BMP5/BMP6/CLCN2/CYP11B1/CYP11B2/CYP21A2/DAB2/DHCR7/DKK3/HSD17B12/HSD17B2/HSD3B2/RDH8/SRD5A2/WNT4	16
cluster5	BP	GO:0044782	cilium organization	99/3710	391/18903	0.00321434	0.038951109	0.033432262	ABLIM3/AHI1/AKAP4/BBOF1/BBS1/CC2D2A/CCDC103/CCDC40/CCNO/CDC14C/CELSR2/CEP131/CFAP126/CFAP206/CFAP221/CFAP43/CFAP46/CFAP47/CFAP52/CFAP54/CFAP61/CFAP65/CFAP69/CFAP74/CFAP91/CFAP97D1/CIBAR1/CIBAR2/CLCN4/DAW1/DCX/DNAAF6/DNAAF8/DNAH2/DNAH5/DNAH7/DNAI1/DNAI3/DRC1/DYNC2I2/DZIP1/EHD3/ENO4/FAM161A/FNBP1L/FOXJ1/FSIP2/GK2/GMNC/GSK3B/HAP1/HDAC6/HOATZ/HYDIN/IFT140/IFT81/IQUB/KIF24/KIF3A/LAMA5/LCA5/MCIDAS/MNS1/NEK1/NEURL1/NME5/ODAD2/ODAD3/PIBF1/PIFO/PKHD1/PLA2G3/RAB8A/RFX3/RFX4/ROPN1/RP1/RSPH1/RSPH6A/RSPH9/SAXO1/SPAG17/SPAG6/SPEF1/SYNE2/TBC1D32/TEKT1/TEKT2/TEKT5/TMEM17/TMEM231/TOGARAM1/TTC29/TTC36/TUB/UBE2B/VANGL2/WDR90/YAP1	99
cluster5	BP	GO:0021884	forebrain neuron development	11/3710	24/18903	0.003226601	0.039009498	0.033482379	DCLK2/FGFR2/FOXG1/GBX2/NDNF/NHLH2/NRP2/PLXNA1/SECISBP2/SEMA3E/SOX1	11
cluster5	BP	GO:0120255	olefinic compound biosynthetic process	11/3710	24/18903	0.003226601	0.039009498	0.033482379	BMP2/BMP5/BMP6/CLCN2/CYP11B1/CYP11B2/DAB2/DKK3/DKKL1/SRD5A2/WNT4	11

cluster5	BP	GO:0010543	regulation of platelet activation	19/3710	52/18903	0.003323891	0.040047158	0.034373016	ADAMTS18/ALOX12/CTSG/DMTN/EMILIN2/F2/GP1BB/GP6/GP9/LYN/MMRN1/NOS3/PDGFA/PDGFRA/PDPN/PRKCD/PRKG1/SELP/SERPINE2	19
cluster5	BP	GO:0048013	ephrin receptor signaling pathway	19/3710	52/18903	0.003323891	0.040047158	0.034373016	CDK5R1/EFNA2/EFNA3/EFNA5/EFNB2/EFNB3/EPHA10/EPHA3/EPHA5/EPHA6/EPHA7/EPHA8/EPHB3/LYN/NCK2/NGEF/PAK3/RASA1/SRC	19
cluster5	BP	GO:0097479	synaptic vesicle localization	19/3710	52/18903	0.003323891	0.040047158	0.034373016	AP3B2/AP3D1/BLOC1S6/BRSK1/BRSK2/CDH2/DNM1/KIF5A/KIF5C/MAP2/NLGN1/NLGN2/PCLO/PRKN/SNAP91/SYN1/SYN2/SYN3/SYNDIG1	19
cluster5	BP	GO:0007127	meiosis I	39/3710	131/18903	0.003466377	0.041321376	0.035466695	ANKLE1/ANKRD31/BRCA2/BRME1/C14orf39/CCNB2/CCNE2/DDX4/DMC1/DMRT1/DMRTC2/EHMT2/ESPL1/FMN2/HFM1/HSF2BP/HSPA2/MAEL/MEIOB/MLH3/MND1/MOV10L1/MRE11/MSH5/P3H4/PLK1/RAD50/RPL10L/SGO1/SLC25A31/SLX4/SPO11/SYCP1/TERB1/TEX15/TEX19/TOP2A/UBE2B/WEE2	39

cluster5	BP	GO:0007368	determination of left/right symmetry	39/3710	131/18903	0.003466377	0.041321376	0.035466695	AHI1/CC2D2A/CCDC103/CCDC40/CFAP52/CFC1/CFC1B/DAW1/DLL1/DNAH11/DNAH5/DNAI1/DRC1/ENKUR/FGF10/FOLR1/FOXJ1/FOXN4/GATA4/HIF1A/IFT140/LBX1/LEFTY1/MMP21/NDRG4/NOTCH2/ODAD2/ODAD3/RFX3/SHH/SMO/SOX18/TBC1D32/TBX20/TDGF1/VANGL2/WNT3A/WNT5A/ZIC3	39
cluster5	BP	GO:0003163	sinoatrial node development	7/3710	12/18903	0.003476956	0.041321376	0.035466695	BMP4/BVES/CACNA1G/HCN4/ISL1/SHOX2/TBX5	7
cluster5	BP	GO:0010749	regulation of nitric oxide mediated signal transduction	7/3710	12/18903	0.003476956	0.041321376	0.035466695	EGFR/GUCY1A1/GUCY1A2/INS/PDE5A/SPINK1/VEGFA	7
cluster5	BP	GO:0030432	peristalsis	7/3710	12/18903	0.003476956	0.041321376	0.035466695	DRD1/DRD2/GDNF/NEUROG1/P2RX2/P2RX3/SSTR2	7

cluster5	BP	GO:0040034	regulation of development, heterochronic	7/3710	12/18903	0.003476956	0.041321376	0.035466695	ASCL1/DLL1/FGF9/HES1/NR2E1/PAX6/SERPINE2	7
cluster5	BP	GO:0046549	retinal cone cell development	7/3710	12/18903	0.003476956	0.041321376	0.035466695	CRB2/HCN1/RORB/RP1/THRB/THY1/USH1C	7
cluster5	BP	GO:0048521	negative regulation of behavior	7/3710	12/18903	0.003476956	0.041321376	0.035466695	ADORA1/CRH/DRD2/GHSR/INS/MC4R/NPSR1	7
cluster5	BP	GO:0099010	modification of postsynaptic structure	7/3710	12/18903	0.003476956	0.041321376	0.035466695	ARHGAP44/CAMKV/CTTNBP2/CYFIP1/PFN2/WASF1/WASF3	7

cluster5	BP	GO:0099149	regulation of postsynaptic neurotransmitter receptor internalization	7/3710	12/18903	0.003476956	0.041321376	0.035466695	ARC/DRD4/EFNB2/GSG1L/HPCA/ITGB3/OPHN1	7
cluster5	BP	GO:0099150	regulation of postsynaptic specialization assembly	7/3710	12/18903	0.003476956	0.041321376	0.035466695	CBLN1/GAP43/GRID2/LRRC4B/LRRTM2/NPTX1/PTPRD	7
cluster5	BP	GO:1904338	regulation of dopaminergic neuron differentiation	7/3710	12/18903	0.003476956	0.041321376	0.035466695	CSNK1E/FERD3L/FOXA1/GSK3B/SFRP2/SHH/WNT3A	7
cluster5	BP	GO:0007413	axonal fasciculation	10/3710	21/18903	0.003507205	0.041352665	0.03549355	CDK5R1/CNTN4/CRTAC1/EPHA3/EPHB3/FEZF2/NCAM2/NRCAM/SEMA5A/TNFRSF21	10

cluster5	BP	GO:0010002	cardioblast differentiation	10/3710	21/18903	0.003507205	0.041352665	0.03549355	EEF1AKMT4-ECE2/GATA4/GREM1/ISL1/ITGB1/MYOCD/PRICKLE1/TBX5/TBXT/WNT3A	10
cluster5	BP	GO:0030502	negative regulation of bone mineralization	10/3710	21/18903	0.003507205	0.041352665	0.03549355	ECM1/ENPP1/FGF23/GREM1/HIF1A/PTH/RFLNA/SOX9/TRPM4/ZNF664-RFLNA	10
cluster5	BP	GO:0060445	branching involved in salivary gland morphogenesis	10/3710	21/18903	0.003507205	0.041352665	0.03549355	BMP7/DAG1/FGF10/FGF7/FGFR2/LAMA1/LAMA5/PDGFA/PLXNA1/SHH	10
cluster5	BP	GO:0099500	vesicle fusion to plasma membrane	10/3710	21/18903	0.003507205	0.041352665	0.03549355	GRIK5/PRRT2/SNAP23/SNAP25/STX11/STX1A/STXBP1/SYT1/SYT9/TRARG1	10

cluster5	BP	GO:0106030	neuron projection fasciculation	10/3710	21/18903	0.003507205	0.041352665	0.03549355	CDK5R1/CNTN4/CRTAC1/EPHA3/EPHB3/FEZF2/NCAM2/NRCAM/SEMA5A/TNFRSF21	10
cluster5	BP	GO:2000047	regulation of cell-cell adhesion mediated by cadherin	10/3710	21/18903	0.003507205	0.041352665	0.03549355	BMP6/EPCAM/FOXA1/FOXA2/NEXMIF/NOTCH4/PTPRU/VEGFA/WNT3A/WNT5A	10
cluster5	BP	GO:0001704	formation of primary germ layer	38/3710	127/18903	0.003524561	0.041510617	0.035629123	AHDC1/APELA/BMP4/BMP7/COL11A1/COL12A1/COL6A1/COL8A1/CRB2/DUSP4/FGFR2/FOXC1/HMGA2/HNF1B/HOXA11/HSBP1/ITGA2/ITGA7/ITGB1/ITGB3/ITGB5/LAMA3/LAMB1/LHX1/MMP15/MMP8/NR0B1/PAX2/PRKAR1A/SFRP2/SIX2/TAF10/TBX20/TBXT/TLX2/WNT11/WNT3A/WNT5A	38
cluster5	BP	GO:0022612	gland morphogenesis	37/3710	123/18903	0.003578573	0.041997441	0.036046971	BMP4/BMP7/BSX/CPB2/DAG1/EGFR/FGF10/FGF7/FGFR2/FOXA1/GLI3/HOXB13/HOXD13/HPN/ID4/LAMA1/LAMA5/LRP5/MDK/MSX2/NOTCH2/NRG3/NTN1/PAX6/PDGFA/PLXNA1/PTCH1/PTN/SHH/SOSTDC1/SOX9/SRC/SULF1/TNC/WNT3A/WNT4/WNT5A	37

cluster5	BP	GO:0006858	extracellular transport	17/3710	45/18903	0.00359165	0.041997441	0.036046971	CABYR/CCDC103/CCDC40/CFAP221/CFAP43/CFAP54/DAW1/DNAH11/DNAH5/DNAH9/DNAI1/NEK10/NME5/ODAD3/RFX3/SPAG17/SPAG6	17
cluster5	BP	GO:0010712	regulation of collagen metabolic process	17/3710	45/18903	0.00359165	0.041997441	0.036046971	AMELX/BMP4/CCN2/CST3/EMILIN1/F2/F2R/FAP/HDAC2/ITGA2/ITGB1/LARP6/MFAP4/NPPC/PDGFRB/SERPINB7/WNT4	17
cluster5	BP	GO:0019098	reproductive behavior	17/3710	45/18903	0.00359165	0.041997441	0.036046971	APP/AVP/BRINP1/DBH/DRD1/EDNRB/HDAC2/MAPK8IP2/MTNR1A/NHLH2/NPAS1/OPRK1/SERPINE2/TAC1/TACR1/TH/THRB	17
cluster5	BP	GO:0021575	hindbrain morphogenesis	17/3710	45/18903	0.00359165	0.041997441	0.036046971	ABL1/CBLN1/DAB1/DLL1/FAIM2/GLI2/GRID2/GSX2/HES1/KNDC1/LHX1/LHX5/OPHN1/SERPINE2/SMO/WHRN/WNT7A	17

cluster5	BP	GO:0030517	negative regulation of axon extension	17/3710	45/18903	0.00359165	0.041997441	0.036046971	CDKL3/DRAXIN/MAP2/MT3/NTN1/PTPRS/SEMA3A/SEMA3E/SEMA4D/SEMA5A/SEMA5B/SEMA6A/SEMA6D/SLIT1/TNR/WNT3A/WNT5A	17
cluster5	BP	GO:0022604	regulation of cell morphogenesis	81/3710	312/18903	0.003593942	0.041997441	0.036046971	ABL1/ARC/ARHGAP18/BCL11A/BRWD3/BVES/CACNG7/CAMK2B/CARMIL1/CDC42SE2/CDKL3/CLDN4/CPNE5/CPNE6/CPNE9/CSPG5/CUL7/CUX1/CYFIP1/DAB2/DAG1/DBN1/DMTN/EFNA5/EPS8/F2/FAM171A1/FBLIM1/FBLN1/FGD1/GRIP1/HAS2/HPN/IL1RAPL1/ILK/ITGA7/ITGB3/ITPKA/MDK/MFSD2A/MYADM/MYH10/MYH9/MYL12A/MYOC/NEDD4L/NTNG1/NTNG2/PAK3/PDPN/PHIP/PKP2/PLEKHO1/PLXNA1/PLXNA2/PLXNA4/PLXNB1/PLXNB3/PRAG1/PRKN/PTPRD/RASA1/RELN/RHOBTB1/RHOD/RND2/RND3/SEMA3E/SEMA4D/SRC/STRIP2/SYT1/SYT2/SYT3/SYT4/TPM1/UNC13A/VEGFA/WASF3/WNT5A/ZNF135	81
cluster5	BP	GO:0090092	regulation of transmembrane receptor protein serine/threonine kinase signaling pathway	76/3710	290/18903	0.003625864	0.042323276	0.036326639	ABL1/ADAMTSL2/ASPN/BMP2/BMP4/BMP5/BMP6/BMP7/BMPER/CDKN2B/CHRD/CHRDL1/CHRDL2/CITED1/CRB2/DAB2/DACT2/DKK3/DLX1/DMRT1/EMILIN1/FERMT1/FGF10/FGF9/FKBP1A/FOLR1/GATA4/GDF1/GDF10/GDF5/GDF6/GIPC1/GPC3/GREM1/GREM2/HDAC2/HES1/HES5/HFE/IGSF1/IL17RD/ILK/INHA/INHBC/LEFTY1/LEFTY2/LOX/LRP2/LTBP1/MAGI2/MSX2/MYOCD/NKX2-1/NOTCH2/NREP/PMEPA1/RASL11B/RBPMS2/RNF111/SFRP2/SHH/SKOR2/SLC2A10/SOSTDC1/SPRY1/SULF1/TBX20/TFAP2B/TGFB1I1/TOB1/VASN/VEPH1/VWC2/VWC2L/WFIKKN2/WNT5A	76
cluster5	BP	GO:0045987	positive regulation of smooth muscle contraction	13/3710	31/18903	0.003670775	0.042704844	0.036654144	ADRA1A/CHRM3/EDN2/F2R/GHSR/ITGA2/MYOCD/NMU/PTGS2/SPX/TACR1/TACR3/TBXA2R	13

cluster5	BP	GO:0061437	renal system vasculature development	13/3710	31/18903	0.003670775	0.042704844	0.036654144	ANGPT1/ANGPT2/BMP4/BMP7/EDNRA/HES1/ITGB3/NOTCH2/PDGFRA/PDGFRB/PECAM1/SERPINB7/WT1	13
cluster5	BP	GO:0061440	kidney vasculature development	13/3710	31/18903	0.003670775	0.042704844	0.036654144	ANGPT1/ANGPT2/BMP4/BMP7/EDNRA/HES1/ITGB3/NOTCH2/PDGFRA/PDGFRB/PECAM1/SERPINB7/WT1	13
cluster5	BP	GO:0003128	heart field specification	9/3710	18/18903	0.003703234	0.042987041	0.036896358	BMP2/BMP4/ISL1/LRP2/ROBO1/ROBO2/WNT11/WNT3A/WNT5A	9
cluster5	BP	GO:0090493	catecholamine uptake	9/3710	18/18903	0.003703234	0.042987041	0.036896358	DRD1/DRD2/DRD4/GDNF/PRKN/RAB3B/SLC29A4/SLC6A2/SLC6A3	9

cluster5	BP	GO:0044065	regulation of respiratory system process	8/3710	15/18903	0.003734269	0.043203756	0.037082367	ADORA1/ATP1A2/GLRA1/GSX2/NLGN2/NLGN3/PHOX2B/TLX3	8
cluster5	BP	GO:0072075	metanephric mesenchyme development	8/3710	15/18903	0.003734269	0.043203756	0.037082367	BASP1/BMP7/PAX2/PDGFRB/SHH/SIX4/WNT4/WT1	8
cluster5	BP	GO:0072148	epithelial cell fate commitment	8/3710	15/18903	0.003734269	0.043203756	0.037082367	ARX/DLL1/NEUROD1/NKX2-2/NR2F2/PDPN/SOSTDC1/SPDEF	8
cluster5	BP	GO:0030901	midbrain development	29/3710	91/18903	0.00375498	0.043316031	0.037178734	BARHL1/BASP1/CMA1/CSNK1E/DLG5/EN1/EN2/FGF2/FGF9/FGFR2/FZD3/HES1/INA/LMX1A/LRP6/MAOB/NR4A2/OTX1/OTX2/PADI2/PLP1/SFRP2/SHH/SIRT2/SYNGR3/TTBK1/WNT3A/WNT5A/WNT9B	29

cluster5	BP	GO:0106027	neuron projection organization	29/3710	91/18903	0.00375498	0.043316031	0.037178734	APP/ARC/ARHGAP44/ATP1A3/CAMK2B/CDK5R1/CHRNA7/CTNND2/DBN1/EPHB3/GRIN2B/GSK3B/HDAC6/HOMER1/INS/ITPKA/KIF1A/LZTS3/MAP1A/NGEF/NLGN1/PAK3/PPFIA2/RELN/SHANK1/SRCIN1/TANC2/WNT7A/ZDHHC15	29
cluster5	BP	GO:0070167	regulation of biomineral tissue development	32/3710	103/18903	0.003757645	0.043316031	0.037178734	ADGRV1/AMELX/ANO6/ASPN/ATF4/BMP2/BMP4/BMP6/BMP7/BMPR1B/CFTR/ECM1/ENPP1/FGF23/GAS6/GPM6B/GREM1/HIF1A/NELL1/NOS3/OMD/PKDCC/PTH/PTN/RFLNA/ROCK1/ROCK2/SOX9/TFAP2A/TRPM4/WNT4/ZNF664-RFLNA	32
cluster5	BP	GO:0030177	positive regulation of Wnt signaling pathway	42/3710	144/18903	0.003760503	0.043316031	0.037178734	ABL1/ASPM/BMP2/CRBN/CSNK1E/DAB2/DDX3X/DEPDC1B/DLX5/EGF/EGFR/FGF10/FGF2/FGF9/FGFR2/GPC3/GPRC5B/ILK/LBX2/LGR4/LGR5/LYPD6/PTK7/RSPO1/RSPO2/RSPO3/RSPO4/SALL1/SCEL/SEMA5A/SFRP2/SHH/SRC/SULF1/SULF2/TMEM198/TRPM4/UBE2B/WNK2/WNT3A/WNT5A/YAP1	42
cluster5	BP	GO:1901379	regulation of potassium ion transmembrane transport	31/3710	99/18903	0.003768352	0.043358795	0.037215439	ABCC8/ABCC9/ACTN2/AKAP6/ANK3/ANO6/ATP1B1/CASQ2/CRBN/DPP10/DPP6/FHL1/GRP/ITGB1/KCNC1/KCNC2/KCNE4/KCNH2/KCNIP1/KCNIP3/KCNK3/KCNN2/KCNRG/KCNS2/LRRC55/NEDD4L/OPRK1/RGS4/RGS7/WNK2/WNK3	31

cluster5	BP	GO:1903046	meiotic cell cycle process	58/3710	212/18903	0.003818823	0.04389134	0.03767253	ANKLE1/ANKRD31/ASPM/ATRX/BRCA2/BRME1/C14orf39/CCNB2/CCNE2/DDX4/DMC1/DMRT1/DMRTC2/EDNRA/EHMT2/ESPL1/FBXO43/FMN2/HFM1/HORMAD2/HSF2BP/HSPA2/HUS1B/KIF18A/MAEL/MEIOB/MLH3/MND1/MOV10L1/MRE11/MSH5/MSX2/MYH9/NPM2/OOEP/OVOL1/P3H4/PDE3A/PLCB1/PLK1/RAD50/RPL10L/RPS6KA2/SGO1/SIRT2/SLC25A31/SLX4/SPO11/SYCP1/TERB1/TEX15/TEX19/TOP2A/UBE2B/USP17L2/WEE2/WNT4/WNT5A	58
cluster5	BP	GO:0035094	response to nicotine	18/3710	49/18903	0.003940633	0.045192251	0.038789119	ATP1A2/AVP/CHRNA2/CHRNA3/CHRNA4/CHRNA7/CHRNB4/DRD2/GRM2/HDAC2/HOMER1/IL13/LYPD1/NKX6-1/PENK/SLC6A3/TACR1/TH	18
cluster5	BP	GO:0055081	anion homeostasis	18/3710	49/18903	0.003940633	0.045192251	0.038789119	ATP6V1B1/CA12/CPS1/ENPP1/FGF23/FGFR4/GCM2/GRM2/PTH/SLC12A5/SLC12A9/SLC17A6/SLC17A7/SLC17A8/SLC1A1/SLC34A1/SLC34A2/TFAP2B	18
cluster5	BP	GO:0030199	collagen fibril organization	22/3710	64/18903	0.003958685	0.045300152	0.038881732	ADAMTS14/ADAMTS2/ADAMTS3/ADAMTS7/CHADL/COL11A1/COL12A1/COL14A1/COL2A1/COL5A3/EMILIN1/FKBP10/FOXC1/GREM1/LOX/LOXL4/OPTC/P3H4/PXDN/SFRP2/TLL2/TNXB	22

cluster5	BP	GO:0098930	axonal transport	22/3710	64/18903	0.003958685	0.045300152	0.038881732	AGBL4/AP3B2/AP3D1/ARMCX3/BLOC1S6/FEZ1/HAP1/HDAC6/HIF1A/HSBP1/KIF1A/KIF3A/KIF5A/KIF5C/MAP1A/MAP2/NDEL1/RAB27B/SYBU/TMEM108/TRAK1/UCHL1	22
cluster5	BP	GO:0042446	hormone biosynthetic process	23/3710	68/18903	0.004105895	0.046933466	0.040283628	BMP2/BMP5/BMP6/CHST8/CHST9/CLCN2/CYP11B1/CYP11B2/CYP21A2/DAB2/DHCR7/DKK3/DUOX2/HFE/HIF1A/HSD17B12/HSD17B2/HSD3B2/LHCGR/RDH8/SRD5A2/STC2/WNT4	23
cluster5	BP	GO:0014072	response to isoquinoline alkaloid	12/3710	28/18903	0.004195634	0.047750962	0.040985295	ADCY8/DRD2/FOSB/GHR/OPRK1/PCSK1/PENK/PRKCG/RGS4/SLC1A1/TACR1/TACR3	12
cluster5	BP	GO:0035116	embryonic hindlimb morphogenesis	12/3710	28/18903	0.004195634	0.047750962	0.040985295	ALX4/BMP4/FGF4/GPC3/MSX2/PITX1/RARB/RSPO2/SHH/TBX4/WNT7A/ZBTB16	12

cluster5	BP	GO:0035640	exploration behavior	12/3710	28/18903	0.004195634	0.047750962	0.040985295	AGTR2/ATP1A2/BRINP1/CHL1/CRBN/CRH/EIF4A3/GAD1/JPH3/NLGN2/PENK/TNR	12
cluster5	BP	GO:0043278	response to morphine	12/3710	28/18903	0.004195634	0.047750962	0.040985295	ADCY8/DRD2/FOSB/GHR/OPRK1/PCSK1/PENK/PRKCG/RGS4/SLC1A1/TACR1/TACR3	12
cluster5	BP	GO:0045600	positive regulation of fat cell differentiation	24/3710	72/18903	0.004224616	0.047781958	0.041011899	ADIG/AKT1/BMP2/BMP7/CCDC3/CDS1/DKKL1/FNDC5/FRZB/HTR2A/HTR2C/INS/LMO3/LRP5/PTGS2/SFRP2/SULT1E1/TRPM4/VSTM2A/WIF1/ZBTB16/ZBTB7C/ZC3H12A/ZFP36	24
cluster5	BP	GO:0007632	visual behavior	19/3710	53/18903	0.004243469	0.047781958	0.041011899	ABCC8/ADAM2/APP/ATP1A2/B4GALT2/DBH/DRD1/DRD2/GRIN1/GRIN2A/HIF1A/ITGB1/NDRG4/NPHP4/NPTN/OPN4/PDE1B/SLC1A2/TTC36	19

cluster5	BP	GO:0007442	hindgut morphogenesis	5/3710	7/18903	0.004275852	0.047781958	0.041011899	GLI2/GLI3/HOXD13/SHH/WNT5A	5
cluster5	BP	GO:0021521	ventral spinal cord interneuron specification	5/3710	7/18903	0.004275852	0.047781958	0.041011899	DMRT3/GLI2/GLI3/LHX3/SOX1	5
cluster5	BP	GO:0021684	cerebellar granular layer formation	5/3710	7/18903	0.004275852	0.047781958	0.041011899	CBLN1/GRID2/KNDC1/OPHN1/WNT7A	5
cluster5	BP	GO:0021707	cerebellar granule cell differentiation	5/3710	7/18903	0.004275852	0.047781958	0.041011899	CBLN1/GRID2/KNDC1/OPHN1/WNT7A	5

cluster5	BP	GO:0021798	forebrain dorsal/ventral pattern formation	5/3710	7/18903	0.004275852	0.047781958	0.041011899	GLI3/GSX2/NKX2-1/PAX6/SIX3	5
cluster5	BP	GO:0021877	forebrain neuron fate commitment	5/3710	7/18903	0.004275852	0.047781958	0.041011899	ASCL1/FEZF2/NKX2-1/PAX6/TBR1	5
cluster5	BP	GO:0033564	anterior/posterior axon guidance	5/3710	7/18903	0.004275852	0.047781958	0.041011899	LHX1/LHX9/NTN1/UNC5B/UNC5C	5
cluster5	BP	GO:0050955	thermoception	5/3710	7/18903	0.004275852	0.047781958	0.041011899	ADRA2A/GRIK2/OPN4/RHO/TRPM8	5

cluster5	BP	GO:0051964	negative regulation of synapse assembly	5/3710	7/18903	0.004275852	0.047781958	0.041011899	CBLN1/EPHA7/ROBO2/SLIT1/WNT5A	5
cluster5	BP	GO:0060449	bud elongation involved in lung branching	5/3710	7/18903	0.004275852	0.047781958	0.041011899	BMP4/FGF10/FGFR2/SPRY1/YAP1	5
cluster5	BP	GO:0060573	cell fate specification involved in pattern specification	5/3710	7/18903	0.004275852	0.047781958	0.041011899	DMRT3/GLI2/GLI3/LHX3/SOX1	5
cluster5	BP	GO:0060592	mammary gland formation	5/3710	7/18903	0.004275852	0.047781958	0.041011899	BMP4/FGF10/FGFR2/GLI3/NRG3	5

cluster5	BP	GO:0060638	mesenchymal-epithelial cell signaling	5/3710	7/18903	0.004275852	0.047781958	0.041011899	FGF10/FGF7/TNC/WNT2B/WNT5A	5
cluster5	BP	GO:1903367	positive regulation of fear response	5/3710	7/18903	0.004275852	0.047781958	0.041011899	GRP/GRPR/NPAS2/PENK/PRKAR1B	5
cluster5	BP	GO:1990384	hyaloid vascular plexus regression	5/3710	7/18903	0.004275852	0.047781958	0.041011899	DRD2/OPN4/SLC17A6/SLC6A3/TH	5
cluster5	BP	GO:0035633	maintenance of blood-brain barrier	14/3710	35/18903	0.004369663	0.048726389	0.041822517	ABCC8/ANGPT1/CLDN1/CLDN5/DMD/ITGB1/MFSD2A/PECAM1/PTGS2/SH3GL2/SLC1A1/TJP2/VEGFA/WNK3	14

cluster5	BP	GO:0060914	heart formation	14/3710	35/18903	0.004369663	0.048726389	0.041822517	BMP2/BMP4/EMP2/FOLR1/HES1/ISL1/LRP2/RBM20/ROBO1/ROBO2/TBX5/WNT11/WNT3A/WNT5A	14
cluster5	BP	GO:0034754	cellular hormone metabolic process	41/3710	141/18903	0.004412746	0.049102341	0.042145202	ADH1A/ADH1C/ADH7/BCO1/BMP2/BMP5/BMP6/CEL/CLCN2/CRABP1/CRABP2/CYP11B1/CYP11B2/CYP21A2/CYP26A1/CYP26C1/CYP27C1/CYP2W1/CYP46A1/DAB2/DKK3/ECE1/EDNRB/HSD17B12/HSD17B2/HSD3B2/LRAT/PDGFRA/RBP1/RDH8/RPE65/SCPEP1/SHH/SRD5A2/SULT1E1/TIPARP/TTR/UGT1A3/UGT1A8/UGT2B7/WNT4	41
cluster5	BP	GO:0090090	negative regulation of canonical Wnt signaling pathway	41/3710	141/18903	0.004412746	0.049102341	0.042145202	APC2/BMP2/CDH2/CTNND1/DAB2/DACT3/DDIT3/DKK3/DKKL1/DRAXIN/FERMT1/FRZB/GLI3/GPC3/GREM1/GSK3B/IGFBP2/IGFBP4/ISL1/LRP4/MCC/MDK/NKD2/NPHP4/PRICKLE1/PRKN/PTPRU/RBX1/SFRP2/SHH/SHISA6/SOSTDC1/SOX10/SOX9/TLE1/TMEM170B/TNN/TPBG/WNT11/WNT5A/ZNRF3	41
cluster5	BP	GO:0008589	regulation of smoothened signaling pathway	27/3710	84/18903	0.00443405	0.049287081	0.042303767	CIBAR1/DLG5/ENPP1/EVC/FGF10/FGF9/FGFR2/FOXA1/GLI3/GPC3/GPR161/HHIP/IFT140/IFT81/KIF7/OTX2/PTCH1/PTCH2/RFX4/SCUBE1/SERPINE2/SHH/SHOX2/SMO/TEDC2/TULP3/ZIC1	27

cluster5	BP	GO:0002028	regulation of sodium ion transport	29/3710	92/18903	0.00447432	0.049629443	0.042597621	AKT1/ANK3/ATP1A2/ATP1B1/CHP2/CNKSR3/DMD/DRD2/DRD4/FGF14/FXYD3/FXYD6/GRP/NEDD4L/NKAIN1/NKAIN4/NOS3/PER1/PKP2/PRSS8/PTPN3/SCN2B/SCN3B/SERPINE2/SIK1/SPTBN4/STOM/WNK2/WNK3	29
cluster5	BP	GO:0090277	positive regulation of peptide hormone secretion	29/3710	92/18903	0.00447432	0.049629443	0.042597621	ADCY8/ADCYAP1/CFTR/CRH/DOC2B/DRD2/GCG/GCK/GPLD1/GRP/HFE/HIF1A/INS/ISL1/KISS1/NKX6-1/NLGN2/NNAT/NR0B2/NR1H4/RASL10B/RFX6/RPH3AL/SLC30A8/SPINK1/TRH/TRPM4/UCN3/VSNL1	29
cluster5	BP	GO:1903053	regulation of extracellular matrix organization	20/3710	57/18903	0.004501658	0.049879892	0.042812585	ABL1/AGT/BMP2/CHADL/CPB2/CST3/DAG1/EMILIN1/FAP/FGFR4/HAS2/ITGB3/LAMA1/LAMB1/LAMB2/NID1/PDPN/SLC2A10/SOX9/TNXB	20
cluster5	BP	GO:0061982	meiosis I cell cycle process	40/3710	137/18903	0.004515225	0.049977389	0.042896268	ANKLE1/ANKRD31/BRCA2/BRME1/C14orf39/CCNB2/CCNE2/DDX4/DMC1/DMRT1/DMRTC2/EHMT2/ESPL1/FMN2/HFM1/HSF2BP/HSPA2/MAEL/MEIOB/MLH3/MND1/MOV10L1/MRE11/MSH5/P3H4/PLK1/RAD50/RPL10L/SGO1/SLC25A31/SLX4/SPO11/SYCP1/TERB1/TEX15/TEX19/TOP2A/UBE2B/USP17L2/WEE2	40

cluster5	CC	GO:0097060	synaptic membrane	191/3954	378/19869	4.31E-41	5.08E-38	4.27E-38	ACP4/ADAM10/ADCY1/ADCY8/ADORA1/ADRA1A/ADRA2A/ANK3/APBA1/ARC/ATP1A3/ATP2B2/ATP2B3/CACNA1C/CACNG2/CACNG3/CACNG4/CACNG5/CACNG7/CADM3/CBLN1/CDH10/CDH2/CDH8/CHRM1/CHRM2/CHRM3/CHRNA2/CHRNA3/CHRNA4/CHRNA6/CHRNA7/CHRNB4/CHRNG/CLSTN2/CNTN5/CNTN6/CNTNAP1/CNTNAP4/CRYAB/CSPG5/DAG1/DAGLA/DBN1/DDN/DGKB/DGKI/DMD/DNM1/DRD1/DRD2/EFNB2/EFNB3/EPHA7/ERBB4/ERC1/F2R/FAIM2/FLRT3/FXYD6/GABBR2/GABRA1/GABRA2/GABRA3/GABRA4/GABRA5/GABRA6/GABRB2/GABRB3/GABRD/GABRE/GABRG1/GABRG2/GABRG3/GABRQ/GAD2/GHSR/GLRA1/GLRA2/GLRB/GPM6A/GRIA1/GRIA2/GRIA3/GRIA4/GRID1/GRID2/GRID2IP/GRIK1/GRIK2/GRIK3/GRIK5/GRIN1/GRIN2A/GRIN2B/GRIN2D/GRIP1/GRIP2/GRM2/GRM3/GRM5/GRM7/GSG1L/HTR2A/IGSF21/IL1RAPL1/ITGB1/ITGB3/KCNB1/KCNC1/KCNC2/KCNC3/KCND2/KCND3/KCNH1/KCNJ3/KCNJ4/KCNJ9/KCNMA1/KCTD8/LHFPL4/LRFN3/LRP4/LRRC4B/LRRC4C/LRRTM2/LRRTM3/LRRTM4/MUSK/NECTIN1/NECTIN3/NLGN1/NLGN2/NLGN3/NLGN4X/NPTN/NRCAM/NRP2/NRXN1/NTNG1/NTNG2/OPRK1/OTOF/P2RX3/PLPPR4/PRKCG/PRR7/PRRT1/PRRT2/PTPRD/PTPRS/RGS7BP/RGS9/RIMS3/RIMS4/SCN10A/SCN2A/SHANK1/SHANK2/SHC4/SHISA6/SHISA7/SHISA9/SLC1A2/SLC1A6/SLC30A1/SLC6A1/SLC6A11/SLC6A2/SLC6A3/SLC6A5/SLITRK1/SLITRK3/SNAP25/SNAP91/SNCAIP/SRC/SRPX2/SSPN/STRN/STX11/STX1A/STXBP1/SYDE2/SYNDIG1/SYP/SYT1/SYT7/TENM2/UNC13A/UNC13C	191
cluster5	CC	GO:0045211	postsynaptic membrane	139/3954	269/19869	1.65E-31	9.75E-29	8.19E-29	ACP4/ADCY1/ADORA1/ADRA1A/ADRA2A/ANK3/ARC/ATP2B2/CACNA1C/CACNG2/CACNG3/CACNG4/CACNG5/CACNG7/CBLN1/CDH10/CDH2/CHRM1/CHRM2/CHRM3/CHRNA2/CHRNA3/CHRNA4/CHRNA6/CHRNA7/CHRNB4/CHRNG/CLSTN2/CSPG5/DAG1/DAGLA/DBN1/DDN/DGKB/DMD/DRD1/DRD2/EFNB2/EFNB3/EPHA7/ERBB4/F2R/FAIM2/FLRT3/FXYD6/GABBR2/GABRA1/GABRA2/GABRA3/GABRA4/GABRA5/GABRA6/GABRB2/GABRB3/GABRD/GABRE/GABRG1/GABRG2/GABRG3/GABRQ/GLRA1/GLRA2/GLRB/GRIA1/GRIA2/GRIA3/GRIA4/GRID1/GRID2/GRID2IP/GRIK1/GRIK2/GRIK3/GRIK5/GRIN1/GRIN2A/GRIN2B/GRIN2D/GRIP1/GRIP2/GRM2/GRM3/GRM5/GRM7/GSG1L/HTR2A/IGSF21/IL1RAPL1/ITGB3/KCNB1/KCNC1/KCNC2/KCNC3/KCND2/KCND3/KCNH1/KCNJ4/KCNMA1/KCTD8/LHFPL4/LRFN3/LRRC4B/LRRC4C/LRRTM2/LRRTM3/LRRTM4/MUSK/NECTIN3/NLGN1/NLGN2/NLGN3/NLGN4X/NPTN/NRCAM/NRP2/OPRK1/PLPPR4/PRR7/PRRT1/PRRT2/PTPRS/RGS7BP/RGS9/SHANK1/SHANK2/SHC4/SHISA6/SHISA7/SHISA9/SLC30A1/SLC6A1/SLC6A11/SLC6A3/SLITRK1/SLITRK3/SSPN/STRN/SYNDIG1/TENM2	139
cluster5	CC	GO:0099240	intrinsic component of synaptic membrane	100/3954	166/19869	3.49E-30	1.37E-27	1.15E-27	ADCY1/ADCY8/ADORA1/ADRA1A/ADRA2A/ATP1A3/ATP2B2/ATP2B3/CACNG2/CACNG3/CACNG4/CACNG5/CACNG7/CADM3/CDH10/CDH2/CHRM1/CHRM2/CHRNA6/CLSTN2/CNTN5/CNTN6/CSPG5/DAGLA/DRD1/DRD2/EFNB2/EFNB3/EPHA7/ERBB4/FLRT3/FXYD6/GABRA2/GABRA3/GABRA4/GABRA5/GABRB2/GABRD/GABRG2/GABRG3/GHSR/GPM6A/GRIA1/GRID1/GRID2/GRIK5/GRIN2A/GRIN2D/GRM2/GRM3/GRM5/GSG1L/HTR2A/ITGB1/ITGB3/KCNC1/KCND2/KCND3/KCNH1/KCNJ3/KCNJ9/LRFN3/LRRC4B/LRRC4C/LRRTM2/LRRTM3/NECTIN1/NECTIN3/NLGN1/NLGN2/NLGN3/NLGN4X/NPTN/NRCAM/NRP2/NTNG1/NTNG2/OPRK1/P2RX3/PLPPR4/PRRT2/PTPRD/PTPRS/RGS7BP/SCN10A/SCN2A/SHISA6/SHISA7/SHISA9/SLC1A2/SLC1A6/SLC30A1/SLC6A1/SLC6A11/SLC6A3/SLC6A5/SLITRK1/SLITRK3/STX1A/SYT7	100
cluster5	CC	GO:0099699	integral component of synaptic membrane	95/3954	155/19869	1.4E-29	4.13E-27	3.47E-27	ADCY1/ADCY8/ADORA1/ADRA1A/ADRA2A/ATP1A3/ATP2B2/ATP2B3/CACNG2/CACNG3/CACNG4/CACNG5/CACNG7/CADM3/CDH10/CDH2/CHRM1/CHRM2/CHRNA6/CLSTN2/CSPG5/DAGLA/DRD1/DRD2/EFNB2/EFNB3/EPHA7/ERBB4/FLRT3/FXYD6/GABRA2/GABRA3/GABRA4/GABRA5/GABRB2/GABRD/GABRG2/GABRG3/GHSR/GPM6A/GRIA1/GRID1/GRID2/GRIK5/GRIN2A/GRIN2D/GRM2/GRM3/GRM5/GSG1L/HTR2A/ITGB1/ITGB3/KCNC1/KCND2/KCND3/KCNH1/KCNJ3/KCNJ9/LRFN3/LRRC4B/LRRC4C/LRRTM2/LRRTM3/NECTIN1/NECTIN3/NLGN1/NLGN2/NLGN3/NLGN4X/NPTN/NRCAM/NRP2/OPRK1/P2RX3/PLPPR4/PRRT2/PTPRD/PTPRS/SCN10A/SCN2A/SHISA6/SHISA7/SHISA9/SLC1A2/SLC1A6/SLC30A1/SLC6A1/SLC6A11/SLC6A3/SLC6A5/SLITRK1/SLITRK3/STX1A/SYT7	95

cluster5	CC	GO:0098978	glutamatergic synapse	152/3954	324/19869	2.35E-28	5.55E-26	4.66E-26	ABLIM3/ACTBL2/ACTC1/ACTN1/ADAM10/ADCY1/ADCY8/ADGRA1/ADGRL3/ADORA2B/ADRA1A/ADRA2A/AP3D1/APBA1/ARC/ARHGAP39/ARHGAP44/ATP2B2/ATP2B3/BCAN/BSN/CACNG2/CACNG3/CACNG4/CACNG5/CACNG7/CADPS/CALB1/CAMKV/CBLN1/CBLN2/CDH10/CDH11/CDH8/CHRM1/CHRM2/CLSTN2/CPT1C/CSPG5/CTBP1/CTNND1/CTTNBP2/DAG1/DBN1/DLGAP2/DLGAP3/DNM1/DRD1/DRD2/DRD4/EFNB2/EFNB3/EIF4A3/ELAVL4/EPHA7/EPS8/ERBB4/FLRT3/FXYD6/GABRG2/GHSR/GIPC1/GPM6A/GRIA1/GRID1/GRID2/GRIK2/GRIK3/GRIK5/GRIN2A/GRIN2D/GRIP2/GRIPAP1/GRM3/GRM5/GSG1L/GSK3B/GUCY1A1/HOMER1/HPCA/HTR2A/IL1RAPL1/IL1RAPL2/ITGB1/ITGB3/KCND2/LGI1/LRFN3/LRRC4B/LRRC4C/LRRTM2/LRRTM3/LYN/MAL2/NLGN1/NLGN3/NLGN4X/NPTN/NPTX1/NPTX2/NPTXR/NRCAM/NRG3/NRP2/NTNG1/NTNG2/OPHN1/PAK3/PCLO/PFN2/PLCB1/PLEKHA5/PLPPR4/PRKAR1A/PRKAR1B/PRR7/PRRT2/PSD2/PTPRD/RAB8A/RGS7BP/RGS9/SCN10A/SCN2A/SH3GL2/SH3GL3/SHANK1/SHANK2/SHISA6/SHISA7/SHISA9/SLC1A2/SLC1A6/SLC30A3/SLC6A17/SLITRK1/SLITRK2/SNAP25/SRC/STX1A/STXBP1/SV2A/SYN2/SYN3/SYT1/SYT4/TNR/WASF3/WNT3A/WNT5A/WNT7A/YWHAZ	152
cluster5	CC	GO:0098984	neuron to neuron synapse	157/3954	351/19869	1.98E-26	3.89E-24	3.27E-24	ADAM10/ADCY1/ADCY8/ADGRA1/ADGRB1/ADORA1/ADRA2A/ARC/ARHGAP32/ARHGAP44/ATP1A3/ATP2B2/BCL11A/BSN/CABP1/CACNA1C/CACNG2/CACNG3/CACNG4/CACNG5/CACNG7/CADM1/CALB1/CAMK2A/CAP2/CDH2/CDK5R1/CHRM1/CHRM2/CHRNA3/CLSTN2/CPEB1/CPEB3/CRYAB/CTNND1/CTNND2/DAGLA/DBN1/DCLK1/DGKI/DLG5/DLGAP2/DLGAP3/DMTN/DRD2/EFNB2/EFNB3/EPHA7/EPS8/ERBB4/FLRT3/GAP43/GNG3/GRIA1/GRIA2/GRID1/GRID2/GRIK2/GRIK5/GRIN1/GRIN2A/GRIN2B/GRIN2D/GRIP1/GRIP2/GRM3/GRM5/GRM7/GSG1L/HOMER1/IGSF11/INSYN1/INSYN2A/KCND2/KCND3/KCNH1/LRFN3/LRP4/LRRC4B/LRRC4C/LRRTM2/LRRTM3/LYN/LZTS1/LZTS3/MAGI2/MAL2/MAPK8IP2/MINK1/MPDZ/MPP2/MT3/NCK2/NECTIN1/NECTIN3/NEFH/NEURL1/NGFR/NLGN1/NLGN2/NLGN3/NLGN4X/NPTN/NRCAM/NTRK2/P2RX3/PAK3/PCLO/PENK/PLEKHA5/PLPPR4/PPP1R9A/PRKAR1B/PRKCG/PRKN/PRR7/PRRT1/PRRT2/PSD/PTCH1/PTPRD/PTPRS/RAB8A/RGS7BP/RGS9/SAMD14/SH2D5/SH3GL3/SHANK1/SHANK2/SHISA6/SHISA7/SHISA9/SLC1A1/SLC30A1/SLC30A3/SLC8A2/SLC8A3/SLITRK1/SLITRK3/SPOCK1/SRC/SRCIN1/STRN/STX1A/SYN1/SYN2/SYN3/SYNDIG1/SYT1/SYT12/SYT7/SYT9/TMEM108/USP50/YWHAZ/ZDHHC15	157
cluster5	CC	GO:0034702	ion channel complex	135/3954	296/19869	7.15E-24	1.2E-21	1.01E-21	ABCC8/ABCC9/AKAP6/ANO6/BEST2/BEST3/C2CD6/CACNA1A/CACNA1B/CACNA1C/CACNA1G/CACNA1S/CACNA2D1/CACNB1/CACNG2/CACNG3/CACNG4/CACNG5/CACNG7/CASQ2/CATSPER4/CATSPERG/CFTR/CHRNA2/CHRNA3/CHRNA4/CHRNA6/CHRNA7/CHRNB4/CLCN2/CLCNKA/CLCNKB/CLDN4/CLIC6/CNGA3/CNGB1/CNTNAP2/CPT1C/DPP10/DPP6/EPS8/FKBP1A/FKBP1B/GABRA1/GABRA2/GABRA3/GABRA4/GABRA5/GABRA6/GABRB2/GABRB3/GABRD/GABRE/GABRG1/GABRG2/GABRG3/GABRQ/GLRA1/GLRA2/GLRB/GRIA1/GRIA2/GRIA3/GRIA4/GRID2/GRIK2/GRIK3/GRIK5/GRIN1/GRIN2A/GRIN2B/GRIN2D/HCN1/HCN3/HCN4/HSPA2/HTR3C/KCNA10/KCNA4/KCNA7/KCNB1/KCNB2/KCNC1/KCNC2/KCNC3/KCND2/KCND3/KCNG3/KCNH1/KCNH2/KCNH4/KCNIP1/KCNIP3/KCNJ16/KCNJ3/KCNJ4/KCNJ5/KCNJ6/KCNK6/KCNMA1/KCNMB2/KCNN1/KCNQ2/KCNQ3/KCNQ4/KCNS2/KCNV1/LRRC55/LRRC8B/MICU3/OLFM3/PKD1L3/RYR2/RYR3/SCN10A/SCN1A/SCN2A/SCN2B/SCN3B/SCN4A/SCN8A/SCN9A/SCNN1G/SHISA6/SHISA7/SHISA9/TMEM249/TRPC3/TRPC4/TRPC7/TRPM4/TTYH1/UNC80/VWC2/VWC2L	135
cluster5	CC	GO:0099572	postsynaptic specialization	147/3954	342/19869	1E-22	1.47E-20	1.24E-20	ADAM10/ADCY1/ADCY8/ADGRA1/ADGRB1/ADORA1/ADRA2A/ARC/ARHGAP32/ARHGAP44/ATP2B2/BCL11A/BSN/CABP1/CACNA1C/CACNG2/CACNG3/CACNG4/CACNG5/CACNG7/CADM1/CAMK2A/CAP2/CDH10/CDH2/CDK5R1/CHRM1/CHRNA3/CLSTN2/CPEB1/CPEB3/CRYAB/CTNND1/CTNND2/DAGLA/DBN1/DCLK1/DGKI/DLG5/DLGAP2/DLGAP3/DMTN/DRD2/EFNB2/EFNB3/EPHA7/EPS8/ERBB4/FLRT3/GABRA2/GABRA3/GABRA4/GABRA5/GABRB2/GABRG2/GAP43/GNG3/GRIA1/GRIA2/GRID1/GRID2/GRIK2/GRIK5/GRIN1/GRIN2A/GRIN2B/GRIN2D/GRIP1/GRIP2/GRM3/GRM5/GSG1L/HOMER1/IGSF11/INSYN1/INSYN2A/KCND2/KCND3/KCNH1/LRFN3/LRP4/LRRC4B/LRRC4C/LRRTM2/LRRTM3/LYN/LZTS1/LZTS3/MAGI2/MAPK8IP2/MINK1/MPDZ/MPP2/MT3/NCK2/NECTIN3/NEFH/NEURL1/NGFR/NLGN1/NLGN2/NLGN3/NLGN4X/NPTN/NRCAM/NTRK2/PAK3/PCLO/PLEKHA5/PLPPR4/PPP1R9A/PRKCG/PRKN/PRR7/PRRT1/PRRT2/PSD/PTCH1/PTPRS/RAB8A/RGS7BP/RGS9/SAMD14/SH2D5/SH3GL3/SHANK1/SHANK2/SHISA6/SHISA7/SHISA9/SLC30A1/SLC8A2/SLC8A3/SLITRK1/SLITRK3/SPOCK1/SRC/SRCIN1/STRN/STX1A/SYN1/SYN2/SYN3/SYNDIG1/TMEM108/USP50/ZDHHC15	147

cluster5	CC	GO:0099055	integral component of postsynaptic membrane	72/3954	118/19869	1.38E-22	1.81E-20	1.52E-20	ADCY1/ADORA1/ADRA1A/ADRA2A/ATP2B2/CACNG2/CACNG3/CACNG4/CACNG5/CACNG7/CDH10/CDH2/CHRM1/CHRM2/CHRNA6/CLSTN2/CSPG5/DAGLA/DRD1/DRD2/EFNB2/EFNB3/EPHA7/ERBB4/FLRT3/FXYD6/GABRA2/GABRA3/GABRA4/GABRA5/GABRB2/GABRD/GABRG2/GRIA1/GRID1/GRID2/GRIK5/GRIN2A/GRIN2D/GRM2/GRM3/GRM5/GSG1L/HTR2A/KCNC1/KCND2/KCND3/LRFN3/LRRC4B/LRRC4C/LRRTM2/LRRTM3/NECTIN3/NLGN1/NLGN2/NLGN3/NLGN4X/NPTN/NRCAM/NRP2/OPRK1/PLPPR4/PTPRS/SHISA6/SHISA7/SHISA9/SLC30A1/SLC6A1/SLC6A11/SLC6A3/SLITRK1/SLITRK3	72
cluster5	CC	GO:0032279	asymmetric synapse	142/3954	327/19869	1.65E-22	1.95E-20	1.64E-20	ADAM10/ADCY1/ADCY8/ADGRA1/ADGRB1/ADORA1/ADRA2A/ARC/ARHGAP32/ARHGAP44/ATP2B2/BCL11A/BSN/CABP1/CACNA1C/CACNG2/CACNG3/CACNG4/CACNG5/CACNG7/CADM1/CAMK2A/CAP2/CDH2/CDK5R1/CHRM1/CHRM2/CHRNA3/CLSTN2/CPEB1/CPEB3/CRYAB/CTNND1/CTNND2/DAGLA/DBN1/DCLK1/DGKI/DLG5/DLGAP2/DLGAP3/DMTN/DRD2/EFNB2/EFNB3/EPHA7/EPS8/ERBB4/FLRT3/GAP43/GNG3/GRIA1/GRIA2/GRID1/GRID2/GRIK2/GRIK5/GRIN1/GRIN2A/GRIN2B/GRIN2D/GRIP1/GRIP2/GRM3/GRM5/GRM7/GSG1L/HOMER1/IGSF11/INSYN1/INSYN2A/KCND2/KCND3/KCNH1/LRFN3/LRP4/LRRC4B/LRRC4C/LRRTM2/LRRTM3/LYN/LZTS1/LZTS3/MAGI2/MAPK8IP2/MINK1/MPDZ/MPP2/MT3/NCK2/NECTIN3/NEFH/NEURL1/NGFR/NLGN1/NLGN3/NLGN4X/NPTN/NRCAM/NTRK2/PAK3/PCLO/PLEKHA5/PLPPR4/PPP1R9A/PRKCG/PRKN/PRR7/PRRT1/PRRT2/PSD/PTCH1/PTPRS/RAB8A/RGS7BP/RGS9/SAMD14/SH2D5/SH3GL3/SHANK1/SHANK2/SHISA6/SHISA7/SHISA9/SLC1A1/SLC30A1/SLC8A2/SLC8A3/SLITRK1/SLITRK3/SPOCK1/SRC/SRCIN1/STRN/STX1A/SYN1/SYN2/SYN3/SYNDIG1/TMEM108/USP50/ZDHHC15	142
cluster5	CC	GO:0098936	intrinsic component of postsynaptic membrane	73/3954	123/19869	7.37E-22	7.91E-20	6.65E-20	ADCY1/ADORA1/ADRA1A/ADRA2A/ATP2B2/CACNG2/CACNG3/CACNG4/CACNG5/CACNG7/CDH10/CDH2/CHRM1/CHRM2/CHRNA6/CLSTN2/CSPG5/DAGLA/DRD1/DRD2/EFNB2/EFNB3/EPHA7/ERBB4/FLRT3/FXYD6/GABRA2/GABRA3/GABRA4/GABRA5/GABRB2/GABRD/GABRG2/GRIA1/GRID1/GRID2/GRIK5/GRIN2A/GRIN2D/GRM2/GRM3/GRM5/GSG1L/HTR2A/KCNC1/KCND2/KCND3/LRFN3/LRRC4B/LRRC4C/LRRTM2/LRRTM3/NECTIN3/NLGN1/NLGN2/NLGN3/NLGN4X/NPTN/NRCAM/NRP2/OPRK1/PLPPR4/PTPRS/RGS7BP/SHISA6/SHISA7/SHISA9/SLC30A1/SLC6A1/SLC6A11/SLC6A3/SLITRK1/SLITRK3	73
cluster5	CC	GO:0014069	postsynaptic density	138/3954	321/19869	2.01E-21	1.98E-19	1.66E-19	ADAM10/ADCY1/ADCY8/ADGRA1/ADGRB1/ADORA1/ADRA2A/ARC/ARHGAP32/ARHGAP44/ATP2B2/BCL11A/BSN/CABP1/CACNA1C/CACNG2/CACNG3/CACNG4/CACNG5/CACNG7/CADM1/CAMK2A/CAP2/CDH2/CDK5R1/CHRM1/CHRNA3/CLSTN2/CPEB1/CPEB3/CRYAB/CTNND1/CTNND2/DAGLA/DBN1/DCLK1/DGKI/DLG5/DLGAP2/DLGAP3/DMTN/DRD2/EFNB2/EFNB3/EPHA7/EPS8/ERBB4/FLRT3/GAP43/GNG3/GRIA1/GRIA2/GRID1/GRID2/GRIK2/GRIK5/GRIN1/GRIN2A/GRIN2B/GRIN2D/GRIP1/GRIP2/GRM3/GRM5/GSG1L/HOMER1/IGSF11/INSYN1/INSYN2A/KCND2/KCND3/KCNH1/LRFN3/LRP4/LRRC4B/LRRC4C/LRRTM2/LRRTM3/LYN/LZTS1/LZTS3/MAGI2/MAPK8IP2/MINK1/MPDZ/MPP2/MT3/NCK2/NECTIN3/NEFH/NEURL1/NGFR/NLGN1/NLGN4X/NPTN/NRCAM/NTRK2/PAK3/PCLO/PLEKHA5/PLPPR4/PPP1R9A/PRKCG/PRKN/PRR7/PRRT1/PRRT2/PSD/PTCH1/PTPRS/RAB8A/RGS7BP/RGS9/SAMD14/SH2D5/SH3GL3/SHANK1/SHANK2/SHISA6/SHISA7/SHISA9/SLC30A1/SLC8A2/SLC8A3/SLITRK1/SLITRK3/SPOCK1/SRC/SRCIN1/STRN/STX1A/SYN1/SYN2/SYN3/SYNDIG1/TMEM108/USP50/ZDHHC15	138

cluster5	CC	GO:0042734	presynaptic membrane	79/3954	148/19869	1.31E-19	1.19E-17	1E-17	ADCY8/ADORA1/ADRA1A/ADRA2A/APBA1/ATP1A3/ATP2B2/ATP2B3/CADM3/CDH10/CDH2/CHRM1/CHRM2/CHRNA6/CNTN5/CNTN6/CNTNAP1/CNTNAP4/DNM1/DRD1/DRD2/EFNB2/EFNB3/ERBB4/ERC1/FXYD6/GABRA5/GAD2/GPM6A/GRIK2/GRIK3/GRIK5/GRIN2A/GRM2/GRM3/HTR2A/IGSF21/KCNC1/KCNC2/KCNC3/KCNH1/KCNJ3/KCNJ9/KCTD8/LRFN3/LRRC4B/NECTIN1/NLGN2/NPTN/NRXN1/NTNG1/NTNG2/OPRK1/OTOF/P2RX3/PRRT2/PTPRD/RGS7BP/RIMS3/SCN10A/SCN2A/SLC1A2/SLC1A6/SLC6A1/SLC6A11/SLC6A2/SLC6A3/SLC6A5/SNAP25/SNAP91/SNCAIP/STX11/STX1A/STXBP1/SYP/SYT1/SYT7/UNC13A/UNC13C	79
cluster5	CC	GO:0099060	integral component of postsynaptic specialization membrane	50/3954	76/19869	3.98E-18	3.36E-16	2.82E-16	ADCY1/ADRA2A/ATP2B2/CACNG2/CACNG3/CACNG4/CACNG7/CDH10/CDH2/CHRM1/CLSTN2/EFNB2/EFNB3/EPHA7/ERBB4/GABRA2/GABRA3/GABRA4/GABRA5/GABRB2/GABRG2/GRIA1/GRID1/GRID2/GRIK5/GRIN2A/GRIN2D/GSG1L/KCND2/KCND3/LRFN3/LRRC4B/LRRC4C/LRRTM2/LRRTM3/NECTIN3/NLGN1/NLGN2/NLGN3/NLGN4X/NPTN/NRCAM/PLPPR4/PTPRS/SHISA6/SHISA7/SHISA9/SLC30A1/SLITRK1/SLITRK3	50
cluster5	CC	GO:0098948	intrinsic component of postsynaptic specialization membrane	51/3954	79/19869	6.28E-18	4.94E-16	4.15E-16	ADCY1/ADRA2A/ATP2B2/CACNG2/CACNG3/CACNG4/CACNG7/CDH10/CDH2/CHRM1/CLSTN2/EFNB2/EFNB3/EPHA7/ERBB4/GABRA2/GABRA3/GABRA4/GABRA5/GABRB2/GABRG2/GRIA1/GRID1/GRID2/GRIK5/GRIN2A/GRIN2D/GSG1L/KCND2/KCND3/LRFN3/LRRC4B/LRRC4C/LRRTM2/LRRTM3/NECTIN3/NLGN1/NLGN2/NLGN3/NLGN4X/NPTN/NRCAM/PLPPR4/PTPRS/RGS7BP/SHISA6/SHISA7/SHISA9/SLC30A1/SLITRK1/SLITRK3	51
cluster5	CC	GO:0043025	neuronal cell body	180/3954	497/19869	7.98E-18	5.89E-16	4.95E-16	ABL1/ADCY8/ADCYAP1/ADORA1/ADRA2A/APOD/APP/ARC/ASCL1/ASIC2/ASTN1/ATP1A2/ATP1A3/ATP2B2/AVPR1B/BRINP1/BRINP2/BRINP3/CACNA1A/CACNA1B/CACNA1C/CACNG7/CALB1/CALCA/CDK5R1/CHRM2/CHRNA3/CHRNA4/CLCN2/CNGA3/CNTNAP2/CPLX2/CPNE5/CPNE6/CRH/CRMP1/CRYAB/CSNK1E/CTNND2/CYFIP1/DDN/DNER/DPYSL5/DRD2/DSCAM/EFNA2/EIF4A3/ELAVL4/EPHA5/EPHA7/EVX1/FEZ1/FLRT1/FZD3/GABRA2/GABRA5/GABRD/GAP43/GDI1/GFRA1/GHR/GLRA1/GNAT1/GPM6A/GRIA1/GRIA2/GRIA4/GRIK2/GRIK3/GRIK5/GRIP1/HCN3/HDAC6/HOMER1/HPCA/HPN/HTR2A/IGF2BP1/INHA/KCNB1/KCNB2/KCNC1/KCNC2/KCNC3/KCND2/KCND3/KCNH1/KCNN1/KCNN2/KCNN3/KIF5A/KIF5C/KISS1/KLHL1/KNDC1/L1CAM/LRP4/LRP6/MAP1A/MAP2/MAPK8IP1/MAPK8IP2/NDEL1/NEURL1/NEUROG1/NGFR/NPY/NRSN1/NRSN2/NRXN1/OLFM1/OPN4/OPRK1/P2RX2/P2RX3/PCSK1/PCSK2/PDE11A/PDE1A/PDE1B/PDE1C/PDYN/PENK/PRKAA2/PRPH/PSD2/PTPRF/PTPRS/PVALB/RAB5A/RAB8A/RET/RGS7BP/RGS8/ROR2/RTN4RL1/RTN4RL2/SCN1A/SEPTIN4/SEZ6L/SEZ6L2/SHANK2/SIRT2/SLC12A5/SLC17A8/SLC1A1/SLC5A7/SLC6A1/SLC6A2/SLC6A3/SLC8A2/SLC8A3/SNCAIP/SNCB/SPTBN4/SRC/SRCIN1/SRD5A2/SST/STMN2/STRN/SV2A/SYT4/SYT5/TAC1/TACR3/TH/THY1/TMPRSS5/TNN/TPX2/TRPM4/TTBK1/TTLL7/TUBB3/TUBB4A/UCHL1/UNC5C/USH2A/WHRN	180

cluster5	CC	GO:0034703	cation channel complex	100/3954	222/19869	1.42E-17	9.85E-16	8.28E-16	ABCC8/ABCC9/AKAP6/C2CD6/CACNA1A/CACNA1B/CACNA1C/CACNA1G/CACNA1S/CACNA2D1/CACNB1/CACNG2/CACNG3/CACNG4/CACNG5/CACNG7/CASQ2/CATSPER4/CATSPERG/CNGA3/CNGB1/CNTNAP2/CPT1C/DPP10/DPP6/EPS8/FKBP1A/FKBP1B/GRIA1/GRIA2/GRIA3/GRIA4/GRIK2/GRIK3/GRIK5/GRIN1/GRIN2A/GRIN2B/GRIN2D/HCN1/HCN3/HCN4/HSPA2/HTR3C/KCNA10/KCNA4/KCNA7/KCNB1/KCNB2/KCNC1/KCNC2/KCNC3/KCND2/KCND3/KCNG3/KCNH1/KCNH2/KCNH4/KCNIP1/KCNIP3/KCNJ16/KCNJ3/KCNJ4/KCNJ5/KCNJ6/KCNK6/KCNMA1/KCNMB2/KCNN1/KCNQ2/KCNQ3/KCNQ4/KCNS2/KCNV1/LRRC55/MICU3/OLFM3/PKD1L3/RYR2/RYR3/SCN10A/SCN1A/SCN2A/SCN2B/SCN3B/SCN4A/SCN8A/SCN9A/SCNN1G/SHISA6/SHISA7/SHISA9/TMEM249/TRPC3/TRPC4/TRPC7/TRPM4/UNC80/VWC2/VWC2L	100
cluster5	CC	GO:0099634	postsynaptic specialization membrane	64/3954	115/19869	2.3E-17	1.51E-15	1.27E-15	ADCY1/ADRA2A/ARC/ATP2B2/CACNA1C/CACNG2/CACNG3/CACNG4/CACNG5/CACNG7/CDH10/CDH2/CHRM1/CLSTN2/DAGLA/EFNB2/EFNB3/EPHA7/ERBB4/GABRA2/GABRA3/GABRA4/GABRA5/GABRB2/GABRG2/GRIA1/GRIA2/GRID1/GRID2/GRIK5/GRIN2A/GRIN2B/GRIN2D/GRM5/GSG1L/KCND2/KCND3/KCNH1/LRFN3/LRRC4B/LRRC4C/LRRTM2/LRRTM3/NECTIN3/NLGN1/NLGN2/NLGN3/NLGN4X/NPTN/NRCAM/PLPPR4/PRR7/PRRT1/PRRT2/PTPRS/RGS7BP/RGS9/SHISA6/SHISA7/SHISA9/SLC30A1/SLITRK1/SLITRK3/SYNDIG1	64
cluster5	CC	GO:0098889	intrinsic component of presynaptic membrane	50/3954	80/19869	9.76E-17	6.06E-15	5.09E-15	ADCY8/ADORA1/ADRA1A/ADRA2A/ATP1A3/ATP2B2/ATP2B3/CADM3/CDH10/CDH2/CHRM1/CHRM2/CHRNA6/CNTN5/CNTN6/DRD1/DRD2/EFNB2/EFNB3/ERBB4/FXYD6/GABRA5/GPM6A/GRIK5/GRM3/HTR2A/KCNC1/KCNH1/KCNJ3/KCNJ9/LRFN3/NECTIN1/NPTN/NTNG1/NTNG2/OPRK1/P2RX3/PRRT2/PTPRD/RGS7BP/SCN10A/SCN2A/SLC1A2/SLC1A6/SLC6A1/SLC6A11/SLC6A3/SLC6A5/STX1A/SYT7	50
cluster5	CC	GO:1902495	transmembrane transporter complex	143/3954	379/19869	4.06E-16	2.4E-14	2.01E-14	ABCC8/ABCC9/AKAP6/ANO6/ATP1A2/ATP1A3/ATP1B1/ATP1B4/BEST2/BEST3/C2CD6/CACNA1A/CACNA1B/CACNA1C/CACNA1G/CACNA1S/CACNA2D1/CACNB1/CACNG2/CACNG3/CACNG4/CACNG5/CACNG7/CASQ2/CATSPER4/CATSPERG/CFTR/CHRNA2/CHRNA3/CHRNA4/CHRNA6/CHRNA7/CHRNB4/CLCN2/CLCNKA/CLCNKB/CLDN4/CLIC6/CNGA3/CNGB1/CNTNAP2/CPT1C/DPP10/DPP6/EPS8/FKBP1A/FKBP1B/GABRA1/GABRA2/GABRA3/GABRA4/GABRA5/GABRA6/GABRB2/GABRB3/GABRD/GABRE/GABRG1/GABRG2/GABRG3/GABRQ/GLRA1/GLRA2/GLRB/GRIA1/GRIA2/GRIA3/GRIA4/GRID2/GRIK2/GRIK3/GRIK5/GRIN1/GRIN2A/GRIN2B/GRIN2D/HCN1/HCN3/HCN4/HSPA2/HTR3C/KCNA10/KCNA4/KCNA7/KCNB1/KCNB2/KCNC1/KCNC2/KCNC3/KCND2/KCND3/KCNG3/KCNH1/KCNH2/KCNH4/KCNIP1/KCNIP3/KCNJ16/KCNJ3/KCNJ4/KCNJ5/KCNJ6/KCNK6/KCNMA1/KCNMB2/KCNN1/KCNQ2/KCNQ3/KCNQ4/KCNS2/KCNV1/LRRC55/LRRC8B/MICU3/NDUFS6/OLFM3/PKD1L3/RYR2/RYR3/SCN10A/SCN1A/SCN2A/SCN2B/SCN3B/SCN4A/SCN8A/SCN9A/SCNN1G/SHISA6/SHISA7/SHISA9/TMEM249/TRPC3/TRPC4/TRPC7/TRPM4/TTYH1/UNC80/UQCR10/UQCRH/UQCRHL/VWC2/VWC2L	143

cluster5	CC	GO:1990351	transporter complex	149/3954	405/19869	1.22E-15	6.87E-14	5.77E-14	ABCC8/ABCC9/AKAP6/ANO6/ATP10B/ATP11A/ATP11B/ATP1A2/ATP1A3/ATP1B1/ATP1B4/BEST2/BEST3/C2CD6/CACNA1A/CACNA1B/CACNA1C/CACNA1G/CACNA1S/CACNA2D1/CACNB1/CACNG2/CACNG3/CACNG4/CACNG5/CACNG7/CASQ2/CATSPER4/CATSPERG/CFTR/CHRNA2/CHRNA3/CHRNA4/CHRNA6/CHRNA7/CHRNB4/CLCN2/CLCNKA/CLCNKB/CLDN4/CLIC6/CNGA3/CNGB1/CNTNAP2/CPT1C/DPP10/DPP6/EPS8/FKBP1A/FKBP1B/GABRA1/GABRA2/GABRA3/GABRA4/GABRA5/GABRA6/GABRB2/GABRB3/GABRD/GABRE/GABRG1/GABRG2/GABRG3/GABRQ/GLRA1/GLRA2/GLRB/GRIA1/GRIA2/GRIA3/GRIA4/GRID2/GRIK2/GRIK3/GRIK5/GRIN1/GRIN2A/GRIN2B/GRIN2D/HCN1/HCN3/HCN4/HSPA2/HTR3C/KCNA10/KCNA4/KCNA7/KCNB1/KCNB2/KCNC1/KCNC2/KCNC3/KCND2/KCND3/KCNG3/KCNH1/KCNH2/KCNH4/KCNIP1/KCNIP3/KCNJ16/KCNJ3/KCNJ4/KCNJ5/KCNJ6/KCNK6/KCNMA1/KCNMB2/KCNN1/KCNQ2/KCNQ3/KCNQ4/KCNS2/KCNV1/LRRC55/LRRC8B/MICU3/NDUFS6/OLFM3/PKD1L3/RYR2/RYR3/SCN10A/SCN1A/SCN2A/SCN2B/SCN3B/SCN4A/SCN8A/SCN9A/SCNN1G/SHISA6/SHISA7/SHISA9/TIMM8B/TIMM9/TMEM249/TMEM30A/TRPC3/TRPC4/TRPC7/TRPM4/TTYH1/UNC80/UQCR10/UQCRH/UQCRHL/VWC2/VWC2L	149
cluster5	CC	GO:0099056	integral component of presynaptic membrane	45/3954	73/19869	7.02E-15	3.77E-13	3.17E-13	ADCY8/ADORA1/ADRA1A/ADRA2A/ATP1A3/ATP2B2/ATP2B3/CADM3/CDH10/CDH2/CHRM1/CHRM2/CHRNA6/DRD1/DRD2/EFNB2/EFNB3/ERBB4/FXYD6/GABRA5/GPM6A/GRIK5/GRM3/HTR2A/KCNC1/KCNH1/KCNJ3/KCNJ9/LRFN3/NECTIN1/NPTN/OPRK1/P2RX3/PRRT2/PTPRD/SCN10A/SCN2A/SLC1A2/SLC1A6/SLC6A1/SLC6A11/SLC6A3/SLC6A5/STX1A/SYT7	45
cluster5	CC	GO:0098839	postsynaptic density membrane	51/3954	89/19869	7.93E-15	4.07E-13	3.42E-13	ADCY1/ADRA2A/ARC/ATP2B2/CACNA1C/CACNG2/CACNG3/CACNG4/CACNG5/CACNG7/CHRM1/CLSTN2/DAGLA/EFNB2/EFNB3/EPHA7/ERBB4/GRIA1/GRIA2/GRID1/GRID2/GRIK5/GRIN2A/GRIN2B/GRIN2D/GRM5/GSG1L/KCNH1/LRFN3/LRRC4B/LRRC4C/LRRTM2/LRRTM3/NECTIN3/NLGN4X/NPTN/NRCAM/PLPPR4/PRR7/PRRT1/PRRT2/PTPRS/RGS7BP/RGS9/SHISA6/SHISA7/SHISA9/SLC30A1/SLITRK1/SLITRK3/SYNDIG1	51
cluster5	CC	GO:0150034	distal axon	111/3954	278/19869	9.37E-15	4.61E-13	3.87E-13	ABL1/ADORA1/ADRA2A/AP3D1/APP/ATCAY/ATP1A3/AUTS2/AVPR1B/BASP1/BOC/BRSK1/BRSK2/CALB1/CALB2/CALCA/CBARP/CDH8/CDK5R1/CDK5R2/CHRM1/CHRM2/CPLX2/CPLX4/CRMP1/CSNK1E/CTNND1/CYFIP1/DBN1/DPYSL3/DRD2/DSCAM/ELAVL4/EPS8/FEZ1/FLRT3/GAD1/GAP43/GPM6A/GPRIN1/GRIK3/GRIK5/GRIN1/HAP1/HCN3/IGF2BP1/ITGA2/KCNC1/KCNC2/KIF20B/KIF21B/KIF5C/L1CAM/LAMP5/MAP2/NDEL1/NECTIN1/NGEF/NGFR/NMU/NPY/NRSN1/NTRK2/NTS/OLFM1/OPHN1/OPRK1/OTX2/P2RX3/PACSIN1/PCDH9/PCDHGB1/PCSK1/PDYN/PENK/PRKCG/PRRT2/PRSS12/PTCH1/PTPRS/RAB5A/RASGRF1/ROR1/SHANK2/SIRT2/SLC17A8/SLC1A1/SLC2A13/SLC6A3/SLC8A2/SLC8A3/SNAP25/SNCB/SRC/STMN2/STMN4/SYP/TENM2/TH/THY1/TNN/TPBG/TRPV4/TUBB3/TULP1/UCN3/UNC13A/UNC13C/UNC5C/USH2A/WHRN	111

cluster5	CC	GO:0044309	neuron spine	80/3954	176/19869	1.3E-14	6.13E-13	5.15E-13	ACTN1/ACTN2/ADGRB1/ADORA1/APBA1/APP/ARC/ARHGAP32/ARHGAP44/ASIC2/ATP1A2/ATP1A3/ATP2B2/CALB1/CAMK2A/CDK5R1/CRYAB/CTNND1/CTTNBP2/CYFIP1/DAGLA/DDN/DGKI/DRD1/DRD2/EEA1/FRMPD4/GIPC1/GPM6A/GRIA1/GRIA2/GRIA3/GRIA4/GRID2/GRIN1/GRIN2A/GRM3/GRM5/HAP1/HOMER1/HPCA/IGF2BP1/ITGB1/ITPKA/KCNA4/KCNC3/KCND2/KCND3/KCNN2/LZTS1/LZTS3/MPP2/MT3/MYL7/NEURL1/NGFR/NLGN1/NTRK2/OPHN1/P2RX3/PPFIA2/PPP1R9A/PRRT2/PSD/RAB8A/RGS7BP/SHANK1/SHANK2/SHISA6/SHISA7/SHISA9/SLC1A1/SLC8A2/SLC8A3/STRN/STRN4/SYNDIG1/TANC2/TENM2/USP50	80
cluster5	CC	GO:0060076	excitatory synapse	36/3954	52/19869	1.73E-14	7.84E-13	6.59E-13	ADCY8/ATP2B2/ATP2B3/BSN/CACNG3/CALB2/CBLN1/CNTN6/CYFIP1/ELFN1/FGFR2/GRIA1/GRIA2/GRIA3/GRID2/GRIN1/HOMER1/IGSF11/KCNJ3/KCNJ9/LRRTM2/NLGN1/NLGN2/NLGN3/NLGN4X/SHANK1/SHISA6/SHISA7/SLC17A6/SLC17A7/SLC17A8/SRPX2/SYNDIG1/SYP/SYT1/UNC13C	36
cluster5	CC	GO:0098982	GABA-ergic synapse	44/3954	72/19869	2.2E-14	9.62E-13	8.08E-13	ADRA1A/ADRA2A/ATP2B2/ATP2B3/BSN/CALB1/CDH10/CNTN5/CSPG5/CTBP1/DAG1/DRD1/DRD2/EFNA5/ERBB4/GABRA1/GABRA2/GABRA3/GABRA4/GABRA5/GABRB2/GABRD/GABRG2/GABRG3/GAP43/GLRB/GUCY1A1/KCND2/KCND3/LRRTM2/NLGN1/NLGN2/NLGN3/NLGN4X/NPTN/PCLO/PLCB1/SLC6A1/SLC6A11/SLC6A17/SLITRK1/SLITRK2/SLITRK3/SV2A	44
cluster5	CC	GO:0043197	dendritic spine	79/3954	175/19869	3.02E-14	1.27E-12	1.07E-12	ACTN1/ACTN2/ADGRB1/ADORA1/APBA1/APP/ARC/ARHGAP32/ARHGAP44/ASIC2/ATP1A2/ATP1A3/ATP2B2/CALB1/CAMK2A/CDK5R1/CRYAB/CTNND1/CTTNBP2/CYFIP1/DAGLA/DDN/DGKI/DRD1/DRD2/FRMPD4/GIPC1/GPM6A/GRIA1/GRIA2/GRIA3/GRIA4/GRID2/GRIN1/GRIN2A/GRM3/GRM5/HAP1/HOMER1/HPCA/IGF2BP1/ITGB1/ITPKA/KCNA4/KCNC3/KCND2/KCND3/KCNN2/LZTS1/LZTS3/MPP2/MT3/MYL7/NEURL1/NGFR/NLGN1/NTRK2/OPHN1/P2RX3/PPFIA2/PPP1R9A/PRRT2/PSD/RAB8A/RGS7BP/SHANK1/SHANK2/SHISA6/SHISA7/SHISA9/SLC1A1/SLC8A2/SLC8A3/STRN/STRN4/SYNDIG1/TANC2/TENM2/USP50	79

cluster5	CC	GO:0099061	integral component of postsynaptic density membrane	35/3954	51/19869	6.07E-14	2.47E-12	2.07E-12	ADCY1/ADRA2A/ATP2B2/CACNG2/CACNG3/CACNG4/CACNG7/CHRM1/CLSTN2/EFNB2/EFNB3/EPHA7/ERBB4/GRIA1/GRID1/GRID2/GRIK5/GRIN2A/GRIN2D/GSG1L/LRFN3/LRRC4B/LRRC4C/LRRTM2/LRRTM3/NECTIN3/NPTN/NRCAM/PLPPR4/PTPRS/SHISA6/SHISA9/SLC30A1/SLITRK1/SLITRK3	35
cluster5	CC	GO:0099146	intrinsic component of postsynaptic density membrane	36/3954	54/19869	1.06E-13	4.15E-12	3.49E-12	ADCY1/ADRA2A/ATP2B2/CACNG2/CACNG3/CACNG4/CACNG7/CHRM1/CLSTN2/EFNB2/EFNB3/EPHA7/ERBB4/GRIA1/GRID1/GRID2/GRIK5/GRIN2A/GRIN2D/GSG1L/LRFN3/LRRC4B/LRRC4C/LRRTM2/LRRTM3/NECTIN3/NPTN/NRCAM/PLPPR4/PTPRS/RGS7BP/SHISA6/SHISA9/SLC30A1/SLITRK1/SLITRK3	36
cluster5	CC	GO:0062023	collagen-containing extracellular matrix	149/3954	433/19869	6.51E-13	2.48E-11	2.08E-11	ACAN/ADAM11/ADAMTS15/ADAMTS2/ADAMTS20/ADAMTS3/ADAMTS8/AGT/AMELX/ANGPT1/ANGPT2/ANGPTL2/ANGPTL4/ANXA7/ASPN/ATRNL1/BCAN/BMP7/CBLN1/CCN2/CDH2/CDON/CHADL/CMA1/COL11A1/COL12A1/COL14A1/COL16A1/COL20A1/COL21A1/COL23A1/COL24A1/COL25A1/COL26A1/COL27A1/COL28A1/COL2A1/COL4A1/COL4A5/COL4A6/COL5A3/COL6A1/COL6A5/COL6A6/COL8A1/COL9A1/CSPG4/CTSG/DAG1/ECM1/ECM2/EDIL3/EFNA5/ELANE/EMILIN1/EMILIN2/EMILIN3/F2/F3/FBLN1/FBN3/FGF10/FGFBP3/FGFR2/FIBCD1/FREM1/FREM2/FREM3/GDF10/GPC2/GPC3/GPC6/GREM1/HAPLN1/HAPLN4/HMCN2/ICAM1/IL7/IMPG1/KAZALD1/KNG1/L1CAM/LAD1/LAMA1/LAMA3/LAMA5/LAMB1/LAMB2/LAMC2/LEFTY2/LOX/LOXL4/LTBP1/MATN2/MATN3/MATN4/MBL2/MDK/MFAP2/MFAP4/MMP8/MMRN1/MXRA5/MYOC/NAV2/NCAM1/NDP/NID1/NTN1/NTN3/NTNG1/NTNG2/OGN/OMD/PF4/PKM/PODN/PRG3/PRTN3/PSAP/PTPRZ1/PXDN/RBP3/SDC2/SERPINB1/SERPINE1/SERPINE2/SFRP2/SHH/SRPX2/SULF1/TGFB1I1/THSD4/TIMP1/TMEFF2/TNC/TNN/TNR/TNXB/USH2A/VIT/VWA1/VWA2/VWC2/VWF/WNT2B/WNT5A/WNT8A/ZP4	149
cluster5	CC	GO:0032589	neuron projection membrane	37/3954	60/19869	1.73E-12	6.38E-11	5.36E-11	ADGRV1/ADORA1/ATF4/ATP2B2/CLRN2/CNTNAP2/DAGLA/DDN/GABRA1/GABRA2/GABRA3/GABRA4/GABRA5/GABRA6/GABRE/GABRG1/GABRG2/GABRG3/GRIA1/HPCA/KCNB1/KCNC1/KCNC2/KCNC3/KCNH1/LAMP5/MPP2/MYO1C/ROBO2/SHISA6/SHISA7/SHISA9/SLC12A5/SLC1A2/TACR3/THY1/USH2A	37

cluster5	CC	GO:0008021	synaptic vesicle	85/3954	208/19869	2.85E-12	1.02E-10	8.56E-11	ABCC8/AMPH/APBA1/APP/ATP6V0A1/ATP6V1B1/BDNF/BRSK1/BSN/CBARP/CLCN4/CTTNBP2/DGKI/DOC2A/DRD2/GABRA2/GAD2/GIPC1/GRIA1/GRIN1/GRIN2A/HAP1/KIRREL3/LAMP1/LAMP5/LGI3/MAL2/MT3/NDEL1/NGF/NTF3/OPRK1/OTOF/PENK/PRRT1/PRRT2/PTPRS/RAB27B/RAB3B/RAB5A/RAB5B/RAB8A/RABAC1/SCAMP1/SCAMP5/SEPTIN4/SEPTIN5/SLC17A6/SLC17A7/SLC17A8/SLC30A3/SLC6A17/SLC9B2/SNAP25/SNAP91/SNCAIP/STON1/STX11/STX1A/STX7/SV2A/SYN1/SYN2/SYN3/SYNDIG1/SYNGR3/SYNGR4/SYNPR/SYP/SYT1/SYT12/SYT2/SYT4/SYT5/SYT6/SYT7/SYT8/SYT9/TH/TMEM163/TRIM9/UNC13A/UNC13C/WFS1/ZNRF1	85
cluster5	CC	GO:0070382	exocytic vesicle	90/3954	226/19869	3.71E-12	1.29E-10	1.08E-10	ABCC8/AMPH/APBA1/APP/ATP6V0A1/ATP6V1B1/BDNF/BRSK1/BSN/CBARP/CLCN4/CTTNBP2/DGKI/DOC2A/DPYSL3/DRD2/GABRA2/GAD2/GIPC1/GRIA1/GRIN1/GRIN2A/HAP1/KIRREL3/LAMP1/LAMP5/LGI3/MAL2/MT3/NDEL1/NGF/NKD2/NTF3/OPRK1/OTOF/PENK/PRRT1/PRRT2/PTPRS/RAB27B/RAB3B/RAB5A/RAB5B/RAB8A/RABAC1/SCAMP1/SCAMP5/SEPTIN4/SEPTIN5/SLC17A6/SLC17A7/SLC17A8/SLC30A3/SLC6A17/SLC9B2/SNAP25/SNAP91/SNCAIP/STON1/STX11/STX1A/STX7/SV2A/SYN1/SYN2/SYN3/SYNDIG1/SYNGR3/SYNGR4/SYNPR/SYP/SYT1/SYT10/SYT12/SYT13/SYT2/SYT3/SYT4/SYT5/SYT6/SYT7/SYT8/SYT9/TH/TMEM163/TRIM9/UNC13A/UNC13C/WFS1/ZNRF1	90
cluster5	CC	GO:0044306	neuron projection terminus	61/3954	131/19869	4.86E-12	1.64E-10	1.38E-10	ADORA1/ADRA2A/AP3D1/ATP1A3/AVPR1B/BSN/CALB1/CALB2/CALCA/CDH8/CHRM1/CHRM2/CPLX2/CPLX4/CYFIP1/DMD/DRD2/FLRT1/FLRT3/GAD1/GRIK3/GRIK5/GRIN1/HCN3/ITGA2/KCNC1/KCNC2/NMU/NPY/NTRK2/NTS/OPHN1/OPRK1/P2RX3/PACSIN1/PCSK1/PDYN/PENK/PRKCG/PRRT2/PRSS12/RAB5A/ROR1/SLC17A8/SLC1A1/SLC1A2/SLC6A3/SLC8A2/SLC8A3/SNCB/SYP/SYT1/TH/TNN/TPBG/TULP1/UCHL1/UCN3/UNC13A/UNC13C/USH2A	61
cluster5	CC	GO:0030672	synaptic vesicle membrane	55/3954	114/19869	9.34E-12	2.98E-10	2.5E-10	ABCC8/AMPH/ATP6V0A1/ATP6V1B1/BSN/CBARP/DGKI/DOC2A/DRD2/GABRA2/GAD2/GRIA1/LAMP5/MAL2/OPRK1/OTOF/PRRT1/PRRT2/PTPRS/RAB27B/RAB3B/RAB5A/RAB5B/SCAMP1/SCAMP5/SLC17A6/SLC17A7/SLC17A8/SLC30A3/SLC6A17/SLC9B2/STX1A/SV2A/SYN1/SYN2/SYN3/SYNDIG1/SYNGR3/SYNGR4/SYNPR/SYP/SYT1/SYT12/SYT2/SYT4/SYT5/SYT6/SYT7/SYT8/SYT9/TMEM163/UNC13A/UNC13C/WFS1/ZNRF1	55

cluster5	CC	GO:0099501	exocytic vesicle membrane	55/3954	114/19869	9.34E-12	2.98E-10	2.5E-10	ABCC8/AMPH/ATP6V0A1/ATP6V1B1/BSN/CBARP/DGKI/DOC2A/DRD2/GABRA2/GAD2/GRIA1/LAMP5/MAL2/OPRK1/OTOF/PRRT1/PRRT2/PTPRS/RAB27B/RAB3B/RAB5A/RAB5B/SCAMP1/SCAMP5/SLC17A6/SLC17A7/SLC17A8/SLC30A3/SLC6A17/SLC9B2/STX1A/SV2A/SYN1/SYN2/SYN3/SYNDIG1/SYNGR3/SYNGR4/SYNPR/SYP/SYT1/SYT12/SYT2/SYT4/SYT5/SYT6/SYT7/SYT8/SYT9/TMEM163/UNC13A/UNC13C/WFS1/ZNRF1	55
cluster5	CC	GO:0043679	axon terminus	55/3954	115/19869	1.44E-11	4.49E-10	3.77E-10	ADORA1/ADRA2A/AP3D1/ATP1A3/AVPR1B/CALB1/CALB2/CALCA/CDH8/CHRM1/CHRM2/CPLX2/CPLX4/CYFIP1/DRD2/FLRT3/GAD1/GRIK3/GRIK5/GRIN1/HCN3/ITGA2/KCNC1/KCNC2/NMU/NPY/NTRK2/NTS/OPHN1/OPRK1/P2RX3/PACSIN1/PCSK1/PDYN/PENK/PRKCG/PRRT2/PRSS12/RAB5A/ROR1/SLC17A8/SLC1A1/SLC6A3/SLC8A2/SLC8A3/SNCB/SYP/TH/TNN/TPBG/TULP1/UCN3/UNC13A/UNC13C/USH2A	55
cluster5	CC	GO:0032590	dendrite membrane	28/3954	41/19869	2.43E-11	7.35E-10	6.18E-10	ATF4/ATP2B2/DAGLA/DDN/GABRA1/GABRA2/GABRA3/GABRA4/GABRA5/GABRA6/GABRE/GABRG1/GABRG2/GABRG3/GRIA1/HPCA/KCNB1/KCNC1/KCNC2/KCNC3/LAMP5/MPP2/SHISA6/SHISA7/SHISA9/SLC12A5/TACR3/THY1	28
cluster5	CC	GO:0098686	hippocampal mossy fiber to CA3 synapse	24/3954	34/19869	2.25E-10	6.63E-09	5.57E-09	ADCY1/ADCY8/CACNG2/CALB1/CTNND1/EFNB3/EPHA7/GRIK2/GRIK5/GRIN2D/LRRTM2/MAL2/NECTIN1/NECTIN3/P2RX3/PRKAR1B/PTPRD/SHANK2/SLC30A3/SYT1/SYT12/SYT7/SYT9/YWHAZ	24

cluster5	CC	GO:0048786	presynaptic active zone	39/3954	75/19869	5.89E-10	1.69E-08	1.42E-08	ADCY8/ADORA1/ADRA2A/APBA1/APP/ARHGAP44/ATP2B2/BRSK1/BSN/CDH10/CDH2/CNTNAP1/CTBP1/CTNND1/ERC1/FZD3/GAD1/GPM6A/GRM7/LRFN3/NECTIN1/NPTN/NTNG1/NTNG2/OTOF/PCLO/PPFIA2/RIMS3/RIMS4/SHANK2/SLC17A7/STX11/STX1A/STXBP1/SV2A/SYN1/SYP/UNC13A/UNC13C	39
cluster5	CC	GO:0044304	main axon	34/3954	63/19869	2.06E-09	0.000000058	4.87E-08	ADORA1/ANK3/APP/CLDN5/CNGA3/CNTNAP1/CNTNAP2/DAG1/DAGLA/HAPLN2/IQCJ-SCHIP1/KCNA4/KCNC1/KCNC2/KCNH1/KCNQ2/KCNQ3/MAP1A/MAP2/MYOC/NFASC/NRCAM/PARD3/ROBO2/SCN1A/SCN2A/SCN8A/SIRT2/SLC1A2/SPOCK1/SPTBN4/THY1/TUBB4A/UCN3	34
cluster5	CC	GO:0008328	ionotropic glutamate receptor complex	25/3954	40/19869	4.76E-09	0.000000131	0.00000011	CACNG2/CACNG3/CACNG4/CACNG5/CACNG7/CPT1C/EPS8/GRIA1/GRIA2/GRIA3/GRIA4/GRID2/GRIK2/GRIK3/GRIK5/GRIN1/GRIN2A/GRIN2B/GRIN2D/OLFM3/SHISA6/SHISA7/SHISA9/VWC2/VWC2L	25
cluster5	CC	GO:0034705	potassium channel complex	42/3954	89/19869	5.96E-09	0.00000016	0.000000134	ABCC8/ABCC9/CNTNAP2/DPP10/DPP6/GRIK2/GRIK3/GRIK5/HCN1/HCN3/HCN4/KCNA10/KCNA4/KCNA7/KCNB1/KCNB2/KCNC1/KCNC2/KCNC3/KCND2/KCND3/KCNG3/KCNH1/KCNH2/KCNH4/KCNIP1/KCNIP3/KCNJ16/KCNJ3/KCNJ4/KCNJ5/KCNJ6/KCNK6/KCNMA1/KCNMB2/KCNN1/KCNQ2/KCNQ3/KCNQ4/KCNS2/KCNV1/LRRC55	42

cluster5	CC	GO:0098685	Schaffer collateral - CA1 synapse	36/3954	72/19869	1.05E-08	0.000000276	0.000000232	ADCY1/ADCY8/ADORA2B/APBA1/BSN/CACNG2/CACNG3/CDH11/CHRM1/CTNND1/EFNB2/EPHA7/GHSR/GIPC1/GRM5/GSG1L/INA/IQSEC2/ITGB1/LRRC4C/LRRTM2/NEFH/NPTN/NTNG1/NTNG2/P2RX3/PFN2/PRKAR1B/PTPRD/SHANK1/SLC30A1/SYN1/SYN2/SYP/TNR/WNT7A	36
cluster5	CC	GO:0043204	perikaryon	61/3954	154/19869	1.33E-08	0.000000342	0.000000287	ADCYAP1/APP/ASTN1/AVPR1B/CACNA1C/CDK5R1/CLCN2/CNGA3/CNTNAP2/CPLX2/CPNE5/CPNE6/CRMP1/CRYAB/CTNND2/DDN/DRD2/EFNA2/ELAVL4/GAP43/GLRA1/GRIK3/GRIK5/GRIP1/HDAC6/HPCA/KCNB1/KCNB2/KCNC2/KCNC3/KCND2/KCNH1/KIF5A/KNDC1/NEURL1/NEUROG1/NGFR/NPY/OLFM1/OPN4/OPRK1/PCSK1/PCSK2/PDE11A/PENK/PRPH/PTPRS/RGS7BP/RGS8/RTN4RL1/RTN4RL2/SEPTIN4/SIRT2/SLC12A5/SLC17A8/SLC1A1/SLC5A7/SLC8A2/SLC8A3/TH/TTLL7	61
cluster5	CC	GO:0098878	neurotransmitter receptor complex	26/3954	45/19869	2.45E-08	0.000000615	0.000000517	CACNG2/CACNG3/CACNG4/CACNG5/CACNG7/CPT1C/EPS8/GRIA1/GRIA2/GRIA3/GRIA4/GRID2/GRIK2/GRIK3/GRIK5/GRIN1/GRIN2A/GRIN2B/GRIN2D/HTR3C/OLFM3/SHISA6/SHISA7/SHISA9/VWC2/VWC2L	26
cluster5	CC	GO:0008076	voltage-gated potassium channel complex	37/3954	79/19869	6.05E-08	0.00000149	0.00000125	CNTNAP2/DPP10/DPP6/HCN1/HCN3/HCN4/KCNA10/KCNA4/KCNA7/KCNB1/KCNB2/KCNC1/KCNC2/KCNC3/KCND2/KCND3/KCNG3/KCNH1/KCNH2/KCNH4/KCNIP1/KCNIP3/KCNJ16/KCNJ3/KCNJ4/KCNJ5/KCNJ6/KCNK6/KCNMA1/KCNMB2/KCNN1/KCNQ2/KCNQ3/KCNQ4/KCNS2/KCNV1/LRRC55	37

cluster5	CC	GO:0030133	transport vesicle	128/3954	423/19869	0.000000196	0.00000473	0.00000397	ABCA12/ABCC8/AMPH/AP1M2/AP1S2/AP1S3/APBA1/APP/ATP6V0A1/ATP6V1B1/BDNF/BET1/BLOC1S6/BRSK1/BSN/CBARP/CHGA/CLCN4/CLRN1/CRISPLD2/CTTNBP2/DBH/DGKI/DOC2A/DPYSL3/DRD2/EEF1AKMT4-ECE2/EXOC3L1/FGFR3/FGFR4/FOLR1/GABRA2/GAD2/GALNT15/GIPC1/GOSR1/GRIA1/GRIN1/GRIN2A/HAP1/HLA-B/HLA-C/INS/KDELR1/KIRREL3/LAMP1/LAMP5/LGI3/MAL2/MAP6/MT3/NDEL1/NGF/NKD2/NPTX1/NRSN1/NRSN2/NTF3/NTS/OPRK1/OTOF/PCSK1/PCSK2/PENK/PRRT1/PRRT2/PTPRS/RAB27B/RAB3B/RAB5A/RAB5B/RAB8A/RABAC1/RPH3AL/SCAMP1/SCAMP5/SCG3/SEPTIN4/SEPTIN5/SLC17A6/SLC17A7/SLC17A8/SLC30A3/SLC30A8/SLC6A17/SLC9B2/SNAP25/SNAP91/SNCAIP/SORT1/SPX/SREBF2/SSPN/STEAP2/STON1/STX11/STX1A/STX7/SV2A/SYN1/SYN2/SYN3/SYNDIG1/SYNGR3/SYNGR4/SYNPR/SYP/SYT1/SYT10/SYT12/SYT13/SYT2/SYT3/SYT4/SYT5/SYT6/SYT7/SYT8/SYT9/TH/TMED10/TMEM163/TMEM30A/TRIM9/UNC13A/UNC13C/WFS1/ZNRF1	128
cluster5	CC	GO:0034707	chloride channel complex	27/3954	52/19869	0.000000267	0.00000631	0.0000053	ANO6/BEST2/BEST3/CFTR/CLCN2/CLCNKA/CLCNKB/CLDN4/CLIC6/GABRA1/GABRA2/GABRA3/GABRA4/GABRA5/GABRA6/GABRB2/GABRB3/GABRD/GABRE/GABRG1/GABRG2/GABRG3/GABRQ/GLRA1/GLRA2/GLRB/TTYH1	27
cluster5	CC	GO:0043195	terminal bouton	25/3954	47/19869	0.000000404	0.00000935	0.00000786	ADORA1/AP3D1/CALB1/CALB2/CALCA/CPLX2/CPLX4/CYFIP1/GRIK3/GRIK5/GRIN1/KCNC2/NMU/NPY/NTRK2/OPHN1/P2RX3/PRSS12/RAB5A/SYP/TH/TNN/UNC13A/UNC13C/USH2A	25
cluster5	CC	GO:0099568	cytoplasmic region	92/3954	285/19869	0.000000467	0.0000105	0.00000882	ABHD13/AGBL4/AK8/AKAP14/AP3B2/AP3D1/ARC/ARMCX3/BBS1/BLOC1S6/BSN/CCDC103/CCDC40/CFAP126/CFAP206/CFAP221/CFAP43/CFAP46/CFAP52/CFAP54/CFAP61/CFAP74/CFAP91/CTBP1/CTNND1/DCX/DNAH10/DNAH11/DNAH12/DNAH14/DNAH2/DNAH3/DNAH5/DNAH7/DNAH9/DNAI1/DNAI3/DNALI1/DRC1/DYNC2I2/EFHC2/ENKUR/ERC1/GLI3/GNAI1/GRIK3/HAP1/HDAC6/HIF1A/HPCA/HSBP1/HYDIN/IFT140/KHDC3L/KIF1A/KIF3A/KIF5A/KIF5C/LCA5/MAP1A/MAP1LC3B/MAP2/MNS1/MYO5B/NDEL1/ODAD2/ODAD3/OOEP/PACRG/PCLO/PRKAR1A/RAB27B/RIMS3/RP1/RSPH6A/RSPH9/SAXO1/SPAG17/SPAG6/SPEF1/SYBU/TEKT1/TEKT2/TMEM108/TOGARAM1/TRAK1/TUBB4A/TULP3/UCHL1/UNC13A/UNC13C/WASF1	92

cluster5	CC	GO:1902710	GABA receptor complex	15/3954	21/19869	0.000000471	0.0000105	0.00000882	GABBR2/GABRA1/GABRA2/GABRA3/GABRA4/GABRA5/GABRA6/GABRB2/GABRB3/GABRD/GABRE/GABRG1/GABRG2/GABRG3/GABRQ	15
cluster5	CC	GO:0098563	intrinsic component of synaptic vesicle membrane	23/3954	42/19869	0.000000591	0.0000129	0.0000109	GABRA2/MAL2/OPRK1/PTPRS/RAB27B/RAB3B/RAB5A/RAB5B/SCAMP1/SLC17A6/SLC17A7/SLC17A8/SLC30A3/SLC6A17/STX1A/SV2A/SYN1/SYP/SYT1/SYT4/SYT9/TMEM163/WFS1	23
cluster5	CC	GO:1902711	GABA-A receptor complex	14/3954	19/19869	0.000000628	0.0000132	0.0000111	GABRA1/GABRA2/GABRA3/GABRA4/GABRA5/GABRA6/GABRB2/GABRB3/GABRD/GABRE/GABRG1/GABRG2/GABRG3/GABRQ	14
cluster5	CC	GO:0031514	motile cilium	81/3954	244/19869	0.000000628	0.0000132	0.0000111	ABHD2/ACE/AK7/AK8/AKAP4/ATP1B1/BBS1/C2CD6/CABCOCO1/CABS1/CABYR/CATSPER4/CATSPERG/CCDC103/CCDC181/CFAP206/CFAP221/CFAP300/CFAP43/CFAP47/CFAP52/CFAP61/CFAP65/CFAP69/CFAP91/CFAP99/DAAM1/DCDC2C/DNAH10/DNAH11/DNAH2/DNAH3/DNAH5/DNAH9/DNAI1/DNALI1/DRC1/DRD2/ENKUR/ENO4/FSCB/FSIP2/GK2/HIF1A/IFT81/IQCA1/IQUB/LDHC/MNS1/NME5/ODF1/ODF3/PACRG/PRKAR1A/RHO/ROPN1/RSPH1/RSPH6A/RSPH9/SAXO1/SEPTIN4/SLC25A31/SLC9B2/SPACA9/SPAG17/SPAG6/SPEF1/SQSTM1/TACR1/TACR3/TCP11/TCP11X1/TCTE1/TEKT1/TEKT2/TEKT5/TMEM249/TSSK1B/TTC29/TTLL9/ZBBX	81

cluster5	CC	GO:0097729	9+2 motile cilium	59/3954	165/19869	0.00000141	0.0000291	0.0000245	ABHD2/ACE/AK8/AKAP4/ATP1B1/C2CD6/CABCOCO1/CABS1/CABYR/CATSPER4/CATSPERG/CCDC181/CFAP221/CFAP43/CFAP47/CFAP52/CFAP65/CFAP69/DCDC2C/DNAH10/DNAH11/DNAH2/DNAH3/DNAH5/DNAH9/DNAI1/DNALI1/DRD2/ENKUR/ENO4/FSCB/FSIP2/GK2/IFT81/MNS1/NME5/ODF1/ODF3/PACRG/PRKAR1A/RHO/RSPH1/RSPH6A/RSPH9/SAXO1/SEPTIN4/SLC9B2/SPACA9/SPAG6/SPEF1/SQSTM1/TACR1/TACR3/TCP11/TCP11X1/TCTE1/TEKT5/TMEM249/TTC29	59
cluster5	CC	GO:0030285	integral component of synaptic vesicle membrane	18/3954	30/19869	0.00000167	0.000034	0.0000286	GABRA2/MAL2/OPRK1/PTPRS/SCAMP1/SLC17A6/SLC17A7/SLC17A8/SLC30A3/SLC6A17/STX1A/SV2A/SYP/SYT1/SYT4/SYT9/TMEM163/WFS1	18
cluster5	CC	GO:0031256	leading edge membrane	62/3954	177/19869	0.00000171	0.0000342	0.0000288	ADGRV1/ADORA1/AMPH/APC2/ARHGAP44/ARHGEF4/ATF4/ATP2B2/CLRN2/CNTNAP2/CSPG4/DAGLA/DDN/EGFR/EPS8/EPS8L3/FAP/FERMT1/GABRA1/GABRA2/GABRA3/GABRA4/GABRA5/GABRA6/GABRE/GABRG1/GABRG2/GABRG3/GRIA1/HPCA/ITGB1/ITGB3/KCNB1/KCNC1/KCNC2/KCNC3/KCNH1/LAMP5/MPP2/MYO1C/MYO6/NCKAP1/PACSIN1/PDPN/PLEKHO1/PSD/PSD2/ROBO2/SHISA6/SHISA7/SHISA9/SLC12A5/SLC1A2/SNTG1/SRC/SYNE2/TACR3/THY1/TPM1/TRPV4/USH2A/WWC1	62
cluster5	CC	GO:0032838	plasma membrane bounded cell projection cytoplasm	80/3954	246/19869	0.00000184	0.0000362	0.0000304	ABHD13/AGBL4/AK8/AKAP14/AP3B2/AP3D1/ARC/ARMCX3/BBS1/BLOC1S6/CCDC103/CCDC40/CFAP126/CFAP206/CFAP221/CFAP43/CFAP46/CFAP52/CFAP54/CFAP61/CFAP74/CFAP91/DCX/DNAH10/DNAH11/DNAH12/DNAH14/DNAH2/DNAH3/DNAH5/DNAH7/DNAH9/DNAI1/DNAI3/DNALI1/DRC1/DYNC2I2/EFHC2/ENKUR/GLI3/GRIK3/HAP1/HDAC6/HIF1A/HPCA/HSBP1/HYDIN/IFT140/KIF1A/KIF3A/KIF5A/KIF5C/LCA5/MAP1A/MAP1LC3B/MAP2/MNS1/NDEL1/ODAD2/ODAD3/PACRG/PRKAR1A/RAB27B/RP1/RSPH6A/RSPH9/SAXO1/SPAG17/SPAG6/SPEF1/SYBU/TEKT1/TEKT2/TMEM108/TOGARAM1/TRAK1/TUBB4A/TULP3/UCHL1/WASF1	80

cluster5	CC	GO:0033268	node of Ranvier	12/3954	16/19869	0.00000309	0.0000597	0.0000502	ANK3/DAG1/HAPLN2/KCNQ2/KCNQ3/MYOC/NFASC/SCN1A/SCN2A/SCN8A/SPOCK1/SPTBN4	12
cluster5	CC	GO:0030658	transport vesicle membrane	73/3954	222/19869	0.00000325	0.0000618	0.0000519	ABCA12/ABCC8/AMPH/AP1M2/AP1S2/AP1S3/ATP6V0A1/ATP6V1B1/BSN/CBARP/DBH/DGKI/DOC2A/DRD2/EEF1AKMT4-ECE2/FOLR1/GABRA2/GAD2/GRIA1/HLA-B/HLA-C/LAMP5/MAL2/MAP6/OPRK1/OTOF/PRRT1/PRRT2/PTPRS/RAB27B/RAB3B/RAB5A/RAB5B/RPH3AL/SCAMP1/SCAMP5/SCG3/SLC17A6/SLC17A7/SLC17A8/SLC30A3/SLC30A8/SLC6A17/SLC9B2/SREBF2/STX1A/SV2A/SYN1/SYN2/SYN3/SYNDIG1/SYNGR3/SYNGR4/SYNPR/SYP/SYT1/SYT10/SYT12/SYT2/SYT3/SYT4/SYT5/SYT6/SYT7/SYT8/SYT9/TMED10/TMEM163/TMEM30A/UNC13A/UNC13C/WFS1/ZNRF1	73
cluster5	CC	GO:0032281	AMPA glutamate receptor complex	16/3954	26/19869	0.000004	0.000075	0.000063	CACNG2/CACNG3/CACNG4/CACNG5/CACNG7/CPT1C/GRIA1/GRIA2/GRIA3/GRIA4/OLFM3/SHISA6/SHISA7/SHISA9/VWC2/VWC2L	16
cluster5	CC	GO:0032809	neuronal cell body membrane	17/3954	29/19869	0.0000051	0.0000941	0.000079	ADCY8/ATP1A3/ATP2B2/FLRT1/GABRA5/HPCA/KCNB1/KCNB2/KCNC1/KCNC2/KCNC3/KCND2/RGS8/SLC6A2/SLC6A3/TACR3/THY1	17

cluster5	CC	GO:0031225	anchored component of membrane	58/3954	169/19869	0.00000749	0.000136033	0.000114311	ALPG/BCAN/CEACAM5/CFC1/CNTFR/CNTN3/CNTN4/CNTN5/CNTN6/EFNA2/EFNA3/EFNA5/ENPP6/FOLR1/GAD2/GFRA1/GFRA2/GFRA3/GPC2/GPC3/GPC6/IGSF21/LY6G6C/LY6G6D/LY6H/LY6K/LYPD1/LYPD5/LYPD6/MDGA2/NCAM1/NTM/NTNG1/NTNG2/OPCML/PKHD1/PRND/PRSS55/PSCA/RAB27B/RAB3B/RAB5A/RAB5B/RAET1G/RAET1L/RGS7BP/RTN4RL1/RTN4RL2/SLURP2/SPACA4/SYN1/TDGF1/TECTB/TFPI/THY1/TREH/ULBP1/ULBP2	58
cluster5	CC	GO:0031253	cell projection membrane	102/3954	344/19869	0.00000873	0.000156078	0.000131156	ABCG2/ACE/ACE2/ADCY3/ADGRV1/ADORA1/APC2/ARHGEF4/ATF4/ATP2B2/ATP6V0A4/B4GALT1/BBS1/BVES/CA9/CLRN2/CLTRN/CNGB1/CNTNAP2/CSPG4/DAGLA/DDN/DMD/DMTN/DRD1/DRD2/EGFR/EHD3/EPS8/EPS8L3/EVC/EVC2/FAP/FERMT1/FOLR1/GABRA1/GABRA2/GABRA3/GABRA4/GABRA5/GABRA6/GABRE/GABRG1/GABRG2/GABRG3/GAP43/GNAT1/GPR161/GRIA1/HHIP/HPCA/ITGB1/ITGB3/KCNB1/KCNC1/KCNC2/KCNC3/KCNH1/LAMP5/LRP2/MCHR1/MPP2/MYO1C/MYO6/NCKAP1/NDRG4/PACSIN1/PDPN/PLEKHO1/PROM1/PROM2/PSD/PSD2/PTCH1/RAB8A/RHO/ROBO2/SHANK2/SHISA6/SHISA7/SHISA9/SLC12A5/SLC1A2/SLC22A12/SLC28A3/SLC34A1/SLC34A2/SLC5A1/SLC7A8/SMO/SNTG1/SRC/SYNE2/TACR3/THY1/TMEM17/TMEM231/TPM1/TRPV4/TTYH1/USH2A/WWC1	102
cluster5	CC	GO:0005930	axoneme	54/3954	156/19869	0.0000115	0.000202184	0.0001699	AK8/AKAP14/BBS1/CCDC103/CCDC40/CFAP126/CFAP206/CFAP221/CFAP43/CFAP46/CFAP52/CFAP54/CFAP61/CFAP74/CFAP91/DCX/DNAH10/DNAH11/DNAH12/DNAH14/DNAH2/DNAH3/DNAH5/DNAH7/DNAH9/DNAI1/DNAI3/DNALI1/DRC1/DYNC2I2/EFHC2/ENKUR/GLI3/HYDIN/IFT140/LCA5/MAP1LC3B/MNS1/ODAD2/ODAD3/PACRG/PRKAR1A/RP1/RSPH6A/RSPH9/SAXO1/SPAG17/SPAG6/SPEF1/TEKT1/TEKT2/TOGARAM1/TUBB4A/TULP3	54
cluster5	CC	GO:0031252	cell leading edge	120/3954	421/19869	0.0000119	0.000206281	0.000173342	ABL1/ABLIM3/ACTA1/ACTC1/ACTN1/ADGRV1/ADORA1/AJUBA/AKT1/AMPH/APC2/APP/ARHGAP18/ARHGAP44/ARHGEF26/ARHGEF4/ASAP3/ATF4/ATP2B2/BRK1/CARMIL1/CARMIL3/CDH2/CLIP1/CLRN1/CLRN2/CNTNAP2/CSPG4/CTNND1/CYFIP1/DAG1/DAGLA/DBN1/DDN/DDX3X/DPYSL3/DUOX2/DUOXA2/EGFR/EPS8/EPS8L3/FAP/FAT1/FER/FERMT1/FGD1/FRMD4B/GABRA1/GABRA2/GABRA3/GABRA4/GABRA5/GABRA6/GABRE/GABRG1/GABRG2/GABRG3/GDPD2/GRIA1/HDAC6/HPCA/IGF2BP1/ILK/ITGB1/ITGB3/KCNB1/KCNC1/KCNC2/KCNC3/KCNH1/KIF18A/KITLG/LAMP5/MCC/MPP2/MYADM/MYH10/MYH9/MYLK/MYO1C/MYO6/NCKAP1/NDEL1/PACSIN1/PDPN/PKN2/PLEKHG5/PLEKHO1/PSD/PSD2/PSTPIP1/RAB5A/RASA1/ROBO2/ROCK1/SH3RF1/SHISA6/SHISA7/SHISA9/SLC12A5/SLC1A2/SNTG1/SNX9/SPEF1/SRC/SRCIN1/STMN2/STON1/SYNE2/TACR3/THY1/TPM1/TRPV4/TUBB3/UNC5C/USH2A/WASF1/WASF3/WIPF1/WWC1	120

cluster5	CC	GO:0045121	membrane raft	97/3954	326/19869	0.0000122	0.000209449	0.000176004	ABCG2/ACE2/ADCY1/ADCY2/ADCY8/ADCYAP1R1/ADRA1A/ADRA1B/AKAP6/ANGPT1/APP/ARC/ARID3C/ATP1A2/ATP1B1/ATP2B2/BCL10/BVES/CAVIN3/CAVIN4/CBLB/CBLC/CDH2/CHRNA3/CHRNA7/CLIP3/DAG1/DLL1/DMD/EDNRB/EFNA5/EGFR/EMP2/F2R/FAIM2/FYB2/GASK1A/GHSR/GP6/GPM6B/HAS2/HDAC6/HTR2A/ICAM1/ITGAM/ITGB1/KCND2/KCNMA1/KIF18A/LAMTOR1/LRP4/LRP6/LY6K/LYN/LYPD6/MAL2/MALL/MYADM/MYO1C/NOS3/P2RX3/PDPN/PECAM1/PGK1/PLPP3/PLVAP/PRKAR1A/PROM2/PRTN3/PTCH1/PTGS2/RAB5A/RET/RGS7/RIT2/RTN4RL1/RTN4RL2/SDC4/SGCA/SHH/SLC1A1/SLC1A2/SLC34A1/SLC6A2/SLC6A3/SMO/SRC/STOM/STOML3/SULF1/TDGF1/TFPI/THY1/TNR/TRPC4/TRPM8/UNC5B	97
cluster5	CC	GO:0098857	membrane microdomain	97/3954	327/19869	0.0000141	0.000228767	0.000192238	ABCG2/ACE2/ADCY1/ADCY2/ADCY8/ADCYAP1R1/ADRA1A/ADRA1B/AKAP6/ANGPT1/APP/ARC/ARID3C/ATP1A2/ATP1B1/ATP2B2/BCL10/BVES/CAVIN3/CAVIN4/CBLB/CBLC/CDH2/CHRNA3/CHRNA7/CLIP3/DAG1/DLL1/DMD/EDNRB/EFNA5/EGFR/EMP2/F2R/FAIM2/FYB2/GASK1A/GHSR/GP6/GPM6B/HAS2/HDAC6/HTR2A/ICAM1/ITGAM/ITGB1/KCND2/KCNMA1/KIF18A/LAMTOR1/LRP4/LRP6/LY6K/LYN/LYPD6/MAL2/MALL/MYADM/MYO1C/NOS3/P2RX3/PDPN/PECAM1/PGK1/PLPP3/PLVAP/PRKAR1A/PROM2/PRTN3/PTCH1/PTGS2/RAB5A/RET/RGS7/RIT2/RTN4RL1/RTN4RL2/SDC4/SGCA/SHH/SLC1A1/SLC1A2/SLC34A1/SLC6A2/SLC6A3/SMO/SRC/STOM/STOML3/SULF1/TDGF1/TFPI/THY1/TNR/TRPC4/TRPM8/UNC5B	97
cluster5	CC	GO:0097014	ciliary plasm	54/3954	157/19869	0.0000141	0.000228767	0.000192238	AK8/AKAP14/BBS1/CCDC103/CCDC40/CFAP126/CFAP206/CFAP221/CFAP43/CFAP46/CFAP52/CFAP54/CFAP61/CFAP74/CFAP91/DCX/DNAH10/DNAH11/DNAH12/DNAH14/DNAH2/DNAH3/DNAH5/DNAH7/DNAH9/DNAI1/DNAI3/DNALI1/DRC1/DYNC2I2/EFHC2/ENKUR/GLI3/HYDIN/IFT140/LCA5/MAP1LC3B/MNS1/ODAD2/ODAD3/PACRG/PRKAR1A/RP1/RSPH6A/RSPH9/SAXO1/SPAG17/SPAG6/SPEF1/TEKT1/TEKT2/TOGARAM1/TUBB4A/TULP3	54
cluster5	CC	GO:0043194	axon initial segment	13/3954	20/19869	0.0000142	0.000228767	0.000192238	ANK3/CNGA3/IQCJ-SCHIP1/KCNA4/KCNQ2/KCNQ3/MAP1A/MAP2/NFASC/NRCAM/SCN1A/SCN8A/SPTBN4	13

cluster5	CC	GO:0060077	inhibitory synapse	13/3954	20/19869	0.0000142	0.000228767	0.000192238	GABRA2/GABRG2/GAD1/GAD2/GLRA1/IGSF21/IGSF9/LHFPL4/NLGN1/NLGN2/NLGN3/NLGN4X/NPTN	13
cluster5	CC	GO:0031594	neuromuscular junction	29/3954	68/19869	0.0000157	0.000249901	0.000209997	ANK3/APP/CDK5R1/CIB2/COL4A5/DES/DLGAP3/EFNA2/EPHA7/F2R/ITGB1/LAMA5/LAMB2/LRP4/MUSK/MYH9/NLGN1/PDZRN3/PRKAR1A/SERPINE2/SLC5A7/SLC8A3/SPOCK1/SV2A/SYNGR3/SYNGR4/SYP/UNC13A/UNC13C	29
cluster5	CC	GO:0005788	endoplasmic reticulum lumen	93/3954	312/19869	0.000017	0.000266761	0.000224165	ACE2/ADAM10/ADAMTS13/ADAMTS7/ADAMTSL1/AMBN/AMELX/APOA5/APP/ARSI/ARSJ/ARSL/BCHE/BMP4/CASQ2/CDH2/CES3/CHGB/CHRDL1/CNPY3/COL11A1/COL12A1/COL14A1/COL16A1/COL20A1/COL21A1/COL22A1/COL23A1/COL24A1/COL25A1/COL26A1/COL27A1/COL28A1/COL2A1/COL4A1/COL4A5/COL4A6/COL5A3/COL6A1/COL8A1/COL9A1/CP/CST3/CYP2W1/DAG1/DBI/ERO1A/EVA1A/F2/F5/FGF23/FKBP10/FKBP7/FOXRED2/GAS6/GCG/GPC3/GPX8/HRC/IGFBP4/INS/KNG1/LAMB1/LAMB2/LIPC/LTBP1/MATN3/MMP24-AS1-EDEM2/PDGFA/PDGFC/PDIA2/PENK/PPIB/PTGS2/QSOX1/SCG2/SCG3/SDC2/SHH/STC2/TIMP1/TMEM132A/TNC/TRDN/TSPAN33/UGGT2/VWA1/WFS1/WNT3A/WNT4/WNT5A/WNT7A/WNT7B	93
cluster5	CC	GO:0005581	collagen trimer	34/3954	86/19869	0.0000215	0.000333536	0.000280277	C1QL4/CCBE1/COL11A1/COL12A1/COL14A1/COL16A1/COL20A1/COL21A1/COL22A1/COL23A1/COL24A1/COL25A1/COL26A1/COL27A1/COL28A1/COL2A1/COL4A1/COL4A5/COL4A6/COL5A3/COL6A1/COL6A5/COL6A6/COL8A1/COL9A1/COLEC10/COLEC12/EMILIN1/EMILIN2/GLDN/LOX/MBL2/SFTPA1/SFTPA2	34

cluster5	CC	GO:0030426	growth cone	56/3954	167/19869	0.0000229	0.000351584	0.000295444	ABL1/APP/ATCAY/AUTS2/BASP1/BOC/CBARP/CDK5R1/CDK5R2/CRMP1/CSNK1E/CTNND1/CYFIP1/DBN1/DPYSL3/DSCAM/ELAVL4/EPS8/FEZ1/FLRT3/GAP43/GPM6A/GPRIN1/HAP1/IGF2BP1/KIF20B/KIF21B/KIF5C/L1CAM/LAMP5/MAP2/NDEL1/NECTIN1/NGEF/NGFR/NRSN1/OLFM1/OTX2/PCDH9/PCDHGB1/PTCH1/PTPRS/RASGRF1/SHANK2/SIRT2/SLC2A13/SNAP25/SRC/STMN2/STMN4/TENM2/THY1/TRPV4/TUBB3/UNC5C/WHRN	56
cluster5	CC	GO:0031045	dense core granule	15/3954	26/19869	0.0000242	0.000365837	0.00030742	CADPS/CHGA/KIF1A/NPY/PENK/SCG2/SLC6A5/SPX/SYT1/SYT2/SYT4/SYT5/SYT7/SYT8/SYT9	15
cluster5	CC	GO:0030017	sarcomere	68/3954	214/19869	0.0000246	0.000367455	0.00030878	ABCC9/ACTA1/ACTC1/ACTN1/ACTN2/ADRA1A/AKAP4/ANK3/C10orf71/CACNA1C/CACNA1S/CASQ2/CAVIN4/CMYA5/CRYAB/CSRP2/CSRP3/DCTN4/DES/DMD/FBP2/FHOD3/FKBP1A/FKBP1B/HOMER1/HRC/ILK/JPH1/KCNN2/KLHL40/LDB3/LMOD2/MYBPHL/MYH1/MYH2/MYH4/MYH6/MYH7/MYH8/MYL1/MYL12A/MYL7/MYO18B/MYOZ1/MYOZ2/MYOZ3/MYPN/NEXN/NRAP/PRICKLE4/PVALEF/RTN2/RYR2/RYR3/SCN1A/SCN3B/SCN8A/SMPX/SQSTM1/STYXL2/SYNE2/SYNPO2L/TNNI1/TNNI3/TNNT2/TPM1/TRIM54/TRIM63	68
cluster5	CC	GO:0048787	presynaptic active zone membrane	16/3954	29/19869	0.0000277	0.000408638	0.000343387	ADRA2A/APBA1/ATP2B2/CDH10/CDH2/CNTNAP1/GPM6A/LRFN3/NECTIN1/NPTN/NTNG1/NTNG2/OTOF/STX11/STX1A/STXBP1	16

cluster5	CC	GO:0044298	cell body membrane	17/3954	32/19869	0.0000301	0.000437891	0.000367969	ADCY8/ATP1A3/ATP2B2/FLRT1/GABRA5/HPCA/KCNB1/KCNB2/KCNC1/KCNC2/KCNC3/KCND2/RGS8/SLC6A2/SLC6A3/TACR3/THY1	17
cluster5	CC	GO:0030427	site of polarized growth	57/3954	173/19869	0.0000346	0.000497404	0.000417979	ABL1/APP/ATCAY/AUTS2/BASP1/BOC/CBARP/CDK5R1/CDK5R2/CRMP1/CSNK1E/CTNND1/CYFIP1/DBN1/DPYSL3/DSCAM/ELAVL4/EPS8/FEZ1/FLRT3/GAP43/GPM6A/GPRIN1/GRM6/HAP1/IGF2BP1/KIF20B/KIF21B/KIF5C/L1CAM/LAMP5/MAP2/NDEL1/NECTIN1/NGEF/NGFR/NRSN1/OLFM1/OTX2/PCDH9/PCDHGB1/PTCH1/PTPRS/RASGRF1/SHANK2/SIRT2/SLC2A13/SNAP25/SRC/STMN2/STMN4/TENM2/THY1/TRPV4/TUBB3/UNC5C/WHRN	57
cluster5	CC	GO:0030016	myofibril	72/3954	233/19869	0.0000407	0.00057799	0.000485697	ABCC9/ACTA1/ACTC1/ACTN1/ACTN2/ADRA1A/AKAP4/ANK3/C10orf71/CACNA1C/CACNA1S/CASQ2/CAVIN4/CMYA5/CRYAB/CSRP2/CSRP3/DAG1/DCTN4/DES/DMD/FBP2/FHOD3/FKBP1A/FKBP1B/HOMER1/HRC/ILK/JPH1/KCNN2/KLHL40/LDB3/LMOD2/MYBPC1/MYBPHL/MYH1/MYH13/MYH2/MYH4/MYH6/MYH7/MYH8/MYL1/MYL12A/MYL7/MYO18B/MYOZ1/MYOZ2/MYOZ3/MYPN/NEXN/NRAP/PRICKLE4/PVALEF/RTN2/RYR2/RYR3/SCN1A/SCN3B/SCN8A/SDC4/SMPX/SQSTM1/STYXL2/SYNE2/SYNPO2L/TNNI1/TNNI3/TNNT2/TPM1/TRIM54/TRIM63	72
cluster5	CC	GO:0089717	spanning component of membrane	14/3954	25/19869	0.0000712	0.000999791	0.000840145	AJAP1/EGFR/ERBB2/ERBB3/ERBB4/NLGN1/NLGN2/NLGN3/NLGN4X/NPC1L1/SLC24A2/SLC24A3/SLC4A4/TRPM4	14

cluster5	CC	GO:0016342	catenin complex	16/3954	31/19869	0.0000816	0.001132769	0.000951889	APC2/CDH10/CDH11/CDH12/CDH17/CDH18/CDH19/CDH2/CDH22/CDH4/CDH6/CDH7/CDH8/CDH9/CTNNA2/CTNND1	16
cluster5	CC	GO:0043292	contractile fiber	73/3954	242/19869	0.0000857	0.001175704	0.000987969	ABCC9/ACTA1/ACTC1/ACTN1/ACTN2/ADRA1A/AKAP4/ANK3/C10orf71/CACNA1C/CACNA1S/CASQ2/CAVIN4/CDK5R1/CMYA5/CRYAB/CSRP2/CSRP3/DAG1/DCTN4/DES/DMD/FBP2/FHOD3/FKBP1A/FKBP1B/HOMER1/HRC/ILK/JPH1/KCNN2/KLHL40/LDB3/LMOD2/MYBPC1/MYBPHL/MYH1/MYH13/MYH2/MYH4/MYH6/MYH7/MYH8/MYL1/MYL12A/MYL7/MYO18B/MYOZ1/MYOZ2/MYOZ3/MYPN/NEXN/NRAP/PRICKLE4/PVALEF/RTN2/RYR2/RYR3/SCN1A/SCN3B/SCN8A/SDC4/SMPX/SQSTM1/STYXL2/SYNE2/SYNPO2L/TNNI1/TNNI3/TNNT2/TPM1/TRIM54/TRIM63	73
cluster5	CC	GO:0005882	intermediate filament	66/3954	215/19869	0.000103428	0.00140282	0.001178819	CLIP1/DES/DLGAP2/EVPL/FER/GFAP/INA/KRT15/KRT20/KRT222/KRT27/KRT28/KRT39/KRT6C/KRT75/KRT76/KRT8/KRT80/KRT83/KRT84/KRTAP1-1/KRTAP1-4/KRTAP10-10/KRTAP10-12/KRTAP10-2/KRTAP10-4/KRTAP10-7/KRTAP10-8/KRTAP12-4/KRTAP13-2/KRTAP13-3/KRTAP15-1/KRTAP19-1/KRTAP19-7/KRTAP2-1/KRTAP21-1/KRTAP21-2/KRTAP21-3/KRTAP22-2/KRTAP24-1/KRTAP25-1/KRTAP27-1/KRTAP29-1/KRTAP3-1/KRTAP3-2/KRTAP4-8/KRTAP5-1/KRTAP5-11/KRTAP5-3/KRTAP5-4/KRTAP5-7/KRTAP9-2/KRTAP9-4/KRTAP9-6/KRTAP9-7/KRTAP9-9/LMNA/LMNTD1/MNS1/NEFH/NEFM/PKP2/PRPH/RTN2/SHANK2/UPP2	66
cluster5	CC	GO:0034706	sodium channel complex	14/3954	26/19869	0.000125992	0.00168944	0.001419672	CACNA1G/GRIK2/GRIK3/GRIK5/SCN10A/SCN1A/SCN2A/SCN2B/SCN3B/SCN4A/SCN8A/SCN9A/SCNN1G/TRPM4	14

cluster5	CC	GO:0030018	Z disc	44/3954	130/19869	0.000129019	0.001710593	0.001437447	ACTN1/ACTN2/ADRA1A/AKAP4/ANK3/C10orf71/CACNA1C/CASQ2/CAVIN4/CRYAB/CSRP2/CSRP3/DES/DMD/FBP2/FHOD3/FKBP1A/FKBP1B/HOMER1/HRC/JPH1/KCNN2/LDB3/MYH6/MYH7/MYL12A/MYO18B/MYOZ1/MYOZ2/MYOZ3/MYPN/NEXN/NRAP/PRICKLE4/RTN2/RYR2/RYR3/SCN1A/SCN3B/SCN8A/SYNE2/SYNPO2L/TRIM54/TRIM63	44
cluster5	CC	GO:0036126	sperm flagellum	49/3954	150/19869	0.000149733	0.001952814	0.001640991	ABHD2/ACE/AK8/AKAP4/ATP1B1/C2CD6/CABCOCO1/CABS1/CABYR/CATSPER4/CATSPERG/CCDC181/CFAP221/CFAP47/CFAP65/CFAP69/DCDC2C/DNAH2/DNALI1/DRD2/ENKUR/ENO4/FSCB/FSIP2/GK2/IFT81/MNS1/NME5/ODF1/ODF3/PACRG/PRKAR1A/RHO/RSPH1/RSPH6A/SAXO1/SEPTIN4/SLC9B2/SPACA9/SPAG6/SQSTM1/TACR1/TACR3/TCP11/TCP11X1/TCTE1/TEKT5/TMEM249/TTC29	49
cluster5	CC	GO:0043083	synaptic cleft	11/3954	18/19869	0.000150598	0.001952814	0.001640991	ADGRB3/CBLN1/CDH8/GRIN1/LAMA5/LAMB2/LGI1/NLGN1/NPTX1/PRSS12/SLC1A1	11
cluster5	CC	GO:0031674	I band	47/3954	143/19869	0.000171864	0.002204337	0.001852351	ACTC1/ACTN1/ACTN2/ADRA1A/AKAP4/ANK3/C10orf71/CACNA1C/CACNA1S/CASQ2/CAVIN4/CRYAB/CSRP2/CSRP3/DES/DMD/FBP2/FHOD3/FKBP1A/FKBP1B/HOMER1/HRC/JPH1/KCNN2/KLHL40/LDB3/MYH6/MYH7/MYL12A/MYO18B/MYOZ1/MYOZ2/MYOZ3/MYPN/NEXN/NRAP/PRICKLE4/RTN2/RYR2/RYR3/SCN1A/SCN3B/SCN8A/SYNE2/SYNPO2L/TRIM54/TRIM63	47

cluster5	CC	GO:0005614	interstitial matrix	8/3954	11/19869	0.00022567	0.002863335	0.002406121	COL14A1/ECM2/KAZALD1/NAV2/TNC/VIT/VWA1/VWC2	8
cluster5	CC	GO:0098688	parallel fiber to Purkinje cell synapse	10/3954	16/19869	0.000235501	0.002956291	0.002484234	ATP2B2/ATP2B3/CALB2/CBLN1/CNTN6/GRIA3/GRID2/KCNJ3/KCNJ9/UNC13C	10
cluster5	CC	GO:0014701	junctional sarcoplasmic reticulum membrane	7/3954	9/19869	0.000302597	0.003750412	0.003151551	AKAP6/CASQ2/JPH1/JPH3/JPH4/RYR2/TRDN	7
cluster5	CC	GO:0005875	microtubule associated complex	52/3954	166/19869	0.00030943	0.003750412	0.003151551	ACTA1/ACTC1/ACTL8/AURKB/BIRC5/CCDC103/CLIP2/DCTN4/DCX/DNAH10/DNAH11/DNAH12/DNAH14/DNAH2/DNAH3/DNAH5/DNAH7/DNAH9/DNAI1/DNAI3/DNALI1/DRC1/DYNC1I1/DYNC2I2/DYNLRB2/EML1/HDAC6/KIF12/KIF18A/KIF1A/KIF20A/KIF20B/KIF21A/KIF21B/KIF25/KIF26B/KIF2B/KIF3A/KIF3C/KIF5A/KIF5C/KIF6/KIF7/KIFC1/KLC4/MAP1A/MAP2/MID1/NDEL1/RP1/TRIM54/TTBK1	52

cluster5	CC	GO:0098966	perisynaptic extracellular matrix	5/3954	5/19869	0.000311475	0.003750412	0.003151551	BCAN/HAPLN4/PTPRZ1/TNC/TNR	5
cluster5	CC	GO:0099535	synapse-associated extracellular matrix	5/3954	5/19869	0.000311475	0.003750412	0.003151551	BCAN/HAPLN4/PTPRZ1/TNC/TNR	5
cluster5	CC	GO:0098858	actin-based cell projection	66/3954	223/19869	0.000329837	0.003931396	0.003303635	ACTA1/ACTC1/ACTN2/ADGRV1/ANGPT1/AOC3/APP/ATP6V1B1/B4GALT1/CA9/CALB1/CEACAM16/CIB2/CLRN1/CLRN2/CYFIP1/DAG1/DMD/DNALI1/DOCK4/DYNC2I2/ENPP7/EPS8/FAT1/FMN2/FOXA1/FZD3/GAP43/GPM6A/GRXCR1/IGF2BP1/ITGB1/ITGB3/KITLG/LHFPL5/LY6G6D/MAP2/MYO1C/MYO3A/MYO3B/MYO6/NFASC/NLGN1/PCDH15/PDGFA/PDGFRA/PDPN/PDZD7/PPP1R9A/PROM1/PROM2/PVALB/SLC7A8/SPEF1/SRC/SRCIN1/SYNE2/TENM2/TMC1/TRPV4/TTYH1/TUBB3/UNC5C/USH1C/USH2A/WHRN	66
cluster5	CC	GO:0098637	protein complex involved in cell-matrix adhesion	9/3954	14/19869	0.000365131	0.004308548	0.003620564	EMILIN1/LAMA1/LAMB1/LAMB2/MMRN1/NID1/TNC/TNN/TNR	9

cluster5	CC	GO:0042383	sarcolemma	44/3954	136/19869	0.000401041	0.004685425	0.003937262	ABCC8/ADRA1A/AKAP6/ALOX12/ANK3/ATP1A2/ATP1B1/BVES/CACNA1C/CACNA1S/CACNA2D1/CACNB1/CACNG4/CACNG7/CAVIN4/CDH2/CIB2/COL6A1/DAG1/DES/DMD/FER1L5/ITGB1/KCNB1/KCND3/KCNJ3/LAMP1/MLIP/POPDC3/RTN2/RYR2/RYR3/SCN1A/SCN2A/SCN2B/SGCA/SGCZ/SLC27A6/SLC30A1/SLC8A2/SLC8A3/SNTG2/SSPN/STAC2	44
cluster5	CC	GO:0097386	glial cell projection	17/3954	38/19869	0.00044939	0.005198827	0.004368684	APP/CNGA3/GFAP/GPR161/GRM2/GRM3/GRM5/ITGB1/MT3/NFASC/SIRT2/SLC17A8/SLC1A1/SLC1A2/SLC2A13/SYT4/WASF3	17
cluster5	CC	GO:0098945	intrinsic component of presynaptic active zone membrane	10/3954	17/19869	0.00046987	0.005334325	0.004482546	ADRA2A/ATP2B2/CDH10/CDH2/GPM6A/LRFN3/NECTIN1/NPTN/NTNG1/NTNG2	10
cluster5	CC	GO:0005796	Golgi lumen	36/3954	106/19869	0.000470144	0.005334325	0.004482546	ACAN/APP/BCAN/CGA/CSPG4/CSPG5/DAG1/ERO1A/F2/FGF23/GAS6/GPC2/GPC3/GPC6/INS/KERA/MMP16/MUC13/MUC16/MUC5AC/MUC5B/NCAN/NGF/OGN/OMD/PDGFA/PODXL2/PROS1/SDC1/SDC2/SDC4/WNT3A/WNT4/WNT5A/WNT7A/WNT7B	36

cluster5	CC	GO:0005604	basement membrane	34/3954	99/19869	0.000529504	0.005950615	0.005000428	ATRNL1/COL28A1/COL2A1/COL4A1/COL4A5/COL4A6/COL8A1/COL9A1/DAG1/EFNA5/FBLN1/FREM1/FREM2/FREM3/LAD1/LAMA1/LAMA3/LAMA5/LAMB1/LAMB2/LAMC2/NID1/NTN1/NTN3/NTNG1/NTNG2/PXDN/TIMP1/TMEFF2/TNC/USH2A/VWA1/VWA2/VWC2	34
cluster5	CC	GO:0043256	laminin complex	8/3954	12/19869	0.000558424	0.006216416	0.005223786	LAMA1/LAMA3/LAMA5/LAMB1/LAMB2/LAMC2/NTNG1/NTNG2	8
cluster5	CC	GO:0045111	intermediate filament cytoskeleton	72/3954	253/19869	0.000639397	0.007051292	0.005925349	ADCY5/CLIP1/DES/DLGAP2/DTNA/EVPL/FER/GFAP/INA/KRT15/KRT20/KRT222/KRT27/KRT28/KRT39/KRT6C/KRT75/KRT76/KRT8/KRT80/KRT83/KRT84/KRTAP1-1/KRTAP1-4/KRTAP10-10/KRTAP10-12/KRTAP10-2/KRTAP10-4/KRTAP10-7/KRTAP10-8/KRTAP12-4/KRTAP13-2/KRTAP13-3/KRTAP15-1/KRTAP19-1/KRTAP19-7/KRTAP2-1/KRTAP21-1/KRTAP21-2/KRTAP21-3/KRTAP22-2/KRTAP24-1/KRTAP25-1/KRTAP27-1/KRTAP29-1/KRTAP3-1/KRTAP3-2/KRTAP4-8/KRTAP5-1/KRTAP5-11/KRTAP5-3/KRTAP5-4/KRTAP5-7/KRTAP9-2/KRTAP9-4/KRTAP9-6/KRTAP9-7/KRTAP9-9/LMNA/LMNTD1/MNS1/NDEL1/NEFH/NEFM/PKN2/PKP2/PRPH/RTN2/SHANK2/SLC1A6/SYNE2/UPP2	72
cluster5	CC	GO:0016323	basolateral plasma membrane	66/3954	229/19869	0.00072835	0.007957902	0.006687194	ABCA8/ABCC3/ADCY8/ADORA1/ADRA2A/AJAP1/ANK3/ATP1B1/ATP2B2/ATP6V0A4/ATP6V1B1/B4GALT1/CA9/CADM1/CDH16/CDH17/CDH2/CEACAM5/CHRM3/CLDN1/CLDN19/CLDN8/CNNM2/DAG1/EGFR/ENPP1/EPCAM/ERBB2/ERBB3/ERBB4/FOLR1/FRMPD2/HEPH/KCNC2/KCNJ16/KCNJ4/LEPR/MARVELD2/MEGF11/NDRG4/NKD2/OTOF/PDPN/PIANP/PLPP3/PROM2/PTH1R/RHCG/SLC16A12/SLC16A8/SLC22A7/SLC22A9/SLC29A4/SLC38A3/SLC39A5/SLC4A11/SLC4A4/SLC7A8/SLC8A2/SLC9A4/SLC9B2/SLCO1B1/SLCO1B3/SLCO1C1/SPEF1/TRPC4	66

cluster5	CC	GO:0001917	photoreceptor inner segment	26/3954	71/19869	0.000779967	0.008443675	0.007095399	ADGRV1/AIPL1/ATP1A3/CIB2/DNM1/FAM161A/GNAT1/GNGT1/IMPG1/INHA/LOC118142757/MAGI2/MYO3A/MYO3B/PCARE/PDC/REEP6/RHO/RP1/SHANK2/SLC24A2/SNAP25/TULP1/USH1C/USH2A/WHRN	26
cluster5	CC	GO:0044853	plasma membrane raft	37/3954	113/19869	0.000867444	0.009237599	0.00776255	ADCY8/ADCYAP1R1/ADRA1A/ADRA1B/AKAP6/ATP1A2/ATP1B1/BVES/CAVIN3/CAVIN4/CDH2/CHRNA3/CHRNA7/DAG1/EFNA5/F2R/GASK1A/HAS2/HDAC6/HTR2A/ITGAM/KCND2/KCNMA1/KIF18A/LRP4/LRP6/NOS3/PLVAP/PRKAR1A/PRTN3/PTCH1/PTGS2/SLC6A3/SMO/SRC/TFPI/TRPC4	37
cluster5	CC	GO:0044214	spanning component of plasma membrane	10/3954	18/19869	0.000868961	0.009237599	0.00776255	AJAP1/EGFR/ERBB2/ERBB3/ERBB4/NPC1L1/SLC24A2/SLC24A3/SLC4A4/TRPM4	10
cluster5	CC	GO:0016324	apical plasma membrane	98/3954	368/19869	0.000976791	0.010291186	0.008647901	ABCB11/ABCG2/ACE2/ADCY8/ADGRG2/AJAP1/ANXA13/AQP6/ASPM/ATP1B1/ATP2B2/ATP6V0A4/ATP6V1B1/CDH2/CEACAM5/CFAP126/CFTR/CLDN1/CLDN4/CNKSR3/CNTFR/CRB2/CSPG4/DLL1/DUOX2/EMP2/EPCAM/ERBB2/ERBB3/FAT1/FOLR1/FZD3/GNAS/GNAT1/HPN/IGFBP2/IGSF5/ITGB3/KCNB1/KCNC2/KCNE4/KCNMA1/KISS1/LCT/LHFPL5/LMO7/LRP2/MAL2/MARVELD2/MFRP/MIP/MPDZ/MUC13/NPC1L1/P2RX2/PAPPA2/PARD3/PDGFRB/PDPN/PKHD1/PROM1/PROM2/PTH1R/RAB27B/RHCG/SCNN1G/SHANK2/SHROOM2/SI/SLC16A2/SLC16A8/SLC17A4/SLC1A1/SLC22A11/SLC22A12/SLC26A9/SLC29A4/SLC2A13/SLC34A1/SLC34A2/SLC38A3/SLC4A11/SLC4A5/SLC5A1/SLC6A20/SLC7A8/SLC9A4/SLC9B2/SPEF1/STC1/TDGF1/THY1/TMEM30A/TRPV4/UPK1A/UPK2/USH2A/VANGL2	98

cluster5	CC	GO:0009925	basal plasma membrane	71/3954	254/19869	0.001182559	0.012348849	0.010376999	ABCA8/ABCC3/ACE/ADCY8/ADORA1/ADRA2A/AJAP1/ANK3/ATP1B1/ATP2B2/ATP6V0A4/ATP6V1B1/B4GALT1/CA9/CADM1/CDH16/CDH17/CDH2/CEACAM5/CHRM3/CLCA2/CLDN1/CLDN19/CLDN4/CLDN8/CNNM2/DAG1/EGFR/ENPP1/EPCAM/ERBB2/ERBB3/ERBB4/FOLR1/FRMPD2/HEPH/KCNC2/KCNJ16/KCNJ4/KCNQ4/LEPR/MARVELD2/MEGF11/MYO1C/NDRG4/NKD2/OTOF/PDPN/PIANP/PLPP3/PROM2/PTH1R/RHCG/SLC16A12/SLC16A8/SLC22A7/SLC22A9/SLC29A4/SLC38A3/SLC39A5/SLC4A11/SLC4A4/SLC7A8/SLC8A2/SLC9A4/SLC9B2/SLCO1B1/SLCO1B3/SLCO1C1/SPEF1/TRPC4	71
cluster5	CC	GO:0098992	neuronal dense core vesicle	8/3954	13/19869	0.001198333	0.012403801	0.010423176	CADPS/CHGA/KIF1A/NPY/PENK/SCG2/SYT4/SYT5	8
cluster5	CC	GO:0045178	basal part of cell	75/3954	272/19869	0.00132511	0.013596779	0.011425661	ABCA8/ABCC3/ACE/ADCY8/ADORA1/ADRA2A/AJAP1/ANK3/ATP1B1/ATP2B2/ATP6V0A4/ATP6V1B1/B4GALT1/CA9/CADM1/CDH16/CDH17/CDH2/CEACAM5/CHRM3/CLCA2/CLDN1/CLDN19/CLDN4/CLDN8/CLRN1/CNNM2/DAG1/EGFR/ENPP1/EPCAM/ERBB2/ERBB3/ERBB4/FAP/FOLR1/FRMPD2/HEPH/HFE/ITGA2/KCNC2/KCNJ16/KCNJ4/KCNQ4/LEPR/MARVELD2/MEGF11/MYO1C/NDRG4/NKD2/OTOF/PDPN/PIANP/PLPP3/PROM2/PTH1R/RHCG/SLC16A12/SLC16A8/SLC22A7/SLC22A9/SLC29A4/SLC38A3/SLC39A5/SLC4A11/SLC4A4/SLC7A8/SLC8A2/SLC9A4/SLC9B2/SLCO1B1/SLCO1B3/SLCO1C1/SPEF1/TRPC4	75
cluster5	CC	GO:0030175	filopodium	35/3954	108/19869	0.001443596	0.014608083	0.012275481	ACTA1/ACTC1/ACTN2/APP/B4GALT1/CYFIP1/DAG1/DMD/DNALI1/DYNC2I2/FAT1/FZD3/GAP43/GPM6A/IGF2BP1/ITGB1/ITGB3/KITLG/LY6G6D/MAP2/MYO3A/MYO3B/MYO6/NLGN1/PDPN/PPP1R9A/SPEF1/SRC/SRCIN1/SYNE2/TENM2/TRPV4/TTYH1/TUBB3/UNC5C	35

cluster5	CC	GO:0043198	dendritic shaft	16/3954	38/19869	0.001460155	0.014608083	0.012275481	APP/GIPC1/GRM5/GRM7/HOMER1/HTR2A/JPH4/KIRREL1/KIRREL3/MAP1A/MAP2/MPP2/NLGN2/RGS7BP/SLC1A1/SYNDIG1	16
cluster5	CC	GO:0097381	photoreceptor disc membrane	12/3954	25/19869	0.001460808	0.014608083	0.012275481	ABCA4/GNAT1/GNGT1/GRK4/GUCA1C/GUCY2F/LOC118142757/OPN1LW/OPN1MW2/OPN1MW3/OPN4/RHO	12
cluster5	CC	GO:0097449	astrocyte projection	10/3954	19/19869	0.001508558	0.014669862	0.012327395	APP/GFAP/GRM2/GRM3/GRM5/MT3/SLC17A8/SLC1A2/SLC2A13/SYT4	10
cluster5	CC	GO:0098636	protein complex involved in cell adhesion	20/3954	52/19869	0.001527307	0.014669862	0.012327395	EMILIN1/ITGA2/ITGA2B/ITGA7/ITGAM/ITGB1/ITGB3/ITGB5/ITGB8/LAMA1/LAMB1/LAMB2/LYN/MMRN1/NID1/NLGN1/PLP1/TNC/TNN/TNR	20

cluster5	CC	GO:0002139	stereocilia coupling link	5/3954	6/19869	0.001559241	0.014669862	0.012327395	ADGRV1/PDZD7/USH1C/USH2A/WHRN	5
cluster5	CC	GO:0002141	stereocilia ankle link	5/3954	6/19869	0.001559241	0.014669862	0.012327395	ADGRV1/PDZD7/USH1C/USH2A/WHRN	5
cluster5	CC	GO:0002142	stereocilia ankle link complex	5/3954	6/19869	0.001559241	0.014669862	0.012327395	ADGRV1/PDZD7/USH1C/USH2A/WHRN	5
cluster5	CC	GO:0098985	asymmetric, glutamatergic, excitatory synapse	5/3954	6/19869	0.001559241	0.014669862	0.012327395	NLGN1/NLGN3/NLGN4X/SHISA6/SHISA7	5

cluster5	CC	GO:0072534	perineuronal net	4/3954	4/19869	0.001566443	0.014669862	0.012327395	BCAN/HAPLN4/PTPRZ1/TNR	4
cluster5	CC	GO:1990696	USH2 complex	4/3954	4/19869	0.001566443	0.014669862	0.012327395	ADGRV1/PDZD7/USH2A/WHRN	4
cluster5	CC	GO:0005871	kinesin complex	19/3954	49/19869	0.001775066	0.016492735	0.013859194	KIF12/KIF18A/KIF1A/KIF20A/KIF20B/KIF21A/KIF21B/KIF25/KIF26B/KIF2B/KIF3A/KIF3C/KIF5A/KIF5C/KIF6/KIF7/KIFC1/KLC4/NDEL1	19
cluster5	CC	GO:0098691	dopaminergic synapse	7/3954	11/19869	0.00189981	0.017513874	0.014717278	ADRA1A/ADRA2A/CHRNA6/DRD2/NLGN2/RAB3B/SLC6A3	7

cluster5	CC	GO:0016460	myosin II complex	12/3954	26/19869	0.002225709	0.020359197	0.017108264	MYH1/MYH10/MYH13/MYH2/MYH4/MYH6/MYH7/MYH8/MYH9/MYL1/MYL12A/MYO18B	12
cluster5	CC	GO:0005874	microtubule	118/3954	466/19869	0.002299726	0.020643469	0.017347144	APC2/ARHGAP18/ASPM/AURKB/BCL10/BIRC5/C4orf47/CAPN6/CCDC181/CDK1/CFAP126/CFAP206/CFAP52/CLIP1/CLIP2/CLIP3/CLMP/DCDC2C/DCX/DNAH10/DNAH11/DNAH12/DNAH14/DNAH2/DNAH3/DNAH5/DNAH7/DNAH9/DNAI1/DNM1/DYNC1I1/DYNLRB2/EFHC2/EML1/EML5/ENKUR/FAM161A/FEZ1/FIGN/FSD1/GABARAPL1/GAS2L3/HDAC6/HID1/KIF12/KIF18A/KIF1A/KIF20A/KIF20B/KIF21A/KIF21B/KIF24/KIF25/KIF26B/KIF2B/KIF3A/KIF3C/KIF5A/KIF5C/KIF6/KIF7/KIFC1/KLC4/KLHL21/MAP1A/MAP1LC3B/MAP1LC3B2/MAP1LC3C/MAP2/MAP6/MAP9/MAPRE2/MAPRE3/MDM1/MID1/MISP/MNS1/MT3/NAV3/NCKAP5/NDEL1/NEK2/NINL/PACRG/PLK1/RADIL/REEP1/REEP2/ROR2/RP1/SAA1/SAXO1/SHROOM2/SIRT2/SKA3/SLAIN1/SPACA9/SPAG17/SPAG6/SPEF1/SYBU/TEKT1/TEKT2/TOGARAM1/TPPP/TPX2/TRIM54/TRIM55/TRIM63/TRPV4/TTLL11/TTLL6/TTLL7/TTLL9/TUBA3E/TUBA4A/TUBB3/TUBB4A	118
cluster5	CC	GO:0030673	axolemma	8/3954	14/19869	0.002309269	0.020643469	0.017347144	ADORA1/CNTNAP2/KCNC1/KCNC2/KCNH1/ROBO2/SLC1A2/THY1	8
cluster5	CC	GO:0099059	integral component of presynaptic active zone membrane	8/3954	14/19869	0.002309269	0.020643469	0.017347144	ADRA2A/ATP2B2/CDH10/CDH2/GPM6A/LRFN3/NECTIN1/NPTN	8

cluster5	CC	GO:0001518	voltage-gated sodium channel complex	9/3954	17/19869	0.002478169	0.021986762	0.018475941	CACNA1G/SCN10A/SCN1A/SCN2A/SCN2B/SCN3B/SCN4A/SCN8A/SCN9A	9
cluster5	CC	GO:0034704	calcium channel complex	24/3954	69/19869	0.002723567	0.023983649	0.020153967	AKAP6/C2CD6/CACNA1A/CACNA1B/CACNA1C/CACNA1G/CACNA1S/CACNA2D1/CACNB1/CACNG2/CACNG3/CACNG4/CACNG7/CASQ2/CATSPER4/CATSPERG/FKBP1A/FKBP1B/HSPA2/MICU3/RYR2/RYR3/TMEM249/TRPC4	24
cluster5	CC	GO:0045177	apical part of cell	110/3954	435/19869	0.003310585	0.028936963	0.024316342	ABCB11/ABCG2/ACE2/ADCY8/ADGRG2/AJAP1/ANXA13/APP/AQP6/ASPM/ATP1B1/ATP2B2/ATP6V0A4/ATP6V1B1/CDH2/CEACAM5/CFAP126/CFTR/CHL1/CLDN1/CLDN4/CNKSR3/CNTFR/CRB2/CSPG4/DLL1/DUOX2/DUOXA2/EMP2/EPB41L4B/EPCAM/ERBB2/ERBB3/FAP/FAT1/FOLR1/FZD3/GNAS/GNAT1/HFE/HOMER1/HPN/IGFBP2/IGSF5/ITGB3/KCNB1/KCNC2/KCNE4/KCNMA1/KHDC3L/KISS1/LCT/LHFPL5/LMO7/LRP2/MAL2/MARVELD2/MFRP/MIP/MPDZ/MUC13/MYO5B/NPC1L1/OOEP/P2RX2/PAPPA2/PARD3/PDGFRB/PDPN/PKHD1/PROM1/PROM2/PTCH1/PTH1R/RAB27B/RHCG/SCNN1G/SHANK2/SHROOM2/SI/SLC16A2/SLC16A8/SLC17A4/SLC1A1/SLC22A11/SLC22A12/SLC26A9/SLC29A4/SLC2A13/SLC34A1/SLC34A2/SLC38A3/SLC4A11/SLC4A5/SLC5A1/SLC6A20/SLC7A8/SLC9A4/SLC9B2/SPEF1/STC1/TDGF1/THY1/TMEM30A/TRPV4/UPK1A/UPK2/USH1C/USH2A/VANGL2	110
cluster5	CC	GO:0045095	keratin filament	32/3954	101/19869	0.003353972	0.029100636	0.024453879	KRT6C/KRT75/KRT76/KRT8/KRT80/KRT83/KRT84/KRTAP1-1/KRTAP1-4/KRTAP10-10/KRTAP10-12/KRTAP10-2/KRTAP10-4/KRTAP10-7/KRTAP10-8/KRTAP12-4/KRTAP2-1/KRTAP24-1/KRTAP29-1/KRTAP3-1/KRTAP3-2/KRTAP4-8/KRTAP5-1/KRTAP5-11/KRTAP5-3/KRTAP5-4/KRTAP5-7/KRTAP9-2/KRTAP9-4/KRTAP9-6/KRTAP9-7/KRTAP9-9	32

cluster5	CC	GO:0005858	axonemal dynein complex	11/3954	24/19869	0.003616544	0.030924069	0.025986149	CCDC103/DNAH12/DNAH14/DNAH2/DNAH3/DNAH5/DNAH7/DNAH9/DNAI1/DNAI3/DRC1	11
cluster5	CC	GO:0032982	myosin filament	11/3954	24/19869	0.003616544	0.030924069	0.025986149	MYBPC1/MYH1/MYH10/MYH13/MYH2/MYH4/MYH6/MYH7/MYH8/MYH9/MYO18B	11
cluster5	CC	GO:0033017	sarcoplasmic reticulum membrane	16/3954	41/19869	0.003698514	0.031397459	0.026383949	AKAP6/CAMK2B/CASQ2/DHRS7C/FKBP1A/FKBP1B/JPH1/JPH3/JPH4/JSRP1/RTN2/RYR2/RYR3/SLN/SYNE2/TRDN	16
cluster5	CC	GO:0005901	caveola	27/3954	82/19869	0.003729161	0.031431501	0.026412555	ADCY8/ADCYAP1R1/ADRA1A/ADRA1B/AKAP6/ATP1A2/ATP1B1/BVES/CAVIN3/CAVIN4/EFNA5/F2R/GASK1A/HDAC6/HTR2A/KCNMA1/KIF18A/LRP6/NOS3/PLVAP/PTCH1/PTGS2/SLC6A3/SMO/SRC/TFPI/TRPC4	27

cluster5	CC	GO:0005859	muscle myosin complex	8/3954	15/19869	0.004089565	0.033983708	0.028557229	MYH1/MYH13/MYH2/MYH4/MYH6/MYH7/MYH8/MYL1	8
cluster5	CC	GO:0032591	dendritic spine membrane	8/3954	15/19869	0.004089565	0.033983708	0.028557229	ATP2B2/DAGLA/DDN/GRIA1/KCNC3/SHISA6/SHISA7/SHISA9	8
cluster5	CC	GO:0005891	voltage-gated calcium channel complex	17/3954	45/19869	0.004174011	0.034442888	0.028943087	C2CD6/CACNA1A/CACNA1B/CACNA1C/CACNA1G/CACNA1S/CACNA2D1/CACNB1/CACNG2/CACNG3/CACNG4/CACNG7/CATSPER4/CATSPERG/FKBP1A/HSPA2/TMEM249	17
cluster5	CC	GO:0032420	stereocilium	20/3954	56/19869	0.004215829	0.034546376	0.02903005	ADGRV1/CALB1/CEACAM16/CIB2/CLRN1/CLRN2/DOCK4/EPS8/GRXCR1/LHFPL5/MYO1C/MYO3A/MYO3B/PCDH15/PDZD7/PVALB/TMC1/USH1C/USH2A/WHRN	20

cluster5	CC	GO:0098690	glycinergic synapse	5/3954	7/19869	0.004558326	0.036841263	0.030958492	GLRA1/GLRA2/GLRB/NLGN2/SLC6A5	5
cluster5	CC	GO:0099026	anchored component of presynaptic membrane	5/3954	7/19869	0.004558326	0.036841263	0.030958492	CNTN5/CNTN6/NTNG1/NTNG2/RGS7BP	5
cluster5	CC	GO:0031091	platelet alpha granule	29/3954	91/19869	0.004621553	0.037098178	0.031174384	ACTN1/ACTN2/APP/EGF/F5/FERMT3/GAS6/ISLR/ITGA2B/ITGB3/KNG1/LEFTY2/LHFPL2/MMRN1/PDGFA/PECAM1/PF4/PPBP/PROS1/QSOX1/SELP/SERPINE1/SERPINE2/STXBP1/TIMP1/TREML1/VEGFA/VEGFD/VWF	29
cluster5	CC	GO:0099091	postsynaptic specialization, intracellular component	6/3954	10/19869	0.00619337	0.049379574	0.0414947	CTNND1/LYN/PRR7/PSD/SH3GL3/SRC	6

cluster5	MF	GO:0022836	gated channel activity	148/3764	341/18432	2.9E-22	6.04E-19	5.59E-19	ABCC8/ABCC9/ANO3/ANO4/ANO6/ASIC1/ASIC2/ASIC3/ASIC4/CACNA1A/CACNA1B/CACNA1C/CACNA1G/CACNA1S/CACNA2D1/CACNB1/CACNG2/CACNG3/CACNG4/CACNG5/CACNG7/CATSPER4/CFTR/CHRNA2/CHRNA3/CHRNA4/CHRNA6/CHRNA7/CHRNB4/CHRNG/CLCA2/CLCN2/CLCN4/CLCNKA/CLCNKB/CLIC6/CNGA3/CNGB1/FKBP1B/GABRA1/GABRA2/GABRA3/GABRA4/GABRA5/GABRA6/GABRB2/GABRB3/GABRD/GABRE/GABRG1/GABRG2/GABRG3/GABRQ/GLRA1/GLRA2/GLRB/GRIA1/GRIA2/GRIA3/GRIA4/GRID1/GRID2/GRIK1/GRIK2/GRIK3/GRIK5/GRIN1/GRIN2A/GRIN2B/GRIN2D/HCN1/HCN3/HCN4/HTR3C/KCNA10/KCNA4/KCNA7/KCNB1/KCNB2/KCNC1/KCNC2/KCNC3/KCND2/KCND3/KCNE4/KCNG3/KCNH1/KCNH2/KCNH4/KCNH5/KCNH6/KCNIP1/KCNIP3/KCNJ13/KCNJ16/KCNJ18/KCNJ3/KCNJ4/KCNJ5/KCNJ6/KCNJ9/KCNK10/KCNK3/KCNK5/KCNK6/KCNMA1/KCNMB2/KCNN1/KCNN2/KCNN3/KCNQ2/KCNQ3/KCNQ4/KCNS2/KCNT1/KCNT2/KCNV1/LRRC55/NALCN/NALF1/NALF2/P2RX2/P2RX3/PEX5L/RYR2/RYR3/SCN10A/SCN1A/SCN2A/SCN2B/SCN3B/SCN4A/SCN8A/SCN9A/SCNN1G/SHROOM2/SNAP25/TMC1/TMC3/TMC7/TMEM37/TMEM63C/TRPM3/TRPM4/TRPM8/TRPV4/TTYH1/ZACN	148
cluster5	MF	GO:0005216	ion channel activity	179/3764	446/18432	4.98E-22	6.04E-19	5.59E-19	ABCC8/ABCC9/ANO3/ANO4/ANO6/ASIC1/ASIC2/ASIC3/ASIC4/ATP5F1E/BEST2/BEST3/CACNA1A/CACNA1B/CACNA1C/CACNA1G/CACNA1S/CACNA2D1/CACNB1/CACNG2/CACNG3/CACNG4/CACNG5/CACNG7/CALHM5/CATSPER4/CFTR/CHRNA2/CHRNA3/CHRNA4/CHRNA6/CHRNA7/CHRNB4/CHRNG/CLCA2/CLCN2/CLCN4/CLCNKA/CLCNKB/CLDN4/CLIC6/CNGA3/CNGB1/FKBP1B/FXYD3/GABRA1/GABRA2/GABRA3/GABRA4/GABRA5/GABRA6/GABRB2/GABRB3/GABRD/GABRE/GABRG1/GABRG2/GABRG3/GABRQ/GJC1/GLRA1/GLRA2/GLRB/GPM6A/GRIA1/GRIA2/GRIA3/GRIA4/GRID1/GRID2/GRIK1/GRIK2/GRIK3/GRIK5/GRIN1/GRIN2A/GRIN2B/GRIN2D/HCN1/HCN3/HCN4/HTR3C/KCNA10/KCNA4/KCNA7/KCNB1/KCNB2/KCNC1/KCNC2/KCNC3/KCND2/KCND3/KCNE4/KCNG3/KCNH1/KCNH2/KCNH4/KCNH5/KCNH6/KCNIP1/KCNIP3/KCNJ13/KCNJ16/KCNJ18/KCNJ3/KCNJ4/KCNJ5/KCNJ6/KCNJ9/KCNK10/KCNK3/KCNK5/KCNK6/KCNMA1/KCNMB2/KCNN1/KCNN2/KCNN3/KCNQ2/KCNQ3/KCNQ4/KCNS2/KCNT1/KCNT2/KCNV1/LRRC55/LRRC8B/NALCN/NALF1/NALF2/OTOP1/P2RX2/P2RX3/PEX5L/PKD1L2/PKD1L3/PKDREJ/RHCE/RHCG/RHD/RYR2/RYR3/SCN10A/SCN1A/SCN2A/SCN2B/SCN3B/SCN4A/SCN8A/SCN9A/SCNN1G/SEC61A1/SHROOM2/SLC12A5/SLC17A6/SLC17A7/SLC17A8/SLC1A1/SLC24A2/SLC24A3/SLC26A9/SLC4A11/SNAP25/TMC1/TMC3/TMC7/TMEM37/TMEM63C/TRPC3/TRPC4/TRPC7/TRPM1/TRPM3/TRPM4/TRPM8/TRPV4/TTYH1/UNC80/ZACN	179
cluster5	MF	GO:0015267	channel activity	187/3764	494/18432	1.24E-19	9.67E-17	8.95E-17	ABCC8/ABCC9/ANO3/ANO4/ANO6/AQP12A/AQP6/ASIC1/ASIC2/ASIC3/ASIC4/ATP5F1E/BEST2/BEST3/CACNA1A/CACNA1B/CACNA1C/CACNA1G/CACNA1S/CACNA2D1/CACNB1/CACNG2/CACNG3/CACNG4/CACNG5/CACNG7/CALHM5/CATSPER4/CFTR/CHRNA2/CHRNA3/CHRNA4/CHRNA6/CHRNA7/CHRNB4/CHRNG/CLCA2/CLCN2/CLCN4/CLCNKA/CLCNKB/CLDN4/CLIC6/CNGA3/CNGB1/FKBP1B/FXYD3/GABRA1/GABRA2/GABRA3/GABRA4/GABRA5/GABRA6/GABRB2/GABRB3/GABRD/GABRE/GABRG1/GABRG2/GABRG3/GABRQ/GJA10/GJB3/GJC1/GJD2/GJE1/GLRA1/GLRA2/GLRB/GPM6A/GRIA1/GRIA2/GRIA3/GRIA4/GRID1/GRID2/GRIK1/GRIK2/GRIK3/GRIK5/GRIN1/GRIN2A/GRIN2B/GRIN2D/HCN1/HCN3/HCN4/HTR3C/KCNA10/KCNA4/KCNA7/KCNB1/KCNB2/KCNC1/KCNC2/KCNC3/KCND2/KCND3/KCNE4/KCNG3/KCNH1/KCNH2/KCNH4/KCNH5/KCNH6/KCNIP1/KCNIP3/KCNJ13/KCNJ16/KCNJ18/KCNJ3/KCNJ4/KCNJ5/KCNJ6/KCNJ9/KCNK10/KCNK3/KCNK5/KCNK6/KCNMA1/KCNMB2/KCNN1/KCNN2/KCNN3/KCNQ2/KCNQ3/KCNQ4/KCNS2/KCNT1/KCNT2/KCNV1/LRRC55/LRRC8B/MIP/NALCN/NALF1/NALF2/OTOP1/P2RX2/P2RX3/PANX2/PEX5L/PKD1L2/PKD1L3/PKDREJ/RHCE/RHCG/RHD/RYR2/RYR3/SCN10A/SCN1A/SCN2A/SCN2B/SCN3B/SCN4A/SCN8A/SCN9A/SCNN1G/SEC61A1/SHROOM2/SLC12A5/SLC17A6/SLC17A7/SLC17A8/SLC1A1/SLC24A2/SLC24A3/SLC26A9/SLC4A11/SNAP25/TMC1/TMC3/TMC7/TMEM37/TMEM63C/TRPC3/TRPC4/TRPC7/TRPM1/TRPM3/TRPM4/TRPM8/TRPV4/TTYH1/UNC80/ZACN	187
cluster5	MF	GO:0022803	passive transmembrane transporter activity	187/3764	495/18432	1.59E-19	9.67E-17	8.95E-17	ABCC8/ABCC9/ANO3/ANO4/ANO6/AQP12A/AQP6/ASIC1/ASIC2/ASIC3/ASIC4/ATP5F1E/BEST2/BEST3/CACNA1A/CACNA1B/CACNA1C/CACNA1G/CACNA1S/CACNA2D1/CACNB1/CACNG2/CACNG3/CACNG4/CACNG5/CACNG7/CALHM5/CATSPER4/CFTR/CHRNA2/CHRNA3/CHRNA4/CHRNA6/CHRNA7/CHRNB4/CHRNG/CLCA2/CLCN2/CLCN4/CLCNKA/CLCNKB/CLDN4/CLIC6/CNGA3/CNGB1/FKBP1B/FXYD3/GABRA1/GABRA2/GABRA3/GABRA4/GABRA5/GABRA6/GABRB2/GABRB3/GABRD/GABRE/GABRG1/GABRG2/GABRG3/GABRQ/GJA10/GJB3/GJC1/GJD2/GJE1/GLRA1/GLRA2/GLRB/GPM6A/GRIA1/GRIA2/GRIA3/GRIA4/GRID1/GRID2/GRIK1/GRIK2/GRIK3/GRIK5/GRIN1/GRIN2A/GRIN2B/GRIN2D/HCN1/HCN3/HCN4/HTR3C/KCNA10/KCNA4/KCNA7/KCNB1/KCNB2/KCNC1/KCNC2/KCNC3/KCND2/KCND3/KCNE4/KCNG3/KCNH1/KCNH2/KCNH4/KCNH5/KCNH6/KCNIP1/KCNIP3/KCNJ13/KCNJ16/KCNJ18/KCNJ3/KCNJ4/KCNJ5/KCNJ6/KCNJ9/KCNK10/KCNK3/KCNK5/KCNK6/KCNMA1/KCNMB2/KCNN1/KCNN2/KCNN3/KCNQ2/KCNQ3/KCNQ4/KCNS2/KCNT1/KCNT2/KCNV1/LRRC55/LRRC8B/MIP/NALCN/NALF1/NALF2/OTOP1/P2RX2/P2RX3/PANX2/PEX5L/PKD1L2/PKD1L3/PKDREJ/RHCE/RHCG/RHD/RYR2/RYR3/SCN10A/SCN1A/SCN2A/SCN2B/SCN3B/SCN4A/SCN8A/SCN9A/SCNN1G/SEC61A1/SHROOM2/SLC12A5/SLC17A6/SLC17A7/SLC17A8/SLC1A1/SLC24A2/SLC24A3/SLC26A9/SLC4A11/SNAP25/TMC1/TMC3/TMC7/TMEM37/TMEM63C/TRPC3/TRPC4/TRPC7/TRPM1/TRPM3/TRPM4/TRPM8/TRPV4/TTYH1/UNC80/ZACN	187

cluster5	MF	GO:0004984	olfactory receptor activity	167/3764	429/18432	4.73E-19	2.3E-16	2.13E-16	OR10A5/OR10A7/OR10G7/OR10H3/OR10H4/OR10J1/OR10J3/OR10K1/OR10Q1/OR10W1/OR10Z1/OR11H2/OR11H4/OR11L1/OR12D1/OR12D2/OR12D3/OR13C8/OR13C9/OR13D1/OR13F1/OR13H1/OR14A16/OR14I1/OR14J1/OR1A1/OR1A2/OR1B1/OR1E1/OR1E2/OR1G1/OR1J4/OR1L1/OR1L3/OR1L4/OR1L6/OR1M1/OR1S2/OR2A2/OR2AG1/OR2AG2/OR2AJ1/OR2AK2/OR2AT4/OR2B6/OR2D3/OR2F1/OR2G2/OR2G3/OR2H1/OR2J1/OR2J2/OR2L13/OR2L2/OR2L8/OR2M4/OR2S2/OR2T11/OR2T27/OR2T29/OR2T34/OR2T5/OR2T6/OR2T7/OR2W1/OR2W3/OR2Y1/OR2Z1/OR3A2/OR3A3/OR4A15/OR4A16/OR4A5/OR4C13/OR4C16/OR4C3/OR4C45/OR4C46/OR4C6/OR4D11/OR4D5/OR4D6/OR4F15/OR4F17/OR4F21/OR4F29/OR4F3/OR4F5/OR4K1/OR4K13/OR4K15/OR4K5/OR4L1/OR4M1/OR4M2/OR4N2/OR4N4/OR4N5/OR4X1/OR51A2/OR51B5/OR51B6/OR51E2/OR51G1/OR51I1/OR51L1/OR51Q1/OR51V1/OR52A5/OR52B2/OR52B6/OR52E4/OR52E5/OR52E6/OR52E8/OR52I2/OR52J3/OR52L1/OR52N5/OR56A5/OR5AC2/OR5AK2/OR5B17/OR5B3/OR5D14/OR5D16/OR5H2/OR5K1/OR5K4/OR5M10/OR5M11/OR5P2/OR5T3/OR5V1/OR5W2/OR6B1/OR6B2/OR6C3/OR6C6/OR6C70/OR6C76/OR6J1/OR6P1/OR6T1/OR7A10/OR7C1/OR7C2/OR7E24/OR7G1/OR7G2/OR7G3/OR8A1/OR8B2/OR8B3/OR8B4/OR8D2/OR8H1/OR8H3/OR8I2/OR8J1/OR8J3/OR8K1/OR9A4/OR9G1/OR9I1/OR9K2/OR9Q1	167
cluster5	MF	GO:0046873	metal ion transmembrane transporter activity	168/3764	436/18432	1.22E-18	4.92E-16	4.56E-16	ABCC8/ABCC9/ASIC1/ASIC2/ASIC3/ASIC4/ATP1A2/ATP1A3/ATP1B1/ATP2B2/ATP2B3/CACNA1A/CACNA1B/CACNA1C/CACNA1G/CACNA1S/CACNA2D1/CACNB1/CACNG2/CACNG3/CACNG4/CACNG5/CACNG7/CATSPER4/CHRNA7/CLDN16/CNNM2/FKBP1B/GPM6A/GRIK1/GRIK2/GRIK3/GRIK5/GRIN1/GRIN2A/GRIN2B/GRIN2D/HCN1/HCN3/HCN4/KCNA10/KCNA4/KCNA7/KCNB1/KCNB2/KCNC1/KCNC2/KCNC3/KCND2/KCND3/KCNE4/KCNG3/KCNH1/KCNH2/KCNH4/KCNH5/KCNH6/KCNIP1/KCNIP3/KCNJ13/KCNJ16/KCNJ18/KCNJ3/KCNJ4/KCNJ5/KCNJ6/KCNJ9/KCNK10/KCNK3/KCNK5/KCNK6/KCNMA1/KCNMB2/KCNN1/KCNN2/KCNN3/KCNQ2/KCNQ3/KCNQ4/KCNS2/KCNT1/KCNT2/KCNV1/LRRC55/MFSD2A/NALCN/NALF1/NALF2/NIPAL2/PKD1L2/PKD1L3/PKDREJ/RYR2/RYR3/SCN10A/SCN1A/SCN2A/SCN2B/SCN3B/SCN4A/SCN8A/SCN9A/SCNN1G/SEC61A1/SHROOM2/SLC10A3/SLC10A5/SLC11A1/SLC12A5/SLC12A9/SLC13A1/SLC13A4/SLC13A5/SLC17A2/SLC17A4/SLC17A6/SLC17A7/SLC17A8/SLC1A1/SLC1A2/SLC1A6/SLC24A2/SLC24A3/SLC28A3/SLC30A1/SLC30A3/SLC30A8/SLC34A1/SLC34A2/SLC38A3/SLC39A12/SLC39A5/SLC41A2/SLC4A11/SLC4A4/SLC4A5/SLC5A1/SLC5A7/SLC6A1/SLC6A11/SLC6A15/SLC6A2/SLC6A20/SLC6A3/SLC6A5/SLC6A7/SLC8A2/SLC8A3/SLC9A2/SLC9A4/SLC9A5/SLC9A6/SLC9A7/SLC9B2/SLC9C2/SNAP25/TMC1/TMEM37/TRPC3/TRPC4/TRPC7/TRPM1/TRPM3/TRPM4/TRPM8/TRPV4/TTYH1/TUSC3	168
cluster5	MF	GO:0015276	ligand-gated ion channel activity	77/3764	146/18432	4.52E-18	1.37E-15	1.27E-15	ABCC8/ABCC9/ASIC1/ASIC2/ASIC3/ASIC4/CFTR/CHRNA2/CHRNA3/CHRNA4/CHRNA6/CHRNA7/CHRNB4/CHRNG/CLCA2/CNGA3/CNGB1/FKBP1B/GABRA1/GABRA2/GABRA3/GABRA4/GABRA5/GABRA6/GABRB2/GABRB3/GABRD/GABRE/GABRG1/GABRG2/GABRG3/GABRQ/GLRA1/GLRA2/GLRB/GRIA1/GRIA2/GRIA3/GRIA4/GRID1/GRID2/GRIK1/GRIK2/GRIK3/GRIK5/GRIN1/GRIN2A/GRIN2B/GRIN2D/HCN1/HCN4/HTR3C/KCNA10/KCNH2/KCNH6/KCNJ13/KCNJ16/KCNJ18/KCNJ3/KCNJ4/KCNJ5/KCNJ6/KCNJ9/KCNK6/KCNN1/KCNN2/KCNN3/P2RX2/P2RX3/PEX5L/RYR2/RYR3/SCNN1G/SHROOM2/TRPM4/TRPM8/ZACN	77
cluster5	MF	GO:0022834	ligand-gated channel activity	77/3764	146/18432	4.52E-18	1.37E-15	1.27E-15	ABCC8/ABCC9/ASIC1/ASIC2/ASIC3/ASIC4/CFTR/CHRNA2/CHRNA3/CHRNA4/CHRNA6/CHRNA7/CHRNB4/CHRNG/CLCA2/CNGA3/CNGB1/FKBP1B/GABRA1/GABRA2/GABRA3/GABRA4/GABRA5/GABRA6/GABRB2/GABRB3/GABRD/GABRE/GABRG1/GABRG2/GABRG3/GABRQ/GLRA1/GLRA2/GLRB/GRIA1/GRIA2/GRIA3/GRIA4/GRID1/GRID2/GRIK1/GRIK2/GRIK3/GRIK5/GRIN1/GRIN2A/GRIN2B/GRIN2D/HCN1/HCN4/HTR3C/KCNA10/KCNH2/KCNH6/KCNJ13/KCNJ16/KCNJ18/KCNJ3/KCNJ4/KCNJ5/KCNJ6/KCNJ9/KCNK6/KCNN1/KCNN2/KCNN3/P2RX2/P2RX3/PEX5L/RYR2/RYR3/SCNN1G/SHROOM2/TRPM4/TRPM8/ZACN	77

cluster5	MF	GO:0005261	cation channel activity	136/3764	346/18432	3.74E-16	1.01E-13	9.33E-14	ABCC8/ABCC9/ANO6/ASIC1/ASIC2/ASIC3/ASIC4/ATP5F1E/CACNA1A/CACNA1B/CACNA1C/CACNA1G/CACNA1S/CACNA2D1/CACNB1/CACNG2/CACNG3/CACNG4/CACNG5/CACNG7/CALHM5/CATSPER4/CHRNA2/CHRNA3/CHRNA4/CHRNA6/CHRNA7/CHRNB4/CHRNG/CNGA3/CNGB1/FKBP1B/GPM6A/GRIA1/GRIA2/GRIA3/GRIA4/GRIK1/GRIK2/GRIK3/GRIK5/GRIN1/GRIN2A/GRIN2B/GRIN2D/HCN1/HCN3/HCN4/HTR3C/KCNA10/KCNA4/KCNA7/KCNB1/KCNB2/KCNC1/KCNC2/KCNC3/KCND2/KCND3/KCNE4/KCNG3/KCNH1/KCNH2/KCNH4/KCNH5/KCNH6/KCNIP1/KCNIP3/KCNJ13/KCNJ16/KCNJ18/KCNJ3/KCNJ4/KCNJ5/KCNJ6/KCNJ9/KCNK10/KCNK3/KCNK5/KCNK6/KCNMA1/KCNMB2/KCNN1/KCNN2/KCNN3/KCNQ2/KCNQ3/KCNQ4/KCNS2/KCNT1/KCNT2/KCNV1/LRRC55/NALCN/NALF1/NALF2/OTOP1/P2RX2/P2RX3/PEX5L/PKD1L2/PKD1L3/PKDREJ/RHCE/RHCG/RHD/RYR2/RYR3/SCN10A/SCN1A/SCN2A/SCN2B/SCN3B/SCN4A/SCN8A/SCN9A/SCNN1G/SEC61A1/SHROOM2/SLC12A5/SLC24A2/SLC24A3/SLC4A11/SNAP25/TMC1/TMEM37/TMEM63C/TRPC3/TRPC4/TRPC7/TRPM1/TRPM3/TRPM4/TRPM8/TRPV4/UNC80	136
cluster5	MF	GO:0030594	neurotransmitter receptor activity	60/3764	111/18432	4.83E-15	1.17E-12	1.09E-12	ADORA1/CHRM1/CHRM2/CHRM3/CHRNA2/CHRNA3/CHRNA4/CHRNA6/CHRNA7/CHRNB4/CHRNG/DRD1/DRD2/DRD4/GABRA1/GABRA2/GABRA3/GABRA4/GABRA5/GABRA6/GABRB2/GABRB3/GABRD/GABRE/GABRG1/GABRG2/GABRG3/GABRQ/GLRA1/GLRA2/GLRB/GRIA1/GRIA2/GRIA3/GRIA4/GRID1/GRID2/GRIK1/GRIK2/GRIK3/GRIK5/GRIN1/GRIN2A/GRIN2B/GRIN2D/GRM5/HRH3/HTR1D/HTR1E/HTR1F/HTR2A/HTR2C/HTR3C/HTR4/OR10H3/OR10H4/OR11H4/OR5T3/OR6T1/ZACN	60
cluster5	MF	GO:0022824	transmitter-gated ion channel activity	40/3764	65/18432	5.74E-13	1.16E-10	1.08E-10	CHRNA2/CHRNA3/CHRNA4/CHRNA6/CHRNA7/CHRNB4/CHRNG/GABRA1/GABRA2/GABRA3/GABRA4/GABRA5/GABRA6/GABRB2/GABRB3/GABRD/GABRE/GABRG1/GABRG2/GABRG3/GABRQ/GLRA1/GLRA2/GLRB/GRIA1/GRIA2/GRIA3/GRIA4/GRID1/GRID2/GRIK1/GRIK2/GRIK3/GRIK5/GRIN1/GRIN2A/GRIN2B/GRIN2D/HTR3C/ZACN	40
cluster5	MF	GO:0022835	transmitter-gated channel activity	40/3764	65/18432	5.74E-13	1.16E-10	1.08E-10	CHRNA2/CHRNA3/CHRNA4/CHRNA6/CHRNA7/CHRNB4/CHRNG/GABRA1/GABRA2/GABRA3/GABRA4/GABRA5/GABRA6/GABRB2/GABRB3/GABRD/GABRE/GABRG1/GABRG2/GABRG3/GABRQ/GLRA1/GLRA2/GLRB/GRIA1/GRIA2/GRIA3/GRIA4/GRID1/GRID2/GRIK1/GRIK2/GRIK3/GRIK5/GRIN1/GRIN2A/GRIN2B/GRIN2D/HTR3C/ZACN	40

cluster5	MF	GO:0098960	postsynaptic neurotransmitter receptor activity	43/3764	75/18432	2.43E-12	4.54E-10	4.2E-10	CHRM1/CHRM2/CHRM3/CHRNA2/CHRNA3/CHRNA4/CHRNA6/CHRNA7/CHRNB4/CHRNG/DRD1/DRD2/DRD4/GABRA1/GABRA2/GABRA3/GABRA4/GABRA5/GABRA6/GABRB2/GABRB3/GABRD/GABRE/GABRG1/GABRG2/GABRG3/GABRQ/GLRA1/GLRA2/GLRB/GRIA1/GRIA3/GRID1/GRID2/GRIK1/GRIK2/GRIK3/GRIK5/GRIN2D/GRM5/HRH3/HTR3C/ZACN	43
cluster5	MF	GO:0005230	extracellular ligand-gated ion channel activity	42/3764	74/18432	6.91E-12	1.2E-09	1.11E-09	CHRNA2/CHRNA3/CHRNA4/CHRNA6/CHRNA7/CHRNB4/CHRNG/GABRA1/GABRA2/GABRA3/GABRA4/GABRA5/GABRA6/GABRB2/GABRB3/GABRD/GABRE/GABRG1/GABRG2/GABRG3/GABRQ/GLRA1/GLRA2/GLRB/GRIA1/GRIA2/GRIA3/GRIA4/GRID1/GRID2/GRIK1/GRIK2/GRIK3/GRIK5/GRIN1/GRIN2A/GRIN2B/GRIN2D/HTR3C/P2RX2/P2RX3/ZACN	42
cluster5	MF	GO:0015079	potassium ion transmembrane transporter activity	70/3764	158/18432	9.07E-12	1.47E-09	1.36E-09	ABCC8/ABCC9/ATP1A2/ATP1A3/ATP1B1/GRIK1/GRIK2/GRIK3/GRIK5/HCN1/HCN3/HCN4/KCNA10/KCNA4/KCNA7/KCNB1/KCNB2/KCNC1/KCNC2/KCNC3/KCND2/KCND3/KCNE4/KCNG3/KCNH1/KCNH2/KCNH4/KCNH5/KCNH6/KCNIP1/KCNIP3/KCNJ13/KCNJ16/KCNJ18/KCNJ3/KCNJ4/KCNJ5/KCNJ6/KCNJ9/KCNK10/KCNK3/KCNK5/KCNK6/KCNMA1/KCNMB2/KCNN1/KCNN2/KCNN3/KCNQ2/KCNQ3/KCNQ4/KCNS2/KCNT1/KCNT2/KCNV1/LRRC55/SCN2B/SLC12A5/SLC12A9/SLC17A6/SLC17A7/SLC24A2/SLC24A3/SLC9A2/SLC9A4/SLC9A5/SLC9A6/SLC9A7/SLC9C2/SNAP25	70
cluster5	MF	GO:0005244	voltage-gated ion channel activity	83/3764	201/18432	1.06E-11	1.52E-09	1.41E-09	ABCC8/ABCC9/ANO6/CACNA1A/CACNA1B/CACNA1C/CACNA1G/CACNA1S/CACNA2D1/CACNB1/CACNG2/CACNG3/CACNG4/CACNG5/CACNG7/CATSPER4/CLCN2/CLCN4/CLCNKA/CLCNKB/CLIC6/GRIN1/GRIN2A/GRIN2B/GRIN2D/HCN1/HCN3/HCN4/KCNA10/KCNA4/KCNA7/KCNB1/KCNB2/KCNC1/KCNC2/KCNC3/KCND2/KCND3/KCNE4/KCNG3/KCNH1/KCNH2/KCNH4/KCNH5/KCNH6/KCNIP1/KCNIP3/KCNJ13/KCNJ16/KCNJ18/KCNJ3/KCNJ4/KCNJ5/KCNJ6/KCNJ9/KCNK10/KCNK3/KCNK5/KCNK6/KCNMA1/KCNN1/KCNN2/KCNN3/KCNQ2/KCNQ3/KCNQ4/KCNS2/KCNT1/KCNT2/KCNV1/LRRC55/NALCN/SCN10A/SCN1A/SCN2A/SCN2B/SCN3B/SCN4A/SCN8A/SCN9A/SNAP25/TMC1/TMEM37	83

cluster5	MF	GO:0022832	voltage-gated channel activity	83/3764	201/18432	1.06E-11	1.52E-09	1.41E-09	ABCC8/ABCC9/ANO6/CACNA1A/CACNA1B/CACNA1C/CACNA1G/CACNA1S/CACNA2D1/CACNB1/CACNG2/CACNG3/CACNG4/CACNG5/CACNG7/CATSPER4/CLCN2/CLCN4/CLCNKA/CLCNKB/CLIC6/GRIN1/GRIN2A/GRIN2B/GRIN2D/HCN1/HCN3/HCN4/KCNA10/KCNA4/KCNA7/KCNB1/KCNB2/KCNC1/KCNC2/KCNC3/KCND2/KCND3/KCNE4/KCNG3/KCNH1/KCNH2/KCNH4/KCNH5/KCNH6/KCNIP1/KCNIP3/KCNJ13/KCNJ16/KCNJ18/KCNJ3/KCNJ4/KCNJ5/KCNJ6/KCNJ9/KCNK10/KCNK3/KCNK5/KCNK6/KCNMA1/KCNN1/KCNN2/KCNN3/KCNQ2/KCNQ3/KCNQ4/KCNS2/KCNT1/KCNT2/KCNV1/LRRC55/NALCN/SCN10A/SCN1A/SCN2A/SCN2B/SCN3B/SCN4A/SCN8A/SCN9A/SNAP25/TMC1/TMEM37	83
cluster5	MF	GO:0015081	sodium ion transmembrane transporter activity	69/3764	156/18432	1.4E-11	1.88E-09	1.75E-09	ASIC1/ASIC2/ASIC3/ASIC4/ATP1A2/ATP1A3/ATP1B1/CACNA1G/GRIK1/GRIK2/GRIK3/GRIK5/HCN1/HCN3/HCN4/MFSD2A/NALCN/SCN10A/SCN1A/SCN2A/SCN2B/SCN3B/SCN4A/SCN8A/SCN9A/SCNN1G/SHROOM2/SLC10A3/SLC10A5/SLC13A1/SLC13A4/SLC13A5/SLC17A2/SLC17A4/SLC17A6/SLC17A7/SLC17A8/SLC1A1/SLC1A2/SLC1A6/SLC24A2/SLC24A3/SLC28A3/SLC34A1/SLC34A2/SLC38A3/SLC4A11/SLC4A4/SLC4A5/SLC5A1/SLC5A7/SLC6A1/SLC6A11/SLC6A15/SLC6A2/SLC6A20/SLC6A3/SLC6A5/SLC6A7/SLC8A2/SLC8A3/SLC9A2/SLC9A4/SLC9A5/SLC9A6/SLC9A7/SLC9B2/SLC9C2/TRPM4	69
cluster5	MF	GO:1904315	transmitter-gated ion channel activity involved in regulation of postsynaptic membrane potential	35/3764	58/18432	3.58E-11	4.58E-09	4.24E-09	CHRNA2/CHRNA3/CHRNA4/CHRNA6/CHRNA7/CHRNB4/CHRNG/GABRA1/GABRA2/GABRA3/GABRA4/GABRA5/GABRA6/GABRB2/GABRB3/GABRD/GABRE/GABRG1/GABRG2/GABRG3/GABRQ/GLRA1/GLRA2/GLRB/GRIA1/GRIA3/GRID1/GRID2/GRIK1/GRIK2/GRIK3/GRIK5/GRIN2D/HTR3C/ZACN	35
cluster5	MF	GO:0099529	neurotransmitter receptor activity involved in regulation of postsynaptic membrane potential	36/3764	61/18432	4.67E-11	5.67E-09	5.25E-09	CHRM1/CHRNA2/CHRNA3/CHRNA4/CHRNA6/CHRNA7/CHRNB4/CHRNG/GABRA1/GABRA2/GABRA3/GABRA4/GABRA5/GABRA6/GABRB2/GABRB3/GABRD/GABRE/GABRG1/GABRG2/GABRG3/GABRQ/GLRA1/GLRA2/GLRB/GRIA1/GRIA3/GRID1/GRID2/GRIK1/GRIK2/GRIK3/GRIK5/GRIN2D/HTR3C/ZACN	36

cluster5	MF	GO:0099094	ligand-gated cation channel activity	55/3764	116/18432	6.22E-11	7.19E-09	6.66E-09	ABCC8/ABCC9/ASIC1/ASIC2/ASIC3/ASIC4/CHRNA2/CHRNA3/CHRNA4/CHRNA6/CHRNA7/CHRNB4/CHRNG/CNGA3/CNGB1/FKBP1B/GRIA1/GRIA2/GRIA3/GRIA4/GRIK1/GRIK2/GRIK3/GRIK5/GRIN1/GRIN2A/GRIN2B/GRIN2D/HCN1/HCN4/HTR3C/KCNA10/KCNH2/KCNH6/KCNJ13/KCNJ16/KCNJ18/KCNJ3/KCNJ4/KCNJ5/KCNJ6/KCNJ9/KCNK6/KCNN1/KCNN2/KCNN3/P2RX2/P2RX3/PEX5L/RYR2/RYR3/SCNN1G/SHROOM2/TRPM4/TRPM8	55
cluster5	MF	GO:0022843	voltage-gated cation channel activity	66/3764	151/18432	7.28E-11	8.03E-09	7.43E-09	ABCC8/ABCC9/CACNA1A/CACNA1B/CACNA1C/CACNA1G/CACNA1S/CACNA2D1/CACNB1/CACNG2/CACNG3/CACNG4/CACNG5/CACNG7/CATSPER4/GRIN1/GRIN2A/GRIN2B/GRIN2D/HCN1/HCN3/HCN4/KCNA10/KCNA4/KCNA7/KCNB1/KCNB2/KCNC1/KCNC2/KCNC3/KCND2/KCND3/KCNE4/KCNG3/KCNH1/KCNH2/KCNH4/KCNH5/KCNH6/KCNJ13/KCNJ16/KCNJ18/KCNJ3/KCNJ4/KCNJ5/KCNJ6/KCNJ9/KCNK10/KCNK3/KCNK5/KCNK6/KCNMA1/KCNN1/KCNN2/KCNN3/KCNQ2/KCNQ3/KCNQ4/KCNS2/KCNT1/KCNT2/KCNV1/LRRC55/SCN2B/SNAP25/TMC1	66
cluster5	MF	GO:0030546	signaling receptor activator activity	161/3764	498/18432	1.76E-10	1.86E-08	1.72E-08	ADCYAP1/AGT/ALKAL2/AMBN/AMELX/ANGPTL8/APELA/APP/ARTN/AVP/BDNF/BMP2/BMP4/BMP5/BMP6/BMP7/BTC/CALCA/CALCB/CCK/CCL22/CCL25/CCN2/CGA/CGB2/CGB7/CHGB/CLEC11A/COLEC10/CRH/CRLF1/CSH1/CSH2/CSPG5/CTSG/CXCL13/CXCL14/CXCL3/CXCL5/DEFB104A/DEFB104B/EDN2/EFNA5/EGF/EGFR/EPHA7/EPO/F2/FGF10/FGF14/FGF16/FGF17/FGF19/FGF2/FGF23/FGF4/FGF5/FGF7/FGF9/FLRT2/FLRT3/FNDC5/GAS6/GAST/GCG/GDF1/GDF10/GDF5/GDF6/GDNF/GH2/GREM1/GREM2/GRP/IAPP/IFNA10/IFNA16/IFNA17/IFNA4/IFNA6/IFNA8/IFNB1/IL13/IL17C/IL17D/IL20/IL31/IL36B/IL36RN/IL5/IL7/IL9/INHA/INHBC/INS/INS-IGF2/INSL4/KITLG/LACRT/LEFTY1/LEFTY2/MDK/MLN/NDP/NGF/NPPC/NPY/NRG2/NRG3/NTF3/NTS/OGN/PDGFA/PDGFC/PDYN/PENK/PF4/PF4V1/PPBP/PTH/PTN/PYY/SCG2/SEMA3A/SEMA3E/SEMA4D/SEMA5A/SEMA5B/SEMA6A/SEMA6D/SFRP2/SHH/SLURP1/SPX/SST/STC1/STC2/TAFA3/TAFA4/TAFA5/TDGF1/THPO/TIMP1/TNFRSF11B/TRH/TTR/UCN2/UCN3/VEGFA/VEGFD/WNT11/WNT2B/WNT3A/WNT4/WNT5A/WNT7A/WNT7B/WNT8A/WNT8B/WNT9A/WNT9B	161
cluster5	MF	GO:0048018	receptor ligand activity	159/3764	491/18432	1.99E-10	2.02E-08	1.87E-08	ADCYAP1/AGT/ALKAL2/AMBN/AMELX/ANGPTL8/APELA/ARTN/AVP/BDNF/BMP2/BMP4/BMP5/BMP6/BMP7/BTC/CALCA/CALCB/CCK/CCL22/CCL25/CCN2/CGA/CGB2/CGB7/CHGB/CLEC11A/COLEC10/CRH/CRLF1/CSH1/CSH2/CSPG5/CTSG/CXCL13/CXCL14/CXCL3/CXCL5/DEFB104A/DEFB104B/EDN2/EFNA5/EGF/EPHA7/EPO/F2/FGF10/FGF14/FGF16/FGF17/FGF19/FGF2/FGF23/FGF4/FGF5/FGF7/FGF9/FLRT2/FLRT3/FNDC5/GAS6/GAST/GCG/GDF1/GDF10/GDF5/GDF6/GDNF/GH2/GREM1/GREM2/GRP/IAPP/IFNA10/IFNA16/IFNA17/IFNA4/IFNA6/IFNA8/IFNB1/IL13/IL17C/IL17D/IL20/IL31/IL36B/IL36RN/IL5/IL7/IL9/INHA/INHBC/INS/INS-IGF2/INSL4/KITLG/LACRT/LEFTY1/LEFTY2/MDK/MLN/NDP/NGF/NPPC/NPY/NRG2/NRG3/NTF3/NTS/OGN/PDGFA/PDGFC/PDYN/PENK/PF4/PF4V1/PPBP/PTH/PTN/PYY/SCG2/SEMA3A/SEMA3E/SEMA4D/SEMA5A/SEMA5B/SEMA6A/SEMA6D/SFRP2/SHH/SLURP1/SPX/SST/STC1/STC2/TAFA3/TAFA4/TAFA5/TDGF1/THPO/TIMP1/TNFRSF11B/TRH/TTR/UCN2/UCN3/VEGFA/VEGFD/WNT11/WNT2B/WNT3A/WNT4/WNT5A/WNT7A/WNT7B/WNT8A/WNT8B/WNT9A/WNT9B	159

cluster5	MF	GO:0008066	glutamate receptor activity	21/3764	27/18432	2.52E-10	2.45E-08	2.27E-08	GRIA1/GRIA2/GRIA3/GRIA4/GRID1/GRID2/GRIK1/GRIK2/GRIK3/GRIK5/GRIN1/GRIN2A/GRIN2B/GRIN2D/GRM2/GRM3/GRM4/GRM5/GRM6/GRM7/GRM8	21
cluster5	MF	GO:0005249	voltage-gated potassium channel activity	48/3764	99/18432	3.87E-10	3.61E-08	3.34E-08	ABCC8/ABCC9/HCN1/HCN3/HCN4/KCNA10/KCNA4/KCNA7/KCNB1/KCNB2/KCNC1/KCNC2/KCNC3/KCND2/KCND3/KCNE4/KCNG3/KCNH1/KCNH2/KCNH4/KCNH5/KCNH6/KCNJ13/KCNJ16/KCNJ18/KCNJ3/KCNJ4/KCNJ5/KCNJ6/KCNJ9/KCNK10/KCNK3/KCNK5/KCNK6/KCNMA1/KCNN1/KCNN2/KCNN3/KCNQ2/KCNQ3/KCNQ4/KCNS2/KCNT1/KCNT2/KCNV1/LRRC55/SCN2B/SNAP25	48
cluster5	MF	GO:0005267	potassium channel activity	55/3764	122/18432	6.73E-10	6.05E-08	0.000000056	ABCC8/ABCC9/GRIK1/GRIK2/GRIK3/GRIK5/HCN1/HCN3/HCN4/KCNA10/KCNA4/KCNA7/KCNB1/KCNB2/KCNC1/KCNC2/KCNC3/KCND2/KCND3/KCNE4/KCNG3/KCNH1/KCNH2/KCNH4/KCNH5/KCNH6/KCNIP1/KCNIP3/KCNJ13/KCNJ16/KCNJ18/KCNJ3/KCNJ4/KCNJ5/KCNJ6/KCNJ9/KCNK10/KCNK3/KCNK5/KCNK6/KCNMA1/KCNMB2/KCNN1/KCNN2/KCNN3/KCNQ2/KCNQ3/KCNQ4/KCNS2/KCNT1/KCNT2/KCNV1/LRRC55/SCN2B/SNAP25	55
cluster5	MF	GO:0005237	inhibitory extracellular ligand-gated ion channel activity	14/3764	15/18432	2.61E-09	0.000000227	0.00000021	GABRA1/GABRA2/GABRA3/GABRA4/GABRA5/GABRA6/GABRB2/GABRE/GABRG1/GABRG2/GABRG3/GLRA1/GLRA2/GLRB	14

cluster5	MF	GO:0005516	calmodulin binding	77/3764	200/18432	2.82E-09	0.000000236	0.000000219	ADCY1/ADCY3/ADCY8/AKT1/ASPM/ATP2B2/ATP2B3/CACNA1C/CACNA1S/CAMK1G/CAMK2A/CAMK2B/CAMKV/CFAP221/CNN1/EEA1/ENKUR/FBXL2/GAP43/GEM/GRIN1/IQCF3/IQCF6/ITPKA/KCNH1/KCNH5/KCNN1/KCNN2/KCNN3/KCNQ2/KCNQ3/KCNQ4/MAP2/MAP6/MIP/MYH1/MYH10/MYH13/MYH2/MYH4/MYH6/MYH7/MYH8/MYH9/MYLK/MYO1C/MYO3A/MYO5B/MYO6/NGFR/NOS2/NOS3/PCP4/PDE1A/PDE1B/PDE1C/PHKB/PLCB1/PNCK/REM1/RGS16/RGS4/RIT2/RYR2/RYR3/SLC8A2/SLC8A3/SPATA17/STRN/STRN4/SYT1/SYT7/TRPM4/TRPV4/UNC13A/UNC13C/WFS1	77
cluster5	MF	GO:0099095	ligand-gated anion channel activity	16/3764	19/18432	4.57E-09	0.00000037	0.000000342	CFTR/GABRA1/GABRA2/GABRA3/GABRA4/GABRA5/GABRA6/GABRB2/GABRB3/GABRE/GABRG1/GABRG2/GABRG3/GLRA1/GLRA2/GLRB	16
cluster5	MF	GO:0005201	extracellular matrix structural constituent	67/3764	173/18432	2.24E-08	0.00000175	0.00000162	ACAN/AMBN/AMELX/ANOS1/ASPN/CHADL/COL11A1/COL12A1/COL14A1/COL16A1/COL21A1/COL22A1/COL23A1/COL24A1/COL25A1/COL27A1/COL28A1/COL2A1/COL4A1/COL4A5/COL4A6/COL5A3/COL6A1/COL6A5/COL6A6/COL8A1/COL9A1/DSPP/ECM1/EDIL3/EMILIN1/EMILIN2/EMILIN3/FBLN1/FBN3/HAPLN1/HAPLN4/HMCN2/IMPG1/LAMA1/LAMA3/LAMA5/LAMB1/LAMB2/LAMC2/LTBP1/MATN2/MATN3/MFAP2/MFAP4/MMRN1/MUC5AC/MXRA5/NID1/OGN/OPTC/PODN/PRG3/PXDN/SRPX2/TECTB/THSD4/TNC/TNXB/VWA1/VWF/ZP4	67
cluster5	MF	GO:0005539	glycosaminoglycan binding	84/3764	237/18432	0.000000049	0.00000372	0.00000344	ACAN/ADAMTS15/ADAMTS3/ADAMTS8/ALK/ANOS1/APOA5/APP/AZU1/BCAN/BMP4/BMP7/CCN2/CEL/CEMIP/COL11A1/COL23A1/COL25A1/COL5A3/CRISPLD2/CTSG/CXCL13/DPYSL3/ECM2/ELANE/EPYC/F2/FGF10/FGF2/FGF4/FGF7/FGF9/FGFBP3/FGFR1/FGFR2/FGFR4/GREM2/HABP2/HAPLN1/HAPLN2/HAPLN4/HSD17B12/IMPG1/KNG1/LAMC2/LIPC/LIPG/LIPI/LRTM1/LRTM2/MDK/NAV2/NCAN/NDNF/NELL1/NRP2/PF4/PF4V1/PGLYRP4/PODXL2/PTCH1/PTN/PTPRF/PTPRS/REG3A/REG3G/RSPO1/RSPO2/RSPO3/RSPO4/RTN4RL1/SAA1/SELP/SEMA5A/SERPINE2/SHH/SLIT1/SLIT3/SPOCK3/SULF1/SULF2/TNXB/VEGFA/VIT	84

cluster5	MF	GO:0022851	GABA-gated chloride ion channel activity	12/3764	13/18432	5.47E-08	0.00000403	0.00000373	GABRA1/GABRA2/GABRA3/GABRA4/GABRA5/GABRA6/GABRB2/GABRB3/GABRE/GABRG1/GABRG2/GABRG3	12
cluster5	MF	GO:0016917	GABA receptor activity	16/3764	22/18432	0.000000186	0.000013	0.000012	GABBR2/GABRA1/GABRA2/GABRA3/GABRA4/GABRA5/GABRA6/GABRB2/GABRB3/GABRD/GABRE/GABRG1/GABRG2/GABRG3/GABRQ/GPR156	16
cluster5	MF	GO:0005231	excitatory extracellular ligand-gated ion channel activity	29/3764	56/18432	0.000000187	0.000013	0.000012	CHRNA2/CHRNA3/CHRNA4/CHRNA6/CHRNA7/CHRNB4/CHRNG/GABRA1/GABRA2/GABRA3/GABRA4/GABRA5/GABRA6/GABRB2/GABRB3/GABRD/GABRE/GABRG1/GABRG2/GABRG3/GABRQ/GLRA1/GLRA2/GLRB/GRIK2/HTR3C/P2RX2/P2RX3/ZACN	29
cluster5	MF	GO:0008201	heparin binding	64/3764	171/18432	0.000000201	0.0000136	0.0000126	ADAMTS15/ADAMTS3/ADAMTS8/ALK/ANOS1/APOA5/APP/AZU1/BMP4/BMP7/CCN2/CEL/COL11A1/COL23A1/COL25A1/COL5A3/CRISPLD2/CTSG/CXCL13/ECM2/ELANE/F2/FGF10/FGF2/FGF4/FGF7/FGF9/FGFBP3/FGFR1/FGFR2/FGFR4/GREM2/HSD17B12/IMPG1/KNG1/LAMC2/LIPC/LIPG/LIPI/LRTM1/LRTM2/MDK/NAV2/NDNF/NELL1/NRP2/PF4/PF4V1/PTCH1/PTN/PTPRF/PTPRS/RSPO1/RSPO2/RSPO3/RSPO4/RTN4RL1/SAA1/SELP/SERPINE2/SLIT1/SLIT3/TNXB/VEGFA	64

cluster5	MF	GO:0005254	chloride channel activity	36/3764	78/18432	0.000000282	0.0000185	0.0000171	ANO3/ANO4/ANO6/BEST2/BEST3/CFTR/CLCA2/CLCN2/CLCN4/CLCNKA/CLCNKB/CLDN4/CLIC6/FXYD3/GABRA1/GABRA2/GABRA3/GABRA4/GABRA5/GABRA6/GABRB2/GABRB3/GABRD/GABRE/GABRG1/GABRG2/GABRG3/GABRQ/GLRA1/GLRA2/GLRB/SLC17A6/SLC17A7/SLC17A8/SLC26A9/TTYH1	36
cluster5	MF	GO:0005272	sodium channel activity	25/3764	46/18432	0.000000391	0.0000249	0.0000231	ASIC1/ASIC2/ASIC3/ASIC4/CACNA1G/GRIK1/GRIK2/GRIK3/GRIK5/HCN1/HCN3/HCN4/NALCN/SCN10A/SCN1A/SCN2A/SCN2B/SCN3B/SCN4A/SCN8A/SCN9A/SCNN1G/SHROOM2/SLC4A11/TRPM4	25
cluster5	MF	GO:0004890	GABA-A receptor activity	14/3764	19/18432	0.000000874	0.000053	0.0000491	GABRA1/GABRA2/GABRA3/GABRA4/GABRA5/GABRA6/GABRB2/GABRB3/GABRD/GABRE/GABRG1/GABRG2/GABRG3/GABRQ	14
cluster5	MF	GO:0004970	ionotropic glutamate receptor activity	14/3764	19/18432	0.000000874	0.000053	0.0000491	GRIA1/GRIA2/GRIA3/GRIA4/GRID1/GRID2/GRIK1/GRIK2/GRIK3/GRIK5/GRIN1/GRIN2A/GRIN2B/GRIN2D	14

cluster5	MF	GO:0003774	cytoskeletal motor activity	45/3764	111/18432	0.000000994	0.0000588	0.0000545	ACTC1/DNAH10/DNAH11/DNAH12/DNAH14/DNAH2/DNAH3/DNAH5/DNAH7/DNAH9/DNAI1/DYNC1I1/DYNLRB2/KIF12/KIF18A/KIF1A/KIF20A/KIF20B/KIF21A/KIF21B/KIF24/KIF25/KIF2B/KIF3A/KIF3C/KIF5A/KIF5C/KIF6/KIF7/KIFC1/MYH1/MYH10/MYH13/MYH2/MYH4/MYH6/MYH7/MYH8/MYH9/MYO18B/MYO1C/MYO3A/MYO3B/MYO5B/MYO6	45
cluster5	MF	GO:0008503	benzodiazepine receptor activity	10/3764	11/18432	0.00000112	0.0000647	0.0000599	GABRA1/GABRA2/GABRA3/GABRA4/GABRA5/GABRA6/GABRE/GABRG1/GABRG2/GABRG3	10
cluster5	MF	GO:0008083	growth factor activity	59/3764	162/18432	0.00000168	0.0000949	0.0000879	AGT/AMBN/AMELX/ARTN/BDNF/BMP2/BMP4/BMP5/BMP6/BMP7/BTC/CCN2/CLEC11A/CSH1/CSH2/CSPG5/EGF/F2/FGF10/FGF14/FGF16/FGF17/FGF19/FGF2/FGF23/FGF4/FGF5/FGF7/FGF9/GDF1/GDF10/GDF5/GDF6/GDNF/GH2/IL5/IL7/IL9/INHA/INHBC/KITLG/LACRT/LEFTY1/LEFTY2/MDK/NGF/NRG2/NRG3/NTF3/OGN/PDGFA/PDGFC/PPBP/PTN/TDGF1/THPO/TIMP1/VEGFA/VEGFD	59
cluster5	MF	GO:0008146	sulfotransferase activity	26/3764	52/18432	0.00000191	0.000105503	0.0000977	CHST1/CHST15/CHST3/CHST4/CHST5/CHST6/CHST8/CHST9/GAL3ST1/GAL3ST2/GAL3ST3/HS3ST3A1/HS3ST4/HS3ST5/HS6ST1/HS6ST2/HS6ST3/NDST3/NDST4/SULT1C2/SULT1C3/SULT1C4/SULT1E1/SULT4A1/TPST2/WSCD1	26

cluster5	MF	GO:0015108	chloride transmembrane transporter activity	43/3764	107/18432	0.00000226	0.000121833	0.000112816	ANO3/ANO4/ANO6/BEST2/BEST3/CFTR/CLCA2/CLCN2/CLCN4/CLCNKA/CLCNKB/CLDN4/CLIC6/FXYD3/GABRA1/GABRA2/GABRA3/GABRA4/GABRA5/GABRA6/GABRB2/GABRB3/GABRD/GABRE/GABRG1/GABRG2/GABRG3/GABRQ/GLRA1/GLRA2/GLRB/SLC12A5/SLC12A9/SLC17A6/SLC17A7/SLC17A8/SLC1A1/SLC26A9/SLC6A1/SLC6A11/SLC6A2/SLC6A3/TTYH1	43
cluster5	MF	GO:0005253	anion channel activity	38/3764	91/18432	0.00000289	0.000152527	0.000141238	ANO3/ANO4/ANO6/BEST2/BEST3/CFTR/CLCA2/CLCN2/CLCN4/CLCNKA/CLCNKB/CLDN4/CLIC6/FXYD3/GABRA1/GABRA2/GABRA3/GABRA4/GABRA5/GABRA6/GABRB2/GABRB3/GABRD/GABRE/GABRG1/GABRG2/GABRG3/GABRQ/GLRA1/GLRA2/GLRB/LRRC8B/SLC17A6/SLC17A7/SLC17A8/SLC1A1/SLC26A9/TTYH1	38
cluster5	MF	GO:0015103	inorganic anion transmembrane transporter activity	56/3764	159/18432	0.00000978	0.000505273	0.000467876	ABCC3/ABCC9/ADAMTS8/ANO3/ANO4/ANO6/AQP6/BEST2/BEST3/CFTR/CLCA2/CLCN2/CLCN4/CLCNKA/CLCNKB/CLDN4/CLIC6/FXYD3/GABRA1/GABRA2/GABRA3/GABRA4/GABRA5/GABRA6/GABRB2/GABRB3/GABRD/GABRE/GABRG1/GABRG2/GABRG3/GABRQ/GLRA1/GLRA2/GLRB/LRRC8B/SLC12A5/SLC12A9/SLC13A1/SLC13A4/SLC17A6/SLC17A7/SLC17A8/SLC1A1/SLC22A11/SLC26A9/SLC37A1/SLC4A11/SLC4A3/SLC4A4/SLC4A5/SLC6A1/SLC6A11/SLC6A2/SLC6A3/TTYH1	56
cluster5	MF	GO:0005242	inward rectifier potassium channel activity	16/3764	27/18432	0.0000113	0.000572993	0.000530583	ABCC8/ABCC9/KCNH2/KCNH6/KCNJ13/KCNJ16/KCNJ18/KCNJ3/KCNJ4/KCNJ5/KCNJ6/KCNJ9/KCNK6/KCNN1/KCNN2/KCNN3	16

cluster5	MF	GO:0004714	transmembrane receptor protein tyrosine kinase activity	27/3764	60/18432	0.0000151	0.00074839	0.000692999	ALK/EFNA3/EFNB3/EGFR/EPHA10/EPHA3/EPHA5/EPHA6/EPHA7/EPHA8/EPHB3/ERBB2/ERBB4/FGFR1/FGFR2/FGFR3/FGFR4/FLT4/MUSK/NRP2/NTRK2/PDGFRA/PDGFRB/PDGFRL/RET/ROR2/TYRO3	27
cluster5	MF	GO:0005544	calcium-dependent phospholipid binding	25/3764	54/18432	0.000017	0.000823289	0.000762354	ANXA13/ANXA7/CPNE2/CPNE4/CPNE5/CPNE6/CPNE9/DOC2A/DOC2B/ESYT3/FER1L5/PCLO/PLA2G4C/PLA2G4E/SYT1/SYT10/SYT12/SYT13/SYT2/SYT3/SYT4/SYT5/SYT7/SYT8/SYT9	25
cluster5	MF	GO:0005179	hormone activity	45/3764	122/18432	0.0000189	0.000900063	0.000833446	ADCYAP1/AGT/ANGPTL8/APELA/AVP/CALCA/CALCB/CCK/CCL25/CGA/CGB2/CGB7/CHGB/CRH/CSH1/CSH2/EDN2/EPO/FNDC5/GAST/GCG/GH2/GRP/IAPP/INHA/INHBC/INS/INS-IGF2/INSL4/MLN/NPPC/NPY/NTS/PENK/PTH/PYY/SPX/SST/STC1/STC2/THPO/TRH/TTR/UCN2/UCN3	45
cluster5	MF	GO:0001640	adenylate cyclase inhibiting G protein-coupled glutamate receptor activity	8/3764	9/18432	0.0000222	0.001014499	0.000939412	GRIK3/GRM2/GRM3/GRM4/GRM5/GRM6/GRM7/GRM8	8

cluster5	MF	GO:0098988	G protein-coupled glutamate receptor activity	8/3764	9/18432	0.0000222	0.001014499	0.000939412	GRIK3/GRM2/GRM3/GRM4/GRM5/GRM6/GRM7/GRM8	8
cluster5	MF	GO:0030020	extracellular matrix structural constituent conferring tensile strength	20/3764	41/18432	0.0000461	0.001997356	0.001849524	COL11A1/COL12A1/COL14A1/COL16A1/COL21A1/COL23A1/COL24A1/COL25A1/COL27A1/COL28A1/COL2A1/COL4A1/COL4A5/COL4A6/COL5A3/COL6A1/COL6A5/COL6A6/COL8A1/COL9A1	20
cluster5	MF	GO:0005501	retinoid binding	19/3764	38/18432	0.0000461	0.001997356	0.001849524	ABCA4/ADH7/CRABP1/CRABP2/CYP26A1/CYP26C1/CYP27C1/CYP2W1/LCN12/LRAT/NR2F2/OPN4/RBP1/RBP2/RBP3/RHO/STRA6/UGT1A3/UGT1A8	19
cluster5	MF	GO:0019840	isoprenoid binding	19/3764	38/18432	0.0000461	0.001997356	0.001849524	ABCA4/ADH7/CRABP1/CRABP2/CYP26A1/CYP26C1/CYP27C1/CYP2W1/LCN12/LRAT/NR2F2/OPN4/RBP1/RBP2/RBP3/RHO/STRA6/UGT1A3/UGT1A8	19

cluster5	MF	GO:0003777	microtubule motor activity	28/3764	67/18432	0.0000555	0.002364407	0.002189408	DNAH10/DNAH11/DNAH12/DNAH14/DNAH2/DNAH3/DNAH5/DNAH7/DNAH9/DYNC1I1/DYNLRB2/KIF12/KIF18A/KIF1A/KIF20A/KIF20B/KIF21A/KIF21B/KIF24/KIF25/KIF2B/KIF3A/KIF3C/KIF5A/KIF5C/KIF6/KIF7/KIFC1	28
cluster5	MF	GO:0008227	G protein-coupled amine receptor activity	23/3764	51/18432	0.0000611	0.002557986	0.00236866	ADRA1A/ADRA1B/ADRA1D/ADRA2A/ADRB3/CHRM1/CHRM2/CHRM3/DRD4/HRH3/HTR1D/HTR1E/HTR1F/HTR2A/HTR2C/HTR4/OR10H3/OR10H4/OR11H4/OR5T3/OR6T1/TAAR2/ZNF219	23
cluster5	MF	GO:0035254	glutamate receptor binding	20/3764	42/18432	0.0000711	0.00292637	0.002709778	ATP2B2/CACNG2/CACNG3/CACNG4/CAMK2A/CDK5R1/DRD2/GNAS/GRIP2/HOMER1/IGSF11/MYL12A/OPHN1/RASGRF1/SHANK1/SHANK2/SHISA6/SHISA7/SQSTM1/SYNDIG1	20
cluster5	MF	GO:0035255	ionotropic glutamate receptor binding	13/3764	22/18432	0.0000805	0.003255585	0.003014627	CACNG2/CACNG3/CACNG4/CDK5R1/DRD2/GNAS/IGSF11/OPHN1/SHANK1/SHANK2/SHISA6/SHISA7/SQSTM1	13

cluster5	MF	GO:0005104	fibroblast growth factor receptor binding	14/3764	25/18432	0.0000957	0.003808424	0.003526548	FGF10/FGF16/FGF17/FGF19/FGF2/FGF23/FGF4/FGF5/FGF7/FGF9/FLRT2/FLRT3/FRS3/NPTN	14
cluster5	MF	GO:0015370	solute:sodium symporter activity	30/3764	76/18432	0.000108601	0.004251185	0.003936539	MFSD2A/SLC10A3/SLC10A5/SLC13A1/SLC13A4/SLC13A5/SLC17A2/SLC17A4/SLC17A6/SLC17A7/SLC17A8/SLC1A1/SLC1A2/SLC1A6/SLC28A3/SLC34A1/SLC34A2/SLC38A3/SLC4A4/SLC4A5/SLC5A1/SLC5A7/SLC6A1/SLC6A11/SLC6A15/SLC6A2/SLC6A20/SLC6A3/SLC6A5/SLC6A7	30
cluster5	MF	GO:1901681	sulfur compound binding	81/3764	270/18432	0.000111021	0.004276952	0.003960398	ACADL/ACBD7/ACOT7/ADAMTS15/ADAMTS3/ADAMTS8/ALK/ANOS1/APOA5/APP/AZU1/BMP4/BMP7/CCN2/CEL/CHST15/COL11A1/COL23A1/COL25A1/COL5A3/CRISPLD2/CTSG/CXCL13/DBI/DPYSL3/ECM2/ELANE/ENPP1/F2/FGF10/FGF2/FGF4/FGF7/FGF9/FGFBP3/FGFR1/FGFR2/FGFR4/GAL3ST3/GLRA1/GREM2/HPSE2/HS3ST5/HSD17B12/IMPG1/ITGA2/KNG1/LAMC2/LIPC/LIPG/LIPI/LRTM1/LRTM2/MDK/NAV2/NDNF/NELL1/NRP2/OGDHL/PF4/PF4V1/PNPLA3/PTCH1/PTN/PTPRF/PTPRS/RSPO1/RSPO2/RSPO3/RSPO4/RTN4RL1/RYR2/SAA1/SELP/SEMA5A/SERPINE2/SLIT1/SLIT3/SULT1C3/TNXB/VEGFA	81
cluster5	MF	GO:0019199	transmembrane receptor protein kinase activity	30/3764	79/18432	0.000244616	0.009186485	0.008506558	ALK/BMPR1B/EFNA3/EFNB3/EGFR/EPHA10/EPHA3/EPHA5/EPHA6/EPHA7/EPHA8/EPHB3/ERBB2/ERBB4/FGFR1/FGFR2/FGFR3/FGFR4/FLT4/LTBP1/MUSK/NRP2/NTRK2/PDGFRA/PDGFRB/PDGFRL/RET/ROR2/SOSTDC1/TYRO3	30

cluster5	MF	GO:0005549	odorant binding	41/3764	119/18432	0.000247117	0.009186485	0.008506558	OR10J1/OR10J3/OR10K1/OR10Q1/OR10W1/OR10Z1/OR11L1/OR12D1/OR12D2/OR12D3/OR14A16/OR14I1/OR14J1/OR5AC2/OR5AK2/OR5B17/OR5B3/OR5D14/OR5D16/OR5H2/OR5K1/OR5K4/OR5M10/OR5M11/OR5P2/OR5W2/OR6B2/OR8A1/OR8B2/OR8B3/OR8B4/OR8D2/OR8H1/OR8H3/OR8I2/OR8J1/OR8J3/OR8K1/OR9I1/OR9K2/OR9Q1	41
cluster5	MF	GO:0001228	DNA-binding transcription activator activity, RNA polymerase II-specific	127/3764	468/18432	0.000249818	0.009186485	0.008506558	ALX1/ALX4/ARNT2/ATF4/ATOH1/BARHL1/BARHL2/BARX2/CASZ1/CDX1/CDX4/CREB3L3/CRX/CSRNP3/DDIT3/DLX2/DLX3/DLX5/DMRT1/EBF2/ELF1/ESRRB/ESRRG/ETV1/ETV4/ETV5/FEV/FEZF2/FOS/FOSB/FOXA1/FOXC1/FOXF2/FOXJ1/FOXN1/FOXR1/GATA4/GBX2/GCM2/GLIS3/GRHL1/GRHL2/GSX1/HEYL/HIF1A/HNF1A/HNF4G/HOXB1/HOXC10/HOXC13/HOXC4/HOXD10/HOXD13/HOXD3/HSF1/ISL1/JUNB/KLF10/KLF15/LHX2/LHX3/LMX1A/MEF2B/MEIS1/MEIS3/MITF/MLXIP/MYCN/MYOG/NEUROD1/NEUROD2/NEUROD6/NEUROG3/NHLH2/NKX2-2/NKX2-6/NKX2-8/NPAS4/NR1H4/NR2E1/NR2E3/NR4A2/NR6A1/ONECUT3/OTX1/OTX2/PAX6/PBX1/PHOX2B/PITX1/POU3F2/POU4F3/PTF1A/RAX/RAX2/RBPJL/RFX4/RFX6/RORB/SALL2/SHOX/SIX2/SIX3/SIX4/SOX1/SOX10/SOX18/SOX9/SP7/STOX2/TBX20/TBX5/TCF4/TFAP2A/TFAP2B/TFAP2C/TLX1/TLX2/TP73/WT1/ZBTB16/ZFAT/ZIC1/ZIC3/ZNF292/ZNF48/ZNF521	127
cluster5	MF	GO:0005248	voltage-gated sodium channel activity	13/3764	24/18432	0.000267101	0.009590399	0.008880577	CACNA1G/HCN1/HCN3/HCN4/NALCN/SCN10A/SCN1A/SCN2A/SCN2B/SCN3B/SCN4A/SCN8A/SCN9A	13
cluster5	MF	GO:0001664	G protein-coupled receptor binding	84/3764	289/18432	0.000268705	0.009590399	0.008880577	ACE/ADCYAP1/ADM5/ADORA1/ADRA2A/ADRB3/AGT/APELA/ATP1A3/AVP/BBS1/CALCA/CALCB/CCKBR/CCL22/CCL25/CLIC6/CXCL13/CXCL14/CXCL3/CXCL5/EDN2/EDNRB/GCG/GNAI1/GNAO1/GNAS/GNAT1/GNAZ/GPRC5B/GRM5/HOMER1/KISS1/LRP6/MAGI2/MLN/MRAP2/MYOC/NDP/NMS/NMU/NPB/NPFFR2/NPY/NTS/PACRG/PDYN/PENK/PF4/PF4V1/PPBP/PRKN/PTCH1/PTCH2/PTH/PYY/REEP1/REEP2/RSPO1/RSPO3/RTP1/S100A14/SAA1/SHANK1/TAC1/TAFA5/TUB/TULP3/UCHL1/UCN2/UCN3/USP4/WNT11/WNT2B/WNT3A/WNT4/WNT5A/WNT7A/WNT7B/WNT8A/WNT8B/WNT9A/WNT9B/ZNRF3	84

cluster5	MF	GO:0016782	transferase activity, transferring sulphur-containing groups	27/3764	69/18432	0.000278581	0.009798768	0.009073524	CHST1/CHST15/CHST3/CHST4/CHST5/CHST6/CHST8/CHST9/GAL3ST1/GAL3ST2/GAL3ST3/HS3ST3A1/HS3ST4/HS3ST5/HS6ST1/HS6ST2/HS6ST3/NDST3/NDST4/NFS1/SULT1C2/SULT1C3/SULT1C4/SULT1E1/SULT4A1/TPST2/WSCD1	27
cluster5	MF	GO:0019841	retinol binding	10/3764	16/18432	0.000294542	0.010212205	0.009456361	ADH7/CRABP1/CRABP2/CYP27C1/CYP2W1/LRAT/RBP1/RBP2/RBP3/STRA6	10
cluster5	MF	GO:0001216	DNA-binding transcription activator activity	127/3764	472/18432	0.000363914	0.012439707	0.011518997	ALX1/ALX4/ARNT2/ATF4/ATOH1/BARHL1/BARHL2/BARX2/CASZ1/CDX1/CDX4/CREB3L3/CRX/CSRNP3/DDIT3/DLX2/DLX3/DLX5/DMRT1/EBF2/ELF1/ESRRB/ESRRG/ETV1/ETV4/ETV5/FEV/FEZF2/FOS/FOSB/FOXA1/FOXC1/FOXF2/FOXJ1/FOXN1/FOXR1/GATA4/GBX2/GCM2/GLIS3/GRHL1/GRHL2/GSX1/HEYL/HIF1A/HNF1A/HNF4G/HOXB1/HOXC10/HOXC13/HOXC4/HOXD10/HOXD13/HOXD3/HSF1/ISL1/JUNB/KLF10/KLF15/LHX2/LHX3/LMX1A/MEF2B/MEIS1/MEIS3/MITF/MLXIP/MYCN/MYOG/NEUROD1/NEUROD2/NEUROD6/NEUROG3/NHLH2/NKX2-2/NKX2-6/NKX2-8/NPAS4/NR1H4/NR2E1/NR2E3/NR4A2/NR6A1/ONECUT3/OTX1/OTX2/PAX6/PBX1/PHOX2B/PITX1/POU3F2/POU4F3/PTF1A/RAX/RAX2/RBPJL/RFX4/RFX6/RORB/SALL2/SHOX/SIX2/SIX3/SIX4/SOX1/SOX10/SOX18/SOX9/SP7/STOX2/TBX20/TBX5/TCF4/TFAP2A/TFAP2B/TFAP2C/TLX1/TLX2/TP73/WT1/ZBTB16/ZFAT/ZIC1/ZIC3/ZNF292/ZNF48/ZNF521	127
cluster5	MF	GO:0016918	retinal binding	11/3764	19/18432	0.000372042	0.012540932	0.011612729	ABCA4/CRABP1/CRABP2/CYP27C1/CYP2W1/OPN4/RBP1/RBP2/RBP3/RHO/STRA6	11

cluster5	MF	GO:0005178	integrin binding	50/3764	156/18432	0.000409695	0.013620958	0.012612819	ACTN1/ACTN2/ADAM10/ADAM11/ADAM2/ADAMTS13/ADAMTS8/ANXA7/CCN2/CDH17/CIB2/COL16A1/ECM2/EDIL3/EMILIN1/EMP2/FAP/FBLN1/FERMT1/FERMT3/FGF2/GFAP/GFRA1/IBSP/ICAM1/ITGA2/ITGA2B/ITGA7/ITGAM/ITGB1/ITGB3/ITGB5/ITGB8/LAMA3/LAMA5/LAMB1/LAMB2/LGALS12/LYN/MYH9/PLPP3/PTN/PTPRZ1/SFRP2/SRC/THY1/TNN/TNXB/TSPAN8/VWF	50
cluster5	MF	GO:0004993	G protein-coupled serotonin receptor activity	16/3764	34/18432	0.000441707	0.014293628	0.013235702	CHRM1/CHRM2/CHRM3/DRD4/HRH3/HTR1D/HTR1E/HTR1F/HTR2A/HTR2C/HTR4/OR10H3/OR10H4/OR11H4/OR5T3/OR6T1	16
cluster5	MF	GO:0099589	serotonin receptor activity	16/3764	34/18432	0.000441707	0.014293628	0.013235702	CHRM1/CHRM2/CHRM3/DRD4/HRH3/HTR1D/HTR1E/HTR1F/HTR2A/HTR2C/HTR4/OR10H3/OR10H4/OR11H4/OR5T3/OR6T1	16
cluster5	MF	GO:0015293	symporter activity	47/3764	145/18432	0.000453913	0.014307088	0.013248165	MFSD2A/SLC10A3/SLC10A5/SLC12A5/SLC12A9/SLC13A1/SLC13A4/SLC13A5/SLC15A5/SLC16A12/SLC16A2/SLC16A8/SLC16A9/SLC17A2/SLC17A4/SLC17A6/SLC17A7/SLC17A8/SLC1A1/SLC1A2/SLC1A6/SLC24A2/SLC24A3/SLC28A3/SLC2A10/SLC2A12/SLC2A13/SLC34A1/SLC34A2/SLC38A3/SLC38A4/SLC45A1/SLC45A4/SLC4A11/SLC4A4/SLC4A5/SLC5A1/SLC5A7/SLC6A1/SLC6A11/SLC6A15/SLC6A17/SLC6A2/SLC6A20/SLC6A3/SLC6A5/SLC6A7	47

cluster5	MF	GO:0016247	channel regulator activity	47/3764	145/18432	0.000453913	0.014307088	0.013248165	ABCC9/AKT1/ANKRD36C/CABP1/CACNG2/CACNG3/CACNG4/CACNG5/CACNG7/CFTR/CHRNA7/CRISP1/DPP10/DPP6/DRD4/FGF14/FKBP1B/FXYD3/FXYD6/GEM/GPLD1/GRM2/GRM3/KCNE4/KCNIP1/KCNIP3/KCNMB2/KCNV1/LRRC55/NEDD4L/NPY/NRXN1/PACSIN3/PKP2/PRKG1/PRSS8/PTPN3/RASA1/REM1/SCN2B/SCN3B/SLC30A1/STX1A/STX7/TNNI3/WNK2/WNK3	47
cluster5	MF	GO:0098631	cell adhesion mediator activity	25/3764	64/18432	0.000476597	0.014660724	0.013575628	CNTN4/CNTN5/CNTN6/DSCAM/DSCAML1/DSG2/EMILIN1/EPCAM/GLDN/IGSF9/ITGA2/ITGB1/IZUMO1/LRRC4C/MYPN/NEXN/NFASC/NPTN/NRCAM/NTNG1/PKP2/PLXNB3/ROBO2/ROBO3/VSTM2L	25
cluster5	MF	GO:0019905	syntaxin binding	27/3764	71/18432	0.000477214	0.014660724	0.013575628	ABL1/BLOC1S6/CACNA1A/CPLX2/CPLX4/DOC2B/PRRT2/SCFD2/SNAP23/SNAP25/STX7/STXBP1/STXBP5L/SYBU/SYT1/SYT10/SYT12/SYT2/SYT3/SYT5/SYT6/SYT7/SYT8/SYT9/TMED10/UNC13A/UNC13C	27
cluster5	MF	GO:0005262	calcium channel activity	40/3764	119/18432	0.000518247	0.015532575	0.01438295	CACNA1A/CACNA1B/CACNA1C/CACNA1G/CACNA1S/CACNA2D1/CACNB1/CACNG2/CACNG3/CACNG4/CACNG5/CACNG7/CATSPER4/CHRNA7/FKBP1B/GPM6A/GRIN1/GRIN2A/GRIN2B/GRIN2D/NALF1/NALF2/PKD1L2/PKD1L3/PKDREJ/RYR2/RYR3/SEC61A1/SLC24A2/SLC24A3/TMC1/TMEM37/TRPC3/TRPC4/TRPC7/TRPM1/TRPM3/TRPM4/TRPM8/TRPV4	40

cluster5	MF	GO:0098632	cell-cell adhesion mediator activity	22/3764	54/18432	0.000518392	0.015532575	0.01438295	CNTN4/CNTN5/CNTN6/DSCAM/DSCAML1/DSG2/EPCAM/GLDN/IGSF9/IZUMO1/LRRC4C/MYPN/NEXN/NFASC/NPTN/NRCAM/NTNG1/PKP2/PLXNB3/ROBO2/ROBO3/VSTM2L	22
cluster5	MF	GO:0015085	calcium ion transmembrane transporter activity	44/3764	135/18432	0.000594778	0.017603988	0.016301049	ATP2B2/ATP2B3/CACNA1A/CACNA1B/CACNA1C/CACNA1G/CACNA1S/CACNA2D1/CACNB1/CACNG2/CACNG3/CACNG4/CACNG5/CACNG7/CATSPER4/CHRNA7/FKBP1B/GPM6A/GRIN1/GRIN2A/GRIN2B/GRIN2D/NALF1/NALF2/PKD1L2/PKD1L3/PKDREJ/RYR2/RYR3/SEC61A1/SLC24A2/SLC24A3/SLC8A2/SLC8A3/TMC1/TMEM37/TRPC3/TRPC4/TRPC7/TRPM1/TRPM3/TRPM4/TRPM8/TRPV4	44
cluster5	MF	GO:0001972	retinoic acid binding	11/3764	20/18432	0.000674499	0.019488216	0.018045818	CRABP1/CRABP2/CYP26A1/CYP26C1/CYP27C1/CYP2W1/LCN12/LRAT/NR2F2/UGT1A3/UGT1A8	11
cluster5	MF	GO:0051139	metal cation:proton antiporter activity	11/3764	20/18432	0.000674499	0.019488216	0.018045818	SLC11A1/SLC17A6/SLC17A7/SLC38A3/SLC9A2/SLC9A4/SLC9A5/SLC9A6/SLC9A7/SLC9B2/SLC9C2	11

cluster5	MF	GO:0140828	metal cation:monoatomic cation antiporter activity	15/3764	32/18432	0.000699218	0.019915975	0.018441917	SLC11A1/SLC17A6/SLC17A7/SLC24A2/SLC24A3/SLC38A3/SLC8A2/SLC8A3/SLC9A2/SLC9A4/SLC9A5/SLC9A6/SLC9A7/SLC9B2/SLC9C2	15
cluster5	MF	GO:0042562	hormone binding	31/3764	87/18432	0.000705716	0.019915975	0.018441917	ABHD2/ADCYAP1R1/ADIPOR2/ATP1A2/ATP1A3/AVPR1B/CALCR/CCKAR/CCKBR/CRHR1/ECE1/EDNRB/EGFR/GHR/GHSR/GLP1R/GLP2R/HCRTR1/HCRTR2/LEPR/LHCGR/LRP2/MC4R/MCHR1/MTNR1A/PDE3A/PTH1R/THRB/TTR/UCN2/VIPR2	31
cluster5	MF	GO:0022853	active ion transmembrane transporter activity	79/3764	278/18432	0.000839548	0.023292292	0.02156834	ABCC3/ABCC8/ABCC9/ATP13A5/ATP1A2/ATP1A3/ATP1B1/ATP2B2/ATP2B3/ATP6V0A1/ATP6V0A4/ATP6V0B/ATP6V1B1/CFTR/CLCN4/COX4I2/COX5B/COX6A2/MFSD2A/SLC10A3/SLC10A5/SLC11A1/SLC12A5/SLC12A9/SLC13A1/SLC13A4/SLC13A5/SLC17A2/SLC17A4/SLC17A6/SLC17A7/SLC17A8/SLC1A1/SLC1A2/SLC1A6/SLC22A11/SLC22A7/SLC22A9/SLC24A2/SLC24A3/SLC25A31/SLC26A9/SLC28A3/SLC2A10/SLC2A13/SLC34A1/SLC34A2/SLC37A1/SLC38A3/SLC45A1/SLC45A4/SLC4A11/SLC4A3/SLC4A4/SLC4A5/SLC5A1/SLC5A7/SLC6A1/SLC6A11/SLC6A15/SLC6A2/SLC6A20/SLC6A3/SLC6A5/SLC6A7/SLC8A2/SLC8A3/SLC9A2/SLC9A4/SLC9A5/SLC9A6/SLC9A7/SLC9B2/SLC9C2/SLCO1B1/SLCO1B3/SLCO1C1/UQCR10/UQCRH	79
cluster5	MF	GO:0008509	anion transmembrane transporter activity	85/3764	303/18432	0.00084455	0.023292292	0.02156834	ABCC3/ABCC9/ABCG2/ADAMTS8/ANO3/ANO4/ANO6/AQP6/ASIC3/BEST2/BEST3/CFTR/CLCA2/CLCN2/CLCN4/CLCNKA/CLCNKB/CLDN4/CLIC6/FXYD3/GABRA1/GABRA2/GABRA3/GABRA4/GABRA5/GABRA6/GABRB2/GABRB3/GABRD/GABRE/GABRG1/GABRG2/GABRG3/GABRQ/GLRA1/GLRA2/GLRB/LRRC8B/MFSD2A/OCA2/SLC12A5/SLC12A9/SLC13A1/SLC13A4/SLC13A5/SLC16A12/SLC16A8/SLC16A9/SLC17A6/SLC17A7/SLC17A8/SLC1A1/SLC1A2/SLC1A6/SLC22A11/SLC22A7/SLC22A9/SLC25A2/SLC25A21/SLC25A23/SLC25A31/SLC26A9/SLC27A6/SLC2A10/SLC37A1/SLC38A3/SLC4A11/SLC4A3/SLC4A4/SLC4A5/SLC66A1/SLC6A1/SLC6A11/SLC6A2/SLC6A20/SLC6A3/SLC6A5/SLC6A7/SLC7A10/SLC7A3/SLC7A8/SLCO1B1/SLCO1B3/SLCO1C1/TTYH1	85

cluster5	MF	GO:0030548	acetylcholine receptor regulator activity	9/3764	15/18432	0.000916466	0.024714033	0.022884853	LY6G6D/LY6H/LYPD1/LYPD6/PATE1/PATE4/SLURP1/SLURP2/TMEM35A	9
cluster5	MF	GO:0099602	neurotransmitter receptor regulator activity	9/3764	15/18432	0.000916466	0.024714033	0.022884853	LY6G6D/LY6H/LYPD1/LYPD6/PATE1/PATE4/SLURP1/SLURP2/TMEM35A	9
cluster5	MF	GO:0015291	secondary active transmembrane transporter activity	71/3764	246/18432	0.000945513	0.025217134	0.023350717	ATP1A3/CDH17/CLCN4/MFSD2A/SLC10A3/SLC10A5/SLC11A1/SLC12A5/SLC12A9/SLC13A1/SLC13A4/SLC13A5/SLC15A5/SLC16A12/SLC16A2/SLC16A8/SLC16A9/SLC17A2/SLC17A4/SLC17A6/SLC17A7/SLC17A8/SLC1A1/SLC1A2/SLC1A6/SLC22A11/SLC22A7/SLC22A9/SLC24A2/SLC24A3/SLC25A31/SLC26A9/SLC28A3/SLC2A10/SLC2A12/SLC2A13/SLC34A1/SLC34A2/SLC37A1/SLC38A3/SLC38A4/SLC45A1/SLC45A4/SLC4A11/SLC4A3/SLC4A4/SLC4A5/SLC5A1/SLC5A7/SLC6A1/SLC6A11/SLC6A15/SLC6A17/SLC6A2/SLC6A20/SLC6A3/SLC6A5/SLC6A7/SLC7A8/SLC8A2/SLC8A3/SLC9A2/SLC9A4/SLC9A5/SLC9A6/SLC9A7/SLC9B2/SLC9C2/SLCO1B1/SLCO1B3/SLCO1C1	71
cluster5	MF	GO:0071855	neuropeptide receptor binding	16/3764	36/18432	0.00096543	0.025468471	0.023583452	ADCYAP1/APELA/CCKBR/EDN2/GNAO1/GNAS/KISS1/MRAP2/NMU/NPY/NTS/PYY/SHANK1/TAC1/UCN2/UCN3	16

cluster5	MF	GO:0005251	delayed rectifier potassium channel activity	15/3764	33/18432	0.001041778	0.027187058	0.02517484	KCNA10/KCNA4/KCNA7/KCNB1/KCNB2/KCNC1/KCNC2/KCNC3/KCNE4/KCNG3/KCNH1/KCNH2/KCNQ2/KCNQ3/KCNQ4	15
cluster5	MF	GO:0008569	minus-end-directed microtubule motor activity	10/3764	18/18432	0.001074763	0.027749471	0.025695627	DNAH10/DNAH11/DNAH12/DNAH14/DNAH2/DNAH3/DNAH5/DNAH7/DNAH9/KIF25	10
cluster5	MF	GO:0015294	solute:cation symporter activity	36/3764	109/18432	0.001372172	0.034569448	0.032010831	MFSD2A/SLC10A3/SLC10A5/SLC12A5/SLC12A9/SLC13A1/SLC13A4/SLC13A5/SLC17A2/SLC17A4/SLC17A6/SLC17A7/SLC17A8/SLC1A1/SLC1A2/SLC1A6/SLC28A3/SLC2A10/SLC2A13/SLC34A1/SLC34A2/SLC38A3/SLC45A1/SLC45A4/SLC4A4/SLC4A5/SLC5A1/SLC5A7/SLC6A1/SLC6A11/SLC6A15/SLC6A2/SLC6A20/SLC6A3/SLC6A5/SLC6A7	36
cluster5	MF	GO:0099106	ion channel regulator activity	44/3764	140/18432	0.001374561	0.034569448	0.032010831	ABCC9/AKT1/ANKRD36C/CABP1/CACNG4/CACNG7/CFTR/CHRNA7/CRISP1/DPP10/DPP6/DRD4/FGF14/FKBP1B/FXYD3/FXYD6/GEM/GPLD1/GRM2/GRM3/KCNE4/KCNIP1/KCNIP3/KCNMB2/KCNV1/LRRC55/NEDD4L/NPY/NRXN1/PACSIN3/PKP2/PRKG1/PRSS8/PTPN3/RASA1/REM1/SCN2B/SCN3B/SLC30A1/STX1A/STX7/TNNI3/WNK2/WNK3	44

cluster5	MF	GO:0005109	frizzled binding	16/3764	37/18432	0.001381638	0.034569448	0.032010831	LRP6/MYOC/NDP/RSPO3/WNT11/WNT2B/WNT3A/WNT4/WNT5A/WNT7A/WNT7B/WNT8A/WNT8B/WNT9A/WNT9B/ZNRF3	16
cluster5	MF	GO:0005436	sodium:phosphate symporter activity	8/3764	13/18432	0.001432786	0.035483383	0.032857121	MFSD2A/SLC17A2/SLC17A4/SLC17A6/SLC17A7/SLC17A8/SLC34A1/SLC34A2	8
cluster5	MF	GO:0022839	ion gated channel activity	18/3764	44/18432	0.001540465	0.037764732	0.03496962	ANO3/ANO4/ANO6/ASIC1/ASIC2/CATSPER4/CLCA2/KCNMA1/KCNMB2/KCNN1/KCNN2/KCNN3/KCNT1/KCNT2/TMEM63C/TRPM3/TRPM4/TTYH1	18
cluster5	MF	GO:0000149	SNARE binding	36/3764	110/18432	0.001645141	0.039927569	0.036972377	ABL1/BLOC1S6/CACNA1A/CPLX2/CPLX4/DOC2B/EXOC3L1/EXOC3L2/EXOC3L4/KCNB1/PRRT2/SCFD2/SNAP23/SNAP25/SNAP91/STX11/STX1A/STX7/STXBP1/STXBP5L/SYBU/SYT1/SYT10/SYT12/SYT13/SYT2/SYT3/SYT4/SYT5/SYT6/SYT7/SYT8/SYT9/TMED10/UNC13A/UNC13C	36

cluster5	MF	GO:0005518	collagen binding	25/3764	69/18432	0.001703798	0.040925128	0.037896102	ASPN/CCBE1/CHADL/COL14A1/COL5A3/COL6A1/DSPP/ECM2/GP6/HSD17B12/ITGA2/ITGB1/LACRT/LOX/MMP13/MRC2/MUSK/NID1/P3H4/PDGFA/PODN/PPIB/TNXB/USH2A/VWF	25
cluster5	MF	GO:0004971	AMPA glutamate receptor activity	4/3764	4/18432	0.001736831	0.040925128	0.037896102	GRIA1/GRIA2/GRIA3/GRIA4	4
cluster5	MF	GO:0015467	G-protein activated inward rectifier potassium channel activity	4/3764	4/18432	0.001736831	0.040925128	0.037896102	KCNJ3/KCNJ5/KCNJ6/KCNJ9	4
cluster5	MF	GO:0005283	amino acid:sodium symporter activity	10/3764	19/18432	0.001855564	0.042680934	0.039521955	SLC1A1/SLC1A2/SLC1A6/SLC38A3/SLC6A1/SLC6A11/SLC6A15/SLC6A20/SLC6A5/SLC6A7	10

cluster5	MF	GO:0022821	solute:potassium antiporter activity	10/3764	19/18432	0.001855564	0.042680934	0.039521955	SLC17A6/SLC17A7/SLC24A2/SLC24A3/SLC9A2/SLC9A4/SLC9A5/SLC9A6/SLC9A7/SLC9C2	10
cluster5	MF	GO:0003779	actin binding	115/3764	439/18432	0.001864103	0.042680934	0.039521955	ABL1/ABLIM2/ABLIM3/ACTN1/ACTN2/ACTR3B/ADCY8/AFAP1/AJUBA/ARPC1B/BLOC1S6/CAMK2B/CAP2/CDK5R1/CDK5R2/CGN/CNN1/CORO2B/CSRP3/CTNNA2/CTNNAL1/CYFIP1/DAAM1/DAG1/DBN1/DIAPH3/DMD/DMTN/EGFR/EPB41L1/EPS8/EPS8L3/FER/FERMT1/FHOD1/FHOD3/FMN2/GAS2L3/GC/GIPC1/HDAC6/HPCA/INF2/ITGB1/KCNMA1/KIF18A/KLHL1/KLHL4/LDB3/LMOD2/MAP1A/MISP/MOBP/MYBPC1/MYH1/MYH10/MYH13/MYH2/MYH4/MYH6/MYH7/MYH8/MYH9/MYLK/MYO18B/MYO1C/MYO3A/MYO3B/MYO5B/MYO6/MYOZ1/MYOZ2/MYOZ3/MYPN/NEXN/NOS3/NRAP/OPHN1/PACRG/PFN2/PKNOX2/PPP1R42/PPP1R9A/PRICKLE4/PRKN/PSTPIP1/PVALEF/SAMD14/SHROOM2/SLC6A2/SMTN/SNTG1/SNTG2/SPATA32/SPEF1/SPTBN4/SYN1/SYNE2/SYNPO2L/TMSB15A/TMSB4Y/TNNI1/TNNI3/TNNT2/TPM1/TRPV4/TULP1/USH1C/VASH2/WASF1/WASF3/WIPF1/WIPF3/XIRP1/ZNF185	115
cluster5	MF	GO:0015464	acetylcholine receptor activity	11/3764	22/18432	0.001888128	0.042826969	0.039657181	CHRM1/CHRM2/CHRM3/CHRNA2/CHRNA3/CHRNA4/CHRNA6/CHRNA7/CHRNB4/CHRNG/HRH3	11
cluster5	KEGG	hsa04740	Olfactory transduction	179/1784	441/8779	1.27E-23	4.28E-21	3.49E-21	ADCY3/CALML3/CAMK2A/CAMK2B/CNGA3/CNGB1/LOC102723532/OR10A5/OR10A7/OR10G7/OR10H3/OR10H4/OR10J1/OR10K1/OR10Q1/OR10W1/OR10Z1/OR11H2/OR11H4/OR11L1/OR12D1/OR12D2/OR12D3/OR13C8/OR13C9/OR13D1/OR13F1/OR13H1/OR14A16/OR14I1/OR14J1/OR1A1/OR1A2/OR1B1/OR1E1/OR1E2/OR1G1/OR1J4/OR1L1/OR1L3/OR1L4/OR1L6/OR1M1/OR1S2/OR2A2/OR2AG1/OR2AG2/OR2AJ1/OR2AK2/OR2AT4/OR2B6/OR2D3/OR2F1/OR2G2/OR2G3/OR2H1/OR2J1/OR2J2/OR2L13/OR2L2/OR2L8/OR2M4/OR2S2/OR2T11/OR2T27/OR2T29/OR2T34/OR2T5/OR2T6/OR2T7/OR2W1/OR2W3/OR2Y1/OR2Z1/OR3A2/OR3A3/OR4A15/OR4A16/OR4A5/OR4C13/OR4C16/OR4C3/OR4C45/OR4C46/OR4C6/OR4D11/OR4D5/OR4D6/OR4F15/OR4F17/OR4F21/OR4F29/OR4F3/OR4F5/OR4K1/OR4K13/OR4K15/OR4K5/OR4L1/OR4M1/OR4M2/OR4N2/OR4N4/OR4N5/OR4X1/OR51A2/OR51B5/OR51B6/OR51E2/OR51G1/OR51I1/OR51L1/OR51Q1/OR51V1/OR52A5/OR52B2/OR52B6/OR52E4/OR52E5/OR52E6/OR52E8/OR52I2/OR52J3/OR52L1/OR52N5/OR56A5/OR5AC2/OR5AK2/OR5B17/OR5B3/OR5D14/OR5D16/OR5H2/OR5K1/OR5K4/OR5M10/OR5M11/OR5P2/OR5T3/OR5V1/OR5W2/OR6B1/OR6B2/OR6C3/OR6C6/OR6C70/OR6C76/OR6J1/OR6P1/OR6T1/OR7A10/OR7C1/OR7C2/OR7E24/OR7G1/OR7G2/OR7G3/OR8A1/OR8B2/OR8B3/OR8B4/OR8D2/OR8H1/OR8H3/OR8I2/OR8J1/OR8J3/OR8K1/OR9A4/OR9G1/OR9I1/OR9K2/OR9Q1/PDE1A/PDE1B/PDE1C/PRKG1/SLC8A2/SLC8A3	179

cluster5	KEGG	hsa04080	Neuroactive ligand-receptor interaction	155/1784	366/8779	9.72E-23	1.63E-20	1.33E-20	ADCYAP1/ADCYAP1R1/ADORA1/ADORA2B/ADRA1A/ADRA1B/ADRA1D/ADRA2A/ADRB3/AGT/AGTR2/APELA/AVP/AVPR1B/CALCA/CALCB/CALCR/CCK/CCKAR/CCKBR/CGA/CHRM1/CHRM2/CHRM3/CHRNA2/CHRNA3/CHRNA4/CHRNA6/CHRNA7/CHRNB4/CHRNG/CRH/CRHR1/CSH1/CSH2/CTSG/DRD1/DRD2/DRD4/EDN2/EDNRA/EDNRB/F2/F2R/F2RL3/GABBR2/GABRA1/GABRA2/GABRA3/GABRA4/GABRA5/GABRA6/GABRB2/GABRB3/GABRD/GABRE/GABRG1/GABRG2/GABRG3/GABRQ/GCG/GH2/GHR/GHSR/GLP1R/GLP2R/GLRA1/GLRA2/GLRB/GPR156/GPR50/GRIA1/GRIA2/GRIA3/GRIA4/GRID1/GRID2/GRIK1/GRIK2/GRIK3/GRIK5/GRIN1/GRIN2A/GRIN2B/GRIN2D/GRM2/GRM3/GRM4/GRM5/GRM6/GRM7/GRM8/GRP/GRPR/HCRTR1/HCRTR2/HRH3/HTR1D/HTR1E/HTR1F/HTR2A/HTR2C/HTR4/IAPP/KISS1/KNG1/LEPR/LHCGR/LYPD6/MC2R/MC4R/MCHR1/MLN/MTNR1A/NMBR/NMS/NMU/NPB/NPFFR2/NPSR1/NPY/NPY5R/NTS/OPRK1/P2RX2/P2RX3/PATE1/PATE2/PATE4/PDYN/PENK/PTGFR/PTH/PTH1R/PTH2/PYY/QRFPR/SLURP1/SLURP2/SPX/SST/SSTR1/SSTR2/SSTR5/TAAR2/TAC1/TAC3/TACR1/TACR3/TBXA2R/THRB/TRH/UCN2/UCN3/VIPR2	155
cluster5	KEGG	hsa05033	Nicotine addiction	30/1784	40/8779	1.4E-13	1.57E-11	1.28E-11	CACNA1A/CACNA1B/CHRNA4/CHRNA6/CHRNA7/GABRA1/GABRA2/GABRA3/GABRA4/GABRA5/GABRA6/GABRB2/GABRB3/GABRD/GABRE/GABRG1/GABRG2/GABRG3/GABRQ/GRIA1/GRIA2/GRIA3/GRIA4/GRIN1/GRIN2A/GRIN2B/GRIN2D/SLC17A6/SLC17A7/SLC17A8	30
cluster5	KEGG	hsa04020	Calcium signaling pathway	99/1784	253/8779	2.54E-12	2.13E-10	1.74E-10	ADCY1/ADCY2/ADCY3/ADCY8/ADORA2B/ADRA1A/ADRA1B/ADRA1D/ADRB3/ATP2B2/ATP2B3/AVPR1B/CACNA1A/CACNA1B/CACNA1C/CACNA1G/CACNA1S/CALML3/CAMK1G/CAMK2A/CAMK2B/CASQ2/CCKAR/CCKBR/CHRM1/CHRM2/CHRM3/CHRNA7/DRD1/EDNRA/EDNRB/EGF/EGFR/ERBB2/ERBB3/ERBB4/F2R/FGF10/FGF16/FGF17/FGF19/FGF2/FGF23/FGF4/FGF5/FGF7/FGF9/FGFR1/FGFR2/FGFR3/FGFR4/FLT4/GDNF/GNAS/GRIN1/GRIN2A/GRIN2B/GRIN2D/GRM5/GRPR/HRC/HTR2A/HTR2C/HTR4/ITPKA/LHCGR/MYLK/NFATC4/NGF/NOS2/NOS3/NTRK2/P2RX2/P2RX3/PDE1A/PDE1B/PDE1C/PDGFA/PDGFC/PDGFRA/PDGFRB/PHKB/PLCB1/PLCD4/PRKCG/PTGFR/RET/RYR2/RYR3/SLC25A31/SLC8A2/SLC8A3/SLN/TACR1/TACR3/TBXA2R/TRDN/VEGFA/VEGFD	99
cluster5	KEGG	hsa04360	Axon guidance	74/1784	182/8779	1.97E-10	1.32E-08	1.08E-08	ABL1/ABLIM2/ABLIM3/BMP7/BMPR1B/BOC/BUB1B-PAK6/CAMK2A/CAMK2B/DPYSL5/EFNA2/EFNA3/EFNA5/EFNB2/EFNB3/EPHA3/EPHA5/EPHA6/EPHA7/EPHA8/EPHB3/FZD3/GNAI1/GSK3B/ILK/ITGB1/L1CAM/LRRC4C/MYL12A/NCK2/NFATC4/NGEF/NTN1/NTN3/NTNG1/NTNG2/PAK3/PAK5/PARD3/PLXNA1/PLXNA2/PLXNA4/PLXNB1/PLXNB3/PTCH1/RASA1/RGS3/RHOD/ROBO1/ROBO2/ROBO3/ROCK1/ROCK2/SEMA3A/SEMA3E/SEMA4D/SEMA5A/SEMA5B/SEMA6A/SEMA6D/SHH/SLIT1/SLIT3/SMO/SRC/SRGAP1/SRGAP3/TRPC3/TRPC4/UNC5B/UNC5C/UNC5D/WNT4/WNT5A	74

cluster5	KEGG	hsa05032	Morphine addiction	43/1784	91/8779	6.45E-09	0.000000361	0.000000294	ADCY1/ADCY2/ADCY3/ADCY5/ADCY8/ADORA1/CACNA1A/CACNA1B/DRD1/GABBR2/GABRA1/GABRA2/GABRA3/GABRA4/GABRA5/GABRA6/GABRB2/GABRB3/GABRD/GABRE/GABRG1/GABRG2/GABRG3/GABRQ/GNAI1/GNAO1/GNAS/GNG11/GNG3/GNG8/GNGT1/GRK4/KCNJ3/KCNJ5/KCNJ6/KCNJ9/PDE10A/PDE11A/PDE1A/PDE1B/PDE1C/PDE3A/PRKCG	43
cluster5	KEGG	hsa04727	GABAergic synapse	41/1784	89/8779	0.000000036	0.00000167	0.00000136	ADCY1/ADCY2/ADCY3/ADCY5/ADCY8/CACNA1A/CACNA1B/CACNA1C/CACNA1S/GABARAPL1/GABBR2/GABRA1/GABRA2/GABRA3/GABRA4/GABRA5/GABRA6/GABRB2/GABRB3/GABRD/GABRE/GABRG1/GABRG2/GABRG3/GABRQ/GAD1/GAD2/GNAI1/GNAO1/GNG11/GNG3/GNG8/GNGT1/HAP1/KCNJ6/PRKCG/SLC12A5/SLC38A3/SLC6A1/SLC6A11/SRC	41
cluster5	KEGG	hsa04724	Glutamatergic synapse	49/1784	115/8779	3.97E-08	0.00000167	0.00000136	ADCY1/ADCY2/ADCY3/ADCY5/ADCY8/CACNA1A/CACNA1C/GNAI1/GNAO1/GNAS/GNG11/GNG3/GNG8/GNGT1/GRIA1/GRIA2/GRIA3/GRIA4/GRIK1/GRIK2/GRIK3/GRIK5/GRIN1/GRIN2A/GRIN2B/GRIN2D/GRM2/GRM3/GRM4/GRM5/GRM6/GRM7/GRM8/HOMER1/KCNJ3/LZTS3/PLA2G4C/PLA2G4E/PLCB1/PRKCG/SHANK1/SHANK2/SLC17A6/SLC17A7/SLC17A8/SLC1A1/SLC1A2/SLC1A6/SLC38A3	49
cluster5	KEGG	hsa04024	cAMP signaling pathway	80/1784	225/8779	5.56E-08	0.00000208	0.00000169	ADCY1/ADCY2/ADCY3/ADCY5/ADCY8/ADCYAP1/ADCYAP1R1/ADORA1/AKT1/ATP1A2/ATP1A3/ATP1B1/ATP1B4/ATP2B2/ATP2B3/BDNF/BVES/CACNA1C/CACNA1S/CALML3/CAMK2A/CAMK2B/CFTR/CGA/CHRM1/CHRM2/CNGA3/CNGB1/CREB3L3/CRH/CRHR1/DRD1/DRD2/EDN2/EDNRA/F2R/FOS/GABBR2/GCG/GHSR/GLI3/GLP1R/GNAI1/GNAS/GRIA1/GRIA2/GRIA3/GRIA4/GRIN1/GRIN2A/GRIN2B/GRIN2D/HCN4/HHIP/HTR1D/HTR1E/HTR1F/HTR4/LHCGR/MAPK10/MC2R/NFKBIA/NPY/PDE10A/PDE3A/POPDC3/PTCH1/RAP1B/ROCK1/ROCK2/RYR2/SOX9/SST/SSTR1/SSTR2/SSTR5/SUCNR1/TNNI3/VAV2/VIPR2	80

cluster5	KEGG	hsa04820	Cytoskeleton in muscle cells	81/1784	232/8779	0.000000114	0.00000382	0.00000311	ACTA1/ACTC1/ACTN2/AMPD2/ANK3/ATP1A2/ATP1A3/ATP1B1/ATP1B4/COL11A1/COL24A1/COL27A1/COL4A1/COL4A5/COL4A6/COL5A3/COL6A1/COL6A5/COL6A6/COL9A1/CSRP2/CSRP3/DAAM1/DAG1/DES/DIAPH3/DMD/DSG2/DTNA/FBLN1/FBN3/FHL1/FMN2/INF2/ITGA2/ITGA2B/ITGA7/ITGB1/ITGB3/ITGB5/ITGB8/LAMA1/LDB3/LMNA/LMOD2/MYBPC1/MYBPHL/MYH1/MYH10/MYH13/MYH2/MYH4/MYH6/MYH7/MYH8/MYH9/MYL1/MYL7/MYOZ1/MYOZ2/MYOZ3/MYPN/NID1/NRAP/PKP2/SDC1/SDC2/SDC4/SGCA/SGCZ/SPTBN4/SSPN/SYNE2/TNNI1/TNNI3/TNNT2/TPM1/TRIM54/TRIM55/TRIM63/XIRP1	81
cluster5	KEGG	hsa04911	Insulin secretion	38/1784	86/8779	0.000000431	0.0000132	0.0000107	ABCC8/ADCY1/ADCY2/ADCY3/ADCY5/ADCY8/ADCYAP1/ADCYAP1R1/ATF4/ATP1A2/ATP1A3/ATP1B1/ATP1B4/CACNA1C/CACNA1S/CAMK2A/CAMK2B/CCK/CCKAR/CHRM3/CREB3L3/GCG/GCK/GLP1R/GNAS/INS/KCNMA1/KCNMB2/KCNN1/KCNN2/KCNN3/PCLO/PLCB1/PRKCG/RYR2/SNAP25/STX1A/TRPM4	38
cluster5	KEGG	hsa04713	Circadian entrainment	41/1784	97/8779	0.000000653	0.0000183	0.0000149	ADCY1/ADCY2/ADCY3/ADCY5/ADCY8/ADCYAP1/ADCYAP1R1/CACNA1C/CACNA1G/CALML3/CAMK2A/CAMK2B/FOS/GNAI1/GNAO1/GNAS/GNG11/GNG3/GNG8/GNGT1/GRIA1/GRIA2/GRIA3/GRIA4/GRIN1/GRIN2A/GRIN2B/GRIN2D/GUCY1A1/GUCY1A2/KCNJ3/KCNJ5/KCNJ6/KCNJ9/MTNR1A/PER1/PLCB1/PRKCG/PRKG1/RYR2/RYR3	41
cluster5	KEGG	hsa04512	ECM-receptor interaction	38/1784	89/8779	0.00000123	0.0000318	0.0000259	COL2A1/COL4A1/COL4A5/COL4A6/COL6A1/COL6A5/COL6A6/COL9A1/DAG1/DSPP/FREM1/FREM2/GP1BB/GP6/GP9/IBSP/ITGA2/ITGA2B/ITGA7/ITGB1/ITGB3/ITGB5/ITGB8/LAMA1/LAMA3/LAMA5/LAMB1/LAMB2/LAMC2/RELN/SDC1/SDC4/SV2A/TNC/TNN/TNR/TNXB/VWF	38

cluster5	KEGG	hsa05414	Dilated cardiomyopathy	41/1784	105/8779	0.00000735	0.000176437	0.000143714	ACTC1/ADCY1/ADCY2/ADCY3/ADCY5/ADCY8/AGT/CACNA1C/CACNA1S/CACNA2D1/CACNB1/CACNG2/CACNG3/CACNG4/CACNG5/CACNG7/DAG1/DES/DMD/DTNA/GNAS/ITGA2/ITGA2B/ITGA7/ITGB1/ITGB3/ITGB5/ITGB8/LAMA1/LMNA/MYH6/MYH7/RYR2/SGCA/SGCZ/SLC8A2/SLC8A3/SSPN/TNNI3/TNNT2/TPM1	41
cluster5	KEGG	hsa04514	Cell adhesion molecules	55/1784	157/8779	0.0000109	0.0002425	0.000197525	CADM1/CADM3/CD276/CDH2/CDH4/CLDN1/CLDN14/CLDN16/CLDN18/CLDN19/CLDN2/CLDN22/CLDN4/CLDN5/CLDN6/CLDN8/CNTNAP1/CNTNAP2/HLA-B/HLA-C/ICAM1/IGSF11/ITGAM/ITGB1/ITGB8/L1CAM/LRRC4B/LRRC4C/NCAM1/NCAM2/NECTIN1/NECTIN3/NFASC/NLGN1/NLGN2/NLGN3/NLGN4X/NRCAM/NRXN1/NRXN3/NTNG1/NTNG2/PECAM1/PTPRD/PTPRF/PTPRS/SDC1/SDC2/SDC4/SELP/SLITRK1/SLITRK2/SLITRK3/SLITRK6/VTCN1	55
cluster5	KEGG	hsa04742	Taste transduction	35/1784	86/8779	0.0000117	0.0002425	0.000197525	ADCY8/ASIC2/CACNA1A/CACNA1C/CHRM3/GABBR2/GABRA1/GABRA2/GABRA3/GABRA4/GABRA5/GABRA6/GRM4/HCN4/HTR1D/HTR1E/HTR1F/HTR3C/KCNK5/P2RX2/P2RX3/PDE1A/PDE1B/PDE1C/PKD1L3/PLCB1/SCN2A/SCN9A/SCNN1G/TAS1R1/TAS2R1/TAS2R13/TAS2R16/TAS2R7/TAS2R8	35
cluster5	KEGG	hsa05217	Basal cell carcinoma	28/1784	63/8779	0.0000123	0.0002425	0.000197525	APC2/BMP2/BMP4/CDKN1A/FZD10/FZD3/GADD45A/GADD45B/GLI2/GLI3/GSK3B/HHIP/KIF7/PTCH1/PTCH2/SHH/SMO/WNT11/WNT2B/WNT3A/WNT4/WNT5A/WNT7A/WNT7B/WNT8A/WNT8B/WNT9A/WNT9B	28

cluster5	KEGG	hsa04151	PI3K-Akt signaling pathway	106/1784	359/8779	0.0000146	0.000273085	0.000222438	AKT1/ANGPT1/ANGPT2/ARTN/ATF4/BDNF/CCND3/CCNE2/CDKN1A/CHRM1/CHRM2/COL2A1/COL4A1/COL4A5/COL4A6/COL6A1/COL6A5/COL6A6/COL9A1/CREB3L3/CSH1/CSH2/EFNA2/EFNA3/EFNA5/EGF/EGFR/EIF4E1B/EPO/ERBB2/ERBB3/ERBB4/F2R/FGF10/FGF16/FGF17/FGF19/FGF2/FGF23/FGF4/FGF5/FGF7/FGF9/FGFR1/FGFR2/FGFR3/FGFR4/FLT4/G6PC1/GDNF/GH2/GHR/GNG11/GNG3/GNG8/GNGT1/GSK3B/IBSP/IFNA10/IFNA16/IFNA17/IFNA4/IFNA6/IFNA8/IFNB1/IKBKG/IL7/INS/ITGA2/ITGA2B/ITGA7/ITGB1/ITGB3/ITGB5/ITGB8/KITLG/LAMA1/LAMA3/LAMA5/LAMB1/LAMB2/LAMC2/MAGI2/NGF/NGFR/NOS3/NTF3/NTRK2/PDGFA/PDGFC/PDGFRA/PDGFRB/PHLPP1/PKN2/PPP2R2C/PRKAA2/RELN/RET/TNC/TNN/TNR/TNXB/VEGFA/VEGFD/VWF/YWHAZ	106
cluster5	KEGG	hsa05030	Cocaine addiction	23/1784	49/8779	0.0000248	0.000438653	0.000357299	ADCY5/ATF4/BDNF/CDK5R1/CREB3L3/DRD1/DRD2/FOSB/GNAI1/GNAS/GPSM1/GRIA2/GRIN1/GRIN2A/GRIN2B/GRIN2D/GRM2/GRM3/MAOB/PDYN/RGS9/SLC6A3/TH	23
cluster5	KEGG	hsa04974	Protein digestion and absorption	39/1784	103/8779	0.0000281	0.000472528	0.000384892	ACE2/ATP1A2/ATP1A3/ATP1B1/ATP1B4/CELA3A/COL11A1/COL12A1/COL14A1/COL16A1/COL20A1/COL21A1/COL22A1/COL23A1/COL24A1/COL25A1/COL26A1/COL27A1/COL28A1/COL2A1/COL4A1/COL4A5/COL4A6/COL5A3/COL6A1/COL6A5/COL6A6/COL8A1/COL9A1/CPA2/CPB1/CPB2/KCNJ13/KCNK5/MEP1B/SLC1A1/SLC7A8/SLC8A2/SLC8A3	39
cluster5	KEGG	hsa04950	Maturity onset diabetes of the young	15/1784	26/8779	0.0000309	0.000493927	0.000402321	FOXA2/GCK/HES1/HNF1A/HNF1B/HNF4G/IAPP/INS/MNX1/NEUROD1/NEUROG3/NKX2-2/NKX6-1/PAX6/RFX6	15

cluster5	KEGG	hsa04015	Rap1 signaling pathway	67/1784	210/8779	0.0000427	0.000652218	0.000531256	ADCY1/ADCY2/ADCY3/ADCY5/ADCY8/ADORA2B/AKT1/ANGPT1/ANGPT2/CALML3/CTNND1/DOCK4/DRD2/EFNA2/EFNA3/EFNA5/EGF/EGFR/F2R/F2RL3/FGF10/FGF16/FGF17/FGF19/FGF2/FGF23/FGF4/FGF5/FGF7/FGF9/FGFR1/FGFR2/FGFR3/FGFR4/FLT4/FYB1/GNAI1/GNAO1/GNAS/GRIN1/GRIN2A/GRIN2B/ID1/INS/ITGA2B/ITGAM/ITGB1/ITGB3/KITLG/MAGI2/MAGI3/MAPK12/NGF/NGFR/PARD3/PDGFA/PDGFC/PDGFRA/PDGFRB/PFN2/PLCB1/PRKCG/RAP1B/SRC/VAV2/VEGFA/VEGFD	67
cluster5	KEGG	hsa05410	Hypertrophic cardiomyopathy	37/1784	99/8779	0.000062	0.000906406	0.000738301	ACE/ACTC1/AGT/CACNA1C/CACNA1S/CACNA2D1/CACNB1/CACNG2/CACNG3/CACNG4/CACNG5/CACNG7/DAG1/DES/DMD/DTNA/ITGA2/ITGA2B/ITGA7/ITGB1/ITGB3/ITGB5/ITGB8/LAMA1/LMNA/MYH6/MYH7/PRKAA2/RYR2/SGCA/SGCZ/SLC8A2/SLC8A3/SSPN/TNNI3/TNNT2/TPM1	37
cluster5	KEGG	hsa04814	Motor proteins	62/1784	193/8779	0.0000651	0.000911736	0.000742642	ACTA1/ACTC1/DCTN4/DNAH10/DNAH11/DNAH12/DNAH14/DNAH2/DNAH3/DNAH5/DNAH7/DNAH9/DNAI1/DNAI3/DNALI1/DYNC1I1/DYNLRB2/KIF12/KIF18A/KIF1A/KIF20A/KIF20B/KIF21A/KIF21B/KIF24/KIF25/KIF26B/KIF2B/KIF3A/KIF3C/KIF5A/KIF5C/KIF6/KIF7/KIFC1/KLC4/MYH1/MYH10/MYH13/MYH2/MYH4/MYH6/MYH7/MYH8/MYH9/MYL1/MYL12A/MYL7/MYO18B/MYO1C/MYO3A/MYO3B/MYO5B/MYO6/TNNI1/TNNI3/TNNT2/TPM1/TUBA3E/TUBA4A/TUBB3/TUBB4A	62
cluster5	KEGG	hsa05412	Arrhythmogenic right ventricular cardiomyopathy	33/1784	86/8779	0.0000839	0.001127599	0.00091847	ACTN2/CACNA1C/CACNA1S/CACNA2D1/CACNB1/CACNG2/CACNG3/CACNG4/CACNG5/CACNG7/CDH2/CTNNA2/DAG1/DES/DMD/DSG2/DTNA/ITGA2/ITGA2B/ITGA7/ITGB1/ITGB3/ITGB5/ITGB8/LAMA1/LMNA/PKP2/RYR2/SGCA/SGCZ/SLC8A2/SLC8A3/SSPN	33

cluster5	KEGG	hsa04260	Cardiac muscle contraction	33/1784	87/8779	0.000109188	0.001411048	0.00114935	ACTC1/ATP1A2/ATP1A3/ATP1B1/ATP1B4/CACNA1C/CACNA1S/CACNA2D1/CACNB1/CACNG2/CACNG3/CACNG4/CACNG5/CACNG7/CASQ2/COX4I2/COX5B/COX6A2/HRC/MYH6/MYH7/RYR2/SLC8A2/SLC8A3/SLC9A6/SLC9A7/TNNI3/TNNT2/TPM1/TRDN/UQCR10/UQCRH/UQCRHL	33
cluster5	KEGG	hsa04972	Pancreatic secretion	37/1784	102/8779	0.000128316	0.001596819	0.001300667	ADCY1/ADCY2/ADCY3/ADCY5/ADCY8/ATP1A2/ATP1A3/ATP1B1/ATP1B4/ATP2B2/ATP2B3/CCK/CCKAR/CEL/CELA3A/CFTR/CHRM3/CLCA2/CPA2/CPB1/CPB2/GNAS/KCNMA1/PLA2G12A/PLA2G12B/PLA2G2E/PLA2G2F/PLA2G3/PLA2G5/PLCB1/PNLIPRP1/PRKCG/RAB27B/RAB8A/RAP1B/RYR2/SLC4A4	37
cluster5	KEGG	hsa05224	Breast cancer	49/1784	147/8779	0.000135709	0.00161937	0.001319036	AKT1/APC2/BRCA2/CDKN1A/DLL1/DLL3/EGF/EGFR/ERBB2/FGF10/FGF16/FGF17/FGF19/FGF2/FGF23/FGF4/FGF5/FGF7/FGF9/FGFR1/FLT4/FOS/FZD10/FZD3/GADD45A/GADD45B/GSK3B/HES1/HES5/HEYL/LRP5/LRP6/NCOA1/NOTCH2/NOTCH4/SHC2/SHC3/SHC4/WNT11/WNT2B/WNT3A/WNT4/WNT5A/WNT7A/WNT7B/WNT8A/WNT8B/WNT9A/WNT9B	49
cluster5	KEGG	hsa04728	Dopaminergic synapse	45/1784	132/8779	0.000139767	0.00161937	0.001319036	ADCY5/AKT1/ATF4/CACNA1A/CACNA1B/CACNA1C/CALML3/CAMK2A/CAMK2B/COMT/CREB3L3/DRD1/DRD2/DRD4/FOS/GNAI1/GNAO1/GNAS/GNG11/GNG3/GNG8/GNGT1/GRIA1/GRIA2/GRIA3/GRIA4/GRIN2A/GRIN2B/GSK3B/KCNJ3/KCNJ5/KCNJ6/KCNJ9/KIF5A/KIF5C/LRTOMT/MAOB/MAPK10/MAPK12/PLCB1/PPP2R2C/PRKCG/SCN1A/SLC6A3/TH	45

cluster5	KEGG	hsa05034	Alcoholism	59/1784	188/8779	0.000198446	0.002222596	0.001810385	ADCY5/ADORA2B/ATF4/BDNF/CALML3/CREB3L3/CRH/DRD1/DRD2/FOSB/GNAI1/GNAO1/GNAS/GNG11/GNG3/GNG8/GNGT1/GRIN1/GRIN2A/GRIN2B/GRIN2D/H2AC1/H2AC13/H2AC15/H2AC16/H2AC20/H2AC21/H2AC4/H2AC8/H2AJ/H2BC13/H2BC14/H2BC17/H2BC3/H2BC6/H2BC8/H2BW1/H3-4/H3C1/H3C12/H3C4/H3C7/H4C1/H4C13/H4C2/H4C6/H4C7/H4C9/HDAC2/HDAC6/MAOB/NPY/NTRK2/PDYN/SHC2/SHC3/SHC4/SLC6A3/TH	59
cluster5	KEGG	hsa05031	Amphetamine addiction	27/1784	69/8779	0.000248493	0.002693343	0.002193826	ADCY5/ARC/ATF4/CACNA1C/CALML3/CAMK2A/CAMK2B/CREB3L3/DRD1/FOS/FOSB/GNAS/GRIA1/GRIA2/GRIA3/GRIA4/GRIN1/GRIN2A/GRIN2B/GRIN2D/HDAC2/MAOB/PDYN/PRKCG/SLC6A3/STX1A/TH	27
cluster5	KEGG	hsa04725	Cholinergic synapse	39/1784	113/8779	0.000285127	0.002993833	0.002438586	ADCY1/ADCY2/ADCY3/ADCY5/ADCY8/AKT1/ATF4/CACNA1A/CACNA1B/CACNA1C/CACNA1S/CAMK2A/CAMK2B/CHRM1/CHRM2/CHRM3/CHRNA3/CHRNA4/CHRNA6/CHRNA7/CHRNB4/CREB3L3/FOS/GNAI1/GNAO1/GNG11/GNG3/GNG8/GNGT1/KCNJ18/KCNJ3/KCNJ4/KCNJ6/KCNQ2/KCNQ3/KCNQ4/PLCB1/PRKCG/SLC5A7	39
cluster5	KEGG	hsa04723	Retrograde endocannabinoid signaling	48/1784	148/8779	0.000327615	0.003335712	0.002717058	ADCY1/ADCY2/ADCY3/ADCY5/ADCY8/CACNA1A/CACNA1B/CACNA1C/CACNA1S/DAGLA/GABRA1/GABRA2/GABRA3/GABRA4/GABRA5/GABRA6/GABRB2/GABRB3/GABRD/GABRE/GABRG1/GABRG2/GABRG3/GABRQ/GNAI1/GNAO1/GNG11/GNG3/GNG8/GNGT1/GRIA1/GRIA2/GRIA3/GRIA4/GRM5/KCNJ3/KCNJ5/KCNJ6/KCNJ9/MAPK10/MAPK12/NDUFS6/PLCB1/PRKCG/PTGS2/SLC17A6/SLC17A7/SLC17A8	48

cluster5	KEGG	hsa04014	Ras signaling pathway	70/1784	236/8779	0.000351515	0.003473796	0.002829533	ABL1/AKT1/ANGPT1/ANGPT2/BDNF/BUB1B-PAK6/CALML3/EFNA2/EFNA3/EFNA5/EGF/EGFR/FGF10/FGF16/FGF17/FGF19/FGF2/FGF23/FGF4/FGF5/FGF7/FGF9/FGFR1/FGFR2/FGFR3/FGFR4/FLT4/GAB1/GNG11/GNG3/GNG8/GNGT1/GRIN1/GRIN2A/GRIN2B/IKBKG/INS/KITLG/KSR2/MAPK10/NGF/NGFR/NTF3/NTRK2/PAK3/PAK5/PDGFA/PDGFC/PDGFRA/PDGFRB/PLA2G12A/PLA2G12B/PLA2G2E/PLA2G2F/PLA2G3/PLA2G4C/PLA2G4E/PLA2G5/PRKCG/RAB5A/RAB5B/RAP1B/RASA1/RASGRF1/SHC2/SHC3/SHC4/SHOC2/VEGFA/VEGFD	70
cluster5	KEGG	hsa04970	Salivary secretion	33/1784	93/8779	0.000462741	0.004442314	0.003618426	ADCY1/ADCY2/ADCY3/ADCY5/ADCY8/ADRA1A/ADRA1B/ADRA1D/ADRB3/ATP1A2/ATP1A3/ATP1B1/ATP1B4/ATP2B2/ATP2B3/BEST2/CALML3/CHRM3/CST1/CST3/CST4/GNAS/GUCY1A1/GUCY1A2/HTN3/KCNMA1/MUC5AC/MUC5B/PLCB1/PRB2/PRKCG/PRKG1/RYR3	33
cluster5	KEGG	hsa04510	Focal adhesion	61/1784	202/8779	0.000493331	0.00460442	0.003750467	ACTN1/AKT1/BUB1B-PAK6/CCND3/COL2A1/COL4A1/COL4A5/COL4A6/COL6A1/COL6A5/COL6A6/COL9A1/EGF/EGFR/EMP2/ERBB2/FLT4/GSK3B/IBSP/ILK/ITGA2/ITGA2B/ITGA7/ITGB1/ITGB3/ITGB5/ITGB8/LAMA1/LAMA3/LAMA5/LAMB1/LAMB2/LAMC2/MAPK10/MYL12A/MYL7/MYLK/PAK3/PAK5/PDGFA/PDGFC/PDGFRA/PDGFRB/PRKCG/RAP1B/RASGRF1/RELN/ROCK1/ROCK2/SHC2/SHC3/SHC4/SRC/TNC/TNN/TNR/TNXB/VAV2/VEGFA/VEGFD/VWF	61
cluster5	KEGG	hsa05226	Gastric cancer	47/1784	149/8779	0.000750891	0.006818901	0.005554242	AKT1/APC2/CCNE2/CDH17/CDKN1A/CDKN2B/CDX2/CTNNA2/EGF/EGFR/ERBB2/FGF10/FGF16/FGF17/FGF19/FGF2/FGF23/FGF4/FGF5/FGF7/FGF9/FGFR2/FZD10/FZD3/GAB1/GADD45A/GADD45B/GSK3B/LRP5/LRP6/RARB/RXRG/SHC2/SHC3/SHC4/SHH/WNT11/WNT2B/WNT3A/WNT4/WNT5A/WNT7A/WNT7B/WNT8A/WNT8B/WNT9A/WNT9B	47

cluster5	KEGG	hsa04270	Vascular smooth muscle contraction	43/1784	134/8779	0.000837642	0.007374552	0.006006841	ADCY1/ADCY2/ADCY3/ADCY5/ADCY8/ADORA2B/ADRA1A/ADRA1B/ADRA1D/AGT/AVP/AVPR1B/CACNA1C/CACNA1S/CALCA/CALCB/CALML3/EDN2/EDNRA/GNAS/GUCY1A1/GUCY1A2/KCNMA1/KCNMB2/MYH10/MYH9/MYLK/NPPC/PLA2G12A/PLA2G12B/PLA2G2E/PLA2G2F/PLA2G3/PLA2G4C/PLA2G4E/PLA2G5/PLCB1/PPP1R14A/PRKCD/PRKCG/PRKG1/ROCK1/ROCK2	43
cluster5	KEGG	hsa04726	Serotonergic synapse	38/1784	115/8779	0.000886951	0.007374552	0.006006841	ADCY5/ALOX12/ALOX12B/APP/CACNA1A/CACNA1B/CACNA1C/CACNA1S/CYP2C19/GABRB2/GABRB3/GNAI1/GNAO1/GNAS/GNG11/GNG3/GNG8/GNGT1/HTR1D/HTR1E/HTR1F/HTR2A/HTR2C/HTR3C/HTR4/KCND2/KCNJ3/KCNJ5/KCNJ6/KCNJ9/KCNN2/MAOB/PLA2G4C/PLA2G4E/PLCB1/PRKCG/PTGS2/TPH2	38
cluster5	KEGG	hsa04261	Adrenergic signaling in cardiomyocytes	48/1784	154/8779	0.000889166	0.007374552	0.006006841	ACTC1/ADCY1/ADCY2/ADCY3/ADCY5/ADCY8/ADRA1A/ADRA1B/ADRA1D/AGT/AGTR2/AKT1/ATF4/ATP1A2/ATP1A3/ATP1B1/ATP1B4/ATP2B2/ATP2B3/BVES/CACNA1C/CACNA1S/CACNA2D1/CACNB1/CACNG2/CACNG3/CACNG4/CACNG5/CACNG7/CALML3/CAMK2A/CAMK2B/CREB3L3/GNAI1/GNAS/MAPK12/MYH6/MYH7/PLCB1/POPDC3/PPP1R1A/PPP2R2C/RYR2/SLC8A2/SLC8A3/TNNI3/TNNT2/TPM1	48
cluster5	KEGG	hsa04022	cGMP-PKG signaling pathway	51/1784	166/8779	0.000899871	0.007374552	0.006006841	ADCY1/ADCY2/ADCY3/ADCY5/ADCY8/ADORA1/ADRA1A/ADRA1B/ADRA1D/ADRA2A/ADRB3/AKT1/ATF4/ATP1A2/ATP1A3/ATP1B1/ATP1B4/ATP2B2/ATP2B3/CACNA1C/CACNA1S/CALML3/CNGB1/CREB3L3/EDNRA/EDNRB/GATA4/GNAI1/GUCY1A1/GUCY1A2/INS/IRS4/KCNMA1/KCNMB2/KNG1/MEF2B/MYH6/MYH7/MYLK/NFATC4/NOS3/NPPC/PDE3A/PDE5A/PLCB1/PRKG1/ROCK1/ROCK2/SLC25A31/SLC8A2/SLC8A3	51

cluster5	KEGG	hsa04978	Mineral absorption	23/1784	60/8779	0.00097872	0.007829757	0.006377621	ATOX1/ATP1A2/ATP1A3/ATP1B1/ATP1B4/ATP2B2/ATP2B3/CLCN2/HEPH/MT1A/MT1B/MT1HL1/MT1X/MT2A/SLC26A9/SLC30A1/SLC34A1/SLC34A2/SLC5A1/SLC8A2/SLC8A3/STEAP1/STEAP2	23
cluster5	KEGG	hsa04934	Cushing syndrome	48/1784	155/8779	0.001039409	0.008121894	0.006615578	ADCY1/ADCY2/ADCY3/ADCY5/ADCY8/AGT/AIPL1/APC2/ATF4/CACNA1C/CACNA1G/CACNA1S/CAMK2A/CAMK2B/CCNE2/CDKN1A/CDKN2B/CREB3L3/CRH/CRHR1/CYP11B1/CYP21A2/EGFR/FZD10/FZD3/GNAI1/GNAS/GSK3B/HSD3B2/KCNA4/KCNK3/MC2R/PBX1/PDE11A/PLCB1/RAP1B/WDR5/WNT11/WNT2B/WNT3A/WNT4/WNT5A/WNT7A/WNT7B/WNT8A/WNT8B/WNT9A/WNT9B	48
cluster5	KEGG	hsa04916	Melanogenesis	34/1784	101/8779	0.001125474	0.008594529	0.007000556	ADCY1/ADCY2/ADCY3/ADCY5/ADCY8/CALML3/CAMK2A/CAMK2B/CREB3L3/DCT/EDNRB/FZD10/FZD3/GNAI1/GNAO1/GNAS/GSK3B/KITLG/MITF/PLCB1/PRKCG/TYR/TYRP1/WNT11/WNT2B/WNT3A/WNT4/WNT5A/WNT7A/WNT7B/WNT8A/WNT8B/WNT9A/WNT9B	34
cluster5	KEGG	hsa04540	Gap junction	30/1784	88/8779	0.00171608	0.012813398	0.010436979	ADCY1/ADCY2/ADCY3/ADCY5/ADCY8/CDK1/DRD1/DRD2/EGF/EGFR/GJD2/GNAI1/GNAS/GRM5/GUCY1A1/GUCY1A2/HTR2A/HTR2C/PDGFA/PDGFC/PDGFRA/PDGFRB/PLCB1/PRKCG/PRKG1/SRC/TUBA3E/TUBA4A/TUBB3/TUBB4A	30

cluster5	KEGG	hsa04721	Synaptic vesicle cycle	27/1784	78/8779	0.002225272	0.016254163	0.013239607	ATP6V0A1/ATP6V0A4/ATP6V0B/ATP6V1B1/CACNA1A/CACNA1B/CPLX2/CPLX4/DNM1/SLC17A6/SLC17A7/SLC17A8/SLC1A1/SLC1A2/SLC1A6/SLC6A1/SLC6A11/SLC6A2/SLC6A3/SLC6A5/SLC6A7/SNAP25/STX1A/STXBP1/SYT1/UNC13A/UNC13C	27
cluster5	KEGG	hsa04925	Aldosterone synthesis and secretion	32/1784	98/8779	0.002701799	0.019314991	0.015732762	ADCY1/ADCY2/ADCY3/ADCY5/ADCY8/AGT/ATF4/ATP1A2/ATP1A3/ATP1B1/ATP1B4/ATP2B2/ATP2B3/CACNA1C/CACNA1G/CACNA1S/CALML3/CAMK1G/CAMK2A/CAMK2B/CREB3L3/CYP11B2/CYP21A2/DAGLA/GNAS/HSD3B2/KCNJ5/KCNK3/MC2R/NR4A2/PLCB1/PRKCG	32
cluster5	KEGG	hsa04310	Wnt signaling pathway	51/1784	174/8779	0.002779978	0.01941547	0.015814606	APC2/APCDD1/CAMK2A/CAMK2B/CCND3/CSNK1E/CTBP1/CTNND2/CXXC4/DAAM1/FRZB/FZD10/FZD3/GSK3B/LGR4/LGR5/LRP5/LRP6/MAPK10/MCC/NFATC4/NKD2/PLCB1/PRICKLE1/PRICKLE4/PRKCG/RBX1/ROCK2/ROR1/ROR2/RSPO1/RSPO2/RSPO3/RSPO4/SFRP2/TBL1Y/TLE1/VANGL2/WIF1/WNT11/WNT2B/WNT3A/WNT4/WNT5A/WNT7A/WNT7B/WNT8A/WNT8B/WNT9A/WNT9B/ZNRF3	51
cluster5	KEGG	hsa00350	Tyrosine metabolism	15/1784	36/8779	0.002831423	0.01941547	0.015814606	ADH1A/ADH1C/ADH7/ALDH3B2/AOC2/AOC3/COMT/DBH/DCT/LRTOMT/MAOB/TAT/TH/TYR/TYRP1	15

cluster5	KEGG	hsa04921	Oxytocin signaling pathway	46/1784	154/8779	0.00292187	0.019634967	0.015993394	ADCY1/ADCY2/ADCY3/ADCY5/ADCY8/CACNA1C/CACNA1S/CACNA2D1/CACNB1/CACNG2/CACNG3/CACNG4/CACNG5/CACNG7/CALML3/CAMK1G/CAMK2A/CAMK2B/CDKN1A/EGFR/FOS/GNAI1/GNAO1/GNAS/GUCY1A1/GUCY1A2/KCNJ18/KCNJ3/KCNJ4/KCNJ5/KCNJ6/KCNJ9/MYLK/NFATC4/NOS3/PLA2G4C/PLA2G4E/PLCB1/PRKAA2/PRKCG/PTGS2/ROCK1/ROCK2/RYR2/RYR3/SRC	46
cluster5	KEGG	hsa04971	Gastric acid secretion	26/1784	76/8779	0.00320296	0.021101856	0.017188228	ADCY1/ADCY2/ADCY3/ADCY5/ADCY8/ATP1A2/ATP1A3/ATP1B1/ATP1B4/CALML3/CAMK2A/CAMK2B/CCKBR/CFTR/CHRM3/GAST/GNAI1/GNAS/KCNJ16/KCNK10/MYLK/PLCB1/PRKCG/SLC9A4/SST/SSTR2	26
cluster5	KEGG	hsa04550	Signaling pathways regulating pluripotency of stem cells	43/1784	143/8779	0.003430005	0.022163111	0.01805266	AKT1/APC2/BMP4/BMPR1B/DLX5/DUSP9/ESRRB/ESX1/FGF2/FGFR1/FGFR2/FGFR3/FGFR4/FZD10/FZD3/GSK3B/HOXB1/ID1/ID4/INHBC/ISL1/LEFTY1/LEFTY2/LHX5/MAPK12/MEIS1/NEUROG1/OTX1/PAX6/PCGF5/SMAD5/WNT11/WNT2B/WNT3A/WNT4/WNT5A/WNT7A/WNT7B/WNT8A/WNT8B/WNT9A/WNT9B/ZIC3	43
cluster5	KEGG	hsa04810	Regulation of actin cytoskeleton	63/1784	229/8779	0.004995125	0.031667205	0.02579409	ACTN1/ACTR3B/AKT1/APC2/ARHGEF4/ARPC1B/BRK1/BUB1B-PAK6/C7/C8B/CHRM1/CHRM2/CHRM3/CYFIP1/DIAPH3/EGF/EGFR/F2/F2R/FGD1/FGF10/FGF16/FGF17/FGF19/FGF2/FGF23/FGF4/FGF5/FGF7/FGF9/FGFR1/FGFR2/FGFR3/FGFR4/INS/ITGA2/ITGA2B/ITGA7/ITGAM/ITGB1/ITGB3/ITGB5/ITGB8/KNG1/MYH10/MYH9/MYL12A/MYL7/MYLK/NCKAP1/PAK3/PAK5/PDGFA/PDGFC/PDGFRA/PDGFRB/PFN2/ROCK1/ROCK2/SRC/TMSB4Y/VAV2/WASF1	63

cluster5	KEGG	hsa04611	Platelet activation	37/1784	124/8779	0.007253489	0.045132821	0.036762323	ADCY1/ADCY2/ADCY3/ADCY5/ADCY8/AKT1/F2/F2R/F2RL3/FERMT3/GNAI1/GNAS/GP1BB/GP6/GP9/GUCY1A1/GUCY1A2/ITGA2/ITGA2B/ITGB1/ITGB3/LYN/MAPK12/MYL12A/MYLK/NOS3/PLA2G4C/PLA2G4E/PLCB1/PRKG1/RAP1B/ROCK1/ROCK2/SNAP23/SRC/TBXA2R/VWF	37
cluster5	KEGG	hsa04390	Hippo signaling pathway	45/1784	157/8779	0.007428367	0.045380572	0.036964125	AJUBA/APC2/BIRC5/BMP2/BMP4/BMP5/BMP6/BMP7/BMPR1B/CCN2/CCND3/CRB2/CSNK1E/CTNNA2/DLG5/FZD10/FZD3/GDF5/GDF6/GLI2/GSK3B/ID1/MOB1B/NKD2/PARD3/PPP2R2C/RASSF6/SERPINE1/TEAD1/TEAD2/TP73/WNT11/WNT2B/WNT3A/WNT4/WNT5A/WNT7A/WNT7B/WNT8A/WNT8B/WNT9A/WNT9B/WWC1/YAP1/YWHAZ	45
cluster5	KEGG	hsa04924	Renin secretion	23/1784	69/8779	0.007672831	0.046036988	0.0374988	ACE/ADCY5/ADCYAP1/ADCYAP1R1/ADORA1/ADRB3/AGT/CACNA1C/CACNA1S/CALML3/CLCA2/EDN2/EDNRA/GNAI1/GNAS/GUCY1A1/GUCY1A2/KCNMA1/PDE1A/PDE1B/PDE1C/PDE3A/PLCB1	23
cluster6	BP	GO:0015671	oxygen transport	8/1149	16/18903	0.00000151	0.009864837	0.009864837	BPGM/HBB/HBD/HBE1/HBG1/HBG2/HBM/HBZ	8

cluster6	BP	GO:0042744	hydrogen peroxide catabolic process	10/1149	30/18903	0.00000649	0.021213318	0.021213318	CAT/HBB/HBD/HBE1/HBG1/HBG2/HBM/HBZ/HP/LPO	10
cluster6	CC	GO:0031838	haptoglobin-hemoglobin complex	8/1226	11/19869	2.87E-08	0.0000242	0.0000236	HBB/HBD/HBE1/HBG1/HBG2/HBM/HBZ/HP	8
cluster6	CC	GO:0005833	hemoglobin complex	7/1226	12/19869	0.00000202	0.000848338	0.000830414	HBB/HBD/HBE1/HBG1/HBG2/HBM/HBZ	7
cluster6	CC	GO:0030667	secretory granule membrane	39/1226	313/19869	0.0000232	0.006515582	0.006377924	ACP3/ADGRE5/AP2A2/C3AR1/CA4/CAV2/CD14/CD36/CKAP4/CYSTM1/DOK3/FAM170B/FCAR/FCGR2A/FLOT2/FPR1/GPR84/IQGAP2/LGALS3/LILRA3/MME/MOSPD2/NFAM1/RAB24/RAB3D/RAB4B/RHOG/SELL/SIGLEC9/SIRPB1/SLCO4C1/SRI/STING1/SYNGR1/TLR2/TMED2/TMEM225/TRIP11/VAMP2	39

cluster6	CC	GO:0101002	ficolin-1-rich granule	25/1226	185/19869	0.000184765	0.035578778	0.034827082	AP2A2/CAT/COTL1/CTSB/CTSS/DOK3/FCAR/FCN1/FGL2/FPR1/FTH1/HBB/HSPA6/LGALS3/LILRA3/LRG1/MAPK1/MIF/PLEKHO2/PNP/PSMB7/PSMC2/PSMD13/PSMD6/SERPINA1	25
cluster6	CC	GO:1904813	ficolin-1-rich granule lumen	19/1226	124/19869	0.000211275	0.035578778	0.034827082	CAT/COTL1/CTSB/CTSS/FCN1/FGL2/FTH1/HBB/HSPA6/LRG1/MAPK1/MIF/PLEKHO2/PNP/PSMB7/PSMC2/PSMD13/PSMD6/SERPINA1	19
cluster6	MF	GO:0031720	haptoglobin binding	7/1192	10/18432	0.000000469	0.000736489	0.000734241	HBB/HBD/HBE1/HBG1/HBG2/HBM/HBZ	7
cluster6	MF	GO:0031721	hemoglobin alpha binding	5/1192	5/18432	0.00000112	0.00088044	0.000877752	HBB/HBD/HBE1/HBG1/HBG2	5

cluster6	MF	GO:0030492	hemoglobin binding	6/1192	9/18432	0.00000512	0.002679374	0.002671195	HBB/HBD/HBE1/HBG1/HBG2/HP	6
cluster6	MF	GO:0005344	oxygen carrier activity	7/1192	14/18432	0.0000107	0.004181008	0.004168245	HBB/HBD/HBE1/HBG1/HBG2/HBM/HBZ	7
cluster6	MF	GO:0140375	immune receptor activity	24/1192	148/18432	0.000027	0.00847278	0.008446916	C3AR1/CCR10/CD74/CRLF2/CSF2RA/CSF3R/CXCR3/EPOR/FCGR1A/FPR1/GFRA4/IL17RA/IL17RC/IL18RAP/IL1RAP/IL3RA/IL5RA/KIR2DL4/KIR3DL1/KIR3DS1/KLRC2/LILRA3/LILRA5/XCR1	24
cluster6	MF	GO:0004601	peroxidase activity	12/1192	55/18432	0.000165528	0.043285484	0.043153353	ALOX5AP/CAT/HBB/HBD/HBE1/HBG1/HBG2/HBM/HBZ/LPO/LTC4S/TXNRD1	12

cluster6	MF	GO:0030292	protein tyrosine kinase inhibitor activity	4/1192	6/18432	0.00023492	0.046429935	0.046288205	CEP43/HYAL2/LILRB4/NCAPG2	4
cluster6	MF	GO:0016684	oxidoreductase activity, acting on peroxide as acceptor	12/1192	57/18432	0.000236736	0.046429935	0.046288205	ALOX5AP/CAT/HBB/HBD/HBE1/HBG1/HBG2/HBM/HBZ/LPO/LTC4S/TXNRD1	12
cluster6	KEGG	hsa04612	Antigen processing and presentation	16/560	78/8779	0.0000257	0.008343976	0.008269657	CD74/CTSB/CTSS/HLA-DRB4/HSPA6/KIR2DL1/KIR2DL2/KIR2DL3/KIR2DL4/KIR2DL5A/KIR2DS1/KIR2DS2/KIR2DS4/KIR3DL1/KIR3DL2/KLRC2	16
cluster7	BP	GO:0009451	RNA modification	30/1202	176/18903	0.000000684	0.004741115	0.004669691	ALKBH8/CDKAL1/DPH3/DTWD1/DTWD2/ELP3/ELP6/FBL/METTL15/METTL25B/METTL5/METTL8/MRM3/NAF1/NSUN6/PARN/PUS7L/RNMT/RPUSD2/SNRPD1/SNRPE/SNRPF/THUMPD1/TPRKB/TRMT61B/TRUB2/TYW1/TYW5/YRDC/ZCCHC4	30

cluster7	BP	GO:0002637	regulation of immunoglobulin production	16/1202	76/18903	0.0000186	0.047840443	0.047119741	CD28/CD40/CD40LG/CGAS/FCGR2B/GPI/HPX/MSH2/NDFIP1/PAXIP1/RBP4/SHLD1/SHLD3/TNFSF4/TP53BP1/ZPBP2	16
cluster7	BP	GO:0034470	ncRNA processing	51/1202	439/18903	0.0000227	0.047840443	0.047119741	ALKBH8/CDKAL1/DDX1/DDX21/DPH3/DTWD1/DTWD2/ELP3/ELP6/FBL/GTF2H5/HELQ/INTS8/KRR1/METTL15/METTL25B/METTL5/METTL8/MRM3/MTERF4/MTREX/NAF1/NCBP2/NPM3/NSUN6/PARN/PPP3CA/RPL26/RPL35/RPL35A/RPS24/RPUSD2/RRP15/SMAD3/THUMPD1/TOE1/TPRKB/TRMT61B/TRUB2/TSR2/TYW1/TYW5/URB1/UTP14C/UTP20/UTP4/YRDC/ZBTB8OS/ZC3H10/ZCCHC4/ZCCHC8	51
cluster7	BP	GO:0022613	ribonucleoprotein complex biogenesis	55/1202	489/18903	0.0000276	0.047840443	0.047119741	CELF6/CLNS1A/DDX1/DDX21/DHX29/EIF3H/FBL/GCFC2/GEMIN2/GPATCH4/GTF2H5/HELQ/KHDC4/KRR1/LUC7L2/LUC7L3/METTL15/METTL17/METTL25B/METTL5/MRM3/MRPL20/MTERF4/MTREX/NAF1/NMD3/NPM3/PRPF19/PRPF31/RBIS/RBM5/RPL26/RPL35/RPL35A/RPL38/RPS24/RPUSD2/RRP15/SART3/SF3B6/SMN1/SMN2/SNRPD1/SNRPE/SNRPF/SURF6/TMA16/TRMT61B/TSR2/URB1/UTP14C/UTP20/UTP4/ZCCHC4/ZRSR2	55
cluster8	BP	GO:0048732	gland development	106/2556	441/18903	1.25E-09	0.0000118	0.0000112	ACADM/ACER1/ALDH1A3/APLN/APOA1/AR/AREG/ARHGAP5/ASS1/ATF2/ATP7B/BAAT/BCL11B/BRAF/BTRC/CAPN1/CAV1/CCL11/CCND1/CDKN1B/COBL/COX2/CPT1A/CRKL/CSMD1/CYP19A1/CYP1A1/DEAF1/E2F8/EAF2/EDN1/ELF3/ELK1/EPHA2/ESR1/FBXW7/FGF1/FGF8/FGL1/FOXF1/GATA2/GATA6/GHRH/HAMP/HAND2/HESX1/HOXA13/HOXB3/HOXD9/IGFBP5/IHH/IQGAP3/IRF6/JARID2/LIMS2/MET/MMP2/NF1/NFIB/NKX2-3/NR5A1/NRP1/NTN4/ONECUT1/ONECUT2/OTC/OXTR/PCK1/PCSK9/PGR/PITX2/PKD2/PLAG1/PRLR/PROP1/PROX1/PSEN1/RGN/RPGRIP1L/RREB1/RXFP1/SEC63/SEMA3C/SERPINA10/SERPINF1/SMAD2/SNAI2/SOCS2/SRSF5/TBX1/TBX2/TBX3/TCF21/TGFB3/TNFAIP3/TP63/TSPO/TWSG1/UGT1A1/UGT1A10/UGT1A7/UGT1A9/VTN/WNT2/ZBTB7B/ZDHHC21	106

cluster8	BP	GO:0050673	epithelial cell proliferation	108/2556	481/18903	4.47E-08	0.00021082	0.000200194	ACVR2A/ADAM17/AGGF1/AGTR1/APLN/APLNR/APOA1/APOE/APOH/AR/AREG/ATF2/ATOH8/BCL11B/BCL2L2/BMPR2/BTK/BTRC/CAV1/CCL11/CCL2/CCND1/CD109/CD34/CDK6/CDKN1B/CNMD/CRNN/CXCL12/DAB2IP/DEAF1/DLG1/DLL4/EAF2/EGR3/EPHA2/ERN1/ERRFI1/ESR1/EYA1/FBXW7/FGF1/FGFBP1/FGL1/FLT1/FSHB/FST/GATA2/IFT74/IGF1/IGFBP5/IHH/IL12B/IQGAP3/IRF6/JCAD/KDR/KIT/KLK8/KRT4/LAMC1/LIMS2/LOXL2/MEF2C/MMRN2/MTA3/MTSS1/NCSTN/NF1/NFIB/NKX2-3/NR4A3/NRAS/NRP1/NUPR1/OSR1/OSR2/OVOL2/PDCD10/PGR/PIK3CB/PRKD1/PROK1/PROX1/PSEN1/PTPRK/PTPRM/RGN/RIDA/RREB1/SERPINF1/SNAI2/SPARC/SRSF6/ST8SIA1/STAT3/STXBP4/TBX1/TBX2/TEK/THBS1/THBS4/TNFAIP3/TNMD/TP63/VEGFC/VIP/WNT2	108
cluster8	BP	GO:0008544	epidermis development	85/2556	362/18903	0.000000161	0.000506608	0.000481074	ACER1/ADAM9/ATP2C1/BCL11B/BNC1/C1orf68/CALML5/CASP14/CD109/CDSN/CLIC4/COL17A1/COL7A1/CYP26B1/DKK1/DSP/ELAPOR2/EPHA2/ERRFI1/FABP5/FGF20/FLG/FLG2/FST/GATA6/GJB5/GRHL3/GSDME/HEY2/HOXA7/HRNR/IFT74/IGFBP5/IL1A/INHBA/IRF6/JAG2/KLK14/KLK7/KRT16/KRT3/KRT34/KRT4/KRT5/KRT6A/KRT6B/KRT71/KRT78/KRT82/KRT85/KRT9/KRTAP6-1/KRTAP6-2/KRTAP6-3/LCE1B/LCE1F/LCE2A/LCE2B/LDB2/MYSM1/NF1/NTF4/OVOL2/PAFAH1B1/PALLD/PITX2/PTHLH/SFRP4/SLC44A4/SLC4A7/SNAI1/SPINK5/SPRR1A/SPRR1B/SPRR2B/SPRR2D/SPRR2E/SPRR3/SRSF6/STRC/TCHH/TGM5/TP63/TPRN/ZDHHC21	85
cluster8	BP	GO:0003007	heart morphogenesis	65/2556	258/18903	0.000000325	0.000767726	0.000729032	ADAMTS1/ADGRG6/ALPK2/ANKRD1/APLNR/ATF2/AXIN2/BBS7/BMPR2/CCM2L/CCN1/COL5A1/CPLANE2/CRKL/DKK1/DLC1/DLL4/DSP/EDN1/ELN/EYA1/FGF8/FOXF1/FOXH1/GAA/GATA5/GATA6/GJA5/HAND1/HAND2/HEG1/HEY1/HEY2/IHH/MEF2C/MIB1/MYL2/MYL3/MYLK2/NPY1R/NPY2R/NRP1/OVOL2/PARVA/PITX2/PKD2/POU4F1/PROX1/PSEN1/RBM15/RYR1/SEMA3C/SETDB2/SMAD6/SNAI1/SNAI2/SOX17/TBX1/TBX2/TBX3/TEK/TMEM100/WNT2/XIRP2/ZFPM2	65
cluster8	BP	GO:0031424	keratinization	29/2556	84/18903	0.000000819	0.001547083	0.001469108	CASP14/CDSN/CYP26B1/HRNR/IL1A/KRT16/KRT3/KRT4/KRT5/KRT6A/KRT6B/KRT71/KRT78/KRT82/KRT85/KRTAP6-1/KRTAP6-2/KRTAP6-3/LCE1B/LCE1F/LCE2A/LCE2B/SPRR1A/SPRR1B/SPRR2B/SPRR2D/SPRR2E/SPRR3/TCHH	29

cluster8	BP	GO:0060021	roof of mouth development	30/2556	91/18903	0.00000162	0.002550916	0.002422346	ACVR2B/ASPH/BBS7/BNC2/COL11A2/CSRNP1/DLG1/GDF11/HAND2/INHBA/INSIG1/INSIG2/IRF6/ITGB6/JAG2/MEOX2/OSR1/OSR2/PRRX1/SATB2/SMAD2/SNAI1/SNAI2/SUMO1/TBX1/TBX2/TBX3/TCF21/TGFB3/TMEM107	30
cluster8	BP	GO:0009913	epidermal cell differentiation	58/2556	235/18903	0.00000277	0.003556843	0.003377573	ACER1/ADAM9/BCL11B/CASP14/CD109/CDSN/CLIC4/CYP26B1/DSP/EPHA2/ERRFI1/FGF20/FLG/GATA6/GSDME/HEY2/HOXA7/HRNR/IFT74/IL1A/IRF6/JAG2/KRT16/KRT3/KRT4/KRT5/KRT6A/KRT6B/KRT71/KRT78/KRT82/KRT85/KRTAP6-1/KRTAP6-2/KRTAP6-3/LCE1B/LCE1F/LCE2A/LCE2B/OVOL2/PAFAH1B1/PALLD/PITX2/SFRP4/SLC44A4/SLC4A7/SPINK5/SPRR1A/SPRR1B/SPRR2B/SPRR2D/SPRR2E/SPRR3/SRSF6/STRC/TCHH/TP63/TPRN	58
cluster8	BP	GO:0043588	skin development	70/2556	302/18903	0.00000316	0.003556843	0.003377573	ACER1/ADAM9/ALOXE3/BCL11B/CASP14/CD109/CDSN/CLIC4/COL1A1/COL1A2/COL5A1/COL5A2/COMP/CYP26B1/DKK1/DSP/EPHA2/ERRFI1/FLG/FLG2/FST/GATA6/GRHL3/HOXA7/HRNR/IFT74/IGFBP5/IL1A/INHBA/IRF6/ITGB4/ITGB6/KRT16/KRT3/KRT4/KRT5/KRT6A/KRT6B/KRT71/KRT78/KRT82/KRT85/KRT9/KRTAP6-1/KRTAP6-2/KRTAP6-3/LCE1B/LCE1F/LCE2A/LCE2B/LDB2/MET/MYSM1/NF1/OVOL2/PALLD/PSEN1/RYR1/SNAI1/SPINK5/SPRR1A/SPRR1B/SPRR2B/SPRR2D/SPRR2E/SPRR3/SRSF6/TCHH/TP63/ZDHHC21	70
cluster8	BP	GO:0001655	urogenital system development	80/2556	360/18903	0.00000369	0.003556843	0.003377573	ACVR2B/ADAMTS1/ADAMTS16/AGTR1/AQP1/AQP2/AR/ARL3/ASS1/C1GALT1/CASP9/CD24/CD34/CDKN1B/CENPF/CEP290/CNTRL/CRKL/CTNNBIP1/CYP19A1/CYP26B1/CYP4A11/CYP4A22/DLG1/DYNC2H1/EAF2/ENPEP/ESR1/EYA1/FBN1/FGF1/FGF8/FOXF1/GATA2/GDF11/GPR4/HOXA13/HPGD/HS2ST1/IRX1/IRX3/ITGA8/KANK2/KCNJ8/LIF/LIN28A/MEF2C/MMP2/MTSS1/NF1/NPNT/NRP1/ODC1/OSR1/OSR2/PCSK9/PKD2/PLAG1/PRLR/PROX1/RDH10/REN/RGN/RIDA/RPGRIP1L/SERPINF1/SIM1/SMAD2/SMAD6/SOX17/SOX8/TBX18/TCF21/TEK/TNS2/TP63/TRAF3IP1/TTC8/WNK4/WWTR1	80

cluster8	BP	GO:0007160	cell-matrix adhesion	58/2556	238/18903	0.00000424	0.003556843	0.003377573	ACER2/ADAM9/ADAMTS12/ADAMTS9/ANGPTL3/ARHGEF7/BCAM/CCL21/CD34/CDK6/CLASP2/COL17A1/CTTN/CX3CL1/DLC1/DUSP22/EPB41L5/FAM107A/FGA/FGB/FGG/FN1/HOXA7/HRG/ITGA1/ITGA8/ITGA9/ITGAV/ITGB4/ITGB6/ITGBL1/KDR/LIMCH1/LYVE1/MUC4/MYF5/NF1/NPNT/NRP1/ONECUT1/ONECUT2/OTOA/PHLDB2/PIK3CB/PTPRA/PTPRK/SGCE/SLC9A1/SORBS1/STRC/TECTA/TEK/THBS1/THSD1/TIAM1/VCAM1/VCL/VTN	58
cluster8	BP	GO:0050678	regulation of epithelial cell proliferation	89/2556	414/18903	0.00000453	0.003556843	0.003377573	ADAM17/AGGF1/AGTR1/APLN/APLNR/APOE/APOH/AR/AREG/ATF2/ATOH8/BCL11B/BMPR2/BTK/CAV1/CCL11/CCL2/CCND1/CD109/CDK6/CDKN1B/CNMD/CRNN/CXCL12/DAB2IP/DEAF1/DLG1/DLL4/EAF2/EGR3/ERRFI1/EYA1/FBXW7/FGF1/FGFBP1/FLT1/GATA2/IFT74/IGF1/IHH/IL12B/IQGAP3/IRF6/JCAD/KDR/KRT4/LAMC1/LIMS2/MEF2C/MMRN2/MTA3/MTSS1/NF1/NFIB/NKX2-3/NR4A3/NRAS/NRP1/NUPR1/OSR1/OSR2/OVOL2/PDCD10/PGR/PRKD1/PROK1/PROX1/PTPRK/PTPRM/RGN/RIDA/RREB1/SERPINF1/SNAI2/SPARC/SRSF6/ST8SIA1/STAT3/STXBP4/TBX1/TEK/THBS1/THBS4/TNFAIP3/TNMD/TP63/VEGFC/VIP/WNT2	89
cluster8	BP	GO:0061008	hepaticobiliary system development	40/2556	144/18903	0.0000047	0.003556843	0.003377573	ACADM/ASS1/ATF2/BAAT/CCND1/COBL/CPT1A/CYP1A1/E2F8/ELK1/FBXW7/FGF1/FGL1/GATA6/HAMP/IHH/JARID2/LIMS2/MET/NF1/ONECUT1/ONECUT2/OTC/PCK1/PCSK9/PKD2/PROX1/RGN/RPGRIP1L/SEC63/SERPINA10/SOX17/SRSF5/TBX3/TNFAIP3/UGT1A1/UGT1A10/UGT1A7/UGT1A9/VTN	40
cluster8	BP	GO:0030216	keratinocyte differentiation	45/2556	170/18903	0.00000506	0.003556843	0.003377573	ACER1/ADAM9/BCL11B/CASP14/CD109/CDSN/CLIC4/CYP26B1/DSP/EPHA2/ERRFI1/FLG/HOXA7/HRNR/IFT74/IL1A/IRF6/KRT16/KRT3/KRT4/KRT5/KRT6A/KRT6B/KRT71/KRT78/KRT82/KRT85/KRTAP6-1/KRTAP6-2/KRTAP6-3/LCE1B/LCE1F/LCE2A/LCE2B/OVOL2/PALLD/SPRR1A/SPRR1B/SPRR2B/SPRR2D/SPRR2E/SPRR3/SRSF6/TCHH/TP63	45

cluster8	BP	GO:0031589	cell-substrate adhesion	81/2556	369/18903	0.00000527	0.003556843	0.003377573	ABI3BP/ACER2/ADAM9/ADAMTS12/ADAMTS9/AKIP1/ANGPTL3/AP1AR/APOA1/ARHGEF7/BCAM/BRAF/CCDC80/CCL21/CCN1/CD34/CDK6/CLASP2/COL17A1/COL1A1/CRKL/CTTN/CX3CL1/DLC1/DOCK1/DUSP22/EGFLAM/EPB41L5/FAM107A/FGA/FGB/FGG/FN1/FOXF1/HOXA7/HRG/ITGA1/ITGA8/ITGA9/ITGAV/ITGB4/ITGB6/ITGBL1/KDR/LAMC1/LIMCH1/LIMS2/LYVE1/MELTF/MERTK/MUC4/MYF5/NF1/NPNT/NRP1/NTN4/ONECUT1/ONECUT2/OTOA/PARVA/PHLDB2/PIK3CB/PTPRA/PTPRK/RREB1/SGCE/SLC9A1/SMAD6/SORBS1/SPRY4/ST6GAL1/STRC/TECTA/TEK/THBS1/THSD1/TIAM1/UNC13D/VCAM1/VCL/VTN	81
cluster8	BP	GO:0043062	extracellular structure organization	72/2556	319/18903	0.00000627	0.00384124	0.003647636	ABI3BP/ADAMTS1/ADAMTS12/ADAMTS16/ADAMTS5/ADAMTS9/BMP1/CAV1/CCDC80/CCN1/CLASP2/COL11A2/COL15A1/COL17A1/COL1A1/COL1A2/COL5A1/COL5A2/COLGALT2/COMP/CREB3L1/CTSV/DDR2/DNAJB6/DPT/EGFLAM/ELF3/ELN/FMOD/FOXF1/GAS2/HMCN1/IHH/IMPG2/ITGA8/KLK4/KLK7/LAMA2/LAMC1/LOXL2/LUM/MATN1/MELTF/MMP1/MMP2/MMP20/MMP7/MYF5/NF1/NOX1/NPNT/NTN4/OLFML2A/OTOL1/PBXIP1/PHLDB1/PHLDB2/POSTN/PRDM5/RAMP2/RXFP1/SCX/SERAC1/SMOC1/SMOC2/SPINK5/TCF15/TEX14/TLL1/TMEM38B/VTN/ZNF469	72
cluster8	BP	GO:0001889	liver development	39/2556	141/18903	0.00000687	0.00384124	0.003647636	ACADM/ASS1/ATF2/BAAT/CCND1/COBL/CPT1A/CYP1A1/E2F8/ELK1/FBXW7/FGF1/FGL1/GATA6/HAMP/IHH/JARID2/LIMS2/MET/NF1/ONECUT1/ONECUT2/OTC/PCK1/PCSK9/PKD2/PROX1/RGN/RPGRIP1L/SEC63/SERPINA10/SRSF5/TBX3/TNFAIP3/UGT1A1/UGT1A10/UGT1A7/UGT1A9/VTN	39
cluster8	BP	GO:0007498	mesoderm development	38/2556	136/18903	0.00000692	0.00384124	0.003647636	ACVR2A/ACVR2B/BMPR2/BTK/DKK1/EPB41L5/EPHA2/EYA1/FGF8/FOXF1/FOXH1/GDF11/GJA1/HAND1/INHBA/IRX3/ITGA8/ITGB4/OSR1/POFUT2/POU4F1/PUS7/SCX/SETD2/SMAD2/SNAI1/TBX1/TBX3/TCF15/TP63/TRIM15/TSPY1/TSPY10/TSPY3/TSPY4/TSPY8/TWSG1/ZFPM2	38

cluster8	BP	GO:0060840	artery development	32/2556	107/18903	0.00000778	0.00384124	0.003647636	ACVR2B/ADAMTS9/ADGRF5/ANGPTL3/APLNR/APOB/APOE/BMPR2/CNTRL/COMP/DLL4/EDN1/EYA1/FGF8/FOXF1/FOXH1/GJA5/HAND2/HEY1/HEY2/HOXA13/HPGD/NDST1/NF1/NRP1/PDE2A/PKD2/PROX1/PRRX1/SMAD6/TBX1/TBX2	32
cluster8	BP	GO:0045229	external encapsulating structure organization	72/2556	321/18903	0.00000789	0.00384124	0.003647636	ABI3BP/ADAMTS1/ADAMTS12/ADAMTS16/ADAMTS5/ADAMTS9/BMP1/CAV1/CCDC80/CCN1/CLASP2/COL11A2/COL15A1/COL17A1/COL1A1/COL1A2/COL5A1/COL5A2/COLGALT2/COMP/CREB3L1/CTSV/DDR2/DNAJB6/DPT/EGFLAM/ELF3/ELN/FMOD/FOXF1/GAS2/HMCN1/HRNR/IHH/IMPG2/ITGA8/KLK4/KLK7/LAMA2/LAMC1/LOXL2/LUM/MATN1/MELTF/MMP1/MMP2/MMP20/MMP7/MYF5/NF1/NOX1/NPNT/NTN4/OLFML2A/OTOL1/PBXIP1/PHLDB1/PHLDB2/POSTN/PRDM5/RAMP2/RXFP1/SCX/SERAC1/SMOC1/SMOC2/SPINK5/TCF15/TLL1/TMEM38B/VTN/ZNF469	72
cluster8	BP	GO:0048844	artery morphogenesis	26/2556	79/18903	0.00000814	0.00384124	0.003647636	ADAMTS9/ADGRF5/ANGPTL3/APOB/APOE/BMPR2/COMP/DLL4/EDN1/EYA1/FGF8/FOXF1/FOXH1/GJA5/HAND2/HEY1/HEY2/HOXA13/HPGD/NF1/NRP1/PKD2/PROX1/PRRX1/TBX1/TBX2	26
cluster8	BP	GO:0060537	muscle tissue development	89/2556	422/18903	0.0000101	0.004053646	0.003849337	ACADM/ADAMTS9/AKIRIN1/ALPK2/ALPK3/ANKRD1/ATF3/CASQ1/CAV1/CENPF/CYP26B1/DKK1/DLG1/DLL4/DSP/EDN1/ELN/EMD/EYA1/FGF20/FGF8/FOXH1/GATA5/GATA6/GJA1/GJA5/HAMP/HAND1/HDAC9/HEG1/HEY1/HEY2/HOXD9/IFRD1/IGF1/IGFBP5/IHH/IRX3/ITGA8/JARID2/KCNK2/KLHL41/MED20/MEF2A/MEF2C/MEOX2/MRTFB/MSTN/MTM1/MTPN/MYF5/MYF6/MYH14/MYL2/MYL3/MYL6B/MYLK2/MYLK3/NDUFV2/NEBL/NF1/NIBAN2/NOX4/NPPB/NUPR1/OSR1/PARP2/PDLIM5/PGM5/PKD2/POU4F1/PROX1/RIPOR2/RYR1/SCX/SEMA3C/SGCB/SLC9A1/SORBS2/SOX8/SRPK3/TBX1/TBX18/TBX2/TBX3/TCF21/WNT2/XIRP2/ZFPM2	89

cluster8	BP	GO:0006805	xenobiotic metabolic process	34/2556	118/18903	0.0000101	0.004053646	0.003849337	AADAC/ABCC4/ACSM2B/AHR/ALDH3A1/AOX1/CYP1A1/CYP26B1/CYP2A6/CYP2C18/CYP2C8/CYP2C9/CYP2E1/CYP3A4/CYP3A7/FMO1/FMO2/GLYAT/GSTA1/GSTA2/GSTA3/NAT2/NCEH1/NOS1/NQO1/NR1I2/PON3/SLCO2B1/SULT2A1/UGT1A1/UGT1A10/UGT1A7/UGT1A9/UGT2B28	34
cluster8	BP	GO:0022612	gland morphogenesis	35/2556	123/18903	0.0000101	0.004053646	0.003849337	AR/AREG/BTRC/CAPN1/CAV1/CCL11/CSMD1/ELF3/EPHA2/ESR1/FBXW7/FGF1/FGF8/FGL1/HOXA13/IGFBP5/LIMS2/MMP2/NFIB/NKX2-3/NRP1/NTN4/PGR/PLAG1/PROP1/PROX1/RXFP1/SEMA3C/SNAI2/TBX2/TBX3/TGFB3/TNFAIP3/TP63/TWSG1	35
cluster8	BP	GO:0003151	outflow tract morphogenesis	26/2556	80/18903	0.0000105	0.004053646	0.003849337	ATF2/BMPR2/CRKL/EDN1/ELN/EYA1/FGF8/FOXH1/GATA6/GJA5/HAND2/HEY2/MEF2C/NPY1R/NPY2R/NRP1/PARVA/PITX2/RYR1/SEMA3C/SMAD6/SOX17/TBX1/TBX2/TBX3/ZFPM2	26
cluster8	BP	GO:0030198	extracellular matrix organization	71/2556	318/18903	0.0000107	0.004053646	0.003849337	ABI3BP/ADAMTS1/ADAMTS12/ADAMTS16/ADAMTS5/ADAMTS9/BMP1/CAV1/CCDC80/CCN1/CLASP2/COL11A2/COL15A1/COL17A1/COL1A1/COL1A2/COL5A1/COL5A2/COLGALT2/COMP/CREB3L1/CTSV/DDR2/DNAJB6/DPT/EGFLAM/ELF3/ELN/FMOD/FOXF1/GAS2/HMCN1/IHH/IMPG2/ITGA8/KLK4/KLK7/LAMA2/LAMC1/LOXL2/LUM/MATN1/MELTF/MMP1/MMP2/MMP20/MMP7/MYF5/NF1/NOX1/NPNT/NTN4/OLFML2A/OTOL1/PBXIP1/PHLDB1/PHLDB2/POSTN/PRDM5/RAMP2/RXFP1/SCX/SERAC1/SMOC1/SMOC2/SPINK5/TCF15/TLL1/TMEM38B/VTN/ZNF469	71

cluster8	BP	GO:0042476	odontogenesis	37/2556	135/18903	0.0000146	0.005312101	0.005044363	ACVR2A/ACVR2B/ADAMTS5/AMTN/AQP1/AQP5/ATF2/AXIN2/BCL11B/CD34/CEMP1/COL1A1/COL1A2/EDN1/FAM20C/FGF8/FST/GATA6/HAND1/HAND2/INHBA/ITGB6/JAG2/KLK4/LHX8/MMP20/NKX2-3/OSR1/OSR2/PAX9/PERP/PHEX/PITX2/SSUH2/TBX1/TGFB3/TP63	37
cluster8	BP	GO:0071466	cellular response to xenobiotic stimulus	46/2556	183/18903	0.0000173	0.006049954	0.005745028	AADAC/ABCC4/ACER2/ACSM2B/AHR/ALDH3A1/ANKRD1/AOX1/BRAF/CRHBP/CRKL/CYP1A1/CYP26B1/CYP2A6/CYP2C18/CYP2C8/CYP2C9/CYP2E1/CYP3A4/CYP3A7/DDC/DPEP1/EDN1/FMO1/FMO2/GLYAT/GSTA1/GSTA2/GSTA3/HSPA5/MEF2C/NAT2/NCEH1/NOS1/NQO1/NR1I2/PON3/REN/SLCO2B1/SULT2A1/UGT1A1/UGT1A10/UGT1A7/UGT1A9/UGT2B28/WNK4	46
cluster8	BP	GO:0060348	bone development	55/2556	233/18903	0.0000199	0.006704245	0.006366342	ADAMTS12/ATF2/AXIN2/BBX/BGN/BMPR2/BNC2/CCN4/COL1A1/COMP/CYP26B1/DDR2/FAM20C/FBN1/FBXW7/FGF18/FGF8/FLI1/GJA1/GLG1/IFITM5/IGF1/IHH/INSIG1/INSIG2/ITGB6/KDR/KIT/MATN1/MCPH1/MEF2C/MIGA2/NOTUM/OSR2/OSTN/PAFAH1B1/PAPSS2/PHEX/PITX2/PLS3/RAB23/RANBP3L/RFLNB/RGN/RIPPLY2/RYR1/SCX/SFRP4/SLC10A7/SLC38A10/SPNS2/TGFB3/TMEM107/TMEM38B/TSKU	55
cluster8	BP	GO:0001667	ameboidal-type cell migration	99/2556	492/18903	0.0000254	0.008254461	0.007838424	ADAM17/ADAM9/ADAMTS9/AKAP12/AMOTL1/ANLN/APOA1/APOE/APOH/APPL1/AQP1/ARHGEF7/ATOH8/BCAR1/BMPR2/BRAF/CAPN7/CARD10/CDH5/CENPV/CLASP2/CYGB/DAB2IP/DCN/DDR2/DLL4/DOCK1/EDN1/EDN3/EFNB1/EGR3/EPB41L5/EPHA2/FBXW7/FGF1/FGF18/FGF8/FGFBP1/FN1/GATA2/GJA1/GNA13/HAND2/HBEGF/HDAC9/HRG/ITGB4/JCAD/KANK2/KDR/KIT/KRT16/LOXL2/MACIR/MEF2C/MEOX2/MET/MMRN2/NANOS1/NF1/NRP1/OVOL2/P2RY12/PAFAH1B1/PDCD10/PIK3C2A/PIK3CB/PIK3R3/PITX2/PLK2/PRKD1/PROX1/PRSS3/PTPRM/PTPRR/RAB11A/RAB13/RHOJ/RREB1/SASH1/SEMA3C/SEMA3D/SEMA3F/SEMA3G/SEMA6B/SERPINF1/SMOC2/SNAI1/SNAI2/SOX17/SOX8/SPARC/STARD13/SYDE1/TBX1/TEK/THBS1/VEGFC/VIL1	99

cluster8	BP	GO:0048880	sensory system development	83/2556	398/18903	0.0000302	0.009507382	0.009028196	ABCB5/ABI2/ACVR2B/ALDH1A3/AQP1/AQP5/ARHGEF15/BBS7/BCL11B/BIRC7/BMPR2/C3/CDKN1B/CEP290/CLIC4/COL5A1/COL5A2/CRYBA2/CRYBA4/CRYBB2/CRYBG3/CRYGC/CYP1A1/DIO3/DLG1/DLL4/DZANK1/EFEMP1/EPHA2/FBN1/FBN2/FLT1/GDF11/GRHL3/HESX1/IFT122/IGFN1/IHH/IMPG2/INHBA/KDR/LENEP/MAX/MERTK/MFAP5/MYF5/NES/NF1/NKD1/NPHP1/NRP1/OSR2/P2RY12/PBX4/PITX2/PLAAT3/POU4F1/POU4F2/PROX1/PSEN1/PTPRM/RCN1/RDH10/RHOJ/RP1L1/RPGRIP1L/SERPINF1/SIX5/SLC44A4/SLC7A11/SMOC1/SOX8/SPRED2/STAT3/STAU2/TBX2/TSKU/TSPAN12/TTC8/TWSG1/USP45/WNT2/WNT5B	83
cluster8	BP	GO:0008202	steroid metabolic process	70/2556	323/18903	0.0000346	0.010545693	0.010014175	ABCA1/AFP/AKR1B15/AKR1C2/AKR1D1/ANGPTL3/APOA1/APOA2/APOB/APOC1/APOE/ATP8B1/BAAT/CHST10/CYP11A1/CYP19A1/CYP1A1/CYP24A1/CYP26B1/CYP2A6/CYP2C8/CYP2C9/CYP2E1/CYP3A4/CYP3A7/CYP51A1/CYP7A1/DHRS2/ESR1/FGF1/FSHB/GNB3/HMGCS1/HSD17B3/HSD17B6/HSD17B8/HSD3B1/IL1A/INSIG1/INSIG2/KIT/LDLRAP1/LIMA1/MALRD1/NR1I2/NR5A1/NR5A2/OSBPL1A/PCSK9/PIP4P1/PON1/PRLR/PROX1/SCNN1B/SDR9C7/SERPINA6/SMPD1/SNAI1/SNAI2/STARD4/SULT2A1/TSKU/TSPO/UGT1A1/UGT1A7/UGT2A1/UGT2B10/UGT2B28/UGT2B4/VLDLR	70
cluster8	BP	GO:0001935	endothelial cell proliferation	48/2556	199/18903	0.0000364	0.010737774	0.010196575	ADAM17/AGGF1/AGTR1/APLN/APLNR/APOA1/APOE/APOH/ATOH8/BMPR2/CAV1/CCL11/CCL2/CD34/CNMD/CXCL12/DLG1/DLL4/EGR3/EPHA2/ERN1/FGFBP1/FLT1/GATA2/IL12B/JCAD/KDR/LOXL2/MEF2C/MMRN2/NF1/NRAS/NRP1/PDCD10/PIK3CB/PRKD1/PROK1/PROX1/PTPRM/SPARC/STAT3/TEK/THBS1/THBS4/TNMD/VEGFC/VIP/WNT2	48
cluster8	BP	GO:0002933	lipid hydroxylation	6/2556	7/18903	0.0000376	0.01076832	0.010225581	CYP1A1/CYP2C8/CYP2E1/CYP3A4/CYP3A7/CYP4A22	6

cluster8	BP	GO:0060485	mesenchyme development	68/2556	313/18903	0.000041	0.011389139	0.01081511	ACTG2/APLF/APLNR/AXIN2/BAMBI/BMPR2/BNC2/CLASP2/COL1A1/CPLANE2/DAB2IP/DPPA4/EDN1/EDN3/EFNB1/EPB41L5/FGF8/FN1/FOXF1/FOXH1/GATA5/HAND1/HAND2/HEY1/HEY2/IGF1/LOXL2/MEF2C/MEOX1/NRP1/OSR1/OVOL2/PEF1/PHLDB1/PHLDB2/PITX2/PKD2/POFUT2/RANBP3L/RDH10/RFLNB/SCX/SEMA3C/SEMA3D/SEMA3F/SEMA3G/SEMA6B/SMAD2/SNAI1/SNAI2/SOX8/SPRED2/TBX1/TBX2/TBX3/TCF15/TCF21/TGFB3/TIAM1/TMEM100/TSPY1/TSPY10/TSPY3/TSPY4/TSPY8/WNT2/WWTR1/ZFPM2	68
cluster8	BP	GO:0003206	cardiac chamber morphogenesis	34/2556	126/18903	0.0000452	0.012195966	0.011581272	ADAMTS1/ADGRG6/APLNR/BMPR2/CCN1/DLL4/DSP/FGF8/FOXF1/FOXH1/GATA6/HAND1/HAND2/HEG1/HEY1/HEY2/MEF2C/MYL2/MYL3/NPY2R/NRP1/OVOL2/PARVA/POU4F1/PROX1/RBM15/SEMA3C/SMAD6/TBX1/TBX2/TBX3/TEK/WNT2/ZFPM2	34
cluster8	BP	GO:0150063	visual system development	81/2556	392/18903	0.0000523	0.013716851	0.013025503	ABCB5/ABI2/ACVR2B/ALDH1A3/AQP1/AQP5/ARHGEF15/BBS7/BCL11B/BIRC7/BMPR2/C3/CDKN1B/CEP290/CLIC4/COL5A1/COL5A2/CRYBA2/CRYBA4/CRYBB2/CRYBG3/CRYGC/CYP1A1/DIO3/DLG1/DLL4/DZANK1/EFEMP1/EPHA2/FBN1/FBN2/FLT1/GDF11/GRHL3/HESX1/IFT122/IGFN1/IHH/IMPG2/INHBA/KDR/LENEP/MAX/MERTK/MFAP5/MYF5/NES/NF1/NKD1/NPHP1/NRP1/OSR2/P2RY12/PBX4/PITX2/PLAAT3/POU4F2/PROX1/PSEN1/PTPRM/RCN1/RDH10/RHOJ/RP1L1/RPGRIP1L/SERPINF1/SIX5/SLC7A11/SMOC1/SOX8/SPRED2/STAT3/STAU2/TBX2/TSKU/TSPAN12/TTC8/TWSG1/USP45/WNT2/WNT5B	81
cluster8	BP	GO:0007389	pattern specification process	94/2556	472/18903	0.0000603	0.015379745	0.014604584	ACVR2A/ACVR2B/APLNR/AR/ARL6/ASPH/ATP6AP2/AXIN2/BBS7/BMI1/BMP1/BMPR2/C3/CFAP53/COBL/CPLANE2/CRKL/CTNNBIP1/CYP26B1/DBX1/DKK1/DLL4/DNAAF4/DYNC2H1/DYNC2LI1/EDN1/EFNB1/EPB41L5/EYA1/FGF1/FGF8/FOXF1/FOXH1/FST/GAS8/GATA5/GDF11/GRHL3/HAND1/HAND2/HES3/HEY1/HEY2/HOXA2/HOXA6/HOXA7/HOXB3/HOXD8/HOXD9/IFT74/IHH/IRX1/IRX3/MEF2C/MEOX1/MEOX2/MIB1/MYF5/MYF6/NKD1/NRP1/NTF4/OSR1/OVOL2/PCDH8/PCGF2/PITX2/PKD2/PRKACB/PROP1/PSEN1/RIPPLY2/RPGRIP1L/SATB2/SEMA3C/SEMA3F/SETDB2/SMAD2/SMAD6/SNAI1/SOX17/TBX1/TBX18/TBX2/TBX3/TCF15/TDRD10/TDRD15/TDRD6/TLL1/TMEM107/TP63/TTC8/WNT2	94

cluster8	BP	GO:0072001	renal system development	68/2556	318/18903	0.0000688	0.017081749	0.016220804	ACVR2B/ADAMTS1/ADAMTS16/AGTR1/AQP1/AQP2/ARL3/ASS1/C1GALT1/CASP9/CD24/CD34/CENPF/CEP290/CNTRL/CTNNBIP1/CYP26B1/CYP4A11/CYP4A22/DLG1/DYNC2H1/ENPEP/EYA1/FBN1/FGF1/FGF8/FOXF1/GDF11/GPR4/HPGD/HS2ST1/IRX1/IRX3/ITGA8/KANK2/KCNJ8/LIF/LIN28A/MEF2C/MTSS1/NF1/NPNT/NRP1/ODC1/OSR1/OSR2/PCSK9/PKD2/PROX1/RDH10/REN/RGN/RIDA/RPGRIP1L/SERPINF1/SIM1/SMAD2/SMAD6/SOX17/SOX8/TBX18/TCF21/TEK/TNS2/TRAF3IP1/TTC8/WNK4/WWTR1	68
cluster8	BP	GO:0045104	intermediate filament cytoskeleton organization	26/2556	89/18903	0.0000829	0.020069846	0.019058297	ATF2/DNAJB6/DSP/DST/ERBIN/KRT16/KRT19/KRT3/KRT34/KRT35/KRT37/KRT38/KRT4/KRT5/KRT6A/KRT6B/KRT71/KRT78/KRT82/KRT85/KRT9/MTM1/NES/PKP1/SYNM/TCHH	26
cluster8	BP	GO:0003205	cardiac chamber development	41/2556	167/18903	0.0000856	0.0201959	0.019177998	ADAMTS1/ADGRG6/APLNR/BMPR2/CCN1/CNTRL/DLL4/DSP/FGF8/FOXF1/FOXH1/GATA6/GJA5/HAND1/HAND2/HEG1/HEY1/HEY2/HOXA13/KCNK2/MEF2C/MYL2/MYL3/NDST1/NPY2R/NRP1/OVOL2/PARVA/PDE2A/POU4F1/PROX1/RBM15/SEMA3C/SMAD6/TBX1/TBX2/TBX3/TEK/WNT2/XIRP2/ZFPM2	41
cluster8	BP	GO:0008210	estrogen metabolic process	15/2556	39/18903	0.0000902	0.02077372	0.019726695	AKR1B15/CHST10/CYP19A1/CYP1A1/CYP2C8/CYP2C9/CYP3A4/CYP3A7/HSD17B8/HSD3B1/UGT1A1/UGT1A7/UGT2B10/UGT2B28/UGT2B4	15

cluster8	BP	GO:0034754	cellular hormone metabolic process	36/2556	141/18903	0.0000964	0.021667647	0.020575567	ADH1B/ADH4/ADH6/AFP/AKR1B15/AKR1C2/AKR1D1/ALDH1A3/CHST10/CYP11A1/CYP19A1/CYP1A1/CYP26B1/CYP2C18/CYP2C8/CYP2C9/CYP3A4/CYP3A7/DHRS2/ESR1/FSHB/HSD17B3/HSD17B6/HSD17B8/HSD3B1/RDH10/RDH11/SCNN1B/SDR9C7/TSPO/UGT1A1/UGT1A7/UGT1A9/UGT2B10/UGT2B28/UGT2B4	36
cluster8	BP	GO:0045103	intermediate filament-based process	26/2556	90/18903	0.000101779	0.022343942	0.021217776	ATF2/DNAJB6/DSP/DST/ERBIN/KRT16/KRT19/KRT3/KRT34/KRT35/KRT37/KRT38/KRT4/KRT5/KRT6A/KRT6B/KRT71/KRT78/KRT82/KRT85/KRT9/MTM1/NES/PKP1/SYNM/TCHH	26
cluster8	BP	GO:0001654	eye development	79/2556	388/18903	0.000107252	0.02301037	0.021850614	ABCB5/ABI2/ACVR2B/ALDH1A3/AQP1/AQP5/ARHGEF15/BBS7/BCL11B/BIRC7/BMPR2/CDKN1B/CEP290/CLIC4/COL5A1/COL5A2/CRYBA2/CRYBA4/CRYBB2/CRYBG3/CRYGC/CYP1A1/DIO3/DLG1/DLL4/DZANK1/EFEMP1/EPHA2/FBN1/FBN2/FLT1/GDF11/GRHL3/HESX1/IFT122/IGFN1/IHH/IMPG2/INHBA/KDR/LENEP/MAX/MERTK/MFAP5/MYF5/NES/NF1/NKD1/NPHP1/NRP1/OSR2/PBX4/PITX2/PLAAT3/POU4F2/PROX1/PSEN1/PTPRM/RCN1/RDH10/RHOJ/RP1L1/RPGRIP1L/SERPINF1/SIX5/SLC7A11/SMOC1/SOX8/SPRED2/STAT3/STAU2/TBX2/TSKU/TSPAN12/TTC8/TWSG1/USP45/WNT2/WNT5B	79
cluster8	BP	GO:0019216	regulation of lipid metabolic process	72/2556	347/18903	0.000116068	0.02434852	0.02312132	AADAC/ACACB/ACER1/ADGRF5/ANGPTL3/APOA1/APOA2/APOB/APOC1/APOC2/APOE/AVPR1A/BCL11B/BRCA1/C1QTNF2/C3/CAPN2/CAV1/CCL21/CCN1/CERS2/CIDEA/CPT1A/CYP7A1/DAB2IP/DNAJC15/FABP1/FABP5/FBXW7/FGF1/FLT1/FMO1/FMO2/FSHB/GNB3/GOLM1/HPGD/IL1A/INSIG1/INSIG2/IRS1/KIT/LDLRAP1/MALRD1/MTMR2/NR4A3/NR5A1/ORMDL3/P2RY12/PCK1/PIK3IP1/PLPP1/PRKD1/PROX1/RAB38/RARRES2/RDH10/RGN/SERPINA3/SNAI1/SNAI2/SORBS1/SPHK1/STARD4/TBL1XR1/TEK/THRSP/TM6SF2/TNFAIP8L3/TSPO/UGT1A1/VAV3	72

cluster8	BP	GO:0035282	segmentation	29/2556	106/18903	0.000122128	0.025062812	0.02379961	AXIN2/BMI1/COBL/DKK1/EPB41L5/FOXF1/HOXA2/HOXD8/IRX1/IRX3/MEOX1/MEOX2/MIB1/MYF5/MYF6/NKD1/NRP1/OSR1/PCDH8/PSEN1/RIPPLY2/SEMA3C/SEMA3F/TBX18/TBX3/TCF15/TDRD10/TDRD15/TDRD6	29
cluster8	BP	GO:0003002	regionalization	74/2556	360/18903	0.000128058	0.025325927	0.024049464	ACVR2A/ACVR2B/AR/ATP6AP2/AXIN2/BMI1/BMP1/BMPR2/C3/COBL/CPLANE2/CRKL/CTNNBIP1/CYP26B1/DBX1/DKK1/DLL4/DYNC2H1/EDN1/EPB41L5/FGF1/FGF8/FOXF1/FOXH1/GATA5/GDF11/HES3/HEY1/HEY2/HOXA2/HOXA6/HOXA7/HOXB3/HOXD8/HOXD9/IRX1/IRX3/MEF2C/MEOX1/MEOX2/MIB1/MYF5/MYF6/NKD1/NRP1/NTF4/OSR1/OVOL2/PCDH8/PCGF2/PITX2/PRKACB/PROP1/PSEN1/RIPPLY2/RPGRIP1L/SEMA3C/SEMA3F/SETDB2/SMAD2/SMAD6/SNAI1/SOX17/TBX1/TBX18/TBX3/TCF15/TDRD10/TDRD15/TDRD6/TLL1/TMEM107/TP63/WNT2	74
cluster8	BP	GO:0007608	sensory perception of smell	90/2556	457/18903	0.000129349	0.025325927	0.024049464	B3GNT2/CNGA2/GFY/GJB4/OR10AG1/OR10C1/OR10G4/OR10G8/OR10G9/OR10H2/OR10P1/OR10R2/OR10S1/OR10X1/OR11H1/OR11H12/OR14C36/OR1C1/OR1N2/OR1Q1/OR2A12/OR2A14/OR2AE1/OR2AP1/OR2B2/OR2F2/OR2H2/OR2J3/OR2K2/OR2L3/OR2M2/OR2M5/OR2M7/OR2T1/OR2T2/OR2T35/OR2V2/OR4B1/OR4C15/OR4C5/OR4D2/OR4P4/OR4Q3/OR4S1/OR4S2/OR51A7/OR51E1/OR51F2/OR51J1/OR51S1/OR51T1/OR52D1/OR52E2/OR52I1/OR52M1/OR52N2/OR56A3/OR5A2/OR5AR1/OR5B12/OR5B2/OR5D13/OR5D18/OR5H15/OR5I1/OR5J2/OR5L1/OR5L2/OR5M9/OR5P3/OR5T2/OR6B3/OR6C1/OR6C2/OR6F1/OR6K2/OR6K6/OR6Q1/OR6S1/OR8G5/OR8K5/OR8U3/OR8U8/OR8U9/OR9A2/OR9G4/OR9G9/OR9Q2/TTC8/UGT2A1	90
cluster8	BP	GO:0090596	sensory organ morphogenesis	59/2556	272/18903	0.000134028	0.025325927	0.024049464	ABI2/ALDH1A3/AQP1/AQP5/CEP290/CHRNA9/COL5A1/COL5A2/CTHRC1/CYP26B1/DIO3/DZANK1/EDN1/EFEMP1/EPHA2/EYA1/FBN1/FBN2/FGF8/FOXI1/GATA2/GDF11/GRHL3/HESX1/HOXA2/IFT122/IGFN1/IHH/IMPG2/INSIG1/INSIG2/ITGA8/KDR/MFAP5/NF1/NKD1/OSR1/OSR2/PITX2/PROX1/PRRX1/PTPRM/RPGRIP1L/SLC44A4/SOX8/STAT3/STAU2/STRC/TBX1/TBX18/TBX2/TBX3/TPRN/TSKU/TSPAN12/TTC39C/TTC8/USH1G/WNT2	59

cluster8	BP	GO:0018149	peptide cross-linking	14/2556	36/18903	0.000134142	0.025325927	0.024049464	BGN/DCN/DSP/EGFLAM/FLG/FN1/SPRR1A/SPRR1B/SPRR2E/SPRR3/TGM5/TGM6/TGM7/THBS1	14
cluster8	BP	GO:0048705	skeletal system morphogenesis	51/2556	227/18903	0.000149856	0.027737982	0.026339948	ACVR2B/ATF2/AXIN2/BMI1/BMP1/BMPR2/COL1A1/COMP/CSRNP1/CYP26B1/DLG1/EYA1/FBN2/FGF18/FGF8/GLG1/HOXA2/HOXA7/HOXB3/HOXD8/HOXD9/IFITM5/IHH/INSIG1/INSIG2/MATN1/MEF2C/MGP/MMP2/MYF5/NDST1/OSR1/OSR2/PCGF2/PRRX1/PSEN1/RAB23/RDH10/RFLNB/RIPPLY2/SATB2/SCX/SETD2/SFRP4/SLC39A1/SMAD2/TBX1/TCF15/TGFB3/TMEM107/TSKU	51
cluster8	BP	GO:0003158	endothelium development	35/2556	139/18903	0.000160778	0.028813555	0.027361311	ACVR2B/ADAMTS12/AFDN/APOLD1/ATOH8/BMPR2/CD34/CDH5/CLIC4/COL15A1/DLL4/FGF1/GJA5/HEG1/HEY1/HEY2/HOXA13/KDR/MET/NRP1/PDCD10/PDE2A/PDE4D/PROX1/RAP2C/RDX/ROBO4/SLC40A1/SOX17/STARD13/TJP1/TMEM100/TNMD/VCL/ZDHHC21	35
cluster8	BP	GO:0001822	kidney development	65/2556	309/18903	0.000161771	0.028813555	0.027361311	ACVR2B/ADAMTS1/ADAMTS16/AGTR1/AQP1/AQP2/ARL3/ASS1/C1GALT1/CASP9/CD24/CD34/CENPF/CEP290/CNTRL/CTNNBIP1/CYP26B1/CYP4A11/CYP4A22/DLG1/DYNC2H1/ENPEP/EYA1/FBN1/FGF1/FGF8/GDF11/GPR4/HPGD/HS2ST1/IRX1/IRX3/ITGA8/KANK2/KCNJ8/LIF/LIN28A/MEF2C/MTSS1/NF1/NPNT/NRP1/ODC1/OSR1/OSR2/PCSK9/PKD2/PROX1/RDH10/REN/RGN/RIDA/RPGRIP1L/SERPINF1/SIM1/SMAD2/SMAD6/SOX17/SOX8/TCF21/TEK/TNS2/TRAF3IP1/WNK4/WWTR1	65

cluster8	BP	GO:0048568	embryonic organ development	88/2556	449/18903	0.000181243	0.031289588	0.029712548	ALDH1A3/APLNR/BBS7/BMI1/CCN1/CEP290/CHRNA9/COBL/CTHRC1/CXCL8/DLG1/DNAJB6/E2F8/EDN1/EFEMP1/EPAS1/EPHA2/EYA1/FBN1/FBN2/FGF8/FOXF1/FOXH1/FOXI1/GATA2/GJB5/GRHL3/HAND1/HAND2/HESX1/HEY1/HEY2/HOXA2/HOXA7/HOXB3/HOXD9/IFT122/IHH/IL3/INSIG1/INSIG2/ITGA8/KDR/KIT/KRT19/LIF/MEF2C/MFAP5/MIB1/MYF5/NDST1/NES/OSR1/OSR2/OVOL2/PBX4/PCGF2/PITX2/PKD2/PLK4/PROX1/PRRX1/PSEN1/RARRES2/RDH10/RNF112/SATB2/SETD2/SETDB2/SLC39A1/SLC44A4/SMAD2/SNAI1/SOX17/STRC/TBX1/TBX18/TBX2/TBX3/TCF21/TEAD4/TGFB3/TPO/TPRN/TTC39C/USH1G/WNT2/ZFPM2	88
cluster8	BP	GO:0042310	vasoconstriction	24/2556	83/18903	0.000182302	0.031289588	0.029712548	ADRA2C/AGTR1/AVPR1A/AVPR2/BDKRB2/BMPR2/CAV1/COMP/EDN1/EDN3/FGA/FGB/FGG/GJA5/HRH1/KCNMB4/MMP2/OXTR/PER2/PIK3C2A/RAP1GDS1/SCNN1B/SMTNL2/ZDHHC21	24
cluster8	BP	GO:0019229	regulation of vasoconstriction	20/2556	64/18903	0.000195561	0.032684432	0.03103709	ADRA2C/AGTR1/AVPR1A/AVPR2/BDKRB2/BMPR2/CAV1/EDN1/EDN3/FGA/FGB/FGG/GJA5/HRH1/KCNMB4/MMP2/OXTR/PER2/SMTNL2/ZDHHC21	20
cluster8	BP	GO:0051216	cartilage development	46/2556	201/18903	0.000197353	0.032684432	0.03103709	ADAMTS12/ARID5A/ATF2/AXIN2/BGN/BMP1/BMPR2/CCN1/CCN4/CHI3L1/CNMD/COL11A2/COL1A1/COMP/EDN1/EFEMP1/FGF18/GLG1/HAND1/HAND2/HOXB3/IHH/LOXL2/MATN1/MBOAT2/MEF2C/MGP/MUSTN1/MYF5/NFIB/OSR1/OSR2/PBXIP1/PRKG2/PRRX1/PTHLH/RFLNB/SATB2/SCIN/SCX/SLC39A14/SNAI1/SNAI2/TSKU/TWSG1/WNT5B	46

cluster8	BP	GO:0045109	intermediate filament organization	21/2556	69/18903	0.00020701	0.033201962	0.031528536	DNAJB6/DSP/KRT16/KRT19/KRT3/KRT34/KRT35/KRT37/KRT38/KRT4/KRT5/KRT6A/KRT6B/KRT71/KRT78/KRT82/KRT85/KRT9/MTM1/PKP1/TCHH	21
cluster8	BP	GO:0048562	embryonic organ morphogenesis	62/2556	294/18903	0.00021044	0.033201962	0.031528536	ALDH1A3/APLNR/BBS7/BMI1/CEP290/CHRNA9/CTHRC1/DLG1/EDN1/EFEMP1/EPHA2/EYA1/FBN1/FBN2/FGF8/FOXF1/FOXH1/FOXI1/GATA2/GRHL3/HAND1/HAND2/HESX1/HOXA2/HOXA7/HOXB3/HOXD9/IHH/INSIG1/INSIG2/ITGA8/MEF2C/MFAP5/MIB1/MYF5/NDST1/OSR1/OSR2/OVOL2/PCGF2/PKD2/PROX1/PRRX1/PSEN1/RDH10/SATB2/SETD2/SETDB2/SLC39A1/SLC44A4/SMAD2/SOX17/STRC/TBX1/TBX18/TBX2/TBX3/TCF21/TGFB3/TPRN/TTC39C/USH1G	62
cluster8	BP	GO:0055078	sodium ion homeostasis	18/2556	55/18903	0.000211029	0.033201962	0.031528536	AGTR1/ATP12A/ATP1A4/ATP4A/ATP4B/AVPR1A/AVPR2/CORIN/CYP4A11/EDN1/IL1A/MC3R/NPPB/NPR1/SCN7A/SCNN1B/SLC12A2/SLC9A1	18
cluster8	BP	GO:0035924	cellular response to vascular endothelial growth factor stimulus	22/2556	74/18903	0.000215192	0.033301858	0.031623396	ADAMTS12/CADM4/DAB2IP/DCN/DLL4/EGR3/ERN1/FLT1/HRG/IL12B/JCAD/KDR/MT1G/NRP1/PIK3CB/PRKD1/RAMP2/SMOC2/SPHK1/TSPAN12/VCAM1/VEGFC	22

cluster8	BP	GO:0003382	epithelial cell morphogenesis	13/2556	34/18903	0.000282854	0.042383201	0.040247027	AR/BCL11B/CLIC4/COL15A1/EPB41L5/FRMD6/HEG1/HOXA13/IHH/MET/PALLD/POF1B/TNMD	13
cluster8	BP	GO:0042573	retinoic acid metabolic process	13/2556	34/18903	0.000282854	0.042383201	0.040247027	ADH1B/ADH6/ALDH1A3/CYP1A1/CYP26B1/CYP2C18/CYP2C8/CYP3A4/CYP3A7/RDH10/UGT1A1/UGT1A7/UGT1A9	13
cluster8	BP	GO:0010259	multicellular organism aging	11/2556	26/18903	0.000294529	0.043442982	0.041253393	AVPR1A/COMP/DDC/EDN1/GNA13/INHBA/NR5A1/SEC63/SERP1/TP63/WRN	11
cluster8	BP	GO:0050911	detection of chemical stimulus involved in sensory perception of smell	84/2556	431/18903	0.000302964	0.043999696	0.041782049	OR10AG1/OR10C1/OR10G4/OR10G8/OR10G9/OR10H2/OR10P1/OR10R2/OR10S1/OR10X1/OR11H1/OR11H12/OR14C36/OR1C1/OR1N2/OR1Q1/OR2A12/OR2A14/OR2AE1/OR2AP1/OR2B2/OR2F2/OR2H2/OR2J3/OR2K2/OR2L3/OR2M2/OR2M5/OR2M7/OR2T1/OR2T2/OR2T35/OR2V2/OR4B1/OR4C15/OR4C5/OR4D2/OR4P4/OR4Q3/OR4S1/OR4S2/OR51A7/OR51E1/OR51F2/OR51J1/OR51S1/OR51T1/OR52D1/OR52E2/OR52I1/OR52M1/OR52N2/OR56A3/OR5A2/OR5AR1/OR5B12/OR5B2/OR5D13/OR5D18/OR5H15/OR5I1/OR5J2/OR5L1/OR5L2/OR5M9/OR5P3/OR5T2/OR6B3/OR6C1/OR6C2/OR6F1/OR6K2/OR6K6/OR6Q1/OR6S1/OR8G5/OR8K5/OR8U3/OR8U8/OR8U9/OR9A2/OR9G4/OR9G9/OR9Q2	84

cluster8	BP	GO:0010810	regulation of cell-substrate adhesion	49/2556	222/18903	0.000310743	0.04403558	0.041816124	ABI3BP/ACER2/AP1AR/APOA1/ARHGEF7/BRAF/CCDC80/CCL21/CCN1/CDK6/CLASP2/COL1A1/CRKL/CX3CL1/DLC1/DOCK1/DUSP22/EGFLAM/EPB41L5/FAM107A/FGA/FGB/FGG/FN1/FOXF1/HOXA7/HRG/KDR/LIMCH1/LIMS2/MELTF/MYF5/NF1/NPNT/NRP1/ONECUT1/ONECUT2/PHLDB2/PIK3CB/PTPRA/RREB1/SLC9A1/SPRY4/ST6GAL1/TEK/THBS1/UNC13D/VCL/VTN	49
cluster8	BP	GO:0043010	camera-type eye development	69/2556	340/18903	0.000312541	0.04403558	0.041816124	ABI2/ACVR2B/ALDH1A3/AQP1/AQP5/ARHGEF15/BCL11B/BIRC7/BMPR2/CDKN1B/CEP290/CLIC4/CRYBA2/CRYBA4/CRYBB2/CRYBG3/CRYGC/CYP1A1/DIO3/DLG1/DLL4/EFEMP1/EPHA2/FBN1/FBN2/FLT1/GDF11/GRHL3/HESX1/IFT122/IGFN1/IHH/IMPG2/INHBA/KDR/LENEP/MAX/MERTK/MYF5/NES/NF1/NPHP1/NRP1/OSR2/PITX2/PLAAT3/POU4F2/PROX1/PSEN1/PTPRM/RCN1/RDH10/RHOJ/RP1L1/RPGRIP1L/SERPINF1/SIX5/SLC7A11/SOX8/SPRED2/STAT3/TBX2/TSKU/TSPAN12/TTC8/TWSG1/USP45/WNT2/WNT5B	69
cluster8	BP	GO:0014706	striated muscle tissue development	55/2556	257/18903	0.000317234	0.044039547	0.041819891	ACADM/ADAMTS9/ALPK2/ALPK3/ANKRD1/CENPF/DKK1/DLL4/DSP/EDN1/EYA1/FGF20/FGF8/FOXH1/GATA5/GATA6/GJA1/GJA5/HAMP/HAND1/HEG1/HEY1/HEY2/IFRD1/IGF1/IRX3/JARID2/KCNK2/MEF2A/MEF2C/MRTFB/MTPN/MYL2/MYL3/MYLK2/MYLK3/NDUFV2/NEBL/NOX4/NPPB/PARP2/PDLIM5/PGM5/POU4F1/PROX1/SEMA3C/SGCB/SLC9A1/SORBS2/TBX18/TBX2/TBX3/WNT2/XIRP2/ZFPM2	55
cluster8	BP	GO:0007265	Ras protein signal transduction	71/2556	353/18903	0.000338648	0.046330955	0.043995808	ABCA1/ABI2/AGTR1/APOA1/APOE/ARFGEF2/ARFGEF3/ARHGAP29/ARHGAP5/ARHGEF10/ARHGEF25/ARHGEF28/BRAP/CADM4/CCNA2/CDC42EP5/CDK2/COL1A2/CRKL/DAB2IP/DENND4A/DLC1/DNMT1/ERAS/ERBIN/FOXM1/GARRE1/GNA13/GPR4/GRAP2/HACE1/HEG1/IGF1/IQGAP3/KANK2/LAT/LPAR4/MCF2L/MET/MRAS/NET1/NF1/NRAS/NRP1/PIK3CB/PLK2/PPP2CB/PRKD1/RAB12/RAB30/RAB39A/RAB4A/RAB9B/RALA/RAP2C/RAPGEF4/RAPGEF5/RASA3/RASIP1/RDX/RERG/RGL1/RHOJ/RIPOR2/SPRY4/SSX2IP/STARD13/STARD8/SYDE1/TIAM1/TNFAIP1	71

cluster8	BP	GO:0043589	skin morphogenesis	7/2556	12/18903	0.00034781	0.04690461	0.04454055	CDSN/COL1A1/COL1A2/ERRFI1/ITGB4/PSEN1/TP63	7
cluster8	BP	GO:0007506	gonadal mesoderm development	6/2556	9/18903	0.000353643	0.047019601	0.044649746	TSPY1/TSPY10/TSPY3/TSPY4/TSPY8/ZFPM2	6
cluster8	BP	GO:0050907	detection of chemical stimulus involved in sensory perception	91/2556	477/18903	0.000369353	0.047817238	0.045407181	AZGP1/GNAT3/OR10AG1/OR10C1/OR10G4/OR10G8/OR10G9/OR10H2/OR10P1/OR10R2/OR10S1/OR10X1/OR11H1/OR11H12/OR14C36/OR1C1/OR1N2/OR1Q1/OR2A12/OR2A14/OR2AE1/OR2AP1/OR2B2/OR2F2/OR2H2/OR2J3/OR2K2/OR2L3/OR2M2/OR2M5/OR2M7/OR2T1/OR2T2/OR2T35/OR2V2/OR4B1/OR4C15/OR4C5/OR4D2/OR4P4/OR4Q3/OR4S1/OR4S2/OR51A7/OR51E1/OR51F2/OR51J1/OR51S1/OR51T1/OR52D1/OR52E2/OR52I1/OR52M1/OR52N2/OR56A3/OR5A2/OR5AR1/OR5B12/OR5B2/OR5D13/OR5D18/OR5H15/OR5I1/OR5J2/OR5L1/OR5L2/OR5M9/OR5P3/OR5T2/OR6B3/OR6C1/OR6C2/OR6F1/OR6K2/OR6K6/OR6Q1/OR6S1/OR8G5/OR8K5/OR8U3/OR8U8/OR8U9/OR9A2/OR9G4/OR9G9/OR9Q2/RTP3/TAS1R2/TAS2R3/TAS2R50/TRPV1	91
cluster8	BP	GO:0010171	body morphogenesis	16/2556	48/18903	0.000369773	0.047817238	0.045407181	ASPH/ATP6AP2/BRAF/CLASP2/COL1A1/CSRNP1/DKK1/IFT122/IHH/MMP2/PAX9/PHLDB1/PHLDB2/SCX/TBX1/TGFB3	16

cluster8	CC	GO:0062023	collagen-containing extracellular matrix	111/2706	433/19869	1.26E-11	1.46E-08	1.41E-08	ABI3BP/ADAMTS1/ADAMTS5/ADAMTS9/AMBP/AMTN/ANGPTL3/APCS/APOA1/APOE/APOH/AZGP1/BCAM/BGN/C1QB/C1QC/CBLN4/CCDC80/CCN1/CLEC3B/COL11A2/COL15A1/COL17A1/COL1A1/COL1A2/COL5A1/COL5A2/COL6A3/COL7A1/COMP/CTHRC1/CTSD/CXCL12/DCN/DLG1/DPT/DST/EFEMP1/EGFLAM/ELN/ERBIN/F7/F9/FBN1/FBN2/FCN2/FCN3/FGA/FGB/FGG/FGL1/FLG/FMOD/FN1/GPC5/HMCN1/HRG/HRNR/IFNA2/IMPG2/INHBE/ITGB4/ITIH5/LAMA2/LAMA4/LAMC1/LMAN1L/LOXL2/LRRC15/LUM/MATN1/MFAP5/MGP/MMP2/MMRN2/MUC17/MUC2/NPNT/NTN4/NTN5/OTOL1/POSTN/PRG4/RARRES2/RTBDN/SCARA3/SERPINA3/SERPINA5/SERPINB8/SERPINF1/SMOC1/SMOC2/SOD3/SOST/SPARC/SPARCL1/SPON1/SRPX/SSC5D/TECTA/TGFB3/THBS1/THBS2/THBS4/TIMP3/TINAG/TINAGL1/TMEFF1/VTN/WNT2/WNT5B	111
cluster8	CC	GO:0045177	apical part of cell	100/2706	435/19869	6.04E-08	0.0000351	0.0000339	ABCC4/ADAM17/ADGRF5/ANO1/AQP1/AQP10/AQP2/AQP5/AQP8/ATP12A/ATP4A/ATP4B/ATP6AP2/ATP6V0D2/ATP6V1E1/ATP8B1/C1QTNF5/CD300LG/CD34/CEACAM7/CETN2/CLCA4/CLCN3/CLIC4/CLIC5/CYP4A11/DDR2/DLG1/DPEP1/DSG1/DYNC2H1/DYNC2LI1/ENPEP/FABP1/FABP2/FN1/FXYD1/GJA1/GNAT3/GPIHBP1/HAMP/ITGA8/KCNA1/KCNK1/KCNK2/KL/MTCL1/MUC17/MUC20/MYO1B/OSMR/OTOA/OTOG/OXTR/PALS1/PARD3B/PARD6G/PDE4D/PFKM/PLAT/PLPP1/PRKG2/PSEN1/PTPRH/RAB17/RDX/REN/RIPOR2/SCNN1B/SHROOM4/SLC10A2/SLC12A2/SLC16A1/SLC17A1/SLC22A2/SLC22A8/SLC26A3/SLC26A4/SLC2A2/SLC30A5/SLC39A14/SLC3A1/SLC44A4/SLC4A7/SLC5A12/SLC5A6/SLC6A18/SLC7A11/SLC7A13/SLC9A1/SLC9A3R2/SLCO2B1/SORBS2/STK39/TEK/TJP1/TMEM235/TNIK/UPK1B/VCAM1	100
cluster8	CC	GO:0005604	basement membrane	34/2706	99/19869	0.000000132	0.0000512	0.0000494	ADAMTS1/AMTN/CCDC80/COL15A1/COL17A1/COL5A1/COL7A1/DLG1/DST/EGFLAM/ERBIN/FBN1/FN1/HMCN1/ITGB4/LAMA2/LAMA4/LAMC1/LOXL2/MMRN2/NPNT/NTN4/NTN5/OTOL1/SERPINF1/SMOC1/SMOC2/SPARC/THBS2/THBS4/TIMP3/TINAG/TMEFF1/VTN	34
cluster8	CC	GO:0016324	apical plasma membrane	84/2706	368/19869	0.00000092	0.000267377	0.000258198	ABCC4/ADAM17/ANO1/AQP1/AQP10/AQP2/AQP5/ATP12A/ATP4A/ATP4B/ATP6AP2/ATP6V0D2/ATP6V1E1/ATP8B1/C1QTNF5/CD300LG/CD34/CEACAM7/CLCA4/CLCN3/CLIC5/CYP4A11/DDR2/DLG1/DPEP1/DSG1/ENPEP/FN1/FXYD1/GJA1/GNAT3/GPIHBP1/KCNA1/KCNK1/KCNK2/KL/MTCL1/MUC17/MUC20/OSMR/OTOA/OTOG/OXTR/PALS1/PARD3B/PARD6G/PDE4D/PFKM/PLPP1/PRKG2/PSEN1/PTPRH/RAB17/RDX/RIPOR2/SCNN1B/SHROOM4/SLC10A2/SLC12A2/SLC16A1/SLC17A1/SLC22A2/SLC22A8/SLC26A3/SLC26A4/SLC2A2/SLC30A5/SLC39A14/SLC3A1/SLC44A4/SLC4A7/SLC5A12/SLC5A6/SLC6A18/SLC7A13/SLC9A1/SLC9A3R2/SLCO2B1/SORBS2/STK39/TEK/TMEM235/TNIK/UPK1B	84

cluster8	CC	GO:0005938	cell cortex	71/2706	312/19869	0.00000696	0.001618151	0.001562596	ADD3/AKAP12/ANLN/AQP7/ARHGEF7/ASPH/C2CD5/CALD1/CAPN2/CAPZA3/CAV1/CLASP2/CLIC5/COBL/CTTN/DLC1/DLG4/DST/ECT2/EMD/ENO2/FABP1/FABP2/FAM110A/FGB/FGF1/FRMPD1/GLRX3/GYS2/HAMP/HMCN1/ITPR2/KRT19/MICAL3/MTSS2/MYO10/MYRIP/MYZAP/PAFAH1B1/PARD3B/PARD6G/PHLDB1/PHLDB2/PKD2/PLEKHH2/PPFIA3/PRKD1/PSEN1/RAI14/RDX/RHOBTB3/RIC8B/RIMS1/RND1/RYR1/SCIN/SELE/SEPTIN10/SEPTIN11/SEPTIN12/SEPTIN2/SEPTIN6/SEPTIN8/SHROOM4/SPINK5/SPIRE1/SPTA1/STXBP6/TLE6/UNC13B/WASL	71
cluster8	CC	GO:0035869	ciliary transition zone	24/2706	71/19869	0.0000122	0.002358449	0.002277477	ARL3/BBS9/CEP290/CETN1/CETN2/CETN3/CPLANE1/CPLANE2/DYNC2LI1/IFT122/KIF17/KIFAP3/MACIR/NPHP1/RP1L1/RPGRIP1L/SEPTIN2/SPATA7/TCTN2/TMEM107/TMEM67/TRAF3IP1/TTC8/USH1G	24
cluster8	CC	GO:0001533	cornified envelope	20/2706	59/19869	0.0000606	0.010062831	0.009717345	CAPN1/CASP14/CDSN/DSC3/DSG1/DSP/FLG/FLG2/HRNR/KLK7/KRT16/PKP1/RPTN/SPRR1A/SPRR1B/SPRR2B/SPRR2D/SPRR2E/SPRR3/TCHH	20
cluster8	CC	GO:0042627	chylomicron	8/2706	13/19869	0.0000795	0.011550573	0.011154009	APOA1/APOA2/APOB/APOC1/APOC2/APOE/APOH/LPL	8

cluster8	CC	GO:0034361	very-low-density lipoprotein particle	10/2706	20/19869	0.000107915	0.012352612	0.011928512	APOA1/APOA2/APOB/APOC1/APOC2/APOE/APOH/APOM/LPL/VLDLR	10
cluster8	CC	GO:0034385	triglyceride-rich plasma lipoprotein particle	10/2706	20/19869	0.000107915	0.012352612	0.011928512	APOA1/APOA2/APOB/APOC1/APOC2/APOE/APOH/APOM/LPL/VLDLR	10
cluster8	CC	GO:0042383	sarcolemma	35/2706	136/19869	0.000116935	0.012352612	0.011928512	AHNAK2/AQP1/AQP4/BGN/CACNA1D/CACNG1/CAMK2D/CASQ1/CAV1/COL6A3/DLG1/FKRP/FLNC/FXYD1/KCNJ11/KCNJ8/KRT19/LAMA2/NCSTN/NOS1/PDE9A/PGM5/PSEN1/RDX/RYR1/SGCB/SGCE/SLC9A1/SLMAP/SNTB1/STAC/SYNM/TGFB3/VCAM1/VCL	35
cluster8	CC	GO:0005577	fibrinogen complex	6/2706	8/19869	0.000138849	0.013445231	0.012983618	FGA/FGB/FGG/FGL1/FN1/THBS1	6

cluster8	CC	GO:0005788	endoplasmic reticulum lumen	66/2706	312/19869	0.000151521	0.013543659	0.013078667	ADAM17/ADAMTS5/AFP/ALB/AMTN/APOA1/APOA2/APOB/APOE/ARSK/BACE1/BMP15/C3/CALU/CASQ1/CCN1/COL11A2/COL15A1/COL17A1/COL1A1/COL1A2/COL5A1/COL5A2/COL6A3/COL7A1/COLGALT2/EDN1/F10/F7/F9/FAM20C/FBN1/FGA/FGG/FKBP14/FMO1/FN1/GOLM1/HSPA5/IGFBP5/IL12B/KTN1/LAMC1/LRPAP1/MELTF/MEPE/MTTP/MXRA8/NOTUM/NUCB1/P4HA3/PCSK9/POGLUT2/POGLUT3/RCN1/SERPINA10/SPARCL1/SPON1/SRL/SUMF2/TGOLN2/THBS1/TSPAN15/TXNDC16/VTN/WNT5B	66
cluster8	CC	GO:0032391	photoreceptor connecting cilium	15/2706	41/19869	0.000189603	0.015737043	0.015196746	ARL3/CEP290/CETN1/CETN2/CETN3/IFT122/KIF17/KIFAP3/NPHP1/RP1L1/RPGRIP1L/SEPTIN2/SPATA7/TTC8/USH1G	15
cluster8	CC	GO:0032432	actin filament bundle	23/2706	79/19869	0.0002457	0.018351964	0.017721889	FAM107A/GAS2L2/LIMA1/LIMCH1/MST1R/MYH14/NEBL/PALLD/PDLIM3/PDLIM4/PDLIM5/PGM5/PLS3/RFLNB/SEPTIN11/SEPTIN12/SHROOM4/SORBS1/SYNPO/SYNPO2/TEK/VIL1/XIRP2	23
cluster8	CC	GO:0045111	intermediate filament cytoskeleton	55/2706	253/19869	0.000252695	0.018351964	0.017721889	CASP14/DSP/DST/HOXA13/HSDL1/KRT16/KRT19/KRT3/KRT34/KRT35/KRT37/KRT38/KRT4/KRT5/KRT6A/KRT6B/KRT71/KRT78/KRT82/KRT85/KRT9/KRTAP1-3/KRTAP12-2/KRTAP13-1/KRTAP13-4/KRTAP19-3/KRTAP19-6/KRTAP19-8/KRTAP20-1/KRTAP20-2/KRTAP20-3/KRTAP23-1/KRTAP26-1/KRTAP3-3/KRTAP4-11/KRTAP4-3/KRTAP4-5/KRTAP4-9/KRTAP5-10/KRTAP5-6/KRTAP6-1/KRTAP6-2/KRTAP6-3/KRTAP9-3/LDLRAP1/LMNB2/MDN1/NES/NR1I2/NRP1/PHLDB2/PJA2/PKP1/SESTD1/SYNM	55

cluster8	CC	GO:0005882	intermediate filament	48/2706	215/19869	0.000319849	0.021862647	0.021112041	CASP14/DSP/DST/KRT16/KRT19/KRT3/KRT34/KRT35/KRT37/KRT38/KRT4/KRT5/KRT6A/KRT6B/KRT71/KRT78/KRT82/KRT85/KRT9/KRTAP1-3/KRTAP12-2/KRTAP13-1/KRTAP13-4/KRTAP19-3/KRTAP19-6/KRTAP19-8/KRTAP20-1/KRTAP20-2/KRTAP20-3/KRTAP23-1/KRTAP26-1/KRTAP3-3/KRTAP4-11/KRTAP4-3/KRTAP4-5/KRTAP4-9/KRTAP5-10/KRTAP5-6/KRTAP6-1/KRTAP6-2/KRTAP6-3/KRTAP9-3/LDLRAP1/LMNB2/NES/NRP1/PKP1/SYNM	48
cluster8	CC	GO:0005581	collagen trimer	24/2706	86/19869	0.000366844	0.023681841	0.022868776	C1QB/C1QC/C1QTNF1/C1QTNF2/C1QTNF3/C1QTNF5/C1QTNF9/C1QTNF9B/COL11A2/COL15A1/COL17A1/COL1A1/COL1A2/COL5A1/COL5A2/COL6A3/COL7A1/COLEC11/CTHRC1/FCN2/FCN3/LUM/OTOL1/SCARA3	24
cluster8	CC	GO:0032994	protein-lipid complex	14/2706	39/19869	0.000387281	0.023685282	0.0228721	APOA1/APOA2/APOB/APOC1/APOC2/APOE/APOH/APOM/LPA/LPL/PEX3/PON1/SAA2/VLDLR	14
cluster8	CC	GO:0042641	actomyosin	22/2706	78/19869	0.000539685	0.030733853	0.029678673	CDC42BPA/CDC42BPB/FAM107A/GAS2L2/LIMA1/LIMCH1/MST1R/MYH14/NEBL/PALLD/PDLIM3/PDLIM4/PDLIM5/PGM5/SEPTIN11/SEPTIN12/SHROOM4/SORBS1/SYNPO/SYNPO2/TEK/XIRP2	22

cluster8	CC	GO:0034358	plasma lipoprotein particle	13/2706	36/19869	0.00058188	0.030733853	0.029678673	APOA1/APOA2/APOB/APOC1/APOC2/APOE/APOH/APOM/LPA/LPL/PON1/SAA2/VLDLR	13
cluster8	CC	GO:1990777	lipoprotein particle	13/2706	36/19869	0.00058188	0.030733853	0.029678673	APOA1/APOA2/APOB/APOC1/APOC2/APOE/APOH/APOM/LPA/LPL/PON1/SAA2/VLDLR	13
cluster8	CC	GO:0001725	stress fiber	20/2706	70/19869	0.00079239	0.038364861	0.037047687	FAM107A/GAS2L2/LIMA1/LIMCH1/MST1R/MYH14/NEBL/PALLD/PDLIM3/PDLIM4/PDLIM5/PGM5/SEPTIN11 /SEPTIN12/SHROOM4/SORBS1/SYNPO/SYNPO2/TEK/XIRP2	20
cluster8	CC	GO:0097517	contractile actin filament bundle	20/2706	70/19869	0.00079239	0.038364861	0.037047687	FAM107A/GAS2L2/LIMA1/LIMCH1/MST1R/MYH14/NEBL/PALLD/PDLIM3/PDLIM4/PDLIM5/PGM5/SEPTIN11 /SEPTIN12/SHROOM4/SORBS1/SYNPO/SYNPO2/TEK/XIRP2	20

cluster8	MF	GO:0005201	extracellular matrix structural constituent	56/2622	173/18432	9.42E-10	0.00000206	0.00000201	ABI3BP/BGN/CCN1/CHI3L1/COL11A2/COL15A1/COL17A1/COL1A1/COL1A2/COL5A1/COL5A2/COL6A3/COL7A1/COMP/CTHRC1/DCN/DPT/EFEMP1/ELN/FBN1/FBN2/FGA/FGB/FGG/FMOD/FN1/HMCN1/IMPG2/LAMA2/LAMA4/LAMC1/LUM/MATN1/MEPE/MFAP5/MGP/MMRN2/MUC17/MUC3A/MUC4/MUC6/NPNT/OIT3/OTOL1/POSTN/PRG4/SPARC/SPON1/SRPX/TECTA/TFPI2/THBS1/THBS2/TINAGL1/VTN/ZPLD1	56
cluster8	MF	GO:0050840	extracellular matrix binding	22/2622	56/18432	0.00000341	0.00372881	0.003646499	ADAM9/ADAMTS5/ADGRG6/BCAM/BGN/CCN1/DCN/DMBT1/ELN/ITGAV/LRRC15/NTN4/OLFML2A/SMOC1/SMOC2/SPARC/SPARCL1/SSC5D/THBS1/THSD1/TINAGL1/VTN	22
cluster8	MF	GO:0008201	heparin binding	45/2622	171/18432	0.0000222	0.016131535	0.01577544	ADA2/ADAMTS1/ADAMTS5/ANGPTL3/APOB/APOE/APOH/CCDC80/CCL15/CCN1/CCN4/CCN5/CLEC3B/COL5A1/COMP/CXCL11/CXCL8/DEFB106A/DEFB106B/FBN1/FGF1/FGFBP1/FN1/GPNMB/HBEGF/HRG/IMPG2/LPA/LPL/LRPAP1/MSTN/NRP1/PAFAH1B1/POSTN/SERPINA10/SERPINA5/SMOC1/SMOC2/SOD3/SOST/THBS1/THBS2/THBS4/TWSG1/VTN	45
cluster8	KEGG	hsa00982	Drug metabolism - cytochrome P450	26/1223	72/8779	0.00000187	0.000339452	0.000323388	ADH1B/ADH4/ADH6/ALDH3A1/AOX1/CYP2A6/CYP2C8/CYP2C9/CYP2E1/CYP3A4/FMO1/FMO2/GSTA1/GSTA2/GSTA3/MAOA/MGST2/UGT1A1/UGT1A10/UGT1A7/UGT1A9/UGT2A1/UGT2A3/UGT2B10/UGT2B28/UGT2B4	26

cluster8	KEGG	hsa00830	Retinol metabolism	25/1223	68/8779	0.00000201	0.000339452	0.000323388	ADH1B/ADH4/ADH6/ALDH1A3/AOX1/CYP1A1/CYP26B1/CYP2A6/CYP2C18/CYP2C8/CYP2C9/CYP3A4/CYP3A7/HSD17B6/RDH10/RDH11/UGT1A1/UGT1A10/UGT1A7/UGT1A9/UGT2A1/UGT2A3/UGT2B10/UGT2B28/UGT2B4	25
cluster8	KEGG	hsa05204	Chemical carcinogenesis - DNA adducts	24/1223	70/8779	0.0000129	0.001337663	0.001274363	AKR1C2/CYP1A1/CYP2A6/CYP2C18/CYP2C8/CYP2C9/CYP2E1/CYP3A4/CYP3A7/GSTA1/GSTA2/GSTA3/MGST2/NAT2/SULT2A1/UGT1A1/UGT1A10/UGT1A7/UGT1A9/UGT2A1/UGT2A3/UGT2B10/UGT2B28/UGT2B4	24
cluster8	KEGG	hsa00140	Steroid hormone biosynthesis	22/1223	62/8779	0.0000159	0.001337663	0.001274363	AKR1C2/AKR1D1/CYP11A1/CYP19A1/CYP1A1/CYP2E1/CYP3A4/CYP3A7/CYP7A1/HSD17B3/HSD17B6/HSD17B8/HSD3B1/UGT1A1/UGT1A10/UGT1A7/UGT1A9/UGT2A1/UGT2A3/UGT2B10/UGT2B28/UGT2B4	22
cluster8	KEGG	hsa04740	Olfactory transduction	92/1223	441/8779	0.0000279	0.001883718	0.001794577	ANO2/CALML5/CAMK2D/CNGA2/GNG13/OR10AG1/OR10C1/OR10G4/OR10G8/OR10G9/OR10H2/OR10P1/OR10R2/OR10S1/OR10X1/OR11H1/OR11H12/OR14C36/OR1C1/OR1N2/OR1Q1/OR2A12/OR2A14/OR2AE1/OR2AP1/OR2B2/OR2F2/OR2H2/OR2J3/OR2K2/OR2L3/OR2M2/OR2M5/OR2M7/OR2T1/OR2T2/OR2T35/OR2V2/OR4B1/OR4C15/OR4C5/OR4D2/OR4N4C/OR4P4/OR4Q3/OR4S1/OR4S2/OR51A7/OR51E1/OR51F2/OR51S1/OR51T1/OR52D1/OR52E2/OR52I1/OR52M1/OR52N2/OR56A3/OR5A2/OR5AR1/OR5B12/OR5B2/OR5D13/OR5D18/OR5H15/OR5I1/OR5J2/OR5L1/OR5L2/OR5M9/OR5P3/OR5T2/OR6B3/OR6C1/OR6C2/OR6F1/OR6K2/OR6K6/OR6Q1/OR6S1/OR8G5/OR8K5/OR8U3/OR8U8/OR8U9/OR9A2/OR9G4/OR9G9/OR9Q2/PDE2A/PRKACB/PRKG2	92

cluster8	KEGG	hsa04979	Cholesterol metabolism	18/1223	51/8779	0.000100553	0.005647745	0.005380485	ABCA1/ANGPTL3/APOA1/APOA2/APOB/APOC1/APOC2/APOE/APOH/CYP7A1/LDLRAP1/LPA/LPL/LRPAP1/NCEH1/PCSK9/TSPO/VDAC3	18
cluster8	KEGG	hsa04820	Cytoskeleton in muscle cells	52/1223	232/8779	0.000262315	0.011335599	0.010799181	ACTG2/AMPD1/ANKRD1/ATP1A4/BGN/CAPZA3/CKM/COL11A2/COL1A1/COL1A2/COL5A1/COL5A2/COL6A3/COMP/DCN/DSP/ELN/EMD/ENO2/FBN1/FBN2/FLNC/FN1/ITGA1/ITGA8/ITGA9/ITGAV/ITGB4/ITGB6/LAMA2/LMNB2/LMOD1/MYBPC2/MYH14/MYH15/MYL2/MYL3/MYOM1/MYOM3/NEBL/PDLIM3/PDLIM4/PDLIM5/SGCB/SGCE/SNTB1/SYNPO2/THBS1/THBS2/THBS4/VCL/XIRP2	52
cluster8	KEGG	hsa00980	Metabolism of xenobiotics by cytochrome P450	23/1223	78/8779	0.000269094	0.011335599	0.010799181	ADH1B/ADH4/ADH6/ALDH3A1/CYP1A1/CYP2A6/CYP2C9/CYP2E1/CYP3A4/GSTA1/GSTA2/GSTA3/MGST2/SULT2A1/UGT1A1/UGT1A10/UGT1A7/UGT1A9/UGT2A1/UGT2A3/UGT2B10/UGT2B28/UGT2B4	23
cluster8	KEGG	hsa04976	Bile secretion	25/1223	89/8779	0.000341264	0.012778433	0.012173738	ABCC4/AQP1/AQP4/AQP8/ATP1A4/BAAT/CYP3A4/CYP7A1/NCEH1/PRKACB/SLC10A2/SLC22A8/SLC51B/SLC9A1/SLCO1A2/SULT2A1/UGT1A1/UGT1A10/UGT1A7/UGT1A9/UGT2A1/UGT2A3/UGT2B10/UGT2B28/UGT2B4	25

cluster8	KEGG	hsa00053	Ascorbate and aldarate metabolism	12/1223	30/8779	0.000385972	0.013007252	0.012391729	KL/RGN/UGDH/UGT1A1/UGT1A10/UGT1A7/UGT1A9/UGT2A1/UGT2A3/UGT2B10/UGT2B28/UGT2B4	12
cluster8	KEGG	hsa00040	Pentose and glucuronate interconversions	13/1223	36/8779	0.00071383	0.021869145	0.020834263	AKR1B15/FGGY/KL/UGDH/UGT1A1/UGT1A10/UGT1A7/UGT1A9/UGT2A1/UGT2A3/UGT2B10/UGT2B28/UGT2B4	13
cluster9	BP	GO:0030218	erythrocyte differentiation	26/1419	128/18903	0.0000026	0.010365855	0.009701756	ABCB10/AHSP/ALAS2/ARID4A/CASP3/DYRK3/EPB42/FAM210B/FLVCR1/FOXO3/HCLS1/HSPA1A/INPP5D/JAK3/MAEA/P4HTM/PKNOX1/PTPN2/SLC4A1/SMAP1/SRF/TGFBR3/TMEM14C/TSPO2/ZBTB7A/ZFPM1	26
cluster9	BP	GO:0010639	negative regulation of organelle organization	53/1419	366/18903	0.00000292	0.010365855	0.009701756	ACD/ADD1/ADD2/ARAP1/ARRB2/BAZ1B/C11orf65/CDCA8/DCP2/GSN/H3-3B/HNRNPC/HSPA1A/INPP5K/KAT2B/MAD2L2/MAK/MPV17L/MUL1/NBDY/NBN/NPM1/PAK2/PATL2/PFN4/PHF23/PIK3CA/PPARG/PSMD10/PTTG1/PTTG2/RAD1/SLC35F6/SLX1A/SLX1B/SNCA/SPTBN2/SSH2/STN1/STYXL1/SWAP70/TAOK1/TBC1D7/TEN1/TENT4B/TIGAR/TMOD1/TMSB10/TRIOBP/WAS/WASHC2C/XRCC3/ZNF207	53

cluster9	BP	GO:0043161	proteasome-mediated ubiquitin-dependent protein catabolic process	61/1419	450/18903	0.00000483	0.010365855	0.009701756	ANKRD9/ARRB2/ASB2/ASCC2/ATXN3/CDC16/CDC27/CDC34/CSNK1A1/CUL3/CUL4A/DCAF11/DDA1/DDB1/ELOB/ERLIN2/FBXL22/FBXO38/FBXO9/FZR1/GABARAP/HECTD1/HECTD3/HSP90B1/HSPA1A/ITCH/KBTBD7/KEAP1/LTN1/MAEA/NEDD4/NUB1/OS9/PANO1/PCBP2/PSMB1/PSMC3/PSMD10/PSMD14/PSMD4/PSMF1/RAD23A/RNF175/RNF5/SGTA/SH3RF2/SH3RF3/SIAH3/SIRT6/SPOPL/SPSB2/SYVN1/TAF1/TBL1X/UBE4B/UBQLN1/UBR2/UCHL5/WAC/YOD1/ZYG11B	61
cluster9	BP	GO:0035303	regulation of dephosphorylation	26/1419	133/18903	0.00000545	0.010365855	0.009701756	BOD1L1/CD300A/CHP1/GNAI2/HSP90B1/INPP5K/IQGAP1/MTMR3/PPIA/PPP1R12A/PPP1R16A/PPP1R16B/PPP1R2B/PPP1R7/PPP1R9B/PPP6R1/PTPA/RCAN1/RIPK3/SMG5/SMG7/STYXL1/SWAP70/SYMPK/TIPRL/TNF	26
cluster9	BP	GO:0002366	leukocyte activation involved in immune response	44/1419	293/18903	0.00000781	0.010539133	0.009863932	ADGRE2/ANXA1/C17orf99/CCR2/CD244/CD300A/CD80/CD81/CD86/CLNK/CR1/DOCK11/DYSF/FCGR3A/FGR/GAPT/IL12RB1/IL21/IL23R/ITGAL/ITGB2/JAK3/KARS1/LFNG/LILRA2/LILRB1/LY9/MAD2L2/NBN/PLCL2/PRKCZ/PTAFR/PTGDS/PTGER4/PTK2B/RAB27A/RAC2/RC3H2/RELB/SLAMF6/SWAP70/SYK/TYROBP/ZFPM1	44
cluster9	BP	GO:0034101	erythrocyte homeostasis	26/1419	137/18903	0.00000955	0.010539133	0.009863932	ABCB10/AHSP/ALAS2/ARID4A/CASP3/DYRK3/EPB42/FAM210B/FLVCR1/FOXO3/HCLS1/HSPA1A/INPP5D/JAK3/MAEA/P4HTM/PKNOX1/PTPN2/SLC4A1/SMAP1/SRF/TGFBR3/TMEM14C/TSPO2/ZBTB7A/ZFPM1	26

cluster9	BP	GO:0045862	positive regulation of proteolysis	52/1419	372/18903	0.00001	0.010539133	0.009863932	ANP32B/ANXA2/ASTL/ATXN3/BCL2L13/CASP8/CFLAR/CIDEB/CLPX/COL4A3/CR1/CSNK1A1/CTSC/CYFIP2/DDA1/ELOB/FADD/FASLG/FZR1/GABARAP/GSN/HECTD1/HSPA1A/KEAP1/KHDC1/MTCH1/MUL1/NUB1/PDCD6/PPARG/PSMC3/PSMD10/PSMD14/PSME1/PSME2/PSME3/PSME4/PTK2B/RAD23A/SGTA/SH3RF2/SH3RF3/SIRT6/SNCA/SYK/TAF1/TANK/TNF/TNIP1/UBQLN1/WDR48/ZYG11B	52
cluster9	BP	GO:0002263	cell activation involved in immune response	44/1419	297/18903	0.0000111	0.010539133	0.009863932	ADGRE2/ANXA1/C17orf99/CCR2/CD244/CD300A/CD80/CD81/CD86/CLNK/CR1/DOCK11/DYSF/FCGR3A/FGR/GAPT/IL12RB1/IL21/IL23R/ITGAL/ITGB2/JAK3/KARS1/LFNG/LILRA2/LILRB1/LY9/MAD2L2/NBN/PLCL2/PRKCZ/PTAFR/PTGDS/PTGER4/PTK2B/RAB27A/RAC2/RC3H2/RELB/SLAMF6/SWAP70/SYK/TYROBP/ZFPM1	44
cluster9	BP	GO:0030099	myeloid cell differentiation	53/1419	407/18903	0.0000596	0.045704089	0.042776011	ABCB10/AHSP/ALAS2/ANXA2/ARID4A/CASP3/CASP8/CD81/CDC73/CUL4A/DYRK3/EPB42/FADD/FAM210B/FARP2/FLVCR1/FOXO3/HCLS1/HMGB3/HSPA1A/IL23R/IL31RA/INPP5D/JAK3/LILRB1/MAEA/NBEAL2/NKAP/P4HTM/PKNOX1/PPARG/PRXL2A/PTK2B/PTPN2/RAB7B/RASGRP4/RELB/SIGLEC15/SLC4A1/SMAP1/SNX10/SRF/TESC/TGFBR3/THOC5/TIRAP/TMEM14C/TNF/TSPO2/TYROBP/VPS33B/ZBTB7A/ZFPM1	53
cluster9	BP	GO:0035304	regulation of protein dephosphorylation	19/1419	94/18903	0.0000601	0.045704089	0.042776011	BOD1L1/CD300A/GNAI2/HSP90B1/PPIA/PPP1R12A/PPP1R16A/PPP1R16B/PPP1R2B/PPP1R7/PPP1R9B/PPP6R1/PTPA/RCAN1/STYXL1/SWAP70/SYMPK/TIPRL/TNF	19

cluster9	BP	GO:0031331	positive regulation of cellular catabolic process	55/1419	429/18903	0.0000668	0.046173195	0.043215063	ABCD1/ADRB2/ATG2A/BTG2/CSNK1A1/DCP2/DDA1/DHRSX/ELOB/EXOSC2/FBXO7/FOXO3/FZR1/GABARAP/HECTD1/HSPA1A/INSR/ITCH/KEAP1/METTL16/NOD2/NUB1/OPTN/PAIP1/PATL2/PNLDC1/PPARA/PSMD10/PTK2B/RAD23A/RC3H2/RNF152/SAMD4A/SESN3/SGTA/SH3RF2/SH3RF3/SIRT6/SNCA/SPTLC1/STK11/TAF1/TENT4B/TLR9/TNF/TRIM14/TRIM27/TRIM5/UBQLN1/ULK1/WAC/WDR45/ZC3H18/ZFP36L2/ZYG11B	55
cluster9	BP	GO:0042176	regulation of protein catabolic process	48/1419	361/18903	0.0000781	0.04951678	0.046344438	ANXA2/ATG7/ATXN3/AZIN2/CD81/CSNK1A1/DDA1/DDB1/EGLN2/ELOB/F8A3/FZR1/GABARAP/HECTD1/HSP90AA1/HSPA1A/ITCH/KEAP1/MAD2L2/MSN/NEDD4/NRG1/NUB1/PANO1/PRKACA/PSMC3/PSMD10/PSMD14/PSME1/PSME2/PSME3/PSMF1/RAD23A/RELA/RILP/SGTA/SH3RF2/SH3RF3/SIRT6/SNCA/SNF8/STX5/TAF1/TNF/UBQLN1/UCHL5/WAC/ZYG11B	48
