# Supplementary material for: RBAD: The first database dedicated alterations of blood RNA in individuals with Alzheimer’s disease and their clinical relevance
Source: Neural Regen Res. 2025 Mar 25;21(6):2553–62. doi: 10.4103/NRR.NRR-D-24-01165 (PMC13211806; doi:10.4103/NRR.NRR-D-24-01165)
Supplement: Supplementary file 12 [file NRR-21-2553_Suppl9.pdf]

Additional Table 12. The expression of olfactory-related gene in blood cells from AD and control evaluated by scRNA-seq.	
Method	scRNA-seq
Description	Data set: scRNA-seq datasets (SRP309935, SRP330776, and SRP215507) in RBAD. Clusters of cf-mRNA expression trend from control to MCI to AD. ●Control/MCI/AD: Normalized average expression of each gene in control, MCI, and AD groups. ● PCT: percentage of cells with gene expressed

Symbol	Celltype	Average_expression	PCT	Project
PDE1B	B cell_1	0.002188389	0.001824818	SRP309935
PDE1B	B cell_2	-	-	SRP309935
PDE1B	B cell_3	0.023160949	0.026548673	SRP309935
PDE1B	CD4+ T cell	0.003776011	0.002403846	SRP309935
PDE1B	CD8+ T cell_1	0.006302678	0.003769897	SRP309935
PDE1B	CD8+ T cell_2	0.009044159	0.004990198	SRP309935
PDE1B	Dendritic cell	0.018862418	0.019011407	SRP309935
PDE1B	Monocyte_1	0.010399697	0.006095308	SRP309935
PDE1B	Monocyte_2	0.009868159	0.007778154	SRP309935
PDE1B	Monocyte_3	0.003923619	0.002717391	SRP309935
PDE1B	Monocyte_4	0.020619351	0.017391304	SRP309935
PDE1B	Naive CD8+ T cell_1	0.004484704	0.002945074	SRP309935
PDE1B	Naive CD8+ T cell_2	0.002057085	0.001263538	SRP309935
PDE1B	Naive CD8+ T cell_3	0.013225428	0.011135857	SRP309935
PDE1B	Natural killer cell_1	0.003733666	0.002124372	SRP309935
PDE1B	Natural killer cell_2	0.003835596	0.002145504	SRP309935
PDE1B	Plasmacytoid dendritic cell_1	0.006843613	0.00619195	SRP309935
PDE1B	Plasmacytoid dendritic cell_2	0.007917291	0.008583691	SRP309935
PRKACB	B cell_1	0.21809294	0.159529807	SRP309935
PRKACB	B cell_2	0.160307865	0.09375	SRP309935
PRKACB	B cell_3	0.054363411	0.084745763	SRP309935
PRKACB	CD4+ T cell	0.211465868	0.131176999	SRP309935
PRKACB	CD8+ T cell_1	0.285039404	0.157447903	SRP309935
PRKACB	CD8+ T cell_2	0.319822953	0.182083266	SRP309935
PRKACB	Dendritic cell	0.204236957	0.189328744	SRP309935
PRKACB	Monocyte_1	0.11290606	0.067463859	SRP309935
PRKACB	Monocyte_2	0.106313718	0.081738566	SRP309935
PRKACB	Monocyte_3	0.197986346	0.112820513	SRP309935
PRKACB	Monocyte_4	0.319956416	0.244274809	SRP309935
PRKACB	Naive CD8+ T cell_1	0.185011123	0.122477191	SRP309935
PRKACB	Naive CD8+ T cell_2	0.181083242	0.114684363	SRP309935
PRKACB	Naive CD8+ T cell_3	0.345678773	0.276391555	SRP309935
PRKACB	Natural killer cell_1	0.349751331	0.182041604	SRP309935
PRKACB	Natural killer cell_2	0.349930615	0.185453902	SRP309935
PRKACB	Plasmacytoid dendritic cell_1	0.380601494	0.295514512	SRP309935
PRKACB	Plasmacytoid dendritic cell_2	0.437777585	0.299270073	SRP309935
RGS2	B cell_1	0.187973462	0.109032847	SRP309935

RGS2	B cell_2	0.056043328	0.032131148	SRP309935
RGS2	B cell_3	0.144319171	0.150442478	SRP309935
RGS2	CD4+ T cell	0.27260865	0.154807692	SRP309935
RGS2	CD8+ T cell_1	0.093331269	0.052080424	SRP309935
RGS2	CD8+ T cell_2	0.126909248	0.071110319	SRP309935
RGS2	Dendritic cell	0.502604322	0.3878327	SRP309935
RGS2	Monocyte_1	0.777199824	0.366642039	SRP309935
RGS2	Monocyte_2	0.702563042	0.443692932	SRP309935
RGS2	Monocyte_3	0.759943226	0.375	SRP309935
RGS2	Monocyte_4	0.708164218	0.426086957	SRP309935
RGS2	Naive CD8+ T cell_1	0.067561416	0.044323369	SRP309935
RGS2	Naive CD8+ T cell_2	0.034308954	0.021119134	SRP309935
RGS2	Naive CD8+ T cell_3	0.09524637	0.076837416	SRP309935
RGS2	Natural killer cell_1	0.136196289	0.070973349	SRP309935
RGS2	Natural killer cell_2	0.193808233	0.098888239	SRP309935
RGS2	Plasmacytoid dendritic cell_1	0.44087413	0.269349845	SRP309935
RGS2	Plasmacytoid dendritic cell_2	0.091192807	0.064377682	SRP309935
CAMK2A	Not detected in any cells	-	-	SRP309935
OR10A2	Not detected in any cells	-	-	SRP309935
OR10A6	Not detected in any cells	-	-	SRP309935
OR10C1	Not detected in any cells	-	-	SRP309935
OR10D3	Not detected in any cells	-	-	SRP309935
OR10G2	Not detected in any cells	-	-	SRP309935
OR10S1	Not detected in any cells	-	-	SRP309935
OR10V1	Not detected in any cells	-	-	SRP309935
OR10Z1	Not detected in any cells	-	-	SRP309935
OR11A1	Not detected in any cells	-	-	SRP309935
OR11H12	Not detected in any cells	-	-	SRP309935
OR11H2	Not detected in any cells	-	-	SRP309935
OR11H4	Not detected in any cells	-	-	SRP309935
OR13C2	Not detected in any cells	-	-	SRP309935
OR13C5	Not detected in any cells	-	-	SRP309935
OR13F1	Not detected in any cells	-	-	SRP309935
OR14J1	Not detected in any cells	-	-	SRP309935
OR14K1	Not detected in any cells	-	-	SRP309935
OR1E2	Not detected in any cells	-	-	SRP309935
OR1Q1	Not detected in any cells	-	-	SRP309935
OR2A12	Not detected in any cells	-	-	SRP309935
OR2A2	Not detected in any cells	-	-	SRP309935
OR2AG2	Not detected in any cells	-	-	SRP309935
OR2B3	Not detected in any cells	-	-	SRP309935
OR2D2	Not detected in any cells	-	-	SRP309935
OR2J3	Not detected in any cells	-	-	SRP309935
OR2K2	Not detected in any cells	-	-	SRP309935
OR2L5	Not detected in any cells	-	-	SRP309935
OR2M3	Not detected in any cells	-	-	SRP309935
OR2T11	Not detected in any cells	-	-	SRP309935
OR2T12	Not detected in any cells	-	-	SRP309935
OR2T27	Not detected in any cells	-	-	SRP309935
OR2T4	Not detected in any cells	-	-	SRP309935
OR2T8	Not detected in any cells	-	-	SRP309935
OR2W3	Not detected in any cells	-	-	SRP309935
OR3A1	Not detected in any cells	-	-	SRP309935
OR4A16	Not detected in any cells	-	-	SRP309935
OR4A47	Not detected in any cells	-	-	SRP309935

OR4C16	Not detected in any cells	-	-	SRP309935
OR4D10	Not detected in any cells	-	-	SRP309935
OR4D11	Not detected in any cells	-	-	SRP309935
OR4F5	Not detected in any cells	-	-	SRP309935
OR4K1	Not detected in any cells	-	-	SRP309935
OR4K14	Not detected in any cells	-	-	SRP309935
OR4L1	Not detected in any cells	-	-	SRP309935
OR4M1	Not detected in any cells	-	-	SRP309935
OR4Q3	Not detected in any cells	-	-	SRP309935
OR4S1	Not detected in any cells	-	-	SRP309935
OR4X2	Not detected in any cells	-	-	SRP309935
OR51B6	Not detected in any cells	-	-	SRP309935
OR51F1	Not detected in any cells	-	-	SRP309935
OR51T1	Not detected in any cells	-	-	SRP309935
OR52D1	Not detected in any cells	-	-	SRP309935
OR52E2	Not detected in any cells	-	-	SRP309935
OR52E4	Not detected in any cells	-	-	SRP309935
OR52E5	Not detected in any cells	-	-	SRP309935
OR52E6	Not detected in any cells	-	-	SRP309935
OR52K2	Not detected in any cells	-	-	SRP309935
OR52N5	Not detected in any cells	-	-	SRP309935
OR52W1	Not detected in any cells	-	-	SRP309935
OR56A1	Not detected in any cells	-	-	SRP309935
OR5D13	Not detected in any cells	-	-	SRP309935
OR5D16	Not detected in any cells	-	-	SRP309935
OR5H15	Not detected in any cells	-	-	SRP309935
OR5H2	Not detected in any cells	-	-	SRP309935
OR5I1	Not detected in any cells	-	-	SRP309935
OR5J2	Not detected in any cells	-	-	SRP309935
OR5K1	Not detected in any cells	-	-	SRP309935
OR5L1	Not detected in any cells	-	-	SRP309935
OR5M1	Not detected in any cells	-	-	SRP309935
OR5M3	Not detected in any cells	-	-	SRP309935
OR5P3	Not detected in any cells	-	-	SRP309935
OR5T2	Not detected in any cells	-	-	SRP309935
OR5V1	Not detected in any cells	-	-	SRP309935
OR6B2	Not detected in any cells	-	-	SRP309935
OR6B3	Not detected in any cells	-	-	SRP309935
OR6C2	Not detected in any cells	-	-	SRP309935
OR6C76	Not detected in any cells	-	-	SRP309935
OR6P1	Not detected in any cells	-	-	SRP309935
OR6T1	Not detected in any cells	-	-	SRP309935
OR6X1	Not detected in any cells	-	-	SRP309935
OR7A5	Not detected in any cells	-	-	SRP309935
OR7E24	Not detected in any cells	-	-	SRP309935
OR7G2	Not detected in any cells	-	-	SRP309935
OR8D1	Not detected in any cells	-	-	SRP309935
OR8D2	Not detected in any cells	-	-	SRP309935
OR8D4	Not detected in any cells	-	-	SRP309935
OR8J3	Not detected in any cells	-	-	SRP309935
OR9G1	Not detected in any cells	-	-	SRP309935
OR9K2	Not detected in any cells	-	-	SRP309935
OR9Q1	Not detected in any cells	-	-	SRP309935
OR10A2	Naive CD8+ T cell_1	0.000496593	0.000273038	SRP330776
OR10G2	CD8+ T cell_1	8.61E-05	8.85E-05	SRP330776

OR10G2	Naive CD8+ T cell_1	0.000780963	0.000409528	SRP330776
OR10Z1	B cell_2	0.00109657	0.000797024	SRP330776
OR2AG2	Natural killer cell_1	0.001252478	0.000778917	SRP330776
OR2L5	Naive CD8+ T cell_1	0.000329467	0.000204785	SRP330776
OR2T8	B cell_1	0.001276973	0.000896158	SRP330776
OR3A1	CD8+ T cell_1	9.93E-05	8.85E-05	SRP330776
OR3A1	Naive CD8+ T cell_1	0.000294572	0.000204785	SRP330776
OR52N5	CD8+ T cell_1	0.000308259	0.000265522	SRP330776
OR6C2	Memory T cell_2	0.000866532	0.000738212	SRP330776
PDE1B	B cell_1	0.012608479	0.009369771	SRP330776
PDE1B	B cell_2	0.006832099	0.004508088	SRP330776
PDE1B	B cell_3	0.015598856	0.014388489	SRP330776
PDE1B	B cell_4	0.060913551	0.053811659	SRP330776
PDE1B	CD8+ T cell_1	0.007445705	0.005649219	SRP330776
PDE1B	CD8+ T cell_2	0.009763322	0.006392045	SRP330776
PDE1B	CD8+ T cell_3	0.015752637	0.010455564	SRP330776
PDE1B	Memory T cell_1	0.010239714	0.005467372	SRP330776
PDE1B	Memory T cell_2	0.007492406	0.0046049	SRP330776
PDE1B	Monocyte_1	0.020855925	0.021052632	SRP330776
PDE1B	Monocyte_2	0.041267767	0.0269179	SRP330776
PDE1B	Naive CD8+ T cell_1	0.00455552	0.00265894	SRP330776
PDE1B	Naive CD8+ T cell_2	0.011724282	0.005149662	SRP330776
PDE1B	Naive CD8+ T cell_3	0.003416677	0.002961208	SRP330776
PDE1B	Natural killer T cell	0.014667243	0.012041598	SRP330776
PDE1B	Natural killer cell_1	0.020422587	0.013415893	SRP330776
PDE1B	Regulatory T cell	0.049758844	0.033684211	SRP330776
PDE1B	T cell	0.012200491	0.008965738	SRP330776
PDE1B	γδ T cell	0.006082442	0.003916252	SRP330776
PRKACB	B cell_1	0.373964574	0.258077111	SRP330776
PRKACB	B cell_2	0.295522456	0.173322005	SRP330776
PRKACB	B cell_3	0.539309428	0.440677966	SRP330776
PRKACB	B cell_4	0.215756997	0.264	SRP330776
PRKACB	CD8+ T cell_1	0.403358514	0.274872108	SRP330776
PRKACB	CD8+ T cell_2	0.638203958	0.367358809	SRP330776
PRKACB	CD8+ T cell_3	0.465933209	0.282951982	SRP330776
PRKACB	Memory T cell_1	0.661273842	0.344095468	SRP330776
PRKACB	Memory T cell_2	0.345679475	0.241681545	SRP330776
PRKACB	Monocyte_1	0.245429691	0.158504007	SRP330776
PRKACB	Monocyte_2	0.063613681	0.047936085	SRP330776
PRKACB	Naive CD8+ T cell_1	0.385661059	0.239581768	SRP330776
PRKACB	Naive CD8+ T cell_2	0.342595049	0.148192411	SRP330776
PRKACB	Naive CD8+ T cell_3	0.441714571	0.295678544	SRP330776
PRKACB	Natural killer T cell	0.522802006	0.412587413	SRP330776
PRKACB	Natural killer cell_1	0.49469656	0.298154492	SRP330776
PRKACB	Natural killer cell_2	0.59523473	0.335974643	SRP330776
PRKACB	Regulatory T cell	0.656921324	0.422297297	SRP330776
PRKACB	T cell	0.413335939	0.277085065	SRP330776
PRKACB	γδ T cell	0.534491099	0.310555839	SRP330776
RGS2	B cell_1	0.6785868	0.374681239	SRP330776
RGS2	B cell_2	0.222413321	0.116395494	SRP330776
RGS2	B cell_3	0.437038291	0.30228471	SRP330776
RGS2	B cell_4	0.36019384	0.337164751	SRP330776
RGS2	CD8+ T cell_1	0.135063327	0.089140731	SRP330776
RGS2	CD8+ T cell_2	0.203344424	0.118758796	SRP330776
RGS2	CD8+ T cell_3	0.215296612	0.126911584	SRP330776

RGS2	Memory T cell_1	0.240601178	0.127493998	SRP330776
RGS2	Memory T cell_2	0.158019084	0.104130207	SRP330776
RGS2	Monocyte_1	0.906766384	0.597966102	SRP330776
RGS2	Monocyte_2	1.185863287	0.659963437	SRP330776
RGS2	Naive CD8+ T cell_1	0.067197079	0.041056503	SRP330776
RGS2	Naive CD8+ T cell_2	0.170558629	0.068256194	SRP330776
RGS2	Naive CD8+ T cell_3	0.083775571	0.053117783	SRP330776
RGS2	Natural killer T cell	0.202984326	0.166582534	SRP330776
RGS2	Natural killer cell_1	0.140908425	0.088855795	SRP330776
RGS2	Natural killer cell_2	0.05970364	0.037383178	SRP330776
RGS2	Regulatory T cell	0.365177332	0.203846154	SRP330776
RGS2	T cell	0.160361864	0.110030395	SRP330776
RGS2	γδ T cell	0.238225355	0.137979486	SRP330776
OR10A2	Not detected in any cells	-	-	SRP330776
OR10G2	Not detected in any cells	-	-	SRP330776
OR10Z1	Not detected in any cells	-	-	SRP330776
OR2AG2	Not detected in any cells	-	-	SRP330776
OR2L5	Not detected in any cells	-	-	SRP330776
OR2T8	Not detected in any cells	-	-	SRP330776
OR3A1	Not detected in any cells	-	-	SRP330776
OR52K2	Not detected in any cells	-	-	SRP330776
OR52N5	Not detected in any cells	-	-	SRP330776
OR6C2	Not detected in any cells	-	-	SRP330776
PDE1B	Not detected in any cells	-	-	SRP330776
CAMK2A	Not detected in any cells	-	-	SRP330776
OR10A6	Not detected in any cells	-	-	SRP330776
OR10C1	Not detected in any cells	-	-	SRP330776
OR10D3	Not detected in any cells	-	-	SRP330776
OR10S1	Not detected in any cells	-	-	SRP330776
OR10V1	Not detected in any cells	-	-	SRP330776
OR11A1	Not detected in any cells	-	-	SRP330776
OR11H12	Not detected in any cells	-	-	SRP330776
OR11H2	Not detected in any cells	-	-	SRP330776
OR11H4	Not detected in any cells	-	-	SRP330776
OR13C2	Not detected in any cells	-	-	SRP330776
OR13C5	Not detected in any cells	-	-	SRP330776
OR13F1	Not detected in any cells	-	-	SRP330776
OR14J1	Not detected in any cells	-	-	SRP330776
OR14K1	Not detected in any cells	-	-	SRP330776
OR1E2	Not detected in any cells	-	-	SRP330776
OR1Q1	Not detected in any cells	-	-	SRP330776
OR2A12	Not detected in any cells	-	-	SRP330776
OR2A2	Not detected in any cells	-	-	SRP330776
OR2B3	Not detected in any cells	-	-	SRP330776
OR2D2	Not detected in any cells	-	-	SRP330776
OR2J3	Not detected in any cells	-	-	SRP330776
OR2K2	Not detected in any cells	-	-	SRP330776
OR2M3	Not detected in any cells	-	-	SRP330776
OR2T11	Not detected in any cells	-	-	SRP330776
OR2T12	Not detected in any cells	-	-	SRP330776
OR2T27	Not detected in any cells	-	-	SRP330776
OR2T4	Not detected in any cells	-	-	SRP330776
OR2W3	Not detected in any cells	-	-	SRP330776
OR4A16	Not detected in any cells	-	-	SRP330776
OR4A47	Not detected in any cells	-	-	SRP330776

OR4C16	Not detected in any cells	-	-	SRP330776
OR4D10	Not detected in any cells	-	-	SRP330776
OR4D11	Not detected in any cells	-	-	SRP330776
OR4F5	Not detected in any cells	-	-	SRP330776
OR4K1	Not detected in any cells	-	-	SRP330776
OR4K14	Not detected in any cells	-	-	SRP330776
OR4L1	Not detected in any cells	-	-	SRP330776
OR4M1	Not detected in any cells	-	-	SRP330776
OR4Q3	Not detected in any cells	-	-	SRP330776
OR4S1	Not detected in any cells	-	-	SRP330776
OR4X2	Not detected in any cells	-	-	SRP330776
OR51B6	Not detected in any cells	-	-	SRP330776
OR51F1	Not detected in any cells	-	-	SRP330776
OR51T1	Not detected in any cells	-	-	SRP330776
OR52D1	Not detected in any cells	-	-	SRP330776
OR52E2	Not detected in any cells	-	-	SRP330776
OR52E4	Not detected in any cells	-	-	SRP330776
OR52E5	Not detected in any cells	-	-	SRP330776
OR52E6	Not detected in any cells	-	-	SRP330776
OR52W1	Not detected in any cells	-	-	SRP330776
OR56A1	Not detected in any cells	-	-	SRP330776
OR5D13	Not detected in any cells	-	-	SRP330776
OR5D16	Not detected in any cells	-	-	SRP330776
OR5H15	Not detected in any cells	-	-	SRP330776
OR5H2	Not detected in any cells	-	-	SRP330776
OR5I1	Not detected in any cells	-	-	SRP330776
OR5J2	Not detected in any cells	-	-	SRP330776
OR5K1	Not detected in any cells	-	-	SRP330776
OR5L1	Not detected in any cells	-	-	SRP330776
OR5M1	Not detected in any cells	-	-	SRP330776
OR5M3	Not detected in any cells	-	-	SRP330776
OR5P3	Not detected in any cells	-	-	SRP330776
OR5T2	Not detected in any cells	-	-	SRP330776
OR5V1	Not detected in any cells	-	-	SRP330776
OR6B2	Not detected in any cells	-	-	SRP330776
OR6B3	Not detected in any cells	-	-	SRP330776
OR6C76	Not detected in any cells	-	-	SRP330776
OR6P1	Not detected in any cells	-	-	SRP330776
OR6T1	Not detected in any cells	-	-	SRP330776
OR6X1	Not detected in any cells	-	-	SRP330776
OR7A5	Not detected in any cells	-	-	SRP330776
OR7E24	Not detected in any cells	-	-	SRP330776
OR7G2	Not detected in any cells	-	-	SRP330776
OR8D1	Not detected in any cells	-	-	SRP330776
OR8D2	Not detected in any cells	-	-	SRP330776
OR8D4	Not detected in any cells	-	-	SRP330776
OR8J3	Not detected in any cells	-	-	SRP330776
OR9G1	Not detected in any cells	-	-	SRP330776
OR9K2	Not detected in any cells	-	-	SRP330776
OR9Q1	Not detected in any cells	-	-	SRP330776
OR10G2	CD8+ T cell_5	0.00057634	0.000343249	SRP215507
OR3A1	CD8+ T cell_1	0.000174523	0.000114074	SRP215507
OR3A1	CD8+ T cell_2	0.000131519	0.000110558	SRP215507
PDE1B	CD8+ T cell_1	0.007072126	0.004905176	SRP215507
PDE1B	CD8+ T cell_10	0.016394645	0.008849558	SRP215507

PDE1B	CD8+ T cell_2	0.00519884	0.003795836	SRP215507
PDE1B	CD8+ T cell_3	0.004335781	0.00315871	SRP215507
PDE1B	CD8+ T cell_4	0.024970007	0.009648956	SRP215507
PDE1B	CD8+ T cell_5	0.004801401	0.003775744	SRP215507
PDE1B	CD8+ T cell_6	0.023084231	0.020456334	SRP215507
PDE1B	CD8+ T cell_7	0.002442511	0.002754821	SRP215507
PDE1B	CD8+ T cell_8	0.011026929	0.008379888	SRP215507
PDE1B	CD8+ T cell_9	0.026556231	0.033333333	SRP215507
PRKACB	CD8+ T cell_1	0.321820283	0.208584058	SRP215507
PRKACB	CD8+ T cell_10	0.176097684	0.088495575	SRP215507
PRKACB	CD8+ T cell_11	0.240454962	0.095238095	SRP215507
PRKACB	CD8+ T cell_2	0.24255917	0.169780726	SRP215507
PRKACB	CD8+ T cell_3	0.252673328	0.187853865	SRP215507
PRKACB	CD8+ T cell_4	0.166882537	0.063733892	SRP215507
PRKACB	CD8+ T cell_5	0.212918717	0.145652174	SRP215507
PRKACB	CD8+ T cell_6	0.379871594	0.294256491	SRP215507
PRKACB	CD8+ T cell_7	0.188845104	0.148760331	SRP215507
PRKACB	CD8+ T cell_8	0.250994717	0.175977654	SRP215507
PRKACB	CD8+ T cell_9	0.05928606	0.066666667	SRP215507
RGS2	CD8+ T cell_1	0.168532158	0.109824611	SRP215507
RGS2	CD8+ T cell_10	0.301014077	0.159292035	SRP215507
RGS2	CD8+ T cell_11	0.14354983	0.047619048	SRP215507
RGS2	CD8+ T cell_2	0.178858459	0.118334255	SRP215507
RGS2	CD8+ T cell_3	0.078885681	0.061326658	SRP215507
RGS2	CD8+ T cell_4	0.073755137	0.029137307	SRP215507
RGS2	CD8+ T cell_5	0.468629695	0.289016018	SRP215507
RGS2	CD8+ T cell_6	0.210529062	0.160503541	SRP215507
RGS2	CD8+ T cell_7	0.522423598	0.360881543	SRP215507
RGS2	CD8+ T cell_8	0.163404737	0.108938547	SRP215507
RGS2	CD8+ T cell_9	1.471816679	0.85	SRP215507
OR10G2	Not detected in any cells	-	-	SRP215507
OR2AG2	Not detected in any cells	-	-	SRP215507
OR3A1	Not detected in any cells	-	-	SRP215507
PDE1B	Not detected in any cells	-	-	SRP215507
CAMK2A	Not detected in any cells	-	-	SRP215507
OR10A2	Not detected in any cells	-	-	SRP215507
OR10A6	Not detected in any cells	-	-	SRP215507
OR10C1	Not detected in any cells	-	-	SRP215507
OR10D3	Not detected in any cells	-	-	SRP215507
OR10S1	Not detected in any cells	-	-	SRP215507
OR10V1	Not detected in any cells	-	-	SRP215507
OR10Z1	Not detected in any cells	-	-	SRP215507
OR11A1	Not detected in any cells	-	-	SRP215507
OR11H12	Not detected in any cells	-	-	SRP215507
OR11H2	Not detected in any cells	-	-	SRP215507
OR11H4	Not detected in any cells	-	-	SRP215507
OR13C2	Not detected in any cells	-	-	SRP215507
OR13C5	Not detected in any cells	-	-	SRP215507
OR13F1	Not detected in any cells	-	-	SRP215507
OR14J1	Not detected in any cells	-	-	SRP215507
OR14K1	Not detected in any cells	-	-	SRP215507
OR1E2	Not detected in any cells	-	-	SRP215507
OR1Q1	Not detected in any cells	-	-	SRP215507
OR2A12	Not detected in any cells	-	-	SRP215507
OR2A2	Not detected in any cells	-	-	SRP215507

OR2B3	Not detected in any cells	-	-	SRP215507
OR2D2	Not detected in any cells	-	-	SRP215507
OR2J3	Not detected in any cells	-	-	SRP215507
OR2K2	Not detected in any cells	-	-	SRP215507
OR2L5	Not detected in any cells	-	-	SRP215507
OR2M3	Not detected in any cells	-	-	SRP215507
OR2T11	Not detected in any cells	-	-	SRP215507
OR2T12	Not detected in any cells	-	-	SRP215507
OR2T27	Not detected in any cells	-	-	SRP215507
OR2T4	Not detected in any cells	-	-	SRP215507
OR2T8	Not detected in any cells	-	-	SRP215507
OR2W3	Not detected in any cells	-	-	SRP215507
OR4A16	Not detected in any cells	-	-	SRP215507
OR4A47	Not detected in any cells	-	-	SRP215507
OR4C16	Not detected in any cells	-	-	SRP215507
OR4D10	Not detected in any cells	-	-	SRP215507
OR4D11	Not detected in any cells	-	-	SRP215507
OR4F5	Not detected in any cells	-	-	SRP215507
OR4K1	Not detected in any cells	-	-	SRP215507
OR4K14	Not detected in any cells	-	-	SRP215507
OR4L1	Not detected in any cells	-	-	SRP215507
OR4M1	Not detected in any cells	-	-	SRP215507
OR4Q3	Not detected in any cells	-	-	SRP215507
OR4S1	Not detected in any cells	-	-	SRP215507
OR4X2	Not detected in any cells	-	-	SRP215507
OR51B6	Not detected in any cells	-	-	SRP215507
OR51F1	Not detected in any cells	-	-	SRP215507
OR51T1	Not detected in any cells	-	-	SRP215507
OR52D1	Not detected in any cells	-	-	SRP215507
OR52E2	Not detected in any cells	-	-	SRP215507
OR52E4	Not detected in any cells	-	-	SRP215507
OR52E5	Not detected in any cells	-	-	SRP215507
OR52E6	Not detected in any cells	-	-	SRP215507
OR52K2	Not detected in any cells	-	-	SRP215507
OR52N5	Not detected in any cells	-	-	SRP215507
OR52W1	Not detected in any cells	-	-	SRP215507
OR56A1	Not detected in any cells	-	-	SRP215507
OR5D13	Not detected in any cells	-	-	SRP215507
OR5D16	Not detected in any cells	-	-	SRP215507
OR5H15	Not detected in any cells	-	-	SRP215507
OR5H2	Not detected in any cells	-	-	SRP215507
OR5I1	Not detected in any cells	-	-	SRP215507
OR5J2	Not detected in any cells	-	-	SRP215507
OR5K1	Not detected in any cells	-	-	SRP215507
OR5L1	Not detected in any cells	-	-	SRP215507
OR5M1	Not detected in any cells	-	-	SRP215507
OR5M3	Not detected in any cells	-	-	SRP215507
OR5P3	Not detected in any cells	-	-	SRP215507
OR5T2	Not detected in any cells	-	-	SRP215507
OR5V1	Not detected in any cells	-	-	SRP215507
OR6B2	Not detected in any cells	-	-	SRP215507
OR6B3	Not detected in any cells	-	-	SRP215507
OR6C2	Not detected in any cells	-	-	SRP215507
OR6C76	Not detected in any cells	-	-	SRP215507
OR6P1	Not detected in any cells	-	-	SRP215507

OR6T1	Not detected in any cells	-	-	SRP215507
OR6X1	Not detected in any cells	-	-	SRP215507
OR7A5	Not detected in any cells	-	-	SRP215507
OR7E24	Not detected in any cells	-	-	SRP215507
OR7G2	Not detected in any cells	-	-	SRP215507
OR8D1	Not detected in any cells	-	-	SRP215507
OR8D2	Not detected in any cells	-	-	SRP215507
OR8D4	Not detected in any cells	-	-	SRP215507
OR8J3	Not detected in any cells	-	-	SRP215507
OR9G1	Not detected in any cells	-	-	SRP215507
OR9K2	Not detected in any cells	-	-	SRP215507
OR9Q1	Not detected in any cells	-	-	SRP215507
