# Supplementary material for: RBAD: The first database dedicated alterations of blood RNA in individuals with Alzheimer’s disease and their clinical relevance
Source: Neural Regen Res. 2025 Mar 25;21(6):2553–62. doi: 10.4103/NRR.NRR-D-24-01165 (PMC13211806; doi:10.4103/NRR.NRR-D-24-01165)
Supplement: Supplementary file 13 [file NRR-21-2553_Suppl17.pdf]

Additional file 2 Methods for detailed information about the participants

HMACS cohort

HMACS, the Hubei Memory & Aging Cohort Study was designed as a prospective study in Central China to determine the prevalence, incidence, and risk factors for dementia and mild cognitive impairment (MCI) among urban and rural older adults (Li et al., 2022). It contains more than 10,000 individuals who completed their baseline clinical evaluation. The diagnoses of MCI and dementia were made via consensus diagnosis using the Diagnostic and Statistical Manual of Mental Disorders fourth edition criteria. We selected 2694 individuals from the Liyuan community (2019-2023) with blood chemical test data and definite cognitive evaluation. Wilcoxon rank-sum test was used to compare the features of red blood cells between dementia, MCI, and control.

Blood chemistry data from HMACS

Venous blood samples (3 mL total) for hematological analyses were drawn from each subject in the morning. The samples were analyzed in the hospital's clinical biochemistry laboratory using a Sysmex-XNL hematology analyzer. Six parameters about erythroid cells were estimated: hematocrit percentage (%), red blood cell count (RCC, $\times 10^{12}$/L), hemoglobin (g/L), mean corpuscular hemoglobin (MCH, pg), and MC volume (MCV, fL), and MCH concentration (MCHC, g/L). The hematocrit percentage and RCC were used to determine the population of erythroid cells, the MCV was used to define red blood cell size, while hemoglobin, MCH, and MCHC were used to determine the hemoglobin content.

External data sets for validation

Multiple external data sets were used to validate the results we got from RBAD, including mass spectrometry-based plasma proteomics data of 199 individuals (AD: 26; MCI: 36; normal: 137) from self-constructed HMACS cohort and public RNA-seq data of blood exosomes from traumatic brain injury (TBI, n = 8) and control (n = 8) individuals (GSE254880).

Plasma samples were collected at time of enrollment, and participants were stratified as normal (N), mild cognitive impairment (MCI), and dementia (D). Plasma samples were processed through the Proteonano^{TM} pipeline (Zhao et al., 2023), an affinity-selective mass spectrometry platform to enrich low abundance proteins, and analyzed by a ThermoFisher Orbitrap Astral mass spectrometer at data independent acquisition mode. Raw data were analyzed by using DIA-NN, normalized, and further processed by using a customized biostastic and bioinformatic pipeline. Quality control was performed for proteome data according to a published study (Geyer et al., 2019) to handle sample-related bias based on erythrocyte quality marker panel. The marker panel comprises 29 proteins, including hemoglobin *HBA1*, *HBB*, and *HBD*. The contamination indices was calculated by summing the intensities of these proteins and dividing by the total intensity of all quantified plasma proteins. Samples with a contamination indices exceeding the mean by more than two standard deviations were defined as abnormal. This analysis identified eight abnormal samples, consisting of two from the AD group, two from the MCI group, and two from the Control group. A chi-square test indicated that the distribution of abnormal samples across different disease states did not exhibit statistically significant differences (P = 0.49). The eight abnormal samples were excluded from downstream analysis, including differential expression analysis performed by limma method.

References

Geyer PE, Voytik E, Treit PV, Doll S, Kleinhempel A, Niu L, Müller JB, Buchholtz ML, Bader JM, Teupser D, Holdt LM, Mann M (2019) Plasma proteome profiling to detect and avoid sample-related biases in biomarker studies. EMBO Mol Med 11:e10427.

Li L, Cheng GR, Liu D, Hu FF, Gan XG, Zhang B, An L, Chen C, Zou MJ, Xu L, Ou YM, Chen YS, Li JQ, Wei Z, Wu Q, Chen XX, Guo MQ, Wu QM, Wang R, Zeng Y (2022) The Hubei Memory and Aging Cohort Study: study design, baseline characteristics, and prevalence of cognitive impairments. J Alzheimers Dis 85:561-571.

Zhao B, Gao X, Ouyang X, Fang J, Deng Z, Wu H, Mao Y (2023) Proteonano™: a novel deep proteomics platform with 1000-plex profiling capacity and picogram sensitivity and its application in diabetic kidney disease. bioRxiv doi: 10.1101/2023.09.12.556305.
