# Supplementary material for: RBAD: The first database dedicated alterations of blood RNA in individuals with Alzheimer’s disease and their clinical relevance
Source: Neural Regen Res. 2025 Mar 25;21(6):2553–62. doi: 10.4103/NRR.NRR-D-24-01165 (PMC13211806; doi:10.4103/NRR.NRR-D-24-01165)
Supplement: Supplementary file 14 [file NRR-21-2553_Suppl10.pdf]

Additional Table 14. Differential expression analysis of olfactory-related genes using HMACS plasma proteome data.

Method	R package limma
Description	Data set: HMACS plasma proteome data<br>Differential expression of protein products of olfactory-related genes between normal, MCI, and AD individuals<br>Olfactory-related genes were collected from KEGG hsa04740.

Protein.Ids	Protein.Group	Protein.Names	Genes	LogFC	AveExpr	t	P value	B	Group
P62873;P62873-2	P62873	GBB1_HUMAN	GNB1	-1.135511799	16.7443974	-5.736617733	3.49597E-08	8.425619443	AD vs control
P17612;P22694;P22694-10;P22694-2;P22694-3;P22694-4;P22694-5;P22694-6;P22694-7;P22694-9;P17612-2	P22694-2	KAPCB_HUMAN	PRKACB	-1.100191581	13.99866741	-4.038488178	7.97386E-05	1.246822931	AD vs control
Q9UQM7;Q9UQM7-2	Q9UQM7;Q9UQM7-2	KCC2A_HUMAN	CAMK2A	-0.531033577	13.65050863	-3.728829763	0.00026621	0.085221284	AD vs control
Q13555;Q13555-5;Q13555-10;Q13555-11;Q13555-2;Q13555-3;Q13555-4;Q13555-6;Q13555-7;Q13555-8;Q13555-9	Q13555-11;Q13555-5;Q13555-6	KCC2G_HUMAN	CAMK2G	-0.84864477	13.43197138	-3.389921087	0.000961706	-0.87026044	AD vs control
P25098	P25098	ARBK1_HUMAN	GRK2	-1.366707578	13.29416777	-2.980761306	0.003384912	-1.908589513	AD vs control
P49407;P49407-2	P49407-2	ARRB1_HUMAN	ARRB1	-0.394609603	15.18759354	-1.265993359	0.207239881	-5.45297465	AD vs control
Q13557;Q13557-8;Q13557-10;Q13557-11;Q13557-12;Q13557-3;Q13557-4;Q13557-5;Q13557-6;Q13557-9	Q13557-12	KCC2D_HUMAN	CAMK2D	-0.409255538	11.91684452	-1.13860194	0.259825883	-5.225378679	AD vs control
P17612;P17612-2	P17612	KAPCA_HUMAN	PRKACA	-0.768798985	14.24274589	-1.045805609	0.298203674	-4.824179883	AD vs control
P32121;P32121-2;P32121-3;P32121-4;P32121-5	P32121;P32121-3	ARRB2_HUMAN	ARRB2	0.381738278	15.64826777	0.995655008	0.321138494	-5.550809068	AD vs control
Q13976;Q13976-2;Q13976-3	Q13976-2	KGP1_HUMAN	PRKG1	-0.02276048	13.53623696	-0.063131805	0.949787869	-6.104111048	AD vs control
**O00408;O00408-3;O00408-2;O00408-4;O00408-6**	O00408;O00408-2;O00408-3;O00408-4	PDE2A_HUMAN	PDE2A	NA	11.54322583	NA	NA	NA	AD vs control
P62873;P62873-2	P62873	GBB1_HUMAN	GNB1	-1.030948778	16.7443974	-4.33064767	2.34133E-05	2.439159309	AD vs MCI

P17612;P22694;P22694-10;P22694-2;P22694-3;P22694-4;P22694-5;P22694-6;P22694-7;P22694-9;P17612-2	P22694-2	KAPCB_HUMAN	PRKACB	-0.989930189	13.99866741	-3.137268939	0.001994697	-1.47429194	AD vs MCI
P25098	P25098	ARBK1_HUMAN	GRK2	-1.583916854	13.29416777	-3.112060079	0.002247418	-1.524861755	AD vs MCI
**Q13555;Q13555-5;Q13555-10;Q13555-11;Q13555-2;Q13555-3;Q13555-4;Q13555-6;Q13555-7;Q13555-8;Q13555-9**	Q13555-11;Q13555-5;Q13555-6	KCC2G_HUMAN	CAMK2G	-0.72587466	13.43197138	-2.563764726	0.011658183	-2.863042017	AD vs MCI
Q9UQM7;Q9UQM7-2	Q9UQM7;Q9UQM7-2	KCC2A_HUMAN	CAMK2A	-0.423557326	13.65050863	-2.380283475	0.018470617	-3.372537	AD vs MCI
**P17612;P17612-2**	P17612	KAPCA_HUMAN	PRKACA	-1.125908226	14.24274589	-1.485303007	0.140648641	-4.393067552	AD vs MCI
P49407;P49407-2	P49407-2	ARRB1_HUMAN	ARRB1	-0.442102581	15.18759354	-1.260119909	0.209345449	-5.146038077	AD vs MCI
Q13976;Q13976-2;Q13976-3	Q13976-2	KGP1_HUMAN	PRKG1	0.327817633	13.53623696	0.806466888	0.421892071	-5.469507546	AD vs MCI
P32121;P32121-2;P32121-3;P32121-4;P32121-5	P32121;P32121-3	ARRB2_HUMAN	ARRB2	0.124594605	15.64826777	0.286743163	0.774733635	-5.642089425	AD vs MCI
Q13557;Q13557-8;Q13557-10;Q13557-11;Q13557-12;Q13557-3;Q13557-4;Q13557-5;Q13557-6;Q13557-9	Q13557-12	KCC2D_HUMAN	CAMK2D	-0.099736711	11.91684452	-0.254707197	0.79990336	-5.500021109	AD vs MCI
O00408;O00408-3;O00408-2;O00408-4;O00408-6	O00408;O00408-2;O00408-3;O00408-4	PDE2A_HUMAN	PDE2A	NA	11.54322583	NA	NA	NA	AD vs MCI
P17612;P17612-2	P17612	KAPCA_HUMAN	PRKACA	0.357109241	14.24274589	1.631938469	0.105877134	-4.376603732	MCI vs control
Q13976;Q13976-2;Q13976-3	Q13976-2	KGP1_HUMAN	PRKG1	-0.350578113	13.53623696	-1.351347734	0.179640598	-4.812439466	MCI vs control
**Q13557;Q13557-8;Q13557-10;Q13557-11;Q13557-12;Q13557-3;Q13557-4;Q13557-5;Q13557-6;Q13557-9**	Q13557-12	KCC2D_HUMAN	CAMK2D	-0.309518827	11.91684452	-1.252711007	0.215634091	-4.763195823	MCI vs control
P32121;P32121-2;P32121-3;P32121-4;P32121-5	P32121;P32121-3	ARRB2_HUMAN	ARRB2	0.257143673	15.64826777	1.051027186	0.29506	-5.144963598	MCI vs control
Q9UQM7;Q9UQM7-2	Q9UQM7;Q9UQM7-2	KCC2A_HUMAN	CAMK2A	-0.107476251	13.65050863	-0.817935323	0.414604781	-5.403531747	MCI vs control
P25098	P25098	ARBK1_HUMAN	GRK2	0.217209276	13.29416777	0.816626376	0.415512168	-5.354758086	MCI vs control
Q13555;Q13555-5;Q13555-10;Q13555-11;Q13555-2;Q13555-3;Q13555-4;Q13555-6;Q13555-7;Q13555-8;Q13555-9	Q13555-11;Q13555-5;Q13555-6	KCC2G_HUMAN	CAMK2G	-0.12277011	13.43197138	-0.701747503	0.48426983	-5.421445479	MCI vs control
O00408;O00408-3;O00408-2;O00408-4;O00408-6	O00408;O00408-2;O00408-3;O00408-4	PDE2A_HUMAN	PDE2A	-0.604972969	11.54322583	-0.6595352	0.520437574	-4.687896653	MCI vs control
P62873;P62873-2	P62873	GBB1_HUMAN	GNB1	-0.104563021	16.7443974	-0.606375897	0.544946815	-5.703088734	MCI vs control

P17612;P22694;P22694-10;P22694-2;P22694-3;P22694-4;P22694-5;P22694-6;P22694-7;P22694-9;P17612-2	P22694-2	KAPCB_HUMAN	PRKACB	-0.110261392	13.99866741	-0.544356373	0.586874626	-5.675330311	MCI vs control
P49407;P49407-2	P49407-2	ARRB1_HUMAN	ARRB1	0.047492978	15.18759354	0.23238977	0.816513579	-5.771835385	MCI vs control
