# Supplementary material for: RBAD: The first database dedicated alterations of blood RNA in individuals with Alzheimer’s disease and their clinical relevance
Source: Neural Regen Res. 2025 Mar 25;21(6):2553–62. doi: 10.4103/NRR.NRR-D-24-01165 (PMC13211806; doi:10.4103/NRR.NRR-D-24-01165)
Supplement: Supplementary file 15 [file NRR-21-2553_Suppl11.pdf]

Additional Table 15. The overlapped genes between gene clusters (from bulk RNA-seq) and cell markers and differentially expressed genes (from scRNA-seq)		
Method	Got gene clusters from bulk RNA-seq data using Time-Series Analysis (detailed results are presented in Table S2)	
	Got **cell markers** of each cell type from scRNA-seq data through CellMarker database and manual annotation from published researches<br>Got **differentially expressed genes (DEGs)** between AD and normal of each cell type from scRNA-seq data by Wilcoxon Rank Sum test	
P value adjustment for multiple test	Benjiamini-Hochberg (FDR)	
Description	bulk RNA-seq	Cluster analysis of expression patterns of circulating mRNAs at different stages of disease (control, MCI, and AD) by Mfuzz R package. Data set: ROSMAP cohort in RAD-Blood.
	scRNA-seq	**Cell markers of each cell types and DEGs between AD and normal of each cell types. Data set: SRP309935 in RAD-Blood.**<br>**Cell marker analysis:** ●Pct. of this cell type (%): The proportion of cells in which the gene expression is detected in the current cell type. ● Percent of other CTs (%): The percentage of cells in which the gene expression is detected in other cell types. ●Log2FC: the log2 value of average gene expression of a selected cell type divided by that of other cell types.<br>**Differential expression analysis:** ●Average expr. (AD): the average expression of a specific genes in a specific cell type of AD samples. ●Average expr. (CT): the average expression of a specific genes in a specific cell type of normal samples. ●# of cell (AD): cell number of a specific cell type with a specific gene expressed, in AD samples. ●# of cell (CT): cell number of a specific cell type with a specific gene expressed, in normal samples. ●Pct. of cell (AD): the percentage of a specific cell type with a specific gene expressed, in AD samples. ●Pct. of cell (CT): the percentage of a specific cell type with a specific gene expressed, in normal samples. ●log2FC (AD vs. CT): the log2 value of average gene expression of a selected cell type in AD divided by that in normal.

Data type	bulk RNA-seq				scRNA-seq														
Method	Time-Series Analysis					Cell marker analysis					Differential expression analysis								
Symbol	Cluster	Control	MCI	AD	Cell type	Pct. of this cell type	Pct. of other cell types	log2FC	P value	FDR	Average expr. (AD)	Average expr. (CT)	# of cell (AD)	# of cell (CT)	Pct. of cell (AD)	Pct.of cell (CT)	log2FC (AD vs. CT)	P value	FDR
IL32	1	-1.04003349	0.08556565	0.95446784	B cell_1	18.20%	66.90%	-2.69821044	2.16E-07	0.00417728	0.41389098	0.2236821	1563	629	20.2815099	12.8775835	0.88780097	9.33E-06	0.00053781
KLRB1	1	-1.12510103	0.33758218	0.78751885	B cell_2	8.70%	40.10%	-2.37285699	2.27E-06	0.04403693	0.23351372	0.08640198	1052	473	10.5513308	4.4397463	1.434371	6.14E-05	0.00738332
S100A4	1	-1.13290465	0.37307371	0.75983095	B cell_2	30.20%	90.80%	-3.10962401	9.42E-15	1.82E-10	0.76088072	0.4726851	1052	473	33.0798479	23.8900634	0.68679094	6.20E-06	0.00112252
KLRB1	1	-1.12510103	0.33758218	0.78751885	CD4+ T cell	95.50%	37.60%	2.32658183	1.38E-14	2.67E-10	3.48351855	3.0415444	1830	250	96.5027322	88.4	0.19574118	6.59E-07	0.00022374
IL32	1	-1.04003349	0.08556565	0.95446784	CD8+ T cell_2	97.20%	62.40%	0.80682022	1.29E-06	0.02499564	3.06337085	2.93094697	4333	1278	97.1613201	97.1830986	0.06375316	1.66E-14	2.40E-12
BTF3	1	-1.00235086	0.00471841	0.99763244	Erythroid cell	81.70%	81.00%	-1.1481687	1.14E-06	0.02211826	1.44033223	1.18588338	172	270	80.8139535	82.2222222	0.28043949	5.21E-05	0.00200434
CA1	2	0.89740863	0.18057567	-1.07798431	Erythroid cell	70.60%	0.10%	3.62233782	7.16E-15	1.39E-10	1.02438805	2.13602676	172	270	48.255814	84.8148148	-1.06016739	1.17E-17	2.67E-15
CLIC1	1	-1.10946076	0.27756146	0.8318993	Erythroid cell	31.40%	61.40%	-1.61570121	4.30E-07	0.00833288	0.69827318	0.24988486	172	270	46.5116279	21.8518519	1.48252808	2.66E-10	2.79E-08
EEF1A1	1	-1.1467895	0.45653848	0.69025102	Erythroid cell	76.00%	98.80%	-2.2607299	4.08E-15	7.90E-11	2.77189198	1.50927278	172	270	86.627907	69.2592593	0.87701747	5.12E-16	9.54E-14

GYPC	1	-0.95981476	-0.07603032	1.03584508	Erythroid cell	87.30%	30.70%	1.05936095	1.97E-08	0.00038237	1.62996566	1.3594157	172	270	84.8837209	88.8888889	0.26185488	0.00011826	0.00409619
IL32	1	-1.04003349	0.08556565	0.95446784	Erythroid cell	37.60%	65.50%	-2.04711975	7.45E-08	0.0014426	1.12262277	0.45355733	172	270	54.6511628	26.6666667	1.30751641	4.60E-11	5.12E-09
NPM1	1	-1.13261951	0.76093706	0.37168245	Erythroid cell	37.60%	67.20%	-1.63938564	3.33E-07	0.00644795	0.80906441	0.35931792	172	270	52.3255814	28.1481481	1.17099368	4.39E-09	3.84E-07
RPL12	1	-1.14807416	0.68101507	0.4670591	Erythroid cell	70.60%	97.30%	-2.00911618	3.03E-12	5.87E-08	2.18412786	1.14223046	172	270	85.4651163	61.1111111	0.93520355	7.25E-17	1.54E-14
RPL15	1	-1.13136824	0.36567303	0.76569521	Erythroid cell	62.70%	97.70%	-2.05715298	3.59E-13	6.96E-09	2.23629658	0.9281418	172	270	84.8837209	48.5185185	1.26869439	3.05E-24	5.90E-21
RPL17	1	-1.12834443	0.35173604	0.7766084	Erythroid cell	64.70%	97.80%	-2.06703619	4.11E-14	7.96E-10	2.31890853	1.03438517	172	270	83.1395349	52.962963	1.16467242	3.63E-22	2.35E-19
RPL18	1	-1.1079059	0.272158	0.83574789	Erythroid cell	58.80%	96.10%	-2.00517495	3.78E-15	7.32E-11	2.02844578	0.83212435	172	270	82.5581395	43.7037037	1.2855037	2.39E-22	1.71E-19
RPL22	1	-1.13096495	0.36376805	0.7671969	Erythroid cell	53.20%	89.80%	-1.85809496	4.04E-12	7.81E-08	1.57591033	0.64927604	172	270	75.5813953	38.8888889	1.27928157	1.14E-18	2.94E-16
RPL23	1	-1.05371975	0.11789042	0.93582933	Erythroid cell	47.10%	85.80%	-1.93342301	2.47E-10	4.78E-06	1.19604342	0.54917561	172	270	64.5348837	35.9259259	1.12293031	4.13E-12	5.22E-10
RPL24	1	-1.15412723	0.54555564	0.60857159	Erythroid cell	58.10%	90.10%	-1.77404977	2.81E-10	5.44E-06	1.60075859	0.71701827	172	270	77.9069767	45.5555556	1.15867396	9.30E-18	2.14E-15
RPL29	1	-1.0186533	0.0384136	0.9802397	Erythroid cell	61.80%	96.00%	-2.07950772	1.79E-13	3.47E-09	1.8820236	0.84953324	172	270	81.3953488	49.2592593	1.14754243	2.63E-19	7.39E-17
RPL34	1	-1.03581509	0.07596261	0.95985248	Erythroid cell	73.10%	98.90%	-2.19516098	2.45E-16	4.75E-12	2.88645454	1.40015971	172	270	87.7906977	63.7037037	1.04370711	2.83E-22	1.96E-19
RPL36	1	-1.15258583	0.51579972	0.63678611	Erythroid cell	60.60%	96.80%	-2.13434844	1.61E-15	3.12E-11	2.05753486	0.87310872	172	270	84.8837209	45.1851852	1.23668365	6.40E-22	3.64E-19
RPL7	1	-1.15205578	0.64367112	0.50838466	Erythroid cell	77.10%	98.00%	-2.0662819	9.17E-15	1.77E-10	2.37106329	1.26992696	172	270	90.1162791	68.8888889	0.90078865	8.11E-20	2.62E-17
RPL7A	1	-1.04943378	0.10756397	0.94186981	Erythroid cell	56.60%	91.70%	-2.00672969	2.26E-12	4.37E-08	1.57872075	0.74627386	172	270	74.4186047	45.1851852	1.08097894	1.19E-14	1.93E-12
RPL9	1	-0.99031039	-0.01910545	1.00941583	Erythroid cell	63.30%	96.10%	-2.08528757	2.21E-12	4.27E-08	1.98292628	0.99302284	172	270	80.2325581	52.5925926	0.99773224	1.95E-15	3.46E-13
RPS10	1	-1.15189217	0.64564771	0.50624446	Erythroid cell	61.50%	94.40%	-2.0965278	1.17E-14	2.27E-10	1.86220662	0.7868377	172	270	84.3023256	47.037037	1.24287516	6.12E-22	3.59E-19
RPS13	1	-1.06199287	0.13840458	0.92358829	Erythroid cell	67.00%	96.40%	-2.05603388	1.49E-11	2.88E-07	2.0689669	0.98231049	172	270	84.8837209	55.5555556	1.07465956	3.39E-20	1.22E-17
RPS19	1	-1.13581007	0.3877617	0.74804837	Erythroid cell	72.20%	98.80%	-2.04260586	8.84E-14	1.71E-09	2.74309357	1.28940721	172	270	88.372093	61.8518519	1.08909588	8.05E-23	6.49E-20
RPS2	1	-1.15439347	0.60025739	0.55413608	Erythroid cell	73.30%	98.50%	-2.12812338	3.20E-15	6.19E-11	2.67024322	1.2990492	172	270	86.627907	64.8148148	1.03951508	9.90E-22	5.33E-19
RPS24	1	-1.12761045	0.34846595	0.7791445	Erythroid cell	66.70%	96.40%	-2.02028889	2.63E-11	5.08E-07	1.98680323	1.00359921	172	270	84.8837209	55.1851852	0.98526575	7.92E-17	1.67E-14
RPS27A	1	-0.89408047	-0.18578287	1.07986334	Erythroid cell	71.50%	98.80%	-2.12749417	4.72E-17	9.13E-13	2.64914727	1.25349247	172	270	88.372093	60.7407407	1.07957472	4.97E-22	3.01E-19
RPS28	1	-1.15230853	0.64048773	0.5118208	Erythroid cell	64.90%	98.40%	-2.1626013	2.67E-14	5.17E-10	2.42433804	1.10593957	172	270	83.7209302	52.962963	1.13231832	3.14E-21	1.48E-18
RPS3A	1	-1.00838926	0.01699516	0.9913941	Erythroid cell	70.40%	97.20%	-2.08165227	1.24E-15	2.41E-11	2.098492	1.21560376	172	270	83.7209302	61.8518519	0.78767992	1.76E-11	2.07E-09
S100A4	1	-1.13290465	0.37307371	0.75983095	Erythroid cell	52.90%	89.70%	-2.2019781	4.28E-09	8.28E-05	1.8376638	0.80673372	172	270	73.255814	40	1.18770838	1.39E-15	2.53E-13
SRGN	1	-1.08593371	0.20302425	0.88290946	Erythroid cell	43.70%	72.20%	-1.70512564	6.11E-07	0.01182225	1.18784994	0.39759551	172	270	66.8604651	28.8888889	1.57897923	4.75E-19	1.26E-16

SRP14	1	-0.91796332	-0.14765457	1.06561789	Erythroid cell	38.50%	71.30%	-1.63723904	7.93E-10	1.54E-05	0.78589468	0.35919377	172	270	51.744186	30	1.12957365	1.44E-08	1.18E-06
TPT1	1	-1.08786577	0.20865532	0.87921045	Erythroid cell	85.10%	96.30%	-1.90493811	9.30E-12	1.80E-07	2.19624134	1.48673312	172	270	91.2790698	81.1111111	0.5628909	8.18E-11	8.95E-09
TRIM58	2	1.0766673	-0.17696038	-0.89970692	Erythroid cell	34.60%	0.80%	0.70948578	1.77E-06	0.03417702	0.2168607	0.47024128	172	270	18.0232558	45.1851852	-1.11663259	2.32E-07	1.58E-05
PF4	2	1.11208718	-0.28688458	-0.8252026	Megakaryocyte_1	80.30%	10.50%	1.65511746	1.15E-09	2.23E-05	2.16450453	2.43547952	1569	765	78.3301466	84.3137255	-0.17016903	1.50E-05	0.00113215
IL32	1	-1.04003349	0.08556565	0.95446784	Megakaryocyte_2	8.40%	66.00%	-2.84735295	2.04E-09	3.96E-05	0.31009428	0.12494712	417	418	11.7505995	5.02392344	1.31138931	0.00038326	0.02821538
ITGA2B	2	1.11407948	-0.29413257	-0.81994692	Megakaryocyte_2	55.70%	1.40%	3.50832969	2.70E-09	5.24E-05	1.34150032	1.84862424	417	418	49.4004796	61.9617225	-0.4626046	2.70E-07	0.00012436
KLRB1	1	-1.12510103	0.33758218	0.78751885	Megakaryocyte_2	4.90%	39.80%	-2.52975955	2.18E-06	0.04220463	0.20289745	0.04366727	417	418	7.91366906	1.9138756	2.21612638	5.27E-05	0.00633717
MYO1F	1	-1.13704511	0.39432063	0.74272449	Megakaryocyte_2	3.60%	43.40%	-1.7237531	1.77E-06	0.03417702	0.1152689	0.02477028	417	418	5.75539568	1.4354067	2.2183211	0.00075391	0.04477673
NPM1	1	-1.13261951	0.76093706	0.37168245	Megakaryocyte_2	5.90%	67.80%	-2.35703868	7.57E-11	1.46E-06	0.16924673	0.0699939	417	418	8.63309353	3.11004785	1.2738268	0.00082079	0.0477243
PARVB	2	1.11026213	-0.28037936	-0.82988277	Megakaryocyte_2	41.10%	3.60%	2.94670355	7.78E-09	0.00015063	0.91211406	1.43208358	417	418	33.0935252	49.0430622	-0.65082955	3.30E-07	0.00014518
PF4	2	1.11208718	-0.28688458	-0.8252026	Megakaryocyte_2	99.60%	11.80%	7.11143003	9.12E-20	1.77E-15	5.39688682	5.85757274	417	418	99.2805755	100	-0.11817553	3.76E-23	1.45E-19
RPL18	1	-1.1079059	0.272158	0.83574789	Megakaryocyte_2	16.20%	96.90%	-3.02485456	1.38E-14	2.68E-10	0.58112474	0.28902629	417	418	21.3429257	11.0047847	1.00764714	3.22E-05	0.00442834
RPL23	1	-1.05371975	0.11789042	0.93582933	Megakaryocyte_2	10.70%	86.50%	-2.69026686	4.14E-15	8.01E-11	0.33956238	0.14706554	417	418	15.1079137	6.22009569	1.20721734	4.44E-05	0.00555203
RPL24	1	-1.15412723	0.54555564	0.60857159	Megakaryocyte_2	12.30%	90.80%	-2.7526658	4.03E-14	7.80E-10	0.39236038	0.20767495	417	418	16.3069544	8.37320574	0.91785216	0.00067736	0.04176639
RPL29	1	-1.0186533	0.0384136	0.9802397	Megakaryocyte_2	20.10%	96.70%	-2.90897207	3.48E-16	6.74E-12	0.6464003	0.38012071	417	418	25.1798561	15.0717703	0.76597024	0.0001916	0.01656103
RPL34	1	-1.03581509	0.07596261	0.95985248	Megakaryocyte_2	29.30%	99.70%	-3.42839591	6.32E-17	1.22E-12	1.06683864	0.63780472	417	418	35.4916067	23.2057416	0.7421553	1.66E-05	0.00276246
RPS10	1	-1.15189217	0.64564771	0.50624446	Megakaryocyte_2	14.00%	95.20%	-3.16034916	2.01E-12	3.90E-08	0.48499987	0.23476968	417	418	18.705036	9.33014354	1.04673825	7.91E-05	0.00827652
RPS13	1	-1.06199287	0.13840458	0.92358829	Megakaryocyte_2	20.60%	97.10%	-2.88505638	5.44E-14	1.05E-09	0.70128271	0.42016212	417	418	25.8992806	15.3110048	0.73905004	0.00020341	0.01727415
RPS19	1	-1.13581007	0.3877617	0.74804837	Megakaryocyte_2	29.90%	99.50%	-3.21087969	3.04E-15	5.89E-11	1.10069123	0.61136904	417	418	37.6498801	22.2488038	0.84829441	3.18E-07	0.00014313
RPS24	1	-1.12761045	0.34846595	0.7791445	Megakaryocyte_2	19.00%	97.20%	-2.92781663	2.21E-16	4.28E-12	0.67373899	0.35348978	417	418	24.940048	13.1578947	0.93052128	1.76E-05	0.00285553
S100A4	1	-1.13290465	0.37307371	0.75983095	Megakaryocyte_2	16.30%	90.40%	-3.13351202	3.22E-13	6.23E-09	0.61193379	0.29314642	417	418	21.5827338	11.0047847	1.06175412	1.82E-05	0.00289895
TREML1	2	0.99522992	0.00947285	-1.00470278	Megakaryocyte_2	71.90%	2.10%	4.15955589	1.46E-12	2.83E-08	1.88784215	2.61953897	417	418	63.5491607	80.1435407	-0.47257478	1.49E-13	2.54E-10
UQCRB	1	-1.14141483	0.72196595	0.41944888	Megakaryocyte_2	18.60%	73.20%	-1.36273995	1.49E-09	2.88E-05	0.55535024	0.33550661	417	418	23.7410072	13.3971292	0.72705675	0.00027585	0.02164308
HLA-B	2	0.96406056	0.06836996	-1.03243052	Monocyte_1	92.50%	98.80%	-0.86456886	4.10E-07	0.00794666	2.81915832	2.9629403	7941	2887	91.80204	94.5964669	-0.07176506	6.40E-12	5.08E-10
HLA-DRA	2	0.83081405	0.27907913	-1.10989318	Monocyte_1	84.30%	31.20%	1.21693089	2.37E-08	0.00045982	2.42630622	2.71162833	7941	2887	82.3196071	89.7817804	-0.1603978	1.55E-23	2.92E-21
IL32	1	-1.04003349	0.08556565	0.95446784	Monocyte_1	18.90%	74.30%	-2.72849697	2.91E-09	5.63E-05	0.4237744	0.34019645	7941	2887	19.7330311	16.6608937	0.31692835	6.95E-05	0.00105761

NFKBIA	1	-1.10321361	0.25632763	0.84688599	Monocyte_1	89.20%	46.10%	2.1804931	2.15E-10	4.16E-06	2.80822393	2.57853701	7941	2887	89.4975444	88.3962591	0.12310523	1.11E-34	3.41E-32
S100A4	1	-1.13290465	0.37307371	0.75983095	Monocyte_1	99.10%	87.60%	0.92566085	6.32E-07	0.01224243	3.97108453	3.78071934	7941	2887	99.206649	98.926221	0.07087231	9.94E-60	5.66E-57
S100A8	1	-1.14861824	0.67681326	0.47180498	Monocyte_1	98.20%	11.70%	5.13298338	4.74E-15	9.17E-11	4.34546401	4.03810504	7941	2887	98.4888553	97.4714236	0.1058318	7.64E-41	2.96E-38
CTSW	1	-1.12500494	0.33717402	0.78783092	Monocyte_2	6.00%	42.60%	-2.37020635	1.11E-07	0.00214128	0.09691123	0.05128387	2139	818	6.82561945	3.78973105	0.91815874	0.0016801	0.027199
IL32	1	-1.04003349	0.08556565	0.95446784	Monocyte_2	15.50%	67.60%	-3.20423579	1.87E-11	3.62E-07	0.27717329	0.17970483	2139	818	16.6900421	12.4694377	0.62515904	0.00112522	0.02019132
NPC2	1	-1.12084353	0.3200422	0.80080133	Monocyte_2	95.00%	28.80%	1.98811569	2.12E-10	4.11E-06	2.38014942	2.29938259	2139	818	95.1846657	94.6210269	0.04980562	0.00027719	0.00665883
RPS19	1	-1.13581007	0.3877617	0.74804837	Monocyte_2	100.00%	98.60%	0.63911497	4.12E-07	0.0079695	4.59036188	4.52366077	2139	818	100	100	0.02111715	3.49E-07	2.41E-05
S100A4	1	-1.13290465	0.37307371	0.75983095	Monocyte_2	100.00%	88.90%	1.16440083	1.01E-09	1.95E-05	4.24421815	4.13267317	2139	818	99.9532492	100	0.03842354	2.01E-08	1.87E-06
MT-CO3	2	0.9358458	0.11786245	-1.05370825	Monocyte_3	100.00%	99.00%	0.73718493	1.52E-06	0.02945215	4.23427526	4.48363526	420	316	100	100	-0.08255386	8.22E-17	1.76E-13
NFKBIA	1	-1.10321361	0.25632763	0.84688599	Monocyte_3	91.60%	52.70%	1.63383651	1.00E-12	1.95E-08	2.97537627	2.69816727	420	316	91.9047619	91.1392405	0.14109233	1.65E-06	0.00066519
PF4	2	1.11208718	-0.28688458	-0.8252026	Monocyte_3	83.20%	12.20%	1.63485667	1.11E-10	2.15E-06	2.09642732	2.65433872	420	316	78.8095238	88.9240506	-0.34041967	1.00E-08	6.47E-06
RPL15	1	-1.13136824	0.36567303	0.76569521	Monocyte_3	93.50%	97.50%	-0.54282236	1.97E-07	0.0038235	3.06519975	2.88076875	420	316	94.047619	92.721519	0.08952724	0.00011954	0.02291567
S100A8	1	-1.14861824	0.67681326	0.47180498	Monocyte_3	98.40%	24.90%	2.12896999	2.42E-16	4.68E-12	4.16617943	3.8017663	420	316	98.8095238	97.7848101	0.13205513	1.38E-05	0.00387773
RPL18	1	-1.1079059	0.272158	0.83574789	Naive CD8+ T cell_1	99.70%	95.40%	0.49322393	1.19E-06	0.02307662	3.6136022	3.58296209	4730	2061	99.7251586	99.7088792	0.01228492	3.25E-06	9.64E-05
RPL36	1	-1.15258583	0.51579972	0.63678611	Naive CD8+ T cell_1	99.80%	96.20%	0.57308912	1.13E-07	0.00218327	3.7951885	3.76582205	4730	2061	99.7674419	99.9029597	0.01120672	6.99E-06	0.00018777
RPS2	1	-1.15439347	0.60025739	0.55413608	Naive CD8+ T cell_1	100.00%	98.10%	0.52547103	2.13E-08	0.00041194	4.44779818	4.41782202	4730	2061	99.9577167	99.9514799	0.00975603	1.26E-06	4.19E-05
RPS27A	1	-0.89408047	-0.18578287	1.07986334	Naive CD8+ T cell_1	99.90%	98.50%	0.46192721	1.05E-07	0.00202415	4.34641354	4.32897269	4730	2061	99.9154334	99.9029597	0.00580075	0.00311639	0.02709031
RPL36	1	-1.15258583	0.51579972	0.63678611	Naive CD8+ T cell_2	99.80%	96.20%	0.70844192	8.65E-08	0.00167449	3.91520519	3.83268793	3748	1792	99.8399146	99.6651786	0.03073139	1.60E-19	3.36E-17
RPS27A	1	-0.89408047	-0.18578287	1.07986334	Naive CD8+ T cell_2	99.90%	98.60%	0.72783037	2.30E-08	0.00044618	4.53943112	4.50062932	3748	1792	99.9199573	99.7767857	0.01238476	1.70E-05	0.00053216
RPS28	1	-1.15230853	0.64048773	0.5118208	Naive CD8+ T cell_2	99.90%	98.10%	0.6577097	1.98E-07	0.00382993	4.32593474	4.30607724	3748	1792	99.8932764	99.7767857	0.00663771	0.00092784	0.01322893
S100A4	1	-1.13290465	0.37307371	0.75983095	Naive CD8+ T cell_2	65.80%	91.60%	-2.20937385	1.14E-12	2.20E-08	1.55891053	1.33684043	3748	1792	66.8356457	63.6160714	0.22171085	1.68E-14	2.44E-12
CTSW	1	-1.12500494	0.33717402	0.78783092	Natural killer cell_1	82.40%	33.40%	1.59317498	2.32E-07	0.00449082	2.24831817	2.01413062	7639	2717	83.2176987	79.9411115	0.15868896	6.08E-33	1.37E-30
CCL4	2	1.08548518	-0.20172728	-0.8837579	Natural killer cell_2	65.70%	30.40%	1.66798416	9.95E-09	0.00019267	1.60434796	2.08152576	3604	1523	60.8490566	77.216021	-0.37565433	2.25E-25	9.08E-23
CD7	2	1.06051229	-0.13467397	-0.92583832	Natural killer cell_2	81.30%	32.70%	1.99091004	7.44E-10	1.44E-05	2.08138194	2.35129306	3604	1523	78.9400666	86.9336835	-0.17591264	2.92E-13	4.55E-11
CTSW	1	-1.12500494	0.33717402	0.78783092	Natural killer cell_2	86.80%	37.20%	1.63106371	2.62E-09	5.08E-05	2.43164443	2.28008539	3604	1523	86.9034406	86.4740643	0.09284443	7.39E-13	1.08E-10
HOPX	2	0.93815547	0.11393018	-1.05208566	Natural killer cell_2	75.10%	28.50%	1.65112623	3.35E-08	0.00064946	1.84676675	2.06774598	3604	1523	72.3917869	81.5495732	-0.1630573	2.93E-06	0.00011615
