# Supplementary material for: RBAD: The first database dedicated alterations of blood RNA in individuals with Alzheimer’s disease and their clinical relevance
Source: Neural Regen Res. 2025 Mar 25;21(6):2553–62. doi: 10.4103/NRR.NRR-D-24-01165 (PMC13211806; doi:10.4103/NRR.NRR-D-24-01165)
Supplement: Supplementary file 18 [file NRR-21-2553_Suppl12.pdf]

Additional Table 17. Circulating mRNAs that significantly associated with OS independent of sex, age, ApoE genotype, education, and race.

Method	Survival analysis (log-rank test, univariate Cox proportional hacard regression, and multivariate Cox proportional hazard regression).
	Differential expression analysis (DEseq2)
P value adjustment for multiple test	Benjiamini-Hochberg (FDR)
Description	**Circulating mRNAs whose expression were associated with OS and their differences between AD and normal samples.**<br>**In survival analysis**, samples were divided into two groups by median expression of each circulating mRNA. The lower expression group was set as the reference group, the hazard ratio is the probability of death in higher expression group relative to the reference group probability. In multivarate Cox proportional hazard regression analysis, the sex, age, ApoE genotypes, education levels, and race were added as covariates to construct multivariate regression model and check whether a circulating mRNA is independently assocaited with OS. Risk: the gene's higher expression has higher risk of death. Protective: the gene's higher expression has lower risk of death. NS: not significant.<br>**In differential expression analysis,** the expression of each gene was compared between AD patients and healthy controls. Set the control group as the reference group to get the fold change. DEG: up, down, and NS mean genes upregulated, downregulated, and not significant in AD compared with normal.

Method	Survival analysis							Differential expression analysis (DEseq2)			
	Log-rank test	Univariate Cox proportional hazard regression		Multivariate Cox proportional hazard regression		Survival risk					
Symbol	P value	Hazard ratio	P value	Hazard ratio	P value	Group with higher risk of death	Risk or protective?	Log2 fold change(AD vs. CT)	P value	FDR	DEG
GOLGA6A	0.02856961	0.432626282	0.033034625	0.308339893	0.025507316	Lower expr.	Protective	-0.473836686	0.000258793	0.007860526	NS
CKMT2	0.042127309	0.470965482	0.046749607	0.312435869	0.023333668	Lower expr.	Protective	0.813990804	0.000485288	0.012025962	Up
KIT	0.000985088	0.254534221	0.002039158	0.359294791	0.049754668	Lower expr.	Protective	-0.685830731	0.000553037	0.013164878	Down
MCM2	0.04010834	2.164170359	0.045038701	3.477998456	0.011924816	Higher expr.	Risk	-0.445510851	0.000605125	0.014032225	NS
NAPSA	0.029073865	2.515431991	0.034474527	4.919487357	0.003052247	Higher expr.	Risk	-0.37286214	0.000636724	0.014542011	NS
TAS2R3	0.028531047	2.296457801	0.03304877	2.606962546	0.044019915	Higher expr.	Risk	1.245853693	0.000661257	0.014910868	Up
ARMH2	0.01412851	2.658058999	0.017710033	5.592223486	0.001216064	Higher expr.	Risk	1.21016307	0.000716221	0.015729139	Up
HRAS	0.022786845	2.479853268	0.027253024	3.033297917	0.029725001	Higher expr.	Risk	-0.329492213	0.001036873	0.020109404	NS
GPHB5	0.045603963	2.419620876	0.05233798	3.275397625	0.032598777	Higher expr.	Risk	1.123264212	0.001493489	0.025877207	Up
OVOL1	0.01872762	2.476653698	0.02267523	4.00357479	0.006267488	Higher expr.	Risk	-0.653173081	0.001518402	0.026210831	Down
MPIG6B	0.048363413	2.206386246	0.053588098	3.316441676	0.011887541	Higher expr.	Risk	-0.428687771	0.002500251	0.036031623	NS
TTC39B	0.008847177	3.068402582	0.012256222	3.278883086	0.024297757	Higher expr.	Risk	-0.289550146	0.002623249	0.037263135	NS
ARL8A	0.013626885	0.399758556	0.016905047	0.232430124	0.003447836	Lower expr.	Protective	0.279691598	0.002675665	0.037708964	NS
NTM	0.044258434	0.478723249	0.04877268	0.207867285	0.003307875	Lower expr.	Protective	0.528049324	0.002688067	0.037816638	NS
ZNF596	0.004684799	2.992716465	0.006886941	3.965130845	0.004897144	Higher expr.	Risk	-0.317053939	0.00282262	0.039066863	NS
DEPDC1	0.010802365	0.335400053	0.014096918	0.119954272	0.00182354	Lower expr.	Protective	0.721937092	0.00286699	0.03951859	Up
CSRNP3	0.001048029	0.266320468	0.002168699	0.120214017	0.000205276	Lower expr.	Protective	0.547556556	0.003174796	0.042116898	NS
OR10D3	0.007121641	0.307899387	0.010775987	0.355307075	0.047774801	Lower expr.	Protective	0.843180394	0.003252662	0.042862255	Up
ACE	0.003582479	0.313543104	0.005541901	0.253074566	0.005258898	Lower expr.	Protective	-0.412563669	0.003648926	0.046085415	NS
IL17F	0.038865304	0.400084818	0.044134226	0.268058065	0.033184187	Lower expr.	Protective	0.896681664	0.003770714	0.047056881	Up
TRIM35	0.020358354	2.40593764	0.023817118	2.966596454	0.022017142	Higher expr.	Risk	-0.619512762	0.003784297	0.047173444	Down
ERCC6L	0.003890771	0.314755708	0.005965095	0.22890736	0.019917249	Lower expr.	Protective	0.646816142	0.004477605	0.05233408	NS
OR8S1	0.012647493	2.542790228	0.016033776	3.556713137	0.008415247	Higher expr.	Risk	0.902597007	0.004812379	0.054762233	NS
OR10G6	0.008658779	2.755620043	0.011748476	2.729857356	0.039933608	Higher expr.	Risk	1.148465913	0.005331683	0.058506314	NS
LTBP1	0.005574523	3.025737375	0.008127874	6.440159241	0.001983683	Higher expr.	Risk	-0.522215673	0.005401361	0.058960969	NS

HNF1A	0.046622746	0.430961376	0.052529853	0.208091693	0.006367505	Lower expr.	Protective	-0.388339448	0.005639246	0.060617265	NS
KCNA7	0.009778515	0.375618238	0.012463239	0.270078967	0.007088344	Lower expr.	Protective	-0.412166357	0.006582897	0.066883007	NS
CD8B	0.010909575	3.009577095	0.014778346	16.49845361	4.41E-05	Higher expr.	Risk	0.416312574	0.006969462	0.069632851	NS
SLC10A5	0.026836858	0.320206059	0.035401171	0.094935803	0.008848697	Lower expr.	Protective	0.987029677	0.007397463	0.072301844	NS
DEFB131B	0.002790548	3.365568605	0.004746306	7.624884961	0.000115835	Higher expr.	Risk	1.118489677	0.008082456	0.076152925	NS
PRRG3	0.018080972	0.405328418	0.021845199	0.332829897	0.045708536	Lower expr.	Protective	-0.293880468	0.009811741	0.085798127	NS
TMEM247	0.001003773	4.030649384	0.002288223	3.632788234	0.022946253	Higher expr.	Risk	0.903158408	0.010835344	0.091350188	NS
DAPK2	0.039746198	2.193307467	0.044681832	2.59353619	0.045496001	Higher expr.	Risk	-0.289754802	0.011086513	0.092694454	NS
SAA1	0.023096359	2.369787035	0.027018137	4.882862663	0.001802384	Higher expr.	Risk	0.745256496	0.01128571	0.093557779	NS
TRHR	0.01526517	0.379041007	0.018843283	0.276652648	0.00907331	Lower expr.	Protective	0.679171993	0.01144264	0.094310161	NS
DKK4	0.00492339	2.995855917	0.007158693	4.510964802	0.01498539	Higher expr.	Risk	0.867987149	0.014691108	0.110424685	NS
RPS4Y1	0.021742499	2.43647154	0.02613989	2.812985597	0.042845075	Higher expr.	Risk	-1.07756187	0.015274388	0.113065866	NS
PTMS	0.037838655	0.461355217	0.041955197	0.108509087	0.000251363	Lower expr.	Protective	-0.385120105	0.015787762	0.115390793	NS
NPC1L1	0.032907775	2.311454415	0.037148449	4.558868838	0.002589823	Higher expr.	Risk	-0.411178964	0.020651913	0.134558192	NS
ZNF470	0.032689142	0.435251591	0.037563427	0.334590638	0.035880764	Lower expr.	Protective	-0.293361221	0.020694252	0.134739289	NS
SLC10A2	0.044879508	2.075108716	0.049520023	2.914444063	0.028730868	Higher expr.	Risk	0.627147206	0.020925701	0.135627948	NS
PMEPA1	0.033233024	0.460787942	0.037529087	0.18972244	0.002173137	Lower expr.	Protective	-0.324331166	0.02438973	0.1485284	NS
TKTL1	0.00107448	0.263236465	0.001955263	0.075592463	0.000105547	Lower expr.	Protective	-0.486442923	0.024643038	0.149535787	NS
RAPGEF3	0.038326553	0.44473189	0.042561117	0.333775253	0.048538845	Lower expr.	Protective	-0.326453869	0.025031047	0.150922894	NS
FABP2	0.004724787	0.300244765	0.007419449	0.270162028	0.020846237	Lower expr.	Protective	0.703393732	0.026657982	0.156125886	NS
LCE6A	0.030186108	2.439376208	0.035788675	3.751763898	0.005719234	Higher expr.	Risk	0.970153155	0.026849211	0.15679623	NS
FAM205C	0.009933205	2.638234446	0.012932697	3.394144739	0.007739056	Higher expr.	Risk	0.648221657	0.030693522	0.169179458	NS
EMSY	0.005914429	3.211324479	0.008802348	3.598727014	0.020557484	Higher expr.	Risk	-0.232848093	0.032678751	0.175912569	NS
IZUMO2	0.00981426	0.373895393	0.012872546	0.280929281	0.029901172	Lower expr.	Protective	-0.346944704	0.03303376	0.177271704	NS
KRT8	0.007338792	3.193100342	0.010439256	4.308731082	0.008239344	Higher expr.	Risk	-0.251039641	0.033180845	0.177714791	NS
OPN1MW	0.000463306	0.223996012	0.00120194	0.285832848	0.036636747	Lower expr.	Protective	-0.465892109	0.033798761	0.179520784	NS
TBC1D23	0.001409939	0.29782071	0.002509393	0.217850571	0.002878744	Lower expr.	Protective	0.254948652	0.034028647	0.180275289	NS
H2BW1	0.013629189	2.976966124	0.018622986	4.758674704	0.004284217	Higher expr.	Risk	0.746710717	0.034800698	0.18267655	NS
ZNF385D	0.001436071	0.296918413	0.002526636	0.239348635	0.002986061	Lower expr.	Protective	0.325904231	0.037473468	0.189891992	NS
OR11H4	0.015371263	2.63909989	0.019513354	4.534950749	0.002027438	Higher expr.	Risk	0.671668156	0.039036794	0.194306544	NS
CDX2	0.006637413	0.333994743	0.009551541	0.294750733	0.016614032	Lower expr.	Protective	-0.500778911	0.040078295	0.19718402	NS
CYP4Z1	0.040713361	0.439826263	0.045616935	0.329779534	0.027372037	Lower expr.	Protective	0.473878677	0.040105843	0.197214444	NS
RIC8B	0.027185471	2.421953884	0.03115868	4.619147001	0.016814285	Higher expr.	Risk	-0.248017971	0.041610629	0.201418201	NS
TRIM42	0.005662709	2.960561434	0.00815686	3.146400455	0.013200934	Higher expr.	Risk	0.554950005	0.04409122	0.207933824	NS
GBP5	0.040615425	2.119166877	0.045047243	4.275312909	0.001975364	Higher expr.	Risk	-0.306075068	0.044295072	0.208440337	NS
BNIP5	0.037905651	2.201046656	0.042351193	2.976748336	0.03221354	Higher expr.	Risk	-0.457259206	0.048870062	0.220201921	NS
UTS2R	0.038668368	0.462214122	0.043313127	0.346204909	0.032420909	Lower expr.	Protective	0.214003522	0.050886659	0.225036521	NS
LCOR	0.003836407	0.332137693	0.00577688	0.330017077	0.016866082	Lower expr.	Protective	-0.146408655	0.050961359	0.225187706	NS
ZFP69B	0.005540645	3.001874579	0.007807199	6.437486724	0.00095376	Higher expr.	Risk	0.439435846	0.051255943	0.226004507	NS
ADAM23	0.018641861	0.413414219	0.022061995	0.412722187	0.047816483	Lower expr.	Protective	0.299558006	0.052758972	0.229608105	NS
FBXW11	0.036763244	0.443911188	0.041382136	0.205663576	0.003999038	Lower expr.	Protective	-0.123369018	0.052894996	0.229920837	NS
DEFB113	8.56E-08	15.13379369	4.21E-05	120.862111	0.000385331	Higher expr.	Risk	1.170584049	0.053308643	0.230951491	NS
GJB5	0.012272032	2.634933884	0.015646963	4.179893454	0.00286939	Higher expr.	Risk	0.534591543	0.054837459	0.234680175	NS
ZEB1	0.000505617	0.269112559	0.001097504	0.161466399	0.00077798	Lower expr.	Protective	-0.276759823	0.055394252	0.235724551	NS
LHCGR	0.02445544	0.437697758	0.028432431	0.236173194	0.004654382	Lower expr.	Protective	0.394540704	0.05602028	0.237352789	NS
OR56B1	0.00136643	0.269222515	0.002533827	0.213781008	0.004369301	Lower expr.	Protective	0.696983729	0.056974095	0.23954978	NS
CCL13	0.000184067	5.642275763	0.000841487	9.223975716	0.000172914	Higher expr.	Risk	0.846038162	0.057027622	0.239702307	NS
OR4D6	0.017050798	2.595234763	0.021353332	3.589026093	0.026942149	Higher expr.	Risk	0.691685677	0.057480582	0.240678015	NS
OR13G1	0.045103967	2.170092761	0.050051552	3.088874468	0.028777023	Higher expr.	Risk	0.682363819	0.057553744	0.240821085	NS
SAR1A	0.04517442	0.463922272	0.050211526	0.244047678	0.030707797	Lower expr.	Protective	-0.175449921	0.057572283	0.240862394	NS
TSPYL6	0.000317794	4.19434919	0.000708476	5.882807323	0.000759158	Higher expr.	Risk	0.538981018	0.057681916	0.241085167	NS
MFHAS1	0.019990796	0.399513894	0.023755672	0.283506974	0.013124732	Lower expr.	Protective	-0.221393992	0.059201361	0.244302942	NS
PYROXD2	0.039062347	2.176774919	0.043441573	2.689980337	0.033408268	Higher expr.	Risk	0.239387949	0.062547945	0.251829198	NS
CENPB	0.031633209	2.293664901	0.036550905	3.667161469	0.008936705	Higher expr.	Risk	-0.173267785	0.062869584	0.252488299	NS
FUCA2	0.008219808	0.347302621	0.010737291	0.13914737	0.001384201	Lower expr.	Protective	0.195035422	0.064623541	0.256319806	NS

MPL	0.048683133	2.185263329	0.053956667	3.938248676	0.006549256	Higher expr.	Risk	-0.254748675	0.066637811	0.260679576	NS
RAB26	0.016161199	0.388588689	0.020183624	0.170889244	0.00304644	Lower expr.	Protective	-0.289925637	0.0671672	0.261902607	NS
MESD	0.034684221	2.238341032	0.039464102	3.989091913	0.020337357	Higher expr.	Risk	-0.154215072	0.070801387	0.269752846	NS
TRAM1	0.023852841	0.409882278	0.028004309	0.079289729	0.000333679	Lower expr.	Protective	-0.148681831	0.073859328	0.276290254	NS
UBE2E3	0.012359459	0.383164165	0.015713472	0.245591077	0.004726271	Lower expr.	Protective	-0.151293124	0.075942907	0.280702923	NS
TPTE2	0.020466135	0.417806559	0.0243441	0.364849399	0.040165045	Lower expr.	Protective	0.312811924	0.076844619	0.282588907	NS
SALL3	0.018868756	0.409382808	0.022950461	0.243125823	0.012280037	Lower expr.	Protective	-0.31318401	0.079238653	0.287737722	NS
ZNF528	0.003874446	0.318105509	0.00582599	0.218087731	0.002714529	Lower expr.	Protective	-0.265696444	0.080999914	0.291015399	NS
AKAIN1	0.024068542	2.412631224	0.028652148	2.990432604	0.019537564	Higher expr.	Risk	0.525565171	0.083386985	0.295541808	NS
SIX5	0.009488107	0.375075347	0.012559437	0.199780189	0.003582133	Lower expr.	Protective	-0.367418632	0.083599498	0.295898783	NS
STAC	0.049987308	0.449459306	0.055004685	0.34642691	0.04388461	Lower expr.	Protective	0.422126628	0.086644046	0.301453594	NS
FGF21	0.006136245	3.530969604	0.009844536	7.315902261	0.00109293	Higher expr.	Risk	-0.473296344	0.087908223	0.30349507	NS
ADAM11	0.018941011	0.399006484	0.023075042	0.288517378	0.008003521	Lower expr.	Protective	0.293589018	0.088664232	0.305211365	NS
COL11A1	0.006034502	0.343994445	0.008238762	0.25930266	0.025468349	Lower expr.	Protective	0.311382596	0.089593982	0.3069264	NS
FAM169A	0.034421908	0.447264464	0.038898998	0.274839164	0.006989119	Lower expr.	Protective	0.299298069	0.090392535	0.308302343	NS
FAM184A	0.002035027	0.313356295	0.003206585	0.374396284	0.033130919	Lower expr.	Protective	0.300016573	0.090669843	0.308842872	NS
WARS1	0.023783004	2.29730551	0.027718213	3.192172748	0.007340473	Higher expr.	Risk	-0.10453244	0.091303211	0.309883777	NS
EOMES	0.015111572	0.402690451	0.018628178	0.401268317	0.042938445	Lower expr.	Protective	0.38794275	0.091287033	0.309883777	NS
ANO5	0.018369441	2.383560045	0.021895234	3.714173212	0.004079228	Higher expr.	Risk	0.269326754	0.091476395	0.31015194	NS
CCBE1	0.032394755	0.447897271	0.0370484	0.280029902	0.015863988	Lower expr.	Protective	0.209004217	0.092653944	0.312542591	NS
HHIP	0.01204305	0.384045947	0.015278149	0.215039854	0.011752456	Lower expr.	Protective	-0.239742331	0.0956803	0.318162136	NS
BUB1	0.005327987	0.359666055	0.007476279	0.266832123	0.014250584	Lower expr.	Protective	0.29058421	0.097047846	0.320669806	NS
EMCN	0.009796326	0.348145318	0.013216718	0.272369083	0.010063811	Lower expr.	Protective	0.268254858	0.097900947	0.321982641	NS
HSD3B7	0.018281488	2.489793708	0.022327738	2.881571461	0.043022353	Higher expr.	Risk	-0.247703194	0.099872616	0.325437887	NS
CYP26C1	0.015591538	0.386338845	0.019641154	0.335785156	0.027120691	Lower expr.	Protective	-0.376712943	0.101842261	0.328562322	NS
COBLL1	0.005169655	0.326089631	0.007647548	0.27963612	0.017196339	Lower expr.	Protective	-0.204531838	0.103416925	0.331315639	NS
TIAM2	0.001204978	0.289855945	0.00216913	0.336105848	0.035911581	Lower expr.	Protective	0.179562652	0.103793659	0.331949114	NS
NUP214	0.003997193	0.348931372	0.005690929	0.122618741	0.00012765	Lower expr.	Protective	-0.071566866	0.105209744	0.33436126	NS
HRH2	0.040948189	2.196719954	0.045647997	4.564694626	0.017640546	Higher expr.	Risk	-0.133595157	0.105263605	0.334455941	NS
MED29	0.032771028	2.472024402	0.038469796	3.781036159	0.014980693	Higher expr.	Risk	0.091295946	0.107602601	0.338556804	NS
GSTCD	0.025638693	2.356739172	0.02956807	2.798096695	0.040820933	Higher expr.	Risk	-0.186528467	0.108524284	0.340264768	NS
LEPROT	0.030461799	0.449078962	0.034634635	0.20209736	0.002314932	Lower expr.	Protective	0.136111385	0.111819465	0.34528142	NS
SYT8	0.014779476	0.401017136	0.018278652	0.227079741	0.007611872	Lower expr.	Protective	-0.303863125	0.113316784	0.34787364	NS
CPNE5	0.019962568	2.746604993	0.025059008	6.676666084	0.002158684	Higher expr.	Risk	-0.218372986	0.114596365	0.349831081	NS
FHDC1	0.003546516	3.084431788	0.005424687	4.515513754	0.00242529	Higher expr.	Risk	0.23338494	0.115466163	0.351024569	NS
NPRL2	0.004584353	2.915727578	0.006694798	2.535124779	0.033302132	Higher expr.	Risk	-0.241326904	0.116778516	0.353150111	NS
FCGBP	0.019253065	0.412786298	0.022981799	0.316700925	0.013889173	Lower expr.	Protective	-0.243642303	0.117916518	0.354841197	NS
LIMA1	0.017263567	0.403065313	0.020992712	0.327912673	0.022247264	Lower expr.	Protective	-0.180442772	0.120424483	0.358807743	NS
TBX3	0.030720352	0.436266767	0.035066919	0.220460957	0.017844605	Lower expr.	Protective	-0.3438263	0.120935885	0.359490858	NS
CSF2	0.017791866	2.5296185	0.022078898	3.407865099	0.043509224	Higher expr.	Risk	0.463579276	0.121119687	0.359726609	NS
GOLT1A	0.010207945	0.223076068	0.017163951	0.247414739	0.040707997	Lower expr.	Protective	0.510036065	0.124047168	0.364277145	NS
CRACR2B	0.025907624	0.432458568	0.030166771	0.288567473	0.006076184	Lower expr.	Protective	-0.238598838	0.124269606	0.364564404	NS
DMRTC1	0.048170938	0.48396004	0.052749685	0.370155518	0.034555595	Lower expr.	Protective	-0.27998342	0.124386831	0.364703855	NS
SCGB3A2	0.046270351	2.103336463	0.051102565	3.270454355	0.008825869	Higher expr.	Risk	0.237480412	0.124461779	0.364745341	NS
IGF1	0.008086622	0.347789974	0.010945166	0.181096476	0.013920572	Lower expr.	Protective	0.194530489	0.124490873	0.364770728	NS
OR11H1	0.042632241	0.470083762	0.047168526	0.310465411	0.032774819	Lower expr.	Protective	0.436601107	0.126032311	0.366908291	NS
ELAVL4	0.007430971	0.337062908	0.010473019	0.318403265	0.019544635	Lower expr.	Protective	0.296730477	0.12884903	0.371142896	NS
C5orf51	0.045138823	2.331299236	0.051039621	3.768313387	0.014634068	Higher expr.	Risk	0.322258745	0.130674269	0.373573963	NS
ART1	0.018416013	2.473222836	0.022050356	3.892577977	0.003541203	Higher expr.	Risk	-0.367369013	0.132877071	0.376636365	NS
KRT39	0.032388712	0.43374831	0.0366288	0.286124457	0.007184559	Lower expr.	Protective	0.3964226	0.133090229	0.376913807	NS
CDYL2	0.000390077	0.250180317	0.000924225	0.174515178	0.000360857	Lower expr.	Protective	-0.238068046	0.133139606	0.376957612	NS
HDX	0.027698846	0.440380705	0.031916607	0.344567752	0.035048305	Lower expr.	Protective	-0.258761012	0.135110031	0.380077734	NS
ASPM	0.02278177	0.436780845	0.02642038	0.357381484	0.018358542	Lower expr.	Protective	0.292199374	0.139449267	0.385303379	NS
CYLD	0.009218274	0.373836038	0.012089362	0.33839979	0.0486438	Lower expr.	Protective	-0.128208581	0.140019297	0.386034056	NS
CLU	0.044360072	2.144851241	0.049216843	2.472248918	0.046092673	Higher expr.	Risk	-0.248504788	0.145174561	0.393724459	NS

TNFSF10	0.000621601	4.732156825	0.001615617	4.477468577	0.009338306	Higher expr.	Risk	-0.194790779	0.145605921	0.394251254	NS
SHC2	0.00823107	0.382226657	0.010643303	0.250885727	0.01041784	Lower expr.	Protective	-0.293422592	0.145915373	0.394658902	NS
PRH2	0.042878255	0.443306665	0.047667467	0.318877835	0.02544948	Lower expr.	Protective	0.472098815	0.149852029	0.400142928	NS
KRTAP10-12	0.005889301	3.428074173	0.009172463	4.779630205	0.010819906	Higher expr.	Risk	0.425073793	0.155966971	0.408930419	NS
POTEB2	0.001675524	0.281484158	0.002947631	0.266218939	0.009251241	Lower expr.	Protective	0.242787764	0.158053314	0.41161545	NS
SULT6B1	0.015315578	2.557793734	0.0189297	2.572507475	0.040651723	Higher expr.	Risk	0.249386618	0.160104025	0.414007811	NS
TAP2	0.011051787	2.671661178	0.014048472	3.247677915	0.032501623	Higher expr.	Risk	-0.092605343	0.166025263	0.42237957	NS
TNKS2	0.016885035	0.412234924	0.020499937	0.158751919	0.001107774	Lower expr.	Protective	-0.105366005	0.166610549	0.423064447	NS
NECTIN1	0.021636062	2.388734336	0.025554379	3.654893383	0.018955535	Higher expr.	Risk	-0.169255904	0.166870794	0.423422718	NS
NID2	0.022458382	0.421296095	0.026381647	0.281780843	0.018139042	Lower expr.	Protective	-0.249136506	0.168556041	0.425555005	NS
PLAA	0.002816882	0.318433896	0.004271213	0.214074818	0.010980913	Lower expr.	Protective	-0.128634909	0.1690238	0.426193927	NS
ABHD3	0.008757492	2.672593697	0.011340025	2.76125885	0.019891543	Higher expr.	Risk	-0.152034016	0.170293558	0.428133982	NS
HMGA2	0.02274774	2.389221416	0.026854942	4.659204838	0.002840943	Higher expr.	Risk	-0.1596218	0.171959366	0.43047151	NS
PREP	0.018785629	0.417436574	0.022102045	0.299663634	0.009234878	Lower expr.	Protective	-0.157894243	0.172019567	0.43051816	NS
OR9A2	0.034145005	2.836305317	0.042519323	3.70664053	0.039307025	Higher expr.	Risk	0.56543948	0.173281463	0.43226267	NS
TXNDC11	0.015843893	0.410664999	0.019133907	0.232144479	0.004476992	Lower expr.	Protective	0.090760651	0.176064562	0.436190614	NS
HORMAD1	0.025733314	2.414690903	0.029983983	3.874984802	0.007308129	Higher expr.	Risk	-0.28454495	0.178632337	0.439169672	NS
TMTC3	0.039626363	2.180591334	0.044421127	2.743105706	0.026515157	Higher expr.	Risk	0.300116938	0.181722838	0.442740973	NS
KCNK1	0.005669314	0.306026274	0.008509559	0.299481435	0.033400303	Lower expr.	Protective	0.274838703	0.182974159	0.444235483	NS
CCR10	0.021072485	0.419002675	0.024987714	0.270675648	0.011023022	Lower expr.	Protective	-0.268937706	0.184491774	0.446081305	NS
ZNF664	0.033443009	0.457526686	0.03783416	0.274942931	0.008333171	Lower expr.	Protective	-0.097587144	0.185260407	0.446821316	NS
TOP3B	0.003360572	3.074608244	0.005138343	2.782139952	0.046768962	Higher expr.	Risk	-0.147891754	0.185935403	0.447372809	NS
DAZ1	0.021742499	2.43647154	0.02613989	2.812985597	0.042845075	Higher expr.	Risk	-0.553974871	0.189141302	0.451521652	NS
TDRD1	0.011620558	0.38593124	0.01486773	0.321348852	0.016601493	Lower expr.	Protective	0.132153379	0.189692404	0.452118803	NS
MTMR14	0.04439378	0.479991106	0.048798713	0.16616312	0.000434149	Lower expr.	Protective	-0.058320412	0.191486137	0.454146527	NS
GPX8	0.002806433	0.330562136	0.004275996	0.217534576	0.003773209	Lower expr.	Protective	-0.222465727	0.191580622	0.454231249	NS
DEFB123	0.032539925	2.6864133	0.03965683	3.252723942	0.0364236	Higher expr.	Risk	0.545347274	0.191929497	0.454769334	NS
BACE1	0.026715148	2.286318265	0.030838969	2.399376165	0.047658321	Higher expr.	Risk	0.154796562	0.192764815	0.455883907	NS
H2AL1Q	0.026043843	2.461811669	0.031113338	3.98788202	0.012594244	Higher expr.	Risk	0.691093001	0.193608262	0.457003636	NS
MRGPRX1	0.014510357	0.372932778	0.018476283	0.219620679	0.006542874	Lower expr.	Protective	0.443297299	0.194204895	0.457934003	NS
PPP1CA	0.034313341	2.24710353	0.039259642	2.620333764	0.04451359	Higher expr.	Risk	0.13417935	0.195479601	0.45928926	NS
EIF4G2	0.002080471	0.306600617	0.003409317	0.186438156	0.00583618	Lower expr.	Protective	0.08962029	0.19869671	0.463107547	NS
GNPTG	0.023602695	0.436801577	0.027578064	0.169699516	0.000953483	Lower expr.	Protective	0.083795122	0.200306142	0.465227168	NS
NKAP	0.002586244	3.258616335	0.004169838	3.538872689	0.007471385	Higher expr.	Risk	-0.102591618	0.200454143	0.465400951	NS
CIP2A	0.013836484	0.363561675	0.017749659	0.135216211	0.000613327	Lower expr.	Protective	0.293026179	0.20455578	0.470203302	NS
OR11H2	0.013695259	0.397693518	0.016855452	0.254660147	0.008723918	Lower expr.	Protective	0.348865818	0.205544262	0.47139214	NS
ANAPC7	0.015563695	2.490763333	0.019043114	2.764488078	0.017599518	Higher expr.	Risk	-0.116445923	0.206603961	0.472601518	NS
SNRPE	0.033361285	0.432209523	0.038293102	0.375092257	0.04750871	Lower expr.	Protective	0.236211794	0.207477173	0.473296835	NS
NEK2	0.022989499	0.416779453	0.027446605	0.2850772	0.011340337	Lower expr.	Protective	0.157130139	0.209305451	0.475290234	NS
XPR1	0.040869815	2.185868081	0.045357134	2.686876287	0.028831453	Higher expr.	Risk	-0.069914971	0.21349016	0.480156427	NS
FAM167B	0.01319238	0.270603535	0.019035226	0.278189104	0.046278358	Lower expr.	Protective	-0.312572344	0.216392614	0.484003343	NS
SMAD9	0.032165509	2.332345794	0.037000781	2.793168449	0.031851607	Higher expr.	Risk	-0.162886585	0.218099675	0.485877791	NS
LHX9	0.028240797	0.437089923	0.032579189	0.364805618	0.042041506	Lower expr.	Protective	0.199288422	0.22422091	0.492846214	NS
NLRP5	0.009722969	2.5840312	0.012392209	6.776049358	0.000304514	Higher expr.	Risk	0.183915112	0.224949743	0.493782129	NS
FAM90A23P	0.033883986	0.450311758	0.038312375	0.323741195	0.013619377	Lower expr.	Protective	-0.196752446	0.22968351	0.499109029	NS
ADCK2	0.033897981	2.241099863	0.038686936	2.915215757	0.03229913	Higher expr.	Risk	-0.161186963	0.240503445	0.511067407	NS
GAL3ST1	0.004377891	0.343138134	0.006282746	0.363953878	0.044493105	Lower expr.	Protective	-0.202035263	0.240544436	0.511082218	NS
GREB1L	0.004906471	0.34392912	0.006954615	0.253145695	0.017960257	Lower expr.	Protective	0.138147913	0.243938291	0.515260692	NS
NRAS	0.018550268	0.397771441	0.022327407	0.276647212	0.012292737	Lower expr.	Protective	-0.142283019	0.245144528	0.516510759	NS
MSX2	0.023221212	2.781700988	0.029458529	5.199317152	0.002389628	Higher expr.	Risk	0.389746482	0.246423882	0.517755625	NS
TMEM79	0.017364442	2.518149711	0.021264637	2.795552676	0.022152814	Higher expr.	Risk	-0.138830371	0.255391666	0.527105065	NS
SDR42E2	0.044959331	0.468085895	0.049617223	0.370061572	0.033632467	Lower expr.	Protective	0.162723705	0.255450304	0.527156106	NS
SLC25A37	0.004814637	0.324676333	0.006845149	0.219292267	0.003658781	Lower expr.	Protective	-0.100668393	0.259500671	0.532057739	NS
FAM174A	0.0201454	2.485483546	0.023846989	4.374046556	0.002595243	Higher expr.	Risk	0.24788213	0.260840592	0.533580929	NS
SNAX-DISC	0.040697993	2.212463159	0.045248565	2.783897069	0.043997496	Higher expr.	Risk	-0.133755767	0.26508171	0.537549939	NS

ZSCAN5A	0.038603337	2.350160654	0.044103634	3.300446075	0.022291299	Higher expr.	Risk	-0.111810687	0.269619896	0.541974613	NS
AHDC1	0.039752976	0.468073193	0.044298618	0.284251483	0.021330289	Lower expr.	Protective	-0.143274489	0.270382746	0.54290296	NS
CHD2	0.01425774	0.395077643	0.017752062	0.311808441	0.036151816	Lower expr.	Protective	0.086816558	0.272152301	0.544593886	NS
RPS27L	0.011392509	2.608419155	0.014203283	3.573842557	0.009346626	Higher expr.	Risk	-0.096679432	0.273995419	0.546452489	NS
DR1	0.024801737	2.352897956	0.028478827	2.638959869	0.034603495	Higher expr.	Risk	-0.082348946	0.275850168	0.548242949	NS
LBP	0.000144916	0.247188735	0.000384554	0.1507538	0.000171686	Lower expr.	Protective	0.22792252	0.276695739	0.549143284	NS
BICD1	0.02787936	0.414503288	0.032607931	0.165841259	0.004259227	Lower expr.	Protective	-0.156567593	0.279255897	0.551680847	NS
CEMIP	0.043311582	2.147250442	0.048197432	3.321047519	0.021391461	Higher expr.	Risk	0.14277481	0.283248761	0.555425778	NS
ZNF705B	0.013413648	2.582624199	0.017015251	3.590403841	0.007600925	Higher expr.	Risk	0.253854091	0.289319364	0.561159665	NS
SAP30BP	0.009964202	0.352046739	0.01276433	0.347349091	0.034541892	Lower expr.	Protective	-0.09996665	0.289344259	0.561162292	NS
NCBP2L	0.043106325	2.226481241	0.048350642	2.819856015	0.026582189	Higher expr.	Risk	0.343819641	0.291316669	0.563160218	NS
F1AKMT4-E(	0.004472062	3.325042133	0.006967906	4.630076618	0.00603098	Higher expr.	Risk	-0.153728996	0.291443121	0.563287125	NS
FCAMR	0.041618003	2.248309676	0.046703331	2.511752255	0.041418805	Higher expr.	Risk	0.238512615	0.292980639	0.564830284	NS
BDKRB1	0.018675625	2.499104773	0.022333025	4.815234619	0.002867707	Higher expr.	Risk	-0.228317837	0.29306523	0.564852992	NS
PPM1N	0.039983645	2.115553376	0.044347426	5.532188077	0.0006682	Higher expr.	Risk	0.182124431	0.29929385	0.571152173	NS
CPEB4	0.022653493	0.432052822	0.026481861	0.217738082	0.00139551	Lower expr.	Protective	0.091971326	0.301790791	0.573620905	NS
FAM118B	0.04302907	2.280120318	0.048270652	3.074306453	0.020679115	Higher expr.	Risk	-0.148763283	0.308469424	0.579576633	NS
MED20	0.000293392	4.552841604	0.000789151	10.47783726	8.40E-05	Higher expr.	Risk	0.17829909	0.308925778	0.579975358	NS
MKRN3	0.0002767	0.253565063	0.00067576	0.240854836	0.013761355	Lower expr.	Protective	0.171034506	0.309881952	0.581026501	NS
TMEM214	0.015251448	2.546850457	0.01901976	7.728075254	0.001387049	Higher expr.	Risk	-0.067703324	0.320445266	0.59036553	NS
KIAA0408	0.010364276	0.393785272	0.013213969	0.354017522	0.021851436	Lower expr.	Protective	0.139423012	0.321578532	0.59140488	NS
SYDE1	0.012331995	0.393305781	0.015429558	0.314790788	0.010064309	Lower expr.	Protective	-0.157634511	0.322009091	0.591781312	NS
CCDC150	0.008779871	0.365605626	0.011477447	0.228092616	0.010055985	Lower expr.	Protective	-0.097711541	0.330741329	0.600072222	NS
C9orf152	0.001466572	0.287698596	0.002681639	0.372263727	0.029819917	Lower expr.	Protective	0.242677039	0.331790403	0.601120806	NS
SSUH2	0.046249921	0.475528137	0.051229707	0.411341731	0.049460237	Lower expr.	Protective	0.126042395	0.33180389	0.601120806	NS
NKX2-5	0.020013492	2.435344175	0.023894557	4.401416267	0.003620582	Higher expr.	Risk	0.261917866	0.33330668	0.602547192	NS
SFTPD	0.000240199	4.122544103	0.000544076	8.533576815	4.92E-05	Higher expr.	Risk	0.20206572	0.333661154	0.602912851	NS
SLC26A8	0.003780044	0.312865202	0.005857464	0.283124745	0.017322262	Lower expr.	Protective	0.113623676	0.334366298	0.603579381	NS
TSEN54	0.037483298	0.456684294	0.041634307	0.368639784	0.041954604	Lower expr.	Protective	-0.099613445	0.339625046	0.608267803	NS
SMIM36	0.017910184	0.412778381	0.021426217	0.09672055	8.66E-05	Lower expr.	Protective	-0.143682316	0.341397234	0.609738358	NS
ZNF341	0.013941009	2.529839067	0.017211825	3.874033694	0.011921899	Higher expr.	Risk	-0.094563019	0.344216119	0.612036959	NS
SCLT1	0.036419195	2.177301724	0.041032959	2.66373265	0.03359156	Higher expr.	Risk	-0.095279569	0.345095826	0.612890319	NS
AZU1	0.049334529	0.480212415	0.053670471	0.254752651	0.007121204	Lower expr.	Protective	-0.08691936	0.348292571	0.615639398	NS
TRAPPC9	0.029556391	2.247921135	0.033864193	2.432317985	0.040744587	Higher expr.	Risk	-0.075877396	0.349372062	0.616353607	NS
PHTF1	0.007463382	0.353316039	0.010031821	0.125687969	0.00032956	Lower expr.	Protective	0.112556212	0.355486978	0.621842296	NS
ZFAND6	0.002081834	0.309492406	0.003451391	0.241834761	0.010685061	Lower expr.	Protective	0.080769507	0.356408476	0.622546827	NS
PCDHB7	0.036744581	2.255435081	0.041563105	3.302462623	0.026962565	Higher expr.	Risk	-0.190825354	0.360570858	0.62613548	NS
TG	0.007411903	0.366228437	0.009465091	0.167484727	0.001571187	Lower expr.	Protective	-0.129810315	0.361177212	0.626783866	NS
METTL9	0.002252544	0.322996368	0.003605159	0.24307487	0.001605204	Lower expr.	Protective	-0.072252212	0.362906031	0.627778191	NS
GBP4	0.013458257	2.514892032	0.016689019	2.322440291	0.049642103	Higher expr.	Risk	-0.108640454	0.364122336	0.628672269	NS
PLAC8L1	0.019771782	2.542190641	0.024393655	4.422089601	0.007603537	Higher expr.	Risk	0.23926389	0.364997026	0.629355579	NS
HCN4	0.017177351	2.614572875	0.020940846	4.237735236	0.006699812	Higher expr.	Risk	-0.149913387	0.367778002	0.63191369	NS
EIF2B5	0.043022453	0.472551841	0.0473329	0.366523045	0.028945509	Lower expr.	Protective	-0.111352118	0.373484235	0.636179372	NS
KCTD1	0.018208799	2.511078222	0.021959753	5.059675777	0.001435238	Higher expr.	Risk	0.179451621	0.377900878	0.640422526	NS
PPP4R4	0.002482483	0.315626105	0.003869937	0.093964791	0.000237217	Lower expr.	Protective	0.155949406	0.380429517	0.642425273	NS
MEAK7	0.006143684	2.919226539	0.008616865	2.77530623	0.032414259	Higher expr.	Risk	-0.108871816	0.391432004	0.651008542	NS
VSIG10L	0.010311203	2.623344882	0.013230091	4.407012525	0.003070327	Higher expr.	Risk	-0.131770368	0.392809633	0.651953737	NS
EHD4	0.037178876	0.43838898	0.041555737	0.246463598	0.008972004	Lower expr.	Protective	-0.088725674	0.398914555	0.656966212	NS
TFF3	0.04259366	2.600172674	0.050526193	3.379599822	0.030292558	Higher expr.	Risk	0.272697947	0.404732831	0.661630855	NS
SLC28A2	0.039637904	0.469783475	0.044280168	0.247740271	0.007617344	Lower expr.	Protective	-0.102215976	0.406040143	0.662416051	NS
ZNF579	0.04041583	0.453291321	0.045033614	0.213558801	0.003388419	Lower expr.	Protective	-0.125774167	0.407418034	0.663402976	NS
LAMA4	0.028975348	0.450055577	0.03300908	0.377533781	0.030937423	Lower expr.	Protective	-0.097816315	0.409480626	0.665076806	NS
CTTNBP2NI	0.010727253	2.767731621	0.013668323	5.260579907	0.002286704	Higher expr.	Risk	-0.077231767	0.412353801	0.667434875	NS
BBS10	0.005380977	2.866557686	0.00766739	3.127060116	0.010540477	Higher expr.	Risk	-0.221248226	0.413945892	0.668801223	NS
TSPY10	0.021742499	2.43647154	0.02613989	2.812985597	0.042845075	Higher expr.	Risk	-0.330024667	0.414159608	0.668818053	NS

POGLUT2	0.022717783	2.462600406	0.026975982	4.176579818	0.005079239	Higher expr.	Risk	0.186009347	0.41558318	0.669987452	NS
APLN	0.002630847	0.305874141	0.004264289	0.303631472	0.040986993	Lower expr.	Protective	-0.173365301	0.41691402	0.670742058	NS
ALKBH5	0.046040063	0.474601869	0.050447049	0.284492476	0.006799663	Lower expr.	Protective	0.074563844	0.419355057	0.672702972	NS
TRPC3	0.002240028	0.312020232	0.003665884	0.21213772	0.002244356	Lower expr.	Protective	0.162656801	0.419399376	0.672704303	NS
ADH7	0.016088234	2.438501818	0.019658644	4.733180491	0.002090011	Higher expr.	Risk	0.186665306	0.420239949	0.673305011	NS
RAB8B	0.01870136	0.407614575	0.02225581	0.208080023	0.006252355	Lower expr.	Protective	-0.084353034	0.422061738	0.674619112	NS
IMP3	0.016037949	2.581559516	0.019580591	4.995089534	0.008644556	Higher expr.	Risk	-0.100044442	0.425908044	0.677348837	NS
TDRP	0.004634539	2.972851933	0.006715652	5.280827827	0.002505935	Higher expr.	Risk	0.17696811	0.427891091	0.678739227	NS
WDR55	0.024284481	0.430957492	0.02787487	0.250716268	0.004289581	Lower expr.	Protective	0.064749778	0.428462778	0.679245634	NS
ICAM2	0.025845037	2.548936188	0.030910003	5.462582285	0.002610943	Higher expr.	Risk	-0.062761794	0.430830235	0.681150868	NS
NLRC5	0.023735481	2.381576617	0.027879049	4.664010702	0.002226501	Higher expr.	Risk	-0.042541031	0.433906714	0.683779507	NS
HMGB1	0.029907987	0.449450682	0.034112326	0.231219004	0.004047246	Lower expr.	Protective	-0.039527228	0.435266852	0.684795301	NS
RNF11	0.013827621	0.378160202	0.016995701	0.282569827	0.023830493	Lower expr.	Protective	0.055260033	0.436037244	0.685317838	NS
SSX7	0.011507919	2.806000088	0.015221928	7.8096969	0.000789302	Higher expr.	Risk	0.146130121	0.440395785	0.688658581	NS
VSNL1	0.041240001	0.463418955	0.045894089	0.339609249	0.024083	Lower expr.	Protective	-0.118757692	0.444170009	0.691406569	NS
CCDC85A	0.014892946	0.367457282	0.018667923	0.351660143	0.036075125	Lower expr.	Protective	-0.191246412	0.447491127	0.694030158	NS
POM121L2	0.019034508	0.424280749	0.022696261	0.236161445	0.0051339	Lower expr.	Protective	0.202135506	0.467712664	0.70994248	NS
PAPOLA	0.038591144	0.468287917	0.04321918	0.155093426	0.000649277	Lower expr.	Protective	-0.046135054	0.468887757	0.710754508	NS
STAG2	0.021233221	0.417185129	0.025334677	0.30906021	0.021014446	Lower expr.	Protective	0.056378088	0.472101777	0.713272769	NS
PATL1	0.006666564	0.342196989	0.008915943	0.143789872	0.000567572	Lower expr.	Protective	-0.0488952	0.480117639	0.719253369	NS
PIP5KL1	0.005751141	0.334896029	0.008074986	0.135506806	0.000973529	Lower expr.	Protective	0.113534211	0.480179015	0.719297968	NS
AOC2	0.011583357	0.397102862	0.01453726	0.219282496	0.002140929	Lower expr.	Protective	-0.172315143	0.480742734	0.71977096	NS
SCRN3	0.03013451	2.561823225	0.036027773	3.430705871	0.020111484	Higher expr.	Risk	0.113144216	0.480847143	0.719852611	NS
IPO11	0.018503467	2.437307965	0.021885133	2.968958591	0.02067962	Higher expr.	Risk	-0.098340568	0.48187996	0.720603059	NS
LYPD3	0.00725768	0.353354545	0.009997284	0.258426095	0.004957048	Lower expr.	Protective	-0.126544043	0.483078522	0.721580044	NS
ZNF175	0.034387965	0.434084018	0.039109583	0.31444284	0.019102209	Lower expr.	Protective	0.071489499	0.484329172	0.722496785	NS
NAV3	0.011036629	0.382486092	0.013990736	0.188563617	0.001010273	Lower expr.	Protective	-0.074067635	0.486115853	0.723877752	NS
AIMP2	0.042959495	2.152512781	0.047368272	2.43037591	0.048965463	Higher expr.	Risk	-0.089735928	0.486648785	0.724108011	NS
RC3H1	0.007083845	0.372274992	0.009232839	0.169474927	0.000382269	Lower expr.	Protective	-0.047663068	0.491379166	0.72760455	NS
G2E3	0.029029942	0.421759566	0.033660862	0.255214689	0.011947301	Lower expr.	Protective	0.080501956	0.492044974	0.728140469	NS
CLINT1	0.030101042	0.441431965	0.034531325	0.129398042	0.000403691	Lower expr.	Protective	0.058534286	0.492668799	0.728388726	NS
OR3A2	0.029643873	0.430798619	0.034410805	0.254336365	0.006757627	Lower expr.	Protective	-0.117109157	0.499854802	0.733649313	NS
PXDN	0.006638839	0.351549751	0.008991937	0.246184251	0.012992225	Lower expr.	Protective	-0.084402291	0.500050995	0.73376015	NS
LTA4H	0.02314603	0.412760989	0.027277706	0.306171572	0.018228683	Lower expr.	Protective	0.062433528	0.503047576	0.735881262	NS
OPCML	0.002352084	0.31090703	0.00359448	0.305229313	0.021429465	Lower expr.	Protective	0.129780069	0.503333954	0.735974771	NS
GNG2	0.027226975	0.433168452	0.031750799	0.370313564	0.032545803	Lower expr.	Protective	-0.061331897	0.510291095	0.740807047	NS
RGS2	0.044309671	2.134483626	0.04884229	3.707064207	0.005762898	Higher expr.	Risk	0.054721012	0.510510026	0.740970057	NS
CRPPA	0.012051185	0.400943074	0.014959354	0.201124899	0.001231963	Lower expr.	Protective	0.070640965	0.516068399	0.745262299	NS
CTPS1	0.018484729	0.388155397	0.022922822	0.198641327	0.004449601	Lower expr.	Protective	0.066587083	0.518160365	0.745976993	NS
SLC25A43	0.026873215	0.406968041	0.031691042	0.309920077	0.021432032	Lower expr.	Protective	-0.09571013	0.5202914	0.747122082	NS
GCAT	0.004134545	0.303095896	0.006362008	0.151965378	0.001036596	Lower expr.	Protective	-0.104053607	0.521168057	0.747661011	NS
TRRAP	0.006414753	0.364365002	0.008518513	0.170846319	0.000592584	Lower expr.	Protective	-0.042518383	0.524084704	0.749671218	NS
SON	0.03000361	0.449537606	0.034214344	0.286212432	0.012263876	Lower expr.	Protective	-0.035500603	0.524241251	0.749721504	NS
FCGR2A	0.036948251	2.270676734	0.041532109	2.947072865	0.044850313	Higher expr.	Risk	-0.034227022	0.525053353	0.750380931	NS
PLA2G4F	0.034262156	2.414651182	0.039351531	6.535225366	0.002103426	Higher expr.	Risk	0.108454205	0.525234582	0.750494949	NS
DTNBP1	0.011804587	0.371657296	0.015164761	0.146799364	0.000398742	Lower expr.	Protective	0.083141965	0.525341333	0.750513227	NS
TRMT61A	0.0251133	2.343465649	0.029219025	2.787896558	0.03237446	Higher expr.	Risk	-0.081561441	0.527141813	0.751915689	NS
KCNE3	0.029687216	2.242209798	0.03403922	2.566136044	0.033164207	Higher expr.	Risk	-0.074053166	0.527925875	0.752534756	NS
LDLRAP1	0.037752642	0.46788489	0.042139262	0.278700802	0.006565116	Lower expr.	Protective	-0.087353892	0.533597449	0.75623357	NS
RBMS3	0.003915688	0.332355746	0.005876667	0.387767655	0.043832576	Lower expr.	Protective	0.062165864	0.535624538	0.757792566	NS
PIGQ	0.008331237	2.807690799	0.010870807	3.670089686	0.031418174	Higher expr.	Risk	-0.04829721	0.536720741	0.758629006	NS
TMEM170A	0.023217042	0.432973028	0.027050669	0.29579828	0.010244545	Lower expr.	Protective	0.051430423	0.537929204	0.759275187	NS
TCAF1	0.016840749	2.45074927	0.020512775	2.519557589	0.045935829	Higher expr.	Risk	-0.113034893	0.541705467	0.761863823	NS
SRRT	0.042318809	0.47541324	0.046802856	0.300005646	0.010604981	Lower expr.	Protective	0.040185865	0.551182254	0.768761296	NS
ATF4	0.002027498	0.290256922	0.003290921	0.195125052	0.005613834	Lower expr.	Protective	-0.048460992	0.554017773	0.770807337	NS

PARVB	0.039240247	0.45835149	0.043986027	0.311111052	0.021516394	Lower expr.	Protective	-0.043194249	0.554453023	0.771026916	NS
MAJIN	0.028945206	2.329190796	0.033687914	6.19773731	0.001407416	Higher expr.	Risk	-0.100080843	0.557238222	0.772535493	NS
TSPY9P	0.021742499	2.43647154	0.02613989	2.812985597	0.042845075	Higher expr.	Risk	0.221120722	0.558242239	0.773256065	NS
CAST	0.033702073	0.453430499	0.038304883	0.170126714	0.00169835	Lower expr.	Protective	-0.032856404	0.560524701	0.77462532	NS
PNKD	0.048435812	0.481702111	0.052857841	0.196583136	0.001612821	Lower expr.	Protective	-0.042664933	0.563209367	0.77638583	NS
SNX11	0.015751111	0.386882093	0.019287865	0.284883419	0.015617822	Lower expr.	Protective	-0.068648858	0.568332924	0.779671416	NS
PPP1R1A	0.039718267	0.465794914	0.0441819	0.318555708	0.017833282	Lower expr.	Protective	-0.109660709	0.573544182	0.783385669	NS
LGALS1	0.007208919	0.338822657	0.009720387	0.371875736	0.039464653	Lower expr.	Protective	-0.044181859	0.577138097	0.785322627	NS
CXCL17	0.030395879	2.222189917	0.034614765	2.859401084	0.019773048	Higher expr.	Risk	0.135146171	0.577794227	0.785792232	NS
GNA11	0.041599007	2.121621307	0.0459082	2.630462673	0.034778548	Higher expr.	Risk	-0.060738964	0.582162986	0.788627106	NS
KRTAP10-9	0.027352293	2.724599463	0.033197923	4.284309208	0.006298154	Higher expr.	Risk	0.164267422	0.589384951	0.793171758	NS
QRFPR	0.001232068	4.1233354	0.002551794	3.683326951	0.016820512	Higher expr.	Risk	-0.084088388	0.592381249	0.794798671	NS
FPGS	0.000682379	0.258006708	0.001399475	0.245238244	0.004602381	Lower expr.	Protective	-0.044913653	0.593082253	0.79526574	NS
GJA3	0.017952922	0.402524122	0.02201358	0.368123423	0.035786522	Lower expr.	Protective	0.1121673	0.595840549	0.79723749	NS
C13orf42	0.028797127	0.442342059	0.033051895	0.336768665	0.036038475	Lower expr.	Protective	0.091773525	0.598686688	0.798910571	NS
ABHD18	0.017658065	2.431590614	0.021434532	2.292472528	0.049956134	Higher expr.	Risk	0.056016304	0.606067786	0.803648004	NS
COL17A1	0.001345683	0.269740208	0.002583949	0.216421872	0.003629291	Lower expr.	Protective	-0.089141288	0.60625304	0.803729554	NS
INO80E	0.013394547	2.582836816	0.016451971	3.399893204	0.020687313	Higher expr.	Risk	-0.039153853	0.611080905	0.806456171	NS
ARL9	0.007529602	2.736775382	0.010156841	3.802902169	0.006551352	Higher expr.	Risk	0.086718043	0.620804681	0.811859414	NS
PFDN6	0.00102589	3.588434427	0.001906774	6.518718324	0.000705955	Higher expr.	Risk	-0.059450981	0.622861919	0.813199532	NS
EMC10	0.040667611	0.468812233	0.044923451	0.237506024	0.003019516	Lower expr.	Protective	0.029745119	0.627876067	0.81619924	NS
BMP3	0.020628291	0.398269276	0.024975515	0.17467329	0.001471539	Lower expr.	Protective	0.088736933	0.628078884	0.816291074	NS
ETFDH	0.021996839	2.379259748	0.025795269	2.617587263	0.027468796	Higher expr.	Risk	-0.044267738	0.631587107	0.818353297	NS
HTR1E	0.035257926	0.330318398	0.043825274	0.156501091	0.010436927	Lower expr.	Protective	0.152921118	0.636691915	0.821206251	NS
C2CD3	0.018852156	2.530892855	0.02290259	2.558191594	0.048711953	Higher expr.	Risk	-0.048090026	0.63741417	0.821716594	NS
NMRK1	0.00901061	0.35390785	0.012186751	0.158931646	0.000607476	Lower expr.	Protective	-0.074974915	0.639464789	0.8229347	NS
UBA2	0.046686426	0.483377803	0.051296858	0.187528127	0.002438249	Lower expr.	Protective	0.03231657	0.639552639	0.822994394	NS
SOX5	0.015343849	0.378670281	0.01909698	0.293631631	0.013508113	Lower expr.	Protective	-0.086556524	0.639720084	0.823061024	NS
DEPTOR	0.019637002	2.392747701	0.023594474	4.116661641	0.00328371	Higher expr.	Risk	0.086544874	0.646514933	0.827329544	NS
SMAGP	0.00862898	2.601627107	0.011092589	5.119452045	0.001988872	Higher expr.	Risk	-0.054269859	0.648199872	0.828132063	NS
SUSD3	0.000473747	0.245037783	0.001061673	0.300246497	0.032402113	Lower expr.	Protective	-0.064509609	0.653502244	0.831218964	NS
COQ10A	0.000865091	0.233629789	0.001961445	0.134848901	0.000927667	Lower expr.	Protective	0.069485962	0.655333774	0.832156629	NS
GCNT2	0.006525906	2.778567886	0.008633108	4.199666824	0.004102489	Higher expr.	Risk	0.05785679	0.657493227	0.83373873	NS
GGA2	0.036831093	0.463486932	0.041069875	0.311792505	0.011778229	Lower expr.	Protective	0.047387582	0.658309178	0.834354646	NS
KIAA0895L	0.007610977	0.369816582	0.010206372	0.147102268	0.005078815	Lower expr.	Protective	0.041844311	0.658967999	0.834751868	NS
CSKMT	0.039580166	0.462574835	0.043545666	0.187559256	0.001335978	Lower expr.	Protective	-0.067843749	0.66006783	0.835261674	NS
AKR1C1	0.005470837	0.354025235	0.007510098	0.292704534	0.01498731	Lower expr.	Protective	-0.053178877	0.666547788	0.839178779	NS
BARD1	0.040066351	2.287926644	0.045084241	2.669354472	0.045642598	Higher expr.	Risk	-0.051582908	0.667753272	0.839969531	NS
MT-CO2	0.003897176	2.976156682	0.005728226	3.788966279	0.02454153	Higher expr.	Risk	-0.028272021	0.669410393	0.840890162	NS
PPP3CC	0.041275986	0.466020435	0.045974028	0.381425217	0.041343024	Lower expr.	Protective	-0.047846267	0.671947498	0.842495934	NS
ZDHHC22	0.029922901	2.454721131	0.034712446	4.783394851	0.004062515	Higher expr.	Risk	0.093143785	0.674607323	0.84358777	NS
VPS72	0.019044375	2.412812063	0.022893106	2.984903925	0.012406913	Higher expr.	Risk	-0.088748448	0.680858793	0.84716855	NS
IGFL3	0.006338045	3.950962985	0.011422986	5.392914919	0.007538057	Higher expr.	Risk	0.186248655	0.685177979	0.849483639	NS
DPYSL4	0.024526794	2.386535541	0.028366604	4.458996882	0.002817752	Higher expr.	Risk	-0.056799407	0.688525453	0.851175313	NS
TRMT11	0.041180176	0.432888784	0.046345018	0.177910505	0.002872115	Lower expr.	Protective	0.076664434	0.700350157	0.857582956	NS
PTPRG	0.004646636	0.335502317	0.006463525	0.222835272	0.018504438	Lower expr.	Protective	0.054057689	0.700440282	0.857617742	NS
KLK3	0.03333698	2.281843605	0.038102187	4.684775483	0.001429493	Higher expr.	Risk	0.078061993	0.702128598	0.858502353	NS
LHPP	0.000610884	3.712047882	0.001239461	3.345904928	0.026690054	Higher expr.	Risk	-0.027516142	0.704672943	0.859771164	NS
FZD4	0.011781744	0.373386059	0.015141741	0.229576513	0.014765066	Lower expr.	Protective	-0.054410422	0.707041643	0.860846006	NS
MRPL58	0.01440331	2.494349012	0.01788734	2.74585341	0.021582331	Higher expr.	Risk	0.077954607	0.710710108	0.862685854	NS
SCAF8	0.001450502	0.307226767	0.002481109	0.213270583	0.001919204	Lower expr.	Protective	0.022036371	0.713281549	0.864203013	NS
PLA2G4A	0.03807771	2.158595582	0.042487432	5.066079672	0.005931116	Higher expr.	Risk	0.073623772	0.71387623	0.864527787	NS
YWHAZ	0.004454574	0.340409419	0.006403494	0.314759974	0.0198648	Lower expr.	Protective	-0.015644475	0.716144654	0.865652933	NS
EDC3	0.026938524	2.297093183	0.030863546	2.811105914	0.033542604	Higher expr.	Risk	0.045261518	0.716574396	0.865918414	NS
NBL1	0.038645226	2.219827099	0.04322872	4.867205404	0.002664081	Higher expr.	Risk	0.029272611	0.717465319	0.866552058	NS

PRKAR1B	0.028779669	0.448644274	0.032773768	0.370383129	0.024850996	Lower expr.	Protective	-0.043338581	0.718495221	0.867230643	NS
OR8A1	0.001142058	0.251163658	0.002430415	0.173688325	0.002338521	Lower expr.	Protective	0.046948551	0.719421084	0.867801682	NS
PKNOX2	0.041948124	0.466521118	0.046630581	0.401153093	0.048188996	Lower expr.	Protective	-0.053987663	0.71955034	0.867826492	NS
AXL	0.002059752	0.311990463	0.003325884	0.17467018	0.003517262	Lower expr.	Protective	-0.057465363	0.721959466	0.869184842	NS
PPP2R2C	0.00438229	3.066140181	0.00664575	4.645923905	0.001937216	Higher expr.	Risk	-0.042092646	0.722732077	0.869540794	NS
AMD1	0.04991671	0.47220431	0.055082373	0.314537508	0.026882173	Lower expr.	Protective	0.031697946	0.723905324	0.870181731	NS
CREBBP	0.024366263	0.440811865	0.028226265	0.265023223	0.006136546	Lower expr.	Protective	0.017855604	0.724233728	0.87037702	NS
SSU72P7	0.005956949	0.331287851	0.008769455	0.303961558	0.029063786	Lower expr.	Protective	0.083108763	0.726400691	0.871650006	NS
RNF214	0.024036324	2.330069511	0.028265353	2.748450178	0.026054694	Higher expr.	Risk	-0.046258187	0.728065235	0.872228334	NS
SHCBP1	0.028169214	0.444383609	0.032263464	0.268558002	0.010142207	Lower expr.	Protective	0.030914414	0.730460363	0.873269942	NS
BOK	0.04146929	2.128199034	0.04573684	4.615186276	0.003892925	Higher expr.	Risk	-0.083129307	0.731817079	0.873860244	NS
C19orf38	0.026296329	2.30509416	0.030616858	3.032114772	0.021359817	Higher expr.	Risk	-0.02871403	0.733403353	0.874889838	NS
MOV10L1	0.0195634	0.426739002	0.023073619	0.204134097	0.001647786	Lower expr.	Protective	-0.048936851	0.735847123	0.876018986	NS
CPD	0.029413313	0.435213843	0.033924014	0.210208691	0.004638559	Lower expr.	Protective	-0.029338941	0.736094636	0.876169996	NS
SLC50A1	0.028581082	2.31360648	0.032858117	4.241354803	0.038675762	Higher expr.	Risk	-0.027653572	0.738622386	0.877443614	NS
FAM89A	0.013831628	2.530199812	0.017134003	2.326634608	0.045917079	Higher expr.	Risk	-0.068123181	0.740419288	0.878470527	NS
PRRT2	0.013036547	2.729936292	0.016456466	4.161448665	0.004850153	Higher expr.	Risk	-0.049367551	0.747330582	0.881946065	NS
PHF24	0.039483795	0.451911922	0.044089264	0.251466705	0.042274689	Lower expr.	Protective	0.051047442	0.754695567	0.885429605	NS
DAZ4	0.021742499	2.43647154	0.02613989	2.812985597	0.042845075	Higher expr.	Risk	-0.128187589	0.757700079	0.886879109	NS
CCDC22	0.014636717	2.485380232	0.017882233	2.422653956	0.043815061	Higher expr.	Risk	-0.034127533	0.759557818	0.88773562	NS
RGMB	0.00133098	0.280363242	0.002435214	0.316892602	0.01508492	Lower expr.	Protective	0.062309369	0.766503631	0.891567536	NS
FGL2	0.01997477	2.419025459	0.023567149	2.788826129	0.02513759	Higher expr.	Risk	-0.032162013	0.768434882	0.892727941	NS
NRXN1	0.014976997	0.375603555	0.01891329	0.311620573	0.015891906	Lower expr.	Protective	-0.02720505	0.777506043	0.897677667	NS
AURKC	0.00730361	0.331111792	0.010372687	0.060622941	1.05E-05	Lower expr.	Protective	0.068638918	0.787341961	0.903665451	NS
TMEM63C	0.017039965	2.562708183	0.020881704	4.214687776	0.005376976	Higher expr.	Risk	0.040109958	0.787358193	0.903665451	NS
ZNF266	0.031229069	2.394572894	0.035807278	3.299147761	0.023782791	Higher expr.	Risk	-0.027947875	0.790604037	0.905595656	NS
NLGN1	0.001645199	0.287073393	0.002736914	0.181875875	0.000715007	Lower expr.	Protective	-0.047352022	0.801378659	0.911493463	NS
ILF3	0.049598503	0.486954308	0.054228053	0.270357656	0.016015506	Lower expr.	Protective	0.017523549	0.80138856	0.911493463	NS
POTEB	0.00951105	0.384131625	0.012007486	0.162347241	0.00090394	Lower expr.	Protective	0.038311334	0.801898869	0.911615806	NS
GPR32	0.001247725	0.26699591	0.002512173	0.182227329	0.000795005	Lower expr.	Protective	-0.065214656	0.803651329	0.912592443	NS
DUX4	0.037390959	2.309065778	0.042187995	4.196492136	0.008785058	Higher expr.	Risk	-0.039958078	0.803921145	0.912742875	NS
ZNF76	0.005807252	2.785204521	0.008085717	3.416957285	0.012436112	Higher expr.	Risk	-0.020668969	0.806211172	0.914035976	NS
KANSL2	0.044257115	0.472791013	0.048790319	0.366730325	0.043536914	Lower expr.	Protective	0.027735817	0.810137113	0.915517856	NS
RAB29	0.005220428	3.272492653	0.007900821	4.146096178	0.006746215	Higher expr.	Risk	-0.022094889	0.823768774	0.92235287	NS
PDK1	0.024919997	0.421852293	0.029058928	0.268193594	0.031266972	Lower expr.	Protective	0.029030125	0.825701048	0.923177453	NS
TTC19	0.011065156	0.390260664	0.014078534	0.268193121	0.003471281	Lower expr.	Protective	-0.018502492	0.834021073	0.926991103	NS
PPP1R42	0.047267534	2.103403188	0.052054543	3.469868477	0.01344747	Higher expr.	Risk	0.025733374	0.838787331	0.928809312	NS
KDM4A	0.03169196	0.455115678	0.035823856	0.150878325	0.000400114	Lower expr.	Protective	-0.024604581	0.840911796	0.929575692	NS
WDR90	0.049301603	0.487105928	0.053949128	0.142619007	0.000299326	Lower expr.	Protective	0.022472327	0.841774457	0.929832898	NS
WAPL	0.047420133	0.483630444	0.051849416	0.234434779	0.002547925	Lower expr.	Protective	0.012275997	0.846245675	0.931738641	NS
TP53TG3	0.003170235	0.32914288	0.004632407	0.247727553	0.014123789	Lower expr.	Protective	0.045603522	0.859374906	0.93788645	NS
PTTG1	0.009925821	0.355018952	0.012859678	0.356234985	0.039008985	Lower expr.	Protective	0.042524172	0.860695362	0.938443104	NS
KLHL24	0.040024955	0.470226393	0.044664647	0.134426208	0.000454061	Lower expr.	Protective	-0.014371598	0.860882346	0.938476645	NS
VSIG10	0.009436571	0.374965686	0.012356222	0.336916819	0.034164116	Lower expr.	Protective	-0.018653009	0.861912788	0.938976674	NS
SLC45A4	0.017414304	2.488132298	0.020884494	4.005679835	0.010441141	Higher expr.	Risk	-0.021514118	0.868366086	0.942025167	NS
SLC39A5	0.004890781	0.33798062	0.006863776	0.183784735	0.000536932	Lower expr.	Protective	-0.025730759	0.869522466	0.942651725	NS
FGD5	0.020876925	2.487138248	0.024824404	10.29385863	0.000197279	Higher expr.	Risk	0.018072828	0.87038702	0.943035205	NS
TMEM269	0.036958947	0.463434045	0.041449756	0.132080726	0.000243944	Lower expr.	Protective	0.01704971	0.874968356	0.945290309	NS
GLRA1	0.02689536	0.41403127	0.031341066	0.282846984	0.017997116	Lower expr.	Protective	0.029634672	0.878054742	0.946984953	NS
ZNF354C	0.007205099	3.279673471	0.010773638	2.946698185	0.036688203	Higher expr.	Risk	0.030230398	0.878064856	0.946984953	NS
CEP295	0.048695724	2.146700616	0.053987195	4.375839936	0.00478338	Higher expr.	Risk	-0.018465221	0.883558063	0.949647314	NS
MIPEP	0.023257799	2.452685808	0.027306336	3.719083691	0.013179833	Higher expr.	Risk	-0.031504524	0.884492623	0.950042436	NS
DYNLT4	0.007484215	0.345515369	0.010109502	0.24285687	0.009207581	Lower expr.	Protective	0.015166245	0.888213769	0.951210585	NS
ABCF3	0.026844707	0.445751815	0.030793656	0.299965731	0.008678828	Lower expr.	Protective	0.01659281	0.889686	0.9519455	NS
EEF2KMT	0.020302433	2.410268707	0.024358242	5.019234301	0.010546977	Higher expr.	Risk	-0.016560372	0.891233611	0.952733461	NS

HGF	0.027824575	2.297758585	0.032149099	2.868631174	0.024876132	Higher expr.	Risk	-0.021494285	0.891827421	0.952982548	NS
DSG3	0.006868122	0.363798484	0.009293701	0.272484525	0.025113889	Lower expr.	Protective	-0.016253459	0.894873048	0.954567888	NS
PSG5	0.047294627	0.456354211	0.052402533	0.372497986	0.03360024	Lower expr.	Protective	-0.02168423	0.895172339	0.954703927	NS
MTUS1	0.00418137	0.316863326	0.006368071	0.261494236	0.029512144	Lower expr.	Protective	0.02374488	0.896994327	0.955343625	NS
DNAJB1	0.007660891	0.372879545	0.009982198	0.285101601	0.007711921	Lower expr.	Protective	0.010953739	0.906590268	0.959799718	NS
SLC2A13	0.0417909	0.445500192	0.046780606	0.364501186	0.044795599	Lower expr.	Protective	0.016374414	0.909719153	0.960801316	NS
USP9X	0.036019843	0.462948242	0.040247843	0.15165288	0.000573027	Lower expr.	Protective	-0.007925654	0.910636747	0.961291679	NS
SUN3	0.001744414	0.287422027	0.003082877	0.161882062	0.002205758	Lower expr.	Protective	0.019257161	0.913705923	0.962685383	NS
TNFRSF11A	0.022229185	0.403718224	0.02647199	0.309465886	0.03348217	Lower expr.	Protective	0.013748766	0.917686694	0.964319348	NS
OR2AG1	0.003221956	0.314987577	0.005030384	0.272332847	0.013421003	Lower expr.	Protective	-0.016908883	0.921237039	0.966097006	NS
MECP2	0.001781749	0.300928567	0.002991537	0.310198078	0.012595433	Lower expr.	Protective	0.007405106	0.922534644	0.966628641	NS
AVPR2	0.00296766	0.334251833	0.004385953	0.165598699	0.000814391	Lower expr.	Protective	-0.011610541	0.922736856	0.966776095	NS
DVL3	0.023656107	0.437312901	0.027408525	0.268148123	0.006977406	Lower expr.	Protective	-0.006359914	0.92391111	0.967094933	NS
MRAS	0.015820783	0.400987778	0.019484454	0.331021776	0.021064011	Lower expr.	Protective	0.011829315	0.925968992	0.968391888	NS
GBP1	0.038543874	2.162156542	0.042787094	3.755877276	0.004788196	Higher expr.	Risk	0.011504313	0.928769309	0.969569223	NS
TACSTD2	0.003650324	0.29385935	0.00601853	0.257931365	0.016272746	Lower expr.	Protective	0.03555383	0.929036874	0.969651661	NS
LANCL2	0.038798375	0.439232731	0.043409019	0.335459966	0.047051162	Lower expr.	Protective	-0.011400648	0.931085946	0.970638632	NS
WASF1	0.042953085	2.145355477	0.047906197	2.9460712	0.037762855	Higher expr.	Risk	-0.020204523	0.931316795	0.970742942	NS
LRATD1	0.020678238	0.410469681	0.024386219	0.309288051	0.019667738	Lower expr.	Protective	0.017198789	0.93174582	0.97088952	NS
TMEM38B	0.012785927	0.381191533	0.016077158	0.372169184	0.027914499	Lower expr.	Protective	0.013247207	0.93425365	0.972026709	NS
FAHD2A	0.035768629	2.216623892	0.040694723	3.649252974	0.005712571	Higher expr.	Risk	0.010929132	0.937663167	0.973332936	NS
PLEKHH2	0.001855948	0.305441111	0.003152377	0.17448692	0.00070735	Lower expr.	Protective	0.007521802	0.941803026	0.974765833	NS
KRBA1	0.015819231	2.440815905	0.019296136	2.65799804	0.022264151	Higher expr.	Risk	-0.010612956	0.943044116	0.975274268	NS
MT-CO3	0.002954737	3.23655765	0.0045548	5.786247471	0.002070364	Higher expr.	Risk	0.003678563	0.943578434	0.975453968	NS
CD1B	0.010470334	0.353014405	0.013832899	0.173830851	0.002526082	Lower expr.	Protective	0.023515298	0.945803639	0.976460177	NS
EME1	0.025428248	0.422993203	0.02989518	0.304492493	0.029573582	Lower expr.	Protective	-0.011517484	0.948857935	0.978249054	NS
GPRC5B	0.020187254	0.406361398	0.023845433	0.149633407	0.001218547	Lower expr.	Protective	-0.007917099	0.950721455	0.979008139	NS
CCDC96	0.02619764	2.374240703	0.030706515	3.625149338	0.013849613	Higher expr.	Risk	0.011362952	0.955081026	0.980677985	NS
PELO	0.041762327	2.333265346	0.04744751	3.270207779	0.045040039	Higher expr.	Risk	-0.010143258	0.957157748	0.981664549	NS
GTF2H1	0.022942168	2.37082299	0.026751234	2.378790573	0.042122752	Higher expr.	Risk	-0.005510921	0.960107298	0.982649511	NS
PLA2G2E	0.035701375	0.288057602	0.046468801	0.251849725	0.048814692	Lower expr.	Protective	-0.017438144	0.960281616	0.982663083	NS
EIF3D	0.004693627	0.352659581	0.006501193	0.150486322	0.000289028	Lower expr.	Protective	0.004382139	0.966896097	0.985928577	NS
PBOV1	0.008999573	0.372148816	0.011589858	0.261631015	0.007312128	Lower expr.	Protective	0.007899428	0.967685758	0.986131368	NS
NCAN	0.004311687	0.334029018	0.00617343	0.15751215	0.001752891	Lower expr.	Protective	0.005805867	0.968961124	0.986553476	NS
RB1CC1	0.005269732	0.344657308	0.007334912	0.120975091	0.000652357	Lower expr.	Protective	0.003582854	0.969416509	0.986815017	NS
PCDHA6	0.02005052	2.495389581	0.024200307	7.046689175	0.000617701	Higher expr.	Risk	-0.004242933	0.982057279	0.992466897	NS
LANCL3	0.00017505	0.215423898	0.000522488	0.272913564	0.014397366	Lower expr.	Protective	0.00050386	0.996857863	0.998778704	NS
OR2H2	0.038658058	2.224128265	0.043867226	3.230100733	0.017715198	Higher expr.	Risk	-0.000600134	0.998135063	0.999357651	NS
RRAGA	0.018092748	0.393737078	0.021802233	0.106261842	8.90E-05	Lower expr.	Protective	0.000123151	0.999428412	0.999725368	NS
