# Supplementary material for: RBAD: The first database dedicated alterations of blood RNA in individuals with Alzheimer’s disease and their clinical relevance
Source: Neural Regen Res. 2025 Mar 25;21(6):2553–62. doi: 10.4103/NRR.NRR-D-24-01165 (PMC13211806; doi:10.4103/NRR.NRR-D-24-01165)
Supplement: Supplementary file 19 [file NRR-21-2553_Suppl13.pdf]

Additional Table 18. Pathway enrichment of OS-related circulating mRNAs.

**Method**	Gene Set Enrichment Analysis (GSEA)
**P value adjustment for multiple test**	Benjiamini-Hochberg (FDR)
**Description**	GSEA of OS-related circulating mRNAs.

ONTOLOGY	ID	Description	SetSize	EnrichmentScore	NES	P value	FDR	Q value	Rank	Leading edge	Symbol
**BP**	GO:0007606	sensory perception of chemical stimulus	15	0.886258241	1.990681062	8.02E-07	0.000252852	0.00022837	51	tags=73%, list=11%, signal=67%	TAS2R3/OR10G6/OR8S1/OR10D3/OR56B1/OR4D6/OR13G1/OR11H4/OR9A2/OR11H1/OR11H2
**BP**	GO:0050907	detection of chemical stimulus involved in sensory perception	15	0.886258241	1.990681062	8.02E-07	0.000252852	0.00022837	51	tags=73%, list=11%, signal=67%	TAS2R3/OR10G6/OR8S1/OR10D3/OR56B1/OR4D6/OR13G1/OR11H4/OR9A2/OR11H1/OR11H2
**BP**	GO:0007608	sensory perception of smell	14	0.880421956	1.947276692	1.55E-06	0.000252852	0.00022837	51	tags=71%, list=11%, signal=65%	OR10G6/OR8S1/OR10D3/OR56B1/OR4D6/OR13G1/OR11H4/OR9A2/OR11H1/OR11H2
**BP**	GO:0050911	detection of chemical stimulus involved in sensory perception of smell	14	0.880421956	1.947276692	1.55E-06	0.000252852	0.00022837	51	tags=71%, list=11%, signal=65%	OR10G6/OR8S1/OR10D3/OR56B1/OR4D6/OR13G1/OR11H4/OR9A2/OR11H1/OR11H2
**BP**	GO:0009593	detection of chemical stimulus	16	0.860244173	1.978167733	3.36E-06	0.000457293	0.000413016	51	tags=69%, list=11%, signal=63%	TAS2R3/OR10G6/OR8S1/OR10D3/OR56B1/OR4D6/OR13G1/OR11H4/OR9A2/OR11H1/OR11H2
**BP**	GO:0007186	G protein-coupled receptor signaling pathway	41	0.718016304	1.944137942	4.15E-06	0.000484047	0.000437179	51	tags=39%, list=11%, signal=38%	TAS2R3/OR10G6/GPHB5/OR8S1/CCL13/OR10D3/OR56B1/OR4D6/OR13G1/TRHR/OR11H4/OR9A2/MRGPRX1/OR11H1/LHCGR/OR11H2
**BP**	GO:0050906	detection of stimulus involved in sensory perception	19	0.786091473	1.884892002	9.78E-05	0.00798825	0.007214795	56	tags=63%, list=12%, signal=58%	TAS2R3/OR10G6/OR8S1/OR10D3/OR56B1/OR4D6/OR13G1/OR11H4/OR9A2/OR11H1/OR11H2/COL11A1
**BP**	GO:0031323	regulation of cellular metabolic process	134	-0.346648243	-1.441548311	0.000556264	0.037872338	0.034205384	62	tags=21%, list=14%, signal=26%	MFHAS1/BDKRB1/EMSY/CDYL2/NPRL2/CLU/HDX/ZNF528/ZEB1/DMRTC1/DAPK2/ZNF470/SALL3/ZNF596/PMEPA1/RAPGEF3/HRAS/TBX3/SIX5/PTMS/HNF1A/ACE/MCM2/FGF21/CDX2/DAZ1/OVOL1/KIT
**BP**	GO:0050877	nervous system process	40	0.620795726	1.673634726	0.001094604	0.055893235	0.050481424	61	tags=38%, list=14%, signal=36%	TAS2R3/OR10G6/OR8S1/OR10D3/OR56B1/OR4D6/OR13G1/OR11H4/OR9A2/OR11H1/STAC/LHCGR/OR11H2/COL11A1/ELAVL4
**BP**	GO:0019222	regulation of metabolic process	164	-0.324747161	-1.412797734	0.001276138	0.05792249	0.052314199	62	tags=20%, list=14%, signal=26%	MFHAS1/BDKRB1/EMSY/CDYL2/NPRL2/CLU/HDX/ZNF528/ZEB1/DMRTC1/TTC39B/DAPK2/RAB26/ZNF470/GBP5/SALL3/ZNF596/PMEPA1/RAPGEF3/HRAS/TBX3/SIX5/PTMS/HNF1A/ACE/MCM2/FGF21/CDX2/LTBP1/DAZ1/OVOL1/KIT
**BP**	GO:0051606	detection of stimulus	23	0.707786232	1.742522444	0.001618722	0.058483046	0.05282048	56	tags=52%, list=12%, signal=48%	TAS2R3/OR10G6/OR8S1/OR10D3/OR56B1/OR4D6/OR13G1/OR11H4/OR9A2/OR11H1/OR11H2/COL11A1
**BP**	GO:0043170	macromolecule metabolic process	208	-0.31304286	-1.427519783	0.001416266	0.058483046	0.05282048	47	tags=15%, list=10%, signal=25%	HDX/ZNF528/ZEB1/DMRTC1/HORMAD1/DAPK2/RAB26/ZNF470/GBP5/SALL3/ZNF596/PMEPA1/RAPGEF3/HRAS/TBX3/ART1/SIX5/NAPSA/PTMS/HNF1A/NPC1L1/ACE/MCM2/FGF21/CDX2/LTBP1/DAZ1/TRIM35/OVOL1/KIT/RPS4Y1

**BP**	GO:0044238	primary metabolic process	223	-0.296151386	-1.319963468	0.001511578	0.058483046	0.05282048	47	tags=17%, list=10%, signal=30%	MFHAS1/BDKRB1/EMSY/CDYL2/HSD3B7/CLU/HDX/ZNF528/ZEB1/DMRTC1/HORMAD1/TTC39B/DAPK2/RAB26/ZNF470/SALL3/ZNF596/PMEPA1/RAPGEF3/HRAS/TBX3/ART1/SIX5/NAPSA/CYP26C1/PTMS/HNF1A/NPC1L1/ACE/MCM2/FGF21/TKTL1/CDX2/DAZ1/TRIM35/OVOL1/KIT/RPS4Y1
**BP**	GO:0051171	regulation of nitrogen compound metabolic process	138	-0.328790936	-1.381522	0.002195649	0.074743558	0.067506582	62	tags=20%, list=14%, signal=24%	MFHAS1/BDKRB1/EMSY/CDYL2/CLU/HDX/ZNF528/ZEB1/DMRTC1/RAB26/ZNF470/SALL3/ZNF596/PMEPA1/RAPGEF3/HRAS/TBX3/SIX5/PTMS/HNF1A/ACE/MCM2/FGF21/CDX2/DAZ1/OVOL1/KIT
**BP**	GO:0006807	nitrogen compound metabolic process	210	-0.299727893	-1.328018312	0.002340921	0.076501314	0.069094145	47	tags=14%, list=10%, signal=24%	HDX/ZNF528/ZEB1/DMRTC1/HORMAD1/DAPK2/RAB26/ZNF470/SALL3/ZNF596/PMEPA1/RAPGEF3/HRAS/TBX3/ART1/SIX5/NAPSA/PTMS/HNF1A/NPC1L1/ACE/MCM2/FGF21/TKTL1/CDX2/DAZ1/TRIM35/OVOL1/KIT/RPS4Y1
**BP**	GO:0080090	regulation of primary metabolic process	142	-0.323348712	-1.36764397	0.002612892	0.082105114	0.074155363	62	tags=20%, list=14%, signal=25%	MFHAS1/BDKRB1/EMSY/CDYL2/CLU/HDX/ZNF528/ZEB1/DMRTC1/TTC39B/RAB26/ZNF470/SALL3/ZNF596/PMEPA1/RAPGEF3/HRAS/TBX3/SIX5/PTMS/HNF1A/ACE/MCM2/FGF21/CDX2/DAZ1/OVOL1/KIT
**BP**	GO:0048523	negative regulation of cellular process	128	-0.338938819	-1.409918664	0.003404302	0.092390057	0.083444476	62	tags=20%, list=14%, signal=24%	MFHAS1/BDKRB1/CDYL2/HHIP/NPRL2/CLU/ZEB1/HORMAD1/ZNF596/PMEPA1/RAPGEF3/HRAS/TBX3/SIX5/CYP26C1/PTMS/ACE/MPIG6B/MCM2/FGF21/CDX2/LTBP1/DAZ1/TRIM35/OVOL1/KIT
**BP**	GO:1901360	organic cyclic compound metabolic process	129	-0.32973613	-1.378014956	0.00350562	0.092390057	0.083444476	59	tags=37%, list=13%, signal=45%	TSEN54/SAP30BP/IMP3/SLC28A2/NKAP/WARS1/TNKS2/ZSCAN5A/FBXW11/ZNF579/DAZ4/CYLD/TG/AHDC1/LCOR/TOP3B/BICD1/HMGA2/SMAD9/AOC2/APLN/LIMA1/EMSY/CDYL2/HSD3B7/CLU/HDX/ZNF528/ZEB1/DMRTC1/HORMAD1/TTC39B/ZNF470/SALL3/ZNF596/HRAS/TBX3/SIX5/CYP26C1/PTMS/HNF1A/NPC1L1/MCM2/TKTL1/CDX2/DAZ1/OVOL1/KIT
**BP**	GO:0060255	regulation of macromolecule metabolic process	153	-0.316216976	-1.366302074	0.004571504	0.113179352	0.102220867	62	tags=19%, list=14%, signal=25%	MFHAS1/BDKRB1/EMSY/CDYL2/CLU/HDX/ZNF528/ZEB1/DMRTC1/RAB26/ZNF470/GBP5/SALL3/ZNF596/PMEPA1/RAPGEF3/HRAS/TBX3/SIX5/PTMS/HNF1A/ACE/MCM2/FGF21/CDX2/LTBP1/DAZ1/OVOL1/KIT
**BP**	GO:0071704	organic substance metabolic process	240	-0.289103199	-1.268569569	0.004724402	0.113524612	0.102532697	47	tags=14%, list=10%, signal=27%	HDX/ZNF528/ZEB1/DMRTC1/HORMAD1/TTC39B/DAPK2/RAB26/ZNF470/GBP5/SALL3/ZNF596/PMEPA1/RAPGEF3/HRAS/TBX3/ART1/SIX5/NAPSA/CYP26C1/PTMS/HNF1A/NPC1L1/ACE/MCM2/FGF21/TKTL1/CDX2/LTBP1/DAZ1/TRIM35/OVOL1/KIT/RPS4Y1
**BP**	GO:0007600	sensory perception	31	0.634958948	1.646842822	0.006326163	0.136965977	0.123704374	56	tags=39%, list=12%, signal=36%	TAS2R3/OR10G6/OR8S1/OR10D3/OR56B1/OR4D6/OR13G1/OR11H4/OR9A2/OR11H1/OR11H2/COL11A1
**BP**	GO:0046483	heterocycle metabolic process	118	-0.324215872	-1.347058762	0.00650181	0.136965977	0.123704374	59	tags=19%, list=13%, signal=23%	EMSY/CDYL2/CLU/HDX/ZNF528/ZEB1/DMRTC1/HORMAD1/ZNF470/SALL3/ZNF596/HRAS/TBX3/SIX5/PTMS/HNF1A/NPC1L1/MCM2/TKTL1/CDX2/DAZ1/OVOL1/KIT

**BP**	GO:0034641	cellular nitrogen compound metabolic process	137	-0.319771666	-1.338688348	0.006538156	0.136965977	0.123704374	59	tags=18%, list=13%, signal=22%	EMSY/CDYL2/CLU/HDX/ZNF528/ZEB1/DMRTC1/HORMAD1/ZNF470/SALL3/ZNF596/HRAS/TBX3/SIX5/PTMS/HNF1A/ACE/MCM2/TKTL1/CDX2/DAZ1/OVOL1/KIT/RPS4Y1
**BP**	GO:0008152	metabolic process	247	-0.280716142	-1.258371128	0.006905445	0.14104372	0.127387293	62	tags=17%, list=14%, signal=32%	MFHAS1/BDKRB1/EMSY/CDYL2/NPRL2/HSD3B7/CLU/HDX/ZNF528/ZEB1/DMRTC1/HORMAD1/TTC39B/DAPK2/RAB26/ZNF470/GBP5/SALL3/ZNF596/PMEPA1/RAPGEF3/HRAS/TBX3/ART1/SIX5/NAPSA/CYP26C1/PTMS/HNF1A/NPC1L1/ACE/MCM2/FGF21/TKTL1/CDX2/LTBP1/DAZ1/TRIM35/OVOL1/KIT/RPS4Y1
**BP**	GO:0042325	regulation of phosphorylation	32	-0.503517827	-1.634584239	0.009262289	0.173436807	0.15664395	60	tags=28%, list=13%, signal=26%	BDKRB1/NPRL2/CLU/PMEPA1/RAPGEF3/HRAS/ACE/FGF21/KIT
**BP**	GO:0042127	regulation of cell population proliferation	39	-0.47207093	-1.603342036	0.0101032	0.175623706	0.158619104	51	tags=28%, list=11%, signal=27%	CLU/MPL/ZEB1/HRAS/TBX3/SIX5/ACE/FGF21/CDX2/OVOL1/KIT
**BP**	GO:0019219	regulation of nucleobase-containing compound metabolic process	95	-0.333492765	-1.325831595	0.01007415	0.175623706	0.158619104	59	tags=21%, list=13%, signal=23%	EMSY/CDYL2/CLU/HDX/ZNF528/ZEB1/DMRTC1/ZNF470/SALL3/ZNF596/HRAS/TBX3/SIX5/PTMS/HNF1A/MCM2/CDX2/DAZ1/OVOL1/KIT
**BP**	GO:0044271	cellular nitrogen compound biosynthetic process	105	-0.324672624	-1.301331418	0.009710869	0.175623706	0.158619104	59	tags=19%, list=13%, signal=22%	EMSY/CDYL2/CLU/HDX/ZNF528/ZEB1/DMRTC1/ZNF470/SALL3/ZNF596/HRAS/TBX3/SIX5/PTMS/HNF1A/CDX2/DAZ1/OVOL1/KIT/RPS4Y1
**BP**	GO:0001932	regulation of protein phosphorylation	28	-0.503028933	-1.641589507	0.012198819	0.203396627	0.183702939	60	tags=32%, list=13%, signal=30%	HMGA2/BDKRB1/CLU/PMEPA1/RAPGEF3/HRAS/ACE/FGF21/KIT
**BP**	GO:0031667	response to nutrient levels	12	-0.671064036	-1.684081333	0.016306296	0.211464192	0.190989369	55	tags=42%, list=12%, signal=38%	MEAK7/NPRL2/ZEB1/ACE/FGF21
**BP**	GO:0007283	spermatogenesis	20	-0.568651524	-1.683769406	0.014572519	0.211464192	0.190989369	42	tags=40%, list=9%, signal=38%	GAL3ST1/HORMAD1/TSPY10/SIX5/ACE/DAZ1/OVOL1/KIT
**BP**	GO:0048232	male gamete generation	20	-0.568651524	-1.683769406	0.014572519	0.211464192	0.190989369	42	tags=40%, list=9%, signal=38%	GAL3ST1/HORMAD1/TSPY10/SIX5/ACE/DAZ1/OVOL1/KIT
**BP**	GO:0009991	response to extracellular stimulus	13	-0.646334472	-1.679725848	0.013039103	0.211464192	0.190989369	55	tags=31%, list=12%, signal=28%	NPRL2/ZEB1/ACE/FGF21
**BP**	GO:0007276	gamete generation	23	-0.528999451	-1.648450266	0.015950738	0.211464192	0.190989369	42	tags=30%, list=9%, signal=29%	HORMAD1/TSPY10/SIX5/ACE/DAZ1/OVOL1/KIT
**BP**	GO:0033554	cellular response to stress	42	-0.441906233	-1.568193119	0.013672925	0.211464192	0.190989369	62	tags=26%, list=14%, signal=25%	MFHAS1/GPX8/EMSY/NPRL2/CLU/MPL/HRAS/TBX3/NPC1L1/MCM2/FGF21
**BP**	GO:0006468	protein phosphorylation	36	-0.45324755	-1.516759809	0.015150766	0.211464192	0.190989369	60	tags=25%, list=13%, signal=24%	BDKRB1/CLU/DAPK2/PMEPA1/RAPGEF3/HRAS/ACE/FGF21/KIT
**BP**	GO:0008283	cell population proliferation	50	-0.415380743	-1.465289134	0.015494381	0.211464192	0.190989369	56	tags=24%, list=12%, signal=24%	HHIP/CLU/MPL/ZEB1/HRAS/TBX3/SIX5/ACE/FGF21/CDX2/OVOL1/KIT
**BP**	GO:0051128	regulation of cellular component organization	63	-0.377821586	-1.40773701	0.014357366	0.211464192	0.190989369	83	tags=30%, list=18%, signal=29%	BICD1/SYDE1/NECTIN1/APLN/SAR1A/LIMA1/TNFSF10/CPNE5/BBS10/BDKRB1/CLU/HORMAD1/GBP5/PMEPA1/RAPGEF3/HRAS/ACE/MCM2/KIT
**BP**	GO:0065008	regulation of biological quality	91	-0.336740295	-1.335421926	0.014319515	0.211464192	0.190989369	107	tags=33%, list=24%, signal=31%	VSNL1/FBXW11/DAZ4/PLAA/TG/HRH2/TMEM79/HCN4/NECTIN1/APLN/LIMA1/BBS10/MFHAS1/BDKRB1/CLU/MPL/CCR10/TTC39B/SYT8/HRAS/TBX3/NAPSA/CYP26C1/HNF1A/NPC1L1/ACE/MPIG6B/FGF21/DAZ1/KIT
**BP**	GO:0010468	regulation of gene expression	120	-0.313145408	-1.307767027	0.016267338	0.211464192	0.190989369	59	tags=19%, list=13%, signal=23%	EMSY/CDYL2/CLU/HDX/ZNF528/ZEB1/DMRTC1/ZNF470/GBP5/SALL3/ZNF596/HRAS/TBX3/SIX5/PTMS/HNF1A/ACE/FGF21/CDX2/LTBP1/DAZ1/OVOL1/KIT
**BP**	GO:0010467	gene expression	139	-0.302336685	-1.271570791	0.015246836	0.211464192	0.190989369	59	tags=17%, list=13%, signal=22%	EMSY/CDYL2/CLU/HDX/ZNF528/ZEB1/DMRTC1/ZNF470/GBP5/SALL3/ZNF596/HRAS/TBX3/SIX5/PTMS/HNF1A/ACE/FGF21/CDX2/LTBP1/DAZ1/OVOL1/KIT/RPS4Y1

**BP**	GO:0051493	regulation of cytoskeleton organization	12	-0.669421373	-1.679958958	0.017302082	0.214178802	0.193441139	102	tags=50%, list=23%, signal=40%	CYLD/BICD1/SYDE1/LIMA1/RAPGEF3/HRAS
**BP**	GO:0031399	regulation of protein modification process	40	-0.436462768	-1.515098117	0.01851757	0.225803799	0.203940556	62	tags=22%, list=14%, signal=21%	MFHAS1/BDKRB1/CLU/PMEPA1/RAPGEF3/HRAS/ACE/FGF21/KIT
**BP**	GO:0044087	regulation of cellular component biogenesis	23	-0.521293926	-1.624438571	0.019900337	0.239096698	0.215946383	77	tags=43%, list=17%, signal=38%	NECTIN1/SAR1A/BBS10/CLU/GBP5/PMEPA1/RAPGEF3/HRAS/ACE/KIT
**BP**	GO:0032504	multicellular organism reproduction	24	-0.522738366	-1.649126607	0.022142437	0.258433869	0.23341125	42	tags=29%, list=9%, signal=28%	HORMAD1/TSPY10/SIX5/ACE/DAZ1/OVOL1/KIT
**BP**	GO:0048609	multicellular organismal reproductive process	24	-0.522738366	-1.649126607	0.022142437	0.258433869	0.23341125	42	tags=29%, list=9%, signal=28%	HORMAD1/TSPY10/SIX5/ACE/DAZ1/OVOL1/KIT
**BP**	GO:0007423	sensory organ development	12	-0.644828795	-1.618242192	0.027269786	0.30213657	0.272882479	44	tags=50%, list=10%, signal=46%	NECTIN1/ZEB1/TBX3/SIX5/MCM2/KIT
**BP**	GO:0019220	regulation of phosphate metabolic process	37	-0.451224371	-1.514267186	0.027456025	0.30213657	0.272882479	62	tags=27%, list=14%, signal=25%	MFHAS1/BDKRB1/NPRL2/CLU/PMEPA1/RAPGEF3/HRAS/ACE/FGF21/KIT
**BP**	GO:0051174	regulation of phosphorus metabolic process	37	-0.451224371	-1.514267186	0.027456025	0.30213657	0.272882479	62	tags=27%, list=14%, signal=25%	MFHAS1/BDKRB1/NPRL2/CLU/PMEPA1/RAPGEF3/HRAS/ACE/FGF21/KIT
**BP**	GO:0003008	system process	69	0.496137394	1.434093794	0.026641491	0.30213657	0.272882479	71	tags=28%, list=16%, signal=27%	TAS2R3/OR10G6/OR8S1/OR10D3/CKMT2/FABP2/OR56B1/OR4D6/OR13G1/OR11H4/OR9A2/OR11H1/STAC/LHCGR/OR11H2/COL11A1/ELAVL4/TFF3/NKX2-5
**BP**	GO:0009056	catabolic process	46	-0.395792578	-1.395225134	0.027735915	0.30213657	0.272882479	55	tags=33%, list=12%, signal=32%	ANAPC7/FBXW11/DAZ4/CYLD/PLAA/ABHD3/NPRL2/CLU/DAPK2/RAB26/CYP26C1/ACE/FGF21/TKTL1/DAZ1
**BP**	GO:0009314	response to radiation	12	-0.63962534	-1.605183764	0.029771534	0.316331055	0.285702596	28	tags=25%, list=6%, signal=24%	HRAS/OPN1MW/KIT
**BP**	GO:0001934	positive regulation of protein phosphorylation	20	-0.53370051	-1.58027993	0.03284891	0.316331055	0.285702596	51	tags=30%, list=11%, signal=28%	CLU/RAPGEF3/HRAS/ACE/FGF21/KIT
**BP**	GO:0019953	sexual reproduction	27	-0.488531077	-1.564361183	0.035420016	0.316331055	0.285702596	42	tags=26%, list=9%, signal=25%	HORMAD1/TSPY10/SIX5/ACE/DAZ1/OVOL1/KIT
**BP**	GO:0031401	positive regulation of protein modification process	27	-0.486270174	-1.55712138	0.037944239	0.316331055	0.285702596	51	tags=22%, list=11%, signal=21%	CLU/RAPGEF3/HRAS/ACE/FGF21/KIT
**BP**	GO:0044248	cellular catabolic process	39	-0.422291899	-1.434272499	0.037852418	0.316331055	0.285702596	118	tags=36%, list=26%, signal=29%	GCAT/ANAPC7/FBXW11/DAZ4/CYLD/PLAA/ABHD3/NPRL2/CLU/DAPK2/CYP26C1/ACE/FGF21/DAZ1
**BP**	GO:0043067	regulation of programmed cell death	41	-0.397842249	-1.391065454	0.033623144	0.316331055	0.285702596	56	tags=27%, list=12%, signal=26%	TNFSF10/HHIP/CLU/DAPK2/HRAS/TBX3/PTMS/ACE/FGF21/TRIM35/KIT
**BP**	GO:0031325	positive regulation of cellular metabolic process	73	-0.35551216	-1.360777013	0.034486806	0.316331055	0.285702596	55	tags=19%, list=12%, signal=20%	NPRL2/CLU/ZEB1/RAPGEF3/HRAS/TBX3/SIX5/PTMS/HNF1A/ACE/FGF21/CDX2/DAZ1/KIT
**BP**	GO:0012501	programmed cell death	51	-0.375397583	-1.332000623	0.034375122	0.316331055	0.285702596	56	tags=22%, list=12%, signal=21%	HHIP/CLU/DAPK2/HRAS/TBX3/PTMS/ACE/MCM2/FGF21/TRIM35/KIT
**BP**	GO:0051173	positive regulation of nitrogen compound metabolic process	77	-0.342156806	-1.331366903	0.032733082	0.316331055	0.285702596	29	tags=14%, list=6%, signal=16%	RAPGEF3/HRAS/TBX3/SIX5/PTMS/HNF1A/ACE/FGF21/CDX2/DAZ1/KIT
**BP**	GO:0006351	DNA-templated transcription	80	-0.32615997	-1.271886237	0.03684615	0.316331055	0.285702596	59	tags=22%, list=13%, signal=24%	EMSY/CDYL2/CLU/HDX/ZNF528/ZEB1/DMRTC1/ZNF470/SALL3/ZNF596/HRAS/TBX3/SIX5/PTMS/HNF1A/CDX2/OVOL1/KIT
**BP**	GO:0006355	regulation of DNA-templated transcription	80	-0.32615997	-1.271886237	0.03684615	0.316331055	0.285702596	59	tags=22%, list=13%, signal=24%	EMSY/CDYL2/CLU/HDX/ZNF528/ZEB1/DMRTC1/ZNF470/SALL3/ZNF596/HRAS/TBX3/SIX5/PTMS/HNF1A/CDX2/OVOL1/KIT
**BP**	GO:0032774	RNA biosynthetic process	80	-0.32615997	-1.271886237	0.03684615	0.316331055	0.285702596	59	tags=22%, list=13%, signal=24%	EMSY/CDYL2/CLU/HDX/ZNF528/ZEB1/DMRTC1/ZNF470/SALL3/ZNF596/HRAS/TBX3/SIX5/PTMS/HNF1A/CDX2/OVOL1/KIT
**BP**	GO:0097659	nucleic acid-templated transcription	80	-0.32615997	-1.271886237	0.03684615	0.316331055	0.285702596	59	tags=22%, list=13%, signal=24%	EMSY/CDYL2/CLU/HDX/ZNF528/ZEB1/DMRTC1/ZNF470/SALL3/ZNF596/HRAS/TBX3/SIX5/PTMS/HNF1A/CDX2/OVOL1/KIT
**BP**	GO:1903506	regulation of nucleic acid-templated transcription	80	-0.32615997	-1.271886237	0.03684615	0.316331055	0.285702596	59	tags=22%, list=13%, signal=24%	EMSY/CDYL2/CLU/HDX/ZNF528/ZEB1/DMRTC1/ZNF470/SALL3/ZNF596/HRAS/TBX3/SIX5/PTMS/HNF1A/CDX2/OVOL1/KIT

**BP**	GO:2001141	regulation of RNA biosynthetic process	80	-0.32615997	-1.271886237	0.03684615	0.316331055	0.285702596	59	tags=22%, list=13%, signal=24%	EMSY/CDYL2/CLU/HDX/ZNF528/ZEB1/DMRTC1/ZNF470/SALL3/ZNF596/HRAS/TBX3/SIX5/PTMS/HNF1A/CDX2/OVOL1/KIT
**BP**	GO:0006725	cellular aromatic compound metabolic process	121	-0.299818452	-1.25516731	0.030227458	0.316331055	0.285702596	59	tags=36%, list=13%, signal=42%	TSEN54/SAP30BP/IMP3/SLC28A2/NKAP/WARS1/TNKS2/ZSCAN5A/FBXW11/ZNF579/DAZ4/CYLD/TG/AHDC1/LCOR/TOP3B/BICD1/HMGA2/SMAD9/AOC2/APLN/EMSY/CDYL2/CLU/HDX/ZNF528/ZEB1/DMRTC1/HORMAD1/ZNF470/SALL3/ZNF596/HRAS/TBX3/SIX5/PTMS/HNF1A/MCM2/TKTL1/CDX2/DAZ1/OVOL1/KIT
**BP**	GO:0009059	macromolecule biosynthetic process	110	-0.303992237	-1.236574317	0.036762963	0.316331055	0.285702596	59	tags=18%, list=13%, signal=21%	EMSY/CDYL2/CLU/HDX/ZNF528/ZEB1/DMRTC1/ZNF470/SALL3/ZNF596/HRAS/TBX3/SIX5/PTMS/HNF1A/CDX2/DAZ1/OVOL1/KIT/RPS4Y1
**BP**	GO:0090304	nucleic acid metabolic process	111	-0.300423813	-1.218694555	0.036218337	0.316331055	0.285702596	59	tags=19%, list=13%, signal=22%	EMSY/CDYL2/CLU/HDX/ZNF528/ZEB1/DMRTC1/HORMAD1/ZNF470/SALL3/ZNF596/HRAS/TBX3/SIX5/PTMS/HNF1A/MCM2/CDX2/DAZ1/OVOL1/KIT
**BP**	GO:0042592	homeostatic process	43	-0.405516929	-1.428323816	0.038571422	0.318311631	0.287491405	72	tags=30%, list=16%, signal=28%	LIMA1/BBS10/MFHAS1/BDKRB1/MPL/TTC39B/NAPSA/HNF1A/NPC1L1/ACE/MPIG6B/FGF21/KIT
**BP**	GO:0042327	positive regulation of phosphorylation	21	-0.524202383	-1.592369595	0.039287828	0.320981554	0.289902814	51	tags=29%, list=11%, signal=27%	CLU/RAPGEF3/HRAS/ACE/FGF21/KIT
**BP**	GO:0050804	modulation of chemical synaptic transmission	15	-0.590244561	-1.626802767	0.041732601	0.327229708	0.295545996	39	tags=27%, list=9%, signal=25%	RAB26/HRAS/ACE/KIT
**BP**	GO:0099177	regulation of trans-synaptic signaling	15	-0.590244561	-1.626802767	0.041732601	0.327229708	0.295545996	39	tags=27%, list=9%, signal=25%	RAB26/HRAS/ACE/KIT
**BP**	GO:0033674	positive regulation of kinase activity	13	-0.595236291	-1.546929378	0.041472797	0.327229708	0.295545996	80	tags=38%, list=18%, signal=33%	HMGA2/CLU/HRAS/ACE/KIT
**BP**	GO:0051252	regulation of RNA metabolic process	90	-0.324997033	-1.288574173	0.042848748	0.327229708	0.295545996	59	tags=21%, list=13%, signal=23%	EMSY/CDYL2/CLU/HDX/ZNF528/ZEB1/DMRTC1/ZNF470/SALL3/ZNF596/HRAS/TBX3/SIX5/PTMS/HNF1A/CDX2/DAZ1/OVOL1/KIT
**BP**	GO:1901362	organic cyclic compound biosynthetic process	90	-0.32398809	-1.284573837	0.042848748	0.327229708	0.295545996	59	tags=22%, list=13%, signal=24%	EMSY/CDYL2/HSD3B7/CLU/HDX/ZNF528/ZEB1/DMRTC1/ZNF470/SALL3/ZNF596/HRAS/TBX3/SIX5/PTMS/HNF1A/NPC1L1/CDX2/OVOL1/KIT
**BP**	GO:0018130	heterocycle biosynthetic process	86	-0.310662091	-1.242692252	0.044151061	0.327921973	0.296171234	59	tags=21%, list=13%, signal=22%	EMSY/CDYL2/CLU/HDX/ZNF528/ZEB1/DMRTC1/ZNF470/SALL3/ZNF596/HRAS/TBX3/SIX5/PTMS/HNF1A/CDX2/OVOL1/KIT
**BP**	GO:0019438	aromatic compound biosynthetic process	86	-0.310662091	-1.242692252	0.044151061	0.327921973	0.296171234	59	tags=21%, list=13%, signal=22%	EMSY/CDYL2/CLU/HDX/ZNF528/ZEB1/DMRTC1/ZNF470/SALL3/ZNF596/HRAS/TBX3/SIX5/PTMS/HNF1A/CDX2/OVOL1/KIT
**BP**	GO:0006412	translation	19	-0.522009692	-1.525871826	0.046352794	0.338127078	0.305388239	6	tags=37%, list=1%, signal=38%	AIMP2/RPS27L/WARS1/EIF2B5/DAZ4/DAZ1/RPS4Y1
**BP**	GO:0043043	peptide biosynthetic process	19	-0.522009692	-1.525871826	0.046352794	0.338127078	0.305388239	6	tags=37%, list=1%, signal=38%	AIMP2/RPS27L/WARS1/EIF2B5/DAZ4/DAZ1/RPS4Y1
**BP**	GO:1901615	organic hydroxy compound metabolic process	17	-0.553915397	-1.588925783	0.047044519	0.340136036	0.307202682	76	tags=47%, list=17%, signal=41%	AOC2/LIMA1/HSD3B7/TTC39B/CYP26C1/NPC1L1/TKTL1/KIT
**BP**	GO:0044089	positive regulation of cellular component biogenesis	12	-0.615624246	-1.544951367	0.047784219	0.341060942	0.308038034	51	tags=42%, list=11%, signal=38%	CLU/GBP5/RAPGEF3/HRAS/KIT
**BP**	GO:0045860	positive regulation of protein kinase activity	12	-0.614389031	-1.54185151	0.048284573	0.341060942	0.308038034	80	tags=42%, list=18%, signal=35%	HMGA2/CLU/HRAS/ACE/KIT
**BP**	GO:0000278	mitotic cell cycle	21	-0.50981781	-1.548673579	0.052488292	0.343633561	0.310361562	14	tags=29%, list=3%, signal=29%	ANAPC7/FBXW11/CYLD/MCM2/TRIM35/OVOL1
**BP**	GO:0051338	regulation of transferase activity	23	-0.476787887	-1.485750352	0.053431167	0.343633561	0.310361562	55	tags=26%, list=12%, signal=24%	HMGA2/NPRL2/CLU/HRAS/ACE/KIT
**BP**	GO:0008202	steroid metabolic process	11	-0.608141306	-1.480000173	0.05119186	0.343633561	0.310361562	72	tags=55%, list=16%, signal=47%	LIMA1/HSD3B7/TTC39B/CYP26C1/NPC1L1/KIT

**BP**	GO:0009617	response to bacterium	22	0.589313862	1.444768533	0.050715215	0.343633561	0.310361562	40	tags=27%, list=9%, signal=26%	DEFB113/DEFB131B/IL17F/SLC10A2/DEFB123/CSF2
**BP**	GO:1901575	organic substance catabolic process	36	-0.403253329	-1.349457801	0.053202796	0.343633561	0.310361562	109	tags=39%, list=24%, signal=32%	GCAT/ANAPC7/FBXW11/DAZ4/CYLD/PLAA/ABHD3/CLU/RAB26/CYP26C1/ACE/FGF21/TKTL1/DAZ1
**BP**	GO:0006357	regulation of transcription by RNA polymerase II	55	-0.366981287	-1.336138479	0.050855157	0.343633561	0.310361562	47	tags=24%, list=10%, signal=24%	HDX/ZNF528/ZEB1/ZNF470/SALL3/ZNF596/HRAS/TBX3/SIX5/PTMS/HNF1A/CDX2/OVOL1
**BP**	GO:0006366	transcription by RNA polymerase II	55	-0.366981287	-1.336138479	0.050855157	0.343633561	0.310361562	47	tags=24%, list=10%, signal=24%	HDX/ZNF528/ZEB1/ZNF470/SALL3/ZNF596/HRAS/TBX3/SIX5/PTMS/HNF1A/CDX2/OVOL1
**BP**	GO:0010941	regulation of cell death	46	-0.376255551	-1.326354338	0.050028392	0.343633561	0.310361562	56	tags=24%, list=12%, signal=23%	TNFSF10/HHIP/CLU/DAPK2/HRAS/TBX3/PTMS/ACE/FGF21/TRIM35/KIT
**BP**	GO:0008219	cell death	57	-0.354189951	-1.30703858	0.053210126	0.343633561	0.310361562	56	tags=19%, list=12%, signal=19%	HHIP/CLU/DAPK2/HRAS/TBX3/PTMS/ACE/MCM2/FGF21/TRIM35/KIT
**BP**	GO:0007165	signal transduction	145	0.414057087	1.284838508	0.054054054	0.343633561	0.310361562	65	tags=19%, list=14%, signal=24%	TAS2R3/OR10G6/GPHB5/OR8S1/IL17F/DKK4/CCL13/OR10D3/DEPDC1/OR56B1/OR4D6/OR13G1/TRHR/OR11H4/OR9A2/CSF2/MRGPRX1/OR11H1/STAC/CD8B/LHCGR/MSX2/OR11H2/TPTE2/ADAM11/ASPM/BUB1
**BP**	GO:0048519	negative regulation of biological process	144	-0.290454054	-1.228994739	0.052704285	0.343633561	0.310361562	62	tags=19%, list=14%, signal=24%	MFHAS1/BDKRB1/CDYL2/HHIP/NPRL2/CLU/ZEB1/HORMAD1/TTC39B/ZNF596/PMEPA1/RAPGEF3/HRAS/TBX3/SIX5/CYP26C1/PTMS/ACE/MPIG6B/MCM2/FGF21/CDX2/LTBP1/DAZ1/TRIM35/OVOL1/KIT
**BP**	GO:0010556	regulation of macromolecule biosynthetic process	98	-0.301530478	-1.203369282	0.054257931	0.343633561	0.310361562	59	tags=19%, list=13%, signal=22%	EMSY/CDYL2/CLU/HDX/ZNF528/ZEB1/DMRTC1/ZNF470/SALL3/ZNF596/HRAS/TBX3/SIX5/PTMS/HNF1A/CDX2/DAZ1/OVOL1/KIT
**BP**	GO:0044249	cellular biosynthetic process	124	-0.281741346	-1.174921131	0.054982891	0.345546326	0.312089125	59	tags=17%, list=13%, signal=20%	EMSY/CDYL2/HSD3B7/CLU/HDX/ZNF528/ZEB1/DMRTC1/ZNF470/SALL3/ZNF596/HRAS/TBX3/SIX5/PTMS/HNF1A/CDX2/DAZ1/OVOL1/KIT/RPS4Y1
**BP**	GO:0080135	regulation of cellular response to stress	17	-0.5459081	-1.565956572	0.056394872	0.351714585	0.317660148	196	tags=76%, list=44%, signal=45%	INO80E/HMGB1/TRRAP/ATF4/BARD1/BOK/VPS72/MEAK7/CYLD/HMGA2/MFHAS1/CLU/HRAS
**BP**	GO:0045786	negative regulation of cell cycle	11	-0.60243978	-1.466124681	0.057648326	0.3568082	0.322260579	42	tags=27%, list=9%, signal=25%	HORMAD1/TRIM35/OVOL1
**BP**	GO:0044237	cellular metabolic process	208	-0.264302977	-1.205259013	0.061805878	0.373110209	0.336984161	62	tags=16%, list=14%, signal=26%	MFHAS1/BDKRB1/EMSY/CDYL2/NPRL2/HSD3B7/CLU/HDX/ZNF528/ZEB1/DMRTC1/HORMAD1/DAPK2/ZNF470/SALL3/ZNF596/PMEPA1/RAPGEF3/HRAS/TBX3/SIX5/CYP26C1/PTMS/HNF1A/NPC1L1/ACE/MCM2/FGF21/TKTL1/CDX2/DAZ1/OVOL1/KIT/RPS4Y1
**BP**	GO:0048731	system development	104	-0.285126899	-1.13974097	0.061214183	0.373110209	0.336984161	88	tags=25%, list=20%, signal=26%	HCN4/EEF1AKMT4-ECE2/HMGA2/NECTIN1/APLN/PCDHB7/TNFSF10/GAL3ST1/CPNE5/MFHAS1/HHIP/CLU/MPL/ZEB1/RAB26/RAPGEF3/TSPY10/TBX3/SIX5/CYP26C1/HNF1A/ACE/MPIG6B/CDX2/OVOL1/KIT
**BP**	GO:0050673	epithelial cell proliferation	12	-0.594819687	-1.492740897	0.062854028	0.374830224	0.338537637	44	tags=50%, list=10%, signal=46%	NRAS/APLN/ZEB1/HRAS/OVOL1/KIT
**BP**	GO:0043549	regulation of kinase activity	22	-0.507217046	-1.562156083	0.065172173	0.37885644	0.342174018	55	tags=27%, list=12%, signal=25%	HMGA2/NPRL2/CLU/HRAS/ACE/KIT
**BP**	GO:0045859	regulation of protein kinase activity	19	-0.504408009	-1.474420842	0.06489366	0.37885644	0.342174018	51	tags=26%, list=11%, signal=24%	HMGA2/CLU/HRAS/ACE/KIT
**BP**	GO:0034654	nucleobase-containing compound biosynthetic process	85	-0.312215752	-1.239743391	0.06467986	0.37885644	0.342174018	59	tags=21%, list=13%, signal=23%	EMSY/CDYL2/CLU/HDX/ZNF528/ZEB1/DMRTC1/ZNF470/SALL3/ZNF596/HRAS/TBX3/SIX5/PTMS/HNF1A/CDX2/OVOL1/KIT

**BP**	GO:0006139	nucleobase-containing compound metabolic process	116	-0.291929858	-1.188642561	0.065847753	0.37885644	0.342174018	59	tags=18%, list=13%, signal=21%	EMSY/CDYL2/CLU/HDX/ZNF528/ZEB1/DMRTC1/HORMAD1/ZNF470/SALL3/ZNF596/HRAS/TBX3/SIX5/PTMS/HNF1A/MCM2/CDX2/DAZ1/OVOL1/KIT
**BP**	GO:0010604	positive regulation of macromolecule metabolic process	88	-0.296468951	-1.178008932	0.065847347	0.37885644	0.342174018	34	tags=14%, list=8%, signal=16%	GBP5/RAPGEF3/HRAS/TBX3/SIX5/PTMS/HNF1A/ACE/FGF21/CDX2/DAZ1/KIT
**BP**	GO:0010562	positive regulation of phosphorus metabolic process	23	-0.463411014	-1.44406579	0.068116516	0.381172557	0.34426588	51	tags=26%, list=11%, signal=24%	CLU/RAPGEF3/HRAS/ACE/FGF21/KIT
**BP**	GO:0045937	positive regulation of phosphate metabolic process	23	-0.463411014	-1.44406579	0.068116516	0.381172557	0.34426588	51	tags=26%, list=11%, signal=24%	CLU/RAPGEF3/HRAS/ACE/FGF21/KIT
**BP**	GO:0051726	regulation of cell cycle	31	-0.431946005	-1.41866749	0.067552253	0.381172557	0.34426588	42	tags=19%, list=9%, signal=19%	HORMAD1/HRAS/TBX3/OPN1MW/TRIM35/OVOL1
**BP**	GO:0015849	organic acid transport	13	0.643635167	1.404548683	0.06779661	0.381172557	0.34426588	48	tags=31%, list=11%, signal=28%	SLC10A5/FABP2/SLC10A2/LHCGR
**BP**	GO:0006518	peptide metabolic process	27	-0.454310239	-1.454780127	0.06902165	0.383610122	0.34646743	16	tags=15%, list=4%, signal=15%	CLU/ACE/DAZ1/RPS4Y1
**BP**	GO:0045087	innate immune response	18	0.609637594	1.442604517	0.070291777	0.388029608	0.350459003	32	tags=28%, list=7%, signal=27%	DEFB113/DEFB131B/IL17F/CCL13/DEFB123
**BP**	GO:0009894	regulation of catabolic process	24	-0.457244699	-1.442508238	0.074221967	0.406975483	0.367570461	55	tags=25%, list=12%, signal=23%	NPRL2/CLU/DAPK2/RAB26/FGF21/DAZ1
**BP**	GO:0019538	protein metabolic process	124	-0.275082713	-1.147153221	0.076154116	0.414786085	0.374624809	40	tags=11%, list=9%, signal=14%	DAPK2/RAB26/PMEPA1/RAPGEF3/HRAS/ART1/NAPSA/NPC1L1/ACE/FGF21/DAZ1/TRIM35/KIT/RPS4Y1
**BP**	GO:0051347	positive regulation of transferase activity	14	-0.549792448	-1.485113167	0.083003643	0.430959627	0.389232363	51	tags=36%, list=11%, signal=33%	HMGA2/CLU/HRAS/ACE/KIT
**BP**	GO:0048863	stem cell differentiation	13	-0.551746467	-1.433905882	0.083355192	0.430959627	0.389232363	26	tags=38%, list=6%, signal=37%	TBX3/CYP26C1/ACE/CDX2/KIT
**BP**	GO:0043603	cellular amide metabolic process	30	-0.424568031	-1.406075077	0.081942746	0.430959627	0.389232363	16	tags=17%, list=4%, signal=17%	GAL3ST1/CLU/ACE/DAZ1/RPS4Y1
**BP**	GO:0098542	defense response to other organism	25	0.551478271	1.371514813	0.083870968	0.430959627	0.389232363	32	tags=20%, list=7%, signal=20%	DEFB113/DEFB131B/IL17F/CCL13/DEFB123
**BP**	GO:0048522	positive regulation of cellular process	145	-0.274080882	-1.167503734	0.082866115	0.430959627	0.389232363	84	tags=22%, list=19%, signal=26%	MESD/BICD1/SYDE1/HMGA2/SMAD9/APLN/SAR1A/TNFSF10/CPNE5/MFHAS1/BDKRB1/NPRL2/CLU/MPL/ZEB1/DAPK2/SHC2/GBP5/RAPGEF3/HRAS/TBX3/SIX5/PTMS/HNF1A/ACE/OPN1MW/FGF21/CDX2/DAZ1/TRIM35/OVOL1/KIT
**BP**	GO:0009889	regulation of biosynthetic process	100	-0.290745555	-1.15327038	0.083166183	0.430959627	0.389232363	59	tags=19%, list=13%, signal=21%	EMSY/CDYL2/CLU/HDX/ZNF528/ZEB1/DMRTC1/ZNF470/SALL3/ZNF596/HRAS/TBX3/SIX5/PTMS/HNF1A/CDX2/DAZ1/OVOL1/KIT
**BP**	GO:0016310	phosphorylation	43	-0.37048395	-1.304929612	0.085684096	0.437524413	0.39516152	60	tags=28%, list=13%, signal=27%	HMGA2/ADCK2/BDKRB1/NPRL2/CLU/DAPK2/PMEPA1/RAPGEF3/HRAS/ACE/FGF21/KIT
**BP**	GO:0043069	negative regulation of programmed cell death	25	-0.44239236	-1.405978998	0.086763949	0.440286625	0.397656283	56	tags=28%, list=12%, signal=26%	HHIP/CLU/HRAS/TBX3/PTMS/FGF21/KIT
**BP**	GO:0071900	regulation of protein serine/threonine kinase activity	11	-0.585958705	-1.426015593	0.088957055	0.443158013	0.400249652	28	tags=27%, list=6%, signal=26%	HMGA2/HRAS/KIT
**BP**	GO:0050896	response to stimulus	213	0.383802536	1.212431362	0.088685015	0.443158013	0.400249652	67	tags=18%, list=15%, signal=29%	TAS2R3/DEFB113/OR10G6/GPHB5/DEFB131B/OR8S1/IL17F/DKK4/CCL13/OR10D3/SAA1/DEPDC1/OR56B1/OR4D6/OR13G1/TRHR/OR11H4/SLC10A2/OR9A2/DEFB123/CSF2/MRGPRX1/OR11H1/STAC/CD8B/LHCGR/MSX2/EOMES/OR11H2/TPTE2/COL11A1/TMTC3/ADAM23/ELAVL4/ADAM11/ASPM/BUB1/KCNK1
**BP**	GO:1901576	organic substance biosynthetic process	129	-0.272918371	-1.140565326	0.089722366	0.444261655	0.401246435	59	tags=17%, list=13%, signal=21%	EMSY/CDYL2/HSD3B7/CLU/HDX/ZNF528/ZEB1/DMRTC1/ZNF470/SALL3/ZNF596/HRAS/TBX3/SIX5/PTMS/HNF1A/NPC1L1/CDX2/DAZ1/OVOL1/KIT/RPS4Y1

**BP**	GO:0043604	amide biosynthetic process	21	-0.485108883	-1.47361527	0.09159761	0.450304705	0.406704372	6	tags=38%, list=1%, signal=39%	AIMP2/RPS27L/WARS1/EIF2B5/DAZ4/GAL3ST1/DAZ1/RPS4Y1
**BP**	GO:0030154	cell differentiation	99	-0.300649934	-1.194102698	0.094440616	0.450304705	0.406704372	64	tags=20%, list=14%, signal=22%	CPNE5/MFHAS1/HHIP/CLU/KRT8/MPL/ZEB1/HORMAD1/RAPGEF3/TSPY10/TBX3/SIX5/CYP26C1/ACE/MPIG6B/FGF21/CDX2/DAZ1/OVOL1/KIT
**BP**	GO:0048869	cellular developmental process	99	-0.300649934	-1.194102698	0.094440616	0.450304705	0.406704372	64	tags=20%, list=14%, signal=22%	CPNE5/MFHAS1/HHIP/CLU/KRT8/MPL/ZEB1/HORMAD1/RAPGEF3/TSPY10/TBX3/SIX5/CYP26C1/ACE/MPIG6B/FGF21/CDX2/DAZ1/OVOL1/KIT
**BP**	GO:0009058	biosynthetic process	131	-0.268244522	-1.119668823	0.094800991	0.450304705	0.406704372	59	tags=17%, list=13%, signal=21%	EMSY/CDYL2/HSD3B7/CLU/HDX/ZNF528/ZEB1/DMRTC1/ZNF470/SALL3/ZNF596/HRAS/TBX3/SIX5/PTMS/HNF1A/NPC1L1/CDX2/DAZ1/OVOL1/KIT/RPS4Y1
**BP**	GO:0031329	regulation of cellular catabolic process	21	-0.480708652	-1.460248688	0.099177987	0.458434047	0.414046598	55	tags=24%, list=12%, signal=22%	NPRL2/CLU/DAPK2/FGF21/DAZ1
**BP**	GO:0007154	cell communication	160	0.386605148	1.208232451	0.101135191	0.458434047	0.414046598	65	tags=18%, list=14%, signal=24%	TAS2R3/OR10G6/GPHB5/OR8S1/IL17F/DKK4/CCL13/OR10D3/DEPDC1/OR56B1/OR4D6/OR13G1/TRHR/OR11H4/OR9A2/GJB5/CSF2/MRGPRX1/OR11H1/STAC/CD8B/LHCGR/MSX2/OR11H2/TPTE2/ELAVL4/ADAM11/ASPM/BUB1
**BP**	GO:0023052	signaling	161	0.385095038	1.20279478	0.101344364	0.458434047	0.414046598	65	tags=18%, list=14%, signal=24%	TAS2R3/OR10G6/GPHB5/OR8S1/IL17F/DKK4/CCL13/OR10D3/DEPDC1/OR56B1/OR4D6/OR13G1/TRHR/OR11H4/OR9A2/GJB5/CSF2/MRGPRX1/OR11H1/STAC/CD8B/LHCGR/MSX2/OR11H2/TPTE2/ELAVL4/ADAM11/ASPM/BUB1
**BP**	GO:0008284	positive regulation of cell population proliferation	21	-0.478205713	-1.452645509	0.102968178	0.46001256	0.415472273	80	tags=43%, list=18%, signal=37%	NRAS/HMGA2/APLN/MPL/HRAS/TBX3/FGF21/CDX2/KIT
**BP**	GO:0006915	apoptotic process	49	-0.339431717	-1.206186132	0.10942987	0.485892411	0.438846331	56	tags=22%, list=12%, signal=22%	TNFSF10/HHIP/CLU/DAPK2/HRAS/TBX3/PTMS/ACE/MCM2/FGF21/TRIM35
**BP**	GO:0051960	regulation of nervous system development	11	-0.558257957	-1.358601802	0.110429448	0.487680318	0.440461126	16	tags=27%, list=4%, signal=27%	TG/ACE/KIT
**BP**	GO:0003006	developmental process involved in reproduction	31	-0.409173145	-1.343873152	0.11122567	0.488555764	0.441251808	42	tags=32%, list=9%, signal=31%	TNFSF10/GAL3ST1/HORMAD1/TSPY10/TBX3/SIX5/ACE/DAZ1/OVOL1/KIT
**BP**	GO:0009628	response to abiotic stimulus	25	-0.421095685	-1.338295466	0.115905544	0.49351643	0.445732162	60	tags=24%, list=13%, signal=22%	BDKRB1/MPL/HRAS/ACE/OPN1MW/KIT
**BP**	GO:0030029	actin filament-based process	17	-0.465392138	-1.334993704	0.1171875	0.49351643	0.445732162	88	tags=35%, list=20%, signal=29%	HCN4/SYDE1/LIMA1/RAPGEF3/HRAS/KIT
**BP**	GO:0002520	immune system development	18	-0.462136268	-1.330509247	0.116935484	0.49351643	0.445732162	62	tags=33%, list=14%, signal=30%	MFHAS1/MPL/ZEB1/ACE/MPIG6B/KIT
**BP**	GO:0030097	hemopoiesis	18	-0.462136268	-1.330509247	0.116935484	0.49351643	0.445732162	62	tags=33%, list=14%, signal=30%	MFHAS1/MPL/ZEB1/ACE/MPIG6B/KIT
**BP**	GO:0048534	hematopoietic or lymphoid organ development	18	-0.462136268	-1.330509247	0.116935484	0.49351643	0.445732162	62	tags=33%, list=14%, signal=30%	MFHAS1/MPL/ZEB1/ACE/MPIG6B/KIT
**BP**	GO:0009607	response to biotic stimulus	41	0.473617537	1.282391248	0.113801453	0.49351643	0.445732162	40	tags=17%, list=9%, signal=17%	DEFB113/DEFB131B/IL17F/CCL13/SLC10A2/DEFB123/CSF2
**BP**	GO:0016070	RNA metabolic process	103	-0.283711892	-1.128000022	0.116042223	0.49351643	0.445732162	59	tags=18%, list=13%, signal=21%	EMSY/CDYL2/CLU/HDX/ZNF528/ZEB1/DMRTC1/ZNF470/SALL3/ZNF596/HRAS/TBX3/SIX5/PTMS/HNF1A/CDX2/DAZ1/OVOL1/KIT
**BP**	GO:0031326	regulation of cellular biosynthetic process	99	-0.293106393	-1.16414173	0.120621411	0.50279435	0.454111756	59	tags=19%, list=13%, signal=21%	EMSY/CDYL2/CLU/HDX/ZNF528/ZEB1/DMRTC1/ZNF470/SALL3/ZNF596/HRAS/TBX3/SIX5/PTMS/HNF1A/CDX2/DAZ1/OVOL1/KIT
**BP**	GO:0008285	negative regulation of cell population proliferation	15	-0.501450701	-1.382073535	0.126315789	0.503414634	0.454671982	44	tags=40%, list=10%, signal=37%	APLN/ZEB1/HRAS/TBX3/SIX5/OVOL1
**BP**	GO:0010817	regulation of hormone levels	11	-0.550303875	-1.339244387	0.125766871	0.503414634	0.454671982	26	tags=55%, list=6%, signal=53%	VSNL1/TG/TBX3/CYP26C1/HNF1A/ACE
**BP**	GO:0006281	DNA repair	11	-0.549464494	-1.337201631	0.125766871	0.503414634	0.454671982	80	tags=73%, list=18%, signal=61%	INO80E/HMGB1/TRRAP/BARD1/VPS72/HMGA2/EMSY/MCM2

**BP**	GO:0009615	response to virus	12	-0.531394798	-1.333571778	0.123745819	0.503414634	0.454671982	155	tags=50%, list=35%, signal=34%	XPR1/AZU1/SAP30BP/HMGA2/CLU/TRIM35
**BP**	GO:0001944	vasculature development	19	-0.450735277	-1.317531589	0.123931624	0.503414634	0.454671982	29	tags=26%, list=6%, signal=26%	RAPGEF3/TBX3/ACE/CDX2/KIT
**BP**	GO:0051129	negative regulation of cellular component organization	27	-0.405836052	-1.299557379	0.125603865	0.503414634	0.454671982	51	tags=26%, list=11%, signal=24%	LIMA1/CLU/HORMAD1/PMEPA1/RAPGEF3/ACE/MCM2
**BP**	GO:0043207	response to external biotic stimulus	39	0.478038278	1.286776154	0.122276029	0.503414634	0.454671982	40	tags=18%, list=9%, signal=18%	DEFB113/DEFB131B/IL17F/CCL13/SLC10A2/DEFB123/CSF2
**BP**	GO:0051707	response to other organism	39	0.478038278	1.286776154	0.122276029	0.503414634	0.454671982	40	tags=18%, list=9%, signal=18%	DEFB113/DEFB131B/IL17F/CCL13/SLC10A2/DEFB123/CSF2
**BP**	GO:0065007	biological regulation	278	-0.233981063	-1.047129591	0.12565659	0.503414634	0.454671982	64	tags=18%, list=14%, signal=40%	CPNE5/BBS10/MFHAS1/BDKRB1/EMSY/CDYL2/CRACR2B/HHIP/NPRL2/RIC8B/CLU/MPL/HDX/ZNF528/CCR10/ZEB1/DMRTC1/HORMAD1/TTC39B/DAPK2/RAB26/ZNF470/SHC2/SYT8/GBP5/SALL3/ZNF596/PMEPA1/RAPGEF3/HRAS/TBX3/SIX5/NAPSA/CYP26C1/PTMS/HNF1A/NPC1L1/KCNA7/ACE/MPIG6B/MCM2/OPN1MW/FGF21/CDX2/LTBP1/DAZ1/TRIM35/OVOL1/KIT
**BP**	GO:0023061	signal release	14	-0.508997471	-1.374916752	0.128472222	0.505013736	0.456116252	35	tags=64%, list=8%, signal=61%	PNKD/NLGN1/PRRT2/FZD4/RAB8B/VSNL1/SYT8/TBX3/HNF1A
**BP**	GO:0061024	membrane organization	14	-0.507940916	-1.372062758	0.131944444	0.507453416	0.458319712	182	tags=71%, list=41%, signal=44%	NLGN1/PRRT2/TRAPPC9/BOK/RAB8B/SCLT1/TRAM1/MESD/SAR1A/CLU
**BP**	GO:0006974	cellular response to DNA damage stimulus	15	-0.49370188	-1.360716619	0.133333333	0.507453416	0.458319712	145	tags=73%, list=32%, signal=51%	INO80E/HMGB1/TRRAP/BARD1/BOK/VPS72/RPS27L/HMGA2/EMSY/CLU/MCM2
**BP**	GO:0060548	negative regulation of cell death	29	-0.412633831	-1.352146859	0.134211289	0.507453416	0.458319712	56	tags=24%, list=12%, signal=23%	HHIP/CLU/HRAS/TBX3/PTMS/FGF21/KIT
**BP**	GO:0099536	synaptic signaling	21	-0.438401347	-1.331731787	0.130252101	0.507453416	0.458319712	39	tags=24%, list=9%, signal=23%	RAB26/SYT8/HRAS/ACE/KIT
**BP**	GO:0007268	chemical synaptic transmission	20	-0.446690669	-1.322644978	0.134782609	0.507453416	0.458319712	39	tags=25%, list=9%, signal=24%	RAB26/SYT8/HRAS/ACE/KIT
**BP**	GO:0098916	anterograde trans-synaptic signaling	20	-0.446690669	-1.322644978	0.134782609	0.507453416	0.458319712	39	tags=25%, list=9%, signal=24%	RAB26/SYT8/HRAS/ACE/KIT
**BP**	GO:0099537	trans-synaptic signaling	20	-0.446690669	-1.322644978	0.134782609	0.507453416	0.458319712	39	tags=25%, list=9%, signal=24%	RAB26/SYT8/HRAS/ACE/KIT
**BP**	GO:0043408	regulation of MAPK cascade	19	-0.42841745	-1.252294977	0.136752137	0.512506861	0.462883862	62	tags=26%, list=14%, signal=24%	MFHAS1/SHC2/HRAS/FGF21/KIT
**BP**	GO:0007346	regulation of mitotic cell cycle	11	-0.542409116	-1.320031345	0.141104294	0.521638953	0.471131748	5	tags=45%, list=1%, signal=46%	RPS27L/ANAPC7/CYLD/TRIM35/OVOL1
**BP**	GO:0018193	peptidyl-amino acid modification	25	-0.399354922	-1.269200564	0.140969163	0.521638953	0.471131748	29	tags=16%, list=6%, signal=16%	RAPGEF3/ART1/ACE/KIT
**BP**	GO:0044419	biological process involved in interspecies interaction between organisms	41	0.466138406	1.262140368	0.140435835	0.521638953	0.471131748	40	tags=17%, list=9%, signal=17%	DEFB113/DEFB131B/IL17F/CCL13/SLC10A2/DEFB123/CSF2
**BP**	GO:0030036	actin cytoskeleton organization	15	-0.47634693	-1.312883768	0.143859649	0.52237037	0.471792346	82	tags=33%, list=18%, signal=28%	SYDE1/LIMA1/RAPGEF3/HRAS/KIT
**BP**	GO:0006914	autophagy	12	-0.519539008	-1.303818857	0.143812709	0.52237037	0.471792346	101	tags=50%, list=22%, signal=40%	MTMR14/BOK/PLAA/NPRL2/CLU/DAPK2
**BP**	GO:0061919	process utilizing autophagic mechanism	12	-0.519539008	-1.303818857	0.143812709	0.52237037	0.471792346	101	tags=50%, list=22%, signal=40%	MTMR14/BOK/PLAA/NPRL2/CLU/DAPK2
**BP**	GO:0032940	secretion by cell	23	-0.412992379	-1.286952937	0.142857143	0.52237037	0.471792346	39	tags=22%, list=9%, signal=21%	RAB26/SYT8/TBX3/HNF1A/KIT
**BP**	GO:0042221	response to chemical	125	0.386888426	1.188320064	0.144834931	0.523584683	0.472889085	51	tags=15%, list=11%, signal=19%	TAS2R3/OR10G6/GPHB5/OR8S1/IL17F/CCL13/OR10D3/SAA1/OR56B1/OR4D6/OR13G1/OR11H4/OR9A2/CSF2/MRGPRX1/OR11H1/LHCGR/MSX2/OR11H2
**BP**	GO:0051051	negative regulation of transport	12	-0.514446839	-1.291039709	0.147157191	0.523969857	0.473236964	195	tags=83%, list=43%, signal=48%	HMGB1/PNKD/PPP3CC/ATF4/PRRT2/BARD1/KCNE3/CTTNBP2NL/VSNL1/ACE
**BP**	GO:0140352	export from cell	24	-0.404112626	-1.274888027	0.147679325	0.523969857	0.473236964	39	tags=21%, list=9%, signal=20%	RAB26/SYT8/TBX3/HNF1A/KIT
**BP**	GO:0042981	regulation of apoptotic process	40	-0.349084899	-1.211782337	0.146892655	0.523969857	0.473236964	68	tags=28%, list=15%, signal=26%	HMGA2/TNFSF10/HHIP/CLU/DAPK2/HRAS/TBX3/PTMS/ACE/FGF21/TRIM35

**BP**	GO:0030031	cell projection assembly	14	-0.48010788	-1.296879462	0.15625	0.537920929	0.485837237	63	tags=43%, list=14%, signal=38%	EHD4/SCLT1/CYLD/BBS10/HRAS/KIT
**BP**	GO:0120031	plasma membrane bounded cell projection assembly	14	-0.48010788	-1.296879462	0.15625	0.537920929	0.485837237	63	tags=43%, list=14%, signal=38%	EHD4/SCLT1/CYLD/BBS10/HRAS/KIT
**BP**	GO:0060284	regulation of cell development	11	-0.532240744	-1.295285135	0.156441718	0.537920929	0.485837237	48	tags=27%, list=11%, signal=25%	MPL/ACE/KIT
**BP**	GO:0044265	cellular macromolecule catabolic process	16	-0.463715319	-1.294940214	0.156133829	0.537920929	0.485837237	109	tags=44%, list=24%, signal=34%	ANAPC7/FBXW11/DAZ4/CYLD/PLAA/CLU/DAZ1
**BP**	GO:0043410	positive regulation of MAPK cascade	15	-0.46437952	-1.279899786	0.157894737	0.537920929	0.485837237	62	tags=33%, list=14%, signal=30%	MFHAS1/SHC2/HRAS/FGF21/KIT
**BP**	GO:0043086	negative regulation of catalytic activity	15	-0.463981688	-1.2788033	0.157894737	0.537920929	0.485837237	117	tags=40%, list=26%, signal=31%	WARS1/PPP1R1A/BICD1/NPRL2/HRAS/MCM2
**BP**	GO:0070647	protein modification by small protein conjugation or removal	18	-0.434578528	-1.251169385	0.153225806	0.537920929	0.485837237	135	tags=44%, list=30%, signal=32%	AIMP2/TNKS2/ANAPC7/FBXW11/CYLD/PLAA/UBE2E3/TRIM35
**BP**	GO:0009892	negative regulation of metabolic process	81	-0.280647223	-1.098846568	0.158018388	0.537920929	0.485837237	62	tags=19%, list=14%, signal=19%	MFHAS1/BDKRB1/CDYL2/NPRL2/CLU/ZEB1/ZNF596/PMEPA1/HRAS/TBX3/SIX5/ACE/CDX2/DAZ1/OVOL1
**BP**	GO:0009893	positive regulation of metabolic process	93	-0.276193239	-1.102341237	0.16632795	0.563858653	0.509263564	55	tags=16%, list=12%, signal=18%	NPRL2/CLU/ZEB1/GBP5/RAPGEF3/HRAS/TBX3/SIX5/PTMS/HNF1A/ACE/FGF21/CDX2/DAZ1/KIT
**BP**	GO:0090407	organophosphate biosynthetic process	12	0.588282002	1.267081143	0.169274538	0.571476435	0.516143762	55	tags=25%, list=12%, signal=23%	CKMT2/LHCGR/TPTE2
**BP**	GO:0045595	regulation of cell differentiation	31	-0.390071355	-1.281135937	0.174215592	0.575001646	0.519327648	48	tags=19%, list=11%, signal=19%	MPL/ZEB1/TBX3/ACE/CDX2/KIT
**BP**	GO:0019827	stem cell population maintenance	10	-0.535840419	-1.275178626	0.176638177	0.575001646	0.519327648	26	tags=40%, list=6%, signal=39%	HMGA2/TBX3/CDX2/KIT
**BP**	GO:0050678	regulation of epithelial cell proliferation	10	-0.534540339	-1.27208473	0.176638177	0.575001646	0.519327648	95	tags=50%, list=21%, signal=40%	NRAS/APLN/ZEB1/HRAS/OVOL1
**BP**	GO:0007548	sex differentiation	13	-0.486981124	-1.26559052	0.173469388	0.575001646	0.519327648	27	tags=38%, list=6%, signal=37%	TNFSF10/TSPY10/TBX3/ACE/KIT
**BP**	GO:0048608	reproductive structure development	13	-0.486981124	-1.26559052	0.173469388	0.575001646	0.519327648	27	tags=38%, list=6%, signal=37%	TNFSF10/TSPY10/TBX3/ACE/KIT
**BP**	GO:0061458	reproductive system development	13	-0.486981124	-1.26559052	0.173469388	0.575001646	0.519327648	27	tags=38%, list=6%, signal=37%	TNFSF10/TSPY10/TBX3/ACE/KIT
**BP**	GO:0006887	exocytosis	11	-0.518006974	-1.260645189	0.174846626	0.575001646	0.519327648	39	tags=45%, list=9%, signal=43%	VSNL1/TMEM79/RAB26/SYT8/KIT
**BP**	GO:0060627	regulation of vesicle-mediated transport	21	-0.405996257	-1.233294844	0.172268908	0.575001646	0.519327648	195	tags=81%, list=43%, signal=48%	HMGB1/PRKAR1B/NLGN1/PPP3CC/PRRT2/AXL/RAB8B/AZU1/LDLRAP1/EHD4/VSNL1/BICD1/APLN/SAR1A/CLU/RAB26/SYT8
**BP**	GO:0007610	behavior	12	-0.497667486	-1.24893077	0.180602007	0.585523172	0.528830437	29	tags=33%, list=6%, signal=32%	APLN/RAPGEF3/ACE/KIT
**BP**	GO:0007169	transmembrane receptor protein tyrosine kinase signaling pathway	15	-0.452977493	-1.248474084	0.18245614	0.586176658	0.52942065	74	tags=33%, list=16%, signal=29%	APLN/HHIP/SHC2/FGF21/KIT
**BP**	GO:0051247	positive regulation of protein metabolic process	43	-0.335558447	-1.181913966	0.181818182	0.586176658	0.52942065	29	tags=44%, list=6%, signal=46%	BOK/AZU1/EHD4/AIMP2/RPS27L/SAP30BP/SLC25A37/EIF2B5/FBXW11/DAZ4/HMGA2/TNFSF10/CLU/RAPGEF3/HRAS/ACE/FGF21/DAZ1/KIT
**BP**	GO:1901564	organonitrogen compound metabolic process	137	-0.261041322	-1.092820325	0.184517121	0.586577774	0.529782928	40	tags=11%, list=9%, signal=14%	DAPK2/RAB26/PMEPA1/RAPGEF3/HRAS/ART1/NAPSA/NPC1L1/ACE/FGF21/TKTL1/DAZ1/TRIM35/KIT/RPS4Y1
**BP**	GO:0015711	organic anion transport	12	0.580445697	1.250202786	0.190611664	0.595512617	0.537852663	48	tags=25%, list=11%, signal=23%	SLC10A5/SLC10A2/LHCGR
**BP**	GO:0046942	carboxylic acid transport	12	0.580445697	1.250202786	0.190611664	0.595512617	0.537852663	48	tags=25%, list=11%, signal=23%	SLC10A5/SLC10A2/LHCGR
**BP**	GO:1901214	regulation of neuron death	13	-0.47678417	-1.239090173	0.19047619	0.595512617	0.537852663	51	tags=62%, list=11%, signal=56%	ATF4/AXL/BOK/AIMP2/MEAK7/CLU/HRAS/FGF21
**BP**	GO:0070997	neuron death	14	-0.458638267	-1.238885204	0.190972222	0.595512617	0.537852663	51	tags=64%, list=11%, signal=59%	ATF4/DPYSL4/AXL/BOK/AIMP2/MEAK7/CLU/HRAS/FGF21
**BP**	GO:0048468	cell development	56	-0.312810235	-1.152961383	0.19379845	0.602027883	0.543737095	51	tags=18%, list=11%, signal=18%	CLU/MPL/HORMAD1/RAPGEF3/TBX3/SIX5/CYP26C1/ACE/MPIG6B/KIT
**BP**	GO:0006952	defense response	43	0.448232279	1.225469396	0.19474313	0.602670975	0.54431792	41	tags=16%, list=9%, signal=16%	DEFB113/DEFB131B/IL17F/CCL13/SAA1/DEFB123/MRGPRX1
**BP**	GO:0051241	negative regulation of multicellular organismal process	25	0.484086733	1.203913481	0.197419355	0.607715814	0.548874297	92	tags=24%, list=20%, signal=20%	IL17F/DKK4/MSX2/LBP/SFTPD/IGF1

**BP**	GO:0002682	regulation of immune system process	36	-0.347661057	-1.163422323	0.197860963	0.607715814	0.548874297	62	tags=25%, list=14%, signal=23%	MFHAS1/BDKRB1/CLU/MPL/ZEB1/DAPK2/GBP5/HRAS/KIT
**BP**	GO:0031346	positive regulation of cell projection organization	10	-0.519839896	-1.237101011	0.202279202	0.608151064	0.549267405	28	tags=20%, list=6%, signal=19%	HRAS/KIT
**BP**	GO:0007167	enzyme-linked receptor protein signaling pathway	29	-0.369370322	-1.210378021	0.204878049	0.608151064	0.549267405	78	tags=31%, list=17%, signal=27%	SMAD9/APLN/HHIP/ZEB1/SHC2/PMEPA1/FGF21/LTBP1/KIT
**BP**	GO:0033043	regulation of organelle organization	30	-0.355664362	-1.177881418	0.199052133	0.608151064	0.549267405	116	tags=43%, list=26%, signal=34%	TNKS2/ANAPC7/CYLD/BICD1/SYDE1/SAR1A/LIMA1/TNFSF10/CLU/HORMAD1/RAPGEF3/HRAS/MCM2
**BP**	GO:0007049	cell cycle	50	-0.323694647	-1.141859019	0.204081633	0.608151064	0.549267405	42	tags=14%, list=9%, signal=14%	HORMAD1/HRAS/TBX3/MCM2/OPN1MW/TRIM35/OVOL1
**BP**	GO:0051716	cellular response to stimulus	179	0.362191777	1.137628243	0.201646091	0.608151064	0.549267405	65	tags=17%, list=14%, signal=25%	TAS2R3/OR10G6/GPHB5/OR8S1/IL17F/DKK4/CCL13/OR10D3/SAA1/DEPDC1/OR56B1/OR4D6/OR13G1/TRHR/OR11H4/OR9A2/CSF2/MRGPRX1/OR11H1/STAC/CD8B/LHCGR/MSX2/OR11H2/TPTE2/TMTC3/ADAM23/ELAVL4/ADAM11/ASPM/BUB1
**BP**	GO:0016567	protein ubiquitination	15	-0.445013093	-1.226523	0.214035088	0.609291521	0.550297438	135	tags=67%, list=30%, signal=48%	RNF214/RC3H1/BARD1/AIMP2/TNKS2/ANAPC7/FBXW11/PLAA/UBE2E3/TRIM35
**BP**	GO:0034330	cell junction organization	13	-0.466201045	-1.211586229	0.210884354	0.609291521	0.550297438	143	tags=85%, list=32%, signal=59%	YWHAZ/WASF1/RAB29/NRXN1/CAST/NLGN1/RAB8B/COL17A1/MESD/NECTIN1/ACE
**BP**	GO:0034645	cellular macromolecule biosynthetic process	29	-0.368687547	-1.208140658	0.209756098	0.609291521	0.550297438	6	tags=31%, list=1%, signal=33%	AIMP2/RPS27L/SLC25A37/WARS1/EIF2B5/DAZ4/GAL3ST1/DAZ1/RPS4Y1
**BP**	GO:0098727	maintenance of cell number	11	-0.495109557	-1.204920999	0.208588957	0.609291521	0.550297438	26	tags=36%, list=6%, signal=35%	HMGA2/TBX3/CDX2/KIT
**BP**	GO:0032446	protein modification by small protein conjugation	16	-0.431390803	-1.204672943	0.211895911	0.609291521	0.550297438	135	tags=44%, list=30%, signal=32%	AIMP2/TNKS2/ANAPC7/FBXW11/PLAA/UBE2E3/TRIM35
**BP**	GO:0000165	MAPK cascade	20	-0.406795231	-1.20451513	0.208695652	0.609291521	0.550297438	62	tags=35%, list=14%, signal=32%	CYLD/NRAS/MFHAS1/SHC2/HRAS/FGF21/KIT
**BP**	GO:0045597	positive regulation of cell differentiation	23	-0.383174443	-1.194035289	0.212244898	0.609291521	0.550297438	48	tags=22%, list=11%, signal=20%	MPL/ZEB1/ACE/CDX2/KIT
**BP**	GO:0040012	regulation of locomotion	30	-0.354876126	-1.175270956	0.208530806	0.609291521	0.550297438	60	tags=33%, list=13%, signal=31%	MEAK7/TCAF1/PLAA/SYDE1/BDKRB1/DAPK2/HRAS/ACE/FGF21/KIT
**BP**	GO:0044057	regulation of system process	19	-0.394056479	-1.151855392	0.213675214	0.609291521	0.550297438	16	tags=26%, list=4%, signal=26%	HRH2/HCN4/APLN/ACE/KIT
**BP**	GO:0032879	regulation of localization	61	-0.303617914	-1.135239336	0.211864407	0.609291521	0.550297438	88	tags=28%, list=20%, signal=26%	HCN4/MESD/BICD1/APLN/SAR1A/MFHAS1/BDKRB1/CRACR2B/CLU/TTC39B/RAB26/SYT8/RAPGEF3/HRAS/KCNA7/ACE/FGF21
**BP**	GO:0051246	regulation of protein metabolic process	71	-0.284385908	-1.095388171	0.213675214	0.609291521	0.550297438	68	tags=17%, list=15%, signal=17%	TNFSF10/MFHAS1/BDKRB1/CLU/RAB26/PMEPA1/RAPGEF3/HRAS/ACE/FGF21/DAZ1/KIT
**BP**	GO:0009887	animal organ morphogenesis	22	-0.388230793	-1.19569541	0.21888412	0.620931688	0.560810556	56	tags=41%, list=12%, signal=38%	FBXW11/AHDC1/NECTIN1/HHIP/ZEB1/HRAS/TBX3/FGF21/CDX2
**BP**	GO:0070661	leukocyte proliferation	10	-0.507560574	-1.207879009	0.222222222	0.626166098	0.565538149	51	tags=40%, list=11%, signal=36%	CLU/MPL/ACE/KIT
**BP**	GO:0030334	regulation of cell migration	29	-0.363648897	-1.191629665	0.224390244	0.626166098	0.565538149	60	tags=34%, list=13%, signal=32%	MEAK7/TCAF1/PLAA/SYDE1/BDKRB1/DAPK2/HRAS/ACE/FGF21/KIT
**BP**	GO:2000145	regulation of cell motility	29	-0.363648897	-1.191629665	0.224390244	0.626166098	0.565538149	60	tags=34%, list=13%, signal=32%	MEAK7/TCAF1/PLAA/SYDE1/BDKRB1/DAPK2/HRAS/ACE/FGF21/KIT
**BP**	GO:0016192	vesicle-mediated transport	37	-0.337172781	-1.131520614	0.224043716	0.626166098	0.565538149	107	tags=35%, list=24%, signal=29%	VSNL1/PLAA/TMEM79/MESD/BICD1/APLN/SAR1A/CLU/RAB26/SYT8/HRAS/CDX2/KIT
**BP**	GO:0010563	negative regulation of phosphorus metabolic process	12	-0.474446526	-1.190656174	0.227424749	0.627723041	0.566944343	62	tags=42%, list=14%, signal=37%	WARS1/MFHAS1/BDKRB1/NPRL2/PMEPA1
**BP**	GO:0045936	negative regulation of phosphate metabolic process	12	-0.474446526	-1.190656174	0.227424749	0.627723041	0.566944343	62	tags=42%, list=14%, signal=37%	WARS1/MFHAS1/BDKRB1/NPRL2/PMEPA1
**BP**	GO:0060070	canonical Wnt signaling pathway	11	0.573272301	1.209756368	0.229289941	0.630740342	0.569669497	116	tags=45%, list=26%, signal=35%	DKK4/ASPM/NKX2-5/PPM1N/PPP1CA
**BP**	GO:1902531	regulation of intracellular signal transduction	42	-0.322634547	-1.144933561	0.23255814	0.635999308	0.574419268	68	tags=21%, list=15%, signal=20%	TNFSF10/MFHAS1/NPRL2/CLU/DAPK2/SHC2/HRAS/FGF21/KIT

**BP**	GO:0031324	negative regulation of cellular metabolic process	60	-0.303827039	-1.122755857	0.232758621	0.635999308	0.574419268	62	tags=22%, list=14%, signal=22%	MFHAS1/BDKRB1/CDYL2/NPRL2/CLU/ZEB1/ZNF596/PMEPA1/TBX3/SIX5/CDX2/DAZ1/OVOL1
**BP**	GO:1901565	organonitrogen compound catabolic process	17	-0.414450631	-1.188866202	0.23828125	0.642494328	0.580285413	118	tags=47%, list=26%, signal=36%	GCAT/ANAPC7/FBXW11/CYLD/PLAA/CLU/RAB26/ACE
**BP**	GO:1902533	positive regulation of intracellular signal transduction	27	-0.361581617	-1.157847009	0.236714976	0.642494328	0.580285413	68	tags=22%, list=15%, signal=20%	TNFSF10/MFHAS1/SHC2/HRAS/FGF21/KIT
**BP**	GO:0031344	regulation of cell projection organization	15	-0.429744219	-1.184439689	0.242105263	0.64852459	0.585731801	28	tags=13%, list=6%, signal=13%	HRAS/KIT
**BP**	GO:0045944	positive regulation of transcription by RNA polymerase II	27	-0.356170705	-1.140520331	0.241545894	0.64852459	0.585731801	44	tags=26%, list=10%, signal=25%	ZEB1/HRAS/TBX3/SIX5/PTMS/HNF1A/CDX2
**BP**	GO:0048513	animal organ development	86	-0.279008514	-1.116073473	0.245098039	0.654395745	0.591034487	28	tags=15%, list=6%, signal=18%	HRAS/TSPY10/TBX3/SIX5/CYP26C1/HNF1A/ACE/MPIG6B/MCM2/FGF21/CDX2/OVOL1/KIT
**BP**	GO:0120035	regulation of plasma membrane bounded cell projection organization	12	-0.466629245	-1.171038169	0.247491639	0.655353636	0.591899631	28	tags=25%, list=6%, signal=24%	CYLD/HRAS/KIT
**BP**	GO:0048878	chemical homeostasis	26	-0.364991871	-1.15937197	0.247706422	0.655353636	0.591899631	72	tags=31%, list=16%, signal=27%	LIMA1/BDKRB1/TTC39B/NAPSA/HNF1A/NPC1L1/ACE/FGF21
**BP**	GO:0010608	post-transcriptional regulation of gene expression	19	-0.384874864	-1.125016872	0.247863248	0.655353636	0.591899631	16	tags=26%, list=4%, signal=26%	RPS27L/EIF2B5/DAZ4/ACE/DAZ1
**BP**	GO:0036211	protein modification process	83	-0.278684662	-1.102659864	0.25	0.658870968	0.595076401	66	tags=16%, list=15%, signal=16%	GAL3ST1/MFHAS1/BDKRB1/CLU/DAPK2/PMEPA1/RAPGEF3/HRAS/ART1/ACE/FGF21/TRIM35/KIT
**BP**	GO:0010038	response to metal ion	11	-0.47971009	-1.16744416	0.254601227	0.662449689	0.598308616	64	tags=36%, list=14%, signal=32%	EIF2B5/CPNE5/SYT8/KIT
**BP**	GO:0022412	cellular process involved in reproduction in multicellular organism	11	-0.478748602	-1.16510424	0.254601227	0.662449689	0.598308616	42	tags=27%, list=9%, signal=25%	HORMAD1/SIX5/KIT
**BP**	GO:0000003	reproduction	45	-0.319728044	-1.134118882	0.253012048	0.662449689	0.598308616	27	tags=18%, list=6%, signal=19%	HORMAD1/TSPY10/TBX3/SIX5/ACE/DAZ1/OVOL1/KIT
**BP**	GO:0022414	reproductive process	45	-0.319728044	-1.134118882	0.253012048	0.662449689	0.598308616	27	tags=18%, list=6%, signal=19%	HORMAD1/TSPY10/TBX3/SIX5/ACE/DAZ1/OVOL1/KIT
**BP**	GO:0000122	negative regulation of transcription by RNA polymerase II	25	-0.357924625	-1.137529828	0.259911894	0.674120691	0.608849584	44	tags=20%, list=10%, signal=19%	ZEB1/ZNF596/TBX3/CDX2/OVOL1
**BP**	GO:0006338	chromatin remodeling	14	0.534516247	1.182218392	0.264705882	0.675495711	0.610091469	50	tags=29%, list=11%, signal=26%	H2BW1/ERCC6L/TSPYL6/EOMES
**BP**	GO:0007281	germ cell development	10	-0.492259157	-1.171465108	0.262108262	0.675495711	0.610091469	42	tags=30%, list=9%, signal=28%	HORMAD1/SIX5/KIT
**BP**	GO:0043066	negative regulation of apoptotic process	24	-0.367465087	-1.159272958	0.261603376	0.675495711	0.610091469	56	tags=29%, list=12%, signal=27%	HMGA2/HHIP/CLU/HRAS/TBX3/PTMS/FGF21
**BP**	GO:0046903	secretion	31	-0.340214304	-1.117387283	0.265306122	0.675495711	0.610091469	39	tags=19%, list=9%, signal=19%	RAB26/SYT8/TBX3/HNF1A/ACE/KIT
**BP**	GO:0002684	positive regulation of immune system process	28	-0.340295471	-1.110523545	0.265402844	0.675495711	0.610091469	60	tags=25%, list=13%, signal=23%	BDKRB1/CLU/MPL/DAPK2/GBP5/HRAS/KIT
**BP**	GO:0030111	regulation of Wnt signaling pathway	10	0.576357463	1.185192517	0.273425499	0.682277306	0.616216442	116	tags=50%, list=26%, signal=38%	DKK4/ASPM/NKX2-5/PPM1N/PPP1CA
**BP**	GO:0060828	regulation of canonical Wnt signaling pathway	10	0.576357463	1.185192517	0.273425499	0.682277306	0.616216442	116	tags=50%, list=26%, signal=38%	DKK4/ASPM/NKX2-5/PPM1N/PPP1CA
**BP**	GO:0098609	cell-cell adhesion	20	-0.384800619	-1.139389384	0.273913043	0.682277306	0.616216442	77	tags=20%, list=17%, signal=17%	NECTIN1/PCDHB7/MPL/KIT
**BP**	GO:0010639	negative regulation of organelle organization	15	-0.404065287	-1.113664691	0.273684211	0.682277306	0.616216442	72	tags=47%, list=16%, signal=41%	NAV3/BOK/TNKS2/LIMA1/CLU/HORMAD1/MCM2
**BP**	GO:0048856	anatomical structure development	142	-0.240102763	-1.015544776	0.273120604	0.682277306	0.616216442	101	tags=28%, list=22%, signal=32%	PLAA/TG/TMEM79/NRAS/AHDC1/HCN4/EEF1AKMT4-ECE2/MESD/BICD1/SYDE1/HMGA2/SMAD9/NECTIN1/APLN/PCDHB7/TNFSF10/GAL3ST1/CPNE5/MFHAS1/HHIP/CLU/KRT8/MPL/ZEB1/HORMAD1/RAB26/RAPGEF3/HRAS/TSPY10/TBX3/SIX5/CYP26C1/HNF1A/ACE/MPIG6B/MCM2/FGF21/CDX2/OVOL1/KIT
**BP**	GO:0006629	lipid metabolic process	31	-0.33713136	-1.107261777	0.275510204	0.682967607	0.616839905	72	tags=29%, list=16%, signal=26%	LIMA1/GAL3ST1/HSD3B7/CLU/TTC39B/CYP26C1/NPC1L1/FGF21/KIT
**BP**	GO:0044085	cellular component biogenesis	69	-0.278123094	-1.06594998	0.275862069	0.682967607	0.616839905	83	tags=23%, list=18%, signal=22%	BICD1/NECTIN1/SAR1A/LIMA1/BBS10/CLU/HORMAD1/GBP5/PMEPA1/RAPGEF3/HRAS/TSPY10/KCNA7/ACE/MCM2/KIT

**BP**	GO:0010948	negative regulation of cell cycle process	10	-0.482951321	-1.149314571	0.279202279	0.685009796	0.618684361	42	tags=20%, list=9%, signal=19%	HORMAD1/OVOL1
**BP**	GO:0042326	negative regulation of phosphorylation	10	-0.482585757	-1.148444612	0.279202279	0.685009796	0.618684361	60	tags=40%, list=13%, signal=35%	WARS1/BDKRB1/NPRL2/PMEPA1
**BP**	GO:0030855	epithelial cell differentiation	17	-0.394186736	-1.130738506	0.28515625	0.697522923	0.629985916	49	tags=35%, list=11%, signal=33%	KRT8/ZEB1/RAPGEF3/TBX3/CDX2/OVOL1
**BP**	GO:0007389	pattern specification process	11	-0.467650315	-1.138094946	0.288343558	0.701121093	0.633235697	56	tags=45%, list=12%, signal=41%	HHIP/ZEB1/TBX3/CYP26C1/CDX2
**BP**	GO:0009968	negative regulation of signal transduction	29	-0.33994791	-1.113964645	0.287804878	0.701121093	0.633235697	83	tags=34%, list=18%, signal=30%	BICD1/APLN/MFHAS1/HHIP/NPRL2/CLU/PMEPA1/CYP26C1/MPIG6B/LTBP1
**BP**	GO:0022607	cellular component assembly	67	-0.285850305	-1.078463031	0.293103448	0.71058017	0.641778908	83	tags=24%, list=18%, signal=23%	BICD1/NECTIN1/SAR1A/LIMA1/BBS10/CLU/HORMAD1/GBP5/PMEPA1/RAPGEF3/HRAS/TSPY10/KCNA7/ACE/MCM2/KIT
**BP**	GO:0044262	cellular carbohydrate metabolic process	10	0.568502926	1.169040842	0.294930876	0.712895045	0.643869647	145	tags=60%, list=32%, signal=42%	LHCGR/TFF3/IGF1/PPP1CA/GNPTG/PLA2G4A
**BP**	GO:0006820	anion transport	18	0.48208452	1.140771686	0.297082228	0.715976933	0.646653134	69	tags=22%, list=15%, signal=20%	SLC10A5/SLC10A2/LHCGR/ANO5
**BP**	GO:0006325	chromatin organization	17	0.484367649	1.127978149	0.305630027	0.7233603	0.653321613	50	tags=24%, list=11%, signal=22%	H2BW1/ERCC6L/TSPYL6/EOMES
**BP**	GO:0006897	endocytosis	15	-0.390855845	-1.077257481	0.305263158	0.7233603	0.653321613	83	tags=33%, list=18%, signal=28%	BICD1/APLN/CLU/SYT8/HRAS
**BP**	GO:0009725	response to hormone	16	-0.396666096	-1.107703065	0.308550186	0.724383626	0.654245857	30	tags=25%, list=7%, signal=24%	PMEPA1/ACE/FGF21/KIT
**BP**	GO:0006955	immune response	45	0.408122632	1.121934019	0.314593301	0.734350649	0.663247832	50	tags=18%, list=11%, signal=18%	DEFB113/DEFB131B/IL17F/CCL13/DEFB123/CSF2/CD8B/EOMES
**BP**	GO:0032870	cellular response to hormone stimulus	11	-0.457955304	-1.114500729	0.32208589	0.741800023	0.669975927	30	tags=36%, list=7%, signal=35%	PMEPA1/ACE/FGF21/KIT
**BP**	GO:0030163	protein catabolic process	12	-0.442339517	-1.110081427	0.321070234	0.741800023	0.669975927	109	tags=50%, list=24%, signal=39%	ANAPC7/FBXW11/CYLD/PLAA/CLU/RAB26
**BP**	GO:0010605	negative regulation of macromolecule metabolic process	77	-0.273928282	-1.065882781	0.323232323	0.741800023	0.669975927	62	tags=18%, list=14%, signal=19%	MFHAS1/BDKRB1/CDYL2/CLU/ZEB1/ZNF596/PMEPA1/HRAS/TBX3/SIX5/ACE/CDX2/DAZ1/OVOL1
**BP**	GO:0010557	positive regulation of macromolecule biosynthetic process	48	-0.310252327	-1.09538512	0.324675325	0.743024483	0.67108183	28	tags=17%, list=6%, signal=17%	ZEB1/HRAS/TBX3/SIX5/PTMS/HNF1A/CDX2/DAZ1
**BP**	GO:0010564	regulation of cell cycle process	18	-0.370421692	-1.066459226	0.326612903	0.745370788	0.673200956	42	tags=22%, list=9%, signal=21%	HMGA2/HORMAD1/OPN1MW/OVOL1
**BP**	GO:0009896	positive regulation of catabolic process	11	-0.455777787	-1.109201427	0.328220859	0.746953877	0.674630764	55	tags=27%, list=12%, signal=25%	NPRL2/CLU/FGF21
**BP**	GO:0008406	gonad development	10	-0.465502196	-1.107789614	0.33048433	0.747938222	0.675519801	27	tags=40%, list=6%, signal=38%	TNFSF10/TSPY10/ACE/KIT
**BP**	GO:0045137	development of primary sexual characteristics	10	-0.465502196	-1.107789614	0.33048433	0.747938222	0.675519801	27	tags=40%, list=6%, signal=38%	TNFSF10/TSPY10/ACE/KIT
**BP**	GO:0032502	developmental process	149	-0.242512726	-1.036742124	0.339822612	0.766947718	0.69268872	85	tags=28%, list=19%, signal=34%	DAZ4/PLAA/TG/TMEM79/NRAS/AHDC1/HCN4/EEF1AKMT4-ECE2/MESD/BICD1/SYDE1/HMGA2/SMAD9/NECTIN1/APLN/PCDHB7/TNFSF10/GAL3ST1/CPNE5/MFHAS1/HHIP/CLU/KRT8/MPL/ZEB1/HORMAD1/RAB26/RAPGEF3/HRAS/TSPY10/TBX3/SIX5/CYP26C1/HNF1A/ACE/MPIG6B/MCM2/FGF21/CDX2/DAZ1/OVOL1/KIT
**BP**	GO:0019637	organophosphate metabolic process	17	0.468269975	1.09049046	0.347184987	0.775000366	0.699961678	150	tags=47%, list=33%, signal=33%	CKMT2/LHCGR/TPTE2/IGF1/PIP5KL1/PLA2G4F/PLA2G4A/CTPS1
**BP**	GO:0022402	cell cycle process	29	-0.328657368	-1.07696702	0.346341463	0.775000366	0.699961678	14	tags=14%, list=3%, signal=14%	HORMAD1/MCM2/OPN1MW/OVOL1
**BP**	GO:2000241	regulation of reproductive process	10	-0.456858667	-1.087219974	0.35042735	0.778678108	0.703283327	42	tags=40%, list=9%, signal=37%	SYDE1/HORMAD1/OVOL1/KIT
**BP**	GO:0051049	regulation of transport	53	-0.298949276	-1.061702264	0.353383459	0.778678108	0.703283327	88	tags=28%, list=20%, signal=26%	HCN4/BICD1/APLN/SAR1A/BDKRB1/CRACR2B/CLU/TTC39B/RAB26/SYT8/RAPGEF3/HRAS/KCNA7/ACE/FGF21

**BP**	GO:0048518	positive regulation of biological process	158	-0.243637359	-1.053759888	0.356920385	0.779689718	0.704196988	146	tags=39%, list=33%, signal=40%	DR1/BOK/RAB8B/SOX5/AZU1/LDLRAP1/EHD4/VPS72/AIMP2/TAP2/ZNF341/RPS27L/SAP30BP/SLC25A37/NKAP/WARS1/TNKS2/MEAK7/EIF2B5/TCAF1/ANAPC7/VSNL1/FBXW11/DAZ4/CYLD/PLAA/HRH2/TMEM79/NRAS/MESD/BICD1/SYDE1/HMGA2/SMAD9/APLN/SAR1A/TNFSF10/CPNE5/MFHAS1/BDKRB1/NPRL2/CLU/MPL/ZEB1/DAPK2/SHC2/GBP5/RAPGEF3/HRAS/TBX3/SIX5/PTMS/HNF1A/ACE/OPN1MW/FGF21/CDX2/DAZ1/TRIM35/OVOL1/KIT
**BP**	GO:0035556	intracellular signal transduction	61	-0.281771812	-1.053555899	0.355932203	0.779689718	0.704196988	82	tags=23%, list=18%, signal=22%	SYDE1/HMGA2/TNFSF10/MFHAS1/NPRL2/CLU/CCR10/HORMAD1/DAPK2/SHC2/RAPGEF3/HRAS/FGF21/KIT
**BP**	GO:0010648	negative regulation of cell communication	32	-0.322228297	-1.046058882	0.361702128	0.783847847	0.70795251	83	tags=31%, list=18%, signal=27%	BICD1/APLN/MFHAS1/HHIP/NPRL2/CLU/PMEPA1/CYP26C1/MPIG6B/LTBP1
**BP**	GO:0023057	negative regulation of signaling	32	-0.322228297	-1.046058882	0.361702128	0.783847847	0.70795251	83	tags=31%, list=18%, signal=27%	BICD1/APLN/MFHAS1/HHIP/NPRL2/CLU/PMEPA1/CYP26C1/MPIG6B/LTBP1
**BP**	GO:0009057	macromolecule catabolic process	22	-0.343995619	-1.059457392	0.364806867	0.788484683	0.712140389	109	tags=36%, list=24%, signal=29%	ANAPC7/FBXW11/DAZ4/CYLD/PLAA/CLU/RAB26/DAZ1
**BP**	GO:0006875	cellular metal ion homeostasis	10	0.537462641	1.10521116	0.367127496	0.789324117	0.712898545	111	tags=40%, list=25%, signal=31%	CCL13/LHCGR/TRPC3/CEMIP
**BP**	GO:0030003	cellular cation homeostasis	10	0.537462641	1.10521116	0.367127496	0.789324117	0.712898545	111	tags=40%, list=25%, signal=31%	CCL13/LHCGR/TRPC3/CEMIP
**BP**	GO:0001568	blood vessel development	18	-0.359413722	-1.034766829	0.379032258	0.806430612	0.72834872	29	tags=33%, list=6%, signal=32%	HMGA2/APLN/RAPGEF3/TBX3/ACE/CDX2
**BP**	GO:0051172	negative regulation of nitrogen compound metabolic process	61	-0.280699874	-1.04954788	0.381355932	0.809267004	0.730910481	62	tags=20%, list=14%, signal=20%	MFHAS1/BDKRB1/CDYL2/CLU/ZEB1/ZNF596/PMEPA1/TBX3/SIX5/CDX2/DAZ1/OVOL1
**BP**	GO:0022603	regulation of anatomical structure morphogenesis	26	0.421704726	1.061593491	0.382653061	0.809915935	0.73149658	13	tags=12%, list=3%, signal=12%	IL17F/DKK4/CCL13
**BP**	GO:0019221	cytokine-mediated signaling pathway	13	0.496244868	1.082911736	0.38700565	0.811119384	0.732583506	40	tags=23%, list=9%, signal=22%	IL17F/CCL13/CSF2
**BP**	GO:0045893	positive regulation of DNA-templated transcription	39	-0.303588203	-1.031107184	0.392045455	0.811119384	0.732583506	44	tags=26%, list=10%, signal=25%	HMGA2/SMAD9/APLN/ZEB1/HRAS/TBX3/SIX5/PTMS/HNF1A/CDX2
**BP**	GO:1902680	positive regulation of RNA biosynthetic process	39	-0.303588203	-1.031107184	0.392045455	0.811119384	0.732583506	44	tags=26%, list=10%, signal=25%	HMGA2/SMAD9/APLN/ZEB1/HRAS/TBX3/SIX5/PTMS/HNF1A/CDX2
**BP**	GO:1903508	positive regulation of nucleic acid-templated transcription	39	-0.303588203	-1.031107184	0.392045455	0.811119384	0.732583506	44	tags=26%, list=10%, signal=25%	HMGA2/SMAD9/APLN/ZEB1/HRAS/TBX3/SIX5/PTMS/HNF1A/CDX2
**BP**	GO:0006397	mRNA processing	11	0.508852921	1.073814418	0.402366864	0.813112307	0.734383466	82	tags=27%, list=18%, signal=23%	NCBP2L/ELAVL4/SNRPE
**BP**	GO:0048511	rhythmic process	12	0.497507442	1.071564822	0.40398293	0.813112307	0.734383466	48	tags=17%, list=11%, signal=15%	CSF2/LHCGR
**BP**	GO:0006869	lipid transport	17	0.452717448	1.054272287	0.396782842	0.813112307	0.734383466	48	tags=24%, list=11%, signal=22%	SLC10A5/FABP2/SLC10A2/LHCGR
**BP**	GO:0010876	lipid localization	17	0.452717448	1.054272287	0.396782842	0.813112307	0.734383466	48	tags=24%, list=11%, signal=22%	SLC10A5/FABP2/SLC10A2/LHCGR
**BP**	GO:0031328	positive regulation of cellular biosynthetic process	49	-0.295760122	-1.050997121	0.405063291	0.813112307	0.734383466	51	tags=24%, list=11%, signal=24%	HMGA2/SMAD9/APLN/CLU/ZEB1/HRAS/TBX3/SIX5/PTMS/HNF1A/CDX2/DAZ1
**BP**	GO:0051254	positive regulation of RNA metabolic process	42	-0.295843975	-1.049861827	0.401162791	0.813112307	0.734383466	44	tags=24%, list=10%, signal=24%	HMGA2/SMAD9/APLN/ZEB1/HRAS/TBX3/SIX5/PTMS/HNF1A/CDX2
**BP**	GO:1904951	positive regulation of establishment of protein localization	10	-0.437211521	-1.040464225	0.398860399	0.813112307	0.734383466	110	tags=50%, list=24%, signal=39%	TCAF1/VSNL1/SAR1A/RAPGEF3/HRAS
**BP**	GO:0002764	immune response-regulating signaling pathway	13	-0.399037343	-1.037037893	0.397959184	0.813112307	0.734383466	62	tags=38%, list=14%, signal=34%	EIF2B5/CYLD/MFHAS1/HRAS/KIT
**BP**	GO:0070848	response to growth factor	24	-0.32044812	-1.010944585	0.405063291	0.813112307	0.734383466	78	tags=29%, list=17%, signal=25%	SMAD9/APLN/HHIP/ZEB1/PMEPA1/FGF21/LTBP1
**BP**	GO:0071363	cellular response to growth factor stimulus	24	-0.32044812	-1.010944585	0.405063291	0.813112307	0.734383466	78	tags=29%, list=17%, signal=25%	SMAD9/APLN/HHIP/ZEB1/PMEPA1/FGF21/LTBP1
**BP**	GO:1903829	positive regulation of protein localization	14	-0.371632625	-1.003863378	0.399305556	0.813112307	0.734383466	114	tags=57%, list=25%, signal=44%	MEAK7/TCAF1/VSNL1/MESD/BICD1/SAR1A/RAPGEF3/HRAS

**BP**	GO:0051052	regulation of DNA metabolic process	13	-0.396070732	-1.029328119	0.408163265	0.815515578	0.736554043	196	tags=62%, list=44%, signal=36%	INO80E/HMGB1/TRRAP/BARD1/VPS72/TNKS2/HMGA2/MCM2
**BP**	GO:0043085	positive regulation of catalytic activity	26	-0.319997718	-1.01645109	0.408256881	0.815515578	0.736554043	82	tags=31%, list=18%, signal=27%	SYDE1/HMGA2/TNFSF10/CLU/RAPGEF3/HRAS/ACE/KIT
**BP**	GO:0006631	fatty acid metabolic process	10	0.520747697	1.070839391	0.413210445	0.8233974	0.743672714	38	tags=20%, list=8%, signal=19%	FABP2/CYP4Z1
**BP**	GO:0006066	alcohol metabolic process	11	-0.425325894	-1.035092322	0.417177914	0.829280671	0.748986343	72	tags=36%, list=16%, signal=31%	LIMA1/TTC39B/NPC1L1/TKTL1
**BP**	GO:0006259	DNA metabolic process	22	-0.333835791	-1.028166572	0.420600858	0.830026332	0.749659806	91	tags=27%, list=20%, signal=23%	TNKS2/TOP3B/HMGA2/EMSY/PTMS/MCM2
**BP**	GO:0034762	regulation of transmembrane transport	18	-0.347748141	-1.001181143	0.419354839	0.830026332	0.749659806	17	tags=28%, list=4%, signal=28%	HCN4/BDKRB1/KCNA7/ACE/FGF21
**BP**	GO:0043412	macromolecule modification	89	-0.253309274	-1.004030821	0.431818182	0.844008264	0.762287951	66	tags=15%, list=15%, signal=16%	GAL3ST1/MFHAS1/BDKRB1/CLU/DAPK2/PMEPA1/RAPGEF3/HRAS/ART1/ACE/FGF21/TRIM35/KIT
**BP**	GO:0006873	cellular ion homeostasis	12	0.483371126	1.041117077	0.440967283	0.846666667	0.764688956	111	tags=33%, list=25%, signal=26%	CCL13/LHCGR/TRPC3/CEMIP
**BP**	GO:0016055	Wnt signaling pathway	13	0.475707525	1.038094889	0.442090395	0.846666667	0.764688956	116	tags=38%, list=26%, signal=29%	DKK4/ASPM/NKX2-5/PPM1N/PPP1CA
**BP**	GO:0198738	cell-cell signaling by wnt	13	0.475707525	1.038094889	0.442090395	0.846666667	0.764688956	116	tags=38%, list=26%, signal=29%	DKK4/ASPM/NKX2-5/PPM1N/PPP1CA
**BP**	GO:0048871	multicellular organismal homeostasis	12	-0.410489055	-1.030150504	0.444816054	0.846666667	0.764688956	127	tags=50%, list=28%, signal=37%	LAMA4/SLC28A2/TMEM79/BBS10/NAPSA/FGF21
**BP**	GO:0009891	positive regulation of biosynthetic process	50	-0.291482508	-1.028228098	0.442176871	0.846666667	0.764688956	51	tags=24%, list=11%, signal=24%	HMGA2/SMAD9/APLN/CLU/ZEB1/HRAS/TBX3/SIX5/PTMS/HNF1A/CDX2/DAZ1
**BP**	GO:0006812	cation transport	27	0.401668649	1.018953286	0.440251572	0.846666667	0.764688956	71	tags=26%, list=16%, signal=23%	SLC10A5/SLC10A2/CSF2/STAC/LHCGR/KCNK1/NKX2-5
**BP**	GO:0030001	metal ion transport	21	0.41792185	1.011182229	0.435863874	0.846666667	0.764688956	71	tags=29%, list=16%, signal=25%	SLC10A5/SLC10A2/STAC/LHCGR/KCNK1/NKX2-5
**BP**	GO:0032386	regulation of intracellular transport	10	-0.422988435	-1.006616508	0.444444444	0.846666667	0.764688956	154	tags=70%, list=34%, signal=47%	BARD1/NUP214/LDLRAP1/TCAF1/SAR1A/RAPGEF3/HRAS
**BP**	GO:0001501	skeletal system development	15	-0.357029909	-0.984028115	0.445614035	0.846666667	0.764688956	56	tags=40%, list=12%, signal=36%	HMGA2/HHIP/ZEB1/TBX3/MPIG6B/KIT
**BP**	GO:0006793	phosphorus metabolic process	65	-0.260479592	-0.987101301	0.45	0.851041667	0.768640351	62	tags=22%, list=14%, signal=22%	ABHD3/HMGA2/ADCK2/MFHAS1/BDKRB1/NPRL2/CLU/DAPK2/PMEPA1/RAPGEF3/HRAS/ACE/FGF21/KIT
**BP**	GO:0006796	phosphate-containing compound metabolic process	65	-0.260479592	-0.987101301	0.45	0.851041667	0.768640351	62	tags=22%, list=14%, signal=22%	ABHD3/HMGA2/ADCK2/MFHAS1/BDKRB1/NPRL2/CLU/DAPK2/PMEPA1/RAPGEF3/HRAS/ACE/FGF21/KIT
**BP**	GO:1905114	cell surface receptor signaling pathway involved in cell-cell signaling	18	0.428125812	1.013087507	0.452254642	0.85249893	0.769956516	116	tags=28%, list=26%, signal=21%	DKK4/ASPM/NKX2-5/PPM1N/PPP1CA
**BP**	GO:0007267	cell-cell signaling	50	-0.286876062	-1.011978486	0.455782313	0.85249893	0.769956516	39	tags=18%, list=9%, signal=18%	RAB26/SYT8/HRAS/TBX3/HNF1A/ACE/FGF21/LTBP1/KIT
**BP**	GO:0051239	regulation of multicellular organismal process	70	-0.260207861	-1.006154875	0.456896552	0.85249893	0.769956516	51	tags=17%, list=11%, signal=18%	CLU/MPL/ZEB1/GBP5/RAPGEF3/HRAS/TBX3/ACE/FGF21/CDX2/LTBP1/KIT
**BP**	GO:0043065	positive regulation of apoptotic process	17	-0.345249032	-0.990358982	0.45703125	0.85249893	0.769956516	68	tags=29%, list=15%, signal=26%	TNFSF10/CLU/DAPK2/ACE/TRIM35
**BP**	GO:0043068	positive regulation of programmed cell death	17	-0.345249032	-0.990358982	0.45703125	0.85249893	0.769956516	68	tags=29%, list=15%, signal=26%	TNFSF10/CLU/DAPK2/ACE/TRIM35
**BP**	GO:0031400	negative regulation of protein modification process	13	-0.378663698	-0.984089861	0.455782313	0.85249893	0.769956516	117	tags=38%, list=26%, signal=29%	WARS1/PLAA/MFHAS1/BDKRB1/PMEPA1
**BP**	GO:0051336	regulation of hydrolase activity	19	-0.343672988	-1.004580829	0.461538462	0.857897008	0.77483193	83	tags=37%, list=18%, signal=31%	PLAA/BICD1/SYDE1/TNFSF10/RAPGEF3/HRAS/KIT
**BP**	GO:0072359	circulatory system development	30	-0.300074894	-0.99378144	0.464454976	0.86045287	0.777140323	29	tags=17%, list=6%, signal=17%	RAPGEF3/TBX3/ACE/CDX2/KIT
**BP**	GO:1903311	regulation of mRNA metabolic process	10	-0.414104539	-0.985474851	0.467236467	0.861697954	0.778264853	6	tags=70%, list=1%, signal=71%	SON/PAPOLA/RC3H1/PATL1/BARD1/DAZ4/DAZ1
**BP**	GO:0044093	positive regulation of molecular function	34	-0.303772361	-0.992539645	0.472527473	0.869493119	0.785305259	82	tags=26%, list=18%, signal=23%	SYDE1/HMGA2/TNFSF10/CLU/RAPGEF3/HRAS/ACE/FGF21/KIT
**BP**	GO:0048598	embryonic morphogenesis	13	0.465889033	1.016668854	0.47740113	0.872185559	0.787737006	71	tags=38%, list=16%, signal=33%	GJB5/MSX2/EOMES/COL11A1/NKX2-5
**BP**	GO:0000902	cell morphogenesis	29	-0.298417164	-0.977873843	0.47804878	0.872185559	0.787737006	77	tags=21%, list=17%, signal=18%	NECTIN1/CPNE5/CLU/MPL/MPIG6B/KIT
**BP**	GO:0045935	positive regulation of nucleobase-containing compound metabolic process	45	-0.280371906	-0.994517306	0.481927711	0.876374363	0.791520233	44	tags=22%, list=10%, signal=22%	HMGA2/SMAD9/APLN/ZEB1/HRAS/TBX3/SIX5/PTMS/HNF1A/CDX2

BP	GO:0008015	blood circulation	15	-0.347973679	-0.959067783	0.484210526	0.876374363	0.791520233	98	tags=53%, list=22%, signal=43%	GNA11/KCNE3/AZU1/HRH2/HCN4/APLN/BDKRB1/ACE
BP	GO:0044255	cellular lipid metabolic process	22	-0.314661178	-0.969111501	0.489270386	0.880471158	0.795220359	21	tags=14%, list=5%, signal=14%	CYP26C1/FGF21/KIT
BP	GO:0048589	developmental growth	12	0.469450609	1.011134137	0.490753912	0.881199881	0.795878524	92	tags=33%, list=20%, signal=27%	MSX2/ASPM/NKX2-5/IGF1
BP	GO:0010942	positive regulation of cell death	18	-0.333065569	-0.958909417	0.495967742	0.888608871	0.802570146	68	tags=28%, list=15%, signal=25%	TNFSF10/CLU/DAPK2/ACE/TRIM35
BP	GO:0033365	protein localization to organelle	23	-0.305334041	-0.951471653	0.497959184	0.890224624	0.804029455	126	tags=48%, list=28%, signal=36%	NUP214/RAB8B/IPO11/TNKS2/MEAK7/TRAM1/BICD1/MFHAS1/CLU/GBP5/HRAS
BP	GO:0030155	regulation of cell adhesion	14	0.447130009	0.988941539	0.504201681	0.895506028	0.808799492	92	tags=21%, list=20%, signal=18%	SAA1/SFTPD/IGF1
BP	GO:0050790	regulation of catalytic activity	47	-0.282422085	-0.993587976	0.509803922	0.897650439	0.810736273	83	tags=26%, list=18%, signal=23%	BICD1/SYDE1/HMGA2/TNFSF10/NPRL2/RIC8B/CLU/RAPGEF3/HRAS/ACE/MCM2/KIT
BP	GO:0045934	negative regulation of nucleobase-containing compound metabolic process	41	-0.280631831	-0.981236275	0.517045455	0.907498475	0.819630781	44	tags=20%, list=10%, signal=19%	CDYL2/ZEB1/ZNF596/TBX3/SIX5/CDX2/DAZ1/OVOL1
BP	GO:0006811	ion transport	38	0.361190286	0.972367325	0.517618469	0.907498475	0.819630781	71	tags=21%, list=16%, signal=19%	SLC10A5/SLC10A2/CSF2/STAC/LHCGR/KCNK1/ANO5/NKX2-5
BP	GO:0031347	regulation of defense response	19	0.403061794	0.966462527	0.51953125	0.908901566	0.820898019	17	tags=11%, list=4%, signal=11%	IL17F/SAA1
BP	GO:0051253	negative regulation of RNA metabolic process	39	-0.286452457	-0.972907324	0.522727273	0.91253885	0.824183127	44	tags=21%, list=10%, signal=20%	CDYL2/ZEB1/ZNF596/TBX3/SIX5/CDX2/DAZ1/OVOL1
BP	GO:0043270	positive regulation of ion transport	12	0.457621466	0.985655737	0.524893314	0.912849162	0.824463393	145	tags=58%, list=32%, signal=41%	STAC/LHCGR/NKX2-5/TRPC3/CEMIP/DTNBP1/PLA2G4A
BP	GO:0051094	positive regulation of developmental process	38	-0.288495736	-0.976695483	0.525139665	0.912849162	0.824463393	101	tags=29%, list=22%, signal=25%	PLAA/TMEM79/SYDE1/HMGA2/CPNE5/MPL/ZEB1/RAPGEF3/ACE/CDX2/KIT
BP	GO:0002250	adaptive immune response	13	0.444414397	0.969806636	0.528248588	0.915677743	0.827018099	80	tags=31%, list=18%, signal=26%	IL17F/CD8B/EOMES/FCAMR
BP	GO:0003013	circulatory system process	17	-0.327340586	-0.938987976	0.53125	0.915677743	0.827018099	164	tags=53%, list=37%, signal=35%	GNA11/KCNE3/AZU1/SLC28A2/HRH2/HCN4/APLN/BDKRB1/ACE
BP	GO:0051656	establishment of organelle localization	12	-0.38026204	-0.954293731	0.535117057	0.916542213	0.827798868	122	tags=42%, list=27%, signal=31%	MAJIN/FBXW11/BICD1/SAR1A/KIT
BP	GO:0050776	regulation of immune response	23	-0.299935971	-0.93465037	0.534693878	0.916542213	0.827798868	62	tags=22%, list=14%, signal=20%	MFHAS1/CLU/GBP5/HRAS/KIT
BP	GO:0010035	response to inorganic substance	14	-0.345011382	-0.93195341	0.534722222	0.916542213	0.827798868	64	tags=21%, list=14%, signal=19%	CPNE5/SYT8/KIT
BP	GO:0051345	positive regulation of hydrolase activity	12	-0.379264909	-0.951791362	0.538461538	0.918419785	0.829494646	29	tags=50%, list=6%, signal=48%	PLAA/SYDE1/TNFSF10/RAPGEF3/HRAS/KIT
BP	GO:0034660	ncRNA metabolic process	13	-0.358086322	-0.930612364	0.537414966	0.918419785	0.829494646	181	tags=62%, list=40%, signal=38%	RC3H1/MOV10L1/TRMT61A/TSEN54/IMP3/WARS1/APLN/HRAS
BP	GO:0030335	positive regulation of cell migration	17	-0.324260796	-0.930153491	0.546875	0.928891632	0.838952566	60	tags=35%, list=13%, signal=32%	PLAA/SYDE1/BDKRB1/DAPK2/HRAS/KIT
BP	GO:2000147	positive regulation of cell motility	17	-0.324260796	-0.930153491	0.546875	0.928891632	0.838952566	60	tags=35%, list=13%, signal=32%	PLAA/SYDE1/BDKRB1/DAPK2/HRAS/KIT
BP	GO:0032102	negative regulation of response to external stimulus	10	0.468485391	0.963369811	0.559139785	0.936405946	0.845739314	115	tags=40%, list=26%, signal=30%	SAA1/IGF1/BACE1/CXCL17
BP	GO:0009653	anatomical structure morphogenesis	66	-0.254631825	-0.962374366	0.559322034	0.936405946	0.845739314	83	tags=27%, list=18%, signal=26%	BICD1/HMGA2/SMAD9/NECTIN1/APLN/CPNE5/HHIP/CLU/MPL/ZEB1/RAPGEF3/HRAS/TBX3/ACE/MPIG6B/FGF21/CDX2/KIT
BP	GO:0140694	non-membrane-bounded organelle assembly	13	0.43301475	0.944930184	0.559322034	0.936405946	0.845739314	106	tags=31%, list=24%, signal=24%	CSF2/ASPM/NKX2-5/NEK2
BP	GO:0030182	neuron differentiation	35	0.355029074	0.940578903	0.558139535	0.936405946	0.845739314	98	tags=23%, list=22%, signal=19%	NTM/EOMES/ELAVL4/ASPM/NKX2-5/TBC1D23/LHX9/TIAM2
BP	GO:0009790	embryo development	29	-0.282441684	-0.925524295	0.556097561	0.936405946	0.845739314	44	tags=21%, list=10%, signal=20%	ZEB1/HORMAD1/TBX3/ACE/CDX2/KIT
BP	GO:0097529	myeloid leukocyte migration	12	0.444227181	0.95680623	0.566145092	0.937590565	0.846809233	17	tags=17%, list=4%, signal=16%	CCL13/SAA1
BP	GO:0051093	negative regulation of developmental process	21	0.387757695	0.93819859	0.568062827	0.937590565	0.846809233	71	tags=24%, list=16%, signal=21%	IL17F/DKK4/MSX2/ASPM/NKX2-5
BP	GO:0051235	maintenance of location	10	-0.393044356	-0.935356393	0.564102564	0.937590565	0.846809233	60	tags=30%, list=13%, signal=27%	BDKRB1/TTC39B/LTBP1

**BP**	GO:0043269	regulation of ion transport	22	-0.303240559	-0.933937625	0.56223176	0.937590565	0.846809233	60	tags=23%, list=13%, signal=21%	BDKRB1/CRACR2B/SYT8/KCNA7/ACE
**BP**	GO:0051603	proteolysis involved in protein catabolic process	10	-0.391428519	-0.931511067	0.566951567	0.937590565	0.846809233	109	tags=50%, list=24%, signal=39%	ANAPC7/FBXW11/CYLD/PLAA/CLU
**BP**	GO:0006954	inflammatory response	25	0.371735331	0.924497916	0.588387097	0.965130261	0.871682423	41	tags=16%, list=9%, signal=15%	IL17F/CCL13/SAA1/MRGPRX1
**BP**	GO:0046649	lymphocyte activation	15	-0.328702218	-0.905952741	0.589473684	0.965130261	0.871682423	48	tags=20%, list=11%, signal=18%	MPL/ZEB1/KIT
**BP**	GO:0061061	muscle structure development	13	-0.347256203	-0.902466517	0.588435374	0.965130261	0.871682423	44	tags=23%, list=10%, signal=21%	ZEB1/TBX3/KIT
**BP**	GO:0007275	multicellular organism development	120	-0.234697243	-0.980149504	0.609375	0.978304476	0.883581058	88	tags=27%, list=20%, signal=29%	PLAA/TG/TMEM79/AHDC1/HCN4/EEF1AKMT4-ECE2/SYDE1/HMGA2/NECTIN1/APLN/PCDHB7/TNFSF10/GAL3ST1/CPNE5/MFHAS1/HHIP/CLU/MPL/ZEB1/HORMAD1/RAB26/RAPGEF3/TSPY10/TBX3/SIX5/CYP26C1/HNF1A/ACE/MPIG6B/CDX2/OVOL1/KIT
**BP**	GO:0032501	multicellular organismal process	189	0.302462433	0.953673182	0.611282051	0.978304476	0.883581058	51	tags=13%, list=11%, signal=19%	TAS2R3/OR10G6/LCE6A/OR8S1/IL17F/DKK4/OR10D3/CKMT2/SAA1/FABP2/OR56B1/OR4D6/OR13G1/OR11H4/OR9A2/GJB5/NTM/CSF2/OR11H1/STAC/LHCGR/MSX2/EOMES/OR11H2
**BP**	GO:1901566	organonitrogen compound biosynthetic process	38	-0.275424576	-0.932443381	0.608938547	0.978304476	0.883581058	6	tags=5%, list=1%, signal=6%	DAZ1/RPS4Y1
**BP**	GO:0015850	organic hydroxy compound transport	12	0.430364783	0.926948469	0.613086771	0.978304476	0.883581058	28	tags=17%, list=6%, signal=16%	SLC10A5/SLC10A2
**BP**	GO:0051248	negative regulation of protein metabolic process	24	-0.28653748	-0.903963842	0.607594937	0.978304476	0.883581058	62	tags=25%, list=14%, signal=23%	WARS1/PLAA/MFHAS1/BDKRB1/CLU/PMEPA1
**BP**	GO:0040017	positive regulation of locomotion	18	-0.312694818	-0.90026119	0.612903226	0.978304476	0.883581058	60	tags=33%, list=13%, signal=30%	PLAA/SYDE1/BDKRB1/DAPK2/HRAS/KIT
**BP**	GO:0050808	synapse organization	10	-0.373799561	-0.889558147	0.603988604	0.978304476	0.883581058	84	tags=80%, list=19%, signal=67%	YWHAZ/WASF1/RAB29/NRXN1/CAST/NLGN1/MESD/NECTIN1
**BP**	GO:0090066	regulation of anatomical structure size	10	-0.37242995	-0.886298784	0.612535613	0.978304476	0.883581058	98	tags=30%, list=22%, signal=24%	HRH2/LIMA1/ACE
**BP**	GO:1901137	carbohydrate derivative biosynthetic process	10	0.445346771	0.915788716	0.619047619	0.983972577	0.88870035	156	tags=70%, list=35%, signal=47%	TMTC3/IGF1/POGLUT2/CEMIP/CRPPA/CTPS1/GCNT2
**BP**	GO:0000280	nuclear division	13	0.413270374	0.901843761	0.624293785	0.98846516	0.892757943	106	tags=38%, list=24%, signal=30%	MSX2/ASPM/BUB1/IGF1/NEK2
**BP**	GO:0048285	organelle fission	13	0.413270374	0.901843761	0.624293785	0.98846516	0.892757943	106	tags=38%, list=24%, signal=30%	MSX2/ASPM/BUB1/IGF1/NEK2
**BP**	GO:0048732	gland development	10	-0.368291524	-0.876450269	0.626780627	0.990483118	0.894580514	100	tags=40%, list=22%, signal=32%	TG/APLN/TBX3/HNF1A
**BP**	GO:0043009	chordate embryonic development	15	0.39843086	0.894940922	0.629009763	0.992086827	0.896028945	107	tags=53%, list=24%, signal=42%	GJB5/CSF2/EOMES/COL11A1/NKX2-5/IGF1/NEK2/PPP4R4
**BP**	GO:0010629	negative regulation of gene expression	32	-0.283665356	-0.920870912	0.632978723	0.994483121	0.89819322	28	tags=22%, list=6%, signal=22%	DAZ4/CYLD/HMGA2/APLN/HRAS/ACE/DAZ1
**BP**	GO:0030595	leukocyte chemotaxis	13	0.410971519	0.89682717	0.634180791	0.994483121	0.89819322	17	tags=15%, list=4%, signal=15%	CCL13/SAA1
**BP**	GO:0022604	regulation of cell morphogenesis	11	-0.366310617	-0.891470076	0.63190184	0.994483121	0.89819322	64	tags=36%, list=14%, signal=32%	PLAA/CPNE5/MPL/KIT
**BP**	GO:0051130	positive regulation of cellular component organization	35	-0.278736868	-0.924913553	0.637837838	0.996392951	0.899918133	83	tags=23%, list=18%, signal=20%	BICD1/APLN/TNFSF10/CLU/GBP5/RAPGEF3/HRAS/KIT
**BP**	GO:0010646	regulation of cell communication	92	-0.229217316	-0.9115412	0.641975309	0.999035861	0.902305146	56	tags=18%, list=12%, signal=20%	MFHAS1/HHIP/NPRL2/RIC8B/CLU/ZEB1/DAPK2/RAB26/SHC2/PMEPA1/HRAS/CYP26C1/ACE/MPIG6B/FGF21/LTBP1/KIT
**BP**	GO:0060485	mesenchyme development	10	-0.363891665	-0.865979602	0.641025641	0.999035861	0.902305146	27	tags=40%, list=6%, signal=38%	HMGA2/TSPY10/TBX3/CYP26C1
**BP**	GO:0044260	cellular macromolecule metabolic process	73	-0.242614274	-0.928643134	0.697247706	1	0.903175932	121	tags=26%, list=27%, signal=23%	SLC25A37/WARS1/TNKS2/PPP1R1A/EIF2B5/ANAPC7/FBXW11/DAZ4/CYLD/PLAA/TOP3B/HMGA2/GAL3ST1/EMSY/CLU/PTMS/MCM2/DAZ1/RPS4Y1

**BP**	GO:0023051	regulation of signaling	91	-0.232572897	-0.922321895	0.662650602	1	0.903175932	56	tags=19%, list=12%, signal=21%	MFHAS1/HHIP/NPRL2/RIC8B/CLU/ZEB1/DAPK2/RAB26/SHC2/PMEPA1/HRAS/CYP26C1/ACE/MPIG6B/FGF21/LTBP1/KIT
**BP**	GO:0035295	tube development	33	-0.27888707	-0.919929454	0.656084656	1	0.903175932	56	tags=27%, list=12%, signal=26%	HMGA2/APLN/HHIP/ZEB1/RAPGEF3/TBX3/ACE/CDX2/KIT
**BP**	GO:0044281	small molecule metabolic process	32	-0.279867661	-0.908542345	0.654255319	1	0.903175932	53	tags=19%, list=12%, signal=18%	HSD3B7/TTC39B/CYP26C1/NPC1L1/TKTL1/KIT
**BP**	GO:0009605	response to external stimulus	74	0.310288185	0.906135509	0.663322185	1	0.903175932	40	tags=11%, list=9%, signal=12%	DEFB113/DEFB131B/IL17F/CCL13/SAA1/SLC10A2/DEFB123/CSF2
**BP**	GO:0002376	immune system process	64	0.312827127	0.898796405	0.675339367	1	0.903175932	50	tags=14%, list=11%, signal=15%	DEFB113/DEFB131B/IL17F/CCL13/SAA1/DEFB123/CSF2/CD8B/EOMES
**BP**	GO:0048585	negative regulation of response to stimulus	35	-0.269663096	-0.894804674	0.697297297	1	0.903175932	83	tags=31%, list=18%, signal=28%	BICD1/HMGA2/APLN/MFHAS1/HHIP/NPRL2/CLU/PMEPA1/CYP26C1/MPIG6B/LTBP1
**BP**	GO:0120036	plasma membrane bounded cell projection organization	37	-0.266183464	-0.893287044	0.677595628	1	0.903175932	102	tags=22%, list=23%, signal=18%	CYLD/PLAA/NECTIN1/LIMA1/CPNE5/BBS10/HRAS/KIT
**BP**	GO:0021700	developmental maturation	11	0.415958316	0.877782201	0.662721893	1	0.903175932	120	tags=36%, list=27%, signal=27%	MSX2/IGF1/TDRD1/SLC26A8
**BP**	GO:0043254	regulation of protein-containing complex assembly	12	-0.348958761	-0.875736	0.64548495	1	0.903175932	73	tags=42%, list=16%, signal=36%	SAR1A/BBS10/CLU/GBP5/PMEPA1
**BP**	GO:0045892	negative regulation of DNA-templated transcription	35	-0.26342105	-0.874092116	0.756756757	1	0.903175932	58	tags=20%, list=13%, signal=19%	CDYL2/ZEB1/ZNF596/TBX3/SIX5/CDX2/OVOL1
**BP**	GO:1902679	negative regulation of RNA biosynthetic process	35	-0.26342105	-0.874092116	0.756756757	1	0.903175932	58	tags=20%, list=13%, signal=19%	CDYL2/ZEB1/ZNF596/TBX3/SIX5/CDX2/OVOL1
**BP**	GO:1903507	negative regulation of nucleic acid-templated transcription	35	-0.26342105	-0.874092116	0.756756757	1	0.903175932	58	tags=20%, list=13%, signal=19%	CDYL2/ZEB1/ZNF596/TBX3/SIX5/CDX2/OVOL1
**BP**	GO:0001819	positive regulation of cytokine production	15	0.387703934	0.870846491	0.666666667	1	0.903175932	40	tags=20%, list=9%, signal=19%	IL17F/SAA1/CSF2
**BP**	GO:0006950	response to stress	88	0.292267793	0.867697635	0.747530187	1	0.903175932	61	tags=14%, list=14%, signal=15%	DEFB113/DEFB131B/IL17F/CCL13/SAA1/DEFB123/CSF2/MRGPRX1/STAC/MSX2/TMTC3/ELAVL4
**BP**	GO:0009888	tissue development	44	0.314896049	0.867050283	0.678953627	1	0.903175932	56	tags=18%, list=12%, signal=18%	LCE6A/IL17F/DKK4/GJB5/KRT39/MSX2/EOMES/COL11A1
**BP**	GO:0034097	response to cytokine	23	0.352118449	0.866892111	0.671070013	1	0.903175932	59	tags=17%, list=13%, signal=16%	IL17F/CCL13/CSF2/ADAM23
**BP**	GO:0040011	locomotion	41	-0.247919537	-0.866856912	0.772727273	1	0.903175932	60	tags=24%, list=13%, signal=23%	SYDE1/NECTIN1/BDKRB1/HSD3B7/CCR10/DAPK2/HRAS/ACE/FGF21/KIT
**BP**	GO:0071345	cellular response to cytokine stimulus	22	0.353496196	0.866635276	0.66579974	1	0.903175932	59	tags=18%, list=13%, signal=17%	IL17F/CCL13/CSF2/ADAM23
**BP**	GO:0030030	cell projection organization	38	-0.255928262	-0.866439072	0.754189944	1	0.903175932	102	tags=21%, list=23%, signal=18%	CYLD/PLAA/NECTIN1/LIMA1/CPNE5/BBS10/HRAS/KIT
**BP**	GO:0002521	leukocyte differentiation	11	-0.353806967	-0.861040629	0.662576687	1	0.903175932	44	tags=18%, list=10%, signal=17%	ZEB1/KIT
**BP**	GO:0071824	protein-DNA complex subunit organization	10	0.418564133	0.860714245	0.678955453	1	0.903175932	33	tags=20%, list=7%, signal=19%	H2BW1/TSPYL6
**BP**	GO:0031349	positive regulation of defense response	11	0.406804214	0.858464621	0.687869822	1	0.903175932	11	tags=9%, list=2%, signal=9%	IL17F
**BP**	GO:0001775	cell activation	23	0.348672918	0.858409442	0.678996037	1	0.903175932	96	tags=35%, list=21%, signal=29%	SAA1/CSF2/CD8B/EOMES/LBP/SFTPD/IGF1/NLRP5
**BP**	GO:0090287	regulation of cellular response to growth factor stimulus	13	-0.329670048	-0.856762751	0.649659864	1	0.903175932	74	tags=38%, list=16%, signal=33%	APLN/HHIP/ZEB1/PMEPA1/LTBP1
**BP**	GO:0006417	regulation of translation	15	-0.310172149	-0.854881082	0.656140351	1	0.903175932	6	tags=47%, list=1%, signal=48%	RC3H1/ATF4/PATL1/RPS27L/EIF2B5/DAZ4/DAZ1
**BP**	GO:0010628	positive regulation of gene expression	33	-0.258395064	-0.852335071	0.798941799	1	0.903175932	34	tags=18%, list=8%, signal=18%	CLU/GBP5/HRAS/FGF21/DAZ1/KIT
**BP**	GO:1904062	regulation of cation transmembrane transport	12	0.395644895	0.852166451	0.688477952	1	0.903175932	111	tags=33%, list=25%, signal=26%	STAC/LHCGR/TRPC3/CEMIP
**BP**	GO:0055085	transmembrane transport	37	0.318016768	0.852157748	0.710622711	1	0.903175932	69	tags=19%, list=15%, signal=17%	SLC10A5/SLC10A2/GJB5/STAC/LHCGR/KCNK1/ANO5
**BP**	GO:0055065	metal ion homeostasis	13	0.38996063	0.850976947	0.692090395	1	0.903175932	48	tags=15%, list=11%, signal=14%	CCL13/LHCGR
**BP**	GO:0055080	cation homeostasis	13	0.38996063	0.850976947	0.692090395	1	0.903175932	48	tags=15%, list=11%, signal=14%	CCL13/LHCGR

**BP**	GO:0051641	cellular localization	79	-0.219001927	-0.850649858	0.822916667	1	0.903175932	116	tags=29%, list=26%, signal=26%	TNKS2/MEAK7/TCAF1/VSNL1/FBXW11/PLAA/TRAM1/MESD/BICD1/NECTIN1/SAR1A/MFHAS1/BDKRB1/CLU/RAB26/SYT8/GBP5/RAPGEF3/HRAS/HNF1A/CDX2/LTBP1/KIT
**BP**	GO:0050900	leukocyte migration	14	0.384141456	0.849626361	0.68907563	1	0.903175932	17	tags=14%, list=4%, signal=14%	CCL13/SAA1
**BP**	GO:1903131	mononuclear cell differentiation	10	-0.356897767	-0.849335711	0.660968661	1	0.903175932	44	tags=20%, list=10%, signal=18%	ZEB1/KIT
**BP**	GO:0032101	regulation of response to external stimulus	24	0.34358509	0.849000618	0.703267974	1	0.903175932	17	tags=8%, list=4%, signal=8%	IL17F/SAA1
**BP**	GO:0051301	cell division	21	-0.279439599	-0.84885368	0.718487395	1	0.903175932	12	tags=10%, list=3%, signal=10%	OPN1MW/KIT
**BP**	GO:0072657	protein localization to membrane	17	-0.29557064	-0.847854768	0.70703125	1	0.903175932	143	tags=47%, list=32%, signal=33%	RAB8B/LDLRAP1/EHD4/TCAF1/TRAM1/MESD/RAB26/HRAS
**BP**	GO:0060326	cell chemotaxis	15	0.37733147	0.847548238	0.708507671	1	0.903175932	17	tags=13%, list=4%, signal=13%	CCL13/SAA1
**BP**	GO:0009890	negative regulation of biosynthetic process	39	-0.24937606	-0.846981025	0.801136364	1	0.903175932	58	tags=18%, list=13%, signal=17%	CDYL2/ZEB1/ZNF596/TBX3/SIX5/CDX2/OVOL1
**BP**	GO:0010558	negative regulation of macromolecule biosynthetic process	39	-0.24937606	-0.846981025	0.801136364	1	0.903175932	58	tags=18%, list=13%, signal=17%	CDYL2/ZEB1/ZNF596/TBX3/SIX5/CDX2/OVOL1
**BP**	GO:0031327	negative regulation of cellular biosynthetic process	39	-0.24937606	-0.846981025	0.801136364	1	0.903175932	58	tags=18%, list=13%, signal=17%	CDYL2/ZEB1/ZNF596/TBX3/SIX5/CDX2/OVOL1
**BP**	GO:0097190	apoptotic signaling pathway	15	-0.306607282	-0.845055775	0.670175439	1	0.903175932	68	tags=47%, list=15%, signal=41%	BOK/RPS27L/CYLD/TNFSF10/CLU/DAPK2/HRAS
**BP**	GO:0051046	regulation of secretion	14	-0.310176378	-0.837856221	0.708333333	1	0.903175932	39	tags=21%, list=9%, signal=20%	RAB26/SYT8/ACE
**BP**	GO:0045321	leukocyte activation	21	-0.275486526	-0.83684543	0.731092437	1	0.903175932	62	tags=24%, list=14%, signal=22%	MFHAS1/CLU/MPL/ZEB1/KIT
**BP**	GO:0048568	embryonic organ development	12	0.386683662	0.832865147	0.706970128	1	0.903175932	72	tags=50%, list=16%, signal=43%	GJB5/CSF2/EOMES/COL11A1/NKX2-5/TBC1D23
**BP**	GO:0046486	glycerolipid metabolic process	11	-0.340694339	-0.829129144	0.702453988	1	0.903175932	86	tags=27%, list=19%, signal=23%	ABHD3/GAL3ST1/FGF21
**BP**	GO:0050727	regulation of inflammatory response	12	0.384667833	0.828523321	0.712660028	1	0.903175932	115	tags=42%, list=26%, signal=32%	SAA1/LBP/IGF1/NLRP5/CXCL17
**BP**	GO:0002252	immune effector process	16	-0.295961738	-0.826482844	0.695167286	1	0.903175932	16	tags=19%, list=4%, signal=19%	CLU/ACE/KIT
**BP**	GO:0048699	generation of neurons	38	0.305453319	0.822316763	0.736330498	1	0.903175932	98	tags=21%, list=22%, signal=18%	NTM/EOMES/ELAVL4/ASPM/NKX2-5/TBC1D23/LHX9/TIAM2
**BP**	GO:0043414	macromolecule methylation	13	0.376652977	0.821936821	0.724576271	1	0.903175932	143	tags=31%, list=32%, signal=22%	EOMES/SNRPE/TDRD1/TRMT11
**BP**	GO:0051668	localization within membrane	18	-0.285338467	-0.821501135	0.77016129	1	0.903175932	143	tags=44%, list=32%, signal=32%	RAB8B/LDLRAP1/EHD4/TCAF1/TRAM1/MESD/RAB26/HRAS
**BP**	GO:0007399	nervous system development	62	-0.218400944	-0.820115874	0.875	1	0.903175932	77	tags=26%, list=17%, signal=25%	EIF2B5/FBXW11/PLAA/TG/EEF1AKMT4-ECE2/NECTIN1/PCDHB7/GAL3ST1/CPNE5/HHIP/CLU/ZEB1/TBX3/CYP26C1/ACE/KIT
**BP**	GO:0051090	regulation of DNA-binding transcription factor activity	10	-0.344014988	-0.818677621	0.706552707	1	0.903175932	51	tags=30%, list=11%, signal=27%	CYLD/CLU/KIT
**BP**	GO:1903046	meiotic cell cycle process	10	-0.343352487	-0.817101019	0.706552707	1	0.903175932	42	tags=20%, list=9%, signal=19%	HORMAD1/OVOL1
**BP**	GO:0002697	regulation of immune effector process	11	0.386733758	0.816110645	0.723372781	1	0.903175932	11	tags=9%, list=2%, signal=9%	IL17F
**BP**	GO:0050801	ion homeostasis	15	0.362534988	0.814312918	0.741980474	1	0.903175932	48	tags=13%, list=11%, signal=12%	CCL13/LHCGR
**BP**	GO:0098771	inorganic ion homeostasis	15	0.362534988	0.814312918	0.741980474	1	0.903175932	48	tags=13%, list=11%, signal=12%	CCL13/LHCGR
**BP**	GO:0001701	in utero embryonic development	11	0.384030803	0.810406694	0.732248521	1	0.903175932	107	tags=55%, list=24%, signal=43%	GJB5/CSF2/EOMES/IGF1/NEK2/PPP4R4
**BP**	GO:0098660	inorganic ion transmembrane transport	18	0.342040854	0.809381976	0.736074271	1	0.903175932	120	tags=39%, list=27%, signal=30%	STAC/LHCGR/KCNK1/ANO5/TRPC3/CEMIP/SLC26A8
**BP**	GO:0051179	localization	128	-0.194352497	-0.808468069	0.964912281	1	0.903175932	41	tags=12%, list=9%, signal=15%	TTC39B/RAB26/SYT8/GBP5/RAPGEF3/HRAS/TBX3/HNF1A/NPC1L1/KCNA7/ACE/FGF21/CDX2/LTBP1/KIT

**BP**	GO:0051649	establishment of localization in cell	53	-0.227331505	-0.807355604	0.857142857	1	0.903175932	156	tags=43%, list=35%, signal=32%	SNX11/NUP214/TRAPPC9/BOK/RAB8B/LDLRAP1/EHD4/TAP2/IPO11/MAJIN/TCAF1/VSNL1/FBXW11/PLAA/TRAM1/BICD1/SAR1A/CLU/SYT8/RAPGEF3/HRAS/CDX2/KIT
**BP**	GO:0045596	negative regulation of cell differentiation	14	0.36346928	0.803904596	0.736694678	1	0.903175932	92	tags=29%, list=20%, signal=23%	MSX2/ASPM/NKX2-5/IGF1
**BP**	GO:1990778	protein localization to cell periphery	10	-0.33762463	-0.803470019	0.752136752	1	0.903175932	39	tags=50%, list=9%, signal=47%	RAB8B/LDLRAP1/EHD4/RAB26/HRAS
**BP**	GO:0040007	growth	14	0.362675666	0.802149317	0.742296919	1	0.903175932	92	tags=29%, list=20%, signal=23%	MSX2/ASPM/NKX2-5/IGF1
**BP**	GO:0035239	tube morphogenesis	25	-0.251832479	-0.800355539	0.81938326	1	0.903175932	80	tags=28%, list=18%, signal=24%	HMGA2/APLN/HHIP/ZEB1/RAPGEF3/TBX3/ACE
**BP**	GO:0051640	organelle localization	14	-0.296026606	-0.799634501	0.760416667	1	0.903175932	3	tags=50%, list=1%, signal=51%	TRAPPC9/RAB8B/MAJIN/FBXW11/BICD1/SAR1A/KIT
**BP**	GO:0033036	macromolecule localization	70	0.273494306	0.792905813	0.849887133	1	0.903175932	48	tags=11%, list=11%, signal=12%	SLC10A5/SAA1/FABP2/SLC10A2/AKAIN1/GOLT1A/STAC/LHCGR
**BP**	GO:0018205	peptidyl-lysine modification	11	0.375566401	0.792544564	0.75295858	1	0.903175932	50	tags=9%, list=11%, signal=8%	EOMES
**BP**	GO:0030900	forebrain development	10	0.384919362	0.791528828	0.737327189	1	0.903175932	64	tags=30%, list=14%, signal=26%	EOMES/ELAVL4/ASPM
**BP**	GO:0002253	activation of immune response	10	-0.332040915	-0.790182045	0.769230769	1	0.903175932	51	tags=30%, list=11%, signal=27%	EIF2B5/CLU/HRAS
**BP**	GO:0001816	cytokine production	22	0.321870509	0.789101383	0.77893368	1	0.903175932	92	tags=32%, list=20%, signal=27%	IL17F/SAA1/CSF2/LBP/CCBE1/SFTPD/IGF1
**BP**	GO:0001817	regulation of cytokine production	22	0.321870509	0.789101383	0.77893368	1	0.903175932	92	tags=32%, list=20%, signal=27%	IL17F/SAA1/CSF2/LBP/CCBE1/SFTPD/IGF1
**BP**	GO:0051050	positive regulation of transport	32	-0.242787552	-0.788168133	0.861702128	1	0.903175932	110	tags=34%, list=24%, signal=28%	TCAF1/VSNL1/PLAA/BICD1/APLN/SAR1A/BDKRB1/CLU/RAPGEF3/HRAS/FGF21
**BP**	GO:0009987	cellular process	369	0.254985099	0.786115092	0.92020202	1	0.903175932	52	tags=11%, list=12%, signal=56%	TAS2R3/OR10G6/GPHB5/SLC10A5/LCE6A/OR8S1/IL17F/DKK4/CCL13/OR10D3/CKMT2/H2BW1/SAA1/DEPDC1/FABP2/OR56B1/OR4D6/OR13G1/TRHR/OR11H4/ERCC6L/SLC10A2/OR9A2/CSRNP3/TSPYL6/GJB5/NTM/AKAIN1/GOLT1A/CYP4Z1/CSF2/MRGPRX1/ZFP69B/OR11H1/STAC/CD8B/KRT39/LHCGR/MSX2/EOMES/OR11H2/NCBP2L
**BP**	GO:0071407	cellular response to organic cyclic compound	15	-0.285001889	-0.785508061	0.775438596	1	0.903175932	30	tags=40%, list=7%, signal=39%	HRH2/LCOR/HCN4/PMEPA1/RAPGEF3/ACE
**BP**	GO:0071702	organic substance transport	59	0.275017145	0.783851504	0.828213879	1	0.903175932	48	tags=12%, list=11%, signal=12%	SLC10A5/SAA1/FABP2/SLC10A2/GOLT1A/CSF2/LHCGR
**BP**	GO:0048584	positive regulation of response to stimulus	62	-0.207403933	-0.778821074	0.958333333	1	0.903175932	68	tags=16%, list=15%, signal=16%	TNFSF10/MFHAS1/CLU/DAPK2/SHC2/GBP5/HRAS/ACE/FGF21/KIT
**BP**	GO:0009792	embryo development ending in birth or egg hatching	16	0.338482703	0.778355241	0.791268759	1	0.903175932	107	tags=50%, list=24%, signal=39%	GJB5/CSF2/EOMES/COL11A1/NKX2-5/IGF1/NEK2/PPP4R4
**BP**	GO:0048646	anatomical structure formation involved in morphogenesis	27	0.306343794	0.777133134	0.812578616	1	0.903175932	71	tags=22%, list=16%, signal=20%	IL17F/DKK4/MSX2/EOMES/COL11A1/NKX2-5
**BP**	GO:0050778	positive regulation of immune response	18	0.328324394	0.776924288	0.777188329	1	0.903175932	46	tags=11%, list=10%, signal=10%	IL17F/CD8B
**BP**	GO:0006508	proteolysis	34	-0.237703921	-0.776668968	0.901098901	1	0.903175932	109	tags=29%, list=24%, signal=24%	ANAPC7/FBXW11/CYLD/PLAA/EEF1AKMT4-ECE2/PREP/TNFSF10/CLU/NAPSA/ACE
**BP**	GO:0060429	epithelium development	25	0.311894254	0.775674421	0.797419355	1	0.903175932	49	tags=16%, list=11%, signal=15%	LCE6A/DKK4/KRT39/MSX2
**BP**	GO:0006996	organelle organization	89	-0.195400762	-0.774501403	0.954545455	1	0.903175932	91	tags=21%, list=20%, signal=21%	TOP3B/TRAM1/FAM118B/BICD1/SYDE1/HMGA2/SAR1A/LIMA1/TNFSF10/BBS10/CLU/KRT8/HORMAD1/RAPGEF3/HRAS/CYP26C1/MCM2/GOLGA6A/KIT

**BP**	GO:0050794	regulation of cellular process	260	0.244289524	0.770108862	0.938196555	1	0.903175932	51	tags=11%, list=11%, signal=23%	TAS2R3/OR10G6/GPHB5/OR8S1/IL17F/DKK4/CCL13/OR10D3/SAA1/DEPDC1/OR56B1/OR4D6/OR13G1/TRHR/OR11H4/OR9A2/CSRNP3/AKAIN1/CSF2/MRGPRX1/ZFP69B/OR11H1/STAC/CD8B/LHCGR/MSX2/EOMES/OR11H2
**BP**	GO:0051249	regulation of lymphocyte activation	10	-0.322125014	-0.766584448	0.792022792	1	0.903175932	48	tags=80%, list=11%, signal=73%	FGL2/HMGB1/LGALS1/RC3H1/AXL/NKAP/MPL/ZEB1
**BP**	GO:0071495	cellular response to endogenous stimulus	44	0.278273145	0.766210975	0.809750297	1	0.903175932	61	tags=14%, list=14%, signal=13%	GPHB5/IL17F/OR11H4/LHCGR/MSX2/ELAVL4
**BP**	GO:0051604	protein maturation	10	-0.321391869	-0.764839731	0.794871795	1	0.903175932	16	tags=20%, list=4%, signal=20%	EEF1AKMT4-ECE2/ACE
**BP**	GO:0006936	muscle contraction	10	0.370865113	0.762628377	0.76344086	1	0.903175932	71	tags=30%, list=16%, signal=26%	CKMT2/STAC/NKX2-5
**BP**	GO:0050793	regulation of developmental process	58	0.268044982	0.762401649	0.860889396	1	0.903175932	98	tags=21%, list=22%, signal=19%	IL17F/DKK4/CCL13/CSF2/MSX2/ELAVL4/ASPM/NKX2-5/CCBE1/IGF1/NLRP5/TIAM2
**BP**	GO:0014070	response to organic cyclic compound	19	0.317868876	0.762186745	0.81640625	1	0.903175932	61	tags=21%, list=14%, signal=19%	OR11H4/MRGPRX1/MSX2/ELAVL4
**BP**	GO:0048666	neuron development	27	0.298963637	0.758411147	0.825157233	1	0.903175932	161	tags=37%, list=36%, signal=25%	NTM/ELAVL4/TBC1D23/LHX9/TIAM2/OPCML/DTNBP1/CRPPA/RGS2/PTPRG
**BP**	GO:2000112	regulation of cellular macromolecule biosynthetic process	18	-0.263174883	-0.757691269	0.846774194	1	0.903175932	6	tags=28%, list=1%, signal=29%	RPS27L/SLC25A37/EIF2B5/DAZ4/DAZ1
**BP**	GO:0002694	regulation of leukocyte activation	13	-0.291229863	-0.756862507	0.806122449	1	0.903175932	62	tags=23%, list=14%, signal=20%	MFHAS1/MPL/ZEB1
**BP**	GO:0050865	regulation of cell activation	13	-0.291229863	-0.756862507	0.806122449	1	0.903175932	62	tags=23%, list=14%, signal=20%	MFHAS1/MPL/ZEB1
**BP**	GO:0048729	tissue morphogenesis	15	0.335091578	0.752670528	0.810320781	1	0.903175932	71	tags=27%, list=16%, signal=23%	MSX2/EOMES/COL11A1/NKX2-5
**BP**	GO:0003012	muscle system process	13	0.344827218	0.752486252	0.807909605	1	0.903175932	105	tags=38%, list=23%, signal=30%	CKMT2/STAC/NKX2-5/IGF1/TRPC3
**BP**	GO:0070925	organelle assembly	22	0.306561457	0.751569539	0.828348505	1	0.903175932	106	tags=23%, list=24%, signal=18%	CSF2/ASPM/NKX2-5/FHDC1/NEK2
**BP**	GO:0007178	transmembrane receptor protein serine/threonine kinase signaling pathway	14	0.339804145	0.751563142	0.806722689	1	0.903175932	49	tags=14%, list=11%, signal=13%	IL17F/MSX2
**BP**	GO:0007017	microtubule-based process	18	0.31749093	0.751288724	0.814323607	1	0.903175932	157	tags=44%, list=35%, signal=30%	ASPM/ARL8A/NLRP5/NEK2/SLC26A8/DTNBP1/AURKC/STAG2
**BP**	GO:0016043	cellular component organization	147	-0.176942366	-0.749101703	1	1	0.903175932	91	tags=21%, list=20%, signal=25%	TOP3B/TRAM1/FAM118B/MESD/BICD1/SYDE1/HMGA2/NECTIN1/APLN/SAR1A/LIMA1/TNFSF10/CPNE5/BBS10/BDKRB1/EMSY/CLU/NID2/KRT8/HORMAD1/GBP5/PMEPA1/RAPGEF3/HRAS/TSPY10/CYP26C1/KCNA7/ACE/MCM2/GOLGA6A/KIT
**BP**	GO:0042110	T cell activation	13	-0.288209153	-0.749012137	0.823129252	1	0.903175932	44	tags=15%, list=10%, signal=14%	ZEB1/KIT
**BP**	GO:0005975	carbohydrate metabolic process	16	0.32547973	0.748454358	0.815825375	1	0.903175932	145	tags=44%, list=32%, signal=31%	LHCGR/TFF3/FUCA2/IGF1/PPP1CA/GNPTG/PLA2G4A
**BP**	GO:0006810	transport	111	-0.182709332	-0.741175827	0.985294118	1	0.903175932	41	tags=28%, list=9%, signal=34%	TCAF1/VSNL1/FBXW11/PLAA/TG/HRH2/TMEM79/TRAM1/HCN4/MESD/BICD1/NECTIN1/APLN/SAR1A/LIMA1/BDKRB1/CRACR2B/CLU/TTC39B/RAB26/SYT8/RAPGEF3/HRAS/TBX3/HNF1A/NPC1L1/KCNA7/ACE/FGF21/CDX2/KIT
**BP**	GO:0010256	endomembrane system organization	13	-0.285111338	-0.740961382	0.829931973	1	0.903175932	51	tags=23%, list=11%, signal=21%	TRAM1/CLU/GOLGA6A
**BP**	GO:0080134	regulation of response to stress	33	0.281544173	0.740298827	0.852398524	1	0.903175932	17	tags=6%, list=4%, signal=6%	IL17F/SAA1

**BP**	GO:0071840	cellular component organization or biogenesis	149	-0.172915744	-0.739214961	1	1	0.903175932	91	tags=21%, list=20%, signal=25%	TOP3B/TRAM1/FAM118B/MESD/BICD1/SYDE1/HMGA2/NECTIN1/APLN/SAR1A/LIMA1/TNFSF10/CPNE5/BBS10/BDKRB1/EMSY/CLU/NID2/KRT8/HORMAD1/GBP5/PMEPA1/RAPGEF3/HRAS/TSPY10/CYP26C1/KCNA7/ACE/MCM2/GOLGA6A/KIT
**BP**	GO:0048870	cell motility	45	0.268397649	0.737828362	0.854066986	1	0.903175932	92	tags=24%, list=20%, signal=22%	CCL13/SAA1/CSF2/MSX2/EOMES/TPTE2/ASPM/LBP/CCBE1/SFTPD/IGF1
**BP**	GO:0006935	chemotaxis	21	0.302374732	0.731610362	0.848167539	1	0.903175932	17	tags=10%, list=4%, signal=10%	CCL13/SAA1
**BP**	GO:0042330	taxis	21	0.302374732	0.731610362	0.848167539	1	0.903175932	17	tags=10%, list=4%, signal=10%	CCL13/SAA1
**BP**	GO:0008104	protein localization	55	-0.200633877	-0.730485865	0.977099237	1	0.903175932	90	tags=31%, list=20%, signal=28%	TNKS2/MEAK7/TCAF1/VSNL1/TRAM1/MESD/BICD1/NECTIN1/SAR1A/MFHAS1/CLU/RAB26/GBP5/RAPGEF3/HRAS/HNF1A/LTBP1
**BP**	GO:0070727	cellular macromolecule localization	55	-0.200633877	-0.730485865	0.977099237	1	0.903175932	90	tags=31%, list=20%, signal=28%	TNKS2/MEAK7/TCAF1/VSNL1/TRAM1/MESD/BICD1/NECTIN1/SAR1A/MFHAS1/CLU/RAB26/GBP5/RAPGEF3/HRAS/HNF1A/LTBP1
**BP**	GO:0051234	establishment of localization	114	-0.180029919	-0.729328426	1	1	0.903175932	41	tags=27%, list=9%, signal=33%	TCAF1/VSNL1/FBXW11/PLAA/TG/HRH2/TMEM79/TRAM1/HCN4/MESD/BICD1/NECTIN1/APLN/SAR1A/LIMA1/BDKRB1/CRACR2B/CLU/TTC39B/RAB26/SYT8/RAPGEF3/HRAS/TBX3/HNF1A/NPC1L1/KCNA7/ACE/FGF21/CDX2/KIT
**BP**	GO:0000904	cell morphogenesis involved in differentiation	17	-0.254163868	-0.729077989	0.859375	1	0.903175932	77	tags=18%, list=17%, signal=15%	NECTIN1/MPL/MPIG6B
**BP**	GO:0034248	regulation of cellular amide metabolic process	19	-0.249373856	-0.728937693	0.888888889	1	0.903175932	51	tags=26%, list=11%, signal=24%	RPS27L/EIF2B5/DAZ4/CLU/DAZ1
**BP**	GO:2000026	regulation of multicellular organismal development	37	0.271146402	0.726563913	0.858363858	1	0.903175932	98	tags=24%, list=22%, signal=21%	IL17F/DKK4/MSX2/ASPM/NKX2-5/CCBE1/IGF1/NLRP5/TIAM2
**BP**	GO:0071310	cellular response to organic substance	72	0.249681597	0.726209295	0.930022573	1	0.903175932	61	tags=12%, list=14%, signal=13%	GPHB5/IL17F/CCL13/OR11H4/CSF2/LHCGR/MSX2/ADAM23/ELAVL4
**BP**	GO:0097435	supramolecular fiber organization	19	0.302046084	0.724246817	0.845052083	1	0.903175932	108	tags=32%, list=24%, signal=25%	KRT39/COL11A1/NKX2-5/FHDC1/NLRP5/BACE1
**BP**	GO:0032787	monocarboxylic acid metabolic process	15	-0.262056902	-0.722268228	0.852631579	1	0.903175932	21	tags=33%, list=5%, signal=33%	PLAA/ABHD3/HSD3B7/CYP26C1/KIT
**BP**	GO:0010243	response to organonitrogen compound	28	0.282486566	0.721188267	0.876106195	1	0.903175932	114	tags=32%, list=25%, signal=26%	OR11H4/MRGPRX1/LHCGR/ELAVL4/IGF1/TRPC3/BACE1/HTR1E/LEPROT
**BP**	GO:1901698	response to nitrogen compound	28	0.282486566	0.721188267	0.876106195	1	0.903175932	114	tags=32%, list=25%, signal=26%	OR11H4/MRGPRX1/LHCGR/ELAVL4/IGF1/TRPC3/BACE1/HTR1E/LEPROT
**BP**	GO:0032880	regulation of protein localization	21	-0.236444889	-0.718248647	0.87394958	1	0.903175932	114	tags=43%, list=25%, signal=34%	MEAK7/TCAF1/VSNL1/MESD/BICD1/SAR1A/MFHAS1/RAPGEF3/HRAS
**BP**	GO:0009966	regulation of signal transduction	80	0.243277244	0.715486376	0.924778761	1	0.903175932	98	tags=20%, list=22%, signal=19%	GPHB5/IL17F/DKK4/CCL13/CSF2/LHCGR/MSX2/TPTE2/ASPM/NKX2-5/LBP/CCBE1/IGF1/ADH7/PPM1N/TIAM2
**BP**	GO:0098662	inorganic cation transmembrane transport	14	0.323432835	0.715353834	0.859943978	1	0.903175932	111	tags=36%, list=25%, signal=28%	STAC/LHCGR/KCNK1/TRPC3/CEMIP
**BP**	GO:0034220	ion transmembrane transport	22	0.29140937	0.71442251	0.872561769	1	0.903175932	120	tags=32%, list=27%, signal=25%	STAC/LHCGR/KCNK1/ANO5/TRPC3/CEMIP/SLC26A8
**BP**	GO:0032103	positive regulation of response to external stimulus	14	0.322035449	0.712263159	0.859943978	1	0.903175932	11	tags=7%, list=2%, signal=7%	IL17F
**BP**	GO:0006396	RNA processing	17	0.305789582	0.712111901	0.879356568	1	0.903175932	82	tags=18%, list=18%, signal=15%	NCBP2L/ELAVL4/SNRPE
**BP**	GO:0050789	regulation of biological process	272	0.225111338	0.70951171	0.977710233	1	0.903175932	51	tags=10%, list=11%, signal=23%	TAS2R3/OR10G6/GPHB5/OR8S1/IL17F/DKK4/CCL13/OR10D3/SAA1/DEPDC1/OR56B1/OR4D6/OR13G1/TRHR/OR11H4/OR9A2/CSRNP3/AKAIN1/CSF2/MRGPRX1/ZFP69B/OR11H1/STAC/CD8B/LHCGR/MSX2/EOMES/OR11H2
**BP**	GO:0009719	response to endogenous stimulus	49	0.255393662	0.708010585	0.898104265	1	0.903175932	61	tags=12%, list=14%, signal=12%	GPHB5/IL17F/OR11H4/LHCGR/MSX2/ELAVL4

**BP**	GO:0070371	ERK1 and ERK2 cascade	11	0.334439102	0.70575507	0.831360947	1	0.903175932	13	tags=9%, list=3%, signal=9%	CCL13
**BP**	GO:0070372	regulation of ERK1 and ERK2 cascade	11	0.334439102	0.70575507	0.831360947	1	0.903175932	13	tags=9%, list=3%, signal=9%	CCL13
**BP**	GO:0008150	biological_process	401	0.234118467	0.705145735	0.96439471	1	0.903175932	52	tags=11%, list=12%, signal=93%	TAS2R3/DEFB113/OR10G6/GPHB5/DEFB131B/SLC10A5/LCE6A/OR8S1/IL17F/DKK4/CCL13/OR10D3/CKMT2/H2BW1/SAA1/DEPDC1/FABP2/OR56B1/OR4D6/OR13G1/TRHR/OR11H4/ERCC6L/SLC10A2/OR9A2/CSRNP3/DEFB123/TSPYL6/GJB5/NTM/AKAIN1/GOLT1A/CYP4Z1/CSF2/MRGPRX1/ZFP69B/OR11H1/STAC/CD8B/KRT39/LHCGR/MSX2/EOMES/OR11H2/NCBP2L
**BP**	GO:0033993	response to lipid	15	-0.255832915	-0.705113984	0.870175439	1	0.903175932	101	tags=33%, list=22%, signal=27%	PLAA/LCOR/BDKRB1/PMEPA1/ACE
**BP**	GO:0006082	organic acid metabolic process	22	-0.227595714	-0.700962303	0.909871245	1	0.903175932	21	tags=32%, list=5%, signal=32%	GCAT/WARS1/PLAA/ABHD3/HSD3B7/CYP26C1/KIT
**BP**	GO:0019752	carboxylic acid metabolic process	22	-0.227595714	-0.700962303	0.909871245	1	0.903175932	21	tags=32%, list=5%, signal=32%	GCAT/WARS1/PLAA/ABHD3/HSD3B7/CYP26C1/KIT
**BP**	GO:0043436	oxoacid metabolic process	22	-0.227595714	-0.700962303	0.909871245	1	0.903175932	21	tags=32%, list=5%, signal=32%	GCAT/WARS1/PLAA/ABHD3/HSD3B7/CYP26C1/KIT
**BP**	GO:0051240	positive regulation of multicellular organismal process	44	0.25368822	0.698517632	0.900118906	1	0.903175932	105	tags=27%, list=23%, signal=23%	IL17F/SAA1/CSF2/MSX2/ASPM/NKX2-5/LBP/CCBE1/IGF1/NLRP5/TIAM2/TRPC3
**BP**	GO:0007166	cell surface receptor signaling pathway	72	0.239745183	0.697308821	0.952595937	1	0.903175932	97	tags=19%, list=22%, signal=18%	IL17F/DKK4/CCL13/CSF2/MRGPRX1/CD8B/MSX2/ADAM11/ASPM/NKX2-5/LBP/CCBE1/IGF1/PPM1N
**BP**	GO:0016071	mRNA metabolic process	19	-0.238421639	-0.696923577	0.923076923	1	0.903175932	6	tags=47%, list=1%, signal=49%	PAPOLA/RC3H1/ATF4/PATL1/BARD1/TRMT61A/TSEN54/DAZ4/DAZ1
**BP**	GO:0010959	regulation of metal ion transport	13	0.319151743	0.69645691	0.855932203	1	0.903175932	111	tags=38%, list=25%, signal=30%	STAC/LHCGR/NKX2-5/TRPC3/CEMIP
**BP**	GO:0001525	angiogenesis	14	0.314856374	0.696384812	0.87394958	1	0.903175932	11	tags=7%, list=2%, signal=7%	IL17F
**BP**	GO:0007010	cytoskeleton organization	32	-0.214016786	-0.694768779	0.968085106	1	0.903175932	83	tags=28%, list=18%, signal=25%	FBXW11/CYLD/BICD1/SYDE1/LIMA1/KRT8/RAPGEF3/HRAS/KIT
**BP**	GO:0016477	cell migration	42	0.255324813	0.694051078	0.906024096	1	0.903175932	92	tags=24%, list=20%, signal=21%	CCL13/SAA1/CSF2/MSX2/EOMES/ASPM/LBP/CCBE1/SFTPD/IGF1
**BP**	GO:0051321	meiotic cell cycle	15	-0.250177288	-0.689526225	0.887719298	1	0.903175932	42	tags=13%, list=9%, signal=13%	HORMAD1/OVOL1
**BP**	GO:0007420	brain development	23	-0.220413012	-0.686843603	0.914285714	1	0.903175932	44	tags=26%, list=10%, signal=25%	EIF2B5/FBXW11/EEF1AKMT4-ECE2/ZEB1/TBX3/ACE
**BP**	GO:0060322	head development	23	-0.220413012	-0.686843603	0.914285714	1	0.903175932	44	tags=26%, list=10%, signal=25%	EIF2B5/FBXW11/EEF1AKMT4-ECE2/ZEB1/TBX3/ACE
**BP**	GO:0006470	protein dephosphorylation	10	0.333071874	0.68491226	0.857142857	1	0.903175932	116	tags=40%, list=26%, signal=30%	TPTE2/PPM1N/PPP4R4/PPP1CA
**BP**	GO:0065003	protein-containing complex assembly	34	0.256441219	0.678555422	0.918292683	1	0.903175932	40	tags=12%, list=9%, signal=12%	H2BW1/TSPYL6/AKAIN1/CSF2
**BP**	GO:0098655	cation transmembrane transport	17	0.290792428	0.677187061	0.912868633	1	0.903175932	111	tags=29%, list=25%, signal=23%	STAC/LHCGR/KCNK1/TRPC3/CEMIP
**BP**	GO:0060341	regulation of cellular localization	24	0.273639786	0.676165392	0.918954248	1	0.903175932	114	tags=29%, list=25%, signal=23%	SAA1/STAC/LHCGR/IGF1/TRPC3/CEMIP/LEPROT
**BP**	GO:0007507	heart development	15	0.300648334	0.675305365	0.89539749	1	0.903175932	92	tags=33%, list=20%, signal=27%	MSX2/EOMES/COL11A1/NKX2-5/IGF1
**BP**	GO:0016570	histone modification	11	0.319487797	0.674203858	0.866863905	1	0.903175932	50	tags=9%, list=11%, signal=8%	EOMES
**BP**	GO:0065009	regulation of molecular function	69	0.232914417	0.673243186	0.963882619	1	0.903175932	50	tags=10%, list=11%, signal=11%	DKK4/CCL13/DEPDC1/STAC/LHCGR/MSX2/EOMES
**BP**	GO:0048583	regulation of response to stimulus	104	0.222407991	0.669813715	0.966522678	1	0.903175932	116	tags=23%, list=26%, signal=22%	GPHB5/IL17F/DKK4/CCL13/SAA1/CSF2/CD8B/LHCGR/MSX2/TPTE2/ASPM/NKX2-5/LBP/CCBE1/IGF1/ADH7/NLRP5/PPM1N/TIAM2/TRPC3/BACE1/LEPROT/CXCL17/PPP1CA

**BP**	GO:0032989	cellular component morphogenesis	17	0.287233028	0.668898057	0.912868633	1	0.903175932	98	tags=24%, list=22%, signal=19%	ELAVL4/NKX2-5/LHX9/TIAM2
**BP**	GO:1903530	regulation of secretion by cell	12	0.309693176	0.667037887	0.880512091	1	0.903175932	17	tags=8%, list=4%, signal=8%	SAA1
**BP**	GO:0010033	response to organic substance	86	0.224715321	0.663209633	0.974444444	1	0.903175932	61	tags=12%, list=14%, signal=12%	GPHB5/IL17F/CCL13/OR11H4/CSF2/MRGPRX1/LHCGR/MSX2/ADAM23/ELAVL4
**BP**	GO:0070887	cellular response to chemical stimulus	87	0.224018017	0.662932082	0.971270718	1	0.903175932	61	tags=11%, list=14%, signal=12%	GPHB5/IL17F/CCL13/SAA1/OR11H4/CSF2/LHCGR/MSX2/ADAM23/ELAVL4
**BP**	GO:0022008	neurogenesis	46	-0.187947244	-0.66254077	0.98089172	1	0.903175932	64	tags=15%, list=14%, signal=15%	NECTIN1/CPNE5/HHIP/CLU/ZEB1/ACE/KIT
**BP**	GO:0007417	central nervous system development	29	0.25821519	0.662075889	0.936010038	1	0.903175932	72	tags=21%, list=16%, signal=19%	LHCGR/EOMES/ADAM23/ELAVL4/ASPM/TBC1D23
**BP**	GO:0006886	intracellular protein transport	23	-0.212006007	-0.660645975	0.946938776	1	0.903175932	156	tags=43%, list=35%, signal=30%	SNX11/NUP214/RAB8B/IPO11/TCAF1/TRAM1/SAR1A/CLU/RAPGEF3/HRAS
**BP**	GO:0032259	methylation	14	0.29755511	0.658118675	0.910364146	1	0.903175932	117	tags=21%, list=26%, signal=16%	EOMES/SNRPE/TDRD1
**BP**	GO:1901700	response to oxygen-containing compound	41	0.241293954	0.653339944	0.94188862	1	0.903175932	114	tags=29%, list=25%, signal=24%	OR11H4/CSF2/LHCGR/MSX2/ELAVL4/LBP/IGF1/ADH7/TRPC3/BACE1/HTR1E/LEPROT
**BP**	GO:0055082	cellular chemical homeostasis	15	0.290663174	0.652877062	0.917712692	1	0.903175932	48	tags=13%, list=11%, signal=12%	CCL13/LHCGR
**BP**	GO:0008610	lipid biosynthetic process	14	-0.241352884	-0.65194847	0.927083333	1	0.903175932	86	tags=29%, list=19%, signal=24%	ABHD3/GAL3ST1/HSD3B7/NPC1L1
**BP**	GO:0046907	intracellular transport	38	-0.191069632	-0.646861715	0.988826816	1	0.903175932	156	tags=47%, list=35%, signal=34%	SNX11/NUP214/TRAPPC9/BOK/RAB8B/LDLRAP1/EHD4/TAP2/IPO11/TCAF1/FBXW11/TRAM1/BICD1/SAR1A/CLU/RAPGEF3/HRAS/CDX2
**BP**	GO:1901701	cellular response to oxygen-containing compound	31	0.249392624	0.646829931	0.946650124	1	0.903175932	114	tags=29%, list=25%, signal=23%	OR11H4/CSF2/LHCGR/MSX2/LBP/IGF1/BACE1/HTR1E/LEPROT
**BP**	GO:0048514	blood vessel morphogenesis	16	0.278772523	0.641049165	0.929058663	1	0.903175932	11	tags=6%, list=2%, signal=6%	IL17F
**BP**	GO:1903047	mitotic cell cycle process	15	-0.231926583	-0.639224538	0.940350877	1	0.903175932	14	tags=27%, list=3%, signal=27%	RPS27L/ANAPC7/FBXW11/MCM2
**BP**	GO:0016311	dephosphorylation	12	0.296362107	0.638324539	0.900426743	1	0.903175932	116	tags=33%, list=26%, signal=25%	TPTE2/PPM1N/PPP4R4/PPP1CA
**BP**	GO:0019725	cellular homeostasis	16	0.274748948	0.631796786	0.938608458	1	0.903175932	48	tags=12%, list=11%, signal=12%	CCL13/LHCGR
**BP**	GO:0051276	chromosome organization	18	-0.219387784	-0.631626415	0.963709677	1	0.903175932	122	tags=39%, list=27%, signal=30%	MAJIN/TNKS2/ANAPC7/TOP3B/HMGA2/HORMAD1/MCM2
**BP**	GO:1901135	carbohydrate derivative metabolic process	15	0.275953527	0.619836787	0.945606695	1	0.903175932	156	tags=67%, list=35%, signal=45%	COL11A1/TMTC3/FUCA2/IGF1/POGLUT2/CEMIP/GNPTG/CRPPA/CTPS1/GCNT2
**BP**	GO:0043933	protein-containing complex organization	42	0.225592337	0.613229096	0.969879518	1	0.903175932	40	tags=10%, list=9%, signal=10%	H2BW1/TSPYL6/AKAIN1/CSF2
**BP**	GO:2001233	regulation of apoptotic signaling pathway	11	-0.250489304	-0.609602093	0.95398773	1	0.903175932	68	tags=36%, list=15%, signal=32%	CYLD/TNFSF10/CLU/DAPK2
**BP**	GO:0070201	regulation of establishment of protein localization	12	0.277310482	0.597289874	0.93314367	1	0.903175932	17	tags=8%, list=4%, signal=8%	SAA1
**BP**	GO:0009967	positive regulation of signal transduction	44	0.215667919	0.593830665	0.977407848	1	0.903175932	116	tags=23%, list=26%, signal=19%	CCL13/LHCGR/MSX2/ASPM/LBP/CCBE1/IGF1/PPM1N/CXCL17/PPP1CA
**BP**	GO:0051223	regulation of protein transport	11	0.279854942	0.590568039	0.928994083	1	0.903175932	17	tags=9%, list=4%, signal=9%	SAA1
**BP**	GO:0071396	cellular response to lipid	13	0.270346642	0.589953812	0.947740113	1	0.903175932	84	tags=23%, list=19%, signal=19%	CSF2/MSX2/LBP
**BP**	GO:0071417	cellular response to organonitrogen compound	19	0.244324532	0.585841943	0.954427083	1	0.903175932	137	tags=42%, list=31%, signal=31%	OR11H4/LHCGR/IGF1/BACE1/HTR1E/LEPROT/CPEB4/DTNBP1
**BP**	GO:1901699	cellular response to nitrogen compound	19	0.244324532	0.585841943	0.954427083	1	0.903175932	137	tags=42%, list=31%, signal=31%	OR11H4/LHCGR/IGF1/BACE1/HTR1E/LEPROT/CPEB4/DTNBP1
**BP**	GO:0051098	regulation of binding	12	0.267805282	0.576816938	0.944523471	1	0.903175932	137	tags=42%, list=31%, signal=30%	MSX2/IGF1/NEK2/PPP1CA/DTNBP1
**BP**	GO:0031175	neuron projection development	23	0.233391294	0.574593781	0.974900925	1	0.903175932	98	tags=17%, list=22%, signal=14%	ELAVL4/TBC1D23/LHX9/TIAM2
**BP**	GO:0002009	morphogenesis of an epithelium	11	0.269918022	0.569598507	0.948224852	1	0.903175932	116	tags=36%, list=26%, signal=28%	MSX2/NKX2-5/GREB1L/PPP1CA

**BP**	GO:0006644	phospholipid metabolic process	11	0.266613296	0.562624658	0.951183432	1	0.903175932	145	tags=36%, list=32%, signal=25%	TPTE2/PIP5KL1/PLA2G4F/PLA2G4A
**BP**	GO:1901652	response to peptide	10	-0.236101411	-0.561867791	0.965811966	1	0.903175932	11	tags=40%, list=2%, signal=40%	MEAK7/EIF2B5/TNFSF10/FGF21
**BP**	GO:0034765	regulation of ion transmembrane transport	14	0.252892896	0.559336848	0.969187675	1	0.903175932	48	tags=14%, list=11%, signal=13%	STAC/LHCGR
**BP**	GO:0071705	nitrogen compound transport	39	0.205607536	0.553451232	0.986682809	1	0.903175932	92	tags=18%, list=20%, signal=16%	SAA1/GOLT1A/CSF2/LHCGR/ARL8A/POM121L2/IGF1
**BP**	GO:0072594	establishment of protein localization to organelle	11	0.261899177	0.55267662	0.957100592	1	0.903175932	139	tags=45%, list=31%, signal=32%	LHCGR/POM121L2/LEPROT/GNPTG/ZFAND6
**BP**	GO:0044092	negative regulation of molecular function	30	0.213994298	0.548109224	0.986093552	1	0.903175932	50	tags=10%, list=11%, signal=10%	DKK4/MSX2/EOMES
**BP**	GO:0007005	mitochondrion organization	12	-0.216460091	-0.54322148	0.969899666	1	0.903175932	68	tags=17%, list=15%, signal=15%	TNFSF10/CLU
**BP**	GO:0015031	protein transport	32	0.205525458	0.538123871	0.981572482	1	0.903175932	114	tags=25%, list=25%, signal=20%	SAA1/GOLT1A/LHCGR/ARL8A/POM121L2/IGF1/CEMIP/LEPROT
**BP**	GO:0010638	positive regulation of organelle organization	16	0.233423041	0.536766121	0.983628922	1	0.903175932	106	tags=25%, list=24%, signal=20%	CSF2/MSX2/IGF1/NEK2
**BP**	GO:0010647	positive regulation of cell communication	48	0.193300771	0.536689372	0.994103774	1	0.903175932	97	tags=17%, list=22%, signal=15%	CCL13/LHCGR/MSX2/ASPM/LBP/CCBE1/IGF1/PPM1N
**BP**	GO:0023056	positive regulation of signaling	48	0.193300771	0.536689372	0.994103774	1	0.903175932	97	tags=17%, list=22%, signal=15%	CCL13/LHCGR/MSX2/ASPM/LBP/CCBE1/IGF1/PPM1N
**BP**	GO:0045184	establishment of protein localization	34	0.200422206	0.5303265	0.987804878	1	0.903175932	114	tags=24%, list=25%, signal=19%	SAA1/GOLT1A/LHCGR/ARL8A/POM121L2/IGF1/CEMIP/LEPROT
**BP**	GO:0006605	protein targeting	14	-0.189308689	-0.511365386	0.993055556	1	0.903175932	110	tags=29%, list=24%, signal=22%	TCAF1/TRAM1/CLU/HRAS
**BP**	GO:0044403	biological process involved in symbiotic interaction	11	-0.209567555	-0.510013076	0.990797546	1	0.903175932	195	tags=73%, list=43%, signal=42%	HMGB1/LGALS1/AXL/XPR1/AZU1/SAP30BP/HMGA2/NECTIN1
**BP**	GO:0000226	microtubule cytoskeleton organization	13	0.232854003	0.508136906	0.978813559	1	0.903175932	106	tags=23%, list=24%, signal=18%	ASPM/NLRP5/NEK2
**BP**	GO:0043543	protein acylation	10	0.2434407	0.50059922	0.978494624	1	0.903175932	50	tags=10%, list=11%, signal=9%	EOMES
**BP**	GO:0030162	regulation of proteolysis	14	-0.184105468	-0.497310313	0.996527778	1	0.903175932	145	tags=64%, list=32%, signal=45%	CAST/CCDC22/HMGB1/C2CD3/BOK/RPS27L/FBXW11/TNFSF10/CLU
**BP**	GO:0007059	chromosome segregation	13	0.225421929	0.491918542	0.984463277	1	0.903175932	106	tags=23%, list=24%, signal=18%	BUB1/ARL8A/NEK2
**BP**	GO:0007155	cell adhesion	37	0.178024121	0.477033443	0.996336996	1	0.903175932	92	tags=14%, list=20%, signal=12%	SAA1/NTM/ADAM23/SFTPD/IGF1
**BP**	GO:0032990	cell part morphogenesis	16	0.20412064	0.469384015	0.994542974	1	0.903175932	98	tags=19%, list=22%, signal=15%	ELAVL4/LHX9/TIAM2
**BP**	GO:0048812	neuron projection morphogenesis	16	0.20412064	0.469384015	0.994542974	1	0.903175932	98	tags=19%, list=22%, signal=15%	ELAVL4/LHX9/TIAM2
**BP**	GO:0048858	cell projection morphogenesis	16	0.20412064	0.469384015	0.994542974	1	0.903175932	98	tags=19%, list=22%, signal=15%	ELAVL4/LHX9/TIAM2
**BP**	GO:0120039	plasma membrane bounded cell projection morphogenesis	16	0.20412064	0.469384015	0.994542974	1	0.903175932	98	tags=19%, list=22%, signal=15%	ELAVL4/LHX9/TIAM2
**BP**	GO:0042391	regulation of membrane potential	11	0.196347032	0.414344234	0.99704142	1	0.903175932	363	tags=100%, list=81%, signal=20%	KCNK1/PIP5KL1/GLRA1/MECP2/NRXN1/PRKAR1B/NLGN1/GNA11/KCNE3/BOK/HCN4
**BP**	GO:0098813	nuclear chromosome segregation	10	-0.170697341	-0.406220944	1	1	0.903175932	122	tags=30%, list=27%, signal=22%	MAJIN/ANAPC7/HORMAD1

**CC**	GO:0043227	membrane-bounded organelle	291	-0.329748903	-1.480081594	3.49E-05	0.003167	0.002860358	143	tags=38%, list=32%, signal=74%	RAB8B/PXDN/SOX5/AZU1/LDLRAP1/EHD4/VPS72/COL17A1/AIMP2/TAP2/ZNF341/SCLT1/SLC25A43/RPS27L/ZNF664/LAMA4/IPO11/TSEN54/SAP30BP/IMP3/MAJIN/SLC25A37/SLC28A2/NKAP/GCAT/WARS1/TNKS2/GBP4/MEAK7/EIF2B5/ZSCAN5A/ANAPC7/FBXW11/ZNF579/DAZ4/CYLD/PLAA/VSIG10L/TMEM79/NRAS/AHDC1/LCOR/TOP3B/TRAM1/FAM118B/UBE2E3/EEF1AKMT4-ECE2/MESD/BICD1/PREP/HMGA2/SMAD9/NECTIN1/CENPB/SAR1A/GSTCD/TNFSF10/GAL3ST1/COBLL1/CPNE5/BBS10/GPX8/BDKRB1/EMSY/CDYL2/HHIP/NPRL2/FCGBP/HSD3B7/CLU/NID2/KRT8/MPL/HDX/ZNF528/CCR10/ZEB1/DMRTC1/HORMAD1/DAPK2/RAB26/ZNF470/SYT8/GBP5/SALL3/ZNF596/PMEPA1/RAPGEF3/HRAS/TSPY10/TBX3/ART1/SIX5/NAPSA/CYP26C1/PTMS/HNF1A/NPC1L1/ACE/MPIG6B/MCM2/OPN1MW/GOLGA6A/TKTL1/CDX2/LTBP1/DAZ1/TRIM35/OVOL1/KIT/RPS4Y1
**CC**	GO:0043231	intracellular membrane-bounded organelle	267	-0.292637921	-1.298939502	0.001002523	0.054604087	0.049317097	61	tags=17%, list=14%, signal=37%	GPX8/BDKRB1/EMSY/CDYL2/HHIP/NPRL2/HSD3B7/CLU/KRT8/MPL/HDX/ZNF528/CCR10/ZEB1/DMRTC1/HORMAD1/DAPK2/RAB26/ZNF470/SYT8/GBP5/SALL3/ZNF596/PMEPA1/HRAS/TSPY10/TBX3/ART1/SIX5/NAPSA/CYP26C1/PTMS/HNF1A/NPC1L1/ACE/MPIG6B/MCM2/GOLGA6A/TKTL1/CDX2/LTBP1/DAZ1/TRIM35/OVOL1/KIT/RPS4Y1
**CC**	GO:0043229	intracellular organelle	289	-0.282433694	-1.275450229	0.00456387	0.113179352	0.102220867	61	tags=17%, list=14%, signal=40%	GPX8/BDKRB1/EMSY/CDYL2/HHIP/NPRL2/HSD3B7/RIC8B/CLU/KRT8/MPL/HDX/ZNF528/CCR10/ZEB1/DMRTC1/HORMAD1/DAPK2/RAB26/ZNF470/SYT8/GBP5/SALL3/ZNF596/PMEPA1/RAPGEF3/HRAS/TSPY10/TBX3/ART1/SIX5/NAPSA/CYP26C1/PTMS/HNF1A/NPC1L1/ACE/MPIG6B/MCM2/GOLGA6A/TKTL1/CDX2/LTBP1/DAZ1/TRIM35/OVOL1/KIT/RPS4Y1
**CC**	GO:0016021	integral component of membrane	124	0.467859133	1.436231214	0.009340538	0.173436807	0.15664395	70	tags=25%, list=16%, signal=29%	TAS2R3/OR10G6/SLC10A5/TMEM247/OR8S1/OR10D3/OR56B1/OR4D6/OR13G1/TRHR/OR11H4/FAM205C/SLC10A2/OR9A2/GJB5/GOLT1A/CYP4Z1/CSF2/MRGPRX1/OR11H1/CD8B/LHCGR/OR11H2/TPTE2/TMTC3/ADAM23/ADAM11/CIP2A/KCNK1/ANO5/EMCN
**CC**	GO:0031224	intrinsic component of membrane	129	0.460127076	1.41675978	0.00909353	0.173436807	0.15664395	70	tags=25%, list=16%, signal=29%	TAS2R3/OR10G6/SLC10A5/TMEM247/OR8S1/OR10D3/OR56B1/OR4D6/OR13G1/TRHR/OR11H4/FAM205C/SLC10A2/OR9A2/GJB5/NTM/GOLT1A/CYP4Z1/CSF2/MRGPRX1/OR11H1/CD8B/LHCGR/OR11H2/TPTE2/TMTC3/ADAM23/ADAM11/CIP2A/KCNK1/ANO5/EMCN
**CC**	GO:0005634	nucleus	169	-0.30128181	-1.339440441	0.01071259	0.182337205	0.164682575	59	tags=18%, list=13%, signal=25%	EMSY/CDYL2/HHIP/CLU/KRT8/MPL/HDX/ZNF528/ZEB1/DMRTC1/HORMAD1/DAPK2/ZNF470/SALL3/ZNF596/HRAS/TSPY10/TBX3/SIX5/PTMS/HNF1A/MPIG6B/MCM2/TKTL1/CDX2/DAZ1/TRIM35/OVOL1/KIT/RPS4Y1

**CC**	GO:0005929	cilium	14	-0.592403578	-1.60021542	0.042856278	0.327229708	0.295545996	63	tags=43%, list=14%, signal=38%	CYLD/BBS10/HHIP/TBX3/ACE/OPN1MW
**CC**	GO:0048471	perinuclear region of cytoplasm	15	-0.571373905	-1.574792401	0.05328111	0.343633561	0.310361562	143	tags=67%, list=32%, signal=47%	RAB8B/EHD4/TNKS2/GBP4/CYLD/HCN4/BICD1/CLU/GBP5/HRAS
**CC**	GO:0031981	nuclear lumen	86	-0.292844681	-1.171420096	0.093138313	0.450304705	0.406704372	126	tags=30%, list=28%, signal=27%	IPO11/TSEN54/SAP30BP/IMP3/NKAP/GCAT/MEAK7/ANAPC7/VSIG10L/LCOR/FAM118B/UBE2E3/HMGA2/SMAD9/CENPB/EMSY/KRT8/ZEB1/HORMAD1/ZNF470/HRAS/MPIG6B/MCM2/CDX2/KIT/RPS4Y1
**CC**	GO:0005886	plasma membrane	141	0.395996714	1.224737568	0.09832636	0.458434047	0.414046598	70	tags=21%, list=16%, signal=26%	TAS2R3/OR10G6/OR8S1/OR10D3/FABP2/OR56B1/OR4D6/OR13G1/TRHR/OR11H4/SLC10A2/OR9A2/GJB5/NTM/CSF2/MRGPRX1/OR11H1/STAC/CD8B/LHCGR/OR11H2/TPTE2/ADAM23/ADAM11/CIP2A/ASPM/ARL8A/KCNK1/ANO5/EMCN
**CC**	GO:0005794	Golgi apparatus	49	-0.343577472	-1.220918262	0.098011405	0.458434047	0.414046598	96	tags=31%, list=21%, signal=27%	TMEM79/NRAS/EEF1AKMT4-ECE2/BICD1/SAR1A/GAL3ST1/CLU/MPL/DAPK2/RAB26/GBP5/PMEPA1/HRAS/MPIG6B/GOLGA6A
**CC**	GO:0005654	nucleoplasm	77	-0.301728164	-1.174054946	0.10049417	0.458434047	0.414046598	126	tags=31%, list=28%, signal=27%	IPO11/TSEN54/SAP30BP/IMP3/NKAP/GCAT/MEAK7/ANAPC7/VSIG10L/LCOR/FAM118B/UBE2E3/HMGA2/SMAD9/CENPB/EMSY/KRT8/ZEB1/ZNF470/HRAS/MPIG6B/MCM2/CDX2/RPS4Y1
**CC**	GO:0009986	cell surface	23	-0.435313729	-1.356509976	0.118435752	0.496215435	0.448169838	56	tags=30%, list=12%, signal=28%	HHIP/CLU/MPL/CCR10/ART1/ACE/KIT
**CC**	GO:0030141	secretory granule	19	-0.432482403	-1.264177129	0.128205128	0.505013736	0.456116252	51	tags=26%, list=11%, signal=24%	CLU/RAB26/SYT8/NAPSA/KIT
**CC**	GO:0016020	membrane	224	0.37422386	1.180096359	0.128571429	0.505013736	0.456116252	70	tags=19%, list=16%, signal=32%	TAS2R3/OR10G6/SLC10A5/TMEM247/OR8S1/OR10D3/CKMT2/H2BW1/FABP2/OR56B1/OR4D6/OR13G1/TRHR/OR11H4/FAM205C/ERCC6L/SLC10A2/OR9A2/GJB5/NTM/GOLT1A/CYP4Z1/CSF2/MRGPRX1/OR11H1/STAC/CD8B/LHCGR/OR11H2/TPTE2/TMTC3/ADAM23/FAM169A/ELAVL4/ADAM11/CIP2A/ASPM/BUB1/ARL8A/KCNK1/ANO5/EMCN
**CC**	GO:0005576	extracellular region	98	0.400469748	1.199658291	0.133841132	0.507453416	0.458319712	94	tags=27%, list=21%, signal=27%	DEFB113/GPHB5/DEFB131B/IL17F/DKK4/CCL13/SAA1/DEFB123/NTM/PRH2/CSF2/CD8B/LHCGR/COL11A1/FAM184A/ADAM23/ARL8A/TFF3/EMCN/SCGB3A2/LBP/CCBE1/SFTPD/FUCA2/IGF1/IGFL3
**CC**	GO:0071944	cell periphery	152	0.372023628	1.16084379	0.176652893	0.575001646	0.519327648	70	tags=20%, list=16%, signal=26%	TAS2R3/OR10G6/OR8S1/OR10D3/FABP2/OR56B1/OR4D6/OR13G1/TRHR/OR11H4/SLC10A2/OR9A2/GJB5/NTM/CSF2/MRGPRX1/OR11H1/STAC/CD8B/LHCGR/OR11H2/TPTE2/COL11A1/ADAM23/ADAM11/CIP2A/ASPM/ARL8A/KCNK1/ANO5/EMCN
**CC**	GO:0098552	side of membrane	14	-0.460873412	-1.244922833	0.1875	0.59375	0.53626071	48	tags=29%, list=11%, signal=26%	MPL/CCR10/ACE/KIT
**CC**	GO:0031974	membrane-enclosed lumen	108	-0.274155429	-1.102156143	0.205446382	0.608151064	0.549267405	61	tags=15%, list=14%, signal=17%	GPX8/EMSY/CLU/KRT8/ZEB1/HORMAD1/DAPK2/ZNF470/HRAS/NAPSA/MPIG6B/MCM2/CDX2/LTBP1/KIT/RPS4Y1
**CC**	GO:0043233	organelle lumen	108	-0.274155429	-1.102156143	0.205446382	0.608151064	0.549267405	61	tags=15%, list=14%, signal=17%	GPX8/EMSY/CLU/KRT8/ZEB1/HORMAD1/DAPK2/ZNF470/HRAS/NAPSA/MPIG6B/MCM2/CDX2/LTBP1/KIT/RPS4Y1

**CC**	GO:0070013	intracellular organelle lumen	108	-0.274155429	-1.102156143	0.205446382	0.608151064	0.549267405	61	tags=15%, list=14%, signal=17%	GPX8/EMSY/CLU/KRT8/ZEB1/HORMAD1/DAPK2/ZNF470/HRAS/NAPSA/MPIG6B/MCM2/CDX2/LTBP1/KIT/RPS4Y1
**CC**	GO:1990904	ribonucleoprotein complex	12	-0.474185935	-1.190002203	0.227424749	0.627723041	0.566944343	2	tags=25%, list=0%, signal=26%	RPS27L/IMP3/RPS4Y1
**CC**	GO:0005615	extracellular space	75	0.383738119	1.121702795	0.263919822	0.675495711	0.610091469	98	tags=28%, list=22%, signal=26%	GPHB5/DEFB131B/IL17F/DKK4/CCL13/SAA1/PRH2/CSF2/LHCGR/COL11A1/FAM184A/ARL8A/TFF3/SCGB3A2/LBP/CCBE1/SFTPD/FUCA2/IGF1/IGFL3/TIAM2
**CC**	GO:0000785	chromatin	32	-0.349170846	-1.133523244	0.271276596	0.682277306	0.616216442	47	tags=38%, list=10%, signal=36%	HMGA2/SMAD9/CENPB/HDX/ZEB1/DMRTC1/TSPY10/TBX3/SIX5/HNF1A/MCM2/CDX2
**CC**	GO:0099503	secretory vesicle	24	-0.361493826	-1.14043492	0.278481013	0.685009796	0.618684361	51	tags=29%, list=11%, signal=27%	NRAS/BICD1/CLU/RAB26/SYT8/NAPSA/KIT
**CC**	GO:0043230	extracellular organelle	45	-0.309638601	-1.098330253	0.307228916	0.7233603	0.653321613	71	tags=24%, list=16%, signal=23%	GSTCD/TNFSF10/COBLL1/CPNE5/FCGBP/CLU/NID2/KRT8/RAPGEF3/NAPSA/ACE
**CC**	GO:0065010	extracellular membrane-bounded organelle	45	-0.309638601	-1.098330253	0.307228916	0.7233603	0.653321613	71	tags=24%, list=16%, signal=23%	GSTCD/TNFSF10/COBLL1/CPNE5/FCGBP/CLU/NID2/KRT8/RAPGEF3/NAPSA/ACE
**CC**	GO:0070062	extracellular exosome	45	-0.309638601	-1.098330253	0.307228916	0.7233603	0.653321613	71	tags=24%, list=16%, signal=23%	GSTCD/TNFSF10/COBLL1/CPNE5/FCGBP/CLU/NID2/KRT8/RAPGEF3/NAPSA/ACE
**CC**	GO:1903561	extracellular vesicle	45	-0.309638601	-1.098330253	0.307228916	0.7233603	0.653321613	71	tags=24%, list=16%, signal=23%	GSTCD/TNFSF10/COBLL1/CPNE5/FCGBP/CLU/NID2/KRT8/RAPGEF3/NAPSA/ACE
**CC**	GO:0099513	polymeric cytoskeletal fiber	17	0.47807333	1.113320164	0.3230563	0.741800023	0.669975927	106	tags=41%, list=24%, signal=33%	SAA1/KRTAP10-12/KRT39/ASPM/FHDC1/KRTAP10-9/NEK2
**CC**	GO:0005635	nuclear envelope	20	0.44991606	1.086486326	0.343264249	0.772580967	0.697776536	88	tags=25%, list=20%, signal=21%	H2BW1/GOLT1A/FAM169A/ELAVL4/POM121L2
**CC**	GO:0099080	supramolecular complex	28	0.425808127	1.087088247	0.346396966	0.775000366	0.699961678	106	tags=36%, list=24%, signal=29%	SAA1/ERCC6L/KRTAP10-12/KRT39/COL11A1/ASPM/BUB1/FHDC1/KRTAP10-9/NEK2
**CC**	GO:0031967	organelle envelope	31	0.417909569	1.083899009	0.353598015	0.778678108	0.703283327	61	tags=16%, list=14%, signal=15%	CKMT2/H2BW1/GOLT1A/FAM169A/ELAVL4
**CC**	GO:0031975	envelope	31	0.417909569	1.083899009	0.353598015	0.778678108	0.703283327	61	tags=16%, list=14%, signal=15%	CKMT2/H2BW1/GOLT1A/FAM169A/ELAVL4
**CC**	GO:0030312	external encapsulating structure	13	-0.407362603	-1.05867399	0.37755102	0.805376459	0.727396634	54	tags=31%, list=12%, signal=28%	FCGBP/CLU/NID2/LTBP1
**CC**	GO:0031012	extracellular matrix	13	-0.407362603	-1.05867399	0.37755102	0.805376459	0.727396634	54	tags=31%, list=12%, signal=28%	FCGBP/CLU/NID2/LTBP1
**CC**	GO:0000139	Golgi membrane	18	-0.354313993	-1.020084499	0.39516129	0.813112307	0.734383466	116	tags=50%, list=26%, signal=39%	TNKS2/GBP4/NRAS/EEF1AKMT4-ECE2/GAL3ST1/RAB26/GBP5/PMEPA1/HRAS
**CC**	GO:0030139	endocytic vesicle	10	0.516340851	1.061777373	0.422427035	0.830898669	0.75044768	111	tags=40%, list=25%, signal=31%	SAA1/SCGB3A2/SFTPD/CEMIP
**CC**	GO:0031965	nuclear membrane	11	0.497218057	1.049261773	0.423076923	0.830898669	0.75044768	60	tags=18%, list=13%, signal=16%	H2BW1/FAM169A
**CC**	GO:0031982	vesicle	94	-0.256274332	-1.017974024	0.430232558	0.842925659	0.761310169	73	tags=20%, list=16%, signal=21%	SAR1A/GSTCD/TNFSF10/COBLL1/CPNE5/FCGBP/CLU/NID2/KRT8/DAPK2/RAB26/SYT8/GBP5/PMEPA1/RAPGEF3/NAPSA/NPC1L1/ACE/KIT
**CC**	GO:0098797	plasma membrane protein complex	11	0.494039704	1.042554606	0.433431953	0.845140586	0.763310636	92	tags=45%, list=20%, signal=37%	GJB5/CSF2/CD8B/KCNK1/IGF1
**CC**	GO:0005737	cytoplasm	266	-0.220224005	-0.992446624	0.443841815	0.846666667	0.764688956	62	tags=15%, list=14%, signal=31%	MFHAS1/GPX8/BDKRB1/CRACR2B/HHIP/NPRL2/HSD3B7/RIC8B/CLU/KRT8/MPL/CCR10/ZEB1/DAPK2/RAB26/SHC2/SYT8/GBP5/PMEPA1/RAPGEF3/HRAS/TSPY10/ART1/SIX5/NAPSA/CYP26C1/HNF1A/NPC1L1/ACE/MPIG6B/MCM2/FGF21/GOLGA6A/TKTL1/LTBP1/DAZ1/TRIM35/KIT/RPS4Y1

**CC**	GO:0098588	bounding membrane of organelle	49	-0.283919646	-1.008921448	0.462025316	0.857897008	0.77483193	145	tags=45%, list=32%, signal=34%	BOK/RAB8B/AZU1/LDLRAP1/EHD4/TAP2/TNKS2/GBP4/MEAK7/TMEM79/NRAS/EEF1AKMT4-ECE2/SAR1A/GAL3ST1/HHIP/NPRL2/RAB26/SYT8/GBP5/PMEPA1/HRAS/ART1
**CC**	GO:0031090	organelle membrane	85	-0.247656205	-0.983390946	0.466019417	0.861397883	0.777993836	56	tags=18%, list=12%, signal=19%	HHIP/NPRL2/HSD3B7/CLU/MPL/RAB26/SYT8/GBP5/PMEPA1/HRAS/ART1/CYP26C1/NPC1L1/OPN1MW/GOLGA6A
**CC**	GO:0005694	chromosome	43	-0.281659244	-0.992068584	0.484848485	0.876374363	0.791520233	47	tags=23%, list=10%, signal=23%	HDX/ZEB1/DMRTC1/HORMAD1/TSPY10/TBX3/SIX5/HNF1A/MCM2/CDX2
**CC**	GO:0062023	collagen-containing extracellular matrix	10	-0.40996777	-0.97563028	0.484330484	0.876374363	0.791520233	51	tags=60%, list=11%, signal=54%	PXDN/COL17A1/LAMA4/CLU/NID2/LTBP1
**CC**	GO:0012506	vesicle membrane	30	-0.297524163	-0.985333985	0.488151659	0.880397142	0.795153509	145	tags=47%, list=32%, signal=34%	BOK/RAB8B/AZU1/LDLRAP1/EHD4/TAP2/SLC28A2/NRAS/EEF1AKMT4-ECE2/SAR1A/RAB26/SYT8/PMEPA1/NPC1L1
**CC**	GO:0099081	supramolecular polymer	20	0.406833763	0.982448416	0.501295337	0.892283857	0.805889304	106	tags=40%, list=24%, signal=32%	SAA1/KRTAP10-12/KRT39/COL11A1/ASPM/FHDC1/KRTAP10-9/NEK2
**CC**	GO:0099512	supramolecular fiber	20	0.406833763	0.982448416	0.501295337	0.892283857	0.805889304	106	tags=40%, list=24%, signal=32%	SAA1/KRTAP10-12/KRT39/COL11A1/ASPM/FHDC1/KRTAP10-9/NEK2
**CC**	GO:0031410	cytoplasmic vesicle	62	-0.258435238	-0.970448374	0.508333333	0.89699424	0.81014361	40	tags=16%, list=9%, signal=17%	CLU/DAPK2/RAB26/SYT8/GBP5/PMEPA1/NAPSA/NPC1L1/ACE/KIT
**CC**	GO:0097708	intracellular vesicle	62	-0.258435238	-0.970448374	0.508333333	0.89699424	0.81014361	40	tags=16%, list=9%, signal=17%	CLU/DAPK2/RAB26/SYT8/GBP5/PMEPA1/NAPSA/NPC1L1/ACE/KIT
**CC**	GO:0043228	non-membrane-bounded organelle	114	-0.24166063	-0.979003754	0.53030303	0.915677743	0.827018099	53	tags=15%, list=12%, signal=18%	HSD3B7/RIC8B/CLU/KRT8/HDX/ZEB1/DMRTC1/HORMAD1/RAPGEF3/TSPY10/TBX3/SIX5/HNF1A/MCM2/CDX2/KIT/RPS4Y1
**CC**	GO:0043232	intracellular non-membrane-bounded organelle	114	-0.24166063	-0.979003754	0.53030303	0.915677743	0.827018099	53	tags=15%, list=12%, signal=18%	HSD3B7/RIC8B/CLU/KRT8/HDX/ZEB1/DMRTC1/HORMAD1/RAPGEF3/TSPY10/TBX3/SIX5/HNF1A/MCM2/CDX2/KIT/RPS4Y1
**CC**	GO:0005874	microtubule	12	0.450243502	0.969764583	0.549075391	0.93069418	0.840580584	106	tags=33%, list=24%, signal=26%	SAA1/ASPM/FHDC1/NEK2
**CC**	GO:0012505	endomembrane system	118	-0.23926635	-0.994108746	0.552238806	0.934118229	0.843673102	61	tags=18%, list=14%, signal=21%	GPX8/BDKRB1/HSD3B7/CLU/MPL/CCR10/DAPK2/RAB26/SYT8/GBP5/PMEPA1/RAPGEF3/HRAS/ART1/NAPSA/CYP26C1/ACE/MPIG6B/GOLGA6A/LTBP1/KIT
**CC**	GO:0030659	cytoplasmic vesicle membrane	28	-0.288087724	-0.940148276	0.563981043	0.937590565	0.846809233	145	tags=46%, list=32%, signal=34%	BOK/RAB8B/AZU1/LDLRAP1/EHD4/TAP2/NRAS/EEF1AKMT4-ECE2/SAR1A/RAB26/SYT8/PMEPA1/NPC1L1
**CC**	GO:0032991	protein-containing complex	109	-0.236057485	-0.956383566	0.6	0.975011513	0.880606932	28	tags=9%, list=6%, signal=11%	HRAS/SIX5/HNF1A/KCNA7/MCM2/CDX2/LTBP1/DAZ1/KIT/RPS4Y1
**CC**	GO:0005740	mitochondrial envelope	13	0.42136421	0.919506229	0.600282486	0.975011513	0.880606932	15	tags=8%, list=3%, signal=8%	CKMT2
**CC**	GO:0031966	mitochondrial membrane	13	0.42136421	0.919506229	0.600282486	0.975011513	0.880606932	15	tags=8%, list=3%, signal=8%	CKMT2
**CC**	GO:0005773	vacuole	19	-0.311586966	-0.910791083	0.606837607	0.978304476	0.883581058	55	tags=21%, list=12%, signal=19%	NPRL2/DAPK2/NAPSA/ACE
**CC**	GO:0005743	mitochondrial inner membrane	12	0.426600075	0.91883979	0.618776671	0.983972577	0.88870035	15	tags=8%, list=3%, signal=8%	CKMT2

**CC**	GO:0005575	cellular_component	420	0.319871242	0.927526411	0.6375	0.996392951	0.899918133	75	tags=17%, list=17%, signal=218%	TAS2R3/DEFB113/OR10G6/GPHB5/DEFB131B/SLC10A5/TMEM247/OR8S1/IL17F/DKK4/CCL13/OR10D3/CKMT2/H2BW1/SAA1/DEPDC1/FABP2/OR56B1/OR4D6/OR13G1/TRHR/OR11H4/FAM205C/ERCC6L/SLC10A2/OR9A2/TRIM42/CSRNP3/DEFB123/TSPYL6/GJB5/NTM/AKAIN1/GOLT1A/CYP4Z1/PRH2/CSF2/MRGPRX1/ZFP69B/OR11H1/KRTAP10-12/STAC/CD8B/KRT39/LHCGR/MSX2/EOMES/OR11H2/NCBP2L/ZNF385D/TPTE2/COL11A1/TMTC3/FAM184A/ADAM23/FAM169A/ELAVL4/ADAM11/CIP2A/ASPM/BUB1/ARL8A/KCNK1/TFF3/ANO5/EMCN/NKX2-5/TBC1D23/ZNF705B/SULT6B1/FAM174A
**CC**	GO:0098796	membrane protein complex	20	0.365679404	0.883066215	0.647668394	1	0.903175932	105	tags=30%, list=23%, signal=24%	GJB5/CSF2/CD8B/KCNK1/IGF1/TRPC3
**CC**	GO:0110165	cellular anatomical entity	417	0.300316084	0.878465041	0.748186528	1	0.903175932	75	tags=17%, list=17%, signal=196%	TAS2R3/DEFB113/OR10G6/GPHB5/DEFB131B/SLC10A5/TMEM247/OR8S1/IL17F/DKK4/CCL13/OR10D3/CKMT2/H2BW1/SAA1/DEPDC1/FABP2/OR56B1/OR4D6/OR13G1/TRHR/OR11H4/FAM205C/ERCC6L/SLC10A2/OR9A2/TRIM42/CSRNP3/DEFB123/TSPYL6/GJB5/NTM/AKAIN1/GOLT1A/CYP4Z1/PRH2/CSF2/MRGPRX1/ZFP69B/OR11H1/KRTAP10-12/STAC/CD8B/KRT39/LHCGR/MSX2/EOMES/OR11H2/ZNF385D/TPTE2/COL11A1/TMTC3/FAM184A/ADAM23/FAM169A/ELAVL4/ADAM11/CIP2A/ASPM/BUB1/ARL8A/KCNK1/TFF3/ANO5/EMCN/NKX2-5/TBC1D23/ZNF705B/SULT6B1/FAM174A
**CC**	GO:0005730	nucleolus	22	-0.278800827	-0.85866674	0.703862661	1	0.903175932	3	tags=18%, list=1%, signal=19%	TSEN54/IMP3/MEAK7/KIT
**CC**	GO:0120025	plasma membrane bounded cell projection	57	-0.227860519	-0.840855277	0.8125	1	0.903175932	77	tags=23%, list=17%, signal=22%	NECTIN1/LIMA1/CPNE5/BBS10/BDKRB1/HHIP/CLU/MPL/SYT8/RAPGEF3/TBX3/ACE/OPN1MW
**CC**	GO:0019866	organelle inner membrane	15	0.37391297	0.839869729	0.719665272	1	0.903175932	60	tags=13%, list=13%, signal=12%	CKMT2/FAM169A
**CC**	GO:0005887	integral component of plasma membrane	47	0.301684206	0.836337864	0.725559482	1	0.903175932	67	tags=19%, list=15%, signal=18%	OR4D6/TRHR/OR11H4/SLC10A2/CSF2/CD8B/LHCGR/ADAM23/KCNK1
**CC**	GO:0005768	endosome	28	-0.253872949	-0.828491448	0.767772512	1	0.903175932	39	tags=14%, list=9%, signal=14%	RAB26/PMEPA1/NAPSA/ACE
**CC**	GO:0005911	cell-cell junction	12	-0.328909892	-0.825421985	0.722408027	1	0.903175932	77	tags=25%, list=17%, signal=21%	NECTIN1/CCDC85A/KIT
**CC**	GO:0030425	dendrite	20	0.341661617	0.825066514	0.71761658	1	0.903175932	137	tags=40%, list=31%, signal=29%	OR11H4/ELAVL4/KCNK1/BACE1/HTR1E/PPP1CA/CPEB4/DTNBP1
**CC**	GO:0097447	dendritic tree	20	0.341661617	0.825066514	0.71761658	1	0.903175932	137	tags=40%, list=31%, signal=29%	OR11H4/ELAVL4/KCNK1/BACE1/HTR1E/PPP1CA/CPEB4/DTNBP1
**CC**	GO:0015629	actin cytoskeleton	12	-0.324621458	-0.814659866	0.742474916	1	0.903175932	72	tags=50%, list=16%, signal=43%	PARVB/PKNOX2/AXL/CTTNBP2NL/LIMA1/RAPGEF3
**CC**	GO:0031226	intrinsic component of plasma membrane	51	0.277323977	0.775922916	0.811188811	1	0.903175932	67	tags=18%, list=15%, signal=17%	OR4D6/TRHR/OR11H4/SLC10A2/CSF2/CD8B/LHCGR/ADAM23/KCNK1
**CC**	GO:0031252	cell leading edge	11	-0.3171236	-0.771766328	0.766871166	1	0.903175932	72	tags=55%, list=16%, signal=47%	PARVB/ATF4/MTMR14/CTTNBP2NL/LIMA1/RAPGEF3
**CC**	GO:0042995	cell projection	60	-0.208549462	-0.770669163	0.931034483	1	0.903175932	77	tags=22%, list=17%, signal=21%	NECTIN1/LIMA1/CPNE5/BBS10/BDKRB1/HHIP/CLU/MPL/SYT8/RAPGEF3/TBX3/ACE/OPN1MW
**CC**	GO:0005789	endoplasmic reticulum membrane	23	-0.243147617	-0.757688412	0.87755102	1	0.903175932	53	tags=26%, list=12%, signal=24%	NRAS/TRAM1/HSD3B7/HRAS/ART1/CYP26C1

**CC**	GO:0098827	endoplasmic reticulum subcompartment	23	-0.243147617	-0.757688412	0.87755102	1	0.903175932	53	tags=26%, list=12%, signal=24%	NRAS/TRAM1/HSD3B7/HRAS/ART1/CYP26C1
**CC**	GO:0042175	nuclear outer membrane-endoplasmic reticulum membrane network	24	-0.235923819	-0.744288678	0.890295359	1	0.903175932	53	tags=25%, list=12%, signal=23%	NRAS/TRAM1/HSD3B7/HRAS/ART1/CYP26C1
**CC**	GO:0005783	endoplasmic reticulum	51	-0.208564149	-0.740035601	0.958333333	1	0.903175932	61	tags=20%, list=14%, signal=19%	GPX8/BDKRB1/HSD3B7/CLU/CCR10/HRAS/ART1/CYP26C1/MPIG6B/LTBP1
**CC**	GO:0005819	spindle	12	0.339947107	0.732200828	0.819345661	1	0.903175932	106	tags=25%, list=24%, signal=20%	ASPM/ARL8A/NEK2
**CC**	GO:0000323	lytic vacuole	17	-0.253274782	-0.726527613	0.875	1	0.903175932	55	tags=29%, list=12%, signal=27%	MEAK7/TMEM79/NPRL2/NAPSA/ACE
**CC**	GO:0005764	lysosome	17	-0.253274782	-0.726527613	0.875	1	0.903175932	55	tags=29%, list=12%, signal=27%	MEAK7/TMEM79/NPRL2/NAPSA/ACE
**CC**	GO:0031984	organelle subcompartment	32	-0.223774079	-0.726444156	0.925531915	1	0.903175932	28	tags=28%, list=6%, signal=28%	TMEM79/NRAS/TRAM1/BICD1/HSD3B7/HRAS/ART1/CYP26C1/GOLGA6A
**CC**	GO:0005813	centrosome	17	-0.248886907	-0.71394084	0.890625	1	0.903175932	132	tags=35%, list=29%, signal=26%	SCLT1/TNKS2/FBXW11/CYLD/BICD1/RIC8B
**CC**	GO:0098793	presynapse	12	-0.284232165	-0.713300159	0.842809365	1	0.903175932	77	tags=25%, list=17%, signal=21%	NECTIN1/RAB26/SYT8
**CC**	GO:0016604	nuclear body	16	-0.253378838	-0.707568703	0.884758364	1	0.903175932	177	tags=56%, list=39%, signal=35%	ATF4/PATL1/BARD1/VPS72/TAP2/GCAT/FAM118B/CENPB/ZNF470
**CC**	GO:0005667	transcription regulator complex	14	0.315624622	0.698083988	0.87254902	1	0.903175932	71	tags=21%, list=16%, signal=19%	DEPDC1/MSX2/NKX2-5
**CC**	GO:0043005	neuron projection	34	-0.213334212	-0.697043875	0.967032967	1	0.903175932	106	tags=29%, list=24%, signal=24%	FBXW11/HRH2/HCN4/NECTIN1/CPNE5/BDKRB1/CLU/MPL/SYT8/OPN1MW
**CC**	GO:0010008	endosome membrane	11	0.322145888	0.679813135	0.859467456	1	0.903175932	114	tags=36%, list=25%, signal=28%	CD8B/ARL8A/BACE1/LEPROT
**CC**	GO:0005815	microtubule organizing center	21	-0.222660856	-0.67637689	0.920168067	1	0.903175932	132	tags=29%, list=29%, signal=21%	SCLT1/TNKS2/FBXW11/CYLD/BICD1/RIC8B
**CC**	GO:0030054	cell junction	44	-0.192245521	-0.671687116	0.98136646	1	0.903175932	82	tags=25%, list=18%, signal=23%	PLAA/HRH2/SYDE1/NECTIN1/LIMA1/CCDC85A/CLU/RAB26/SYT8/HRAS/KIT
**CC**	GO:1902494	catalytic complex	33	-0.202718938	-0.668683286	0.947089947	1	0.903175932	196	tags=52%, list=44%, signal=31%	INO80E/PPP2R2C/TRRAP/PRKAR1B/PPP3CC/PIGQ/BARD1/GNA11/GNG2/TRMT61A/DR1/VPS72/AIMP2/TSEN54/ANAPC7/FBXW11/HRAS
**CC**	GO:0000793	condensed chromosome	11	-0.274732616	-0.668601714	0.886503067	1	0.903175932	106	tags=45%, list=24%, signal=36%	FBXW11/TOP3B/CENPB/HORMAD1/CDX2
**CC**	GO:0005739	mitochondrion	26	0.264415073	0.665634753	0.920918367	1	0.903175932	15	tags=4%, list=3%, signal=4%	CKMT2
**CC**	GO:0015630	microtubule cytoskeleton	33	0.253077793	0.665448662	0.93603936	1	0.903175932	83	tags=12%, list=18%, signal=11%	SAA1/ASPM/ARL8A/FHDC1
**CC**	GO:0005856	cytoskeleton	54	0.235848728	0.664508185	0.948275862	1	0.903175932	66	tags=11%, list=15%, signal=11%	SAA1/KRTAP10-12/KRT39/ELAVL4/ASPM/ARL8A
**CC**	GO:0043025	neuronal cell body	14	0.299268253	0.661907724	0.908963585	1	0.903175932	116	tags=36%, list=26%, signal=27%	ELAVL4/KCNK1/TIAM2/BACE1/PPP1CA
**CC**	GO:0044297	cell body	15	0.293882706	0.660108658	0.909344491	1	0.903175932	116	tags=33%, list=26%, signal=26%	ELAVL4/KCNK1/TIAM2/BACE1/PPP1CA
**CC**	GO:0045202	synapse	32	0.244376152	0.639845992	0.943488943	1	0.903175932	116	tags=28%, list=26%, signal=22%	OR11H4/ADAM23/ELAVL4/ARL8A/KCNK1/TIAM2/BACE1/HTR1E/PPP1CA
**CC**	GO:0070161	anchoring junction	25	-0.200487288	-0.637174016	0.986784141	1	0.903175932	3	tags=16%, list=1%, signal=17%	NECTIN1/LIMA1/CCDC85A/KIT
**CC**	GO:0036477	somatodendritic compartment	26	0.246683004	0.620996293	0.947704082	1	0.903175932	137	tags=35%, list=31%, signal=26%	OR11H4/ELAVL4/KCNK1/TIAM2/BACE1/HTR1E/PPP1CA/CPEB4/DTNBP1
**CC**	GO:0005829	cytosol	120	0.202557921	0.619426784	0.990405117	1	0.903175932	107	tags=20%, list=24%, signal=21%	FABP2/ERCC6L/AKAIN1/GOLT1A/KRTAP10-12/STAC/KRT39/MSX2/TPTE2/ELAVL4/CIP2A/BUB1/TBC1D23/SULT6B1/SNRPE/ADH7/POGLUT2/NLRP5/PPM1N/TIAM2/TDRP/KRTAP10-9/NEK2/PPP4R4
**CC**	GO:0098978	glutamatergic synapse	12	0.287284677	0.618772963	0.918918919	1	0.903175932	61	tags=17%, list=14%, signal=15%	ADAM23/ELAVL4
**CC**	GO:0016607	nuclear speck	10	0.295939987	0.608556115	0.921658986	1	0.903175932	49	tags=10%, list=11%, signal=9%	MSX2

**CC**	GO:0140535	intracellular protein-containing complex	21	0.242990654	0.587927699	0.971204188	1	0.903175932	345	tags=100%, list=77%, signal=24%	NCBP2L/DTNBP1/RNF11/KANSL2/CREBBP/GTF2H1/EME1/KLHL24/TRRAP/PRKAR1B/PPP3CC/BARD1/PFDN6/TRAPPC9/DR1/VPS72/AIMP2/SCLT1/TSEN54/ANAPC7/FBXW11
**CC**	GO:0098687	chromosomal region	13	0.268612735	0.586170057	0.950564972	1	0.903175932	116	tags=31%, list=26%, signal=24%	ERCC6L/BUB1/NEK2/PPP1CA
**CC**	GO:0098590	plasma membrane region	25	0.229672162	0.571189816	0.978064516	1	0.903175932	67	tags=16%, list=15%, signal=14%	SLC10A2/ADAM23/ASPM/KCNK1
**CC**	GO:1990234	transferase complex	17	0.240740741	0.560628473	0.974530831	1	0.903175932	345	tags=100%, list=77%, signal=24%	SNRPE/GNPTG/RNF11/KANSL2/CREBBP/RB1CC1/GTF2H1/KLHL24/TRRAP/PRKAR1B/PIGQ/BARD1/TRMT61A/DR1/VPS72/ANAPC7/FBXW11
**CC**	GO:0030424	axon	15	0.239024572	0.53688831	0.981868898	1	0.903175932	137	tags=40%, list=31%, signal=29%	ELAVL4/ARL8A/TIAM2/BACE1/CPEB4/DTNBP1
**CC**	GO:0140513	nuclear protein-containing complex	23	-0.164319249	-0.512046107	1	1	0.903175932	380	tags=43%, list=85%, signal=7%	ATF4/BARD1/NUP214/DR1/VPS72/TSEN54/IMP3/ANAPC7/SMAD9/MCM2
**CC**	GO:0098791	Golgi apparatus subcompartment	10	0.240496098	0.494544089	0.978494624	1	0.903175932	108	tags=30%, list=24%, signal=23%	GOLT1A/TBC1D23/BACE1
**CC**	GO:0030135	coated vesicle	10	-0.200455581	-0.477038803	0.994301994	1	0.903175932	362	tags=50%, list=81%, signal=10%	FZD4/RAB8B/LDLRAP1/SLC28A2/SAR1A
**CC**	GO:0005769	early endosome	10	0.218360558	0.449025675	0.993855607	1	0.903175932	46	tags=10%, list=10%, signal=9%	CD8B
**KEGG**	hsa04740	Olfactory transduction	15	0.871497524	1.986903042	4.73984E-06	5.21382E-05	2.99358E-05	51	tags=67%, list=12%, signal=61%	OR10G6/OR8S1/OR10D3/OR56B1/OR4D6/OR13G1/OR11H4/OR9A2/OR11H1/OR11H2
**KEGG**	hsa05200	Pathways in cancer	16	-0.629382171	-1.70622542	0.010361834	0.056990086	0.03272158	60	tags=38%, list=14%, signal=34%	BDKRB1/HHIP/DAPK2/HRAS/FGF21/KIT
**KEGG**	hsa04014	Ras signaling pathway	12	-0.664262944	-1.669305447	0.020421589	0.074879159	0.042992819	37	tags=33%, list=8%, signal=31%	SHC2/HRAS/FGF21/KIT
**KEGG**	hsa04151	PI3K-Akt signaling pathway	11	-0.64919189	-1.586591724	0.04284637	0.105719165	0.060699999	28	tags=36%, list=6%, signal=35%	NRAS/HRAS/FGF21/KIT
**KEGG**	hsa04010	MAPK signaling pathway	11	-0.640991302	-1.566549906	0.048054166	0.105719165	0.060699999	28	tags=36%, list=6%, signal=35%	NRAS/HRAS/FGF21/KIT
**KEGG**	hsa04080	Neuroactive ligand-receptor interaction	11	0.577169276	1.225972123	0.225947522	0.414237123	0.237839497	48	tags=27%, list=11%, signal=25%	GPHB5/TRHR/LHCGR
**KEGG**	hsa05010	Alzheimer disease	10	0.490962821	1.009058265	0.483180428	0.753164557	0.43243898	12	tags=10%, list=3%, signal=10%	DKK4
**KEGG**	hsa01100	Metabolic pathways	23	-0.300336601	-0.899221715	0.62173913	0.753164557	0.43243898	75	tags=26%, list=17%, signal=23%	AOC2/GAL3ST1/GPX8/HSD3B7/CYP26C1/TKTL1
**KEGG**	hsa05022	Pathways of neurodegeneration - multiple diseases	10	0.434933297	0.893902795	0.636085627	0.753164557	0.43243898	12	tags=10%, list=3%, signal=10%	DKK4
**KEGG**	hsa05166	Human T-cell leukemia virus 1 infection	10	0.411536968	0.84581716	0.703363914	0.753164557	0.43243898	49	tags=20%, list=11%, signal=18%	CSF2/MSX2
**KEGG**	hsa05168	Herpes simplex virus 1 infection	11	-0.315501587	-0.771069716	0.753164557	0.753164557	0.43243898	76	tags=55%, list=17%, signal=46%	TAP2/EIF2B5/NECTIN1/ZNF528/ZNF470/ZNF596
**MF**	GO:0004984	olfactory receptor activity	14	0.880421956	1.947276692	1.55E-06	0.000252852	0.00022837	51	tags=71%, list=11%, signal=65%	OR10G6/OR8S1/OR10D3/OR56B1/OR4D6/OR13G1/OR11H4/OR9A2/OR11H1/OR11H2
**MF**	GO:0004930	G protein-coupled receptor activity	30	0.745865712	1.910405467	1.27E-05	0.001301829	0.001175781	51	tags=47%, list=11%, signal=44%	TAS2R3/OR10G6/OR8S1/OR10D3/OR56B1/OR4D6/OR13G1/TRHR/OR11H4/OR9A2/MRGPRX1/OR11H1/LHCGR/OR11H2
**MF**	GO:0046982	protein heterodimerization activity	10	0.857412241	1.763139437	0.000458953	0.034087692	0.030787183	16	tags=30%, list=4%, signal=30%	GPHB5/IL17F/H2BW1
**MF**	GO:0004888	transmembrane signaling receptor activity	40	0.625465035	1.686222954	0.000939028	0.054604087	0.049317097	51	tags=35%, list=11%, signal=34%	TAS2R3/OR10G6/OR8S1/OR10D3/OR56B1/OR4D6/OR13G1/TRHR/OR11H4/OR9A2/MRGPRX1/OR11H1/LHCGR/OR11H2
**MF**	GO:1901363	heterocyclic compound binding	133	-0.34134746	-1.416655842	0.000891351	0.054604087	0.049317097	47	tags=18%, list=10%, signal=23%	HDX/ZNF528/ZEB1/DAPK2/RAB26/ZNF470/GBP5/SALL3/ZNF596/RAPGEF3/HRAS/TBX3/SIX5/CYP26C1/HNF1A/NPC1L1/ACE/MCM2/TKTL1/CDX2/DAZ1/OVOL1/KIT/RPS4Y1

**MF**	GO:0046872	metal ion binding	92	-0.382582794	-1.521438193	0.001261627	0.05792249	0.052314199	78	tags=26%, list=17%, signal=27%	SMAD9/AOC2/LIMA1/PCDHB7/TNFSF10/CPNE5/CRACR2B/HHIP/NID2/ZNF528/ZEB1/ZNF470/PRRG3/SYT8/SALL3/ZNF596/CYP26C1/ACE/MCM2/TKTL1/LTBP1/TRIM35/OVOL1/KIT
**MF**	GO:0038023	signaling receptor activity	46	0.612290751	1.690891998	0.001646402	0.058483046	0.05282048	51	tags=33%, list=11%, signal=32%	TAS2R3/OR10G6/OR8S1/OR10D3/OR56B1/OR4D6/OR13G1/TRHR/OR11H4/OR9A2/MRGPRX1/OR11H1/CD8B/LHCGR/OR11H2
**MF**	GO:0060089	molecular transducer activity	46	0.612290751	1.690891998	0.001646402	0.058483046	0.05282048	51	tags=33%, list=11%, signal=32%	TAS2R3/OR10G6/OR8S1/OR10D3/OR56B1/OR4D6/OR13G1/TRHR/OR11H4/OR9A2/MRGPRX1/OR11H1/CD8B/LHCGR/OR11H2
**MF**	GO:0000978	RNA polymerase II cis-regulatory region sequence-specific DNA binding	26	-0.590617166	-1.876055446	0.002964895	0.086511395	0.07813501	47	tags=46%, list=10%, signal=44%	HMGA2/SMAD9/HDX/ZNF528/ZEB1/ZNF470/SALL3/TBX3/SIX5/HNF1A/CDX2/OVOL1
**MF**	GO:0000987	cis-regulatory region sequence-specific DNA binding	26	-0.590617166	-1.876055446	0.002964895	0.086511395	0.07813501	47	tags=46%, list=10%, signal=44%	HMGA2/SMAD9/HDX/ZNF528/ZEB1/ZNF470/SALL3/TBX3/SIX5/HNF1A/CDX2/OVOL1
**MF**	GO:0043169	cation binding	94	-0.375405617	-1.491187834	0.003208969	0.090404412	0.081651089	78	tags=26%, list=17%, signal=27%	SMAD9/AOC2/LIMA1/PCDHB7/TNFSF10/CPNE5/CRACR2B/HHIP/NID2/ZNF528/ZEB1/ZNF470/PRRG3/SYT8/SALL3/ZNF596/CYP26C1/ACE/MCM2/TKTL1/LTBP1/TRIM35/OVOL1/KIT
**MF**	GO:0097159	organic cyclic compound binding	132	-0.32822192	-1.363396078	0.005232325	0.122137405	0.110311565	47	tags=17%, list=10%, signal=22%	HDX/ZNF528/ZEB1/DAPK2/RAB26/ZNF470/GBP5/SALL3/ZNF596/RAPGEF3/HRAS/TBX3/SIX5/CYP26C1/HNF1A/NPC1L1/MCM2/TKTL1/CDX2/DAZ1/OVOL1/KIT/RPS4Y1
**MF**	GO:0043167	ion binding	136	-0.317553441	-1.32982126	0.005999377	0.136152538	0.122969695	88	tags=26%, list=20%, signal=31%	HCN4/UBE2E3/EEF1AKMT4-ECE2/ADCK2/SMAD9/AOC2/SAR1A/LIMA1/PCDHB7/TNFSF10/CPNE5/BBS10/MFHAS1/CRACR2B/HHIP/NID2/ZNF528/ZEB1/DAPK2/RAB26/ZNF470/PRRG3/SYT8/GBP5/SALL3/ZNF596/RAPGEF3/HRAS/CYP26C1/ACE/MCM2/TKTL1/LTBP1/TRIM35/OVOL1/KIT
**MF**	GO:1990837	sequence-specific double-stranded DNA binding	38	-0.45428205	-1.537961126	0.009110347	0.173436807	0.15664395	47	tags=32%, list=10%, signal=31%	HDX/ZNF528/ZEB1/ZNF470/SALL3/ZNF596/TBX3/SIX5/HNF1A/MCM2/CDX2/OVOL1
**MF**	GO:0000977	RNA polymerase II transcription regulatory region sequence-specific DNA binding	32	-0.480719704	-1.560574044	0.015858149	0.211464192	0.190989369	47	tags=34%, list=10%, signal=33%	HDX/ZNF528/ZEB1/ZNF470/SALL3/ZNF596/TBX3/SIX5/HNF1A/CDX2/OVOL1
**MF**	GO:0003690	double-stranded DNA binding	40	-0.444231667	-1.542066382	0.014123624	0.211464192	0.190989369	47	tags=30%, list=10%, signal=29%	HDX/ZNF528/ZEB1/ZNF470/SALL3/ZNF596/TBX3/SIX5/HNF1A/MCM2/CDX2/OVOL1
**MF**	GO:0000976	transcription cis-regulatory region binding	35	-0.460938267	-1.529500038	0.017116247	0.214178802	0.193441139	47	tags=31%, list=10%, signal=31%	HDX/ZNF528/ZEB1/ZNF470/SALL3/ZNF596/TBX3/SIX5/HNF1A/CDX2/OVOL1
**MF**	GO:0001067	transcription regulatory region nucleic acid binding	35	-0.460938267	-1.529500038	0.017116247	0.214178802	0.193441139	47	tags=31%, list=10%, signal=31%	HDX/ZNF528/ZEB1/ZNF470/SALL3/ZNF596/TBX3/SIX5/HNF1A/CDX2/OVOL1
**MF**	GO:0019904	protein domain specific binding	14	-0.603574639	-1.630390973	0.031428102	0.316331055	0.285702596	30	tags=71%, list=7%, signal=69%	NLGN1/ATF4/FZD4/BOK/RAB8B/WARS1/HMGA2/PMEPA1/RAPGEF3/KIT
**MF**	GO:0005509	calcium ion binding	16	-0.583230122	-1.628689214	0.031979477	0.316331055	0.285702596	70	tags=38%, list=16%, signal=33%	PCDHB7/CRACR2B/NID2/PRRG3/SYT8/LTBP1
**MF**	GO:0043565	sequence-specific DNA binding	40	-0.41998469	-1.457897579	0.033853721	0.316331055	0.285702596	47	tags=30%, list=10%, signal=29%	HDX/ZNF528/ZEB1/ZNF470/SALL3/ZNF596/TBX3/SIX5/HNF1A/MCM2/CDX2/OVOL1
**MF**	GO:0019899	enzyme binding	42	-0.403266907	-1.431073701	0.037001655	0.316331055	0.285702596	117	tags=33%, list=26%, signal=27%	IPO11/WARS1/TNKS2/ANAPC7/CYLD/LCOR/BICD1/MFHAS1/CLU/SHC2/NPC1L1/ACE/MCM2/KIT
**MF**	GO:0036094	small molecule binding	58	-0.365476406	-1.361691416	0.037813702	0.316331055	0.285702596	40	tags=29%, list=9%, signal=31%	HCN4/UBE2E3/ADCK2/SAR1A/BBS10/MFHAS1/DAPK2/RAB26/GBP5/RAPGEF3/HRAS/CYP26C1/NPC1L1/MCM2/TKTL1/CDX2/KIT

**MF**	GO:0000981	DNA-binding transcription factor activity, RNA polymerase II-specific	31	-0.451711283	-1.48358384	0.042363625	0.327229708	0.295545996	47	tags=35%, list=10%, signal=34%	HDX/ZNF528/ZEB1/ZNF470/SALL3/ZNF596/TBX3/SIX5/HNF1A/CDX2/OVOL1
**MF**	GO:0030545	signaling receptor regulator activity	16	0.64121075	1.474491146	0.043656207	0.327921973	0.296171234	40	tags=31%, list=9%, signal=30%	GPHB5/IL17F/DKK4/CCL13/CSF2
**MF**	GO:0019900	kinase binding	11	-0.611858978	-1.489047668	0.048424809	0.341060942	0.308038034	117	tags=73%, list=26%, signal=55%	PRKAR1B/ATF4/BARD1/WARS1/CYLD/BICD1/SHC2/ACE
**MF**	GO:0019901	protein kinase binding	10	-0.649194958	-1.54493671	0.062108921	0.373110209	0.336984161	117	tags=50%, list=26%, signal=38%	WARS1/CYLD/BICD1/SHC2/ACE
**MF**	GO:0003700	DNA-binding transcription factor activity	33	-0.440665852	-1.453568635	0.061346987	0.373110209	0.336984161	47	tags=33%, list=10%, signal=32%	HDX/ZNF528/ZEB1/ZNF470/SALL3/ZNF596/TBX3/SIX5/HNF1A/CDX2/OVOL1
**MF**	GO:0030546	signaling receptor activator activity	13	0.629730557	1.374205869	0.077683616	0.417549435	0.3771206	40	tags=31%, list=9%, signal=29%	GPHB5/IL17F/CCL13/CSF2
**MF**	GO:0048018	receptor ligand activity	13	0.629730557	1.374205869	0.077683616	0.417549435	0.3771206	40	tags=31%, list=9%, signal=29%	GPHB5/IL17F/CCL13/CSF2
**MF**	GO:0140110	transcription regulator activity	43	-0.374662664	-1.319648003	0.08205729	0.430959627	0.389232363	58	tags=37%, list=13%, signal=36%	AHDC1/LCOR/HMGA2/SMAD9/CDYL2/HDX/ZNF528/ZEB1/ZNF470/SALL3/ZNF596/TBX3/SIX5/HNF1A/CDX2/OVOL1
**MF**	GO:0003676	nucleic acid binding	84	-0.307554614	-1.212288191	0.087402847	0.4407909	0.398111732	47	tags=17%, list=10%, signal=18%	HDX/ZNF528/ZEB1/ZNF470/SALL3/ZNF596/TBX3/SIX5/HNF1A/MCM2/CDX2/DAZ1/OVOL1/RPS4Y1
**MF**	GO:0004672	protein kinase activity	10	-0.592878266	-1.41091576	0.094017094	0.450304705	0.406704372	79	tags=50%, list=18%, signal=42%	ADCK2/NPRL2/DAPK2/LTBP1/KIT
**MF**	GO:0005488	binding	371	-0.261695941	-1.106141832	0.092591008	0.450304705	0.406704372	81	tags=20%, list=18%, signal=93%	PREP/HMGA2/ADCK2/SMAD9/NECTIN1/AOC2/CENPB/APLN/SAR1A/LIMA1/GSTCD/PCDHB7/CCDC85A/TNFSF10/COBLL1/CPNE5/BBS10/MFHAS1/GPX8/BDKRB1/EMSY/CDYL2/CRACR2B/HHIP/NPRL2/FCGBP/HSD3B7/RIC8B/CLU/NID2/KRT8/MPL/HDX/ZNF528/CCR10/ZEB1/HORMAD1/TTC39B/DAPK2/RAB26/ZNF470/SHC2/PRRG3/SYT8/GBP5/SALL3/ZNF596/PMEPA1/RAPGEF3/HRAS/TSPY10/TBX3/SIX5/NAPSA/CYP26C1/PTMS/HNF1A/NPC1L1/ACE/MPIG6B/MCM2/BNIP5/OPN1MW/FGF21/GOLGA6A/TKTL1/CDX2/LTBP1/DAZ1/TRIM35/OVOL1/KIT/RPS4Y1
**MF**	GO:0005525	GTP binding	17	-0.475086833	-1.362803277	0.1015625	0.458434047	0.414046598	143	tags=53%, list=32%, signal=38%	RAB8B/EHD4/GBP4/NRAS/SAR1A/MFHAS1/RAB26/GBP5/HRAS
**MF**	GO:0019001	guanyl nucleotide binding	17	-0.475086833	-1.362803277	0.1015625	0.458434047	0.414046598	143	tags=53%, list=32%, signal=38%	RAB8B/EHD4/GBP4/NRAS/SAR1A/MFHAS1/RAB26/GBP5/HRAS
**MF**	GO:0032561	guanyl ribonucleotide binding	17	-0.475086833	-1.362803277	0.1015625	0.458434047	0.414046598	143	tags=53%, list=32%, signal=38%	RAB8B/EHD4/GBP4/NRAS/SAR1A/MFHAS1/RAB26/GBP5/HRAS
**MF**	GO:0005215	transporter activity	23	0.536776405	1.321507668	0.103038309	0.46001256	0.415472273	69	tags=26%, list=15%, signal=23%	SLC10A5/FABP2/SLC10A2/GJB5/KCNK1/ANO5
**MF**	GO:0016773	phosphotransferase activity, alcohol group as acceptor	12	-0.538949704	-1.352531333	0.117056856	0.49351643	0.445732162	79	tags=42%, list=18%, signal=35%	ADCK2/NPRL2/DAPK2/LTBP1/KIT
**MF**	GO:0005102	signaling receptor binding	40	0.471798049	1.271944322	0.134545455	0.507453416	0.458319712	94	tags=32%, list=21%, signal=28%	GPHB5/IL17F/DKK4/CCL13/SAA1/CSF2/CD8B/ADAM23/ADAM11/LBP/IGF1/ADH7/IGFL3
**MF**	GO:0001217	DNA-binding transcription repressor activity	10	-0.552275918	-1.314291384	0.148148148	0.523969857	0.473236964	44	tags=50%, list=10%, signal=46%	ZEB1/ZNF596/TBX3/CDX2/OVOL1
**MF**	GO:0001227	DNA-binding transcription repressor activity, RNA polymerase II-specific	10	-0.552275918	-1.314291384	0.148148148	0.523969857	0.473236964	44	tags=50%, list=10%, signal=46%	ZEB1/ZNF596/TBX3/CDX2/OVOL1
**MF**	GO:0003723	RNA binding	35	-0.357550206	-1.186434482	0.156756757	0.537920929	0.485837237	6	tags=6%, list=1%, signal=6%	DAZ1/RPS4Y1
**MF**	GO:0000166	nucleotide binding	50	-0.328571378	-1.159062082	0.183673469	0.586176658	0.52942065	95	tags=30%, list=21%, signal=27%	NRAS/HCN4/UBE2E3/ADCK2/SAR1A/BBS10/MFHAS1/DAPK2/RAB26/GBP5/RAPGEF3/HRAS/MCM2/CDX2/KIT
**MF**	GO:1901265	nucleoside phosphate binding	50	-0.328571378	-1.159062082	0.183673469	0.586176658	0.52942065	95	tags=30%, list=21%, signal=27%	NRAS/HCN4/UBE2E3/ADCK2/SAR1A/BBS10/MFHAS1/DAPK2/RAB26/GBP5/RAPGEF3/HRAS/MCM2/CDX2/KIT

**MF**	GO:0098772	molecular function regulator activity	43	0.444367837	1.214903991	0.200716846	0.608151064	0.549267405	18	tags=12%, list=4%, signal=12%	GPHB5/IL17F/DKK4/CCL13/DEPDC1
**MF**	GO:0140096	catalytic activity, acting on a protein	51	-0.3229247	-1.145814254	0.201388889	0.608151064	0.549267405	24	tags=24%, list=5%, signal=25%	UBE2E3/EEF1AKMT4-ECE2/PREP/ADCK2/NPRL2/DAPK2/ART1/NAPSA/ACE/LTBP1/TRIM35/KIT
**MF**	GO:0003924	GTPase activity	15	-0.439601014	-1.211606498	0.224561404	0.626166098	0.565538149	115	tags=40%, list=26%, signal=31%	GBP4/NRAS/SAR1A/RAB26/GBP5/HRAS
**MF**	GO:0022857	transmembrane transporter activity	22	0.482679351	1.183342161	0.237971391	0.642494328	0.580285413	69	tags=23%, list=15%, signal=20%	SLC10A5/SLC10A2/GJB5/KCNK1/ANO5
**MF**	GO:0003677	DNA binding	55	-0.309609105	-1.127252681	0.236641221	0.642494328	0.580285413	47	tags=22%, list=10%, signal=22%	HDX/ZNF528/ZEB1/ZNF470/SALL3/ZNF596/TBX3/SIX5/HNF1A/MCM2/CDX2/OVOL1
**MF**	GO:0043168	anion binding	60	-0.298412417	-1.102746779	0.267241379	0.678062754	0.61240996	40	tags=27%, list=9%, signal=28%	HCN4/UBE2E3/ADCK2/SAR1A/BBS10/MFHAS1/DAPK2/RAB26/GBP5/RAPGEF3/HRAS/CYP26C1/ACE/MCM2/TKTL1/KIT
**MF**	GO:0019955	cytokine binding	10	-0.473253271	-1.126235414	0.304843305	0.7233603	0.653321613	45	tags=30%, list=10%, signal=28%	CCR10/LTBP1/KIT
**MF**	GO:0003824	catalytic activity	118	-0.252970132	-1.051045501	0.30293719	0.7233603	0.653321613	87	tags=22%, list=19%, signal=24%	UBE2E3/ABHD3/EEF1AKMT4-ECE2/PREP/HMGA2/ADCK2/AOC2/SAR1A/GAL3ST1/GPX8/CDYL2/NPRL2/HSD3B7/DAPK2/RAB26/GBP5/HRAS/ART1/NAPSA/CYP26C1/ACE/MCM2/TKTL1/LTBP1/TRIM35/KIT
**MF**	GO:0008270	zinc ion binding	17	-0.3869199	-1.109893331	0.3125	0.731554441	0.660722365	68	tags=29%, list=15%, signal=26%	TNFSF10/HHIP/ZEB1/ACE/TRIM35
**MF**	GO:0015075	ion transmembrane transporter activity	18	0.467594687	1.10648394	0.322281167	0.741800023	0.669975927	69	tags=22%, list=15%, signal=20%	SLC10A5/SLC10A2/KCNK1/ANO5
**MF**	GO:0015318	inorganic molecular entity transmembrane transporter activity	18	0.467594687	1.10648394	0.322281167	0.741800023	0.669975927	69	tags=22%, list=15%, signal=20%	SLC10A5/SLC10A2/KCNK1/ANO5
**MF**	GO:0046914	transition metal ion binding	22	-0.345924939	-1.065399419	0.35193133	0.778678108	0.703283327	76	tags=32%, list=17%, signal=28%	AOC2/TNFSF10/HHIP/ZEB1/CYP26C1/ACE/TRIM35
**MF**	GO:0001216	DNA-binding transcription activator activity	10	-0.453312853	-1.078781741	0.356125356	0.779689718	0.704196988	26	tags=30%, list=6%, signal=29%	TBX3/SIX5/HNF1A
**MF**	GO:0005198	structural molecule activity	15	-0.375214616	-1.034147903	0.357894737	0.779733333	0.70423638	50	tags=27%, list=11%, signal=25%	NID2/KRT8/LTBP1/RPS4Y1
**MF**	GO:0016740	transferase activity	45	-0.30095221	-1.067518443	0.373493976	0.800904405	0.723357583	87	tags=24%, list=19%, signal=22%	UBE2E3/EEF1AKMT4-ECE2/ADCK2/GAL3ST1/NPRL2/DAPK2/ART1/TKTL1/LTBP1/TRIM35/KIT
**MF**	GO:0008324	cation transmembrane transporter activity	14	0.480940479	1.063721979	0.386554622	0.811119384	0.732583506	67	tags=21%, list=15%, signal=19%	SLC10A5/SLC10A2/KCNK1
**MF**	GO:0022890	inorganic cation transmembrane transporter activity	14	0.480940479	1.063721979	0.386554622	0.811119384	0.732583506	67	tags=21%, list=15%, signal=19%	SLC10A5/SLC10A2/KCNK1
**MF**	GO:0017076	purine nucleotide binding	47	-0.300644136	-1.0576949	0.392156863	0.811119384	0.732583506	95	tags=30%, list=21%, signal=26%	NRAS/HCN4/UBE2E3/ADCK2/SAR1A/BBS10/MFHAS1/DAPK2/RAB26/GBP5/RAPGEF3/HRAS/MCM2/KIT
**MF**	GO:0032553	ribonucleotide binding	47	-0.300644136	-1.0576949	0.392156863	0.811119384	0.732583506	95	tags=30%, list=21%, signal=26%	NRAS/HCN4/UBE2E3/ADCK2/SAR1A/BBS10/MFHAS1/DAPK2/RAB26/GBP5/RAPGEF3/HRAS/MCM2/KIT
**MF**	GO:0032555	purine ribonucleotide binding	47	-0.300644136	-1.0576949	0.392156863	0.811119384	0.732583506	95	tags=30%, list=21%, signal=26%	NRAS/HCN4/UBE2E3/ADCK2/SAR1A/BBS10/MFHAS1/DAPK2/RAB26/GBP5/RAPGEF3/HRAS/MCM2/KIT
**MF**	GO:0046873	metal ion transmembrane transporter activity	11	0.498669638	1.052324994	0.420118343	0.830026332	0.749659806	67	tags=27%, list=15%, signal=24%	SLC10A5/SLC10A2/KCNK1
**MF**	GO:0016301	kinase activity	13	-0.381412064	-0.99123245	0.442176871	0.846666667	0.764688956	55	tags=38%, list=12%, signal=35%	ADCK2/NPRL2/DAPK2/LTBP1/KIT
**MF**	GO:0046983	protein dimerization activity	25	0.404817406	1.006772337	0.47483871	0.87178253	0.787372999	71	tags=24%, list=16%, signal=21%	GPHB5/IL17F/H2BW1/CIP2A/ANO5/NKX2-5
**MF**	GO:0003729	mRNA binding	12	-0.396118382	-0.994086312	0.47826087	0.872185559	0.787737006	6	tags=17%, list=1%, signal=17%	DAZ4/DAZ1
**MF**	GO:0016772	transferase activity, transferring phosphorus-containing groups	17	-0.331964985	-0.952253227	0.5078125	0.89699424	0.81014361	55	tags=29%, list=12%, signal=27%	ADCK2/NPRL2/DAPK2/LTBP1/KIT
**MF**	GO:0008092	cytoskeletal protein binding	22	-0.301880921	-0.929750135	0.566523605	0.937590565	0.846809233	83	tags=27%, list=18%, signal=23%	FBXW11/BICD1/LIMA1/COBLL1/CLU/NPC1L1
**MF**	GO:0042803	protein homodimerization activity	17	-0.318164992	-0.912667462	0.5859375	0.965130261	0.871682423	34	tags=18%, list=8%, signal=17%	GBP5/NPC1L1/KIT

**MF**	GO:0035639	purine ribonucleoside triphosphate binding	44	-0.267403269	-0.934280959	0.596273292	0.974310559	0.879973848	95	tags=27%, list=21%, signal=24%	NRAS/UBE2E3/ADCK2/SAR1A/BBS10/MFHAS1/DAPK2/RAB26/GBP5/HRAS/MCM2/KIT
**MF**	GO:0003674	molecular_function	411	-0.222312398	-0.932897219	0.71589716	1	0.903175932	92	tags=21%, list=20%, signal=199%	LCOR/TOP3B/TRAM1/FAM118B/HCN4/UBE2E3/ABHD3/EEF1AKMT4-ECE2/MESD/BICD1/SYDE1/PREP/HMGA2/ADCK2/SMAD9/NECTIN1/AOC2/CENPB/APLN/SAR1A/LIMA1/GSTCD/PCDHB7/CCDC85A/TNFSF10/GAL3ST1/COBLL1/CPNE5/BBS10/MFHAS1/GPX8/BDKRB1/EMSY/CDYL2/CRACR2B/HHIP/NPRL2/FCGBP/HSD3B7/RIC8B/CLU/NID2/KRT8/MPL/HDX/ZNF528/CCR10/ZEB1/HORMAD1/TTC39B/DAPK2/RAB26/ZNF470/SHC2/PRRG3/SYT8/GBP5/SALL3/ZNF596/PMEPA1/RAPGEF3/HRAS/TSPY10/TBX3/ART1/SIX5/NAPSA/CYP26C1/PTMS/HNF1A/NPC1L1/KCNA7/ACE/MPIG6B/MCM2/BNIP5/OPN1MW/FGF21/GOLGA6A/TKTL1/CDX2/LTBP1/DAZ1/TRIM35/OVOL1/KIT/RPS4Y1
**MF**	GO:0097367	carbohydrate derivative binding	55	-0.242115552	-0.881516081	0.755725191	1	0.903175932	88	tags=27%, list=20%, signal=25%	NRAS/HCN4/UBE2E3/ADCK2/SAR1A/BBS10/MFHAS1/DAPK2/RAB26/GBP5/RAPGEF3/HRAS/MPIG6B/MCM2/KIT
**MF**	GO:0015267	channel activity	12	0.401058379	0.863826375	0.679943101	1	0.903175932	124	tags=50%, list=28%, signal=37%	GJB5/KCNK1/ANO5/TRPC3/SLC26A8/GJA3
**MF**	GO:0022803	passive transmembrane transporter activity	12	0.401058379	0.863826375	0.679943101	1	0.903175932	124	tags=50%, list=28%, signal=37%	GJB5/KCNK1/ANO5/TRPC3/SLC26A8/GJA3
**MF**	GO:0004175	endopeptidase activity	11	-0.352653061	-0.858232432	0.665644172	1	0.903175932	85	tags=36%, list=19%, signal=30%	EEF1AKMT4-ECE2/PREP/NAPSA/ACE
**MF**	GO:0008289	lipid binding	18	-0.296716813	-0.854259859	0.689516129	1	0.903175932	64	tags=22%, list=14%, signal=20%	CPNE5/SYT8/CYP26C1/NPC1L1
**MF**	GO:0005543	phospholipid binding	12	-0.338619403	-0.849788671	0.672240803	1	0.903175932	64	tags=42%, list=14%, signal=37%	AXL/SNX11/LDLRAP1/CPNE5/SYT8
**MF**	GO:0042802	identical protein binding	42	-0.235477687	-0.83563992	0.796511628	1	0.903175932	100	tags=29%, list=22%, signal=24%	TG/TMEM79/FAM118B/HCN4/MESD/NECTIN1/TNFSF10/DAPK2/GBP5/NPC1L1/OPN1MW/KIT
**MF**	GO:0016788	hydrolase activity, acting on ester bonds	15	0.364788662	0.81937504	0.736401674	1	0.903175932	161	tags=47%, list=36%, signal=31%	TPTE2/PPM1N/PPP1CA/PLA2G4F/MRPL58/PLA2G4A/PTPRG
**MF**	GO:0140297	DNA-binding transcription factor binding	11	-0.329964267	-0.803015958	0.726993865	1	0.903175932	92	tags=36%, list=20%, signal=30%	LCOR/HMGA2/BBS10/TBX3
**MF**	GO:0061629	RNA polymerase II-specific DNA-binding transcription factor binding	10	-0.335482879	-0.798373138	0.763532764	1	0.903175932	92	tags=40%, list=20%, signal=33%	LCOR/HMGA2/BBS10/TBX3
**MF**	GO:0008233	peptidase activity	16	-0.28570888	-0.797851403	0.758364312	1	0.903175932	102	tags=31%, list=23%, signal=25%	CYLD/EEF1AKMT4-ECE2/PREP/NAPSA/ACE
**MF**	GO:0030554	adenyl nucleotide binding	32	-0.239576714	-0.7777447	0.888297872	1	0.903175932	88	tags=25%, list=20%, signal=22%	HCN4/UBE2E3/ADCK2/BBS10/DAPK2/RAPGEF3/MCM2/KIT
**MF**	GO:0032559	adenyl ribonucleotide binding	32	-0.239576714	-0.7777447	0.888297872	1	0.903175932	88	tags=25%, list=20%, signal=22%	HCN4/UBE2E3/ADCK2/BBS10/DAPK2/RAPGEF3/MCM2/KIT
**MF**	GO:0016787	hydrolase activity	54	-0.212782596	-0.766312071	0.939393939	1	0.903175932	39	tags=22%, list=9%, signal=23%	CYLD/NRAS/ABHD3/EEF1AKMT4-ECE2/PREP/SAR1A/RAB26/GBP5/HRAS/NAPSA/ACE/MCM2
**MF**	GO:0030234	enzyme regulator activity	20	-0.255307402	-0.755961734	0.852173913	1	0.903175932	130	tags=45%, list=29%, signal=33%	RPS27L/WARS1/PPP1R1A/EIF2B5/PLAA/SYDE1/NPRL2/RIC8B/RAPGEF3
**MF**	GO:0003682	chromatin binding	14	0.328220599	0.725943187	0.844537815	1	0.903175932	71	tags=21%, list=16%, signal=19%	TSPYL6/EOMES/NKX2-5
**MF**	GO:0017111	ribonucleoside triphosphate phosphatase activity	19	-0.2467613	-0.721301005	0.893162393	1	0.903175932	39	tags=32%, list=9%, signal=30%	GBP4/NRAS/SAR1A/RAB26/GBP5/HRAS
**MF**	GO:0016462	pyrophosphatase activity	20	-0.243400831	-0.72070654	0.882608696	1	0.903175932	39	tags=30%, list=9%, signal=29%	GBP4/NRAS/SAR1A/RAB26/GBP5/HRAS
**MF**	GO:0016817	hydrolase activity, acting on acid anhydrides	20	-0.243400831	-0.72070654	0.882608696	1	0.903175932	39	tags=30%, list=9%, signal=29%	GBP4/NRAS/SAR1A/RAB26/GBP5/HRAS
**MF**	GO:0016818	hydrolase activity, acting on acid anhydrides, in phosphorus-containing anhydrides	20	-0.243400831	-0.72070654	0.882608696	1	0.903175932	39	tags=30%, list=9%, signal=29%	GBP4/NRAS/SAR1A/RAB26/GBP5/HRAS

**MF**	GO:0003712	transcription coregulator activity	11	-0.288645824	-0.702461527	0.846625767	1	0.903175932	92	tags=27%, list=20%, signal=22%	LCOR/HMGA2/CDYL2
**MF**	GO:0016491	oxidoreductase activity	13	-0.268494555	-0.697776868	0.87414966	1	0.903175932	76	tags=31%, list=17%, signal=26%	AOC2/GPX8/HSD3B7/CYP26C1
**MF**	GO:0042277	peptide binding	15	0.304000672	0.682835266	0.891213389	1	0.903175932	111	tags=33%, list=25%, signal=26%	LHCGR/LBP/POM121L2/BACE1/CEMIP
**MF**	GO:0050839	cell adhesion molecule binding	20	0.281393195	0.67952644	0.901554404	1	0.903175932	131	tags=30%, list=29%, signal=22%	ADAM23/ADAM11/CIP2A/IGF1/PPP1CA/EIF4G2
**MF**	GO:0044877	protein-containing complex binding	29	-0.19990933	-0.655076613	0.980487805	1	0.903175932	106	tags=48%, list=24%, signal=39%	PKNOX2/FZD4/GNA11/ICAM2/BOK/LDLRAP1/FBXW11/NRAS/BICD1/HMGA2/NECTIN1/LIMA1/CLU/NID2
**MF**	GO:0033218	amide binding	16	0.284401539	0.653993327	0.919508868	1	0.903175932	111	tags=31%, list=25%, signal=24%	LHCGR/LBP/POM121L2/BACE1/CEMIP
**MF**	GO:0008134	transcription factor binding	14	-0.235321481	-0.635656293	0.947916667	1	0.903175932	92	tags=29%, list=20%, signal=23%	LCOR/HMGA2/BBS10/TBX3
**MF**	GO:0005524	ATP binding	29	0.224289165	0.575087965	0.974905897	1	0.903175932	27	tags=7%, list=6%, signal=7%	CKMT2/ERCC6L
**MF**	GO:0005216	ion channel activity	10	0.268668761	0.552476935	0.952380952	1	0.903175932	120	tags=40%, list=27%, signal=30%	KCNK1/ANO5/TRPC3/SLC26A8
**MF**	GO:0140640	catalytic activity, acting on a nucleic acid	12	0.244291387	0.526171137	0.965860597	1	0.903175932	27	tags=8%, list=6%, signal=8%	ERCC6L
**MF**	GO:0045296	cadherin binding	11	-0.184632614	-0.449330277	1	1	0.903175932	72	tags=27%, list=16%, signal=23%	EHD4/LIMA1/COBLL1
