# Supplementary material for: RBAD: The first database dedicated alterations of blood RNA in individuals with Alzheimer’s disease and their clinical relevance
Source: Neural Regen Res. 2025 Mar 25;21(6):2553–62. doi: 10.4103/NRR.NRR-D-24-01165 (PMC13211806; doi:10.4103/NRR.NRR-D-24-01165)
Supplement: Supplementary file 21 [file NRR-21-2553_Suppl14.pdf]

Additional Table 19. Correlation between RNA expression and cognitive function in total population (N = 608; healthy: 322, MCI: 137, and AD: 149)

Method	Spearman correlation analysis
P value adjustment for multiple test	Benjiamini-Hochberg (FDR)
Description	**Correltion analysis**: Correlation between RNA expression and cognitive function defined by MMSE score through the Spearman correlation analysis. Negative: correlation coefficient <=-0.2 and FDR<=0.05. Positive: correlation coefficient>=0.2 and FDR<=0.05. NS: not significant.<br>**Survival analysis**: The association of each RNA with OS are also presented in colum 'Survival risk'. Detailed survival results are showing in Table S4. Risk: the gene's higher expression has higher risk of death. Protective: the gene's higher expression has lower risk of death. NS: not significant.

Symbol	Survival analysis	Correlation analysis			
	Survival risk	P value	Correlation coefficient	FDR	Correlation
RAC3	NS	6.31E-53	0.303374314	1.22E-48	Positive
SCRT2	NS	1.10E-48	0.291097363	1.06E-44	Positive
SOX3	NS	1.11E-47	0.288094543	7.18E-44	Positive
BARX1	NS	3.63E-46	0.283506166	1.75E-42	Positive
HES5	NS	5.46E-45	0.279873883	2.11E-41	Positive
ARHGDIG	NS	1.71E-43	0.275179582	5.52E-40	Positive
FOXQ1	NS	2.24E-41	0.268379618	5.41E-38	Positive
CTU1	NS	1.92E-40	0.26531763	4.12E-37	Positive
ZIC2	NS	2.48E-40	0.264947038	4.65E-37	Positive
SOX18	NS	6.04E-40	0.263664639	9.74E-37	Positive
SLC34A3	NS	7.86E-40	0.263283368	1.17E-36	Positive
UNCX	NS	2.02E-39	0.261912164	2.80E-36	Positive
HRH3	NS	3.05E-39	0.261312187	3.94E-36	Positive
FOXL3	NS	1.31E-38	0.25917749	1.58E-35	Positive
TMEM121	NS	1.45E-37	0.255604039	1.40E-34	Positive
ENTPD2	NS	3.05E-37	0.254487113	2.57E-34	Positive
DUSP9	NS	3.04E-37	0.254492847	2.57E-34	Positive
SCRT1	NS	2.97E-37	0.254529099	2.57E-34	Positive
POU3F1	NS	4.47E-37	0.253914545	3.60E-34	Positive
FOXD1	NS	7.89E-37	0.253054869	6.11E-34	Positive
NTN3	NS	9.33E-36	0.249284343	6.95E-33	Positive
DIO3	NS	2.47E-35	0.247783395	1.77E-32	Positive
PRKACG	NS	8.98E-35	0.24577039	6.21E-32	Positive
KISS1R	NS	2.38E-34	0.244238205	1.54E-31	Positive
FIGNL2	NS	2.76E-34	0.244006817	1.72E-31	Positive
PKP3	NS	2.28E-32	0.236934509	1.30E-29	Positive
TMEM151A	NS	1.01E-31	0.234492711	5.30E-29	Positive
MAFA	NS	3.48E-31	0.232446651	1.77E-28	Positive
DIRAS1	NS	5.45E-31	0.231701293	2.70E-28	Positive

NKX2-4	NS	6.78E-31	0.231337011	3.20E-28	Positive
SHISA8	NS	8.85E-31	0.230890645	4.08E-28	Positive
ZNF853	NS	1.67E-30	0.229820142	7.53E-28	Positive
KCNF1	NS	1.77E-30	0.229727369	7.78E-28	Positive
TMEM210	NS	2.50E-30	0.229139831	1.08E-27	Positive
PDIA2	NS	3.41E-30	0.228619038	1.43E-27	Positive
ADRA2C	NS	1.34E-29	0.226283806	5.21E-27	Positive
LCNL1	NS	1.34E-29	0.226279484	5.21E-27	Positive
NXPH4	NS	1.45E-29	0.226151541	5.50E-27	Positive
FBLL1	NS	1.84E-29	0.225743693	6.84E-27	Positive
C2CD4C	NS	2.89E-29	0.2249652	1.05E-26	Positive
FOXO6	NS	5.97E-29	0.223709232	2.03E-26	Positive
SRMS	NS	6.91E-29	0.223456664	2.30E-26	Positive
BHLHA9	NS	7.28E-29	0.223366111	2.39E-26	Positive
PCSK1N	NS	7.74E-29	0.223259509	2.50E-26	Positive
H2AB3	NS	8.05E-29	0.223189449	2.56E-26	Positive
IL11	NS	1.21E-28	0.222483975	3.77E-26	Positive
SLC8A2	NS	2.76E-28	0.221035084	8.35E-26	Positive
FZD8	NS	8.17E-28	0.21911955	2.29E-25	Positive
RNF225	NS	8.12E-28	0.219129277	2.29E-25	Positive
CXorf51B	NS	9.19E-28	0.218910731	2.51E-25	Positive
NOTUM	NS	1.15E-27	0.218518171	3.08E-25	Positive
C2CD4B	NS	1.18E-27	0.218459426	3.14E-25	Positive
CBLN1	NS	1.99E-27	0.217535657	5.13E-25	Positive
FAM171A2	NS	2.21E-27	0.217349882	5.62E-25	Positive
PNMA6A	NS	2.87E-27	0.216874958	7.23E-25	Positive
FGF17	NS	3.53E-27	0.216506588	8.54E-25	Positive
RNF224	NS	4.83E-27	0.215941311	1.11E-24	Positive
HPCA	NS	5.88E-27	0.215586947	1.32E-24	Positive
SCYGR3	NS	7.11E-27	0.215242524	1.58E-24	Positive
ESX1	NS	8.51E-27	0.214916948	1.87E-24	Positive
CXorf51A	NS	9.13E-27	0.214790676	1.99E-24	Positive
LCN6	NS	1.32E-26	0.214121546	2.84E-24	Positive
PTGER1	NS	1.70E-26	0.213661599	3.61E-24	Positive
PRSS33	NS	2.05E-26	0.213321979	4.30E-24	Positive
SOX1	NS	2.17E-26	0.213211135	4.52E-24	Positive
AMH	NS	2.26E-26	0.213142474	4.65E-24	Positive
H2AB2	NS	2.50E-26	0.21295738	5.09E-24	Positive
LRRC10B	NS	2.97E-26	0.21263957	5.93E-24	Positive
SHD	NS	3.80E-26	0.212188235	7.43E-24	Positive
TMEM271	NS	5.13E-26	0.211635891	9.93E-24	Positive
RBMXL2	NS	6.16E-26	0.211299102	1.18E-23	Positive
TCF15	NS	8.86E-26	0.210625946	1.64E-23	Positive
TMDD1	NS	8.89E-26	0.210621016	1.64E-23	Positive
B4GALNT4	NS	1.01E-25	0.210378189	1.85E-23	Positive
LY6H	NS	1.19E-25	0.210074122	2.14E-23	Positive
NAT8L	NS	1.54E-25	0.209601277	2.73E-23	Positive
P2RX2	NS	2.17E-25	0.208960932	3.78E-23	Positive
HOXD11	NS	2.58E-25	0.208637747	4.42E-23	Positive
PNCK	NS	6.38E-25	0.206935978	1.07E-22	Positive
H2AB1	NS	1.02E-24	0.206049274	1.67E-22	Positive
POU3F3	NS	1.51E-24	0.205302353	2.46E-22	Positive
ATOH7	NS	2.26E-24	0.204531031	3.65E-22	Positive
HBM	NS	3.08E-24	0.203939445	4.89E-22	Positive
FOXE3	NS	7.02E-24	0.202356414	1.10E-21	Positive

TAF11L2	NS	1.13E-23	0.201425671	1.73E-21	Positive
ITPKA	NS	2.13E-23	0.200198289	3.20E-21	Positive
HSPB9	NS	4.23E-42	-0.270725911	1.17E-38	Negative
CSN1S1	NS	2.64E-40	-0.264857058	4.65E-37	Negative
H2BC9	NS	3.61E-38	-0.257676021	4.11E-35	Negative
GPR22	NS	5.50E-38	-0.257051461	5.91E-35	Negative
H1-3	NS	1.15E-37	-0.255948497	1.17E-34	Negative
C1orf53	NS	2.16E-34	-0.244393008	1.44E-31	Negative
H2AC8	NS	2.46E-33	-0.240532978	1.49E-30	Negative
LRRC18	NS	5.83E-33	-0.239143636	3.42E-30	Negative
CETN3	NS	7.80E-32	-0.234922536	4.31E-29	Negative
HGD	NS	9.87E-32	-0.23453369	5.30E-29	Negative
H2AC11	NS	6.41E-31	-0.231429523	3.10E-28	Negative
TRAPPC3L	NS	4.71E-30	-0.228069397	1.94E-27	Negative
TRUB1	NS	9.88E-30	-0.226806462	3.98E-27	Negative
APOBEC2	NS	3.06E-29	-0.224866032	1.10E-26	Negative
H4C3	NS	4.29E-29	-0.224282854	1.51E-26	Negative
S100P	NS	4.66E-29	-0.224139567	1.61E-26	Negative
CELA1	NS	2.64E-28	-0.221111724	8.12E-26	Negative
STX19	NS	2.83E-28	-0.220993627	8.42E-26	Negative
METTL7B	NS	5.92E-28	-0.219691133	1.73E-25	Negative
H2BC8	NS	6.00E-28	-0.219666002	1.73E-25	Negative
SLC27A6	NS	9.13E-28	-0.218921747	2.51E-25	Negative
GPRC6A	NS	1.45E-27	-0.218094009	3.80E-25	Negative
KLRB1	NS	3.26E-27	-0.21664892	8.09E-25	Negative
CNTF	NS	3.51E-27	-0.216516422	8.54E-25	Negative
MRPS28	NS	3.59E-27	-0.216474517	8.58E-25	Negative
PDE6H	NS	4.31E-27	-0.216145425	1.02E-24	Negative
PMCH	NS	4.53E-27	-0.216056969	1.06E-24	Negative
PFDN4	NS	4.94E-27	-0.215899876	1.13E-24	Negative
RAD51AP1	NS	2.68E-26	-0.212830354	5.40E-24	Negative
SLCO1B1	NS	3.05E-26	-0.212588616	6.03E-24	Negative
GZMA	NS	7.17E-26	-0.211019013	1.36E-23	Negative
H3C11	NS	7.79E-26	-0.210864915	1.46E-23	Negative
PTTG2	NS	1.13E-25	-0.210171823	2.05E-23	Negative
MCF2	NS	1.55E-25	-0.209587067	2.73E-23	Negative
LRC4-KLRK	NS	2.27E-25	-0.20887483	3.93E-23	Negative
TMEM60	NS	3.18E-25	-0.20824529	5.40E-23	Negative
RTP4	NS	7.29E-25	-0.206684908	1.22E-22	Negative
OR14A2	NS	8.74E-25	-0.206340082	1.45E-22	Negative
GABRG1	NS	2.49E-24	-0.20434819	3.98E-22	Negative
N6AMT1	NS	3.14E-24	-0.203907064	4.94E-22	Negative
MAGEH1	NS	9.88E-24	-0.201695039	1.53E-21	Negative
C14orf39	NS	1.09E-23	-0.201500588	1.68E-21	Negative
ADAM7	NS	1.43E-23	-0.200977364	2.16E-21	Negative
POSTN	NS	2.31E-23	-0.200042726	3.43E-21	Negative
FUCA2	Protective	1.85E-02	-0.047751646	4.53E-02	NS
SNX11	Protective	2.17E-03	-0.062147331	7.47E-03	NS
CREBBP	Protective	3.09E-01	0.020634505	4.32E-01	NS
TKTL1	Protective	6.07E-04	0.069484948	2.50E-03	NS
PIGQ	Risk	4.75E-01	0.014491983	0.595988766	NS
TTC19	Protective	5.94E-02	0.038238569	0.117984986	NS
NPC1L1	Risk	6.18E-02	0.03789273	0.121882069	NS
IGF1	Protective	1.24E-02	-0.050737216	0.032616747	NS
HGF	Risk	1.15E-01	-0.031965767	0.200099996	NS

SLC45A4	Risk	3.57E-02	-0.042593552	0.07762489	NS
RB1CC1	Protective	6.51E-02	0.037421904	0.12689736	NS
DEPDC1	Protective	3.98E-17	-0.169494782	2.30E-15	NS
MIPEP	Risk	5.02E-14	-0.15193948	1.63E-12	NS
DAPK2	Risk	6.68E-05	0.08078418	0.000367103	NS
TBC1D23	Protective	3.12E-01	0.020511518	0.435456909	NS
TG	Protective	6.63E-02	0.037255724	0.12880038	NS
DTNBP1	Protective	1.68E-02	-0.048498484	0.041881264	NS
LTBP1	Risk	3.62E-01	-0.018512966	0.48711524	NS
LIMA1	Protective	7.56E-03	0.054158767	0.021619242	NS
CCDC85A	Protective	1.13E-01	0.032156219	0.197017409	NS
COL11A1	Protective	9.98E-07	-0.099027817	8.50E-06	NS
WAPL	Protective	4.02E-01	0.01699435	0.526627878	NS
MED29	Risk	6.52E-01	-0.009140935	0.748104891	NS
ZNF76	Risk	3.39E-01	0.019384122	0.464143298	NS
COL17A1	Protective	3.33E-01	-0.019640649	0.457464641	NS
KDM4A	Protective	5.12E-01	-0.013308434	0.629422086	NS
ASPM	Protective	2.74E-06	-0.094938706	2.13E-05	NS
TRMT11	Protective	1.49E-08	-0.114523687	1.85E-07	NS
TRAM1	Protective	3.41E-04	0.072585814	0.001515935	NS
NAV3	Protective	6.31E-01	0.009749801	0.730043324	NS
FBXW11	Protective	7.63E-03	0.054103336	0.021760863	NS
MCM2	Risk	5.33E-01	-0.012651724	0.647821033	NS
MOV10L1	Protective	8.32E-01	0.004311182	0.88477633	NS
ADAM11	Protective	8.20E-01	-0.004617851	0.876543463	NS
PPP2R2C	Risk	9.42E-06	0.089713233	6.44E-05	NS
SLC25A43	Protective	9.08E-01	-0.002332857	0.93989163	NS
SAR1A	Protective	5.73E-06	0.091850413	4.12E-05	NS
RAPGEF3	Protective	5.27E-01	0.012830463	0.642743415	NS
PCDHA6	Risk	3.65E-01	-0.018363227	0.49123734	NS
COBLL1	Protective	4.28E-02	0.041086086	0.089919539	NS
CYLD	Protective	1.61E-06	0.097110609	1.31E-05	NS
PREP	Protective	2.99E-01	-0.02105531	0.422238511	NS
IPO11	Risk	2.75E-01	-0.022161109	0.396076107	NS
ZFAND6	Protective	7.80E-02	0.035757074	0.146697833	NS
SRRT	Protective	3.51E-01	0.018930648	0.47630267	NS
NID2	Protective	1.68E-01	-0.027980548	0.269976606	NS
GNA11	Risk	4.53E-03	-0.057549678	0.013988445	NS
PAPOLA	Protective	4.21E-02	0.041227461	0.088667836	NS
GNPTG	Protective	1.79E-02	-0.048037663	0.043908654	NS
ALKBH5	Protective	4.38E-01	0.01573243	0.560628221	NS
G2E3	Protective	6.92E-03	-0.054762797	0.019989143	NS
TMEM38B	Protective	5.55E-01	-0.011984768	0.66667399	NS
TDRD1	Protective	2.67E-01	-0.022505542	0.387699867	NS
HSD3B7	Risk	1.07E-01	-0.032712091	0.188631627	NS
TOP3B	Risk	7.38E-02	0.036270007	0.140341006	NS
LGALS1	Protective	4.76E-01	-0.01445953	0.596959222	NS
GCAT	Protective	7.01E-01	-0.007797871	0.786004072	NS
EIF3D	Protective	6.06E-01	0.010461794	0.710386298	NS
BDKRB1	Risk	3.82E-05	0.083421928	0.000224516	NS
NKAP	Risk	6.91E-01	0.008060928	0.778552012	NS
STAG2	Protective	6.55E-01	-0.009079529	0.749708638	NS
CCDC22	Risk	4.37E-01	-0.015784279	0.559512103	NS
GGA2	Protective	1.29E-04	0.077572366	0.000652021	NS
CEMIP	Risk	3.98E-03	-0.058380867	0.01253622	NS

EHD4	Protective	3.30E-04	0.072761358	0.001473744	NS
TRIM35	Risk	1.65E-02	-0.048638153	0.041308771	NS
DKK4	Risk	1.64E-01	-0.028264463	0.264685795	NS
KCNA7	Protective	4.79E-05	0.082357256	0.000274362	NS
SYDE1	Protective	9.97E-04	0.066712892	0.003824329	NS
AURKC	Protective	4.08E-01	-0.016772606	0.532506663	NS
ZNF175	Protective	1.13E-01	-0.032122951	0.197525999	NS
FGF21	Risk	5.38E-15	0.157644324	2.11E-13	NS
AIMP2	Risk	2.74E-01	-0.022202697	0.395102417	NS
NMRK1	Protective	2.42E-02	-0.045708342	0.056480916	NS
TNKS2	Protective	6.58E-04	0.069041308	0.002676648	NS
LHPP	Risk	2.73E-02	0.044762019	0.062181734	NS
CPD	Protective	2.48E-03	0.061336069	0.008357852	NS
ICAM2	Risk	5.39E-03	0.05642927	0.01613307	NS
EIF4G2	Protective	3.34E-01	-0.01959454	0.4586675	NS
NECTIN1	Risk	7.54E-01	-0.006371248	0.82699196	NS
GTF2H1	Risk	8.51E-01	-0.00381155	0.899477462	NS
LTA4H	Protective	5.28E-01	-0.012796501	0.643915124	NS
RIC8B	Risk	4.24E-03	0.057988907	0.013211835	NS
GCNT2	Risk	4.23E-03	-0.058004313	0.013186118	NS
FAM184A	Protective	2.25E-02	-0.046266011	0.053320676	NS
SLC26A8	Protective	1.35E-01	-0.030336751	0.227485975	NS
IL17F	Protective	7.25E-05	-0.080385144	0.000394091	NS
WASF1	Risk	1.39E-06	-0.09770969	1.15E-05	NS
LAMA4	Protective	5.60E-03	-0.056170787	0.016671013	NS
PCDHB7	Risk	2.85E-01	0.021705447	0.407156848	NS
CLINT1	Protective	2.01E-01	0.025951635	0.311674939	NS
CPEB4	Protective	8.93E-02	0.034464701	0.163390032	NS
HRH2	Risk	2.02E-02	0.047106447	0.048645337	NS
NPRL2	Risk	6.59E-01	-0.008950886	0.753332688	NS
KLHL24	Protective	6.72E-04	0.068927793	0.002723641	NS
ADAM23	Protective	8.59E-05	-0.079567007	0.000457734	NS
FAHD2A	Risk	1.77E-05	-0.086939086	0.000112689	NS
PLA2G4A	Risk	5.77E-04	-0.069769008	0.002390288	NS
RGS2	Risk	4.29E-04	0.071365593	0.001850337	NS
PHTF1	Protective	1.10E-02	-0.051556068	0.029632921	NS
GBP1	Risk	3.13E-01	0.020473251	0.436466368	NS
RAB29	Risk	1.05E-02	-0.051882551	0.028494301	NS
MPL	Risk	8.55E-03	0.053322874	0.023976179	NS
DR1	Risk	8.15E-01	0.004754403	0.873124803	NS
NEK2	Protective	6.57E-08	0.109304044	7.16E-07	NS
MESD	Risk	3.16E-01	0.02032635	0.440059102	NS
EEF2KMT	Risk	9.86E-01	0.000352718	0.990019211	NS
PPP4R4	Protective	2.08E-02	-0.046898289	0.049756543	NS
TMEM214	Risk	1.27E-04	0.077655161	0.00064192	NS
PYROXD2	Risk	2.71E-05	-0.085007106	0.00016566	NS
MSX2	Risk	2.33E-01	-0.024208698	0.349661935	NS
WDR55	Protective	2.56E-01	-0.023053356	0.375420329	NS
SMAD9	Risk	7.44E-01	0.006615347	0.819903311	NS
CLU	Risk	8.21E-01	0.004597613	0.876985022	NS
PPP3CC	Protective	4.15E-01	0.016531258	0.538677548	NS
GJA3	Protective	6.33E-02	0.037669772	0.124299971	NS
TNFSF10	Risk	8.23E-01	0.004546348	0.878212241	NS
PHF24	Protective	8.33E-01	0.004271157	0.886023879	NS
RNF11	Protective	1.32E-03	0.065104289	0.00488103	NS

AMD1	Protective	6.17E-01	0.010154777	0.71894792	NS
H2BW1	Risk	5.11E-01	-0.013329656	0.628845403	NS
PMEPA1	Protective	4.00E-01	0.017079396	0.524294756	NS
LYPD3	Protective	8.61E-03	0.053275591	0.024116575	NS
USP9X	Protective	4.38E-05	0.082780925	0.000253851	NS
MED20	Risk	3.51E-04	-0.07243599	0.001554894	NS
CPNE5	Risk	8.18E-02	0.035304134	0.152217221	NS
SSUH2	Protective	2.07E-02	-0.046904033	0.049731449	NS
SLC10A2	Risk	2.94E-05	-0.084627879	0.000178162	NS
CENPB	Risk	1.83E-02	0.047842835	0.044869565	NS
UBA2	Protective	3.48E-02	0.042799596	0.075960013	NS
AHDC1	Protective	4.67E-03	0.057355119	0.014326463	NS
NUP214	Protective	1.87E-04	0.075702648	0.00089684	NS
AVPR2	Protective	7.29E-05	0.080360369	0.000396013	NS
TAS2R3	Risk	3.26E-09	-0.119659672	4.62E-08	NS
PNKD	Protective	1.36E-01	0.030273652	0.228520039	NS
FGL2	Risk	3.38E-02	-0.043048026	0.074112779	NS
GAL3ST1	Protective	1.83E-03	0.063165926	0.006455777	NS
ATF4	Protective	2.13E-02	0.046709748	0.05081565	NS
ILF3	Protective	9.39E-02	0.033981491	0.170084039	NS
MTUS1	Protective	7.93E-01	-0.005329728	0.856782576	NS
ART1	Risk	1.17E-05	0.088777124	7.78E-05	NS
RPS4Y1	Risk	1.30E-07	-0.106812718	1.34E-06	NS
SHC2	Protective	2.45E-04	0.074314989	0.001136711	NS
LBP	Protective	3.59E-04	-0.072323769	0.001583866	NS
PRRG3	Protective	2.10E-04	0.075111922	0.000990906	NS
NCAN	Protective	4.10E-01	0.016706523	0.534018358	NS
PXDN	Protective	6.24E-01	0.009950359	0.724704478	NS
OR11H1	Protective	1.41E-01	-0.02988413	0.235158188	NS
ZNF341	Risk	1.55E-03	0.064146807	0.005613963	NS
NAPSA	Risk	1.58E-05	0.087442734	0.000101708	NS
AOC2	Protective	7.40E-01	-0.006727034	0.816907375	NS
CKMT2	Protective	2.77E-12	-0.14110793	6.75E-11	NS
ZSCAN5A	Risk	1.64E-03	0.063846053	0.00586709	NS
DNAJB1	Protective	1.08E-03	0.066271928	0.004089501	NS
LANCL2	Protective	5.73E-02	0.038559004	0.114282693	NS
TPTE2	Protective	3.49E-03	-0.059223177	0.011199131	NS
ADCK2	Risk	3.27E-02	-0.0433307	0.071942949	NS
KRBA1	Risk	6.52E-02	-0.037399826	0.127142716	NS
SFTPD	Risk	3.16E-01	-0.020336961	0.439806116	NS
KCTD1	Risk	6.84E-04	-0.068826627	0.002765805	NS
SOX5	Protective	2.16E-04	-0.074961612	0.001017178	NS
PRH2	Protective	4.64E-16	-0.163673432	2.25E-14	NS
DSG3	Protective	5.48E-01	-0.012187909	0.660932645	NS
POGLUT2	Risk	6.07E-05	-0.081236388	0.000337707	NS
HNF1A	Protective	1.76E-01	0.027425638	0.280836649	NS
TBX3	Protective	2.25E-01	0.024629812	0.340375925	NS
PPP1R1A	Protective	3.37E-01	-0.019491209	0.461421145	NS
COQ10A	Protective	7.18E-03	-0.054509569	0.020663624	NS
KCNK1	Protective	2.43E-01	-0.023667547	0.361368551	NS
RC3H1	Protective	1.71E-01	0.027743104	0.274641867	NS
FPGS	Protective	2.65E-05	0.085099587	0.00016296	NS
PLAA	Protective	6.48E-06	0.091330948	4.60E-05	NS
FHDC1	Risk	1.36E-02	-0.050066159	0.035231467	NS
SLC28A2	Protective	7.82E-01	0.005627765	0.848408384	NS

LHCGR	Protective	9.82E-04	-0.06679917	0.003778712	NS
SULT6B1	Risk	3.42E-06	-0.094023454	2.60E-05	NS
BARD1	Risk	9.69E-02	-0.03367669	0.174400209	NS
HCN4	Risk	4.56E-08	0.110609745	5.17E-07	NS
TRPC3	Protective	1.10E-05	-0.089021542	7.41E-05	NS
GSTCD	Risk	1.36E-02	0.050052624	0.035292573	NS
TMTC3	Risk	4.57E-11	-0.133015585	9.13E-10	NS
SLC39A5	Protective	1.30E-01	0.030735216	0.220656622	NS
KANSL2	Protective	2.48E-01	0.023417851	0.367257051	NS
WARS1	Risk	1.14E-05	0.088871491	7.64E-05	NS
NLRC5	Risk	2.41E-01	0.023771248	0.358999116	NS
MEAK7	Risk	7.58E-01	-0.00626344	0.829721063	NS
GREB1L	Protective	8.46E-01	-0.003945012	0.895741501	NS
TNFRSF11A	Protective	6.34E-01	-0.009653848	0.732842571	NS
GPR32	Protective	3.27E-08	0.111794026	3.81E-07	NS
KLK3	Risk	6.77E-01	-0.008447244	0.767913132	NS
CTTNBP2NL	Risk	8.78E-02	0.034632711	0.16117851	NS
FCGR2A	Risk	8.93E-01	-0.0027185	0.929401487	NS
XPR1	Risk	4.90E-05	0.082257199	0.000279261	NS
LHX9	Protective	6.42E-03	-0.055260235	0.018733083	NS
HORMAD1	Risk	3.09E-01	0.020627387	0.432557122	NS
ARL8A	Protective	6.86E-01	0.008195499	0.774813287	NS
SCRN3	Risk	1.51E-02	-0.049279379	0.038459096	NS
CCDC150	Protective	3.55E-01	0.018757978	0.480735161	NS
RBMS3	Protective	3.30E-02	-0.043245475	0.072585457	NS
STAC	Protective	6.30E-06	-0.091451015	4.49E-05	NS
PTPRG	Protective	8.92E-01	-0.002763103	0.928382608	NS
EIF2B5	Protective	1.61E-04	-0.076449773	0.00079295	NS
FABP2	Protective	5.99E-05	-0.081305665	0.000333312	NS
GLRA1	Protective	2.49E-03	-0.061323524	0.00837209	NS
TIAM2	Protective	7.97E-03	-0.053805646	0.022549227	NS
LANCL3	Protective	6.60E-01	0.008916677	0.754537311	NS
MFHAS1	Protective	2.87E-01	-0.021581192	0.410132889	NS
SLC25A37	Protective	9.77E-04	0.066830353	0.003761766	NS
ZEB1	Protective	6.10E-03	0.055603573	0.017930494	NS
SYT8	Protective	3.36E-07	0.103254353	3.16E-06	NS
HMGA2	Risk	5.66E-02	0.038673708	0.113076931	NS
SLC2A13	Protective	6.55E-01	-0.009061166	0.750152531	NS
SCLT1	Risk	1.67E-01	0.028014875	0.269362186	NS
DPYSL4	Risk	1.03E-01	-0.033089777	0.182973479	NS
BICD1	Protective	5.27E-01	-0.012832545	0.642702148	NS
ZNF385D	Protective	1.62E-02	-0.048760554	0.040753609	NS
PDK1	Protective	1.85E-04	0.075743692	0.000890884	NS
PLEKHH2	Protective	2.34E-02	-0.045987227	0.05480118	NS
PELO	Risk	3.69E-02	-0.042317722	0.079746125	NS
BMP3	Protective	9.49E-01	0.001289526	0.966828141	NS
TXNDC11	Protective	1.26E-01	0.031065665	0.215200286	NS
CAST	Protective	3.78E-03	0.058722358	0.011966667	NS
GBP5	Risk	6.79E-01	-0.008388111	0.769588736	NS
FGD5	Risk	6.95E-01	-0.007961863	0.781217556	NS
EME1	Protective	6.04E-01	-0.010520222	0.708931942	NS
TTC39B	Risk	1.57E-02	0.048983129	0.039782381	NS
DEPTOR	Risk	6.46E-08	-0.1093651	7.05E-07	NS
RRAGA	Protective	7.57E-02	-0.036033503	0.143185003	NS
TRIM42	Risk	1.34E-02	-0.050150656	0.03491192	NS

SUSD3	Protective	4.98E-01	-0.013736598	0.617718497	NS
KIT	Protective	7.19E-01	0.007298694	0.800609321	NS
LDLRAP1	Protective	3.38E-02	-0.04304466	0.074126545	NS
MRAS	Protective	1.88E-01	-0.026717597	0.295564602	NS
ABHD3	Risk	6.12E-01	-0.010283534	0.715213433	NS
CD1B	Protective	2.36E-08	-0.11293037	2.84E-07	NS
POM121L2	Protective	5.23E-01	-0.012965929	0.639373432	NS
EMSY	Risk	4.62E-01	0.014921373	0.584090346	NS
NBL1	Risk	9.86E-02	-0.033507583	0.176832316	NS
SON	Protective	5.79E-03	0.055949771	0.017153961	NS
GOLGA6A	Protective	5.92E-01	0.010886917	0.698650273	NS
PTMS	Protective	2.70E-05	0.085014403	0.000165448	NS
ACE	Protective	8.22E-01	0.004555878	0.877967443	NS
TFF3	Risk	9.59E-02	0.033781052	0.172914485	NS
DVL3	Protective	6.60E-04	0.069024307	0.002682087	NS
ABCF3	Protective	7.02E-01	-0.007772911	0.786662133	NS
SAP30BP	Protective	2.77E-01	0.02205319	0.398713882	NS
IZUMO2	Protective	7.53E-12	0.138275983	1.72E-10	NS
EMC10	Protective	9.12E-01	-0.002246433	0.942126081	NS
WDR90	Protective	6.81E-01	-0.008340572	0.770857177	NS
ELAVL4	Protective	5.69E-03	-0.056072283	0.016890596	NS
GBP4	Risk	8.20E-02	0.035279204	0.15253123	NS
FCAMR	Risk	8.09E-04	-0.067893308	0.003206246	NS
LRATD1	Protective	8.63E-01	-0.00348942	0.908086289	NS
VSNL1	Protective	4.99E-04	0.070557437	0.002110358	NS
VPS72	Risk	2.93E-02	-0.044211927	0.065741904	NS
TMEM79	Risk	1.06E-01	0.032747082	0.188073062	NS
CIP2A	Protective	3.09E-04	-0.073108213	0.001392486	NS
EOMES	Protective	2.36E-01	-0.024060867	0.352789742	NS
MTMR14	Protective	3.97E-09	0.119007748	5.53E-08	NS
EMCN	Protective	3.96E-02	-0.041741252	0.084425179	NS
ABHD18	Risk	1.33E-01	-0.030514379	0.224477749	NS
HHIP	Protective	7.24E-01	-0.007174172	0.804503792	NS
SCGB3A2	Risk	2.92E-01	-0.021388722	0.41475073	NS
GPX8	Protective	4.35E-01	0.015833555	0.558196271	NS
CSF2	Risk	3.26E-01	-0.019923946	0.450250028	NS
PTTG1	Protective	5.95E-06	-0.091693827	4.26E-05	NS
SUN3	Protective	9.11E-03	0.052880672	0.02533349	NS
YWHAZ	Protective	9.82E-05	0.078914779	0.000514726	NS
HDX	Protective	2.64E-03	0.060957244	0.008796993	NS
PKNOX2	Protective	3.82E-01	0.017728241	0.507719042	NS
TMEM63C	Risk	7.65E-01	0.006060433	0.835325218	NS
CDX2	Protective	5.79E-08	0.109758921	6.41E-07	NS
CEP295	Risk	2.19E-01	-0.024950235	0.333282987	NS
RAB8B	Protective	1.65E-03	0.063808707	0.005888983	NS
TRMT61A	Risk	7.60E-01	0.006191473	0.831360632	NS
CDYL2	Protective	8.60E-02	-0.034826988	0.158514544	NS
TMEM170A	Protective	6.00E-01	-0.010649027	0.705583808	NS
OR4D6	Risk	8.74E-04	-0.067460138	0.003423312	NS
PATL1	Protective	2.29E-04	0.074668009	0.001069975	NS
PIP5KL1	Protective	4.17E-02	0.041308016	0.08799702	NS
GPRC5B	Protective	9.70E-01	0.000771631	0.979893285	NS
RNF214	Risk	1.94E-02	-0.047417497	0.047008124	NS
PRRT2	Risk	2.32E-03	0.061740077	0.007914644	NS
ZNF528	Protective	4.49E-01	-0.015346448	0.571487795	NS

AXL	Protective	2.80E-01	-0.021930483	0.401827091	NS
TRAPPC9	Risk	1.43E-01	0.029692686	0.238534906	NS
MRPL58	Risk	7.82E-10	-0.124285655	1.24E-08	NS
RAB26	Protective	1.62E-01	-0.028370084	0.262850292	NS
C2CD3	Risk	9.76E-03	-0.052404036	0.026768375	NS
MAJIN	Risk	4.18E-04	0.071507172	0.001806483	NS
HTR1E	Protective	1.48E-02	0.049412395	0.037857467	NS
PLA2G4F	Risk	4.06E-01	0.016876282	0.529843628	NS
MECP2	Protective	1.39E-02	0.04988337	0.035937402	NS
SLC50A1	Risk	5.10E-01	0.01337892	0.627484724	NS
INO80E	Risk	9.30E-01	0.001784689	0.954069445	NS
BUB1	Protective	5.06E-01	-0.013503007	0.624248738	NS
NLGN1	Protective	8.18E-01	-0.004659867	0.875451527	NS
UBE2E3	Protective	2.92E-01	0.021384408	0.414783998	NS
MRGPRX1	Protective	4.10E-01	-0.016702639	0.534088208	NS
KRT8	Risk	1.52E-02	0.049234057	0.038661557	NS
SMAGP	Risk	6.94E-01	-0.007974864	0.780847203	NS
NCBP2L	Risk	2.81E-04	-0.073609021	0.00127721	NS
SHCBP1	Protective	3.13E-03	0.059900108	0.010206195	NS
APLN	Protective	2.04E-07	0.105131353	2.02E-06	NS
NLRP5	Risk	1.28E-03	-0.065267817	0.004770988	NS
ETFDH	Risk	4.94E-01	0.013866814	0.613546986	NS
ANO5	Risk	1.00E-01	-0.033354135	0.178971362	NS
CTPS1	Protective	6.73E-03	-0.054950745	0.019525682	NS
CD8B	Risk	6.09E-01	0.010392582	0.712250878	NS
AZU1	Protective	2.15E-03	0.062208319	0.00741138	NS
PPP1CA	Risk	1.23E-02	-0.050774665	0.032486682	NS
ZNF596	Risk	2.35E-06	0.095577805	1.85E-05	NS
OVOL1	Risk	1.82E-04	0.075844994	0.000877206	NS
CCDC96	Risk	3.06E-01	0.020767937	0.429199258	NS
PLAC8L1	Risk	2.71E-02	-0.044830014	0.061715301	NS
SAA1	Risk	2.57E-01	-0.022989875	0.376742213	NS
CHD2	Protective	4.22E-01	0.016279173	0.545957487	NS
FAM174A	Risk	6.33E-09	-0.117443561	8.44E-08	NS
RGMB	Protective	1.74E-04	-0.076067376	0.000844813	NS
TRHR	Protective	1.94E-16	-0.165766157	1.00E-14	NS
GOLT1A	Protective	8.21E-01	0.004582923	0.877209014	NS
ZNF266	Risk	3.67E-04	0.072208563	0.001614986	NS
HRAS	Risk	4.16E-03	0.058105369	0.013009316	NS
FZD4	Protective	4.50E-01	0.015342029	0.57161414	NS
KCNE3	Risk	4.43E-01	0.01555307	0.565800081	NS
OR11H4	Risk	8.61E-04	-0.067544209	0.003380099	NS
BOK	Risk	4.86E-05	0.082290673	0.000277612	NS
VSIG10	Protective	7.26E-03	-0.054440892	0.020849176	NS
SIX5	Protective	1.08E-10	0.130420199	2.01E-09	NS
ZDHHC22	Risk	7.89E-04	0.06803689	0.003131447	NS
CRACR2B	Protective	5.02E-03	0.056883262	0.015226459	NS
ZNF354C	Risk	1.23E-01	-0.031317139	0.210975128	NS
IMP3	Risk	3.77E-01	-0.017935474	0.502694416	NS
TSPYL6	Risk	6.02E-08	-0.109615998	6.64E-07	NS
PPP1R42	Risk	2.57E-02	-0.045256692	0.059005579	NS
CSRNP3	Protective	2.12E-07	-0.104986631	2.08E-06	NS
EDC3	Risk	2.46E-02	-0.045571658	0.057265156	NS
ZNF664	Protective	4.65E-08	0.110542447	5.24E-07	NS
MKRN3	Protective	2.31E-01	-0.024300228	0.347770793	NS

OR9A2	Risk	7.16E-02	-0.03654171	0.137234208	NS
GPHB5	Risk	4.99E-03	-0.056926792	0.015139389	NS
NRXN1	Protective	5.63E-01	0.011741626	0.673848694	NS
BBS10	Risk	4.98E-10	-0.125713782	8.17E-09	NS
TDRP	Risk	6.65E-01	-0.008781567	0.758624111	NS
DEFB123	Risk	1.56E-01	-0.028759069	0.255627149	NS
OR56B1	Protective	7.52E-06	-0.090690967	5.26E-05	NS
CCL13	Risk	4.42E-03	-0.057712698	0.013720372	NS
UTS2R	Protective	2.12E-01	0.025296549	0.325667049	NS
SNRPE	Protective	1.29E-05	-0.088354113	8.47E-05	NS
FAM89A	Risk	3.50E-05	-0.08382098	0.000207983	NS
TSEN54	Protective	3.35E-08	-0.111704155	3.90E-07	NS
NTM	Protective	1.73E-12	-0.142423406	4.37E-11	NS
NKX2-5	Risk	4.64E-01	-0.014849379	0.586063008	NS
CCBE1	Protective	3.65E-02	-0.042407836	0.079030307	NS
FAM167B	Protective	1.36E-05	0.088106711	8.90E-05	NS
TP53TG3	Protective	2.53E-01	-0.023200182	0.372123113	NS
OPCML	Protective	7.23E-05	-0.080399043	0.000393175	NS
SDR42E2	Protective	2.91E-01	-0.021425062	0.414030693	NS
TACSTD2	Protective	3.24E-04	-0.072857437	0.001450774	NS
CCR10	Protective	3.95E-01	0.017273143	0.519707228	NS
RPS27L	Risk	6.48E-03	-0.055205468	0.018867248	NS
CYP4Z1	Protective	1.48E-06	-0.097457063	1.21E-05	NS
BACE1	Risk	3.55E-01	-0.018756749	0.480735161	NS
GNG2	Protective	4.90E-06	0.092519708	3.57E-05	NS
VSIG10L	Risk	1.70E-02	0.048399961	0.042313933	NS
QRFPR	Risk	1.13E-01	0.032117608	0.197595056	NS
ERCC6L	Protective	4.65E-15	-0.158009872	1.85E-13	NS
AKR1C1	Protective	9.81E-03	0.052365134	0.026883567	NS
CYP26C1	Protective	2.77E-01	0.02207636	0.398165478	NS
SSX7	Risk	2.37E-01	0.023992415	0.35422472	NS
FAM205C	Risk	1.16E-05	-0.088815865	7.72E-05	NS
ZFP69B	Risk	3.51E-03	-0.059185689	0.011254694	NS
DAZ1	Risk	1.09E-05	-0.089088155	7.31E-05	NS
PRKAR1B	Protective	1.25E-02	-0.050665894	0.032889757	NS
DYNLT4	Protective	7.09E-04	0.068632005	0.002849787	NS
IGFL3	Risk	6.93E-01	-0.008001806	0.780016533	NS
PARVB	Protective	5.36E-05	0.081831993	0.00030244	NS
PLA2G2E	Protective	3.61E-05	0.08368458	0.000213538	NS
C9orf152	Protective	3.04E-02	0.043902055	0.06791408	NS
KRTAP10-12	Risk	1.84E-02	0.047809596	0.045034163	NS
GJB5	Risk	0.079616158	-0.035559453	0.149109855	NS
BNIP5	Risk	0.547315955	-0.012211177	0.660458499	NS
KIAA0408	Protective	0.183570757	-0.026980594	0.290151525	NS
CXCL17	Risk	0.000340796	-0.072593809	0.001514336	NS
HMGB1	Protective	0.00037388	0.072103251	0.00164361	NS
OR8A1	Protective	0.049535846	0.03983722	0.101386891	NS
KIAA0895L	Protective	0.682663064	0.008295989	0.772049446	NS
LCOR	Protective	5.44E-12	0.139207227	1.26E-10	NS
ADH7	Risk	0.207143176	0.025593712	0.319504607	NS
TRRAP	Protective	0.057330549	0.038556871	0.114294996	NS
ARL9	Risk	0.387231077	-0.017544368	0.51256546	NS
ANAPC7	Risk	0.029960157	0.044025416	0.067049231	NS
KRT39	Protective	0.001775203	-0.063359228	0.006281687	NS
METTL9	Protective	0.316542202	0.020322427	0.440121393	NS

ZNF470	Protective	0.215455002	0.025132171	0.32951541	NS
OR10D3	Protective	8.58E-14	-0.150539922	2.66E-12	NS
OR13G1	Risk	3.20E-05	-0.084236642	0.0001918	NS
FAM118B	Risk	0.36171082	0.01850721	0.487248565	NS
TCAF1	Risk	2.72E-05	-0.084986931	0.000166182	NS
OR10G6	Risk	8.08E-11	-0.131309338	1.54E-09	NS
MT-CO2	Risk	0.352462107	-0.018866578	0.477916396	NS
FAM169A	Protective	1.19E-05	-0.088678198	7.94E-05	NS
MT-CO3	Risk	0.71625956	0.00737538	0.798199093	NS
PFDN6	Risk	0.931054876	-0.001755641	0.954811832	NS
TAP2	Risk	0.324348187	0.019997301	0.448595917	NS
MPIG6B	Risk	0.000606682	0.069490204	0.002494782	NS
OR2H2	Risk	0.094790649	-0.033891585	0.171377527	NS
PSG5	Protective	0.608555746	-0.010392131	0.712250878	NS
C5orf51	Risk	4.66E-05	-0.08248826	0.000268017	NS
DAZ4	Risk	1.29E-05	-0.088344317	8.48E-05	NS
SCAF8	Protective	0.046855082	0.040316705	0.09681758	NS
NRAS	Protective	0.566721747	0.011624777	0.677340494	NS
LEPROT	Protective	2.70E-05	-0.085027739	0.000165071	NS
PPM1N	Risk	0.791411929	-0.005366816	0.855690387	NS
C19orf38	Risk	0.287593713	-0.021576611	0.410248649	NS
DEFB113	Risk	1.76E-15	-0.160422637	7.54E-14	NS
CSKMT	Protective	0.034544652	-0.042871192	0.075433921	NS
CRPPA	Protective	0.061484541	-0.03793281	0.121425852	NS
ZNF705B	Risk	0.015158259	-0.049251442	0.038585384	NS
ZNF579	Protective	0.000461806	0.070973643	0.001971922	NS
KRTAP10-9	Risk	0.072055063	0.036488999	0.137775146	NS
OR3A2	Protective	0.075269706	0.036084513	0.142667211	NS
DEFB131B	Risk	0.06665476	-0.037203062	0.129340304	NS
C13orf42	Protective	0.850363587	-0.003828217	0.898942522	NS
POTEB2	Protective	0.006061217	-0.055648029	0.017824508	NS
AKAIN1	Risk	0.011549909	-0.051216669	0.030835868	NS
POTEB	Protective	0.400076092	-0.017074415	0.524439444	NS
LCE6A	Risk	1.43E-07	-0.106461589	1.46E-06	NS
TSPY10	Risk	8.18E-07	-0.099810198	7.12E-06	NS
TSPY9P	Risk	5.24E-05	-0.081936368	0.000296276	NS
H2AL1Q	Risk	0.00193657	-0.062839678	0.006769776	NS
SLC10A5	Protective	0.217742264	-0.025007422	0.33210578	NS
PBOV1	Protective	0.002882292	0.060416178	0.009488036	NS
SALL3	Protective	1.32E-06	0.097900914	1.10E-05	NS
OR11H2	Protective	0.003303135	-0.059566238	0.010686192	NS
ARMH2	Risk	7.91E-18	-0.17320966	5.39E-16	NS
DUX4	Risk	0.000183265	0.075804869	0.000882189	NS
SMIM36	Protective	0.096864203	0.033680704	0.174344756	NS
OPN1MW	Protective	0.024071198	0.045755234	0.056182577	NS
DMRTC1	Protective	2.14E-10	0.128346997	3.80E-09	NS
TSNAX-DISC	Risk	0.385366542	0.017613367	0.510936008	NS
TMEM269	Protective	0.002984782	-0.060199249	0.009778849	NS
FCGBP	Protective	0.585413133	-0.011069	0.693467356	NS
OR2AG1	Protective	0.241693815	-0.023754116	0.359283118	NS
SSU72P7	Protective	2.29E-12	0.141648223	5.62E-11	NS
TMEM247	Risk	0.522340422	-0.012981277	0.638889176	NS
OR8S1	Risk	0.000594659	-0.069599983	0.002456298	NS
F1AKMT4-EC	Risk	1.07E-06	-0.098752055	9.09E-06	NS
FAM90A23P	Protective	0.089408895	0.034456699	0.163479932	NS

NUDT12	NS	2.44E-23	-0.199933783	3.60E-21	NS
SI	NS	2.66E-23	-0.199761512	3.90E-21	NS
COL6A1	NS	2.98E-23	0.199539425	4.33E-21	NS
KRTAP5-4	NS	3.00E-23	0.199528266	4.33E-21	NS
KRTAP10-4	NS	3.11E-23	0.199456602	4.46E-21	NS
C9orf50	NS	3.20E-23	0.199400983	4.55E-21	NS
RNASE1	NS	3.97E-23	-0.198974953	5.61E-21	NS
LCN12	NS	4.04E-23	0.198940815	5.67E-21	NS
CMTM2	NS	4.14E-23	-0.19889418	5.77E-21	NS
SLC25A41	NS	4.31E-23	0.19881662	5.95E-21	NS
BCHE	NS	4.42E-23	-0.198765434	6.07E-21	NS
LRRN4CL	NS	4.69E-23	0.19864863	6.39E-21	NS
HOXD10	NS	5.17E-23	0.19845639	7.00E-21	NS
H3C4	NS	5.25E-23	-0.198425138	7.06E-21	NS
OR11A1	NS	5.67E-23	-0.198274141	7.57E-21	NS
FJX1	NS	5.99E-23	0.198164482	7.95E-21	NS
IRX1	NS	6.15E-23	0.198112047	8.10E-21	NS
LMTK3	NS	6.82E-23	0.197908869	8.92E-21	NS
ANGPTL7	NS	8.59E-23	-0.197451928	1.12E-20	NS
PRG4	NS	8.93E-23	-0.197374525	1.15E-20	NS
DNASE1L2	NS	1.18E-22	0.196828688	1.51E-20	NS
NYAP1	NS	1.20E-22	0.196794119	1.52E-20	NS
TOX	NS	1.20E-22	-0.19678743	1.52E-20	NS
NTF4	NS	1.46E-22	0.196398028	1.83E-20	NS
IL12A	NS	1.62E-22	-0.196188789	2.02E-20	NS
MOCS2	NS	1.76E-22	-0.19602241	2.18E-20	NS
FOLH1	NS	1.90E-22	-0.195868416	2.34E-20	NS
GLMN	NS	2.12E-22	-0.195647314	2.60E-20	NS
CCDC166	NS	2.86E-22	0.195050481	3.48E-20	NS
EPS8L2	NS	2.90E-22	0.195020039	3.51E-20	NS
C19orf73	NS	3.11E-22	-0.194882503	3.74E-20	NS
CLIC2	NS	3.56E-22	-0.194612123	4.25E-20	NS
CCNA2	NS	3.91E-22	-0.194419831	4.64E-20	NS
DEFB114	NS	4.52E-22	-0.194126479	5.34E-20	NS
ADAM2	NS	4.96E-22	-0.19393885	5.80E-20	NS
CFAP161	NS	4.98E-22	-0.193933704	5.80E-20	NS
AARD	NS	5.24E-22	0.193827507	6.08E-20	NS
C6	NS	5.53E-22	-0.193718829	6.37E-20	NS
AKR1C4	NS	6.69E-22	-0.193335313	7.66E-20	NS
TMEM238	NS	6.76E-22	0.193312284	7.70E-20	NS
LIPI	NS	9.19E-22	-0.192688356	1.04E-19	NS
GPR174	NS	9.48E-22	-0.192624576	1.07E-19	NS
CGB5	NS	1.09E-21	0.192332504	1.22E-19	NS
PABPC1L2B	NS	1.11E-21	0.192294781	1.24E-19	NS
PHOX2B	NS	1.56E-21	0.191609458	1.72E-19	NS
IL2	NS	1.63E-21	-0.1915201	1.79E-19	NS
C11orf45	NS	1.71E-21	-0.191418704	1.87E-19	NS
SCYGR6	NS	2.04E-21	0.191057919	2.22E-19	NS
LRRC70	NS	2.48E-21	-0.190657941	2.68E-19	NS
RAB38	NS	2.53E-21	-0.190616123	2.72E-19	NS
MAGEA4	NS	2.98E-21	0.190277901	3.19E-19	NS
KCTD4	NS	3.58E-21	-0.189899619	3.81E-19	NS
SLC18A3	NS	3.60E-21	0.189886666	3.81E-19	NS
RASL10B	NS	3.71E-21	0.189827566	3.90E-19	NS
SLC24A5	NS	3.84E-21	-0.189755142	4.02E-19	NS

P2RY10	NS	4.15E-21	-0.189592123	4.32E-19	NS
C1QL1	NS	5.24E-21	0.189111267	5.42E-19	NS
KCNG2	NS	5.33E-21	0.189072898	5.49E-19	NS
ANGPTL1	NS	5.75E-21	-0.18891667	5.89E-19	NS
KLK10	NS	6.02E-21	0.188820512	6.13E-19	NS
TMEM117	NS	6.57E-21	-0.188638857	6.66E-19	NS
LMOD2	NS	7.13E-21	-0.188468415	7.19E-19	NS
LAMP5	NS	7.59E-21	-0.18833747	7.62E-19	NS
JRKL	NS	7.97E-21	-0.188237484	7.95E-19	NS
ACVR1C	NS	8.38E-21	-0.188131947	8.32E-19	NS
H1-5	NS	8.73E-21	-0.188047397	8.62E-19	NS
METTL18	NS	9.05E-21	-0.187970359	8.90E-19	NS
TDRD15	NS	9.64E-21	-0.18783921	9.42E-19	NS
PBDC1	NS	9.76E-21	-0.187813529	9.49E-19	NS
TPBGL	NS	1.10E-20	0.187559047	1.07E-18	NS
SERPINI2	NS	1.26E-20	-0.187283402	1.21E-18	NS
PRDM12	NS	1.29E-20	0.18722716	1.23E-18	NS
LPO	NS	1.29E-20	0.187221109	1.23E-18	NS
OTOR	NS	1.55E-20	-0.186836668	1.48E-18	NS
OR52K2	NS	1.76E-20	-0.1865794	1.66E-18	NS
SPOCK3	NS	1.79E-20	-0.186543121	1.68E-18	NS
AQP5	NS	1.96E-20	0.186346345	1.83E-18	NS
FGFBP2	NS	1.98E-20	-0.186322257	1.85E-18	NS
P2RY14	NS	2.04E-20	-0.186267627	1.89E-18	NS
TAF11L12	NS	2.07E-20	0.186238338	1.90E-18	NS
ZNF837	NS	2.19E-20	0.186110072	2.01E-18	NS
GAS2	NS	2.51E-20	-0.18582605	2.29E-18	NS
FAM81B	NS	3.45E-20	-0.185153704	3.13E-18	NS
STATH	NS	3.52E-20	-0.185112079	3.18E-18	NS
CAPNS2	NS	3.56E-20	-0.185086591	3.20E-18	NS
FZD9	NS	3.58E-20	0.18507665	3.20E-18	NS
OR52N4	NS	3.66E-20	-0.185028744	3.26E-18	NS
CLDN9	NS	4.95E-20	0.184385008	4.39E-18	NS
LHX2	NS	5.04E-20	0.184345269	4.46E-18	NS
MAGEA12	NS	5.11E-20	0.184317255	4.49E-18	NS
LRRC26	NS	5.24E-20	0.184263097	4.59E-18	NS
UCN2	NS	5.55E-20	0.184139389	4.84E-18	NS
LYVE1	NS	6.14E-20	-0.183922527	5.33E-18	NS
HS3ST6	NS	6.75E-20	0.183719934	5.84E-18	NS
ITM2A	NS	7.40E-20	-0.18352364	6.37E-18	NS
PDGFD	NS	7.75E-20	-0.183424493	6.64E-18	NS
HCN2	NS	7.83E-20	0.183404707	6.67E-18	NS
CD200R1	NS	8.22E-20	-0.183299901	6.98E-18	NS
TMEM54	NS	9.27E-20	0.183040648	7.84E-18	NS
C1QTNF4	NS	9.78E-20	0.182926796	8.19E-18	NS
MEX3D	NS	9.77E-20	0.182928014	8.19E-18	NS
PPIAL4D	NS	1.03E-19	0.18281783	8.58E-18	NS
TDO2	NS	1.10E-19	-0.182682045	9.10E-18	NS
ANTD3-TME	NS	1.17E-19	-0.18253914	9.68E-18	NS
C18orf54	NS	1.23E-19	-0.182432924	1.01E-17	NS
OR6M1	NS	1.28E-19	-0.182340019	1.05E-17	NS
LY6D	NS	1.33E-19	0.182256057	1.09E-17	NS
NRARP	NS	1.66E-19	0.181786242	1.35E-17	NS
CETN2	NS	1.96E-19	-0.181427006	1.59E-17	NS
HOXD13	NS	1.97E-19	0.181417558	1.59E-17	NS

CRISPLD1	NS	2.03E-19	-0.181346654	1.63E-17	NS
POF1B	NS	2.42E-19	-0.180970094	1.93E-17	NS
GLIPR1L1	NS	2.50E-19	-0.180894195	1.99E-17	NS
CCDC194	NS	2.67E-19	0.180754993	2.12E-17	NS
TAF11L11	NS	3.03E-19	0.180475241	2.40E-17	NS
LRRC39	NS	3.07E-19	-0.180448236	2.42E-17	NS
CPLX1	NS	3.66E-19	0.180061886	2.87E-17	NS
PTGFR	NS	3.87E-19	-0.179940604	3.02E-17	NS
O1B3-SLCO	NS	4.03E-19	-0.179851864	3.14E-17	NS
P2RY12	NS	4.23E-19	-0.179748569	3.27E-17	NS
PVALEF	NS	4.98E-19	0.1793906	3.84E-17	NS
TBXA2R	NS	5.66E-19	0.179107297	4.35E-17	NS
MICU3	NS	5.97E-19	-0.178990881	4.57E-17	NS
KCNH8	NS	6.04E-19	-0.178967943	4.60E-17	NS
TMEM240	NS	6.08E-19	0.178950146	4.62E-17	NS
ERP27	NS	6.21E-19	-0.178905844	4.69E-17	NS
KRTAP10-5	NS	6.77E-19	0.178714256	5.10E-17	NS
HAPLN4	NS	7.88E-19	0.178381065	5.91E-17	NS
INSM1	NS	9.72E-19	0.177915787	7.27E-17	NS
ZNF223	NS	9.93E-19	-0.177869733	7.39E-17	NS
IRX5	NS	1.00E-18	0.177850484	7.43E-17	NS
CGB2	NS	1.17E-18	0.177507159	8.64E-17	NS
H2BC7	NS	1.40E-18	-0.177108756	1.03E-16	NS
GNRH1	NS	2.08E-18	-0.176225565	1.52E-16	NS
TAS2R10	NS	2.18E-18	-0.176115667	1.59E-16	NS
ARMCX1	NS	2.26E-18	-0.176041448	1.64E-16	NS
MIS18A	NS	2.43E-18	-0.175880147	1.76E-16	NS
ODF3L2	NS	2.90E-18	0.17548209	2.09E-16	NS
PLPPR5	NS	2.92E-18	-0.175460186	2.10E-16	NS
SLC6A4	NS	2.94E-18	0.175450049	2.11E-16	NS
HUS1B	NS	2.99E-18	-0.175411258	2.14E-16	NS
IL17D	NS	3.13E-18	0.175310441	2.22E-16	NS
DC169-SOHI	NS	3.44E-18	-0.175092569	2.44E-16	NS
H4C1	NS	3.46E-18	-0.17508427	2.44E-16	NS
AASDH	NS	3.53E-18	-0.175038123	2.48E-16	NS
CSN3	NS	3.57E-18	-0.175009323	2.51E-16	NS
CD2	NS	4.48E-18	-0.174498134	3.13E-16	NS
TCEAL8	NS	5.41E-18	-0.174073077	3.77E-16	NS
OPN1SW	NS	5.59E-18	-0.173999082	3.88E-16	NS
IRX2	NS	6.77E-18	0.173565711	4.68E-16	NS
GPR88	NS	6.95E-18	0.173503951	4.79E-16	NS
PLPPR4	NS	7.51E-18	-0.173327912	5.16E-16	NS
RSPO2	NS	7.64E-18	-0.17328935	5.23E-16	NS
TPRX1	NS	7.95E-18	0.173198083	5.40E-16	NS
C10orf88	NS	8.61E-18	-0.173018172	5.83E-16	NS
DPEP1	NS	8.85E-18	0.172956203	5.97E-16	NS
FCER1A	NS	9.12E-18	-0.172887001	6.13E-16	NS
FOXF2	NS	9.94E-18	0.172689785	6.66E-16	NS
LRRN3	NS	1.02E-17	-0.172635247	6.80E-16	NS
HOXD12	NS	1.08E-17	0.172498098	7.19E-16	NS
TNNI3	NS	1.11E-17	0.172447289	7.33E-16	NS
TMEM190	NS	1.19E-17	0.172282662	7.85E-16	NS
ADRA1D	NS	1.21E-17	0.172249655	7.93E-16	NS
GRB14	NS	1.22E-17	-0.17223046	7.97E-16	NS
CLDN3	NS	1.27E-17	0.17213288	8.28E-16	NS

LIPJ	NS	1.27E-17	-0.172128641	8.28E-16	NS
EXTL2	NS	1.32E-17	-0.172045131	8.56E-16	NS
SLITRK6	NS	1.36E-17	-0.171968598	8.82E-16	NS
SAAL1	NS	1.45E-17	-0.171826432	9.35E-16	NS
GPR150	NS	1.52E-17	0.17171742	9.78E-16	NS
F1E1-BLOC1	NS	1.54E-17	-0.171689905	9.86E-16	NS
TRIB2	NS	1.77E-17	-0.171369894	1.13E-15	NS
MYCT1	NS	1.79E-17	-0.171343833	1.14E-15	NS
CAMK2N2	NS	1.91E-17	0.171193835	1.21E-15	NS
SDHAF3	NS	1.91E-17	-0.171195062	1.21E-15	NS
GPX2	NS	1.93E-17	-0.171168779	1.22E-15	NS
MS4A1	NS	1.94E-17	-0.171152463	1.22E-15	NS
FOXD3	NS	2.00E-17	0.171086359	1.25E-15	NS
NAALAD2	NS	2.05E-17	-0.171026065	1.28E-15	NS
LRCOL1	NS	2.10E-17	0.170970843	1.31E-15	NS
KCNE2	NS	2.12E-17	-0.170948403	1.32E-15	NS
LANCL1	NS	2.15E-17	-0.17091535	1.33E-15	NS
DNAJC5G	NS	2.21E-17	0.170856597	1.36E-15	NS
TCEAL9	NS	2.35E-17	-0.170714361	1.44E-15	NS
DOC2B	NS	2.36E-17	0.170701454	1.45E-15	NS
PANO1	NS	2.45E-17	0.170621492	1.49E-15	NS
MPZL2	NS	2.62E-17	-0.170458473	1.60E-15	NS
PRRT4	NS	2.68E-17	0.1704084	1.63E-15	NS
ZC4H2	NS	2.72E-17	-0.170376089	1.65E-15	NS
TEKT4	NS	2.76E-17	0.17034378	1.66E-15	NS
C4orf45	NS	2.81E-17	-0.170297757	1.69E-15	NS
TMEFF1	NS	3.08E-17	-0.170086733	1.85E-15	NS
PABPC1L2A	NS	3.11E-17	0.170064362	1.86E-15	NS
CSH2	NS	3.25E-17	0.169959724	1.94E-15	NS
CACNA1H	NS	3.28E-17	0.169943189	1.95E-15	NS
KLK11	NS	3.37E-17	0.169878482	1.99E-15	NS
LCN10	NS	3.36E-17	0.169883579	1.99E-15	NS
SLC22A12	NS	3.53E-17	0.169773769	2.07E-15	NS
CYP26A1	NS	3.78E-17	0.169610603	2.22E-15	NS
TIMM8B	NS	3.80E-17	-0.169597421	2.22E-15	NS
OR5I1	NS	3.88E-17	-0.169549983	2.26E-15	NS
KLRC1	NS	3.90E-17	-0.169539296	2.27E-15	NS
ARL4D	NS	3.97E-17	-0.169498101	2.30E-15	NS
SUPT6H	NS	4.09E-17	0.169426826	2.35E-15	NS
KLRC4	NS	4.08E-17	-0.169433216	2.35E-15	NS
EFNA5	NS	4.38E-17	-0.169271551	2.51E-15	NS
FMO2	NS	4.41E-17	-0.169255769	2.52E-15	NS
SLCO1C1	NS	4.49E-17	-0.169212159	2.55E-15	NS
SPINK13	NS	4.49E-17	-0.169211289	2.55E-15	NS
CGB1	NS	4.69E-17	0.16911042	2.65E-15	NS
HAND2	NS	4.81E-17	0.169050206	2.71E-15	NS
FAM171B	NS	5.35E-17	-0.168802875	3.01E-15	NS
FOXC2	NS	5.59E-17	0.168697888	3.13E-15	NS
GJC2	NS	5.59E-17	0.168699699	3.13E-15	NS
NR2F2	NS	5.69E-17	0.16865758	3.17E-15	NS
C3orf14	NS	6.11E-17	-0.168493247	3.40E-15	NS
IHH	NS	6.49E-17	0.168348791	3.60E-15	NS
UGT2A3	NS	7.34E-17	-0.168062732	4.06E-15	NS
CNBD1	NS	7.45E-17	-0.168027804	4.11E-15	NS
OR5L1	NS	7.70E-17	-0.167947583	4.24E-15	NS

LGALS7	NS	7.93E-17	0.167878427	4.35E-15	NS
PPIAL4H	NS	8.36E-17	0.167754916	4.57E-15	NS
KLK7	NS	8.92E-17	0.167604018	4.86E-15	NS
KRTAP4-8	NS	9.06E-17	0.167567515	4.92E-15	NS
CCR9	NS	9.12E-17	-0.167550796	4.94E-15	NS
KCNK15	NS	9.40E-17	0.167478396	5.08E-15	NS
MAGEB10	NS	9.56E-17	0.167439838	5.15E-15	NS
SOX8	NS	1.00E-16	0.167327838	5.39E-15	NS
NKD2	NS	1.05E-16	0.167227611	5.61E-15	NS
HMX2	NS	1.05E-16	0.167213157	5.63E-15	NS
TMPRSS11A	NS	1.08E-16	-0.167154845	5.75E-15	NS
RNF150	NS	1.09E-16	-0.167122395	5.82E-15	NS
SPACA4	NS	1.17E-16	0.166957102	6.22E-15	NS
C1QL4	NS	1.19E-16	0.16692142	6.30E-15	NS
CDCA2	NS	1.27E-16	-0.166760224	6.72E-15	NS
ZNF680	NS	1.30E-16	-0.166708401	6.85E-15	NS
EN2	NS	1.44E-16	0.166473826	7.55E-15	NS
DMRTA2	NS	1.50E-16	0.1663805	7.83E-15	NS
RTN4RL1	NS	1.68E-16	0.166107715	8.76E-15	NS
LINGO1	NS	1.71E-16	0.166060557	8.91E-15	NS
EMX2	NS	1.89E-16	0.165826364	9.80E-15	NS
SLC6A18	NS	2.05E-16	0.165627494	1.06E-14	NS
ETDC	NS	2.07E-16	-0.165607095	1.07E-14	NS
SMIM32	NS	2.12E-16	0.165554551	1.09E-14	NS
DMRTB1	NS	2.14E-16	0.165529965	1.10E-14	NS
SULT4A1	NS	2.17E-16	0.165493869	1.11E-14	NS
KRTAP10-6	NS	2.50E-16	0.165162495	1.27E-14	NS
BBS12	NS	2.54E-16	-0.165121056	1.29E-14	NS
T5C1B-RDH	NS	2.56E-16	-0.165097008	1.30E-14	NS
MRPS31	NS	2.59E-16	-0.165074177	1.31E-14	NS
KIR2DL4	NS	2.59E-16	-0.165073903	1.31E-14	NS
GAS1	NS	2.62E-16	0.165041474	1.32E-14	NS
LINGO2	NS	2.65E-16	-0.165020544	1.33E-14	NS
BCEL-TECT	NS	2.90E-16	-0.164804249	1.45E-14	NS
CLEC3B	NS	3.06E-16	-0.164677257	1.52E-14	NS
MORC1	NS	3.16E-16	-0.164593113	1.57E-14	NS
HFM1	NS	3.34E-16	-0.164466685	1.66E-14	NS
ACMSD	NS	3.56E-16	-0.164311322	1.76E-14	NS
MNX1	NS	3.83E-16	0.164131682	1.89E-14	NS
SCYGR4	NS	3.87E-16	0.164108031	1.91E-14	NS
ARX	NS	3.94E-16	0.16406554	1.94E-14	NS
GYS2	NS	4.14E-16	-0.163946142	2.03E-14	NS
FAM43B	NS	4.16E-16	0.163933522	2.04E-14	NS
SCX	NS	4.53E-16	0.163732125	2.21E-14	NS
CISD1	NS	4.65E-16	-0.163669199	2.25E-14	NS
FMO4	NS	4.72E-16	-0.163630286	2.28E-14	NS
BTG4	NS	4.82E-16	-0.163582097	2.32E-14	NS
GSPT2	NS	4.81E-16	-0.16358361	2.32E-14	NS
HBZ	NS	4.83E-16	0.163576032	2.32E-14	NS
SAPCD1	NS	4.95E-16	-0.163516678	2.37E-14	NS
GDF6	NS	5.03E-16	0.163479393	2.40E-14	NS
RAG2	NS	5.05E-16	-0.16346888	2.41E-14	NS
TSHB	NS	5.17E-16	-0.163412719	2.46E-14	NS
GAL3ST3	NS	5.23E-16	0.163382617	2.48E-14	NS
TMEM145	NS	5.44E-16	0.163288509	2.57E-14	NS

MBIP	NS	5.65E-16	-0.163196886	2.67E-14	NS
NR5A1	NS	5.66E-16	0.163190444	2.67E-14	NS
WNT9A	NS	5.69E-16	0.163178564	2.67E-14	NS
NALF2	NS	5.80E-16	0.163132958	2.72E-14	NS
STEAP1	NS	5.90E-16	-0.163090588	2.76E-14	NS
C1QL2	NS	5.93E-16	0.163078205	2.77E-14	NS
EGR4	NS	5.98E-16	0.163060278	2.78E-14	NS
GPR62	NS	6.07E-16	0.163021496	2.82E-14	NS
TRMT10A	NS	6.13E-16	-0.162998815	2.84E-14	NS
FAM13C	NS	6.14E-16	-0.162994667	2.84E-14	NS
GCGR	NS	6.53E-16	0.162845655	3.01E-14	NS
PRR35	NS	7.17E-16	0.162619905	3.30E-14	NS
NUDT2	NS	7.19E-16	-0.162612334	3.30E-14	NS
SPAM1	NS	7.31E-16	-0.162572708	3.34E-14	NS
SPEM2	NS	7.76E-16	0.162428266	3.54E-14	NS
IGIP	NS	7.80E-16	-0.162413597	3.55E-14	NS
CYP2W1	NS	7.83E-16	0.16240401	3.56E-14	NS
CXCL9	NS	8.58E-16	-0.162183001	3.89E-14	NS
ARL14EPL	NS	8.62E-16	-0.162170402	3.90E-14	NS
FAM71E2	NS	9.09E-16	0.162041846	4.10E-14	NS
BEND5	NS	9.32E-16	-0.16197972	4.20E-14	NS
LIPF	NS	9.47E-16	-0.161941755	4.25E-14	NS
KREMEN2	NS	9.49E-16	0.161935375	4.25E-14	NS
NKX2-3	NS	9.72E-16	0.161877173	4.35E-14	NS
H2BE1	NS	1.08E-15	-0.161612511	4.83E-14	NS
TAAR2	NS	1.11E-15	-0.161559242	4.93E-14	NS
GRPR	NS	1.13E-15	-0.161502757	5.03E-14	NS
NMRK2	NS	1.14E-15	0.16149191	5.04E-14	NS
GPT	NS	1.19E-15	0.161389828	5.25E-14	NS
PFN3	NS	1.21E-15	0.161347034	5.33E-14	NS
FXYD2	NS	1.22E-15	0.161317565	5.38E-14	NS
SLC25A27	NS	1.30E-15	-0.161161484	5.72E-14	NS
PACSIN1	NS	1.36E-15	0.161048461	5.97E-14	NS
GSC2	NS	1.49E-15	0.160834486	6.50E-14	NS
KRTAP24-1	NS	1.50E-15	-0.160819389	6.53E-14	NS
ABCB1	NS	1.59E-15	-0.160674132	6.91E-14	NS
PRTN3	NS	1.59E-15	0.160666312	6.92E-14	NS
KIF6	NS	1.67E-15	-0.16054892	7.24E-14	NS
PRR4	NS	1.68E-15	-0.160539625	7.25E-14	NS
FGFR3	NS	1.69E-15	0.16051586	7.31E-14	NS
REC114	NS	1.70E-15	-0.160501415	7.33E-14	NS
MAGEA2	NS	1.72E-15	0.160475302	7.39E-14	NS
CCT8L2	NS	1.79E-15	0.160386455	7.63E-14	NS
BOD1L2	NS	1.83E-15	0.160332059	7.78E-14	NS
C1QC	NS	1.85E-15	-0.160298299	7.87E-14	NS
OR13C2	NS	2.02E-15	-0.160088403	8.56E-14	NS
OR5D3P	NS	2.04E-15	-0.16006161	8.63E-14	NS
EFCAB7	NS	2.11E-15	-0.159974335	8.92E-14	NS
RDH14	NS	2.15E-15	-0.159931999	9.04E-14	NS
OR13C5	NS	2.15E-15	-0.159934323	9.04E-14	NS
NKAIN1	NS	2.24E-15	0.159830059	9.40E-14	NS
EID3	NS	2.24E-15	-0.159823534	9.40E-14	NS
TRDN	NS	2.33E-15	-0.159725927	9.76E-14	NS
NKG7	NS	2.34E-15	-0.159719824	9.76E-14	NS
ACKR4	NS	2.39E-15	-0.159668854	9.94E-14	NS

SCYGR7	NS	2.55E-15	0.159510482	1.06E-13	NS
MYCN	NS	2.55E-15	0.159503725	1.06E-13	NS
DAZL	NS	2.66E-15	-0.159401989	1.10E-13	NS
SIX3	NS	2.67E-15	0.159397154	1.10E-13	NS
KRTAP10-2	NS	2.75E-15	0.159319005	1.13E-13	NS
GABRB1	NS	2.96E-15	-0.159141038	1.21E-13	NS
ARMCX2	NS	3.09E-15	-0.159029511	1.27E-13	NS
ZNF35	NS	3.20E-15	-0.158941953	1.31E-13	NS
KLRG1	NS	3.22E-15	-0.158929298	1.31E-13	NS
LRRCC1	NS	3.31E-15	-0.158862404	1.35E-13	NS
GIMAP7	NS	3.38E-15	-0.158809469	1.37E-13	NS
LIPM	NS	3.44E-15	-0.158761281	1.40E-13	NS
KRTAP10-1	NS	3.58E-15	0.158664131	1.45E-13	NS
KCNH4	NS	3.82E-15	0.158500502	1.54E-13	NS
SOWAHA	NS	3.86E-15	0.158474177	1.56E-13	NS
TAF11L13	NS	3.99E-15	0.158390743	1.61E-13	NS
DLL3	NS	4.02E-15	0.158376505	1.61E-13	NS
SPTA1	NS	4.03E-15	-0.158369272	1.61E-13	NS
MRFAP1L1	NS	4.20E-15	-0.158266921	1.68E-13	NS
NYX	NS	4.37E-15	0.158168792	1.74E-13	NS
TEX12	NS	4.63E-15	-0.15802059	1.84E-13	NS
GBX2	NS	4.81E-15	0.157924234	1.91E-13	NS
KRTAP4-9	NS	4.98E-15	0.157840993	1.97E-13	NS
TLX3	NS	5.12E-15	0.157768537	2.02E-13	NS
CENPW	NS	5.13E-15	-0.157764244	2.02E-13	NS
ETAA1	NS	5.21E-15	-0.15772562	2.05E-13	NS
GJA8	NS	5.33E-15	-0.157670222	2.09E-13	NS
SNX7	NS	5.35E-15	-0.157662137	2.09E-13	NS
DCAF12L2	NS	5.63E-15	0.157532819	2.20E-13	NS
CNTN6	NS	5.72E-15	-0.157490621	2.22E-13	NS
DSG1	NS	5.71E-15	-0.157497982	2.22E-13	NS
HPGD	NS	5.72E-15	-0.157492377	2.22E-13	NS
FGF3	NS	5.72E-15	0.157493821	2.22E-13	NS
SERPINA9	NS	5.79E-15	-0.157461503	2.24E-13	NS
FOXL2	NS	5.93E-15	0.157400988	2.29E-13	NS
CAMK2N1	NS	6.21E-15	0.157284005	2.39E-13	NS
RAB39B	NS	6.35E-15	-0.157230546	2.44E-13	NS
ZNF723	NS	6.44E-15	0.1571934	2.47E-13	NS
CLVS2	NS	6.49E-15	-0.157174146	2.48E-13	NS
SMIM45	NS	7.02E-15	0.156975888	2.68E-13	NS
NOX3	NS	7.06E-15	-0.156964708	2.69E-13	NS
PRSS56	NS	7.07E-15	0.156959206	2.69E-13	NS
IL1RAPL1	NS	7.41E-15	-0.156840353	2.81E-13	NS
TLL1	NS	7.43E-15	-0.156835052	2.81E-13	NS
CFAP43	NS	7.58E-15	0.15678488	2.86E-13	NS
OBP2B	NS	7.99E-15	0.156650514	3.02E-13	NS
KITLG	NS	8.10E-15	-0.156616712	3.05E-13	NS
OR2J1	NS	8.58E-15	-0.156470046	3.23E-13	NS
HSPB6	NS	8.88E-15	0.156385418	3.33E-13	NS
AM47E-STBI	NS	9.03E-15	-0.156341285	3.38E-13	NS
WNT3A	NS	9.25E-15	0.156281175	3.45E-13	NS
BGLAP	NS	9.25E-15	-0.156280016	3.45E-13	NS
SSTR3	NS	9.52E-15	0.156208327	3.54E-13	NS
PAK5	NS	9.93E-15	-0.15610243	3.69E-13	NS
MMRN1	NS	1.02E-14	-0.156023118	3.80E-13	NS

TBC1D19	NS	1.04E-14	-0.155981141	3.85E-13	NS
KHDRBS2	NS	1.05E-14	-0.155965944	3.87E-13	NS
PLSCR5	NS	1.08E-14	-0.155898514	3.97E-13	NS
POPDC2	NS	1.10E-14	-0.155834408	4.06E-13	NS
VIP	NS	1.12E-14	-0.155803184	4.10E-13	NS
TRIM77	NS	1.16E-14	-0.155715139	4.24E-13	NS
SAA4	NS	1.16E-14	-0.1557083	4.24E-13	NS
IZUMO3	NS	1.21E-14	-0.155592634	4.43E-13	NS
ZNF184	NS	1.26E-14	-0.155495531	4.60E-13	NS
CCDC177	NS	1.27E-14	0.155483239	4.61E-13	NS
PPFIA2	NS	1.31E-14	-0.155407716	4.74E-13	NS
MACIR	NS	1.32E-14	-0.155386632	4.77E-13	NS
ALPP	NS	1.34E-14	0.155345202	4.84E-13	NS
CACNG7	NS	1.37E-14	0.155287983	4.93E-13	NS
CA10	NS	1.37E-14	-0.155286653	4.93E-13	NS
HES3	NS	1.39E-14	0.1552492	5.00E-13	NS
SLC2A12	NS	1.47E-14	-0.155101178	5.29E-13	NS
NDUFAF4	NS	1.52E-14	-0.155027079	5.43E-13	NS
GATA5	NS	1.56E-14	0.154953483	5.56E-13	NS
EPHX4	NS	1.56E-14	0.154954624	5.56E-13	NS
HOXD8	NS	1.56E-14	0.154959327	5.56E-13	NS
FGA	NS	1.56E-14	-0.154947279	5.56E-13	NS
HES4	NS	1.59E-14	0.15490437	5.65E-13	NS
PPCDC	NS	1.61E-14	0.15486535	5.72E-13	NS
OVCH1	NS	1.64E-14	-0.154823811	5.81E-13	NS
SOX21	NS	1.64E-14	0.154818331	5.81E-13	NS
CCDC188	NS	1.68E-14	0.154771332	5.91E-13	NS
LBX1	NS	1.73E-14	0.154692588	6.07E-13	NS
PRR36	NS	1.73E-14	0.154695436	6.07E-13	NS
CYSRT1	NS	1.74E-14	0.15466765	6.12E-13	NS
GPR171	NS	1.76E-14	-0.154645567	6.16E-13	NS
CD248	NS	1.79E-14	0.154594987	6.27E-13	NS
TCF24	NS	1.87E-14	0.154496567	6.51E-13	NS
INSL6	NS	1.95E-14	-0.154388337	6.77E-13	NS
OLFM3	NS	1.99E-14	-0.154333599	6.91E-13	NS
SLC5A5	NS	2.04E-14	0.154261842	7.09E-13	NS
CCNB3	NS	2.08E-14	-0.154212733	7.22E-13	NS
HMMR	NS	2.19E-14	-0.154087433	7.56E-13	NS
NXNL2	NS	2.22E-14	0.154053613	7.65E-13	NS
CYP39A1	NS	2.29E-14	-0.153971805	7.88E-13	NS
C1orf210	NS	2.33E-14	0.15392878	8.00E-13	NS
SMIM30	NS	2.42E-14	-0.153830683	8.30E-13	NS
RRS1	NS	2.52E-14	-0.153721402	8.64E-13	NS
MKS1	NS	2.56E-14	0.153684257	8.75E-13	NS
IL1RL1	NS	2.56E-14	-0.153679114	8.76E-13	NS
EBF4	NS	2.62E-14	0.153627107	8.92E-13	NS
TGFBR3L	NS	2.63E-14	0.153615432	8.94E-13	NS
LHB	NS	2.64E-14	0.153605986	8.96E-13	NS
FABP3	NS	2.75E-14	-0.153500237	9.32E-13	NS
EN1	NS	2.81E-14	0.153444751	9.51E-13	NS
ABCD2	NS	2.82E-14	-0.153437604	9.52E-13	NS
ESF1	NS	2.94E-14	-0.153323244	9.93E-13	NS
H2AC16	NS	2.95E-14	-0.15331904	9.93E-13	NS
SSU72P2	NS	3.03E-14	0.153245912	1.02E-12	NS
ADORA3	NS	3.13E-14	-0.15316912	1.05E-12	NS

NUF2	NS	3.29E-14	-0.153038817	1.10E-12	NS
TSBP1	NS	3.53E-14	-0.152855442	1.18E-12	NS
CENPQ	NS	3.60E-14	-0.152804459	1.20E-12	NS
C1QTNF8	NS	3.80E-14	0.15266445	1.27E-12	NS
OR4C16	NS	3.83E-14	-0.152643271	1.27E-12	NS
ELFN1	NS	3.86E-14	0.15262619	1.28E-12	NS
SOX9	NS	3.88E-14	0.152608482	1.29E-12	NS
PARD6G	NS	3.88E-14	0.152607353	1.29E-12	NS
FAM180B	NS	4.04E-14	0.152502674	1.34E-12	NS
OR4F6	NS	4.17E-14	-0.152422222	1.38E-12	NS
MSLNL	NS	4.26E-14	0.152368617	1.40E-12	NS
TENT5C	NS	4.37E-14	-0.152301129	1.44E-12	NS
HSD11B1	NS	4.65E-14	-0.152137721	1.53E-12	NS
CALML6	NS	4.79E-14	0.152063741	1.57E-12	NS
AFP	NS	4.90E-14	-0.152004075	1.60E-12	NS
SCG3	NS	4.94E-14	-0.151982513	1.61E-12	NS
BARHL1	NS	4.95E-14	0.151975675	1.61E-12	NS
KRTAP3-2	NS	5.09E-14	-0.151904989	1.65E-12	NS
OR6C2	NS	5.16E-14	-0.151869417	1.67E-12	NS
OVCH2	NS	5.28E-14	-0.151810888	1.71E-12	NS
EVX2	NS	5.45E-14	0.151727591	1.76E-12	NS
VHLL	NS	5.48E-14	0.151711725	1.77E-12	NS
HOXC6	NS	5.52E-14	0.151691999	1.78E-12	NS
CITED4	NS	5.56E-14	0.151677232	1.79E-12	NS
DDR2	NS	5.58E-14	-0.151665683	1.79E-12	NS
MAPK15	NS	5.61E-14	0.151650934	1.80E-12	NS
CATSPERZ	NS	5.64E-14	0.151638947	1.80E-12	NS
MMP3	NS	5.78E-14	-0.151575447	1.84E-12	NS
COL21A1	NS	5.86E-14	-0.151540028	1.87E-12	NS
DKK2	NS	5.89E-14	-0.151524758	1.87E-12	NS
AMER3	NS	6.01E-14	0.151473175	1.91E-12	NS
FAM24B	NS	6.24E-14	-0.151371719	1.98E-12	NS
ZNF404	NS	6.41E-14	-0.151302858	2.03E-12	NS
ALPI	NS	6.51E-14	0.151262977	2.06E-12	NS
COL3A1	NS	6.61E-14	-0.15122215	2.09E-12	NS
CLDN6	NS	6.72E-14	0.151178373	2.12E-12	NS
GP9	NS	7.03E-14	-0.151063445	2.21E-12	NS
CCN6	NS	7.09E-14	-0.151041074	2.23E-12	NS
HTR1F	NS	7.33E-14	-0.150951782	2.30E-12	NS
IFNK	NS	7.44E-14	-0.150914842	2.33E-12	NS
FGF10	NS	7.59E-14	-0.150860198	2.37E-12	NS
TMEM52	NS	7.83E-14	0.150779193	2.44E-12	NS
GPR34	NS	7.89E-14	-0.150758338	2.46E-12	NS
ACR	NS	7.91E-14	0.150753598	2.46E-12	NS
SCOC	NS	8.17E-14	-0.150666362	2.54E-12	NS
KRTAP10-7	NS	8.87E-14	0.150452057	2.75E-12	NS
ODAM	NS	8.99E-14	-0.150416625	2.78E-12	NS
RAB33A	NS	9.15E-14	-0.150370774	2.82E-12	NS
MTERF2	NS	9.29E-14	-0.150330716	2.86E-12	NS
ATP1B2	NS	9.36E-14	0.150311112	2.88E-12	NS
OVOL3	NS	9.41E-14	0.150296769	2.89E-12	NS
ZIC3	NS	9.46E-14	0.150283131	2.90E-12	NS
IMPG1	NS	9.91E-14	-0.150160248	3.03E-12	NS
HORMAD2	NS	1.01E-13	-0.150109441	3.09E-12	NS
THEMIS	NS	1.03E-13	-0.150052263	3.15E-12	NS

ADM2	NS	1.04E-13	0.150021763	3.18E-12	NS
APOE	NS	1.05E-13	0.149999833	3.21E-12	NS
RBM45	NS	1.06E-13	-0.149988921	3.21E-12	NS
TAS2R50	NS	1.06E-13	-0.14998735	3.21E-12	NS
GCSAM	NS	1.11E-13	-0.149851625	3.37E-12	NS
MXRA8	NS	1.12E-13	0.149830417	3.40E-12	NS
TMEFF2	NS	1.17E-13	-0.149721161	3.53E-12	NS
ATP6V1G3	NS	1.19E-13	-0.149682597	3.58E-12	NS
CDKL4	NS	1.24E-13	-0.149558221	3.75E-12	NS
NDFIP2	NS	1.30E-13	-0.149450364	3.90E-12	NS
GRIN1	NS	1.32E-13	0.149407813	3.95E-12	NS
RIPPLY3	NS	1.38E-13	0.149282606	4.14E-12	NS
OIP5	NS	1.46E-13	-0.149138001	4.36E-12	NS
CCNP	NS	1.47E-13	0.149109135	4.41E-12	NS
CHST1	NS	1.49E-13	0.149075218	4.45E-12	NS
KRTAP4-16	NS	1.50E-13	0.149062341	4.47E-12	NS
KDR	NS	1.52E-13	-0.149027522	4.52E-12	NS
GPIHBP1	NS	1.55E-13	0.14897861	4.60E-12	NS
TAAR1	NS	1.57E-13	-0.148938743	4.66E-12	NS
OR6A2	NS	1.70E-13	-0.148732513	5.03E-12	NS
LHX3	NS	1.74E-13	0.148668273	5.14E-12	NS
TINAG	NS	1.78E-13	-0.148613192	5.24E-12	NS
ARTN	NS	1.82E-13	0.148546302	5.37E-12	NS
TECRL	NS	1.90E-13	-0.148434953	5.59E-12	NS
OTUD6A	NS	2.00E-13	0.148302622	5.87E-12	NS
ANGPTL3	NS	2.05E-13	-0.148230004	6.02E-12	NS
FAM110D	NS	2.09E-13	0.148182278	6.12E-12	NS
DCSTAMP	NS	2.13E-13	-0.148133963	6.22E-12	NS
ZNF300	NS	2.15E-13	-0.148101319	6.29E-12	NS
B4GALT2	NS	2.17E-13	0.148081285	6.32E-12	NS
TAS2R41	NS	2.18E-13	-0.148066446	6.35E-12	NS
TPM4	NS	2.21E-13	0.148031106	6.43E-12	NS
SYT4	NS	2.22E-13	-0.148019576	6.43E-12	NS
ACOT4	NS	2.22E-13	0.148020306	6.43E-12	NS
SULT1E1	NS	2.37E-13	-0.147844531	6.86E-12	NS
LXN	NS	2.48E-13	-0.147725237	7.16E-12	NS
HOMER1	NS	2.64E-13	-0.147555909	7.62E-12	NS
CDH15	NS	2.69E-13	0.147502698	7.76E-12	NS
C5orf63	NS	2.80E-13	-0.147394413	8.06E-12	NS
PPIAL4E	NS	2.82E-13	0.147380874	8.09E-12	NS
RBBP8NL	NS	2.83E-13	0.147370164	8.11E-12	NS
LGALS14	NS	2.86E-13	-0.147338256	8.20E-12	NS
RFC3	NS	2.90E-13	-0.147305462	8.29E-12	NS
TMEM244	NS	2.92E-13	-0.14728515	8.34E-12	NS
PDCL3	NS	2.95E-13	-0.147260542	8.40E-12	NS
WLS	NS	3.01E-13	-0.147205455	8.56E-12	NS
MUCL1	NS	3.02E-13	-0.147194816	8.58E-12	NS
REN	NS	3.03E-13	0.147190416	8.59E-12	NS
TPD52L2	NS	3.05E-13	0.147172461	8.63E-12	NS
C8orf88	NS	3.06E-13	-0.147161439	8.65E-12	NS
OSTN	NS	3.12E-13	-0.147109267	8.81E-12	NS
C1QL3	NS	3.16E-13	-0.147070053	8.93E-12	NS
CYTL1	NS	3.37E-13	-0.14690178	9.47E-12	NS
C2CD4A	NS	3.37E-13	0.14690351	9.47E-12	NS
BRINP3	NS	3.43E-13	-0.146854256	9.63E-12	NS

MRPL36	NS	3.46E-13	-0.146828069	9.71E-12	NS
MMP8	NS	3.49E-13	-0.146806436	9.77E-12	NS
STAC2	NS	3.59E-13	0.146728851	1.00E-11	NS
SPATA24	NS	3.59E-13	-0.146731399	1.00E-11	NS
GNRHR	NS	3.76E-13	-0.146606677	1.05E-11	NS
LRFN3	NS	3.85E-13	0.146539189	1.07E-11	NS
MAGEA2B	NS	3.87E-13	0.146526503	1.08E-11	NS
SRCIN1	NS	3.89E-13	0.146514393	1.08E-11	NS
NMUR2	NS	4.00E-13	-0.146437478	1.11E-11	NS
ERH	NS	4.14E-13	-0.146345329	1.15E-11	NS
ZNRF4	NS	4.20E-13	0.1463038	1.16E-11	NS
NAT16	NS	4.57E-13	0.146075298	1.26E-11	NS
RAI2	NS	4.85E-13	0.145916834	1.34E-11	NS
CD164L2	NS	4.91E-13	0.145880567	1.35E-11	NS
LMO3	NS	4.98E-13	-0.145846062	1.37E-11	NS
P3A7-CYP3A	NS	5.17E-13	-0.145744555	1.42E-11	NS
PDE1A	NS	5.29E-13	-0.145679508	1.45E-11	NS
FIBCD1	NS	5.46E-13	0.145593773	1.50E-11	NS
ZNF79	NS	5.54E-13	-0.145555346	1.51E-11	NS
LEAP2	NS	5.56E-13	-0.145544137	1.52E-11	NS
ANKRD65	NS	5.56E-13	0.145546149	1.52E-11	NS
COX6B2	NS	5.61E-13	0.145519301	1.53E-11	NS
DRD4	NS	5.63E-13	0.145512156	1.53E-11	NS
C12orf40	NS	5.69E-13	-0.145481619	1.54E-11	NS
ZWINT	NS	6.14E-13	-0.145275592	1.66E-11	NS
CDKN3	NS	6.18E-13	-0.145256092	1.67E-11	NS
CD1C	NS	6.20E-13	-0.145250275	1.67E-11	NS
MYL10	NS	6.30E-13	0.145203652	1.70E-11	NS
KCNK13	NS	6.30E-13	-0.145204218	1.70E-11	NS
RHOXF2	NS	6.57E-13	-0.14509159	1.77E-11	NS
GALR3	NS	6.81E-13	0.144991883	1.83E-11	NS
HOGA1	NS	6.85E-13	0.144978294	1.84E-11	NS
IL33	NS	6.93E-13	-0.144944457	1.86E-11	NS
KRTAP10-3	NS	6.93E-13	0.144944767	1.86E-11	NS
GABRA3	NS	7.06E-13	-0.144894171	1.89E-11	NS
ZPBP	NS	7.79E-13	-0.144624719	2.08E-11	NS
MSI1	NS	7.86E-13	0.144601558	2.09E-11	NS
THEG	NS	8.38E-13	0.144424437	2.23E-11	NS
SLC28A3	NS	8.49E-13	-0.144391132	2.26E-11	NS
ERICH6B	NS	8.54E-13	-0.144374731	2.27E-11	NS
KLK5	NS	9.06E-13	0.14421148	2.40E-11	NS
ZNF449	NS	9.53E-13	-0.144071329	2.52E-11	NS
CFAP300	NS	9.69E-13	0.144027139	2.56E-11	NS
GNA14	NS	9.73E-13	-0.14401448	2.57E-11	NS
SLC35F4	NS	9.87E-13	-0.143976176	2.60E-11	NS
TAFA1	NS	9.90E-13	-0.143967803	2.61E-11	NS
TIMM10	NS	1.06E-12	-0.143780023	2.78E-11	NS
ACTL7B	NS	1.06E-12	0.143782424	2.78E-11	NS
ADRA2A	NS	1.06E-12	0.143772196	2.79E-11	NS
MEX3A	NS	1.07E-12	0.143761688	2.79E-11	NS
CRISP1	NS	1.12E-12	-0.143616597	2.94E-11	NS
GKN1	NS	1.14E-12	-0.143569274	2.99E-11	NS
OR4F29	NS	1.15E-12	-0.143561992	2.99E-11	NS
LAG3	NS	1.17E-12	0.143508832	3.05E-11	NS
PRSS51	NS	1.18E-12	-0.143475656	3.08E-11	NS

C14orf180	NS	1.23E-12	0.143360742	3.21E-11	NS
STAP1	NS	1.33E-12	-0.143161032	3.44E-11	NS
SERPINI1	NS	1.33E-12	-0.14315782	3.44E-11	NS
TMEM86B	NS	1.33E-12	-0.143152327	3.44E-11	NS
SSU72P5	NS	1.35E-12	0.143118859	3.48E-11	NS
CPEB2	NS	1.37E-12	0.14306407	3.55E-11	NS
SLCO1B3	NS	1.38E-12	-0.143045883	3.56E-11	NS
FUT1	NS	1.39E-12	0.143024395	3.59E-11	NS
C19orf67	NS	1.43E-12	0.14295128	3.68E-11	NS
RAB23	NS	1.48E-12	-0.142854524	3.80E-11	NS
SSBP4	NS	1.49E-12	0.142844243	3.81E-11	NS
DDX53	NS	1.50E-12	-0.14282251	3.84E-11	NS
RTN4IP1	NS	1.53E-12	-0.142773325	3.90E-11	NS
FAM177B	NS	1.52E-12	-0.14277554	3.90E-11	NS
ISOC1	NS	1.54E-12	-0.142741566	3.94E-11	NS
TUSC1	NS	1.60E-12	-0.142639795	4.08E-11	NS
MMP10	NS	1.61E-12	-0.142628173	4.09E-11	NS
ZNF132	NS	1.62E-12	-0.142613265	4.11E-11	NS
MTERF1	NS	1.65E-12	-0.142548374	4.20E-11	NS
HOXA11	NS	1.66E-12	0.142535722	4.21E-11	NS
FOXJ1	NS	1.69E-12	0.142485484	4.28E-11	NS
SMCO2	NS	1.70E-12	-0.142481688	4.28E-11	NS
NPSR1	NS	1.79E-12	-0.14233417	4.51E-11	NS
RMI1	NS	1.82E-12	-0.14227963	4.59E-11	NS
CWH43	NS	1.87E-12	-0.142206332	4.70E-11	NS
CTRB1	NS	1.89E-12	0.142183019	4.74E-11	NS
PPIAL4F	NS	1.90E-12	0.142160053	4.77E-11	NS
ERBB4	NS	1.95E-12	-0.142096075	4.88E-11	NS
ONECUT3	NS	1.99E-12	0.142036239	4.98E-11	NS
NXPE2	NS	2.04E-12	-0.141967954	5.09E-11	NS
CALY	NS	2.11E-12	0.141872033	5.26E-11	NS
SBK2	NS	2.12E-12	0.141861327	5.28E-11	NS
OLFM4	NS	2.15E-12	-0.141813621	5.36E-11	NS
FOXA1	NS	2.18E-12	0.141782159	5.42E-11	NS
FEV	NS	2.19E-12	0.1417729	5.43E-11	NS
ADAM22	NS	2.20E-12	-0.141750462	5.46E-11	NS
FSTL5	NS	2.21E-12	-0.14174045	5.46E-11	NS
EXOC3L2	NS	2.21E-12	0.141742356	5.46E-11	NS
SMIM44	NS	2.21E-12	0.141738661	5.46E-11	NS
CD1E	NS	2.22E-12	-0.141732734	5.47E-11	NS
LRRIQ3	NS	2.26E-12	-0.14168645	5.55E-11	NS
HOXA6	NS	2.31E-12	0.141617609	5.68E-11	NS
SMCO3	NS	2.36E-12	-0.141564976	5.78E-11	NS
GBP7	NS	2.50E-12	-0.141399663	6.12E-11	NS
C10orf82	NS	2.56E-12	0.141336133	6.26E-11	NS
MIX23	NS	2.62E-12	-0.141271382	6.39E-11	NS
MARS2	NS	2.76E-12	-0.141123584	6.73E-11	NS
BEX3	NS	2.78E-12	-0.141103601	6.75E-11	NS
COMMD1	NS	2.77E-12	-0.141107001	6.75E-11	NS
ZNF454	NS	2.81E-12	0.141066044	6.84E-11	NS
DZIP1L	NS	2.86E-12	-0.141022313	6.93E-11	NS
TBC1D15	NS	2.94E-12	0.140945551	7.10E-11	NS
SYTL5	NS	2.94E-12	-0.140948233	7.10E-11	NS
CATSPER3	NS	2.94E-12	-0.140944827	7.10E-11	NS
CYP2U1	NS	2.96E-12	-0.140927034	7.14E-11	NS

SEMA3G	NS	2.99E-12	0.140892092	7.22E-11	NS
COLEC10	NS	3.00E-12	-0.140883062	7.23E-11	NS
ELOVL6	NS	3.02E-12	-0.140867158	7.27E-11	NS
KLF14	NS	3.03E-12	0.140862604	7.27E-11	NS
NANOGNB	NS	3.08E-12	0.140814359	7.38E-11	NS
STBD1	NS	3.14E-12	-0.140757901	7.52E-11	NS
MIOX	NS	3.21E-12	0.140693357	7.68E-11	NS
ZSWIM2	NS	3.22E-12	-0.140692421	7.68E-11	NS
DBNDD1	NS	3.24E-12	0.140669953	7.74E-11	NS
C1orf105	NS	3.30E-12	-0.140616377	7.87E-11	NS
CWF19L2	NS	3.35E-12	-0.140579703	7.97E-11	NS
SPEM1	NS	3.47E-12	0.140480523	8.24E-11	NS
CEACAM21	NS	3.50E-12	-0.140451428	8.32E-11	NS
GNG3	NS	3.52E-12	-0.140439838	8.34E-11	NS
RNF223	NS	3.53E-12	0.140427471	8.37E-11	NS
SYT1	NS	3.62E-12	-0.140361858	8.56E-11	NS
KRTAP10-11	NS	3.67E-12	0.140322664	8.66E-11	NS
OR8B4	NS	4.12E-12	-0.139994498	9.72E-11	NS
CSN2	NS	4.18E-12	-0.139954952	9.85E-11	NS
PHOSPHO2	NS	4.20E-12	-0.139940217	9.88E-11	NS
ZBTB12	NS	4.26E-12	0.139898148	1.00E-10	NS
MANEA	NS	4.28E-12	-0.139883504	1.01E-10	NS
LYG1	NS	4.37E-12	-0.139823774	1.03E-10	NS
BET1	NS	4.46E-12	-0.139771173	1.04E-10	NS
TAS2R30	NS	4.50E-12	-0.139742322	1.05E-10	NS
SLFN12	NS	4.51E-12	-0.13973633	1.05E-10	NS
METTL13	NS	4.57E-12	-0.139699038	1.07E-10	NS
RNF180	NS	4.65E-12	-0.139652111	1.08E-10	NS
MRPS14	NS	5.02E-12	-0.139433837	1.17E-10	NS
KLK12	NS	5.18E-12	0.139342261	1.21E-10	NS
FASLG	NS	5.28E-12	-0.139291918	1.23E-10	NS
TACR1	NS	5.39E-12	-0.139229761	1.25E-10	NS
RTL6	NS	5.61E-12	0.139119071	1.30E-10	NS
APOC4	NS	5.63E-12	0.139107953	1.30E-10	NS
CSPG5	NS	5.93E-12	0.138962137	1.37E-10	NS
MRPL35	NS	6.19E-12	-0.138839123	1.43E-10	NS
GRM8	NS	6.42E-12	-0.138735019	1.48E-10	NS
HESX1	NS	6.67E-12	-0.138623649	1.54E-10	NS
GOT1	NS	6.73E-12	-0.138600553	1.55E-10	NS
KCNH7	NS	6.89E-12	-0.138529848	1.58E-10	NS
OR12D3	NS	6.91E-12	-0.13852529	1.58E-10	NS
HOXD9	NS	6.93E-12	0.138514633	1.59E-10	NS
TBCEL	NS	7.11E-12	-0.138441517	1.63E-10	NS
PCSK4	NS	7.21E-12	0.138400319	1.65E-10	NS
CRISP3	NS	7.33E-12	-0.138355258	1.67E-10	NS
MEIKIN	NS	7.57E-12	-0.138264244	1.72E-10	NS
OR6C1	NS	7.83E-12	-0.1381674	1.78E-10	NS
NKX2-2	NS	7.84E-12	0.138161477	1.78E-10	NS
SMIM17	NS	8.04E-12	0.138090426	1.82E-10	NS
PTPRR	NS	8.07E-12	-0.138079018	1.83E-10	NS
AIRE	NS	8.37E-12	0.137975309	1.89E-10	NS
LGR5	NS	8.47E-12	-0.137938998	1.92E-10	NS
PRPH	NS	8.81E-12	0.137826941	1.99E-10	NS
MEP1A	NS	9.02E-12	-0.137761437	2.03E-10	NS
DYNLT2	NS	9.09E-12	-0.137739323	2.05E-10	NS

CSAG1	NS	9.13E-12	0.137725974	2.05E-10	NS
SFN	NS	9.15E-12	0.137719499	2.06E-10	NS
PPIL1	NS	9.35E-12	-0.137657274	2.10E-10	NS
GSC	NS	9.36E-12	0.13765274	2.10E-10	NS
DRICH1	NS	9.68E-12	-0.137556371	2.17E-10	NS
BGN	NS	1.04E-11	0.137360933	2.32E-10	NS
CCDC92B	NS	1.04E-11	0.137341085	2.33E-10	NS
CCDC175	NS	1.07E-11	-0.137279633	2.38E-10	NS
ZIC5	NS	1.07E-11	0.137257655	2.39E-10	NS
CDH2	NS	1.09E-11	-0.137224084	2.42E-10	NS
RARRES1	NS	1.09E-11	-0.137214012	2.43E-10	NS
TMEM26	NS	1.15E-11	-0.137058822	2.56E-10	NS
LGI2	NS	1.16E-11	-0.137031903	2.58E-10	NS
SOWAHD	NS	1.22E-11	-0.136896226	2.70E-10	NS
CRY1	NS	1.22E-11	0.13688855	2.70E-10	NS
CDKN2A	NS	1.22E-11	-0.136888688	2.70E-10	NS
SSTR5	NS	1.22E-11	0.136878967	2.71E-10	NS
DNAJB7	NS	1.27E-11	-0.136780273	2.80E-10	NS
PMVK	NS	1.28E-11	-0.136750158	2.82E-10	NS
MKKS	NS	1.30E-11	-0.136707017	2.86E-10	NS
PEX12	NS	1.32E-11	-0.136670164	2.89E-10	NS
CPA3	NS	1.36E-11	-0.136570355	2.99E-10	NS
RPA4	NS	1.38E-11	-0.136524339	3.04E-10	NS
ADIPOQ	NS	1.45E-11	0.136389379	3.18E-10	NS
FNDC4	NS	1.46E-11	0.136374456	3.19E-10	NS
CYP2C18	NS	1.48E-11	-0.136322576	3.24E-10	NS
TMIGD3	NS	1.49E-11	-0.136319738	3.24E-10	NS
C8B	NS	1.50E-11	-0.136292514	3.27E-10	NS
OR8G1	NS	1.50E-11	-0.136293001	3.27E-10	NS
SEC14L1	NS	1.53E-11	0.136242398	3.32E-10	NS
PLA2G3	NS	1.57E-11	0.136157203	3.42E-10	NS
SSU72P4	NS	1.59E-11	0.136129734	3.44E-10	NS
GZMK	NS	1.59E-11	-0.136112614	3.46E-10	NS
H2AC14	NS	1.59E-11	0.13611526	3.46E-10	NS
AADACL3	NS	1.62E-11	-0.136074372	3.50E-10	NS
EEF1AKMT1	NS	1.63E-11	-0.136054664	3.52E-10	NS
PROX2	NS	1.65E-11	-0.13601077	3.57E-10	NS
FOXG1	NS	1.68E-11	0.135955499	3.63E-10	NS
FRMD5	NS	1.70E-11	-0.135922673	3.67E-10	NS
NME1	NS	1.70E-11	-0.135919458	3.67E-10	NS
TEX30	NS	1.71E-11	-0.135905185	3.68E-10	NS
SYCP2	NS	1.74E-11	-0.135857895	3.74E-10	NS
SH2D7	NS	1.75E-11	0.135846881	3.75E-10	NS
KLHL1	NS	1.77E-11	-0.135808421	3.80E-10	NS
MACROD1	NS	1.78E-11	0.135787788	3.82E-10	NS
RGS11	NS	1.85E-11	0.135686678	3.95E-10	NS
TMEM225	NS	1.86E-11	-0.135669013	3.97E-10	NS
C11orf52	NS	1.86E-11	0.135658813	3.98E-10	NS
ANKRD22	NS	1.87E-11	-0.135649187	3.99E-10	NS
OR9K2	NS	1.90E-11	-0.135606935	4.04E-10	NS
OXGR1	NS	1.90E-11	-0.135596108	4.05E-10	NS
KRT20	NS	1.92E-11	-0.135566698	4.09E-10	NS
MOGAT3	NS	1.99E-11	0.135461619	4.23E-10	NS
LRRC63	NS	2.00E-11	-0.135459954	4.23E-10	NS
MTARC2	NS	2.00E-11	-0.1354566	4.23E-10	NS

COX7A1	NS	2.06E-11	-0.135366641	4.36E-10	NS
TXNDC16	NS	2.07E-11	-0.135349761	4.38E-10	NS
DEFB124	NS	2.09E-11	0.135323585	4.41E-10	NS
GRIFIN	NS	2.24E-11	0.135117667	4.73E-10	NS
DZIP3	NS	2.26E-11	-0.135089565	4.77E-10	NS
LIN9	NS	2.29E-11	-0.135057456	4.82E-10	NS
OR4F5	NS	2.29E-11	-0.1350534	4.82E-10	NS
TMPRSS11E	NS	2.42E-11	-0.134898043	5.07E-10	NS
PGBD1	NS	2.42E-11	-0.13489226	5.08E-10	NS
TMEM81	NS	2.43E-11	-0.134878776	5.10E-10	NS
SUB1	NS	2.46E-11	0.134845501	5.15E-10	NS
CDCA8	NS	2.48E-11	-0.13482153	5.19E-10	NS
PLA1A	NS	2.48E-11	-0.134818756	5.19E-10	NS
PYDC1	NS	2.51E-11	0.134793373	5.23E-10	NS
FYB2	NS	2.54E-11	-0.134756253	5.29E-10	NS
PLEKHO1	NS	2.60E-11	0.13467934	5.42E-10	NS
CAMKV	NS	2.62E-11	0.134663061	5.44E-10	NS
SULT1C3	NS	2.62E-11	-0.134662304	5.44E-10	NS
PRR23C	NS	2.64E-11	0.134640642	5.47E-10	NS
PTPRE	NS	2.71E-11	0.134563222	5.61E-10	NS
THY1	NS	2.76E-11	0.134504017	5.71E-10	NS
CYP3A7	NS	2.77E-11	-0.134502536	5.71E-10	NS
H2AC4	NS	2.76E-11	-0.134507728	5.71E-10	NS
CADM2	NS	2.83E-11	-0.134436834	5.84E-10	NS
OR2F1	NS	2.85E-11	-0.134419112	5.86E-10	NS
RDH12	NS	2.86E-11	-0.134401828	5.89E-10	NS
CLDN19	NS	2.89E-11	0.134370431	5.95E-10	NS
CXorf49B	NS	2.90E-11	0.134359959	5.96E-10	NS
TMCO3	NS	2.95E-11	0.134310353	6.06E-10	NS
MDFIC2	NS	2.97E-11	-0.134293242	6.09E-10	NS
CCL16	NS	3.15E-11	0.134122116	6.45E-10	NS
SCT	NS	3.16E-11	0.134106462	6.47E-10	NS
CDRT4	NS	3.23E-11	-0.134041163	6.61E-10	NS
CFAP126	NS	3.34E-11	-0.133943697	6.83E-10	NS
RANGRF	NS	3.41E-11	-0.133885687	6.95E-10	NS
AMELX	NS	3.46E-11	-0.133843381	7.05E-10	NS
SGCZ	NS	3.53E-11	-0.133786425	7.18E-10	NS
LIPT2	NS	3.54E-11	-0.13377403	7.20E-10	NS
CTAGE1	NS	3.70E-11	-0.133641611	7.52E-10	NS
KNCN	NS	3.76E-11	0.133593645	7.63E-10	NS
POMC	NS	3.77E-11	-0.13358969	7.64E-10	NS
KLHL4	NS	3.79E-11	-0.133569179	7.68E-10	NS
SMIM10L2A	NS	3.81E-11	0.133560171	7.70E-10	NS
PIK3CG	NS	3.82E-11	0.133545948	7.73E-10	NS
STPG2	NS	3.84E-11	-0.133530181	7.75E-10	NS
OR4M1	NS	3.85E-11	-0.133529242	7.75E-10	NS
SNCB	NS	4.03E-11	0.133391683	8.11E-10	NS
HOXC5	NS	4.16E-11	0.133298986	8.36E-10	NS
SMIM21	NS	4.22E-11	-0.133256311	8.47E-10	NS
TAF1A	NS	4.26E-11	-0.13322474	8.56E-10	NS
IL12B	NS	4.31E-11	-0.133192612	8.64E-10	NS
MSH4	NS	4.38E-11	-0.133142888	8.78E-10	NS
MOGAT1	NS	4.47E-11	-0.133079261	8.96E-10	NS
DYNAP	NS	4.56E-11	-0.133023155	9.12E-10	NS
AFM	NS	4.57E-11	-0.133014364	9.13E-10	NS

PRRX1	NS	4.61E-11	-0.132989014	9.19E-10	NS
MTRNR2L13	NS	4.75E-11	-0.132902098	9.46E-10	NS
PRG2	NS	4.79E-11	-0.132876229	9.53E-10	NS
ALX1	NS	4.85E-11	-0.132842115	9.63E-10	NS
RNF208	NS	4.87E-11	0.132828328	9.66E-10	NS
OR2A25	NS	4.98E-11	0.132761981	9.86E-10	NS
IQCF3	NS	4.98E-11	-0.132762398	9.86E-10	NS
HYLS1	NS	5.09E-11	-0.132696424	1.01E-09	NS
SPINK6	NS	5.22E-11	-0.132619891	1.03E-09	NS
ZNF750	NS	5.26E-11	-0.132600414	1.04E-09	NS
RGS21	NS	5.28E-11	-0.132585677	1.04E-09	NS
RDH8	NS	5.31E-11	0.132568523	1.05E-09	NS
TPSG1	NS	5.32E-11	0.132566153	1.05E-09	NS
FKBPL	NS	5.47E-11	-0.132481399	1.08E-09	NS
PIGK	NS	5.57E-11	-0.132428468	1.09E-09	NS
RIDA	NS	5.62E-11	-0.132400992	1.10E-09	NS
CHCHD1	NS	5.79E-11	-0.132308846	1.14E-09	NS
CTLA4	NS	5.92E-11	-0.132243193	1.16E-09	NS
LMBRD2	NS	6.00E-11	-0.132202749	1.17E-09	NS
DMRTC1B	NS	6.06E-11	0.132176483	1.18E-09	NS
EIF2AK3	NS	6.24E-11	0.132084936	1.22E-09	NS
SULT2B1	NS	6.38E-11	0.132021417	1.24E-09	NS
APOO	NS	6.62E-11	-0.13190852	1.29E-09	NS
CDC42EP5	NS	6.65E-11	0.131895499	1.30E-09	NS
FEZF2	NS	6.74E-11	0.131853767	1.31E-09	NS
FOXC1	NS	6.81E-11	0.131822832	1.32E-09	NS
KNDC1	NS	7.06E-11	0.131717213	1.37E-09	NS
PRAP1	NS	7.20E-11	0.131658386	1.39E-09	NS
CLEC9A	NS	7.20E-11	-0.131657757	1.39E-09	NS
DPRX	NS	7.19E-11	0.131661148	1.39E-09	NS
GDF3	NS	7.28E-11	0.131624189	1.41E-09	NS
FCRLB	NS	7.29E-11	-0.131621083	1.41E-09	NS
TNNT3	NS	7.36E-11	0.131592412	1.42E-09	NS
SIAH3	NS	7.46E-11	-0.131549163	1.44E-09	NS
IRX4	NS	7.47E-11	0.13154607	1.44E-09	NS
ASNS	NS	7.55E-11	-0.13151634	1.45E-09	NS
BHLHE23	NS	7.58E-11	0.131503726	1.45E-09	NS
TEX47	NS	7.57E-11	-0.131507271	1.45E-09	NS
FOXA3	NS	7.57E-11	0.131505968	1.45E-09	NS
SERPINC1	NS	7.65E-11	-0.131474546	1.47E-09	NS
RAD23A	NS	7.86E-11	0.131392408	1.51E-09	NS
THPO	NS	8.04E-11	0.13132477	1.54E-09	NS
SULF2	NS	8.09E-11	0.131307195	1.54E-09	NS
OR6C68	NS	8.15E-11	-0.131284883	1.55E-09	NS
BMP6	NS	8.25E-11	-0.131248953	1.57E-09	NS
MAIP1	NS	8.29E-11	-0.131231312	1.58E-09	NS
ADAMTS19	NS	8.35E-11	-0.131210297	1.59E-09	NS
SLC15A5	NS	8.48E-11	-0.131164166	1.61E-09	NS
NR2F1	NS	8.66E-11	0.131101822	1.64E-09	NS
MRPL17	NS	8.73E-11	-0.131078098	1.65E-09	NS
FOXF1	NS	8.85E-11	0.131037195	1.68E-09	NS
CADPS2	NS	9.07E-11	-0.130960734	1.71E-09	NS
LITAF	NS	9.07E-11	0.130961045	1.71E-09	NS
NPM3	NS	9.15E-11	-0.130934569	1.73E-09	NS
IFNG	NS	9.39E-11	-0.130856133	1.77E-09	NS

CLEC6A	NS	9.42E-11	-0.130848589	1.77E-09	NS
CLEC2L	NS	9.49E-11	0.130823264	1.79E-09	NS
OR2T2	NS	9.59E-11	-0.13079439	1.80E-09	NS
OR2M7	NS	9.66E-11	-0.130771203	1.82E-09	NS
RIN2	NS	9.67E-11	0.130768233	1.82E-09	NS
TMEM275	NS	9.78E-11	0.130734382	1.83E-09	NS
OR51I1	NS	9.99E-11	-0.130669114	1.87E-09	NS
CAMSAP3	NS	1.00E-10	0.130651356	1.88E-09	NS
OR4F3	NS	1.01E-10	-0.130630858	1.89E-09	NS
RNMT	NS	1.02E-10	0.130601373	1.91E-09	NS
SKA3	NS	1.02E-10	-0.130596906	1.91E-09	NS
KBTBD13	NS	1.04E-10	0.130548582	1.94E-09	NS
ZKSCAN8P1	NS	1.04E-10	-0.13054516	1.94E-09	NS
EEF1A2	NS	1.04E-10	0.130541793	1.94E-09	NS
PRR18	NS	1.06E-10	0.130497634	1.97E-09	NS
WNT3	NS	1.06E-10	0.130482731	1.97E-09	NS
DEFB112	NS	1.13E-10	-0.130300887	2.09E-09	NS
TMPRSS15	NS	1.17E-10	-0.130199744	2.16E-09	NS
HSD17B8	NS	1.19E-10	-0.130139248	2.20E-09	NS
C5orf34	NS	1.21E-10	-0.130092238	2.23E-09	NS
IFNL3	NS	1.22E-10	0.130053438	2.26E-09	NS
AGXT	NS	1.24E-10	0.130015726	2.29E-09	NS
USH1G	NS	1.25E-10	0.129987568	2.31E-09	NS
OR51A7	NS	1.28E-10	-0.129917869	2.36E-09	NS
AGO1	NS	1.32E-10	0.129815154	2.43E-09	NS
FBXL16	NS	1.35E-10	0.1297605	2.47E-09	NS
TPSB2	NS	1.35E-10	0.129762854	2.47E-09	NS
GLYATL1B	NS	1.37E-10	-0.129711192	2.51E-09	NS
DNAAF3	NS	1.41E-10	0.129621666	2.58E-09	NS
HOXA4	NS	1.41E-10	0.129616738	2.59E-09	NS
AOX1	NS	1.42E-10	-0.129596893	2.60E-09	NS
SERPINA10	NS	1.43E-10	0.12958041	2.61E-09	NS
TUBB8B	NS	1.43E-10	0.129571621	2.62E-09	NS
OR5D13	NS	1.45E-10	-0.129544453	2.64E-09	NS
H1-2	NS	1.45E-10	-0.12952615	2.65E-09	NS
DEFB104B	NS	1.46E-10	-0.129515862	2.66E-09	NS
ILDR2	NS	1.49E-10	-0.129457043	2.71E-09	NS
EXOG	NS	1.51E-10	-0.129405775	2.75E-09	NS
CTRB2	NS	1.53E-10	0.129379858	2.77E-09	NS
GNG11	NS	1.53E-10	-0.12936322	2.78E-09	NS
SMIM10L2B	NS	1.57E-10	0.129286091	2.85E-09	NS
CACNG6	NS	1.58E-10	0.129268174	2.87E-09	NS
KIF12	NS	1.60E-10	0.1292376	2.89E-09	NS
CBR3	NS	1.62E-10	-0.129198644	2.93E-09	NS
GPR85	NS	1.62E-10	-0.129194362	2.93E-09	NS
SLC39A2	NS	1.62E-10	-0.12918994	2.93E-09	NS
PCDH11Y	NS	1.64E-10	0.129158635	2.96E-09	NS
SRRM3	NS	1.65E-10	0.129141674	2.97E-09	NS
OR8K3	NS	1.68E-10	-0.129083646	3.02E-09	NS
HNF4G	NS	1.75E-10	-0.12896728	3.14E-09	NS
TAAR8	NS	1.75E-10	-0.128952376	3.15E-09	NS
C1orf198	NS	1.77E-10	-0.128922419	3.18E-09	NS
FUOM	NS	1.77E-10	-0.128919255	3.18E-09	NS
SPDL1	NS	1.84E-10	-0.128813872	3.29E-09	NS
TMEM106C	NS	1.87E-10	-0.128763663	3.34E-09	NS

TFRC	NS	1.89E-10	0.128728433	3.37E-09	NS
CROT	NS	1.97E-10	-0.12859575	3.52E-09	NS
VWA1	NS	1.98E-10	0.128574534	3.54E-09	NS
QRFP	NS	2.02E-10	-0.12852468	3.59E-09	NS
PHYHIPL	NS	2.05E-10	-0.128468202	3.66E-09	NS
KCNT2	NS	2.14E-10	-0.128334691	3.81E-09	NS
SPATA16	NS	2.15E-10	-0.128329031	3.82E-09	NS
XK	NS	2.16E-10	-0.128313803	3.83E-09	NS
CTXN3	NS	2.17E-10	-0.12830481	3.84E-09	NS
CSTF2	NS	2.17E-10	-0.128292819	3.85E-09	NS
MRPL1	NS	2.19E-10	-0.128275964	3.87E-09	NS
PRSS37	NS	2.22E-10	-0.128233314	3.92E-09	NS
GPR87	NS	2.24E-10	-0.128196893	3.96E-09	NS
RGS9BP	NS	2.28E-10	0.128143559	4.03E-09	NS
TPSAB1	NS	2.30E-10	0.128121895	4.05E-09	NS
KDELR3	NS	2.31E-10	-0.128104988	4.07E-09	NS
P2RX1	NS	2.32E-10	0.128098562	4.07E-09	NS
SOX2	NS	2.36E-10	0.128045293	4.14E-09	NS
SOHLH1	NS	2.37E-10	0.128023324	4.17E-09	NS
ABHD10	NS	2.40E-10	-0.127993156	4.20E-09	NS
H2BC1	NS	2.41E-10	0.12797272	4.23E-09	NS
ACTL6B	NS	2.43E-10	0.127955	4.25E-09	NS
DMAC1	NS	2.47E-10	-0.127896686	4.33E-09	NS
ARL15	NS	2.51E-10	-0.127853622	4.38E-09	NS
C7orf57	NS	2.52E-10	-0.127837188	4.40E-09	NS
KIF26A	NS	2.61E-10	0.127729707	4.55E-09	NS
UGT1A10	NS	2.69E-10	-0.12763184	4.70E-09	NS
CATSPERE	NS	2.70E-10	-0.127625953	4.70E-09	NS
FLRT3	NS	2.70E-10	-0.127622894	4.70E-09	NS
PRLHR	NS	2.73E-10	0.12759211	4.74E-09	NS
MAPK8IP2	NS	2.77E-10	0.127544241	4.81E-09	NS
ATG4C	NS	2.87E-10	-0.127436993	4.98E-09	NS
EXO5	NS	2.89E-10	-0.127409282	5.02E-09	NS
NANOS3	NS	2.91E-10	0.127395491	5.04E-09	NS
MAPK11	NS	2.91E-10	0.127389647	5.04E-09	NS
SELENBP1	NS	2.97E-10	-0.12732892	5.14E-09	NS
DYNLT5	NS	3.09E-10	-0.127202053	5.34E-09	NS
SLC26A7	NS	3.11E-10	-0.127188118	5.36E-09	NS
SLC17A3	NS	3.17E-10	-0.127123809	5.47E-09	NS
ZNF287	NS	3.17E-10	-0.127120107	5.47E-09	NS
C6orf226	NS	3.18E-10	-0.127113157	5.48E-09	NS
KLHL41	NS	3.19E-10	-0.127102089	5.49E-09	NS
ZFP62	NS	3.24E-10	-0.127059177	5.57E-09	NS
CNGA1	NS	3.25E-10	-0.127044004	5.59E-09	NS
PROX1	NS	3.27E-10	0.127025823	5.62E-09	NS
OR4C5	NS	3.30E-10	-0.126998139	5.66E-09	NS
PRL	NS	3.33E-10	-0.126968698	5.71E-09	NS
HMGCLL1	NS	3.38E-10	-0.126927903	5.77E-09	NS
LRRN1	NS	3.38E-10	-0.12692763	5.77E-09	NS
MYLPF	NS	3.37E-10	0.126931428	5.77E-09	NS
CCDC102A	NS	3.40E-10	0.126905022	5.81E-09	NS
PJA1	NS	3.41E-10	-0.126895295	5.82E-09	NS
BCL6B	NS	3.42E-10	0.126889405	5.83E-09	NS
ADAM18	NS	3.44E-10	-0.126874475	5.85E-09	NS
CCN3	NS	3.44E-10	-0.126868227	5.85E-09	NS

GNMT	NS	3.51E-10	-0.126810752	5.96E-09	NS
HSFX3	NS	3.53E-10	-0.126788776	6.00E-09	NS
MMUT	NS	3.54E-10	-0.126784928	6.00E-09	NS
RBM7	NS	3.55E-10	0.126774376	6.01E-09	NS
PRRG1	NS	3.58E-10	-0.126748147	6.06E-09	NS
TCP11	NS	3.58E-10	-0.1267454	6.06E-09	NS
NKX1-1	NS	3.59E-10	0.126734546	6.07E-09	NS
DEFB125	NS	3.62E-10	-0.12671245	6.11E-09	NS
TNNI3K	NS	3.64E-10	-0.126695056	6.14E-09	NS
NEUROD4	NS	3.68E-10	-0.126656749	6.21E-09	NS
CAPSL	NS	3.73E-10	-0.126615513	6.29E-09	NS
OR52E5	NS	3.73E-10	-0.126614235	6.29E-09	NS
MMP12	NS	3.87E-10	-0.126503065	6.51E-09	NS
CHI3L2	NS	3.89E-10	-0.126489968	6.53E-09	NS
IRGC	NS	3.98E-10	0.12641776	6.68E-09	NS
LRRC14B	NS	3.99E-10	0.126409878	6.69E-09	NS
KRT25	NS	4.01E-10	-0.126393741	6.72E-09	NS
XRCC4	NS	4.05E-10	-0.126363802	6.77E-09	NS
SYNGR4	NS	4.07E-10	0.126346591	6.81E-09	NS
NEK11	NS	4.08E-10	-0.126340465	6.81E-09	NS
NFRKB	NS	4.09E-10	0.126331923	6.83E-09	NS
SSPN	NS	4.13E-10	-0.126299294	6.89E-09	NS
CCL25	NS	4.18E-10	0.126260331	6.97E-09	NS
OR10G3	NS	4.25E-10	0.12621281	7.07E-09	NS
WFDC9	NS	4.35E-10	-0.126137725	7.24E-09	NS
ETS2	NS	4.44E-10	0.126074657	7.38E-09	NS
PTPRD	NS	4.44E-10	-0.126069007	7.38E-09	NS
ATP5MK	NS	4.47E-10	-0.126049141	7.42E-09	NS
TXNDC8	NS	4.49E-10	-0.126036224	7.45E-09	NS
IRX6	NS	4.51E-10	0.126020084	7.48E-09	NS
NKX3-2	NS	4.57E-10	0.12598053	7.57E-09	NS
KLKB1	NS	4.61E-10	-0.125954719	7.63E-09	NS
DDX4	NS	4.62E-10	-0.125948857	7.63E-09	NS
IFNE	NS	4.66E-10	-0.125917999	7.70E-09	NS
USP26	NS	4.69E-10	-0.125899939	7.74E-09	NS
CERS1	NS	4.74E-10	0.125866158	7.82E-09	NS
BRS3	NS	4.80E-10	0.125824587	7.91E-09	NS
KIR3DL3	NS	4.81E-10	-0.125823405	7.91E-09	NS
C7orf31	NS	4.86E-10	-0.125786121	8.00E-09	NS
OR2AP1	NS	4.88E-10	0.125772369	8.03E-09	NS
ELL	NS	5.02E-10	0.125683621	8.24E-09	NS
ZNF311	NS	5.03E-10	-0.125680638	8.24E-09	NS
INSL4	NS	5.19E-10	-0.125582207	8.50E-09	NS
CXorf49	NS	5.26E-10	0.125541042	8.60E-09	NS
INKA1	NS	5.29E-10	-0.125523208	8.64E-09	NS
TCAP	NS	5.33E-10	-0.125496411	8.71E-09	NS
BATF	NS	5.34E-10	-0.12549	8.72E-09	NS
KCNJ18	NS	5.37E-10	0.125472909	8.76E-09	NS
CPO	NS	5.42E-10	-0.125442635	8.84E-09	NS
RBP2	NS	5.44E-10	-0.125432318	8.86E-09	NS
GFRA4	NS	5.45E-10	0.125427343	8.87E-09	NS
GDF1	NS	5.54E-10	0.125377644	9.00E-09	NS
WDR38	NS	5.58E-10	-0.125355483	9.05E-09	NS
SHANK1	NS	5.61E-10	0.125335339	9.10E-09	NS
FAM181B	NS	5.67E-10	0.125304877	9.18E-09	NS

HS3ST5	NS	5.66E-10	-0.125307474	9.18E-09	NS
KRTAP8-1	NS	5.71E-10	-0.125282638	9.23E-09	NS
ORMDL2	NS	5.76E-10	-0.125251405	9.32E-09	NS
SP8	NS	5.78E-10	0.125241944	9.34E-09	NS
CALCR	NS	5.99E-10	-0.125130605	9.67E-09	NS
DDX31	NS	6.08E-10	-0.125081846	9.81E-09	NS
OR6C70	NS	6.20E-10	-0.125020725	9.99E-09	NS
ELFN2	NS	6.40E-10	0.12492013	1.03E-08	NS
OR1C1	NS	6.40E-10	-0.12492102	1.03E-08	NS
NXPE4	NS	6.41E-10	-0.124914981	1.03E-08	NS
TRMT6	NS	6.43E-10	0.124903637	1.03E-08	NS
CYP2A7	NS	6.45E-10	0.124893567	1.04E-08	NS
LTB	NS	6.57E-10	-0.124838722	1.05E-08	NS
MBOAT4	NS	6.58E-10	-0.124830433	1.05E-08	NS
YBX2	NS	6.80E-10	0.124726393	1.09E-08	NS
CD300LD	NS	7.11E-10	-0.124585779	1.14E-08	NS
MYOZ2	NS	7.23E-10	-0.124533578	1.16E-08	NS
ARL6	NS	7.28E-10	-0.12451293	1.16E-08	NS
CDO1	NS	7.28E-10	-0.124510364	1.16E-08	NS
TMCO5A	NS	7.37E-10	-0.12447149	1.18E-08	NS
ENPEP	NS	7.48E-10	-0.124425733	1.19E-08	NS
RTL9	NS	7.50E-10	-0.124418439	1.19E-08	NS
TMEM88B	NS	7.73E-10	0.124322687	1.23E-08	NS
TAS2R1	NS	7.74E-10	-0.12431489	1.23E-08	NS
AKAP3	NS	7.80E-10	-0.12429057	1.24E-08	NS
NODAL	NS	7.83E-10	-0.124280263	1.24E-08	NS
COA6	NS	7.92E-10	-0.124243629	1.25E-08	NS
TSPO2	NS	8.06E-10	-0.124185823	1.28E-08	NS
LKAAEAR1	NS	8.25E-10	0.124114984	1.30E-08	NS
XCL2	NS	8.39E-10	-0.12406068	1.33E-08	NS
NCMAP	NS	8.41E-10	0.124051823	1.33E-08	NS
NEIL3	NS	8.43E-10	-0.124046298	1.33E-08	NS
KIF25	NS	8.54E-10	0.124002891	1.35E-08	NS
BPY2	NS	8.64E-10	-0.123967148	1.36E-08	NS
BPY2B	NS	8.64E-10	-0.123967148	1.36E-08	NS
CTXN1	NS	8.69E-10	0.123946913	1.37E-08	NS
ERN1	NS	8.71E-10	0.123942044	1.37E-08	NS
OR4L1	NS	8.77E-10	-0.123918824	1.38E-08	NS
AGRP	NS	8.78E-10	-0.123916138	1.38E-08	NS
TXLNB	NS	8.88E-10	-0.123880122	1.39E-08	NS
MMP7	NS	8.99E-10	-0.123840241	1.41E-08	NS
ITGA10	NS	9.07E-10	-0.123809923	1.42E-08	NS
PANK1	NS	9.07E-10	-0.123810973	1.42E-08	NS
OR5H6	NS	9.09E-10	-0.123803589	1.42E-08	NS
LIPK	NS	9.14E-10	-0.123787701	1.43E-08	NS
TMEM267	NS	9.14E-10	-0.123785114	1.43E-08	NS
SGCD	NS	9.23E-10	-0.123756399	1.44E-08	NS
C7orf25	NS	9.37E-10	-0.123707371	1.46E-08	NS
FAM83E	NS	9.43E-10	0.123688316	1.47E-08	NS
ELAVL3	NS	9.76E-10	0.123577431	1.52E-08	NS
OR8U1	NS	9.97E-10	-0.123509033	1.55E-08	NS
RRAGB	NS	1.03E-09	-0.123399633	1.60E-08	NS
C1QTNF2	NS	1.05E-09	0.123353022	1.62E-08	NS
CCNH	NS	1.06E-09	0.123320454	1.64E-08	NS
OBP2A	NS	1.07E-09	0.123292173	1.65E-08	NS

ZG16B	NS	1.07E-09	-0.123269118	1.66E-08	NS
KCNG3	NS	1.08E-09	0.123261231	1.67E-08	NS
FCN3	NS	1.10E-09	0.123184042	1.70E-08	NS
VPS50	NS	1.11E-09	-0.123165498	1.71E-08	NS
GPR149	NS	1.11E-09	-0.123158876	1.71E-08	NS
CRHR1	NS	1.12E-09	0.123146005	1.72E-08	NS
GTF2H4	NS	1.13E-09	-0.123110071	1.74E-08	NS
KRTAP13-3	NS	1.13E-09	0.123093909	1.75E-08	NS
TOMM20L	NS	1.14E-09	-0.123081401	1.75E-08	NS
C3orf80	NS	1.15E-09	0.123037124	1.77E-08	NS
UTS2	NS	1.16E-09	-0.123020693	1.78E-08	NS
CCDC8	NS	1.17E-09	0.122989019	1.80E-08	NS
OR52H1	NS	1.17E-09	-0.122985619	1.80E-08	NS
NTN5	NS	1.18E-09	0.122958029	1.81E-08	NS
LNP1	NS	1.18E-09	-0.12296008	1.81E-08	NS
PPBP	NS	1.19E-09	-0.12293046	1.82E-08	NS
CMTM8	NS	1.19E-09	-0.122929277	1.82E-08	NS
HMX1	NS	1.20E-09	0.122923931	1.83E-08	NS
CDH20	NS	1.21E-09	-0.122877741	1.85E-08	NS
PPP1R17	NS	1.21E-09	-0.122876092	1.85E-08	NS
TREM2	NS	1.22E-09	-0.122870461	1.85E-08	NS
PKIA	NS	1.23E-09	-0.122846465	1.86E-08	NS
NDUFA8	NS	1.24E-09	-0.122810305	1.88E-08	NS
CCDC34	NS	1.27E-09	-0.122737534	1.93E-08	NS
RPTN	NS	1.28E-09	-0.122716983	1.94E-08	NS
MDH1B	NS	1.29E-09	-0.122678538	1.96E-08	NS
CLDND2	NS	1.29E-09	0.122672461	1.96E-08	NS
IL13RA2	NS	1.30E-09	-0.122650747	1.97E-08	NS
ZNF728	NS	1.30E-09	-0.122645392	1.97E-08	NS
LACTB2	NS	1.33E-09	-0.12257217	2.02E-08	NS
RASGEF1C	NS	1.35E-09	0.122538719	2.04E-08	NS
FOS	NS	1.36E-09	0.122510273	2.05E-08	NS
KRTAP10-8	NS	1.36E-09	0.122508987	2.05E-08	NS
PRSS22	NS	1.42E-09	0.12236185	2.15E-08	NS
PTER	NS	1.42E-09	-0.122361006	2.15E-08	NS
C11orf98	NS	1.44E-09	-0.122335446	2.16E-08	NS
CLYBL	NS	1.48E-09	-0.122232617	2.23E-08	NS
TMEM255A	NS	1.48E-09	-0.122234449	2.23E-08	NS
CCDC74A	NS	1.52E-09	0.122161176	2.28E-08	NS
CARNMT1	NS	1.57E-09	-0.122056233	2.35E-08	NS
OVGP1	NS	1.57E-09	-0.122038443	2.36E-08	NS
WARS2	NS	1.58E-09	-0.12202466	2.37E-08	NS
ALDH1A1	NS	1.61E-09	-0.121960815	2.41E-08	NS
PAM16	NS	1.61E-09	0.12195955	2.41E-08	NS
ZNF256	NS	1.62E-09	-0.121945131	2.42E-08	NS
RFPL3	NS	1.65E-09	-0.121889262	2.46E-08	NS
RBP5	NS	1.66E-09	-0.121867894	2.48E-08	NS
MROH2B	NS	1.66E-09	-0.121860173	2.48E-08	NS
HIGD2B	NS	1.66E-09	-0.121856966	2.48E-08	NS
SLC35B3	NS	1.67E-09	-0.121852023	2.48E-08	NS
FBXL4	NS	1.69E-09	-0.121801807	2.52E-08	NS
ZNF582	NS	1.72E-09	-0.121758129	2.55E-08	NS
GZMB	NS	1.72E-09	-0.121755184	2.55E-08	NS
NKX2-8	NS	1.74E-09	0.12171044	2.58E-08	NS
MRPS9	NS	1.74E-09	-0.121705664	2.59E-08	NS

GRIA4	NS	1.76E-09	-0.121679573	2.61E-08	NS
HOXC9	NS	1.76E-09	0.121675121	2.61E-08	NS
DEPDC1B	NS	1.77E-09	-0.121657416	2.62E-08	NS
HIGD1C	NS	1.80E-09	-0.121611757	2.65E-08	NS
TMEM229A	NS	1.81E-09	0.121584794	2.67E-08	NS
PLEKHG5	NS	1.83E-09	0.121544902	2.71E-08	NS
PLPP6	NS	1.84E-09	-0.121523883	2.72E-08	NS
H2AC7	NS	1.85E-09	-0.121508217	2.73E-08	NS
PTPN20	NS	1.87E-09	-0.121471691	2.76E-08	NS
TAF4B	NS	1.92E-09	-0.121389087	2.83E-08	NS
CDKN2B	NS	1.98E-09	-0.121297861	2.91E-08	NS
STC1	NS	1.98E-09	-0.121289028	2.91E-08	NS
SERINC1	NS	2.00E-09	0.121264544	2.93E-08	NS
PTBP2	NS	2.00E-09	0.121263949	2.93E-08	NS
CT47A5	NS	2.02E-09	0.121230666	2.96E-08	NS
CNFN	NS	2.06E-09	-0.121159986	3.02E-08	NS
LYN	NS	2.07E-09	0.12114005	3.04E-08	NS
ISLR2	NS	2.14E-09	0.121035419	3.13E-08	NS
ERICH6	NS	2.25E-09	-0.120879426	3.29E-08	NS
SEC14L4	NS	2.26E-09	0.120857931	3.30E-08	NS
SLC35F3	NS	2.28E-09	-0.120830754	3.33E-08	NS
PRRT1	NS	2.31E-09	0.120793168	3.37E-08	NS
EDIL3	NS	2.39E-09	-0.120681587	3.48E-08	NS
KRT1	NS	2.41E-09	-0.120650194	3.51E-08	NS
SYCE3	NS	2.42E-09	0.120638978	3.52E-08	NS
SPEM3	NS	2.43E-09	0.120620178	3.54E-08	NS
ADGRA1	NS	2.45E-09	0.120594885	3.56E-08	NS
BPY2C	NS	2.48E-09	-0.120556688	3.60E-08	NS
ZNF138	NS	2.48E-09	-0.120552062	3.60E-08	NS
FAM246A	NS	2.50E-09	0.120534757	3.62E-08	NS
CACNA2D1	NS	2.56E-09	-0.120452138	3.71E-08	NS
VGF	NS	2.57E-09	0.120443137	3.72E-08	NS
EFNB3	NS	2.57E-09	0.120440639	3.72E-08	NS
ALOX5AP	NS	2.59E-09	0.120420367	3.74E-08	NS
PTGFRN	NS	2.60E-09	-0.12039573	3.76E-08	NS
CLGN	NS	2.65E-09	-0.120339187	3.82E-08	NS
CYP26B1	NS	2.67E-09	0.120312599	3.85E-08	NS
GIP	NS	2.68E-09	0.120307775	3.86E-08	NS
HSD17B6	NS	2.68E-09	-0.120298676	3.86E-08	NS
OR52N5	NS	2.68E-09	-0.120299263	3.86E-08	NS
PLSCR4	NS	2.70E-09	-0.120273103	3.89E-08	NS
GCFC2	NS	2.72E-09	-0.120247754	3.92E-08	NS
RTN4R	NS	2.73E-09	0.12024515	3.92E-08	NS
GLIS1	NS	2.79E-09	0.120165997	4.01E-08	NS
MIXL1	NS	2.81E-09	0.120148587	4.03E-08	NS
OR51F1	NS	2.83E-09	-0.120119256	4.06E-08	NS
PFKFB4	NS	2.86E-09	0.120085644	4.10E-08	NS
SULT2A1	NS	2.87E-09	0.120076891	4.11E-08	NS
NCAPG	NS	2.89E-09	-0.12005922	4.12E-08	NS
OR4F21	NS	2.90E-09	-0.120047262	4.14E-08	NS
ANKRD63	NS	2.91E-09	0.120033191	4.15E-08	NS
TBX2	NS	2.93E-09	0.120010702	4.18E-08	NS
MAOB	NS	3.01E-09	-0.119919556	4.29E-08	NS
STPG3	NS	3.02E-09	0.119910631	4.30E-08	NS
GNAT1	NS	3.15E-09	0.119774848	4.48E-08	NS

CCDC185	NS	3.16E-09	0.119758907	4.50E-08	NS
NCR3	NS	3.18E-09	-0.11973971	4.52E-08	NS
SOX30	NS	3.23E-09	0.119689544	4.58E-08	NS
RERGL	NS	3.25E-09	-0.119668537	4.61E-08	NS
IFIT5	NS	3.26E-09	-0.119656647	4.62E-08	NS
IRX3	NS	3.27E-09	0.11965026	4.63E-08	NS
NKAIN4	NS	3.31E-09	0.119607608	4.68E-08	NS
TMCO2	NS	3.31E-09	0.119605752	4.68E-08	NS
LCA5	NS	3.31E-09	-0.119602223	4.68E-08	NS
OR10S1	NS	3.33E-09	-0.119581927	4.71E-08	NS
PTPRN2	NS	3.36E-09	-0.11955843	4.74E-08	NS
OR51G2	NS	3.49E-09	-0.119435212	4.91E-08	NS
POP1	NS	3.55E-09	-0.119375882	5.00E-08	NS
SUCNR1	NS	3.55E-09	-0.119377826	5.00E-08	NS
FAM163B	NS	3.59E-09	0.119339711	5.05E-08	NS
CTTNBP2	NS	3.62E-09	-0.119308834	5.09E-08	NS
EXOC3L4	NS	3.64E-09	0.119288661	5.12E-08	NS
GIMD1	NS	3.68E-09	-0.119253214	5.17E-08	NS
ANKRD33	NS	3.75E-09	0.119197327	5.25E-08	NS
CYLC1	NS	3.75E-09	-0.119196698	5.25E-08	NS
CGB7	NS	3.75E-09	0.119193453	5.25E-08	NS
SCYGR2	NS	3.82E-09	0.119136411	5.34E-08	NS
SNX24	NS	3.84E-09	-0.119112238	5.37E-08	NS
HPX	NS	3.87E-09	-0.119085388	5.41E-08	NS
TTR	NS	3.92E-09	-0.119047164	5.47E-08	NS
NABP1	NS	3.93E-09	0.119035394	5.49E-08	NS
RCOR2	NS	4.05E-09	0.118940271	5.64E-08	NS
IFNGR1	NS	4.09E-09	0.118907339	5.69E-08	NS
SGO1	NS	4.13E-09	-0.118876291	5.74E-08	NS
WNT6	NS	4.16E-09	0.118851022	5.78E-08	NS
PLPPR3	NS	4.18E-09	0.118837037	5.80E-08	NS
KRTAP19-8	NS	4.24E-09	-0.11878524	5.89E-08	NS
SYT7	NS	4.24E-09	0.118782178	5.89E-08	NS
CCDC83	NS	4.25E-09	-0.118774555	5.90E-08	NS
GIPC3	NS	4.26E-09	0.118771555	5.90E-08	NS
XPO6	NS	4.28E-09	0.118754136	5.93E-08	NS
ASPN	NS	4.34E-09	-0.118711338	6.00E-08	NS
ZBTB49	NS	4.44E-09	-0.118629159	6.14E-08	NS
SLC32A1	NS	4.49E-09	0.11859287	6.21E-08	NS
GC	NS	4.50E-09	-0.118590233	6.21E-08	NS
USP17L17	NS	4.50E-09	-0.118584059	6.21E-08	NS
CDCA7L	NS	4.51E-09	-0.118578683	6.22E-08	NS
GSTA5	NS	4.55E-09	-0.118547513	6.27E-08	NS
COCH	NS	4.66E-09	-0.118473292	6.41E-08	NS
SOD2	NS	4.67E-09	0.118464263	6.42E-08	NS
OR6B1	NS	4.68E-09	-0.118459325	6.43E-08	NS
YY1AP1	NS	4.73E-09	0.118418099	6.50E-08	NS
MMP20	NS	4.76E-09	-0.118402367	6.53E-08	NS
C14orf28	NS	4.80E-09	-0.118374037	6.58E-08	NS
BEX1	NS	4.82E-09	-0.11836126	6.60E-08	NS
FKBP10	NS	4.82E-09	0.118355321	6.61E-08	NS
MADCAM1	NS	4.88E-09	0.118316826	6.68E-08	NS
OR10A6	NS	4.90E-09	-0.118304088	6.70E-08	NS
TC2N	NS	4.94E-09	-0.118277184	6.75E-08	NS
FAM228A	NS	4.95E-09	-0.118267247	6.77E-08	NS

PIKFYVE	NS	4.97E-09	0.118255915	6.78E-08	NS
LRRC8A	NS	4.98E-09	0.118249548	6.79E-08	NS
SRSF3	NS	4.98E-09	0.118247001	6.79E-08	NS
PALM	NS	5.02E-09	0.118219887	6.84E-08	NS
MIB1	NS	5.06E-09	-0.118194983	6.89E-08	NS
DUSP26	NS	5.18E-09	0.118116551	7.05E-08	NS
GRIA3	NS	5.19E-09	-0.118112924	7.05E-08	NS
RBM46	NS	5.26E-09	-0.118066838	7.14E-08	NS
UNC5B	NS	5.29E-09	-0.118047148	7.18E-08	NS
PTX4	NS	5.32E-09	0.118028412	7.22E-08	NS
PRKCQ	NS	5.35E-09	-0.118009074	7.25E-08	NS
VCP	NS	5.40E-09	0.117979679	7.31E-08	NS
NOBOX	NS	5.43E-09	0.117961455	7.35E-08	NS
CLEC4D	NS	5.43E-09	-0.117957068	7.35E-08	NS
SOX17	NS	5.46E-09	0.117944632	7.37E-08	NS
FOXB2	NS	5.45E-09	0.117946021	7.37E-08	NS
PGLYRP4	NS	5.49E-09	-0.117926342	7.40E-08	NS
TMEM272	NS	5.61E-09	-0.117851104	7.57E-08	NS
CAPS2	NS	5.62E-09	-0.117843228	7.58E-08	NS
IFNA21	NS	5.65E-09	-0.117828351	7.61E-08	NS
PLLP	NS	5.66E-09	-0.117818963	7.62E-08	NS
OR5K1	NS	5.70E-09	-0.117799792	7.66E-08	NS
KIF7	NS	5.71E-09	0.117791795	7.67E-08	NS
ZSCAN16	NS	5.75E-09	-0.117771029	7.72E-08	NS
IFI30	NS	5.84E-09	0.117717904	7.83E-08	NS
CT45A7	NS	5.86E-09	-0.117703022	7.86E-08	NS
H2BC14	NS	5.86E-09	0.117702908	7.86E-08	NS
KPNB1	NS	5.93E-09	-0.117667358	7.94E-08	NS
A1CF	NS	5.94E-09	-0.117660682	7.95E-08	NS
LELP1	NS	5.99E-09	-0.117633447	8.01E-08	NS
LCN8	NS	6.05E-09	0.117596373	8.09E-08	NS
PERP	NS	6.12E-09	-0.117558649	8.18E-08	NS
GGT6	NS	6.25E-09	0.117488881	8.34E-08	NS
RSPO4	NS	6.32E-09	0.117451886	8.43E-08	NS
SEMA4G	NS	6.56E-09	-0.117323439	8.74E-08	NS
ARL2BP	NS	6.67E-09	-0.117271212	8.87E-08	NS
OR6B3	NS	6.69E-09	-0.117258389	8.90E-08	NS
CCDC54	NS	6.74E-09	-0.117235908	8.96E-08	NS
PLK5	NS	6.82E-09	0.117192269	9.07E-08	NS
PROB1	NS	6.83E-09	0.117187851	9.07E-08	NS
TMEM158	NS	6.87E-09	0.117172086	9.11E-08	NS
ALOX12	NS	6.91E-09	0.117150893	9.16E-08	NS
PLS1	NS	6.98E-09	-0.117117306	9.24E-08	NS
QTNF3-AMA	NS	7.04E-09	-0.117086663	9.32E-08	NS
H2AC15	NS	7.06E-09	-0.117077728	9.34E-08	NS
ACSL1	NS	7.14E-09	0.117040407	9.44E-08	NS
OR2L5	NS	7.21E-09	-0.117007477	9.52E-08	NS
NPFF	NS	7.25E-09	-0.116988163	9.57E-08	NS
UPP2	NS	7.26E-09	-0.116985537	9.57E-08	NS
ZNF691	NS	7.31E-09	-0.11695859	9.64E-08	NS
OR5D14	NS	7.32E-09	-0.11695442	9.65E-08	NS
FSD1	NS	7.51E-09	0.116870456	9.88E-08	NS
KLHL38	NS	7.57E-09	0.116840291	9.97E-08	NS
AMTN	NS	7.71E-09	-0.116778502	1.01E-07	NS
TAGLN3	NS	7.78E-09	-0.116751529	1.02E-07	NS

COL5A2	NS	7.82E-09	-0.116732217	1.03E-07	NS
HTR4	NS	7.84E-09	-0.116725775	1.03E-07	NS
ZNF202	NS	7.89E-09	-0.116702134	1.03E-07	NS
PGBD2	NS	7.93E-09	-0.116687157	1.04E-07	NS
KLHL32	NS	7.93E-09	-0.116683855	1.04E-07	NS
SPRR2D	NS	8.05E-09	-0.116634951	1.05E-07	NS
OR5H2	NS	8.05E-09	-0.116633413	1.05E-07	NS
PFKFB3	NS	8.12E-09	0.116603532	1.06E-07	NS
C1QTNF12	NS	8.12E-09	0.116605447	1.06E-07	NS
CDH26	NS	8.16E-09	-0.116588815	1.06E-07	NS
RAB25	NS	8.20E-09	0.116573742	1.07E-07	NS
WNT7B	NS	8.24E-09	0.116555775	1.07E-07	NS
CCDC181	NS	8.31E-09	-0.11652471	1.08E-07	NS
ABRACL	NS	8.33E-09	-0.116517041	1.08E-07	NS
ANKRD53	NS	8.39E-09	-0.116494911	1.09E-07	NS
GPR4	NS	8.68E-09	0.11638042	1.13E-07	NS
TTLL8	NS	8.69E-09	0.116373323	1.13E-07	NS
MEPE	NS	8.85E-09	-0.116314591	1.15E-07	NS
SLAMF6	NS	8.93E-09	-0.116281819	1.16E-07	NS
FAM246B	NS	8.93E-09	0.116282264	1.16E-07	NS
FNDC11	NS	9.00E-09	0.116256439	1.17E-07	NS
TXLNG	NS	9.04E-09	-0.116240086	1.17E-07	NS
SPB2-C11orf5	NS	9.04E-09	0.116241537	1.17E-07	NS
HENMT1	NS	9.06E-09	-0.116234507	1.17E-07	NS
TYRP1	NS	9.18E-09	-0.116189745	1.19E-07	NS
MPPED1	NS	9.24E-09	0.116168221	1.19E-07	NS
FGF22	NS	9.25E-09	0.116164612	1.19E-07	NS
GKAP1	NS	9.36E-09	-0.11612151	1.21E-07	NS
CBLC	NS	9.41E-09	0.116103114	1.21E-07	NS
MAB21L4	NS	9.52E-09	0.116065933	1.23E-07	NS
FAM83C	NS	9.57E-09	0.116048148	1.23E-07	NS
TNFRSF4	NS	9.71E-09	0.115998818	1.25E-07	NS
KLK1	NS	9.74E-09	0.115986396	1.25E-07	NS
RNF212B	NS	9.74E-09	-0.115985756	1.25E-07	NS
UTF1	NS	9.75E-09	0.115982378	1.25E-07	NS
TLK1	NS	9.77E-09	0.115978182	1.25E-07	NS
SPAAR	NS	9.80E-09	0.115965044	1.26E-07	NS
ERAS	NS	9.98E-09	0.115905102	1.28E-07	NS
AMBN	NS	9.99E-09	-0.115899242	1.28E-07	NS
EPOP	NS	9.99E-09	0.115900622	1.28E-07	NS
ARHGEF16	NS	1.01E-08	0.115869576	1.29E-07	NS
HOXA13	NS	1.01E-08	0.115852855	1.29E-07	NS
SPON1	NS	1.02E-08	-0.11584388	1.30E-07	NS
SBK1	NS	1.03E-08	0.115805393	1.31E-07	NS
SP9	NS	1.03E-08	0.115796233	1.31E-07	NS
WDR64	NS	1.04E-08	-0.115778064	1.32E-07	NS
LHFPL1	NS	1.07E-08	-0.115669596	1.36E-07	NS
UPP1	NS	1.07E-08	0.115651488	1.37E-07	NS
C16orf92	NS	1.09E-08	0.115612911	1.38E-07	NS
HBD	NS	1.09E-08	-0.115607358	1.38E-07	NS
CRYBA1	NS	1.11E-08	-0.11554278	1.41E-07	NS
ZBTB32	NS	1.12E-08	0.115507904	1.42E-07	NS
PLAAT1	NS	1.14E-08	-0.115459773	1.44E-07	NS
ERVH48-1	NS	1.14E-08	-0.115436139	1.45E-07	NS
INHBB	NS	1.15E-08	0.1154163	1.46E-07	NS

OR9Q1	NS	1.15E-08	-0.115416319	1.46E-07	NS
FAM216B	NS	1.16E-08	-0.115397253	1.46E-07	NS
SMPX	NS	1.17E-08	-0.115358266	1.48E-07	NS
MPZ	NS	1.17E-08	0.11535795	1.48E-07	NS
EDNRB	NS	1.17E-08	-0.115349706	1.48E-07	NS
AIPL1	NS	1.19E-08	0.115317134	1.50E-07	NS
UTP3	NS	1.19E-08	-0.115311412	1.50E-07	NS
RAB27B	NS	1.21E-08	-0.115239724	1.53E-07	NS
MUCL3	NS	1.22E-08	-0.115217921	1.54E-07	NS
TDP1	NS	1.26E-08	0.115118418	1.58E-07	NS
SYCN	NS	1.28E-08	0.115058161	1.61E-07	NS
BARHL2	NS	1.28E-08	0.115051172	1.61E-07	NS
SPATC1L	NS	1.29E-08	-0.115037145	1.62E-07	NS
COMTD1	NS	1.30E-08	-0.115010673	1.63E-07	NS
DNAJC19	NS	1.30E-08	-0.115005539	1.63E-07	NS
TMEM128	NS	1.30E-08	-0.114996697	1.63E-07	NS
TAC4	NS	1.31E-08	-0.114980817	1.64E-07	NS
DYDC1	NS	1.32E-08	0.114938569	1.66E-07	NS
PLAG1	NS	1.33E-08	-0.114919203	1.67E-07	NS
TPSD1	NS	1.35E-08	0.114875011	1.69E-07	NS
ACER2	NS	1.36E-08	-0.11484905	1.70E-07	NS
KCNJ2	NS	1.36E-08	-0.114832678	1.70E-07	NS
TTC30A	NS	1.37E-08	-0.114819731	1.71E-07	NS
OR4A47	NS	1.40E-08	-0.114753239	1.74E-07	NS
SERPINB3	NS	1.40E-08	-0.114744971	1.75E-07	NS
ANKRD61	NS	1.41E-08	-0.114714962	1.76E-07	NS
RWDD3	NS	1.43E-08	-0.114678765	1.78E-07	NS
C9	NS	1.44E-08	-0.114640403	1.80E-07	NS
CGB3	NS	1.46E-08	0.114606498	1.81E-07	NS
TOP1MT	NS	1.46E-08	0.114599812	1.81E-07	NS
KTI12	NS	1.47E-08	-0.114583227	1.82E-07	NS
SAMD13	NS	1.49E-08	-0.114541065	1.84E-07	NS
RIMBP3	NS	1.53E-08	-0.114432343	1.90E-07	NS
SLURP1	NS	1.54E-08	0.11441699	1.91E-07	NS
IL20RA	NS	1.55E-08	-0.11439045	1.92E-07	NS
LCD4-RWDD	NS	1.56E-08	-0.114369198	1.93E-07	NS
RBM33	NS	1.58E-08	0.114323186	1.96E-07	NS
NXNL1	NS	1.60E-08	0.11428522	1.98E-07	NS
NUP35	NS	1.60E-08	-0.114276502	1.98E-07	NS
NOTO	NS	1.60E-08	0.114275052	1.98E-07	NS
SPINK5	NS	1.62E-08	-0.114236954	2.00E-07	NS
TSPAN13	NS	1.65E-08	-0.114180103	2.03E-07	NS
TRMT13	NS	1.66E-08	-0.114149621	2.05E-07	NS
AKR1B10	NS	1.67E-08	0.114134816	2.06E-07	NS
LMX1B	NS	1.70E-08	0.114077803	2.09E-07	NS
ADGRG6	NS	1.70E-08	-0.11407495	2.09E-07	NS
OR5M3	NS	1.73E-08	-0.11402268	2.12E-07	NS
GPC5	NS	1.74E-08	-0.114001364	2.13E-07	NS
TGFB3	NS	1.81E-08	-0.11386345	2.22E-07	NS
GZMH	NS	1.81E-08	-0.113849548	2.22E-07	NS
LRRC4C	NS	1.82E-08	-0.113844712	2.23E-07	NS
BRAF	NS	1.85E-08	0.113788011	2.26E-07	NS
DCLRE1A	NS	1.85E-08	-0.11378079	2.26E-07	NS
GUCY2C	NS	1.88E-08	-0.113725474	2.30E-07	NS
ALK	NS	1.90E-08	-0.113695163	2.32E-07	NS

TRIM47	NS	1.91E-08	0.113671395	2.33E-07	NS
C1orf232	NS	1.91E-08	0.113672873	2.33E-07	NS
MMP27	NS	1.92E-08	-0.113656207	2.34E-07	NS
PFDN1	NS	1.92E-08	0.113651276	2.34E-07	NS
ACTL9	NS	1.94E-08	0.11362439	2.36E-07	NS
CBLL1	NS	1.95E-08	0.113602138	2.37E-07	NS
LDB2	NS	1.95E-08	-0.11359209	2.38E-07	NS
ACSM4	NS	1.96E-08	-0.113585136	2.38E-07	NS
TNFSF18	NS	1.96E-08	-0.113576355	2.39E-07	NS
SOX7	NS	1.98E-08	0.113539126	2.41E-07	NS
MTERF3	NS	2.00E-08	-0.113510981	2.43E-07	NS
HPDL	NS	2.01E-08	-0.113498562	2.44E-07	NS
BMP10	NS	2.02E-08	-0.113481281	2.45E-07	NS
TCEAL6	NS	2.02E-08	-0.113470149	2.45E-07	NS
VWDE	NS	2.03E-08	-0.113462629	2.46E-07	NS
DEFB103B	NS	2.07E-08	-0.113396161	2.50E-07	NS
HBG2	NS	2.12E-08	-0.113311814	2.56E-07	NS
GAP43	NS	2.13E-08	-0.113301003	2.57E-07	NS
COLGALT2	NS	2.13E-08	-0.113298348	2.57E-07	NS
CXCL11	NS	2.20E-08	-0.113181954	2.65E-07	NS
DLX1	NS	2.22E-08	0.113154169	2.67E-07	NS
PTH2	NS	2.30E-08	0.113029615	2.77E-07	NS
SFTPC	NS	2.30E-08	0.113024008	2.77E-07	NS
TMEM45A	NS	2.30E-08	0.113020589	2.77E-07	NS
IZUMO1	NS	2.31E-08	0.11300585	2.78E-07	NS
SCGN	NS	2.34E-08	-0.112964022	2.81E-07	NS
SRD5A2	NS	2.34E-08	-0.112962809	2.81E-07	NS
UBE2L5	NS	2.37E-08	0.112919965	2.84E-07	NS
KCNJ3	NS	2.40E-08	-0.112874595	2.88E-07	NS
LGALS7B	NS	2.44E-08	0.112819	2.92E-07	NS
NFKBIL1	NS	2.45E-08	-0.11279828	2.94E-07	NS
KRT16	NS	2.47E-08	0.112777196	2.96E-07	NS
TMEM239	NS	2.51E-08	0.112724708	3.00E-07	NS
TNFSF11	NS	2.54E-08	-0.112679584	3.03E-07	NS
APOC1	NS	2.54E-08	0.112678958	3.03E-07	NS
BSX	NS	2.58E-08	0.112619657	3.08E-07	NS
CLDN20	NS	2.61E-08	-0.112587839	3.11E-07	NS
ADGRF5	NS	2.61E-08	-0.112579856	3.12E-07	NS
TNKS	NS	2.63E-08	0.112551666	3.14E-07	NS
TAS2R20	NS	2.67E-08	-0.112509754	3.17E-07	NS
THSD8	NS	2.72E-08	-0.112436869	3.24E-07	NS
PARS2	NS	2.75E-08	-0.112400535	3.27E-07	NS
ASRGL1	NS	2.76E-08	-0.112384501	3.28E-07	NS
AICDA	NS	2.80E-08	0.112339717	3.32E-07	NS
SLC38A11	NS	2.80E-08	-0.112330492	3.33E-07	NS
FRA10AC1	NS	2.86E-08	-0.112265443	3.39E-07	NS
NOL4L	NS	2.86E-08	0.112261167	3.39E-07	NS
PRIMPOL	NS	2.90E-08	-0.112207079	3.44E-07	NS
PAX3	NS	2.91E-08	-0.112203112	3.45E-07	NS
SAMD15	NS	2.92E-08	0.112188003	3.46E-07	NS
TEX36	NS	2.92E-08	-0.112183916	3.46E-07	NS
NPHS2	NS	2.93E-08	-0.112179229	3.46E-07	NS
SMG7	NS	2.93E-08	0.112175434	3.46E-07	NS
DDIT4L	NS	2.97E-08	-0.112125174	3.51E-07	NS
MAP9	NS	2.98E-08	-0.112114663	3.52E-07	NS

PRELID3A	NS	2.99E-08	0.112103102	3.53E-07	NS
ZNF385B	NS	3.00E-08	-0.112093475	3.54E-07	NS
POLR3G	NS	3.02E-08	-0.112064979	3.56E-07	NS
LYPD6B	NS	3.04E-08	-0.112050343	3.58E-07	NS
ATP6V1G2	NS	3.06E-08	-0.112022386	3.60E-07	NS
SEPTIN12	NS	3.07E-08	0.112008419	3.61E-07	NS
GAR1	NS	3.08E-08	-0.111998555	3.62E-07	NS
HTR2B	NS	3.08E-08	-0.112000565	3.62E-07	NS
MAP1LC3C	NS	3.09E-08	0.111994384	3.62E-07	NS
ZNF322	NS	3.10E-08	-0.111979965	3.63E-07	NS
TMPRSS11F	NS	3.11E-08	-0.111971079	3.64E-07	NS
SPATA31D1	NS	3.13E-08	-0.111947943	3.66E-07	NS
MORN2	NS	3.15E-08	-0.111923622	3.69E-07	NS
CHERP	NS	3.16E-08	0.111909589	3.70E-07	NS
FOXD2	NS	3.16E-08	0.11190755	3.70E-07	NS
SP5	NS	3.18E-08	0.11188757	3.72E-07	NS
INS	NS	3.22E-08	0.111841425	3.76E-07	NS
MAGEA9	NS	3.25E-08	0.111813505	3.79E-07	NS
CDY1	NS	3.25E-08	-0.111815416	3.79E-07	NS
ITIH6	NS	3.25E-08	-0.11180674	3.80E-07	NS
GRK4	NS	3.28E-08	-0.111782561	3.82E-07	NS
A2M	NS	3.30E-08	-0.111756944	3.84E-07	NS
PYHIN1	NS	3.32E-08	-0.111732173	3.87E-07	NS
KRTAP9-6	NS	3.39E-08	0.111665691	3.94E-07	NS
MCC	NS	3.40E-08	-0.111656255	3.94E-07	NS
TAS2R5	NS	3.52E-08	-0.111532491	4.08E-07	NS
EID2	NS	3.52E-08	-0.111534197	4.08E-07	NS
NUFIP1	NS	3.53E-08	0.111519636	4.09E-07	NS
OR8H3	NS	3.53E-08	-0.111521698	4.09E-07	NS
OLR1	NS	3.54E-08	0.111511864	4.10E-07	NS
C19orf33	NS	3.57E-08	-0.111478524	4.13E-07	NS
LHFPL6	NS	3.64E-08	-0.111415008	4.20E-07	NS
SSU72P3	NS	3.65E-08	0.111402907	4.22E-07	NS
TNC	NS	3.69E-08	-0.111366886	4.26E-07	NS
TTC23L	NS	3.72E-08	-0.111334294	4.29E-07	NS
DAND5	NS	3.72E-08	0.111331555	4.29E-07	NS
OTOP3	NS	3.74E-08	0.111311893	4.32E-07	NS
LCE1C	NS	3.75E-08	0.11130796	4.32E-07	NS
G6PC2	NS	3.78E-08	-0.111281327	4.35E-07	NS
RGP1	NS	3.81E-08	0.111252912	4.38E-07	NS
CALHM4	NS	3.82E-08	-0.111239332	4.39E-07	NS
ADGRF4	NS	3.88E-08	-0.111186929	4.46E-07	NS
ENPP3	NS	3.89E-08	-0.111174339	4.47E-07	NS
RTP1	NS	3.92E-08	-0.111152959	4.49E-07	NS
MED19	NS	3.94E-08	-0.111134759	4.52E-07	NS
HEMGN	NS	4.00E-08	-0.111079625	4.58E-07	NS
CYP46A1	NS	4.00E-08	-0.111075613	4.58E-07	NS
CCDC68	NS	4.00E-08	-0.111074625	4.58E-07	NS
WFIKKN1	NS	4.03E-08	0.111050212	4.61E-07	NS
H4C7	NS	4.04E-08	0.111042121	4.62E-07	NS
NDUFB1	NS	4.09E-08	-0.111000325	4.67E-07	NS
DCTN5	NS	4.10E-08	0.110987243	4.69E-07	NS
SPINDOC	NS	4.17E-08	0.110928684	4.76E-07	NS
AK5	NS	4.19E-08	-0.110911224	4.78E-07	NS
OLFML1	NS	4.20E-08	-0.110904802	4.79E-07	NS

KRTAP12-4	NS	4.21E-08	0.110897535	4.80E-07	NS
ADAMTSL5	NS	4.24E-08	0.110869082	4.83E-07	NS
RHOBTB1	NS	4.25E-08	-0.110863999	4.84E-07	NS
MAGEA9B	NS	4.26E-08	0.110855934	4.84E-07	NS
HAS2	NS	4.29E-08	-0.110830556	4.87E-07	NS
CT45A6	NS	4.29E-08	-0.110831882	4.87E-07	NS
MBD3L5	NS	4.30E-08	0.110822462	4.88E-07	NS
NBPF3	NS	4.31E-08	-0.110812016	4.89E-07	NS
KCNJ12	NS	4.37E-08	0.110764243	4.96E-07	NS
MMP15	NS	4.40E-08	0.110738672	4.99E-07	NS
RBM11	NS	4.40E-08	-0.110738491	4.99E-07	NS
PCGF5	NS	4.58E-08	0.110597133	5.18E-07	NS
PLCXD3	NS	4.59E-08	-0.110585388	5.20E-07	NS
ZNF649	NS	4.61E-08	-0.11057114	5.21E-07	NS
OR14C36	NS	4.62E-08	-0.110568045	5.21E-07	NS
BMP5	NS	4.62E-08	-0.110563605	5.22E-07	NS
ZMYM2	NS	4.62E-08	0.110561903	5.22E-07	NS
C1orf226	NS	4.63E-08	-0.110559043	5.22E-07	NS
TMEM225B	NS	4.67E-08	-0.110526569	5.26E-07	NS
CLDN22	NS	4.73E-08	-0.110483681	5.32E-07	NS
SFXN1	NS	4.75E-08	-0.110464961	5.34E-07	NS
FRMPD4	NS	4.75E-08	-0.110463582	5.34E-07	NS
SPINK14	NS	4.77E-08	-0.110447261	5.37E-07	NS
ZNHIT2	NS	4.81E-08	-0.110422713	5.40E-07	NS
HOXA7	NS	4.82E-08	0.11041434	5.41E-07	NS
LCE3B	NS	4.85E-08	0.110393609	5.44E-07	NS
SPATA31D4	NS	4.86E-08	-0.110383649	5.45E-07	NS
PRR22	NS	4.97E-08	0.110303716	5.57E-07	NS
GYPE	NS	5.04E-08	-0.11025332	5.65E-07	NS
CDK11A	NS	5.11E-08	0.110202001	5.72E-07	NS
GCG	NS	5.13E-08	-0.11018791	5.74E-07	NS
KRT81	NS	5.21E-08	0.110135873	5.82E-07	NS
WDR35	NS	5.23E-08	-0.1101197	5.85E-07	NS
TRIB1	NS	5.26E-08	0.110099799	5.88E-07	NS
RPP25L	NS	5.29E-08	-0.110081163	5.90E-07	NS
ALDH18A1	NS	5.30E-08	-0.110071007	5.92E-07	NS
HCAR1	NS	5.33E-08	0.110052262	5.94E-07	NS
SEL1L2	NS	5.35E-08	-0.110040025	5.96E-07	NS
CT47A12	NS	5.40E-08	0.110006894	6.01E-07	NS
CLEC4F	NS	5.45E-08	-0.109971629	6.07E-07	NS
GPR52	NS	5.53E-08	-0.109918933	6.16E-07	NS
AGPAT1	NS	5.54E-08	0.109911568	6.16E-07	NS
SLC30A2	NS	5.57E-08	0.10989245	6.19E-07	NS
RGS9	NS	5.67E-08	-0.109829161	6.30E-07	NS
CD177	NS	5.68E-08	0.109823955	6.31E-07	NS
CENPE	NS	5.74E-08	-0.109785908	6.37E-07	NS
KLF15	NS	5.75E-08	0.109779629	6.38E-07	NS
C1orf94	NS	5.78E-08	-0.109765309	6.40E-07	NS
GDF9	NS	5.81E-08	-0.109745911	6.43E-07	NS
TREML2	NS	5.81E-08	0.109740884	6.43E-07	NS
TLE2	NS	5.83E-08	0.109728994	6.45E-07	NS
MYL1	NS	5.83E-08	-0.109728519	6.45E-07	NS
LEFTY2	NS	5.88E-08	0.109699449	6.50E-07	NS
SLC38A4	NS	5.91E-08	-0.109681017	6.52E-07	NS
RTP3	NS	5.99E-08	0.109633189	6.61E-07	NS

SLAMF8	NS	6.03E-08	-0.109611303	6.64E-07	NS
LECT2	NS	6.03E-08	-0.109608273	6.64E-07	NS
FMN1	NS	6.06E-08	-0.109589917	6.67E-07	NS
IL6	NS	6.07E-08	-0.109584532	6.68E-07	NS
NOM1	NS	6.10E-08	-0.109571606	6.70E-07	NS
SLC22A3	NS	6.17E-08	-0.109525464	6.78E-07	NS
EEF1E1	NS	6.21E-08	-0.10950417	6.82E-07	NS
CACNA2D4	NS	6.21E-08	0.109501786	6.82E-07	NS
SPAG6	NS	6.25E-08	-0.10947875	6.86E-07	NS
SMC2	NS	6.26E-08	-0.1094759	6.86E-07	NS
ZDHHC5	NS	6.29E-08	0.109458957	6.89E-07	NS
EEF1AKMT4	NS	6.31E-08	-0.109446743	6.91E-07	NS
IFT172	NS	6.32E-08	-0.109442596	6.91E-07	NS
NAMPT	NS	6.40E-08	0.109398409	6.99E-07	NS
MAB21L1	NS	6.41E-08	0.109391421	7.00E-07	NS
STRIT1	NS	6.45E-08	-0.109370616	7.04E-07	NS
TEK	NS	6.46E-08	-0.109361528	7.05E-07	NS
TRAT1	NS	6.55E-08	-0.109315195	7.14E-07	NS
LEFTY1	NS	6.61E-08	0.109280282	7.20E-07	NS
CEACAM6	NS	6.74E-08	-0.109211272	7.33E-07	NS
TTC29	NS	6.74E-08	-0.109209915	7.33E-07	NS
CTAGE6	NS	6.74E-08	-0.109208534	7.33E-07	NS
GSTM3	NS	6.78E-08	-0.109190124	7.36E-07	NS
CDSN	NS	6.81E-08	0.109169825	7.40E-07	NS
FADD	NS	6.90E-08	-0.109123316	7.49E-07	NS
C17orf99	NS	6.92E-08	0.109116543	7.50E-07	NS
SSX5	NS	6.99E-08	0.109077159	7.58E-07	NS
VSIG8	NS	7.01E-08	0.109066603	7.60E-07	NS
PLN	NS	7.17E-08	-0.108987179	7.76E-07	NS
GNPDA2	NS	7.19E-08	-0.108975374	7.78E-07	NS
SHH	NS	7.19E-08	0.108973907	7.78E-07	NS
RNLS	NS	7.20E-08	-0.108968429	7.79E-07	NS
FSHR	NS	7.22E-08	-0.108960772	7.80E-07	NS
NAT10	NS	7.27E-08	-0.108937462	7.84E-07	NS
GMFB	NS	7.26E-08	0.108939311	7.84E-07	NS
NMS	NS	7.34E-08	-0.108900421	7.92E-07	NS
STYXL2	NS	7.37E-08	-0.108885623	7.95E-07	NS
RPL3L	NS	7.38E-08	0.108880042	7.96E-07	NS
UTP23	NS	7.39E-08	0.108874831	7.96E-07	NS
UBQLNL	NS	7.52E-08	-0.108812456	8.10E-07	NS
UBL4B	NS	7.54E-08	0.108803885	8.11E-07	NS
S100A5	NS	7.66E-08	-0.108744873	8.24E-07	NS
H2AC17	NS	7.71E-08	-0.108722866	8.29E-07	NS
MRGPRF	NS	7.72E-08	0.108716775	8.30E-07	NS
XKR3	NS	7.73E-08	-0.108714182	8.30E-07	NS
FAM240B	NS	7.81E-08	0.108676199	8.38E-07	NS
HMBS	NS	7.82E-08	-0.108673871	8.38E-07	NS
MRNIP	NS	7.87E-08	0.108648907	8.43E-07	NS
DLX6	NS	8.04E-08	0.108572734	8.61E-07	NS
PRKAR2B	NS	8.11E-08	-0.108539101	8.68E-07	NS
NDP	NS	8.14E-08	-0.108528259	8.70E-07	NS
MEP1B	NS	8.24E-08	-0.108483155	8.81E-07	NS
SASS6	NS	8.31E-08	-0.108453189	8.88E-07	NS
ZNF37A	NS	8.44E-08	-0.10839621	9.01E-07	NS
DAW1	NS	8.44E-08	-0.108394648	9.01E-07	NS

SLC39A12	NS	8.57E-08	-0.108339296	9.14E-07	NS
TEX52	NS	8.65E-08	-0.10830636	9.22E-07	NS
AP1S1	NS	8.66E-08	-0.108302638	9.23E-07	NS
CMTM4	NS	8.66E-08	-0.108300747	9.23E-07	NS
BBIP1	NS	8.67E-08	0.108297645	9.23E-07	NS
FOXI2	NS	8.70E-08	0.108283709	9.26E-07	NS
FETUB	NS	8.73E-08	-0.108270533	9.29E-07	NS
ANKRD45	NS	8.86E-08	0.108217572	9.42E-07	NS
TMX2	NS	8.91E-08	-0.108197321	9.47E-07	NS
GON7	NS	8.98E-08	-0.108168516	9.54E-07	NS
MNAT1	NS	9.07E-08	-0.108132346	9.62E-07	NS
NELL2	NS	9.07E-08	-0.108132292	9.62E-07	NS
ICOS	NS	9.21E-08	-0.108078009	9.76E-07	NS
OR5V1	NS	9.21E-08	-0.108075051	9.76E-07	NS
FAM3C	NS	9.24E-08	-0.108063428	9.79E-07	NS
CPSF4L	NS	9.40E-08	-0.108003944	9.94E-07	NS
ECEL1	NS	9.47E-08	0.107974418	1.00E-06	NS
BIRC6	NS	9.51E-08	0.107959895	1.01E-06	NS
HAPLN1	NS	9.52E-08	-0.107957041	1.01E-06	NS
TEX50	NS	9.56E-08	-0.10794164	1.01E-06	NS
DBNDD2	NS	9.63E-08	-0.107912621	1.02E-06	NS
GLRB	NS	9.65E-08	-0.107905173	1.02E-06	NS
ALDH3A1	NS	9.67E-08	0.107898841	1.02E-06	NS
NKX1-2	NS	9.71E-08	0.107883864	1.02E-06	NS
AQP6	NS	9.72E-08	0.107881342	1.02E-06	NS
USP15	NS	9.76E-08	0.10786483	1.03E-06	NS
PRKCG	NS	9.82E-08	0.10784248	1.03E-06	NS
EP300	NS	9.87E-08	0.107822495	1.04E-06	NS
OR4C12	NS	9.89E-08	-0.107817286	1.04E-06	NS
RPRD1A	NS	9.91E-08	0.107810081	1.04E-06	NS
SKIDA1	NS	1.00E-07	0.107763785	1.05E-06	NS
SIRT3	NS	1.01E-07	0.107757281	1.05E-06	NS
ANKRD31	NS	1.01E-07	-0.107753852	1.06E-06	NS
TMEM178A	NS	1.01E-07	-0.10774266	1.06E-06	NS
SPOCK1	NS	1.01E-07	-0.107723556	1.06E-06	NS
RTCB	NS	1.04E-07	-0.107627962	1.09E-06	NS
ACER1	NS	1.05E-07	0.107583	1.10E-06	NS
GDF2	NS	1.07E-07	0.107543399	1.11E-06	NS
PGM3	NS	1.07E-07	-0.107531953	1.12E-06	NS
TGFA	NS	1.07E-07	-0.107519851	1.12E-06	NS
ZMAT2	NS	1.08E-07	-0.107497711	1.13E-06	NS
PSAPL1	NS	1.08E-07	0.107485016	1.13E-06	NS
BIRC7	NS	1.09E-07	0.107458812	1.14E-06	NS
MYLK4	NS	1.10E-07	-0.107439543	1.14E-06	NS
DEFB106A	NS	1.11E-07	-0.107409894	1.15E-06	NS
THAP8	NS	1.12E-07	-0.107370006	1.16E-06	NS
ODAPH	NS	1.12E-07	0.107346812	1.17E-06	NS
GSTT4	NS	1.16E-07	0.107238604	1.20E-06	NS
FOXH1	NS	1.16E-07	-0.10722596	1.21E-06	NS
RBMY1J	NS	1.18E-07	-0.107178484	1.22E-06	NS
KRT40	NS	1.18E-07	0.107157958	1.23E-06	NS
LRRC53	NS	1.20E-07	-0.107097685	1.25E-06	NS
ARHGEF7	NS	1.22E-07	0.107044016	1.27E-06	NS
OR56B2P	NS	1.22E-07	-0.107035881	1.27E-06	NS
AARSD1	NS	1.24E-07	-0.106986967	1.29E-06	NS

GNAT2	NS	1.26E-07	0.106938853	1.30E-06	NS
C2CD4D	NS	1.27E-07	0.106902872	1.31E-06	NS
PLOD2	NS	1.27E-07	-0.10690057	1.31E-06	NS
TUBB8	NS	1.27E-07	0.106897534	1.31E-06	NS
DUOXA2	NS	1.27E-07	0.106893819	1.31E-06	NS
C1QTNF7	NS	1.27E-07	-0.106885336	1.32E-06	NS
BUD13	NS	1.31E-07	-0.106793401	1.35E-06	NS
KCNK16	NS	1.31E-07	0.106776922	1.35E-06	NS
SELENOV	NS	1.32E-07	0.106765875	1.36E-06	NS
RILP	NS	1.32E-07	0.106753709	1.36E-06	NS
DNTT	NS	1.33E-07	-0.106731697	1.37E-06	NS
PPP1R14C	NS	1.33E-07	-0.106720645	1.37E-06	NS
NCAM1	NS	1.34E-07	-0.106711049	1.37E-06	NS
HIPK4	NS	1.34E-07	-0.106705396	1.38E-06	NS
GZMM	NS	1.35E-07	0.106664585	1.39E-06	NS
OR2B6	NS	1.36E-07	-0.106658667	1.39E-06	NS
CEP162	NS	1.36E-07	-0.106636102	1.40E-06	NS
SPATA21	NS	1.37E-07	0.106620125	1.41E-06	NS
SNURF	NS	1.37E-07	-0.106612761	1.41E-06	NS
SLC35D1	NS	1.37E-07	-0.106608799	1.41E-06	NS
EDNRA	NS	1.38E-07	-0.106602303	1.41E-06	NS
FGF4	NS	1.38E-07	0.106596083	1.41E-06	NS
RNGTT	NS	1.40E-07	-0.106548368	1.43E-06	NS
NAT1	NS	1.40E-07	-0.106533581	1.43E-06	NS
FAM240C	NS	1.40E-07	0.106532366	1.43E-06	NS
PET117	NS	1.41E-07	-0.106525742	1.44E-06	NS
CCL7	NS	1.41E-07	-0.106521277	1.44E-06	NS
MMP13	NS	1.41E-07	0.106520053	1.44E-06	NS
HCRTR2	NS	1.41E-07	-0.106511106	1.44E-06	NS
ZNF502	NS	1.43E-07	-0.106452475	1.46E-06	NS
CAVIN1	NS	1.44E-07	0.10644264	1.46E-06	NS
CFAP97D2	NS	1.44E-07	0.106433165	1.47E-06	NS
ZNF133	NS	1.48E-07	-0.106345276	1.50E-06	NS
CHST13	NS	1.48E-07	-0.106322634	1.51E-06	NS
SPR	NS	1.49E-07	-0.106310559	1.51E-06	NS
DDAH1	NS	1.49E-07	-0.106311691	1.51E-06	NS
CADM4	NS	1.50E-07	0.10628595	1.52E-06	NS
SPRR3	NS	1.53E-07	0.106202217	1.56E-06	NS
FDX2	NS	1.54E-07	-0.106197905	1.56E-06	NS
GKN2	NS	1.55E-07	-0.106170823	1.57E-06	NS
ASB15	NS	1.55E-07	-0.106164097	1.57E-06	NS
IGSF11	NS	1.55E-07	-0.106157224	1.57E-06	NS
ZMAT4	NS	1.58E-07	-0.106100211	1.60E-06	NS
ZNF678	NS	1.60E-07	-0.106038752	1.62E-06	NS
RBBP5	NS	1.60E-07	-0.106036393	1.62E-06	NS
RHOD	NS	1.61E-07	0.106032188	1.62E-06	NS
IL7	NS	1.62E-07	-0.105988747	1.64E-06	NS
ADAD1	NS	1.62E-07	-0.105988819	1.64E-06	NS
FABP4	NS	1.64E-07	-0.105961106	1.65E-06	NS
TMED4	NS	1.64E-07	0.105959085	1.65E-06	NS
TRIAP1	NS	1.64E-07	-0.105942911	1.66E-06	NS
FUCA1	NS	1.65E-07	-0.105935014	1.66E-06	NS
RPL10L	NS	1.65E-07	-0.10592749	1.66E-06	NS
OR8U3	NS	1.67E-07	-0.105884154	1.68E-06	NS
TMEM14A	NS	1.69E-07	-0.105845558	1.70E-06	NS

PEMT	NS	1.70E-07	0.105827096	1.71E-06	NS
SLC46A3	NS	1.72E-07	-0.105777277	1.73E-06	NS
CDK1	NS	1.72E-07	-0.105777176	1.73E-06	NS
CTAGE15	NS	1.73E-07	-0.10574714	1.74E-06	NS
FZD7	NS	1.75E-07	0.105714744	1.75E-06	NS
RFX6	NS	1.78E-07	-0.105646823	1.79E-06	NS
TUBB4A	NS	1.79E-07	0.105635945	1.79E-06	NS
ET1-SH3BG	NS	1.79E-07	-0.105635013	1.79E-06	NS
SGCG	NS	1.79E-07	-0.105631	1.79E-06	NS
MEIS3	NS	1.79E-07	0.10563073	1.79E-06	NS
OR14L1P	NS	1.81E-07	-0.10558335	1.81E-06	NS
SAA2-SAA4	NS	1.81E-07	-0.105581851	1.81E-06	NS
DRAXIN	NS	1.82E-07	-0.105568031	1.82E-06	NS
PRR30	NS	1.82E-07	0.105564908	1.82E-06	NS
FGF19	NS	1.84E-07	0.105531982	1.83E-06	NS
OR52L1	NS	1.84E-07	-0.105521605	1.84E-06	NS
ACOT12	NS	1.85E-07	-0.105513174	1.84E-06	NS
IL4I1	NS	1.85E-07	0.105497105	1.85E-06	NS
H3C12	NS	1.86E-07	-0.105493061	1.85E-06	NS
CFAP44	NS	1.88E-07	-0.105451632	1.87E-06	NS
RPRM	NS	1.89E-07	0.105420054	1.88E-06	NS
CT47A8	NS	1.91E-07	0.105386068	1.90E-06	NS
BEX2	NS	1.92E-07	-0.105366726	1.91E-06	NS
FAM181A	NS	1.92E-07	0.10536866	1.91E-06	NS
YIPF7	NS	1.92E-07	-0.105364196	1.91E-06	NS
GDPD4	NS	1.92E-07	-0.105364749	1.91E-06	NS
ORC5	NS	1.94E-07	-0.105333384	1.92E-06	NS
TBX19	NS	1.94E-07	-0.105329043	1.92E-06	NS
CA7	NS	1.94E-07	0.105318229	1.93E-06	NS
PTGDR	NS	1.95E-07	-0.105313937	1.93E-06	NS
SOHLH2	NS	1.95E-07	-0.1053082	1.93E-06	NS
LY6G5B	NS	1.95E-07	0.105304046	1.93E-06	NS
PLA2G2D	NS	1.96E-07	0.105283693	1.94E-06	NS
LRRC56	NS	1.97E-07	0.105278529	1.94E-06	NS
CROCC2	NS	1.99E-07	0.105239478	1.96E-06	NS
CDK17	NS	2.04E-07	0.105142135	2.01E-06	NS
FAM24A	NS	2.04E-07	-0.10513437	2.02E-06	NS
ELAPOR2	NS	2.05E-07	-0.105116995	2.02E-06	NS
ANOS1	NS	2.08E-07	-0.105071077	2.05E-06	NS
HES1	NS	2.08E-07	0.105069866	2.05E-06	NS
DEFA5	NS	2.08E-07	-0.105071028	2.05E-06	NS
ADAM20	NS	2.10E-07	-0.105034659	2.06E-06	NS
NDUFB6	NS	2.11E-07	-0.105017039	2.07E-06	NS
RBMY1A1	NS	2.11E-07	-0.105017393	2.07E-06	NS
OR10R2	NS	2.12E-07	-0.105002542	2.08E-06	NS
MRPL19	NS	2.12E-07	-0.104988591	2.08E-06	NS
FOXE1	NS	2.16E-07	0.104933414	2.11E-06	NS
HMX3	NS	2.16E-07	0.104920029	2.12E-06	NS
ASB10	NS	2.18E-07	0.104896079	2.13E-06	NS
OR5B21	NS	2.18E-07	-0.104897903	2.13E-06	NS
LRIG3	NS	2.23E-07	-0.104808206	2.18E-06	NS
TMPRSS13	NS	2.23E-07	-0.104799416	2.18E-06	NS
OR5AN1	NS	2.27E-07	-0.104746576	2.21E-06	NS
PLK4	NS	2.27E-07	-0.104739725	2.22E-06	NS
TIFA	NS	2.27E-07	-0.104741168	2.22E-06	NS

CASP6	NS	2.30E-07	-0.104684514	2.25E-06	NS
OR4E2	NS	2.31E-07	0.104677892	2.25E-06	NS
FER1L6	NS	2.32E-07	-0.104660501	2.26E-06	NS
ZBTB9	NS	2.33E-07	-0.104635644	2.27E-06	NS
MMP16	NS	2.36E-07	-0.104598309	2.30E-06	NS
RCOR3	NS	2.36E-07	0.104593284	2.30E-06	NS
GRB7	NS	2.37E-07	0.104574556	2.31E-06	NS
PLSCR1	NS	2.38E-07	0.104556584	2.32E-06	NS
MOGS	NS	2.39E-07	-0.104548589	2.32E-06	NS
ZFP41	NS	2.39E-07	-0.104540663	2.33E-06	NS
TMEM59L	NS	2.39E-07	0.104538048	2.33E-06	NS
SH3BGR	NS	2.41E-07	-0.104514514	2.34E-06	NS
CASKIN2	NS	2.42E-07	0.104497908	2.35E-06	NS
SLC39A10	NS	2.43E-07	-0.104488151	2.35E-06	NS
TLCD3A	NS	2.45E-07	0.104446368	2.38E-06	NS
C21orf140	NS	2.46E-07	-0.104437854	2.38E-06	NS
TCEAL7	NS	2.47E-07	-0.104423084	2.39E-06	NS
CDK2	NS	2.48E-07	-0.104412781	2.40E-06	NS
CSNK1A1	NS	2.49E-07	0.104388198	2.41E-06	NS
PHLDB2	NS	2.50E-07	-0.104378907	2.41E-06	NS
KLRC3	NS	2.52E-07	-0.104351093	2.43E-06	NS
ATXN7L2	NS	2.52E-07	0.104346574	2.43E-06	NS
GFRA2	NS	2.52E-07	-0.104345754	2.43E-06	NS
DNAJB12	NS	2.54E-07	0.104322854	2.45E-06	NS
ZNF613	NS	2.54E-07	-0.104315791	2.45E-06	NS
TAS2R42	NS	2.56E-07	-0.104286937	2.47E-06	NS
ZNF420	NS	2.57E-07	-0.104272434	2.47E-06	NS
PDCD1LG2	NS	2.57E-07	-0.104271817	2.47E-06	NS
TP53RK	NS	2.57E-07	-0.104268688	2.48E-06	NS
SLC26A3	NS	2.58E-07	-0.104259651	2.48E-06	NS
HELB	NS	2.58E-07	-0.104253071	2.48E-06	NS
GMNN	NS	2.60E-07	-0.104231208	2.50E-06	NS
RAD51	NS	2.60E-07	-0.104223321	2.50E-06	NS
ZNF615	NS	2.61E-07	-0.104206937	2.51E-06	NS
PRM2	NS	2.63E-07	0.104180838	2.53E-06	NS
FAM71B	NS	2.70E-07	0.104084885	2.59E-06	NS
ZNF729	NS	2.72E-07	-0.104060371	2.61E-06	NS
RESP18	NS	2.73E-07	-0.104048146	2.61E-06	NS
CYP2C8	NS	2.75E-07	-0.104022581	2.63E-06	NS
ZNF823	NS	2.75E-07	-0.104012352	2.63E-06	NS
POMGNT1	NS	2.76E-07	-0.104005944	2.64E-06	NS
SYNPR	NS	2.77E-07	-0.103990404	2.65E-06	NS
AP3B2	NS	2.77E-07	-0.103983333	2.65E-06	NS
PROM2	NS	2.79E-07	0.103955293	2.67E-06	NS
DPT	NS	2.81E-07	-0.10393399	2.68E-06	NS
TAS2R31	NS	2.85E-07	-0.103878141	2.72E-06	NS
REXO2	NS	2.87E-07	-0.103850937	2.74E-06	NS
DDX39A	NS	2.89E-07	0.103823197	2.76E-06	NS
LCN15	NS	2.89E-07	0.103821998	2.76E-06	NS
VSTM2B	NS	2.90E-07	0.103819203	2.76E-06	NS
TMEM95	NS	2.91E-07	0.103795929	2.77E-06	NS
BECN2	NS	2.93E-07	0.103771993	2.79E-06	NS
LARGE2	NS	2.94E-07	0.10376914	2.79E-06	NS
LLPH	NS	2.96E-07	0.103736561	2.81E-06	NS
VAX1	NS	3.03E-07	0.103653098	2.87E-06	NS

SOSTDC1	NS	3.03E-07	-0.103653377	2.87E-06	NS
MCEMP1	NS	3.03E-07	0.103648665	2.88E-06	NS
BRD2	NS	3.05E-07	0.103617272	2.90E-06	NS
SLC25A38	NS	3.08E-07	-0.103586509	2.92E-06	NS
MAGEB4	NS	3.09E-07	-0.103574286	2.93E-06	NS
CTAG1A	NS	3.12E-07	0.103536737	2.96E-06	NS
GIPC1	NS	3.12E-07	0.103534792	2.96E-06	NS
IQCF2	NS	3.13E-07	-0.103528671	2.96E-06	NS
C6orf163	NS	3.17E-07	-0.103475227	3.00E-06	NS
GRXCR1	NS	3.18E-07	-0.10346762	3.00E-06	NS
MAPK7	NS	3.20E-07	0.103439562	3.03E-06	NS
KRTAP5-1	NS	3.21E-07	0.103433624	3.03E-06	NS
PAK1IP1	NS	3.21E-07	-0.10342703	3.03E-06	NS
TEX53	NS	3.24E-07	-0.103392001	3.06E-06	NS
MYADML2	NS	3.25E-07	0.103378959	3.07E-06	NS
RHBDD2	NS	3.27E-07	0.103358238	3.08E-06	NS
SMG1	NS	3.28E-07	0.103343054	3.09E-06	NS
HOOK3	NS	3.28E-07	0.103344165	3.09E-06	NS
PIK3C2G	NS	3.31E-07	-0.103316569	3.11E-06	NS
MRPL30	NS	3.31E-07	-0.103311699	3.12E-06	NS
LACTBL1	NS	3.38E-07	0.103231142	3.18E-06	NS
ZNF57	NS	3.39E-07	-0.103226002	3.18E-06	NS
C19orf71	NS	3.39E-07	0.103222678	3.18E-06	NS
ADGRG7	NS	3.39E-07	-0.103215956	3.19E-06	NS
ZC2HC1A	NS	3.42E-07	-0.103190234	3.21E-06	NS
F13B	NS	3.43E-07	-0.103179837	3.21E-06	NS
BTBD19	NS	3.44E-07	0.103167436	3.22E-06	NS
DLX3	NS	3.45E-07	0.103151815	3.23E-06	NS
GORASP1	NS	3.50E-07	0.103102535	3.28E-06	NS
XIRP2	NS	3.52E-07	-0.10307839	3.29E-06	NS
KIR3DL1	NS	3.56E-07	-0.103034223	3.33E-06	NS
HMGB3	NS	3.57E-07	0.103026033	3.34E-06	NS
GPN3	NS	3.60E-07	-0.102990909	3.36E-06	NS
EDA2R	NS	3.60E-07	-0.102991898	3.36E-06	NS
MRPL53	NS	3.63E-07	-0.102960919	3.39E-06	NS
HOATZ	NS	3.63E-07	0.102956403	3.39E-06	NS
CENPJ	NS	3.66E-07	-0.102928144	3.42E-06	NS
CXCL10	NS	3.69E-07	-0.102899027	3.44E-06	NS
VP23C-CDRT	NS	3.69E-07	-0.102895078	3.44E-06	NS
PRDM4	NS	3.70E-07	0.10289031	3.44E-06	NS
CAPN11	NS	3.78E-07	0.102808709	3.52E-06	NS
PDS5B	NS	3.79E-07	0.102797244	3.53E-06	NS
C1orf87	NS	3.80E-07	-0.102785938	3.53E-06	NS
ZFP37	NS	3.81E-07	-0.102771652	3.55E-06	NS
CILP2	NS	3.85E-07	0.102739804	3.57E-06	NS
CXorf65	NS	3.85E-07	-0.102738222	3.57E-06	NS
SYNPO2L	NS	3.87E-07	0.102712017	3.60E-06	NS
VSIG1	NS	3.88E-07	0.102706702	3.60E-06	NS
RAPGEFL1	NS	3.92E-07	0.102661959	3.64E-06	NS
CCDC178	NS	3.93E-07	-0.102653176	3.65E-06	NS
UGT2B17	NS	3.93E-07	-0.102652399	3.65E-06	NS
C17orf50	NS	3.94E-07	0.102648415	3.65E-06	NS
NDRG4	NS	3.94E-07	0.102644908	3.65E-06	NS
TMEM19	NS	3.95E-07	-0.102635034	3.66E-06	NS
RND1	NS	3.95E-07	0.102632972	3.66E-06	NS

ZNF254	NS	3.96E-07	0.102628607	3.66E-06	NS
DSCC1	NS	3.97E-07	-0.102618782	3.67E-06	NS
LRRC73	NS	3.98E-07	0.102612658	3.67E-06	NS
RTBDN	NS	4.00E-07	0.102589419	3.69E-06	NS
FAM131C	NS	4.01E-07	0.10258321	3.70E-06	NS
CPLX3	NS	4.05E-07	0.102544455	3.73E-06	NS
C19orf84	NS	4.05E-07	0.102545808	3.73E-06	NS
CARMIL2	NS	4.07E-07	0.10252617	3.75E-06	NS
INHA	NS	4.12E-07	0.102471261	3.80E-06	NS
TGDS	NS	4.19E-07	-0.102410964	3.86E-06	NS
FZD10	NS	4.28E-07	0.102327165	3.94E-06	NS
TRPC1	NS	4.32E-07	-0.102293964	3.97E-06	NS
DAB1	NS	4.33E-07	-0.102280694	3.98E-06	NS
NT5E	NS	4.45E-07	-0.102181326	4.09E-06	NS
SLC22A18AS	NS	4.47E-07	0.102161341	4.11E-06	NS
GAL	NS	4.49E-07	0.102142861	4.12E-06	NS
OR6C65	NS	4.56E-07	-0.102085835	4.18E-06	NS
NOXA1	NS	4.57E-07	0.102079821	4.19E-06	NS
SLC6A16	NS	4.64E-07	0.102021386	4.25E-06	NS
GALNTL6	NS	4.65E-07	-0.102010354	4.26E-06	NS
DCUN1D4	NS	4.67E-07	0.101998183	4.27E-06	NS
SLCO4C1	NS	4.68E-07	-0.101982969	4.29E-06	NS
NECTIN3	NS	4.69E-07	-0.101976665	4.29E-06	NS
ADAMTSL2	NS	4.70E-07	0.101965652	4.30E-06	NS
CHIC1	NS	4.71E-07	-0.101964071	4.30E-06	NS
TOMM34	NS	4.74E-07	-0.101933745	4.33E-06	NS
UBAP2L	NS	4.83E-07	0.101864309	4.41E-06	NS
FTCD	NS	4.85E-07	0.101851865	4.42E-06	NS
TCF7L2	NS	4.88E-07	0.101825187	4.45E-06	NS
PDZK1IP1	NS	4.88E-07	-0.10182533	4.45E-06	NS
GLIPR1L2	NS	4.89E-07	-0.101813273	4.46E-06	NS
E2F7	NS	4.92E-07	-0.101791405	4.48E-06	NS
GNG8	NS	4.95E-07	0.101770328	4.51E-06	NS
ARGFX	NS	5.01E-07	0.101722991	4.56E-06	NS
VSIG4	NS	5.14E-07	-0.101627117	4.67E-06	NS
TRMT10C	NS	5.14E-07	-0.101623861	4.67E-06	NS
HSPA14	NS	5.14E-07	-0.101622715	4.67E-06	NS
OR5D16	NS	5.18E-07	-0.101595475	4.70E-06	NS
ACSL3	NS	5.26E-07	0.101535127	4.77E-06	NS
GJA4	NS	5.26E-07	0.101534572	4.77E-06	NS
CRYZ	NS	5.27E-07	-0.101530152	4.78E-06	NS
SPRYD3	NS	5.27E-07	0.101524116	4.78E-06	NS
PITX3	NS	5.28E-07	0.101518219	4.79E-06	NS
FBXO27	NS	5.31E-07	0.101500595	4.81E-06	NS
PCDH18	NS	5.32E-07	-0.101493559	4.81E-06	NS
ACOT9	NS	5.34E-07	0.101473077	4.84E-06	NS
TP53BP1	NS	5.36E-07	-0.101462313	4.85E-06	NS
DPYS	NS	5.37E-07	-0.101457042	4.85E-06	NS
CHRNG	NS	5.37E-07	-0.101453474	4.85E-06	NS
RNASEH2A	NS	5.42E-07	-0.101416665	4.90E-06	NS
LRCH2	NS	5.47E-07	-0.101379895	4.94E-06	NS
GPR19	NS	5.50E-07	-0.101362427	4.96E-06	NS
FAM131B	NS	5.57E-07	0.101316093	5.02E-06	NS
MAGOHB	NS	5.57E-07	-0.101313492	5.02E-06	NS
GPR135	NS	5.61E-07	-0.101283132	5.06E-06	NS

OPALIN	NS	5.62E-07	-0.101276305	5.06E-06	NS
CCDC88C	NS	5.64E-07	0.101263504	5.08E-06	NS
TFPI2	NS	5.67E-07	-0.101245872	5.10E-06	NS
METTL4	NS	5.71E-07	-0.10121409	5.14E-06	NS
RASD2	NS	5.72E-07	0.101210577	5.14E-06	NS
AQP8	NS	5.73E-07	-0.10120583	5.14E-06	NS
MT1H	NS	5.73E-07	0.101204483	5.14E-06	NS
FAM111B	NS	5.76E-07	-0.101184649	5.17E-06	NS
TMEM252	NS	5.78E-07	-0.101170444	5.19E-06	NS
TEX33	NS	5.80E-07	0.101153341	5.21E-06	NS
VIT	NS	5.83E-07	-0.101135905	5.23E-06	NS
FSCN2	NS	5.84E-07	0.10112901	5.23E-06	NS
FMNL2	NS	5.86E-07	-0.101113203	5.25E-06	NS
RARRES2	NS	5.90E-07	0.101090432	5.28E-06	NS
USP27X	NS	6.01E-07	0.101014712	5.38E-06	NS
CTAG1B	NS	6.04E-07	0.101000905	5.40E-06	NS
HTT	NS	6.08E-07	0.100970679	5.44E-06	NS
SERPINB4	NS	6.14E-07	-0.100937286	5.48E-06	NS
TMEM82	NS	6.16E-07	0.100924273	5.50E-06	NS
OR51F2	NS	6.17E-07	-0.100913489	5.51E-06	NS
NOTCH4	NS	6.26E-07	0.100860112	5.58E-06	NS
BMPR1B	NS	6.27E-07	-0.100849626	5.60E-06	NS
ZNF222	NS	6.32E-07	-0.100820769	5.64E-06	NS
VAPA	NS	6.35E-07	0.100806241	5.65E-06	NS
C1QTNF5	NS	6.41E-07	0.100769192	5.71E-06	NS
FCRL4	NS	6.45E-07	-0.100740352	5.75E-06	NS
CNR1	NS	6.47E-07	-0.100732827	5.75E-06	NS
MS4A10	NS	6.47E-07	0.100730473	5.75E-06	NS
ACVR2A	NS	6.48E-07	-0.100725902	5.76E-06	NS
RPA2	NS	6.54E-07	0.100690057	5.81E-06	NS
GREP1	NS	6.55E-07	0.100680667	5.82E-06	NS
SMAD1	NS	6.61E-07	-0.100644178	5.87E-06	NS
ADAM33	NS	6.66E-07	0.100615951	5.91E-06	NS
SEMA6A	NS	6.72E-07	-0.100583241	5.96E-06	NS
HSPA13	NS	6.74E-07	0.100568874	5.98E-06	NS
GEMIN7	NS	6.89E-07	-0.10048607	6.10E-06	NS
RPL39L	NS	6.93E-07	-0.100460805	6.14E-06	NS
RAD51AP2	NS	6.93E-07	-0.100459742	6.14E-06	NS
GSTA1	NS	6.96E-07	-0.100446545	6.16E-06	NS
CDCA7	NS	6.97E-07	-0.100442062	6.16E-06	NS
RUBCN	NS	6.96E-07	0.100443153	6.16E-06	NS
AMT	NS	6.98E-07	-0.100433497	6.17E-06	NS
GPCPD1	NS	7.02E-07	0.100412846	6.20E-06	NS
RPRML	NS	7.06E-07	0.100387618	6.24E-06	NS
PSG4	NS	7.10E-07	-0.100368267	6.27E-06	NS
HLA-DOB	NS	7.17E-07	-0.100326535	6.33E-06	NS
CDC27	NS	7.18E-07	0.100321906	6.33E-06	NS
PNOC	NS	7.18E-07	-0.100322345	6.33E-06	NS
COL20A1	NS	7.19E-07	0.100318634	6.33E-06	NS
SLC44A2	NS	7.23E-07	0.100296393	6.37E-06	NS
CHAC2	NS	7.24E-07	-0.10028906	6.38E-06	NS
THRB	NS	7.25E-07	-0.100287143	6.38E-06	NS
AGTR1	NS	7.31E-07	-0.100252501	6.43E-06	NS
FAM120B	NS	7.35E-07	0.100232827	6.46E-06	NS
NR4A1	NS	7.35E-07	0.100234228	6.46E-06	NS

PRR15L	NS	7.46E-07	-0.100171097	6.56E-06	NS
LRRFIP2	NS	7.47E-07	0.100167636	6.56E-06	NS
RND3	NS	7.47E-07	-0.10016735	6.56E-06	NS
UGT1A9	NS	7.56E-07	-0.100123123	6.63E-06	NS
TNNC2	NS	7.61E-07	-0.100096775	6.67E-06	NS
CLDN25	NS	7.61E-07	0.100094192	6.67E-06	NS
DNAAF11	NS	7.64E-07	-0.100078414	6.69E-06	NS
CNTNAP4	NS	7.64E-07	-0.10007879	6.69E-06	NS
TGFBR2	NS	7.69E-07	0.100052881	6.73E-06	NS
MYBL1	NS	7.70E-07	-0.100049932	6.73E-06	NS
NFIC	NS	7.73E-07	0.100033768	6.76E-06	NS
PRR5L	NS	7.85E-07	-0.099975323	6.86E-06	NS
SLC22A9	NS	7.95E-07	-0.099921936	6.95E-06	NS
ARV1	NS	7.98E-07	-0.099911417	6.96E-06	NS
SLC22A1	NS	7.98E-07	-0.099910979	6.96E-06	NS
ANKRD46	NS	7.97E-07	-0.099913935	6.96E-06	NS
DEUP1	NS	8.00E-07	-0.099901464	6.97E-06	NS
BTBD7	NS	8.04E-07	0.099881607	7.00E-06	NS
PPP1R14D	NS	8.06E-07	0.099867692	7.03E-06	NS
FRZB	NS	8.11E-07	-0.099843222	7.07E-06	NS
MBLAC1	NS	8.17E-07	-0.099814284	7.11E-06	NS
F9	NS	8.23E-07	-0.099788517	7.16E-06	NS
SLPI	NS	8.24E-07	-0.099785174	7.16E-06	NS
ALAS2	NS	8.26E-07	-0.099772442	7.18E-06	NS
ZCCHC17	NS	8.29E-07	0.09976008	7.20E-06	NS
HSPB2	NS	8.30E-07	0.099752323	7.21E-06	NS
BEND6	NS	8.35E-07	-0.099729434	7.25E-06	NS
MARK3	NS	8.37E-07	0.099721476	7.26E-06	NS
WDR75	NS	8.44E-07	-0.099688094	7.32E-06	NS
NKX2-1	NS	8.45E-07	0.09968284	7.32E-06	NS
UCN	NS	8.54E-07	-0.099641949	7.40E-06	NS
SEMG2	NS	8.57E-07	-0.09962936	7.42E-06	NS
BAAT	NS	8.57E-07	-0.099628076	7.42E-06	NS
POC1A	NS	8.59E-07	-0.099621284	7.43E-06	NS
TSPY8	NS	8.61E-07	-0.099611951	7.44E-06	NS
GPR12	NS	8.64E-07	0.099594762	7.47E-06	NS
OR4N5	NS	8.67E-07	-0.099584748	7.48E-06	NS
NEUROG3	NS	8.70E-07	0.09956929	7.51E-06	NS
DEFB104A	NS	8.76E-07	-0.099544109	7.56E-06	NS
HSPA1B	NS	8.77E-07	-0.099538553	7.56E-06	NS
RRH	NS	8.77E-07	-0.099536181	7.56E-06	NS
GPM6B	NS	8.78E-07	-0.099532169	7.57E-06	NS
PCLO	NS	8.86E-07	-0.099497292	7.63E-06	NS
KRTAP19-7	NS	8.88E-07	-0.099488299	7.65E-06	NS
PGPEP1L	NS	8.93E-07	0.099467156	7.68E-06	NS
TOMT	NS	8.95E-07	-0.09945693	7.70E-06	NS
REPS1	NS	8.96E-07	0.099452753	7.70E-06	NS
LGALS16	NS	8.97E-07	-0.099446344	7.71E-06	NS
MMP23B	NS	9.00E-07	0.099436495	7.73E-06	NS
ENDOG	NS	9.05E-07	-0.099412645	7.77E-06	NS
ENKUR	NS	9.11E-07	-0.09938872	7.82E-06	NS
CYP19A1	NS	9.11E-07	-0.099385168	7.82E-06	NS
CCNL1	NS	9.19E-07	0.099351019	7.88E-06	NS
RPRD1B	NS	9.24E-07	0.099330627	7.92E-06	NS
MOSPD1	NS	9.24E-07	-0.099331228	7.92E-06	NS

MLNR	NS	9.25E-07	0.099327141	7.92E-06	NS
SLC17A1	NS	9.30E-07	-0.099304841	7.96E-06	NS
TLK2	NS	9.36E-07	0.099279146	8.01E-06	NS
HRCT1	NS	9.39E-07	0.099269333	8.03E-06	NS
GJB7	NS	9.45E-07	-0.099242064	8.08E-06	NS
CAPN6	NS	9.63E-07	-0.099168037	8.23E-06	NS
KRTAP5-8	NS	9.63E-07	-0.099169276	8.23E-06	NS
PRKG2	NS	9.71E-07	-0.099137107	8.29E-06	NS
UNG	NS	9.82E-07	-0.099088573	8.38E-06	NS
OXA1L	NS	9.87E-07	0.099069757	8.42E-06	NS
CRIM1	NS	9.94E-07	-0.099044014	8.47E-06	NS
CD27	NS	1.01E-06	-0.098966109	8.63E-06	NS
HSPA1A	NS	1.02E-06	-0.098945261	8.67E-06	NS
SESTD1	NS	1.04E-06	0.098875418	8.82E-06	NS
ERVFRD-1	NS	1.05E-06	-0.098842626	8.89E-06	NS
NDUFAB1	NS	1.05E-06	-0.098826326	8.93E-06	NS
OR2M2	NS	1.06E-06	-0.098769239	9.05E-06	NS
CCDC157	NS	1.07E-06	0.098743112	9.10E-06	NS
DNTTIP2	NS	1.07E-06	0.098739258	9.11E-06	NS
CCDC172	NS	1.07E-06	-0.098735218	9.11E-06	NS
STRADB	NS	1.08E-06	-0.098712786	9.16E-06	NS
BHMT2	NS	1.08E-06	0.098706872	9.17E-06	NS
SHLD1	NS	1.08E-06	-0.098704999	9.17E-06	NS
DPY19L2	NS	1.08E-06	-0.098695692	9.19E-06	NS
THEMIS2	NS	1.09E-06	0.098690981	9.20E-06	NS
C18orf25	NS	1.09E-06	0.098686077	9.20E-06	NS
KRT6A	NS	1.09E-06	-0.098686221	9.20E-06	NS
STXBP5L	NS	1.09E-06	-0.098672134	9.23E-06	NS
PNMA8A	NS	1.09E-06	0.098669958	9.23E-06	NS
CXXC1	NS	1.10E-06	0.098652943	9.26E-06	NS
HOXB8	NS	1.10E-06	0.098640635	9.29E-06	NS
BCAR3	NS	1.10E-06	0.098635914	9.29E-06	NS
TAS2R13	NS	1.10E-06	-0.098634248	9.29E-06	NS
REEP2	NS	1.11E-06	0.098607539	9.35E-06	NS
SLC16A11	NS	1.12E-06	0.098578942	9.42E-06	NS
CDC26	NS	1.12E-06	-0.098563398	9.45E-06	NS
ZNF621	NS	1.12E-06	-0.09855914	9.46E-06	NS
OR8H2	NS	1.12E-06	-0.098554288	9.46E-06	NS
KIAA2012	NS	1.14E-06	0.098512742	9.55E-06	NS
C2orf72	NS	1.14E-06	0.098513835	9.55E-06	NS
OR51Q1	NS	1.14E-06	-0.098482015	9.62E-06	NS
POTEG	NS	1.15E-06	0.098463389	9.66E-06	NS
ETV5	NS	1.15E-06	0.09846256	9.66E-06	NS
SRSF4	NS	1.16E-06	0.09843505	9.72E-06	NS
SDR16C5	NS	1.16E-06	-0.098435846	9.72E-06	NS
TTLL10	NS	1.16E-06	0.098429518	9.73E-06	NS
MGAT4C	NS	1.17E-06	0.098400167	9.80E-06	NS
PCDHB11	NS	1.18E-06	0.098372459	9.86E-06	NS
NLGN2	NS	1.20E-06	0.098285213	1.01E-05	NS
MUC7	NS	1.22E-06	-0.098232766	1.02E-05	NS
POLR2K	NS	1.22E-06	-0.098225081	1.02E-05	NS
C12orf54	NS	1.22E-06	-0.098221202	1.02E-05	NS
ZNF787	NS	1.22E-06	0.098214292	1.02E-05	NS
CIB1	NS	1.23E-06	0.098184195	1.03E-05	NS
SDHAF1	NS	1.23E-06	-0.098182911	1.03E-05	NS

TARM1	NS	1.23E-06	0.098184159	1.03E-05	NS
PRR15	NS	1.24E-06	0.098165835	1.03E-05	NS
FN3KRP	NS	1.25E-06	-0.098127896	1.04E-05	NS
PDPN	NS	1.26E-06	0.098083548	1.06E-05	NS
IL18RAP	NS	1.27E-06	-0.098054041	1.06E-05	NS
KCNJ15	NS	1.28E-06	-0.098045084	1.06E-05	NS
RNF222	NS	1.28E-06	0.098038848	1.07E-05	NS
IQANK1	NS	1.28E-06	0.098020432	1.07E-05	NS
KCNIP2	NS	1.29E-06	-0.098015682	1.07E-05	NS
MYO3A	NS	1.29E-06	-0.097999667	1.07E-05	NS
ZNF843	NS	1.30E-06	0.097972	1.08E-05	NS
ACOT1	NS	1.30E-06	-0.097971037	1.08E-05	NS
ZFR	NS	1.31E-06	0.097930194	1.09E-05	NS
CMTM5	NS	1.32E-06	-0.097908543	1.10E-05	NS
CD101	NS	1.33E-06	-0.097886244	1.10E-05	NS
PCDH20	NS	1.34E-06	-0.097864593	1.11E-05	NS
HHATL	NS	1.34E-06	0.097861245	1.11E-05	NS
CCDC15	NS	1.34E-06	0.097848109	1.11E-05	NS
MALSU1	NS	1.34E-06	-0.097844332	1.11E-05	NS
RAB22A	NS	1.35E-06	0.097830034	1.12E-05	NS
H2BC5	NS	1.36E-06	-0.097804698	1.12E-05	NS
HPR	NS	1.36E-06	-0.09780161	1.12E-05	NS
C2CD6	NS	1.37E-06	-0.097770157	1.13E-05	NS
CDK5R2	NS	1.37E-06	0.097770596	1.13E-05	NS
YBEY	NS	1.37E-06	-0.097765612	1.13E-05	NS
TTC4	NS	1.37E-06	-0.097764641	1.13E-05	NS
JMJD6	NS	1.38E-06	0.097733672	1.14E-05	NS
KCNU1	NS	1.38E-06	-0.097725688	1.14E-05	NS
PARD6A	NS	1.39E-06	0.097706831	1.15E-05	NS
NEGR1	NS	1.41E-06	0.09764123	1.16E-05	NS
NEURL2	NS	1.42E-06	-0.097622772	1.17E-05	NS
EEF2K	NS	1.42E-06	-0.097615222	1.17E-05	NS
HNRNPUL1	NS	1.43E-06	0.097604984	1.17E-05	NS
POC4-APOC	NS	1.42E-06	0.097606202	1.17E-05	NS
PPP1R2	NS	1.43E-06	0.097602418	1.17E-05	NS
RGPD2	NS	1.43E-06	0.097595785	1.17E-05	NS
MCM3	NS	1.43E-06	-0.097583447	1.18E-05	NS
EMILIN3	NS	1.43E-06	0.097584222	1.18E-05	NS
RNASE9	NS	1.43E-06	-0.0975849	1.18E-05	NS
CYB5D2	NS	1.44E-06	0.097561136	1.18E-05	NS
ANKRD34A	NS	1.44E-06	0.097561955	1.18E-05	NS
NPPA	NS	1.45E-06	-0.097532409	1.19E-05	NS
FAM104B	NS	1.46E-06	-0.097516352	1.19E-05	NS
MDM2	NS	1.47E-06	0.09747755	1.21E-05	NS
UGT2B11	NS	1.48E-06	-0.097458937	1.21E-05	NS
MCTS2P	NS	1.48E-06	-0.097452461	1.21E-05	NS
INSYN1	NS	1.48E-06	0.097451639	1.21E-05	NS
ZDHHC2	NS	1.48E-06	-0.097440692	1.21E-05	NS
MAPK8IP1	NS	1.49E-06	0.09743774	1.21E-05	NS
MUC21	NS	1.49E-06	0.097438117	1.21E-05	NS
WDR91	NS	1.49E-06	0.09742597	1.22E-05	NS
SLC6A19	NS	1.49E-06	0.097420833	1.22E-05	NS
CNDP1	NS	1.51E-06	0.097362011	1.24E-05	NS
FNDC5	NS	1.52E-06	0.097349527	1.24E-05	NS
PRM1	NS	1.52E-06	0.097336046	1.24E-05	NS

RELT	NS	1.53E-06	0.097318432	1.25E-05	NS
PDCD6IP	NS	1.53E-06	0.097310355	1.25E-05	NS
TBX4	NS	1.54E-06	0.097300351	1.25E-05	NS
DHDH	NS	1.58E-06	0.09720072	1.28E-05	NS
GXYLT1	NS	1.58E-06	-0.097188928	1.29E-05	NS
PANX3	NS	1.58E-06	-0.097184916	1.29E-05	NS
IL22RA2	NS	1.58E-06	-0.097183197	1.29E-05	NS
MYL3	NS	1.60E-06	0.097147697	1.30E-05	NS
ZP4	NS	1.60E-06	-0.097135014	1.30E-05	NS
LY6G6F	NS	1.65E-06	-0.097015675	1.34E-05	NS
PLEKHO2	NS	1.66E-06	0.096989982	1.35E-05	NS
KCNG1	NS	1.68E-06	0.096942744	1.36E-05	NS
CRYAA	NS	1.68E-06	0.096943904	1.36E-05	NS
OTX1	NS	1.69E-06	0.096911499	1.37E-05	NS
ITIH2	NS	1.70E-06	-0.096899981	1.37E-05	NS
SQSTM1	NS	1.70E-06	0.096886623	1.38E-05	NS
SOX10	NS	1.71E-06	0.096863466	1.39E-05	NS
KCNH5	NS	1.71E-06	-0.09686151	1.39E-05	NS
SERPINF1	NS	1.73E-06	-0.096813384	1.40E-05	NS
INSM2	NS	1.75E-06	0.096785039	1.41E-05	NS
C3orf85	NS	1.76E-06	-0.096746445	1.42E-05	NS
ANKS4B	NS	1.76E-06	0.096742058	1.43E-05	NS
PRR32	NS	1.78E-06	-0.0967004	1.44E-05	NS
PSORS1C2	NS	1.83E-06	0.096605241	1.47E-05	NS
RAX2	NS	1.84E-06	0.096577213	1.48E-05	NS
UQCR10	NS	1.84E-06	-0.096576239	1.48E-05	NS
DEFB128	NS	1.85E-06	-0.096541795	1.49E-05	NS
CLCA2	NS	1.87E-06	-0.096505017	1.51E-05	NS
MND1	NS	1.87E-06	0.096497347	1.51E-05	NS
ADGRG3	NS	1.88E-06	0.096493198	1.51E-05	NS
JUND	NS	1.88E-06	0.096484413	1.51E-05	NS
KIAA1958	NS	1.89E-06	0.096472219	1.52E-05	NS
NOD1	NS	1.90E-06	-0.096446753	1.53E-05	NS
IL17C	NS	1.90E-06	-0.096445196	1.53E-05	NS
FNDC10	NS	1.91E-06	-0.096418652	1.53E-05	NS
MOS	NS	1.91E-06	-0.096415022	1.54E-05	NS
GPR182	NS	1.92E-06	0.096405294	1.54E-05	NS
DAZ2	NS	1.93E-06	-0.096386224	1.55E-05	NS
TBCE	NS	1.94E-06	-0.09635607	1.56E-05	NS
CA5A	NS	1.95E-06	0.096340404	1.56E-05	NS
ESYT2	NS	1.97E-06	0.096293128	1.58E-05	NS
SYN1	NS	1.98E-06	0.096272399	1.59E-05	NS
CCDC190	NS	1.98E-06	-0.096268017	1.59E-05	NS
CIAO2B	NS	2.00E-06	-0.096227322	1.60E-05	NS
OR4F16	NS	2.02E-06	-0.096198485	1.61E-05	NS
SH2D1A	NS	2.03E-06	-0.0961792	1.62E-05	NS
TSKU	NS	2.03E-06	0.096167627	1.62E-05	NS
CABS1	NS	2.05E-06	-0.096133229	1.64E-05	NS
REC8	NS	2.06E-06	0.096109219	1.65E-05	NS
CAGE1	NS	2.07E-06	-0.096092082	1.65E-05	NS
MED15	NS	2.08E-06	0.096069179	1.66E-05	NS
CFAP53	NS	2.08E-06	-0.096069716	1.66E-05	NS
TSPY3	NS	2.08E-06	-0.096070223	1.66E-05	NS
SLAMF1	NS	2.10E-06	-0.09603835	1.67E-05	NS
ITK	NS	2.10E-06	-0.096030436	1.67E-05	NS

OOSP1	NS	2.11E-06	-0.096023638	1.68E-05	NS
MAPRE2	NS	2.11E-06	0.096013222	1.68E-05	NS
PTTG1IP2	NS	2.11E-06	-0.096012078	1.68E-05	NS
NKIRAS1	NS	2.14E-06	-0.095956129	1.70E-05	NS
CTF1	NS	2.15E-06	0.095941187	1.71E-05	NS
SERPINB10	NS	2.17E-06	0.095894164	1.73E-05	NS
SEC61B	NS	2.18E-06	0.095883466	1.73E-05	NS
ANO4	NS	2.19E-06	-0.095872869	1.73E-05	NS
FOXS1	NS	2.19E-06	0.095866681	1.74E-05	NS
HAND1	NS	2.19E-06	0.095862952	1.74E-05	NS
POU4F1	NS	2.22E-06	0.095806833	1.76E-05	NS
ANXA9	NS	2.23E-06	-0.095781623	1.77E-05	NS
SNRNP40	NS	2.25E-06	-0.09575689	1.78E-05	NS
CREB3L1	NS	2.26E-06	0.095743254	1.79E-05	NS
TNPO2	NS	2.26E-06	0.095728259	1.79E-05	NS
TMEM237	NS	2.27E-06	-0.095718404	1.80E-05	NS
C6orf89	NS	2.28E-06	0.095698171	1.80E-05	NS
VKORC1	NS	2.29E-06	-0.095684091	1.81E-05	NS
GPA33	NS	2.32E-06	-0.095623327	1.84E-05	NS
IL18BP	NS	2.33E-06	0.095616205	1.84E-05	NS
ZNF749	NS	2.33E-06	-0.095616307	1.84E-05	NS
PDX1	NS	2.33E-06	0.09560282	1.84E-05	NS
SPINK9	NS	2.34E-06	-0.095601266	1.84E-05	NS
DOP1A	NS	2.38E-06	-0.095521807	1.88E-05	NS
HECW2	NS	2.39E-06	-0.095498903	1.89E-05	NS
TMEM235	NS	2.40E-06	0.095495395	1.89E-05	NS
USP43	NS	2.40E-06	-0.095490465	1.89E-05	NS
SLC25A42	NS	2.41E-06	-0.095467243	1.90E-05	NS
KDM2A	NS	2.42E-06	0.095455711	1.90E-05	NS
H4C4	NS	2.42E-06	-0.09545658	1.90E-05	NS
LHFPL3	NS	2.44E-06	-0.095418395	1.92E-05	NS
POU1F1	NS	2.45E-06	-0.095403052	1.93E-05	NS
HSFX4	NS	2.46E-06	-0.095382454	1.93E-05	NS
MYCBPAP	NS	2.47E-06	0.09537263	1.94E-05	NS
CHODL	NS	2.47E-06	-0.095364489	1.94E-05	NS
BET1L	NS	2.48E-06	0.095356984	1.94E-05	NS
GPR143	NS	2.49E-06	-0.095335901	1.95E-05	NS
SNAP25	NS	2.49E-06	0.095333276	1.95E-05	NS
MAP4K4	NS	2.50E-06	0.095315086	1.96E-05	NS
GPBP1L1	NS	2.52E-06	0.09529528	1.97E-05	NS
MYOC	NS	2.53E-06	0.095270647	1.98E-05	NS
LPAR2	NS	2.56E-06	0.095222056	2.00E-05	NS
AP3M2	NS	2.58E-06	-0.095193071	2.02E-05	NS
SLC18A2	NS	2.58E-06	-0.095190902	2.02E-05	NS
GP1BB	NS	2.58E-06	0.095189083	2.02E-05	NS
NXPH3	NS	2.59E-06	0.095176879	2.02E-05	NS
UBAP1L	NS	2.59E-06	0.095176013	2.02E-05	NS
KRTAP4-7	NS	2.60E-06	0.095157688	2.03E-05	NS
CCDC27	NS	2.61E-06	0.0951387	2.04E-05	NS
CCDC102B	NS	2.62E-06	-0.095128546	2.04E-05	NS
GLYATL3	NS	2.63E-06	-0.095118217	2.05E-05	NS
ATXN1	NS	2.64E-06	0.095091502	2.06E-05	NS
SCEL	NS	2.65E-06	-0.09508268	2.06E-05	NS
WNT10A	NS	2.65E-06	0.095080014	2.06E-05	NS
CDK2AP2	NS	2.66E-06	-0.095059899	2.07E-05	NS

FAM237A	NS	2.67E-06	-0.095050508	2.08E-05	NS
FMO5	NS	2.69E-06	-0.095023245	2.09E-05	NS
SURF2	NS	2.69E-06	-0.095023522	2.09E-05	NS
JAKMIP1	NS	2.69E-06	-0.095019222	2.09E-05	NS
DCTPP1	NS	2.70E-06	-0.095007533	2.10E-05	NS
C7orf33	NS	2.72E-06	-0.094974634	2.11E-05	NS
SMIM34	NS	2.73E-06	-0.094964412	2.12E-05	NS
RIPK2	NS	2.73E-06	0.094953899	2.12E-05	NS
CT45A5	NS	2.74E-06	-0.094944886	2.13E-05	NS
COX6A2	NS	2.77E-06	0.094892058	2.15E-05	NS
EDARADD	NS	2.77E-06	-0.094891595	2.15E-05	NS
NFAT5	NS	2.79E-06	0.094872854	2.16E-05	NS
RSF1	NS	2.80E-06	0.094860985	2.16E-05	NS
POU4F3	NS	2.81E-06	0.094842717	2.17E-05	NS
OR52A5	NS	2.82E-06	-0.094820014	2.18E-05	NS
PRSS3	NS	2.83E-06	-0.094813228	2.19E-05	NS
SMN2	NS	2.85E-06	0.094786901	2.20E-05	NS
TAS2R7	NS	2.86E-06	-0.094764893	2.21E-05	NS
UGT2A2	NS	2.86E-06	-0.094760824	2.21E-05	NS
PDIK1L	NS	2.87E-06	-0.094757117	2.21E-05	NS
EFCC1	NS	2.89E-06	0.094729043	2.23E-05	NS
ZNF469	NS	2.92E-06	0.094679912	2.25E-05	NS
HES2	NS	2.94E-06	0.09465513	2.27E-05	NS
TKT	NS	2.94E-06	0.09464593	2.27E-05	NS
FSHB	NS	2.96E-06	-0.094629171	2.28E-05	NS
OR10A7	NS	2.96E-06	-0.094617778	2.28E-05	NS
KAT5	NS	2.98E-06	0.09459722	2.29E-05	NS
P2RY4	NS	3.00E-06	-0.094566891	2.31E-05	NS
MOSMO	NS	3.02E-06	-0.094540628	2.32E-05	NS
UPK1A	NS	3.03E-06	0.094524408	2.33E-05	NS
GEMIN5	NS	3.04E-06	-0.094514728	2.34E-05	NS
PODXL2	NS	3.07E-06	0.094468906	2.36E-05	NS
AGPAT5	NS	3.08E-06	-0.094464918	2.36E-05	NS
NUDT9	NS	3.09E-06	-0.094440055	2.38E-05	NS
PP2D1	NS	3.10E-06	-0.094430871	2.38E-05	NS
OR52I1	NS	3.11E-06	-0.094415916	2.39E-05	NS
CPA6	NS	3.12E-06	-0.09440697	2.39E-05	NS
ALPG	NS	3.12E-06	0.094403683	2.39E-05	NS
MBD3L2	NS	3.13E-06	0.094395377	2.40E-05	NS
QTRT2	NS	3.14E-06	0.094375454	2.41E-05	NS
RAF1	NS	3.15E-06	0.094361227	2.42E-05	NS
XPA	NS	3.19E-06	-0.09430839	2.44E-05	NS
SMN1	NS	3.19E-06	0.094308993	2.44E-05	NS
HOXB1	NS	3.21E-06	0.094290458	2.45E-05	NS
GLYAT	NS	3.21E-06	-0.094287867	2.45E-05	NS
PCDHB4	NS	3.24E-06	0.094248128	2.48E-05	NS
PTP4A1	NS	3.24E-06	0.094249942	2.48E-05	NS
ADH1A	NS	3.24E-06	-0.094247179	2.48E-05	NS
WNT16	NS	3.25E-06	-0.094231027	2.48E-05	NS
TSGA10	NS	3.28E-06	0.094203982	2.50E-05	NS
TIMP1	NS	3.28E-06	0.094197309	2.50E-05	NS
BTNL8	NS	3.29E-06	-0.094183232	2.51E-05	NS
OLIG3	NS	3.30E-06	0.094174486	2.51E-05	NS
ITGBL1	NS	3.35E-06	-0.094115732	2.55E-05	NS
CNEP1R1	NS	3.39E-06	0.094063592	2.58E-05	NS

TBL1X	NS	3.39E-06	0.0940606	2.58E-05	NS
PSMD10	NS	3.40E-06	-0.094043993	2.59E-05	NS
SST	NS	3.44E-06	-0.093995924	2.62E-05	NS
HIVEP1	NS	3.45E-06	0.093989812	2.62E-05	NS
ZNF330	NS	3.46E-06	0.093976846	2.63E-05	NS
KRTAP5-11	NS	3.46E-06	0.093973997	2.63E-05	NS
CPLANE2	NS	3.48E-06	-0.09395414	2.64E-05	NS
FAM214B	NS	3.48E-06	0.093947635	2.64E-05	NS
APLF	NS	3.49E-06	-0.093945434	2.64E-05	NS
TOPAZ1	NS	3.49E-06	-0.093939997	2.65E-05	NS
HBP1	NS	3.49E-06	0.093937435	2.65E-05	NS
MCOLN3	NS	3.50E-06	-0.093925874	2.65E-05	NS
DPM3	NS	3.50E-06	0.09392464	2.65E-05	NS
LINGO4	NS	3.51E-06	0.093912581	2.66E-05	NS
SIGLEC11	NS	3.54E-06	-0.093887304	2.67E-05	NS
CDKL3	NS	3.55E-06	-0.093869157	2.69E-05	NS
PRDX4	NS	3.55E-06	-0.093866494	2.69E-05	NS
OR51S1	NS	3.56E-06	-0.093863303	2.69E-05	NS
CBARP	NS	3.57E-06	0.093844398	2.70E-05	NS
MED11	NS	3.57E-06	0.093845559	2.70E-05	NS
BOD1	NS	3.59E-06	-0.093819784	2.71E-05	NS
SELP	NS	3.60E-06	-0.093814452	2.71E-05	NS
NRG2	NS	3.61E-06	0.093804339	2.72E-05	NS
TSTD1	NS	3.62E-06	-0.093786778	2.73E-05	NS
KIF15	NS	3.64E-06	-0.093765779	2.74E-05	NS
CYP11B2	NS	3.64E-06	0.093765524	2.74E-05	NS
TMOD4	NS	3.65E-06	-0.093752627	2.75E-05	NS
SLF1	NS	3.67E-06	-0.093730023	2.76E-05	NS
FPGT	NS	3.67E-06	-0.093726549	2.76E-05	NS
MGAT4D	NS	3.69E-06	-0.09370862	2.78E-05	NS
GSDMA	NS	3.69E-06	0.093705946	2.78E-05	NS
UBE3D	NS	3.70E-06	-0.093700157	2.78E-05	NS
CHST6	NS	3.72E-06	0.093678483	2.79E-05	NS
EQTN	NS	3.77E-06	-0.093620483	2.83E-05	NS
CRADD	NS	3.77E-06	0.093617485	2.83E-05	NS
SLC4A11	NS	3.82E-06	0.093561484	2.87E-05	NS
ELOC	NS	3.87E-06	0.093509673	2.90E-05	NS
CCDC187	NS	3.87E-06	0.093509675	2.90E-05	NS
ERICH3	NS	3.87E-06	-0.09350623	2.90E-05	NS
C1orf21	NS	3.88E-06	-0.093501596	2.90E-05	NS
CLEC2A	NS	3.88E-06	-0.093500845	2.90E-05	NS
ZNF284	NS	3.88E-06	-0.093495908	2.91E-05	NS
NCKAP1L	NS	3.89E-06	0.093492037	2.91E-05	NS
NUDT7	NS	3.91E-06	-0.093460983	2.93E-05	NS
DAOA	NS	3.94E-06	-0.093432003	2.95E-05	NS
CSNK2B	NS	3.95E-06	0.093428071	2.95E-05	NS
FGF18	NS	3.97E-06	0.093406452	2.96E-05	NS
ZNF443	NS	3.98E-06	-0.093391953	2.97E-05	NS
EHD2	NS	4.04E-06	0.093331535	3.02E-05	NS
OR4P4	NS	4.05E-06	0.093323445	3.02E-05	NS
C17orf97	NS	4.06E-06	0.093310747	3.03E-05	NS
SEMG1	NS	4.06E-06	-0.093307179	3.03E-05	NS
OR6N1	NS	4.07E-06	-0.093300256	3.03E-05	NS
GPC3	NS	4.08E-06	0.093282925	3.04E-05	NS
C11orf86	NS	4.08E-06	0.093283642	3.04E-05	NS

RAD54B	NS	4.12E-06	-0.093246029	3.07E-05	NS
R3URF-PIK3	NS	4.12E-06	-0.093247267	3.07E-05	NS
ROPN1L	NS	4.16E-06	-0.093204384	3.10E-05	NS
SAMD9	NS	4.20E-06	-0.093164087	3.13E-05	NS
PRRC2C	NS	4.21E-06	0.093158829	3.13E-05	NS
PDSS2	NS	4.22E-06	-0.093149201	3.13E-05	NS
FCRLA	NS	4.23E-06	-0.093141338	3.14E-05	NS
RBP1	NS	4.23E-06	0.093136564	3.14E-05	NS
RAB11FIP1	NS	4.24E-06	0.093126292	3.15E-05	NS
GJA10	NS	4.25E-06	0.09311913	3.15E-05	NS
LITAFD	NS	4.25E-06	0.093120545	3.15E-05	NS
LURAP1L	NS	4.27E-06	-0.093098236	3.17E-05	NS
OR2A2	NS	4.29E-06	-0.093073988	3.18E-05	NS
FGF8	NS	4.30E-06	0.093067617	3.19E-05	NS
BYSL	NS	4.32E-06	-0.093048112	3.20E-05	NS
CLDN4	NS	4.32E-06	0.093046845	3.20E-05	NS
LBHD2	NS	4.32E-06	0.093047766	3.20E-05	NS
MAK16	NS	4.34E-06	-0.093027148	3.21E-05	NS
HOXC8	NS	4.35E-06	0.093017499	3.22E-05	NS
PAFAH1B1	NS	4.38E-06	0.092985779	3.24E-05	NS
HMGB4	NS	4.39E-06	-0.092979716	3.25E-05	NS
TAF9B	NS	4.39E-06	0.09297613	3.25E-05	NS
TLCD3B	NS	4.40E-06	0.092967261	3.25E-05	NS
H4C6	NS	4.40E-06	-0.09296784	3.25E-05	NS
C7orf61	NS	4.43E-06	-0.092942977	3.27E-05	NS
CD5L	NS	4.43E-06	-0.092940009	3.27E-05	NS
KERA	NS	4.46E-06	-0.092910665	3.29E-05	NS
PPP1R15B	NS	4.48E-06	0.092895142	3.30E-05	NS
OR1L6	NS	4.49E-06	-0.092888069	3.31E-05	NS
TCL1A	NS	4.50E-06	-0.092875976	3.31E-05	NS
C12orf42	NS	4.50E-06	-0.092872547	3.32E-05	NS
FAM166B	NS	4.52E-06	-0.092860333	3.32E-05	NS
KCNJ10	NS	4.53E-06	-0.092847732	3.33E-05	NS
DPM1	NS	4.55E-06	0.092828438	3.35E-05	NS
ARHGAP18	NS	4.56E-06	-0.092817598	3.35E-05	NS
OR2AK2	NS	4.57E-06	-0.092814125	3.36E-05	NS
OR2D3	NS	4.58E-06	-0.092800494	3.37E-05	NS
RPP21	NS	4.58E-06	-0.092798927	3.37E-05	NS
KATNBL1	NS	4.61E-06	-0.092778675	3.38E-05	NS
LARP6	NS	4.60E-06	0.09277949	3.38E-05	NS
KCNK4	NS	4.65E-06	0.092740129	3.41E-05	NS
ACOT2	NS	4.66E-06	-0.092728243	3.42E-05	NS
GIN1	NS	4.67E-06	-0.092715595	3.43E-05	NS
OR4K13	NS	4.69E-06	0.092704171	3.43E-05	NS
KLRF1	NS	4.69E-06	-0.092699694	3.44E-05	NS
C21orf91	NS	4.70E-06	-0.092696208	3.44E-05	NS
BHLHE22	NS	4.70E-06	0.092694415	3.44E-05	NS
SPHKAP	NS	4.72E-06	-0.092677541	3.45E-05	NS
CT45A1	NS	4.72E-06	-0.092674409	3.45E-05	NS
RBMY1B	NS	4.76E-06	-0.092636648	3.48E-05	NS
TMEM243	NS	4.77E-06	-0.092629133	3.49E-05	NS
EPB42	NS	4.77E-06	-0.092629705	3.49E-05	NS
SLC1A1	NS	4.79E-06	-0.092615228	3.50E-05	NS
FGFRL1	NS	4.82E-06	0.092590516	3.51E-05	NS
PDE3A	NS	4.83E-06	-0.092575209	3.53E-05	NS

ANKMY2	NS	4.84E-06	-0.092565833	3.53E-05	NS
GINS1	NS	4.88E-06	-0.092536373	3.56E-05	NS
UQCC2	NS	4.88E-06	-0.092532678	3.56E-05	NS
SSX3	NS	4.90E-06	0.092517635	3.57E-05	NS
CD79B	NS	4.91E-06	-0.092504518	3.58E-05	NS
TEX55	NS	4.94E-06	-0.092479072	3.60E-05	NS
H3C3	NS	4.95E-06	-0.09247467	3.60E-05	NS
OR9I1	NS	4.95E-06	-0.092472531	3.60E-05	NS
WDR49	NS	4.95E-06	-0.092469909	3.60E-05	NS
EFNA1	NS	4.97E-06	-0.092457378	3.61E-05	NS
CCDC74B	NS	4.98E-06	0.092446076	3.62E-05	NS
MCTP2	NS	5.01E-06	-0.092426263	3.63E-05	NS
SCG5	NS	5.08E-06	-0.092365105	3.69E-05	NS
ZNF334	NS	5.13E-06	-0.092323673	3.72E-05	NS
SLC16A1	NS	5.13E-06	-0.092318972	3.72E-05	NS
CBLL2	NS	5.18E-06	-0.09228202	3.76E-05	NS
ZSCAN20	NS	5.18E-06	-0.092279172	3.76E-05	NS
PCDHB1	NS	5.20E-06	0.092262078	3.77E-05	NS
GPRIN1	NS	5.24E-06	0.092236996	3.79E-05	NS
PDC	NS	5.25E-06	-0.092225097	3.80E-05	NS
PRAC1	NS	5.25E-06	0.092225202	3.80E-05	NS
OR2T35	NS	5.26E-06	-0.092218117	3.80E-05	NS
OR56A1	NS	5.30E-06	-0.092182032	3.83E-05	NS
AHSG	NS	5.32E-06	-0.092166955	3.85E-05	NS
ZNF556	NS	5.39E-06	0.092115223	3.89E-05	NS
ILDR1	NS	5.39E-06	-0.092113077	3.89E-05	NS
TPCN2	NS	5.39E-06	0.092111107	3.89E-05	NS
TAS2R46	NS	5.41E-06	-0.092097705	3.90E-05	NS
GPR153	NS	5.49E-06	0.092033287	3.96E-05	NS
EXOC6B	NS	5.52E-06	-0.092010833	3.98E-05	NS
PCBP2	NS	5.53E-06	0.092005763	3.99E-05	NS
GPR179	NS	5.61E-06	-0.091940692	4.05E-05	NS
MINAR2	NS	5.62E-06	0.091936467	4.05E-05	NS
RABIF	NS	5.65E-06	-0.091913211	4.07E-05	NS
UBASH3A	NS	5.68E-06	-0.091887388	4.09E-05	NS
CHRNA1	NS	5.69E-06	-0.091880434	4.10E-05	NS
GPR25	NS	5.72E-06	0.091859705	4.12E-05	NS
TEX9	NS	5.73E-06	-0.09185464	4.12E-05	NS
IPO8	NS	5.77E-06	-0.091825219	4.14E-05	NS
SERINC2	NS	5.77E-06	0.091826493	4.14E-05	NS
UBE2G1	NS	5.79E-06	0.091807592	4.16E-05	NS
ZNF878	NS	5.80E-06	-0.091800869	4.16E-05	NS
TGIF2	NS	5.81E-06	0.091796644	4.17E-05	NS
PPP6C	NS	5.82E-06	0.091789343	4.17E-05	NS
OR7C2	NS	5.85E-06	0.091762193	4.20E-05	NS
IFITM1	NS	5.86E-06	0.091760476	4.20E-05	NS
TBX15	NS	5.96E-06	-0.091689088	4.26E-05	NS
ATP6V0B	NS	5.97E-06	0.091679889	4.27E-05	NS
SLC22A2	NS	5.98E-06	-0.091672936	4.28E-05	NS
PRKAA1	NS	6.00E-06	0.091658082	4.29E-05	NS
FAM149B1	NS	6.07E-06	-0.091610064	4.34E-05	NS
AHI1	NS	6.10E-06	0.091586437	4.36E-05	NS
FBXL17	NS	6.17E-06	-0.091537697	4.41E-05	NS
SYCP2L	NS	6.18E-06	-0.091528902	4.42E-05	NS
NKAPL	NS	6.20E-06	-0.091519161	4.42E-05	NS

S100A4	NS	6.20E-06	-0.091517862	4.42E-05	NS
CALML5	NS	6.21E-06	0.091511115	4.43E-05	NS
ITGB3BP	NS	6.23E-06	-0.091494396	4.45E-05	NS
GLTP	NS	6.26E-06	0.091473682	4.47E-05	NS
TMEM135	NS	6.28E-06	-0.091464131	4.47E-05	NS
TH	NS	6.35E-06	0.091413729	4.52E-05	NS
KIF20A	NS	6.41E-06	-0.091372477	4.57E-05	NS
PAIP2B	NS	6.42E-06	-0.091369654	4.57E-05	NS
HGS	NS	6.44E-06	0.091357411	4.58E-05	NS
SUPT20HL1	NS	6.45E-06	0.091345998	4.59E-05	NS
KCNJ4	NS	6.47E-06	0.091332462	4.60E-05	NS
PRAMEF19	NS	6.50E-06	0.091314744	4.62E-05	NS
ADGRL4	NS	6.54E-06	-0.091289166	4.64E-05	NS
AADACL4	NS	6.54E-06	-0.091290665	4.64E-05	NS
GIPC2	NS	6.55E-06	-0.091282077	4.65E-05	NS
BICC1	NS	6.63E-06	-0.091231322	4.70E-05	NS
RBMY1E	NS	6.63E-06	-0.091232011	4.70E-05	NS
NDOR1	NS	6.70E-06	0.091184938	4.75E-05	NS
EPHA8	NS	6.71E-06	0.091178811	4.75E-05	NS
AKAP12	NS	6.71E-06	-0.091177978	4.75E-05	NS
AMACR	NS	6.72E-06	-0.091174737	4.76E-05	NS
MAB21L3	NS	6.80E-06	0.091125065	4.81E-05	NS
TYMS	NS	6.81E-06	-0.091118549	4.81E-05	NS
C16orf89	NS	6.85E-06	0.091087853	4.85E-05	NS
MRC1	NS	6.88E-06	-0.091073924	4.86E-05	NS
RGPD1	NS	6.90E-06	0.0910592	4.88E-05	NS
DST	NS	6.92E-06	-0.091049843	4.89E-05	NS
OR10Q1	NS	6.95E-06	-0.091028701	4.91E-05	NS
ITPRID1	NS	6.97E-06	-0.09101453	4.92E-05	NS
XIRP1	NS	7.01E-06	0.090990042	4.95E-05	NS
C8G	NS	7.03E-06	0.09097656	4.96E-05	NS
OGFOD2	NS	7.06E-06	-0.090959697	4.98E-05	NS
MRAP	NS	7.10E-06	-0.090936145	5.01E-05	NS
PIBF1	NS	7.11E-06	-0.090933475	5.01E-05	NS
RD3L	NS	7.13E-06	-0.090919104	5.02E-05	NS
IBA57	NS	7.14E-06	0.090911032	5.03E-05	NS
DQX1	NS	7.15E-06	-0.090906819	5.03E-05	NS
MMP1	NS	7.16E-06	-0.090903131	5.04E-05	NS
RAP1B	NS	7.17E-06	0.09089588	5.04E-05	NS
UGT2B7	NS	7.17E-06	-0.090893636	5.04E-05	NS
LGALS3	NS	7.21E-06	0.090873279	5.06E-05	NS
SOBP	NS	7.22E-06	0.090863148	5.07E-05	NS
RBMXL3	NS	7.22E-06	0.090864339	5.07E-05	NS
B3GLCT	NS	7.23E-06	-0.090860718	5.07E-05	NS
ATPSCKMT	NS	7.28E-06	-0.090826924	5.11E-05	NS
NES	NS	7.31E-06	0.090812059	5.13E-05	NS
TIMD4	NS	7.33E-06	-0.090800221	5.14E-05	NS
TIMM23	NS	7.33E-06	0.090799229	5.14E-05	NS
SPATA31D3	NS	7.35E-06	-0.090789417	5.15E-05	NS
TM2D3	NS	7.39E-06	0.090766887	5.17E-05	NS
ERBB3	NS	7.40E-06	0.090761229	5.18E-05	NS
ARHGEF2	NS	7.40E-06	0.090757023	5.18E-05	NS
ZFC3H1	NS	7.44E-06	0.090735467	5.21E-05	NS
CNTNAP3C	NS	7.48E-06	-0.090714653	5.23E-05	NS
ADGRL2	NS	7.53E-06	0.090682416	5.27E-05	NS

BCAM	NS	7.54E-06	0.090680889	5.27E-05	NS
C5orf49	NS	7.56E-06	-0.090668742	5.28E-05	NS
SLC15A3	NS	7.59E-06	0.090651268	5.30E-05	NS
TRPM4	NS	7.60E-06	0.090644325	5.30E-05	NS
KCNA2	NS	7.61E-06	-0.090640581	5.31E-05	NS
FARSB	NS	7.62E-06	0.090635475	5.31E-05	NS
TUBGCP4	NS	7.72E-06	-0.090577799	5.38E-05	NS
CENPO	NS	7.76E-06	0.090555521	5.41E-05	NS
DLX2	NS	7.78E-06	0.090544247	5.42E-05	NS
SPATA13	NS	7.90E-06	0.09047857	5.50E-05	NS
LRRTM3	NS	7.92E-06	-0.090467913	5.51E-05	NS
TMEM186	NS	7.93E-06	-0.090463364	5.52E-05	NS
NEURL1B	NS	7.93E-06	0.090462167	5.52E-05	NS
GFY	NS	8.01E-06	0.090419517	5.57E-05	NS
EPYC	NS	8.08E-06	-0.090379953	5.62E-05	NS
PLCH1	NS	8.12E-06	-0.090361304	5.64E-05	NS
CBS	NS	8.18E-06	0.090328814	5.68E-05	NS
MARCOL	NS	8.18E-06	-0.090327327	5.68E-05	NS
ZFP3	NS	8.28E-06	-0.090272622	5.75E-05	NS
SLC25A40	NS	8.29E-06	-0.090269684	5.75E-05	NS
ITGAX	NS	8.29E-06	0.090269157	5.75E-05	NS
MYO16	NS	8.32E-06	-0.090254918	5.77E-05	NS
H4C12	NS	8.33E-06	-0.090246554	5.78E-05	NS
CTAGE9	NS	8.39E-06	-0.090217644	5.81E-05	NS
SHISAL2B	NS	8.39E-06	-0.090214871	5.82E-05	NS
ZFY	NS	8.41E-06	0.090207395	5.82E-05	NS
RPLP1	NS	8.47E-06	0.090177338	5.86E-05	NS
ZNF135	NS	8.47E-06	-0.090175871	5.86E-05	NS
ZDHHC19	NS	8.51E-06	0.090158163	5.88E-05	NS
IBSP	NS	8.52E-06	-0.090153104	5.89E-05	NS
MTTP	NS	8.57E-06	-0.090123779	5.93E-05	NS
CD81	NS	8.63E-06	0.09009365	5.97E-05	NS
OCEL1	NS	8.67E-06	-0.090072967	5.99E-05	NS
EPHA4	NS	8.68E-06	-0.090069363	6.00E-05	NS
FAM50B	NS	8.74E-06	-0.090041033	6.03E-05	NS
PRSS45P	NS	8.74E-06	0.090039192	6.03E-05	NS
SHISA2	NS	8.78E-06	0.090022939	6.05E-05	NS
NPAS4	NS	8.80E-06	0.09001313	6.06E-05	NS
ECRG4	NS	8.81E-06	-0.090007949	6.07E-05	NS
IQCA1	NS	8.84E-06	-0.089991559	6.09E-05	NS
TLE6	NS	8.90E-06	0.089962782	6.13E-05	NS
ATF1	NS	8.92E-06	-0.089952152	6.14E-05	NS
FIS1	NS	8.92E-06	-0.089949935	6.14E-05	NS
NME8	NS	8.96E-06	-0.089934448	6.16E-05	NS
BTBD17	NS	8.96E-06	0.089934235	6.16E-05	NS
SUGCT	NS	8.98E-06	-0.089921191	6.17E-05	NS
CALCB	NS	8.98E-06	-0.08992113	6.17E-05	NS
PITX1	NS	9.00E-06	0.089912218	6.19E-05	NS
LDHAL6A	NS	9.01E-06	-0.089908615	6.19E-05	NS
OR4F4	NS	9.04E-06	-0.089893531	6.21E-05	NS
KRTAP6-3	NS	9.06E-06	0.089884688	6.22E-05	NS
BORA	NS	9.11E-06	-0.089862483	6.25E-05	NS
FMR1	NS	9.11E-06	0.089859402	6.25E-05	NS
PLCXD2	NS	9.12E-06	-0.089855213	6.25E-05	NS
TEX46	NS	9.14E-06	0.089846313	6.26E-05	NS

RXFP3	NS	9.19E-06	0.089824758	6.29E-05	NS
PFN4	NS	9.20E-06	-0.089819171	6.30E-05	NS
CDS1	NS	9.21E-06	-0.089812097	6.31E-05	NS
IFT46	NS	9.24E-06	-0.089798885	6.32E-05	NS
INSL3	NS	9.28E-06	-0.089780132	6.35E-05	NS
PRPH2	NS	9.39E-06	-0.08972709	6.43E-05	NS
ISCA2	NS	9.41E-06	-0.089719546	6.43E-05	NS
TBX22	NS	9.49E-06	-0.08968129	6.49E-05	NS
ARHGAP42	NS	9.56E-06	-0.089652805	6.52E-05	NS
CPHXL	NS	9.55E-06	-0.089654189	6.52E-05	NS
PRUNE2	NS	9.56E-06	-0.089649137	6.53E-05	NS
SMIM18	NS	9.57E-06	-0.089647522	6.53E-05	NS
CLCA4	NS	9.61E-06	-0.089628341	6.55E-05	NS
GSS	NS	9.66E-06	-0.089603748	6.58E-05	NS
GRIK5	NS	9.66E-06	0.089605047	6.58E-05	NS
CIBAR2	NS	9.66E-06	-0.089605326	6.58E-05	NS
PLEKHA1	NS	9.69E-06	-0.089593011	6.60E-05	NS
TMEM126B	NS	9.75E-06	-0.089564744	6.64E-05	NS
ZNF670	NS	9.79E-06	-0.089549286	6.66E-05	NS
S1PR1	NS	9.81E-06	-0.089538575	6.67E-05	NS
PLEKHG7	NS	9.86E-06	-0.089514545	6.71E-05	NS
AKR1C3	NS	9.88E-06	-0.089508535	6.72E-05	NS
GPR142	NS	9.88E-06	0.089506005	6.72E-05	NS
PROC	NS	1.00E-05	-0.089453632	6.80E-05	NS
PSMA5	NS	1.00E-05	-0.089447151	6.80E-05	NS
CSRP2	NS	1.00E-05	-0.089436657	6.82E-05	NS
RECQL4	NS	1.01E-05	0.089428335	6.83E-05	NS
S100A13	NS	1.01E-05	-0.089429314	6.83E-05	NS
OR5AR1	NS	1.01E-05	-0.089424538	6.83E-05	NS
SAV1	NS	1.01E-05	-0.089409767	6.85E-05	NS
MAGEA8	NS	1.02E-05	0.089373606	6.90E-05	NS
AMPD1	NS	1.02E-05	-0.089358556	6.93E-05	NS
RAB6A	NS	1.02E-05	0.089356103	6.93E-05	NS
RFX2	NS	1.02E-05	0.08934845	6.94E-05	NS
ADRA1B	NS	1.03E-05	0.089305076	7.00E-05	NS
IQCJ	NS	1.04E-05	-0.089296171	7.02E-05	NS
TANGO6	NS	1.04E-05	-0.08929114	7.02E-05	NS
LDHAL6B	NS	1.04E-05	-0.089289739	7.02E-05	NS
SLC7A10	NS	1.04E-05	0.089267668	7.05E-05	NS
KCNN3	NS	1.04E-05	0.089264502	7.06E-05	NS
NR2E1	NS	1.05E-05	0.089260195	7.06E-05	NS
C21orf62	NS	1.05E-05	0.089231745	7.11E-05	NS
OPN5	NS	1.06E-05	-0.089212794	7.13E-05	NS
GCSAML	NS	1.06E-05	-0.089192637	7.16E-05	NS
ARHGEF12	NS	1.06E-05	0.089190653	7.16E-05	NS
C2orf16	NS	1.06E-05	-0.089192035	7.16E-05	NS
OR5BS1P	NS	1.07E-05	-0.089164965	7.20E-05	NS
CRYGN	NS	1.08E-05	0.089117689	7.28E-05	NS
CRIP2	NS	1.08E-05	0.089115899	7.28E-05	NS
ANXA10	NS	1.08E-05	-0.089100534	7.30E-05	NS
BLOC1S5	NS	1.08E-05	-0.089101099	7.30E-05	NS
ALPL	NS	1.09E-05	0.089088156	7.31E-05	NS
HGFAC	NS	1.09E-05	0.089078038	7.33E-05	NS
ADAMTS10	NS	1.09E-05	-0.08907054	7.34E-05	NS
CD24	NS	1.09E-05	0.089066928	7.34E-05	NS

IL17RB	NS	1.09E-05	-0.08906477	7.34E-05	NS
VPS53	NS	1.10E-05	0.089044048	7.38E-05	NS
GNGT2	NS	1.10E-05	-0.089030473	7.39E-05	NS
DAZ3	NS	1.10E-05	-0.089031245	7.39E-05	NS
C4BPA	NS	1.11E-05	-0.089015379	7.41E-05	NS
DUSP10	NS	1.11E-05	0.089014278	7.41E-05	NS
ANKRD13C	NS	1.11E-05	-0.088994275	7.44E-05	NS
FAM71C	NS	1.12E-05	-0.088946598	7.52E-05	NS
HPF1	NS	1.13E-05	-0.088934031	7.54E-05	NS
TEX54	NS	1.13E-05	0.088933433	7.54E-05	NS
TRNT1	NS	1.13E-05	-0.088928697	7.55E-05	NS
SDR39U1	NS	1.13E-05	0.088909485	7.58E-05	NS
RASGEF1B	NS	1.14E-05	0.088884169	7.62E-05	NS
NUP54	NS	1.15E-05	0.088860102	7.65E-05	NS
ZNF735	NS	1.15E-05	-0.088836314	7.69E-05	NS
UPK2	NS	1.15E-05	0.088825781	7.71E-05	NS
SERPIND1	NS	1.16E-05	-0.088800262	7.75E-05	NS
ZNF549	NS	1.17E-05	0.088785011	7.77E-05	NS
TAS2R40	NS	1.17E-05	-0.088772951	7.79E-05	NS
CHAD	NS	1.17E-05	-0.088764544	7.80E-05	NS
OR7A17	NS	1.18E-05	0.088747115	7.83E-05	NS
ZNF543	NS	1.18E-05	0.088730577	7.86E-05	NS
CPT1C	NS	1.19E-05	0.088692869	7.92E-05	NS
OR1S1	NS	1.19E-05	0.088687284	7.93E-05	NS
OR1N2	NS	1.19E-05	-0.088681996	7.94E-05	NS
ZP2	NS	1.19E-05	-0.088674731	7.94E-05	NS
PERCC1	NS	1.20E-05	0.088656828	7.97E-05	NS
NKIRAS2	NS	1.21E-05	0.088620447	8.04E-05	NS
ALDOB	NS	1.21E-05	-0.088618323	8.04E-05	NS
LTK	NS	1.21E-05	0.088613533	8.04E-05	NS
KCTD16	NS	1.22E-05	-0.08859766	8.07E-05	NS
MYH1	NS	1.22E-05	-0.088574845	8.11E-05	NS
ING2	NS	1.23E-05	-0.088532693	8.18E-05	NS
IGF1R	NS	1.24E-05	-0.088526619	8.19E-05	NS
SCYGR5	NS	1.24E-05	0.088527106	8.19E-05	NS
NAPA	NS	1.24E-05	0.088501595	8.23E-05	NS
CCNYL1	NS	1.25E-05	0.088485349	8.26E-05	NS
CYGB	NS	1.25E-05	-0.088470757	8.29E-05	NS
TLX2	NS	1.26E-05	0.088455511	8.31E-05	NS
ZBTB18	NS	1.26E-05	0.088452287	8.31E-05	NS
HHIPL1	NS	1.26E-05	0.088449332	8.32E-05	NS
PADI6	NS	1.26E-05	-0.088426149	8.36E-05	NS
PHKB	NS	1.26E-05	0.088424507	8.36E-05	NS
VXN	NS	1.27E-05	-0.088403944	8.39E-05	NS
NPNT	NS	1.27E-05	-0.088397612	8.40E-05	NS
SPDYE13	NS	1.27E-05	0.088398586	8.40E-05	NS
SDF2	NS	1.27E-05	0.088392	8.41E-05	NS
CACNG4	NS	1.28E-05	0.088372706	8.44E-05	NS
C22orf23	NS	1.28E-05	-0.088363282	8.46E-05	NS
TAFA4	NS	1.29E-05	-0.088353455	8.47E-05	NS
WDR89	NS	1.29E-05	-0.088349492	8.47E-05	NS
ARPC2	NS	1.29E-05	0.088347674	8.47E-05	NS
PRAF2	NS	1.29E-05	0.088347135	8.47E-05	NS
GABRA6	NS	1.29E-05	-0.088322392	8.52E-05	NS
GRIN3A	NS	1.31E-05	-0.08827732	8.60E-05	NS

UBE2T	NS	1.31E-05	-0.088268748	8.61E-05	NS
TFCP2L1	NS	1.31E-05	-0.088258277	8.63E-05	NS
PSME4	NS	1.32E-05	0.088243846	8.66E-05	NS
C3AR1	NS	1.33E-05	0.08820815	8.73E-05	NS
KHNYN	NS	1.33E-05	0.088197973	8.74E-05	NS
OR9G1	NS	1.34E-05	-0.088177364	8.78E-05	NS
TFPI	NS	1.34E-05	0.088159285	8.81E-05	NS
GPR15	NS	1.35E-05	-0.088151043	8.83E-05	NS
NPAS3	NS	1.35E-05	0.088141249	8.84E-05	NS
OR5B12	NS	1.36E-05	-0.088120492	8.88E-05	NS
HRH1	NS	1.36E-05	-0.088110344	8.90E-05	NS
LOXHD1	NS	1.36E-05	-0.088102996	8.91E-05	NS
PSMB5	NS	1.37E-05	-0.088084627	8.94E-05	NS
TSPOAP1	NS	1.37E-05	0.088065848	8.98E-05	NS
SAYSD1	NS	1.37E-05	-0.088065225	8.98E-05	NS
ETF1	NS	1.37E-05	0.088055837	8.99E-05	NS
RHOXF2B	NS	1.38E-05	-0.088032617	9.04E-05	NS
ARL2	NS	1.39E-05	-0.088022164	9.05E-05	NS
SLC9A1	NS	1.39E-05	0.088015222	9.06E-05	NS
DENND11	NS	1.39E-05	-0.088013088	9.07E-05	NS
TNMD	NS	1.39E-05	-0.088010399	9.07E-05	NS
ARPP21	NS	1.39E-05	-0.088009987	9.07E-05	NS
EGR2	NS	1.39E-05	0.087995811	9.09E-05	NS
MGAT4B	NS	1.40E-05	0.087975194	9.13E-05	NS
CFAP36	NS	1.41E-05	-0.087959049	9.16E-05	NS
SLX4	NS	1.41E-05	-0.087956217	9.16E-05	NS
GRM3	NS	1.41E-05	-0.087952641	9.17E-05	NS
MSTN	NS	1.41E-05	-0.087943913	9.18E-05	NS
CT47A1	NS	1.42E-05	0.087923629	9.22E-05	NS
FAM20B	NS	1.42E-05	-0.087918144	9.23E-05	NS
CD99	NS	1.43E-05	0.087878765	9.31E-05	NS
LRRC40	NS	1.43E-05	-0.087876469	9.31E-05	NS
CEMP1	NS	1.43E-05	0.087880142	9.31E-05	NS
AATF	NS	1.43E-05	-0.0878762	9.31E-05	NS
NOC3L	NS	1.44E-05	-0.087859193	9.34E-05	NS
SPDYE10	NS	1.44E-05	0.087856592	9.34E-05	NS
SIM2	NS	1.45E-05	0.087829692	9.40E-05	NS
RNF113A	NS	1.45E-05	-0.08781914	9.41E-05	NS
PRORP	NS	1.46E-05	-0.087802219	9.45E-05	NS
ZIM2	NS	1.46E-05	-0.087788982	9.47E-05	NS
SEPTIN6	NS	1.46E-05	0.087786119	9.48E-05	NS
ASCL4	NS	1.46E-05	0.08778422	9.48E-05	NS
SFRP2	NS	1.46E-05	-0.087777116	9.49E-05	NS
RHOV	NS	1.48E-05	0.087727766	9.59E-05	NS
CRYBB1	NS	1.48E-05	-0.087715323	9.61E-05	NS
ME1	NS	1.49E-05	-0.087708824	9.63E-05	NS
PLAAT4	NS	1.49E-05	-0.08768791	9.66E-05	NS
KRTAP25-1	NS	1.49E-05	-0.08768781	9.66E-05	NS
ARG1	NS	1.50E-05	-0.087680137	9.68E-05	NS
PARPBP	NS	1.50E-05	-0.087660081	9.72E-05	NS
GPR17	NS	1.51E-05	0.087642755	9.75E-05	NS
TAFA5	NS	1.52E-05	0.087621493	9.80E-05	NS
TEX22	NS	1.54E-05	0.08756232	9.92E-05	NS
CYFIP2	NS	1.55E-05	0.087509735	0.000100393	NS
PBX1	NS	1.56E-05	0.087496578	0.000100657	NS

SCGB1C2	NS	1.57E-05	0.08748092	0.000100978	NS
ADH6	NS	1.57E-05	-0.087476756	0.000101039	NS
CEP350	NS	1.57E-05	-0.087473103	0.000101088	NS
ZC2HC1B	NS	1.58E-05	-0.087444649	0.000101702	NS
P4HTM	NS	1.58E-05	0.087441401	0.000101708	NS
OR51E2	NS	1.59E-05	0.087423992	0.000102073	NS
UFC2-KCTD	NS	1.59E-05	-0.087420786	0.000102112	NS
SLC52A3	NS	1.60E-05	0.08738749	0.000102844	NS
USP8	NS	1.60E-05	0.087371511	0.000103116	NS
LYSMD1	NS	1.60E-05	-0.087373653	0.000103116	NS
CMTR2	NS	1.60E-05	-0.087370794	0.000103116	NS
ALKBH2	NS	1.60E-05	-0.087369784	0.000103116	NS
DEPDC7	NS	1.61E-05	-0.087363654	0.000103223	NS
PTGS1	NS	1.61E-05	0.087349154	0.000103525	NS
KRT19	NS	1.62E-05	0.087338923	0.000103729	NS
GALNT18	NS	1.63E-05	-0.087309798	0.000104374	NS
CLDN1	NS	1.64E-05	-0.087269721	0.00010528	NS
EFL1	NS	1.65E-05	-0.087255722	0.000105576	NS
MRGPRG	NS	1.65E-05	0.087253675	0.00010559	NS
ARL4A	NS	1.65E-05	0.087250485	0.00010563	NS
CIPC	NS	1.66E-05	0.08723112	0.000106054	NS
HAO2	NS	1.69E-05	-0.087134849	0.000108329	NS
FIGNL1	NS	1.69E-05	-0.087131725	0.000108369	NS
APBA3	NS	1.70E-05	-0.087113895	0.000108715	NS
FOXN4	NS	1.70E-05	0.087113029	0.000108715	NS
HBB	NS	1.70E-05	-0.087115909	0.000108715	NS
GSX1	NS	1.70E-05	0.087104323	0.000108891	NS
KRT17	NS	1.71E-05	0.08708407	0.000109349	NS
ZNF716	NS	1.74E-05	0.087015959	0.00011099	NS
PTH1R	NS	1.74E-05	0.087013621	0.000111011	NS
RDM1	NS	1.74E-05	0.087006995	0.000111139	NS
RPUSD4	NS	1.75E-05	-0.086994415	0.000111415	NS
H3C1	NS	1.76E-05	-0.086960785	0.000112218	NS
KAT2B	NS	1.76E-05	-0.086947982	0.000112503	NS
TCAF2C	NS	1.77E-05	-0.086930272	0.000112874	NS
NAA11	NS	1.77E-05	-0.086923722	0.000113002	NS
THSD7B	NS	1.78E-05	-0.086913927	0.000113212	NS
C5orf22	NS	1.78E-05	-0.086901557	0.000113488	NS
EFNA2	NS	1.79E-05	0.08688533	0.000113862	NS
BRINP1	NS	1.79E-05	-0.086881492	0.000113922	NS
ALOX12B	NS	1.79E-05	0.086870082	0.000114175	NS
TRIM32	NS	1.84E-05	-0.086758748	0.000117006	NS
CA6	NS	1.85E-05	0.086742913	0.000117381	NS
SH3GL2	NS	1.85E-05	-0.086734012	0.000117576	NS
FER	NS	1.86E-05	-0.086707238	0.00011824	NS
SLFNL1	NS	1.87E-05	-0.086696175	0.000118493	NS
CDH22	NS	1.87E-05	0.086688795	0.000118649	NS
CSH1	NS	1.87E-05	0.086683372	0.000118753	NS
TMEM177	NS	1.88E-05	-0.086672077	0.000119013	NS
DDX18	NS	1.88E-05	0.086657626	0.000119358	NS
DYRK4	NS	1.89E-05	-0.086647074	0.000119599	NS
CD209	NS	1.89E-05	-0.086641644	0.000119705	NS
SERPINE3	NS	1.90E-05	-0.086618094	0.000120294	NS
TREML1	NS	1.90E-05	-0.086614845	0.000120342	NS
ZNF32	NS	1.90E-05	-0.086611468	0.000120393	NS

XYLT1	NS	1.91E-05	0.086599787	0.000120667	NS
HMHB1	NS	1.91E-05	-0.086597898	0.000120678	NS
ESR1	NS	1.92E-05	-0.086577769	0.00012118	NS
SLC3A1	NS	1.92E-05	-0.086574841	0.000121219	NS
SUMO2	NS	1.92E-05	0.08656605	0.000121417	NS
ERP29	NS	1.92E-05	-0.086564403	0.000121422	NS
FAF2	NS	1.92E-05	0.086560795	0.000121479	NS
LRRC8E	NS	1.93E-05	0.086545505	0.000121853	NS
PPP2R2B	NS	1.95E-05	-0.086507523	0.000122846	NS
OR8D1	NS	1.95E-05	-0.086502281	0.00012295	NS
HEPACAM2	NS	1.96E-05	-0.086469339	0.000123812	NS
MSLN	NS	1.96E-05	0.086465494	0.000123878	NS
NPTX2	NS	1.99E-05	0.086397508	0.000125721	NS
MINPP1	NS	2.01E-05	-0.086362789	0.000126653	NS
UTP6	NS	2.01E-05	-0.086350261	0.000126964	NS
PNMA3	NS	2.02E-05	0.086348776	0.000126964	NS
GSKIP	NS	2.02E-05	-0.086341683	0.000127123	NS
ACHE	NS	2.02E-05	0.086337363	0.000127203	NS
RRP9	NS	2.03E-05	-0.086322476	0.000127583	NS
CSDE1	NS	2.04E-05	0.086298049	0.000128234	NS
TMEM102	NS	2.05E-05	-0.086271243	0.000128956	NS
CASD1	NS	2.06E-05	-0.086238959	0.00012984	NS
FOXR1	NS	2.07E-05	0.086217225	0.000130425	NS
STK10	NS	2.08E-05	0.086211133	0.000130559	NS
SLC5A12	NS	2.09E-05	-0.08617339	0.000131612	NS
DEFB107A	NS	2.10E-05	-0.086164676	0.000131823	NS
SHB	NS	2.10E-05	-0.086155734	0.000132042	NS
GPR31	NS	2.10E-05	-0.086153897	0.000132053	NS
YME1L1	NS	2.10E-05	0.086152333	0.000132056	NS
POTEH	NS	2.11E-05	0.086134793	0.000132526	NS
EPHA5	NS	2.14E-05	-0.086080043	0.000134098	NS
LCE1B	NS	2.14E-05	0.086077946	0.000134117	NS
ZNF207	NS	2.16E-05	0.086041721	0.000135152	NS
FRMD6	NS	2.16E-05	-0.086035098	0.000135307	NS
ADAMTS12	NS	2.16E-05	-0.086028749	0.000135453	NS
TMEM201	NS	2.17E-05	-0.086023801	0.000135557	NS
TRIM51GP	NS	2.17E-05	-0.08600637	0.000136037	NS
FOXI3	NS	2.18E-05	0.085990336	0.000136476	NS
SLC25A12	NS	2.19E-05	-0.085974172	0.00013692	NS
HNRNPK	NS	2.19E-05	0.085971497	0.000136956	NS
CFAP141	NS	2.19E-05	-0.08596881	0.000136993	NS
NFE2L1	NS	2.21E-05	0.085939296	0.000137845	NS
METTL26	NS	2.22E-05	0.08591648	0.000138497	NS
COX17	NS	2.23E-05	0.085890523	0.000139248	NS
MEDAG	NS	2.25E-05	-0.085860001	0.000140144	NS
NPS	NS	2.25E-05	-0.085855504	0.000140238	NS
STMN2	NS	2.26E-05	-0.085835641	0.000140809	NS
PAQR5	NS	2.26E-05	0.085823608	0.000141138	NS
REELD1	NS	2.31E-05	-0.085725553	0.000144176	NS
PIM2	NS	2.32E-05	0.0857119	0.000144564	NS
HIC1	NS	2.33E-05	0.085700434	0.000144883	NS
ZFP42	NS	2.33E-05	0.085697655	0.000144925	NS
PHB	NS	2.33E-05	-0.08568974	0.000145085	NS
FZD2	NS	2.33E-05	-0.085689898	0.000145085	NS
CSRP3	NS	2.35E-05	-0.085648335	0.000146323	NS

GALNT8	NS	2.35E-05	-0.085648223	0.000146323	NS
FERD3L	NS	2.36E-05	0.085624323	0.000147048	NS
OR1M1	NS	2.37E-05	0.085605124	0.000147624	NS
CAPZA1	NS	2.38E-05	0.0855917	0.000148013	NS
SCTR	NS	2.40E-05	0.085565962	0.000148806	NS
WIPF2	NS	2.41E-05	0.085538219	0.000149669	NS
PER2	NS	2.42E-05	0.085512873	0.000150457	NS
XCL1	NS	2.43E-05	-0.085509364	0.000150525	NS
IQGAP3	NS	2.46E-05	-0.08545258	0.000152366	NS
APOC3	NS	2.46E-05	0.085442354	0.00015266	NS
LSM11	NS	2.46E-05	-0.085440818	0.000152663	NS
IL18R1	NS	2.46E-05	-0.08543833	0.000152697	NS
FAM47B	NS	2.47E-05	0.085433157	0.000152773	NS
PPT2-EGFL8	NS	2.47E-05	0.085433988	0.000152773	NS
NDUFS4	NS	2.48E-05	-0.085400831	0.000153813	NS
OR4C6	NS	2.49E-05	-0.085384432	0.000154318	NS
GLT8D2	NS	2.50E-05	-0.085377933	0.00015444	NS
LINC02218	NS	2.50E-05	-0.085378906	0.00015444	NS
TRIM26	NS	2.50E-05	0.085373932	0.000154526	NS
TRPC5OS	NS	2.51E-05	-0.085357934	0.00015502	NS
UMPS	NS	2.51E-05	-0.085347303	0.000155333	NS
DTL	NS	2.52E-05	-0.085336014	0.000155668	NS
CA3	NS	2.53E-05	-0.085322141	0.000156093	NS
CYP11A1	NS	2.53E-05	0.085315462	0.000156272	NS
DTHD1	NS	2.55E-05	-0.085280028	0.000157442	NS
ZDHHC16	NS	2.56E-05	-0.08525959	0.000158048	NS
DEFB107B	NS	2.56E-05	-0.085259583	0.000158048	NS
REG1B	NS	2.57E-05	-0.085254508	0.000158174	NS
CASP14	NS	2.58E-05	0.085229433	0.000158995	NS
SETSIP	NS	2.58E-05	-0.085221274	0.000159229	NS
MAP1LC3A	NS	2.60E-05	0.085185559	0.000160429	NS
L3HYPDH	NS	2.61E-05	-0.0851704	0.000160868	NS
TRA2A	NS	2.61E-05	0.085167285	0.000160868	NS
KRT79	NS	2.61E-05	0.085169238	0.000160868	NS
CT47A3	NS	2.61E-05	0.085167538	0.000160868	NS
GPATCH2	NS	2.63E-05	-0.085148264	0.000161488	NS
ASB17	NS	2.63E-05	-0.085143306	0.000161612	NS
ZNF510	NS	2.64E-05	-0.085127477	0.000162122	NS
DXO	NS	2.64E-05	0.085124039	0.000162193	NS
EXOC1L	NS	2.64E-05	-0.085119517	0.000162302	NS
ARL2-SNX15	NS	2.66E-05	-0.085094953	0.000163073	NS
PTRHD1	NS	2.66E-05	0.08508116	0.000163515	NS
GNG7	NS	2.68E-05	0.085050539	0.00016451	NS
KRTAP9-3	NS	2.68E-05	0.085051804	0.00016451	NS
MGAT3	NS	2.68E-05	-0.085048201	0.000164542	NS
ZNF682	NS	2.69E-05	-0.085045947	0.000164571	NS
THOP1	NS	2.69E-05	-0.085042721	0.000164635	NS
GAST	NS	2.69E-05	0.085037072	0.000164786	NS
ELOVL2	NS	2.70E-05	-0.085021567	0.000165241	NS
FAM166C	NS	2.71E-05	0.084998524	0.000165919	NS
ZNF738	NS	2.71E-05	0.084996685	0.000165933	NS
C5AR1	NS	2.72E-05	0.084994455	0.000165962	NS
SPDYE11	NS	2.73E-05	0.084968069	0.000166816	NS
GRIA2	NS	2.74E-05	-0.084953757	0.000167286	NS
PLAUR	NS	2.75E-05	0.084944597	0.000167569	NS

PAGE4	NS	2.76E-05	-0.084922964	0.000168309	NS
TRIM68	NS	2.76E-05	-0.08491344	0.000168607	NS
WAC	NS	2.78E-05	0.084882562	0.000169694	NS
H4C11	NS	2.79E-05	-0.084876765	0.000169856	NS
ICAM1	NS	2.79E-05	0.084874398	0.00016989	NS
ARHGEF17	NS	2.81E-05	0.084840389	0.000171058	NS
SRRM1	NS	2.81E-05	0.084840139	0.000171058	NS
PLCH2	NS	2.82E-05	0.084819171	0.000171789	NS
KRT83	NS	2.83E-05	0.084805339	0.000172254	NS
ZNF488	NS	2.87E-05	0.084748546	0.000174346	NS
GLMP	NS	2.87E-05	0.084744504	0.000174445	NS
SEPTIN2	NS	2.88E-05	0.084732011	0.000174866	NS
GPRASP1	NS	2.88E-05	-0.084720542	0.000175249	NS
SCGB1C1	NS	2.89E-05	0.084715949	0.000175369	NS
C6orf118	NS	2.90E-05	-0.084693066	0.000176191	NS
PRDM6	NS	2.90E-05	-0.084688647	0.000176264	NS
JDP2	NS	2.90E-05	0.084686849	0.000176264	NS
LRRC43	NS	2.90E-05	0.084688252	0.000176264	NS
WDR45B	NS	2.91E-05	0.084677636	0.000176562	NS
OR2J3	NS	2.92E-05	-0.084658514	0.000177244	NS
PIDD1	NS	2.92E-05	0.084655316	0.000177312	NS
NKAPD1	NS	2.94E-05	-0.084633265	0.00017811	NS
PLCB2	NS	2.94E-05	-0.084629114	0.000178162	NS
MOG	NS	2.94E-05	0.084627601	0.000178162	NS
CYP20A1	NS	2.97E-05	-0.084588072	0.000179646	NS
PPIF	NS	2.97E-05	0.084576329	0.00018005	NS
IFITM2	NS	2.98E-05	0.08457394	0.000180087	NS
NAP1L1	NS	2.99E-05	0.084558617	0.000180632	NS
ACSM1	NS	2.99E-05	-0.084554024	0.000180649	NS
PIFO	NS	2.99E-05	0.084553874	0.000180649	NS
RAB6C	NS	2.99E-05	0.084554301	0.000180649	NS
VWA5A	NS	3.00E-05	-0.084532611	0.00018143	NS
SRF	NS	3.01E-05	0.08452351	0.000181715	NS
MYBBP1A	NS	3.01E-05	-0.084522529	0.000181715	NS
SLC6A6	NS	3.01E-05	0.084517358	0.000181863	NS
CYP11B1	NS	3.02E-05	0.084511703	0.00018203	NS
LRRC72	NS	3.02E-05	-0.084506657	0.000182173	NS
VCAN	NS	3.03E-05	-0.084489589	0.000182794	NS
NCAM2	NS	3.04E-05	-0.084472404	0.000183421	NS
TCEAL1	NS	3.05E-05	-0.084457977	0.00018394	NS
FAM104A	NS	3.06E-05	0.084447204	0.000184313	NS
COA3	NS	3.06E-05	-0.084441572	0.000184482	NS
TRIM58	NS	3.08E-05	0.08442093	0.000185254	NS
CTAG2	NS	3.08E-05	0.084418883	0.000185278	NS
CCL8	NS	3.09E-05	-0.084405219	0.000185714	NS
GABRQ	NS	3.09E-05	-0.084405467	0.000185714	NS
SLC16A2	NS	3.11E-05	0.084367246	0.000187194	NS
CD8A	NS	3.12E-05	-0.08436334	0.000187294	NS
ERV3-1	NS	3.13E-05	0.084340189	0.00018818	NS
CENPH	NS	3.15E-05	-0.084316941	0.000189073	NS
OTP	NS	3.17E-05	0.0842904	0.000190106	NS
MSANTD4	NS	3.17E-05	-0.084288661	0.000190119	NS
PXT1	NS	3.17E-05	-0.084282505	0.000190314	NS
CRB3	NS	3.18E-05	-0.084267992	0.000190855	NS
CERS3	NS	3.18E-05	-0.084264954	0.000190922	NS

GABRR1	NS	3.19E-05	-0.084254462	0.000191297	NS
TXNDC12	NS	3.19E-05	-0.08425275	0.000191306	NS
RBMY1F	NS	3.19E-05	-0.084251394	0.000191306	NS
C5orf46	NS	3.20E-05	-0.084239904	0.000191724	NS
MRM1	NS	3.22E-05	-0.084217996	0.000192518	NS
HOXB3	NS	3.22E-05	0.084213311	0.000192654	NS
SPAG8	NS	3.23E-05	-0.084203729	0.000192995	NS
ANKRD40CL	NS	3.26E-05	0.084156898	0.000194903	NS
CNTN1	NS	3.26E-05	-0.084155237	0.000194913	NS
ARHGAP11A	NS	3.27E-05	-0.084136358	0.000195652	NS
ADA	NS	3.28E-05	0.084132778	0.000195743	NS
PLXNA1	NS	3.28E-05	0.084119708	0.000196237	NS
PROSER2	NS	3.30E-05	-0.084099998	0.000196955	NS
CXCL5	NS	3.30E-05	-0.084100675	0.000196955	NS
MRPS22	NS	3.30E-05	-0.08409456	0.000197126	NS
IGSF5	NS	3.31E-05	-0.084085312	0.00019746	NS
ITLN1	NS	3.32E-05	-0.084072527	0.000197947	NS
FAM133A	NS	3.33E-05	-0.084062595	0.000198312	NS
CCDC93	NS	3.34E-05	0.084046631	0.000198937	NS
OR52B4	NS	3.36E-05	-0.084020324	0.000200011	NS
DENND5A	NS	3.36E-05	0.084012484	0.000200289	NS
TECR	NS	3.40E-05	0.083958193	0.000202592	NS
CHCHD6	NS	3.42E-05	-0.083937937	0.000203404	NS
ZNF746	NS	3.42E-05	0.083936832	0.000203404	NS
SEMA3A	NS	3.42E-05	-0.083931136	0.000203592	NS
TMEM200C	NS	3.44E-05	0.083906712	0.000204606	NS
OR5T2	NS	3.46E-05	-0.0838788	0.000205781	NS
LRRC9	NS	3.47E-05	-0.083872171	0.000206012	NS
MMP19	NS	3.47E-05	0.08386845	0.000206115	NS
ALDOA	NS	3.49E-05	0.083841204	0.000207267	NS
MORF4L1	NS	3.49E-05	0.083836832	0.000207336	NS
KLRG2	NS	3.49E-05	0.083838094	0.000207336	NS
SLC25A53	NS	3.51E-05	-0.083815058	0.000208185	NS
DEFB106B	NS	3.52E-05	-0.083800381	0.000208757	NS
CD55	NS	3.52E-05	0.083799506	0.000208757	NS
FGL1	NS	3.55E-05	-0.083760261	0.000210468	NS
CREBRF	NS	3.56E-05	0.083748303	0.000210947	NS
ATIC	NS	3.57E-05	-0.083740465	0.000211239	NS
STAP2	NS	3.57E-05	0.083733841	0.000211476	NS
SDCBP2	NS	3.58E-05	0.083726473	0.000211748	NS
BANF2	NS	3.59E-05	0.083711679	0.000212359	NS
OR5H15	NS	3.61E-05	-0.083682479	0.000213569	NS
USP9Y	NS	3.61E-05	0.083677974	0.000213712	NS
ARC	NS	3.62E-05	0.083672582	0.000213895	NS
LVRN	NS	3.62E-05	-0.083669354	0.000213978	NS
NEURL1	NS	3.65E-05	-0.083635557	0.000215414	NS
PALM3	NS	3.65E-05	0.083635482	0.000215414	NS
FAM78B	NS	3.65E-05	-0.083633025	0.000215462	NS
PCDHB2	NS	3.68E-05	0.083596293	0.000217107	NS
GDF5	NS	3.68E-05	0.083590513	0.000217312	NS
USP17L5	NS	3.69E-05	-0.083585933	0.00021746	NS
TSPAN33	NS	3.71E-05	0.083552108	0.000218982	NS
TMX3	NS	3.72E-05	-0.083539743	0.000219499	NS
MED30	NS	3.73E-05	0.083525468	0.000220107	NS
MTMR11	NS	3.75E-05	0.083507908	0.000220873	NS

HOXC12	NS	3.75E-05	0.083505006	0.000220944	NS
RIOK3	NS	3.75E-05	0.083500897	0.000221072	NS
GP6	NS	3.78E-05	0.083473112	0.000222329	NS
NEXMIF	NS	3.79E-05	-0.08346114	0.000222834	NS
CITED1	NS	3.80E-05	0.083445322	0.000223525	NS
ZBTB26	NS	3.81E-05	-0.083427926	0.000224295	NS
OR52N2	NS	3.82E-05	-0.083420462	0.000224518	NS
SGK2	NS	3.82E-05	0.083416388	0.000224647	NS
TXNRD3	NS	3.84E-05	-0.083397675	0.000225483	NS
B3GNT7	NS	3.84E-05	-0.083393749	0.000225605	NS
DOCK9	NS	3.86E-05	-0.083374409	0.000226476	NS
AADAC	NS	3.87E-05	-0.083364392	0.000226895	NS
TUBGCP3	NS	3.88E-05	0.083347851	0.000227633	NS
RERG	NS	3.89E-05	-0.083332951	0.000228224	NS
SERHL2	NS	3.89E-05	-0.083333601	0.000228224	NS
GPAT4	NS	3.90E-05	0.083327912	0.000228333	NS
SMCR8	NS	3.90E-05	0.08332877	0.000228333	NS
GPR180	NS	3.90E-05	-0.083323177	0.000228496	NS
KPNA1	NS	3.92E-05	0.083294844	0.000229821	NS
PRYP3	NS	3.93E-05	-0.083287071	0.000230135	NS
TM4SF18	NS	3.94E-05	-0.083273536	0.000230734	NS
SLC29A2	NS	3.96E-05	0.083246688	0.000231997	NS
CPNE4	NS	3.97E-05	-0.08324431	0.000232045	NS
WDR48	NS	3.97E-05	0.083242076	0.000232086	NS
SFSWAP	NS	3.98E-05	0.083226276	0.000232804	NS
TRIM64C	NS	3.99E-05	-0.083220925	0.000233001	NS
OR2T8	NS	3.99E-05	-0.083218745	0.000233039	NS
PRDX3	NS	4.00E-05	-0.083205714	0.000233621	NS
H2AP	NS	4.01E-05	-0.083196196	0.000234027	NS
CHRM2	NS	4.01E-05	-0.083191667	0.000234184	NS
VRTN	NS	4.02E-05	0.08318315	0.000234541	NS
OR4Q3	NS	4.03E-05	0.083166663	0.0002353	NS
RASAL3	NS	4.04E-05	0.083162609	0.000235434	NS
MMP24OS	NS	4.04E-05	0.083153504	0.000235823	NS
ECT2	NS	4.05E-05	-0.083150783	0.000235837	NS
TNFSF12	NS	4.05E-05	0.083150409	0.000235837	NS
EDEM3	NS	4.07E-05	-0.083121052	0.000237254	NS
RBMY1D	NS	4.09E-05	-0.083106911	0.000237902	NS
PTGES3L	NS	4.11E-05	0.083076526	0.000239383	NS
POLR1F	NS	4.15E-05	0.083030316	0.00024169	NS
NUP62	NS	4.17E-05	0.083012017	0.000242565	NS
OR51B6	NS	4.19E-05	-0.08299082	0.000243594	NS
PIP5K1A	NS	4.20E-05	0.082979585	0.000244107	NS
CPA2	NS	4.20E-05	-0.08297572	0.000244162	NS
OR10AD1	NS	4.20E-05	-0.082975802	0.000244162	NS
FDFT1	NS	4.21E-05	0.08296368	0.000244718	NS
SKAP1	NS	4.21E-05	-0.082961677	0.000244749	NS
GTF3C6	NS	4.24E-05	-0.082929592	0.00024636	NS
CCDC169	NS	4.25E-05	-0.082922737	0.000246647	NS
NHLRC3	NS	4.27E-05	-0.082898474	0.000247855	NS
TRHDE	NS	4.31E-05	-0.082860245	0.000249813	NS
NUP37	NS	4.31E-05	-0.082853636	0.000250091	NS
IL5RA	NS	4.38E-05	-0.082786068	0.000253648	NS
FAM20C	NS	4.39E-05	0.082772818	0.000254214	NS
SNCA	NS	4.41E-05	-0.082746314	0.000255579	NS

OR52B2	NS	4.42E-05	-0.082735091	0.000256115	NS
HPS4	NS	4.43E-05	0.082725027	0.000256589	NS
PEX11G	NS	4.44E-05	0.082722647	0.000256642	NS
VPS37C	NS	4.45E-05	0.082708755	0.00025725	NS
OR6S1	NS	4.45E-05	0.082710113	0.00025725	NS
ERLIN1	NS	4.46E-05	-0.082698546	0.000257734	NS
ABCB4	NS	4.47E-05	-0.082682294	0.000258474	NS
COQ7	NS	4.47E-05	-0.082682945	0.000258474	NS
CT45A2	NS	4.50E-05	-0.082659418	0.00025966	NS
ZBTB41	NS	4.50E-05	-0.08265494	0.00025983	NS
NIPAL3	NS	4.51E-05	-0.082641722	0.000260485	NS
UGT2B28	NS	4.53E-05	-0.08262129	0.000261543	NS
PUM2	NS	4.54E-05	0.08261766	0.000261668	NS
EPS8	NS	4.54E-05	-0.082608074	0.000262124	NS
NEFL	NS	4.55E-05	-0.082602379	0.000262364	NS
SLC41A1	NS	4.55E-05	0.082600927	0.000262367	NS
IL1RL2	NS	4.56E-05	-0.08259733	0.00026249	NS
CD79A	NS	4.58E-05	-0.082572836	0.000263748	NS
KEL	NS	4.58E-05	-0.082572092	0.000263748	NS
RNF151	NS	4.62E-05	0.082527791	0.000266167	NS
HCFC2	NS	4.63E-05	-0.0825159	0.000266683	NS
EIF1AY	NS	4.63E-05	0.082517262	0.000266683	NS
ALDH3B2	NS	4.64E-05	0.082511132	0.000266874	NS
ATG14	NS	4.66E-05	0.082491726	0.000267899	NS
PDGFRL	NS	4.68E-05	-0.082467412	0.000269128	NS
ZNF655	NS	4.69E-05	0.082460148	0.000269464	NS
MTRF1	NS	4.72E-05	-0.082425637	0.000271368	NS
HEPHL1	NS	4.73E-05	-0.08242318	0.000271429	NS
NPFFR2	NS	4.73E-05	0.082418554	0.000271615	NS
EPGN	NS	4.73E-05	-0.082415503	0.000271711	NS
ARL8B	NS	4.75E-05	0.08240391	0.0002723	NS
BPNT2	NS	4.75E-05	-0.08239608	0.000272547	NS
C3orf52	NS	4.75E-05	-0.082398083	0.000272547	NS
BICDL1	NS	4.76E-05	0.082395471	0.000272547	NS
BCAS4	NS	4.76E-05	-0.082389493	0.000272768	NS
EXOSC4	NS	4.76E-05	0.082388871	0.000272768	NS
LCP2	NS	4.77E-05	0.082378934	0.000273263	NS
HNMT	NS	4.79E-05	-0.082359465	0.000274315	NS
HPCAL4	NS	4.80E-05	0.082352471	0.00027456	NS
PRIM2	NS	4.80E-05	-0.08234854	0.000274627	NS
SORCS2	NS	4.80E-05	0.082349059	0.000274627	NS
ZC3H18	NS	4.81E-05	0.08234461	0.000274775	NS
ACTA1	NS	4.82E-05	0.082335453	0.000275229	NS
CASP5	NS	4.82E-05	-0.082329708	0.000275483	NS
NUBPL	NS	4.83E-05	-0.082320729	0.000275927	NS
GAPDH	NS	4.86E-05	0.082288372	0.000277665	NS
PSMB11	NS	4.89E-05	0.082263072	0.000279078	NS
GMCL2	NS	4.90E-05	0.082258304	0.000279261	NS
RSPH1	NS	4.93E-05	-0.082229064	0.00028085	NS
CEP43	NS	4.95E-05	0.082204564	0.00028223	NS
ZNF597	NS	4.96E-05	-0.082201409	0.000282335	NS
MT4	NS	4.97E-05	0.08218448	0.000283184	NS
CRISP2	NS	4.97E-05	-0.082184836	0.000283184	NS
TMEM132B	NS	4.97E-05	0.082182653	0.00028321	NS
SYT13	NS	4.99E-05	0.082163671	0.000284268	NS

PHF23	NS	5.01E-05	-0.082144746	0.000285327	NS
C15orf62	NS	5.02E-05	-0.082143063	0.000285345	NS
HPSE2	NS	5.02E-05	-0.082137182	0.000285617	NS
OR3A1	NS	5.04E-05	-0.082123026	0.00028639	NS
TTBK2	NS	5.07E-05	0.082097539	0.000287795	NS
GOLGA2	NS	5.07E-05	-0.082097162	0.000287795	NS
ZNF507	NS	5.08E-05	-0.082086862	0.000288338	NS
PRPF39	NS	5.09E-05	-0.082078965	0.000288736	NS
LILRB5	NS	5.09E-05	0.082070008	0.000289199	NS
DBI	NS	5.10E-05	-0.082064052	0.000289479	NS
GADL1	NS	5.13E-05	-0.082041928	0.000290745	NS
HAP1	NS	5.13E-05	0.082040677	0.000290745	NS
PPP6R3	NS	5.15E-05	0.082023187	0.000291737	NS
BHMT	NS	5.16E-05	0.082006427	0.000292688	NS
SMIM3	NS	5.17E-05	0.082004898	0.000292697	NS
RBM22	NS	5.17E-05	0.081998797	0.000292989	NS
ADIRF	NS	5.19E-05	-0.081985328	0.000293739	NS
LSAMP	NS	5.19E-05	-0.081981726	0.000293877	NS
KLHL40	NS	5.20E-05	0.08197615	0.000294138	NS
IGSF21	NS	5.21E-05	-0.081966174	0.000294673	NS
C3orf18	NS	5.21E-05	-0.081960851	0.000294919	NS
CYP27B1	NS	5.23E-05	-0.081945899	0.000295766	NS
ODAD2	NS	5.28E-05	-0.081901535	0.000298378	NS
RNASE4	NS	5.34E-05	-0.081850482	0.000301526	NS
OR52W1	NS	5.35E-05	-0.081842189	0.000301966	NS
STOX2	NS	5.35E-05	-0.081838382	0.000302121	NS
ATOH8	NS	5.36E-05	-0.081825874	0.000302743	NS
LYG2	NS	5.39E-05	-0.081806557	0.000303892	NS
OR52E2	NS	5.41E-05	-0.081784125	0.000305245	NS
OR2L13	NS	5.43E-05	-0.081771622	0.000305962	NS
MYO9B	NS	5.43E-05	0.081767338	0.00030615	NS
MRPL52	NS	5.43E-05	-0.081765135	0.000306203	NS
MCL1	NS	5.44E-05	0.081755646	0.000306727	NS
MYL7	NS	5.45E-05	0.081748416	0.000307106	NS
FAM53A	NS	5.47E-05	-0.081733607	0.000307977	NS
ADAMTS2	NS	5.50E-05	-0.081704679	0.000309768	NS
SMIM42	NS	5.51E-05	-0.081703357	0.000309768	NS
TMEM101	NS	5.51E-05	-0.081696025	0.000310157	NS
CDC14C	NS	5.52E-05	0.0816926	0.000310291	NS
ESGALNACT	NS	5.52E-05	0.08168704	0.000310565	NS
GRID2	NS	5.53E-05	-0.081683256	0.000310722	NS
AGBL3	NS	5.53E-05	-0.081680327	0.000310824	NS
OR2A4	NS	5.53E-05	-0.081678609	0.000310846	NS
CEP44	NS	5.56E-05	0.081659608	0.000312003	NS
GRHL3	NS	5.57E-05	-0.081644156	0.00031293	NS
LONRF1	NS	5.59E-05	0.081632146	0.000313632	NS
TMEM179	NS	5.59E-05	0.081627738	0.000313832	NS
MAGED2	NS	5.64E-05	-0.081585591	0.000316447	NS
OTOL1	NS	5.64E-05	-0.081586431	0.000316447	NS
H3C15	NS	5.65E-05	0.081582506	0.000316561	NS
ZNF107	NS	5.65E-05	0.081579467	0.000316672	NS
SNX30	NS	5.66E-05	0.081574931	0.000316883	NS
GABRD	NS	5.66E-05	0.081570503	0.000317087	NS
CD96	NS	5.68E-05	-0.081557398	0.000317871	NS
IDI2	NS	5.69E-05	-0.081550696	0.000318227	NS

ZNF438	NS	5.69E-05	-0.081546445	0.00031842	NS
EIF1	NS	5.72E-05	0.081521602	0.000319997	NS
FBXO4	NS	5.73E-05	-0.081511953	0.000320554	NS
PKD2L1	NS	5.75E-05	-0.081497118	0.000321463	NS
LMAN2L	NS	5.80E-05	-0.081458605	0.000323984	NS
SPRR2A	NS	5.80E-05	-0.081456299	0.000324048	NS
CNNM3	NS	5.82E-05	-0.081440282	0.000325047	NS
ATP1B4	NS	5.83E-05	0.081434236	0.000325367	NS
ZNHIT1	NS	5.83E-05	-0.081429906	0.000325475	NS
OR4A15	NS	5.83E-05	-0.081430625	0.000325475	NS
SNIP1	NS	5.85E-05	0.081417915	0.000326202	NS
COL10A1	NS	5.89E-05	-0.081385039	0.000328369	NS
IGHMBP2	NS	5.89E-05	0.08137992	0.000328628	NS
BNIP2	NS	5.91E-05	0.081362254	0.000329659	NS
XPC	NS	5.91E-05	0.081363358	0.000329659	NS
ZFYVE1	NS	5.92E-05	0.081357151	0.000329918	NS
WFIKKN2	NS	5.95E-05	0.081330308	0.000331687	NS
PAEP	NS	5.99E-05	0.081301342	0.000333519	NS
FBXO34	NS	6.03E-05	0.081269807	0.000335637	NS
PSKH1	NS	6.06E-05	-0.081247501	0.000337114	NS
CT47A2	NS	6.06E-05	0.081244471	0.000337231	NS
ANKRD29	NS	6.08E-05	-0.081231129	0.000337982	NS
VAPB	NS	6.09E-05	0.081223801	0.000338405	NS
ARIH2	NS	6.10E-05	0.08121409	0.000338997	NS
COA5	NS	6.16E-05	-0.081171526	0.000341938	NS
GPC2	NS	6.16E-05	0.081169877	0.000341958	NS
MYO1A	NS	6.16E-05	-0.081166349	0.000342112	NS
GABRA4	NS	6.18E-05	-0.08115313	0.000342963	NS
LGR6	NS	6.19E-05	0.081144586	0.000343479	NS
MRPL41	NS	6.22E-05	-0.081120543	0.000345115	NS
GPC1	NS	6.26E-05	-0.081091985	0.000347086	NS
EGR1	NS	6.27E-05	0.081087743	0.000347295	NS
MYOD1	NS	6.27E-05	0.081081864	0.000347571	NS
TKTL2	NS	6.27E-05	-0.081081211	0.000347571	NS
NT5C1B	NS	6.28E-05	-0.08107455	0.000347956	NS
KRT85	NS	6.29E-05	0.081066906	0.000348414	NS
ZFAND1	NS	6.31E-05	-0.081055773	0.000349123	NS
ZNF879	NS	6.31E-05	-0.081054468	0.000349123	NS
YAE1	NS	6.32E-05	-0.081049651	0.000349375	NS
ADRB3	NS	6.33E-05	0.081041567	0.000349867	NS
MOCS1	NS	6.35E-05	-0.081024846	0.000350993	NS
CT47A11	NS	6.35E-05	0.081020933	0.00035118	NS
SPEF1	NS	6.36E-05	0.081013181	0.000351649	NS
NR4A2	NS	6.37E-05	0.081005182	0.000352138	NS
RIMBP3C	NS	6.38E-05	-0.081002854	0.000352207	NS
ZAR1L	NS	6.38E-05	-0.08100151	0.000352207	NS
VIM	NS	6.40E-05	0.080988681	0.000353053	NS
TLR4	NS	6.41E-05	0.080976328	0.000353865	NS
SLC51A	NS	6.47E-05	-0.080933191	0.00035697	NS
INS-IGF2	NS	6.47E-05	0.080931267	0.000357012	NS
KRT14	NS	6.48E-05	0.080925654	0.000357329	NS
CYP4F2	NS	6.50E-05	-0.080914789	0.000358039	NS
LRRC4B	NS	6.56E-05	0.080869122	0.000361369	NS
CCDC152	NS	6.57E-05	-0.08086127	0.000361859	NS
OMG	NS	6.59E-05	-0.080844816	0.000363001	NS

SH2D5	NS	6.59E-05	0.080843404	0.000363005	NS
PEX3	NS	6.63E-05	-0.080818027	0.00036483	NS
PMP2	NS	6.64E-05	-0.080807537	0.000365525	NS
PCDHGB1	NS	6.67E-05	-0.080785334	0.000367103	NS
SDF2L1	NS	6.70E-05	-0.080766251	0.000368374	NS
DIO1	NS	6.73E-05	-0.080747222	0.000369734	NS
NOL4	NS	6.74E-05	-0.080740629	0.000370138	NS
SCNN1B	NS	6.75E-05	0.080728796	0.000370947	NS
APEX2	NS	6.76E-05	0.080726001	0.000371058	NS
FAN1	NS	6.77E-05	0.080719016	0.000371494	NS
BSDC1	NS	6.77E-05	0.080717529	0.000371504	NS
DDIT3	NS	6.78E-05	0.080708443	0.000372103	NS
NR2C2AP	NS	6.80E-05	-0.08069864	0.000372758	NS
SPDYE15	NS	6.80E-05	0.080696748	0.0003728	NS
FABP12	NS	6.82E-05	-0.080681746	0.000373755	NS
PLEKHM1	NS	6.82E-05	0.08068203	0.000373755	NS
OPN1LW	NS	6.85E-05	0.080660195	0.000375332	NS
KLHL13	NS	6.87E-05	-0.080645136	0.000376405	NS
ELANE	NS	6.88E-05	-0.080640922	0.000376629	NS
TRAF3IP3	NS	6.91E-05	0.080621021	0.000378086	NS
GDPD3	NS	6.95E-05	-0.08059174	0.000380291	NS
SOWAHB	NS	6.96E-05	0.080587685	0.000380505	NS
PIP4K2A	NS	6.99E-05	-0.0805655	0.000382158	NS
FMO1	NS	7.00E-05	-0.080555513	0.000382734	NS
TENT5D	NS	7.00E-05	-0.080554194	0.000382734	NS
ZPBP2	NS	7.00E-05	-0.080555924	0.000382734	NS
FOXA2	NS	7.01E-05	0.080550007	0.00038296	NS
ZNF718	NS	7.01E-05	-0.080548102	0.000383004	NS
RNF112	NS	7.02E-05	0.08054333	0.000383276	NS
LGMN	NS	7.02E-05	0.080541462	0.000383297	NS
KLHL14	NS	7.02E-05	-0.080540354	0.000383297	NS
GOLGA8A	NS	7.09E-05	0.080492154	0.000387051	NS
TEPP	NS	7.11E-05	0.080480775	0.000387859	NS
TBXT	NS	7.12E-05	0.080477091	0.000388047	NS
SLC2A11	NS	7.12E-05	-0.080475569	0.00038806	NS
ACP7	NS	7.12E-05	0.080473877	0.000388087	NS
C12orf71	NS	7.14E-05	-0.080462605	0.000388889	NS
SLC27A2	NS	7.14E-05	-0.080459748	0.00038901	NS
BANK1	NS	7.16E-05	-0.080451375	0.000389578	NS
PTCD3	NS	7.17E-05	-0.080440776	0.000390304	NS
SAC3D1	NS	7.17E-05	-0.080439712	0.000390304	NS
DPPA5	NS	7.18E-05	0.080432491	0.000390781	NS
KIR2DL3	NS	7.20E-05	-0.080424098	0.000391353	NS
TRPA1	NS	7.23E-05	-0.08039985	0.000393175	NS
APCS	NS	7.24E-05	-0.080397441	0.000393195	NS
KRTAP10-10	NS	7.30E-05	0.08035592	0.000396267	NS
FGF7	NS	7.30E-05	-0.08035361	0.000396346	NS
PDK3	NS	7.35E-05	0.080324873	0.00039848	NS
RGL1	NS	7.35E-05	-0.080324514	0.00039848	NS
OR4D5	NS	7.35E-05	-0.080323696	0.00039848	NS
TREM1	NS	7.40E-05	0.080286132	0.000401487	NS
PPAT	NS	7.42E-05	-0.080278999	0.000401969	NS
FOCAD	NS	7.43E-05	-0.080272594	0.000402391	NS
HEBP1	NS	7.44E-05	-0.080266365	0.000402798	NS
PDPR	NS	7.45E-05	0.080259712	0.000403242	NS

DES	NS	7.45E-05	-0.080255734	0.000403462	NS
ZNF780B	NS	7.53E-05	-0.080207771	0.000407381	NS
A4GALT	NS	7.54E-05	0.080197326	0.000408149	NS
AVP	NS	7.55E-05	0.080192989	0.000408402	NS
DMP1	NS	7.58E-05	-0.080175222	0.000409794	NS
TAX1BP1	NS	7.58E-05	0.080171387	0.000409891	NS
ESPN	NS	7.58E-05	0.080171436	0.000409891	NS
SESN3	NS	7.62E-05	-0.080147549	0.000411806	NS
TRIM51	NS	7.62E-05	-0.080145657	0.000411852	NS
SYCE2	NS	7.64E-05	-0.080133413	0.000412783	NS
SOAT1	NS	7.65E-05	0.080131987	0.00041279	NS
SLC27A5	NS	7.71E-05	0.080088906	0.000416373	NS
DAPK1	NS	7.73E-05	0.080079079	0.000417104	NS
PXN	NS	7.73E-05	0.080076465	0.000417106	NS
TRIM59	NS	7.73E-05	-0.080076371	0.000417106	NS
TMPRSS11D	NS	7.76E-05	-0.080061239	0.000418298	NS
APLP2	NS	7.77E-05	0.080051796	0.000418999	NS
KDM6A	NS	7.78E-05	0.080046954	0.000419302	NS
ANKIB1	NS	7.81E-05	-0.080029961	0.000420662	NS
OR13C4	NS	7.83E-05	-0.080018762	0.000421521	NS
AMZ1	NS	7.84E-05	0.080011577	0.00042203	NS
NLRP11	NS	7.85E-05	0.080006465	0.000422359	NS
UBA3	NS	7.87E-05	-0.07999498	0.000423246	NS
LRRC17	NS	7.88E-05	-0.079988648	0.000423683	NS
MCM6	NS	7.93E-05	-0.079954984	0.000426286	NS
TRIM56	NS	7.93E-05	-0.079957587	0.000426286	NS
OR51E1	NS	7.93E-05	-0.079955924	0.000426286	NS
KRBOX1	NS	7.95E-05	-0.079942611	0.000427259	NS
AFMID	NS	7.96E-05	-0.079939989	0.000427372	NS
MPV17L	NS	7.96E-05	0.079936835	0.000427532	NS
NUDT15	NS	8.00E-05	0.079910592	0.000429738	NS
LEUTX	NS	8.01E-05	-0.07990666	0.000429968	NS
SSMEM1	NS	8.03E-05	-0.079896801	0.000430725	NS
PLK3	NS	8.04E-05	0.07988882	0.000431316	NS
LRRC7	NS	8.05E-05	0.079885639	0.00043148	NS
UGT2B10	NS	8.11E-05	-0.079846432	0.000434867	NS
PTHLH	NS	8.13E-05	0.079834916	0.000435782	NS
TTC21B	NS	8.16E-05	0.079814629	0.000437489	NS
DDIT4	NS	8.17E-05	0.079809742	0.000437809	NS
PDZD7	NS	8.18E-05	0.079807919	0.000437853	NS
ARHGEF28	NS	8.18E-05	0.079802428	0.000438228	NS
AVPR1B	NS	8.32E-05	0.079721811	0.000445453	NS
OR52J3	NS	8.33E-05	-0.079715366	0.000445922	NS
ACVR1B	NS	8.37E-05	0.079692254	0.000447927	NS
GARS1	NS	8.40E-05	0.079678386	0.000449085	NS
OR1L4	NS	8.40E-05	-0.079675939	0.000449188	NS
ZNF165	NS	8.42E-05	0.079663171	0.000450246	NS
CLN5	NS	8.44E-05	0.079655551	0.000450829	NS
CRACD	NS	8.44E-05	-0.079650933	0.000451134	NS
PCGF2	NS	8.45E-05	-0.079645688	0.000451497	NS
ASB4	NS	8.48E-05	-0.079631085	0.000452659	NS
OR2W3	NS	8.48E-05	-0.079630527	0.000452659	NS
SLC23A3	NS	8.49E-05	-0.079625144	0.000453036	NS
PYM1	NS	8.52E-05	0.079606863	0.00045462	NS
TSPAN14	NS	8.54E-05	0.07959804	0.000455321	NS

TPBG	NS	8.56E-05	-0.079586213	0.000456305	NS
OR8K5	NS	8.56E-05	-0.079582168	0.000456559	NS
KCNK2	NS	8.58E-05	0.079570654	0.000457516	NS
SUCO	NS	8.62E-05	0.079550779	0.000458886	NS
LRP2BP	NS	8.62E-05	0.079551233	0.000458886	NS
PRRX2	NS	8.62E-05	0.079551131	0.000458886	NS
RAB3IL1	NS	8.63E-05	0.079547427	0.000459076	NS
TRIM74	NS	8.64E-05	-0.079539186	0.000459729	NS
MMD2	NS	8.64E-05	0.079536889	0.00045982	NS
MOGAT2	NS	8.65E-05	0.079535036	0.000459869	NS
CDH24	NS	8.69E-05	0.079512575	0.000461872	NS
TBC1D26	NS	8.70E-05	0.079506753	0.000462298	NS
OR8J1	NS	8.71E-05	-0.079498015	0.000463002	NS
MARS1	NS	8.73E-05	0.079491795	0.000463468	NS
SLC9A9	NS	8.78E-05	-0.079462822	0.000466109	NS
CES5A	NS	8.78E-05	-0.079461411	0.000466116	NS
OMD	NS	8.80E-05	-0.079451636	0.000466926	NS
UGT1A4	NS	8.80E-05	-0.079449302	0.000467021	NS
REM1	NS	8.81E-05	0.079443576	0.000467443	NS
POLA1	NS	8.82E-05	-0.079438104	0.000467841	NS
OR51G1	NS	8.85E-05	-0.079420936	0.000469366	NS
MAP3K1	NS	8.86E-05	0.079419054	0.000469419	NS
GALNS	NS	8.91E-05	0.0793911	0.000471993	NS
POLD1	NS	8.92E-05	-0.079386377	0.000472321	NS
SCAPER	NS	8.96E-05	-0.079361157	0.000474514	NS
CCL18	NS	8.96E-05	-0.079362184	0.000474514	NS
SLC25A52	NS	9.00E-05	0.079341136	0.000476338	NS
ZSCAN31	NS	9.01E-05	-0.079334736	0.000476834	NS
TYROBP	NS	9.02E-05	0.079331115	0.000477058	NS
KLHDC3	NS	9.02E-05	-0.079328518	0.000477182	NS
NNAT	NS	9.05E-05	-0.079316491	0.000478121	NS
CCL21	NS	9.05E-05	0.079316275	0.000478121	NS
CAPZB	NS	9.05E-05	0.079312577	0.000478353	NS
DCST2	NS	9.11E-05	0.079280593	0.000481371	NS
OIT3	NS	9.13E-05	-0.079273607	0.00048193	NS
TUBGCP2	NS	9.16E-05	0.079257071	0.000483435	NS
PRADC1	NS	9.22E-05	-0.079225178	0.000486474	NS
NLRP7	NS	9.27E-05	0.079194524	0.000489407	NS
CD1A	NS	9.35E-05	-0.079156483	0.000493102	NS
TMEM263	NS	9.35E-05	-0.079153103	0.000493309	NS
PPARGC1A	NS	9.36E-05	-0.079147279	0.000493763	NS
CFHR1	NS	9.41E-05	0.079123538	0.000496034	NS
CT47A6	NS	9.43E-05	0.079114252	0.000496843	NS
PDE10A	NS	9.44E-05	0.07910832	0.000497176	NS
CD200R1L	NS	9.44E-05	-0.079109238	0.000497176	NS
CT47B1	NS	9.44E-05	0.079106052	0.000497271	NS
RPUSD2	NS	9.47E-05	-0.079091125	0.000498658	NS
OR6N2	NS	9.49E-05	-0.079082976	0.000499354	NS
OR52E4	NS	9.52E-05	-0.079065771	0.000500979	NS
RACGAP1	NS	9.54E-05	-0.079054886	0.00050196	NS
STRBP	NS	9.58E-05	-0.079038615	0.000503497	NS
ZNF576	NS	9.61E-05	0.079018493	0.000505432	NS
PSORS1C1	NS	9.62E-05	0.079017201	0.000505432	NS
GJA9	NS	9.68E-05	-0.078987751	0.000508347	NS
IL31RA	NS	9.69E-05	0.078980557	0.000508819	NS

TRAM1L1	NS	9.69E-05	-0.078981393	0.000508819	NS
PRTFDC1	NS	9.71E-05	-0.078972313	0.000509538	NS
ZZEF1	NS	9.73E-05	0.078960427	0.000510639	NS
C16orf54	NS	9.74E-05	0.078954329	0.000511137	NS
TPGS2	NS	9.78E-05	-0.078933527	0.000513176	NS
STMN3	NS	9.79E-05	0.078931852	0.000513212	NS
SAA2	NS	9.81E-05	0.078920566	0.000514257	NS
DCAF4L2	NS	9.83E-05	-0.078910689	0.000515017	NS
PLEKHA2	NS	9.85E-05	0.078901648	0.000515829	NS
TAAR6	NS	9.85E-05	-0.078900167	0.000515846	NS
SLC4A10	NS	9.88E-05	-0.078886807	0.000517115	NS
SLC16A9	NS	9.89E-05	-0.078882095	0.000517473	NS
HBE1	NS	9.90E-05	-0.078874787	0.000518106	NS
BBS9	NS	9.91E-05	-0.078868328	0.000518649	NS
DUSP14	NS	9.93E-05	-0.078862839	0.00051909	NS
KAT8	NS	9.94E-05	0.078857671	0.000519357	NS
SENP5	NS	9.94E-05	0.078857772	0.000519357	NS
UBE4A	NS	9.99E-05	0.078830817	0.000522071	NS
GALNT14	NS	0.000100107	-0.078820905	0.000522987	NS
LCN9	NS	0.000100138	0.078819374	0.000523009	NS
GES3L-AARS	NS	0.000100273	-0.078812752	0.000523433	NS
CKMT1A	NS	0.000100269	-0.078812981	0.000523433	NS
KRT5	NS	0.000100439	-0.07880465	0.000524158	NS
TUT4	NS	0.000100878	0.078783292	0.000526305	NS
CTRC	NS	0.000100992	0.07877772	0.000526553	NS
ZEB2	NS	0.000101002	0.078777273	0.000526553	NS
MAGEL2	NS	0.000101007	0.078777015	0.000526553	NS
ASAP2	NS	0.000101103	-0.078772363	0.000526911	NS
UBA6	NS	0.000101711	0.078742964	0.000529794	NS
NANOG	NS	0.000101696	0.078743666	0.000529794	NS
C1QTNF3	NS	0.000101779	-0.078739682	0.000530005	NS
MICAL3	NS	0.000102008	0.078728668	0.000531054	NS
CASR	NS	0.000102405	-0.07870961	0.000532802	NS
S100A10	NS	0.000102423	0.078708744	0.000532802	NS
C9orf57	NS	0.000102426	0.078708587	0.000532802	NS
BUD23	NS	0.00010253	0.078703616	0.000533198	NS
RPL27A	NS	0.000102696	0.078695658	0.00053392	NS
IFI35	NS	0.000102831	-0.078689194	0.00053448	NS
OOSP4B	NS	0.000103173	-0.07867291	0.000536112	NS
UGT2A1	NS	0.000103373	-0.078663418	0.000537006	NS
ENC1	NS	0.000103439	-0.078660284	0.000537204	NS
OR5AU1	NS	0.000103547	0.078655149	0.000537622	NS
SLA	NS	0.000103647	0.078650386	0.000538	NS
CLIC3	NS	0.00010408	-0.07862994	0.000540099	NS
GCLM	NS	0.000104332	0.078618043	0.000541264	NS
POLDIP2	NS	0.000104885	-0.078592078	0.000543986	NS
ZC3H11B	NS	0.000105054	0.078584179	0.000544715	NS
HYKK	NS	0.000105425	0.078566855	0.000546492	NS
ANKRD33B	NS	0.000105488	-0.07856391	0.000546673	NS
TENT2	NS	0.000105993	0.078540441	0.000549142	NS
ZFP91	NS	0.000106161	0.078532636	0.000549867	NS
KRTAP9-1	NS	0.000106345	0.078524114	0.000550674	NS
HTR2A	NS	0.00010653	-0.078515586	0.000551482	NS
R3HDM4	NS	0.000106712	0.078507179	0.000552278	NS
MS4A18	NS	0.000106744	-0.078505705	0.000552295	NS

SH2B3	NS	0.000106846	0.07850102	0.000552674	NS
ACTR1A	NS	0.000106883	0.07849929	0.000552721	NS
XAGE3	NS	0.000107113	0.078488736	0.00055376	NS
WDR53	NS	0.000107266	-0.078481713	0.000554403	NS
ZNF608	NS	0.000108354	-0.078432031	0.000559878	NS
DOLK	NS	0.000108697	-0.078416461	0.000561501	NS
CDNF	NS	0.000108804	-0.078411642	0.000561901	NS
FOXL1	NS	0.00010899	0.078403221	0.000562713	NS
APOC2	NS	0.00010917	0.078395097	0.000563492	NS
TCP11X1	NS	0.000109345	-0.078387213	0.000564244	NS
TRPV1	NS	0.000109471	-0.078381524	0.000564745	NS
SLC35D3	NS	0.000109719	0.078370376	0.000565874	NS
PLEKHA5	NS	0.000110303	0.078344212	0.000568584	NS
CACNG8	NS	0.000110285	0.078345013	0.000568584	NS
CTXN2	NS	0.000110435	-0.078338315	0.000569113	NS
OR4C11	NS	0.000110611	-0.078330475	0.000569867	NS
NAP1L5	NS	0.000110646	0.078328904	0.000569897	NS
OR5C1	NS	0.000111019	-0.078312335	0.000571512	NS
JPT1	NS	0.000111007	0.078312858	0.000571512	NS
ARAP2	NS	0.000111109	0.078308353	0.00057167	NS
WIPI2	NS	0.000111109	0.078308349	0.00057167	NS
PIGBOS1	NS	0.00011117	-0.078305644	0.000571832	NS
CNPPD1	NS	0.000111661	0.078283906	0.000574206	NS
WDR83OS	NS	0.000111727	-0.078280975	0.000574394	NS
ADH5	NS	0.000112009	-0.078268538	0.000575692	NS
OR4N4	NS	0.000112263	0.07825738	0.000576842	NS
PRKN	NS	0.000112404	0.078251179	0.000577415	NS
NPR1	NS	0.00011258	0.07824349	0.000578162	NS
TUBB2B	NS	0.000112906	0.078229206	0.000579684	NS
TCL1B	NS	0.000113373	0.078208823	0.000581929	NS
PEX2	NS	0.000113698	-0.078194697	0.000583442	NS
GALNT13	NS	0.000114202	-0.078172865	0.000585872	NS
MS4A8	NS	0.000114416	-0.078163632	0.000586658	NS
PYCR1	NS	0.000114391	0.078164682	0.000586658	NS
KCTD3	NS	0.000114654	-0.07815338	0.000587721	NS
AMFR	NS	0.000114719	0.078150575	0.000587899	NS
UGGT2	NS	0.000114835	-0.078145586	0.000588334	NS
KCNMB1	NS	0.000114864	0.078144304	0.000588334	NS
EIF3H	NS	0.000115081	0.078134996	0.000589132	NS
GLYATL1	NS	0.000115066	-0.078135645	0.000589132	NS
OLFML2B	NS	0.000115284	-0.078126278	0.000590017	NS
NKX3-1	NS	0.000116313	-0.078082381	0.000595124	NS
APOF	NS	0.00011659	0.078070615	0.000596384	NS
TMSB15B	NS	0.000116973	-0.078054404	0.000598185	NS
EPS15L1	NS	0.000117488	0.078032691	0.000600658	NS
TIGD2	NS	0.000117627	-0.078026813	0.000601214	NS
PPP1R3B	NS	0.000117683	0.078024482	0.000601338	NS
LZTS3	NS	0.000118538	0.077988666	0.000605388	NS
LGR4	NS	0.000118517	-0.077989559	0.000605388	NS
KRT26	NS	0.000118742	-0.077980172	0.000606268	NS
NIPBL	NS	0.000119151	0.077963139	0.000608197	NS
C1QB	NS	0.000119458	-0.077950409	0.000609603	NS
GMNC	NS	0.000119978	-0.077928887	0.000612097	NS
ARHGAP30	NS	0.000120204	0.077919558	0.000613089	NS
LACTB	NS	0.000120388	0.077911994	0.000613865	NS

MRPL14	NS	0.000120441	-0.077909832	0.000613971	NS
PACSIN3	NS	0.000120551	0.077905296	0.000614372	NS
CT47A10	NS	0.000121046	0.077884973	0.000616735	NS
SYT9	NS	0.000121469	-0.077867705	0.000618725	NS
PIH1D2	NS	0.000121631	-0.0778611	0.000619387	NS
SLC22A31	NS	0.000121871	0.07785132	0.000620448	NS
GPX7	NS	0.000122403	-0.077829731	0.000622992	NS
ELAVL2	NS	0.00012249	-0.077826238	0.000623267	NS
ZFHX2	NS	0.000122591	-0.077822117	0.000623621	NS
CD3E	NS	0.000122832	-0.077812396	0.000624681	NS
C2orf73	NS	0.000123356	-0.077791283	0.000627181	NS
BCAR1	NS	0.000123489	0.077785948	0.000627691	NS
GLRX3	NS	0.00012365	-0.07777947	0.00062819	NS
MRPS6	NS	0.000123652	-0.077779401	0.00062819	NS
ACTRT2	NS	0.000123828	0.077772336	0.000628921	NS
DYRK2	NS	0.000123906	-0.077769227	0.00062915	NS
MYRIP	NS	0.000124092	-0.077761775	0.00062993	NS
DMRT2	NS	0.000124462	0.077747015	0.000631641	NS
DUSP18	NS	0.000124659	0.077739162	0.000632476	NS
PHKA1	NS	0.000124888	0.077730027	0.000633475	NS
ZDHHC21	NS	0.000125028	-0.077724499	0.000634015	NS
SLC4A4	NS	0.000125189	-0.077718098	0.000634666	NS
KHDC4	NS	0.000125704	0.077697709	0.000637111	NS
OCM	NS	0.000125939	-0.077688455	0.000638133	NS
TIMP4	NS	0.000126354	-0.077672088	0.000640072	NS
BLOC1S4	NS	0.000126432	-0.077669042	0.000640297	NS
OR6P1	NS	0.000127498	-0.077627323	0.000645358	NS
MRPL37	NS	0.000127702	0.077619365	0.000646224	NS
GLCCI1	NS	0.000128646	-0.077582752	0.000650831	NS
CUL3	NS	0.000129904	0.077534366	0.000656734	NS
EPN3	NS	0.000129915	0.077533944	0.000656734	NS
RPE65	NS	0.00013015	-0.077524936	0.000657753	NS
TIMM23B	NS	0.00013019	0.07752343	0.00065778	NS
LCE1E	NS	0.000130415	0.077514831	0.000658746	NS
CREB3L3	NS	0.000130827	0.07749913	0.000660655	NS
EYA2	NS	0.000131081	-0.077489486	0.000661764	NS
RLBP1	NS	0.000131456	-0.077475277	0.000663483	NS
MROH9	NS	0.000132591	-0.077432452	0.000669039	NS
TMPRSS6	NS	0.000132688	-0.077428805	0.000669354	NS
ACOT7	NS	0.000132846	0.077422896	0.000669974	NS
SIGLECL1	NS	0.000132999	-0.07741715	0.000670573	NS
SSX2	NS	0.000133075	-0.07741432	0.000670779	NS
MIA3	NS	0.000133208	0.077409335	0.000671276	NS
CCN5	NS	0.000133514	0.077397879	0.000672625	NS
SLC25A29	NS	0.000133545	0.077396735	0.000672625	NS
NFATC2IP	NS	0.000133642	0.077393115	0.000672939	NS
TDRKH	NS	0.000134031	-0.077378639	0.000674721	NS
ANG	NS	0.0001343	-0.07736864	0.0006759	NS
PIM1	NS	0.00013465	0.077355655	0.000677487	NS
FBN2	NS	0.000134708	-0.077353514	0.000677602	NS
KCNJ13	NS	0.000135772	-0.077314277	0.000682777	NS
FAP	NS	0.000135815	-0.077312706	0.000682815	NS
ABCA13	NS	0.000136239	0.077297165	0.000684768	NS
TFB1M	NS	0.000136335	-0.077293635	0.000685075	NS
LNX1	NS	0.000136421	-0.0772905	0.000685283	NS

CST8	NS	0.000136448	-0.077289527	0.000685283	NS
EPHA6	NS	0.000136976	0.077270256	0.000687757	NS
MRPL12	NS	0.000137252	-0.077260185	0.000688967	NS
ZNF236	NS	0.000137525	0.077250271	0.000690158	NS
MMP9	NS	0.000138071	0.077230491	0.000692719	NS
FNDC9	NS	0.000138536	-0.077213725	0.000694864	NS
LRRC69	NS	0.000138571	-0.077212463	0.000694864	NS
TGM7	NS	0.000139011	0.077196619	0.000696886	NS
CGB8	NS	0.000139046	0.077195367	0.000696886	NS
APOOL	NS	0.000139159	-0.0771913	0.000697273	NS
VMO1	NS	0.000140197	-0.077154165	0.000702293	NS
CRABP2	NS	0.000140469	-0.077144482	0.000703473	NS
MCF2L2	NS	0.000140568	0.077140974	0.000703785	NS
SLC26A5	NS	0.000141046	-0.077124009	0.000705995	NS
ACTA2	NS	0.000141083	-0.077122704	0.000705997	NS
APOL6	NS	0.000141248	-0.077116857	0.000706641	NS
ZCCHC2	NS	0.000141989	0.077090698	0.000710164	NS
CHP2	NS	0.000142185	0.077083779	0.000710963	NS
C19orf85	NS	0.000142426	0.077075314	0.000711984	NS
EPX	NS	0.000142607	-0.077068977	0.000712702	NS
SLC19A1	NS	0.000143233	0.077047044	0.000715649	NS
ASAH2	NS	0.000143475	-0.077038614	0.000716671	NS
RAB11FIP2	NS	0.000144018	0.077019694	0.000719014	NS
C10orf62	NS	0.000144001	0.077020289	0.000719014	NS
GPR21	NS	0.000144376	-0.077007265	0.000720616	NS
GCM1	NS	0.000144611	-0.076999123	0.000721603	NS
NRAP	NS	0.000145123	-0.076981428	0.000723971	NS
CENPVL3	NS	0.000145464	0.076969681	0.000725485	NS
MRPL57	NS	0.000145724	-0.076960756	0.000726592	NS
TNFRSF18	NS	0.000146307	0.076940735	0.000729144	NS
MUC12	NS	0.000146332	0.076939896	0.000729144	NS
GIF2-RAB5I	NS	0.000146349	0.07693932	0.000729144	NS
EPHB1	NS	0.000146796	-0.076924043	0.000731183	NS
PLAC9	NS	0.000146977	-0.076917862	0.000731897	NS
ADGRL1	NS	0.000147381	0.076904116	0.000733718	NS
MOB3A	NS	0.000148076	0.076880531	0.000736989	NS
GUCD1	NS	0.000148256	0.076874417	0.00073742	NS
CDK20	NS	0.000148276	0.07687374	0.00073742	NS
IFITM5	NS	0.000148229	0.076875341	0.00073742	NS
RLIM	NS	0.000148352	0.076871183	0.000737606	NS
OR2B11	NS	0.000148687	0.076859861	0.000739084	NS
TXNDC2	NS	0.000148877	-0.076853454	0.000739839	NS
WASF3	NS	0.00014896	-0.076850678	0.000740058	NS
GNE	NS	0.000149244	0.076841118	0.00074128	NS
SLC7A2	NS	0.000149635	-0.076827985	0.000743033	NS
PLCG1	NS	0.000149755	-0.076823968	0.000743438	NS
C9orf64	NS	0.000149885	-0.07681963	0.00074389	NS
PRSS38	NS	0.000150065	0.076813591	0.000744595	NS
ZBBX	NS	0.000150635	-0.076794594	0.000747228	NS
CD14	NS	0.000150729	-0.076791435	0.000747507	NS
MDFI	NS	0.000151128	0.076778181	0.000749291	NS
ZNF781	NS	0.000151236	0.076774605	0.000749634	NS
GRPEL2	NS	0.000151355	-0.076770642	0.000750033	NS
ANGPT1	NS	0.000151421	-0.076768465	0.000750167	NS
PRSS55	NS	0.000151827	0.076755033	0.000751984	NS

CCDC82	NS	0.000151975	0.076750141	0.000752525	NS
KIF17	NS	0.000152097	0.07674611	0.000752937	NS
CRYBB2	NS	0.00015216	-0.076744025	0.000753057	NS
FAM114A2	NS	0.000152262	-0.07674065	0.00075337	NS
KRTAP6-2	NS	0.000152732	0.076725174	0.000755502	NS
LSM6	NS	0.000152814	-0.076722485	0.000755521	NS
POU3F2	NS	0.000152806	0.076722756	0.000755521	NS
WEE1	NS	0.000153039	0.076715082	0.000756442	NS
ST3GAL6	NS	0.000153388	0.07670365	0.000757972	NS
STRN4	NS	0.000153906	0.076686712	0.000759983	NS
GID4	NS	0.000153899	0.076686946	0.000759983	NS
MBL2	NS	0.000153913	-0.076686488	0.000759983	NS
CARTPT	NS	0.000154098	-0.076680443	0.000760631	NS
USP17L11	NS	0.000154123	-0.076679645	0.000760631	NS
TCEA3	NS	0.000154645	-0.076662635	0.000763015	NS
PRSS53	NS	0.000155981	-0.0766194	0.00076941	NS
KCNJ1	NS	0.000156266	-0.076610223	0.000770619	NS
NIFK	NS	0.000156906	-0.076589668	0.000773577	NS
C1QTNF6	NS	0.000157591	0.076567724	0.000776761	NS
MYZAP	NS	0.000157705	-0.076564085	0.000777125	NS
ARL13B	NS	0.000157979	-0.076555361	0.000778275	NS
SPECC1	NS	0.000158383	0.076542521	0.000780065	NS
HIP1	NS	0.00015877	0.076530222	0.000781774	NS
CD274	NS	0.000158969	-0.076523932	0.000782553	NS
FAM13A	NS	0.000159502	0.076507067	0.000784979	NS
EIF4E1B	NS	0.000159568	0.076504984	0.000785104	NS
IL31	NS	0.000159697	0.07650091	0.00078554	NS
AS3MT	NS	0.000160738	0.076468187	0.000790459	NS
CCNT1	NS	0.000161004	0.076459866	0.000791565	NS
PLIN2	NS	0.000161702	0.076438073	0.000794592	NS
C1QBP	NS	0.00016206	-0.076426914	0.000796151	NS
SLC13A2	NS	0.000163431	0.076384435	0.000802684	NS
PCIF1	NS	0.000163581	0.076379819	0.000803012	NS
DSC2	NS	0.000163572	-0.076380091	0.000803012	NS
FOSB	NS	0.000163685	0.076376617	0.000803318	NS
TAS2R38	NS	0.000164049	-0.076365413	0.000804901	NS
CCDC186	NS	0.000164119	0.076363255	0.000805041	NS
TAF11L14	NS	0.000164195	0.076360934	0.000805208	NS
MT-ND4	NS	0.000164618	0.076347941	0.00080708	NS
PCDH8	NS	0.000164879	0.076339947	0.000808155	NS
IFNB1	NS	0.000164952	-0.076337733	0.000808305	NS
KRT33A	NS	0.000165014	-0.076335815	0.000808408	NS
ACTR3	NS	0.000165116	0.076332715	0.000808495	NS
MPV17L2	NS	0.000165113	0.076332813	0.000808495	NS
MTFR2	NS	0.000165354	-0.076325432	0.000809458	NS
SIGIRR	NS	0.000165421	0.076323394	0.000809581	NS
TTC30B	NS	0.00016563	-0.076317029	0.000810397	NS
GFAP	NS	0.000165943	0.076307486	0.000811726	NS
C20orf141	NS	0.00016609	0.076303023	0.000812239	NS
TMEM52B	NS	0.000166323	0.076295953	0.000812966	NS
FCAR	NS	0.000166307	0.076296446	0.000812966	NS
SLC35E1	NS	0.000166406	0.076293434	0.000813167	NS
TFDP2	NS	0.000166593	0.076287771	0.000813874	NS
UIMC1	NS	0.000166648	0.07628609	0.00081394	NS
FYCO1	NS	0.000166947	0.076277056	0.000815192	NS

RTL10	NS	0.000167142	-0.076271153	0.00081594	NS
OPN1MW3	NS	0.000167661	0.076255496	0.000818267	NS
DC1B-GALNT	NS	0.000167798	0.076251357	0.000818731	NS
PRAMEF13	NS	0.000168074	-0.076243068	0.000819869	NS
ZNF326	NS	0.000168169	0.076240215	0.000820126	NS
SPRY1	NS	0.000168225	-0.076238522	0.000820194	NS
DZANK1	NS	0.000169383	0.07620387	0.000825215	NS
HAAO	NS	0.000169379	0.076204001	0.000825215	NS
UBTFL1	NS	0.000169367	-0.076204354	0.000825215	NS
PTH2R	NS	0.000169489	-0.076200714	0.000825523	NS
CLPTM1L	NS	0.000169803	0.076191368	0.000826843	NS
DCAF15	NS	0.000170131	0.076181619	0.000828231	NS
TMEM265	NS	0.000170391	-0.076173874	0.000829293	NS
EHD1	NS	0.000170743	0.07616344	0.000830797	NS
THRAP3	NS	0.000170893	0.076159016	0.000831315	NS
CCND2	NS	0.000171157	0.076151201	0.000832392	NS
NDUFAF6	NS	0.000172824	-0.076102177	0.000840288	NS
DOCK10	NS	0.000173183	0.076091677	0.000841822	NS
ARHGAP17	NS	0.000173401	0.076085315	0.000842669	NS
PRDM8	NS	0.000173533	0.076081464	0.000842888	NS
TMEM125	NS	0.00017349	0.076082729	0.000842888	NS
SLC16A8	NS	0.000173686	0.076076997	0.00084342	NS
WRNIP1	NS	0.000174739	-0.07604641	0.000848107	NS
HIBADH	NS	0.000174999	-0.076038893	0.000848976	NS
LSM5	NS	0.000175006	-0.076038686	0.000848976	NS
SAMD7	NS	0.000175185	0.076033517	0.00084963	NS
C8orf89	NS	0.000175262	-0.076031279	0.000849793	NS
SH2D6	NS	0.000175545	0.076023122	0.000850949	NS
LIM2	NS	0.000175979	0.07601061	0.000852841	NS
TANK	NS	0.000176334	0.076000409	0.000854346	NS
COG7	NS	0.000177289	-0.075973035	0.000858759	NS
GULP1	NS	0.000177912	-0.075955255	0.000861562	NS
ELL2	NS	0.000178292	0.075944437	0.000863187	NS
RAB42	NS	0.000178849	0.075928629	0.000865666	NS
TAS2R8	NS	0.000178982	-0.075924852	0.000866095	NS
TEX44	NS	0.000179123	0.075920854	0.000866561	NS
MAP3K2	NS	0.000179458	0.075911398	0.000867962	NS
ANXA13	NS	0.000179517	0.075909714	0.000868033	NS
PODXL	NS	0.000179706	-0.075904383	0.000868636	NS
CSAG2	NS	0.000179732	0.075903664	0.000868636	NS
CHMP4C	NS	0.000179906	0.075898753	0.00086926	NS
RBMS2	NS	0.000180229	0.075889659	0.000870603	NS
FBXL19	NS	0.000180396	0.075884958	0.000871193	NS
ARNTL	NS	0.00018087	0.075871644	0.000873264	NS
AQP9	NS	0.000181071	0.075866003	0.000874018	NS
GLP2R	NS	0.000181392	-0.075857027	0.000875347	NS
RASGRF2	NS	0.00018247	-0.075826937	0.000880113	NS
TIGD7	NS	0.000182577	-0.075823957	0.000880411	NS
CHL1	NS	0.000182716	-0.075820111	0.000880639	NS
SYAP1	NS	0.000182708	0.075820333	0.000880639	NS
DNAJC10	NS	0.000182977	0.075812863	0.000881239	NS
FBXW8	NS	0.000182972	-0.075812993	0.000881239	NS
KLRF2	NS	0.000182906	-0.075814828	0.000881239	NS
KRTAP3-3	NS	0.000183214	-0.07580628	0.000882163	NS
LYPD8	NS	0.000183428	-0.075800356	0.000882754	NS

CD80	NS	0.000183633	-0.075794686	0.000883081	NS
IGSF1	NS	0.00018359	0.07579587	0.000883081	NS
ZSCAN23	NS	0.000183556	-0.075796809	0.000883081	NS
CUEDC1	NS	0.000183812	0.075789748	0.000883721	NS
PRKD3	NS	0.000183886	0.075787701	0.000883857	NS
ATRNL1	NS	0.000184003	-0.075784471	0.0008842	NS
TSPY4	NS	0.000184572	-0.075768773	0.000886717	NS
CYP4A22	NS	0.000186771	0.075708593	0.000896558	NS
GPR146	NS	0.000186788	-0.075708133	0.000896558	NS
JMJD7	NS	0.000186806	-0.075707645	0.000896558	NS
RALYL	NS	0.000186894	-0.075705255	0.000896758	NS
YTHDF2	NS	0.000187004	0.075702266	0.00089684	NS
GRINA	NS	0.000187195	0.07569706	0.000897537	NS
CKMT1B	NS	0.000187297	-0.075694301	0.000897802	NS
CARD16	NS	0.00018742	-0.075690972	0.000898167	NS
LSM1	NS	0.000187759	-0.075681767	0.000899572	NS
DNASE2B	NS	0.000187815	-0.075680267	0.000899615	NS
GUCY2F	NS	0.00018794	-0.075676873	0.000899993	NS
GPR173	NS	0.000188028	0.075674488	0.000900192	NS
HEPACAM	NS	0.00018873	-0.075655548	0.000903327	NS
CCDC42	NS	0.000188963	0.075649264	0.00090422	NS
NHLRC4	NS	0.000189618	0.075631668	0.000907129	NS
ZNF485	NS	0.000190188	-0.075616387	0.000909633	NS
JADE2	NS	0.000190357	-0.075611875	0.000910215	NS
AMDHD2	NS	0.000190838	0.075599029	0.00091229	NS
HMGXB3	NS	0.000191626	0.075578055	0.000915722	NS
ZNF410	NS	0.000191651	0.075577396	0.000915722	NS
NEUROG2	NS	0.000191724	0.075575456	0.000915845	NS
CD300LF	NS	0.000191922	0.075570195	0.000916566	NS
KLC2	NS	0.000192294	0.075560323	0.000918118	NS
LHX5	NS	0.000192791	0.075547197	0.000920261	NS
ADGRB3	NS	0.000193549	-0.0755272	0.000923653	NS
LONP2	NS	0.000193712	0.075522919	0.000924201	NS
ZNF736	NS	0.00019489	-0.075492029	0.000929592	NS
OSBPL3	NS	0.000195304	-0.075481218	0.000931336	NS
GPC6	NS	0.00019536	-0.07547974	0.000931377	NS
RRP12	NS	0.000195956	0.075464214	0.000933528	NS
WDFY1	NS	0.000195947	0.075464444	0.000933528	NS
TCF25	NS	0.000195883	0.075466106	0.000933528	NS
FBXW2	NS	0.000196069	0.075461274	0.000933837	NS
AFAP1	NS	0.000196634	-0.075446594	0.000936069	NS
TMEM191B	NS	0.0001966	-0.075447492	0.000936069	NS
TRPC7	NS	0.000197503	-0.075424111	0.000939974	NS
LDLRAD1	NS	0.000197631	0.075420818	0.000940349	NS
ATP4B	NS	0.000198891	0.075388398	0.000946111	NS
TMPO	NS	0.000199127	-0.075382347	0.000947001	NS
DHDDS	NS	0.000200366	-0.075350682	0.000952661	NS
CCDC153	NS	0.000200538	-0.075346286	0.000953247	NS
UBTD2	NS	0.000201509	-0.075321639	0.000957625	NS
TOMM6	NS	0.000202002	-0.075309148	0.000959734	NS
MS4A5	NS	0.000202529	-0.075295857	0.000961998	NS
DOC2A	NS	0.000203067	0.075282294	0.000964319	NS
PLOD3	NS	0.000203417	0.0752735	0.000965743	NS
MYOT	NS	0.000203597	-0.07526898	0.000966361	NS
FN3K	NS	0.000203742	0.075265336	0.000966813	NS

RNASEH1	NS	0.000203825	-0.075263251	0.000966971	NS
DYNLRB1	NS	0.000203956	-0.075259965	0.000967355	NS
ADORA2A	NS	0.000204278	0.075251917	0.000968405	NS
MAGEA1	NS	0.000204228	0.075253161	0.000968405	NS
DCTN4	NS	0.000205268	0.075227188	0.000972863	NS
ANKRD28	NS	0.000206208	0.075203834	0.000976838	NS
USP17L10	NS	0.000206205	-0.075203902	0.000976838	NS
METTL16	NS	0.000206884	-0.075187098	0.000979417	NS
TCF19	NS	0.000206841	-0.075188164	0.000979417	NS
SLC25A46	NS	0.000206904	-0.075186597	0.000979417	NS
EPCAM	NS	0.000207212	0.075178995	0.000980634	NS
LY6G6C	NS	0.000207375	-0.075174959	0.000980928	NS
DGKK	NS	0.000207368	-0.075175138	0.000980928	NS
TLR1	NS	0.000207802	0.07516444	0.000982707	NS
KLF10	NS	0.000208391	0.075149973	0.000985109	NS
SH2B2	NS	0.000208412	0.075149446	0.000985109	NS
METTL25	NS	0.000208539	-0.075146323	0.00098547	NS
RGS4	NS	0.000208919	-0.075137014	0.000987023	NS
EVX1	NS	0.000209127	0.075131923	0.000987764	NS
GALM	NS	0.000209284	-0.075128071	0.000988026	NS
TMEM132E	NS	0.000209276	0.075128262	0.000988026	NS
IL2RG	NS	0.000210163	0.07510661	0.000991624	NS
IRF7	NS	0.0002102	0.075105715	0.000991624	NS
MRGPRX3	NS	0.000211819	0.075066415	0.00099902	NS
SARAF	NS	0.000212026	0.075061431	0.000999749	NS
TCEAL4	NS	0.000212209	-0.075057013	0.001000365	NS
ADH4	NS	0.00021226	-0.075055781	0.001000365	NS
SRPRA	NS	0.000212724	0.075044583	0.001002309	NS
FAF1	NS	0.000212935	-0.075039493	0.001002817	NS
CIBAR1	NS	0.00021293	-0.075039615	0.001002817	NS
C3orf38	NS	0.000214217	-0.075008743	0.001008607	NS
HACD3	NS	0.000214956	-0.074991089	0.00101184	NS
PHF20L1	NS	0.00021577	0.074971713	0.001015424	NS
FBL	NS	0.000216896	-0.074945016	0.001020227	NS
MYRFL	NS	0.000217131	-0.074939464	0.001021083	NS
HDAC1	NS	0.00021738	0.07493358	0.001022007	NS
SAXO2	NS	0.000218468	0.074907947	0.001026626	NS
L1CAM	NS	0.000218464	0.074908058	0.001026626	NS
UQCRQ	NS	0.000218866	-0.074898606	0.001028247	NS
ZNF565	NS	0.000219234	0.074889982	0.001029726	NS
RNASEL	NS	0.000219758	-0.074877745	0.001031933	NS
CNBD2	NS	0.000220213	-0.074867114	0.001033821	NS
TRIM15	NS	0.000220983	-0.074849193	0.001037183	NS
LIPT1	NS	0.000221202	0.074844102	0.00103796	NS
PLVAP	NS	0.000221335	0.074841017	0.001038332	NS
ZBTB8A	NS	0.000222194	0.074821128	0.001042108	NS
DMRT1	NS	0.000223659	0.074787342	0.001048728	NS
ATL1	NS	0.00022395	0.074780669	0.001049836	NS
TGIF2LX	NS	0.000224424	-0.074769793	0.001051805	NS
CPB1	NS	0.000224549	-0.074766946	0.001052133	NS
VTCN1	NS	0.000224793	-0.074761347	0.001053022	NS
ZNF229	NS	0.000224847	-0.074760114	0.001053022	NS
VEZF1	NS	0.000225956	0.074734828	0.001057957	NS
CNTNAP3B	NS	0.000226547	0.074721385	0.001060469	NS
UNC5C	NS	0.000226672	-0.074718539	0.0010608	NS

CIART	NS	0.000228032	0.074687766	0.001066906	NS
PPP1R3E	NS	0.000228171	0.074684635	0.001067297	NS
HJV	NS	0.000228645	0.074673947	0.001069258	NS
LMO7	NS	0.000228821	-0.074670001	0.001069819	NS
CNOT3	NS	0.000229418	0.074656576	0.001072095	NS
OR2Y1	NS	0.000229546	-0.074653707	0.001072434	NS
CALML3	NS	0.000229961	0.07464441	0.001074113	NS
CFAP210	NS	0.000230544	-0.074631362	0.001076579	NS
SLC30A5	NS	0.000230781	-0.074626075	0.001077425	NS
RING1	NS	0.000231371	0.074612922	0.001079919	NS
LUZP2	NS	0.000232036	0.074598147	0.00108276	NS
SLC37A4	NS	0.000233249	-0.074571285	0.001088158	NS
RIMS4	NS	0.000233357	0.074568893	0.001088401	NS
FOXR2	NS	0.000234617	-0.074541152	0.001094011	NS
KCND2	NS	0.000235436	0.074523177	0.001097568	NS
PANX1	NS	0.000235759	-0.074516104	0.00109881	NS
PTK7	NS	0.000235982	-0.07451123	0.001099584	NS
MYO5A	NS	0.000236386	0.07450241	0.001101201	NS
PTF1A	NS	0.000237255	0.074483483	0.001104984	NS
RPS16	NS	0.000238976	0.074446202	0.001112729	NS
MARK4	NS	0.000239297	0.074439265	0.001113958	NS
PCP2	NS	0.000239722	-0.074430111	0.001115667	NS
TBC1D2	NS	0.000239984	0.074424479	0.001116616	NS
TMEM47	NS	0.000240042	-0.074423224	0.001116619	NS
SNAPC1	NS	0.000240229	0.074419203	0.001117221	NS
CLRN1	NS	0.00024071	-0.074408873	0.00111919	NS
INTS14	NS	0.000240887	-0.074405074	0.001119744	NS
OPA3	NS	0.000241369	-0.074394767	0.001121712	NS
BBS4	NS	0.000243537	-0.074348565	0.001131519	NS
SHISAL2A	NS	0.000243617	0.074346877	0.001131617	NS
PDZRN3	NS	0.000243748	-0.074344089	0.001131684	NS
ZNF221	NS	0.000243748	0.074344088	0.001131684	NS
ZNF45	NS	0.000243972	-0.074339349	0.001132451	NS
PNPLA8	NS	0.00024429	0.074332618	0.001133655	NS
PRPF18	NS	0.000244555	-0.074327004	0.001134615	NS
ATL2	NS	0.000244859	0.074320598	0.00113575	NS
PCGF1	NS	0.000245662	-0.074303667	0.001138931	NS
UBE2V1	NS	0.000245992	0.074296738	0.001140185	NS
PRDM2	NS	0.000247004	0.074275504	0.001144603	NS
LIN28A	NS	0.000247384	0.074267551	0.00114609	NS
ASIP	NS	0.000247708	-0.074260773	0.001147318	NS
OTOP2	NS	0.000248004	0.074254606	0.001148412	NS
THSD7A	NS	0.000248234	-0.074249812	0.001149202	NS
C14orf119	NS	0.000248347	-0.074247457	0.00114945	NS
FGD3	NS	0.000248522	0.074243818	0.001149984	NS
FAAH2	NS	0.000248861	-0.074236747	0.001151282	NS
ADAT2	NS	0.000248989	0.074234099	0.001151596	NS
ZNF280B	NS	0.000249522	-0.074223041	0.001153784	NS
GSTA2	NS	0.000250022	-0.074212666	0.001155823	NS
KCNB2	NS	0.000250218	-0.074208618	0.001156451	NS
IQUB	NS	0.000250739	-0.074197849	0.001158583	NS
RSC1A1	NS	0.000251197	-0.074188393	0.001160424	NS
LAD1	NS	0.000252192	0.074167931	0.001164742	NS
DNM1	NS	0.000252782	0.074155843	0.001167185	NS
SYNCRIP	NS	0.000254848	0.074113675	0.001176444	NS

RXFP4	NS	0.000255068	0.074109202	0.001177179	NS
ELK3	NS	0.000256047	0.074089344	0.001181417	NS
PRODH2	NS	0.000257069	0.074068685	0.001185852	NS
RPAP1	NS	0.000257983	0.074050285	0.001189784	NS
MED13	NS	0.000258202	0.074045896	0.001190508	NS
MFNG	NS	0.0002588	0.074033887	0.001192984	NS
PLPPR1	NS	0.000259669	-0.074016496	0.001196705	NS
F7	NS	0.000260496	0.074000004	0.001200229	NS
MDGA1	NS	0.000261026	0.07398946	0.001202384	NS
ARSK	NS	0.000261312	-0.073983772	0.001203416	NS
MFAP1	NS	0.000261494	0.073980159	0.001203968	NS
SHF	NS	0.000262364	0.073962925	0.001207398	NS
DNMT3L	NS	0.000262303	0.073964131	0.001207398	NS
ZNF593	NS	0.000262618	-0.073957896	0.001208281	NS
RAG1	NS	0.00026294	-0.073951537	0.001209474	NS
PRY2	NS	0.000263201	-0.073946382	0.001210388	NS
MRPL47	NS	0.000263983	-0.073930989	0.001213693	NS
TUT7	NS	0.000264489	0.07392103	0.001215734	NS
ALG1L2	NS	0.0002648	0.073914933	0.001216873	NS
HAUS1	NS	0.000264994	-0.073911122	0.001217477	NS
BMX	NS	0.000265461	-0.073901977	0.001219277	NS
PKN1	NS	0.000265512	0.073900983	0.001219277	NS
TOP2B	NS	0.000265602	0.073899224	0.0012194	NS
SPPL2A	NS	0.000265732	0.073896687	0.001219707	NS
CNRIP1	NS	0.000265988	-0.073891672	0.001220595	NS
MFGE8	NS	0.000266543	-0.073880854	0.001222849	NS
TNPO1	NS	0.00026683	0.073875262	0.001223876	NS
CADM1	NS	0.000266985	-0.07387224	0.001224297	NS
HSPBAP1	NS	0.000267111	0.073869781	0.001224587	NS
ADAMTS7	NS	0.000269241	0.073828492	0.00123406	NS
CDK15	NS	0.000269637	-0.073820863	0.001235579	NS
RREB1	NS	0.000269752	0.073818635	0.001235816	NS
RNF138	NS	0.000270216	0.073809705	0.001237647	NS
ZNF574	NS	0.000270829	0.073797908	0.001239943	NS
ZNF600	NS	0.000270845	-0.073797602	0.001239943	NS
TYK2	NS	0.000271784	0.0737796	0.001243948	NS
PIMREG	NS	0.000271904	0.073777297	0.001244204	NS
PTGER3	NS	0.000272077	-0.073773992	0.001244692	NS
ZNF827	NS	0.00027214	-0.073772799	0.001244692	NS
EIF5	NS	0.000272213	0.073771397	0.001244733	NS
TTI1	NS	0.000273241	-0.073751773	0.001249141	NS
RBM8A	NS	0.000273941	0.073738462	0.001252044	NS
PLEKHN1	NS	0.000274037	-0.073736633	0.001252188	NS
AGAP3	NS	0.000274266	0.073732295	0.001252641	NS
KIRREL1	NS	0.000274236	0.073732853	0.001252641	NS
OSTC	NS	0.000274458	-0.073728658	0.00125322	NS
GPC4	NS	0.000275599	-0.073707056	0.001258134	NS
TMPRSS2	NS	0.000276014	-0.073699211	0.001259734	NS
HSD17B1	NS	0.000276571	0.073688707	0.001261889	NS
COL6A5	NS	0.000276617	-0.07368785	0.001261889	NS
SH2D3C	NS	0.000276721	0.073685882	0.001262069	NS
ECSCR	NS	0.000277161	0.073677617	0.001263775	NS
RBAK	NS	0.000277248	-0.073675984	0.001263874	NS
PNMA6E	NS	0.000277671	0.073668033	0.001265506	NS
GCDH	NS	0.000278469	-0.073653084	0.001268843	NS

CERT1	NS	0.00027872	0.073648381	0.00126969	NS
RIBC1	NS	0.000279009	0.073642982	0.001270707	NS
TMPRSS5	NS	0.000279188	-0.07363965	0.001271221	NS
RXRA	NS	0.000279612	0.073631741	0.001272852	NS
ACOXL	NS	0.000280376	-0.073617519	0.00127603	NS
TNFRSF11B	NS	0.000280566	0.073613987	0.001276594	NS
DIPK1C	NS	0.000280814	0.07360937	0.00127721	NS
KRTAP20-4	NS	0.000281323	-0.073599927	0.00127914	NS
OR2M4	NS	0.000281777	0.073591531	0.0012809	NS
CENATAC	NS	0.000281975	0.07358786	0.001281501	NS
RAB40A	NS	0.00028401	-0.07355036	0.001290444	NS
ZFAND3	NS	0.000284262	0.073545728	0.001291287	NS
GOLGA6B	NS	0.00028654	0.073504074	0.001301024	NS
GTF2A1L	NS	0.000286516	-0.073504503	0.001301024	NS
MAP10	NS	0.000287407	-0.073488294	0.001304657	NS
PTCRA	NS	0.000288816	-0.073462763	0.001310744	NS
ZNF804B	NS	0.000289196	-0.073455889	0.001312162	NS
UPK3A	NS	0.000290017	-0.073441078	0.001315271	NS
ZNF589	NS	0.000289996	-0.07344147	0.001315271	NS
BAIAP3	NS	0.000291072	0.073422113	0.001319745	NS
RSPH4A	NS	0.000291463	-0.073415106	0.001321206	NS
OR4N2	NS	0.00029157	-0.073413183	0.001321383	NS
IFT20	NS	0.000292746	0.07339215	0.001326401	NS
ABITRAM	NS	0.000293414	-0.07338022	0.00132912	NS
CYSLTR1	NS	0.00029355	-0.073377798	0.001329424	NS
TUBB6	NS	0.000294178	0.073366633	0.001331643	NS
ARPIN-AP3S2	NS	0.000294125	0.073367569	0.001331643	NS
TCHH	NS	0.000294685	0.073357621	0.001333628	NS
LSM10	NS	0.000295059	-0.073350992	0.001335008	NS
CCDC183	NS	0.000295466	-0.073343789	0.001336535	NS
OR1L3	NS	0.000296236	-0.073330168	0.001339707	NS
GSDMC	NS	0.000296607	-0.073323615	0.001341073	NS
KLC1	NS	0.000297201	0.073313146	0.001343445	NS
CST11	NS	0.000297697	-0.07330443	0.00134537	NS
RTL8A	NS	0.000298508	0.073290186	0.001348722	NS
DDTL	NS	0.000299016	0.073281299	0.001350699	NS
TNIP3	NS	0.000300704	0.073251811	0.001358011	NS
RAD23B	NS	0.000301417	0.073239423	0.00136091	NS
TMEM176B	NS	0.000301588	0.07323645	0.001361095	NS
MRPL32	NS	0.000301598	-0.073236269	0.001361095	NS
NEDD1	NS	0.000302049	-0.07322845	0.001362811	NS
OR10H2	NS	0.000302915	0.07321345	0.0013664	NS
CHST14	NS	0.000303474	-0.073203785	0.001368604	NS
OR7D2	NS	0.000303561	0.073202289	0.001368676	NS
ZNF703	NS	0.000303873	0.073196914	0.001369762	NS
PHOSPHO1	NS	0.000308743	-0.073113559	0.001391391	NS
INTS4	NS	0.000309237	-0.073105163	0.001392971	NS
IDS	NS	0.000309339	0.073103436	0.001393106	NS
OR4A16	NS	0.000309813	-0.073095405	0.001394916	NS
GDI1	NS	0.000310324	0.073086759	0.001396891	NS
BFSP2	NS	0.000310457	-0.073084504	0.001397167	NS
NXF1	NS	0.000310956	0.073076086	0.001399085	NS
PPP2R5C	NS	0.000311462	0.073067544	0.001401038	NS
ARFIP2	NS	0.000311613	-0.073065012	0.001401388	NS
ELOVL4	NS	0.000311706	-0.07306345	0.00140148	NS

CCDC198	NS	0.000312709	0.073046581	0.001405665	NS
RNF128	NS	0.000313671	-0.073030445	0.001409664	NS
SCFD2	NS	0.00031381	-0.073028127	0.001409959	NS
SPANXB1	NS	0.000314585	-0.073015171	0.001413115	NS
PPP4R3B	NS	0.000314815	0.073011334	0.001413819	NS
DDA1	NS	0.000315318	0.073002943	0.001415751	NS
TXN	NS	0.000315944	-0.072992531	0.001418232	NS
ARMC1	NS	0.000316116	-0.072989679	0.001418673	NS
FBXO28	NS	0.000316871	0.072977147	0.001421731	NS
ARPC1B	NS	0.00031839	0.072952006	0.001428219	NS
CHRM1	NS	0.000319039	0.072941303	0.001430799	NS
FAM216A	NS	0.00031915	-0.072939482	0.001430963	NS
SPDYE14	NS	0.000319668	0.072930962	0.001432952	NS
YY1	NS	0.000320444	0.07291821	0.001436099	NS
TRIR	NS	0.000320678	0.072914373	0.001436815	NS
IFT74	NS	0.00032108	0.072907777	0.001438286	NS
DYRK1A	NS	0.000322012	0.072892539	0.001442126	NS
CTDP1	NS	0.000323727	0.072864599	0.00144941	NS
AAGAB	NS	0.000323788	0.072863601	0.00144941	NS
PRKAG1	NS	0.000325224	0.072840327	0.001455162	NS
CCDC148	NS	0.000325814	-0.072830781	0.001457467	NS
ABHD5	NS	0.000325996	0.072827844	0.001457943	NS
FAM102A	NS	0.00032608	0.072826496	0.00145798	NS
TMEM208	NS	0.000326186	0.072824771	0.001458121	NS
NRCAM	NS	0.000327439	-0.07280459	0.001463383	NS
TSPY1	NS	0.00032866	-0.072784998	0.001468499	NS
SLC4A9	NS	0.000329091	-0.072778092	0.001470087	NS
TGFB2	NS	0.000329764	-0.072767338	0.001472751	NS
SCYGR1	NS	0.000329989	0.072763733	0.00147342	NS
OPN3	NS	0.000330472	-0.072756037	0.001474014	NS
TTLL12	NS	0.000330579	-0.072754322	0.001474014	NS
TMIGD2	NS	0.000330537	0.072755001	0.001474014	NS
ABHD15	NS	0.000330438	-0.072756571	0.001474014	NS
CTXND1	NS	0.000330389	-0.072757353	0.001474014	NS
MAMDC2	NS	0.000332042	-0.072731062	0.001479513	NS
PJA2	NS	0.000331944	0.072732617	0.001479513	NS
GOLGA8B	NS	0.000331966	0.072732261	0.001479513	NS
KRTAP11-1	NS	0.000332349	-0.072726194	0.001480539	NS
CD86	NS	0.00033302	0.072715568	0.001483186	NS
SMG8	NS	0.000333828	-0.072702791	0.001486443	NS
CT45A8	NS	0.0003342	-0.07269691	0.00148776	NS
CYSTM1	NS	0.000335388	0.072678209	0.001492703	NS
PTPN6	NS	0.000337007	0.072652805	0.001499565	NS
WIPF1	NS	0.000337241	0.072649143	0.001500262	NS
MICAL1	NS	0.000338299	0.072632621	0.001504622	NS
BEND3	NS	0.000340231	-0.072602573	0.001512866	NS
FFAR4	NS	0.000340309	-0.072601351	0.001512869	NS
CYRIA	NS	0.000340459	0.072599034	0.001513185	NS
PRR23A	NS	0.000341165	0.072588102	0.001515626	NS
SAMD11	NS	0.000341405	-0.072584378	0.001516	NS
SLC10A6	NS	0.000341537	-0.07258234	0.001516237	NS
BAALC	NS	0.000343445	-0.072552925	0.001524358	NS
GNAT3	NS	0.000343877	-0.072546285	0.001525925	NS
PCK2	NS	0.00034441	-0.072538105	0.00152794	NS
RNF25	NS	0.000347704	0.072487803	0.001542201	NS

ADAMTS5	NS	0.000347923	-0.072484487	0.001542815	NS
LRRC51	NS	0.000348052	-0.072482526	0.001543035	NS
LRRC8B	NS	0.000348326	0.072478361	0.001543897	NS
UHRF1BP1L	NS	0.000349333	0.072463103	0.001548005	NS
SMIM15	NS	0.000350325	-0.072448111	0.001552045	NS
SLCO6A1	NS	0.000350665	-0.072442972	0.001553198	NS
SPDYE9	NS	0.000353808	0.072395765	0.001566403	NS
ZHX1	NS	0.0003542	-0.072389918	0.001567776	NS
SEPTIN14	NS	0.000354671	0.072382871	0.001569148	NS
US4L-BCAP2	NS	0.00035461	0.072383794	0.001569148	NS
KRTAP20-2	NS	0.000355235	-0.072374464	0.001571283	NS
MELTF	NS	0.000356017	0.072362824	0.001574023	NS
MLLT3	NS	0.000355949	-0.072363841	0.001574023	NS
POLR2A	NS	0.000356277	0.072358963	0.001574811	NS
ENTHD1	NS	0.000356798	-0.072351224	0.001576755	NS
TBL1XR1	NS	0.00035715	0.072346001	0.001577951	NS
NSD3	NS	0.000357622	0.072339006	0.001579676	NS
SLC25A10	NS	0.000359271	0.072314655	0.001586232	NS
PNMT	NS	0.000362256	0.072270802	0.001599051	NS
OR4D2	NS	0.000362551	-0.07226649	0.001599987	NS
PDE6D	NS	0.000362998	-0.072259961	0.001601594	NS
CDHR1	NS	0.000364091	0.072244026	0.001606049	NS
CA5B	NS	0.000364917	0.072232012	0.001609326	NS
ZNF671	NS	0.000365202	-0.072227871	0.001610216	NS
ENOX1	NS	0.000365885	-0.072217961	0.001612861	NS
SP140L	NS	0.000366344	-0.072211312	0.001614517	NS
OR5P2	NS	0.000366884	-0.072203507	0.001616067	NS
NEK5	NS	0.000366946	0.072202598	0.001616067	NS
TNS4	NS	0.000367672	0.072192122	0.001618894	NS
TREML4	NS	0.000369337	0.072168143	0.001625857	NS
ANKRD60	NS	0.000370497	0.072151509	0.00163059	NS
CACNA2D2	NS	0.000370812	-0.072146993	0.001631607	NS
CLN8	NS	0.000371764	0.072133379	0.001635425	NS
ZSCAN25	NS	0.000371938	0.072130901	0.001635817	NS
METTL21A	NS	0.00037217	0.072127586	0.001636466	NS
CLDN24	NS	0.000374014	-0.072101344	0.001643827	NS
PDZK1	NS	0.000374645	0.072092392	0.001646226	NS
ERGIC1	NS	0.000374761	0.072090739	0.001646365	NS
MAGEB2	NS	0.000375328	-0.072082721	0.001648478	NS
ZUP1	NS	0.00037567	-0.072077876	0.001649607	NS
KCNIP4	NS	0.000376025	-0.072072858	0.001650792	NS
EIF2AK1	NS	0.000376429	0.072067147	0.001652192	NS
BCL2L12	NS	0.000377951	-0.072045718	0.001658492	NS
ERLIN2	NS	0.000378553	0.072037246	0.001660762	NS
RIOK1	NS	0.000378853	-0.072033035	0.001661701	NS
MT-ATP6	NS	0.000379537	0.072023458	0.00166432	NS
MAU2	NS	0.000379944	0.072017762	0.001665727	NS
ANKRD7	NS	0.000380385	-0.072011591	0.001667284	NS
RUNDC3B	NS	0.000382165	-0.071986765	0.001674707	NS
TMBIM1	NS	0.000382269	0.071985309	0.001674786	NS
THEGL	NS	0.000383166	-0.071972854	0.001678333	NS
ABCF2	NS	0.000383911	-0.071962511	0.0016806	NS
NUP107	NS	0.000383944	-0.071962062	0.0016806	NS
ANKRD66	NS	0.000383812	-0.071963884	0.0016806	NS
MALL	NS	0.000384041	0.07196071	0.001680647	NS

ANKRD24	NS	0.000385011	0.071947294	0.001684509	NS
BPIFA3	NS	0.000385192	-0.071944788	0.001684922	NS
SLC31A2	NS	0.000386124	0.071931923	0.001688618	NS
ARHGEF40	NS	0.000386681	0.07192426	0.001690287	NS
ERFE	NS	0.000386594	0.071925458	0.001690287	NS
SETDB2	NS	0.000387734	0.071909783	0.001694508	NS
IRS1	NS	0.000387908	-0.071907395	0.001694886	NS
COL8A2	NS	0.000388307	-0.071901912	0.001696249	NS
LYAR	NS	0.000389699	-0.07188286	0.001701561	NS
SOX14	NS	0.000389617	0.071883979	0.001701561	NS
RAB11FIP5	NS	0.00039139	-0.071859808	0.001708556	NS
GAGE2E	NS	0.000392611	0.071843214	0.0017135	NS
WASL	NS	0.000393778	0.0718274	0.001717818	NS
TRPC4	NS	0.000393723	-0.071828139	0.001717818	NS
CS	NS	0.000394773	0.07181394	0.001721667	NS
ASPA	NS	0.000394838	0.071813071	0.001721667	NS
FAM217A	NS	0.000396024	-0.07179708	0.001726451	NS
STK33	NS	0.000397588	-0.071776071	0.001732876	NS
KIF1A	NS	0.000397801	0.071773209	0.001733417	NS
NPVF	NS	0.000397924	-0.071771564	0.001733561	NS
LPIN3	NS	0.000398215	0.071767657	0.001734441	NS
SLN	NS	0.000398836	-0.071759352	0.001736753	NS
POM121L12	NS	0.000399517	0.071750252	0.001739328	NS
PADI2	NS	0.000399958	0.071744364	0.001740857	NS
GADD45GIP1	NS	0.00040094	0.071731278	0.00174474	NS
LBHD1	NS	0.000401519	-0.071723586	0.001746472	NS
DHFR2	NS	0.000401519	-0.071723588	0.001746472	NS
GORAB	NS	0.000401692	-0.071721291	0.001746831	NS
NUAK1	NS	0.000405301	-0.071673537	0.001762133	NS
UBE4B	NS	0.000406383	0.071659304	0.001766439	NS
AIMP1	NS	0.00040725	0.071647931	0.00176941	NS
ARID5A	NS	0.000407189	0.071648724	0.00176941	NS
SLF2	NS	0.000407378	-0.071646253	0.001769569	NS
SPDYE8	NS	0.000408023	0.071637796	0.001771975	NS
B4GALNT2	NS	0.000408363	0.071633351	0.001773041	NS
NPIPB8	NS	0.000408452	0.071632189	0.001773041	NS
BST1	NS	0.000408991	-0.071625142	0.001774983	NS
LRRN4	NS	0.000409312	-0.071620953	0.001775977	NS
PKHD1L1	NS	0.000409734	0.07161544	0.001777412	NS
TCIM	NS	0.00041087	-0.071600644	0.001781941	NS
C20orf204	NS	0.000411069	0.071598053	0.001782406	NS
EPHB6	NS	0.000411366	-0.071594204	0.001782897	NS
BTC	NS	0.000411367	-0.071594187	0.001782897	NS
OMP	NS	0.00041159	0.071591295	0.001783463	NS
ACD	NS	0.000412699	0.071576905	0.00178747	NS
STYXL1	NS	0.000412647	-0.071577577	0.00178747	NS
CSF1R	NS	0.000413497	-0.071566585	0.001790523	NS
SUPT5H	NS	0.000413984	0.071560286	0.001792233	NS
TEDC2	NS	0.000414127	0.071558435	0.001792453	NS
FKTN	NS	0.000415601	-0.071539446	0.001798025	NS
ABR	NS	0.000415513	0.071540576	0.001798025	NS
GUCY1B1	NS	0.000416082	-0.071533257	0.001799705	NS
EFCAB5	NS	0.00041678	0.071524281	0.001802324	NS
B3GALT2	NS	0.00041696	-0.071521973	0.001802699	NS
NQO1	NS	0.000417394	-0.071516416	0.00180417	NS

CENPI	NS	0.000417783	0.071511431	0.001805449	NS
HIRIP3	NS	0.000419165	-0.071493761	0.001810208	NS
CFL1	NS	0.000419106	0.071494508	0.001810208	NS
TSPYL2	NS	0.000419638	0.071487727	0.001811846	NS
ZNF142	NS	0.000420396	0.071478059	0.001814718	NS
COMP	NS	0.000421334	0.071466139	0.001818358	NS
P3H1	NS	0.000421767	0.071460631	0.001819824	NS
FGFR4	NS	0.000422807	0.071447455	0.001823901	NS
MGAT5B	NS	0.000426867	0.071396269	0.001841005	NS
TXNDC15	NS	0.000428699	-0.071373323	0.001848082	NS
ARRDC4	NS	0.000428611	0.071374419	0.001848082	NS
EBF3	NS	0.000429494	0.071363389	0.001850686	NS
DDT	NS	0.000430225	0.071354268	0.001853208	NS
CFAP107	NS	0.000430271	0.071353703	0.001853208	NS
XAGE1B	NS	0.000431749	-0.071335318	0.001859161	NS
OR12D2	NS	0.000433066	-0.071318979	0.001864419	NS
NUDT17	NS	0.000433356	-0.071315391	0.001865252	NS
GGT5	NS	0.000433808	0.071309804	0.001866781	NS
PSG9	NS	0.000434873	-0.071296644	0.001870951	NS
ROCK2	NS	0.000435784	-0.071285423	0.001874035	NS
ANGPTL4	NS	0.000435761	-0.071285705	0.001874035	NS
PLIN4	NS	0.000435986	0.071282935	0.001874488	NS
SFXN4	NS	0.000436121	-0.071281275	0.001874652	NS
SLC16A5	NS	0.000437021	0.071270214	0.001878103	NS
SSC5D	NS	0.000438589	0.071250995	0.001884422	NS
KRT24	NS	0.000439947	-0.071234397	0.001889839	NS
CIB4	NS	0.000441076	-0.071220631	0.00189427	NS
TENT4B	NS	0.000441575	0.071214569	0.001895568	NS
MEIG1	NS	0.000441562	-0.071214721	0.001895568	NS
NMB	NS	0.000442311	-0.07120562	0.001898309	NS
IL1RN	NS	0.000443139	0.071195575	0.001901061	NS
ZNF394	NS	0.000443149	0.071195458	0.001901061	NS
C1orf146	NS	0.000444091	-0.071184053	0.001904679	NS
CLNK	NS	0.000446141	-0.071159303	0.00191305	NS
NUPR2	NS	0.000447066	0.07114817	0.001916168	NS
PCDHGA6	NS	0.000447032	-0.071148583	0.001916168	NS
ZNF783	NS	0.000448669	-0.071128939	0.00192261	NS
SLC25A17	NS	0.000449905	-0.071114139	0.001927484	NS
BTN2A2	NS	0.000451449	-0.071095724	0.001933668	NS
PLD2	NS	0.000453683	0.07106917	0.001942808	NS
IKBKG	NS	0.000454667	0.071057511	0.001946592	NS
FN1	NS	0.000455181	-0.07105143	0.001948362	NS
EIF2B2	NS	0.000455884	-0.071043127	0.001950939	NS
C7orf26	NS	0.000456891	-0.071031255	0.001954383	NS
IL20	NS	0.000456852	-0.071031718	0.001954383	NS
ISLR	NS	0.000457524	0.071023801	0.001956659	NS
GPR37	NS	0.000457691	-0.071021836	0.001956941	NS
PPM1L	NS	0.000458689	0.071010118	0.001960354	NS
OR5L2	NS	0.000458692	-0.071010079	0.001960354	NS
NXPH2	NS	0.000459507	-0.071000524	0.001963403	NS
LEF1	NS	0.000460179	-0.070992649	0.001965842	NS
GAGE12B	NS	0.000460496	-0.070988941	0.001966762	NS
FGFBP3	NS	0.0004626	-0.070964397	0.001974874	NS
NDUFS6	NS	0.000464354	-0.070943998	0.001981928	NS
LYZ	NS	0.000464724	-0.070939714	0.001983067	NS

MCAT	NS	0.000466089	-0.070923903	0.001988456	NS
BBS7	NS	0.000468019	-0.070901634	0.001996248	NS
ATP11C	NS	0.00046878	-0.070892869	0.001999056	NS
TAB1	NS	0.000469184	-0.070888232	0.001999894	NS
ADAMTS3	NS	0.000469184	-0.070888235	0.001999894	NS
ARMC10	NS	0.000470483	0.070873315	0.002004551	NS
PNP	NS	0.00047043	0.070873926	0.002004551	NS
PRDM16	NS	0.00047188	0.070857327	0.00201006	NS
IQCF6	NS	0.000472417	0.07085119	0.002011905	NS
SKA1	NS	0.000473102	0.070843373	0.002014379	NS
SBK3	NS	0.000473269	0.070841472	0.002014646	NS
GPAA1	NS	0.000475283	0.070818557	0.002022775	NS
INTS6L	NS	0.000477079	0.070798201	0.002029972	NS
MRPL43	NS	0.000477231	-0.070796482	0.002030173	NS
DEFB126	NS	0.000477989	-0.070787919	0.002032949	NS
ARMT1	NS	0.000478225	-0.070785253	0.002033506	NS
ADGRD2	NS	0.000478334	0.070784015	0.002033525	NS
SPAG11A	NS	0.000480148	-0.070763577	0.002040789	NS
PZP	NS	0.000480784	-0.070756438	0.00204304	NS
EGLN1	NS	0.000482088	0.070741802	0.002048134	NS
VSX2	NS	0.000482461	0.07073762	0.00204927	NS
CLK1	NS	0.000483439	0.070726681	0.002052878	NS
TSPAN10	NS	0.000483523	0.070725745	0.002052878	NS
TSPY2	NS	0.000485143	-0.070707674	0.002059304	NS
RAPGEF1	NS	0.000485482	0.070703894	0.002060293	NS
CRIP1	NS	0.000485916	0.070699063	0.002061683	NS
NLRP10	NS	0.000487502	0.070681457	0.002067956	NS
GUCA1C	NS	0.000490186	-0.07065177	0.002078886	NS
TIMMDC1	NS	0.000491069	-0.070642034	0.002082175	NS
DGAT2L6	NS	0.000493567	-0.07061459	0.002092307	NS
NABP2	NS	0.000494026	0.070609557	0.00209288	NS
ARF1	NS	0.000493925	0.07061066	0.00209288	NS
SPATA31E1	NS	0.00049388	0.070611151	0.00209288	NS
AGAP5	NS	0.000494185	-0.070607816	0.002093095	NS
KIAA0232	NS	0.000495677	0.070591501	0.002098956	NS
EBLN2	NS	0.000495963	-0.070588384	0.002099705	NS
BEX4	NS	0.000497544	-0.070571148	0.002105941	NS
DNAAF9	NS	0.000497851	0.070567811	0.002106779	NS
TM4SF19	NS	0.000499638	0.07054841	0.002113418	NS
NRBF2	NS	0.000500161	0.070542747	0.002115167	NS
ZNF77	NS	0.000500351	-0.070540687	0.002115509	NS
SLC22A17	NS	0.000501381	0.070529555	0.002118937	NS
IL10RB	NS	0.000501328	0.070530132	0.002118937	NS
CAMTA1	NS	0.000502104	0.070521748	0.00212153	NS
CDK5	NS	0.000502928	-0.070512865	0.002124548	NS
OR7G2	NS	0.00050371	0.070504451	0.002127385	NS
ACAD10	NS	0.000504772	-0.070493037	0.002131407	NS
OR51I2	NS	0.0005053	-0.070487367	0.002133173	NS
EWSR1	NS	0.000508214	0.070456207	0.002145003	NS
OR4C13	NS	0.000508442	-0.070453769	0.0021455	NS
NFATC2	NS	0.000509458	-0.070442948	0.002149318	NS
CFC1	NS	0.000509679	0.070440602	0.00214978	NS
CCNJ	NS	0.000509808	0.070439227	0.002149856	NS
NACAD	NS	0.000511332	0.070423038	0.002155814	NS
CLK2	NS	0.000511875	0.070417286	0.002157632	NS

RNASE6	NS	0.000512266	-0.070413138	0.002158812	NS
IQCF1	NS	0.000512761	0.070407904	0.002160426	NS
LOX	NS	0.000513484	-0.070400252	0.002163004	NS
HTR1D	NS	0.000513711	0.070397861	0.002163487	NS
FAM124A	NS	0.000513902	0.070395845	0.00216382	NS
NDEL1	NS	0.000514936	0.070384941	0.002167702	NS
IRAK4	NS	0.000517051	-0.070362687	0.002176136	NS
TPD52L3	NS	0.000517418	-0.070358844	0.002177203	NS
GLIS2	NS	0.000519565	0.070336362	0.002185763	NS
C19orf12	NS	0.000520155	-0.070330203	0.002187769	NS
MFSD6L	NS	0.000520459	0.070327026	0.002188574	NS
IPPK	NS	0.000520775	0.07032373	0.002189427	NS
RIMKLB	NS	0.000522016	0.070310807	0.002194052	NS
HELZ	NS	0.000522102	0.07030991	0.002194052	NS
RSPH6A	NS	0.000522425	0.070306553	0.002194932	NS
JAGN1	NS	0.000522562	-0.070305126	0.002195032	NS
RXFP2	NS	0.000524177	-0.070288361	0.00220134	NS
SIRPG	NS	0.000524537	0.070284638	0.002202371	NS
SNX9	NS	0.000524871	0.070281175	0.00220282	NS
FNDC1	NS	0.000524806	-0.070281844	0.00220282	NS
ZSCAN32	NS	0.0005257	-0.070272599	0.002205821	NS
MYBL2	NS	0.000526217	0.070267262	0.002207509	NS
SLC9A3R2	NS	0.000529057	0.070238	0.002218946	NS
KLHL31	NS	0.00052978	-0.070230575	0.002221497	NS
ZNF140	NS	0.00053034	-0.070224833	0.002223363	NS
NGFR	NS	0.000530472	0.070223479	0.002223435	NS
NEBL	NS	0.000530887	-0.070219233	0.002224208	NS
POP7	NS	0.000530822	-0.070219891	0.002224208	NS
GPATCH1	NS	0.000531954	-0.070208313	0.002228197	NS
C19orf44	NS	0.00053216	0.070206206	0.002228578	NS
RWDD4	NS	0.000534045	-0.070186978	0.002235987	NS
SMTNL1	NS	0.000534418	-0.070183171	0.002237068	NS
FAM151A	NS	0.000536088	-0.070166206	0.00224357	NS
USP17L20	NS	0.000536803	-0.070158945	0.00224608	NS
ZNF74	NS	0.000536979	-0.070157164	0.00224633	NS
BTNL3	NS	0.000537312	-0.070153791	0.00224679	NS
SHARPIN	NS	0.000537321	0.070153696	0.00224679	NS
CDC42SE2	NS	0.000537807	0.070148775	0.002247851	NS
EGFLAM	NS	0.000537771	-0.070149139	0.002247851	NS
MDM4	NS	0.000539068	0.07013603	0.002252635	NS
C1orf115	NS	0.000539423	-0.070132446	0.002253632	NS
HNRNPA1	NS	0.000539561	0.070131052	0.002253723	NS
CAPN8	NS	0.000541515	-0.070111378	0.002261394	NS
NEDD9	NS	0.000545541	0.070071035	0.002277716	NS
TGFBRAP1	NS	0.00054782	-0.070048318	0.002286739	NS
RASL12	NS	0.000549167	0.070034939	0.002291865	NS
SAMSN1	NS	0.000549501	0.070031621	0.002292766	NS
INSYN2A	NS	0.000550343	0.070023279	0.002295783	NS
BLOC1S2	NS	0.000550677	0.070019972	0.002296681	NS
PMP22	NS	0.000551243	-0.070014367	0.002298549	NS
SRPK3	NS	0.000552503	0.070001925	0.002303304	NS
USP6	NS	0.000552816	0.069998835	0.002304114	NS
SHLD3	NS	0.00055307	-0.069996332	0.002304675	NS
DNAH17	NS	0.000554987	0.06997746	0.002312167	NS
PPARGC1B	NS	0.000559542	0.069932864	0.002330642	NS

USP31	NS	0.000560636	-0.069922201	0.002334698	NS
SAMD12	NS	0.000561519	0.06991361	0.002337873	NS
KIAA1755	NS	0.000564462	-0.06988507	0.002349357	NS
ZNF474	NS	0.000564521	-0.069884508	0.002349357	NS
MAGEC3	NS	0.000566026	0.069869965	0.002355115	NS
DDX3X	NS	0.000566555	0.06986486	0.002356811	NS
SCCPDH	NS	0.000566862	-0.069861904	0.00235758	NS
KCTD7	NS	0.000567033	0.069860252	0.002357787	NS
RPL31	NS	0.000567264	0.069858025	0.002358241	NS
TRNAU1AP	NS	0.000567612	-0.069854679	0.00235918	NS
PLA2G1B	NS	0.000567924	0.06985168	0.002359968	NS
NFILZ	NS	0.000568297	0.06984809	0.002361013	NS
DDI1	NS	0.000568607	-0.069845112	0.002361793	NS
UTP11	NS	0.000570618	-0.069825825	0.002369636	NS
ADPRM	NS	0.000571172	-0.069820521	0.002371429	NS
KLHDC2	NS	0.000571372	-0.069818604	0.002371752	NS
NFYA	NS	0.00057366	-0.069796759	0.002380739	NS
VTA1	NS	0.000574816	-0.069785757	0.002385023	NS
CHRNA4	NS	0.000575491	0.069779336	0.002387314	NS
PDE3B	NS	0.000575812	-0.069776291	0.002388132	NS
FIBIN	NS	0.000575964	0.069774846	0.002388251	NS
SLC5A7	NS	0.000576702	-0.069767843	0.002390288	NS
CDIP1	NS	0.000576957	0.069765422	0.002390834	NS
PNO1	NS	0.000579602	-0.069740405	0.00240128	NS
TNFRSF1B	NS	0.000579922	0.06973739	0.00240209	NS
POLR3A	NS	0.000581336	-0.069724066	0.002407431	NS
CSTB	NS	0.000583375	-0.069704901	0.00241536	NS
WFDC8	NS	0.000583634	0.069702476	0.002415914	NS
PARM1	NS	0.000584632	-0.069693122	0.002419529	NS
GRM5	NS	0.000585298	-0.069686887	0.002421768	NS
CCDC191	NS	0.000586841	-0.069672475	0.002427633	NS
PNPLA5	NS	0.0005883	0.069658873	0.002433151	NS
C6orf58	NS	0.000588785	-0.069654361	0.002434636	NS
RPTOR	NS	0.000589054	0.06965186	0.002435228	NS
TMC2	NS	0.000590192	0.069641294	0.002439409	NS
VOPP1	NS	0.000591713	0.069627194	0.002445174	NS
PCDHGA2	NS	0.000592987	-0.069615409	0.002449916	NS
PHYHIP	NS	0.000595459	0.069592615	0.002459078	NS
MTCH1	NS	0.000596464	0.06958337	0.002462178	NS
SLC9C1	NS	0.000596453	-0.069583471	0.002462178	NS
CD83	NS	0.000596789	0.069580385	0.002462994	NS
ARRB2	NS	0.00059723	0.069576331	0.002464291	NS
RIIAD1	NS	0.000598277	0.06956673	0.002468084	NS
ACYP1	NS	0.000598665	-0.06956318	0.002469156	NS
DGKA	NS	0.000598937	0.069560684	0.002469754	NS
TYW5	NS	0.000599179	0.069558473	0.00247012	NS
SLC35E3	NS	0.000599281	0.069557537	0.00247012	NS
C5orf52	NS	0.000599883	0.069552033	0.002472074	NS
ZFYVE21	NS	0.000601659	-0.069535817	0.002478866	NS
SYVN1	NS	0.000602184	0.06953103	0.002480493	NS
FAM170B	NS	0.00060231	0.069529885	0.002480493	NS
CFC1B	NS	0.000602809	0.069525342	0.00248202	NS
CFAP206	NS	0.000603479	-0.069519252	0.002484248	NS
ACTL6A	NS	0.000603898	-0.069515442	0.002485264	NS
CYP4A11	NS	0.000603982	0.069514677	0.002485264	NS

CYP4F22	NS	0.000605279	-0.069502911	0.002490069	NS
YKT6	NS	0.000606327	0.069493417	0.002493851	NS
TMEM196	NS	0.000607607	-0.069481843	0.002497524	NS
CT45A10	NS	0.000608931	-0.069469894	0.002502435	NS
THAP3	NS	0.00060948	-0.069464946	0.00250416	NS
TLE4	NS	0.000610392	0.069456737	0.002507375	NS
PDIA4	NS	0.000612431	-0.06943843	0.002515214	NS
SLC25A31	NS	0.000612966	-0.069433629	0.00251688	NS
USP17L15	NS	0.000613208	-0.069431464	0.002517338	NS
TFF2	NS	0.000613486	0.06942897	0.002517948	NS
IP6K3	NS	0.000616421	0.069402759	0.002529454	NS
LRGUK	NS	0.000616984	-0.069397738	0.002531231	NS
SVBP	NS	0.000618647	-0.069382947	0.002537515	NS
ING1	NS	0.000621111	0.069361098	0.002547083	NS
OC90	NS	0.000622191	-0.069351546	0.002550972	NS
HMCN2	NS	0.000623185	0.069342769	0.002553965	NS
H3-5	NS	0.000623124	0.069343313	0.002553965	NS
EMD	NS	0.000624377	0.069332261	0.002558309	NS
CELF6	NS	0.000626205	0.069316186	0.002565254	NS
RRAGC	NS	0.000626861	-0.069310427	0.002567398	NS
MTNR1B	NS	0.000628486	-0.069296187	0.002573508	NS
CLCN1	NS	0.000629286	-0.069289184	0.002576241	NS
RHCE	NS	0.000630953	0.069274631	0.002582517	NS
CCND1	NS	0.000631528	0.069269614	0.002583779	NS
BICRAL	NS	0.000631488	-0.069269962	0.002583779	NS
ELOVL3	NS	0.000632388	-0.069262128	0.00258675	NS
CHRDL1	NS	0.000633149	-0.069255507	0.002589119	NS
FFAR2	NS	0.000633235	0.069254763	0.002589119	NS
PGS1	NS	0.000635297	0.069236859	0.002597004	NS
CCR3	NS	0.000635476	-0.069235309	0.002597187	NS
C1GALT1C1	NS	0.000636293	-0.069228233	0.002599977	NS
MRPL45	NS	0.000636497	0.069226474	0.002600259	NS
DHRS11	NS	0.000637844	-0.069214831	0.002605213	NS
MRGPRD	NS	0.000638265	-0.069211197	0.002606383	NS
TEX26	NS	0.000639567	-0.069199968	0.002611151	NS
CHRM4	NS	0.000640381	-0.069192968	0.00261392	NS
GABARAPL1	NS	0.000641037	0.06918732	0.002616049	NS
RIOK2	NS	0.000641353	-0.069184612	0.002616783	NS
ZSCAN5C	NS	0.000641877	0.069180106	0.002618372	NS
VAC14	NS	0.000642703	0.069173024	0.002620635	NS
GNG5	NS	0.000642616	0.069173769	0.002620635	NS
PCDHGB3	NS	0.000643759	-0.069163975	0.002624389	NS
BRAP	NS	0.000644995	0.069153405	0.002628874	NS
HMGCS2	NS	0.000645871	-0.069145926	0.002631889	NS
PCDHB5	NS	0.000647779	0.069129663	0.002639109	NS
PCNA	NS	0.000649798	-0.069112504	0.002646777	NS
GLA	NS	0.000650918	0.069103005	0.002650782	NS
DENND3	NS	0.000651648	0.069096819	0.002653199	NS
HAGH	NS	0.000652297	-0.069091333	0.00265528	NS
CHMP5	NS	0.000655673	0.069062849	0.002668464	NS
TMUB1	NS	0.000655861	0.06906127	0.002668666	NS
SPRY4	NS	0.000656339	0.069057252	0.002670048	NS
ADAMTS16	NS	0.000656966	-0.069051981	0.002672038	NS
CC1L-ADOR	NS	0.000658398	0.069039961	0.002676739	NS
ANKRD49	NS	0.000658658	0.069037785	0.002677232	NS

OSBPL7	NS	0.000659228	-0.069033009	0.002678987	NS
ADAP1	NS	0.000659772	0.069028458	0.002680634	NS
FXN	NS	0.000662291	-0.069007415	0.00268974	NS
TM4SF5	NS	0.000663159	0.069000185	0.002692699	NS
FBXO30	NS	0.000663944	-0.068993646	0.002695324	NS
SINHCAF	NS	0.000664677	0.068987554	0.002697732	NS
PGM2	NS	0.000665556	-0.068980252	0.002700735	NS
RNF181	NS	0.000666566	0.068971874	0.002704267	NS
C1orf174	NS	0.000668393	-0.068956751	0.002711111	NS
GBA2	NS	0.000668978	0.068951916	0.002712915	NS
INPP5K	NS	0.00067028	0.068941172	0.002717626	NS
CALB2	NS	0.000672876	-0.068919806	0.002726437	NS
TMEM211	NS	0.000672811	0.068920345	0.002726437	NS
MED21	NS	0.000673504	0.068914653	0.002728409	NS
SNX15	NS	0.000673789	-0.068912313	0.002728993	NS
FARS2	NS	0.000675579	-0.068897645	0.002735097	NS
NCOA6	NS	0.000675465	0.068898577	0.002735097	NS
POC5	NS	0.000675902	-0.068894996	0.002735835	NS
CUZD1	NS	0.000677493	-0.068881998	0.0027417	NS
QPCTL	NS	0.000680208	0.068859868	0.002752114	NS
C9orf40	NS	0.000681306	-0.068850945	0.00275598	NS
CLIP1	NS	0.000682405	0.06884203	0.002759847	NS
CYP2A6	NS	0.000682924	0.068837819	0.002761371	NS
NGB	NS	0.000683743	0.068831186	0.002764105	NS
SRA1	NS	0.000684588	0.068824357	0.002766363	NS
TEX13B	NS	0.000684901	0.068821824	0.002767051	NS
AP4E1	NS	0.000685234	-0.068819129	0.002767821	NS
ZNF224	NS	0.000686984	-0.068805014	0.002774308	NS
SALL1	NS	0.00068802	-0.068796666	0.002777915	NS
KBTBD3	NS	0.000689201	-0.068787175	0.00278152	NS
TOP1	NS	0.000689143	0.068787636	0.00278152	NS
FANCF	NS	0.000689372	-0.068785796	0.002781632	NS
IL36G	NS	0.000689604	-0.068783935	0.002781987	NS
TSPYL5	NS	0.000691918	-0.068765378	0.002790742	NS
RAB40AL	NS	0.00069313	-0.068755684	0.002795048	NS
CMPK1	NS	0.000693525	0.068752527	0.002796059	NS
PABPC4L	NS	0.00069369	-0.068751215	0.002796139	NS
CRLF2	NS	0.000695719	0.068735032	0.002803733	NS
MS4A12	NS	0.000698248	-0.068714921	0.00281334	NS
TSNAXIP1	NS	0.000700416	0.068697737	0.002820901	NS
ZNF473	NS	0.0007004	-0.068697863	0.002820901	NS
YWHAQ	NS	0.000701139	0.068692014	0.002823226	NS
FGF6	NS	0.000701452	-0.068689541	0.002823899	NS
FBXL13	NS	0.000701822	-0.06868662	0.002824799	NS
OPRK1	NS	0.000704085	0.068668761	0.002833322	NS
PCDHGC4	NS	0.000705706	-0.068656009	0.002839253	NS
IFNA5	NS	0.000706897	-0.068646655	0.002843273	NS
ZNRD2	NS	0.000706999	0.068645854	0.002843273	NS
ZNF383	NS	0.000710525	-0.068618254	0.002856265	NS
BMP7	NS	0.00071099	0.068614619	0.002857542	NS
MBD3L1	NS	0.000711515	0.068610522	0.002859059	NS
PELP1	NS	0.000712633	-0.068601811	0.002862955	NS
ASB6	NS	0.000713046	0.06859859	0.002864022	NS
LIMK2	NS	0.00071656	0.068571301	0.002877537	NS
SSU72P8	NS	0.00071755	-0.068563634	0.002880915	NS

UBQLN1	NS	0.000719869	0.068545713	0.002889626	NS
KL	NS	0.000720528	-0.068540624	0.002891675	NS
OSGEPL1	NS	0.000721727	-0.068531386	0.002895887	NS
KASH5	NS	0.000723248	0.06851969	0.002901205	NS
SPOUT1	NS	0.000723353	-0.068518889	0.002901205	NS
RNF175	NS	0.000724468	-0.068510326	0.002905076	NS
NPTXR	NS	0.000725624	0.068501463	0.00290911	NS
THOC7	NS	0.00072593	-0.068499124	0.002909732	NS
HOXD4	NS	0.000726562	0.068494285	0.002911663	NS
GABRA5	NS	0.000727996	0.068483323	0.002916806	NS
SPATS2L	NS	0.000728643	-0.068478381	0.002918796	NS
ISOC2	NS	0.00073057	-0.068463703	0.002925301	NS
KCNK12	NS	0.000730567	0.068463721	0.002925301	NS
NPAT	NS	0.000732113	-0.068451965	0.002930876	NS
ZNF33A	NS	0.000733263	-0.068443236	0.002934872	NS
CAMK1D	NS	0.00073449	-0.068433938	0.002939174	NS
THAP1	NS	0.000738547	-0.068403287	0.002954799	NS
COL19A1	NS	0.000742102	-0.068376563	0.002967794	NS
SHC4	NS	0.000742072	-0.068376784	0.002967794	NS
FBXO2	NS	0.000742751	0.068371691	0.002969778	NS
TIMM8A	NS	0.00074317	-0.068368553	0.002970811	NS
CDC42BPA	NS	0.000743317	-0.068367456	0.002970811	NS
RUNX1T1	NS	0.000744283	-0.068360225	0.002974057	NS
ABCA6	NS	0.000744504	-0.06835857	0.002974327	NS
KCNC1	NS	0.000744735	-0.068356839	0.002974638	NS
TPRKB	NS	0.000745133	-0.068353868	0.002975612	NS
TESPA1	NS	0.000746473	-0.068343861	0.002980348	NS
YPEL5	NS	0.000748587	0.068328111	0.00298712	NS
AGR3	NS	0.000748632	-0.068327774	0.00298712	NS
DMWD	NS	0.000748535	-0.068328496	0.00298712	NS
ZNF384	NS	0.000749387	0.068322158	0.002988616	NS
FAM71D	NS	0.00074947	0.068321542	0.002988616	NS
MAGEE1	NS	0.000749276	0.068322982	0.002988616	NS
VPS37B	NS	0.000753484	0.068291777	0.003003385	NS
AQP2	NS	0.000753362	0.068292684	0.003003385	NS
SCML1	NS	0.000754707	-0.068282742	0.003007639	NS
FERMT1	NS	0.000755573	-0.068276344	0.003010473	NS
AQP1	NS	0.000756809	0.068267239	0.003014774	NS
SFXN3	NS	0.00075813	0.068257514	0.003019416	NS
ASIC2	NS	0.000759045	0.068250786	0.003022439	NS
PPIC	NS	0.000760331	-0.06824135	0.003026936	NS
ADAMTS6	NS	0.000764798	-0.068208674	0.003044094	NS
MMP11	NS	0.000766247	0.06819811	0.003049235	NS
DCUN1D5	NS	0.00076741	-0.068189648	0.003053235	NS
NAT9	NS	0.000768224	0.068183736	0.003055844	NS
SBSN	NS	0.000772142	0.068155336	0.0030708	NS
PACSIN2	NS	0.000781968	0.068084708	0.003109238	NS
COPB1	NS	0.000782288	0.068082422	0.003109871	NS
LRRC3B	NS	0.000784808	-0.068064443	0.00311925	NS
COLEC12	NS	0.00078517	-0.068061871	0.003119726	NS
TEAD4	NS	0.00078525	0.068061297	0.003119726	NS
ATG4A	NS	0.000787792	0.068043233	0.00312918	NS
CDH9	NS	0.000788261	-0.0680399	0.003130404	NS
ZMYND11	NS	0.000788967	-0.068034895	0.003131922	NS
SPATA9	NS	0.000789647	-0.068030081	0.003133977	NS

CTXND2	NS	0.00079312	0.068005536	0.003147116	NS
SLC8A3	NS	0.00079644	-0.067982168	0.003159365	NS
CLDN5	NS	0.000796533	-0.067981509	0.003159365	NS
THEM5	NS	0.000799603	0.067959986	0.003170889	NS
TET2	NS	0.000799865	0.06795815	0.00317128	NS
CNMD	NS	0.000803992	-0.067929343	0.003186988	NS
OR4K14	NS	0.00080808	-0.067900934	0.00320254	NS
ARG2	NS	0.00080957	-0.067890616	0.003207131	NS
RPL10	NS	0.000811112	0.067879958	0.00321258	NS
PGF	NS	0.000811819	-0.067875077	0.003214382	NS
RPP40	NS	0.000811899	-0.067874523	0.003214382	NS
BORCS7	NS	0.000813075	0.06786641	0.003218382	NS
ARHGAP21	NS	0.000813605	0.067862762	0.003219161	NS
FARSA	NS	0.000813571	0.067862992	0.003219161	NS
TMEM42	NS	0.000814824	-0.067854373	0.003223324	NS
SLC5A8	NS	0.000815727	-0.06784816	0.003226239	NS
ZNHIT6	NS	0.000817052	-0.067839066	0.003230349	NS
FAM71A	NS	0.0008171	-0.067838734	0.003230349	NS
CDC45	NS	0.000817286	-0.067837458	0.003230425	NS
EIF3A	NS	0.000819632	0.067821387	0.003239037	NS
ZSCAN9	NS	0.000820804	-0.067813378	0.003243004	NS
S1PR3	NS	0.000821817	0.067806461	0.003246343	NS
OR52K1	NS	0.000823202	-0.067797012	0.003251154	NS
GNPAT	NS	0.000823753	-0.067793265	0.003252663	NS
SKOR2	NS	0.000824158	0.067790507	0.003253599	NS
ZSCAN1	NS	0.000824893	0.067785503	0.003255838	NS
KDM5B	NS	0.000825337	0.067782483	0.003256927	NS
LHX1	NS	0.000827674	0.06776662	0.003265483	NS
STARD7	NS	0.000828651	0.067759998	0.003268673	NS
HMG20A	NS	0.00082986	-0.067751823	0.003272771	NS
CFHR5	NS	0.000831359	-0.067741692	0.003278016	NS
ZC3H14	NS	0.000831844	0.067738415	0.003278595	NS
SYT5	NS	0.000831763	0.067738962	0.003278595	NS
DEFA6	NS	0.000832806	-0.067731927	0.003281718	NS
UNC119B	NS	0.000833871	-0.067724754	0.003285245	NS
INHBC	NS	0.000834414	0.067721101	0.003286714	NS
KRTAP2-1	NS	0.000838031	-0.067696809	0.003300292	NS
IQCJ-SCHIP1	NS	0.000838843	0.067691371	0.003302817	NS
VBP1	NS	0.000841158	-0.067675893	0.003311256	NS
SIGLEC8	NS	0.000843682	0.06765906	0.003320517	NS
ZFPM1	NS	0.00084424	0.067655341	0.00332204	NS
NSUN4	NS	0.000845534	0.067646736	0.003325778	NS
C20orf144	NS	0.000845514	0.06764687	0.003325778	NS
ZNF214	NS	0.000847039	-0.067636739	0.003331023	NS
SNX32	NS	0.000848705	0.067625698	0.003336894	NS
FNIP1	NS	0.00084898	0.067623878	0.003337296	NS
OC1S5-TXNI	NS	0.000853308	-0.067595286	0.003353629	NS
BLOC1S6	NS	0.000856962	0.067571253	0.003367306	NS
NNMT	NS	0.000857472	-0.067567903	0.003368628	NS
RAN	NS	0.000857737	0.067566168	0.003368984	NS
CELF4	NS	0.000858739	0.067559595	0.003372237	NS
PTDSS2	NS	0.000859696	0.06755333	0.00337531	NS
THAP6	NS	0.000865799	0.06751352	0.003397891	NS
ASF1B	NS	0.000866439	-0.067509359	0.003399714	NS
OR2T1	NS	0.000867801	-0.067500516	0.003404368	NS

SYNE1	NS	0.00086799	-0.06749929	0.003404419	NS
TRAPPC10	NS	0.000868535	0.067495753	0.003405868	NS
USP3	NS	0.000868911	0.067493316	0.003406652	NS
PLEKHF2	NS	0.000869718	-0.067488087	0.003409126	NS
H3C6	NS	0.000872611	-0.067469387	0.003419772	NS
SPTBN1	NS	0.000873633	-0.067462794	0.003422391	NS
HMGB1P1	NS	0.000873529	0.067463462	0.003422391	NS
DHX15	NS	0.00087601	0.067447485	0.003430315	NS
STAT3	NS	0.000877143	0.067440197	0.003434059	NS
GTPBP6	NS	0.000877881	0.067435461	0.003436252	NS
OR4D10	NS	0.00088046	-0.067418925	0.003445651	NS
PAK1	NS	0.000881831	0.067410157	0.00345009	NS
SFTA2	NS	0.000881951	-0.067409389	0.00345009	NS
ABCF2-H2BE	NS	0.000882451	-0.067406194	0.003451349	NS
CCDC92	NS	0.000884923	0.067390424	0.003460317	NS
OSBP2	NS	0.00088591	0.067384138	0.003463476	NS
PAGE1	NS	0.000888466	-0.067367887	0.003472069	NS
BAIAP2L2	NS	0.000888433	0.067368096	0.003472069	NS
PON2	NS	0.000889165	-0.067363456	0.003473919	NS
PCDHB12	NS	0.000889341	0.067362334	0.003473919	NS
PCDHGC3	NS	0.000889478	-0.067361466	0.003473919	NS
NBDY	NS	0.000889684	-0.067360164	0.00347402	NS
TUBB1	NS	0.000889896	0.067358815	0.00347415	NS
MRGPRX2	NS	0.000891936	-0.0673459	0.00348141	NS
AFF1	NS	0.00089271	0.067341004	0.00348373	NS
NTHL1	NS	0.000893343	-0.067337006	0.003485497	NS
CTNNAL1	NS	0.000895645	0.067322482	0.003493775	NS
RNASE12	NS	0.000897419	0.067311313	0.00349999	NS
GAK	NS	0.000899274	0.06729966	0.003506516	NS
EHBP1	NS	0.000899646	0.067297325	0.003507261	NS
TIMM22	NS	0.000900463	-0.067292198	0.00350974	NS
TPH2	NS	0.000901406	0.067286289	0.003512707	NS
SHISA7	NS	0.000902827	0.067277397	0.003517536	NS
H1-7	NS	0.000903937	0.067270457	0.003521152	NS
ALB	NS	0.000904184	-0.067268915	0.003521405	NS
HEXIM2	NS	0.000905942	0.067257943	0.003527543	NS
SPRR2E	NS	0.000908202	-0.067243869	0.003535631	NS
PSG11	NS	0.000910171	-0.067231633	0.003542584	NS
DLX4	NS	0.000910632	0.067228769	0.003543667	NS
OXT	NS	0.000910984	0.067226589	0.003544322	NS
TASOR2	NS	0.000912524	-0.06721704	0.003549603	NS
ANGPT4	NS	0.000912962	0.067214331	0.003550591	NS
L3MBTL1	NS	0.000913945	-0.06720825	0.003553699	NS
CRKL	NS	0.000914591	-0.067204255	0.003555498	NS
HIP1R	NS	0.000917443	0.067186653	0.00356587	NS
FAM210B	NS	0.000919755	-0.067172423	0.003574137	NS
OR10AG1	NS	0.000919954	-0.067171203	0.003574191	NS
MC4R	NS	0.000924488	-0.067143395	0.003591087	NS
LIG3	NS	0.000926133	0.067133334	0.003596757	NS
SLC6A12	NS	0.000926696	-0.067129896	0.003597519	NS
SELENOM	NS	0.000926701	-0.067129866	0.003597519	NS
ASCL3	NS	0.000930981	-0.067103794	0.003613407	NS
ADIPOR1	NS	0.000932033	0.067097399	0.003616767	NS
ZNF416	NS	0.000933551	-0.06708819	0.003621203	NS
HCAR3	NS	0.000933549	0.067088201	0.003621203	NS

ULK1	NS	0.000935625	0.067075624	0.003628523	NS
TRPM1	NS	0.000938016	-0.067061171	0.003636478	NS
ZFAND2B	NS	0.000938052	-0.067060954	0.003636478	NS
UBE2C	NS	0.000938622	-0.067057515	0.003637959	NS
DNAI1	NS	0.000940261	-0.067047635	0.003643581	NS
CPNE7	NS	0.00094304	0.067030918	0.003653618	NS
OLFM1	NS	0.000945664	0.067015171	0.003663054	NS
DIO2	NS	0.000946185	-0.067012051	0.003664338	NS
DERA	NS	0.000947546	-0.067003907	0.003668873	NS
MRGPRE	NS	0.000949402	0.066992817	0.003674589	NS
ARHGEF35	NS	0.000949274	-0.066993582	0.003674589	NS
AMBRA1	NS	0.00094962	-0.066991514	0.003674699	NS
OR2S2	NS	0.000950672	0.066985239	0.003678034	NS
MPHOSPH6	NS	0.000951718	-0.066979007	0.003681344	NS
PRY	NS	0.00095249	-0.066974407	0.003683272	NS
OR5W2	NS	0.000952597	-0.066973773	0.003683272	NS
TRIL	NS	0.000952992	0.066971421	0.003684065	NS
FBXO39	NS	0.000953518	-0.066968289	0.003685364	NS
COX8C	NS	0.000955443	0.066956858	0.003692064	NS
MRPL51	NS	0.000956213	-0.066952289	0.003694302	NS
RNF133	NS	0.000956477	-0.066950721	0.003694586	NS
TET3	NS	0.000957423	0.066945113	0.003697503	NS
SH3BGRL3	NS	0.000958666	0.066937759	0.003701562	NS
TUBA8	NS	0.000959711	-0.066931579	0.003704858	NS
RIN1	NS	0.000960242	0.06692844	0.00370617	NS
DLGAP5	NS	0.000963169	-0.066911174	0.003716725	NS
C12orf60	NS	0.000963702	-0.066908032	0.003718042	NS
TULP1	NS	0.000964829	0.066901402	0.003720906	NS
RBM4	NS	0.000964762	0.066901794	0.003720906	NS
RAC1	NS	0.000967003	0.066888627	0.003728547	NS
FBLN1	NS	0.000967621	-0.066884998	0.003730189	NS
KRT2	NS	0.000968076	-0.066882331	0.003731199	NS
ABCA7	NS	0.000968639	-0.066879029	0.003732627	NS
HM13	NS	0.000970415	0.06686863	0.003738726	NS
TNFRSF13C	NS	0.000973767	0.066849054	0.003750892	NS
PRPS1L1	NS	0.000975489	-0.066839018	0.003756778	NS
DGKQ	NS	0.000977393	-0.066827943	0.003762614	NS
ADAM30	NS	0.000978054	-0.066824105	0.003764408	NS
ZNF480	NS	0.000980164	-0.066811861	0.00377178	NS
AQP12A	NS	0.000982335	0.066799289	0.003778712	NS
NLRP14	NS	0.000982882	-0.066796126	0.003779985	NS
G0S2	NS	0.000983439	0.066792907	0.003781375	NS
GNRH2	NS	0.000983811	0.066790759	0.003782053	NS
GRK1	NS	0.000986687	0.066774167	0.003791842	NS
B3GALT4	NS	0.000986749	-0.066773811	0.003791842	NS
CLVS1	NS	0.000987418	-0.066769955	0.003793662	NS
OR1L8	NS	0.000988086	0.066766113	0.003795473	NS
FAM117B	NS	0.000990407	0.066752772	0.003803635	NS
SSH1	NS	0.000991683	0.066745449	0.003806271	NS
COX6A1	NS	0.00099135	-0.066747362	0.003806271	NS
MIER1	NS	0.00099153	0.066746326	0.003806271	NS
MRPS33	NS	0.000995054	-0.066726149	0.003818452	NS
SH2D1B	NS	0.000995848	-0.066721613	0.003820741	NS
SAMD3	NS	0.000997223	-0.066713767	0.003824329	NS
ZNF219	NS	0.000997127	0.066714311	0.003824329	NS

CATSPERG	NS	0.000999328	0.066701769	0.003831054	NS
TNFSF4	NS	0.000999752	-0.066699354	0.003831922	NS
DCLRE1B	NS	0.001000463	-0.06669531	0.003833887	NS
LAMP2	NS	0.001005051	0.066669271	0.003849182	NS
TMEM140	NS	0.00100505	0.066669279	0.003849182	NS
GJD3	NS	0.001005043	0.066669315	0.003849182	NS
CTNNB1	NS	0.001007579	0.066654971	0.0038581	NS
NEURL3	NS	0.001010961	0.066635891	0.003870284	NS
SRRM4	NS	0.001011198	0.066634557	0.003870426	NS
SIGLEC15	NS	0.001011843	-0.066630923	0.00387213	NS
SP3	NS	0.001013364	0.066622367	0.003877185	NS
TMEM67	NS	0.001023163	-0.066567537	0.003913902	NS
RNF17	NS	0.001026615	-0.066548334	0.003926333	NS
MYB	NS	0.001028185	-0.066539623	0.00393156	NS
RNF20	NS	0.001031804	-0.066519586	0.003944619	NS
DEFB105A	NS	0.001032097	0.066517969	0.003944958	NS
PYURF	NS	0.001033151	-0.066512146	0.003947429	NS
PIGY	NS	0.001033151	-0.066512146	0.003947429	NS
MTF1	NS	0.001038508	0.066482643	0.003967112	NS
UBE2D3	NS	0.001040007	0.066474412	0.003972054	NS
BAK-RBAKD	NS	0.001040529	-0.066471544	0.003973267	NS
SETD4	NS	0.001041053	-0.066468672	0.003974483	NS
ITGA6	NS	0.001043058	-0.066457689	0.003981353	NS
DDN	NS	0.001044721	-0.066448596	0.003986612	NS
MAGEB3	NS	0.001044848	-0.066447902	0.003986612	NS
NCOR1	NS	0.001045151	0.066446247	0.003986982	NS
H2BC3	NS	0.001047453	-0.066433685	0.003994977	NS
CCDC105	NS	0.001048148	0.066429899	0.003996839	NS
LRBA	NS	0.001048476	-0.066428111	0.003997303	NS
TFIP11	NS	0.001049797	0.066420925	0.004001549	NS
FSIP2	NS	0.001050432	0.066417469	0.004003182	NS
CCDC85B	NS	0.001052174	0.066408005	0.004009032	NS
IL6ST	NS	0.001052469	0.066406402	0.004009368	NS
TRIP4	NS	0.001053462	-0.066401019	0.004011569	NS
FAM236D	NS	0.001053257	0.066402131	0.004011569	NS
MAGOH	NS	0.001054828	-0.066393617	0.00401598	NS
AMELY	NS	0.001055195	-0.066391627	0.004016589	NS
ABCC2	NS	0.00105574	-0.06638868	0.004017575	NS
TTL	NS	0.001055869	-0.066387978	0.004017575	NS
NATD1	NS	0.001057087	-0.066381391	0.004021419	NS
SCMH1	NS	0.001058928	-0.066371448	0.004027631	NS
B4GALNT1	NS	0.001059277	0.066369568	0.004028165	NS
SLC47A1	NS	0.001060304	-0.066364029	0.004031279	NS
CELF5	NS	0.001061838	0.066355762	0.004036321	NS
ARHGAP22	NS	0.001067109	-0.066327459	0.004055558	NS
MFSD14A	NS	0.0010691	0.066316796	0.004062329	NS
TMEM61	NS	0.001069659	0.06631381	0.004063653	NS
GLB1L	NS	0.001073241	-0.066294687	0.004076463	NS
CTIF	NS	0.001074727	-0.066286774	0.004081304	NS
TMEM30B	NS	0.001074975	0.066285452	0.004081446	NS
CD68	NS	0.001077288	0.066273156	0.004089426	NS
NTRK2	NS	0.001078002	0.066269365	0.004090531	NS
GPI	NS	0.001078746	0.066265412	0.004092555	NS
C2CD5	NS	0.001079682	0.066260453	0.004095301	NS
STYK1	NS	0.001080169	-0.066257872	0.004096345	NS

SHBG	NS	0.001085012	0.066232261	0.004113908	NS
SERPINB2	NS	0.001087815	0.066217491	0.004123726	NS
PCDHB15	NS	0.001088138	0.066215793	0.004124142	NS
SPAG11B	NS	0.001090048	-0.066205747	0.004130574	NS
TCTE1	NS	0.001090678	0.066202441	0.00413215	NS
C6orf52	NS	0.001097427	-0.0661671	0.004156907	NS
NDUFB10	NS	0.001098128	-0.06616344	0.004158749	NS
LAMTOR2	NS	0.001098356	-0.066162249	0.0041588	NS
IFFO2	NS	0.001099976	0.066153804	0.004164119	NS
VAMP8	NS	0.001101074	-0.066148088	0.004167459	NS
GNG4	NS	0.001102537	0.066140476	0.004172181	NS
MUSK	NS	0.001107388	-0.06611531	0.004189717	NS
MUC5AC	NS	0.001108657	-0.066108739	0.004193701	NS
FOXRED1	NS	0.001110516	-0.066099131	0.004199912	NS
IRF1	NS	0.00111078	0.066097769	0.004200089	NS
NSUN7	NS	0.001111607	-0.066093501	0.004202395	NS
SF3B5	NS	0.001114268	-0.066079784	0.004211634	NS
TOP2A	NS	0.001116915	-0.066066172	0.00421999	NS
CEND1	NS	0.001116713	0.066067209	0.00421999	NS
IFT88	NS	0.001120498	-0.066047791	0.004232652	NS
SLC35E4	NS	0.001120704	0.06604674	0.004232652	NS
FYTTD1	NS	0.001128	0.06600949	0.004259375	NS
ZNF292	NS	0.001128867	0.066005074	0.004261821	NS
MROH2A	NS	0.001130089	0.065998861	0.004265603	NS
CAPG	NS	0.001134006	0.065978988	0.004279553	NS
MFAP3L	NS	0.001135498	-0.065971437	0.004284347	NS
H4C14	NS	0.001136438	-0.065966681	0.004287059	NS
STAT5B	NS	0.001138174	0.065957912	0.004292771	NS
SREBF1	NS	0.001142258	0.065937329	0.004307334	NS
TRAF4	NS	0.001150578	0.065895607	0.004337862	NS
CD82	NS	0.001157551	0.065860851	0.004363302	NS
DNMBP	NS	0.001158002	0.065858611	0.004363303	NS
TWIST2	NS	0.001157965	-0.065858796	0.004363303	NS
HJURP	NS	0.001160775	0.065844846	0.004372902	NS
CLASP1	NS	0.001162297	0.065837307	0.004377782	NS
HERPUD2	NS	0.001163236	0.06583266	0.004380466	NS
PTPN23	NS	0.001163726	0.065830234	0.004381461	NS
SYPL2	NS	0.001166004	0.065818982	0.00438918	NS
KRT10	NS	0.001166301	-0.065817511	0.004389448	NS
LUZP4	NS	0.001167394	-0.065812122	0.004392087	NS
DSPP	NS	0.001167457	-0.065811812	0.004392087	NS
QRICH2	NS	0.001168787	0.065805256	0.004395514	NS
FHOD3	NS	0.001168889	-0.065804752	0.004395514	NS
PAQR7	NS	0.001169049	0.065803967	0.004395514	NS
RCAN1	NS	0.001170357	-0.065797524	0.00439958	NS
STPG1	NS	0.00117114	-0.065793677	0.004401665	NS
SHOC2	NS	0.001171602	0.065791402	0.004402472	NS
USP45	NS	0.001171809	-0.065790386	0.004402472	NS
NFE2L3	NS	0.001176771	0.06576605	0.004417683	NS
ETFB	NS	0.001176746	-0.065766171	0.004417683	NS
SCAP	NS	0.001176275	-0.065768476	0.004417683	NS
ATP5MC1	NS	0.001176682	-0.065766486	0.004417683	NS
GRIK1	NS	0.001177448	-0.065762736	0.004419368	NS
CT45A9	NS	0.001181994	-0.065740531	0.004435573	NS
GPR137	NS	0.001184776	0.065726986	0.004445149	NS

C2orf81	NS	0.001186331	-0.065719427	0.004450119	NS
ADGRE5	NS	0.001188882	0.065707043	0.004458826	NS
KCNK5	NS	0.001194889	-0.065677981	0.004480487	NS
POLR2G	NS	0.001195163	-0.065676661	0.004480645	NS
TM6SF2	NS	0.001196271	0.065671317	0.00448393	NS
NPTN	NS	0.001197123	0.065667206	0.004486258	NS
TMEM132A	NS	0.001200468	0.065651111	0.004496312	NS
SPICE1	NS	0.001200117	-0.065652798	0.004496312	NS
FAM183A	NS	0.001200593	0.065650511	0.004496312	NS
ANKRD35	NS	0.001200736	-0.065649826	0.004496312	NS
PANX2	NS	0.001205257	0.065628137	0.004511499	NS
MTX2	NS	0.001205158	-0.065628611	0.004511499	NS
BTG2	NS	0.001211432	0.065598641	0.004533735	NS
CTSL	NS	0.001212992	0.06559121	0.004538696	NS
PAM	NS	0.001213311	-0.065589691	0.004539012	NS
PHACTR4	NS	0.001216621	0.06557396	0.004550515	NS
CFHR4	NS	0.001218611	-0.065564522	0.004557076	NS
PARVA	NS	0.001220567	0.065555255	0.004563511	NS
MAPK1	NS	0.001224187	0.065538147	0.004576161	NS
LRRC1	NS	0.001225525	-0.065531834	0.004580279	NS
RNF148	NS	0.00122629	-0.065528229	0.004582252	NS
TP53INP2	NS	0.001226895	0.065525378	0.004582744	NS
DENND2B	NS	0.001226726	0.065526175	0.004582744	NS
LRRC74A	NS	0.001227547	-0.065522307	0.004583411	NS
CACNG1	NS	0.001227509	0.065522487	0.004583411	NS
ZFP91-CNTF	NS	0.001227837	0.065520944	0.004583608	NS
EIF4EBP1	NS	0.001229809	-0.065511667	0.004590086	NS
GOLGA6C	NS	0.001230511	0.065508369	0.00459182	NS
CYP2J2	NS	0.001232641	-0.065498369	0.004598884	NS
CDC42	NS	0.001234024	0.065491888	0.004602267	NS
C8orf82	NS	0.001233898	-0.065492478	0.004602267	NS
JAKMIP3	NS	0.001236269	-0.06548138	0.004609751	NS
HCN3	NS	0.001237372	-0.06547622	0.004612978	NS
JARID2	NS	0.00123856	0.065470674	0.004616515	NS
RAB6D	NS	0.001239493	0.065466316	0.004619105	NS
ZFR2	NS	0.001242455	0.065452512	0.004629251	NS
LMBRD1	NS	0.001244462	0.065443174	0.004635837	NS
IFNA2	NS	0.001248047	-0.065426529	0.004648298	NS
GRIA1	NS	0.001250556	-0.06541491	0.004656743	NS
ZW10	NS	0.001258722	0.065377231	0.00468625	NS
USO1	NS	0.001259826	-0.06537215	0.004689462	NS
NRIP2	NS	0.001264176	-0.065352192	0.004704746	NS
PGK1	NS	0.001280383	0.065278367	0.004764145	NS
FCN1	NS	0.001281882	-0.06527158	0.004768809	NS
HMGN2	NS	0.001283193	0.065265655	0.00477185	NS
ARR3	NS	0.001284562	-0.06525947	0.004775107	NS
NLRP3	NS	0.001284328	0.065260527	0.004775107	NS
SERPINF2	NS	0.001285289	-0.065256189	0.004776891	NS
TBX1	NS	0.001288433	0.065242017	0.004787657	NS
TDGF1P3	NS	0.001290117	0.06523444	0.004792995	NS
KIFC3	NS	0.001292139	0.065225354	0.004799586	NS
IQCD	NS	0.001293815	-0.065217833	0.004804889	NS
THYN1	NS	0.001295905	-0.065208464	0.004811729	NS
FCF1	NS	0.001298014	0.065199029	0.004818633	NS
HHAT	NS	0.001299566	-0.065192092	0.00482347	NS

ECHS1	NS	0.001300182	-0.065189341	0.004823907	NS
TNFRSF14	NS	0.001300126	0.065189593	0.004823907	NS
OR6B2	NS	0.001300977	-0.065185792	0.004825932	NS
SECISBP2L	NS	0.00130237	0.06517958	0.004830173	NS
BOC	NS	0.001303057	-0.065176519	0.004831364	NS
ERC2	NS	0.00130319	-0.065175924	0.004831364	NS
PEX26	NS	0.001307601	-0.065156304	0.004846787	NS
KRT27	NS	0.001309042	0.065149908	0.004851198	NS
MUC15	NS	0.001310309	-0.065144288	0.004854965	NS
ZNF692	NS	0.001313145	0.065131729	0.004864542	NS
HS3ST4	NS	0.001313565	0.065129869	0.004865168	NS
HLA-DQA2	NS	0.001315746	-0.065120233	0.004872312	NS
SEPTIN1	NS	0.001317308	0.065113338	0.004877164	NS
ZNF112	NS	0.001317918	-0.065110646	0.004877558	NS
UBAP1	NS	0.001317773	0.065111289	0.004877558	NS
CD300LB	NS	0.001318353	0.065108729	0.004878234	NS
RPS9	NS	0.001320932	0.06509737	0.00488591	NS
SLC9A4	NS	0.00132231	0.065091311	0.004890072	NS
MRPL23	NS	0.001322765	0.065089312	0.004890819	NS
AQP12B	NS	0.001323996	0.065083905	0.004894435	NS
DDX5	NS	0.00132501	0.065079452	0.004897251	NS
RADIL	NS	0.001332942	0.065044746	0.004925627	NS
ECM2	NS	0.001334963	-0.065035935	0.00493121	NS
NR3C2	NS	0.001334854	-0.065036409	0.00493121	NS
PABPC1L	NS	0.001338016	-0.065022646	0.004941545	NS
SPEG	NS	0.00133853	0.065020413	0.004942499	NS
ITPRIPL2	NS	0.001339343	-0.065016879	0.004944559	NS
SMR3A	NS	0.001340105	-0.065013571	0.004946427	NS
RPL8	NS	0.001341403	0.065007935	0.004950276	NS
RBFOX2	NS	0.001341702	-0.065006638	0.004950436	NS
TCTN1	NS	0.001346644	0.064985243	0.004967721	NS
APELA	NS	0.001349373	0.064973454	0.004976842	NS
CSTL1	NS	0.001351083	0.064966083	0.004982198	NS
SLC38A1	NS	0.001352388	-0.06496046	0.004986063	NS
ATP6V0A1	NS	0.001353344	0.064956346	0.004988635	NS
AMOTL2	NS	0.001354096	0.064953111	0.004990458	NS
HAPLN2	NS	0.001359383	0.064930413	0.00500899	NS
CCDC85C	NS	0.001362442	0.064917318	0.005019306	NS
HOMER2	NS	0.001363034	-0.06491479	0.005019574	NS
PAAF1	NS	0.001362826	-0.064915677	0.005019574	NS
POP5	NS	0.00136548	-0.06490434	0.005027628	NS
SPX	NS	0.001365875	-0.064902658	0.005028123	NS
ACBD5	NS	0.001368522	0.064891372	0.005036912	NS
CDRT15L2	NS	0.001372782	0.064873259	0.005051627	NS
USP17L8	NS	0.001373584	0.064869853	0.005053619	NS
FRMD7	NS	0.001375596	-0.06486132	0.005060059	NS
TMEM115	NS	0.001378703	0.064848165	0.005070524	NS
ZSCAN26	NS	0.001382305	-0.064832947	0.005082805	NS
CAVIN4	NS	0.001383533	0.064827764	0.005086358	NS
CDH19	NS	0.001387492	-0.064811096	0.005099942	NS
BEST2	NS	0.001389051	0.064804545	0.00510422	NS
ATP13A5	NS	0.001389183	-0.064803988	0.00510422	NS
SLC7A4	NS	0.001390924	0.064796681	0.005109646	NS
HLA-B	NS	0.001391426	0.064794576	0.00511052	NS
TFAP4	NS	0.001392869	-0.064788527	0.005114848	NS

ERCC6L2	NS	0.00139397	0.064783914	0.005117922	NS
TNS2	NS	0.00139501	0.064779559	0.0051198	NS
RTKN2	NS	0.00139485	-0.06478023	0.0051198	NS
ERVK3-1	NS	0.00139716	0.06477057	0.005126372	NS
COL18A1	NS	0.001397331	0.064769857	0.005126372	NS
POLR3GL	NS	0.001399583	-0.064760454	0.005133663	NS
MT-ND5	NS	0.001401891	0.064750835	0.005141152	NS
SFRP4	NS	0.001406792	-0.064730456	0.005158147	NS
ASGR2	NS	0.001408376	-0.06472388	0.005162979	NS
CNTN5	NS	0.001408702	-0.064722527	0.005163197	NS
B3GALNT1	NS	0.001409244	0.064720283	0.005164203	NS
IFT57	NS	0.001411035	-0.06471286	0.005168648	NS
IRAG2	NS	0.001411258	0.06471194	0.005168648	NS
GPANK1	NS	0.001411102	0.064712584	0.005168648	NS
OR9A4	NS	0.001411958	-0.064709042	0.005170234	NS
GHDC	NS	0.001413158	0.064704078	0.00517365	NS
FBP1	NS	0.001413539	0.064702504	0.005174065	NS
TBC1D8	NS	0.001415603	0.064693976	0.005180642	NS
C5orf15	NS	0.001420141	-0.06467527	0.005196268	NS
CYYR1	NS	0.001420464	-0.064673942	0.005196466	NS
MAP4K1	NS	0.001422973	0.064663625	0.005204661	NS
ZNF699	NS	0.001425896	-0.06465163	0.005214365	NS
LYPLA2	NS	0.001426508	0.06464912	0.005215618	NS
AGO2	NS	0.001426836	0.064647773	0.005215835	NS
HOOK2	NS	0.001429978	0.06463491	0.005226333	NS
H2BC6	NS	0.001432748	-0.064623592	0.005235468	NS
OSR1	NS	0.001438481	0.06460023	0.005255425	NS
LRRC41	NS	0.001438883	0.064598595	0.005255902	NS
ZNF652	NS	0.001439668	0.064595403	0.005257778	NS
EAF1	NS	0.001441698	0.064587161	0.005264195	NS
PLD5	NS	0.001444784	-0.064574643	0.005274471	NS
TRMT112	NS	0.001447268	-0.064564589	0.005282542	NS
WFDC1	NS	0.001456421	0.064527674	0.005314949	NS
GSX2	NS	0.001457073	0.064525052	0.005316327	NS
CCL14	NS	0.001457923	-0.064521639	0.005318422	NS
SMARCB1	NS	0.001458846	0.064517929	0.005318707	NS
S100A1	NS	0.0014591	-0.064516912	0.005318707	NS
APEH	NS	0.00145898	-0.064517392	0.005318707	NS
ZNF695	NS	0.001459074	-0.064517014	0.005318707	NS
ID3	NS	0.001467666	-0.064482616	0.005347917	NS
WWC2	NS	0.001467416	-0.064483614	0.005347917	NS
FER1L5	NS	0.001468745	-0.064478309	0.005350841	NS
CDC5L	NS	0.001469548	0.064475105	0.00535276	NS
HOXB4	NS	0.001470245	0.064472324	0.005354293	NS
GABBR1	NS	0.001473937	0.064457622	0.005366729	NS
KRTAP7-1	NS	0.001475296	-0.064452219	0.005370667	NS
RNASE11	NS	0.001476235	0.06444849	0.005373074	NS
STXBP2	NS	0.001479157	0.064436895	0.005382697	NS
ATP2B1	NS	0.00148063	0.064431058	0.005387045	NS
AKNAD1	NS	0.00148144	0.064427852	0.005388978	NS
FAIM	NS	0.00148354	0.064419543	0.005395604	NS
CRYGA	NS	0.001485795	0.064410636	0.005402788	NS
SUV39H2	NS	0.001488336	-0.064400608	0.005411014	NS
KCNH2	NS	0.001490858	0.064390675	0.005419166	NS
AQR	NS	0.001495769	0.064371377	0.005435994	NS

SYT3	NS	0.00149795	0.064362825	0.005442899	NS
F8A3	NS	0.001499476	0.064356849	0.005447419	NS
PPIL2	NS	0.001501877	0.064347455	0.00545512	NS
ZNF248	NS	0.001506181	-0.064330653	0.005469726	NS
EVC	NS	0.001506557	-0.064329186	0.005470066	NS
CDK8	NS	0.001510472	-0.064313945	0.005483251	NS
CCNK	NS	0.001511097	0.064311516	0.005483642	NS
NEMP2	NS	0.001511146	-0.064311323	0.005483642	NS
DNAJA2	NS	0.001512876	0.064304603	0.00548889	NS
U2AF1	NS	0.001519075	0.064280577	0.005510348	NS
FAM162A	NS	0.001519594	0.064278571	0.005511197	NS
FBXL14	NS	0.001521047	0.064272955	0.005515432	NS
TTC27	NS	0.001522966	-0.064265545	0.005521356	NS
ARHGAP36	NS	0.001523652	-0.064262895	0.005522812	NS
NECAP1	NS	0.001524454	0.064259803	0.005524684	NS
EDS1-UBE2V	NS	0.001525758	0.064254778	0.005528215	NS
LBR	NS	0.001526	0.064253845	0.005528215	NS
DEGS1	NS	0.00152724	0.064249072	0.005531528	NS
ATP13A2	NS	0.001527486	-0.064248123	0.005531528	NS
UBE2Q2	NS	0.00152854	0.064244066	0.005532735	NS
P2RY13	NS	0.001528677	0.064243542	0.005532735	NS
KRTAP1-1	NS	0.001528332	-0.064244868	0.005532735	NS
PDAP1	NS	0.001532775	0.064227799	0.005546531	NS
ZNF780A	NS	0.001537701	-0.064208927	0.005563318	NS
ARHGEF9	NS	0.001539447	-0.064202252	0.005568593	NS
ZNF569	NS	0.001540044	-0.064199972	0.005569711	NS
CXCL16	NS	0.001543789	0.064185683	0.005582211	NS
PLEK	NS	0.001544628	0.064182487	0.005584201	NS
DACH2	NS	0.001545958	-0.064177422	0.005587966	NS
PYGL	NS	0.001548338	-0.064168368	0.005595525	NS
TTLL7	NS	0.001548885	0.064166288	0.005596458	NS
JMJD8	NS	0.001557483	0.064133701	0.005625424	NS
FLII	NS	0.001558744	0.064128936	0.005628927	NS
RMND5A	NS	0.001560891	0.064120833	0.005635627	NS
PTGER2	NS	0.001561794	0.064117425	0.005637838	NS
CENPC	NS	0.001567074	0.064097546	0.005655842	NS
AKT1	NS	0.001568348	0.06409276	0.005659384	NS
CLDN23	NS	0.001568657	-0.064091599	0.005659444	NS
POLR3F	NS	0.001570561	-0.064084449	0.005664205	NS
COL14A1	NS	0.001570491	-0.064084714	0.005664205	NS
KIF20B	NS	0.001572281	-0.064078	0.005669352	NS
CUL1	NS	0.001575154	-0.064067243	0.005678651	NS
FLT1	NS	0.001578012	-0.064056558	0.005687897	NS
PARD3	NS	0.001580162	-0.064048535	0.005694584	NS
EHHADH	NS	0.001583257	-0.064036996	0.005704678	NS
FAM209B	NS	0.001586359	-0.064025456	0.005714791	NS
ZG16	NS	0.001587371	0.064021694	0.005717373	NS
DCT	NS	0.001593683	-0.06399829	0.005736904	NS
PLD4	NS	0.001593215	0.064000024	0.005736904	NS
TEN1-CDK3	NS	0.001593514	0.063998915	0.005736904	NS
SLC22A24	NS	0.001595504	-0.063991554	0.00574239	NS
ARID3C	NS	0.001598091	0.063981996	0.005750631	NS
ZNF551	NS	0.001602427	-0.063966006	0.005765163	NS
NFX1	NS	0.001603216	0.063963103	0.005766928	NS
CARMIL1	NS	0.001607383	-0.06394778	0.005780845	NS

AUNIP	NS	0.001609856	-0.063938706	0.005788661	NS
LPL	NS	0.00161067	-0.063935719	0.005790516	NS
ATG9A	NS	0.001613229	0.063926347	0.005798638	NS
PRMT3	NS	0.001615573	-0.063917772	0.005805985	NS
ROPN1	NS	0.00161912	-0.063904822	0.005817651	NS
KCTD14	NS	0.001620498	-0.063899797	0.005821522	NS
PHF1	NS	0.001622401	0.063892866	0.005826859	NS
IF816-ZNF32	NS	0.001622586	-0.063892191	0.005826859	NS
PSMG3	NS	0.001625298	-0.063882324	0.005834648	NS
CD34	NS	0.001625358	0.063882108	0.005834648	NS
OR4S2	NS	0.001632056	-0.063857805	0.005857607	NS
SEMA3D	NS	0.001632698	-0.063855481	0.005858824	NS
CHRFAM7A	NS	0.001635965	0.063843666	0.005868371	NS
OSBPL9	NS	0.001636826	0.063840554	0.005869286	NS
CLTC	NS	0.001636584	0.063841429	0.005869286	NS
IL1RAPL2	NS	0.001638236	-0.063835463	0.005873255	NS
TP63	NS	0.001639622	-0.063830464	0.005876683	NS
C1orf216	NS	0.0016398	-0.063829823	0.005876683	NS
LRRC3C	NS	0.001641335	0.063824291	0.005881096	NS
NOSIP	NS	0.001643005	-0.063818279	0.005885829	NS
ADAM9	NS	0.001643264	0.063817345	0.005885829	NS
B4GALT6	NS	0.00164412	-0.063814266	0.005886716	NS
JUNB	NS	0.001643996	0.06381471	0.005886716	NS
OPN1MW2	NS	0.001644783	0.06381188	0.005888002	NS
ALDH1L1	NS	0.00164536	0.063809807	0.005888977	NS
C16orf74	NS	0.001648722	0.063797736	0.005898827	NS
MEOX1	NS	0.001649372	0.063795402	0.005900064	NS
KRTAP15-1	NS	0.001650305	-0.063792059	0.005902309	NS
MBD3L2B	NS	0.001651787	0.063786748	0.005906519	NS
FRK	NS	0.001653145	0.063781885	0.005910285	NS
GPR119	NS	0.00165584	0.063772249	0.005918824	NS
IL27RA	NS	0.001656263	-0.063770738	0.005919243	NS
F3	NS	0.001656824	-0.063768733	0.005920156	NS
TMEM253	NS	0.001666092	0.063735719	0.005952172	NS
MCPH1	NS	0.001667841	0.063729504	0.005957324	NS
ACAT1	NS	0.001668564	-0.063726938	0.005958807	NS
SELENOO	NS	0.001671904	0.0637151	0.00596743	NS
CD40LG	NS	0.001671768	-0.06371558	0.00596743	NS
SMIM1	NS	0.001671398	0.063716892	0.00596743	NS
CFAP221	NS	0.00167638	0.063699267	0.005982301	NS
C16orf46	NS	0.001680039	-0.06368635	0.005994256	NS
TRIM13	NS	0.001681656	0.063680652	0.005998918	NS
SLC2A4RG	NS	0.001683184	-0.063675269	0.006003265	NS
PODNL1	NS	0.001687284	-0.063660854	0.006016779	NS
CUTC	NS	0.00168798	-0.063658412	0.00601815	NS
ZKSCAN3	NS	0.001689206	-0.063654108	0.006021413	NS
FAM78A	NS	0.001694369	-0.06363602	0.006037593	NS
KRTAP5-5	NS	0.001694259	0.063636404	0.006037593	NS
RAB11FIP4	NS	0.001697479	0.063625149	0.006047561	NS
TEP1	NS	0.001700624	-0.063614173	0.006057651	NS
USP16	NS	0.001701327	-0.063611721	0.006059041	NS
FXYD1	NS	0.001702561	0.063607421	0.006062321	NS
SACM1L	NS	0.001702931	-0.063606133	0.006062523	NS
MT-ATP8	NS	0.001709364	0.063583771	0.006084303	NS
KRT32	NS	0.00171086	-0.06357858	0.00608851	NS

MATN2	NS	0.001712994	-0.063571183	0.006094985	NS
TMEM132D	NS	0.001713331	-0.063570016	0.006095064	NS
FIGN	NS	0.001713983	-0.063567759	0.006096262	NS
CELSR2	NS	0.001714349	-0.063566494	0.006096442	NS
GINS3	NS	0.00171585	-0.063561301	0.006100659	NS
XRCC2	NS	0.001719295	0.063549396	0.006111786	NS
GPR176	NS	0.001719919	-0.063547243	0.006112882	NS
ITGB5	NS	0.001721259	-0.063542619	0.006116522	NS
EIF2A	NS	0.001725127	0.063529295	0.006129141	NS
TEX29	NS	0.001725781	-0.063527046	0.006130339	NS
ZNFX1	NS	0.001727399	0.063521482	0.00613496	NS
C1QA	NS	0.001732366	-0.063504432	0.006151473	NS
USP36	NS	0.001734324	0.063497722	0.006157297	NS
SLC23A1	NS	0.001734648	-0.063496615	0.006157315	NS
PTK6	NS	0.001735756	-0.063492823	0.006160118	NS
TRIM21	NS	0.001738241	0.063484323	0.006166679	NS
COL4A6	NS	0.001737961	-0.063485281	0.006166679	NS
CPB2	NS	0.001749913	-0.06344456	0.006206947	NS
BRAT1	NS	0.001750463	0.063442692	0.006207761	NS
PHACTR1	NS	0.001752544	0.063435628	0.006214004	NS
TTLL9	NS	0.001752992	-0.063434108	0.006214454	NS
ANKLE2	NS	0.001756797	0.063421221	0.0062268	NS
AEN	NS	0.001760998	-0.063407019	0.006240548	NS
MAGEF1	NS	0.001763645	-0.063398086	0.006248785	NS
DLA2-SMG1	NS	0.001765613	0.063391455	0.006254611	NS
TRIB3	NS	0.001766141	0.063389676	0.006255337	NS
ZNF620	NS	0.0017682	-0.063382744	0.006261486	NS
MRPL18	NS	0.001769072	-0.063379813	0.006262899	NS
ANXA1	NS	0.001769246	0.063379225	0.006262899	NS
NAXE	NS	0.00176963	-0.063377936	0.00626311	NS
SVIL	NS	0.001777222	0.063352463	0.006287683	NS
GLDC	NS	0.001777818	-0.063350469	0.00628864	NS
DBF4	NS	0.001779351	0.063345338	0.006291765	NS
B4GALT1	NS	0.001779339	0.063345378	0.006291765	NS
AGBL5	NS	0.001783803	-0.063330465	0.006306354	NS
OR52R1	NS	0.001787719	-0.06331741	0.006319045	NS
GREM2	NS	0.001790508	-0.063308129	0.006327747	NS
CCDC144A	NS	0.001792799	0.063300513	0.006334688	NS
COX4I1	NS	0.001793731	0.063297417	0.006336825	NS
PCDHGA7	NS	0.001795854	-0.063290372	0.006343168	NS
CIZ1	NS	0.001796276	0.063288974	0.006343499	NS
OR2A7	NS	0.001797513	-0.063284872	0.006346711	NS
MAB21L2	NS	0.001799609	0.063277931	0.006352953	NS
DDX56	NS	0.001800671	-0.063274416	0.006355135	NS
TAT	NS	0.001800884	-0.063273712	0.006355135	NS
GJD2	NS	0.001801923	-0.063270276	0.006357643	NS
BDP1	NS	0.001806995	-0.06325353	0.006374373	NS
MCOLN2	NS	0.001810196	-0.06324298	0.006383342	NS
PPM1K	NS	0.001810177	-0.063243042	0.006383342	NS
HLA-DRB5	NS	0.001812692	-0.063234769	0.006390977	NS
TCF7	NS	0.001816758	0.063221412	0.006404147	NS
GATB	NS	0.00182325	-0.063200144	0.00642586	NS
LY6G6D	NS	0.00182412	-0.063197301	0.006427755	NS
LCE2C	NS	0.001826895	-0.063188233	0.006436364	NS
ZNF71	NS	0.001828445	-0.063183173	0.006440653	NS

H4C15	NS	0.001829663	-0.063179204	0.006443768	NS
U2AF2	NS	0.001831915	0.063171866	0.006450526	NS
OR4D11	NS	0.00183458	-0.063163193	0.006457561	NS
CNDP2	NS	0.001843284	-0.063134946	0.006487019	NS
TRAPPC6A	NS	0.001847504	-0.063121297	0.006499506	NS
FAM236C	NS	0.00184735	0.063121794	0.006499506	NS
FCHO2	NS	0.001847974	0.063119776	0.006499981	NS
PLEKHG6	NS	0.00184833	0.063118629	0.006500048	NS
EGLN3	NS	0.001849944	-0.063113416	0.006504545	NS
GABRA2	NS	0.001853887	0.063100703	0.006517224	NS
HSPE1	NS	0.001864116	-0.063067837	0.006551995	NS
TBC1D7	NS	0.001864873	0.063065413	0.006553464	NS
RASGRF1	NS	0.001874497	-0.063034652	0.006586091	NS
NRTN	NS	0.00187641	0.063028558	0.006591612	NS
MGAT1	NS	0.001877477	0.063025157	0.006594167	NS
BNC2	NS	0.001883282	-0.063006701	0.006613355	NS
CLRN3	NS	0.001884011	0.063004389	0.006614713	NS
CAB39L	NS	0.001884468	-0.06300294	0.006615117	NS
PSD	NS	0.001891457	0.062980799	0.006637769	NS
PPP3R2	NS	0.001891606	-0.062980327	0.006637769	NS
MPC1	NS	0.001892031	-0.062978986	0.006638054	NS
XRCC5	NS	0.001892881	-0.062976297	0.006639835	NS
ZDHHC9	NS	0.001895023	0.062969531	0.006646145	NS
INTS5	NS	0.001899128	-0.062956587	0.006659333	NS
ATP6V1A	NS	0.00190225	0.062946756	0.006669074	NS
C4orf17	NS	0.00190303	-0.062944306	0.006670597	NS
YTHDC2	NS	0.0019062	-0.062934344	0.006680335	NS
CHMP6	NS	0.001906498	-0.062933409	0.006680335	NS
NEFH	NS	0.001906866	-0.062932252	0.006680337	NS
INPP5B	NS	0.001907189	-0.062931241	0.006680337	NS
SPG21	NS	0.001913667	0.062910941	0.006701816	NS
SLC12A1	NS	0.001915076	-0.062906533	0.006705538	NS
GUCA1ANB	NS	0.001919366	-0.062893136	0.006719342	NS
VN1R4	NS	0.001921442	-0.06288666	0.006725396	NS
DDX28	NS	0.001923783	-0.062879367	0.006732372	NS
EVPL	NS	0.001924211	0.062878037	0.00673265	NS
OR10A5	NS	0.001926162	-0.062871965	0.006737044	NS
NSG1	NS	0.001926037	0.062872356	0.006737044	NS
SLC25A14	NS	0.001926563	-0.062870717	0.006737229	NS
ZSCAN10	NS	0.001935815	0.062842015	0.006768358	NS
VWF	NS	0.00194169	-0.062823852	0.006786448	NS
IL3RA	NS	0.001943043	0.062819678	0.00678995	NS
FLT4	NS	0.001946024	0.062810486	0.006799141	NS
MSS51	NS	0.001947008	-0.062807456	0.006800975	NS
TMEM207	NS	0.001947252	-0.062806705	0.006800975	NS
TRIM48	NS	0.001948264	-0.06280359	0.006803282	NS
GH2	NS	0.001950931	0.062795386	0.006811368	NS
PARD3B	NS	0.001952503	-0.062790556	0.006815626	NS
BTBD16	NS	0.001953488	-0.06278753	0.006817837	NS
TOX2	NS	0.001955472	0.062781442	0.006823531	NS
ASEK-C17or	NS	0.001956006	0.062779806	0.006824161	NS
SLC45A2	NS	0.001956958	-0.062776887	0.006826253	NS
STX1B	NS	0.001960216	0.062766908	0.006836387	NS
NIF3L1	NS	0.001962948	-0.062758552	0.006843449	NS
ADH1B	NS	0.001962759	-0.06275913	0.006843449	NS

GTPBP1	NS	0.001963644	0.062756425	0.006844643	NS
BCOR	NS	0.001964252	0.062754569	0.006845528	NS
NXPE1	NS	0.001971577	-0.062732232	0.006867347	NS
DLST	NS	0.001971387	0.06273281	0.006867347	NS
NYNRIN	NS	0.001971558	-0.062732289	0.006867347	NS
PIGX	NS	0.001975213	0.062721173	0.006878114	NS
SPDYE12	NS	0.001975461	0.06272042	0.006878114	NS
AK4P3	NS	0.001975734	-0.062719589	0.006878114	NS
REG4	NS	0.001979507	-0.062708138	0.006888768	NS
ZNF124	NS	0.001979177	0.062709137	0.006888768	NS
PTPN11	NS	0.001980858	0.06270404	0.006892233	NS
MBLAC2	NS	0.001982087	-0.062700316	0.006895269	NS
SLC25A48	NS	0.001989622	-0.062677531	0.006917772	NS
OR5AK2	NS	0.001989628	-0.062677512	0.006917772	NS
CCDC192	NS	0.001989146	-0.062678968	0.006917772	NS
MYBPC3	NS	0.001994993	-0.062661335	0.006935181	NS
CEP170B	NS	0.001996127	0.062657923	0.006937875	NS
CLK4	NS	0.001998215	0.062651641	0.006943886	NS
SHROOM3	NS	0.00200095	0.06264342	0.006950897	NS
SLC39A4	NS	0.002000693	0.062644194	0.006950897	NS
TRARG1	NS	0.002001411	0.062642038	0.006951248	NS
PLEK2	NS	0.002004386	-0.06263311	0.006959085	NS
E2F6	NS	0.002004208	0.062633645	0.006959085	NS
HLA-F	NS	0.002010548	0.062614659	0.006979228	NS
ATP6V1B2	NS	0.002013908	0.062604621	0.006989638	NS
AGAP2	NS	0.002014662	0.062602373	0.006990998	NS
KRTAP5-7	NS	0.002018106	-0.0625921	0.007001696	NS
TBL1Y	NS	0.002028143	0.06256226	0.007035259	NS
KLLN	NS	0.002040718	0.062525067	0.00707761	NS
ITLN2	NS	0.002045639	0.06251057	0.007093405	NS
SMIM4	NS	0.002046253	-0.062508763	0.007094263	NS
PPIAL4G	NS	0.002046642	0.062507618	0.007094342	NS
DLEU7	NS	0.002049651	-0.062498774	0.007103498	NS
RPS8	NS	0.002052365	0.062490806	0.007111631	NS
KAT14	NS	0.002055357	-0.062482033	0.007120723	NS
GPN1	NS	0.002055902	0.062480435	0.007121338	NS
DISP2	NS	0.002057103	0.062476918	0.007124223	NS
NFKB1	NS	0.002058126	0.062473924	0.007126491	NS
TRIM9	NS	0.00205952	-0.062469846	0.007128853	NS
EIF3B	NS	0.002059545	-0.062469773	0.007128853	NS
RAB24	NS	0.002060052	-0.06246829	0.007129334	NS
ERN2	NS	0.002065477	0.062452446	0.00714683	NS
APOA4	NS	0.002067062	0.062447823	0.00714976	NS
NAF1	NS	0.002066969	0.062448094	0.00714976	NS
C15orf61	NS	0.002069345	0.062441172	0.007156377	NS
MKNK2	NS	0.002070619	0.062437465	0.007159502	NS
ALDH4A1	NS	0.002071111	-0.062436032	0.007159925	NS
DOK6	NS	0.002077286	0.062418089	0.00717999	NS
MBD5	NS	0.002091149	-0.062377985	0.007226615	NS
SLC9A8	NS	0.002097389	0.06236001	0.007246888	NS
GOLGA8O	NS	0.002101722	0.062347561	0.007260561	NS
GRID1	NS	0.002103141	-0.062343486	0.007264169	NS
ST3GAL3	NS	0.002107701	0.062330416	0.007278621	NS
CASC3	NS	0.002110645	0.062321992	0.007287487	NS
TMED5	NS	0.002115891	0.062307007	0.007304297	NS

BEST1	NS	0.002120174	0.062294798	0.007317777	NS
CARS1	NS	0.002121833	-0.062290076	0.007322197	NS
SLC19A2	NS	0.002124751	0.062281777	0.007330959	NS
MAEA	NS	0.002134333	0.062254598	0.007362707	NS
DEFB136	NS	0.002134811	0.062253246	0.007363043	NS
COL27A1	NS	0.002136767	0.062247712	0.007368477	NS
PLIN1	NS	0.002145927	0.062221861	0.007398746	NS
ZNF711	NS	0.00214758	-0.062217207	0.007401809	NS
CABP2	NS	0.002147459	0.062217547	0.007401809	NS
SLC35G1	NS	0.0021562	-0.062192988	0.007428874	NS
ERVMER34-1	NS	0.002160281	0.062181555	0.007441608	NS
TRIM34	NS	0.002160812	-0.062180068	0.007442114	NS
CCRL2	NS	0.002164118	0.062170822	0.007452174	NS
CLOCK	NS	0.00216596	-0.062165676	0.007455864	NS
GTF2B	NS	0.002165841	0.062166008	0.007455864	NS
ANKRD20A2	NS	0.002166455	-0.062164293	0.007456242	NS
UNC13C	NS	0.002170575	-0.062152799	0.007469094	NS
SLC10A1	NS	0.002175586	-0.062138848	0.007483675	NS
SALL2	NS	0.002185666	0.06211087	0.007517014	NS
ZGRF1	NS	0.002188621	-0.06210269	0.00752584	NS
CCDC73	NS	0.002189623	-0.062099919	0.007527949	NS
C10orf95	NS	0.002197868	0.062077162	0.007553609	NS
PPME1	NS	0.002197765	-0.062077444	0.007553609	NS
RIC1	NS	0.002201795	0.062066348	0.007565764	NS
SLC17A4	NS	0.002203219	-0.062062432	0.007569313	NS
CEP104	NS	0.002204071	-0.062060091	0.007570895	NS
COL22A1	NS	0.002207648	-0.062050267	0.007581836	NS
IL4R	NS	0.002208397	0.062048212	0.007583064	NS
SIRPB1	NS	0.002218875	0.062019525	0.007616342	NS
TSPAN8	NS	0.002218786	-0.062019771	0.007616342	NS
PHC3	NS	0.002220265	0.06201573	0.007619761	NS
RILPL2	NS	0.002221756	0.062011661	0.007622175	NS
OR14K1	NS	0.002221511	-0.062012331	0.007622175	NS
ZNF117	NS	0.002224052	0.0620054	0.007628699	NS
WSCD2	NS	0.002228345	-0.061993708	0.00764207	NS
FBXL20	NS	0.002230348	0.061988259	0.007644878	NS
TRIM24	NS	0.002230247	-0.061988535	0.007644878	NS
ZNF501	NS	0.002230218	-0.061988614	0.007644878	NS
CFAP99	NS	0.002232764	0.061981695	0.007651803	NS
RGS13	NS	0.002233381	-0.061980019	0.007652563	NS
ALAS1	NS	0.002234213	0.061977759	0.007654048	NS
OR5H1	NS	0.002234606	-0.061976695	0.007654048	NS
C16orf86	NS	0.002235009	-0.061975599	0.007654077	NS
SLAIN1	NS	0.002235564	-0.061974095	0.007654621	NS
MYOZ3	NS	0.00223635	0.061971962	0.007655959	NS
SPG11	NS	0.002238512	0.061966101	0.007662005	NS
CYB5R4	NS	0.002239901	0.061962339	0.007665403	NS
GNLY	NS	0.002248386	-0.0619394	0.007693081	NS
CFL2	NS	0.002249032	-0.061937656	0.007693932	NS
ANAPC4	NS	0.002252164	-0.061929212	0.007703284	NS
ELMOD2	NS	0.002255649	-0.061919827	0.007713842	NS
HIVEP2	NS	0.002257525	-0.06191478	0.007718337	NS
ZNF700	NS	0.002257761	-0.061914146	0.007718337	NS
STX4	NS	0.002263839	0.061897824	0.00773775	NS
VPS36	NS	0.002266657	0.061890273	0.007746011	NS

SPIN2B	NS	0.002270141	-0.061880944	0.00775655	NS
GGN	NS	0.002281106	0.061851677	0.007792638	NS
CPXCR1	NS	0.002282221	0.061848709	0.007795071	NS
ATP8B3	NS	0.00228305	-0.061846503	0.007795151	NS
NFKBIZ	NS	0.002282648	0.061847571	0.007795151	NS
VN1R2	NS	0.002283848	0.061844378	0.007796502	NS
RPS6KB1	NS	0.002287248	0.061835339	0.007806732	NS
TYR	NS	0.002287757	-0.061833988	0.007807091	NS
LETM1	NS	0.002298881	-0.061804505	0.007843669	NS
RXYLT1	NS	0.0022995	-0.061802868	0.007843703	NS
HEY2	NS	0.002299701	-0.061802336	0.007843703	NS
INHBE	NS	0.002310486	0.061773887	0.007879098	NS
SERPINA3	NS	0.002318614	0.061752525	0.007905423	NS
AKAP11	NS	0.002319804	-0.061749403	0.007908089	NS
FUT6	NS	0.002321849	0.061744042	0.00791242	NS
OR8H1	NS	0.002321892	0.061743929	0.00791242	NS
HYAL2	NS	0.002322318	-0.061742812	0.007912479	NS
CTNNA2	NS	0.002326813	-0.061731046	0.007925004	NS
CLSPN	NS	0.002328333	0.061727072	0.00792739	NS
MED6	NS	0.002328005	0.061727929	0.00792739	NS
TAS2R19	NS	0.002331705	-0.061718263	0.007937476	NS
OASL	NS	0.002334439	0.061711132	0.007945384	NS
HTR3B	NS	0.002335722	0.061707787	0.007948353	NS
CARNS1	NS	0.002336598	0.061705505	0.007949936	NS
CYTIP	NS	0.002338973	0.061699319	0.007956618	NS
FBXO7	NS	0.002340682	0.061694873	0.007961032	NS
CCN2	NS	0.002341213	0.061693491	0.00796144	NS
ABCA2	NS	0.002342118	-0.061691137	0.007961721	NS
PAFAH2	NS	0.002341885	-0.061691742	0.007961721	NS
SPATA33	NS	0.002343129	-0.06168851	0.007963758	NS
SPATC1	NS	0.002348913	0.061673495	0.007982016	NS
MALRD1	NS	0.002352903	-0.061663159	0.00799417	NS
CD99L2	NS	0.002357383	-0.06165157	0.008007986	NS
ARHGAP11B	NS	0.00235827	-0.061649277	0.008009595	NS
CYP21A2	NS	0.002366513	0.061628017	0.008036179	NS
PBK	NS	0.002369829	-0.061619482	0.008046029	NS
BCL10	NS	0.002371316	0.06161566	0.008049044	NS
STK19	NS	0.002371549	0.061615061	0.008049044	NS
AFDN	NS	0.002372814	-0.061611809	0.008051426	NS
NRSN1	NS	0.002373082	0.06161112	0.008051426	NS
MAP1LC3B	NS	0.002378475	0.061597283	0.008067636	NS
P4HB	NS	0.002378694	0.061596721	0.008067636	NS
AGRN	NS	0.002379597	-0.061594407	0.008068296	NS
RAMACL	NS	0.002379722	-0.061594086	0.008068296	NS
FLACC1	NS	0.00238046	-0.061592195	0.008069385	NS
NCKAP5	NS	0.002384826	-0.061581023	0.008082768	NS
SYF2	NS	0.002385998	0.061578027	0.008085324	NS
SPACA5B	NS	0.002387061	0.06157531	0.008087511	NS
TIGD6	NS	0.002392327	0.061561868	0.008103936	NS
PDRG1	NS	0.002394245	-0.061556979	0.008109014	NS
NEU1	NS	0.002395339	0.061554193	0.008111299	NS
C1orf131	NS	0.002396788	-0.061550505	0.008114785	NS
MAOA	NS	0.002400405	-0.061541304	0.008125609	NS
MAGEB18	NS	0.002401334	-0.061538943	0.008127333	NS
RHOQ	NS	0.002402688	0.061535504	0.008130493	NS

TRIM40	NS	0.002403924	-0.061532364	0.008133255	NS
PLG	NS	0.002407943	-0.06152217	0.008144664	NS
WWP1	NS	0.002408138	-0.061521676	0.008144664	NS
FAM50A	NS	0.002409759	0.061517568	0.008148724	NS
CHST8	NS	0.002417624	0.061497676	0.008173893	NS
TDRD12	NS	0.002418855	-0.061494569	0.008176625	NS
OR2D2	NS	0.002422896	-0.061484376	0.008188855	NS
PPEF2	NS	0.002424808	-0.061479559	0.008193887	NS
LRRC28	NS	0.002427237	-0.061473444	0.008200664	NS
HTR2C	NS	0.002430315	-0.061465703	0.008209631	NS
COG4	NS	0.002433928	-0.061456629	0.008219155	NS
ZNF554	NS	0.002433984	-0.061456488	0.008219155	NS
GLYR1	NS	0.002440328	-0.061440585	0.00823914	NS
COG5	NS	0.002446013	0.061426366	0.008256894	NS
YLPM1	NS	0.002448107	0.061421137	0.00826252	NS
CXCR1	NS	0.002450004	-0.061416402	0.008267209	NS
TUB	NS	0.00245035	-0.061415538	0.008267209	NS
SMURF1	NS	0.00245199	0.061411449	0.0082713	NS
ZNF619	NS	0.002456252	-0.061400832	0.008284234	NS
KLF3	NS	0.002461786	0.061387073	0.008301188	NS
FAM72D	NS	0.002462137	-0.061386201	0.008301188	NS
CLEC10A	NS	0.00246365	-0.061382444	0.008304842	NS
NBPF12	NS	0.002466815	0.061374592	0.008314064	NS
RYR2	NS	0.002467513	0.061372861	0.00831497	NS
ITM2B	NS	0.002468708	0.0613699	0.008317548	NS
KRT15	NS	0.002473424	0.061358226	0.008331987	NS
LGI3	NS	0.002474458	0.061355669	0.008334021	NS
RAB3A	NS	0.002475535	0.061353007	0.008336197	NS
LZTFL1	NS	0.00248666	-0.06132557	0.008370747	NS
ZMAT3	NS	0.002491049	-0.061314777	0.008382605	NS
USP33	NS	0.002495531	-0.061303772	0.008396229	NS
CLEC18C	NS	0.002498162	-0.061297322	0.00840175	NS
SPATA2	NS	0.002498474	0.061296556	0.00840175	NS
RBPJ	NS	0.002497729	0.061298382	0.00840175	NS
TULP3	NS	0.002502493	-0.061286716	0.008413801	NS
R3HCC1	NS	0.002504014	-0.061282994	0.008415993	NS
PDF	NS	0.002503699	-0.061283765	0.008415993	NS
PLGRKT	NS	0.00250562	-0.061279069	0.008417645	NS
PPP2R1B	NS	0.002505811	-0.061278603	0.008417645	NS
PCDHB16	NS	0.002505223	0.061280038	0.008417645	NS
CHMP4B	NS	0.002510918	0.061266132	0.008433338	NS
SFTPB	NS	0.002511954	0.061263605	0.008435355	NS
ACKR2	NS	0.00251282	-0.061261493	0.008436799	NS
TBP	NS	0.002526369	0.061228545	0.00848082	NS
USP17L25	NS	0.002531629	-0.061215799	0.008497001	NS
FKBP8	NS	0.002536327	0.061204433	0.00850982	NS
CMC1	NS	0.002536251	-0.061204616	0.00850982	NS
SCN2A	NS	0.002537102	0.061202559	0.008510945	NS
DGAT2	NS	0.0025435	0.061187117	0.008527973	NS
IVD	NS	0.002542765	0.06118889	0.008527973	NS
SNRPF	NS	0.002543341	-0.061187501	0.008527973	NS
PDYN	NS	0.002545619	-0.061182012	0.008533598	NS
ISYNA1	NS	0.002547816	0.061176719	0.008539487	NS
WDR61	NS	0.002551568	0.061167695	0.008548278	NS
ABCA12	NS	0.002550932	-0.061169224	0.008548278	NS

ZFAND2A	NS	0.002551873	0.061166961	0.008548278	NS
CENPS-CORT	NS	0.002552206	-0.061166162	0.008548278	NS
TMEM232	NS	0.002555198	-0.061158975	0.008556818	NS
CHIT1	NS	0.002557059	0.061154508	0.008561569	NS
TFCP2	NS	0.002558151	-0.061151889	0.008562263	NS
DUSP7	NS	0.002557917	0.06115245	0.008562263	NS
CDC42EP4	NS	0.002562389	0.061141733	0.008574382	NS
CAPN14	NS	0.002562658	-0.06114109	0.008574382	NS
ARHGEF15	NS	0.002563538	0.061138983	0.008575844	NS
ACOT6	NS	0.002565645	0.06113394	0.008581412	NS
DDX6	NS	0.002571533	0.061119873	0.008599618	NS
CRYM	NS	0.002583923	-0.061090365	0.008639559	NS
C1orf43	NS	0.002588292	0.06107999	0.008652674	NS
AASDHPPT	NS	0.002595652	-0.061062549	0.008675781	NS
KCNT1	NS	0.002596717	0.06106003	0.008677395	NS
GRAP	NS	0.002597032	-0.061059285	0.008677395	NS
LMNA	NS	0.00260295	0.061045301	0.008695668	NS
SLC7A9	NS	0.002608506	0.061032199	0.008711807	NS
NDC1	NS	0.002608681	-0.061031786	0.008711807	NS
STAU1	NS	0.002611937	0.061024121	0.008719671	NS
SLC17A8	NS	0.002611488	-0.061025177	0.008719671	NS
SPDYE3	NS	0.002612449	0.061022916	0.008719876	NS
EBNA1BP2	NS	0.00261502	-0.06101687	0.008726953	NS
SEC23B	NS	0.002616769	0.06101276	0.008731284	NS
LPAR4	NS	0.00261796	-0.061009963	0.008733753	NS
TCP11X2	NS	0.002618974	-0.061007582	0.008735631	NS
KRT33B	NS	0.002620027	-0.061005111	0.008737637	NS
ARMC2	NS	0.002626463	-0.060990027	0.008757591	NS
MARVELD1	NS	0.002627888	-0.06098669	0.008760835	NS
POLR1H	NS	0.002631034	-0.060979333	0.008769812	NS
FZR1	NS	0.002637418	0.060964428	0.008789577	NS
MOCS3	NS	0.002640552	0.060957122	0.008796993	NS
RAB15	NS	0.002647964	-0.060939875	0.008820169	NS
APTX	NS	0.002657846	-0.060916951	0.008851561	NS
CD163L1	NS	0.002663003	-0.060905019	0.00886721	NS
STAU2	NS	0.002668591	-0.060892113	0.008882093	NS
VWC2L	NS	0.002668421	-0.060892505	0.008882093	NS
LYSMD4	NS	0.00266885	-0.060891517	0.008882093	NS
SPIRE2	NS	0.002670398	-0.060887946	0.008885717	NS
ZNF658	NS	0.002671419	-0.060885591	0.008887588	NS
MAPKBP1	NS	0.002674764	0.060877885	0.008897186	NS
ERGIC3	NS	0.002676063	-0.060874894	0.008899978	NS
PADI1	NS	0.002680259	0.060865244	0.008912402	NS
MSTO1	NS	0.002685272	-0.060853733	0.008927538	NS
DNASE2	NS	0.002693219	-0.060835525	0.008952421	NS
NELL1	NS	0.002694604	0.060832356	0.008955488	NS
SCARB2	NS	0.002700336	-0.060819261	0.008972995	NS
ST7	NS	0.00270082	-0.060818156	0.008973064	NS
SDAD1	NS	0.002707843	-0.060802146	0.008994854	NS
PRPSAP1	NS	0.00270865	0.060800309	0.008995532	NS
TRIM33	NS	0.002708977	0.060799566	0.008995532	NS
DEFB105B	NS	0.002710917	-0.060795152	0.009000429	NS
APOH	NS	0.002712046	0.060792583	0.009002635	NS
SYT6	NS	0.002713925	-0.060788312	0.009007328	NS
NKX6-1	NS	0.002716856	0.060781656	0.00901551	NS

LRRTM2	NS	0.002720771	-0.060772776	0.009026953	NS
OR2C1	NS	0.002727375	0.060757822	0.009047311	NS
IQCG	NS	0.002729909	-0.060752092	0.009054167	NS
4SF19-DYNL	NS	0.002734504	0.060741715	0.009067854	NS
TPT1	NS	0.002743253	0.060722002	0.009095307	NS
STX5	NS	0.002745671	0.060716564	0.009100206	NS
NEUROD6	NS	0.002745214	0.060717591	0.009100206	NS
MRPL24	NS	0.002750988	-0.060704619	0.00911627	NS
CLDN7	NS	0.002752886	-0.060700362	0.009120996	NS
HDC	NS	0.002758736	-0.060687256	0.009138813	NS
CNST	NS	0.00275944	0.060685678	0.009139583	NS
GABPB1	NS	0.00276055	0.060683195	0.009140131	NS
GSTA3	NS	0.002760085	-0.060684237	0.009140131	NS
MEGF10	NS	0.002763467	-0.060676674	0.009147945	NS
LPAR3	NS	0.002763855	-0.060675805	0.009147945	NS
FAM124B	NS	0.002765371	0.060672419	0.009151396	NS
IER3	NS	0.002766063	0.060670874	0.009152121	NS
POU4F2	NS	0.002767937	0.060666689	0.009156759	NS
ERMARD	NS	0.002783981	-0.060630979	0.009208261	NS
DUSP11	NS	0.002786231	-0.060625986	0.009214128	NS
MRPL46	NS	0.002789198	-0.060619408	0.009222365	NS
PCDHB3	NS	0.002790478	0.060616574	0.009223948	NS
LCE2B	NS	0.00279063	-0.060616236	0.009223948	NS
MUC5B	NS	0.002791291	0.060614772	0.009224557	NS
GTSF1	NS	0.002792618	-0.060611835	0.009227365	NS
ZNF415	NS	0.002798292	0.060599286	0.009244535	NS
SLAMF9	NS	0.002809068	-0.060575516	0.009275388	NS
AKAP13	NS	0.002809009	0.060575647	0.009275388	NS
FAM220A	NS	0.002808269	-0.060577276	0.009275388	NS
DDX41	NS	0.002809838	0.060573823	0.009276346	NS
KCNAB3	NS	0.002819073	-0.060553523	0.009305249	NS
NTS	NS	0.002820252	-0.060550937	0.009307552	NS
EIF4A3	NS	0.002821761	0.060547627	0.009310945	NS
DHX58	NS	0.002829578	-0.060530507	0.009335149	NS
IFNA14	NS	0.002832188	-0.060524803	0.009342165	NS
FHIP2A	NS	0.002841778	0.060503876	0.00936901	NS
TASOR	NS	0.0028416	-0.060504264	0.00936901	NS
PAGE2	NS	0.002841547	-0.060504379	0.00936901	NS
ZSCAN4	NS	0.002846032	0.060494615	0.009381435	NS
ZBED8	NS	0.002846976	0.060492561	0.009382951	NS
NXT1	NS	0.002849231	0.060487658	0.009387648	NS
RAB8A	NS	0.002849371	0.060487353	0.009387648	NS
FTSJ3	NS	0.002852287	-0.060481019	0.009395655	NS
AIP	NS	0.002857901	-0.06046884	0.009412545	NS
PSMA8	NS	0.002873054	-0.060436075	0.009460844	NS
GYG2	NS	0.002873954	0.060434134	0.009462198	NS
ITPRIP	NS	0.002882787	0.060415115	0.009488049	NS
UBASH3B	NS	0.002883998	0.06041251	0.009490424	NS
CFAP74	NS	0.002886841	0.060406402	0.009498163	NS
GRAMD2A	NS	0.002895208	0.060388457	0.009522455	NS
C8orf76	NS	0.002895158	-0.060388563	0.009522455	NS
SIN3A	NS	0.002901195	0.060375644	0.009540526	NS
PON3	NS	0.002914856	-0.060346501	0.009583822	NS
ALG2	NS	0.002919435	-0.06033676	0.009597247	NS
ZFAND4	NS	0.002922368	-0.060330529	0.009605256	NS

MRPL15	NS	0.002928891	-0.060316689	0.009625063	NS
Y6G6F-LY6G	NS	0.002934606	-0.060304588	0.009642206	NS
HNRNPA2B1	NS	0.002935758	0.06030215	0.009644355	NS
HPS3	NS	0.002936997	0.06029953	0.009646788	NS
FLNC	NS	0.002938172	0.060297048	0.009649007	NS
LCN1	NS	0.002940078	0.060293021	0.009653629	NS
CAB39	NS	0.002944432	0.060283831	0.009666286	NS
RTF1	NS	0.00294544	0.060281705	0.009667956	NS
IDH1	NS	0.002948702	-0.060274829	0.009677023	NS
KCTD8	NS	0.00295982	-0.060251449	0.009711864	NS
NCAPG2	NS	0.002960772	-0.060249451	0.00971334	NS
PTAR1	NS	0.002962176	0.060246506	0.009716299	NS
ZYG11A	NS	0.002969617	0.060230915	0.009739055	NS
NFIB	NS	0.00297446	0.060220785	0.009753275	NS
RNF220	NS	0.002974961	-0.06021974	0.009753275	NS
TMEM200A	NS	0.002976275	-0.060216994	0.009755932	NS
DAB2	NS	0.002977837	-0.060213733	0.009759399	NS
MLF2	NS	0.002983986	0.060200907	0.009777897	NS
UGT1A8	NS	0.002991328	-0.060185624	0.009798638	NS
FBXO17	NS	0.003000265	0.060167069	0.00982625	NS
LAMTOR3	NS	0.003010359	-0.060146174	0.009857638	NS
ADCY8	NS	0.003015472	-0.060135611	0.009872713	NS
GIMAP6	NS	0.003019606	-0.060127085	0.009884575	NS
RPS6KA6	NS	0.003024417	0.060117174	0.009898651	NS
BRMS1L	NS	0.003027133	-0.060111586	0.009905865	NS
CHIA	NS	0.003035432	-0.06009454	0.009931342	NS
CHD1L	NS	0.00304009	-0.06008499	0.009942576	NS
BCL2A1	NS	0.003040406	0.060084342	0.009942576	NS
ABCA10	NS	0.003039629	-0.060085935	0.009942576	NS
KATNB1	NS	0.003041297	0.060082519	0.009943807	NS
RPL19	NS	0.00304732	0.060070195	0.009961819	NS
TRPT1	NS	0.003054367	0.060055804	0.00998317	NS
TMEM258	NS	0.00305857	-0.060047235	0.00999522	NS
CMTM6	NS	0.003065311	0.060033515	0.010015557	NS
MTMR10	NS	0.003066437	0.060031227	0.010017544	NS
EBAG9	NS	0.003073669	0.060016542	0.010039477	NS
NUTF2	NS	0.00308813	-0.059987274	0.010085008	NS
KIAA1586	NS	0.003092421	-0.059978614	0.010097317	NS
GSTA4	NS	0.003099785	-0.059963776	0.010119655	NS
BTN3A1	NS	0.003100558	-0.05996222	0.010120474	NS
ARPP19	NS	0.003102338	0.05995864	0.010123232	NS
IGBP1P2	NS	0.00310245	0.059958416	0.010123232	NS
TRIM65	NS	0.003111848	-0.05993954	0.010152188	NS
EIF5A	NS	0.003117784	0.059927646	0.010169839	NS
VPS4B	NS	0.003121111	0.059920989	0.010178975	NS
OOSP4A	NS	0.003122898	-0.059917414	0.010183089	NS
PRPF8	NS	0.003127692	0.059907838	0.010197002	NS
FUBP3	NS	0.003129743	0.059903744	0.010201972	NS
RUFY2	NS	0.003132369	0.059898507	0.010207094	NS
CCNA1	NS	0.003139094	-0.059885114	0.010227284	NS
LCE3A	NS	0.003143252	0.059876846	0.010239108	NS
GEMIN6	NS	0.003154025	-0.059855469	0.010272473	NS
PPP1R7	NS	0.003159001	-0.059845618	0.010286949	NS
CHST2	NS	0.00316098	0.059841705	0.010290516	NS
HGAP11A-S(	NS	0.00316116	-0.059841349	0.010290516	NS

ASPRV1	NS	0.003161925	-0.059839836	0.010291277	NS
TBC1D3L	NS	0.003166842	0.059830124	0.010305546	NS
ZBED6	NS	0.00316987	0.059824149	0.010313668	NS
TOLLIP	NS	0.003177022	0.05981006	0.010334074	NS
MT-ND1	NS	0.00317721	0.05980969	0.010334074	NS
GMPR	NS	0.003178066	-0.059808006	0.010335121	NS
CHRNB1	NS	0.003182457	-0.059799371	0.010347664	NS
SPRR2B	NS	0.003184589	-0.059795185	0.010352854	NS
ATG12	NS	0.003187297	0.059789868	0.010355812	NS
FAM222B	NS	0.003187183	0.059790091	0.010355812	NS
FAM53B	NS	0.00318638	0.059791668	0.010355812	NS
RPE	NS	0.003187638	-0.059789198	0.010355812	NS
PARP16	NS	0.003188538	-0.059787432	0.010356998	NS
CYP17A1	NS	0.003192066	-0.059780516	0.010366717	NS
ISY1	NS	0.003194894	0.059774976	0.010374162	NS
FAM168A	NS	0.003200856	0.059763314	0.010391775	NS
SRGAP2	NS	0.003213945	0.059737774	0.010432522	NS
WFDC10A	NS	0.003220653	-0.059724724	0.010452542	NS
NDUFB3	NS	0.003222355	-0.059721417	0.010456313	NS
VAT1	NS	0.003228065	0.059710332	0.010473086	NS
MYMX	NS	0.003232889	0.059700982	0.010486978	NS
DDX21	NS	0.003247999	0.059671774	0.010534228	NS
PRKD2	NS	0.003248863	0.059670108	0.010535264	NS
PPP2R5E	NS	0.00324983	0.059668244	0.010536635	NS
R3HDM1	NS	0.003254696	0.05965887	0.010550643	NS
TMEM176A	NS	0.003259422	0.059649777	0.010564195	NS
SYBU	NS	0.003264164	-0.059640666	0.010577793	NS
RPS6KA2	NS	0.00326656	-0.059636065	0.010583787	NS
AGTR2	NS	0.003267941	-0.059633416	0.010586489	NS
EOLA1	NS	0.003272746	0.059624207	0.010600278	NS
PANK4	NS	0.003274168	0.059621483	0.01060311	NS
MED28	NS	0.003279433	0.059611408	0.010618386	NS
DYNLRB2	NS	0.003283655	-0.059603341	0.010630277	NS
RAP1GAP	NS	0.003287708	0.059595605	0.010641619	NS
NSDHL	NS	0.003288822	-0.05959348	0.010643445	NS
DPP7	NS	0.00329658	-0.059578702	0.010666766	NS
APLNR	NS	0.003303925	0.059564738	0.010686962	NS
CENPV	NS	0.00330761	-0.059557744	0.010697092	NS
RNASEK	NS	0.003309527	0.059554108	0.010701504	NS
ZBTB47	NS	0.003310093	-0.059553035	0.010701546	NS
SMAD3	NS	0.003311326	0.059550698	0.010703744	NS
LEKR1	NS	0.003312864	-0.059547783	0.01070693	NS
PCDHGA10	NS	0.003319348	-0.05953551	0.010726095	NS
SHCBP1L	NS	0.003323275	0.059528088	0.010736991	NS
KRTAP19-5	NS	0.003331068	-0.059513383	0.010760373	NS
APBB1IP	NS	0.003334762	-0.059506424	0.010770493	NS
RBM14-RBM	NS	0.003335314	0.059505384	0.010770493	NS
PTGS2	NS	0.003361285	0.059456658	0.010850738	NS
GRPEL1	NS	0.003361173	0.059456867	0.010850738	NS
ADAMDEC1	NS	0.003375075	-0.059430924	0.010892043	NS
COLQ	NS	0.003375205	-0.059430681	0.010892043	NS
MFRP	NS	0.003377635	0.059426157	0.010898067	NS
LIG4	NS	0.003379509	-0.059422671	0.010900478	NS
DNAJC22	NS	0.00337943	0.059422816	0.010900478	NS
ANKDD1A	NS	0.00338291	0.059416346	0.010909631	NS

NUP62CL	NS	0.003386822	-0.059409077	0.010920429	NS
SLC24A3	NS	0.003390193	-0.059402822	0.010929476	NS
GPR137B	NS	0.00339792	0.059388502	0.010952564	NS
PHF12	NS	0.003401552	0.059381782	0.010962444	NS
TRIM14	NS	0.003405286	0.05937488	0.01097265	NS
MCIDAS	NS	0.003406623	0.05937241	0.010975132	NS
CD163	NS	0.003412291	-0.05936195	0.010991563	NS
USP20	NS	0.003418373	0.059350742	0.011007492	NS
ZNF785	NS	0.003418045	-0.059351347	0.011007492	NS
CYP4F11	NS	0.003421442	-0.059345094	0.011015542	NS
PLEKHH3	NS	0.003429513	0.059330263	0.011039689	NS
THAP11	NS	0.003435807	-0.059318718	0.011058112	NS
C4orf48	NS	0.003438286	-0.059314177	0.011064249	NS
ZNF774	NS	0.003443096	0.059305373	0.011077886	NS
PDS5A	NS	0.003445384	-0.059301189	0.011083406	NS
COX19	NS	0.003447678	0.059296997	0.011088942	NS
B4GALT4	NS	0.003459446	0.059275531	0.011124944	NS
CYB5R1	NS	0.003460759	0.05927314	0.011127319	NS
MFSD12	NS	0.003462912	0.059269222	0.01113239	NS
SUSD1	NS	0.003465534	-0.059264452	0.011137813	NS
TTPA	NS	0.003465749	0.059264061	0.011137813	NS
KBTBD4	NS	0.003469743	-0.059256804	0.011148795	NS
DIRAS2	NS	0.003472115	0.059252496	0.011154566	NS
CA2	NS	0.003479956	0.059238277	0.011177902	NS
RBM12B	NS	0.003482277	-0.059234074	0.011183501	NS
PHF3	NS	0.003484635	0.059229807	0.011189216	NS
PRDX2	NS	0.003498845	-0.059204145	0.011231119	NS
NAA80	NS	0.003500065	0.059201946	0.011233172	NS
PSMC6	NS	0.003503747	0.059195315	0.011243123	NS
GRIN2D	NS	0.003505731	0.059191742	0.011245763	NS
TUBA3E	NS	0.003505234	0.059192638	0.011245763	NS
PROM1	NS	0.003510922	-0.059182409	0.011258682	NS
IFNA16	NS	0.003516744	-0.059171955	0.011275484	NS
KPNA6	NS	0.003518814	0.059168241	0.011280027	NS
PCDHGB4	NS	0.003519327	-0.059167323	0.011280027	NS
DEPDC5	NS	0.003520917	0.059164471	0.011283258	NS
HOXB7	NS	0.003527101	0.059153397	0.011301203	NS
MT-CO1	NS	0.003530078	0.059148072	0.011308869	NS
RPS27AP5	NS	0.00353143	-0.059145655	0.011309499	NS
RASSF5	NS	0.003531443	0.059145631	0.011309499	NS
FA2H	NS	0.003532904	-0.05914302	0.011310864	NS
C17orf114	NS	0.003533038	0.059142781	0.011310864	NS
AQP7	NS	0.003540365	0.059129703	0.011331823	NS
RPF2	NS	0.003540756	-0.059129005	0.011331823	NS
OBI1	NS	0.003549271	-0.059113838	0.011357197	NS
APRT	NS	0.003554683	-0.059104214	0.011372637	NS
CCDC57	NS	0.003560137	0.059094531	0.011386319	NS
TEDC1	NS	0.003559767	0.059095187	0.011386319	NS
OR5B17	NS	0.003562062	-0.059091115	0.011390597	NS
NOX1	NS	0.003573457	-0.059070937	0.011424401	NS
FSD1L	NS	0.003573814	-0.059070305	0.011424401	NS
ASPG	NS	0.003579828	0.059059679	0.011440016	NS
RGS7	NS	0.003579881	0.059059586	0.011440016	NS
GNG13	NS	0.003584265	-0.059051851	0.011452135	NS
BCO2	NS	0.003588346	-0.059044657	0.011463283	NS

SMIM38	NS	0.0035907	0.059040512	0.011468909	NS
OR8B12	NS	0.003595057	-0.059032844	0.011480932	NS
TMEM87B	NS	0.003601819	-0.059020964	0.011500627	NS
CEBPA	NS	0.003606872	0.059012097	0.011514862	NS
CYP3A5	NS	0.003609024	-0.059008325	0.011517933	NS
APOL5	NS	0.003608792	-0.059008731	0.011517933	NS
PRMT6	NS	0.003610639	-0.059005495	0.011521189	NS
HDAC8	NS	0.00361536	-0.05899723	0.011533602	NS
CALM3	NS	0.0036162	0.058995759	0.011533602	NS
MSANTD1	NS	0.003616317	0.058995555	0.011533602	NS
BABAM2	NS	0.003617865	0.058992849	0.011536637	NS
MCM5	NS	0.003620314	0.058988567	0.011542547	NS
STK31	NS	0.003625534	-0.058979451	0.011557285	NS
FOXL2NB	NS	0.003630198	0.058971316	0.011570247	NS
FOXN1	NS	0.003641614	0.058951444	0.011604722	NS
KIAA2013	NS	0.003648378	0.058939697	0.011624363	NS
CNOT11	NS	0.003658842	0.058921563	0.011655784	NS
IL36RN	NS	0.003662022	-0.05891606	0.011663996	NS
PDPK1	NS	0.003664802	0.058911254	0.011669927	NS
ZNF792	NS	0.00366509	-0.058910757	0.011669927	NS
CDKN1A	NS	0.003675689	0.058892465	0.011701749	NS
NWD2	NS	0.003686462	-0.058873921	0.011734116	NS
PDZD4	NS	0.003689293	0.058869057	0.011739265	NS
ETV6	NS	0.003688787	0.058869925	0.011739265	NS
NTMT2	NS	0.003690716	-0.058866613	0.011741863	NS
RAB9B	NS	0.003693553	-0.058861742	0.011748959	NS
C17orf67	NS	0.003694212	-0.058860612	0.011749122	NS
ADM5	NS	0.003700648	-0.058849578	0.011767658	NS
KRTAP9-9	NS	0.003707798	0.05883734	0.011788459	NS
TAF7	NS	0.003715037	0.058824974	0.011809533	NS
DZIP1	NS	0.003716785	0.058821989	0.011813153	NS
LRRC20	NS	0.00372484	-0.058808259	0.011836809	NS
NDST4	NS	0.003731858	-0.058796318	0.01185522	NS
MAF1	NS	0.003731431	0.058797044	0.01185522	NS
KRT23	NS	0.003736282	-0.058788802	0.011866207	NS
MAP3K12	NS	0.003736543	0.058788358	0.011866207	NS
GTF3C2	NS	0.003748832	-0.058767523	0.011903279	NS
JAM3	NS	0.003755262	-0.058756644	0.011921742	NS
EFNB2	NS	0.003758694	-0.058750847	0.011930678	NS
ZFAND5	NS	0.003760892	0.058747135	0.01193529	NS
DENND2D	NS	0.00376138	-0.058746311	0.01193529	NS
NOL8	NS	0.003764814	-0.058740517	0.01194423	NS
ZBTB1	NS	0.003770243	0.058731368	0.011957532	NS
SHOC1	NS	0.003770186	-0.058731464	0.011957532	NS
ATP6V1H	NS	0.003771058	0.058729995	0.011958157	NS
MCM4	NS	0.003772009	-0.058728394	0.011959214	NS
SERPINB13	NS	0.003774181	-0.058724737	0.011964143	NS
SEMA3B	NS	0.003779445	0.058715885	0.011972986	NS
ZNRF2	NS	0.003779338	-0.058716065	0.011972986	NS
EDDM3A	NS	0.00377828	-0.058717845	0.011972986	NS
CT47A7	NS	0.003784692	0.058707074	0.011987644	NS
NOX4	NS	0.003791633	-0.058695433	0.012007665	NS
PRR3	NS	0.003799661	-0.058681995	0.01203112	NS
ABCD4	NS	0.003809957	0.058664796	0.012059775	NS
NPPC	NS	0.003809777	0.058665096	0.012059775	NS

TWIST1	NS	0.003822954	0.058643147	0.012098936	NS
PIANP	NS	0.003826851	0.058636668	0.012108846	NS
ARID5B	NS	0.003827336	0.058635862	0.012108846	NS
ARFGAP1	NS	0.00383891	0.05861666	0.01213895	NS
PALLD	NS	0.003839028	0.058616463	0.01213895	NS
PRPS1	NS	0.003838107	-0.058617991	0.01213895	NS
TVP23A	NS	0.00383936	-0.058615914	0.01213895	NS
CLEC1B	NS	0.003849088	-0.058599816	0.012167719	NS
EVPLL	NS	0.003850174	0.058598021	0.012169167	NS
BAZ2A	NS	0.003855517	0.058589197	0.012184064	NS
NXF3	NS	0.003859367	-0.058582846	0.01219424	NS
PTPN18	NS	0.003861098	-0.058579993	0.012197716	NS
RMC1	NS	0.003863034	0.058576802	0.012201842	NS
GPR63	NS	0.00387033	-0.058564792	0.012222894	NS
CTAGE8	NS	0.003871895	-0.058562219	0.012225841	NS
MTRNR2L4	NS	0.003873488	-0.058559602	0.012228874	NS
FPR3	NS	0.003878003	0.058552185	0.012241131	NS
C3orf20	NS	0.00388675	-0.05853784	0.012264741	NS
OR4C3	NS	0.003886302	-0.058538573	0.012264741	NS
SOCS1	NS	0.003890107	0.058532341	0.012273336	NS
ZNF768	NS	0.003893137	-0.058527382	0.012280894	NS
TTYH1	NS	0.003897862	0.058519658	0.012293793	NS
SIX4	NS	0.003901347	-0.058513965	0.012302781	NS
CCL3	NS	0.003902591	0.058511934	0.0123047	NS
TSC1	NS	0.003912857	-0.058495197	0.012335016	NS
ADAL	NS	0.003913481	-0.058494181	0.012335016	NS
SCPEP1	NS	0.003930217	0.058466984	0.01238575	NS
OR4K5	NS	0.003932714	-0.058462936	0.012391602	NS
CACNA1A	NS	0.003935949	0.058457694	0.012399776	NS
SH3BP2	NS	0.003955682	0.058425803	0.01245789	NS
CCDC78	NS	0.003955638	0.058425874	0.01245789	NS
CTDSPL2	NS	0.003956977	0.058423715	0.012459941	NS
CTSG	NS	0.003964132	-0.058412192	0.012480438	NS
ATF5	NS	0.003972408	0.058398885	0.012504462	NS
PATE1	NS	0.003983793	-0.058380623	0.01253622	NS
TBCCD1	NS	0.003986274	-0.058376648	0.01254199	NS
ABCG8	NS	0.003989174	-0.058372006	0.012549075	NS
CD46	NS	0.00399038	0.058370077	0.012550829	NS
MAP1B	NS	0.003998373	-0.058357302	0.012573925	NS
G6PC1	NS	0.004002264	0.058351092	0.012584116	NS
NDC80	NS	0.00400496	0.058346792	0.012590548	NS
PCP4	NS	0.004010676	-0.058337685	0.012606469	NS
PPIAL4A	NS	0.004015097	0.058330647	0.012618319	NS
CLIP3	NS	0.004022596	0.05831873	0.012639831	NS
COL8A1	NS	0.004023747	0.058316901	0.012641398	NS
CASP9	NS	0.00402523	0.058314547	0.012644004	NS
PKHD1	NS	0.004026371	-0.058312737	0.012645535	NS
SMIM20	NS	0.004047115	-0.058279899	0.012708623	NS
C19orf81	NS	0.004050833	0.05827403	0.012718235	NS
GGT2	NS	0.00405777	-0.05826309	0.012737951	NS
PCBP1	NS	0.004064183	0.058252994	0.012754281	NS
UGT1A7	NS	0.00406429	-0.058252825	0.012754281	NS
PITPNM2	NS	0.004069918	0.058243977	0.01276987	NS
ENPP7	NS	0.004073712	0.058238018	0.012779704	NS
SNX22	NS	0.004080943	-0.058226674	0.012800314	NS

LAMB2	NS	0.004084542	-0.058221036	0.012809525	NS
ZNF646	NS	0.004087778	0.058215969	0.012817598	NS
RBL1	NS	0.004090782	-0.058211269	0.012822862	NS
ELOVL7	NS	0.004090734	0.058211345	0.012822862	NS
TMEM215	NS	0.004098939	-0.058198522	0.012846352	NS
CYB561D1	NS	0.004103437	-0.058191505	0.012858365	NS
METTL1	NS	0.004117997	0.058168832	0.012899768	NS
IAPP	NS	0.004119259	0.05816687	0.012899768	NS
FGF2	NS	0.004119315	-0.058166783	0.012899768	NS
CYP2B6	NS	0.004117761	0.058169199	0.012899768	NS
HOOK1	NS	0.004120352	0.058165172	0.012900927	NS
ATP4A	NS	0.004123174	0.058160787	0.012903503	NS
PRF1	NS	0.004122887	-0.058161234	0.012903503	NS
KRTAP2-3	NS	0.004122699	-0.058161525	0.012903503	NS
TRAIP	NS	0.004125119	-0.058157768	0.012907503	NS
CHURC1	NS	0.004158299	-0.058106452	0.01300922	NS
TRIM46	NS	0.004165149	0.058095905	0.013026438	NS
TUBB2A	NS	0.004170438	0.058087772	0.013040871	NS
OLFM2	NS	0.004180656	-0.058072084	0.013070713	NS
FNDC3A	NS	0.004183692	0.058067431	0.013075979	NS
MCM8	NS	0.004183123	0.058068303	0.013075979	NS
VPS37D	NS	0.004194073	0.058051539	0.01310631	NS
POLR3B	NS	0.004194751	-0.058050503	0.013106311	NS
EFS	NS	0.004197154	0.058046829	0.013111704	NS
NPIPB15	NS	0.004197846	0.058045772	0.013111749	NS
RPS5	NS	0.004198749	0.058044393	0.013112453	NS
AGFG1	NS	0.004201666	0.058039939	0.013119445	NS
FAM3D	NS	0.004205748	-0.05803371	0.013130075	NS
EIF4E	NS	0.004212399	0.058023574	0.013148716	NS
AKAP8	NS	0.00423535	0.057988704	0.013211835	NS
OR1F1	NS	0.004234672	0.057989731	0.013211835	NS
PIAS2	NS	0.004237261	-0.057985809	0.013215665	NS
UTP25	NS	0.004240455	0.057980971	0.013223498	NS
THAP4	NS	0.004244127	0.057975414	0.013232817	NS
CFAP46	NS	0.004246516	0.057971801	0.013236002	NS
SGSH	NS	0.004245983	0.057972608	0.013236002	NS
MBD2	NS	0.00424739	0.05797048	0.013236594	NS
USF1	NS	0.004254483	0.057959766	0.013256564	NS
NOTCH2NLR	NS	0.004263563	0.057946074	0.013282719	NS
GCLC	NS	0.004272394	-0.057932783	0.013308088	NS
PAX9	NS	0.004279973	-0.057921396	0.013329552	NS
CTSO	NS	0.004291714	-0.057903793	0.013363966	NS
SAP130	NS	0.004293171	0.057901611	0.013366354	NS
SLC38A3	NS	0.004302744	0.057887295	0.013394002	NS
PPM1G	NS	0.004321863	-0.057858787	0.013451357	NS
ANKRA2	NS	0.004327127	0.057850958	0.013465575	NS
GCC1	NS	0.004335396	-0.057838678	0.013489139	NS
SPTY2D1	NS	0.00435162	0.057814646	0.01353744	NS
ZNF544	NS	0.004359364	0.057803202	0.013559353	NS
TMEM121B	NS	0.004360511	-0.05780151	0.013560741	NS
OR2T34	NS	0.004364052	-0.057796285	0.013569571	NS
DPEP3	NS	0.004367899	-0.057790613	0.013579352	NS
EIF3J	NS	0.004378473	-0.057775046	0.013608767	NS
TMEM249	NS	0.004378766	0.057774615	0.013608767	NS
KCNH6	NS	0.00438928	0.057759172	0.013639251	NS

RAB5IF	NS	0.004393598	0.057752838	0.013650478	NS
CEP72	NS	0.004399362	-0.057744393	0.013664001	NS
CIDEB	NS	0.004398755	0.057745282	0.013664001	NS
ZNF41	NS	0.004400109	-0.057743299	0.013664128	NS
EIF5A2	NS	0.004403236	-0.057738722	0.013671647	NS
SLCO1A2	NS	0.004410911	0.057727502	0.013693279	NS
ODF3	NS	0.004420949	0.057712855	0.013720372	NS
PAMR1	NS	0.004422352	-0.057710809	0.013722194	NS
MYO15B	NS	0.004427953	0.05770265	0.013737372	NS
PNMA8B	NS	0.004429287	0.057700709	0.013739308	NS
NCAPD2	NS	0.004431814	-0.057697032	0.013744945	NS
MYO1H	NS	0.004432921	-0.057695422	0.013746175	NS
SRPRB	NS	0.004434368	-0.057693319	0.013746255	NS
PET100	NS	0.004434268	-0.057693463	0.013746255	NS
DDX20	NS	0.004440871	0.057683871	0.013762008	NS
PDLIM5	NS	0.004440612	0.057684246	0.013762008	NS
GK5	NS	0.004443468	-0.057680102	0.013767851	NS
PVALB	NS	0.004445071	-0.057677776	0.013770614	NS
USP19	NS	0.004450286	0.057670214	0.013784564	NS
TYSND1	NS	0.004458323	-0.057658578	0.013805106	NS
IL15	NS	0.004458345	-0.057658547	0.013805106	NS
RBM19	NS	0.004465176	-0.057648671	0.013824047	NS
CYP51A1	NS	0.004466727	-0.057646431	0.013826637	NS
USP38	NS	0.004473126	0.057637195	0.013844231	NS
ZNF91	NS	0.004476029	0.057633009	0.013851002	NS
FCER1G	NS	0.004478184	0.057629903	0.013855455	NS
OR10G9	NS	0.004479168	0.057628485	0.013856285	NS
ATP6V1G1	NS	0.004496559	0.057603475	0.013902327	NS
LRPPRC	NS	0.004494791	-0.057606013	0.013902327	NS
TPM3	NS	0.004496924	0.057602951	0.013902327	NS
LCE1A	NS	0.004496747	0.057603206	0.013902327	NS
CNTN3	NS	0.00449829	-0.05760099	0.013904278	NS
AXIN2	NS	0.004498992	0.057599983	0.013904278	NS
MESP2	NS	0.004502139	0.057595469	0.013911783	NS
NPBWR2	NS	0.004509821	0.057584462	0.013931432	NS
DENND5B	NS	0.004510067	-0.05758411	0.013931432	NS
SERINC4	NS	0.004510657	0.057583265	0.013931432	NS
NPY	NS	0.004513421	-0.05757931	0.013937744	NS
ZNF717	NS	0.004517211	-0.057573889	0.013947224	NS
PCDH19	NS	0.004522777	0.057565937	0.013959954	NS
TRIM49B	NS	0.004522365	-0.057566525	0.013959954	NS
PPP2R5D	NS	0.004526189	0.057561065	0.013968259	NS
COX7B2	NS	0.004533287	-0.057550944	0.013987933	NS
CRP	NS	0.004542235	-0.057538205	0.014008842	NS
SAMD1	NS	0.004541522	-0.057539219	0.014008842	NS
VIPAS39	NS	0.004546767	-0.05753176	0.014020588	NS
CYP4V2	NS	0.004549364	-0.057528071	0.014026359	NS
PRR16	NS	0.004554772	-0.057520392	0.014040798	NS
SEC31B	NS	0.004556376	0.057518118	0.014043504	NS
C1RL	NS	0.004562652	0.057509219	0.014060612	NS
SIX6	NS	0.004563649	0.057507807	0.014060991	NS
EZHIP	NS	0.004564871	0.057506077	0.014060991	NS
HP	NS	0.004564955	-0.057505958	0.014060991	NS
MS4A15	NS	0.00456828	-0.057501251	0.014068994	NS
GMCL1	NS	0.004575725	-0.057490723	0.014089681	NS

MRPL13	NS	0.004577165	-0.057488689	0.014091872	NS
PTCH1	NS	0.004580507	-0.057483969	0.014099919	NS
STARD6	NS	0.004584178	0.057478788	0.014107581	NS
PHF2	NS	0.004584454	-0.0574784	0.014107581	NS
ABCC6	NS	0.004588122	0.057473226	0.014116626	NS
IGLL1	NS	0.004599603	0.057457062	0.014142957	NS
BAG3	NS	0.004599169	0.057457671	0.014142957	NS
ANAPC2	NS	0.004599337	0.057457435	0.014142957	NS
OR51A4	NS	0.004599521	-0.057457176	0.014142957	NS
SETMAR	NS	0.00460057	-0.057455701	0.014143684	NS
SNU13	NS	0.004612165	-0.057439416	0.014175378	NS
ITGB1BP2	NS	0.004612344	-0.057439165	0.014175378	NS
GPR82	NS	0.00462859	-0.05741641	0.014223048	NS
GLO1	NS	0.004630264	0.057414068	0.01422474	NS
RAPH1	NS	0.00463061	-0.057413585	0.01422474	NS
LGALS13	NS	0.004638581	-0.057402451	0.014246965	NS
ZNF552	NS	0.004640801	-0.057399353	0.014251523	NS
SPACA9	NS	0.004647558	0.057389933	0.014270007	NS
GTF2H2	NS	0.004650143	-0.057386331	0.014275682	NS
PERM1	NS	0.004651874	0.057383921	0.014278731	NS
AIM2	NS	0.004657729	-0.057375775	0.014294436	NS
CFHR3	NS	0.004659315	-0.05737357	0.014297036	NS
GAGE10	NS	0.004666446	0.057363665	0.014316646	NS
GYPB	NS	0.004667787	-0.057361804	0.014318491	NS
H4C13	NS	0.00467009	0.057358607	0.014323288	NS
TIMM10B	NS	0.004672471	0.057355306	0.014326463	NS
XPNPEP2	NS	0.004682071	-0.057342007	0.014351293	NS
CCDC87	NS	0.004682187	0.057341846	0.014351293	NS
PRSS27	NS	0.004683477	0.057340062	0.014352973	NS
SCD5	NS	0.004684708	-0.057338359	0.014353104	NS
TMEM141	NS	0.004685003	-0.057337951	0.014353104	NS
NAT14	NS	0.004688157	0.057333589	0.014355951	NS
MRPS36	NS	0.004687909	-0.057333932	0.014355951	NS
GLDN	NS	0.004686781	-0.057335491	0.014355951	NS
HOXB2	NS	0.004690408	-0.057330478	0.014360572	NS
C2orf49	NS	0.004698725	0.057318996	0.014382717	NS
SPDYE17	NS	0.00469975	0.057317581	0.014382717	NS
AP5Z1	NS	0.00469987	0.057317416	0.014382717	NS
SHE	NS	0.004703199	0.057312826	0.014390628	NS
IL2RB	NS	0.004707173	-0.05730735	0.014400513	NS
PRAMEF17	NS	0.004709818	0.057303708	0.014406327	NS
KCTD10	NS	0.004715321	0.057296137	0.014420879	NS
SPINT1	NS	0.004719032	-0.057291035	0.014429951	NS
GLRA2	NS	0.004722363	-0.057286459	0.014433997	NS
ST3GAL5	NS	0.004722593	0.057286144	0.014433997	NS
SUMO3	NS	0.004722072	0.057286859	0.014433997	NS
WWC3	NS	0.004725961	0.05728152	0.01444201	NS
RASSF10	NS	0.004731739	0.057273595	0.014457386	NS
PPP1R15A	NS	0.004750003	0.057248601	0.0145109	NS
KANK4	NS	0.00475627	-0.057240046	0.014527751	NS
ELMO2	NS	0.004758437	0.057237089	0.014529785	NS
LIN7C	NS	0.00475824	-0.057237357	0.014529785	NS
ATP7A	NS	0.004764454	0.057228887	0.014545864	NS
ABCB6	NS	0.004791087	-0.057192697	0.014624866	NS
EED	NS	0.004796598	-0.057185231	0.014639076	NS

SEPHS2	NS	0.004797255	-0.057184341	0.014639076	NS
SWAP70	NS	0.004805532	0.057173143	0.014662024	NS
GEM	NS	0.004816216	-0.057158715	0.014692306	NS
GCNT3	NS	0.00482086	0.057152453	0.014701838	NS
NVL	NS	0.004820471	-0.057152977	0.014701838	NS
PLA2G10	NS	0.004821946	0.057150989	0.014702834	NS
LRRC52	NS	0.004822846	-0.057149777	0.014703261	NS
KCTD6	NS	0.004828468	0.057142205	0.014718083	NS
EFHC1	NS	0.004832994	0.057136117	0.014727951	NS
SYDE2	NS	0.004833697	0.05713517	0.014727951	NS
APOL2	NS	0.004833988	-0.05713478	0.014727951	NS
NRP1	NS	0.00484145	-0.057124753	0.014748366	NS
MAPKAPK3	NS	0.004847917	0.057116075	0.014765741	NS
ZC3HAV1	NS	0.004859973	0.057099925	0.014800132	NS
UCK2	NS	0.00486454	-0.057093816	0.01481171	NS
ERVW-1	NS	0.004866795	-0.057090802	0.014816246	NS
NIPSNAP3A	NS	0.004887576	-0.057063085	0.014877169	NS
EPB41L2	NS	0.004899131	-0.057047718	0.014907653	NS
DPPA4	NS	0.004898981	0.057047918	0.014907653	NS
FBP2	NS	0.004902727	-0.057042943	0.014916249	NS
PRRG4	NS	0.004908369	-0.057035456	0.01493107	NS
NBPF26	NS	0.004913704	0.057028385	0.014944949	NS
BLK	NS	0.004920903	0.057018854	0.014962674	NS
ALOX15	NS	0.004921078	0.057018623	0.014962674	NS
PRPS2	NS	0.004931987	-0.057004204	0.014991134	NS
ZC3H12B	NS	0.004931496	-0.057004852	0.014991134	NS
BBLN	NS	0.004946847	0.056984609	0.01503394	NS
TUBAL3	NS	0.00495101	-0.056979129	0.01504423	NS
HOXA5	NS	0.004954315	-0.056974782	0.015051911	NS
ATE1	NS	0.004965369	0.05696026	0.015080761	NS
TRIQK	NS	0.004964782	-0.056961031	0.015080761	NS
HMGN1	NS	0.004966733	0.05695847	0.015082536	NS
CRNKL1	NS	0.004968264	-0.056956462	0.015084818	NS
TSSK6	NS	0.004978858	-0.056942579	0.015114614	NS
HMGCR	NS	0.004982458	0.056937867	0.015122188	NS
CTRL	NS	0.004982916	-0.056937269	0.015122188	NS
CEP152	NS	0.004985681	-0.056933652	0.015128152	NS
ATP1A3	NS	0.004986444	0.056932655	0.015128152	NS
GNPTAB	NS	0.005005297	0.05690805	0.015180589	NS
HTRA4	NS	0.005008345	-0.056904079	0.015187454	NS
IPO5	NS	0.005009244	-0.056902908	0.015187802	NS
LRTM1	NS	0.005013223	-0.05689773	0.015197484	NS
CFAP299	NS	0.005018072	-0.056891423	0.015209804	NS
MYEOV	NS	0.00503074	0.056874974	0.015243427	NS
SLC11A2	NS	0.005032306	0.056872944	0.015245783	NS
DNAJC14	NS	0.005036157	0.056867953	0.015255063	NS
CCDC18	NS	0.005049659	0.056850478	0.01529357	NS
PCDH17	NS	0.005051403	-0.056848225	0.015294064	NS
FAM228B	NS	0.005050979	0.056848773	0.015294064	NS
TAF1C	NS	0.005057973	0.056839741	0.01531156	NS
RNASET2	NS	0.005070119	0.056824081	0.01534593	NS
CRB1	NS	0.005073851	-0.056819277	0.015354825	NS
ZCCHC12	NS	0.005076064	-0.056816428	0.015359122	NS
PPIN-WFDC	NS	0.005077046	-0.056815166	0.01535969	NS
NECTIN2	NS	0.005078396	-0.05681343	0.015361373	NS

FNDC3B	NS	0.005079666	0.056811797	0.015362815	NS
OR4K15	NS	0.005089707	-0.056798899	0.015390777	NS
SP4	NS	0.005096527	-0.056790151	0.015408444	NS
RAP2A	NS	0.005097141	-0.056789364	0.015408444	NS
DTNB	NS	0.005103087	0.056781747	0.01542401	NS
ARRB1	NS	0.00510788	0.056775613	0.015436085	NS
HCAR2	NS	0.005109045	0.056774123	0.015437197	NS
CDON	NS	0.005111208	0.056771357	0.015438912	NS
GJB3	NS	0.005110877	0.056771781	0.015438912	NS
TBATA	NS	0.005115509	0.05676586	0.015447083	NS
MARCHF10	NS	0.005114748	0.056766833	0.015447083	NS
PPM1D	NS	0.005125721	0.056752826	0.015475504	NS
RIPK4	NS	0.005128266	0.056749581	0.015480774	NS
PKP4	NS	0.005139242	-0.056735605	0.015511486	NS
DCTN6	NS	0.005140995	0.056733375	0.015514359	NS
USP17L27	NS	0.00514229	-0.056731727	0.015515849	NS
PRX	NS	0.005149181	-0.05672297	0.01553422	NS
SEC61A2	NS	0.005160778	-0.056708257	0.015559745	NS
NECAB3	NS	0.005160858	-0.056708157	0.015559745	NS
XRRA1	NS	0.005159257	-0.056710186	0.015559745	NS
NPTX1	NS	0.005159268	0.056710172	0.015559745	NS
NOL3	NS	0.005170671	-0.056695731	0.015586903	NS
YOD1	NS	0.005174348	0.05669108	0.015595558	NS
DRG2	NS	0.005177971	-0.056686501	0.015601861	NS
MTRNR2L1	NS	0.005178051	0.056686399	0.015601861	NS
APOBEC3A	NS	0.00517961	0.056684429	0.015601883	NS
EPHB2	NS	0.00517967	-0.056684353	0.015601883	NS
ARHGEF3	NS	0.005182826	0.056680368	0.01560896	NS
ALDH1A2	NS	0.005186202	0.056676107	0.015616697	NS
CLEC5A	NS	0.005205421	0.056651897	0.01567213	NS
UXT	NS	0.005211587	0.056644147	0.015688254	NS
XAGE1A	NS	0.005213557	-0.056641672	0.015691745	NS
GLI2	NS	0.00521907	0.056634752	0.015705896	NS
TSPAN11	NS	0.005221301	0.056631954	0.015710167	NS
ZNF586	NS	0.005229346	0.056621872	0.015729482	NS
SMIM10	NS	0.005229101	-0.056622179	0.015729482	NS
TMEM167B	NS	0.005237502	0.056611664	0.015751568	NS
ZFTA	NS	0.005246288	0.056600685	0.015775538	NS
YY2	NS	0.005264176	-0.056578381	0.01582687	NS
STXBP4	NS	0.005267402	-0.056574367	0.015834109	NS
METTL14	NS	0.005269921	0.056571233	0.015839222	NS
PCOLCE2	NS	0.005282171	-0.056556014	0.015873576	NS
ZNF572	NS	0.005289243	-0.056547243	0.01589236	NS
ZMYND15	NS	0.005296575	0.05653816	0.01591192	NS
CHCHD5	NS	0.00531622	0.056513879	0.015965983	NS
ZNF518B	NS	0.005316001	0.056514149	0.015965983	NS
CEACAM7	NS	0.005324415	-0.056503774	0.015988115	NS
RWDD2B	NS	0.005330025	-0.056496865	0.016002476	NS
LIPG	NS	0.005332386	0.05649396	0.016007081	NS
PCDHB13	NS	0.005333471	-0.056492624	0.016007856	NS
SP140	NS	0.005335616	-0.056489986	0.016009329	NS
CAVIN3	NS	0.005335137	-0.056490575	0.016009329	NS
TTC16	NS	0.00534337	-0.056480455	0.016030111	NS
HAO1	NS	0.00534802	-0.056474747	0.016036053	NS
OR5AP2	NS	0.005347773	-0.05647505	0.016036053	NS

SRARP	NS	0.005346832	0.056476205	0.016036053	NS
ZNF763	NS	0.005348665	-0.056473955	0.016036053	NS
C12orf4	NS	0.005365102	-0.056453813	0.016082841	NS
BBS2	NS	0.005369578	-0.056448337	0.016093768	NS
SHOX2	NS	0.00537048	-0.056447234	0.01609398	NS
EIF1B	NS	0.005382398	0.056432677	0.016127198	NS
OR2M3	NS	0.005390862	-0.056422357	0.016147559	NS
NOVA1	NS	0.005391807	-0.056421205	0.016147892	NS
CXCR3	NS	0.005395907	0.056416212	0.01615767	NS
TBC1D8B	NS	0.005398952	0.056412506	0.016164288	NS
CD93	NS	0.005414918	0.056393104	0.016208244	NS
CAMKMT	NS	0.005415309	-0.05639263	0.016208244	NS
PRSS41	NS	0.005418831	0.056388357	0.016213773	NS
RTL1	NS	0.005418548	0.056388701	0.016213773	NS
ANKRD10	NS	0.005420351	0.056386514	0.016215813	NS
PRAMEF33	NS	0.005422487	-0.056383924	0.016219697	NS
APBA1	NS	0.005424616	-0.056381344	0.016221052	NS
SLC2A9	NS	0.005424179	-0.056381873	0.016221052	NS
FAM83G	NS	0.005434177	-0.056369769	0.016247133	NS
ASB2	NS	0.005435565	0.05636809	0.016248771	NS
SPCS1	NS	0.005443312	-0.056358727	0.016267648	NS
IP6K1	NS	0.00544356	0.056358427	0.016267648	NS
MAP3K20	NS	0.005446767	0.056354555	0.016274719	NS
GABRA1	NS	0.005459817	-0.056338818	0.016311194	NS
NECAB1	NS	0.005466796	-0.056330417	0.016327002	NS
GFM2	NS	0.005466173	0.056331166	0.016327002	NS
FILIP1	NS	0.00546957	-0.05632708	0.016332766	NS
INSR	NS	0.00548807	-0.056304865	0.016385482	NS
SOX4	NS	0.005496204	0.056295119	0.016404706	NS
ST8SIA6	NS	0.005495915	-0.056295465	0.016404706	NS
SNRPD3	NS	0.005519259	-0.056267565	0.01646844	NS
CFAP157	NS	0.005518581	-0.056268373	0.01646844	NS
SDHC	NS	0.005522263	-0.056263983	0.016474865	NS
CHD8	NS	0.005524665	0.056261119	0.016479492	NS
RANBP3L	NS	0.005525896	0.056259652	0.016480623	NS
KLHL30	NS	0.005527961	0.056257192	0.016482994	NS
MAGEB5	NS	0.005528394	-0.056256676	0.016482994	NS
ARMS2	NS	0.005531844	0.056252568	0.016490741	NS
GOLGA8M	NS	0.005536568	0.056246947	0.016502279	NS
IFNL2	NS	0.005546392	0.05623527	0.016529017	NS
PSME1	NS	0.00555308	-0.056227333	0.016546399	NS
PPP1R8	NS	0.005573043	-0.056203689	0.016603326	NS
BTF3L4	NS	0.005583146	0.056191751	0.01662575	NS
HSPG2	NS	0.005583007	0.056191916	0.01662575	NS
C17orf58	NS	0.005581865	-0.056193264	0.01662575	NS
NLK	NS	0.005594386	0.056178494	0.016656658	NS
TTC13	NS	0.005596255	-0.056176292	0.01665966	NS
UGP2	NS	0.005615943	-0.056153135	0.016713129	NS
ZNF92	NS	0.005621072	0.056147114	0.016725821	NS
SNAPIN	NS	0.005624801	-0.05614274	0.016734344	NS
DOHH	NS	0.005642359	-0.056122179	0.016784003	NS
ZBTB40	NS	0.005653446	-0.056109226	0.016814397	NS
IGF2BP2	NS	0.005656058	0.056106177	0.016818913	NS
CCDC127	NS	0.005656702	-0.056105426	0.016818913	NS
DSCAM	NS	0.005658647	-0.056103157	0.016822112	NS

RNF44	NS	0.00567087	0.056088915	0.01685586	NS
COX5B	NS	0.005674883	-0.056084246	0.016865197	NS
CD200	NS	0.005676181	0.056082735	0.016866466	NS
ZNF623	NS	0.005691005	-0.056065513	0.016905325	NS
OR7A10	NS	0.005694606	0.056061337	0.016913424	NS
C4orf19	NS	0.005706675	-0.056047353	0.016946672	NS
PPM1H	NS	0.005714076	-0.056038792	0.016966046	NS
PI3	NS	0.005728474	-0.056022165	0.017006187	NS
PACRG	NS	0.005730785	-0.0560195	0.017010438	NS
OR4B1	NS	0.005747045	-0.056000774	0.017056088	NS
GLUD1	NS	0.005749925	0.055997462	0.01706202	NS
DDIAS	NS	0.005760139	0.05598573	0.017089706	NS
SPHK2	NS	0.005767027	0.055977827	0.017107115	NS
RNF169	NS	0.005768142	-0.05597655	0.017107115	NS
DYNC1H1	NS	0.005768658	0.055975958	0.017107115	NS
ASTN1	NS	0.005770565	-0.055973772	0.017108599	NS
TVP23B	NS	0.005770926	0.055973359	0.017108599	NS
USP4	NS	0.005775401	0.055968232	0.017116624	NS
ASCL2	NS	0.005775067	-0.055968615	0.017116624	NS
DUXA	NS	0.00578093	0.055961904	0.017130386	NS
RNF130	NS	0.005786917	0.055955057	0.017145503	NS
HID1	NS	0.005788901	0.05595279	0.017148757	NS
NDRG1	NS	0.00579819	0.055942184	0.017171017	NS
HSD11B1L	NS	0.00580637	0.055932857	0.01719261	NS
SPTBN2	NS	0.005811587	0.055926913	0.017205427	NS
TUBE1	NS	0.0058149	-0.055923143	0.017212601	NS
NMBR	NS	0.005829628	-0.055906401	0.017250856	NS
CLEC3A	NS	0.005831315	-0.055904485	0.017250856	NS
SPANXN2	NS	0.00582881	-0.055907329	0.017250856	NS
SLURP2	NS	0.005831388	0.055904402	0.017250856	NS
CLN3	NS	0.005841748	0.055892651	0.017278863	NS
RIMBP3B	NS	0.005848011	-0.055885556	0.017294745	NS
PCDHAC1	NS	0.005853215	-0.055879666	0.017306141	NS
FAM90A9P	NS	0.005853653	0.055879171	0.017306141	NS
TRIM41	NS	0.005857132	0.055875236	0.017313783	NS
SDC1	NS	0.005867774	0.055863214	0.017342592	NS
GCHFR	NS	0.005870956	-0.055859623	0.017349347	NS
TRPV5	NS	0.005876973	-0.055852837	0.017364478	NS
TPCN1	NS	0.005890935	-0.055837116	0.017403073	NS
ALDH3A2	NS	0.00589194	-0.055835985	0.017403387	NS
MRPL9	NS	0.005893125	-0.055834653	0.017404231	NS
S100A7	NS	0.005918132	-0.055806588	0.017475417	NS
SERP2	NS	0.005923611	0.055800453	0.017488928	NS
RNF167	NS	0.005926239	0.055797512	0.01749402	NS
YIPF3	NS	0.005928357	0.055795144	0.017497601	NS
MEX3B	NS	0.005938386	0.055783935	0.01752453	NS
KXD1	NS	0.005939767	0.055782393	0.017525935	NS
IL5	NS	0.005949525	-0.055771506	0.017552052	NS
RIM6-TRIM3	NS	0.005951125	-0.055769723	0.017554095	NS
FAM222A	NS	0.005962144	0.055757452	0.017583918	NS
DYNC1I1	NS	0.00597312	-0.055745248	0.017613607	NS
KRT82	NS	0.005978471	0.055739307	0.0176267	NS
TRIM63	NS	0.005986205	0.055730727	0.017646816	NS
RNPEP	NS	0.005994124	0.055721953	0.017667468	NS
UBXN8	NS	0.006000869	-0.055714487	0.017681965	NS

SREK1	NS	0.006000793	0.055714572	0.017681965	NS
MLXIP	NS	0.006002546	0.055712633	0.017684213	NS
CCL3L1	NS	0.006009098	0.05570539	0.017700821	NS
RSRP1	NS	0.006018974	0.055694487	0.017727215	NS
C5	NS	0.006039823	-0.05567152	0.017785915	NS
SLC18A1	NS	0.00604144	-0.055669743	0.017787969	NS
CDKN2C	NS	0.006045105	-0.055665714	0.017793348	NS
OR4N4C	NS	0.006044266	0.055666636	0.017793348	NS
FUBP1	NS	0.00604942	0.055660973	0.017803341	NS
NLGN4Y	NS	0.006053013	0.055657028	0.017811208	NS
TRIM17	NS	0.006055969	-0.055653784	0.017817198	NS
SPCS3	NS	0.006059318	0.055650111	0.017821631	NS
FAM47C	NS	0.006059123	0.055650325	0.017821631	NS
TM9SF4	NS	0.006078797	0.05562878	0.017871879	NS
C6orf15	NS	0.006079172	0.05562837	0.017871879	NS
TMED10	NS	0.006092121	0.055614226	0.017907227	NS
RBM5	NS	0.00610119	0.055604335	0.017930494	NS
GHSR	NS	0.006104859	-0.055600337	0.017936499	NS
DYTN	NS	0.006115253	-0.055589024	0.01796431	NS
LRRC38	NS	0.006116598	0.055587562	0.017965533	NS
APPBP2	NS	0.006118922	-0.055585035	0.017969635	NS
ICA1	NS	0.00613325	-0.055569477	0.01800898	NS
TMEM192	NS	0.006138253	-0.055564052	0.018020936	NS
IRF2BP2	NS	0.006141031	0.055561042	0.018025923	NS
ANXA4	NS	0.006141814	-0.055560193	0.018025923	NS
DCLK3	NS	0.006145465	-0.055556239	0.018033903	NS
TNFAIP3	NS	0.006153503	0.055547541	0.018054752	NS
BTNL2	NS	0.006158489	-0.05554215	0.018066644	NS
H2AC13	NS	0.006159583	-0.055540968	0.018067115	NS
TMEM242	NS	0.006160635	-0.055539832	0.018067461	NS
RABGEF1	NS	0.006168443	0.055531401	0.018087619	NS
ZNF425	NS	0.006170514	0.055529166	0.018090951	NS
RBM15	NS	0.006185227	-0.05551331	0.018131344	NS
TEX43	NS	0.006193123	-0.055504815	0.018151741	NS
GRTP1	NS	0.006194913	0.05550289	0.018154237	NS
SMARCC2	NS	0.006198521	0.055499013	0.018162059	NS
ZNF578	NS	0.006201139	0.0554962	0.018166982	NS
SLC38A9	NS	0.006211305	-0.05548529	0.018194009	NS
ACOT8	NS	0.006221442	0.055474426	0.018220947	NS
IRF8	NS	0.006225312	0.055470284	0.018229522	NS
KCNJ16	NS	0.006229038	-0.055466297	0.018237673	NS
RPL35A	NS	0.006236933	-0.055457857	0.018258026	NS
OXSM	NS	0.00624213	-0.055452306	0.018268572	NS
ENPP6	NS	0.006242423	0.055451993	0.018268572	NS
SMIM2	NS	0.006245005	-0.055449237	0.018273366	NS
PDE4DIP	NS	0.00625206	0.055441711	0.018291244	NS
UNC45A	NS	0.006273944	-0.055418416	0.018350276	NS
MRTFA	NS	0.006274133	0.055418215	0.018350276	NS
VEGFC	NS	0.006275976	0.055416257	0.018352891	NS
PHC2	NS	0.006277072	0.055415092	0.018353324	NS
CHRNA10	NS	0.006299726	-0.055391066	0.018416778	NS
SCYL1	NS	0.006306331	0.055384074	0.018433305	NS
SLC35G5	NS	0.006311751	-0.055378343	0.018446362	NS
SH3BGRL2	NS	0.006312942	-0.055377084	0.018447057	NS
RAB3IP	NS	0.006322337	0.055367161	0.018471722	NS

SFRP5	NS	0.006325564	0.055363756	0.018472784	NS
ELP3	NS	0.00632479	-0.055364572	0.018472784	NS
KDM4D	NS	0.006324022	-0.055365383	0.018472784	NS
NUP50	NS	0.006340022	0.055348519	0.018512213	NS
MRPL54	NS	0.006347583	0.055340562	0.018531495	NS
MAMLD1	NS	0.006367544	-0.055319599	0.018586967	NS
ZKSCAN4	NS	0.006372286	-0.055314627	0.018598004	NS
RBM4B	NS	0.006392095	0.055293895	0.018653007	NS
ATP1A1	NS	0.006399068	0.055286611	0.01867054	NS
MCRIP2	NS	0.006410578	-0.055274603	0.018701303	NS
MARCHF5	NS	0.00642085	0.055263903	0.018728448	NS
TXLNA	NS	0.006423924	-0.055260704	0.018733083	NS
FHIP2B	NS	0.006428095	0.055256366	0.018741105	NS
C19orf47	NS	0.006440311	0.055243674	0.018773895	NS
PRB3	NS	0.006453553	-0.05522994	0.018809663	NS
PCDHGB5	NS	0.006460645	-0.055222596	0.018827497	NS
POLN	NS	0.006474027	-0.055208756	0.018860815	NS
GFRAL	NS	0.006473719	0.055209074	0.018860815	NS
GUSB	NS	0.006502971	0.05517891	0.01893944	NS
KRTAP3-1	NS	0.006531373	-0.055149739	0.019019296	NS
SLC13A4	NS	0.00654542	-0.055135354	0.019057334	NS
RETSAT	NS	0.006568502	-0.055111777	0.019121662	NS
C16orf96	NS	0.006577862	0.055102237	0.019146031	NS
EXOC8	NS	0.006586056	-0.055093896	0.019164116	NS
RALB	NS	0.006585967	0.055093986	0.019164116	NS
ARHGAP26	NS	0.006601096	0.05507861	0.019204991	NS
MED1	NS	0.00660259	0.055077093	0.019206451	NS
ZNF75D	NS	0.006604198	0.055075461	0.019208243	NS
CD70	NS	0.006622797	0.055056609	0.019259443	NS
ADAD2	NS	0.006627641	0.055051706	0.019270636	NS
ZNF512B	NS	0.006640982	0.055038222	0.019306524	NS
NSUN2	NS	0.006646834	-0.055032315	0.019315097	NS
PAIP2	NS	0.006646924	0.055032224	0.019315097	NS
KLHL34	NS	0.006645841	0.055033316	0.019315097	NS
TRIOBP	NS	0.006661247	0.055017785	0.019353813	NS
TEKT5	NS	0.006672092	0.055006871	0.019382412	NS
CT55	NS	0.006715652	-0.054963195	0.019506027	NS
ADGRE2	NS	0.006720873	0.054957977	0.019518263	NS
SPATA12	NS	0.006722873	-0.054955979	0.019521142	NS
SERTAD4	NS	0.006724368	-0.054954486	0.019522553	NS
TCF20	NS	0.006728472	0.054950389	0.019525682	NS
F2RL2	NS	0.006727389	-0.05495147	0.019525682	NS
CLP1	NS	0.006741395	-0.054937502	0.019560252	NS
HEYL	NS	0.006752982	0.054925967	0.019590936	NS
TNFRSF9	NS	0.006754785	-0.054924173	0.01959323	NS
LORICRIN	NS	0.006759194	0.05491979	0.019603081	NS
ZAP70	NS	0.006766841	-0.054912193	0.01961644	NS
GUCY2D	NS	0.006766166	-0.054912863	0.01961644	NS
USF3	NS	0.006766706	0.054912327	0.01961644	NS
TGM1	NS	0.006781629	-0.054897523	0.019656367	NS
SNAP29	NS	0.006783978	-0.054895196	0.019657287	NS
FSBP	NS	0.006783619	-0.054895552	0.019657287	NS
TUBB	NS	0.006787373	0.054891834	0.019664179	NS
PCDHB10	NS	0.00679346	0.054885808	0.01967887	NS
CPED1	NS	0.006798094	-0.054881224	0.019689347	NS

ZFP1	NS	0.00680277	-0.054876601	0.019699944	NS
ANKRD37	NS	0.00681999	0.054859604	0.019746855	NS
KLF4	NS	0.006841528	0.054838397	0.019806254	NS
TYW1	NS	0.006842777	0.054837169	0.019806908	NS
ELMO3	NS	0.006845171	-0.054834816	0.019810876	NS
TRAF3IP1	NS	0.006849192	-0.054830866	0.019819547	NS
CMYA5	NS	0.006862882	-0.054817431	0.019856194	NS
THOC6	NS	0.00686602	-0.054814355	0.019862306	NS
TEC	NS	0.006872534	-0.054807973	0.019878178	NS
DBH	NS	0.006874762	0.054805792	0.019879617	NS
SLC22A4	NS	0.006875085	0.054805475	0.019879617	NS
DOLPP1	NS	0.006880825	-0.054799859	0.019893241	NS
OSM	NS	0.00688662	0.054794192	0.019907022	NS
CLEC18A	NS	0.006891144	-0.054789772	0.019917125	NS
MCHR1	NS	0.006900862	0.054780285	0.019942234	NS
ZNF532	NS	0.00691916	-0.054762454	0.019989143	NS
GLI4	NS	0.006939988	0.05474221	0.020046322	NS
GNG10	NS	0.006956138	0.054726549	0.020089975	NS
ZNF888	NS	0.006992971	-0.054690955	0.020193338	NS
ADAM8	NS	0.007007866	0.054676609	0.02023333	NS
CHD3	NS	0.007028031	0.054657231	0.020288525	NS
RCCD1	NS	0.007034455	-0.054651068	0.020304042	NS
P2RX6	NS	0.007061012	0.054625643	0.020377656	NS
MARCHF8	NS	0.007066816	0.054620098	0.020391366	NS
RND2	NS	0.007073628	0.054613595	0.020407981	NS
DNAJC6	NS	0.007086298	-0.054601515	0.020435396	NS
NKX6-3	NS	0.007084805	0.054602938	0.020435396	NS
AMER1	NS	0.007085961	-0.054601836	0.020435396	NS
SERPINB8	NS	0.007098475	0.054589923	0.020467464	NS
IGFL2	NS	0.00710175	-0.054586809	0.020473857	NS
OR1D5	NS	0.007108848	-0.054580063	0.020491269	NS
OVOL2	NS	0.007111191	-0.054577838	0.020494968	NS
DCX	NS	0.007119927	-0.054569546	0.020517091	NS
TBR1	NS	0.007122939	0.054566689	0.020520116	NS
ALDH1B1	NS	0.007123097	-0.054566539	0.020520116	NS
CKB	NS	0.007129238	0.054560718	0.020534751	NS
ZDHHC18	NS	0.007133758	0.054556437	0.020544712	NS
SGPL1	NS	0.00713593	0.054554379	0.020547913	NS
MTHFD2	NS	0.007156105	-0.054535304	0.02060294	NS
CD44	NS	0.007160797	0.054530874	0.020613383	NS
GADD45G	NS	0.007174613	-0.054517847	0.020650082	NS
SOD1	NS	0.007178974	-0.054513739	0.020659563	NS
UMODL1	NS	0.007180265	-0.054512523	0.020660207	NS
RFPL4A	NS	0.007183588	-0.054509395	0.020663624	NS
SLC35G4	NS	0.007188878	-0.054504418	0.020675769	NS
TMEM150C	NS	0.007195921	0.054497798	0.020692949	NS
MEGF8	NS	0.007219574	-0.054475605	0.020757883	NS
FCER2	NS	0.007226097	-0.054469496	0.020771363	NS
MAPK1IP1L	NS	0.007226408	0.054469205	0.020771363	NS
ZNF331	NS	0.007227938	0.054467773	0.020772676	NS
GRIK3	NS	0.007230649	-0.054465236	0.020777381	NS
TAGAP	NS	0.007258437	0.054439282	0.020851041	NS
VPS13B	NS	0.007268503	-0.054429903	0.020876857	NS
HNRNPL	NS	0.007273649	0.054425113	0.020888538	NS
MTHFD1L	NS	0.007279667	0.054419513	0.020902721	NS

EPG5	NS	0.007291629	0.054408398	0.020933961	NS
MED22	NS	0.007297396	-0.054403045	0.020944639	NS
SECISBP2	NS	0.007297512	0.054402937	0.020944639	NS
BLZF1	NS	0.007302966	0.054397878	0.020957184	NS
PTH	NS	0.007319175	-0.054382863	0.021000585	NS
DRD1	NS	0.007347307	0.054356873	0.021078179	NS
CISD2	NS	0.007358893	-0.054346195	0.021108289	NS
C16orf82	NS	0.007361383	0.054343902	0.021112302	NS
OTC	NS	0.007370756	-0.054335277	0.021136053	NS
BCAN	NS	0.007373571	0.054332689	0.021140993	NS
NAA40	NS	0.007402441	-0.054306195	0.021220622	NS
NSUN6	NS	0.007408709	-0.054300455	0.021235447	NS
C17orf107	NS	0.007414164	0.054295463	0.021247936	NS
CDY2A	NS	0.00742428	-0.054286215	0.021273777	NS
FAM89B	NS	0.007428808	0.054282079	0.0212836	NS
UFD1	NS	0.007456468	0.054256862	0.021359686	NS
SPTBN5	NS	0.007472718	0.054242086	0.021403068	NS
KDM7A	NS	0.007485873	0.054230146	0.021437573	NS
EXOSC1	NS	0.007499393	-0.054217894	0.021473113	NS
TENM2	NS	0.007503757	0.054213944	0.021482431	NS
VPREB1	NS	0.007507354	0.054210689	0.021489552	NS
MISP3	NS	0.007517752	0.054201288	0.021516134	NS
AZGP1	NS	0.007522085	0.054197375	0.02152535	NS
ATP6V0A2	NS	0.00752881	0.054191305	0.02154141	NS
SFT2D2	NS	0.007535902	0.054184908	0.021558515	NS
LIMD1	NS	0.007543674	0.054177904	0.021574433	NS
MCFD2	NS	0.007543695	0.054177885	0.021574433	NS
RRP15	NS	0.00755021	-0.054172019	0.021589875	NS
RFWD3	NS	0.0075588	-0.054164292	0.021611245	NS
DIPK2A	NS	0.007563318	0.054160231	0.021619242	NS
ATP2A1	NS	0.007564894	0.054158815	0.021619242	NS
TMEM147	NS	0.007566142	-0.054157694	0.021619463	NS
LAMTOR5	NS	0.007576759	-0.054148163	0.021646604	NS
LEO1	NS	0.007582699	-0.054142835	0.021660378	NS
CLEC14A	NS	0.007584512	-0.05414121	0.021662359	NS
HUS1	NS	0.007592934	0.054133664	0.021683215	NS
LPCAT1	NS	0.00759623	0.054130714	0.021686227	NS
DHRS3	NS	0.007595892	0.054131016	0.021686227	NS
FAM185A	NS	0.007598515	-0.054128668	0.021689552	NS
ABCC9	NS	0.007613849	0.054114958	0.021726915	NS
LSM14A	NS	0.007612815	0.054115882	0.021726915	NS
ECE1	NS	0.007629283	0.054101184	0.02176454	NS
MID1	NS	0.007636078	-0.054095128	0.021777504	NS
FAM9B	NS	0.007634974	0.054096112	0.021777504	NS
RAVER1	NS	0.007638662	0.054092826	0.021781664	NS
IRAK3	NS	0.00764033	0.05409134	0.021783213	NS
MINDY4	NS	0.007648988	-0.054083634	0.021804686	NS
BLVRB	NS	0.007657359	-0.054076192	0.021822119	NS
LRRC55	NS	0.007656849	-0.054076645	0.021822119	NS
ERBB2	NS	0.007684755	0.054051883	0.02189697	NS
TNP1	NS	0.00768791	-0.054049088	0.021902735	NS
IFNAR1	NS	0.007690727	0.054046594	0.021907537	NS
SUFU	NS	0.007693873	-0.054043809	0.021910051	NS
ZNF141	NS	0.007693517	0.054044124	0.021910051	NS
USP17L26	NS	0.007699315	-0.054038995	0.021922321	NS

RUFY4	NS	0.007713306	-0.054026632	0.021958927	NS
PLBD1	NS	0.007729963	-0.054011939	0.022003112	NS
PTCHD1	NS	0.00773699	0.054005748	0.022019877	NS
MTMR8	NS	0.007738474	-0.054004442	0.022020862	NS
RASD1	NS	0.007745353	-0.053998388	0.022034209	NS
UNC93A	NS	0.007746495	-0.053997384	0.022034209	NS
LRTOMT	NS	0.007746579	-0.053997309	0.022034209	NS
RPL5	NS	0.007760314	0.053985239	0.022069564	NS
ZCCHC9	NS	0.00776129	0.053984382	0.022069564	NS
ADCY6	NS	0.007762754	0.053983096	0.022070486	NS
PRAMEF18	NS	0.00776438	0.053981669	0.022071867	NS
CACUL1	NS	0.007769301	0.053977351	0.022082612	NS
TACR3	NS	0.007775503	-0.053971912	0.022096994	NS
KCP	NS	0.007781715	0.053966468	0.022111404	NS
DBR1	NS	0.007813059	-0.05393906	0.022196022	NS
UBE3B	NS	0.007813789	-0.053938424	0.022196022	NS
FNBP1L	NS	0.00782993	-0.053924349	0.022238611	NS
TRIM60	NS	0.007834589	0.053920291	0.022248579	NS
POLR2J	NS	0.007850671	-0.053906302	0.022290976	NS
MLC1	NS	0.007863921	-0.053894794	0.022325324	NS
USP17L29	NS	0.007867848	-0.053891387	0.022333198	NS
BEGAIN	NS	0.00787318	-0.053886764	0.022345054	NS
OTUD5	NS	0.007875753	0.053884534	0.022349081	NS
ATP2B3	NS	0.007881278	-0.053879747	0.02236148	NS
TMSB4X	NS	0.007887175	-0.053874642	0.022374932	NS
CIR1	NS	0.007903029	0.053860932	0.022416622	NS
CAPRIN1	NS	0.007908966	0.053855805	0.022430175	NS
URB2	NS	0.007927159	-0.053840114	0.022468602	NS
TTC5	NS	0.007926592	-0.053840602	0.022468602	NS
C1orf35	NS	0.007926662	0.053840542	0.022468602	NS
PDE6G	NS	0.007925628	0.053841433	0.022468602	NS
UNC45B	NS	0.007932208	-0.053835765	0.022479621	NS
XAGE5	NS	0.007935061	0.053833308	0.022484416	NS
DENR	NS	0.007938069	0.05383072	0.022489646	NS
PHACTR3	NS	0.007947164	-0.053822897	0.022510252	NS
CDKN2D	NS	0.007947791	0.053822358	0.022510252	NS
DEFB116	NS	0.007949994	-0.053820465	0.022510252	NS
ASCL5	NS	0.007949694	0.053820722	0.022510252	NS
DEFB127	NS	0.007954344	-0.053816727	0.022517168	NS
MGP	NS	0.007954763	0.053816367	0.022517168	NS
ELAC1	NS	0.007969105	-0.053804058	0.02255117	NS
PHKG1	NS	0.007978377	0.053796112	0.022574106	NS
SIN3B	NS	0.007987139	0.053788609	0.022595596	NS
KRTAP2-2	NS	0.007995229	-0.053781689	0.022615176	NS
PRAMEF12	NS	0.008014883	-0.053764903	0.022667457	NS
GGA1	NS	0.008023071	0.05375792	0.0226873	NS
FOSL2	NS	0.008025041	0.053756241	0.022689556	NS
WDR5	NS	0.008034042	0.053748575	0.022711685	NS
MTMR1	NS	0.008035496	0.053747337	0.02271248	NS
HURC1-FNT	NS	0.008040091	-0.053743427	0.02272215	NS
ACP6	NS	0.008042354	-0.053741502	0.022725228	NS
POLR2E	NS	0.008050647	0.053734452	0.022738701	NS
LIX1	NS	0.008048738	-0.053736074	0.022738701	NS
ROBO4	NS	0.008050338	0.053734714	0.022738701	NS
RGS3	NS	0.008052332	-0.05373302	0.022740142	NS

PLCZ1	NS	0.008059539	0.053726899	0.022757176	NS
TIGIT	NS	0.008074602	-0.053714123	0.022796381	NS
ACSM3	NS	0.008079918	-0.053709619	0.022808064	NS
OR10K1	NS	0.008086613	0.053703951	0.022823633	NS
TRIM22	NS	0.008089517	0.053701493	0.022828501	NS
KRR1	NS	0.008105989	0.053687569	0.02287165	NS
EDA	NS	0.008115746	0.053679333	0.022895842	NS
VIPR2	NS	0.008129993	0.053667322	0.022932694	NS
FBXO6	NS	0.008132303	0.053665377	0.022935868	NS
PCDHGC5	NS	0.008134353	-0.053663651	0.022938307	NS
FGFR1	NS	0.008165233	0.053637696	0.023022031	NS
PRR23B	NS	0.008177712	0.053627233	0.023053857	NS
RLF	NS	0.008195549	0.053612301	0.023100778	NS
CHI3L1	NS	0.008197834	-0.05361039	0.023103853	NS
RD3	NS	0.008213958	0.05359692	0.023145928	NS
HSPBP1	NS	0.008218876	-0.053592817	0.023156413	NS
ASB18	NS	0.008220132	0.05359177	0.023156581	NS
BBOX1	NS	0.008234032	-0.053580185	0.023192362	NS
ARHGDIA	NS	0.00824447	0.053571496	0.023218386	NS
AKIRIN2	NS	0.008254484	0.053563171	0.023243205	NS
CHGB	NS	0.008267673	0.053552219	0.023276956	NS
SPAG17	NS	0.008269714	-0.053550526	0.023279316	NS
OR10A2	NS	0.00828733	0.053535925	0.023325514	NS
SLC2A1	NS	0.008300267	-0.053525221	0.023358529	NS
RCVRN	NS	0.008305848	-0.053520608	0.023370837	NS
DMKN	NS	0.008339705	0.05349268	0.023459284	NS
LEMD2	NS	0.008339486	0.053492861	0.023459284	NS
CRACR2A	NS	0.008341707	0.053491032	0.023461504	NS
CACNA1C	NS	0.008355228	-0.053479909	0.023496121	NS
IFI44	NS	0.008371956	-0.053466172	0.023536526	NS
NUDT4	NS	0.008372154	0.053466009	0.023536526	NS
C13orf46	NS	0.008373244	0.053465114	0.023536526	NS
ARPIN	NS	0.008390006	0.053451376	0.023580216	NS
FBXO43	NS	0.00841867	0.053427936	0.023657344	NS
ATXN7	NS	0.008426736	0.053421354	0.023676571	NS
KREMEN1	NS	0.008428167	0.053420187	0.023677155	NS
RNF113B	NS	0.008437483	0.053412592	0.023699886	NS
DCAF12L1	NS	0.008448017	0.053404013	0.023726034	NS
ARL13A	NS	0.008450074	-0.053402339	0.023728368	NS
FFAR1	NS	0.008463304	0.053391581	0.023762071	NS
WDR82	NS	0.008468018	0.053387752	0.023771856	NS
PAFAH1B3	NS	0.008497331	-0.05336398	0.023850686	NS
LRCH1	NS	0.00850242	-0.05335986	0.023861512	NS
TBX10	NS	0.008532542	0.053335522	0.023939106	NS
RPS26	NS	0.008531558	0.053336316	0.023939106	NS
TSPAN3	NS	0.008547424	0.053323526	0.023976179	NS
PF4	NS	0.008555422	-0.053317087	0.023992863	NS
LMNTD2	NS	0.008565374	0.053309082	0.024017293	NS
COL9A3	NS	0.00857036	0.053305074	0.024027794	NS
ADAM17	NS	0.00857882	0.053298279	0.024048029	NS
STRC	NS	0.008587839	-0.053291042	0.024069825	NS
FAM186B	NS	0.008603736	-0.053278302	0.024110891	NS
MLH3	NS	0.008608257	-0.053274683	0.024116575	NS
KRTAP4-11	NS	0.008610766	0.053272675	0.024120113	NS
DBX2	NS	0.008617111	-0.0532676	0.024134394	NS

TLE7	NS	0.008629375	0.053257799	0.024165246	NS
HIPK1	NS	0.00866699	0.053227817	0.02426707	NS
MMGT1	NS	0.008674448	0.053221886	0.02428444	NS
ALOX5	NS	0.008685716	0.053212935	0.024312468	NS
IRF3	NS	0.008693569	-0.053206702	0.024327413	NS
TSPAN2	NS	0.008692624	-0.053207452	0.024327413	NS
TUBG1	NS	0.008698839	-0.053202522	0.024338642	NS
PCDHGA5	NS	0.008712723	-0.053191521	0.024373967	NS
DSG4	NS	0.008716104	-0.053188845	0.024379899	NS
MRPS2	NS	0.00872935	-0.053178367	0.024413424	NS
FAM170A	NS	0.00873131	-0.053176818	0.024415376	NS
CLIP2	NS	0.008736627	-0.053172617	0.024426717	NS
CACNG3	NS	0.008744909	-0.053166078	0.024446341	NS
RS1	NS	0.008747325	0.053164172	0.024449562	NS
EIF1AD	NS	0.008749495	0.05316246	0.024452097	NS
FSF12-TNFS	NS	0.008756826	0.05315668	0.024469051	NS
AXDND1	NS	0.00875869	-0.05315521	0.024470727	NS
ZNF668	NS	0.008762242	0.053152411	0.024477118	NS
BLOC1S1	NS	0.008765893	-0.053149536	0.024480251	NS
MFSD5	NS	0.008765852	0.053149568	0.024480251	NS
ALDH16A1	NS	0.008768992	-0.053147096	0.024485372	NS
SLC43A2	NS	0.008770522	0.053145891	0.024486113	NS
GRK6	NS	0.008783924	0.053135349	0.024519993	NS
CGREF1	NS	0.00878901	-0.053131352	0.024530654	NS
FHL5	NS	0.008796607	-0.053125386	0.024548317	NS
TET1	NS	0.008817415	0.053109068	0.024595745	NS
OR5B2	NS	0.008816749	-0.05310959	0.024595745	NS
LIX1L	NS	0.008815432	0.053110621	0.024595745	NS
JAK2	NS	0.008834056	-0.053096042	0.024638616	NS
CPEB3	NS	0.008839341	0.05309191	0.024649804	NS
CASP1	NS	0.008844309	-0.053088028	0.024656249	NS
FANCD2	NS	0.008845474	0.053087118	0.024656249	NS
MRPL21	NS	0.008845364	0.053087204	0.024656249	NS
CES1	NS	0.008855321	-0.05307943	0.024680142	NS
BMP4	NS	0.008860594	0.053075315	0.024691285	NS
ZNF324B	NS	0.008866829	-0.053070454	0.024705101	NS
ZSWIM3	NS	0.008892793	0.053050241	0.024773878	NS
SF3B2	NS	0.008926819	0.053023833	0.024861512	NS
SKI	NS	0.008925878	0.053024562	0.024861512	NS
STMN4	NS	0.008933456	-0.053018692	0.024876417	NS
H2AZ2	NS	0.008937343	0.053015683	0.02488366	NS
ELOA2	NS	0.008950574	0.053005449	0.024916915	NS
TMCO1	NS	0.008954588	-0.053002346	0.024924506	NS
CKS1B	NS	0.008966915	-0.052992828	0.024955228	NS
SSH2	NS	0.008976858	0.052985159	0.024975716	NS
C16orf72	NS	0.008976384	0.052985524	0.024975716	NS
B4GALT7	NS	0.0089905	0.052974648	0.025010078	NS
DUOX1	NS	0.009022266	-0.052950229	0.025094838	NS
TMEM151B	NS	0.009024303	0.052948666	0.025096898	NS
ABCB10	NS	0.009038616	-0.052937691	0.025133093	NS
DPYSL3	NS	0.00910351	-0.052888124	0.025309903	NS
MAG	NS	0.009120771	0.052874993	0.025350608	NS
PVR	NS	0.009127727	-0.052869707	0.025366301	NS
TRIM43	NS	0.009132362	-0.052866188	0.025375539	NS
DUSP2	NS	0.009134316	0.052864704	0.025377325	NS

ZNF777	NS	0.009139543	0.052860737	0.025388203	NS
PPP1R1B	NS	0.009141357	0.052859361	0.025389599	NS
GVQW3	NS	0.009153554	-0.052850115	0.025419827	NS
HMCES	NS	0.009156464	-0.05284791	0.025424261	NS
CTNNA3	NS	0.0091605	-0.052844854	0.025431817	NS
GLI3	NS	0.009173254	0.052835203	0.025459924	NS
MUC1	NS	0.009172699	0.052835623	0.025459924	NS
MRPL44	NS	0.009184106	-0.052827001	0.025486388	NS
GADD45A	NS	0.00918764	-0.052824332	0.025492539	NS
IL1R1	NS	0.009193902	0.052819605	0.025506257	NS
HECTD2	NS	0.009223287	-0.052797459	0.02558115	NS
CDPF1	NS	0.00922354	-0.052797268	0.02558115	NS
SMYD5	NS	0.009229607	-0.052792704	0.025594309	NS
TASP1	NS	0.009237059	-0.052787102	0.025611304	NS
PNMA5	NS	0.009249565	-0.052777709	0.025642305	NS
ABHD11	NS	0.009254521	0.052773989	0.025652371	NS
GFI1	NS	0.009261747	0.052768569	0.025668725	NS
MSRB2	NS	0.009271425	-0.052761316	0.02568531	NS
SERPINB9	NS	0.009271712	0.052761101	0.02568531	NS
C11orf54	NS	0.009271339	-0.052761381	0.02568531	NS
NOG	NS	0.009287069	0.052749607	0.025722258	NS
KRTAP27-1	NS	0.009287707	-0.052749129	0.025722258	NS
PRSS36	NS	0.009295675	-0.052743173	0.02574064	NS
CCDC90B	NS	0.009302213	-0.052738288	0.025755062	NS
BRMS1	NS	0.009307266	0.052734515	0.025765368	NS
RNF103	NS	0.009309578	-0.05273279	0.025768081	NS
POLR2C	NS	0.009316686	0.052727487	0.025780381	NS
HVCN1	NS	0.009315627	0.052728276	0.025780381	NS
TSACC	NS	0.009318411	-0.0527262	0.025781471	NS
CCT5	NS	0.009323159	-0.05272266	0.025790919	NS
LSR	NS	0.009328986	0.052718318	0.025803352	NS
HSDL2	NS	0.009342473	-0.052708277	0.025836965	NS
VSTM2A	NS	0.009359901	0.052695322	0.025881464	NS
DHH	NS	0.009373355	0.052685336	0.025914963	NS
TOB1	NS	0.009384659	0.052676954	0.025938416	NS
MPC2	NS	0.009385858	0.052676066	0.025938416	NS
AMZ2	NS	0.009383901	0.052677517	0.025938416	NS
CCNI2	NS	0.009393453	-0.052670441	0.025955699	NS
DDX43	NS	0.009397133	-0.052667717	0.025962162	NS
RAMP2	NS	0.009416244	0.052653585	0.026007538	NS
TMEM199	NS	0.009415362	0.052654237	0.026007538	NS
HSPB11	NS	0.009427158	-0.052645526	0.026033967	NS
SLC22A6	NS	0.00943744	0.052637942	0.026058642	NS
IQSEC3	NS	0.009439734	0.05263625	0.02606126	NS
TAAR9	NS	0.009442577	-0.052634154	0.026065392	NS
CFB	NS	0.009447382	-0.052630614	0.026074937	NS
ZNF707	NS	0.009482166	-0.052605034	0.026167208	NS
CAV3	NS	0.009490425	-0.052598972	0.026186268	NS
TMEM254	NS	0.009491998	-0.052597819	0.026186874	NS
HHLA2	NS	0.009505001	-0.052588287	0.02621901	NS
RFXAP	NS	0.009506425	-0.052587243	0.026219202	NS
SLC25A25	NS	0.009518908	0.052578104	0.02624989	NS
OR10J5	NS	0.009537486	-0.052564523	0.026297376	NS
S100A11	NS	0.009539275	0.052563216	0.026298563	NS
RGS17	NS	0.009554835	-0.052551861	0.026328378	NS

BRD1	NS	0.00955686	0.052550385	0.026328378	NS
TAS1R3	NS	0.009556891	0.052550362	0.026328378	NS
OR6K6	NS	0.009554045	-0.052552438	0.026328378	NS
PLXNB2	NS	0.009556624	0.052550556	0.026328378	NS
MTNR1A	NS	0.009568104	-0.052542191	0.026355517	NS
ATP10A	NS	0.009569615	0.052541091	0.026355928	NS
TIGD3	NS	0.009580429	0.052533219	0.026381959	NS
ZNF106	NS	0.00959574	0.052522089	0.026416604	NS
C20orf203	NS	0.009595601	0.05252219	0.026416604	NS
SNX16	NS	0.009598893	0.052519799	0.026421526	NS
TAS2R43	NS	0.009601543	-0.052517874	0.026425063	NS
TXNL4B	NS	0.00961021	-0.052511584	0.026445156	NS
FOXJ2	NS	0.009618194	0.052505794	0.026463363	NS
CNGB3	NS	0.009622118	-0.05250295	0.026470397	NS
CLDND1	NS	0.009625091	0.052500796	0.026474813	NS
SLC12A9	NS	0.009638035	0.052491423	0.02650665	NS
PRDX5	NS	0.009639576	0.052490309	0.026507121	NS
NUP85	NS	0.009645682	0.052485892	0.026520144	NS
NOD2	NS	0.009651157	0.052481934	0.026531429	NS
LCAT	NS	0.009654767	-0.052479325	0.026537084	NS
MEF2B	NS	0.009655956	0.052478466	0.026537084	NS
DCBLD2	NS	0.009685477	-0.052457169	0.026614436	NS
SIK1	NS	0.009690474	0.052453569	0.026617865	NS
TRMT10B	NS	0.009691897	-0.052452544	0.026617865	NS
RPS6KB2	NS	0.00969013	0.052453817	0.026617865	NS
SRGAP2B	NS	0.009692225	0.052452308	0.026617865	NS
PFDN2	NS	0.009696843	-0.052448984	0.026622993	NS
PDCL2	NS	0.009696005	0.052449587	0.026622993	NS
SMIM22	NS	0.009718734	-0.052433245	0.02667931	NS
PITPNA	NS	0.009720939	0.052431661	0.026681579	NS
PCDHGA9	NS	0.009726654	-0.052427558	0.02669348	NS
EP400	NS	0.009732982	0.052423017	0.026707061	NS
FRAT1	NS	0.009741414	0.052416971	0.026726408	NS
ATG9B	NS	0.009753799	0.0524081	0.026756594	NS
UBR3	NS	0.00976093	-0.052402995	0.02676857	NS
ZNF14	NS	0.009767003	-0.052398651	0.026777636	NS
PSMB3	NS	0.009766805	-0.052398793	0.026777636	NS
CCS	NS	0.009770691	-0.052396015	0.026783953	NS
ZNF385C	NS	0.009785799	0.052385222	0.026821569	NS
ANKFN1	NS	0.009797906	0.052376584	0.02685095	NS
EIF4G3	NS	0.009799764	0.052375259	0.02685224	NS
KRT36	NS	0.009811921	-0.052366596	0.026881746	NS
YPEL2	NS	0.009823798	0.052358142	0.02690667	NS
SELENOI	NS	0.00982855	-0.052354762	0.026912069	NS
OPRPN	NS	0.009828418	-0.052354856	0.026912069	NS
FKBP1B	NS	0.009840743	-0.052346096	0.026941644	NS
SMG6	NS	0.009873395	0.052322937	0.027025307	NS
KRTAP13-2	NS	0.009874094	-0.052322442	0.027025307	NS
ZNRF1	NS	0.009881901	0.052316915	0.027042848	NS
PCDHB8	NS	0.009893292	-0.052308857	0.02706637	NS
OR2T4	NS	0.009892758	-0.052309235	0.02706637	NS
CMSS1	NS	0.00989981	-0.052304251	0.027080375	NS
LRIF1	NS	0.009914179	0.052294105	0.027115848	NS
CDK6	NS	0.009919027	-0.052290685	0.027117914	NS
KIRREL2	NS	0.009918733	0.052290893	0.027117914	NS

RAB20	NS	0.009919138	0.052290607	0.027117914	NS
PTPRA	NS	0.009922684	0.052288106	0.027123777	NS
GNA12	NS	0.009924794	0.052286619	0.027125714	NS
DUXB	NS	0.009956886	-0.052264027	0.027209585	NS
PHETA1	NS	0.009961894	-0.052260508	0.027219426	NS
SGF29	NS	0.009966737	-0.052257105	0.027228817	NS
SLC26A11	NS	0.009987227	0.052242728	0.027280943	NS
ITGA11	NS	0.010002978	-0.052231694	0.027316259	NS
SLC39A1	NS	0.010002394	0.052232103	0.027316259	NS
RFPL4AL1	NS	0.010018897	-0.052220558	0.02735587	NS
SNX31	NS	0.010020483	-0.052219449	0.027356342	NS
OSER1	NS	0.01002857	0.052213798	0.02737456	NS
EFHD2	NS	0.010032676	0.052210931	0.027381905	NS
SUCLA2	NS	0.010037975	0.052207232	0.027392505	NS
STK32C	NS	0.010044015	-0.052203018	0.027401262	NS
ACTL10	NS	0.010043465	0.052203401	0.027401262	NS
GALNT5	NS	0.010058946	0.05219261	0.0274377	NS
DENND4A	NS	0.010060617	0.052191446	0.0274377	NS
RPSAP58	NS	0.010061624	-0.052190745	0.0274377	NS
BCS1L	NS	0.010067853	0.052186408	0.02745082	NS
ZNF154	NS	0.010076259	-0.052180559	0.02746987	NS
HMG20B	NS	0.010086028	0.052173767	0.02749263	NS
MAP2	NS	0.010102235	-0.052162513	0.02753199	NS
CCDC28B	NS	0.010103313	-0.052161765	0.02753199	NS
ADGRE1	NS	0.010111133	-0.052156341	0.02754942	NS
PTPRC	NS	0.010133375	0.052140933	0.027606137	NS
ZNF30	NS	0.01013601	-0.05213911	0.027609431	NS
DMAC2	NS	0.010143569	0.052133881	0.027626133	NS
HLA-DRA	NS	0.010161771	0.052121307	0.027671811	NS
OR13C8	NS	0.01017325	-0.052113386	0.027699174	NS
TSPEAR	NS	0.010181019	0.052108031	0.027712529	NS
CALHM5	NS	0.010179916	-0.052108791	0.027712529	NS
OR13H1	NS	0.01020902	-0.052088757	0.027784372	NS
TRIM61	NS	0.010210283	0.052087889	0.027784372	NS
PXMP2	NS	0.010226342	-0.052076858	0.027824159	NS
KDM5D	NS	0.010239755	-0.052067656	0.027856739	NS
PEG3	NS	0.010252994	-0.052058584	0.027888834	NS
SLC34A1	NS	0.010259094	-0.052054408	0.027901505	NS
EDEM1	NS	0.010262141	0.052052322	0.027905872	NS
SH3GL3	NS	0.010277398	-0.052041888	0.027943436	NS
KNSTRN	NS	0.010280704	-0.05203963	0.027948498	NS
RPL36AL	NS	0.010284508	0.052037031	0.027954912	NS
DIABLO	NS	0.010300275	0.05202627	0.027993838	NS
KRTAP26-1	NS	0.010352736	-0.05199057	0.028132466	NS
FAM76B	NS	0.010357155	-0.051987569	0.0281392	NS
TSSK2	NS	0.010358122	-0.051986914	0.0281392	NS
GAGE12D	NS	0.010377467	0.051973796	0.028187798	NS
TMEM69	NS	0.010399654	-0.051958777	0.028240138	NS
CENPVL1	NS	0.010399208	0.051959079	0.028240138	NS
TOR2A	NS	0.010402909	0.051956576	0.028245015	NS
MAD2L1	NS	0.010410646	0.051951348	0.028258092	NS
KRTAP9-7	NS	0.010409194	0.051952328	0.028258092	NS
OR8G5	NS	0.010426666	-0.051940531	0.028297609	NS
GCC2	NS	0.010461459	0.051917092	0.028388055	NS
PPT1	NS	0.0104783	0.05190577	0.028425786	NS

GNG12	NS	0.010478	-0.051905972	0.028425786	NS
CD244	NS	0.010481261	-0.051903782	0.028429834	NS
C17orf75	NS	0.010484805	0.051901402	0.028435462	NS
P4HA1	NS	0.010486944	0.051899966	0.028437278	NS
TTYH2	NS	0.010502955	-0.051889226	0.028476706	NS
KRTAP2-4	NS	0.0105075	-0.05188618	0.028485038	NS
UQCRHL	NS	0.010513861	-0.051881919	0.028494301	NS
BPIFB1	NS	0.010525803	0.051873925	0.028522672	NS
TMC6	NS	0.01053004	0.051871091	0.028526166	NS
RSKR	NS	0.010529001	0.051871786	0.028526166	NS
GPATCH4	NS	0.010537618	-0.051866024	0.028534714	NS
PASD1	NS	0.010537329	0.051866217	0.028534714	NS
TMEM221	NS	0.010536513	-0.051866763	0.028534714	NS
LOXL2	NS	0.010560525	0.051850729	0.028592744	NS
HEXB	NS	0.010565401	0.051847477	0.028601943	NS
B3GNT6	NS	0.010578473	0.051838765	0.028633326	NS
MTFP1	NS	0.010593604	0.051828693	0.028670275	NS
SERTAD3	NS	0.010597626	-0.051826018	0.02867715	NS
NOP9	NS	0.0106133	0.051815601	0.028715549	NS
CCDC115	NS	0.010618158	-0.051812376	0.028724677	NS
INTS2	NS	0.010636758	-0.051800037	0.028766954	NS
CCNF	NS	0.010635692	-0.051800744	0.028766954	NS
SRSF10	NS	0.010641468	0.051796916	0.028773583	NS
LTC4S	NS	0.010642182	0.051796443	0.028773583	NS
ANKRD13B	NS	0.01065398	0.05178863	0.028801457	NS
OR4F15	NS	0.01067743	0.051773124	0.028860822	NS
ADAMTS9	NS	0.010679574	-0.051771708	0.028862584	NS
IKZF1	NS	0.010713745	0.051749171	0.028950893	NS
BCL2L14	NS	0.01072571	-0.051741295	0.028975135	NS
XKR4	NS	0.010724782	0.051741906	0.028975135	NS
IFI27L1	NS	0.010732059	-0.051737119	0.02898824	NS
ADPRH	NS	0.010737792	0.05173335	0.028999679	NS
TEKT1	NS	0.010749167	-0.051725876	0.02902635	NS
STARD10	NS	0.010801542	0.051691557	0.029163712	NS
PHF21A	NS	0.010812452	0.051684427	0.029180957	NS
PHLDB3	NS	0.010812064	0.051684681	0.029180957	NS
NALF1	NS	0.010811008	-0.051685371	0.029180957	NS
UBE3C	NS	0.010829171	-0.051673513	0.029222003	NS
TRIM29	NS	0.010832586	-0.051671286	0.02922307	NS
SMPD1	NS	0.010831328	-0.051672106	0.02922307	NS
WDR86	NS	0.01083891	-0.051667162	0.029236056	NS
SH2D4A	NS	0.010856309	0.051655829	0.029278908	NS
NFKBIE	NS	0.010858438	0.051654443	0.02928057	NS
TBC1D17	NS	0.010864372	0.051650582	0.02929249	NS
TRIM62	NS	0.010880561	0.051640059	0.029332053	NS
RBKS	NS	0.010886226	0.05163638	0.029343237	NS
AP5S1	NS	0.010899043	-0.051628062	0.029373694	NS
USP29	NS	0.010948953	0.051595753	0.029504098	NS
POLR1E	NS	0.010954895	-0.051591916	0.029516	NS
CPNE3	NS	0.010963809	-0.051586162	0.029529133	NS
LRRC8C	NS	0.010962774	0.05158683	0.029529133	NS
FAM205A	NS	0.010964346	-0.051585816	0.029529133	NS
XYLT2	NS	0.010989288	-0.051569738	0.029588549	NS
ANTXR1	NS	0.010989465	-0.051569625	0.029588549	NS
RRAS	NS	0.011003944	0.051560307	0.029623413	NS

TNFRSF6B	NS	0.011009859	0.051556504	0.029632921	NS
ARL6IP6	NS	0.011018777	0.051550773	0.029650972	NS
DRCS8-MEF2	NS	0.011020894	0.051549413	0.029652547	NS
MYPOP	NS	0.011034692	0.051540556	0.029685545	NS
CT47A9	NS	0.011042031	0.051535849	0.029701161	NS
TGFBR3	NS	0.011045718	-0.051533485	0.029702824	NS
GALNT9	NS	0.011045431	0.05153367	0.029702824	NS
OR6K2	NS	0.011047916	0.051532077	0.029704606	NS
CSNK1G1	NS	0.011061151	-0.051523599	0.029736061	NS
HTRA3	NS	0.011070288	0.051517752	0.029756493	NS
RHOG	NS	0.011074052	0.051515345	0.029762474	NS
NMI	NS	0.011105199	-0.05149545	0.029842043	NS
TUSC3	NS	0.011122993	0.051484106	0.029879587	NS
ELF2	NS	0.011123802	0.051483591	0.029879587	NS
KLF2	NS	0.011123304	0.051483908	0.029879587	NS
CTAGE4	NS	0.011133399	-0.05147748	0.029901218	NS
TBC1D3K	NS	0.011138542	0.051474207	0.02991088	NS
SRSF6	NS	0.011153076	0.051464966	0.029933296	NS
AMDHD1	NS	0.011151572	-0.051465921	0.029933296	NS
TNFAIP2	NS	0.011150831	0.051466392	0.029933296	NS
PNLIPRP3	NS	0.011152691	-0.05146521	0.029933296	NS
SSX2B	NS	0.011171148	-0.051453489	0.029977643	NS
FBRS	NS	0.011189678	0.051441738	0.030023206	NS
GFOD1	NS	0.011196985	0.05143711	0.030035031	NS
MEI4	NS	0.011197189	-0.051436981	0.030035031	NS
PWWP3A	NS	0.011203493	-0.051432989	0.030047778	NS
BPHL	NS	0.011211498	0.051427924	0.030065081	NS
NPY1R	NS	0.011228716	-0.05141704	0.030107082	NS
PDCD2L	NS	0.011230455	-0.051415941	0.030107573	NS
HGSNAT	NS	0.011240832	0.05140939	0.030127048	NS
NANP	NS	0.011240186	-0.051409797	0.030127048	NS
TMEM181	NS	0.011248309	0.051404672	0.030142913	NS
TGFB1	NS	0.011256432	0.05139955	0.03015655	NS
RBM47	NS	0.011256514	0.051399499	0.03015655	NS
FRRS1L	NS	0.011259078	0.051397883	0.030159247	NS
MUC16	NS	0.011263374	0.051395176	0.030166578	NS
TMEM234	NS	0.011301343	0.051371292	0.030264083	NS
KLRC2	NS	0.011305527	-0.051368664	0.0302711	NS
PECR	NS	0.011315851	-0.051362184	0.030294553	NS
CHRNE	NS	0.011319004	0.051360207	0.030298802	NS
CSNK2A3	NS	0.011339875	0.051347126	0.030350473	NS
SKAP2	NS	0.011345046	-0.051343889	0.030360114	NS
DHX9	NS	0.011357436	-0.051336137	0.030389069	NS
PEBP4	NS	0.011362136	0.051333198	0.030397444	NS
RSRC2	NS	0.011375288	0.051324981	0.030428424	NS
RGN	NS	0.011417432	0.051298706	0.030536937	NS
JPH2	NS	0.011425127	0.051293918	0.030551542	NS
SENP2	NS	0.011426049	0.051293344	0.030551542	NS
UTY	NS	0.011444654	0.05128178	0.030597062	NS
SLC9B2	NS	0.01144757	-0.051279969	0.03060063	NS
RBM27	NS	0.011454181	0.051275865	0.030613854	NS
RLN2	NS	0.01145568	-0.051274935	0.030613854	NS
TMEM43	NS	0.01146686	0.051268	0.0306395	NS
MGAM	NS	0.011490386	0.051253425	0.030698125	NS
NUBP1	NS	0.011502309	-0.05124605	0.030725737	NS

MRPL11	NS	0.011504764	-0.051244531	0.030728055	NS
ADAMTS20	NS	0.011518216	-0.051236219	0.030759739	NS
NAA50	NS	0.011539971	0.051222794	0.030813586	NS
KCNMB3	NS	0.0115659	0.051206824	0.030874301	NS
NBPF19	NS	0.011584215	-0.051195561	0.030918929	NS
MCEE	NS	0.011598803	-0.051186602	0.030953596	NS
RRAD	NS	0.011629692	0.051167666	0.031027472	NS
ATCAY	NS	0.011628656	0.0511683	0.031027472	NS
LIPE	NS	0.011657364	-0.051150738	0.031097015	NS
SPINK4	NS	0.011659288	-0.051149563	0.031097861	NS
P2RX5	NS	0.011669057	-0.051143597	0.031115341	NS
ST3GAL2	NS	0.011668975	0.051143647	0.031115341	NS
UHMK1	NS	0.011683429	0.051134827	0.031149374	NS
CDA	NS	0.011699893	0.051124793	0.031188972	NS
DDB2	NS	0.011701506	-0.05112381	0.031188976	NS
LCMT2	NS	0.011707884	-0.051119927	0.03120168	NS
RXRG	NS	0.011711931	-0.051117463	0.03120817	NS
OR4F17	NS	0.011738332	0.051101414	0.031274212	NS
SLC47A2	NS	0.011749534	0.051094614	0.031299749	NS
BMF	NS	0.011752042	0.051093092	0.031302123	NS
ZNF274	NS	0.011758098	0.051089418	0.031313945	NS
MAN2A1	NS	0.011781721	-0.051075106	0.031368223	NS
PPP4R1	NS	0.011781402	0.051075299	0.031368223	NS
NEDD8	NS	0.011788629	-0.051070925	0.031382301	NS
CCNQ	NS	0.011792052	0.051068855	0.031387096	NS
RIMS3	NS	0.011801153	-0.051063351	0.031407001	NS
FAM111A	NS	0.011805867	-0.051060503	0.031415226	NS
CACNA2D3	NS	0.01181446	-0.051055312	0.031433772	NS
SMARCA2	NS	0.011820567	0.051051625	0.031444844	NS
STN1	NS	0.011821871	-0.051050838	0.031444844	NS
VPS35L	NS	0.011835376	0.051042692	0.031476442	NS
OR2T29	NS	0.011886406	-0.051011984	0.031607814	NS
YNJ2BP-COX	NS	0.011898308	-0.051004839	0.031635117	NS
GPR35	NS	0.011911936	0.050996666	0.031667	NS
SYT10	NS	0.011915872	-0.050994306	0.031673115	NS
FSTL1	NS	0.011925126	-0.050988762	0.031693359	NS
MON1B	NS	0.011930188	0.050985732	0.031702459	NS
PLAC1	NS	0.011942196	-0.050978546	0.031729744	NS
LRRC25	NS	0.011943734	0.050977626	0.031729744	NS
GDAP1L1	NS	0.011967883	-0.050963196	0.031789535	NS
EPHX2	NS	0.011984486	-0.050953291	0.03182927	NS
SIGLEC5	NS	0.012001302	0.05094327	0.031869557	NS
RNF38	NS	0.012004706	0.050941243	0.031874225	NS
IL34	NS	0.012030154	0.050926107	0.031937412	NS
KIF16B	NS	0.012033468	-0.050924138	0.031941828	NS
VARS1	NS	0.012065225	-0.050905293	0.032021732	NS
CLPS	NS	0.012069245	0.050902911	0.03202346	NS
TMEM164	NS	0.012068252	0.050903499	0.03202346	NS
CFAP95	NS	0.012070839	-0.050901966	0.03202346	NS
ACTR6	NS	0.012090647	-0.050890239	0.032071613	NS
SPDYE16	NS	0.012100978	0.050884129	0.03209462	NS
TCTN3	NS	0.012112819	0.050877133	0.032121622	NS
OR10Z1	NS	0.012116789	-0.050874788	0.032127748	NS
PLAGL2	NS	0.01211848	0.050873789	0.032127833	NS
NR0B2	NS	0.012125297	0.050869766	0.032141501	NS

CHRNA2	NS	0.012132032	-0.050865793	0.032154951	NS
CLEC12B	NS	0.012144494	-0.050858445	0.032183575	NS
AKR7A3	NS	0.012165921	-0.050845828	0.032235945	NS
PPARD	NS	0.012169545	0.050843697	0.032241131	NS
RBPJL	NS	0.012171892	0.050842316	0.032242936	NS
G3BP1	NS	0.012174501	-0.050840782	0.032245435	NS
EIPR1	NS	0.0121932	-0.050829794	0.032290542	NS
RUVBL2	NS	0.012197724	-0.050827138	0.032298106	NS
COASY	NS	0.012207201	-0.050821577	0.032314357	NS
LRP4	NS	0.012205767	0.050822418	0.032314357	NS
H2AC18	NS	0.012214488	0.050817304	0.032329226	NS
RAD9A	NS	0.012222379	-0.050812678	0.032345688	NS
LTB4R2	NS	0.012226701	0.050810146	0.032352703	NS
INSRR	NS	0.012230865	0.050807708	0.032359297	NS
ADAT3	NS	0.012250257	-0.050796359	0.032406175	NS
PGAM5	NS	0.012257029	-0.0507924	0.032419659	NS
OR10H1	NS	0.012281925	0.050777862	0.032481068	NS
EDN1	NS	0.012287121	-0.050774831	0.032486682	NS
USP2	NS	0.012302375	0.050765939	0.032521821	NS
DNAJB8	NS	0.012306026	0.050763812	0.03252703	NS
PLXDC1	NS	0.012312891	-0.050759815	0.032540731	NS
NOTCH2NLA	NS	0.01231838	0.05075662	0.032550793	NS
GNL1	NS	0.012326995	0.050751609	0.032569112	NS
AKT2	NS	0.012335143	0.050746871	0.032586193	NS
TNRC6B	NS	0.012340401	0.050743816	0.032595633	NS
YPEL4	NS	0.012344975	0.050741159	0.032603266	NS
LYPD5	NS	0.012355435	0.050735086	0.032621989	NS
C2orf92	NS	0.012383038	-0.050719082	0.032690409	NS
PLPP7	NS	0.012393015	-0.050713305	0.032712287	NS
NUP93	NS	0.012417294	-0.050699264	0.032771904	NS
RELL2	NS	0.012428815	-0.05069261	0.032797838	NS
NRIP1	NS	0.012452621	0.050678877	0.032856181	NS
SYT16	NS	0.012465427	0.0506715	0.032885486	NS
ARID4A	NS	0.012474685	0.050666171	0.032889757	NS
PANK2	NS	0.012475272	-0.050665833	0.032889757	NS
KCNH3	NS	0.012475542	0.050665677	0.032889757	NS
TPGT-TNNI3I	NS	0.012469524	-0.050669141	0.032889757	NS
ALG8	NS	0.012481566	0.050662212	0.032901156	NS
TEDDM1	NS	0.012489993	0.050657366	0.032918887	NS
OXER1	NS	0.012500431	-0.050651368	0.03293352	NS
DNAJC28	NS	0.01250065	0.050651243	0.03293352	NS
STUM	NS	0.012499129	-0.050652116	0.03293352	NS
H3Y1	NS	0.01251046	-0.05064561	0.03295488	NS
MT1E	NS	0.012523158	-0.050638324	0.032983841	NS
TLDC2	NS	0.012534023	-0.050632096	0.033007966	NS
KSR1	NS	0.012558298	0.050618197	0.033067393	NS
EPB41L4B	NS	0.012575567	-0.050608324	0.033107928	NS
GAPVD1	NS	0.012578425	0.050606691	0.033107928	NS
ZNF721	NS	0.012578823	0.050606463	0.033107928	NS
ZC3H11A	NS	0.012593071	0.050598329	0.033136417	NS
PSEN1	NS	0.0125914	0.050599282	0.033136417	NS
PDE8B	NS	0.012600029	-0.050594359	0.033150219	NS
IL24	NS	0.012603415	-0.050592428	0.033154621	NS
ZNF296	NS	0.012622784	0.05058139	0.03320106	NS
DAP	NS	0.012632799	0.050575689	0.033222888	NS

TEX10	NS	0.012659843	-0.050560313	0.033289488	NS
RLN3	NS	0.012667523	0.050555952	0.033305159	NS
MNT	NS	0.012685198	0.050545924	0.033339102	NS
GALR1	NS	0.012682335	0.050547548	0.033339102	NS
PAGE2B	NS	0.012685601	-0.050545696	0.033339102	NS
RBM18	NS	0.012697605	0.050538893	0.033366119	NS
NEU2	NS	0.01271127	0.050531155	0.033397495	NS
ZNF267	NS	0.012717338	0.050527721	0.033408902	NS
KARS1	NS	0.012726195	-0.050522712	0.033427635	NS
ZNF350	NS	0.012728683	-0.050521305	0.033429633	NS
VTI1A	NS	0.012735675	0.050517354	0.03344245	NS
TMEM89	NS	0.012737019	0.050516595	0.03344245	NS
SWI5	NS	0.012757977	-0.050504763	0.033492935	NS
BEX5	NS	0.012765821	-0.050500339	0.033508983	NS
SH3PXD2B	NS	0.012772459	0.050496597	0.03352186	NS
ACP5	NS	0.012776229	-0.050494473	0.033527208	NS
KLHL23	NS	0.012780107	-0.050492288	0.03353284	NS
CAV1	NS	0.012785475	-0.050489265	0.033542377	NS
RELA	NS	0.012822746	0.050468306	0.033635596	NS
ARMH3	NS	0.012828014	0.050465348	0.033644855	NS
ABCA4	NS	0.012836496	-0.050460587	0.033662541	NS
DKKL1	NS	0.012859883	0.050447476	0.033719303	NS
GALNT2	NS	0.012870067	0.050441773	0.033741437	NS
CD247	NS	0.012877105	-0.050437834	0.033755314	NS
STK32A	NS	0.012884664	-0.050433606	0.033767936	NS
FAM210A	NS	0.012885409	0.050433189	0.033767936	NS
DNAJC9	NS	0.012901295	-0.05042431	0.033804991	NS
LYZL4	NS	0.012917429	-0.050415303	0.033842685	NS
LRRC14	NS	0.012931119	-0.050407668	0.033873966	NS
STARD13	NS	0.01293494	0.050405538	0.033879392	NS
ANKRD36	NS	0.012947851	0.050398346	0.033905894	NS
BPIFA1	NS	0.012948562	-0.05039795	0.033905894	NS
TEX19	NS	0.012963537	0.050389617	0.033940514	NS
NME9	NS	0.012969114	-0.050386515	0.033950522	NS
LGALS3BP	NS	0.012973024	-0.050384341	0.033956167	NS
DNAJC8	NS	0.012982652	-0.050378991	0.033976774	NS
ZBTB8B	NS	0.012993836	0.050372781	0.034001444	NS
DERL1	NS	0.012997477	0.05037076	0.034003989	NS
NSMF	NS	0.012998321	0.050370292	0.034003989	NS
FBXL6	NS	0.013004091	0.050367091	0.034014485	NS
NUFIP2	NS	0.013031524	0.050351888	0.034081636	NS
TK2	NS	0.013033517	0.050350785	0.034082243	NS
MAGEB16	NS	0.01305711	-0.050337735	0.034139325	NS
TARBP2	NS	0.013076199	-0.050327191	0.034184619	NS
NRSN2	NS	0.013093306	-0.050317754	0.034224717	NS
ZPR1	NS	0.013122223	0.050301826	0.034292235	NS
PLAGL1	NS	0.01312268	0.050301575	0.034292235	NS
BRPF3	NS	0.013143407	0.050290178	0.034337553	NS
PTGES	NS	0.01314357	0.050290088	0.034337553	NS
BTK	NS	0.013149918	0.0502866	0.034347655	NS
DCP1A	NS	0.013150985	0.050286014	0.034347655	NS
BCLAF1	NS	0.013167442	0.050276981	0.034384096	NS
CASP8	NS	0.01316849	0.050276406	0.034384096	NS
PDCD6-AHRI	NS	0.013186515	0.050266525	0.034426514	NS
MON2	NS	0.013200314	0.050258967	0.034448799	NS

CPLX4	NS	0.013200384	-0.050258929	0.034448799	NS
ZNF461	NS	0.01320039	-0.050258926	0.034448799	NS
IZUMO1R	NS	0.013222111	0.050247045	0.034500833	NS
COL1A1	NS	0.013236837	0.050238999	0.034534603	NS
MSMP	NS	0.013249178	0.050232263	0.034562142	NS
ABCA9	NS	0.013303567	-0.050202642	0.034699346	NS
DNAJB5	NS	0.013333075	0.050186616	0.03476594	NS
KRT6B	NS	0.013334488	0.050185849	0.03476594	NS
RACK1	NS	0.013331789	0.050187314	0.03476594	NS
CEBPD	NS	0.013337334	0.050184305	0.034768678	NS
SCN3A	NS	0.013350075	-0.050177397	0.034797205	NS
KBTBD7	NS	0.013374977	-0.050163912	0.03485742	NS
TACC1	NS	0.013382915	0.050159618	0.034873412	NS
MTFR1	NS	0.013415982	-0.050141756	0.034950168	NS
CYB561	NS	0.013430683	-0.050133827	0.034983758	NS
RBBP4	NS	0.013433564	-0.050132273	0.034986554	NS
PAWR	NS	0.013440945	0.050128296	0.035001067	NS
OSBPL5	NS	0.013482613	0.05010588	0.035104761	NS
PRKG1	NS	0.013484392	0.050104923	0.035104761	NS
OGN	NS	0.013488962	-0.050102469	0.035111934	NS
WASF2	NS	0.013495011	0.050099221	0.035122957	NS
TSR3	NS	0.013498039	-0.050097596	0.035126115	NS
MLXIPL	NS	0.013520554	0.050085521	0.035179975	NS
VCY1B	NS	0.013524631	-0.050083336	0.035185856	NS
CCNY	NS	0.013527844	-0.050081615	0.035189483	NS
CROCC	NS	0.013531577	-0.050079615	0.035192385	NS
DYNC1I2	NS	0.013532596	-0.05007907	0.035192385	NS
AMPD2	NS	0.013536702	0.050076871	0.035193608	NS
NBAS	NS	0.013536538	0.050076959	0.035193608	NS
GPR27	NS	0.013544154	-0.050072882	0.035208253	NS
OR13C9	NS	0.013555711	-0.050066701	0.035231467	NS
FAM240A	NS	0.01361142	-0.050036967	0.035364113	NS
FAM110B	NS	0.013620275	-0.050032251	0.035382372	NS
PALMD	NS	0.013635209	-0.050024303	0.035413727	NS
DMBX1	NS	0.013636005	0.05002388	0.035413727	NS
CNIH2	NS	0.013640982	0.050021232	0.035421902	NS
CALHM3	NS	0.013670305	0.050005655	0.035493283	NS
CPZ	NS	0.013676574	0.050002328	0.035501027	NS
INSC	NS	0.013676956	-0.050002126	0.035501027	NS
PTN	NS	0.013691245	-0.049994549	0.035533353	NS
KCNK3	NS	0.013693338	0.04999344	0.035534019	NS
DPCD	NS	0.013700323	-0.049989739	0.035542614	NS
GK3P	NS	0.013698622	0.04999064	0.035542614	NS
HSD17B4	NS	0.013704958	-0.049987284	0.035549876	NS
ARVCF	NS	0.013717737	0.04998052	0.035578255	NS
PDE9A	NS	0.013727285	0.049975469	0.035598248	NS
MDFIC	NS	0.013732221	0.049972859	0.035605202	NS
CDH12	NS	0.013733645	0.049972107	0.035605202	NS
MUC2	NS	0.013735988	0.049970868	0.035606507	NS
AARS1	NS	0.013775175	-0.049950184	0.035684634	NS
ZMYND8	NS	0.013773115	-0.04995127	0.035684634	NS
NT5DC4	NS	0.013775345	-0.049950094	0.035684634	NS
TMEM256	NS	0.013768526	-0.04995369	0.035684634	NS
B3GALT9	NS	0.013771232	0.049952263	0.035684634	NS
ENTPD8	NS	0.013783293	0.049945906	0.035700445	NS

NCBP2AS2	NS	0.013791524	-0.04994157	0.035716985	NS
PHF13	NS	0.01380769	-0.049933061	0.035754066	NS
EIF4E2	NS	0.013811867	0.049930864	0.0357601	NS
DRAM1	NS	0.013825626	0.049923631	0.035786148	NS
DEFB119	NS	0.013824619	-0.04992416	0.035786148	NS
SH3TC1	NS	0.013854268	0.049908594	0.03585549	NS
CDK7	NS	0.013858327	-0.049906465	0.035861201	NS
FAM90A17P	NS	0.013878238	0.049896031	0.035907923	NS
HSF2	NS	0.013881127	-0.049894518	0.035910599	NS
KLK15	NS	0.013884115	0.049892954	0.035913527	NS
ZNF638	NS	0.013897815	0.049885785	0.035937402	NS
CACHD1	NS	0.013899668	-0.049884815	0.035937402	NS
ARID3B	NS	0.013897931	0.049885724	0.035937402	NS
LAGE3	NS	0.013902628	-0.049883268	0.035937402	NS
DHX29	NS	0.013911607	-0.049878574	0.035955811	NS
RERE	NS	0.013923425	0.0498724	0.03598155	NS
KRTAP1-4	NS	0.013931188	0.049868347	0.035996805	NS
FABP9	NS	0.013937482	-0.049865062	0.036008263	NS
TGIF2LY	NS	0.013941458	-0.049862988	0.0360131	NS
IGSF23	NS	0.013943076	-0.049862144	0.0360131	NS
SCNN1D	NS	0.013961641	-0.049852467	0.036056241	NS
DLK1	NS	0.013970892	0.049847649	0.036075318	NS
YAP1	NS	0.013976492	-0.049844734	0.036084618	NS
GAGE12G	NS	0.013978222	0.049843834	0.036084618	NS
KIFC1	NS	0.013981426	0.049842167	0.036088075	NS
C10orf99	NS	0.013999346	0.049832848	0.036129511	NS
RNASE7	NS	0.014017625	-0.049823354	0.036163704	NS
TMEM50A	NS	0.0140182	-0.049823055	0.036163704	NS
HRNR	NS	0.014016836	-0.049823763	0.036163704	NS
DPP6	NS	0.014026111	-0.049818949	0.036179292	NS
UTS2B	NS	0.014045313	-0.049808993	0.036223994	NS
DNAAF6	NS	0.014049604	0.049806769	0.036230233	NS
MXRA7	NS	0.014057703	0.049802574	0.036246291	NS
ZNF766	NS	0.014062217	0.049800237	0.0362531	NS
MDN1	NS	0.01406462	-0.049798994	0.036254465	NS
DNAH5	NS	0.014100714	0.049780333	0.036337827	NS
ZNF517	NS	0.014099666	-0.049780874	0.036337827	NS
CDK16	NS	0.014112693	-0.049774149	0.036362753	NS
AURKB	NS	0.014114143	-0.049773401	0.036362753	NS
TINCR	NS	0.014119017	0.049770886	0.036370469	NS
CCL11	NS	0.014142312	-0.049758879	0.036420781	NS
CCDC71	NS	0.014141748	0.049759169	0.036420781	NS
EVA1B	NS	0.014194905	-0.049731833	0.036551362	NS
VPS9D1	NS	0.014203636	-0.049727352	0.036568979	NS
DOK7	NS	0.01420705	0.049725601	0.036572903	NS
ANKRD30BL	NS	0.014218804	-0.049719573	0.036598294	NS
PDE4D	NS	0.014222021	0.049717924	0.036601707	NS
HDAC4	NS	0.014227923	-0.049714899	0.036607163	NS
ENOPH1	NS	0.014227261	-0.049715239	0.036607163	NS
COPS2	NS	0.01423069	0.049713482	0.036609414	NS
DDX51	NS	0.014239963	-0.049708733	0.036628403	NS
MPPED2	NS	0.014245993	-0.049705647	0.036639044	NS
RPL28	NS	0.014253003	-0.04970206	0.036652203	NS
KANSL3	NS	0.014266903	0.049694953	0.036683073	NS
COX18	NS	0.014279731	0.049688399	0.036711179	NS

MYOM2	NS	0.014294677	-0.049680769	0.036744722	NS
ACSS3	NS	0.014297485	-0.049679337	0.036747061	NS
PIRT	NS	0.014308405	-0.049673767	0.036770246	NS
GLYATL2	NS	0.014314201	-0.049670813	0.036780257	NS
MTMR3	NS	0.014341362	0.049656983	0.036840265	NS
PRR13	NS	0.014341105	0.049657114	0.036840265	NS
RRN3	NS	0.014344927	0.04965517	0.036842696	NS
INPP4B	NS	0.014347074	-0.049654078	0.036842696	NS
S100A14	NS	0.014349922	0.049652629	0.036842696	NS
FITM2	NS	0.014348074	0.049653569	0.036842696	NS
USP17L19	NS	0.014363882	-0.049645534	0.036873648	NS
SNTA1	NS	0.014375708	0.049639527	0.036899112	NS
ARL1	NS	0.014379727	-0.049637487	0.036904534	NS
HOXD3	NS	0.014395276	0.049629599	0.036939542	NS
UBE2R2	NS	0.014400861	0.049626767	0.036948975	NS
PDCD1	NS	0.014405528	-0.049624402	0.03695605	NS
DACT2	NS	0.01443409	0.04960994	0.037024415	NS
THAP5	NS	0.014456211	-0.049598756	0.037076246	NS
UBE2G2	NS	0.014464507	-0.049594567	0.037092607	NS
ERAP2	NS	0.014474126	-0.049589711	0.037112357	NS
ERAP1	NS	0.014504684	0.049574304	0.037185784	NS
RPAP3	NS	0.014509888	0.049571683	0.037194199	NS
LATS1	NS	0.014512709	0.049570262	0.037196505	NS
LDAH	NS	0.014536776	-0.049558155	0.037253254	NS
LMTK2	NS	0.014554828	-0.049549084	0.037290881	NS
CD3D	NS	0.014555311	-0.049548841	0.037290881	NS
ZSCAN29	NS	0.014575482	-0.049538718	0.037337616	NS
TAB2	NS	0.014587201	0.049532842	0.037361057	NS
SOST	NS	0.014588493	0.049532195	0.037361057	NS
CYP4X1	NS	0.014629254	0.049511792	0.03746049	NS
TIPRL	NS	0.014634444	-0.049509198	0.037468821	NS
DNAAF5	NS	0.014649665	-0.049501594	0.037502832	NS
ZNF24	NS	0.014653038	0.04949991	0.037506506	NS
SLC6A2	NS	0.014666491	-0.049493197	0.037535978	NS
SS18L1	NS	0.014680601	0.049486162	0.037567122	NS
SHISA9	NS	0.0146842	0.049484368	0.037571365	NS
OTUD3	NS	0.014702866	-0.049475072	0.037614152	NS
DPP10	NS	0.014711446	-0.049470803	0.037631128	NS
MMP25	NS	0.014718361	0.049467363	0.037638869	NS
MCCC2	NS	0.014717485	-0.049467799	0.037638869	NS
PNLDC1	NS	0.014725129	-0.049463999	0.037651202	NS
CSMD3	NS	0.014733198	0.049459989	0.037666858	NS
NPHS1	NS	0.014738758	0.049457227	0.037676096	NS
MED13L	NS	0.014748748	0.049452267	0.037696655	NS
FBXO24	NS	0.014780581	0.049436481	0.037773028	NS
GIMAP1	NS	0.014789545	0.049432041	0.037790948	NS
PLEKHA8	NS	0.014799853	0.049426938	0.037802317	NS
CDC123	NS	0.014797966	-0.049427872	0.037802317	NS
TNFAIP8L3	NS	0.014796258	0.049428717	0.037802317	NS
GOT2	NS	0.014807745	0.049423034	0.037817485	NS
WDR24	NS	0.014814731	-0.049419579	0.037830334	NS
GPR20	NS	0.014820282	0.049416835	0.037839518	NS
USP17L30	NS	0.014862703	-0.049395895	0.037937818	NS
REEP5	NS	0.014875762	-0.049389459	0.037966145	NS
DMRTA1	NS	0.01488325	-0.049385771	0.037980248	NS

PRDM7	NS	0.014887966	-0.049383449	0.037987276	NS
SELENOH	NS	0.014918088	-0.049368634	0.038059115	NS
KLRK1	NS	0.014928172	-0.04936368	0.038079823	NS
CYP1A2	NS	0.014940426	0.049357664	0.038106059	NS
ROMO1	NS	0.014955156	-0.049350439	0.038138604	NS
NPBWR1	NS	0.014975415	0.049340511	0.038185236	NS
ACTN2	NS	0.014989115	-0.049333804	0.038215135	NS
NR2C1	NS	0.014995936	-0.049330467	0.03822749	NS
ATP13A3	NS	0.015015538	0.049320884	0.038267381	NS
FAM180A	NS	0.015014572	0.049321356	0.038267381	NS
IGSF3	NS	0.015043769	-0.049307102	0.038329233	NS
MYO18A	NS	0.015042936	0.049307508	0.038329233	NS
ST8SIA5	NS	0.015051808	0.049303182	0.038344668	NS
RASSF9	NS	0.015088084	0.049285514	0.038432024	NS
ADGRA2	NS	0.015107114	0.04927626	0.038470373	NS
PSTK	NS	0.015116107	0.049271891	0.038483148	NS
NR2E3	NS	0.015114643	0.049272603	0.038483148	NS
ACCSL	NS	0.01516732	-0.049247053	0.038603371	NS
SMIM11	NS	0.015193774	-0.049234251	0.038661557	NS
ARHGAP44	NS	0.015220281	-0.049221444	0.038722891	NS
EFCAB10	NS	0.015232201	-0.049215691	0.03874489	NS
TMEM116	NS	0.015232931	-0.049215338	0.03874489	NS
BIVM	NS	0.015240373	-0.049211749	0.038758725	NS
MEIS1	NS	0.015245394	-0.049209328	0.038766402	NS
FBLN2	NS	0.015257296	-0.049203592	0.038791571	NS
OAZ3	NS	0.015262347	-0.049201159	0.038799316	NS
PCDHGA1	NS	0.015288576	-0.049188536	0.03886089	NS
TP53I13	NS	0.015292218	-0.049186784	0.038865043	NS
TENT5A	NS	0.015312323	0.049177123	0.038911031	NS
BMP8B	NS	0.015316669	-0.049175036	0.038916966	NS
SLC7A13	NS	0.015318881	0.049173974	0.038917478	NS
IL1RAP	NS	0.015352734	0.049157738	0.038998363	NS
KLHDC8B	NS	0.015375477	-0.049146849	0.039051007	NS
MED31	NS	0.015378832	-0.049145244	0.039054404	NS
TP53I11	NS	0.01539705	0.049136532	0.039095539	NS
PRRC1	NS	0.015418829	0.04912613	0.039145704	NS
ZBTB17	NS	0.015441565	0.049115284	0.039198286	NS
OR8D2	NS	0.015448055	-0.049112191	0.039209618	NS
NGEF	NS	0.015467095	-0.049103123	0.03925172	NS
DNAL4	NS	0.015468698	-0.04910236	0.03925172	NS
MPP1	NS	0.015473445	0.049100101	0.039258617	NS
LSMEM2	NS	0.015490071	-0.049092193	0.039295652	NS
USHBP1	NS	0.015516148	0.049079805	0.039356645	NS
KPNA7	NS	0.015521989	0.049077033	0.039366302	NS
PHRF1	NS	0.015535612	0.049070571	0.039393594	NS
PCDHGB2	NS	0.015536821	-0.049069998	0.039393594	NS
SCN11A	NS	0.015561679	0.04905822	0.039451455	NS
TPX2	NS	0.015568149	-0.049055157	0.039462689	NS
FLRT2	NS	0.015573381	0.049052681	0.039470781	NS
DDR1	NS	0.015626256	-0.0490277	0.039599608	NS
SASH3	NS	0.015630672	0.049025617	0.039605614	NS
AKTIP	NS	0.015656704	-0.049013349	0.039666382	NS
MINAR1	NS	0.01566502	-0.049009433	0.039682256	NS
PNPLA4	NS	0.015671354	0.049006452	0.039682722	NS
CLUAP1	NS	0.015667682	-0.04900818	0.039682722	NS

HELLS	NS	0.015670374	-0.049006913	0.039682722	NS
GRB10	NS	0.015674615	-0.049004917	0.039685789	NS
OR8B8	NS	0.015682476	-0.04900122	0.039700497	NS
TMEM92	NS	0.01568983	0.048997762	0.039713921	NS
LIPA	NS	0.015702839	0.048991649	0.039741652	NS
ZNF687	NS	0.0157408	0.048973837	0.039827311	NS
HECTD1	NS	0.015754312	0.048967506	0.03985629	NS
DDAH2	NS	0.01576538	0.048962323	0.039879077	NS
FAM219B	NS	0.015773974	-0.048958302	0.039895602	NS
CHST9	NS	0.015782306	-0.048954404	0.03991146	NS
MC5R	NS	0.015816027	0.04893865	0.03999151	NS
SRI	NS	0.015823958	-0.048934949	0.040006338	NS
ZC3H12A	NS	0.015829121	0.04893254	0.04000894	NS
HINT1	NS	0.015827473	0.048933308	0.04000894	NS
RPL32	NS	0.015864153	0.048916216	0.040092249	NS
GAS8	NS	0.015874978	0.048911178	0.04011437	NS
SRPX2	NS	0.015882391	0.04890773	0.040118911	NS
TENT5B	NS	0.015882993	0.04890745	0.040118911	NS
METTL23	NS	0.015879805	-0.048908932	0.040118911	NS
FZD5	NS	0.015891646	0.048903427	0.04013553	NS
CRTC2	NS	0.015898535	0.048900225	0.040147689	NS
TRIM50	NS	0.015909828	0.04889498	0.040170967	NS
COMMD8	NS	0.015913417	-0.048893313	0.040174788	NS
CT47A4	NS	0.015922886	0.048888918	0.040193451	NS
EMC9	NS	0.015933138	-0.048884163	0.040208843	NS
ARPC5	NS	0.015931975	0.048884702	0.040208843	NS
KDM2B	NS	0.015944634	0.048878833	0.040232609	NS
CLEC1A	NS	0.01600216	-0.048852214	0.040372498	NS
NRROS	NS	0.016011675	0.048847819	0.040391239	NS
STAT5A	NS	0.016019392	0.048844257	0.040405441	NS
ZNF625	NS	0.016025287	-0.048841536	0.040415043	NS
B3GNT9	NS	0.016048777	-0.048830705	0.040469011	NS
MT1G	NS	0.016059355	0.048825832	0.04049041	NS
TELO2	NS	0.016064368	-0.048823523	0.04049416	NS
KHDC1L	NS	0.016065027	-0.04882322	0.04049416	NS
STON1	NS	0.016070542	-0.048820681	0.040502787	NS
SLC25A36	NS	0.016075686	0.048818314	0.040510478	NS
POM121C	NS	0.016079169	0.048816711	0.04051398	NS
RAD50	NS	0.016087287	-0.048812977	0.040522773	NS
TUFT1	NS	0.01608894	0.048812217	0.040522773	NS
DHCR7	NS	0.016086398	0.048813386	0.040522773	NS
SMIM24	NS	0.016092621	0.048810525	0.040526772	NS
SMS	NS	0.016112202	0.048801528	0.040570804	NS
TEX13D	NS	0.016132097	0.048792397	0.040615618	NS
KRTAP19-6	NS	0.016153082	-0.048782776	0.040663162	NS
SPDYE6	NS	0.016156565	0.04878118	0.040666642	NS
EFNA4	NS	0.01616006	-0.048779579	0.040670151	NS
GBP6	NS	0.01616595	0.048776881	0.040679685	NS
BLID	NS	0.016196222	-0.048763032	0.040750562	NS
YIPF5	NS	0.016200096	0.048761261	0.040753609	NS
TEAD1	NS	0.016215859	-0.04875406	0.040784065	NS
NOVA2	NS	0.016218576	0.048752819	0.0407856	NS
RAB3C	NS	0.016245513	-0.04874053	0.04084497	NS
ALG3	NS	0.016246405	0.048740123	0.04084497	NS
GPAM	NS	0.016257526	-0.048735055	0.04086652	NS

PCDHGA4	NS	0.0162592	-0.048734292	0.04086652	NS
YEATS4	NS	0.01626238	-0.048732843	0.040869205	NS
USP17L21	NS	0.01628475	-0.04872266	0.040920112	NS
GLT8D1	NS	0.016287877	-0.048721238	0.040922656	NS
TNFRSF10C	NS	0.016291153	-0.048719747	0.040925575	NS
IER3IP1	NS	0.016305184	-0.048713369	0.040955508	NS
NR4A3	NS	0.016355776	0.048690409	0.041061529	NS
PAQR3	NS	0.016351092	-0.048692532	0.041061529	NS
CXCL8	NS	0.016355385	0.048690586	0.041061529	NS
CFAP54	NS	0.016355879	0.048690362	0.041061529	NS
TESMIN	NS	0.016363142	-0.048687071	0.041074436	NS
ZNF114	NS	0.016396069	0.048672167	0.041151751	NS
TTC9C	NS	0.016416979	-0.048662716	0.041198891	NS
TMEM44	NS	0.016428258	-0.048657623	0.041215528	NS
CABCOCO1	NS	0.016429421	-0.048657098	0.041215528	NS
SPRR2F	NS	0.016429997	-0.048656838	0.041215528	NS
MACROH2A1	NS	0.016449783	0.048647911	0.041259815	NS
C12orf29	NS	0.016476367	-0.048635931	0.041314098	NS
KRTAP13-1	NS	0.016477828	-0.048635274	0.041314098	NS
PDE6C	NS	0.016485524	-0.048631809	0.041328042	NS
EFR3A	NS	0.016501424	0.048624656	0.041362543	NS
AUH	NS	0.016513327	-0.048619305	0.041381663	NS
GLB1L2	NS	0.016512578	0.048619641	0.041381663	NS
RAP1GAP2	NS	0.0165248	0.04861415	0.041405051	NS
PATE3	NS	0.016540691	-0.048607016	0.041439506	NS
FAM189A1	NS	0.016560679	0.048598051	0.041484213	NS
TCF23	NS	0.016564674	0.04859626	0.041488849	NS
CNTNAP1	NS	0.016574924	0.048591667	0.041509151	NS
TJP3	NS	0.016579782	0.048589492	0.041510575	NS
FCRL3	NS	0.016578885	-0.048589893	0.041510575	NS
RELL1	NS	0.016589479	0.04858515	0.041529481	NS
PABIR1	NS	0.016613386	-0.048574456	0.041583952	NS
DHRSX	NS	0.016622561	0.048570355	0.041601538	NS
MICAL2	NS	0.0166286	-0.048567657	0.041611272	NS
CAMK2D	NS	0.016634575	-0.048564989	0.041620841	NS
SMAD7	NS	0.016644732	0.048560455	0.041630892	NS
SLC17A2	NS	0.016644912	-0.048560375	0.041630892	NS
PXDNL	NS	0.016645044	0.048560315	0.041630892	NS
KCNC2	NS	0.016650389	0.04855793	0.041638881	NS
RAB36	NS	0.016656776	0.048555081	0.04164947	NS
FAM71E1	NS	0.016661512	-0.048552969	0.041655933	NS
PLEKHH1	NS	0.0166782	0.048545531	0.041692269	NS
TTLL1	NS	0.016684822	-0.048542582	0.041703436	NS
FAM98A	NS	0.016689234	-0.048540617	0.041703694	NS
ALKBH8	NS	0.016687192	-0.048541526	0.041703694	NS
LRRC24	NS	0.016704065	-0.048534016	0.041735365	NS
WDR13	NS	0.016736197	0.048519732	0.041810251	NS
C11orf97	NS	0.016741877	-0.04851721	0.041819044	NS
MYH7	NS	0.016747504	-0.048514712	0.041827275	NS
MTHFS	NS	0.016749494	0.048513828	0.041827275	NS
TMEM134	NS	0.016757329	0.048510351	0.041841442	NS
CLCNKA	NS	0.016763777	0.048507491	0.041847085	NS
AP298-TCP1	NS	0.016763913	-0.048507431	0.041847085	NS
SMDT1	NS	0.016767627	0.048505784	0.04185096	NS
KRTAP20-3	NS	0.016775953	-0.048502093	0.041866342	NS

SRSF11	NS	0.016809019	0.048487449	0.041928792	NS
TBC1D14	NS	0.016809642	-0.048487174	0.041928792	NS
YDJC	NS	0.016808274	-0.048487779	0.041928792	NS
CENPS	NS	0.016813006	-0.048485685	0.041931781	NS
SEM1	NS	0.016815585	-0.048484545	0.041932808	NS
TOMM70	NS	0.016828017	-0.048479047	0.041958405	NS
RPS14	NS	0.016839838	0.048473824	0.041977065	NS
H3C14	NS	0.016839608	0.048473925	0.041977065	NS
TFPT	NS	0.016849005	0.048469775	0.041994506	NS
LGALS8	NS	0.016869754	0.048460618	0.042040808	NS
SEMA6B	NS	0.016873255	0.048459074	0.04204412	NS
C2CD2	NS	0.016904681	-0.048445227	0.042115276	NS
ARSI	NS	0.016906163	-0.048444575	0.042115276	NS
FBXL15	NS	0.016929003	-0.048434526	0.042155897	NS
POLE4	NS	0.016928495	-0.048434749	0.042155897	NS
LCK	NS	0.016927579	-0.048435152	0.042155897	NS
PKDREJ	NS	0.016936403	0.048431272	0.042168898	NS
HBS1L	NS	0.016951745	0.048424532	0.042201668	NS
RAMP3	NS	0.016956061	-0.048422636	0.042206985	NS
TTC31	NS	0.016974229	-0.048414662	0.042246777	NS
OR2T6	NS	0.016976512	-0.048413661	0.042247026	NS
SNRK	NS	0.017004333	0.048401466	0.04231082	NS
IGLL5	NS	0.017018573	-0.048395231	0.042335369	NS
PTPRK	NS	0.017030176	-0.048390154	0.042353346	NS
ARMCX6	NS	0.017029413	-0.048390488	0.042353346	NS
ATP6V1C2	NS	0.017038671	-0.048386439	0.042364952	NS
NCCRP1	NS	0.017039221	0.048386199	0.042364952	NS
OR8D4	NS	0.017048869	0.048381982	0.042380744	NS
DMD	NS	0.017049951	0.048381509	0.042380744	NS
MLANA	NS	0.017056882	-0.04837848	0.042392528	NS
FBXL18	NS	0.017059113	0.048377506	0.042392629	NS
SMYD1	NS	0.017063044	-0.048375789	0.042396955	NS
PTPRQ	NS	0.017087587	-0.048365078	0.04244704	NS
BOLL	NS	0.017086518	0.048365545	0.04244704	NS
ALPK3	NS	0.017105593	0.048357229	0.042486317	NS
GAL3ST4	NS	0.017113663	-0.048353713	0.042500906	NS
SCAMP5	NS	0.017129799	0.048346688	0.042535523	NS
AK9	NS	0.017146084	-0.048339604	0.042561691	NS
SUPV3L1	NS	0.017142729	-0.048341063	0.042561691	NS
LYPD6	NS	0.017146934	-0.048339234	0.042561691	NS
MAGEE2	NS	0.017158149	-0.048334359	0.042584066	NS
SRRM2	NS	0.017160378	0.048333391	0.042584139	NS
HSD11B2	NS	0.017168449	0.048329885	0.042598705	NS
ZNF180	NS	0.017174832	-0.048327113	0.042609082	NS
TX16-NPEPL	NS	0.017184837	0.04832277	0.042628437	NS
HOXD1	NS	0.017189154	0.048320897	0.042633683	NS
NUPR1	NS	0.017191852	0.048319726	0.042634912	NS
IP6K2	NS	0.017228296	0.048303931	0.042719818	NS
CP	NS	0.017248836	-0.048295041	0.04276527	NS
SORBS1	NS	0.017253355	-0.048293087	0.042770996	NS
ATG16L2	NS	0.017279635	0.048281729	0.042830658	NS
RPS12	NS	0.017284965	0.048279428	0.042838385	NS
ARSB	NS	0.017292349	0.04827624	0.042848314	NS
SLC9A6	NS	0.017293399	0.048275787	0.042848314	NS
PPFIBP2	NS	0.017312076	0.04826773	0.0428891	NS

PHF5A	NS	0.01731854	-0.048264944	0.042899623	NS
USP40	NS	0.017329149	-0.048260372	0.042904476	NS
FAM110A	NS	0.01732366	-0.048262737	0.042904476	NS
MAML2	NS	0.017329242	-0.048260332	0.042904476	NS
OR10G8	NS	0.017329365	0.048260279	0.042904476	NS
ODF2L	NS	0.017348105	0.048252209	0.04294538	NS
DNAJC27	NS	0.017356242	0.048248708	0.042954534	NS
GPR84	NS	0.017355562	0.048249001	0.042954534	NS
STS	NS	0.017360655	-0.04824681	0.042959965	NS
C1orf162	NS	0.01736729	0.048243956	0.04297089	NS
SCML2	NS	0.017374856	-0.048240704	0.042982845	NS
ILVBL	NS	0.017376563	-0.04823997	0.042982845	NS
GTDC1	NS	0.017400292	-0.048229778	0.043036039	NS
DNAH7	NS	0.017409323	0.048225903	0.043041876	NS
STARD3	NS	0.017406266	0.048227214	0.043041876	NS
FCSK	NS	0.017408405	-0.048226296	0.043041876	NS
ARFGEF2	NS	0.017426871	0.048218377	0.043079759	NS
GSTP1	NS	0.017451908	0.048207651	0.043136142	NS
TBX18	NS	0.017471199	-0.048199395	0.043173343	NS
MMS22L	NS	0.01747142	-0.048199301	0.043173343	NS
NPAS1	NS	0.017476332	0.0481972	0.043179971	NS
ATP5PF	NS	0.017484512	-0.048193703	0.043194668	NS
VPS35	NS	0.017503783	-0.048185469	0.043236757	NS
MAPK8IP3	NS	0.017513619	0.04818127	0.043255533	NS
REST	NS	0.017529232	0.048174609	0.043284177	NS
LARP7	NS	0.017529689	-0.048174414	0.043284177	NS
PTRH2	NS	0.017533425	-0.04817282	0.04328788	NS
LAPTM5	NS	0.017544754	0.048167991	0.043310326	NS
ERF	NS	0.017552271	-0.048164788	0.043323357	NS
ANKDD1B	NS	0.017554782	-0.048163719	0.04332403	NS
CA4	NS	0.017566931	0.048158545	0.043348486	NS
MBD3L4	NS	0.017575714	0.048154807	0.043364631	NS
CLEC20A	NS	0.017588036	-0.048149566	0.043389501	NS
OTUB2	NS	0.017609517	-0.048140436	0.043434023	NS
KCNIP3	NS	0.017615059	0.048138083	0.043434023	NS
INA	NS	0.017614223	0.048138438	0.043434023	NS
DMBT1	NS	0.01761292	0.048138991	0.043434023	NS
KLHL9	NS	0.017617394	-0.048137091	0.043434248	NS
ACTL7A	NS	0.017620338	-0.048135841	0.043435974	NS
SNAPC5	NS	0.01762624	-0.048133336	0.04344499	NS
PITX2	NS	0.017682066	0.048109674	0.043573281	NS
CCDC179	NS	0.017682792	-0.048109367	0.043573281	NS
MDP1	NS	0.017702097	0.0481012	0.043615299	NS
RHBDL1	NS	0.017719751	0.048093739	0.043653239	NS
AKT1S1	NS	0.017734016	0.048087714	0.043682822	NS
MUC13	NS	0.017740275	-0.048085072	0.043692678	NS
KRTAP21-2	NS	0.017742815	-0.048084	0.043693375	NS
PHLDA1	NS	0.017746186	0.048082578	0.043696116	NS
OR7A5	NS	0.017764616	0.048074805	0.043735934	NS
ZNF98	NS	0.017768154	0.048073314	0.043739081	NS
CHRNB2	NS	0.017774889	-0.048070476	0.043750096	NS
FAM187A	NS	0.017786222	-0.048065703	0.043772424	NS
BRWD3	NS	0.017789323	0.048064397	0.043774489	NS
C1orf116	NS	0.017801683	-0.048059195	0.043799336	NS
EGR3	NS	0.017843126	0.048041774	0.043895724	NS

PTPN21	NS	0.01785153	-0.048038246	0.043908654	NS
NPLOC4	NS	0.01786485	0.048032657	0.043932414	NS
DCAF7	NS	0.017868283	0.048031217	0.043932631	NS
NAP1L3	NS	0.017869477	0.048030717	0.043932631	NS
OGFOD1	NS	0.017897722	-0.04801888	0.043996484	NS
RPUSD1	NS	0.017923316	-0.048008168	0.044048984	NS
HTR7	NS	0.017923631	-0.048008036	0.044048984	NS
TCEAL2	NS	0.017928123	0.048006158	0.044049069	NS
HLA-DQB2	NS	0.017928217	-0.048006118	0.044049069	NS
ZFHX4	NS	0.017973655	-0.047987139	0.044155105	NS
HSPA6	NS	0.017987431	-0.047981393	0.04418334	NS
TOM1	NS	0.018004657	0.047974213	0.044220041	NS
SERTAD1	NS	0.018019054	0.047968217	0.044249786	NS
ZBTB33	NS	0.018032541	0.047962605	0.044271672	NS
HYPK	NS	0.018030266	0.047963551	0.044271672	NS
TRPS1	NS	0.018049591	-0.047955514	0.044307911	NS
MRPL40	NS	0.018113153	-0.047929132	0.044458304	NS
MMAB	NS	0.018116555	-0.047927722	0.044461015	NS
STAB1	NS	0.018124327	-0.047924502	0.044474451	NS
SPINK7	NS	0.018139404	-0.047918259	0.044505807	NS
JADE3	NS	0.018149092	-0.04791425	0.044523932	NS
PSME3	NS	0.018159371	0.047909999	0.044543502	NS
IFI27L2	NS	0.018177273	-0.0479026	0.044576239	NS
AFAP1L2	NS	0.018177322	-0.047902579	0.044576239	NS
MTRNR2L7	NS	0.018200042	-0.047893198	0.044626299	NS
MTPAP	NS	0.018203289	-0.047891858	0.044628609	NS
CD59	NS	0.018231807	-0.047880099	0.044692864	NS
ABCC5	NS	0.018235962	-0.047878387	0.044697389	NS
PCDHGB7	NS	0.01824229	-0.04787578	0.044707239	NS
NRDC	NS	0.018253054	-0.047871348	0.044727953	NS
WDR19	NS	0.018263831	-0.047866913	0.044745143	NS
CCL15	NS	0.018264692	0.047866559	0.044745143	NS
TMEM185B	NS	0.018276782	-0.047861586	0.044769095	NS
AQP11	NS	0.018290832	-0.047855811	0.044797843	NS
THSD1	NS	0.01833054	-0.047839511	0.044883735	NS
CEP131	NS	0.01836821	-0.047824075	0.044970285	NS
GALNT16	NS	0.018387335	-0.047816249	0.045000031	NS
SIL1	NS	0.018386906	-0.047816424	0.045000031	NS
FOXO1	NS	0.018384589	0.047817372	0.045000031	NS
HNRNPH2	NS	0.01841751	0.047803916	0.045062485	NS
LMOD3	NS	0.018433569	-0.04779736	0.045096077	NS
RCAN3	NS	0.018451288	-0.047790131	0.045128018	NS
SF3B4	NS	0.018450878	0.047790298	0.045128018	NS
ANK3	NS	0.018457727	0.047787506	0.045132359	NS
OR52I2	NS	0.01845641	-0.047788043	0.045132359	NS
KIAA0825	NS	0.018465621	0.047784289	0.045145959	NS
ZC3H12D	NS	0.01847282	0.047781355	0.045157857	NS
GP5	NS	0.018482536	0.047777399	0.045175901	NS
TFEB	NS	0.018486527	0.047775774	0.045179952	NS
MEIOSIN	NS	0.018490429	0.047774185	0.045183782	NS
CBX2	NS	0.018501645	0.047769621	0.045205483	NS
MAX	NS	0.018512435	0.047765233	0.045226137	NS
ZNF365	NS	0.018524796	-0.047760209	0.045250625	NS
GUCY1A1	NS	0.018546882	-0.047751239	0.045293142	NS
NAAA	NS	0.018557959	-0.047746743	0.045314477	NS

CD160	NS	0.01859244	-0.047732765	0.045392946	NS
SNPH	NS	0.018599615	-0.047729859	0.04539901	NS
DHTKD1	NS	0.018598932	-0.047730135	0.04539901	NS
OPHN1	NS	0.018617381	-0.047722668	0.045430916	NS
SYN3	NS	0.018615356	0.047723487	0.045430916	NS
IL1R2	NS	0.018628953	-0.047717987	0.045439837	NS
CAMP	NS	0.018628711	-0.047718085	0.045439837	NS
ZNF316	NS	0.018629935	0.04771759	0.045439837	NS
CSAG3	NS	0.018630427	0.047717391	0.045439837	NS
GABRB2	NS	0.018633471	-0.047716161	0.045441535	NS
CCDC38	NS	0.018644191	-0.047711828	0.04546195	NS
PRMT5	NS	0.018657429	-0.04770648	0.0454885	NS
PLPP4	NS	0.018676412	0.047698818	0.045529047	NS
OR4C15	NS	0.018682146	-0.047696505	0.045537289	NS
IGF2	NS	0.018690089	0.047693301	0.045550915	NS
TSPO	NS	0.018704218	0.047687606	0.045579609	NS
TFF1	NS	0.018717019	-0.047682449	0.045605064	NS
RHOXF1	NS	0.018723084	-0.047680008	0.045614098	NS
THBD	NS	0.018744664	0.047671324	0.045660926	NS
SPANXN4	NS	0.018760377	-0.047665007	0.04569345	NS
NACC1	NS	0.018831687	-0.047636395	0.045861365	NS
MAP6	NS	0.018850721	0.047628774	0.045901945	NS
DEFB103A	NS	0.018859231	-0.047625369	0.04591689	NS
OR3A3	NS	0.01887173	0.047620371	0.045941542	NS
SLC10A4	NS	0.018886626	0.047614417	0.045968817	NS
RASGRP1	NS	0.018890058	-0.047613046	0.045968817	NS
ATXN1L	NS	0.018889459	0.047613285	0.045968817	NS
SMIM14	NS	0.018924199	-0.047599418	0.046046108	NS
DYNC1LI1	NS	0.018933469	-0.047595721	0.046057086	NS
HLA-E	NS	0.018931314	0.047596581	0.046057086	NS
TOX4	NS	0.018972149	0.047580314	0.046141711	NS
PXDC1	NS	0.018973025	-0.047579965	0.046141711	NS
KIFC2	NS	0.018993533	-0.047571808	0.046185782	NS
EMILIN1	NS	0.019028586	0.047557883	0.046265206	NS
SMIM23	NS	0.019060034	-0.047545409	0.046335848	NS
FTMT	NS	0.019069904	0.047541498	0.046354019	NS
VSIG10L2	NS	0.01909023	0.047533449	0.046397601	NS
HPS6	NS	0.019099511	0.047529776	0.04641433	NS
IPO4	NS	0.019115862	0.047523309	0.046448232	NS
NDUFA4L2	NS	0.019123339	0.047520354	0.046460568	NS
OR5T1	NS	0.019137556	-0.047514737	0.046489274	NS
EPN2	NS	0.019151977	-0.047509043	0.046517501	NS
IGFN1	NS	0.019153983	0.047508251	0.046517501	NS
TP53TG3D	NS	0.019162104	-0.047505047	0.046531386	NS
ORC1	NS	0.019188879	-0.04749449	0.046590559	NS
INTS11	NS	0.01921962	-0.047482385	0.046659345	NS
GJD4	NS	0.019258663	0.047467036	0.046748268	NS
FRMD8	NS	0.019278687	-0.047459174	0.046791005	NS
METTL21C	NS	0.019283944	0.047457111	0.046797897	NS
LIN37	NS	0.019301193	-0.047450347	0.046833884	NS
ALX4	NS	0.019316522	-0.047444339	0.046865205	NS
PITPNM3	NS	0.019337296	-0.047436205	0.046909725	NS
AK8	NS	0.019346182	-0.047432727	0.046925402	NS
TTC9	NS	0.019369208	0.047423723	0.046975365	NS
CAPZA3	NS	0.019394857	-0.047413705	0.047025785	NS

ACAP3	NS	0.019400656	-0.047411441	0.047028599	NS
OR6C76	NS	0.019400877	-0.047411355	0.047028599	NS
GRID2IP	NS	0.019406561	0.047409137	0.047036488	NS
LYST	NS	0.019415218	0.04740576	0.047051579	NS
TSPAN9	NS	0.01943155	-0.047399392	0.047085264	NS
MMP2	NS	0.019456741	-0.04738958	0.047140404	NS
ZNF169	NS	0.019481769	-0.047379842	0.047195134	NS
SYPL1	NS	0.019494021	0.047375079	0.047218906	NS
VPS4A	NS	0.019499317	-0.047373021	0.047225824	NS
CWC25	NS	0.019525983	0.047362666	0.047284491	NS
CORO6	NS	0.019531661	-0.047360463	0.047292322	NS
TCEA2	NS	0.019553085	0.047352154	0.047338276	NS
NSL1	NS	0.019560324	-0.047349349	0.047349878	NS
ZNF771	NS	0.019579883	-0.047341773	0.0473913	NS
SLC40A1	NS	0.019585319	-0.047339669	0.047398528	NS
DPPA2	NS	0.019597	0.047335149	0.047420868	NS
DRD3	NS	0.019609237	-0.047330416	0.047444549	NS
RPP38	NS	0.019614692	-0.047328307	0.047451816	NS
TMIGD1	NS	0.019634149	-0.047320789	0.04749295	NS
MT3	NS	0.019697367	-0.047296407	0.047639914	NS
FOXO4	NS	0.019707755	0.047292407	0.047659083	NS
VEGFD	NS	0.019711986	-0.047290778	0.04766336	NS
ANAPC1	NS	0.019731911	0.047283112	0.04770558	NS
KRTAP4-4	NS	0.019752996	0.047275008	0.047750593	NS
SKA2	NS	0.019759517	-0.047272503	0.04776039	NS
CSRNP2	NS	0.01983766	-0.047242541	0.047943282	NS
COL7A1	NS	0.019880563	0.047226135	0.048016993	NS
TCF21	NS	0.01987929	0.047226621	0.048016993	NS
FOXK2	NS	0.019880116	0.047226306	0.048016993	NS
ZNF155	NS	0.019872286	-0.047229298	0.048016993	NS
ORCS7-ASM	NS	0.019875364	0.047228122	0.048016993	NS
CKM	NS	0.01988442	-0.047224661	0.048020317	NS
LSM14B	NS	0.019893811	-0.047221075	0.048037002	NS
ABO	NS	0.019917859	0.047211898	0.04808907	NS
MIP	NS	0.019927801	-0.047208107	0.048107073	NS
TGM2	NS	0.019932425	0.047206344	0.048112235	NS
HPRT1	NS	0.019935226	-0.047205277	0.048112996	NS
SPATA20	NS	0.019976769	-0.047189457	0.048207249	NS
MYH2	NS	0.019980586	-0.047188006	0.048210449	NS
C15orf39	NS	0.020001655	0.047179995	0.048255271	NS
C3	NS	0.020014619	0.04717507	0.048280528	NS
RPIA	NS	0.020027071	-0.047170342	0.048304548	NS
LMLN2	NS	0.020045167	-0.047163475	0.04834217	NS
NEFM	NS	0.02004982	-0.04716171	0.048347367	NS
MLN	NS	0.020071761	0.047153394	0.048394245	NS
ATP1A2	NS	0.020121391	0.047134611	0.048507865	NS
TEFM	NS	0.020133285	-0.047130116	0.048530493	NS
MFSD4B	NS	0.02016287	-0.047118944	0.048595756	NS
SAMD4B	NS	0.020165953	0.047117781	0.048597134	NS
TRIM54	NS	0.020176141	0.047113937	0.048615633	NS
CLDN2	NS	0.020179239	-0.047112769	0.048617047	NS
ANTXR2	NS	0.020190925	0.047108363	0.048639147	NS
GP1BA	NS	0.020205622	0.047102825	0.048662438	NS
FAM72C	NS	0.020209447	-0.047101385	0.048665594	NS
ODR4	NS	0.020219364	-0.04709765	0.048683421	NS

OTOS	NS	0.020223629	0.047096045	0.048687631	NS
TMF1	NS	0.020247895	0.047086916	0.04873999	NS
SRBD1	NS	0.020250965	-0.047085762	0.048741318	NS
RNF216	NS	0.020259757	0.047082458	0.048756416	NS
RPS15A	NS	0.020283247	0.047073634	0.048806879	NS
MYC	NS	0.020309927	-0.047063624	0.048861924	NS
C8orf44-SGK[illegible]	NS	0.020311172	0.047063157	0.048861924	NS
GGTLC2	NS	0.020329039	-0.04705646	0.048898829	NS
CCNE2	NS	0.020348652	-0.047049115	0.04893785	NS
ZNF593OS	NS	0.020350318	-0.047048491	0.04893785	NS
EOLA2	NS	0.020356231	0.047046278	0.048945989	NS
LCE1D	NS	0.020358891	0.047045283	0.048946303	NS
MTMR12	NS	0.020387785	0.047034477	0.049009682	NS
RPL26	NS	0.020414112	0.047024643	0.049066876	NS
NEU3	NS	0.020445067	-0.047013094	0.049129078	NS
PRAMEF27	NS	0.020442997	-0.047013866	0.049129078	NS
INTS13	NS	0.020457696	-0.047008387	0.049147221	NS
MPP3	NS	0.020456008	-0.047009016	0.049147221	NS
OTOG	NS	0.020490372	-0.04699622	0.049219613	NS
IFIT2	NS	0.020492984	-0.046995248	0.049219779	NS
KRTCAP3	NS	0.020514159	-0.046987373	0.049264524	NS
GOLPH3L	NS	0.020545277	-0.046975813	0.049333132	NS
ITPA	NS	0.020564693	-0.046968608	0.04937363	NS
GSG1L2	NS	0.020610371	0.046951681	0.04947716	NS
MARCHF6	NS	0.020616401	0.046949449	0.049485497	NS
MAP7	NS	0.020630965	-0.04694406	0.049514316	NS
INTS12	NS	0.0206458	0.046938574	0.049543777	NS
SHC1	NS	0.020649125	0.046937345	0.049545613	NS
ZCCHC7	NS	0.020666527	0.046930915	0.049581222	NS
CDH4	NS	0.02068099	0.046925575	0.049609771	NS
PUS3	NS	0.020700001	-0.046918561	0.049649221	NS
HSP90AB1	NS	0.020706499	0.046916164	0.049658655	NS
PUDP	NS	0.020754214	-0.046898588	0.049756543	NS
ANKRD62	NS	0.020766797	0.046893959	0.049778591	NS
LOXL4	NS	0.020794344	-0.046883833	0.049838451	NS
NAP1L2	NS	0.020844374	-0.046865474	0.049952173	NS
ROM1	NS	0.020855107	0.04686154	0.049971706	NS
PSMA7	NS	0.02085943	0.046859956	0.04997588	NS
ASTE1	NS	0.02087124	-0.04685563	0.049997985	NS
OR2G3	NS	0.020885065	-0.046850569	0.050024913	NS
DDHD2	NS	0.020909394	0.04684167	0.05007699	NS
RFPL4B	NS	0.020913654	-0.046840112	0.050080995	NS
LRRC23	NS	0.020926007	-0.046835598	0.050104377	NS
RFFL	NS	0.020954081	0.046825347	0.050159188	NS
CBY2	NS	0.020952813	0.04682581	0.050159188	NS
ALG5	NS	0.020957039	0.046824268	0.050160064	NS
VLDLR	NS	0.020964523	0.046821537	0.050171774	NS
RGS22	NS	0.020988696	-0.046812724	0.050223414	NS
PUS7L	NS	0.020991773	0.046811603	0.050224569	NS
GOLGA4	NS	0.020997133	0.04680965	0.050231184	NS
OLIG1	NS	0.021032898	0.046796632	0.050310525	NS
HMGCS1	NS	0.021124804	0.046763266	0.050524119	NS
CCR4	NS	0.021155244	0.046752243	0.050590673	NS
DYRK1B	NS	0.021159724	0.046750622	0.050595136	NS
FBXW9	NS	0.021179158	-0.046743593	0.05063535	NS

S1PR5	NS	0.021181972	-0.046742575	0.050635822	NS
MGST2	NS	0.021188078	-0.046740368	0.050644164	NS
SLC9B1	NS	0.021199267	-0.046736325	0.050664652	NS
KIF23	NS	0.021211383	-0.04673195	0.050687348	NS
CNPY1	NS	0.021219307	0.046729089	0.050700026	NS
OR14I1	NS	0.021257585	-0.046715283	0.050785216	NS
TMEM37	NS	0.021281164	0.04670679	0.050829	NS
FKBP15	NS	0.021291558	0.046703049	0.05084755	NS
EEF1AKMT3	NS	0.021298008	-0.046700728	0.050856678	NS
NEK7	NS	0.021302231	0.046699209	0.050860488	NS
MCAM	NS	0.021324199	-0.04669131	0.050906659	NS
KLHL29	NS	0.021339404	-0.046685846	0.050936677	NS
GPR156	NS	0.021394277	-0.046666159	0.051061359	NS
MIGA1	NS	0.021423767	-0.046655597	0.051125438	NS
RHBDL3	NS	0.021429801	-0.046653437	0.051127231	NS
ELMO1	NS	0.021427844	0.046654138	0.051127231	NS
F8A1	NS	0.021448316	0.046646814	0.051165097	NS
TFE3	NS	0.021466072	0.046640467	0.051201145	NS
EFCAB2	NS	0.021474768	-0.04663736	0.051215575	NS
STAT1	NS	0.021491158	0.046631508	0.051248349	NS
IDE	NS	0.021506292	0.046626107	0.05127812	NS
PNPLA6	NS	0.021519482	0.046621402	0.051298166	NS
SERPINA11	NS	0.021519999	-0.046621218	0.051298166	NS
ALDH9A1	NS	0.021531869	-0.046616986	0.051320141	NS
RICTOR	NS	0.021555944	0.046608411	0.051371195	NS
CLPSL2	NS	0.021562185	0.046606189	0.051379744	NS
GGH	NS	0.021615403	-0.046587266	0.051500215	NS
SLC45A3	NS	0.021633983	-0.04658067	0.051531795	NS
HEXIM1	NS	0.021633357	0.046580892	0.051531795	NS
CCDC168	NS	0.021637737	-0.046579337	0.051534397	NS
TCF7L1	NS	0.021654788	-0.046573289	0.051568661	NS
ADAMTS17	NS	0.021660975	-0.046571095	0.05157705	NS
RASSF8	NS	0.021672638	0.046566961	0.051598473	NS
ZNF90	NS	0.021676365	-0.04656564	0.051600999	NS
OPRM1	NS	0.021685543	0.046562389	0.051616499	NS
ZNF641	NS	0.021704271	0.046555758	0.051654725	NS
TPRN	NS	0.021717343	0.046551133	0.051679481	NS
XPNPEP1	NS	0.021749285	0.046539841	0.051742769	NS
OXLD1	NS	0.02174772	-0.046540394	0.051742769	NS
N4BP3	NS	0.021758092	0.04653673	0.051750998	NS
PCED1B	NS	0.021756752	-0.046537204	0.051750998	NS
VPREB3	NS	0.021762056	0.046535331	0.051754067	NS
CPNE8	NS	0.021783766	0.046527668	0.051799334	NS
SAMD5	NS	0.021799646	-0.046522067	0.051830728	NS
CD8B2	NS	0.021802748	-0.046520973	0.051831737	NS
GPR158	NS	0.021822983	-0.046513843	0.051873469	NS
LHFPL5	NS	0.02184376	0.046506527	0.051916481	NS
MT2A	NS	0.021849752	0.046504419	0.051924347	NS
PLD1	NS	0.021854159	0.046502868	0.051928444	NS
PCDHGA12	NS	0.021856862	-0.046501917	0.051928492	NS
CHAC1	NS	0.021906932	0.046484322	0.052041061	NS
RAI14	NS	0.021943956	-0.046471333	0.052122617	NS
CCDC3	NS	0.021990926	0.046454883	0.052227775	NS
STX12	NS	0.021994406	0.046453665	0.052229631	NS
CELA3B	NS	0.022017475	-0.046445598	0.052275424	NS

OVCA2	NS	0.022019092	0.046445033	0.052275424	NS
COPA	NS	0.022043297	0.046436577	0.052326473	NS
KIF3C	NS	0.022055832	-0.046432201	0.052349807	NS
RASL10A	NS	0.022059855	0.046430797	0.052352936	NS
FBXO5	NS	0.022069697	-0.046427363	0.052369874	NS
HRK	NS	0.022079929	0.046423795	0.052387731	NS
DBF4B	NS	0.022108489	-0.046413842	0.052449064	NS
GPBAR1	NS	0.022118393	0.046410393	0.052466129	NS
KDM3A	NS	0.022139947	0.046402893	0.052506915	NS
OXTR	NS	0.022141013	-0.046402522	0.052506915	NS
AIFM1	NS	0.022158569	-0.046396417	0.052542112	NS
SYNJ1	NS	0.022196265	0.046383324	0.052625049	NS
TEX38	NS	0.022212073	-0.046377839	0.052656078	NS
TFAP2B	NS	0.022255347	-0.04636284	0.052752205	NS
CDC37L1	NS	0.022281962	-0.046353629	0.052808822	NS
CLCC1	NS	0.022322768	0.046339524	0.052899058	NS
ANO10	NS	0.022334064	-0.046335623	0.052919347	NS
PDK4	NS	0.022363858	0.046325344	0.052983457	NS
SLAIN2	NS	0.022375051	0.046321485	0.053003487	NS
MRPL38	NS	0.022391869	-0.04631569	0.053036835	NS
TAAR5	NS	0.022398001	-0.046313578	0.053044869	NS
MYBPC1	NS	0.022418685	-0.046306458	0.053087359	NS
NRBP1	NS	0.022426299	0.046303838	0.053098893	NS
KICS2	NS	0.022451524	0.046295165	0.053152115	NS
SOGA3	NS	0.022468318	0.046289396	0.053185368	NS
PINLYP	NS	0.022489487	0.046282129	0.053228969	NS
SLC27A4	NS	0.022522049	-0.046270962	0.053295974	NS
ZNF526	NS	0.022523304	0.046270532	0.053295974	NS
TBPL1	NS	0.022539821	0.046264873	0.053322022	NS
POLR3K	NS	0.022543523	-0.046263605	0.053324261	NS
SLC5A10	NS	0.022554683	-0.046259784	0.053344141	NS
SLC41A2	NS	0.022563304	-0.046256834	0.053358011	NS
DUSP1	NS	0.022566255	0.046255824	0.05335847	NS
KRTAP4-5	NS	0.022584271	0.046249662	0.053394546	NS
ARHGAP5	NS	0.022595797	0.046245723	0.053415271	NS
GSTZ1	NS	0.022611882	-0.046240227	0.053425073	NS
HMBOX1	NS	0.022609832	0.046240927	0.053425073	NS
UROS	NS	0.022613251	-0.04623976	0.053425073	NS
VPS52	NS	0.022613744	0.046239591	0.053425073	NS
ST20-MTHFS	NS	0.022610297	0.046240768	0.053425073	NS
SIX2	NS	0.022627344	0.046234948	0.053450681	NS
CLEC11A	NS	0.022639796	-0.046230699	0.053473567	NS
AP2A1	NS	0.022649815	0.046227281	0.053490704	NS
APOBEC4	NS	0.022671346	-0.046219941	0.053535021	NS
TMEM245	NS	0.022689204	0.046213858	0.053563874	NS
ATP9B	NS	0.022691306	0.046213142	0.053563874	NS
DDX52	NS	0.022691866	-0.046212951	0.053563874	NS
RRP1	NS	0.022700573	0.046209987	0.053577892	NS
MFSD3	NS	0.022720059	-0.046203357	0.053617344	NS
LAMC2	NS	0.022745219	0.046194803	0.053670176	NS
TPM2	NS	0.022750338	-0.046193064	0.053675712	NS
RGS14	NS	0.022767502	-0.046187235	0.053709661	NS
PABIR3	NS	0.022820664	0.046169204	0.053828513	NS
RHAG	NS	0.0228871	0.046146722	0.053965491	NS
TRIM27	NS	0.022885875	-0.046147136	0.053965491	NS

TMEM35B	NS	0.022883258	-0.046148021	0.053965491	NS
RALA	NS	0.022896239	-0.046143634	0.05397389	NS
HLA-DOA	NS	0.022894353	0.046144271	0.05397389	NS
NOS1	NS	0.022901748	0.046141773	0.053978548	NS
NSMCE2	NS	0.022905377	0.046140547	0.053978548	NS
PTX3	NS	0.022906582	0.04614014	0.053978548	NS
DOCK7	NS	0.022912803	0.046138039	0.053980065	NS
HLA-DRB1	NS	0.022911368	-0.046138524	0.053980065	NS
IFT22	NS	0.022932718	0.046131318	0.05401891	NS
PGRMC2	NS	0.022934873	-0.046130591	0.05401891	NS
ATP5MC3	NS	0.02294282	-0.04612791	0.054031053	NS
NDUFAF2	NS	0.022958859	-0.046122502	0.054062249	NS
INCENP	NS	0.022971181	-0.046118351	0.054084682	NS
MYDGF	NS	0.022999426	-0.04610884	0.0541446	NS
WASHC1	NS	0.023023221	0.046100836	0.054194024	NS
GDNF	NS	0.023062766	0.046087549	0.054280509	NS
NUDT13	NS	0.023078152	-0.046082385	0.054310116	NS
DCAF13	NS	0.023081531	-0.046081251	0.054311465	NS
ESPNL	NS	0.023111178	0.04607131	0.054374615	NS
ALG12	NS	0.023128412	0.046065536	0.05440855	NS
PHLDB1	NS	0.02314623	0.046059571	0.054434819	NS
SENP1	NS	0.023150828	0.046058032	0.054434819	NS
ADCY4	NS	0.023148391	0.046058848	0.054434819	NS
ZNF761	NS	0.023149566	-0.046058455	0.054434819	NS
TIMP3	NS	0.023157117	-0.046055928	0.054442992	NS
SYT11	NS	0.023166224	-0.046052882	0.054454815	NS
FBXL7	NS	0.023167772	0.046052364	0.054454815	NS
ASPSCR1	NS	0.023170777	0.046051359	0.054455266	NS
DHX34	NS	0.023187177	0.046045877	0.054487192	NS
ASIC4	NS	0.023209521	0.046038414	0.054530317	NS
MED10	NS	0.023211163	-0.046037865	0.054530317	NS
MIF4GD	NS	0.023223239	0.046033835	0.054552064	NS
XRN1	NS	0.023228198	0.04603218	0.054557092	NS
ASCL1	NS	0.023264473	-0.046020084	0.054634705	NS
BTN3A2	NS	0.023266887	-0.04601928	0.054634705	NS
LY6K	NS	0.023285921	0.046012941	0.054672766	NS
BHLHE41	NS	0.023299105	0.046008552	0.054690454	NS
ZNF527	NS	0.023297192	-0.046009189	0.054690454	NS
NDUFC2	NS	0.023302881	-0.046007296	0.054692683	NS
PF4V1	NS	0.02330945	-0.04600511	0.054701469	NS
TDGF1	NS	0.023323479	0.046000445	0.054721122	NS
SMIM33	NS	0.023322343	0.046000823	0.054721122	NS
MOB3B	NS	0.023328895	0.045998644	0.054727195	NS
CSNKA2IP	NS	0.023415054	-0.04597005	0.054916004	NS
HOXC4	NS	0.023427306	0.045965991	0.054938083	NS
MOCOS	NS	0.02344795	-0.045959157	0.054979834	NS
UACA	NS	0.023462613	-0.045954305	0.05500755	NS
MAP3K11	NS	0.023466987	0.045952859	0.055011142	NS
VPS33A	NS	0.023493961	-0.045943942	0.055061039	NS
SURF6	NS	0.023493168	0.045944205	0.055061039	NS
AGXT2	NS	0.023558921	-0.045922507	0.055206594	NS
PLEKHA4	NS	0.023563715	-0.045920927	0.055211145	NS
OBSCN	NS	0.023575558	-0.045917025	0.055232208	NS
CPXM1	NS	0.023600954	0.045908664	0.055285013	NS
ERRFI1	NS	0.023649758	-0.045892617	0.055392633	NS

SCRIB	NS	0.023660602	-0.045889056	0.055411327	NS
CEACAM4	NS	0.023701588	0.045875608	0.055493883	NS
PPIH	NS	0.023700048	-0.045876113	0.055493883	NS
CTDSPL	NS	0.023718392	-0.0458701	0.055526512	NS
ZFP2	NS	0.023736666	0.045864114	0.055562571	NS
AHRR	NS	0.023743382	-0.045861915	0.055564854	NS
AHR	NS	0.02374319	0.045861978	0.055564854	NS
AKAP1	NS	0.023749924	-0.045859774	0.055573444	NS
T6GALNAC	NS	0.023755726	-0.045857875	0.055580301	NS
CLDN15	NS	0.023764035	0.045855156	0.055593022	NS
CNTNAP3	NS	0.023774747	0.045851653	0.055597326	NS
CUTA	NS	0.023770403	-0.045853073	0.055597326	NS
SH3BP5	NS	0.023772439	0.045852408	0.055597326	NS
B4GALT3	NS	0.023777364	0.045850797	0.055597326	NS
ZNF530	NS	0.023790751	0.045846421	0.055621907	NS
CYTH1	NS	0.023794483	0.045845202	0.055623915	NS
CALU	NS	0.023810976	-0.045839814	0.055655748	NS
DBX1	NS	0.023842764	0.045829439	0.05572332	NS
TAS2R60	NS	0.023861579	-0.045823304	0.055760561	NS
USB1	NS	0.023875154	0.04581888	0.055778814	NS
OR1E1	NS	0.023875072	-0.045818907	0.055778814	NS
TLE3	NS	0.023895414	0.045812282	0.05581941	NS
NCLN	NS	0.023976905	0.04578579	0.056003014	NS
PTPN1	NS	0.023980841	0.045784513	0.056005449	NS
RPL3	NS	0.023992446	0.045780747	0.056025792	NS
ADI1	NS	0.024001011	-0.045777969	0.056039032	NS
DAGLB	NS	0.024038495	-0.04576582	0.056119782	NS
PPP1R13B	NS	0.024067669	0.045756376	0.056181114	NS
JPT2	NS	0.024134316	-0.045734839	0.056323104	NS
SRP9	NS	0.024168688	0.045723751	0.05638972	NS
CEP120	NS	0.024166368	0.045724499	0.05638972	NS
CCN1	NS	0.024213722	-0.045709245	0.056480916	NS
DEFB4B	NS	0.024215864	-0.045708556	0.056480916	NS
PIGU	NS	0.024220252	0.045707144	0.056482794	NS
MINDY2	NS	0.02424255	-0.045699972	0.056527984	NS
COL13A1	NS	0.024283548	-0.0456868	0.056616762	NS
SPP1	NS	0.024291184	0.045684349	0.056627744	NS
MST1R	NS	0.024301061	0.045681179	0.056643946	NS
CTSZ	NS	0.024308566	0.045678771	0.056654617	NS
IQCA1L	NS	0.024315762	0.045676463	0.056664567	NS
ZSWIM4	NS	0.024334702	0.045670392	0.056701877	NS
PSMD3	NS	0.024342174	0.045667998	0.05671246	NS
HMCN1	NS	0.024404283	0.045648121	0.056837948	NS
FABP7	NS	0.024401378	-0.04564905	0.056837948	NS
PPDPFL	NS	0.024404845	-0.045647941	0.056837948	NS
SARDH	NS	0.024410308	-0.045646195	0.05684383	NS
RHBDF2	NS	0.024418834	0.045643471	0.056856844	NS
CYS1	NS	0.024434623	0.045638427	0.056886765	NS
GPR55	NS	0.024442436	-0.045635933	0.056898111	NS
AAR2	NS	0.024459803	0.04563039	0.056931691	NS
HLF	NS	0.02448507	-0.045622332	0.056983651	NS
SLC24A4	NS	0.024505246	0.045615903	0.057023749	NS
IFIH1	NS	0.024541268	0.045604436	0.057100707	NS
INTS9	NS	0.024554802	-0.045600132	0.057120376	NS
C19orf53	NS	0.024555623	-0.045599871	0.057120376	NS

USP17L12	NS	0.024567634	-0.045596053	0.057141447	NS
KRT6C	NS	0.024591052	-0.045588613	0.057182653	NS
STRADA	NS	0.024591259	0.045588547	0.057182653	NS
CD40	NS	0.024608976	-0.045582922	0.057210106	NS
RAB12	NS	0.024608479	-0.04558308	0.057210106	NS
PPP1R14A	NS	0.024625575	-0.045577656	0.057234946	NS
NDNF	NS	0.024625428	0.045577702	0.057234946	NS
MLX	NS	0.024641025	0.045572757	0.057263978	NS
ZNF66	NS	0.024689451	-0.045557418	0.057362743	NS
ANK2	NS	0.024697614	0.045554835	0.057367937	NS
TRPV6	NS	0.024697248	-0.045554951	0.057367937	NS
PRPF4	NS	0.024732486	0.045543809	0.057442045	NS
USP17L28	NS	0.02474109	-0.04554109	0.057455133	NS
KCND1	NS	0.02474962	0.045538396	0.057468047	NS
CFAP410	NS	0.024752652	0.045537439	0.057468193	NS
PDE12	NS	0.024780096	0.045528777	0.05752501	NS
FLOT1	NS	0.024841569	0.045509404	0.057658003	NS
PARP6	NS	0.024843343	0.045508846	0.057658003	NS
MRC2	NS	0.024851236	0.045506362	0.057662493	NS
ZNF772	NS	0.024849858	-0.045506796	0.057662493	NS
H1-1	NS	0.02485507	-0.045505155	0.057664477	NS
ANHX	NS	0.024863633	0.045502462	0.057677429	NS
OR6C4	NS	0.024869737	0.045500542	0.057684676	NS
NOS3	NS	0.024880878	0.045497039	0.057703603	NS
NSF	NS	0.024898136	0.045491615	0.05773321	NS
TAP1	NS	0.024899609	-0.045491152	0.05773321	NS
WBP11	NS	0.024915212	0.045486252	0.057762467	NS
PAG1	NS	0.024921265	0.045484351	0.057769581	NS
PLAAT5	NS	0.024948495	-0.045475807	0.057825778	NS
OR6T1	NS	0.024957616	-0.045472947	0.057837055	NS
TUBB4B	NS	0.024959337	0.045472407	0.057837055	NS
OOSP3	NS	0.024966678	-0.045470106	0.057847142	NS
PSMC5	NS	0.024978151	-0.045466511	0.057866798	NS
OTOF	NS	0.02498659	-0.045463867	0.05787942	NS
PON1	NS	0.025009789	-0.045456604	0.05788355	NS
RASAL2	NS	0.025010633	-0.04545634	0.05788355	NS
MYH9	NS	0.024997367	0.045460492	0.05788355	NS
GMDS	NS	0.025007118	-0.04545744	0.05788355	NS
SRD5A3	NS	0.025004025	-0.045458408	0.05788355	NS
QRSL1	NS	0.025015287	-0.045454883	0.05788355	NS
DNAJC7	NS	0.025002612	0.04545885	0.05788355	NS
PLSCR3	NS	0.025012572	0.045455733	0.05788355	NS
WDSUB1	NS	0.025014996	0.045454974	0.05788355	NS
ARHGAP33	NS	0.025040575	0.045446974	0.05793514	NS
SMIM9	NS	0.025045595	0.045445405	0.057939827	NS
RGCC	NS	0.025058751	0.045441294	0.057945704	NS
WNT5A	NS	0.02506011	-0.045440869	0.057945704	NS
VAMP5	NS	0.025057363	-0.045441727	0.057945704	NS
RFESD	NS	0.025058061	-0.045441509	0.057945704	NS
PRSS23	NS	0.025103971	-0.045427176	0.058040189	NS
ARAP1	NS	0.025116101	0.045423393	0.058061299	NS
ZNF713	NS	0.02513897	-0.045416265	0.058107224	NS
SDCCAG8	NS	0.025156213	-0.045410894	0.058140138	NS
CDCP2	NS	0.025166557	0.045407674	0.058152608	NS
SERTM1	NS	0.025167617	-0.045407344	0.058152608	NS

KCNE5	NS	0.025174329	-0.045405255	0.058161173	NS
ARSL	NS	0.02519945	0.045397441	0.058212263	NS
QDPR	NS	0.02522694	-0.045388897	0.058268814	NS
IL17REL	NS	0.025233086	0.045386989	0.058276054	NS
PDGFRA	NS	0.025245109	-0.045383256	0.058296866	NS
LHX4	NS	0.025260603	-0.045378447	0.058325686	NS
CD320	NS	0.025266136	-0.04537673	0.058331504	NS
ADCY1	NS	0.025280534	-0.045372265	0.058357784	NS
C2orf69	NS	0.025286593	0.045370387	0.058364809	NS
WDR70	NS	0.025289668	-0.045369433	0.058364947	NS
GALNT15	NS	0.025298619	0.045366659	0.058378645	NS
HIBCH	NS	0.025312743	-0.045362284	0.058404274	NS
WSB1	NS	0.025325279	0.045358402	0.058424778	NS
TNS3	NS	0.025327666	0.045357663	0.058424778	NS
SPRYD7	NS	0.025343925	-0.045352631	0.058448352	NS
ALKBH3	NS	0.025343677	-0.045352708	0.058448352	NS
ABCC4	NS	0.025347883	-0.045351407	0.058450516	NS
ZNF3	NS	0.025363358	0.045346621	0.058479233	NS
ODAD1	NS	0.025368561	0.045345012	0.058484262	NS
MFSD8	NS	0.025408411	0.045332702	0.058569157	NS
N4BP2L2	NS	0.025434191	0.045324747	0.0586216	NS
XRCC3	NS	0.025459337	0.045316994	0.058672571	NS
CD63	NS	0.025466026	0.045314933	0.058681001	NS
KIAA0930	NS	0.025483851	-0.045309442	0.058715083	NS
REEP1	NS	0.025501891	-0.045303889	0.058739037	NS
FEN1	NS	0.025501307	-0.045304069	0.058739037	NS
ALYREF	NS	0.025503351	0.04530344	0.058739037	NS
SEC24C	NS	0.025543867	0.045290981	0.058825353	NS
IGFLR1	NS	0.025587793	-0.045277493	0.058908548	NS
GTPBP8	NS	0.025588463	-0.045277287	0.058908548	NS
WDR87	NS	0.025589123	0.045277085	0.058908548	NS
PLEKHB2	NS	0.0256023	0.045273043	0.058931873	NS
NDUFB4	NS	0.02560588	-0.045271945	0.058933106	NS
CYBRD1	NS	0.025622737	-0.045266777	0.058957881	NS
HRG	NS	0.025619749	0.045267693	0.058957881	NS
UBR4	NS	0.025627246	0.045265395	0.058961247	NS
STAMBP	NS	0.025632659	0.045263737	0.05896258	NS
CD3G	NS	0.025633918	-0.045263351	0.05896258	NS
SLC52A2	NS	0.025679466	-0.045249407	0.059053315	NS
P2RX3	NS	0.025693921	0.045244987	0.059079537	NS
OSBPL2	NS	0.025704784	0.045241666	0.059097494	NS
DCAKD	NS	0.025733784	-0.045232807	0.05915714	NS
MRAP2	NS	0.025784541	-0.045217323	0.059266782	NS
ACTC1	NS	0.025800597	-0.04521243	0.059296646	NS
TRIM3	NS	0.02581211	0.045208923	0.059315586	NS
GLOD4	NS	0.025814967	-0.045208053	0.059315586	NS
AFG3L2	NS	0.025820468	0.045206378	0.059321184	NS
RGS1	NS	0.025836433	0.045201519	0.059350818	NS
MSX1	NS	0.025846729	-0.045198386	0.059367425	NS
KRT7	NS	0.025859247	-0.04519458	0.059382254	NS
MAP2K1	NS	0.025864443	0.045193	0.059382254	NS
PLEKHD1	NS	0.025865457	0.045192692	0.059382254	NS
PSMG4	NS	0.025861782	-0.045193809	0.059382254	NS
SERTM2	NS	0.025873857	-0.045190139	0.059394493	NS
HSPA4L	NS	0.02590655	0.045180208	0.059455441	NS

PRCD	NS	0.025905002	0.045180678	0.059455441	NS
CENPVL2	NS	0.025914031	0.045177938	0.059465558	NS
AP1G1	NS	0.025926911	0.045174029	0.059488061	NS
SERPINA12	NS	0.025959504	-0.045164147	0.059548728	NS
GLB1L3	NS	0.025959113	0.045164266	0.059548728	NS
MYBPHL	NS	0.025973854	-0.045159799	0.059574585	NS
SOCS5	NS	0.025979651	-0.045158044	0.05958082	NS
C2	NS	0.026008138	-0.045149421	0.059639086	NS
BPIFB4	NS	0.02601525	0.045147269	0.059648328	NS
UNC13A	NS	0.026028803	0.045143171	0.059672332	NS
XKR8	NS	0.02604114	0.045139442	0.059693546	NS
HDAC9	NS	0.026051121	-0.045136426	0.059702284	NS
EBI3	NS	0.026049919	0.045136789	0.059702284	NS
TCF4	NS	0.02606586	-0.045131974	0.059728991	NS
GSTT2B	NS	0.026072055	-0.045130104	0.059736114	NS
FNTA	NS	0.026077808	0.045128367	0.059742223	NS
FBXL8	NS	0.026090841	-0.045124434	0.059765008	NS
SCYL2	NS	0.026124565	0.045114264	0.059829877	NS
TMEM171	NS	0.026125342	0.04511403	0.059829877	NS
GNAI3	NS	0.026130557	0.045112458	0.059834741	NS
SNX17	NS	0.026188841	-0.045094913	0.059954812	NS
VCAM1	NS	0.026189189	-0.045094809	0.059954812	NS
PKD1L1	NS	0.026201422	0.04509113	0.059975723	NS
RP2	NS	0.026211025	0.045088244	0.059990612	NS
C22orf42	NS	0.026300332	-0.045061444	0.060187896	NS
ZNF7	NS	0.026405654	0.04502994	0.060421781	NS
H3C8	NS	0.026409645	0.045028748	0.060423768	NS
SDR42E1	NS	0.02643083	0.045022425	0.060465091	NS
PRRC2B	NS	0.026472256	0.045010073	0.060552705	NS
CDYL	NS	0.026487159	-0.045005634	0.060579634	NS
INPP5F	NS	0.026529203	0.044993121	0.060668626	NS
TJAP1	NS	0.026542591	0.04498914	0.060692073	NS
ADD3	NS	0.026556334	-0.044985056	0.060716324	NS
FUT2	NS	0.026570248	0.044980922	0.060740961	NS
SALL4	NS	0.026574483	0.044979664	0.06074347	NS
ABHD14B	NS	0.026589825	0.044975109	0.060771361	NS
KIAA0319	NS	0.026617661	0.044966851	0.060827797	NS
IL12RB1	NS	0.026631677	-0.044962695	0.060852644	NS
CCR8	NS	0.026635643	0.04496152	0.060854521	NS
CYP7B1	NS	0.026643047	-0.044959326	0.060864252	NS
RRP7A	NS	0.026656307	-0.044955397	0.060887358	NS
OR1S2	NS	0.026682737	0.044947572	0.060940538	NS
CPVL	NS	0.026687279	-0.044946228	0.06094372	NS
MGST1	NS	0.026701176	-0.044942117	0.060968262	NS
ZNF644	NS	0.026755886	0.044925951	0.061085979	NS
CCDC9	NS	0.026774705	0.044920397	0.061121736	NS
S100A9	NS	0.026795883	0.04491415	0.061162868	NS
NF103-CHMI	NS	0.026801845	-0.044912392	0.061169263	NS
MLLT11	NS	0.026813516	-0.044908952	0.061188684	NS
BAMBI	NS	0.026854847	0.044896781	0.061275776	NS
NANOS2	NS	0.0268968	0.044884443	0.061364269	NS
ADCY9	NS	0.02692548	-0.044876018	0.061422463	NS
CYTH3	NS	0.02693526	0.044873146	0.061437531	NS
OR1B1	NS	0.02695063	-0.044868636	0.061465347	NS
CBR4	NS	0.026960783	-0.044865657	0.061481258	NS

ESCO1	NS	0.026974035	0.044861772	0.061504232	NS
CD84	NS	0.027011797	0.044850708	0.061583079	NS
LETMD1	NS	0.027030791	-0.044845148	0.061619124	NS
UGT3A1	NS	0.027042536	-0.044841711	0.06163864	NS
ZNF28	NS	0.027057554	-0.044837319	0.061665608	NS
MAP3K8	NS	0.027089543	0.04482797	0.061723978	NS
FEM1B	NS	0.027126837	-0.044817084	0.061801678	NS
KPNA4	NS	0.02714931	0.04481053	0.061838931	NS
DNLZ	NS	0.027149578	-0.044810451	0.061838931	NS
ACSM2B	NS	0.027156849	-0.044808332	0.061848213	NS
ZFYVE16	NS	0.027247237	0.044782024	0.062046766	NS
TP53AIP1	NS	0.027252471	0.044780503	0.062051385	NS
TNK2	NS	0.027257647	0.044778999	0.062055872	NS
LIN7A	NS	0.027329389	-0.044758179	0.06220457	NS
EIF5B	NS	0.027338992	-0.044755396	0.062211794	NS
ITGA5	NS	0.027337385	0.044755862	0.062211794	NS
IARS2	NS	0.027342838	-0.044754281	0.062213232	NS
ZC3H6	NS	0.027360972	0.044749028	0.062241468	NS
ARID2	NS	0.027361678	0.044748824	0.062241468	NS
INAVA	NS	0.027383032	0.044742642	0.062282723	NS
UCHL3	NS	0.027392759	-0.044739828	0.062297526	NS
GAPDHS	NS	0.027397792	-0.044738372	0.062301652	NS
GK2	NS	0.027404371	0.044736469	0.062309293	NS
ANO1	NS	0.027461714	0.0447199	0.062421933	NS
TMEM127	NS	0.027466811	0.044718429	0.062421933	NS
ANKH	NS	0.027465754	-0.044718734	0.062421933	NS
C6orf141	NS	0.027459914	0.04472042	0.062421933	NS
RNASE8	NS	0.027477144	0.044715447	0.062438084	NS
SPTLC2	NS	0.027494497	-0.044710442	0.062467737	NS
CASZ1	NS	0.027496648	0.044709822	0.062467737	NS
SNX2	NS	0.027506255	0.044707052	0.06248223	NS
LOXL1	NS	0.027532038	0.044699622	0.062533458	NS
RTP5	NS	0.027556481	0.044692584	0.062581632	NS
THEM6	NS	0.027566816	-0.04468961	0.062597757	NS
AGL	NS	0.027629712	-0.044671531	0.062733221	NS
KYNU	NS	0.027638408	0.044669034	0.062745605	NS
AGGF1	NS	0.027701971	-0.044650804	0.062882531	NS
RBSN	NS	0.027711991	0.044647934	0.0628979	NS
HAUS4	NS	0.027729934	-0.044642796	0.062931248	NS
STOX1	NS	0.027750108	-0.044637023	0.062969647	NS
AIF1L	NS	0.027768497	0.044631763	0.063003989	NS
ACSM5	NS	0.027781986	-0.044627907	0.063027205	NS
ADCYAP1	NS	0.02779749	0.044623477	0.06305499	NS
HDAC5	NS	0.027812058	0.044619316	0.063079745	NS
ARHGEF19	NS	0.027817191	-0.044617851	0.063079745	NS
OR10A4	NS	0.027818181	-0.044617568	0.063079745	NS
FANCL	NS	0.027835252	0.044612696	0.063111063	NS
FPR1	NS	0.02784028	0.044611262	0.06311507	NS
AURKA	NS	0.027856319	-0.044606687	0.063144034	NS
NOCT	NS	0.027861625	0.044605174	0.063148666	NS
TMEM138	NS	0.027980754	0.044571273	0.063411249	NS
OR56A5	NS	0.02800046	-0.044565677	0.063448478	NS
LAIR2	NS	0.02801908	0.044560392	0.063476545	NS
BRSK2	NS	0.028019405	0.0445603	0.063476545	NS
LRRC32	NS	0.028031632	0.044556832	0.063496812	NS

YIPF2	NS	0.02805833	0.044549263	0.063549851	NS
ATP5PD	NS	0.02806585	0.044547132	0.063559446	NS
STT3B	NS	0.028074437	0.0445447	0.063571457	NS
CCN4	NS	0.02807926	-0.044543334	0.063574938	NS
SLC12A2	NS	0.028093697	-0.044539246	0.063600187	NS
FBN3	NS	0.028109105	0.044534886	0.063627627	NS
GPR157	NS	0.028146887	-0.044524202	0.0637057	NS
ARSJ	NS	0.028155417	-0.044521792	0.063717556	NS
ATAD3A	NS	0.028160143	0.044520457	0.0637208	NS
PHYKPL	NS	0.028207637	0.044507049	0.063820809	NS
RAD18	NS	0.028240922	-0.044497665	0.06388865	NS
CCNB1IP1	NS	0.02826163	0.044491831	0.063920555	NS
DNPEP	NS	0.028260873	-0.044492045	0.063920555	NS
SLC18B1	NS	0.028279648	-0.044486758	0.063953834	NS
CX3CL1	NS	0.028286619	0.044484797	0.063962125	NS
THAP12	NS	0.028321598	0.044474958	0.064033741	NS
OOSP2	NS	0.028348228	-0.044467476	0.064086463	NS
SPDYA	NS	0.028351614	0.044466524	0.064086634	NS
EDN3	NS	0.028360995	0.04446389	0.064100352	NS
MTMR7	NS	0.028381365	0.044458173	0.064138902	NS
TMCC1	NS	0.028431993	0.044443978	0.064245816	NS
RSPH3	NS	0.028454719	-0.044437613	0.064289664	NS
TMEM179B	NS	0.028488293	-0.044428218	0.064358006	NS
KIF5B	NS	0.02852866	0.044416934	0.064441679	NS
HSPA2	NS	0.0285433	0.044412845	0.064467225	NS
SELENON	NS	0.028558342	-0.044408646	0.064493673	NS
ANKRD40	NS	0.028575749	-0.044403789	0.064523103	NS
ZNF726	NS	0.028578041	-0.04440315	0.064523103	NS
TNRC6A	NS	0.028587255	0.04440058	0.064528852	NS
TRAF6	NS	0.02858419	0.044401435	0.064528852	NS
OLFML3	NS	0.028646992	-0.044383937	0.064656155	NS
HAUS8	NS	0.028665514	-0.044378783	0.064690415	NS
CSNK1E	NS	0.028672358	-0.044376879	0.064698317	NS
ACTR5	NS	0.028680836	-0.044374521	0.064702606	NS
KPNA3	NS	0.028680944	0.044374491	0.064702606	NS
UBE2L6	NS	0.028701138	-0.044368877	0.064740616	NS
SPON2	NS	0.028712205	-0.044365802	0.064758034	NS
TTLL4	NS	0.028716776	0.044364532	0.064760796	NS
BEND2	NS	0.028748249	-0.044355794	0.06482422	NS
CD37	NS	0.028774849	0.044348415	0.064869086	NS
SAG	NS	0.028773406	0.044348815	0.064869086	NS
TRPV4	NS	0.028798763	0.044341786	0.064915438	NS
H3-2	NS	0.028821597	-0.044335461	0.064959343	NS
SNF8	NS	0.028839822	0.044330416	0.064992781	NS
ALKBH4	NS	0.028843149	-0.044329495	0.064992781	NS
MOB1A	NS	0.028854264	0.04432642	0.065002693	NS
REEP6	NS	0.028851721	0.044327123	0.065002693	NS
PRPF40A	NS	0.028861192	0.044324504	0.065010733	NS
SLC7A11	NS	0.028882424	0.044318633	0.065050988	NS
KRBOX5	NS	0.028933987	-0.044304392	0.065159542	NS
ZNF669	NS	0.028947889	-0.044300556	0.065175682	NS
SPANXN3	NS	0.028945032	-0.044301344	0.065175682	NS
OR51C1P	NS	0.028973115	-0.0442936	0.065224892	NS
MAP3K7CL	NS	0.029006516	-0.044284397	0.065284899	NS
GRM7	NS	0.029003565	0.04428521	0.065284899	NS

SMTN	NS	0.02903119	-0.044277605	0.065332836	NS
ENSA	NS	0.029050089	0.044272405	0.065367766	NS
FAM151B	NS	0.029058316	-0.044270143	0.065371078	NS
PIK3R6	NS	0.029056147	0.044270739	0.065371078	NS
ASB5	NS	0.02907119	-0.044266604	0.065392441	NS
GLT1D1	NS	0.029092803	-0.044260665	0.065433453	NS
PI4KB	NS	0.029096262	0.044259715	0.065433628	NS
STX11	NS	0.029128757	0.044250794	0.065488071	NS
LRATD2	NS	0.029130621	-0.044250283	0.065488071	NS
PRR20A	NS	0.029129292	0.044250648	0.065488071	NS
CST9L	NS	0.029135894	-0.044248836	0.065492319	NS
CEACAM3	NS	0.029172812	-0.044238714	0.06556769	NS
HBG1	NS	0.029177963	-0.044237302	0.065571654	NS
FBXL3	NS	0.029221241	0.044225452	0.065661288	NS
RTL5	NS	0.029229675	-0.044223145	0.065672617	NS
YIF1A	NS	0.02924072	-0.044220123	0.065689807	NS
NF559-ZNF17	NS	0.029265867	-0.044213249	0.06573867	NS
NCBP2	NS	0.029331744	0.044195263	0.065848439	NS
MMEL1	NS	0.029330388	0.044195633	0.065848439	NS
TRIM44	NS	0.029331479	-0.044195335	0.065848439	NS
COX8A	NS	0.029321715	-0.044197999	0.065848439	NS
KRTAP17-1	NS	0.02933686	-0.044193867	0.065852286	NS
SNRPN	NS	0.02934842	-0.044190715	0.065870597	NS
SNRNP27	NS	0.029363529	-0.044186597	0.065896866	NS
PIGG	NS	0.029389056	0.044179644	0.065946507	NS
CH25H	NS	0.029443171	-0.04416492	0.066060278	NS
MOSPD3	NS	0.029456358	0.044161335	0.066082206	NS
MPDZ	NS	0.029460543	-0.044160198	0.066083934	NS
KCNK18	NS	0.02948545	0.044153432	0.06613214	NS
RAD51D	NS	0.02951345	-0.044145832	0.066179604	NS
EML6	NS	0.029512143	-0.044146187	0.066179604	NS
DECR1	NS	0.029567904	-0.04413107	0.066290359	NS
CXXC5	NS	0.029569692	0.044130586	0.066290359	NS
GPR148	NS	0.029671551	0.044103036	0.066511007	NS
EIF4EBP2	NS	0.029694569	0.044096822	0.06654719	NS
BAZ1A	NS	0.029692621	0.044097347	0.06654719	NS
REV3L	NS	0.029733742	0.044086255	0.066627264	NS
SELENOF	NS	0.029767873	-0.044077059	0.066696023	NS
ZNF239	NS	0.02978011	-0.044073763	0.066715718	NS
HELQ	NS	0.029800409	0.0440683	0.066753467	NS
CACNB3	NS	0.029811154	-0.044065409	0.066769809	NS
NEDD8-MDP	NS	0.029817323	0.04406375	0.0667759	NS
GLRX5	NS	0.029855459	-0.0440535	0.06685357	NS
OR8J3	NS	0.029889815	-0.044044275	0.06692276	NS
CD300LG	NS	0.029895846	0.044042656	0.0669259	NS
MYH4	NS	0.029898133	-0.044042043	0.0669259	NS
ZNF704	NS	0.029922191	0.04403559	0.066972008	NS
FBXO42	NS	0.030007409	0.044012769	0.067147214	NS
PYGO2	NS	0.030051214	0.04400106	0.067237463	NS
ARHGAP45	NS	0.030060558	0.043998564	0.067250597	NS
CCT6A	NS	0.030096113	-0.043989073	0.067322358	NS
ZNF490	NS	0.030099871	-0.043988071	0.067322983	NS
BTN1A1	NS	0.030128028	-0.043980562	0.067378177	NS
BASP1	NS	0.0301445	0.043976173	0.067407226	NS
RALGAPA2	NS	0.030155417	0.043973264	0.067423849	NS

NT5DC1	NS	0.03016335	-0.043971152	0.067433797	NS
ARF6	NS	0.030174332	0.043968228	0.067450557	NS
NDN	NS	0.030236806	0.043951612	0.067582403	NS
EHMT2	NS	0.030270816	-0.043942579	0.067642797	NS
MRLN	NS	0.030267372	0.043943494	0.067642797	NS
ERICH1	NS	0.030304449	0.043933655	0.067710138	NS
ZFP36L2	NS	0.030311478	0.043931791	0.067718025	NS
ST7L	NS	0.030326344	0.04392785	0.067735601	NS
OR4M2B	NS	0.030325488	-0.043928077	0.067735601	NS
MAMDC4	NS	0.030353372	-0.043920688	0.067788147	NS
U2AF1L4	NS	0.030384051	0.043912567	0.067848833	NS
EPOR	NS	0.030412116	0.043905143	0.06790367	NS
NUDT5	NS	0.030422499	-0.043902398	0.06791408	NS
ON1-GTF2A	NS	0.030444821	-0.043896499	0.067937509	NS
CPOX	NS	0.030440953	-0.043897521	0.067937509	NS
TIMM9	NS	0.03044473	-0.043896523	0.067937509	NS
ABHD14A	NS	0.030515064	0.043877962	0.068086407	NS
DLG2	NS	0.030531234	-0.0438737	0.068114634	NS
PAGE5	NS	0.030537737	-0.043871986	0.068121291	NS
RPL13A	NS	0.030574722	0.043862247	0.068195934	NS
CNIH3	NS	0.030591603	-0.043857804	0.068217866	NS
EFCAB6	NS	0.030588131	-0.043858718	0.068217866	NS
RBBP8	NS	0.030631977	0.043847189	0.06829216	NS
ZNF48	NS	0.030628449	0.043848116	0.06829216	NS
ZFP64	NS	0.030635559	-0.043846248	0.068292281	NS
MAGI3	NS	0.030640482	-0.043844954	0.068295391	NS
UPK1B	NS	0.030659856	-0.043839866	0.068329946	NS
POTEJ	NS	0.030663046	0.043839028	0.068329946	NS
PRAMEF2	NS	0.030698114	0.043829825	0.068400218	NS
LMAN1	NS	0.030751206	-0.04381591	0.068510628	NS
EPM2AIP1	NS	0.030761711	0.043813159	0.068526145	NS
TMEM53	NS	0.030770934	-0.043810744	0.068538803	NS
FCN2	NS	0.030778705	-0.04380871	0.068548223	NS
NUDT10	NS	0.030828929	-0.043795576	0.068652181	NS
PTGER4	NS	0.030912969	0.043773638	0.068825843	NS
MT-ND4L	NS	0.030914025	0.043773363	0.068825843	NS
AGPAT3	NS	0.030951392	-0.043763626	0.068901109	NS
ZBTB25	NS	0.03099941	0.043751127	0.068992133	NS
SBDS	NS	0.030998056	0.04375148	0.068992133	NS
MAN1A1	NS	0.03102122	0.043745456	0.069032735	NS
NME4	NS	0.031044303	0.043739458	0.069076162	NS
NR1H4	NS	0.031054905	-0.043736704	0.069090315	NS
LIMD2	NS	0.031061372	0.043735024	0.069090315	NS
RAB3B	NS	0.03105832	0.043735817	0.069090315	NS
ATP8A2	NS	0.031076481	0.043731102	0.069115979	NS
UTP20	NS	0.031096254	0.043725971	0.069144211	NS
GPR101	NS	0.03109632	0.043725954	0.069144211	NS
TRIM45	NS	0.03110624	-0.043723381	0.069150383	NS
AHSP	NS	0.031103666	0.043724049	0.069150383	NS
THAP10	NS	0.031118844	-0.043720113	0.069165551	NS
BAG5	NS	0.03112021	0.043719759	0.069165551	NS
TBL2	NS	0.031191152	-0.043701386	0.069315263	NS
CDC16	NS	0.031216033	-0.043694951	0.069362591	NS
LGALSL	NS	0.031260417	0.043683482	0.069449795	NS
LIPC	NS	0.031264203	-0.043682504	0.069449795	NS

NPIPB9	NS	0.031266042	0.043682029	0.069449795	NS
MECOM	NS	0.031286828	0.043676664	0.06948799	NS
COMMD4	NS	0.031334703	-0.043664317	0.069578354	NS
PCDHGA11	NS	0.03133391	-0.043664522	0.069578354	NS
MIS12	NS	0.031410286	-0.043644858	0.069738184	NS
HACL1	NS	0.031416929	0.04364315	0.069744934	NS
AMMECR1	NS	0.031443143	0.043636412	0.069795123	NS
MS4A7	NS	0.031477259	0.04362765	0.069862839	NS
COQ9	NS	0.03151443	-0.043618112	0.069937321	NS
FAM25A	NS	0.031530531	0.043613983	0.069965031	NS
IRGM	NS	0.031592504	0.04359811	0.070076504	NS
C4orf54	NS	0.031595249	-0.043597408	0.070076504	NS
DCANP1	NS	0.031593464	-0.043597865	0.070076504	NS
UHRF1	NS	0.031592597	-0.043598087	0.070076504	NS
OSBP	NS	0.031643053	0.043585183	0.070166448	NS
SAGE1	NS	0.031639865	-0.043585998	0.070166448	NS
SMARCD3	NS	0.031651535	0.043583016	0.07016918	NS
DRAP1	NS	0.031648965	0.043583672	0.07016918	NS
DLD	NS	0.03166478	0.043579632	0.070182467	NS
NIPSNAP3B	NS	0.031661821	-0.043580388	0.070182467	NS
SCN7A	NS	0.031676379	0.04357667	0.070200137	NS
USP30	NS	0.031686135	0.043574179	0.070213719	NS
HSFY2	NS	0.03170408	0.0435696	0.070245442	NS
OR10A3	NS	0.031718242	0.043565987	0.070268776	NS
RAB4B	NS	0.031728746	0.043563309	0.070284001	NS
CLIC5	NS	0.031735671	-0.043561543	0.070291296	NS
HSPB3	NS	0.03176089	-0.043555116	0.070339106	NS
SLC41A3	NS	0.031771138	-0.043552506	0.070353752	NS
PRKAG3	NS	0.031838167	-0.04353545	0.070494117	NS
ANO2	NS	0.031843172	-0.043534178	0.070495414	NS
H3C13	NS	0.031846037	-0.04353345	0.070495414	NS
ZNF217	NS	0.03187793	-0.043525347	0.070557944	NS
OR4K1	NS	0.031916493	-0.043515559	0.070635221	NS
CHST4	NS	0.031921598	-0.043514264	0.070638442	NS
FREM1	NS	0.031965031	-0.043503254	0.070726468	NS
S100A3	NS	0.031970415	-0.04350189	0.070730296	NS
MANF	NS	0.03198896	-0.043497193	0.070763235	NS
PUM3	NS	0.032021629	-0.043488925	0.070827409	NS
CCNDBP1	NS	0.032030161	-0.043486767	0.070838184	NS
TRAF5	NS	0.032047645	0.043482346	0.070860659	NS
UBXN2A	NS	0.03204598	0.043482767	0.070860659	NS
PDP1	NS	0.03210075	0.043468931	0.070969974	NS
SURF4	NS	0.03217758	-0.043449556	0.071131708	NS
DNAJB6	NS	0.032182607	0.04344829	0.071133335	NS
ABCC11	NS	0.032185997	0.043447436	0.071133335	NS
CACNA1D	NS	0.032189341	0.043446593	0.071133335	NS
DEFB121	NS	0.032202214	-0.043443352	0.07115366	NS
PEX5	NS	0.032247531	-0.04343195	0.071245659	NS
NPIPB7	NS	0.032264696	0.043427635	0.071275445	NS
PML	NS	0.03228142	0.043423432	0.071304252	NS
PC	NS	0.03229418	0.043420227	0.071324299	NS
SSU72	NS	0.032316088	0.043414727	0.07136454	NS
OR2T27	NS	0.032331401	-0.043410884	0.071390211	NS
CHRNA7	NS	0.032367435	0.043401848	0.071461625	NS
PSD4	NS	0.032402357	0.043393098	0.071530569	NS

ZNF226	NS	0.032411553	-0.043390795	0.07154271	NS
ZNF398	NS	0.032417974	0.043389188	0.071548724	NS
ARHGEF11	NS	0.032429408	0.043386326	0.071552136	NS
BTBD9	NS	0.03242516	0.043387389	0.071552136	NS
POTEI	NS	0.03243061	0.043386025	0.071552136	NS
TOR1AIP2	NS	0.032437684	0.043384256	0.071559586	NS
FBXW10	NS	0.032481961	0.043373184	0.071649098	NS
C9orf72	NS	0.032504469	0.043367561	0.071690578	NS
RPL26L1	NS	0.032513025	-0.043365425	0.071701276	NS
TBK1	NS	0.032552428	0.043355591	0.071779994	NS
HBA1	NS	0.032564351	0.043352617	0.071798107	NS
LRRC2	NS	0.032591616	-0.043345821	0.071850035	NS
LY6L	NS	0.032616893	0.043339525	0.071897572	NS
SPDYE5	NS	0.032630371	0.043336169	0.07191909	NS
CELSR1	NS	0.032642895	0.043333052	0.071938504	NS
MFSD9	NS	0.032651012	-0.043331032	0.071942949	NS
MCRIP1	NS	0.032665191	-0.043327505	0.071963058	NS
CAAP1	NS	0.032690545	-0.043321202	0.072010719	NS
PSMD1	NS	0.032729481	-0.04331153	0.072088284	NS
SPDYE1	NS	0.032733457	0.043310543	0.072088837	NS
MYADM	NS	0.032750431	0.04330633	0.072118015	NS
MT1A	NS	0.032755452	-0.043305084	0.072120867	NS
ADARB2	NS	0.032785476	0.043297638	0.072178762	NS
ALOX15B	NS	0.032836579	-0.043284977	0.072283046	NS
ASAH2B	NS	0.032868246	-0.04327714	0.072344526	NS
EYA3	NS	0.032903853	0.043268335	0.072414665	NS
F8A2	NS	0.032956713	0.043255279	0.072522753	NS
OR13F1	NS	0.032982505	0.043248916	0.07257126	NS
ZNF628	NS	0.032994528	0.043245951	0.072585457	NS
RBX1	NS	0.033004143	-0.04324358	0.072588017	NS
OR52M1	NS	0.033005122	-0.043243339	0.072588017	NS
SLC26A2	NS	0.033009737	-0.043242201	0.072589919	NS
FGF12	NS	0.033031668	-0.043236797	0.072629895	NS
CHPF	NS	0.033045433	0.043233407	0.072651908	NS
OLIG2	NS	0.033049388	0.043232433	0.07265235	NS
NLE1	NS	0.033055005	-0.04323105	0.072656448	NS
PRKACB	NS	0.03306424	-0.043228777	0.072668495	NS
ZNF571	NS	0.033084195	-0.043223867	0.072704096	NS
POLR3H	NS	0.033124503	-0.043213956	0.072776149	NS
RNF183	NS	0.033122357	0.043214483	0.072776149	NS
CCSER1	NS	0.03319483	-0.043196689	0.072922384	NS
ACAT2	NS	0.03322098	0.043190277	0.072971549	NS
GPX4	NS	0.033231643	0.043187663	0.072986689	NS
LDAF1	NS	0.033238342	-0.043186022	0.072993118	NS
APOD	NS	0.033251218	0.043182867	0.073013112	NS
PDCD2	NS	0.033353701	0.043157798	0.073221531	NS
XXYLT1	NS	0.033350559	-0.043158565	0.073221531	NS
SLC1A7	NS	0.033394418	0.043147856	0.073302604	NS
TDRD10	NS	0.033400525	0.043146365	0.073307695	NS
CLNS1A	NS	0.033567189	-0.043105785	0.073665138	NS
ARHGEF5	NS	0.033608849	-0.043095668	0.073748202	NS
NPEPPS	NS	0.033642632	0.043087472	0.073813963	NS
TTLL6	NS	0.033658545	-0.043083614	0.073840507	NS
CASP10	NS	0.033717657	0.043069295	0.073961806	NS
GATAD2A	NS	0.033750108	0.043061444	0.074016212	NS

CARMIL3	NS	0.033749908	0.043061492	0.074016212	NS
SHC3	NS	0.033769598	-0.043056731	0.074050564	NS
RAD51C	NS	0.033784254	0.043053189	0.074074312	NS
SLC22A5	NS	0.033813705	0.043046075	0.074122093	NS
CGRRF1	NS	0.033849051	-0.043037545	0.074182772	NS
TMUB2	NS	0.033898975	0.043025509	0.074283771	NS
EDAR	NS	0.033914719	0.043021716	0.074309859	NS
GALNT10	NS	0.033920578	-0.043020305	0.074314284	NS
ORO7-PAM1	NS	0.03394313	0.043014876	0.074355276	NS
SESN1	NS	0.033974468	0.043007337	0.074415504	NS
PSRC1	NS	0.034014261	-0.042997772	0.074485806	NS
GFPT1	NS	0.034012567	0.042998179	0.074485806	NS
B3GNT8	NS	0.034022772	-0.042995728	0.074496015	NS
REV1	NS	0.034030677	-0.042993829	0.074504897	NS
CAPN7	NS	0.034048197	0.042989623	0.074534822	NS
CLCN6	NS	0.034065917	0.04298537	0.07456518	NS
KMT5C	NS	0.034075072	-0.042983174	0.074576785	NS
RABGAP1	NS	0.03410091	0.042976978	0.074618964	NS
CLHC1	NS	0.034102054	-0.042976704	0.074618964	NS
SLC22A16	NS	0.034110127	-0.042974769	0.074628193	NS
NFIX	NS	0.034125672	0.042971044	0.074653765	NS
CLPB	NS	0.034161891	-0.042962371	0.074721014	NS
UGT1A6	NS	0.034164503	-0.042961746	0.074721014	NS
CORO7	NS	0.034167994	0.04296091	0.074721014	NS
SF3A2	NS	0.034247776	0.042941836	0.074887025	NS
HECTD3	NS	0.034287283	0.042932405	0.074956478	NS
CALR3	NS	0.034283663	0.042933269	0.074956478	NS
LCE3D	NS	0.034293645	0.042930887	0.074961918	NS
SUMO1	NS	0.034303739	-0.042928479	0.074975516	NS
SH2D3A	NS	0.034338069	0.042920295	0.075042077	NS
T6GALNAC	NS	0.034372849	0.04291201	0.075101124	NS
VENTX	NS	0.034371478	-0.042912337	0.075101124	NS
KAZALD1	NS	0.034397755	-0.042906082	0.075147058	NS
SPANXN5	NS	0.034403336	0.042904754	0.07515077	NS
SPATA31A3	NS	0.034420947	-0.042900565	0.075180755	NS
ZNF831	NS	0.034536475	-0.04287313	0.075424574	NS
LRP3	NS	0.034598158	-0.042858514	0.075542238	NS
OR5H14	NS	0.034621354	-0.042853024	0.075584359	NS
NIPA1	NS	0.034627785	-0.042851502	0.075589873	NS
IQCE	NS	0.034667041	0.042842219	0.075667033	NS
COL4A5	NS	0.034682695	-0.042838519	0.075692666	NS
SLAMF7	NS	0.034690602	0.042836651	0.075701386	NS
VMAC	NS	0.034707405	-0.042832682	0.075729515	NS
LCP1	NS	0.034718602	0.042830039	0.075745408	NS
C10orf53	NS	0.034731375	-0.042827024	0.075761542	NS
FUT4	NS	0.034733826	-0.042826446	0.075761542	NS
KCTD20	NS	0.034752992	0.042821924	0.075794806	NS
ZNF426	NS	0.034813896	-0.042807569	0.075919082	NS
EGFR	NS	0.03483903	-0.042801651	0.075956776	NS
ZNF25	NS	0.034838231	-0.042801839	0.075956776	NS
PRR5	NS	0.034848363	0.042799455	0.075960013	NS
NET1	NS	0.034854369	-0.042798041	0.07596455	NS
CTNNA1	NS	0.034872574	0.042793759	0.07599567	NS
NRIP3	NS	0.034898264	0.042787719	0.076043094	NS
PAN3	NS	0.034932628	0.042779646	0.076109405	NS

TBC1D1	NS	0.034987984	0.042766655	0.076221431	NS
CLC	NS	0.035013326	-0.042760714	0.076268054	NS
CRELD1	NS	0.035019314	0.04275931	0.076272514	NS
CCNG2	NS	0.035046875	-0.042752854	0.076323955	NS
RIOX1	NS	0.03507524	-0.042746213	0.076377134	NS
GPER1	NS	0.035083973	-0.04274417	0.076387556	NS
CXCL13	NS	0.035097985	-0.042740892	0.076409469	NS
TMEM33	NS	0.035123934	-0.042734825	0.076457362	NS
HOXB9	NS	0.035131557	0.042733043	0.076465357	NS
GPR6	NS	0.035167413	0.042724667	0.076534792	NS
CCDC134	NS	0.035187695	0.042719932	0.076570323	NS
ZNF324	NS	0.035226208	-0.042710948	0.076629832	NS
TMEM106A	NS	0.035220601	-0.042712256	0.076629832	NS
PSMD12	NS	0.035226919	0.042710782	0.076629832	NS
RPL24	NS	0.035311642	0.042691048	0.0768055	NS
BRIP1	NS	0.035344012	-0.042683519	0.07686727	NS
GGCT	NS	0.035349901	-0.04268215	0.076871439	NS
LIMK1	NS	0.035411307	0.042667886	0.076996322	NS
CACNB2	NS	0.035443653	0.04266038	0.077057998	NS
MIEN1	NS	0.035494984	0.042648482	0.07716093	NS
CSNK1G2	NS	0.035634558	0.042616202	0.077455644	NS
ZBTB46	NS	0.035668681	0.042608327	0.077521107	NS
RAPGEF4	NS	0.035685086	-0.042604543	0.077548055	NS
SMIM41	NS	0.035697788	-0.042601614	0.077566949	NS
H4C9	NS	0.035724393	-0.042595483	0.077616045	NS
NLRP8	NS	0.035736484	0.042592697	0.07762489	NS
HEATR5B	NS	0.035741237	-0.042591603	0.077626501	NS
ZNF146	NS	0.035756737	-0.042588034	0.077642743	NS
COPS8	NS	0.035754207	0.042588616	0.077642743	NS
C17orf80	NS	0.035791394	0.042580059	0.07770928	NS
FOLR2	NS	0.035875127	-0.042560817	0.077882341	NS
ARHGAP9	NS	0.035888743	0.042557692	0.077903163	NS
ADIG	NS	0.035939508	0.042546049	0.078004611	NS
CD72	NS	0.035955697	0.042542339	0.078030998	NS
GUCA1A	NS	0.035964181	0.042540395	0.07804066	NS
GPSM3	NS	0.035982059	0.042536301	0.078061953	NS
IQCF5	NS	0.03598127	0.042536481	0.078061953	NS
CLCN3	NS	0.035989427	0.042534614	0.078069187	NS
SMOC1	NS	0.036015043	0.042528751	0.078115999	NS
MMP28	NS	0.036030887	0.042525126	0.078141607	NS
XIAP	NS	0.036037658	0.042523578	0.078147536	NS
CLEC18B	NS	0.036055979	-0.042519389	0.078178508	NS
ADAMTS1	NS	0.036086992	0.042512303	0.078236986	NS
SIRT1	NS	0.036101282	0.042509039	0.078242942	NS
MISP	NS	0.036101866	0.042508906	0.078242942	NS
EVC2	NS	0.036101173	-0.042509064	0.078242942	NS
SYCE1	NS	0.036131568	0.042502126	0.078298547	NS
PAX5	NS	0.036140369	-0.042500118	0.078308854	NS
EPHB4	NS	0.036207007	-0.042484928	0.078444463	NS
RETREG2	NS	0.036257899	0.042473343	0.078545931	NS
CBX8	NS	0.036308391	0.042461863	0.078641074	NS
RNF149	NS	0.036309944	0.04246151	0.078641074	NS
GOT1L1	NS	0.03633842	0.042455042	0.078693943	NS
AMHR2	NS	0.036388519	0.042443673	0.07879362	NS
RFX1	NS	0.036399706	0.042441136	0.078809029	NS

DOCK5	NS	0.036420078	0.042436518	0.078844317	NS
ZNF584	NS	0.036432152	0.042433782	0.078861635	NS
PHKG2	NS	0.036439931	0.042432019	0.078869654	NS
PRSS54	NS	0.036470279	-0.042425147	0.078926512	NS
LENG1	NS	0.036496124	-0.042419298	0.078973615	NS
ACIN1	NS	0.036517731	0.042414411	0.078993876	NS
ADAP2	NS	0.036516179	0.042414762	0.078993876	NS
H2BC12	NS	0.036517461	-0.042414472	0.078993876	NS
PGC	NS	0.036545195	0.042408203	0.079030307	NS
ASB8	NS	0.03654495	-0.042408258	0.079030307	NS
PAF1	NS	0.036561314	0.042404561	0.079043984	NS
NOTCH2NLB	NS	0.036557797	0.042405356	0.079043984	NS
ZFP69	NS	0.036568444	-0.042402951	0.07905057	NS
KRT13	NS	0.036588592	0.042398401	0.079085292	NS
NEMP1	NS	0.036708763	0.042371311	0.079336179	NS
DEFB115	NS	0.036722873	-0.042368135	0.079357814	NS
ANKRD34C	NS	0.036731235	-0.042366254	0.079367021	NS
ATXN3	NS	0.036773996	0.042356637	0.079432817	NS
SMOC2	NS	0.036767842	-0.04235802	0.079432817	NS
CIDEA	NS	0.036773556	0.042356736	0.079432817	NS
MACROH2A2	NS	0.036784164	0.042354352	0.079445913	NS
LRRC8D	NS	0.036844234	-0.042340862	0.079566773	NS
LMOD1	NS	0.036891319	-0.042330301	0.079659568	NS
MAP4K5	NS	0.036925368	0.042322672	0.079715304	NS
PROCA1	NS	0.036923571	0.042323074	0.079715304	NS
TSPAN15	NS	0.036947884	-0.042317629	0.079746125	NS
ASL	NS	0.036960603	-0.042314783	0.079764681	NS
GNB5	NS	0.03698117	0.04231018	0.07979391	NS
GPLD1	NS	0.036982391	-0.042309907	0.07979391	NS
RBM12	NS	0.036996488	0.042306754	0.079815428	NS
TERT	NS	0.037024797	0.042300426	0.079867599	NS
KRT75	NS	0.037037287	-0.042297635	0.079885639	NS
ZNF622	NS	0.03709292	-0.042285214	0.079996719	NS
LAS1L	NS	0.037156373	0.042271065	0.080124639	NS
HLA-DMB	NS	0.037171964	-0.042267592	0.08014933	NS
DPEP2	NS	0.037270643	0.042245639	0.080353148	NS
KLHDC4	NS	0.03730118	-0.042238855	0.080410027	NS
CNNM1	NS	0.037327996	0.042232902	0.080440959	NS
PLPP2	NS	0.037325195	0.042233523	0.080440959	NS
PLB1	NS	0.037326003	0.042233344	0.080440959	NS
ITGB2	NS	0.03734754	0.042228565	0.080474116	NS
TLR3	NS	0.037363096	0.042225115	0.080498673	NS
HCFC1	NS	0.037383551	0.04222058	0.080533779	NS
LLGL2	NS	0.037452706	-0.042205263	0.080664802	NS
ARPC3	NS	0.037449449	0.042205984	0.080664802	NS
RBBP6	NS	0.037464296	0.042202699	0.080680787	NS
OR4X2	NS	0.037491941	-0.042196584	0.080731339	NS
AKR1C8P	NS	0.037508363	-0.042192954	0.080757718	NS
ELF1	NS	0.037512735	0.042191987	0.080758148	NS
PCLAF	NS	0.037534463	0.042187187	0.080795937	NS
LAMB4	NS	0.037555163	0.042182615	0.080831507	NS
LRAT	NS	0.037581761	-0.042176744	0.080879761	NS
FSCN1	NS	0.037586216	0.042175761	0.080880356	NS
POLR2F	NS	0.037610434	-0.042170419	0.080923473	NS
IFNA17	NS	0.037651916	-0.042161276	0.081003721	NS

ADNP2	NS	0.03768321	0.042154384	0.081062038	NS
KNG1	NS	0.037708556	0.042148806	0.081107547	NS
VN1R1	NS	0.037738137	0.042142299	0.081162154	NS
IJD7-PLA2G4	NS	0.037767439	-0.042135858	0.081216148	NS
AFF3	NS	0.037776564	0.042133853	0.081226747	NS
PRICKLE2	NS	0.037783092	-0.042132419	0.081229144	NS
H2BS1	NS	0.037786072	-0.042131765	0.081229144	NS
RETREG1	NS	0.037804991	0.04212761	0.08126079	NS
RNF4	NS	0.037829852	0.042122154	0.081304788	NS
STXBP3	NS	0.037833861	0.042121274	0.081304788	NS
GOLIM4	NS	0.037840389	-0.042119842	0.081309788	NS
SLC25A6	NS	0.037865731	0.042114284	0.08135521	NS
TMEM31	NS	0.03788482	-0.0421101	0.081384546	NS
SLFN12L	NS	0.037887794	-0.042109448	0.081384546	NS
CCDC182	NS	0.03789541	-0.042107779	0.081391872	NS
ITGB4	NS	0.03791	0.042104583	0.081414175	NS
B3GNT3	NS	0.037938789	0.042098279	0.081466144	NS
EM256-PLSC	NS	0.037942616	0.042097441	0.081466144	NS
ABCG2	NS	0.037950965	-0.042095614	0.081475031	NS
LALBA	NS	0.038027812	-0.042078812	0.081630956	NS
MOB3C	NS	0.038113038	-0.042060211	0.081804831	NS
CEP85	NS	0.038123838	0.042057857	0.081814646	NS
PDE2A	NS	0.038126065	0.042057371	0.081814646	NS
ITPR1	NS	0.038141863	0.042053928	0.081839475	NS
KCTD21	NS	0.038181161	0.042045369	0.081914714	NS
PRR27	NS	0.038207975	-0.042039533	0.081963156	NS
LTO1	NS	0.038230033	0.042034734	0.081992302	NS
PDP2	NS	0.038229724	-0.042034802	0.081992302	NS
BIK	NS	0.038253019	-0.042029737	0.082018064	NS
ACSBG1	NS	0.038249211	-0.042030565	0.082018064	NS
C12orf50	NS	0.038254757	0.042029359	0.082018064	NS
MED18	NS	0.03826231	0.042027718	0.08202517	NS
ACLY	NS	0.038273479	0.042025291	0.082040029	NS
ERG28	NS	0.03828344	-0.042023127	0.082052293	NS
FAM53C	NS	0.038312215	0.042016878	0.082104875	NS
VAX2	NS	0.038324857	-0.042014134	0.082113784	NS
CAMSAP2	NS	0.038323525	-0.042014424	0.082113784	NS
TMCC3	NS	0.038345636	0.042009626	0.082149211	NS
PPP1R35	NS	0.038406779	0.041996372	0.082271096	NS
ANK1	NS	0.038428777	0.041991608	0.082290896	NS
HTATIP2	NS	0.038424519	-0.04199253	0.082290896	NS
LAMTOR4	NS	0.038426651	-0.041992068	0.082290896	NS
TP5MF-PTCI	NS	0.038459954	-0.04198486	0.082348547	NS
LSM3	NS	0.038490564	-0.041978239	0.082404973	NS
SYNGR1	NS	0.038524721	0.041970856	0.082468978	NS
MBTPS1	NS	0.03852946	-0.041969832	0.082470003	NS
ERCC5	NS	0.038552677	-0.041964818	0.082510572	NS
RBBP7	NS	0.038585072	-0.041957825	0.082561646	NS
ARHGAP25	NS	0.038582886	0.041958297	0.082561646	NS
ADAM21	NS	0.038608677	0.041952733	0.082603024	NS
LCORL	NS	0.038732094	-0.041926153	0.082848759	NS
MB21D2	NS	0.038728088	-0.041927015	0.082848759	NS
ITPRIPL1	NS	0.038765737	0.04191892	0.082911558	NS
OR2T33	NS	0.038792957	-0.041913071	0.082960609	NS
PPP2R3B	NS	0.038885032	0.041893314	0.08314833	NS

MAGED4	NS	0.038923849	0.041884997	0.083212947	NS
L1TD1	NS	0.038922054	-0.041885382	0.083212947	NS
SULT1A4	NS	0.038970291	-0.041875055	0.083303033	NS
TMED6	NS	0.038989576	-0.04187093	0.083325296	NS
BRD3	NS	0.038993621	0.041870065	0.083325296	NS
RARG	NS	0.038990521	0.041870728	0.083325296	NS
TMEM248	NS	0.039050812	0.041857842	0.083429087	NS
KIAA1328	NS	0.039047629	-0.041858522	0.083429087	NS
NIPAL1	NS	0.039122179	-0.041842611	0.083572332	NS
RNH1	NS	0.039167635	0.041832922	0.083660202	NS
CSTF2T	NS	0.039194205	-0.041827263	0.083707715	NS
ZSWIM6	NS	0.039226615	-0.041820364	0.083767691	NS
MAPK10	NS	0.039267762	0.041811613	0.08384631	NS
GLTPD2	NS	0.03931053	-0.041802525	0.083928371	NS
G6PD	NS	0.039319848	0.041800546	0.083939008	NS
RIT1	NS	0.039324257	0.04179961	0.083939161	NS
CDK11B	NS	0.039387998	0.041786086	0.084065948	NS
SLC25A11	NS	0.039399956	-0.041783551	0.084073265	NS
ATAD3B	NS	0.039400113	0.041783517	0.084073265	NS
SPIC	NS	0.039410773	-0.041781258	0.084086742	NS
TSPAN12	NS	0.039426409	-0.041777945	0.084110831	NS
RTN3	NS	0.039441487	0.041774751	0.084133725	NS
INIP	NS	0.039453634	-0.041772178	0.084150363	NS
WASHC3	NS	0.039540511	-0.041753801	0.084326369	NS
RNF157	NS	0.039548874	0.041752033	0.084334913	NS
SSX4B	NS	0.039573244	0.041746885	0.084377585	NS
CD48	NS	0.039609652	0.041739199	0.08443661	NS
DKC1	NS	0.03971788	0.041716387	0.084657999	NS
RMDN3	NS	0.039754237	-0.041708735	0.084726163	NS
STOML3	NS	0.039789639	-0.04170129	0.084792276	NS
PLEKHA6	NS	0.039801707	-0.041698754	0.084808657	NS
SLC13A1	NS	0.039807668	-0.041697501	0.084812023	NS
FGG	NS	0.039831216	-0.041692554	0.084852852	NS
OR10P1	NS	0.039863602	-0.041685754	0.0849125	NS
COL6A3	NS	0.039901368	0.041677831	0.084983592	NS
MRPS34	NS	0.039913672	-0.041675251	0.085000443	NS
FAM25C	NS	0.039922995	0.041673296	0.085010944	NS
NDUFB2	NS	0.039959273	-0.041665694	0.085069478	NS
ARCN1	NS	0.0399573	0.041666108	0.085069478	NS
PYDC2	NS	0.039973293	-0.041662758	0.085089967	NS
FAM227B	NS	0.039986668	-0.041659958	0.085109078	NS
RNF19B	NS	0.040015964	-0.041653827	0.085162066	NS
PARK7	NS	0.040049935	-0.041646723	0.085224993	NS
CSF3R	NS	0.040086478	0.041639086	0.085293379	NS
BTBD11	NS	0.040093153	-0.041637692	0.085298205	NS
HSF1	NS	0.040101183	0.041636014	0.085305913	NS
F13A1	NS	0.040141035	0.041627696	0.085381304	NS
NANOGP8	NS	0.040148544	0.041626129	0.085387894	NS
C11orf91	NS	0.040179626	0.041619647	0.08544461	NS
SIM1	NS	0.040224163	-0.041610366	0.085520529	NS
VIL1	NS	0.040219911	-0.041611251	0.085520529	NS
CNTNAP5	NS	0.04023575	-0.041607952	0.085535769	NS
PABPN1	NS	0.040247844	-0.041605434	0.085546295	NS
NT5C1A	NS	0.040252059	0.041604557	0.085546295	NS
MARVELD2	NS	0.040256267	0.041603681	0.085546295	NS

ID4	NS	0.04025838	0.041603241	0.085546295	NS
CELA2B	NS	0.040278182	-0.04159912	0.085578978	NS
ST6GALNAC	NS	0.040289249	0.041596818	0.085593096	NS
GOLGA1	NS	0.040298204	0.041594956	0.085593333	NS
ENTPD3	NS	0.040296127	0.041595387	0.085593333	NS
ZNF479	NS	0.040310399	0.04159242	0.085609841	NS
YTHDF1	NS	0.040318771	-0.041590679	0.085618227	NS
SLC6A17	NS	0.040380139	-0.04157793	0.085734739	NS
JRK	NS	0.040382497	-0.041577441	0.085734739	NS
SLC2A3	NS	0.040411655	0.04157139	0.085787234	NS
IVNS1ABP	NS	0.040421645	0.041569317	0.085799031	NS
RNFT1	NS	0.040470333	-0.041559223	0.085892957	NS
PM20D1	NS	0.040508003	-0.04155142	0.08596348	NS
FTCDNL1	NS	0.040566304	0.041539356	0.086077765	NS
TEAD2	NS	0.04059424	0.041533581	0.086127602	NS
FTSJ1	NS	0.04060422	-0.041531518	0.086139333	NS
FAM166A	NS	0.040680192	0.041515832	0.086291047	NS
B4GALT5	NS	0.040712394	0.041509191	0.08634989	NS
APOBEC1	NS	0.040775273	0.041496235	0.08647378	NS
PROKR2	NS	0.04081327	0.041488415	0.086525921	NS
NCF2	NS	0.040804712	0.041490176	0.086525921	NS
ICE1	NS	0.040809301	-0.041489231	0.086525921	NS
WHRN	NS	0.040823397	0.041486332	0.086537911	NS
SVOP	NS	0.040833968	0.041484157	0.086550842	NS
CLEC4M	NS	0.040842476	0.041482408	0.086559396	NS
ZNF148	NS	0.040848191	0.041481233	0.08656203	NS
C2orf88	NS	0.040861308	-0.041478536	0.086580347	NS
SPACA6	NS	0.040869884	0.041476774	0.08658904	NS
KRTAP12-3	NS	0.040876515	0.041475411	0.08659361	NS
CUX2	NS	0.040912255	0.04146807	0.08665984	NS
GRWD1	NS	0.040930489	0.041464327	0.086688975	NS
ALDH1A3	NS	0.040953939	0.041459516	0.086729152	NS
OR2AJ1	NS	0.040982792	-0.041453598	0.086779065	NS
PIM3	NS	0.040986475	0.041452843	0.086779065	NS
ADAMTSL1	NS	0.041002141	0.041449632	0.086802738	NS
RP1L1	NS	0.041057189	0.041438357	0.086909771	NS
TAF8	NS	0.041125033	0.041424479	0.087034348	NS
CXCL1	NS	0.041121833	0.041425133	0.087034348	NS
ASXL1	NS	0.041141522	-0.041421109	0.087059724	NS
PSTPIP2	NS	0.041156432	0.041418063	0.087081755	NS
CHEK2	NS	0.041230445	0.041402954	0.087228821	NS
FBXO11	NS	0.041262683	0.04139638	0.087287486	NS
IGSF9	NS	0.041269294	0.041395033	0.08729193	NS
SPRYD4	NS	0.041311845	0.041386364	0.087372385	NS
GARRE1	NS	0.04132688	0.041383303	0.08738513	NS
GREB1	NS	0.0413269	0.041383299	0.08738513	NS
ADAMTS4	NS	0.04133839	0.04138096	0.087390331	NS
CPAMD8	NS	0.041335429	-0.041381563	0.087390331	NS
IKBKE	NS	0.041375323	0.041373446	0.087458857	NS
GAA	NS	0.041401049	0.041368216	0.087494127	NS
LMNB2	NS	0.041397141	-0.04136901	0.087494127	NS
PCNX2	NS	0.041406966	0.041367013	0.087497077	NS
BECN1	NS	0.04142984	0.041362365	0.087535857	NS
OR10G4	NS	0.041447361	0.041358806	0.087563319	NS
PUS10	NS	0.041473114	0.041353578	0.087608163	NS

SLC2A14	NS	0.04152276	0.041343507	0.087703465	NS
ITFG1	NS	0.041553687	0.041337238	0.087749636	NS
COP1	NS	0.041550716	-0.04133784	0.087749636	NS
NUSAP1	NS	0.041572936	0.041333338	0.087780708	NS
PACRGL	NS	0.041614002	-0.041325024	0.087857833	NS
SNAPC4	NS	0.041673492	-0.041312991	0.087973837	NS
KLK4	NS	0.041686259	0.041310411	0.087991192	NS
GUF1	NS	0.041691495	-0.041309353	0.087992649	NS
PRAMEF4	NS	0.041703473	0.041306933	0.087998738	NS
TPO	NS	0.04178655	-0.041290164	0.088164429	NS
SLC25A3	NS	0.041796884	0.04128808	0.088176621	NS
FDX1	NS	0.041854833	0.041276402	0.088288139	NS
CRYL1	NS	0.041858868	0.04127559	0.088288139	NS
RPL6	NS	0.041870419	0.041273264	0.0882994	NS
EFCAB13	NS	0.041873331	-0.041272678	0.0882994	NS
ZBTB48	NS	0.041895574	0.041268201	0.08833668	NS
PCDHA7	NS	0.041967636	-0.04125371	0.088478986	NS
SOAT2	NS	0.041972243	0.041252784	0.088479061	NS
IGDCC4	NS	0.041985325	0.041250156	0.088496999	NS
C1orf50	NS	0.042024534	-0.041242283	0.088561193	NS
H3Y2	NS	0.04202493	-0.041242204	0.088561193	NS
HELZ2	NS	0.042039894	0.041239201	0.088583082	NS
NHLH1	NS	0.042056618	-0.041235846	0.088608675	NS
CHPF2	NS	0.042065054	0.041234154	0.088616804	NS
MEOX2	NS	0.042071652	-0.041232831	0.088621057	NS
PCDHGB6	NS	0.04216526	-0.041214078	0.088798909	NS
OGFOD3	NS	0.04217177	-0.041212775	0.088802958	NS
SEMA6D	NS	0.042245451	-0.041198041	0.088948434	NS
RPH3A	NS	0.042305348	0.04118608	0.089064859	NS
CLDN16	NS	0.042361807	0.041174818	0.089174022	NS
B9D2	NS	0.042374532	-0.041172281	0.089191109	NS
PPP1R13L	NS	0.042385713	0.041170053	0.089198949	NS
FEZF1	NS	0.042387473	0.041169702	0.089198949	NS
TMEM38A	NS	0.042431772	-0.041160879	0.089282463	NS
AJM1	NS	0.042441724	-0.041158898	0.089293697	NS
FXYD4	NS	0.042467882	0.041153693	0.089339019	NS
GABRP	NS	0.042486698	0.04114995	0.089368891	NS
VARS2	NS	0.04249726	-0.04114785	0.089381393	NS
DNAJC1	NS	0.042534682	0.041140413	0.089450381	NS
WFS1	NS	0.042555782	-0.041136222	0.089475312	NS
IFNA4	NS	0.042555276	-0.041136322	0.089475312	NS
FAM72A	NS	0.042562429	0.041134902	0.089479567	NS
NAB2	NS	0.042590771	-0.041129276	0.089526895	NS
ZBTB43	NS	0.042594192	0.041128597	0.089526895	NS
SEMA4A	NS	0.042606461	0.041126162	0.089542961	NS
RINL	NS	0.04262394	-0.041122695	0.089569968	NS
CDIN1	NS	0.042654514	-0.041116634	0.089624486	NS
WNT1	NS	0.042742881	0.041099135	0.089800414	NS
KLHL7	NS	0.042773168	-0.041093144	0.08985429	NS
RPS3A	NS	0.042826723	0.04108256	0.089947271	NS
MTCL1	NS	0.042847138	-0.041078528	0.089980385	NS
OR5K4	NS	0.042911642	-0.0410658	0.090106069	NS
DLGAP3	NS	0.042918567	-0.041064434	0.090110834	NS
TFB2M	NS	0.042923416	-0.041063478	0.090111241	NS
LRRC61	NS	0.04301102	-0.041046222	0.090285359	NS

MAP2K3	NS	0.043087756	0.041031131	0.090436631	NS
RPL34	NS	0.043099714	0.041028781	0.090451921	NS
RTN1	NS	0.043128895	0.041023049	0.09050335	NS
KIAA1191	NS	0.043218794	-0.041005413	0.090682165	NS
SH3D19	NS	0.043227705	-0.041003666	0.090689396	NS
DSE	NS	0.043231611	0.041002901	0.090689396	NS
GPKOW	NS	0.043278575	-0.040993702	0.090776195	NS
CCDC138	NS	0.043282368	-0.040992959	0.090776195	NS
TSC22D3	NS	0.04329163	0.040991146	0.090778416	NS
STAT2	NS	0.043292806	-0.040990916	0.090778416	NS
FANCB	NS	0.043335588	-0.040982545	0.090857876	NS
HNRNPCL2	NS	0.043340089	0.040981665	0.090857876	NS
ZSCAN21	NS	0.043433094	-0.040963495	0.09104299	NS
MTFR1L	NS	0.043439526	-0.040962239	0.091046613	NS
CXCL14	NS	0.043496487	-0.040951128	0.091156128	NS
VCPKMT	NS	0.043502019	0.04095005	0.091157853	NS
COPS9	NS	0.043535104	-0.040943602	0.091207433	NS
SUPT4H1	NS	0.043534621	0.040943696	0.091207433	NS
CTDNEP1	NS	0.043540498	0.040942552	0.091208861	NS
LTBP4	NS	0.043567311	-0.04093733	0.091228638	NS
TXNL1	NS	0.043573226	0.040936179	0.091228638	NS
IFIT3	NS	0.043560819	-0.040938594	0.091228638	NS
ACP2	NS	0.043573505	-0.040936124	0.091228638	NS
SOX11	NS	0.043565529	0.040937677	0.091228638	NS
PGAP4	NS	0.043613334	-0.040928374	0.091302152	NS
SYNE3	NS	0.043703818	0.040910789	0.091481681	NS
TRAF3	NS	0.043734092	0.040904912	0.091530574	NS
MACC1	NS	0.043736633	0.040904419	0.091530574	NS
CCDC91	NS	0.043791814	0.040893717	0.091636146	NS
ACVRL1	NS	0.043818006	-0.040888641	0.091675553	NS
TMC7	NS	0.043820119	0.040888232	0.091675553	NS
ZNF835	NS	0.043842839	0.040883831	0.091713173	NS
GCM2	NS	0.043917288	-0.040869424	0.091858982	NS
PLXNA4	NS	0.043936899	-0.040865632	0.091890072	NS
STOML1	NS	0.043974232	0.040858418	0.091949861	NS
RPS7	NS	0.043974988	0.040858272	0.091949861	NS
OR13D1	NS	0.043984331	-0.040856467	0.091959464	NS
DNAI7	NS	0.044010081	-0.040851495	0.092003362	NS
PHC1	NS	0.044039797	0.040845761	0.092055541	NS
ETNK2	NS	0.044063006	-0.040841284	0.092094109	NS
HACD2	NS	0.044107123	-0.04083278	0.092176364	NS
GPHA2	NS	0.044131027	0.040828176	0.09220918	NS
MYD88	NS	0.044132354	0.04082792	0.09220918	NS
MBD3L3	NS	0.044156172	0.040823334	0.092248987	NS
ZMAT5	NS	0.044177326	-0.040819263	0.092283221	NS
MRPL34	NS	0.044191443	-0.040816547	0.09229279	NS
PDXDC1	NS	0.044191196	-0.040816595	0.09229279	NS
LIPN	NS	0.04421696	0.04081164	0.092336118	NS
KRT4	NS	0.044251553	-0.040804991	0.092398388	NS
NAA10	NS	0.044269541	-0.040801535	0.092425977	NS
TULP2	NS	0.044303836	-0.04079495	0.092487602	NS
RNASE10	NS	0.04431774	0.040792281	0.09250665	NS
SRPK1	NS	0.044324421	0.040790999	0.09251062	NS
TNNI2	NS	0.044333052	-0.040789343	0.092518659	NS
SDC2	NS	0.04434714	-0.040786641	0.092538082	NS

LY9	NS	0.044367457	0.040782745	0.092570496	NS
MRRF	NS	0.044424061	0.040771898	0.092678608	NS
CHKA	NS	0.044490872	0.040759111	0.092807987	NS
SPIB	NS	0.044510356	0.040755385	0.092838625	NS
SGSM3	NS	0.044548173	-0.040748157	0.092897483	NS
PKIG	NS	0.044546253	0.040748524	0.092897483	NS
ATAD2B	NS	0.04456445	0.040745047	0.092920774	NS
IL36B	NS	0.044568943	-0.040744189	0.092920774	NS
WDR26	NS	0.044618781	0.040734675	0.09301466	NS
TANGO2	NS	0.044639313	0.040730758	0.093047442	NS
GOLGB1	NS	0.044663766	0.040726095	0.093088388	NS
PLCL1	NS	0.044702825	0.040718651	0.093159763	NS
NEIL2	NS	0.044709147	-0.040717447	0.093162908	NS
PIK3IP1	NS	0.044739973	0.040711577	0.093217107	NS
BMERB1	NS	0.044749626	-0.040709739	0.093227184	NS
VAV1	NS	0.044786738	0.040702678	0.093294459	NS
PCTP	NS	0.044831336	-0.040694199	0.093377309	NS
MM23B-AGA	NS	0.044844904	0.04069162	0.09339552	NS
ATPAF1	NS	0.044860399	0.040688677	0.093417738	NS
PFKFB1	NS	0.044873512	-0.040686186	0.093434994	NS
UGT2B4	NS	0.044932711	0.040674951	0.093538133	NS
CFAP77	NS	0.044930012	-0.040675463	0.093538133	NS
GORASP2	NS	0.04496605	0.040668629	0.093587405	NS
ADAM15	NS	0.044963661	0.040669082	0.093587405	NS
PMFBP1	NS	0.045001793	-0.040661855	0.093651727	NS
GDAP2	NS	0.045069652	0.040649008	0.093772782	NS
RGCP-MRPS	NS	0.045066078	-0.040649685	0.093772782	NS
SMOX	NS	0.045107173	0.040641912	0.09384076	NS
PCNX3	NS	0.045125341	-0.040638477	0.093868469	NS
YRDC	NS	0.045138057	-0.040636074	0.09388483	NS
NKD1	NS	0.04514304	0.040635133	0.093885106	NS
CALD1	NS	0.045149515	-0.040633909	0.093888484	NS
WDR73	NS	0.045173535	-0.040629373	0.093928342	NS
PSMD8	NS	0.045181274	-0.040627911	0.093934343	NS
DIAPH2	NS	0.045186844	-0.04062686	0.093935834	NS
MRPS35	NS	0.045213223	-0.040621881	0.093980579	NS
CCL24	NS	0.045227517	0.040619184	0.094000195	NS
GRIN2A	NS	0.04526938	-0.04061129	0.094077101	NS
SPDYE18	NS	0.045373497	0.040591683	0.09428335	NS
HSPB1	NS	0.045388074	-0.040588941	0.094299683	NS
SLU7	NS	0.045391101	0.040588372	0.094299683	NS
TPD52	NS	0.0454345	-0.040580213	0.094369797	NS
PROP1	NS	0.045434601	0.040580194	0.094369797	NS
ABHD17C	NS	0.045486764	-0.040570396	0.094468006	NS
NUB1	NS	0.045502307	-0.040567479	0.094490146	NS
NR1H2	NS	0.045515452	0.040565012	0.094507305	NS
FAM81A	NS	0.045533762	0.040561577	0.094525042	NS
ZSCAN12	NS	0.045530966	-0.040562101	0.094525042	NS
CRTAM	NS	0.045548964	-0.040558726	0.094535914	NS
SPN	NS	0.045548741	0.040558768	0.094535914	NS
CL2L2-PABP	NS	0.045558535	-0.040556931	0.094535914	NS
MIA-RAB4B	NS	0.045554007	0.04055778	0.094535914	NS
PRR23D1	NS	0.045596143	-0.040549883	0.094603809	NS
CENPT	NS	0.045617022	0.040545971	0.094636987	NS
PYCR3	NS	0.045626917	-0.040544118	0.09464737	NS

ZNF182	NS	0.04571401	-0.040527823	0.094817871	NS
ZCCHC14	NS	0.045770802	0.040517211	0.094925494	NS
ORC3	NS	0.04580339	-0.040511127	0.094982904	NS
CNTNAP2	NS	0.045822168	0.040507623	0.095011664	NS
BHD14A-AC	NS	0.045845495	-0.040503272	0.095045154	NS
EIF3M	NS	0.04584814	-0.040502778	0.095045154	NS
VANGL1	NS	0.04587206	-0.040498318	0.095084556	NS
ZNF710	NS	0.045905158	-0.04049215	0.095142975	NS
PCDHGA3	NS	0.045957615	-0.040482383	0.095241499	NS
TMEM70	NS	0.046007891	0.04047303	0.095335483	NS
CLDN18	NS	0.04608623	0.040458473	0.095485636	NS
ELP1	NS	0.04609022	-0.040457733	0.095485636	NS
PCGF6	NS	0.046096941	-0.040456485	0.09548934	NS
PYGB	NS	0.046113329	-0.040453443	0.095513066	NS
CCDC13	NS	0.046129211	0.040450496	0.095535739	NS
PRLH	NS	0.046222124	0.040433272	0.095712967	NS
CHMP2B	NS	0.046224675	0.040432799	0.095712967	NS
TMEM268	NS	0.046230441	-0.040431731	0.095714667	NS
CYSLTR2	NS	0.046385125	0.040403125	0.096024651	NS
FATE1	NS	0.046392472	0.040401769	0.096029589	NS
ARMCX4	NS	0.046459402	0.040389418	0.096157847	NS
ZKSCAN2	NS	0.046493475	-0.040383136	0.096207794	NS
ZXDB	NS	0.046493068	0.040383211	0.096207794	NS
COLEC11	NS	0.046509908	0.040380107	0.096231511	NS
OR1I1	NS	0.046519582	0.040378325	0.096241239	NS
RASAL1	NS	0.04653053	-0.040376309	0.0962536	NS
NSMCE4A	NS	0.046587926	-0.040365742	0.096362033	NS
GJB1	NS	0.046610938	0.040361509	0.09638903	NS
ARRDC1	NS	0.046609785	-0.040361721	0.09638903	NS
EAF2	NS	0.046622108	-0.040359455	0.096391532	NS
C9orf85	NS	0.046618865	-0.040360051	0.096391532	NS
CLMN	NS	0.046719224	0.040341613	0.096582004	NS
NASP	NS	0.046748367	0.040336265	0.09663193	NS
GTF2H2C	NS	0.046825287	-0.040322162	0.096780594	NS
ATAD1	NS	0.046858798	-0.040316024	0.09681758	NS
ATP6AP2	NS	0.046856782	-0.040316394	0.09681758	NS
ZNF10	NS	0.04686319	-0.04031522	0.09681758	NS
NTNG2	NS	0.046928967	0.040303185	0.096943125	NS
ELMOD3	NS	0.047018293	-0.040286863	0.097117286	NS
ATP23	NS	0.047041082	-0.040282703	0.09715399	NS
CCDC97	NS	0.047046959	0.04028163	0.09715576	NS
SLC31A1	NS	0.047088432	0.040274065	0.097222215	NS
COPS6	NS	0.047089185	-0.040273927	0.097222215	NS
COL23A1	NS	0.047107892	-0.040270517	0.097250464	NS
C16orf95	NS	0.047125494	-0.040267309	0.097276427	NS
SGCB	NS	0.04716427	-0.040260245	0.097346087	NS
F10	NS	0.047226349	0.040248946	0.097463826	NS
OR1L1	NS	0.047267475	-0.040241468	0.0975383	NS
GPRASP2	NS	0.047274644	-0.040240165	0.097542693	NS
CELF2	NS	0.047305465	0.040234565	0.097595884	NS
PLA2G15	NS	0.047316022	0.040232648	0.097597983	NS
SMC4	NS	0.047316567	-0.040232549	0.097597983	NS
GGTLC1	NS	0.04733214	0.040229721	0.097619702	NS
THUMPD3	NS	0.047407975	0.040215962	0.097765689	NS
MEGF6	NS	0.047416692	0.040214381	0.09777325	NS

NEK9	NS	0.047434846	0.040211091	0.097789847	NS
RASGRP4	NS	0.047433571	0.040211322	0.097789847	NS
SMPD2	NS	0.047444461	0.040209348	0.097799252	NS
HDGFL2	NS	0.047449966	0.040208351	0.097800185	NS
EPHX1	NS	0.047475686	0.040203692	0.097842779	NS
TAC1	NS	0.047509336	-0.0401976	0.097901704	NS
TTK	NS	0.047554024	-0.040189515	0.097981952	NS
PTEP2-CSNK	NS	0.047558402	-0.040188723	0.097981952	NS
SP110	NS	0.047582562	0.040184355	0.098021292	NS
SGPP1	NS	0.047595057	0.040182097	0.098026168	NS
BAHCC1	NS	0.04759494	0.040182118	0.098026168	NS
SUN2	NS	0.047670425	0.040168485	0.098170948	NS
SLC25A13	NS	0.047677054	0.040167289	0.098174154	NS
SMPDL3B	NS	0.047712822	-0.040160837	0.098237355	NS
STXBP6	NS	0.047766778	-0.040151111	0.098337986	NS
TMEM88	NS	0.047862009	0.040133967	0.098523562	NS
LRP1	NS	0.047892783	0.040128433	0.098576425	NS
RNF166	NS	0.047928593	0.040121998	0.098639643	NS
CYP4F8	NS	0.04793984	0.040119977	0.0986523	NS
C18orf63	NS	0.047970065	-0.040114549	0.098704006	NS
KEAP1	NS	0.048090948	-0.04009287	0.098942218	NS
CMA1	NS	0.048101462	-0.040090987	0.098953332	NS
TPI1	NS	0.048178427	0.04007721	0.09910113	NS
GCSH	NS	0.048204685	-0.040072514	0.099144606	NS
ITGA1	NS	0.048249159	0.040064566	0.099225534	NS
TWNK	NS	0.048286114	-0.040057966	0.099290983	NS
SNUPN	NS	0.048457191	-0.040027466	0.099632185	NS
FBXO9	NS	0.048466928	-0.040025733	0.099641621	NS
PID1	NS	0.048475722	0.040024168	0.099649116	NS
ETV4	NS	0.04853128	-0.040014286	0.099752729	NS
TTLL2	NS	0.048548664	0.040011196	0.099777865	NS
ETHE1	NS	0.048600104	0.040002057	0.099872982	NS
ZNF367	NS	0.048632143	-0.03999637	0.099928212	NS
NAJC25-GNG	NS	0.048675918	0.039988604	0.100007543	NS
EPS8L3	NS	0.048692291	0.0399857	0.100030565	NS
PRDM5	NS	0.048714434	-0.039981775	0.100065433	NS
CEP70	NS	0.04878817	-0.039968716	0.100206262	NS
OR5M1	NS	0.048794785	-0.039967545	0.100209216	NS
NR1D1	NS	0.048805305	0.039965683	0.100220188	NS
RAB11A	NS	0.048853129	-0.039957225	0.10030775	NS
RORA	NS	0.048866789	-0.03995481	0.100325156	NS
EVL	NS	0.048872745	0.039953757	0.100326742	NS
BCL7B	NS	0.048886727	0.039951286	0.100334162	NS
ACBD4	NS	0.048884422	-0.039951693	0.100334162	NS
REEP4	NS	0.048895066	0.039949813	0.100340639	NS
UROD	NS	0.04891446	-0.039946387	0.100369796	NS
COL2A1	NS	0.04896566	0.039937348	0.100464206	NS
NRN1	NS	0.049002212	-0.0399309	0.100528545	NS
SLC23A2	NS	0.049054689	0.03992165	0.100625537	NS
OR11L1	NS	0.049101118	0.039913473	0.100710103	NS
METTL24	NS	0.049179698	-0.039899648	0.100831292	NS
MX2	NS	0.049181041	0.039899412	0.100831292	NS
RYR1	NS	0.049170882	0.039901198	0.100831292	NS
CYB5RL	NS	0.0491807	-0.039899472	0.100831292	NS
WDR62	NS	0.04920348	-0.039895467	0.100834575	NS

EDN2	NS	0.049191576	-0.03989756	0.100834575	NS
THRSP	NS	0.049199114	-0.039896235	0.100834575	NS
SAP30L	NS	0.049194319	-0.039897077	0.100834575	NS
KRTAP12-1	NS	0.049236587	0.03988965	0.100891741	NS
PNPO	NS	0.049250493	-0.039887208	0.100909553	NS
ZC3H7A	NS	0.049307147	0.039877264	0.101014939	NS
UBTD1	NS	0.049326494	0.039873871	0.10104388	NS
UXS1	NS	0.049370314	0.039866189	0.101112241	NS
ANKRD39	NS	0.049369015	0.039866416	0.101112241	NS
C2orf42	NS	0.049416024	-0.039858181	0.101195149	NS
FEZ2	NS	0.049483426	-0.039846385	0.101311741	NS
ZNF408	NS	0.04947849	-0.039847248	0.101311741	NS
MAT1A	NS	0.049489009	-0.039845408	0.101312455	NS
HIRA	NS	0.049494723	0.039844409	0.101313437	NS
SPZ1	NS	0.04961576	0.039823264	0.101539719	NS
BEAN1	NS	0.049685077	0.039811174	0.101670827	NS
CLEC17A	NS	0.049720867	-0.039804937	0.101733309	NS
CYB5B	NS	0.04974478	-0.039800772	0.101771478	NS
ZNF740	NS	0.049871038	-0.039778809	0.102008551	NS
MFAP5	NS	0.049871199	0.039778781	0.102008551	NS
OR2A1	NS	0.049915795	-0.039771034	0.102088983	NS
TPRA1	NS	0.049974487	0.039760848	0.102198223	NS
NDUFA13	NS	0.049994106	-0.039757445	0.102227543	NS
OR13J1	NS	0.049999609	0.039756491	0.102227996	NS
MVP	NS	0.050058644	0.03974626	0.102337886	NS
SULT1A3	NS	0.050066963	-0.039744819	0.102344085	NS
GHRHR	NS	0.050084117	0.039741848	0.102368339	NS
USP37	NS	0.050120634	0.039735527	0.102432161	NS
GMIP	NS	0.050126939	0.039734436	0.102434231	NS
COX20	NS	0.050143326	0.039731601	0.102456901	NS
RFLNA	NS	0.050185796	0.039724257	0.102532854	NS
BRPF1	NS	0.050217578	-0.039718765	0.10258696	NS
MAGI1	NS	0.050231987	-0.039716276	0.102605566	NS
TCF3	NS	0.050246737	0.039713728	0.102624865	NS
HNRNPD	NS	0.050261901	0.03971111	0.102645005	NS
SEC61G	NS	0.050267724	-0.039710105	0.102646067	NS
TSSC4	NS	0.05029974	-0.03970458	0.102700608	NS
UTP4	NS	0.050326239	-0.039700009	0.102743875	NS
DIMT1	NS	0.050347024	0.039696425	0.102775238	NS
FKBP14	NS	0.050352221	-0.039695529	0.102775238	NS
SLC24A1	NS	0.050365196	0.039693293	0.102790884	NS
SSR2	NS	0.050464138	0.039676255	0.102981957	NS
CFAP276	NS	0.050490295	-0.039671755	0.103013612	NS
ZBTB20	NS	0.050489242	0.039671936	0.103013612	NS
NCKIPSD	NS	0.050508849	-0.039668565	0.103040607	NS
ENO3	NS	0.050587351	0.039655077	0.103189879	NS
BATF3	NS	0.050600678	-0.039652789	0.103206187	NS
OPRL1	NS	0.050607725	-0.039651579	0.103209685	NS
OR51M1	NS	0.050622331	0.039649072	0.103228596	NS
PPP1R21	NS	0.050630726	-0.039647632	0.10323484	NS
SCD	NS	0.050667337	-0.039641352	0.103287728	NS
WDR5B	NS	0.050666964	-0.039641416	0.103287728	NS
ZNF688	NS	0.050739398	0.039629002	0.103423736	NS
CNN1	NS	0.05077048	0.03962368	0.103454408	NS
RELN	NS	0.050761479	0.039625221	0.103454408	NS

RPL39	NS	0.050768365	-0.039624042	0.103454408	NS
NTNG1	NS	0.050834885	-0.03961266	0.103574741	NS
ZNF865	NS	0.050900677	0.039601415	0.103697874	NS
ILRUN	NS	0.050941501	0.039594444	0.103770121	NS
GNA13	NS	0.050960059	0.039591276	0.103797	NS
PHAX	NS	0.050970849	-0.039589435	0.103808054	NS
SLC12A5	NS	0.050999425	0.039584561	0.103855325	NS
CCDC136	NS	0.051006069	-0.039583428	0.103857928	NS
ZNF566	NS	0.051016373	0.03958167	0.103867984	NS
PALB2	NS	0.051027091	-0.039579843	0.103878879	NS
ZNF181	NS	0.051074345	-0.039571791	0.103964141	NS
ZNF100	NS	0.05110078	0.039567289	0.104007014	NS
LRG1	NS	0.051134322	0.03956158	0.10406434	NS
IGFBP1	NS	0.051148377	0.039559188	0.104082001	NS
CD74	NS	0.051154457	0.039558154	0.104083429	NS
IL13	NS	0.05118623	-0.03955275	0.104137132	NS
FAM118A	NS	0.05121736	0.039547459	0.104189514	NS
MAGEB6B	NS	0.051296909	0.039533949	0.10434037	NS
ADGRA3	NS	0.051311482	0.039531476	0.104348082	NS
ZNF747	NS	0.051311036	-0.039531552	0.104348082	NS
ERG	NS	0.051328065	0.039528663	0.104370839	NS
NPIPB2	NS	0.051346509	0.039525535	0.104397377	NS
FBXO40	NS	0.051394782	-0.039517352	0.10448455	NS
WASHC4	NS	0.051401507	0.039516212	0.104487247	NS
TES	NS	0.051474745	0.039503811	0.10461415	NS
PDLIM7	NS	0.051471238	0.039504405	0.10461415	NS
MAL	NS	0.05150479	-0.039498728	0.104664223	NS
AK1	NS	0.051524699	-0.039495361	0.1046827	NS
CC2D1B	NS	0.051520175	0.039496126	0.1046827	NS
PRSS58	NS	0.051581538	-0.039485755	0.104787179	NS
ZNF442	NS	0.051626602	-0.039478144	0.104867721	NS
USP35	NS	0.051646883	0.039474721	0.104886902	NS
TMPRSS3	NS	0.051646505	-0.039474785	0.104886902	NS
RBPMS2	NS	0.051658747	0.039472719	0.104899991	NS
SPPL2C	NS	0.051693971	0.039466778	0.104960505	NS
DLX5	NS	0.05170001	-0.03946576	0.104961757	NS
H2BW2	NS	0.051720201	0.039462356	0.104985807	NS
JUP	NS	0.051722704	0.039461934	0.104985807	NS
SPEF2	NS	0.051748604	0.03945757	0.105020613	NS
MAF	NS	0.051755673	-0.039456379	0.105020613	NS
HLA-DPB1	NS	0.051756129	0.039456302	0.105020613	NS
PAXX	NS	0.051803188	0.039448377	0.105105085	NS
KRTAP13-4	NS	0.051889119	0.039433923	0.105268397	NS
PLEKHG3	NS	0.051903703	-0.039431472	0.10528695	NS
RPL11	NS	0.051922224	0.03942836	0.105313483	NS
GPR152	NS	0.051932711	-0.039426598	0.105323717	NS
RANBP2	NS	0.052001518	0.039415046	0.105452214	NS
CA8	NS	0.052057646	-0.039405633	0.105554975	NS
CHRD	NS	0.052078709	0.039402102	0.105586623	NS
PTRH1	NS	0.052153772	-0.03938953	0.105727735	NS
MUC6	NS	0.052247059	-0.039373927	0.105905757	NS
CST4	NS	0.052266751	0.039370637	0.105923488	NS
TAS2R16	NS	0.052261782	-0.039371467	0.105923488	NS
UNC119	NS	0.052433652	0.039342788	0.106250605	NS
C6orf136	NS	0.052496237	-0.039332364	0.10636629	NS

CCDC159	NS	0.05251252	0.039329654	0.106388146	NS
KCNE1	NS	0.052532219	0.039326376	0.106413812	NS
TOMM7	NS	0.052536185	-0.039325716	0.106413812	NS
ACBD3	NS	0.052601784	0.039314809	0.106535537	NS
ZNF143	NS	0.052641643	-0.039308187	0.106605109	NS
AGMO	NS	0.052669037	0.039303638	0.106649428	NS
HNRNPF	NS	0.052732812	0.039293056	0.106767396	NS
CORO1A	NS	0.052775275	0.039286016	0.106842194	NS
PCCB	NS	0.052782581	0.039284805	0.106845809	NS
PTMA	NS	0.052809834	0.03928029	0.106889799	NS
HDHD3	NS	0.052824431	-0.039277872	0.106908166	NS
RPS6KA3	NS	0.052885914	-0.039267695	0.107021406	NS
FBXO32	NS	0.052948913	-0.039257278	0.107134409	NS
MPLKIP	NS	0.052952825	-0.039256631	0.107134409	NS
AKIP1	NS	0.05301364	-0.039246585	0.107246239	NS
ZC3H3	NS	0.053032154	0.039243528	0.107261271	NS
RAB33B	NS	0.05303086	-0.039243742	0.107261271	NS
MAML3	NS	0.053080916	-0.039235483	0.107348679	NS
SF3B6	NS	0.053098068	-0.039232654	0.107372148	NS
RASSF3	NS	0.053113645	-0.039230086	0.107392429	NS
CHP1	NS	0.05315092	0.039223943	0.107456572	NS
GFRA1	NS	0.053197062	-0.039216344	0.107538624	NS
PDXK	NS	0.053250327	0.039207578	0.107635059	NS
NADK	NS	0.053336204	0.039193461	0.107797386	NS
SPAG16	NS	0.053376333	-0.03918687	0.107862998	NS
WHAMM	NS	0.053379813	0.039186299	0.107862998	NS
CXXC4	NS	0.053424496	-0.039178966	0.107942019	NS
KMT2A	NS	0.053440907	0.039176274	0.107963907	NS
GOLGA6L6	NS	0.053459715	0.03917319	0.107990633	NS
LRPAP1	NS	0.05349329	0.039167687	0.108047179	NS
NR1H3	NS	0.053516821	-0.039163831	0.108072152	NS
ZNF534	NS	0.053515717	0.039164012	0.108072152	NS
MTG2	NS	0.053525119	0.039162472	0.108077633	NS
PIK3AP1	NS	0.053589523	0.039151928	0.108196391	NS
PPP1R3A	NS	0.05359689	-0.039150723	0.10819998	NS
CACTIN	NS	0.053618821	-0.039147135	0.108232964	NS
LARP4B	NS	0.05374358	-0.03912675	0.108473487	NS
DPH1	NS	0.053798143	0.039117847	0.108572292	NS
SLC25A19	NS	0.053808553	-0.03911615	0.108581981	NS
HERC1	NS	0.053869163	0.03910627	0.1086703	NS
BCL7A	NS	0.053860857	-0.039107624	0.1086703	NS
ALX3	NS	0.053866645	-0.039106681	0.1086703	NS
DLG1	NS	0.053896356	-0.039101841	0.108713825	NS
FAM136A	NS	0.053921579	-0.039097734	0.108753368	NS
MRPL28	NS	0.053936557	-0.039095296	0.108772243	NS
CARD6	NS	0.053942329	-0.039094357	0.108772552	NS
AMPH	NS	0.053953803	-0.03909249	0.108784355	NS
MEPCE	NS	0.053977257	0.039088675	0.108808975	NS
AWAT1	NS	0.053976344	0.039088823	0.108808975	NS
RPP30	NS	0.053997918	-0.039085315	0.10883929	NS
CD1D	NS	0.054032223	0.039079738	0.108897096	NS
SCP2D1	NS	0.054072009	0.039073275	0.108965936	NS
ARL5B	NS	0.05412683	0.039064375	0.109065055	NS
CSNK1D	NS	0.054140524	0.039062153	0.109071381	NS
PLEKHS1	NS	0.054141239	-0.039062037	0.109071381	NS

GPR65	NS	0.054202556	0.039052094	0.109183544	NS
TARS3	NS	0.054210241	-0.039050848	0.109187661	NS
TCEANC	NS	0.054242752	-0.039045581	0.10923041	NS
NPIPB6	NS	0.054237275	0.039046468	0.10923041	NS
ADH1C	NS	0.054262444	-0.039042391	0.109258699	NS
BCAS3	NS	0.054294279	-0.039037237	0.109311427	NS
AFF4	NS	0.054317892	0.039033416	0.109315699	NS
MTCH2	NS	0.054312822	-0.039034237	0.109315699	NS
OR2T10	NS	0.054303516	0.039035742	0.109315699	NS
CHSY3	NS	0.054318991	0.039033238	0.109315699	NS
NT5DC2	NS	0.054397962	0.039020469	0.109463244	NS
ANAPC11	NS	0.054408722	-0.039018731	0.109473516	NS
EIF4A1	NS	0.054424446	0.03901619	0.109493772	NS
TOP3A	NS	0.054527738	0.038999518	0.109678781	NS
MIA	NS	0.054522256	0.039000403	0.109678781	NS
CCDC163	NS	0.054546713	-0.038996459	0.109705547	NS
IFITM10	NS	0.054554346	0.038995228	0.109709498	NS
NONO	NS	0.054626142	0.03898366	0.109842468	NS
DHX37	NS	0.054677723	-0.038975357	0.109911934	NS
TAF3	NS	0.054671227	0.038976402	0.109911934	NS
ZWILCH	NS	0.054676377	-0.038975573	0.109911934	NS
UFL1	NS	0.05472515	0.038967728	0.109995847	NS
SEL1L	NS	0.054736255	0.038965943	0.110002923	NS
CDRT15	NS	0.054740037	-0.038965335	0.110002923	NS
FGGY	NS	0.054762737	0.038961686	0.110037116	NS
ASAH1	NS	0.054829267	0.038951	0.110149487	NS
SMIM5	NS	0.054830043	-0.038950876	0.110149487	NS
PRMT9	NS	0.054862041	0.03894574	0.110202331	NS
TRIML2	NS	0.054872639	0.03894404	0.110212183	NS
ETNPPL	NS	0.054927728	0.038935206	0.110311382	NS
KRTAP5-2	NS	0.05506508	0.038913211	0.110575753	NS
PREX1	NS	0.055152195	0.038899285	0.110724229	NS
AGO3	NS	0.055164802	0.038897271	0.110724229	NS
SLC49A3	NS	0.055172186	-0.038896092	0.110724229	NS
BPGM	NS	0.055173341	-0.038895907	0.110724229	NS
ZFP90	NS	0.055147931	-0.038899966	0.110724229	NS
ZNF665	NS	0.05516894	0.03889661	0.110724229	NS
PTGDS	NS	0.055181545	-0.038894597	0.110729213	NS
ZNF200	NS	0.055234491	-0.038886146	0.11081248	NS
ANKRD52	NS	0.055232819	0.038886413	0.11081248	NS
CPLX2	NS	0.05526337	-0.038881539	0.110858928	NS
TSC22D1	NS	0.055273722	-0.038879888	0.110868203	NS
FGD6	NS	0.055310753	0.038873985	0.110930984	NS
NOXRED1	NS	0.055363361	-0.038865604	0.111024991	NS
DDX24	NS	0.055385621	0.03886206	0.111058126	NS
CHRND	NS	0.055421475	-0.038856354	0.111118508	NS
DGLUCY	NS	0.055452071	-0.038851487	0.111168336	NS
MAPKAP1	NS	0.055469048	-0.038848787	0.111190855	NS
GPR75	NS	0.05547504	0.038847835	0.11119135	NS
SLC37A3	NS	0.055488803	-0.038845647	0.111207422	NS
ZNF418	NS	0.05549743	-0.038844276	0.111213196	NS
PRDX1	NS	0.055626878	-0.038823723	0.111455922	NS
NBPF14	NS	0.055630072	0.038823216	0.111455922	NS
TDRD6	NS	0.055668137	-0.03881718	0.111520644	NS
HSF4	NS	0.05567473	-0.038816135	0.11152231	NS

ADGRL3	NS	0.055702542	-0.038811728	0.111554931	NS
FOXD4	NS	0.05569921	0.038812256	0.111554931	NS
ADGRG4	NS	0.055731088	-0.038807206	0.111600553	NS
OMA1	NS	0.055754163	-0.038803552	0.111635211	NS
INPP5E	NS	0.055814568	-0.038793994	0.111744598	NS
LINGO3	NS	0.05591657	-0.038777872	0.111937235	NS
OPTC	NS	0.055945591	-0.038773289	0.11198375	NS
ZFAT	NS	0.055991347	-0.038766069	0.112063749	NS
C11orf94	NS	0.056002433	0.03876432	0.112074348	NS
CDC34	NS	0.056025899	0.038760619	0.112109718	NS
CPSF2	NS	0.056106621	-0.038747899	0.112259639	NS
ELOA3DP	NS	0.056113154	0.03874687	0.112261107	NS
FGF1	NS	0.056132592	-0.038743809	0.112288389	NS
KCNA4	NS	0.056154361	-0.038740383	0.112320327	NS
C7orf50	NS	0.056329807	-0.038712808	0.112659615	NS
C20orf173	NS	0.056347172	-0.038710082	0.112682695	NS
TPPP2	NS	0.056352991	0.038709169	0.112682695	NS
SIT1	NS	0.056376952	-0.03870541	0.112718963	NS
SUSD5	NS	0.056415314	-0.038699394	0.112784013	NS
ZXDA	NS	0.056435153	0.038696285	0.112811774	NS
TICAM2	NS	0.056440856	-0.038695391	0.112811774	NS
NIM1K	NS	0.056655876	0.038661748	0.113218164	NS
ZNF724	NS	0.056718034	0.038652042	0.113330678	NS
INSIG1	NS	0.056764957	0.038644721	0.113412728	NS
IGF2BP3	NS	0.056789499	-0.038640894	0.113450051	NS
OR10K2	NS	0.056859827	-0.038629935	0.113578826	NS
ATP6V0D1	NS	0.056869193	0.038628476	0.113584291	NS
RNF135	NS	0.0568743	0.038627681	0.113584291	NS
HEXA	NS	0.056890467	0.038625164	0.113604856	NS
RHPN2	NS	0.056903422	0.038623147	0.113619005	NS
SRP19	NS	0.056931915	-0.038618713	0.113664173	NS
TIPARP	NS	0.056988222	0.038609956	0.113764853	NS
DOK5	NS	0.057023046	-0.038604544	0.113822634	NS
GOLGA8N	NS	0.057042063	0.038601589	0.113848852	NS
NOL7	NS	0.057051702	0.038600092	0.11385635	NS
PHACTR2	NS	0.057111041	-0.03859088	0.113963022	NS
SBF2	NS	0.057127855	0.038588271	0.113984822	NS
ACSM2A	NS	0.057149834	-0.038584862	0.114016923	NS
OSBPL8	NS	0.057166219	-0.038582321	0.114037859	NS
TMEM203	NS	0.057175191	-0.03858093	0.114044002	NS
ZNF500	NS	0.057270426	-0.038566175	0.114222191	NS
RNF40	NS	0.057301196	0.038561412	0.114271785	NS
HBEGF	NS	0.057318474	0.038558739	0.114282693	NS
C2orf74	NS	0.05735104	-0.038553701	0.114324071	NS
KRT31	NS	0.057443219	-0.038539456	0.11449603	NS
PFN1	NS	0.057491757	0.038531963	0.114580978	NS
C2orf80	NS	0.0575681	0.038520188	0.114721316	NS
PPIB	NS	0.057623429	-0.038511662	0.114819754	NS
WDR93	NS	0.057642766	0.038508684	0.114846462	NS
CDCP1	NS	0.057676659	0.038503466	0.114902162	NS
DGKG	NS	0.057756449	0.038491192	0.115049278	NS
RPL23A	NS	0.057788274	-0.0384863	0.115100827	NS
SEPTIN4	NS	0.05784908	-0.038476961	0.115210082	NS
SLC44A5	NS	0.057972383	-0.038458046	0.11544377	NS
FBXO3	NS	0.058017449	-0.038451141	0.115521628	NS

MS4A14	NS	0.058049979	0.03844616	0.115574511	NS
PRSS21	NS	0.058167021	-0.038428257	0.115795625	NS
FAM8A1	NS	0.058304445	-0.038407274	0.116057264	NS
HEATR1	NS	0.058334815	0.038402642	0.116105776	NS
MS4A4E	NS	0.058390339	-0.03839418	0.11620434	NS
ARAF	NS	0.058439989	0.038386618	0.116279237	NS
VTI1B	NS	0.058439633	-0.038386673	0.116279237	NS
CENPF	NS	0.058522361	-0.038374085	0.116431166	NS
SCHIP1	NS	0.058583361	-0.038364813	0.116540549	NS
POLR1A	NS	0.058645229	-0.038355417	0.116651634	NS
TM2D2	NS	0.05868263	-0.038349741	0.116714036	NS
FAM107B	NS	0.058727132	0.038342991	0.116790545	NS
GPRC5D	NS	0.058803322	-0.038331445	0.11693005	NS
TNFRSF25	NS	0.058834193	0.038326771	0.116979419	NS
MDGA2	NS	0.058975862	-0.038305344	0.117249054	NS
SGSM2	NS	0.059029582	0.03829723	0.117343802	NS
FOXO3	NS	0.059046014	0.038294749	0.117364412	NS
LIMCH1	NS	0.059090703	-0.038288006	0.117441179	NS
NUP98	NS	0.059109358	0.038285192	0.117466194	NS
CRHBP	NS	0.059166826	-0.038276529	0.117568328	NS
UCHL1	NS	0.05917452	0.038275369	0.117571548	NS
JPH3	NS	0.059240169	0.038265482	0.117689901	NS
COG2	NS	0.059272729	-0.038260582	0.117742502	NS
ATP5PO	NS	0.059314466	-0.038254304	0.117813319	NS
QKI	NS	0.059335104	0.038251201	0.117842219	NS
MRPL39	NS	0.059371897	-0.038245671	0.117903194	NS
MUSTN1	NS	0.059484596	-0.03822875	0.118102763	NS
LRIG1	NS	0.059524665	-0.038222741	0.118170197	NS
MFSD2B	NS	0.059542203	-0.038220111	0.11819289	NS
ARIH1	NS	0.059578822	0.038214624	0.118253453	NS
HACD1	NS	0.059667295	-0.038201376	0.118406157	NS
MROH7	NS	0.059667992	-0.038201272	0.118406157	NS
TUSC2	NS	0.059725078	-0.038192733	0.11850729	NS
USP48	NS	0.059752171	-0.038188683	0.118548895	NS
STK3	NS	0.059804875	-0.038180808	0.118641299	NS
TTC33	NS	0.059851668	-0.038173822	0.118721959	NS
DPYD	NS	0.059895641	0.03816726	0.118797011	NS
MEF2A	NS	0.059914196	0.038164493	0.118821636	NS
C2orf78	NS	0.059933302	0.038161644	0.118847352	NS
PDCD7	NS	0.059939557	0.038160711	0.118847579	NS
PASK	NS	0.059959595	0.038157725	0.118875133	NS
IKZF3	NS	0.060030463	-0.038147168	0.119003445	NS
S100A8	NS	0.060096537	-0.038137335	0.119122229	NS
CBL	NS	0.060125073	0.038133091	0.11916659	NS
HSD3B2	NS	0.060177732	-0.038125263	0.119258746	NS
GSTM4	NS	0.060207737	0.038120806	0.119305995	NS
GIMAP4	NS	0.060244309	0.038115375	0.119366245	NS
JADE1	NS	0.060340993	-0.038101032	0.119545575	NS
KISS1	NS	0.060385818	0.038094388	0.119622137	NS
CLEC12A	NS	0.060409797	-0.038090836	0.119654066	NS
PCCA	NS	0.0604143	-0.038090169	0.119654066	NS
ESRRB	NS	0.060453127	-0.03808442	0.119714122	NS
ZNF208	NS	0.060456992	-0.038083848	0.119714122	NS
UGT1A1	NS	0.060560788	0.038068495	0.119907387	NS
ANKRD54	NS	0.060606357	0.038061762	0.11998105	NS

BCAP31	NS	0.06061039	0.038061166	0.11998105	NS
CPN1	NS	0.060722804	0.038044574	0.120191286	NS
OR2V2	NS	0.060739472	0.038042116	0.120211985	NS
METTL27	NS	0.060753218	0.038040089	0.120226897	NS
FGFR2	NS	0.060764893	0.038038368	0.120237708	NS
FCGR2B	NS	0.060778126	-0.038036418	0.120251601	NS
DDX46	NS	0.060836843	-0.038027768	0.120355472	NS
SLC25A24	NS	0.060920022	-0.038015527	0.120490826	NS
SLC12A4	NS	0.060923937	0.038014951	0.120490826	NS
FMC1	NS	0.060915981	-0.038016121	0.120490826	NS
SMCO1	NS	0.060977781	0.038007034	0.120584994	NS
PCDH11X	NS	0.061031509	0.037999141	0.120678914	NS
C14orf93	NS	0.061039875	-0.037997912	0.120683128	NS
SPRTN	NS	0.061095574	0.037989736	0.120780915	NS
SPSB3	NS	0.061131481	0.037984468	0.120839557	NS
LYPLAL1	NS	0.061148648	-0.037981951	0.120861149	NS
VCY	NS	0.061155785	-0.037980904	0.120862913	NS
PFKM	NS	0.061250835	-0.037966977	0.121038403	NS
CBR1	NS	0.061325025	-0.037956119	0.121160271	NS
CLPSL1	NS	0.061324746	-0.03795616	0.121160271	NS
C10orf67	NS	0.061337939	-0.03795423	0.121173417	NS
YTHDC1	NS	0.06135304	0.037952022	0.121182256	NS
UCKL1	NS	0.061354934	-0.037951745	0.121182256	NS
WASHC2C	NS	0.061608545	-0.037914724	0.121658335	NS
OR51B5	NS	0.061653173	-0.037908223	0.121734042	NS
NUDT4B	NS	0.061664026	0.037906642	0.121743052	NS
MGRN1	NS	0.06168953	0.037902929	0.121780983	NS
BCL2L2	NS	0.061731089	-0.037896881	0.121844934	NS
OR10W1	NS	0.061734515	-0.037896383	0.121844934	NS
ITGB3	NS	0.061788813	0.037888486	0.121927237	NS
CT45A3	NS	0.061796509	-0.037887368	0.121929992	NS
YTHDF3	NS	0.061843976	0.03788047	0.121996914	NS
SPDYE2	NS	0.061849335	0.037879692	0.121996914	NS
KIR3DL2	NS	0.061842496	-0.037880685	0.121996914	NS
ASB9	NS	0.061859462	-0.037878221	0.122004458	NS
PARP2	NS	0.061887384	-0.037874166	0.122034657	NS
LMAN2	NS	0.061886818	-0.037874248	0.122034657	NS
FURIN	NS	0.061920711	0.037869329	0.122087937	NS
CFAP20	NS	0.061963039	0.037863188	0.122158952	NS
NCK2	NS	0.061970377	0.037862124	0.122160976	NS
SEC23IP	NS	0.061988595	0.037859482	0.122184444	NS
SSBP2	NS	0.062099132	0.037843467	0.122365339	NS
NSA2	NS	0.062099335	0.037843438	0.122365339	NS
ALKAL1	NS	0.062094399	-0.037844153	0.122365339	NS
POLR2J3	NS	0.062127443	0.037839369	0.122408265	NS
DLG3	NS	0.062168237	-0.037833468	0.122476172	NS
CYTH4	NS	0.062267188	0.037819165	0.12265243	NS
BRDT	NS	0.062270377	0.037818704	0.12265243	NS
GLIS3	NS	0.062323474	-0.037811037	0.122744523	NS
PRSS8	NS	0.062362043	-0.037805472	0.122807987	NS
CIB2	NS	0.062387841	0.03780175	0.122834986	NS
OTUB1	NS	0.062388445	0.037801663	0.122834986	NS
KCNK10	NS	0.062427627	-0.037796014	0.12288713	NS
PCMTD1	NS	0.062424941	0.037796401	0.12288713	NS
SYP	NS	0.06263026	-0.037766845	0.123270955	NS

EIF3F	NS	0.06263535	0.037766113	0.123270955	NS
RBIS	NS	0.062642525	-0.037765082	0.12327254	NS
SPOPL	NS	0.062664891	0.037761868	0.123290534	NS
TRIM11	NS	0.062669798	0.037761163	0.123290534	NS
ZFYVE28	NS	0.06267427	0.03776052	0.123290534	NS
MLLT6	NS	0.062677147	0.037760107	0.123290534	NS
C11orf42	NS	0.06268609	-0.037758822	0.123295596	NS
LIMS4	NS	0.06272938	-0.037752605	0.123368205	NS
EXOC6	NS	0.062887429	0.037729938	0.123665613	NS
CHAMP1	NS	0.062893381	-0.037729085	0.123665613	NS
POLR2J2	NS	0.062938679	0.037722598	0.12374211	NS
DBP	NS	0.062993036	-0.037714818	0.123832853	NS
PDE4C	NS	0.063004026	0.037713245	0.123832853	NS
PXK	NS	0.063003011	0.037713391	0.123832853	NS
BCAP29	NS	0.063010601	0.037712305	0.1238332	NS
CCR1	NS	0.063046295	0.037707201	0.12389077	NS
FNBP4	NS	0.063073864	0.03770326	0.123932364	NS
MFN1	NS	0.063107776	0.037698414	0.123977651	NS
ARL16	NS	0.063109723	-0.037698136	0.123977651	NS
ZNF280C	NS	0.063225703	-0.03768158	0.124180284	NS
SLC5A6	NS	0.063221967	-0.037682113	0.124180284	NS
TSHZ3	NS	0.063266888	0.037675707	0.124248569	NS
BAK1	NS	0.063318749	0.037668316	0.124299971	NS
BNIP3L	NS	0.063314332	0.037668946	0.124299971	NS
ALDH5A1	NS	0.063309364	0.037669654	0.124299971	NS
ASCC2	NS	0.063333041	-0.03766628	0.124315418	NS
ARSD	NS	0.063378633	-0.037659788	0.124343003	NS
BCL11B	NS	0.063372084	-0.037660721	0.124343003	NS
SKP2	NS	0.063379214	-0.037659706	0.124343003	NS
MEAF6	NS	0.063377196	-0.037659993	0.124343003	NS
BRCC3	NS	0.063359925	-0.037662452	0.124343003	NS
LONRF3	NS	0.06338701	0.037658596	0.124345694	NS
PLCB4	NS	0.06347759	0.037645711	0.124510765	NS
GNAL	NS	0.063496128	0.037643075	0.124534507	NS
LACRT	NS	0.063540639	0.037636751	0.124609181	NS
PRR11	NS	0.063563712	-0.037633473	0.124629175	NS
SREK1IP1	NS	0.0635637	-0.037633475	0.124629175	NS
TMEM17	NS	0.063612774	0.037626508	0.124712738	NS
ROS1	NS	0.063719214	-0.037611413	0.124908761	NS
NOP58	NS	0.063742097	-0.03760817	0.124940966	NS
ALPK1	NS	0.063759842	0.037605656	0.124963092	NS
FYB1	NS	0.063778592	0.037603001	0.124974532	NS
INHBA	NS	0.063776612	0.037603281	0.124974532	NS
CTBP2	NS	0.063790152	0.037601364	0.124984531	NS
GAGE2A	NS	0.063882474	0.037588299	0.12515275	NS
MCCC1	NS	0.063894581	-0.037586587	0.125163801	NS
SPDYE2B	NS	0.063972593	0.037575561	0.125298536	NS
RBM20	NS	0.063976308	0.037575037	0.125298536	NS
DCLRE1C	NS	0.064033365	0.03756698	0.125397593	NS
COQ8A	NS	0.064051602	0.037564406	0.125420618	NS
TMOD3	NS	0.064072146	0.037561507	0.125448155	NS
TCTN2	NS	0.064100764	0.037557471	0.125491492	NS
KCTD17	NS	0.064161891	0.037548854	0.125566673	NS
CD52	NS	0.064165115	-0.037548399	0.125566673	NS
ZNF709	NS	0.064161593	-0.037548896	0.125566673	NS

TBC1D3	NS	0.064146079	0.037551082	0.125566673	NS
UBE2L3	NS	0.064179734	0.03754634	0.125582584	NS
TRMT61B	NS	0.064269792	-0.037533659	0.125742807	NS
SLC6A9	NS	0.064274609	0.037532981	0.125742807	NS
VPS37A	NS	0.064287006	0.037531237	0.125754349	NS
APOBEC3C	NS	0.064329138	0.037525311	0.125811337	NS
TIFAB	NS	0.064325353	-0.037525843	0.125811337	NS
BACH2	NS	0.064344998	-0.037523081	0.12582964	NS
COL25A1	NS	0.064357553	-0.037521316	0.125841478	NS
UGDH	NS	0.064397587	-0.03751569	0.125868987	NS
LAMA5	NS	0.064380816	0.037518047	0.125868987	NS
CHMP7	NS	0.064391159	0.037516593	0.125868987	NS
APOA2	NS	0.064397633	-0.037515684	0.125868987	NS
NRXN2	NS	0.064434206	0.037510547	0.125915042	NS
ARID3A	NS	0.064431683	0.037510901	0.125915042	NS
RAB11FIP3	NS	0.064491333	-0.037502528	0.126001236	NS
DHRS4L2	NS	0.064487971	-0.037502999	0.126001236	NS
METTL2B	NS	0.064583136	-0.037489653	0.12615513	NS
CCL5	NS	0.064578502	0.037490303	0.12615513	NS
SEPTIN9	NS	0.064597143	0.03748769	0.126169758	NS
VGLL4	NS	0.064628847	0.037483248	0.126218945	NS
ALKBH7	NS	0.064647829	-0.037480589	0.126243279	NS
TEKT3	NS	0.064671384	0.037477291	0.126276537	NS
RENBP	NS	0.064729496	-0.037469158	0.126377258	NS
INO80D	NS	0.064761591	0.037464669	0.126427167	NS
COMMD10	NS	0.064786909	0.03746113	0.126459756	NS
PRICKLE4	NS	0.064791352	-0.037460509	0.126459756	NS
HCRT	NS	0.064808692	0.037458085	0.126480847	NS
SMIM35	NS	0.06482203	-0.037456221	0.126494123	NS
HIPK3	NS	0.064843234	0.037453258	0.126515138	NS
UBE3A	NS	0.064845871	0.03745289	0.126515138	NS
KIF5A	NS	0.064858574	0.037451116	0.126527168	NS
ATN1	NS	0.064901484	0.037445124	0.126598118	NS
CD36	NS	0.065011612	0.037429762	0.126800158	NS
CCDC17	NS	0.065088126	0.037419101	0.126923813	NS
PTP4A3	NS	0.06515193	-0.03741022	0.127035435	NS
TJP1	NS	0.065206629	-0.037402611	0.12712659	NS
RHOT1	NS	0.065211816	0.03740189	0.12712659	NS
CADM3	NS	0.065290268	-0.037390987	0.127253895	NS
TUBA1B	NS	0.06534406	-0.037383517	0.127345916	NS
SLC25A51	NS	0.065383479	0.037378047	0.127409909	NS
NOMO3	NS	0.065441507	-0.037369999	0.127509451	NS
H3-4	NS	0.065447737	-0.037369135	0.127509451	NS
STRAP	NS	0.065518924	0.037359271	0.127622452	NS
CT83	NS	0.065516256	-0.03735964	0.127622452	NS
DNMT3A	NS	0.065532405	-0.037357404	0.127635865	NS
E4F1	NS	0.06558396	-0.037350267	0.127710577	NS
C1orf141	NS	0.065581778	-0.037350569	0.127710577	NS
CDHR2	NS	0.065621523	0.037345069	0.127770868	NS
PURA	NS	0.065678678	-0.037337166	0.127861319	NS
WWP2	NS	0.065681189	0.037336819	0.127861319	NS
OR52D1	NS	0.065694876	-0.037334927	0.127875103	NS
KCNC4	NS	0.065722224	-0.037331149	0.127915471	NS
FSCN3	NS	0.06576633	-0.037325057	0.127988445	NS
TJP2	NS	0.065785288	-0.03732244	0.128012469	NS

TIGD1	NS	0.065803671	-0.037319902	0.128035369	NS
KATNAL1	NS	0.065831845	0.037316014	0.128064441	NS
TENM3	NS	0.065829938	0.037316278	0.128064441	NS
DNAI3	NS	0.065863534	-0.037311643	0.128113211	NS
HOXA1	NS	0.065932753	-0.037302102	0.128234965	NS
RHEX	NS	0.065961231	-0.037298178	0.128277462	NS
RTL8C	NS	0.065990386	0.037294163	0.128321268	NS
C11orf65	NS	0.066129981	0.037274959	0.128579801	NS
KDM8	NS	0.066157387	-0.037271193	0.128620166	NS
MAN2B2	NS	0.066191451	-0.037266513	0.128660546	NS
PCBD1	NS	0.066186919	-0.037267136	0.128660546	NS
POLR1B	NS	0.066298581	-0.037251809	0.128842905	NS
HMOX1	NS	0.066312095	0.037249955	0.128856231	NS
S100B	NS	0.066344812	-0.03724547	0.128906864	NS
SLA2	NS	0.066382214	0.037240344	0.128958366	NS
STX6	NS	0.066397969	-0.037238185	0.128958366	NS
PABIR2	NS	0.06639178	0.037239033	0.128958366	NS
TOMM5	NS	0.066390152	-0.037239256	0.128958366	NS
ESD	NS	0.066433279	-0.037233349	0.129014002	NS
DNAJB13	NS	0.066454049	0.037230505	0.129041389	NS
TREX2	NS	0.066486926	-0.037226005	0.12909228	NS
FAM9C	NS	0.066505998	0.037223396	0.129116359	NS
MPHOSPH8	NS	0.066536818	-0.03721918	0.129163238	NS
RPL35	NS	0.066627417	-0.037206797	0.129319807	NS
HSBP1	NS	0.066630835	0.03720633	0.129319807	NS
MMACHC	NS	0.06665395	-0.037203173	0.129340304	NS
ARL6IP1	NS	0.066729783	0.037192822	0.129472904	NS
RTEL1	NS	0.066751734	-0.037189827	0.129502513	NS
RPSA	NS	0.066822034	-0.037180243	0.129625907	NS
TMEM255B	NS	0.066889204	-0.037171093	0.129743203	NS
DEAF1	NS	0.067015772	-0.037153872	0.12997568	NS
CHCHD4	NS	0.067022495	-0.037152958	0.129975696	NS
APEX1	NS	0.067055424	0.037148482	0.130026526	NS
H2AC19	NS	0.067080015	0.037145141	0.13006118	NS
TENM1	NS	0.067132816	-0.037137971	0.130150519	NS
KIAA1143	NS	0.067190015	0.037130208	0.130248366	NS
SLC36A1	NS	0.067213729	0.037126991	0.130274145	NS
TMEM41B	NS	0.067216775	-0.037126578	0.130274145	NS
PDE6A	NS	0.067256918	-0.037121136	0.130325848	NS
GOLGA8Q	NS	0.067253477	0.037121602	0.130325848	NS
TMEM129	NS	0.067346507	-0.037108998	0.130486385	NS
GART	NS	0.067371771	0.037105578	0.130522271	NS
CHD7	NS	0.067431073	0.037097553	0.130624085	NS
PDE5A	NS	0.067465068	0.037092956	0.13067686	NS
ASH1L	NS	0.067507023	0.037087285	0.130745041	NS
RTKN	NS	0.067521626	-0.037085311	0.130760239	NS
UPRT	NS	0.067545202	0.037082126	0.130792811	NS
FAM83A	NS	0.067604823	-0.037074076	0.130895163	NS
SPAG4	NS	0.067639327	0.03706942	0.13093687	NS
SMC6	NS	0.067646657	-0.037068431	0.13093687	NS
PTEN	NS	0.067644963	0.037068659	0.13093687	NS
SUPT3H	NS	0.067827224	0.037044097	0.131273248	NS
ZDHHC4	NS	0.067858541	-0.037039882	0.131302757	NS
RSAD1	NS	0.067869606	0.037038393	0.131302757	NS
CTDSP1	NS	0.067867452	0.037038683	0.131302757	NS

CASTOR2	NS	0.067865318	-0.03703897	0.131302757	NS
EDDM3B	NS	0.067921096	-0.037031468	0.13138924	NS
OGFR	NS	0.067958161	0.037026485	0.131445708	NS
RNPC3	NS	0.067963869	-0.037025718	0.131445708	NS
HMGXB4	NS	0.067977169	0.03702393	0.131458296	NS
PITHD1	NS	0.068069445	-0.037011538	0.131613813	NS
ZNF558	NS	0.068071186	0.037011304	0.131613813	NS
ANGEL1	NS	0.06810987	-0.037006114	0.131675454	NS
CBLIF	NS	0.068125639	-0.037003998	0.131692786	NS
HEY1	NS	0.068137674	0.037002384	0.131702898	NS
MAGEA6	NS	0.068266721	0.036985091	0.131939157	NS
TACC3	NS	0.068338817	-0.036975441	0.132063787	NS
POLRMT	NS	0.068344852	-0.036974634	0.132063787	NS
DCTN2	NS	0.068378254	0.036970166	0.132115141	NS
MTF2	NS	0.068421974	-0.036964322	0.132186418	NS
TBC1D3C	NS	0.068434235	0.036962683	0.13219691	NS
H2BU1	NS	0.068456675	-0.036959685	0.132213871	NS
GAGE1	NS	0.068450197	0.03696055	0.132213871	NS
CALB1	NS	0.068573483	0.03694409	0.132426253	NS
VAMP2	NS	0.06859083	0.036941776	0.13244654	NS
SLC12A8	NS	0.068632449	0.036936227	0.132513683	NS
CC2D1A	NS	0.068657114	-0.036932939	0.132548086	NS
TPR	NS	0.068724077	-0.036924018	0.132658601	NS
IFNA7	NS	0.068728066	-0.036923486	0.132658601	NS
FOXN3	NS	0.068799317	0.036914003	0.132782887	NS
SLC66A1	NS	0.068888791	0.036902104	0.132921049	NS
CLEC4G	NS	0.068887788	0.036902238	0.132921049	NS
GH1	NS	0.068891504	-0.036901744	0.132921049	NS
SMUG1	NS	0.068966521	-0.036891778	0.133052524	NS
CPT1A	NS	0.068995445	0.036887938	0.133095059	NS
CD69	NS	0.069070953	0.03687792	0.13321569	NS
CNN3	NS	0.06907214	-0.036877762	0.13321569	NS
TEX14	NS	0.069078626	0.036876902	0.13321569	NS
CCDC86	NS	0.069126808	0.036870515	0.133295328	NS
FRMD1	NS	0.06917731	0.036863823	0.133379422	NS
SEC14L6	NS	0.069186357	0.036862625	0.133383578	NS
SHPK	NS	0.06924902	0.036854329	0.133491089	NS
RFLNB	NS	0.069266318	0.03685204	0.133511138	NS
KBTBD6	NS	0.069376702	-0.036837445	0.133708928	NS
GTF2I	NS	0.069382748	0.036836646	0.133708928	NS
SNCAIP	NS	0.069411979	-0.036832784	0.133751942	NS
RHOC	NS	0.069431348	0.036830226	0.133775948	NS
CHRM3	NS	0.06946298	-0.03682605	0.133823573	NS
CLN6	NS	0.069473188	-0.036824702	0.133829919	NS
DIPK1B	NS	0.06954053	-0.036815817	0.133946314	NS
COX10	NS	0.069597881	-0.036808256	0.134043441	NS
ELP4	NS	0.069689974	-0.036796125	0.134207456	NS
DGUOK	NS	0.069706331	-0.036793972	0.13421225	NS
PHP3-ACAD	NS	0.069705671	0.036794059	0.13421225	NS
TPH1	NS	0.069718516	0.036792368	0.13422236	NS
BHLHA15	NS	0.069769015	0.036785724	0.134306222	NS
NR2F6	NS	0.069778127	0.036784525	0.134310405	NS
ADAM12	NS	0.069799527	0.036781711	0.134324879	NS
NDUFAF8	NS	0.069794421	-0.036782383	0.134324879	NS
IFNW1	NS	0.069813687	-0.03677985	0.134338774	NS

AQP3	NS	0.069916881	-0.036766292	0.134510599	NS
EIF4EBP3	NS	0.069915102	-0.036766525	0.134510599	NS
ANTXRL	NS	0.069999916	0.036755394	0.134656965	NS
PCF11	NS	0.070080251	0.036744862	0.134784711	NS
TMEM119	NS	0.070078601	0.036745078	0.134784711	NS
CIT	NS	0.070132367	0.036738034	0.134869248	NS
PTCHD4	NS	0.070138141	0.036737278	0.134869248	NS
KLHL2	NS	0.070207598	0.036728185	0.134989398	NS
MAD2L1BP	NS	0.070242847	0.036723573	0.135043757	NS
FRS3	NS	0.070265577	-0.036720601	0.135061663	NS
DACT1	NS	0.070266116	0.03672053	0.135061663	NS
CDY2B	NS	0.070279316	-0.036718804	0.135066101	NS
RAX	NS	0.070282381	0.036718403	0.135066101	NS
DIXDC1	NS	0.070300987	0.036715971	0.135088445	NS
STPG4	NS	0.070312647	-0.036714447	0.135097438	NS
ANXA7	NS	0.070335338	0.036711482	0.135114212	NS
NUTM2E	NS	0.070333363	-0.03671174	0.135114212	NS
INAFM1	NS	0.070344865	0.036710237	0.135119104	NS
LYRM1	NS	0.070361468	0.036708068	0.135132398	NS
ACTR8	NS	0.070365749	-0.036707509	0.135132398	NS
SAR1B	NS	0.070376365	0.036706123	0.135139376	NS
TOM1L1	NS	0.070384335	-0.036705082	0.135141274	NS
HRH4	NS	0.070440359	0.036697768	0.135235429	NS
TRIM73	NS	0.070454349	0.036695943	0.135248872	NS
PRXL2A	NS	0.070490079	-0.036691281	0.135304044	NS
MIPOL1	NS	0.070504937	0.036689344	0.135319143	NS
SRGAP3	NS	0.070548336	-0.036683686	0.135389014	NS
KRT222	NS	0.070604255	0.0366764	0.135482895	NS
ATP11B	NS	0.070617409	0.036674687	0.135494704	NS
BTG3	NS	0.070641098	-0.036671602	0.135526722	NS
ZNF260	NS	0.070702656	0.03666359	0.135631378	NS
WDR25	NS	0.070722353	0.036661028	0.13565572	NS
H4C5	NS	0.07076201	-0.03665587	0.135718339	NS
MRPS5	NS	0.070770942	-0.036654709	0.135722021	NS
GYS1	NS	0.070846172	-0.036644934	0.135852833	NS
CRNN	NS	0.070860228	-0.036643108	0.135866328	NS
BZW1	NS	0.070947125	0.036631828	0.136019468	NS
USP17L24	NS	0.070957296	-0.036630509	0.136025494	NS
CHPT1	NS	0.071053898	0.036617985	0.136197193	NS
DOK3	NS	0.07106518	0.036616523	0.136204833	NS
WIF1	NS	0.071071958	-0.036615645	0.136204833	NS
RIPOR2	NS	0.071196921	-0.036599467	0.136430809	NS
TMEM150A	NS	0.071229019	0.036595316	0.136478805	NS
ARMC6	NS	0.071319392	-0.036583635	0.136638439	NS
UFM1	NS	0.071622585	0.036544536	0.137205737	NS
AREL1	NS	0.071668303	0.036538652	0.137266146	NS
TAF6	NS	0.071683402	0.03653671	0.137274402	NS
GPS2	NS	0.071686798	0.036536273	0.137274402	NS
TESK2	NS	0.071714613	-0.036532695	0.137314082	NS
DKK3	NS	0.071755133	-0.036527485	0.137368863	NS
TCAIM	NS	0.071757418	-0.036527192	0.137368863	NS
ARL5A	NS	0.071809875	0.036520451	0.13745569	NS
ADAM19	NS	0.07187193	0.036512482	0.137560871	NS
KIF22	NS	0.071881634	-0.036511237	0.13756584	NS
MTMR9	NS	0.07190782	-0.036507876	0.137602349	NS

IL12RB2	NS	0.071928338	-0.036505244	0.137614403	NS
PRB4	NS	0.071925957	-0.036505549	0.137614403	NS
KDM4F	NS	0.071950697	-0.036502376	0.137643574	NS
CARD8	NS	0.071963845	0.03650069	0.137655123	NS
NADK2	NS	0.071978812	-0.036498771	0.137670147	NS
HS3ST3A1	NS	0.071987217	-0.036497693	0.137672619	NS
SEC11C	NS	0.072041407	-0.036490748	0.137762643	NS
LRTM2	NS	0.072086987	0.03648491	0.137822573	NS
DAO	NS	0.072121857	0.036480445	0.137848392	NS
NRDE2	NS	0.07211534	0.03648128	0.137848392	NS
CXorf66	NS	0.072117392	0.036481017	0.137848392	NS
OR10V1	NS	0.072138879	-0.036478267	0.137867313	NS
DMC1	NS	0.072152772	0.036476489	0.137880251	NS
CWC27	NS	0.072175989	-0.036473518	0.137897388	NS
UQCRB	NS	0.072171093	-0.036474145	0.137897388	NS
GRK3	NS	0.072227224	-0.036466966	0.137981657	NS
PCDH10	NS	0.07223907	-0.036465452	0.137990669	NS
SMARCD1	NS	0.072252339	0.036463756	0.138002395	NS
RBFOX3	NS	0.072415623	0.036442905	0.138300621	NS
KLF6	NS	0.072457958	0.036437505	0.13832688	NS
SLC22A11	NS	0.072447519	0.036438836	0.13832688	NS
GNB2	NS	0.072438546	0.036439981	0.13832688	NS
C9orf131	NS	0.072452412	0.036438212	0.13832688	NS
MYH8	NS	0.072506315	0.03643134	0.138405545	NS
ARHGAP35	NS	0.072514679	-0.036430274	0.138407861	NS
SRP14	NS	0.072558485	-0.036424693	0.138477818	NS
ENAM	NS	0.072664939	-0.036411142	0.138667314	NS
KATNAL2	NS	0.072717831	-0.036404415	0.138754568	NS
OR4D1	NS	0.072742489	0.03640128	0.138787936	NS
IFNAR2	NS	0.072794766	0.036394637	0.138873988	NS
USP42	NS	0.072824104	0.036390911	0.138916266	NS
CMTM7	NS	0.072926735	-0.036377885	0.139098332	NS
C11orf21	NS	0.072963732	0.036373193	0.139155188	NS
DNAJC13	NS	0.073012804	-0.036366973	0.139235057	NS
HCRTR1	NS	0.073021325	0.036365893	0.139237589	NS
PLEKHA3	NS	0.07307466	0.036359137	0.139311841	NS
PRKX	NS	0.073073864	0.036359238	0.139311841	NS
NPY2R	NS	0.073124473	-0.03635283	0.139393077	NS
NIT2	NS	0.073133585	-0.036351677	0.139396719	NS
PBXIP1	NS	0.073173264	-0.036346657	0.139458615	NS
ZBED3	NS	0.073182601	0.036345476	0.139462679	NS
NUP155	NS	0.073203846	-0.036342789	0.139475701	NS
OR51L1	NS	0.073197816	-0.036343551	0.139475701	NS
UBE2O	NS	0.073214734	0.036341412	0.139482715	NS
PAPLN	NS	0.073245051	-0.036337579	0.139522708	NS
SLC39A6	NS	0.073264559	0.036335114	0.139522708	NS
FSIP1	NS	0.07325699	0.036336071	0.139522708	NS
IER2	NS	0.073261015	0.036335562	0.139522708	NS
PEX14	NS	0.073325874	0.036327368	0.139625737	NS
CLTRN	NS	0.073343628	0.036325127	0.139645806	NS
ERC1	NS	0.073407692	0.036317041	0.139754036	NS
ANKRD44	NS	0.073421943	-0.036315243	0.13976742	NS
DDX17	NS	0.073531833	0.036301389	0.139962844	NS
SGK1	NS	0.073582674	0.036294985	0.140045845	NS
DHRS13	NS	0.073606744	-0.036291955	0.140077882	NS

MMP21	NS	0.073642361	-0.036287472	0.14011811	NS
LGALS9C	NS	0.073635833	-0.036288294	0.14011811	NS
NAGS	NS	0.073750201	-0.03627391	0.140295713	NS
RNF123	NS	0.073745953	0.036274444	0.140295713	NS
SLFN14	NS	0.073813424	-0.036265966	0.14038839	NS
AKAP6	NS	0.073834548	-0.036263313	0.140414767	NS
KCNN1	NS	0.073853586	0.036260923	0.140431921	NS
EPC1	NS	0.073858078	0.036260359	0.140431921	NS
IFITM3	NS	0.073979284	0.036245153	0.140648563	NS
RPAP2	NS	0.074054164	0.036235769	0.140777097	NS
TAF1	NS	0.074067806	0.036234061	0.140777774	NS
FAM133B	NS	0.074069066	0.036233903	0.140777774	NS
PDE8A	NS	0.074141796	0.036224797	0.14090217	NS
TMEM74B	NS	0.074159743	-0.036222552	0.14092244	NS
CRYBG3	NS	0.074178355	-0.036220223	0.140943971	NS
SFMBT1	NS	0.074219468	-0.036215081	0.140995452	NS
FIBP	NS	0.074220018	-0.036215013	0.140995452	NS
KRT3	NS	0.074280706	0.036207427	0.141096893	NS
FANCA	NS	0.074649566	-0.036161429	0.141783633	NS
TTLL3	NS	0.074703147	0.036154763	0.141871478	NS
MANBA	NS	0.074741851	0.03614995	0.141913016	NS
SLC44A3	NS	0.074738987	-0.036150306	0.141913016	NS
CMKLR2	NS	0.074747014	0.036149308	0.141913016	NS
OR6F1	NS	0.074781177	-0.036145062	0.141963954	NS
OPN4	NS	0.074788539	0.036144147	0.141964006	NS
CARS2	NS	0.074844164	0.036137237	0.142051299	NS
SLC66A3	NS	0.074849204	-0.036136611	0.142051299	NS
ZNF304	NS	0.074870418	-0.036133977	0.142077628	NS
PKNOX1	NS	0.07492434	0.036127285	0.142166016	NS
DEF8	NS	0.074969689	-0.03612166	0.14223812	NS
FST	NS	0.074996191	0.036118373	0.142274455	NS
PRAMEF9	NS	0.075031152	-0.03611404	0.14232683	NS
HNRNPA0	NS	0.075081268	-0.03610783	0.14240794	NS
SULF1	NS	0.075155838	-0.036098598	0.142521446	NS
TBC1D3I	NS	0.075154855	0.036098719	0.142521446	NS
MARVELD3	NS	0.075175685	-0.036096141	0.142545118	NS
CACNA1S	NS	0.075219804	-0.036090684	0.142614803	NS
LPIN2	NS	0.075240616	0.03608811	0.14264029	NS
SMAP2	NS	0.075276928	0.036083621	0.142667211	NS
REXO4	NS	0.075267287	-0.036084812	0.142667211	NS
EVA1A	NS	0.07530118	-0.036080623	0.142699201	NS
STARD8	NS	0.075323535	0.036077861	0.14270195	NS
KIF21A	NS	0.075319237	-0.036078392	0.14270195	NS
OTX2	NS	0.075324748	-0.036077712	0.14270195	NS
BLACAT1	NS	0.075354093	0.036074087	0.142743573	NS
KCTD11	NS	0.075429061	0.036064832	0.142871602	NS
AADACL2	NS	0.075447616	-0.036062543	0.142892766	NS
CREBL2	NS	0.075465603	0.036060324	0.142912847	NS
ENDOV	NS	0.07547688	0.036058933	0.14292022	NS
NDUFA6	NS	0.075489433	-0.036057385	0.142930008	NS
PARP9	NS	0.075527852	0.036052648	0.142975971	NS
GABPB2	NS	0.075528482	-0.03605257	0.142975971	NS
THOC1	NS	0.075588737	-0.036045145	0.143076042	NS
FBRSL1	NS	0.075650325	0.036037561	0.143157907	NS
GJB2	NS	0.075645431	-0.036038163	0.143157907	NS

TK1	NS	0.075661572	-0.036036176	0.143157907	NS
C1orf100	NS	0.075656191	-0.036036839	0.143157907	NS
SCYGR8	NS	0.075704366	0.03603091	0.143210878	NS
PDE4B	NS	0.075780881	0.0360215	0.143341614	NS
PAXIP1	NS	0.075811979	0.036017678	0.143372416	NS
PRAMEF25	NS	0.075806078	0.036018403	0.143372416	NS
LMCD1	NS	0.075823763	-0.03601623	0.143380691	NS
DUT	NS	0.075842812	0.036013889	0.143393255	NS
NARS1	NS	0.075845223	-0.036013593	0.143393255	NS
KHK	NS	0.075930997	-0.036003061	0.143541399	NS
INTS7	NS	0.075988402	-0.035996018	0.14363589	NS
NT5C	NS	0.076037976	0.035989939	0.143715562	NS
CFAP298	NS	0.076052456	-0.035988164	0.143728894	NS
TMEM39B	NS	0.076063217	0.035986845	0.143735197	NS
HNRNPR	NS	0.076084874	-0.035984191	0.143762088	NS
UBE2S	NS	0.076236089	0.035965677	0.144033747	NS
CTNND2	NS	0.076309937	0.035956647	0.144159198	NS
H3C2	NS	0.07637889	-0.035948221	0.144275378	NS
DKK1	NS	0.076405931	0.035944919	0.144312373	NS
IL3	NS	0.076432872	-0.035941629	0.144349172	NS
HMGCL	NS	0.076455381	0.035938882	0.144377595	NS
VPS26A	NS	0.076521729	0.035930787	0.144488789	NS
WDR1	NS	0.076569218	0.035924997	0.144564357	NS
TSG101	NS	0.076583803	0.035923219	0.144577791	NS
CEP78	NS	0.076643271	-0.035915973	0.144675947	NS
AKAP10	NS	0.076721414	0.035906459	0.144809332	NS
DDX19A	NS	0.076824994	0.03589386	0.144990696	NS
SOCS6	NS	0.076889799	0.035885985	0.145098854	NS
ELAVL1	NS	0.076898361	0.035884945	0.145100864	NS
SLC33A1	NS	0.07691026	0.035883499	0.145109172	NS
SNAP91	NS	0.076945304	-0.035879244	0.14516114	NS
PPP5C	NS	0.076964604	-0.035876901	0.1451834	NS
CA12	NS	0.077005024	-0.035871995	0.14521719	NS
EXPH5	NS	0.076996896	0.035872982	0.14521719	NS
MTRFR	NS	0.077000086	-0.035872594	0.14521719	NS
DNASE1L1	NS	0.077041487	0.035867572	0.1452718	NS
ARHGAP20	NS	0.07705848	-0.035865511	0.145275537	NS
EFNA3	NS	0.077057057	-0.035865683	0.145275537	NS
NOMO1	NS	0.077127799	-0.035857108	0.145392061	NS
ZNF496	NS	0.077139733	0.035855662	0.14539662	NS
DEFB110	NS	0.077145241	0.035854994	0.14539662	NS
LST1	NS	0.077284036	0.035838191	0.145644026	NS
CCR7	NS	0.07730576	0.035835563	0.145656603	NS
KCTD18	NS	0.077301036	0.035836134	0.145656603	NS
ZNF547	NS	0.077350458	-0.035830158	0.145726637	NS
GNB1	NS	0.077386766	-0.035825769	0.145780851	NS
RPL18A	NS	0.077520576	0.03580961	0.146018711	NS
HEPN1	NS	0.077551367	0.035805894	0.146062495	NS
PDILT	NS	0.077573008	0.035803284	0.146089039	NS
SNAPC2	NS	0.077599763	-0.035800057	0.146125208	NS
FAM189A2	NS	0.077704741	-0.035787406	0.146308655	NS
ZBTB42	NS	0.077739654	-0.035783202	0.146360155	NS
AMOTL1	NS	0.077801631	-0.035775742	0.146462591	NS
ZCCHC10	NS	0.077861057	0.035768594	0.146545956	NS
OTUD7A	NS	0.077860434	-0.035768669	0.146545956	NS

UFSP2	NS	0.077903349	-0.035763509	0.146611301	NS
HNRNPA1P4[illegible]	NS	0.07796697	0.035755865	0.146702506	NS
PIWIL1	NS	0.078028188	0.035748514	0.146789153	NS
FUT10	NS	0.07802124	-0.035749348	0.146789153	NS
ZNF99	NS	0.078170177	0.035731482	0.147041978	NS
PIN4	NS	0.078217185	-0.035725849	0.147102738	NS
SLC43A3	NS	0.078217678	0.03572579	0.147102738	NS
SPRED2	NS	0.078269699	0.03571956	0.147186272	NS
UBR2	NS	0.078287245	-0.035717459	0.147199782	NS
ELN	NS	0.078292093	0.035716878	0.147199782	NS
KHDC3L	NS	0.078316082	0.035714007	0.147230583	NS
ABCB7	NS	0.078333671	0.035711902	0.147249347	NS
RNF207	NS	0.078349412	0.035710019	0.147264637	NS
TAGLN2	NS	0.078415481	0.035702117	0.147374508	NS
CENPL	NS	0.078487	0.03569357	0.147489563	NS
SDC3	NS	0.07849194	-0.035692979	0.147489563	NS
NINJ1	NS	0.078620723	0.035677605	0.147717212	NS
MED8	NS	0.078660752	-0.03567283	0.147778076	NS
AHCTF1	NS	0.078670026	0.035671724	0.147781155	NS
KRTAP4-6	NS	0.078707795	0.035667222	0.147837756	NS
OR2T5	NS	0.078736092	-0.035663849	0.147876556	NS
GALE	NS	0.078755762	0.035661506	0.14789364	NS
TGOLN2	NS	0.078761415	0.035660832	0.14789364	NS
LPAR1	NS	0.07876811	-0.035660035	0.14789364	NS
SNAP23	NS	0.078777936	0.035658864	0.147897742	NS
BPIFC	NS	0.078851902	-0.035650057	0.148022249	NS
ERMP1	NS	0.078869755	0.035647933	0.148041405	NS
PSTPIP1	NS	0.078955254	-0.035637763	0.148187521	NS
CACNB4	NS	0.079000319	0.035632406	0.148257726	NS
KCNJ6	NS	0.079035341	-0.035628245	0.148309071	NS
PSEN2	NS	0.079122262	-0.035617924	0.148457785	NS
ST3GAL1	NS	0.079156339	0.03561388	0.148469134	NS
KCNN2	NS	0.07917251	-0.035611962	0.148469134	NS
SH3GLB1	NS	0.079150961	0.035614519	0.148469134	NS
ADNP	NS	0.079166503	0.035612675	0.148469134	NS
PACS2	NS	0.079174334	-0.035611746	0.148469134	NS
TECPR1	NS	0.079165923	-0.035612743	0.148469134	NS
NOL6	NS	0.07923944	-0.035604025	0.148576829	NS
NBPF10	NS	0.079298168	0.035597065	0.148672544	NS
NIBAN2	NS	0.079314045	-0.035595184	0.148687908	NS
MRPL2	NS	0.07935045	-0.035590873	0.148741751	NS
VAT1L	NS	0.079376006	-0.035587847	0.148775247	NS
ATXN7L1	NS	0.079466769	0.035577107	0.148930943	NS
GALC	NS	0.079530793	-0.035569538	0.14900765	NS
CST9	NS	0.079526963	-0.035569991	0.14900765	NS
KBTBD12	NS	0.07952694	0.035569993	0.14900765	NS
ZBED5	NS	0.079557771	0.03556635	0.149043768	NS
PMS2	NS	0.079581969	0.035563491	0.149072294	NS
PAFAH1B2	NS	0.079588401	0.035562731	0.149072294	NS
NSUN3	NS	0.079687311	-0.035551053	0.149228676	NS
G3BP2	NS	0.079710488	0.035548319	0.149257637	NS
JPH1	NS	0.079751266	-0.035543509	0.14931955	NS
SLC25A28	NS	0.079832068	0.035533984	0.14945638	NS
DNAJC15	NS	0.079913309	-0.035524415	0.149579538	NS
OR10C1	NS	0.079907429	-0.035525108	0.149579538	NS

SLC4A1	NS	0.079937787	0.035521534	0.149610889	NS
SMU1	NS	0.080072315	0.03550571	0.149833694	NS
GFI1B	NS	0.08006798	0.03550622	0.149833694	NS
TUBA4A	NS	0.080101215	0.035502314	0.149873283	NS
VANGL2	NS	0.080184851	-0.03549249	0.150015269	NS
GDF10	NS	0.080197422	-0.035491014	0.150024287	NS
B3GALNT2	NS	0.080291264	-0.035480003	0.150185321	NS
TTPAL	NS	0.080302536	0.035478681	0.150191891	NS
BPIFA2	NS	0.080348173	-0.03547333	0.150262728	NS
ZNF525	NS	0.080413231	-0.035465707	0.150369867	NS
CARD18	NS	0.080443559	0.035462155	0.15041205	NS
GID8	NS	0.080454536	0.03546087	0.150418044	NS
DTX2	NS	0.080477129	0.035458225	0.150429299	NS
RDH5	NS	0.080483871	-0.035457435	0.150429299	NS
RABEP2	NS	0.080469771	-0.035459086	0.150429299	NS
PPP3CB	NS	0.080496289	-0.035455982	0.150435244	NS
CKAP4	NS	0.080502597	0.035455244	0.150435244	NS
MIDEAS	NS	0.080521824	0.035452994	0.15045665	NS
TMEM175	NS	0.080532005	0.035451803	0.150461148	NS
MYLK3	NS	0.080592156	0.035444767	0.150558997	NS
CPT1B	NS	0.080675021	-0.035435082	0.150699258	NS
SLC14A2	NS	0.080693315	-0.035432945	0.150711942	NS
FES	NS	0.080697384	-0.03543247	0.150711942	NS
PROCR	NS	0.08074965	0.035426366	0.150795004	NS
PGAP1	NS	0.080775055	0.035423401	0.150827895	NS
BAHD1	NS	0.080816621	0.035418551	0.150890953	NS
KRT76	NS	0.080855586	0.035414006	0.150949144	NS
ZNF627	NS	0.080901984	-0.035408596	0.151021198	NS
SYTL4	NS	0.080914377	0.035407152	0.15102916	NS
KCNV1	NS	0.080921855	0.03540628	0.15102916	NS
CAMLG	NS	0.080951617	0.035402812	0.15106012	NS
ZNF662	NS	0.080954052	0.035402529	0.15106012	NS
ATP6V1F	NS	0.081003155	0.035396809	0.151137177	NS
SMARCA1	NS	0.081042126	-0.035392272	0.151195314	NS
TNFSF13B	NS	0.081091037	0.03538658	0.151271985	NS
PHF6	NS	0.081115188	0.035383771	0.151302456	NS
FBXL22	NS	0.081126399	0.035382467	0.151308785	NS
LDLRAD2	NS	0.081140969	-0.035380773	0.151321379	NS
SSTR4	NS	0.081167513	-0.035377687	0.151341719	NS
CARD19	NS	0.081159963	0.035378564	0.151341719	NS
RHOU	NS	0.081179788	-0.03537626	0.151350026	NS
BOLA1	NS	0.081218089	-0.035371808	0.151406852	NS
PARP3	NS	0.081241214	0.035369122	0.151408379	NS
DHRS9	NS	0.081234626	0.035369887	0.151408379	NS
ZNF648	NS	0.081242376	0.035368987	0.151408379	NS
COX11	NS	0.081281111	-0.035364488	0.151465985	NS
BCCIP	NS	0.081337018	-0.035357998	0.151555576	NS
CDHR5	NS	0.08136864	0.035354328	0.151599904	NS
E2F2	NS	0.08143177	0.035347006	0.151690974	NS
SLC25A26	NS	0.081433195	0.035346841	0.151690974	NS
PCID2	NS	0.081442909	0.035345715	0.151694471	NS
MOV10	NS	0.081552317	0.035333037	0.151883638	NS
NOTCH3	NS	0.081610281	-0.035326326	0.151976967	NS
DFFA	NS	0.081620787	-0.035325111	0.151981909	NS
SCNN1G	NS	0.081634506	0.035323523	0.151992833	NS

YBX1	NS	0.081642385	0.035322611	0.151992884	NS
CYP1A1	NS	0.081711814	-0.035314581	0.152094305	NS
FAM163A	NS	0.08171818	0.035313845	0.152094305	NS
CLK3	NS	0.081720437	0.035313584	0.152094305	NS
GATA2	NS	0.081785194	-0.035306099	0.152200194	NS
PICALM	NS	0.081821911	0.035301858	0.152239249	NS
PPP1R2B	NS	0.081890451	0.035293944	0.152352131	NS
ZNF548	NS	0.081922078	-0.035290294	0.152390624	NS
CEACAM16	NS	0.081926888	0.035289739	0.152390624	NS
VPS51	NS	0.081955093	0.035286485	0.15242844	NS
CDR2L	NS	0.082034311	-0.035277351	0.152544634	NS
CEP112	NS	0.082041759	0.035276493	0.152544634	NS
CEBPB	NS	0.08204909	0.035275648	0.152544634	NS
H2AW	NS	0.082153698	-0.035263599	0.15272445	NS
MYH6	NS	0.082182029	-0.035260338	0.152762446	NS
RAB10	NS	0.082242252	0.035253409	0.152859711	NS
APOA5	NS	0.082261154	0.035251235	0.152880163	NS
PEX11B	NS	0.082271535	0.035250042	0.152884777	NS
CBY1	NS	0.082280414	-0.035249021	0.152886598	NS
TMEM131	NS	0.08247786	0.035226341	0.153238766	NS
SNX20	NS	0.082508106	0.035222871	0.153280247	NS
IRF5	NS	0.082605902	0.035211657	0.153447201	NS
FAM83H	NS	0.082657992	0.035205688	0.153529229	NS
KIF2A	NS	0.08274009	0.035196288	0.153666972	NS
PAPSS1	NS	0.082759548	-0.035194061	0.153688363	NS
MATR3	NS	0.082811322	0.035188137	0.153769758	NS
DUSP13	NS	0.0828369	0.035185212	0.153802497	NS
TRA2B	NS	0.082865545	0.035181937	0.153840925	NS
RAPSN	NS	0.082917193	0.035176034	0.153922048	NS
SLC16A14	NS	0.083047086	0.035161202	0.154148388	NS
CAPZA2	NS	0.083075692	0.035157938	0.1541867	NS
RBM39	NS	0.08309884	0.035155297	0.154214876	NS
PPP4R3A	NS	0.083121456	0.035152718	0.154242058	NS
PGLYRP1	NS	0.083171	0.03514707	0.154312802	NS
PTPRZ1	NS	0.083175525	-0.035146554	0.154312802	NS
HNRNPCL3	NS	0.083193361	-0.035144521	0.154331099	NS
PHGDH	NS	0.083220524	-0.035141426	0.154366695	NS
HABP2	NS	0.083248176	-0.035138276	0.15440319	NS
TBXAS1	NS	0.083298772	-0.035132515	0.15448223	NS
LARS1	NS	0.083413601	-0.035119451	0.154680367	NS
ZNF157	NS	0.083442301	0.035116188	0.154718765	NS
CHN1	NS	0.0835207	-0.035107279	0.154849297	NS
MATN4	NS	0.083529008	0.035106335	0.154849868	NS
CHMP4A	NS	0.083574892	0.035101125	0.154920093	NS
MBD1	NS	0.083591001	0.035099296	0.154935116	NS
DERL3	NS	0.083748985	-0.035081376	0.155213075	NS
SPTY2D1OS	NS	0.083784394	-0.035077363	0.155263833	NS
PCMT1	NS	0.083839964	-0.035071069	0.155351938	NS
KLK6	NS	0.08385304	0.035069588	0.155361294	NS
MYH7B	NS	0.083907958	0.035063371	0.155448165	NS
AFF2	NS	0.083931724	-0.035060682	0.155477314	NS
CBFA2T2	NS	0.083959927	-0.035057492	0.155514674	NS
TAF1L	NS	0.084004544	0.035052446	0.155582428	NS
KRTAP19-3	NS	0.084020655	0.035050625	0.155597377	NS
URI1	NS	0.08408238	-0.035043649	0.155681896	NS

LIF	NS	0.084082192	0.03504367	0.155681896	NS
RAB19	NS	0.084104713	0.035041126	0.155708353	NS
ING4	NS	0.084122254	-0.035039145	0.155719307	NS
DAGLA	NS	0.08412672	0.035038641	0.155719307	NS
SUPT16H	NS	0.084197389	0.035030663	0.155835213	NS
METTL6	NS	0.084214588	0.035028722	0.155852142	NS
PRKCE	NS	0.084258482	0.03502377	0.155918468	NS
IL1B	NS	0.084276661	0.03502172	0.1559372	NS
TTLL5	NS	0.084326957	-0.035016049	0.156009889	NS
RPL4	NS	0.084332066	0.035015474	0.156009889	NS
JCAD	NS	0.084341031	-0.035014463	0.156011563	NS
APMAP	NS	0.084444909	0.035002762	0.156179094	NS
TMA16	NS	0.084447737	0.035002444	0.156179094	NS
PHOX2A	NS	0.084620866	-0.034982969	0.15648433	NS
IZUMO4	NS	0.084649224	0.034979782	0.156506865	NS
S100G	NS	0.084648646	-0.034979847	0.156506865	NS
GLRX2	NS	0.084682255	-0.034976071	0.156552982	NS
OR2B3	NS	0.084698159	-0.034974284	0.156567431	NS
CHST10	NS	0.084764271	-0.034966861	0.156674678	NS
UBE2B	NS	0.084872695	0.034954698	0.156860105	NS
DRD2	NS	0.084885146	0.034953302	0.156868139	NS
SYNPO	NS	0.084940636	0.034947082	0.156952257	NS
ACTN3	NS	0.084946882	0.034946383	0.156952257	NS
ECT2L	NS	0.084964509	-0.034944408	0.156969842	NS
NOXO1	NS	0.084983237	0.03494231	0.156989457	NS
NME3	NS	0.085009707	-0.034939345	0.157023369	NS
SELENOK	NS	0.085046153	0.034935265	0.157075701	NS
PHAF1	NS	0.085089746	0.034930386	0.15714122	NS
SELE	NS	0.085146548	-0.034924032	0.157231119	NS
SAP25	NS	0.085177646	0.034920554	0.15727354	NS
AP5B1	NS	0.085237994	0.034913809	0.157369956	NS
MROH7-TTC	NS	0.085279444	-0.034909179	0.157431465	NS
PSMC1	NS	0.085293322	-0.034907629	0.157442069	NS
VPS13A	NS	0.085306437	-0.034906164	0.157451263	NS
ZC3H12C	NS	0.085316781	-0.034905009	0.157455341	NS
BTBD2	NS	0.08533176	0.034903337	0.157467972	NS
DMRT3	NS	0.085382407	0.034897684	0.157546413	NS
GMPPB	NS	0.08539376	-0.034896417	0.157552341	NS
ARHGEF4	NS	0.085434736	0.034891846	0.157610156	NS
ABCG5	NS	0.085441381	-0.034891105	0.157610156	NS
ZBTB44	NS	0.085517628	-0.034882605	0.157735774	NS
CHD5	NS	0.085641968	0.034868756	0.157950066	NS
PLGLB2	NS	0.085652557	-0.034867577	0.157954543	NS
RPL23	NS	0.085697795	-0.034862543	0.158022914	NS
VCX3B	NS	0.08589365	-0.034840774	0.158368973	NS
EMILIN2	NS	0.085929698	0.034836771	0.158405261	NS
ANKRD18B	NS	0.085928471	0.034836908	0.158405261	NS
PI4K2B	NS	0.085955996	0.034833852	0.15843865	NS
AP1B1	NS	0.086019316	0.034826827	0.158514544	NS
SHOX	NS	0.086021738	0.034826558	0.158514544	NS
DYSF	NS	0.086031887	0.034825433	0.158518153	NS
HEPH	NS	0.086332884	-0.034792098	0.159057614	NS
PARN	NS	0.086369817	0.034788014	0.15911035	NS
MTA1	NS	0.086377948	0.034787115	0.15911035	NS
ULBP2	NS	0.086443876	-0.034779829	0.159216639	NS

SIRT7	NS	0.086540753	-0.034769131	0.159379906	NS
DIAPH1	NS	0.086588664	0.034763844	0.159437803	NS
HARS1	NS	0.086586812	-0.034764049	0.159437803	NS
ZSWIM7	NS	0.086662999	-0.034755646	0.159559498	NS
FAXDC2	NS	0.086698804	0.034751699	0.159604201	NS
SGTB	NS	0.08670377	0.034751152	0.159604201	NS
CRTAC1	NS	0.086770175	0.034743835	0.159711251	NS
MAP3K9	NS	0.086795545	-0.034741041	0.159727568	NS
MYO10	NS	0.086794751	0.034741129	0.159727568	NS
RSPRY1	NS	0.08684462	0.034735639	0.159802688	NS
UBP1	NS	0.086877756	0.034731992	0.159848464	NS
MSH3	NS	0.086933563	-0.034725853	0.159927142	NS
DYDC2	NS	0.086961829	-0.034722745	0.159927142	NS
GET1	NS	0.086961117	-0.034722823	0.159927142	NS
DPP4	NS	0.086954828	0.034723515	0.159927142	NS
CEACAM18	NS	0.086954913	-0.034723505	0.159927142	NS
ALG14	NS	0.087061366	-0.034711806	0.160094984	NS
UBE2U	NS	0.087096003	-0.034708002	0.160143463	NS
IL17RD	NS	0.087114095	-0.034706016	0.160161514	NS
EFCAB14	NS	0.087166583	0.034700254	0.160242794	NS
TAF4	NS	0.08717536	0.034699291	0.16024371	NS
SLC66A2	NS	0.087195172	0.034697117	0.160264909	NS
PPP1R18	NS	0.0873025	0.034685348	0.160446942	NS
PGRMC1	NS	0.087327702	0.034682587	0.160478021	NS
YPEL3	NS	0.087420309	-0.034672443	0.16063295	NS
MASP2	NS	0.087464164	-0.034667643	0.160698277	NS
ZFP82	NS	0.087512705	0.034662332	0.160772201	NS
KLK2	NS	0.087551843	0.034658052	0.160828838	NS
DLK2	NS	0.087607018	0.03465202	0.160914922	NS
OR51T1	NS	0.087700201	-0.03464184	0.161070793	NS
S100A12	NS	0.087730587	-0.034638522	0.161111313	NS
CNOT9	NS	0.087745766	0.034636865	0.161123901	NS
USP46	NS	0.087802842	0.034630637	0.161182836	NS
PSMB4	NS	0.087794599	-0.034631536	0.161182836	NS
REM2	NS	0.087884563	-0.034621725	0.161317554	NS
RPS10-NUDT	NS	0.087914192	0.034618495	0.161356637	NS
TOR1B	NS	0.087960149	0.034613488	0.161422178	NS
MUC20	NS	0.087966581	-0.034612787	0.161422178	NS
PLET1	NS	0.087999461	-0.034609206	0.161467207	NS
ADM	NS	0.088008037	-0.034608272	0.161467636	NS
UBC	NS	0.088026771	0.034606232	0.161486701	NS
FUT5	NS	0.088151231	-0.03459269	0.1616997	NS
CDS2	NS	0.088191877	0.03458827	0.161758928	NS
RNF152	NS	0.088205418	-0.034586798	0.161768436	NS
TNK1	NS	0.088277382	-0.034578979	0.161885078	NS
GSDMB	NS	0.08834269	-0.034571887	0.161989496	NS
TP53TG5	NS	0.088362203	-0.034569769	0.162009928	NS
DTD2	NS	0.088382898	-0.034567523	0.162032523	NS
VTN	NS	0.088404393	-0.03456519	0.16205658	NS
NTN4	NS	0.088446817	-0.034560588	0.162103644	NS
AVIL	NS	0.088441992	0.034561112	0.162103644	NS
COMMD2	NS	0.088519218	-0.034552739	0.162220979	NS
ADRA1A	NS	0.08863162	0.034540562	0.162411592	NS
PYY	NS	0.088640543	0.034539596	0.162412566	NS
LRFN2	NS	0.08869482	0.034533721	0.162496634	NS

TMEM205	NS	0.088749107	0.034527848	0.162580703	NS
KCNQ2	NS	0.088807832	0.034521498	0.162657494	NS
ASCC1	NS	0.08880172	0.034522159	0.162657494	NS
PNISR	NS	0.088883619	-0.034513308	0.162765499	NS
SATL1	NS	0.088882887	-0.034513388	0.162765499	NS
ZBTB38	NS	0.088917509	-0.034509648	0.162799298	NS
ZNF594	NS	0.088918898	-0.034509498	0.162799298	NS
GHRH	NS	0.089015101	0.034499113	0.162960021	NS
TRIM5	NS	0.089047039	-0.034495668	0.163003072	NS
GOLGA6L2	NS	0.089056278	0.034494671	0.163004568	NS
SNRNP48	NS	0.089104384	-0.034489484	0.163077199	NS
WDR6	NS	0.089112977	-0.034488557	0.163077505	NS
DEFB4A	NS	0.089206429	-0.034478487	0.163233092	NS
RETNLB	NS	0.089216045	-0.034477452	0.163235255	NS
TMEM100	NS	0.089233414	-0.034475581	0.163251604	NS
OR4M2	NS	0.089296756	-0.034468762	0.163352047	NS
OR6J1	NS	0.089321472	0.034466102	0.163381819	NS
RTN4	NS	0.089351286	0.034462895	0.163390032	NS
FAM90A16P	NS	0.089350364	0.034462994	0.163390032	NS
ZNF775	NS	0.089444144	0.03445291	0.163528936	NS
DEPDC4	NS	0.089498666	0.034447051	0.163613164	NS
ITIH3	NS	0.089531735	-0.034443499	0.163658161	NS
HMGB2	NS	0.089575624	0.034438787	0.163707466	NS
DSCAML1	NS	0.089574134	-0.034438947	0.163707466	NS
ARHGEF6	NS	0.089588837	0.034437368	0.163716156	NS
MYH15	NS	0.0896042	-0.034435719	0.163728773	NS
CYP2A13	NS	0.089636073	0.034432299	0.163771552	NS
PNPLA2	NS	0.089645599	0.034431277	0.163773499	NS
OR2A42	NS	0.089669722	-0.034428689	0.163802109	NS
CLASRP	NS	0.089710332	0.034424334	0.163860828	NS
DPH5	NS	0.089768663	-0.034418081	0.163938506	NS
LILRB4	NS	0.089769799	0.034417959	0.163938506	NS
PGR	NS	0.089918168	-0.03440207	0.164187643	NS
EFCAB8	NS	0.089923187	0.034401533	0.164187643	NS
IGSF8	NS	0.089955575	-0.034398067	0.164231287	NS
PRAMEF1	NS	0.0899845	0.034394973	0.164268602	NS
CRTAP	NS	0.090003452	0.034392946	0.164287704	NS
SLK	NS	0.090149202	0.034377369	0.164538232	NS
MYL2	NS	0.090195839	-0.034372389	0.164607832	NS
TMEM139	NS	0.090297581	-0.034361532	0.164777974	NS
SLC51B	NS	0.090343986	-0.034356583	0.164847114	NS
CORIN	NS	0.090505255	0.034339401	0.16512581	NS
CCDC117	NS	0.090549728	-0.034334667	0.16519138	NS
SERGEF	NS	0.0905669	-0.03433284	0.165207135	NS
R3HCC1L	NS	0.090622601	-0.034326914	0.165293165	NS
CD164	NS	0.090668102	0.034322076	0.165360575	NS
NUGGC	NS	0.090704712	0.034318184	0.165411759	NS
DCAF4	NS	0.090793873	-0.034308712	0.165558757	NS
DNAJB2	NS	0.090827276	0.034305166	0.165604064	NS
DNAJC30	NS	0.090840733	-0.034303737	0.165613001	NS
BTBD6	NS	0.090864764	0.034301186	0.165641211	NS
MGME1	NS	0.090910858	-0.034296295	0.16570963	NS
UROC1	NS	0.090931811	-0.034294073	0.165732216	NS
CNTLN	NS	0.090951352	0.034292	0.165752224	NS
CALM1	NS	0.090967159	0.034290324	0.165765423	NS

CFAP45	NS	0.091012969	0.034285468	0.165833288	NS
BCL2L13	NS	0.091024631	0.034284232	0.165838927	NS
SEZ6L2	NS	0.091075385	0.034278854	0.165915779	NS
BOD1L1	NS	0.091127134	0.034273374	0.16599443	NS
SLMAP	NS	0.091202307	0.034265417	0.16611573	NS
CALHM1	NS	0.091279588	0.034257243	0.166240845	NS
KDM5C	NS	0.091315394	0.034253457	0.16629041	NS
ROPN1B	NS	0.091326741	-0.034252258	0.166295428	NS
ASB14	NS	0.091411737	-0.034243277	0.166434539	NS
TERB2	NS	0.091472132	0.0342369	0.166528836	NS
GDI2	NS	0.091501861	-0.034233762	0.166567292	NS
FNDC7	NS	0.091605261	-0.034222855	0.166739838	NS
YS1-DBNDD	NS	0.091814339	-0.034200831	0.167104686	NS
EXOC3	NS	0.091845909	0.034197509	0.167146429	NS
SLFN11	NS	0.091854776	-0.034196576	0.16714685	NS
DSC1	NS	0.091947389	-0.034186836	0.167299649	NS
TMEM80	NS	0.092025639	0.034178613	0.167426288	NS
ANKRD23	NS	0.092035958	0.034177529	0.167429324	NS
GPD1L	NS	0.092081045	-0.034172794	0.167495602	NS
PSMA1	NS	0.092105223	-0.034170255	0.16752384	NS
CARD10	NS	0.092143479	0.03416624	0.167577674	NS
AASS	NS	0.092208147	0.034159456	0.167679527	NS
PDLIM1	NS	0.092280029	-0.034151919	0.16779448	NS
GEMIN2	NS	0.092317973	-0.034147942	0.167847707	NS
MR1	NS	0.092426053	0.034136622	0.168028429	NS
MYL6B	NS	0.092563282	-0.034122265	0.168262104	NS
IPP	NS	0.092577702	-0.034120757	0.168272513	NS
MET	NS	0.09259683	0.034118758	0.168291477	NS
BOP1	NS	0.092666517	-0.034111476	0.16840232	NS
SNX10	NS	0.092714945	0.034106418	0.168474509	NS
PPIL6	NS	0.092764076	0.034101288	0.168547963	NS
TRMT2A	NS	0.092800133	-0.034097525	0.168597652	NS
RPS24	NS	0.092822198	-0.034095223	0.168621912	NS
FCRL5	NS	0.092910667	-0.034085997	0.168766786	NS
CYP4F3	NS	0.092963785	-0.034080461	0.168847427	NS
NOL12	NS	0.092999017	-0.03407679	0.16889557	NS
PER1	NS	0.093071824	0.034069208	0.169011936	NS
AMY2A	NS	0.093093423	-0.03406696	0.169035299	NS
CLEC2B	NS	0.093173394	-0.034058639	0.169164638	NS
FLT3LG	NS	0.093193786	-0.034056519	0.169176523	NS
TAPT1	NS	0.093202493	0.034055613	0.169176523	NS
MPC1L	NS	0.09320616	-0.034055232	0.169176523	NS
POLDIP3	NS	0.093229725	0.034052782	0.169203427	NS
CD19	NS	0.093249345	-0.034050742	0.169223169	NS
PUM1	NS	0.093354496	0.034039817	0.169398109	NS
ADGRV1	NS	0.093386588	0.034036485	0.169440457	NS
SNN	NS	0.093446887	0.034030226	0.169533971	NS
LIMS3	NS	0.093468788	-0.034027954	0.169557813	NS
SCN1B	NS	0.093499077	0.034024812	0.169596864	NS
CHN2	NS	0.093516357	-0.03402302	0.169612313	NS
SMTNL2	NS	0.093608425	0.034013476	0.169763391	NS
VGLL1	NS	0.09363583	-0.034010636	0.169797182	NS
NECTIN4	NS	0.093663599	0.03400776	0.169831498	NS
GGTA1	NS	0.093672302	-0.034006858	0.169831498	NS
DNAH9	NS	0.093741189	0.033999726	0.169900474	NS

STK25	NS	0.093728665	-0.034001022	0.169900474	NS
ACTR10	NS	0.093745457	-0.033999284	0.169900474	NS
CHCHD10	NS	0.093728978	-0.03400099	0.169900474	NS
CLPP	NS	0.09377216	0.033996521	0.169932958	NS
PKDCC	NS	0.093798904	0.033993754	0.169949601	NS
MRPS11	NS	0.093793113	-0.033994353	0.169949601	NS
MRPL22	NS	0.093833757	-0.033990149	0.169996837	NS
CHKB-CPT1B	NS	0.093868048	0.033986603	0.170043045	NS
ELOA3CP	NS	0.093883529	0.033985002	0.170055174	NS
SPACA3	NS	0.093925826	0.03398063	0.170084039	NS
SLC6A3	NS	0.093917601	0.03398148	0.170084039	NS
OR4X1	NS	0.094065772	-0.033966176	0.170321524	NS
CDC42EP1	NS	0.094083193	0.033964378	0.170332993	NS
PSD2	NS	0.094089707	0.033963706	0.170332993	NS
S100Z	NS	0.094099642	-0.033962681	0.170335049	NS
ARFIP1	NS	0.09411215	0.03396139	0.17034176	NS
UGT2B15	NS	0.094125827	-0.033959979	0.170350584	NS
TUBB3	NS	0.094246568	-0.033947529	0.170553158	NS
RAMP1	NS	0.094273194	-0.033944785	0.170585392	NS
AANAT	NS	0.09428604	-0.033943462	0.170592689	NS
AP4B1	NS	0.094334809	0.033938438	0.170664974	NS
CYBA	NS	0.094429473	0.033928693	0.170820269	NS
DIS3L	NS	0.094478426	0.033923657	0.170892853	NS
TCEA1	NS	0.094518491	0.033919537	0.170949347	NS
ANKS1B	NS	0.09466565	0.033904415	0.171199507	NS
CCL19	NS	0.094704871	-0.033900388	0.171254436	NS
H1-10	NS	0.094740932	-0.033896686	0.171303642	NS
ZCCHC18	NS	0.094804758	-0.033890138	0.171387028	NS
SPAST	NS	0.094819708	-0.033888604	0.171398046	NS
NFKB2	NS	0.094905326	0.033879826	0.17153679	NS
PHLPP1	NS	0.094945892	0.03387567	0.171594089	NS
DLG4	NS	0.094965476	0.033873663	0.171604525	NS
PCDHA4	NS	0.094969398	0.033873262	0.171604525	NS
TGM3	NS	0.094993945	-0.033870747	0.171632857	NS
CABP7	NS	0.095041671	-0.033865861	0.171671655	NS
EBP	NS	0.095042026	-0.033865824	0.171671655	NS
NBPF6	NS	0.095034028	-0.033866643	0.171671655	NS
RANBP1	NS	0.095178146	-0.033851898	0.171901482	NS
CCT6B	NS	0.095274176	-0.033842083	0.172058868	NS
PDZD3	NS	0.095294279	-0.033840029	0.172079117	NS
SPATA5	NS	0.095345987	-0.033834748	0.172156429	NS
TMEM98	NS	0.095375806	0.033831703	0.172179645	NS
ZNF555	NS	0.095376636	-0.033831619	0.172179645	NS
USP54	NS	0.095453547	0.03382377	0.172302421	NS
RIF1	NS	0.095518261	0.03381717	0.172403157	NS
UCK1	NS	0.095533561	0.03381561	0.172414695	NS
ZNF493	NS	0.095601655	0.03380867	0.172521502	NS
TRAF1	NS	0.095685594	0.033800121	0.17265688	NS
NDUFV1	NS	0.095706996	-0.033797942	0.172679402	NS
C14orf132	NS	0.095752269	0.033793334	0.172744982	NS
GSTM5	NS	0.095763486	0.033792192	0.172749118	NS
GSN	NS	0.095786857	0.033789815	0.172775175	NS
MAST2	NS	0.095920089	-0.033776268	0.172966891	NS
ATF3	NS	0.095928889	0.033775374	0.172966891	NS
BACE2	NS	0.095911337	0.033777158	0.172966891	NS

ZNF599	NS	0.096028174	0.03376529	0.173129781	NS
PRSS57	NS	0.096052283	0.033762842	0.173157119	NS
NISCH	NS	0.096086085	0.033759412	0.173201925	NS
LTB4R	NS	0.096097681	-0.033758235	0.173206696	NS
PRCC	NS	0.096149795	0.033752948	0.173284491	NS
COL4A4	NS	0.09618987	-0.033748884	0.173324438	NS
CES3	NS	0.096185061	0.033749372	0.173324438	NS
CETP	NS	0.096258905	-0.033741887	0.173416545	NS
SCFD1	NS	0.09625034	0.033742755	0.173416545	NS
RTN2	NS	0.096283789	0.033739365	0.173445232	NS
JTB	NS	0.096430145	0.033724547	0.173682296	NS
RSL1D1	NS	0.096433335	0.033724224	0.173682296	NS
OTUD7B	NS	0.096485863	-0.03371891	0.173760734	NS
CGNL1	NS	0.096608046	-0.033706559	0.173964587	NS
HTR3C	NS	0.096698793	0.033697393	0.174111799	NS
MTMR2	NS	0.096708259	0.033696437	0.174112646	NS
ZNF235	NS	0.096744766	-0.033692752	0.174162174	NS
NLRP9	NS	0.096839125	-0.033683233	0.174315829	NS
PDE1C	NS	0.096929369	-0.033674135	0.174429608	NS
GIT2	NS	0.096977932	0.033669243	0.174500776	NS
KCNN4	NS	0.097051544	0.03366183	0.174607317	NS
DNAH12	NS	0.097055183	-0.033661463	0.174607317	NS
GNPNAT1	NS	0.097105103	-0.033656439	0.17468089	NS
ANKRD13D	NS	0.097171104	0.033649799	0.174783374	NS
TRPM8	NS	0.097319774	-0.033634857	0.175018262	NS
ZGLP1	NS	0.097319271	-0.033634907	0.175018262	NS
MEF2D	NS	0.09734976	-0.033631845	0.175055923	NS
MFAP4	NS	0.097404632	0.033626336	0.175138324	NS
FAM234B	NS	0.097426468	-0.033624144	0.175145046	NS
DUSP6	NS	0.097423794	0.033624412	0.175145046	NS
CACFD1	NS	0.097441251	0.033622661	0.175155354	NS
SIPA1	NS	0.097493288	-0.03361744	0.17523262	NS
NOL10	NS	0.097554251	-0.033611326	0.175325912	NS
DSP	NS	0.097633949	0.033603339	0.175378982	NS
CPQ	NS	0.097611335	-0.033605605	0.175378982	NS
BHLHE40	NS	0.097623156	0.03360442	0.175378982	NS
FADS6	NS	0.097638143	0.033602919	0.175378982	NS
PLCB1	NS	0.097631732	-0.033603561	0.175378982	NS
PTTG1IP	NS	0.097606016	0.033606138	0.175378982	NS
ADAM28	NS	0.097709709	0.033595751	0.175491243	NS
COA7	NS	0.097734557	0.033593263	0.175519584	NS
MCM3AP	NS	0.097876559	-0.033579056	0.175758296	NS
RFK	NS	0.097897846	-0.033576927	0.175780214	NS
ASAP1	NS	0.09803841	0.033562883	0.176016275	NS
ZNF12	NS	0.09809257	0.033557475	0.176097179	NS
FBH1	NS	0.09818641	-0.033548112	0.176249295	NS
CXCL6	NS	0.098203637	-0.033546394	0.176263872	NS
PSCA	NS	0.098272612	-0.033539517	0.176371319	NS
HSPB7	NS	0.098282366	0.033538545	0.176372471	NS
SRPX	NS	0.098322752	-0.033534521	0.176428588	NS
OR5A2	NS	0.098373134	-0.033529503	0.176502631	NS
ACTN4	NS	0.098400043	0.033526823	0.176534548	NS
SERPINB12	NS	0.098469848	0.033519875	0.176643408	NS
GTPBP2	NS	0.098587368	0.033508186	0.176832316	NS
MTR	NS	0.09860297	-0.033506635	0.176833048	NS

VPS33B	NS	0.098614924	-0.033505447	0.176838101	NS
SIRPA	NS	0.098639841	-0.03350297	0.176866398	NS
B4GALNT3	NS	0.098670442	-0.03349993	0.17690488	NS
THOC3	NS	0.098790933	-0.033487965	0.177103851	NS
NPY4R2	NS	0.09879972	0.033487093	0.177103851	NS
TMEM59	NS	0.098819255	-0.033485154	0.177122465	NS
EPHX3	NS	0.098985268	-0.033468692	0.177403596	NS
C16orf91	NS	0.098997568	-0.033467473	0.177409215	NS
ZNF414	NS	0.099012324	0.033466011	0.17741774	NS
RIPK1	NS	0.099020658	0.033465185	0.17741774	NS
TDG	NS	0.099039425	-0.033463326	0.17741852	NS
BAG6	NS	0.099036755	0.03346359	0.17741852	NS
VWC2	NS	0.099057505	0.033461535	0.177434488	NS
SEZ6L	NS	0.099081697	0.03345914	0.177461397	NS
CASTOR1	NS	0.099178489	0.033449559	0.177618324	NS
SPATA7	NS	0.099230771	-0.033444387	0.177682275	NS
ZNF366	NS	0.099232558	-0.03344421	0.177682275	NS
VPS45	NS	0.099308258	-0.033436726	0.177801373	NS
SLC45A1	NS	0.09935335	0.03343227	0.177865654	NS
GOLGA8H	NS	0.099448768	-0.033422846	0.178020009	NS
KIDINS220	NS	0.099490825	0.033418694	0.178062358	NS
TMEM168	NS	0.099483111	-0.033419455	0.178062358	NS
HINFP	NS	0.099514325	-0.033416375	0.178087951	NS
THBS3	NS	0.09952801	-0.033415025	0.178095974	NS
NPHP4	NS	0.09954875	-0.033412978	0.178116621	NS
PILRA	NS	0.099641886	0.033403794	0.178250309	NS
AAK1	NS	0.099635883	0.033404386	0.178250309	NS
SMARCC1	NS	0.099668406	0.03340118	0.178281274	NS
CXCR4	NS	0.099738175	0.033394306	0.178389588	NS
GPX5	NS	0.099830905	-0.033385175	0.178538944	NS
ENKD1	NS	0.099920292	0.03337638	0.17866579	NS
BTLA	NS	0.099919108	-0.033376497	0.17866579	NS
ZNF570	NS	0.099946703	0.033373783	0.178696507	NS
TXNL4A	NS	0.100000447	0.033368499	0.178771823	NS
STK11IP	NS	0.1000073	0.033367826	0.178771823	NS
CHKB	NS	0.10005285	0.033363349	0.178836731	NS
RPL15	NS	0.100113702	-0.033357372	0.178928976	NS
SERPINB5	NS	0.100174312	0.033351421	0.179004245	NS
CDC23	NS	0.10018908	-0.033349972	0.179014107	NS
CLDN17	NS	0.100305285	-0.033338572	0.179188657	NS
FAM153A	NS	0.100298312	0.033339255	0.179188657	NS
DNAL1	NS	0.100344788	-0.033334699	0.179242684	NS
EFHD1	NS	0.100389564	0.03333031	0.179306118	NS
KDF1	NS	0.100456119	-0.03332379	0.179408438	NS
SNCG	NS	0.100471274	0.033322306	0.179418947	NS
CEP55	NS	0.100542046	-0.033315378	0.179521694	NS
GPAT3	NS	0.100547359	0.033314858	0.179521694	NS
SGMS1	NS	0.10057281	-0.033312367	0.179550572	NS
ZMPSTE24	NS	0.100686396	0.033301258	0.179736778	NS
AKR7A2	NS	0.100792256	-0.033290913	0.179909158	NS
ZNF343	NS	0.100842367	-0.03328602	0.179970659	NS
TNFAIP6	NS	0.100845307	0.033285732	0.179970659	NS
SSRP1	NS	0.100858615	-0.033284433	0.179977815	NS
MATN3	NS	0.100905223	-0.033279884	0.180044385	NS
SGPP2	NS	0.10091806	-0.033278631	0.180050693	NS

MST1	NS	0.101014954	-0.03326918	0.180206954	NS
EZR	NS	0.101040844	0.033266655	0.18023653	NS
MAGED1	NS	0.101062001	-0.033264593	0.180257657	NS
FKBP6	NS	0.101143719	-0.033256631	0.180386789	NS
GARNL3	NS	0.101181732	-0.033252928	0.180437958	NS
CCNJL	NS	0.101215398	-0.03324965	0.180481367	NS
LUC7L	NS	0.101360694	0.033235513	0.180723802	NS
KNL1	NS	0.101383512	0.033233295	0.180747837	NS
CD300C	NS	0.101411274	0.033230596	0.18078068	NS
PRPSAP2	NS	0.101444077	0.033227408	0.180822503	NS
H3C10	NS	0.101518173	-0.033220209	0.180937914	NS
C11orf24	NS	0.101586866	-0.033213539	0.181043678	NS
CNTFR	NS	0.101716685	0.033200944	0.181249919	NS
ZNF385A	NS	0.10172132	0.033200495	0.181249919	NS
ENPP1	NS	0.101747354	0.033197971	0.181264349	NS
MRPL33	NS	0.101748148	0.033197894	0.181264349	NS
TLNRD1	NS	0.101965791	0.033176812	0.181635363	NS
MTUS2	NS	0.102139226	-0.033160038	0.181927565	NS
DNAJC25	NS	0.102230857	-0.033151184	0.182074022	NS
SGK3	NS	0.102272946	0.03314712	0.182132225	NS
RBM28	NS	0.102412504	-0.033133653	0.182330429	NS
PDCD11	NS	0.10240586	-0.033134294	0.182330429	NS
CREB3L2	NS	0.102396438	0.033135202	0.182330429	NS
TMEM160	NS	0.102446578	-0.033130367	0.182374318	NS
BRF2	NS	0.102508109	-0.033124435	0.182467074	NS
SEC62	NS	0.102585637	0.033116966	0.182588284	NS
PCDH1	NS	0.102634991	-0.033112213	0.182659331	NS
CISD3	NS	0.102659095	-0.033109893	0.182685433	NS
CNIH4	NS	0.1026789	0.033107986	0.182703878	NS
USP17L3	NS	0.102711742	-0.033104826	0.182745517	NS
ACSF3	NS	0.10279233	-0.033097074	0.182855284	NS
OR4E1	NS	0.102783623	0.033097911	0.182855284	NS
VDAC2	NS	0.102998962	0.033077219	0.18318756	NS
LRRC57	NS	0.103007512	-0.033076398	0.18318756	NS
AQP4	NS	0.103016994	-0.033075488	0.183187593	NS
TBC1D10A	NS	0.103028336	0.033074399	0.183190931	NS
PRKACA	NS	0.103181694	0.033059687	0.183446759	NS
KDM6B	NS	0.103234171	0.033054657	0.183523201	NS
ZNF891	NS	0.103254019	-0.033052755	0.183541629	NS
TOX3	NS	0.103264214	0.033051778	0.183542894	NS
DTX3L	NS	0.103290554	0.033049255	0.183572855	NS
FAM25G	NS	0.103310872	0.033047309	0.183592108	NS
HNRNPU	NS	0.103330768	0.033045403	0.183610607	NS
IL23A	NS	0.103363518	-0.033042267	0.183651942	NS
HEBP2	NS	0.103411703	-0.033037655	0.183720691	NS
FAM156B	NS	0.103428777	-0.033036021	0.18373416	NS
ATP1A4	NS	0.103516026	0.033027674	0.183872277	NS
AUP1	NS	0.103584808	0.033021099	0.183977569	NS
RIOX2	NS	0.103617627	-0.033017962	0.184018974	NS
C22orf39	NS	0.103757905	0.033004565	0.184251193	NS
TIMM21	NS	0.103777002	0.033002743	0.184251298	NS
CST6	NS	0.103770061	-0.033003405	0.184251298	NS
PDZRN4	NS	0.103837347	-0.032996985	0.18434153	NS
C3orf49	NS	0.103866245	-0.032994228	0.18437592	NS
PRAMEF14	NS	0.103912982	-0.032989772	0.18444197	NS

OR7G3	NS	0.103939638	0.032987231	0.184472367	NS
LRRTM4	NS	0.104181487	0.032964199	0.184884648	NS
RIM39-RPP2	NS	0.104362928	-0.032946948	0.185189662	NS
PES1	NS	0.104396198	0.032943788	0.185209283	NS
RAD9B	NS	0.104408779	-0.032942592	0.185209283	NS
COL26A1	NS	0.104401907	0.032943245	0.185209283	NS
OSR2	NS	0.10441226	-0.032942262	0.185209283	NS
P3H2	NS	0.104463291	-0.032937416	0.185282824	NS
IFNL1	NS	0.104506154	0.032933347	0.185341865	NS
SOCS4	NS	0.104527921	-0.032931282	0.185363485	NS
SAT1	NS	0.104565712	0.032927696	0.185413515	NS
SPAG5	NS	0.10458508	0.032925859	0.185413886	NS
PXYLP1	NS	0.104583726	0.032925987	0.185413886	NS
ZNF23	NS	0.10463328	-0.032921288	0.185482348	NS
PRR7	NS	0.104805317	0.032904986	0.185770303	NS
TRAPPC11	NS	0.104868863	0.03289897	0.18586592	NS
HSCB	NS	0.104930375	-0.03289315	0.185957915	NS
GGCX	NS	0.105033394	-0.032883408	0.186123443	NS
YWHAH	NS	0.105072797	-0.032879683	0.186138914	NS
CKAP5	NS	0.105080591	0.032878947	0.186138914	NS
OR52N1	NS	0.105065685	0.032880355	0.186138914	NS
KLK9	NS	0.105074894	0.032879485	0.186138914	NS
MGAT5	NS	0.105179116	-0.03286964	0.186296393	NS
RNF6	NS	0.105200499	0.032867621	0.186317217	NS
UBD	NS	0.105291008	-0.032859079	0.186460453	NS
TYMP	NS	0.105356504	-0.032852901	0.186543584	NS
NADSYN1	NS	0.105357226	-0.032852833	0.186543584	NS
DPP3	NS	0.105380461	-0.032850642	0.186567658	NS
ARHGAP40	NS	0.105421576	-0.032846766	0.186618727	NS
ZNF568	NS	0.105428589	-0.032846105	0.186618727	NS
RAET1E	NS	0.105535407	0.032836042	0.186790722	NS
FSTL4	NS	0.105599527	-0.032830005	0.186852953	NS
KYAT3	NS	0.105593101	-0.03283061	0.186852953	NS
PPOX	NS	0.105585385	0.032831336	0.186852953	NS
EPAS1	NS	0.105651087	0.032825152	0.186927096	NS
TRAPPC3	NS	0.10578185	-0.032812855	0.187141349	NS
PRAME	NS	0.105791668	0.032811932	0.187141612	NS
MS4A6E	NS	0.105893696	0.032802346	0.187304979	NS
BICD2	NS	0.105907231	-0.032801075	0.187311802	NS
CENPP	NS	0.105954757	-0.032796612	0.187378737	NS
CDH13	NS	0.106017171	-0.032790754	0.187471986	NS
UCP2	NS	0.106066106	0.032786163	0.187541385	NS
ITPR3	NS	0.106079405	-0.032784916	0.187547768	NS
GNL3L	NS	0.106091981	-0.032783736	0.187552871	NS
HSD17B12	NS	0.106169771	0.032776443	0.187656112	NS
PNMA1	NS	0.106166558	-0.032776744	0.187656112	NS
KLF7	NS	0.106229227	0.032770872	0.187744057	NS
T6GALNAC	NS	0.10631407	0.032762926	0.187876851	NS
ARMCX3	NS	0.106346511	-0.032759889	0.187899869	NS
NDUFA5	NS	0.106340956	0.032760409	0.187899869	NS
KLF16	NS	0.106454656	0.03274977	0.188073062	NS
UVSSA	NS	0.106481291	0.032747279	0.188073062	NS
TNIP2	NS	0.106473531	0.032748005	0.188073062	NS
AOPEP	NS	0.106526003	0.032743099	0.188131142	NS
AKR1E2	NS	0.106627399	-0.032733624	0.188293033	NS

DNAJC3	NS	0.106692892	0.032727508	0.188391499	NS
RNF26	NS	0.106775046	-0.03271984	0.188519365	NS
NELFB	NS	0.106807425	-0.032716819	0.188559332	NS
EMC4	NS	0.107004329	-0.032698465	0.188872497	NS
SFRP1	NS	0.107043178	0.032694847	0.18892384	NS
TM7SF2	NS	0.107062202	-0.032693075	0.188940189	NS
CYLC2	NS	0.107108175	-0.032688795	0.189004087	NS
NCALD	NS	0.107139721	-0.03268586	0.189042519	NS
ZRANB1	NS	0.10718675	0.032681484	0.189108261	NS
TRIM2	NS	0.107273364	0.03267343	0.189243823	NS
RPS4Y2	NS	0.107291269	-0.032671765	0.18925816	NS
CCNO	NS	0.107313174	0.032669729	0.189279551	NS
PGGT1B	NS	0.107327701	0.032668379	0.189287926	NS
LAP3	NS	0.107359957	0.032665382	0.189327563	NS
CCT7	NS	0.10754651	-0.032648063	0.189639268	NS
ZFHX3	NS	0.107569351	-0.032645944	0.189662266	NS
OR51A2	NS	0.107628828	-0.032640428	0.189749849	NS
OR6C3	NS	0.107739939	-0.03263013	0.189928439	NS
USP14	NS	0.107812228	0.032623435	0.190007759	NS
DYNLT1	NS	0.107814384	-0.032623235	0.190007759	NS
ACY1	NS	0.107808935	-0.032623739	0.190007759	NS
RETN	NS	0.107953495	-0.032610361	0.190235602	NS
RNFT2	NS	0.1079923	0.032606772	0.19028666	NS
SOS2	NS	0.108042228	0.032602156	0.190357306	NS
ATP13A4	NS	0.108082275	0.032598454	0.190410532	NS
GPR107	NS	0.108096966	0.032597097	0.190419083	NS
DDX27	NS	0.108171127	0.032590246	0.190515044	NS
PRR23D2	NS	0.108162287	-0.032591062	0.190515044	NS
ITGA7	NS	0.108400095	-0.032569118	0.19090094	NS
STEAP4	NS	0.108414536	-0.032567787	0.19090594	NS
ADSS1	NS	0.10842266	0.032567038	0.19090594	NS
MRPS12	NS	0.108452806	-0.032564259	0.190941651	NS
ZSCAN2	NS	0.108599091	-0.032550784	0.19118181	NS
GIMAP2	NS	0.108622278	0.03254865	0.19120524	NS
CDC20	NS	0.108685696	0.032542814	0.191299476	NS
NCOA1	NS	0.108701626	0.032541348	0.191310117	NS
BUD31	NS	0.108871772	-0.032525705	0.191592146	NS
USP11	NS	0.108891563	-0.032523887	0.191609554	NS
PRKAG2	NS	0.108929729	0.032520381	0.191659288	NS
SH3GL1	NS	0.10896547	0.032517099	0.191691595	NS
GOLGA6L9	NS	0.10896931	-0.032516747	0.191691595	NS
PRAMEF15	NS	0.108977801	0.032515967	0.191691595	NS
PCDH9	NS	0.109039304	0.032510322	0.19178235	NS
SMAD2	NS	0.109053376	-0.03250903	0.191789673	NS
ABCD1	NS	0.109144471	-0.032500674	0.191932441	NS
ZNF653	NS	0.109177454	0.03249765	0.191973002	NS
RGS5	NS	0.109272547	0.032488935	0.192122755	NS
INO80	NS	0.109283936	-0.032487892	0.192125329	NS
RGL2	NS	0.109301697	0.032486265	0.192139102	NS
SV2B	NS	0.109332119	-0.032483478	0.192175126	NS
NMU	NS	0.109450133	-0.032472676	0.192365093	NS
GOLPH3	NS	0.109479554	0.032469984	0.192399332	NS
NUP58	NS	0.109501863	0.032467944	0.192403601	NS
ICOSLG	NS	0.109497935	0.032468303	0.192403601	NS
VAMP3	NS	0.109538065	0.032464633	0.192449741	NS

S100A16	NS	0.109605198	0.032458496	0.192550209	NS
FLNB	NS	0.109672381	0.032452357	0.19265075	NS
KLHDC10	NS	0.109812098	-0.0324396	0.192861171	NS
NGRN	NS	0.109806639	0.032440098	0.192861171	NS
ISM2	NS	0.109850315	0.032436113	0.192910787	NS
PTBP1	NS	0.109864146	0.032434851	0.192917573	NS
ANGPTL6	NS	0.109901151	0.032431476	0.192943455	NS
SHMT1	NS	0.10989905	-0.032431667	0.192943455	NS
EMP2	NS	0.10990879	0.032430779	0.192943455	NS
FAM102B	NS	0.109944198	0.032427551	0.192982831	NS
PSMD2	NS	0.109961131	0.032426007	0.192982831	NS
TAF11L6	NS	0.109956786	0.032426403	0.192982831	NS
CAMK2A	NS	0.110089307	0.032414327	0.193172751	NS
FKBP1A	NS	0.110086246	0.032414606	0.193172751	NS
PAX2	NS	0.110162404	0.032407672	0.193283493	NS
BCAT2	NS	0.110403931	-0.032385704	0.193689702	NS
OCLN	NS	0.110490821	0.032377811	0.193824573	NS
CST1	NS	0.110534666	0.03237383	0.193866346	NS
TEX13C	NS	0.110527789	0.032374454	0.193866346	NS
SMIM29	NS	0.110578374	-0.032369862	0.193925433	NS
KIAA0513	NS	0.110592477	0.032368582	0.193925877	NS
TEX45	NS	0.110598665	0.032368021	0.193925877	NS
ADO	NS	0.11067259	0.032361314	0.194020346	NS
TUBA3C	NS	0.110672203	0.032361349	0.194020346	NS
IVL	NS	0.110705696	0.032358312	0.194060808	NS
IGFALS	NS	0.110742507	0.032354974	0.194107758	NS
MTCP1	NS	0.110759824	0.032353405	0.194120532	NS
URB1	NS	0.110821759	-0.032347792	0.194211496	NS
TMIE	NS	0.11085807	-0.032344502	0.194257542	NS
BMS1	NS	0.110868134	-0.032343591	0.19425759	NS
WFDC11	NS	0.110899286	-0.03234077	0.194277	NS
ZNF433	NS	0.110893857	-0.032341261	0.194277	NS
FOXK1	NS	0.110970058	0.032334363	0.194383388	NS
TAMALIN	NS	0.111021073	0.032329746	0.194455152	NS
MANSC4	NS	0.111069433	-0.032325372	0.194522253	NS
RIPOR1	NS	0.111176774	0.032315667	0.194692629	NS
ADGRE3	NS	0.111204065	0.032313201	0.194722804	NS
CRYBB3	NS	0.111272951	0.032306978	0.194800061	NS
NUCB1	NS	0.111278377	0.032306488	0.194800061	NS
DNA2	NS	0.111263501	-0.032307832	0.194800061	NS
COMMD9	NS	0.111316154	-0.032303077	0.194824077	NS
LNX2	NS	0.111322293	-0.032302523	0.194824077	NS
OR4A5	NS	0.111315574	-0.03230313	0.194824077	NS
ARRDC2	NS	0.111524651	0.032284267	0.195160577	NS
CCDC25	NS	0.111576183	-0.032279622	0.195233104	NS
CFAP92	NS	0.111680911	0.032270188	0.19539869	NS
MPPE1	NS	0.111706092	0.03226792	0.195425083	NS
DAPP1	NS	0.111749185	-0.032264041	0.195482803	NS
MID2	NS	0.111817823	-0.032257864	0.195567524	NS
ANKK1	NS	0.11181733	0.032257909	0.195567524	NS
ATP8A1	NS	0.111892339	0.032251162	0.195662492	NS
CBWD3	NS	0.111884641	-0.032251855	0.195662492	NS
REXO5	NS	0.111905638	0.032249967	0.195668069	NS
DHX8	NS	0.111936192	0.03224722	0.195703814	NS
RABGGTA	NS	0.11197544	-0.032243692	0.19573979	NS

KRT80	NS	0.111976994	-0.032243553	0.19573979	NS
ATR	NS	0.112072589	-0.032234965	0.195889202	NS
SYT14	NS	0.112189023	0.032224513	0.19607501	NS
MYL5	NS	0.112224474	-0.032221332	0.196119259	NS
PRRT1B	NS	0.112237594	0.032220156	0.19612448	NS
TM9SF2	NS	0.112291431	-0.032215327	0.196183133	NS
ORAI3	NS	0.112288658	-0.032215576	0.196183133	NS
LILRA1	NS	0.112358969	0.032209273	0.196283413	NS
MTRNR2L11	NS	0.112391006	-0.032206402	0.196321659	NS
SEMA3C	NS	0.112491001	-0.032197445	0.196478597	NS
IFI27	NS	0.112504345	-0.03219625	0.196484175	NS
COX7A2	NS	0.112544645	-0.032192643	0.196536823	NS
ENO4	NS	0.112566384	-0.032190697	0.196557052	NS
DEF6	NS	0.112596028	0.032188045	0.196585297	NS
KIF18B	NS	0.112602872	0.032187432	0.196585297	NS
BBOF1	NS	0.112644837	-0.032183678	0.196640824	NS
PICK1	NS	0.112696568	-0.032179052	0.19671339	NS
BTBD8	NS	0.112710079	-0.032177844	0.196719233	NS
DCN	NS	0.112744501	-0.032174767	0.196761569	NS
PPWD1	NS	0.11277341	0.032172183	0.196792211	NS
A2ML1	NS	0.112782393	0.03217138	0.196792211	NS
LSG1	NS	0.112802742	-0.032169562	0.196809976	NS
MAP3K21	NS	0.112867564	0.032163772	0.196905325	NS
SLC39A9	NS	0.112920442	0.032159051	0.19697982	NS
EREG	NS	0.112978055	0.032153908	0.197044804	NS
RTN4RL2	NS	0.112998682	0.032152068	0.197063022	NS
VAV2	NS	0.113044049	0.032148021	0.19712438	NS
CD300A	NS	0.113081163	0.032144711	0.197171335	NS
TRDMT1	NS	0.113126227	0.032140693	0.197232143	NS
DSC3	NS	0.11319188	0.032134842	0.197328833	NS
MARCHF9	NS	0.113324797	-0.032123004	0.197525999	NS
MORC4	NS	0.11337262	0.032118747	0.19759053	NS
CYP2F1	NS	0.113463755	0.03211064	0.197713759	NS
MACROD2	NS	0.113479864	0.032109207	0.19772403	NS
GLUL	NS	0.113656107	0.032093544	0.198013285	NS
TRUB2	NS	0.113702217	-0.032089449	0.198075792	NS
LINS1	NS	0.113750179	-0.032085192	0.19814151	NS
H1-0	NS	0.113761153	0.032084218	0.198142795	NS
WDR11	NS	0.113777794	0.032082741	0.198153949	NS
GTF2F2	NS	0.113935114	0.032068787	0.198410083	NS
REG3A	NS	0.113950681	-0.032067408	0.19841934	NS
ZBTB34	NS	0.113990081	0.032063916	0.198470091	NS
WNT8B	NS	0.11416805	-0.032048155	0.198762078	NS
MBTD1	NS	0.114232201	0.032042479	0.198855876	NS
TCP11L1	NS	0.114319168	0.032034788	0.198989371	NS
PARP8	NS	0.114411114	-0.032026661	0.19913151	NS
MTHFD2L	NS	0.114457812	0.032022536	0.199194876	NS
PPAN	NS	0.114476214	-0.032020911	0.199208991	NS
CD2BP2	NS	0.114523678	-0.03201672	0.199273671	NS
GCN1	NS	0.114623106	-0.032007944	0.199428749	NS
SPANXA2	NS	0.114660008	0.032004689	0.199475025	NS
OR5D18	NS	0.114678766	-0.032003035	0.199489727	NS
FAM47E	NS	0.114720908	0.031999318	0.199545103	NS
LPAR6	NS	0.114758405	0.031996013	0.199592388	NS
TP73	NS	0.11488156	-0.031985162	0.199782236	NS

TNFSF8	NS	0.114888204	0.031984577	0.199782236	NS
SLC26A6	NS	0.115006333	0.031974178	0.19996969	NS
CYB5A	NS	0.115051598	-0.031970195	0.200030426	NS
MT-CYB	NS	0.115207971	0.031956447	0.200266321	NS
RPN2	NS	0.115234457	0.03195412	0.200294375	NS
MLPH	NS	0.115271138	-0.031950897	0.20034014	NS
CELA2A	NS	0.115367678	-0.03194242	0.200489925	NS
PDHA2	NS	0.115548372	-0.031926569	0.200767889	NS
ZSWIM8	NS	0.115542103	0.031927119	0.200767889	NS
RBCK1	NS	0.115707686	0.031912609	0.201023758	NS
RBM44	NS	0.115716404	-0.031911846	0.201023758	NS
KPTN	NS	0.11584142	0.031900903	0.201222877	NS
SPAG9	NS	0.115861454	0.03189915	0.201239619	NS
LRFN5	NS	0.115903122	0.031895505	0.201293927	NS
EPHA2	NS	0.115945608	0.03189179	0.20134965	NS
IQCK	NS	0.115975567	-0.031889171	0.201383608	NS
HUNK	NS	0.11603771	0.031883739	0.201448399	NS
RPN1	NS	0.116042962	-0.03188328	0.201448399	NS
FBXW12	NS	0.116044102	-0.031883181	0.201448399	NS
MYLK2	NS	0.116135093	0.031875233	0.201588275	NS
STIMATE	NS	0.116167539	0.0318724	0.201626514	NS
CSTA	NS	0.116190918	-0.031870359	0.201649009	NS
TTC21A	NS	0.116216861	0.031868095	0.201675952	NS
NBR1	NS	0.116328359	-0.031858367	0.201851341	NS
ASB3	NS	0.116354102	0.031856122	0.201877913	NS
COL9A1	NS	0.11639111	0.031852896	0.201924022	NS
YARS2	NS	0.11643385	-0.031849171	0.201980069	NS
SRSF5	NS	0.116517199	0.031841909	0.202106542	NS
PRPF38B	NS	0.116571221	0.031837205	0.202182128	NS
RCOR1	NS	0.116701932	0.031825829	0.202390699	NS
NUCB2	NS	0.116725674	0.031823764	0.202413738	NS
RPS6KA1	NS	0.116939509	0.031805179	0.202766384	NS
DGKD	NS	0.11695436	0.031803889	0.202773969	NS
FUZ	NS	0.117075202	0.031793399	0.202965303	NS
PMPCB	NS	0.117117211	0.031789755	0.203015494	NS
RNASE3	NS	0.117133295	0.03178836	0.203015494	NS
FOXN2	NS	0.117135619	0.031788158	0.203015494	NS
SLC2A5	NS	0.117265838	-0.031776868	0.203209895	NS
SERPINH1	NS	0.117268781	-0.031776613	0.203209895	NS
MID1IP1	NS	0.117281259	0.031775532	0.203213324	NS
PGAM1	NS	0.117302194	0.031773718	0.203231406	NS
TMEM14C	NS	0.117369847	-0.031767858	0.203330417	NS
KMO	NS	0.117449063	-0.031760999	0.203449442	NS
NCL	NS	0.117540359	0.0317531	0.203553097	NS
CHST7	NS	0.117540451	-0.031753092	0.203553097	NS
CLASP2	NS	0.117535436	-0.031753526	0.203553097	NS
RRP36	NS	0.117563864	-0.031751067	0.203575429	NS
ERCC6	NS	0.117616274	0.031746534	0.203647965	NS
EIF2B1	NS	0.117759921	0.031734121	0.203878446	NS
PRAC2	NS	0.117849957	-0.031726346	0.204016078	NS
KRTAP19-4	NS	0.117890981	0.031722805	0.204053123	NS
MYT1	NS	0.11789244	0.031722679	0.204053123	NS
RPL18	NS	0.1182441	-0.031692367	0.204643491	NS
UPK3BL2	NS	0.118266421	0.031690445	0.204663821	NS
COL1A2	NS	0.11830238	0.03168735	0.204707747	NS

GLCE	NS	0.118368422	0.031681667	0.204803717	NS
C10orf120	NS	0.118563694	-0.031664879	0.205123245	NS
ATAD5	NS	0.118684652	-0.031654491	0.205314159	NS
NRN1L	NS	0.118805604	-0.031644112	0.205505028	NS
ADAMTS8	NS	0.118829609	0.031642053	0.205528184	NS
USPL1	NS	0.118872405	0.031638383	0.205565467	NS
MRPS17	NS	0.118864462	-0.031639064	0.205565467	NS
POU2F3	NS	0.118904356	-0.031635644	0.205583986	NS
PGAM2	NS	0.118896386	-0.031636327	0.205583986	NS
SLC39A11	NS	0.118939719	-0.031632613	0.205622375	NS
RCE1	NS	0.118947806	0.03163192	0.205622375	NS
TPM1	NS	0.119022187	-0.031625547	0.205732582	NS
UPK3B	NS	0.119089319	0.031619798	0.205830241	NS
UBE2E2	NS	0.119100094	0.031618875	0.205830484	NS
LSMEM1	NS	0.119138186	-0.031615614	0.205877933	NS
TP53TG3E	NS	0.11916651	-0.03161319	0.205908496	NS
CSNK1G3	NS	0.119185051	-0.031611604	0.205922151	NS
DESI2	NS	0.119258264	0.031605341	0.206030254	NS
CABP4	NS	0.119283563	0.031603177	0.206055569	NS
ZNF346	NS	0.119630084	0.03157358	0.206629792	NS
ETS1	NS	0.119637326	0.031572962	0.206629792	NS
DYRK3	NS	0.119918656	0.031548983	0.207086781	NS
GFM1	NS	0.119923317	-0.031548586	0.207086781	NS
CCDC142	NS	0.119939101	-0.031547242	0.207095562	NS
KCNK6	NS	0.119965301	-0.031545012	0.207117786	NS
XKR9	NS	0.119973373	0.031544325	0.207117786	NS
CPSF3	NS	0.119999354	-0.031542113	0.207144163	NS
ZNF674	NS	0.120270797	-0.031519031	0.207594217	NS
ZNF121	NS	0.120290323	0.031517372	0.207609407	NS
KANK3	NS	0.120327534	-0.031514212	0.207655113	NS
NUDT1	NS	0.120351159	-0.031512205	0.207677367	NS
PTPN5	NS	0.120394735	-0.031508505	0.207734042	NS
ANAPC5	NS	0.120424275	0.031505998	0.207766491	NS
RELCH	NS	0.120477919	0.031501446	0.207803475	NS
PI16	NS	0.120456536	-0.03150326	0.207803475	NS
CSPG4	NS	0.120474287	-0.031501754	0.207803475	NS
MS4A3	NS	0.120514734	-0.031498323	0.207848452	NS
C17orf49	NS	0.120581955	-0.031492622	0.20794586	NS
RIMKLA	NS	0.120610052	-0.03149024	0.207975784	NS
EEF1D	NS	0.120635186	0.031488109	0.208000594	NS
SYS1	NS	0.120705198	0.031482176	0.208102772	NS
ADRB2	NS	0.120771316	0.031476576	0.208198218	NS
PLEKHG2	NS	0.120804785	0.031473742	0.208237369	NS
TNFRSF10B	NS	0.120850072	0.031469908	0.208296883	NS
PRR20B	NS	0.120897565	0.031465889	0.208360187	NS
LRRK1	NS	0.121087513	0.031449826	0.208664216	NS
FASTK	NS	0.121095533	0.031449148	0.208664216	NS
TPST1	NS	0.121174675	0.031442462	0.208782002	NS
TMEM8B	NS	0.121193502	-0.031440872	0.208795854	NS
MAVS	NS	0.121361309	0.031426708	0.209066348	NS
ASNSD1	NS	0.121406392	0.031422905	0.209106792	NS
NUDT16L1	NS	0.121400077	0.031423438	0.209106792	NS
DNAJA1	NS	0.121423919	0.031421427	0.209118372	NS
USP17L7	NS	0.121513871	-0.031413844	0.209254669	NS
LHX8	NS	0.121656963	-0.031401789	0.209482446	NS

DNMT1	NS	0.121892105	-0.031382005	0.209850004	NS
LDLRAD4	NS	0.121887488	0.031382393	0.209850004	NS
MAP3K19	NS	0.122054507	0.031368358	0.210110906	NS
MAP3K15	NS	0.122120527	0.031362814	0.210205863	NS
C9orf24	NS	0.12221742	0.031354682	0.210353937	NS
SRFBP1	NS	0.122306056	-0.031347248	0.210487777	NS
CGGBP1	NS	0.12237957	0.031341085	0.210595569	NS
UPF1	NS	0.122395449	0.031339754	0.210604171	NS
DVL1	NS	0.122450193	-0.031335167	0.210679639	NS
RFTN2	NS	0.122463445	0.031334057	0.210683713	NS
RRP1B	NS	0.122513746	0.031329844	0.210751516	NS
PRXL2C	NS	0.122575384	0.031324683	0.210838811	NS
QARS1	NS	0.122753537	0.031309779	0.211107726	NS
EPB41L3	NS	0.122775521	0.031307941	0.211126775	NS
ZPLD1	NS	0.12284104	-0.031302464	0.211220678	NS
UBQLN4	NS	0.122883478	-0.031298918	0.21127488	NS
CDC73	NS	0.122928309	0.031295173	0.211333187	NS
NRBP2	NS	0.122941372	-0.031294082	0.211336873	NS
HDAC11	NS	0.122989418	-0.03129007	0.211397542	NS
FOXD4L1	NS	0.122998508	0.031289312	0.211397542	NS
MAN1C1	NS	0.123025872	0.031287027	0.211425799	NS
ID1	NS	0.123087514	-0.031281883	0.211512955	NS
SLC38A2	NS	0.123123546	-0.031278877	0.211556091	NS
NOP53	NS	0.123277813	0.031266015	0.21176502	NS
TM4SF4	NS	0.123277963	-0.031266002	0.21176502	NS
ZNF492	NS	0.123277893	0.031266008	0.21176502	NS
MYO1G	NS	0.123341645	0.031260696	0.211855611	NS
PKD1L3	NS	0.123378824	0.0312576	0.211900666	NS
NPHP1	NS	0.123398451	-0.031255965	0.211915573	NS
ANAPC15	NS	0.123487331	0.031248566	0.212049394	NS
HIKESHI	NS	0.123862001	0.03121742	0.212673901	NS
PLEKHM3	NS	0.123945152	-0.031210518	0.212797797	NS
CPNE6	NS	0.124052434	-0.031201618	0.21294421	NS
CEP97	NS	0.124044217	-0.0312023	0.21294421	NS
OR2K2	NS	0.124130212	-0.03119517	0.213058827	NS
FCMR	NS	0.124162828	-0.031192466	0.213095912	NS
CCDC200	NS	0.124250751	0.031185182	0.213227904	NS
WFDC10B	NS	0.124269236	-0.031183651	0.213240721	NS
CDK12	NS	0.12430465	0.031180719	0.213263677	NS
P6V1G2-DDX	NS	0.12430426	0.031180751	0.213263677	NS
TAB3	NS	0.124506793	0.031163992	0.213591552	NS
MICB	NS	0.124666083	0.031150826	0.213845862	NS
PREX2	NS	0.124865087	0.031134396	0.21415481	NS
PRPF6	NS	0.124872176	0.031133811	0.21415481	NS
GATA1	NS	0.124879383	-0.031133217	0.21415481	NS
PRR19	NS	0.124929852	0.031129054	0.214222379	NS
RAB11B	NS	0.124958297	0.031126708	0.214252174	NS
HAUS6	NS	0.124979179	-0.031124986	0.214268997	NS
PEX11A	NS	0.125030438	-0.03112076	0.214337893	NS
CSGALNACT	NS	0.125138896	-0.031111824	0.214504824	NS
SCG2	NS	0.125252307	-0.031102486	0.214661205	NS
SOWAHC	NS	0.125245891	0.031103014	0.214661205	NS
C1orf127	NS	0.125267322	-0.03110125	0.214667932	NS
ABHD12B	NS	0.125303956	-0.031098235	0.214692694	NS
PRNP	NS	0.12530143	0.031098443	0.214692694	NS

VASP	NS	0.125354158	0.031094105	0.214759699	NS
ADAT1	NS	0.125419464	0.031088734	0.214814543	NS
MOXD1	NS	0.12541627	-0.031088997	0.214814543	NS
TAFAZZIN	NS	0.125407523	0.031089716	0.214814543	NS
B3GNT5	NS	0.125513457	0.031081008	0.214956509	NS
CYP27A1	NS	0.125551481	-0.031077883	0.215002607	NS
TRIP6	NS	0.125690898	-0.031066434	0.215200286	NS
ZNF358	NS	0.12569015	0.031066495	0.215200286	NS
BPIFB3	NS	0.125732738	0.031063	0.215236833	NS
MRPL27	NS	0.125805754	-0.031057009	0.215342782	NS
GTPBP3	NS	0.125828086	0.031055178	0.215361963	NS
RBM23	NS	0.126053369	0.031036713	0.215709398	NS
GBF1	NS	0.126043966	0.031037484	0.215709398	NS
SLC35A4	NS	0.126142187	0.031029441	0.215842307	NS
ZNF793	NS	0.126169075	0.03102724	0.215869232	NS
SORCS3	NS	0.126182422	-0.031026148	0.215872985	NS
NT5M	NS	0.126249405	-0.031020667	0.215968492	NS
USP24	NS	0.12635668	0.031011895	0.216132901	NS
PPIP5K1	NS	0.126373219	-0.031010543	0.21614209	NS
ZZZ3	NS	0.126400155	-0.031008341	0.216169059	NS
WDR36	NS	0.126482692	-0.031001597	0.216286629	NS
FRG2B	NS	0.12649125	0.031000898	0.216286629	NS
TMEM260	NS	0.126603608	-0.030991724	0.216421393	NS
TTYH3	NS	0.126593976	0.03099251	0.216421393	NS
RAB31	NS	0.126597381	0.030992232	0.216421393	NS
BAG4	NS	0.126655594	0.030987481	0.216491141	NS
CD6	NS	0.126793429	-0.030976238	0.216707604	NS
TMEM71	NS	0.12683992	0.030972449	0.216767922	NS
TNFSF14	NS	0.126906191	0.030967048	0.216858758	NS
RPL30	NS	0.126915479	0.030966291	0.216858758	NS
CATIP	NS	0.127014032	0.030958265	0.217007998	NS
EPO	NS	0.127120418	0.030949606	0.217170593	NS
SNX19	NS	0.127200245	-0.030943112	0.21728779	NS
RAB2B	NS	0.127243721	0.030939576	0.217342875	NS
MARCHF2	NS	0.127370281	-0.03092929	0.217539853	NS
SCNN1A	NS	0.127382658	0.030928285	0.217540825	NS
COQ10B	NS	0.127404483	0.030926512	0.217540825	NS
DGKE	NS	0.127404566	0.030926505	0.217540825	NS
SPTB	NS	0.127467219	-0.030921417	0.217628605	NS
GABRB3	NS	0.127523086	0.030916882	0.217666389	NS
TLN2	NS	0.127516055	-0.030917452	0.217666389	NS
SLC8A1	NS	0.127507549	0.030918143	0.217666389	NS
TMC4	NS	0.127538835	0.030915603	0.21766821	NS
ZNF286A	NS	0.127546644	-0.03091497	0.21766821	NS
TAF2	NS	0.127681637	0.030904019	0.217879376	NS
DCAF10	NS	0.127702237	-0.030902348	0.21789532	NS
DHRS7B	NS	0.127766769	-0.030897117	0.217953898	NS
GPR68	NS	0.127779891	-0.030896054	0.217953898	NS
SPRR1A	NS	0.127753691	-0.030898177	0.217953898	NS
PCSK1	NS	0.12778161	-0.030895915	0.217953898	NS
TACC2	NS	0.127834159	0.030891657	0.218024318	NS
BCL11A	NS	0.127891433	0.030887017	0.218102782	NS
OMMD3-BM	NS	0.12792307	-0.030884456	0.218137516	NS
CDH10	NS	0.127963437	-0.030881187	0.218187128	NS
ANGPTL2	NS	0.128018054	-0.030876767	0.218256662	NS

METTL15	NS	0.128026769	0.030876062	0.218256662	NS
TTN	NS	0.128077138	-0.030871986	0.218323301	NS
GPR83	NS	0.128190544	0.030862816	0.218497373	NS
CSHL1	NS	0.128268481	0.030856517	0.218610963	NS
XPO4	NS	0.128433075	0.030843224	0.218872213	NS
OTUD6B	NS	0.128450283	-0.030841835	0.218882267	NS
HMGN5	NS	0.128488434	-0.030838756	0.218928005	NS
SMIM40	NS	0.12855959	0.030833016	0.219029964	NS
ABCB8	NS	0.128579734	-0.030831391	0.219045004	NS
ALDOC	NS	0.128614169	-0.030828614	0.219084384	NS
RAB27A	NS	0.128638472	0.030826655	0.219094843	NS
ZSCAN18	NS	0.128642947	0.030826294	0.219094843	NS
SAMHD1	NS	0.128691356	-0.030822392	0.219158005	NS
C9orf116	NS	0.128744091	-0.030818142	0.219228524	NS
WDR20	NS	0.128809444	0.030812878	0.219320514	NS
PLIN3	NS	0.128852047	0.030809447	0.219373755	NS
NAPG	NS	0.129044841	0.030793933	0.219668345	NS
RFNG	NS	0.129047776	0.030793697	0.219668345	NS
PPP2R5A	NS	0.12926481	0.030776254	0.220018437	NS
ERFL	NS	0.129326669	0.030771287	0.220104371	NS
SNRPG	NS	0.129341949	-0.03077006	0.220111021	NS
DOP1B	NS	0.129358338	-0.030768745	0.22011956	NS
CHTOP	NS	0.129394555	0.030765838	0.220161832	NS
LARGE1	NS	0.129421038	0.030763713	0.220187538	NS
MTIF3	NS	0.129611101	-0.03074847	0.220490034	NS
MTURN	NS	0.129621621	-0.030747627	0.220490034	NS
DMAP1	NS	0.129641819	-0.030746009	0.220505013	NS
SDHAF4	NS	0.129670813	-0.030743685	0.220534949	NS
TRAM2	NS	0.129730591	0.030738897	0.22060767	NS
BANF1	NS	0.129736366	-0.030738434	0.22060767	NS
COX7A2L	NS	0.129801425	-0.030733225	0.220679525	NS
OS9	NS	0.129877127	0.030727165	0.220788835	NS
ITIH1	NS	0.129913153	-0.030724283	0.220830684	NS
CHRNA5	NS	0.129930297	-0.030722911	0.22084043	NS
F2RL3	NS	0.129945548	-0.030721691	0.220846959	NS
PREB	NS	0.129983968	0.030718618	0.220892861	NS
ABHD1	NS	0.130057987	0.0307127	0.220999245	NS
ADRA2B	NS	0.130075764	0.030711279	0.22101005	NS
LY6G5C	NS	0.130088405	0.030710269	0.221012127	NS
CCDC66	NS	0.130146478	0.030705628	0.221091385	NS
INF2	NS	0.130280498	0.030694925	0.221299634	NS
LRIG2	NS	0.130342148	0.030690005	0.221384926	NS
C1orf68	NS	0.130501211	-0.030677317	0.221635645	NS
IST1	NS	0.130550168	0.030673415	0.221699337	NS
GTPBP4	NS	0.130577815	0.030671211	0.221714531	NS
PGP	NS	0.130582024	-0.030670876	0.221714531	NS
NME1-NME2	NS	0.130610214	-0.03066863	0.221742943	NS
FOXP4	NS	0.130697169	0.030661704	0.221871111	NS
TTC12	NS	0.130719427	-0.030659931	0.221889435	NS
CYTH2	NS	0.130756379	0.030656989	0.221913238	NS
NLRP12	NS	0.130746414	0.030657783	0.221913238	NS
TSPAN6	NS	0.130848294	-0.030649674	0.222034446	NS
PDCD5	NS	0.13085074	-0.03064948	0.222034446	NS
ANKEF1	NS	0.130863781	-0.030648442	0.22203711	NS
OR10H3	NS	0.131019897	0.030636028	0.222282507	NS

CALN1	NS	0.131037514	-0.030634628	0.222292912	NS
TMED9	NS	0.13119603	-0.030622036	0.222522814	NS
KRTAP22-1	NS	0.131192587	-0.03062231	0.222522814	NS
GABPA	NS	0.131310908	-0.030612918	0.222698145	NS
RAPGEF2	NS	0.131323229	0.030611941	0.222699528	NS
MAML1	NS	0.1313804	0.030607406	0.222776962	NS
C1orf112	NS	0.131475896	0.030599834	0.222919363	NS
CCDC80	NS	0.131578286	0.03059172	0.223073426	NS
ZNF575	NS	0.131615432	0.030588778	0.22311686	NS
ALOXE3	NS	0.131847931	0.030570376	0.223489679	NS
ANKRD34B	NS	0.131858449	-0.030569544	0.223489679	NS
OCM2	NS	0.131878942	-0.030567923	0.223504842	NS
TMEM51	NS	0.131916981	-0.030564916	0.223549736	NS
HTR3A	NS	0.132206424	0.030542051	0.22402062	NS
SAFB	NS	0.132230658	0.030540139	0.22402246	NS
CCL23	NS	0.132220657	-0.030540928	0.22402246	NS
STKLD1	NS	0.132428453	-0.030524538	0.224337925	NS
SLIT1	NS	0.132470513	0.030521223	0.224389538	NS
NEK6	NS	0.132504706	-0.030518529	0.224427817	NS
PLEKHG4B	NS	0.132534339	-0.030516194	0.224458365	NS
MIIP	NS	0.132603814	0.030510722	0.224536736	NS
GRHL2	NS	0.132749095	-0.030499287	0.224763076	NS
FAM229B	NS	0.132769838	-0.030497655	0.224778534	NS
ICAM3	NS	0.132826822	0.030493173	0.224842983	NS
DYNC2I1	NS	0.132831138	0.030492834	0.224842983	NS
GABRE	NS	0.132843227	0.030491883	0.224843783	NS
SLC17A7	NS	0.132919575	0.030485881	0.224953335	NS
CIRBP	NS	0.132982168	0.030480962	0.225019918	NS
KLHDC7A	NS	0.132974576	0.030481558	0.225019918	NS
FANCI	NS	0.133033391	0.030476938	0.225086916	NS
WIZ	NS	0.13309313	0.030472246	0.225146923	NS
CDC14B	NS	0.133087163	0.030472715	0.225146923	NS
POLH	NS	0.133103753	0.030471412	0.225146923	NS
PLCD4	NS	0.133185137	-0.030465024	0.2252649	NS
FDPS	NS	0.133228774	0.0304616	0.225319018	NS
DDX1	NS	0.133261587	-0.030459025	0.22535482	NS
SH3KBP1	NS	0.133388208	-0.030449096	0.22554924	NS
OXSR1	NS	0.133445675	0.030444593	0.225626702	NS
RALY	NS	0.133460373	0.030443441	0.225631844	NS
AP4S1	NS	0.133530176	-0.030437973	0.225730139	NS
DYNLL1	NS	0.133558418	-0.030435761	0.225758164	NS
SNRNP70	NS	0.133590189	0.030433273	0.225792149	NS
OR14A16	NS	0.133609555	-0.030431757	0.225805164	NS
TUBA4B	NS	0.133646633	0.030428855	0.225848107	NS
HAUS3	NS	0.133670251	-0.030427006	0.2258683	NS
MATK	NS	0.133695721	0.030425013	0.225891618	NS
UNC93B1	NS	0.133742833	0.030421327	0.225931775	NS
VWA5B2	NS	0.133741228	-0.030421453	0.225931775	NS
ALPK2	NS	0.13376718	-0.030419423	0.225953185	NS
MORF4L2	NS	0.134186107	0.030386696	0.226641038	NS
DNAJA3	NS	0.134236674	0.030382751	0.226706663	NS
URGCP	NS	0.13430012	-0.030377803	0.226794025	NS
DOCK2	NS	0.134314399	0.03037669	0.226798352	NS
IPO7	NS	0.134448313	0.030366253	0.227004671	NS
TIMM44	NS	0.134491467	0.030362891	0.227057727	NS

IFRD1	NS	0.134510887	0.030361379	0.227064791	NS
POPDC3	NS	0.134519114	0.030360738	0.227064791	NS
TLCD1	NS	0.134533455	-0.030359621	0.227069198	NS
RRM2	NS	0.134547461	-0.030358531	0.227073037	NS
LCTL	NS	0.134761365	-0.030341886	0.22741421	NS
REG3G	NS	0.134788335	-0.030339788	0.227439894	NS
SPATS1	NS	0.134862424	0.030334029	0.227525239	NS
DCUN1D2	NS	0.134876345	-0.030332947	0.227528894	NS
ADAMTS15	NS	0.134893511	0.030331613	0.227538021	NS
FGF14	NS	0.134952589	-0.030327023	0.227617839	NS
CEP83	NS	0.134995348	0.030323701	0.227670118	NS
UPF3A	NS	0.135009989	-0.030322564	0.227674974	NS
SULT1A2	NS	0.13505309	-0.030319218	0.227727818	NS
PRRT3	NS	0.135126554	0.030313515	0.227812001	NS
GLT6D1	NS	0.135123789	-0.03031373	0.227812001	NS
PMEL	NS	0.13532027	-0.03029849	0.228098855	NS
ORM2	NS	0.135314068	-0.030298971	0.228098855	NS
GINS2	NS	0.135364866	-0.030295033	0.228140977	NS
CYP2R1	NS	0.135368832	-0.030294726	0.228140977	NS
SPC25	NS	0.135381627	0.030293735	0.228142676	NS
AVEN	NS	0.13559372	0.030277309	0.228480199	NS
GAS2L1	NS	0.135633329	-0.030274243	0.228520039	NS
MXD4	NS	0.135667033	0.030271636	0.228524157	NS
TDRD5	NS	0.135659693	-0.030272203	0.228524157	NS
CABIN1	NS	0.135687144	0.03027008	0.228538145	NS
EDF1	NS	0.135705921	-0.030268627	0.228549884	NS
RLN1	NS	0.135734647	-0.030266406	0.228578374	NS
PNPT1	NS	0.135883245	0.030254918	0.228808708	NS
CBFA2T3	NS	0.135933515	0.030251034	0.228873445	NS
NT5C2	NS	0.13599813	0.030246044	0.22896232	NS
SDHA	NS	0.136022287	-0.030244178	0.228983074	NS
BAZ1B	NS	0.136175974	0.030232317	0.229221858	NS
VHL	NS	0.136230754	0.030228092	0.229274192	NS
C10orf105	NS	0.136221935	-0.030228772	0.229274192	NS
COPG2	NS	0.136273417	0.030224802	0.229326053	NS
CILP	NS	0.136373949	0.030217053	0.229475281	NS
BAG1	NS	0.13643755	0.030212153	0.229542392	NS
HS6ST1	NS	0.136435976	0.030212275	0.229542392	NS
RTTN	NS	0.136455796	-0.030210748	0.229553136	NS
LAMB1	NS	0.136490395	0.030208083	0.229571435	NS
POTEE	NS	0.136483158	0.030208641	0.229571435	NS
ARHGEF37	NS	0.13650409	-0.030207029	0.229574521	NS
AGBL1	NS	0.136547808	-0.030203663	0.229628095	NS
ASIC5	NS	0.136562422	-0.030202538	0.22963272	NS
SYNGAP1	NS	0.136582465	-0.030200995	0.229646472	NS
NAA20	NS	0.13666573	-0.030194588	0.229766513	NS
ZNF660	NS	0.136834537	0.030181608	0.230030336	NS
STK16	NS	0.136847967	0.030180576	0.230032935	NS
SLCO3A1	NS	0.136891331	0.030177244	0.230085845	NS
PPDPF	NS	0.136932548	-0.030174077	0.230135139	NS
H2AC20	NS	0.137010381	0.0301681	0.230245957	NS
BPTF	NS	0.137072344	0.030163343	0.230330089	NS
DHX30	NS	0.137102159	0.030161055	0.230360189	NS
UPK3BL1	NS	0.137165571	0.030156189	0.230446732	NS
ROR1	NS	0.13724177	-0.030150345	0.230534731	NS

TMPRSS11B	NS	0.137232519	-0.030151054	0.230534731	NS
ZC3HAV1L	NS	0.137644645	-0.030119487	0.231191405	NS
TAS2R4	NS	0.137773567	-0.030109627	0.231387867	NS
EML5	NS	0.137786644	0.030108627	0.231389753	NS
C8orf48	NS	0.137887247	0.030100938	0.231538609	NS
NUP133	NS	0.137975292	0.030094213	0.231666355	NS
CAPN9	NS	0.138007664	-0.030091741	0.231700611	NS
MYL9	NS	0.138167762	0.030079522	0.231909052	NS
SLITRK4	NS	0.13816575	-0.030079676	0.231909052	NS
BORCS6	NS	0.138151318	-0.030080777	0.231909052	NS
ARMC8	NS	0.138208117	0.030076444	0.231956674	NS
IFIT1	NS	0.138331542	-0.030067034	0.23214369	NS
LFNG	NS	0.13839398	0.030062276	0.232228339	NS
ZNF197	NS	0.138532035	0.030051762	0.232439847	NS
RNF43	NS	0.138586838	-0.030047591	0.232501019	NS
FHOD1	NS	0.138592517	-0.030047158	0.232501019	NS
TTC17	NS	0.138730695	0.030036646	0.232712655	NS
CCDC39	NS	0.138762158	-0.030034254	0.232745263	NS
SP7	NS	0.138862169	0.030026652	0.232872652	NS
CXCR2	NS	0.138860851	-0.030026752	0.232872652	NS
NUDCD3	NS	0.138938914	-0.030020821	0.232935207	NS
ODF1	NS	0.138947608	-0.030020161	0.232935207	NS
CCDC60	NS	0.138929527	-0.030021534	0.232935207	NS
PRKD1	NS	0.138941742	0.030020606	0.232935207	NS
ZNF460	NS	0.139065281	0.030011226	0.233112287	NS
SPTLC3	NS	0.139159821	0.030004051	0.233250563	NS
HES7	NS	0.139200775	0.030000945	0.233299005	NS
DCST1	NS	0.139216694	0.029999737	0.233305483	NS
TMEM187	NS	0.139239643	0.029997997	0.233323742	NS
SLC35A3	NS	0.139258384	-0.029996576	0.233334945	NS
IL6R	NS	0.139319305	0.029991957	0.233416815	NS
ARHGEF38	NS	0.139339651	-0.029990415	0.233430699	NS
KCNJ11	NS	0.13937644	-0.029987627	0.233472122	NS
UBA7	NS	0.139426339	0.029983847	0.233535498	NS
CINP	NS	0.139521208	0.029976662	0.233653963	NS
MRPS7	NS	0.139513131	0.029977273	0.233653963	NS
IDH3G	NS	0.13957072	0.029972914	0.233704846	NS
SLC25A20	NS	0.13957574	-0.029972534	0.233704846	NS
COLGALT1	NS	0.139622184	0.029969019	0.233762391	NS
SLC22A13	NS	0.139702321	0.029962956	0.233856103	NS
PLIN5	NS	0.139691352	0.029963785	0.233856103	NS
SUGP2	NS	0.139757249	0.029958802	0.23392782	NS
FCRL1	NS	0.139780297	-0.029957059	0.233946168	NS
SFTPA2	NS	0.140037153	0.029937653	0.234355797	NS
STC2	NS	0.14011465	-0.029931803	0.234414663	NS
CHMP3	NS	0.140120772	0.029931341	0.234414663	NS
CAMK2G	NS	0.140102223	-0.029932741	0.234414663	NS
CLMP	NS	0.140110272	0.029932133	0.234414663	NS
TTC28	NS	0.140174462	-0.02992729	0.23442344	NS
HK1	NS	0.140172168	0.029927463	0.23442344	NS
FAAP20	NS	0.140156751	0.029928626	0.23442344	NS
H2AC21	NS	0.14013832	0.029930017	0.23442344	NS
RBFA	NS	0.140255776	-0.029921156	0.234539161	NS
APOL1	NS	0.140378586	-0.029911898	0.234723466	NS
CHEK1	NS	0.140390244	-0.02991102	0.234723466	NS

IL17RC	NS	0.140447487	0.029906707	0.234798891	NS
CERS4	NS	0.140467051	-0.029905233	0.234811317	NS
RPL41	NS	0.140503711	0.029902472	0.234852317	NS
RGS12	NS	0.140616632	0.029893971	0.235020769	NS
PTOV1	NS	0.140683464	0.029888942	0.23509187	NS
NME7	NS	0.140681795	-0.029889068	0.23509187	NS
DPH2	NS	0.140747448	-0.029884129	0.235158188	NS
ODC1	NS	0.140796058	0.029880474	0.235212841	NS
SIPA1L2	NS	0.140804463	0.029879842	0.235212841	NS
ZC3H10	NS	0.140919243	0.029871215	0.235384266	NS
ANXA3	NS	0.140938643	-0.029869758	0.235396356	NS
ENPP2	NS	0.140968954	-0.029867481	0.235426667	NS
TREH	NS	0.141194404	0.029850556	0.235782839	NS
TOGARAM2	NS	0.141237856	-0.029847297	0.235835054	NS
MSANTD2	NS	0.141255206	-0.029845995	0.235843679	NS
SP100	NS	0.141350538	0.029838847	0.235982492	NS
RAP2C	NS	0.14146018	0.029830631	0.236134583	NS
TMEM169	NS	0.141466038	-0.029830192	0.236134583	NS
RPL7	NS	0.141510654	0.02982685	0.236188688	NS
NGLY1	NS	0.141556149	-0.029823443	0.236244251	NS
COIL	NS	0.141574174	-0.029822094	0.236253963	NS
XDH	NS	0.141615081	-0.029819031	0.236301854	NS
ACP3	NS	0.141642678	-0.029816966	0.236303614	NS
SCUBE3	NS	0.141645856	0.029816728	0.236303614	NS
ZNF524	NS	0.141652979	-0.029816195	0.236303614	NS
C11orf71	NS	0.141664969	0.029815297	0.236303614	NS
KIAA1614	NS	0.141836695	-0.029802453	0.236569675	NS
C1orf109	NS	0.141900002	-0.02979772	0.23664437	NS
BSND	NS	0.141905931	-0.029797277	0.23664437	NS
SUV39H1	NS	0.142013006	0.029789277	0.236802528	NS
GPSM2	NS	0.142034055	-0.029787705	0.236817225	NS
GRAMD1B	NS	0.142239736	-0.029772353	0.237102487	NS
ARHGEF26	NS	0.14223821	-0.029772467	0.237102487	NS
UBE2J1	NS	0.142241893	0.029772192	0.237102487	NS
PRND	NS	0.142320997	0.029766292	0.237213918	NS
DEFB135	NS	0.142486553	0.029753952	0.237469409	NS
SYCP1	NS	0.14255907	0.029748551	0.237569811	NS
TFAP2E	NS	0.142603784	-0.029745221	0.237623868	NS
SEC24A	NS	0.14270927	0.02973737	0.23777917	NS
ARHGAP10	NS	0.142807938	-0.02973003	0.237888781	NS
KRT71	NS	0.142821994	-0.029728984	0.237888781	NS
TRIM43B	NS	0.142817669	-0.029729306	0.237888781	NS
CD7	NS	0.142835903	0.02972795	0.237888781	NS
B4GAT1	NS	0.142836506	0.029727905	0.237888781	NS
WDR77	NS	0.142925543	-0.029721286	0.238016589	NS
FCRL6	NS	0.143036521	-0.02971304	0.23818091	NS
INTS8	NS	0.143143022	-0.029705131	0.23833775	NS
COQ6	NS	0.143284031	-0.029694666	0.238531497	NS
DONSON	NS	0.143280757	-0.029694909	0.238531497	NS
DUSP29	NS	0.143298993	0.029693557	0.238534906	NS
VRK3	NS	0.143339207	0.029690574	0.238541286	NS
FITM1	NS	0.143331493	-0.029691146	0.238541286	NS
ZNF714	NS	0.143378722	0.029687644	0.238586532	NS
PM20D2	NS	0.143421708	-0.029684457	0.238637547	NS
SLC5A4	NS	0.143532196	-0.029676269	0.238800858	NS

SFT2D3	NS	0.143676714	0.029665567	0.239020755	NS
CERKL	NS	0.143759133	-0.029659467	0.239137313	NS
SVOPL	NS	0.143854096	-0.029652442	0.239274717	NS
CERCAM	NS	0.143877803	-0.029650688	0.239293586	NS
JAK3	NS	0.143930491	0.029646793	0.239319524	NS
UCP1	NS	0.143918958	-0.029647645	0.239319524	NS
PPFIA4	NS	0.143922418	-0.02964739	0.239319524	NS
MTAP	NS	0.143987944	-0.029642546	0.239394489	NS
EPC2	NS	0.144125464	-0.029632386	0.23960255	NS
JMJD1C	NS	0.144166109	0.029629385	0.239649537	NS
UBXN2B	NS	0.144198452	0.029626997	0.239682717	NS
GINS4	NS	0.144241861	-0.029623793	0.239734282	NS
MYT1L	NS	0.144268227	0.029621847	0.239736933	NS
C2orf15	NS	0.144262176	0.029622294	0.239736933	NS
GLIPR1	NS	0.144445827	0.029608747	0.240011453	NS
NCS1	NS	0.144543503	-0.029601548	0.240153137	NS
RPL29	NS	0.144590356	-0.029598096	0.240210362	NS
PAX8	NS	0.144637276	0.02959464	0.24026769	NS
AZI2	NS	0.144664824	-0.029592611	0.24027221	NS
HNRNPCL1	NS	0.144663119	0.029592737	0.24027221	NS
CPPED1	NS	0.144693962	0.029590466	0.240298373	NS
AGAP9	NS	0.144705406	-0.029589623	0.240298373	NS
LYNX1	NS	0.144754228	0.029586029	0.240358826	NS
CCAR2	NS	0.144806872	0.029582155	0.240404993	NS
MAGED4B	NS	0.144794483	0.029583066	0.240404993	NS
CBWD5	NS	0.144899768	-0.029575321	0.240538586	NS
TAL1	NS	0.14505001	0.029564275	0.240746699	NS
SPRN	NS	0.145040835	-0.029564949	0.240746699	NS
ANP32E	NS	0.145160209	-0.029556179	0.240908943	NS
OR1R1P	NS	0.145204626	0.029552917	0.240961997	NS
ISG20	NS	0.145287773	-0.029546813	0.241079309	NS
CABYR	NS	0.145310486	-0.029545146	0.241096328	NS
MORN4	NS	0.145360402	0.029541483	0.241158476	NS
DUSP23	NS	0.145419998	0.029537111	0.241236671	NS
PRAM1	NS	0.145547571	0.029527757	0.24142761	NS
CCER2	NS	0.145678534	0.029518161	0.24162414	NS
MECR	NS	0.145704028	-0.029516294	0.241645718	NS
SLC27A1	NS	0.145743906	-0.029513374	0.241691145	NS
TNFAIP1	NS	0.145874876	0.029503787	0.241849986	NS
ATP7B	NS	0.145856234	0.029505151	0.241849986	NS
IQSEC1	NS	0.145877174	0.029503619	0.241849986	NS
ZNF333	NS	0.145895758	0.029502259	0.24186008	NS
PGLS	NS	0.145965332	0.02949717	0.241954694	NS
DOCK3	NS	0.146100638	0.029487277	0.242158242	NS
THEM4	NS	0.146130371	-0.029485104	0.242186784	NS
PARP14	NS	0.14616889	0.02948229	0.242229884	NS
QPCT	NS	0.146218054	0.029478698	0.242290613	NS
SETD1A	NS	0.146268119	0.029475042	0.242352825	NS
DGKZ	NS	0.146305519	-0.029472311	0.242394044	NS
MAP3K3	NS	0.146395219	-0.029465764	0.242521898	NS
INMT	NS	0.146453718	0.029461496	0.242598046	NS
H3-3A	NS	0.146499162	0.029458181	0.242652557	NS
PRRC2A	NS	0.146528195	0.029456064	0.242679878	NS
CCDC65	NS	0.146544921	-0.029454844	0.242686815	NS
PCSK5	NS	0.146565285	0.02945336	0.242699773	NS

WDR47	NS	0.146711794	-0.029442682	0.242921599	NS
NEK4	NS	0.14683662	-0.029433591	0.243094676	NS
TBX6	NS	0.146841442	-0.02943324	0.243094676	NS
OR2T3	NS	0.146877285	-0.029430631	0.243112428	NS
CLIC1	NS	0.146874886	0.029430805	0.243112428	NS
PRKAB2	NS	0.146964126	-0.029424311	0.243214571	NS
LRRC74B	NS	0.14696265	-0.029424418	0.243214571	NS
CCDC170	NS	0.146982947	-0.029422942	0.243224923	NS
PLCD1	NS	0.147054149	0.029417763	0.243321945	NS
TSPAN4	NS	0.147109714	0.029413722	0.243393078	NS
TRIP12	NS	0.147189142	0.029407949	0.243503678	NS
LDHB	NS	0.147496922	-0.0293856	0.243971152	NS
MLIP	NS	0.147491174	-0.029386017	0.243971152	NS
ZNF536	NS	0.147632963	-0.029375733	0.244175309	NS
OR2M5	NS	0.147650752	-0.029374443	0.244183864	NS
FCGR1A	NS	0.147733986	-0.02936841	0.244300644	NS
PKMYT1	NS	0.14790154	-0.029356273	0.244556827	NS
EIF2S2	NS	0.147954946	-0.029352407	0.244603343	NS
DCC	NS	0.147947885	-0.029352918	0.244603343	NS
USP17L13	NS	0.147978755	-0.029350684	0.24462181	NS
UBA5	NS	0.147996251	0.029349417	0.244629841	NS
TAGLN	NS	0.148051948	-0.029345387	0.244701008	NS
ZC2HC1C	NS	0.148446628	-0.029316862	0.245311443	NS
ZFYVE9	NS	0.148443905	-0.029317059	0.245311443	NS
DHCR24	NS	0.148622634	-0.02930416	0.24558133	NS
COQ3	NS	0.148736068	-0.02929598	0.245747787	NS
KRTAP12-2	NS	0.148818676	0.029290025	0.245863289	NS
ANXA2R	NS	0.148911831	-0.029283314	0.245996194	NS
PTPN14	NS	0.148935671	-0.029281597	0.24601458	NS
ATP5MJ	NS	0.149037828	-0.029274241	0.246162318	NS
TNFRSF21	NS	0.149142063	0.02926674	0.246313462	NS
TECTB	NS	0.149207045	-0.029262066	0.246399757	NS
UNC79	NS	0.149224277	-0.029260827	0.246407192	NS
CAND2	NS	0.149254787	-0.029258633	0.246436548	NS
OXNAD1	NS	0.149303122	-0.029255158	0.246495328	NS
SLC35G3	NS	0.149342156	0.029252352	0.246538743	NS
ACYP2	NS	0.149417421	0.029246944	0.246641958	NS
IFNA8	NS	0.149523189	-0.029239347	0.246795501	NS
INTS10	NS	0.149606315	-0.029233379	0.246894268	NS
CTBP1	NS	0.149608538	-0.02923322	0.246894268	NS
POGZ	NS	0.149763961	0.029222069	0.247129687	NS
XRN2	NS	0.149841303	0.029216523	0.247236235	NS
BMT2	NS	0.150093807	0.029198434	0.247631753	NS
RPA3	NS	0.150112807	-0.029197073	0.247641992	NS
ACAA2	NS	0.150183861	0.029191988	0.247738096	NS
PQBP1	NS	0.150211768	0.029189991	0.247744013	NS
PRMT7	NS	0.150213047	-0.029189899	0.247744013	NS
PFKFB2	NS	0.150255952	0.029186829	0.247793661	NS
TNFAIP8L1	NS	0.150319318	-0.029182297	0.247877043	NS
WNK1	NS	0.150396814	0.029176756	0.247962584	NS
PIGM	NS	0.15039593	-0.029176819	0.247962584	NS
CDH1	NS	0.150570699	0.029164331	0.248206988	NS
GRIP2	NS	0.150567648	-0.029164549	0.248206988	NS
NDUFB9	NS	0.150628189	0.029160226	0.248280611	NS
CNOT6L	NS	0.150663946	0.029157673	0.248318404	NS

FZD6	NS	0.150868372	0.029143086	0.248620222	NS
CST2	NS	0.150872759	0.029142774	0.248620222	NS
ZNF875	NS	0.150919802	-0.029139419	0.248676572	NS
ERCC8	NS	0.150979859	-0.029135138	0.24874929	NS
SLC5A3	NS	0.150989637	-0.029134441	0.24874929	NS
TSEN15	NS	0.151007316	-0.029133181	0.248757244	NS
OSBPL6	NS	0.151064634	0.029129097	0.248830487	NS
GRP	NS	0.151358996	-0.02910814	0.249294139	NS
PRAMEF10	NS	0.151419737	-0.02910382	0.249372963	NS
NLRP1	NS	0.151459824	0.029100969	0.249417761	NS
STXBP1	NS	0.15149249	0.029098647	0.249450331	NS
CCL4	NS	0.15150926	-0.029097455	0.249456723	NS
OR1K1	NS	0.151538087	0.029095406	0.249482964	NS
EXOSC10	NS	0.151573918	0.029092859	0.24952073	NS
PSMD14	NS	0.151623319	0.029089349	0.249538386	NS
FRS2	NS	0.151617788	0.029089742	0.249538386	NS
RNF41	NS	0.151600124	0.029090997	0.249538386	NS
OXCT1	NS	0.151752768	-0.029080155	0.249730197	NS
EPHA3	NS	0.151861118	-0.029072465	0.249887257	NS
IFNLR1	NS	0.151948388	-0.029066273	0.250009605	NS
NANS	NS	0.151994005	0.029063038	0.250042153	NS
GAB1	NS	0.151987372	0.029063508	0.250042153	NS
SPDYE4	NS	0.152045933	-0.029059356	0.250106322	NS
RIMS1	NS	0.152059878	-0.029058367	0.250108004	NS
M6PR	NS	0.152176995	-0.029050067	0.250274008	NS
OR5K2	NS	0.1521906	-0.029049104	0.250274008	NS
TAF9	NS	0.152199594	0.029048466	0.250274008	NS
ZNF16	NS	0.152425551	0.029032469	0.250624275	NS
CAPN13	NS	0.152474999	-0.02902897	0.250684285	NS
FAM236B	NS	0.152500366	0.029027176	0.250704696	NS
KHDRBS1	NS	0.152555594	0.02902327	0.250774188	NS
NEUROG1	NS	0.152597076	0.029020336	0.250821077	NS
SORD	NS	0.152818184	-0.029004713	0.251163179	NS
MFN2	NS	0.152850424	0.029002436	0.251194838	NS
SLC9A3R1	NS	0.152902485	0.02899876	0.251237733	NS
COQ8B	NS	0.152891058	0.028999567	0.251237733	NS
TRAFD1	NS	0.15299249	0.028992408	0.251364284	NS
FAU	NS	0.153064339	0.028987339	0.251460986	NS
TSGA13	NS	0.153119355	-0.028983459	0.25153002	NS
ARL6IP4	NS	0.153208982	0.02897714	0.251655894	NS
MEST	NS	0.153439841	0.028960878	0.252013709	NS
CNGA2	NS	0.15348277	0.028957855	0.252062829	NS
DPY19L4	NS	0.153569862	-0.028951726	0.252184462	NS
GALNTL5	NS	0.153649	-0.028946159	0.252293013	NS
EAPP	NS	0.153698474	-0.02894268	0.252331439	NS
TRIM10	NS	0.153688534	-0.028943379	0.252331439	NS
ASF1A	NS	0.153711656	0.028941753	0.252331678	NS
KIAA0040	NS	0.153781381	-0.028936852	0.252424729	NS
ATP5MGL	NS	0.153999912	0.0289215	0.252762002	NS
TMEM216	NS	0.154062922	-0.028917077	0.252843982	NS
EXOC2	NS	0.154122141	-0.028912921	0.252898284	NS
RBM10	NS	0.154113565	0.028913523	0.252898284	NS
SF1	NS	0.154191426	0.028908061	0.252983397	NS
TTLL11	NS	0.154200151	0.028907449	0.252983397	NS
RFC5	NS	0.154232764	-0.028905161	0.253009738	NS

HPGDS	NS	0.154242349	0.028904489	0.253009738	NS
NEXN	NS	0.154268492	-0.028902656	0.253031178	NS
CREM	NS	0.154299439	0.028900486	0.253039053	NS
ZNF592	NS	0.154290469	0.028901115	0.253039053	NS
GGA3	NS	0.154388591	0.028894237	0.25315198	NS
MSRA	NS	0.154394458	-0.028893826	0.25315198	NS
PHF11	NS	0.154544341	0.028883327	0.253376272	NS
CDC37	NS	0.154582279	0.028880671	0.253417006	NS
HIPK2	NS	0.154621844	0.028877901	0.253460401	NS
TRAP1	NS	0.154678742	-0.028873919	0.253532198	NS
CHADL	NS	0.154871726	-0.028860421	0.253827022	NS
SNAI2	NS	0.154892505	-0.028858969	0.253839584	NS
ZNF513	NS	0.154941239	-0.028855563	0.253897954	NS
ZNF213	NS	0.155115618	0.028843381	0.254162187	NS
KLHL33	NS	0.155165638	-0.028839889	0.254222625	NS
SWT1	NS	0.155304581	0.028830193	0.254428732	NS
TMEM251	NS	0.155359143	0.028826387	0.25445351	NS
FANK1	NS	0.155345827	0.028827316	0.25445351	NS
TBC1D3F	NS	0.155351496	0.02882692	0.25445351	NS
THBS4	NS	0.155379432	-0.028824972	0.254465207	NS
KLK14	NS	0.155435277	0.028821078	0.254535129	NS
SEC61A1	NS	0.15558481	0.028810657	0.254758446	NS
SIPA1L3	NS	0.15563989	0.02880682	0.254822692	NS
MINDY3	NS	0.155650377	0.02880609	0.254822692	NS
PRSS12	NS	0.155664642	-0.028805096	0.254824493	NS
SRM	NS	0.155804197	-0.028795381	0.255009812	NS
GCNT7	NS	0.155796053	-0.028795948	0.255009812	NS
METRN	NS	0.155838356	0.028793004	0.255022591	NS
KRT38	NS	0.155832061	0.028793442	0.255022591	NS
AGTRAP	NS	0.155891808	0.028789285	0.255088497	NS
TRAPPC2L	NS	0.155927315	-0.028786815	0.25512503	NS
H2BC4	NS	0.156009106	0.028781128	0.255237279	NS
NMD3	NS	0.156066616	0.02877713	0.255309788	NS
FAM227A	NS	0.156153062	-0.028771123	0.255429618	NS
SRD5A1	NS	0.156289414	-0.028761653	0.255609454	NS
LYPD4	NS	0.156276936	-0.02876252	0.255609454	NS
ZNF33B	NS	0.156316293	-0.028759787	0.255627149	NS
SVIP	NS	0.156437869	-0.02875135	0.255787413	NS
DECR2	NS	0.156464361	-0.028749512	0.25580912	NS
RCHY1	NS	0.156478005	0.028748566	0.25580982	NS
NCDN	NS	0.156621532	-0.028738614	0.256022834	NS
ANKRD13A	NS	0.15667447	-0.028734945	0.256066119	NS
FBLN5	NS	0.156662875	0.028735748	0.256066119	NS
ATP5F1C	NS	0.156735846	-0.028730692	0.256123177	NS
POU5F1	NS	0.156732251	0.028730941	0.256123177	NS
IMMT	NS	0.156750352	-0.028729687	0.256125258	NS
SGMS2	NS	0.156792516	-0.028726766	0.256149602	NS
GYPA	NS	0.156804951	0.028725905	0.256149602	NS
BOLA2	NS	0.156792365	-0.028726777	0.256149602	NS
SEPSECS	NS	0.156875578	0.028721015	0.256221728	NS
ZNF791	NS	0.156865305	0.028721726	0.256221728	NS
SCAMP4	NS	0.156899932	0.028719329	0.256239882	NS
C7	NS	0.157104598	0.028705168	0.256552485	NS
ST20	NS	0.15719241	-0.028699097	0.256674228	NS
ADCYAP1R1	NS	0.157260487	-0.028694392	0.256742071	NS

ANKRD16	NS	0.157249134	-0.028695176	0.256742071	NS
PCYT1B	NS	0.157389096	-0.028685507	0.256930365	NS
BCORL1	NS	0.157439357	-0.028682036	0.256990741	NS
AIF1	NS	0.157454294	-0.028681005	0.25699345	NS
MEA1	NS	0.157503793	-0.028677588	0.257052565	NS
FASTKD1	NS	0.15755776	-0.028673864	0.257118961	NS
PEDS1	NS	0.15762822	0.028669003	0.257212259	NS
CYC1	NS	0.157663574	0.028666564	0.257248263	NS
MTFMT	NS	0.15779802	-0.028657294	0.257445927	NS
TRPM3	NS	0.157893939	-0.028650685	0.257559621	NS
RPS19	NS	0.157907627	-0.028649742	0.257559621	NS
MED7	NS	0.157905004	-0.028649922	0.257559621	NS
KCNIP1	NS	0.158021165	0.028641922	0.25770138	NS
RASA3	NS	0.158011204	-0.028642608	0.25770138	NS
OSGEP	NS	0.158055593	-0.028639552	0.257735809	NS
WDR97	NS	0.158135547	-0.028634049	0.257844465	NS
WAS	NS	0.158233976	0.028627277	0.257983225	NS
NCBP3	NS	0.158381519	0.028617133	0.258182349	NS
PSAT1	NS	0.158382786	-0.028617046	0.258182349	NS
GABARAP	NS	0.158421693	-0.028614372	0.258224024	NS
BRSK1	NS	0.158613757	0.028601179	0.258515315	NS
LRP5	NS	0.158723927	-0.028593618	0.258673093	NS
NCEH1	NS	0.158826232	-0.028586599	0.258818029	NS
TAF11L5	NS	0.158870474	0.028583565	0.25886833	NS
ZNF407	NS	0.158901445	0.028581441	0.258897001	NS
ZNF614	NS	0.158977115	0.028576254	0.258998488	NS
CXCR5	NS	0.159027178	-0.028572823	0.259019098	NS
C3orf33	NS	0.159023511	-0.028573075	0.259019098	NS
XYD6-FXYD	NS	0.159029911	0.028572636	0.259019098	NS
KIAA1217	NS	0.159105224	0.028567477	0.259119959	NS
TBC1D3D	NS	0.159141342	0.028565003	0.259156977	NS
ACSS2	NS	0.159168621	0.028563135	0.259157791	NS
SIAH1	NS	0.159167088	0.02856324	0.259157791	NS
MYO1E	NS	0.159442237	-0.028544411	0.259581457	NS
MED24	NS	0.15962272	-0.028532074	0.259853438	NS
DNAI2	NS	0.159689109	0.028527538	0.259920133	NS
IRAK1	NS	0.159690547	0.02852744	0.259920133	NS
UNC80	NS	0.159736426	0.028524306	0.259972948	NS
CRYBA2	NS	0.159778992	0.0285214	0.26002036	NS
REPS2	NS	0.159962704	-0.028508862	0.260297445	NS
NID1	NS	0.160077847	0.028501009	0.26044102	NS
SLC2A8	NS	0.160069363	-0.028501588	0.26044102	NS
ZNF814	NS	0.160109034	-0.028498883	0.260469865	NS
ATP8B2	NS	0.160140435	0.028496743	0.260499055	NS
CAMKK2	NS	0.160167916	0.02849487	0.260521864	NS
ZNF26	NS	0.160219331	0.028491366	0.260583596	NS
TRPC6	NS	0.160259046	-0.02848866	0.26062629	NS
TUBGCP5	NS	0.160331203	-0.028483745	0.260721731	NS
RGPD3	NS	0.160436696	0.028476563	0.260871362	NS
GIT1	NS	0.16057623	-0.028467069	0.261076313	NS
HTR3E	NS	0.1606222	-0.028463942	0.261129121	NS
MTA3	NS	0.160702313	-0.028458495	0.261237421	NS
INPP5J	NS	0.160870521	0.028447065	0.261488899	NS
LZTR1	NS	0.160942171	0.028442198	0.261583396	NS
TXNDC9	NS	0.160957497	-0.028441158	0.261586341	NS

UBN1	NS	0.160994689	-0.028438633	0.261624817	NS
CARD11	NS	0.161054793	-0.028434553	0.261700519	NS
SLC30A1	NS	0.161207419	0.028424198	0.261926534	NS
SMC1B	NS	0.161288791	-0.028418681	0.26203675	NS
BRD8	NS	0.1613696	-0.028413203	0.262141598	NS
CBLB	NS	0.161380414	0.02841247	0.262141598	NS
C1orf167	NS	0.161482085	0.028405582	0.26228474	NS
ID2	NS	0.161534921	-0.028402004	0.262340498	NS
ABL2	NS	0.161543521	-0.028401422	0.262340498	NS
ZNF491	NS	0.161569282	0.028399677	0.26236032	NS
FAAH	NS	0.161586156	-0.028398535	0.26236571	NS
OR4C46	NS	0.161613073	0.028396713	0.262387406	NS
JSRP1	NS	0.161639341	-0.028394935	0.262408041	NS
IGFBP3	NS	0.161660527	0.028393501	0.262420425	NS
CAMK4	NS	0.161804789	0.028383741	0.26262898	NS
SLC29A4	NS	0.16181614	-0.028382973	0.26262898	NS
PHTF2	NS	0.161854308	0.028380392	0.262668901	NS
SCP2	NS	0.161924061	-0.028375677	0.26276007	NS
ZNF335	NS	0.161938327	0.028374713	0.262761192	NS
SCYL3	NS	0.162062237	0.02836634	0.262896326	NS
FRMPD1	NS	0.162062356	0.028366332	0.262896326	NS
MPND	NS	0.16220119	0.028356957	0.262999213	NS
USP22	NS	0.162213468	0.028356128	0.262999213	NS
GAD2	NS	0.162234481	-0.02835471	0.262999213	NS
NRG4	NS	0.16222901	0.028355079	0.262999213	NS
TANC2	NS	0.162196384	0.028357281	0.262999213	NS
FAM110C	NS	0.162215997	0.028355957	0.262999213	NS
NCR1	NS	0.162227654	0.02835517	0.262999213	NS
ANKRD20A3	NS	0.162156007	0.028360007	0.262999213	NS
AREG	NS	0.162304609	0.028349977	0.263090865	NS
ARHGAP39	NS	0.162371306	0.028345477	0.263176938	NS
AHCY	NS	0.162441273	-0.028340759	0.263268298	NS
ANAPC16	NS	0.162538587	-0.028334198	0.26340396	NS
PRPF40B	NS	0.162568812	-0.028332161	0.263408834	NS
ECHDC3	NS	0.162558787	-0.028332837	0.263408834	NS
BARX2	NS	0.162796074	0.028316854	0.263742599	NS
SYNJ2	NS	0.162815681	-0.028315534	0.263742599	NS
BDNF	NS	0.162802449	-0.028316425	0.263742599	NS
ALDH2	NS	0.162830754	0.028314519	0.263744943	NS
PSMC3IP	NS	0.162923611	0.02830827	0.263873267	NS
GABARAPL2	NS	0.162988445	-0.028303909	0.263956187	NS
SRGAP2C	NS	0.163013407	0.02830223	0.263974526	NS
SLC7A6OS	NS	0.16321697	-0.028288547	0.264259947	NS
ZDHHC24	NS	0.163215066	0.028288674	0.264259947	NS
NDUFAF7	NS	0.163235827	-0.02828728	0.264268373	NS
KCNA6	NS	0.163307861	0.028282441	0.26436288	NS
CYP4F12	NS	0.163365559	-0.028278566	0.264434167	NS
RANBP3	NS	0.163416602	0.02827514	0.264494668	NS
ZYG11B	NS	0.163447203	0.028273086	0.264522079	NS
NPIPB4	NS	0.163467478	0.028271725	0.264532773	NS
NPAS2	NS	0.163627185	-0.02826101	0.264746953	NS
CCDC28A	NS	0.163689991	0.028256799	0.264826435	NS
ZXDC	NS	0.163846873	0.028246285	0.265058092	NS
DPYSL5	NS	0.163862639	-0.028245229	0.265061444	NS
ANKRD1	NS	0.163921041	0.028241317	0.265133757	NS

SH2B1	NS	0.163948716	0.028239464	0.265156363	NS
HTR6	NS	0.163968132	-0.028238164	0.265165609	NS
DEFB130B	NS	0.164018045	-0.028234822	0.265224167	NS
HELT	NS	0.164104389	0.028229043	0.265341621	NS
MYO5B	NS	0.164148668	0.028226081	0.265358865	NS
GABRR3	NS	0.164156182	-0.028225578	0.265358865	NS
MANEAL	NS	0.164152664	-0.028225813	0.265358865	NS
MSGN1	NS	0.16425395	-0.028219039	0.265494735	NS
HSD17B14	NS	0.164465442	0.028204903	0.265814387	NS
AKR1C2	NS	0.164551093	-0.028199182	0.265930613	NS
ATP6V1C1	NS	0.164584346	-0.028196962	0.265962147	NS
RELB	NS	0.164658673	0.028192	0.266060044	NS
FEZ1	NS	0.164686402	-0.028190149	0.266082637	NS
FAM32A	NS	0.164757694	-0.028185392	0.266175605	NS
MINDY1	NS	0.164862449	0.028178405	0.266322614	NS
STAG3	NS	0.164935855	0.028173511	0.266418962	NS
AATK	NS	0.165110076	0.028161902	0.266678123	NS
RAB41	NS	0.165284881	-0.028150263	0.266938185	NS
HTR1B	NS	0.16532467	-0.028147615	0.266980169	NS
ARHGAP8	NS	0.165399847	0.028142613	0.26707929	NS
TSFM	NS	0.165416015	-0.028141538	0.267083115	NS
AK7	NS	0.165433519	-0.028140374	0.267089098	NS
ZFYVE26	NS	0.165501327	0.028135864	0.267166068	NS
CSNK1A1L	NS	0.1655088	0.028135368	0.267166068	NS
PNKP	NS	0.165538508	-0.028133392	0.267191741	NS
PIGH	NS	0.16555236	0.028132472	0.267191819	NS
KCNC3	NS	0.165838682	0.028113451	0.267631609	NS
P2RY6	NS	0.165935744	-0.028107008	0.267765924	NS
TFDP3	NS	0.166014962	-0.028101753	0.267826771	NS
ADAM32	NS	0.16601067	-0.028102037	0.267826771	NS
FBXO48	NS	0.166009972	-0.028102084	0.267826771	NS
MAT2A	NS	0.166064178	-0.028098488	0.267883843	NS
TACR2	NS	0.166087122	-0.028096967	0.267898527	NS
DMAC2L	NS	0.166152261	-0.028092648	0.267981266	NS
CLBA1	NS	0.166172968	0.028091275	0.267992333	NS
RYR3	NS	0.166259549	0.028085537	0.268109625	NS
SCGB1D4	NS	0.166305879	-0.028082467	0.268161995	NS
MSMB	NS	0.16636599	0.028078485	0.268236577	NS
RGPD4	NS	0.166419634	0.028074933	0.268300719	NS
PTPRU	NS	0.166443198	0.028073373	0.268313877	NS
MAPK3	NS	0.16645552	0.028072557	0.268313877	NS
NRGN	NS	0.166548967	-0.028066372	0.268442152	NS
BST2	NS	0.16659815	-0.028063118	0.268462876	NS
ZSWIM5	NS	0.166590428	0.028063628	0.268462876	NS
SYT12	NS	0.166603434	0.028062768	0.268462876	NS
DIPK2B	NS	0.166748542	0.028053171	0.268674335	NS
NCSTN	NS	0.166847739	0.028046614	0.26881179	NS
KLF1	NS	0.167124706	-0.028028322	0.269190797	NS
PSMD9	NS	0.167123186	0.028028422	0.269190797	NS
ASB12	NS	0.167122731	0.028028452	0.269190797	NS
ENDOD1	NS	0.16716211	-0.028025853	0.26922864	NS
ZNF232	NS	0.167201199	0.028023274	0.269247401	NS
PDXP	NS	0.167205091	0.028023017	0.269247401	NS
ZNF486	NS	0.167215489	0.028022331	0.269247401	NS
OR5M11	NS	0.16723422	0.028021095	0.269255163	NS

RIMS2	NS	0.167316592	0.028015662	0.269362186	NS
BRD4	NS	0.167414102	0.028009233	0.269455127	NS
JAML	NS	0.167403797	0.028009912	0.269455127	NS
ORC6	NS	0.167454324	0.028006581	0.269497456	NS
GOLM2	NS	0.16755367	-0.028000035	0.269634921	NS
POLQ	NS	0.167628762	0.027995089	0.269733338	NS
A4GNT	NS	0.167721495	-0.027988984	0.26983769	NS
PKIB	NS	0.167710288	-0.027989721	0.26983769	NS
SYTL3	NS	0.16779819	0.027983936	0.269916211	NS
IGFL4	NS	0.167789238	0.027984525	0.269916211	NS
CCDC121	NS	0.167898651	0.027977327	0.270032927	NS
ZNF22	NS	0.167936651	-0.027974827	0.270071604	NS
XYLB	NS	0.167985027	0.027971646	0.270126957	NS
DUSP22	NS	0.168044655	-0.027967726	0.270200394	NS
PLA2G7	NS	0.168058948	-0.027966787	0.270200931	NS
WDR74	NS	0.168189713	0.027958194	0.270388711	NS
PEBP1	NS	0.168233568	-0.027955314	0.270436753	NS
SPDYE21	NS	0.168318622	0.027949729	0.270551009	NS
NHLRC1	NS	0.16837436	-0.02794607	0.270618128	NS
CCDC32	NS	0.168410606	-0.027943691	0.27065391	NS
PDE7B	NS	0.168473849	-0.027939541	0.27073307	NS
GPATCH8	NS	0.16849313	0.027938276	0.270741576	NS
RPL37	NS	0.168563373	0.027933669	0.270831963	NS
CX3CR1	NS	0.168601721	0.027931154	0.270871092	NS
LAMA3	NS	0.168640234	0.027928629	0.270910479	NS
LEPR	NS	0.168656018	-0.027927594	0.270913352	NS
KRTAP5-6	NS	0.168777729	-0.027919618	0.27108636	NS
TBCA	NS	0.169060764	-0.027901086	0.271518433	NS
EXOC1	NS	0.169167365	-0.027894112	0.271667096	NS
B3GALT1	NS	0.169480463	0.027873648	0.272147324	NS
SSX2IP	NS	0.169680667	-0.027860577	0.272446205	NS
SLX4IP	NS	0.169759585	-0.027855428	0.27255031	NS
GATAD1	NS	0.169800574	-0.027852755	0.2725709	NS
PSENEN	NS	0.169789015	-0.027853509	0.2725709	NS
SELENOP	NS	0.169816563	-0.027851712	0.272573961	NS
VEPH1	NS	0.170018878	-0.027838523	0.27287607	NS
DNAJB9	NS	0.170070588	0.027835154	0.272935042	NS
TNNI1	NS	0.170083823	0.027834292	0.272935042	NS
HLA-DQB1	NS	0.17012353	0.027831706	0.272953502	NS
KIF19	NS	0.17011513	-0.027832253	0.272953502	NS
MGST3	NS	0.170348213	0.027817079	0.273291339	NS
AGER	NS	0.170475973	-0.027808769	0.27347364	NS
NXT2	NS	0.170513046	0.027806358	0.273510443	NS
DNAJC5	NS	0.170688245	-0.027794972	0.273728672	NS
SYNGR3	NS	0.170675597	0.027795793	0.273728672	NS
SETD2	NS	0.17069152	0.027794759	0.273728672	NS
CXCR6	NS	0.170757539	-0.02779047	0.273811857	NS
ALG10	NS	0.170798666	-0.0277878	0.273850717	NS
FHIT	NS	0.170810069	-0.027787059	0.273850717	NS
CLCN4	NS	0.170909607	-0.027780597	0.273969815	NS
RAET1L	NS	0.170912663	-0.027780399	0.273969815	NS
DDO	NS	0.171042603	-0.027771968	0.274155401	NS
ZNF790	NS	0.171131743	0.027766186	0.274275567	NS
RUVBL1	NS	0.171146576	0.027765225	0.274276629	NS
PIGZ	NS	0.171230278	-0.027759799	0.27438805	NS

HOMER3	NS	0.17137725	0.027750276	0.274532653	NS
MFSD4A	NS	0.171375604	0.027750382	0.274532653	NS
PLAAT3	NS	0.17134903	0.027752104	0.274532653	NS
FAM200A	NS	0.171348737	-0.027752123	0.274532653	NS
E2F1	NS	0.171413514	0.027747927	0.274568022	NS
TMEM174	NS	0.171484379	0.027743338	0.274641867	NS
CREB3	NS	0.171528113	0.027740507	0.274683392	NS
MREG	NS	0.171648572	0.027732712	0.274853553	NS
TYRO3	NS	0.171837295	0.027720507	0.275132988	NS
RTL4	NS	0.171962539	0.027712413	0.275310744	NS
LSM2	NS	0.172195407	-0.027697376	0.275660765	NS
PARD6B	NS	0.172225599	-0.027695427	0.275686296	NS
ZNF467	NS	0.172362582	0.02768659	0.275882754	NS
MYCBP2	NS	0.172432656	-0.027682071	0.275926462	NS
XRCC1	NS	0.172411672	0.027683424	0.275926462	NS
MS4A2	NS	0.172428375	0.027682347	0.275926462	NS
CILK1	NS	0.172462588	0.027680142	0.275951546	NS
CR2	NS	0.172576538	-0.027672797	0.27610414	NS
ZNF561	NS	0.172586484	0.027672156	0.27610414	NS
USP13	NS	0.172853931	-0.027654934	0.276509148	NS
SHFL	NS	0.173067829	0.027641174	0.276826882	NS
SPATA31A7	NS	0.17308116	-0.027640317	0.276826882	NS
ABRAXAS2	NS	0.173321019	0.027624903	0.277187609	NS
GNAZ	NS	0.173367632	-0.02762191	0.27723925	NS
OPRD1	NS	0.173383247	0.027620907	0.277241316	NS
SPIDR	NS	0.173461349	0.027615893	0.277333332	NS
FAM71F2	NS	0.173469449	-0.027615373	0.277333332	NS
ZC3H15	NS	0.173547393	0.027610371	0.277435029	NS
UNK	NS	0.173647362	0.027603957	0.277571917	NS
MAP2K6	NS	0.173708739	-0.027600021	0.277647098	NS
ACTBL2	NS	0.173853768	-0.027590725	0.277855959	NS
ZDHHC8	NS	0.173880264	-0.027589027	0.277875363	NS
TMED3	NS	0.173934186	-0.027585572	0.277938587	NS
FAT1	NS	0.17397433	-0.027583001	0.277969113	NS
DOCK1	NS	0.173982011	-0.027582509	0.277969113	NS
RBM41	NS	0.174014306	-0.027580441	0.277997763	NS
SLCO4A1	NS	0.174132738	0.027572859	0.278141053	NS
DEK	NS	0.174120086	0.027573668	0.278141053	NS
ULK2	NS	0.174165116	-0.027570786	0.278146864	NS
CDIPT	NS	0.174163568	0.027570885	0.278146864	NS
PI4KA	NS	0.1742714	0.027563986	0.27829364	NS
GLRX	NS	0.174287985	-0.027562925	0.278297166	NS
ADIPOR2	NS	0.174318863	0.02756095	0.27832351	NS
LMAN1L	NS	0.174368221	0.027557794	0.278379353	NS
FAM187B	NS	0.174481124	-0.027550577	0.27853663	NS
ZNF430	NS	0.174556736	0.027545745	0.278589446	NS
ACKR3	NS	0.174556004	-0.027545792	0.278589446	NS
SNORC	NS	0.174557388	-0.027545704	0.278589446	NS
CTC1	NS	0.174591774	0.027543507	0.278621353	NS
IQCC	NS	0.174658864	-0.027539222	0.278705439	NS
JOSD2	NS	0.174774386	-0.027531846	0.278866789	NS
OR6C75	NS	0.174847912	0.027527154	0.278961111	NS
R3HDM2	NS	0.174907271	0.027523367	0.279032814	NS
DHX57	NS	0.174925173	0.027522225	0.279038376	NS
CEACAM8	NS	0.174964412	0.027519722	0.279077969	NS

PRDM15	NS	0.175070286	0.027512971	0.279223834	NS
PLPP3	NS	0.175208398	0.027504169	0.279398069	NS
SSX4	NS	0.175202395	0.027504551	0.279398069	NS
NR5A2	NS	0.175246079	0.027501768	0.279435136	NS
DNAJC12	NS	0.175286241	0.02749921	0.279449388	NS
NR6A1	NS	0.17529833	-0.02749844	0.279449388	NS
FNTB	NS	0.17527409	-0.027499984	0.279449388	NS
PCDHB9	NS	0.175343951	0.027495535	0.27947608	NS
H2AC12	NS	0.175334713	-0.027496123	0.27947608	NS
ATP5MC2	NS	0.175512231	0.027484824	0.279682674	NS
STRA6	NS	0.17551447	0.027484681	0.279682674	NS
RNPS1	NS	0.175516917	0.027484526	0.279682674	NS
APCDD1	NS	0.175555613	-0.027482064	0.279721307	NS
CCT4	NS	0.175872799	-0.027461899	0.280180567	NS
PTPN9	NS	0.175872036	-0.027461947	0.280180567	NS
TMEM200B	NS	0.175905562	-0.027459817	0.280209699	NS
PPP4C	NS	0.175953687	0.027456761	0.280261601	NS
IMMP2L	NS	0.175967102	-0.027455909	0.280261601	NS
JCHAIN	NS	0.17599722	-0.027453996	0.280286506	NS
RAB4A	NS	0.176015676	-0.027452824	0.280292836	NS
IGSF9B	NS	0.176058421	-0.02745011	0.280337842	NS
ZNF789	NS	0.176080587	0.027448703	0.280350074	NS
SLC2A7	NS	0.176172483	0.027442871	0.280473317	NS
C2CD2L	NS	0.176301726	0.027434673	0.280655993	NS
CCT3	NS	0.176442654	0.027425738	0.280836649	NS
OSTM1	NS	0.176508665	-0.027421555	0.280916108	NS
PMM1	NS	0.17675951	-0.02740567	0.281292203	NS
C4A	NS	0.176792573	-0.027403577	0.28132169	NS
SFPQ	NS	0.176912552	-0.027395986	0.281489465	NS
STUB1	NS	0.176939622	0.027394274	0.281508452	NS
TBC1D31	NS	0.176953572	0.027393392	0.281508452	NS
CHCHD7	NS	0.17703882	-0.027388001	0.281620924	NS
ANKRD27	NS	0.177070776	-0.027385981	0.28164861	NS
PRSS16	NS	0.177146111	0.02738122	0.281745286	NS
MFAP3	NS	0.177251149	-0.027374584	0.281889183	NS
BRK1	NS	0.177275679	0.027373034	0.281905031	NS
SLC17A6	NS	0.177329513	-0.027369635	0.281944312	NS
PHLDA3	NS	0.177321134	-0.027370164	0.281944312	NS
NEPRO	NS	0.177370404	-0.027367053	0.281986163	NS
UHRF1BP1	NS	0.177516426	0.027357837	0.282195133	NS
MT1F	NS	0.1775413	0.027356268	0.282211497	NS
RASIP1	NS	0.177585293	0.027353493	0.282235071	NS
LRRC59	NS	0.177580627	0.027353787	0.282235071	NS
USP12	NS	0.177616657	0.027351514	0.282261742	NS
CES4A	NS	0.177819199	0.027338746	0.282560414	NS
ITGB8	NS	0.177851995	0.02733668	0.28258933	NS
KRT35	NS	0.177950249	-0.027330491	0.282722237	NS
AP3S2	NS	0.178041349	0.027324754	0.282843759	NS
NELFA	NS	0.178142043	-0.027318416	0.2829805	NS
SCAF1	NS	0.178179903	0.027316034	0.283017414	NS
NOS2	NS	0.17820877	-0.027314218	0.283038325	NS
DNAJC2	NS	0.178222314	0.027313366	0.283038325	NS
ATG2A	NS	0.178262652	0.027310829	0.283079161	NS
FLYWCH1	NS	0.178291258	-0.027309029	0.283101361	NS
NME5	NS	0.178321815	-0.027307108	0.283126655	NS

DUOX2	NS	0.178554738	-0.027292468	0.283473221	NS
PDIA5	NS	0.178692797	-0.027283798	0.283669137	NS
SH3PXD2A	NS	0.178756035	0.027279828	0.283746254	NS
UEVLD	NS	0.178890651	0.027271381	0.28393665	NS
NEK1	NS	0.179162284	0.02725435	0.28434447	NS
GGACT	NS	0.179248571	-0.027248944	0.284434771	NS
RAD21	NS	0.179237088	0.027249664	0.284434771	NS
C1orf74	NS	0.179367069	0.027241524	0.284599475	NS
FAM219A	NS	0.17944015	-0.027236949	0.284692094	NS
SLC27A3	NS	0.179545521	0.027230355	0.28481258	NS
PIGW	NS	0.179544869	-0.027230396	0.28481258	NS
CD47	NS	0.179587651	0.027227719	0.284856066	NS
STEAP1B	NS	0.179609884	-0.027226329	0.284867987	NS
C1orf52	NS	0.17966611	-0.027222813	0.284933816	NS
ANKRD20A4	NS	0.17978698	-0.027215256	0.285102145	NS
ZNF514	NS	0.179844345	-0.027211671	0.28516975	NS
ESPL1	NS	0.179910696	-0.027207526	0.285251592	NS
PRR20G	NS	0.179948347	-0.027205174	0.285287919	NS
SHISA4	NS	0.180059494	-0.027198234	0.285440751	NS
RNF227	NS	0.180101293	-0.027195624	0.285460254	NS
NTF3	NS	0.180088171	0.027196443	0.285460254	NS
ZNF250	NS	0.180319451	-0.027182013	0.285782633	NS
TAS2R9	NS	0.180347224	-0.027180281	0.285803248	NS
ARMC5	NS	0.180414544	0.027176084	0.285886526	NS
CYFIP1	NS	0.180723759	0.02715682	0.286353069	NS
MKNK1	NS	0.18079738	-0.027152237	0.286446271	NS
MRGPRX4	NS	0.180916055	0.027144853	0.286610833	NS
XNDC1N	NS	0.181033298	-0.027137561	0.286749633	NS
GGNBP2	NS	0.181024355	0.027138117	0.286749633	NS
TRIM4	NS	0.181148671	-0.027130389	0.286908901	NS
IL26	NS	0.181188695	-0.027127901	0.286948812	NS
TAL2	NS	0.1812368	-0.027124912	0.287001513	NS
SEZ6	NS	0.181277486	0.027122385	0.287042459	NS
NDUFA10	NS	0.181368962	-0.027116704	0.287163814	NS
MED27	NS	0.18175954	0.02709247	0.287758683	NS
FAM83B	NS	0.181867781	0.027085761	0.287906499	NS
BBS1	NS	0.181950452	-0.027080639	0.288013817	NS
PLSCR2	NS	0.182015554	-0.027076606	0.288093308	NS
BIRC5	NS	0.182374516	0.027054392	0.288637869	NS
UNC50	NS	0.182568962	-0.027042372	0.288914248	NS
ART3	NS	0.182578998	0.027041751	0.288914248	NS
ZNF629	NS	0.182699253	0.027034323	0.289032509	NS
LYL1	NS	0.182703787	-0.027034043	0.289032509	NS
GNAI2	NS	0.18270655	0.027033872	0.289032509	NS
RBMX2	NS	0.182713463	-0.027033445	0.289032509	NS
ZKSCAN1	NS	0.182749744	-0.027031205	0.289066277	NS
TAC3	NS	0.182820443	-0.02702684	0.289154477	NS
LONRF2	NS	0.182836686	0.027025837	0.28915654	NS
PIWIL3	NS	0.18291643	0.027020916	0.289259021	NS
FEM1C	NS	0.182933863	0.02701984	0.289262957	NS
GUCA2B	NS	0.182995153	0.027016059	0.289336234	NS
ATP9A	NS	0.183157228	-0.027006065	0.289568839	NS
CDKN2AIPNL	NS	0.183285743	-0.026998144	0.289748354	NS
TMEM131L	NS	0.18331044	0.026996623	0.28976373	NS
CD22	NS	0.183638425	-0.02697643	0.290234781	NS

ZBTB21	NS	0.183814882	0.026965577	0.290489946	NS
GSG1	NS	0.183868684	-0.02696227	0.290551249	NS
NSG2	NS	0.183930134	-0.026958493	0.290624626	NS
HAL	NS	0.184043722	-0.026951514	0.290694263	NS
CWF19L1	NS	0.184011694	0.026953482	0.290694263	NS
KRT37	NS	0.184010923	0.026953529	0.290694263	NS
CNOT1	NS	0.184049297	0.026951172	0.290694263	NS
BCL2L1	NS	0.184043581	0.026951523	0.290694263	NS
AMBP	NS	0.184147338	-0.026945151	0.290825382	NS
USP1	NS	0.184276841	0.026937202	0.290994959	NS
HLA-C	NS	0.18428478	0.026936714	0.290994959	NS
HROB	NS	0.184352459	0.026932562	0.291069629	NS
SRSF2	NS	0.184392219	-0.026930123	0.291069629	NS
IDH3A	NS	0.184380419	-0.026930847	0.291069629	NS
DEFB130A	NS	0.184382806	-0.0269307	0.291069629	NS
ZNF81	NS	0.184414481	-0.026928757	0.291081032	NS
MPZL3	NS	0.184455362	-0.02692625	0.291098083	NS
KRTAP20-1	NS	0.184440625	0.026927154	0.291098083	NS
C19orf18	NS	0.184478835	-0.026924811	0.291111391	NS
ZNF354B	NS	0.184494758	-0.026923834	0.291112785	NS
ABI2	NS	0.184591716	-0.026917891	0.291242033	NS
CDAN1	NS	0.184622671	0.026915994	0.291267131	NS
BCL7C	NS	0.184746046	-0.026908435	0.291438016	NS
RABL6	NS	0.184769907	0.026906973	0.291451905	NS
ARID1B	NS	0.185049924	-0.026889833	0.291865958	NS
PRR14L	NS	0.185062559	0.02688906	0.291865958	NS
TRIM75P	NS	0.185147448	0.026883868	0.291976048	NS
ZNF607	NS	0.185181432	-0.026881789	0.292005849	NS
TPP2	NS	0.185207752	0.02688018	0.292023563	NS
PRODH	NS	0.185259347	-0.026877026	0.292081121	NS
ACRV1	NS	0.185384344	-0.026869387	0.292254388	NS
COL24A1	NS	0.185526359	-0.026860713	0.292454452	NS
FNIP2	NS	0.185684787	0.026851042	0.292680353	NS
RAB5A	NS	0.185971205	0.026833574	0.293084077	NS
SPHK1	NS	0.185958341	0.026834358	0.293084077	NS
SLC26A9	NS	0.186017157	-0.026830773	0.29313263	NS
DAP3	NS	0.186141741	0.026823183	0.293305074	NS
GPR89A	NS	0.186214347	0.026818761	0.293395597	NS
PPARG	NS	0.186572154	-0.026796988	0.293935424	NS
FAM90A18P	NS	0.186690138	0.026789815	0.294097364	NS
SIRT2	NS	0.186750644	0.026786138	0.29416874	NS
TUG1	NS	0.186782577	0.026784197	0.294195098	NS
FBXO25	NS	0.187151434	-0.026761803	0.294752087	NS
LMO2	NS	0.187183096	0.026759882	0.294777968	NS
MATE-MUST	NS	0.18719992	0.026758861	0.294780479	NS
PPP1R3D	NS	0.187307635	0.026752329	0.294926103	NS
ABCA8	NS	0.187491523	-0.026741183	0.295191632	NS
CCDC146	NS	0.187516919	-0.026739645	0.295194661	NS
ZCCHC13	NS	0.187523949	0.026739219	0.295194661	NS
BTRC	NS	0.187553671	-0.026737418	0.29520481	NS
DEFB131A	NS	0.187560899	0.026736981	0.29520481	NS
EPRS1	NS	0.187697312	-0.02672872	0.295391621	NS
TIGD4	NS	0.187710112	0.026727945	0.295391621	NS
ATRIP	NS	0.187787044	0.026723289	0.295464642	NS
COMMD5	NS	0.187777252	0.026723881	0.295464642	NS

SHISA6	NS	0.187846132	0.026719713	0.295533588	NS
MPI	NS	0.188100835	-0.02670431	0.295886206	NS
SLC16A4	NS	0.188278221	-0.026693592	0.29614117	NS
WDFY3	NS	0.188310175	0.026691662	0.296167364	NS
HLA-A	NS	0.188352692	0.026689095	0.296210165	NS
SYT2	NS	0.188459805	0.026682628	0.296354535	NS
CEP68	NS	0.188478336	-0.02668151	0.296359599	NS
ANGPTL8	NS	0.188564517	-0.026676309	0.296471026	NS
SRXN1	NS	0.188818133	-0.026661015	0.296845662	NS
TBC1D20	NS	0.188968522	-0.026651953	0.297057964	NS
WFDC5	NS	0.189030503	-0.02664822	0.297131267	NS
CGAS	NS	0.189065845	-0.026646091	0.297162687	NS
KMT2E	NS	0.189178009	0.026639339	0.297314838	NS
SNX3	NS	0.189286252	0.026632825	0.297460801	NS
GDPD1	NS	0.18932	0.026630795	0.297489684	NS
CCDC71L	NS	0.189343823	-0.026629362	0.297502966	NS
MXD1	NS	0.189381595	0.02662709	0.297538161	NS
SLC38A10	NS	0.18946291	0.0266222	0.297641757	NS
CERS5	NS	0.189504007	-0.026619729	0.297682158	NS
PSME2	NS	0.189588786	-0.026614634	0.297791166	NS
RANBP9	NS	0.189645505	-0.026611226	0.297856085	NS
THG1L	NS	0.189703137	-0.026607764	0.297922428	NS
UNC5D	NS	0.189790721	-0.026602504	0.298035794	NS
SYNC	NS	0.189937344	0.026593702	0.298241846	NS
OR2B2	NS	0.189962058	-0.026592219	0.298256456	NS
GATC	NS	0.189993666	-0.026590322	0.298281888	NS
LCE5A	NS	0.190162762	-0.02658018	0.298523149	NS
MAGEB1	NS	0.190312225	-0.026571221	0.298733552	NS
SPIN4	NS	0.19037154	-0.026567667	0.298802427	NS
DYNC2I2	NS	0.19044905	0.026563024	0.298899848	NS
GABRG3	NS	0.190522198	0.026558643	0.298990405	NS
RAD51B	NS	0.190583528	-0.026554971	0.299062406	NS
LRIT3	NS	0.190619791	-0.026552801	0.299095062	NS
GON4L	NS	0.190646667	0.026551192	0.299112984	NS
ELF4	NS	0.190705299	0.026547683	0.299148465	NS
SEC16A	NS	0.190730492	0.026546176	0.299148465	NS
ZFYVE19	NS	0.190731102	-0.02654614	0.299148465	NS
BRINP2	NS	0.190709404	-0.026547438	0.299148465	NS
LUC7L3	NS	0.190811441	-0.026541334	0.299250222	NS
ATP2C1	NS	0.190843069	-0.026539442	0.299275576	NS
H2BC13	NS	0.190925169	0.026534533	0.299380069	NS
CAND1	NS	0.191288247	0.026512842	0.299898027	NS
CUL9	NS	0.191301971	0.026512023	0.299898027	NS
TRAF2	NS	0.191291905	-0.026512624	0.299898027	NS
PTPA	NS	0.191376255	-0.026507589	0.299990183	NS
BMPR1A	NS	0.191460309	0.026502573	0.300073339	NS
AGAP1	NS	0.191448458	-0.02650328	0.300073339	NS
PNMA2	NS	0.191484667	0.02650112	0.300087216	NS
ILK	NS	0.191539003	0.026497879	0.300148069	NS
SESN2	NS	0.191605979	0.026493885	0.300212223	NS
KLB	NS	0.191610963	-0.026493588	0.300212223	NS
RPL12	NS	0.191658629	-0.026490746	0.3002626	NS
C16orf78	NS	0.19171576	-0.02648734	0.300327796	NS
ZBTB10	NS	0.191784377	0.026483251	0.300410974	NS
CREBZF	NS	0.192032576	-0.026468469	0.300775412	NS

RCL1	NS	0.1920597	-0.026466854	0.300793555	NS
RAB3D	NS	0.192171862	0.026460179	0.300944868	NS
AB4B-EGLN	NS	0.192190358	0.026459079	0.300949484	NS
C20orf202	NS	0.192304343	-0.026452299	0.301103613	NS
ACTL8	NS	0.19235646	0.0264492	0.301160855	NS
TLR9	NS	0.192551845	-0.026437587	0.301442374	NS
GNB3	NS	0.192611717	-0.026434031	0.301511719	NS
AUTS2	NS	0.192674641	-0.026430293	0.301585828	NS
CLEC4E	NS	0.19279182	0.026423336	0.301744842	NS
HIGD1A	NS	0.19281003	-0.026422256	0.301748944	NS
ANXA11	NS	0.19282883	0.02642114	0.301753968	NS
BID	NS	0.192891138	0.026417442	0.301827071	NS
GAB2	NS	0.192955577	0.026413619	0.301903497	NS
SLC39A13	NS	0.19298003	0.026412169	0.30191735	NS
KRT28	NS	0.193043108	-0.026408428	0.301961954	NS
ZNF676	NS	0.193037884	-0.026408737	0.301961954	NS
MAS1L	NS	0.193055341	-0.026407702	0.301961954	NS
USP5	NS	0.193213051	-0.026398353	0.302184213	NS
ABHD17B	NS	0.193790548	-0.026364166	0.303038443	NS
NAP1L4	NS	0.193787765	0.026364331	0.303038443	NS
CFAP70	NS	0.193832278	-0.026361699	0.303079213	NS
FBXO8	NS	0.193895838	0.026357942	0.303154107	NS
ICMT	NS	0.193955182	-0.026354434	0.303222398	NS
TSTD3	NS	0.19413969	0.026343534	0.303486339	NS
ANAPC13	NS	0.19417282	-0.026341578	0.303513616	NS
COL5A3	NS	0.194293393	0.02633446	0.303677561	NS
MXD3	NS	0.194310829	-0.026333431	0.303680291	NS
PTPN4	NS	0.194432826	-0.026326233	0.303846422	NS
ATG2B	NS	0.194462987	0.026324453	0.303853263	NS
RIT2	NS	0.1944686	-0.026324122	0.303853263	NS
ING3	NS	0.194824418	-0.02630315	0.304384651	NS
SRPK2	NS	0.19484452	0.026301966	0.304391487	NS
C11orf53	NS	0.194929451	-0.026296964	0.304499592	NS
MSL3	NS	0.194980579	0.026293954	0.304530306	NS
TCOF1	NS	0.194969173	0.026294625	0.304530306	NS
GRAMD1A	NS	0.195026359	0.026291259	0.304577231	NS
ZNF706	NS	0.195147437	0.026284134	0.304741733	NS
KCNAB1	NS	0.195167495	0.026282954	0.30474847	NS
ACBD7	NS	0.195272551	0.026276776	0.304887916	NS
ZGPAT	NS	0.195352344	-0.026272084	0.304963302	NS
DACH1	NS	0.195347997	-0.02627234	0.304963302	NS
TRPM2	NS	0.195564589	-0.026259612	0.305270014	NS
CCL17	NS	0.195648499	0.026254684	0.305376368	NS
SLC25A44	NS	0.195996484	0.026234263	0.305894851	NS
PRC1	NS	0.196065543	-0.026230214	0.305977961	NS
SAP30	NS	0.196095183	-0.026228476	0.305999545	NS
STX8	NS	0.196246431	-0.026219612	0.306210877	NS
RESF1	NS	0.196262925	0.026218646	0.306211927	NS
TNFRSF12A	NS	0.196476922	0.026206113	0.306521102	NS
CPS1	NS	0.196665234	0.026195094	0.306790157	NS
SC5D	NS	0.196738252	-0.026190823	0.306854602	NS
P4HA3	NS	0.196732129	-0.026191181	0.306854602	NS
EVI2A	NS	0.196812732	-0.026186468	0.30692192	NS
USP6NL	NS	0.196813126	0.026186445	0.30692192	NS
WDCP	NS	0.196917055	-0.026180369	0.307059254	NS

AMY1A	NS	0.197014558	-0.026174672	0.307186546	NS
KRT78	NS	0.197041357	0.026173106	0.307203585	NS
CEBPZ	NS	0.197132174	0.026167802	0.307320422	NS
EMC8	NS	0.197254665	0.026160651	0.307474292	NS
OR9Q2	NS	0.197262645	-0.026160185	0.307474292	NS
MAD2L2	NS	0.197285726	-0.026158838	0.307485507	NS
GALNT3	NS	0.197552823	0.026143257	0.307877008	NS
ZBTB39	NS	0.197588676	-0.026141166	0.307908092	NS
VILL	NS	0.197695491	0.02613494	0.308049744	NS
KDM4B	NS	0.197778109	0.026130126	0.308153673	NS
GAGE12E	NS	0.197829166	0.026127152	0.308208414	NS
SSTR1	NS	0.197851768	0.026125836	0.308218819	NS
TRIM8	NS	0.198144309	0.026108807	0.308649705	NS
ESRP2	NS	0.198182907	0.026106562	0.308678099	NS
GOLGA6L10	NS	0.198194432	-0.026105891	0.308678099	NS
ZKSCAN8	NS	0.19837417	0.02609544	0.308933176	NS
PLA2G4C	NS	0.198421426	0.026092693	0.308981909	NS
KMT2D	NS	0.19854515	0.026085504	0.3091497	NS
KRT18	NS	0.198630972	0.026080519	0.30923358	NS
B3GNT4	NS	0.19862315	-0.026080973	0.30923358	NS
HAUS2	NS	0.198704344	0.026076258	0.309273181	NS
TMEM150B	NS	0.198698078	-0.026076622	0.309273181	NS
BORCS8	NS	0.198696592	0.026076709	0.309273181	NS
RABGGTB	NS	0.198721976	0.026075235	0.309275754	NS
CCNI	NS	0.198777302	-0.026072023	0.309336988	NS
SPATA3	NS	0.19886015	-0.026067215	0.309441038	NS
PPP1R9B	NS	0.198918291	-0.026063842	0.309501054	NS
DIAPH3	NS	0.198930699	-0.026063122	0.309501054	NS
DAPL1	NS	0.198968685	0.026060919	0.309535272	NS
SPATA48	NS	0.199007041	-0.026058694	0.309547086	NS
SNX33	NS	0.199008263	-0.026058623	0.309547086	NS
SMIM8	NS	0.199129406	-0.026051599	0.309663526	NS
NXPE3	NS	0.19914041	-0.026050961	0.309663526	NS
ZNF829	NS	0.199117477	0.026052291	0.309663526	NS
WDR45	NS	0.199147116	-0.026050573	0.309663526	NS
UBFD1	NS	0.199265506	-0.026043712	0.309822727	NS
DOCK11	NS	0.199302277	0.026041581	0.309846112	NS
DGKI	NS	0.199312562	0.026040985	0.309846112	NS
RIPK3	NS	0.19951731	0.026029129	0.310114596	NS
ARL14	NS	0.199506	0.026029784	0.310114596	NS
KCNA10	NS	0.199749236	-0.026015709	0.310450154	NS
GSR	NS	0.19977236	0.026014372	0.310461164	NS
DEDD	NS	0.199832552	0.026010891	0.310513812	NS
FCHSD1	NS	0.199838322	-0.026010558	0.310513812	NS
MTRNR2L3	NS	0.199949154	0.026004151	0.310661087	NS
ZNF319	NS	0.200217108	0.025988672	0.31105244	NS
CER1	NS	0.200283324	-0.025984849	0.311093575	NS
SLC13A3	NS	0.200291803	-0.02598436	0.311093575	NS
ZNF616	NS	0.200267483	0.025985764	0.311093575	NS
SUN5	NS	0.200329108	-0.025982207	0.311126552	NS
FMN2	NS	0.200397179	-0.025978279	0.3112073	NS
DOCK4	NS	0.200467788	0.025974205	0.311267006	NS
ADGRF3	NS	0.200459294	-0.025974695	0.311267006	NS
WTAP	NS	0.200517904	0.025971314	0.311319849	NS
CFAP97	NS	0.20059734	-0.025966733	0.311418199	NS

RNF170	NS	0.200669976	-0.025962546	0.311505979	NS
DLGAP4	NS	0.200729193	-0.025959133	0.311563943	NS
MYOCD	NS	0.200739509	-0.025958538	0.311563943	NS
NR2C2	NS	0.200795883	-0.02595529	0.311601468	NS
CBX6	NS	0.200793625	0.02595542	0.311601468	NS
FAM217B	NS	0.200895034	-0.025949578	0.311705354	NS
SERTAD2	NS	0.201008799	0.025943027	0.31185687	NS
TRIM55	NS	0.201047632	-0.025940792	0.311892118	NS
ARRDC3	NS	0.201251097	-0.025929084	0.31218274	NS
CNPY3	NS	0.201283659	0.025927211	0.312208231	NS
RPA1	NS	0.201401062	0.02592046	0.3123653	NS
TRIT1	NS	0.201475135	-0.025916202	0.312441407	NS
EIF2AK2	NS	0.20149601	0.025915002	0.312441407	NS
MTRES1	NS	0.201498558	-0.025914855	0.312441407	NS
PPM1A	NS	0.201547908	0.02591202	0.312453321	NS
TANC1	NS	0.201554669	0.025911631	0.312453321	NS
SPATA17	NS	0.20153397	0.02591282	0.312453321	NS
SEPHS1	NS	0.20166011	-0.025905574	0.312575067	NS
H4-16	NS	0.201665501	-0.025905264	0.312575067	NS
ASB11	NS	0.201757361	0.025899989	0.312692408	NS
SPDEF	NS	0.201804871	0.025897261	0.31271681	NS
AP3M1	NS	0.201805418	-0.02589723	0.31271681	NS
GBGT1	NS	0.201832057	-0.025895701	0.312733053	NS
PDCD4	NS	0.201907274	0.025891384	0.312799519	NS
CATSPER1	NS	0.201891521	-0.025892288	0.312799519	NS
AHNAK	NS	0.201935944	-0.025889739	0.312818898	NS
TMEM18	NS	0.202286229	-0.025869652	0.313329661	NS
SLC16A12	NS	0.202309173	-0.025868337	0.313329661	NS
NANOS1	NS	0.202314223	0.025868048	0.313329661	NS
PDHB	NS	0.202381588	0.025864188	0.313408915	NS
LATS2	NS	0.202400412	0.02586311	0.313412989	NS
HCCS	NS	0.202446652	0.025860461	0.313434442	NS
IL20RB	NS	0.202444104	-0.025860607	0.313434442	NS
NOP2	NS	0.202564457	-0.025853716	0.313591748	NS
TTC37	NS	0.202654111	0.025848584	0.313705452	NS
PDE11A	NS	0.202796585	0.025840432	0.313900896	NS
KIF18A	NS	0.203014069	-0.025827996	0.314212404	NS
MRPS18B	NS	0.203125168	-0.025821647	0.314359219	NS
GPS1	NS	0.203239876	-0.025815095	0.314511595	NS
LAYN	NS	0.203456346	-0.025802737	0.314821412	NS
DIS3	NS	0.203591695	0.025795015	0.315005664	NS
ZNF431	NS	0.203674135	0.025790313	0.315108029	NS
JOSD1	NS	0.203814181	0.025782329	0.315299496	NS
PRKAA2	NS	0.203894719	-0.02577774	0.31539888	NS
KIN	NS	0.204133288	-0.025764152	0.315742681	NS
GLIPR2	NS	0.20426306	0.025756766	0.315918161	NS
MRTFB	NS	0.204291918	-0.025755124	0.315937549	NS
PLD5P1	NS	0.204471846	-0.02574489	0.316190545	NS
KMT5A	NS	0.204511721	0.025742623	0.316226944	NS
DIP2C	NS	0.204635614	-0.025735581	0.316393238	NS
PRR29	NS	0.204720759	-0.025730743	0.316499602	NS
PIP4K2B	NS	0.204799088	-0.025726294	0.316595412	NS
EYA4	NS	0.204817133	0.025725269	0.316598021	NS
GPR3	NS	0.204914704	-0.025719729	0.31672355	NS
TFG	NS	0.204993906	0.025715233	0.316816873	NS

RNF213	NS	0.205007818	-0.025714443	0.316816873	NS
PHF10	NS	0.205224975	-0.025702124	0.317101829	NS
CYP4B1	NS	0.205219154	-0.025702454	0.317101829	NS
PACC1	NS	0.205329103	-0.02569622	0.317212078	NS
EIF2B3	NS	0.205315903	0.025696968	0.317212078	NS
SPPL3	NS	0.205538423	-0.025684358	0.317484776	NS
DMXL1	NS	0.205529955	-0.025684838	0.317484776	NS
HIF1A	NS	0.205687221	0.025675932	0.317689263	NS
TMEM65	NS	0.20610214	-0.025652458	0.318304718	NS
ATAT1	NS	0.206161822	0.025649084	0.318371487	NS
COX16	NS	0.206355364	-0.025638148	0.318606337	NS
CKAP2	NS	0.206335909	-0.025639247	0.318606337	NS
MOAP1	NS	0.20636328	0.025637701	0.318606337	NS
CFAP91	NS	0.206393499	0.025635994	0.318627578	NS
MORN5	NS	0.206440343	0.025633349	0.318674479	NS
SERPINB7	NS	0.206488372	-0.025630637	0.318723201	NS
SNTB1	NS	0.206511012	0.025629359	0.318732728	NS
SLC16A10	NS	0.206589094	0.025624952	0.318827819	NS
ADCY10	NS	0.206714345	0.025617885	0.318995684	NS
DUOXA1	NS	0.20681693	0.025612099	0.319128547	NS
DAAM2	NS	0.20690959	-0.025606874	0.319246076	NS
DPH6	NS	0.206961742	-0.025603935	0.319300078	NS
LONP1	NS	0.206977582	0.025603042	0.319300078	NS
RNF32	NS	0.207091958	-0.025596597	0.319451063	NS
CRYGD	NS	0.207410699	0.02557865	0.319866264	NS
HYOU1	NS	0.207398191	-0.025579354	0.319866264	NS
CBX4	NS	0.207499435	0.025573657	0.319977618	NS
COG3	NS	0.207532608	-0.025571791	0.319977789	NS
CXorf58	NS	0.207520882	-0.02557245	0.319977789	NS
SLC25A2	NS	0.207606915	-0.025567612	0.32004137	NS
CRYZL1	NS	0.207591475	0.02556848	0.32004137	NS
ELK1	NS	0.207656926	0.025564799	0.320067483	NS
RR5-ARHGA	NS	0.207643403	0.02556556	0.320067483	NS
CIAPIN1	NS	0.207770173	-0.025558433	0.320216535	NS
RAB6B	NS	0.207857848	-0.025553506	0.320300653	NS
TEX51	NS	0.207841531	0.025554423	0.320300653	NS
IL10RA	NS	0.207892521	0.025551558	0.320328581	NS
PAK4	NS	0.207913061	-0.025550404	0.32033473	NS
TP53BP2	NS	0.207986313	0.02554629	0.320422085	NS
INTS6	NS	0.20811825	0.025538882	0.320589051	NS
DPH7	NS	0.208129939	-0.025538225	0.320589051	NS
SRGAP1	NS	0.208144379	0.025537415	0.320589051	NS
MCM10	NS	0.208221489	-0.025533087	0.320682299	NS
WDR7	NS	0.208238439	0.025532136	0.320682889	NS
FAM95C	NS	0.208328325	-0.025527093	0.320795789	NS
HS2ST1	NS	0.208376755	-0.025524377	0.320844838	NS
ZNF506	NS	0.208411812	0.025522411	0.320873292	NS
ZNF581	NS	0.208473684	0.025518941	0.320933223	NS
CEP290	NS	0.208483899	-0.025518369	0.320933223	NS
ACSL4	NS	0.208576993	0.02551315	0.321050996	NS
ABHD2	NS	0.20862739	0.025510326	0.321103034	NS
GAGE12C	NS	0.208674352	0.025507694	0.321149778	NS
GPR139	NS	0.208698906	-0.025506319	0.321162031	NS
VPS28	NS	0.208789497	0.025501244	0.321275897	NS
EMC1	NS	0.208820967	0.025499482	0.321298779	NS

MAP7D2	NS	0.209035799	-0.025487456	0.321603761	NS
MIER2	NS	0.209136207	0.025481839	0.321732668	NS
SAXO1	NS	0.20922244	-0.025477016	0.321839748	NS
OR5J2	NS	0.209348156	-0.025469987	0.321981956	NS
EPPK1	NS	0.209337769	0.025470568	0.321981956	NS
D2HGDH	NS	0.209481524	0.025462534	0.322161479	NS
HS6ST2	NS	0.20955604	-0.025458371	0.322250473	NS
OCRL	NS	0.209590289	0.025456458	0.322277536	NS
UGGT1	NS	0.209706634	-0.025449962	0.322430821	NS
VDR	NS	0.209749991	0.025447541	0.322457859	NS
PLCXD1	NS	0.209757538	0.02544712	0.322457859	NS
FBLN7	NS	0.209790938	0.025445256	0.322483593	NS
PLAC8	NS	0.209838771	-0.025442586	0.322518153	NS
CALR	NS	0.209846746	0.025442141	0.322518153	NS
AK2	NS	0.210057667	0.025430376	0.32281669	NS
DRC7	NS	0.210336776	-0.02541482	0.323219961	NS
STIM2	NS	0.210381767	0.025412314	0.323263434	NS
SLC7A3	NS	0.210462412	-0.025407823	0.323361681	NS
NTRK1	NS	0.210485263	-0.025406551	0.32337112	NS
UBE2F	NS	0.210641546	-0.025397851	0.323585537	NS
CBX7	NS	0.210866993	-0.02538531	0.32390616	NS
PRR20C	NS	0.21096682	0.025379761	0.324033786	NS
OR2Z1	NS	0.211003861	0.025377702	0.324064964	NS
FANCD2OS	NS	0.211165414	-0.025368725	0.324261623	NS
TMEM184B	NS	0.211161735	0.02536893	0.324261623	NS
CD4	NS	0.211273848	0.025362703	0.324402396	NS
IDO1	NS	0.211355774	0.025358154	0.324476709	NS
ZNF587B	NS	0.211346165	-0.025358688	0.324476709	NS
AURKAIP1	NS	0.211385071	-0.025356528	0.32449595	NS
PAN2	NS	0.211538183	0.025348032	0.32470524	NS
FERMT3	NS	0.211616218	0.025343703	0.324799264	NS
NBEAL1	NS	0.211653135	-0.025341656	0.324830168	NS
CRMP1	NS	0.211706987	-0.025338669	0.324867794	NS
PGAP2	NS	0.211711219	-0.025338435	0.324867794	NS
RUSC1	NS	0.211748354	0.025336376	0.32489902	NS
ARHGAP27	NS	0.211787954	0.025334181	0.324934024	NS
TTC7A	NS	0.211805397	0.025333214	0.324935029	NS
DHFR	NS	0.211885711	-0.025328763	0.325032479	NS
USP32	NS	0.21196617	0.025324305	0.325130136	NS
HINT2	NS	0.212016511	-0.025321517	0.325181584	NS
SLC25A4	NS	0.212057662	-0.025319237	0.32521893	NS
ZBTB22	NS	0.212168506	0.0253131	0.325363144	NS
SNAPC3	NS	0.212200773	0.025311314	0.325386847	NS
NKX2-6	NS	0.212244871	0.025308873	0.325428686	NS
MUC3A	NS	0.212281592	0.025306841	0.325459207	NS
GRM4	NS	0.212427727	-0.025298756	0.325631669	NS
UBE2V2	NS	0.212426798	-0.025298808	0.325631669	NS
PHF7	NS	0.212489316	-0.02529535	0.325674495	NS
ADAMTS14	NS	0.212602733	0.02528908	0.32579673	NS
SEC24D	NS	0.212586923	0.025289954	0.32579673	NS
TATDN2	NS	0.212655197	0.02528618	0.32585133	NS
SLC4A7	NS	0.212792022	-0.02527862	0.32600937	NS
GDPGP1	NS	0.21278532	0.02527899	0.32600937	NS
SPSB2	NS	0.212901915	0.02527255	0.326151916	NS
CLTA	NS	0.21293233	0.025270871	0.326172695	NS

HADHB	NS	0.213012781	0.025266429	0.326270111	NS
VRK1	NS	0.213156283	-0.02525851	0.326438248	NS
NNT	NS	0.2131524	-0.025258724	0.326438248	NS
TRMT5	NS	0.213197157	0.025256255	0.326468669	NS
NBEA	NS	0.21320988	0.025255553	0.326468669	NS
MFAP2	NS	0.213376008	0.025246391	0.326697201	NS
PSAP	NS	0.213443553	0.025242668	0.32677477	NS
ANKRD30B	NS	0.213463642	-0.02524156	0.326779679	NS
CAP2	NS	0.213665303	-0.025230449	0.327062522	NS
KIF11	NS	0.213686302	-0.025229293	0.327068802	NS
KCNS1	NS	0.214030792	-0.025210331	0.327570176	NS
BABAM1	NS	0.214097474	-0.025206663	0.327646325	NS
ZCCHC3	NS	0.214139606	0.025204346	0.327684894	NS
TRABD2A	NS	0.214639145	-0.025176899	0.328423344	NS
USH2A	NS	0.214697324	0.025173706	0.328460434	NS
NRK	NS	0.214693001	-0.025173943	0.328460434	NS
SKOR1	NS	0.214718665	-0.025172534	0.328467121	NS
PPP4R3C	NS	0.214912907	-0.025161877	0.328738282	NS
GRSF1	NS	0.215308129	0.025140214	0.329316803	NS
TBRG4	NS	0.215495146	-0.025129974	0.329550767	NS
GUCY1A2	NS	0.215606855	-0.02512386	0.329695552	NS
OR1N1	NS	0.215679205	-0.025119901	0.329780133	NS
FAM90A22P	NS	0.215760698	0.025115443	0.329878678	NS
ASB13	NS	0.215856758	0.02511019	0.32999948	NS
KRTAP6-1	NS	0.215928611	-0.025106262	0.330083257	NS
BPI	NS	0.21599855	0.02510244	0.33015991	NS
DOCK8	NS	0.216012869	0.025101657	0.33015991	NS
DARS2	NS	0.216162006	-0.025093509	0.330361768	NS
CELF3	NS	0.216193494	0.02509179	0.330383805	NS
ST8SIA2	NS	0.216293992	-0.025086302	0.33051129	NS
MAN2A2	NS	0.21635605	0.025082914	0.330553927	NS
SULT1C2	NS	0.21635299	-0.025083081	0.330553927	NS
NOP56	NS	0.216418384	-0.025079512	0.330613768	NS
TEX261	NS	0.216446459	-0.02507798	0.330613768	NS
SLC25A22	NS	0.216444604	-0.025078081	0.330613768	NS
MYLIP	NS	0.216487829	0.025075723	0.330650865	NS
ENOX2	NS	0.216752279	-0.0250613	0.33102865	NS
PHKA2	NS	0.216915061	0.025052429	0.331251118	NS
SLC4A1AP	NS	0.217110039	-0.02504181	0.331494942	NS
GLOD5	NS	0.217126104	-0.025040935	0.331494942	NS
ZNF727	NS	0.217099673	-0.025042374	0.331494942	NS
GHR	NS	0.217261047	-0.02503359	0.331622492	NS
MIER3	NS	0.217256715	0.025033825	0.331622492	NS
LENG8	NS	0.217258522	0.025033727	0.331622492	NS
PLA2G2C	NS	0.217322235	-0.02503026	0.331689731	NS
ADTRP	NS	0.217551518	0.025017789	0.331993616	NS
ZIC1	NS	0.217555643	0.025017565	0.331993616	NS
UNC5CL	NS	0.217598362	0.025015243	0.332006455	NS
TCN1	NS	0.217584608	-0.02501599	0.332006455	NS
NUAK2	NS	0.21762151	0.025013985	0.332015603	NS
LRIT1	NS	0.217695978	0.025009937	0.332080426	NS
PRTG	NS	0.217698312	-0.02500981	0.332080426	NS
ATP13A1	NS	0.217755955	0.025006678	0.33210578	NS
CLDN34	NS	0.217766406	0.02500611	0.33210578	NS
MAN1B1	NS	0.21786299	0.025000864	0.3322269	NS

TST	NS	0.217881185	-0.024999876	0.332228471	NS
SSX1	NS	0.217996763	-0.0249936	0.332363799	NS
GAB3	NS	0.218004278	0.024993192	0.332363799	NS
TBCD	NS	0.218027441	-0.024991935	0.332372934	NS
MTX3	NS	0.218058247	-0.024990262	0.332393717	NS
EMC6	NS	0.218192089	-0.024982999	0.332571548	NS
SHROOM1	NS	0.218310022	-0.024976602	0.332725101	NS
DIS3L2	NS	0.218348717	0.024974504	0.332757875	NS
AGT	NS	0.218411519	-0.024971099	0.332781916	NS
RBP4	NS	0.21841607	0.024970852	0.332781916	NS
ZNF83	NS	0.218383646	0.02497261	0.332781916	NS
USF2	NS	0.218472686	0.024967783	0.332841976	NS
CIAO3	NS	0.21878613	-0.024950802	0.333282987	NS
LEP	NS	0.218878423	0.024945805	0.333381393	NS
CCDC24	NS	0.218929529	0.024943039	0.333432998	NS
CFLAR	NS	0.218974003	0.024940632	0.333448258	NS
GPX6	NS	0.218972918	0.024940691	0.333448258	NS
XAGE2	NS	0.219066659	0.024935619	0.33356311	NS
COA4	NS	0.219104468	-0.024933573	0.333594438	NS
IPCEF1	NS	0.219185965	-0.024929166	0.333692271	NS
NDUFA4	NS	0.21922842	-0.02492687	0.333730656	NS
UBXN10	NS	0.219350281	-0.024920282	0.333889905	NS
SETD6	NS	0.219468883	-0.024913873	0.33402875	NS
LRRN2	NS	0.21947601	-0.024913488	0.33402875	NS
TP53I3	NS	0.21955445	0.024909251	0.334105964	NS
PCDHA10	NS	0.219561266	0.024908883	0.334105964	NS
GATM	NS	0.219704135	-0.024901168	0.334297087	NS
PAGE3	NS	0.219756373	0.024898348	0.334350288	NS
DEFA3	NS	0.219821852	-0.024894814	0.334423624	NS
TSPAN7	NS	0.219872691	-0.024892071	0.334474679	NS
TMEM218	NS	0.220034996	-0.024883316	0.334695275	NS
FIZ1	NS	0.220105335	0.024879523	0.334775959	NS
MAGEA10	NS	0.220127132	0.024878348	0.334782806	NS
VGLL3	NS	0.220211805	-0.024873784	0.334885269	NS
RPS11	NS	0.220430707	0.02486199	0.335191826	NS
GMPPA	NS	0.220479434	-0.024859366	0.335239585	NS
RRP8	NS	0.220811567	-0.02484149	0.335718223	NS
ANKRD12	NS	0.22087149	0.024838267	0.335782953	NS
CMKLR1	NS	0.221065582	0.024827832	0.33605163	NS
FUT3	NS	0.221085116	0.024826782	0.336054932	NS
ARMCX5	NS	0.221203888	-0.0248204	0.336209067	NS
CMBL	NS	0.22123439	-0.024818762	0.336229024	NS
ALDH7A1	NS	0.221331567	0.024813542	0.336350303	NS
BLMH	NS	0.221488892	-0.024805096	0.336562961	NS
ACY3	NS	0.221536981	0.024802515	0.336583187	NS
C1D	NS	0.221529815	0.0248029	0.336583187	NS
SHLD2	NS	0.221690228	0.024794293	0.336769811	NS
CSTF3	NS	0.221694613	-0.024794058	0.336769811	NS
ZNF782	NS	0.221848665	0.024785797	0.336950938	NS
TRIM39	NS	0.221847068	0.024785883	0.336950938	NS
CCL2	NS	0.222012107	0.024777037	0.337172722	NS
HNRNPA3	NS	0.222062434	0.024774341	0.337222696	NS
CLCF1	NS	0.222144054	-0.024769968	0.337303022	NS
TOGARAM1	NS	0.222150182	-0.02476964	0.337303022	NS
BIRC2	NS	0.222295541	0.024761857	0.337497254	NS

ZDBF2	NS	0.222408239	-0.024755825	0.337641873	NS
PPP6R1	NS	0.222454169	-0.024753367	0.337685114	NS
RPS10	NS	0.222546656	0.024748419	0.337799017	NS
MYRF	NS	0.222614788	-0.024744775	0.337875937	NS
CPSF7	NS	0.222654559	0.024742648	0.337909804	NS
STX2	NS	0.222912647	-0.024728853	0.338260257	NS
KLHDC1	NS	0.22292043	-0.024728438	0.338260257	NS
POLI	NS	0.223126396	-0.024717437	0.338546251	NS
PDGFB	NS	0.22337093	0.024704386	0.338890713	NS
ASB7	NS	0.223438624	-0.024700775	0.338966848	NS
KDM1B	NS	0.223485762	-0.024698261	0.339011788	NS
SH3BGRL	NS	0.223526873	0.024696069	0.339027052	NS
PATJ	NS	0.223548371	0.024694923	0.339027052	NS
SEMA4D	NS	0.223535754	-0.024695595	0.339027052	NS
RBM34	NS	0.22357922	0.024693278	0.339047272	NS
OSBPL1A	NS	0.223648099	-0.024689606	0.3391042	NS
DCK	NS	0.223651799	-0.024689409	0.3391042	NS
NPIPB5	NS	0.223745072	0.024684438	0.33921905	NS
MAPK4	NS	0.223809531	0.024681003	0.3392902	NS
RILPL1	NS	0.223844187	0.024679157	0.339316163	NS
SELENOW	NS	0.223890989	-0.024676664	0.339360531	NS
CLUL1	NS	0.223935165	0.024674312	0.339400912	NS
RMND1	NS	0.223993803	-0.024671189	0.339463205	NS
TXK	NS	0.224429058	-0.024648031	0.340096206	NS
SERPINA7	NS	0.224503944	-0.02464405	0.340154339	NS
PRRG2	NS	0.22452014	0.024643189	0.340154339	NS
ZNF799	NS	0.224502056	-0.02464415	0.340154339	NS
CMTM3	NS	0.224656618	-0.024635936	0.340293827	NS
ANGEL2	NS	0.224681256	0.024634627	0.340293827	NS
PAK2	NS	0.224682533	0.024634559	0.340293827	NS
NKAIN3	NS	0.224670791	-0.024635183	0.340293827	NS
IRAG1	NS	0.224727528	0.024632169	0.340335344	NS
BTN2A1	NS	0.22485222	-0.024625547	0.340470904	NS
TLX1	NS	0.224914759	0.024622226	0.340538961	NS
ZCRB1	NS	0.224949982	0.024620356	0.340565651	NS
TIGAR	NS	0.225007149	0.024617322	0.340625557	NS
H3-3B	NS	0.225233561	0.02460531	0.340941645	NS
HDLBP	NS	0.225389039	0.024597067	0.341144847	NS
CXCL3	NS	0.225404533	0.024596245	0.341144847	NS
PPID	NS	0.225420675	-0.02459539	0.341144847	NS
PAOX	NS	0.225467883	-0.024592888	0.341189613	NS
RCBTB1	NS	0.225632817	0.02458415	0.341412508	NS
GOLGA8K	NS	0.225750797	0.024577902	0.341564327	NS
SYNJ2BP	NS	0.225772143	0.024576772	0.341569924	NS
ARHGEF33	NS	0.225790229	-0.024575814	0.341570587	NS
GTF2H3	NS	0.225882511	-0.024570929	0.341604211	NS
CEP85L	NS	0.225894194	0.024570311	0.341604211	NS
SLC9A2	NS	0.225850407	-0.024572628	0.341604211	NS
ZNF764	NS	0.225871919	-0.02457149	0.341604211	NS
ASPH	NS	0.225900698	0.024569967	0.341604211	NS
LGI1	NS	0.225957087	0.024566983	0.341662789	NS
CCND3	NS	0.225983562	0.024565582	0.34167613	NS
ACTG1	NS	0.22605661	0.024561718	0.341759879	NS
SIPA1L1	NS	0.226126337	0.02455803	0.341838595	NS
KRT73	NS	0.226336513	-0.024546918	0.342129601	NS

OR5P3	NS	0.226381142	-0.02454456	0.342170341	NS
ACOT11	NS	0.22642711	-0.024542131	0.342213098	NS
MRPS10	NS	0.226591717	-0.024533436	0.342435141	NS
FSD2	NS	0.226871229	-0.024518683	0.342830784	NS
UBQLN2	NS	0.226944195	0.024514833	0.342914274	NS
NBPF11	NS	0.227069291	0.024508236	0.343076513	NS
DUSP8	NS	0.227124374	0.024505332	0.343132952	NS
CACNG5	NS	0.227159165	0.024503498	0.343158729	NS
MILR1	NS	0.227234008	0.024499553	0.343245002	NS
HS1BP3	NS	0.227304267	-0.024495851	0.343301866	NS
PEG10	NS	0.227307125	0.0244957	0.343301866	NS
SPSB4	NS	0.227331902	0.024494395	0.343312499	NS
HS3ST1	NS	0.227440194	-0.02448869	0.343449243	NS
CCDC113	NS	0.227547382	0.024483046	0.343584298	NS
SPRR1B	NS	0.227642246	-0.024478052	0.343700727	NS
IGBP1	NS	0.2277229	0.024473808	0.343776389	NS
SMYD4	NS	0.227727881	-0.024473546	0.343776389	NS
ZC3HC1	NS	0.227840317	0.02446763	0.343899895	NS
DMXL2	NS	0.227845229	0.024467372	0.343899895	NS
SERP1	NS	0.22808837	0.024454588	0.344240039	NS
TPPP	NS	0.228179641	-0.024449791	0.344350939	NS
SRGN	NS	0.228204713	-0.024448474	0.344361927	NS
H4C2	NS	0.228486815	-0.024433659	0.344760741	NS
PAQR4	NS	0.228595025	-0.024427979	0.344884227	NS
PARG	NS	0.22860429	-0.024427493	0.344884227	NS
MRPL55	NS	0.228854366	-0.024414375	0.345234596	NS
ZBTB7C	NS	0.229035224	0.024404895	0.345480501	NS
STRIP1	NS	0.22912334	0.024400277	0.345575288	NS
GJE1	NS	0.22913377	-0.024399731	0.345575288	NS
HACD4	NS	0.229161118	-0.024398298	0.345589606	NS
RBMXL1	NS	0.229244057	-0.024393954	0.34568775	NS
NPFFR1	NS	0.229429104	-0.024384266	0.345939841	NS
RNF14	NS	0.229493912	-0.024380874	0.345983656	NS
SLC6A1	NS	0.229479373	0.024381635	0.345983656	NS
CDY1B	NS	0.229673917	-0.024371457	0.346174143	NS
TLR6	NS	0.229670138	0.024371654	0.346174143	NS
FAM114A1	NS	0.22966336	0.024372009	0.346174143	NS
GADD45B	NS	0.229736151	-0.024368202	0.346240983	NS
LRRC75A	NS	0.229876013	-0.02436089	0.346424799	NS
SAMM50	NS	0.229939271	-0.024357584	0.346493152	NS
PDGFC	NS	0.230036811	-0.024352487	0.346590746	NS
NAXD	NS	0.230039849	-0.024352329	0.346590746	NS
ATP6V0A4	NS	0.230085826	-0.024349927	0.346633036	NS
RFX8	NS	0.230147286	-0.024346717	0.346698643	NS
EIF5AL1	NS	0.230264021	0.024340622	0.346847501	NS
ANKRD50	NS	0.230299891	0.024338749	0.346874537	NS
PITPNM1	NS	0.230433476	0.024331777	0.347048736	NS
TIMP2	NS	0.230489508	0.024328854	0.347106117	NS
TERB1	NS	0.230576536	0.024324314	0.347210162	NS
CD2AP	NS	0.230797683	-0.024312784	0.347516137	NS
SLC2A10	NS	0.230833727	-0.024310905	0.347543374	NS
CASP3	NS	0.230858039	0.024309638	0.347552944	NS
MRPL48	NS	0.230932155	-0.024305777	0.347637486	NS
HLTF	NS	0.231073595	0.024298409	0.347796307	NS
STX7	NS	0.231201401	0.024291755	0.347879394	NS

C11orf58	NS	0.231189143	-0.024292393	0.347879394	NS
PHF19	NS	0.231171267	0.024293324	0.347879394	NS
HERC3	NS	0.231178872	0.024292928	0.347879394	NS
OR2H1	NS	0.231218661	-0.024290857	0.347879394	NS
PAX7	NS	0.231374479	0.024282748	0.34805972	NS
CFAP52	NS	0.231366869	-0.024283144	0.34805972	NS
EYS	NS	0.231470133	0.024277772	0.348176554	NS
GET3	NS	0.231574508	-0.024272344	0.348306486	NS
SPACA5	NS	0.231693629	0.024266151	0.348458577	NS
ATP12A	NS	0.231719828	0.024264789	0.348470903	NS
ADK	NS	0.231879941	0.02425647	0.348684597	NS
RBM3	NS	0.231945371	0.024253071	0.348755892	NS
SNRPC	NS	0.231972811	-0.024251646	0.348770058	NS
KATNIP	NS	0.231992489	-0.024250624	0.348772552	NS
SERPINA4	NS	0.232028252	-0.024248767	0.348799227	NS
STK40	NS	0.232046437	0.024247823	0.348799474	NS
ZSWIM9	NS	0.23214103	0.024242912	0.348914566	NS
ATP2A3	NS	0.232190526	0.024240343	0.348961862	NS
FAM241B	NS	0.232337949	0.024232694	0.349156315	NS
TRIM6	NS	0.232402138	0.024229365	0.349225665	NS
PLCG2	NS	0.232517502	0.024223382	0.349371896	NS
POMT2	NS	0.232627676	-0.024217671	0.349439727	NS
NDUFS2	NS	0.232585799	0.024219842	0.349439727	NS
APOBR	NS	0.232611856	-0.024218491	0.349439727	NS
ZNF395	NS	0.232634859	-0.024217299	0.349439727	NS
KRTAP4-12	NS	0.232821085	0.02420765	0.349665186	NS
CCL27	NS	0.232875599	-0.024204826	0.349719924	NS
CAMK1	NS	0.232905247	-0.024203291	0.349737313	NS
VPS25	NS	0.232971967	0.024199836	0.349808426	NS
NCOA4	NS	0.232988749	0.024198967	0.349808426	NS
ZNF227	NS	0.233028981	-0.024196884	0.349841694	NS
MAPK6	NS	0.233145666	0.024190845	0.349989725	NS
KCND3	NS	0.233195082	0.024188288	0.350036759	NS
MMP26	NS	0.233352494	0.024180146	0.350245881	NS
PSMC4	NS	0.233469038	-0.02417412	0.350370757	NS
RFX4	NS	0.233471896	0.024173972	0.350370757	NS
GBP3	NS	0.233545012	0.024170193	0.350438142	NS
IL2RA	NS	0.233553009	-0.024169779	0.350438142	NS
YJU2	NS	0.233729739	0.024160648	0.350676136	NS
SLC38A7	NS	0.233781462	0.024157976	0.350726553	NS
ZNF816	NS	0.233868118	-0.024153501	0.350829365	NS
PTPN22	NS	0.233956179	-0.024148955	0.350934268	NS
GTF2IRD1	NS	0.23405048	-0.024144088	0.351039596	NS
SLC30A9	NS	0.2340746	-0.024142843	0.351039596	NS
FXYD7	NS	0.234080805	-0.024142523	0.351039596	NS
HILPDA	NS	0.234142601	-0.024139334	0.351105066	NS
MAGEC2	NS	0.234201185	0.024136312	0.351165709	NS
POLR3E	NS	0.234266927	-0.024132921	0.351237075	NS
TRPC5	NS	0.234340898	-0.024129107	0.351320767	NS
ANXA5	NS	0.234529006	0.024119411	0.351575545	NS
STON2	NS	0.234596219	0.024115948	0.351649069	NS
OR2AT4	NS	0.234647466	-0.024113307	0.351698649	NS
METTL7A	NS	0.234726097	-0.024109257	0.351789263	NS
ZBTB8OS	NS	0.234749366	-0.024108059	0.351796898	NS
ADRB1	NS	0.234780808	-0.02410644	0.351816778	NS

KIF1C	NS	0.234825876	0.02410412	0.351857072	NS
CNPY4	NS	0.234899578	-0.024100325	0.351940261	NS
PARL	NS	0.235069459	0.024091583	0.352167527	NS
FUT7	NS	0.235125131	0.024088719	0.352196411	NS
S100A2	NS	0.235117091	0.024089133	0.352196411	NS
SETD9	NS	0.23527563	-0.02408098	0.352394575	NS
CNGB1	NS	0.235350893	-0.02407711	0.352480028	NS
TFR2	NS	0.235407403	-0.024074206	0.352537384	NS
ACAA1	NS	0.235458539	0.024071578	0.352563601	NS
RMDN2	NS	0.235461339	-0.024071434	0.352563601	NS
NMT-MINDY	NS	0.235588373	-0.024064907	0.352726528	NS
DCAF12	NS	0.235648347	-0.024061827	0.352789033	NS
TMC3	NS	0.235726868	-0.024057795	0.352852004	NS
IL1F10	NS	0.235766907	-0.024055739	0.352884647	NS
MED4	NS	0.236100038	-0.024038645	0.353355937	NS
ADPRHL1	NS	0.236125174	0.024037356	0.353363534	NS
PDGFA	NS	0.236141626	-0.024036512	0.353363534	NS
TAX1BP3	NS	0.236242494	0.02403134	0.353487146	NS
CDH23	NS	0.236322471	0.02402724	0.353579482	NS
NACC2	NS	0.236406225	0.024022948	0.353677453	NS
AK4	NS	0.236530472	-0.024016583	0.353825607	NS
NOMO2	NS	0.236541814	-0.024016002	0.353825607	NS
DOK1	NS	0.236595294	0.024013263	0.353870297	NS
STRN3	NS	0.236608255	-0.024012599	0.353870297	NS
ZMYM6	NS	0.236770239	0.024004306	0.354057846	NS
POU5F1B	NS	0.23676136	0.02400476	0.354057846	NS
CALCRL	NS	0.236803609	0.024002598	0.35408039	NS
TMEM184C	NS	0.236854086	-0.024000014	0.35412851	NS
TMC5	NS	0.236872442	-0.023999075	0.3541286	NS
IMPDH2	NS	0.236907679	-0.023997272	0.354153925	NS
NDST2	NS	0.237009937	0.023992041	0.35422472	NS
C1orf122	NS	0.236994242	-0.023992844	0.35422472	NS
ZDHHC11B	NS	0.23708505	-0.023988199	0.354309622	NS
ILF2	NS	0.237139033	-0.023985439	0.354362938	NS
CFHR2	NS	0.237382324	0.023973004	0.354699109	NS
NDRG3	NS	0.23768861	-0.023957363	0.35512935	NS
NPIPA7	NS	0.23783181	-0.023950054	0.355315878	NS
ZCWPW1	NS	0.23796574	0.023943222	0.355488529	NS
STK38	NS	0.238087779	-0.023936999	0.355643391	NS
RAB17	NS	0.238133626	0.023934661	0.355684425	NS
CEP295NL	NS	0.238185912	0.023931996	0.35573507	NS
ZNF277	NS	0.238280924	0.023927154	0.355849515	NS
ATG4B	NS	0.238343558	0.023923963	0.355915592	NS
CDC14A	NS	0.238395775	0.023921302	0.355950275	NS
C11orf68	NS	0.238418568	0.023920141	0.355950275	NS
MICA	NS	0.238421953	-0.023919969	0.355950275	NS
GPR26	NS	0.238464141	-0.02391782	0.355985802	NS
PEX6	NS	0.238504844	-0.023915747	0.356018703	NS
TMEM236	NS	0.238522967	0.023914825	0.356018703	NS
TEX2	NS	0.238542167	-0.023913847	0.356019907	NS
AEBP1	NS	0.238650241	0.023908345	0.356118727	NS
MRPS27	NS	0.238663573	0.023907666	0.356118727	NS
DCXR	NS	0.238648502	0.023908434	0.356118727	NS
IKBKB	NS	0.238795956	-0.02390093	0.35628258	NS
RHNO1	NS	0.238810198	0.023900205	0.35628258	NS

SIGLEC14	NS	0.238833782	0.023899005	0.356290303	NS
PSMB6	NS	0.238892502	0.023896018	0.356326626	NS
FLYWCH2	NS	0.238895333	-0.023895874	0.356326626	NS
ENTPD7	NS	0.238913358	-0.023894957	0.356326626	NS
PYCR2	NS	0.239033796	-0.023888833	0.356478785	NS
CAPN3	NS	0.239153639	0.023882741	0.356630033	NS
SLC46A2	NS	0.239173335	0.02388174	0.356631929	NS
HTATSF1	NS	0.239311665	-0.023874711	0.356783634	NS
NAA60	NS	0.239321189	0.023874227	0.356783634	NS
BCL9L	NS	0.239330373	0.023873761	0.356783634	NS
MARK1	NS	0.239370301	-0.023871732	0.356788199	NS
FMR1NB	NS	0.239369459	-0.023871775	0.356788199	NS
IQSEC2	NS	0.239466578	0.023866843	0.356849264	NS
GAPT	NS	0.239460274	-0.023867163	0.356849264	NS
FOXO3B	NS	0.239442227	0.023868079	0.356849264	NS
CUL4B	NS	0.239650268	0.023857518	0.357082414	NS
SSNA1	NS	0.239659932	-0.023857027	0.357082414	NS
KRT84	NS	0.239749892	0.023852463	0.357188956	NS
TM4SF20	NS	0.23989452	-0.023845126	0.357376921	NS
SPPL2B	NS	0.240067911	-0.023836335	0.357580183	NS
RHBG	NS	0.24006258	0.023836605	0.357580183	NS
CIDEC	NS	0.240105341	0.023834438	0.357608417	NS
KCTD9	NS	0.240299926	0.023824579	0.357858956	NS
EXT2	NS	0.240310535	0.023824041	0.357858956	NS
OR52E6	NS	0.240384494	-0.023820295	0.357941554	NS
FCHO1	NS	0.240624063	-0.023808167	0.358270721	NS
TMEM233	NS	0.240664002	0.023806146	0.358302624	NS
SPRED3	NS	0.240708648	-0.023803887	0.358313973	NS
F286A-TBC1	NS	0.240707838	-0.023803928	0.358313973	NS
GUCA1B	NS	0.240815949	-0.02379846	0.358404893	NS
SLC30A8	NS	0.240798073	-0.023799364	0.358404893	NS
NUDT6	NS	0.240825276	-0.023797988	0.358404893	NS
RFC4	NS	0.240864668	-0.023795996	0.358435959	NS
MGLL	NS	0.240984628	0.02378993	0.358543066	NS
BCL2L10	NS	0.241010738	-0.02378861	0.358543066	NS
SFXN2	NS	0.241010528	0.023788621	0.358543066	NS
KLHL15	NS	0.241001027	0.023789101	0.358543066	NS
ZDHHC13	NS	0.241312221	-0.023773377	0.358963983	NS
OLA1	NS	0.241420643	0.023767902	0.359046084	NS
LLCFC1	NS	0.241423062	-0.02376778	0.359046084	NS
TM7SF3	NS	0.241498242	-0.023763985	0.359130299	NS
CNTROB	NS	0.241549699	0.023761388	0.359179225	NS
RNF111	NS	0.241584675	0.023759623	0.359203639	NS
MYO15A	NS	0.241656385	-0.023756004	0.359255069	NS
SLC30A3	NS	0.241645012	0.023756578	0.359255069	NS
DTX3	NS	0.241734078	-0.023752085	0.359315375	NS
BTBD10	NS	0.241909524	-0.023743237	0.359548549	NS
ANGPT2	NS	0.242038061	0.023736758	0.359686443	NS
TADA3	NS	0.242039467	0.023736687	0.359686443	NS
EBLN1	NS	0.242127577	0.023732247	0.359789758	NS
STAT4	NS	0.242167934	-0.023730214	0.359822102	NS
INPP4A	NS	0.242370175	0.023720028	0.360001697	NS
MAP2K5	NS	0.242381799	-0.023719443	0.360001697	NS
WASHC5	NS	0.24231204	0.023722955	0.360001697	NS
OR2G6	NS	0.242360463	-0.023720517	0.360001697	NS

MAGIX	NS	0.24234785	0.023721152	0.360001697	NS
HOXB6	NS	0.242678585	0.023704507	0.360414847	NS
PROZ	NS	0.242885106	0.023694121	0.360693887	NS
POMT1	NS	0.242983649	-0.023689167	0.360812545	NS
IFNA1	NS	0.24321586	0.0236775	0.361129655	NS
HCST	NS	0.243266546	-0.023674955	0.361177208	NS
AKAP9	NS	0.243465437	-0.02366497	0.361417056	NS
TADA2B	NS	0.243625694	0.023656928	0.361627219	NS
CHID1	NS	0.243758947	-0.023650244	0.361797269	NS
SH2D2A	NS	0.243784271	-0.023648975	0.361807112	NS
MMP14	NS	0.243864582	0.023644948	0.361898555	NS
SEMA4B	NS	0.244035748	0.023636369	0.362124803	NS
UBE2Q1	NS	0.244076493	-0.023634328	0.3621575	NS
SCGB1D1	NS	0.244120512	-0.023632122	0.36216729	NS
OR6C6	NS	0.244108004	0.023632749	0.36216729	NS
FASN	NS	0.244187593	-0.023628762	0.362239044	NS
MTX1	NS	0.244343187	0.023620971	0.362442083	NS
TMEM9	NS	0.244481395	-0.023614053	0.362619302	NS
C17orf98	NS	0.24462274	-0.023606981	0.362801146	NS
GOLGA6D	NS	0.244858184	0.023595207	0.363122511	NS
CYP1B1	NS	0.244901611	0.023593036	0.363159089	NS
GNAI1	NS	0.245017543	0.023587243	0.363303169	NS
SYNE2	NS	0.24504274	0.023585984	0.363312698	NS
LDB1	NS	0.245085543	0.023583845	0.3633205	NS
ZNF564	NS	0.245067176	0.023584763	0.3633205	NS
ADGRG1	NS	0.245161921	-0.02358003	0.363405892	NS
RASSF7	NS	0.245221351	-0.023577062	0.363466152	NS
KRTAP29-1	NS	0.245547611	-0.023560777	0.363921865	NS
FAM214A	NS	0.245583114	0.023559006	0.363946617	NS
DENND6B	NS	0.245707583	0.023552798	0.364103198	NS
RPGRIP1L	NS	0.245876062	0.023544398	0.364324967	NS
KLHDC9	NS	0.245920008	-0.023542207	0.364362192	NS
BRI3	NS	0.245967338	0.023539849	0.364392204	NS
DISP3	NS	0.245977916	-0.023539322	0.364392204	NS
ZMYND10	NS	0.246082659	0.023534103	0.364519472	NS
ALAD	NS	0.246129078	-0.023531791	0.364560333	NS
APCDD1L	NS	0.246253998	-0.02352557	0.364717452	NS
CANT1	NS	0.246362447	0.023520171	0.364850155	NS
MAGEA11	NS	0.246764972	-0.023500147	0.365418315	NS
DENND2A	NS	0.246800268	-0.023498392	0.365442625	NS
CAMK1G	NS	0.246923017	0.02349229	0.36556845	NS
ANLN	NS	0.246922721	0.023492305	0.36556845	NS
ZNF131	NS	0.247088921	0.023484048	0.365786093	NS
FLAD1	NS	0.247200885	-0.023478487	0.365923856	NS
MRTO4	NS	0.247329822	-0.023472085	0.36608672	NS
SLC1A6	NS	0.247406507	-0.023468279	0.366172224	NS
MYBPC2	NS	0.247563973	-0.023460466	0.366377266	NS
ABRA	NS	0.247603395	0.023458511	0.366407593	NS
ZFP57	NS	0.247694643	-0.023453985	0.366514601	NS
PSG3	NS	0.247838335	-0.023446861	0.36669919	NS
RPS6KL1	NS	0.247872014	0.023445192	0.366720989	NS
TARBP1	NS	0.247892339	-0.023444185	0.366723029	NS
CFTR	NS	0.247936246	-0.023442009	0.366759952	NS
AGR2	NS	0.247957408	-0.02344096	0.366761111	NS
EMP1	NS	0.247974925	0.023440092	0.366761111	NS

TBC1D3E	NS	0.248026825	0.023437521	0.366809842	NS
BRF1	NS	0.24804928	0.023436408	0.366815026	NS
TIMM29	NS	0.24807013	-0.023435376	0.366817832	NS
ITGAD	NS	0.24812732	0.023432543	0.366874373	NS
PRAMEF8	NS	0.248221656	0.023427871	0.366985821	NS
CCDC120	NS	0.248755556	-0.023401455	0.367716027	NS
VPS39	NS	0.248772544	-0.023400615	0.367716027	NS
SH3RF1	NS	0.248894988	-0.023394563	0.36786892	NS
NBPF4	NS	0.248928044	-0.02339293	0.367889687	NS
PSMG2	NS	0.24897471	-0.023390624	0.367930561	NS
C18orf32	NS	0.249071465	0.023385844	0.368045445	NS
OSTF1	NS	0.249194513	-0.023379767	0.36819916	NS
STARD4	NS	0.249302343	-0.023374443	0.368330367	NS
FTHL17	NS	0.249495809	0.023364895	0.368588069	NS
ZNHIT3	NS	0.249621531	-0.023358693	0.368745657	NS
FRYL	NS	0.249684482	0.023355589	0.368810503	NS
TRADD	NS	0.24986302	-0.023346787	0.36904606	NS
TIMM13	NS	0.249904377	-0.023344749	0.369078981	NS
WIPI1	NS	0.250098797	0.02333517	0.369260408	NS
SEMA5B	NS	0.250103531	0.023334937	0.369260408	NS
NLRC4	NS	0.250075529	-0.023336316	0.369260408	NS
TEN1	NS	0.250088934	0.023335656	0.369260408	NS
OR4K2	NS	0.250153608	-0.023332471	0.369306173	NS
TRAPPC2	NS	0.250258728	-0.023327295	0.369433186	NS
ACCS	NS	0.250321093	-0.023324225	0.369497069	NS
SCML4	NS	0.250353361	0.023322636	0.369516521	NS
MOB4	NS	0.250441403	0.023318303	0.369618285	NS
LRSAM1	NS	0.250489037	0.02331596	0.369660399	NS
MOBP	NS	0.250523744	0.023314252	0.369683433	NS
BCAS2	NS	0.250669459	0.023307085	0.369870259	NS
PRR20E	NS	0.250722287	0.023304487	0.369920008	NS
SPECC1L	NS	0.25080309	0.023300515	0.370011022	NS
GBE1	NS	0.250965155	-0.02329255	0.370221899	NS
SUGP1	NS	0.251043033	-0.023288724	0.37029886	NS
C12orf56	NS	0.251055587	-0.023288107	0.37029886	NS
TNFRSF13B	NS	0.251342337	0.023274026	0.370693559	NS
AFG1L	NS	0.25143681	0.02326939	0.37080464	NS
TOR1AIP1	NS	0.251467814	0.023267868	0.37082211	NS
DCHS1	NS	0.251493807	-0.023266593	0.370832188	NS
RNF145	NS	0.251612215	-0.023260785	0.370907073	NS
RPL7A	NS	0.251602558	0.023261258	0.370907073	NS
YEATS2	NS	0.251621243	0.023260342	0.370907073	NS
ZNF732	NS	0.25160686	-0.023261047	0.370907073	NS
ABCA3	NS	0.251702826	-0.023256341	0.370970829	NS
C20orf96	NS	0.25170082	0.023256439	0.370970829	NS
PPT2	NS	0.251785255	0.023252299	0.371064062	NS
CNOT2	NS	0.25183354	0.023249932	0.371090782	NS
RNF2	NS	0.251841729	-0.023249531	0.371090782	NS
GRHL1	NS	0.252250995	-0.023229482	0.371639582	NS
PI4K2A	NS	0.252260126	0.023229035	0.371639582	NS
TMEM259	NS	0.252271775	-0.023228464	0.371639582	NS
IDO2	NS	0.252305271	0.023226824	0.371660641	NS
NKPD1	NS	0.252351395	-0.023224567	0.371700298	NS
THAP9	NS	0.252417122	-0.02322135	0.371768818	NS
TBC1D12	NS	0.252452029	-0.023219642	0.371791941	NS

ISL1	NS	0.252497863	0.023217399	0.37183115	NS
CFAP61	NS	0.252577706	-0.023213493	0.371892141	NS
HAPLN3	NS	0.252573401	0.023213703	0.371892141	NS
SLC6A8	NS	0.252652005	-0.023209859	0.371973241	NS
PRKAB1	NS	0.252719998	0.023206534	0.372045047	NS
FHAD1	NS	0.252759766	0.023204589	0.372075293	NS
MTREX	NS	0.252796034	-0.023202816	0.372100383	NS
ITGAL	NS	0.252848713	0.023200241	0.372123113	NS
PCYT1A	NS	0.25289246	-0.023198103	0.372157413	NS
PLA2G12A	NS	0.252915889	-0.023196958	0.372163597	NS
RIPPLY1	NS	0.253267928	0.023179761	0.372624963	NS
PSMB10	NS	0.253262213	-0.02318004	0.372624963	NS
MYOG	NS	0.253292841	0.023178545	0.372633292	NS
SNAI1	NS	0.253427349	-0.023171979	0.372717861	NS
RAB13	NS	0.253413961	-0.023172633	0.372717861	NS
SCN8A	NS	0.253371673	0.023174697	0.372717861	NS
ACKR1	NS	0.253389452	-0.023173829	0.372717861	NS
FUT9	NS	0.253471202	-0.023169839	0.372754034	NS
ENPP4	NS	0.253494874	-0.023168684	0.372760525	NS
SPATA5L1	NS	0.253529717	-0.023166984	0.37278344	NS
BBC3	NS	0.253600973	-0.023163508	0.372859889	NS
PMM2	NS	0.253729653	0.023157232	0.373020748	NS
ECHDC1	NS	0.25381878	-0.023152887	0.373123438	NS
TMEM35A	NS	0.253940101	0.023146973	0.37326849	NS
FTH1	NS	0.253956021	0.023146198	0.37326849	NS
STK17A	NS	0.253976156	0.023145216	0.37326974	NS
TMPRSS4	NS	0.254162955	0.023136116	0.373487561	NS
ZSCAN30	NS	0.25414711	0.023136888	0.373487561	NS
ZFP14	NS	0.254206884	0.023133977	0.373523756	NS
SAE1	NS	0.254397541	0.023124694	0.373775528	NS
ENOSF1	NS	0.254444887	-0.02312239	0.373816716	NS
LAPTM4B	NS	0.254550462	-0.023117253	0.373943439	NS
NUMB	NS	0.254684781	-0.023110719	0.374083974	NS
H1-6	NS	0.25467306	-0.023111289	0.374083974	NS
HP1BP3	NS	0.254751921	0.023107454	0.37412581	NS
OR4S1	NS	0.254735397	-0.023108257	0.37412581	NS
SEMA6C	NS	0.2548087	-0.023104693	0.374180806	NS
SIK2	NS	0.25484027	0.023103158	0.374198777	NS
ABCA5	NS	0.254899811	-0.023100264	0.374257812	NS
SPRED1	NS	0.254933214	-0.023098641	0.374278466	NS
PRMT8	NS	0.254989678	-0.023095897	0.37433297	NS
DEFB132	NS	0.255037859	-0.023093555	0.374375307	NS
HAVCR2	NS	0.255155108	0.023087859	0.374519016	NS
CHRNA6	NS	0.25523647	-0.023083908	0.374610033	NS
MLEC	NS	0.255299403	0.023080852	0.374673989	NS
FOXRED2	NS	0.255539146	0.023069215	0.3749974	NS
CFAP97D1	NS	0.255811243	0.023056018	0.375368237	NS
STK38L	NS	0.255983105	0.023047687	0.375563479	NS
SPATA46	NS	0.256041384	-0.023044862	0.375620512	NS
TNPO3	NS	0.256087922	-0.023042608	0.375639167	NS
PPP2R3A	NS	0.256092914	-0.023042366	0.375639167	NS
BRI3BP	NS	0.256212697	-0.023036563	0.375786389	NS
IL11RA	NS	0.256288445	-0.023032895	0.375869006	NS
SYNE4	NS	0.256333901	-0.023030694	0.375907189	NS
SQOR	NS	0.256458066	-0.023024683	0.376060782	NS

PTPN7	NS	0.256524459	-0.02302147	0.376129644	NS
PRSS48	NS	0.256577875	0.023018885	0.376179469	NS
SMLR1	NS	0.256671628	-0.02301435	0.376288422	NS
RPF1	NS	0.256731577	0.02301145	0.376347805	NS
LHX6	NS	0.256772586	0.023009467	0.376379415	NS
USP53	NS	0.256792035	-0.023008526	0.376379421	NS
LCLAT1	NS	0.256837728	-0.023006317	0.37641789	NS
UBAC2	NS	0.256886634	0.023003952	0.37643256	NS
LY75-CD302	NS	0.256872111	0.023004654	0.37643256	NS
SLC39A3	NS	0.256958106	-0.023000497	0.376508789	NS
TAFA2	NS	0.257079822	-0.022994615	0.376658621	NS
HOXB5	NS	0.257126973	0.022992337	0.37669919	NS
MYH13	NS	0.257214731	-0.022988097	0.376742213	NS
TAF10	NS	0.257202699	0.022988678	0.376742213	NS
RHOBTB2	NS	0.25723715	0.022987014	0.37674336	NS
NALCN	NS	0.257254442	0.022986179	0.37674336	NS
PTGDR2	NS	0.25737353	0.022980428	0.376889245	NS
SPINK2	NS	0.257408838	0.022978724	0.376912434	NS
WDR72	NS	0.25745212	0.022976634	0.376947294	NS
RRBP1	NS	0.257539271	-0.022972428	0.376966431	NS
GDPD2	NS	0.257499045	0.022974369	0.376966431	NS
NECAP2	NS	0.257560229	-0.022971417	0.376966431	NS
SRP72	NS	0.257546196	-0.022972094	0.376966431	NS
SLC30A10	NS	0.257562567	0.022971304	0.376966431	NS
GTPBP10	NS	0.257698484	-0.022964746	0.377129	NS
SHISA5	NS	0.257713242	0.022964034	0.377129	NS
STK39	NS	0.257732094	-0.022963125	0.377129	NS
ACER3	NS	0.257957658	-0.022952248	0.377430526	NS
KANTR	NS	0.258032608	-0.022948635	0.377511652	NS
IDI1	NS	0.258179847	0.02294154	0.377616426	NS
FKBP5	NS	0.258182258	0.022941424	0.377616426	NS
MRPS18A	NS	0.258154229	0.022942775	0.377616426	NS
GSTM1	NS	0.258129089	0.022943986	0.377616426	NS
RDH10	NS	0.258248305	-0.022938243	0.37767259	NS
NLRP4	NS	0.258259682	-0.022937695	0.37767259	NS
SS18	NS	0.2583265	0.022934476	0.377741764	NS
SLC4A3	NS	0.258430238	-0.022929481	0.37786491	NS
NDUFS5	NS	0.258518532	-0.022925231	0.377965459	NS
EVI2B	NS	0.258587769	0.022921899	0.378038131	NS
ZHX1-C8orf7	NS	0.258611974	-0.022920734	0.378044963	NS
TRIP11	NS	0.258692289	0.022916869	0.378133812	NS
SLC35B1	NS	0.258717981	-0.022915633	0.37814281	NS
CNKSR1	NS	0.258843167	0.022909612	0.378297217	NS
CRABP1	NS	0.258994183	-0.022902351	0.378489348	NS
ZMYM5	NS	0.259087868	-0.022897848	0.378597672	NS
CCIN	NS	0.259232277	-0.022890909	0.378780098	NS
IL23R	NS	0.259272095	-0.022888996	0.378809682	NS
EPM2A	NS	0.259340136	-0.022885728	0.378851899	NS
C4B	NS	0.25932078	-0.022886658	0.378851899	NS
PLTP	NS	0.259425676	-0.02288162	0.37894826	NS
ABLIM1	NS	0.259450192	-0.022880443	0.378955471	NS
HEMK1	NS	0.259539707	-0.022876146	0.37903072	NS
NPY5R	NS	0.259540874	0.02287609	0.37903072	NS
ATP6V1D	NS	0.259568673	-0.022874755	0.379042719	NS
TRMU	NS	0.259752602	-0.022865929	0.379282692	NS

NFE2L2	NS	0.259833073	0.022862069	0.379371574	NS
TCF12	NS	0.259918321	0.02285798	0.379467418	NS
ANKZF1	NS	0.260011363	-0.022853519	0.379574624	NS
PGD	NS	0.260106576	0.022848955	0.379684983	NS
HYI	NS	0.260355823	0.022837013	0.380020158	NS
CLRN2	NS	0.260445166	-0.022832734	0.3801219	NS
MYNN	NS	0.260542112	0.022828092	0.380234722	NS
CACNG2	NS	0.26060697	-0.022824987	0.380300702	NS
ATP5MG	NS	0.260653954	-0.022822739	0.38034059	NS
IL17RE	NS	0.260681823	-0.022821405	0.380352583	NS
PBX2	NS	0.260758687	-0.022817727	0.380436055	NS
SENP8	NS	0.26093165	-0.022809453	0.380659709	NS
ZFP92	NS	0.260965544	0.022807832	0.380680464	NS
VPS54	NS	0.260998012	0.022806279	0.380699135	NS
INTU	NS	0.261070903	-0.022802794	0.380759954	NS
HAUS7	NS	0.261079051	-0.022802405	0.380759954	NS
MAPK9	NS	0.261101974	0.022801309	0.380764696	NS
VPS26C	NS	0.261144915	-0.022799256	0.380798627	NS
GPAT2	NS	0.261184132	-0.022797382	0.380827124	NS
CDC40	NS	0.261207815	-0.02279625	0.380832967	NS
FEM1A	NS	0.261330574	-0.022790384	0.380983248	NS
PHPT1	NS	0.261477001	-0.02278339	0.381168009	NS
RPS6KC1	NS	0.261550346	0.022779888	0.381246216	NS
SLC43A1	NS	0.261576658	-0.022778632	0.381255858	NS
COPG1	NS	0.261689427	0.022773249	0.381391502	NS
SLC7A1	NS	0.261742095	0.022770735	0.381439541	NS
BTG1	NS	0.261814442	0.022767283	0.381488523	NS
MZB1	NS	0.261815124	-0.02276725	0.381488523	NS
SHROOM2	NS	0.262118441	0.022752784	0.381901734	NS
ACO2	NS	0.262170763	-0.022750289	0.381949217	NS
HSD17B7	NS	0.262468279	-0.022736113	0.382353881	NS
PWWP3B	NS	0.262564704	-0.022731521	0.382426987	NS
CYBB	NS	0.262569362	-0.022731299	0.382426987	NS
KCTD13	NS	0.262577736	0.0227309	0.382426987	NS
DCAF5	NS	0.262630098	-0.022728407	0.38247447	NS
SLC8B1	NS	0.262749441	0.022722726	0.382573146	NS
ZNF563	NS	0.26275715	0.022722359	0.382573146	NS
NXF2	NS	0.262755748	-0.022722425	0.382573146	NS
FAM161A	NS	0.2628507	-0.022717907	0.382680568	NS
PBX3	NS	0.262981966	0.022711662	0.38281565	NS
TLR10	NS	0.262983039	-0.022711611	0.38281565	NS
POTEM	NS	0.263010477	0.022710306	0.382826801	NS
ZBED9	NS	0.263107948	-0.02270567	0.382939878	NS
ZNF302	NS	0.263176247	0.022702423	0.383010485	NS
PTBP3	NS	0.263255948	0.022698634	0.383046521	NS
SEC31A	NS	0.263272221	0.02269786	0.383046521	NS
SUSD4	NS	0.26327924	-0.022697527	0.383046521	NS
SPSB1	NS	0.263280167	0.022697483	0.383046521	NS
PIP4K2C	NS	0.263364481	-0.022693476	0.383140391	NS
CDK9	NS	0.263413941	0.022691126	0.383183544	NS
RGSL1	NS	0.26347065	-0.022688431	0.383237236	NS
NAA16	NS	0.263543231	0.022684984	0.383314005	NS
AGO4	NS	0.263568626	-0.022683778	0.383322137	NS
SIDT2	NS	0.263614794	0.022681585	0.383331678	NS
CENPU	NS	0.263604321	-0.022682082	0.383331678	NS

CHGA	NS	0.263753442	-0.022675002	0.383504479	NS
FRMPD2	NS	0.263819805	-0.022671852	0.383572158	NS
ACVR1	NS	0.263902939	-0.022667907	0.38366421	NS
POLB	NS	0.264001374	-0.022663236	0.38377849	NS
L3MBTL4	NS	0.264031193	-0.022661822	0.383793013	NS
APPL2	NS	0.264291151	-0.022649494	0.384142038	NS
IL19	NS	0.264370945	-0.022645712	0.384229164	NS
CASP7	NS	0.264458881	-0.022641545	0.38432811	NS
LILRB2	NS	0.264498711	0.022639658	0.384357136	NS
COL4A1	NS	0.264550272	0.022637215	0.384403203	NS
SYNGR2	NS	0.264781464	0.022626266	0.384710255	NS
ZNF577	NS	0.264843899	0.02262331	0.384772087	NS
CRTC3	NS	0.265007517	0.022615567	0.3849809	NS
IPO13	NS	0.265109589	-0.022610738	0.385100278	NS
TMEM219	NS	0.265165254	0.022608105	0.385152232	NS
FAXC	NS	0.265187573	-0.022607049	0.385155749	NS
OR10J1	NS	0.265331	0.022600267	0.385335147	NS
ZNF611	NS	0.265450618	0.022594613	0.385479943	NS
CSMD1	NS	0.26549196	-0.022592659	0.385482137	NS
ATP5MF	NS	0.26548752	-0.022592869	0.385482137	NS
SLC6A13	NS	0.265556356	-0.022589617	0.385546716	NS
VPS16	NS	0.265577642	0.022588611	0.385548702	NS
ATXN7L3B	NS	0.265684237	-0.022583575	0.385674523	NS
HSFX1	NS	0.265796268	-0.022578284	0.385808216	NS
IL32	NS	0.265920646	-0.022572412	0.38595981	NS
DCD	NS	0.266024246	-0.022567523	0.386081226	NS
SART1	NS	0.266130089	0.022562528	0.386205879	NS
INPP1	NS	0.266412645	0.022549203	0.386586937	NS
DCDC2	NS	0.266462196	0.022546867	0.386629854	NS
H2AJ	NS	0.266549453	-0.022542754	0.386727473	NS
IL13RA1	NS	0.266621294	-0.022539369	0.386802711	NS
DMPK	NS	0.266831156	0.022529483	0.387078158	NS
SQLE	NS	0.267162511	0.022513885	0.387529794	NS
GLS	NS	0.267193585	0.022512423	0.387535757	NS
SPIRE1	NS	0.267206666	0.022511807	0.387535757	NS
RPL9	NS	0.267377495	-0.022503772	0.387725411	NS
SLBP	NS	0.267603776	-0.022493134	0.388024472	NS
IL22	NS	0.267729279	-0.022487236	0.388177373	NS
USE1	NS	0.267798348	-0.022483991	0.388224145	NS
ABCA1	NS	0.267802252	-0.022483808	0.388224145	NS
ROBO2	NS	0.267821709	-0.022482894	0.388224145	NS
ALKBH1	NS	0.267923206	0.022478127	0.388342188	NS
ZMYM3	NS	0.268112665	-0.022469232	0.388587701	NS
C1S	NS	0.268135753	-0.022468148	0.388592066	NS
HNRNPDL	NS	0.268206565	0.022464825	0.388665588	NS
RGS18	NS	0.268492489	0.022451412	0.388992561	NS
GBP2	NS	0.268457036	0.022453074	0.388992561	NS
CDK10	NS	0.268488181	-0.022451614	0.388992561	NS
CEP95	NS	0.268528218	0.022449736	0.389015207	NS
PIGA	NS	0.26888122	0.022433192	0.3894683	NS
INCA1	NS	0.268861296	0.022434125	0.3894683	NS
STMND1	NS	0.268907478	0.022431962	0.389477189	NS
CYP2C19	NS	0.268963931	-0.022429318	0.389500662	NS
SCN9A	NS	0.2689551	-0.022429731	0.389500662	NS
TRIM38	NS	0.269047512	0.022425403	0.389592553	NS

UBE2QL1	NS	0.26910618	-0.022422656	0.389648355	NS
CSNK2A1	NS	0.26928299	0.02241438	0.389875201	NS
CRTC1	NS	0.2694334	0.022407342	0.390063792	NS
SKP1	NS	0.269781886	-0.022391047	0.390480685	NS
PCDHA11	NS	0.269756875	-0.022392216	0.390480685	NS
COA8	NS	0.269762054	-0.022391974	0.390480685	NS
PDCL	NS	0.269811893	-0.022389645	0.390494916	NS
LIG1	NS	0.269994646	-0.022381106	0.390730195	NS
FAM167A	NS	0.2700493	0.022378553	0.390780073	NS
PARP4	NS	0.270132111	-0.022374685	0.390870684	NS
PYROXD1	NS	0.270166161	0.022373095	0.390875228	NS
SLC25A32	NS	0.270175639	0.022372653	0.390875228	NS
CCNB2	NS	0.270418638	-0.02236131	0.391197545	NS
ADPGK	NS	0.270524729	0.02235636	0.391321774	NS
BCKDK	NS	0.270597096	0.022352984	0.391397205	NS
FAM156A	NS	0.270784944	-0.022344225	0.391639645	NS
BROX	NS	0.270977863	0.022335233	0.391889384	NS
HLX	NS	0.271613134	0.022305654	0.392778769	NS
SDSL	NS	0.271660443	-0.022303454	0.392817835	NS
ITPKB	NS	0.271986591	0.022288288	0.39323082	NS
EID2B	NS	0.271986682	-0.022288284	0.39323082	NS
TOMM40L	NS	0.272076779	0.022284097	0.393331701	NS
ATG4D	NS	0.272120125	0.022282083	0.393364984	NS
MTMR4	NS	0.272176149	-0.02227948	0.39341506	NS
DPF2	NS	0.272195417	0.022278585	0.39341506	NS
SSTR2	NS	0.272417712	0.022268261	0.393706953	NS
FBXO16	NS	0.272465746	0.022266031	0.393746975	NS
URM1	NS	0.272536229	-0.022262759	0.393819429	NS
TEAD3	NS	0.27258221	0.022260625	0.39385647	NS
FLVCR1	NS	0.272644637	0.022257728	0.393858467	NS
OXR1	NS	0.272628672	-0.022258469	0.393858467	NS
WT1	NS	0.272635092	-0.022258171	0.393858467	NS
PCYT2	NS	0.272711302	-0.022254635	0.39392537	NS
PUS7	NS	0.272893588	-0.02224618	0.394159263	NS
CFI	NS	0.272946464	0.022243728	0.39420622	NS
SERF2	NS	0.273242556	0.022230004	0.39460441	NS
WDFY2	NS	0.273293023	0.022227665	0.394647848	NS
ADCK1	NS	0.27337854	0.022223704	0.394741889	NS
USP44	NS	0.273413383	-0.02222209	0.3947477	NS
RPLP2	NS	0.273423352	0.022221629	0.3947477	NS
LEXM	NS	0.273455369	-0.022220146	0.394764478	NS
ARFRP1	NS	0.273498009	-0.022218172	0.394768846	NS
G6PC3	NS	0.273499185	0.022218117	0.394768846	NS
ITPRID2	NS	0.273547294	0.02221589	0.394808846	NS
ANKRD9	NS	0.273593597	0.022213746	0.394846233	NS
NOP16	NS	0.273678056	0.022209837	0.394909233	NS
KLHL12	NS	0.273676899	0.02220989	0.394909233	NS
EBPL	NS	0.273892525	-0.022199913	0.395159788	NS
THBS2	NS	0.274010467	-0.022194458	0.395300485	NS
NIPA2	NS	0.274055465	0.022192377	0.395335935	NS
FCGR1B	NS	0.274264774	-0.022182701	0.395608388	NS
HGH1	NS	0.274391787	0.022176832	0.395762103	NS
COPZ1	NS	0.274500335	-0.022171818	0.39585967	NS
BRICD5	NS	0.274482058	-0.022172662	0.39585967	NS
PPP1R27	NS	0.274611586	-0.02216668	0.395961104	NS

SERPINB11	NS	0.274594399	-0.022167474	0.395961104	NS
SH3RF2	NS	0.274681856	0.022163436	0.396032925	NS
SETD1B	NS	0.275053227	0.022146299	0.396488105	NS
AMY1B	NS	0.275059014	-0.022146032	0.396488105	NS
CDK5R1	NS	0.275081981	0.022144972	0.396491684	NS
ZNF18	NS	0.27516091	-0.022141332	0.396575918	NS
PAPPA2	NS	0.275251116	0.022137173	0.39667639	NS
ENPP5	NS	0.275470038	0.022127083	0.396962333	NS
PMPCA	NS	0.275497702	-0.022125809	0.396972644	NS
RNF7	NS	0.27556803	0.022122569	0.397044424	NS
TAPBPL	NS	0.275608858	-0.022120688	0.397073692	NS
PCDHA12	NS	0.275700662	-0.02211646	0.397176393	NS
RAB3GAP1	NS	0.275789354	0.022112376	0.397274595	NS
HSP90B1	NS	0.275832855	0.022110373	0.39730769	NS
PCGF3	NS	0.275968433	0.022104133	0.397473398	NS
PRAG1	NS	0.276025803	-0.022101493	0.397526447	NS
SLC16A3	NS	0.276177239	0.022094526	0.397714949	NS
PSKH2	NS	0.276503882	-0.022079507	0.398126099	NS
BTD	NS	0.276485725	0.022080342	0.398126099	NS
FGD4	NS	0.276525889	-0.022078496	0.398128169	NS
DPPA3	NS	0.276622242	-0.022074068	0.398207654	NS
RNF13	NS	0.276693516	0.022070794	0.398280635	NS
HDDC2	NS	0.276763822	-0.022067565	0.398352211	NS
CEP135	NS	0.276923077	-0.022060251	0.398551793	NS
GPRC5A	NS	0.276988876	-0.022057231	0.398616854	NS
KLF18	NS	0.277173245	0.022048769	0.398822876	NS
OR51B4	NS	0.277215312	-0.022046839	0.398853756	NS
PDE4A	NS	0.277520517	0.022032842	0.399263203	NS
PIGS	NS	0.277606463	0.022028902	0.399357168	NS
UBE2I	NS	0.277676237	-0.022025704	0.399386904	NS
TSTD2	NS	0.277689035	0.022025118	0.399386904	NS
MCTS1	NS	0.277676065	-0.022025712	0.399386904	NS
FRAT2	NS	0.277814252	0.02201938	0.399537311	NS
PAPOLG	NS	0.278029209	-0.022009535	0.399816743	NS
CYREN	NS	0.278099464	0.022006319	0.399888064	NS
RPL21	NS	0.278140754	0.022004429	0.399917727	NS
NUDT3	NS	0.278255859	-0.02199916	0.40005351	NS
TNFAIP8L2	NS	0.278594566	0.021983667	0.400510726	NS
SRCAP	NS	0.278715326	0.021978146	0.400611772	NS
BCKDHB	NS	0.278715497	-0.021978138	0.400611772	NS
PCDHA13	NS	0.278726944	-0.021977615	0.400611772	NS
MAP6D1	NS	0.278843427	0.021972291	0.400749434	NS
MSC	NS	0.278944196	0.021967687	0.400864494	NS
ATP6AP1	NS	0.279061149	-0.021962345	0.401002791	NS
MEIOB	NS	0.279191228	-0.021956404	0.40115993	NS
ECM1	NS	0.279236191	0.021954352	0.401181728	NS
CD151	NS	0.279247852	-0.021953819	0.401181728	NS
IRS4	NS	0.279406193	0.021946592	0.401379418	NS
LRRC37B	NS	0.279535898	0.021940674	0.401535944	NS
MSH6	NS	0.279814604	0.021927964	0.40187664	NS
C12orf57	NS	0.279884219	-0.02192479	0.401946798	NS
CTCF	NS	0.280116965	0.021914185	0.402221363	NS
SYT15	NS	0.280104053	-0.021914773	0.402221363	NS
VAMP4	NS	0.280204344	-0.021910204	0.402287144	NS
LRRC75B	NS	0.280195956	-0.021910586	0.402287144	NS

LARS2	NS	0.28023897	-0.021908627	0.402307016	NS
NUDT8	NS	0.280370768	-0.021902626	0.402466374	NS
TDP2	NS	0.280854151	0.021880632	0.403130364	NS
TRIM36	NS	0.280934675	-0.02187697	0.403210577	NS
COPB2	NS	0.280951697	-0.021876196	0.403210577	NS
UQCRH	NS	0.281051616	0.021871654	0.403324071	NS
MYLK	NS	0.28111932	0.021868577	0.40336992	NS
ATP5F1D	NS	0.281125244	0.021868308	0.40336992	NS
HSPD1	NS	0.281283001	0.02186114	0.403566361	NS
PPARA	NS	0.281372693	-0.021857066	0.403635207	NS
ELOA3BP	NS	0.281358017	0.021857733	0.403635207	NS
TIA1	NS	0.281448218	-0.021853636	0.403713629	NS
DHRS12	NS	0.281498076	-0.021851372	0.403726965	NS
NAA35	NS	0.281499231	-0.02185132	0.403726965	NS
ZNF337	NS	0.28153799	-0.02184956	0.403752636	NS
MTSS1	NS	0.281663159	0.021843878	0.403902216	NS
MTHFR	NS	0.28169442	0.02184246	0.403917119	NS
POLR2I	NS	0.281990576	-0.021829024	0.404302574	NS
CCZ1	NS	0.282005014	0.021828369	0.404302574	NS
PPM1F	NS	0.282167725	0.021820992	0.404505887	NS
NTN1	NS	0.282210305	0.021819062	0.404536967	NS
DTYMK	NS	0.282330192	0.021813629	0.40467885	NS
BCLAF3	NS	0.282769644	-0.021793726	0.405278729	NS
ZFP28	NS	0.282892376	-0.021788172	0.405424614	NS
CCL28	NS	0.282945989	-0.021785746	0.405471427	NS
ZNF44	NS	0.283002603	0.021783184	0.405522533	NS
NOC4L	NS	0.283052591	-0.021780923	0.405564138	NS
GJC1	NS	0.283146507	0.021776675	0.405668675	NS
CHST11	NS	0.283179541	-0.021775181	0.405685974	NS
BBS5	NS	0.283208747	0.021773861	0.405697788	NS
GGT1	NS	0.283264753	0.021771328	0.405747987	NS
PSPN	NS	0.283319138	0.02176887	0.405795858	NS
EPHA7	NS	0.283346676	0.021767625	0.405805273	NS
ZNF540	NS	0.283375898	0.021766304	0.405817097	NS
ARSF	NS	0.283465459	-0.021762256	0.405861649	NS
ACAP2	NS	0.283469913	0.021762055	0.405861649	NS
MITF	NS	0.283430136	0.021763853	0.405861649	NS
UBALD1	NS	0.283636914	0.02175451	0.406070718	NS
PLAT	NS	0.283999057	-0.021738158	0.40649839	NS
ADGRD1	NS	0.283995822	0.021738304	0.40649839	NS
STAB2	NS	0.284013884	-0.021737489	0.40649839	NS
MC1R	NS	0.284019644	-0.021737229	0.40649839	NS
YNX1-SLURI	NS	0.284090651	0.021734025	0.406569955	NS
SGIP1	NS	0.284186492	0.021729701	0.406677047	NS
DNM2	NS	0.284235105	0.021727508	0.406716544	NS
MSANTD3	NS	0.284277879	0.021725579	0.406719083	NS
CTNND1	NS	0.284278905	0.021725532	0.406719083	NS
GAL3ST2	NS	0.284353254	0.021722179	0.406795386	NS
PLEKHG4	NS	0.28445358	0.021717656	0.406878768	NS
HSD17B11	NS	0.284436124	0.021718443	0.406878768	NS
BNIP3	NS	0.284631853	-0.02170962	0.407103683	NS
RARS2	NS	0.284732129	0.021705102	0.407156848	NS
KCNMB2	NS	0.284728619	0.02170526	0.407156848	NS
FOXP3	NS	0.284844668	-0.021700032	0.407287685	NS
PPP3R1	NS	0.284900573	-0.021697514	0.407337531	NS

PGM2L1	NS	0.28514537	0.021686493	0.407657419	NS
F2	NS	0.285181848	-0.021684851	0.407679457	NS
GAREM2	NS	0.285287728	-0.021680086	0.407800699	NS
ACADVL	NS	0.28543986	-0.021673242	0.407988032	NS
EIF1AX	NS	0.285463098	0.021672197	0.407991119	NS
HOXA2	NS	0.285688818	0.021662048	0.408283576	NS
NDUFV3	NS	0.285731807	0.021660116	0.408314866	NS
PPP2R3C	NS	0.285798059	-0.021657138	0.40836491	NS
BCR	NS	0.285809023	0.021656645	0.40836491	NS
SLITRK3	NS	0.28592384	0.021651486	0.408498808	NS
ENO1	NS	0.285945373	-0.021650519	0.40849942	NS
SMR3B	NS	0.286092276	-0.02164392	0.408679122	NS
POLR2M	NS	0.286251819	-0.021636757	0.408876851	NS
PYGM	NS	0.286361487	0.021631834	0.409003316	NS
IGLON5	NS	0.286472987	-0.021626831	0.40913238	NS
MTA2	NS	0.286582684	-0.021621909	0.409258849	NS
TFAP2D	NS	0.286913668	-0.021607068	0.409701288	NS
SLC7A14	NS	0.287022839	0.021602175	0.409796715	NS
PPIAL4C	NS	0.287016474	0.021602461	0.409796715	NS
ZIM3	NS	0.287049183	0.021600995	0.4098041	NS
HARS2	NS	0.287079753	-0.021599625	0.409817516	NS
YBX3	NS	0.287161381	0.021595968	0.409903812	NS
ACAD8	NS	0.287216263	-0.02159351	0.409951921	NS
MPV17	NS	0.28724707	0.02159213	0.40996566	NS
MFSD10	NS	0.287339424	-0.021587994	0.409976552	NS
GCNA	NS	0.287319122	-0.021588903	0.409976552	NS
USP25	NS	0.287322006	-0.021588774	0.409976552	NS
HBA2	NS	0.287287232	0.021590332	0.409976552	NS
TMEM30A	NS	0.287446787	0.021583188	0.410099506	NS
CRIP3	NS	0.287649085	0.021574133	0.410297398	NS
DBNL	NS	0.287902245	-0.021562809	0.410628241	NS
IL4	NS	0.288061799	-0.021555676	0.410825537	NS
CPNE1	NS	0.288218036	0.021548693	0.411018073	NS
LY6E	NS	0.288281889	0.02154584	0.411078844	NS
AXIN1	NS	0.288452591	0.021538214	0.41129196	NS
AVL9	NS	0.288795593	0.021522902	0.4117507	NS
TBC1D25	NS	0.2888713	-0.021519524	0.411828304	NS
CASP4	NS	0.288906683	-0.021517945	0.411848413	NS
TMEM126A	NS	0.289026512	0.0215126	0.411988892	NS
GPATCH3	NS	0.289063136	-0.021510966	0.412010756	NS
DHX40	NS	0.289152038	0.021507002	0.412107124	NS
MFSD2A	NS	0.289225636	0.021503721	0.412164907	NS
SYNPO2	NS	0.289235169	0.021503296	0.412164907	NS
OAZ1	NS	0.289298511	-0.021500472	0.412194478	NS
DPY30	NS	0.289298459	0.021500474	0.412194478	NS
AGPS	NS	0.289330352	-0.021499053	0.412209503	NS
TTC26	NS	0.289403243	-0.021495805	0.412283003	NS
RCC2	NS	0.289462731	0.021493154	0.412307058	NS
PLA2G4B	NS	0.28944157	-0.021494097	0.412307058	NS
XPO1	NS	0.289568956	0.021488421	0.412428015	NS
AGMAT	NS	0.28964537	0.021485018	0.412506496	NS
SIRT4	NS	0.289713216	0.021481996	0.412572765	NS
UBE2N	NS	0.28996943	0.021470591	0.412907253	NS
SAFB2	NS	0.289999112	0.02146927	0.412919142	NS
SPA17	NS	0.290044835	0.021467235	0.412923495	NS

KRTCAP2	NS	0.29002975	0.021467906	0.412923495	NS
MDH1	NS	0.290298034	-0.021455972	0.413253567	NS
YIPF6	NS	0.290648037	0.021440414	0.413721385	NS
HOXA3	NS	0.290694099	-0.021438367	0.413746453	NS
NACA	NS	0.290708398	0.021437732	0.413746453	NS
MXI1	NS	0.290796123	0.021433835	0.413820815	NS
IRAK1BP1	NS	0.290803406	0.021433512	0.413820815	NS
BSCL2	NS	0.290848271	-0.021431519	0.413854233	NS
ADGRF1	NS	0.29105172	0.021422485	0.414082844	NS
IFI44L	NS	0.291089815	-0.021420794	0.414106604	NS
HCN1	NS	0.291229777	-0.021414583	0.414275268	NS
REPIN1	NS	0.291351628	0.021409176	0.414418145	NS
TMEM161B	NS	0.291463935	-0.021404195	0.414547427	NS
GOLGA8S	NS	0.291583197	-0.021398906	0.414686581	NS
GAGE12J	NS	0.291607177	-0.021397843	0.414690216	NS
GCKR	NS	0.29167446	-0.02139486	0.414694494	NS
TIRAP	NS	0.291647568	0.021396052	0.414694494	NS
UBL5	NS	0.291671878	-0.021394975	0.414694494	NS
FAM221B	NS	0.291735269	-0.021392165	0.414750486	NS
ATG5	NS	0.291861462	-0.021386573	0.41475073	NS
TTC1	NS	0.291864006	-0.02138646	0.41475073	NS
MYOM3	NS	0.291769907	-0.02139063	0.41475073	NS
HNRNPAB	NS	0.291805895	-0.021389035	0.41475073	NS
KRTAP16-1	NS	0.291845133	0.021387296	0.41475073	NS
METTL22	NS	0.291993828	0.021380708	0.414783998	NS
FIG4	NS	0.292037421	-0.021378778	0.414783998	NS
SLIRP	NS	0.292017286	-0.021379669	0.414783998	NS
ARMC9	NS	0.292004085	0.021380254	0.414783998	NS
AIG1	NS	0.292008892	-0.021380041	0.414783998	NS
CHD1	NS	0.291931707	0.02138346	0.414783998	NS
SSR3	NS	0.29208662	-0.021376599	0.414820302	NS
RGPD8	NS	0.292105844	0.021375747	0.414820302	NS
PALS2	NS	0.292150064	-0.021373789	0.414852662	NS
GTF3C3	NS	0.29221688	-0.021370831	0.414917102	NS
TRIM67	NS	0.292303809	0.021366982	0.414985939	NS
DNASE1L3	NS	0.29230824	0.021366786	0.414985939	NS
INTS1	NS	0.292374652	-0.021363847	0.415049781	NS
HSPB8	NS	0.292574357	-0.021355011	0.41530282	NS
ERICH5	NS	0.292604516	0.021353676	0.415315173	NS
SUOX	NS	0.292710455	0.021348991	0.415374162	NS
MOB1B	NS	0.29270073	0.021349421	0.415374162	NS
APOBEC3B	NS	0.292671407	0.021350718	0.415374162	NS
TBC1D3H	NS	0.292731933	0.021348041	0.415374188	NS
CRELD2	NS	0.292789539	0.021345494	0.415425474	NS
SEC14L3	NS	0.292862807	-0.021342255	0.415498974	NS
MALT1	NS	0.293131054	0.0213304	0.415849067	NS
CRAMP1	NS	0.293298398	0.021323008	0.416055976	NS
OR10T2	NS	0.293376469	-0.02131956	0.416136226	NS
WBP2	NS	0.293537668	0.021312444	0.416334367	NS
DNAJC24	NS	0.293761034	-0.021302587	0.416559603	NS
PHETA2	NS	0.29374855	0.021303138	0.416559603	NS
ZNF177	NS	0.293735645	-0.021303707	0.416559603	NS
MTIF2	NS	0.294074274	-0.021288773	0.416942693	NS
HDHD2	NS	0.294068934	-0.021289008	0.416942693	NS
YAF2	NS	0.294122532	0.021286645	0.416946556	NS

NUCKS1	NS	0.294108413	0.021287268	0.416946556	NS
SNAI3	NS	0.294141621	0.021285804	0.416946556	NS
MARK2	NS	0.294189552	0.021283691	0.41698396	NS
ZNF860	NS	0.294228932	0.021281956	0.416999475	NS
RPS28	NS	0.294243585	0.02128131	0.416999475	NS
MAGEB17	NS	0.294291522	-0.021279198	0.417036877	NS
KCNJ5	NS	0.294373941	-0.021275566	0.4170926	NS
COL28A1	NS	0.294367039	0.02127587	0.4170926	NS
PLPP1	NS	0.294405468	0.021274177	0.417106737	NS
TCHP	NS	0.294440039	0.021272654	0.417125185	NS
BATF2	NS	0.294576022	0.021266666	0.417287287	NS
FAM43A	NS	0.294670079	-0.021262524	0.417389978	NS
GPR50	NS	0.294728528	0.021259951	0.417442221	NS
MYO1F	NS	0.294781539	0.021257618	0.417456209	NS
CATSPERD	NS	0.294773138	-0.021257987	0.417456209	NS
TNFRSF10D	NS	0.294873402	-0.021253575	0.417555751	NS
SPIN2A	NS	0.294895955	-0.021252582	0.41755714	NS
LILRA2	NS	0.29504476	0.021246036	0.417737282	NS
EFCAB11	NS	0.295137168	0.021241971	0.417837553	NS
KAAG1	NS	0.295181594	-0.021240017	0.417839325	NS
TTC8	NS	0.295177725	0.021240188	0.417839325	NS
TNFRSF17	NS	0.295243193	0.021237309	0.417865403	NS
PRSS50	NS	0.295232521	0.021237778	0.417865403	NS
COQ4	NS	0.295285665	-0.021235442	0.417894957	NS
E2F5	NS	0.295405907	-0.021230156	0.418034561	NS
OTOGL	NS	0.295431118	-0.021229048	0.418039675	NS
SIRPD	NS	0.295575121	-0.02122272	0.418182299	NS
RHOH	NS	0.295562613	-0.02122327	0.418182299	NS
RHOJ	NS	0.295613285	-0.021221043	0.418205726	NS
TSSK4	NS	0.29581136	-0.021212343	0.418455359	NS
MAP4K2	NS	0.295840609	0.021211059	0.418466152	NS
KCNA3	NS	0.295927241	0.021207256	0.418527523	NS
TNP2	NS	0.295926506	0.021207288	0.418527523	NS
PTGIR	NS	0.295972852	0.021205253	0.418530868	NS
AP4M1	NS	0.295957195	0.021205941	0.418530868	NS
RANBP17	NS	0.296037043	0.021202436	0.418591058	NS
TTF1	NS	0.2961	-0.021199673	0.418618917	NS
RNF187	NS	0.29607887	0.0212006	0.418618917	NS
ARMC12	NS	0.296399112	-0.021186551	0.419011191	NS
EBF1	NS	0.296433894	0.021185026	0.419029757	NS
RAI1	NS	0.296525385	-0.021181015	0.419067272	NS
KCNB1	NS	0.296498995	0.021182172	0.419067272	NS
IKBIP	NS	0.296525289	-0.021181019	0.419067272	NS
TRAPPC1	NS	0.296599974	0.021177745	0.419142082	NS
OR1Q1	NS	0.296633234	-0.021176287	0.419158482	NS
TRAPPC14	NS	0.296780404	0.021169837	0.419335828	NS
CFAP57	NS	0.296999083	-0.021160258	0.419614179	NS
SPI1	NS	0.297046569	-0.021158179	0.419620011	NS
PAH	NS	0.297044913	-0.021158251	0.419620011	NS
MCRS1	NS	0.297244382	0.021149518	0.419868806	NS
PEX16	NS	0.297363501	0.021144305	0.419976739	NS
GGT7	NS	0.297364187	0.021144275	0.419976739	NS
SND1	NS	0.29748633	0.021138931	0.420118591	NS
B3GALT5	NS	0.297646396	0.02113193	0.420313974	NS
EXOSC5	NS	0.29769138	-0.021129963	0.420346831	NS

LCE2A	NS	0.297923978	-0.021119795	0.420639923	NS
CCDC196	NS	0.297942413	-0.021118989	0.420639923	NS
PTPN13	NS	0.298058383	-0.021113922	0.42077296	NS
PIN1	NS	0.298241411	-0.021105927	0.421000638	NS
ACADM	NS	0.298300701	-0.021103338	0.421049998	NS
PARVG	NS	0.298319884	0.0211025	0.421049998	NS
ACOX3	NS	0.298407613	0.02109867	0.42114311	NS
TRIM31	NS	0.298553175	-0.021092316	0.421317822	NS
ARMC7	NS	0.298633206	-0.021088824	0.421338606	NS
DTX1	NS	0.298592476	0.021090601	0.421338606	NS
CRYBG2	NS	0.298631547	-0.021088896	0.421338606	NS
PTS	NS	0.298788329	-0.021082056	0.421504417	NS
WDR88	NS	0.298794281	0.021081797	0.421504417	NS
FKBP3	NS	0.298921733	-0.021076238	0.421651331	NS
HES6	NS	0.298941993	-0.021075355	0.421651331	NS
NSUN5	NS	0.299158591	0.021065912	0.421926091	NS
NCAPD3	NS	0.29947756	0.021052016	0.422314415	NS
ABCC3	NS	0.299550712	0.02104883	0.422386799	NS
KLK13	NS	0.299732935	0.021040897	0.422612958	NS
RHBDD1	NS	0.299783362	0.021038702	0.42265327	NS
NEUROD2	NS	0.299895801	0.021033809	0.422781	NS
GALNT1	NS	0.3000448	0.021027327	0.422960247	NS
ZNF362	NS	0.300252335	0.021018302	0.423221978	NS
MC3R	NS	0.300294258	-0.021016479	0.423250248	NS
CALCA	NS	0.300471766	-0.021008764	0.423469602	NS
FKBP4	NS	0.300625968	0.021002064	0.423656078	NS
GDE1	NS	0.301286561	-0.020973388	0.424524865	NS
FHIP1B	NS	0.301308255	0.020972447	0.424524865	NS
NFATC3	NS	0.301304969	0.020972589	0.424524865	NS
KCNE4	NS	0.301406065	0.020968205	0.424569959	NS
HDAC3	NS	0.301400521	-0.020968445	0.424569959	NS
ORM1	NS	0.301371135	-0.02096972	0.424569959	NS
TRMT9B	NS	0.301590441	-0.020960211	0.424798762	NS
EPHA1	NS	0.301632619	0.020958382	0.424827255	NS
OCA2	NS	0.301691775	-0.020955818	0.424879657	NS
KIAA0319L	NS	0.301780964	0.020951953	0.424974343	NS
RAB14	NS	0.302026728	-0.020941307	0.425289492	NS
RAB39A	NS	0.302152714	0.020935852	0.425435944	NS
ATRN	NS	0.302234754	-0.0209323	0.425489556	NS
DUS3L	NS	0.302226106	0.020932675	0.425489556	NS
KMT5B	NS	0.302282226	0.020930246	0.425525437	NS
NUP205	NS	0.302344615	-0.020927545	0.425551365	NS
UBN2	NS	0.302331704	-0.020928104	0.425551365	NS
PIF1	NS	0.30248585	0.020921434	0.425719197	NS
CDKL1	NS	0.302531197	0.020919472	0.425721109	NS
TUBA1C	NS	0.302529787	-0.020919533	0.425721109	NS
BRD9	NS	0.302554849	-0.020918449	0.425723441	NS
OR51B2	NS	0.302586433	-0.020917083	0.425736934	NS
GAD1	NS	0.302640303	-0.020914753	0.425781778	NS
RBM24	NS	0.302735612	0.020910632	0.425884913	NS
CCT2	NS	0.302850122	0.020905681	0.426015041	NS
AIDA	NS	0.30298291	0.020899941	0.42617086	NS
LGALS12	NS	0.303015592	-0.020898529	0.42618586	NS
NBN	NS	0.30309334	0.02089517	0.426257366	NS
CEP126	NS	0.303132499	0.020893478	0.426257366	NS

SOS1	NS	0.30313244	0.02089348	0.426257366	NS
MPO	NS	0.303209484	-0.020890152	0.426322345	NS
SOD3	NS	0.303222759	0.020889579	0.426322345	NS
SCN3B	NS	0.303300583	-0.020886218	0.426400791	NS
CNIH1	NS	0.303326124	0.020885115	0.426405728	NS
ING5	NS	0.303577637	-0.020874256	0.426728303	NS
UBE2A	NS	0.303705245	0.02086875	0.426845681	NS
NAB1	NS	0.303686827	0.020869544	0.426845681	NS
EHMT1	NS	0.303727965	-0.020867769	0.42684662	NS
HSPA8	NS	0.304008528	-0.020855668	0.427132648	NS
DISP1	NS	0.304027689	-0.020854842	0.427132648	NS
HTR1A	NS	0.303995278	0.020856239	0.427132648	NS
SPG7	NS	0.304041828	0.020854232	0.427132648	NS
ECI2	NS	0.304007751	-0.020855701	0.427132648	NS
ITIH5	NS	0.304099326	0.020851753	0.42718242	NS
DNAAF4	NS	0.304195365	-0.020847613	0.42728632	NS
KRTAP5-9	NS	0.304240191	-0.020845681	0.427318274	NS
C4orf47	NS	0.304306075	-0.020842842	0.4273798	NS
PRR12	NS	0.30444291	0.020836947	0.427540953	NS
TM2D1	NS	0.304520564	-0.020833602	0.427618981	NS
MCUR1	NS	0.304591053	0.020830566	0.427666503	NS
KHDC1	NS	0.304610183	-0.020829742	0.427666503	NS
SLC36A3	NS	0.30462069	0.02082929	0.427666503	NS
ARHGAP23	NS	0.304730138	-0.020824577	0.427789132	NS
ACAN	NS	0.304840322	-0.020819834	0.427912776	NS
SCRN1	NS	0.304888717	0.020817751	0.427949674	NS
CD276	NS	0.305026241	0.020811833	0.428111662	NS
RNF31	NS	0.305083398	0.020809375	0.428160837	NS
SSR4	NS	0.305200772	0.020804326	0.428294508	NS
HOXA9	NS	0.305301791	-0.020799982	0.42840521	NS
MAFK	NS	0.305429724	0.020794482	0.428553659	NS
C4orf3	NS	0.305476102	-0.020792488	0.428557748	NS
LENG9	NS	0.305476919	-0.020792453	0.428557748	NS
LSP1	NS	0.305525339	0.020790372	0.428594612	NS
TGFB1I1	NS	0.30588335	-0.020774992	0.429065739	NS
CCDC137	NS	0.306067233	-0.020767097	0.429199258	NS
ZNF583	NS	0.306030835	0.020768659	0.429199258	NS
SPANXN1	NS	0.306030029	-0.020768694	0.429199258	NS
CENPX	NS	0.306154515	-0.02076335	0.429290554	NS
PPP1R3G	NS	0.306282874	-0.020757842	0.429439429	NS
PELI3	NS	0.306372594	-0.020753993	0.429503	NS
SEBOX	NS	0.306350602	-0.020754936	0.429503	NS
TRANK1	NS	0.30643479	-0.020751325	0.429559081	NS
DNALI1	NS	0.306566541	-0.020745675	0.429681533	NS
UBXN7	NS	0.306548955	0.020746429	0.429681533	NS
TBC1D30	NS	0.306615019	-0.020743596	0.429687251	NS
TBC1D3G	NS	0.306605286	0.020744013	0.429687251	NS
HSPA4	NS	0.306712134	-0.020739433	0.429773445	NS
SHISA3	NS	0.306720933	0.020739055	0.429773445	NS
MTG1	NS	0.307010642	-0.020726641	0.430148244	NS
PIH1D1	NS	0.307712627	-0.020696591	0.431100579	NS
APBB1	NS	0.307787209	0.020693401	0.431142655	NS
CBWD6	NS	0.307775062	-0.02069392	0.431142655	NS
CDH3	NS	0.307899315	-0.020688607	0.431174876	NS
SYNDIG1	NS	0.30785138	-0.020690656	0.431174876	NS

TEX37	NS	0.307880526	-0.02068941	0.431174876	NS
PRAMEF20	NS	0.307872386	0.020689758	0.431174876	NS
CDH6	NS	0.308010641	-0.020683847	0.431280672	NS
TINAGL1	NS	0.308035664	0.020682778	0.431280672	NS
IRS2	NS	0.308041707	0.020682519	0.431280672	NS
KCNJ8	NS	0.308176985	0.020676738	0.431438863	NS
OR2AG2	NS	0.308226363	-0.020674628	0.431476783	NS
PGM5	NS	0.308269617	-0.02067278	0.431506125	NS
MAP2K4	NS	0.308418891	-0.020666403	0.431652643	NS
KCTD15	NS	0.308408682	-0.020666839	0.431652643	NS
REP15	NS	0.308643946	-0.020656794	0.431936391	NS
INAFM2	NS	0.308683653	0.020655099	0.431960728	NS
GTF2IRD2B	NS	0.309042552	-0.020639785	0.432431695	NS
TPRG1L	NS	0.30908296	0.020638061	0.432456973	NS
EHF	NS	0.309148599	-0.020635262	0.432479882	NS
DNER	NS	0.309135682	-0.020635813	0.432479882	NS
MGARP	NS	0.309232701	0.020631676	0.432541419	NS
MTHFD1	NS	0.309302553	0.020628698	0.432545352	NS
IGSF10	NS	0.309300934	-0.020628767	0.432545352	NS
ZNF701	NS	0.309263394	-0.020630367	0.432545352	NS
OR4D9	NS	0.30941613	0.020623857	0.432641669	NS
SERAC1	NS	0.309451322	0.020622357	0.432659621	NS
ZNF559	NS	0.309491349	-0.020620651	0.43268433	NS
OR5B3	NS	0.309652261	-0.020613796	0.432878027	NS
TNS1	NS	0.3098271	0.02060635	0.433059889	NS
NMNAT3	NS	0.309809312	-0.020607107	0.433059889	NS
IGFBP2	NS	0.309898012	0.02060333	0.43312773	NS
DENND1A	NS	0.309929801	-0.020601977	0.433140882	NS
GML	NS	0.310210385	0.020590035	0.433501712	NS
DNM1L	NS	0.310264736	-0.020587723	0.433546364	NS
TRIM49C	NS	0.310389861	-0.020582401	0.433689897	NS
TSLP	NS	0.310573978	-0.020574572	0.43391583	NS
DCP1B	NS	0.310714032	-0.020568618	0.434080173	NS
ABCE1	NS	0.310756428	-0.020566816	0.43410807	NS
FAM83F	NS	0.310874913	0.020561782	0.434210912	NS
NIBAN1	NS	0.310864771	-0.020562213	0.434210912	NS
FAAP24	NS	0.31095129	-0.020558537	0.434286253	NS
HDAC6	NS	0.311024085	-0.020555445	0.434356579	NS
BPIFB2	NS	0.311095981	-0.020552392	0.434425641	NS
SLC25A5	NS	0.311239069	-0.020546316	0.434594101	NS
FERMT2	NS	0.311317039	0.020543006	0.434671615	NS
GPR89B	NS	0.311341615	0.020541963	0.434674574	NS
CMPK2	NS	0.311484198	-0.020535912	0.434842275	NS
GASK1B	NS	0.311697466	-0.020526865	0.435108622	NS
FHL1	NS	0.311805061	0.020522302	0.435164667	NS
PRKAR2A	NS	0.311780855	-0.020523328	0.435164667	NS
SPNS2	NS	0.311803525	-0.020522367	0.435164667	NS
NR3C1	NS	0.312035724	0.020512523	0.435455189	NS
FABP1	NS	0.312299122	-0.020501363	0.435759934	NS
CRLF3	NS	0.312361062	-0.020498739	0.435814944	NS
PHYHD1	NS	0.312443216	0.02049526	0.435898146	NS
CR1L	NS	0.312679277	-0.020485266	0.436196042	NS
SLC19A3	NS	0.312720052	0.020483541	0.436215206	NS
NPIPB3	NS	0.312738087	0.020482777	0.436215206	NS
CCDC9B	NS	0.312867248	-0.020477312	0.436363917	NS

POLG2	NS	0.313064715	0.020468959	0.436576413	NS
ARHGAP15	NS	0.313243565	0.020461397	0.436755047	NS
L2HGDH	NS	0.313254479	-0.020460936	0.436755047	NS
GOLGA8F	NS	0.313260762	-0.02046067	0.436755047	NS
ATOX1	NS	0.313283068	-0.020459727	0.436755047	NS
TMED8	NS	0.313344029	-0.02045715	0.436777115	NS
NFATC4	NS	0.313331983	-0.020457659	0.436777115	NS
KIF4B	NS	0.313556353	0.020448178	0.437041605	NS
ERI3	NS	0.313631808	-0.020444991	0.437104547	NS
RDH13	NS	0.313646676	0.020444363	0.437104547	NS
CUEDC2	NS	0.313769002	-0.020439196	0.437243542	NS
CNBP	NS	0.313859497	0.020435375	0.437338164	NS
PYGO1	NS	0.314094398	0.02042546	0.437633974	NS
PIAS4	NS	0.314137282	0.020423651	0.43766222	NS
N4BP1	NS	0.314356272	0.020414412	0.437809756	NS
HMOX2	NS	0.314309354	0.020416391	0.437809756	NS
ONECUT2	NS	0.314324788	-0.02041574	0.437809756	NS
APC	NS	0.314353866	0.020414514	0.437809756	NS
RSPO3	NS	0.314354154	0.020414502	0.437809756	NS
DMTN	NS	0.314444569	-0.020410689	0.437901221	NS
KRTAP9-4	NS	0.314495023	0.020408561	0.437939976	NS
CCR2	NS	0.31454548	-0.020406434	0.437978729	NS
ADGRG5	NS	0.314899014	-0.020391534	0.438439456	NS
SEC13	NS	0.314993758	0.020387543	0.438513352	NS
TALDO1	NS	0.314997399	-0.02038739	0.438513352	NS
RYBP	NS	0.315022037	0.020386352	0.438516112	NS
PLXNB1	NS	0.315187182	0.020379398	0.438714446	NS
CACYBP	NS	0.315272522	0.020375805	0.438801678	NS
ALS2	NS	0.315445384	-0.02036853	0.438981169	NS
CEL	NS	0.315446842	0.020368468	0.438981169	NS
PHYH	NS	0.315633157	-0.02036063	0.439177298	NS
KIF13B	NS	0.31561474	-0.020361404	0.439177298	NS
TEX101	NS	0.315692218	0.020358145	0.439227902	NS
TSPAN31	NS	0.315828399	-0.020352418	0.43938579	NS
RABL2A	NS	0.316064287	-0.020342502	0.439682359	NS
ATG101	NS	0.316198693	-0.020336855	0.439806116	NS
TASL	NS	0.316284039	0.020333269	0.439893214	NS
ZC3H4	NS	0.316358956	-0.020330122	0.439965796	NS
FGD1	NS	0.316565302	-0.020321457	0.440121393	NS
POMZP3	NS	0.316560324	0.020321666	0.440121393	NS
OSCAR	NS	0.31658453	-0.02032065	0.440121393	NS
WNT2B	NS	0.316634108	-0.020318569	0.440137608	NS
MGAM2	NS	0.316641671	-0.020318251	0.440137608	NS
CHRM5	NS	0.317008152	-0.020302874	0.44061538	NS
RPS13	NS	0.31706298	-0.020300574	0.440659943	NS
ISX	NS	0.317114806	-0.020298401	0.44070033	NS
CPA4	NS	0.317299919	0.020290639	0.440925926	NS
MAT2B	NS	0.317370322	-0.020287688	0.440960445	NS
SNX14	NS	0.317361024	-0.020288078	0.440960445	NS
DDX25	NS	0.31751705	0.02028154	0.441132645	NS
NSD1	NS	0.317540083	-0.020280574	0.441132982	NS
SIGMAR1	NS	0.317933236	-0.020264109	0.441647461	NS
GRAMD1C	NS	0.318019406	-0.020260501	0.441735459	NS
EFCAB1	NS	0.318338594	0.020247146	0.442147089	NS
EIF3E	NS	0.318364566	-0.020246059	0.442151435	NS

USP17L2	NS	0.318388637	0.020245052	0.442153139	NS
PIGT	NS	0.318417906	-0.020243828	0.442162062	NS
RNF115	NS	0.318440942	0.020242865	0.44216233	NS
DUSP19	NS	0.31847626	0.020241388	0.442179649	NS
ATP1B3	NS	0.318579625	0.020237066	0.442263445	NS
EPB41L5	NS	0.318582311	-0.020236954	0.442263445	NS
MROH8	NS	0.318697711	-0.020232129	0.442297282	NS
EYA1	NS	0.318633796	0.020234801	0.442297282	NS
TBCB	NS	0.318683575	-0.02023272	0.442297282	NS
CDH18	NS	0.318698088	-0.020232114	0.442297282	NS
TXNRD2	NS	0.31899817	-0.020219574	0.442682003	NS
CAMKK1	NS	0.319290291	0.020207375	0.443055622	NS
FAM168B	NS	0.319314554	0.020206362	0.443057527	NS
ZBED2	NS	0.319338078	-0.02020538	0.443058407	NS
INSL5	NS	0.319393481	-0.020203068	0.443103513	NS
EME2	NS	0.319475626	0.020199639	0.44318571	NS
SLC22A7	NS	0.319546586	-0.020196678	0.443252381	NS
ACTR1B	NS	0.319596639	0.02019459	0.443290042	NS
RGL4	NS	0.319659573	0.020191964	0.443345564	NS
LACC1	NS	0.31975779	0.020187868	0.443450009	NS
MSMO1	NS	0.320118335	0.020172835	0.44385462	NS
ULBP1	NS	0.320114266	-0.020173005	0.44385462	NS
PDHA1	NS	0.320101736	0.020173527	0.44385462	NS
HCK	NS	0.320193889	0.020169687	0.443927578	NS
RETREG3	NS	0.320362156	-0.020162676	0.444129057	NS
C17orf64	NS	0.320515686	0.020156282	0.444310078	NS
LRRC66	NS	0.320598356	-0.020152839	0.444392852	NS
ODF4	NS	0.32064375	0.020150949	0.444423948	NS
COPZ2	NS	0.32070675	-0.020148327	0.444447616	NS
TUT1	NS	0.32068746	-0.02014913	0.444447616	NS
PIK3R5	NS	0.320805912	0.020144199	0.44455321	NS
PER3	NS	0.321148534	0.020129944	0.444996136	NS
MYOZ1	NS	0.321182096	-0.020128549	0.445010784	NS
SNX6	NS	0.321205557	0.020127573	0.445011435	NS
THOC5	NS	0.321373202	-0.020120603	0.445204657	NS
RIN3	NS	0.321391025	0.020119862	0.445204657	NS
F2R	NS	0.32174862	0.020105002	0.445668118	NS
CFAP251	NS	0.321990206	-0.020094969	0.445970836	NS
STAM	NS	0.322076097	-0.020091403	0.44605788	NS
TDRD9	NS	0.322130763	0.020089134	0.446076163	NS
SMIM10L1	NS	0.32213539	0.020088942	0.446076163	NS
RIC8A	NS	0.322337585	0.020080551	0.446324222	NS
RAB28	NS	0.322701271	0.020065468	0.446763879	NS
RNASEH2C	NS	0.322680337	0.020066336	0.446763879	NS
ABCC8	NS	0.322927962	-0.020056071	0.447013777	NS
IFT122	NS	0.322923666	-0.020056249	0.447013777	NS
TCTA	NS	0.323061348	0.020050545	0.447166437	NS
VWA7	NS	0.323123218	0.020047982	0.447220093	NS
RUFY3	NS	0.323238386	0.020043211	0.447315521	NS
CCDC201	NS	0.323218368	-0.02004404	0.447315521	NS
ZNF683	NS	0.323319122	-0.020039868	0.447395263	NS
PIK3CA	NS	0.323372884	0.020037642	0.447426654	NS
BCO1	NS	0.323388039	-0.020037014	0.447426654	NS
MCM7	NS	0.323479972	0.020033209	0.44752186	NS
ANP32D	NS	0.323666037	0.020025508	0.447747271	NS

CRYAB	NS	0.323815009	0.020019345	0.44792134	NS
SLC38A8	NS	0.324349031	-0.019997266	0.448595917	NS
ARL14EP	NS	0.32442176	-0.019994261	0.448664445	NS
CDC42BPB	NS	0.324560841	-0.019988515	0.448824723	NS
RCC1	NS	0.324658816	-0.019984469	0.448928136	NS
SV2A	NS	0.324734019	0.019981364	0.449000048	NS
PGK2	NS	0.32476701	-0.019980001	0.449013589	NS
CELA3A	NS	0.324859004	-0.019976204	0.449108698	NS
PECAM1	NS	0.324958963	0.019972078	0.449214804	NS
TEX49	NS	0.3253594	0.019955558	0.449736238	NS
CEP170	NS	0.325485387	0.019950363	0.449846137	NS
GOLGA7B	NS	0.325484564	-0.019950397	0.449846137	NS
CAPN15	NS	0.325516274	-0.019949089	0.449856704	NS
PLCB3	NS	0.325584091	0.019946294	0.449918302	NS
SPOCK2	NS	0.325646737	0.019943712	0.449972747	NS
NKTR	NS	0.325704953	0.019941313	0.450019534	NS
ZNF529	NS	0.325750346	0.019939442	0.450019534	NS
ZNF468	NS	0.325746234	-0.019939611	0.450019534	NS
DUSP12	NS	0.326067188	-0.01992639	0.450250028	NS
ECPAS	NS	0.326072908	0.019926155	0.450250028	NS
DDX60	NS	0.326009715	-0.019928757	0.450250028	NS
SLC24A2	NS	0.325980505	-0.01992996	0.450250028	NS
UCMA	NS	0.326104557	-0.019924851	0.450250028	NS
POLR1C	NS	0.326054585	0.019926909	0.450250028	NS
TGIF1	NS	0.326096096	0.0199252	0.450250028	NS
UMAD1	NS	0.326038475	0.019927573	0.450250028	NS
GSK3B	NS	0.326194844	0.019921134	0.450290789	NS
ADAM10	NS	0.326202596	0.019920815	0.450290789	NS
PCK1	NS	0.326247637	0.019918961	0.450320848	NS
TULP4	NS	0.326354837	0.019914548	0.450436697	NS
RHBDD3	NS	0.326481854	-0.019909321	0.450579876	NS
KLHL26	NS	0.326620945	0.019903599	0.450739699	NS
DDX49	NS	0.326793468	-0.019896503	0.450888973	NS
SETBP1	NS	0.326822292	0.019895318	0.450888973	NS
ACOX1	NS	0.326806321	-0.019895975	0.450888973	NS
CHRNA9	NS	0.326821929	0.019895333	0.450888973	NS
NCOA2	NS	0.326937144	0.019890596	0.451015277	NS
THBS1	NS	0.327002363	0.019887915	0.4510731	NS
SNRPD2	NS	0.327047703	-0.019886052	0.451085047	NS
THAP2	NS	0.327057633	0.019885643	0.451085047	NS
CDK14	NS	0.327258304	0.019877398	0.451329656	NS
ADD1	NS	0.327283432	-0.019876365	0.451332154	NS
SLC22A14	NS	0.327338896	-0.019874087	0.451376481	NS
IL21	NS	0.327393751	-0.019871834	0.451419963	NS
MYG1	NS	0.327530198	0.01986623	0.451563729	NS
DLGAP2	NS	0.327544676	-0.019865636	0.451563729	NS
SERPINB1	NS	0.327687672	0.019859765	0.451728694	NS
ZNF85	NS	0.327759468	0.019856819	0.45179549	NS
AFAP1L1	NS	0.327806151	-0.019854903	0.451827662	NS
SLC30A7	NS	0.327854566	-0.019852916	0.451862217	NS
PRKCI	NS	0.327960441	0.019848572	0.451975956	NS
DCAF6	NS	0.328032124	-0.019845631	0.452010379	NS
KIAA1522	NS	0.328025059	0.019845921	0.452010379	NS
LRRC30	NS	0.328066855	0.019844207	0.452026056	NS
CRIPT	NS	0.328196892	0.019838873	0.452173041	NS

ZNF516	NS	0.328398107	-0.019830624	0.452385862	NS
MYEF2	NS	0.32838005	-0.019831364	0.452385862	NS
PRPF31	NS	0.328729717	-0.019817036	0.452810448	NS
POFUT1	NS	0.328775209	-0.019815172	0.452840884	NS
APOBEC3H	NS	0.328830769	0.019812896	0.452852961	NS
LRRIQ1	NS	0.328808089	0.019813825	0.452852961	NS
JC02210-CRH	NS	0.32895213	-0.019807927	0.452987864	NS
KCTD2	NS	0.329066926	-0.019803227	0.45311371	NS
TOB2	NS	0.32913533	0.019800427	0.453175661	NS
HSPH1	NS	0.329185113	-0.019798389	0.453211968	NS
HYAL1	NS	0.329657758	0.019779054	0.45383041	NS
RRAGD	NS	0.329770631	-0.01977444	0.453938329	NS
OPTN	NS	0.329787609	0.019773745	0.453938329	NS
CNKSR3	NS	0.329806505	0.019772973	0.453938329	NS
ZNF320	NS	0.329896425	0.019769298	0.454029807	NS
P3R3URF	NS	0.330065638	-0.019762383	0.454230394	NS
FAM153B	NS	0.330090147	0.019761382	0.454231827	NS
TMEM183A	NS	0.330204936	0.019756693	0.454357484	NS
HLA-G	NS	0.330385924	0.019749302	0.454574207	NS
SFR1	NS	0.330495029	0.019744847	0.454692003	NS
PCMTD2	NS	0.330648431	-0.019738586	0.45487072	NS
ARID4B	NS	0.330780772	0.019733186	0.455020441	NS
CDADC1	NS	0.33085266	0.019730253	0.455086988	NS
KCNMA1	NS	0.330927204	-0.019727213	0.455157177	NS
PNPLA7	NS	0.33098727	-0.019724763	0.455207447	NS
NTSR2	NS	0.331033	0.019722898	0.455237993	NS
ERAL1	NS	0.33107996	0.019720983	0.455263918	NS
SRSF1	NS	0.331098893	-0.019720211	0.455263918	NS
TMEM198	NS	0.331288514	0.019712481	0.455492291	NS
PLAAT2	NS	0.331619715	-0.019698986	0.455850522	NS
LRRC36	NS	0.331609734	-0.019699392	0.455850522	NS
PUSL1	NS	0.331577916	-0.019700688	0.455850522	NS
PNRC1	NS	0.331655751	0.019697518	0.455867683	NS
NIPSNAP2	NS	0.331782194	0.019692369	0.456009099	NS
PPEF1	NS	0.332089767	-0.019679848	0.456399428	NS
UBE2E1	NS	0.33224174	-0.019673665	0.456546741	NS
TMEM222	NS	0.33224413	-0.019673567	0.456546741	NS
RRAS2	NS	0.332691987	-0.019655355	0.457129701	NS
PPM1B	NS	0.332835393	0.019649527	0.457272096	NS
CDKAL1	NS	0.332842868	-0.019649223	0.457272096	NS
DOT1L	NS	0.333052913	0.01964069	0.457464641	NS
PSMD11	NS	0.333041038	-0.019641172	0.457464641	NS
DDX55	NS	0.333118313	-0.019638033	0.457520617	NS
TP53TG3B	NS	0.333146479	-0.019636889	0.457526837	NS
RNF168	NS	0.333242193	0.019633003	0.457593352	NS
ABCF1	NS	0.333237897	0.019633177	0.457593352	NS
HSD17B13	NS	0.333314636	0.019630061	0.457627898	NS
ESCO2	NS	0.333310818	0.019630216	0.457627898	NS
SPART	NS	0.333427953	0.019625461	0.457751008	NS
CDC25B	NS	0.33349978	-0.019622546	0.457817144	NS
ATP6V1B1	NS	0.333966835	-0.0196036	0.458425789	NS
GPR161	NS	0.334153884	-0.019596017	0.45865002	NS
ZNF689	NS	0.334322241	-0.019589194	0.45881603	NS
UST	NS	0.334348359	-0.019588136	0.458819343	NS
LUZP1	NS	0.334392763	0.019586337	0.458847747	NS

MGAT4A	NS	0.334508034	0.019581667	0.458973382	NS
YARS1	NS	0.334584639	0.019578564	0.45904595	NS
TMEM217	NS	0.334624924	0.019576933	0.459068681	NS
ATP5F1B	NS	0.334723944	-0.019572923	0.459171981	NS
BDKRB2	NS	0.334974324	-0.019562788	0.459450328	NS
AP3S1	NS	0.334963005	0.019563246	0.459450328	NS
MUTYH	NS	0.335670963	-0.019534615	0.460351803	NS
AWAT2	NS	0.335702919	-0.019533324	0.460351803	NS
C10orf143	NS	0.335685766	0.019534017	0.460351803	NS
ATL3	NS	0.33580167	-0.019529333	0.460438775	NS
NUDT16	NS	0.335813917	-0.019528838	0.460438775	NS
TRIM71	NS	0.335861481	-0.019526917	0.460471371	NS
SPTSSB	NS	0.335974531	-0.01952235	0.460593741	NS
SDK2	NS	0.336192495	-0.019513548	0.460794642	NS
DGKH	NS	0.336155563	0.019515039	0.460794642	NS
INTS3	NS	0.336191904	-0.019513572	0.460794642	NS
DUSP16	NS	0.336275643	0.019510191	0.460875971	NS
TMPRSS9	NS	0.336537389	-0.019499628	0.461202047	NS
PAQR8	NS	0.336687283	-0.019493581	0.461374801	NS
TNIK	NS	0.336768781	-0.019490294	0.461421145	NS
RAB30	NS	0.336882169	0.019485721	0.461543833	NS
CHRDL2	NS	0.337263505	0.019470351	0.4620281	NS
SDCBP	NS	0.337283377	0.01946955	0.4620281	NS
POU5F2	NS	0.337453926	0.01946268	0.462229014	NS
DUSP5	NS	0.337517565	-0.019460117	0.46228347	NS
SASH1	NS	0.337589166	-0.019457233	0.462348822	NS
ANKUB1	NS	0.337678681	0.019453629	0.462438697	NS
GTF3A	NS	0.338041567	-0.019439024	0.462902905	NS
ZNF550	NS	0.338109483	0.019436292	0.462963154	NS
KLHL28	NS	0.338167767	0.019433948	0.462977458	NS
HEATR4	NS	0.338144837	0.01943487	0.462977458	NS
AGPAT2	NS	0.338332477	-0.019427324	0.463170198	NS
NCF4	NS	0.338426145	-0.019423558	0.463234257	NS
TAF5L	NS	0.338427135	-0.019423518	0.463234257	NS
CHMP1B	NS	0.338454909	-0.019422401	0.463239514	NS
HKDC1	NS	0.338489018	-0.01942103	0.463253442	NS
RHOBTB3	NS	0.338579367	0.019417399	0.463344331	NS
RNF8	NS	0.338628752	-0.019415414	0.463379154	NS
HDGFL1	NS	0.338838446	-0.019406988	0.463633321	NS
WNK4	NS	0.338891487	0.019404858	0.463645401	NS
CNOT8	NS	0.338895181	0.019404709	0.463645401	NS
USP47	NS	0.339136646	0.019395013	0.463942958	NS
GRAP2	NS	0.339207259	-0.019392178	0.464006763	NS
CCDC40	NS	0.339273362	0.019389524	0.464048654	NS
FAM237B	NS	0.339285831	-0.019389024	0.464048654	NS
LRMDA	NS	0.339366436	0.019385789	0.464126103	NS
MDK	NS	0.339426968	-0.01938336	0.464143298	NS
GYG1	NS	0.339560623	-0.019377997	0.464276112	NS
ATG13	NS	0.339572066	0.019377538	0.464276112	NS
TMEM120B	NS	0.339715621	0.01937178	0.464439579	NS
STYX	NS	0.339881926	0.019365111	0.464634124	NS
DNAH3	NS	0.340044812	0.019358582	0.464823968	NS
RPL10A	NS	0.340361005	-0.019345913	0.465223333	NS
FBXO36	NS	0.340563599	0.019337799	0.465467379	NS
BUB1B-PAK6	NS	0.340632816	-0.019335028	0.46552911	NS

ECH1	NS	0.340669373	-0.019333564	0.465546201	NS
LRRC42	NS	0.340870161	-0.019325527	0.465787705	NS
NPAP1	NS	0.340962052	0.019321851	0.465880381	NS
H2AZ1	NS	0.341112526	0.019315831	0.466053085	NS
TMEM63B	NS	0.341155834	0.019314098	0.466075743	NS
TLE1	NS	0.341177268	-0.019313241	0.466075743	NS
MRS2	NS	0.341565651	-0.019297714	0.466573376	NS
DGKB	NS	0.341758082	0.019290024	0.466803291	NS
KRTAP4-2	NS	0.341850244	0.019286343	0.466896226	NS
GSAP	NS	0.342010858	-0.019279928	0.46704968	NS
KMT2B	NS	0.342004568	-0.019280179	0.46704968	NS
ATP5ME	NS	0.342138628	-0.019274826	0.467191201	NS
ITSN2	NS	0.34223577	0.019270948	0.467290883	NS
EEA1	NS	0.342341024	-0.019266748	0.467401626	NS
SHKBP1	NS	0.342370442	0.019265574	0.467408822	NS
RFX3	NS	0.34263479	-0.019255027	0.467703738	NS
GNL2	NS	0.342617168	-0.01925573	0.467703738	NS
CMIP	NS	0.342676219	-0.019253375	0.467727304	NS
WDR44	NS	0.34272274	-0.019251519	0.467757817	NS
PRPF19	NS	0.342940247	-0.019242847	0.467945775	NS
HIGD2A	NS	0.342931533	0.019243194	0.467945775	NS
EXOC3L1	NS	0.342957159	-0.019242173	0.467945775	NS
DENND1B	NS	0.342894407	-0.019244675	0.467945775	NS
AGBL2	NS	0.34315281	-0.019234375	0.468179728	NS
NPIPA8	NS	0.343193063	-0.019232771	0.468201644	NS
GPBP1	NS	0.343263885	0.01922995	0.468220437	NS
LRRC31	NS	0.343231637	0.019231234	0.468220437	NS
USP7	NS	0.343279408	0.019229331	0.468220437	NS
GRIN2C	NS	0.343543895	0.019218797	0.46854817	NS
DUSP3	NS	0.343943955	-0.019202873	0.469027701	NS
GPR18	NS	0.343929432	-0.019203451	0.469027701	NS
CREB3L4	NS	0.343979691	-0.019201451	0.469043388	NS
SLIT3	NS	0.344143428	-0.019194938	0.469212567	NS
ACAD11	NS	0.344152243	0.019194587	0.469212567	NS
ZNF329	NS	0.344248454	-0.019190761	0.469310683	NS
RSPH10B	NS	0.344304067	0.019188549	0.469320389	NS
NKAIN2	NS	0.344292146	0.019189023	0.469320389	NS
EPS15	NS	0.344364553	0.019186145	0.469353787	NS
FOLR1	NS	0.344377065	-0.019185647	0.469353787	NS
KCNRG	NS	0.344583231	0.019177452	0.469601705	NS
ERICH2	NS	0.34461186	-0.019176314	0.469607657	NS
ACSL6	NS	0.344751213	-0.019170777	0.469764483	NS
KLF8	NS	0.344812714	0.019168334	0.469782142	NS
PDE7A	NS	0.344792904	-0.019169121	0.469782142	NS
RABEPK	NS	0.345131338	0.01915568	0.470183149	NS
SPDYC	NS	0.345249093	-0.019151006	0.470277372	NS
ASIC3	NS	0.34523318	-0.019151637	0.470277372	NS
OR52E8	NS	0.345334111	-0.019147631	0.470360076	NS
RAB34	NS	0.345932014	0.019123915	0.471108145	NS
EXOSC9	NS	0.345910553	-0.019124766	0.471108145	NS
MEI1	NS	0.346009383	0.019120848	0.471180359	NS
NSRP1	NS	0.346124545	0.019116284	0.471304023	NS
C4orf50	NS	0.346149624	-0.01911529	0.471305018	NS
FAM178B	NS	0.346204536	-0.019113114	0.471346628	NS
CTSK	NS	0.346244893	-0.019111515	0.471368417	NS

MTPN	NS	0.346314283	0.019108766	0.471399509	NS
MRPL42	NS	0.346340794	-0.019107716	0.471399509	NS
ZNF808	NS	0.34632867	-0.019108196	0.471399509	NS
ANXA2	NS	0.346367862	0.019106644	0.471403202	NS
JAZF1	NS	0.34639591	-0.019105533	0.471408228	NS
GSG1L	NS	0.346653143	-0.019095346	0.47172513	NS
TBC1D2B	NS	0.347152713	0.019075577	0.472371734	NS
PDIA6	NS	0.347226944	-0.019072641	0.472439528	NS
ZNF846	NS	0.347350834	-0.019067742	0.472541661	NS
LTA	NS	0.347343715	0.019068024	0.472541661	NS
CHST3	NS	0.347615946	0.019057262	0.472869087	NS
LRP11	NS	0.347679269	-0.01905476	0.47288876	NS
TBC1D5	NS	0.347676204	-0.019054881	0.47288876	NS
CSAD	NS	0.347813206	-0.019049468	0.473037691	NS
CAMSAP1	NS	0.347863575	-0.019047478	0.473072955	NS
SMG5	NS	0.347996561	0.019042225	0.473220559	NS
OR13C3	NS	0.348291898	-0.019030564	0.473588898	NS
FLG2	NS	0.348331065	-0.019029018	0.473608886	NS
MTRF1L	NS	0.348443147	-0.019024595	0.473728002	NS
ABHD17A	NS	0.348534543	0.019020989	0.473803291	NS
HADH	NS	0.348547482	0.019020478	0.473803291	NS
ACOD1	NS	0.348678072	-0.019015327	0.473914245	NS
CCM2	NS	0.348677712	0.019015341	0.473914245	NS
ANKRD11	NS	0.348890129	-0.019006964	0.474169171	NS
MT-ND2	NS	0.348997316	0.019002738	0.474281545	NS
HAMP	NS	0.349052388	-0.019000567	0.474323085	NS
FSTL3	NS	0.349263758	0.018992237	0.474543682	NS
CMTR1	NS	0.34926217	0.018992299	0.474543682	NS
CHDH	NS	0.349760793	-0.018972661	0.475171388	NS
CCPG1	NS	0.349774847	-0.018972108	0.475171388	NS
FBXO41	NS	0.349872723	0.018968255	0.475270996	NS
IQCN	NS	0.350016965	0.018962579	0.47543357	NS
RPP14	NS	0.350234664	0.018954015	0.475662414	NS
TMEM167A	NS	0.350259165	-0.018953051	0.475662414	NS
MVB12B	NS	0.35021094	-0.018954948	0.475662414	NS
DHRS1	NS	0.350646114	0.018937837	0.476114183	NS
TOR4A	NS	0.350651167	0.018937638	0.476114183	NS
ZNF813	NS	0.350665622	0.01893707	0.476114183	NS
TPPP3	NS	0.350869089	0.018929075	0.476323613	NS
TTC7B	NS	0.350951589	0.018925834	0.476402199	NS
ZBTB16	NS	0.351063406	0.018921442	0.476477847	NS
STK11	NS	0.351048559	0.018922025	0.476477847	NS
ADAR	NS	0.351081166	0.018920744	0.476477847	NS
NUDCD2	NS	0.35139223	-0.018908531	0.476866579	NS
TMEM266	NS	0.351417921	0.018907523	0.476868009	NS
ORMDL3	NS	0.351445421	-0.018906444	0.476871895	NS
MYO1B	NS	0.351512137	-0.018903826	0.476928987	NS
ARL11	NS	0.351655224	-0.018898211	0.477089683	NS
RPS18	NS	0.351749324	0.01889452	0.477183902	NS
SUPT7L	NS	0.351913997	-0.018888061	0.47737384	NS
MRPS15	NS	0.352017927	-0.018883986	0.477447901	NS
KIF27	NS	0.352005631	-0.018884468	0.477447901	NS
TRIM25	NS	0.352179423	0.018877655	0.477633471	NS
OFD1	NS	0.352262384	-0.018874404	0.477712513	NS
SHROOM4	NS	0.352369859	0.018870192	0.477824785	NS

HFE	NS	0.352495086	-0.018865286	0.477927633	NS
SP6	NS	0.352574905	0.01886216	0.477986635	NS
MYO19	NS	0.352587992	-0.018861647	0.477986635	NS
BAD	NS	0.352839073	0.018851815	0.478260021	NS
FKRP	NS	0.352822211	-0.018852475	0.478260021	NS
ASTL	NS	0.352972259	-0.018846602	0.478407048	NS
BEND7	NS	0.353111695	-0.018841145	0.478562524	NS
RCN1	NS	0.353172301	-0.018838774	0.478611151	NS
RTRAF	NS	0.353547641	0.018824093	0.479061657	NS
KIF4A	NS	0.353554235	-0.018823835	0.479061657	NS
KLHDC7B	NS	0.35360615	-0.018821806	0.479098463	NS
DVL2	NS	0.353710684	-0.018817719	0.479206551	NS
MMD	NS	0.353902539	0.018810221	0.479368474	NS
CDK4	NS	0.353929265	-0.018809177	0.479368474	NS
TMEM9B	NS	0.353925674	0.018809317	0.479368474	NS
RUNDC1	NS	0.353891189	-0.018810665	0.479368474	NS
EHD3	NS	0.354190832	-0.018798959	0.479689179	NS
STK17B	NS	0.354250191	0.018796641	0.479736004	NS
CCDC47	NS	0.354383463	-0.018791437	0.479882909	NS
OR8I2	NS	0.354456874	0.018788572	0.479948741	NS
MVB12A	NS	0.354506256	0.018786644	0.479982029	NS
TMEM212	NS	0.354669797	0.018780261	0.480169868	NS
ZNF705G	NS	0.354772348	0.01877626	0.480275113	NS
NDUFB7	NS	0.354801066	-0.01877514	0.4802804	NS
PILRB	NS	0.354847188	0.01877334	0.480309243	NS
NUMA1	NS	0.35490599	0.018771047	0.480355243	NS
MED7-TICAM	NS	0.355054711	0.018765247	0.480522933	NS
ELOB	NS	0.355286035	-0.018756228	0.480735161	NS
SLC35F1	NS	0.355387959	-0.018752256	0.480839461	NS
RTF2	NS	0.355464022	0.018749292	0.480908759	NS
MT-ND3	NS	0.355608155	-0.018743676	0.481070133	NS
ZP1	NS	0.355670965	0.018741229	0.481087855	NS
OR2A5	NS	0.355649663	-0.018742059	0.481087855	NS
CCSER2	NS	0.355701129	0.018740054	0.481095035	NS
ZNF654	NS	0.355743883	0.018738389	0.481119243	NS
TBC1D28	NS	0.355791064	0.018736552	0.481149433	NS
ANKRD2	NS	0.355834856	0.018734846	0.481175036	NS
SLC10A3	NS	0.356002904	0.018728303	0.481368648	NS
H2AC1	NS	0.356065334	0.018725873	0.481419434	NS
TBC1D22A	NS	0.356147859	0.018722661	0.481497378	NS
NLRC3	NS	0.356187545	0.018721117	0.481517399	NS
OR10X1	NS	0.356302338	-0.01871665	0.481638945	NS
SPIN1	NS	0.356406093	0.018712613	0.481745554	NS
MTM1	NS	0.356481502	0.01870968	0.481813836	NS
SLC7A8	NS	0.356709137	-0.018700828	0.481993468	NS
NAGPA	NS	0.356738915	-0.01869967	0.481993468	NS
SMAD4	NS	0.356681745	-0.018701893	0.481993468	NS
DRC1	NS	0.356726451	-0.018700154	0.481993468	NS
CDK3	NS	0.356659661	0.018702751	0.481993468	NS
BTAF1	NS	0.356889504	0.018693816	0.482163276	NS
TWF1	NS	0.357052046	-0.018687499	0.482349205	NS
ROBO1	NS	0.357093031	0.018685907	0.482370906	NS
TNFRSF8	NS	0.35712078	0.018684829	0.482374726	NS
APOM	NS	0.357336971	0.018676431	0.482633063	NS
GDAP1	NS	0.357653065	-0.018664158	0.48299262	NS

MYL12B	NS	0.357702997	0.01866222	0.48299262	NS
OR5AC2	NS	0.357653798	-0.018664129	0.48299262	NS
PAPSS2	NS	0.357701667	-0.018662271	0.48299262	NS
DYNC2LI1	NS	0.357987725	-0.018651171	0.48334336	NS
KIAA2026	NS	0.358060351	0.018648354	0.483407697	NS
ABI1	NS	0.358139817	-0.018645272	0.483481259	NS
PLRG1	NS	0.358319645	-0.018638299	0.483656558	NS
TMEM229B	NS	0.358308878	0.018638716	0.483656558	NS
CDKN1C	NS	0.358346344	0.018637264	0.483658869	NS
RIBC2	NS	0.358622927	-0.018626544	0.483998422	NS
MTARC1	NS	0.35886863	-0.018617025	0.484296256	NS
FAM162B	NS	0.35900181	-0.018611867	0.484442208	NS
SCO1	NS	0.359031193	0.018610729	0.484445811	NS
WNT4	NS	0.359054536	-0.018609825	0.484445811	NS
SUCLG1	NS	0.359196206	-0.01860434	0.484603175	NS
EEF1AKMT2	NS	0.359401678	0.018596388	0.484846591	NS
CHAF1A	NS	0.359457198	-0.018594239	0.484887695	NS
ST6GAL2	NS	0.359780305	0.018581741	0.485289726	NS
ATP2B2	NS	0.359923092	0.01857622	0.485414672	NS
KAZN	NS	0.35991438	-0.018576557	0.485414672	NS
BSPH1	NS	0.359962548	-0.018574694	0.485434062	NS
ZFYVE27	NS	0.360037383	0.018571802	0.485501156	NS
SLC4A8	NS	0.36009254	0.01856967	0.485541707	NS
ZNF397	NS	0.360181572	-0.018566229	0.485627926	NS
DUS4L	NS	0.360383872	-0.018558412	0.485838587	NS
BEND4	NS	0.360388016	-0.018558252	0.485838587	NS
UBQLN3	NS	0.360450005	0.018555858	0.485888314	NS
ZNF773	NS	0.360717279	0.018545536	0.486214739	NS
ARHGEF10L	NS	0.360799853	0.018542348	0.486284393	NS
ECHDC2	NS	0.360819201	-0.018541602	0.486284393	NS
KLHL21	NS	0.360961514	0.018536109	0.486442321	NS
PCNX4	NS	0.361018898	0.018533894	0.486485783	NS
NACA2	NS	0.361231712	0.018525684	0.486738672	NS
BICRA	NS	0.361493778	0.018515577	0.487057885	NS
CAP1	NS	0.361652376	0.018509463	0.487203743	NS
C11orf96	NS	0.361799037	0.01850381	0.487333483	NS
FRY	NS	0.362081555	0.018492926	0.48768009	NS
MRPS25	NS	0.362237591	-0.018486917	0.487848689	NS
MFF	NS	0.36225714	0.018486164	0.487848689	NS
ZDHHC3	NS	0.36271873	0.018468397	0.488436326	NS
EFEMP2	NS	0.362807418	0.018464985	0.488521765	NS
ABLIM2	NS	0.363098962	-0.018453773	0.488860312	NS
ZIK1	NS	0.363109356	0.018453373	0.488860312	NS
WFDC3	NS	0.363231905	0.018448662	0.488991289	NS
RGMA	NS	0.36341405	-0.018441661	0.489168453	NS
SMKR1	NS	0.363392854	-0.018442476	0.489168453	NS
ARMH1	NS	0.363713358	-0.018430162	0.48953729	NS
ZNF212	NS	0.364098038	-0.018415392	0.490020972	NS
ZNF786	NS	0.364211995	-0.018411018	0.490140261	NS
ZNF639	NS	0.36427586	-0.018408567	0.490192126	NS
C8orf74	NS	0.364589982	0.018396517	0.490546621	NS
SRC	NS	0.364568063	0.018397358	0.490546621	NS
MRPL16	NS	0.364694048	-0.018392526	0.490607308	NS
TBL3	NS	0.364711126	-0.018391871	0.490607308	NS
HLA-DQA1	NS	0.364660866	-0.018393799	0.490607308	NS

GSK3A	NS	0.364801402	-0.01838841	0.49066271	NS
NOTCH2	NS	0.364853709	0.018386405	0.49066271	NS
TOM1L2	NS	0.364832106	0.018387233	0.49066271	NS
SAPCD2	NS	0.364825887	-0.018387471	0.49066271	NS
DNAAF8	NS	0.365005181	0.018380599	0.490832312	NS
POFUT2	NS	0.365145201	0.018375234	0.49098649	NS
MAPK8	NS	0.365184816	0.018373716	0.491005647	NS
CPXM2	NS	0.365275221	-0.018370252	0.491093087	NS
ADAMTSL3	NS	0.365317084	0.018368649	0.491115257	NS
UTP14C	NS	0.365347839	-0.018367471	0.491122492	NS
KIF14	NS	0.365546932	0.018359847	0.491321881	NS
CBFB	NS	0.365733424	0.018352707	0.491538409	NS
PLXNB3	NS	0.366062615	0.018340111	0.491946676	NS
FBLIM1	NS	0.366159583	0.018336401	0.49200867	NS
LDHC	NS	0.366149829	0.018336774	0.49200867	NS
RABL3	NS	0.366239858	-0.018333331	0.492035475	NS
P2RY1	NS	0.366255793	-0.018332722	0.492035475	NS
DNAH10	NS	0.366232553	0.018333611	0.492035475	NS
PTPRS	NS	0.366373713	-0.018328213	0.492159733	NS
TGM5	NS	0.366402508	0.018327112	0.492164257	NS
EEPD1	NS	0.36643768	0.018325767	0.492177346	NS
SORBS2	NS	0.36647306	0.018324414	0.492190712	NS
TMEM11	NS	0.366595119	0.018319749	0.492320482	NS
MYL12A	NS	0.366766894	-0.018313185	0.492516997	NS
ANPEP	NS	0.366965306	0.018305605	0.492749251	NS
DCLK2	NS	0.367019781	-0.018303524	0.492788213	NS
DLGAP1	NS	0.367061787	-0.01830192	0.492810429	NS
POC1B	NS	0.367134173	-0.018299155	0.492873425	NS
NUP43	NS	0.367187823	-0.018297107	0.492888396	NS
PSMC3	NS	0.367196253	-0.018296785	0.492888396	NS
KCNQ1	NS	0.367347227	0.018291021	0.493056856	NS
CEP57	NS	0.367500588	0.018285168	0.493228496	NS
PSMB7	NS	0.367900636	0.018269907	0.493731173	NS
ALKBH6	NS	0.368102114	-0.018262225	0.493933069	NS
CLEC19A	NS	0.368080117	0.018263063	0.493933069	NS
CCDC81	NS	0.368131884	-0.01826109	0.493938774	NS
TRMO	NS	0.368275705	-0.018255608	0.494089925	NS
SPRR2G	NS	0.36829559	-0.01825485	0.494089925	NS
KIAA1109	NS	0.368545441	0.01824533	0.494356587	NS
SYNRG	NS	0.368531904	-0.018245846	0.494356587	NS
LOXL3	NS	0.368673221	-0.018240463	0.49449372	NS
UNKL	NS	0.368824953	0.018234685	0.494662957	NS
RASSF6	NS	0.368855829	-0.018233509	0.494670092	NS
C19orf54	NS	0.368959452	-0.018229564	0.494774778	NS
PBLD	NS	0.369084217	0.018224815	0.494907801	NS
CDC42EP3	NS	0.369118701	0.018223503	0.494919755	NS
FAM90A19P	NS	0.369256382	0.018218264	0.495070065	NS
MAP7D3	NS	0.369329866	-0.018215468	0.495134291	NS
EDEM2	NS	0.369420324	0.018212027	0.495221261	NS
STX3	NS	0.369500187	-0.018208989	0.495284031	NS
PWWP4	NS	0.369518325	0.018208299	0.495284031	NS
HDAC10	NS	0.369621292	0.018204383	0.495289971	NS
ITM2C	NS	0.36962511	-0.018204238	0.495289971	NS
PIGR	NS	0.369624585	0.018204258	0.495289971	NS
DUS2	NS	0.36955631	0.018206855	0.495289971	NS

ABCG1	NS	0.369870988	-0.018194891	0.495585133	NS
CCDC160	NS	0.369941589	0.018192207	0.495645421	NS
KANSL1L	NS	0.370006891	0.018189726	0.495664293	NS
FMNL1	NS	0.369994711	0.018190189	0.495664293	NS
KDM1A	NS	0.370072758	0.018187223	0.495718222	NS
TERF1	NS	0.370168375	0.01818359	0.495808318	NS
C15orf48	NS	0.370191249	0.018182721	0.495808318	NS
WNT8A	NS	0.37031488	0.018178025	0.495905273	NS
PPP3CA	NS	0.370297704	-0.018178677	0.495905273	NS
DDX11	NS	0.370444743	-0.018173093	0.496044859	NS
SCIN	NS	0.370562761	-0.018168612	0.496134246	NS
HADHA	NS	0.370538166	-0.018169546	0.496134246	NS
RUNX2	NS	0.370769871	0.01816075	0.496377205	NS
GHV3OR16-1	NS	0.37082228	-0.018158761	0.496413033	NS
NMT2	NS	0.370852834	0.018157602	0.496419603	NS
PSMF1	NS	0.371309727	0.018140271	0.496996824	NS
UBIAD1	NS	0.371464966	0.018134385	0.497101485	NS
TMEM165	NS	0.371445846	-0.01813511	0.497101485	NS
ZFP36L1	NS	0.371462989	0.01813446	0.497101485	NS
PARP15	NS	0.371596309	0.018129407	0.497242874	NS
RGS20	NS	0.371775785	-0.018122606	0.497448645	NS
ZNF722P	NS	0.371862296	-0.018119328	0.497530006	NS
MAPT	NS	0.372036885	0.018112715	0.497729192	NS
ZC3H13	NS	0.37228255	-0.018103413	0.498023432	NS
SLC1A4	NS	0.372335653	0.018101403	0.498041762	NS
BRWD1	NS	0.372347713	-0.018100947	0.498041762	NS
NFKBIA	NS	0.372392068	0.018099268	0.498066672	NS
ZFPM2	NS	0.372526345	-0.018094186	0.498188171	NS
CEACAM19	NS	0.372534387	0.018093882	0.498188171	NS
RBM42	NS	0.372564343	0.018092748	0.498193812	NS
BCAS1	NS	0.373123401	-0.018071604	0.498906918	NS
POLK	NS	0.373373632	-0.018062146	0.49920702	NS
KLHL18	NS	0.37341786	0.018060475	0.499231669	NS
UFC1	NS	0.373536519	-0.018055992	0.499355817	NS
C8orf34	NS	0.373642054	-0.018052005	0.499415048	NS
SCN10A	NS	0.373658231	-0.018051394	0.499415048	NS
BLOC1S3	NS	0.373613938	0.018053067	0.499415048	NS
DTWD1	NS	0.373719356	0.018049086	0.499462256	NS
PIGC	NS	0.373750141	0.018047923	0.499468912	NS
COPS7B	NS	0.373845608	0.018044318	0.499562001	NS
FTO	NS	0.373891	0.018042604	0.499588167	NS
SLC7A7	NS	0.374009398	0.018038135	0.499711873	NS
OSMR	NS	0.3741754	0.018031869	0.499899161	NS
TFEC	NS	0.374311925	-0.018026718	0.500047044	NS
SPARCL1	NS	0.374603542	0.018015718	0.500402081	NS
PAPOLB	NS	0.374774679	0.018009265	0.500596141	NS
ZDHHC11	NS	0.374815517	0.018007725	0.500616144	NS
AGA	NS	0.375184746	0.01799381	0.501074722	NS
KCNAB2	NS	0.375270593	0.017990576	0.501121085	NS
MACF1	NS	0.375271241	0.017990552	0.501121085	NS
DHRS4	NS	0.375343597	-0.017987826	0.501183131	NS
ARHGAP6	NS	0.375434731	-0.017984394	0.501270239	NS
RHD	NS	0.375657096	0.017976022	0.50153254	NS
C11orf80	NS	0.375753748	-0.017972383	0.501626977	NS
CCL22	NS	0.375790947	-0.017970983	0.50164204	NS

PPP1R12B	NS	0.375931182	-0.017965706	0.501794632	NS
CDK5RAP1	NS	0.375988309	-0.017963556	0.501836278	NS
SLC5A1	NS	0.376155491	-0.017957267	0.5020248	NS
TOR3A	NS	0.376375851	-0.017948979	0.502284264	NS
KLF9	NS	0.37671296	-0.017936307	0.502694416	NS
SOX15	NS	0.37676341	-0.017934411	0.502697496	NS
ARAP3	NS	0.376809871	-0.017932665	0.502724831	NS
SPATA1	NS	0.376853952	0.017931009	0.50274899	NS
C4orf33	NS	0.377051096	0.017923603	0.502977327	NS
OR4K17	NS	0.377096933	0.017921882	0.503003807	NS
CEBPZOS	NS	0.377170807	-0.017919107	0.503067678	NS
PPFIA1	NS	0.377215906	-0.017917414	0.503093163	NS
WWTR1	NS	0.377316494	0.017913637	0.503192645	NS
LIMS2	NS	0.377412813	0.017910021	0.503286421	NS
NCR2	NS	0.377784126	0.017896087	0.50368643	NS
ATP6V0E2	NS	0.377788962	0.017895906	0.50368643	NS
ANKRD20A1	NS	0.377790844	0.017895835	0.50368643	NS
DCUN1D1	NS	0.377898595	0.017891793	0.503795386	NS
CNR2	NS	0.378108971	0.017883904	0.504041133	NS
CDC25C	NS	0.378150556	0.017882345	0.504061853	NS
LDHA	NS	0.378213736	0.017879976	0.504111354	NS
VWA5B1	NS	0.378311093	0.017876326	0.504181176	NS
UBXN1	NS	0.378318217	-0.017876059	0.504181176	NS
ZNF347	NS	0.378366768	-0.01787424	0.504211164	NS
NFYB	NS	0.378475723	-0.017870156	0.504294967	NS
KLRD1	NS	0.378481762	-0.01786993	0.504294967	NS
FAM135B	NS	0.37855848	0.017867055	0.504327754	NS
ZNF624	NS	0.3785481	-0.017867444	0.504327754	NS
RASSF2	NS	0.378949915	0.017852394	0.504814491	NS
AHCYL1	NS	0.378996749	-0.01785064	0.504842136	NS
CCSAP	NS	0.379104352	0.017846612	0.504950718	NS
DDX50	NS	0.379170638	0.017844131	0.505004257	NS
ETNK1	NS	0.37930435	0.017839126	0.505147585	NS
RNF139	NS	0.379443363	0.017833925	0.505228433	NS
GCNT1	NS	0.37944056	-0.01783403	0.505228433	NS
WDR46	NS	0.379421416	-0.017834746	0.505228433	NS
CORO2B	NS	0.379596646	-0.01782819	0.505397763	NS
MRPS24	NS	0.379670324	-0.017825435	0.505433511	NS
CFAP58	NS	0.379679358	0.017825097	0.505433511	NS
WDR41	NS	0.379727946	-0.01782328	0.505433511	NS
LILRA4	NS	0.37971062	-0.017823928	0.505433511	NS
CCDC30	NS	0.379879512	0.017817612	0.505600483	NS
ASIC1	NS	0.380001566	0.017813049	0.505722408	NS
UMOD	NS	0.380023374	0.017812234	0.505722408	NS
POT1	NS	0.380086764	0.017809865	0.505771992	NS
MRGBP	NS	0.380178202	-0.017806448	0.50585889	NS
SDK1	NS	0.380243945	-0.017803991	0.505911589	NS
SH3YL1	NS	0.380469745	-0.017795555	0.506177221	NS
ZNF804A	NS	0.38052536	0.017793478	0.506216417	NS
GRM1	NS	0.380603962	-0.017790543	0.506286185	NS
HNRNPH3	NS	0.380755598	0.017784881	0.506453089	NS
PKN2	NS	0.380891661	0.017779801	0.506506156	NS
C22orf31	NS	0.380854797	-0.017781177	0.506506156	NS
MAS1	NS	0.380952501	0.017777531	0.506506156	NS
CCDC89	NS	0.380940948	0.017777962	0.506506156	NS

RXRB	NS	0.380878763	0.017780283	0.506506156	NS
XKR7	NS	0.380945627	0.017777787	0.506506156	NS
NUDC	NS	0.381026935	-0.017774753	0.506570325	NS
ARFGEF1	NS	0.381326979	0.017763558	0.50693441	NS
LIN52	NS	0.381380926	-0.017761546	0.506971307	NS
CAV2	NS	0.381602982	0.017753265	0.507196822	NS
SIGLEC7	NS	0.381577977	0.017754198	0.507196822	NS
CST5	NS	0.381630405	0.017752243	0.507198443	NS
PRDM1	NS	0.381748662	0.017747834	0.507320776	NS
ATP8B1	NS	0.38185361	-0.017743923	0.507355743	NS
NPC1	NS	0.381814067	0.017745397	0.507355743	NS
NR0B1	NS	0.381848512	0.017744113	0.507355743	NS
TINF2	NS	0.38188808	-0.017742638	0.507366715	NS
NDUFAF1	NS	0.382099405	-0.017734764	0.507612635	NS
PFKP	NS	0.382197415	-0.017731113	0.507707993	NS
NAT8	NS	0.382310655	0.017726896	0.507719042	NS
SNED1	NS	0.382294548	0.017727495	0.507719042	NS
UGT1A3	NS	0.38225407	-0.017729003	0.507719042	NS
GBA	NS	0.382359438	0.017725079	0.50774899	NS
HSPA12A	NS	0.382500265	0.017719835	0.507901155	NS
SPRR5	NS	0.382608903	0.017715791	0.508010559	NS
RAB1B	NS	0.382723593	0.017711522	0.508127984	NS
PCDHB6	NS	0.382879524	0.01770572	0.508271401	NS
HCLS1	NS	0.382884134	-0.017705548	0.508271401	NS
CADPS	NS	0.383004351	0.017701076	0.50839612	NS
DAPK3	NS	0.383092868	-0.017697783	0.508478746	NS
TNRC6C	NS	0.38333385	-0.017688821	0.508728832	NS
CRLS1	NS	0.383308801	-0.017689753	0.508728832	NS
ALS2CL	NS	0.383369596	0.017687492	0.50874139	NS
POMK	NS	0.383485007	0.017683202	0.508859655	NS
RMDN1	NS	0.383691028	-0.017675545	0.509098132	NS
E2F8	NS	0.38382557	0.017670546	0.509206836	NS
PCDHA2	NS	0.383823683	0.017670616	0.509206836	NS
TRMT1L	NS	0.383917507	-0.017667131	0.509293899	NS
WDR31	NS	0.384076276	0.017661234	0.5094696	NS
TAF11L8	NS	0.384336165	0.017651584	0.509779402	NS
CREB5	NS	0.38451176	0.017645067	0.509977362	NS
SDF4	NS	0.384583677	-0.017642398	0.510037797	NS
PTGIS	NS	0.384967191	0.017628172	0.510511438	NS
UPF3B	NS	0.385143393	-0.017621638	0.510710112	NS
CDC25A	NS	0.385339062	-0.017614385	0.510934573	NS
CAD	NS	0.385411705	-0.017611693	0.510960888	NS
APLP1	NS	0.385444249	0.017610487	0.510969036	NS
RFPL2	NS	0.385501782	0.017608355	0.510975313	NS
NUDT14	NS	0.385499902	-0.017608425	0.510975313	NS
GOPC	NS	0.385661352	-0.017602443	0.51113827	NS
MBOAT2	NS	0.385677539	0.017601844	0.51113827	NS
NAGLU	NS	0.385882633	-0.017594248	0.511329365	NS
SHMT2	NS	0.38590098	-0.017593569	0.511329365	NS
THNSL1	NS	0.385870512	-0.017594697	0.511329365	NS
TPST2	NS	0.385946387	0.017591887	0.511354525	NS
BAZ2B	NS	0.386066856	0.017587427	0.511479128	NS
CSE1L	NS	0.386130711	0.017585063	0.511528714	NS
DACT3	NS	0.386160269	0.017583969	0.511532861	NS
KRTAP1-5	NS	0.386192223	0.017582786	0.511540182	NS

SPRING1	NS	0.386341414	0.017577265	0.511702778	NS
CFP	NS	0.386415003	-0.017574542	0.511765227	NS
CASP8AP2	NS	0.386489352	-0.017571792	0.511828674	NS
SARM1	NS	0.386642948	0.01756611	0.511997052	NS
GJB4	NS	0.386722161	0.01756318	0.512066914	NS
WNT11	NS	0.386822727	-0.017559462	0.512165039	NS
MIA2	NS	0.386955019	0.017554571	0.512305154	NS
DPP9	NS	0.386992798	-0.017553174	0.512320128	NS
SMCHD1	NS	0.387025462	0.017551967	0.51232833	NS
CD9	NS	0.387267677	-0.017543015	0.512578854	NS
CCNT2	NS	0.387388772	0.017538541	0.512704074	NS
POU3F4	NS	0.387508814	0.017534107	0.512827882	NS
ACP4	NS	0.387629918	-0.017529634	0.512936476	NS
PPP2R2A	NS	0.387643871	0.017529119	0.512936476	NS
PGAP3	NS	0.387709084	-0.017526711	0.512987698	NS
UQCRC1	NS	0.387829839	0.017522253	0.513112397	NS
CXorf38	NS	0.387883102	0.017520287	0.51314779	NS
GK	NS	0.388153973	0.01751029	0.513471043	NS
LY96	NS	0.388212843	0.017508118	0.513513825	NS
TMEM230	NS	0.388298841	-0.017504945	0.513576543	NS
SSC4D	NS	0.388339858	0.017503432	0.513576543	NS
CNPY2	NS	0.388338144	-0.017503495	0.513576543	NS
MYO6	NS	0.388460733	-0.017498974	0.513631118	NS
CNOT7	NS	0.388455505	0.017499166	0.513631118	NS
USP51	NS	0.388451696	0.017499307	0.513631118	NS
TMC1	NS	0.388495637	0.017497686	0.513642183	NS
LCT	NS	0.388598952	0.017493877	0.513743687	NS
SLC25A39	NS	0.389027657	-0.017478075	0.514205089	NS
SLC30A6	NS	0.388976956	-0.017479943	0.514205089	NS
KCTD19	NS	0.389022165	0.017478277	0.514205089	NS
BAG2	NS	0.389101692	-0.017475347	0.514267828	NS
SRP68	NS	0.389246888	0.017469998	0.514424605	NS
CAPN1	NS	0.38946295	0.01746204	0.514639873	NS
PNMA8C	NS	0.389448773	0.017462563	0.514639873	NS
CCDC110	NS	0.389511255	0.017460262	0.514668567	NS
APBB2	NS	0.389748527	-0.017451527	0.514946927	NS
CSF3	NS	0.389834312	0.01744837	0.515025114	NS
HTN1	NS	0.389911166	0.017445542	0.515091491	NS
ZNF396	NS	0.39000246	0.017442183	0.515176935	NS
EXT1	NS	0.390176433	0.017435783	0.515371574	NS
GPR108	NS	0.390260129	0.017432705	0.51544695	NS
ACP1	NS	0.390313988	0.017430724	0.515482912	NS
TCERG1	NS	0.390438081	0.017426161	0.51557368	NS
C1GALT1C1L	NS	0.390462625	0.017425259	0.51557368	NS
PRB1	NS	0.39041097	-0.017427158	0.51557368	NS
SNRNP200	NS	0.390665476	0.017417802	0.515797126	NS
SLC5A11	NS	0.390711793	-0.0174161	0.515797126	NS
JUN	NS	0.390687955	0.017416976	0.515797126	NS
PCED1A	NS	0.390766928	0.017414073	0.515834731	NS
EBF2	NS	0.390942342	-0.017407628	0.516031095	NS
TSPAN17	NS	0.391211981	0.017397724	0.516281388	NS
DDX19B	NS	0.391193963	0.017398386	0.516281388	NS
LGALS9	NS	0.391189187	0.017398561	0.516281388	NS
EEF1G	NS	0.391548459	-0.017385371	0.516690207	NS
H2BC18	NS	0.391738699	-0.01737839	0.51690601	NS

PPP1CB	NS	0.391778374	0.017376934	0.516923122	NS
PFDN5	NS	0.391944256	0.017370848	0.517071498	NS
KIRREL3	NS	0.391933279	0.017371251	0.517071498	NS
NDUFA9	NS	0.392152258	0.01736322	0.517310645	NS
SAMD10	NS	0.392186725	-0.017361956	0.517320856	NS
RBL2	NS	0.39234945	-0.01735599	0.517482349	NS
FAM174B	NS	0.392362624	-0.017355507	0.517482349	NS
ZNF101	NS	0.392421657	-0.017353343	0.517524943	NS
OR5K3	NS	0.392522172	0.017349659	0.517622234	NS
ENTPD1	NS	0.392831002	0.017338344	0.517994201	NS
GDPD5	NS	0.392869643	0.017336929	0.518009865	NS
TMEM178B	NS	0.392922861	-0.017334979	0.518044745	NS
AMN	NS	0.393144791	0.017326853	0.518302041	NS
MED25	NS	0.393229156	0.017323764	0.518377957	NS
KCTD12	NS	0.393261748	0.017322571	0.518385617	NS
IFTAP	NS	0.3932979	0.017321247	0.518397967	NS
CEACAM5	NS	0.39343146	-0.017316359	0.5185387	NS
LRRC4	NS	0.39349252	0.017314125	0.518583865	NS
MT1X	NS	0.393545122	0.0173122	0.518617877	NS
CLCA1	NS	0.393915768	-0.017298642	0.519070978	NS
TMEM86A	NS	0.394063571	-0.017293237	0.519230393	NS
NPIPB11	NS	0.394156582	-0.017289837	0.519317596	NS
RNF24	NS	0.394252178	0.017286342	0.519408192	NS
SEC22C	NS	0.394342327	-0.017283048	0.519456246	NS
KLF11	NS	0.394329087	0.017283531	0.519456246	NS
TSSK1B	NS	0.394422037	-0.017280135	0.51952589	NS
ZNF618	NS	0.394572555	-0.017274635	0.519688784	NS
MME	NS	0.394679479	-0.017270729	0.519758879	NS
KCNS2	NS	0.394709671	-0.017269626	0.519763276	NS
CNGA4	NS	0.395022849	-0.01725819	0.520140291	NS
TXNIP	NS	0.3950796	0.017256119	0.52017963	NS
PPTC7	NS	0.395297976	0.017248148	0.520431752	NS
SCO2	NS	0.395399682	0.017244437	0.520530249	NS
STARD3NL	NS	0.395443629	-0.017242834	0.520552699	NS
PRIMA1	NS	0.395525796	0.017239836	0.520625455	NS
SEC24B	NS	0.395602396	-0.017237042	0.520655469	NS
LARP4	NS	0.395576939	0.01723797	0.520655469	NS
MARCHF4	NS	0.395672708	0.017234477	0.520709229	NS
SMIM7	NS	0.395697048	-0.01723359	0.520709229	NS
IMP4	NS	0.395779198	0.017230594	0.520781928	NS
TF	NS	0.395969843	0.017223643	0.520891144	NS
EPB41L4A	NS	0.3959433	0.01722461	0.520891144	NS
PLEKHA7	NS	0.395958408	0.01722406	0.520891144	NS
GSTM2	NS	0.395942523	0.017224639	0.520891144	NS
SMCP	NS	0.396159198	-0.017216741	0.521104821	NS
MLST8	NS	0.396231481	0.017214107	0.521164484	NS
PLEKHM2	NS	0.396280285	0.017212328	0.521170468	NS
ZNF318	NS	0.396316497	0.017211009	0.521170468	NS
ATF6B	NS	0.396316807	-0.017210998	0.521170468	NS
IL10	NS	0.396391786	0.017208266	0.521223369	NS
NHSL2	NS	0.396410892	-0.01720757	0.521223369	NS
RIC3	NS	0.39657381	-0.017201635	0.521402164	NS
KLHL3	NS	0.39666225	-0.017198415	0.521483021	NS
IQGAP2	NS	0.396805211	-0.017193209	0.521600113	NS
ZNF696	NS	0.396789559	0.017193779	0.521600113	NS

TPTE	NS	0.396962292	0.017187491	0.521771161	NS
JHY	NS	0.397095648	0.017182637	0.521911005	NS
GMPR2	NS	0.397247466	0.017177113	0.522075092	NS
CYP2S1	NS	0.397286677	0.017175686	0.522091175	NS
SLC13A5	NS	0.397344098	0.017173597	0.522131186	NS
CEP250	NS	0.397447552	-0.017169834	0.522231676	NS
HTD2	NS	0.397503834	0.017167787	0.522270175	NS
ATF7IP2	NS	0.397577788	-0.017165097	0.522331885	NS
SLC4A2	NS	0.397677388	-0.017161475	0.522427279	NS
SAT2	NS	0.397807191	0.017156756	0.522557586	NS
MAFG	NS	0.397830573	0.017155906	0.522557586	NS
EFR3B	NS	0.398060188	-0.017147561	0.5227173	NS
LEPROTL1	NS	0.398035221	-0.017148468	0.5227173	NS
PHF14	NS	0.398031862	0.01714859	0.5227173	NS
SLC20A1	NS	0.398027935	0.017148733	0.5227173	NS
C4orf36	NS	0.398093792	0.017146339	0.522725964	NS
MKRN1	NS	0.398160895	0.017143901	0.52276353	NS
CDCA5	NS	0.398176417	-0.017143337	0.52276353	NS
LAMA2	NS	0.398286408	-0.017139341	0.522872471	NS
IYD	NS	0.398379973	0.017135943	0.522932065	NS
PRELP	NS	0.398385835	0.01713573	0.522932065	NS
FIGLA	NS	0.398544697	-0.01712996	0.523105117	NS
DPYSL2	NS	0.398763702	-0.017122009	0.52335708	NS
CSF2RB	NS	0.398848138	-0.017118944	0.523432407	NS
CHM	NS	0.398945728	-0.017115402	0.523524983	NS
FAM3B	NS	0.399006478	-0.017113198	0.523569209	NS
AHSA1	NS	0.399034299	-0.017112188	0.523570221	NS
PBRM1	NS	0.399074504	-0.017110729	0.52358748	NS
PIGF	NS	0.399156705	-0.017107747	0.523588858	NS
CDKN2AIP	NS	0.399156052	-0.017107771	0.523588858	NS
SERPINA5	NS	0.39914871	-0.017108037	0.523588858	NS
CRCP	NS	0.39923304	-0.017104978	0.523653501	NS
UCP3	NS	0.399384982	-0.017099467	0.523781808	NS
GPR132	NS	0.399365189	0.017100184	0.523781808	NS
NRG3	NS	0.399420523	0.017098178	0.523792929	NS
LTV1	NS	0.399547259	0.017093582	0.523852655	NS
GPT2	NS	0.399509427	-0.017094954	0.523852655	NS
LDLRAD3	NS	0.39954127	0.017093799	0.523852655	NS
TRABD2B	NS	0.399589552	-0.017092049	0.523872621	NS
HSD17B10	NS	0.400537798	-0.017057694	0.525009116	NS
TTF2	NS	0.400579042	0.017056201	0.525027624	NS
GPD2	NS	0.400750044	0.017050011	0.525182889	NS
ELOF1	NS	0.400766439	-0.017049418	0.525182889	NS
EIF3G	NS	0.400791934	-0.017048495	0.525182889	NS
ABCC12	NS	0.400806036	-0.017047985	0.525182889	NS
LTBP3	NS	0.400980606	-0.017041668	0.525376065	NS
ANKS3	NS	0.401150011	-0.01703554	0.525562448	NS
CACNA1G	NS	0.401217392	0.017033102	0.52561515	NS
POU2F2	NS	0.401299365	0.017030138	0.525686959	NS
SIAE	NS	0.401416789	0.017025892	0.525805195	NS
CDK2AP1	NS	0.401760354	-0.017013473	0.526096746	NS
NPPB	NS	0.401775269	0.017012934	0.526096746	NS
PGAP6	NS	0.401693614	0.017015885	0.526096746	NS
ADRM1	NS	0.401748284	-0.017013909	0.526096746	NS
TBPL2	NS	0.401766851	-0.017013238	0.526096746	NS

ENY2	NS	0.402098195	0.017001267	0.526448367	NS
THTPA	NS	0.402094988	-0.017001383	0.526448367	NS
ITGB1	NS	0.402268679	0.01699511	0.526627878	NS
STAC3	NS	0.402346397	-0.016992304	0.526666454	NS
PLA2G4D	NS	0.40262877	0.016982111	0.527000437	NS
CEP128	NS	0.402780679	-0.016976629	0.52712798	NS
C1orf159	NS	0.402754761	0.016977564	0.52712798	NS
DUSP28	NS	0.402999309	0.016968741	0.527378449	NS
POMGNT2	NS	0.403059678	0.016966564	0.52742179	NS
PORCN	NS	0.403131121	0.016963987	0.527479618	NS
IKZF4	NS	0.403237582	0.016960148	0.527532894	NS
TRPV2	NS	0.403244281	-0.016959906	0.527532894	NS
FUT11	NS	0.403253601	-0.01695957	0.527532894	NS
ENO2	NS	0.403677647	0.016944285	0.52804988	NS
PGAM4	NS	0.403703354	-0.016943359	0.52804988	NS
BOLA2B	NS	0.403731131	0.016942358	0.528050528	NS
RPL22	NS	0.403811821	-0.01693945	0.528120378	NS
C10orf71	NS	0.404119	-0.016928386	0.528486411	NS
MYF6	NS	0.404216531	-0.016924874	0.528542536	NS
MAGEB6	NS	0.404213215	0.016924993	0.528542536	NS
WRAP53	NS	0.404536073	0.016913371	0.52892463	NS
IFT43	NS	0.404642034	-0.016909558	0.529027438	NS
INO80B	NS	0.404712964	-0.016907006	0.529084434	NS
BIN1	NS	0.404768076	-0.016905023	0.529120745	NS
FGF20	NS	0.404914152	0.016899768	0.529275955	NS
DCTN3	NS	0.404945119	-0.016898654	0.52928069	NS
VMP1	NS	0.404990436	-0.016897025	0.529304179	NS
HNRNPM	NS	0.405079412	0.016893825	0.529384721	NS
ABHD6	NS	0.405226216	0.016888546	0.529540821	NS
SPINK8	NS	0.405272839	-0.01688687	0.529565994	NS
NME2	NS	0.405369953	0.016883379	0.529657135	NS
DEFA1	NS	0.405415421	-0.016881745	0.529680788	NS
LSM7	NS	0.405634668	0.016873866	0.529871022	NS
PWP2	NS	0.405643149	-0.016873561	0.529871022	NS
SETX	NS	0.405762568	0.016869271	0.52994835	NS
MYO1C	NS	0.405738039	0.016870152	0.52994835	NS
SPANXA1	NS	0.405784485	-0.016868483	0.52994835	NS
FAM193A	NS	0.40584629	0.016866263	0.529993306	NS
MEGF11	NS	0.405959463	-0.016862198	0.530105334	NS
CRB2	NS	0.406084421	-0.016857711	0.530232734	NS
EOGT	NS	0.406213011	0.016853094	0.530364859	NS
FAM76A	NS	0.406314956	0.016849435	0.530462181	NS
ZNF257	NS	0.406464857	-0.016844055	0.530622093	NS
B3GNTL1	NS	0.406579093	-0.016839955	0.530735428	NS
DNHD1	NS	0.406645472	0.016837574	0.530786281	NS
SDHB	NS	0.407061864	0.016822639	0.531186505	NS
APH1A	NS	0.407042995	0.016823316	0.531186505	NS
SOX13	NS	0.407028054	0.016823851	0.531186505	NS
RAD54L2	NS	0.407040016	0.016823422	0.531186505	NS
ARFGEF3	NS	0.407245057	0.016816071	0.531318099	NS
GNB4	NS	0.407231744	-0.016816549	0.531318099	NS
RBM14	NS	0.407206419	0.016817456	0.531318099	NS
SLC35F5	NS	0.407306541	0.016813868	0.5313625	NS
RBM38	NS	0.407354072	0.016812164	0.531373028	NS
GP2	NS	0.407369517	-0.01681161	0.531373028	NS

ITGA2	NS	0.407732318	-0.016798611	0.531810428	NS
RER1	NS	0.407860906	-0.016794006	0.531942302	NS
PIK3R1	NS	0.407920439	-0.016791874	0.5319841	NS
MAN2C1	NS	0.408062212	0.016786797	0.532133138	NS
TERF2	NS	0.408180216	-0.016782573	0.532251163	NS
COL6A2	NS	0.408260154	-0.016779711	0.532319538	NS
STIP1	NS	0.408345125	-0.01677667	0.532394466	NS
IDUA	NS	0.408634263	0.016766324	0.532663802	NS
P3H4	NS	0.408608782	0.016767236	0.532663802	NS
MASP1	NS	0.408662856	-0.016765301	0.532665201	NS
LCE2D	NS	0.408760235	0.016761818	0.532756253	NS
ARHGAP28	NS	0.409070385	0.016750728	0.533124587	NS
C20orf85	NS	0.409178175	-0.016746874	0.533157372	NS
GDF11	NS	0.409145275	0.01674805	0.533157372	NS
ODF3B	NS	0.409168435	-0.016747223	0.533157372	NS
GBX1	NS	0.409221328	0.016745332	0.533177707	NS
UBR5	NS	0.40953497	0.016734124	0.53351453	NS
FKBP9	NS	0.409513699	-0.016734884	0.53351453	NS
NOA1	NS	0.409597002	0.016731908	0.533523524	NS
MED9	NS	0.40959273	-0.016732061	0.533523524	NS
TCEAL3	NS	0.409672906	-0.016729197	0.533586486	NS
SLC25A1	NS	0.40971606	0.016727655	0.533606786	NS
RECQL	NS	0.409919341	0.016720396	0.533835616	NS
DHX16	NS	0.409959286	-0.016718969	0.533851718	NS
PRKCH	NS	0.410055613	-0.01671553	0.533905318	NS
NRP2	NS	0.410037348	-0.016716182	0.533905318	NS
GIPR	NS	0.410134131	0.016712727	0.533928832	NS
IL9R	NS	0.410156427	0.016711931	0.533928832	NS
TBX20	NS	0.410147004	-0.016712268	0.533928832	NS
NOTCH1	NS	0.41019637	0.016710506	0.533944918	NS
TMEM156	NS	0.410289913	0.016707167	0.534018358	NS
ZNF805	NS	0.410407502	0.016702971	0.534088208	NS
OTUD4	NS	0.41050142	-0.01669962	0.534162385	NS
NHP2	NS	0.410573985	-0.016697031	0.534184987	NS
PRMT2	NS	0.410549324	-0.016697911	0.534184987	NS
CIC	NS	0.41068155	0.016693195	0.534253113	NS
NGF	NS	0.410680711	0.016693224	0.534253113	NS
GNB1L	NS	0.410837839	-0.016687621	0.534420512	NS
TAF7L	NS	0.411087115	-0.016678733	0.534708837	NS
TICAM1	NS	0.411299549	0.016671162	0.534877323	NS
SIGLEC10	NS	0.411268732	0.01667226	0.534877323	NS
UQCC3	NS	0.411297502	-0.016671235	0.534877323	NS
ATP2C2	NS	0.41135056	0.016669344	0.534907721	NS
ANO8	NS	0.411412097	-0.016667152	0.534927161	NS
ZBTB2	NS	0.411420781	0.016666842	0.534927161	NS
PJVK	NS	0.411487108	-0.016664479	0.534977462	NS
POTEC	NS	0.411972322	0.016647199	0.53557232	NS
FCGRT	NS	0.412094265	0.016642858	0.535694869	NS
NDST3	NS	0.412142627	0.016641137	0.535721758	NS
BMP2	NS	0.412471102	0.016629448	0.536092211	NS
NFKBID	NS	0.412483018	-0.016629024	0.536092211	NS
CNNM4	NS	0.412516629	0.016627829	0.536099897	NS
GCOM1	NS	0.412657204	-0.016622828	0.536206229	NS
GFRA3	NS	0.412681556	0.016621962	0.536206229	NS
KIAA0895	NS	0.412661169	0.016622687	0.536206229	NS

CEP41	NS	0.413136777	-0.016605778	0.536725649	NS
DNAJC4	NS	0.413127892	-0.016606094	0.536725649	NS
OR5A1	NS	0.413210039	0.016603174	0.536784799	NS
GPM6A	NS	0.413238629	-0.016602158	0.536785915	NS
RUBCNL	NS	0.413351436	0.01659815	0.536896416	NS
H6PD	NS	0.413476065	-0.016593722	0.537022258	NS
RARB	NS	0.413512167	0.016592439	0.537033113	NS
RPS17	NS	0.413815294	0.016581674	0.53739073	NS
LRRC58	NS	0.413911544	-0.016578257	0.537479662	NS
ABRAXAS1	NS	0.414149776	0.0165698	0.537730363	NS
CRACDL	NS	0.41416017	-0.016569431	0.537730363	NS
SPOCD1	NS	0.414312087	0.016564041	0.537891525	NS
NCOA3	NS	0.414436387	-0.016559631	0.537921083	NS
ARF3	NS	0.414413922	0.016560428	0.537921083	NS
PKLR	NS	0.414363701	-0.016562209	0.537921083	NS
PPP1R14B	NS	0.414461632	0.016558735	0.537921083	NS
ZNF609	NS	0.414473808	-0.016558303	0.537921083	NS
ALG13	NS	0.414517209	0.016556763	0.537930345	NS
CIAO1	NS	0.414536527	-0.016556078	0.537930345	NS
DDC	NS	0.414566066	0.016555031	0.537932612	NS
STAG1	NS	0.41466575	-0.016551495	0.538025892	NS
FBXO44	NS	0.41527485	0.016529903	0.538677548	NS
SMIM6	NS	0.41523169	-0.016531433	0.538677548	NS
SEC22B	NS	0.415279313	0.016529745	0.538677548	NS
BTBD1	NS	0.415595929	0.016518529	0.539016001	NS
PCBD2	NS	0.415572434	0.016519361	0.539016001	NS
GATA3	NS	0.415771214	0.016512322	0.539184563	NS
CYP3A4	NS	0.415781607	-0.016511954	0.539184563	NS
ZDHHC6	NS	0.415975622	-0.016505085	0.539400023	NS
AP2S1	NS	0.416121008	0.016499939	0.5395524	NS
OBSL1	NS	0.416220447	0.01649642	0.53960904	NS
ZDHHC23	NS	0.416197242	-0.016497241	0.53960904	NS
PPIP5K2	NS	0.416259433	-0.016495041	0.53962344	NS
CHRAC1	NS	0.416314866	-0.016493079	0.539659158	NS
KCNQ5	NS	0.416444792	-0.016488483	0.539791428	NS
MKRN2OS	NS	0.416517196	0.016485922	0.539849127	NS
ALDH1L2	NS	0.416684075	-0.01648002	0.540029257	NS
PPIE	NS	0.416962703	0.016470169	0.540354183	NS
BTN3A3	NS	0.417038704	-0.016467482	0.540416493	NS
SH2D4B	NS	0.41728671	-0.016458718	0.54070167	NS
TXN2	NS	0.417355914	-0.016456273	0.540755143	NS
CPSF6	NS	0.41755187	-0.016449351	0.540972825	NS
CDH16	NS	0.417602943	-0.016447547	0.541002783	NS
LRRC46	NS	0.417760327	-0.01644199	0.541134236	NS
FAM90A1	NS	0.417746091	-0.016442492	0.541134236	NS
FKBP1C	NS	0.417904346	0.016436905	0.541284564	NS
DCAF16	NS	0.418024853	0.016432651	0.541404419	NS
IGFBP6	NS	0.41827557	0.016423804	0.541683454	NS
TRNP1	NS	0.418296269	0.016423073	0.541683454	NS
S100A6	NS	0.418628123	0.016411368	0.54207693	NS
IL7R	NS	0.418919918	-0.016401079	0.542418485	NS
XBP1	NS	0.41901793	0.016397625	0.542479252	NS
LIN54	NS	0.419022902	-0.016397449	0.542479252	NS
APOBEC3G	NS	0.4190539	-0.016396357	0.542483098	NS
ENTPD6	NS	0.419171717	0.016392205	0.542599328	NS

FXYD5	NS	0.419245497	-0.016389605	0.542658542	NS
FANCM	NS	0.419324921	-0.016386806	0.542704581	NS
CORT	NS	0.419337141	-0.016386376	0.542704581	NS
ZNF280A	NS	0.419424252	0.016383307	0.542781027	NS
AKNA	NS	0.419813151	0.016369611	0.543247984	NS
GTSF1L	NS	0.419844975	-0.016368491	0.543252847	NS
RBP7	NS	0.419947848	-0.01636487	0.543349635	NS
JAM2	NS	0.420330632	-0.016351399	0.543786783	NS
WFDC6	NS	0.420341901	-0.016351002	0.543786783	NS
MFSD11	NS	0.420454627	-0.016347037	0.543896261	NS
ZBTB6	NS	0.420499184	-0.01634547	0.543917549	NS
SPATA31A6	NS	0.420616234	0.016341353	0.544032598	NS
EPHB3	NS	0.420846598	-0.016333253	0.544294183	NS
OTUD1	NS	0.420894828	0.016331557	0.54432019	NS
HACE1	NS	0.421553737	0.016308405	0.545135899	NS
ADSS2	NS	0.421923884	0.016295408	0.545578109	NS
NOSTRIN	NS	0.421995724	0.016292886	0.545634552	NS
ZNF444	NS	0.422176915	-0.016286527	0.545759459	NS
OR2G2	NS	0.422148961	-0.016287508	0.545759459	NS
KNTC1	NS	0.422159975	0.016287121	0.545759459	NS
CLDN8	NS	0.422297824	-0.016282285	0.545879302	NS
CHAF1B	NS	0.422519941	0.016274493	0.546093481	NS
BNIP1	NS	0.422764335	-0.016265922	0.546372869	NS
PSMD13	NS	0.422799423	-0.016264692	0.546381736	NS
FBXO22	NS	0.422843216	0.016263156	0.546401848	NS
GGPS1	NS	0.422896609	-0.016261285	0.546434364	NS
SLC9A5	NS	0.423000449	-0.016257645	0.546532053	NS
RPUSD3	NS	0.423090655	0.016254483	0.546612115	NS
SORL1	NS	0.423122506	0.016253367	0.546616781	NS
DERPC	NS	0.423202977	-0.016250547	0.546684251	NS
ZNF862	NS	0.423339694	-0.016245756	0.546824366	NS
CASKIN1	NS	0.423626135	-0.016235723	0.547157845	NS
NCAPH	NS	0.423656147	-0.016234672	0.547160098	NS
ART5	NS	0.423805582	0.016229439	0.547316577	NS
PA2G4	NS	0.424098534	0.016219184	0.547658365	NS
EMX1	NS	0.424239795	0.016214241	0.547804234	NS
PIK3R3	NS	0.424547807	-0.016203465	0.548164884	NS
UGT8	NS	0.424575736	0.016202488	0.548164884	NS
FAM3A	NS	0.424607214	-0.016201388	0.548168961	NS
EXD1	NS	0.424686409	-0.016198618	0.548206348	NS
SIGLEC12	NS	0.424692818	-0.016198394	0.548206348	NS
TTC34	NS	0.424728079	-0.016197161	0.548215304	NS
LILRA6	NS	0.424811864	0.016194231	0.548286887	NS
IFT140	NS	0.425333222	-0.016176009	0.548923179	NS
ATM	NS	0.425527906	-0.016169208	0.549137819	NS
TMEM68	NS	0.425838807	-0.01615835	0.549477481	NS
NDUFA12	NS	0.425847886	0.016158033	0.549477481	NS
RASSF1	NS	0.425962836	-0.01615402	0.549589165	NS
SIGLEC6	NS	0.42612734	0.016148278	0.549764767	NS
LMBR1L	NS	0.426299208	0.016142281	0.549949844	NS
PAK3	NS	0.42641711	-0.016138167	0.550055846	NS
PCDH15	NS	0.426438212	-0.016137431	0.550055846	NS
PLCD3	NS	0.426513429	0.016134807	0.550116208	NS
MCF2L	NS	0.426582334	0.016132404	0.550166508	NS
RDX	NS	0.426609274	0.016131464	0.550166508	NS

FBXW4	NS	0.4268498	0.016123076	0.550440022	NS
CRISPLD2	NS	0.426929032	-0.016120314	0.550505519	NS
SMIM12	NS	0.426998152	-0.016117904	0.550557969	NS
TRIM49D1	NS	0.427131952	-0.016113241	0.550693803	NS
DHX32	NS	0.427186175	-0.016111351	0.550727028	NS
GALP	NS	0.427336151	0.016106125	0.550883686	NS
EIF2S3B	NS	0.427578171	0.016097693	0.551158969	NS
BIRC3	NS	0.427614684	0.016096421	0.55116933	NS
TAF13	NS	0.427760293	0.01609135	0.551320298	NS
TSEN2	NS	0.427789787	-0.016090323	0.551321601	NS
DCDC1	NS	0.427938894	0.016085132	0.551477046	NS
HEG1	NS	0.428019845	0.016082313	0.551544646	NS
FRAS1	NS	0.428078296	0.016080279	0.551583245	NS
SNRPB2	NS	0.428239464	-0.016074669	0.551717456	NS
TMEM273	NS	0.428219479	0.016075365	0.551717456	NS
NT5C3A	NS	0.428565589	0.016063323	0.552089817	NS
PEX19	NS	0.428585534	0.016062629	0.552089817	NS
NPY4R	NS	0.428624225	-0.016061283	0.552102915	NS
PCM1	NS	0.428752755	0.016056813	0.552206215	NS
LUM	NS	0.42876148	-0.016056509	0.552206215	NS
ZNF519	NS	0.428792718	-0.016055423	0.552209704	NS
TMEM62	NS	0.429087788	0.016045164	0.552552939	NS
ST6GAL1	NS	0.42919797	-0.016041335	0.552638672	NS
TCP11L2	NS	0.429211467	0.016040865	0.552638672	NS
NFS1	NS	0.429259893	-0.016039182	0.55266426	NS
HHEX	NS	0.429321477	-0.016037042	0.552706784	NS
FZD1	NS	0.429358388	0.01603576	0.552717542	NS
ROCK1	NS	0.429508644	0.016030539	0.552837433	NS
DLC1	NS	0.429493273	-0.016031073	0.552837433	NS
TTI2	NS	0.429557044	-0.016028858	0.552862966	NS
ATP10B	NS	0.429622687	-0.016026578	0.552910687	NS
LAMC1	NS	0.429766984	-0.016021566	0.55305962	NS
RP9	NS	0.42997601	-0.016014308	0.553291826	NS
GALNT17	NS	0.430031993	0.016012364	0.553327079	NS
PRR20D	NS	0.430173599	0.016007448	0.553472493	NS
MYL4	NS	0.430301204	0.01600302	0.553599874	NS
CFAP119	NS	0.430330279	-0.016002011	0.553600484	NS
ADCY5	NS	0.430412502	0.015999158	0.553669462	NS
FOXM1	NS	0.430643291	-0.015991151	0.553929528	NS
SHISAL1	NS	0.430845508	-0.015984137	0.554152811	NS
SCGB1D2	NS	0.431063603	-0.015976575	0.55435965	NS
WDR43	NS	0.431055554	-0.015976854	0.55435965	NS
KY	NS	0.431112039	-0.015974896	0.554385107	NS
WDR37	NS	0.431189105	-0.015972225	0.554447373	NS
MRPS16	NS	0.431380368	-0.015965596	0.554656464	NS
DNTTIP1	NS	0.431573344	0.01595891	0.554867728	NS
ARF5	NS	0.431831464	0.015949969	0.555097525	NS
C12orf76	NS	0.431838114	0.015949738	0.555097525	NS
AP2A2	NS	0.43181796	0.015950436	0.555097525	NS
SCAMP3	NS	0.43188441	0.015948135	0.55512017	NS
MAP3K6	NS	0.432052326	-0.015942321	0.555298337	NS
SLC28A1	NS	0.432080401	-0.015941349	0.555298337	NS
CLEC16A	NS	0.432199524	-0.015937225	0.555414552	NS
OR6C74	NS	0.432234857	0.015936002	0.555423083	NS
ZNF215	NS	0.432444369	-0.015928751	0.555655417	NS

ZDHHC14	NS	0.432580382	-0.015924045	0.555793288	NS
KIF13A	NS	0.432818241	0.015915817	0.556061985	NS
METTL2A	NS	0.432891024	-0.0159133	0.556118581	NS
C17orf78	NS	0.432973566	-0.015910445	0.556187706	NS
SETD7	NS	0.43303659	0.015908266	0.556231749	NS
FANCG	NS	0.433127737	0.015905115	0.556311909	NS
ZNF705D	NS	0.433278306	0.01589991	0.556468376	NS
DELE1	NS	0.433798874	0.015881922	0.557099987	NS
WRN	NS	0.433873444	0.015879347	0.557158786	NS
LCE4A	NS	0.433996886	-0.015875084	0.557280333	NS
RPL22L1	NS	0.434062915	0.015872804	0.557328145	NS
CAT	NS	0.434166	-0.015869244	0.557423527	NS
PODN	NS	0.434249323	0.015866368	0.557493526	NS
FMNL3	NS	0.434288045	-0.015865031	0.55750626	NS
LYSMD2	NS	0.434319862	-0.015863933	0.55751013	NS
IRAK2	NS	0.434363071	0.015862442	0.557528621	NS
OR6Q1	NS	0.43444271	0.015859693	0.557593866	NS
DNAJC16	NS	0.434476828	-0.015858515	0.557600682	NS
TMEM270	NS	0.43451589	0.015857167	0.557613841	NS
ZNF451	NS	0.434656846	0.015852304	0.557757751	NS
RSPH10B2	NS	0.4349259	-0.015843022	0.558064238	NS
OGDHL	NS	0.434982184	0.015841081	0.558064238	NS
VDAC1	NS	0.434959768	-0.015841854	0.558064238	NS
ABHD12	NS	0.435058574	0.015838447	0.558118952	NS
TMEM184A	NS	0.4350825	0.015837622	0.558118952	NS
C10orf90	NS	0.435195528	-0.015833725	0.558196271	NS
CASQ2	NS	0.435279914	-0.015830815	0.5582612	NS
MDM1	NS	0.435538564	-0.0158219	0.55838929	NS
GOLT1B	NS	0.435498547	0.01582328	0.55838929	NS
IL17A	NS	0.435460468	0.015824592	0.55838929	NS
KRI1	NS	0.435439663	0.015825309	0.55838929	NS
KCNV2	NS	0.435552878	-0.015821407	0.55838929	NS
CFAP65	NS	0.435525148	0.015822363	0.55838929	NS
LRRC10	NS	0.435637328	-0.015818497	0.558460568	NS
RRM2B	NS	0.435697123	-0.015816437	0.558463249	NS
PTPRB	NS	0.435679135	0.015817057	0.558463249	NS
SUSD2	NS	0.435957315	-0.015807474	0.558759753	NS
CCDC61	NS	0.436385119	-0.015792743	0.559244236	NS
THAP7	NS	0.436393105	-0.015792468	0.559244236	NS
FAM126A	NS	0.436775934	0.015779293	0.559660729	NS
ENTR1	NS	0.436848006	-0.015776814	0.559716026	NS
SNRPD1	NS	0.436888019	0.015775437	0.559721581	NS
ADAM29	NS	0.436910176	-0.015774675	0.559721581	NS
SLC25A16	NS	0.437080513	-0.015768816	0.559848476	NS
LRP8	NS	0.437057607	0.015769604	0.559848476	NS
NHEJ1	NS	0.437096	-0.015768284	0.559848476	NS
PAX6	NS	0.437192698	-0.015764958	0.559925342	NS
ARL17A	NS	0.437213867	0.01576423	0.559925342	NS
UPF2	NS	0.437350005	-0.01575955	0.560062633	NS
ATP8B4	NS	0.437486418	-0.01575486	0.5601632	NS
CA13	NS	0.437463435	-0.01575565	0.5601632	NS
PRIM1	NS	0.43753885	-0.015753058	0.560193278	NS
FARP2	NS	0.437725897	0.01574663	0.560283984	NS
INPP5A	NS	0.437662508	0.015748808	0.560283984	NS
TNIP1	NS	0.437768006	0.015745183	0.560283984	NS

EML3	NS	0.437711207	0.015747135	0.560283984	NS
ENAH	NS	0.437762583	0.015745369	0.560283984	NS
FGF11	NS	0.43781232	0.01574366	0.560283984	NS
KCNK7	NS	0.437790058	0.015744425	0.560283984	NS
AADAT	NS	0.437984306	-0.015737752	0.560467025	NS
USP17L22	NS	0.438328371	-0.015725936	0.560833154	NS
CBY3	NS	0.438384329	-0.015724014	0.560867677	NS
P2RY11	NS	0.438687701	-0.015713601	0.561218714	NS
UBR7	NS	0.438786622	-0.015710206	0.561271071	NS
POGK	NS	0.438760371	-0.015711107	0.561271071	NS
QSER1	NS	0.439057777	0.015700903	0.561570522	NS
DBT	NS	0.43907875	0.015700184	0.561570522	NS
SNX25	NS	0.439523052	-0.015684948	0.562101631	NS
SMIM27	NS	0.43955427	-0.015683878	0.562104416	NS
TECTA	NS	0.439924287	-0.015671197	0.562540431	NS
ZNF697	NS	0.440021109	0.015667879	0.56262707	NS
DEFB129	NS	0.440064832	0.015666381	0.562645808	NS
WDR54	NS	0.440111253	0.015664791	0.562667993	NS
FASTKD2	NS	0.440186137	-0.015662226	0.562726562	NS
VEZT	NS	0.440351114	0.015656576	0.562827766	NS
KIF3B	NS	0.440309997	-0.015657984	0.562827766	NS
ZNF345	NS	0.440352536	-0.015656527	0.562827766	NS
PDLIM4	NS	0.440674385	-0.015645508	0.563201942	NS
SRRD	NS	0.440731713	-0.015643546	0.563238019	NS
LMBR1	NS	0.440821627	0.015640468	0.563315734	NS
EMG1	NS	0.441010546	-0.015634004	0.563497011	NS
PEAR1	NS	0.44102171	0.015633622	0.563497011	NS
BMI1	NS	0.441332953	0.015622975	0.563846527	NS
NLRP6	NS	0.44135352	0.015622271	0.563846527	NS
UCN3	NS	0.441546043	-0.015615688	0.564055254	NS
CCDC149	NS	0.44170978	-0.01561009	0.564227183	NS
MAP1S	NS	0.442049295	0.015598486	0.564623608	NS
MAP3K4	NS	0.442179736	0.01559403	0.564752951	NS
ELSPBP1	NS	0.442418868	0.015585861	0.565021088	NS
TBC1D21	NS	0.442594884	0.015579851	0.565208588	NS
APOB	NS	0.442633645	0.015578527	0.565220796	NS
GALR2	NS	0.442834784	-0.01557166	0.565440338	NS
MADD	NS	0.442978984	0.015566739	0.565488637	NS
ACTG2	NS	0.442989471	-0.015566381	0.565488637	NS
ZNF562	NS	0.442928383	0.015568466	0.565488637	NS
SPATA31A1	NS	0.44295983	0.015567392	0.565488637	NS
CISH	NS	0.443137536	-0.015561328	0.565624666	NS
PRDX6	NS	0.443154477	-0.01556075	0.565624666	NS
AP1AR	NS	0.443289039	-0.015556159	0.565759109	NS
MEF2C	NS	0.443378546	0.015553106	0.565800081	NS
ATF7IP	NS	0.443480252	-0.015549637	0.56589121	NS
SERF1B	NS	0.443510671	-0.015548599	0.565892719	NS
PIP5K1C	NS	0.443561109	0.015546879	0.56591977	NS
EVI5L	NS	0.443771482	0.015539706	0.566150858	NS
CIB3	NS	0.443805693	0.015538539	0.566157187	NS
CEP76	NS	0.443947378	-0.01553371	0.56630061	NS
ZDHHC1	NS	0.444113769	0.015528039	0.566475527	NS
ITGB6	NS	0.444206974	0.015524862	0.566525953	NS
TAMM41	NS	0.44421184	-0.015524697	0.566525953	NS
PRUNE1	NS	0.444512991	-0.015514437	0.566764649	NS

CRYGC	NS	0.444496263	0.015515007	0.566764649	NS
EFHC2	NS	0.444516126	-0.01551433	0.566764649	NS
ATP6V1E2	NS	0.444473688	-0.015515776	0.566764649	NS
THSD4	NS	0.444557498	-0.015512921	0.56677007	NS
TBC1D9B	NS	0.44457894	0.015512191	0.56677007	NS
PCNX1	NS	0.444674678	0.015508931	0.566817456	NS
FAM90A14P	NS	0.444663389	0.015509315	0.566817456	NS
POP4	NS	0.445041616	-0.015496438	0.567173131	NS
ELF5	NS	0.445023309	-0.015497061	0.567173131	NS
SCRN2	NS	0.444988503	-0.015498246	0.567173131	NS
SNTG1	NS	0.445185685	0.015491534	0.567282035	NS
GHRL	NS	0.445181873	-0.015491664	0.567282035	NS
KANK2	NS	0.445779166	0.015471345	0.568000891	NS
STX16	NS	0.445880107	0.015467913	0.568092111	NS
SMARCA5	NS	0.446314747	0.015453138	0.568608455	NS
XAB2	NS	0.446380122	0.015450917	0.568654314	NS
ARHGDIB	NS	0.446412882	-0.015449804	0.568658621	NS
AIFM2	NS	0.446830155	-0.015435629	0.568967676	NS
NYAP2	NS	0.446767626	0.015437753	0.568967676	NS
NAPRT	NS	0.446705708	0.015439856	0.568967676	NS
SUZ12	NS	0.446778287	0.015437391	0.568967676	NS
AJAP1	NS	0.446816867	-0.01543608	0.568967676	NS
C3orf56	NS	0.446831868	0.015435571	0.568967676	NS
FHL2	NS	0.44694699	-0.015431662	0.569060821	NS
BACH1	NS	0.446963818	0.01543109	0.569060821	NS
SLC26A1	NS	0.447428828	0.015415306	0.56961539	NS
FAR2	NS	0.447524432	0.015412062	0.569662167	NS
AMIGO3	NS	0.447519212	-0.015412239	0.569662167	NS
DHX38	NS	0.447556488	-0.015410974	0.569665508	NS
PDIA3	NS	0.447669562	-0.015407138	0.569771964	NS
MZF1	NS	0.447892656	0.015399571	0.56998095	NS
KBTBD2	NS	0.447868733	0.015400382	0.56998095	NS
PCSK2	NS	0.448005284	0.015395751	0.570086798	NS
DSG2	NS	0.448174733	0.015390006	0.570264932	NS
TERF2IP	NS	0.448427522	-0.015381437	0.570549078	NS
LRRC19	NS	0.448462739	-0.015380243	0.570556381	NS
KIAA1549L	NS	0.448710454	0.01537185	0.570777443	NS
ZRSR2	NS	0.448722235	0.015371451	0.570777443	NS
ALG10B	NS	0.448724961	0.015371358	0.570777443	NS
NDUFB11	NS	0.448906016	-0.015365225	0.570970222	NS
DOK2	NS	0.4490802	-0.015359326	0.571154238	NS
KCNQ4	NS	0.449259723	0.015353247	0.571307483	NS
ARMH4	NS	0.449242522	0.01535383	0.571307483	NS
NEDD4L	NS	0.449398957	0.015348534	0.571446999	NS
RPS15	NS	0.449618996	0.015341086	0.57161414	NS
STX18	NS	0.449775914	0.015335777	0.57177608	NS
RNF185	NS	0.449949614	0.0153299	0.57195933	NS
ARL3	NS	0.450131011	-0.015323765	0.572144298	NS
METAP1	NS	0.450154243	0.015322979	0.572144298	NS
SLC38A5	NS	0.450224022	0.01532062	0.572195415	NS
IRF2BPL	NS	0.450266013	-0.0153192	0.572196762	NS
CNNM2	NS	0.450284205	0.015318585	0.572196762	NS
IARS1	NS	0.450376317	-0.01531547	0.572276242	NS
KLF12	NS	0.45054106	-0.015309901	0.572342642	NS
EIF2S3	NS	0.45054685	0.015309706	0.572342642	NS

SMPD4	NS	0.450504492	0.015311137	0.572342642	NS
TAF11L4	NS	0.450524324	0.015310467	0.572342642	NS
ZNF281	NS	0.450849482	-0.015299478	0.572689498	NS
CACNB1	NS	0.45107628	0.015291816	0.572902393	NS
TMEM40	NS	0.451047693	0.015292782	0.572902393	NS
MAL2	NS	0.451577076	-0.015274905	0.573500813	NS
KATNA1	NS	0.451920749	0.015263306	0.573899621	NS
ACVR2B	NS	0.452085055	0.015257763	0.573995298	NS
CSRNP1	NS	0.452078231	0.015257993	0.573995298	NS
PRAMEF11	NS	0.452043224	-0.015259174	0.573995298	NS
ZNF263	NS	0.4525401	-0.015242416	0.574535365	NS
SLC7A5	NS	0.452635859	-0.015239187	0.574581564	NS
CES2	NS	0.452621157	0.015239683	0.574581564	NS
RPS29	NS	0.452721376	0.015236304	0.574652433	NS
RPL13	NS	0.452905091	0.015230112	0.574772551	NS
TLCD5	NS	0.452853658	0.015231845	0.574772551	NS
TTC3	NS	0.452890108	-0.015230617	0.574772551	NS
SORT1	NS	0.452977403	0.015227675	0.574826632	NS
NF2	NS	0.453250372	-0.015218478	0.575135321	NS
IQCM	NS	0.453729375	-0.015202347	0.575705394	NS
SUPT20H	NS	0.453927927	0.015195663	0.575919569	NS
ADORA1	NS	0.453970497	-0.01519423	0.575935828	NS
POGLUT3	NS	0.454088406	0.015190262	0.576047659	NS
GOLM1	NS	0.454189485	-0.015186861	0.576138127	NS
ERICH4	NS	0.454621356	0.015172333	0.576648163	NS
SCN1A	NS	0.454894025	-0.015163165	0.576956211	NS
UBOX5	NS	0.455016069	-0.015159062	0.57707319	NS
H2BC17	NS	0.45551581	0.015142269	0.577669136	NS
PCDHA1	NS	0.455723308	-0.0151353	0.577894415	NS
C1orf185	NS	0.455913173	-0.015128924	0.578097306	NS
VPS41	NS	0.456038959	-0.015124701	0.578218924	NS
MUL1	NS	0.456287139	0.015116371	0.578495701	NS
CSF1	NS	0.456468877	0.015110272	0.57868821	NS
-ZNF705EP-/	NS	0.456628939	-0.015104902	0.578853216	NS
ATP5F1E	NS	0.456747296	0.015100932	0.578965334	NS
PRR9	NS	0.456892197	0.015096072	0.579111084	NS
LCE3E	NS	0.457119455	-0.015088452	0.579361195	NS
OTOP1	NS	0.457635182	-0.015071167	0.57997686	NS
MCM9	NS	0.457883116	0.015062861	0.580253083	NS
CLEC7A	NS	0.457959898	0.015060289	0.580312392	NS
NAIF1	NS	0.458003633	0.015058825	0.58032982	NS
TNRC18	NS	0.458174341	-0.015053108	0.58050812	NS
ZNF800	NS	0.458252539	-0.01505049	0.580569194	NS
FLRT1	NS	0.458348211	-0.015047287	0.580652397	NS
DCDC2B	NS	0.458559819	0.015040204	0.580882451	NS
ICAM4	NS	0.458628251	0.015037914	0.580931119	NS
CALHM6	NS	0.458694091	0.015035711	0.580976497	NS
AAAS	NS	0.459220901	-0.015018089	0.581567637	NS
PMIS2	NS	0.459200475	0.015018772	0.581567637	NS
RANBP10	NS	0.459278954	-0.015016148	0.581603104	NS
CPSF1	NS	0.459509776	-0.01500843	0.581857335	NS
HK2	NS	0.459710797	0.015001711	0.5820738	NS
KIFAP3	NS	0.459861713	-0.014996668	0.582158304	NS
ARID1A	NS	0.45986297	-0.014996626	0.582158304	NS
OR10G7	NS	0.459867765	0.014996466	0.582158304	NS

TMEM104	NS	0.459943502	0.014993935	0.582216103	NS
ANP32A	NS	0.460004931	0.014991883	0.582217711	NS
LGSN	NS	0.459975393	0.01499287	0.582217711	NS
KCNJ9	NS	0.460089215	0.014989068	0.582248241	NS
ZNF626	NS	0.460069431	0.014989728	0.582248241	NS
TMEM154	NS	0.460202497	0.014985284	0.582353526	NS
SLC9A3	NS	0.46028207	-0.014982626	0.582416143	NS
LMNTD1	NS	0.460327371	0.014981113	0.58243539	NS
ARHGEF10	NS	0.460418268	-0.014978078	0.582474249	NS
ANKAR	NS	0.460390758	-0.014978996	0.582474249	NS
DPEP2NB	NS	0.460840859	0.01496397	0.582970765	NS
RAB43	NS	0.460919673	-0.01496134	0.583032361	NS
EXOC5	NS	0.461126844	-0.014954427	0.583238042	NS
DEFA4	NS	0.46114254	0.014953904	0.583238042	NS
FAM91A1	NS	0.461249579	-0.014950333	0.583335306	NS
KMT2C	NS	0.461330391	0.014947637	0.583399389	NS
S100PBP	NS	0.46136875	-0.014946358	0.583409781	NS
NFXL1	NS	0.461517802	0.014941387	0.583560137	NS
GRN	NS	0.461606072	-0.014938444	0.583633623	NS
PIEZO1	NS	0.46167339	0.014936199	0.583660107	NS
IWS1	NS	0.461687327	0.014935734	0.583660107	NS
AVPR1A	NS	0.462026887	-0.014924416	0.58405123	NS
IQGAP1	NS	0.462107745	0.014921721	0.584090346	NS
AARS2	NS	0.462227927	-0.014917717	0.584114628	NS
LGI4	NS	0.462219716	-0.01491799	0.584114628	NS
SRL	NS	0.462178763	0.014919355	0.584114628	NS
TRIM49D2	NS	0.462431729	-0.014910927	0.584334022	NS
SERF1A	NS	0.46254821	-0.014907047	0.584443054	NS
TMED1	NS	0.462862064	-0.014896596	0.584799249	NS
SPATA32	NS	0.46289054	-0.014895648	0.584799249	NS
WDR83	NS	0.463241405	0.01488397	0.585166131	NS
HSFX2	NS	0.46321641	-0.014884801	0.585166131	NS
LEMD3	NS	0.463419026	0.014878059	0.585352302	NS
CHTF8	NS	0.463479029	-0.014876063	0.585389891	NS
ST8SIA1	NS	0.463668698	0.014869754	0.585580374	NS
TXNDC5	NS	0.463690349	-0.014869034	0.585580374	NS
STT3A	NS	0.463757157	-0.014866812	0.585626536	NS
ITGA9	NS	0.463935322	0.014860887	0.585813301	NS
TENM4	NS	0.464025831	-0.014857878	0.585889367	NS
APBB3	NS	0.464158361	0.014853472	0.585990245	NS
C1QTNF1	NS	0.464166276	0.014853209	0.585990245	NS
KRTAP5-3	NS	0.464284468	-0.014849281	0.586063008	NS
TUBGCP6	NS	0.464387961	-0.014845841	0.5861172	NS
EVA1C	NS	0.464359895	-0.014846774	0.5861172	NS
PANK3	NS	0.464461736	0.014843389	0.586149474	NS
SLC35B2	NS	0.464474098	0.014842979	0.586149474	NS
EXOC7	NS	0.464507721	0.014841861	0.58615369	NS
KAT7	NS	0.464656785	0.014836909	0.58626535	NS
APP	NS	0.464651474	-0.014837085	0.58626535	NS
PITRM1	NS	0.464815275	0.014831644	0.586427094	NS
SMG9	NS	0.46491366	0.014828376	0.586512989	NS
NCOR2	NS	0.465007225	-0.014825269	0.586592794	NS
GINM1	NS	0.4651066	0.014821969	0.586679918	NS
CYP3A43	NS	0.465243612	-0.014817421	0.586776266	NS
MRI1	NS	0.465234294	0.01481773	0.586776266	NS

TCN2	NS	0.465284967	0.014816048	0.586790189	NS
EIF3K	NS	0.46534103	0.014814187	0.586822658	NS
WWC1	NS	0.465502361	0.014808832	0.586987863	NS
MAN1A2	NS	0.465801889	-0.014798893	0.587327299	NS
DNAJC11	NS	0.466100325	-0.014788994	0.587665314	NS
MAP1A	NS	0.466536999	0.014774516	0.588177564	NS
ADGB	NS	0.466610017	-0.014772095	0.588192998	NS
SIRT5	NS	0.46660303	0.014772327	0.588192998	NS
SYK	NS	0.466868409	-0.014763533	0.588480393	NS
PIP4P2	NS	0.467134526	-0.014754716	0.588777489	NS
LRCH4	NS	0.467243304	0.014751114	0.588837906	NS
MEN1	NS	0.467214175	0.014752078	0.588837906	NS
SLFN5	NS	0.467332164	-0.014748171	0.588911547	NS
ACSL5	NS	0.467489914	-0.014742947	0.58903364	NS
DAXX	NS	0.467472235	0.014743533	0.58903364	NS
SLC11A1	NS	0.46761105	0.014738937	0.589147919	NS
MN1	NS	0.46771387	-0.014735533	0.589239108	NS
ACTR3B	NS	0.467928202	0.01472844	0.589470763	NS
MT1HL1	NS	0.467968153	-0.014727117	0.589482727	NS
CD53	NS	0.468005482	-0.014725882	0.589491384	NS
SVEP1	NS	0.468188278	0.014719834	0.589683258	NS
SRP54	NS	0.468343367	0.014714704	0.589801835	NS
TMSB15A	NS	0.468320963	-0.014715445	0.589801835	NS
RPP25	NS	0.468512241	-0.014709119	0.58997612	NS
IL21R	NS	0.468587288	0.014706637	0.590032237	NS
FH	NS	0.468786163	-0.014700062	0.590244258	NS
TMCC2	NS	0.469060015	-0.01469101	0.590512237	NS
DRGX	NS	0.469055544	0.014691158	0.590512237	NS
TBCC	NS	0.469166547	-0.01468749	0.59060794	NS
NXPH1	NS	0.469511036	0.014676109	0.591003161	NS
PPP1R2C	NS	0.469559949	0.014674493	0.591026296	NS
RALGAPA1	NS	0.469642838	0.014671756	0.591092189	NS
IREB2	NS	0.469786408	-0.014667015	0.591234441	NS
PPFIBP1	NS	0.469845823	-0.014665053	0.591270773	NS
PNMA6F	NS	0.470284503	0.014650572	0.591784348	NS
PRKCD	NS	0.470455891	-0.014644917	0.59194289	NS
NOTCH2NLC	NS	0.470471659	0.014644397	0.59194289	NS
GSDME	NS	0.470641882	0.014638781	0.592118574	NS
BTF3	NS	0.470691332	0.01463715	0.5921423	NS
RBBP9	NS	0.470954511	-0.01462847	0.592299594	NS
CSPP1	NS	0.470900011	0.014630268	0.592299594	NS
FLT3	NS	0.47089468	-0.014630443	0.592299594	NS
EMP3	NS	0.470916913	-0.01462971	0.592299594	NS
NDUFAF3	NS	0.470969366	-0.014627981	0.592299594	NS
PPP1R12C	NS	0.471148106	-0.014622087	0.592393485	NS
SSB	NS	0.471096318	0.014623795	0.592393485	NS
UGCG	NS	0.471166445	0.014621483	0.592393485	NS
PAGR1	NS	0.47115111	-0.014621988	0.592393485	NS
GDF7	NS	0.471318208	0.01461648	0.592519126	NS
PABPN1L	NS	0.471327598	-0.014616171	0.592519126	NS
FLVCR2	NS	0.471402768	-0.014613693	0.592536657	NS
SMAD6	NS	0.471374896	-0.014614612	0.592536657	NS
ZFP30	NS	0.471450384	-0.014612124	0.592558028	NS
LYZL6	NS	0.471564246	0.014608372	0.592662655	NS
ANXA8L1	NS	0.471829954	0.014599618	0.592958095	NS

GRIN3B	NS	0.471885884	0.014597775	0.592989883	NS
NSFL1C	NS	0.47213105	0.014589701	0.593107833	NS
SERPINE1	NS	0.472152109	0.014589008	0.593107833	NS
CALM2	NS	0.472128879	0.014589773	0.593107833	NS
CBWD1	NS	0.472164305	-0.014588606	0.593107833	NS
P2RY2	NS	0.472037332	-0.014592787	0.593107833	NS
TP53TG3C	NS	0.472194239	-0.01458762	0.593107833	NS
ETDB	NS	0.472163685	-0.014588626	0.593107833	NS
DLL4	NS	0.472592631	0.014574506	0.59356972	NS
TMEM204	NS	0.472647156	-0.014572711	0.593599686	NS
CIAO2A	NS	0.472936152	-0.014563202	0.593885569	NS
PGA4	NS	0.472934636	0.014563252	0.593885569	NS
SF3B3	NS	0.473020008	0.014560443	0.593952337	NS
PRICKLE1	NS	0.473287098	-0.014551659	0.594249161	NS
PRPF3	NS	0.473505747	-0.014544469	0.594408019	NS
ATOH1	NS	0.473484594	-0.014545165	0.594408019	NS
SETD3	NS	0.473487615	-0.014545066	0.594408019	NS
UBE2D1	NS	0.473869611	0.014532509	0.594826213	NS
DRD5	NS	0.474032999	-0.01452714	0.594992719	NS
SORBS3	NS	0.474205802	-0.014521463	0.595171022	NS
SYCE1L	NS	0.474310329	-0.01451803	0.595263615	NS
CCP110	NS	0.47438026	0.014515733	0.595274186	NS
TAS2R39	NS	0.474363322	-0.014516289	0.595274186	NS
AP3D1	NS	0.474578192	-0.014509233	0.595445355	NS
ZDHHC17	NS	0.474569861	0.014509506	0.595445355	NS
CCDC59	NS	0.474729729	-0.014504257	0.595596878	NS
RRM1	NS	0.474858054	-0.014500045	0.595719262	NS
SPINT4	NS	0.475643577	-0.014474271	0.596627379	NS
SLCO2A1	NS	0.475695166	0.01447258	0.596653424	NS
ATAD2	NS	0.475745397	0.014470932	0.596677762	NS
EFTUD2	NS	0.476017866	0.014461999	0.596947865	NS
SNRPA1	NS	0.476053278	0.014460838	0.596947865	NS
ABHD13	NS	0.476035865	-0.014461409	0.596947865	NS
HECW1	NS	0.47618381	-0.01445656	0.59703419	NS
ZFPL1	NS	0.476351094	-0.014451078	0.597205245	NS
NCR3LG1	NS	0.476591489	-0.014443201	0.597467931	NS
FBXO46	NS	0.476670152	-0.014440624	0.597527846	NS
GTF2A1	NS	0.476907863	-0.014432839	0.597787112	NS
CPNE2	NS	0.476944737	-0.014431631	0.59779462	NS
COX4I2	NS	0.477233916	-0.014422163	0.598118343	NS
DDHD1	NS	0.477272165	-0.014420911	0.598127551	NS
SUMF2	NS	0.477416246	-0.014416195	0.598269381	NS
FHIP1A	NS	0.477491124	-0.014413744	0.598324477	NS
MIEF1	NS	0.477565514	0.01441131	0.598378955	NS
MYO5C	NS	0.477625605	0.014409344	0.59841551	NS
MESP1	NS	0.477707968	0.014406649	0.598479963	NS
NT5C3B	NS	0.477800908	-0.014403608	0.598518922	NS
MAGEA3	NS	0.477785384	-0.014404116	0.598518922	NS
KPRP	NS	0.477985361	0.014397575	0.598711231	NS
ZNF580	NS	0.478021791	0.014396383	0.598718117	NS
DNAH6	NS	0.478065753	-0.014394945	0.598734436	NS
OPA1	NS	0.478107864	0.014393568	0.598748435	NS
LMO1	NS	0.478222338	-0.014389825	0.598853049	NS
GNPDA1	NS	0.478328603	0.01438635	0.598908625	NS
SP1	NS	0.47830257	0.014387201	0.598908625	NS

COG6	NS	0.478369187	-0.014385023	0.598909117	NS
YIF1B	NS	0.478390879	0.014384314	0.598909117	NS
AP1S2	NS	0.478491705	0.014381018	0.598996601	NS
DLEC1	NS	0.478567271	0.014378548	0.599020208	NS
PHLPP2	NS	0.478648999	0.014375877	0.599020208	NS
PSG8	NS	0.478665301	-0.014375344	0.599020208	NS
AMIGO2	NS	0.478663289	-0.01437541	0.599020208	NS
VWCE	NS	0.47860804	-0.014377216	0.599020208	NS
ACACB	NS	0.47879009	-0.014371266	0.599098906	NS
PDHX	NS	0.478770584	0.014371903	0.599098906	NS
SEPTIN7	NS	0.47889436	-0.014367859	0.599190643	NS
CC2D2B	NS	0.47916627	-0.014358976	0.599492102	NS
EGFL6	NS	0.479423337	-0.014350581	0.599736193	NS
GOLGA6L7	NS	0.479412739	0.014350927	0.599736193	NS
PINX1	NS	0.479612161	0.014344415	0.59993363	NS
CD302	NS	0.479856771	-0.014336431	0.600200818	NS
CABLES1	NS	0.480133145	-0.014327412	0.600460326	NS
CCDC12	NS	0.480157312	-0.014326623	0.600460326	NS
AR	NS	0.480112075	0.014328099	0.600460326	NS
MYO3B	NS	0.48033846	0.014320714	0.600624768	NS
C8orf33	NS	0.480350869	0.014320309	0.600624768	NS
PIAS3	NS	0.480440208	0.014317395	0.600697672	NS
EPS8L1	NS	0.480561196	-0.014313449	0.600778789	NS
TVP23C	NS	0.4805982	0.014312242	0.600778789	NS
URAD	NS	0.480594949	-0.014312348	0.600778789	NS
ERVV-2	NS	0.480659043	-0.014310258	0.600816044	NS
BRD7	NS	0.480829911	0.014304687	0.600952009	NS
PTPN2	NS	0.480828047	0.014304748	0.600952009	NS
LENEP	NS	0.481047355	-0.014297599	0.60115317	NS
ARHGAP19	NS	0.481052979	0.014297416	0.60115317	NS
SLITRK5	NS	0.481103586	0.014295766	0.6011776	NS
NLN	NS	0.48114203	-0.014294513	0.601186826	NS
RHOA	NS	0.481195395	-0.014292774	0.601214695	NS
NUP188	NS	0.481315548	0.014288859	0.601326001	NS
FOXJ3	NS	0.481545072	0.014281381	0.601573927	NS
MAP3K13	NS	0.481646776	0.014278068	0.601662149	NS
ZSWIM1	NS	0.481708229	0.014276067	0.601700082	NS
DDX10	NS	0.481770344	-0.014274044	0.601738838	NS
CR1	NS	0.48189845	-0.014269872	0.601860008	NS
DIRAS3	NS	0.481962074	-0.0142678	0.601900632	NS
CCNB1	NS	0.482218085	0.014259466	0.6021815	NS
CALML4	NS	0.482253668	-0.014258307	0.602187084	NS
FBXL5	NS	0.482390416	0.014253857	0.602318985	NS
TLR5	NS	0.48286546	-0.014238401	0.60287324	NS
PWWP2A	NS	0.482955716	0.014235465	0.602947035	NS
TNR	NS	0.483026379	-0.014233167	0.602996362	NS
GPSM1	NS	0.483117352	-0.014230209	0.60300408	NS
NDUFV2	NS	0.483110519	0.014230431	0.60300408	NS
DDX39B	NS	0.483126022	0.014229927	0.60300408	NS
BLVRA	NS	0.483310757	-0.01422392	0.603195758	NS
KIFBP	NS	0.483455506	-0.014219215	0.603337509	NS
SMC5	NS	0.483879613	0.014205432	0.60382785	NS
NIPSNAP1	NS	0.484098147	-0.014198333	0.604061613	NS
ZAN	NS	0.48414797	0.014196714	0.604084839	NS
TMED7	NS	0.484236537	0.014193838	0.604156401	NS

CMC2	NS	0.484284424	0.014192283	0.604177203	NS
SLC25A18	NS	0.484348417	-0.014190205	0.604218095	NS
NAGK	NS	0.484424356	0.014187739	0.604273883	NS
ZNF497	NS	0.484760075	0.01417684	0.604653693	NS
HIGD1B	NS	0.484902431	-0.01417222	0.604792284	NS
RNF144A	NS	0.484981031	0.014169669	0.604851343	NS
OR7C1	NS	0.485161888	-0.014163801	0.605037916	NS
HPS5	NS	0.485219852	-0.01416192	0.605071218	NS
SYTL1	NS	0.485445891	0.014154588	0.605275101	NS
ZNF845	NS	0.485435334	-0.014154931	0.605275101	NS
AQP10	NS	0.485546645	0.014151321	0.605341755	NS
WDR81	NS	0.485561897	0.014150826	0.605341755	NS
SLC15A2	NS	0.485844082	-0.014141676	0.60565454	NS
ODF2	NS	0.48594853	0.014138291	0.605745733	NS
ZNF417	NS	0.486363709	-0.014124836	0.606224222	NS
PLCE1	NS	0.486495586	0.014120563	0.606349554	NS
DRAM2	NS	0.486538118	0.014119185	0.606363519	NS
MRPS21	NS	0.486875716	0.014108251	0.606745195	NS
TXNRD1	NS	0.487006684	0.01410401	0.606869334	NS
TCFL5	NS	0.487071501	-0.014101912	0.606911032	NS
VSX1	NS	0.487197054	-0.014097848	0.607028399	NS
TRIM64	NS	0.487312036	-0.014094126	0.60713258	NS
CBLN3	NS	0.487560555	-0.014086083	0.607403109	NS
KCNQ3	NS	0.487717546	0.014081004	0.607559584	NS
SPATA18	NS	0.487778513	0.014079032	0.607596429	NS
HSPA12B	NS	0.487812814	-0.014077922	0.607600054	NS
RAD54L	NS	0.487911501	0.01407473	0.607659377	NS
KRTAP19-2	NS	0.48792323	-0.01407435	0.607659377	NS
COX5A	NS	0.487987773	0.014072263	0.607700659	NS
CEP192	NS	0.488087941	0.014069024	0.607786296	NS
TTC39C	NS	0.488120254	0.014067979	0.607787433	NS
LRRK2	NS	0.488309746	0.014061852	0.607984269	NS
UBE2W	NS	0.488413028	-0.014058513	0.608066723	NS
LRRC47	NS	0.488470215	-0.014056664	0.608066723	NS
ZNF585B	NS	0.48844887	-0.014057354	0.608066723	NS
GTF2H5	NS	0.48854819	-0.014054144	0.608124679	NS
F11	NS	0.488617389	-0.014051907	0.608171704	NS
FGB	NS	0.48872069	-0.014048569	0.608237514	NS
MED12	NS	0.488733109	0.014048167	0.608237514	NS
NDUFS8	NS	0.488807721	0.014045756	0.608291259	NS
BPNT1	NS	0.488844973	0.014044553	0.608298509	NS
KRBOX4	NS	0.488879779	-0.014043428	0.608299692	NS
ZNF770	NS	0.488908778	-0.014042491	0.608299692	NS
EXOC4	NS	0.489311227	0.014029491	0.608761288	NS
PPIL4	NS	0.489693326	-0.014017154	0.609197507	NS
ESR2	NS	0.48995785	-0.014008616	0.609487412	NS
VPS29	NS	0.490271646	-0.013998491	0.609838569	NS
ARHGEF1	NS	0.490345294	0.013996115	0.609890985	NS
CNP	NS	0.490634548	-0.013986786	0.610202015	NS
OAF	NS	0.490658409	0.013986016	0.610202015	NS
DGCR6L	NS	0.490806213	-0.013981251	0.610346614	NS
SLCO5A1	NS	0.490889379	-0.013978569	0.61041082	NS
MICALL2	NS	0.491012199	-0.01397461	0.610524323	NS
COPS5	NS	0.491049765	-0.013973399	0.610531813	NS
MBD6	NS	0.491236518	0.01396738	0.610724777	NS

NAALADL1	NS	0.491296755	-0.013965439	0.610760437	NS
BMP1	NS	0.491385187	-0.013962589	0.61083114	NS
FAM177A1	NS	0.491486176	0.013959335	0.610917443	NS
KRIT1	NS	0.491544261	0.013957464	0.610950409	NS
MCUB	NS	0.491689023	-0.013952801	0.61105186	NS
LRP10	NS	0.491683721	-0.013952972	0.61105186	NS
EZH1	NS	0.492300296	0.013933118	0.611693705	NS
MMP17	NS	0.492278054	0.013933834	0.611693705	NS
EFCAB9	NS	0.492248633	-0.013934781	0.611693705	NS
DAB2IP	NS	0.492484393	0.013927193	0.61188317	NS
OR5M8	NS	0.492539352	-0.013925424	0.611912176	NS
FBXO21	NS	0.492795492	-0.013917182	0.612171807	NS
TCAF2	NS	0.492811588	-0.013916664	0.612171807	NS
SPINT2	NS	0.492918664	0.01391322	0.612265524	NS
AKAP8L	NS	0.493221509	-0.01390348	0.612327338	NS
TLE5	NS	0.493073388	-0.013908243	0.612327338	NS
WDTC1	NS	0.493058839	0.013908711	0.612327338	NS
NLGN4X	NS	0.493203243	0.013904067	0.612327338	NS
H1-4	NS	0.493120086	-0.013906741	0.612327338	NS
ACTRT3	NS	0.493050723	-0.013908972	0.612327338	NS
FOXD4L4	NS	0.493199688	0.013904181	0.612327338	NS
FAM90A7P	NS	0.493144377	0.01390596	0.612327338	NS
FGR	NS	0.493580204	0.013891947	0.612733352	NS
B3GALT6	NS	0.493670747	-0.013889037	0.61280645	NS
GPATCH11	NS	0.49386224	0.013882883	0.613004843	NS
ZNF839	NS	0.49412729	0.013874367	0.613294507	NS
PLK2	NS	0.494822751	0.013852033	0.614078936	NS
IQCB1	NS	0.494941151	-0.013848232	0.614186493	NS
PACS1	NS	0.495226563	0.013839073	0.614501272	NS
BNC1	NS	0.495411512	0.013833139	0.61469136	NS
SERPINE2	NS	0.49547733	-0.013831027	0.614694219	NS
FAM161B	NS	0.495473654	-0.013831145	0.614694219	NS
TMEM223	NS	0.495518306	-0.013829713	0.614705655	NS
TRIM28	NS	0.495712309	0.013823491	0.614906912	NS
TMED2	NS	0.496213099	0.013807434	0.615488673	NS
DGCR8	NS	0.49640298	0.013801349	0.615684742	NS
SCGB2A2	NS	0.496768681	0.013789631	0.61609884	NS
USP17L18	NS	0.496934368	-0.013784324	0.61626484	NS
PROSER1	NS	0.497068425	-0.013780031	0.616391596	NS
CNOT6	NS	0.497112379	-0.013778623	0.616406612	NS
PCSK7	NS	0.497658947	0.013761126	0.617027183	NS
CCDC167	NS	0.497676605	0.013760561	0.617027183	NS
PSMG1	NS	0.497941301	-0.013752091	0.617315815	NS
ABHD16B	NS	0.498096438	-0.013747128	0.617429053	NS
HMSD	NS	0.498083334	0.013747547	0.617429053	NS
LYRM2	NS	0.498255642	-0.013742036	0.617547301	NS
SLC22A8	NS	0.498255195	0.01374205	0.617547301	NS
NHSL1	NS	0.498465554	0.013735323	0.61772836	NS
MRPL10	NS	0.498613127	-0.013730604	0.617871683	NS
QRICH1	NS	0.498681203	-0.013728428	0.617876928	NS
NF625-ZNF2	NS	0.498656209	-0.013729227	0.617876928	NS
SOCS2	NS	0.498743135	0.013726448	0.617914109	NS
DNAH2	NS	0.498963014	0.01371942	0.61814696	NS
IBTK	NS	0.499079425	-0.0137157	0.618249901	NS
SLC30A4	NS	0.49910999	-0.013714723	0.618249901	NS

TSPYL1	NS	0.499416664	0.013704925	0.618550611	NS
ODAD4	NS	0.499394767	0.013705625	0.618550611	NS
NREP	NS	0.499462723	0.013703454	0.618568076	NS
SLC6A11	NS	0.499753406	0.01369417	0.618816388	NS
ANKRD6	NS	0.499722837	-0.013695146	0.618816388	NS
POTEF	NS	0.499786249	0.013693121	0.618816388	NS
NF1	NS	0.499791104	0.013692966	0.618816388	NS
CHSY1	NS	0.499857276	0.013690854	0.618858734	NS
STK4	NS	0.499936837	0.013688313	0.618878065	NS
DGCR6	NS	0.499922022	-0.013688786	0.618878065	NS
ECSIT	NS	0.500015453	0.013685804	0.618912419	NS
BAIAP2	NS	0.50002854	0.013685386	0.618912419	NS
KLHL20	NS	0.500214433	-0.013679452	0.619082293	NS
RCN2	NS	0.500229751	0.013678963	0.619082293	NS
UBTF	NS	0.500452243	-0.013671863	0.619293118	NS
NCOA5	NS	0.500524456	0.013669559	0.619293118	NS
TBC1D24	NS	0.500528081	-0.013669443	0.619293118	NS
SMIM26	NS	0.50050574	0.013670156	0.619293118	NS
GATD3	NS	0.500943843	-0.013656181	0.619767914	NS
BHLHB9	NS	0.500981211	-0.013654989	0.619774531	NS
TEKT2	NS	0.501039124	-0.013653142	0.619806562	NS
MICALL1	NS	0.50137856	-0.01364232	0.620186823	NS
BCL6	NS	0.501489927	0.01363877	0.62025583	NS
VSTM2L	NS	0.501498437	0.013638499	0.62025583	NS
OLAH	NS	0.501625884	0.013634437	0.620373818	NS
P3H3	NS	0.501713964	-0.01363163	0.620427314	NS
OR1D2	NS	0.501733248	-0.013631015	0.620427314	NS
MAP2K2	NS	0.502222605	-0.013615426	0.620992765	NS
TRPM6	NS	0.5022819	-0.013613537	0.620996016	NS
IPMK	NS	0.5022894	-0.013613298	0.620996016	NS
KRBA2	NS	0.502373111	0.013610633	0.621059841	NS
WDR3	NS	0.502422267	-0.013609067	0.621080942	NS
VPS13D	NS	0.502614195	0.013602956	0.621238847	NS
KLC4	NS	0.502582436	-0.013603968	0.621238847	NS
ZNF234	NS	0.502868395	-0.013594865	0.621513354	NS
TEX28	NS	0.502955572	-0.01359209	0.621581411	NS
CCAR1	NS	0.503128914	0.013586574	0.621674129	NS
LMF2	NS	0.503213393	0.013583886	0.621674129	NS
RBM25	NS	0.503072013	-0.013588385	0.621674129	NS
ACE2	NS	0.503134661	0.013586391	0.621674129	NS
ASTN2	NS	0.50322082	-0.01358365	0.621674129	NS
CCDC126	NS	0.503223303	-0.013583571	0.621674129	NS
ESRP1	NS	0.503330368	-0.013580165	0.621766711	NS
KRTAP21-3	NS	0.503401165	0.013577913	0.621814483	NS
SENP7	NS	0.503472397	-0.013575647	0.621862786	NS
ZNF75A	NS	0.50353586	0.013573628	0.621901488	NS
ACTRT1	NS	0.50404151	0.01355755	0.622446566	NS
MCU	NS	0.504032485	-0.013557837	0.622446566	NS
RPS27	NS	0.504119985	0.013555056	0.622503759	NS
SLC9A7	NS	0.504240997	0.01355121	0.622573754	NS
TSR2	NS	0.504232501	-0.01355148	0.622573754	NS
NRL	NS	0.504577887	-0.013540505	0.622949967	NS
STX17	NS	0.504807278	-0.013533218	0.623193422	NS
RHOF	NS	0.505252723	0.013519072	0.623703553	NS
TRIO	NS	0.505655985	0.013506272	0.624161549	NS

MANSC1	NS	0.505894968	0.013498689	0.624376905	NS
ATG10	NS	0.505980808	0.013495965	0.624443032	NS
NTMT1	NS	0.506023181	0.013494621	0.624455511	NS
RYK	NS	0.506068186	-0.013493194	0.624471236	NS
ZRANB3	NS	0.506178664	0.013489689	0.624527934	NS
NQO2	NS	0.506168496	-0.013490012	0.624527934	NS
KLHL35	NS	0.506617509	-0.013475773	0.625029543	NS
NAPB	NS	0.506730698	0.013472185	0.625129343	NS
FBXO33	NS	0.506795723	-0.013470124	0.625169716	NS
ZNF225	NS	0.506905084	0.013466658	0.625264772	NS
ESAM	NS	0.507101021	0.013460448	0.625466601	NS
SLC22A25	NS	0.507206514	0.013457106	0.625556855	NS
HHIPL2	NS	0.507411639	-0.013450607	0.625730102	NS
LAMTOR1	NS	0.507386796	0.013451394	0.625730102	NS
ZNF43	NS	0.507534416	0.013446718	0.625841636	NS
RC3H2	NS	0.507635261	-0.013443524	0.625926112	NS
DAG1	NS	0.507791245	-0.013438585	0.626078561	NS
POMP	NS	0.507855682	0.013436544	0.626118126	NS
SYNM	NS	0.507897045	-0.013435235	0.626129241	NS
ZNF189	NS	0.508091864	0.013429067	0.62632952	NS
H2BC15	NS	0.508165195	-0.013426746	0.626380023	NS
TPD52L1	NS	0.508288668	-0.013422838	0.626492324	NS
MYOF	NS	0.508355886	-0.013420711	0.626535277	NS
KRTAP9-8	NS	0.508406603	-0.013419106	0.62655789	NS
RIPOR3	NS	0.508643322	-0.013411616	0.626729909	NS
WFDC2	NS	0.508620265	-0.013412345	0.626729909	NS
SLC1A2	NS	0.508633535	0.013411925	0.626729909	NS
TNN	NS	0.508699345	-0.013409843	0.62675904	NS
ATRAID	NS	0.508735497	0.0134087	0.626763687	NS
CGN	NS	0.508844071	0.013405265	0.626857551	NS
ZDHHC7	NS	0.508891216	-0.013403774	0.626874634	NS
KCNK9	NS	0.508922711	0.013402778	0.626874634	NS
RPS6KA4	NS	0.508957376	-0.013401682	0.62687744	NS
TRO	NS	0.509038702	0.01339911	0.626893693	NS
SLC7A6	NS	0.509067734	0.013398192	0.626893693	NS
PPA1	NS	0.509017682	0.013399774	0.626893693	NS
NLRP2	NS	0.50923183	0.013393003	0.627055875	NS
RGS8	NS	0.509363587	-0.013388837	0.627178219	NS
OR10H5	NS	0.509606872	0.013381147	0.627437861	NS
NARS2	NS	0.509907083	0.01337166	0.627567972	NS
DUSP15	NS	0.509883622	-0.013372401	0.627567972	NS
TMEM74	NS	0.509877146	-0.013372606	0.627567972	NS
PVRIG	NS	0.509808741	0.013374767	0.627567972	NS
COLCA2	NS	0.509830157	0.013374091	0.627567972	NS
IFRD2	NS	0.510122047	-0.013364869	0.627792621	NS
MYF5	NS	0.510295946	-0.013359376	0.627966707	NS
GOLGA6L22	NS	0.510378037	0.013356784	0.628027799	NS
ZBTB14	NS	0.510696088	0.013346741	0.628379218	NS
ZNF511	NS	0.510779529	0.013344107	0.628441938	NS
PRELID3B	NS	0.510906695	0.013340093	0.628518494	NS
CYP27C1	NS	0.510897692	0.013340377	0.628518494	NS
PCYOX1	NS	0.51119574	-0.013330971	0.628834111	NS
SDC4	NS	0.511378278	0.013325211	0.628963101	NS
GPR155	NS	0.511398083	0.013324587	0.628963101	NS
BRIX1	NS	0.511697061	0.013315156	0.629290826	NS

ATXN3L	NS	0.511815863	-0.013311409	0.62939694	NS
DUSP4	NS	0.511936535	-0.013307604	0.629422086	NS
FAHD1	NS	0.511932737	-0.013307724	0.629422086	NS
KDM4E	NS	0.511966383	0.013306663	0.629422086	NS
GNA15	NS	0.512174313	-0.013300108	0.629637726	NS
C11orf16	NS	0.512211955	-0.013298921	0.629644011	NS
XCR1	NS	0.512551135	-0.013288232	0.630020941	NS
PGLYRP3	NS	0.512662324	-0.013284728	0.630117599	NS
MAST4	NS	0.512756547	-0.01328176	0.630193392	NS
LRP2	NS	0.51294651	0.013275776	0.630258738	NS
ITPK1	NS	0.512942635	-0.013275898	0.630258738	NS
SMARCA4	NS	0.512935962	0.013276108	0.630258738	NS
BLM	NS	0.512972523	-0.013274956	0.630258738	NS
FAR1	NS	0.512888968	0.013277588	0.630258738	NS
RAB1A	NS	0.513768863	-0.013249884	0.631116971	NS
PRKCA	NS	0.513748387	-0.013250528	0.631116971	NS
UBB	NS	0.51373545	-0.013250936	0.631116971	NS
SEL1L3	NS	0.513846556	-0.013247439	0.631132301	NS
METRNL	NS	0.513832475	0.013247882	0.631132301	NS
KDM3B	NS	0.513948987	0.013244216	0.631137964	NS
OR5F1	NS	0.51394159	-0.013244448	0.631137964	NS
ABTB2	NS	0.513923741	0.01324501	0.631137964	NS
TROAP	NS	0.514027958	-0.013241731	0.631194896	NS
CHD9	NS	0.514144631	0.01323806	0.631298115	NS
RPL27	NS	0.514252601	0.013234664	0.631390634	NS
USH1C	NS	0.514450605	0.013228436	0.631570696	NS
TAF1B	NS	0.514497145	-0.013226972	0.631570696	NS
TRAPPC6B	NS	0.51447365	0.013227711	0.631570696	NS
CATSPER2	NS	0.514844582	0.013216048	0.631957114	NS
RALBP1	NS	0.514901949	0.013214244	0.631987453	NS
CCDC88A	NS	0.515033607	-0.013210106	0.632065762	NS
TSEN34	NS	0.51501431	0.013210712	0.632065762	NS
TCERG1L	NS	0.515063715	0.013209159	0.632065762	NS
DOK4	NS	0.51549669	0.013195554	0.632556989	NS
SFMBT2	NS	0.515626333	-0.013191481	0.632675962	NS
NPIPB13	NS	0.515787426	0.013186421	0.632833508	NS
GTSE1	NS	0.515851018	0.013184424	0.632855698	NS
NCKAP5L	NS	0.515870903	0.013183799	0.632855698	NS
AKAP5	NS	0.515940693	-0.013181608	0.632901201	NS
MLLT1	NS	0.516254676	0.013171749	0.63324623	NS
TMC8	NS	0.516333508	-0.013169274	0.633302794	NS
PSG2	NS	0.516452088	-0.013165552	0.63339031	NS
GPR162	NS	0.516470307	-0.01316498	0.63339031	NS
ALMS1	NS	0.516748972	0.013156235	0.63369191	NS
ELK4	NS	0.516902494	0.013151419	0.633840017	NS
YES1	NS	0.516992914	0.013148582	0.633910734	NS
KLHL17	NS	0.517134209	0.01314415	0.634043817	NS
ASDURF	NS	0.517420459	0.013135173	0.634354599	NS
RGS10	NS	0.517498861	0.013132715	0.634410536	NS
CUL5	NS	0.517532063	-0.013131674	0.634411058	NS
CRX	NS	0.517618412	-0.013128966	0.634476726	NS
RAC2	NS	0.517765744	0.013124348	0.634617131	NS
PIGO	NS	0.518022685	0.013116295	0.634891857	NS
DEXI	NS	0.518505169	-0.013101179	0.635442956	NS
SLC6A15	NS	0.518549071	0.013099804	0.635456525	NS

PRKCZ	NS	0.518770656	0.013092864	0.635687821	NS
SLC2A6	NS	0.518893036	-0.013089032	0.635797531	NS
SNX29	NS	0.519021232	-0.013085019	0.635914354	NS
EML2	NS	0.51926108	-0.013077511	0.636167951	NS
SMYD2	NS	0.519350152	-0.013074723	0.636236806	NS
JPH4	NS	0.519480319	-0.013070649	0.636355994	NS
GLG1	NS	0.519595689	-0.013067039	0.636457041	NS
GTF2IRD2	NS	0.519644778	-0.013065504	0.636476891	NS
MORN3	NS	0.519915099	-0.013057047	0.636765061	NS
TRIM66	NS	0.519945845	-0.013056086	0.636765061	NS
ME2	NS	0.520247227	0.01304666	0.637053541	NS
RNF182	NS	0.520221154	-0.013047476	0.637053541	NS
EPDR1	NS	0.520357403	0.013043216	0.637112416	NS
BSG	NS	0.520361138	-0.013043099	0.637112416	NS
ARGLU1	NS	0.520541065	0.013037474	0.637252094	NS
OPLAH	NS	0.520519503	-0.013038148	0.637252094	NS
TMSB4Y	NS	0.520615785	-0.013035138	0.637303259	NS
KLHL6	NS	0.521011005	-0.013022787	0.637746728	NS
TOMM20	NS	0.521109604	0.013019707	0.637824117	NS
S100A7L2	NS	0.521140133	0.013018753	0.637824117	NS
FAM90A26	NS	0.52141235	0.01301025	0.638116935	NS
C11orf49	NS	0.521445953	0.0130092	0.6381173	NS
ARMC3	NS	0.521478583	0.013008181	0.6381173	NS
NUTM1	NS	0.521576962	0.013005109	0.638197337	NS
NUS1	NS	0.521707237	-0.013001041	0.638276043	NS
CDK19	NS	0.521688941	0.013001613	0.638276043	NS
PHGR1	NS	0.521796601	-0.012998251	0.638345027	NS
CALHM2	NS	0.521997934	0.012991966	0.638550971	NS
KIF2C	NS	0.52227088	-0.012983447	0.638844486	NS
SYNDIG1L	NS	0.522775797	0.012967695	0.639373432	NS
SZT2	NS	0.522835435	-0.012965835	0.639373432	NS
NFIA	NS	0.522886407	0.012964245	0.639395368	NS
NHS	NS	0.52294263	-0.012962492	0.639423723	NS
TARS1	NS	0.523060385	-0.01295882	0.639460799	NS
FLOT2	NS	0.523072062	-0.012958456	0.639460799	NS
S1PR2	NS	0.523044295	-0.012959321	0.639460799	NS
MYPN	NS	0.523140446	-0.012956323	0.639504009	NS
RUSF1	NS	0.523450628	0.012946654	0.639842776	NS
CHMP2A	NS	0.523514082	-0.012944676	0.639868768	NS
WFDC12	NS	0.523538008	-0.01294393	0.639868768	NS
TMEM161A	NS	0.523618415	-0.012941424	0.639901254	NS
CLTB	NS	0.523655073	-0.012940282	0.639901254	NS
COL6A6	NS	0.523663766	-0.012940011	0.639901254	NS
HSPA9	NS	0.52390253	0.012932571	0.640152602	NS
NMUR1	NS	0.523988085	-0.012929905	0.640216726	NS
RBM48	NS	0.524090078	0.012926728	0.640260512	NS
ZNF672	NS	0.524082241	-0.012926972	0.640260512	NS
ZFP36	NS	0.524159032	0.01292458	0.64026393	NS
MMRN2	NS	0.524155921	0.012924677	0.64026393	NS
TTC22	NS	0.524250668	-0.012921726	0.640335453	NS
ZNF708	NS	0.524334169	-0.012919126	0.640397032	NS
AEBP2	NS	0.524410292	0.012916755	0.640449593	NS
ERO1A	NS	0.524582311	0.012911399	0.640619256	NS
GATAD2B	NS	0.524661401	0.012908936	0.640675419	NS
GTF2A2	NS	0.524881647	-0.01290208	0.640903934	NS

HNRNPC	NS	0.525024743	0.012897626	0.641004778	NS
ZNF776	NS	0.525048298	-0.012896893	0.641004778	NS
TNFSF13	NS	0.525096702	0.012895387	0.641004778	NS
PEAK1	NS	0.525079529	-0.012895921	0.641004778	NS
CARD14	NS	0.525366524	-0.012886991	0.641253276	NS
EIF4E3	NS	0.525366123	-0.012887004	0.641253276	NS
APAF1	NS	0.525401757	-0.012885895	0.641255843	NS
IFNGR2	NS	0.525463844	-0.012883964	0.641291183	NS
MYH10	NS	0.525666792	0.012877651	0.641498419	NS
SCN4A	NS	0.525807385	0.012873278	0.641629539	NS
PEA15	NS	0.525850519	-0.012871937	0.641641723	NS
NECAB2	NS	0.525979137	-0.012867938	0.641645098	NS
PIK3R2	NS	0.525932305	-0.012869394	0.641645098	NS
SLC10A7	NS	0.525985884	-0.012867728	0.641645098	NS
DCAF8	NS	0.525925512	0.012869605	0.641645098	NS
PITPNC1	NS	0.526122133	0.012863492	0.641762215	NS
ZHX2	NS	0.526148202	-0.012862681	0.641762215	NS
CRYGB	NS	0.526649426	0.012847103	0.642333099	NS
RAB40B	NS	0.526780197	-0.01284304	0.642452113	NS
OR2L8	NS	0.526880524	-0.012839923	0.642533985	NS
LRRC3	NS	0.526968828	0.01283718	0.642601187	NS
AGTPBP1	NS	0.527091873	0.012833358	0.642702148	NS
OR1A2	NS	0.527692703	-0.012814702	0.643321784	NS
POLG	NS	0.527736342	0.012813347	0.643334465	NS
CYRIB	NS	0.528163793	0.012800082	0.643815	NS
F11R	NS	0.528657415	0.01278477	0.644294984	NS
HOPX	NS	0.528640208	-0.012785303	0.644294984	NS
CNTN4	NS	0.528829836	0.012779423	0.644342837	NS
FAM107A	NS	0.528806829	-0.012780136	0.644342837	NS
TMEM94	NS	0.528763959	0.012781466	0.644342837	NS
ZNF546	NS	0.528771212	-0.012781241	0.644342837	NS
UBL3	NS	0.528874504	-0.012778038	0.6443567	NS
CCDC184	NS	0.529021166	-0.012773491	0.644494819	NS
XPO5	NS	0.52909872	0.012771086	0.644548733	NS
CACNA1B	NS	0.529333701	0.012763803	0.644794407	NS
ROGDI	NS	0.529416915	0.012761224	0.644855189	NS
NDUFA3	NS	0.529711833	-0.012752086	0.645173813	NS
NDUFA2	NS	0.52977874	-0.012750013	0.645214704	NS
PLXND1	NS	0.529911356	0.012745905	0.645218268	NS
SPATS2	NS	0.529948338	0.012744759	0.645218268	NS
RNF122	NS	0.52990229	-0.012746186	0.645218268	NS
ZNF852	NS	0.529933382	-0.012745223	0.645218268	NS
E2F4	NS	0.529856315	-0.01274761	0.645218268	NS
RAB32	NS	0.530029089	-0.012742258	0.645235412	NS
MAP3K10	NS	0.530020807	0.012742515	0.645235412	NS
MBNL2	NS	0.530230087	-0.012736034	0.645414682	NS
FGD2	NS	0.530243039	-0.012735633	0.645414682	NS
TMOD2	NS	0.53037031	-0.012731692	0.645500264	NS
POLR3D	NS	0.530380048	-0.01273139	0.645500264	NS
OR8B3	NS	0.530532449	-0.012726672	0.645645148	NS
DDX23	NS	0.530650949	-0.012723004	0.645748759	NS
IL25	NS	0.530727331	-0.01272064	0.645801108	NS
PRDM13	NS	0.530829519	0.012717477	0.64584425	NS
ATP6V1FNB	NS	0.530818258	0.012717826	0.64584425	NS
GUK1	NS	0.53094209	0.012713993	0.645940609	NS

MYO7A	NS	0.531124367	-0.012708353	0.646121756	NS
ZMYND12	NS	0.531238642	-0.012704818	0.646220158	NS
SMIM19	NS	0.531440381	0.012698578	0.646422013	NS
MTRNR2L5	NS	0.531471374	-0.012697619	0.646422013	NS
SLITRK1	NS	0.531535976	-0.012695621	0.646459966	NS
CEP164	NS	0.531686184	0.012690976	0.646595724	NS
TMEM209	NS	0.531714411	0.012690103	0.646595724	NS
DPM2	NS	0.532214195	-0.012674652	0.647162832	NS
GPHN	NS	0.532356406	-0.012670257	0.647295093	NS
YPEL1	NS	0.532490382	0.012666117	0.647417327	NS
PROS1	NS	0.532536515	0.012664692	0.647432748	NS
CCDC69	NS	0.532754541	-0.012657956	0.647657135	NS
AGPAT4	NS	0.532860283	-0.012654689	0.647745001	NS
LHFPL4	NS	0.533128036	0.01264642	0.64798909	NS
BRME1	NS	0.533510026	0.012634626	0.648412663	NS
RSPO1	NS	0.533570647	-0.012632755	0.648426186	NS
BRD3OS	NS	0.533588153	-0.012632215	0.648426186	NS
NEK8	NS	0.53367438	0.012629553	0.648490257	NS
TMEM143	NS	0.533728939	0.012627869	0.648515841	NS
TRABD	NS	0.533766912	0.012626697	0.64852127	NS
SLC52A1	NS	0.534129746	0.012615501	0.648921375	NS
PDCD10	NS	0.534190586	-0.012613625	0.648954559	NS
DESI1	NS	0.534521754	-0.01260341	0.649316121	NS
SRSF9	NS	0.534625444	-0.012600212	0.649401324	NS
RGS19	NS	0.534687561	-0.012598297	0.649436021	NS
ZNF280D	NS	0.534895496	-0.012591886	0.649620256	NS
SH3RF3	NS	0.534906367	0.01259155	0.649620256	NS
SURF1	NS	0.53510569	0.012585406	0.649821553	NS
MANBAL	NS	0.535480616	-0.012573852	0.650236061	NS
GAB4	NS	0.53561103	0.012569834	0.650353622	NS
MPHOSPH9	NS	0.535824461	0.012563259	0.650531159	NS
PEAK3	NS	0.535793982	0.012564198	0.650531159	NS
RSL24D1	NS	0.536319059	-0.012548028	0.651075406	NS
SEPTIN8	NS	0.536340016	-0.012547383	0.651075406	NS
HSF2BP	NS	0.536773386	-0.012534043	0.65147891	NS
ZNF483	NS	0.536756787	-0.012534554	0.65147891	NS
MUC22	NS	0.536706184	-0.012536112	0.65147891	NS
FRMPD3	NS	0.536829495	0.012532317	0.651506157	NS
FOLR3	NS	0.537079632	-0.01252462	0.651684888	NS
GFOD2	NS	0.537111439	-0.012523642	0.651684888	NS
SMPDL3A	NS	0.537020747	0.012526432	0.651684888	NS
ENTPD4	NS	0.53706632	0.01252503	0.651684888	NS
ANKS1A	NS	0.537265406	-0.012518905	0.651749135	NS
CHORDC1	NS	0.537232716	0.012519911	0.651749135	NS
CITED2	NS	0.537216051	-0.012520423	0.651749135	NS
ICA1L	NS	0.537415447	-0.01251429	0.651890292	NS
PLK1	NS	0.53754251	0.012510382	0.652003561	NS
GPR151	NS	0.537660338	-0.012506759	0.652105614	NS
MED23	NS	0.537746626	0.012504106	0.652128545	NS
ARL10	NS	0.537720838	0.012504899	0.652128545	NS
PSG6	NS	0.537972523	-0.012497161	0.652321072	NS
SLC35C1	NS	0.537972788	-0.012497153	0.652321072	NS
ICAM5	NS	0.538367108	0.012485034	0.652758315	NS
DUS1L	NS	0.538566719	-0.0124789	0.652959438	NS
MCMBP	NS	0.538752798	0.012473184	0.653144129	NS

SLC35A5	NS	0.538789322	0.012472062	0.6531475	NS
GPALPP1	NS	0.538952204	0.012467059	0.653304037	NS
TPRG1	NS	0.539106327	-0.012462326	0.653449939	NS
MBNL3	NS	0.539187873	-0.012459822	0.653507856	NS
SPEGNB	NS	0.539234969	-0.012458376	0.653524016	NS
CD207	NS	0.539393033	-0.012453523	0.653674652	NS
FAM90A10P	NS	0.539567731	0.01244816	0.653845427	NS
ITGB7	NS	0.539731667	0.012443129	0.654003139	NS
SLC29A1	NS	0.540041148	0.012433632	0.654337181	NS
PTP4A2	NS	0.54018072	0.01242935	0.654443827	NS
SRY	NS	0.540196788	-0.012428857	0.654443827	NS
CSRP1	NS	0.540305302	0.012425529	0.654534324	NS
MTDH	NS	0.540426652	0.012421807	0.65458718	NS
KRTAP4-1	NS	0.540415973	0.012422134	0.65458718	NS
SHANK3	NS	0.540450389	0.012421079	0.65458718	NS
SARS2	NS	0.540756845	0.012411681	0.654917375	NS
MSR1	NS	0.540919408	0.012406697	0.65503582	NS
ERBIN	NS	0.540922326	0.012406608	0.65503582	NS
ADORA2B	NS	0.540962172	0.012405386	0.65504309	NS
CHCHD3	NS	0.541159786	-0.012399329	0.655241388	NS
ADHFE1	NS	0.541242655	-0.012396789	0.655300734	NS
HRC	NS	0.54136152	0.012393146	0.655379367	NS
CETN1	NS	0.54137532	0.012392724	0.655379367	NS
METTL25B	NS	0.541538181	-0.012387733	0.655535525	NS
VEGFA	NS	0.541694087	0.012382957	0.655683243	NS
SULT1C4	NS	0.542027251	0.012372752	0.656045489	NS
ZNF595	NS	0.542116359	-0.012370023	0.656112314	NS
MBD3	NS	0.542228006	-0.012366605	0.656165382	NS
RORC	NS	0.542211347	-0.012367115	0.656165382	NS
ADA2	NS	0.542293082	-0.012364612	0.656188327	NS
LZTS2	NS	0.542314769	-0.012363948	0.656188327	NS
EGFL7	NS	0.542382209	0.012361883	0.656228905	NS
RAD17	NS	0.54245486	0.012359659	0.656245458	NS
QTRT1	NS	0.542463697	-0.012359389	0.656245458	NS
R3HDML	NS	0.542520424	0.012357652	0.656273065	NS
MLH1	NS	0.542746598	0.01235073	0.656505634	NS
ASXL3	NS	0.542784625	0.012349566	0.656510605	NS
IRF2	NS	0.542873478	0.012346847	0.656577045	NS
FADS3	NS	0.543061113	-0.012341105	0.656762943	NS
FDXR	NS	0.543164652	0.012337938	0.656838192	NS
NETO2	NS	0.543191204	0.012337125	0.656838192	NS
POLL	NS	0.543322604	-0.012333106	0.656956042	NS
NAIP	NS	0.543522502	0.012326992	0.657156696	NS
ATF7	NS	0.54360522	0.012324462	0.657215655	NS
GTF2F1	NS	0.543677796	-0.012322243	0.65722849	NS
MAFB	NS	0.543683746	-0.012322061	0.65722849	NS
A3GALT2	NS	0.543828008	-0.01231765	0.657361826	NS
JMJD4	NS	0.54396207	-0.012313551	0.657441763	NS
C11orf1	NS	0.543951662	-0.012313869	0.657441763	NS
C1QTNF9	NS	0.54408079	-0.012309922	0.657544191	NS
MKLN1	NS	0.544165582	-0.01230733	0.657605607	NS
HS3ST2	NS	0.544357235	-0.012301473	0.657755081	NS
HPD	NS	0.544349451	-0.012301711	0.657755081	NS
USP21	NS	0.54445143	0.012298594	0.657786772	NS
H2BC10	NS	0.544442421	0.01229887	0.657786772	NS

CHRNA3	NS	0.544587852	0.012294426	0.657910527	NS
NUBP2	NS	0.544785101	0.0122884	0.658107747	NS
SLC25A23	NS	0.5451481	0.012277313	0.658500461	NS
POLE3	NS	0.545178233	0.012276393	0.658500461	NS
FKBP7	NS	0.545477001	-0.012267271	0.658820219	NS
REG1A	NS	0.545571076	0.012264399	0.658878362	NS
FAM236A	NS	0.545593221	0.012263723	0.658878362	NS
FYN	NS	0.545949137	0.01225286	0.659267047	NS
ZNF445	NS	0.546270553	0.012243053	0.659614026	NS
BRCA1	NS	0.546323611	-0.012241435	0.659636942	NS
LLGL1	NS	0.546364695	-0.012240182	0.659645399	NS
EIF6	NS	0.546569777	0.012233926	0.659825704	NS
C17orf100	NS	0.546582214	-0.012233547	0.659825704	NS
EFCAB3	NS	0.546658749	0.012231213	0.659876941	NS
CEP20	NS	0.546777024	-0.012227606	0.659978553	NS
CTSW	NS	0.546976484	-0.012221525	0.660178139	NS
TMEM50B	NS	0.547345257	-0.012210284	0.660458499	NS
IMMP1L	NS	0.547312761	0.012211274	0.660458499	NS
POLD4	NS	0.547264996	-0.01221273	0.660458499	NS
FCHSD2	NS	0.547566608	-0.012203538	0.660643226	NS
H2BC21	NS	0.54754053	-0.012204333	0.660643226	NS
OTOA	NS	0.547650678	0.012200977	0.660662295	NS
MC2R	NS	0.547638307	-0.012201354	0.660662295	NS
CYB5R3	NS	0.547695126	0.012199623	0.660674738	NS
PLAU	NS	0.547884794	0.012193845	0.660862347	NS
SRRM5	NS	0.54794449	-0.012192026	0.660893167	NS
COL16A1	NS	0.547987641	-0.012190712	0.660893492	NS
PRELID2	NS	0.548013048	0.012189938	0.660893492	NS
TSC22D2	NS	0.548313448	0.012180789	0.66117338	NS
SGSM1	NS	0.54842961	-0.012177252	0.661272256	NS
RAD21L1	NS	0.548490778	0.01217539	0.661304815	NS
FASTKD5	NS	0.548592999	-0.012172278	0.661386864	NS
CPA5	NS	0.548809435	0.01216569	0.661606591	NS
BCL2	NS	0.549197141	0.012153891	0.662032749	NS
TAS2R14	NS	0.549430218	0.012146801	0.662231228	NS
MTRNR2L6	NS	0.549404612	-0.012147579	0.662231228	NS
FMO3	NS	0.549656934	-0.012139905	0.662462352	NS
CASK	NS	0.549724649	-0.012137845	0.662462352	NS
NWD1	NS	0.549714155	0.012138164	0.662462352	NS
RNF217	NS	0.549762319	-0.0121367	0.662466504	NS
ST14	NS	0.549892605	0.012132738	0.662541006	NS
LDOC1	NS	0.549884376	0.012132988	0.662541006	NS
TAFA3	NS	0.550030432	-0.012128547	0.662665819	NS
ZNF598	NS	0.550082756	-0.012126956	0.662666397	NS
SOCS7	NS	0.550099383	0.012126451	0.662666397	NS
PIWIL4	NS	0.550142393	0.012125143	0.662676966	NS
RFPL1	NS	0.550342889	-0.012119049	0.662877223	NS
F5	NS	0.550403489	-0.012117207	0.662908962	NS
GDF15	NS	0.550498779	-0.012114311	0.662982477	NS
PHIP	NS	0.551023825	0.012098358	0.663573518	NS
OR2C3	NS	0.551083466	-0.012096546	0.663604054	NS
FAS	NS	0.551152365	-0.012094454	0.663622248	NS
DCUN1D3	NS	0.551229416	-0.012092113	0.663622248	NS
PPP1R26	NS	0.551179466	-0.012093631	0.663622248	NS
GGTLC3	NS	0.551235715	0.012091922	0.663622248	NS

SCN5A	NS	0.551373137	0.012087749	0.663746405	NS
TSGA10IP	NS	0.55149319	-0.012084103	0.663849638	NS
COPE	NS	0.551583556	0.01208136	0.66386103	NS
PATE2	NS	0.551605546	-0.012080692	0.66386103	NS
AKAP17A	NS	0.551580706	0.012081446	0.66386103	NS
SPO11	NS	0.552098994	0.012065714	0.664413587	NS
EXOSC7	NS	0.552362194	-0.012057727	0.664689003	NS
TBX5	NS	0.552884406	0.012041887	0.665276051	NS
MPZL1	NS	0.553009986	0.012038079	0.665385795	NS
AKT3	NS	0.553063474	-0.012036457	0.665408789	NS
PDZD2	NS	0.553184547	0.012032786	0.665513089	NS
PGA5	NS	0.553480006	0.01202383	0.66582716	NS
SLC16A7	NS	0.553760349	-0.012015333	0.666081613	NS
STARD9	NS	0.553758495	0.01201539	0.666081613	NS
COPRS	NS	0.553851716	-0.012012565	0.666150116	NS
RTCA	NS	0.554012022	-0.012007708	0.666218732	NS
KRT86	NS	0.553985086	0.012008524	0.666218732	NS
ZNF730	NS	0.553964354	0.012009152	0.666218732	NS
IKZF2	NS	0.554128308	0.012004185	0.666294199	NS
UBE2M	NS	0.554169717	-0.012002931	0.666294199	NS
DIPK1A	NS	0.554178049	-0.012002678	0.666294199	NS
MAP2K7	NS	0.554213529	-0.012001604	0.66629547	NS
SS18L2	NS	0.554270573	0.011999876	0.666322664	NS
NSD2	NS	0.554376887	-0.011996656	0.666405993	NS
SPOP	NS	0.554408747	-0.011995691	0.666405993	NS
VIPR1	NS	0.554549798	-0.011991419	0.666534146	NS
LAMA1	NS	0.554688151	0.01198723	0.66665904	NS
GRIP1	NS	0.554736819	-0.011985756	0.66667399	NS
TOPBP1	NS	0.55483433	0.011982804	0.666710535	NS
ZNF160	NS	0.555521471	-0.011962008	0.667494791	NS
SEH1L	NS	0.555829105	-0.011952702	0.667781525	NS
VASH2	NS	0.555823153	-0.011952882	0.667781525	NS
AP1G2	NS	0.555936427	0.011949456	0.66786901	NS
TAF6L	NS	0.556064693	0.011945576	0.667981642	NS
TPGS1	NS	0.556107348	-0.011944287	0.667991426	NS
FUT8	NS	0.556146599	-0.0119431	0.667997118	NS
ZNF471	NS	0.55625466	-0.011939832	0.668085455	NS
NAA25	NS	0.556391511	-0.011935695	0.6681935	NS
POU6F1	NS	0.556413662	-0.011935025	0.6681935	NS
DTWD2	NS	0.556521721	0.011931758	0.668281806	NS
LRRC37A2	NS	0.556689734	0.01192668	0.66844209	NS
RCAN2	NS	0.556802227	-0.01192328	0.668535693	NS
HS3ST3B1	NS	0.557004738	-0.01191716	0.66873736	NS
TATDN1	NS	0.55713758	0.011913146	0.668826098	NS
NEB	NS	0.557147758	-0.011912839	0.668826098	NS
CCDC62	NS	0.557293746	-0.011908428	0.66895986	NS
ZCCHC4	NS	0.557450908	-0.011903681	0.669107018	NS
SPATA4	NS	0.557632399	0.0118982	0.669283358	NS
DNAJB4	NS	0.557730517	-0.011895237	0.669359616	NS
SBF1	NS	0.557769325	0.011894065	0.669364688	NS
ARNTL2	NS	0.557993535	0.011887295	0.669592242	NS
SLC22A23	NS	0.558272917	0.011878861	0.66988597	NS
NCBP1	NS	0.5583112	0.011877706	0.669890379	NS
GALNT4	NS	0.558574422	0.011869762	0.670164664	NS
ETV1	NS	0.558669467	-0.011866894	0.670237152	NS

KIF1B	NS	0.558717204	0.011865454	0.670252879	NS
TMEM130	NS	0.559015563	0.011856453	0.670569239	NS
GALNT11	NS	0.559118972	-0.011853334	0.670651721	NS
STIM1	NS	0.559336282	-0.011846781	0.670870807	NS
NIP7	NS	0.559463467	-0.011842946	0.670940203	NS
SH3TC2	NS	0.55944196	0.011843594	0.670940203	NS
PISD	NS	0.559536982	-0.011840729	0.670986793	NS
AMER2	NS	0.55965899	-0.011837051	0.671091526	NS
TTC36	NS	0.559797002	0.011832891	0.67119329	NS
DNM3	NS	0.559813209	-0.011832402	0.67119329	NS
C12orf75	NS	0.55990674	-0.011829583	0.67126385	NS
PPP1R36	NS	0.560206562	-0.011820547	0.671581705	NS
PCP4L1	NS	0.560354239	0.011816098	0.671717139	NS
VRK2	NS	0.560779778	-0.011803279	0.672143996	NS
TNKS1BP1	NS	0.560772262	-0.011803506	0.672143996	NS
HSD17B3	NS	0.561019233	0.011796068	0.672389367	NS
TKFC	NS	0.561068677	0.011794579	0.672406991	NS
CCNE1	NS	0.561228083	0.01178978	0.672556388	NS
CXCL12	NS	0.561315511	-0.011787148	0.672619515	NS
NEU4	NS	0.561482597	-0.011782118	0.672778083	NS
ANKRD17	NS	0.561561808	0.011779734	0.672789697	NS
FOSL1	NS	0.561558345	0.011779839	0.672789697	NS
SCLY	NS	0.561738153	-0.011774427	0.672917669	NS
DCTN1	NS	0.561724212	-0.011774847	0.672917669	NS
SGCA	NS	0.561920182	-0.01176895	0.673094068	NS
NFATC1	NS	0.562044989	0.011765195	0.673201906	NS
HNF4A	NS	0.562102982	-0.01176345	0.673229708	NS
PLOD1	NS	0.562391306	-0.011754778	0.673533358	NS
IDNK	NS	0.562567215	-0.011749488	0.673629473	NS
VGLL2	NS	0.562575968	-0.011749224	0.673629473	NS
SLC35F6	NS	0.562572098	0.011749341	0.673629473	NS
OR10G2	NS	0.562777548	0.011743163	0.673829161	NS
RASA2	NS	0.562890415	-0.01173977	0.67388093	NS
KAT6B	NS	0.563005066	0.011736323	0.673976502	NS
FAM126B	NS	0.563127645	-0.011732639	0.674081553	NS
PARP1	NS	0.563531624	-0.011720499	0.674523413	NS
PCDH7	NS	0.563691245	0.011715703	0.674672751	NS
PPP1R37	NS	0.564039305	0.011705248	0.675047597	NS
CAPRIN2	NS	0.564129176	0.011702549	0.675113413	NS
PAICS	NS	0.564322368	-0.011696748	0.67530286	NS
DSTN	NS	0.564603038	-0.011688321	0.675596959	NS
SPP2	NS	0.564745793	0.011684036	0.675684237	NS
HDDC3	NS	0.564726954	-0.011684602	0.675684237	NS
NBPF20	NS	0.564820764	-0.011681786	0.675732166	NS
PLP1	NS	0.564867881	-0.011680372	0.675746767	NS
UQCR11	NS	0.565034252	-0.011675379	0.675904022	NS
EIF4G1	NS	0.565072522	0.011674231	0.67590803	NS
TRPC4AP	NS	0.565259231	-0.011668628	0.676006035	NS
TBC1D10B	NS	0.565226432	-0.011669613	0.676006035	NS
HNRNPCL4	NS	0.565237618	-0.011669277	0.676006035	NS
GIMAP8	NS	0.565386697	-0.011664804	0.6761167	NS
HSPE1-MOB4	NS	0.565578374	-0.011659055	0.676304133	NS
SCAF4	NS	0.565743156	0.011654113	0.676459385	NS
NUP160	NS	0.566198322	-0.011640465	0.676961808	NS
ASZ1	NS	0.566472239	-0.011632254	0.677247478	NS

PCDHA9	NS	0.56657341	0.011629222	0.677326596	NS
STRA8	NS	0.566739627	-0.011624241	0.677340494	NS
OCSTAMP	NS	0.566760004	-0.011623631	0.677340494	NS
C9orf43	NS	0.566701108	0.011625395	0.677340494	NS
SUMO4	NS	0.566758689	0.01162367	0.677340494	NS
PCDHA5	NS	0.566949512	-0.011617953	0.677525144	NS
NFYC	NS	0.567177594	0.01161112	0.677755865	NS
RAD1	NS	0.567248134	-0.011609007	0.67776401	NS
EID1	NS	0.567254441	-0.011608818	0.67776401	NS
HEATR6	NS	0.567540488	-0.011600252	0.677834448	NS
TGFBR1	NS	0.567348985	0.011605987	0.677834448	NS
FGFR1OP2	NS	0.567663587	0.011596566	0.677834448	NS
EXOSC8	NS	0.567577702	-0.011599138	0.677834448	NS
IGFBPL1	NS	0.567595549	-0.011598603	0.677834448	NS
MDH2	NS	0.567532881	-0.01160048	0.677834448	NS
PRDM14	NS	0.567620362	0.01159786	0.677834448	NS
CAPN2	NS	0.56750567	-0.011601295	0.677834448	NS
POLR2H	NS	0.567640465	-0.011597258	0.677834448	NS
WNK3	NS	0.56763849	0.011597317	0.677834448	NS
LCN2	NS	0.567738629	0.011594319	0.677882235	NS
C4BPB	NS	0.567807408	-0.01159226	0.677922538	NS
SPNS3	NS	0.567868021	-0.011590446	0.677953088	NS
RAPGEF5	NS	0.567991654	-0.011586745	0.678003875	NS
BTNL9	NS	0.567986501	0.011586899	0.678003875	NS
NME6	NS	0.568015646	0.011586027	0.678003875	NS
LYPD2	NS	0.568245221	-0.011579155	0.678236079	NS
PRB2	NS	0.568505138	-0.011571378	0.678504468	NS
IFFO1	NS	0.568692167	0.011565782	0.678643994	NS
GABRR2	NS	0.56865795	0.011566806	0.678643994	NS
NRF1	NS	0.569024763	0.011555834	0.678957176	NS
TUBD1	NS	0.56900344	-0.011556472	0.678957176	NS
FCGR3B	NS	0.56911065	-0.011553266	0.679017799	NS
RPS6	NS	0.569234247	0.01154957	0.679123403	NS
STING1	NS	0.569355162	-0.011545954	0.679225794	NS
CENPK	NS	0.56940428	0.011544486	0.679242527	NS
RTL3	NS	0.569558432	0.011539877	0.679384545	NS
SLC25A21	NS	0.569708213	0.0115354	0.679521331	NS
TMEM202	NS	0.569863329	0.011530764	0.679664465	NS
ITGAE	NS	0.56993065	0.011528752	0.679702876	NS
CABP1	NS	0.57004318	0.011525389	0.679795194	NS
C5AR2	NS	0.570171587	0.011521553	0.679891266	NS
AK3	NS	0.570196786	0.0115208	0.679891266	NS
SLC22A15	NS	0.570264244	-0.011518784	0.679891266	NS
USP17L4	NS	0.570243901	0.011519392	0.679891266	NS
SCAND1	NS	0.570362561	0.011515847	0.679966601	NS
PARP12	NS	0.570618444	0.011508204	0.680229759	NS
METTL3	NS	0.57071071	0.011505448	0.680297851	NS
ERI2	NS	0.570798494	-0.011502827	0.680360592	NS
REL	NS	0.571087053	0.011494211	0.680662623	NS
SAMD8	NS	0.571224265	0.011490114	0.680784242	NS
ATP6V0E1	NS	0.571304493	0.011487719	0.680837936	NS
DISC1	NS	0.571426562	-0.011484076	0.680941484	NS
C12orf43	NS	0.571462062	-0.011483016	0.680941866	NS
GDA	NS	0.571570953	0.011479766	0.681029693	NS
DEDD2	NS	0.571777511	0.011473603	0.681191943	NS

HASPIN	NS	0.571745379	-0.011474561	0.681191943	NS
SH3GLB2	NS	0.571925222	-0.011469195	0.681325984	NS
FSCB	NS	0.572100299	0.011463972	0.681492608	NS
PSME3IP1	NS	0.572141703	-0.011462737	0.681499988	NS
PRAMEF26	NS	0.572238447	0.011459852	0.681573281	NS
ZBTB5	NS	0.572284402	-0.011458481	0.681586075	NS
HSPA1L	NS	0.57239017	-0.011455327	0.681670099	NS
CCDC125	NS	0.572818057	0.011442568	0.682137709	NS
PAX1	NS	0.572924907	0.011439383	0.682222977	NS
KCTD5	NS	0.57303511	0.011436098	0.682312228	NS
MRPS18C	NS	0.573143448	-0.011432868	0.682399248	NS
COL5A1	NS	0.573184628	-0.011431641	0.682406303	NS
EPN1	NS	0.573326845	-0.011427403	0.682533637	NS
HLA-DMA	NS	0.573370454	0.011426103	0.682543574	NS
SPEN	NS	0.57364771	0.011417842	0.682789637	NS
LRP12	NS	0.573622035	0.011418607	0.682789637	NS
TTC14	NS	0.573744679	-0.011414953	0.682863064	NS
ABHD8	NS	0.57392096	-0.011409703	0.682988878	NS
SELL	NS	0.573896088	0.011410443	0.682988878	NS
SLC35F2	NS	0.574019892	0.011406756	0.683064616	NS
SCUBE2	NS	0.57408614	-0.011404783	0.683101452	NS
TEX264	NS	0.574324417	-0.011397687	0.683342969	NS
MKRN2	NS	0.574591523	0.011389735	0.683576738	NS
DNAAF1	NS	0.574576325	-0.011390187	0.683576738	NS
ZNF275	NS	0.575190993	0.011371894	0.684247856	NS
PHLDA2	NS	0.575292201	0.011368883	0.684326194	NS
RFT1	NS	0.575623924	0.011359015	0.684678711	NS
CAPS	NS	0.575698558	-0.011356795	0.684725408	NS
MORN1	NS	0.57583495	-0.011352738	0.684845548	NS
HECTD4	NS	0.575894052	0.011350981	0.684873757	NS
MSN	NS	0.576078365	0.0113455	0.685050859	NS
KIZ	NS	0.576302998	-0.011338822	0.685275884	NS
CC2D2A	NS	0.576498475	-0.011333012	0.685466213	NS
CHAT	NS	0.576536428	0.011331884	0.685469232	NS
MSANTD5	NS	0.577089625	0.011315445	0.68608481	NS
DAZAP2	NS	0.577557091	0.011301561	0.686598394	NS
CTNNBL1	NS	0.577801577	0.011294301	0.686846854	NS
ATP5PB	NS	0.577855338	0.011292705	0.686868577	NS
AAMP	NS	0.578227249	-0.011281665	0.687268445	NS
PRM3	NS	0.578299146	-0.011279531	0.687311696	NS
LPCAT2	NS	0.578412283	-0.011276173	0.68731955	NS
TARDBP	NS	0.578400205	-0.011276531	0.68731955	NS
MIS18BP1	NS	0.578393493	0.011276731	0.68731955	NS
DDOST	NS	0.578521477	0.011272933	0.687407104	NS
LRRC71	NS	0.57859859	0.011270645	0.687456528	NS
TSC2	NS	0.578951754	0.011260167	0.687820854	NS
ISG15	NS	0.579011831	0.011258385	0.687820854	NS
C5orf58	NS	0.578987911	0.011259095	0.687820854	NS
IFI16	NS	0.579219259	0.011252233	0.687982817	NS
RNF186	NS	0.579213319	0.011252409	0.687982817	NS
FAM193B	NS	0.579367102	0.011247849	0.688116195	NS
PLA2G2A	NS	0.579591851	-0.011241185	0.688340892	NS
ZNF821	NS	0.579792255	0.011235244	0.688536652	NS
SUGT1	NS	0.579964364	0.011230142	0.688698788	NS
ZRANB2	NS	0.58007069	-0.011226991	0.688782792	NS

TOMM40	NS	0.580597431	0.011211384	0.689365959	NS
SSBP1	NS	0.580684176	-0.011208814	0.689426664	NS
SPAG7	NS	0.580872047	0.01120325	0.689561698	NS
RPGRIP1	NS	0.580853485	-0.011203799	0.689561698	NS
NDUFAF5	NS	0.580904786	0.01120228	0.689561698	NS
F8	NS	0.580979422	0.01120007	0.689608002	NS
ELAC2	NS	0.581098211	-0.011196552	0.68963687	NS
AMMECR1L	NS	0.581062507	-0.011197609	0.68963687	NS
RAVER2	NS	0.581178659	-0.01119417	0.68963687	NS
GOLGA8R	NS	0.581150146	0.011195014	0.68963687	NS
DENND1C	NS	0.581181888	-0.011194074	0.68963687	NS
ATXN10	NS	0.581264614	-0.011191625	0.689692752	NS
GNAQ	NS	0.581515341	-0.011184202	0.689947955	NS
BAP1	NS	0.581723109	-0.011178052	0.69015216	NS
HEATR9	NS	0.581911228	0.011172485	0.690333031	NS
USP39	NS	0.582114673	0.011166465	0.690532059	NS
FBN1	NS	0.582179399	-0.01116455	0.690566519	NS
KCNA5	NS	0.582566887	0.011153088	0.690941462	NS
HABP4	NS	0.582559245	0.011153314	0.690941462	NS
DLG5	NS	0.582611024	-0.011151782	0.690951472	NS
OSCP1	NS	0.582773417	-0.01114698	0.691059381	NS
SFXN5	NS	0.582754745	0.011147532	0.691059381	NS
HDAC7	NS	0.58306513	-0.011138354	0.691362941	NS
SATB1	NS	0.583569569	0.011123443	0.691918688	NS
KRTAP4-3	NS	0.583622921	-0.011121867	0.69193956	NS
HHLA1	NS	0.583763492	0.011117713	0.692063829	NS
SEMA7A	NS	0.583970496	0.011111597	0.692266838	NS
CEP89	NS	0.584045563	-0.011109379	0.692313425	NS
ASMTL	NS	0.584092242	0.011108	0.692326359	NS
CDKL5	NS	0.584252606	-0.011103263	0.692474034	NS
MBP	NS	0.584573142	-0.011093796	0.692811519	NS
LPGAT1	NS	0.584869736	0.011085039	0.693120591	NS
MXRA5	NS	0.58492835	-0.011083309	0.693147615	NS
ANKRD18A	NS	0.584991758	0.011081437	0.693180316	NS
KIAA1549	NS	0.585070518	-0.011079112	0.693231203	NS
NUP88	NS	0.585189337	-0.011075605	0.693329547	NS
SMAP1	NS	0.58531461	-0.011071908	0.693393083	NS
CCHCR1	NS	0.585289238	0.011072656	0.693393083	NS
DAZAP1	NS	0.585664384	0.011061587	0.693609436	NS
UCHL5	NS	0.585610484	0.011063177	0.693609436	NS
SLC2A4	NS	0.585676411	-0.011061232	0.693609436	NS
PSG1	NS	0.585600507	-0.011063471	0.693609436	NS
TSNARE1	NS	0.585779787	-0.011058182	0.693689419	NS
NUDT22	NS	0.586310174	-0.011042538	0.694275036	NS
AOC3	NS	0.586363522	-0.011040965	0.694295732	NS
NAA38	NS	0.586412318	0.011039526	0.694311037	NS
SEPTIN3	NS	0.586724822	0.011030313	0.694596065	NS
MED16	NS	0.586705324	-0.011030887	0.694596065	NS
HOXB13	NS	0.586770959	0.011028953	0.694608201	NS
POU6F2	NS	0.586858478	0.011026373	0.694626839	NS
PIK3R4	NS	0.586852945	0.011026536	0.694626839	NS
KRTAP9-2	NS	0.586936789	-0.011024065	0.69467705	NS
RAPGEF6	NS	0.587006773	-0.011022002	0.694717402	NS
MCTP1	NS	0.587050799	0.011020704	0.694727027	NS
SHTN1	NS	0.587154856	-0.011017638	0.694807691	NS

ZMIZ2	NS	0.58723803	-0.011015187	0.694863633	NS
FAT3	NS	0.58754006	-0.011006288	0.69517852	NS
MAFIP	NS	0.587726004	0.01100081	0.695356022	NS
HSP90AA1	NS	0.58784688	0.01099725	0.695371522	NS
SERBP1	NS	0.587823908	0.010997926	0.695371522	NS
TMEM72	NS	0.587810915	-0.010998309	0.695371522	NS
SCGB2B2	NS	0.588088357	-0.010990138	0.695614657	NS
GNAO1	NS	0.588162915	0.010987943	0.695660336	NS
CARD9	NS	0.588307824	-0.010983676	0.695789212	NS
MBTPS2	NS	0.588364541	-0.010982006	0.695813775	NS
PPP4R2	NS	0.588479493	0.010978622	0.695907201	NS
CRY2	NS	0.588655937	0.010973428	0.696073329	NS
CLDN11	NS	0.588821587	-0.010968553	0.696141622	NS
BBX	NS	0.588818223	0.010968652	0.696141622	NS
TBC1D3B	NS	0.588766194	0.010970183	0.696141622	NS
ORMDL1	NS	0.588951271	0.010964736	0.696209893	NS
SNTG2	NS	0.588942282	0.010965001	0.696209893	NS
CCDC50	NS	0.589012843	0.010962924	0.696240158	NS
ETFA	NS	0.589360302	-0.010952701	0.69660833	NS
COPS3	NS	0.589423287	0.010950848	0.696640235	NS
NDUFS7	NS	0.589470361	-0.010949464	0.696653334	NS
LINC02203	NS	0.589731603	0.01094178	0.696919525	NS
ITGA3	NS	0.590815892	0.010909905	0.698036894	NS
CST3	NS	0.59082137	-0.010909744	0.698036894	NS
SNX21	NS	0.590752829	0.010911758	0.698036894	NS
MGAT2	NS	0.590767353	-0.010911331	0.698036894	NS
ERCC3	NS	0.591049861	0.01090303	0.698264227	NS
ATP10D	NS	0.591108012	-0.010901322	0.698290307	NS
POLD3	NS	0.59124195	-0.010897387	0.698405907	NS
SNX27	NS	0.59132294	0.010895008	0.698458951	NS
NPIPA9	NS	0.591359396	-0.010893937	0.698459389	NS
TMEM108	NS	0.591423233	0.010892062	0.698492165	NS
RAB3GAP2	NS	0.591629295	0.010886011	0.698650273	NS
STAT6	NS	0.591697033	0.010884021	0.698687639	NS
VWA8	NS	0.591872131	-0.01087888	0.698851764	NS
RHBDF1	NS	0.592494592	0.010860609	0.699530483	NS
LHFPL2	NS	0.592519232	0.010859885	0.699530483	NS
SNRPB	NS	0.592580288	0.010858094	0.699559896	NS
ARHGAP4	NS	0.592717787	0.010854059	0.699670051	NS
TSPAN18	NS	0.592745893	-0.010853234	0.699670051	NS
P4HA2	NS	0.592810221	0.010851347	0.699703313	NS
CYP24A1	NS	0.592891898	-0.010848951	0.699757047	NS
CTDSP2	NS	0.593054135	0.010844191	0.699863781	NS
INSYN2B	NS	0.593054647	-0.010844176	0.699863781	NS
SEMA4F	NS	0.593127648	-0.010842035	0.699907258	NS
SATB2	NS	0.593292769	0.010837192	0.700059427	NS
MAK	NS	0.593799096	0.010822345	0.700614161	NS
AKR1A1	NS	0.593873449	0.010820165	0.700616473	NS
SKIL	NS	0.593865458	0.0108204	0.700616473	NS
GEMIN8	NS	0.593950387	0.01081791	0.700621835	NS
TADA2A	NS	0.593944169	-0.010818092	0.700621835	NS
LRR1	NS	0.594105897	0.010813352	0.700756565	NS
OR11G2	NS	0.594137011	-0.01081244	0.700756565	NS
NCKAP1	NS	0.594220755	0.010809985	0.700770271	NS
TLN1	NS	0.59422104	-0.010809977	0.700770271	NS

ORC4	NS	0.594521333	-0.010801177	0.701081694	NS
MBNL1	NS	0.594603507	0.010798769	0.701135881	NS
RBP3	NS	0.595102409	0.010784155	0.701681422	NS
RNF39	NS	0.59526892	0.010779279	0.701835001	NS
SNX4	NS	0.595544159	-0.010771219	0.702086524	NS
SFTPA1	NS	0.595591048	0.010769846	0.702086524	NS
LARP1B	NS	0.595663613	0.010767722	0.702086524	NS
HR	NS	0.595637437	-0.010768488	0.702086524	NS
NDUFA7	NS	0.595653016	0.010768032	0.702086524	NS
SULT1A1	NS	0.595852095	-0.010762205	0.702265917	NS
LARP1	NS	0.596020489	0.010757276	0.702421614	NS
HMGN3	NS	0.596171554	-0.010752855	0.70255687	NS
STRN	NS	0.59634316	-0.010747833	0.702629359	NS
FASTKD3	NS	0.596329186	-0.010748242	0.702629359	NS
TBC1D16	NS	0.596351063	-0.010747602	0.702629359	NS
TMEM170B	NS	0.596378267	0.010746806	0.702629359	NS
SMARCAL1	NS	0.59649417	0.010743415	0.702723138	NS
ATXN2L	NS	0.597058118	0.010726919	0.70334471	NS
ACBD6	NS	0.5972051	-0.010722621	0.703475044	NS
RAB5B	NS	0.597246974	-0.010721397	0.703481557	NS
H4C8	NS	0.597338277	-0.010718727	0.703546288	NS
GFER	NS	0.597566261	0.010712062	0.703694328	NS
TTC6	NS	0.597609391	-0.010710801	0.703694328	NS
FAM98B	NS	0.597509453	0.010713723	0.703694328	NS
ITSN1	NS	0.597605735	0.010710908	0.703694328	NS
SPANXC	NS	0.597771053	-0.010706076	0.70384187	NS
ELP2	NS	0.59802946	-0.010698524	0.704103299	NS
SRSF8	NS	0.598230413	-0.010692652	0.704297054	NS
SLC6A7	NS	0.598333684	-0.010689635	0.704332956	NS
RMND5B	NS	0.59832474	0.010689896	0.704332956	NS
NBPF15	NS	0.598445871	-0.010686357	0.704422177	NS
PREPL	NS	0.598637924	-0.010680747	0.704605391	NS
MPP4	NS	0.598695863	0.010679055	0.704630738	NS
TMBIM6	NS	0.59907614	-0.01066795	0.705035433	NS
AOC1	NS	0.599191964	-0.010664568	0.705075182	NS
EXD2	NS	0.599158288	-0.010665551	0.705075182	NS
TAF11L3	NS	0.599219196	0.010663773	0.705075182	NS
SMARCD2	NS	0.599688854	-0.010650063	0.705583808	NS
ARHGAP31	NS	0.599776001	0.01064752	0.705600965	NS
SCAF11	NS	0.599811856	-0.010646473	0.705600965	NS
NUMBL	NS	0.599878669	0.010644524	0.705636677	NS
CCDC197	NS	0.600122993	0.010637394	0.705881178	NS
RPS4X	NS	0.600294007	-0.010632405	0.706039423	NS
CACNA1E	NS	0.600460399	0.010627552	0.706192215	NS
MPP7	NS	0.600529709	0.01062553	0.706230818	NS
CBLN4	NS	0.600664633	0.010621595	0.706346576	NS
SPATA2L	NS	0.600831754	-0.010616721	0.706457261	NS
DEFB134	NS	0.600824014	-0.010616947	0.706457261	NS
MAST3	NS	0.600961434	-0.01061294	0.706566817	NS
ETV2	NS	0.601034595	-0.010610807	0.70660069	NS
ELP6	NS	0.601063255	-0.010609971	0.70660069	NS
MORC3	NS	0.60118031	-0.010606558	0.706695377	NS
UBAC1	NS	0.601349979	0.010601612	0.706851898	NS
EML1	NS	0.6014091	0.010599889	0.706878464	NS
PDE6B	NS	0.601522043	-0.010596597	0.706968283	NS

IK	NS	0.601559857	0.010595495	0.706969799	NS
PLXNA2	NS	0.601643835	-0.010593047	0.707025565	NS
NGDN	NS	0.601700742	0.010591389	0.707032529	NS
MGMT	NS	0.601722817	-0.010590746	0.707032529	NS
NDUFA1	NS	0.601917257	-0.01058508	0.707218067	NS
BLCAP	NS	0.602075604	0.010580467	0.707342085	NS
CTSF	NS	0.602095898	0.010579875	0.707342085	NS
FBXO10	NS	0.602167298	-0.010577795	0.707383032	NS
TMEM220	NS	0.602508534	0.010567856	0.707697991	NS
TYW1B	NS	0.602475028	-0.010568832	0.707697991	NS
COL4A3	NS	0.602550478	-0.010566634	0.707704312	NS
DRC3	NS	0.602598447	-0.010565237	0.707717708	NS
PBX4	NS	0.602717087	-0.010561782	0.707814096	NS
DMTF1	NS	0.60278662	0.010559758	0.707816002	NS
ADCK5	NS	0.602791846	0.010559606	0.707816002	NS
SUSD6	NS	0.602941805	-0.010555239	0.707949141	NS
DCDC2C	NS	0.602988396	-0.010553883	0.707960899	NS
TTBK1	NS	0.60309665	-0.010550731	0.708045051	NS
NHLRC2	NS	0.603200547	-0.010547707	0.708124078	NS
RPLP0	NS	0.603406563	-0.01054171	0.708322969	NS
NEDD4	NS	0.603617727	0.010535565	0.708527879	NS
ANKLE1	NS	0.603740349	-0.010531997	0.708587575	NS
GCNT4	NS	0.6037418	-0.010531955	0.708587575	NS
OR2F2	NS	0.603812424	-0.0105299	0.708627496	NS
FAT4	NS	0.603985433	0.010524866	0.708787562	NS
KIF21B	NS	0.604345159	-0.010514403	0.709123721	NS
CHRNB4	NS	0.604771995	0.01050199	0.709390235	NS
HOXC13	NS	0.604727671	0.010503279	0.709390235	NS
HNRNPA1L2	NS	0.604797602	0.010501246	0.709390235	NS
MIGA2	NS	0.60490214	0.010498207	0.709390235	NS
CAPN5	NS	0.604656286	-0.010505355	0.709390235	NS
CNKSR2	NS	0.604889842	0.010498564	0.709390235	NS
CTHRC1	NS	0.604754528	-0.010502498	0.709390235	NS
AGAP4	NS	0.604865286	0.010499278	0.709390235	NS
WDHD1	NS	0.6048524	0.010499653	0.709390235	NS
DGCR2	NS	0.605126521	0.010491684	0.709524408	NS
SLC36A2	NS	0.605109917	0.010492167	0.709524408	NS
FAM120AOS	NS	0.605096415	0.010492559	0.709524408	NS
OSBPL11	NS	0.605238253	0.010488436	0.709612431	NS
SCARA5	NS	0.60539921	0.010483758	0.709758153	NS
WNT9B	NS	0.605484966	0.010481266	0.709815699	NS
TMEM14B	NS	0.605568712	0.010478833	0.709827892	NS
LCE1F	NS	0.605535145	0.010479808	0.709827892	NS
ERVV-1	NS	0.605735509	0.010473986	0.709980411	NS
MAST1	NS	0.60605198	0.010464792	0.710308333	NS
SHANK2	NS	0.60625576	0.010458874	0.710461131	NS
ZNF17	NS	0.606480641	-0.010452343	0.710681638	NS
GLE1	NS	0.60656252	-0.010449965	0.710734556	NS
ENGASE	NS	0.606608758	-0.010448623	0.710745709	NS
CREG1	NS	0.606834187	0.010442078	0.710966802	NS
PGBD5	NS	0.606928032	-0.010439354	0.711033712	NS
DNAAF10	NS	0.606965261	0.010438273	0.711034291	NS
ZBP1	NS	0.607086556	0.010434752	0.711133344	NS
GALNT7	NS	0.607130952	0.010433463	0.711142312	NS
SEC14L5	NS	0.607349782	-0.010427113	0.711269505	NS

ABLIM3	NS	0.607332569	0.010427612	0.711269505	NS
OR7E24	NS	0.607314489	0.010428137	0.711269505	NS
CD226	NS	0.607445035	0.010424348	0.711318305	NS
SPANXD	NS	0.607464951	0.010423771	0.711318305	NS
SIKE1	NS	0.607709254	-0.010416682	0.71147525	NS
NDFIP1	NS	0.607702008	-0.010416892	0.71147525	NS
ZNF487	NS	0.607666378	0.010417926	0.71147525	NS
GPR37L1	NS	0.607940372	0.010409977	0.711702784	NS
EXOSC2	NS	0.608031309	-0.01040734	0.711766194	NS
OAS2	NS	0.608505789	0.01039358	0.712250878	NS
SIGLEC1	NS	0.608737435	-0.010386864	0.712420448	NS
CLCN5	NS	0.608861271	0.010383274	0.712522295	NS
KIAA0586	NS	0.608999966	-0.010379254	0.712641518	NS
MYH14	NS	0.609156139	0.010374727	0.712776514	NS
HNRNPH1	NS	0.609188978	0.010373775	0.712776514	NS
MRPL49	NS	0.609307153	-0.010370351	0.712871691	NS
RALGPS2	NS	0.609407034	0.010367457	0.712945455	NS
LYRM4	NS	0.609610828	-0.010361552	0.71314077	NS
TTLL13P	NS	0.609652237	0.010360352	0.713146111	NS
GPRIN3	NS	0.609729702	0.010358108	0.713150529	NS
OR2W1	NS	0.60972569	-0.010358224	0.713150529	NS
MNDA	NS	0.609779035	-0.010356679	0.713165136	NS
LRRTM1	NS	0.610407026	-0.010338491	0.713856468	NS
ATP1B1	NS	0.610473469	0.010336567	0.713878877	NS
MEIOC	NS	0.610499951	-0.010335801	0.713878877	NS
PCYOX1L	NS	0.61055247	0.01033428	0.713897162	NS
CDH17	NS	0.61062096	-0.010332297	0.713934116	NS
RBM26	NS	0.610705231	-0.010329858	0.713989518	NS
UAP1L1	NS	0.610762995	0.010328186	0.714013924	NS
CD180	NS	0.610805717	-0.010326949	0.714020742	NS
TNFRSF10A	NS	0.611004436	-0.010321197	0.714209908	NS
PDCD6	NS	0.61109078	-0.010318698	0.714267702	NS
ENDOU	NS	0.611236018	-0.010314495	0.714394322	NS
IFT52	NS	0.611341868	0.010311432	0.714431757	NS
BMPER	NS	0.61133774	-0.010311552	0.714431757	NS
PSMD6	NS	0.611382507	-0.010310256	0.714436115	NS
PEX1	NS	0.6114313	-0.010308844	0.714449999	NS
ZMYND19	NS	0.611691624	-0.010301313	0.714711039	NS
ARL5C	NS	0.611760569	-0.010299319	0.71474845	NS
ANXA8	NS	0.611833113	0.01029722	0.714790062	NS
ACRBP	NS	0.611911953	0.01029494	0.714839022	NS
N4BP2	NS	0.611988949	0.010292713	0.714885823	NS
SLC36A4	NS	0.612469875	0.010278806	0.715361266	NS
ETFRF1	NS	0.612649793	0.010273605	0.715528233	NS
ACSBG2	NS	0.612835019	-0.01026825	0.715658198	NS
TMEM262	NS	0.612816646	-0.010268782	0.715658198	NS
SNX18	NS	0.613157412	0.010258933	0.715991486	NS
PTDSS1	NS	0.613213161	0.010257322	0.716013388	NS
FAM149A	NS	0.613675905	-0.010243953	0.716467267	NS
MUC4	NS	0.613673458	-0.010244023	0.716467267	NS
ARSG	NS	0.613760695	-0.010241503	0.716518734	NS
PAQR6	NS	0.61379405	0.01024054	0.716518734	NS
LIMS1	NS	0.613868059	0.010238402	0.716518734	NS
TMPRSS12	NS	0.613844007	0.010239097	0.716518734	NS
KIF26B	NS	0.613941711	0.010236275	0.71656149	NS

SCGB3A1	NS	0.613988181	0.010234933	0.716572519	NS
LPAR5	NS	0.614339474	-0.010224789	0.716939278	NS
RAE1	NS	0.61438487	0.010223478	0.716949029	NS
CCDC63	NS	0.614465235	0.010221158	0.716999583	NS
GSPT1	NS	0.614575282	0.010217981	0.717084765	NS
PSMD5	NS	0.615139757	-0.010201689	0.717570369	NS
SIGLEC9	NS	0.61510913	0.010202573	0.717570369	NS
PI15	NS	0.615062475	-0.010203919	0.717570369	NS
SERPINA1	NS	0.61511354	-0.010202446	0.717570369	NS
UBE2H	NS	0.615208612	0.010199702	0.717607442	NS
VCX3A	NS	0.615456247	-0.010192557	0.717853035	NS
COX6B1	NS	0.615538178	0.010190194	0.717862082	NS
HARBI1	NS	0.615526644	0.010190526	0.717862082	NS
RSAD2	NS	0.615638554	0.010187298	0.717935886	NS
SIX1	NS	0.615895801	-0.010179878	0.71819261	NS
PLPP5	NS	0.616271243	0.010169052	0.718557167	NS
ZNF518A	NS	0.616282678	0.010168722	0.718557167	NS
TMPRSS7	NS	0.616442822	0.010164105	0.718700595	NS
PPP1R32	NS	0.616684824	-0.010157128	0.718939436	NS
TADA1	NS	0.616755176	0.010155101	0.71894792	NS
SLC34A2	NS	0.616874048	-0.010151675	0.719030115	NS
SLC6A14	NS	0.616923862	-0.010150239	0.719044876	NS
OR10H4	NS	0.617136065	-0.010144124	0.719248896	NS
OR5M10	NS	0.617234289	0.010141293	0.719320061	NS
ZNF573	NS	0.617350661	0.01013794	0.719412366	NS
GEN1	NS	0.61739522	-0.010136657	0.719420979	NS
TBCK	NS	0.617450552	0.010135063	0.719442144	NS
COX15	NS	0.617633613	-0.010129789	0.719490971	NS
HSPA5	NS	0.617752658	-0.01012636	0.719490971	NS
KANK1	NS	0.617539411	0.010132503	0.719490971	NS
ZNF557	NS	0.61770612	0.010127701	0.719490971	NS
NFIL3	NS	0.617743795	0.010126615	0.719490971	NS
CLEC4C	NS	0.617574235	0.0101315	0.719490971	NS
TCP10L	NS	0.617715799	-0.010127422	0.719490971	NS
DNAJC5B	NS	0.617851511	-0.010123513	0.719562807	NS
ANKS6	NS	0.618013305	0.010118853	0.719621339	NS
FUNDC2	NS	0.618006656	-0.010119045	0.719621339	NS
L36A-HNRNI	NS	0.61795528	-0.010120524	0.719621339	NS
TMEM41A	NS	0.61819554	0.010113606	0.719790235	NS
ECD	NS	0.618243254	0.010112232	0.719802492	NS
POLE2	NS	0.618486356	-0.010105233	0.720042218	NS
RNF34	NS	0.618943842	0.010092065	0.720494849	NS
ANGPTL5	NS	0.618949595	0.0100919	0.720494849	NS
DCBLD1	NS	0.618987826	0.010090799	0.720496023	NS
SAMD14	NS	0.619521046	0.010075457	0.721073323	NS
DTNA	NS	0.619655287	-0.010071596	0.721142842	NS
HDGF	NS	0.619648803	0.010071782	0.721142842	NS
MRO	NS	0.619764647	0.01006845	0.721213656	NS
MAPK12	NS	0.619790657	0.010067702	0.721213656	NS
SREBF2	NS	0.620100204	0.0100588	0.721530481	NS
RFTN1	NS	0.620227794	0.010055131	0.72163556	NS
UGT3A2	NS	0.620359604	0.010051342	0.721745537	NS
CLIC6	NS	0.620399061	0.010050207	0.721748061	NS
SIMC1	NS	0.620651105	0.010042962	0.721997884	NS
TRIM72	NS	0.620727692	0.010040761	0.722043583	NS

OGA	NS	0.620780913	-0.010039231	0.722062097	NS
FAM72B	NS	0.620859202	0.010036981	0.722109766	NS
OR6X1	NS	0.620973967	-0.010033683	0.722199851	NS
MROH1	NS	0.621142592	-0.010028837	0.72235256	NS
BFSP1	NS	0.621258998	0.010025492	0.722444528	NS
TRERF1	NS	0.62131138	0.010023987	0.722462037	NS
POLA2	NS	0.621822278	-0.010009311	0.723012676	NS
MCMDC2	NS	0.621911762	-0.010006741	0.723073286	NS
CDX4	NS	0.622070736	0.010002176	0.723214677	NS
CORO1C	NS	0.622153895	0.009999788	0.723267915	NS
FAM86B2	NS	0.622316806	-0.009995111	0.723413855	NS
GJA1	NS	0.622505175	-0.009989703	0.723589369	NS
NETO1	NS	0.622637755	0.009985897	0.723700017	NS
GSTO2	NS	0.622753331	0.00998258	0.723727325	NS
CYP2C9	NS	0.62277342	0.009982003	0.723727325	NS
DYNLT3	NS	0.622767886	0.009982162	0.723727325	NS
FGF16	NS	0.623001946	0.009975445	0.723905971	NS
ORAI1	NS	0.622970431	0.009976349	0.723905971	NS
PRKCB	NS	0.62307523	0.009973342	0.723947665	NS
INKA2	NS	0.623211989	0.009969417	0.724019642	NS
VSTM5	NS	0.623183633	0.009970231	0.724019642	NS
USP10	NS	0.623836405	0.009951505	0.724701563	NS
IMPG2	NS	0.624086056	-0.009944346	0.724904567	NS
GJB6	NS	0.624261692	0.009939309	0.72502156	NS
CBWD2	NS	0.6242257	-0.009940341	0.72502156	NS
FAM184B	NS	0.624304151	-0.009938092	0.725027368	NS
ITGA8	NS	0.624393087	0.009935542	0.72504365	NS
TMA7	NS	0.624388097	-0.009935685	0.72504365	NS
FAH	NS	0.624535405	0.009931462	0.725138904	NS
DNAJB14	NS	0.624550045	0.009931043	0.725138904	NS
FTL	NS	0.624783908	-0.009924339	0.725359249	NS
ESM1	NS	0.624814774	-0.009923454	0.725359249	NS
IGFBP7	NS	0.625150334	-0.009913838	0.725705281	NS
DYM	NS	0.625207012	-0.009912214	0.725727552	NS
C2orf68	NS	0.62540714	0.00990648	0.725916323	NS
OR51D1	NS	0.625519406	0.009903264	0.726003096	NS
ABAT	NS	0.625654585	-0.009899392	0.72611645	NS
STARD5	NS	0.625888181	-0.009892701	0.726344004	NS
ETV3	NS	0.625993133	0.009889695	0.726422247	NS
B9D1	NS	0.626147501	-0.009885275	0.726557822	NS
GPR61	NS	0.626263853	-0.009881943	0.726649271	NS
UBE2Z	NS	0.626356809	0.009879282	0.726713564	NS
MFSD1	NS	0.626417491	-0.009877544	0.726740407	NS
FAM117A	NS	0.626573352	-0.009873082	0.726877663	NS
UBE2F-SCLY	NS	0.626764186	-0.00986762	0.72705547	NS
SLC6A5	NS	0.626983481	0.009861344	0.72726627	NS
CRYBG1	NS	0.627034782	-0.009859875	0.727282193	NS
TBC1D13	NS	0.627369793	-0.009850289	0.727627163	NS
TBX21	NS	0.627552619	-0.009845059	0.727751991	NS
AJUBA	NS	0.627546501	0.009845234	0.727751991	NS
ZNF93	NS	0.627674503	-0.009841572	0.727849729	NS
NDE1	NS	0.628062918	-0.009830463	0.728095799	NS
EPPIN	NS	0.628105308	-0.009829251	0.728095799	NS
GPN2	NS	0.628122241	-0.009828767	0.728095799	NS
TMEM45B	NS	0.628177172	0.009827196	0.728095799	NS

NEK10	NS	0.62799111	0.009832517	0.728095799	NS
OR9G4	NS	0.628048679	0.00983087	0.728095799	NS
ZNF587	NS	0.628187634	-0.009826897	0.728095799	NS
C4orf51	NS	0.628123575	-0.009828729	0.728095799	NS
IL15RA	NS	0.628364652	0.009821835	0.72821376	NS
OAZ2	NS	0.628329283	0.009822847	0.72821376	NS
TSKS	NS	0.628525321	-0.009817242	0.728356349	NS
VDAC3	NS	0.628572149	-0.009815903	0.728367009	NS
PPRC1	NS	0.628646191	0.009813787	0.728409199	NS
CLPTM1	NS	0.628726879	0.00981148	0.728459085	NS
FPR2	NS	0.62894305	0.009805302	0.728665929	NS
ONECUT1	NS	0.629033264	0.009802723	0.728726829	NS
JAK1	NS	0.62920946	0.009797688	0.728887324	NS
GATA4	NS	0.629366027	0.009793215	0.729025064	NS
BEST3	NS	0.629486083	-0.009789785	0.729120497	NS
TRPM7	NS	0.629615569	0.009786085	0.72922684	NS
ARL17B	NS	0.629686805	0.00978405	0.729265709	NS
MTBP	NS	0.629738381	-0.009782577	0.729281806	NS
RBFOX1	NS	0.629890327	0.009778237	0.729403075	NS
SMPD3	NS	0.629993832	-0.009775281	0.729403075	NS
DET1	NS	0.629990824	-0.009775367	0.729403075	NS
MIOS	NS	0.629960013	0.009776247	0.729403075	NS
USP28	NS	0.6301165	-0.009771778	0.729457834	NS
HPCAL1	NS	0.630093917	0.009772423	0.729457834	NS
LILRB1	NS	0.630287997	0.00976688	0.729531167	NS
N4BP2L1	NS	0.630292917	-0.00976674	0.729531167	NS
ABCG4	NS	0.630253311	-0.009767871	0.729531167	NS
VSTM1	NS	0.630352539	0.009765038	0.72955655	NS
MARF1	NS	0.630575159	-0.009758682	0.72977057	NS
ZNF230	NS	0.630653145	0.009756455	0.729817187	NS
RNF146	NS	0.6309459	-0.009748099	0.730068677	NS
DDX3Y	NS	0.631246349	0.009739525	0.73032901	NS
PPIL3	NS	0.631240956	0.009739678	0.73032901	NS
RAB35	NS	0.631415444	0.0097347	0.730480983	NS
HYAL3	NS	0.631465665	0.009733267	0.730495422	NS
ARNT	NS	0.63166058	0.009727706	0.730633572	NS
APIP	NS	0.63162872	-0.009728615	0.730633572	NS
ELAPOR1	NS	0.6317762	0.009724408	0.730680809	NS
DDX54	NS	0.631776918	-0.009724387	0.730680809	NS
TM6SF1	NS	0.631946837	-0.009719541	0.730833661	NS
RFX7	NS	0.632017598	0.009717522	0.730871826	NS
TUBA3D	NS	0.632135903	-0.009714148	0.730964964	NS
TRAF3IP2	NS	0.632336969	-0.009708415	0.731153786	NS
ASH2L	NS	0.632493663	-0.009703947	0.731291282	NS
HSD17B2	NS	0.632614065	-0.009700514	0.731386802	NS
ADAMTS18	NS	0.632794527	-0.00969537	0.731551745	NS
TMEM106B	NS	0.632990732	0.009689777	0.731647475	NS
MNS1	NS	0.63297136	-0.009690329	0.731647475	NS
ATP2A2	NS	0.632939498	0.009691237	0.731647475	NS
ADCY7	NS	0.633427294	0.009677336	0.73210836	NS
UQCRC2	NS	0.633509683	0.009674988	0.732159865	NS
COL15A1	NS	0.633625888	-0.009671678	0.732250444	NS
OTULINL	NS	0.633914362	-0.00966346	0.732540083	NS
HTRA1	NS	0.634218932	0.009654785	0.732842571	NS
PLP2	NS	0.634349755	-0.00965106	0.732911947	NS

NELFCD	NS	0.634695549	-0.009641214	0.733223936	NS
EHBP1L1	NS	0.63467138	-0.009641902	0.733223936	NS
MBOAT1	NS	0.634815991	-0.009637785	0.733319308	NS
GLS2	NS	0.634891409	-0.009635638	0.733325395	NS
NPB	NS	0.634897033	0.009635478	0.733325395	NS
GATA6	NS	0.635095069	0.009629841	0.733510362	NS
TMEM163	NS	0.6353906	-0.009621431	0.733790049	NS
MYCBP	NS	0.63541305	-0.009620792	0.733790049	NS
ANKFY1	NS	0.635597645	0.009615539	0.733959433	NS
KLHL5	NS	0.635662012	0.009613708	0.733989972	NS
ZCCHC8	NS	0.636277624	0.009596197	0.734646873	NS
BCL3	NS	0.636306821	-0.009595366	0.734646873	NS
FRRS1	NS	0.636348368	-0.009594185	0.73465102	NS
FREM3	NS	0.63649013	-0.009590153	0.734770857	NS
CCDC124	NS	0.636548023	-0.009588507	0.734793865	NS
CHST5	NS	0.636821046	0.009580745	0.73506519	NS
CDK13	NS	0.637171148	-0.009570793	0.735425447	NS
RNF121	NS	0.637325518	0.009566405	0.735559759	NS
C4orf46	NS	0.637489298	-0.009561751	0.735704916	NS
CTNNBIP1	NS	0.63753592	0.009560426	0.735714855	NS
NSMCE1	NS	0.637574196	0.009559338	0.735715163	NS
WDFY4	NS	0.637653625	0.009557081	0.735719097	NS
NSMCE3	NS	0.637640642	0.00955745	0.735719097	NS
ZNF737	NS	0.637746724	-0.009554436	0.735782654	NS
IFNA6	NS	0.637874954	-0.009550793	0.735886733	NS
SCRG1	NS	0.637955076	-0.009548517	0.735935303	NS
FAM186A	NS	0.638043867	0.009545995	0.735993867	NS
DHRS2	NS	0.638196278	0.009541665	0.736125806	NS
DSTYK	NS	0.638274832	0.009539434	0.736132997	NS
SLIT2	NS	0.638278574	-0.009539328	0.736132997	NS
NAT2	NS	0.638542624	-0.009531829	0.73639365	NS
CPNE9	NS	0.638625778	0.009529468	0.736401797	NS
SH3D21	NS	0.638612444	0.009529846	0.736401797	NS
EFNB1	NS	0.6386823	0.009527863	0.736423101	NS
CBX1	NS	0.638844097	-0.009523269	0.736565781	NS
PHB2	NS	0.639059751	0.009517147	0.736770537	NS
NOC2L	NS	0.639599837	-0.009501818	0.737349282	NS
NPM1	NS	0.639673795	-0.009499719	0.737390624	NS
SENP3	NS	0.639743885	-0.00949773	0.737401815	NS
VCX2	NS	0.639759696	-0.009497281	0.737401815	NS
TM9SF3	NS	0.639945012	0.009492023	0.737567664	NS
ATAD3C	NS	0.639979795	0.009491037	0.737567664	NS
ZNF276	NS	0.640338236	0.009480869	0.737936823	NS
MS4A6A	NS	0.640479155	0.009476872	0.738011343	NS
APPL1	NS	0.640477106	-0.00947693	0.738011343	NS
CCDC116	NS	0.640650946	-0.009472	0.73816535	NS
DOCK6	NS	0.640911823	0.009464603	0.738378028	NS
AHCYL2	NS	0.64090939	-0.009464672	0.738378028	NS
XPO7	NS	0.641012268	-0.009461755	0.738449795	NS
SPATA45	NS	0.6410638	0.009460294	0.738465209	NS
KNOP1	NS	0.641469462	-0.009448795	0.738888532	NS
ERLEC1	NS	0.641605595	-0.009444936	0.739001363	NS
SNX13	NS	0.641765828	-0.009440395	0.739141936	NS
CAPN10	NS	0.642136409	-0.009429895	0.739490278	NS
ESRRG	NS	0.642144688	0.009429661	0.739490278	NS

SLC35C2	NS	0.642279193	-0.00942585	0.739587959	NS
ITPKC	NS	0.642305931	0.009425093	0.739587959	NS
LIFR	NS	0.642768774	0.009411984	0.739812831	NS
GCA	NS	0.642652607	-0.009415274	0.739812831	NS
CTBS	NS	0.642739363	-0.009412817	0.739812831	NS
RPS2	NS	0.642765501	-0.009412076	0.739812831	NS
DNAJC21	NS	0.642720675	0.009413346	0.739812831	NS
MYO7B	NS	0.642737027	-0.009412883	0.739812831	NS
RGPD6	NS	0.642539532	0.009418476	0.739812831	NS
EML4	NS	0.642920294	-0.009407693	0.739899233	NS
MZT1	NS	0.642907578	0.009408053	0.739899233	NS
WNT2	NS	0.643006384	-0.009405255	0.739954314	NS
CFH	NS	0.64322725	-0.009399002	0.740140837	NS
PTPRO	NS	0.643244946	-0.009398501	0.740140837	NS
DHODH	NS	0.643472946	0.009392047	0.740359172	NS
HSBP1L1	NS	0.643511338	-0.00939096	0.740359336	NS
DLL1	NS	0.643784434	-0.009383231	0.74062951	NS
FBXO45	NS	0.644128331	0.009373499	0.740981099	NS
KLF5	NS	0.644265462	0.009369619	0.741075983	NS
TMEM132C	NS	0.644287386	-0.009368999	0.741075983	NS
ANKRD36C	NS	0.644461991	0.00936406	0.741232771	NS
ABCB11	NS	0.645032044	0.009347937	0.74181346	NS
EI24	NS	0.645043518	-0.009347612	0.74181346	NS
ZNF264	NS	0.645408869	0.009337282	0.742189524	NS
NR1I2	NS	0.645512222	-0.009334361	0.742264277	NS
ZNF880	NS	0.645705571	-0.009328895	0.742442499	NS
TTC9B	NS	0.64586915	0.009324272	0.742586473	NS
WDR17	NS	0.645984321	0.009321017	0.742674775	NS
AP1M1	NS	0.64610102	0.009317719	0.742764824	NS
HAS1	NS	0.64631021	-0.009311808	0.742828836	NS
PTGES2	NS	0.646281429	0.009312621	0.742828836	NS
GPR78	NS	0.646254285	-0.009313388	0.742828836	NS
KIAA1671	NS	0.646204031	0.009314808	0.742828836	NS
VCL	NS	0.646362881	-0.00931032	0.742845264	NS
HPN	NS	0.646491486	0.009306686	0.742948952	NS
TRIML1	NS	0.646530835	-0.009305575	0.742950062	NS
PLA2G12B	NS	0.646820657	-0.009297388	0.743238982	NS
FGF9	NS	0.647070499	-0.009290331	0.743481929	NS
LMNB1	NS	0.647263424	-0.009284883	0.743659455	NS
EFCAB12	NS	0.64757123	0.009276193	0.743968942	NS
BSN	NS	0.647701498	-0.009272515	0.744074437	NS
MRPL50	NS	0.648658899	0.009245496	0.745130068	NS
ZNF268	NS	0.648934436	-0.009237723	0.74527498	NS
IGF2BP1	NS	0.648932721	0.009237772	0.74527498	NS
TRAPPC5	NS	0.648939064	0.009237593	0.74527498	NS
WEE2	NS	0.64888211	0.009239199	0.74527498	NS
SLC26A4	NS	0.64926813	0.009228311	0.745578823	NS
MAN2B1	NS	0.64928067	0.009227958	0.745578823	NS
RBM43	NS	0.649336648	-0.009226379	0.74559887	NS
OSBPL10	NS	0.649455825	-0.009223018	0.745672557	NS
KSR2	NS	0.64947787	0.009222397	0.745672557	NS
ZNF423	NS	0.6495456	-0.009220487	0.745706087	NS
PRAMEF7	NS	0.649675555	0.009216823	0.745811046	NS
CMTM1	NS	0.649947654	0.009209152	0.746079159	NS
RECQL5	NS	0.649991908	-0.009207904	0.746085713	NS

PDSS1	NS	0.650067242	-0.009205781	0.746127938	NS
AZIN2	NS	0.650225324	-0.009201325	0.746265127	NS
AGK	NS	0.650448925	-0.009195023	0.746477493	NS
TBC1D32	NS	0.650518499	-0.009193063	0.746513077	NS
FECH	NS	0.65063188	0.009189868	0.746598925	NS
MLYCD	NS	0.65120909	0.009173607	0.747216975	NS
HAUS5	NS	0.651256846	-0.009172261	0.747227476	NS
ATP11A	NS	0.651423437	-0.009167569	0.747330017	NS
RGR	NS	0.65138505	-0.009168651	0.747330017	NS
CASS4	NS	0.651515114	-0.009164988	0.747390894	NS
LRRC27	NS	0.651564338	-0.009163601	0.747403066	NS
ASPHD1	NS	0.651874928	-0.009154856	0.747715029	NS
PELI1	NS	0.652012542	-0.009150981	0.747828559	NS
LRIT2	NS	0.652140421	0.009147381	0.747886597	NS
TRIM16	NS	0.652117594	0.009148024	0.747886597	NS
ENP3-EIF4A	NS	0.652419242	0.009139533	0.748117704	NS
IHO1	NS	0.652521138	0.009136665	0.748145904	NS
TRIM69	NS	0.65248566	0.009137664	0.748145904	NS
PPL	NS	0.652617385	0.009133956	0.748211935	NS
VASH1	NS	0.652983844	-0.009123645	0.748370135	NS
CACNA1F	NS	0.652891731	-0.009126236	0.748370135	NS
VNN2	NS	0.652987353	-0.009123546	0.748370135	NS
ATP6V1E1	NS	0.652926059	-0.00912527	0.748370135	NS
KLF13	NS	0.652826492	-0.009128072	0.748370135	NS
EMID1	NS	0.652914831	-0.009125586	0.748370135	NS
SLC2A2	NS	0.653479244	0.009109708	0.748889535	NS
KRT34	NS	0.653550608	0.009107701	0.748926977	NS
ZNF844	NS	0.653711255	0.009103183	0.749066722	NS
PATZ1	NS	0.653872581	0.009098646	0.749204843	NS
NEMF	NS	0.653909207	-0.009097616	0.749204843	NS
ZCWPW2	NS	0.654029336	0.009094238	0.749298125	NS
ERCC4	NS	0.654122501	0.009091619	0.749360508	NS
FGF23	NS	0.654373008	0.009084576	0.749603122	NS
FKBP11	NS	0.654581317	-0.00907872	0.749708638	NS
POTED	NS	0.654519217	0.009080466	0.749708638	NS
IER5	NS	0.655013701	0.009066568	0.750115087	NS
RNF212	NS	0.655001449	-0.009066913	0.750115087	NS
BLNK	NS	0.655102667	0.009064069	0.750152531	NS
PTK2B	NS	0.655240175	0.009060205	0.750152531	NS
VCPIP1	NS	0.655236898	0.009060297	0.750152531	NS
PGBD4	NS	0.655165551	0.009062302	0.750152531	NS
ZHX3	NS	0.65545373	-0.009054205	0.750352638	NS
NXN	NS	0.655525723	0.009052183	0.750390674	NS
NCAPH2	NS	0.655676967	0.009047934	0.750519419	NS
DNAJC17	NS	0.65586842	-0.009042557	0.750687877	NS
PLGLB1	NS	0.655901704	-0.009041622	0.750687877	NS
DNAH11	NS	0.656001993	0.009038805	0.750758268	NS
EIF3L	NS	0.656063219	-0.009037086	0.750783946	NS
STK35	NS	0.656344816	0.009029179	0.751017395	NS
TGM6	NS	0.656308596	-0.009030196	0.751017395	NS
NMNAT2	NS	0.65650094	-0.009024795	0.751151634	NS
PKN3	NS	0.656630229	-0.009021166	0.751238283	NS
STH	NS	0.656654294	-0.00902049	0.751238283	NS
DDX59	NS	0.656698816	-0.00901924	0.751244816	NS
FAM86B1	NS	0.656819632	0.009015849	0.751338621	NS

EARS2	NS	0.656881744	-0.009014106	0.751365266	NS
C12orf73	NS	0.657162739	-0.009006219	0.75164226	NS
RGPD5	NS	0.657468398	0.008997642	0.75194743	NS
SSR1	NS	0.657612209	0.008993607	0.752040356	NS
OR52B6	NS	0.657627354	0.008993183	0.752040356	NS
TOR1A	NS	0.657683847	-0.008991598	0.752060528	NS
DYNC2H1	NS	0.658362681	0.008972557	0.752787846	NS
PRSS1	NS	0.658397678	-0.008971576	0.752787846	NS
KBTBD8	NS	0.658485276	0.00896912	0.752807135	NS
ZNF8	NS	0.658492333	-0.008968922	0.752807135	NS
SOX6	NS	0.658736791	0.008962067	0.753042129	NS
PUS1	NS	0.658990271	-0.008954961	0.753287409	NS
C15orf65	NS	0.659042069	0.008953509	0.753302131	NS
LSS	NS	0.659146642	0.008950578	0.753332688	NS
ZNF419	NS	0.659891501	-0.008929705	0.754139452	NS
PPP2R1A	NS	0.660043465	0.008925448	0.754268586	NS
TSC22D4	NS	0.660338163	0.008917193	0.754537311	NS
DCAF11	NS	0.660425876	0.008914736	0.754571941	NS
PARP11	NS	0.660537103	-0.008911621	0.754654478	NS
ACOT13	NS	0.660580664	-0.008910401	0.754659702	NS
UQCC1	NS	0.660814721	0.008903847	0.75479816	NS
PSPC1	NS	0.660818848	0.008903731	0.75479816	NS
LRRC34	NS	0.66081694	0.008903784	0.75479816	NS
ASMT	NS	0.660923387	0.008900804	0.75487302	NS
ZNF462	NS	0.66104414	-0.008897423	0.75496639	NS
NPIPB12	NS	0.66109944	0.008895875	0.754984999	NS
NOP14	NS	0.661496833	0.008884751	0.755363953	NS
GAMT	NS	0.661509318	0.008884401	0.755363953	NS
SMO	NS	0.661633696	0.00888092	0.75546141	NS
DPF3	NS	0.661673659	0.008879802	0.755462475	NS
IRF2BP1	NS	0.661864178	-0.00887447	0.755635427	NS
OOEP	NS	0.661963391	-0.008871694	0.755704123	NS
TLL2	NS	0.662142276	0.008866689	0.755819183	NS
MCHR2	NS	0.66214128	0.008866716	0.755819183	NS
IFNA10	NS	0.662325526	-0.008861562	0.755983777	NS
MSRB1	NS	0.662375081	0.008860176	0.755995758	NS
ZNF80	NS	0.662508734	0.008856437	0.756103718	NS
MCCD1	NS	0.662817189	-0.008847809	0.756411149	NS
UBAP2	NS	0.663305354	-0.008834159	0.756923619	NS
ITGB1BP1	NS	0.663465945	-0.008829669	0.757058082	NS
VPS26B	NS	0.66350141	-0.008828678	0.757058082	NS
MSL2	NS	0.663622993	-0.008825279	0.757152176	NS
CHST15	NS	0.663807076	-0.008820133	0.757317562	NS
ZNF836	NS	0.664182126	0.008809651	0.757700786	NS
LRRFIP1	NS	0.664463096	0.008801801	0.757931975	NS
DCAF4L1	NS	0.664451149	-0.008802134	0.757931975	NS
MSL1	NS	0.665042225	0.008785623	0.758547867	NS
SBSPON	NS	0.665112307	-0.008783665	0.758583102	NS
ZNF784	NS	0.665292819	0.008778624	0.75869957	NS
KLHL8	NS	0.665668792	0.008768126	0.759046541	NS
HSD3B1	NS	0.665714718	-0.008766844	0.759046541	NS
USP17L23	NS	0.665693881	-0.008767425	0.759046541	NS
WBP2NL	NS	0.665852271	-0.008763004	0.759158659	NS
IFI6	NS	0.665934368	-0.008760712	0.759207541	NS
ZMYM1	NS	0.666110309	0.008755801	0.759363398	NS

PPP6R2	NS	0.666324498	-0.008749822	0.759424743	NS
SLC14A1	NS	0.66628066	-0.008751046	0.759424743	NS
FBXO38	NS	0.666348515	0.008749152	0.759424743	NS
GM2A	NS	0.666205558	0.008753142	0.759424743	NS
TSPAN19	NS	0.666360293	-0.008748823	0.759424743	NS
GMEB2	NS	0.666525215	-0.008744221	0.759567975	NS
XKR5	NS	0.666785959	0.008736945	0.759820383	NS
SEC63	NS	0.666902678	0.008733689	0.75990865	NS
XPOT	NS	0.667014214	0.008730577	0.759991001	NS
TOMM22	NS	0.667183187	-0.008725864	0.760138783	NS
DIP2A	NS	0.667327769	0.008721831	0.760258757	NS
TRAPPC8	NS	0.667419649	-0.008719268	0.760318682	NS
RASEF	NS	0.667647081	-0.008712925	0.76053301	NS
CD38	NS	0.667804299	0.008708541	0.760667334	NS
ASB1	NS	0.668027512	0.008702317	0.76087681	NS
PEF1	NS	0.66817063	-0.008698327	0.760993897	NS
AKAP14	NS	0.668208942	0.008697259	0.760993897	NS
PTCD2	NS	0.668584022	0.008686804	0.761376262	NS
FADS1	NS	0.668858657	0.00867915	0.761644203	NS
PIEZO2	NS	0.668978151	0.00867582	0.761735461	NS
BCL2L15	NS	0.669963297	0.008648377	0.762812328	NS
CDH11	NS	0.670006028	-0.008647187	0.762816109	NS
BOLA3	NS	0.670205277	0.008641639	0.762998079	NS
PAQR9	NS	0.670374737	0.00863692	0.763146116	NS
NTAQ1	NS	0.670535245	-0.008632452	0.763248653	NS
ALCAM	NS	0.670543673	0.008632217	0.763248653	NS
HSH2D	NS	0.670592735	0.008630851	0.763259613	NS
RTL8B	NS	0.670745636	0.008626595	0.763388754	NS
C2orf66	NS	0.670857789	-0.008623473	0.763471506	NS
B3GAT1	NS	0.671008933	-0.008619266	0.763561724	NS
MTRNR2L8	NS	0.67101596	0.00861907	0.763561724	NS
LAT2	NS	0.671253615	-0.008612456	0.763744075	NS
SLC25A15	NS	0.671265811	0.008612117	0.763744075	NS
ZNF185	NS	0.671294583	0.008611316	0.763744075	NS
GALNT12	NS	0.671543806	-0.008604382	0.763982715	NS
TCEAL5	NS	0.671708822	0.008599791	0.764125533	NS
PRLR	NS	0.671820522	0.008596683	0.76416278	NS
APOL3	NS	0.671792319	-0.008597468	0.76416278	NS
PIK3C2B	NS	0.671922543	0.008593845	0.764233913	NS
OR1E2	NS	0.672104371	-0.008588787	0.764395805	NS
IL18	NS	0.672228286	-0.008585341	0.764446902	NS
SLC4A5	NS	0.672193531	-0.008586308	0.764446902	NS
ART4	NS	0.672446969	0.008579259	0.764560827	NS
ZNF841	NS	0.67240592	0.008580401	0.764560827	NS
ARSH	NS	0.672440596	-0.008579436	0.764560827	NS
IRF4	NS	0.672530978	0.008576923	0.764611431	NS
TYW3	NS	0.672649077	0.008573639	0.764700783	NS
ISG20L2	NS	0.672757401	0.008570627	0.764779013	NS
LETM2	NS	0.672904396	-0.00856654	0.764870157	NS
PCDHGA8	NS	0.67291661	-0.008566201	0.764870157	NS
RALGDS	NS	0.672976447	0.008564537	0.764893254	NS
NOL9	NS	0.673045386	0.008562621	0.764926692	NS
HECA	NS	0.673091092	-0.00856135	0.764933723	NS
ZNF446	NS	0.67334765	-0.008554219	0.765090526	NS
SET	NS	0.673291835	-0.00855577	0.765090526	NS

IRF9	NS	0.673320183	0.008554982	0.765090526	NS
PIK3C2A	NS	0.673408991	0.008552514	0.765115311	NS
ZNF174	NS	0.673524353	-0.008549308	0.765171132	NS
DDX58	NS	0.67369531	-0.008544557	0.765171132	NS
ZBTB45	NS	0.673663904	0.00854543	0.765171132	NS
SACS	NS	0.673676846	-0.00854507	0.765171132	NS
UBXN6	NS	0.673617852	0.008546709	0.765171132	NS
MARCKSL1	NS	0.673657391	0.008545611	0.765171132	NS
CFAP20DC	NS	0.674231254	-0.008529666	0.765734915	NS
TNFAIP8	NS	0.674285426	-0.008528161	0.765751508	NS
GOLGA6L25P	NS	0.674403946	-0.008524869	0.765841172	NS
RAB40C	NS	0.674821015	-0.008513285	0.766224882	NS
COL11A2	NS	0.674807602	0.008513657	0.766224882	NS
PABPC1	NS	0.674925005	0.008510397	0.766298005	NS
TECPR2	NS	0.675080557	-0.008506078	0.76642966	NS
MON1A	NS	0.675223465	0.00850211	0.766546943	NS
GET4	NS	0.675271496	0.008500776	0.766556511	NS
PLEKHF1	NS	0.675426966	-0.00849646	0.766688034	NS
MOSPD2	NS	0.675506488	-0.008494253	0.766733336	NS
GPR42	NS	0.675795931	0.008486218	0.76701487	NS
RPL14	NS	0.675833778	0.008485168	0.76701487	NS
PRPF38A	NS	0.675975785	-0.008481227	0.767099576	NS
ZNF679	NS	0.675987677	-0.008480897	0.767099576	NS
TAS1R1	NS	0.676055903	-0.008479003	0.767132024	NS
RECK	NS	0.676157007	-0.008476198	0.767148551	NS
PLA2G2F	NS	0.676189369	-0.0084753	0.767148551	NS
GIMAP5	NS	0.676146507	-0.008476489	0.767148551	NS
PCOLCE	NS	0.676430199	-0.008468617	0.767376798	NS
EEF1B2	NS	0.676569962	0.00846474	0.767419857	NS
DYNC1LI2	NS	0.676574106	-0.008464625	0.767419857	NS
MS4A13	NS	0.676587098	-0.008464264	0.767419857	NS
AMOT	NS	0.677041706	-0.008451654	0.767803794	NS
NUDT21	NS	0.677044594	0.008451574	0.767803794	NS
KPNA5	NS	0.676987274	-0.008453164	0.767803794	NS
SEC14L2	NS	0.677220353	0.008446699	0.767913132	NS
COQ5	NS	0.677426027	-0.008440996	0.768101352	NS
LPCAT3	NS	0.677532874	0.008438033	0.768156944	NS
GPRIN2	NS	0.677554428	0.008437435	0.768156944	NS
ACADS	NS	0.677799502	0.00843064	0.768344783	NS
KIF5C	NS	0.677790754	0.008430883	0.768344783	NS
DDX60L	NS	0.67807309	0.008423056	0.768564896	NS
ZNF84	NS	0.678045411	0.008423823	0.768564896	NS
ATP6V0C	NS	0.678159828	0.008420652	0.768618201	NS
RDH16	NS	0.678308074	0.008416543	0.768741208	NS
CARM1	NS	0.678579226	0.008409028	0.769003483	NS
PLEKHG1	NS	0.678727943	-0.008404907	0.769081961	NS
KIR2DL1	NS	0.678705548	-0.008405528	0.769081961	NS
FOXD4L5	NS	0.678815221	0.008402489	0.769135832	NS
GOLGA3	NS	0.678923873	-0.008399479	0.769200541	NS
NHLH2	NS	0.678951811	-0.008398705	0.769200541	NS
PFAS	NS	0.679390645	-0.008386548	0.769607616	NS
ZSCAN22	NS	0.67966402	0.008378976	0.769872236	NS
RNF215	NS	0.679813602	-0.008374834	0.769964707	NS
C6orf132	NS	0.679884141	0.008372881	0.769964707	NS
PTPRT	NS	0.679827232	-0.008374457	0.769964707	NS

ATXN2	NS	0.679904773	0.008372309	0.769964707	NS
ZBTB3	NS	0.679948807	-0.00837109	0.769969526	NS
SLC12A7	NS	0.680008749	-0.00836943	0.769992357	NS
ZBTB11	NS	0.680099448	0.008366919	0.77005001	NS
GABRG2	NS	0.680275853	0.008362035	0.770204691	NS
CD28	NS	0.680419415	-0.008358061	0.770322173	NS
DNAAF2	NS	0.680760632	-0.008348616	0.770663399	NS
METTL5	NS	0.68088084	-0.008345289	0.770754404	NS
PDZD11	NS	0.681049397	-0.008340624	0.770857177	NS
GRM2	NS	0.681102731	-0.008339148	0.770870335	NS
CD109	NS	0.681196879	0.008336543	0.770931812	NS
GOSR1	NS	0.681265121	0.008334655	0.770943211	NS
C5orf24	NS	0.68128661	0.00833406	0.770943211	NS
PRDM11	NS	0.68145404	0.008329428	0.771087595	NS
SLCO2B1	NS	0.681670304	-0.008323445	0.771287216	NS
TMOD1	NS	0.681777842	0.00832047	0.771363801	NS
PALS1	NS	0.681832055	-0.008318971	0.771380048	NS
GRAPL	NS	0.681995226	-0.008314457	0.771519556	NS
ZNF484	NS	0.682081701	-0.008312066	0.771572288	NS
BCAT1	NS	0.68224107	-0.008307658	0.771617451	NS
EIF4H	NS	0.68224122	0.008307654	0.771617451	NS
ITGAV	NS	0.682166179	0.008309729	0.771617451	NS
SANBR	NS	0.682827989	0.00829143	0.772190848	NS
PRICKLE3	NS	0.682954678	0.008287928	0.772288996	NS
ZNF667	NS	0.683115388	-0.008283485	0.772425602	NS
IMPA1	NS	0.683204483	-0.008281023	0.772472327	NS
JAKMIP2	NS	0.683236528	-0.008280137	0.772472327	NS
PMF1-BGLAP	NS	0.683296146	-0.008278489	0.77249461	NS
CDK5RAP3	NS	0.683386083	0.008276003	0.772551163	NS
DNPH1	NS	0.683628366	-0.008269308	0.772734796	NS
KRT77	NS	0.683599817	0.008270097	0.772734796	NS
LY6G6E	NS	0.683730731	-0.008266479	0.772805373	NS
LZTS1	NS	0.683915735	0.008261368	0.772969343	NS
DIDO1	NS	0.684043271	0.008257844	0.773068346	NS
UNC13B	NS	0.684177487	0.008254136	0.773174885	NS
C6orf47	NS	0.684302792	-0.008250675	0.773271344	NS
ASCC3	NS	0.684354075	-0.008249258	0.773284149	NS
CAPNS1	NS	0.684907679	0.008233969	0.773864517	NS
EMC3	NS	0.685010015	0.008231143	0.773934966	NS
GNGT1	NS	0.685229605	0.00822508	0.774092694	NS
HTR3D	NS	0.685202294	0.008225834	0.774092694	NS
METTL17	NS	0.685327263	0.008222384	0.774157835	NS
GOLGA5	NS	0.685512628	-0.008217267	0.774322037	NS
CCDC107	NS	0.685765703	0.008210282	0.774562698	NS
LGALS2	NS	0.686004683	0.008203686	0.774705866	NS
CHST12	NS	0.686019598	-0.008203274	0.774705866	NS
ZMYM4	NS	0.686092579	-0.00820126	0.774705866	NS
PROSER3	NS	0.686078891	-0.008201638	0.774705866	NS
ZNF134	NS	0.686057537	0.008202227	0.774705866	NS
HSFY1	NS	0.686168969	-0.008199152	0.774746926	NS
SEMA3E	NS	0.686307801	-0.008195322	0.774813287	NS
DHRS7C	NS	0.686647219	0.008185957	0.775151263	NS
GXYLT2	NS	0.6868701	0.008179809	0.775357652	NS
ZNF521	NS	0.686966193	-0.008177158	0.775418063	NS
FAM174C	NS	0.687003739	-0.008176123	0.775418063	NS

KLF17	NS	0.687132123	0.008172582	0.775517747	NS
FDXACB1	NS	0.687400308	0.008165186	0.775775195	NS
PINK1	NS	0.687482013	0.008162933	0.775822169	NS
MARCHF3	NS	0.687647829	-0.008158361	0.775964052	NS
PTPRM	NS	0.687807736	-0.008153952	0.776099251	NS
RPS21	NS	0.688078469	-0.008146488	0.776359478	NS
LNPEP	NS	0.688134208	0.008144952	0.776377111	NS
CAVIN2	NS	0.688222173	-0.008142527	0.776378228	NS
CRCT1	NS	0.688190641	0.008143396	0.776378228	NS
EPHA10	NS	0.688255529	-0.008141608	0.776378228	NS
ITPR2	NS	0.688326302	0.008139657	0.776394789	NS
LRWD1	NS	0.688350433	-0.008138992	0.776394789	NS
GPRC5C	NS	0.68842612	-0.008136906	0.776413215	NS
HRURF	NS	0.688446994	0.008136331	0.776413215	NS
APBA2	NS	0.689136834	-0.008117322	0.776874348	NS
RDH11	NS	0.689010714	0.008120797	0.776874348	NS
C1orf54	NS	0.689120391	0.008117775	0.776874348	NS
WIPF3	NS	0.68895167	0.008122424	0.776874348	NS
DNAI4	NS	0.688913609	-0.008123473	0.776874348	NS
PCDHA8	NS	0.68902624	0.008120369	0.776874348	NS
PCDHAC2	NS	0.689102396	0.008118271	0.776874348	NS
SPTSSA	NS	0.689567886	-0.008105448	0.777276222	NS
MTRNR2L10	NS	0.689573636	-0.00810529	0.777276222	NS
ZNRF3	NS	0.689633107	-0.008103652	0.777297992	NS
BAIAP2L1	NS	0.68980508	-0.008098916	0.777356028	NS
EXOSC3	NS	0.689764974	-0.00810002	0.777356028	NS
TMEM109	NS	0.689804429	0.008098934	0.777356028	NS
NR1I3	NS	0.689879234	0.008096874	0.777394332	NS
RASSF4	NS	0.690294504	-0.008085439	0.777771723	NS
PDZD9	NS	0.690267772	0.008086175	0.777771723	NS
EGF	NS	0.690393148	0.008082723	0.777837589	NS
ARFGAP2	NS	0.690526153	0.008079061	0.777942159	NS
TGFBI	NS	0.691175657	-0.008061184	0.778552012	NS
CXADR	NS	0.691223965	-0.008059855	0.778552012	NS
C3orf62	NS	0.69122837	-0.008059734	0.778552012	NS
CCDC180	NS	0.691315398	0.008057339	0.778604727	NS
MITD1	NS	0.691403785	0.008054907	0.778613665	NS
MICOS10	NS	0.691398332	0.008055057	0.778613665	NS
KLC3	NS	0.691646439	-0.00804823	0.778841613	NS
PSMA2	NS	0.691770194	0.008044826	0.778890342	NS
SLC35D2	NS	0.69175781	0.008045166	0.778890342	NS
TAOK1	NS	0.691983996	0.008038944	0.779085751	NS
TEX35	NS	0.692026293	0.008037781	0.779088055	NS
PELI2	NS	0.692102147	-0.008035694	0.779128136	NS
LASP1	NS	0.692311	-0.00802995	0.779260062	NS
SPACA1	NS	0.692261705	-0.008031306	0.779260062	NS
ZADH2	NS	0.692340116	0.008029149	0.779260062	NS
CDK5RAP2	NS	0.692627675	0.008021241	0.779538393	NS
PRH1	NS	0.692762352	0.008017538	0.779644636	NS
STEEP1	NS	0.693020739	-0.008010434	0.779871878	NS
FUNDC1	NS	0.69305845	-0.008009397	0.779871878	NS
CBLN2	NS	0.69309118	-0.008008497	0.779871878	NS
ZNF610	NS	0.693125435	-0.008007556	0.779871878	NS
SLC25A35	NS	0.693309881	-0.008002485	0.780016533	NS
GNG5P2	NS	0.6935275	-0.007996504	0.780188207	NS

GRK2	NS	0.693601182	0.007994479	0.78022575	NS
CCM2L	NS	0.693809096	-0.007988765	0.780414276	NS
TIMM17A	NS	0.693934686	-0.007985314	0.780510185	NS
ANKRD36B	NS	0.694162909	0.007979043	0.780721515	NS
NTAN1	NS	0.694404185	0.007972414	0.780902126	NS
U2SURP	NS	0.694805757	0.007961383	0.781217556	NS
PPP2R2D	NS	0.69476871	0.007962401	0.781217556	NS
CCDC195	NS	0.695003451	-0.007955954	0.781394447	NS
FAM221A	NS	0.69539363	-0.007945239	0.781787716	NS
LGALS9B	NS	0.695446468	0.007943788	0.781801709	NS
HMGN4	NS	0.695768024	-0.00793496	0.782117769	NS
PSMA4	NS	0.695863513	0.007932339	0.782179684	NS
ZNF705A	NS	0.695912259	0.007931001	0.782189054	NS
PSIP1	NS	0.696063162	-0.007926859	0.782313237	NS
GSTO1	NS	0.696110991	0.007925546	0.782321566	NS
RWDD2A	NS	0.6963555	-0.007918835	0.782505489	NS
TAF5	NS	0.696351949	0.007918933	0.782505489	NS
PPP2CB	NS	0.696511133	0.007914564	0.782634941	NS
TWSG1	NS	0.696682886	0.007909851	0.782772747	NS
PCDHA3	NS	0.696714657	-0.00790898	0.782772747	NS
OR6Y1	NS	0.696905732	0.007903737	0.782941979	NS
ARHGEF18	NS	0.697060189	0.0078995	0.783070053	NS
ACAP1	NS	0.69740572	-0.007890022	0.783140054	NS
TENT4A	NS	0.697163271	0.007896672	0.783140054	NS
FOXP1	NS	0.697374789	-0.00789087	0.783140054	NS
ILKAP	NS	0.697267287	0.007893819	0.783140054	NS
SLC15A4	NS	0.69736191	0.007891223	0.783140054	NS
RNF165	NS	0.697402335	0.007890114	0.783140054	NS
CTSB	NS	0.697260458	-0.007894006	0.783140054	NS
CASP2	NS	0.697687071	-0.007882305	0.783365099	NS
PIK3CD	NS	0.697652597	0.007883251	0.783365099	NS
KCNMB4	NS	0.697733105	0.007881043	0.783371345	NS
SERINC3	NS	0.697786385	0.007879582	0.783385724	NS
UFSP1	NS	0.698103933	0.007870874	0.783696771	NS
KLHL42	NS	0.698566095	-0.007858204	0.784170118	NS
TAOK2	NS	0.69866994	0.007855358	0.784241206	NS
SECTM1	NS	0.698746298	-0.007853265	0.784281435	NS
EMB	NS	0.698860391	0.007850138	0.784364011	NS
ACACA	NS	0.699328641	-0.007837306	0.784844041	NS
NCK1	NS	0.699387716	0.007835687	0.784864832	NS
MUC17	NS	0.699608868	-0.007829628	0.785067497	NS
GSDMD	NS	0.699701719	0.007827084	0.785126172	NS
ARPC4-TTLL	NS	0.699851364	0.007822984	0.785248565	NS
AHNAK2	NS	0.699978546	-0.007819501	0.785345742	NS
YJEFN3	NS	0.700200643	0.007813417	0.785549391	NS
CALCOCO2	NS	0.700502647	0.007805147	0.785842659	NS
EEFSEC	NS	0.700632448	0.007801592	0.785942722	NS
RPEL1	NS	0.700719204	0.007799217	0.78599449	NS
TSHZ1	NS	0.70095079	-0.007792876	0.786163141	NS
PTPN12	NS	0.701031014	0.00779068	0.786207562	NS
SNRPA	NS	0.7011207	0.007788225	0.786262588	NS
SORCS1	NS	0.701348763	-0.007781982	0.78647278	NS
MT1M	NS	0.701435014	-0.007779621	0.786523933	NS
PNPLA3	NS	0.701599084	-0.00777513	0.786629084	NS
FANCE	NS	0.701610069	0.00777483	0.786629084	NS

GYPC	NS	0.702032767	-0.007763262	0.78696625	NS
SLC12A6	NS	0.702013787	0.007763782	0.78696625	NS
CAMTA2	NS	0.702479633	-0.007751036	0.787421576	NS
SPC24	NS	0.702534855	-0.007749526	0.787437875	NS
MKI67	NS	0.702609745	-0.007747477	0.787476214	NS
CDH7	NS	0.702705012	0.007744871	0.787537387	NS
CLEC2D	NS	0.702751857	-0.00774359	0.787544288	NS
SIK3	NS	0.702945426	0.007738296	0.787715606	NS
GPD1	NS	0.703230054	0.007730512	0.787988939	NS
C3orf22	NS	0.703315374	-0.007728179	0.788027007	NS
LYRM9	NS	0.703345452	-0.007727356	0.788027007	NS
SPNS1	NS	0.703538032	0.007722091	0.788197149	NS
TAF12	NS	0.703873437	-0.007712921	0.788527275	NS
PITPNB	NS	0.704000485	-0.007709448	0.788623959	NS
SAP18	NS	0.704164766	-0.007704958	0.788762338	NS
DEFB1	NS	0.704377372	-0.007699147	0.78895483	NS
PIK3CB	NS	0.704665445	0.007691275	0.789231822	NS
TBC1D22B	NS	0.704966847	0.007683039	0.789523711	NS
LIN28B	NS	0.70519357	-0.007676845	0.789731934	NS
WNT5B	NS	0.705445411	-0.007669966	0.78996826	NS
ST8SIA3	NS	0.705665837	0.007663945	0.790169382	NS
MTO1	NS	0.705827972	0.007659517	0.790271356	NS
CHUK	NS	0.705838562	-0.007659228	0.790271356	NS
ZMIZ1	NS	0.705998309	0.007654866	0.790404492	NS
TRMT1	NS	0.706215726	0.007648929	0.790602174	NS
PLXNC1	NS	0.706314231	-0.00764624	0.79066672	NS
SLC5A9	NS	0.70637027	-0.00764471	0.790683724	NS
CCDC171	NS	0.706673951	-0.007636419	0.79097791	NS
CTTN	NS	0.707264895	-0.00762029	0.791410527	NS
GFUS	NS	0.707254361	-0.007620578	0.791410527	NS
MERTK	NS	0.707149948	-0.007623427	0.791410527	NS
MAPK13	NS	0.707243677	0.007620869	0.791410527	NS
TRMT12	NS	0.707131486	0.007623931	0.791410527	NS
DERL2	NS	0.707388922	0.007616906	0.791503554	NS
EVI5	NS	0.707475051	-0.007614556	0.791549184	NS
SLC20A2	NS	0.707525121	0.007613189	0.791549184	NS
CCL15-CCL1[illegible]	NS	0.707552386	-0.007612445	0.791549184	NS
RHOT2	NS	0.70772398	-0.007607764	0.791695391	NS
STEAP2	NS	0.707862187	0.007603993	0.791786293	NS
RPL38	NS	0.707887053	0.007603315	0.791786293	NS
CPM	NS	0.70811218	0.007597173	0.791992335	NS
TBC1D9	NS	0.708297569	0.007592117	0.792108143	NS
TSPAN16	NS	0.708288513	-0.007592364	0.792108143	NS
PEPD	NS	0.708482957	0.00758706	0.792269693	NS
KDSR	NS	0.708668233	-0.007582008	0.792431099	NS
PLXDC2	NS	0.708892559	-0.00757589	0.792636149	NS
EIF4A2	NS	0.708952555	-0.007574255	0.792657443	NS
TRAK2	NS	0.709156504	-0.007568694	0.792820326	NS
ODF3L1	NS	0.709180158	0.007568049	0.792820326	NS
AMN1	NS	0.709286364	-0.007565154	0.792893264	NS
POLR1D	NS	0.709432921	0.007561158	0.793011296	NS
SPATA6	NS	0.709502324	0.007559266	0.793043078	NS
COX7C	NS	0.709622917	-0.007555979	0.793086275	NS
LPA	NS	0.709595983	-0.007556714	0.793086275	NS
CD33	NS	0.709673473	0.007554601	0.793096983	NS

SLC39A8	NS	0.709763449	-0.007552149	0.793151742	NS
CIITA	NS	0.709941016	0.00754731	0.793261387	NS
IDH2	NS	0.709943532	0.007547241	0.793261387	NS
KIF3A	NS	0.710339193	-0.007536459	0.793513021	NS
STAMBPL1	NS	0.710290422	0.007537788	0.793513021	NS
SLC3A2	NS	0.710379008	0.007535374	0.793513021	NS
METAP1D	NS	0.710414711	-0.007534402	0.793513021	NS
MAGI2	NS	0.710331495	-0.007536669	0.793513021	NS
RPL37A	NS	0.710291671	0.007537754	0.793513021	NS
CLEC4A	NS	0.710813393	-0.00752354	0.793912523	NS
CERK	NS	0.71097898	0.007519029	0.794051649	NS
SLC6A20	NS	0.711032228	0.007517579	0.794065301	NS
OR14J1	NS	0.711260174	-0.007511371	0.794274039	NS
TMEM185A	NS	0.711341877	-0.007509145	0.794319451	NS
SPATA31A5	NS	0.711450474	0.007506188	0.794394887	NS
PRR33	NS	0.711581873	-0.00750261	0.794495774	NS
CRBN	NS	0.711727806	0.007498636	0.794612875	NS
CNN2	NS	0.711830275	0.007495846	0.79468144	NS
PWWP2B	NS	0.711946121	0.007492692	0.794719096	NS
ZNF765	NS	0.711944464	-0.007492737	0.794719096	NS
TUBA1A	NS	0.711994499	0.007491375	0.794727266	NS
GAS7	NS	0.712213702	0.007485407	0.794926099	NS
DICER1	NS	0.712403552	0.00748024	0.795054496	NS
ALKAL2	NS	0.71241089	0.00748004	0.795054496	NS
SUCLG2	NS	0.712857571	0.007467883	0.795507129	NS
EEF2	NS	0.713037183	-0.007462995	0.795661692	NS
CENPA	NS	0.713537043	-0.007449395	0.796037126	NS
FGF5	NS	0.713470773	0.007451198	0.796037126	NS
GRAMD2B	NS	0.713538134	-0.007449365	0.796037126	NS
VCX	NS	0.713475796	0.007451061	0.796037126	NS
CATSPERB	NS	0.71361154	0.007447369	0.796073136	NS
PRCP	NS	0.713703161	0.007444876	0.79612946	NS
PALD1	NS	0.713826056	-0.007441533	0.79617478	NS
XAF1	NS	0.713808278	-0.007442017	0.79617478	NS
MARCO	NS	0.713952019	-0.007438107	0.796269391	NS
SOGA1	NS	0.714086271	0.007434456	0.796373235	NS
PUF60	NS	0.714169234	0.0074322	0.79641987	NS
MEX3C	NS	0.714285972	-0.007429025	0.796504164	NS
CNTD1	NS	0.715030177	-0.00740879	0.797242172	NS
PL17-C18orf3	NS	0.715004004	-0.007409502	0.797242172	NS
HMGA1	NS	0.715279079	0.007402025	0.797473754	NS
ACADSB	NS	0.715376487	0.007399377	0.797536418	NS
GAGE13	NS	0.715572498	-0.00739405	0.797708995	NS
GALNT6	NS	0.715894135	-0.007385309	0.797929683	NS
PAPPA	NS	0.71588697	0.007385504	0.797929683	NS
CHML	NS	0.715833269	0.007386963	0.797929683	NS
PLPBP	NS	0.71593695	-0.007384146	0.797931457	NS
TARS2	NS	0.716072281	0.007380469	0.798036335	NS
COX7B	NS	0.716376607	-0.007372201	0.798262247	NS
CENPN	NS	0.716398713	-0.0073716	0.798262247	NS
DNAJA4	NS	0.716497401	0.007368919	0.798326255	NS
CDC6	NS	0.716634993	0.007365181	0.798429055	NS
CKS2	NS	0.716672164	-0.007364172	0.798429055	NS
YWHAE	NS	0.716797695	-0.007360762	0.798449852	NS
TONSL	NS	0.716814583	0.007360303	0.798449852	NS

PLA2G4E	NS	0.716745221	0.007362187	0.798449852	NS
ITGA4	NS	0.716920841	-0.007357417	0.798522258	NS
SLC38A6	NS	0.717439643	-0.007343329	0.79905413	NS
HTN3	NS	0.71776559	-0.007334479	0.79937116	NS
SLC16A6	NS	0.718119255	0.007324879	0.79971902	NS
ANTKMT	NS	0.718191981	-0.007322905	0.799753997	NS
CDH5	NS	0.718247819	-0.007321389	0.799770165	NS
MB	NS	0.718934818	-0.007302746	0.80048909	NS
LMLN	NS	0.719168228	0.007296413	0.800656863	NS
OCIAD2	NS	0.719254349	0.007294077	0.800706688	NS
SART3	NS	0.719303537	-0.007292742	0.800709356	NS
REEP3	NS	0.719339481	-0.007291767	0.800709356	NS
C9orf78	NS	0.719673304	0.007282712	0.801034875	NS
RASL11B	NS	0.719855307	0.007277776	0.801169517	NS
STOML2	NS	0.719877053	-0.007277186	0.801169517	NS
MRM3	NS	0.720022791	-0.007273234	0.801285641	NS
WWOX	NS	0.72020389	-0.007268323	0.801441101	NS
GATD1	NS	0.720476486	0.007260931	0.801698354	NS
ACSF2	NS	0.720726283	0.007254159	0.80193021	NS
OAT	NS	0.721178081	-0.007241912	0.802386787	NS
RABEP1	NS	0.721395782	0.007236012	0.802582869	NS
ZNF285	NS	0.721450316	0.007234534	0.802597409	NS
MGA	NS	0.721504788	-0.007233058	0.802611878	NS
C8orf58	NS	0.721629887	-0.007229668	0.802704907	NS
PFN2	NS	0.721731924	-0.007226903	0.802726146	NS
UBALD2	NS	0.721715478	-0.007227348	0.802726146	NS
CREG2	NS	0.721857716	-0.007223494	0.802819924	NS
SIVA1	NS	0.722012903	-0.00721929	0.80294638	NS
PTGR1	NS	0.722125292	0.007216245	0.80302523	NS
KCNS3	NS	0.722376792	-0.007209432	0.803258757	NS
IMPA2	NS	0.722900889	0.007195236	0.803795358	NS
TCP1	NS	0.723056605	0.00719102	0.803922319	NS
COL9A2	NS	0.723116117	0.007189408	0.803942307	NS
LAMP1	NS	0.723704279	0.007173483	0.804503792	NS
MICOS13	NS	0.723752795	-0.00717217	0.80451152	NS
IGFBP5	NS	0.724078481	0.007163354	0.804827328	NS
LRRC15	NS	0.72420168	-0.00716002	0.804918042	NS
ARSA	NS	0.72443644	-0.007153666	0.805063474	NS
E2F3	NS	0.72442527	0.007153968	0.805063474	NS
WBP1L	NS	0.724457305	-0.007153102	0.805063474	NS
RP1	NS	0.724513833	0.007151572	0.80508007	NS
TSPYL4	NS	0.724684329	-0.007146958	0.805223299	NS
AGBL4	NS	0.724741739	0.007145405	0.805240864	NS
MMS19	NS	0.7248083	-0.007143604	0.805268594	NS
CFAP69	NS	0.725009668	-0.007138155	0.805446085	NS
CYB561A3	NS	0.725103759	-0.00713561	0.805458155	NS
VIRMA	NS	0.725092019	-0.007135928	0.805458155	NS
STK24	NS	0.725182471	0.007133481	0.805499363	NS
MYOM1	NS	0.725343747	0.007129118	0.805539822	NS
OSGIN2	NS	0.725306952	-0.007130113	0.805539822	NS
ARHGAP12	NS	0.725310353	0.007130021	0.805539822	NS
CDHR4	NS	0.726068499	0.007109516	0.806298443	NS
AMY2B	NS	0.726179574	-0.007106513	0.806375528	NS
LCE3C	NS	0.726613184	-0.007094789	0.806810738	NS
ELL3	NS	0.726824869	0.007089066	0.806941221	NS

GMPS	NS	0.726855765	0.007088231	0.806941221	NS
XRCC6	NS	0.72681618	0.007089301	0.806941221	NS
MUS81	NS	0.727027035	-0.007083602	0.80708507	NS
COL12A1	NS	0.727176114	-0.007079572	0.807204271	NS
MX1	NS	0.727246747	0.007077663	0.807236383	NS
CALCOCO1	NS	0.727703649	0.007065316	0.8076046	NS
METAP2	NS	0.727651207	-0.007066733	0.8076046	NS
FFAR3	NS	0.727668357	0.00706627	0.8076046	NS
RFX5	NS	0.727763732	0.007063693	0.807624974	NS
H1-8	NS	0.727938524	-0.00705897	0.807772635	NS
NLRP2B	NS	0.728051266	0.007055924	0.807851427	NS
DGAT1	NS	0.728097846	-0.007054666	0.807856801	NS
ERCC1	NS	0.728236707	0.007050914	0.807964559	NS
NPM2	NS	0.728337311	-0.007048197	0.807986466	NS
AGAP6	NS	0.728339939	0.007048126	0.807986466	NS
CABLES2	NS	0.728470821	-0.00704459	0.808085347	NS
GJA5	NS	0.728689057	-0.007038696	0.808281111	NS
IMPDH1	NS	0.728905655	0.007032846	0.808475036	NS
LAMC3	NS	0.728965985	0.007031217	0.808495623	NS
MTMR6	NS	0.729337573	-0.007021183	0.808775823	NS
PNRC2	NS	0.729343975	0.00702101	0.808775823	NS
OXCT2	NS	0.729315352	0.007021783	0.808775823	NS
LMO4	NS	0.729501646	0.007016753	0.808857986	NS
KLHL11	NS	0.729481464	-0.007017298	0.808857986	NS
GPR137C	NS	0.729879837	-0.007006543	0.809230961	NS
RAP1GDS1	NS	0.730352129	-0.006993796	0.809607558	NS
GMEB1	NS	0.730289082	0.006995497	0.809607558	NS
TSR1	NS	0.730386814	-0.00699286	0.809607558	NS
NPIPA3	NS	0.730334795	0.006994263	0.809607558	NS
CLDN10	NS	0.730527415	0.006989065	0.809717039	NS
PIK3C3	NS	0.730693823	0.006984575	0.80980874	NS
PIP5K1B	NS	0.730661511	0.006985447	0.80980874	NS
ZNF567	NS	0.730877923	0.006979607	0.809966396	NS
KRAS	NS	0.731231671	0.006970063	0.81031203	NS
ZP3	NS	0.731358313	0.006966647	0.810405972	NS
ELOVL1	NS	0.731492134	0.006963037	0.810445949	NS
CSNK2A2	NS	0.731526605	0.006962107	0.810445949	NS
CLCN2	NS	0.731561874	0.006961156	0.810445949	NS
CTH	NS	0.731531576	0.006961973	0.810445949	NS
GRK7	NS	0.731673036	0.006958158	0.810522708	NS
NPRL3	NS	0.731906121	-0.006951871	0.810734513	NS
CCDC43	NS	0.732033902	0.006948425	0.810829654	NS
PRXL2B	NS	0.732190587	0.0069442	0.810956798	NS
BIN2	NS	0.732289812	-0.006941525	0.811020289	NS
ZIC4	NS	0.732614005	0.006932784	0.811332915	NS
SCAMP1	NS	0.732832287	-0.006926899	0.81152822	NS
IL9	NS	0.732938513	-0.006924036	0.811552993	NS
SNX12	NS	0.732907632	0.006924868	0.811552993	NS
SRSF7	NS	0.7331371	-0.006918683	0.811680015	NS
BZW2	NS	0.733134982	-0.00691874	0.811680015	NS
WDR76	NS	0.73322852	-0.006916219	0.8117348	NS
P2RX7	NS	0.733557414	-0.006907355	0.812023933	NS
NCOA7	NS	0.733588652	0.006906513	0.812023933	NS
SEPTIN10	NS	0.733615546	-0.006905788	0.812023933	NS
ZC3H8	NS	0.73366025	-0.006904584	0.812026979	NS

ZNF253	NS	0.734143196	-0.006891571	0.81251505	NS
IGSF22	NS	0.734199597	0.006890051	0.812531012	NS
COMMD6	NS	0.734293634	0.006887518	0.812588622	NS
TRIM64B	NS	0.734464603	0.006882912	0.812731355	NS
UAP1	NS	0.734602129	0.006879208	0.812837067	NS
C18orf21	NS	0.734730274	0.006875756	0.812932387	NS
AACS	NS	0.734860173	0.006872258	0.812977354	NS
NPEPL1	NS	0.734892742	-0.00687138	0.812977354	NS
GAS2L2	NS	0.734896919	0.006871268	0.812977354	NS
ACTB	NS	0.73520157	-0.006863063	0.813267893	NS
ESRRA	NS	0.735594273	-0.006852489	0.813655795	NS
F2RL1	NS	0.735681835	-0.006850132	0.813706149	NS
KHD1-EIF4E	NS	0.735740673	0.006848548	0.813724728	NS
NEO1	NS	0.735806573	0.006846774	0.813726298	NS
RTP2	NS	0.735826173	-0.006846246	0.813726298	NS
GNL3	NS	0.735875363	0.006844922	0.813734205	NS
ATP2B4	NS	0.736152453	0.006837463	0.813947611	NS
FAM71F1	NS	0.736116688	-0.006838426	0.813947611	NS
LRRC37A3	NS	0.736239294	0.006835125	0.813967878	NS
OR11H12	NS	0.736254888	-0.006834706	0.813967878	NS
MT-ND6	NS	0.736384401	0.00683122	0.814064565	NS
ARPC5L	NS	0.736426997	0.006830073	0.81406516	NS
RANBP6	NS	0.736564914	-0.006826362	0.814124628	NS
SMIM31	NS	0.73653786	0.00682709	0.814124628	NS
AKIRIN1	NS	0.736698891	-0.006822756	0.814226216	NS
FILIP1L	NS	0.736745153	0.006821511	0.814230854	NS
RPS20	NS	0.736950785	-0.006815978	0.814411613	NS
PWP1	NS	0.736998255	0.006814701	0.814417574	NS
METTL8	NS	0.737072457	-0.006812704	0.814453073	NS
CNTN2	NS	0.737654104	0.006797056	0.815049254	NS
RHEBL1	NS	0.737761793	-0.00679416	0.815121712	NS
IDH3B	NS	0.737848546	-0.006791826	0.815171031	NS
AKR1B1	NS	0.738040144	0.006786673	0.815293702	NS
CEP57L1	NS	0.738043823	0.006786574	0.815293702	NS
RO60	NS	0.738228493	0.006781608	0.815451162	NS
OR2V1	NS	0.73837124	0.006777769	0.815562299	NS
TTC39A	NS	0.738500511	-0.006774293	0.815574183	NS
HYAL4	NS	0.738599366	-0.006771635	0.815574183	NS
CCKBR	NS	0.738449715	-0.006775659	0.815574183	NS
KHDRBS3	NS	0.738634812	0.006770682	0.815574183	NS
FADS2	NS	0.738600562	-0.006771603	0.815574183	NS
C11orf87	NS	0.738576191	-0.006772258	0.815574183	NS
ALG1	NS	0.738845283	0.006765023	0.815760042	NS
HERPUD1	NS	0.73926351	0.00675378	0.816175251	NS
RAB5C	NS	0.739466943	-0.006748312	0.81633743	NS
ZNF283	NS	0.739494756	0.006747564	0.81633743	NS
LRRD1	NS	0.739559627	-0.006745821	0.816362483	NS
SULT1B1	NS	0.739813664	-0.006738993	0.816596333	NS
TNFSF15	NS	0.73988723	-0.006737017	0.816630965	NS
OGT	NS	0.740004598	0.006733863	0.816713935	NS
DCAF1	NS	0.740264278	-0.006726885	0.816907375	NS
CFDP1	NS	0.740309394	-0.006725673	0.816910588	NS
YWHAB	NS	0.740392398	0.006723443	0.816955607	NS
CPSF4	NS	0.740489155	-0.006720843	0.816969225	NS
YWHAG	NS	0.740471202	-0.006721326	0.816969225	NS

TMLHE	NS	0.740598823	-0.006717897	0.817043649	NS
TMEM213	NS	0.740724215	-0.006714529	0.81713541	NS
TTC23	NS	0.740833784	0.006711585	0.817206073	NS
DCHS2	NS	0.74087271	-0.00671054	0.817206073	NS
PCBP3	NS	0.740916526	0.006709363	0.817207834	NS
GTF3C4	NS	0.741009651	-0.006706861	0.817263978	NS
MSRB3	NS	0.741159977	-0.006702824	0.817336629	NS
RPH3AL	NS	0.741119932	0.006703899	0.817336629	NS
NOB1	NS	0.741749597	0.00668699	0.817940249	NS
CDX1	NS	0.74189041	-0.006683209	0.817969267	NS
INO80C	NS	0.741840558	0.006684548	0.817969267	NS
CDC42BPG	NS	0.741902689	0.00668288	0.817969267	NS
CRLF1	NS	0.741965213	-0.006681201	0.817991608	NS
POM121	NS	0.742350628	0.006670854	0.818369903	NS
EFHB	NS	0.742485164	0.006667243	0.818471601	NS
C3orf70	NS	0.74267118	0.00666225	0.818630032	NS
NBEAL2	NS	0.74286057	0.006657167	0.818792164	NS
RINT1	NS	0.742933303	-0.006655215	0.818825704	NS
SLC17A5	NS	0.743028023	-0.006652673	0.818883472	NS
PPP1R16B	NS	0.743330829	-0.006644547	0.81917055	NS
SLITRK2	NS	0.743375733	-0.006643342	0.819173396	NS
COMT	NS	0.743464302	0.006640966	0.819224355	NS
ERI1	NS	0.74356444	0.006638279	0.819288057	NS
COPS4	NS	0.743703402	-0.006634551	0.819394526	NS
SPRY3	NS	0.743758705	-0.006633067	0.819408817	NS
FLG	NS	0.743932496	0.006628405	0.819506994	NS
ITGAM	NS	0.743901722	0.006629231	0.819506994	NS
ISM1	NS	0.744009808	-0.006626331	0.819545518	NS
GOLGA6L1	NS	0.744103784	-0.006623811	0.819602392	NS
PDK2	NS	0.744619406	0.006609981	0.820076997	NS
IER5L	NS	0.744695906	-0.00660793	0.820114586	NS
FXR2	NS	0.744875752	0.006603107	0.820265976	NS
NARF	NS	0.744966094	0.006600685	0.820318791	NS
DENND6A	NS	0.745030861	-0.006598948	0.820343441	NS
OR51V1	NS	0.745105136	-0.006596957	0.820378556	NS
A1BG	NS	0.745311245	0.006591431	0.820558811	NS
C6orf62	NS	0.745358644	-0.00659016	0.820564322	NS
PSMB1	NS	0.745460516	0.006587429	0.82058313	NS
ZBTB7A	NS	0.745428199	0.006588295	0.82058313	NS
RHPN1	NS	0.74551254	-0.006586034	0.820593729	NS
GJC3	NS	0.745580827	0.006584204	0.820622227	NS
RBM6	NS	0.745708447	0.006580783	0.820716023	NS
ST8SIA4	NS	0.74578803	0.00657865	0.820756942	NS
LY86	NS	0.745879848	-0.006576189	0.820811321	NS
PPIG	NS	0.745929525	0.006574857	0.820819322	NS
TNNT1	NS	0.74599484	0.006573107	0.820822027	NS
TEX15	NS	0.746016795	0.006572518	0.820822027	NS
COMMD3	NS	0.746204777	0.00656748	0.82098219	NS
USP50	NS	0.746292377	0.006565133	0.821031901	NS
RABAC1	NS	0.746341687	-0.006563811	0.821039482	NS
PTGES3	NS	0.746571201	0.006557661	0.821245292	NS
SCARA3	NS	0.746754494	-0.00655275	0.821400238	NS
SPTLC1	NS	0.746842961	0.00655038	0.821450867	NS
ZKSCAN7	NS	0.747137214	-0.006542497	0.82172782	NS
CEPT1	NS	0.747181164	0.006541319	0.821729466	NS

FAM189B	NS	0.747388327	-0.00653577	0.8219106	NS
CYCS	NS	0.747728631	-0.006526655	0.82223812	NS
RITA1	NS	0.747896974	0.006522147	0.822376517	NS
EL1-TNFRSF	NS	0.748002616	-0.006519318	0.822427499	NS
GPR183	NS	0.748070808	-0.006517492	0.822427499	NS
TRAK1	NS	0.748064886	-0.00651765	0.822427499	NS
EPB41	NS	0.748301012	0.006511328	0.822633861	NS
ANO9	NS	0.748597926	0.006503378	0.822913531	NS
CEBPE	NS	0.748694061	0.006500805	0.822959773	NS
FMOD	NS	0.748725026	-0.006499976	0.822959773	NS
OR2J2	NS	0.749105899	0.00648978	0.823331656	NS
TMCO4	NS	0.74920584	0.006487105	0.823394745	NS
UBL4A	NS	0.749370676	0.006482694	0.823529143	NS
RNASE2	NS	0.749723804	0.006473243	0.823870441	NS
PLXNA3	NS	0.749989941	-0.006466122	0.824069329	NS
DPY19L1	NS	0.749958812	-0.006466955	0.824069329	NS
ADCY2	NS	0.750191039	-0.006460742	0.8242435	NS
C8A	NS	0.75064638	-0.006448561	0.824650169	NS
MINDY4B	NS	0.750635485	-0.006448852	0.824650169	NS
SLC16A13	NS	0.750715467	0.006446713	0.824679261	NS
MMAA	NS	0.750862471	-0.006442781	0.824793938	NS
PARP10	NS	0.751160525	0.006434809	0.825074516	NS
NUDCD1	NS	0.751445584	-0.006427186	0.825340789	NS
OR5M9	NS	0.751563667	-0.006424029	0.825423646	NS
YIPF4	NS	0.751607509	0.006422857	0.825424962	NS
PPP1R3F	NS	0.751857763	-0.006416166	0.825564779	NS
LDLR	NS	0.751806813	0.006417528	0.825564779	NS
UBLCP1	NS	0.751862777	0.006416032	0.825564779	NS
CCL4L2	NS	0.751986285	0.00641273	0.825653556	NS
MSH2	NS	0.752625788	-0.006395635	0.826272827	NS
PTCH2	NS	0.75263568	0.006395371	0.826272827	NS
ACADL	NS	0.753094136	-0.006383119	0.826729248	NS
STK26	NS	0.753179149	0.006380848	0.826775682	NS
TUBG2	NS	0.753348676	-0.006376318	0.826867988	NS
ANKRD30A	NS	0.753311159	0.00637732	0.826867988	NS
ZAR1	NS	0.753547076	-0.006371017	0.82699196	NS
WDR27	NS	0.753632868	0.006368725	0.827039221	NS
BIVM-ERCC5	NS	0.753709577	-0.006366676	0.827076511	NS
CSTF1	NS	0.753860452	0.006362645	0.827195176	NS
HYDIN	NS	0.753988878	0.006359215	0.827197984	NS
PURG	NS	0.753991219	-0.006359152	0.827197984	NS
ARHGEF25	NS	0.753920349	0.006361045	0.827197984	NS
PMS1	NS	0.754231434	-0.006352736	0.82737845	NS
H3C7	NS	0.754241205	-0.006352475	0.82737845	NS
MCX5-GPRA	NS	0.754479414	-0.006346113	0.827592856	NS
GRK5	NS	0.754536653	0.006344584	0.827608742	NS
TCP10L2	NS	0.754736726	-0.006339241	0.827734379	NS
NOS1AP	NS	0.754710253	0.006339948	0.827734379	NS
TMEM191C	NS	0.754821936	-0.006336966	0.827780928	NS
REX1BD	NS	0.754943983	-0.006333707	0.827820969	NS
INPP5D	NS	0.754931164	-0.006334049	0.827820969	NS
TRMT44	NS	0.75513754	-0.006328539	0.827986304	NS
LY75	NS	0.755328288	0.006323446	0.82806065	NS
HOXC11	NS	0.75531785	-0.006323725	0.82806065	NS
UBR1	NS	0.755333686	-0.006323302	0.82806065	NS

CBX3	NS	0.755585073	0.006316591	0.828289328	NS
MAPKAPK5	NS	0.755663469	0.006314499	0.828318447	NS
SCAI	NS	0.755697224	0.006313598	0.828318447	NS
POLE	NS	0.755797911	-0.00631091	0.828381901	NS
LDHD	NS	0.755879257	0.006308739	0.828424149	NS
KIAA1210	NS	0.755940403	-0.006307107	0.828444256	NS
FBXW7	NS	0.756037552	0.006304514	0.82845691	NS
RALGAPB	NS	0.756017724	-0.006305043	0.82845691	NS
LAT	NS	0.756195285	0.006300304	0.828582844	NS
HOMEZ	NS	0.756393723	-0.006295008	0.828753363	NS
FAM209A	NS	0.756642388	-0.006288373	0.828978891	NS
NRM	NS	0.756763712	-0.006285136	0.829064886	NS
DHX33	NS	0.756951765	-0.006280119	0.829177043	NS
SLC35A2	NS	0.756923033	-0.006280885	0.829177043	NS
ANKMY1	NS	0.757166809	-0.006274382	0.82936567	NS
PRKDC	NS	0.757478792	-0.006266059	0.829660452	NS
IFT81	NS	0.757978143	-0.006252741	0.83011344	NS
PRPF4B	NS	0.758026601	-0.006251449	0.830119541	NS
TNFSF9	NS	0.758294131	-0.006244315	0.830365535	NS
ZNF205	NS	0.758513668	-0.006238461	0.83046499	NS
STXBP5	NS	0.758432452	0.006240627	0.83046499	NS
TEPSIN	NS	0.758507288	0.006238631	0.83046499	NS
LTF	NS	0.758605572	-0.006236011	0.830471662	NS
FAIM2	NS	0.758586461	-0.00623652	0.830471662	NS
OSGIN1	NS	0.758779417	-0.006231376	0.830614998	NS
MAP4K3	NS	0.759003907	0.006225391	0.830719049	NS
CYP2D6	NS	0.758933274	-0.006227274	0.830719049	NS
MED26	NS	0.759061764	-0.006223849	0.830719049	NS
TNFRSF19	NS	0.759131977	0.006221977	0.830719049	NS
RPL36	NS	0.758988378	0.006225805	0.830719049	NS
TPK1	NS	0.759111892	0.006222513	0.830719049	NS
TBRG1	NS	0.759284965	0.0062179	0.830839492	NS
ERGIC2	NS	0.759416347	0.006214398	0.830841322	NS
SCAMP2	NS	0.759587107	-0.006209847	0.830841322	NS
RGS16	NS	0.759569429	-0.006210318	0.830841322	NS
CELF1	NS	0.759521507	0.006211595	0.830841322	NS
OR1A1	NS	0.759438463	-0.006213808	0.830841322	NS
LPCAT4	NS	0.75941039	0.006214556	0.830841322	NS
NBPF1	NS	0.75947289	-0.006212891	0.830841322	NS
ARF4	NS	0.759667061	0.006207716	0.830881824	NS
DEGS2	NS	0.759814498	0.006203787	0.830996125	NS
HNF1B	NS	0.759996108	0.006198947	0.831147786	NS
PPP1CC	NS	0.760101999	0.006196126	0.831216627	NS
HEATR5A	NS	0.760468852	-0.006186351	0.831523845	NS
RNPUL2-BS(	NS	0.760958098	-0.006173318	0.832011802	NS
MLKL	NS	0.761014702	0.006171811	0.832026693	NS
SLC17A9	NS	0.761395825	-0.00616166	0.832375029	NS
GOLGA7	NS	0.761462319	-0.006159889	0.832375029	NS
SNRNP25	NS	0.761449411	-0.006160233	0.832375029	NS
RBM17	NS	0.761581499	0.006156715	0.832458295	NS
MARCKS	NS	0.761677898	0.006154148	0.832516652	NS
TXNDC17	NS	0.761751109	0.006152199	0.832549659	NS
NR1D2	NS	0.761836914	0.006149914	0.832596426	NS
RNF144B	NS	0.762061276	-0.00614394	0.832794606	NS
DENND4C	NS	0.762236539	0.006139274	0.832901477	NS

TMEM114	NS	0.762245132	-0.006139045	0.832901477	NS
APOBEC3F	NS	0.762742012	0.006125818	0.833350327	NS
ULBP3	NS	0.762718766	0.006126437	0.833350327	NS
CYP8B1	NS	0.76289327	-0.006121792	0.83346854	NS
ORAI2	NS	0.763261872	0.006111982	0.833824179	NS
CTSH	NS	0.76375466	0.006098869	0.834315436	NS
NRG1	NS	0.764024253	0.006091697	0.834562835	NS
WDPCP	NS	0.764127087	-0.006088961	0.834628063	NS
PSMA3	NS	0.764218744	-0.006086523	0.834681075	NS
JAG2	NS	0.764368021	0.006082552	0.83479701	NS
B2M	NS	0.764499513	-0.006079054	0.83489351	NS
MEFV	NS	0.764774851	-0.006071731	0.835147082	NS
CACNA1I	NS	0.764900227	-0.006068396	0.835189756	NS
ENG	NS	0.764879225	-0.006068955	0.835189756	NS
NEIL1	NS	0.765030389	-0.006064935	0.835284759	NS
C16orf87	NS	0.765219159	-0.006059915	0.835325218	NS
SGO2	NS	0.765111819	-0.006062769	0.835325218	NS
CCK	NS	0.765240068	0.006059359	0.835325218	NS
NUTM2F	NS	0.765328251	-0.006057014	0.835342223	NS
ASB16	NS	0.765385117	-0.006055502	0.835342223	NS
BPIFB6	NS	0.765364469	-0.006056051	0.835342223	NS
NAALADL2	NS	0.765582462	0.006050255	0.835510495	NS
CELSR3	NS	0.765907616	-0.00604161	0.835818222	NS
FRG2C	NS	0.766029559	-0.006038368	0.835904168	NS
SLX1A	NS	0.76655773	-0.006024329	0.836386213	NS
MZT2B	NS	0.766541876	0.006024751	0.836386213	NS
GNAS	NS	0.766798129	-0.00601794	0.83650705	NS
MPDU1	NS	0.766791661	0.006018112	0.83650705	NS
SLX9	NS	0.766768932	0.006018716	0.83650705	NS
CORO1B	NS	0.766865119	-0.00601616	0.836532983	NS
TMTC1	NS	0.767600063	-0.005996632	0.837287507	NS
CTU2	NS	0.767655273	0.005995165	0.837300545	NS
RCBTB2	NS	0.76781916	0.005990812	0.837432111	NS
CANX	NS	0.767936813	0.005987686	0.837466052	NS
KALRN	NS	0.767903147	-0.00598858	0.837466052	NS
OR56A4	NS	0.768244808	0.005979505	0.837754732	NS
GRIK2	NS	0.768594476	0.005970219	0.838088822	NS
TPP1	NS	0.768680052	0.005967946	0.838134919	NS
NMRAL1	NS	0.769101133	-0.005956765	0.83854681	NS
SEC22A	NS	0.769217569	-0.005953673	0.838626522	NS
POLR1G	NS	0.769413831	0.005948462	0.838746008	NS
ABT1	NS	0.769381216	0.005949328	0.838746008	NS
VPS11	NS	0.769613084	0.005943173	0.838915968	NS
IL37	NS	0.769689253	-0.005941151	0.838951751	NS
PLD3	NS	0.76994136	0.005934459	0.839179288	NS
ADGRB2	NS	0.770014723	0.005932512	0.839211992	NS
ZNF70	NS	0.77028789	-0.005925261	0.839462441	NS
HAGHL	NS	0.770376715	-0.005922904	0.839511974	NS
SPIN3	NS	0.770731165	-0.005913498	0.839850951	NS
TMEM250	NS	0.771040211	0.005905298	0.840140415	NS
CPEB1	NS	0.771516794	0.005892654	0.840612388	NS
LYRM7	NS	0.771896108	-0.005882593	0.840978333	NS
ISCU	NS	0.772069668	-0.00587799	0.841120081	NS
KDM4C	NS	0.772133928	0.005876286	0.841142746	NS
HSDL1	NS	0.772425591	-0.005868551	0.841413121	NS

SIAH2	NS	0.772513143	-0.005866229	0.841413786	NS
HNRNPUL2	NS	0.772498988	0.005866605	0.841413786	NS
NFAM1	NS	0.772658344	-0.005862379	0.841524584	NS
WNT7A	NS	0.772853633	0.005857201	0.841689918	NS
PRR14	NS	0.773149598	0.005849354	0.841908994	NS
BNIPL	NS	0.773157343	0.005849149	0.841908994	NS
TRIM49	NS	0.77318528	-0.005848408	0.841908994	NS
IFT80	NS	0.773364596	0.005843655	0.841914799	NS
SMNDC1	NS	0.773289284	0.005845651	0.841914799	NS
SCN4B	NS	0.77326469	0.005846303	0.841914799	NS
SLX1B	NS	0.773328682	-0.005844607	0.841914799	NS
SETD5	NS	0.773561482	-0.005838436	0.842077456	NS
VWA3A	NS	0.773601019	-0.005837388	0.842077456	NS
PLEKHB1	NS	0.773694238	-0.005834917	0.842131568	NS
TMEM231	NS	0.77421312	0.005821165	0.842648963	NS
THUMPD1	NS	0.774905795	0.005802811	0.843201394	NS
CST7	NS	0.774853898	-0.005804186	0.843201394	NS
POU2AF1	NS	0.774823259	0.005804998	0.843201394	NS
AVPI1	NS	0.7749385	-0.005801945	0.843201394	NS
OR2I1P	NS	0.774774222	-0.005806297	0.843201394	NS
ZNF317	NS	0.774988087	-0.005800631	0.843207949	NS
ETV3L	NS	0.775256565	-0.005793519	0.843452648	NS
PKD2L2	NS	0.776038557	0.005772807	0.844255975	NS
FABP5	NS	0.776251351	-0.005767172	0.844440013	NS
INSIG2	NS	0.776320069	-0.005765352	0.844447613	NS
MAP1LC3B2	NS	0.776345592	0.005764676	0.844447613	NS
NT5DC3	NS	0.776772816	-0.005753365	0.844627528	NS
ATG3	NS	0.776710563	0.005755013	0.844627528	NS
OR7D4	NS	0.776593527	0.005758112	0.844627528	NS
LAMB3	NS	0.776679725	-0.005755829	0.844627528	NS
OR6K3	NS	0.776689109	-0.005755581	0.844627528	NS
MTRNR2L12	NS	0.77675357	-0.005753874	0.844627528	NS
NDUFA11	NS	0.776869252	-0.005750812	0.844684937	NS
DCLK1	NS	0.777035904	-0.0057464	0.844694899	NS
MICU2	NS	0.777140254	-0.005743638	0.844694899	NS
ZBTB4	NS	0.777029464	0.00574657	0.844694899	NS
COX14	NS	0.777133158	-0.005743825	0.844694899	NS
TCHHL1	NS	0.776955295	-0.005748534	0.844694899	NS
SLC35B4	NS	0.777136523	0.005743736	0.844694899	NS
DDX42	NS	0.777553196	0.005732707	0.845096281	NS
CCDC14	NS	0.778341737	-0.00571184	0.84590582	NS
CMAS	NS	0.778521019	-0.005707096	0.846025872	NS
ELF3	NS	0.778539617	-0.005706604	0.846025872	NS
OAS3	NS	0.778622753	0.005704404	0.846068715	NS
SMIM39	NS	0.778769683	0.005700517	0.846180868	NS
RUSC2	NS	0.779072026	0.005692519	0.846461866	NS
NF511-PRAP	NS	0.779221218	-0.005688573	0.846576443	NS
SIRPB2	NS	0.779310121	0.005686221	0.84662551	NS
CCR6	NS	0.779367854	0.005684694	0.846640711	NS
RSPH9	NS	0.779461723	0.005682211	0.846695163	NS
CSF2RA	NS	0.779616945	0.005678106	0.84681625	NS
CSMD2	NS	0.779695266	-0.005676035	0.8468538	NS
FANCC	NS	0.779779685	0.005673802	0.846897968	NS
BRCA2	NS	0.780152021	-0.005663956	0.847254812	NS
FBXO15	NS	0.780331627	0.005659207	0.847378739	NS

ISY1-RAB43	NS	0.78035369	-0.005658624	0.847378739	NS
KRTAP5-10	NS	0.780406704	-0.005657222	0.847388768	NS
CTSD	NS	0.780482282	-0.005655224	0.847423293	NS
NDUFC1	NS	0.781338994	0.005632576	0.848258318	NS
ST6GALNAC	NS	0.781322085	-0.005633023	0.848258318	NS
HDAC2	NS	0.781739964	0.005621979	0.848598438	NS
ALG11	NS	0.781827543	0.005619665	0.848645913	NS
SLC15A1	NS	0.781884425	-0.005618162	0.848660064	NS
EFEMP1	NS	0.78199331	0.005615284	0.848699471	NS
CASQ1	NS	0.782008425	0.005614885	0.848699471	NS
GRM6	NS	0.782183032	0.005610271	0.84882842	NS
PPP1R1C	NS	0.782214948	-0.005609428	0.84882842	NS
FAHD2B	NS	0.782627958	-0.005598516	0.849228992	NS
TLR2	NS	0.782835438	-0.005593035	0.84940651	NS
KRT74	NS	0.783179424	0.005583948	0.849732115	NS
TRAF7	NS	0.783230548	-0.005582598	0.849739952	NS
ANO7	NS	0.783483328	-0.005575922	0.849966557	NS
FARP1	NS	0.78369093	0.005570439	0.8501308	NS
ZNF429	NS	0.783722567	-0.005569604	0.8501308	NS
VSIG2	NS	0.783959213	-0.005563354	0.850196914	NS
KRT72	NS	0.783895064	0.005565048	0.850196914	NS
L3MBTL3	NS	0.783843265	0.005566416	0.850196914	NS
OR13A1	NS	0.783921192	-0.005564358	0.850196914	NS
DARS1	NS	0.784144853	-0.005558453	0.850350595	NS
PSMB2	NS	0.784486176	0.005549441	0.850673077	NS
SGCE	NS	0.784625821	0.005545754	0.850773391	NS
DMRTC2	NS	0.784666593	0.005544678	0.850773391	NS
SFT2D1	NS	0.784772826	0.005541873	0.850840913	NS
PPP1R10	NS	0.784871074	0.00553928	0.85089977	NS
PSMA6	NS	0.785158862	0.005531683	0.851164096	NS
SDHAF2	NS	0.785370433	-0.005526099	0.851345771	NS
MMP24	NS	0.78544463	0.005524141	0.851378521	NS
MPEG1	NS	0.785569426	0.005520847	0.851466111	NS
ZNF354A	NS	0.785847821	0.005513501	0.851720165	NS
ELOA	NS	0.785916243	0.005511695	0.85174663	NS
NPIPA5	NS	0.786104023	-0.00550674	0.85190244	NS
C2orf50	NS	0.786163751	0.005505164	0.85191947	NS
RPRD2	NS	0.786309056	-0.00550133	0.85197415	NS
MAMSTR	NS	0.786329054	-0.005500803	0.85197415	NS
USP18	NS	0.786346259	0.005500349	0.85197415	NS
VPS8	NS	0.786922611	0.005485144	0.852503165	NS
IFIT1B	NS	0.786897072	0.005485818	0.852503165	NS
UBA1	NS	0.787035663	-0.005482162	0.852577921	NS
CTCFL	NS	0.787139615	-0.00547942	0.852642811	NS
DDI2	NS	0.787239567	-0.005476784	0.852703361	NS
BIN3	NS	0.787467973	0.00547076	0.852903033	NS
OGG1	NS	0.787728157	-0.005463898	0.853137097	NS
PIR	NS	0.78786832	-0.005460202	0.853162976	NS
NUP153	NS	0.787928361	0.005458619	0.853162976	NS
SAMD9L	NS	0.787884751	0.005459769	0.853162976	NS
SCARF2	NS	0.787893665	-0.005459534	0.853162976	NS
YJU2B	NS	0.788043463	0.005455584	0.853189637	NS
TSPAN1	NS	0.788033221	0.005455854	0.853189637	NS
ZER1	NS	0.78808522	-0.005454483	0.853189637	NS
TIAL1	NS	0.788205648	-0.005451307	0.853272289	NS

SBNO1	NS	0.788292544	0.005449016	0.853318633	NS
ACSM6	NS	0.788366064	-0.005447078	0.853350494	NS
KCNH1	NS	0.788479063	-0.005444099	0.853387337	NS
SWSAP1	NS	0.78848828	0.005443856	0.853387337	NS
LAMP3	NS	0.788810732	-0.005435355	0.853593142	NS
RHEB	NS	0.788729903	-0.005437486	0.853593142	NS
CHD4	NS	0.788799242	-0.005435658	0.853593142	NS
SLC25A47	NS	0.788911844	-0.00543269	0.853654833	NS
GOLGA8J	NS	0.789152339	0.00542635	0.8538558	NS
RAP2B	NS	0.789185796	-0.005425469	0.8538558	NS
NOPCHAP1	NS	0.789610856	-0.005414266	0.854254551	NS
RBM15B	NS	0.789642613	0.005413429	0.854254551	NS
WDR18	NS	0.789850017	0.005407963	0.85443117	NS
FOXD4L6	NS	0.790254514	0.005397305	0.854820966	NS
CREB1	NS	0.790350232	-0.005394783	0.85487673	NS
TM9SF1	NS	0.790521519	-0.00539027	0.855014223	NS
MS4A4A	NS	0.790768734	0.005383758	0.855199279	NS
ESYT1	NS	0.790780983	-0.005383435	0.855199279	NS
RNASEH2B	NS	0.790993757	0.00537783	0.855381594	NS
TMEM91	NS	0.791182367	-0.005372862	0.855537759	NS
ZNF391	NS	0.791247759	0.00537114	0.855560674	NS
BDH2	NS	0.791645784	-0.005360657	0.855895425	NS
PLPPR2	NS	0.792154678	-0.005347256	0.856397786	NS
DNAJC18	NS	0.79222831	-0.005345318	0.856429555	NS
COPS7A	NS	0.792275971	0.005344063	0.856433245	NS
ANO3	NS	0.79238841	-0.005341102	0.856506957	NS
CA11	NS	0.792635111	-0.005334607	0.856725776	NS
KPNA2	NS	0.792795851	0.005330376	0.856782576	NS
RORB	NS	0.792815765	0.005329852	0.856782576	NS
TEX11	NS	0.793030582	-0.005324197	0.85691397	NS
C1QTNF9B	NS	0.792990076	-0.005325263	0.85691397	NS
NLRX1	NS	0.793322444	-0.005316515	0.857181491	NS
CTNS	NS	0.793929463	-0.005300539	0.857410884	NS
DPP8	NS	0.793762481	0.005304934	0.857410884	NS
PLEKHJ1	NS	0.793691952	0.00530679	0.857410884	NS
TRIM23	NS	0.794016177	-0.005298258	0.857410884	NS
DNAH1	NS	0.794022014	0.005298104	0.857410884	NS
TOE1	NS	0.793940762	0.005300242	0.857410884	NS
NF670-ZNF6	NS	0.793712304	0.005306254	0.857410884	NS
CKAP2L	NS	0.793669655	0.005307376	0.857410884	NS
C15orf40	NS	0.793635067	0.005308287	0.857410884	NS
XPNPEP3	NS	0.793970694	0.005299454	0.857410884	NS
DND1	NS	0.793919995	-0.005300789	0.857410884	NS
TIE1	NS	0.794194759	-0.005293559	0.857538308	NS
ABCC10	NS	0.794228624	0.005292668	0.857538308	NS
SDE2	NS	0.794401708	0.005288113	0.857677346	NS
ZNF69	NS	0.794603551	0.005282803	0.857847416	NS
SMAD5	NS	0.7947631	-0.005278606	0.85797181	NS
TESC	NS	0.795184623	-0.005267517	0.858283252	NS
SEPTIN5	NS	0.795158138	0.005268214	0.858283252	NS
PSMB8	NS	0.795129723	0.005268961	0.858283252	NS
POLR2B	NS	0.795280996	0.005264982	0.858322604	NS
TLR7	NS	0.79530977	-0.005264226	0.858322604	NS
ARRDC5	NS	0.795377638	-0.005262441	0.858347991	NS
HBQ1	NS	0.795450673	0.00526052	0.85837895	NS

M1AP	NS	0.795669714	0.005254759	0.858567454	NS
NUP42	NS	0.795973112	-0.00524678	0.858842536	NS
CYB5D1	NS	0.796013385	0.005245721	0.858842536	NS
PRSS2	NS	0.796318485	0.005237698	0.859123828	NS
RHBDL2	NS	0.796692167	0.005227874	0.859479076	NS
EMC2	NS	0.796869129	-0.005223221	0.859622073	NS
SDHD	NS	0.797021491	0.005219216	0.859738519	NS
ANKRD55	NS	0.797808278	-0.005198536	0.860491308	NS
MYMK	NS	0.797781112	0.00519925	0.860491308	NS
SNX8	NS	0.7978847	0.005196528	0.860522668	NS
H2AL3	NS	0.797926268	0.005195435	0.860522668	NS
NOP10	NS	0.798208143	0.005188028	0.860778696	NS
CLPX	NS	0.798516594	-0.005179923	0.861015386	NS
FAM229A	NS	0.798507246	-0.005180169	0.861015386	NS
NOL11	NS	0.798701573	0.005175063	0.86116687	NS
LIME1	NS	0.798766947	-0.005173346	0.861189385	NS
SARS1	NS	0.798931336	-0.005169027	0.86120734	NS
SYT17	NS	0.798897368	0.00516992	0.86120734	NS
SEPTIN11	NS	0.799050559	0.005165895	0.86120734	NS
KLHDC8A	NS	0.799038213	-0.005166219	0.86120734	NS
MMADHC	NS	0.798994003	-0.005167381	0.86120734	NS
SLC44A4	NS	0.799032118	-0.00516638	0.86120734	NS
ABHD4	NS	0.799158858	0.00516305	0.861276106	NS
CLCN7	NS	0.79931725	0.00515889	0.861398847	NS
CYBC1	NS	0.799509822	0.005153831	0.861558408	NS
PLEC	NS	0.799779891	0.005146738	0.861801457	NS
OR2L2	NS	0.800027948	0.005140224	0.862020761	NS
PPIA	NS	0.800188809	0.005135999	0.862146095	NS
CEP19	NS	0.800450635	0.005129124	0.86238019	NS
ELMOD1	NS	0.800596229	0.005125301	0.862489042	NS
COA1	NS	0.800740646	-0.005121509	0.862573209	NS
NPR3	NS	0.800763484	-0.00512091	0.862573209	NS
BCL2L11	NS	0.800902853	0.005117251	0.862621036	NS
FLNA	NS	0.800941582	0.005116234	0.862621036	NS
GANC	NS	0.80092002	0.0051168	0.862621036	NS
SCARB1	NS	0.801047502	-0.005113453	0.862687111	NS
ITCH	NS	0.801344031	0.005105669	0.862862431	NS
FOXI1	NS	0.801310683	0.005106544	0.862862431	NS
PCSK9	NS	0.801255112	0.005108003	0.862862431	NS
SLC22A18	NS	0.801403349	-0.005104112	0.862878302	NS
PSMD7	NS	0.801633574	0.005098069	0.863078177	NS
APOA1	NS	0.801736236	0.005095374	0.863140696	NS
RSU1	NS	0.801898579	0.005091113	0.863232328	NS
GRIK4	NS	0.801910544	-0.005090799	0.863232328	NS
CDK18	NS	0.802004691	0.005088329	0.863285663	NS
LSM4	NS	0.802323607	0.005079959	0.863554584	NS
ZNF830	NS	0.802343751	0.005079431	0.863554584	NS
LAX1	NS	0.802514631	-0.005074947	0.863690475	NS
CDV3	NS	0.802911428	0.005064535	0.864069476	NS
NAV2	NS	0.803113012	0.005059247	0.864238364	NS
TGM4	NS	0.803293172	0.00505452	0.86438418	NS
CSK	NS	0.803363269	0.005052681	0.864411553	NS
NTPCR	NS	0.803495881	-0.005049203	0.864497694	NS
ZNF677	NS	0.803532652	-0.005048238	0.864497694	NS
EIF2B4	NS	0.803589163	-0.005046756	0.864510441	NS

HIVEP3	NS	0.803724036	-0.005043218	0.864607483	NS
AP1S3	NS	0.80380882	-0.005040994	0.864650635	NS
KDELR2	NS	0.803939855	-0.005037557	0.864689499	NS
RUNX1	NS	0.803978968	0.005036531	0.864689499	NS
ZACN	NS	0.803911685	-0.005038296	0.864689499	NS
KANSL1	NS	0.804291607	-0.005028332	0.864977684	NS
ZDHHC15	NS	0.804348744	-0.005026834	0.864991072	NS
CRYBA4	NS	0.804529971	0.005022081	0.865137895	NS
IRF6	NS	0.804752402	-0.005016249	0.865329009	NS
ELP5	NS	0.804958831	0.005010836	0.865502896	NS
FRG1	NS	0.805046409	0.00500854	0.86554898	NS
NO80B-WBP	NS	0.80509582	0.005007244	0.865554027	NS
UBE2K	NS	0.805340081	-0.00500084	0.865768542	NS
ZBED4	NS	0.805505062	-0.004996515	0.865897811	NS
IL36A	NS	0.80565197	0.004992664	0.865966939	NS
IMPACT	NS	0.805658847	0.004992484	0.865966939	NS
SCGB2A1	NS	0.805914072	-0.004985794	0.866193169	NS
PMF1	NS	0.806003399	0.004983452	0.866241077	NS
FAM172A	NS	0.806120225	0.00498039	0.866270434	NS
CCDC122	NS	0.806110562	0.004980644	0.866270434	NS
SERPINA6	NS	0.806324868	0.004975027	0.866442245	NS
ZNF441	NS	0.806407748	-0.004972855	0.866483201	NS
MPP2	NS	0.806469573	0.004971235	0.866501529	NS
C1GALT1	NS	0.806640373	-0.004966759	0.866626276	NS
HERC5	NS	0.806675223	-0.004965845	0.866626276	NS
GPR160	NS	0.806844548	-0.004961408	0.866760077	NS
EIF3I	NS	0.8074346	0.004945948	0.867345809	NS
AP1M2	NS	0.807484375	0.004944644	0.867351142	NS
PATL2	NS	0.807529368	0.004943465	0.867351337	NS
CDKN1B	NS	0.807656401	-0.004940137	0.867439646	NS
PTPDC1	NS	0.807757343	0.004937493	0.867451793	NS
TSHZ2	NS	0.807740912	-0.004937923	0.867451793	NS
NPR2	NS	0.807871085	0.004934513	0.867477683	NS
C22orf15	NS	0.807828429	0.004935631	0.867477683	NS
ETV7	NS	0.807921915	-0.004933182	0.86748414	NS
NKX6-2	NS	0.808822131	-0.004909604	0.86840255	NS
SSH3	NS	0.80897451	0.004905614	0.868517978	NS
SLC46A1	NS	0.809186316	0.004900068	0.86869719	NS
PNLIPRP1	NS	0.809272774	0.004897804	0.868741823	NS
TRIP13	NS	0.809358245	-0.004895566	0.868785392	NS
TMPPE	NS	0.809410241	0.004894204	0.868793025	NS
UNC5A	NS	0.809563126	0.004890202	0.868908942	NS
ROR2	NS	0.809766749	0.004884871	0.869079301	NS
ECE2	NS	0.809936236	0.004880434	0.869213006	NS
NXF2B	NS	0.810097088	0.004876223	0.869337431	NS
NRXN3	NS	0.810395521	-0.004868411	0.869561268	NS
ZDHHC20	NS	0.810377104	0.004868893	0.869561268	NS
IGFBP4	NS	0.810735214	0.00485952	0.869829322	NS
CYHR1	NS	0.810705827	-0.004860289	0.869829322	NS
CA14	NS	0.810831085	-0.004857011	0.869883964	NS
FNBP1	NS	0.811618997	-0.004836393	0.870681002	NS
LTBP2	NS	0.811709465	-0.004834026	0.870681541	NS
FAM98C	NS	0.811666348	0.004835154	0.870681541	NS
PIGL	NS	0.812071135	-0.004824564	0.871021218	NS
PPHLN1	NS	0.812514755	-0.004812959	0.871448751	NS

ADAMTSL4	NS	0.812591069	0.004810963	0.87148231	NS
NDRG2	NS	0.812710763	-0.004807832	0.871562387	NS
ZNF20	NS	0.812973647	-0.004800956	0.871699419	NS
RPS27A	NS	0.812937463	0.004801902	0.871699419	NS
SUMF1	NS	0.812907727	0.00480268	0.871699419	NS
ALG9	NS	0.813117081	-0.004797205	0.87180492	NS
COBL	NS	0.813188429	-0.004795339	0.871833125	NS
POU2F1	NS	0.813795488	-0.004779464	0.872435639	NS
RPS19BP1	NS	0.814014524	0.004773737	0.872622127	NS
CLUH	NS	0.814164171	0.004769825	0.872734214	NS
CCDC6	NS	0.814211014	-0.0047686	0.872736094	NS
GNG14	NS	0.814573606	0.004759121	0.873062939	NS
TAF11L7	NS	0.814606152	-0.00475827	0.873062939	NS
ASS1	NS	0.81467927	0.004756359	0.87309296	NS
AKR1D1	NS	0.814898603	0.004750626	0.873182981	NS
NMNAT1	NS	0.814881632	-0.004751069	0.873182981	NS
COTL1	NS	0.81509129	-0.004745589	0.873216259	NS
CCNG1	NS	0.815110114	0.004745097	0.873216259	NS
CCZ1B	NS	0.815057834	0.004746464	0.873216259	NS
AIFM3	NS	0.81509204	0.004745569	0.873216259	NS
RPS23	NS	0.815158495	0.004743833	0.873219759	NS
KDM5A	NS	0.815427027	0.004736814	0.873381988	NS
CCDC141	NS	0.815445303	0.004736337	0.873381988	NS
TRH	NS	0.815367361	-0.004738374	0.873381988	NS
SKIV2L	NS	0.815566247	-0.004733176	0.873463193	NS
TMEM241	NS	0.815685286	0.004730065	0.87349402	NS
FICD	NS	0.815668016	-0.004730516	0.87349402	NS
MLF1	NS	0.815790989	-0.004727303	0.873558884	NS
LGALS4	NS	0.815908784	0.004724225	0.873588363	NS
OST4	NS	0.815901126	-0.004724425	0.873588363	NS
FAM83D	NS	0.816015452	-0.004721437	0.873654245	NS
PAK6	NS	0.816161006	0.004717634	0.873761749	NS
ZSCAN5B	NS	0.816292944	0.004714187	0.873854667	NS
NAE1	NS	0.816370265	-0.004712167	0.873889108	NS
TMEM25	NS	0.816611257	-0.00470587	0.874098738	NS
FBXL2	NS	0.816740396	-0.004702497	0.874188626	NS
APOL4	NS	0.816959132	-0.004696783	0.874374396	NS
ABCB5	NS	0.817083226	-0.004693541	0.874410511	NS
CRH	NS	0.817068306	0.004693931	0.874410511	NS
AP2M1	NS	0.817372639	-0.004685981	0.874644356	NS
B3GNT2	NS	0.817437301	0.004684292	0.874644356	NS
STMP1	NS	0.817429611	0.004684493	0.874644356	NS
PROK1	NS	0.818154913	-0.004665551	0.875359023	NS
CNOT10	NS	0.818195673	0.004664487	0.875359023	NS
NENF	NS	0.818337865	0.004660774	0.875451527	NS
XG	NS	0.818798585	-0.004648744	0.875762028	NS
CYP7A1	NS	0.818788254	0.004649014	0.875762028	NS
OR6V1	NS	0.81876743	0.004649558	0.875762028	NS
ALG6	NS	0.81908756	-0.0046412	0.875974298	NS
MTLN	NS	0.819081048	-0.00464137	0.875974298	NS
SENP6	NS	0.819231818	0.004637434	0.876080169	NS
FXR1	NS	0.819471379	0.00463118	0.876260501	NS
FABP6	NS	0.819490989	0.004630669	0.876260501	NS
SPARC	NS	0.819602454	-0.004627759	0.876331276	NS
TMEM144	NS	0.819733704	-0.004624333	0.876423198	NS

MAEL	NS	0.819861983	-0.004620985	0.876477178	NS
OR1J4	NS	0.819874756	-0.004620652	0.876477178	NS
RNF10	NS	0.820076541	0.004615385	0.876588478	NS
XKRX	NS	0.820114731	0.004614388	0.876588478	NS
UBXN11	NS	0.82023766	0.00461118	0.87667146	NS
BEST4	NS	0.820284608	-0.004609955	0.87667323	NS
CLTCL1	NS	0.820434411	-0.004606046	0.876736513	NS
LRFN4	NS	0.820413504	0.004606591	0.876736513	NS
CHIC2	NS	0.820750225	0.004597805	0.876985022	NS
MAP1-GIMA	NS	0.820869481	0.004594693	0.877056176	NS
PPP1R3C	NS	0.821026461	-0.004590597	0.877064551	NS
MDC1	NS	0.821000657	-0.00459127	0.877064551	NS
CLSTN2	NS	0.821058569	-0.004589759	0.877064551	NS
KLHL25	NS	0.820935423	0.004592972	0.877064551	NS
ABCB9	NS	0.821171312	0.004586818	0.877136578	NS
FAM135A	NS	0.821329767	0.004582684	0.877209014	NS
MYCL	NS	0.821473303	-0.004578939	0.877217341	NS
ASPHD2	NS	0.821473524	-0.004578933	0.877217341	NS
DDRGK1	NS	0.821471369	0.004578989	0.877217341	NS
FUS	NS	0.821870883	0.004568568	0.877593248	NS
NUP210L	NS	0.821984417	0.004565606	0.877666063	NS
NUTM2D	NS	0.822123028	-0.004561991	0.877765643	NS
LRP6	NS	0.822658179	-0.004548033	0.878212241	NS
BDH1	NS	0.822682176	-0.004547407	0.878212241	NS
ZC3H7B	NS	0.8229868	0.004539464	0.878348727	NS
NAA30	NS	0.822941381	0.004540648	0.878348727	NS
GHITM	NS	0.822984775	-0.004539516	0.878348727	NS
RUNDC3A	NS	0.82312365	-0.004535895	0.878446346	NS
NDUFS3	NS	0.823269818	0.004532084	0.878553898	NS
CDC7	NS	0.823396635	0.004528777	0.878640788	NS
DNAH14	NS	0.823459972	0.004527126	0.878659935	NS
CENPM	NS	0.823506552	-0.004525911	0.878661199	NS
LAIR1	NS	0.8236185	0.004522992	0.878732206	NS
HSF5	NS	0.823674836	-0.004521524	0.878743875	NS
ANP32B	NS	0.823851857	-0.004516909	0.878835853	NS
SMIM43	NS	0.823820343	0.00451773	0.878835853	NS
POLR2L	NS	0.823898685	0.004515688	0.878837371	NS
CCDC174	NS	0.824031025	-0.004512238	0.8789301	NS
FAM13B	NS	0.824717568	-0.004494342	0.879613911	NS
GRHPR	NS	0.825205167	-0.004481634	0.880085471	NS
FRMD3	NS	0.825253757	0.004480368	0.8800888	NS
CHMP1A	NS	0.825336596	-0.004478209	0.880102241	NS
CPTP	NS	0.825357299	0.00447767	0.880102241	NS
MAZ	NS	0.82556575	-0.004472238	0.880227535	NS
PDLIM2	NS	0.825552296	-0.004472589	0.880227535	NS
GTF3C1	NS	0.825665986	0.004469626	0.880237432	NS
RUFY1	NS	0.825658099	-0.004469832	0.880237432	NS
DCAF17	NS	0.825845977	-0.004464936	0.880332346	NS
TMEM64	NS	0.825825216	0.004465477	0.880332346	NS
ERP44	NS	0.82596146	-0.004461927	0.880361257	NS
DEFB118	NS	0.825964065	-0.00446186	0.880361257	NS
SPATA19	NS	0.826181857	-0.004456185	0.880544905	NS
GAREM1	NS	0.82639678	0.004450586	0.880725475	NS
ABCD3	NS	0.826635267	0.004444373	0.880882638	NS
OR11H6	NS	0.826600713	-0.004445274	0.880882638	NS

NLGN3	NS	0.826786477	0.004440435	0.880995268	NS
RNF126	NS	0.826895267	-0.004437601	0.881062688	NS
HAS3	NS	0.827094199	-0.004432419	0.881226142	NS
PSMB9	NS	0.82715774	0.004430764	0.881245333	NS
CFAP100	NS	0.827934849	-0.004410526	0.882024709	NS
RASA4	NS	0.82805311	0.004407447	0.882043966	NS
MRPS23	NS	0.828073841	0.004406907	0.882043966	NS
NUDT19	NS	0.828089634	0.004406495	0.882043966	NS
MROH6	NS	0.82815184	-0.004404876	0.882061686	NS
MFSD6	NS	0.828428252	-0.004397679	0.882307541	NS
THADA	NS	0.828552498	-0.004394444	0.882391315	NS
RNASE13	NS	0.828690904	0.00439084	0.882490159	NS
CDC42SE1	NS	0.828963994	0.004383731	0.882732413	NS
PAN-P2RY1	NS	0.829075561	0.004380826	0.88280265	NS
PEX5L	NS	0.82925136	0.00437625	0.882929661	NS
DENND10	NS	0.829286074	-0.004375347	0.882929661	NS
ITIH4	NS	0.829816205	0.004361548	0.883445491	NS
PCNT	NS	0.829956524	0.004357896	0.88354628	NS
NIN	NS	0.830020762	0.004356224	0.883566071	NS
PPA2	NS	0.830230702	0.004350761	0.883692354	NS
ZYX	NS	0.830195021	0.004351689	0.883692354	NS
GRIN2B	NS	0.830298818	-0.004348988	0.883716261	NS
QPRT	NS	0.83049613	0.004343854	0.883877665	NS
CCL20	NS	0.830701345	0.004338514	0.883995289	NS
HAX1	NS	0.830743661	0.004337413	0.883995289	NS
OR56B4	NS	0.830719678	0.004338037	0.883995289	NS
KIF9	NS	0.830855212	-0.00433451	0.884054436	NS
VAV3	NS	0.830890592	0.00433359	0.884054436	NS
GREM1	NS	0.831146225	-0.004326939	0.884277817	NS
RNF141	NS	0.831258098	0.004324028	0.884348232	NS
OR2T11	NS	0.831656704	0.004313658	0.88472367	NS
PIGB	NS	0.831976685	0.004305335	0.884966792	NS
MOB2	NS	0.832072092	-0.004302853	0.88501964	NS
DPAGT1	NS	0.832219078	-0.00429903	0.885127341	NS
ZFX	NS	0.832575902	0.00428975	0.885458195	NS
HERC6	NS	0.832979048	-0.004279266	0.885838275	NS
WSCD1	NS	0.833082068	0.004276587	0.88589916	NS
PIAS1	NS	0.833183869	-0.00427394	0.885958741	NS
RBMS1	NS	0.833491738	-0.004265935	0.886188744	NS
SELENOS	NS	0.833773576	0.004258607	0.886439709	NS
MICU1	NS	0.833979812	0.004253245	0.886512891	NS
RB1	NS	0.833959287	0.004253779	0.886512891	NS
HNRNPLL	NS	0.833977775	-0.004253298	0.886512891	NS
LTN1	NS	0.834134536	-0.004249223	0.88662867	NS
CCDC51	NS	0.834350156	-0.004243618	0.886793824	NS
CYB5R2	NS	0.834381542	-0.004242802	0.886793824	NS
KRTDAP	NS	0.834949121	-0.004228049	0.887348332	NS
APOBEC3D	NS	0.835029012	-0.004225973	0.887384515	NS
FAM241A	NS	0.835379964	0.004216852	0.887708733	NS
F12	NS	0.835722517	-0.004207951	0.888023992	NS
GAS2L3	NS	0.835939466	-0.004202313	0.888205758	NS
CATSPER4	NS	0.836012214	-0.004200423	0.888234297	NS
MRFAP1	NS	0.836100089	-0.00419814	0.888278902	NS
POGLUT1	NS	0.836391063	0.00419058	0.888516958	NS
CDCA4	NS	0.836415969	-0.004189933	0.888516958	NS

ZNF585A	NS	0.836833723	-0.00417908	0.888911949	NS
TCIRG1	NS	0.837017298	-0.004174312	0.889009373	NS
PENK	NS	0.83698005	-0.004175279	0.889009373	NS
GRIPAP1	NS	0.837173331	-0.004170258	0.889102552	NS
GSE1	NS	0.837196896	-0.004169646	0.889102552	NS
TNNT2	NS	0.837364396	0.004165296	0.889176268	NS
LYZL2	NS	0.837397759	-0.004164429	0.889176268	NS
SLFN13	NS	0.83745006	-0.004163071	0.889176268	NS
GAGE12H	NS	0.837420532	-0.004163838	0.889176268	NS
TNNC1	NS	0.837534213	0.004160885	0.889216841	NS
MAPRE1	NS	0.838015877	0.004148376	0.889679427	NS
PRKRA	NS	0.838186975	0.004143933	0.889763469	NS
IFNA13	NS	0.83817814	0.004144162	0.889763469	NS
VWA2	NS	0.838412511	0.004138076	0.889954075	NS
DALRD3	NS	0.838610255	0.004132941	0.890115163	NS
VPS13C	NS	0.838695595	-0.004130725	0.89015693	NS
SLC35E2B	NS	0.838777301	0.004128604	0.890194837	NS
AP5M1	NS	0.839043128	0.004121702	0.890428136	NS
KBTBD11	NS	0.839237716	-0.00411665	0.890585813	NS
ALDH6A1	NS	0.839317004	-0.004114592	0.890621125	NS
CCDC70	NS	0.839806082	-0.004101896	0.891042401	NS
FBF1	NS	0.839786209	-0.004102412	0.891042401	NS
KCNA1	NS	0.84000428	-0.004096752	0.89120384	NS
LRFN1	NS	0.840431255	-0.00408567	0.891578701	NS
FRMD4A	NS	0.840495792	-0.004083995	0.891578701	NS
OR5T3	NS	0.840460693	-0.004084906	0.891578701	NS
GPR39	NS	0.840706112	-0.004078537	0.891752932	NS
FAM234A	NS	0.841019779	0.004070397	0.892036761	NS
ZNF675	NS	0.84136104	0.004061542	0.892349824	NS
P2RX4	NS	0.841585966	0.004055706	0.892539475	NS
EMC7	NS	0.842299916	-0.004037184	0.893180025	NS
ASXL2	NS	0.842438141	0.004033598	0.893180025	NS
API5	NS	0.842454228	0.004033181	0.893180025	NS
DHX36	NS	0.842286872	0.004037522	0.893180025	NS
DNASE1	NS	0.842386761	-0.004034931	0.893180025	NS
ETDA	NS	0.842466818	-0.004032854	0.893180025	NS
ITGA2B	NS	0.842640516	-0.004028349	0.893235792	NS
MYH11	NS	0.842624603	0.004028762	0.893235792	NS
ACTR2	NS	0.842657861	0.004027899	0.893235792	NS
GPATCH2L	NS	0.84286231	0.004022596	0.893377508	NS
TMEM97	NS	0.842883863	0.004022037	0.893377508	NS
STRIP2	NS	0.842988187	-0.004019331	0.893439158	NS
CTSA	NS	0.843243506	-0.004012709	0.893611897	NS
PCBP4	NS	0.843203812	0.004013739	0.893611897	NS
BFAR	NS	0.843935755	-0.003994758	0.894296533	NS
STAR	NS	0.844160191	0.003988939	0.894485392	NS
RCSD1	NS	0.844246708	-0.003986695	0.894528097	NS
ICE2	NS	0.844525994	-0.003979454	0.894726061	NS
BTBD18	NS	0.844508713	0.003979902	0.894726061	NS
SF3A3	NS	0.84469196	-0.003975151	0.894852914	NS
ZNF19	NS	0.844836822	0.003971396	0.894908418	NS
PPM1M	NS	0.844815575	0.003971947	0.894908418	NS
TNFRSF1A	NS	0.844907705	-0.003969558	0.894934526	NS
TSN	NS	0.845022756	0.003966576	0.895007412	NS
TCEANC2	NS	0.845282362	-0.003959846	0.895233388	NS

RADX	NS	0.84572024	-0.003948497	0.895648135	NS
RASGRP3	NS	0.845992108	0.003941451	0.895838024	NS
MRPL3	NS	0.846098045	0.003938705	0.89590119	NS
DDX47	NS	0.846317336	0.003933022	0.89608437	NS
LTBR	NS	0.846372458	0.003931594	0.896093715	NS
PTPMT1	NS	0.846841298	-0.003919445	0.896541058	NS
PPM1J	NS	0.846895054	-0.003918052	0.896548931	NS
SUDS3	NS	0.846994577	0.003915473	0.896605252	NS
MIB2	NS	0.847349475	-0.003906278	0.896931884	NS
TPMT	NS	0.847544409	-0.003901228	0.897040113	NS
BUB3	NS	0.847528802	-0.003901632	0.897040113	NS
PLBD2	NS	0.847860366	0.003893043	0.897325455	NS
CHCHD2	NS	0.847912626	-0.003891689	0.8973317	NS
ZNF233	NS	0.848146505	0.003885631	0.897435151	NS
PIP4P1	NS	0.848149473	0.003885554	0.897435151	NS
ULK4	NS	0.848058871	-0.003887901	0.897435151	NS
TNF	NS	0.848222476	-0.003883663	0.897463335	NS
TRIM52	NS	0.848341216	0.003880587	0.897539905	NS
SBNO2	NS	0.84858147	0.003874365	0.897548794	NS
CHTF18	NS	0.848501207	0.003876443	0.897548794	NS
FGFBP1	NS	0.84857756	-0.003874466	0.897548794	NS
ZBTB7B	NS	0.848511554	-0.003876175	0.897548794	NS
HS6ST3	NS	0.848549342	0.003875197	0.897548794	NS
CCR5	NS	0.848732713	0.003870447	0.897659712	NS
ATRX	NS	0.84900151	-0.003863486	0.897894941	NS
CCDC88B	NS	0.849124308	0.003860306	0.897975747	NS
ISH5-SAPCD	NS	0.849461804	-0.003851566	0.898283581	NS
UBL7	NS	0.849530039	0.003849799	0.898306662	NS
NPDC1	NS	0.849677002	0.003845994	0.898412982	NS
TMTC2	NS	0.849841677	0.00384173	0.898538018	NS
LPXN	NS	0.850212741	0.003832123	0.898881244	NS
TDRD7	NS	0.85029227	0.003830064	0.898916226	NS
WDR4	NS	0.85070636	0.003819344	0.899255764	NS
SPINT3	NS	0.85104131	-0.003810673	0.899477462	NS
SPAG1	NS	0.851065559	-0.003810046	0.899477462	NS
MPST	NS	0.851137799	-0.003808176	0.899477462	NS
TBC1D10C	NS	0.851148439	-0.0038079	0.899477462	NS
NUP210	NS	0.85153528	0.003797888	0.89983714	NS
RFXANK	NS	0.851977299	-0.003786448	0.90021453	NS
ACSS1	NS	0.85202147	-0.003785305	0.90021453	NS
NDUFB8	NS	0.852031938	-0.003785034	0.90021453	NS
BORCS5	NS	0.85213379	0.003782398	0.900273	NS
MFSD14B	NS	0.852461056	-0.00377393	0.9005696	NS
TAPBP	NS	0.852892495	-0.003762766	0.900976212	NS
AMY1C	NS	0.852974901	-0.003760634	0.90101409	NS
PFKL	NS	0.853279613	-0.003752751	0.901286777	NS
WDR33	NS	0.853493823	0.003747209	0.901463846	NS
ZNF436	NS	0.853762182	-0.003740267	0.901648887	NS
PLCL2	NS	0.853733692	0.003741004	0.901648887	NS
FOXB1	NS	0.853873983	-0.003737375	0.901717759	NS
ATG16L1	NS	0.853928524	0.003735964	0.90172616	NS
ACO1	NS	0.854409542	0.003723522	0.902184883	NS
SLC35A1	NS	0.854507233	-0.003720996	0.902189603	NS
MIDN	NS	0.854498436	0.003721223	0.902189603	NS
NPIPA2	NS	0.854648887	0.003717332	0.902289945	NS

PPFIA3	NS	0.854928626	0.003710098	0.902536051	NS
OR8B2	NS	0.855150384	-0.003704363	0.902720922	NS
ABTB1	NS	0.855263527	-0.003701437	0.902741893	NS
FBXW5	NS	0.855223359	0.003702476	0.902741893	NS
CCL1	NS	0.855555491	0.003693887	0.903000822	NS
CLDN12	NS	0.855658745	-0.003691218	0.90306056	NS
C20orf27	NS	0.855798843	0.003687595	0.903159174	NS
MAPRE3	NS	0.855919466	-0.003684476	0.903237226	NS
XKR6	NS	0.856261174	0.003675642	0.903548562	NS
BUB1B	NS	0.856519486	0.003668964	0.90377187	NS
MTRR	NS	0.856566517	0.003667748	0.903772227	NS
IL27	NS	0.856685788	-0.003664665	0.903848802	NS
SCIMP	NS	0.857210752	-0.003651095	0.904304083	NS
C3orf84	NS	0.857198496	0.003651412	0.904304083	NS
NUDT18	NS	0.857355525	-0.003647353	0.904407517	NS
OARD1	NS	0.857529138	0.003642866	0.904541362	NS
EXO1	NS	0.857894153	-0.003633432	0.904877076	NS
RPS6KA5	NS	0.857992994	0.003630878	0.904882711	NS
LMF1	NS	0.857973559	0.00363138	0.904882711	NS
TATDN3	NS	0.858049843	0.003629409	0.904893362	NS
SHPRH	NS	0.858311687	-0.003622642	0.90508052	NS
FAM47A	NS	0.858320832	-0.003622406	0.90508052	NS
HTR5A	NS	0.858544028	-0.003616639	0.905266558	NS
GASK1A	NS	0.858707837	0.003612406	0.905339784	NS
DLAT	NS	0.858753794	0.003611219	0.905339784	NS
CDRT1	NS	0.858731456	0.003611796	0.905339784	NS
MSI2	NS	0.858895769	0.00360755	0.905440146	NS
PROKR1	NS	0.859105227	-0.003602138	0.905519377	NS
MAFF	NS	0.859111275	0.003601982	0.905519377	NS
C5orf47	NS	0.859058346	0.00360335	0.905519377	NS
MRPL20	NS	0.859169759	-0.003600471	0.90553171	NS
MRPL4	NS	0.859611637	-0.003589056	0.905850708	NS
SMCO4	NS	0.859597898	-0.00358941	0.905850708	NS
PIPOX	NS	0.859612823	0.003589025	0.905850708	NS
GTF2E1	NS	0.859671641	0.003587505	0.905863373	NS
RNF125	NS	0.859733067	-0.003585919	0.905878783	NS
MLLT10	NS	0.859846998	0.003582976	0.905899968	NS
PTCD1	NS	0.859977233	-0.003579611	0.905899968	NS
HAVCR1	NS	0.860058749	0.003577506	0.905899968	NS
CHD6	NS	0.860127589	0.003575728	0.905899968	NS
EIF2D	NS	0.860026517	0.003578338	0.905899968	NS
NICN1	NS	0.859869383	-0.003582397	0.905899968	NS
DFFB	NS	0.860084834	-0.003576832	0.905899968	NS
PLD6	NS	0.860044751	-0.003577867	0.905899968	NS
SMC1A	NS	0.860211632	-0.003573557	0.905939189	NS
DNMT3B	NS	0.860285711	-0.003571643	0.905967912	NS
TFAP2C	NS	0.861042271	-0.003552103	0.906487963	NS
NINL	NS	0.861066322	0.003551482	0.906487963	NS
NFKBIB	NS	0.861198559	-0.003548067	0.906487963	NS
SMURF2	NS	0.861247863	0.003546794	0.906487963	NS
VNN1	NS	0.860971164	0.00355394	0.906487963	NS
PPP2CA	NS	0.861209163	0.003547794	0.906487963	NS
VAMP1	NS	0.860845581	0.003557183	0.906487963	NS
NOLC1	NS	0.861090228	0.003550865	0.906487963	NS
TFDP1	NS	0.861111876	-0.003550306	0.906487963	NS

NAGA	NS	0.860991314	0.003553419	0.906487963	NS
SLC9C2	NS	0.861796522	-0.003532627	0.90701612	NS
AGFG2	NS	0.861931882	0.003529132	0.90710926	NS
GALK2	NS	0.862138155	0.003523806	0.90722769	NS
ZNF503	NS	0.862128774	-0.003524048	0.90722769	NS
LYPD1	NS	0.862215712	-0.003521803	0.90725998	NS
DCPS	NS	0.862337763	0.003518652	0.907339082	NS
RSBN1L	NS	0.862517927	0.003514001	0.907479317	NS
REXO1	NS	0.862710141	0.003509039	0.907513845	NS
PPCS	NS	0.862719456	0.003508798	0.907513845	NS
LYSMD3	NS	0.862785171	0.003507102	0.907513845	NS
MIEF2	NS	0.862778806	0.003507266	0.907513845	NS
TOPORS	NS	0.862698745	0.003509333	0.907513845	NS
GCK	NS	0.862896698	0.003504223	0.907581834	NS
CDHR3	NS	0.86296111	0.00350256	0.907600263	NS
TMCO6	NS	0.864031298	-0.003474937	0.908577704	NS
ACAD9	NS	0.864001446	0.003475708	0.908577704	NS
CRHR2	NS	0.864249223	0.003469313	0.90870813	NS
EXD3	NS	0.864237325	0.00346962	0.90870813	NS
LRCH3	NS	0.864299111	0.003468026	0.908711222	NS
SH3BP5L	NS	0.864554753	0.003461429	0.908930628	NS
NPC2	NS	0.864604697	-0.00346014	0.908933767	NS
APH1B	NS	0.864865683	0.003453405	0.909158756	NS
L3MBTL2	NS	0.865264202	0.003443123	0.90934702	NS
ERMAP	NS	0.865227617	0.003444067	0.90934702	NS
ZNF440	NS	0.865194548	0.00344492	0.90934702	NS
LBX2	NS	0.865279676	-0.003442723	0.90934702	NS
EGLN2	NS	0.865263999	-0.003443128	0.90934702	NS
PTPRH	NS	0.865497762	0.003437097	0.909477453	NS
TP53	NS	0.865454064	-0.003438224	0.909477453	NS
NEURL4	NS	0.865571154	-0.003435203	0.909488781	NS
KLF-CMTM	NS	0.865602517	0.003434394	0.909488781	NS
CCDC77	NS	0.865746417	0.003430682	0.909590601	NS
BCKDHA	NS	0.866052376	-0.003422789	0.909862668	NS
ATXN7L3	NS	0.866285938	0.003416764	0.910011497	NS
MYOCOS	NS	0.866288068	-0.003416709	0.910011497	NS
PLS3	NS	0.866544491	0.003410094	0.910231463	NS
MYBPH	NS	0.866709836	-0.00340583	0.910324908	NS
ASPDH	NS	0.866727513	0.003405374	0.910324908	NS
OAS1	NS	0.86695928	-0.003399396	0.910469526	NS
PIP	NS	0.866957317	0.003399446	0.910469526	NS
NELFE	NS	0.867484379	0.003385853	0.910971551	NS
BCL9	NS	0.86794029	-0.003374096	0.911400871	NS
WNK2	NS	0.868012895	-0.003372224	0.911427666	NS
TREX1	NS	0.868103724	-0.003369882	0.911473593	NS
CD300E	NS	0.868433432	0.003361381	0.911720861	NS
UBA52	NS	0.868413382	0.003361898	0.911720861	NS
C1R	NS	0.868539291	-0.003358651	0.911761809	NS
TUNAR	NS	0.868566646	-0.003357946	0.911761809	NS
RABL2B	NS	0.868689976	0.003354766	0.911841821	NS
MAP4	NS	0.86898822	0.003347077	0.912055958	NS
DUSP21	NS	0.868988154	-0.003347079	0.912055958	NS
SEC23A	NS	0.869050645	-0.003345468	0.912072021	NS
IL22RA1	NS	0.869134328	-0.00334331	0.912110391	NS
GIGYF1	NS	0.869358074	0.003337542	0.912295737	NS

C19orf25	NS	0.869564272	0.003332227	0.91245627	NS
MRPS26	NS	0.869605333	-0.003331168	0.91245627	NS
HIF1AN	NS	0.869859516	0.003324616	0.912673503	NS
CFAP47	NS	0.869986345	0.003321347	0.912757097	NS
HDGFL3	NS	0.8701001	0.003318415	0.912819607	NS
CDT1	NS	0.870140244	-0.003317381	0.912819607	NS
DHPS	NS	0.870223849	-0.003315226	0.912857838	NS
AP3B1	NS	0.870451682	-0.003309354	0.91304735	NS
EIF3CL	NS	0.87054037	0.003307068	0.913090896	NS
SYTL2	NS	0.870747337	-0.003301734	0.913258491	NS
TMX4	NS	0.871231674	0.003289253	0.913716964	NS
NPW	NS	0.871305246	-0.003287357	0.913744614	NS
ZNF382	NS	0.871464929	0.003283242	0.913862562	NS
LDB3	NS	0.872203845	0.003264204	0.914477184	NS
CERS2	NS	0.872240018	0.003263272	0.914477184	NS
CNGA3	NS	0.872111085	-0.003266593	0.914477184	NS
KRTAP19-1	NS	0.872189201	-0.003264581	0.914477184	NS
DNAJB11	NS	0.872410254	0.003258886	0.91457647	NS
EIF3C	NS	0.872429218	0.003258397	0.91457647	NS
NFASC	NS	0.872483406	0.003257001	0.914583742	NS
FDCSP	NS	0.872540136	0.00325554	0.914593679	NS
CARHSP1	NS	0.87269815	0.003251469	0.914660244	NS
TRAPPC12	NS	0.872668413	0.003252235	0.914660244	NS
RAB21	NS	0.872851501	-0.003247519	0.914721909	NS
LNPK	NS	0.872844029	-0.003247711	0.914721909	NS
CNTRL	NS	0.873109106	0.003240883	0.914942334	NS
CLSTN1	NS	0.87317925	0.003239076	0.914966304	NS
SCARF1	NS	0.873639059	0.003227233	0.915398562	NS
ENTPD5	NS	0.873891504	-0.003220731	0.915613508	NS
WDR59	NS	0.874465424	0.003205951	0.916165236	NS
CBX5	NS	0.874543243	-0.003203947	0.916197175	NS
RPL7L1	NS	0.874720211	0.00319939	0.916332977	NS
FXYD3	NS	0.874852554	-0.003195982	0.916422017	NS
TDRD3	NS	0.875014083	-0.003191822	0.916519107	NS
CECR2	NS	0.875039941	0.003191157	0.916519107	NS
SPATA22	NS	0.875102184	-0.003189554	0.916534705	NS
PIWIL2	NS	0.875601946	-0.003176686	0.917008509	NS
FHL3	NS	0.875782945	-0.003172026	0.917148444	NS
GNS	NS	0.876024759	0.003165801	0.917212891	NS
TFAP2A	NS	0.875985152	0.003166821	0.917212891	NS
SCUBE1	NS	0.876067645	0.003164697	0.917212891	NS
SPTAN1	NS	0.87597002	0.00316721	0.917212891	NS
PTPRCAP	NS	0.876081418	-0.003164343	0.917212891	NS
SERPINB6	NS	0.876151107	-0.003162549	0.917236239	NS
SF3B1	NS	0.876346224	0.003157526	0.917390887	NS
POLD2	NS	0.876585103	-0.003151377	0.917564639	NS
DPY19L3	NS	0.876607011	-0.003150813	0.917564639	NS
PKP2	NS	0.876959964	0.003141728	0.917830804	NS
DCTD	NS	0.876929095	0.003142522	0.917830804	NS
PSMD4	NS	0.87700355	0.003140606	0.917830804	NS
GLYCTK	NS	0.877300488	0.003132963	0.918091925	NS
DBN1	NS	0.877373456	-0.003131085	0.918118647	NS
ACTN1	NS	0.877578483	0.003125808	0.91828355	NS
TIGD5	NS	0.877647818	-0.003124024	0.918306458	NS
RAET1G	NS	0.878139965	-0.003111359	0.918771739	NS

ABL1	NS	0.878187986	0.003110123	0.918772318	NS
ARNT2	NS	0.878463035	0.003103046	0.918960736	NS
OR2T12	NS	0.878435002	0.003103767	0.918960736	NS
STEAP3	NS	0.878574541	-0.003100176	0.919027714	NS
IGSF6	NS	0.878847343	-0.003093157	0.919263397	NS
FNDC8	NS	0.879027879	0.003088512	0.919402552	NS
KLK8	NS	0.879345873	-0.003080331	0.919685456	NS
TSNAX	NS	0.879421137	0.003078395	0.919714477	NS
MACO1	NS	0.879487486	0.003076688	0.919734171	NS
CCNC	NS	0.879708921	-0.003070991	0.919916039	NS
SPTBN4	NS	0.879950017	-0.003064789	0.920118445	NS
C6orf120	NS	0.88006379	-0.003061862	0.920187701	NS
LCMT1	NS	0.88046959	0.003051424	0.920562275	NS
PIGP	NS	0.880594182	-0.003048219	0.92064281	NS
CEP63	NS	0.88085844	0.003041422	0.920869347	NS
DROSHA	NS	0.881042058	0.0030367	0.921011562	NS
ZNF681	NS	0.881210245	0.003032374	0.92113763	NS
KTN1	NS	0.881568126	-0.003023171	0.921461964	NS
CTSV	NS	0.881653192	-0.003020983	0.921501117	NS
TMSB10	NS	0.88173254	0.003018943	0.921534289	NS
SEMA3F	NS	0.881876155	0.00301525	0.921544847	NS
UPB1	NS	0.881885472	0.00301501	0.921544847	NS
CCT8	NS	0.881807053	0.003017027	0.921544847	NS
SERPING1	NS	0.882005353	-0.003011927	0.921620364	NS
ARPC4	NS	0.88213029	-0.003008715	0.921701155	NS
EZH2	NS	0.882228191	-0.003006197	0.92175369	NS
TAF15	NS	0.882431525	-0.003000969	0.92191637	NS
MYH3	NS	0.882508358	-0.002998994	0.92194688	NS
KLHL22	NS	0.882557346	0.002997734	0.921948297	NS
DEFA1B	NS	0.882823583	-0.002990889	0.922176647	NS
TTC32	NS	0.883077815	-0.002984353	0.92234266	NS
ZBTB37	NS	0.883064485	0.002984695	0.92234266	NS
RNF5	NS	0.883137465	0.002982819	0.922355192	NS
MBD4	NS	0.883349649	-0.002977364	0.922453685	NS
UTRN	NS	0.883374742	-0.002976719	0.922453685	NS
UTP14A	NS	0.883303071	-0.002978562	0.922453685	NS
CDR2	NS	0.883424529	-0.002975439	0.922455909	NS
DHRS7	NS	0.884045451	0.002959478	0.923050862	NS
SGTA	NS	0.884089684	0.002958341	0.923050862	NS
CTSS	NS	0.884147787	-0.002956848	0.923061735	NS
PCDH12	NS	0.884216271	-0.002955087	0.923083444	NS
ENHO	NS	0.884327468	-0.002952229	0.92314974	NS
NDST1	NS	0.884409937	-0.002950109	0.92318604	NS
DCAF8L1	NS	0.884652184	0.002943883	0.923389111	NS
TRIP10	NS	0.884735391	-0.002941745	0.923426165	NS
RHOB	NS	0.884948397	-0.00293627	0.923598682	NS
PRAMEF6	NS	0.885069738	-0.002933152	0.923675519	NS
CUL7	NS	0.885228524	-0.002929071	0.923746767	NS
ZNF432	NS	0.885233456	0.002928944	0.923746767	NS
DPH3	NS	0.885826707	-0.002913699	0.924315996	NS
PKP1	NS	0.886025738	-0.002908585	0.924473838	NS
SYCP3	NS	0.886464853	0.002897303	0.924882152	NS
THRA	NS	0.886726789	-0.002890573	0.925055715	NS
ROBO3	NS	0.886692118	0.002891464	0.925055715	NS
OR2AE1	NS	0.887026703	0.002882868	0.925318721	NS

AKR1B15	NS	0.887290291	-0.002876096	0.925543807	NS
ZNF2	NS	0.887486802	0.002871048	0.925698903	NS
LIPH	NS	0.887620319	0.002867618	0.92578828	NS
MTHFSD	NS	0.887857166	0.002861534	0.925985415	NS
CUBN	NS	0.88815308	-0.002853934	0.926144328	NS
PGLYRP2	NS	0.888136188	0.002854367	0.926144328	NS
CKLF	NS	0.88811725	-0.002854854	0.926144328	NS
IAH1	NS	0.888250036	0.002851443	0.926195534	NS
LINC00672	NS	0.888396602	0.002847679	0.926298461	NS
TNXB	NS	0.888570868	-0.002843203	0.926430257	NS
VAMP7	NS	0.888644226	0.002841319	0.926456837	NS
C16orf90	NS	0.888698825	0.002839917	0.926463859	NS
RNPEPL1	NS	0.889025972	0.002831515	0.926750098	NS
C1orf56	NS	0.889069155	-0.002830406	0.926750098	NS
SUN1	NS	0.889315677	0.002824075	0.926957149	NS
CA9	NS	0.889445925	-0.00282073	0.927042988	NS
MINK1	NS	0.889509495	0.002819098	0.927059325	NS
PTPRF	NS	0.889734287	0.002813326	0.927237944	NS
OTULIN	NS	0.889776688	-0.002812237	0.927237944	NS
RPS3	NS	0.889969809	0.002807278	0.927389266	NS
SV2C	NS	0.890095419	0.002804053	0.927420301	NS
ZNF282	NS	0.89004962	0.002805229	0.927420301	NS
HOXC10	NS	0.890184043	0.002801777	0.927462716	NS
CEBPG	NS	0.89023443	-0.002800483	0.92746529	NS
CLDN14	NS	0.890335736	-0.002797882	0.927470992	NS
SLC22A10	NS	0.890306221	0.00279864	0.927470992	NS
ATF2	NS	0.890440443	0.002795194	0.927527387	NS
TP53TG3F	NS	0.890485712	-0.002794032	0.927527387	NS
LIAS	NS	0.890566435	0.002791959	0.927561554	NS
GABBR2	NS	0.890820493	-0.002785437	0.927745862	NS
PRSS35	NS	0.890839253	0.002784955	0.927745862	NS
TMEM39A	NS	0.890988776	0.002781116	0.927851657	NS
OR2L3	NS	0.891157111	-0.002776795	0.927977031	NS
VSIR	NS	0.891207792	-0.002775494	0.927979882	NS
NUDT11	NS	0.891602358	-0.002765365	0.928340787	NS
FBXO31	NS	0.891807346	0.002760103	0.928454335	NS
ARHGAP1	NS	0.891920236	-0.002757205	0.928521922	NS
CPT2	NS	0.892228674	0.002749289	0.928749309	NS
TRAPPC4	NS	0.892234625	-0.002749136	0.928749309	NS
PHF20	NS	0.892362595	-0.002745851	0.928750627	NS
MASTL	NS	0.892290538	0.002747701	0.928750627	NS
THNSL2	NS	0.892415469	-0.002744494	0.928750627	NS
NIPAL4	NS	0.892427821	-0.002744177	0.928750627	NS
NAPEPLD	NS	0.892659476	0.002738232	0.928941764	NS
LYPLA1	NS	0.893004093	-0.002729387	0.929150525	NS
POLR3C	NS	0.893002072	-0.002729439	0.929150525	NS
SFI1	NS	0.892929175	0.00273131	0.929150525	NS
AMIGO1	NS	0.893091729	-0.002727138	0.929191761	NS
AZIN1	NS	0.893263818	-0.002722722	0.929320852	NS
MYSM1	NS	0.893485372	0.002717036	0.929401487	NS
KRTAP21-1	NS	0.893459524	-0.0027177	0.929401487	NS
ZNF195	NS	0.893679952	0.002712043	0.929503984	NS
STX10	NS	0.893634528	-0.002713209	0.929503984	NS
DDB1	NS	0.893907636	0.002706201	0.929690839	NS
RAB9A	NS	0.893982773	0.002704273	0.929719028	NS

TUFM	NS	0.894360127	0.002694591	0.930020061	NS
RAB7B	NS	0.894368331	0.00269438	0.930020061	NS
PPP2R5B	NS	0.894525029	0.00269036	0.930133036	NS
SLC1A3	NS	0.895023578	0.002677569	0.930601438	NS
KRTAP23-1	NS	0.89507944	-0.002676136	0.930609531	NS
RNF114	NS	0.895151171	0.002674296	0.930634121	NS
CDCA3	NS	0.895307989	-0.002670273	0.930747163	NS
FRMD4B	NS	0.895365597	0.002668795	0.930757062	NS
NBPF9	NS	0.895422858	0.002667326	0.930766599	NS
SLC12A3	NS	0.895627041	0.002662088	0.930916763	NS
PEX10	NS	0.895707852	-0.002660015	0.930916763	NS
DENND4B	NS	0.895711603	-0.002659919	0.930916763	NS
BTBD3	NS	0.895868157	-0.002655903	0.930979494	NS
HIC2	NS	0.89584028	0.002656618	0.930979494	NS
IQCH	NS	0.896201805	-0.002647345	0.93127622	NS
TIPIN	NS	0.896318515	-0.002644351	0.931312056	NS
GPX3	NS	0.896332521	0.002643992	0.931312056	NS
IKZF5	NS	0.896495097	0.002639822	0.931350279	NS
JAG1	NS	0.89651366	0.002639346	0.931350279	NS
ZMAT1	NS	0.89646526	-0.002640587	0.931350279	NS
TEF	NS	0.896566729	0.002637985	0.931355424	NS
RPL17	NS	0.896735466	-0.002633657	0.931480717	NS
DAAM1	NS	0.89720313	-0.002621663	0.931816482	NS
GTF3C5	NS	0.897146648	-0.002623111	0.931816482	NS
ADGRB1	NS	0.897175292	-0.002622377	0.931816482	NS
PROK2	NS	0.897313467	0.002618833	0.931881074	NS
SNTN	NS	0.897462177	-0.00261502	0.931985509	NS
DYNLL2	NS	0.897917468	0.002603344	0.932408289	NS
HLA-DPA1	NS	0.89826192	0.002594512	0.932715934	NS
PADI4	NS	0.898369663	-0.002591749	0.932777771	NS
ABI3	NS	0.898503062	-0.002588329	0.932866237	NS
MRM2	NS	0.898619595	-0.002585341	0.932937185	NS
PSMC2	NS	0.898963476	-0.002576524	0.933213338	NS
PNPLA1	NS	0.898982016	0.002576049	0.933213338	NS
ST13	NS	0.899069159	-0.002573815	0.933253748	NS
MTERF4	NS	0.899282023	0.002568357	0.933424648	NS
TTC24	NS	0.899569254	-0.002560994	0.933672716	NS
ISCA1	NS	0.900071652	-0.002548115	0.934043904	NS
RAB37	NS	0.900040788	-0.002548906	0.934043904	NS
MED14	NS	0.900063284	0.002548329	0.934043904	NS
PRELID1	NS	0.900176867	-0.002545418	0.934080107	NS
KCMF1	NS	0.900251312	-0.00254351	0.934080107	NS
KIAA0753	NS	0.900237597	-0.002543861	0.934080107	NS
LILRA5	NS	0.900420152	-0.002539182	0.934205213	NS
ERCC2	NS	0.90051156	-0.002536839	0.934249974	NS
SELPLG	NS	0.90063699	-0.002533624	0.934330023	NS
STX1A	NS	0.900847462	0.002528229	0.934498284	NS
BMPR2	NS	0.90091027	-0.002526619	0.934513354	NS
SCGB1A1	NS	0.901100586	0.002521742	0.93466068	NS
CDKL2	NS	0.901401716	-0.002514024	0.934910666	NS
RABGAP1L	NS	0.901438198	0.002513089	0.934910666	NS
PLA2R1	NS	0.901552111	0.00251017	0.934978711	NS
UQCRFS1	NS	0.901927551	0.002500548	0.935286243	NS
SCART1	NS	0.901945289	-0.002500094	0.935286243	NS
ZNF273	NS	0.902200245	0.00249356	0.935500506	NS

FAM200B	NS	0.902841235	0.002477135	0.936115007	NS
RSBN1	NS	0.903051337	-0.002471752	0.936182727	NS
TIMM17B	NS	0.903051647	-0.002471744	0.936182727	NS
SERINC5	NS	0.903032098	0.002472245	0.936182727	NS
SUPT20HL2	NS	0.903149115	-0.002469247	0.936233627	NS
ZNF630	NS	0.903213883	-0.002467588	0.936250625	NS
SCNM1	NS	0.903292501	-0.002465573	0.936281978	NS
PTPRN	NS	0.903372302	-0.002463529	0.936295054	NS
SLC5A2	NS	0.9034435	0.002461705	0.936295054	NS
CCKAR	NS	0.903450234	0.002461532	0.936295054	NS
PRAMEF5	NS	0.903578548	0.002458245	0.936377898	NS
SLC25A34	NS	0.90368328	0.002455561	0.936436296	NS
KLHL10	NS	0.903768781	-0.002453371	0.93647476	NS
LMX1A	NS	0.903954046	0.002448625	0.93656706	NS
RAB44	NS	0.90395463	-0.00244861	0.93656706	NS
ESYT3	NS	0.904268288	0.002440575	0.936841888	NS
SNW1	NS	0.904349701	-0.002438489	0.936876087	NS
CWC15	NS	0.904458197	-0.00243571	0.936938339	NS
PHEX	NS	0.90468659	0.002429859	0.936974351	NS
CLIP4	NS	0.904684168	-0.002429921	0.936974351	NS
IGF2R	NS	0.904574082	0.002432741	0.936974351	NS
MAP3K5	NS	0.904618103	0.002431614	0.936974351	NS
PCARE	NS	0.90476384	-0.00242788	0.937004221	NS
LPIN1	NS	0.904931231	-0.002423593	0.937127436	NS
ATP6V0D2	NS	0.905029792	-0.002421068	0.937129227	NS
NOX5	NS	0.905001186	-0.002421801	0.937129227	NS
CFAP73	NS	0.905331021	-0.002413353	0.937390994	NS
DTX4	NS	0.905625805	-0.002405802	0.937646059	NS
GLB1	NS	0.905799981	-0.002401342	0.937776232	NS
CCDC112	NS	0.905903188	0.002398698	0.93783292	NS
OR8K1	NS	0.906275777	-0.002389156	0.938168464	NS
LRRC45	NS	0.906466408	-0.002384274	0.938315621	NS
TLR8	NS	0.90674114	0.002377239	0.938466183	NS
PYCARD	NS	0.906776815	0.002376325	0.938466183	NS
SMC3	NS	0.906701352	-0.002378258	0.938466183	NS
EEF1A1	NS	0.906854282	-0.002374342	0.938466183	NS
ADSL	NS	0.906827446	-0.002375029	0.938466183	NS
BSPRY	NS	0.9069738	-0.002371281	0.938539689	NS
PPM1E	NS	0.907252975	0.002364133	0.938778391	NS
WASHC2A	NS	0.907999394	-0.002345021	0.939500522	NS
TAF11	NS	0.908225985	0.00233922	0.939684743	NS
TAS1R2	NS	0.908619236	0.002329152	0.939991124	NS
RALGPS1	NS	0.908900408	0.002321954	0.940181504	NS
ME3	NS	0.908893333	0.002322135	0.940181504	NS
FAAP100	NS	0.909117688	0.002316392	0.940356008	NS
RASGRP2	NS	0.909394593	0.002309304	0.940592163	NS
SH3BP1	NS	0.909578539	0.002304595	0.940681887	NS
MARCHF11	NS	0.909554573	-0.002305208	0.940681887	NS
PADI3	NS	0.909705008	-0.002301358	0.940762415	NS
NAV1	NS	0.909759592	0.002299961	0.9407686	NS
FAM120A	NS	0.91002656	-0.002293127	0.940793361	NS
PDGFRB	NS	0.909924666	0.002295735	0.940793361	NS
PCSK6	NS	0.910014966	0.002293424	0.940793361	NS
STK36	NS	0.909859223	0.00229741	0.940793361	NS
SLC48A1	NS	0.909957735	0.002294889	0.940793361	NS

LRRC49	NS	0.910142147	-0.002290169	0.940862604	NS
RHO	NS	0.910277689	-0.0022867	0.940952467	NS
CLSTN3	NS	0.910407531	-0.002283377	0.941015263	NS
DCP2	NS	0.91043567	-0.002282656	0.941015263	NS
LAPTM4A	NS	0.9106158	-0.002278046	0.941151186	NS
SLC29A3	NS	0.910912935	-0.002270442	0.941408019	NS
EPSTI1	NS	0.911047528	0.002266997	0.941496848	NS
SMARCAD1	NS	0.911395071	0.002258103	0.941805723	NS
PRMT1	NS	0.911605908	0.002252708	0.941973305	NS
GLUD2	NS	0.911765609	0.002248621	0.942088033	NS
SIDT1	NS	0.911961882	-0.002243599	0.942139955	NS
RCN3	NS	0.911919456	-0.002244684	0.942139955	NS
CCL26	NS	0.912028394	0.002241897	0.942158381	NS
AAMDC	NS	0.912160015	-0.002238529	0.942244063	NS
CEACAM1	NS	0.912404492	0.002232273	0.942446307	NS
RBMX	NS	0.912616563	0.002226847	0.942615059	NS
CPN2	NS	0.912779223	0.002222686	0.942732759	NS
CTSE	NS	0.913172219	-0.002212631	0.94308833	NS
EIF4B	NS	0.913297338	0.00220943	0.943167224	NS
ANKRD26	NS	0.913395107	-0.002206928	0.943217867	NS
COG1	NS	0.913692095	-0.002199331	0.943474216	NS
DCAF8L2	NS	0.913826364	-0.002195896	0.943521045	NS
MIF	NS	0.913834936	-0.002195676	0.943521045	NS
SRSF12	NS	0.914158742	-0.002187393	0.943805025	NS
H2AX	NS	0.914526611	-0.002177983	0.944134465	NS
GTF2E2	NS	0.914641064	0.002175055	0.944202263	NS
TRPM5	NS	0.914996491	0.002165964	0.944518803	NS
RAB2A	NS	0.915272263	0.002158911	0.944753089	NS
PPY	NS	0.915398625	-0.002155679	0.944833135	NS
MAPK14	NS	0.91545077	-0.002154345	0.944836574	NS
CSDC2	NS	0.915503937	0.002152985	0.944841067	NS
KYAT1	NS	0.915666082	0.002148838	0.944958023	NS
SRR	NS	0.91573603	-0.002147049	0.944979826	NS
ARHGAP29	NS	0.915860021	0.002143878	0.945007014	NS
EIF4ENIF1	NS	0.915826097	-0.002144746	0.945007014	NS
TMEM123	NS	0.915915808	0.002142451	0.945014199	NS
T6GALNAC	NS	0.916007111	0.002140116	0.945058027	NS
PDE1B	NS	0.916340746	0.002131584	0.945351854	NS
HTRA2	NS	0.916513553	-0.002127165	0.945479738	NS
PRKRIP1	NS	0.916796833	0.002119921	0.945627222	NS
GPR45	NS	0.916764566	0.002120746	0.945627222	NS
TLCD4	NS	0.916803082	0.002119761	0.945627222	NS
COX6C	NS	0.917207387	-0.002109423	0.945993828	NS
PIGN	NS	0.917517234	-0.0021015	0.946262979	NS
TSSK3	NS	0.917570765	-0.002100131	0.946267768	NS
UBE2D2	NS	0.917844069	0.002093143	0.946499191	NS
C2orf76	NS	0.917906807	0.002091539	0.94651346	NS
IL16	NS	0.918146939	0.002085399	0.946710641	NS
CXCL2	NS	0.918474854	-0.002077016	0.946998311	NS
CHFR	NS	0.918651344	0.002072504	0.947129829	NS
KDELR1	NS	0.918824382	0.00206808	0.947207325	NS
SPESP1	NS	0.91879196	0.002068909	0.947207325	NS
SDS	NS	0.918958729	-0.002064645	0.94729537	NS
NFU1	NS	0.919109715	0.002060785	0.947400556	NS
SHQ1	NS	0.919244287	0.002057345	0.947438361	NS

PHF8	NS	0.919200121	0.002058474	0.947438361	NS
MCOLN1	NS	0.919299654	-0.00205593	0.947444976	NS
PRDM10	NS	0.919571087	-0.002048991	0.94767426	NS
CTR9	NS	0.91965929	0.002046737	0.947714701	NS
LSM8	NS	0.919870561	0.002041336	0.947881952	NS
CD58	NS	0.920007726	0.00203783	0.947972827	NS
PMAIP1	NS	0.920536306	0.002024319	0.948416498	NS
FREM2	NS	0.920505472	-0.002025108	0.948416498	NS
GPR141	NS	0.920787269	-0.002017905	0.94857408	NS
CCNL2	NS	0.920758821	-0.002018632	0.94857408	NS
TACO1	NS	0.92119711	0.00200743	0.948895283	NS
LBH	NS	0.921186876	-0.002007692	0.948895283	NS
NMT1	NS	0.921642953	0.001996036	0.949238673	NS
PDLIM3	NS	0.921583246	0.001997562	0.949238673	NS
RPGR	NS	0.9216776	-0.001995151	0.949238673	NS
CRAT	NS	0.9218484	0.001990786	0.949322075	NS
ARPC1A	NS	0.921856671	-0.001990574	0.949322075	NS
NIPAL2	NS	0.921960849	-0.001987912	0.949378848	NS
PHF21B	NS	0.922055863	0.001985484	0.949426178	NS
MYO1D	NS	0.922214834	-0.001981422	0.949539354	NS
SLC25A45	NS	0.922509116	0.001973902	0.949791833	NS
KCNK17	NS	0.92261234	0.001971264	0.949847586	NS
DIP2B	NS	0.92277193	0.001967186	0.94996136	NS
TSHR	NS	0.92297487	0.001962001	0.95009073	NS
INPPL1	NS	0.922995768	-0.001961467	0.95009073	NS
GOLGA8T	NS	0.923081585	0.001959274	0.950128538	NS
TMEM238L	NS	0.923143189	0.0019577	0.950141421	NS
GANAB	NS	0.923480938	0.00194907	0.950374669	NS
TRAPPC13	NS	0.923517108	-0.001948146	0.950374669	NS
DSN1	NS	0.923458609	0.001949641	0.950374669	NS
ZBED1	NS	0.923930441	-0.001937586	0.950749475	NS
HDHD5	NS	0.924092759	0.001933439	0.950865953	NS
GSTK1	NS	0.924168845	0.001931495	0.950893694	NS
HLCS	NS	0.924334614	-0.00192726	0.950963155	NS
GIGYF2	NS	0.924289429	0.001928414	0.950963155	NS
TRIM37	NS	0.924461153	-0.001924027	0.950992245	NS
SLC25A33	NS	0.924441908	0.001924519	0.950992245	NS
MOK	NS	0.92470599	0.001917772	0.951193556	NS
COMMD7	NS	0.924823976	-0.001914758	0.951264368	NS
ST18	NS	0.924994431	-0.001910404	0.951389139	NS
GAS6	NS	0.92506014	0.001908725	0.951406168	NS
SIRT6	NS	0.925142953	-0.00190661	0.951440784	NS
ARL4C	NS	0.925255	-0.001903747	0.951505461	NS
PKM	NS	0.925562339	-0.001895897	0.951686146	NS
PAX4	NS	0.92554648	-0.001896302	0.951686146	NS
HGAP19-SL	NS	0.925578203	0.001895491	0.951686146	NS
CABP5	NS	0.926040952	0.001883671	0.952111371	NS
ISL2	NS	0.926110086	0.001881906	0.952131875	NS
KRT9	NS	0.926214726	-0.001879233	0.95218888	NS
TTC38	NS	0.926289558	-0.001877322	0.952215236	NS
RIMBP2	NS	0.926466943	-0.001872791	0.952331574	NS
RASL11A	NS	0.92650113	-0.001871918	0.952331574	NS
LCA5L	NS	0.926648626	0.001868151	0.952432604	NS
MC1-LUC7L	NS	0.926701413	-0.001866802	0.952436285	NS
NSMAF	NS	0.927020196	0.001858661	0.952499338	NS

APC2	NS	0.926965024	-0.00186007	0.952499338	NS
ZNF605	NS	0.926863134	0.001862672	0.952499338	NS
PSG7	NS	0.927053022	0.001857822	0.952499338	NS
SMIM13	NS	0.926871405	0.001862461	0.952499338	NS
GAN	NS	0.92705802	0.001857695	0.952499338	NS
TAF11L10	NS	0.92713712	-0.001855675	0.952530047	NS
MVK	NS	0.927415334	-0.001848569	0.952629689	NS
MEIS2	NS	0.927343662	0.0018504	0.952629689	NS
PPP1R11	NS	0.92743097	-0.00184817	0.952629689	NS
ZNF850	NS	0.927405963	0.001848809	0.952629689	NS
ELOVL5	NS	0.92799326	0.001833811	0.953100613	NS
CYB561D2	NS	0.928278779	-0.001826519	0.953100613	NS
MATN1	NS	0.928238028	-0.00182756	0.953100613	NS
LRRIQ4	NS	0.928190593	0.001828771	0.953100613	NS
ZNF34	NS	0.928135358	-0.001830182	0.953100613	NS
GALT	NS	0.928019154	0.001833149	0.953100613	NS
C17orf113	NS	0.928065183	-0.001831974	0.953100613	NS
FAM90A8P	NS	0.928283362	0.001826402	0.953100613	NS
COQ2	NS	0.928334141	0.001825106	0.953102193	NS
SLTM	NS	0.928421793	-0.001822868	0.953141627	NS
MAP3K14	NS	0.928521835	0.001820313	0.953143224	NS
CAPN12	NS	0.928506716	-0.001820699	0.953143224	NS
CORO2A	NS	0.928782552	0.001813656	0.953319024	NS
ABI3BP	NS	0.928791597	0.001813425	0.953319024	NS
RAB7A	NS	0.928923915	0.001810046	0.953404279	NS
RAP1A	NS	0.9291838	0.00180341	0.953620447	NS
SNX1	NS	0.929262646	0.001801397	0.953650802	NS
OCIAD1	NS	0.929341695	-0.001799379	0.953654266	NS
NFE2	NS	0.92936456	-0.001798795	0.953654266	NS
ZNF606	NS	0.929456904	-0.001796437	0.953698464	NS
IGDCC3	NS	0.929755721	0.001788808	0.953954505	NS
AK6	NS	0.930351436	-0.001773599	0.95445306	NS
DNAH8	NS	0.930389561	0.001772625	0.95445306	NS
MVD	NS	0.93046221	0.001770771	0.954477002	NS
HAT1	NS	0.930622989	-0.001766666	0.954490175	NS
USP49	NS	0.930544582	0.001768668	0.954490175	NS
C9orf153	NS	0.930585719	-0.001767618	0.954490175	NS
CFAP418	NS	0.930930525	-0.001758815	0.954755007	NS
SH3BP4	NS	0.931084591	-0.001754882	0.954811832	NS
FGF13	NS	0.931198973	0.001751962	0.954827956	NS
WASH6P	NS	0.931164126	0.001752852	0.954827956	NS
RFC2	NS	0.932179973	0.001726921	0.955724374	NS
RWDD1	NS	0.932369464	-0.001722084	0.955724374	NS
ORC2	NS	0.932325303	-0.001723212	0.955724374	NS
FXYD6	NS	0.932364753	-0.001722205	0.955724374	NS
ODAD3	NS	0.932212211	-0.001726098	0.955724374	NS
ARFGAP3	NS	0.932349298	-0.001722599	0.955724374	NS
ZBTB24	NS	0.932710166	0.001713388	0.955769917	NS
COL4A2	NS	0.932473231	0.001719436	0.955769917	NS
MAPKAPK2	NS	0.932658944	-0.001714696	0.955769917	NS
VWA3B	NS	0.932670004	0.001714413	0.955769917	NS
OLFML2A	NS	0.932669356	-0.00171443	0.955769917	NS
POTEB3	NS	0.932660696	-0.001714651	0.955769917	NS
MFSD13A	NS	0.932892854	0.001708726	0.955906515	NS
MPG	NS	0.933145524	-0.001702277	0.955982949	NS

KLHL36	NS	0.933461342	-0.001694217	0.955982949	NS
NEK3	NS	0.9334523	-0.001694447	0.955982949	NS
SPACA7	NS	0.933454226	-0.001694398	0.955982949	NS
NCF1	NS	0.933234813	0.001699998	0.955982949	NS
WNT10B	NS	0.933355342	0.001696922	0.955982949	NS
ZNF778	NS	0.933221447	-0.001700339	0.955982949	NS
LURAP1	NS	0.933162293	0.001701849	0.955982949	NS
RX5-TAX1B	NS	0.933332621	0.001697502	0.955982949	NS
RCC1L	NS	0.933141459	-0.001702381	0.955982949	NS
NDUFB5	NS	0.933582927	-0.001691114	0.956056883	NS
NTSR1	NS	0.933732288	-0.001687302	0.956159251	NS
HOXA10	NS	0.934065828	0.00167879	0.956450202	NS
TRIM7	NS	0.934244448	0.001674231	0.956582498	NS
CTSC	NS	0.934512575	-0.001667389	0.956806421	NS
MYL6	NS	0.934839895	0.001659037	0.957040303	NS
ANKRD42	NS	0.93479886	-0.001660084	0.957040303	NS
VASN	NS	0.934910509	-0.001657235	0.957061974	NS
ZCCHC24	NS	0.935119017	0.001651914	0.957210537	NS
KRTAP1-3	NS	0.935154539	0.001651008	0.957210537	NS
ADD2	NS	0.935317322	0.001646854	0.957326534	NS
ST3GAL4	NS	0.935518169	-0.001641729	0.957481476	NS
PURB	NS	0.935640019	-0.00163862	0.957555555	NS
CMC4	NS	0.935752158	0.001635759	0.957619688	NS
ASAP3	NS	0.935970834	-0.001630179	0.957703506	NS
ACTR3C	NS	0.935970777	-0.001630181	0.957703506	NS
ZNF428	NS	0.935982497	0.001629882	0.957703506	NS
CFD	NS	0.936079954	-0.001627395	0.957752595	NS
ARL6IP5	NS	0.936494693	-0.001616813	0.95812629	NS
KAT6A	NS	0.936620785	0.001613596	0.958204646	NS
UTP18	NS	0.936796396	0.001609116	0.958290348	NS
ERMN	NS	0.936901337	-0.001606439	0.958290348	NS
ARHGEF39	NS	0.936902591	0.001606407	0.958290348	NS
ULK3	NS	0.936860122	-0.00160749	0.958290348	NS
EGFL8	NS	0.936983039	-0.001604354	0.958321992	NS
STAM2	NS	0.937159669	0.001599848	0.958452	NS
ADPRS	NS	0.937348228	-0.001595038	0.95857978	NS
ATP5F1A	NS	0.937383657	-0.001594134	0.95857978	NS
NTRK3	NS	0.937445423	-0.001592558	0.958592298	NS
GLI1	NS	0.937935858	-0.001580047	0.959043131	NS
GLP1R	NS	0.938376776	-0.0015688	0.959291268	NS
ATF6	NS	0.938240627	0.001572273	0.959291268	NS
FAM204A	NS	0.938300761	-0.001570739	0.959291268	NS
CPLANE1	NS	0.938342782	-0.001569667	0.959291268	NS
UGT1A5	NS	0.93843887	-0.001567216	0.959304081	NS
CDC20B	NS	0.938526753	-0.001564974	0.959343253	NS
DTD1	NS	0.938652787	0.001561759	0.959421415	NS
ANXA6	NS	0.938755755	-0.001559133	0.959475995	NS
MAP3K7	NS	0.938827603	-0.0015573	0.959498764	NS
SNX5	NS	0.938952893	0.001554104	0.959537285	NS
MKX	NS	0.938964441	0.001553809	0.959537285	NS
GOLGA8G	NS	0.939040951	0.001551858	0.95956481	NS
SPATA6L	NS	0.939334709	-0.001544365	0.959763652	NS
SEMA4C	NS	0.93932216	-0.001544685	0.959763652	NS
ITFG2	NS	0.939690955	0.001535279	0.960055968	NS
EXOSC6	NS	0.939720003	-0.001534538	0.960055968	NS

FAM199X	NS	0.939813403	-0.001532156	0.960100714	NS
HERC4	NS	0.940002178	-0.001527341	0.960242884	NS
EDRF1	NS	0.940168012	-0.001523111	0.960321185	NS
POR	NS	0.940178056	-0.001522855	0.960321185	NS
WBP4	NS	0.940269847	0.001520514	0.960364264	NS
KHSRP	NS	0.940467379	0.001515476	0.960515333	NS
ATP5IF1	NS	0.940774931	0.001507633	0.960778746	NS
TMBIM4	NS	0.942071578	0.001474567	0.962052203	NS
DMGDH	NS	0.94263552	0.001460187	0.962577322	NS
UBXN4	NS	0.942751582	0.001457227	0.962594272	NS
SDR9C7	NS	0.942748733	-0.0014573	0.962594272	NS
DEPP1	NS	0.942961585	-0.001451873	0.962757909	NS
MSH5	NS	0.943411456	0.001440402	0.963166419	NS
RNF19A	NS	0.943473284	0.001438826	0.963178739	NS
TEX13A	NS	0.943561045	-0.001436588	0.96321753	NS
TFAM	NS	0.944052302	0.001424063	0.963617379	NS
H2BC11	NS	0.944045387	-0.001424239	0.963617379	NS
PRKCSH	NS	0.944193845	0.001420454	0.963711035	NS
HUWE1	NS	0.944320524	-0.001417225	0.963763517	NS
HPS1	NS	0.944344847	0.001416605	0.963763517	NS
NDUFS1	NS	0.944501671	0.001412607	0.963816841	NS
TSPAN32	NS	0.944447111	-0.001413998	0.963816841	NS
GUCA2A	NS	0.94454648	0.001411464	0.963816841	NS
TAF1D	NS	0.944783733	0.001405416	0.964008115	NS
PTPRJ	NS	0.944834906	-0.001404111	0.964009511	NS
TMEM87A	NS	0.94505482	-0.001398505	0.964183064	NS
TIMM50	NS	0.94515536	-0.001395942	0.964234816	NS
RPAIN	NS	0.945219483	0.001394307	0.96424941	NS
TMEM63A	NS	0.945488897	0.001387439	0.964473417	NS
SELENOT	NS	0.945585746	0.00138497	0.964521379	NS
CGA	NS	0.945663341	-0.001382992	0.964532221	NS
SLC35G2	NS	0.945696037	0.001382159	0.964532221	NS
HINT3	NS	0.945863093	0.0013779	0.96455013	NS
PTGR2	NS	0.945788259	0.001379808	0.96455013	NS
RAMAC	NS	0.945814248	0.001379145	0.96455013	NS
RPL36A	NS	0.945916391	0.001376542	0.964553665	NS
GRAMD4	NS	0.946183351	-0.001369736	0.964775059	NS
RARS1	NS	0.946518563	-0.001361192	0.965015186	NS
P2RY8	NS	0.94647508	-0.0013623	0.965015186	NS
RIPPLY2	NS	0.946885185	0.001351847	0.965338125	NS
TESK1	NS	0.947100401	-0.001346361	0.965422709	NS
CHRNB3	NS	0.947092073	0.001346574	0.965422709	NS
PRG3	NS	0.947117784	-0.001345918	0.965422709	NS
PLA2G5	NS	0.947200854	0.001343801	0.965456542	NS
EIF2AK4	NS	0.947324947	0.001340638	0.965532182	NS
NIT1	NS	0.947591477	0.001333845	0.965752982	NS
SLC39A7	NS	0.947818219	0.001328066	0.96593321	NS
CCDC103	NS	0.948011546	-0.001323138	0.966079366	NS
S1PR4	NS	0.948163354	0.001319269	0.966132337	NS
PAXBP1	NS	0.948150709	-0.001319592	0.966132337	NS
GPR33	NS	0.948375338	-0.001313867	0.96629747	NS
PRDM9	NS	0.948515273	-0.0013103	0.966389179	NS
CEMIP2	NS	0.948602796	0.00130807	0.966427481	NS
STIL	NS	0.948716573	-0.00130517	0.966492526	NS
WTIP	NS	0.948790463	0.001303287	0.966516931	NS

TAOK3	NS	0.949079397	-0.001295924	0.966709509	NS
CDC42EP2	NS	0.949032977	0.001297106	0.966709509	NS
SPCS2	NS	0.949345714	-0.001289137	0.966828141	NS
ATPAF2	NS	0.949326434	-0.001289628	0.966828141	NS
HCFC1R1	NS	0.949673024	0.001280796	0.96710175	NS
FLCN	NS	0.949714304	-0.001279744	0.96710175	NS
CYP2E1	NS	0.949797111	-0.001277633	0.967135193	NS
IPO9	NS	0.949922229	-0.001274445	0.967211713	NS
VMA21	NS	0.950210661	0.001267095	0.967405268	NS
CERS6	NS	0.950212283	-0.001267054	0.967405268	NS
SZRD1	NS	0.950350302	0.001263537	0.967494895	NS
DAD1	NS	0.950916935	0.001249098	0.968020837	NS
ALDH3B1	NS	0.951144097	-0.00124331	0.968201164	NS
ALDH8A1	NS	0.951332739	0.001238503	0.968342265	NS
ERO1B	NS	0.951534312	0.001233367	0.968496511	NS
MBOAT7	NS	0.951780991	0.001227082	0.968696648	NS
ZNF684	NS	0.951885183	-0.001224427	0.96870082	NS
WBP1	NS	0.951871079	-0.001224787	0.96870082	NS
KIAA0100	NS	0.952022373	0.001220932	0.968789498	NS
GOSR2	NS	0.952079247	-0.001219483	0.968796441	NS
FOXD4L3	NS	0.952284808	0.001214246	0.968954673	NS
FRG2	NS	0.952364162	-0.001212224	0.968984479	NS
OGFRL1	NS	0.95268387	-0.001204078	0.969258816	NS
AOAH	NS	0.952829729	-0.001200362	0.969306016	NS
LSM12	NS	0.952830418	0.001200345	0.969306016	NS
STK32B	NS	0.953300308	-0.001188373	0.969631149	NS
NKRF	NS	0.953296197	0.001188478	0.969631149	NS
CCDC154	NS	0.953294824	0.001188513	0.969631149	NS
SF3A1	NS	0.953437344	-0.001184882	0.969683061	NS
PKD2	NS	0.953468837	-0.00118408	0.969683061	NS
PSD3	NS	0.953501638	0.001183244	0.969683061	NS
NUTM2G	NS	0.953605242	0.001180605	0.969737474	NS
UNC13D	NS	0.954075709	0.00116862	0.970066727	NS
ZNF541	NS	0.95407937	-0.001168526	0.970066727	NS
USP17L1	NS	0.954045692	-0.001169384	0.970066727	NS
RBPMS	NS	0.954522401	-0.00115724	0.970466204	NS
CCDC33	NS	0.954638035	0.001154295	0.970532791	NS
RUNX3	NS	0.954894648	-0.001147758	0.970609697	NS
CUL2	NS	0.954943146	0.001146523	0.970609697	NS
MARCHF1	NS	0.954964407	0.001145981	0.970609697	NS
SYN2	NS	0.954929063	-0.001146881	0.970609697	NS
NPIPA1	NS	0.954861898	-0.001148592	0.970609697	NS
MAP7D1	NS	0.955262927	-0.001138377	0.970862128	NS
DHX35	NS	0.955396313	0.001134979	0.97094671	NS
VPS18	NS	0.95566178	-0.001128217	0.971165507	NS
ICOS10-NBI	NS	0.956084579	-0.001117448	0.971544157	NS
PGA3	NS	0.95635633	0.001110526	0.971769285	NS
MAGT1	NS	0.956742795	0.001100683	0.971918972	NS
AFTPH	NS	0.956799111	0.001099248	0.971918972	NS
TGS1	NS	0.956717028	0.001101339	0.971918972	NS
HEATR3	NS	0.956773103	-0.001099911	0.971918972	NS
PABPC5	NS	0.956734155	0.001100903	0.971918972	NS
RGL3	NS	0.956804919	0.0010991	0.971918972	NS
WRAP73	NS	0.956883494	-0.001097099	0.97194778	NS
ZNF439	NS	0.957204847	0.001088914	0.972223174	NS

TSPAN5	NS	0.957565555	-0.001079728	0.972538508	NS
VSTM4	NS	0.957707681	0.001076108	0.972631821	NS
SP2	NS	0.957979261	-0.001069191	0.972856589	NS
LPP	NS	0.95808365	-0.001066533	0.972911554	NS
SLC49A4	NS	0.958292363	-0.001061217	0.973065633	NS
SOCS3	NS	0.958335925	0.001060108	0.973065633	NS
ALM2AKAP	NS	0.958417766	0.001058023	0.973097686	NS
SAMD4A	NS	0.958480055	-0.001056437	0.973109884	NS
RAB18	NS	0.958574948	0.001054021	0.973155182	NS
SYMPK	NS	0.95878162	0.001048757	0.973313947	NS
POLR2D	NS	0.958989072	0.001043474	0.973473489	NS
RANGAP1	NS	0.959201102	0.001038075	0.973631643	NS
ANAPC10	NS	0.959245476	-0.001036945	0.973631643	NS
KRTAP22-2	NS	0.959341711	0.001034494	0.973678263	NS
SLC37A1	NS	0.959462325	-0.001031422	0.973749621	NS
IL1A	NS	0.95965499	-0.001026516	0.973894091	NS
AMPD3	NS	0.959823926	0.001022214	0.974014466	NS
ARHGAP24	NS	0.960128219	0.001014465	0.974120155	NS
PPP1R9A	NS	0.960129382	-0.001014436	0.974120155	NS
NEUROD1	NS	0.960002363	0.00101767	0.974120155	NS
PLA2G6	NS	0.960060419	0.001016192	0.974120155	NS
ATMIN	NS	0.960244651	-0.001011501	0.97418604	NS
ADARB1	NS	0.960452322	-0.001006212	0.974345657	NS
OR5AS1	NS	0.960697596	0.000999967	0.974543403	NS
AKAP4	NS	0.960783641	0.000997776	0.974579612	NS
LEMD1	NS	0.961124677	-0.000989092	0.974831066	NS
SLC35G6	NS	0.961132262	0.000988899	0.974831066	NS
QSOX1	NS	0.961542732	0.000978447	0.974900957	NS
GFPT2	NS	0.961372282	-0.000982787	0.974900957	NS
MTSS2	NS	0.96131703	0.000984194	0.974900957	NS
ARHGAP32	NS	0.961298588	0.000984664	0.974900957	NS
LZIC	NS	0.961440915	0.00098104	0.974900957	NS
DENND2C	NS	0.961489377	0.000979806	0.974900957	NS
OR52A1	NS	0.961553739	0.000978167	0.974900957	NS
THOC2	NS	0.961842624	-0.000970812	0.975142773	NS
MEMO1	NS	0.962260456	0.000960173	0.975515287	NS
CLIC4	NS	0.962422734	-0.000956041	0.975628702	NS
PABPC4	NS	0.962482703	-0.000954514	0.975638398	NS
SMYD3	NS	0.962545983	0.000952903	0.975651448	NS
RET	NS	0.962722369	0.000948412	0.975779137	NS
FIP1L1	NS	0.96278201	-0.000946894	0.97578849	NS
ABHD16A	NS	0.962855632	-0.000945019	0.975812012	NS
TMTC4	NS	0.962962053	-0.00094231	0.97586877	NS
RASA1	NS	0.963141165	-0.00093775	0.97595564	NS
MZT2A	NS	0.963184466	0.000936647	0.97595564	NS
FAM131A	NS	0.963199038	0.000936276	0.97595564	NS
BMP2K	NS	0.963252639	-0.000934912	0.975958861	NS
SLC1A5	NS	0.963312729	0.000933382	0.975968657	NS
OR1J2	NS	0.963603853	-0.00092597	0.976212508	NS
CPE	NS	0.963675042	-0.000924158	0.976233533	NS
INVS	NS	0.963776666	0.00092157	0.976285386	NS
PPP1R12A	NS	0.964116556	-0.000912917	0.976578579	NS
RPS25	NS	0.964276546	-0.000908844	0.976587318	NS
LYZL1	NS	0.964182834	0.00091123	0.976587318	NS
ALLC	NS	0.964268807	-0.000909041	0.976587318	NS

COG8	NS	0.964394704	-0.000905836	0.976655883	NS
OR7G1	NS	0.964916852	-0.000892543	0.977133546	NS
PGPEP1	NS	0.965076688	0.000888474	0.977244278	NS
GMFG	NS	0.965294307	-0.000882935	0.977413507	NS
NLRP13	NS	0.965657715	-0.000873684	0.977730331	NS
FKBP2	NS	0.965715131	0.000872222	0.97773732	NS
WDR12	NS	0.965903103	-0.000867437	0.977763147	NS
KCNJ14	NS	0.9659427	-0.000866429	0.977763147	NS
PATE4	NS	0.965837574	-0.000869105	0.977763147	NS
GOLGA6L24I	NS	0.965905596	-0.000867373	0.977763147	NS
MAD1L1	NS	0.966525061	-0.000851605	0.978071211	NS
THUMPD2	NS	0.966625901	0.000849038	0.978071211	NS
CCDC158	NS	0.966472184	0.000852951	0.978071211	NS
PDZD8	NS	0.966466076	-0.000853106	0.978071211	NS
RGS6	NS	0.966570811	-0.00085044	0.978071211	NS
NUTM2B	NS	0.966491316	-0.000852464	0.978071211	NS
DYNLT2B	NS	0.966578782	-0.000850237	0.978071211	NS
CDR1	NS	0.966651284	0.000848392	0.978071211	NS
OR2A12	NS	0.966827825	-0.000843898	0.978198702	NS
TAF11L9	NS	0.966966609	-0.000840365	0.978287983	NS
TRAPPC2B	NS	0.967122438	0.000836399	0.978394497	NS
KRCC1	NS	0.967273668	0.000832549	0.978496348	NS
CD5	NS	0.96741083	-0.000829058	0.978583957	NS
QSOX2	NS	0.967508127	-0.000826581	0.978631235	NS
RASA4B	NS	0.967585578	0.00082461	0.978644268	NS
APOLD1	NS	0.967622132	-0.00082368	0.978644268	NS
RXFP1	NS	0.967829469	0.000818402	0.978802821	NS
MAGEC1	NS	0.967930435	-0.000815832	0.978853788	NS
DRP2	NS	0.96809702	0.000811592	0.978971105	NS
UBE2D4	NS	0.968399702	0.000803888	0.979072584	NS
USP34	NS	0.968318714	0.000805949	0.979072584	NS
HK3	NS	0.968371223	-0.000804613	0.979072584	NS
BICDL2	NS	0.968386887	0.000804214	0.979072584	NS
GEMIN4	NS	0.968546624	-0.000800149	0.979169981	NS
ATG7	NS	0.968872585	0.000791852	0.97944836	NS
SPRR4	NS	0.968927721	0.000790449	0.979452944	NS
MPHOSPH10	NS	0.969093539	-0.000786228	0.979518253	NS
FCGR3A	NS	0.969054601	0.000787219	0.979518253	NS
BAX	NS	0.969201633	0.000783477	0.979576357	NS
ASGR1	NS	0.969368135	-0.00077924	0.979693485	NS
PTK2	NS	0.969643342	-0.000772235	0.979893285	NS
PAIP1	NS	0.969902753	-0.000765633	0.980080276	NS
SEMA5A	NS	0.970165073	-0.000758957	0.980294172	NS
SETDB1	NS	0.970466212	0.000751293	0.980547267	NS
ADAMTS13	NS	0.970628359	-0.000747166	0.980659908	NS
RGS7BP	NS	0.970702912	-0.000745269	0.980684042	NS
ADCY3	NS	0.970858866	-0.0007413	0.980763055	NS
EXTL1	NS	0.971016869	0.000737279	0.980763055	NS
NAA15	NS	0.97103447	-0.000736831	0.980763055	NS
CRK	NS	0.970952791	0.000738909	0.980763055	NS
GOLGA6L4	NS	0.970966148	-0.000738569	0.980763055	NS
SMARCE1	NS	0.97109727	0.000735233	0.980775307	NS
SLC44A1	NS	0.971287413	-0.000730394	0.980916162	NS
DRG1	NS	0.971420163	-0.000727015	0.980999044	NS
TMEM120A	NS	0.971873759	-0.000715472	0.98140591	NS

PNN	NS	0.972012977	0.000711929	0.981495288	NS
SEC16B	NS	0.972273089	0.00070531	0.981706724	NS
OR1J1	NS	0.972558736	0.00069804	0.98194392	NS
DPF1	NS	0.972614611	0.000696619	0.981949113	NS
MYO9A	NS	0.972917159	0.000688919	0.982203334	NS
RFC1	NS	0.973042788	-0.000685723	0.982227703	NS
NUTM2A	NS	0.973037211	-0.000685865	0.982227703	NS
KAT2A	NS	0.973097069	-0.000684341	0.982231271	NS
HEXD	NS	0.973177784	0.000682287	0.982261521	NS
CZIB	NS	0.973244986	-0.000680577	0.982278129	NS
SPRY2	NS	0.973474689	0.000674732	0.982407512	NS
FAM9A	NS	0.973459919	-0.000675108	0.982407512	NS
CLCNKB	NS	0.973610818	-0.000671268	0.982493665	NS
B3GAT3	NS	0.973675049	-0.000669634	0.98250726	NS
IFT27	NS	0.973928594	-0.000663182	0.982558213	NS
TIMELESS	NS	0.973919327	-0.000663418	0.982558213	NS
BVES	NS	0.973836846	-0.000665516	0.982558213	NS
FAM171A1	NS	0.973836159	-0.000665534	0.982558213	NS
AP2B1	NS	0.974033773	-0.000660505	0.982561898	NS
H2AC6	NS	0.974027325	-0.000660669	0.982561898	NS
EXTL3	NS	0.974438496	0.000650207	0.982905508	NS
CRYGS	NS	0.974475961	-0.000649254	0.982905508	NS
SMIM28	NS	0.974617001	0.000645665	0.982996544	NS
CAMK2B	NS	0.97480484	0.000640885	0.983083545	NS
GPX1	NS	0.974804754	-0.000640887	0.983083545	NS
TMX1	NS	0.974993459	-0.000636086	0.983222538	NS
NPHP3	NS	0.975106657	-0.000633205	0.983285463	NS
ZNF211	NS	0.975248073	-0.000629607	0.983376834	NS
SPATA31C2	NS	0.975424397	0.00062512	0.983452163	NS
LILRB3	NS	0.975407407	0.000625553	0.983452163	NS
TIAM1	NS	0.975569663	0.000621424	0.983496166	NS
FBXO47	NS	0.975546728	0.000622008	0.983496166	NS
FCRL2	NS	0.975904768	0.000612898	0.983782756	NS
TLCD2	NS	0.976074502	0.000608579	0.983902618	NS
DEFB108B	NS	0.976551686	0.000596438	0.984332366	NS
PTPN3	NS	0.976879151	-0.000588106	0.984595615	NS
OR2A14	NS	0.976914589	-0.000587204	0.984595615	NS
SARNP	NS	0.97698188	0.000585492	0.984612167	NS
CWC22	NS	0.977048279	-0.000583803	0.984627817	NS
FAT2	NS	0.977289937	0.000577654	0.984820076	NS
PCNP	NS	0.977347584	-0.000576187	0.984826896	NS
GCH1	NS	0.97745	0.000573582	0.984878824	NS
PABPC3	NS	0.977739836	0.000566207	0.985068305	NS
SNRNP35	NS	0.977703696	-0.000567127	0.985068305	NS
CPA1	NS	0.977841096	-0.000563631	0.985119047	NS
DSEL	NS	0.978106055	0.00055689	0.985283414	NS
VEGFB	NS	0.978098109	0.000557092	0.985283414	NS
EIF2S1	NS	0.978161098	0.00055549	0.985287584	NS
UHRF2	NS	0.97861803	-0.000543864	0.98569655	NS
RMI2	NS	0.978872877	0.000537381	0.985901936	NS
RHCG	NS	0.979025367	-0.000533501	0.986004214	NS
ZDHHC12	NS	0.979166819	0.000529902	0.986095367	NS
CNOT4	NS	0.979315122	-0.000526129	0.986188038	NS
VKORC1L1	NS	0.97936074	-0.000524969	0.986188038	NS
ETFBKMT	NS	0.979524618	0.000520799	0.986301748	NS

CUL4A	NS	0.979649803	-0.000517615	0.986376488	NS
TRMT2B	NS	0.979839444	0.00051279	0.986516113	NS
PCDHB14	NS	0.979990013	0.000508959	0.986579531	NS
ZNF560	NS	0.980004372	0.000508594	0.986579531	NS
SLC39A14	NS	0.980406809	0.000498356	0.986719915	NS
PRKAR1A	NS	0.980504654	-0.000495867	0.986719915	NS
MORC2	NS	0.980425767	0.000497874	0.986719915	NS
MARCHF7	NS	0.980371987	0.000499242	0.986719915	NS
PPP1R16A	NS	0.980551641	-0.000494671	0.986719915	NS
ACOX2	NS	0.980250792	0.000502325	0.986719915	NS
NINJ2	NS	0.980230449	0.000502843	0.986719915	NS
TWF2	NS	0.98048469	-0.000496375	0.986719915	NS
TBC1D4	NS	0.980629155	0.000492699	0.986746617	NS
PXMP4	NS	0.981040571	-0.000482233	0.987006668	NS
ZKSCAN5	NS	0.980962611	-0.000484216	0.987006668	NS
CCER1	NS	0.981034423	0.000482389	0.987006668	NS
RSPH14	NS	0.981284511	0.000476027	0.987150239	NS
IL17RA	NS	0.981285273	-0.000476008	0.987150239	NS
SLC37A2	NS	0.981370375	-0.000473843	0.987179163	NS
IRGQ	NS	0.981416028	-0.000472682	0.987179163	NS
GZF1	NS	0.981659892	-0.000466478	0.987351385	NS
BMP15	NS	0.981689264	0.000465731	0.987351385	NS
GLRA3	NS	0.98179302	-0.000463091	0.987402366	NS
GAGE12F	NS	0.981841979	-0.000461846	0.987402366	NS
OR56A3	NS	0.982091202	0.000455506	0.987601688	NS
PGGHG	NS	0.982151867	-0.000453962	0.987610334	NS
BCDIN3D	NS	0.982201846	0.000452691	0.987610334	NS
TP53INP1	NS	0.982273788	-0.000450861	0.987631367	NS
EDDM13	NS	0.982439398	0.000446648	0.987746572	NS
HPSE	NS	0.982700365	-0.000440009	0.98795763	NS
RAD52	NS	0.982800597	-0.00043746	0.988007082	NS
LIN7B	NS	0.98288012	-0.000435437	0.98803571	NS
SPATA25	NS	0.982997	-0.000432463	0.988101887	NS
FBXL12	NS	0.983080724	0.000430334	0.98813473	NS
S100A7A	NS	0.983193883	-0.000427455	0.988197154	NS
MED12L	NS	0.983328935	0.000424019	0.988281575	NS
UTP15	NS	0.98343913	-0.000421216	0.988289694	NS
SLC25A30	NS	0.983415114	-0.000421827	0.988289694	NS
IL17B	NS	0.983642025	0.000416055	0.988442271	NS
ATF7-NPFF	NS	0.983726066	-0.000413917	0.988475405	NS
CCDC106	NS	0.984046489	-0.000405766	0.988746047	NS
RARA	NS	0.984416865	0.000396345	0.988964179	NS
MYORG	NS	0.9843767	0.000397367	0.988964179	NS
LRRC37A	NS	0.98434872	0.000398078	0.988964179	NS
MRPS30	NS	0.984589303	-0.000391959	0.989072092	NS
TMEM107	NS	0.98464098	0.000390644	0.989072092	NS
CEACAM20	NS	0.984677579	0.000389713	0.989072092	NS
KIF2B	NS	0.985029994	0.000380749	0.989374737	NS
GPNMB	NS	0.98535091	-0.000372585	0.989645714	NS
FAM120C	NS	0.985474493	0.000369442	0.989667131	NS
IGFL1	NS	0.985441018	0.000370293	0.989667131	NS
MTOR	NS	0.98561358	0.000365904	0.989755458	NS
WFDC13	NS	0.98576604	0.000362026	0.989794805	NS
SNTB2	NS	0.985806172	-0.000361005	0.989794805	NS
RASGEF1A	NS	0.985773876	-0.000361827	0.989794805	NS

YIPF1	NS	0.986059688	-0.000354557	0.989997994	NS
FOXP2	NS	0.986193865	-0.000351144	0.990030001	NS
KRT12	NS	0.986337215	0.000347497	0.990122557	NS
BMP8A	NS	0.986747181	0.000337069	0.990482727	NS
TEX48	NS	0.987115323	-0.000327705	0.990800881	NS
GALK1	NS	0.987430351	0.000319692	0.991065692	NS
POLM	NS	0.987568513	0.000316178	0.991101578	NS
PNLIP	NS	0.987555429	0.000316511	0.991101578	NS
MEGF9	NS	0.987976215	0.000305808	0.991459333	NS
EDC4	NS	0.988502334	-0.000292426	0.991899997	NS
SNAP47	NS	0.988517821	-0.000292032	0.991899997	NS
STOM	NS	0.988827364	-0.000284159	0.992159166	NS
SSBP3	NS	0.988915422	-0.000281919	0.992196087	NS
CCDC7	NS	0.989044488	0.000278636	0.992274147	NS
GRB2	NS	0.989109779	-0.000276975	0.992288218	NS
UVRAG	NS	0.989272313	0.000272841	0.992399839	NS
FLI1	NS	0.989438677	-0.00026861	0.992509168	NS
UBE2J2	NS	0.989502044	0.000266998	0.992509168	NS
PTAFR	NS	0.989535128	0.000266157	0.992509168	NS
BANP	NS	0.98997614	-0.00025494	0.992900056	NS
HERC2	NS	0.990444722	0.000243021	0.993254419	NS
ANKHD1	NS	0.990483405	0.000242038	0.993254419	NS
MYO18B	NS	0.990394728	-0.000244293	0.993254419	NS
FAM20A	NS	0.990839425	-0.000232982	0.99355996	NS
TBKBP1	NS	0.990916157	0.000231031	0.99358543	NS
PEX7	NS	0.991122937	-0.000225772	0.993741288	NS
B3GAT2	NS	0.991304385	-0.000221157	0.993825788	NS
GRXCR2	NS	0.991309904	0.000221016	0.993825788	NS
LUC7L2	NS	0.991486371	-0.000216528	0.993951223	NS
ECI1	NS	0.991674507	-0.000211743	0.99408834	NS
TRPV3	NS	0.991783766	-0.000208964	0.994094898	NS
ZNF512	NS	0.991744737	-0.000209957	0.994094898	NS
JKAMP	NS	0.992518907	-0.000190266	0.99472873	NS
CUX1	NS	0.99251059	0.000190478	0.99472873	NS
SCN2B	NS	0.992672194	0.000186368	0.994830848	NS
ANO6	NS	0.993239425	0.000171941	0.995341952	NS
ZNF136	NS	0.993285035	0.000170781	0.995341952	NS
EPB41L1	NS	0.993341689	-0.00016934	0.995347193	NS
MPRIP	NS	0.993419737	-0.000167355	0.995373871	NS
FZD3	NS	0.993925766	0.000154485	0.995777801	NS
ADGRG2	NS	0.993885165	0.000155518	0.995777801	NS
JMY	NS	0.993998582	-0.000152633	0.995799211	NS
TMEM182	NS	0.994078897	-0.00015059	0.99582813	NS
WSB2	NS	0.994142452	-0.000148974	0.995840258	NS
MED17	NS	0.994513764	-0.00013953	0.99608849	NS
CARF	NS	0.994452486	-0.000141089	0.99608849	NS
KCNG4	NS	0.994544646	-0.000138745	0.99608849	NS
LRP1B	NS	0.994726292	0.000134125	0.996218871	NS
KIF24	NS	0.99486443	-0.000130612	0.996305666	NS
MRE11	NS	0.994974401	-0.000127815	0.996315677	NS
MT1B	NS	0.994977373	0.000127739	0.996315677	NS
CDH8	NS	0.995298571	0.00011957	0.99658575	NS
PGM1	NS	0.995698711	0.000109394	0.996934836	NS
SEC11A	NS	0.996303637	9.40E-05	0.997437323	NS
C21orf58	NS	0.996279449	-9.46E-05	0.997437323	NS

NPL	NS	0.996726192	-8.33E-05	0.99780875	NS
OR1G1	NS	0.996825821	-8.07E-05	0.997856878	NS
STMN1	NS	0.997294234	-6.88E-05	0.998274148	NS
PKD1	NS	0.99777283	-5.66E-05	0.998701567	NS
ZNF251	NS	0.998016569	-5.04E-05	0.998893878	NS
CTPS2	NS	0.998209645	-4.55E-05	0.998932162	NS
HIF3A	NS	0.998118716	-4.78E-05	0.998932162	NS
RSRC1	NS	0.998184964	4.62E-05	0.998932162	NS
ABCC1	NS	0.998395861	4.08E-05	0.999015213	NS
TM4SF1	NS	0.998371668	4.14E-05	0.999015213	NS
CA1	NS	0.998542068	3.71E-05	0.999022715	NS
PSPH	NS	0.998558198	-3.67E-05	0.999022715	NS
PEX13	NS	0.998503488	3.81E-05	0.999022715	NS
OGDH	NS	0.998961484	2.64E-05	0.999322884	NS
SOX12	NS	0.998959597	-2.65E-05	0.999322884	NS
PIGV	NS	0.999142414	2.18E-05	0.999406429	NS
ESS2	NS	0.999148264	-2.17E-05	0.999406429	NS
AKAP7	NS	0.999222541	-1.98E-05	0.999429077	NS
MELK	NS	0.999289287	1.81E-05	0.999444192	NS
TICRR	NS	0.999431988	1.44E-05	0.999535268	NS
SPINK1	NS	0.999507175	1.25E-05	0.999558816	NS
NIBAN3	NS	0.999777071	5.67E-06	0.999777071	NS
