# Supplementary material for: RBAD: The first database dedicated alterations of blood RNA in individuals with Alzheimer’s disease and their clinical relevance
Source: Neural Regen Res. 2025 Mar 25;21(6):2553–62. doi: 10.4103/NRR.NRR-D-24-01165 (PMC13211806; doi:10.4103/NRR.NRR-D-24-01165)
Supplement: Supplementary file 22 [file NRR-21-2553_Suppl15.pdf]

Additional Table 20. Correlation between RNA expression and cognitive function in AD population (N = 149)

Method	Spearman correlation analysis
P value adjustment for multiple test	Benjiamini-Hochberg (FDR)
Description	**Correltion analysis:** Correlation between RNA expression and cognitive function defined by MMSE score through the Spearman correlation analysis. Negative: correlation coefficient <=-0.2 and FDR<=0.05. Positive: correlation coefficient>=0.2 and FDR<=0.05. NS: not significant. **Survival analysis:** The association of each RNA with OS are presented in colum 'Survival risk'. Risk: the gene's higher expression has higher risk of death. Protective: the gene's higher expression has lower risk of death. NS: not significant. Detailed survival results are showing in Table S4.

Symbol	Survival analysis	Correlation analysis			
	Survival risk	P value	Correlation coefficient	FDR	Correlation
FGF21	Risk	5.97E-08	2.20E-01	3.12E-06	Positive
DMRTC1	Protective	2.22E-09	2.42E-01	2.36E-07	Positive
SSU72P7	Protective	3.03E-08	2.25E-01	1.86E-06	Positive
NXPH4	NS	1.68E-20	3.68E-01	3.26E-16	Positive
MADCAM1	NS	1.63E-15	3.19E-01	2.43E-12	Positive
SCRT1	NS	2.52E-12	2.82E-01	1.19E-09	Positive
NKX2-8	NS	6.44E-12	2.77E-01	2.54E-09	Positive
RAC3	NS	1.21E-11	2.73E-01	4.34E-09	Positive
SYCE1L	NS	3.71E-11	2.67E-01	1.09E-08	Positive
MAFF	NS	4.89E-11	2.65E-01	1.26E-08	Positive
SYNGR4	NS	5.91E-11	2.64E-01	1.40E-08	Positive
KRTAP10-6	NS	5.95E-11	2.64E-01	1.40E-08	Positive

FAM246B	NS	6.49E-11	2.64E-01	1.43E-08	Positive
NR0B2	NS	9.24E-11	2.62E-01	1.84E-08	Positive
KLK11	NS	1.01E-10	2.61E-01	1.99E-08	Positive
KRTAP10-8	NS	1.07E-10	2.61E-01	2.04E-08	Positive
ADRA2C	NS	1.92E-10	2.57E-01	3.34E-08	Positive
FZD8	NS	2.11E-10	2.57E-01	3.61E-08	Positive
PAX2	NS	2.77E-10	2.55E-01	4.65E-08	Positive
C1QTNF4	NS	3.47E-10	2.54E-01	5.73E-08	Positive
MAFA	NS	3.53E-10	2.54E-01	5.79E-08	Positive
TMEM151A	NS	4.00E-10	2.53E-01	6.35E-08	Positive
NEUROG1	NS	4.98E-10	2.51E-01	7.55E-08	Positive
KRT83	NS	6.67E-10	2.50E-01	9.22E-08	Positive
CTU1	NS	6.72E-10	2.50E-01	9.22E-08	Positive
FOXD1	NS	8.43E-10	2.48E-01	1.09E-07	Positive
LCNL1	NS	9.04E-10	2.48E-01	1.14E-07	Positive
FBLL1	NS	2.40E-09	2.42E-01	2.54E-07	Positive
DMRTC1B	NS	4.11E-09	2.38E-01	4.01E-07	Positive
NAT8L	NS	4.25E-09	2.38E-01	4.09E-07	Positive
FSD1	NS	4.39E-09	2.38E-01	4.19E-07	Positive
MND1	NS	4.60E-09	2.37E-01	4.28E-07	Positive
PDIA2	NS	5.02E-09	2.37E-01	4.63E-07	Positive
NKX2-2	NS	5.31E-09	2.36E-01	4.82E-07	Positive
DUSP9	NS	5.97E-09	2.36E-01	5.27E-07	Positive
CITED4	NS	6.36E-09	2.35E-01	5.47E-07	Positive
LRRC38	NS	7.23E-09	2.34E-01	6.06E-07	Positive
ANO8	NS	1.34E-08	2.30E-01	1.02E-06	Positive
DEFB105A	NS	1.54E-08	2.29E-01	1.14E-06	Positive
SOX30	NS	1.65E-08	2.29E-01	1.20E-06	Positive
MMP15	NS	1.81E-08	2.28E-01	1.27E-06	Positive
TAF11L2	NS	1.88E-08	2.28E-01	1.30E-06	Positive
KRTAP10-7	NS	1.95E-08	2.28E-01	1.33E-06	Positive
STX18	NS	2.33E-08	2.26E-01	1.53E-06	Positive
TRIB3	NS	2.87E-08	2.25E-01	1.80E-06	Positive
FOXO6	NS	2.98E-08	2.25E-01	1.86E-06	Positive
KRTAP10-1	NS	3.06E-08	2.25E-01	1.87E-06	Positive
GRIN1	NS	3.30E-08	2.24E-01	1.96E-06	Positive
KIF26A	NS	3.59E-08	2.23E-01	2.09E-06	Positive

ZIC2	NS	3.68E-08	2.23E-01	2.13E-06	Positive
CSPG5	NS	5.39E-08	2.21E-01	2.87E-06	Positive
CHST1	NS	5.71E-08	2.20E-01	3.02E-06	Positive
WIPI2	NS	6.04E-08	2.20E-01	3.14E-06	Positive
PEAK3	NS	6.18E-08	2.20E-01	3.15E-06	Positive
FBXO17	NS	7.36E-08	2.18E-01	3.64E-06	Positive
RNF151	NS	8.39E-08	2.18E-01	4.02E-06	Positive
TMEM271	NS	9.17E-08	2.17E-01	4.31E-06	Positive
SLC9A1	NS	9.47E-08	2.17E-01	4.41E-06	Positive
C1QL1	NS	9.97E-08	2.16E-01	4.62E-06	Positive
FOXQ1	NS	1.00E-07	2.16E-01	4.62E-06	Positive
CYSRT1	NS	1.02E-07	2.16E-01	4.67E-06	Positive
OR1M1	NS	1.05E-07	2.16E-01	4.76E-06	Positive
NR5A1	NS	1.05E-07	2.16E-01	4.78E-06	Positive
FBXL14	NS	1.37E-07	2.14E-01	5.91E-06	Positive
HBM	NS	1.41E-07	2.14E-01	6.03E-06	Positive
UTF1	NS	1.58E-07	2.13E-01	6.68E-06	Positive
HAPLN4	NS	1.83E-07	2.12E-01	7.51E-06	Positive
FAM71D	NS	1.87E-07	2.12E-01	7.64E-06	Positive
KCNF1	NS	1.92E-07	2.11E-01	7.82E-06	Positive
GJC2	NS	1.97E-07	2.11E-01	7.98E-06	Positive
COLGALT1	NS	2.07E-07	2.11E-01	8.24E-06	Positive
HRH3	NS	2.37E-07	2.10E-01	9.19E-06	Positive
MTRNR2L8	NS	2.48E-07	2.10E-01	9.51E-06	Positive
OR2AP1	NS	2.52E-07	2.09E-01	9.64E-06	Positive
UNCX	NS	3.02E-07	2.08E-01	1.11E-05	Positive
H1-4	NS	3.26E-07	2.08E-01	1.17E-05	Positive
EN1	NS	3.39E-07	2.07E-01	1.21E-05	Positive
SCGB3A1	NS	3.43E-07	2.07E-01	1.22E-05	Positive
ITPKA	NS	3.47E-07	2.07E-01	1.23E-05	Positive
TMEM52	NS	3.49E-07	2.07E-01	1.23E-05	Positive
FOXL3	NS	3.91E-07	2.06E-01	1.35E-05	Positive
DEFB4A	NS	4.27E-07	2.06E-01	1.45E-05	Positive
RGS9BP	NS	4.36E-07	2.05E-01	1.47E-05	Positive
USP30	NS	4.93E-07	2.04E-01	1.63E-05	Positive
DIO3	NS	5.59E-07	2.03E-01	1.80E-05	Positive
ANKRD54	NS	6.01E-07	2.03E-01	1.90E-05	Positive

SLC66A2	NS	6.19E-07	2.03E-01	1.95E-05	Positive
CDC27	NS	6.26E-07	2.03E-01	1.96E-05	Positive
PITX3	NS	6.67E-07	2.02E-01	2.06E-05	Positive
SBNO2	NS	7.24E-07	2.01E-01	2.21E-05	Positive
DEF6	NS	7.40E-07	2.01E-01	2.24E-05	Positive
C11orf52	NS	7.46E-07	2.01E-01	2.26E-05	Positive
DIRAS1	NS	7.52E-07	2.01E-01	2.27E-05	Positive
ACTRT2	NS	7.92E-07	2.01E-01	2.35E-05	Positive
SLC22A12	NS	8.01E-07	2.01E-01	2.37E-05	Positive
ARX	NS	8.05E-07	2.01E-01	2.38E-05	Positive
BARX1	NS	8.22E-07	2.00E-01	2.41E-05	Positive
HRCT1	NS	8.34E-07	2.00E-01	2.43E-05	Positive
HSD3B7	Risk	8.22E-07	-0.2	2.41E-05	Negative
FAM184A	Protective	4.60E-07	-0.205	1.52E-05	Negative
ADAM23	Protective	3.52E-08	-0.224	2.06E-06	Negative
TBX3	Protective	5.34E-08	-0.221	2.85E-06	Negative
BICD1	Protective	4.61E-10	-0.252	7.13E-08	Negative
CD1B	Protective	6.16E-08	-0.22	3.14E-06	Negative
PTTG1	Protective	1.27E-09	-0.246	1.50E-07	Negative
PKNOX2	Protective	8.05E-08	-0.218	3.89E-06	Negative
RAB26	Protective	5.50E-08	-0.221	2.92E-06	Negative
UBE2E3	Protective	9.38E-08	-0.217	4.39E-06	Negative
FAM174A	Risk	1.78E-07	-0.212	7.34E-06	Negative
ERCC6L	Protective	1.05E-10	-0.261	2.03E-08	Negative
FAM169A	Protective	3.74E-08	-0.223	2.15E-06	Negative
CRPPA	Protective	6.93E-09	-0.235	5.90E-07	Negative
FETUB	NS	3.75E-19	-0.355	2.28E-15	Negative
GPR22	NS	2.68E-19	-0.357	2.28E-15	Negative
HIGD1C	NS	4.70E-19	-0.354	2.28E-15	Negative
CCER2	NS	2.40E-18	-0.348	9.30E-15	Negative
MAGOHB	NS	3.58E-18	-0.346	1.16E-14	Negative
FCRLA	NS	7.17E-17	-0.333	1.98E-13	Negative
WDR75	NS	2.09E-16	-0.328	5.02E-13	Negative
OR51G2	NS	2.33E-16	-0.328	5.02E-13	Negative
PBDC1	NS	7.77E-16	-0.322	1.37E-12	Negative
GPR149	NS	7.21E-16	-0.323	1.37E-12	Negative
DEPDC1B	NS	1.55E-15	-0.319	2.43E-12	Negative

DNAJB7	NS	2.68E-15	-0.316	3.70E-12	Negative
TCHHL1	NS	8.14E-15	-0.311	1.05E-11	Negative
TRUB1	NS	9.28E-15	-0.31	1.12E-11	Negative
POPDC2	NS	9.94E-15	-0.31	1.13E-11	Negative
BORCS5	NS	1.74E-14	-0.307	1.87E-11	Negative
LEAP2	NS	4.07E-14	-0.303	4.15E-11	Negative
PAPOLG	NS	7.54E-14	-0.3	7.30E-11	Negative
GLYATL1	NS	8.16E-14	-0.3	7.52E-11	Negative
MIX23	NS	2.53E-13	-0.294	2.13E-10	Negative
CLEC3B	NS	2.47E-13	-0.294	2.13E-10	Negative
CD1E	NS	3.81E-13	-0.292	2.84E-10	Negative
LIPM	NS	3.68E-13	-0.292	2.84E-10	Negative
IFIT1	NS	3.57E-13	-0.292	2.84E-10	Negative
SMIM5	NS	4.30E-13	-0.291	3.08E-10	Negative
HENMT1	NS	4.81E-13	-0.291	3.33E-10	Negative
LYVE1	NS	6.16E-13	-0.289	4.11E-10	Negative
OMD	NS	8.30E-13	-0.288	5.36E-10	Negative
JRKL	NS	9.38E-13	-0.287	5.67E-10	Negative
TMEM225	NS	9.34E-13	-0.287	5.67E-10	Negative
RND3	NS	1.32E-12	-0.285	7.74E-10	Negative
POLR3G	NS	1.55E-12	-0.284	7.89E-10	Negative
CISD1	NS	1.50E-12	-0.285	7.89E-10	Negative
MRPS28	NS	1.45E-12	-0.285	7.89E-10	Negative
ZNF749	NS	1.40E-12	-0.285	7.89E-10	Negative
TOMM20L	NS	1.53E-12	-0.285	7.89E-10	Negative
MRPL47	NS	1.69E-12	-0.284	8.39E-10	Negative
FRMD5	NS	2.10E-12	-0.283	1.02E-09	Negative
ESRP2	NS	2.62E-12	-0.282	1.21E-09	Negative
GABRR3	NS	2.79E-12	-0.281	1.26E-09	Negative
ARL4D	NS	3.70E-12	-0.28	1.62E-09	Negative
CD300LD	NS	3.76E-12	-0.28	1.62E-09	Negative
TNFSF18	NS	4.34E-12	-0.279	1.82E-09	Negative
ODF2L	NS	4.88E-12	-0.278	2.01E-09	Negative
TREM2	NS	5.11E-12	-0.278	2.06E-09	Negative
TBC1D19	NS	6.75E-12	-0.276	2.61E-09	Negative
ACKR4	NS	8.61E-12	-0.275	3.24E-09	Negative
IL1RAPL1	NS	8.71E-12	-0.275	3.24E-09	Negative

YEATS4	NS	1.11E-11	-0.274	4.06E-09	Negative
TMEM265	NS	1.85E-11	-0.271	6.52E-09	Negative
SPINK14	NS	1.98E-11	-0.27	6.84E-09	Negative
KHDRBS2	NS	2.03E-11	-0.27	6.88E-09	Negative
PMCH	NS	2.10E-11	-0.27	7.02E-09	Negative
ARL14EPL	NS	2.16E-11	-0.27	7.09E-09	Negative
HSFX4	NS	2.32E-11	-0.27	7.47E-09	Negative
NCAPG	NS	2.46E-11	-0.269	7.81E-09	Negative
MIS12	NS	3.00E-11	-0.268	9.37E-09	Negative
ZNF37A	NS	3.49E-11	-0.267	1.07E-08	Negative
CDKN3	NS	3.60E-11	-0.267	1.09E-08	Negative
HGD	NS	3.84E-11	-0.267	1.09E-08	Negative
LMO2	NS	3.79E-11	-0.267	1.09E-08	Negative
GAGE12G	NS	3.78E-11	-0.267	1.09E-08	Negative
NOX3	NS	3.95E-11	-0.266	1.11E-08	Negative
SLFN12L	NS	4.04E-11	-0.266	1.12E-08	Negative
ARHGEF26	NS	4.21E-11	-0.266	1.12E-08	Negative
SYT4	NS	4.16E-11	-0.266	1.12E-08	Negative
ZUP1	NS	4.22E-11	-0.266	1.12E-08	Negative
IDI2	NS	4.48E-11	-0.266	1.17E-08	Negative
COG6	NS	5.06E-11	-0.265	1.29E-08	Negative
OPN1SW	NS	5.39E-11	-0.265	1.36E-08	Negative
GRIA3	NS	5.52E-11	-0.265	1.37E-08	Negative
ITM2A	NS	5.81E-11	-0.264	1.40E-08	Negative
FRMD7	NS	5.79E-11	-0.264	1.40E-08	Negative
UTP6	NS	6.27E-11	-0.264	1.43E-08	Negative
SSPN	NS	6.45E-11	-0.264	1.43E-08	Negative
PTPRD	NS	6.18E-11	-0.264	1.43E-08	Negative
SH2D4B	NS	6.28E-11	-0.264	1.43E-08	Negative
TAS2R19	NS	6.45E-11	-0.264	1.43E-08	Negative
ST8SIA1	NS	6.98E-11	-0.263	1.51E-08	Negative
METTL18	NS	7.18E-11	-0.263	1.51E-08	Negative
PLD5	NS	7.03E-11	-0.263	1.51E-08	Negative
CCDC71L	NS	7.12E-11	-0.263	1.51E-08	Negative
CAMK1D	NS	7.31E-11	-0.263	1.52E-08	Negative
ZNHIT6	NS	7.83E-11	-0.262	1.61E-08	Negative
BTBD3	NS	8.35E-11	-0.262	1.70E-08	Negative

BEND6	NS	8.65E-11	-0.262	1.74E-08	Negative
KCNE2	NS	1.02E-10	-0.261	2.00E-08	Negative
TMEM244	NS	1.07E-10	-0.261	2.04E-08	Negative
ITGB1BP2	NS	1.14E-10	-0.26	2.15E-08	Negative
PFAS	NS	1.30E-10	-0.26	2.42E-08	Negative
MRPS9	NS	1.44E-10	-0.259	2.66E-08	Negative
SERPINB4	NS	1.46E-10	-0.259	2.67E-08	Negative
ARR3	NS	1.54E-10	-0.259	2.78E-08	Negative
SLC27A4	NS	1.57E-10	-0.258	2.81E-08	Negative
MTMR9	NS	1.65E-10	-0.258	2.92E-08	Negative
PLPPR5	NS	1.83E-10	-0.257	3.21E-08	Negative
GPR174	NS	2.04E-10	-0.257	3.53E-08	Negative
MAGEH1	NS	2.62E-10	-0.255	4.45E-08	Negative
SLC22A1	NS	2.79E-10	-0.255	4.65E-08	Negative
IL18R1	NS	3.76E-10	-0.253	6.12E-08	Negative
CASTOR2	NS	3.84E-10	-0.253	6.19E-08	Negative
OR13C4	NS	3.98E-10	-0.253	6.35E-08	Negative
WLS	NS	4.32E-10	-0.252	6.79E-08	Negative
DRP2	NS	4.54E-10	-0.252	7.08E-08	Negative
LGR5	NS	4.88E-10	-0.252	7.50E-08	Negative
C6orf163	NS	4.99E-10	-0.251	7.55E-08	Negative
PROX2	NS	5.04E-10	-0.251	7.56E-08	Negative
FAM171B	NS	5.14E-10	-0.251	7.66E-08	Negative
LYPD6B	NS	5.37E-10	-0.251	7.94E-08	Negative
BET1	NS	5.47E-10	-0.251	8.02E-08	Negative
MXRA5	NS	5.98E-10	-0.25	8.64E-08	Negative
UTP3	NS	5.98E-10	-0.25	8.64E-08	Negative
FAM71A	NS	6.15E-10	-0.25	8.81E-08	Negative
SH2D1A	NS	6.19E-10	-0.25	8.81E-08	Negative
ANO4	NS	6.39E-10	-0.25	9.03E-08	Negative
DNER	NS	6.51E-10	-0.25	9.13E-08	Negative
C4orf54	NS	6.60E-10	-0.25	9.20E-08	Negative
ERICH6	NS	6.91E-10	-0.249	9.42E-08	Negative
PTPRO	NS	7.44E-10	-0.249	1.01E-07	Negative
CNGA1	NS	7.53E-10	-0.249	1.01E-07	Negative
RNF148	NS	7.66E-10	-0.249	1.02E-07	Negative
WBP4	NS	7.78E-10	-0.249	1.03E-07	Negative

CYP8B1	NS	7.80E-10	-0.249	1.03E-07	Negative
REELD1	NS	8.10E-10	-0.248	1.06E-07	Negative
PGGHG	NS	8.18E-10	-0.248	1.06E-07	Negative
CD79B	NS	8.92E-10	-0.248	1.14E-07	Negative
CELA1	NS	8.88E-10	-0.248	1.14E-07	Negative
APOBEC2	NS	9.49E-10	-0.247	1.19E-07	Negative
GSTT2B	NS	9.43E-10	-0.247	1.19E-07	Negative
HTR2A	NS	1.03E-09	-0.247	1.28E-07	Negative
DUSP21	NS	1.06E-09	-0.247	1.30E-07	Negative
PLCL2	NS	1.07E-09	-0.247	1.31E-07	Negative
AFM	NS	1.10E-09	-0.247	1.34E-07	Negative
CDH2	NS	1.13E-09	-0.246	1.37E-07	Negative
EXOG	NS	1.15E-09	-0.246	1.38E-07	Negative
GLIPR1L1	NS	1.18E-09	-0.246	1.41E-07	Negative
DCLRE1A	NS	1.21E-09	-0.246	1.44E-07	Negative
PGBD2	NS	1.28E-09	-0.246	1.50E-07	Negative
C4orf45	NS	1.31E-09	-0.245	1.53E-07	Negative
KHK	NS	1.33E-09	-0.245	1.54E-07	Negative
ILVBL	NS	1.42E-09	-0.245	1.64E-07	Negative
ZNF608	NS	1.50E-09	-0.245	1.72E-07	Negative
KBTBD3	NS	1.62E-09	-0.244	1.84E-07	Negative
KLHL4	NS	1.65E-09	-0.244	1.87E-07	Negative
KLRC4	NS	1.67E-09	-0.244	1.88E-07	Negative
ZNF225	NS	1.70E-09	-0.244	1.90E-07	Negative
FAP	NS	1.75E-09	-0.244	1.95E-07	Negative
LMCD1	NS	1.80E-09	-0.243	1.99E-07	Negative
CCR5	NS	1.83E-09	-0.243	2.01E-07	Negative
RASSF6	NS	1.85E-09	-0.243	2.03E-07	Negative
RPP25L	NS	1.92E-09	-0.243	2.09E-07	Negative
RTL9	NS	2.03E-09	-0.243	2.19E-07	Negative
CLEC1A	NS	2.09E-09	-0.242	2.25E-07	Negative
KCNJ1	NS	2.13E-09	-0.242	2.28E-07	Negative
SPAG8	NS	2.45E-09	-0.241	2.58E-07	Negative
PLPPR4	NS	2.57E-09	-0.241	2.68E-07	Negative
OR5J2	NS	2.76E-09	-0.241	2.87E-07	Negative
FAM234B	NS	2.94E-09	-0.24	3.04E-07	Negative
MMAA	NS	2.97E-09	-0.24	3.05E-07	Negative

RPSAP58	NS	2.99E-09	-0.24	3.07E-07	Negative
LRRN3	NS	3.04E-09	-0.24	3.10E-07	Negative
GAGE12B	NS	3.06E-09	-0.24	3.10E-07	Negative
SAV1	NS	3.21E-09	-0.24	3.23E-07	Negative
CENPP	NS	3.28E-09	-0.24	3.29E-07	Negative
PLA1A	NS	3.33E-09	-0.239	3.33E-07	Negative
CEACAM3	NS	3.86E-09	-0.238	3.84E-07	Negative
ERMP1	NS	4.03E-09	-0.238	3.98E-07	Negative
MLIP	NS	4.09E-09	-0.238	4.01E-07	Negative
RBX1	NS	4.15E-09	-0.238	4.02E-07	Negative
LMOD2	NS	4.16E-09	-0.238	4.02E-07	Negative
PPP1R17	NS	4.27E-09	-0.238	4.09E-07	Negative
P2RY14	NS	4.50E-09	-0.237	4.27E-07	Negative
OR52I2	NS	4.52E-09	-0.237	4.27E-07	Negative
SNTG1	NS	4.58E-09	-0.237	4.28E-07	Negative
UMODL1	NS	4.60E-09	-0.237	4.28E-07	Negative
PRRX1	NS	4.99E-09	-0.237	4.62E-07	Negative
LRIG1	NS	5.09E-09	-0.237	4.67E-07	Negative
STX19	NS	5.22E-09	-0.237	4.77E-07	Negative
NMUR2	NS	5.56E-09	-0.236	5.03E-07	Negative
PKD2L2	NS	5.65E-09	-0.236	5.08E-07	Negative
TCEANC	NS	5.67E-09	-0.236	5.08E-07	Negative
ERCC5	NS	5.99E-09	-0.236	5.27E-07	Negative
FAM3C	NS	5.92E-09	-0.236	5.27E-07	Negative
OR2W3	NS	5.95E-09	-0.236	5.27E-07	Negative
FMNL3	NS	6.05E-09	-0.236	5.30E-07	Negative
TMEM9	NS	6.08E-09	-0.236	5.30E-07	Negative
IL18RAP	NS	6.21E-09	-0.235	5.39E-07	Negative
TCEAL9	NS	6.25E-09	-0.235	5.40E-07	Negative
ZNF707	NS	6.72E-09	-0.235	5.76E-07	Negative
INPP4B	NS	6.96E-09	-0.235	5.90E-07	Negative
MYBBP1A	NS	6.98E-09	-0.235	5.90E-07	Negative
NDP	NS	7.22E-09	-0.234	6.06E-07	Negative
ATP6V1C2	NS	7.60E-09	-0.234	6.34E-07	Negative
ZNF711	NS	7.64E-09	-0.234	6.34E-07	Negative
HAS2	NS	8.27E-09	-0.233	6.84E-07	Negative
TEX29	NS	8.37E-09	-0.233	6.89E-07	Negative

ADAMTS12	NS	8.58E-09	-0.233	7.04E-07	Negative
DES	NS	8.64E-09	-0.233	7.06E-07	Negative
ZNF300	NS	8.82E-09	-0.233	7.17E-07	Negative
HPDL	NS	8.93E-09	-0.233	7.24E-07	Negative
WDR4	NS	9.13E-09	-0.233	7.36E-07	Negative
HECA	NS	9.31E-09	-0.233	7.47E-07	Negative
DAB1	NS	9.46E-09	-0.233	7.56E-07	Negative
GCG	NS	9.62E-09	-0.232	7.66E-07	Negative
KRTAP8-1	NS	9.65E-09	-0.232	7.66E-07	Negative
SPOCK3	NS	9.74E-09	-0.232	7.69E-07	Negative
TNMD	NS	1.05E-08	-0.232	8.29E-07	Negative
GAS2	NS	1.06E-08	-0.232	8.30E-07	Negative
OLFM3	NS	1.14E-08	-0.231	8.87E-07	Negative
SPSB2	NS	1.16E-08	-0.231	9.05E-07	Negative
MTERF3	NS	1.23E-08	-0.231	9.55E-07	Negative
KCNQ1	NS	1.26E-08	-0.231	9.71E-07	Negative
CAV3	NS	1.27E-08	-0.231	9.78E-07	Negative
GNRH1	NS	1.33E-08	-0.23	1.02E-06	Negative
OTOR	NS	1.39E-08	-0.23	1.06E-06	Negative
APLF	NS	1.40E-08	-0.23	1.06E-06	Negative
GRID1	NS	1.44E-08	-0.23	1.08E-06	Negative
MCOLN2	NS	1.45E-08	-0.23	1.09E-06	Negative
TMEM14A	NS	1.46E-08	-0.23	1.09E-06	Negative
TRIM17	NS	1.47E-08	-0.23	1.09E-06	Negative
HYLS1	NS	1.49E-08	-0.23	1.10E-06	Negative
PPP1R3F	NS	1.54E-08	-0.229	1.14E-06	Negative
LONRF2	NS	1.58E-08	-0.229	1.16E-06	Negative
CPB2	NS	1.64E-08	-0.229	1.20E-06	Negative
MMP27	NS	1.66E-08	-0.229	1.20E-06	Negative
NDFIP2	NS	1.68E-08	-0.229	1.21E-06	Negative
CLDN20	NS	1.69E-08	-0.229	1.21E-06	Negative
PGRMC2	NS	1.72E-08	-0.229	1.23E-06	Negative
SAMD3	NS	1.72E-08	-0.229	1.23E-06	Negative
H3Y1	NS	1.73E-08	-0.228	1.23E-06	Negative
TMEM106A	NS	1.76E-08	-0.228	1.25E-06	Negative
AIM2	NS	1.77E-08	-0.228	1.25E-06	Negative
SEBOX	NS	1.77E-08	-0.228	1.25E-06	Negative

GOLM1	NS	1.81E-08	-0.228	1.27E-06	Negative
CBR3	NS	1.84E-08	-0.228	1.28E-06	Negative
KLRB1	NS	1.86E-08	-0.228	1.29E-06	Negative
DDAH2	NS	1.88E-08	-0.228	1.30E-06	Negative
POC5	NS	1.89E-08	-0.228	1.30E-06	Negative
PHLDB2	NS	1.95E-08	-0.228	1.33E-06	Negative
FAM186B	NS	1.99E-08	-0.228	1.35E-06	Negative
SIGLEC12	NS	1.99E-08	-0.228	1.35E-06	Negative
C15orf62	NS	2.08E-08	-0.227	1.40E-06	Negative
GABRB1	NS	2.09E-08	-0.227	1.40E-06	Negative
LNX1	NS	2.19E-08	-0.227	1.47E-06	Negative
MYRFL	NS	2.23E-08	-0.227	1.49E-06	Negative
ZNF613	NS	2.23E-08	-0.227	1.49E-06	Negative
LYSMD1	NS	2.28E-08	-0.227	1.51E-06	Negative
PRPF40B	NS	2.30E-08	-0.227	1.52E-06	Negative
PLAAT5	NS	2.32E-08	-0.227	1.53E-06	Negative
LURAP1L	NS	2.36E-08	-0.226	1.54E-06	Negative
CCT8	NS	2.36E-08	-0.226	1.54E-06	Negative
ST20	NS	2.39E-08	-0.226	1.55E-06	Negative
TIMM10	NS	2.45E-08	-0.226	1.59E-06	Negative
SRPX	NS	2.49E-08	-0.226	1.61E-06	Negative
OVCH2	NS	2.53E-08	-0.226	1.63E-06	Negative
OR5AN1	NS	2.63E-08	-0.226	1.69E-06	Negative
IFNK	NS	2.66E-08	-0.226	1.70E-06	Negative
SEMA3E	NS	2.79E-08	-0.225	1.77E-06	Negative
SLC9C1	NS	2.79E-08	-0.225	1.77E-06	Negative
NPHS2	NS	2.80E-08	-0.225	1.77E-06	Negative
GOLGA8H	NS	2.82E-08	-0.225	1.78E-06	Negative
RPP14	NS	2.89E-08	-0.225	1.81E-06	Negative
LAMC1	NS	3.02E-08	-0.225	1.86E-06	Negative
ZNF189	NS	3.02E-08	-0.225	1.86E-06	Negative
SESN3	NS	2.99E-08	-0.225	1.86E-06	Negative
CETN3	NS	3.02E-08	-0.225	1.86E-06	Negative
ETV7	NS	3.04E-08	-0.225	1.86E-06	Negative
LRRC69	NS	3.12E-08	-0.224	1.90E-06	Negative
GABRG1	NS	3.13E-08	-0.224	1.90E-06	Negative
NMS	NS	3.15E-08	-0.224	1.91E-06	Negative

CLSTN3	NS	3.18E-08	-0.224	1.92E-06	Negative
KIF21A	NS	3.24E-08	-0.224	1.95E-06	Negative
METTL4	NS	3.27E-08	-0.224	1.96E-06	Negative
PTH	NS	3.28E-08	-0.224	1.96E-06	Negative
CCDC172	NS	3.39E-08	-0.224	2.01E-06	Negative
KLRC4-KLRK1	NS	3.40E-08	-0.224	2.01E-06	Negative
FAM216A	NS	3.42E-08	-0.224	2.02E-06	Negative
TEX30	NS	3.48E-08	-0.224	2.05E-06	Negative
CYP39A1	NS	3.52E-08	-0.224	2.06E-06	Negative
SPDL1	NS	3.60E-08	-0.223	2.09E-06	Negative
CYLC1	NS	3.71E-08	-0.223	2.14E-06	Negative
MYH10	NS	3.76E-08	-0.223	2.16E-06	Negative
ZNF510	NS	3.79E-08	-0.223	2.17E-06	Negative
CETN2	NS	3.88E-08	-0.223	2.21E-06	Negative
MVD	NS	3.87E-08	-0.223	2.21E-06	Negative
PHPT1	NS	3.93E-08	-0.223	2.23E-06	Negative
IL1RL1	NS	3.95E-08	-0.223	2.24E-06	Negative
SDHAF1	NS	3.98E-08	-0.223	2.25E-06	Negative
ADAM32	NS	4.05E-08	-0.223	2.28E-06	Negative
H2AW	NS	4.12E-08	-0.223	2.31E-06	Negative
NXPE1	NS	4.15E-08	-0.222	2.32E-06	Negative
ANKRD22	NS	4.27E-08	-0.222	2.38E-06	Negative
KLRF2	NS	4.31E-08	-0.222	2.40E-06	Negative
NUDT14	NS	4.44E-08	-0.222	2.46E-06	Negative
CFAP47	NS	4.61E-08	-0.222	2.55E-06	Negative
SPHKAP	NS	4.72E-08	-0.222	2.60E-06	Negative
PFKFB1	NS	4.77E-08	-0.222	2.62E-06	Negative
OR51Q1	NS	4.80E-08	-0.221	2.63E-06	Negative
IL12A	NS	4.83E-08	-0.221	2.64E-06	Negative
HACD3	NS	4.86E-08	-0.221	2.65E-06	Negative
MSTN	NS	4.88E-08	-0.221	2.65E-06	Negative
HOMER1	NS	4.87E-08	-0.221	2.65E-06	Negative
REXO5	NS	5.01E-08	-0.221	2.71E-06	Negative
SLC9A4	NS	5.03E-08	-0.221	2.71E-06	Negative
GSPT2	NS	5.03E-08	-0.221	2.71E-06	Negative
NUDT1	NS	5.27E-08	-0.221	2.83E-06	Negative
FSBP	NS	5.50E-08	-0.221	2.92E-06	Negative

SPATA9	NS	5.83E-08	-0.22	3.08E-06	Negative
NYNRIN	NS	5.88E-08	-0.22	3.09E-06	Negative
HNF4G	NS	5.91E-08	-0.22	3.10E-06	Negative
ZNF14	NS	6.08E-08	-0.22	3.14E-06	Negative
CCL7	NS	6.09E-08	-0.22	3.14E-06	Negative
CCDC175	NS	6.05E-08	-0.22	3.14E-06	Negative
GPR171	NS	6.07E-08	-0.22	3.14E-06	Negative
ETDB	NS	6.12E-08	-0.22	3.14E-06	Negative
HSFX3	NS	6.12E-08	-0.22	3.14E-06	Negative
AKR1C4	NS	6.16E-08	-0.22	3.14E-06	Negative
PJA1	NS	6.19E-08	-0.22	3.15E-06	Negative
DGCR8	NS	6.29E-08	-0.22	3.19E-06	Negative
SLC41A2	NS	6.48E-08	-0.219	3.27E-06	Negative
KIF2C	NS	6.49E-08	-0.219	3.27E-06	Negative
PPIL1	NS	6.51E-08	-0.219	3.27E-06	Negative
VWDE	NS	6.58E-08	-0.219	3.30E-06	Negative
SPAM1	NS	6.65E-08	-0.219	3.33E-06	Negative
EID3	NS	6.70E-08	-0.219	3.34E-06	Negative
BRPF3	NS	6.77E-08	-0.219	3.37E-06	Negative
PDP2	NS	6.86E-08	-0.219	3.40E-06	Negative
LMAN1	NS	7.14E-08	-0.219	3.53E-06	Negative
GLRA2	NS	7.46E-08	-0.218	3.68E-06	Negative
HAO2	NS	7.49E-08	-0.218	3.68E-06	Negative
PEX12	NS	7.59E-08	-0.218	3.71E-06	Negative
SPIN3	NS	7.60E-08	-0.218	3.71E-06	Negative
LTB	NS	7.63E-08	-0.218	3.72E-06	Negative
ASB15	NS	7.84E-08	-0.218	3.81E-06	Negative
C12orf4	NS	7.97E-08	-0.218	3.87E-06	Negative
GPR19	NS	8.06E-08	-0.218	3.89E-06	Negative
AQP8	NS	8.14E-08	-0.218	3.92E-06	Negative
MSMO1	NS	8.34E-08	-0.218	4.00E-06	Negative
LNPK	NS	8.42E-08	-0.217	4.02E-06	Negative
PRB2	NS	8.63E-08	-0.217	4.12E-06	Negative
BCDIN3D	NS	8.70E-08	-0.217	4.14E-06	Negative
C18orf54	NS	8.73E-08	-0.217	4.14E-06	Negative
GLIPR1L2	NS	8.92E-08	-0.217	4.22E-06	Negative
DDX1	NS	9.12E-08	-0.217	4.30E-06	Negative

GALNT5	NS	9.17E-08	-0.217	4.31E-06	Negative
CEACAM21	NS	9.25E-08	-0.217	4.33E-06	Negative
TOMM34	NS	9.45E-08	-0.217	4.41E-06	Negative
EIF5B	NS	9.68E-08	-0.216	4.49E-06	Negative
SLCO1B1	NS	1.00E-07	-0.216	4.62E-06	Negative
ZNF611	NS	1.02E-07	-0.216	4.67E-06	Negative
NRP2	NS	1.03E-07	-0.216	4.69E-06	Negative
TGM1	NS	1.04E-07	-0.216	4.75E-06	Negative
FUOM	NS	1.06E-07	-0.216	4.78E-06	Negative
TMEM207	NS	1.06E-07	-0.216	4.78E-06	Negative
TPGS2	NS	1.06E-07	-0.216	4.80E-06	Negative
FGD1	NS	1.07E-07	-0.216	4.83E-06	Negative
SLC4A7	NS	1.07E-07	-0.216	4.83E-06	Negative
INKA1	NS	1.08E-07	-0.216	4.84E-06	Negative
INHBA	NS	1.10E-07	-0.216	4.92E-06	Negative
CDNF	NS	1.11E-07	-0.215	4.95E-06	Negative
TOMM20	NS	1.12E-07	-0.215	4.99E-06	Negative
PRKCA	NS	1.14E-07	-0.215	5.04E-06	Negative
LGR6	NS	1.15E-07	-0.215	5.07E-06	Negative
ZC3H6	NS	1.15E-07	-0.215	5.07E-06	Negative
RTN4IP1	NS	1.17E-07	-0.215	5.14E-06	Negative
SUV39H2	NS	1.17E-07	-0.215	5.14E-06	Negative
SMIM20	NS	1.19E-07	-0.215	5.24E-06	Negative
RIDA	NS	1.20E-07	-0.215	5.27E-06	Negative
OR2A1	NS	1.21E-07	-0.215	5.31E-06	Negative
METTL14	NS	1.22E-07	-0.215	5.34E-06	Negative
DUSP14	NS	1.23E-07	-0.215	5.35E-06	Negative
SERPINA7	NS	1.27E-07	-0.214	5.53E-06	Negative
ROBO2	NS	1.28E-07	-0.214	5.55E-06	Negative
TIGD7	NS	1.31E-07	-0.214	5.67E-06	Negative
ZNF582	NS	1.35E-07	-0.214	5.84E-06	Negative
OR5H14	NS	1.41E-07	-0.214	6.04E-06	Negative
PNPT1	NS	1.45E-07	-0.214	6.19E-06	Negative
OR6C1	NS	1.49E-07	-0.213	6.34E-06	Negative
MBD2	NS	1.52E-07	-0.213	6.47E-06	Negative
TBC1D12	NS	1.53E-07	-0.213	6.48E-06	Negative
MUSK	NS	1.58E-07	-0.213	6.68E-06	Negative

JMJD7	NS	1.58E-07	-0.213	6.68E-06	Negative
CPO	NS	1.59E-07	-0.213	6.70E-06	Negative
ANAPC16	NS	1.64E-07	-0.213	6.87E-06	Negative
OCRL	NS	1.65E-07	-0.213	6.91E-06	Negative
GRK7	NS	1.67E-07	-0.213	6.96E-06	Negative
RRM2	NS	1.67E-07	-0.212	6.98E-06	Negative
MRFAP1L1	NS	1.68E-07	-0.212	6.98E-06	Negative
ZNF214	NS	1.73E-07	-0.212	7.18E-06	Negative
C5orf63	NS	1.75E-07	-0.212	7.27E-06	Negative
CMTM4	NS	1.76E-07	-0.212	7.27E-06	Negative
TEN1-CDK3	NS	1.78E-07	-0.212	7.34E-06	Negative
RAB38	NS	1.81E-07	-0.212	7.44E-06	Negative
AACS	NS	1.90E-07	-0.212	7.74E-06	Negative
NEK11	NS	1.96E-07	-0.211	7.98E-06	Negative
LAMP5	NS	1.97E-07	-0.211	7.98E-06	Negative
CMTM6	NS	1.98E-07	-0.211	8.00E-06	Negative
THSD8	NS	1.99E-07	-0.211	8.02E-06	Negative
TTC30B	NS	2.02E-07	-0.211	8.14E-06	Negative
RPAIN	NS	2.04E-07	-0.211	8.19E-06	Negative
TTC23L	NS	2.05E-07	-0.211	8.23E-06	Negative
TOM1L1	NS	2.06E-07	-0.211	8.24E-06	Negative
CADPS2	NS	2.08E-07	-0.211	8.28E-06	Negative
ENDOU	NS	2.10E-07	-0.211	8.35E-06	Negative
NUDT11	NS	2.11E-07	-0.211	8.36E-06	Negative
MDC1	NS	2.14E-07	-0.211	8.48E-06	Negative
PPIC	NS	2.15E-07	-0.211	8.48E-06	Negative
BCKDHB	NS	2.18E-07	-0.211	8.58E-06	Negative
MTRNR2L13	NS	2.27E-07	-0.21	8.91E-06	Negative
ALG2	NS	2.30E-07	-0.21	9.03E-06	Negative
TRPV6	NS	2.32E-07	-0.21	9.10E-06	Negative
CCDC39	NS	2.33E-07	-0.21	9.10E-06	Negative
FAM83D	NS	2.34E-07	-0.21	9.14E-06	Negative
ICA1	NS	2.36E-07	-0.21	9.19E-06	Negative
LGR4	NS	2.36E-07	-0.21	9.19E-06	Negative
H2BC15	NS	2.41E-07	-0.21	9.32E-06	Negative
TTC21A	NS	2.42E-07	-0.21	9.33E-06	Negative
C1orf115	NS	2.44E-07	-0.21	9.43E-06	Negative

LIN7C	NS	2.45E-07	-0.21	9.43E-06	Negative
CGREF1	NS	2.48E-07	-0.21	9.51E-06	Negative
NAA11	NS	2.49E-07	-0.21	9.51E-06	Negative
CYP7B1	NS	2.55E-07	-0.209	9.73E-06	Negative
AMN1	NS	2.56E-07	-0.209	9.75E-06	Negative
ZNF419	NS	2.58E-07	-0.209	9.78E-06	Negative
BAIAP2	NS	2.64E-07	-0.209	1.00E-05	Negative
PRG4	NS	2.67E-07	-0.209	1.01E-05	Negative
STC1	NS	2.66E-07	-0.209	1.01E-05	Negative
FOLR2	NS	2.67E-07	-0.209	1.01E-05	Negative
ZNF74	NS	2.69E-07	-0.209	1.01E-05	Negative
DEFB107B	NS	2.69E-07	-0.209	1.01E-05	Negative
HAGH	NS	2.70E-07	-0.209	1.01E-05	Negative
CMTR2	NS	2.70E-07	-0.209	1.01E-05	Negative
GTF2F2	NS	2.71E-07	-0.209	1.01E-05	Negative
CSNK1A1L	NS	2.73E-07	-0.209	1.02E-05	Negative
CD200R1	NS	2.75E-07	-0.209	1.02E-05	Negative
DCUN1D5	NS	2.78E-07	-0.209	1.03E-05	Negative
ARL13A	NS	2.78E-07	-0.209	1.03E-05	Negative
SLC38A1	NS	2.87E-07	-0.209	1.06E-05	Negative
ICOS	NS	2.87E-07	-0.209	1.06E-05	Negative
CST7	NS	2.97E-07	-0.208	1.09E-05	Negative
MICU2	NS	2.97E-07	-0.208	1.09E-05	Negative
CD3D	NS	3.03E-07	-0.208	1.11E-05	Negative
GOLGA8J	NS	3.04E-07	-0.208	1.11E-05	Negative
RHOXF2B	NS	3.07E-07	-0.208	1.12E-05	Negative
SKAP1	NS	3.08E-07	-0.208	1.12E-05	Negative
HEPH	NS	3.09E-07	-0.208	1.12E-05	Negative
PRKCQ	NS	3.13E-07	-0.208	1.13E-05	Negative
OR2T2	NS	3.16E-07	-0.208	1.14E-05	Negative
RNF150	NS	3.17E-07	-0.208	1.14E-05	Negative
C8orf82	NS	3.18E-07	-0.208	1.15E-05	Negative
CFAP161	NS	3.26E-07	-0.208	1.17E-05	Negative
STATH	NS	3.30E-07	-0.207	1.18E-05	Negative
NKG7	NS	3.33E-07	-0.207	1.19E-05	Negative
TMEM185B	NS	3.34E-07	-0.207	1.19E-05	Negative
BLZF1	NS	3.35E-07	-0.207	1.20E-05	Negative

PAPLN	NS	3.53E-07	-0.207	1.25E-05	Negative
TUBA8	NS	3.55E-07	-0.207	1.25E-05	Negative
TMEM177	NS	3.57E-07	-0.207	1.26E-05	Negative
HTD2	NS	3.62E-07	-0.207	1.27E-05	Negative
MMP8	NS	3.65E-07	-0.207	1.28E-05	Negative
SMARCC1	NS	3.67E-07	-0.207	1.28E-05	Negative
LRRC18	NS	3.72E-07	-0.207	1.30E-05	Negative
SBF2	NS	3.74E-07	-0.207	1.30E-05	Negative
RAB23	NS	3.84E-07	-0.206	1.33E-05	Negative
CCDC142	NS	3.84E-07	-0.206	1.33E-05	Negative
C7orf25	NS	3.82E-07	-0.206	1.33E-05	Negative
PDIK1L	NS	3.84E-07	-0.206	1.33E-05	Negative
CDK18	NS	3.88E-07	-0.206	1.34E-05	Negative
HEG1	NS	3.91E-07	-0.206	1.35E-05	Negative
ZNF141	NS	3.93E-07	-0.206	1.35E-05	Negative
CD209	NS	4.01E-07	-0.206	1.38E-05	Negative
CACNA2D1	NS	4.19E-07	-0.206	1.43E-05	Negative
CDRT4	NS	4.18E-07	-0.206	1.43E-05	Negative
PERP	NS	4.21E-07	-0.206	1.44E-05	Negative
NHLH1	NS	4.23E-07	-0.206	1.44E-05	Negative
MCM3	NS	4.23E-07	-0.206	1.44E-05	Negative
SLC4A10	NS	4.26E-07	-0.206	1.45E-05	Negative
CDKL4	NS	4.30E-07	-0.205	1.46E-05	Negative
ALKBH2	NS	4.35E-07	-0.205	1.47E-05	Negative
C22orf23	NS	4.39E-07	-0.205	1.48E-05	Negative
GRID2	NS	4.44E-07	-0.205	1.49E-05	Negative
SPRR2D	NS	4.45E-07	-0.205	1.49E-05	Negative
TCP11X2	NS	4.44E-07	-0.205	1.49E-05	Negative
CRIM1	NS	4.53E-07	-0.205	1.51E-05	Negative
SYTL5	NS	4.54E-07	-0.205	1.51E-05	Negative
BRMS1L	NS	4.56E-07	-0.205	1.52E-05	Negative
FA2H	NS	4.56E-07	-0.205	1.52E-05	Negative
SLC13A1	NS	4.60E-07	-0.205	1.52E-05	Negative
RABEP1	NS	4.68E-07	-0.205	1.55E-05	Negative
CDO1	NS	4.83E-07	-0.205	1.59E-05	Negative
CFAP126	NS	5.01E-07	-0.204	1.65E-05	Negative
PASK	NS	5.04E-07	-0.204	1.66E-05	Negative

RTL4	NS	5.07E-07	-0.204	1.66E-05	Negative
ZNF32	NS	5.11E-07	-0.204	1.67E-05	Negative
TNFSF4	NS	5.17E-07	-0.204	1.69E-05	Negative
FDPS	NS	5.19E-07	-0.204	1.69E-05	Negative
LCTL	NS	5.20E-07	-0.204	1.69E-05	Negative
OR5I1	NS	5.22E-07	-0.204	1.70E-05	Negative
GLI1	NS	5.28E-07	-0.204	1.72E-05	Negative
SLC25A48	NS	5.31E-07	-0.204	1.72E-05	Negative
OSGEPL1	NS	5.40E-07	-0.204	1.75E-05	Negative
RRP15	NS	5.43E-07	-0.204	1.75E-05	Negative
CDK1	NS	5.46E-07	-0.204	1.76E-05	Negative
KIAA0319	NS	5.56E-07	-0.204	1.79E-05	Negative
SNX8	NS	5.63E-07	-0.203	1.81E-05	Negative
SNRNP35	NS	5.72E-07	-0.203	1.83E-05	Negative
NMB	NS	5.74E-07	-0.203	1.84E-05	Negative
ZNF649	NS	5.75E-07	-0.203	1.84E-05	Negative
RBM12B	NS	5.76E-07	-0.203	1.84E-05	Negative
THEMIS	NS	5.84E-07	-0.203	1.86E-05	Negative
POLR3F	NS	5.86E-07	-0.203	1.86E-05	Negative
ABCG2	NS	5.99E-07	-0.203	1.90E-05	Negative
ZACN	NS	6.00E-07	-0.203	1.90E-05	Negative
NANOGP8	NS	5.99E-07	-0.203	1.90E-05	Negative
PPP2R1B	NS	6.07E-07	-0.203	1.91E-05	Negative
NDST4	NS	6.20E-07	-0.203	1.95E-05	Negative
CEP19	NS	6.23E-07	-0.203	1.95E-05	Negative
ABCB7	NS	6.31E-07	-0.203	1.97E-05	Negative
ENKUR	NS	6.32E-07	-0.203	1.97E-05	Negative
MAN2A1	NS	6.44E-07	-0.202	2.01E-05	Negative
THAP1	NS	6.45E-07	-0.202	2.01E-05	Negative
RP2	NS	6.54E-07	-0.202	2.03E-05	Negative
OR9K2	NS	6.60E-07	-0.202	2.05E-05	Negative
YIPF7	NS	6.67E-07	-0.202	2.06E-05	Negative
GUCY2F	NS	6.78E-07	-0.202	2.09E-05	Negative
TLCD3A	NS	6.83E-07	-0.202	2.11E-05	Negative
TAAR8	NS	6.85E-07	-0.202	2.11E-05	Negative
ST3GAL2	NS	6.98E-07	-0.202	2.14E-05	Negative
GLTPD2	NS	7.01E-07	-0.202	2.15E-05	Negative

LZTFL1	NS	7.24E-07	-0.201	2.21E-05	Negative
RPGRIP1	NS	7.28E-07	-0.201	2.22E-05	Negative
CCNB1	NS	7.29E-07	-0.201	2.22E-05	Negative
UMPS	NS	7.33E-07	-0.201	2.23E-05	Negative
MGAT5	NS	7.34E-07	-0.201	2.23E-05	Negative
PROKR1	NS	7.37E-07	-0.201	2.23E-05	Negative
TSTD1	NS	7.52E-07	-0.201	2.27E-05	Negative
CLCN5	NS	7.56E-07	-0.201	2.28E-05	Negative
POLR3B	NS	7.59E-07	-0.201	2.28E-05	Negative
RRP9	NS	7.62E-07	-0.201	2.28E-05	Negative
MRPL17	NS	7.62E-07	-0.201	2.28E-05	Negative
MRPL42	NS	7.74E-07	-0.201	2.32E-05	Negative
WASF3	NS	7.78E-07	-0.201	2.32E-05	Negative
MTO1	NS	7.80E-07	-0.201	2.33E-05	Negative
PBK	NS	7.88E-07	-0.201	2.35E-05	Negative
TMIGD1	NS	7.89E-07	-0.201	2.35E-05	Negative
BUD13	NS	8.06E-07	-0.201	2.38E-05	Negative
DEUP1	NS	8.15E-07	-0.201	2.40E-05	Negative
APOL6	NS	8.16E-07	-0.201	2.41E-05	Negative
SURF6	NS	8.24E-07	-0.2	2.41E-05	Negative
GTF2H4	NS	8.24E-07	-0.2	2.41E-05	Negative
ZNF597	NS	8.28E-07	-0.2	2.42E-05	Negative
PLAAT1	NS	8.40E-07	-0.2	2.45E-05	Negative
KCNJ15	NS	8.49E-07	-0.2	2.47E-05	Negative
ITK	NS	8.64E-07	-0.2	2.51E-05	Negative
FUCA2	Protective	1.15E-01	-0.0646	2.21E-01	NS
SNX11	Protective	9.04E-05	-0.16	9.33E-04	NS
CREBBP	Protective	4.40E-01	-0.0317	5.82E-01	NS
TKTL1	Protective	3.78E-04	-0.145	2.83E-03	NS
PIGQ	Risk	6.20E-01	2.04E-02	7.35E-01	NS
TTC19	Protective	2.16E-05	-0.173	3.03E-04	NS
NPC1L1	Risk	1.26E-01	6.28E-02	2.36E-01	NS
IGF1	Protective	9.48E-03	-0.106	3.36E-02	NS
HGF	Risk	9.61E-01	-0.002	9.76E-01	NS
SLC45A4	Risk	4.35E-01	3.21E-02	5.78E-01	NS
RB1CC1	Protective	6.10E-03	-0.112	2.39E-02	NS
DEPDC1	Protective	9.68E-04	-0.135	5.78E-03	NS

MIPEP	Risk	4.03E-01	-0.0343	5.48E-01	NS
DAPK2	Risk	3.48E-03	1.20E-01	1.56E-02	NS
TBC1D23	Protective	7.81E-01	1.14E-02	8.55E-01	NS
TG	Protective	2.93E-01	-0.0432	4.37E-01	NS
DTNBP1	Protective	6.30E-04	1.40E-01	4.18E-03	NS
LTBP1	Risk	5.47E-01	2.48E-02	6.76E-01	NS
LIMA1	Protective	8.22E-02	-0.0713	1.73E-01	NS
CCDC85A	Protective	6.86E-01	1.66E-02	7.85E-01	NS
COL11A1	Protective	2.65E-05	-0.171	3.56E-04	NS
WAPL	Protective	4.57E-02	-0.0819	1.12E-01	NS
MED29	Risk	6.58E-01	-0.0182	7.64E-01	NS
ZNF76	Risk	5.44E-02	7.89E-02	1.27E-01	NS
COL17A1	Protective	3.97E-06	-0.188	8.10E-05	NS
KDM4A	Protective	9.80E-04	-0.135	5.84E-03	NS
ASPM	Protective	1.20E-01	-0.0638	2.28E-01	NS
TRMT11	Protective	7.01E-04	-0.139	4.56E-03	NS
TRAM1	Protective	3.24E-01	4.05E-02	4.71E-01	NS
NAV3	Protective	9.52E-01	-0.00248	9.70E-01	NS
FBXW11	Protective	8.00E-03	-0.109	2.95E-02	NS
MCM2	Risk	4.11E-03	-0.117	1.77E-02	NS
MOV10L1	Protective	6.51E-04	-0.139	4.29E-03	NS
ADAM11	Protective	8.96E-02	-0.0697	1.84E-01	NS
PPP2R2C	Risk	2.44E-01	4.79E-02	3.82E-01	NS
SLC25A43	Protective	8.42E-01	-0.00817	8.99E-01	NS
SAR1A	Protective	6.52E-05	-0.163	7.18E-04	NS
RAPGEF3	Protective	1.04E-02	-0.105	3.63E-02	NS
PCDHA6	Risk	1.97E-01	5.29E-02	3.28E-01	NS
COBLL1	Protective	1.08E-01	-0.066	2.11E-01	NS
CYLD	Protective	1.89E-01	-0.0539	3.20E-01	NS
PREP	Protective	9.79E-01	1.10E-03	9.87E-01	NS
IPO11	Risk	6.93E-02	-0.0745	1.53E-01	NS
ZFAND6	Protective	1.33E-03	-0.131	7.43E-03	NS
SRRT	Protective	1.34E-02	1.01E-01	4.39E-02	NS
NID2	Protective	8.19E-03	-0.108	3.00E-02	NS
GNA11	Risk	3.31E-02	-0.0874	8.71E-02	NS
PAPOLA	Protective	1.53E-01	5.86E-02	2.74E-01	NS
GNPTG	Protective	2.78E-01	-0.0446	4.20E-01	NS

ALKBH5	Protective	2.46E-06	1.92E-01	5.64E-05	NS
G2E3	Protective	3.30E-02	-0.0874	8.71E-02	NS
TMEM38B	Protective	3.87E-04	-0.145	2.88E-03	NS
TDRD1	Protective	1.54E-01	-0.0585	2.75E-01	NS
TOP3B	Risk	3.59E-01	-0.0376	5.06E-01	NS
LGALS1	Protective	2.98E-01	-0.0427	4.42E-01	NS
GCAT	Protective	3.12E-01	-0.0415	4.57E-01	NS
EIF3D	Protective	2.89E-01	-0.0435	4.33E-01	NS
BDKRB1	Risk	5.79E-02	7.78E-02	1.34E-01	NS
NKAP	Risk	2.75E-01	-0.0449	4.17E-01	NS
STAG2	Protective	1.20E-02	-0.103	4.04E-02	NS
CCDC22	Risk	4.74E-01	-0.0294	6.13E-01	NS
GGA2	Protective	5.55E-01	2.42E-02	6.82E-01	NS
CEMIP	Risk	3.01E-01	-0.0425	4.45E-01	NS
EHD4	Protective	1.19E-02	-0.103	4.01E-02	NS
TRIM35	Risk	1.03E-01	-0.0669	2.04E-01	NS
DKK4	Risk	6.22E-05	1.63E-01	6.91E-04	NS
KCNA7	Protective	9.05E-01	-0.00492	9.41E-01	NS
SYDE1	Protective	6.92E-01	1.63E-02	7.89E-01	NS
AURKC	Protective	2.79E-03	-0.122	1.32E-02	NS
ZNF175	Protective	3.51E-05	-0.169	4.44E-04	NS
AIMP2	Risk	3.69E-02	-0.0856	9.47E-02	NS
NMRK1	Protective	2.29E-01	-0.0494	3.67E-01	NS
TNKS2	Protective	6.73E-06	-0.183	1.23E-04	NS
LHPP	Risk	7.38E-04	1.38E-01	4.72E-03	NS
CPD	Protective	9.46E-01	2.81E-03	9.67E-01	NS
ICAM2	Risk	1.48E-01	5.94E-02	2.66E-01	NS
EIF4G2	Protective	2.52E-06	-0.192	5.73E-05	NS
NECTIN1	Risk	4.11E-01	3.38E-02	5.55E-01	NS
GTF2H1	Risk	5.43E-01	2.50E-02	6.72E-01	NS
LTA4H	Protective	6.28E-01	-0.0199	7.41E-01	NS
RIC8B	Risk	4.32E-01	-0.0322	5.76E-01	NS
GCNT2	Risk	4.05E-03	-0.118	1.75E-02	NS
SLC26A8	Protective	7.07E-01	-0.0155	8.00E-01	NS
IL17F	Protective	2.77E-03	-0.122	1.31E-02	NS
WASF1	Risk	8.44E-01	-0.0081	9.00E-01	NS
LAMA4	Protective	3.97E-01	3.48E-02	5.42E-01	NS

PCDHB7	Risk	8.30E-02	7.11E-02	1.74E-01	NS
CLINT1	Protective	3.14E-02	8.83E-02	8.38E-02	NS
CPEB4	Protective	4.26E-02	-0.0832	1.06E-01	NS
HRH2	Risk	2.63E-01	4.60E-02	4.04E-01	NS
NPRL2	Risk	4.54E-01	-0.0307	5.95E-01	NS
KLHL24	Protective	4.95E-03	1.15E-01	2.04E-02	NS
FAHD2A	Risk	6.55E-01	1.84E-02	7.61E-01	NS
PLA2G4A	Risk	3.80E-01	-0.036	5.26E-01	NS
RGS2	Risk	1.81E-01	-0.0549	3.09E-01	NS
PHTF1	Protective	2.61E-04	-0.149	2.12E-03	NS
GBP1	Risk	7.93E-01	1.08E-02	8.65E-01	NS
RAB29	Risk	8.69E-02	-0.0702	1.80E-01	NS
MPL	Risk	4.99E-01	2.77E-02	6.35E-01	NS
DR1	Risk	2.89E-01	-0.0435	4.33E-01	NS
NEK2	Protective	2.83E-03	1.22E-01	1.33E-02	NS
MESD	Risk	3.88E-02	8.47E-02	9.85E-02	NS
EEF2KMT	Risk	6.30E-01	-0.0198	7.42E-01	NS
PPP4R4	Protective	4.24E-01	-0.0329	5.67E-01	NS
TMEM214	Risk	1.47E-03	1.30E-01	8.06E-03	NS
PYROXD2	Risk	2.48E-01	4.75E-02	3.87E-01	NS
MSX2	Risk	2.02E-01	-0.0524	3.34E-01	NS
WDR55	Protective	2.69E-02	-0.0907	7.44E-02	NS
SMAD9	Risk	1.19E-01	-0.0641	2.26E-01	NS
CLU	Risk	3.00E-04	1.48E-01	2.36E-03	NS
PPP3CC	Protective	6.09E-01	-0.021	7.26E-01	NS
GJA3	Protective	7.50E-01	1.31E-02	8.33E-01	NS
TNFSF10	Risk	2.20E-01	5.04E-02	3.56E-01	NS
PHF24	Protective	8.10E-01	9.88E-03	8.77E-01	NS
RNF11	Protective	1.08E-01	6.60E-02	2.11E-01	NS
AMD1	Protective	2.23E-01	-0.0501	3.59E-01	NS
H2BW1	Risk	1.20E-02	1.03E-01	4.05E-02	NS
PMEPA1	Protective	3.30E-02	-0.0874	8.69E-02	NS
LYPD3	Protective	7.12E-01	-0.0151	8.05E-01	NS
USP9X	Protective	5.21E-01	2.64E-02	6.54E-01	NS
MED20	Risk	3.39E-01	-0.0393	4.85E-01	NS
CPNE5	Risk	6.07E-01	2.11E-02	7.25E-01	NS
SSUH2	Protective	1.60E-06	-0.195	4.02E-05	NS

SLC10A2	Risk	1.06E-04	-0.158	1.05E-03	NS
CENPB	Risk	1.66E-03	-0.129	8.83E-03	NS
UBA2	Protective	4.46E-01	3.13E-02	5.88E-01	NS
AHDC1	Protective	1.91E-01	-0.0537	3.21E-01	NS
NUP214	Protective	3.81E-02	-0.085	9.71E-02	NS
AVPR2	Protective	3.70E-03	-0.119	1.63E-02	NS
TAS2R3	Risk	2.84E-01	-0.044	4.27E-01	NS
PNKD	Protective	7.91E-01	1.09E-02	8.63E-01	NS
FGL2	Risk	7.10E-01	1.53E-02	8.03E-01	NS
GAL3ST1	Protective	3.85E-01	-0.0357	5.31E-01	NS
ATF4	Protective	9.92E-05	-0.159	1.00E-03	NS
ILF3	Protective	9.95E-03	-0.106	3.50E-02	NS
MTUS1	Protective	9.16E-03	-0.107	3.27E-02	NS
ART1	Risk	6.67E-02	7.52E-02	1.48E-01	NS
RPS4Y1	Risk	8.79E-02	7.00E-02	1.82E-01	NS
SHC2	Protective	1.11E-01	6.54E-02	2.15E-01	NS
LBP	Protective	4.04E-04	-0.145	2.98E-03	NS
PRRG3	Protective	4.58E-03	-0.116	1.93E-02	NS
NCAN	Protective	4.31E-01	-0.0323	5.75E-01	NS
PXDN	Protective	9.90E-01	-0.000539	9.93E-01	NS
OR11H1	Protective	1.99E-03	-0.126	1.02E-02	NS
ZNF341	Risk	6.87E-01	1.66E-02	7.85E-01	NS
NAPSA	Risk	9.34E-01	3.38E-03	9.60E-01	NS
AOC2	Protective	2.63E-02	-0.0911	7.30E-02	NS
CKMT2	Protective	1.93E-05	-0.174	2.82E-04	NS
ZSCAN5A	Risk	8.24E-01	-0.00916	8.86E-01	NS
DNAJB1	Protective	6.36E-01	-0.0194	7.47E-01	NS
LANCL2	Protective	1.94E-04	-0.152	1.67E-03	NS
TPTE2	Protective	8.21E-01	-0.00928	8.85E-01	NS
ADCK2	Risk	2.60E-01	-0.0462	4.01E-01	NS
KRBA1	Risk	1.83E-01	5.46E-02	3.12E-01	NS
SFTPD	Risk	7.27E-01	1.44E-02	8.15E-01	NS
KCTD1	Risk	1.65E-05	-0.176	2.50E-04	NS
SOX5	Protective	2.11E-05	-0.173	2.99E-04	NS
PRH2	Protective	8.24E-06	-0.182	1.43E-04	NS
DSG3	Protective	7.18E-01	-0.0148	8.09E-01	NS
POGLUT2	Risk	4.01E-01	3.45E-02	5.46E-01	NS

HNF1A	Protective	2.00E-06	-0.193	4.77E-05	NS
PPP1R1A	Protective	2.31E-03	-0.125	1.14E-02	NS
COQ10A	Protective	9.48E-01	-0.00266	9.68E-01	NS
KCNK1	Protective	8.35E-05	-0.161	8.77E-04	NS
RC3H1	Protective	8.42E-02	-0.0709	1.76E-01	NS
FPGS	Protective	8.92E-01	5.56E-03	9.34E-01	NS
PLAA	Protective	1.18E-01	-0.0641	2.26E-01	NS
FHDC1	Risk	1.81E-03	-0.128	9.43E-03	NS
SLC28A2	Protective	7.25E-02	-0.0737	1.58E-01	NS
LHCGR	Protective	1.25E-05	-0.178	1.99E-04	NS
SULT6B1	Risk	4.65E-01	3.00E-02	6.05E-01	NS
BARD1	Risk	6.39E-01	-0.0192	7.49E-01	NS
HCN4	Risk	9.20E-01	-0.0041	9.51E-01	NS
TRPC3	Protective	3.90E-04	-0.145	2.89E-03	NS
GSTCD	Risk	2.40E-01	4.83E-02	3.78E-01	NS
TMTC3	Risk	5.48E-03	-0.114	2.20E-02	NS
SLC39A5	Protective	4.85E-01	-0.0287	6.23E-01	NS
KANSL2	Protective	6.18E-01	-0.0205	7.33E-01	NS
WARS1	Risk	1.42E-02	-0.101	4.57E-02	NS
NLRC5	Risk	3.29E-02	8.75E-02	8.68E-02	NS
MEAK7	Risk	8.91E-01	-0.00563	9.32E-01	NS
GREB1L	Protective	9.37E-01	-0.00323	9.62E-01	NS
TNFRSF11A	Protective	1.60E-05	-0.176	2.44E-04	NS
GPR32	Protective	3.70E-03	1.19E-01	1.63E-02	NS
KLK3	Risk	1.61E-01	5.76E-02	2.83E-01	NS
CTTNBP2NL	Risk	8.44E-01	-0.00808	9.00E-01	NS
FCGR2A	Risk	6.12E-01	-0.0208	7.28E-01	NS
XPR1	Risk	7.24E-01	-0.0145	8.13E-01	NS
LHX9	Protective	1.01E-03	-0.134	6.00E-03	NS
HORMAD1	Risk	7.18E-01	-0.0148	8.09E-01	NS
ARL8A	Protective	8.17E-01	9.53E-03	8.81E-01	NS
SCRN3	Risk	8.98E-01	5.29E-03	9.37E-01	NS
CCDC150	Protective	3.21E-02	-0.0879	8.53E-02	NS
RBMS3	Protective	2.85E-01	-0.0439	4.28E-01	NS
STAC	Protective	2.09E-01	-0.0516	3.43E-01	NS
PTPRG	Protective	2.22E-01	-0.0501	3.59E-01	NS
EIF2B5	Protective	1.20E-02	-0.103	4.04E-02	NS

FABP2	Protective	3.02E-06	-0.19	6.57E-05	NS
GLRA1	Protective	9.51E-01	-0.00254	9.70E-01	NS
TIAM2	Protective	8.83E-07	-0.2	2.55E-05	NS
LANCL3	Protective	8.90E-05	-0.16	9.22E-04	NS
MFHAS1	Protective	1.02E-04	-0.159	1.02E-03	NS
SLC25A37	Protective	8.67E-01	-0.00688	9.16E-01	NS
ZEB1	Protective	1.55E-01	-0.0584	2.76E-01	NS
SYT8	Protective	8.81E-01	6.13E-03	9.26E-01	NS
HMGA2	Risk	8.64E-02	-0.0704	1.80E-01	NS
SLC2A13	Protective	5.43E-01	-0.025	6.73E-01	NS
SCLT1	Risk	8.62E-01	7.16E-03	9.12E-01	NS
DPYSL4	Risk	1.00E+00	1.51E-05	1.00E+00	NS
ZNF385D	Protective	4.85E-03	-0.115	2.01E-02	NS
PDK1	Protective	1.54E-02	-0.0993	4.89E-02	NS
PLEKHH2	Protective	1.42E-03	-0.131	7.83E-03	NS
PELO	Risk	9.74E-02	-0.068	1.95E-01	NS
BMP3	Protective	7.39E-03	-0.11	2.77E-02	NS
TXNDC11	Protective	2.11E-02	9.45E-02	6.19E-02	NS
CAST	Protective	2.96E-01	-0.0429	4.40E-01	NS
GBP5	Risk	7.56E-01	-0.0128	8.38E-01	NS
FGD5	Risk	7.99E-02	-0.0718	1.70E-01	NS
EME1	Protective	4.52E-01	-0.0309	5.93E-01	NS
TTC39B	Risk	3.04E-03	-0.121	1.40E-02	NS
DEPTOR	Risk	1.77E-04	-0.153	1.56E-03	NS
RRAGA	Protective	3.56E-06	-0.189	7.44E-05	NS
TRIM42	Risk	5.49E-02	7.87E-02	1.28E-01	NS
SUSD3	Protective	1.66E-04	-0.154	1.48E-03	NS
KIT	Protective	1.02E-01	-0.0671	2.02E-01	NS
LDLRAP1	Protective	1.95E-01	-0.0532	3.26E-01	NS
MRAS	Protective	3.79E-02	8.51E-02	9.67E-02	NS
ABHD3	Risk	3.53E-05	-0.169	4.46E-04	NS
POM121L2	Protective	9.92E-01	4.01E-04	9.95E-01	NS
EMSY	Risk	2.37E-01	-0.0486	3.75E-01	NS
NBL1	Risk	1.20E-01	-0.0639	2.28E-01	NS
SON	Protective	5.09E-01	2.72E-02	6.43E-01	NS
GOLGA6A	Protective	1.37E-01	-0.061	2.52E-01	NS
PTMS	Protective	1.95E-01	-0.0532	3.26E-01	NS

ACE	Protective	3.64E-01	-0.0373	5.10E-01	NS
TFF3	Risk	6.89E-05	1.62E-01	7.50E-04	NS
DVL3	Protective	9.68E-01	-0.00165	9.80E-01	NS
ABCF3	Protective	7.27E-01	1.43E-02	8.16E-01	NS
SAP30BP	Protective	1.73E-02	-0.0976	5.32E-02	NS
IZUMO2	Protective	4.24E-02	8.33E-02	1.05E-01	NS
EMC10	Protective	4.23E-02	8.33E-02	1.05E-01	NS
WDR90	Protective	1.24E-02	-0.102	4.14E-02	NS
ELAVL4	Protective	1.16E-01	-0.0646	2.22E-01	NS
GBP4	Risk	6.33E-02	-0.0762	1.43E-01	NS
FCAMR	Risk	1.53E-01	-0.0587	2.73E-01	NS
LRATD1	Protective	5.22E-04	-0.142	3.62E-03	NS
VSNL1	Protective	7.68E-01	1.21E-02	8.46E-01	NS
VPS72	Risk	3.36E-01	-0.0395	4.83E-01	NS
TMEM79	Risk	5.45E-01	2.49E-02	6.74E-01	NS
CIP2A	Protective	4.89E-01	-0.0284	6.26E-01	NS
EOMES	Protective	8.77E-01	6.35E-03	9.23E-01	NS
MTMR14	Protective	7.67E-01	1.22E-02	8.45E-01	NS
EMCN	Protective	1.67E-04	-0.154	1.49E-03	NS
ABHD18	Risk	2.62E-01	-0.0461	4.03E-01	NS
HHIP	Protective	2.32E-02	-0.0931	6.62E-02	NS
SCGB3A2	Risk	9.07E-01	-0.0048	9.42E-01	NS
GPX8	Protective	9.69E-02	-0.0681	1.95E-01	NS
CSF2	Risk	4.79E-02	-0.0811	1.16E-01	NS
SUN3	Protective	3.28E-01	-0.0402	4.75E-01	NS
YWHAZ	Protective	7.49E-02	-0.0731	1.62E-01	NS
HDX	Protective	2.65E-04	-0.149	2.14E-03	NS
TMEM63C	Risk	6.92E-01	-0.0163	7.89E-01	NS
CDX2	Protective	9.21E-01	4.10E-03	9.51E-01	NS
CEP295	Risk	5.15E-02	-0.0799	1.22E-01	NS
RAB8B	Protective	6.66E-03	-0.111	2.56E-02	NS
TRMT61A	Risk	2.78E-01	-0.0446	4.20E-01	NS
CDYL2	Protective	3.54E-02	-0.0863	9.17E-02	NS
TMEM170A	Protective	1.96E-01	5.31E-02	3.27E-01	NS
OR4D6	Risk	6.38E-01	1.93E-02	7.48E-01	NS
PATL1	Protective	5.83E-01	-0.0226	7.05E-01	NS
PIP5KL1	Protective	7.64E-01	-0.0123	8.43E-01	NS

GPRC5B	Protective	9.74E-04	-0.135	5.81E-03	NS
RNF214	Risk	9.99E-01	-0.0000263	1.00E+00	NS
PRRT2	Risk	1.03E-01	6.68E-02	2.04E-01	NS
ZNF528	Protective	7.48E-01	-0.0132	8.32E-01	NS
AXL	Protective	5.90E-06	-0.185	1.11E-04	NS
TRAPPC9	Risk	3.77E-01	-0.0363	5.23E-01	NS
MRPL58	Risk	3.03E-03	-0.121	1.40E-02	NS
C2CD3	Risk	2.16E-01	-0.0508	3.51E-01	NS
MAJIN	Risk	6.74E-01	-0.0173	7.75E-01	NS
HTR1E	Protective	2.99E-02	8.90E-02	8.08E-02	NS
PLA2G4F	Risk	5.58E-01	-0.0241	6.84E-01	NS
MECP2	Protective	4.08E-01	-0.034	5.52E-01	NS
SLC50A1	Risk	1.44E-02	1.00E-01	4.64E-02	NS
INO80E	Risk	8.19E-01	9.39E-03	8.83E-01	NS
BUB1	Protective	2.15E-01	5.09E-02	3.50E-01	NS
NLGN1	Protective	8.26E-04	-0.137	5.13E-03	NS
MRGPRX1	Protective	8.87E-01	5.86E-03	9.29E-01	NS
KRT8	Risk	3.54E-01	-0.038	5.01E-01	NS
SMAGP	Risk	4.06E-01	3.41E-02	5.51E-01	NS
NCBP2L	Risk	9.24E-01	3.92E-03	9.53E-01	NS
SHCBP1	Protective	4.45E-02	-0.0824	1.09E-01	NS
APLN	Protective	7.26E-01	1.44E-02	8.15E-01	NS
NLRP5	Risk	8.25E-01	-0.00907	8.88E-01	NS
ETFDH	Risk	3.22E-02	-0.0878	8.54E-02	NS
ANO5	Risk	9.03E-01	5.02E-03	9.40E-01	NS
CTPS1	Protective	4.28E-04	-0.144	3.10E-03	NS
CD8B	Risk	1.13E-01	6.51E-02	2.18E-01	NS
AZU1	Protective	5.75E-01	-0.023	6.99E-01	NS
PPP1CA	Risk	1.43E-01	6.02E-02	2.59E-01	NS
ZNF596	Risk	3.67E-01	-0.037	5.13E-01	NS
OVOL1	Risk	5.51E-01	-0.0245	6.79E-01	NS
CCDC96	Risk	9.88E-02	-0.0677	1.97E-01	NS
PLAC8L1	Risk	5.37E-02	-0.0791	1.26E-01	NS
SAA1	Risk	1.97E-01	-0.053	3.28E-01	NS
CHD2	Protective	7.99E-01	-0.0104	8.69E-01	NS
RGMB	Protective	7.63E-05	-0.161	8.16E-04	NS
TRHR	Protective	8.24E-04	-0.137	5.12E-03	NS

GOLT1A	Protective	3.59E-01	3.76E-02	5.06E-01	NS
ZNF266	Risk	3.04E-01	-0.0422	4.48E-01	NS
HRAS	Risk	2.90E-02	-0.0895	7.88E-02	NS
FZD4	Protective	3.36E-01	-0.0395	4.82E-01	NS
KCNE3	Risk	2.54E-01	4.69E-02	3.94E-01	NS
OR11H4	Risk	8.29E-02	-0.0711	1.74E-01	NS
BOK	Risk	3.62E-01	3.74E-02	5.08E-01	NS
VSIG10	Protective	2.97E-01	4.28E-02	4.41E-01	NS
SIX5	Protective	4.16E-01	-0.0334	5.61E-01	NS
ZDHHC22	Risk	6.67E-01	-0.0177	7.70E-01	NS
CRACR2B	Protective	9.26E-01	3.82E-03	9.55E-01	NS
ZNF354C	Risk	8.44E-06	-0.181	1.46E-04	NS
IMP3	Risk	1.49E-01	5.92E-02	2.68E-01	NS
TSPYL6	Risk	6.01E-01	-0.0215	7.20E-01	NS
PPP1R42	Risk	7.89E-02	-0.0721	1.68E-01	NS
CSRNP3	Protective	1.36E-04	-0.156	1.26E-03	NS
EDC3	Risk	4.25E-01	-0.0328	5.68E-01	NS
ZNF664	Protective	9.93E-01	-0.000362	9.95E-01	NS
MKRN3	Protective	8.28E-02	-0.0712	1.74E-01	NS
OR9A2	Risk	1.11E-02	-0.104	3.80E-02	NS
GPHB5	Risk	4.49E-02	8.23E-02	1.10E-01	NS
NRXN1	Protective	3.62E-01	-0.0374	5.08E-01	NS
BBS10	Risk	1.90E-04	-0.152	1.64E-03	NS
TDRP	Risk	7.21E-01	-0.0147	8.11E-01	NS
DEFB123	Risk	1.72E-05	1.75E-01	2.59E-04	NS
OR56B1	Protective	7.08E-04	-0.138	4.59E-03	NS
CCL13	Risk	2.39E-04	1.50E-01	1.97E-03	NS
UTS2R	Protective	2.24E-01	-0.0499	3.61E-01	NS
SNRPE	Protective	7.59E-04	-0.138	4.83E-03	NS
FAM89A	Risk	1.61E-02	-0.0986	5.05E-02	NS
TSEN54	Protective	1.91E-01	-0.0536	3.21E-01	NS
NTM	Protective	1.38E-02	-0.101	4.49E-02	NS
NKX2-5	Risk	4.48E-01	3.12E-02	5.90E-01	NS
CCBE1	Protective	1.07E-02	-0.105	3.71E-02	NS
FAM167B	Protective	1.95E-01	5.32E-02	3.26E-01	NS
TP53TG3	Protective	7.61E-01	-0.0125	8.41E-01	NS
OPCML	Protective	1.16E-02	-0.103	3.93E-02	NS

SDR42E2	Protective	7.59E-01	-0.0126	8.40E-01	NS
TACSTD2	Protective	3.44E-03	-0.12	1.55E-02	NS
CCR10	Protective	7.74E-01	1.18E-02	8.50E-01	NS
RPS27L	Risk	1.44E-01	-0.0599	2.62E-01	NS
CYP4Z1	Protective	7.77E-04	-0.137	4.93E-03	NS
BACE1	Risk	2.80E-01	4.43E-02	4.23E-01	NS
GNG2	Protective	1.57E-02	-0.099	4.95E-02	NS
VSIG10L	Risk	3.47E-01	3.86E-02	4.93E-01	NS
QRFPR	Risk	7.51E-01	-0.0131	8.34E-01	NS
AKR1C1	Protective	3.05E-01	4.22E-02	4.49E-01	NS
CYP26C1	Protective	2.26E-01	4.97E-02	3.63E-01	NS
SSX7	Risk	9.23E-01	3.98E-03	9.52E-01	NS
FAM205C	Risk	4.19E-04	-0.144	3.06E-03	NS
ZFP69B	Risk	3.60E-01	3.76E-02	5.06E-01	NS
DAZ1	Risk	4.15E-01	3.35E-02	5.60E-01	NS
PRKAR1B	Protective	2.54E-05	-0.172	3.44E-04	NS
DYNLT4	Protective	8.83E-01	6.02E-03	9.27E-01	NS
IGFL3	Risk	2.93E-03	-0.122	1.37E-02	NS
PARVB	Protective	3.61E-01	-0.0375	5.07E-01	NS
PLA2G2E	Protective	8.66E-02	-0.0703	1.80E-01	NS
C9orf152	Protective	1.92E-01	-0.0536	3.22E-01	NS
KRTAP10-12	Risk	2.35E-05	1.72E-01	3.23E-04	NS
GJB5	Risk	9.90E-02	-0.0677	1.98E-01	NS
BNIP5	Risk	5.43E-01	-0.025	6.72E-01	NS
KIAA0408	Protective	4.43E-01	-0.0315	5.86E-01	NS
CXCL17	Risk	2.11E-03	-0.126	1.07E-02	NS
HMGB1	Protective	3.74E-02	-0.0854	9.56E-02	NS
OR8A1	Protective	3.47E-04	-0.146	2.65E-03	NS
KIAA0895L	Protective	3.95E-01	3.49E-02	5.41E-01	NS
LCOR	Protective	5.11E-06	-0.186	9.80E-05	NS
ADH7	Risk	8.50E-01	7.77E-03	9.05E-01	NS
TRRAP	Protective	6.63E-02	-0.0753	1.48E-01	NS
ARL9	Risk	8.30E-02	-0.0711	1.74E-01	NS
ANAPC7	Risk	1.14E-02	1.04E-01	3.88E-02	NS
KRT39	Protective	8.10E-05	-0.161	8.56E-04	NS
METTL9	Protective	8.03E-02	-0.0718	1.70E-01	NS
ZNF470	Protective	1.33E-01	-0.0616	2.47E-01	NS

OR10D3	Protective	1.05E-01	-0.0665	2.07E-01	NS
OR13G1	Risk	8.27E-01	8.97E-03	8.89E-01	NS
FAM118B	Risk	8.71E-01	-0.00665	9.19E-01	NS
TCAF1	Risk	5.60E-03	-0.113	2.24E-02	NS
OR10G6	Risk	1.40E-02	-0.101	4.54E-02	NS
MT-CO2	Risk	1.23E-02	1.03E-01	4.12E-02	NS
MT-CO3	Risk	1.03E-01	6.68E-02	2.04E-01	NS
PFDN6	Risk	7.77E-02	-0.0724	1.66E-01	NS
TAP2	Risk	5.49E-01	2.46E-02	6.78E-01	NS
MPIG6B	Risk	1.38E-02	1.01E-01	4.49E-02	NS
OR2H2	Risk	2.21E-06	-0.193	5.20E-05	NS
PSG5	Protective	1.82E-01	-0.0548	3.11E-01	NS
C5orf51	Risk	3.74E-01	-0.0365	5.21E-01	NS
DAZ4	Risk	3.37E-01	3.94E-02	4.84E-01	NS
SCAF8	Protective	8.79E-01	6.25E-03	9.24E-01	NS
NRAS	Protective	4.90E-05	-0.166	5.74E-04	NS
LEPROT	Protective	1.88E-01	-0.054	3.18E-01	NS
PPM1N	Risk	1.25E-01	6.30E-02	2.35E-01	NS
C19orf38	Risk	9.95E-01	-0.000236	9.97E-01	NS
DEFB113	Risk	3.02E-04	-0.148	2.37E-03	NS
CSKMT	Protective	3.19E-02	-0.088	8.48E-02	NS
ZNF705B	Risk	1.08E-04	-0.158	1.06E-03	NS
ZNF579	Protective	5.42E-01	2.51E-02	6.72E-01	NS
KRTAP10-9	Risk	1.06E-04	1.58E-01	1.05E-03	NS
OR3A2	Protective	2.62E-02	9.11E-02	7.29E-02	NS
DEFB131B	Risk	9.75E-01	-0.00128	9.85E-01	NS
C13orf42	Protective	2.95E-01	-0.043	4.39E-01	NS
POTEB2	Protective	2.66E-05	-0.171	3.57E-04	NS
AKAIN1	Risk	1.44E-02	-0.1	4.64E-02	NS
POTEB	Protective	3.04E-03	-0.121	1.40E-02	NS
LCE6A	Risk	3.48E-01	-0.0386	4.94E-01	NS
TSPY10	Risk	7.80E-02	7.23E-02	1.67E-01	NS
TSPY9P	Risk	4.02E-02	8.41E-02	1.01E-01	NS
H2AL1Q	Risk	3.66E-01	-0.0371	5.12E-01	NS
SLC10A5	Protective	2.61E-02	-0.0912	7.27E-02	NS
PBOV1	Protective	5.82E-02	7.77E-02	1.34E-01	NS
SALL3	Protective	2.21E-01	5.02E-02	3.57E-01	NS

OR11H2	Protective	5.13E-01	-0.0269	6.47E-01	NS
ARMH2	Risk	3.73E-01	-0.0366	5.19E-01	NS
DUX4	Risk	2.14E-02	9.43E-02	6.25E-02	NS
SMIM36	Protective	1.68E-02	-0.098	5.20E-02	NS
OPN1MW	Protective	6.32E-01	1.97E-02	7.43E-01	NS
TSNAX-DISC1	Risk	7.12E-01	1.52E-02	8.05E-01	NS
TMEM269	Protective	6.20E-01	2.03E-02	7.35E-01	NS
FCGBP	Protective	1.24E-03	-0.132	7.08E-03	NS
OR2AG1	Protective	2.80E-05	-0.171	3.72E-04	NS
TMEM247	Risk	1.40E-03	1.31E-01	7.77E-03	NS
OR8S1	Risk	3.28E-01	4.02E-02	4.74E-01	NS
EEF1AKMT4-ECE2	Risk	1.54E-05	-0.176	2.37E-04	NS
FAM90A23P	Protective	2.18E-01	-0.0506	3.54E-01	NS
ZNF773	NS	8.75E-07	-0.2	2.54E-05	NS
RNF32	NS	8.78E-07	-0.2	2.54E-05	NS
ARHGAP42	NS	8.77E-07	-0.2	2.54E-05	NS
PLXDC1	NS	8.83E-07	-0.2	2.55E-05	NS
ACRV1	NS	8.88E-07	-0.2	2.56E-05	NS
WNT4	NS	8.92E-07	-0.2	2.56E-05	NS
COL19A1	NS	8.99E-07	-0.2	2.58E-05	NS
C8orf89	NS	9.11E-07	-0.2	2.61E-05	NS
FEN1	NS	9.21E-07	-0.2	2.63E-05	NS
NTF4	NS	9.20E-07	2.00E-01	2.63E-05	NS
ZZZ3	NS	9.23E-07	-0.2	2.64E-05	NS
PANK3	NS	9.35E-07	-0.199	2.66E-05	NS
C7orf57	NS	9.34E-07	-0.199	2.66E-05	NS
KLHL7	NS	9.36E-07	-0.199	2.66E-05	NS
RBM11	NS	9.48E-07	-0.199	2.69E-05	NS
EPX	NS	9.53E-07	-0.199	2.70E-05	NS
TRAPPC6B	NS	9.52E-07	-0.199	2.70E-05	NS
SSX2B	NS	9.54E-07	-0.199	2.70E-05	NS
KLHL26	NS	9.66E-07	-0.199	2.72E-05	NS
SAAL1	NS	9.68E-07	-0.199	2.73E-05	NS
OR2M7	NS	9.69E-07	-0.199	2.73E-05	NS
GP1BB	NS	9.87E-07	1.99E-01	2.77E-05	NS
DIXDC1	NS	9.93E-07	1.99E-01	2.78E-05	NS
PPM1J	NS	9.94E-07	-0.199	2.78E-05	NS

LSAMP	NS	9.92E-07	-0.199	2.78E-05	NS
PARP14	NS	9.98E-07	-0.199	2.79E-05	NS
PDE8B	NS	1.01E-06	-0.199	2.81E-05	NS
ABCB4	NS	1.02E-06	-0.199	2.85E-05	NS
CTTNBP2	NS	1.03E-06	-0.199	2.86E-05	NS
TIMELESS	NS	1.03E-06	-0.199	2.86E-05	NS
TXNDC9	NS	1.03E-06	-0.199	2.86E-05	NS
KCNH3	NS	1.04E-06	-0.199	2.88E-05	NS
ARHGDIG	NS	1.06E-06	1.99E-01	2.92E-05	NS
TTF1	NS	1.06E-06	-0.199	2.92E-05	NS
ACTR6	NS	1.07E-06	-0.198	2.94E-05	NS
TRMT13	NS	1.07E-06	-0.198	2.94E-05	NS
HOXD11	NS	1.07E-06	1.98E-01	2.94E-05	NS
MAB21L1	NS	1.07E-06	1.98E-01	2.94E-05	NS
DEFB107A	NS	1.07E-06	-0.198	2.94E-05	NS
PKP3	NS	1.09E-06	1.98E-01	2.97E-05	NS
TGDS	NS	1.09E-06	-0.198	2.97E-05	NS
XDH	NS	1.11E-06	-0.198	3.04E-05	NS
TMEM81	NS	1.13E-06	-0.198	3.07E-05	NS
SLC44A5	NS	1.14E-06	-0.198	3.10E-05	NS
ZCCHC4	NS	1.15E-06	-0.198	3.13E-05	NS
ARL13B	NS	1.16E-06	-0.198	3.16E-05	NS
B4GALNT4	NS	1.16E-06	1.98E-01	3.16E-05	NS
FADD	NS	1.17E-06	-0.198	3.16E-05	NS
TADA1	NS	1.19E-06	-0.198	3.21E-05	NS
PRR35	NS	1.19E-06	1.98E-01	3.22E-05	NS
GIMAP7	NS	1.20E-06	-0.198	3.23E-05	NS
CCNB2	NS	1.21E-06	-0.197	3.25E-05	NS
N4BP2	NS	1.21E-06	-0.197	3.26E-05	NS
E2F7	NS	1.21E-06	-0.197	3.26E-05	NS
GSR	NS	1.22E-06	1.97E-01	3.27E-05	NS
XK	NS	1.22E-06	-0.197	3.27E-05	NS
VAMP2	NS	1.23E-06	1.97E-01	3.28E-05	NS
H2BC9	NS	1.23E-06	-0.197	3.29E-05	NS
C14orf39	NS	1.24E-06	-0.197	3.31E-05	NS
RNF224	NS	1.25E-06	1.97E-01	3.33E-05	NS
TSSK6	NS	1.27E-06	-0.197	3.37E-05	NS

FOXA3	NS	1.27E-06	1.97E-01	3.38E-05	NS
WFDC6	NS	1.28E-06	-0.197	3.39E-05	NS
MYC	NS	1.28E-06	-0.197	3.40E-05	NS
LIPI	NS	1.29E-06	-0.197	3.40E-05	NS
CLOCK	NS	1.31E-06	-0.197	3.45E-05	NS
CNGB3	NS	1.31E-06	-0.197	3.45E-05	NS
SHISAL2B	NS	1.32E-06	-0.197	3.48E-05	NS
TMEM117	NS	1.32E-06	-0.197	3.48E-05	NS
IQCH	NS	1.33E-06	-0.197	3.48E-05	NS
MBIP	NS	1.33E-06	-0.197	3.49E-05	NS
TCEAL7	NS	1.34E-06	-0.197	3.52E-05	NS
TIGIT	NS	1.35E-06	-0.197	3.52E-05	NS
TMEM126B	NS	1.35E-06	-0.197	3.52E-05	NS
OR2J1	NS	1.35E-06	-0.197	3.53E-05	NS
SLC5A5	NS	1.37E-06	1.96E-01	3.56E-05	NS
FAM13C	NS	1.37E-06	-0.196	3.57E-05	NS
MMP2	NS	1.43E-06	-0.196	3.71E-05	NS
TASP1	NS	1.43E-06	-0.196	3.71E-05	NS
SRD5A2	NS	1.44E-06	-0.196	3.72E-05	NS
CHRM2	NS	1.44E-06	-0.196	3.72E-05	NS
SLC9C2	NS	1.45E-06	-0.196	3.74E-05	NS
KCNRG	NS	1.45E-06	-0.196	3.74E-05	NS
ZSCAN12	NS	1.45E-06	-0.196	3.74E-05	NS
AMTN	NS	1.47E-06	-0.196	3.78E-05	NS
PTPDC1	NS	1.47E-06	-0.196	3.78E-05	NS
CCDC166	NS	1.48E-06	1.96E-01	3.79E-05	NS
SPRYD7	NS	1.48E-06	-0.196	3.80E-05	NS
FDX2	NS	1.48E-06	-0.196	3.80E-05	NS
ZNF443	NS	1.49E-06	-0.196	3.80E-05	NS
ENPEP	NS	1.49E-06	-0.196	3.81E-05	NS
C12orf40	NS	1.50E-06	-0.196	3.82E-05	NS
ZNF577	NS	1.51E-06	-0.196	3.86E-05	NS
EIF3J	NS	1.52E-06	-0.196	3.86E-05	NS
OR10C1	NS	1.53E-06	-0.196	3.87E-05	NS
SMIM9	NS	1.54E-06	-0.196	3.90E-05	NS
C2orf76	NS	1.54E-06	-0.196	3.90E-05	NS
MAGEB18	NS	1.54E-06	-0.196	3.91E-05	NS

MUL1	NS	1.59E-06	-0.195	4.01E-05	NS
TCEAL4	NS	1.59E-06	-0.195	4.01E-05	NS
IFT43	NS	1.59E-06	-0.195	4.01E-05	NS
KRT36	NS	1.60E-06	-0.195	4.02E-05	NS
SPINT2	NS	1.60E-06	-0.195	4.02E-05	NS
NDUFAF4	NS	1.62E-06	-0.195	4.07E-05	NS
ATXN7L2	NS	1.63E-06	1.95E-01	4.09E-05	NS
SEC23A	NS	1.66E-06	-0.195	4.16E-05	NS
ANKRD30A	NS	1.66E-06	-0.195	4.16E-05	NS
EFNB1	NS	1.67E-06	-0.195	4.17E-05	NS
MPZL3	NS	1.69E-06	-0.195	4.21E-05	NS
CHIC1	NS	1.69E-06	-0.195	4.21E-05	NS
ASPHD2	NS	1.71E-06	-0.195	4.24E-05	NS
PEX16	NS	1.71E-06	-0.195	4.25E-05	NS
ACOT2	NS	1.72E-06	-0.195	4.26E-05	NS
NPFF	NS	1.73E-06	-0.195	4.27E-05	NS
ANKRD46	NS	1.73E-06	-0.195	4.27E-05	NS
CCNA2	NS	1.73E-06	-0.195	4.27E-05	NS
KRTAP6-2	NS	1.73E-06	1.95E-01	4.27E-05	NS
ZNF446	NS	1.74E-06	-0.195	4.29E-05	NS
ISOC1	NS	1.75E-06	-0.194	4.30E-05	NS
ATP1B3	NS	1.75E-06	-0.194	4.30E-05	NS
UTP11	NS	1.75E-06	-0.195	4.30E-05	NS
OR14A2	NS	1.75E-06	-0.195	4.30E-05	NS
GPR26	NS	1.76E-06	-0.194	4.31E-05	NS
GPR62	NS	1.76E-06	1.94E-01	4.31E-05	NS
CCL23	NS	1.77E-06	-0.194	4.31E-05	NS
TRIM32	NS	1.79E-06	-0.194	4.36E-05	NS
MSX1	NS	1.80E-06	-0.194	4.38E-05	NS
RBP2	NS	1.81E-06	-0.194	4.40E-05	NS
OR52K1	NS	1.82E-06	-0.194	4.42E-05	NS
C1orf68	NS	1.82E-06	-0.194	4.42E-05	NS
GOLGA6L1	NS	1.83E-06	-0.194	4.42E-05	NS
CAPNS2	NS	1.83E-06	-0.194	4.44E-05	NS
TMPRSS13	NS	1.84E-06	-0.194	4.45E-05	NS
KRT23	NS	1.87E-06	-0.194	4.51E-05	NS
HAVCR1	NS	1.87E-06	-0.194	4.51E-05	NS

RGS11	NS	1.88E-06	1.94E-01	4.52E-05	NS
CSTF2	NS	1.90E-06	-0.194	4.57E-05	NS
CD93	NS	1.90E-06	-0.194	4.57E-05	NS
KCNU1	NS	1.96E-06	-0.194	4.70E-05	NS
SPX	NS	1.96E-06	-0.194	4.70E-05	NS
PDE3A	NS	1.98E-06	-0.194	4.74E-05	NS
C1orf146	NS	2.02E-06	-0.193	4.82E-05	NS
KRTAP12-4	NS	2.02E-06	1.93E-01	4.82E-05	NS
CAPN8	NS	2.05E-06	-0.193	4.89E-05	NS
OR2F1	NS	2.06E-06	-0.193	4.90E-05	NS
BBS7	NS	2.08E-06	-0.193	4.95E-05	NS
SLC35E1	NS	2.11E-06	1.93E-01	5.01E-05	NS
GOT1	NS	2.11E-06	-0.193	5.01E-05	NS
C21orf91	NS	2.13E-06	-0.193	5.04E-05	NS
CCNB3	NS	2.14E-06	-0.193	5.06E-05	NS
GAS1	NS	2.15E-06	1.93E-01	5.08E-05	NS
CPNE9	NS	2.18E-06	-0.193	5.14E-05	NS
SUPT20HL2	NS	2.19E-06	-0.193	5.15E-05	NS
SPDYE4	NS	2.19E-06	-0.193	5.16E-05	NS
ARMC3	NS	2.23E-06	-0.193	5.24E-05	NS
ZNF350	NS	2.25E-06	-0.192	5.27E-05	NS
SLC2A12	NS	2.27E-06	-0.192	5.30E-05	NS
CD59	NS	2.27E-06	-0.192	5.31E-05	NS
ANKEF1	NS	2.28E-06	-0.192	5.32E-05	NS
PCNA	NS	2.29E-06	-0.192	5.33E-05	NS
CUL3	NS	2.29E-06	-0.192	5.33E-05	NS
MIA	NS	2.32E-06	1.92E-01	5.39E-05	NS
MBTPS1	NS	2.33E-06	-0.192	5.40E-05	NS
ZDHHC7	NS	2.32E-06	1.92E-01	5.40E-05	NS
MRPL39	NS	2.33E-06	-0.192	5.40E-05	NS
PP2D1	NS	2.34E-06	-0.192	5.43E-05	NS
GRB14	NS	2.36E-06	-0.192	5.44E-05	NS
RSAD2	NS	2.36E-06	-0.192	5.44E-05	NS
DYRK1B	NS	2.39E-06	-0.192	5.50E-05	NS
ERFL	NS	2.38E-06	1.92E-01	5.50E-05	NS
THAP9	NS	2.40E-06	-0.192	5.52E-05	NS
SLC24A3	NS	2.40E-06	-0.192	5.53E-05	NS

IL15	NS	2.43E-06	-0.192	5.57E-05	NS
TAF1B	NS	2.45E-06	-0.192	5.61E-05	NS
LDLRAD4	NS	2.46E-06	-0.192	5.64E-05	NS
MAOA	NS	2.47E-06	-0.192	5.65E-05	NS
TYR	NS	2.50E-06	-0.192	5.69E-05	NS
DGKI	NS	2.50E-06	-0.192	5.70E-05	NS
DYNLT1	NS	2.56E-06	-0.191	5.81E-05	NS
TMPRSS5	NS	2.56E-06	-0.191	5.81E-05	NS
ZNF484	NS	2.57E-06	-0.191	5.82E-05	NS
MROH2B	NS	2.57E-06	-0.191	5.82E-05	NS
RTTN	NS	2.57E-06	-0.191	5.82E-05	NS
CDC34	NS	2.59E-06	1.91E-01	5.85E-05	NS
MYLK2	NS	2.60E-06	-0.191	5.87E-05	NS
COX7A2	NS	2.60E-06	-0.191	5.87E-05	NS
REC114	NS	2.61E-06	-0.191	5.88E-05	NS
CFHR4	NS	2.64E-06	-0.191	5.95E-05	NS
NCAM2	NS	2.65E-06	-0.191	5.95E-05	NS
SELENBP1	NS	2.66E-06	-0.191	5.97E-05	NS
NOTO	NS	2.70E-06	1.91E-01	6.05E-05	NS
RNASEH2A	NS	2.71E-06	-0.191	6.07E-05	NS
BMP5	NS	2.72E-06	-0.191	6.09E-05	NS
UCN2	NS	2.73E-06	1.91E-01	6.09E-05	NS
TMEM26	NS	2.73E-06	-0.191	6.09E-05	NS
CRYZ	NS	2.73E-06	-0.191	6.09E-05	NS
BMX	NS	2.74E-06	-0.191	6.09E-05	NS
TAF11L11	NS	2.74E-06	1.91E-01	6.09E-05	NS
GNB1	NS	2.74E-06	-0.191	6.10E-05	NS
IL17C	NS	2.76E-06	-0.191	6.14E-05	NS
RSPO2	NS	2.77E-06	-0.191	6.14E-05	NS
ZGRF1	NS	2.80E-06	-0.191	6.19E-05	NS
HOXD10	NS	2.81E-06	1.91E-01	6.21E-05	NS
GP2	NS	2.83E-06	-0.191	6.25E-05	NS
CLEC14A	NS	2.84E-06	-0.191	6.26E-05	NS
PNPLA3	NS	2.84E-06	-0.191	6.27E-05	NS
NIPSNAP3B	NS	2.87E-06	-0.19	6.33E-05	NS
MFN1	NS	2.89E-06	-0.19	6.36E-05	NS
ANKRD20A2P	NS	2.89E-06	-0.19	6.36E-05	NS

SCARB1	NS	2.90E-06	-0.19	6.37E-05	NS
ACYP1	NS	2.93E-06	-0.19	6.43E-05	NS
NEIL2	NS	2.94E-06	-0.19	6.44E-05	NS
UGT1A6	NS	2.95E-06	-0.19	6.44E-05	NS
ZNF497	NS	2.94E-06	-0.19	6.44E-05	NS
FBXW7	NS	2.97E-06	-0.19	6.48E-05	NS
GPR143	NS	2.99E-06	-0.19	6.52E-05	NS
TPRKB	NS	3.02E-06	-0.19	6.57E-05	NS
MYLIP	NS	3.06E-06	1.90E-01	6.65E-05	NS
NDUFAF7	NS	3.07E-06	-0.19	6.65E-05	NS
NME7	NS	3.08E-06	-0.19	6.67E-05	NS
PLD5P1	NS	3.09E-06	-0.19	6.69E-05	NS
ACTA2	NS	3.11E-06	-0.19	6.72E-05	NS
PCDHGB7	NS	3.12E-06	-0.19	6.75E-05	NS
RPP30	NS	3.14E-06	-0.19	6.77E-05	NS
KCTD20	NS	3.16E-06	-0.19	6.80E-05	NS
OR10AD1	NS	3.16E-06	-0.19	6.80E-05	NS
BTG4	NS	3.16E-06	-0.19	6.80E-05	NS
RGL4	NS	3.20E-06	-0.19	6.87E-05	NS
DNAJC18	NS	3.20E-06	-0.19	6.87E-05	NS
ZNF658	NS	3.20E-06	-0.19	6.87E-05	NS
GNG8	NS	3.21E-06	1.90E-01	6.88E-05	NS
ACSF2	NS	3.22E-06	-0.19	6.89E-05	NS
CBLC	NS	3.24E-06	1.90E-01	6.92E-05	NS
MSS51	NS	3.25E-06	-0.189	6.93E-05	NS
SIMC1	NS	3.25E-06	-0.189	6.93E-05	NS
FAM180B	NS	3.26E-06	1.89E-01	6.93E-05	NS
VBP1	NS	3.27E-06	-0.189	6.95E-05	NS
TK1	NS	3.27E-06	-0.189	6.95E-05	NS
RAD51AP1	NS	3.28E-06	-0.189	6.95E-05	NS
EFHC2	NS	3.28E-06	-0.189	6.95E-05	NS
BTN2A2	NS	3.28E-06	-0.189	6.95E-05	NS
VWC2L	NS	3.33E-06	-0.189	7.04E-05	NS
LRRN2	NS	3.35E-06	-0.189	7.08E-05	NS
DMAC2L	NS	3.36E-06	-0.189	7.08E-05	NS
GYPE	NS	3.37E-06	-0.189	7.11E-05	NS
FNDC1	NS	3.40E-06	-0.189	7.17E-05	NS

IWS1	NS	3.42E-06	-0.189	7.19E-05	NS
AADACL2	NS	3.46E-06	-0.189	7.27E-05	NS
ZFYVE26	NS	3.49E-06	-0.189	7.32E-05	NS
ZNF555	NS	3.49E-06	-0.189	7.32E-05	NS
OR14L1P	NS	3.49E-06	-0.189	7.32E-05	NS
TESPA1	NS	3.51E-06	-0.189	7.35E-05	NS
GJC3	NS	3.57E-06	1.89E-01	7.44E-05	NS
CHFR	NS	3.63E-06	-0.189	7.57E-05	NS
H2AC11	NS	3.63E-06	-0.189	7.57E-05	NS
BBS4	NS	3.64E-06	-0.189	7.58E-05	NS
TST	NS	3.67E-06	-0.188	7.63E-05	NS
SYPL2	NS	3.68E-06	1.88E-01	7.63E-05	NS
A2M	NS	3.68E-06	-0.188	7.63E-05	NS
FAM24B	NS	3.70E-06	-0.188	7.66E-05	NS
ACSM5	NS	3.70E-06	-0.188	7.67E-05	NS
MKI67	NS	3.71E-06	-0.188	7.67E-05	NS
CRYBA1	NS	3.74E-06	-0.188	7.74E-05	NS
ZBTB26	NS	3.77E-06	-0.188	7.78E-05	NS
GIMAP8	NS	3.78E-06	-0.188	7.79E-05	NS
ZNF100	NS	3.81E-06	-0.188	7.85E-05	NS
IL1R2	NS	3.84E-06	-0.188	7.90E-05	NS
H3C11	NS	3.85E-06	-0.188	7.92E-05	NS
LHX4	NS	3.89E-06	-0.188	7.99E-05	NS
MRPL22	NS	3.90E-06	-0.188	8.00E-05	NS
PRPF19	NS	3.94E-06	1.88E-01	8.06E-05	NS
ST14	NS	3.94E-06	1.88E-01	8.06E-05	NS
BOD1L2	NS	3.96E-06	1.88E-01	8.08E-05	NS
MOGAT3	NS	3.99E-06	1.88E-01	8.14E-05	NS
TMEM68	NS	4.03E-06	-0.188	8.20E-05	NS
SHARPIN	NS	4.03E-06	1.88E-01	8.20E-05	NS
APOOL	NS	4.04E-06	-0.188	8.21E-05	NS
GRM8	NS	4.06E-06	-0.188	8.23E-05	NS
TTC4	NS	4.06E-06	-0.188	8.23E-05	NS
TMEM225B	NS	4.06E-06	-0.188	8.23E-05	NS
OR5D16	NS	4.07E-06	-0.188	8.23E-05	NS
UNC13C	NS	4.08E-06	-0.188	8.26E-05	NS
B4GALT2	NS	4.10E-06	1.88E-01	8.28E-05	NS

GFRA2	NS	4.13E-06	-0.187	8.34E-05	NS
GSTA4	NS	4.15E-06	-0.187	8.36E-05	NS
GLRB	NS	4.22E-06	-0.187	8.49E-05	NS
OR2A42	NS	4.23E-06	-0.187	8.50E-05	NS
EPHA5	NS	4.24E-06	-0.187	8.52E-05	NS
SLCO1B3-SLCO1B7	NS	4.24E-06	-0.187	8.52E-05	NS
TMEM54	NS	4.25E-06	1.87E-01	8.52E-05	NS
CILP	NS	4.29E-06	-0.187	8.60E-05	NS
DMRTB1	NS	4.32E-06	1.87E-01	8.64E-05	NS
GPRASP2	NS	4.33E-06	-0.187	8.65E-05	NS
PGBD1	NS	4.34E-06	-0.187	8.66E-05	NS
TULP2	NS	4.36E-06	-0.187	8.68E-05	NS
NR1D1	NS	4.36E-06	1.87E-01	8.68E-05	NS
TMPRSS11E	NS	4.37E-06	-0.187	8.70E-05	NS
FCRLB	NS	4.38E-06	-0.187	8.71E-05	NS
GRAMD1C	NS	4.41E-06	-0.187	8.76E-05	NS
VTI1B	NS	4.45E-06	-0.187	8.83E-05	NS
SNURF	NS	4.45E-06	-0.187	8.83E-05	NS
HEMK1	NS	4.48E-06	-0.187	8.88E-05	NS
EDNRB	NS	4.49E-06	-0.187	8.88E-05	NS
HTR4	NS	4.49E-06	-0.187	8.88E-05	NS
C6orf62	NS	4.52E-06	-0.187	8.92E-05	NS
COL6A1	NS	4.52E-06	1.87E-01	8.92E-05	NS
TRPA1	NS	4.54E-06	-0.187	8.94E-05	NS
CHAD	NS	4.55E-06	-0.187	8.95E-05	NS
GPR25	NS	4.55E-06	1.87E-01	8.95E-05	NS
LRCH2	NS	4.62E-06	-0.187	9.07E-05	NS
EID2	NS	4.65E-06	-0.186	9.13E-05	NS
ASB4	NS	4.66E-06	-0.186	9.15E-05	NS
ZDHHC2	NS	4.70E-06	-0.186	9.20E-05	NS
ABCA12	NS	4.71E-06	-0.186	9.20E-05	NS
ADAM12	NS	4.71E-06	1.86E-01	9.20E-05	NS
OR51I1	NS	4.71E-06	-0.186	9.20E-05	NS
TRIM33	NS	4.70E-06	-0.186	9.20E-05	NS
STK38L	NS	4.75E-06	-0.186	9.26E-05	NS
CDH8	NS	4.77E-06	-0.186	9.29E-05	NS
MTARC2	NS	4.78E-06	-0.186	9.29E-05	NS

DAZL	NS	4.83E-06	-0.186	9.39E-05	NS
ZNF17	NS	4.85E-06	-0.186	9.41E-05	NS
OR2D2	NS	4.88E-06	-0.186	9.46E-05	NS
DOLK	NS	4.89E-06	-0.186	9.47E-05	NS
FAM131C	NS	4.91E-06	1.86E-01	9.51E-05	NS
MEDAG	NS	4.98E-06	-0.186	9.62E-05	NS
CNBD1	NS	4.97E-06	-0.186	9.62E-05	NS
C1orf53	NS	4.99E-06	-0.186	9.64E-05	NS
NLRP14	NS	5.03E-06	-0.186	9.69E-05	NS
PLEKHM3	NS	5.05E-06	-0.186	9.73E-05	NS
KANSL1	NS	5.06E-06	-0.186	9.73E-05	NS
P2RY12	NS	5.11E-06	-0.186	9.80E-05	NS
CLRN2	NS	5.12E-06	1.86E-01	9.81E-05	NS
FMO2	NS	5.13E-06	-0.186	9.83E-05	NS
KDELR3	NS	5.16E-06	-0.186	9.88E-05	NS
EMC2	NS	5.17E-06	-0.186	9.89E-05	NS
F3	NS	5.17E-06	-0.186	9.89E-05	NS
TBC1D30	NS	5.20E-06	-0.186	9.93E-05	NS
CREB3L3	NS	5.21E-06	1.86E-01	9.94E-05	NS
HES4	NS	5.28E-06	1.85E-01	1.01E-04	NS
FFAR4	NS	5.31E-06	-0.185	1.01E-04	NS
ELF3	NS	5.36E-06	-0.185	1.02E-04	NS
SAPCD1	NS	5.38E-06	-0.185	1.02E-04	NS
HAPLN2	NS	5.48E-06	1.85E-01	1.04E-04	NS
ODAD2	NS	5.55E-06	-0.185	1.05E-04	NS
PCDH10	NS	5.56E-06	-0.185	1.05E-04	NS
SS18L1	NS	5.58E-06	1.85E-01	1.06E-04	NS
GOLGA8T	NS	5.59E-06	-0.185	1.06E-04	NS
VGLL1	NS	5.67E-06	-0.185	1.07E-04	NS
TSTD3	NS	5.70E-06	-0.185	1.08E-04	NS
KRT4	NS	5.71E-06	-0.185	1.08E-04	NS
TLX3	NS	5.76E-06	1.85E-01	1.08E-04	NS
OR8G5	NS	5.80E-06	-0.185	1.09E-04	NS
OR13C3	NS	5.87E-06	-0.185	1.10E-04	NS
IVD	NS	5.88E-06	-0.185	1.10E-04	NS
NHLH2	NS	5.90E-06	-0.185	1.11E-04	NS
LYSMD3	NS	5.93E-06	-0.184	1.11E-04	NS

EBLN2	NS	5.92E-06	-0.184	1.11E-04	NS
PARP11	NS	5.96E-06	-0.184	1.11E-04	NS
GINS3	NS	6.01E-06	-0.184	1.12E-04	NS
FAM177B	NS	6.03E-06	-0.184	1.12E-04	NS
GCNT4	NS	6.06E-06	-0.184	1.13E-04	NS
FSTL5	NS	6.07E-06	-0.184	1.13E-04	NS
ARHGAP30	NS	6.09E-06	-0.184	1.13E-04	NS
ZNF286A	NS	6.11E-06	-0.184	1.14E-04	NS
TIMM8A	NS	6.13E-06	-0.184	1.14E-04	NS
ZKSCAN8P1	NS	6.13E-06	-0.184	1.14E-04	NS
C3orf49	NS	6.14E-06	-0.184	1.14E-04	NS
NCBP3	NS	6.20E-06	1.84E-01	1.15E-04	NS
TSPYL5	NS	6.20E-06	-0.184	1.15E-04	NS
CMTM2	NS	6.22E-06	-0.184	1.15E-04	NS
TEX55	NS	6.30E-06	-0.184	1.16E-04	NS
SERPINE2	NS	6.35E-06	-0.184	1.17E-04	NS
TCL1B	NS	6.40E-06	1.84E-01	1.18E-04	NS
TMEM232	NS	6.52E-06	-0.184	1.20E-04	NS
RDH12	NS	6.56E-06	-0.184	1.20E-04	NS
NPPC	NS	6.62E-06	-0.184	1.21E-04	NS
FZD2	NS	6.61E-06	-0.184	1.21E-04	NS
SOCS4	NS	6.63E-06	-0.184	1.21E-04	NS
TMEM170B	NS	6.63E-06	-0.184	1.21E-04	NS
HSD11B1	NS	6.67E-06	-0.183	1.22E-04	NS
MUC5AC	NS	6.69E-06	-0.183	1.22E-04	NS
FAM114A1	NS	6.71E-06	-0.183	1.22E-04	NS
TMX3	NS	6.75E-06	-0.183	1.23E-04	NS
C9	NS	6.76E-06	-0.183	1.23E-04	NS
GADL1	NS	6.76E-06	-0.183	1.23E-04	NS
PSMD10	NS	6.82E-06	-0.183	1.24E-04	NS
TRA2B	NS	6.81E-06	1.83E-01	1.24E-04	NS
TCEAL3	NS	6.82E-06	-0.183	1.24E-04	NS
FASLG	NS	6.88E-06	-0.183	1.25E-04	NS
FMN2	NS	6.90E-06	-0.183	1.25E-04	NS
ALX3	NS	6.90E-06	-0.183	1.25E-04	NS
KCTD7	NS	6.96E-06	1.83E-01	1.26E-04	NS
SLC35B2	NS	6.99E-06	-0.183	1.26E-04	NS

PHEX	NS	7.01E-06	-0.183	1.26E-04	NS
DEFB108B	NS	7.04E-06	-0.183	1.27E-04	NS
CXCR6	NS	7.06E-06	-0.183	1.27E-04	NS
ANKRD35	NS	7.11E-06	-0.183	1.28E-04	NS
GAGE13	NS	7.13E-06	-0.183	1.28E-04	NS
PGP	NS	7.15E-06	-0.183	1.28E-04	NS
ARMCX6	NS	7.19E-06	-0.183	1.29E-04	NS
DNAJB2	NS	7.20E-06	1.83E-01	1.29E-04	NS
HOATZ	NS	7.21E-06	1.83E-01	1.29E-04	NS
CLYBL	NS	7.23E-06	-0.183	1.29E-04	NS
ARIH1	NS	7.22E-06	-0.183	1.29E-04	NS
ARMCX4	NS	7.25E-06	-0.183	1.29E-04	NS
FKBP10	NS	7.28E-06	1.83E-01	1.30E-04	NS
PARPBP	NS	7.28E-06	-0.183	1.30E-04	NS
PRG2	NS	7.28E-06	-0.183	1.30E-04	NS
KRTAP10-4	NS	7.40E-06	1.83E-01	1.32E-04	NS
METTL25	NS	7.42E-06	-0.183	1.32E-04	NS
NIPAL3	NS	7.45E-06	-0.183	1.32E-04	NS
SHLD3	NS	7.48E-06	-0.182	1.33E-04	NS
GRSF1	NS	7.64E-06	1.82E-01	1.35E-04	NS
TMEM208	NS	7.65E-06	-0.182	1.35E-04	NS
RORB	NS	7.64E-06	-0.182	1.35E-04	NS
OR1A1	NS	7.67E-06	-0.182	1.36E-04	NS
SDHAF3	NS	7.68E-06	-0.182	1.36E-04	NS
GPR156	NS	7.76E-06	-0.182	1.37E-04	NS
GAPDHS	NS	7.80E-06	-0.182	1.38E-04	NS
ENO1	NS	7.84E-06	-0.182	1.38E-04	NS
PPM1L	NS	7.88E-06	1.82E-01	1.39E-04	NS
MAFIP	NS	7.90E-06	-0.182	1.39E-04	NS
SCYGR2	NS	7.94E-06	1.82E-01	1.39E-04	NS
ARHGAP11A	NS	7.98E-06	-0.182	1.40E-04	NS
NXNL2	NS	8.00E-06	1.82E-01	1.40E-04	NS
ZNF816	NS	8.00E-06	-0.182	1.40E-04	NS
AKAP3	NS	8.13E-06	-0.182	1.42E-04	NS
TRPC4	NS	8.14E-06	-0.182	1.42E-04	NS
FBXO21	NS	8.13E-06	-0.182	1.42E-04	NS
LSMEM2	NS	8.12E-06	-0.182	1.42E-04	NS

GALNT1	NS	8.17E-06	-0.182	1.42E-04	NS
SSH3	NS	8.21E-06	-0.182	1.43E-04	NS
TMEFF2	NS	8.22E-06	-0.182	1.43E-04	NS
ARHGAP35	NS	8.25E-06	-0.182	1.43E-04	NS
HIGD1B	NS	8.29E-06	-0.182	1.44E-04	NS
PSKH2	NS	8.31E-06	-0.182	1.44E-04	NS
IFT57	NS	8.32E-06	-0.182	1.44E-04	NS
MYL12A	NS	8.36E-06	-0.182	1.45E-04	NS
EML5	NS	8.37E-06	-0.182	1.45E-04	NS
ARL1	NS	8.44E-06	-0.181	1.46E-04	NS
HDAC3	NS	8.52E-06	-0.181	1.47E-04	NS
HSPA4	NS	8.60E-06	-0.181	1.48E-04	NS
PRAG1	NS	8.65E-06	-0.181	1.49E-04	NS
ALDH18A1	NS	8.67E-06	-0.181	1.49E-04	NS
PRTG	NS	8.70E-06	-0.181	1.50E-04	NS
THRB	NS	8.71E-06	-0.181	1.50E-04	NS
TRMO	NS	8.75E-06	-0.181	1.50E-04	NS
HSPA12B	NS	8.79E-06	-0.181	1.51E-04	NS
NECTIN3	NS	8.83E-06	-0.181	1.51E-04	NS
DUSP6	NS	8.88E-06	-0.181	1.52E-04	NS
CCDC125	NS	8.91E-06	-0.181	1.52E-04	NS
CEP295NL	NS	8.93E-06	1.81E-01	1.53E-04	NS
BRINP2	NS	8.96E-06	-0.181	1.53E-04	NS
SLC4A1AP	NS	8.99E-06	-0.181	1.53E-04	NS
ADNP2	NS	9.02E-06	-0.181	1.54E-04	NS
KCNK13	NS	9.08E-06	-0.181	1.55E-04	NS
BCL7A	NS	9.09E-06	-0.181	1.55E-04	NS
TELO2	NS	9.15E-06	-0.181	1.56E-04	NS
RTN4RL1	NS	9.20E-06	1.81E-01	1.56E-04	NS
CALY	NS	9.35E-06	1.81E-01	1.59E-04	NS
HNRNPL	NS	9.45E-06	1.80E-01	1.60E-04	NS
CCT6B	NS	9.46E-06	-0.18	1.60E-04	NS
ADAD1	NS	9.59E-06	-0.18	1.62E-04	NS
PUS3	NS	9.64E-06	-0.18	1.63E-04	NS
SLC9B2	NS	9.64E-06	-0.18	1.63E-04	NS
TMEM135	NS	9.81E-06	-0.18	1.66E-04	NS
SLC18A3	NS	9.84E-06	1.80E-01	1.66E-04	NS

ZNF177	NS	9.90E-06	-0.18	1.67E-04	NS
DNAI7	NS	9.97E-06	-0.18	1.68E-04	NS
IPO9	NS	9.98E-06	-0.18	1.68E-04	NS
IQCF3	NS	9.98E-06	-0.18	1.68E-04	NS
CBX4	NS	1.00E-05	1.80E-01	1.68E-04	NS
SOX15	NS	1.00E-05	-0.18	1.68E-04	NS
CDC42EP2	NS	1.00E-05	-0.18	1.68E-04	NS
AGO2	NS	1.01E-05	-0.18	1.69E-04	NS
RFXAP	NS	1.01E-05	-0.18	1.69E-04	NS
CFAP70	NS	1.01E-05	-0.18	1.69E-04	NS
XPNPEP3	NS	1.01E-05	-0.18	1.69E-04	NS
CPS1	NS	1.02E-05	-0.18	1.70E-04	NS
RORA	NS	1.02E-05	-0.18	1.70E-04	NS
TGFBR3	NS	1.02E-05	-0.18	1.70E-04	NS
AMIGO2	NS	1.02E-05	-0.18	1.70E-04	NS
TMEM60	NS	1.03E-05	-0.18	1.71E-04	NS
BRINP1	NS	1.03E-05	-0.18	1.71E-04	NS
ZNF728	NS	1.03E-05	-0.18	1.71E-04	NS
CXCL11	NS	1.03E-05	-0.18	1.71E-04	NS
PABIR1	NS	1.03E-05	-0.18	1.71E-04	NS
RANGRF	NS	1.04E-05	-0.18	1.72E-04	NS
DMC1	NS	1.05E-05	1.80E-01	1.73E-04	NS
TMEM94	NS	1.06E-05	-0.179	1.75E-04	NS
SPOCD1	NS	1.06E-05	-0.179	1.75E-04	NS
HMGCR	NS	1.06E-05	-0.179	1.75E-04	NS
EPS8	NS	1.08E-05	-0.179	1.78E-04	NS
PCNT	NS	1.08E-05	-0.179	1.78E-04	NS
ACR	NS	1.08E-05	1.79E-01	1.78E-04	NS
DUSP19	NS	1.08E-05	-0.179	1.78E-04	NS
LRRFIP1	NS	1.09E-05	1.79E-01	1.79E-04	NS
ATP6V0C	NS	1.10E-05	1.79E-01	1.80E-04	NS
HECW2	NS	1.10E-05	-0.179	1.81E-04	NS
DUSP10	NS	1.10E-05	1.79E-01	1.81E-04	NS
GPR132	NS	1.10E-05	-0.179	1.81E-04	NS
MS4A1	NS	1.11E-05	-0.179	1.81E-04	NS
OPRK1	NS	1.11E-05	1.79E-01	1.81E-04	NS
ZMYND12	NS	1.12E-05	-0.179	1.83E-04	NS

GLT8D2	NS	1.12E-05	-0.179	1.83E-04	NS
ANLN	NS	1.14E-05	-0.179	1.85E-04	NS
TIGD2	NS	1.14E-05	-0.179	1.86E-04	NS
AATK	NS	1.14E-05	-0.179	1.86E-04	NS
ANAPC11	NS	1.15E-05	-0.179	1.87E-04	NS
KLRC1	NS	1.15E-05	-0.179	1.87E-04	NS
RGS8	NS	1.15E-05	-0.179	1.87E-04	NS
GIN1	NS	1.15E-05	-0.179	1.87E-04	NS
TNPO2	NS	1.16E-05	-0.179	1.88E-04	NS
AFP	NS	1.16E-05	-0.179	1.88E-04	NS
FAM72D	NS	1.17E-05	-0.179	1.90E-04	NS
ATP2C2	NS	1.18E-05	-0.179	1.91E-04	NS
PKM	NS	1.19E-05	-0.179	1.91E-04	NS
ZNF226	NS	1.19E-05	-0.178	1.93E-04	NS
ARMCX1	NS	1.20E-05	-0.178	1.93E-04	NS
HRC	NS	1.20E-05	1.78E-01	1.94E-04	NS
CXCL5	NS	1.21E-05	-0.178	1.94E-04	NS
RASAL1	NS	1.21E-05	-0.178	1.95E-04	NS
AEN	NS	1.22E-05	-0.178	1.95E-04	NS
GAS8	NS	1.22E-05	-0.178	1.96E-04	NS
CDKN2A	NS	1.22E-05	-0.178	1.96E-04	NS
ZNF574	NS	1.23E-05	1.78E-01	1.97E-04	NS
LRRC4C	NS	1.23E-05	-0.178	1.97E-04	NS
MFSD9	NS	1.24E-05	-0.178	1.98E-04	NS
CCL8	NS	1.24E-05	-0.178	1.98E-04	NS
ZNF235	NS	1.24E-05	-0.178	1.99E-04	NS
ZNF777	NS	1.24E-05	1.78E-01	1.99E-04	NS
C6orf132	NS	1.25E-05	1.78E-01	1.99E-04	NS
MTTP	NS	1.25E-05	-0.178	1.99E-04	NS
SCYGR3	NS	1.26E-05	1.78E-01	2.00E-04	NS
CDC42EP5	NS	1.28E-05	1.78E-01	2.03E-04	NS
UNG	NS	1.28E-05	-0.178	2.03E-04	NS
SLC19A3	NS	1.29E-05	-0.178	2.05E-04	NS
MYF6	NS	1.31E-05	-0.178	2.08E-04	NS
CHRNA7	NS	1.31E-05	-0.178	2.08E-04	NS
FAF2	NS	1.33E-05	-0.177	2.11E-04	NS
ZNF543	NS	1.33E-05	-0.177	2.11E-04	NS

TENT5D	NS	1.35E-05	-0.177	2.13E-04	NS
SLC28A3	NS	1.35E-05	-0.177	2.14E-04	NS
BMP8A	NS	1.36E-05	-0.177	2.14E-04	NS
KRTAP13-2	NS	1.36E-05	-0.177	2.14E-04	NS
SPAG11B	NS	1.36E-05	-0.177	2.14E-04	NS
SPACA5	NS	1.36E-05	1.77E-01	2.14E-04	NS
TUBD1	NS	1.37E-05	-0.177	2.15E-04	NS
C3orf14	NS	1.37E-05	-0.177	2.15E-04	NS
TRIM47	NS	1.37E-05	1.77E-01	2.15E-04	NS
HIPK4	NS	1.37E-05	-0.177	2.15E-04	NS
EPB41L1	NS	1.38E-05	-0.177	2.16E-04	NS
DLK1	NS	1.38E-05	1.77E-01	2.16E-04	NS
PRR5	NS	1.38E-05	-0.177	2.16E-04	NS
NUTM2E	NS	1.39E-05	-0.177	2.17E-04	NS
TPGS1	NS	1.41E-05	-0.177	2.20E-04	NS
ASTE1	NS	1.41E-05	-0.177	2.20E-04	NS
ZNF202	NS	1.41E-05	-0.177	2.21E-04	NS
ZNF256	NS	1.42E-05	-0.177	2.21E-04	NS
TTC32	NS	1.42E-05	-0.177	2.21E-04	NS
ZNF527	NS	1.44E-05	-0.177	2.24E-04	NS
MAP3K7CL	NS	1.46E-05	-0.177	2.27E-04	NS
HOXA2	NS	1.46E-05	-0.177	2.27E-04	NS
CD3E	NS	1.47E-05	-0.177	2.28E-04	NS
ONECUT3	NS	1.47E-05	1.77E-01	2.29E-04	NS
EPGN	NS	1.48E-05	-0.177	2.29E-04	NS
BORA	NS	1.49E-05	-0.176	2.31E-04	NS
MYH2	NS	1.49E-05	-0.176	2.31E-04	NS
C21orf140	NS	1.50E-05	-0.176	2.32E-04	NS
RFX4	NS	1.50E-05	-0.176	2.33E-04	NS
ZNF823	NS	1.51E-05	-0.176	2.33E-04	NS
SLC16A11	NS	1.51E-05	1.76E-01	2.34E-04	NS
TRIM59	NS	1.52E-05	-0.176	2.34E-04	NS
SPRED1	NS	1.53E-05	-0.176	2.35E-04	NS
FEZF1	NS	1.53E-05	-0.176	2.36E-04	NS
ACO2	NS	1.54E-05	-0.176	2.37E-04	NS
TRIML1	NS	1.54E-05	-0.176	2.37E-04	NS
ZNF776	NS	1.54E-05	-0.176	2.37E-04	NS

DPY19L4	NS	1.54E-05	-0.176	2.37E-04	NS
TMEM121	NS	1.55E-05	1.76E-01	2.37E-04	NS
VPS26A	NS	1.55E-05	-0.176	2.38E-04	NS
LMO4	NS	1.57E-05	-0.176	2.40E-04	NS
ZNF132	NS	1.57E-05	-0.176	2.40E-04	NS
SPATA4	NS	1.59E-05	-0.176	2.43E-04	NS
ZNF580	NS	1.62E-05	1.76E-01	2.47E-04	NS
RAVER1	NS	1.63E-05	1.76E-01	2.48E-04	NS
BEND2	NS	1.63E-05	-0.176	2.48E-04	NS
BMP10	NS	1.64E-05	-0.176	2.49E-04	NS
OTUD3	NS	1.64E-05	-0.176	2.49E-04	NS
NACAD	NS	1.64E-05	1.76E-01	2.50E-04	NS
LRRTM1	NS	1.65E-05	-0.176	2.50E-04	NS
PCDHGB1	NS	1.65E-05	-0.176	2.50E-04	NS
EDN2	NS	1.66E-05	-0.176	2.51E-04	NS
PLK4	NS	1.66E-05	-0.176	2.51E-04	NS
RTP4	NS	1.67E-05	-0.176	2.52E-04	NS
MDGA2	NS	1.67E-05	-0.176	2.52E-04	NS
BEX2	NS	1.67E-05	-0.175	2.52E-04	NS
HAGHL	NS	1.68E-05	-0.175	2.53E-04	NS
SLAMF6	NS	1.68E-05	-0.175	2.54E-04	NS
MAF1	NS	1.71E-05	1.75E-01	2.57E-04	NS
NOL4	NS	1.71E-05	-0.175	2.58E-04	NS
SCRT2	NS	1.71E-05	1.75E-01	2.58E-04	NS
ZFP62	NS	1.72E-05	-0.175	2.58E-04	NS
LYPD8	NS	1.72E-05	-0.175	2.58E-04	NS
KRTAP25-1	NS	1.72E-05	-0.175	2.58E-04	NS
TMEM191B	NS	1.73E-05	-0.175	2.59E-04	NS
KEAP1	NS	1.74E-05	-0.175	2.59E-04	NS
ADCY6	NS	1.74E-05	1.75E-01	2.59E-04	NS
OR8G1	NS	1.73E-05	-0.175	2.59E-04	NS
ZNF311	NS	1.73E-05	-0.175	2.59E-04	NS
SLC9A3R2	NS	1.74E-05	-0.175	2.59E-04	NS
ACADSB	NS	1.74E-05	-0.175	2.60E-04	NS
LPAR4	NS	1.75E-05	-0.175	2.61E-04	NS
SLC24A5	NS	1.76E-05	-0.175	2.62E-04	NS
OR1D5	NS	1.77E-05	-0.175	2.63E-04	NS

FRA10AC1	NS	1.77E-05	-0.175	2.63E-04	NS
HSD3B1	NS	1.77E-05	-0.175	2.63E-04	NS
CTAGE1	NS	1.78E-05	-0.175	2.64E-04	NS
HELB	NS	1.79E-05	-0.175	2.65E-04	NS
AIFM3	NS	1.79E-05	-0.175	2.65E-04	NS
SH3BGRL2	NS	1.80E-05	-0.175	2.67E-04	NS
SERPIND1	NS	1.81E-05	-0.175	2.67E-04	NS
OPALIN	NS	1.81E-05	-0.175	2.67E-04	NS
TMPRSS11D	NS	1.81E-05	-0.175	2.68E-04	NS
PRAMEF8	NS	1.82E-05	-0.175	2.69E-04	NS
EXOC1L	NS	1.83E-05	-0.175	2.70E-04	NS
TAL2	NS	1.83E-05	-0.175	2.70E-04	NS
ADGRF3	NS	1.84E-05	-0.175	2.71E-04	NS
ELFN1	NS	1.84E-05	1.75E-01	2.71E-04	NS
SVIL	NS	1.85E-05	-0.175	2.72E-04	NS
KRIT1	NS	1.86E-05	-0.175	2.73E-04	NS
GNG13	NS	1.86E-05	-0.175	2.73E-04	NS
SLITRK4	NS	1.86E-05	-0.175	2.73E-04	NS
BICRAL	NS	1.87E-05	-0.174	2.75E-04	NS
AGBL2	NS	1.89E-05	-0.174	2.77E-04	NS
ABI2	NS	1.90E-05	-0.174	2.78E-04	NS
TPSD1	NS	1.91E-05	1.74E-01	2.80E-04	NS
CIAO2B	NS	1.92E-05	-0.174	2.80E-04	NS
ZNF75A	NS	1.93E-05	-0.174	2.82E-04	NS
MCF2L2	NS	1.93E-05	-0.174	2.82E-04	NS
CLDN10	NS	1.93E-05	-0.174	2.82E-04	NS
STC2	NS	1.95E-05	1.74E-01	2.84E-04	NS
C4orf17	NS	1.96E-05	-0.174	2.86E-04	NS
ZSCAN26	NS	1.96E-05	-0.174	2.86E-04	NS
GABRA3	NS	1.97E-05	-0.174	2.86E-04	NS
CYP27B1	NS	1.97E-05	-0.174	2.86E-04	NS
CHL1	NS	1.97E-05	-0.174	2.86E-04	NS
P2RY4	NS	1.97E-05	-0.174	2.86E-04	NS
DPY19L3	NS	1.98E-05	-0.174	2.86E-04	NS
ACOT1	NS	1.98E-05	-0.174	2.86E-04	NS
TCEAL1	NS	1.98E-05	-0.174	2.87E-04	NS
ANKFN1	NS	1.99E-05	-0.174	2.87E-04	NS

IFNG	NS	1.99E-05	-0.174	2.87E-04	NS
COL14A1	NS	2.00E-05	-0.174	2.89E-04	NS
ADH6	NS	2.00E-05	-0.174	2.89E-04	NS
M6PR	NS	2.01E-05	-0.174	2.90E-04	NS
SH3PXD2A	NS	2.01E-05	-0.174	2.90E-04	NS
DAAM2	NS	2.01E-05	-0.174	2.90E-04	NS
GPRIN1	NS	2.02E-05	1.74E-01	2.90E-04	NS
BBS12	NS	2.01E-05	-0.174	2.90E-04	NS
GDF9	NS	2.04E-05	-0.174	2.93E-04	NS
TPRX1	NS	2.04E-05	1.74E-01	2.93E-04	NS
FREM3	NS	2.04E-05	-0.174	2.93E-04	NS
FAM110B	NS	2.05E-05	-0.174	2.93E-04	NS
PCDHGA9	NS	2.05E-05	-0.174	2.94E-04	NS
NANOS3	NS	2.06E-05	1.74E-01	2.95E-04	NS
GCC2	NS	2.07E-05	-0.174	2.96E-04	NS
RFESD	NS	2.07E-05	-0.174	2.96E-04	NS
EMC9	NS	2.08E-05	-0.174	2.97E-04	NS
ASXL1	NS	2.08E-05	-0.174	2.97E-04	NS
HNRNPH2	NS	2.09E-05	-0.174	2.97E-04	NS
TRIM49	NS	2.09E-05	1.73E-01	2.98E-04	NS
POLR2K	NS	2.09E-05	-0.173	2.98E-04	NS
INHBB	NS	2.10E-05	1.73E-01	2.98E-04	NS
PYGO2	NS	2.10E-05	-0.173	2.99E-04	NS
ADIPOR2	NS	2.10E-05	-0.173	2.99E-04	NS
SLC1A1	NS	2.11E-05	-0.173	2.99E-04	NS
TOR1B	NS	2.12E-05	-0.173	3.00E-04	NS
NDUFS4	NS	2.12E-05	-0.173	3.00E-04	NS
RHEBL1	NS	2.12E-05	-0.173	3.01E-04	NS
OXSM	NS	2.13E-05	-0.173	3.01E-04	NS
KIAA1958	NS	2.13E-05	1.73E-01	3.01E-04	NS
MATN4	NS	2.13E-05	-0.173	3.01E-04	NS
AADACL3	NS	2.13E-05	-0.173	3.01E-04	NS
CLGN	NS	2.14E-05	-0.173	3.01E-04	NS
FLT1	NS	2.15E-05	-0.173	3.03E-04	NS
MIGA1	NS	2.15E-05	-0.173	3.03E-04	NS
TAS2R46	NS	2.16E-05	-0.173	3.03E-04	NS
ENTPD2	NS	2.16E-05	1.73E-01	3.03E-04	NS

GFOD2	NS	2.16E-05	-0.173	3.03E-04	NS
C5orf34	NS	2.16E-05	-0.173	3.03E-04	NS
TSPAN13	NS	2.17E-05	-0.173	3.04E-04	NS
C2CD4C	NS	2.17E-05	1.73E-01	3.04E-04	NS
SAMSN1	NS	2.21E-05	-0.173	3.10E-04	NS
HEPACAM2	NS	2.22E-05	-0.173	3.11E-04	NS
IQUB	NS	2.23E-05	-0.173	3.12E-04	NS
GAL3ST3	NS	2.24E-05	1.73E-01	3.12E-04	NS
C7orf33	NS	2.25E-05	-0.173	3.13E-04	NS
SESN1	NS	2.25E-05	1.73E-01	3.14E-04	NS
SLC38A11	NS	2.26E-05	-0.173	3.14E-04	NS
C16orf46	NS	2.27E-05	-0.173	3.15E-04	NS
HIF1AN	NS	2.27E-05	-0.173	3.16E-04	NS
C1orf167	NS	2.27E-05	-0.173	3.16E-04	NS
ACADM	NS	2.29E-05	-0.173	3.17E-04	NS
SIL1	NS	2.28E-05	-0.173	3.17E-04	NS
CCT8L2	NS	2.29E-05	1.73E-01	3.17E-04	NS
LY6G6F	NS	2.29E-05	-0.173	3.17E-04	NS
CFAP119	NS	2.29E-05	-0.173	3.18E-04	NS
ATP6V1G3	NS	2.31E-05	-0.173	3.19E-04	NS
NOX1	NS	2.31E-05	-0.173	3.19E-04	NS
PCDHGC3	NS	2.32E-05	-0.173	3.21E-04	NS
SLC4A4	NS	2.32E-05	-0.173	3.21E-04	NS
HEPHL1	NS	2.33E-05	-0.173	3.21E-04	NS
PCDH11Y	NS	2.33E-05	1.73E-01	3.21E-04	NS
EXOC2	NS	2.33E-05	-0.173	3.21E-04	NS
PTK2B	NS	2.34E-05	1.72E-01	3.23E-04	NS
DNMT3A	NS	2.36E-05	1.72E-01	3.25E-04	NS
CNTN5	NS	2.36E-05	-0.172	3.25E-04	NS
PCDHGA4	NS	2.37E-05	-0.172	3.26E-04	NS
RNF212B	NS	2.39E-05	-0.172	3.27E-04	NS
IFI27L1	NS	2.39E-05	-0.172	3.28E-04	NS
CRIP2	NS	2.39E-05	1.72E-01	3.28E-04	NS
TAS2R30	NS	2.42E-05	-0.172	3.32E-04	NS
SPAAR	NS	2.43E-05	1.72E-01	3.32E-04	NS
PDE1C	NS	2.43E-05	-0.172	3.33E-04	NS
HGS	NS	2.43E-05	1.72E-01	3.33E-04	NS

RPTN	NS	2.44E-05	-0.172	3.33E-04	NS
MNAT1	NS	2.45E-05	-0.172	3.34E-04	NS
MT-ATP8	NS	2.45E-05	1.72E-01	3.34E-04	NS
CROT	NS	2.45E-05	-0.172	3.34E-04	NS
OTC	NS	2.46E-05	-0.172	3.35E-04	NS
OR13C5	NS	2.46E-05	-0.172	3.35E-04	NS
DNAJC19	NS	2.49E-05	-0.172	3.38E-04	NS
TMCO5A	NS	2.50E-05	-0.172	3.40E-04	NS
FBXO24	NS	2.50E-05	1.72E-01	3.40E-04	NS
STK31	NS	2.51E-05	-0.172	3.40E-04	NS
CCDC105	NS	2.51E-05	1.72E-01	3.40E-04	NS
WSB2	NS	2.52E-05	-0.172	3.41E-04	NS
EPPIN-WFDC6	NS	2.55E-05	-0.172	3.45E-04	NS
EPOP	NS	2.55E-05	1.72E-01	3.45E-04	NS
PGBD4	NS	2.56E-05	-0.172	3.46E-04	NS
MID1	NS	2.56E-05	-0.172	3.46E-04	NS
CDCA7	NS	2.57E-05	-0.172	3.47E-04	NS
UBOX5	NS	2.58E-05	-0.172	3.48E-04	NS
FAM86B2	NS	2.60E-05	-0.172	3.50E-04	NS
MCAM	NS	2.60E-05	-0.172	3.50E-04	NS
TRNT1	NS	2.61E-05	-0.171	3.50E-04	NS
SLC7A4	NS	2.60E-05	1.71E-01	3.50E-04	NS
CFAP53	NS	2.63E-05	-0.171	3.53E-04	NS
CREBRF	NS	2.63E-05	1.71E-01	3.53E-04	NS
SP8	NS	2.64E-05	1.71E-01	3.54E-04	NS
RAB33A	NS	2.66E-05	-0.171	3.57E-04	NS
SPOCK2	NS	2.67E-05	-0.171	3.57E-04	NS
STX3	NS	2.67E-05	1.71E-01	3.57E-04	NS
HSD11B1L	NS	2.68E-05	1.71E-01	3.58E-04	NS
OR2AK2	NS	2.69E-05	-0.171	3.59E-04	NS
C2CD4D	NS	2.70E-05	1.71E-01	3.60E-04	NS
WDR35	NS	2.72E-05	-0.171	3.63E-04	NS
SLC35A2	NS	2.73E-05	-0.171	3.63E-04	NS
TRAPPC4	NS	2.74E-05	-0.171	3.65E-04	NS
RCVRN	NS	2.75E-05	-0.171	3.66E-04	NS
ZFY	NS	2.77E-05	1.71E-01	3.68E-04	NS
MTCP1	NS	2.77E-05	-0.171	3.69E-04	NS

IL5RA	NS	2.78E-05	-0.171	3.69E-04	NS
ZNF598	NS	2.80E-05	1.71E-01	3.72E-04	NS
CTXN1	NS	2.81E-05	1.71E-01	3.73E-04	NS
KCTD16	NS	2.82E-05	-0.171	3.73E-04	NS
VXN	NS	2.83E-05	-0.171	3.74E-04	NS
OR5B21	NS	2.83E-05	-0.171	3.75E-04	NS
SORCS1	NS	2.84E-05	-0.171	3.75E-04	NS
MEPE	NS	2.84E-05	-0.171	3.75E-04	NS
GPR85	NS	2.85E-05	-0.171	3.76E-04	NS
ACVR1B	NS	2.85E-05	1.71E-01	3.77E-04	NS
MYL10	NS	2.87E-05	1.71E-01	3.78E-04	NS
BCAT2	NS	2.89E-05	-0.171	3.80E-04	NS
REXO2	NS	2.95E-05	-0.17	3.88E-04	NS
CNOT6L	NS	2.95E-05	-0.17	3.88E-04	NS
NLRP9	NS	2.95E-05	-0.17	3.88E-04	NS
URI1	NS	2.97E-05	-0.17	3.89E-04	NS
LDB2	NS	2.96E-05	-0.17	3.89E-04	NS
MYOT	NS	2.98E-05	-0.17	3.91E-04	NS
SPINK9	NS	2.98E-05	-0.17	3.91E-04	NS
SLC2A2	NS	3.01E-05	-0.17	3.95E-04	NS
DMAC1	NS	3.02E-05	-0.17	3.95E-04	NS
NCAPG2	NS	3.03E-05	-0.17	3.97E-04	NS
CIRBP	NS	3.03E-05	-0.17	3.97E-04	NS
MMP12	NS	3.05E-05	-0.17	3.98E-04	NS
SLC44A4	NS	3.06E-05	-0.17	4.00E-04	NS
ILRUN	NS	3.07E-05	-0.17	4.00E-04	NS
TLCD4	NS	3.07E-05	-0.17	4.01E-04	NS
AARD	NS	3.08E-05	1.70E-01	4.01E-04	NS
TEN1	NS	3.08E-05	-0.17	4.01E-04	NS
SEPTIN4	NS	3.09E-05	-0.17	4.02E-04	NS
ZNF134	NS	3.09E-05	-0.17	4.02E-04	NS
ACAD9	NS	3.10E-05	-0.17	4.03E-04	NS
BPHL	NS	3.12E-05	-0.17	4.05E-04	NS
OPHN1	NS	3.14E-05	-0.17	4.07E-04	NS
PGAP3	NS	3.15E-05	-0.17	4.08E-04	NS
SLC26A5	NS	3.15E-05	-0.17	4.08E-04	NS
NMBR	NS	3.15E-05	-0.17	4.08E-04	NS

DSG2	NS	3.18E-05	-0.17	4.12E-04	NS
C1QB	NS	3.18E-05	-0.17	4.12E-04	NS
GNMT	NS	3.20E-05	-0.17	4.14E-04	NS
GKN2	NS	3.22E-05	-0.17	4.16E-04	NS
CC2D1B	NS	3.23E-05	-0.17	4.17E-04	NS
XPNPEP2	NS	3.23E-05	-0.17	4.17E-04	NS
CCDC148	NS	3.24E-05	-0.169	4.18E-04	NS
BCL11B	NS	3.25E-05	-0.169	4.18E-04	NS
OR2T4	NS	3.25E-05	-0.169	4.18E-04	NS
OR5D3P	NS	3.27E-05	-0.169	4.21E-04	NS
HERC6	NS	3.28E-05	-0.169	4.22E-04	NS
TXNDC16	NS	3.28E-05	-0.169	4.22E-04	NS
GUCY1B1	NS	3.30E-05	-0.169	4.23E-04	NS
FBXO31	NS	3.29E-05	1.69E-01	4.23E-04	NS
LCE1C	NS	3.30E-05	1.69E-01	4.23E-04	NS
MELTF	NS	3.31E-05	-0.169	4.24E-04	NS
IGSF1	NS	3.32E-05	-0.169	4.26E-04	NS
FCER2	NS	3.33E-05	-0.169	4.27E-04	NS
CACNG3	NS	3.34E-05	-0.169	4.27E-04	NS
SOX8	NS	3.35E-05	1.69E-01	4.28E-04	NS
DYNLT5	NS	3.36E-05	-0.169	4.29E-04	NS
NDUFB3	NS	3.37E-05	-0.169	4.30E-04	NS
VPS54	NS	3.37E-05	-0.169	4.30E-04	NS
SLC25A19	NS	3.41E-05	-0.169	4.34E-04	NS
SELENOI	NS	3.42E-05	-0.169	4.35E-04	NS
TLR10	NS	3.43E-05	-0.169	4.36E-04	NS
NOL8	NS	3.43E-05	-0.169	4.36E-04	NS
KRTAP4-16	NS	3.43E-05	1.69E-01	4.37E-04	NS
PTCD2	NS	3.45E-05	-0.169	4.38E-04	NS
ZNF180	NS	3.45E-05	-0.169	4.38E-04	NS
MTF2	NS	3.45E-05	-0.169	4.38E-04	NS
EFCAB10	NS	3.47E-05	-0.169	4.40E-04	NS
FOXR2	NS	3.47E-05	-0.169	4.40E-04	NS
HHIPL2	NS	3.48E-05	-0.169	4.41E-04	NS
OR5H15	NS	3.51E-05	-0.169	4.44E-04	NS
SHBG	NS	3.52E-05	1.69E-01	4.45E-04	NS
MICB	NS	3.52E-05	-0.169	4.45E-04	NS

PODXL	NS	3.55E-05	-0.169	4.48E-04	NS
SLC41A1	NS	3.55E-05	-0.169	4.48E-04	NS
GPR65	NS	3.55E-05	-0.169	4.48E-04	NS
ACTR5	NS	3.56E-05	-0.169	4.48E-04	NS
FOXL1	NS	3.56E-05	1.69E-01	4.48E-04	NS
MDH1B	NS	3.60E-05	-0.169	4.52E-04	NS
PMP2	NS	3.60E-05	-0.169	4.53E-04	NS
EIF2AK2	NS	3.62E-05	-0.168	4.54E-04	NS
INSL6	NS	3.62E-05	-0.168	4.54E-04	NS
DCUN1D3	NS	3.61E-05	-0.168	4.54E-04	NS
FAF1	NS	3.63E-05	-0.168	4.55E-04	NS
PSMB5	NS	3.64E-05	-0.168	4.56E-04	NS
UBR5	NS	3.64E-05	-0.168	4.56E-04	NS
OR3A1	NS	3.66E-05	-0.168	4.57E-04	NS
DEFB119	NS	3.66E-05	-0.168	4.57E-04	NS
PJVK	NS	3.65E-05	-0.168	4.57E-04	NS
C1orf131	NS	3.66E-05	-0.168	4.57E-04	NS
KPNA1	NS	3.68E-05	-0.168	4.59E-04	NS
TRPM8	NS	3.69E-05	-0.168	4.59E-04	NS
LMLN	NS	3.68E-05	-0.168	4.59E-04	NS
RBBP8NL	NS	3.69E-05	1.68E-01	4.60E-04	NS
POMT1	NS	3.70E-05	-0.168	4.61E-04	NS
LTO1	NS	3.71E-05	-0.168	4.61E-04	NS
ROCK2	NS	3.72E-05	-0.168	4.62E-04	NS
PTPRR	NS	3.74E-05	-0.168	4.64E-04	NS
GNB5	NS	3.75E-05	-0.168	4.65E-04	NS
LRRK1	NS	3.77E-05	-0.168	4.68E-04	NS
PCLO	NS	3.79E-05	-0.168	4.70E-04	NS
CREG1	NS	3.80E-05	1.68E-01	4.71E-04	NS
SCAF1	NS	3.85E-05	1.68E-01	4.76E-04	NS
TBC1D10C	NS	3.85E-05	-0.168	4.76E-04	NS
VSIG8	NS	3.85E-05	1.68E-01	4.76E-04	NS
NUAK1	NS	3.87E-05	-0.168	4.78E-04	NS
MYL2	NS	3.88E-05	-0.168	4.79E-04	NS
CHIC2	NS	3.90E-05	-0.168	4.81E-04	NS
ARL17B	NS	3.91E-05	-0.168	4.83E-04	NS
ABHD16A	NS	3.94E-05	-0.168	4.85E-04	NS

GNG11	NS	3.94E-05	-0.168	4.85E-04	NS
PI4K2A	NS	3.96E-05	1.68E-01	4.87E-04	NS
DDR2	NS	3.96E-05	-0.168	4.87E-04	NS
DDX4	NS	3.97E-05	-0.168	4.88E-04	NS
H1-10	NS	3.97E-05	1.68E-01	4.88E-04	NS
C12orf54	NS	3.98E-05	-0.168	4.89E-04	NS
MAP7D2	NS	4.00E-05	-0.168	4.90E-04	NS
PRTN3	NS	4.01E-05	1.68E-01	4.92E-04	NS
EPHA4	NS	4.02E-05	-0.167	4.93E-04	NS
IFT80	NS	4.03E-05	-0.167	4.93E-04	NS
ARPC2	NS	4.04E-05	-0.167	4.95E-04	NS
TCP11X1	NS	4.04E-05	-0.167	4.95E-04	NS
POLQ	NS	4.05E-05	-0.167	4.95E-04	NS
ZNF827	NS	4.05E-05	-0.167	4.95E-04	NS
DUSP16	NS	4.07E-05	-0.167	4.97E-04	NS
RDH10	NS	4.09E-05	-0.167	4.99E-04	NS
ANGPTL1	NS	4.09E-05	-0.167	4.99E-04	NS
ACVR1C	NS	4.09E-05	-0.167	4.99E-04	NS
GML	NS	4.11E-05	-0.167	5.00E-04	NS
GABRP	NS	4.11E-05	-0.167	5.00E-04	NS
G3BP1	NS	4.12E-05	-0.167	5.01E-04	NS
CCDC83	NS	4.13E-05	-0.167	5.01E-04	NS
CKAP2L	NS	4.13E-05	-0.167	5.01E-04	NS
SELP	NS	4.14E-05	-0.167	5.02E-04	NS
CASC3	NS	4.14E-05	1.67E-01	5.03E-04	NS
TGFB2	NS	4.17E-05	-0.167	5.05E-04	NS
QRICH1	NS	4.17E-05	1.67E-01	5.06E-04	NS
PDLIM4	NS	4.18E-05	-0.167	5.06E-04	NS
MSTO1	NS	4.23E-05	-0.167	5.12E-04	NS
SOX2	NS	4.23E-05	1.67E-01	5.12E-04	NS
NDUFB6	NS	4.24E-05	-0.167	5.12E-04	NS
SLC7A1	NS	4.24E-05	-0.167	5.13E-04	NS
TMX2	NS	4.25E-05	-0.167	5.13E-04	NS
SERINC1	NS	4.28E-05	-0.167	5.16E-04	NS
PKIB	NS	4.29E-05	-0.167	5.18E-04	NS
LY6D	NS	4.29E-05	1.67E-01	5.18E-04	NS
C3orf38	NS	4.30E-05	-0.167	5.18E-04	NS

ATXN2L	NS	4.32E-05	1.67E-01	5.20E-04	NS
TAF11L13	NS	4.33E-05	1.67E-01	5.21E-04	NS
RAG2	NS	4.33E-05	-0.167	5.21E-04	NS
MANSC1	NS	4.36E-05	-0.167	5.23E-04	NS
TCP11	NS	4.35E-05	-0.167	5.23E-04	NS
UBE2J2	NS	4.39E-05	-0.167	5.26E-04	NS
FABP3	NS	4.39E-05	-0.167	5.27E-04	NS
DLL3	NS	4.40E-05	1.67E-01	5.27E-04	NS
CENPQ	NS	4.41E-05	-0.167	5.27E-04	NS
CACNA1C	NS	4.41E-05	-0.167	5.27E-04	NS
PPIAL4D	NS	4.40E-05	1.67E-01	5.27E-04	NS
FGF22	NS	4.44E-05	1.67E-01	5.31E-04	NS
PRKCD	NS	4.46E-05	1.67E-01	5.33E-04	NS
CANX	NS	4.46E-05	-0.167	5.33E-04	NS
DKK2	NS	4.47E-05	-0.167	5.33E-04	NS
ZBTB37	NS	4.47E-05	-0.167	5.33E-04	NS
VPS13A	NS	4.47E-05	-0.167	5.33E-04	NS
MAP10	NS	4.50E-05	-0.166	5.36E-04	NS
ACE2	NS	4.55E-05	-0.166	5.42E-04	NS
ANGPTL6	NS	4.58E-05	-0.166	5.45E-04	NS
GFM2	NS	4.58E-05	-0.166	5.45E-04	NS
PLAC1	NS	4.58E-05	-0.166	5.45E-04	NS
SP9	NS	4.61E-05	1.66E-01	5.48E-04	NS
FOLH1	NS	4.62E-05	-0.166	5.48E-04	NS
CPA2	NS	4.63E-05	-0.166	5.49E-04	NS
TTC14	NS	4.63E-05	-0.166	5.49E-04	NS
LDHD	NS	4.63E-05	-0.166	5.49E-04	NS
PRPSAP2	NS	4.65E-05	-0.166	5.51E-04	NS
CCDC88C	NS	4.66E-05	1.66E-01	5.51E-04	NS
MROH9	NS	4.69E-05	-0.166	5.54E-04	NS
TP53RK	NS	4.69E-05	-0.166	5.54E-04	NS
ADH4	NS	4.69E-05	-0.166	5.54E-04	NS
PPP2CB	NS	4.71E-05	-0.166	5.56E-04	NS
FPR1	NS	4.71E-05	-0.166	5.56E-04	NS
TBX18	NS	4.72E-05	-0.166	5.57E-04	NS
MICU3	NS	4.73E-05	-0.166	5.58E-04	NS
PLA2G15	NS	4.75E-05	1.66E-01	5.59E-04	NS

KIF20A	NS	4.78E-05	-0.166	5.62E-04	NS
THUMPD1	NS	4.79E-05	-0.166	5.63E-04	NS
HMG20B	NS	4.80E-05	1.66E-01	5.64E-04	NS
GYPA	NS	4.81E-05	-0.166	5.65E-04	NS
FAM71F2	NS	4.84E-05	-0.166	5.68E-04	NS
DHX33	NS	4.86E-05	-0.166	5.70E-04	NS
TMEM89	NS	4.87E-05	-0.166	5.71E-04	NS
SSU72P2	NS	4.88E-05	1.66E-01	5.71E-04	NS
BTNL2	NS	4.92E-05	-0.166	5.75E-04	NS
ADGRE3	NS	4.93E-05	-0.166	5.76E-04	NS
POFUT1	NS	4.93E-05	-0.166	5.76E-04	NS
SLC5A9	NS	4.93E-05	-0.166	5.76E-04	NS
CD27	NS	4.96E-05	-0.166	5.79E-04	NS
MYORG	NS	4.96E-05	-0.166	5.79E-04	NS
CNBD2	NS	4.98E-05	-0.166	5.80E-04	NS
OR2A7	NS	4.97E-05	-0.166	5.80E-04	NS
PCDHB13	NS	4.99E-05	-0.165	5.81E-04	NS
EDIL3	NS	5.00E-05	-0.165	5.82E-04	NS
IFITM3	NS	5.01E-05	1.65E-01	5.83E-04	NS
STUB1	NS	5.02E-05	-0.165	5.83E-04	NS
NEBL	NS	5.04E-05	-0.165	5.85E-04	NS
ZNF182	NS	5.07E-05	-0.165	5.88E-04	NS
CYP4F12	NS	5.07E-05	-0.165	5.88E-04	NS
CEP55	NS	5.08E-05	-0.165	5.89E-04	NS
PRELID1	NS	5.13E-05	-0.165	5.95E-04	NS
TMEM150C	NS	5.17E-05	1.65E-01	5.99E-04	NS
DAW1	NS	5.17E-05	-0.165	5.99E-04	NS
XRCC4	NS	5.18E-05	-0.165	5.99E-04	NS
ADK	NS	5.18E-05	-0.165	5.99E-04	NS
SPR	NS	5.22E-05	-0.165	6.03E-04	NS
BHLHA9	NS	5.22E-05	1.65E-01	6.03E-04	NS
CTLA4	NS	5.23E-05	-0.165	6.03E-04	NS
DHX58	NS	5.23E-05	-0.165	6.04E-04	NS
OR6M1	NS	5.26E-05	-0.165	6.06E-04	NS
GFRA4	NS	5.26E-05	1.65E-01	6.06E-04	NS
MEF2C	NS	5.27E-05	-0.165	6.07E-04	NS
IDH2	NS	5.27E-05	-0.165	6.07E-04	NS

CRCP	NS	5.28E-05	1.65E-01	6.07E-04	NS
KLHL12	NS	5.29E-05	-0.165	6.08E-04	NS
PDZD2	NS	5.29E-05	-0.165	6.08E-04	NS
OR52W1	NS	5.30E-05	-0.165	6.08E-04	NS
KRTAP7-1	NS	5.29E-05	-0.165	6.08E-04	NS
INTS5	NS	5.30E-05	-0.165	6.08E-04	NS
PDZD9	NS	5.32E-05	-0.165	6.09E-04	NS
NAB1	NS	5.32E-05	-0.165	6.09E-04	NS
ZNF93	NS	5.33E-05	-0.165	6.10E-04	NS
ZNF347	NS	5.36E-05	-0.165	6.13E-04	NS
CYTH2	NS	5.37E-05	-0.165	6.14E-04	NS
TMEM65	NS	5.37E-05	-0.165	6.14E-04	NS
OMG	NS	5.39E-05	-0.165	6.15E-04	NS
DUSP1	NS	5.40E-05	1.65E-01	6.16E-04	NS
MRGPRX3	NS	5.41E-05	1.65E-01	6.17E-04	NS
CEACAM18	NS	5.42E-05	-0.165	6.18E-04	NS
KLHL21	NS	5.43E-05	1.65E-01	6.18E-04	NS
REG1B	NS	5.43E-05	-0.165	6.18E-04	NS
FAM72C	NS	5.44E-05	-0.165	6.19E-04	NS
MYO7A	NS	5.45E-05	-0.165	6.20E-04	NS
CTAG2	NS	5.46E-05	-0.165	6.21E-04	NS
LHX8	NS	5.46E-05	-0.165	6.21E-04	NS
CCDC127	NS	5.49E-05	-0.165	6.22E-04	NS
ANKK1	NS	5.49E-05	-0.165	6.22E-04	NS
FABP12	NS	5.48E-05	-0.165	6.22E-04	NS
CTSV	NS	5.51E-05	1.65E-01	6.24E-04	NS
MARVELD3	NS	5.51E-05	-0.165	6.24E-04	NS
PRR18	NS	5.51E-05	1.65E-01	6.24E-04	NS
MME	NS	5.51E-05	-0.165	6.24E-04	NS
DYNLT2	NS	5.62E-05	-0.164	6.36E-04	NS
CLDN22	NS	5.63E-05	-0.164	6.36E-04	NS
MYCN	NS	5.66E-05	1.64E-01	6.39E-04	NS
ZNF530	NS	5.66E-05	-0.164	6.39E-04	NS
C1orf35	NS	5.68E-05	1.64E-01	6.41E-04	NS
AMPH	NS	5.69E-05	-0.164	6.41E-04	NS
DCHS2	NS	5.69E-05	-0.164	6.41E-04	NS
RAB44	NS	5.71E-05	-0.164	6.43E-04	NS

UTP14C	NS	5.78E-05	-0.164	6.50E-04	NS
SPIN4	NS	5.80E-05	1.64E-01	6.52E-04	NS
ZNF549	NS	5.80E-05	-0.164	6.52E-04	NS
TRIAP1	NS	5.82E-05	-0.164	6.54E-04	NS
ZNF610	NS	5.95E-05	-0.164	6.68E-04	NS
CCN2	NS	5.96E-05	1.64E-01	6.68E-04	NS
PSMA5	NS	5.96E-05	-0.164	6.68E-04	NS
EEF1AKMT4	NS	5.96E-05	-0.164	6.68E-04	NS
ATG2B	NS	5.99E-05	-0.164	6.71E-04	NS
SLA	NS	6.01E-05	-0.164	6.73E-04	NS
NEFH	NS	6.02E-05	-0.164	6.74E-04	NS
HDHD3	NS	6.03E-05	-0.164	6.74E-04	NS
DHFR2	NS	6.03E-05	-0.164	6.74E-04	NS
RD3L	NS	6.03E-05	-0.164	6.74E-04	NS
ACVR2A	NS	6.07E-05	-0.164	6.77E-04	NS
LRRC75B	NS	6.07E-05	-0.164	6.78E-04	NS
ITIH4	NS	6.09E-05	-0.164	6.79E-04	NS
PACRGL	NS	6.10E-05	-0.164	6.80E-04	NS
PLEKHH3	NS	6.11E-05	1.64E-01	6.80E-04	NS
APH1B	NS	6.12E-05	-0.164	6.81E-04	NS
ICE2	NS	6.14E-05	-0.164	6.83E-04	NS
MS4A12	NS	6.16E-05	-0.163	6.85E-04	NS
ORC5	NS	6.21E-05	-0.163	6.90E-04	NS
IGSF10	NS	6.25E-05	-0.163	6.94E-04	NS
CER1	NS	6.27E-05	-0.163	6.96E-04	NS
SLC22A3	NS	6.30E-05	-0.163	6.98E-04	NS
RAC1	NS	6.31E-05	1.63E-01	6.98E-04	NS
ZNF280B	NS	6.30E-05	-0.163	6.98E-04	NS
PPIAL4F	NS	6.32E-05	1.63E-01	6.99E-04	NS
MBD3L5	NS	6.33E-05	1.63E-01	7.00E-04	NS
ASCC3	NS	6.34E-05	-0.163	7.01E-04	NS
ACO1	NS	6.35E-05	-0.163	7.01E-04	NS
FAM47E-STBD1	NS	6.37E-05	-0.163	7.03E-04	NS
S100G	NS	6.39E-05	-0.163	7.05E-04	NS
ZNF222	NS	6.42E-05	-0.163	7.08E-04	NS
SLC47A1	NS	6.44E-05	-0.163	7.10E-04	NS
CDK16	NS	6.47E-05	1.63E-01	7.13E-04	NS

VPS16	NS	6.50E-05	-0.163	7.16E-04	NS
CCDC194	NS	6.54E-05	1.63E-01	7.20E-04	NS
TRMT10B	NS	6.59E-05	-0.163	7.24E-04	NS
TASOR2	NS	6.59E-05	-0.163	7.24E-04	NS
TPBGL	NS	6.62E-05	1.63E-01	7.27E-04	NS
ADAM17	NS	6.63E-05	-0.163	7.28E-04	NS
CCDC152	NS	6.64E-05	-0.163	7.28E-04	NS
SPANXC	NS	6.71E-05	-0.163	7.35E-04	NS
ZNF480	NS	6.72E-05	-0.163	7.36E-04	NS
MED6	NS	6.76E-05	-0.163	7.41E-04	NS
TGFA	NS	6.79E-05	-0.163	7.42E-04	NS
TMEM267	NS	6.79E-05	-0.163	7.42E-04	NS
FASTKD3	NS	6.81E-05	-0.163	7.45E-04	NS
CPSF2	NS	6.82E-05	-0.163	7.45E-04	NS
DEFB125	NS	6.83E-05	-0.163	7.46E-04	NS
OR1L8	NS	6.84E-05	-0.162	7.46E-04	NS
TAS2R31	NS	6.86E-05	-0.162	7.48E-04	NS
DCC	NS	6.87E-05	-0.162	7.48E-04	NS
MT-CYB	NS	6.88E-05	1.62E-01	7.49E-04	NS
SDR16C5	NS	6.91E-05	-0.162	7.52E-04	NS
SUMO1	NS	6.93E-05	-0.162	7.54E-04	NS
CDHR2	NS	6.99E-05	-0.162	7.60E-04	NS
KIF6	NS	7.00E-05	-0.162	7.61E-04	NS
CADM3	NS	7.01E-05	-0.162	7.61E-04	NS
HOXA10	NS	7.05E-05	1.62E-01	7.65E-04	NS
MEX3D	NS	7.07E-05	1.62E-01	7.67E-04	NS
ZBTB46	NS	7.10E-05	-0.162	7.70E-04	NS
HMGCLL1	NS	7.10E-05	-0.162	7.70E-04	NS
C9orf50	NS	7.12E-05	1.62E-01	7.71E-04	NS
PDPR	NS	7.18E-05	-0.162	7.76E-04	NS
MMRN1	NS	7.18E-05	-0.162	7.76E-04	NS
DOCK10	NS	7.22E-05	-0.162	7.80E-04	NS
ZNF83	NS	7.22E-05	-0.162	7.80E-04	NS
ALCAM	NS	7.31E-05	-0.162	7.89E-04	NS
RNF128	NS	7.33E-05	-0.162	7.90E-04	NS
ZNF287	NS	7.33E-05	-0.162	7.90E-04	NS
MICAL3	NS	7.33E-05	-0.162	7.90E-04	NS

ATP6V1C1	NS	7.34E-05	-0.162	7.91E-04	NS
TTC9	NS	7.36E-05	-0.162	7.92E-04	NS
IAH1	NS	7.38E-05	-0.162	7.94E-04	NS
ABHD12B	NS	7.43E-05	-0.162	7.99E-04	NS
TBL1XR1	NS	7.48E-05	-0.162	8.04E-04	NS
DPP4	NS	7.48E-05	-0.162	8.04E-04	NS
ABCB8	NS	7.53E-05	-0.162	8.08E-04	NS
HES3	NS	7.57E-05	1.62E-01	8.12E-04	NS
CDK3	NS	7.57E-05	-0.162	8.12E-04	NS
ZMAT5	NS	7.59E-05	-0.161	8.13E-04	NS
MTNR1A	NS	7.61E-05	-0.161	8.15E-04	NS
PRDX2	NS	7.63E-05	-0.161	8.16E-04	NS
RNASEH1	NS	7.71E-05	-0.161	8.25E-04	NS
NR3C1	NS	7.74E-05	-0.161	8.27E-04	NS
CLEC10A	NS	7.75E-05	-0.161	8.28E-04	NS
NKAPL	NS	7.76E-05	-0.161	8.28E-04	NS
RNF166	NS	7.77E-05	1.61E-01	8.29E-04	NS
NIP7	NS	7.78E-05	-0.161	8.30E-04	NS
NANOG	NS	7.85E-05	-0.161	8.37E-04	NS
MYL12B	NS	7.85E-05	-0.161	8.37E-04	NS
KRAS	NS	7.88E-05	-0.161	8.39E-04	NS
PRSS36	NS	7.92E-05	-0.161	8.43E-04	NS
CDC42BPB	NS	7.93E-05	-0.161	8.43E-04	NS
MSANTD3-TMEFF1	NS	7.94E-05	-0.161	8.44E-04	NS
ESPL1	NS	7.95E-05	-0.161	8.44E-04	NS
CNTD1	NS	7.98E-05	-0.161	8.48E-04	NS
ZNF19	NS	7.99E-05	-0.161	8.48E-04	NS
SLC38A4	NS	8.02E-05	-0.161	8.51E-04	NS
CLIP1	NS	8.04E-05	-0.161	8.52E-04	NS
STRIT1	NS	8.04E-05	-0.161	8.52E-04	NS
S100B	NS	8.07E-05	-0.161	8.54E-04	NS
YAP1	NS	8.09E-05	-0.161	8.56E-04	NS
APOO	NS	8.10E-05	-0.161	8.56E-04	NS
CAB39L	NS	8.13E-05	-0.161	8.58E-04	NS
KLRC2	NS	8.18E-05	-0.161	8.64E-04	NS
DISP1	NS	8.19E-05	-0.161	8.64E-04	NS
SFT2D1	NS	8.22E-05	-0.161	8.67E-04	NS

FBXO4	NS	8.26E-05	-0.161	8.71E-04	NS
TMEM216	NS	8.27E-05	-0.161	8.71E-04	NS
PARP4	NS	8.29E-05	-0.161	8.72E-04	NS
KRTAP20-4	NS	8.29E-05	-0.161	8.72E-04	NS
SLC25A41	NS	8.29E-05	1.61E-01	8.72E-04	NS
KLF17	NS	8.31E-05	-0.161	8.74E-04	NS
POSTN	NS	8.34E-05	-0.161	8.76E-04	NS
AUNIP	NS	8.35E-05	-0.161	8.77E-04	NS
MRPL53	NS	8.36E-05	-0.161	8.77E-04	NS
CYB561D1	NS	8.37E-05	-0.161	8.77E-04	NS
AHSG	NS	8.38E-05	-0.161	8.77E-04	NS
MRPL52	NS	8.38E-05	-0.161	8.77E-04	NS
ATL1	NS	8.46E-05	-0.16	8.85E-04	NS
TCAIM	NS	8.47E-05	-0.16	8.86E-04	NS
SRP54	NS	8.52E-05	-0.16	8.91E-04	NS
TLNRD1	NS	8.54E-05	-0.16	8.92E-04	NS
MAEA	NS	8.56E-05	-0.16	8.94E-04	NS
METTL24	NS	8.65E-05	-0.16	9.01E-04	NS
SLC26A3	NS	8.63E-05	-0.16	9.01E-04	NS
DTX4	NS	8.64E-05	-0.16	9.01E-04	NS
NAA20	NS	8.65E-05	-0.16	9.01E-04	NS
GPR152	NS	8.65E-05	-0.16	9.01E-04	NS
DICER1	NS	8.67E-05	-0.16	9.03E-04	NS
APBA1	NS	8.67E-05	-0.16	9.03E-04	NS
DNASE1L2	NS	8.70E-05	1.60E-01	9.04E-04	NS
PRB4	NS	8.70E-05	-0.16	9.04E-04	NS
GIPC3	NS	8.72E-05	1.60E-01	9.06E-04	NS
GNG3	NS	8.75E-05	-0.16	9.08E-04	NS
GP9	NS	8.75E-05	-0.16	9.08E-04	NS
MSH6	NS	8.86E-05	-0.16	9.19E-04	NS
SCG5	NS	8.91E-05	-0.16	9.23E-04	NS
ACOD1	NS	8.95E-05	-0.16	9.27E-04	NS
DSPP	NS	9.00E-05	-0.16	9.31E-04	NS
OR8H1	NS	8.99E-05	1.60E-01	9.31E-04	NS
PRSS8	NS	9.01E-05	-0.16	9.31E-04	NS
MAP1LC3C	NS	9.01E-05	1.60E-01	9.31E-04	NS
PLEKHG4	NS	9.03E-05	-0.16	9.33E-04	NS

UCP3	NS	9.05E-05	-0.16	9.34E-04	NS
PGK2	NS	9.08E-05	-0.16	9.36E-04	NS
LSM11	NS	9.09E-05	-0.16	9.37E-04	NS
WDR36	NS	9.11E-05	-0.16	9.38E-04	NS
TLCD3B	NS	9.11E-05	1.60E-01	9.38E-04	NS
MTERF1	NS	9.14E-05	-0.16	9.40E-04	NS
OR8B8	NS	9.14E-05	-0.16	9.40E-04	NS
FBXO45	NS	9.18E-05	-0.16	9.44E-04	NS
OR4F21	NS	9.19E-05	-0.16	9.44E-04	NS
SRGAP2	NS	9.24E-05	-0.16	9.48E-04	NS
SNX19	NS	9.25E-05	-0.16	9.49E-04	NS
TPRA1	NS	9.25E-05	-0.16	9.49E-04	NS
DERL3	NS	9.26E-05	-0.16	9.49E-04	NS
NEXMIF	NS	9.27E-05	-0.16	9.50E-04	NS
BTN2A1	NS	9.28E-05	-0.16	9.50E-04	NS
CNTN6	NS	9.35E-05	-0.159	9.57E-04	NS
USP26	NS	9.37E-05	-0.159	9.58E-04	NS
TSPO2	NS	9.37E-05	-0.159	9.58E-04	NS
OR8B12	NS	9.39E-05	-0.159	9.60E-04	NS
OR5M1	NS	9.44E-05	-0.159	9.64E-04	NS
GREP1	NS	9.44E-05	1.59E-01	9.64E-04	NS
IQCK	NS	9.48E-05	-0.159	9.67E-04	NS
EFNB3	NS	9.50E-05	1.59E-01	9.68E-04	NS
CBX8	NS	9.50E-05	1.59E-01	9.68E-04	NS
ARSJ	NS	9.51E-05	-0.159	9.69E-04	NS
NKTR	NS	9.54E-05	-0.159	9.71E-04	NS
LRRN1	NS	9.54E-05	-0.159	9.71E-04	NS
GAS2L2	NS	9.56E-05	-0.159	9.73E-04	NS
PSMA4	NS	9.62E-05	-0.159	9.77E-04	NS
FUT1	NS	9.63E-05	1.59E-01	9.77E-04	NS
ATG9B	NS	9.62E-05	1.59E-01	9.77E-04	NS
PIGBOS1	NS	9.63E-05	-0.159	9.77E-04	NS
STMND1	NS	9.63E-05	1.59E-01	9.77E-04	NS
NUDT12	NS	9.66E-05	-0.159	9.79E-04	NS
RTKN2	NS	9.67E-05	-0.159	9.79E-04	NS
RLBP1	NS	9.78E-05	-0.159	9.91E-04	NS
ERG28	NS	9.80E-05	-0.159	9.92E-04	NS

CDH20	NS	9.85E-05	-0.159	9.97E-04	NS
PTGDR	NS	9.91E-05	-0.159	1.00E-03	NS
PRKACG	NS	9.91E-05	1.59E-01	1.00E-03	NS
LHX2	NS	9.94E-05	1.59E-01	1.00E-03	NS
RGS9	NS	9.94E-05	-0.159	1.00E-03	NS
MUCL3	NS	9.95E-05	-0.159	1.00E-03	NS
NKX1-1	NS	1.00E-04	1.59E-01	1.01E-03	NS
SLC26A7	NS	1.00E-04	-0.159	1.01E-03	NS
ASB5	NS	1.01E-04	-0.159	1.01E-03	NS
RRS1	NS	1.00E-04	-0.159	1.01E-03	NS
PLPP4	NS	1.00E-04	1.59E-01	1.01E-03	NS
HES5	NS	1.01E-04	1.59E-01	1.01E-03	NS
OTOP3	NS	1.01E-04	1.59E-01	1.02E-03	NS
CEP152	NS	1.01E-04	-0.159	1.02E-03	NS
TAOK2	NS	1.01E-04	-0.159	1.02E-03	NS
KCTD4	NS	1.01E-04	-0.159	1.02E-03	NS
MAP7D3	NS	1.02E-04	-0.159	1.02E-03	NS
SAT1	NS	1.02E-04	-0.159	1.02E-03	NS
GABRG2	NS	1.02E-04	-0.159	1.02E-03	NS
ZNHIT2	NS	1.02E-04	-0.159	1.02E-03	NS
FAM246A	NS	1.02E-04	1.59E-01	1.02E-03	NS
GLMN	NS	1.02E-04	-0.159	1.02E-03	NS
SNORC	NS	1.02E-04	-0.159	1.02E-03	NS
TPX2	NS	1.03E-04	-0.159	1.03E-03	NS
UBAC2	NS	1.03E-04	-0.159	1.03E-03	NS
STK32C	NS	1.03E-04	-0.159	1.03E-03	NS
SLAMF9	NS	1.03E-04	-0.159	1.03E-03	NS
ZFHX2	NS	1.03E-04	-0.159	1.03E-03	NS
AKR1C3	NS	1.03E-04	-0.159	1.03E-03	NS
VARS1	NS	1.04E-04	-0.158	1.04E-03	NS
ZNF628	NS	1.05E-04	1.58E-01	1.04E-03	NS
CFHR2	NS	1.05E-04	-0.158	1.04E-03	NS
MOSPD1	NS	1.06E-04	-0.158	1.05E-03	NS
EGR4	NS	1.05E-04	1.58E-01	1.05E-03	NS
UBASH3B	NS	1.05E-04	-0.158	1.05E-03	NS
FGG	NS	1.06E-04	-0.158	1.05E-03	NS
SLC39A10	NS	1.06E-04	-0.158	1.05E-03	NS

AJM1	NS	1.06E-04	-0.158	1.05E-03	NS
MAIP1	NS	1.06E-04	-0.158	1.05E-03	NS
UQCRHL	NS	1.06E-04	-0.158	1.05E-03	NS
SARDH	NS	1.06E-04	-0.158	1.05E-03	NS
STYK1	NS	1.06E-04	-0.158	1.05E-03	NS
MT-ND4	NS	1.06E-04	1.58E-01	1.05E-03	NS
SNU13	NS	1.06E-04	-0.158	1.05E-03	NS
GPAM	NS	1.07E-04	-0.158	1.05E-03	NS
GPT	NS	1.07E-04	1.58E-01	1.05E-03	NS
SPEF1	NS	1.07E-04	1.58E-01	1.05E-03	NS
CCNE2	NS	1.07E-04	-0.158	1.05E-03	NS
GOLGA8K	NS	1.07E-04	-0.158	1.05E-03	NS
KIAA1586	NS	1.07E-04	-0.158	1.06E-03	NS
GALNTL6	NS	1.08E-04	-0.158	1.06E-03	NS
IL36G	NS	1.08E-04	-0.158	1.06E-03	NS
HRNR	NS	1.08E-04	-0.158	1.06E-03	NS
PM20D1	NS	1.08E-04	-0.158	1.06E-03	NS
LRRC51	NS	1.08E-04	-0.158	1.06E-03	NS
DDIT3	NS	1.09E-04	1.58E-01	1.07E-03	NS
CMTM5	NS	1.09E-04	-0.158	1.07E-03	NS
TMTC4	NS	1.09E-04	-0.158	1.07E-03	NS
ACAD11	NS	1.09E-04	-0.158	1.07E-03	NS
DBNDD1	NS	1.11E-04	1.58E-01	1.09E-03	NS
FAM107A	NS	1.11E-04	-0.158	1.09E-03	NS
CFC1B	NS	1.12E-04	1.58E-01	1.09E-03	NS
GNAT3	NS	1.12E-04	-0.158	1.09E-03	NS
SOWAHC	NS	1.12E-04	-0.158	1.09E-03	NS
ZNF507	NS	1.12E-04	-0.158	1.09E-03	NS
KLHL20	NS	1.12E-04	-0.158	1.10E-03	NS
RBM44	NS	1.12E-04	-0.158	1.10E-03	NS
NKX6-2	NS	1.13E-04	-0.158	1.10E-03	NS
LHFPL4	NS	1.13E-04	-0.158	1.10E-03	NS
NKX2-4	NS	1.13E-04	1.58E-01	1.10E-03	NS
ANG	NS	1.13E-04	-0.158	1.10E-03	NS
SGIP1	NS	1.13E-04	-0.158	1.10E-03	NS
ERICH6B	NS	1.13E-04	-0.158	1.10E-03	NS
DSC3	NS	1.14E-04	-0.158	1.11E-03	NS

CMTM7	NS	1.14E-04	-0.158	1.11E-03	NS
OMA1	NS	1.15E-04	-0.157	1.11E-03	NS
PTCRA	NS	1.15E-04	-0.157	1.11E-03	NS
ATPSCKMT	NS	1.15E-04	-0.157	1.11E-03	NS
OMP	NS	1.15E-04	1.57E-01	1.11E-03	NS
PCBP1	NS	1.15E-04	1.57E-01	1.11E-03	NS
KCNK15	NS	1.15E-04	1.57E-01	1.12E-03	NS
CLEC4F	NS	1.15E-04	-0.157	1.12E-03	NS
TRIM77	NS	1.16E-04	-0.157	1.12E-03	NS
MLH1	NS	1.16E-04	-0.157	1.12E-03	NS
PTPRJ	NS	1.16E-04	-0.157	1.12E-03	NS
DHX36	NS	1.16E-04	-0.157	1.12E-03	NS
ADAM20	NS	1.16E-04	-0.157	1.12E-03	NS
ZNF721	NS	1.16E-04	-0.157	1.12E-03	NS
KIF25	NS	1.17E-04	1.57E-01	1.13E-03	NS
SURF2	NS	1.17E-04	-0.157	1.13E-03	NS
EHBP1L1	NS	1.17E-04	1.57E-01	1.13E-03	NS
ODF3L2	NS	1.17E-04	1.57E-01	1.13E-03	NS
DMXL2	NS	1.18E-04	-0.157	1.13E-03	NS
RETREG3	NS	1.18E-04	-0.157	1.14E-03	NS
SNX31	NS	1.18E-04	-0.157	1.14E-03	NS
NDUFA7	NS	1.19E-04	-0.157	1.14E-03	NS
DLX4	NS	1.19E-04	1.57E-01	1.14E-03	NS
FBXO39	NS	1.19E-04	-0.157	1.14E-03	NS
UBA1	NS	1.19E-04	-0.157	1.14E-03	NS
GMNC	NS	1.19E-04	-0.157	1.14E-03	NS
LIM2	NS	1.19E-04	1.57E-01	1.14E-03	NS
AFG1L	NS	1.20E-04	-0.157	1.15E-03	NS
FYCO1	NS	1.20E-04	-0.157	1.15E-03	NS
TEC	NS	1.20E-04	-0.157	1.15E-03	NS
SEZ6L	NS	1.20E-04	-0.157	1.15E-03	NS
CEBPB	NS	1.21E-04	1.57E-01	1.16E-03	NS
BRD2	NS	1.21E-04	1.57E-01	1.16E-03	NS
CABP4	NS	1.21E-04	-0.157	1.16E-03	NS
BAAT	NS	1.22E-04	-0.157	1.16E-03	NS
PATJ	NS	1.22E-04	-0.157	1.16E-03	NS
EIF5A2	NS	1.22E-04	-0.157	1.16E-03	NS

VWA1	NS	1.22E-04	1.57E-01	1.17E-03	NS
COMTD1	NS	1.23E-04	-0.157	1.17E-03	NS
KCNG3	NS	1.23E-04	1.57E-01	1.17E-03	NS
RNASE12	NS	1.23E-04	1.57E-01	1.17E-03	NS
DRD1	NS	1.24E-04	1.57E-01	1.18E-03	NS
TAS2R40	NS	1.24E-04	-0.157	1.18E-03	NS
DNLZ	NS	1.24E-04	-0.157	1.18E-03	NS
BST2	NS	1.24E-04	-0.157	1.18E-03	NS
TECPR2	NS	1.24E-04	-0.157	1.18E-03	NS
KDR	NS	1.24E-04	-0.157	1.18E-03	NS
AMN	NS	1.24E-04	-0.157	1.18E-03	NS
OR4F29	NS	1.24E-04	-0.157	1.18E-03	NS
GSKIP	NS	1.25E-04	-0.157	1.18E-03	NS
SMCO3	NS	1.25E-04	-0.157	1.18E-03	NS
STK35	NS	1.25E-04	-0.157	1.18E-03	NS
PRSS56	NS	1.25E-04	1.57E-01	1.18E-03	NS
CHST7	NS	1.25E-04	-0.157	1.18E-03	NS
NOXRED1	NS	1.25E-04	-0.157	1.18E-03	NS
MYCBP2	NS	1.25E-04	-0.157	1.19E-03	NS
HHLA2	NS	1.27E-04	-0.156	1.20E-03	NS
GABRA1	NS	1.28E-04	-0.156	1.20E-03	NS
ZNF416	NS	1.28E-04	-0.156	1.21E-03	NS
MRPL9	NS	1.28E-04	-0.156	1.21E-03	NS
GNGT2	NS	1.29E-04	-0.156	1.21E-03	NS
POMC	NS	1.29E-04	-0.156	1.22E-03	NS
GRIA2	NS	1.29E-04	-0.156	1.22E-03	NS
MMP3	NS	1.29E-04	-0.156	1.22E-03	NS
PGLYRP4	NS	1.30E-04	-0.156	1.22E-03	NS
TMEM200A	NS	1.30E-04	-0.156	1.22E-03	NS
EPHA3	NS	1.30E-04	-0.156	1.22E-03	NS
TENT2	NS	1.31E-04	1.56E-01	1.23E-03	NS
PDZK1IP1	NS	1.31E-04	-0.156	1.23E-03	NS
FAM120A	NS	1.32E-04	-0.156	1.24E-03	NS
SOHLH2	NS	1.32E-04	-0.156	1.24E-03	NS
STRIP2	NS	1.32E-04	-0.156	1.24E-03	NS
SLC22A9	NS	1.32E-04	-0.156	1.24E-03	NS
TSACC	NS	1.32E-04	-0.156	1.24E-03	NS

PRSS38	NS	1.32E-04	1.56E-01	1.24E-03	NS
C1QTNF3-AMACR	NS	1.32E-04	-0.156	1.24E-03	NS
ZNF284	NS	1.32E-04	-0.156	1.24E-03	NS
IL10	NS	1.32E-04	-0.156	1.24E-03	NS
XAGE1B	NS	1.32E-04	-0.156	1.24E-03	NS
HNRNPCL2	NS	1.33E-04	1.56E-01	1.24E-03	NS
OLFM4	NS	1.33E-04	-0.156	1.24E-03	NS
EGR3	NS	1.33E-04	-0.156	1.24E-03	NS
CCDC192	NS	1.33E-04	-0.156	1.24E-03	NS
ADRA2A	NS	1.34E-04	1.56E-01	1.25E-03	NS
MEX3A	NS	1.34E-04	1.56E-01	1.25E-03	NS
ABCA4	NS	1.34E-04	-0.156	1.25E-03	NS
ZNF680	NS	1.35E-04	-0.156	1.25E-03	NS
THBS1	NS	1.35E-04	-0.156	1.26E-03	NS
CD1C	NS	1.35E-04	-0.156	1.26E-03	NS
NEDD1	NS	1.36E-04	-0.156	1.26E-03	NS
MCC	NS	1.36E-04	-0.156	1.26E-03	NS
ZNF24	NS	1.36E-04	-0.156	1.26E-03	NS
FAM221B	NS	1.36E-04	-0.156	1.26E-03	NS
TOMM6	NS	1.37E-04	-0.156	1.27E-03	NS
USP29	NS	1.38E-04	1.56E-01	1.28E-03	NS
SEMA3A	NS	1.39E-04	-0.156	1.28E-03	NS
CATSPERG	NS	1.39E-04	1.56E-01	1.28E-03	NS
CLDN8	NS	1.39E-04	-0.156	1.28E-03	NS
PRM1	NS	1.39E-04	1.56E-01	1.28E-03	NS
TCEA3	NS	1.39E-04	-0.156	1.28E-03	NS
CD248	NS	1.39E-04	1.56E-01	1.29E-03	NS
RNGTT	NS	1.39E-04	-0.156	1.29E-03	NS
BIVM-ERCC5	NS	1.39E-04	-0.156	1.29E-03	NS
RFC3	NS	1.39E-04	-0.156	1.29E-03	NS
RAB5IF	NS	1.40E-04	1.56E-01	1.29E-03	NS
SLC25A31	NS	1.40E-04	-0.155	1.29E-03	NS
AQP7	NS	1.41E-04	1.55E-01	1.30E-03	NS
ERP27	NS	1.41E-04	-0.155	1.30E-03	NS
ZCCHC9	NS	1.41E-04	-0.155	1.30E-03	NS
RAB27B	NS	1.42E-04	-0.155	1.31E-03	NS
TMEM18	NS	1.42E-04	-0.155	1.31E-03	NS

H1-8	NS	1.42E-04	1.55E-01	1.31E-03	NS
PTPRU	NS	1.42E-04	-0.155	1.31E-03	NS
KCNH7	NS	1.42E-04	-0.155	1.31E-03	NS
SMIM45	NS	1.42E-04	1.55E-01	1.31E-03	NS
ERICH2	NS	1.43E-04	-0.155	1.31E-03	NS
MRPS5	NS	1.43E-04	1.55E-01	1.31E-03	NS
TEX13D	NS	1.43E-04	-0.155	1.32E-03	NS
SMIM30	NS	1.44E-04	-0.155	1.32E-03	NS
SUN2	NS	1.45E-04	-0.155	1.33E-03	NS
SLFNL1	NS	1.45E-04	-0.155	1.33E-03	NS
ZNF77	NS	1.45E-04	-0.155	1.33E-03	NS
H2BC6	NS	1.45E-04	-0.155	1.33E-03	NS
DAND5	NS	1.45E-04	1.55E-01	1.33E-03	NS
UBE2Q1	NS	1.45E-04	-0.155	1.33E-03	NS
PLOD2	NS	1.45E-04	-0.155	1.33E-03	NS
INO80B	NS	1.46E-04	-0.155	1.33E-03	NS
CLDN24	NS	1.45E-04	-0.155	1.33E-03	NS
WDR5	NS	1.45E-04	1.55E-01	1.33E-03	NS
SMIM21	NS	1.46E-04	-0.155	1.33E-03	NS
ZNF662	NS	1.46E-04	1.55E-01	1.33E-03	NS
CHMP6	NS	1.46E-04	-0.155	1.33E-03	NS
TRARG1	NS	1.46E-04	1.55E-01	1.33E-03	NS
H3C12	NS	1.47E-04	-0.155	1.33E-03	NS
ATP1B1	NS	1.47E-04	-0.155	1.34E-03	NS
IFIT5	NS	1.47E-04	-0.155	1.34E-03	NS
NAALAD2	NS	1.48E-04	-0.155	1.34E-03	NS
BEX3	NS	1.48E-04	-0.155	1.35E-03	NS
CRYBG3	NS	1.48E-04	-0.155	1.35E-03	NS
SLC2A5	NS	1.49E-04	-0.155	1.35E-03	NS
TMEM31	NS	1.49E-04	-0.155	1.35E-03	NS
LCE3B	NS	1.49E-04	1.55E-01	1.35E-03	NS
LRRC40	NS	1.51E-04	-0.155	1.37E-03	NS
NFXL1	NS	1.52E-04	-0.155	1.38E-03	NS
EPB41L5	NS	1.52E-04	-0.155	1.38E-03	NS
RPP40	NS	1.52E-04	-0.155	1.38E-03	NS
TENT5C	NS	1.53E-04	-0.155	1.38E-03	NS
ACOT13	NS	1.53E-04	-0.155	1.38E-03	NS

PPM1F	NS	1.54E-04	-0.155	1.39E-03	NS
TOPAZ1	NS	1.54E-04	-0.155	1.39E-03	NS
CCR7	NS	1.54E-04	-0.155	1.39E-03	NS
KIAA0930	NS	1.54E-04	-0.155	1.39E-03	NS
KLHL5	NS	1.56E-04	-0.154	1.40E-03	NS
PMF1	NS	1.56E-04	-0.154	1.40E-03	NS
CMC4	NS	1.56E-04	-0.154	1.41E-03	NS
GPR87	NS	1.57E-04	-0.154	1.41E-03	NS
USP50	NS	1.57E-04	-0.154	1.41E-03	NS
CT55	NS	1.58E-04	-0.154	1.42E-03	NS
TAF2	NS	1.58E-04	-0.154	1.42E-03	NS
OPA1	NS	1.58E-04	-0.154	1.42E-03	NS
NUS1	NS	1.58E-04	-0.154	1.42E-03	NS
PEX3	NS	1.59E-04	-0.154	1.43E-03	NS
POGLUT1	NS	1.59E-04	-0.154	1.43E-03	NS
P3R3URF-PIK3R3	NS	1.59E-04	-0.154	1.43E-03	NS
MFAP3	NS	1.59E-04	-0.154	1.43E-03	NS
VPS37B	NS	1.59E-04	1.54E-01	1.43E-03	NS
XIRP2	NS	1.59E-04	-0.154	1.43E-03	NS
OR6C68	NS	1.60E-04	-0.154	1.43E-03	NS
CLDND2	NS	1.60E-04	1.54E-01	1.44E-03	NS
SGMS2	NS	1.61E-04	-0.154	1.44E-03	NS
RHOH	NS	1.61E-04	-0.154	1.44E-03	NS
POU2F3	NS	1.62E-04	-0.154	1.45E-03	NS
COX8C	NS	1.63E-04	-0.154	1.46E-03	NS
ABHD10	NS	1.63E-04	-0.154	1.46E-03	NS
FCRL4	NS	1.64E-04	-0.154	1.46E-03	NS
HSPB2-C11orf52	NS	1.64E-04	1.54E-01	1.46E-03	NS
PUSL1	NS	1.64E-04	-0.154	1.47E-03	NS
TRIP6	NS	1.64E-04	-0.154	1.47E-03	NS
SPC24	NS	1.65E-04	-0.154	1.47E-03	NS
N6AMT1	NS	1.67E-04	-0.154	1.49E-03	NS
CHSY1	NS	1.67E-04	-0.154	1.49E-03	NS
DOLPP1	NS	1.68E-04	-0.154	1.49E-03	NS
CCN1	NS	1.68E-04	-0.154	1.50E-03	NS
ZNF746	NS	1.68E-04	1.54E-01	1.50E-03	NS
GCLC	NS	1.69E-04	-0.154	1.50E-03	NS

HSPB6	NS	1.69E-04	1.54E-01	1.50E-03	NS
RAB9B	NS	1.69E-04	-0.154	1.50E-03	NS
RBFOX1	NS	1.69E-04	1.54E-01	1.50E-03	NS
URGCP	NS	1.69E-04	-0.154	1.50E-03	NS
PGLYRP2	NS	1.69E-04	-0.154	1.50E-03	NS
TMEM145	NS	1.69E-04	1.54E-01	1.50E-03	NS
CTNNBIP1	NS	1.69E-04	-0.154	1.50E-03	NS
PRKAA1	NS	1.70E-04	-0.154	1.50E-03	NS
CAPRIN1	NS	1.70E-04	-0.154	1.50E-03	NS
C6orf226	NS	1.70E-04	-0.154	1.50E-03	NS
WBP2NL	NS	1.70E-04	-0.154	1.50E-03	NS
CHODL	NS	1.70E-04	-0.154	1.51E-03	NS
PROSER2	NS	1.71E-04	1.54E-01	1.51E-03	NS
ZNF517	NS	1.71E-04	-0.153	1.51E-03	NS
EPHB1	NS	1.72E-04	-0.153	1.52E-03	NS
NRP1	NS	1.73E-04	-0.153	1.52E-03	NS
ACVR2B	NS	1.73E-04	-0.153	1.53E-03	NS
LRRC26	NS	1.73E-04	1.53E-01	1.53E-03	NS
GOLGA2	NS	1.74E-04	-0.153	1.53E-03	NS
EIF2B3	NS	1.75E-04	-0.153	1.54E-03	NS
AVL9	NS	1.76E-04	-0.153	1.54E-03	NS
RFX6	NS	1.76E-04	-0.153	1.54E-03	NS
PTGS2	NS	1.76E-04	-0.153	1.55E-03	NS
KLHL13	NS	1.78E-04	-0.153	1.56E-03	NS
GTF2A1L	NS	1.78E-04	-0.153	1.56E-03	NS
NUP210L	NS	1.79E-04	-0.153	1.57E-03	NS
RTN2	NS	1.79E-04	-0.153	1.57E-03	NS
MOCS2	NS	1.79E-04	-0.153	1.57E-03	NS
RSPH1	NS	1.80E-04	-0.153	1.57E-03	NS
SOX18	NS	1.80E-04	1.53E-01	1.57E-03	NS
TINAG	NS	1.80E-04	-0.153	1.58E-03	NS
TRIM51	NS	1.81E-04	-0.153	1.58E-03	NS
TRIM44	NS	1.81E-04	-0.153	1.58E-03	NS
TP53BP2	NS	1.82E-04	1.53E-01	1.59E-03	NS
GHRL	NS	1.83E-04	-0.153	1.59E-03	NS
CNTN3	NS	1.83E-04	-0.153	1.60E-03	NS
GPR27	NS	1.83E-04	-0.153	1.60E-03	NS

PCDHB4	NS	1.83E-04	1.53E-01	1.60E-03	NS
PLBD2	NS	1.83E-04	1.53E-01	1.60E-03	NS
ZNF879	NS	1.83E-04	-0.153	1.60E-03	NS
TYRO3	NS	1.84E-04	1.53E-01	1.60E-03	NS
TTN	NS	1.84E-04	-0.153	1.60E-03	NS
HUS1B	NS	1.84E-04	-0.153	1.60E-03	NS
SNX25	NS	1.84E-04	-0.153	1.60E-03	NS
TMEM114	NS	1.84E-04	-0.153	1.60E-03	NS
SPNS1	NS	1.84E-04	1.53E-01	1.60E-03	NS
CA13	NS	1.85E-04	-0.153	1.61E-03	NS
CSTF2T	NS	1.85E-04	-0.153	1.61E-03	NS
CPED1	NS	1.85E-04	-0.153	1.61E-03	NS
PGR	NS	1.86E-04	-0.153	1.61E-03	NS
SCAF4	NS	1.87E-04	1.53E-01	1.62E-03	NS
SMC6	NS	1.87E-04	-0.153	1.62E-03	NS
ZNF267	NS	1.87E-04	-0.153	1.62E-03	NS
ARFGEF1	NS	1.87E-04	-0.153	1.62E-03	NS
RGS10	NS	1.87E-04	-0.153	1.62E-03	NS
AP1S3	NS	1.87E-04	-0.153	1.62E-03	NS
KLK12	NS	1.87E-04	1.53E-01	1.62E-03	NS
RHOU	NS	1.88E-04	-0.153	1.63E-03	NS
KRTAP16-1	NS	1.88E-04	-0.153	1.63E-03	NS
MAGEB3	NS	1.89E-04	-0.152	1.64E-03	NS
RIT1	NS	1.90E-04	1.52E-01	1.64E-03	NS
COX6A2	NS	1.90E-04	1.52E-01	1.64E-03	NS
ZNF785	NS	1.91E-04	-0.152	1.64E-03	NS
PPIAL4A	NS	1.91E-04	1.52E-01	1.65E-03	NS
SOX21	NS	1.91E-04	1.52E-01	1.65E-03	NS
GIMAP6	NS	1.91E-04	-0.152	1.65E-03	NS
CCL21	NS	1.92E-04	1.52E-01	1.65E-03	NS
ARL15	NS	1.92E-04	-0.152	1.65E-03	NS
PREX1	NS	1.93E-04	-0.152	1.66E-03	NS
CRYGB	NS	1.93E-04	1.52E-01	1.66E-03	NS
WNT9A	NS	1.94E-04	1.52E-01	1.66E-03	NS
GPR101	NS	1.95E-04	-0.152	1.67E-03	NS
GFPT2	NS	1.95E-04	1.52E-01	1.67E-03	NS
ASB7	NS	1.95E-04	-0.152	1.67E-03	NS

OR6N2	NS	1.95E-04	-0.152	1.67E-03	NS
CDK5R1	NS	1.95E-04	1.52E-01	1.67E-03	NS
EPB41L2	NS	1.96E-04	-0.152	1.68E-03	NS
LRRC74A	NS	1.96E-04	-0.152	1.68E-03	NS
FTO	NS	1.96E-04	-0.152	1.68E-03	NS
MYO5A	NS	1.96E-04	-0.152	1.68E-03	NS
DMKN	NS	1.97E-04	1.52E-01	1.69E-03	NS
PSMB1	NS	1.98E-04	-0.152	1.70E-03	NS
C2orf78	NS	1.99E-04	-0.152	1.70E-03	NS
ALPI	NS	1.99E-04	1.52E-01	1.70E-03	NS
CUEDC2	NS	2.00E-04	-0.152	1.70E-03	NS
RGS21	NS	2.00E-04	-0.152	1.70E-03	NS
MMD2	NS	2.01E-04	1.52E-01	1.71E-03	NS
GNA14	NS	2.01E-04	-0.152	1.71E-03	NS
DUSP8	NS	2.01E-04	1.52E-01	1.72E-03	NS
SAGE1	NS	2.02E-04	-0.152	1.72E-03	NS
BTN3A1	NS	2.02E-04	-0.152	1.72E-03	NS
ZFP3	NS	2.03E-04	-0.152	1.73E-03	NS
OAZ3	NS	2.04E-04	-0.152	1.74E-03	NS
GJD4	NS	2.04E-04	-0.152	1.74E-03	NS
LDAF1	NS	2.04E-04	-0.152	1.74E-03	NS
SLC41A3	NS	2.05E-04	-0.152	1.74E-03	NS
UNC50	NS	2.05E-04	-0.152	1.74E-03	NS
DCST1	NS	2.05E-04	1.52E-01	1.74E-03	NS
TXNDC5	NS	2.05E-04	-0.152	1.74E-03	NS
SEL1L2	NS	2.06E-04	-0.152	1.75E-03	NS
ZNF502	NS	2.07E-04	-0.152	1.75E-03	NS
CASP9	NS	2.07E-04	-0.152	1.75E-03	NS
PCDHGB3	NS	2.09E-04	-0.151	1.77E-03	NS
ACSM2B	NS	2.10E-04	-0.151	1.77E-03	NS
PEBP1	NS	2.10E-04	-0.151	1.78E-03	NS
RECK	NS	2.10E-04	-0.151	1.78E-03	NS
ZC4H2	NS	2.11E-04	-0.151	1.78E-03	NS
SERPINE3	NS	2.11E-04	-0.151	1.78E-03	NS
USP18	NS	2.11E-04	-0.151	1.78E-03	NS
ZCCHC10	NS	2.12E-04	-0.151	1.79E-03	NS
PLEKHF2	NS	2.12E-04	-0.151	1.79E-03	NS

CD99L2	NS	2.12E-04	-0.151	1.79E-03	NS
SLC16A8	NS	2.12E-04	1.51E-01	1.79E-03	NS
CRHR1	NS	2.12E-04	1.51E-01	1.79E-03	NS
NHLRC3	NS	2.12E-04	-0.151	1.79E-03	NS
NUP58	NS	2.13E-04	-0.151	1.79E-03	NS
PRPF6	NS	2.13E-04	-0.151	1.80E-03	NS
ZNF529	NS	2.15E-04	-0.151	1.81E-03	NS
CCNI2	NS	2.15E-04	-0.151	1.81E-03	NS
OR5K1	NS	2.17E-04	-0.151	1.82E-03	NS
TTC21B	NS	2.17E-04	-0.151	1.82E-03	NS
MTRNR2L12	NS	2.18E-04	1.51E-01	1.83E-03	NS
FBXO9	NS	2.18E-04	-0.151	1.83E-03	NS
MEIKIN	NS	2.18E-04	-0.151	1.83E-03	NS
GALNT6	NS	2.19E-04	-0.151	1.84E-03	NS
DMWD	NS	2.19E-04	1.51E-01	1.84E-03	NS
TBX4	NS	2.21E-04	1.51E-01	1.85E-03	NS
SHC3	NS	2.22E-04	-0.151	1.86E-03	NS
TNS1	NS	2.22E-04	1.51E-01	1.86E-03	NS
NEU3	NS	2.22E-04	-0.151	1.86E-03	NS
ACTRT1	NS	2.23E-04	-0.151	1.86E-03	NS
NELL2	NS	2.23E-04	-0.151	1.87E-03	NS
DPH3	NS	2.24E-04	-0.151	1.88E-03	NS
TRIQK	NS	2.24E-04	-0.151	1.88E-03	NS
SOCS2	NS	2.25E-04	-0.151	1.88E-03	NS
HSPB11	NS	2.25E-04	-0.151	1.88E-03	NS
KRT28	NS	2.25E-04	1.51E-01	1.88E-03	NS
TVP23C-CDRT4	NS	2.26E-04	-0.151	1.88E-03	NS
NAA10	NS	2.26E-04	-0.151	1.88E-03	NS
PPFIBP1	NS	2.26E-04	-0.151	1.88E-03	NS
NINL	NS	2.26E-04	-0.151	1.89E-03	NS
FLYWCH2	NS	2.27E-04	-0.151	1.89E-03	NS
PRSS22	NS	2.27E-04	1.51E-01	1.89E-03	NS
MESP1	NS	2.28E-04	-0.151	1.89E-03	NS
DDX19A	NS	2.27E-04	1.51E-01	1.89E-03	NS
PRF1	NS	2.28E-04	-0.151	1.89E-03	NS
LGALS14	NS	2.29E-04	-0.151	1.90E-03	NS
DDA1	NS	2.28E-04	1.51E-01	1.90E-03	NS

KRTAP9-6	NS	2.28E-04	1.51E-01	1.90E-03	NS
PPIAL4E	NS	2.29E-04	1.51E-01	1.90E-03	NS
CEP63	NS	2.29E-04	-0.151	1.90E-03	NS
NRN1L	NS	2.29E-04	-0.151	1.90E-03	NS
PFKFB4	NS	2.29E-04	-0.151	1.90E-03	NS
FAM163A	NS	2.30E-04	1.50E-01	1.90E-03	NS
TMA16	NS	2.30E-04	-0.15	1.90E-03	NS
ZPLD1	NS	2.31E-04	-0.15	1.91E-03	NS
NEB	NS	2.31E-04	-0.15	1.91E-03	NS
GLCCI1	NS	2.32E-04	-0.15	1.92E-03	NS
MOSMO	NS	2.32E-04	-0.15	1.92E-03	NS
CCDC190	NS	2.32E-04	-0.15	1.92E-03	NS
FANCD2OS	NS	2.33E-04	-0.15	1.92E-03	NS
C16orf91	NS	2.34E-04	-0.15	1.93E-03	NS
ZNF548	NS	2.34E-04	-0.15	1.93E-03	NS
G0S2	NS	2.35E-04	-0.15	1.94E-03	NS
MAP3K7	NS	2.36E-04	-0.15	1.95E-03	NS
MFSD4A	NS	2.37E-04	-0.15	1.95E-03	NS
TAS2R50	NS	2.37E-04	-0.15	1.95E-03	NS
OR5D13	NS	2.36E-04	-0.15	1.95E-03	NS
ATP2C1	NS	2.37E-04	-0.15	1.95E-03	NS
CASP3	NS	2.37E-04	-0.15	1.95E-03	NS
MMUT	NS	2.38E-04	-0.15	1.96E-03	NS
C4orf46	NS	2.39E-04	-0.15	1.96E-03	NS
ZNF594	NS	2.41E-04	-0.15	1.98E-03	NS
ZNF804A	NS	2.41E-04	1.50E-01	1.98E-03	NS
IFIT3	NS	2.42E-04	-0.15	1.98E-03	NS
DENND1B	NS	2.44E-04	-0.15	2.00E-03	NS
CLTRN	NS	2.44E-04	-0.15	2.00E-03	NS
RAP1B	NS	2.45E-04	-0.15	2.01E-03	NS
PLA2R1	NS	2.45E-04	-0.15	2.01E-03	NS
AGR3	NS	2.45E-04	1.50E-01	2.01E-03	NS
UBL4B	NS	2.46E-04	1.50E-01	2.01E-03	NS
PCDHGA2	NS	2.46E-04	-0.15	2.02E-03	NS
RAB11FIP1	NS	2.47E-04	1.50E-01	2.02E-03	NS
MAPK9	NS	2.47E-04	-0.15	2.02E-03	NS
FAM133A	NS	2.48E-04	-0.15	2.02E-03	NS

SPANXB1	NS	2.48E-04	-0.15	2.03E-03	NS
UCHL5	NS	2.51E-04	-0.15	2.05E-03	NS
SNRPF	NS	2.51E-04	-0.15	2.05E-03	NS
HEPACAM	NS	2.51E-04	-0.15	2.05E-03	NS
ADAMTSL2	NS	2.51E-04	1.50E-01	2.05E-03	NS
FSD1L	NS	2.52E-04	-0.15	2.05E-03	NS
ZNF487	NS	2.52E-04	1.50E-01	2.05E-03	NS
MEIS1	NS	2.52E-04	-0.15	2.06E-03	NS
EBI3	NS	2.53E-04	-0.149	2.06E-03	NS
RAP2C	NS	2.53E-04	-0.149	2.06E-03	NS
POMGNT2	NS	2.54E-04	-0.149	2.07E-03	NS
SLC22A10	NS	2.55E-04	-0.149	2.07E-03	NS
PTCH2	NS	2.55E-04	-0.149	2.07E-03	NS
DESI1	NS	2.56E-04	-0.149	2.08E-03	NS
SLC24A1	NS	2.56E-04	-0.149	2.08E-03	NS
NXNL1	NS	2.60E-04	1.49E-01	2.11E-03	NS
USP34	NS	2.60E-04	-0.149	2.11E-03	NS
ZNF155	NS	2.60E-04	-0.149	2.11E-03	NS
PBX3	NS	2.60E-04	1.49E-01	2.11E-03	NS
PM20D2	NS	2.60E-04	-0.149	2.11E-03	NS
ENDOD1	NS	2.60E-04	-0.149	2.11E-03	NS
PRPS2	NS	2.61E-04	-0.149	2.11E-03	NS
F9	NS	2.62E-04	-0.149	2.12E-03	NS
MAB21L3	NS	2.62E-04	-0.149	2.12E-03	NS
ZFAT	NS	2.64E-04	-0.149	2.13E-03	NS
ACTL6A	NS	2.64E-04	-0.149	2.13E-03	NS
FAM170B	NS	2.64E-04	-0.149	2.14E-03	NS
SCX	NS	2.65E-04	1.49E-01	2.14E-03	NS
ETHE1	NS	2.65E-04	1.49E-01	2.14E-03	NS
CCDC82	NS	2.66E-04	-0.149	2.14E-03	NS
TLCD4-RWDD3	NS	2.66E-04	-0.149	2.14E-03	NS
BACH1	NS	2.67E-04	-0.149	2.15E-03	NS
HELQ	NS	2.67E-04	-0.149	2.15E-03	NS
RBM3	NS	2.68E-04	-0.149	2.15E-03	NS
TCL1A	NS	2.68E-04	-0.149	2.15E-03	NS
ARL17A	NS	2.68E-04	-0.149	2.16E-03	NS
OR4D5	NS	2.68E-04	-0.149	2.16E-03	NS

UBE2G2	NS	2.69E-04	-0.149	2.17E-03	NS
OR2T5	NS	2.70E-04	-0.149	2.17E-03	NS
AKR7A3	NS	2.70E-04	-0.149	2.17E-03	NS
THBS2	NS	2.70E-04	-0.149	2.17E-03	NS
GIGYF2	NS	2.71E-04	-0.149	2.17E-03	NS
SLC9A7	NS	2.71E-04	-0.149	2.17E-03	NS
ZNF532	NS	2.71E-04	-0.149	2.17E-03	NS
AFAP1	NS	2.72E-04	-0.149	2.18E-03	NS
RBM41	NS	2.72E-04	-0.149	2.18E-03	NS
SLAIN2	NS	2.72E-04	1.49E-01	2.18E-03	NS
P4HTM	NS	2.73E-04	1.49E-01	2.18E-03	NS
PECAM1	NS	2.73E-04	-0.149	2.19E-03	NS
FAM216B	NS	2.74E-04	-0.149	2.19E-03	NS
FRG2	NS	2.74E-04	-0.149	2.19E-03	NS
PPP1R13B	NS	2.75E-04	-0.149	2.20E-03	NS
SLX1A	NS	2.75E-04	1.49E-01	2.20E-03	NS
MTMR12	NS	2.75E-04	-0.149	2.20E-03	NS
DDX28	NS	2.75E-04	-0.149	2.20E-03	NS
KLRK1	NS	2.76E-04	-0.149	2.20E-03	NS
CELA2B	NS	2.77E-04	-0.149	2.21E-03	NS
SMIM18	NS	2.77E-04	-0.149	2.21E-03	NS
THTPA	NS	2.77E-04	-0.149	2.21E-03	NS
KITLG	NS	2.77E-04	-0.149	2.21E-03	NS
IL2	NS	2.81E-04	-0.148	2.24E-03	NS
PPP4C	NS	2.81E-04	1.48E-01	2.24E-03	NS
SCAND1	NS	2.81E-04	-0.148	2.24E-03	NS
DGAT2L6	NS	2.81E-04	-0.148	2.24E-03	NS
ARHGEF17	NS	2.82E-04	-0.148	2.24E-03	NS
MAGI3	NS	2.82E-04	-0.148	2.24E-03	NS
PTGFR	NS	2.82E-04	-0.148	2.24E-03	NS
LANCL1	NS	2.83E-04	-0.148	2.25E-03	NS
YAE1	NS	2.83E-04	-0.148	2.25E-03	NS
TYRP1	NS	2.84E-04	-0.148	2.25E-03	NS
FNDC4	NS	2.84E-04	1.48E-01	2.25E-03	NS
PRSS53	NS	2.84E-04	-0.148	2.25E-03	NS
CLDN14	NS	2.85E-04	-0.148	2.26E-03	NS
HEPN1	NS	2.86E-04	-0.148	2.26E-03	NS

OR8K3	NS	2.87E-04	-0.148	2.27E-03	NS
PLAC8	NS	2.87E-04	-0.148	2.27E-03	NS
CABP7	NS	2.87E-04	-0.148	2.27E-03	NS
STAC2	NS	2.90E-04	1.48E-01	2.29E-03	NS
XIRP1	NS	2.90E-04	1.48E-01	2.29E-03	NS
ZNF672	NS	2.90E-04	-0.148	2.29E-03	NS
NHLRC2	NS	2.90E-04	-0.148	2.29E-03	NS
MORC1	NS	2.90E-04	-0.148	2.29E-03	NS
HTR2B	NS	2.90E-04	-0.148	2.29E-03	NS
OR52B2	NS	2.91E-04	-0.148	2.29E-03	NS
CLDN17	NS	2.91E-04	1.48E-01	2.30E-03	NS
CKLF	NS	2.94E-04	-0.148	2.32E-03	NS
NT5C1B-RDH14	NS	2.96E-04	-0.148	2.33E-03	NS
HLA-DOB	NS	2.97E-04	-0.148	2.34E-03	NS
CENPX	NS	2.97E-04	-0.148	2.34E-03	NS
PNMT	NS	2.98E-04	1.48E-01	2.35E-03	NS
FRMD3	NS	2.98E-04	-0.148	2.35E-03	NS
DZIP3	NS	2.99E-04	-0.148	2.35E-03	NS
SYNJ1	NS	3.00E-04	-0.148	2.35E-03	NS
RAB18	NS	3.00E-04	-0.148	2.36E-03	NS
RAD51D	NS	3.00E-04	-0.148	2.36E-03	NS
PRY	NS	3.02E-04	1.48E-01	2.37E-03	NS
ROPN1L	NS	3.02E-04	-0.148	2.37E-03	NS
SPATA7	NS	3.03E-04	-0.148	2.38E-03	NS
LRRC14B	NS	3.04E-04	1.48E-01	2.38E-03	NS
MGAM2	NS	3.04E-04	-0.148	2.38E-03	NS
CKLF-CMTM1	NS	3.04E-04	-0.148	2.38E-03	NS
OR5BS1P	NS	3.05E-04	-0.148	2.39E-03	NS
SEPHS1	NS	3.06E-04	-0.148	2.39E-03	NS
PRPF18	NS	3.06E-04	-0.147	2.39E-03	NS
SCGB1D2	NS	3.07E-04	1.47E-01	2.40E-03	NS
ZNF215	NS	3.07E-04	-0.147	2.40E-03	NS
NDE1	NS	3.09E-04	1.47E-01	2.41E-03	NS
OPN1LW	NS	3.09E-04	-0.147	2.41E-03	NS
URB1	NS	3.09E-04	-0.147	2.41E-03	NS
SLC7A13	NS	3.09E-04	1.47E-01	2.41E-03	NS
FPGT	NS	3.09E-04	-0.147	2.41E-03	NS

TEX12	NS	3.11E-04	-0.147	2.42E-03	NS
ZNF460	NS	3.11E-04	-0.147	2.43E-03	NS
MBD5	NS	3.13E-04	-0.147	2.43E-03	NS
RCAN1	NS	3.13E-04	-0.147	2.44E-03	NS
TSPY3	NS	3.13E-04	1.47E-01	2.44E-03	NS
SRPRA	NS	3.15E-04	-0.147	2.45E-03	NS
SH2D1B	NS	3.15E-04	-0.147	2.45E-03	NS
RPUSD4	NS	3.16E-04	-0.147	2.45E-03	NS
PRDM6	NS	3.16E-04	-0.147	2.45E-03	NS
SOCS3	NS	3.16E-04	-0.147	2.46E-03	NS
TTC9B	NS	3.17E-04	-0.147	2.46E-03	NS
ECT2L	NS	3.17E-04	-0.147	2.46E-03	NS
DYNC2H1	NS	3.18E-04	-0.147	2.46E-03	NS
FILIP1	NS	3.19E-04	-0.147	2.47E-03	NS
TRIM48	NS	3.20E-04	-0.147	2.48E-03	NS
LYG1	NS	3.20E-04	-0.147	2.48E-03	NS
ACBD7	NS	3.21E-04	1.47E-01	2.49E-03	NS
CCDC169-SOHLH2	NS	3.21E-04	-0.147	2.49E-03	NS
PICALM	NS	3.21E-04	-0.147	2.49E-03	NS
SPINK5	NS	3.24E-04	-0.147	2.50E-03	NS
C6orf89	NS	3.23E-04	-0.147	2.50E-03	NS
PDE12	NS	3.24E-04	1.47E-01	2.50E-03	NS
LIPA	NS	3.25E-04	-0.147	2.51E-03	NS
KLHDC3	NS	3.24E-04	-0.147	2.51E-03	NS
MTSS2	NS	3.25E-04	1.47E-01	2.51E-03	NS
SPANXN4	NS	3.27E-04	-0.147	2.52E-03	NS
SLC23A3	NS	3.27E-04	-0.147	2.52E-03	NS
PMF1-BGLAP	NS	3.27E-04	-0.147	2.52E-03	NS
CDK5RAP3	NS	3.27E-04	-0.147	2.52E-03	NS
C12orf60	NS	3.28E-04	-0.147	2.53E-03	NS
CHCHD6	NS	3.29E-04	-0.147	2.53E-03	NS
RAPSN	NS	3.30E-04	-0.147	2.54E-03	NS
TACR1	NS	3.33E-04	-0.147	2.56E-03	NS
NRG2	NS	3.34E-04	1.47E-01	2.57E-03	NS
PSKH1	NS	3.34E-04	-0.147	2.57E-03	NS
L3MBTL1	NS	3.34E-04	-0.147	2.57E-03	NS
OR4K1	NS	3.35E-04	-0.147	2.57E-03	NS

CCSER1	NS	3.35E-04	-0.147	2.57E-03	NS
EIF4E1B	NS	3.35E-04	1.47E-01	2.57E-03	NS
ZNF699	NS	3.36E-04	-0.147	2.58E-03	NS
NEIL3	NS	3.37E-04	-0.146	2.58E-03	NS
TNP1	NS	3.37E-04	-0.146	2.58E-03	NS
MRPS17	NS	3.38E-04	-0.146	2.59E-03	NS
TMEM161B	NS	3.41E-04	-0.146	2.61E-03	NS
TRIT1	NS	3.42E-04	-0.146	2.62E-03	NS
HPX	NS	3.43E-04	-0.146	2.63E-03	NS
STMN1	NS	3.44E-04	-0.146	2.63E-03	NS
POTEE	NS	3.45E-04	1.46E-01	2.64E-03	NS
MSRA	NS	3.46E-04	1.46E-01	2.64E-03	NS
TAS2R16	NS	3.48E-04	-0.146	2.66E-03	NS
WFDC9	NS	3.48E-04	-0.146	2.66E-03	NS
IBTK	NS	3.48E-04	-0.146	2.66E-03	NS
TNNI2	NS	3.50E-04	-0.146	2.67E-03	NS
GLO1	NS	3.51E-04	-0.146	2.68E-03	NS
TSHB	NS	3.51E-04	-0.146	2.68E-03	NS
LYN	NS	3.51E-04	-0.146	2.68E-03	NS
LIN28B	NS	3.52E-04	-0.146	2.68E-03	NS
RAI14	NS	3.52E-04	-0.146	2.68E-03	NS
ZNF878	NS	3.52E-04	-0.146	2.68E-03	NS
PRRG4	NS	3.55E-04	-0.146	2.70E-03	NS
CCN3	NS	3.55E-04	-0.146	2.70E-03	NS
SMARCC2	NS	3.54E-04	1.46E-01	2.70E-03	NS
KCNN3	NS	3.55E-04	-0.146	2.70E-03	NS
CLDN16	NS	3.56E-04	1.46E-01	2.70E-03	NS
MBL2	NS	3.56E-04	-0.146	2.70E-03	NS
IARS1	NS	3.56E-04	-0.146	2.70E-03	NS
GC	NS	3.57E-04	-0.146	2.71E-03	NS
KRTAP4-8	NS	3.57E-04	1.46E-01	2.71E-03	NS
FIGNL1	NS	3.58E-04	-0.146	2.71E-03	NS
COL4A5	NS	3.58E-04	-0.146	2.71E-03	NS
MRAP	NS	3.58E-04	-0.146	2.72E-03	NS
MMACHC	NS	3.59E-04	-0.146	2.72E-03	NS
EFHB	NS	3.60E-04	-0.146	2.73E-03	NS
TMEM237	NS	3.61E-04	-0.146	2.73E-03	NS

C10orf90	NS	3.62E-04	-0.146	2.73E-03	NS
KLF10	NS	3.62E-04	1.46E-01	2.73E-03	NS
METRN	NS	3.63E-04	-0.146	2.74E-03	NS
CYP1B1	NS	3.63E-04	-0.146	2.74E-03	NS
PMEL	NS	3.63E-04	-0.146	2.74E-03	NS
PAX3	NS	3.63E-04	-0.146	2.74E-03	NS
ZMAT2	NS	3.63E-04	-0.146	2.74E-03	NS
YY1AP1	NS	3.64E-04	1.46E-01	2.74E-03	NS
WFDC10B	NS	3.64E-04	-0.146	2.74E-03	NS
MAP2K4	NS	3.65E-04	-0.146	2.75E-03	NS
CPSF4L	NS	3.65E-04	-0.146	2.75E-03	NS
KDM4B	NS	3.65E-04	1.46E-01	2.75E-03	NS
CRISPLD1	NS	3.66E-04	-0.146	2.76E-03	NS
STK39	NS	3.66E-04	-0.146	2.76E-03	NS
FAM20B	NS	3.67E-04	-0.146	2.76E-03	NS
C19orf73	NS	3.68E-04	-0.146	2.77E-03	NS
IKZF4	NS	3.70E-04	-0.145	2.78E-03	NS
ITGBL1	NS	3.70E-04	-0.145	2.78E-03	NS
TUBGCP4	NS	3.72E-04	-0.145	2.79E-03	NS
FAM221A	NS	3.73E-04	-0.145	2.80E-03	NS
AGGF1	NS	3.73E-04	-0.145	2.80E-03	NS
TAF11	NS	3.74E-04	-0.145	2.80E-03	NS
FZD6	NS	3.74E-04	-0.145	2.80E-03	NS
SLC51A	NS	3.75E-04	-0.145	2.81E-03	NS
PER1	NS	3.75E-04	1.45E-01	2.81E-03	NS
C2CD4A	NS	3.75E-04	1.45E-01	2.81E-03	NS
PFKL	NS	3.76E-04	-0.145	2.82E-03	NS
CWH43	NS	3.78E-04	-0.145	2.83E-03	NS
MDN1	NS	3.79E-04	-0.145	2.83E-03	NS
THRA	NS	3.80E-04	1.45E-01	2.84E-03	NS
TAFA1	NS	3.80E-04	-0.145	2.84E-03	NS
CDKL5	NS	3.80E-04	-0.145	2.84E-03	NS
KATNAL2	NS	3.82E-04	-0.145	2.85E-03	NS
ZNF26	NS	3.82E-04	-0.145	2.85E-03	NS
GRHL3	NS	3.82E-04	-0.145	2.85E-03	NS
RXRG	NS	3.83E-04	-0.145	2.86E-03	NS
BMP8B	NS	3.83E-04	-0.145	2.86E-03	NS

WSB1	NS	3.84E-04	-0.145	2.86E-03	NS
COL3A1	NS	3.85E-04	-0.145	2.87E-03	NS
DRICH1	NS	3.86E-04	-0.145	2.87E-03	NS
UBE2V2	NS	3.87E-04	-0.145	2.88E-03	NS
DENND5B	NS	3.88E-04	-0.145	2.88E-03	NS
PAPSS1	NS	3.89E-04	-0.145	2.89E-03	NS
CFAP300	NS	3.89E-04	1.45E-01	2.89E-03	NS
ACSS1	NS	3.89E-04	-0.145	2.89E-03	NS
ASPRV1	NS	3.90E-04	-0.145	2.89E-03	NS
ZNF239	NS	3.90E-04	-0.145	2.89E-03	NS
CUL5	NS	3.90E-04	-0.145	2.90E-03	NS
KRTAP26-1	NS	3.92E-04	-0.145	2.91E-03	NS
UGT8	NS	3.93E-04	-0.145	2.91E-03	NS
ALAS1	NS	3.94E-04	1.45E-01	2.92E-03	NS
FBXO10	NS	3.94E-04	-0.145	2.92E-03	NS
NKX2-6	NS	3.94E-04	1.45E-01	2.92E-03	NS
HYAL2	NS	3.94E-04	-0.145	2.92E-03	NS
VWA3B	NS	3.95E-04	-0.145	2.92E-03	NS
PYROXD1	NS	3.96E-04	-0.145	2.93E-03	NS
NES	NS	3.96E-04	1.45E-01	2.93E-03	NS
RBMXL2	NS	3.98E-04	1.45E-01	2.94E-03	NS
ANKRD11	NS	3.98E-04	-0.145	2.94E-03	NS
MYADML2	NS	4.01E-04	1.45E-01	2.96E-03	NS
NOTUM	NS	4.01E-04	1.45E-01	2.96E-03	NS
KCTD2	NS	4.02E-04	-0.145	2.97E-03	NS
OR6N1	NS	4.02E-04	-0.145	2.97E-03	NS
TPRG1	NS	4.03E-04	-0.145	2.97E-03	NS
KCNT2	NS	4.04E-04	-0.145	2.97E-03	NS
AMBN	NS	4.03E-04	-0.145	2.97E-03	NS
MAPK14	NS	4.04E-04	-0.145	2.97E-03	NS
EZH2	NS	4.04E-04	-0.145	2.98E-03	NS
FOXD4	NS	4.04E-04	-0.145	2.98E-03	NS
GPR6	NS	4.06E-04	1.45E-01	2.98E-03	NS
ZNHIT1	NS	4.08E-04	-0.144	3.00E-03	NS
TENT5A	NS	4.09E-04	1.44E-01	3.01E-03	NS
PLS1	NS	4.09E-04	-0.144	3.01E-03	NS
ATR	NS	4.09E-04	-0.144	3.01E-03	NS

C6orf15	NS	4.10E-04	1.44E-01	3.01E-03	NS
ATOH7	NS	4.10E-04	1.44E-01	3.01E-03	NS
SCFD2	NS	4.11E-04	-0.144	3.01E-03	NS
SP5	NS	4.11E-04	1.44E-01	3.02E-03	NS
CT45A2	NS	4.11E-04	-0.144	3.02E-03	NS
RHOB	NS	4.12E-04	1.44E-01	3.02E-03	NS
PARP16	NS	4.12E-04	-0.144	3.02E-03	NS
FGF18	NS	4.12E-04	1.44E-01	3.02E-03	NS
FHL1	NS	4.13E-04	-0.144	3.02E-03	NS
CLEC2D	NS	4.14E-04	-0.144	3.03E-03	NS
CLMN	NS	4.15E-04	1.44E-01	3.03E-03	NS
PTGER1	NS	4.15E-04	1.44E-01	3.03E-03	NS
SYCP2L	NS	4.16E-04	-0.144	3.04E-03	NS
FAM240B	NS	4.17E-04	1.44E-01	3.05E-03	NS
MRPS18C	NS	4.18E-04	-0.144	3.05E-03	NS
SIAH1	NS	4.19E-04	1.44E-01	3.06E-03	NS
METTL23	NS	4.20E-04	-0.144	3.06E-03	NS
PELP1	NS	4.21E-04	-0.144	3.07E-03	NS
PZP	NS	4.22E-04	-0.144	3.07E-03	NS
EMC1	NS	4.22E-04	-0.144	3.07E-03	NS
PTGFRN	NS	4.22E-04	-0.144	3.07E-03	NS
APPL2	NS	4.22E-04	-0.144	3.07E-03	NS
CD8A	NS	4.23E-04	-0.144	3.08E-03	NS
KIF24	NS	4.25E-04	-0.144	3.09E-03	NS
MB21D2	NS	4.26E-04	-0.144	3.10E-03	NS
LIN7B	NS	4.27E-04	-0.144	3.10E-03	NS
C1QL3	NS	4.27E-04	-0.144	3.10E-03	NS
CTSO	NS	4.27E-04	-0.144	3.10E-03	NS
RHOA	NS	4.27E-04	-0.144	3.10E-03	NS
KRT27	NS	4.29E-04	1.44E-01	3.12E-03	NS
KMT5A	NS	4.30E-04	-0.144	3.12E-03	NS
MN1	NS	4.30E-04	-0.144	3.12E-03	NS
NRG4	NS	4.31E-04	-0.144	3.13E-03	NS
OR2B11	NS	4.31E-04	-0.144	3.13E-03	NS
B3GALT4	NS	4.32E-04	-0.144	3.13E-03	NS
CABP1	NS	4.33E-04	-0.144	3.13E-03	NS
RFPL3	NS	4.34E-04	-0.144	3.14E-03	NS

FBXO5	NS	4.34E-04	-0.144	3.14E-03	NS
FAM102B	NS	4.37E-04	-0.144	3.16E-03	NS
NBPF14	NS	4.37E-04	-0.144	3.16E-03	NS
PKN2	NS	4.38E-04	-0.144	3.16E-03	NS
IMPG1	NS	4.38E-04	-0.144	3.16E-03	NS
C1orf74	NS	4.39E-04	-0.144	3.17E-03	NS
CIAPIN1	NS	4.43E-04	-0.144	3.20E-03	NS
CERS4	NS	4.43E-04	-0.144	3.20E-03	NS
SLC9A8	NS	4.44E-04	-0.144	3.20E-03	NS
PHOSPHO1	NS	4.45E-04	-0.144	3.21E-03	NS
ZPR1	NS	4.46E-04	1.43E-01	3.22E-03	NS
SNAPC4	NS	4.48E-04	-0.143	3.23E-03	NS
CCDC34	NS	4.48E-04	-0.143	3.23E-03	NS
SDF4	NS	4.49E-04	1.43E-01	3.23E-03	NS
OR52K2	NS	4.49E-04	-0.143	3.23E-03	NS
H2AC7	NS	4.49E-04	-0.143	3.23E-03	NS
LRRC14	NS	4.50E-04	-0.143	3.24E-03	NS
TDRD6	NS	4.54E-04	-0.143	3.26E-03	NS
RMDN1	NS	4.54E-04	-0.143	3.26E-03	NS
TRPC5	NS	4.55E-04	-0.143	3.27E-03	NS
GAGE2A	NS	4.56E-04	-0.143	3.27E-03	NS
FBXO48	NS	4.56E-04	-0.143	3.27E-03	NS
TTPA	NS	4.58E-04	1.43E-01	3.28E-03	NS
SLX1B	NS	4.58E-04	1.43E-01	3.29E-03	NS
IL1A	NS	4.59E-04	-0.143	3.29E-03	NS
LMNB1	NS	4.60E-04	1.43E-01	3.30E-03	NS
NADSYN1	NS	4.61E-04	-0.143	3.30E-03	NS
SHISA8	NS	4.62E-04	1.43E-01	3.31E-03	NS
NEFL	NS	4.63E-04	-0.143	3.32E-03	NS
SIGLEC1	NS	4.64E-04	-0.143	3.32E-03	NS
GVQW3	NS	4.65E-04	-0.143	3.33E-03	NS
CEP76	NS	4.66E-04	-0.143	3.33E-03	NS
KIF3A	NS	4.66E-04	-0.143	3.33E-03	NS
STAMBPL1	NS	4.66E-04	-0.143	3.33E-03	NS
CDSN	NS	4.66E-04	1.43E-01	3.33E-03	NS
CEP120	NS	4.67E-04	-0.143	3.33E-03	NS
TNFSF9	NS	4.67E-04	-0.143	3.34E-03	NS

IFI35	NS	4.69E-04	-0.143	3.34E-03	NS
FAT1	NS	4.68E-04	-0.143	3.34E-03	NS
BTRC	NS	4.69E-04	-0.143	3.34E-03	NS
ANKRD20A4P	NS	4.70E-04	-0.143	3.35E-03	NS
EPS8L2	NS	4.70E-04	1.43E-01	3.35E-03	NS
FKBP14	NS	4.70E-04	-0.143	3.35E-03	NS
FZD9	NS	4.70E-04	1.43E-01	3.35E-03	NS
FHIP2A	NS	4.71E-04	1.43E-01	3.35E-03	NS
CAPN14	NS	4.72E-04	-0.143	3.35E-03	NS
PLAAT4	NS	4.74E-04	-0.143	3.37E-03	NS
S100A2	NS	4.75E-04	1.43E-01	3.38E-03	NS
DRC3	NS	4.75E-04	-0.143	3.38E-03	NS
ARRDC2	NS	4.76E-04	1.43E-01	3.38E-03	NS
XCL1	NS	4.76E-04	-0.143	3.38E-03	NS
TEX9	NS	4.77E-04	-0.143	3.38E-03	NS
TMEM243	NS	4.78E-04	-0.143	3.39E-03	NS
SEH1L	NS	4.78E-04	-0.143	3.39E-03	NS
OR7G3	NS	4.78E-04	-0.143	3.39E-03	NS
CD14	NS	4.78E-04	-0.143	3.39E-03	NS
CDHR5	NS	4.79E-04	-0.143	3.40E-03	NS
POLR2G	NS	4.81E-04	-0.143	3.41E-03	NS
ABRA	NS	4.83E-04	-0.143	3.42E-03	NS
HAUS8	NS	4.84E-04	-0.143	3.42E-03	NS
MAGEC2	NS	4.85E-04	-0.143	3.43E-03	NS
CCDC195	NS	4.86E-04	1.43E-01	3.44E-03	NS
HILPDA	NS	4.86E-04	-0.143	3.44E-03	NS
BMT2	NS	4.87E-04	-0.143	3.44E-03	NS
YY2	NS	4.87E-04	-0.143	3.44E-03	NS
ZYX	NS	4.88E-04	1.43E-01	3.44E-03	NS
C1GALT1C1	NS	4.88E-04	-0.143	3.44E-03	NS
PCGF3	NS	4.87E-04	-0.143	3.44E-03	NS
ACADVL	NS	4.88E-04	1.43E-01	3.45E-03	NS
ZKSCAN1	NS	4.89E-04	-0.143	3.45E-03	NS
USP46	NS	4.89E-04	-0.143	3.45E-03	NS
KCNG2	NS	4.90E-04	1.42E-01	3.45E-03	NS
ACY1	NS	4.90E-04	-0.142	3.45E-03	NS
KIFC2	NS	4.91E-04	-0.142	3.45E-03	NS

BCL9	NS	4.92E-04	1.42E-01	3.46E-03	NS
TRMT10C	NS	4.93E-04	-0.142	3.47E-03	NS
CD2AP	NS	4.93E-04	-0.142	3.47E-03	NS
GKAP1	NS	4.94E-04	-0.142	3.47E-03	NS
ZSCAN16	NS	4.94E-04	-0.142	3.47E-03	NS
HNMT	NS	4.95E-04	-0.142	3.47E-03	NS
MAGED2	NS	4.96E-04	-0.142	3.48E-03	NS
CLPTM1	NS	4.96E-04	1.42E-01	3.48E-03	NS
TCEAL8	NS	4.97E-04	-0.142	3.49E-03	NS
NPIPB7	NS	4.97E-04	1.42E-01	3.49E-03	NS
QKI	NS	4.97E-04	-0.142	3.49E-03	NS
MC4R	NS	4.98E-04	-0.142	3.49E-03	NS
INSL3	NS	4.98E-04	-0.142	3.49E-03	NS
GOLGA8Q	NS	4.99E-04	-0.142	3.50E-03	NS
PI15	NS	5.00E-04	-0.142	3.50E-03	NS
RUNX1T1	NS	5.00E-04	-0.142	3.50E-03	NS
ZNF556	NS	5.02E-04	1.42E-01	3.51E-03	NS
ANKLE1	NS	5.02E-04	1.42E-01	3.51E-03	NS
FCRL3	NS	5.03E-04	-0.142	3.52E-03	NS
TNFRSF8	NS	5.05E-04	-0.142	3.53E-03	NS
LRRTM3	NS	5.04E-04	-0.142	3.53E-03	NS
CD5	NS	5.06E-04	-0.142	3.53E-03	NS
MDFIC2	NS	5.07E-04	-0.142	3.54E-03	NS
MED26	NS	5.07E-04	1.42E-01	3.54E-03	NS
GPR180	NS	5.07E-04	-0.142	3.54E-03	NS
GPA33	NS	5.08E-04	-0.142	3.55E-03	NS
ATG4C	NS	5.10E-04	-0.142	3.56E-03	NS
LGI2	NS	5.10E-04	-0.142	3.56E-03	NS
ZNF366	NS	5.10E-04	-0.142	3.56E-03	NS
ERICH4	NS	5.13E-04	1.42E-01	3.57E-03	NS
TAS2R9	NS	5.13E-04	-0.142	3.57E-03	NS
VGF	NS	5.13E-04	1.42E-01	3.57E-03	NS
FOXE1	NS	5.13E-04	1.42E-01	3.57E-03	NS
H3-4	NS	5.16E-04	-0.142	3.59E-03	NS
CARMIL2	NS	5.18E-04	1.42E-01	3.60E-03	NS
GAGE12E	NS	5.18E-04	-0.142	3.60E-03	NS
CRTC3	NS	5.18E-04	1.42E-01	3.60E-03	NS

H4C5	NS	5.19E-04	1.42E-01	3.61E-03	NS
SLC2A11	NS	5.21E-04	-0.142	3.62E-03	NS
CFAP206	NS	5.21E-04	-0.142	3.62E-03	NS
CCDC115	NS	5.23E-04	-0.142	3.62E-03	NS
YPEL1	NS	5.24E-04	-0.142	3.63E-03	NS
HYOU1	NS	5.25E-04	-0.142	3.64E-03	NS
APCDD1	NS	5.26E-04	-0.142	3.64E-03	NS
TFIP11	NS	5.26E-04	1.42E-01	3.64E-03	NS
RBAK	NS	5.27E-04	-0.142	3.64E-03	NS
ABCG1	NS	5.27E-04	1.42E-01	3.64E-03	NS
CHRM4	NS	5.27E-04	-0.142	3.64E-03	NS
DPRX	NS	5.27E-04	1.42E-01	3.65E-03	NS
GSAP	NS	5.28E-04	-0.142	3.65E-03	NS
CIBAR1	NS	5.28E-04	-0.142	3.65E-03	NS
LILRA5	NS	5.28E-04	-0.142	3.65E-03	NS
ZNF181	NS	5.29E-04	1.42E-01	3.65E-03	NS
SLC38A6	NS	5.30E-04	-0.142	3.66E-03	NS
GARRE1	NS	5.30E-04	-0.142	3.66E-03	NS
ZNF98	NS	5.30E-04	-0.142	3.66E-03	NS
PRDM13	NS	5.31E-04	-0.142	3.66E-03	NS
IER3IP1	NS	5.31E-04	-0.142	3.66E-03	NS
IDNK	NS	5.32E-04	-0.142	3.66E-03	NS
DEFA1	NS	5.34E-04	-0.142	3.68E-03	NS
MECR	NS	5.35E-04	-0.142	3.68E-03	NS
PDCL	NS	5.36E-04	-0.142	3.69E-03	NS
ARHGAP39	NS	5.37E-04	1.41E-01	3.69E-03	NS
METAP1D	NS	5.37E-04	-0.141	3.69E-03	NS
ZNF547	NS	5.39E-04	-0.141	3.70E-03	NS
CGB1	NS	5.39E-04	1.41E-01	3.70E-03	NS
EPHA2	NS	5.40E-04	-0.141	3.71E-03	NS
TGIF1	NS	5.41E-04	-0.141	3.71E-03	NS
TUBE1	NS	5.41E-04	-0.141	3.72E-03	NS
PCGF2	NS	5.43E-04	-0.141	3.73E-03	NS
C4B	NS	5.44E-04	-0.141	3.73E-03	NS
ZNF740	NS	5.45E-04	-0.141	3.74E-03	NS
PPP1R8	NS	5.48E-04	-0.141	3.76E-03	NS
KDSR	NS	5.49E-04	-0.141	3.76E-03	NS

LZTR1	NS	5.50E-04	1.41E-01	3.77E-03	NS
VSTM2L	NS	5.51E-04	1.41E-01	3.77E-03	NS
PGGT1B	NS	5.54E-04	-0.141	3.79E-03	NS
ATF7IP	NS	5.54E-04	-0.141	3.79E-03	NS
FAM171A2	NS	5.55E-04	1.41E-01	3.80E-03	NS
ADNP	NS	5.55E-04	-0.141	3.80E-03	NS
ESRRG	NS	5.57E-04	-0.141	3.81E-03	NS
WNT7B	NS	5.58E-04	1.41E-01	3.81E-03	NS
TCF7	NS	5.59E-04	1.41E-01	3.82E-03	NS
DBN1	NS	5.60E-04	-0.141	3.82E-03	NS
KCNH8	NS	5.61E-04	-0.141	3.83E-03	NS
ZHX1	NS	5.62E-04	-0.141	3.83E-03	NS
SLC5A7	NS	5.62E-04	-0.141	3.83E-03	NS
NFAT5	NS	5.63E-04	-0.141	3.84E-03	NS
H1-3	NS	5.66E-04	-0.141	3.86E-03	NS
UGT2A3	NS	5.66E-04	-0.141	3.86E-03	NS
GAD1	NS	5.69E-04	-0.141	3.88E-03	NS
OR10AG1	NS	5.70E-04	-0.141	3.88E-03	NS
ARPIN-AP3S2	NS	5.71E-04	1.41E-01	3.89E-03	NS
DTL	NS	5.72E-04	-0.141	3.89E-03	NS
AMH	NS	5.72E-04	1.41E-01	3.89E-03	NS
F2RL3	NS	5.75E-04	-0.141	3.91E-03	NS
HOXD12	NS	5.75E-04	1.41E-01	3.91E-03	NS
RAPH1	NS	5.76E-04	-0.141	3.91E-03	NS
FAM167A	NS	5.76E-04	-0.141	3.91E-03	NS
PRDM1	NS	5.76E-04	-0.141	3.91E-03	NS
RPSA	NS	5.77E-04	-0.141	3.92E-03	NS
TMEM238L	NS	5.78E-04	-0.141	3.92E-03	NS
TMED2	NS	5.79E-04	-0.141	3.93E-03	NS
ZIK1	NS	5.81E-04	-0.141	3.94E-03	NS
AHCY	NS	5.82E-04	-0.141	3.94E-03	NS
ACTR2	NS	5.81E-04	-0.141	3.94E-03	NS
MT-CO1	NS	5.81E-04	1.41E-01	3.94E-03	NS
ASPN	NS	5.83E-04	-0.141	3.95E-03	NS
CLIP3	NS	5.86E-04	-0.141	3.96E-03	NS
LMLN2	NS	5.86E-04	-0.141	3.96E-03	NS
CCDC81	NS	5.87E-04	-0.141	3.97E-03	NS

MYLK4	NS	5.88E-04	-0.141	3.98E-03	NS
DHRS7B	NS	5.89E-04	-0.14	3.98E-03	NS
ZNF45	NS	5.89E-04	-0.14	3.98E-03	NS
STRA8	NS	5.89E-04	-0.14	3.98E-03	NS
TAGLN2	NS	5.90E-04	1.40E-01	3.98E-03	NS
MRPS14	NS	5.90E-04	-0.14	3.98E-03	NS
IL2RB	NS	5.91E-04	-0.14	3.99E-03	NS
ITSN2	NS	5.92E-04	-0.14	3.99E-03	NS
ADAMTS18	NS	5.92E-04	-0.14	3.99E-03	NS
ZNF575	NS	5.95E-04	1.40E-01	4.01E-03	NS
GPR148	NS	5.97E-04	1.40E-01	4.02E-03	NS
CCDC102A	NS	5.97E-04	1.40E-01	4.02E-03	NS
NEMP2	NS	5.98E-04	-0.14	4.03E-03	NS
F13B	NS	5.98E-04	-0.14	4.03E-03	NS
CETN1	NS	5.99E-04	-0.14	4.03E-03	NS
TMEM107	NS	5.99E-04	-0.14	4.03E-03	NS
POTEI	NS	5.98E-04	1.40E-01	4.03E-03	NS
OR5M9	NS	5.99E-04	-0.14	4.03E-03	NS
EXOC3	NS	6.00E-04	1.40E-01	4.03E-03	NS
ESR1	NS	6.00E-04	-0.14	4.03E-03	NS
GYS1	NS	6.00E-04	-0.14	4.03E-03	NS
IL1RAPL2	NS	6.02E-04	-0.14	4.04E-03	NS
NDUFAF8	NS	6.05E-04	-0.14	4.06E-03	NS
CRK	NS	6.06E-04	-0.14	4.06E-03	NS
OTOL1	NS	6.09E-04	-0.14	4.09E-03	NS
VPS8	NS	6.09E-04	-0.14	4.09E-03	NS
CASS4	NS	6.11E-04	-0.14	4.10E-03	NS
MINPP1	NS	6.12E-04	-0.14	4.10E-03	NS
SLC36A1	NS	6.13E-04	-0.14	4.11E-03	NS
GALNT10	NS	6.14E-04	-0.14	4.11E-03	NS
GLOD5	NS	6.16E-04	-0.14	4.12E-03	NS
SPART	NS	6.17E-04	-0.14	4.13E-03	NS
ANKRD37	NS	6.17E-04	1.40E-01	4.13E-03	NS
FAM237A	NS	6.17E-04	-0.14	4.13E-03	NS
MAOB	NS	6.18E-04	-0.14	4.13E-03	NS
MFSD3	NS	6.20E-04	1.40E-01	4.14E-03	NS
GPR150	NS	6.20E-04	1.40E-01	4.14E-03	NS

CCSER2	NS	6.21E-04	-0.14	4.15E-03	NS
ZNF606	NS	6.21E-04	-0.14	4.15E-03	NS
MT-ATP6	NS	6.21E-04	1.40E-01	4.15E-03	NS
IPO4	NS	6.22E-04	1.40E-01	4.15E-03	NS
ENPP4	NS	6.22E-04	1.40E-01	4.15E-03	NS
ERAP2	NS	6.23E-04	-0.14	4.15E-03	NS
LYSMD4	NS	6.24E-04	-0.14	4.16E-03	NS
GJB3	NS	6.25E-04	1.40E-01	4.16E-03	NS
IL36B	NS	6.25E-04	-0.14	4.16E-03	NS
EIF3L	NS	6.26E-04	-0.14	4.16E-03	NS
DCUN1D4	NS	6.26E-04	-0.14	4.17E-03	NS
CYB5R2	NS	6.26E-04	-0.14	4.17E-03	NS
HIVEP1	NS	6.26E-04	-0.14	4.17E-03	NS
SPATA5	NS	6.27E-04	-0.14	4.17E-03	NS
SLC29A1	NS	6.27E-04	-0.14	4.17E-03	NS
REM2	NS	6.30E-04	-0.14	4.18E-03	NS
VRK1	NS	6.31E-04	-0.14	4.18E-03	NS
PRCC	NS	6.31E-04	-0.14	4.18E-03	NS
RASEF	NS	6.31E-04	-0.14	4.19E-03	NS
TRIOBP	NS	6.32E-04	-0.14	4.19E-03	NS
AP2B1	NS	6.34E-04	-0.14	4.20E-03	NS
SBK2	NS	6.37E-04	1.40E-01	4.22E-03	NS
AXIN1	NS	6.39E-04	-0.14	4.23E-03	NS
IFRD2	NS	6.41E-04	-0.14	4.25E-03	NS
OR1E2	NS	6.43E-04	-0.14	4.26E-03	NS
MPDZ	NS	6.46E-04	-0.139	4.28E-03	NS
PHAF1	NS	6.47E-04	1.39E-01	4.28E-03	NS
C5orf47	NS	6.47E-04	-0.139	4.28E-03	NS
WDHD1	NS	6.48E-04	-0.139	4.28E-03	NS
TNS3	NS	6.49E-04	-0.139	4.29E-03	NS
SLC35B4	NS	6.49E-04	-0.139	4.29E-03	NS
FASTKD2	NS	6.50E-04	-0.139	4.29E-03	NS
FGF12	NS	6.53E-04	-0.139	4.31E-03	NS
ATXN2	NS	6.53E-04	-0.139	4.31E-03	NS
SGSH	NS	6.53E-04	1.39E-01	4.31E-03	NS
MEI4	NS	6.54E-04	-0.139	4.31E-03	NS
TECTA	NS	6.55E-04	-0.139	4.31E-03	NS

PLA2G12A	NS	6.55E-04	-0.139	4.32E-03	NS
CNTNAP3C	NS	6.56E-04	-0.139	4.32E-03	NS
TRIM16	NS	6.57E-04	-0.139	4.33E-03	NS
ARMC5	NS	6.58E-04	1.39E-01	4.33E-03	NS
MT4	NS	6.60E-04	1.39E-01	4.34E-03	NS
DNAJC17	NS	6.61E-04	-0.139	4.34E-03	NS
OR52I1	NS	6.62E-04	-0.139	4.35E-03	NS
MYOG	NS	6.63E-04	1.39E-01	4.36E-03	NS
SETD2	NS	6.63E-04	1.39E-01	4.36E-03	NS
RBM45	NS	6.64E-04	-0.139	4.36E-03	NS
MDGA1	NS	6.66E-04	-0.139	4.37E-03	NS
HDAC11	NS	6.66E-04	-0.139	4.37E-03	NS
DIAPH2	NS	6.67E-04	-0.139	4.37E-03	NS
FAS	NS	6.69E-04	-0.139	4.38E-03	NS
COL8A2	NS	6.69E-04	-0.139	4.38E-03	NS
ZNF112	NS	6.69E-04	-0.139	4.39E-03	NS
EPPIN	NS	6.71E-04	-0.139	4.39E-03	NS
ZBTB40	NS	6.72E-04	-0.139	4.40E-03	NS
NKIRAS2	NS	6.72E-04	1.39E-01	4.40E-03	NS
CACUL1	NS	6.73E-04	-0.139	4.41E-03	NS
MRGPRD	NS	6.74E-04	-0.139	4.41E-03	NS
IFITM2	NS	6.75E-04	1.39E-01	4.42E-03	NS
PTPN5	NS	6.76E-04	-0.139	4.42E-03	NS
HIC2	NS	6.77E-04	-0.139	4.42E-03	NS
SPAG4	NS	6.79E-04	1.39E-01	4.44E-03	NS
ZNF678	NS	6.79E-04	-0.139	4.44E-03	NS
TREH	NS	6.85E-04	1.39E-01	4.47E-03	NS
TMSB15A	NS	6.85E-04	1.39E-01	4.47E-03	NS
AGAP6	NS	6.85E-04	-0.139	4.47E-03	NS
ST3GAL1	NS	6.87E-04	-0.139	4.48E-03	NS
MYH11	NS	6.87E-04	1.39E-01	4.48E-03	NS
LZIC	NS	6.89E-04	1.39E-01	4.49E-03	NS
SNX24	NS	6.91E-04	-0.139	4.50E-03	NS
FIBCD1	NS	6.91E-04	1.39E-01	4.50E-03	NS
FIBP	NS	6.94E-04	-0.139	4.52E-03	NS
ADAL	NS	6.96E-04	-0.139	4.53E-03	NS
KBTBD13	NS	6.98E-04	1.39E-01	4.54E-03	NS

TTC30A	NS	6.99E-04	-0.139	4.55E-03	NS
ATP10A	NS	6.99E-04	-0.139	4.55E-03	NS
ZWILCH	NS	7.02E-04	-0.139	4.56E-03	NS
GABRA4	NS	7.02E-04	-0.139	4.56E-03	NS
CELA2A	NS	7.04E-04	-0.139	4.57E-03	NS
SMU1	NS	7.04E-04	-0.139	4.57E-03	NS
ALKBH4	NS	7.06E-04	-0.138	4.58E-03	NS
HS3ST1	NS	7.06E-04	-0.138	4.58E-03	NS
ZGPAT	NS	7.07E-04	-0.138	4.59E-03	NS
AUTS2	NS	7.07E-04	-0.138	4.59E-03	NS
KLHL15	NS	7.08E-04	1.38E-01	4.59E-03	NS
IL34	NS	7.10E-04	1.38E-01	4.59E-03	NS
PROK2	NS	7.10E-04	-0.138	4.59E-03	NS
NLRC3	NS	7.09E-04	-0.138	4.59E-03	NS
POTEH	NS	7.10E-04	-0.138	4.59E-03	NS
EPPK1	NS	7.10E-04	1.38E-01	4.60E-03	NS
SLC27A2	NS	7.11E-04	-0.138	4.60E-03	NS
STEAP4	NS	7.12E-04	-0.138	4.61E-03	NS
SALL1	NS	7.14E-04	-0.138	4.61E-03	NS
FLNA	NS	7.14E-04	-0.138	4.61E-03	NS
KYNU	NS	7.15E-04	-0.138	4.62E-03	NS
SRSF7	NS	7.16E-04	1.38E-01	4.62E-03	NS
DNASE2B	NS	7.17E-04	-0.138	4.62E-03	NS
CACNB3	NS	7.17E-04	-0.138	4.62E-03	NS
ZSWIM2	NS	7.17E-04	-0.138	4.62E-03	NS
EXTL2	NS	7.18E-04	-0.138	4.63E-03	NS
ATRAID	NS	7.19E-04	1.38E-01	4.63E-03	NS
P2RY10	NS	7.20E-04	-0.138	4.64E-03	NS
TMEM82	NS	7.20E-04	1.38E-01	4.64E-03	NS
RAB39A	NS	7.21E-04	-0.138	4.64E-03	NS
KLHL38	NS	7.23E-04	1.38E-01	4.65E-03	NS
RUBCNL	NS	7.23E-04	-0.138	4.65E-03	NS
HJV	NS	7.25E-04	1.38E-01	4.66E-03	NS
CEP170B	NS	7.27E-04	1.38E-01	4.67E-03	NS
GLYR1	NS	7.26E-04	-0.138	4.67E-03	NS
SRPRB	NS	7.26E-04	-0.138	4.67E-03	NS
ACER2	NS	7.28E-04	-0.138	4.68E-03	NS

USP45	NS	7.29E-04	-0.138	4.68E-03	NS
CXXC4	NS	7.30E-04	-0.138	4.69E-03	NS
CAMTA1	NS	7.30E-04	1.38E-01	4.69E-03	NS
SLC32A1	NS	7.31E-04	1.38E-01	4.69E-03	NS
IGSF5	NS	7.32E-04	-0.138	4.70E-03	NS
FAM227B	NS	7.33E-04	-0.138	4.70E-03	NS
GMPR	NS	7.34E-04	-0.138	4.70E-03	NS
PTBP1	NS	7.34E-04	1.38E-01	4.70E-03	NS
CPOX	NS	7.35E-04	-0.138	4.71E-03	NS
CLEC12B	NS	7.37E-04	-0.138	4.72E-03	NS
BFAR	NS	7.43E-04	-0.138	4.75E-03	NS
NPR3	NS	7.43E-04	-0.138	4.75E-03	NS
GCC1	NS	7.43E-04	-0.138	4.75E-03	NS
MAT2B	NS	7.44E-04	-0.138	4.75E-03	NS
OTUD6A	NS	7.44E-04	1.38E-01	4.76E-03	NS
RASGRF2	NS	7.45E-04	-0.138	4.76E-03	NS
WDR19	NS	7.45E-04	-0.138	4.76E-03	NS
SMYD3	NS	7.49E-04	-0.138	4.78E-03	NS
CALCR	NS	7.52E-04	-0.138	4.80E-03	NS
CAD	NS	7.52E-04	-0.138	4.80E-03	NS
MUC7	NS	7.52E-04	-0.138	4.80E-03	NS
C2orf72	NS	7.52E-04	1.38E-01	4.80E-03	NS
TPMT	NS	7.53E-04	-0.138	4.80E-03	NS
C10orf88	NS	7.58E-04	-0.138	4.83E-03	NS
SSB	NS	7.60E-04	-0.138	4.84E-03	NS
CCAR2	NS	7.60E-04	1.38E-01	4.84E-03	NS
RAX2	NS	7.62E-04	1.38E-01	4.85E-03	NS
FAM43B	NS	7.62E-04	1.38E-01	4.85E-03	NS
CCNO	NS	7.63E-04	1.38E-01	4.85E-03	NS
TMEM19	NS	7.64E-04	-0.138	4.86E-03	NS
FAM161A	NS	7.69E-04	-0.138	4.89E-03	NS
UGDH	NS	7.72E-04	-0.137	4.90E-03	NS
PUM1	NS	7.72E-04	1.37E-01	4.90E-03	NS
OXGR1	NS	7.74E-04	-0.137	4.91E-03	NS
FEM1A	NS	7.75E-04	-0.137	4.92E-03	NS
C19orf18	NS	7.76E-04	-0.137	4.92E-03	NS
UBE2W	NS	7.80E-04	-0.137	4.94E-03	NS

SLC25A12	NS	7.82E-04	-0.137	4.95E-03	NS
CHIT1	NS	7.82E-04	-0.137	4.96E-03	NS
WNT6	NS	7.84E-04	1.37E-01	4.97E-03	NS
COA3	NS	7.85E-04	-0.137	4.97E-03	NS
DYRK2	NS	7.86E-04	-0.137	4.98E-03	NS
WDCP	NS	7.87E-04	-0.137	4.98E-03	NS
SERGEF	NS	7.88E-04	-0.137	4.98E-03	NS
ZNF518B	NS	7.88E-04	-0.137	4.98E-03	NS
MAGOH	NS	7.88E-04	-0.137	4.98E-03	NS
CSNKA2IP	NS	7.88E-04	-0.137	4.98E-03	NS
UGT2B28	NS	7.89E-04	-0.137	4.98E-03	NS
FSTL1	NS	7.89E-04	-0.137	4.98E-03	NS
TRMT10A	NS	7.91E-04	-0.137	4.99E-03	NS
ABRAXAS1	NS	7.91E-04	-0.137	5.00E-03	NS
GRM1	NS	7.92E-04	1.37E-01	5.00E-03	NS
PIPOX	NS	7.92E-04	-0.137	5.00E-03	NS
KIF21B	NS	7.93E-04	-0.137	5.00E-03	NS
ZNHIT3	NS	7.95E-04	-0.137	5.01E-03	NS
FRMD1	NS	7.95E-04	1.37E-01	5.01E-03	NS
SIGLEC6	NS	7.96E-04	-0.137	5.01E-03	NS
OR4M1	NS	7.96E-04	-0.137	5.01E-03	NS
TEX11	NS	7.97E-04	-0.137	5.02E-03	NS
FOXP3	NS	8.00E-04	-0.137	5.04E-03	NS
CPQ	NS	8.02E-04	-0.137	5.04E-03	NS
EFR3A	NS	8.03E-04	-0.137	5.05E-03	NS
POP4	NS	8.04E-04	-0.137	5.05E-03	NS
GOLPH3L	NS	8.04E-04	-0.137	5.05E-03	NS
FOXF2	NS	8.06E-04	1.37E-01	5.06E-03	NS
PEF1	NS	8.06E-04	-0.137	5.06E-03	NS
MACIR	NS	8.06E-04	-0.137	5.06E-03	NS
NUP37	NS	8.06E-04	-0.137	5.06E-03	NS
RALYL	NS	8.07E-04	-0.137	5.07E-03	NS
ADAM28	NS	8.08E-04	-0.137	5.07E-03	NS
SLC12A2	NS	8.08E-04	-0.137	5.07E-03	NS
MDH1	NS	8.09E-04	-0.137	5.07E-03	NS
CCNC	NS	8.09E-04	-0.137	5.07E-03	NS
GPN3	NS	8.09E-04	-0.137	5.07E-03	NS

NKX2-1	NS	8.10E-04	1.37E-01	5.07E-03	NS
MRPS23	NS	8.10E-04	-0.137	5.07E-03	NS
CCT6A	NS	8.11E-04	-0.137	5.08E-03	NS
HCLS1	NS	8.11E-04	-0.137	5.08E-03	NS
INTS14	NS	8.12E-04	-0.137	5.08E-03	NS
C16orf86	NS	8.12E-04	-0.137	5.08E-03	NS
SEC24A	NS	8.13E-04	1.37E-01	5.08E-03	NS
TRAPPC13	NS	8.16E-04	-0.137	5.10E-03	NS
MYCT1	NS	8.16E-04	-0.137	5.10E-03	NS
CEP83	NS	8.16E-04	-0.137	5.10E-03	NS
REC8	NS	8.18E-04	1.37E-01	5.10E-03	NS
ARL2BP	NS	8.17E-04	-0.137	5.10E-03	NS
NUDT22	NS	8.18E-04	-0.137	5.10E-03	NS
BNIP3	NS	8.18E-04	-0.137	5.10E-03	NS
MORN1	NS	8.19E-04	1.37E-01	5.10E-03	NS
SNX27	NS	8.19E-04	-0.137	5.10E-03	NS
DPPA3	NS	8.20E-04	-0.137	5.11E-03	NS
EPAS1	NS	8.21E-04	-0.137	5.11E-03	NS
SHH	NS	8.21E-04	1.37E-01	5.11E-03	NS
MYH8	NS	8.22E-04	-0.137	5.12E-03	NS
ENGASE	NS	8.22E-04	-0.137	5.12E-03	NS
SLC52A2	NS	8.23E-04	-0.137	5.12E-03	NS
INS	NS	8.23E-04	1.37E-01	5.12E-03	NS
CCDC57	NS	8.26E-04	-0.137	5.13E-03	NS
SCOC	NS	8.28E-04	-0.137	5.14E-03	NS
UBB	NS	8.29E-04	-0.137	5.14E-03	NS
GEN1	NS	8.29E-04	-0.137	5.14E-03	NS
USP22	NS	8.29E-04	1.37E-01	5.14E-03	NS
EVL	NS	8.30E-04	-0.137	5.15E-03	NS
ZNF224	NS	8.31E-04	-0.137	5.15E-03	NS
INPP5F	NS	8.32E-04	-0.137	5.15E-03	NS
ZNF227	NS	8.32E-04	-0.137	5.15E-03	NS
OXLD1	NS	8.33E-04	-0.137	5.16E-03	NS
MCOLN1	NS	8.35E-04	1.37E-01	5.17E-03	NS
PDLIM2	NS	8.35E-04	1.37E-01	5.17E-03	NS
UBL5	NS	8.36E-04	-0.137	5.17E-03	NS
REL	NS	8.40E-04	-0.137	5.20E-03	NS

ITM2C	NS	8.41E-04	-0.137	5.20E-03	NS
CALM3	NS	8.43E-04	1.37E-01	5.21E-03	NS
ADH1B	NS	8.44E-04	-0.136	5.22E-03	NS
PET117	NS	8.46E-04	-0.136	5.23E-03	NS
SPRR2E	NS	8.47E-04	-0.136	5.23E-03	NS
C8orf33	NS	8.47E-04	-0.136	5.23E-03	NS
KRT1	NS	8.48E-04	-0.136	5.23E-03	NS
MGAM	NS	8.48E-04	-0.136	5.23E-03	NS
HPS5	NS	8.50E-04	-0.136	5.24E-03	NS
KCNB2	NS	8.51E-04	-0.136	5.25E-03	NS
MYH15	NS	8.53E-04	-0.136	5.26E-03	NS
RGS20	NS	8.53E-04	-0.136	5.26E-03	NS
DECR2	NS	8.56E-04	-0.136	5.27E-03	NS
PCCA	NS	8.58E-04	-0.136	5.29E-03	NS
ZNF621	NS	8.59E-04	-0.136	5.29E-03	NS
VPS33B	NS	8.61E-04	-0.136	5.30E-03	NS
NOP53	NS	8.62E-04	1.36E-01	5.30E-03	NS
LINGO4	NS	8.62E-04	-0.136	5.30E-03	NS
CDIPT	NS	8.64E-04	1.36E-01	5.31E-03	NS
KLK10	NS	8.64E-04	1.36E-01	5.31E-03	NS
MEIS2	NS	8.64E-04	-0.136	5.31E-03	NS
NTN3	NS	8.64E-04	1.36E-01	5.31E-03	NS
RILP	NS	8.63E-04	1.36E-01	5.31E-03	NS
STOX2	NS	8.64E-04	-0.136	5.31E-03	NS
CREBZF	NS	8.66E-04	1.36E-01	5.31E-03	NS
H3Y2	NS	8.66E-04	-0.136	5.31E-03	NS
IQCD	NS	8.66E-04	-0.136	5.31E-03	NS
EBNA1BP2	NS	8.67E-04	-0.136	5.32E-03	NS
E2F1	NS	8.72E-04	-0.136	5.35E-03	NS
MXD3	NS	8.73E-04	-0.136	5.35E-03	NS
IFT122	NS	8.75E-04	-0.136	5.36E-03	NS
NOC2L	NS	8.77E-04	-0.136	5.37E-03	NS
PLEKHB1	NS	8.79E-04	-0.136	5.38E-03	NS
EEF1AKMT1	NS	8.79E-04	-0.136	5.38E-03	NS
MXRA7	NS	8.79E-04	1.36E-01	5.38E-03	NS
ARFGAP3	NS	8.82E-04	-0.136	5.39E-03	NS
SDHAF4	NS	8.82E-04	-0.136	5.39E-03	NS

FRG2B	NS	8.89E-04	-0.136	5.43E-03	NS
ZNF85	NS	8.90E-04	-0.136	5.44E-03	NS
COX11	NS	8.93E-04	-0.136	5.45E-03	NS
ITGA9	NS	8.94E-04	-0.136	5.46E-03	NS
GJA10	NS	8.95E-04	1.36E-01	5.46E-03	NS
PPP1R12A	NS	8.98E-04	1.36E-01	5.47E-03	NS
MED22	NS	8.97E-04	-0.136	5.47E-03	NS
NODAL	NS	8.98E-04	-0.136	5.48E-03	NS
OGFR	NS	9.00E-04	1.36E-01	5.48E-03	NS
CCNA1	NS	8.99E-04	-0.136	5.48E-03	NS
LCA5	NS	9.00E-04	-0.136	5.48E-03	NS
NPBWR2	NS	9.02E-04	1.36E-01	5.49E-03	NS
RILPL1	NS	9.02E-04	-0.136	5.49E-03	NS
LCN1	NS	9.02E-04	1.36E-01	5.49E-03	NS
FMO5	NS	9.03E-04	-0.136	5.49E-03	NS
GCHFR	NS	9.04E-04	-0.136	5.50E-03	NS
PNPLA1	NS	9.05E-04	-0.136	5.50E-03	NS
CFAP36	NS	9.06E-04	-0.136	5.50E-03	NS
SLC15A5	NS	9.06E-04	-0.136	5.50E-03	NS
ANOS1	NS	9.08E-04	-0.136	5.52E-03	NS
CTU2	NS	9.09E-04	-0.136	5.52E-03	NS
UBTD2	NS	9.10E-04	-0.136	5.52E-03	NS
LCMT2	NS	9.11E-04	-0.136	5.53E-03	NS
ZNF433	NS	9.11E-04	-0.136	5.53E-03	NS
DDN	NS	9.12E-04	-0.136	5.53E-03	NS
CRYBB1	NS	9.13E-04	-0.136	5.54E-03	NS
LGALS4	NS	9.13E-04	-0.136	5.54E-03	NS
CSNK1G1	NS	9.15E-04	-0.136	5.54E-03	NS
ZNF564	NS	9.15E-04	-0.136	5.54E-03	NS
SSR2	NS	9.18E-04	-0.136	5.56E-03	NS
DHRS7	NS	9.19E-04	-0.136	5.56E-03	NS
ATF7IP2	NS	9.19E-04	-0.136	5.56E-03	NS
GLRX3	NS	9.21E-04	-0.136	5.57E-03	NS
ZNF382	NS	9.21E-04	1.35E-01	5.57E-03	NS
OR51A7	NS	9.22E-04	-0.135	5.58E-03	NS
MRO	NS	9.23E-04	-0.135	5.58E-03	NS
ATP8B1	NS	9.24E-04	-0.135	5.58E-03	NS

VIT	NS	9.24E-04	-0.135	5.58E-03	NS
COL4A6	NS	9.27E-04	-0.135	5.60E-03	NS
LAMA5	NS	9.28E-04	-0.135	5.60E-03	NS
CDK2AP1	NS	9.31E-04	-0.135	5.62E-03	NS
LHX3	NS	9.34E-04	1.35E-01	5.63E-03	NS
ZNF8	NS	9.34E-04	-0.135	5.63E-03	NS
OR2V1	NS	9.34E-04	-0.135	5.63E-03	NS
SFT2D3	NS	9.38E-04	-0.135	5.65E-03	NS
DENND5A	NS	9.39E-04	-0.135	5.66E-03	NS
SSNA1	NS	9.42E-04	-0.135	5.68E-03	NS
CEBPZ	NS	9.44E-04	1.35E-01	5.68E-03	NS
CCDC70	NS	9.45E-04	-0.135	5.69E-03	NS
RTP1	NS	9.45E-04	-0.135	5.69E-03	NS
SMTNL1	NS	9.46E-04	-0.135	5.69E-03	NS
OR12D2	NS	9.46E-04	-0.135	5.69E-03	NS
AIG1	NS	9.47E-04	-0.135	5.69E-03	NS
BHMT	NS	9.49E-04	-0.135	5.71E-03	NS
GPX7	NS	9.52E-04	-0.135	5.72E-03	NS
MINAR1	NS	9.52E-04	-0.135	5.72E-03	NS
FKBP1B	NS	9.55E-04	-0.135	5.73E-03	NS
ZNF682	NS	9.55E-04	-0.135	5.73E-03	NS
LIG3	NS	9.57E-04	-0.135	5.74E-03	NS
CSRP3	NS	9.57E-04	-0.135	5.74E-03	NS
SLC25A27	NS	9.57E-04	-0.135	5.74E-03	NS
SSTR4	NS	9.58E-04	-0.135	5.74E-03	NS
ELMOD3	NS	9.59E-04	-0.135	5.75E-03	NS
NR4A1	NS	9.60E-04	-0.135	5.75E-03	NS
CBWD3	NS	9.60E-04	-0.135	5.75E-03	NS
HDAC8	NS	9.61E-04	-0.135	5.75E-03	NS
ANAPC2	NS	9.61E-04	-0.135	5.75E-03	NS
UGT1A10	NS	9.61E-04	-0.135	5.75E-03	NS
SYPL1	NS	9.64E-04	-0.135	5.77E-03	NS
SAMM50	NS	9.64E-04	-0.135	5.77E-03	NS
CCDC17	NS	9.66E-04	-0.135	5.77E-03	NS
ZBTB44	NS	9.66E-04	-0.135	5.77E-03	NS
TOX	NS	9.66E-04	-0.135	5.77E-03	NS
ZNF492	NS	9.68E-04	-0.135	5.78E-03	NS

PLLP	NS	9.69E-04	-0.135	5.78E-03	NS
TTC26	NS	9.68E-04	-0.135	5.78E-03	NS
SERPINI1	NS	9.71E-04	-0.135	5.80E-03	NS
GLYAT	NS	9.73E-04	-0.135	5.80E-03	NS
C17orf49	NS	9.74E-04	-0.135	5.81E-03	NS
CHTOP	NS	9.77E-04	1.35E-01	5.83E-03	NS
AHSA1	NS	9.81E-04	-0.135	5.84E-03	NS
PRM2	NS	9.84E-04	1.35E-01	5.86E-03	NS
TSPOAP1	NS	9.86E-04	-0.135	5.86E-03	NS
ZDHHC11B	NS	9.86E-04	-0.135	5.86E-03	NS
RPL13A	NS	9.86E-04	-0.135	5.86E-03	NS
SOX10	NS	9.87E-04	1.35E-01	5.87E-03	NS
GAN	NS	9.87E-04	-0.135	5.87E-03	NS
NTF3	NS	9.89E-04	-0.135	5.88E-03	NS
HADH	NS	9.93E-04	-0.135	5.90E-03	NS
LY6G6C	NS	9.93E-04	-0.135	5.90E-03	NS
CDY2B	NS	9.95E-04	1.35E-01	5.91E-03	NS
ASB16	NS	9.95E-04	-0.135	5.91E-03	NS
ADAMTSL5	NS	9.96E-04	1.35E-01	5.91E-03	NS
INSC	NS	9.95E-04	-0.135	5.91E-03	NS
USP17L19	NS	9.97E-04	1.35E-01	5.91E-03	NS
SLC22A13	NS	1.00E-03	-0.135	5.94E-03	NS
MYOF	NS	1.01E-03	-0.135	5.96E-03	NS
PPM1H	NS	1.01E-03	-0.135	5.96E-03	NS
THEG	NS	1.01E-03	1.34E-01	5.97E-03	NS
NDRG3	NS	1.01E-03	-0.134	5.98E-03	NS
PVALEF	NS	1.01E-03	1.34E-01	5.98E-03	NS
SOX1	NS	1.01E-03	1.34E-01	6.00E-03	NS
PPP6R1	NS	1.02E-03	-0.134	6.01E-03	NS
DRD2	NS	1.02E-03	-0.134	6.02E-03	NS
ACBD5	NS	1.02E-03	-0.134	6.03E-03	NS
DGKQ	NS	1.02E-03	-0.134	6.04E-03	NS
MELK	NS	1.02E-03	-0.134	6.04E-03	NS
BIK	NS	1.02E-03	-0.134	6.04E-03	NS
C9orf64	NS	1.02E-03	-0.134	6.05E-03	NS
BAG2	NS	1.03E-03	-0.134	6.07E-03	NS
PNLIPRP3	NS	1.03E-03	-0.134	6.08E-03	NS

SERPINI2	NS	1.03E-03	-0.134	6.08E-03	NS
CST11	NS	1.04E-03	-0.134	6.10E-03	NS
CIAO3	NS	1.04E-03	-0.134	6.11E-03	NS
FAM3B	NS	1.04E-03	-0.134	6.12E-03	NS
LRP4	NS	1.04E-03	-0.134	6.12E-03	NS
CLEC6A	NS	1.04E-03	-0.134	6.13E-03	NS
MLLT11	NS	1.04E-03	-0.134	6.13E-03	NS
RGL1	NS	1.04E-03	-0.134	6.14E-03	NS
NUDT2	NS	1.04E-03	-0.134	6.15E-03	NS
WWC1	NS	1.05E-03	-0.134	6.15E-03	NS
COPB1	NS	1.05E-03	-0.134	6.20E-03	NS
DHTKD1	NS	1.06E-03	-0.134	6.22E-03	NS
LRRC71	NS	1.06E-03	-0.134	6.22E-03	NS
UBE4B	NS	1.06E-03	-0.134	6.23E-03	NS
HSPB9	NS	1.06E-03	-0.134	6.23E-03	NS
SQLE	NS	1.07E-03	-0.134	6.25E-03	NS
ABITRAM	NS	1.07E-03	-0.134	6.25E-03	NS
RTF1	NS	1.07E-03	1.34E-01	6.25E-03	NS
EID1	NS	1.07E-03	-0.134	6.26E-03	NS
CYP20A1	NS	1.07E-03	-0.134	6.29E-03	NS
USP51	NS	1.07E-03	-0.134	6.30E-03	NS
PEX1	NS	1.08E-03	-0.134	6.32E-03	NS
RNF180	NS	1.08E-03	-0.134	6.32E-03	NS
TAT	NS	1.08E-03	-0.134	6.32E-03	NS
NCAPD2	NS	1.08E-03	-0.134	6.33E-03	NS
LRRIQ1	NS	1.08E-03	-0.134	6.35E-03	NS
GRIK1	NS	1.09E-03	-0.134	6.35E-03	NS
DKKL1	NS	1.09E-03	1.34E-01	6.36E-03	NS
ANGPT4	NS	1.09E-03	1.34E-01	6.36E-03	NS
PTPRF	NS	1.09E-03	-0.134	6.38E-03	NS
LY9	NS	1.09E-03	-0.134	6.39E-03	NS
TTC27	NS	1.09E-03	-0.134	6.40E-03	NS
OR13H1	NS	1.10E-03	-0.134	6.40E-03	NS
ZNF30	NS	1.10E-03	-0.133	6.42E-03	NS
GREM2	NS	1.10E-03	-0.133	6.42E-03	NS
XPO7	NS	1.10E-03	-0.133	6.43E-03	NS
FBXO25	NS	1.10E-03	-0.133	6.44E-03	NS

KDM5D	NS	1.10E-03	1.33E-01	6.44E-03	NS
SMTNL2	NS	1.10E-03	1.33E-01	6.44E-03	NS
VMO1	NS	1.11E-03	-0.133	6.45E-03	NS
HSPB7	NS	1.11E-03	1.33E-01	6.49E-03	NS
NOTCH3	NS	1.12E-03	-0.133	6.51E-03	NS
MSLNL	NS	1.12E-03	1.33E-01	6.51E-03	NS
TYW5	NS	1.12E-03	-0.133	6.52E-03	NS
SSU72P8	NS	1.12E-03	-0.133	6.52E-03	NS
RIMBP2	NS	1.12E-03	-0.133	6.52E-03	NS
RAD51C	NS	1.12E-03	-0.133	6.52E-03	NS
FBXL16	NS	1.12E-03	1.33E-01	6.52E-03	NS
IFNE	NS	1.13E-03	-0.133	6.54E-03	NS
AKAP14	NS	1.13E-03	-0.133	6.54E-03	NS
COPB2	NS	1.13E-03	-0.133	6.55E-03	NS
KDM4D	NS	1.13E-03	-0.133	6.55E-03	NS
SLC25A2	NS	1.13E-03	-0.133	6.56E-03	NS
EXD1	NS	1.13E-03	-0.133	6.56E-03	NS
SAMD11	NS	1.13E-03	-0.133	6.56E-03	NS
CA10	NS	1.13E-03	-0.133	6.56E-03	NS
CHIA	NS	1.13E-03	-0.133	6.58E-03	NS
ADGRE2	NS	1.14E-03	-0.133	6.60E-03	NS
DCAF1	NS	1.14E-03	-0.133	6.60E-03	NS
C3	NS	1.14E-03	-0.133	6.61E-03	NS
MFSD2B	NS	1.14E-03	-0.133	6.61E-03	NS
RPL23A	NS	1.14E-03	-0.133	6.61E-03	NS
KLF15	NS	1.14E-03	1.33E-01	6.62E-03	NS
C21orf62	NS	1.15E-03	1.33E-01	6.64E-03	NS
ZNF296	NS	1.15E-03	1.33E-01	6.64E-03	NS
SORT1	NS	1.15E-03	-0.133	6.65E-03	NS
LCN8	NS	1.15E-03	1.33E-01	6.65E-03	NS
IGFBP4	NS	1.15E-03	-0.133	6.65E-03	NS
CRB3	NS	1.15E-03	-0.133	6.66E-03	NS
RDH13	NS	1.15E-03	1.33E-01	6.66E-03	NS
DEFB114	NS	1.15E-03	-0.133	6.66E-03	NS
CFLAR	NS	1.16E-03	-0.133	6.67E-03	NS
ADIG	NS	1.16E-03	1.33E-01	6.68E-03	NS
SIRPB2	NS	1.16E-03	-0.133	6.69E-03	NS

PRR16	NS	1.16E-03	-0.133	6.70E-03	NS
LSM4	NS	1.16E-03	-0.133	6.71E-03	NS
CHEK1	NS	1.17E-03	1.33E-01	6.74E-03	NS
SLC25A20	NS	1.17E-03	-0.133	6.74E-03	NS
NAT1	NS	1.17E-03	-0.133	6.76E-03	NS
ZNF264	NS	1.18E-03	-0.133	6.77E-03	NS
TMEM204	NS	1.18E-03	-0.133	6.79E-03	NS
PRAMEF33	NS	1.19E-03	1.33E-01	6.83E-03	NS
SV2C	NS	1.19E-03	-0.133	6.83E-03	NS
SSX5	NS	1.19E-03	1.33E-01	6.83E-03	NS
MTHFD1	NS	1.19E-03	-0.133	6.84E-03	NS
ADRB1	NS	1.19E-03	-0.133	6.86E-03	NS
DLD	NS	1.19E-03	-0.133	6.86E-03	NS
SNAP23	NS	1.19E-03	-0.133	6.86E-03	NS
SPTA1	NS	1.19E-03	-0.133	6.86E-03	NS
SPACA5B	NS	1.19E-03	1.33E-01	6.86E-03	NS
KRTAP19-7	NS	1.20E-03	-0.133	6.87E-03	NS
KIAA1328	NS	1.20E-03	-0.133	6.87E-03	NS
COX7A1	NS	1.20E-03	-0.132	6.88E-03	NS
ITPR3	NS	1.20E-03	-0.132	6.88E-03	NS
KLHDC10	NS	1.20E-03	-0.132	6.88E-03	NS
PSMD11	NS	1.20E-03	-0.132	6.89E-03	NS
FKBP4	NS	1.20E-03	-0.132	6.89E-03	NS
SERPINB13	NS	1.21E-03	-0.132	6.92E-03	NS
BRCA1	NS	1.21E-03	-0.132	6.92E-03	NS
SCYGR8	NS	1.21E-03	-0.132	6.94E-03	NS
RHOBTB1	NS	1.21E-03	-0.132	6.94E-03	NS
BATF	NS	1.21E-03	-0.132	6.94E-03	NS
VSIG1	NS	1.21E-03	1.32E-01	6.94E-03	NS
MRPL49	NS	1.21E-03	-0.132	6.95E-03	NS
ASIC4	NS	1.22E-03	-0.132	6.96E-03	NS
TRIM43	NS	1.22E-03	-0.132	6.96E-03	NS
FAM184B	NS	1.22E-03	-0.132	6.96E-03	NS
HLA-DRB1	NS	1.22E-03	-0.132	6.97E-03	NS
ZNF668	NS	1.22E-03	-0.132	6.98E-03	NS
NIPA2	NS	1.23E-03	-0.132	7.01E-03	NS
ZNF875	NS	1.23E-03	-0.132	7.01E-03	NS

SH3YL1	NS	1.23E-03	-0.132	7.01E-03	NS
PRADC1	NS	1.23E-03	-0.132	7.02E-03	NS
SPRR2B	NS	1.23E-03	-0.132	7.03E-03	NS
PAQR5	NS	1.24E-03	1.32E-01	7.04E-03	NS
RABIF	NS	1.24E-03	-0.132	7.05E-03	NS
TEKT3	NS	1.24E-03	-0.132	7.05E-03	NS
RPL4	NS	1.24E-03	-0.132	7.05E-03	NS
MMP16	NS	1.24E-03	-0.132	7.05E-03	NS
COL6A5	NS	1.24E-03	-0.132	7.06E-03	NS
RHCE	NS	1.24E-03	-0.132	7.08E-03	NS
CCDC32	NS	1.25E-03	-0.132	7.08E-03	NS
HMMR	NS	1.25E-03	-0.132	7.09E-03	NS
PGM2L1	NS	1.25E-03	-0.132	7.10E-03	NS
PYHIN1	NS	1.25E-03	-0.132	7.11E-03	NS
BEST4	NS	1.25E-03	-0.132	7.12E-03	NS
DCAF12L1	NS	1.26E-03	-0.132	7.13E-03	NS
RNF115	NS	1.26E-03	-0.132	7.15E-03	NS
MAGT1	NS	1.26E-03	-0.132	7.15E-03	NS
RNASE4	NS	1.26E-03	-0.132	7.15E-03	NS
LRRC4	NS	1.26E-03	-0.132	7.15E-03	NS
DXO	NS	1.26E-03	1.32E-01	7.15E-03	NS
HCST	NS	1.26E-03	-0.132	7.15E-03	NS
B3GNT2	NS	1.26E-03	1.32E-01	7.16E-03	NS
ACSM1	NS	1.26E-03	-0.132	7.16E-03	NS
CHORDC1	NS	1.26E-03	-0.132	7.16E-03	NS
ANXA2R	NS	1.27E-03	-0.132	7.17E-03	NS
SLC25A23	NS	1.27E-03	1.32E-01	7.17E-03	NS
NRAP	NS	1.27E-03	-0.132	7.19E-03	NS
ELF5	NS	1.27E-03	-0.132	7.20E-03	NS
CASP5	NS	1.27E-03	-0.132	7.21E-03	NS
ZEB2	NS	1.28E-03	-0.132	7.21E-03	NS
SNTN	NS	1.28E-03	1.32E-01	7.23E-03	NS
COL5A2	NS	1.28E-03	-0.132	7.25E-03	NS
TRIL	NS	1.29E-03	1.32E-01	7.26E-03	NS
PEAK1	NS	1.29E-03	1.32E-01	7.27E-03	NS
BLOC1S2	NS	1.29E-03	-0.132	7.29E-03	NS
KRTAP5-7	NS	1.29E-03	-0.132	7.30E-03	NS

THSD1	NS	1.29E-03	-0.132	7.30E-03	NS
ANXA3	NS	1.29E-03	-0.132	7.30E-03	NS
INPP5B	NS	1.30E-03	-0.132	7.30E-03	NS
NUBP1	NS	1.30E-03	-0.132	7.30E-03	NS
NUDT3	NS	1.30E-03	-0.132	7.31E-03	NS
HOXD8	NS	1.30E-03	1.32E-01	7.31E-03	NS
SQOR	NS	1.30E-03	-0.132	7.32E-03	NS
KLF12	NS	1.30E-03	-0.132	7.32E-03	NS
TMEM255A	NS	1.30E-03	-0.132	7.33E-03	NS
CLDND1	NS	1.30E-03	-0.132	7.34E-03	NS
PDP1	NS	1.31E-03	-0.131	7.35E-03	NS
ZNF700	NS	1.31E-03	-0.131	7.35E-03	NS
SLCO4A1	NS	1.31E-03	-0.131	7.36E-03	NS
VIL1	NS	1.31E-03	-0.131	7.36E-03	NS
CEPT1	NS	1.31E-03	-0.131	7.36E-03	NS
CFD	NS	1.31E-03	-0.131	7.36E-03	NS
IL7	NS	1.31E-03	-0.131	7.38E-03	NS
H6PD	NS	1.32E-03	-0.131	7.39E-03	NS
IL6	NS	1.32E-03	-0.131	7.39E-03	NS
BNIP1	NS	1.32E-03	-0.131	7.39E-03	NS
USP31	NS	1.32E-03	-0.131	7.40E-03	NS
HERC2	NS	1.32E-03	-0.131	7.40E-03	NS
HFM1	NS	1.33E-03	-0.131	7.43E-03	NS
KTI12	NS	1.33E-03	-0.131	7.43E-03	NS
C1N-ZNF705EP-ALG	NS	1.33E-03	-0.131	7.43E-03	NS
L2HGDH	NS	1.33E-03	-0.131	7.45E-03	NS
KPNA4	NS	1.33E-03	-0.131	7.45E-03	NS
PLPPR1	NS	1.33E-03	-0.131	7.47E-03	NS
GGPS1	NS	1.34E-03	-0.131	7.48E-03	NS
ABHD4	NS	1.34E-03	1.31E-01	7.49E-03	NS
VWA8	NS	1.34E-03	-0.131	7.49E-03	NS
PCDHGB2	NS	1.34E-03	-0.131	7.51E-03	NS
AKT2	NS	1.35E-03	-0.131	7.52E-03	NS
NTAN1	NS	1.35E-03	1.31E-01	7.53E-03	NS
VCAN	NS	1.35E-03	-0.131	7.53E-03	NS
SPATA16	NS	1.35E-03	-0.131	7.53E-03	NS
NCMAP	NS	1.35E-03	1.31E-01	7.54E-03	NS

RNF113B	NS	1.35E-03	1.31E-01	7.56E-03	NS
RMND5B	NS	1.36E-03	-0.131	7.56E-03	NS
CXCR4	NS	1.36E-03	1.31E-01	7.57E-03	NS
CLDN1	NS	1.36E-03	-0.131	7.57E-03	NS
ADPRS	NS	1.36E-03	-0.131	7.57E-03	NS
MEFV	NS	1.37E-03	-0.131	7.61E-03	NS
PIH1D1	NS	1.37E-03	-0.131	7.62E-03	NS
SPOPL	NS	1.37E-03	-0.131	7.62E-03	NS
FAM25G	NS	1.37E-03	-0.131	7.64E-03	NS
LEKR1	NS	1.37E-03	-0.131	7.65E-03	NS
ANKHD1-EIF4EBP3	NS	1.37E-03	-0.131	7.65E-03	NS
SLC5A11	NS	1.38E-03	-0.131	7.67E-03	NS
ENPP3	NS	1.38E-03	-0.131	7.67E-03	NS
EXOC6	NS	1.38E-03	-0.131	7.68E-03	NS
WDR89	NS	1.38E-03	-0.131	7.69E-03	NS
DOP1A	NS	1.39E-03	-0.131	7.70E-03	NS
LARP1	NS	1.39E-03	-0.131	7.70E-03	NS
PHC3	NS	1.39E-03	1.31E-01	7.70E-03	NS
CCZ1B	NS	1.39E-03	-0.131	7.72E-03	NS
ADAM7	NS	1.39E-03	-0.131	7.73E-03	NS
WDR64	NS	1.40E-03	-0.131	7.75E-03	NS
ACTL8	NS	1.40E-03	1.31E-01	7.77E-03	NS
S100A7	NS	1.40E-03	-0.131	7.77E-03	NS
DDX53	NS	1.40E-03	-0.131	7.78E-03	NS
STOML3	NS	1.41E-03	-0.131	7.81E-03	NS
GSK3A	NS	1.41E-03	1.31E-01	7.81E-03	NS
CORIN	NS	1.41E-03	-0.131	7.83E-03	NS
GUF1	NS	1.41E-03	-0.131	7.83E-03	NS
MGA	NS	1.41E-03	-0.131	7.83E-03	NS
TUBAL3	NS	1.42E-03	-0.131	7.83E-03	NS
MCTP1	NS	1.42E-03	-0.131	7.83E-03	NS
OR6C70	NS	1.42E-03	1.31E-01	7.83E-03	NS
SPEGNB	NS	1.42E-03	-0.131	7.83E-03	NS
KCNJ13	NS	1.42E-03	-0.131	7.84E-03	NS
C3orf56	NS	1.42E-03	-0.13	7.86E-03	NS
HARS2	NS	1.43E-03	-0.13	7.88E-03	NS
C2orf73	NS	1.43E-03	-0.13	7.90E-03	NS

INAFM1	NS	1.43E-03	1.30E-01	7.90E-03	NS
KCNK5	NS	1.43E-03	-0.13	7.91E-03	NS
TUBGCP6	NS	1.44E-03	-0.13	7.94E-03	NS
EGFLAM	NS	1.44E-03	-0.13	7.94E-03	NS
CRISP3	NS	1.45E-03	-0.13	7.97E-03	NS
SLC18A2	NS	1.45E-03	-0.13	7.99E-03	NS
HTR1D	NS	1.45E-03	1.30E-01	7.99E-03	NS
SSU72P5	NS	1.45E-03	1.30E-01	8.00E-03	NS
MTERF2	NS	1.45E-03	-0.13	8.00E-03	NS
MSL2	NS	1.45E-03	1.30E-01	8.00E-03	NS
AIFM2	NS	1.46E-03	-0.13	8.02E-03	NS
SRR	NS	1.46E-03	-0.13	8.02E-03	NS
KCNG1	NS	1.46E-03	1.30E-01	8.03E-03	NS
CFHR5	NS	1.46E-03	-0.13	8.03E-03	NS
OXCT1	NS	1.46E-03	-0.13	8.05E-03	NS
GOLGA8S	NS	1.46E-03	-0.13	8.05E-03	NS
GLRX2	NS	1.47E-03	-0.13	8.06E-03	NS
GRHL2	NS	1.47E-03	-0.13	8.06E-03	NS
TMC6	NS	1.47E-03	1.30E-01	8.06E-03	NS
SLC39A9	NS	1.47E-03	-0.13	8.06E-03	NS
PGS1	NS	1.47E-03	1.30E-01	8.06E-03	NS
C19orf53	NS	1.47E-03	-0.13	8.06E-03	NS
CST8	NS	1.47E-03	-0.13	8.07E-03	NS
MSI2	NS	1.47E-03	1.30E-01	8.07E-03	NS
GPT2	NS	1.47E-03	-0.13	8.07E-03	NS
SH3PXD2B	NS	1.47E-03	1.30E-01	8.07E-03	NS
FCN2	NS	1.48E-03	-0.13	8.09E-03	NS
GNPDA2	NS	1.48E-03	-0.13	8.10E-03	NS
JUNB	NS	1.48E-03	1.30E-01	8.11E-03	NS
ALDH1L2	NS	1.48E-03	1.30E-01	8.11E-03	NS
SRBD1	NS	1.48E-03	-0.13	8.11E-03	NS
CCR9	NS	1.48E-03	-0.13	8.11E-03	NS
SORCS2	NS	1.49E-03	-0.13	8.14E-03	NS
CACNG4	NS	1.49E-03	1.30E-01	8.16E-03	NS
SLC16A4	NS	1.50E-03	-0.13	8.18E-03	NS
ZSCAN9	NS	1.50E-03	-0.13	8.20E-03	NS
LCN6	NS	1.50E-03	1.30E-01	8.20E-03	NS

RPRD1A	NS	1.50E-03	-0.13	8.21E-03	NS
GFRA3	NS	1.50E-03	1.30E-01	8.21E-03	NS
FSHB	NS	1.51E-03	-0.13	8.22E-03	NS
EHF	NS	1.51E-03	-0.13	8.22E-03	NS
NCAPH2	NS	1.51E-03	-0.13	8.23E-03	NS
TIMM50	NS	1.51E-03	-0.13	8.23E-03	NS
DDB1	NS	1.51E-03	-0.13	8.26E-03	NS
FKBP2	NS	1.52E-03	-0.13	8.26E-03	NS
CT47A11	NS	1.52E-03	1.30E-01	8.27E-03	NS
CD4	NS	1.52E-03	-0.13	8.27E-03	NS
SNF8	NS	1.52E-03	-0.13	8.28E-03	NS
SLC30A4	NS	1.52E-03	-0.13	8.29E-03	NS
EMILIN2	NS	1.52E-03	-0.13	8.29E-03	NS
FANCE	NS	1.52E-03	-0.13	8.29E-03	NS
OR2L8	NS	1.53E-03	-0.13	8.30E-03	NS
LHFPL1	NS	1.53E-03	-0.13	8.32E-03	NS
GPR183	NS	1.54E-03	-0.13	8.34E-03	NS
CD5L	NS	1.54E-03	-0.13	8.34E-03	NS
CABP5	NS	1.54E-03	-0.13	8.34E-03	NS
GSE1	NS	1.54E-03	-0.13	8.34E-03	NS
ZNF852	NS	1.54E-03	-0.13	8.35E-03	NS
ZNF696	NS	1.54E-03	-0.13	8.35E-03	NS
ZBTB16	NS	1.54E-03	1.30E-01	8.35E-03	NS
PNPLA2	NS	1.54E-03	1.30E-01	8.37E-03	NS
GSDMD	NS	1.55E-03	-0.13	8.38E-03	NS
CLMP	NS	1.55E-03	-0.13	8.39E-03	NS
SPANXN5	NS	1.55E-03	1.29E-01	8.40E-03	NS
ZYG11B	NS	1.55E-03	1.29E-01	8.41E-03	NS
MYO5C	NS	1.55E-03	-0.129	8.42E-03	NS
PRAMEF7	NS	1.55E-03	-0.129	8.42E-03	NS
FAM151A	NS	1.56E-03	-0.129	8.42E-03	NS
NBAS	NS	1.56E-03	-0.129	8.43E-03	NS
LRCH1	NS	1.56E-03	-0.129	8.43E-03	NS
SLC22A14	NS	1.56E-03	-0.129	8.45E-03	NS
PTN	NS	1.57E-03	-0.129	8.47E-03	NS
SLC25A5	NS	1.57E-03	-0.129	8.48E-03	NS
MYH13	NS	1.57E-03	-0.129	8.48E-03	NS

ETFB	NS	1.57E-03	-0.129	8.49E-03	NS
ECRG4	NS	1.57E-03	-0.129	8.49E-03	NS
TYMP	NS	1.58E-03	1.29E-01	8.50E-03	NS
LRRC70	NS	1.58E-03	-0.129	8.50E-03	NS
SPATA2L	NS	1.59E-03	-0.129	8.56E-03	NS
LOXL3	NS	1.59E-03	-0.129	8.56E-03	NS
ZNF234	NS	1.59E-03	-0.129	8.56E-03	NS
TMEM91	NS	1.59E-03	1.29E-01	8.56E-03	NS
QPCTL	NS	1.59E-03	-0.129	8.59E-03	NS
MDK	NS	1.59E-03	-0.129	8.59E-03	NS
ZNF101	NS	1.59E-03	-0.129	8.59E-03	NS
TFCP2L1	NS	1.60E-03	-0.129	8.59E-03	NS
HAPLN1	NS	1.60E-03	-0.129	8.60E-03	NS
ERCC6L2	NS	1.60E-03	-0.129	8.61E-03	NS
MBD6	NS	1.60E-03	1.29E-01	8.63E-03	NS
PRR32	NS	1.61E-03	-0.129	8.64E-03	NS
POTEJ	NS	1.61E-03	1.29E-01	8.67E-03	NS
CCL16	NS	1.61E-03	1.29E-01	8.67E-03	NS
ANO1	NS	1.61E-03	1.29E-01	8.68E-03	NS
SMG9	NS	1.62E-03	-0.129	8.70E-03	NS
TMPRSS11A	NS	1.62E-03	-0.129	8.70E-03	NS
TAAR5	NS	1.62E-03	-0.129	8.71E-03	NS
SHC4	NS	1.62E-03	-0.129	8.72E-03	NS
PSPN	NS	1.62E-03	-0.129	8.72E-03	NS
WFDC11	NS	1.63E-03	-0.129	8.74E-03	NS
COPZ2	NS	1.63E-03	-0.129	8.75E-03	NS
NDUFB1	NS	1.63E-03	-0.129	8.75E-03	NS
FAM86B1	NS	1.63E-03	-0.129	8.75E-03	NS
SLC25A53	NS	1.63E-03	-0.129	8.75E-03	NS
TNNI1	NS	1.63E-03	-0.129	8.75E-03	NS
SCAF11	NS	1.63E-03	-0.129	8.76E-03	NS
SLC16A3	NS	1.64E-03	1.29E-01	8.76E-03	NS
REEP5	NS	1.64E-03	-0.129	8.79E-03	NS
FAM126B	NS	1.64E-03	-0.129	8.79E-03	NS
EGFL8	NS	1.64E-03	-0.129	8.79E-03	NS
NUDT21	NS	1.64E-03	-0.129	8.80E-03	NS
GMEB2	NS	1.65E-03	-0.129	8.81E-03	NS

VAX1	NS	1.65E-03	1.29E-01	8.81E-03	NS
CHRNG	NS	1.65E-03	-0.129	8.81E-03	NS
RNF133	NS	1.65E-03	-0.129	8.82E-03	NS
HIP1	NS	1.65E-03	-0.129	8.82E-03	NS
DNAJC5G	NS	1.65E-03	1.29E-01	8.82E-03	NS
SPTY2D1OS	NS	1.65E-03	-0.129	8.82E-03	NS
GK5	NS	1.65E-03	-0.129	8.82E-03	NS
OR6T1	NS	1.65E-03	-0.129	8.82E-03	NS
PSMD1	NS	1.66E-03	-0.129	8.83E-03	NS
GORAB	NS	1.66E-03	-0.129	8.85E-03	NS
TOMT	NS	1.66E-03	-0.129	8.85E-03	NS
RGR	NS	1.66E-03	-0.129	8.86E-03	NS
MEGF11	NS	1.66E-03	-0.129	8.86E-03	NS
UQCC2	NS	1.66E-03	-0.129	8.86E-03	NS
MAGEB16	NS	1.66E-03	-0.129	8.86E-03	NS
SLITRK6	NS	1.67E-03	-0.129	8.86E-03	NS
CENPE	NS	1.67E-03	-0.129	8.88E-03	NS
SYT14	NS	1.67E-03	-0.129	8.88E-03	NS
HOXC5	NS	1.67E-03	1.29E-01	8.89E-03	NS
MORC2	NS	1.68E-03	-0.129	8.91E-03	NS
MED7	NS	1.68E-03	-0.129	8.91E-03	NS
PRR23C	NS	1.68E-03	1.29E-01	8.91E-03	NS
SERPINB3	NS	1.68E-03	-0.129	8.91E-03	NS
GDAP2	NS	1.68E-03	-0.129	8.94E-03	NS
PCMT1	NS	1.68E-03	-0.128	8.95E-03	NS
MTFP1	NS	1.69E-03	-0.128	8.96E-03	NS
KLHDC7A	NS	1.69E-03	1.28E-01	8.97E-03	NS
GOLGA6L22	NS	1.69E-03	-0.128	8.97E-03	NS
KCNJ11	NS	1.69E-03	-0.128	8.97E-03	NS
NEO1	NS	1.69E-03	-0.128	8.98E-03	NS
ANXA5	NS	1.69E-03	-0.128	8.98E-03	NS
ZNF208	NS	1.70E-03	-0.128	9.00E-03	NS
TBX10	NS	1.70E-03	1.28E-01	9.00E-03	NS
FAM9C	NS	1.70E-03	-0.128	9.00E-03	NS
UTP25	NS	1.70E-03	-0.128	9.00E-03	NS
ADCK5	NS	1.70E-03	-0.128	9.02E-03	NS
DHODH	NS	1.72E-03	-0.128	9.09E-03	NS

KNCN	NS	1.72E-03	1.28E-01	9.09E-03	NS
LIPF	NS	1.72E-03	-0.128	9.09E-03	NS
AOC1	NS	1.72E-03	-0.128	9.10E-03	NS
VPREB3	NS	1.72E-03	1.28E-01	9.11E-03	NS
NEDD4	NS	1.73E-03	-0.128	9.12E-03	NS
RUFY3	NS	1.73E-03	-0.128	9.13E-03	NS
NDUFAF6	NS	1.73E-03	-0.128	9.13E-03	NS
OR14J1	NS	1.73E-03	-0.128	9.13E-03	NS
C1QC	NS	1.73E-03	-0.128	9.14E-03	NS
LAMB4	NS	1.74E-03	-0.128	9.17E-03	NS
MYH7	NS	1.74E-03	-0.128	9.17E-03	NS
DENND1C	NS	1.74E-03	1.28E-01	9.17E-03	NS
PRR23A	NS	1.74E-03	1.28E-01	9.17E-03	NS
LRIG2	NS	1.74E-03	-0.128	9.18E-03	NS
C5orf15	NS	1.74E-03	-0.128	9.19E-03	NS
VEGFB	NS	1.74E-03	-0.128	9.19E-03	NS
MCF2	NS	1.75E-03	-0.128	9.20E-03	NS
IL17D	NS	1.75E-03	1.28E-01	9.21E-03	NS
ZNF184	NS	1.75E-03	-0.128	9.21E-03	NS
CACTIN	NS	1.75E-03	1.28E-01	9.21E-03	NS
NPHP3	NS	1.75E-03	-0.128	9.21E-03	NS
TRMT5	NS	1.75E-03	-0.128	9.21E-03	NS
TMEM86A	NS	1.75E-03	-0.128	9.21E-03	NS
CCDC74A	NS	1.75E-03	1.28E-01	9.21E-03	NS
ELMOD2	NS	1.75E-03	-0.128	9.21E-03	NS
GP6	NS	1.76E-03	1.28E-01	9.22E-03	NS
NOS2	NS	1.76E-03	-0.128	9.23E-03	NS
KIN	NS	1.76E-03	-0.128	9.23E-03	NS
ICA1L	NS	1.76E-03	-0.128	9.23E-03	NS
BLOC1S5-TXNDC5	NS	1.76E-03	-0.128	9.23E-03	NS
HFE	NS	1.76E-03	-0.128	9.23E-03	NS
TSTD2	NS	1.76E-03	-0.128	9.25E-03	NS
NME6	NS	1.76E-03	-0.128	9.25E-03	NS
KCNJ6	NS	1.77E-03	-0.128	9.26E-03	NS
RESP18	NS	1.77E-03	-0.128	9.26E-03	NS
OR51M1	NS	1.77E-03	1.28E-01	9.27E-03	NS
SCYGR6	NS	1.77E-03	1.28E-01	9.29E-03	NS

KLRG2	NS	1.77E-03	1.28E-01	9.29E-03	NS
OR5A1	NS	1.78E-03	-0.128	9.31E-03	NS
MAGEB2	NS	1.78E-03	-0.128	9.32E-03	NS
GOLGA6L25P	NS	1.78E-03	-0.128	9.34E-03	NS
EEF1A1	NS	1.80E-03	-0.128	9.41E-03	NS
NUP62CL	NS	1.80E-03	-0.128	9.41E-03	NS
SCRN2	NS	1.80E-03	-0.128	9.42E-03	NS
SERTM2	NS	1.80E-03	-0.128	9.42E-03	NS
SYNE2	NS	1.80E-03	-0.128	9.42E-03	NS
CCR6	NS	1.80E-03	-0.128	9.42E-03	NS
OR4F16	NS	1.81E-03	-0.128	9.43E-03	NS
HIGD1A	NS	1.81E-03	-0.128	9.47E-03	NS
HSP90AB1	NS	1.82E-03	-0.128	9.47E-03	NS
MS4A18	NS	1.82E-03	-0.128	9.49E-03	NS
SMURF2	NS	1.82E-03	-0.128	9.49E-03	NS
PDE6D	NS	1.82E-03	-0.128	9.51E-03	NS
ENDOG	NS	1.82E-03	-0.128	9.51E-03	NS
TMEM186	NS	1.83E-03	-0.128	9.52E-03	NS
KLHL25	NS	1.83E-03	-0.128	9.53E-03	NS
ZNF816-ZNF321P	NS	1.83E-03	-0.128	9.53E-03	NS
RAD9A	NS	1.83E-03	-0.128	9.53E-03	NS
ICAM5	NS	1.83E-03	-0.127	9.54E-03	NS
PDZD7	NS	1.84E-03	1.27E-01	9.57E-03	NS
IMMP2L	NS	1.84E-03	-0.127	9.58E-03	NS
PNMA6A	NS	1.84E-03	1.27E-01	9.58E-03	NS
TBXA2R	NS	1.84E-03	1.27E-01	9.59E-03	NS
C12orf75	NS	1.84E-03	-0.127	9.59E-03	NS
SLC37A4	NS	1.85E-03	-0.127	9.61E-03	NS
SDR42E1	NS	1.85E-03	-0.127	9.63E-03	NS
OR9Q1	NS	1.85E-03	-0.127	9.63E-03	NS
ABCG8	NS	1.86E-03	-0.127	9.64E-03	NS
GPC1	NS	1.86E-03	-0.127	9.64E-03	NS
AK7	NS	1.86E-03	-0.127	9.65E-03	NS
UBALD2	NS	1.86E-03	1.27E-01	9.65E-03	NS
RPS27A	NS	1.86E-03	-0.127	9.66E-03	NS
NR1I2	NS	1.86E-03	-0.127	9.66E-03	NS
FAM71C	NS	1.86E-03	-0.127	9.67E-03	NS

TSPY8	NS	1.87E-03	1.27E-01	9.71E-03	NS
HEATR9	NS	1.87E-03	-0.127	9.72E-03	NS
TRAT1	NS	1.88E-03	-0.127	9.73E-03	NS
SLC34A3	NS	1.88E-03	1.27E-01	9.76E-03	NS
TNPO3	NS	1.88E-03	-0.127	9.76E-03	NS
ZFAND5	NS	1.89E-03	1.27E-01	9.79E-03	NS
HSPB2	NS	1.89E-03	1.27E-01	9.79E-03	NS
INTS6L	NS	1.89E-03	-0.127	9.79E-03	NS
RGS7BP	NS	1.90E-03	-0.127	9.82E-03	NS
RARRES2	NS	1.90E-03	1.27E-01	9.82E-03	NS
CHRNB4	NS	1.90E-03	1.27E-01	9.83E-03	NS
TMEM88B	NS	1.90E-03	1.27E-01	9.83E-03	NS
C7orf31	NS	1.91E-03	-0.127	9.86E-03	NS
BFSP2	NS	1.91E-03	-0.127	9.88E-03	NS
FZD10	NS	1.92E-03	1.27E-01	9.90E-03	NS
PRMT6	NS	1.92E-03	-0.127	9.90E-03	NS
SPDEF	NS	1.92E-03	-0.127	9.91E-03	NS
MATN3	NS	1.92E-03	-0.127	9.91E-03	NS
RELN	NS	1.92E-03	-0.127	9.91E-03	NS
DEFA3	NS	1.92E-03	-0.127	9.91E-03	NS
TMEM223	NS	1.93E-03	-0.127	9.93E-03	NS
FCER1A	NS	1.93E-03	-0.127	9.94E-03	NS
UBL3	NS	1.93E-03	-0.127	9.96E-03	NS
HIF3A	NS	1.93E-03	-0.127	9.96E-03	NS
PERCC1	NS	1.93E-03	1.27E-01	9.96E-03	NS
MAP2	NS	1.94E-03	-0.127	9.97E-03	NS
UBAP2	NS	1.94E-03	1.27E-01	1.00E-02	NS
TRIR	NS	1.95E-03	1.27E-01	1.00E-02	NS
SLC7A7	NS	1.95E-03	-0.127	1.00E-02	NS
ADH1A	NS	1.95E-03	-0.127	1.00E-02	NS
ODAM	NS	1.95E-03	-0.127	1.00E-02	NS
MYO18B	NS	1.96E-03	-0.127	1.01E-02	NS
CDH17	NS	1.96E-03	-0.127	1.01E-02	NS
TAF11L12	NS	1.96E-03	1.27E-01	1.01E-02	NS
ATRN	NS	1.96E-03	-0.127	1.01E-02	NS
FAM71E2	NS	1.96E-03	1.27E-01	1.01E-02	NS
CCDC66	NS	1.96E-03	-0.127	1.01E-02	NS

ADAT3	NS	1.97E-03	1.27E-01	1.01E-02	NS
ABCA2	NS	1.97E-03	1.27E-01	1.01E-02	NS
CAVIN3	NS	1.98E-03	-0.127	1.01E-02	NS
ONECUT1	NS	1.98E-03	1.27E-01	1.02E-02	NS
MPP4	NS	1.98E-03	-0.127	1.02E-02	NS
GTF2E1	NS	1.99E-03	-0.127	1.02E-02	NS
IRAK4	NS	1.99E-03	-0.127	1.02E-02	NS
LAMA3	NS	1.99E-03	-0.126	1.02E-02	NS
ACLY	NS	2.00E-03	-0.126	1.02E-02	NS
ABCA10	NS	2.00E-03	-0.126	1.02E-02	NS
NR2F6	NS	2.00E-03	-0.126	1.02E-02	NS
WDSUB1	NS	2.00E-03	1.26E-01	1.02E-02	NS
EARS2	NS	2.00E-03	-0.126	1.02E-02	NS
E2F3	NS	2.00E-03	-0.126	1.02E-02	NS
MAK16	NS	2.00E-03	-0.126	1.03E-02	NS
RAD54B	NS	2.01E-03	-0.126	1.03E-02	NS
SMC1B	NS	2.01E-03	-0.126	1.03E-02	NS
EXOC3L1	NS	2.01E-03	-0.126	1.03E-02	NS
ENOPH1	NS	2.01E-03	-0.126	1.03E-02	NS
TIGD1	NS	2.02E-03	-0.126	1.03E-02	NS
CALHM4	NS	2.02E-03	-0.126	1.03E-02	NS
PPP5C	NS	2.02E-03	-0.126	1.03E-02	NS
TMEM252	NS	2.02E-03	-0.126	1.03E-02	NS
CSMD2	NS	2.03E-03	-0.126	1.03E-02	NS
MS4A5	NS	2.03E-03	-0.126	1.04E-02	NS
CLEC9A	NS	2.03E-03	-0.126	1.04E-02	NS
CYB561D2	NS	2.03E-03	-0.126	1.04E-02	NS
IQCN	NS	2.04E-03	-0.126	1.04E-02	NS
ADGRV1	NS	2.04E-03	-0.126	1.04E-02	NS
UBE2Z	NS	2.04E-03	1.26E-01	1.04E-02	NS
IGSF22	NS	2.04E-03	-0.126	1.04E-02	NS
RPL3L	NS	2.04E-03	1.26E-01	1.04E-02	NS
EIF2S3B	NS	2.05E-03	-0.126	1.04E-02	NS
IGFBPL1	NS	2.05E-03	-0.126	1.04E-02	NS
MTX2	NS	2.05E-03	-0.126	1.04E-02	NS
ZNF614	NS	2.05E-03	-0.126	1.04E-02	NS
PRR36	NS	2.05E-03	1.26E-01	1.04E-02	NS

PTGES3L	NS	2.05E-03	1.26E-01	1.04E-02	NS
BDH2	NS	2.06E-03	-0.126	1.05E-02	NS
SLBP	NS	2.06E-03	1.26E-01	1.05E-02	NS
ARHGAP8	NS	2.06E-03	-0.126	1.05E-02	NS
GMPR2	NS	2.06E-03	-0.126	1.05E-02	NS
ETNPPL	NS	2.06E-03	-0.126	1.05E-02	NS
CDC123	NS	2.07E-03	-0.126	1.05E-02	NS
SPACA4	NS	2.07E-03	1.26E-01	1.05E-02	NS
RAB35	NS	2.07E-03	1.26E-01	1.05E-02	NS
TRIM49B	NS	2.08E-03	-0.126	1.05E-02	NS
UBXN6	NS	2.08E-03	-0.126	1.05E-02	NS
SIX1	NS	2.08E-03	1.26E-01	1.05E-02	NS
KLK15	NS	2.08E-03	1.26E-01	1.06E-02	NS
APOBEC3C	NS	2.09E-03	1.26E-01	1.06E-02	NS
MAST1	NS	2.09E-03	-0.126	1.06E-02	NS
PLIN4	NS	2.10E-03	-0.126	1.06E-02	NS
DYNLRB2	NS	2.10E-03	-0.126	1.06E-02	NS
UGT2B11	NS	2.10E-03	-0.126	1.06E-02	NS
CASD1	NS	2.10E-03	-0.126	1.06E-02	NS
UGT1A4	NS	2.10E-03	-0.126	1.06E-02	NS
TM9SF2	NS	2.10E-03	-0.126	1.06E-02	NS
PRELID2	NS	2.10E-03	-0.126	1.06E-02	NS
RSBN1L	NS	2.11E-03	-0.126	1.07E-02	NS
PRKD1	NS	2.11E-03	-0.126	1.07E-02	NS
EPM2AIP1	NS	2.12E-03	-0.126	1.07E-02	NS
ZNF133	NS	2.12E-03	-0.126	1.07E-02	NS
PRICKLE2	NS	2.12E-03	-0.126	1.07E-02	NS
FYB2	NS	2.13E-03	-0.126	1.08E-02	NS
KCNV2	NS	2.13E-03	-0.126	1.08E-02	NS
CEACAM5	NS	2.14E-03	-0.126	1.08E-02	NS
KRTCAP3	NS	2.14E-03	-0.126	1.08E-02	NS
CXCL13	NS	2.14E-03	-0.126	1.08E-02	NS
MRPL46	NS	2.14E-03	-0.126	1.08E-02	NS
RPA2	NS	2.15E-03	-0.126	1.08E-02	NS
TSC22D3	NS	2.14E-03	1.26E-01	1.08E-02	NS
MT1X	NS	2.14E-03	-0.126	1.08E-02	NS
GABBR1	NS	2.15E-03	-0.126	1.08E-02	NS

RSPH6A	NS	2.15E-03	1.26E-01	1.08E-02	NS
DPEP3	NS	2.16E-03	-0.126	1.08E-02	NS
SLC52A3	NS	2.16E-03	1.26E-01	1.08E-02	NS
PDCD2L	NS	2.16E-03	-0.126	1.08E-02	NS
MPRIP	NS	2.16E-03	-0.126	1.08E-02	NS
HMHB1	NS	2.16E-03	1.26E-01	1.08E-02	NS
FAM234A	NS	2.16E-03	-0.126	1.08E-02	NS
KCNAB1	NS	2.16E-03	1.26E-01	1.08E-02	NS
C2CD4B	NS	2.16E-03	1.26E-01	1.09E-02	NS
MED8	NS	2.16E-03	-0.125	1.09E-02	NS
KRTAP5-4	NS	2.17E-03	1.25E-01	1.09E-02	NS
C18orf25	NS	2.17E-03	1.25E-01	1.09E-02	NS
TBCEL	NS	2.17E-03	-0.125	1.09E-02	NS
UPK3A	NS	2.18E-03	-0.125	1.09E-02	NS
PSMA8	NS	2.18E-03	-0.125	1.09E-02	NS
ATP6AP2	NS	2.19E-03	-0.125	1.10E-02	NS
GRPR	NS	2.19E-03	-0.125	1.10E-02	NS
CTPS2	NS	2.20E-03	-0.125	1.10E-02	NS
PSMA7	NS	2.20E-03	1.25E-01	1.10E-02	NS
ADAR	NS	2.20E-03	-0.125	1.10E-02	NS
HROB	NS	2.20E-03	1.25E-01	1.10E-02	NS
TACC1	NS	2.20E-03	1.25E-01	1.10E-02	NS
SNX30	NS	2.20E-03	-0.125	1.10E-02	NS
FOXH1	NS	2.20E-03	-0.125	1.10E-02	NS
PPIAL4H	NS	2.20E-03	1.25E-01	1.10E-02	NS
CRISP1	NS	2.21E-03	-0.125	1.10E-02	NS
MLANA	NS	2.21E-03	-0.125	1.11E-02	NS
SCN7A	NS	2.21E-03	-0.125	1.11E-02	NS
LZTS1	NS	2.21E-03	-0.125	1.11E-02	NS
NCF1	NS	2.22E-03	1.25E-01	1.11E-02	NS
B3GNTL1	NS	2.22E-03	-0.125	1.11E-02	NS
MAGEL2	NS	2.22E-03	1.25E-01	1.11E-02	NS
C4A	NS	2.22E-03	-0.125	1.11E-02	NS
INPP5A	NS	2.22E-03	-0.125	1.11E-02	NS
PTAR1	NS	2.22E-03	-0.125	1.11E-02	NS
SNX14	NS	2.23E-03	-0.125	1.11E-02	NS
SLC15A3	NS	2.23E-03	1.25E-01	1.11E-02	NS

PABPC5	NS	2.23E-03	-0.125	1.11E-02	NS
AKAP1	NS	2.24E-03	-0.125	1.12E-02	NS
TNS4	NS	2.24E-03	1.25E-01	1.12E-02	NS
DNAH6	NS	2.25E-03	-0.125	1.12E-02	NS
ENOSF1	NS	2.25E-03	-0.125	1.12E-02	NS
CLEC7A	NS	2.25E-03	-0.125	1.12E-02	NS
GPR18	NS	2.25E-03	-0.125	1.12E-02	NS
PANX3	NS	2.26E-03	-0.125	1.12E-02	NS
NGB	NS	2.26E-03	1.25E-01	1.12E-02	NS
ATP6V0E2	NS	2.25E-03	-0.125	1.12E-02	NS
ZNF703	NS	2.26E-03	1.25E-01	1.12E-02	NS
PIGP	NS	2.26E-03	-0.125	1.12E-02	NS
VN1R1	NS	2.26E-03	-0.125	1.12E-02	NS
FAM240A	NS	2.26E-03	-0.125	1.12E-02	NS
CAPN13	NS	2.27E-03	-0.125	1.13E-02	NS
OTUD6B	NS	2.29E-03	-0.125	1.13E-02	NS
NCR1	NS	2.29E-03	-0.125	1.13E-02	NS
SMIM17	NS	2.29E-03	1.25E-01	1.14E-02	NS
STRN3	NS	2.29E-03	-0.125	1.14E-02	NS
IFNLR1	NS	2.29E-03	-0.125	1.14E-02	NS
DCLK1	NS	2.29E-03	-0.125	1.14E-02	NS
SERPINB7	NS	2.30E-03	-0.125	1.14E-02	NS
OR13D1	NS	2.30E-03	1.25E-01	1.14E-02	NS
NTMT2	NS	2.30E-03	-0.125	1.14E-02	NS
C9orf85	NS	2.31E-03	-0.125	1.14E-02	NS
OAF	NS	2.31E-03	1.25E-01	1.14E-02	NS
PPP1R1C	NS	2.31E-03	-0.125	1.14E-02	NS
HNRNPDL	NS	2.31E-03	1.25E-01	1.15E-02	NS
UBALD1	NS	2.31E-03	1.25E-01	1.15E-02	NS
LXN	NS	2.32E-03	-0.125	1.15E-02	NS
TTC33	NS	2.32E-03	-0.125	1.15E-02	NS
VEGFD	NS	2.32E-03	-0.125	1.15E-02	NS
PRR15L	NS	2.32E-03	-0.125	1.15E-02	NS
FAM83G	NS	2.32E-03	-0.125	1.15E-02	NS
IGFL2	NS	2.32E-03	-0.125	1.15E-02	NS
CLEC18B	NS	2.33E-03	-0.125	1.15E-02	NS
NBPF11	NS	2.33E-03	-0.125	1.15E-02	NS

ATP6V1A	NS	2.33E-03	-0.125	1.15E-02	NS
C7orf61	NS	2.33E-03	-0.125	1.15E-02	NS
CDCA7L	NS	2.33E-03	-0.125	1.15E-02	NS
NDUFS6	NS	2.33E-03	-0.125	1.15E-02	NS
NUP107	NS	2.34E-03	-0.125	1.15E-02	NS
EIF1	NS	2.34E-03	-0.125	1.15E-02	NS
PLXNB1	NS	2.34E-03	-0.125	1.15E-02	NS
FHL5	NS	2.34E-03	-0.125	1.15E-02	NS
PRCD	NS	2.34E-03	-0.125	1.15E-02	NS
ANKRD34A	NS	2.34E-03	1.25E-01	1.15E-02	NS
NUDT15	NS	2.35E-03	-0.125	1.16E-02	NS
COL4A2	NS	2.35E-03	-0.124	1.16E-02	NS
MAD2L1	NS	2.35E-03	-0.124	1.16E-02	NS
ABHD6	NS	2.36E-03	-0.124	1.16E-02	NS
BCAS1	NS	2.36E-03	-0.124	1.16E-02	NS
GGNBP2	NS	2.36E-03	1.24E-01	1.16E-02	NS
LRR1	NS	2.37E-03	-0.124	1.16E-02	NS
CAAP1	NS	2.37E-03	-0.124	1.16E-02	NS
PAAF1	NS	2.37E-03	1.24E-01	1.17E-02	NS
ZNF114	NS	2.37E-03	1.24E-01	1.17E-02	NS
SECISBP2	NS	2.37E-03	-0.124	1.17E-02	NS
LRRC37A	NS	2.37E-03	-0.124	1.17E-02	NS
WDR46	NS	2.38E-03	-0.124	1.17E-02	NS
PDGFC	NS	2.38E-03	-0.124	1.17E-02	NS
CDKN2AIPNL	NS	2.38E-03	-0.124	1.17E-02	NS
SYT10	NS	2.38E-03	-0.124	1.17E-02	NS
ZNF22	NS	2.38E-03	-0.124	1.17E-02	NS
SLAMF7	NS	2.39E-03	-0.124	1.17E-02	NS
DOCK6	NS	2.39E-03	1.24E-01	1.17E-02	NS
SEPTIN12	NS	2.39E-03	1.24E-01	1.17E-02	NS
ZSCAN32	NS	2.39E-03	-0.124	1.17E-02	NS
RAB39B	NS	2.39E-03	-0.124	1.17E-02	NS
TMSB15B	NS	2.39E-03	-0.124	1.17E-02	NS
SREK1IP1	NS	2.39E-03	-0.124	1.17E-02	NS
ACBD3	NS	2.39E-03	-0.124	1.17E-02	NS
IL10RB	NS	2.39E-03	-0.124	1.17E-02	NS
FAM135A	NS	2.39E-03	-0.124	1.17E-02	NS

NEDD9	NS	2.39E-03	-0.124	1.17E-02	NS
OPLAH	NS	2.40E-03	-0.124	1.17E-02	NS
TSG101	NS	2.40E-03	-0.124	1.17E-02	NS
SLC46A3	NS	2.40E-03	-0.124	1.17E-02	NS
IFNA7	NS	2.41E-03	-0.124	1.18E-02	NS
TRAM2	NS	2.41E-03	-0.124	1.18E-02	NS
ADA2	NS	2.41E-03	-0.124	1.18E-02	NS
SLC22A4	NS	2.41E-03	-0.124	1.18E-02	NS
METTL1	NS	2.41E-03	-0.124	1.18E-02	NS
TUBGCP3	NS	2.41E-03	-0.124	1.18E-02	NS
NUP62	NS	2.41E-03	1.24E-01	1.18E-02	NS
KCNC3	NS	2.41E-03	-0.124	1.18E-02	NS
TM4SF19-DYNLT2B	NS	2.42E-03	-0.124	1.18E-02	NS
DNMT3B	NS	2.42E-03	-0.124	1.18E-02	NS
TMEM140	NS	2.42E-03	1.24E-01	1.18E-02	NS
CACNG7	NS	2.43E-03	1.24E-01	1.18E-02	NS
PRYP3	NS	2.43E-03	1.24E-01	1.18E-02	NS
PSMD3	NS	2.43E-03	1.24E-01	1.18E-02	NS
ALAS2	NS	2.43E-03	-0.124	1.18E-02	NS
SLC9A3R1	NS	2.44E-03	1.24E-01	1.19E-02	NS
MRPL28	NS	2.44E-03	-0.124	1.19E-02	NS
PNPO	NS	2.45E-03	-0.124	1.19E-02	NS
IL1R1	NS	2.45E-03	-0.124	1.19E-02	NS
ASB14	NS	2.45E-03	-0.124	1.19E-02	NS
MRPL20	NS	2.45E-03	-0.124	1.19E-02	NS
PCNP	NS	2.45E-03	-0.124	1.19E-02	NS
ZNF441	NS	2.45E-03	-0.124	1.19E-02	NS
NPAS4	NS	2.45E-03	-0.124	1.19E-02	NS
RNF121	NS	2.45E-03	-0.124	1.19E-02	NS
RNLS	NS	2.45E-03	-0.124	1.19E-02	NS
SMARCD3	NS	2.46E-03	1.24E-01	1.19E-02	NS
RNF152	NS	2.46E-03	-0.124	1.19E-02	NS
PACRG	NS	2.46E-03	-0.124	1.19E-02	NS
AP3B1	NS	2.46E-03	-0.124	1.19E-02	NS
ITPK1	NS	2.47E-03	1.24E-01	1.20E-02	NS
CCDC12	NS	2.47E-03	-0.124	1.20E-02	NS
OSBPL1A	NS	2.47E-03	-0.124	1.20E-02	NS

EIF2B2	NS	2.48E-03	-0.124	1.20E-02	NS
TNS2	NS	2.48E-03	-0.124	1.20E-02	NS
HLA-DMB	NS	2.48E-03	-0.124	1.20E-02	NS
SFXN3	NS	2.48E-03	-0.124	1.20E-02	NS
OR4K15	NS	2.48E-03	-0.124	1.20E-02	NS
CDC42EP3	NS	2.48E-03	1.24E-01	1.20E-02	NS
DNAH8	NS	2.49E-03	-0.124	1.20E-02	NS
SPATA19	NS	2.49E-03	-0.124	1.20E-02	NS
BEX5	NS	2.49E-03	-0.124	1.20E-02	NS
RCAN2	NS	2.49E-03	-0.124	1.20E-02	NS
ZNF600	NS	2.49E-03	-0.124	1.20E-02	NS
GSTZ1	NS	2.49E-03	-0.124	1.20E-02	NS
PAIP2B	NS	2.49E-03	-0.124	1.20E-02	NS
GALK1	NS	2.50E-03	-0.124	1.21E-02	NS
OR8J1	NS	2.50E-03	-0.124	1.21E-02	NS
BORCS7-ASMT	NS	2.50E-03	-0.124	1.21E-02	NS
CCDC60	NS	2.50E-03	-0.124	1.21E-02	NS
XNDC1N	NS	2.50E-03	-0.124	1.21E-02	NS
TTLL2	NS	2.51E-03	-0.124	1.21E-02	NS
PMFBP1	NS	2.51E-03	-0.124	1.21E-02	NS
TMIGD3	NS	2.51E-03	-0.124	1.21E-02	NS
ZNF10	NS	2.53E-03	-0.124	1.22E-02	NS
NANOS1	NS	2.53E-03	-0.124	1.22E-02	NS
SLC25A46	NS	2.53E-03	-0.124	1.22E-02	NS
THY1	NS	2.54E-03	1.24E-01	1.22E-02	NS
RRAD	NS	2.54E-03	-0.124	1.22E-02	NS
STAP1	NS	2.55E-03	-0.123	1.23E-02	NS
LUZP1	NS	2.55E-03	-0.123	1.23E-02	NS
PLS3	NS	2.55E-03	-0.123	1.23E-02	NS
COL21A1	NS	2.55E-03	-0.123	1.23E-02	NS
TRIM23	NS	2.56E-03	-0.123	1.23E-02	NS
BCAR1	NS	2.56E-03	1.23E-01	1.23E-02	NS
TOP2A	NS	2.56E-03	-0.123	1.23E-02	NS
RNF215	NS	2.56E-03	-0.123	1.23E-02	NS
GRIA1	NS	2.57E-03	-0.123	1.23E-02	NS
USP11	NS	2.57E-03	-0.123	1.23E-02	NS
MICALL2	NS	2.57E-03	1.23E-01	1.23E-02	NS

SP140	NS	2.57E-03	-0.123	1.24E-02	NS
TRAF6	NS	2.58E-03	1.23E-01	1.24E-02	NS
AASDHPPT	NS	2.58E-03	-0.123	1.24E-02	NS
SHD	NS	2.59E-03	1.23E-01	1.24E-02	NS
NSMCE2	NS	2.59E-03	-0.123	1.24E-02	NS
SPPL3	NS	2.59E-03	-0.123	1.24E-02	NS
IL25	NS	2.59E-03	1.23E-01	1.24E-02	NS
LAMA2	NS	2.59E-03	-0.123	1.24E-02	NS
PLSCR1	NS	2.59E-03	-0.123	1.24E-02	NS
CHKB	NS	2.59E-03	-0.123	1.24E-02	NS
SNAI2	NS	2.61E-03	1.23E-01	1.25E-02	NS
SLC8A3	NS	2.61E-03	-0.123	1.25E-02	NS
PCDHGB6	NS	2.61E-03	-0.123	1.25E-02	NS
PAK5	NS	2.61E-03	-0.123	1.25E-02	NS
VAMP5	NS	2.61E-03	-0.123	1.25E-02	NS
SUN1	NS	2.62E-03	-0.123	1.25E-02	NS
TTF2	NS	2.62E-03	-0.123	1.25E-02	NS
ZNF670	NS	2.63E-03	-0.123	1.26E-02	NS
HSD17B8	NS	2.64E-03	-0.123	1.26E-02	NS
SETSIP	NS	2.64E-03	1.23E-01	1.26E-02	NS
LAMP1	NS	2.64E-03	1.23E-01	1.26E-02	NS
PHACTR2	NS	2.64E-03	-0.123	1.26E-02	NS
COL5A3	NS	2.65E-03	-0.123	1.26E-02	NS
MRPS36	NS	2.65E-03	-0.123	1.26E-02	NS
MAP3K11	NS	2.65E-03	1.23E-01	1.26E-02	NS
DDX6	NS	2.65E-03	1.23E-01	1.27E-02	NS
DCAF4	NS	2.65E-03	-0.123	1.27E-02	NS
PHRF1	NS	2.66E-03	1.23E-01	1.27E-02	NS
TRIM41	NS	2.66E-03	1.23E-01	1.27E-02	NS
TOM1L2	NS	2.66E-03	-0.123	1.27E-02	NS
GPM6B	NS	2.67E-03	-0.123	1.27E-02	NS
ERVH48-1	NS	2.67E-03	-0.123	1.27E-02	NS
GINS1	NS	2.67E-03	-0.123	1.27E-02	NS
CCDC8	NS	2.67E-03	1.23E-01	1.27E-02	NS
KDM2A	NS	2.68E-03	1.23E-01	1.27E-02	NS
PTPRC	NS	2.68E-03	-0.123	1.28E-02	NS
KAT14	NS	2.68E-03	-0.123	1.28E-02	NS

RIPOR1	NS	2.69E-03	1.23E-01	1.28E-02	NS
RNF213	NS	2.69E-03	-0.123	1.28E-02	NS
SLC4A5	NS	2.70E-03	-0.123	1.28E-02	NS
PANK1	NS	2.70E-03	-0.123	1.28E-02	NS
OR2A2	NS	2.70E-03	-0.123	1.29E-02	NS
TWSG1	NS	2.71E-03	-0.123	1.29E-02	NS
TAAR1	NS	2.71E-03	-0.123	1.29E-02	NS
TNFRSF13B	NS	2.71E-03	-0.123	1.29E-02	NS
BCO1	NS	2.72E-03	-0.123	1.29E-02	NS
RD3	NS	2.72E-03	1.23E-01	1.29E-02	NS
SUCLA2	NS	2.73E-03	-0.123	1.29E-02	NS
CALHM6	NS	2.73E-03	-0.123	1.30E-02	NS
PRSS58	NS	2.73E-03	-0.123	1.30E-02	NS
ACADS	NS	2.75E-03	-0.123	1.30E-02	NS
DHX37	NS	2.75E-03	-0.123	1.30E-02	NS
PEX2	NS	2.75E-03	-0.123	1.30E-02	NS
FLT3LG	NS	2.75E-03	-0.123	1.30E-02	NS
SEC22C	NS	2.75E-03	-0.123	1.30E-02	NS
PPP1R15B	NS	2.76E-03	1.23E-01	1.31E-02	NS
TOPBP1	NS	2.76E-03	-0.123	1.31E-02	NS
PENK	NS	2.76E-03	-0.122	1.31E-02	NS
PSMG4	NS	2.76E-03	-0.122	1.31E-02	NS
CAMK2N1	NS	2.77E-03	1.22E-01	1.31E-02	NS
XPO4	NS	2.78E-03	-0.122	1.31E-02	NS
PSIP1	NS	2.77E-03	-0.122	1.31E-02	NS
HELT	NS	2.78E-03	1.22E-01	1.31E-02	NS
TLK1	NS	2.78E-03	1.22E-01	1.31E-02	NS
SLC35F4	NS	2.78E-03	-0.122	1.31E-02	NS
EEF2K	NS	2.78E-03	-0.122	1.31E-02	NS
TSC22D2	NS	2.79E-03	1.22E-01	1.32E-02	NS
LSS	NS	2.79E-03	-0.122	1.32E-02	NS
KEL	NS	2.79E-03	-0.122	1.32E-02	NS
ENPP2	NS	2.80E-03	-0.122	1.32E-02	NS
DET1	NS	2.81E-03	-0.122	1.32E-02	NS
NCR3	NS	2.81E-03	-0.122	1.33E-02	NS
BBOX1	NS	2.81E-03	-0.122	1.33E-02	NS
ECHDC3	NS	2.81E-03	1.22E-01	1.33E-02	NS

FAM217A	NS	2.82E-03	-0.122	1.33E-02	NS
RSPH10B2	NS	2.82E-03	-0.122	1.33E-02	NS
RTCB	NS	2.82E-03	-0.122	1.33E-02	NS
NDUFA8	NS	2.82E-03	-0.122	1.33E-02	NS
MROH2A	NS	2.83E-03	-0.122	1.33E-02	NS
GZMA	NS	2.83E-03	-0.122	1.33E-02	NS
WRN	NS	2.83E-03	-0.122	1.33E-02	NS
MUC17	NS	2.83E-03	-0.122	1.33E-02	NS
SLC26A2	NS	2.84E-03	-0.122	1.33E-02	NS
TCF21	NS	2.84E-03	1.22E-01	1.33E-02	NS
IL23R	NS	2.84E-03	-0.122	1.33E-02	NS
PIN4	NS	2.85E-03	-0.122	1.34E-02	NS
FOXE3	NS	2.85E-03	1.22E-01	1.34E-02	NS
TMEM169	NS	2.85E-03	-0.122	1.34E-02	NS
MTCL1	NS	2.85E-03	-0.122	1.34E-02	NS
OR4M2	NS	2.86E-03	-0.122	1.34E-02	NS
PRSS3	NS	2.87E-03	-0.122	1.35E-02	NS
PDZD11	NS	2.87E-03	-0.122	1.35E-02	NS
FOXC2	NS	2.87E-03	1.22E-01	1.35E-02	NS
H4C11	NS	2.87E-03	-0.122	1.35E-02	NS
NNMT	NS	2.88E-03	-0.122	1.35E-02	NS
FDX1	NS	2.88E-03	1.22E-01	1.35E-02	NS
DPY19L2	NS	2.88E-03	-0.122	1.35E-02	NS
GPR142	NS	2.88E-03	1.22E-01	1.35E-02	NS
PLXNA2	NS	2.89E-03	1.22E-01	1.35E-02	NS
ELP6	NS	2.89E-03	-0.122	1.35E-02	NS
LY6G6F-LY6G6D	NS	2.89E-03	-0.122	1.36E-02	NS
LRGUK	NS	2.90E-03	-0.122	1.36E-02	NS
MECOM	NS	2.90E-03	-0.122	1.36E-02	NS
CBLN1	NS	2.90E-03	1.22E-01	1.36E-02	NS
XKR8	NS	2.90E-03	-0.122	1.36E-02	NS
SETD1A	NS	2.92E-03	1.22E-01	1.36E-02	NS
IL11	NS	2.92E-03	1.22E-01	1.36E-02	NS
RAB32	NS	2.92E-03	1.22E-01	1.37E-02	NS
ARMC2	NS	2.92E-03	-0.122	1.37E-02	NS
PIGL	NS	2.93E-03	-0.122	1.37E-02	NS
PDCD4	NS	2.93E-03	-0.122	1.37E-02	NS

SRP19	NS	2.93E-03	-0.122	1.37E-02	NS
CASR	NS	2.93E-03	-0.122	1.37E-02	NS
MUCL1	NS	2.93E-03	-0.122	1.37E-02	NS
DCAF4L1	NS	2.93E-03	-0.122	1.37E-02	NS
OPTC	NS	2.94E-03	-0.122	1.37E-02	NS
ALDOC	NS	2.94E-03	-0.122	1.37E-02	NS
IL3	NS	2.94E-03	-0.122	1.37E-02	NS
ALS2	NS	2.94E-03	-0.122	1.37E-02	NS
SHOC2	NS	2.95E-03	-0.122	1.37E-02	NS
POLD3	NS	2.95E-03	-0.122	1.37E-02	NS
CRB1	NS	2.95E-03	-0.122	1.37E-02	NS
BRCA2	NS	2.95E-03	-0.122	1.37E-02	NS
UCN3	NS	2.95E-03	1.22E-01	1.37E-02	NS
SPCS1	NS	2.96E-03	-0.122	1.38E-02	NS
ANKRD13C	NS	2.96E-03	-0.122	1.38E-02	NS
RALGAPA2	NS	2.96E-03	-0.122	1.38E-02	NS
CXorf65	NS	2.96E-03	-0.122	1.38E-02	NS
CFAP107	NS	2.97E-03	1.22E-01	1.38E-02	NS
HAUS1	NS	2.97E-03	-0.122	1.38E-02	NS
PPAN-P2RY11	NS	2.97E-03	-0.122	1.38E-02	NS
PPARA	NS	2.97E-03	-0.122	1.38E-02	NS
CSNK1G2	NS	2.98E-03	-0.122	1.38E-02	NS
IQCC	NS	2.99E-03	-0.121	1.39E-02	NS
MLLT3	NS	2.99E-03	-0.122	1.39E-02	NS
PDS5A	NS	3.00E-03	-0.121	1.39E-02	NS
FBXL6	NS	3.00E-03	-0.121	1.39E-02	NS
SPINK13	NS	3.00E-03	-0.121	1.39E-02	NS
POLL	NS	3.00E-03	-0.121	1.39E-02	NS
KIAA1143	NS	3.01E-03	-0.121	1.39E-02	NS
PPP6R3	NS	3.01E-03	-0.121	1.39E-02	NS
NDUFAF1	NS	3.02E-03	-0.121	1.40E-02	NS
GPNMB	NS	3.02E-03	-0.121	1.40E-02	NS
BRICD5	NS	3.02E-03	-0.121	1.40E-02	NS
KIR2DL4	NS	3.02E-03	-0.121	1.40E-02	NS
HDGFL2	NS	3.02E-03	-0.121	1.40E-02	NS
SRRD	NS	3.04E-03	-0.121	1.40E-02	NS
FOLR3	NS	3.04E-03	-0.121	1.40E-02	NS

ADAM19	NS	3.03E-03	-0.121	1.40E-02	NS
C7orf50	NS	3.04E-03	-0.121	1.40E-02	NS
ADORA2A	NS	3.04E-03	-0.121	1.41E-02	NS
AK9	NS	3.04E-03	-0.121	1.41E-02	NS
SEPTIN2	NS	3.04E-03	-0.121	1.41E-02	NS
PSMC4	NS	3.05E-03	-0.121	1.41E-02	NS
MYPN	NS	3.05E-03	-0.121	1.41E-02	NS
ASCL3	NS	3.05E-03	-0.121	1.41E-02	NS
VPS37D	NS	3.05E-03	1.21E-01	1.41E-02	NS
MYL5	NS	3.06E-03	-0.121	1.41E-02	NS
ARHGAP29	NS	3.06E-03	-0.121	1.41E-02	NS
GAD2	NS	3.06E-03	-0.121	1.41E-02	NS
ZNF44	NS	3.06E-03	-0.121	1.41E-02	NS
OR5K3	NS	3.06E-03	-0.121	1.41E-02	NS
RUFY4	NS	3.07E-03	-0.121	1.41E-02	NS
AP1M2	NS	3.07E-03	1.21E-01	1.41E-02	NS
SNX15	NS	3.07E-03	-0.121	1.41E-02	NS
GRXCR1	NS	3.07E-03	-0.121	1.41E-02	NS
ZSCAN5C	NS	3.08E-03	1.21E-01	1.42E-02	NS
ADPRH	NS	3.09E-03	-0.121	1.42E-02	NS
FFAR2	NS	3.09E-03	-0.121	1.42E-02	NS
MED19	NS	3.09E-03	-0.121	1.42E-02	NS
PROM2	NS	3.09E-03	1.21E-01	1.42E-02	NS
MED15	NS	3.11E-03	1.21E-01	1.43E-02	NS
ZNF277	NS	3.11E-03	-0.121	1.43E-02	NS
RNF6	NS	3.11E-03	-0.121	1.43E-02	NS
HNRNPA1	NS	3.11E-03	-0.121	1.43E-02	NS
CADM1	NS	3.11E-03	-0.121	1.43E-02	NS
MEGF10	NS	3.12E-03	-0.121	1.43E-02	NS
UNC93A	NS	3.12E-03	-0.121	1.43E-02	NS
CNPY3	NS	3.13E-03	-0.121	1.43E-02	NS
PDE11A	NS	3.13E-03	1.21E-01	1.44E-02	NS
METTL2B	NS	3.13E-03	-0.121	1.44E-02	NS
SEC24D	NS	3.14E-03	1.21E-01	1.44E-02	NS
FAM47A	NS	3.14E-03	-0.121	1.44E-02	NS
NFE2L2	NS	3.14E-03	-0.121	1.44E-02	NS
TREML1	NS	3.14E-03	-0.121	1.44E-02	NS

ZSCAN21	NS	3.14E-03	-0.121	1.44E-02	NS
FAM24A	NS	3.14E-03	-0.121	1.44E-02	NS
BCL2L14	NS	3.15E-03	-0.121	1.44E-02	NS
ERCC3	NS	3.15E-03	-0.121	1.44E-02	NS
AMER1	NS	3.15E-03	-0.121	1.44E-02	NS
ZNF345	NS	3.15E-03	-0.121	1.44E-02	NS
ANKAR	NS	3.15E-03	-0.121	1.44E-02	NS
C19orf12	NS	3.15E-03	-0.121	1.44E-02	NS
HSD17B7	NS	3.15E-03	-0.121	1.44E-02	NS
CALCOCO2	NS	3.15E-03	-0.121	1.44E-02	NS
VEGFC	NS	3.15E-03	1.21E-01	1.44E-02	NS
OR52N5	NS	3.15E-03	-0.121	1.44E-02	NS
POU5F1	NS	3.15E-03	-0.121	1.44E-02	NS
PIGS	NS	3.16E-03	1.21E-01	1.44E-02	NS
SRMS	NS	3.16E-03	1.21E-01	1.44E-02	NS
PPP2CA	NS	3.16E-03	1.21E-01	1.44E-02	NS
C19orf81	NS	3.17E-03	1.21E-01	1.45E-02	NS
CEACAM4	NS	3.18E-03	-0.121	1.45E-02	NS
CACNB2	NS	3.18E-03	-0.121	1.45E-02	NS
EPS15	NS	3.18E-03	-0.121	1.45E-02	NS
DNAH10	NS	3.18E-03	-0.121	1.45E-02	NS
CCN4	NS	3.18E-03	-0.121	1.45E-02	NS
DHX30	NS	3.18E-03	-0.121	1.45E-02	NS
AQP2	NS	3.19E-03	1.21E-01	1.45E-02	NS
PHB	NS	3.19E-03	-0.121	1.46E-02	NS
SGSM3	NS	3.19E-03	-0.121	1.46E-02	NS
OLFML3	NS	3.20E-03	-0.121	1.46E-02	NS
LSM14B	NS	3.20E-03	1.21E-01	1.46E-02	NS
NCOA6	NS	3.20E-03	-0.121	1.46E-02	NS
ZWINT	NS	3.21E-03	-0.121	1.46E-02	NS
BAK1	NS	3.22E-03	-0.121	1.46E-02	NS
PIWIL1	NS	3.22E-03	-0.121	1.46E-02	NS
SOX3	NS	3.22E-03	1.21E-01	1.46E-02	NS
PLA2G2F	NS	3.22E-03	-0.121	1.47E-02	NS
TCP1	NS	3.23E-03	1.21E-01	1.47E-02	NS
PHYHIPL	NS	3.23E-03	-0.121	1.47E-02	NS
TUBG1	NS	3.24E-03	-0.121	1.47E-02	NS

S100P	NS	3.25E-03	-0.12	1.48E-02	NS
RRAGC	NS	3.25E-03	-0.12	1.48E-02	NS
SYNM	NS	3.26E-03	-0.12	1.48E-02	NS
IFT52	NS	3.26E-03	-0.12	1.48E-02	NS
LIPN	NS	3.26E-03	-0.12	1.48E-02	NS
KLRF1	NS	3.27E-03	-0.12	1.48E-02	NS
SMIM26	NS	3.27E-03	-0.12	1.48E-02	NS
EFNA4	NS	3.27E-03	-0.12	1.48E-02	NS
DNAH11	NS	3.27E-03	-0.12	1.48E-02	NS
MTRNR2L7	NS	3.28E-03	-0.12	1.49E-02	NS
PCDHB10	NS	3.28E-03	1.20E-01	1.49E-02	NS
GHDC	NS	3.29E-03	-0.12	1.49E-02	NS
MEF2D	NS	3.29E-03	1.20E-01	1.49E-02	NS
HOXA11	NS	3.30E-03	1.20E-01	1.50E-02	NS
CD86	NS	3.31E-03	-0.12	1.50E-02	NS
JAGN1	NS	3.31E-03	-0.12	1.50E-02	NS
PLAC9	NS	3.31E-03	-0.12	1.50E-02	NS
DNAJC7	NS	3.31E-03	1.20E-01	1.50E-02	NS
DSN1	NS	3.32E-03	-0.12	1.50E-02	NS
ZFX	NS	3.33E-03	-0.12	1.50E-02	NS
CAMK2B	NS	3.33E-03	1.20E-01	1.50E-02	NS
TFAP2A	NS	3.33E-03	-0.12	1.51E-02	NS
FAM162A	NS	3.35E-03	-0.12	1.51E-02	NS
STOML1	NS	3.35E-03	-0.12	1.51E-02	NS
MID2	NS	3.35E-03	-0.12	1.51E-02	NS
TBL1Y	NS	3.35E-03	1.20E-01	1.51E-02	NS
TAMM41	NS	3.35E-03	-0.12	1.51E-02	NS
EIF2S2	NS	3.36E-03	-0.12	1.52E-02	NS
GPR34	NS	3.36E-03	-0.12	1.52E-02	NS
RIPK4	NS	3.36E-03	1.20E-01	1.52E-02	NS
GET4	NS	3.36E-03	-0.12	1.52E-02	NS
SLFN5	NS	3.37E-03	-0.12	1.52E-02	NS
TMCO2	NS	3.37E-03	1.20E-01	1.52E-02	NS
PRSS41	NS	3.38E-03	1.20E-01	1.52E-02	NS
STX12	NS	3.38E-03	-0.12	1.52E-02	NS
TLR4	NS	3.38E-03	-0.12	1.52E-02	NS
SHFL	NS	3.38E-03	-0.12	1.52E-02	NS

SRGAP3	NS	3.38E-03	-0.12	1.52E-02	NS
FKTN	NS	3.39E-03	-0.12	1.53E-02	NS
BMPER	NS	3.40E-03	1.20E-01	1.53E-02	NS
SHPRH	NS	3.41E-03	-0.12	1.53E-02	NS
LBH	NS	3.41E-03	-0.12	1.53E-02	NS
UBN2	NS	3.43E-03	-0.12	1.54E-02	NS
GLYCTK	NS	3.43E-03	-0.12	1.54E-02	NS
DOK5	NS	3.43E-03	-0.12	1.54E-02	NS
TDG	NS	3.44E-03	-0.12	1.55E-02	NS
GON7	NS	3.45E-03	-0.12	1.55E-02	NS
TAAR6	NS	3.45E-03	-0.12	1.55E-02	NS
GRM2	NS	3.45E-03	-0.12	1.55E-02	NS
NRSN2	NS	3.45E-03	-0.12	1.55E-02	NS
DYNAP	NS	3.45E-03	-0.12	1.55E-02	NS
ZBTB38	NS	3.46E-03	-0.12	1.55E-02	NS
CCDC77	NS	3.47E-03	-0.12	1.56E-02	NS
CT45A9	NS	3.47E-03	-0.12	1.56E-02	NS
ZNF404	NS	3.47E-03	-0.12	1.56E-02	NS
AKR1E2	NS	3.48E-03	-0.12	1.56E-02	NS
KHDC1L	NS	3.48E-03	-0.12	1.56E-02	NS
POC1A	NS	3.48E-03	-0.12	1.56E-02	NS
ZNF440	NS	3.50E-03	-0.12	1.57E-02	NS
PDCD5	NS	3.50E-03	-0.12	1.57E-02	NS
DCAF15	NS	3.50E-03	-0.12	1.57E-02	NS
GIPC2	NS	3.50E-03	-0.12	1.57E-02	NS
S100A3	NS	3.50E-03	-0.12	1.57E-02	NS
SELENOK	NS	3.50E-03	-0.12	1.57E-02	NS
PTPA	NS	3.51E-03	-0.12	1.57E-02	NS
OR2J3	NS	3.51E-03	-0.12	1.57E-02	NS
MSL3	NS	3.51E-03	-0.119	1.57E-02	NS
ABCC4	NS	3.51E-03	-0.119	1.57E-02	NS
LRRC8A	NS	3.51E-03	1.19E-01	1.57E-02	NS
ZNF223	NS	3.51E-03	-0.12	1.57E-02	NS
PIGK	NS	3.52E-03	-0.119	1.57E-02	NS
FAM241B	NS	3.52E-03	-0.119	1.57E-02	NS
GDF10	NS	3.52E-03	-0.119	1.57E-02	NS
SLX4	NS	3.52E-03	-0.119	1.57E-02	NS

APOL5	NS	3.53E-03	-0.119	1.57E-02	NS
OR4L1	NS	3.53E-03	-0.119	1.57E-02	NS
KLK1	NS	3.53E-03	1.19E-01	1.58E-02	NS
UBQLN4	NS	3.53E-03	-0.119	1.58E-02	NS
INTS13	NS	3.54E-03	-0.119	1.58E-02	NS
ZSCAN30	NS	3.54E-03	-0.119	1.58E-02	NS
OR2S2	NS	3.54E-03	1.19E-01	1.58E-02	NS
PPHLN1	NS	3.55E-03	-0.119	1.58E-02	NS
TCERG1L	NS	3.55E-03	-0.119	1.58E-02	NS
RASA4	NS	3.55E-03	1.19E-01	1.58E-02	NS
PUDP	NS	3.56E-03	-0.119	1.58E-02	NS
ARFIP2	NS	3.56E-03	-0.119	1.58E-02	NS
MRPS33	NS	3.57E-03	-0.119	1.59E-02	NS
LRRC7	NS	3.59E-03	-0.119	1.60E-02	NS
ADSS2	NS	3.59E-03	-0.119	1.60E-02	NS
VGLL4	NS	3.59E-03	-0.119	1.60E-02	NS
PLAAT3	NS	3.59E-03	-0.119	1.60E-02	NS
OR10G7	NS	3.59E-03	1.19E-01	1.60E-02	NS
VIP	NS	3.59E-03	-0.119	1.60E-02	NS
SPATS2L	NS	3.59E-03	-0.119	1.60E-02	NS
CNTLN	NS	3.60E-03	-0.119	1.60E-02	NS
CDK5RAP2	NS	3.60E-03	-0.119	1.60E-02	NS
SHOC1	NS	3.61E-03	-0.119	1.60E-02	NS
C1orf112	NS	3.62E-03	-0.119	1.60E-02	NS
CES1	NS	3.61E-03	-0.119	1.60E-02	NS
IL20RA	NS	3.62E-03	-0.119	1.61E-02	NS
ANK1	NS	3.62E-03	-0.119	1.61E-02	NS
CLEC18A	NS	3.62E-03	-0.119	1.61E-02	NS
ERGIC1	NS	3.62E-03	1.19E-01	1.61E-02	NS
SOST	NS	3.62E-03	1.19E-01	1.61E-02	NS
ZNF584	NS	3.63E-03	-0.119	1.61E-02	NS
LKAAEAR1	NS	3.64E-03	1.19E-01	1.61E-02	NS
GAL	NS	3.64E-03	1.19E-01	1.61E-02	NS
IFNA10	NS	3.64E-03	-0.119	1.61E-02	NS
CD40LG	NS	3.64E-03	-0.119	1.61E-02	NS
H4C15	NS	3.65E-03	-0.119	1.62E-02	NS
MAGEA9	NS	3.65E-03	1.19E-01	1.62E-02	NS

KLK13	NS	3.66E-03	-0.119	1.62E-02	NS
MRPL51	NS	3.66E-03	-0.119	1.62E-02	NS
C14orf119	NS	3.66E-03	-0.119	1.62E-02	NS
PPFIA1	NS	3.66E-03	-0.119	1.62E-02	NS
DZIP1L	NS	3.66E-03	-0.119	1.62E-02	NS
CFAP298-TCP10L	NS	3.67E-03	-0.119	1.62E-02	NS
C17orf99	NS	3.68E-03	1.19E-01	1.62E-02	NS
POU2F1	NS	3.68E-03	-0.119	1.63E-02	NS
BEST1	NS	3.69E-03	-0.119	1.63E-02	NS
NME8	NS	3.69E-03	-0.119	1.63E-02	NS
ADCY9	NS	3.69E-03	-0.119	1.63E-02	NS
IRS2	NS	3.71E-03	1.19E-01	1.64E-02	NS
NCAM1	NS	3.71E-03	-0.119	1.64E-02	NS
FCRL5	NS	3.72E-03	-0.119	1.64E-02	NS
STK26	NS	3.72E-03	-0.119	1.64E-02	NS
PHOX2B	NS	3.72E-03	1.19E-01	1.64E-02	NS
PNMA1	NS	3.73E-03	1.19E-01	1.64E-02	NS
PTH2R	NS	3.73E-03	-0.119	1.65E-02	NS
NPPA	NS	3.74E-03	-0.119	1.65E-02	NS
NME1	NS	3.74E-03	-0.119	1.65E-02	NS
NPHP3-ACAD11	NS	3.74E-03	-0.119	1.65E-02	NS
CRTAC1	NS	3.74E-03	-0.119	1.65E-02	NS
PHF12	NS	3.74E-03	1.19E-01	1.65E-02	NS
FAM183A	NS	3.74E-03	1.19E-01	1.65E-02	NS
HTN3	NS	3.75E-03	-0.119	1.65E-02	NS
PPFIA2	NS	3.75E-03	-0.119	1.65E-02	NS
IFT81	NS	3.75E-03	-0.119	1.65E-02	NS
SPRY3	NS	3.75E-03	1.19E-01	1.65E-02	NS
TMIE	NS	3.75E-03	1.19E-01	1.65E-02	NS
HS3ST3A1	NS	3.75E-03	-0.119	1.65E-02	NS
TSPAN19	NS	3.75E-03	-0.119	1.65E-02	NS
OR10G9	NS	3.75E-03	1.19E-01	1.65E-02	NS
ATP2B4	NS	3.76E-03	-0.119	1.65E-02	NS
LAMP3	NS	3.76E-03	-0.119	1.65E-02	NS
RFX3	NS	3.76E-03	1.19E-01	1.65E-02	NS
APEX1	NS	3.76E-03	1.19E-01	1.65E-02	NS
ZCCHC12	NS	3.76E-03	-0.119	1.65E-02	NS

NRBP2	NS	3.76E-03	-0.119	1.65E-02	NS
CMTM1	NS	3.78E-03	-0.119	1.66E-02	NS
DKC1	NS	3.78E-03	-0.119	1.66E-02	NS
LCAT	NS	3.79E-03	-0.119	1.66E-02	NS
AGPAT3	NS	3.80E-03	-0.118	1.66E-02	NS
PRSS33	NS	3.80E-03	1.18E-01	1.67E-02	NS
PSMA1	NS	3.80E-03	-0.118	1.67E-02	NS
AHSP	NS	3.80E-03	-0.118	1.67E-02	NS
TNR	NS	3.81E-03	-0.118	1.67E-02	NS
CLIC2	NS	3.81E-03	-0.118	1.67E-02	NS
NBPF20	NS	3.81E-03	-0.118	1.67E-02	NS
FAM209B	NS	3.81E-03	-0.118	1.67E-02	NS
PPP1R10	NS	3.81E-03	-0.118	1.67E-02	NS
CYP4V2	NS	3.81E-03	-0.118	1.67E-02	NS
HSF1	NS	3.82E-03	1.18E-01	1.67E-02	NS
ARL2-SNX15	NS	3.82E-03	-0.118	1.67E-02	NS
KIF26B	NS	3.82E-03	-0.118	1.67E-02	NS
MPEG1	NS	3.82E-03	-0.118	1.67E-02	NS
LIPE	NS	3.83E-03	-0.118	1.67E-02	NS
PAGE3	NS	3.83E-03	-0.118	1.67E-02	NS
U2AF2	NS	3.83E-03	1.18E-01	1.67E-02	NS
ZCCHC7	NS	3.83E-03	-0.118	1.67E-02	NS
NUMB	NS	3.84E-03	-0.118	1.67E-02	NS
CPN1	NS	3.84E-03	-0.118	1.68E-02	NS
TCF19	NS	3.85E-03	-0.118	1.68E-02	NS
CORO2B	NS	3.85E-03	-0.118	1.68E-02	NS
NEPRO	NS	3.86E-03	-0.118	1.68E-02	NS
ITIH6	NS	3.87E-03	-0.118	1.69E-02	NS
SLC30A7	NS	3.87E-03	-0.118	1.69E-02	NS
ATP7B	NS	3.89E-03	-0.118	1.69E-02	NS
TRPC7	NS	3.89E-03	-0.118	1.70E-02	NS
EXOSC5	NS	3.89E-03	1.18E-01	1.70E-02	NS
CUTC	NS	3.90E-03	-0.118	1.70E-02	NS
PHLPP2	NS	3.90E-03	-0.118	1.70E-02	NS
PRKDC	NS	3.90E-03	-0.118	1.70E-02	NS
PCID2	NS	3.91E-03	-0.118	1.70E-02	NS
TAF1D	NS	3.91E-03	-0.118	1.70E-02	NS

SRM	NS	3.91E-03	-0.118	1.70E-02	NS
PDX1	NS	3.91E-03	1.18E-01	1.70E-02	NS
B3GALNT2	NS	3.91E-03	-0.118	1.70E-02	NS
NUDCD1	NS	3.92E-03	-0.118	1.70E-02	NS
ZCWPW1	NS	3.92E-03	-0.118	1.70E-02	NS
LPO	NS	3.92E-03	1.18E-01	1.70E-02	NS
PMIS2	NS	3.92E-03	1.18E-01	1.70E-02	NS
NARS2	NS	3.93E-03	-0.118	1.71E-02	NS
DSG4	NS	3.93E-03	-0.118	1.71E-02	NS
GRAPL	NS	3.94E-03	-0.118	1.71E-02	NS
CD160	NS	3.94E-03	-0.118	1.71E-02	NS
GNRH2	NS	3.95E-03	-0.118	1.71E-02	NS
ARRB1	NS	3.95E-03	-0.118	1.72E-02	NS
ADCY10	NS	3.95E-03	-0.118	1.72E-02	NS
EFCAB7	NS	3.96E-03	-0.118	1.72E-02	NS
LDHAL6A	NS	3.96E-03	-0.118	1.72E-02	NS
GPRIN3	NS	3.96E-03	-0.118	1.72E-02	NS
ZNF880	NS	3.96E-03	-0.118	1.72E-02	NS
DEFB4B	NS	3.97E-03	1.18E-01	1.72E-02	NS
TGFB3	NS	3.97E-03	-0.118	1.72E-02	NS
SNAP25	NS	3.98E-03	-0.118	1.72E-02	NS
CFC1	NS	3.98E-03	1.18E-01	1.72E-02	NS
RIPPLY2	NS	3.98E-03	-0.118	1.72E-02	NS
ATPAF2	NS	3.98E-03	-0.118	1.72E-02	NS
MPC2	NS	3.99E-03	1.18E-01	1.73E-02	NS
BAZ1B	NS	3.99E-03	-0.118	1.73E-02	NS
CCDC197	NS	3.99E-03	-0.118	1.73E-02	NS
ALDH3A1	NS	3.99E-03	1.18E-01	1.73E-02	NS
PATE3	NS	4.01E-03	-0.118	1.73E-02	NS
HBZ	NS	4.01E-03	1.18E-01	1.73E-02	NS
GALNT4	NS	4.02E-03	-0.118	1.73E-02	NS
FAM178B	NS	4.03E-03	1.18E-01	1.74E-02	NS
PTPRM	NS	4.03E-03	-0.118	1.74E-02	NS
FUT9	NS	4.03E-03	-0.118	1.74E-02	NS
IL9R	NS	4.05E-03	-0.118	1.75E-02	NS
SCGB1A1	NS	4.05E-03	-0.118	1.75E-02	NS
CYP3A7-CYP3A51P	NS	4.05E-03	-0.118	1.75E-02	NS

DAPL1	NS	4.07E-03	1.18E-01	1.75E-02	NS
CDH5	NS	4.07E-03	-0.118	1.75E-02	NS
ATRX	NS	4.07E-03	-0.118	1.76E-02	NS
LSM7	NS	4.08E-03	-0.118	1.76E-02	NS
CLEC17A	NS	4.08E-03	-0.118	1.76E-02	NS
SCHIP1	NS	4.09E-03	-0.118	1.76E-02	NS
MTRNR2L1	NS	4.09E-03	1.18E-01	1.76E-02	NS
PSG4	NS	4.09E-03	-0.118	1.76E-02	NS
SIRT3	NS	4.10E-03	-0.118	1.77E-02	NS
UBXN10	NS	4.11E-03	-0.117	1.77E-02	NS
HOXC10	NS	4.11E-03	-0.117	1.77E-02	NS
RBMX2	NS	4.12E-03	-0.117	1.77E-02	NS
PPP2R3A	NS	4.12E-03	-0.117	1.77E-02	NS
HIRA	NS	4.12E-03	-0.117	1.77E-02	NS
RNF43	NS	4.12E-03	-0.117	1.77E-02	NS
MAML2	NS	4.13E-03	-0.117	1.77E-02	NS
CT47B1	NS	4.13E-03	1.17E-01	1.78E-02	NS
CMTR1	NS	4.15E-03	-0.117	1.78E-02	NS
GEMIN4	NS	4.15E-03	-0.117	1.78E-02	NS
MTREX	NS	4.15E-03	-0.117	1.78E-02	NS
ACSBG1	NS	4.15E-03	-0.117	1.78E-02	NS
KY	NS	4.15E-03	-0.117	1.78E-02	NS
KRTAP11-1	NS	4.15E-03	-0.117	1.78E-02	NS
ATP6V1H	NS	4.16E-03	-0.117	1.79E-02	NS
C11orf42	NS	4.17E-03	1.17E-01	1.79E-02	NS
ITGB2	NS	4.17E-03	-0.117	1.79E-02	NS
ASAP1	NS	4.18E-03	-0.117	1.79E-02	NS
SERPINB9	NS	4.18E-03	-0.117	1.79E-02	NS
CCN5	NS	4.19E-03	1.17E-01	1.79E-02	NS
ELANE	NS	4.19E-03	-0.117	1.79E-02	NS
C6orf47	NS	4.19E-03	-0.117	1.79E-02	NS
OR9A4	NS	4.19E-03	-0.117	1.80E-02	NS
GPATCH3	NS	4.20E-03	-0.117	1.80E-02	NS
RASL11A	NS	4.21E-03	-0.117	1.80E-02	NS
CEMIP2	NS	4.21E-03	-0.117	1.80E-02	NS
NEK1	NS	4.22E-03	-0.117	1.80E-02	NS
NADK2	NS	4.22E-03	-0.117	1.80E-02	NS

SATL1	NS	4.21E-03	-0.117	1.80E-02	NS
SSBP2	NS	4.22E-03	-0.117	1.80E-02	NS
XAGE1A	NS	4.23E-03	-0.117	1.81E-02	NS
TTLL10	NS	4.23E-03	1.17E-01	1.81E-02	NS
NPL	NS	4.24E-03	-0.117	1.81E-02	NS
TECTB	NS	4.24E-03	-0.117	1.81E-02	NS
ZNF831	NS	4.24E-03	-0.117	1.81E-02	NS
IGF2R	NS	4.25E-03	1.17E-01	1.81E-02	NS
PGAP1	NS	4.25E-03	-0.117	1.81E-02	NS
KCND1	NS	4.25E-03	1.17E-01	1.81E-02	NS
OR14K1	NS	4.25E-03	-0.117	1.82E-02	NS
EXOC3L2	NS	4.26E-03	1.17E-01	1.82E-02	NS
PVR	NS	4.26E-03	-0.117	1.82E-02	NS
DSG1	NS	4.27E-03	-0.117	1.82E-02	NS
CYP2C8	NS	4.27E-03	-0.117	1.82E-02	NS
PTPRQ	NS	4.27E-03	-0.117	1.82E-02	NS
CD99	NS	4.28E-03	1.17E-01	1.82E-02	NS
ANKDD1B	NS	4.28E-03	-0.117	1.83E-02	NS
FAM72B	NS	4.29E-03	-0.117	1.83E-02	NS
CD3G	NS	4.31E-03	-0.117	1.83E-02	NS
CPEB1	NS	4.31E-03	-0.117	1.83E-02	NS
CYB561	NS	4.31E-03	-0.117	1.84E-02	NS
KCNIP1	NS	4.32E-03	-0.117	1.84E-02	NS
TUBGCP5	NS	4.32E-03	-0.117	1.84E-02	NS
HLA-DPB1	NS	4.32E-03	-0.117	1.84E-02	NS
B4GALT1	NS	4.34E-03	-0.117	1.84E-02	NS
SP2	NS	4.34E-03	-0.117	1.84E-02	NS
EIF4B	NS	4.34E-03	-0.117	1.85E-02	NS
CDCA8	NS	4.34E-03	-0.117	1.85E-02	NS
GPATCH4	NS	4.36E-03	-0.117	1.85E-02	NS
DNAJC5	NS	4.36E-03	-0.117	1.85E-02	NS
ABCB1	NS	4.36E-03	-0.117	1.85E-02	NS
OAS3	NS	4.38E-03	-0.117	1.86E-02	NS
SUMO3	NS	4.38E-03	-0.117	1.86E-02	NS
DYTN	NS	4.38E-03	-0.117	1.86E-02	NS
GSTM2	NS	4.38E-03	-0.117	1.86E-02	NS
ARGFX	NS	4.39E-03	1.17E-01	1.86E-02	NS

MT1M	NS	4.39E-03	-0.117	1.86E-02	NS
TPST1	NS	4.40E-03	1.17E-01	1.86E-02	NS
CFAP92	NS	4.40E-03	-0.117	1.87E-02	NS
ZSWIM3	NS	4.40E-03	1.17E-01	1.87E-02	NS
ADGRF5	NS	4.41E-03	-0.117	1.87E-02	NS
NBPF12	NS	4.41E-03	-0.117	1.87E-02	NS
GSTM1	NS	4.41E-03	-0.117	1.87E-02	NS
RABGAP1	NS	4.43E-03	-0.117	1.88E-02	NS
LRRC30	NS	4.43E-03	-0.117	1.88E-02	NS
APELA	NS	4.43E-03	1.17E-01	1.88E-02	NS
NDUFAF2	NS	4.44E-03	-0.116	1.88E-02	NS
ADAM22	NS	4.45E-03	-0.116	1.88E-02	NS
ZBTB33	NS	4.46E-03	1.16E-01	1.89E-02	NS
OR7A10	NS	4.46E-03	1.16E-01	1.89E-02	NS
UBR7	NS	4.47E-03	-0.116	1.89E-02	NS
FTSJ3	NS	4.47E-03	-0.116	1.89E-02	NS
KDM2B	NS	4.48E-03	1.16E-01	1.89E-02	NS
NSUN7	NS	4.48E-03	-0.116	1.89E-02	NS
CABIN1	NS	4.49E-03	1.16E-01	1.90E-02	NS
CHRNE	NS	4.49E-03	-0.116	1.90E-02	NS
CACNA1H	NS	4.49E-03	1.16E-01	1.90E-02	NS
GALE	NS	4.49E-03	-0.116	1.90E-02	NS
SLC40A1	NS	4.50E-03	-0.116	1.90E-02	NS
LSMEM1	NS	4.50E-03	-0.116	1.90E-02	NS
DGKK	NS	4.50E-03	-0.116	1.90E-02	NS
METAP2	NS	4.51E-03	-0.116	1.90E-02	NS
MT1B	NS	4.51E-03	-0.116	1.90E-02	NS
PRR12	NS	4.51E-03	1.16E-01	1.90E-02	NS
ULK3	NS	4.51E-03	1.16E-01	1.90E-02	NS
OLFML1	NS	4.52E-03	-0.116	1.90E-02	NS
ADGRE5	NS	4.53E-03	1.16E-01	1.91E-02	NS
SCEL	NS	4.55E-03	-0.116	1.92E-02	NS
SF3B6	NS	4.56E-03	-0.116	1.92E-02	NS
NPFFR2	NS	4.57E-03	-0.116	1.92E-02	NS
GNL3L	NS	4.57E-03	-0.116	1.92E-02	NS
GNL1	NS	4.57E-03	1.16E-01	1.92E-02	NS
MARCOL	NS	4.57E-03	-0.116	1.92E-02	NS

MARCHF7	NS	4.57E-03	-0.116	1.92E-02	NS
HPRT1	NS	4.57E-03	-0.116	1.92E-02	NS
CYP4F2	NS	4.58E-03	-0.116	1.92E-02	NS
ZNF385A	NS	4.59E-03	1.16E-01	1.93E-02	NS
KRT25	NS	4.59E-03	-0.116	1.93E-02	NS
MLH3	NS	4.60E-03	-0.116	1.93E-02	NS
GPAT3	NS	4.61E-03	-0.116	1.94E-02	NS
ADM5	NS	4.62E-03	-0.116	1.94E-02	NS
TM2D1	NS	4.62E-03	-0.116	1.94E-02	NS
OR2T34	NS	4.62E-03	-0.116	1.94E-02	NS
HSPA6	NS	4.62E-03	-0.116	1.94E-02	NS
TICAM2	NS	4.63E-03	-0.116	1.94E-02	NS
LIN7A	NS	4.63E-03	-0.116	1.94E-02	NS
ASCL5	NS	4.64E-03	1.16E-01	1.95E-02	NS
ACAP1	NS	4.64E-03	-0.116	1.95E-02	NS
EFHD2	NS	4.64E-03	1.16E-01	1.95E-02	NS
HNRNPU	NS	4.64E-03	1.16E-01	1.95E-02	NS
PRDM10	NS	4.65E-03	-0.116	1.95E-02	NS
SLC7A2	NS	4.65E-03	-0.116	1.95E-02	NS
IKBIP	NS	4.65E-03	-0.116	1.95E-02	NS
AARS1	NS	4.65E-03	-0.116	1.95E-02	NS
OTOA	NS	4.66E-03	1.16E-01	1.95E-02	NS
ABCA6	NS	4.66E-03	-0.116	1.95E-02	NS
EPHA6	NS	4.67E-03	1.16E-01	1.95E-02	NS
SULT1A1	NS	4.68E-03	-0.116	1.96E-02	NS
POLR3H	NS	4.68E-03	-0.116	1.96E-02	NS
UGT1A9	NS	4.68E-03	-0.116	1.96E-02	NS
RIT2	NS	4.68E-03	-0.116	1.96E-02	NS
PCDHB16	NS	4.68E-03	1.16E-01	1.96E-02	NS
GM2A	NS	4.69E-03	1.16E-01	1.96E-02	NS
DHX9	NS	4.69E-03	-0.116	1.96E-02	NS
DNAJC1	NS	4.69E-03	1.16E-01	1.96E-02	NS
IDH3A	NS	4.69E-03	-0.116	1.96E-02	NS
CYSLTR1	NS	4.69E-03	-0.116	1.96E-02	NS
PPP3R2	NS	4.69E-03	-0.116	1.96E-02	NS
SLC4A11	NS	4.70E-03	1.16E-01	1.96E-02	NS
ATP12A	NS	4.70E-03	-0.116	1.96E-02	NS

NKX2-3	NS	4.70E-03	1.16E-01	1.96E-02	NS
ACTN1	NS	4.70E-03	1.16E-01	1.96E-02	NS
DEPDC7	NS	4.70E-03	-0.116	1.96E-02	NS
OR4A16	NS	4.70E-03	-0.116	1.96E-02	NS
RFTN1	NS	4.71E-03	1.16E-01	1.96E-02	NS
AMDHD1	NS	4.71E-03	-0.116	1.96E-02	NS
ATP6V0D2	NS	4.71E-03	-0.116	1.96E-02	NS
DUSP26	NS	4.72E-03	1.16E-01	1.96E-02	NS
SPANXN3	NS	4.72E-03	-0.116	1.97E-02	NS
PLK1	NS	4.73E-03	-0.116	1.97E-02	NS
SLC8A2	NS	4.73E-03	1.16E-01	1.97E-02	NS
RAD21L1	NS	4.73E-03	-0.116	1.97E-02	NS
ZNF576	NS	4.74E-03	-0.116	1.97E-02	NS
ETS1	NS	4.74E-03	-0.116	1.97E-02	NS
ORC1	NS	4.75E-03	-0.116	1.98E-02	NS
ZMAT4	NS	4.75E-03	-0.116	1.98E-02	NS
C5orf52	NS	4.75E-03	1.16E-01	1.98E-02	NS
MGST2	NS	4.75E-03	-0.116	1.98E-02	NS
OR2B6	NS	4.76E-03	-0.116	1.98E-02	NS
OR13C2	NS	4.77E-03	-0.116	1.98E-02	NS
TKFC	NS	4.79E-03	-0.116	1.99E-02	NS
PSMD9	NS	4.79E-03	-0.116	1.99E-02	NS
MAGEA9B	NS	4.79E-03	1.15E-01	1.99E-02	NS
MFSD2A	NS	4.80E-03	1.15E-01	1.99E-02	NS
POLR2F	NS	4.81E-03	-0.115	2.00E-02	NS
VPS33A	NS	4.81E-03	-0.115	2.00E-02	NS
RPTOR	NS	4.81E-03	-0.115	2.00E-02	NS
POLR3C	NS	4.81E-03	-0.115	2.00E-02	NS
UGT2B17	NS	4.81E-03	1.15E-01	2.00E-02	NS
SYT7	NS	4.82E-03	1.15E-01	2.00E-02	NS
FGFR3	NS	4.82E-03	1.15E-01	2.00E-02	NS
SRF	NS	4.82E-03	1.15E-01	2.00E-02	NS
ZNF79	NS	4.82E-03	-0.115	2.00E-02	NS
SSU72P4	NS	4.82E-03	1.15E-01	2.00E-02	NS
PNN	NS	4.83E-03	1.15E-01	2.00E-02	NS
STN1	NS	4.83E-03	-0.115	2.00E-02	NS
ALKBH6	NS	4.83E-03	1.15E-01	2.00E-02	NS

SLC30A1	NS	4.84E-03	1.15E-01	2.00E-02	NS
OR7A5	NS	4.84E-03	1.15E-01	2.00E-02	NS
CCNL2	NS	4.84E-03	1.15E-01	2.00E-02	NS
ANKRD13D	NS	4.85E-03	1.15E-01	2.00E-02	NS
KCNJ2	NS	4.85E-03	-0.115	2.01E-02	NS
GRIFIN	NS	4.86E-03	1.15E-01	2.01E-02	NS
UBE2U	NS	4.86E-03	-0.115	2.01E-02	NS
TM2D2	NS	4.90E-03	-0.115	2.02E-02	NS
TMEM106C	NS	4.90E-03	-0.115	2.03E-02	NS
RAMACL	NS	4.90E-03	-0.115	2.03E-02	NS
TRAPPC2B	NS	4.90E-03	-0.115	2.03E-02	NS
CAMK1	NS	4.91E-03	-0.115	2.03E-02	NS
CYTH4	NS	4.92E-03	1.15E-01	2.03E-02	NS
SLC25A11	NS	4.92E-03	-0.115	2.03E-02	NS
VPS26B	NS	4.92E-03	-0.115	2.03E-02	NS
TBCE	NS	4.92E-03	-0.115	2.03E-02	NS
SELENOM	NS	4.92E-03	-0.115	2.03E-02	NS
LEPR	NS	4.93E-03	-0.115	2.03E-02	NS
NIT1	NS	4.93E-03	1.15E-01	2.03E-02	NS
ARL11	NS	4.94E-03	-0.115	2.04E-02	NS
SLC44A1	NS	4.94E-03	-0.115	2.04E-02	NS
TRIM36	NS	4.95E-03	-0.115	2.04E-02	NS
C6orf58	NS	4.95E-03	-0.115	2.04E-02	NS
MPC1	NS	4.95E-03	-0.115	2.04E-02	NS
CYP2U1	NS	4.95E-03	-0.115	2.04E-02	NS
ROMO1	NS	4.96E-03	-0.115	2.04E-02	NS
HLA-B	NS	4.96E-03	-0.115	2.04E-02	NS
ETV4	NS	4.97E-03	-0.115	2.04E-02	NS
IQCA1	NS	4.97E-03	-0.115	2.05E-02	NS
EBAG9	NS	4.98E-03	1.15E-01	2.05E-02	NS
SIRT5	NS	4.98E-03	1.15E-01	2.05E-02	NS
WARS2	NS	4.99E-03	-0.115	2.05E-02	NS
SORD	NS	4.99E-03	-0.115	2.05E-02	NS
ASB9	NS	5.00E-03	-0.115	2.05E-02	NS
TM9SF3	NS	5.00E-03	-0.115	2.06E-02	NS
NOL10	NS	5.00E-03	-0.115	2.06E-02	NS
EEF1AKMT2	NS	5.01E-03	-0.115	2.06E-02	NS

AGMO	NS	5.01E-03	-0.115	2.06E-02	NS
PATE4	NS	5.02E-03	-0.115	2.06E-02	NS
ZNF268	NS	5.02E-03	-0.115	2.06E-02	NS
LPXN	NS	5.02E-03	-0.115	2.06E-02	NS
EIF2A	NS	5.03E-03	-0.115	2.06E-02	NS
UNC45B	NS	5.03E-03	-0.115	2.06E-02	NS
LIX1	NS	5.03E-03	-0.115	2.06E-02	NS
NAIF1	NS	5.03E-03	-0.115	2.06E-02	NS
SIVA1	NS	5.04E-03	-0.115	2.06E-02	NS
NOTCH2	NS	5.04E-03	-0.115	2.06E-02	NS
RSL1D1	NS	5.04E-03	1.15E-01	2.07E-02	NS
RPL39L	NS	5.06E-03	-0.115	2.07E-02	NS
MRPL35	NS	5.06E-03	-0.115	2.07E-02	NS
SLC16A13	NS	5.06E-03	-0.115	2.07E-02	NS
NPW	NS	5.06E-03	-0.115	2.07E-02	NS
TEAD2	NS	5.06E-03	-0.115	2.07E-02	NS
SLC22A7	NS	5.07E-03	-0.115	2.07E-02	NS
CDK13	NS	5.07E-03	-0.115	2.07E-02	NS
PSME1	NS	5.07E-03	-0.115	2.07E-02	NS
SLC25A16	NS	5.07E-03	-0.115	2.07E-02	NS
BRD9	NS	5.09E-03	-0.115	2.08E-02	NS
AURKB	NS	5.09E-03	-0.115	2.08E-02	NS
CTDSP1	NS	5.09E-03	-0.115	2.08E-02	NS
CERKL	NS	5.11E-03	-0.115	2.09E-02	NS
NMI	NS	5.11E-03	-0.115	2.09E-02	NS
PROK1	NS	5.11E-03	1.15E-01	2.09E-02	NS
TBX22	NS	5.11E-03	-0.115	2.09E-02	NS
CCDC178	NS	5.11E-03	-0.115	2.09E-02	NS
ADGRG6	NS	5.12E-03	-0.115	2.09E-02	NS
OR52A5	NS	5.12E-03	-0.115	2.09E-02	NS
KRTAP24-1	NS	5.12E-03	-0.115	2.09E-02	NS
NEK9	NS	5.14E-03	-0.115	2.10E-02	NS
PANX2	NS	5.14E-03	-0.115	2.10E-02	NS
OR4N2	NS	5.14E-03	-0.115	2.10E-02	NS
EPM2A	NS	5.15E-03	-0.115	2.10E-02	NS
ITM2B	NS	5.15E-03	-0.115	2.10E-02	NS
CTSZ	NS	5.16E-03	-0.115	2.10E-02	NS

MARK1	NS	5.15E-03	-0.115	2.10E-02	NS
COIL	NS	5.16E-03	-0.115	2.10E-02	NS
HS3ST3B1	NS	5.16E-03	-0.115	2.10E-02	NS
CD300E	NS	5.16E-03	-0.115	2.10E-02	NS
RUNDC3B	NS	5.17E-03	-0.115	2.10E-02	NS
EREG	NS	5.17E-03	-0.114	2.10E-02	NS
GK	NS	5.17E-03	-0.114	2.10E-02	NS
H2AC1	NS	5.18E-03	-0.114	2.11E-02	NS
CEND1	NS	5.18E-03	1.14E-01	2.11E-02	NS
POU3F1	NS	5.19E-03	1.14E-01	2.11E-02	NS
HTN1	NS	5.20E-03	-0.114	2.11E-02	NS
ZNF808	NS	5.21E-03	-0.114	2.12E-02	NS
PCDHA8	NS	5.22E-03	1.14E-01	2.12E-02	NS
PDE5A	NS	5.22E-03	1.14E-01	2.12E-02	NS
IGSF11	NS	5.22E-03	-0.114	2.12E-02	NS
CCL11	NS	5.22E-03	-0.114	2.12E-02	NS
ACKR2	NS	5.24E-03	-0.114	2.13E-02	NS
AFF2	NS	5.24E-03	-0.114	2.13E-02	NS
SARAF	NS	5.25E-03	-0.114	2.13E-02	NS
COMMD8	NS	5.25E-03	-0.114	2.13E-02	NS
MAP1LC3B2	NS	5.25E-03	1.14E-01	2.13E-02	NS
ANXA9	NS	5.25E-03	-0.114	2.13E-02	NS
DLX1	NS	5.27E-03	1.14E-01	2.14E-02	NS
EIF3M	NS	5.28E-03	-0.114	2.14E-02	NS
CRYGD	NS	5.29E-03	1.14E-01	2.14E-02	NS
FANCF	NS	5.29E-03	-0.114	2.14E-02	NS
PTTG2	NS	5.29E-03	-0.114	2.14E-02	NS
TIMMDC1	NS	5.30E-03	-0.114	2.15E-02	NS
NPVF	NS	5.30E-03	-0.114	2.15E-02	NS
TGFBI	NS	5.31E-03	-0.114	2.15E-02	NS
MRGPRG	NS	5.32E-03	1.14E-01	2.15E-02	NS
ACBD6	NS	5.32E-03	-0.114	2.15E-02	NS
MMRN2	NS	5.32E-03	-0.114	2.15E-02	NS
DOCK2	NS	5.33E-03	-0.114	2.15E-02	NS
MAGEE2	NS	5.32E-03	-0.114	2.15E-02	NS
SCNN1D	NS	5.33E-03	-0.114	2.16E-02	NS
CCDC170	NS	5.33E-03	-0.114	2.16E-02	NS

MIPOL1	NS	5.34E-03	-0.114	2.16E-02	NS
ANTXR2	NS	5.34E-03	-0.114	2.16E-02	NS
CDRT15	NS	5.34E-03	-0.114	2.16E-02	NS
KDM1A	NS	5.35E-03	1.14E-01	2.16E-02	NS
RFPL4A	NS	5.35E-03	-0.114	2.16E-02	NS
MAST4	NS	5.36E-03	1.14E-01	2.16E-02	NS
ZNF723	NS	5.35E-03	1.14E-01	2.16E-02	NS
CALCA	NS	5.36E-03	-0.114	2.16E-02	NS
P3H3	NS	5.36E-03	1.14E-01	2.16E-02	NS
MANBAL	NS	5.37E-03	-0.114	2.17E-02	NS
SLC22A15	NS	5.37E-03	-0.114	2.17E-02	NS
GSX1	NS	5.39E-03	1.14E-01	2.17E-02	NS
DCAF8L2	NS	5.39E-03	-0.114	2.17E-02	NS
SHISA3	NS	5.39E-03	1.14E-01	2.17E-02	NS
CHST10	NS	5.43E-03	-0.114	2.19E-02	NS
ZNF81	NS	5.43E-03	1.14E-01	2.19E-02	NS
C1QTNF6	NS	5.43E-03	-0.114	2.19E-02	NS
RNF10	NS	5.44E-03	1.14E-01	2.19E-02	NS
NR1H2	NS	5.44E-03	-0.114	2.19E-02	NS
SEC11C	NS	5.45E-03	-0.114	2.19E-02	NS
HSPBAP1	NS	5.45E-03	-0.114	2.19E-02	NS
ISG15	NS	5.45E-03	-0.114	2.20E-02	NS
POLR2E	NS	5.46E-03	1.14E-01	2.20E-02	NS
CBWD1	NS	5.46E-03	-0.114	2.20E-02	NS
CCT7	NS	5.47E-03	-0.114	2.20E-02	NS
C9orf40	NS	5.47E-03	-0.114	2.20E-02	NS
INTS4	NS	5.48E-03	-0.114	2.20E-02	NS
GUK1	NS	5.48E-03	1.14E-01	2.20E-02	NS
PSMB7	NS	5.48E-03	-0.114	2.20E-02	NS
LRFN3	NS	5.49E-03	1.14E-01	2.21E-02	NS
CEBPD	NS	5.49E-03	1.14E-01	2.21E-02	NS
TEX101	NS	5.50E-03	-0.114	2.21E-02	NS
DOC2B	NS	5.50E-03	1.14E-01	2.21E-02	NS
CD53	NS	5.51E-03	-0.114	2.21E-02	NS
ZNRF2	NS	5.51E-03	-0.114	2.21E-02	NS
COPS4	NS	5.52E-03	-0.114	2.22E-02	NS
RAB30	NS	5.52E-03	-0.114	2.22E-02	NS

TRMT12	NS	5.53E-03	-0.114	2.22E-02	NS
C1R	NS	5.54E-03	1.14E-01	2.22E-02	NS
SDK2	NS	5.55E-03	-0.114	2.22E-02	NS
SIAH3	NS	5.56E-03	-0.114	2.23E-02	NS
CUBN	NS	5.56E-03	-0.114	2.23E-02	NS
TRMU	NS	5.58E-03	-0.114	2.23E-02	NS
SIGLEC15	NS	5.58E-03	-0.114	2.23E-02	NS
HTR6	NS	5.58E-03	1.13E-01	2.23E-02	NS
PLA2G6	NS	5.59E-03	1.13E-01	2.24E-02	NS
FTSJ1	NS	5.60E-03	-0.113	2.24E-02	NS
SHTN1	NS	5.60E-03	-0.113	2.24E-02	NS
EPS8L3	NS	5.61E-03	1.13E-01	2.24E-02	NS
SPIDR	NS	5.61E-03	-0.113	2.24E-02	NS
HYDIN	NS	5.61E-03	1.13E-01	2.24E-02	NS
NEURL3	NS	5.62E-03	-0.113	2.25E-02	NS
OR4C16	NS	5.62E-03	-0.113	2.25E-02	NS
ATP6V0B	NS	5.63E-03	1.13E-01	2.25E-02	NS
PLK3	NS	5.64E-03	1.13E-01	2.25E-02	NS
SLC24A4	NS	5.65E-03	-0.113	2.26E-02	NS
TPST2	NS	5.65E-03	-0.113	2.26E-02	NS
SKIL	NS	5.66E-03	-0.113	2.26E-02	NS
PCGF5	NS	5.66E-03	1.13E-01	2.26E-02	NS
TANC2	NS	5.67E-03	-0.113	2.26E-02	NS
RALGAPA1	NS	5.68E-03	-0.113	2.27E-02	NS
MAP3K9	NS	5.68E-03	-0.113	2.27E-02	NS
HOXA9	NS	5.68E-03	-0.113	2.27E-02	NS
MRPL3	NS	5.68E-03	-0.113	2.27E-02	NS
SLC4A3	NS	5.68E-03	1.13E-01	2.27E-02	NS
SCRG1	NS	5.69E-03	-0.113	2.27E-02	NS
BRWD1	NS	5.69E-03	1.13E-01	2.27E-02	NS
TMEM131	NS	5.71E-03	-0.113	2.28E-02	NS
BCCIP	NS	5.72E-03	-0.113	2.28E-02	NS
PINLYP	NS	5.72E-03	1.13E-01	2.28E-02	NS
CDYL	NS	5.72E-03	-0.113	2.28E-02	NS
MON2	NS	5.72E-03	-0.113	2.28E-02	NS
EFR3B	NS	5.73E-03	-0.113	2.28E-02	NS
KMT2A	NS	5.73E-03	-0.113	2.28E-02	NS

INAFM2	NS	5.75E-03	-0.113	2.29E-02	NS
CHEK2	NS	5.75E-03	-0.113	2.29E-02	NS
TMEM201	NS	5.75E-03	-0.113	2.29E-02	NS
JAG2	NS	5.75E-03	-0.113	2.29E-02	NS
PTPN7	NS	5.76E-03	-0.113	2.29E-02	NS
TSR2	NS	5.76E-03	-0.113	2.29E-02	NS
OR10A4	NS	5.77E-03	-0.113	2.29E-02	NS
SLC16A2	NS	5.77E-03	1.13E-01	2.29E-02	NS
AGA	NS	5.78E-03	-0.113	2.29E-02	NS
RAD23B	NS	5.80E-03	1.13E-01	2.30E-02	NS
RXFP1	NS	5.80E-03	-0.113	2.30E-02	NS
NR2C2AP	NS	5.81E-03	-0.113	2.31E-02	NS
OR10A5	NS	5.81E-03	1.13E-01	2.31E-02	NS
PSMD5	NS	5.83E-03	-0.113	2.31E-02	NS
C18orf32	NS	5.83E-03	-0.113	2.31E-02	NS
TDGF1	NS	5.83E-03	-0.113	2.31E-02	NS
ATRNL1	NS	5.83E-03	-0.113	2.31E-02	NS
PHOSPHO2	NS	5.84E-03	-0.113	2.32E-02	NS
C14orf180	NS	5.84E-03	1.13E-01	2.32E-02	NS
N4BP1	NS	5.85E-03	-0.113	2.32E-02	NS
NDUFB5	NS	5.85E-03	-0.113	2.32E-02	NS
FAM50B	NS	5.86E-03	-0.113	2.32E-02	NS
CYS1	NS	5.86E-03	1.13E-01	2.32E-02	NS
PRPF31	NS	5.87E-03	-0.113	2.32E-02	NS
DAB2IP	NS	5.87E-03	1.13E-01	2.32E-02	NS
RASD2	NS	5.88E-03	1.13E-01	2.33E-02	NS
ZFYVE21	NS	5.88E-03	-0.113	2.33E-02	NS
NT5E	NS	5.88E-03	-0.113	2.33E-02	NS
FUT5	NS	5.90E-03	-0.113	2.33E-02	NS
SHROOM2	NS	5.90E-03	-0.113	2.33E-02	NS
QRSL1	NS	5.90E-03	-0.113	2.33E-02	NS
NDUFV1	NS	5.90E-03	-0.113	2.33E-02	NS
SLC6A7	NS	5.92E-03	-0.113	2.34E-02	NS
CLASP2	NS	5.92E-03	-0.113	2.34E-02	NS
ATOH8	NS	5.92E-03	-0.113	2.34E-02	NS
CALB1	NS	5.92E-03	1.13E-01	2.34E-02	NS
NAE1	NS	5.92E-03	-0.113	2.34E-02	NS

JMJD7-PLA2G4B	NS	5.93E-03	-0.113	2.34E-02	NS
IL1B	NS	5.93E-03	-0.113	2.34E-02	NS
CTSS	NS	5.94E-03	-0.113	2.34E-02	NS
TGFBR2	NS	5.94E-03	-0.113	2.35E-02	NS
SF3B5	NS	5.95E-03	-0.113	2.35E-02	NS
HS3ST5	NS	5.95E-03	-0.113	2.35E-02	NS
ITGAX	NS	5.96E-03	-0.113	2.35E-02	NS
PLPP1	NS	5.96E-03	-0.113	2.35E-02	NS
CYP2J2	NS	5.97E-03	-0.113	2.35E-02	NS
ANKRD6	NS	5.97E-03	-0.113	2.35E-02	NS
LIPJ	NS	5.97E-03	-0.113	2.35E-02	NS
BGLAP	NS	5.97E-03	-0.113	2.35E-02	NS
SETX	NS	5.98E-03	-0.113	2.36E-02	NS
CTIF	NS	5.98E-03	-0.113	2.36E-02	NS
PAPPA	NS	5.99E-03	-0.113	2.36E-02	NS
HAS1	NS	6.01E-03	-0.113	2.36E-02	NS
RAP1GDS1	NS	6.02E-03	1.12E-01	2.37E-02	NS
PLA2G7	NS	6.02E-03	-0.112	2.37E-02	NS
IQCG	NS	6.02E-03	-0.112	2.37E-02	NS
LRG1	NS	6.03E-03	-0.112	2.37E-02	NS
MED13	NS	6.04E-03	-0.112	2.37E-02	NS
LEUTX	NS	6.04E-03	1.12E-01	2.38E-02	NS
ZFYVE16	NS	6.05E-03	-0.112	2.38E-02	NS
MOV10	NS	6.05E-03	-0.112	2.38E-02	NS
PSMC3	NS	6.05E-03	-0.112	2.38E-02	NS
UGT1A7	NS	6.05E-03	-0.112	2.38E-02	NS
CSNK1G3	NS	6.06E-03	1.12E-01	2.38E-02	NS
HSD17B13	NS	6.06E-03	-0.112	2.38E-02	NS
ARG1	NS	6.07E-03	-0.112	2.38E-02	NS
CSTF3	NS	6.07E-03	-0.112	2.38E-02	NS
ISX	NS	6.07E-03	1.12E-01	2.38E-02	NS
OR1A2	NS	6.08E-03	-0.112	2.39E-02	NS
SLC2A8	NS	6.09E-03	-0.112	2.39E-02	NS
LRRC53	NS	6.11E-03	-0.112	2.40E-02	NS
ROPN1	NS	6.12E-03	-0.112	2.40E-02	NS
RTKN	NS	6.13E-03	-0.112	2.40E-02	NS
TTC37	NS	6.13E-03	-0.112	2.40E-02	NS

GRPEL2	NS	6.13E-03	-0.112	2.40E-02	NS
ZBED2	NS	6.14E-03	-0.112	2.40E-02	NS
CEBPZOS	NS	6.14E-03	1.12E-01	2.40E-02	NS
ABCD4	NS	6.14E-03	-0.112	2.41E-02	NS
PRXL2C	NS	6.17E-03	-0.112	2.42E-02	NS
SLC39A12	NS	6.18E-03	-0.112	2.42E-02	NS
STAG1	NS	6.18E-03	-0.112	2.42E-02	NS
CCDC68	NS	6.18E-03	-0.112	2.42E-02	NS
NDUFS1	NS	6.18E-03	-0.112	2.42E-02	NS
ACOT7	NS	6.20E-03	-0.112	2.42E-02	NS
HOXD13	NS	6.20E-03	1.12E-01	2.43E-02	NS
MRGPRE	NS	6.21E-03	1.12E-01	2.43E-02	NS
GDF2	NS	6.21E-03	1.12E-01	2.43E-02	NS
PDC	NS	6.21E-03	-0.112	2.43E-02	NS
CLDN2	NS	6.21E-03	-0.112	2.43E-02	NS
DDX25	NS	6.22E-03	-0.112	2.43E-02	NS
KRTAP3-2	NS	6.22E-03	-0.112	2.43E-02	NS
HS6ST1	NS	6.23E-03	-0.112	2.43E-02	NS
CDX1	NS	6.23E-03	1.12E-01	2.43E-02	NS
GAGE12H	NS	6.24E-03	-0.112	2.43E-02	NS
AHNAK	NS	6.24E-03	-0.112	2.44E-02	NS
UPF3A	NS	6.24E-03	-0.112	2.44E-02	NS
RAP1GAP2	NS	6.24E-03	-0.112	2.44E-02	NS
ISG20	NS	6.26E-03	-0.112	2.44E-02	NS
SMAD6	NS	6.27E-03	1.12E-01	2.44E-02	NS
MLNR	NS	6.27E-03	1.12E-01	2.45E-02	NS
RPL10L	NS	6.27E-03	-0.112	2.45E-02	NS
SETD4	NS	6.27E-03	-0.112	2.45E-02	NS
ARHGEF12	NS	6.27E-03	-0.112	2.45E-02	NS
CFAP61	NS	6.29E-03	-0.112	2.45E-02	NS
CKAP4	NS	6.28E-03	-0.112	2.45E-02	NS
ZNF691	NS	6.29E-03	-0.112	2.45E-02	NS
GGTLC2	NS	6.31E-03	-0.112	2.46E-02	NS
LMO7	NS	6.31E-03	-0.112	2.46E-02	NS
INTS3	NS	6.31E-03	-0.112	2.46E-02	NS
RAB37	NS	6.31E-03	-0.112	2.46E-02	NS
LEP	NS	6.31E-03	-0.112	2.46E-02	NS

ANXA10	NS	6.31E-03	-0.112	2.46E-02	NS
DLGAP5	NS	6.32E-03	-0.112	2.46E-02	NS
GMNN	NS	6.34E-03	-0.112	2.47E-02	NS
FANCG	NS	6.34E-03	-0.112	2.47E-02	NS
C19orf85	NS	6.35E-03	1.12E-01	2.47E-02	NS
SHCBP1L	NS	6.35E-03	1.12E-01	2.47E-02	NS
RBM15	NS	6.35E-03	1.12E-01	2.47E-02	NS
TSHR	NS	6.36E-03	-0.112	2.47E-02	NS
SRI	NS	6.37E-03	-0.112	2.47E-02	NS
GNA13	NS	6.37E-03	-0.112	2.47E-02	NS
METTL13	NS	6.39E-03	-0.112	2.48E-02	NS
RBM23	NS	6.39E-03	-0.112	2.48E-02	NS
PCDHGA6	NS	6.38E-03	-0.112	2.48E-02	NS
TXK	NS	6.39E-03	-0.112	2.48E-02	NS
SOCS1	NS	6.39E-03	1.12E-01	2.48E-02	NS
TM9SF1	NS	6.40E-03	-0.112	2.48E-02	NS
IL5	NS	6.41E-03	-0.112	2.48E-02	NS
MGAT3	NS	6.41E-03	-0.112	2.48E-02	NS
AIPL1	NS	6.41E-03	1.12E-01	2.49E-02	NS
SLC6A18	NS	6.42E-03	1.12E-01	2.49E-02	NS
CCNG1	NS	6.43E-03	-0.112	2.49E-02	NS
OXER1	NS	6.43E-03	-0.112	2.49E-02	NS
SEC14L2	NS	6.43E-03	-0.112	2.49E-02	NS
TSLP	NS	6.45E-03	-0.112	2.50E-02	NS
PRORP	NS	6.46E-03	-0.112	2.50E-02	NS
GID4	NS	6.46E-03	1.12E-01	2.50E-02	NS
CDCA3	NS	6.46E-03	-0.112	2.50E-02	NS
ULBP3	NS	6.47E-03	1.12E-01	2.50E-02	NS
FKBP11	NS	6.47E-03	-0.112	2.50E-02	NS
CHRNA1	NS	6.48E-03	-0.112	2.50E-02	NS
TRIM60	NS	6.48E-03	-0.111	2.51E-02	NS
LURAP1	NS	6.48E-03	-0.111	2.51E-02	NS
NFYB	NS	6.49E-03	-0.111	2.51E-02	NS
WDR11	NS	6.49E-03	-0.111	2.51E-02	NS
DUOX1	NS	6.50E-03	-0.111	2.51E-02	NS
VMA21	NS	6.50E-03	-0.111	2.51E-02	NS
SDC3	NS	6.50E-03	-0.111	2.51E-02	NS

RPS23	NS	6.50E-03	-0.111	2.51E-02	NS
NMNAT3	NS	6.51E-03	-0.111	2.51E-02	NS
FPR3	NS	6.52E-03	-0.111	2.52E-02	NS
ZKSCAN4	NS	6.52E-03	-0.111	2.52E-02	NS
SERPINA3	NS	6.52E-03	1.11E-01	2.52E-02	NS
ZFP91-CNTF	NS	6.52E-03	1.11E-01	2.52E-02	NS
CUL7	NS	6.54E-03	-0.111	2.52E-02	NS
PPP2R5D	NS	6.54E-03	1.11E-01	2.52E-02	NS
ZRANB3	NS	6.54E-03	-0.111	2.52E-02	NS
MOCS1	NS	6.54E-03	-0.111	2.52E-02	NS
FBXW12	NS	6.54E-03	-0.111	2.52E-02	NS
OR1J2	NS	6.54E-03	-0.111	2.52E-02	NS
PCDHGA12	NS	6.54E-03	-0.111	2.52E-02	NS
APLP2	NS	6.55E-03	-0.111	2.52E-02	NS
TMCO3	NS	6.55E-03	1.11E-01	2.52E-02	NS
OS9	NS	6.56E-03	-0.111	2.52E-02	NS
MIS18A	NS	6.58E-03	-0.111	2.53E-02	NS
SCARF2	NS	6.59E-03	-0.111	2.53E-02	NS
BZW2	NS	6.60E-03	-0.111	2.54E-02	NS
ANTXR1	NS	6.60E-03	-0.111	2.54E-02	NS
AP4E1	NS	6.63E-03	-0.111	2.55E-02	NS
DHX34	NS	6.63E-03	1.11E-01	2.55E-02	NS
DNAJB8	NS	6.63E-03	1.11E-01	2.55E-02	NS
GOLGA6L24P	NS	6.63E-03	-0.111	2.55E-02	NS
NBPF26	NS	6.63E-03	-0.111	2.55E-02	NS
ILDR1	NS	6.64E-03	-0.111	2.55E-02	NS
ZBP1	NS	6.64E-03	-0.111	2.55E-02	NS
JCAD	NS	6.65E-03	-0.111	2.55E-02	NS
HNRNPF	NS	6.65E-03	-0.111	2.55E-02	NS
GPC4	NS	6.66E-03	-0.111	2.56E-02	NS
MRNIP	NS	6.66E-03	1.11E-01	2.56E-02	NS
CORO6	NS	6.66E-03	-0.111	2.56E-02	NS
AQP11	NS	6.66E-03	-0.111	2.56E-02	NS
ZNF195	NS	6.66E-03	-0.111	2.56E-02	NS
BUD31	NS	6.67E-03	-0.111	2.56E-02	NS
DQX1	NS	6.66E-03	-0.111	2.56E-02	NS
TAS2R60	NS	6.67E-03	-0.111	2.56E-02	NS

HLA-DRB5	NS	6.67E-03	-0.111	2.56E-02	NS
TNFAIP8L1	NS	6.69E-03	-0.111	2.56E-02	NS
ERVW-1	NS	6.70E-03	-0.111	2.57E-02	NS
TMEM275	NS	6.70E-03	1.11E-01	2.57E-02	NS
KMT5B	NS	6.70E-03	-0.111	2.57E-02	NS
KCNQ4	NS	6.71E-03	-0.111	2.57E-02	NS
CXXC1	NS	6.71E-03	1.11E-01	2.57E-02	NS
CDRT15L2	NS	6.73E-03	1.11E-01	2.57E-02	NS
CDKL1	NS	6.74E-03	-0.111	2.58E-02	NS
MYBPC3	NS	6.74E-03	-0.111	2.58E-02	NS
RSL24D1	NS	6.74E-03	-0.111	2.58E-02	NS
ZBTB7A	NS	6.74E-03	1.11E-01	2.58E-02	NS
PCDHGA1	NS	6.74E-03	-0.111	2.58E-02	NS
GPC6	NS	6.75E-03	-0.111	2.58E-02	NS
POLR3GL	NS	6.78E-03	-0.111	2.59E-02	NS
CPSF7	NS	6.78E-03	1.11E-01	2.59E-02	NS
GABRA5	NS	6.78E-03	1.11E-01	2.59E-02	NS
CDT1	NS	6.79E-03	-0.111	2.59E-02	NS
RFK	NS	6.79E-03	-0.111	2.59E-02	NS
RAX	NS	6.81E-03	1.11E-01	2.60E-02	NS
RSPRY1	NS	6.81E-03	-0.111	2.60E-02	NS
PDE6H	NS	6.82E-03	-0.111	2.60E-02	NS
HINT2	NS	6.83E-03	-0.111	2.60E-02	NS
TAS2R20	NS	6.83E-03	-0.111	2.61E-02	NS
RAB3A	NS	6.84E-03	1.11E-01	2.61E-02	NS
ATG2A	NS	6.86E-03	1.11E-01	2.61E-02	NS
BEND5	NS	6.85E-03	-0.111	2.61E-02	NS
TLR2	NS	6.86E-03	-0.111	2.61E-02	NS
INO80D	NS	6.87E-03	-0.111	2.62E-02	NS
GTF2A1	NS	6.88E-03	-0.111	2.62E-02	NS
LINGO1	NS	6.88E-03	1.11E-01	2.62E-02	NS
CDK17	NS	6.88E-03	1.11E-01	2.62E-02	NS
AKAP5	NS	6.88E-03	-0.111	2.62E-02	NS
ZNF23	NS	6.89E-03	-0.111	2.62E-02	NS
RFX5	NS	6.90E-03	-0.111	2.63E-02	NS
GSTM3	NS	6.91E-03	-0.111	2.63E-02	NS
TBC1D13	NS	6.93E-03	-0.111	2.64E-02	NS

AQP5	NS	6.94E-03	1.11E-01	2.64E-02	NS
SCN9A	NS	6.94E-03	-0.111	2.64E-02	NS
APBA3	NS	6.95E-03	-0.111	2.64E-02	NS
FAM110D	NS	6.95E-03	1.11E-01	2.64E-02	NS
PLEKHS1	NS	6.95E-03	-0.111	2.64E-02	NS
USP16	NS	6.97E-03	-0.111	2.65E-02	NS
BAG6	NS	6.98E-03	-0.11	2.65E-02	NS
H4C14	NS	6.99E-03	-0.11	2.65E-02	NS
CBLIF	NS	7.01E-03	-0.11	2.66E-02	NS
MRPL50	NS	7.01E-03	-0.11	2.66E-02	NS
CHRND	NS	7.01E-03	-0.11	2.66E-02	NS
GLB1L3	NS	7.02E-03	-0.11	2.66E-02	NS
SLC25A18	NS	7.02E-03	-0.11	2.67E-02	NS
TTC23	NS	7.03E-03	-0.11	2.67E-02	NS
JAK3	NS	7.04E-03	-0.11	2.67E-02	NS
PRMT3	NS	7.06E-03	-0.11	2.68E-02	NS
LPAR1	NS	7.06E-03	-0.11	2.68E-02	NS
TMEM47	NS	7.06E-03	-0.11	2.68E-02	NS
TEX47	NS	7.08E-03	-0.11	2.69E-02	NS
ARHGAP15	NS	7.09E-03	1.10E-01	2.69E-02	NS
FAM71B	NS	7.10E-03	1.10E-01	2.69E-02	NS
ZNF451	NS	7.10E-03	-0.11	2.69E-02	NS
TMEM217	NS	7.10E-03	-0.11	2.69E-02	NS
NOL7	NS	7.11E-03	1.10E-01	2.69E-02	NS
FOXK2	NS	7.11E-03	-0.11	2.69E-02	NS
INIP	NS	7.11E-03	-0.11	2.69E-02	NS
RNF14	NS	7.11E-03	-0.11	2.69E-02	NS
BEST2	NS	7.12E-03	1.10E-01	2.69E-02	NS
TBC1D21	NS	7.12E-03	-0.11	2.70E-02	NS
CCL26	NS	7.13E-03	-0.11	2.70E-02	NS
HPSE2	NS	7.14E-03	-0.11	2.70E-02	NS
PDHA1	NS	7.14E-03	-0.11	2.70E-02	NS
VSTM2B	NS	7.15E-03	1.10E-01	2.70E-02	NS
VAT1L	NS	7.15E-03	-0.11	2.70E-02	NS
WDR53	NS	7.15E-03	-0.11	2.70E-02	NS
IPO8	NS	7.15E-03	-0.11	2.70E-02	NS
ZNF589	NS	7.16E-03	-0.11	2.70E-02	NS

CACNA1E	NS	7.16E-03	-0.11	2.70E-02	NS
C11orf45	NS	7.16E-03	-0.11	2.70E-02	NS
ALLC	NS	7.18E-03	-0.11	2.71E-02	NS
PRY2	NS	7.22E-03	1.10E-01	2.72E-02	NS
SPP1	NS	7.22E-03	-0.11	2.73E-02	NS
MIA3	NS	7.23E-03	-0.11	2.73E-02	NS
MAP2K2	NS	7.24E-03	-0.11	2.73E-02	NS
PRKCB	NS	7.24E-03	-0.11	2.73E-02	NS
ADD1	NS	7.25E-03	-0.11	2.73E-02	NS
TBX19	NS	7.25E-03	-0.11	2.73E-02	NS
ASXL2	NS	7.25E-03	-0.11	2.73E-02	NS
TRPV1	NS	7.25E-03	-0.11	2.73E-02	NS
ANKRD1	NS	7.26E-03	1.10E-01	2.73E-02	NS
DAOA	NS	7.26E-03	-0.11	2.74E-02	NS
MMP10	NS	7.27E-03	-0.11	2.74E-02	NS
DHX35	NS	7.28E-03	-0.11	2.74E-02	NS
PFN4	NS	7.28E-03	-0.11	2.74E-02	NS
TCIRG1	NS	7.31E-03	1.10E-01	2.75E-02	NS
SPRED3	NS	7.31E-03	-0.11	2.75E-02	NS
IFNA8	NS	7.32E-03	-0.11	2.75E-02	NS
PTK7	NS	7.33E-03	-0.11	2.76E-02	NS
EIF5A	NS	7.33E-03	1.10E-01	2.76E-02	NS
SMPX	NS	7.34E-03	-0.11	2.76E-02	NS
CBWD2	NS	7.34E-03	-0.11	2.76E-02	NS
NOS3	NS	7.35E-03	1.10E-01	2.76E-02	NS
RRP36	NS	7.36E-03	-0.11	2.77E-02	NS
ALDH4A1	NS	7.36E-03	-0.11	2.77E-02	NS
AMBP	NS	7.37E-03	-0.11	2.77E-02	NS
FAT4	NS	7.38E-03	-0.11	2.77E-02	NS
ALAD	NS	7.39E-03	-0.11	2.77E-02	NS
SP7	NS	7.39E-03	1.10E-01	2.77E-02	NS
MFAP3L	NS	7.39E-03	-0.11	2.77E-02	NS
ZMYM1	NS	7.39E-03	-0.11	2.77E-02	NS
SORBS3	NS	7.41E-03	-0.11	2.78E-02	NS
MSH4	NS	7.41E-03	-0.11	2.78E-02	NS
ZNF318	NS	7.41E-03	1.10E-01	2.78E-02	NS
UTS2	NS	7.42E-03	-0.11	2.78E-02	NS

OR4A47	NS	7.42E-03	-0.11	2.78E-02	NS
ATP4B	NS	7.43E-03	1.10E-01	2.78E-02	NS
GBX1	NS	7.43E-03	-0.11	2.79E-02	NS
KHSRP	NS	7.44E-03	-0.11	2.79E-02	NS
CDADC1	NS	7.46E-03	1.10E-01	2.79E-02	NS
AGPAT5	NS	7.46E-03	-0.11	2.79E-02	NS
AFTPH	NS	7.46E-03	-0.11	2.79E-02	NS
AKAP4	NS	7.47E-03	-0.11	2.80E-02	NS
C4orf3	NS	7.47E-03	-0.11	2.80E-02	NS
PAFAH1B3	NS	7.49E-03	-0.11	2.80E-02	NS
SRGN	NS	7.51E-03	-0.11	2.81E-02	NS
DDX58	NS	7.51E-03	-0.109	2.81E-02	NS
ZCCHC24	NS	7.52E-03	1.09E-01	2.81E-02	NS
OR4M2B	NS	7.52E-03	-0.109	2.81E-02	NS
NT5M	NS	7.53E-03	-0.109	2.82E-02	NS
OSTM1	NS	7.53E-03	-0.109	2.82E-02	NS
EMC4	NS	7.54E-03	-0.109	2.82E-02	NS
OR52E5	NS	7.54E-03	-0.109	2.82E-02	NS
ABHD8	NS	7.55E-03	-0.109	2.82E-02	NS
ARPC5	NS	7.55E-03	-0.109	2.82E-02	NS
PCDHGA5	NS	7.55E-03	-0.109	2.82E-02	NS
WDR24	NS	7.56E-03	-0.109	2.82E-02	NS
HNRNPR	NS	7.56E-03	-0.109	2.82E-02	NS
OSBPL9	NS	7.57E-03	-0.109	2.82E-02	NS
ZP1	NS	7.58E-03	-0.109	2.83E-02	NS
WFDC13	NS	7.58E-03	-0.109	2.83E-02	NS
GABRQ	NS	7.58E-03	-0.109	2.83E-02	NS
ZSCAN23	NS	7.59E-03	-0.109	2.83E-02	NS
OR52H1	NS	7.60E-03	-0.109	2.84E-02	NS
CCDC54	NS	7.61E-03	-0.109	2.84E-02	NS
NPIPB6	NS	7.61E-03	1.09E-01	2.84E-02	NS
LRRC46	NS	7.62E-03	-0.109	2.84E-02	NS
ZNF425	NS	7.62E-03	-0.109	2.84E-02	NS
CDC45	NS	7.62E-03	-0.109	2.84E-02	NS
DNAJA2	NS	7.63E-03	1.09E-01	2.84E-02	NS
LINC00672	NS	7.64E-03	1.09E-01	2.85E-02	NS
TSC22D4	NS	7.65E-03	1.09E-01	2.85E-02	NS

UVRAG	NS	7.67E-03	1.09E-01	2.85E-02	NS
NLRP4	NS	7.68E-03	-0.109	2.86E-02	NS
MON1A	NS	7.68E-03	1.09E-01	2.86E-02	NS
POPDC3	NS	7.69E-03	-0.109	2.86E-02	NS
PGAP4	NS	7.69E-03	-0.109	2.86E-02	NS
SLC26A1	NS	7.71E-03	1.09E-01	2.87E-02	NS
DYRK4	NS	7.73E-03	-0.109	2.87E-02	NS
DCUN1D2	NS	7.73E-03	-0.109	2.87E-02	NS
REG3A	NS	7.74E-03	-0.109	2.88E-02	NS
OXTR	NS	7.74E-03	-0.109	2.88E-02	NS
ASH1L	NS	7.75E-03	-0.109	2.88E-02	NS
MAP1LC3A	NS	7.76E-03	-0.109	2.88E-02	NS
CDC25B	NS	7.76E-03	-0.109	2.88E-02	NS
CLTC	NS	7.77E-03	-0.109	2.88E-02	NS
TMEM44	NS	7.77E-03	-0.109	2.88E-02	NS
ZNF512	NS	7.77E-03	-0.109	2.88E-02	NS
L3HYPDH	NS	7.78E-03	-0.109	2.89E-02	NS
RPS17	NS	7.78E-03	-0.109	2.89E-02	NS
MT-ND4L	NS	7.78E-03	1.09E-01	2.89E-02	NS
EFNA5	NS	7.78E-03	-0.109	2.89E-02	NS
NOC3L	NS	7.79E-03	-0.109	2.89E-02	NS
CYP1A1	NS	7.80E-03	1.09E-01	2.89E-02	NS
SPEF2	NS	7.81E-03	1.09E-01	2.89E-02	NS
ACADL	NS	7.83E-03	-0.109	2.90E-02	NS
TMEM128	NS	7.83E-03	-0.109	2.90E-02	NS
DLEU7	NS	7.85E-03	-0.109	2.91E-02	NS
TSPY4	NS	7.85E-03	1.09E-01	2.91E-02	NS
REXO4	NS	7.87E-03	-0.109	2.91E-02	NS
TLR9	NS	7.87E-03	-0.109	2.91E-02	NS
FAM177A1	NS	7.87E-03	-0.109	2.91E-02	NS
COL6A6	NS	7.87E-03	1.09E-01	2.91E-02	NS
ASIC1	NS	7.88E-03	1.09E-01	2.92E-02	NS
ZNF569	NS	7.89E-03	-0.109	2.92E-02	NS
EXOSC7	NS	7.90E-03	-0.109	2.92E-02	NS
GRM3	NS	7.91E-03	-0.109	2.92E-02	NS
YRDC	NS	7.92E-03	-0.109	2.93E-02	NS
DIDO1	NS	7.92E-03	-0.109	2.93E-02	NS

PRAMEF9	NS	7.93E-03	-0.109	2.93E-02	NS
SELE	NS	7.94E-03	-0.109	2.93E-02	NS
TTC39A	NS	7.94E-03	-0.109	2.93E-02	NS
PHF5A	NS	7.95E-03	-0.109	2.94E-02	NS
PABPN1	NS	7.95E-03	1.09E-01	2.94E-02	NS
MYOCD	NS	7.95E-03	-0.109	2.94E-02	NS
TFCP2	NS	7.96E-03	-0.109	2.94E-02	NS
TNFAIP8	NS	7.96E-03	-0.109	2.94E-02	NS
ZNF41	NS	7.96E-03	-0.109	2.94E-02	NS
RPA4	NS	7.96E-03	-0.109	2.94E-02	NS
C1QL4	NS	7.97E-03	1.09E-01	2.94E-02	NS
ZKSCAN2	NS	7.98E-03	-0.109	2.94E-02	NS
ANGPT1	NS	7.99E-03	-0.109	2.95E-02	NS
TM4SF20	NS	7.99E-03	-0.109	2.95E-02	NS
TMEM199	NS	7.99E-03	-0.109	2.95E-02	NS
GNB3	NS	8.00E-03	-0.109	2.95E-02	NS
STEEP1	NS	8.01E-03	-0.109	2.95E-02	NS
EEFSEC	NS	8.02E-03	1.09E-01	2.95E-02	NS
SH3RF2	NS	8.02E-03	-0.109	2.95E-02	NS
OR6B1	NS	8.02E-03	-0.109	2.95E-02	NS
PROM1	NS	8.03E-03	-0.109	2.96E-02	NS
CARNMT1	NS	8.04E-03	-0.109	2.96E-02	NS
ANKHD1	NS	8.06E-03	-0.109	2.97E-02	NS
CYP2D6	NS	8.07E-03	1.09E-01	2.97E-02	NS
F2RL2	NS	8.07E-03	1.09E-01	2.97E-02	NS
HTR1F	NS	8.07E-03	-0.109	2.97E-02	NS
FASN	NS	8.08E-03	1.09E-01	2.97E-02	NS
HMGN3	NS	8.08E-03	-0.108	2.97E-02	NS
KCNJ10	NS	8.08E-03	-0.108	2.97E-02	NS
OBI1	NS	8.10E-03	-0.108	2.98E-02	NS
MRM3	NS	8.10E-03	-0.108	2.98E-02	NS
SH2D3C	NS	8.11E-03	-0.108	2.98E-02	NS
COQ10B	NS	8.11E-03	-0.108	2.98E-02	NS
CDK2	NS	8.11E-03	-0.108	2.98E-02	NS
MAN2C1	NS	8.12E-03	1.08E-01	2.98E-02	NS
MAPRE1	NS	8.14E-03	-0.108	2.99E-02	NS
ZNF384	NS	8.14E-03	1.08E-01	2.99E-02	NS

ZNF599	NS	8.15E-03	-0.108	2.99E-02	NS
EID2B	NS	8.15E-03	-0.108	2.99E-02	NS
EMP3	NS	8.15E-03	1.08E-01	2.99E-02	NS
CWC22	NS	8.16E-03	-0.108	2.99E-02	NS
FTH1	NS	8.18E-03	-0.108	3.00E-02	NS
FZR1	NS	8.18E-03	1.08E-01	3.00E-02	NS
DNAJC14	NS	8.18E-03	-0.108	3.00E-02	NS
HMGB4	NS	8.19E-03	-0.108	3.00E-02	NS
ERV3-1	NS	8.21E-03	-0.108	3.01E-02	NS
PREB	NS	8.23E-03	1.08E-01	3.01E-02	NS
AP1G2	NS	8.22E-03	1.08E-01	3.01E-02	NS
MFSD14A	NS	8.25E-03	-0.108	3.02E-02	NS
GAL3ST4	NS	8.26E-03	-0.108	3.02E-02	NS
TMEM95	NS	8.28E-03	1.08E-01	3.03E-02	NS
RELL2	NS	8.29E-03	-0.108	3.03E-02	NS
APOM	NS	8.29E-03	-0.108	3.03E-02	NS
ETNK1	NS	8.30E-03	-0.108	3.04E-02	NS
RFLNB	NS	8.30E-03	-0.108	3.04E-02	NS
VEPH1	NS	8.31E-03	-0.108	3.04E-02	NS
PLCXD3	NS	8.31E-03	-0.108	3.04E-02	NS
HCRTR1	NS	8.32E-03	-0.108	3.04E-02	NS
OR4S2	NS	8.33E-03	-0.108	3.04E-02	NS
TAFAZZIN	NS	8.34E-03	1.08E-01	3.05E-02	NS
ACTR10	NS	8.38E-03	-0.108	3.06E-02	NS
SNTG2	NS	8.38E-03	-0.108	3.06E-02	NS
ZNF705G	NS	8.38E-03	-0.108	3.06E-02	NS
SUPT4H1	NS	8.39E-03	1.08E-01	3.06E-02	NS
CYB5B	NS	8.39E-03	-0.108	3.06E-02	NS
TET2	NS	8.41E-03	-0.108	3.07E-02	NS
DLL1	NS	8.42E-03	-0.108	3.07E-02	NS
BCAP29	NS	8.43E-03	-0.108	3.07E-02	NS
RGS18	NS	8.44E-03	1.08E-01	3.08E-02	NS
SMIM6	NS	8.44E-03	1.08E-01	3.08E-02	NS
SPICE1	NS	8.46E-03	-0.108	3.09E-02	NS
NLRC4	NS	8.47E-03	-0.108	3.09E-02	NS
H2BC7	NS	8.48E-03	-0.108	3.09E-02	NS
ABRAXAS2	NS	8.48E-03	-0.108	3.09E-02	NS

PLRG1	NS	8.49E-03	-0.108	3.09E-02	NS
OR10K2	NS	8.49E-03	1.08E-01	3.09E-02	NS
ZNF804B	NS	8.50E-03	-0.108	3.10E-02	NS
SRRM5	NS	8.50E-03	-0.108	3.10E-02	NS
PRR33	NS	8.51E-03	-0.108	3.10E-02	NS
RAB2A	NS	8.51E-03	1.08E-01	3.10E-02	NS
GPR63	NS	8.52E-03	-0.108	3.10E-02	NS
ATP13A3	NS	8.52E-03	-0.108	3.10E-02	NS
PGM2	NS	8.52E-03	-0.108	3.10E-02	NS
AGXT	NS	8.52E-03	1.08E-01	3.10E-02	NS
NCAPD3	NS	8.52E-03	-0.108	3.10E-02	NS
BHLHE40	NS	8.53E-03	1.08E-01	3.10E-02	NS
CD2BP2	NS	8.54E-03	-0.108	3.10E-02	NS
UBE2L3	NS	8.54E-03	-0.108	3.10E-02	NS
TMEFF1	NS	8.54E-03	-0.108	3.10E-02	NS
HOOK2	NS	8.55E-03	1.08E-01	3.11E-02	NS
ZNF500	NS	8.55E-03	-0.108	3.11E-02	NS
KLF7	NS	8.57E-03	-0.108	3.11E-02	NS
H4C4	NS	8.57E-03	-0.108	3.11E-02	NS
RERGL	NS	8.58E-03	-0.108	3.11E-02	NS
NBPF15	NS	8.59E-03	-0.108	3.12E-02	NS
LCE2D	NS	8.60E-03	-0.108	3.12E-02	NS
FNIP1	NS	8.62E-03	-0.108	3.13E-02	NS
DNAH5	NS	8.62E-03	-0.108	3.13E-02	NS
MAP1B	NS	8.62E-03	-0.108	3.13E-02	NS
TMEM266	NS	8.63E-03	1.08E-01	3.13E-02	NS
CABCOCO1	NS	8.63E-03	-0.108	3.13E-02	NS
CDH11	NS	8.64E-03	-0.108	3.13E-02	NS
CCDC74B	NS	8.64E-03	1.08E-01	3.13E-02	NS
TFRC	NS	8.65E-03	1.08E-01	3.13E-02	NS
TPRG1L	NS	8.65E-03	-0.108	3.13E-02	NS
TUSC2	NS	8.65E-03	-0.108	3.13E-02	NS
PLIN5	NS	8.66E-03	-0.108	3.14E-02	NS
SUDS3	NS	8.66E-03	-0.108	3.14E-02	NS
OSBPL3	NS	8.68E-03	-0.108	3.14E-02	NS
SRSF12	NS	8.69E-03	-0.107	3.14E-02	NS
IL24	NS	8.69E-03	-0.107	3.14E-02	NS

TGFB1	NS	8.70E-03	1.07E-01	3.15E-02	NS
CES2	NS	8.73E-03	-0.107	3.16E-02	NS
GSTM5	NS	8.73E-03	-0.107	3.16E-02	NS
IKZF5	NS	8.74E-03	1.07E-01	3.16E-02	NS
SLC37A3	NS	8.74E-03	-0.107	3.16E-02	NS
KRT7	NS	8.75E-03	-0.107	3.16E-02	NS
PFDN4	NS	8.76E-03	-0.107	3.17E-02	NS
CWC27	NS	8.78E-03	-0.107	3.17E-02	NS
CCDC92B	NS	8.78E-03	1.07E-01	3.17E-02	NS
PPP1R16A	NS	8.79E-03	-0.107	3.17E-02	NS
SOD1	NS	8.79E-03	-0.107	3.17E-02	NS
PTGER3	NS	8.79E-03	-0.107	3.17E-02	NS
CIZ1	NS	8.79E-03	-0.107	3.17E-02	NS
MRPS12	NS	8.80E-03	-0.107	3.18E-02	NS
ZNF625	NS	8.80E-03	-0.107	3.18E-02	NS
GTF3C1	NS	8.81E-03	1.07E-01	3.18E-02	NS
C16orf96	NS	8.81E-03	1.07E-01	3.18E-02	NS
SARS2	NS	8.82E-03	-0.107	3.18E-02	NS
SLC49A3	NS	8.82E-03	-0.107	3.18E-02	NS
PCDHGA3	NS	8.82E-03	-0.107	3.18E-02	NS
MED30	NS	8.83E-03	-0.107	3.18E-02	NS
RPS20	NS	8.84E-03	-0.107	3.18E-02	NS
KLHL14	NS	8.84E-03	-0.107	3.19E-02	NS
GNAT1	NS	8.85E-03	1.07E-01	3.19E-02	NS
GPR52	NS	8.85E-03	-0.107	3.19E-02	NS
OR13C8	NS	8.86E-03	-0.107	3.19E-02	NS
RYBP	NS	8.87E-03	-0.107	3.19E-02	NS
SPARC	NS	8.89E-03	-0.107	3.20E-02	NS
TAL1	NS	8.88E-03	-0.107	3.20E-02	NS
KCTD21	NS	8.89E-03	1.07E-01	3.20E-02	NS
ZRSR2	NS	8.89E-03	-0.107	3.20E-02	NS
PRDM8	NS	8.89E-03	1.07E-01	3.20E-02	NS
PAG1	NS	8.90E-03	1.07E-01	3.20E-02	NS
MED11	NS	8.92E-03	-0.107	3.21E-02	NS
MYO3A	NS	8.92E-03	-0.107	3.21E-02	NS
UBR1	NS	8.93E-03	-0.107	3.21E-02	NS
RPL3	NS	8.94E-03	-0.107	3.21E-02	NS

GTF2B	NS	8.94E-03	-0.107	3.21E-02	NS
CHST6	NS	8.95E-03	1.07E-01	3.21E-02	NS
SAPCD2	NS	8.95E-03	-0.107	3.21E-02	NS
SLC17A1	NS	8.96E-03	-0.107	3.22E-02	NS
MORN2	NS	8.97E-03	-0.107	3.22E-02	NS
EFNA3	NS	8.99E-03	-0.107	3.23E-02	NS
ZNF843	NS	9.00E-03	1.07E-01	3.23E-02	NS
ING4	NS	9.01E-03	-0.107	3.23E-02	NS
NETO2	NS	9.01E-03	1.07E-01	3.23E-02	NS
ZNF165	NS	9.01E-03	1.07E-01	3.23E-02	NS
ADAM33	NS	9.02E-03	1.07E-01	3.23E-02	NS
TMEM200B	NS	9.02E-03	-0.107	3.24E-02	NS
UXS1	NS	9.04E-03	-0.107	3.24E-02	NS
RCCD1	NS	9.04E-03	-0.107	3.24E-02	NS
B9D1	NS	9.06E-03	-0.107	3.25E-02	NS
C1QTNF7	NS	9.08E-03	-0.107	3.25E-02	NS
ABCB6	NS	9.09E-03	-0.107	3.25E-02	NS
UBR4	NS	9.09E-03	-0.107	3.26E-02	NS
RC3H2	NS	9.12E-03	-0.107	3.26E-02	NS
TDRD3	NS	9.11E-03	-0.107	3.26E-02	NS
PYDC1	NS	9.12E-03	1.07E-01	3.26E-02	NS
DSE	NS	9.12E-03	-0.107	3.26E-02	NS
ZNF615	NS	9.13E-03	-0.107	3.27E-02	NS
KRTAP12-1	NS	9.14E-03	1.07E-01	3.27E-02	NS
NFS1	NS	9.14E-03	-0.107	3.27E-02	NS
NIPAL1	NS	9.14E-03	-0.107	3.27E-02	NS
IZUMO3	NS	9.14E-03	-0.107	3.27E-02	NS
SIDT1	NS	9.15E-03	-0.107	3.27E-02	NS
TCF15	NS	9.15E-03	1.07E-01	3.27E-02	NS
PEAR1	NS	9.19E-03	-0.107	3.28E-02	NS
CYP4A11	NS	9.20E-03	-0.107	3.29E-02	NS
ADH1C	NS	9.20E-03	-0.107	3.29E-02	NS
ESCO2	NS	9.20E-03	-0.107	3.29E-02	NS
DTYMK	NS	9.24E-03	-0.107	3.30E-02	NS
IFRD1	NS	9.25E-03	1.07E-01	3.30E-02	NS
GLP2R	NS	9.25E-03	-0.107	3.30E-02	NS
UNC5C	NS	9.25E-03	-0.107	3.30E-02	NS

KRTAP4-5	NS	9.27E-03	-0.107	3.31E-02	NS
TTR	NS	9.27E-03	-0.107	3.31E-02	NS
CELSR2	NS	9.27E-03	-0.107	3.31E-02	NS
KLHL8	NS	9.27E-03	-0.107	3.31E-02	NS
SYBU	NS	9.28E-03	-0.107	3.31E-02	NS
PIP4P2	NS	9.29E-03	-0.107	3.31E-02	NS
DMTN	NS	9.30E-03	-0.107	3.31E-02	NS
CCDC25	NS	9.31E-03	-0.107	3.32E-02	NS
CENPVL3	NS	9.31E-03	1.07E-01	3.32E-02	NS
RAP2B	NS	9.31E-03	1.07E-01	3.32E-02	NS
NEUROD4	NS	9.33E-03	-0.106	3.32E-02	NS
TMEM14C	NS	9.34E-03	-0.106	3.33E-02	NS
PDSS2	NS	9.34E-03	-0.106	3.33E-02	NS
RPGRIP1L	NS	9.35E-03	-0.106	3.33E-02	NS
PCDH19	NS	9.35E-03	-0.106	3.33E-02	NS
CDKN2D	NS	9.35E-03	1.06E-01	3.33E-02	NS
SPDYE2B	NS	9.37E-03	1.06E-01	3.33E-02	NS
GOLGA8M	NS	9.37E-03	-0.106	3.33E-02	NS
APOH	NS	9.39E-03	1.06E-01	3.34E-02	NS
ADPRHL1	NS	9.40E-03	-0.106	3.34E-02	NS
PPP2R2D	NS	9.40E-03	1.06E-01	3.34E-02	NS
ZNF607	NS	9.41E-03	-0.106	3.34E-02	NS
PLEKHO1	NS	9.41E-03	-0.106	3.35E-02	NS
FTMT	NS	9.42E-03	-0.106	3.35E-02	NS
SMPD4	NS	9.43E-03	1.06E-01	3.35E-02	NS
C19orf33	NS	9.43E-03	-0.106	3.35E-02	NS
OR5T3	NS	9.43E-03	-0.106	3.35E-02	NS
APOBEC3G	NS	9.44E-03	-0.106	3.35E-02	NS
HABP4	NS	9.45E-03	-0.106	3.36E-02	NS
MMAB	NS	9.46E-03	-0.106	3.36E-02	NS
RNASE13	NS	9.47E-03	1.06E-01	3.36E-02	NS
VAPB	NS	9.48E-03	-0.106	3.36E-02	NS
ABL2	NS	9.49E-03	-0.106	3.37E-02	NS
CLDN3	NS	9.51E-03	1.06E-01	3.37E-02	NS
CT83	NS	9.51E-03	-0.106	3.37E-02	NS
PON1	NS	9.52E-03	-0.106	3.37E-02	NS
TEX53	NS	9.52E-03	-0.106	3.37E-02	NS

FHOD3	NS	9.55E-03	-0.106	3.38E-02	NS
RLN2	NS	9.56E-03	-0.106	3.38E-02	NS
MSI1	NS	9.56E-03	1.06E-01	3.39E-02	NS
NIF3L1	NS	9.56E-03	-0.106	3.39E-02	NS
NUP133	NS	9.57E-03	-0.106	3.39E-02	NS
C20orf27	NS	9.57E-03	-0.106	3.39E-02	NS
ATP5PD	NS	9.58E-03	-0.106	3.39E-02	NS
ALX1	NS	9.59E-03	-0.106	3.39E-02	NS
MARCHF9	NS	9.59E-03	-0.106	3.39E-02	NS
CLIC6	NS	9.59E-03	-0.106	3.39E-02	NS
MAP1A	NS	9.60E-03	-0.106	3.39E-02	NS
ZNF546	NS	9.60E-03	-0.106	3.39E-02	NS
PPID	NS	9.60E-03	-0.106	3.40E-02	NS
CHST2	NS	9.63E-03	-0.106	3.40E-02	NS
TRAPPC14	NS	9.65E-03	-0.106	3.41E-02	NS
OR5AC2	NS	9.66E-03	-0.106	3.41E-02	NS
FICD	NS	9.66E-03	-0.106	3.41E-02	NS
GNL3	NS	9.67E-03	-0.106	3.42E-02	NS
MAP3K10	NS	9.71E-03	-0.106	3.43E-02	NS
KRTAP27-1	NS	9.71E-03	-0.106	3.43E-02	NS
RDH8	NS	9.76E-03	1.06E-01	3.45E-02	NS
NOD1	NS	9.77E-03	1.06E-01	3.45E-02	NS
SERAC1	NS	9.77E-03	-0.106	3.45E-02	NS
UGT2A1	NS	9.79E-03	-0.106	3.46E-02	NS
G6PC2	NS	9.82E-03	-0.106	3.47E-02	NS
PGF	NS	9.83E-03	-0.106	3.47E-02	NS
METTL16	NS	9.83E-03	-0.106	3.47E-02	NS
STRAP	NS	9.84E-03	-0.106	3.47E-02	NS
MACF1	NS	9.85E-03	1.06E-01	3.47E-02	NS
TMEM240	NS	9.87E-03	1.06E-01	3.48E-02	NS
AGT	NS	9.87E-03	-0.106	3.48E-02	NS
PDZRN4	NS	9.90E-03	-0.106	3.49E-02	NS
KRTAP4-6	NS	9.93E-03	1.06E-01	3.50E-02	NS
ZNF799	NS	9.93E-03	-0.106	3.50E-02	NS
KIF12	NS	9.94E-03	1.06E-01	3.50E-02	NS
FBLN1	NS	9.95E-03	-0.106	3.50E-02	NS
BCAT1	NS	9.95E-03	-0.106	3.50E-02	NS

WDR83	NS	9.96E-03	-0.106	3.50E-02	NS
NOC4L	NS	9.96E-03	-0.106	3.50E-02	NS
ZNF503	NS	9.96E-03	-0.106	3.50E-02	NS
ENHO	NS	9.97E-03	-0.106	3.51E-02	NS
RBM18	NS	9.98E-03	1.06E-01	3.51E-02	NS
LPAR3	NS	9.98E-03	-0.106	3.51E-02	NS
OFD1	NS	9.99E-03	-0.106	3.51E-02	NS
PYCR3	NS	1.00E-02	1.06E-01	3.52E-02	NS
ANKRD24	NS	1.00E-02	1.06E-01	3.52E-02	NS
COTL1	NS	1.00E-02	1.06E-01	3.52E-02	NS
ZIC3	NS	1.00E-02	1.05E-01	3.53E-02	NS
HGFAC	NS	1.00E-02	1.05E-01	3.53E-02	NS
GP5	NS	1.00E-02	1.05E-01	3.53E-02	NS
LYZL6	NS	1.01E-02	1.05E-01	3.53E-02	NS
NTMT1	NS	1.01E-02	1.05E-01	3.54E-02	NS
DAB2	NS	1.01E-02	-0.105	3.54E-02	NS
ADRA1D	NS	1.01E-02	1.05E-01	3.54E-02	NS
NIPSNAP1	NS	1.01E-02	-0.105	3.54E-02	NS
ODF4	NS	1.01E-02	-0.105	3.54E-02	NS
TNNI3	NS	1.01E-02	1.05E-01	3.54E-02	NS
KCNH5	NS	1.01E-02	-0.105	3.54E-02	NS
TBPL1	NS	1.01E-02	-0.105	3.55E-02	NS
SPANXA1	NS	1.01E-02	-0.105	3.55E-02	NS
TTC39C	NS	1.01E-02	-0.105	3.55E-02	NS
PHF21B	NS	1.02E-02	-0.105	3.56E-02	NS
GATA5	NS	1.01E-02	1.05E-01	3.56E-02	NS
ADGRL4	NS	1.02E-02	-0.105	3.56E-02	NS
PPIL6	NS	1.02E-02	1.05E-01	3.56E-02	NS
UNC119	NS	1.02E-02	1.05E-01	3.56E-02	NS
PCOLCE	NS	1.02E-02	-0.105	3.57E-02	NS
TMED6	NS	1.02E-02	-0.105	3.57E-02	NS
DZANK1	NS	1.02E-02	-0.105	3.57E-02	NS
LAMB1	NS	1.02E-02	-0.105	3.57E-02	NS
BTNL3	NS	1.02E-02	1.05E-01	3.57E-02	NS
NELFE	NS	1.02E-02	-0.105	3.57E-02	NS
AKAP11	NS	1.02E-02	-0.105	3.58E-02	NS
CEP70	NS	1.02E-02	-0.105	3.58E-02	NS

ADAD2	NS	1.02E-02	1.05E-01	3.58E-02	NS
IL33	NS	1.02E-02	-0.105	3.58E-02	NS
TMEM167A	NS	1.03E-02	-0.105	3.58E-02	NS
IGSF9B	NS	1.03E-02	-0.105	3.58E-02	NS
IGDCC4	NS	1.03E-02	-0.105	3.58E-02	NS
GSTK1	NS	1.03E-02	-0.105	3.60E-02	NS
IQCA1L	NS	1.03E-02	-0.105	3.60E-02	NS
LMBR1	NS	1.03E-02	1.05E-01	3.60E-02	NS
LRRC9	NS	1.03E-02	-0.105	3.60E-02	NS
SPARCL1	NS	1.03E-02	-0.105	3.60E-02	NS
NLRP6	NS	1.03E-02	1.05E-01	3.60E-02	NS
CTAGE6	NS	1.03E-02	-0.105	3.60E-02	NS
FGF11	NS	1.03E-02	-0.105	3.60E-02	NS
SLC45A2	NS	1.03E-02	-0.105	3.60E-02	NS
AGTR2	NS	1.03E-02	-0.105	3.60E-02	NS
UBE2D2	NS	1.03E-02	1.05E-01	3.60E-02	NS
TTC5	NS	1.03E-02	-0.105	3.61E-02	NS
OR2G6	NS	1.04E-02	-0.105	3.61E-02	NS
VSIG4	NS	1.04E-02	-0.105	3.62E-02	NS
TPK1	NS	1.04E-02	-0.105	3.62E-02	NS
IFITM1	NS	1.04E-02	1.05E-01	3.62E-02	NS
DCST2	NS	1.04E-02	1.05E-01	3.62E-02	NS
CYP3A5	NS	1.04E-02	-0.105	3.62E-02	NS
MNX1	NS	1.04E-02	1.05E-01	3.63E-02	NS
LPAR5	NS	1.04E-02	-0.105	3.63E-02	NS
CBWD5	NS	1.04E-02	-0.105	3.63E-02	NS
GTF3C6	NS	1.04E-02	-0.105	3.63E-02	NS
KLHL41	NS	1.04E-02	-0.105	3.63E-02	NS
MAP3K4	NS	1.05E-02	-0.105	3.64E-02	NS
RAG1	NS	1.05E-02	-0.105	3.64E-02	NS
ZNF117	NS	1.05E-02	-0.105	3.64E-02	NS
WDR44	NS	1.05E-02	-0.105	3.64E-02	NS
AMFR	NS	1.05E-02	1.05E-01	3.64E-02	NS
DDX10	NS	1.05E-02	-0.105	3.64E-02	NS
TMEM210	NS	1.05E-02	1.05E-01	3.64E-02	NS
OR11H12	NS	1.05E-02	-0.105	3.64E-02	NS
ZG16	NS	1.05E-02	1.05E-01	3.65E-02	NS

FGF16	NS	1.05E-02	-0.105	3.65E-02	NS
SPATA31D1	NS	1.05E-02	-0.105	3.65E-02	NS
TBC1D5	NS	1.05E-02	-0.105	3.66E-02	NS
GFOD1	NS	1.05E-02	1.05E-01	3.66E-02	NS
ERI1	NS	1.06E-02	-0.105	3.66E-02	NS
C20orf173	NS	1.06E-02	-0.105	3.66E-02	NS
ADGRL2	NS	1.06E-02	-0.105	3.67E-02	NS
DEFA1B	NS	1.06E-02	-0.105	3.67E-02	NS
CCDC120	NS	1.06E-02	1.05E-01	3.67E-02	NS
OR51C1P	NS	1.06E-02	-0.105	3.67E-02	NS
BEND3	NS	1.06E-02	-0.105	3.67E-02	NS
PCDH7	NS	1.06E-02	-0.105	3.68E-02	NS
ZNF706	NS	1.06E-02	-0.105	3.68E-02	NS
MZB1	NS	1.06E-02	-0.105	3.68E-02	NS
CBWD6	NS	1.06E-02	-0.105	3.68E-02	NS
GRAP2	NS	1.06E-02	-0.105	3.69E-02	NS
VGLL2	NS	1.07E-02	1.05E-01	3.69E-02	NS
GBP2	NS	1.07E-02	-0.105	3.69E-02	NS
ARV1	NS	1.07E-02	-0.105	3.69E-02	NS
STMN2	NS	1.07E-02	-0.105	3.69E-02	NS
ZNF572	NS	1.07E-02	-0.105	3.69E-02	NS
TTC29	NS	1.07E-02	-0.105	3.69E-02	NS
FGFBP2	NS	1.07E-02	-0.105	3.69E-02	NS
CTTN	NS	1.07E-02	-0.105	3.69E-02	NS
TIMP4	NS	1.07E-02	-0.105	3.70E-02	NS
ASMTL	NS	1.07E-02	-0.105	3.70E-02	NS
TRIM6	NS	1.07E-02	-0.105	3.70E-02	NS
RPS7	NS	1.07E-02	-0.105	3.71E-02	NS
SGO2	NS	1.07E-02	-0.105	3.71E-02	NS
KREMEN2	NS	1.08E-02	1.04E-01	3.71E-02	NS
ENTPD3	NS	1.08E-02	-0.105	3.71E-02	NS
HBG2	NS	1.07E-02	-0.105	3.71E-02	NS
FOXK1	NS	1.08E-02	-0.104	3.71E-02	NS
PPP4R1	NS	1.08E-02	-0.104	3.72E-02	NS
TNFRSF21	NS	1.08E-02	1.04E-01	3.72E-02	NS
TAX1BP3	NS	1.08E-02	1.04E-01	3.72E-02	NS
SLC29A2	NS	1.08E-02	-0.104	3.72E-02	NS

MGMT	NS	1.08E-02	-0.104	3.73E-02	NS
OCIAD2	NS	1.08E-02	-0.104	3.73E-02	NS
DCAF8	NS	1.08E-02	-0.104	3.73E-02	NS
PAQR6	NS	1.08E-02	1.04E-01	3.73E-02	NS
TRABD2B	NS	1.08E-02	-0.104	3.73E-02	NS
PRODH	NS	1.08E-02	1.04E-01	3.73E-02	NS
SLC38A2	NS	1.08E-02	-0.104	3.73E-02	NS
CNOT10	NS	1.08E-02	-0.104	3.74E-02	NS
ERMN	NS	1.09E-02	-0.104	3.74E-02	NS
CCDC6	NS	1.09E-02	1.04E-01	3.74E-02	NS
BMP6	NS	1.09E-02	-0.104	3.74E-02	NS
GRIN2B	NS	1.09E-02	-0.104	3.74E-02	NS
TENM1	NS	1.09E-02	-0.104	3.74E-02	NS
PCSK1N	NS	1.09E-02	1.04E-01	3.74E-02	NS
SCG3	NS	1.09E-02	-0.104	3.75E-02	NS
HOXC8	NS	1.09E-02	1.04E-01	3.75E-02	NS
ACTL9	NS	1.09E-02	1.04E-01	3.75E-02	NS
PDE8A	NS	1.09E-02	-0.104	3.75E-02	NS
CLVS2	NS	1.09E-02	-0.104	3.75E-02	NS
TUBG2	NS	1.09E-02	-0.104	3.76E-02	NS
RNASE9	NS	1.09E-02	-0.104	3.76E-02	NS
AP1S1	NS	1.09E-02	-0.104	3.76E-02	NS
RABL3	NS	1.10E-02	-0.104	3.76E-02	NS
DMXL1	NS	1.09E-02	-0.104	3.76E-02	NS
SCGB2B2	NS	1.10E-02	-0.104	3.76E-02	NS
STEAP3	NS	1.10E-02	-0.104	3.76E-02	NS
ADGRE1	NS	1.10E-02	-0.104	3.77E-02	NS
IRS1	NS	1.10E-02	-0.104	3.77E-02	NS
FXYD6	NS	1.10E-02	-0.104	3.77E-02	NS
VPS18	NS	1.10E-02	1.04E-01	3.78E-02	NS
SPECC1L-ADORA2A	NS	1.10E-02	-0.104	3.78E-02	NS
MOGS	NS	1.10E-02	-0.104	3.79E-02	NS
EPYC	NS	1.11E-02	-0.104	3.79E-02	NS
SSTR5	NS	1.11E-02	1.04E-01	3.80E-02	NS
SERPINE1	NS	1.11E-02	1.04E-01	3.80E-02	NS
KRTAP15-1	NS	1.11E-02	-0.104	3.80E-02	NS
IL17RB	NS	1.11E-02	-0.104	3.80E-02	NS

DPP10	NS	1.11E-02	-0.104	3.80E-02	NS
LYZL4	NS	1.11E-02	1.04E-01	3.80E-02	NS
PARN	NS	1.11E-02	-0.104	3.81E-02	NS
RHOV	NS	1.12E-02	1.04E-01	3.82E-02	NS
TBC1D22B	NS	1.12E-02	-0.104	3.83E-02	NS
MAGEA11	NS	1.12E-02	-0.104	3.83E-02	NS
DLG2	NS	1.12E-02	-0.104	3.83E-02	NS
CDC25C	NS	1.12E-02	1.04E-01	3.83E-02	NS
DARS2	NS	1.12E-02	-0.104	3.84E-02	NS
ALKBH1	NS	1.12E-02	-0.104	3.84E-02	NS
RNF217	NS	1.12E-02	-0.104	3.84E-02	NS
RNASE11	NS	1.13E-02	1.04E-01	3.85E-02	NS
ZNF566	NS	1.13E-02	-0.104	3.85E-02	NS
CALM2	NS	1.13E-02	-0.104	3.85E-02	NS
TREML2	NS	1.13E-02	1.04E-01	3.85E-02	NS
PIK3CA	NS	1.13E-02	-0.104	3.85E-02	NS
BBIP1	NS	1.13E-02	-0.104	3.85E-02	NS
H2AB3	NS	1.13E-02	1.04E-01	3.85E-02	NS
CCER1	NS	1.13E-02	-0.104	3.85E-02	NS
BTBD11	NS	1.13E-02	-0.104	3.86E-02	NS
TAF7	NS	1.13E-02	-0.104	3.87E-02	NS
SHMT2	NS	1.13E-02	-0.104	3.87E-02	NS
ERBB4	NS	1.13E-02	-0.104	3.87E-02	NS
TUT1	NS	1.14E-02	-0.104	3.87E-02	NS
ARHGAP45	NS	1.14E-02	1.04E-01	3.87E-02	NS
PKHD1	NS	1.14E-02	-0.104	3.88E-02	NS
ALG10B	NS	1.14E-02	-0.104	3.88E-02	NS
FNDC8	NS	1.14E-02	1.04E-01	3.88E-02	NS
SMIM2	NS	1.14E-02	-0.104	3.88E-02	NS
AMMECR1L	NS	1.14E-02	-0.104	3.89E-02	NS
DGAT2	NS	1.14E-02	-0.104	3.89E-02	NS
NFKBIE	NS	1.14E-02	1.04E-01	3.90E-02	NS
LSM6	NS	1.14E-02	-0.104	3.90E-02	NS
FSCB	NS	1.14E-02	1.04E-01	3.90E-02	NS
ZNF674	NS	1.14E-02	-0.104	3.90E-02	NS
ARHGEF10	NS	1.15E-02	-0.104	3.90E-02	NS
ORC4	NS	1.15E-02	-0.104	3.91E-02	NS

INSL5	NS	1.15E-02	-0.104	3.91E-02	NS
FN3KRP	NS	1.15E-02	-0.104	3.91E-02	NS
TIPRL	NS	1.15E-02	-0.104	3.91E-02	NS
SLC6A6	NS	1.15E-02	-0.104	3.91E-02	NS
MEN1	NS	1.15E-02	1.04E-01	3.91E-02	NS
HOXB1	NS	1.15E-02	1.04E-01	3.92E-02	NS
H2AB2	NS	1.15E-02	1.04E-01	3.92E-02	NS
COMMD2	NS	1.15E-02	-0.103	3.92E-02	NS
NSUN3	NS	1.16E-02	-0.103	3.93E-02	NS
PAPSS2	NS	1.16E-02	-0.103	3.93E-02	NS
NAV2	NS	1.16E-02	-0.103	3.93E-02	NS
IGIP	NS	1.16E-02	-0.103	3.93E-02	NS
OST4	NS	1.16E-02	1.03E-01	3.93E-02	NS
TRIM29	NS	1.16E-02	-0.103	3.93E-02	NS
LIG1	NS	1.16E-02	-0.103	3.94E-02	NS
SRL	NS	1.16E-02	-0.103	3.94E-02	NS
STARD3	NS	1.16E-02	-0.103	3.94E-02	NS
ZNF669	NS	1.16E-02	-0.103	3.95E-02	NS
ZMAT3	NS	1.17E-02	-0.103	3.95E-02	NS
RELCH	NS	1.17E-02	-0.103	3.95E-02	NS
GALNT13	NS	1.17E-02	-0.103	3.95E-02	NS
PCDHA5	NS	1.17E-02	1.03E-01	3.95E-02	NS
CACNG6	NS	1.17E-02	1.03E-01	3.97E-02	NS
PROP1	NS	1.17E-02	-0.103	3.97E-02	NS
OR51I2	NS	1.17E-02	-0.103	3.97E-02	NS
CHMP2B	NS	1.17E-02	-0.103	3.97E-02	NS
NSUN6	NS	1.17E-02	-0.103	3.97E-02	NS
KRTAP3-1	NS	1.17E-02	-0.103	3.97E-02	NS
EDA2R	NS	1.17E-02	-0.103	3.98E-02	NS
RRN3	NS	1.18E-02	-0.103	3.98E-02	NS
CALML6	NS	1.18E-02	1.03E-01	3.98E-02	NS
CEP85	NS	1.18E-02	-0.103	3.98E-02	NS
PAK4	NS	1.18E-02	1.03E-01	3.98E-02	NS
KLF8	NS	1.18E-02	-0.103	3.99E-02	NS
CH25H	NS	1.18E-02	-0.103	3.99E-02	NS
FAM181A	NS	1.18E-02	1.03E-01	3.99E-02	NS
KCNA2	NS	1.18E-02	1.03E-01	3.99E-02	NS

KRT73	NS	1.18E-02	-0.103	3.99E-02	NS
PAX1	NS	1.18E-02	1.03E-01	4.00E-02	NS
ENDOV	NS	1.19E-02	-0.103	4.01E-02	NS
CLEC18C	NS	1.19E-02	-0.103	4.01E-02	NS
C19orf71	NS	1.19E-02	1.03E-01	4.02E-02	NS
TMBIM4	NS	1.19E-02	-0.103	4.02E-02	NS
LY75	NS	1.19E-02	-0.103	4.02E-02	NS
TMEM45B	NS	1.19E-02	-0.103	4.02E-02	NS
SLC27A6	NS	1.19E-02	-0.103	4.02E-02	NS
CENPL	NS	1.19E-02	-0.103	4.02E-02	NS
IFI44	NS	1.19E-02	-0.103	4.02E-02	NS
OR51A4	NS	1.19E-02	-0.103	4.02E-02	NS
ARHGAP36	NS	1.19E-02	1.03E-01	4.02E-02	NS
MRPL11	NS	1.20E-02	-0.103	4.03E-02	NS
SEC14L4	NS	1.20E-02	1.03E-01	4.03E-02	NS
DRAP1	NS	1.20E-02	-0.103	4.03E-02	NS
THRSP	NS	1.20E-02	-0.103	4.04E-02	NS
BEND4	NS	1.20E-02	-0.103	4.04E-02	NS
SYT6	NS	1.20E-02	-0.103	4.04E-02	NS
C3orf80	NS	1.20E-02	1.03E-01	4.04E-02	NS
LRFN1	NS	1.20E-02	-0.103	4.04E-02	NS
OR52L1	NS	1.20E-02	-0.103	4.05E-02	NS
ANAPC13	NS	1.20E-02	-0.103	4.05E-02	NS
LINGO3	NS	1.20E-02	1.03E-01	4.05E-02	NS
SLC22A23	NS	1.21E-02	-0.103	4.05E-02	NS
CSMD1	NS	1.21E-02	-0.103	4.05E-02	NS
BSPRY	NS	1.21E-02	-0.103	4.06E-02	NS
EIF4ENIF1	NS	1.21E-02	-0.103	4.06E-02	NS
EPN3	NS	1.21E-02	1.03E-01	4.06E-02	NS
ZNRF4	NS	1.21E-02	1.03E-01	4.06E-02	NS
CEP95	NS	1.21E-02	1.03E-01	4.07E-02	NS
AQP12B	NS	1.21E-02	1.03E-01	4.07E-02	NS
CCDC126	NS	1.21E-02	-0.103	4.07E-02	NS
FCRL6	NS	1.21E-02	-0.103	4.07E-02	NS
TAC4	NS	1.21E-02	-0.103	4.07E-02	NS
RTL3	NS	1.21E-02	1.03E-01	4.07E-02	NS
SGO1	NS	1.21E-02	-0.103	4.07E-02	NS

MTFR2	NS	1.21E-02	-0.103	4.07E-02	NS
OSTF1	NS	1.22E-02	-0.103	4.08E-02	NS
HIVEP2	NS	1.22E-02	-0.103	4.08E-02	NS
CELF2	NS	1.22E-02	1.03E-01	4.08E-02	NS
KLLN	NS	1.22E-02	1.03E-01	4.08E-02	NS
CTRB2	NS	1.22E-02	1.03E-01	4.08E-02	NS
GKN1	NS	1.22E-02	-0.103	4.08E-02	NS
RAB3IL1	NS	1.22E-02	1.03E-01	4.09E-02	NS
FBXL4	NS	1.22E-02	-0.103	4.09E-02	NS
PNCK	NS	1.22E-02	1.03E-01	4.09E-02	NS
ADPGK	NS	1.22E-02	-0.103	4.09E-02	NS
KIF5C	NS	1.22E-02	-0.103	4.09E-02	NS
FGF2	NS	1.22E-02	-0.103	4.09E-02	NS
AKT1S1	NS	1.22E-02	1.03E-01	4.10E-02	NS
ASPA	NS	1.23E-02	-0.103	4.10E-02	NS
LRPPRC	NS	1.23E-02	-0.103	4.10E-02	NS
RXFP2	NS	1.23E-02	-0.103	4.10E-02	NS
B2M	NS	1.23E-02	-0.103	4.11E-02	NS
ICE1	NS	1.23E-02	-0.103	4.11E-02	NS
TMEM220	NS	1.23E-02	-0.103	4.11E-02	NS
ZIC1	NS	1.23E-02	-0.103	4.11E-02	NS
ZBBX	NS	1.23E-02	-0.103	4.12E-02	NS
CFAP99	NS	1.23E-02	1.03E-01	4.12E-02	NS
AATF	NS	1.23E-02	-0.103	4.12E-02	NS
RUNDC1	NS	1.23E-02	-0.103	4.12E-02	NS
SNX3	NS	1.23E-02	1.03E-01	4.12E-02	NS
TAAR2	NS	1.24E-02	-0.103	4.13E-02	NS
GZMK	NS	1.24E-02	-0.103	4.13E-02	NS
LACTBL1	NS	1.24E-02	1.03E-01	4.13E-02	NS
FBLN5	NS	1.24E-02	-0.102	4.13E-02	NS
GLDN	NS	1.24E-02	-0.102	4.13E-02	NS
NOPCHAP1	NS	1.24E-02	-0.102	4.13E-02	NS
RASGEF1B	NS	1.24E-02	-0.102	4.13E-02	NS
PRAC2	NS	1.24E-02	1.02E-01	4.13E-02	NS
CSF3	NS	1.24E-02	-0.102	4.13E-02	NS
PHTF2	NS	1.24E-02	-0.102	4.14E-02	NS
DPY19L1	NS	1.24E-02	-0.102	4.14E-02	NS

URM1	NS	1.24E-02	1.02E-01	4.15E-02	NS
FAM25C	NS	1.25E-02	-0.102	4.15E-02	NS
CHAF1A	NS	1.25E-02	-0.102	4.15E-02	NS
ACP2	NS	1.25E-02	-0.102	4.15E-02	NS
VWA5B2	NS	1.25E-02	1.02E-01	4.15E-02	NS
TIRAP	NS	1.25E-02	-0.102	4.16E-02	NS
HTR3D	NS	1.25E-02	-0.102	4.16E-02	NS
BCL2L10	NS	1.25E-02	-0.102	4.16E-02	NS
MVB12B	NS	1.25E-02	-0.102	4.16E-02	NS
MPHOSPH8	NS	1.25E-02	-0.102	4.16E-02	NS
STOX1	NS	1.25E-02	-0.102	4.16E-02	NS
KCNK7	NS	1.25E-02	-0.102	4.16E-02	NS
MAD1L1	NS	1.25E-02	-0.102	4.16E-02	NS
ALG14	NS	1.25E-02	-0.102	4.16E-02	NS
TCIM	NS	1.25E-02	-0.102	4.17E-02	NS
FBXO34	NS	1.25E-02	-0.102	4.17E-02	NS
PLEKHG7	NS	1.25E-02	-0.102	4.17E-02	NS
KRTAP2-4	NS	1.25E-02	-0.102	4.17E-02	NS
NUMBL	NS	1.26E-02	1.02E-01	4.17E-02	NS
ADAMTS16	NS	1.26E-02	1.02E-01	4.17E-02	NS
KRT5	NS	1.26E-02	-0.102	4.17E-02	NS
ART4	NS	1.26E-02	-0.102	4.17E-02	NS
KL	NS	1.26E-02	-0.102	4.18E-02	NS
ANGPTL7	NS	1.26E-02	-0.102	4.18E-02	NS
CAVIN1	NS	1.26E-02	1.02E-01	4.18E-02	NS
LPIN3	NS	1.26E-02	-0.102	4.18E-02	NS
NCALD	NS	1.26E-02	-0.102	4.18E-02	NS
PIGC	NS	1.27E-02	-0.102	4.20E-02	NS
CRABP2	NS	1.27E-02	-0.102	4.20E-02	NS
ATXN10	NS	1.27E-02	-0.102	4.20E-02	NS
TRAPPC2	NS	1.27E-02	-0.102	4.20E-02	NS
ATP2B3	NS	1.27E-02	-0.102	4.21E-02	NS
TMX1	NS	1.27E-02	-0.102	4.21E-02	NS
MS4A4A	NS	1.27E-02	1.02E-01	4.21E-02	NS
NCOA5	NS	1.27E-02	-0.102	4.21E-02	NS
TBC1D2	NS	1.27E-02	1.02E-01	4.21E-02	NS
TBX2	NS	1.27E-02	1.02E-01	4.21E-02	NS

TIMD4	NS	1.28E-02	-0.102	4.23E-02	NS
RING1	NS	1.28E-02	1.02E-01	4.23E-02	NS
MTRNR2L6	NS	1.28E-02	1.02E-01	4.23E-02	NS
PARM1	NS	1.28E-02	-0.102	4.23E-02	NS
RAB20	NS	1.28E-02	1.02E-01	4.24E-02	NS
ZBTB49	NS	1.28E-02	-0.102	4.24E-02	NS
NGFR	NS	1.28E-02	1.02E-01	4.24E-02	NS
C22orf15	NS	1.28E-02	-0.102	4.24E-02	NS
PSG9	NS	1.28E-02	-0.102	4.24E-02	NS
NOSTRIN	NS	1.28E-02	-0.102	4.24E-02	NS
PHAX	NS	1.28E-02	-0.102	4.24E-02	NS
BCL11A	NS	1.28E-02	-0.102	4.24E-02	NS
YIF1A	NS	1.28E-02	-0.102	4.24E-02	NS
ELP2	NS	1.29E-02	-0.102	4.26E-02	NS
FOXL2	NS	1.29E-02	1.02E-01	4.26E-02	NS
OR5H1	NS	1.29E-02	-0.102	4.26E-02	NS
SLX9	NS	1.29E-02	-0.102	4.27E-02	NS
PDRG1	NS	1.29E-02	-0.102	4.27E-02	NS
TXNDC12	NS	1.29E-02	-0.102	4.27E-02	NS
NOSIP	NS	1.29E-02	-0.102	4.27E-02	NS
DNAI2	NS	1.29E-02	1.02E-01	4.27E-02	NS
TBRG1	NS	1.30E-02	-0.102	4.28E-02	NS
S100A4	NS	1.30E-02	-0.102	4.28E-02	NS
ZNF395	NS	1.30E-02	-0.102	4.29E-02	NS
SPRR2A	NS	1.30E-02	-0.102	4.29E-02	NS
GUCY2D	NS	1.30E-02	1.02E-01	4.29E-02	NS
EIF2S3	NS	1.30E-02	-0.102	4.30E-02	NS
TAS2R41	NS	1.31E-02	-0.102	4.30E-02	NS
GEMIN7	NS	1.31E-02	-0.102	4.31E-02	NS
PLGLB2	NS	1.31E-02	-0.102	4.31E-02	NS
SLC46A2	NS	1.31E-02	-0.102	4.31E-02	NS
FCGR2B	NS	1.31E-02	1.02E-01	4.32E-02	NS
LACTB	NS	1.31E-02	1.02E-01	4.32E-02	NS
SPTBN1	NS	1.31E-02	1.02E-01	4.32E-02	NS
TRIM49D2	NS	1.31E-02	-0.102	4.32E-02	NS
RAVER2	NS	1.31E-02	-0.102	4.33E-02	NS
RHBDL2	NS	1.32E-02	-0.102	4.34E-02	NS

EXPH5	NS	1.32E-02	-0.102	4.34E-02	NS
NIN	NS	1.32E-02	-0.102	4.34E-02	NS
CFAP20	NS	1.32E-02	-0.102	4.34E-02	NS
GTF2F1	NS	1.32E-02	-0.102	4.34E-02	NS
GABPB1	NS	1.32E-02	-0.102	4.35E-02	NS
DEFB105B	NS	1.32E-02	1.02E-01	4.35E-02	NS
ADGRA2	NS	1.32E-02	-0.102	4.35E-02	NS
IRF5	NS	1.33E-02	1.01E-01	4.35E-02	NS
TMC7	NS	1.33E-02	1.01E-01	4.35E-02	NS
TREML4	NS	1.33E-02	-0.101	4.35E-02	NS
ARG2	NS	1.33E-02	-0.101	4.36E-02	NS
TMEM108	NS	1.33E-02	-0.101	4.36E-02	NS
RPE	NS	1.33E-02	-0.101	4.36E-02	NS
NMRAL1	NS	1.33E-02	1.01E-01	4.36E-02	NS
CCDC179	NS	1.33E-02	-0.101	4.36E-02	NS
BAG3	NS	1.33E-02	1.01E-01	4.37E-02	NS
RNASEK-C17orf49	NS	1.33E-02	-0.101	4.37E-02	NS
RNASET2	NS	1.33E-02	1.01E-01	4.37E-02	NS
FGFR4	NS	1.33E-02	1.01E-01	4.37E-02	NS
ZNF430	NS	1.34E-02	-0.101	4.38E-02	NS
MYOCOS	NS	1.34E-02	-0.101	4.38E-02	NS
BAIAP2L2	NS	1.34E-02	1.01E-01	4.39E-02	NS
TRMT44	NS	1.34E-02	-0.101	4.39E-02	NS
STOML2	NS	1.34E-02	-0.101	4.39E-02	NS
BPIFA1	NS	1.34E-02	-0.101	4.39E-02	NS
OR2J2	NS	1.34E-02	-0.101	4.39E-02	NS
PTCHD4	NS	1.34E-02	-0.101	4.39E-02	NS
UEVLD	NS	1.34E-02	-0.101	4.40E-02	NS
ZNF213	NS	1.34E-02	1.01E-01	4.40E-02	NS
CARHSP1	NS	1.34E-02	-0.101	4.40E-02	NS
HAND2	NS	1.34E-02	1.01E-01	4.40E-02	NS
GJB2	NS	1.34E-02	-0.101	4.40E-02	NS
ADAMTS9	NS	1.35E-02	-0.101	4.40E-02	NS
IFNA4	NS	1.35E-02	-0.101	4.40E-02	NS
PHKB	NS	1.35E-02	-0.101	4.40E-02	NS
RPS24	NS	1.35E-02	-0.101	4.40E-02	NS
TMPRSS6	NS	1.35E-02	-0.101	4.41E-02	NS

TMEM245	NS	1.35E-02	-0.101	4.41E-02	NS
C8orf88	NS	1.35E-02	-0.101	4.42E-02	NS
IRX5	NS	1.35E-02	1.01E-01	4.42E-02	NS
CYP2A6	NS	1.35E-02	-0.101	4.42E-02	NS
EPB41L4A	NS	1.35E-02	-0.101	4.43E-02	NS
SCGB2A1	NS	1.36E-02	1.01E-01	4.43E-02	NS
RIOK2	NS	1.36E-02	-0.101	4.43E-02	NS
TCEAL5	NS	1.36E-02	-0.101	4.43E-02	NS
NEDD4L	NS	1.36E-02	-0.101	4.43E-02	NS
FLI1	NS	1.36E-02	1.01E-01	4.44E-02	NS
WDR77	NS	1.36E-02	-0.101	4.44E-02	NS
C6	NS	1.36E-02	-0.101	4.44E-02	NS
ESS2	NS	1.36E-02	1.01E-01	4.45E-02	NS
PI4KB	NS	1.36E-02	-0.101	4.45E-02	NS
GJB7	NS	1.36E-02	1.01E-01	4.45E-02	NS
CKS2	NS	1.36E-02	-0.101	4.45E-02	NS
CCND3	NS	1.37E-02	1.01E-01	4.46E-02	NS
ATP11A	NS	1.37E-02	-0.101	4.46E-02	NS
CD34	NS	1.37E-02	1.01E-01	4.46E-02	NS
FAM133B	NS	1.37E-02	-0.101	4.46E-02	NS
ROS1	NS	1.37E-02	-0.101	4.46E-02	NS
KLHDC4	NS	1.37E-02	-0.101	4.47E-02	NS
BEX1	NS	1.37E-02	-0.101	4.47E-02	NS
RANBP10	NS	1.37E-02	1.01E-01	4.47E-02	NS
DYNLRB1	NS	1.37E-02	-0.101	4.48E-02	NS
RSPH14	NS	1.38E-02	-0.101	4.48E-02	NS
MSMP	NS	1.38E-02	-0.101	4.48E-02	NS
PRSS54	NS	1.38E-02	-0.101	4.49E-02	NS
MAGEB4	NS	1.38E-02	1.01E-01	4.49E-02	NS
IL20	NS	1.38E-02	-0.101	4.49E-02	NS
LNX2	NS	1.38E-02	-0.101	4.49E-02	NS
TGM2	NS	1.38E-02	-0.101	4.50E-02	NS
SYT11	NS	1.39E-02	-0.101	4.51E-02	NS
PSENEN	NS	1.39E-02	-0.101	4.51E-02	NS
NAPRT	NS	1.39E-02	1.01E-01	4.51E-02	NS
HNRNPH3	NS	1.39E-02	-0.101	4.52E-02	NS
MOG	NS	1.39E-02	1.01E-01	4.52E-02	NS

MAMSTR	NS	1.39E-02	-0.101	4.52E-02	NS
GALNT18	NS	1.39E-02	-0.101	4.52E-02	NS
VHLL	NS	1.39E-02	1.01E-01	4.52E-02	NS
ECH1	NS	1.39E-02	-0.101	4.52E-02	NS
SLCO1C1	NS	1.39E-02	-0.101	4.52E-02	NS
CD151	NS	1.39E-02	-0.101	4.53E-02	NS
BCL2L2-PABPN1	NS	1.40E-02	1.01E-01	4.53E-02	NS
ZNF426	NS	1.40E-02	-0.101	4.53E-02	NS
UMOD	NS	1.40E-02	-0.101	4.53E-02	NS
ARPIN	NS	1.40E-02	1.01E-01	4.53E-02	NS
ACOT6	NS	1.40E-02	1.01E-01	4.54E-02	NS
TMEM92	NS	1.40E-02	1.01E-01	4.54E-02	NS
CCDC158	NS	1.40E-02	-0.101	4.55E-02	NS
ASB17	NS	1.40E-02	-0.101	4.55E-02	NS
SH3GLB1	NS	1.41E-02	-0.101	4.55E-02	NS
TIMM17A	NS	1.41E-02	-0.101	4.55E-02	NS
AP5S1	NS	1.41E-02	-0.101	4.55E-02	NS
GAGE12D	NS	1.41E-02	-0.101	4.56E-02	NS
TMEM272	NS	1.41E-02	-0.101	4.56E-02	NS
RAD1	NS	1.41E-02	-0.101	4.56E-02	NS
C10orf99	NS	1.41E-02	1.01E-01	4.56E-02	NS
ZNF358	NS	1.41E-02	-0.101	4.56E-02	NS
LILRA6	NS	1.41E-02	1.01E-01	4.56E-02	NS
RSRC2	NS	1.41E-02	1.01E-01	4.56E-02	NS
IRX2	NS	1.41E-02	1.01E-01	4.56E-02	NS
ACSM6	NS	1.41E-02	-0.101	4.57E-02	NS
LRCH4	NS	1.41E-02	1.01E-01	4.57E-02	NS
HRH1	NS	1.41E-02	-0.101	4.57E-02	NS
NUP188	NS	1.41E-02	-0.101	4.57E-02	NS
TRANK1	NS	1.41E-02	-0.101	4.57E-02	NS
AQP3	NS	1.41E-02	-0.101	4.57E-02	NS
ANKRD20A1	NS	1.42E-02	-0.101	4.58E-02	NS
SWAP70	NS	1.42E-02	-0.101	4.58E-02	NS
CCDC168	NS	1.42E-02	-0.1	4.58E-02	NS
OR6Q1	NS	1.42E-02	1.00E-01	4.58E-02	NS
LTV1	NS	1.42E-02	-0.1	4.58E-02	NS
BCL6B	NS	1.42E-02	1.00E-01	4.58E-02	NS

CLTB	NS	1.42E-02	1.00E-01	4.59E-02	NS
TBP	NS	1.42E-02	1.00E-01	4.59E-02	NS
ZC3HAV1	NS	1.42E-02	-0.1	4.59E-02	NS
CRH	NS	1.43E-02	-0.1	4.60E-02	NS
OSM	NS	1.43E-02	-0.1	4.60E-02	NS
B4GALT7	NS	1.43E-02	-0.1	4.61E-02	NS
NPAS3	NS	1.43E-02	-0.1	4.62E-02	NS
ADM2	NS	1.43E-02	1.00E-01	4.62E-02	NS
SLC4A8	NS	1.43E-02	-0.1	4.62E-02	NS
PABPC1	NS	1.44E-02	-0.1	4.63E-02	NS
SERTAD2	NS	1.44E-02	1.00E-01	4.64E-02	NS
ASCL4	NS	1.44E-02	1.00E-01	4.64E-02	NS
PPARG	NS	1.44E-02	-0.1	4.64E-02	NS
MRPS18B	NS	1.44E-02	-0.1	4.64E-02	NS
UBA52	NS	1.44E-02	-0.1	4.64E-02	NS
TNRC18	NS	1.44E-02	1.00E-01	4.64E-02	NS
FAM185A	NS	1.45E-02	-0.1	4.65E-02	NS
RRAS2	NS	1.45E-02	-0.1	4.66E-02	NS
DHRS13	NS	1.45E-02	-0.1	4.66E-02	NS
TXN2	NS	1.45E-02	-0.1	4.66E-02	NS
CHRD	NS	1.45E-02	1.00E-01	4.67E-02	NS
CTAGE15	NS	1.45E-02	-0.1	4.67E-02	NS
S100A5	NS	1.45E-02	-0.1	4.67E-02	NS
PKDREJ	NS	1.45E-02	-0.1	4.68E-02	NS
WNT16	NS	1.46E-02	-0.1	4.68E-02	NS
SFSWAP	NS	1.46E-02	1.00E-01	4.68E-02	NS
GAST	NS	1.46E-02	1.00E-01	4.68E-02	NS
MEAF6	NS	1.46E-02	-0.1	4.68E-02	NS
ACOT4	NS	1.46E-02	1.00E-01	4.69E-02	NS
PHLPP1	NS	1.46E-02	-0.1	4.70E-02	NS
BIRC2	NS	1.46E-02	-0.1	4.70E-02	NS
ARHGAP44	NS	1.47E-02	-0.1	4.70E-02	NS
SOX11	NS	1.47E-02	-0.1	4.70E-02	NS
SS18L2	NS	1.47E-02	-0.1	4.70E-02	NS
TAF1L	NS	1.47E-02	1.00E-01	4.70E-02	NS
HPR	NS	1.47E-02	-0.1	4.71E-02	NS
MDFI	NS	1.47E-02	1.00E-01	4.71E-02	NS

NECAB1	NS	1.47E-02	-0.1	4.72E-02	NS
SHKBP1	NS	1.47E-02	-0.0999	4.73E-02	NS
ARHGAP10	NS	1.48E-02	-0.0999	4.73E-02	NS
ACAA2	NS	1.48E-02	-0.0999	4.73E-02	NS
MRPS7	NS	1.48E-02	-0.0999	4.73E-02	NS
SPATA48	NS	1.48E-02	-0.0999	4.73E-02	NS
COMMD6	NS	1.48E-02	-0.0999	4.74E-02	NS
MUC15	NS	1.48E-02	-0.0999	4.74E-02	NS
TAF1A	NS	1.48E-02	-0.0999	4.74E-02	NS
ADAMTSL3	NS	1.48E-02	-0.0999	4.74E-02	NS
OR2T29	NS	1.48E-02	-0.0999	4.74E-02	NS
EDC4	NS	1.48E-02	9.99E-02	4.74E-02	NS
ESX1	NS	1.48E-02	9.99E-02	4.74E-02	NS
YJEFN3	NS	1.48E-02	9.99E-02	4.74E-02	NS
FBN3	NS	1.48E-02	-0.0999	4.74E-02	NS
ZNF813	NS	1.48E-02	-0.0999	4.74E-02	NS
ST8SIA4	NS	1.49E-02	-0.0998	4.75E-02	NS
ANKRD40	NS	1.49E-02	-0.0998	4.75E-02	NS
ZNF485	NS	1.49E-02	-0.0998	4.75E-02	NS
MTIF3	NS	1.49E-02	-0.0998	4.75E-02	NS
KIF7	NS	1.49E-02	9.98E-02	4.76E-02	NS
PNPLA4	NS	1.49E-02	-0.0998	4.76E-02	NS
FAM180A	NS	1.49E-02	-0.0998	4.76E-02	NS
MAPKAPK3	NS	1.49E-02	9.98E-02	4.76E-02	NS
MRPL18	NS	1.49E-02	-0.0998	4.76E-02	NS
CAMK2D	NS	1.49E-02	-0.0998	4.76E-02	NS
USP43	NS	1.49E-02	-0.0998	4.76E-02	NS
SKA2	NS	1.49E-02	-0.0998	4.76E-02	NS
PLAT	NS	1.49E-02	9.97E-02	4.76E-02	NS
SERPINC1	NS	1.49E-02	-0.0997	4.77E-02	NS
SCGB1C2	NS	1.49E-02	9.97E-02	4.77E-02	NS
CA12	NS	1.50E-02	-0.0997	4.77E-02	NS
IL20RB	NS	1.50E-02	-0.0997	4.77E-02	NS
CETP	NS	1.50E-02	-0.0997	4.77E-02	NS
TCEAL2	NS	1.50E-02	9.97E-02	4.77E-02	NS
GNAT2	NS	1.50E-02	-0.0997	4.77E-02	NS
ZNF71	NS	1.50E-02	-0.0997	4.77E-02	NS

DCAF16	NS	1.50E-02	9.97E-02	4.78E-02	NS
SREBF1	NS	1.50E-02	9.97E-02	4.78E-02	NS
TAB1	NS	1.50E-02	9.97E-02	4.78E-02	NS
FASTK	NS	1.50E-02	-0.0997	4.78E-02	NS
KIF23	NS	1.50E-02	-0.0996	4.79E-02	NS
SULT4A1	NS	1.51E-02	9.96E-02	4.79E-02	NS
FCRL1	NS	1.51E-02	9.96E-02	4.80E-02	NS
TEX52	NS	1.51E-02	-0.0996	4.80E-02	NS
TIMM21	NS	1.51E-02	-0.0996	4.80E-02	NS
CHCHD1	NS	1.51E-02	-0.0996	4.80E-02	NS
TMEM203	NS	1.51E-02	-0.0996	4.80E-02	NS
TBKBP1	NS	1.51E-02	-0.0996	4.80E-02	NS
SNRPG	NS	1.51E-02	-0.0996	4.80E-02	NS
GULP1	NS	1.51E-02	-0.0996	4.80E-02	NS
FGGY	NS	1.51E-02	-0.0996	4.80E-02	NS
LRCOL1	NS	1.51E-02	9.96E-02	4.81E-02	NS
MOS	NS	1.51E-02	-0.0996	4.81E-02	NS
PRR4	NS	1.51E-02	-0.0995	4.81E-02	NS
ACOT12	NS	1.51E-02	-0.0996	4.81E-02	NS
GMCL1	NS	1.51E-02	-0.0995	4.81E-02	NS
DAZAP1	NS	1.51E-02	9.95E-02	4.81E-02	NS
B3GAT2	NS	1.52E-02	-0.0995	4.81E-02	NS
CHD7	NS	1.52E-02	9.95E-02	4.82E-02	NS
PECR	NS	1.52E-02	-0.0994	4.84E-02	NS
DNAAF3	NS	1.53E-02	9.94E-02	4.84E-02	NS
COA7	NS	1.53E-02	-0.0994	4.84E-02	NS
KRT3	NS	1.53E-02	-0.0994	4.84E-02	NS
CBX1	NS	1.53E-02	-0.0994	4.85E-02	NS
LYSMD2	NS	1.53E-02	9.94E-02	4.85E-02	NS
ARID1B	NS	1.53E-02	-0.0994	4.85E-02	NS
SMIM12	NS	1.53E-02	-0.0994	4.86E-02	NS
SPDYE2	NS	1.53E-02	9.94E-02	4.86E-02	NS
DNAJC16	NS	1.53E-02	-0.0994	4.86E-02	NS
LACTB2	NS	1.53E-02	-0.0994	4.86E-02	NS
PRRT1B	NS	1.54E-02	-0.0993	4.88E-02	NS
GNAO1	NS	1.54E-02	-0.0993	4.88E-02	NS
DDAH1	NS	1.54E-02	-0.0993	4.88E-02	NS

ZFYVE9	NS	1.54E-02	9.93E-02	4.89E-02	NS
EIF3F	NS	1.55E-02	-0.0992	4.89E-02	NS
MASP1	NS	1.55E-02	-0.0992	4.90E-02	NS
PRR27	NS	1.55E-02	9.92E-02	4.90E-02	NS
PIM1	NS	1.55E-02	9.92E-02	4.90E-02	NS
PMVK	NS	1.55E-02	-0.0992	4.90E-02	NS
ZNF764	NS	1.55E-02	-0.0992	4.91E-02	NS
ERO1B	NS	1.55E-02	-0.0992	4.91E-02	NS
IGSF8	NS	1.55E-02	-0.0992	4.91E-02	NS
DPM3	NS	1.55E-02	9.92E-02	4.91E-02	NS
PRPF39	NS	1.55E-02	-0.0992	4.91E-02	NS
IPP	NS	1.55E-02	-0.0992	4.91E-02	NS
TTI2	NS	1.55E-02	-0.0992	4.91E-02	NS
PUM3	NS	1.55E-02	-0.0991	4.91E-02	NS
TMEM59	NS	1.56E-02	-0.0991	4.92E-02	NS
MDM1	NS	1.56E-02	-0.0991	4.92E-02	NS
SMIM34	NS	1.56E-02	-0.0991	4.92E-02	NS
CXCL14	NS	1.56E-02	9.91E-02	4.93E-02	NS
PRR5-ARHGAP8	NS	1.56E-02	-0.0991	4.93E-02	NS
GCA	NS	1.56E-02	-0.0991	4.93E-02	NS
WDR59	NS	1.56E-02	9.91E-02	4.93E-02	NS
FTHL17	NS	1.56E-02	9.91E-02	4.94E-02	NS
NOMO2	NS	1.57E-02	-0.099	4.95E-02	NS
FOXRED1	NS	1.57E-02	-0.099	4.95E-02	NS
HCRTR2	NS	1.57E-02	-0.099	4.95E-02	NS
HMCN2	NS	1.57E-02	9.90E-02	4.95E-02	NS
CP	NS	1.57E-02	-0.099	4.95E-02	NS
ARMCX2	NS	1.57E-02	-0.099	4.95E-02	NS
C2orf15	NS	1.57E-02	-0.099	4.96E-02	NS
ABO	NS	1.57E-02	-0.099	4.96E-02	NS
ASS1	NS	1.57E-02	-0.099	4.96E-02	NS
EXT2	NS	1.58E-02	-0.099	4.96E-02	NS
NDUFV2	NS	1.58E-02	-0.0989	4.96E-02	NS
GET1	NS	1.58E-02	-0.0989	4.97E-02	NS
PPP1R3E	NS	1.58E-02	9.89E-02	4.97E-02	NS
ZNF474	NS	1.58E-02	-0.0989	4.97E-02	NS
RNF34	NS	1.58E-02	-0.0989	4.97E-02	NS

ECM2	NS	1.58E-02	-0.0989	4.98E-02	NS
C16orf82	NS	1.58E-02	9.89E-02	4.98E-02	NS
RASSF4	NS	1.58E-02	-0.0989	4.99E-02	NS
TNRC6B	NS	1.59E-02	-0.0989	4.99E-02	NS
TDO2	NS	1.59E-02	-0.0989	4.99E-02	NS
LPCAT1	NS	1.59E-02	-0.0988	4.99E-02	NS
AGL	NS	1.59E-02	-0.0988	4.99E-02	NS
PKIG	NS	1.59E-02	-0.0988	5.00E-02	NS
DNAH1	NS	1.59E-02	-0.0988	5.00E-02	NS
VAMP7	NS	1.59E-02	-0.0988	5.00E-02	NS
NPNT	NS	1.59E-02	-0.0988	5.00E-02	NS
ZNF763	NS	1.59E-02	-0.0988	5.00E-02	NS
WFS1	NS	1.60E-02	9.88E-02	5.01E-02	NS
ZNF667	NS	1.60E-02	-0.0987	5.02E-02	NS
TBC1D20	NS	1.60E-02	-0.0987	5.02E-02	NS
EXOSC1	NS	1.60E-02	-0.0987	5.03E-02	NS
KRTAP6-3	NS	1.60E-02	9.87E-02	5.03E-02	NS
C3orf18	NS	1.61E-02	-0.0987	5.04E-02	NS
ZBTB34	NS	1.61E-02	-0.0987	5.04E-02	NS
TMEM127	NS	1.61E-02	9.87E-02	5.04E-02	NS
MAGED1	NS	1.61E-02	-0.0987	5.04E-02	NS
COQ9	NS	1.61E-02	-0.0986	5.05E-02	NS
AGBL4	NS	1.61E-02	9.86E-02	5.06E-02	NS
SBK1	NS	1.61E-02	9.86E-02	5.06E-02	NS
APH1A	NS	1.62E-02	-0.0986	5.07E-02	NS
LRRC56	NS	1.62E-02	-0.0986	5.07E-02	NS
VCY1B	NS	1.62E-02	9.86E-02	5.07E-02	NS
CLRN1	NS	1.62E-02	-0.0985	5.07E-02	NS
HSD17B1	NS	1.62E-02	9.85E-02	5.08E-02	NS
MRPS30	NS	1.62E-02	-0.0985	5.08E-02	NS
TNFRSF11B	NS	1.62E-02	9.85E-02	5.08E-02	NS
MORF4L2	NS	1.62E-02	-0.0985	5.08E-02	NS
TMEM259	NS	1.62E-02	9.85E-02	5.08E-02	NS
APOA1	NS	1.62E-02	-0.0985	5.08E-02	NS
LENG1	NS	1.63E-02	-0.0985	5.09E-02	NS
ILKAP	NS	1.63E-02	9.85E-02	5.09E-02	NS
LRRC73	NS	1.62E-02	9.85E-02	5.09E-02	NS

NOL9	NS	1.63E-02	-0.0985	5.09E-02	NS
ART5	NS	1.63E-02	9.85E-02	5.09E-02	NS
MOSPD3	NS	1.63E-02	9.85E-02	5.09E-02	NS
TLCD2	NS	1.63E-02	-0.0985	5.09E-02	NS
MRPS25	NS	1.63E-02	-0.0984	5.10E-02	NS
PRAMEF11	NS	1.63E-02	-0.0984	5.10E-02	NS
GOSR1	NS	1.63E-02	-0.0984	5.10E-02	NS
DUSP2	NS	1.63E-02	9.84E-02	5.10E-02	NS
GPS1	NS	1.63E-02	-0.0984	5.10E-02	NS
DEFB104B	NS	1.64E-02	-0.0984	5.11E-02	NS
TIMP1	NS	1.64E-02	-0.0984	5.12E-02	NS
NUCB1	NS	1.64E-02	-0.0984	5.12E-02	NS
RAPGEF6	NS	1.64E-02	-0.0984	5.12E-02	NS
SPATA31C2	NS	1.64E-02	-0.0984	5.12E-02	NS
DUS3L	NS	1.64E-02	9.84E-02	5.12E-02	NS
SPATC1	NS	1.64E-02	9.83E-02	5.12E-02	NS
CXCL3	NS	1.64E-02	-0.0983	5.12E-02	NS
ARL16	NS	1.64E-02	-0.0983	5.12E-02	NS
PLSCR5	NS	1.64E-02	-0.0983	5.12E-02	NS
HPN	NS	1.64E-02	9.83E-02	5.12E-02	NS
LAP3	NS	1.64E-02	9.83E-02	5.13E-02	NS
HCN2	NS	1.64E-02	9.83E-02	5.13E-02	NS
NAPA	NS	1.64E-02	9.83E-02	5.13E-02	NS
SUGT1	NS	1.65E-02	-0.0983	5.13E-02	NS
SRRM2	NS	1.65E-02	9.83E-02	5.13E-02	NS
LARS1	NS	1.65E-02	-0.0983	5.13E-02	NS
ZBTB22	NS	1.65E-02	9.83E-02	5.13E-02	NS
SPDYE8	NS	1.65E-02	-0.0983	5.13E-02	NS
SPECC1L	NS	1.65E-02	-0.0983	5.14E-02	NS
SSH1	NS	1.65E-02	9.82E-02	5.15E-02	NS
CASK	NS	1.66E-02	-0.0982	5.15E-02	NS
SPATA31D4	NS	1.65E-02	-0.0982	5.15E-02	NS
ZNF782	NS	1.65E-02	-0.0982	5.15E-02	NS
RNASEK	NS	1.65E-02	-0.0982	5.15E-02	NS
KLHDC8A	NS	1.66E-02	-0.0982	5.15E-02	NS
SMPDL3A	NS	1.66E-02	-0.0982	5.15E-02	NS
UST	NS	1.66E-02	-0.0982	5.15E-02	NS

SCGN	NS	1.66E-02	-0.0982	5.15E-02	NS
IL2RA	NS	1.66E-02	-0.0982	5.16E-02	NS
TNKS	NS	1.66E-02	9.82E-02	5.16E-02	NS
FFAR3	NS	1.66E-02	-0.0982	5.16E-02	NS
SPACA7	NS	1.66E-02	-0.0981	5.17E-02	NS
GGN	NS	1.66E-02	-0.0981	5.17E-02	NS
C18orf63	NS	1.66E-02	-0.0981	5.17E-02	NS
NPIPB9	NS	1.67E-02	9.81E-02	5.17E-02	NS
PTHLH	NS	1.67E-02	-0.0981	5.18E-02	NS
DCAF6	NS	1.67E-02	9.81E-02	5.18E-02	NS
LRRC37A3	NS	1.67E-02	-0.0981	5.18E-02	NS
CMC1	NS	1.67E-02	-0.0981	5.18E-02	NS
URB2	NS	1.67E-02	-0.0981	5.18E-02	NS
ELL	NS	1.67E-02	9.81E-02	5.19E-02	NS
CFAP141	NS	1.67E-02	-0.0981	5.19E-02	NS
OR52A1	NS	1.67E-02	-0.0981	5.19E-02	NS
PLET1	NS	1.67E-02	-0.0981	5.19E-02	NS
PCF11	NS	1.67E-02	-0.098	5.19E-02	NS
TRUB2	NS	1.67E-02	-0.098	5.19E-02	NS
HEMGN	NS	1.68E-02	-0.098	5.19E-02	NS
EFCAB3	NS	1.68E-02	-0.098	5.21E-02	NS
KCNIP4	NS	1.68E-02	-0.098	5.21E-02	NS
CNN1	NS	1.68E-02	-0.098	5.22E-02	NS
FAM236D	NS	1.68E-02	9.80E-02	5.22E-02	NS
GZMB	NS	1.69E-02	-0.0979	5.22E-02	NS
WDR12	NS	1.69E-02	-0.0979	5.22E-02	NS
ST13	NS	1.69E-02	-0.0979	5.23E-02	NS
RAMP1	NS	1.69E-02	9.79E-02	5.23E-02	NS
PAX8	NS	1.69E-02	-0.0979	5.24E-02	NS
ABCF2	NS	1.69E-02	-0.0979	5.24E-02	NS
SPANXA2	NS	1.69E-02	-0.0979	5.24E-02	NS
RPP21	NS	1.69E-02	-0.0979	5.24E-02	NS
MXRA8	NS	1.69E-02	9.79E-02	5.24E-02	NS
KIAA0825	NS	1.70E-02	-0.0979	5.24E-02	NS
ASGR1	NS	1.70E-02	-0.0979	5.24E-02	NS
AIFM1	NS	1.70E-02	-0.0978	5.25E-02	NS
THOP1	NS	1.70E-02	-0.0978	5.25E-02	NS

SLFN14	NS	1.70E-02	-0.0978	5.25E-02	NS
PSORS1C2	NS	1.70E-02	-0.0978	5.25E-02	NS
CT47A12	NS	1.70E-02	9.78E-02	5.25E-02	NS
TTC24	NS	1.70E-02	-0.0978	5.25E-02	NS
ANO2	NS	1.70E-02	-0.0978	5.25E-02	NS
ENPP6	NS	1.70E-02	9.78E-02	5.25E-02	NS
GNS	NS	1.70E-02	-0.0978	5.26E-02	NS
DUS1L	NS	1.70E-02	-0.0978	5.26E-02	NS
ZDHHC19	NS	1.70E-02	9.78E-02	5.26E-02	NS
C10orf120	NS	1.71E-02	-0.0978	5.26E-02	NS
P2RX6	NS	1.71E-02	9.78E-02	5.27E-02	NS
RESF1	NS	1.71E-02	-0.0977	5.27E-02	NS
NPEPPS	NS	1.71E-02	-0.0977	5.27E-02	NS
ANKRD18A	NS	1.71E-02	-0.0977	5.28E-02	NS
TEX10	NS	1.71E-02	-0.0977	5.28E-02	NS
OR5B12	NS	1.71E-02	-0.0977	5.28E-02	NS
GOLGA6L2	NS	1.71E-02	-0.0977	5.28E-02	NS
ITGA11	NS	1.71E-02	-0.0977	5.28E-02	NS
TSPAN12	NS	1.71E-02	-0.0977	5.28E-02	NS
ZFYVE28	NS	1.72E-02	-0.0977	5.28E-02	NS
MYO1D	NS	1.72E-02	9.77E-02	5.28E-02	NS
ACACA	NS	1.72E-02	-0.0977	5.28E-02	NS
CDK14	NS	1.72E-02	9.77E-02	5.29E-02	NS
GCSAM	NS	1.72E-02	-0.0977	5.29E-02	NS
PCDHGB4	NS	1.72E-02	-0.0976	5.30E-02	NS
SLCO4C1	NS	1.72E-02	-0.0976	5.30E-02	NS
RGS3	NS	1.72E-02	-0.0976	5.30E-02	NS
MAZ	NS	1.72E-02	-0.0976	5.31E-02	NS
RET	NS	1.72E-02	-0.0976	5.31E-02	NS
FOXA1	NS	1.73E-02	9.76E-02	5.31E-02	NS
TRIP12	NS	1.73E-02	-0.0976	5.32E-02	NS
PTPRB	NS	1.73E-02	-0.0975	5.33E-02	NS
DMRT2	NS	1.73E-02	-0.0975	5.33E-02	NS
ETFRF1	NS	1.73E-02	-0.0975	5.33E-02	NS
PPA1	NS	1.74E-02	-0.0975	5.33E-02	NS
TTC34	NS	1.74E-02	-0.0975	5.33E-02	NS
OR10W1	NS	1.74E-02	-0.0975	5.34E-02	NS

AADAC	NS	1.74E-02	-0.0975	5.34E-02	NS
IP6K1	NS	1.74E-02	-0.0975	5.34E-02	NS
RPL28	NS	1.74E-02	-0.0975	5.35E-02	NS
DENR	NS	1.74E-02	-0.0975	5.35E-02	NS
STK25	NS	1.74E-02	-0.0974	5.35E-02	NS
RIC3	NS	1.75E-02	-0.0974	5.36E-02	NS
VCPIP1	NS	1.75E-02	-0.0974	5.36E-02	NS
MEOX2	NS	1.75E-02	-0.0974	5.36E-02	NS
PDE1A	NS	1.75E-02	-0.0974	5.36E-02	NS
BRDT	NS	1.75E-02	9.74E-02	5.37E-02	NS
ANGPTL5	NS	1.75E-02	9.74E-02	5.37E-02	NS
HMGN4	NS	1.75E-02	-0.0974	5.38E-02	NS
ADAMTS20	NS	1.75E-02	-0.0974	5.38E-02	NS
PARD6G	NS	1.75E-02	9.74E-02	5.38E-02	NS
SLC10A1	NS	1.75E-02	-0.0973	5.38E-02	NS
H2AC17	NS	1.75E-02	-0.0973	5.38E-02	NS
HECTD2	NS	1.76E-02	-0.0973	5.38E-02	NS
RBM43	NS	1.76E-02	-0.0973	5.38E-02	NS
PCDHGA7	NS	1.76E-02	-0.0973	5.38E-02	NS
KYAT3	NS	1.76E-02	-0.0973	5.38E-02	NS
PRPSAP1	NS	1.76E-02	-0.0973	5.39E-02	NS
UPP2	NS	1.76E-02	-0.0973	5.40E-02	NS
THAP5	NS	1.77E-02	-0.0973	5.40E-02	NS
NELL1	NS	1.77E-02	-0.0972	5.42E-02	NS
ALKBH8	NS	1.77E-02	-0.0972	5.43E-02	NS
CLEC2A	NS	1.78E-02	-0.0972	5.43E-02	NS
ZNF770	NS	1.77E-02	-0.0972	5.43E-02	NS
DCAF12	NS	1.77E-02	-0.0972	5.43E-02	NS
MT-ND1	NS	1.77E-02	9.72E-02	5.43E-02	NS
DGCR6L	NS	1.78E-02	-0.0972	5.43E-02	NS
HIVEP3	NS	1.78E-02	-0.0972	5.43E-02	NS
RNF2	NS	1.78E-02	-0.0971	5.43E-02	NS
USH2A	NS	1.78E-02	-0.0971	5.43E-02	NS
MBTD1	NS	1.78E-02	-0.0971	5.44E-02	NS
ARAF	NS	1.78E-02	-0.0971	5.44E-02	NS
SEMG2	NS	1.78E-02	-0.0971	5.44E-02	NS
SUSD1	NS	1.78E-02	9.71E-02	5.44E-02	NS

DEFB110	NS	1.78E-02	-0.0971	5.44E-02	NS
DONSON	NS	1.78E-02	9.71E-02	5.45E-02	NS
NPB	NS	1.79E-02	-0.0971	5.45E-02	NS
CATSPER3	NS	1.79E-02	-0.0971	5.46E-02	NS
STK33	NS	1.79E-02	-0.097	5.46E-02	NS
P2RX5	NS	1.79E-02	-0.097	5.46E-02	NS
WASHC3	NS	1.79E-02	-0.097	5.47E-02	NS
PTGES	NS	1.79E-02	-0.097	5.47E-02	NS
LRRC58	NS	1.79E-02	9.70E-02	5.47E-02	NS
KRTAP9-9	NS	1.80E-02	9.70E-02	5.47E-02	NS
PRSS23	NS	1.80E-02	-0.097	5.48E-02	NS
CCDC141	NS	1.80E-02	-0.097	5.48E-02	NS
FIGN	NS	1.80E-02	-0.097	5.48E-02	NS
KPNA7	NS	1.80E-02	9.70E-02	5.48E-02	NS
MICOS10-NBL1	NS	1.80E-02	-0.097	5.48E-02	NS
C2	NS	1.80E-02	-0.097	5.48E-02	NS
AK5	NS	1.80E-02	-0.097	5.48E-02	NS
OR4F5	NS	1.80E-02	-0.0969	5.49E-02	NS
SUGCT	NS	1.80E-02	-0.0969	5.49E-02	NS
COL16A1	NS	1.80E-02	-0.0969	5.49E-02	NS
IL12RB2	NS	1.81E-02	-0.0969	5.49E-02	NS
CEP85L	NS	1.80E-02	-0.0969	5.49E-02	NS
ESRRA	NS	1.81E-02	-0.0969	5.49E-02	NS
PYCR1	NS	1.80E-02	9.69E-02	5.49E-02	NS
DUSP5	NS	1.81E-02	-0.0969	5.50E-02	NS
SNAPIN	NS	1.81E-02	-0.0969	5.50E-02	NS
ARL10	NS	1.81E-02	-0.0969	5.50E-02	NS
FKBPL	NS	1.81E-02	-0.0969	5.50E-02	NS
IQCJ	NS	1.81E-02	-0.0969	5.50E-02	NS
P2RX5-TAX1BP3	NS	1.81E-02	9.69E-02	5.51E-02	NS
RPL27	NS	1.81E-02	-0.0969	5.51E-02	NS
ZNF35	NS	1.81E-02	-0.0968	5.51E-02	NS
BDP1	NS	1.82E-02	-0.0968	5.52E-02	NS
LSM10	NS	1.82E-02	-0.0968	5.52E-02	NS
DCDC2C	NS	1.82E-02	-0.0968	5.53E-02	NS
LGALS3BP	NS	1.82E-02	-0.0968	5.53E-02	NS
RFC5	NS	1.82E-02	-0.0968	5.53E-02	NS

MAPK8IP1	NS	1.82E-02	-0.0968	5.53E-02	NS
CELF6	NS	1.82E-02	9.68E-02	5.53E-02	NS
STXBP5L	NS	1.83E-02	-0.0967	5.54E-02	NS
PARD3	NS	1.83E-02	-0.0967	5.54E-02	NS
PHOX2A	NS	1.83E-02	-0.0967	5.54E-02	NS
SHMT1	NS	1.83E-02	-0.0967	5.54E-02	NS
CDPF1	NS	1.83E-02	-0.0967	5.55E-02	NS
SEPTIN3	NS	1.83E-02	-0.0967	5.55E-02	NS
SUSD6	NS	1.83E-02	-0.0967	5.56E-02	NS
FDFT1	NS	1.83E-02	9.67E-02	5.56E-02	NS
SOD3	NS	1.83E-02	-0.0967	5.56E-02	NS
ACTR3C	NS	1.84E-02	9.67E-02	5.56E-02	NS
PES1	NS	1.84E-02	-0.0966	5.57E-02	NS
TFDP1	NS	1.84E-02	-0.0966	5.58E-02	NS
TMSB4X	NS	1.84E-02	-0.0966	5.58E-02	NS
ITGA6	NS	1.84E-02	-0.0966	5.58E-02	NS
NLRP8	NS	1.84E-02	9.66E-02	5.58E-02	NS
GOT1L1	NS	1.84E-02	-0.0966	5.59E-02	NS
RBMXL3	NS	1.85E-02	9.66E-02	5.59E-02	NS
TMEM238	NS	1.85E-02	9.66E-02	5.59E-02	NS
FBXO40	NS	1.85E-02	-0.0966	5.59E-02	NS
CCDC28A	NS	1.85E-02	-0.0965	5.60E-02	NS
THAP2	NS	1.85E-02	-0.0965	5.60E-02	NS
OR5K4	NS	1.85E-02	-0.0965	5.60E-02	NS
SCUBE3	NS	1.85E-02	-0.0965	5.60E-02	NS
SPAG7	NS	1.85E-02	-0.0965	5.60E-02	NS
COQ6	NS	1.85E-02	-0.0965	5.60E-02	NS
EPSTI1	NS	1.86E-02	-0.0965	5.61E-02	NS
CLEC4E	NS	1.86E-02	-0.0965	5.61E-02	NS
LTB4R	NS	1.86E-02	9.65E-02	5.61E-02	NS
WWTR1	NS	1.86E-02	-0.0965	5.62E-02	NS
SIPA1L3	NS	1.86E-02	9.64E-02	5.63E-02	NS
NT5C1B	NS	1.86E-02	-0.0964	5.63E-02	NS
PRPF38A	NS	1.86E-02	-0.0964	5.63E-02	NS
COPS9	NS	1.86E-02	-0.0964	5.63E-02	NS
BCLAF3	NS	1.86E-02	-0.0964	5.63E-02	NS
GOLGA8F	NS	1.87E-02	-0.0964	5.63E-02	NS

OTUB1	NS	1.87E-02	9.64E-02	5.63E-02	NS
SART1	NS	1.87E-02	9.64E-02	5.63E-02	NS
PCBD2	NS	1.87E-02	-0.0964	5.64E-02	NS
CENPS	NS	1.87E-02	-0.0964	5.64E-02	NS
CHRFAM7A	NS	1.87E-02	-0.0964	5.64E-02	NS
SAP30	NS	1.87E-02	-0.0964	5.65E-02	NS
CKS1B	NS	1.87E-02	-0.0964	5.65E-02	NS
ELAC1	NS	1.87E-02	-0.0964	5.65E-02	NS
LILRB5	NS	1.87E-02	-0.0964	5.65E-02	NS
PPP3R1	NS	1.88E-02	9.63E-02	5.65E-02	NS
CAMKMT	NS	1.88E-02	-0.0963	5.66E-02	NS
PALMD	NS	1.88E-02	-0.0963	5.66E-02	NS
HUNK	NS	1.88E-02	-0.0963	5.67E-02	NS
CAMSAP3	NS	1.88E-02	9.63E-02	5.67E-02	NS
ATP13A2	NS	1.88E-02	-0.0963	5.67E-02	NS
CLDN12	NS	1.88E-02	-0.0963	5.68E-02	NS
ZNF436	NS	1.89E-02	-0.0962	5.68E-02	NS
CYP2W1	NS	1.89E-02	9.62E-02	5.68E-02	NS
GDPD2	NS	1.89E-02	-0.0962	5.68E-02	NS
RNASEL	NS	1.89E-02	-0.0962	5.68E-02	NS
NDUFB8	NS	1.89E-02	-0.0962	5.68E-02	NS
ERICH5	NS	1.89E-02	-0.0962	5.69E-02	NS
ZHX3	NS	1.89E-02	-0.0962	5.69E-02	NS
FBXO41	NS	1.89E-02	-0.0962	5.69E-02	NS
LACC1	NS	1.89E-02	-0.0962	5.69E-02	NS
ASB2	NS	1.89E-02	-0.0962	5.69E-02	NS
L3MBTL4	NS	1.89E-02	-0.0962	5.69E-02	NS
HK2	NS	1.90E-02	9.62E-02	5.70E-02	NS
USP6	NS	1.90E-02	9.61E-02	5.71E-02	NS
MSRB2	NS	1.90E-02	-0.0961	5.71E-02	NS
KLK2	NS	1.90E-02	-0.0961	5.71E-02	NS
MPV17L2	NS	1.90E-02	-0.0961	5.71E-02	NS
C1orf116	NS	1.90E-02	-0.0961	5.72E-02	NS
DCDC2	NS	1.90E-02	9.61E-02	5.72E-02	NS
KCNK2	NS	1.90E-02	9.61E-02	5.72E-02	NS
ANKRD20A3P	NS	1.91E-02	-0.0961	5.73E-02	NS
COPS6	NS	1.91E-02	-0.0961	5.73E-02	NS

KRTAP6-1	NS	1.91E-02	-0.096	5.74E-02	NS
AMY1A	NS	1.91E-02	-0.096	5.74E-02	NS
METTL21C	NS	1.91E-02	-0.096	5.74E-02	NS
CAVIN2	NS	1.91E-02	-0.096	5.74E-02	NS
C8orf44-SGK3	NS	1.91E-02	-0.096	5.74E-02	NS
SCML1	NS	1.91E-02	-0.096	5.74E-02	NS
TP53BP1	NS	1.91E-02	-0.096	5.74E-02	NS
TAF9B	NS	1.92E-02	9.60E-02	5.74E-02	NS
OR56B2P	NS	1.92E-02	-0.096	5.74E-02	NS
GLRA3	NS	1.92E-02	9.60E-02	5.74E-02	NS
TRIM68	NS	1.92E-02	-0.096	5.74E-02	NS
DTWD1	NS	1.92E-02	-0.096	5.75E-02	NS
RBMXL1	NS	1.92E-02	-0.0959	5.77E-02	NS
OCLN	NS	1.93E-02	-0.0959	5.77E-02	NS
ARHGAP24	NS	1.93E-02	9.59E-02	5.77E-02	NS
WNT3A	NS	1.93E-02	9.59E-02	5.77E-02	NS
CYP4F3	NS	1.93E-02	-0.0959	5.77E-02	NS
FGF9	NS	1.93E-02	-0.0959	5.77E-02	NS
MPHOSPH10	NS	1.93E-02	-0.0959	5.77E-02	NS
ZNF420	NS	1.93E-02	-0.0959	5.77E-02	NS
KLHL10	NS	1.93E-02	-0.0959	5.78E-02	NS
BANF2	NS	1.93E-02	-0.0959	5.78E-02	NS
FOXJ2	NS	1.93E-02	9.59E-02	5.78E-02	NS
KLHL31	NS	1.93E-02	-0.0959	5.78E-02	NS
ALYREF	NS	1.94E-02	-0.0959	5.79E-02	NS
SEMA6A	NS	1.94E-02	-0.0959	5.79E-02	NS
DNAJC22	NS	1.94E-02	9.58E-02	5.79E-02	NS
NDST3	NS	1.94E-02	-0.0958	5.80E-02	NS
IL1RL2	NS	1.94E-02	-0.0958	5.80E-02	NS
NPDC1	NS	1.94E-02	-0.0958	5.80E-02	NS
GOLGA8O	NS	1.94E-02	-0.0958	5.81E-02	NS
FAM156A	NS	1.95E-02	-0.0958	5.81E-02	NS
APOBEC3D	NS	1.95E-02	9.57E-02	5.83E-02	NS
ZNF302	NS	1.95E-02	9.57E-02	5.83E-02	NS
ZNF491	NS	1.95E-02	-0.0957	5.83E-02	NS
DEFB115	NS	1.95E-02	-0.0957	5.83E-02	NS
CFAP210	NS	1.95E-02	-0.0957	5.83E-02	NS

FNDC5	NS	1.95E-02	9.57E-02	5.83E-02	NS
POU3F3	NS	1.96E-02	9.57E-02	5.85E-02	NS
DDX41	NS	1.97E-02	9.56E-02	5.86E-02	NS
RBM34	NS	1.97E-02	9.56E-02	5.86E-02	NS
DENND3	NS	1.97E-02	9.56E-02	5.86E-02	NS
HCAR3	NS	1.97E-02	9.56E-02	5.86E-02	NS
ZGLP1	NS	1.97E-02	-0.0956	5.87E-02	NS
OR6K3	NS	1.97E-02	-0.0956	5.87E-02	NS
STRBP	NS	1.97E-02	9.56E-02	5.88E-02	NS
NLRP7	NS	1.97E-02	-0.0956	5.88E-02	NS
FAM166B	NS	1.97E-02	-0.0956	5.88E-02	NS
CNRIP1	NS	1.98E-02	-0.0955	5.89E-02	NS
FOXP2	NS	1.98E-02	-0.0955	5.89E-02	NS
ZSWIM1	NS	1.98E-02	9.55E-02	5.89E-02	NS
ETAA1	NS	1.98E-02	-0.0955	5.90E-02	NS
HORMAD2	NS	1.98E-02	-0.0955	5.90E-02	NS
ZNF585A	NS	1.98E-02	-0.0955	5.91E-02	NS
OR10X1	NS	1.99E-02	-0.0955	5.91E-02	NS
RERG	NS	1.99E-02	-0.0955	5.91E-02	NS
CPHXL	NS	1.99E-02	-0.0954	5.92E-02	NS
COX5A	NS	1.99E-02	-0.0954	5.92E-02	NS
MTIF2	NS	1.99E-02	-0.0954	5.92E-02	NS
RNPEPL1	NS	1.99E-02	-0.0954	5.93E-02	NS
ZNF282	NS	1.99E-02	9.54E-02	5.93E-02	NS
ZFP37	NS	1.99E-02	-0.0954	5.93E-02	NS
GLUD1	NS	2.00E-02	9.54E-02	5.94E-02	NS
PCBP4	NS	2.00E-02	9.54E-02	5.94E-02	NS
AMELX	NS	2.00E-02	-0.0954	5.94E-02	NS
GRIA4	NS	2.00E-02	-0.0954	5.94E-02	NS
LINC02210-CRHR1	NS	2.00E-02	-0.0953	5.94E-02	NS
SPESP1	NS	2.00E-02	-0.0953	5.95E-02	NS
LRMDA	NS	2.01E-02	9.53E-02	5.96E-02	NS
CLIC5	NS	2.01E-02	-0.0953	5.97E-02	NS
PXYLP1	NS	2.01E-02	-0.0953	5.97E-02	NS
OVCH1	NS	2.01E-02	-0.0953	5.97E-02	NS
HOXD1	NS	2.01E-02	9.53E-02	5.98E-02	NS
SF3B4	NS	2.02E-02	9.52E-02	5.99E-02	NS

RPS28	NS	2.02E-02	-0.0952	6.00E-02	NS
GDPD3	NS	2.02E-02	-0.0952	6.00E-02	NS
JMJD8	NS	2.02E-02	-0.0952	6.00E-02	NS
SULT1C3	NS	2.02E-02	-0.0952	6.00E-02	NS
NUCKS1	NS	2.02E-02	-0.0952	6.00E-02	NS
ANO6	NS	2.03E-02	-0.0951	6.01E-02	NS
RCC2	NS	2.03E-02	9.51E-02	6.01E-02	NS
ROR1	NS	2.03E-02	-0.0951	6.01E-02	NS
APC	NS	2.03E-02	-0.0951	6.02E-02	NS
SMYD4	NS	2.03E-02	-0.0951	6.02E-02	NS
SND1	NS	2.03E-02	-0.0951	6.02E-02	NS
MLYCD	NS	2.03E-02	9.51E-02	6.02E-02	NS
UGGT2	NS	2.03E-02	-0.0951	6.02E-02	NS
NDUFC1	NS	2.03E-02	-0.0951	6.02E-02	NS
PRR23B	NS	2.03E-02	9.51E-02	6.02E-02	NS
LRRC39	NS	2.04E-02	-0.0951	6.03E-02	NS
PTAFR	NS	2.04E-02	-0.0951	6.03E-02	NS
ATP13A5	NS	2.04E-02	-0.0951	6.03E-02	NS
TSFM	NS	2.04E-02	-0.0951	6.03E-02	NS
MCM10	NS	2.04E-02	-0.0951	6.03E-02	NS
ADIRF	NS	2.04E-02	-0.0951	6.03E-02	NS
CHAF1B	NS	2.04E-02	-0.095	6.03E-02	NS
COPS2	NS	2.04E-02	-0.095	6.05E-02	NS
PCDHB2	NS	2.05E-02	-0.095	6.05E-02	NS
GRPEL1	NS	2.05E-02	9.50E-02	6.05E-02	NS
EVC	NS	2.05E-02	-0.095	6.05E-02	NS
TRA2A	NS	2.05E-02	9.50E-02	6.05E-02	NS
USP36	NS	2.05E-02	9.50E-02	6.05E-02	NS
ITGB1BP1	NS	2.05E-02	-0.095	6.06E-02	NS
ANKRD66	NS	2.05E-02	-0.095	6.06E-02	NS
PCDHGA11	NS	2.05E-02	-0.095	6.06E-02	NS
LSG1	NS	2.06E-02	9.49E-02	6.08E-02	NS
PRRT1	NS	2.06E-02	9.49E-02	6.09E-02	NS
PRUNE2	NS	2.06E-02	-0.0949	6.09E-02	NS
ATP6V1D	NS	2.06E-02	-0.0949	6.09E-02	NS
MYEF2	NS	2.06E-02	-0.0949	6.09E-02	NS
SSBP3	NS	2.06E-02	9.49E-02	6.09E-02	NS

FAM53C	NS	2.06E-02	9.49E-02	6.09E-02	NS
CYB5D2	NS	2.06E-02	9.49E-02	6.09E-02	NS
EMG1	NS	2.07E-02	-0.0949	6.09E-02	NS
PITHD1	NS	2.07E-02	9.49E-02	6.09E-02	NS
NSMCE3	NS	2.07E-02	-0.0948	6.10E-02	NS
SLC17A9	NS	2.07E-02	-0.0948	6.11E-02	NS
RASGRP1	NS	2.07E-02	-0.0948	6.11E-02	NS
FABP5	NS	2.08E-02	-0.0948	6.12E-02	NS
LSM5	NS	2.08E-02	-0.0948	6.12E-02	NS
ARL8B	NS	2.08E-02	-0.0948	6.13E-02	NS
UBQLN1	NS	2.08E-02	9.47E-02	6.13E-02	NS
HOXC11	NS	2.08E-02	9.47E-02	6.13E-02	NS
FLOT1	NS	2.08E-02	-0.0947	6.13E-02	NS
ATP5PB	NS	2.08E-02	-0.0947	6.14E-02	NS
EXOC1	NS	2.09E-02	-0.0947	6.14E-02	NS
BSND	NS	2.09E-02	-0.0947	6.14E-02	NS
SPTBN2	NS	2.09E-02	-0.0947	6.14E-02	NS
OR6S1	NS	2.09E-02	9.47E-02	6.14E-02	NS
RASA2	NS	2.09E-02	-0.0947	6.14E-02	NS
EIF1AX	NS	2.09E-02	-0.0947	6.14E-02	NS
C3orf85	NS	2.09E-02	-0.0947	6.14E-02	NS
SMIM11	NS	2.09E-02	-0.0947	6.15E-02	NS
PRPS1L1	NS	2.09E-02	-0.0947	6.15E-02	NS
HPF1	NS	2.09E-02	-0.0947	6.15E-02	NS
HAND1	NS	2.09E-02	9.47E-02	6.15E-02	NS
MAP3K21	NS	2.09E-02	-0.0947	6.15E-02	NS
LSM2	NS	2.09E-02	-0.0947	6.15E-02	NS
TMEM125	NS	2.09E-02	9.47E-02	6.15E-02	NS
ST20-MTHFS	NS	2.09E-02	-0.0947	6.15E-02	NS
MAP4K4	NS	2.09E-02	-0.0946	6.15E-02	NS
DYNC1H1	NS	2.10E-02	-0.0946	6.15E-02	NS
IQCF5	NS	2.10E-02	9.46E-02	6.16E-02	NS
CRTAM	NS	2.10E-02	-0.0946	6.17E-02	NS
GLYATL1B	NS	2.10E-02	-0.0946	6.17E-02	NS
DFFA	NS	2.10E-02	-0.0946	6.17E-02	NS
SMR3A	NS	2.10E-02	-0.0946	6.17E-02	NS
CFAP46	NS	2.10E-02	-0.0946	6.17E-02	NS

SDF2L1	NS	2.11E-02	-0.0946	6.18E-02	NS
DIPK1A	NS	2.11E-02	-0.0946	6.18E-02	NS
OR10Z1	NS	2.11E-02	-0.0946	6.18E-02	NS
SIRPB1	NS	2.11E-02	9.45E-02	6.18E-02	NS
SLC35A4	NS	2.11E-02	-0.0945	6.18E-02	NS
TMEM64	NS	2.11E-02	-0.0945	6.18E-02	NS
ARHGEF28	NS	2.11E-02	-0.0945	6.18E-02	NS
FBXO47	NS	2.11E-02	-0.0945	6.19E-02	NS
CCDC62	NS	2.11E-02	-0.0945	6.19E-02	NS
LIX1L	NS	2.11E-02	9.45E-02	6.19E-02	NS
GPC3	NS	2.11E-02	-0.0945	6.19E-02	NS
ALDH3A2	NS	2.11E-02	-0.0945	6.19E-02	NS
IL22RA2	NS	2.11E-02	-0.0945	6.19E-02	NS
SLAMF1	NS	2.12E-02	-0.0945	6.20E-02	NS
FARS2	NS	2.12E-02	-0.0945	6.20E-02	NS
ITGAV	NS	2.12E-02	-0.0945	6.20E-02	NS
RFLNA	NS	2.12E-02	-0.0945	6.20E-02	NS
CSAG3	NS	2.12E-02	9.45E-02	6.20E-02	NS
PSME3	NS	2.12E-02	-0.0944	6.21E-02	NS
SGTA	NS	2.13E-02	-0.0944	6.22E-02	NS
POR	NS	2.13E-02	9.44E-02	6.22E-02	NS
ZNF704	NS	2.13E-02	-0.0944	6.22E-02	NS
PSMD12	NS	2.13E-02	-0.0944	6.22E-02	NS
RTL5	NS	2.13E-02	-0.0944	6.22E-02	NS
YJU2	NS	2.13E-02	-0.0944	6.22E-02	NS
RANBP6	NS	2.13E-02	9.44E-02	6.22E-02	NS
GNPNAT1	NS	2.13E-02	-0.0944	6.22E-02	NS
KCNQ5	NS	2.13E-02	-0.0944	6.23E-02	NS
DLX5	NS	2.13E-02	9.44E-02	6.24E-02	NS
SUCO	NS	2.14E-02	-0.0943	6.24E-02	NS
SESN2	NS	2.14E-02	9.43E-02	6.24E-02	NS
N4BP2L1	NS	2.14E-02	9.43E-02	6.24E-02	NS
CCDC7	NS	2.14E-02	-0.0943	6.24E-02	NS
ZNF675	NS	2.14E-02	-0.0943	6.25E-02	NS
FAR2	NS	2.14E-02	-0.0943	6.25E-02	NS
ABI3BP	NS	2.14E-02	-0.0943	6.25E-02	NS
SYT16	NS	2.14E-02	9.43E-02	6.26E-02	NS

EVPL	NS	2.15E-02	9.43E-02	6.26E-02	NS
PAGE1	NS	2.15E-02	-0.0943	6.26E-02	NS
CARD6	NS	2.15E-02	-0.0943	6.26E-02	NS
AMACR	NS	2.15E-02	-0.0943	6.26E-02	NS
STAB2	NS	2.15E-02	-0.0942	6.27E-02	NS
TBC1D32	NS	2.15E-02	-0.0942	6.27E-02	NS
CSGALNACT1	NS	2.15E-02	-0.0942	6.27E-02	NS
LENG8	NS	2.15E-02	9.42E-02	6.27E-02	NS
LIN37	NS	2.15E-02	-0.0942	6.28E-02	NS
GOSR2	NS	2.15E-02	9.42E-02	6.28E-02	NS
FAM81B	NS	2.16E-02	-0.0942	6.28E-02	NS
ZNF568	NS	2.16E-02	-0.0942	6.28E-02	NS
SPTSSA	NS	2.16E-02	9.42E-02	6.28E-02	NS
PPP1R2B	NS	2.16E-02	-0.0942	6.28E-02	NS
C8A	NS	2.16E-02	-0.0942	6.28E-02	NS
NAGS	NS	2.16E-02	-0.0942	6.29E-02	NS
H2AC8	NS	2.16E-02	-0.0942	6.29E-02	NS
LRRC1	NS	2.16E-02	-0.0941	6.30E-02	NS
HLCS	NS	2.17E-02	-0.0941	6.31E-02	NS
ZNF322	NS	2.17E-02	-0.0941	6.31E-02	NS
PICK1	NS	2.17E-02	9.41E-02	6.32E-02	NS
PCDH1	NS	2.17E-02	-0.0941	6.32E-02	NS
PCGF6	NS	2.18E-02	-0.0941	6.32E-02	NS
BTN3A2	NS	2.18E-02	-0.0941	6.32E-02	NS
RAD51AP2	NS	2.18E-02	-0.094	6.33E-02	NS
MRPL33	NS	2.18E-02	-0.094	6.33E-02	NS
CLEC1B	NS	2.18E-02	-0.094	6.33E-02	NS
FAM135B	NS	2.18E-02	-0.094	6.35E-02	NS
HHIPL1	NS	2.19E-02	9.40E-02	6.35E-02	NS
POLR1A	NS	2.19E-02	-0.094	6.35E-02	NS
DNAL4	NS	2.19E-02	-0.094	6.35E-02	NS
PSD3	NS	2.19E-02	-0.094	6.35E-02	NS
CCDC188	NS	2.19E-02	9.40E-02	6.35E-02	NS
KIF5A	NS	2.19E-02	-0.094	6.35E-02	NS
ZNF229	NS	2.19E-02	-0.094	6.35E-02	NS
DNTTIP2	NS	2.19E-02	9.40E-02	6.36E-02	NS
FEZ2	NS	2.19E-02	-0.094	6.36E-02	NS

UIMC1	NS	2.19E-02	9.39E-02	6.36E-02	NS
MFSD12	NS	2.19E-02	9.39E-02	6.36E-02	NS
PTGDS	NS	2.19E-02	-0.0939	6.36E-02	NS
SPINT3	NS	2.20E-02	-0.0939	6.37E-02	NS
SLC35G3	NS	2.20E-02	9.39E-02	6.37E-02	NS
ZCRB1	NS	2.20E-02	-0.0939	6.37E-02	NS
NBR1	NS	2.20E-02	-0.0939	6.37E-02	NS
KRBOX4	NS	2.20E-02	-0.0939	6.37E-02	NS
PGM5	NS	2.20E-02	-0.0939	6.38E-02	NS
CCL25	NS	2.20E-02	9.39E-02	6.38E-02	NS
ADGRF4	NS	2.20E-02	-0.0939	6.38E-02	NS
SOCS6	NS	2.21E-02	-0.0938	6.39E-02	NS
AWAT2	NS	2.21E-02	-0.0938	6.39E-02	NS
SH3RF3	NS	2.21E-02	-0.0938	6.39E-02	NS
GABRG3	NS	2.21E-02	-0.0938	6.39E-02	NS
SAP130	NS	2.21E-02	9.38E-02	6.39E-02	NS
IFT140	NS	2.21E-02	-0.0938	6.39E-02	NS
MRPL38	NS	2.21E-02	-0.0938	6.39E-02	NS
CTAGE4	NS	2.21E-02	-0.0938	6.39E-02	NS
AURKAIP1	NS	2.21E-02	-0.0938	6.39E-02	NS
CACNA1A	NS	2.21E-02	9.38E-02	6.39E-02	NS
NBPF9	NS	2.21E-02	-0.0938	6.39E-02	NS
CD28	NS	2.21E-02	-0.0938	6.39E-02	NS
RNF182	NS	2.21E-02	-0.0938	6.39E-02	NS
ZNF445	NS	2.21E-02	9.38E-02	6.39E-02	NS
ASMT	NS	2.21E-02	9.38E-02	6.39E-02	NS
CYP4F22	NS	2.22E-02	-0.0938	6.40E-02	NS
DCTPP1	NS	2.22E-02	-0.0938	6.41E-02	NS
PI4K2B	NS	2.22E-02	-0.0938	6.41E-02	NS
JUND	NS	2.22E-02	9.37E-02	6.41E-02	NS
KALRN	NS	2.22E-02	-0.0937	6.41E-02	NS
GNAZ	NS	2.22E-02	9.37E-02	6.41E-02	NS
HINT1	NS	2.22E-02	-0.0937	6.41E-02	NS
RNF207	NS	2.22E-02	-0.0937	6.42E-02	NS
PIR	NS	2.23E-02	9.37E-02	6.43E-02	NS
ALDH2	NS	2.23E-02	9.37E-02	6.43E-02	NS
THADA	NS	2.23E-02	-0.0937	6.43E-02	NS

ARMC9	NS	2.23E-02	9.37E-02	6.43E-02	NS
PLSCR2	NS	2.23E-02	9.37E-02	6.43E-02	NS
SLC39A2	NS	2.23E-02	-0.0937	6.43E-02	NS
DALRD3	NS	2.23E-02	-0.0937	6.44E-02	NS
CCL3	NS	2.23E-02	9.36E-02	6.44E-02	NS
ZNF501	NS	2.24E-02	-0.0936	6.44E-02	NS
HOXC6	NS	2.24E-02	9.36E-02	6.45E-02	NS
PLEKHA2	NS	2.24E-02	9.36E-02	6.46E-02	NS
CISD2	NS	2.24E-02	-0.0936	6.46E-02	NS
LRRC74B	NS	2.24E-02	9.36E-02	6.46E-02	NS
KRTAP10-3	NS	2.24E-02	9.36E-02	6.46E-02	NS
SGSM1	NS	2.25E-02	-0.0936	6.47E-02	NS
NUP155	NS	2.25E-02	-0.0936	6.47E-02	NS
KLK5	NS	2.25E-02	9.35E-02	6.48E-02	NS
INSM1	NS	2.25E-02	9.35E-02	6.49E-02	NS
JAKMIP2	NS	2.25E-02	-0.0935	6.49E-02	NS
MRPS6	NS	2.26E-02	-0.0935	6.49E-02	NS
SMARCD1	NS	2.26E-02	-0.0935	6.50E-02	NS
CWF19L2	NS	2.26E-02	-0.0935	6.50E-02	NS
F8A3	NS	2.26E-02	9.35E-02	6.50E-02	NS
SERPINB5	NS	2.26E-02	-0.0935	6.50E-02	NS
PGK1	NS	2.26E-02	-0.0934	6.51E-02	NS
GARNL3	NS	2.26E-02	-0.0934	6.51E-02	NS
ERH	NS	2.26E-02	-0.0934	6.51E-02	NS
RPUSD2	NS	2.27E-02	-0.0934	6.52E-02	NS
OC90	NS	2.27E-02	-0.0934	6.52E-02	NS
GSC2	NS	2.27E-02	9.34E-02	6.52E-02	NS
PEX5	NS	2.27E-02	-0.0934	6.52E-02	NS
TMEM229B	NS	2.27E-02	-0.0934	6.52E-02	NS
SAMHD1	NS	2.27E-02	-0.0934	6.52E-02	NS
MANEA	NS	2.27E-02	-0.0934	6.52E-02	NS
CPNE4	NS	2.27E-02	-0.0934	6.52E-02	NS
BBS1	NS	2.27E-02	-0.0934	6.53E-02	NS
C16orf90	NS	2.27E-02	-0.0934	6.53E-02	NS
PDIA3	NS	2.27E-02	-0.0934	6.53E-02	NS
HSCB	NS	2.28E-02	-0.0934	6.53E-02	NS
CHD9	NS	2.28E-02	-0.0933	6.54E-02	NS

OR10A2	NS	2.28E-02	-0.0933	6.54E-02	NS
FBXO27	NS	2.28E-02	9.33E-02	6.54E-02	NS
H2BC17	NS	2.28E-02	-0.0933	6.54E-02	NS
CTSD	NS	2.28E-02	9.33E-02	6.55E-02	NS
H3C7	NS	2.28E-02	-0.0933	6.55E-02	NS
RGS1	NS	2.28E-02	9.33E-02	6.55E-02	NS
GYG1	NS	2.28E-02	-0.0933	6.55E-02	NS
MICA	NS	2.28E-02	-0.0933	6.55E-02	NS
TBX5	NS	2.29E-02	-0.0933	6.55E-02	NS
SENP6	NS	2.29E-02	9.33E-02	6.56E-02	NS
OR2Z1	NS	2.29E-02	-0.0933	6.56E-02	NS
FLRT2	NS	2.29E-02	-0.0933	6.56E-02	NS
TMEM39B	NS	2.29E-02	-0.0932	6.56E-02	NS
LRRCC1	NS	2.29E-02	-0.0932	6.56E-02	NS
FAM76B	NS	2.30E-02	-0.0932	6.58E-02	NS
FSIP1	NS	2.30E-02	-0.0932	6.58E-02	NS
RNF223	NS	2.30E-02	9.32E-02	6.58E-02	NS
SIGLEC8	NS	2.30E-02	-0.0932	6.59E-02	NS
ANKRD31	NS	2.30E-02	-0.0932	6.59E-02	NS
TMPRSS11F	NS	2.30E-02	-0.0932	6.59E-02	NS
SORL1	NS	2.30E-02	-0.0932	6.59E-02	NS
KLF13	NS	2.31E-02	-0.0932	6.59E-02	NS
BCL2	NS	2.31E-02	-0.0932	6.59E-02	NS
GPR139	NS	2.30E-02	-0.0932	6.59E-02	NS
TTBK2	NS	2.31E-02	9.31E-02	6.60E-02	NS
TRPS1	NS	2.31E-02	-0.0931	6.60E-02	NS
COL15A1	NS	2.31E-02	-0.0931	6.60E-02	NS
SETMAR	NS	2.31E-02	-0.0931	6.61E-02	NS
SLC25A3	NS	2.32E-02	-0.0931	6.62E-02	NS
CHRAC1	NS	2.32E-02	-0.0931	6.62E-02	NS
CARS1	NS	2.32E-02	-0.0931	6.62E-02	NS
CCDC88A	NS	2.32E-02	-0.0931	6.62E-02	NS
ZNF365	NS	2.32E-02	-0.0931	6.62E-02	NS
GFRA1	NS	2.32E-02	9.31E-02	6.62E-02	NS
CSN3	NS	2.32E-02	-0.0931	6.62E-02	NS
B3GNT6	NS	2.32E-02	-0.0931	6.62E-02	NS
GAGE10	NS	2.32E-02	-0.093	6.63E-02	NS

CNPY2	NS	2.32E-02	-0.093	6.63E-02	NS
IDI1	NS	2.33E-02	9.30E-02	6.64E-02	NS
LMNB2	NS	2.34E-02	-0.093	6.66E-02	NS
NPAS1	NS	2.34E-02	-0.0929	6.68E-02	NS
CCDC92	NS	2.34E-02	-0.0929	6.69E-02	NS
OR51B2	NS	2.35E-02	-0.0929	6.69E-02	NS
METTL21A	NS	2.35E-02	9.29E-02	6.69E-02	NS
LAGE3	NS	2.35E-02	-0.0928	6.70E-02	NS
OR13C9	NS	2.36E-02	-0.0928	6.71E-02	NS
ALKAL2	NS	2.36E-02	-0.0928	6.71E-02	NS
SSR3	NS	2.36E-02	-0.0928	6.72E-02	NS
UCN	NS	2.36E-02	-0.0928	6.72E-02	NS
CNTN2	NS	2.36E-02	-0.0928	6.72E-02	NS
FAM111A	NS	2.36E-02	9.28E-02	6.73E-02	NS
TTLL6	NS	2.37E-02	9.28E-02	6.74E-02	NS
FILIP1L	NS	2.37E-02	-0.0927	6.74E-02	NS
AGAP4	NS	2.37E-02	-0.0927	6.74E-02	NS
NCR2	NS	2.37E-02	-0.0927	6.74E-02	NS
ZNF786	NS	2.37E-02	-0.0927	6.74E-02	NS
EXOC3L4	NS	2.37E-02	9.27E-02	6.74E-02	NS
VPS13C	NS	2.37E-02	-0.0927	6.74E-02	NS
GPR137B	NS	2.37E-02	9.27E-02	6.74E-02	NS
CD96	NS	2.37E-02	-0.0927	6.74E-02	NS
NBPF19	NS	2.37E-02	-0.0927	6.74E-02	NS
VPS13D	NS	2.37E-02	-0.0927	6.74E-02	NS
PNLDC1	NS	2.37E-02	-0.0927	6.75E-02	NS
PCDHA13	NS	2.37E-02	9.27E-02	6.75E-02	NS
NEURL2	NS	2.38E-02	-0.0927	6.75E-02	NS
SASH1	NS	2.38E-02	-0.0927	6.76E-02	NS
TVP23B	NS	2.38E-02	-0.0927	6.76E-02	NS
STAT4	NS	2.38E-02	-0.0926	6.76E-02	NS
ELOVL4	NS	2.38E-02	-0.0926	6.76E-02	NS
AIMP1	NS	2.38E-02	-0.0926	6.76E-02	NS
SLC25A33	NS	2.38E-02	9.26E-02	6.76E-02	NS
TSGA10IP	NS	2.38E-02	-0.0926	6.77E-02	NS
GOLM2	NS	2.39E-02	-0.0926	6.77E-02	NS
TATDN2	NS	2.39E-02	-0.0926	6.77E-02	NS

NXT1	NS	2.39E-02	9.26E-02	6.78E-02	NS
ZNF853	NS	2.39E-02	9.26E-02	6.78E-02	NS
OR12D3	NS	2.39E-02	-0.0926	6.78E-02	NS
KATNBL1	NS	2.39E-02	-0.0926	6.79E-02	NS
TRPC6	NS	2.40E-02	-0.0926	6.80E-02	NS
SRGAP2B	NS	2.40E-02	-0.0925	6.80E-02	NS
CXCL10	NS	2.40E-02	-0.0925	6.80E-02	NS
HRURF	NS	2.40E-02	-0.0925	6.80E-02	NS
CHRM3	NS	2.40E-02	-0.0925	6.81E-02	NS
GAGE2E	NS	2.40E-02	-0.0925	6.81E-02	NS
GPC5	NS	2.41E-02	-0.0924	6.83E-02	NS
CBL	NS	2.41E-02	-0.0924	6.84E-02	NS
ABT1	NS	2.41E-02	-0.0924	6.84E-02	NS
SH3KBP1	NS	2.42E-02	9.24E-02	6.84E-02	NS
EIF2AK3	NS	2.42E-02	9.24E-02	6.85E-02	NS
FDCSP	NS	2.42E-02	-0.0924	6.85E-02	NS
DDOST	NS	2.42E-02	-0.0924	6.85E-02	NS
FBXL3	NS	2.42E-02	-0.0924	6.86E-02	NS
PPCDC	NS	2.42E-02	-0.0924	6.86E-02	NS
NPM3	NS	2.42E-02	-0.0924	6.86E-02	NS
MAGEB17	NS	2.43E-02	-0.0923	6.88E-02	NS
PEDS1	NS	2.43E-02	9.23E-02	6.88E-02	NS
POMK	NS	2.43E-02	9.23E-02	6.88E-02	NS
STBD1	NS	2.44E-02	-0.0923	6.89E-02	NS
ABCD2	NS	2.44E-02	-0.0923	6.89E-02	NS
CASP4	NS	2.44E-02	-0.0923	6.91E-02	NS
GFRAL	NS	2.44E-02	-0.0922	6.91E-02	NS
COA1	NS	2.44E-02	-0.0922	6.91E-02	NS
APC2	NS	2.45E-02	-0.0922	6.91E-02	NS
BRIP1	NS	2.45E-02	-0.0922	6.91E-02	NS
KCNB1	NS	2.45E-02	-0.0922	6.91E-02	NS
SYNJ2BP	NS	2.45E-02	-0.0922	6.91E-02	NS
KPNB1	NS	2.45E-02	-0.0922	6.91E-02	NS
NMT2	NS	2.45E-02	9.22E-02	6.91E-02	NS
IDUA	NS	2.45E-02	9.22E-02	6.92E-02	NS
KCTD17	NS	2.45E-02	9.22E-02	6.93E-02	NS
UGP2	NS	2.46E-02	-0.0922	6.93E-02	NS

SMN2	NS	2.46E-02	-0.0922	6.93E-02	NS
MMP28	NS	2.46E-02	9.22E-02	6.93E-02	NS
NEU2	NS	2.46E-02	9.22E-02	6.93E-02	NS
ZMAT1	NS	2.46E-02	9.22E-02	6.93E-02	NS
SF1	NS	2.46E-02	9.22E-02	6.93E-02	NS
DDX52	NS	2.46E-02	-0.0921	6.95E-02	NS
NT5C1A	NS	2.47E-02	-0.0921	6.95E-02	NS
ZNF486	NS	2.47E-02	-0.0921	6.95E-02	NS
CENPA	NS	2.47E-02	-0.0921	6.95E-02	NS
POMP	NS	2.47E-02	-0.0921	6.96E-02	NS
PTPRS	NS	2.47E-02	-0.0921	6.96E-02	NS
CEP131	NS	2.47E-02	-0.0921	6.97E-02	NS
TMEM129	NS	2.47E-02	-0.0921	6.97E-02	NS
PTPRT	NS	2.47E-02	-0.0921	6.97E-02	NS
PTER	NS	2.48E-02	-0.092	6.98E-02	NS
LNPEP	NS	2.48E-02	-0.092	6.98E-02	NS
THPO	NS	2.48E-02	9.20E-02	6.98E-02	NS
WHRN	NS	2.48E-02	9.20E-02	6.98E-02	NS
RMDN2	NS	2.48E-02	-0.092	6.98E-02	NS
CNR1	NS	2.48E-02	-0.092	6.98E-02	NS
ATP8A2	NS	2.48E-02	-0.092	6.98E-02	NS
INPP4A	NS	2.48E-02	-0.092	6.99E-02	NS
CARD10	NS	2.48E-02	9.20E-02	6.99E-02	NS
GANC	NS	2.48E-02	9.20E-02	6.99E-02	NS
OR52J3	NS	2.49E-02	-0.092	6.99E-02	NS
H2BE1	NS	2.49E-02	-0.092	6.99E-02	NS
TTLL4	NS	2.49E-02	-0.092	6.99E-02	NS
ZNF724	NS	2.49E-02	-0.092	6.99E-02	NS
ZNF830	NS	2.49E-02	-0.092	6.99E-02	NS
PCYOX1	NS	2.49E-02	-0.0919	7.00E-02	NS
KLHL1	NS	2.49E-02	-0.0919	7.00E-02	NS
ACOT11	NS	2.49E-02	-0.0919	7.01E-02	NS
C2orf92	NS	2.50E-02	-0.0919	7.02E-02	NS
CELF3	NS	2.50E-02	-0.0919	7.02E-02	NS
SLC14A2	NS	2.50E-02	-0.0919	7.03E-02	NS
NSUN5	NS	2.51E-02	-0.0919	7.03E-02	NS
SEPTIN5	NS	2.51E-02	9.18E-02	7.05E-02	NS

TCP10L	NS	2.51E-02	-0.0918	7.05E-02	NS
TMCC3	NS	2.51E-02	-0.0918	7.05E-02	NS
OPA3	NS	2.52E-02	-0.0918	7.07E-02	NS
VAMP4	NS	2.52E-02	-0.0918	7.07E-02	NS
ZC2HC1A	NS	2.52E-02	-0.0918	7.07E-02	NS
PNOC	NS	2.52E-02	-0.0917	7.07E-02	NS
HSD17B11	NS	2.52E-02	-0.0917	7.07E-02	NS
TRPV4	NS	2.52E-02	-0.0917	7.07E-02	NS
FAM72A	NS	2.52E-02	-0.0917	7.08E-02	NS
NHLRC4	NS	2.53E-02	9.17E-02	7.09E-02	NS
PTPRN	NS	2.53E-02	-0.0917	7.09E-02	NS
NLN	NS	2.53E-02	-0.0917	7.09E-02	NS
C2orf16	NS	2.53E-02	-0.0917	7.09E-02	NS
LETMD1	NS	2.53E-02	-0.0917	7.09E-02	NS
PSMB10	NS	2.53E-02	-0.0917	7.09E-02	NS
PKD2	NS	2.53E-02	-0.0917	7.09E-02	NS
SFMBT1	NS	2.53E-02	-0.0917	7.09E-02	NS
KCTD18	NS	2.53E-02	-0.0917	7.09E-02	NS
ADCY8	NS	2.54E-02	-0.0917	7.10E-02	NS
CNTROB	NS	2.54E-02	9.17E-02	7.10E-02	NS
SDR39U1	NS	2.54E-02	-0.0917	7.10E-02	NS
CHMP3	NS	2.54E-02	-0.0916	7.10E-02	NS
UCK1	NS	2.54E-02	-0.0917	7.10E-02	NS
RFFL	NS	2.54E-02	-0.0916	7.11E-02	NS
CTSL	NS	2.54E-02	9.16E-02	7.11E-02	NS
YME1L1	NS	2.54E-02	9.16E-02	7.11E-02	NS
PCSK2	NS	2.55E-02	-0.0916	7.12E-02	NS
CD320	NS	2.55E-02	-0.0916	7.12E-02	NS
PA2G4	NS	2.55E-02	-0.0916	7.12E-02	NS
SPATA33	NS	2.55E-02	-0.0916	7.13E-02	NS
PCDHGC4	NS	2.55E-02	-0.0916	7.14E-02	NS
ABHD12	NS	2.55E-02	9.15E-02	7.14E-02	NS
SRGAP2C	NS	2.56E-02	-0.0915	7.14E-02	NS
MS4A8	NS	2.56E-02	-0.0915	7.15E-02	NS
C22orf39	NS	2.56E-02	-0.0915	7.15E-02	NS
FAM120B	NS	2.56E-02	-0.0915	7.15E-02	NS
TMPRSS11B	NS	2.56E-02	-0.0915	7.16E-02	NS

SPATA31D3	NS	2.57E-02	-0.0915	7.16E-02	NS
FBXL13	NS	2.57E-02	-0.0915	7.17E-02	NS
DDX20	NS	2.57E-02	9.15E-02	7.17E-02	NS
MYO1C	NS	2.57E-02	9.15E-02	7.17E-02	NS
SLC22A17	NS	2.57E-02	9.14E-02	7.18E-02	NS
LEMD1	NS	2.57E-02	-0.0914	7.18E-02	NS
TFAM	NS	2.57E-02	-0.0914	7.18E-02	NS
GZMM	NS	2.57E-02	9.14E-02	7.18E-02	NS
KLHDC2	NS	2.58E-02	-0.0914	7.19E-02	NS
RHOXF2	NS	2.58E-02	-0.0914	7.19E-02	NS
MAPK8IP2	NS	2.59E-02	9.14E-02	7.21E-02	NS
NCBP2	NS	2.59E-02	-0.0914	7.21E-02	NS
METRNL	NS	2.59E-02	-0.0914	7.21E-02	NS
DRC7	NS	2.59E-02	9.13E-02	7.21E-02	NS
HSPA4L	NS	2.59E-02	-0.0913	7.21E-02	NS
MTLN	NS	2.59E-02	-0.0913	7.21E-02	NS
ZNF625-ZNF20	NS	2.59E-02	-0.0913	7.21E-02	NS
ABCC9	NS	2.59E-02	-0.0913	7.22E-02	NS
LPGAT1	NS	2.59E-02	-0.0913	7.22E-02	NS
FAAH2	NS	2.60E-02	-0.0913	7.23E-02	NS
SYN3	NS	2.60E-02	-0.0913	7.23E-02	NS
ABHD14A-ACY1	NS	2.60E-02	-0.0913	7.23E-02	NS
KIR3DL3	NS	2.60E-02	-0.0913	7.23E-02	NS
SNX17	NS	2.60E-02	-0.0913	7.24E-02	NS
PIK3R3	NS	2.61E-02	-0.0912	7.26E-02	NS
GDI1	NS	2.61E-02	9.12E-02	7.26E-02	NS
LRRC37A2	NS	2.61E-02	-0.0912	7.26E-02	NS
FAM90A22P	NS	2.61E-02	-0.0912	7.27E-02	NS
CHRNA5	NS	2.62E-02	-0.0912	7.28E-02	NS
TCERG1	NS	2.62E-02	9.11E-02	7.29E-02	NS
ERAL1	NS	2.63E-02	9.11E-02	7.30E-02	NS
ANKRD13A	NS	2.63E-02	9.11E-02	7.30E-02	NS
PRMT5	NS	2.63E-02	-0.0911	7.30E-02	NS
RPS18	NS	2.63E-02	-0.0911	7.30E-02	NS
NR4A3	NS	2.63E-02	-0.0911	7.31E-02	NS
CTRL	NS	2.63E-02	-0.0911	7.31E-02	NS
SPSB1	NS	2.63E-02	-0.0911	7.31E-02	NS

BTC	NS	2.64E-02	-0.091	7.32E-02	NS
MRPL24	NS	2.64E-02	-0.091	7.32E-02	NS
NT5DC1	NS	2.64E-02	-0.091	7.33E-02	NS
COL9A2	NS	2.64E-02	-0.091	7.33E-02	NS
ARHGAP5	NS	2.64E-02	-0.091	7.33E-02	NS
MDM2	NS	2.64E-02	9.10E-02	7.33E-02	NS
SSX2IP	NS	2.64E-02	-0.091	7.33E-02	NS
PTOV1	NS	2.65E-02	9.10E-02	7.34E-02	NS
CYP11B1	NS	2.65E-02	9.10E-02	7.34E-02	NS
PLCXD2	NS	2.65E-02	-0.091	7.35E-02	NS
LAG3	NS	2.65E-02	-0.0909	7.36E-02	NS
ADCY7	NS	2.65E-02	9.09E-02	7.36E-02	NS
TMEM221	NS	2.65E-02	-0.0909	7.36E-02	NS
C6orf118	NS	2.66E-02	-0.0909	7.36E-02	NS
EIF4E3	NS	2.66E-02	-0.0909	7.36E-02	NS
SLC5A1	NS	2.66E-02	-0.0909	7.37E-02	NS
PLIN1	NS	2.66E-02	9.09E-02	7.37E-02	NS
SLFN13	NS	2.66E-02	-0.0909	7.37E-02	NS
ENOX2	NS	2.67E-02	-0.0909	7.38E-02	NS
NRIP2	NS	2.67E-02	-0.0909	7.38E-02	NS
LPIN2	NS	2.67E-02	-0.0909	7.38E-02	NS
SLC30A6	NS	2.67E-02	-0.0909	7.38E-02	NS
FHIP1A	NS	2.67E-02	-0.0908	7.39E-02	NS
SCGB1C1	NS	2.67E-02	9.08E-02	7.39E-02	NS
FCAR	NS	2.67E-02	-0.0908	7.40E-02	NS
GPR155	NS	2.67E-02	-0.0908	7.40E-02	NS
ITFG1	NS	2.68E-02	-0.0908	7.41E-02	NS
IFNA13	NS	2.68E-02	9.08E-02	7.41E-02	NS
HEATR5B	NS	2.68E-02	9.08E-02	7.41E-02	NS
SEMG1	NS	2.68E-02	-0.0908	7.41E-02	NS
TMBIM6	NS	2.68E-02	-0.0908	7.41E-02	NS
CDK5	NS	2.68E-02	9.08E-02	7.41E-02	NS
KNL1	NS	2.69E-02	-0.0907	7.42E-02	NS
CFL1	NS	2.69E-02	9.07E-02	7.42E-02	NS
MMP21	NS	2.69E-02	-0.0907	7.43E-02	NS
GAGE12C	NS	2.69E-02	-0.0907	7.43E-02	NS
C6orf52	NS	2.69E-02	-0.0907	7.44E-02	NS

KRTAP4-1	NS	2.69E-02	-0.0907	7.44E-02	NS
TRIB2	NS	2.70E-02	-0.0907	7.45E-02	NS
EXOC6B	NS	2.70E-02	-0.0907	7.46E-02	NS
ADAMTS6	NS	2.71E-02	-0.0906	7.47E-02	NS
FAM3A	NS	2.71E-02	-0.0906	7.47E-02	NS
UQCRQ	NS	2.71E-02	-0.0906	7.47E-02	NS
PDHB	NS	2.71E-02	-0.0906	7.47E-02	NS
NFKBIA	NS	2.71E-02	9.06E-02	7.47E-02	NS
LMTK3	NS	2.71E-02	9.06E-02	7.47E-02	NS
SPIRE2	NS	2.71E-02	-0.0906	7.47E-02	NS
KLHL28	NS	2.71E-02	-0.0906	7.47E-02	NS
TRIM6-TRIM34	NS	2.71E-02	-0.0906	7.47E-02	NS
TSPAN14	NS	2.71E-02	9.06E-02	7.48E-02	NS
LMO3	NS	2.72E-02	-0.0906	7.48E-02	NS
NKAIN3	NS	2.72E-02	-0.0906	7.49E-02	NS
ZNF281	NS	2.72E-02	9.06E-02	7.49E-02	NS
COLGALT2	NS	2.72E-02	-0.0905	7.49E-02	NS
BRK1	NS	2.73E-02	-0.0905	7.51E-02	NS
GSTA3	NS	2.73E-02	-0.0905	7.52E-02	NS
TAFA5	NS	2.73E-02	9.05E-02	7.52E-02	NS
ITGA2B	NS	2.73E-02	-0.0905	7.52E-02	NS
FCGR3B	NS	2.74E-02	-0.0905	7.53E-02	NS
STX17	NS	2.74E-02	-0.0904	7.53E-02	NS
SERP2	NS	2.74E-02	-0.0904	7.53E-02	NS
CACNA1B	NS	2.74E-02	-0.0904	7.54E-02	NS
SEPTIN8	NS	2.74E-02	-0.0904	7.54E-02	NS
FMO1	NS	2.74E-02	-0.0904	7.54E-02	NS
DBX2	NS	2.74E-02	-0.0904	7.54E-02	NS
KRTAP13-3	NS	2.75E-02	9.04E-02	7.55E-02	NS
HMGN2	NS	2.75E-02	-0.0904	7.56E-02	NS
CPT1A	NS	2.76E-02	-0.0903	7.57E-02	NS
CD7	NS	2.76E-02	-0.0903	7.58E-02	NS
ST3GAL6	NS	2.76E-02	-0.0903	7.58E-02	NS
TDRD7	NS	2.76E-02	9.03E-02	7.60E-02	NS
NCBP2AS2	NS	2.77E-02	-0.0903	7.61E-02	NS
NPY1R	NS	2.77E-02	-0.0903	7.61E-02	NS
ATG4A	NS	2.77E-02	-0.0903	7.61E-02	NS

OR2A4	NS	2.77E-02	-0.0902	7.62E-02	NS
SPCS3	NS	2.78E-02	-0.0902	7.63E-02	NS
E2F6	NS	2.78E-02	-0.0902	7.64E-02	NS
ATF3	NS	2.78E-02	-0.0902	7.64E-02	NS
RPL35A	NS	2.78E-02	-0.0902	7.64E-02	NS
PCMTD2	NS	2.79E-02	-0.0902	7.64E-02	NS
H2AJ	NS	2.79E-02	-0.0902	7.65E-02	NS
NBPF1	NS	2.79E-02	-0.0901	7.65E-02	NS
ARF4	NS	2.79E-02	-0.0901	7.65E-02	NS
PIGR	NS	2.80E-02	9.01E-02	7.67E-02	NS
TMPRSS9	NS	2.80E-02	-0.0901	7.67E-02	NS
SUMF2	NS	2.80E-02	-0.0901	7.67E-02	NS
UNK	NS	2.80E-02	9.01E-02	7.68E-02	NS
CASP7	NS	2.80E-02	-0.0901	7.68E-02	NS
SMAP1	NS	2.80E-02	-0.0901	7.68E-02	NS
ARMCX5	NS	2.81E-02	-0.0901	7.69E-02	NS
MANEAL	NS	2.81E-02	-0.09	7.70E-02	NS
GRM4	NS	2.81E-02	9.00E-02	7.70E-02	NS
TBX6	NS	2.81E-02	-0.09	7.70E-02	NS
SMIM10L1	NS	2.81E-02	-0.09	7.70E-02	NS
GRB10	NS	2.81E-02	-0.09	7.70E-02	NS
LY86	NS	2.81E-02	-0.09	7.70E-02	NS
COX17	NS	2.81E-02	-0.09	7.70E-02	NS
PPP1CB	NS	2.81E-02	-0.09	7.70E-02	NS
TTC6	NS	2.82E-02	9.00E-02	7.71E-02	NS
GTF3C2	NS	2.82E-02	-0.09	7.71E-02	NS
SNPH	NS	2.82E-02	-0.09	7.71E-02	NS
CIAO1	NS	2.82E-02	-0.09	7.71E-02	NS
PTH2	NS	2.82E-02	9.00E-02	7.72E-02	NS
USP19	NS	2.82E-02	9.00E-02	7.72E-02	NS
TSC1	NS	2.82E-02	-0.09	7.72E-02	NS
ECM1	NS	2.82E-02	8.99E-02	7.72E-02	NS
RNFT1	NS	2.83E-02	-0.0899	7.73E-02	NS
KRTAP21-3	NS	2.83E-02	8.99E-02	7.73E-02	NS
DNA2	NS	2.83E-02	-0.0899	7.73E-02	NS
BPNT2	NS	2.83E-02	-0.0899	7.73E-02	NS
MYCL	NS	2.83E-02	-0.0899	7.73E-02	NS

PELI1	NS	2.83E-02	8.99E-02	7.74E-02	NS
PSMF1	NS	2.84E-02	8.99E-02	7.75E-02	NS
HERC5	NS	2.84E-02	-0.0899	7.75E-02	NS
CABP2	NS	2.84E-02	-0.0898	7.76E-02	NS
TMEM213	NS	2.84E-02	8.98E-02	7.76E-02	NS
C2orf74	NS	2.85E-02	-0.0898	7.77E-02	NS
OSGEP	NS	2.85E-02	-0.0898	7.78E-02	NS
HERC3	NS	2.85E-02	-0.0898	7.78E-02	NS
SCML4	NS	2.85E-02	-0.0898	7.78E-02	NS
AP3S2	NS	2.85E-02	8.98E-02	7.78E-02	NS
SPNS3	NS	2.85E-02	-0.0898	7.78E-02	NS
TLR7	NS	2.85E-02	-0.0898	7.78E-02	NS
DSTYK	NS	2.86E-02	-0.0898	7.79E-02	NS
CHST13	NS	2.86E-02	-0.0898	7.79E-02	NS
HTRA1	NS	2.86E-02	-0.0897	7.80E-02	NS
CPVL	NS	2.86E-02	-0.0897	7.80E-02	NS
MDFIC	NS	2.86E-02	-0.0897	7.80E-02	NS
SMN1	NS	2.86E-02	-0.0897	7.80E-02	NS
KRT13	NS	2.87E-02	-0.0897	7.81E-02	NS
PKMYT1	NS	2.87E-02	-0.0897	7.81E-02	NS
MIF	NS	2.87E-02	-0.0897	7.81E-02	NS
POLR1G	NS	2.87E-02	-0.0897	7.81E-02	NS
OR6C6	NS	2.87E-02	8.97E-02	7.81E-02	NS
SLC17A8	NS	2.87E-02	-0.0897	7.81E-02	NS
KHNYN	NS	2.87E-02	-0.0897	7.82E-02	NS
GSDMA	NS	2.87E-02	8.97E-02	7.82E-02	NS
ZNF729	NS	2.87E-02	-0.0897	7.82E-02	NS
SELENOV	NS	2.87E-02	8.97E-02	7.82E-02	NS
OR52E4	NS	2.87E-02	-0.0897	7.82E-02	NS
THAP12	NS	2.88E-02	8.97E-02	7.83E-02	NS
C17orf50	NS	2.88E-02	8.96E-02	7.84E-02	NS
PBX1	NS	2.88E-02	-0.0896	7.84E-02	NS
CSDE1	NS	2.88E-02	-0.0896	7.84E-02	NS
DEFB132	NS	2.88E-02	-0.0896	7.85E-02	NS
CD200R1L	NS	2.89E-02	-0.0896	7.85E-02	NS
IDE	NS	2.89E-02	-0.0896	7.85E-02	NS
CGA	NS	2.89E-02	-0.0896	7.85E-02	NS

CREB1	NS	2.89E-02	-0.0896	7.86E-02	NS
CDH16	NS	2.89E-02	-0.0896	7.86E-02	NS
TRMT1	NS	2.89E-02	-0.0896	7.86E-02	NS
PEX5L	NS	2.90E-02	-0.0895	7.88E-02	NS
CABLES1	NS	2.90E-02	-0.0895	7.88E-02	NS
STRN4	NS	2.90E-02	-0.0895	7.89E-02	NS
CDC14A	NS	2.91E-02	8.95E-02	7.90E-02	NS
PLD1	NS	2.91E-02	8.95E-02	7.90E-02	NS
CLN8	NS	2.91E-02	8.94E-02	7.91E-02	NS
CNGA2	NS	2.91E-02	-0.0894	7.91E-02	NS
PLAAT2	NS	2.92E-02	-0.0894	7.92E-02	NS
TMEM132B	NS	2.92E-02	-0.0894	7.92E-02	NS
IFNGR1	NS	2.92E-02	-0.0894	7.92E-02	NS
FNBP1L	NS	2.92E-02	-0.0894	7.93E-02	NS
CFAP251	NS	2.92E-02	-0.0894	7.93E-02	NS
IRF7	NS	2.92E-02	8.94E-02	7.93E-02	NS
WEE2	NS	2.93E-02	8.94E-02	7.93E-02	NS
PNO1	NS	2.93E-02	-0.0894	7.95E-02	NS
OR10T2	NS	2.93E-02	-0.0893	7.95E-02	NS
ZBTB2	NS	2.93E-02	8.93E-02	7.95E-02	NS
SCO1	NS	2.94E-02	-0.0893	7.95E-02	NS
GRAMD2A	NS	2.94E-02	8.93E-02	7.96E-02	NS
GRK4	NS	2.94E-02	-0.0893	7.96E-02	NS
AZI2	NS	2.94E-02	-0.0893	7.96E-02	NS
OTUB2	NS	2.94E-02	-0.0893	7.97E-02	NS
CA11	NS	2.94E-02	-0.0893	7.97E-02	NS
RTBDN	NS	2.94E-02	8.93E-02	7.97E-02	NS
NRGN	NS	2.94E-02	8.93E-02	7.97E-02	NS
SERPINH1	NS	2.95E-02	-0.0893	7.98E-02	NS
ZXDC	NS	2.95E-02	-0.0892	7.98E-02	NS
BUD23	NS	2.95E-02	8.92E-02	7.98E-02	NS
PAQR4	NS	2.95E-02	-0.0892	7.99E-02	NS
STK40	NS	2.95E-02	-0.0892	7.99E-02	NS
PHGDH	NS	2.96E-02	-0.0892	8.02E-02	NS
RIPPLY3	NS	2.96E-02	8.92E-02	8.02E-02	NS
BRD8	NS	2.97E-02	-0.0892	8.02E-02	NS
COL22A1	NS	2.96E-02	-0.0892	8.02E-02	NS

GAK	NS	2.97E-02	8.91E-02	8.04E-02	NS
PRDM2	NS	2.97E-02	8.91E-02	8.04E-02	NS
GRK3	NS	2.98E-02	-0.0891	8.04E-02	NS
SCN5A	NS	2.98E-02	-0.0891	8.04E-02	NS
TAS2R10	NS	2.98E-02	-0.0891	8.04E-02	NS
ESPN	NS	2.98E-02	8.91E-02	8.05E-02	NS
PTPRZ1	NS	2.98E-02	-0.0891	8.05E-02	NS
IFI44L	NS	2.98E-02	-0.0891	8.06E-02	NS
MRGPRX4	NS	2.98E-02	-0.0891	8.06E-02	NS
VCAM1	NS	2.99E-02	8.91E-02	8.06E-02	NS
BIRC3	NS	2.99E-02	-0.089	8.08E-02	NS
HMOX1	NS	2.99E-02	8.90E-02	8.08E-02	NS
INTU	NS	2.99E-02	-0.089	8.08E-02	NS
ZNF317	NS	2.99E-02	8.90E-02	8.08E-02	NS
ELOVL2	NS	3.00E-02	-0.089	8.08E-02	NS
NNT	NS	3.00E-02	-0.089	8.09E-02	NS
OR4C12	NS	3.00E-02	-0.089	8.09E-02	NS
AKR1B15	NS	3.00E-02	8.90E-02	8.09E-02	NS
SPI1	NS	3.00E-02	8.90E-02	8.09E-02	NS
PRR22	NS	3.00E-02	-0.089	8.09E-02	NS
PAPOLB	NS	3.00E-02	8.90E-02	8.09E-02	NS
SSX2	NS	3.00E-02	-0.089	8.09E-02	NS
CSNK2A3	NS	3.00E-02	8.90E-02	8.10E-02	NS
SDCCAG8	NS	3.01E-02	-0.0889	8.10E-02	NS
TIMM22	NS	3.01E-02	-0.0889	8.10E-02	NS
IMMP1L	NS	3.01E-02	-0.0889	8.10E-02	NS
CD79A	NS	3.01E-02	-0.0889	8.11E-02	NS
KIF5B	NS	3.01E-02	-0.0889	8.11E-02	NS
UBE3D	NS	3.02E-02	-0.0889	8.12E-02	NS
NISCH	NS	3.02E-02	-0.0889	8.12E-02	NS
CD2	NS	3.02E-02	-0.0889	8.13E-02	NS
GPR37	NS	3.02E-02	-0.0889	8.13E-02	NS
RNF144A	NS	3.02E-02	-0.0889	8.13E-02	NS
ZNF630	NS	3.02E-02	-0.0889	8.13E-02	NS
TMEM190	NS	3.02E-02	8.89E-02	8.13E-02	NS
RASGEF1A	NS	3.02E-02	-0.0888	8.13E-02	NS
CSNK1A1	NS	3.03E-02	-0.0888	8.14E-02	NS

RMND5A	NS	3.03E-02	-0.0888	8.14E-02	NS
PGAM5	NS	3.03E-02	-0.0888	8.14E-02	NS
GMCL2	NS	3.03E-02	-0.0888	8.14E-02	NS
ECHDC2	NS	3.03E-02	-0.0888	8.16E-02	NS
THAP7	NS	3.04E-02	-0.0888	8.16E-02	NS
FMC1	NS	3.04E-02	-0.0888	8.16E-02	NS
PSG7	NS	3.04E-02	8.88E-02	8.16E-02	NS
MRPL32	NS	3.04E-02	-0.0887	8.18E-02	NS
AKAP8L	NS	3.05E-02	8.87E-02	8.18E-02	NS
TMEM191C	NS	3.05E-02	-0.0887	8.19E-02	NS
MPO	NS	3.05E-02	-0.0887	8.19E-02	NS
HRG	NS	3.05E-02	-0.0887	8.19E-02	NS
SLC30A9	NS	3.05E-02	-0.0887	8.19E-02	NS
TFPT	NS	3.05E-02	-0.0887	8.20E-02	NS
ZNF526	NS	3.05E-02	8.87E-02	8.20E-02	NS
PTPN14	NS	3.06E-02	-0.0887	8.20E-02	NS
UBXN1	NS	3.06E-02	-0.0887	8.21E-02	NS
FLCN	NS	3.06E-02	-0.0887	8.21E-02	NS
TRPM1	NS	3.06E-02	-0.0886	8.21E-02	NS
PEX10	NS	3.07E-02	-0.0886	8.22E-02	NS
RMI1	NS	3.07E-02	-0.0886	8.22E-02	NS
SOS2	NS	3.07E-02	8.86E-02	8.22E-02	NS
PLPPR3	NS	3.07E-02	8.86E-02	8.22E-02	NS
VPS41	NS	3.07E-02	-0.0886	8.23E-02	NS
FBXO38	NS	3.07E-02	-0.0886	8.23E-02	NS
APOLD1	NS	3.07E-02	-0.0886	8.24E-02	NS
TGFBR3L	NS	3.08E-02	8.85E-02	8.25E-02	NS
COPS8	NS	3.08E-02	8.85E-02	8.25E-02	NS
DCBLD2	NS	3.09E-02	-0.0885	8.26E-02	NS
SLC25A42	NS	3.08E-02	-0.0885	8.26E-02	NS
NDUFB2	NS	3.09E-02	-0.0885	8.28E-02	NS
LMO1	NS	3.09E-02	-0.0885	8.28E-02	NS
PRIMPOL	NS	3.10E-02	-0.0885	8.29E-02	NS
EEF1E1	NS	3.10E-02	-0.0884	8.30E-02	NS
ZNF521	NS	3.11E-02	-0.0884	8.31E-02	NS
UROC1	NS	3.11E-02	8.84E-02	8.33E-02	NS
SENP2	NS	3.11E-02	-0.0884	8.33E-02	NS

PGAM2	NS	3.11E-02	-0.0884	8.33E-02	NS
SLC25A24	NS	3.12E-02	-0.0884	8.33E-02	NS
SMIM27	NS	3.12E-02	-0.0884	8.33E-02	NS
MARCHF8	NS	3.12E-02	-0.0883	8.35E-02	NS
SLURP2	NS	3.12E-02	8.83E-02	8.35E-02	NS
SLC35F2	NS	3.13E-02	8.83E-02	8.36E-02	NS
VPS50	NS	3.13E-02	-0.0883	8.36E-02	NS
SMIM29	NS	3.13E-02	8.83E-02	8.36E-02	NS
QTRT1	NS	3.13E-02	-0.0883	8.36E-02	NS
NSL1	NS	3.14E-02	-0.0883	8.38E-02	NS
SIX3	NS	3.14E-02	8.83E-02	8.38E-02	NS
RAD52	NS	3.14E-02	-0.0883	8.38E-02	NS
MANF	NS	3.14E-02	-0.0882	8.38E-02	NS
PSORS1C1	NS	3.14E-02	8.82E-02	8.39E-02	NS
OR1N2	NS	3.14E-02	8.82E-02	8.39E-02	NS
RNF181	NS	3.15E-02	8.82E-02	8.40E-02	NS
SNAPC2	NS	3.15E-02	-0.0882	8.41E-02	NS
TRIM64B	NS	3.15E-02	-0.0882	8.41E-02	NS
WDR3	NS	3.15E-02	-0.0882	8.41E-02	NS
APOE	NS	3.15E-02	8.82E-02	8.41E-02	NS
THEM5	NS	3.15E-02	-0.0882	8.41E-02	NS
FOXI1	NS	3.16E-02	8.82E-02	8.42E-02	NS
LEO1	NS	3.16E-02	-0.0881	8.42E-02	NS
EEF1B2	NS	3.16E-02	-0.0881	8.43E-02	NS
CYP2E1	NS	3.16E-02	-0.0881	8.43E-02	NS
CCNJ	NS	3.16E-02	-0.0881	8.43E-02	NS
RNASE10	NS	3.17E-02	-0.0881	8.44E-02	NS
MFNG	NS	3.17E-02	-0.0881	8.44E-02	NS
MICALL1	NS	3.17E-02	-0.0881	8.45E-02	NS
HSFY1	NS	3.17E-02	8.81E-02	8.45E-02	NS
OSGIN2	NS	3.17E-02	-0.0881	8.45E-02	NS
NRIP3	NS	3.17E-02	8.81E-02	8.45E-02	NS
PDGFD	NS	3.17E-02	-0.0881	8.45E-02	NS
MEIG1	NS	3.17E-02	-0.0881	8.45E-02	NS
URAD	NS	3.18E-02	8.81E-02	8.45E-02	NS
CCDC71	NS	3.18E-02	8.81E-02	8.45E-02	NS
IL36A	NS	3.18E-02	-0.088	8.46E-02	NS

BAZ2A	NS	3.18E-02	-0.088	8.47E-02	NS
ADAMTS15	NS	3.18E-02	8.80E-02	8.47E-02	NS
MRPL40	NS	3.19E-02	8.80E-02	8.48E-02	NS
STAM2	NS	3.19E-02	8.80E-02	8.48E-02	NS
TGM7	NS	3.19E-02	8.80E-02	8.48E-02	NS
MRPL23	NS	3.19E-02	8.80E-02	8.48E-02	NS
IGF2BP1	NS	3.19E-02	8.80E-02	8.48E-02	NS
LRRC8B	NS	3.19E-02	-0.088	8.48E-02	NS
LELP1	NS	3.19E-02	-0.088	8.49E-02	NS
MYH7B	NS	3.20E-02	-0.088	8.49E-02	NS
ACMSD	NS	3.20E-02	-0.088	8.49E-02	NS
ZBTB3	NS	3.20E-02	-0.0879	8.49E-02	NS
MALT1	NS	3.20E-02	-0.0879	8.50E-02	NS
CHRNB1	NS	3.20E-02	-0.0879	8.50E-02	NS
PNMA5	NS	3.20E-02	-0.0879	8.51E-02	NS
NR1D2	NS	3.21E-02	8.79E-02	8.52E-02	NS
TPSG1	NS	3.22E-02	8.78E-02	8.54E-02	NS
ATAD1	NS	3.22E-02	-0.0878	8.54E-02	NS
CRYGA	NS	3.22E-02	-0.0878	8.54E-02	NS
NCCRP1	NS	3.22E-02	8.78E-02	8.54E-02	NS
PIGG	NS	3.22E-02	-0.0878	8.54E-02	NS
APOBEC4	NS	3.22E-02	-0.0878	8.55E-02	NS
TRIM71	NS	3.22E-02	-0.0878	8.55E-02	NS
SP3	NS	3.23E-02	-0.0878	8.57E-02	NS
RNF165	NS	3.23E-02	8.78E-02	8.57E-02	NS
MASP2	NS	3.24E-02	-0.0877	8.58E-02	NS
SLC16A12	NS	3.24E-02	8.77E-02	8.58E-02	NS
CDX4	NS	3.24E-02	-0.0877	8.58E-02	NS
DEFB121	NS	3.24E-02	-0.0877	8.59E-02	NS
FGF4	NS	3.24E-02	8.77E-02	8.59E-02	NS
NKAIN4	NS	3.24E-02	8.77E-02	8.59E-02	NS
AANAT	NS	3.24E-02	-0.0877	8.59E-02	NS
C5orf49	NS	3.24E-02	-0.0877	8.59E-02	NS
PEX11B	NS	3.25E-02	-0.0877	8.60E-02	NS
OR52N4	NS	3.25E-02	-0.0877	8.60E-02	NS
C1orf226	NS	3.25E-02	-0.0877	8.60E-02	NS
CCL17	NS	3.25E-02	-0.0877	8.61E-02	NS

CUL1	NS	3.26E-02	-0.0876	8.62E-02	NS
OR5T2	NS	3.26E-02	-0.0876	8.62E-02	NS
SYVN1	NS	3.26E-02	8.76E-02	8.62E-02	NS
LPAR6	NS	3.26E-02	-0.0876	8.64E-02	NS
OR8D1	NS	3.27E-02	-0.0876	8.64E-02	NS
PSMA2	NS	3.27E-02	-0.0876	8.65E-02	NS
ACSL6	NS	3.27E-02	-0.0876	8.65E-02	NS
PTPN21	NS	3.27E-02	-0.0876	8.65E-02	NS
CPE	NS	3.27E-02	-0.0876	8.65E-02	NS
IRF6	NS	3.27E-02	-0.0876	8.65E-02	NS
PSMD13	NS	3.27E-02	-0.0876	8.65E-02	NS
SCN8A	NS	3.27E-02	-0.0876	8.65E-02	NS
N4BP2L2	NS	3.27E-02	-0.0876	8.65E-02	NS
SAA4	NS	3.28E-02	-0.0875	8.67E-02	NS
MEGF6	NS	3.28E-02	-0.0875	8.67E-02	NS
PALS2	NS	3.28E-02	-0.0875	8.68E-02	NS
TIMM23B-AGAP6	NS	3.28E-02	-0.0875	8.68E-02	NS
STARD13	NS	3.29E-02	8.75E-02	8.68E-02	NS
CEP135	NS	3.29E-02	-0.0875	8.68E-02	NS
SPHK1	NS	3.29E-02	8.75E-02	8.68E-02	NS
NUDT18	NS	3.29E-02	-0.0875	8.68E-02	NS
OR4D10	NS	3.29E-02	-0.0875	8.68E-02	NS
SLC35G5	NS	3.29E-02	-0.0875	8.68E-02	NS
TARM1	NS	3.29E-02	8.75E-02	8.68E-02	NS
LETM2	NS	3.29E-02	8.75E-02	8.68E-02	NS
TUBA4B	NS	3.29E-02	-0.0875	8.68E-02	NS
NDUFB7	NS	3.31E-02	-0.0874	8.71E-02	NS
KLHL22	NS	3.31E-02	-0.0874	8.71E-02	NS
SYNGR1	NS	3.31E-02	8.74E-02	8.71E-02	NS
ADGRA3	NS	3.31E-02	8.74E-02	8.71E-02	NS
CHSY3	NS	3.31E-02	-0.0874	8.71E-02	NS
CATSPERZ	NS	3.31E-02	8.74E-02	8.71E-02	NS
CSAD	NS	3.32E-02	8.73E-02	8.73E-02	NS
RBM15B	NS	3.32E-02	-0.0873	8.74E-02	NS
CHST14	NS	3.32E-02	-0.0873	8.75E-02	NS
PARD6B	NS	3.32E-02	-0.0873	8.75E-02	NS
CYTH3	NS	3.33E-02	-0.0873	8.75E-02	NS

ESF1	NS	3.33E-02	-0.0873	8.75E-02	NS
LILRA4	NS	3.33E-02	-0.0873	8.76E-02	NS
CCS	NS	3.33E-02	-0.0873	8.76E-02	NS
SUB1	NS	3.34E-02	-0.0872	8.78E-02	NS
HMG20A	NS	3.34E-02	-0.0872	8.78E-02	NS
INPP5E	NS	3.34E-02	-0.0872	8.78E-02	NS
ANKRD23	NS	3.34E-02	8.72E-02	8.78E-02	NS
DDRGK1	NS	3.34E-02	8.72E-02	8.78E-02	NS
ITGB3	NS	3.34E-02	-0.0872	8.78E-02	NS
CCL3L1	NS	3.34E-02	8.72E-02	8.78E-02	NS
ZNF585B	NS	3.34E-02	-0.0872	8.78E-02	NS
CCDC174	NS	3.34E-02	-0.0872	8.78E-02	NS
ECSIT	NS	3.34E-02	-0.0872	8.79E-02	NS
MMS22L	NS	3.34E-02	-0.0872	8.79E-02	NS
FRAT2	NS	3.35E-02	-0.0872	8.80E-02	NS
DNAJB6	NS	3.36E-02	-0.0871	8.82E-02	NS
TBCEL-TECTA	NS	3.36E-02	-0.0871	8.82E-02	NS
CLUH	NS	3.36E-02	-0.0871	8.82E-02	NS
ATP5F1D	NS	3.36E-02	8.71E-02	8.82E-02	NS
ATXN3L	NS	3.36E-02	-0.0871	8.82E-02	NS
TBCA	NS	3.36E-02	-0.0871	8.82E-02	NS
RDH16	NS	3.36E-02	-0.0871	8.82E-02	NS
IQCE	NS	3.36E-02	-0.0871	8.83E-02	NS
DHDDS	NS	3.37E-02	-0.0871	8.83E-02	NS
CREB5	NS	3.37E-02	8.71E-02	8.83E-02	NS
CAVIN4	NS	3.37E-02	-0.0871	8.83E-02	NS
OR2T33	NS	3.37E-02	-0.0871	8.83E-02	NS
SLC5A3	NS	3.37E-02	-0.0871	8.83E-02	NS
APCDD1L	NS	3.37E-02	-0.0871	8.83E-02	NS
COPS3	NS	3.37E-02	-0.0871	8.83E-02	NS
ANP32E	NS	3.37E-02	-0.0871	8.84E-02	NS
RPS27AP5	NS	3.38E-02	-0.0871	8.85E-02	NS
ITGA7	NS	3.38E-02	-0.087	8.86E-02	NS
NOTCH2NLC	NS	3.39E-02	-0.087	8.87E-02	NS
HYAL1	NS	3.39E-02	-0.087	8.87E-02	NS
OR4F15	NS	3.39E-02	8.70E-02	8.88E-02	NS
BRF2	NS	3.40E-02	-0.087	8.89E-02	NS

TMF1	NS	3.40E-02	-0.087	8.89E-02	NS
VENTX	NS	3.40E-02	-0.0869	8.90E-02	NS
PLEKHN1	NS	3.40E-02	-0.0869	8.91E-02	NS
GPR20	NS	3.40E-02	8.69E-02	8.91E-02	NS
FAM110A	NS	3.41E-02	8.69E-02	8.91E-02	NS
TEX35	NS	3.41E-02	-0.0869	8.92E-02	NS
SLCO1B3	NS	3.41E-02	-0.0869	8.92E-02	NS
CYSTM1	NS	3.41E-02	8.69E-02	8.92E-02	NS
IRF4	NS	3.41E-02	-0.0869	8.92E-02	NS
NPHP1	NS	3.41E-02	8.69E-02	8.92E-02	NS
PAMR1	NS	3.41E-02	-0.0869	8.92E-02	NS
PQBP1	NS	3.41E-02	-0.0869	8.92E-02	NS
CBLN2	NS	3.41E-02	-0.0869	8.92E-02	NS
RCN1	NS	3.41E-02	-0.0869	8.93E-02	NS
SI	NS	3.42E-02	-0.0869	8.93E-02	NS
F8A2	NS	3.42E-02	8.69E-02	8.93E-02	NS
OR8H3	NS	3.42E-02	8.68E-02	8.93E-02	NS
CALR	NS	3.42E-02	-0.0868	8.94E-02	NS
CCDC65	NS	3.42E-02	-0.0868	8.94E-02	NS
LAMC3	NS	3.43E-02	8.68E-02	8.96E-02	NS
ZMYND8	NS	3.43E-02	-0.0868	8.96E-02	NS
APLP1	NS	3.43E-02	8.68E-02	8.96E-02	NS
RCOR3	NS	3.44E-02	8.68E-02	8.97E-02	NS
MYZAP	NS	3.44E-02	-0.0868	8.97E-02	NS
SPINK4	NS	3.44E-02	-0.0867	8.99E-02	NS
ANKRD62	NS	3.45E-02	8.67E-02	9.00E-02	NS
KAT8	NS	3.45E-02	8.67E-02	9.02E-02	NS
IDS	NS	3.46E-02	-0.0867	9.02E-02	NS
HSD17B6	NS	3.46E-02	-0.0866	9.02E-02	NS
RBFOX2	NS	3.46E-02	-0.0866	9.02E-02	NS
SLC22A2	NS	3.46E-02	-0.0866	9.02E-02	NS
FNDC11	NS	3.46E-02	8.67E-02	9.02E-02	NS
ATM	NS	3.46E-02	-0.0867	9.02E-02	NS
LMNTD1	NS	3.46E-02	-0.0866	9.02E-02	NS
TGIF2LX	NS	3.46E-02	-0.0866	9.02E-02	NS
RMND1	NS	3.46E-02	-0.0866	9.03E-02	NS
HOXA4	NS	3.47E-02	8.66E-02	9.03E-02	NS

OSTN	NS	3.47E-02	-0.0866	9.03E-02	NS
CLCA4	NS	3.47E-02	-0.0866	9.04E-02	NS
PLA2G10	NS	3.47E-02	-0.0866	9.04E-02	NS
C5AR1	NS	3.47E-02	-0.0866	9.04E-02	NS
BAHCC1	NS	3.47E-02	-0.0866	9.04E-02	NS
PFDN1	NS	3.47E-02	8.66E-02	9.05E-02	NS
ZNF506	NS	3.48E-02	-0.0866	9.06E-02	NS
CYFIP2	NS	3.48E-02	-0.0866	9.06E-02	NS
ECHS1	NS	3.48E-02	-0.0865	9.06E-02	NS
UGT2B7	NS	3.48E-02	-0.0865	9.07E-02	NS
MTMR8	NS	3.49E-02	-0.0865	9.07E-02	NS
TJP2	NS	3.49E-02	8.65E-02	9.08E-02	NS
ATP7A	NS	3.49E-02	-0.0865	9.08E-02	NS
SH2B1	NS	3.49E-02	8.65E-02	9.08E-02	NS
MGAT2	NS	3.49E-02	-0.0865	9.08E-02	NS
PLPP6	NS	3.49E-02	-0.0865	9.09E-02	NS
ARHGEF25	NS	3.50E-02	8.65E-02	9.09E-02	NS
DDX39A	NS	3.50E-02	8.65E-02	9.09E-02	NS
SLC35B3	NS	3.50E-02	-0.0865	9.09E-02	NS
DEFB106A	NS	3.50E-02	-0.0865	9.09E-02	NS
NUDT4B	NS	3.50E-02	8.65E-02	9.09E-02	NS
TMEM80	NS	3.51E-02	8.64E-02	9.12E-02	NS
TM4SF1	NS	3.51E-02	-0.0864	9.12E-02	NS
FAM136A	NS	3.52E-02	-0.0864	9.14E-02	NS
USP44	NS	3.52E-02	-0.0864	9.14E-02	NS
DPY30	NS	3.52E-02	-0.0864	9.14E-02	NS
XPO5	NS	3.52E-02	-0.0864	9.14E-02	NS
METTL3	NS	3.52E-02	-0.0864	9.14E-02	NS
CNNM4	NS	3.52E-02	8.63E-02	9.15E-02	NS
AMMECR1	NS	3.53E-02	-0.0863	9.16E-02	NS
KRT81	NS	3.53E-02	8.63E-02	9.16E-02	NS
DDX31	NS	3.53E-02	-0.0863	9.16E-02	NS
CFAP20DC	NS	3.53E-02	-0.0863	9.16E-02	NS
IL17REL	NS	3.53E-02	8.63E-02	9.16E-02	NS
BASP1	NS	3.54E-02	-0.0863	9.17E-02	NS
ARPC1A	NS	3.54E-02	-0.0863	9.17E-02	NS
SPATA20	NS	3.54E-02	-0.0863	9.17E-02	NS

PELI3	NS	3.54E-02	-0.0863	9.18E-02	NS
PSD	NS	3.54E-02	8.63E-02	9.18E-02	NS
TMSB4Y	NS	3.54E-02	-0.0863	9.18E-02	NS
GGH	NS	3.55E-02	-0.0862	9.19E-02	NS
RCOR1	NS	3.55E-02	-0.0862	9.20E-02	NS
ANKRD36	NS	3.56E-02	-0.0862	9.21E-02	NS
MNS1	NS	3.56E-02	-0.0862	9.21E-02	NS
SMIM7	NS	3.56E-02	-0.0862	9.21E-02	NS
DMBT1	NS	3.56E-02	-0.0862	9.22E-02	NS
THEM6	NS	3.56E-02	-0.0862	9.22E-02	NS
WRNIP1	NS	3.56E-02	-0.0862	9.22E-02	NS
TRIM8	NS	3.56E-02	8.62E-02	9.22E-02	NS
HLA-DOA	NS	3.56E-02	-0.0862	9.23E-02	NS
ESPNL	NS	3.56E-02	-0.0861	9.23E-02	NS
DMPK	NS	3.57E-02	-0.0861	9.23E-02	NS
TRAP1	NS	3.57E-02	-0.0861	9.23E-02	NS
F8A1	NS	3.57E-02	8.61E-02	9.24E-02	NS
ELL3	NS	3.57E-02	8.61E-02	9.24E-02	NS
ZNF174	NS	3.57E-02	-0.0861	9.24E-02	NS
ARID3C	NS	3.57E-02	-0.0861	9.24E-02	NS
SPAG9	NS	3.58E-02	-0.0861	9.25E-02	NS
IFT88	NS	3.58E-02	-0.0861	9.25E-02	NS
NOXO1	NS	3.58E-02	-0.0861	9.25E-02	NS
XPOT	NS	3.58E-02	-0.0861	9.25E-02	NS
ASAH2	NS	3.58E-02	-0.0861	9.25E-02	NS
LCE1B	NS	3.58E-02	8.61E-02	9.25E-02	NS
KIF13B	NS	3.58E-02	-0.0861	9.25E-02	NS
DNAJC10	NS	3.58E-02	-0.0861	9.26E-02	NS
GDPD4	NS	3.59E-02	-0.086	9.27E-02	NS
SNX18	NS	3.59E-02	-0.086	9.27E-02	NS
F11	NS	3.59E-02	-0.086	9.28E-02	NS
TAC1	NS	3.60E-02	8.60E-02	9.29E-02	NS
USP28	NS	3.60E-02	-0.086	9.29E-02	NS
TRIM14	NS	3.60E-02	8.60E-02	9.29E-02	NS
RPL15	NS	3.60E-02	-0.086	9.29E-02	NS
NSD1	NS	3.61E-02	-0.086	9.30E-02	NS
B3GALT2	NS	3.61E-02	-0.086	9.31E-02	NS

PPP1R3A	NS	3.61E-02	-0.0859	9.31E-02	NS
DECR1	NS	3.61E-02	-0.0859	9.31E-02	NS
MFSD10	NS	3.61E-02	-0.0859	9.31E-02	NS
MTRNR2L3	NS	3.62E-02	8.59E-02	9.32E-02	NS
TAB2	NS	3.62E-02	-0.0859	9.33E-02	NS
RARB	NS	3.62E-02	-0.0859	9.34E-02	NS
PNMA8A	NS	3.62E-02	8.59E-02	9.34E-02	NS
LRRC17	NS	3.62E-02	-0.0859	9.34E-02	NS
CASP6	NS	3.63E-02	-0.0859	9.35E-02	NS
ZNF48	NS	3.63E-02	-0.0858	9.36E-02	NS
KMO	NS	3.64E-02	-0.0858	9.37E-02	NS
HOXC4	NS	3.64E-02	8.58E-02	9.37E-02	NS
SLC25A47	NS	3.64E-02	-0.0858	9.38E-02	NS
ANKIB1	NS	3.64E-02	-0.0858	9.38E-02	NS
GOLGA3	NS	3.64E-02	-0.0858	9.38E-02	NS
ADAMTS3	NS	3.65E-02	-0.0858	9.39E-02	NS
PRRC2A	NS	3.65E-02	8.58E-02	9.39E-02	NS
SLC17A6	NS	3.65E-02	-0.0857	9.40E-02	NS
PIWIL2	NS	3.65E-02	-0.0857	9.40E-02	NS
PPIP5K1	NS	3.65E-02	-0.0857	9.40E-02	NS
CACFD1	NS	3.66E-02	-0.0857	9.41E-02	NS
MSANTD5	NS	3.66E-02	8.57E-02	9.41E-02	NS
THSD7B	NS	3.66E-02	-0.0857	9.42E-02	NS
QRFP	NS	3.66E-02	-0.0857	9.42E-02	NS
ZBED5	NS	3.66E-02	-0.0857	9.42E-02	NS
TTC16	NS	3.67E-02	-0.0857	9.44E-02	NS
DISP2	NS	3.67E-02	8.56E-02	9.45E-02	NS
STS	NS	3.68E-02	-0.0856	9.45E-02	NS
PSG6	NS	3.68E-02	-0.0856	9.45E-02	NS
PRKG1	NS	3.68E-02	8.56E-02	9.45E-02	NS
XRN2	NS	3.68E-02	8.56E-02	9.45E-02	NS
FAM83C	NS	3.68E-02	8.56E-02	9.46E-02	NS
DYDC1	NS	3.68E-02	8.56E-02	9.46E-02	NS
SYNJ2BP-COX16	NS	3.68E-02	-0.0856	9.46E-02	NS
CDC14C	NS	3.69E-02	8.56E-02	9.47E-02	NS
PCP4L1	NS	3.69E-02	-0.0856	9.47E-02	NS
TMEM256-PLSCR3	NS	3.69E-02	8.56E-02	9.47E-02	NS

NFX1	NS	3.69E-02	-0.0856	9.47E-02	NS
C14orf93	NS	3.69E-02	-0.0856	9.47E-02	NS
PKD2L1	NS	3.69E-02	-0.0856	9.47E-02	NS
RAPGEF2	NS	3.69E-02	-0.0856	9.47E-02	NS
NPHP4	NS	3.69E-02	-0.0856	9.47E-02	NS
ODAD1	NS	3.70E-02	8.56E-02	9.48E-02	NS
SECTM1	NS	3.70E-02	-0.0855	9.49E-02	NS
TEFM	NS	3.70E-02	-0.0855	9.49E-02	NS
RBP3	NS	3.70E-02	-0.0855	9.49E-02	NS
FGF14	NS	3.71E-02	-0.0855	9.50E-02	NS
EEF1D	NS	3.71E-02	-0.0855	9.50E-02	NS
RNF8	NS	3.71E-02	-0.0855	9.50E-02	NS
COX6B2	NS	3.71E-02	8.55E-02	9.50E-02	NS
DHRS4L2	NS	3.71E-02	-0.0855	9.50E-02	NS
PROB1	NS	3.71E-02	8.55E-02	9.50E-02	NS
OR5K2	NS	3.71E-02	-0.0855	9.50E-02	NS
ZNF593OS	NS	3.71E-02	8.55E-02	9.51E-02	NS
FAM240C	NS	3.71E-02	8.55E-02	9.51E-02	NS
SLC30A2	NS	3.71E-02	8.55E-02	9.51E-02	NS
GPS2	NS	3.72E-02	8.55E-02	9.52E-02	NS
GSTA1	NS	3.72E-02	-0.0855	9.52E-02	NS
TRIM27	NS	3.72E-02	-0.0854	9.53E-02	NS
SLC37A1	NS	3.72E-02	8.54E-02	9.53E-02	NS
BOLA2B	NS	3.72E-02	8.54E-02	9.53E-02	NS
MLF2	NS	3.73E-02	8.54E-02	9.55E-02	NS
PAPPA2	NS	3.74E-02	-0.0854	9.56E-02	NS
TEX51	NS	3.74E-02	8.54E-02	9.57E-02	NS
RASL10B	NS	3.74E-02	8.53E-02	9.57E-02	NS
INS-IGF2	NS	3.75E-02	8.53E-02	9.59E-02	NS
DCAF8L1	NS	3.75E-02	-0.0853	9.59E-02	NS
FAM126A	NS	3.76E-02	-0.0853	9.61E-02	NS
CMPK1	NS	3.76E-02	-0.0853	9.61E-02	NS
LRRIQ4	NS	3.77E-02	-0.0852	9.62E-02	NS
ITPA	NS	3.77E-02	-0.0852	9.63E-02	NS
CD1A	NS	3.78E-02	-0.0852	9.65E-02	NS
ATP6V1G2	NS	3.78E-02	8.52E-02	9.65E-02	NS
TP53I13	NS	3.78E-02	8.52E-02	9.65E-02	NS

RPL21	NS	3.78E-02	-0.0852	9.65E-02	NS
SERPINB12	NS	3.78E-02	-0.0852	9.65E-02	NS
SLC25A39	NS	3.78E-02	-0.0852	9.65E-02	NS
TCN1	NS	3.78E-02	-0.0852	9.66E-02	NS
RFC1	NS	3.79E-02	8.51E-02	9.66E-02	NS
SLC38A10	NS	3.79E-02	8.51E-02	9.67E-02	NS
SHOX2	NS	3.79E-02	-0.0851	9.67E-02	NS
ATG9A	NS	3.79E-02	8.51E-02	9.67E-02	NS
ME1	NS	3.79E-02	-0.0851	9.67E-02	NS
FGFR2	NS	3.80E-02	-0.0851	9.68E-02	NS
GALNT11	NS	3.80E-02	-0.0851	9.68E-02	NS
GPI	NS	3.80E-02	8.51E-02	9.70E-02	NS
PLGRKT	NS	3.81E-02	-0.0851	9.71E-02	NS
ZBTB12	NS	3.81E-02	8.51E-02	9.71E-02	NS
AOC3	NS	3.81E-02	-0.085	9.71E-02	NS
HPSE	NS	3.81E-02	-0.085	9.71E-02	NS
MTOR	NS	3.81E-02	-0.085	9.71E-02	NS
SLC33A1	NS	3.81E-02	-0.085	9.71E-02	NS
FIBIN	NS	3.81E-02	8.50E-02	9.71E-02	NS
RGL3	NS	3.81E-02	-0.085	9.71E-02	NS
FAM174C	NS	3.81E-02	8.50E-02	9.71E-02	NS
ALPP	NS	3.82E-02	8.50E-02	9.71E-02	NS
IGFBP7	NS	3.82E-02	8.50E-02	9.71E-02	NS
KLHL32	NS	3.82E-02	-0.085	9.71E-02	NS
H2AC16	NS	3.82E-02	-0.085	9.71E-02	NS
ANKRD17	NS	3.82E-02	-0.085	9.72E-02	NS
LAMTOR5	NS	3.82E-02	8.50E-02	9.72E-02	NS
OGFOD2	NS	3.82E-02	-0.085	9.72E-02	NS
SVIP	NS	3.82E-02	-0.085	9.72E-02	NS
ZNF850	NS	3.83E-02	-0.0849	9.75E-02	NS
CHRNA9	NS	3.83E-02	8.49E-02	9.75E-02	NS
RNF103-CHMP3	NS	3.83E-02	-0.0849	9.75E-02	NS
NXPH1	NS	3.84E-02	-0.0849	9.75E-02	NS
SGCB	NS	3.84E-02	-0.0849	9.75E-02	NS
NAALADL1	NS	3.84E-02	-0.0849	9.75E-02	NS
PYURF	NS	3.84E-02	-0.0849	9.77E-02	NS
PIGY	NS	3.84E-02	-0.0849	9.77E-02	NS

CHRNA2	NS	3.85E-02	8.49E-02	9.78E-02	NS
LMX1A	NS	3.85E-02	8.49E-02	9.78E-02	NS
UBL7	NS	3.85E-02	-0.0849	9.78E-02	NS
C1orf162	NS	3.85E-02	8.49E-02	9.78E-02	NS
LRATD2	NS	3.85E-02	-0.0849	9.78E-02	NS
TM4SF4	NS	3.85E-02	-0.0849	9.78E-02	NS
H4C3	NS	3.86E-02	-0.0848	9.80E-02	NS
LHX5	NS	3.86E-02	8.48E-02	9.80E-02	NS
CT45A5	NS	3.86E-02	-0.0848	9.80E-02	NS
USHBP1	NS	3.87E-02	8.48E-02	9.81E-02	NS
PPIL4	NS	3.87E-02	-0.0848	9.81E-02	NS
CADPS	NS	3.87E-02	-0.0848	9.82E-02	NS
NPIPB15	NS	3.87E-02	8.48E-02	9.82E-02	NS
PFDN2	NS	3.88E-02	-0.0848	9.83E-02	NS
CCM2L	NS	3.88E-02	-0.0847	9.83E-02	NS
MMD	NS	3.88E-02	8.47E-02	9.85E-02	NS
ST8SIA3	NS	3.89E-02	-0.0847	9.86E-02	NS
LRP3	NS	3.89E-02	-0.0847	9.86E-02	NS
RNF185	NS	3.89E-02	-0.0847	9.86E-02	NS
AKR1D1	NS	3.89E-02	-0.0847	9.86E-02	NS
PAXX	NS	3.89E-02	-0.0847	9.86E-02	NS
PDE4DIP	NS	3.89E-02	-0.0847	9.86E-02	NS
ADAMTSL4	NS	3.90E-02	-0.0846	9.89E-02	NS
MAP3K13	NS	3.91E-02	-0.0846	9.89E-02	NS
CPNE3	NS	3.90E-02	-0.0846	9.89E-02	NS
P4HA1	NS	3.91E-02	-0.0846	9.89E-02	NS
PLP1	NS	3.91E-02	-0.0846	9.89E-02	NS
TMEM62	NS	3.91E-02	8.46E-02	9.89E-02	NS
FERD3L	NS	3.91E-02	8.46E-02	9.89E-02	NS
LRP12	NS	3.91E-02	-0.0846	9.89E-02	NS
DNAJC24	NS	3.91E-02	-0.0846	9.89E-02	NS
MYO15B	NS	3.91E-02	-0.0846	9.89E-02	NS
GNPDA1	NS	3.91E-02	-0.0846	9.89E-02	NS
MARCHF3	NS	3.91E-02	8.46E-02	9.89E-02	NS
WDTC1	NS	3.92E-02	-0.0846	9.90E-02	NS
FAM210B	NS	3.93E-02	-0.0845	9.93E-02	NS
WSCD2	NS	3.93E-02	-0.0845	9.93E-02	NS

AASDH	NS	3.93E-02	-0.0845	9.94E-02	NS
TRIM43B	NS	3.93E-02	-0.0845	9.94E-02	NS
GPR179	NS	3.95E-02	-0.0844	9.98E-02	NS
C1GALT1	NS	3.95E-02	-0.0844	9.99E-02	NS
MTMR7	NS	3.95E-02	-0.0844	9.99E-02	NS
PTPN4	NS	3.96E-02	8.44E-02	1.00E-01	NS
ENSA	NS	3.97E-02	-0.0844	1.00E-01	NS
MAPK8IP3	NS	3.97E-02	8.44E-02	1.00E-01	NS
ACER1	NS	3.97E-02	8.44E-02	1.00E-01	NS
FPGT-TNNI3K	NS	3.97E-02	-0.0844	1.00E-01	NS
H3-3A	NS	3.97E-02	-0.0843	1.00E-01	NS
DEFB124	NS	3.97E-02	8.43E-02	1.00E-01	NS
ZNF140	NS	3.98E-02	-0.0843	1.00E-01	NS
FNDC3A	NS	3.99E-02	-0.0843	1.01E-01	NS
MRPL34	NS	3.99E-02	-0.0843	1.01E-01	NS
MBD3L2	NS	3.99E-02	8.43E-02	1.01E-01	NS
ABCA8	NS	3.99E-02	-0.0843	1.01E-01	NS
AOPEP	NS	3.99E-02	8.43E-02	1.01E-01	NS
TFDP2	NS	4.00E-02	-0.0842	1.01E-01	NS
CACNA2D3	NS	4.00E-02	-0.0842	1.01E-01	NS
SMIM15	NS	4.00E-02	-0.0842	1.01E-01	NS
MMEL1	NS	4.01E-02	-0.0842	1.01E-01	NS
SPRTN	NS	4.01E-02	-0.0842	1.01E-01	NS
MGRN1	NS	4.01E-02	8.42E-02	1.01E-01	NS
TM9SF4	NS	4.01E-02	-0.0842	1.01E-01	NS
FERMT2	NS	4.01E-02	-0.0842	1.01E-01	NS
PHIP	NS	4.01E-02	-0.0842	1.01E-01	NS
MCUB	NS	4.01E-02	8.42E-02	1.01E-01	NS
C17orf107	NS	4.01E-02	-0.0842	1.01E-01	NS
MIER1	NS	4.01E-02	-0.0842	1.01E-01	NS
CDK4	NS	4.02E-02	8.41E-02	1.01E-01	NS
PIDD1	NS	4.02E-02	-0.0841	1.01E-01	NS
COQ3	NS	4.03E-02	-0.0841	1.01E-01	NS
BPTF	NS	4.03E-02	-0.0841	1.01E-01	NS
ABCC10	NS	4.04E-02	8.41E-02	1.02E-01	NS
UAP1	NS	4.04E-02	8.41E-02	1.02E-01	NS
TPH2	NS	4.04E-02	-0.0841	1.02E-01	NS

EEF1G	NS	4.04E-02	-0.084	1.02E-01	NS
BBC3	NS	4.04E-02	8.40E-02	1.02E-01	NS
WFDC5	NS	4.04E-02	8.40E-02	1.02E-01	NS
PIMREG	NS	4.05E-02	8.40E-02	1.02E-01	NS
NAT8	NS	4.05E-02	-0.084	1.02E-01	NS
CHADL	NS	4.05E-02	-0.084	1.02E-01	NS
SYCE3	NS	4.05E-02	-0.084	1.02E-01	NS
TEK	NS	4.05E-02	-0.084	1.02E-01	NS
ZNF197	NS	4.05E-02	-0.084	1.02E-01	NS
TCF23	NS	4.05E-02	8.40E-02	1.02E-01	NS
MORN4	NS	4.05E-02	-0.084	1.02E-01	NS
HLA-DMA	NS	4.05E-02	-0.084	1.02E-01	NS
FXN	NS	4.06E-02	-0.084	1.02E-01	NS
SPRN	NS	4.06E-02	-0.084	1.02E-01	NS
TMEM42	NS	4.06E-02	-0.084	1.02E-01	NS
PDE4B	NS	4.07E-02	-0.0839	1.02E-01	NS
MDM4	NS	4.07E-02	8.39E-02	1.02E-01	NS
ZNF681	NS	4.07E-02	-0.0839	1.02E-01	NS
PACC1	NS	4.07E-02	-0.0839	1.02E-01	NS
RIOK1	NS	4.07E-02	-0.0839	1.02E-01	NS
PHLDA1	NS	4.07E-02	-0.0839	1.02E-01	NS
CD47	NS	4.07E-02	-0.0839	1.02E-01	NS
EPHA7	NS	4.08E-02	-0.0839	1.02E-01	NS
RACGAP1	NS	4.09E-02	-0.0839	1.02E-01	NS
TEF	NS	4.09E-02	-0.0839	1.03E-01	NS
NFIL3	NS	4.09E-02	-0.0838	1.03E-01	NS
CUX2	NS	4.09E-02	-0.0838	1.03E-01	NS
UBE3C	NS	4.09E-02	-0.0838	1.03E-01	NS
CHCHD2	NS	4.09E-02	-0.0838	1.03E-01	NS
USH1C	NS	4.10E-02	8.38E-02	1.03E-01	NS
MUTYH	NS	4.10E-02	-0.0838	1.03E-01	NS
RALBP1	NS	4.11E-02	-0.0838	1.03E-01	NS
ZBTB4	NS	4.11E-02	-0.0838	1.03E-01	NS
EIF3B	NS	4.12E-02	-0.0837	1.03E-01	NS
C20orf204	NS	4.12E-02	-0.0837	1.03E-01	NS
SLF1	NS	4.12E-02	-0.0837	1.03E-01	NS
CHD8	NS	4.12E-02	-0.0837	1.03E-01	NS

MRPL48	NS	4.13E-02	-0.0837	1.03E-01	NS
B3GAT3	NS	4.13E-02	8.37E-02	1.03E-01	NS
GPAT2	NS	4.13E-02	8.37E-02	1.03E-01	NS
RPS6KA5	NS	4.13E-02	-0.0837	1.03E-01	NS
TAF5	NS	4.14E-02	-0.0837	1.04E-01	NS
COQ7	NS	4.14E-02	-0.0837	1.04E-01	NS
TNN	NS	4.14E-02	-0.0836	1.04E-01	NS
HSPE1-MOB4	NS	4.14E-02	-0.0836	1.04E-01	NS
PLEKHG6	NS	4.14E-02	-0.0836	1.04E-01	NS
IPPK	NS	4.14E-02	-0.0836	1.04E-01	NS
HCAR1	NS	4.15E-02	8.36E-02	1.04E-01	NS
TJP1	NS	4.15E-02	-0.0836	1.04E-01	NS
FAM90A26	NS	4.15E-02	-0.0836	1.04E-01	NS
FIGNL2	NS	4.15E-02	8.36E-02	1.04E-01	NS
ALDOA	NS	4.15E-02	8.36E-02	1.04E-01	NS
SLC17A4	NS	4.16E-02	-0.0836	1.04E-01	NS
CCDC122	NS	4.16E-02	-0.0836	1.04E-01	NS
PRLHR	NS	4.16E-02	8.36E-02	1.04E-01	NS
CORT	NS	4.17E-02	-0.0835	1.04E-01	NS
IL10RA	NS	4.17E-02	-0.0835	1.04E-01	NS
ARHGEF1	NS	4.17E-02	8.35E-02	1.04E-01	NS
DERL1	NS	4.18E-02	8.35E-02	1.04E-01	NS
PDCL3	NS	4.18E-02	-0.0835	1.04E-01	NS
UQCRC2	NS	4.18E-02	-0.0835	1.04E-01	NS
S1PR1	NS	4.18E-02	-0.0835	1.04E-01	NS
ETDA	NS	4.18E-02	-0.0835	1.04E-01	NS
RASSF3	NS	4.19E-02	-0.0834	1.04E-01	NS
GOLGB1	NS	4.19E-02	-0.0834	1.05E-01	NS
PSEN2	NS	4.20E-02	-0.0834	1.05E-01	NS
PAM	NS	4.20E-02	-0.0834	1.05E-01	NS
OLAH	NS	4.20E-02	-0.0834	1.05E-01	NS
NAA15	NS	4.20E-02	-0.0834	1.05E-01	NS
PIK3R4	NS	4.20E-02	-0.0834	1.05E-01	NS
GAB4	NS	4.20E-02	-0.0834	1.05E-01	NS
CDK10	NS	4.21E-02	-0.0834	1.05E-01	NS
FAM163B	NS	4.21E-02	8.34E-02	1.05E-01	NS
PLCD4	NS	4.21E-02	-0.0833	1.05E-01	NS

VPREB1	NS	4.22E-02	-0.0833	1.05E-01	NS
DDX60	NS	4.22E-02	-0.0833	1.05E-01	NS
MAGEF1	NS	4.22E-02	-0.0833	1.05E-01	NS
RAP1A	NS	4.22E-02	-0.0833	1.05E-01	NS
CYREN	NS	4.22E-02	8.33E-02	1.05E-01	NS
CENPS-CORT	NS	4.22E-02	-0.0833	1.05E-01	NS
KCNMA1	NS	4.22E-02	-0.0833	1.05E-01	NS
OR5B3	NS	4.22E-02	-0.0833	1.05E-01	NS
PALM	NS	4.23E-02	8.33E-02	1.05E-01	NS
DCANP1	NS	4.23E-02	-0.0833	1.05E-01	NS
PELI2	NS	4.23E-02	-0.0833	1.05E-01	NS
ITGAD	NS	4.23E-02	-0.0833	1.05E-01	NS
SF3B3	NS	4.23E-02	-0.0833	1.05E-01	NS
CENPM	NS	4.23E-02	-0.0833	1.05E-01	NS
ATG10	NS	4.23E-02	-0.0833	1.05E-01	NS
SNCA	NS	4.24E-02	-0.0833	1.05E-01	NS
SGCZ	NS	4.24E-02	-0.0833	1.05E-01	NS
ADARB1	NS	4.24E-02	-0.0832	1.05E-01	NS
MRC2	NS	4.24E-02	-0.0832	1.05E-01	NS
TRIM37	NS	4.24E-02	-0.0832	1.05E-01	NS
WDR49	NS	4.24E-02	8.32E-02	1.05E-01	NS
NR0B1	NS	4.25E-02	8.32E-02	1.06E-01	NS
CPA6	NS	4.25E-02	-0.0832	1.06E-01	NS
FHIP1B	NS	4.25E-02	8.32E-02	1.06E-01	NS
CDKN2C	NS	4.26E-02	-0.0832	1.06E-01	NS
KRTAP5-5	NS	4.26E-02	8.32E-02	1.06E-01	NS
SCYL1	NS	4.26E-02	8.31E-02	1.06E-01	NS
SORBS1	NS	4.27E-02	-0.0831	1.06E-01	NS
LAD1	NS	4.27E-02	8.31E-02	1.06E-01	NS
COL28A1	NS	4.27E-02	-0.0831	1.06E-01	NS
ADCY1	NS	4.27E-02	-0.0831	1.06E-01	NS
CLASRP	NS	4.28E-02	8.31E-02	1.06E-01	NS
ATP6V1E1	NS	4.28E-02	8.31E-02	1.06E-01	NS
PIK3C2G	NS	4.28E-02	-0.0831	1.06E-01	NS
SEC61B	NS	4.28E-02	8.31E-02	1.06E-01	NS
ACSBG2	NS	4.29E-02	-0.083	1.06E-01	NS
BPIFA2	NS	4.29E-02	-0.0831	1.06E-01	NS

DCD	NS	4.29E-02	-0.0831	1.06E-01	NS
NLRP13	NS	4.29E-02	-0.0831	1.06E-01	NS
CBY1	NS	4.29E-02	-0.083	1.06E-01	NS
HOXB7	NS	4.29E-02	8.30E-02	1.06E-01	NS
WDR1	NS	4.29E-02	-0.083	1.06E-01	NS
SENP1	NS	4.29E-02	-0.083	1.06E-01	NS
HSF4	NS	4.30E-02	-0.083	1.06E-01	NS
ZNF449	NS	4.30E-02	-0.083	1.06E-01	NS
PRMT2	NS	4.30E-02	8.30E-02	1.07E-01	NS
PERM1	NS	4.30E-02	8.30E-02	1.07E-01	NS
ZNRD2	NS	4.31E-02	-0.083	1.07E-01	NS
BAHD1	NS	4.31E-02	8.30E-02	1.07E-01	NS
RFXANK	NS	4.31E-02	-0.083	1.07E-01	NS
KLF6	NS	4.31E-02	8.29E-02	1.07E-01	NS
EYA1	NS	4.31E-02	-0.0829	1.07E-01	NS
RPS6KB1	NS	4.31E-02	-0.083	1.07E-01	NS
KIF11	NS	4.31E-02	-0.083	1.07E-01	NS
GCNT3	NS	4.32E-02	8.29E-02	1.07E-01	NS
MRPS27	NS	4.32E-02	-0.0829	1.07E-01	NS
VSIR	NS	4.33E-02	8.29E-02	1.07E-01	NS
ASB12	NS	4.33E-02	-0.0829	1.07E-01	NS
CD58	NS	4.33E-02	-0.0829	1.07E-01	NS
AGTR1	NS	4.33E-02	-0.0829	1.07E-01	NS
NAGLU	NS	4.33E-02	-0.0829	1.07E-01	NS
GPATCH1	NS	4.33E-02	-0.0829	1.07E-01	NS
KRBOX5	NS	4.33E-02	-0.0829	1.07E-01	NS
HDDC2	NS	4.33E-02	-0.0829	1.07E-01	NS
C2orf42	NS	4.34E-02	-0.0829	1.07E-01	NS
ZNF559	NS	4.34E-02	-0.0828	1.07E-01	NS
CD247	NS	4.34E-02	-0.0828	1.07E-01	NS
ZNF705D	NS	4.34E-02	-0.0828	1.07E-01	NS
TMED5	NS	4.35E-02	-0.0828	1.07E-01	NS
ACY3	NS	4.35E-02	8.28E-02	1.07E-01	NS
RBPJ	NS	4.35E-02	-0.0828	1.07E-01	NS
NSF	NS	4.36E-02	-0.0828	1.07E-01	NS
LPL	NS	4.36E-02	-0.0828	1.08E-01	NS
FRRS1	NS	4.36E-02	-0.0828	1.08E-01	NS

HOMEZ	NS	4.36E-02	8.27E-02	1.08E-01	NS
HTRA3	NS	4.36E-02	8.27E-02	1.08E-01	NS
SNRPA	NS	4.37E-02	8.27E-02	1.08E-01	NS
LDHC	NS	4.37E-02	-0.0827	1.08E-01	NS
RAB42	NS	4.37E-02	8.27E-02	1.08E-01	NS
GIPC1	NS	4.38E-02	-0.0827	1.08E-01	NS
TTLL3	NS	4.38E-02	-0.0827	1.08E-01	NS
BPIFB2	NS	4.38E-02	-0.0827	1.08E-01	NS
DOK4	NS	4.38E-02	8.27E-02	1.08E-01	NS
PPIAL4G	NS	4.38E-02	8.27E-02	1.08E-01	NS
PPM1G	NS	4.38E-02	-0.0827	1.08E-01	NS
APRT	NS	4.39E-02	-0.0827	1.08E-01	NS
MPZL2	NS	4.39E-02	-0.0826	1.08E-01	NS
MRTFB	NS	4.39E-02	-0.0826	1.08E-01	NS
TAGAP	NS	4.40E-02	8.26E-02	1.08E-01	NS
KCNK3	NS	4.40E-02	-0.0826	1.08E-01	NS
LSM1	NS	4.40E-02	-0.0826	1.08E-01	NS
KANSL3	NS	4.41E-02	8.26E-02	1.08E-01	NS
C12orf73	NS	4.41E-02	-0.0826	1.08E-01	NS
MPP7	NS	4.41E-02	-0.0826	1.08E-01	NS
CHD1	NS	4.41E-02	-0.0826	1.08E-01	NS
SPAG11A	NS	4.41E-02	-0.0826	1.09E-01	NS
AKR7A2	NS	4.41E-02	-0.0825	1.09E-01	NS
RNF139	NS	4.41E-02	8.25E-02	1.09E-01	NS
CCDC85B	NS	4.41E-02	-0.0825	1.09E-01	NS
EPB41	NS	4.42E-02	-0.0825	1.09E-01	NS
RNF31	NS	4.42E-02	-0.0825	1.09E-01	NS
SNTA1	NS	4.43E-02	-0.0825	1.09E-01	NS
PRDM7	NS	4.43E-02	-0.0825	1.09E-01	NS
VDAC1	NS	4.43E-02	-0.0825	1.09E-01	NS
ITPKC	NS	4.43E-02	-0.0825	1.09E-01	NS
BORCS7	NS	4.43E-02	-0.0825	1.09E-01	NS
SYCP3	NS	4.44E-02	-0.0825	1.09E-01	NS
RAET1L	NS	4.44E-02	8.25E-02	1.09E-01	NS
NR2F2	NS	4.44E-02	8.24E-02	1.09E-01	NS
TMEM176B	NS	4.44E-02	8.24E-02	1.09E-01	NS
SIRPD	NS	4.44E-02	8.24E-02	1.09E-01	NS

OR52B4	NS	4.44E-02	-0.0824	1.09E-01	NS
UBR2	NS	4.45E-02	-0.0824	1.09E-01	NS
RAPGEF4	NS	4.45E-02	-0.0824	1.09E-01	NS
CHID1	NS	4.45E-02	-0.0824	1.09E-01	NS
SLC25A17	NS	4.46E-02	-0.0824	1.09E-01	NS
ZBTB9	NS	4.46E-02	-0.0824	1.09E-01	NS
SLC17A2	NS	4.46E-02	-0.0824	1.09E-01	NS
TPPP3	NS	4.46E-02	-0.0824	1.09E-01	NS
GRAMD2B	NS	4.46E-02	-0.0824	1.09E-01	NS
RRP12	NS	4.47E-02	8.23E-02	1.10E-01	NS
MMP11	NS	4.47E-02	8.23E-02	1.10E-01	NS
FLRT3	NS	4.47E-02	-0.0823	1.10E-01	NS
VKORC1	NS	4.47E-02	-0.0823	1.10E-01	NS
THSD7A	NS	4.48E-02	-0.0823	1.10E-01	NS
DDX24	NS	4.48E-02	-0.0823	1.10E-01	NS
TTLL8	NS	4.48E-02	8.23E-02	1.10E-01	NS
TMEM100	NS	4.48E-02	-0.0823	1.10E-01	NS
LAIR1	NS	4.48E-02	-0.0823	1.10E-01	NS
PBRM1	NS	4.49E-02	-0.0823	1.10E-01	NS
TCP11L1	NS	4.49E-02	-0.0822	1.10E-01	NS
TNNC1	NS	4.50E-02	-0.0822	1.10E-01	NS
ANAPC4	NS	4.50E-02	8.22E-02	1.10E-01	NS
XYLT2	NS	4.51E-02	-0.0822	1.10E-01	NS
MUC12	NS	4.51E-02	-0.0822	1.10E-01	NS
DEPP1	NS	4.52E-02	-0.0822	1.11E-01	NS
PDCD10	NS	4.52E-02	-0.0821	1.11E-01	NS
CSTL1	NS	4.52E-02	8.21E-02	1.11E-01	NS
NCAPH	NS	4.53E-02	-0.0821	1.11E-01	NS
RGMA	NS	4.53E-02	-0.0821	1.11E-01	NS
SHISA6	NS	4.54E-02	8.21E-02	1.11E-01	NS
DISP3	NS	4.54E-02	-0.0821	1.11E-01	NS
CENPVL2	NS	4.54E-02	8.21E-02	1.11E-01	NS
DACH2	NS	4.54E-02	-0.0821	1.11E-01	NS
TAOK1	NS	4.54E-02	-0.0821	1.11E-01	NS
PLA2G2C	NS	4.55E-02	-0.082	1.11E-01	NS
MAPK13	NS	4.55E-02	-0.082	1.11E-01	NS
TARBP2	NS	4.55E-02	-0.082	1.11E-01	NS

ZC3H12A	NS	4.55E-02	8.20E-02	1.11E-01	NS
OR8D2	NS	4.55E-02	-0.082	1.11E-01	NS
MROH6	NS	4.55E-02	8.20E-02	1.11E-01	NS
EHHADH	NS	4.56E-02	8.20E-02	1.11E-01	NS
RETNLB	NS	4.56E-02	8.20E-02	1.11E-01	NS
GZMH	NS	4.56E-02	-0.082	1.11E-01	NS
FLT3	NS	4.56E-02	8.20E-02	1.11E-01	NS
BIN2	NS	4.57E-02	-0.082	1.11E-01	NS
THOC5	NS	4.57E-02	-0.082	1.11E-01	NS
STYX	NS	4.57E-02	-0.0819	1.12E-01	NS
C7	NS	4.57E-02	8.19E-02	1.12E-01	NS
STK16	NS	4.58E-02	-0.0819	1.12E-01	NS
CHMP5	NS	4.58E-02	-0.0819	1.12E-01	NS
RABAC1	NS	4.58E-02	-0.0819	1.12E-01	NS
RETREG1	NS	4.58E-02	-0.0819	1.12E-01	NS
RACK1	NS	4.58E-02	-0.0819	1.12E-01	NS
OR5F1	NS	4.58E-02	-0.0819	1.12E-01	NS
NKX6-1	NS	4.59E-02	-0.0819	1.12E-01	NS
UBE2N	NS	4.59E-02	-0.0819	1.12E-01	NS
SYT1	NS	4.60E-02	-0.0819	1.12E-01	NS
PDCL2	NS	4.60E-02	-0.0819	1.12E-01	NS
GMPPB	NS	4.60E-02	-0.0818	1.12E-01	NS
NR2E1	NS	4.60E-02	-0.0818	1.12E-01	NS
ADPRM	NS	4.60E-02	-0.0818	1.12E-01	NS
TRDN	NS	4.61E-02	-0.0818	1.12E-01	NS
BPNT1	NS	4.61E-02	-0.0818	1.12E-01	NS
TSEN34	NS	4.61E-02	8.18E-02	1.12E-01	NS
OR4K13	NS	4.61E-02	-0.0818	1.12E-01	NS
NIPSNAP3A	NS	4.62E-02	-0.0818	1.12E-01	NS
RPL36AL	NS	4.62E-02	-0.0818	1.13E-01	NS
PRDX1	NS	4.62E-02	-0.0817	1.13E-01	NS
RNF125	NS	4.63E-02	-0.0817	1.13E-01	NS
NRM	NS	4.63E-02	8.17E-02	1.13E-01	NS
OR11L1	NS	4.63E-02	-0.0817	1.13E-01	NS
FGFBP3	NS	4.64E-02	-0.0817	1.13E-01	NS
HECW1	NS	4.64E-02	-0.0817	1.13E-01	NS
MC3R	NS	4.65E-02	8.17E-02	1.13E-01	NS

FAM90A19P	NS	4.65E-02	-0.0817	1.13E-01	NS
ZNF316	NS	4.65E-02	8.16E-02	1.13E-01	NS
SVEP1	NS	4.65E-02	-0.0816	1.13E-01	NS
TEX46	NS	4.65E-02	8.16E-02	1.13E-01	NS
PAICS	NS	4.66E-02	-0.0816	1.13E-01	NS
TXNL4A	NS	4.66E-02	8.16E-02	1.13E-01	NS
AMZ2	NS	4.66E-02	8.16E-02	1.13E-01	NS
SEL1L3	NS	4.66E-02	-0.0816	1.13E-01	NS
JCHAIN	NS	4.67E-02	-0.0816	1.13E-01	NS
OSER1	NS	4.66E-02	8.16E-02	1.13E-01	NS
MFGE8	NS	4.67E-02	-0.0816	1.13E-01	NS
CDY1B	NS	4.67E-02	8.16E-02	1.13E-01	NS
ZNF418	NS	4.67E-02	-0.0816	1.13E-01	NS
DIO2	NS	4.67E-02	-0.0816	1.13E-01	NS
HINFP	NS	4.67E-02	-0.0816	1.13E-01	NS
C10orf95	NS	4.67E-02	-0.0816	1.13E-01	NS
FRG2C	NS	4.68E-02	-0.0815	1.14E-01	NS
SPIRE1	NS	4.68E-02	8.15E-02	1.14E-01	NS
SLC22A18	NS	4.68E-02	-0.0815	1.14E-01	NS
ATP5MC3	NS	4.68E-02	-0.0815	1.14E-01	NS
FBXO22	NS	4.68E-02	-0.0815	1.14E-01	NS
TAPT1	NS	4.68E-02	8.15E-02	1.14E-01	NS
TMC5	NS	4.69E-02	-0.0815	1.14E-01	NS
BLNK	NS	4.69E-02	-0.0815	1.14E-01	NS
FAM120AOS	NS	4.69E-02	-0.0815	1.14E-01	NS
C1D	NS	4.69E-02	-0.0815	1.14E-01	NS
GPR42	NS	4.70E-02	-0.0815	1.14E-01	NS
NOP14	NS	4.70E-02	-0.0815	1.14E-01	NS
OR5P2	NS	4.70E-02	-0.0815	1.14E-01	NS
CHCHD7	NS	4.71E-02	-0.0814	1.14E-01	NS
CD164L2	NS	4.71E-02	8.14E-02	1.14E-01	NS
CLEC2L	NS	4.71E-02	8.14E-02	1.14E-01	NS
DARS1	NS	4.72E-02	-0.0814	1.14E-01	NS
SLC11A2	NS	4.72E-02	-0.0814	1.14E-01	NS
HYAL4	NS	4.73E-02	-0.0814	1.14E-01	NS
TYMS	NS	4.73E-02	-0.0814	1.14E-01	NS
RABEP2	NS	4.73E-02	-0.0814	1.14E-01	NS

TAS2R14	NS	4.73E-02	-0.0814	1.14E-01	NS
SPEM3	NS	4.73E-02	8.14E-02	1.14E-01	NS
SPATA17	NS	4.73E-02	-0.0814	1.14E-01	NS
LRRC63	NS	4.73E-02	-0.0814	1.14E-01	NS
FBXO11	NS	4.74E-02	-0.0813	1.15E-01	NS
TMEM263	NS	4.74E-02	-0.0813	1.15E-01	NS
TMEM33	NS	4.74E-02	-0.0813	1.15E-01	NS
MYO1H	NS	4.74E-02	-0.0813	1.15E-01	NS
ZNF343	NS	4.75E-02	-0.0813	1.15E-01	NS
SCAMP1	NS	4.75E-02	-0.0813	1.15E-01	NS
PAGE4	NS	4.75E-02	-0.0813	1.15E-01	NS
NQO1	NS	4.75E-02	-0.0813	1.15E-01	NS
ODF3B	NS	4.76E-02	8.12E-02	1.15E-01	NS
FGB	NS	4.76E-02	-0.0812	1.15E-01	NS
LIPG	NS	4.77E-02	8.12E-02	1.15E-01	NS
PRKAR1A	NS	4.77E-02	-0.0812	1.15E-01	NS
ZNF385C	NS	4.77E-02	-0.0812	1.15E-01	NS
ANXA8L1	NS	4.77E-02	-0.0812	1.15E-01	NS
ZNF304	NS	4.77E-02	-0.0812	1.15E-01	NS
DND1	NS	4.78E-02	-0.0812	1.15E-01	NS
RFT1	NS	4.78E-02	-0.0812	1.15E-01	NS
PRSS37	NS	4.78E-02	-0.0812	1.15E-01	NS
GRHL1	NS	4.79E-02	8.11E-02	1.16E-01	NS
POLH	NS	4.79E-02	8.11E-02	1.16E-01	NS
RARA	NS	4.79E-02	8.11E-02	1.16E-01	NS
STUM	NS	4.79E-02	-0.0811	1.16E-01	NS
H1-2	NS	4.79E-02	-0.0811	1.16E-01	NS
OSBPL8	NS	4.80E-02	-0.0811	1.16E-01	NS
CTSA	NS	4.80E-02	-0.0811	1.16E-01	NS
MBD3	NS	4.80E-02	-0.0811	1.16E-01	NS
TM4SF19	NS	4.80E-02	-0.0811	1.16E-01	NS
SLAMF8	NS	4.80E-02	-0.0811	1.16E-01	NS
SHANK1	NS	4.80E-02	8.11E-02	1.16E-01	NS
RSPH4A	NS	4.80E-02	-0.0811	1.16E-01	NS
TRAF1	NS	4.80E-02	-0.0811	1.16E-01	NS
WDFY4	NS	4.81E-02	-0.0811	1.16E-01	NS
NPTX2	NS	4.81E-02	-0.0811	1.16E-01	NS

FOXI2	NS	4.82E-02	8.10E-02	1.16E-01	NS
SSTR1	NS	4.83E-02	-0.081	1.16E-01	NS
COCH	NS	4.83E-02	-0.081	1.16E-01	NS
MYO7B	NS	4.83E-02	-0.081	1.16E-01	NS
CMTM8	NS	4.84E-02	-0.081	1.16E-01	NS
FGF3	NS	4.84E-02	8.10E-02	1.16E-01	NS
OXA1L	NS	4.84E-02	8.10E-02	1.17E-01	NS
BAG5	NS	4.84E-02	-0.0809	1.17E-01	NS
GPCPD1	NS	4.84E-02	8.09E-02	1.17E-01	NS
CELF4	NS	4.85E-02	-0.0809	1.17E-01	NS
NRROS	NS	4.85E-02	8.09E-02	1.17E-01	NS
MARK2	NS	4.85E-02	8.09E-02	1.17E-01	NS
SLC26A9	NS	4.85E-02	-0.0809	1.17E-01	NS
HSPA1L	NS	4.85E-02	-0.0809	1.17E-01	NS
CTDSPL	NS	4.86E-02	-0.0809	1.17E-01	NS
NCOA7	NS	4.86E-02	-0.0809	1.17E-01	NS
MRPL15	NS	4.86E-02	-0.0809	1.17E-01	NS
IQSEC1	NS	4.86E-02	-0.0809	1.17E-01	NS
UROS	NS	4.86E-02	-0.0809	1.17E-01	NS
PDK2	NS	4.86E-02	8.09E-02	1.17E-01	NS
PRSS12	NS	4.87E-02	-0.0808	1.17E-01	NS
IVNS1ABP	NS	4.88E-02	-0.0808	1.17E-01	NS
IKZF3	NS	4.88E-02	-0.0808	1.17E-01	NS
TNFAIP8L3	NS	4.88E-02	-0.0808	1.17E-01	NS
GOLGA6B	NS	4.88E-02	-0.0808	1.17E-01	NS
DNAH9	NS	4.89E-02	-0.0808	1.17E-01	NS
ZNF66	NS	4.90E-02	-0.0808	1.18E-01	NS
CCDC153	NS	4.90E-02	-0.0808	1.18E-01	NS
METTL5	NS	4.90E-02	-0.0807	1.18E-01	NS
GPR173	NS	4.91E-02	8.07E-02	1.18E-01	NS
GLA	NS	4.91E-02	8.07E-02	1.18E-01	NS
TRIM26	NS	4.91E-02	-0.0807	1.18E-01	NS
BAG4	NS	4.92E-02	8.07E-02	1.18E-01	NS
HPS3	NS	4.92E-02	-0.0807	1.18E-01	NS
RHOT1	NS	4.92E-02	-0.0807	1.18E-01	NS
CCL2	NS	4.93E-02	-0.0806	1.18E-01	NS
LAMTOR3	NS	4.93E-02	-0.0806	1.18E-01	NS

SNRNP48	NS	4.93E-02	-0.0807	1.18E-01	NS
CUTA	NS	4.93E-02	-0.0806	1.18E-01	NS
CR2	NS	4.93E-02	-0.0806	1.18E-01	NS
TNFAIP1	NS	4.94E-02	-0.0806	1.18E-01	NS
R3HDM2	NS	4.94E-02	8.06E-02	1.18E-01	NS
ZC2HC1C	NS	4.94E-02	-0.0806	1.18E-01	NS
XIAP	NS	4.94E-02	8.06E-02	1.18E-01	NS
SERPINF1	NS	4.94E-02	-0.0806	1.18E-01	NS
BTBD2	NS	4.95E-02	-0.0806	1.19E-01	NS
POFUT2	NS	4.95E-02	8.06E-02	1.19E-01	NS
TMEM53	NS	4.96E-02	-0.0805	1.19E-01	NS
DCUN1D1	NS	4.97E-02	-0.0805	1.19E-01	NS
PLD6	NS	4.96E-02	-0.0805	1.19E-01	NS
FAM156B	NS	4.97E-02	-0.0805	1.19E-01	NS
RBM20	NS	4.97E-02	-0.0805	1.19E-01	NS
ITCH	NS	4.98E-02	-0.0805	1.19E-01	NS
HMBOX1	NS	4.98E-02	-0.0805	1.19E-01	NS
PRPH2	NS	4.98E-02	-0.0805	1.19E-01	NS
INHBE	NS	4.99E-02	8.04E-02	1.19E-01	NS
CYBB	NS	4.99E-02	-0.0804	1.19E-01	NS
CDC42BPA	NS	4.99E-02	-0.0804	1.19E-01	NS
FRMPD4	NS	5.00E-02	-0.0804	1.20E-01	NS
LGI1	NS	5.01E-02	8.04E-02	1.20E-01	NS
VCY	NS	5.01E-02	8.04E-02	1.20E-01	NS
ATP6V0A2	NS	5.01E-02	-0.0804	1.20E-01	NS
SEPTIN1	NS	5.01E-02	-0.0803	1.20E-01	NS
PWWP3A	NS	5.02E-02	-0.0803	1.20E-01	NS
CYB5A	NS	5.02E-02	-0.0803	1.20E-01	NS
RFPL4B	NS	5.02E-02	-0.0803	1.20E-01	NS
PPP4R3C	NS	5.02E-02	-0.0803	1.20E-01	NS
FCMR	NS	5.03E-02	-0.0803	1.20E-01	NS
IP6K3	NS	5.03E-02	-0.0803	1.20E-01	NS
UGT2B15	NS	5.04E-02	-0.0803	1.20E-01	NS
BICD2	NS	5.04E-02	8.03E-02	1.20E-01	NS
TEPP	NS	5.04E-02	8.02E-02	1.20E-01	NS
KLK7	NS	5.05E-02	-0.0802	1.20E-01	NS
CCDC137	NS	5.04E-02	8.02E-02	1.20E-01	NS

IFNL3	NS	5.05E-02	8.02E-02	1.20E-01	NS
PAOX	NS	5.05E-02	-0.0802	1.20E-01	NS
MYO19	NS	5.05E-02	-0.0802	1.20E-01	NS
OR2T35	NS	5.05E-02	-0.0802	1.21E-01	NS
KLRC3	NS	5.06E-02	-0.0802	1.21E-01	NS
SLC6A12	NS	5.06E-02	8.02E-02	1.21E-01	NS
SEC61G	NS	5.06E-02	-0.0802	1.21E-01	NS
FHOD1	NS	5.06E-02	8.02E-02	1.21E-01	NS
OR2Y1	NS	5.06E-02	-0.0802	1.21E-01	NS
HKDC1	NS	5.07E-02	8.02E-02	1.21E-01	NS
HNRNPH1	NS	5.07E-02	-0.0802	1.21E-01	NS
SYK	NS	5.07E-02	-0.0801	1.21E-01	NS
KCTD11	NS	5.07E-02	-0.0801	1.21E-01	NS
GABRA2	NS	5.07E-02	-0.0801	1.21E-01	NS
KHDC4	NS	5.08E-02	8.01E-02	1.21E-01	NS
H4C2	NS	5.08E-02	8.01E-02	1.21E-01	NS
FGD6	NS	5.08E-02	-0.0801	1.21E-01	NS
DNAJB12	NS	5.08E-02	-0.0801	1.21E-01	NS
MRPS11	NS	5.08E-02	-0.0801	1.21E-01	NS
CT47A6	NS	5.09E-02	8.01E-02	1.21E-01	NS
ZDHHC11	NS	5.09E-02	-0.0801	1.21E-01	NS
CYB5R1	NS	5.09E-02	8.01E-02	1.21E-01	NS
WDR43	NS	5.09E-02	8.01E-02	1.21E-01	NS
CCDC186	NS	5.09E-02	8.01E-02	1.21E-01	NS
NAMPT	NS	5.09E-02	-0.0801	1.21E-01	NS
ASTL	NS	5.10E-02	-0.0801	1.21E-01	NS
CLCN3	NS	5.11E-02	-0.08	1.21E-01	NS
ENAH	NS	5.11E-02	8.00E-02	1.22E-01	NS
TLR5	NS	5.11E-02	8.00E-02	1.22E-01	NS
PHLDB1	NS	5.12E-02	-0.08	1.22E-01	NS
ACOX1	NS	5.12E-02	-0.08	1.22E-01	NS
PARP1	NS	5.12E-02	-0.08	1.22E-01	NS
HNRNPA2B1	NS	5.12E-02	-0.08	1.22E-01	NS
BTBD7	NS	5.12E-02	-0.08	1.22E-01	NS
SHF	NS	5.12E-02	-0.08	1.22E-01	NS
SSU72P3	NS	5.13E-02	7.99E-02	1.22E-01	NS
CIART	NS	5.13E-02	-0.0799	1.22E-01	NS

MYBPC2	NS	5.13E-02	7.99E-02	1.22E-01	NS
CRYBG1	NS	5.13E-02	7.99E-02	1.22E-01	NS
SNAP29	NS	5.14E-02	7.99E-02	1.22E-01	NS
POLR2A	NS	5.14E-02	7.99E-02	1.22E-01	NS
PRRC1	NS	5.14E-02	7.99E-02	1.22E-01	NS
DIABLO	NS	5.14E-02	7.99E-02	1.22E-01	NS
SESTD1	NS	5.14E-02	-0.0799	1.22E-01	NS
KCNC2	NS	5.15E-02	-0.0799	1.22E-01	NS
SLC25A40	NS	5.15E-02	-0.0799	1.22E-01	NS
C14orf28	NS	5.15E-02	-0.0799	1.22E-01	NS
DHPS	NS	5.16E-02	-0.0798	1.22E-01	NS
AMY1C	NS	5.16E-02	-0.0798	1.22E-01	NS
OCA2	NS	5.16E-02	-0.0798	1.22E-01	NS
DEFB126	NS	5.17E-02	-0.0798	1.23E-01	NS
APTX	NS	5.17E-02	-0.0798	1.23E-01	NS
WDR88	NS	5.17E-02	7.98E-02	1.23E-01	NS
C15orf61	NS	5.17E-02	7.98E-02	1.23E-01	NS
CREB3L1	NS	5.17E-02	7.98E-02	1.23E-01	NS
SERPINB8	NS	5.17E-02	-0.0798	1.23E-01	NS
LRRD1	NS	5.18E-02	-0.0798	1.23E-01	NS
DDIAS	NS	5.18E-02	7.98E-02	1.23E-01	NS
CIBAR2	NS	5.18E-02	-0.0798	1.23E-01	NS
WFDC12	NS	5.18E-02	-0.0798	1.23E-01	NS
OR10J1	NS	5.19E-02	7.97E-02	1.23E-01	NS
NKX3-2	NS	5.19E-02	7.97E-02	1.23E-01	NS
ZNF43	NS	5.19E-02	-0.0797	1.23E-01	NS
METTL2A	NS	5.19E-02	-0.0797	1.23E-01	NS
TRIM3	NS	5.19E-02	7.97E-02	1.23E-01	NS
ATXN7L1	NS	5.19E-02	7.97E-02	1.23E-01	NS
C20orf96	NS	5.20E-02	-0.0797	1.23E-01	NS
RASD1	NS	5.20E-02	-0.0797	1.23E-01	NS
CRISP2	NS	5.20E-02	-0.0797	1.23E-01	NS
USP9Y	NS	5.21E-02	-0.0797	1.23E-01	NS
EDRF1	NS	5.21E-02	7.97E-02	1.23E-01	NS
F11R	NS	5.21E-02	-0.0797	1.23E-01	NS
ELF2	NS	5.22E-02	7.96E-02	1.23E-01	NS
OTOG	NS	5.22E-02	-0.0796	1.23E-01	NS

MYSM1	NS	5.22E-02	-0.0796	1.23E-01	NS
BRI3	NS	5.22E-02	7.96E-02	1.23E-01	NS
KSR2	NS	5.22E-02	-0.0796	1.23E-01	NS
SLC6A14	NS	5.22E-02	-0.0796	1.23E-01	NS
TRAPPC3L	NS	5.22E-02	-0.0796	1.23E-01	NS
SPRY2	NS	5.22E-02	-0.0796	1.23E-01	NS
GDF3	NS	5.22E-02	7.96E-02	1.23E-01	NS
TMEM256	NS	5.22E-02	-0.0796	1.23E-01	NS
LCE1A	NS	5.22E-02	7.96E-02	1.23E-01	NS
C16orf92	NS	5.23E-02	7.96E-02	1.24E-01	NS
UGCG	NS	5.24E-02	-0.0796	1.24E-01	NS
OR51B4	NS	5.24E-02	-0.0796	1.24E-01	NS
DYNC1I1	NS	5.24E-02	-0.0796	1.24E-01	NS
EED	NS	5.24E-02	-0.0796	1.24E-01	NS
CFAP73	NS	5.25E-02	-0.0795	1.24E-01	NS
TNNT3	NS	5.25E-02	7.95E-02	1.24E-01	NS
SEZ6	NS	5.26E-02	-0.0795	1.24E-01	NS
BTG2	NS	5.26E-02	-0.0795	1.24E-01	NS
CHAC2	NS	5.26E-02	-0.0795	1.24E-01	NS
CPEB2	NS	5.27E-02	-0.0795	1.24E-01	NS
DDI2	NS	5.27E-02	7.95E-02	1.24E-01	NS
PEBP4	NS	5.27E-02	7.95E-02	1.24E-01	NS
MCTS2P	NS	5.28E-02	-0.0794	1.24E-01	NS
ZSCAN29	NS	5.28E-02	-0.0794	1.25E-01	NS
TRIM52	NS	5.28E-02	-0.0794	1.25E-01	NS
ARHGAP20	NS	5.29E-02	-0.0794	1.25E-01	NS
C11orf87	NS	5.29E-02	-0.0794	1.25E-01	NS
OR8B3	NS	5.29E-02	-0.0794	1.25E-01	NS
RRM1	NS	5.29E-02	7.94E-02	1.25E-01	NS
ERCC4	NS	5.30E-02	-0.0794	1.25E-01	NS
CCR8	NS	5.30E-02	7.94E-02	1.25E-01	NS
ALPK3	NS	5.30E-02	-0.0794	1.25E-01	NS
SERPINF2	NS	5.30E-02	-0.0794	1.25E-01	NS
C17orf78	NS	5.30E-02	-0.0794	1.25E-01	NS
GSDMC	NS	5.31E-02	-0.0793	1.25E-01	NS
ETV3	NS	5.31E-02	7.93E-02	1.25E-01	NS
ZNF829	NS	5.31E-02	-0.0793	1.25E-01	NS

ZNF136	NS	5.32E-02	-0.0793	1.25E-01	NS
SLC25A38	NS	5.32E-02	-0.0793	1.25E-01	NS
LRRC2	NS	5.32E-02	-0.0793	1.25E-01	NS
TTC28	NS	5.32E-02	-0.0793	1.25E-01	NS
HSBP1L1	NS	5.32E-02	-0.0793	1.25E-01	NS
GARS1	NS	5.33E-02	7.93E-02	1.25E-01	NS
RNF112	NS	5.33E-02	7.93E-02	1.25E-01	NS
GPR158	NS	5.33E-02	-0.0793	1.25E-01	NS
ANKS3	NS	5.33E-02	7.93E-02	1.25E-01	NS
ADRB2	NS	5.33E-02	7.93E-02	1.25E-01	NS
SCMH1	NS	5.33E-02	-0.0793	1.25E-01	NS
KDM5A	NS	5.34E-02	-0.0792	1.25E-01	NS
OTOS	NS	5.34E-02	7.92E-02	1.26E-01	NS
ARHGAP25	NS	5.34E-02	-0.0792	1.26E-01	NS
SEMA7A	NS	5.34E-02	-0.0792	1.26E-01	NS
ICAM3	NS	5.35E-02	-0.0792	1.26E-01	NS
SLC27A5	NS	5.35E-02	-0.0792	1.26E-01	NS
NR1H4	NS	5.35E-02	-0.0792	1.26E-01	NS
RASA4B	NS	5.35E-02	7.92E-02	1.26E-01	NS
ZC3H7B	NS	5.35E-02	7.92E-02	1.26E-01	NS
P2RY2	NS	5.35E-02	-0.0792	1.26E-01	NS
SPDYE16	NS	5.36E-02	7.92E-02	1.26E-01	NS
OR51B5	NS	5.36E-02	-0.0792	1.26E-01	NS
HRK	NS	5.36E-02	-0.0792	1.26E-01	NS
JADE1	NS	5.36E-02	-0.0792	1.26E-01	NS
G6PC1	NS	5.36E-02	-0.0792	1.26E-01	NS
SNRK	NS	5.36E-02	-0.0792	1.26E-01	NS
FAU	NS	5.37E-02	-0.0791	1.26E-01	NS
PTPRK	NS	5.37E-02	-0.0791	1.26E-01	NS
FHL3	NS	5.37E-02	7.91E-02	1.26E-01	NS
CT47A7	NS	5.37E-02	7.91E-02	1.26E-01	NS
KIAA1671	NS	5.37E-02	7.91E-02	1.26E-01	NS
HAUS2	NS	5.38E-02	-0.0791	1.26E-01	NS
JADE3	NS	5.39E-02	-0.0791	1.26E-01	NS
PRB3	NS	5.39E-02	-0.0791	1.26E-01	NS
DMAP1	NS	5.40E-02	-0.079	1.27E-01	NS
MICOS10	NS	5.41E-02	-0.079	1.27E-01	NS

USF2	NS	5.41E-02	7.90E-02	1.27E-01	NS
SIT1	NS	5.41E-02	-0.079	1.27E-01	NS
SC5D	NS	5.41E-02	-0.079	1.27E-01	NS
NHS	NS	5.41E-02	-0.079	1.27E-01	NS
MCRIP1	NS	5.41E-02	-0.079	1.27E-01	NS
PCCB	NS	5.41E-02	-0.079	1.27E-01	NS
EMILIN3	NS	5.41E-02	-0.079	1.27E-01	NS
PXK	NS	5.42E-02	7.90E-02	1.27E-01	NS
MARCHF6	NS	5.42E-02	-0.079	1.27E-01	NS
INSYN1	NS	5.42E-02	7.90E-02	1.27E-01	NS
SNX2	NS	5.43E-02	7.89E-02	1.27E-01	NS
ZNF138	NS	5.44E-02	-0.0789	1.27E-01	NS
HNF1B	NS	5.44E-02	-0.0789	1.27E-01	NS
FYTTD1	NS	5.44E-02	7.89E-02	1.27E-01	NS
HSDL2	NS	5.45E-02	-0.0789	1.27E-01	NS
VPS39	NS	5.45E-02	-0.0789	1.28E-01	NS
ST7	NS	5.45E-02	-0.0789	1.28E-01	NS
BOLA3	NS	5.45E-02	-0.0789	1.28E-01	NS
FAAP24	NS	5.45E-02	-0.0789	1.28E-01	NS
GALNS	NS	5.45E-02	7.89E-02	1.28E-01	NS
GMPS	NS	5.46E-02	-0.0789	1.28E-01	NS
CGRRF1	NS	5.46E-02	-0.0788	1.28E-01	NS
SEPTIN10	NS	5.46E-02	-0.0788	1.28E-01	NS
GNB2	NS	5.47E-02	7.88E-02	1.28E-01	NS
PDCD1LG2	NS	5.47E-02	-0.0788	1.28E-01	NS
LRRC10B	NS	5.48E-02	7.88E-02	1.28E-01	NS
ZNF839	NS	5.49E-02	-0.0788	1.28E-01	NS
PDIA6	NS	5.49E-02	-0.0788	1.28E-01	NS
PSCA	NS	5.49E-02	-0.0787	1.28E-01	NS
SYNGAP1	NS	5.49E-02	-0.0787	1.28E-01	NS
CAP1	NS	5.49E-02	-0.0787	1.28E-01	NS
SRD5A3	NS	5.50E-02	-0.0787	1.28E-01	NS
BCHE	NS	5.50E-02	-0.0787	1.28E-01	NS
CLEC11A	NS	5.51E-02	-0.0787	1.29E-01	NS
OR2B3	NS	5.51E-02	7.87E-02	1.29E-01	NS
DUXB	NS	5.51E-02	-0.0787	1.29E-01	NS
PHKA2	NS	5.51E-02	7.87E-02	1.29E-01	NS

RETREG2	NS	5.52E-02	-0.0787	1.29E-01	NS
SRPK1	NS	5.52E-02	7.87E-02	1.29E-01	NS
TCAP	NS	5.52E-02	-0.0786	1.29E-01	NS
SLC22A6	NS	5.52E-02	7.86E-02	1.29E-01	NS
KIF16B	NS	5.52E-02	-0.0786	1.29E-01	NS
CES4A	NS	5.53E-02	-0.0786	1.29E-01	NS
FAT2	NS	5.54E-02	-0.0786	1.29E-01	NS
PRAMEF6	NS	5.54E-02	-0.0786	1.29E-01	NS
ZSCAN1	NS	5.55E-02	7.85E-02	1.29E-01	NS
RANBP2	NS	5.56E-02	7.85E-02	1.30E-01	NS
YWHAB	NS	5.57E-02	-0.0785	1.30E-01	NS
TINF2	NS	5.57E-02	7.85E-02	1.30E-01	NS
ZDHHC18	NS	5.57E-02	-0.0785	1.30E-01	NS
XRCC6	NS	5.57E-02	-0.0785	1.30E-01	NS
C15orf48	NS	5.58E-02	-0.0785	1.30E-01	NS
TEX28	NS	5.59E-02	-0.0784	1.30E-01	NS
ANXA7	NS	5.59E-02	-0.0784	1.30E-01	NS
ARPP21	NS	5.59E-02	-0.0784	1.30E-01	NS
ARVCF	NS	5.60E-02	-0.0784	1.30E-01	NS
CFAP77	NS	5.60E-02	-0.0784	1.30E-01	NS
CTNNA1	NS	5.60E-02	-0.0784	1.30E-01	NS
CCDC9B	NS	5.60E-02	-0.0784	1.30E-01	NS
CA7	NS	5.61E-02	7.84E-02	1.30E-01	NS
CES3	NS	5.61E-02	7.84E-02	1.30E-01	NS
SULT1E1	NS	5.61E-02	-0.0784	1.30E-01	NS
ZFHX4	NS	5.61E-02	-0.0783	1.31E-01	NS
DEFB136	NS	5.62E-02	7.83E-02	1.31E-01	NS
NME9	NS	5.63E-02	-0.0783	1.31E-01	NS
PRDX4	NS	5.63E-02	-0.0783	1.31E-01	NS
PRR23D1	NS	5.63E-02	-0.0783	1.31E-01	NS
DAP	NS	5.63E-02	7.83E-02	1.31E-01	NS
IL11RA	NS	5.64E-02	7.83E-02	1.31E-01	NS
GRK1	NS	5.64E-02	-0.0783	1.31E-01	NS
DCP2	NS	5.64E-02	-0.0783	1.31E-01	NS
KCNH4	NS	5.64E-02	7.83E-02	1.31E-01	NS
CAPZA3	NS	5.65E-02	7.82E-02	1.31E-01	NS
TMEM161A	NS	5.65E-02	-0.0782	1.31E-01	NS

STAR	NS	5.65E-02	-0.0782	1.31E-01	NS
SLA2	NS	5.65E-02	7.82E-02	1.31E-01	NS
RETN	NS	5.65E-02	7.82E-02	1.31E-01	NS
CLP1	NS	5.65E-02	-0.0782	1.31E-01	NS
SNRPD1	NS	5.67E-02	-0.0782	1.32E-01	NS
INTS11	NS	5.67E-02	-0.0782	1.32E-01	NS
OR4C11	NS	5.67E-02	-0.0782	1.32E-01	NS
ZNF276	NS	5.67E-02	-0.0782	1.32E-01	NS
JAK1	NS	5.67E-02	-0.0782	1.32E-01	NS
GLYATL3	NS	5.67E-02	-0.0782	1.32E-01	NS
ZSWIM6	NS	5.68E-02	-0.0781	1.32E-01	NS
GHSR	NS	5.68E-02	-0.0781	1.32E-01	NS
SEMA4B	NS	5.68E-02	7.81E-02	1.32E-01	NS
IMPA1	NS	5.68E-02	-0.0781	1.32E-01	NS
RUSF1	NS	5.68E-02	7.81E-02	1.32E-01	NS
ARHGAP4	NS	5.69E-02	-0.0781	1.32E-01	NS
SGK3	NS	5.69E-02	-0.0781	1.32E-01	NS
SNRNP27	NS	5.69E-02	-0.0781	1.32E-01	NS
HS3ST2	NS	5.69E-02	-0.0781	1.32E-01	NS
KIF13A	NS	5.69E-02	7.81E-02	1.32E-01	NS
KCNJ18	NS	5.69E-02	7.81E-02	1.32E-01	NS
PIM2	NS	5.70E-02	-0.0781	1.32E-01	NS
SPIN1	NS	5.70E-02	-0.0781	1.32E-01	NS
AFDN	NS	5.70E-02	-0.0781	1.32E-01	NS
CTAGE9	NS	5.70E-02	-0.0781	1.32E-01	NS
KRT37	NS	5.71E-02	-0.0781	1.32E-01	NS
RWDD3	NS	5.70E-02	-0.0781	1.32E-01	NS
NEUROD2	NS	5.71E-02	7.81E-02	1.32E-01	NS
STK3	NS	5.71E-02	-0.078	1.32E-01	NS
ZNF248	NS	5.71E-02	-0.078	1.32E-01	NS
BCKDHA	NS	5.71E-02	7.80E-02	1.32E-01	NS
PCSK1	NS	5.71E-02	-0.078	1.32E-01	NS
BOP1	NS	5.72E-02	-0.078	1.32E-01	NS
ZNF717	NS	5.72E-02	-0.078	1.32E-01	NS
SFN	NS	5.72E-02	7.80E-02	1.32E-01	NS
ADGRL1	NS	5.72E-02	-0.078	1.32E-01	NS
CCDC163	NS	5.72E-02	-0.078	1.32E-01	NS

PRKAR2A	NS	5.73E-02	-0.078	1.33E-01	NS
ZNF774	NS	5.73E-02	7.80E-02	1.33E-01	NS
KLHL40	NS	5.73E-02	7.80E-02	1.33E-01	NS
UNKL	NS	5.74E-02	-0.078	1.33E-01	NS
ACSM2A	NS	5.74E-02	-0.078	1.33E-01	NS
UCMA	NS	5.74E-02	-0.0779	1.33E-01	NS
PRH1	NS	5.74E-02	-0.0779	1.33E-01	NS
COX7B	NS	5.74E-02	-0.0779	1.33E-01	NS
SPATA5L1	NS	5.75E-02	7.79E-02	1.33E-01	NS
EMC7	NS	5.75E-02	-0.0779	1.33E-01	NS
CC2D2B	NS	5.75E-02	-0.0779	1.33E-01	NS
FNIP2	NS	5.75E-02	-0.0779	1.33E-01	NS
RNF175	NS	5.75E-02	-0.0779	1.33E-01	NS
HCAR2	NS	5.75E-02	7.79E-02	1.33E-01	NS
ARRB2	NS	5.75E-02	7.79E-02	1.33E-01	NS
POLR3A	NS	5.75E-02	-0.0779	1.33E-01	NS
UPF2	NS	5.75E-02	7.79E-02	1.33E-01	NS
NF2	NS	5.75E-02	-0.0779	1.33E-01	NS
SLC38A5	NS	5.76E-02	-0.0779	1.33E-01	NS
PABPC1L	NS	5.77E-02	-0.0779	1.33E-01	NS
TRHDE	NS	5.77E-02	-0.0779	1.33E-01	NS
CHP1	NS	5.78E-02	7.78E-02	1.33E-01	NS
ABRACL	NS	5.78E-02	-0.0778	1.33E-01	NS
TMEM168	NS	5.79E-02	-0.0778	1.33E-01	NS
TFAP2D	NS	5.79E-02	-0.0778	1.34E-01	NS
GPX4	NS	5.80E-02	-0.0778	1.34E-01	NS
PATZ1	NS	5.80E-02	-0.0778	1.34E-01	NS
CD6	NS	5.81E-02	-0.0777	1.34E-01	NS
DLST	NS	5.82E-02	7.77E-02	1.34E-01	NS
UBE2E1	NS	5.82E-02	-0.0777	1.34E-01	NS
MINDY3	NS	5.82E-02	-0.0777	1.34E-01	NS
UGT1A1	NS	5.82E-02	-0.0777	1.34E-01	NS
DNAJC6	NS	5.83E-02	-0.0777	1.34E-01	NS
DDX55	NS	5.83E-02	-0.0777	1.34E-01	NS
SLC9A9	NS	5.83E-02	-0.0777	1.34E-01	NS
FANCB	NS	5.83E-02	-0.0777	1.34E-01	NS
RANBP9	NS	5.84E-02	-0.0776	1.34E-01	NS

CEACAM1	NS	5.85E-02	-0.0776	1.35E-01	NS
KRT222	NS	5.85E-02	-0.0776	1.35E-01	NS
LRFN2	NS	5.85E-02	7.76E-02	1.35E-01	NS
NUP160	NS	5.85E-02	-0.0776	1.35E-01	NS
TEX54	NS	5.85E-02	-0.0776	1.35E-01	NS
ESYT3	NS	5.86E-02	7.76E-02	1.35E-01	NS
OR6C3	NS	5.86E-02	-0.0776	1.35E-01	NS
PUS7	NS	5.86E-02	-0.0776	1.35E-01	NS
RPS6KA2	NS	5.87E-02	-0.0776	1.35E-01	NS
TEX264	NS	5.87E-02	-0.0776	1.35E-01	NS
C11orf91	NS	5.87E-02	7.76E-02	1.35E-01	NS
AHCYL1	NS	5.87E-02	-0.0775	1.35E-01	NS
ECPAS	NS	5.88E-02	-0.0775	1.35E-01	NS
PPP2R5E	NS	5.88E-02	7.75E-02	1.35E-01	NS
USP5	NS	5.88E-02	-0.0775	1.35E-01	NS
CXorf51A	NS	5.88E-02	7.75E-02	1.35E-01	NS
FRYL	NS	5.89E-02	-0.0775	1.35E-01	NS
TRIM54	NS	5.89E-02	7.75E-02	1.35E-01	NS
DNASE2	NS	5.89E-02	-0.0775	1.35E-01	NS
CNPPD1	NS	5.89E-02	7.75E-02	1.35E-01	NS
PLAGL1	NS	5.89E-02	-0.0775	1.35E-01	NS
C1orf43	NS	5.89E-02	-0.0775	1.35E-01	NS
C1RL	NS	5.90E-02	-0.0775	1.35E-01	NS
SSRP1	NS	5.90E-02	-0.0775	1.35E-01	NS
MEX3B	NS	5.90E-02	7.75E-02	1.35E-01	NS
CLK4	NS	5.90E-02	-0.0775	1.35E-01	NS
EXOSC4	NS	5.90E-02	7.75E-02	1.35E-01	NS
SHROOM1	NS	5.90E-02	-0.0774	1.35E-01	NS
PDZD4	NS	5.90E-02	7.74E-02	1.35E-01	NS
PEG3	NS	5.91E-02	-0.0774	1.35E-01	NS
F2	NS	5.91E-02	-0.0774	1.36E-01	NS
ETV1	NS	5.91E-02	-0.0774	1.36E-01	NS
GUCY1A1	NS	5.91E-02	-0.0774	1.36E-01	NS
ACSF3	NS	5.91E-02	7.74E-02	1.36E-01	NS
MUSTN1	NS	5.91E-02	-0.0774	1.36E-01	NS
CTR9	NS	5.92E-02	-0.0774	1.36E-01	NS
DDX18	NS	5.92E-02	-0.0774	1.36E-01	NS

TNNT2	NS	5.93E-02	7.74E-02	1.36E-01	NS
PCSK5	NS	5.93E-02	7.74E-02	1.36E-01	NS
IL15RA	NS	5.93E-02	-0.0774	1.36E-01	NS
ZNF688	NS	5.93E-02	-0.0774	1.36E-01	NS
SWT1	NS	5.93E-02	-0.0774	1.36E-01	NS
BLOC1S4	NS	5.94E-02	-0.0773	1.36E-01	NS
EVA1A	NS	5.94E-02	-0.0773	1.36E-01	NS
REG3G	NS	5.94E-02	-0.0773	1.36E-01	NS
PLAU	NS	5.94E-02	-0.0773	1.36E-01	NS
CCDC181	NS	5.94E-02	-0.0773	1.36E-01	NS
FLG	NS	5.95E-02	-0.0773	1.36E-01	NS
RIPK1	NS	5.95E-02	-0.0773	1.36E-01	NS
KIF1A	NS	5.95E-02	-0.0773	1.36E-01	NS
PYGB	NS	5.98E-02	-0.0772	1.37E-01	NS
TBATA	NS	5.98E-02	7.72E-02	1.37E-01	NS
CPTP	NS	5.98E-02	7.72E-02	1.37E-01	NS
ELMO3	NS	5.98E-02	-0.0772	1.37E-01	NS
CFAP410	NS	5.98E-02	7.72E-02	1.37E-01	NS
SPDYE12	NS	5.98E-02	-0.0772	1.37E-01	NS
LYST	NS	5.99E-02	-0.0772	1.37E-01	NS
STAU2	NS	6.01E-02	-0.0771	1.37E-01	NS
COA5	NS	6.01E-02	-0.0771	1.37E-01	NS
C11orf24	NS	6.01E-02	7.71E-02	1.37E-01	NS
SEC16B	NS	6.01E-02	-0.0771	1.37E-01	NS
NPM1	NS	6.01E-02	-0.0771	1.37E-01	NS
TRPM6	NS	6.01E-02	-0.0771	1.37E-01	NS
DDC	NS	6.01E-02	7.71E-02	1.37E-01	NS
CCDC144A	NS	6.02E-02	-0.0771	1.38E-01	NS
MYL1	NS	6.02E-02	-0.0771	1.38E-01	NS
AREG	NS	6.02E-02	-0.0771	1.38E-01	NS
PIAS1	NS	6.03E-02	7.71E-02	1.38E-01	NS
LRRC55	NS	6.03E-02	-0.0771	1.38E-01	NS
ITSN1	NS	6.03E-02	-0.0771	1.38E-01	NS
TATDN1	NS	6.03E-02	-0.0771	1.38E-01	NS
KDF1	NS	6.03E-02	-0.0771	1.38E-01	NS
BCAM	NS	6.03E-02	7.71E-02	1.38E-01	NS
TLL1	NS	6.04E-02	-0.077	1.38E-01	NS

HOXD3	NS	6.04E-02	-0.077	1.38E-01	NS
ACP4	NS	6.04E-02	-0.077	1.38E-01	NS
RNF220	NS	6.04E-02	-0.077	1.38E-01	NS
ZNF554	NS	6.04E-02	-0.077	1.38E-01	NS
KISS1R	NS	6.04E-02	7.70E-02	1.38E-01	NS
RND2	NS	6.05E-02	-0.077	1.38E-01	NS
PRELID3B	NS	6.05E-02	-0.077	1.38E-01	NS
ELOC	NS	6.05E-02	-0.077	1.38E-01	NS
GOLGA8G	NS	6.05E-02	-0.077	1.38E-01	NS
TEAD1	NS	6.05E-02	-0.077	1.38E-01	NS
FGD4	NS	6.06E-02	-0.077	1.38E-01	NS
CINP	NS	6.06E-02	-0.077	1.38E-01	NS
FLNB	NS	6.06E-02	-0.077	1.38E-01	NS
CIB3	NS	6.06E-02	7.70E-02	1.38E-01	NS
CDC20B	NS	6.06E-02	-0.077	1.38E-01	NS
GRINA	NS	6.06E-02	7.70E-02	1.38E-01	NS
CARM1	NS	6.07E-02	-0.077	1.38E-01	NS
XAF1	NS	6.07E-02	-0.0769	1.38E-01	NS
RSRP1	NS	6.07E-02	7.69E-02	1.38E-01	NS
CCN6	NS	6.07E-02	-0.0769	1.38E-01	NS
SUPT7L	NS	6.07E-02	-0.0769	1.38E-01	NS
PABPC1L2A	NS	6.07E-02	7.69E-02	1.38E-01	NS
RPS6KL1	NS	6.07E-02	-0.0769	1.38E-01	NS
ZNF536	NS	6.07E-02	-0.0769	1.38E-01	NS
SCN2A	NS	6.08E-02	7.69E-02	1.38E-01	NS
CHST9	NS	6.08E-02	-0.0769	1.38E-01	NS
ST6GALNAC5	NS	6.08E-02	-0.0769	1.38E-01	NS
MRPL30	NS	6.08E-02	-0.0769	1.38E-01	NS
ABCA7	NS	6.09E-02	-0.0769	1.38E-01	NS
TCP11L2	NS	6.09E-02	-0.0769	1.38E-01	NS
KRT72	NS	6.09E-02	-0.0769	1.38E-01	NS
HTR1A	NS	6.09E-02	7.69E-02	1.39E-01	NS
LBHD2	NS	6.10E-02	7.69E-02	1.39E-01	NS
EHMT2	NS	6.10E-02	7.69E-02	1.39E-01	NS
SNX6	NS	6.11E-02	7.68E-02	1.39E-01	NS
ADGRF1	NS	6.11E-02	-0.0768	1.39E-01	NS
HMBS	NS	6.11E-02	-0.0768	1.39E-01	NS

CLCN1	NS	6.12E-02	-0.0768	1.39E-01	NS
PDCD1	NS	6.13E-02	-0.0768	1.39E-01	NS
SUPT6H	NS	6.13E-02	-0.0768	1.39E-01	NS
ANXA6	NS	6.14E-02	7.67E-02	1.39E-01	NS
ADGRB3	NS	6.14E-02	7.67E-02	1.39E-01	NS
RECQL	NS	6.14E-02	-0.0767	1.39E-01	NS
LRP6	NS	6.14E-02	-0.0767	1.39E-01	NS
GORASP1	NS	6.14E-02	-0.0767	1.39E-01	NS
SAMD7	NS	6.14E-02	-0.0767	1.39E-01	NS
FOXG1	NS	6.15E-02	7.67E-02	1.40E-01	NS
ZNF429	NS	6.15E-02	-0.0767	1.40E-01	NS
TGOLN2	NS	6.16E-02	-0.0767	1.40E-01	NS
OR4F3	NS	6.17E-02	-0.0767	1.40E-01	NS
LUZP4	NS	6.17E-02	-0.0766	1.40E-01	NS
DRAXIN	NS	6.17E-02	-0.0766	1.40E-01	NS
HEATR4	NS	6.17E-02	-0.0766	1.40E-01	NS
ABLIM2	NS	6.17E-02	7.66E-02	1.40E-01	NS
RPRM	NS	6.18E-02	7.66E-02	1.40E-01	NS
NUDT17	NS	6.19E-02	-0.0766	1.40E-01	NS
CD300LF	NS	6.19E-02	7.66E-02	1.40E-01	NS
FAM124B	NS	6.20E-02	7.66E-02	1.40E-01	NS
ZFP36L1	NS	6.20E-02	7.66E-02	1.40E-01	NS
NDUFA5	NS	6.20E-02	-0.0766	1.40E-01	NS
NKD2	NS	6.21E-02	7.65E-02	1.41E-01	NS
MS4A7	NS	6.21E-02	-0.0765	1.41E-01	NS
RGS6	NS	6.21E-02	-0.0765	1.41E-01	NS
PIGA	NS	6.22E-02	-0.0765	1.41E-01	NS
SOX7	NS	6.22E-02	7.65E-02	1.41E-01	NS
FAM90A8P	NS	6.22E-02	-0.0765	1.41E-01	NS
GNG4	NS	6.22E-02	7.65E-02	1.41E-01	NS
PARD6A	NS	6.23E-02	7.65E-02	1.41E-01	NS
NAV1	NS	6.23E-02	-0.0765	1.41E-01	NS
KBTBD6	NS	6.24E-02	7.64E-02	1.41E-01	NS
LTBP3	NS	6.24E-02	7.64E-02	1.41E-01	NS
PIK3C2B	NS	6.25E-02	-0.0764	1.41E-01	NS
BABAM2	NS	6.26E-02	-0.0764	1.42E-01	NS
DCLK3	NS	6.26E-02	-0.0764	1.42E-01	NS

CFAP69	NS	6.26E-02	-0.0764	1.42E-01	NS
NADK	NS	6.26E-02	-0.0764	1.42E-01	NS
SLIT1	NS	6.26E-02	-0.0764	1.42E-01	NS
CLK3	NS	6.27E-02	7.64E-02	1.42E-01	NS
ANKRD34B	NS	6.27E-02	-0.0764	1.42E-01	NS
GRHPR	NS	6.27E-02	-0.0764	1.42E-01	NS
KRTAP9-1	NS	6.27E-02	-0.0764	1.42E-01	NS
SSBP4	NS	6.28E-02	-0.0763	1.42E-01	NS
C12orf57	NS	6.28E-02	-0.0763	1.42E-01	NS
BMF	NS	6.29E-02	-0.0763	1.42E-01	NS
PEX19	NS	6.29E-02	-0.0763	1.42E-01	NS
ICOSLG	NS	6.29E-02	-0.0763	1.42E-01	NS
ACTC1	NS	6.29E-02	-0.0763	1.42E-01	NS
MYO5B	NS	6.30E-02	-0.0763	1.42E-01	NS
PSME2	NS	6.30E-02	-0.0763	1.42E-01	NS
ERICH1	NS	6.30E-02	-0.0763	1.42E-01	NS
CDY2A	NS	6.30E-02	7.63E-02	1.42E-01	NS
ADRB3	NS	6.30E-02	7.63E-02	1.42E-01	NS
ACP3	NS	6.30E-02	7.63E-02	1.42E-01	NS
ZNF283	NS	6.31E-02	-0.0762	1.42E-01	NS
P2RY6	NS	6.31E-02	-0.0762	1.42E-01	NS
OR6B3	NS	6.31E-02	-0.0762	1.42E-01	NS
TMEM158	NS	6.31E-02	7.62E-02	1.42E-01	NS
EPDR1	NS	6.32E-02	-0.0762	1.43E-01	NS
SLC6A2	NS	6.32E-02	7.62E-02	1.43E-01	NS
ARID5A	NS	6.32E-02	-0.0762	1.43E-01	NS
SCYGR1	NS	6.33E-02	7.62E-02	1.43E-01	NS
PIK3R2	NS	6.34E-02	-0.0762	1.43E-01	NS
OR1K1	NS	6.34E-02	-0.0762	1.43E-01	NS
TMEM67	NS	6.34E-02	-0.0762	1.43E-01	NS
ZC2HC1B	NS	6.34E-02	-0.0762	1.43E-01	NS
GSTM4	NS	6.34E-02	-0.0762	1.43E-01	NS
ZHX2	NS	6.35E-02	-0.0761	1.43E-01	NS
NT5C3B	NS	6.35E-02	-0.0761	1.43E-01	NS
OR4C5	NS	6.35E-02	-0.0761	1.43E-01	NS
MST1R	NS	6.36E-02	7.61E-02	1.43E-01	NS
CAMSAP1	NS	6.36E-02	-0.0761	1.43E-01	NS

IRAK1BP1	NS	6.36E-02	-0.0761	1.43E-01	NS
CCDC117	NS	6.36E-02	7.61E-02	1.43E-01	NS
ZFP69	NS	6.37E-02	-0.0761	1.43E-01	NS
CBLL1	NS	6.37E-02	7.61E-02	1.43E-01	NS
PCED1A	NS	6.37E-02	7.61E-02	1.43E-01	NS
ARMH4	NS	6.37E-02	-0.0761	1.43E-01	NS
SPANXN1	NS	6.37E-02	-0.0761	1.43E-01	NS
RNF122	NS	6.37E-02	7.61E-02	1.43E-01	NS
ZNF544	NS	6.38E-02	-0.076	1.43E-01	NS
RDX	NS	6.38E-02	-0.076	1.44E-01	NS
NET1	NS	6.38E-02	7.60E-02	1.44E-01	NS
NKD1	NS	6.38E-02	7.60E-02	1.44E-01	NS
ZNF737	NS	6.39E-02	-0.076	1.44E-01	NS
CARD11	NS	6.39E-02	-0.076	1.44E-01	NS
UBE2C	NS	6.40E-02	-0.076	1.44E-01	NS
EDN3	NS	6.40E-02	7.60E-02	1.44E-01	NS
STYXL1	NS	6.40E-02	-0.076	1.44E-01	NS
SIGIRR	NS	6.40E-02	-0.076	1.44E-01	NS
RRH	NS	6.42E-02	-0.0759	1.44E-01	NS
ATXN1L	NS	6.42E-02	7.59E-02	1.44E-01	NS
DEF8	NS	6.43E-02	-0.0759	1.44E-01	NS
OGFRL1	NS	6.43E-02	-0.0759	1.44E-01	NS
B9D2	NS	6.43E-02	-0.0759	1.44E-01	NS
ZNF90	NS	6.43E-02	-0.0759	1.44E-01	NS
AMY2B	NS	6.43E-02	-0.0759	1.44E-01	NS
BTD	NS	6.43E-02	-0.0759	1.44E-01	NS
SOWAHD	NS	6.44E-02	7.59E-02	1.45E-01	NS
CUL2	NS	6.44E-02	-0.0759	1.45E-01	NS
HOXB3	NS	6.44E-02	7.59E-02	1.45E-01	NS
B3GNT8	NS	6.44E-02	7.59E-02	1.45E-01	NS
FAM83E	NS	6.45E-02	7.58E-02	1.45E-01	NS
GMPPA	NS	6.45E-02	-0.0758	1.45E-01	NS
TAF11L8	NS	6.46E-02	-0.0758	1.45E-01	NS
PPP1R18	NS	6.46E-02	7.58E-02	1.45E-01	NS
FOXB1	NS	6.46E-02	-0.0758	1.45E-01	NS
WWC2	NS	6.46E-02	-0.0758	1.45E-01	NS
PRSS48	NS	6.46E-02	7.58E-02	1.45E-01	NS

CCNB1IP1	NS	6.47E-02	-0.0758	1.45E-01	NS
TVP23A	NS	6.48E-02	-0.0758	1.45E-01	NS
RIN3	NS	6.48E-02	7.57E-02	1.45E-01	NS
TMEM230	NS	6.48E-02	-0.0757	1.45E-01	NS
SYT3	NS	6.49E-02	7.57E-02	1.45E-01	NS
DNM3	NS	6.49E-02	-0.0757	1.45E-01	NS
PRKCZ	NS	6.49E-02	7.57E-02	1.45E-01	NS
ATP6V0E1	NS	6.49E-02	-0.0757	1.45E-01	NS
LEPROTL1	NS	6.49E-02	-0.0757	1.45E-01	NS
PARK7	NS	6.49E-02	-0.0757	1.45E-01	NS
SH3D19	NS	6.50E-02	-0.0757	1.46E-01	NS
AMBRA1	NS	6.50E-02	-0.0757	1.46E-01	NS
TMEM141	NS	6.50E-02	-0.0757	1.46E-01	NS
GLTP	NS	6.51E-02	7.57E-02	1.46E-01	NS
OR6C76	NS	6.51E-02	-0.0757	1.46E-01	NS
WDR76	NS	6.51E-02	7.57E-02	1.46E-01	NS
LY6L	NS	6.53E-02	-0.0756	1.46E-01	NS
DRD5	NS	6.53E-02	-0.0756	1.46E-01	NS
ZNF789	NS	6.53E-02	-0.0756	1.46E-01	NS
ZDHHC24	NS	6.54E-02	-0.0756	1.46E-01	NS
PLPP7	NS	6.54E-02	-0.0756	1.46E-01	NS
BDKRB2	NS	6.55E-02	-0.0756	1.46E-01	NS
DHX38	NS	6.55E-02	7.56E-02	1.46E-01	NS
NUDT13	NS	6.55E-02	-0.0756	1.46E-01	NS
CSN1S1	NS	6.55E-02	-0.0755	1.47E-01	NS
RLN3	NS	6.55E-02	7.55E-02	1.47E-01	NS
KLF14	NS	6.55E-02	7.55E-02	1.47E-01	NS
GGT1	NS	6.56E-02	-0.0755	1.47E-01	NS
MC1R	NS	6.56E-02	-0.0755	1.47E-01	NS
CENPK	NS	6.57E-02	7.55E-02	1.47E-01	NS
BTG3	NS	6.58E-02	-0.0755	1.47E-01	NS
CAMLG	NS	6.58E-02	-0.0755	1.47E-01	NS
GFM1	NS	6.59E-02	-0.0755	1.47E-01	NS
RPL39	NS	6.59E-02	-0.0754	1.47E-01	NS
DKK1	NS	6.59E-02	-0.0754	1.47E-01	NS
MARCKSL1	NS	6.59E-02	7.54E-02	1.47E-01	NS
PTPN20	NS	6.60E-02	-0.0754	1.47E-01	NS

TMEM184C	NS	6.60E-02	-0.0754	1.47E-01	NS
TEKT4	NS	6.60E-02	7.54E-02	1.47E-01	NS
PRPH	NS	6.61E-02	7.54E-02	1.47E-01	NS
APIP	NS	6.61E-02	-0.0754	1.47E-01	NS
MYL7	NS	6.61E-02	7.54E-02	1.47E-01	NS
ELP4	NS	6.61E-02	-0.0754	1.47E-01	NS
CDA	NS	6.61E-02	7.54E-02	1.48E-01	NS
TXNDC17	NS	6.62E-02	-0.0754	1.48E-01	NS
NALF2	NS	6.62E-02	7.54E-02	1.48E-01	NS
OBP2B	NS	6.62E-02	7.54E-02	1.48E-01	NS
SLITRK1	NS	6.62E-02	-0.0754	1.48E-01	NS
SST	NS	6.62E-02	-0.0754	1.48E-01	NS
IQCF2	NS	6.63E-02	-0.0753	1.48E-01	NS
TDRD5	NS	6.63E-02	-0.0753	1.48E-01	NS
ALDOB	NS	6.63E-02	-0.0753	1.48E-01	NS
ANKRD33	NS	6.64E-02	7.53E-02	1.48E-01	NS
DDX59	NS	6.64E-02	-0.0753	1.48E-01	NS
CAPN10	NS	6.64E-02	7.53E-02	1.48E-01	NS
GATA6	NS	6.64E-02	-0.0753	1.48E-01	NS
RNMT	NS	6.65E-02	7.53E-02	1.48E-01	NS
TOX3	NS	6.65E-02	-0.0753	1.48E-01	NS
STXBP2	NS	6.65E-02	7.53E-02	1.48E-01	NS
P3H4	NS	6.65E-02	7.53E-02	1.48E-01	NS
MIER3	NS	6.65E-02	-0.0753	1.48E-01	NS
TDRD15	NS	6.66E-02	-0.0753	1.48E-01	NS
HEXB	NS	6.66E-02	7.53E-02	1.48E-01	NS
AIF1L	NS	6.66E-02	7.52E-02	1.48E-01	NS
APCS	NS	6.66E-02	-0.0752	1.48E-01	NS
FAM95C	NS	6.67E-02	-0.0752	1.48E-01	NS
MKRN1	NS	6.67E-02	7.52E-02	1.48E-01	NS
NTSR2	NS	6.68E-02	7.52E-02	1.49E-01	NS
AP4S1	NS	6.68E-02	-0.0752	1.49E-01	NS
BAD	NS	6.69E-02	7.52E-02	1.49E-01	NS
CD48	NS	6.69E-02	-0.0752	1.49E-01	NS
TRPV5	NS	6.69E-02	-0.0752	1.49E-01	NS
WDR45	NS	6.70E-02	-0.0752	1.49E-01	NS
ASIP	NS	6.70E-02	-0.0751	1.49E-01	NS

ATF5	NS	6.70E-02	7.51E-02	1.49E-01	NS
ZNF695	NS	6.70E-02	-0.0751	1.49E-01	NS
RAD21	NS	6.70E-02	7.51E-02	1.49E-01	NS
IGFL4	NS	6.70E-02	-0.0751	1.49E-01	NS
LIN9	NS	6.71E-02	-0.0751	1.49E-01	NS
SLC35C1	NS	6.71E-02	7.51E-02	1.49E-01	NS
ZBTB47	NS	6.71E-02	7.51E-02	1.49E-01	NS
DENND11	NS	6.71E-02	-0.0751	1.49E-01	NS
DNASE1L3	NS	6.71E-02	-0.0751	1.49E-01	NS
SMC2	NS	6.72E-02	-0.0751	1.49E-01	NS
GAGE12J	NS	6.72E-02	-0.0751	1.49E-01	NS
CTSW	NS	6.72E-02	7.51E-02	1.49E-01	NS
KIZ	NS	6.72E-02	-0.0751	1.49E-01	NS
CHRNA10	NS	6.72E-02	-0.0751	1.49E-01	NS
BORCS8	NS	6.72E-02	-0.0751	1.49E-01	NS
POLR1B	NS	6.73E-02	-0.0751	1.49E-01	NS
HEATR3	NS	6.73E-02	7.51E-02	1.49E-01	NS
ZNF821	NS	6.73E-02	-0.0751	1.49E-01	NS
C16orf72	NS	6.73E-02	7.51E-02	1.49E-01	NS
ZDHHC13	NS	6.74E-02	-0.075	1.49E-01	NS
PSMC1	NS	6.74E-02	-0.075	1.50E-01	NS
CNIH4	NS	6.75E-02	7.50E-02	1.50E-01	NS
MSC	NS	6.75E-02	-0.075	1.50E-01	NS
ELF4	NS	6.75E-02	7.50E-02	1.50E-01	NS
ZNF714	NS	6.76E-02	-0.075	1.50E-01	NS
TRIM39-RPP21	NS	6.76E-02	-0.075	1.50E-01	NS
C12orf29	NS	6.77E-02	-0.075	1.50E-01	NS
MATK	NS	6.77E-02	7.50E-02	1.50E-01	NS
PPIB	NS	6.77E-02	-0.075	1.50E-01	NS
MTNR1B	NS	6.77E-02	-0.0749	1.50E-01	NS
LCN9	NS	6.77E-02	7.49E-02	1.50E-01	NS
ING1	NS	6.77E-02	7.49E-02	1.50E-01	NS
STING1	NS	6.77E-02	-0.0749	1.50E-01	NS
MAPK11	NS	6.78E-02	7.49E-02	1.50E-01	NS
PCDHB1	NS	6.78E-02	7.49E-02	1.50E-01	NS
HOXB5	NS	6.78E-02	-0.0749	1.50E-01	NS
MTRR	NS	6.79E-02	-0.0749	1.50E-01	NS

MSR1	NS	6.79E-02	-0.0749	1.50E-01	NS
SF3B2	NS	6.79E-02	7.49E-02	1.50E-01	NS
IP6K2	NS	6.80E-02	-0.0749	1.50E-01	NS
ADAMTS2	NS	6.80E-02	-0.0749	1.50E-01	NS
RTN1	NS	6.80E-02	-0.0749	1.50E-01	NS
MDP1	NS	6.80E-02	7.49E-02	1.50E-01	NS
CDK6	NS	6.80E-02	-0.0749	1.50E-01	NS
API5	NS	6.81E-02	-0.0748	1.51E-01	NS
AOX1	NS	6.82E-02	-0.0748	1.51E-01	NS
MBOAT4	NS	6.83E-02	-0.0748	1.51E-01	NS
BPIFB6	NS	6.83E-02	-0.0748	1.51E-01	NS
F2R	NS	6.83E-02	-0.0748	1.51E-01	NS
ANXA4	NS	6.83E-02	-0.0748	1.51E-01	NS
SNRPD3	NS	6.84E-02	-0.0748	1.51E-01	NS
FOXN1	NS	6.84E-02	-0.0748	1.51E-01	NS
EVA1C	NS	6.84E-02	-0.0748	1.51E-01	NS
SERTAD3	NS	6.85E-02	-0.0747	1.51E-01	NS
PSD2	NS	6.85E-02	7.47E-02	1.51E-01	NS
B3GNT9	NS	6.85E-02	-0.0747	1.51E-01	NS
IL12RB1	NS	6.85E-02	-0.0747	1.51E-01	NS
TMEM35B	NS	6.86E-02	-0.0747	1.51E-01	NS
C20orf202	NS	6.86E-02	7.47E-02	1.51E-01	NS
SULT1C4	NS	6.86E-02	-0.0747	1.51E-01	NS
CYP19A1	NS	6.86E-02	-0.0747	1.51E-01	NS
PMPCB	NS	6.87E-02	-0.0747	1.52E-01	NS
VNN2	NS	6.87E-02	-0.0747	1.52E-01	NS
NUDT6	NS	6.86E-02	-0.0747	1.52E-01	NS
RARS1	NS	6.87E-02	-0.0747	1.52E-01	NS
OR7E24	NS	6.87E-02	7.47E-02	1.52E-01	NS
SPINK6	NS	6.87E-02	-0.0747	1.52E-01	NS
NPSR1	NS	6.88E-02	-0.0747	1.52E-01	NS
SMAD4	NS	6.88E-02	-0.0747	1.52E-01	NS
FAM114A2	NS	6.88E-02	-0.0747	1.52E-01	NS
FKBP6	NS	6.88E-02	7.47E-02	1.52E-01	NS
CXCL2	NS	6.88E-02	-0.0746	1.52E-01	NS
GOLGA6D	NS	6.89E-02	-0.0746	1.52E-01	NS
EPS15L1	NS	6.89E-02	7.46E-02	1.52E-01	NS

PPY	NS	6.90E-02	-0.0746	1.52E-01	NS
PPP1R12C	NS	6.90E-02	7.46E-02	1.52E-01	NS
TRIM28	NS	6.90E-02	-0.0746	1.52E-01	NS
POU2AF1	NS	6.91E-02	7.46E-02	1.52E-01	NS
C11orf94	NS	6.91E-02	7.46E-02	1.52E-01	NS
GRN	NS	6.92E-02	-0.0746	1.52E-01	NS
ETV2	NS	6.92E-02	-0.0746	1.52E-01	NS
MLF1	NS	6.92E-02	-0.0745	1.52E-01	NS
ACKR1	NS	6.92E-02	-0.0745	1.52E-01	NS
PRMT9	NS	6.93E-02	7.45E-02	1.52E-01	NS
ST6GAL2	NS	6.93E-02	7.45E-02	1.53E-01	NS
SLC26A4	NS	6.93E-02	-0.0745	1.53E-01	NS
ZNF660	NS	6.93E-02	-0.0745	1.53E-01	NS
UGT3A2	NS	6.94E-02	-0.0745	1.53E-01	NS
MAP3K5	NS	6.94E-02	-0.0745	1.53E-01	NS
MTHFD2	NS	6.95E-02	-0.0745	1.53E-01	NS
NBN	NS	6.96E-02	-0.0744	1.53E-01	NS
KLF3	NS	6.96E-02	-0.0745	1.53E-01	NS
PTGIR	NS	6.96E-02	-0.0745	1.53E-01	NS
MFSD4B	NS	6.96E-02	-0.0744	1.53E-01	NS
NRARP	NS	6.96E-02	7.44E-02	1.53E-01	NS
TMEM154	NS	6.98E-02	-0.0744	1.53E-01	NS
RAB5A	NS	6.98E-02	-0.0744	1.54E-01	NS
DCAF5	NS	6.99E-02	-0.0744	1.54E-01	NS
ALDH3B1	NS	6.99E-02	-0.0744	1.54E-01	NS
DERA	NS	6.99E-02	-0.0744	1.54E-01	NS
HOXB6	NS	6.99E-02	-0.0744	1.54E-01	NS
KLK4	NS	7.00E-02	-0.0743	1.54E-01	NS
KRTAP20-1	NS	7.00E-02	7.43E-02	1.54E-01	NS
RPS16	NS	7.01E-02	-0.0743	1.54E-01	NS
RCN2	NS	7.01E-02	-0.0743	1.54E-01	NS
ST18	NS	7.01E-02	-0.0743	1.54E-01	NS
RTP3	NS	7.01E-02	-0.0743	1.54E-01	NS
FRMD6	NS	7.02E-02	-0.0743	1.54E-01	NS
PAX5	NS	7.01E-02	-0.0743	1.54E-01	NS
CMPK2	NS	7.02E-02	-0.0743	1.54E-01	NS
IL4R	NS	7.02E-02	-0.0743	1.54E-01	NS

COQ2	NS	7.02E-02	7.43E-02	1.54E-01	NS
FOXN4	NS	7.03E-02	-0.0743	1.54E-01	NS
CYB5R3	NS	7.03E-02	7.43E-02	1.54E-01	NS
SLC16A5	NS	7.04E-02	7.42E-02	1.54E-01	NS
ITGB8	NS	7.05E-02	-0.0742	1.55E-01	NS
JDP2	NS	7.06E-02	7.42E-02	1.55E-01	NS
LIG4	NS	7.06E-02	-0.0742	1.55E-01	NS
FKBP8	NS	7.06E-02	7.42E-02	1.55E-01	NS
CT47A3	NS	7.07E-02	7.42E-02	1.55E-01	NS
SIGLEC7	NS	7.07E-02	-0.0741	1.55E-01	NS
TMEM251	NS	7.07E-02	7.41E-02	1.55E-01	NS
TMEM30B	NS	7.07E-02	7.41E-02	1.55E-01	NS
SETD5	NS	7.08E-02	-0.0741	1.55E-01	NS
MKLN1	NS	7.08E-02	-0.0741	1.55E-01	NS
BRME1	NS	7.08E-02	-0.0741	1.55E-01	NS
KIF17	NS	7.09E-02	7.41E-02	1.55E-01	NS
EPN1	NS	7.10E-02	7.41E-02	1.55E-01	NS
ATP23	NS	7.10E-02	-0.0741	1.55E-01	NS
VTN	NS	7.10E-02	-0.0741	1.55E-01	NS
CPA3	NS	7.10E-02	-0.0741	1.56E-01	NS
FSTL3	NS	7.10E-02	-0.0741	1.56E-01	NS
PITX1	NS	7.11E-02	7.41E-02	1.56E-01	NS
HES7	NS	7.11E-02	7.40E-02	1.56E-01	NS
S100A6	NS	7.11E-02	-0.074	1.56E-01	NS
P2RY11	NS	7.11E-02	-0.0741	1.56E-01	NS
ANXA8	NS	7.11E-02	-0.074	1.56E-01	NS
FAM214A	NS	7.11E-02	7.40E-02	1.56E-01	NS
IFIH1	NS	7.12E-02	7.40E-02	1.56E-01	NS
ADGB	NS	7.12E-02	-0.074	1.56E-01	NS
PPP1R26	NS	7.12E-02	-0.074	1.56E-01	NS
IFI27L2	NS	7.13E-02	-0.074	1.56E-01	NS
GRB2	NS	7.13E-02	-0.074	1.56E-01	NS
CXorf51B	NS	7.13E-02	7.40E-02	1.56E-01	NS
OBSCN	NS	7.13E-02	7.40E-02	1.56E-01	NS
C1QL2	NS	7.13E-02	7.40E-02	1.56E-01	NS
TRIM75P	NS	7.13E-02	7.40E-02	1.56E-01	NS
SIX6	NS	7.14E-02	-0.074	1.56E-01	NS

ZNRF3	NS	7.14E-02	-0.074	1.56E-01	NS
GPN1	NS	7.14E-02	-0.074	1.56E-01	NS
PRNP	NS	7.14E-02	-0.074	1.56E-01	NS
OR5L1	NS	7.14E-02	-0.074	1.56E-01	NS
GAS6	NS	7.15E-02	7.40E-02	1.56E-01	NS
RTN4R	NS	7.15E-02	7.39E-02	1.56E-01	NS
ALX4	NS	7.15E-02	-0.0739	1.56E-01	NS
SNX12	NS	7.16E-02	-0.0739	1.56E-01	NS
RCN3	NS	7.16E-02	-0.0739	1.56E-01	NS
PADI6	NS	7.16E-02	-0.0739	1.56E-01	NS
CHD6	NS	7.17E-02	7.39E-02	1.56E-01	NS
AMPD3	NS	7.17E-02	-0.0739	1.56E-01	NS
EIF3H	NS	7.17E-02	7.39E-02	1.56E-01	NS
DCSTAMP	NS	7.18E-02	-0.0739	1.57E-01	NS
PWWP2A	NS	7.18E-02	-0.0739	1.57E-01	NS
CDK11B	NS	7.19E-02	-0.0738	1.57E-01	NS
ALG3	NS	7.19E-02	7.38E-02	1.57E-01	NS
DCAF10	NS	7.20E-02	-0.0738	1.57E-01	NS
PYGO1	NS	7.20E-02	-0.0738	1.57E-01	NS
OSBPL5	NS	7.20E-02	7.38E-02	1.57E-01	NS
RXYLT1	NS	7.21E-02	7.38E-02	1.57E-01	NS
EGLN1	NS	7.21E-02	7.38E-02	1.57E-01	NS
UTRN	NS	7.22E-02	-0.0738	1.57E-01	NS
FAAP20	NS	7.22E-02	-0.0738	1.57E-01	NS
PASD1	NS	7.23E-02	7.37E-02	1.58E-01	NS
CAND2	NS	7.23E-02	7.37E-02	1.58E-01	NS
ASTN2	NS	7.24E-02	-0.0737	1.58E-01	NS
BBS2	NS	7.25E-02	-0.0737	1.58E-01	NS
AVPR1B	NS	7.25E-02	-0.0737	1.58E-01	NS
FGFRL1	NS	7.25E-02	-0.0737	1.58E-01	NS
CELA3A	NS	7.25E-02	-0.0737	1.58E-01	NS
RTL8B	NS	7.25E-02	-0.0737	1.58E-01	NS
DTX2	NS	7.26E-02	-0.0737	1.58E-01	NS
SOHLH1	NS	7.26E-02	7.37E-02	1.58E-01	NS
DPP9	NS	7.26E-02	7.36E-02	1.58E-01	NS
KANK3	NS	7.27E-02	-0.0736	1.58E-01	NS
SCARF1	NS	7.27E-02	7.36E-02	1.58E-01	NS

SLC39A7	NS	7.27E-02	-0.0736	1.58E-01	NS
DNAJC5B	NS	7.27E-02	-0.0736	1.58E-01	NS
FAM3D	NS	7.28E-02	-0.0736	1.58E-01	NS
C5orf46	NS	7.28E-02	-0.0736	1.58E-01	NS
SYCN	NS	7.28E-02	7.36E-02	1.58E-01	NS
VIM	NS	7.29E-02	-0.0736	1.58E-01	NS
TLK2	NS	7.29E-02	-0.0736	1.58E-01	NS
ELFN2	NS	7.29E-02	7.36E-02	1.58E-01	NS
ABHD5	NS	7.29E-02	7.36E-02	1.59E-01	NS
COX16	NS	7.29E-02	-0.0736	1.59E-01	NS
FXR2	NS	7.30E-02	-0.0736	1.59E-01	NS
CELF5	NS	7.30E-02	7.36E-02	1.59E-01	NS
GBP7	NS	7.30E-02	-0.0736	1.59E-01	NS
TYW3	NS	7.31E-02	-0.0735	1.59E-01	NS
CDC42SE1	NS	7.31E-02	-0.0735	1.59E-01	NS
PCDHGB5	NS	7.31E-02	-0.0735	1.59E-01	NS
ZBED3	NS	7.31E-02	7.35E-02	1.59E-01	NS
KRT18	NS	7.32E-02	-0.0735	1.59E-01	NS
MAMDC2	NS	7.32E-02	-0.0735	1.59E-01	NS
GRM5	NS	7.32E-02	-0.0735	1.59E-01	NS
ELK3	NS	7.32E-02	-0.0735	1.59E-01	NS
BACH2	NS	7.33E-02	-0.0735	1.59E-01	NS
SPRR2F	NS	7.33E-02	-0.0735	1.59E-01	NS
KDM3A	NS	7.33E-02	7.35E-02	1.59E-01	NS
RAB9A	NS	7.33E-02	7.35E-02	1.59E-01	NS
USP17L7	NS	7.33E-02	-0.0735	1.59E-01	NS
OR10H3	NS	7.34E-02	7.35E-02	1.59E-01	NS
DAZAP2	NS	7.34E-02	7.35E-02	1.59E-01	NS
KIFC1	NS	7.34E-02	-0.0734	1.59E-01	NS
UHRF2	NS	7.34E-02	-0.0734	1.59E-01	NS
TAF11L7	NS	7.35E-02	-0.0734	1.59E-01	NS
KCNE5	NS	7.35E-02	-0.0734	1.59E-01	NS
CELSR1	NS	7.35E-02	-0.0734	1.59E-01	NS
NOP56	NS	7.35E-02	-0.0734	1.59E-01	NS
SAFB2	NS	7.35E-02	7.34E-02	1.59E-01	NS
P2RX3	NS	7.35E-02	7.34E-02	1.59E-01	NS
MRPS22	NS	7.36E-02	-0.0734	1.59E-01	NS

ONECUT2	NS	7.36E-02	-0.0734	1.59E-01	NS
LDHB	NS	7.37E-02	-0.0734	1.60E-01	NS
CTAG1A	NS	7.37E-02	-0.0734	1.60E-01	NS
OR10A7	NS	7.37E-02	7.34E-02	1.60E-01	NS
ACBD4	NS	7.37E-02	7.34E-02	1.60E-01	NS
THBS4	NS	7.38E-02	-0.0734	1.60E-01	NS
ATXN7L3B	NS	7.38E-02	-0.0734	1.60E-01	NS
ASPSCR1	NS	7.39E-02	7.33E-02	1.60E-01	NS
CTDP1	NS	7.39E-02	7.33E-02	1.60E-01	NS
TMPRSS2	NS	7.39E-02	-0.0733	1.60E-01	NS
TMEM40	NS	7.40E-02	7.33E-02	1.60E-01	NS
CERS6	NS	7.40E-02	-0.0733	1.60E-01	NS
DSCAML1	NS	7.40E-02	-0.0733	1.60E-01	NS
IMMT	NS	7.40E-02	-0.0733	1.60E-01	NS
ZNF383	NS	7.42E-02	-0.0733	1.60E-01	NS
YBX2	NS	7.42E-02	7.33E-02	1.60E-01	NS
PTF1A	NS	7.42E-02	7.32E-02	1.61E-01	NS
OOSP4A	NS	7.43E-02	-0.0732	1.61E-01	NS
SLC35F5	NS	7.44E-02	-0.0732	1.61E-01	NS
CCDC85C	NS	7.44E-02	7.32E-02	1.61E-01	NS
NRDC	NS	7.44E-02	-0.0732	1.61E-01	NS
ATP2A1	NS	7.44E-02	7.32E-02	1.61E-01	NS
NEU4	NS	7.44E-02	7.32E-02	1.61E-01	NS
TMEM165	NS	7.44E-02	-0.0732	1.61E-01	NS
TBCK	NS	7.45E-02	-0.0732	1.61E-01	NS
ACAT1	NS	7.45E-02	-0.0732	1.61E-01	NS
NMRK2	NS	7.45E-02	7.32E-02	1.61E-01	NS
CASKIN2	NS	7.45E-02	-0.0732	1.61E-01	NS
KAT6B	NS	7.46E-02	-0.0731	1.61E-01	NS
ARHGAP1	NS	7.47E-02	7.31E-02	1.61E-01	NS
TRMT112	NS	7.47E-02	-0.0731	1.61E-01	NS
HDGFL1	NS	7.47E-02	7.31E-02	1.61E-01	NS
HLA-DPA1	NS	7.47E-02	-0.0731	1.61E-01	NS
SCT	NS	7.47E-02	7.31E-02	1.61E-01	NS
CCDC185	NS	7.48E-02	7.31E-02	1.61E-01	NS
NCKAP5	NS	7.48E-02	-0.0731	1.62E-01	NS
ANKDD1A	NS	7.49E-02	7.31E-02	1.62E-01	NS

CATSPERE	NS	7.49E-02	-0.0731	1.62E-01	NS
NLRP10	NS	7.49E-02	7.31E-02	1.62E-01	NS
SYNJ2	NS	7.50E-02	-0.0731	1.62E-01	NS
DPYS	NS	7.50E-02	7.30E-02	1.62E-01	NS
CD163L1	NS	7.50E-02	-0.073	1.62E-01	NS
MISP3	NS	7.51E-02	7.30E-02	1.62E-01	NS
TUBB4A	NS	7.51E-02	7.30E-02	1.62E-01	NS
FNBP1	NS	7.51E-02	-0.073	1.62E-01	NS
MYO1G	NS	7.51E-02	-0.073	1.62E-01	NS
ANAPC15	NS	7.52E-02	7.30E-02	1.62E-01	NS
AMPD2	NS	7.52E-02	-0.073	1.62E-01	NS
PRDM12	NS	7.53E-02	7.30E-02	1.62E-01	NS
FAM204A	NS	7.53E-02	-0.073	1.62E-01	NS
TNFRSF10B	NS	7.54E-02	-0.073	1.62E-01	NS
LIMD1	NS	7.54E-02	-0.073	1.62E-01	NS
SLC35F3	NS	7.54E-02	-0.073	1.62E-01	NS
SIRT4	NS	7.54E-02	-0.0729	1.62E-01	NS
EIF3E	NS	7.54E-02	-0.0729	1.62E-01	NS
GEMIN2	NS	7.55E-02	-0.0729	1.63E-01	NS
TBC1D14	NS	7.55E-02	-0.0729	1.63E-01	NS
OSBPL7	NS	7.56E-02	-0.0729	1.63E-01	NS
CAMK2A	NS	7.56E-02	-0.0729	1.63E-01	NS
LYL1	NS	7.56E-02	7.29E-02	1.63E-01	NS
MAP2K5	NS	7.56E-02	7.29E-02	1.63E-01	NS
ALDH9A1	NS	7.56E-02	7.29E-02	1.63E-01	NS
ATP5MK	NS	7.56E-02	-0.0729	1.63E-01	NS
OR4N5	NS	7.56E-02	-0.0729	1.63E-01	NS
PCDHA12	NS	7.56E-02	7.29E-02	1.63E-01	NS
RBM22	NS	7.57E-02	7.29E-02	1.63E-01	NS
FSD2	NS	7.57E-02	-0.0729	1.63E-01	NS
AGO1	NS	7.57E-02	-0.0729	1.63E-01	NS
MGAT1	NS	7.57E-02	7.29E-02	1.63E-01	NS
MIB2	NS	7.57E-02	7.29E-02	1.63E-01	NS
BHLHB9	NS	7.58E-02	-0.0728	1.63E-01	NS
ZG16B	NS	7.59E-02	-0.0728	1.63E-01	NS
ANXA2	NS	7.59E-02	-0.0728	1.63E-01	NS
CBFB	NS	7.60E-02	-0.0728	1.63E-01	NS

VAMP8	NS	7.62E-02	-0.0727	1.64E-01	NS
ASTN1	NS	7.62E-02	-0.0727	1.64E-01	NS
NR2C2	NS	7.62E-02	-0.0727	1.64E-01	NS
RBBP7	NS	7.63E-02	7.27E-02	1.64E-01	NS
TBC1D10A	NS	7.63E-02	7.27E-02	1.64E-01	NS
CCSAP	NS	7.64E-02	7.27E-02	1.64E-01	NS
LYPD6	NS	7.64E-02	-0.0727	1.64E-01	NS
KRT33A	NS	7.65E-02	-0.0727	1.64E-01	NS
ARNTL2	NS	7.65E-02	-0.0727	1.64E-01	NS
TSC22D1	NS	7.65E-02	-0.0727	1.64E-01	NS
SLU7	NS	7.65E-02	-0.0727	1.64E-01	NS
IDH1	NS	7.66E-02	-0.0727	1.64E-01	NS
ZBED4	NS	7.66E-02	-0.0726	1.64E-01	NS
AK1	NS	7.66E-02	-0.0726	1.64E-01	NS
DYNC2I2	NS	7.66E-02	-0.0726	1.64E-01	NS
C10orf105	NS	7.66E-02	-0.0726	1.64E-01	NS
GTPBP10	NS	7.67E-02	-0.0726	1.64E-01	NS
CFAP43	NS	7.67E-02	7.26E-02	1.64E-01	NS
PHACTR4	NS	7.67E-02	7.26E-02	1.64E-01	NS
KICS2	NS	7.67E-02	-0.0726	1.64E-01	NS
PITPNA	NS	7.67E-02	7.26E-02	1.64E-01	NS
FAM90A18P	NS	7.67E-02	-0.0726	1.65E-01	NS
RPAP2	NS	7.68E-02	7.26E-02	1.65E-01	NS
SLC51B	NS	7.68E-02	-0.0726	1.65E-01	NS
TYW1B	NS	7.69E-02	-0.0726	1.65E-01	NS
ROR2	NS	7.69E-02	7.26E-02	1.65E-01	NS
MYO16	NS	7.69E-02	-0.0726	1.65E-01	NS
USP53	NS	7.69E-02	-0.0726	1.65E-01	NS
POP1	NS	7.70E-02	-0.0726	1.65E-01	NS
TUBB	NS	7.70E-02	-0.0726	1.65E-01	NS
SIK1	NS	7.71E-02	7.25E-02	1.65E-01	NS
ALG11	NS	7.72E-02	-0.0725	1.65E-01	NS
TLL2	NS	7.72E-02	7.25E-02	1.65E-01	NS
RNF145	NS	7.72E-02	-0.0725	1.65E-01	NS
CERK	NS	7.74E-02	-0.0725	1.66E-01	NS
ZMPSTE24	NS	7.74E-02	-0.0724	1.66E-01	NS
SEC23B	NS	7.74E-02	-0.0724	1.66E-01	NS

IQCB1	NS	7.74E-02	-0.0724	1.66E-01	NS
SPATA45	NS	7.75E-02	-0.0724	1.66E-01	NS
DEXI	NS	7.75E-02	-0.0724	1.66E-01	NS
MKS1	NS	7.76E-02	7.24E-02	1.66E-01	NS
ZKSCAN3	NS	7.76E-02	-0.0724	1.66E-01	NS
TNNI3K	NS	7.76E-02	-0.0724	1.66E-01	NS
FBN2	NS	7.76E-02	-0.0724	1.66E-01	NS
MACROD2	NS	7.77E-02	7.24E-02	1.66E-01	NS
USP3	NS	7.77E-02	7.24E-02	1.66E-01	NS
B3GNT5	NS	7.77E-02	-0.0724	1.66E-01	NS
ZFP42	NS	7.77E-02	7.24E-02	1.66E-01	NS
TNFSF8	NS	7.78E-02	-0.0724	1.66E-01	NS
LYAR	NS	7.78E-02	-0.0724	1.66E-01	NS
OTX2	NS	7.78E-02	7.23E-02	1.66E-01	NS
CHST15	NS	7.79E-02	-0.0723	1.66E-01	NS
SULF1	NS	7.79E-02	-0.0723	1.66E-01	NS
PON2	NS	7.79E-02	-0.0723	1.66E-01	NS
FAM236A	NS	7.80E-02	-0.0723	1.66E-01	NS
SLC17A3	NS	7.80E-02	-0.0723	1.67E-01	NS
GPX2	NS	7.80E-02	-0.0723	1.67E-01	NS
DMRT3	NS	7.80E-02	-0.0723	1.67E-01	NS
PLTP	NS	7.81E-02	-0.0723	1.67E-01	NS
CCDC73	NS	7.81E-02	-0.0723	1.67E-01	NS
PPP1R35	NS	7.81E-02	-0.0723	1.67E-01	NS
SH2D5	NS	7.82E-02	7.23E-02	1.67E-01	NS
HSD17B4	NS	7.82E-02	-0.0723	1.67E-01	NS
SLC39A13	NS	7.82E-02	-0.0723	1.67E-01	NS
C15orf40	NS	7.82E-02	-0.0723	1.67E-01	NS
PALLD	NS	7.83E-02	-0.0722	1.67E-01	NS
MTG2	NS	7.84E-02	-0.0722	1.67E-01	NS
PIK3IP1	NS	7.84E-02	7.22E-02	1.67E-01	NS
WFDC1	NS	7.84E-02	7.22E-02	1.67E-01	NS
CNMD	NS	7.85E-02	-0.0722	1.67E-01	NS
HMCES	NS	7.85E-02	-0.0722	1.67E-01	NS
RGP1	NS	7.85E-02	-0.0722	1.67E-01	NS
FUNDC1	NS	7.86E-02	-0.0722	1.67E-01	NS
GASK1A	NS	7.86E-02	-0.0722	1.67E-01	NS

KIR3DL1	NS	7.86E-02	-0.0722	1.67E-01	NS
SPATA2	NS	7.86E-02	-0.0722	1.68E-01	NS
ARSI	NS	7.86E-02	-0.0722	1.68E-01	NS
SIGLEC10	NS	7.87E-02	-0.0721	1.68E-01	NS
PKD1L3	NS	7.87E-02	-0.0721	1.68E-01	NS
GIT1	NS	7.88E-02	-0.0721	1.68E-01	NS
PDE4C	NS	7.89E-02	7.21E-02	1.68E-01	NS
CCDC40	NS	7.90E-02	7.21E-02	1.68E-01	NS
GRB7	NS	7.89E-02	7.21E-02	1.68E-01	NS
PATE2	NS	7.90E-02	-0.0721	1.68E-01	NS
DYNC1LI1	NS	7.90E-02	7.21E-02	1.68E-01	NS
AKT1	NS	7.90E-02	-0.0721	1.68E-01	NS
TIMM8B	NS	7.91E-02	-0.0721	1.68E-01	NS
ZCCHC17	NS	7.91E-02	7.20E-02	1.68E-01	NS
GALP	NS	7.91E-02	7.20E-02	1.68E-01	NS
UQCRB	NS	7.92E-02	-0.072	1.68E-01	NS
SLMAP	NS	7.92E-02	7.20E-02	1.68E-01	NS
GLI4	NS	7.92E-02	-0.072	1.68E-01	NS
OR2H1	NS	7.92E-02	-0.072	1.68E-01	NS
ZNF783	NS	7.92E-02	-0.072	1.68E-01	NS
OPN1MW3	NS	7.93E-02	7.20E-02	1.68E-01	NS
ZBTB5	NS	7.93E-02	-0.072	1.69E-01	NS
TCAF2C	NS	7.93E-02	-0.072	1.69E-01	NS
ZNF471	NS	7.94E-02	-0.072	1.69E-01	NS
GRTP1	NS	7.94E-02	7.20E-02	1.69E-01	NS
ASIC5	NS	7.94E-02	7.20E-02	1.69E-01	NS
PPP1R14C	NS	7.94E-02	-0.072	1.69E-01	NS
SUOX	NS	7.94E-02	7.20E-02	1.69E-01	NS
LMAN1L	NS	7.95E-02	-0.072	1.69E-01	NS
NDFIP1	NS	7.95E-02	-0.0719	1.69E-01	NS
RCOR2	NS	7.95E-02	7.19E-02	1.69E-01	NS
RRBP1	NS	7.95E-02	-0.0719	1.69E-01	NS
SPAG17	NS	7.96E-02	-0.0719	1.69E-01	NS
ZFPM1	NS	7.96E-02	-0.0719	1.69E-01	NS
PCSK9	NS	7.96E-02	-0.0719	1.69E-01	NS
SYNCRIP	NS	7.97E-02	-0.0719	1.69E-01	NS
FBXL2	NS	7.97E-02	-0.0719	1.69E-01	NS

MYOM2	NS	7.98E-02	-0.0719	1.69E-01	NS
POLD2	NS	7.98E-02	-0.0719	1.69E-01	NS
OR10J5	NS	7.99E-02	-0.0719	1.69E-01	NS
DHH	NS	7.99E-02	-0.0719	1.69E-01	NS
PIAS3	NS	7.99E-02	-0.0719	1.69E-01	NS
DPEP1	NS	8.00E-02	7.18E-02	1.70E-01	NS
SEC14L3	NS	8.00E-02	-0.0718	1.70E-01	NS
TMEM176A	NS	8.01E-02	7.18E-02	1.70E-01	NS
IQANK1	NS	8.02E-02	7.18E-02	1.70E-01	NS
FOXM1	NS	8.02E-02	-0.0718	1.70E-01	NS
TAS2R42	NS	8.02E-02	-0.0718	1.70E-01	NS
WDR20	NS	8.03E-02	-0.0718	1.70E-01	NS
ZHX1-C8orf76	NS	8.03E-02	-0.0718	1.70E-01	NS
TCEAL6	NS	8.03E-02	-0.0718	1.70E-01	NS
DPYSL5	NS	8.03E-02	-0.0718	1.70E-01	NS
ANGEL1	NS	8.03E-02	-0.0717	1.70E-01	NS
VAC14	NS	8.04E-02	7.17E-02	1.70E-01	NS
JRK	NS	8.04E-02	-0.0717	1.70E-01	NS
DEFB127	NS	8.05E-02	-0.0717	1.70E-01	NS
MTARC1	NS	8.05E-02	7.17E-02	1.70E-01	NS
CCR1	NS	8.05E-02	-0.0717	1.70E-01	NS
GPR137	NS	8.05E-02	7.17E-02	1.70E-01	NS
C17orf64	NS	8.05E-02	-0.0717	1.70E-01	NS
CLEC12A	NS	8.06E-02	7.17E-02	1.70E-01	NS
OR4C3	NS	8.06E-02	-0.0717	1.70E-01	NS
KRTAP4-3	NS	8.06E-02	-0.0717	1.70E-01	NS
IYD	NS	8.07E-02	-0.0717	1.71E-01	NS
NAT16	NS	8.07E-02	7.17E-02	1.71E-01	NS
MRPS2	NS	8.07E-02	7.17E-02	1.71E-01	NS
ZW10	NS	8.07E-02	-0.0717	1.71E-01	NS
FUT3	NS	8.07E-02	-0.0717	1.71E-01	NS
PRR20E	NS	8.07E-02	-0.0717	1.71E-01	NS
HADHA	NS	8.08E-02	-0.0716	1.71E-01	NS
ZNF280C	NS	8.08E-02	-0.0716	1.71E-01	NS
XPNPEP1	NS	8.08E-02	7.16E-02	1.71E-01	NS
TRIM62	NS	8.09E-02	7.16E-02	1.71E-01	NS
POLR2I	NS	8.10E-02	-0.0716	1.71E-01	NS

PCSK4	NS	8.10E-02	7.16E-02	1.71E-01	NS
NXPH2	NS	8.10E-02	-0.0716	1.71E-01	NS
SYT9	NS	8.11E-02	-0.0716	1.71E-01	NS
NBPF10	NS	8.11E-02	-0.0716	1.71E-01	NS
EGFL6	NS	8.12E-02	-0.0716	1.71E-01	NS
HGH1	NS	8.12E-02	7.16E-02	1.71E-01	NS
AGAP9	NS	8.12E-02	-0.0715	1.71E-01	NS
MEF2A	NS	8.12E-02	-0.0715	1.71E-01	NS
SNRPA1	NS	8.13E-02	-0.0715	1.72E-01	NS
SLC44A2	NS	8.13E-02	-0.0715	1.72E-01	NS
SF3A2	NS	8.14E-02	7.15E-02	1.72E-01	NS
ZDHHC23	NS	8.14E-02	-0.0715	1.72E-01	NS
RPL19	NS	8.14E-02	-0.0715	1.72E-01	NS
CCDC169	NS	8.15E-02	-0.0715	1.72E-01	NS
GPR153	NS	8.15E-02	-0.0715	1.72E-01	NS
SPDYE10	NS	8.16E-02	-0.0715	1.72E-01	NS
NBPF3	NS	8.16E-02	-0.0714	1.72E-01	NS
PDF	NS	8.17E-02	-0.0714	1.72E-01	NS
MRPL4	NS	8.17E-02	-0.0714	1.72E-01	NS
GMFG	NS	8.17E-02	-0.0714	1.72E-01	NS
TFPI2	NS	8.18E-02	-0.0714	1.72E-01	NS
TANC1	NS	8.18E-02	-0.0714	1.72E-01	NS
MRM2	NS	8.19E-02	-0.0714	1.73E-01	NS
CYP2R1	NS	8.19E-02	-0.0714	1.73E-01	NS
FAM25A	NS	8.19E-02	-0.0714	1.73E-01	NS
KRTAP9-8	NS	8.19E-02	7.14E-02	1.73E-01	NS
PHACTR3	NS	8.20E-02	-0.0714	1.73E-01	NS
TP53TG3D	NS	8.20E-02	-0.0714	1.73E-01	NS
ERI3	NS	8.21E-02	-0.0713	1.73E-01	NS
MED16	NS	8.21E-02	-0.0713	1.73E-01	NS
SOX17	NS	8.21E-02	7.13E-02	1.73E-01	NS
TMEM41B	NS	8.21E-02	-0.0713	1.73E-01	NS
TMEM183A	NS	8.23E-02	7.13E-02	1.73E-01	NS
NKX3-1	NS	8.23E-02	-0.0713	1.73E-01	NS
DYM	NS	8.24E-02	-0.0713	1.73E-01	NS
FIP1L1	NS	8.24E-02	-0.0713	1.74E-01	NS
TMEM218	NS	8.25E-02	-0.0713	1.74E-01	NS

NPAS2	NS	8.25E-02	-0.0712	1.74E-01	NS
MALL	NS	8.25E-02	-0.0712	1.74E-01	NS
PPIAL4C	NS	8.26E-02	7.12E-02	1.74E-01	NS
MTSS1	NS	8.26E-02	-0.0712	1.74E-01	NS
SLC18B1	NS	8.27E-02	-0.0712	1.74E-01	NS
GLS	NS	8.28E-02	-0.0712	1.74E-01	NS
NOP58	NS	8.28E-02	-0.0712	1.74E-01	NS
RADX	NS	8.28E-02	-0.0712	1.74E-01	NS
PRAMEF17	NS	8.28E-02	7.12E-02	1.74E-01	NS
EHBP1	NS	8.29E-02	7.12E-02	1.74E-01	NS
DPH7	NS	8.29E-02	7.12E-02	1.74E-01	NS
HCN1	NS	8.30E-02	-0.0711	1.74E-01	NS
OR5A2	NS	8.30E-02	-0.0711	1.74E-01	NS
SLC29A3	NS	8.30E-02	-0.0711	1.74E-01	NS
USP17L24	NS	8.31E-02	7.11E-02	1.74E-01	NS
HACL1	NS	8.31E-02	-0.0711	1.75E-01	NS
METTL27	NS	8.32E-02	7.11E-02	1.75E-01	NS
ST3GAL3	NS	8.33E-02	-0.0711	1.75E-01	NS
SPTSSB	NS	8.33E-02	-0.0711	1.75E-01	NS
SAMD12	NS	8.33E-02	-0.0711	1.75E-01	NS
CCT5	NS	8.33E-02	-0.0711	1.75E-01	NS
C11orf54	NS	8.33E-02	-0.0711	1.75E-01	NS
CNTN4	NS	8.33E-02	-0.071	1.75E-01	NS
DHRS12	NS	8.34E-02	-0.071	1.75E-01	NS
TTLL1	NS	8.36E-02	-0.071	1.75E-01	NS
TUBB2B	NS	8.37E-02	7.10E-02	1.76E-01	NS
DENND2A	NS	8.37E-02	-0.071	1.76E-01	NS
DTWD2	NS	8.37E-02	-0.071	1.76E-01	NS
F8	NS	8.38E-02	-0.071	1.76E-01	NS
TMCC1	NS	8.39E-02	-0.0709	1.76E-01	NS
SERINC2	NS	8.39E-02	7.09E-02	1.76E-01	NS
SLC49A4	NS	8.40E-02	-0.0709	1.76E-01	NS
PRKG2	NS	8.40E-02	-0.0709	1.76E-01	NS
NPIPA8	NS	8.40E-02	7.09E-02	1.76E-01	NS
TRERF1	NS	8.40E-02	7.09E-02	1.76E-01	NS
ERN2	NS	8.40E-02	7.09E-02	1.76E-01	NS
LGMN	NS	8.42E-02	7.09E-02	1.76E-01	NS

KRT17	NS	8.42E-02	-0.0708	1.76E-01	NS
C1QTNF2	NS	8.42E-02	7.08E-02	1.76E-01	NS
NDUFB11	NS	8.42E-02	-0.0708	1.76E-01	NS
MMP20	NS	8.43E-02	-0.0708	1.76E-01	NS
MX2	NS	8.43E-02	-0.0708	1.77E-01	NS
PCDHA9	NS	8.43E-02	7.08E-02	1.77E-01	NS
NSMCE4A	NS	8.44E-02	-0.0708	1.77E-01	NS
SDE2	NS	8.44E-02	7.08E-02	1.77E-01	NS
LY6G6D	NS	8.44E-02	-0.0708	1.77E-01	NS
SMCP	NS	8.44E-02	-0.0708	1.77E-01	NS
HSFY2	NS	8.45E-02	7.08E-02	1.77E-01	NS
C17orf67	NS	8.46E-02	-0.0708	1.77E-01	NS
UBD	NS	8.46E-02	-0.0708	1.77E-01	NS
HSD17B3	NS	8.46E-02	7.08E-02	1.77E-01	NS
NME2	NS	8.47E-02	-0.0708	1.77E-01	NS
LEFTY2	NS	8.47E-02	-0.0707	1.77E-01	NS
KANK1	NS	8.48E-02	-0.0707	1.77E-01	NS
SLC25A21	NS	8.48E-02	7.07E-02	1.77E-01	NS
KCNMB2	NS	8.48E-02	-0.0707	1.77E-01	NS
TUB	NS	8.49E-02	-0.0707	1.78E-01	NS
COA6	NS	8.49E-02	-0.0707	1.78E-01	NS
EVX1	NS	8.50E-02	-0.0707	1.78E-01	NS
ADH5	NS	8.50E-02	-0.0707	1.78E-01	NS
TJP3	NS	8.50E-02	7.07E-02	1.78E-01	NS
GPR68	NS	8.50E-02	-0.0707	1.78E-01	NS
INSYN2B	NS	8.50E-02	-0.0707	1.78E-01	NS
PRPF8	NS	8.51E-02	-0.0707	1.78E-01	NS
AMIGO3	NS	8.51E-02	-0.0706	1.78E-01	NS
HIC1	NS	8.51E-02	-0.0706	1.78E-01	NS
KRTAP17-1	NS	8.52E-02	-0.0706	1.78E-01	NS
RRAS	NS	8.52E-02	-0.0706	1.78E-01	NS
RHOBTB2	NS	8.53E-02	-0.0706	1.78E-01	NS
SEC63	NS	8.53E-02	-0.0706	1.78E-01	NS
PDE6C	NS	8.53E-02	-0.0706	1.78E-01	NS
SLC25A32	NS	8.53E-02	-0.0706	1.78E-01	NS
ARHGAP11A-SCG5	NS	8.54E-02	-0.0706	1.78E-01	NS
ZFP41	NS	8.54E-02	-0.0706	1.78E-01	NS

PDCD11	NS	8.54E-02	-0.0706	1.78E-01	NS
NCKAP1	NS	8.55E-02	-0.0706	1.78E-01	NS
THAP10	NS	8.55E-02	-0.0706	1.78E-01	NS
CRB2	NS	8.55E-02	-0.0706	1.78E-01	NS
IRX1	NS	8.55E-02	7.06E-02	1.78E-01	NS
ZNF524	NS	8.56E-02	7.05E-02	1.78E-01	NS
UPK3B	NS	8.56E-02	-0.0705	1.78E-01	NS
SLC14A1	NS	8.56E-02	7.05E-02	1.78E-01	NS
MAGIX	NS	8.56E-02	-0.0705	1.78E-01	NS
PROC	NS	8.56E-02	-0.0705	1.78E-01	NS
RAET1E	NS	8.57E-02	-0.0705	1.79E-01	NS
RAB6D	NS	8.57E-02	7.05E-02	1.79E-01	NS
COG3	NS	8.58E-02	-0.0705	1.79E-01	NS
PRR15	NS	8.58E-02	-0.0705	1.79E-01	NS
SNAP47	NS	8.58E-02	-0.0705	1.79E-01	NS
SNX33	NS	8.59E-02	7.05E-02	1.79E-01	NS
ZNF646	NS	8.59E-02	-0.0705	1.79E-01	NS
CD276	NS	8.60E-02	7.05E-02	1.79E-01	NS
DGKE	NS	8.60E-02	-0.0704	1.79E-01	NS
SLC35D3	NS	8.60E-02	-0.0704	1.79E-01	NS
EBF4	NS	8.61E-02	7.04E-02	1.79E-01	NS
POP5	NS	8.61E-02	-0.0704	1.79E-01	NS
LRRC34	NS	8.61E-02	-0.0704	1.79E-01	NS
FSCN3	NS	8.62E-02	-0.0704	1.79E-01	NS
OR51E2	NS	8.62E-02	7.04E-02	1.79E-01	NS
DEFB103B	NS	8.62E-02	-0.0704	1.79E-01	NS
LCN10	NS	8.62E-02	7.04E-02	1.79E-01	NS
DCTN1	NS	8.62E-02	-0.0704	1.79E-01	NS
GEMIN5	NS	8.63E-02	-0.0704	1.79E-01	NS
SGSM2	NS	8.63E-02	-0.0704	1.79E-01	NS
ASZ1	NS	8.63E-02	-0.0704	1.80E-01	NS
CHD3	NS	8.63E-02	7.04E-02	1.80E-01	NS
JAKMIP3	NS	8.64E-02	-0.0704	1.80E-01	NS
ASB11	NS	8.64E-02	-0.0704	1.80E-01	NS
TMEM69	NS	8.64E-02	-0.0704	1.80E-01	NS
GAS2L3	NS	8.65E-02	7.03E-02	1.80E-01	NS
TXNL1	NS	8.65E-02	7.03E-02	1.80E-01	NS

NLRP12	NS	8.65E-02	-0.0703	1.80E-01	NS
SERF1A	NS	8.65E-02	7.03E-02	1.80E-01	NS
KRTAP9-7	NS	8.65E-02	7.03E-02	1.80E-01	NS
TIA1	NS	8.67E-02	-0.0703	1.80E-01	NS
EAF2	NS	8.67E-02	-0.0703	1.80E-01	NS
CYP11B2	NS	8.68E-02	7.03E-02	1.80E-01	NS
MAP7D1	NS	8.68E-02	7.03E-02	1.80E-01	NS
C1orf122	NS	8.68E-02	-0.0703	1.80E-01	NS
CDC42SE2	NS	8.68E-02	-0.0703	1.80E-01	NS
AADAT	NS	8.69E-02	7.03E-02	1.80E-01	NS
ISLR	NS	8.69E-02	7.02E-02	1.80E-01	NS
GATD1	NS	8.69E-02	-0.0702	1.80E-01	NS
PHKA1	NS	8.69E-02	-0.0702	1.80E-01	NS
ZNF154	NS	8.70E-02	7.02E-02	1.80E-01	NS
EIF3CL	NS	8.70E-02	-0.0702	1.80E-01	NS
KRT24	NS	8.70E-02	7.02E-02	1.80E-01	NS
LRSAM1	NS	8.70E-02	7.02E-02	1.80E-01	NS
OTUD7B	NS	8.70E-02	-0.0702	1.80E-01	NS
LYRM1	NS	8.71E-02	-0.0702	1.80E-01	NS
PLXDC2	NS	8.71E-02	-0.0702	1.80E-01	NS
CPLX4	NS	8.71E-02	-0.0702	1.80E-01	NS
FANK1	NS	8.71E-02	-0.0702	1.80E-01	NS
UGT1A3	NS	8.71E-02	-0.0702	1.80E-01	NS
ZNF16	NS	8.71E-02	-0.0702	1.80E-01	NS
PABPC1L2B	NS	8.71E-02	7.02E-02	1.80E-01	NS
EEF1A2	NS	8.72E-02	7.02E-02	1.81E-01	NS
GPRC5D	NS	8.72E-02	7.02E-02	1.81E-01	NS
OR5H2	NS	8.72E-02	-0.0702	1.81E-01	NS
EFCAB8	NS	8.72E-02	-0.0702	1.81E-01	NS
FAM90A16P	NS	8.72E-02	-0.0702	1.81E-01	NS
MT1F	NS	8.73E-02	7.02E-02	1.81E-01	NS
PLSCR3	NS	8.74E-02	7.01E-02	1.81E-01	NS
LHB	NS	8.74E-02	7.01E-02	1.81E-01	NS
SH3GL2	NS	8.75E-02	-0.0701	1.81E-01	NS
LRRC20	NS	8.75E-02	-0.0701	1.81E-01	NS
MB	NS	8.75E-02	7.01E-02	1.81E-01	NS
OTP	NS	8.75E-02	7.01E-02	1.81E-01	NS

PRIMA1	NS	8.76E-02	-0.0701	1.81E-01	NS
CA5A	NS	8.76E-02	7.01E-02	1.81E-01	NS
IL9	NS	8.77E-02	-0.0701	1.81E-01	NS
KLKB1	NS	8.78E-02	-0.07	1.82E-01	NS
FRK	NS	8.78E-02	-0.07	1.82E-01	NS
HS6ST3	NS	8.78E-02	7.00E-02	1.82E-01	NS
CHST12	NS	8.79E-02	-0.07	1.82E-01	NS
LITAF	NS	8.79E-02	-0.07	1.82E-01	NS
ZFHX3	NS	8.80E-02	-0.07	1.82E-01	NS
EXOSC9	NS	8.80E-02	-0.07	1.82E-01	NS
SBSPON	NS	8.81E-02	-0.07	1.82E-01	NS
RELA	NS	8.81E-02	7.00E-02	1.82E-01	NS
ZNF479	NS	8.81E-02	7.00E-02	1.82E-01	NS
DCN	NS	8.82E-02	-0.07	1.82E-01	NS
MARCHF2	NS	8.82E-02	6.99E-02	1.82E-01	NS
CBFA2T3	NS	8.82E-02	-0.07	1.82E-01	NS
SH2D6	NS	8.82E-02	-0.07	1.82E-01	NS
NPTX1	NS	8.82E-02	7.00E-02	1.82E-01	NS
ARHGAP11B	NS	8.82E-02	-0.0699	1.82E-01	NS
EIF3G	NS	8.83E-02	-0.0699	1.82E-01	NS
CSPP1	NS	8.84E-02	-0.0699	1.82E-01	NS
ADAMTS5	NS	8.84E-02	6.99E-02	1.82E-01	NS
MAP3K3	NS	8.84E-02	-0.0699	1.82E-01	NS
KLRD1	NS	8.85E-02	-0.0699	1.83E-01	NS
CTC1	NS	8.85E-02	6.99E-02	1.83E-01	NS
RPGR	NS	8.86E-02	-0.0699	1.83E-01	NS
TRPC4AP	NS	8.87E-02	6.99E-02	1.83E-01	NS
TUBA3E	NS	8.87E-02	-0.0699	1.83E-01	NS
CT45A10	NS	8.87E-02	6.99E-02	1.83E-01	NS
PPP1R2C	NS	8.87E-02	-0.0698	1.83E-01	NS
SPACA3	NS	8.87E-02	-0.0698	1.83E-01	NS
OR56A1	NS	8.87E-02	-0.0698	1.83E-01	NS
PON3	NS	8.88E-02	-0.0698	1.83E-01	NS
TMED9	NS	8.88E-02	-0.0698	1.83E-01	NS
PROS1	NS	8.88E-02	-0.0698	1.83E-01	NS
KRTAP10-11	NS	8.88E-02	6.98E-02	1.83E-01	NS
GIMAP4	NS	8.89E-02	-0.0698	1.83E-01	NS

PPP1R9A	NS	8.89E-02	-0.0698	1.83E-01	NS
ATP2B1	NS	8.90E-02	-0.0698	1.83E-01	NS
FOXD4L1	NS	8.91E-02	6.98E-02	1.83E-01	NS
PPCS	NS	8.91E-02	-0.0698	1.84E-01	NS
RPS6	NS	8.91E-02	-0.0698	1.84E-01	NS
GRAP	NS	8.91E-02	6.98E-02	1.84E-01	NS
CNTF	NS	8.92E-02	-0.0697	1.84E-01	NS
RASGRP3	NS	8.92E-02	-0.0697	1.84E-01	NS
PFN2	NS	8.92E-02	-0.0697	1.84E-01	NS
BAIAP3	NS	8.92E-02	-0.0697	1.84E-01	NS
FXYD2	NS	8.93E-02	6.97E-02	1.84E-01	NS
ALOX15	NS	8.93E-02	-0.0697	1.84E-01	NS
RHBDD3	NS	8.93E-02	6.97E-02	1.84E-01	NS
DENND2B	NS	8.93E-02	-0.0697	1.84E-01	NS
SERPINA5	NS	8.94E-02	-0.0697	1.84E-01	NS
ZNF92	NS	8.94E-02	-0.0697	1.84E-01	NS
OR4F17	NS	8.94E-02	-0.0697	1.84E-01	NS
MACC1	NS	8.95E-02	-0.0697	1.84E-01	NS
LACRT	NS	8.96E-02	6.97E-02	1.84E-01	NS
SLC48A1	NS	8.96E-02	6.96E-02	1.84E-01	NS
KRT34	NS	8.97E-02	-0.0696	1.84E-01	NS
DCTN6	NS	8.97E-02	-0.0696	1.84E-01	NS
ALPG	NS	8.97E-02	6.96E-02	1.84E-01	NS
TGM6	NS	8.98E-02	6.96E-02	1.85E-01	NS
POU4F1	NS	8.98E-02	6.96E-02	1.85E-01	NS
KDM7A	NS	8.98E-02	6.96E-02	1.85E-01	NS
CLN5	NS	8.98E-02	-0.0696	1.85E-01	NS
SAMD9	NS	8.98E-02	-0.0696	1.85E-01	NS
MKNK2	NS	8.99E-02	6.96E-02	1.85E-01	NS
SIGMAR1	NS	8.99E-02	6.96E-02	1.85E-01	NS
CYP3A4	NS	8.99E-02	-0.0696	1.85E-01	NS
NOS1	NS	9.00E-02	6.96E-02	1.85E-01	NS
FAM20A	NS	9.00E-02	-0.0696	1.85E-01	NS
LCN12	NS	9.00E-02	6.96E-02	1.85E-01	NS
AMOT	NS	9.00E-02	-0.0696	1.85E-01	NS
BCS1L	NS	9.01E-02	-0.0696	1.85E-01	NS
NAALADL2	NS	9.01E-02	-0.0695	1.85E-01	NS

HMGCS2	NS	9.01E-02	-0.0695	1.85E-01	NS
OIP5	NS	9.02E-02	-0.0695	1.85E-01	NS
CNTN1	NS	9.02E-02	-0.0695	1.85E-01	NS
GLP1R	NS	9.02E-02	-0.0695	1.85E-01	NS
ORAI3	NS	9.02E-02	-0.0695	1.85E-01	NS
OR2AT4	NS	9.02E-02	-0.0695	1.85E-01	NS
WASHC2C	NS	9.03E-02	-0.0695	1.85E-01	NS
CYP2A13	NS	9.03E-02	6.95E-02	1.85E-01	NS
MAMDC4	NS	9.03E-02	-0.0695	1.85E-01	NS
FAM210A	NS	9.03E-02	-0.0695	1.85E-01	NS
CLSTN1	NS	9.04E-02	-0.0695	1.85E-01	NS
NSMF	NS	9.05E-02	6.95E-02	1.85E-01	NS
WDR83OS	NS	9.05E-02	-0.0695	1.85E-01	NS
HPCAL1	NS	9.05E-02	6.95E-02	1.85E-01	NS
UTP23	NS	9.05E-02	6.95E-02	1.85E-01	NS
PITPNM1	NS	9.05E-02	-0.0695	1.86E-01	NS
MACROD1	NS	9.06E-02	6.94E-02	1.86E-01	NS
GPR107	NS	9.06E-02	-0.0694	1.86E-01	NS
UBA6	NS	9.06E-02	-0.0694	1.86E-01	NS
MLPH	NS	9.06E-02	6.94E-02	1.86E-01	NS
STON1-GTF2A1L	NS	9.07E-02	-0.0694	1.86E-01	NS
ATP11B	NS	9.07E-02	-0.0694	1.86E-01	NS
CNTNAP5	NS	9.07E-02	-0.0694	1.86E-01	NS
RXRA	NS	9.07E-02	-0.0694	1.86E-01	NS
DOP1B	NS	9.07E-02	-0.0694	1.86E-01	NS
NUDC	NS	9.08E-02	-0.0694	1.86E-01	NS
RPA3	NS	9.09E-02	-0.0694	1.86E-01	NS
OR2A12	NS	9.10E-02	-0.0694	1.86E-01	NS
DPP8	NS	9.10E-02	-0.0694	1.86E-01	NS
IER5L	NS	9.10E-02	6.93E-02	1.86E-01	NS
NUDT4	NS	9.11E-02	6.93E-02	1.86E-01	NS
ACAP3	NS	9.11E-02	6.93E-02	1.86E-01	NS
GOLT1B	NS	9.12E-02	-0.0693	1.87E-01	NS
OPTN	NS	9.12E-02	-0.0693	1.87E-01	NS
POLK	NS	9.13E-02	-0.0693	1.87E-01	NS
HS1BP3	NS	9.13E-02	-0.0693	1.87E-01	NS
KMT2E	NS	9.14E-02	-0.0693	1.87E-01	NS

IQSEC3	NS	9.14E-02	6.93E-02	1.87E-01	NS
ZFP30	NS	9.14E-02	-0.0693	1.87E-01	NS
GABRR1	NS	9.14E-02	-0.0693	1.87E-01	NS
NUDCD2	NS	9.14E-02	-0.0693	1.87E-01	NS
EPHB4	NS	9.14E-02	-0.0693	1.87E-01	NS
GPAA1	NS	9.14E-02	-0.0693	1.87E-01	NS
DCLRE1B	NS	9.15E-02	-0.0693	1.87E-01	NS
RARRES1	NS	9.15E-02	-0.0692	1.87E-01	NS
DHRS11	NS	9.16E-02	6.92E-02	1.87E-01	NS
ZNF561	NS	9.16E-02	-0.0692	1.87E-01	NS
NUPR2	NS	9.16E-02	-0.0692	1.87E-01	NS
OR5D14	NS	9.16E-02	-0.0692	1.87E-01	NS
DLC1	NS	9.17E-02	-0.0692	1.87E-01	NS
CENPU	NS	9.17E-02	-0.0692	1.87E-01	NS
APOB	NS	9.18E-02	-0.0692	1.87E-01	NS
GAB3	NS	9.18E-02	6.92E-02	1.87E-01	NS
EIF1AY	NS	9.19E-02	6.92E-02	1.87E-01	NS
H3C4	NS	9.19E-02	-0.0692	1.88E-01	NS
C9orf72	NS	9.20E-02	-0.0691	1.88E-01	NS
GRIP1	NS	9.20E-02	-0.0691	1.88E-01	NS
SFMBT2	NS	9.20E-02	-0.0691	1.88E-01	NS
SRD5A1	NS	9.21E-02	-0.0691	1.88E-01	NS
PSG11	NS	9.21E-02	-0.0691	1.88E-01	NS
CYP17A1	NS	9.21E-02	-0.0691	1.88E-01	NS
CR1	NS	9.21E-02	-0.0691	1.88E-01	NS
ZBTB45	NS	9.22E-02	6.91E-02	1.88E-01	NS
SERPING1	NS	9.22E-02	-0.0691	1.88E-01	NS
BOLA1	NS	9.23E-02	-0.0691	1.88E-01	NS
NFE2	NS	9.23E-02	6.91E-02	1.88E-01	NS
FAM149A	NS	9.23E-02	-0.0691	1.88E-01	NS
TOMM40	NS	9.24E-02	6.91E-02	1.88E-01	NS
KLHL30	NS	9.24E-02	-0.069	1.88E-01	NS
EIF4G1	NS	9.24E-02	6.90E-02	1.88E-01	NS
EIF3C	NS	9.25E-02	-0.069	1.88E-01	NS
IFT27	NS	9.25E-02	-0.069	1.88E-01	NS
CEP20	NS	9.25E-02	6.90E-02	1.88E-01	NS
DNALI1	NS	9.26E-02	-0.069	1.89E-01	NS

TCEA1	NS	9.26E-02	-0.069	1.89E-01	NS
SRCIN1	NS	9.26E-02	6.90E-02	1.89E-01	NS
P2RX1	NS	9.26E-02	-0.069	1.89E-01	NS
CDH19	NS	9.27E-02	-0.069	1.89E-01	NS
BNC1	NS	9.27E-02	6.90E-02	1.89E-01	NS
RNF168	NS	9.28E-02	-0.069	1.89E-01	NS
AGPAT2	NS	9.28E-02	-0.069	1.89E-01	NS
CDC73	NS	9.28E-02	6.90E-02	1.89E-01	NS
RUBCN	NS	9.28E-02	-0.069	1.89E-01	NS
LBX2	NS	9.29E-02	-0.069	1.89E-01	NS
CR1L	NS	9.29E-02	-0.069	1.89E-01	NS
DBT	NS	9.29E-02	-0.0689	1.89E-01	NS
TMOD4	NS	9.29E-02	-0.0689	1.89E-01	NS
C1QBP	NS	9.29E-02	-0.0689	1.89E-01	NS
SLC16A1	NS	9.30E-02	-0.0689	1.89E-01	NS
MYH14	NS	9.30E-02	6.89E-02	1.89E-01	NS
PLCD3	NS	9.30E-02	-0.0689	1.89E-01	NS
ARSG	NS	9.30E-02	-0.0689	1.89E-01	NS
RIOK3	NS	9.31E-02	6.89E-02	1.89E-01	NS
ADAT2	NS	9.31E-02	6.89E-02	1.89E-01	NS
SLC9A6	NS	9.31E-02	-0.0689	1.89E-01	NS
MAPK7	NS	9.31E-02	6.89E-02	1.89E-01	NS
MAFG	NS	9.31E-02	-0.0689	1.89E-01	NS
OLIG1	NS	9.32E-02	-0.0689	1.89E-01	NS
KCTD14	NS	9.33E-02	-0.0689	1.90E-01	NS
SLC16A6	NS	9.34E-02	-0.0688	1.90E-01	NS
CDK5R2	NS	9.34E-02	-0.0688	1.90E-01	NS
GABPA	NS	9.34E-02	-0.0688	1.90E-01	NS
PDK3	NS	9.34E-02	6.88E-02	1.90E-01	NS
THEM4	NS	9.35E-02	-0.0688	1.90E-01	NS
AKR1C8P	NS	9.35E-02	-0.0688	1.90E-01	NS
AGRP	NS	9.36E-02	-0.0688	1.90E-01	NS
CXCR3	NS	9.36E-02	-0.0688	1.90E-01	NS
FHL2	NS	9.36E-02	-0.0688	1.90E-01	NS
ZNF99	NS	9.37E-02	-0.0688	1.90E-01	NS
RMC1	NS	9.37E-02	6.88E-02	1.90E-01	NS
BACE2	NS	9.38E-02	-0.0688	1.90E-01	NS

LDHA	NS	9.38E-02	6.88E-02	1.90E-01	NS
IRF8	NS	9.39E-02	6.87E-02	1.90E-01	NS
PAH	NS	9.40E-02	-0.0687	1.91E-01	NS
SCAMP5	NS	9.41E-02	-0.0687	1.91E-01	NS
TMEM231	NS	9.42E-02	-0.0687	1.91E-01	NS
LYPD4	NS	9.42E-02	-0.0687	1.91E-01	NS
SAMD4A	NS	9.43E-02	-0.0687	1.91E-01	NS
CDH9	NS	9.43E-02	-0.0687	1.91E-01	NS
DHRS2	NS	9.43E-02	-0.0687	1.91E-01	NS
CTNNA2	NS	9.43E-02	-0.0687	1.91E-01	NS
CLNK	NS	9.44E-02	-0.0686	1.91E-01	NS
KIF20B	NS	9.44E-02	-0.0686	1.91E-01	NS
MTUS2	NS	9.44E-02	-0.0686	1.91E-01	NS
KRT86	NS	9.44E-02	6.86E-02	1.91E-01	NS
USP17L29	NS	9.44E-02	6.86E-02	1.91E-01	NS
CLCN4	NS	9.45E-02	-0.0686	1.91E-01	NS
OR52N1	NS	9.45E-02	6.86E-02	1.91E-01	NS
IL7R	NS	9.45E-02	-0.0686	1.91E-01	NS
AHCYL2	NS	9.46E-02	-0.0686	1.91E-01	NS
STYXL2	NS	9.46E-02	6.86E-02	1.92E-01	NS
STMN3	NS	9.46E-02	-0.0686	1.92E-01	NS
CCDC88B	NS	9.47E-02	6.86E-02	1.92E-01	NS
CWC25	NS	9.47E-02	6.86E-02	1.92E-01	NS
TRO	NS	9.47E-02	-0.0686	1.92E-01	NS
EHMT1	NS	9.47E-02	6.86E-02	1.92E-01	NS
RPS11	NS	9.48E-02	-0.0685	1.92E-01	NS
PRAMEF1	NS	9.49E-02	-0.0685	1.92E-01	NS
GOLGA5	NS	9.49E-02	6.85E-02	1.92E-01	NS
SRP72	NS	9.50E-02	-0.0685	1.92E-01	NS
THAP8	NS	9.50E-02	-0.0685	1.92E-01	NS
SLF2	NS	9.50E-02	6.85E-02	1.92E-01	NS
ASF1B	NS	9.50E-02	-0.0685	1.92E-01	NS
MAPRE3	NS	9.51E-02	-0.0685	1.92E-01	NS
ZC3HC1	NS	9.51E-02	-0.0685	1.92E-01	NS
ALG13	NS	9.51E-02	-0.0685	1.92E-01	NS
WNT3	NS	9.51E-02	-0.0685	1.92E-01	NS
TNFAIP3	NS	9.51E-02	6.85E-02	1.92E-01	NS

MAT2A	NS	9.51E-02	-0.0685	1.92E-01	NS
HBB	NS	9.51E-02	-0.0685	1.92E-01	NS
NFE2L1	NS	9.51E-02	6.85E-02	1.92E-01	NS
RNH1	NS	9.52E-02	-0.0685	1.92E-01	NS
RIMKLB	NS	9.52E-02	6.85E-02	1.92E-01	NS
EDDM3B	NS	9.52E-02	6.85E-02	1.92E-01	NS
CRHBP	NS	9.53E-02	-0.0685	1.92E-01	NS
F2RL1	NS	9.53E-02	-0.0684	1.92E-01	NS
XKR9	NS	9.53E-02	-0.0684	1.92E-01	NS
CST5	NS	9.53E-02	-0.0684	1.92E-01	NS
NLGN4Y	NS	9.54E-02	6.84E-02	1.93E-01	NS
SPRR1A	NS	9.54E-02	-0.0684	1.93E-01	NS
DDX47	NS	9.54E-02	-0.0684	1.93E-01	NS
PROKR2	NS	9.55E-02	-0.0684	1.93E-01	NS
DEFB131A	NS	9.55E-02	-0.0684	1.93E-01	NS
NEURL1	NS	9.55E-02	-0.0684	1.93E-01	NS
FAAP100	NS	9.55E-02	-0.0684	1.93E-01	NS
ACACB	NS	9.56E-02	-0.0684	1.93E-01	NS
ELMO1	NS	9.57E-02	-0.0684	1.93E-01	NS
FOXF1	NS	9.57E-02	6.84E-02	1.93E-01	NS
IFIT2	NS	9.58E-02	-0.0683	1.93E-01	NS
ITGB5	NS	9.60E-02	-0.0683	1.93E-01	NS
GPR21	NS	9.60E-02	-0.0683	1.94E-01	NS
ACSL4	NS	9.60E-02	-0.0683	1.94E-01	NS
RGS22	NS	9.60E-02	-0.0683	1.94E-01	NS
TAS2R38	NS	9.60E-02	-0.0683	1.94E-01	NS
MAPK15	NS	9.61E-02	6.83E-02	1.94E-01	NS
RAE1	NS	9.61E-02	6.83E-02	1.94E-01	NS
GNG10	NS	9.61E-02	-0.0683	1.94E-01	NS
GJD3	NS	9.61E-02	6.83E-02	1.94E-01	NS
FUNDC2	NS	9.61E-02	-0.0683	1.94E-01	NS
ZNF732	NS	9.61E-02	-0.0683	1.94E-01	NS
TLE4	NS	9.62E-02	-0.0683	1.94E-01	NS
JAK2	NS	9.63E-02	6.82E-02	1.94E-01	NS
TBC1D3	NS	9.63E-02	-0.0682	1.94E-01	NS
RAB3GAP1	NS	9.64E-02	-0.0682	1.94E-01	NS
TRAF2	NS	9.64E-02	-0.0682	1.94E-01	NS

IQCM	NS	9.64E-02	-0.0682	1.94E-01	NS
OR3A3	NS	9.65E-02	-0.0682	1.94E-01	NS
AP1G1	NS	9.65E-02	6.82E-02	1.94E-01	NS
C1orf216	NS	9.65E-02	-0.0682	1.94E-01	NS
CCDC184	NS	9.65E-02	-0.0682	1.94E-01	NS
ABHD17C	NS	9.66E-02	-0.0682	1.94E-01	NS
OOEP	NS	9.66E-02	-0.0682	1.94E-01	NS
PHKG1	NS	9.66E-02	-0.0682	1.94E-01	NS
COMMD7	NS	9.66E-02	-0.0682	1.94E-01	NS
PIAS2	NS	9.67E-02	6.82E-02	1.94E-01	NS
IL6ST	NS	9.67E-02	6.82E-02	1.95E-01	NS
MAF	NS	9.68E-02	-0.0681	1.95E-01	NS
CTNNAL1	NS	9.69E-02	-0.0681	1.95E-01	NS
TOB1	NS	9.69E-02	6.81E-02	1.95E-01	NS
EBP	NS	9.69E-02	-0.0681	1.95E-01	NS
GDF6	NS	9.69E-02	6.81E-02	1.95E-01	NS
LRCH3	NS	9.69E-02	-0.0681	1.95E-01	NS
NWD1	NS	9.70E-02	-0.0681	1.95E-01	NS
ITGAE	NS	9.71E-02	6.81E-02	1.95E-01	NS
CAMK1G	NS	9.71E-02	-0.0681	1.95E-01	NS
OR8B4	NS	9.71E-02	-0.0681	1.95E-01	NS
SMARCAD1	NS	9.72E-02	-0.0681	1.95E-01	NS
SINHCAF	NS	9.72E-02	-0.0681	1.95E-01	NS
WNT5A	NS	9.73E-02	-0.068	1.95E-01	NS
PIGV	NS	9.73E-02	-0.068	1.95E-01	NS
ANKRD12	NS	9.73E-02	-0.068	1.95E-01	NS
KANSL1L	NS	9.73E-02	-0.068	1.95E-01	NS
GYS2	NS	9.74E-02	-0.068	1.95E-01	NS
ITIH3	NS	9.74E-02	6.80E-02	1.95E-01	NS
TICRR	NS	9.74E-02	-0.068	1.95E-01	NS
SPTBN4	NS	9.75E-02	6.80E-02	1.96E-01	NS
ARHGEF5	NS	9.75E-02	-0.068	1.96E-01	NS
NARS1	NS	9.76E-02	-0.068	1.96E-01	NS
IRGQ	NS	9.76E-02	6.80E-02	1.96E-01	NS
ZNF738	NS	9.76E-02	-0.068	1.96E-01	NS
BROX	NS	9.77E-02	6.80E-02	1.96E-01	NS
MARS2	NS	9.77E-02	-0.068	1.96E-01	NS

SS18	NS	9.77E-02	-0.068	1.96E-01	NS
PPAT	NS	9.78E-02	-0.0679	1.96E-01	NS
IL17RD	NS	9.78E-02	6.79E-02	1.96E-01	NS
PCDHB12	NS	9.78E-02	6.79E-02	1.96E-01	NS
FCHO2	NS	9.79E-02	6.79E-02	1.96E-01	NS
CCNL1	NS	9.79E-02	6.79E-02	1.96E-01	NS
CSF2RB	NS	9.80E-02	6.79E-02	1.96E-01	NS
S1PR2	NS	9.81E-02	6.79E-02	1.97E-01	NS
FUT11	NS	9.81E-02	-0.0679	1.97E-01	NS
XPO1	NS	9.82E-02	-0.0678	1.97E-01	NS
SCRN1	NS	9.82E-02	-0.0679	1.97E-01	NS
HID1	NS	9.82E-02	6.79E-02	1.97E-01	NS
PYM1	NS	9.82E-02	-0.0679	1.97E-01	NS
GMFB	NS	9.83E-02	-0.0678	1.97E-01	NS
SYAP1	NS	9.83E-02	-0.0678	1.97E-01	NS
GON4L	NS	9.84E-02	-0.0678	1.97E-01	NS
C10orf53	NS	9.84E-02	-0.0678	1.97E-01	NS
PRKAR2B	NS	9.85E-02	-0.0678	1.97E-01	NS
IL17RA	NS	9.85E-02	6.78E-02	1.97E-01	NS
DSCAM	NS	9.85E-02	-0.0678	1.97E-01	NS
CC2D1A	NS	9.86E-02	-0.0678	1.97E-01	NS
GZF1	NS	9.86E-02	-0.0678	1.97E-01	NS
SPOUT1	NS	9.86E-02	-0.0678	1.97E-01	NS
SLC15A1	NS	9.87E-02	-0.0677	1.97E-01	NS
FBH1	NS	9.88E-02	-0.0677	1.98E-01	NS
MAPKAPK2	NS	9.88E-02	6.77E-02	1.98E-01	NS
TLE1	NS	9.89E-02	6.77E-02	1.98E-01	NS
PLA2G4B	NS	9.89E-02	-0.0677	1.98E-01	NS
GINM1	NS	9.89E-02	-0.0677	1.98E-01	NS
TSPYL2	NS	9.89E-02	6.77E-02	1.98E-01	NS
TPD52L1	NS	9.90E-02	-0.0677	1.98E-01	NS
EPB42	NS	9.90E-02	-0.0677	1.98E-01	NS
MT-ND2	NS	9.90E-02	6.77E-02	1.98E-01	NS
GCLM	NS	9.90E-02	-0.0677	1.98E-01	NS
OR2G2	NS	9.91E-02	-0.0677	1.98E-01	NS
SIDT2	NS	9.91E-02	6.77E-02	1.98E-01	NS
HDAC2	NS	9.91E-02	-0.0677	1.98E-01	NS

SCYGR4	NS	9.92E-02	6.77E-02	1.98E-01	NS
CHTF18	NS	9.92E-02	-0.0676	1.98E-01	NS
CFAP91	NS	9.93E-02	-0.0676	1.98E-01	NS
THOC3	NS	9.93E-02	-0.0676	1.98E-01	NS
ADAM9	NS	9.93E-02	6.76E-02	1.98E-01	NS
TOR1AIP1	NS	9.94E-02	-0.0676	1.98E-01	NS
H3C6	NS	9.95E-02	-0.0676	1.98E-01	NS
CLEC20A	NS	9.95E-02	-0.0676	1.98E-01	NS
OSCP1	NS	9.95E-02	-0.0676	1.99E-01	NS
NOXA1	NS	9.96E-02	6.76E-02	1.99E-01	NS
ABHD14B	NS	9.97E-02	6.76E-02	1.99E-01	NS
WDR48	NS	9.98E-02	-0.0675	1.99E-01	NS
PRRG1	NS	9.98E-02	-0.0675	1.99E-01	NS
CEACAM7	NS	9.99E-02	-0.0675	1.99E-01	NS
ITIH1	NS	9.99E-02	-0.0675	1.99E-01	NS
MSANTD3	NS	9.99E-02	-0.0675	1.99E-01	NS
STAM	NS	9.99E-02	-0.0675	1.99E-01	NS
XCL2	NS	9.99E-02	-0.0675	1.99E-01	NS
LALBA	NS	9.99E-02	-0.0675	1.99E-01	NS
UGT2B10	NS	9.99E-02	-0.0675	1.99E-01	NS
CXCL8	NS	1.00E-01	-0.0675	1.99E-01	NS
SUMF1	NS	1.00E-01	-0.0675	1.99E-01	NS
ZSCAN4	NS	1.00E-01	-0.0674	2.00E-01	NS
LGALS9B	NS	1.00E-01	6.74E-02	2.00E-01	NS
SPATS1	NS	1.00E-01	6.74E-02	2.00E-01	NS
LSP1	NS	1.00E-01	-0.0674	2.00E-01	NS
CCDC182	NS	1.00E-01	-0.0674	2.00E-01	NS
HBQ1	NS	1.00E-01	-0.0674	2.00E-01	NS
SLC39A1	NS	1.01E-01	-0.0674	2.00E-01	NS
RAB40AL	NS	1.01E-01	-0.0674	2.00E-01	NS
NEUROD1	NS	1.01E-01	-0.0674	2.00E-01	NS
LMBRD1	NS	1.01E-01	6.74E-02	2.00E-01	NS
NPIPB2	NS	1.01E-01	-0.0674	2.00E-01	NS
C4orf48	NS	1.01E-01	6.74E-02	2.00E-01	NS
ERVV-2	NS	1.01E-01	6.74E-02	2.00E-01	NS
RIBC2	NS	1.01E-01	-0.0674	2.00E-01	NS
CTBS	NS	1.01E-01	6.74E-02	2.00E-01	NS

FBXL22	NS	1.01E-01	-0.0674	2.00E-01	NS
STK11IP	NS	1.01E-01	-0.0673	2.00E-01	NS
ELOVL3	NS	1.01E-01	6.73E-02	2.00E-01	NS
POLR2L	NS	1.01E-01	-0.0673	2.01E-01	NS
TLN1	NS	1.01E-01	-0.0673	2.01E-01	NS
RAB10	NS	1.01E-01	-0.0673	2.01E-01	NS
PLCB3	NS	1.01E-01	6.73E-02	2.01E-01	NS
BHLHE23	NS	1.01E-01	6.73E-02	2.01E-01	NS
BCL2L11	NS	1.01E-01	6.73E-02	2.01E-01	NS
NRG3	NS	1.01E-01	-0.0673	2.01E-01	NS
RIC1	NS	1.01E-01	-0.0673	2.01E-01	NS
CYRIA	NS	1.01E-01	-0.0673	2.01E-01	NS
SPATA18	NS	1.01E-01	-0.0672	2.01E-01	NS
PNRC2	NS	1.01E-01	-0.0672	2.01E-01	NS
ISLR2	NS	1.01E-01	6.72E-02	2.01E-01	NS
IRX6	NS	1.01E-01	6.72E-02	2.01E-01	NS
ARRDC3	NS	1.01E-01	6.72E-02	2.01E-01	NS
PRR30	NS	1.01E-01	6.72E-02	2.01E-01	NS
ETF1	NS	1.01E-01	-0.0672	2.01E-01	NS
CYP51A1	NS	1.01E-01	-0.0672	2.01E-01	NS
RFTN2	NS	1.02E-01	-0.0672	2.01E-01	NS
BSCL2	NS	1.02E-01	6.72E-02	2.02E-01	NS
CLUAP1	NS	1.02E-01	-0.0672	2.02E-01	NS
PCMTD1	NS	1.02E-01	-0.0672	2.02E-01	NS
AIP	NS	1.02E-01	-0.0672	2.02E-01	NS
CFP	NS	1.02E-01	-0.0671	2.02E-01	NS
NKAIN1	NS	1.02E-01	6.71E-02	2.02E-01	NS
PANO1	NS	1.02E-01	-0.0671	2.02E-01	NS
NF1	NS	1.02E-01	-0.0671	2.02E-01	NS
GABARAPL2	NS	1.02E-01	-0.0671	2.02E-01	NS
CHRNB3	NS	1.02E-01	6.71E-02	2.02E-01	NS
DTD1	NS	1.02E-01	-0.0671	2.02E-01	NS
DENND6B	NS	1.02E-01	-0.0671	2.03E-01	NS
SLC25A4	NS	1.02E-01	6.71E-02	2.03E-01	NS
SEC14L6	NS	1.02E-01	6.70E-02	2.03E-01	NS
PRAMEF4	NS	1.02E-01	-0.067	2.03E-01	NS
CFAP57	NS	1.02E-01	-0.067	2.03E-01	NS

UPF1	NS	1.03E-01	6.70E-02	2.03E-01	NS
RAB17	NS	1.03E-01	6.70E-02	2.04E-01	NS
FAM228B	NS	1.03E-01	-0.067	2.04E-01	NS
CYP2S1	NS	1.03E-01	6.70E-02	2.04E-01	NS
ZCCHC13	NS	1.03E-01	-0.067	2.04E-01	NS
DNAAF2	NS	1.03E-01	6.69E-02	2.04E-01	NS
ABI3	NS	1.03E-01	6.69E-02	2.04E-01	NS
SPTB	NS	1.03E-01	6.69E-02	2.04E-01	NS
TMDD1	NS	1.03E-01	6.69E-02	2.04E-01	NS
JPH4	NS	1.03E-01	-0.0669	2.04E-01	NS
TATDN3	NS	1.03E-01	-0.0669	2.04E-01	NS
ATG5	NS	1.03E-01	-0.0669	2.04E-01	NS
GPANK1	NS	1.03E-01	-0.0669	2.04E-01	NS
AKTIP	NS	1.03E-01	-0.0669	2.04E-01	NS
EFCAB13	NS	1.03E-01	6.69E-02	2.04E-01	NS
RBBP9	NS	1.03E-01	-0.0669	2.04E-01	NS
FAM168A	NS	1.03E-01	6.69E-02	2.04E-01	NS
PITRM1	NS	1.03E-01	6.69E-02	2.04E-01	NS
YIPF5	NS	1.03E-01	-0.0669	2.04E-01	NS
DUXA	NS	1.03E-01	6.69E-02	2.04E-01	NS
ZNF726	NS	1.03E-01	-0.0668	2.04E-01	NS
NXN	NS	1.03E-01	-0.0668	2.04E-01	NS
IRGC	NS	1.03E-01	6.68E-02	2.04E-01	NS
ERCC8	NS	1.03E-01	-0.0668	2.04E-01	NS
CDRT1	NS	1.04E-01	-0.0668	2.05E-01	NS
HMGN5	NS	1.04E-01	6.68E-02	2.05E-01	NS
H2AL3	NS	1.04E-01	6.68E-02	2.05E-01	NS
NFIC	NS	1.04E-01	6.68E-02	2.05E-01	NS
TC2N	NS	1.04E-01	-0.0668	2.05E-01	NS
MTRES1	NS	1.04E-01	-0.0668	2.05E-01	NS
PREPL	NS	1.04E-01	6.68E-02	2.05E-01	NS
HMGA1	NS	1.04E-01	6.68E-02	2.05E-01	NS
MORC4	NS	1.04E-01	-0.0667	2.05E-01	NS
MOBP	NS	1.04E-01	6.67E-02	2.05E-01	NS
SMIM32	NS	1.04E-01	6.67E-02	2.05E-01	NS
ORMDL2	NS	1.04E-01	-0.0667	2.05E-01	NS
ID4	NS	1.04E-01	-0.0667	2.05E-01	NS

TAS1R2	NS	1.04E-01	6.67E-02	2.05E-01	NS
HS3ST4	NS	1.04E-01	-0.0667	2.06E-01	NS
HHAT	NS	1.04E-01	-0.0667	2.06E-01	NS
SIPA1L2	NS	1.04E-01	-0.0667	2.06E-01	NS
UGT1A8	NS	1.04E-01	-0.0667	2.06E-01	NS
DERPC	NS	1.04E-01	6.67E-02	2.06E-01	NS
RNF114	NS	1.04E-01	-0.0667	2.06E-01	NS
GSK3B	NS	1.04E-01	-0.0667	2.06E-01	NS
UBE2Q2	NS	1.04E-01	6.67E-02	2.06E-01	NS
KRTAP19-3	NS	1.04E-01	6.66E-02	2.06E-01	NS
PFKM	NS	1.04E-01	-0.0666	2.06E-01	NS
RBMS2	NS	1.04E-01	-0.0666	2.06E-01	NS
IMPACT	NS	1.04E-01	-0.0666	2.06E-01	NS
POTEC	NS	1.04E-01	-0.0666	2.06E-01	NS
SEPSECS	NS	1.05E-01	-0.0666	2.06E-01	NS
RAB7A	NS	1.05E-01	6.66E-02	2.06E-01	NS
RHOF	NS	1.05E-01	-0.0666	2.06E-01	NS
MPG	NS	1.05E-01	-0.0666	2.06E-01	NS
RAPGEF1	NS	1.05E-01	-0.0666	2.06E-01	NS
POLR1F	NS	1.05E-01	-0.0666	2.06E-01	NS
TNFRSF4	NS	1.05E-01	6.66E-02	2.06E-01	NS
SMIM24	NS	1.05E-01	-0.0666	2.06E-01	NS
DESI2	NS	1.05E-01	6.65E-02	2.06E-01	NS
RAB4A	NS	1.05E-01	-0.0665	2.06E-01	NS
GOLGA6L4	NS	1.05E-01	6.65E-02	2.07E-01	NS
DOCK1	NS	1.05E-01	-0.0665	2.07E-01	NS
ITFG2	NS	1.05E-01	-0.0665	2.07E-01	NS
MTFMT	NS	1.05E-01	-0.0665	2.07E-01	NS
SEMA6B	NS	1.05E-01	-0.0665	2.07E-01	NS
QARS1	NS	1.05E-01	-0.0665	2.07E-01	NS
PLCB2	NS	1.05E-01	6.65E-02	2.07E-01	NS
CHTF8	NS	1.05E-01	6.65E-02	2.07E-01	NS
GRIP2	NS	1.05E-01	-0.0665	2.07E-01	NS
SULT1A2	NS	1.05E-01	-0.0665	2.07E-01	NS
ARL14EP	NS	1.05E-01	-0.0665	2.07E-01	NS
ORM2	NS	1.05E-01	-0.0664	2.07E-01	NS
ID3	NS	1.05E-01	-0.0664	2.07E-01	NS

TPBG	NS	1.05E-01	-0.0664	2.07E-01	NS
LSM8	NS	1.05E-01	6.64E-02	2.07E-01	NS
CAPN6	NS	1.06E-01	-0.0664	2.07E-01	NS
GTPBP1	NS	1.06E-01	-0.0664	2.07E-01	NS
CEP192	NS	1.06E-01	-0.0664	2.07E-01	NS
SIKE1	NS	1.06E-01	-0.0664	2.08E-01	NS
VPS13B	NS	1.06E-01	-0.0664	2.08E-01	NS
STK36	NS	1.06E-01	-0.0664	2.08E-01	NS
KRTDAP	NS	1.06E-01	-0.0664	2.08E-01	NS
ITGA1	NS	1.06E-01	-0.0664	2.08E-01	NS
RBM12	NS	1.06E-01	-0.0664	2.08E-01	NS
ADORA3	NS	1.06E-01	-0.0664	2.08E-01	NS
LMTK2	NS	1.06E-01	-0.0664	2.08E-01	NS
PLA2G3	NS	1.06E-01	6.64E-02	2.08E-01	NS
IRF3	NS	1.06E-01	-0.0663	2.08E-01	NS
TMEM241	NS	1.06E-01	6.63E-02	2.08E-01	NS
DDX54	NS	1.06E-01	-0.0663	2.08E-01	NS
H1-6	NS	1.06E-01	-0.0663	2.08E-01	NS
SAMD15	NS	1.06E-01	6.63E-02	2.08E-01	NS
KDELR1	NS	1.06E-01	-0.0663	2.08E-01	NS
NAPEPLD	NS	1.06E-01	-0.0663	2.08E-01	NS
DEFA5	NS	1.06E-01	-0.0663	2.08E-01	NS
POLM	NS	1.06E-01	-0.0663	2.08E-01	NS
MED4	NS	1.06E-01	-0.0663	2.08E-01	NS
GEMIN6	NS	1.06E-01	-0.0663	2.08E-01	NS
CASP10	NS	1.06E-01	-0.0663	2.09E-01	NS
GFI1	NS	1.06E-01	-0.0662	2.09E-01	NS
TCF20	NS	1.07E-01	-0.0662	2.09E-01	NS
RPL41	NS	1.07E-01	-0.0662	2.09E-01	NS
KIFAP3	NS	1.07E-01	-0.0662	2.09E-01	NS
SPSB3	NS	1.07E-01	-0.0662	2.09E-01	NS
CA5B	NS	1.07E-01	6.62E-02	2.09E-01	NS
OR56B4	NS	1.07E-01	6.62E-02	2.09E-01	NS
ZNF346	NS	1.07E-01	-0.0662	2.09E-01	NS
SKA1	NS	1.07E-01	6.62E-02	2.09E-01	NS
BABAM1	NS	1.07E-01	-0.0662	2.09E-01	NS
POLR1C	NS	1.07E-01	-0.0662	2.09E-01	NS

OR7C2	NS	1.07E-01	-0.0661	2.10E-01	NS
MARS1	NS	1.07E-01	6.61E-02	2.10E-01	NS
PHF14	NS	1.07E-01	-0.0661	2.10E-01	NS
CGGBP1	NS	1.07E-01	6.61E-02	2.10E-01	NS
CTSG	NS	1.07E-01	6.61E-02	2.10E-01	NS
CATIP	NS	1.07E-01	6.61E-02	2.10E-01	NS
C11orf1	NS	1.07E-01	6.61E-02	2.10E-01	NS
CDC25A	NS	1.07E-01	-0.0661	2.10E-01	NS
ADAMTS7	NS	1.08E-01	6.61E-02	2.10E-01	NS
ZNF559-ZNF177	NS	1.08E-01	-0.066	2.10E-01	NS
UGT2A2	NS	1.08E-01	-0.066	2.10E-01	NS
SHB	NS	1.08E-01	-0.066	2.11E-01	NS
PRND	NS	1.08E-01	6.60E-02	2.11E-01	NS
PLD2	NS	1.08E-01	-0.066	2.11E-01	NS
TFB2M	NS	1.08E-01	-0.066	2.11E-01	NS
NPBWR1	NS	1.08E-01	-0.066	2.11E-01	NS
EFNA1	NS	1.08E-01	-0.066	2.11E-01	NS
NRK	NS	1.08E-01	-0.066	2.11E-01	NS
LPCAT3	NS	1.08E-01	-0.066	2.11E-01	NS
C22orf42	NS	1.08E-01	-0.066	2.11E-01	NS
NDUFA4	NS	1.08E-01	-0.0659	2.11E-01	NS
C2orf66	NS	1.08E-01	6.59E-02	2.11E-01	NS
MEIS3	NS	1.08E-01	-0.0659	2.12E-01	NS
DACH1	NS	1.08E-01	6.59E-02	2.12E-01	NS
CCDC159	NS	1.08E-01	-0.0659	2.12E-01	NS
FOS	NS	1.08E-01	-0.0659	2.12E-01	NS
CA14	NS	1.08E-01	-0.0659	2.12E-01	NS
TGFBR1	NS	1.08E-01	-0.0659	2.12E-01	NS
UBR3	NS	1.08E-01	-0.0659	2.12E-01	NS
MRGPRX2	NS	1.09E-01	-0.0659	2.12E-01	NS
EP400	NS	1.09E-01	6.59E-02	2.12E-01	NS
OR10H1	NS	1.09E-01	-0.0658	2.12E-01	NS
ABCC12	NS	1.09E-01	-0.0658	2.12E-01	NS
CFAP97D1	NS	1.09E-01	6.58E-02	2.12E-01	NS
HOXA7	NS	1.09E-01	6.58E-02	2.12E-01	NS
OR2AE1	NS	1.09E-01	-0.0658	2.12E-01	NS
SLC36A2	NS	1.09E-01	-0.0658	2.12E-01	NS

RHBDF2	NS	1.09E-01	-0.0658	2.12E-01	NS
SH3GLB2	NS	1.09E-01	-0.0658	2.12E-01	NS
ZCCHC18	NS	1.09E-01	-0.0658	2.12E-01	NS
EOLA2	NS	1.09E-01	6.58E-02	2.12E-01	NS
YDJC	NS	1.09E-01	-0.0658	2.12E-01	NS
EPHA8	NS	1.09E-01	6.58E-02	2.12E-01	NS
LAMTOR2	NS	1.09E-01	-0.0658	2.12E-01	NS
ARHGEF6	NS	1.09E-01	-0.0658	2.12E-01	NS
OR5M8	NS	1.09E-01	-0.0658	2.12E-01	NS
RPL10A	NS	1.09E-01	-0.0658	2.12E-01	NS
UFD1	NS	1.09E-01	-0.0658	2.13E-01	NS
NR2F1	NS	1.09E-01	6.58E-02	2.13E-01	NS
CAMK2N2	NS	1.09E-01	6.58E-02	2.13E-01	NS
PPIA	NS	1.09E-01	6.57E-02	2.13E-01	NS
SNRNP200	NS	1.09E-01	-0.0657	2.13E-01	NS
B4GALT4	NS	1.09E-01	-0.0657	2.13E-01	NS
PUS1	NS	1.09E-01	-0.0657	2.13E-01	NS
OR7G1	NS	1.09E-01	6.57E-02	2.13E-01	NS
INSYN2A	NS	1.09E-01	-0.0657	2.13E-01	NS
ZNF516	NS	1.09E-01	-0.0657	2.13E-01	NS
SEC14L1	NS	1.09E-01	6.57E-02	2.13E-01	NS
PRAC1	NS	1.09E-01	-0.0657	2.13E-01	NS
ZNF677	NS	1.09E-01	6.57E-02	2.13E-01	NS
IARS2	NS	1.09E-01	-0.0657	2.13E-01	NS
VCL	NS	1.09E-01	6.57E-02	2.13E-01	NS
ATP5ME	NS	1.09E-01	-0.0657	2.13E-01	NS
LRRIQ3	NS	1.09E-01	-0.0657	2.13E-01	NS
ZNF326	NS	1.09E-01	-0.0657	2.13E-01	NS
SERPINA6	NS	1.10E-01	-0.0657	2.13E-01	NS
POT1	NS	1.10E-01	-0.0657	2.13E-01	NS
SEMA3D	NS	1.10E-01	-0.0657	2.13E-01	NS
IL4I1	NS	1.10E-01	6.56E-02	2.13E-01	NS
ARHGEF2	NS	1.10E-01	6.56E-02	2.13E-01	NS
RNF146	NS	1.10E-01	-0.0656	2.13E-01	NS
RWDD2B	NS	1.10E-01	-0.0656	2.14E-01	NS
CX3CL1	NS	1.10E-01	6.56E-02	2.14E-01	NS
RASL11B	NS	1.10E-01	6.56E-02	2.14E-01	NS

HARS1	NS	1.10E-01	-0.0656	2.14E-01	NS
C3orf52	NS	1.10E-01	-0.0656	2.14E-01	NS
PPOX	NS	1.10E-01	-0.0656	2.14E-01	NS
ATL3	NS	1.10E-01	-0.0656	2.14E-01	NS
RPL6	NS	1.10E-01	-0.0656	2.14E-01	NS
MAP4K3	NS	1.10E-01	-0.0656	2.14E-01	NS
GCOM1	NS	1.10E-01	-0.0656	2.14E-01	NS
GTF3C5	NS	1.10E-01	6.56E-02	2.14E-01	NS
KRT6B	NS	1.10E-01	-0.0655	2.14E-01	NS
NUDT16	NS	1.10E-01	6.55E-02	2.14E-01	NS
ELK1	NS	1.10E-01	-0.0655	2.14E-01	NS
ARRDC5	NS	1.10E-01	-0.0655	2.14E-01	NS
FREM2	NS	1.10E-01	-0.0655	2.14E-01	NS
CTSH	NS	1.10E-01	-0.0655	2.14E-01	NS
PCOLCE2	NS	1.10E-01	-0.0655	2.14E-01	NS
CLTCL1	NS	1.10E-01	6.55E-02	2.14E-01	NS
GDAP1L1	NS	1.11E-01	-0.0655	2.14E-01	NS
LMOD3	NS	1.11E-01	-0.0655	2.15E-01	NS
HEXA	NS	1.11E-01	-0.0654	2.15E-01	NS
SPC25	NS	1.11E-01	-0.0654	2.15E-01	NS
PLCE1	NS	1.11E-01	-0.0654	2.15E-01	NS
PINK1	NS	1.11E-01	-0.0654	2.15E-01	NS
SGPL1	NS	1.11E-01	-0.0654	2.15E-01	NS
AGK	NS	1.11E-01	-0.0654	2.15E-01	NS
MNDA	NS	1.11E-01	-0.0654	2.15E-01	NS
TMCO1	NS	1.11E-01	-0.0654	2.15E-01	NS
GTPBP6	NS	1.11E-01	6.54E-02	2.16E-01	NS
TRIM5	NS	1.11E-01	-0.0654	2.16E-01	NS
PRKCI	NS	1.11E-01	-0.0654	2.16E-01	NS
COX7B2	NS	1.11E-01	-0.0654	2.16E-01	NS
CACNA1F	NS	1.11E-01	-0.0653	2.16E-01	NS
CHAC1	NS	1.11E-01	6.53E-02	2.16E-01	NS
TSSK4	NS	1.11E-01	-0.0653	2.16E-01	NS
C8B	NS	1.11E-01	-0.0653	2.16E-01	NS
BTBD10	NS	1.11E-01	-0.0653	2.16E-01	NS
DLAT	NS	1.12E-01	-0.0653	2.16E-01	NS
USP39	NS	1.12E-01	6.53E-02	2.16E-01	NS

ZMYM6	NS	1.12E-01	6.53E-02	2.16E-01	NS
RBM46	NS	1.12E-01	-0.0653	2.16E-01	NS
FBN1	NS	1.12E-01	-0.0653	2.16E-01	NS
ZSCAN20	NS	1.12E-01	-0.0653	2.16E-01	NS
SDSL	NS	1.12E-01	-0.0653	2.16E-01	NS
TMEM35A	NS	1.12E-01	-0.0653	2.16E-01	NS
FAXDC2	NS	1.12E-01	6.52E-02	2.16E-01	NS
BICRA	NS	1.12E-01	6.52E-02	2.16E-01	NS
ACTN2	NS	1.12E-01	-0.0652	2.16E-01	NS
RFX8	NS	1.12E-01	-0.0652	2.17E-01	NS
SH3GL3	NS	1.12E-01	6.52E-02	2.17E-01	NS
IFNW1	NS	1.12E-01	6.52E-02	2.17E-01	NS
JAM2	NS	1.12E-01	6.52E-02	2.17E-01	NS
TMED7-TICAM2	NS	1.12E-01	-0.0652	2.17E-01	NS
TCP10L2	NS	1.12E-01	6.52E-02	2.17E-01	NS
LUM	NS	1.12E-01	-0.0652	2.17E-01	NS
CYHR1	NS	1.12E-01	6.51E-02	2.17E-01	NS
INO80	NS	1.13E-01	-0.0651	2.17E-01	NS
ITPRIPL2	NS	1.13E-01	-0.0651	2.18E-01	NS
RDM1	NS	1.13E-01	6.51E-02	2.18E-01	NS
TP53INP2	NS	1.13E-01	6.51E-02	2.18E-01	NS
EPHX1	NS	1.13E-01	6.51E-02	2.18E-01	NS
OR10G3	NS	1.13E-01	-0.0651	2.18E-01	NS
NUCB2	NS	1.13E-01	-0.0651	2.18E-01	NS
RPS14	NS	1.13E-01	-0.0651	2.18E-01	NS
TNFSF15	NS	1.13E-01	6.51E-02	2.18E-01	NS
RAET1G	NS	1.13E-01	-0.0651	2.18E-01	NS
VAPA	NS	1.13E-01	6.51E-02	2.18E-01	NS
VCX3B	NS	1.13E-01	6.50E-02	2.18E-01	NS
SCN11A	NS	1.13E-01	6.50E-02	2.18E-01	NS
PKN3	NS	1.13E-01	-0.065	2.19E-01	NS
CCDC146	NS	1.13E-01	-0.065	2.19E-01	NS
TBC1D10B	NS	1.13E-01	-0.065	2.19E-01	NS
ARHGAP18	NS	1.13E-01	-0.065	2.19E-01	NS
PPT2	NS	1.13E-01	-0.065	2.19E-01	NS
YIPF6	NS	1.14E-01	-0.0649	2.19E-01	NS
RAB12	NS	1.14E-01	-0.0649	2.19E-01	NS

CBR4	NS	1.14E-01	-0.0649	2.19E-01	NS
DOK3	NS	1.14E-01	6.49E-02	2.19E-01	NS
SPHK2	NS	1.14E-01	-0.0649	2.19E-01	NS
RASSF7	NS	1.14E-01	6.49E-02	2.19E-01	NS
RABGGTB	NS	1.14E-01	6.49E-02	2.19E-01	NS
BTBD16	NS	1.14E-01	6.49E-02	2.19E-01	NS
CT47A8	NS	1.14E-01	6.49E-02	2.19E-01	NS
LMAN2L	NS	1.14E-01	-0.0649	2.19E-01	NS
DNAH7	NS	1.14E-01	-0.0649	2.19E-01	NS
GOLGA7	NS	1.14E-01	-0.0649	2.20E-01	NS
NPY2R	NS	1.14E-01	-0.0648	2.20E-01	NS
PATE1	NS	1.14E-01	6.48E-02	2.20E-01	NS
TMEM205	NS	1.14E-01	-0.0648	2.20E-01	NS
BYSL	NS	1.14E-01	-0.0648	2.20E-01	NS
LMF2	NS	1.14E-01	-0.0648	2.20E-01	NS
NKIRAS1	NS	1.14E-01	-0.0648	2.20E-01	NS
HS2ST1	NS	1.14E-01	-0.0648	2.20E-01	NS
PDXDC1	NS	1.14E-01	-0.0648	2.20E-01	NS
HMX1	NS	1.14E-01	6.48E-02	2.20E-01	NS
SEMA4A	NS	1.14E-01	-0.0648	2.20E-01	NS
H4C13	NS	1.14E-01	6.48E-02	2.20E-01	NS
PIF1	NS	1.14E-01	6.48E-02	2.20E-01	NS
SLC38A8	NS	1.14E-01	-0.0648	2.20E-01	NS
IPCEF1	NS	1.15E-01	-0.0648	2.20E-01	NS
HNRNPD	NS	1.15E-01	6.48E-02	2.20E-01	NS
SFXN1	NS	1.15E-01	-0.0648	2.20E-01	NS
LINGO2	NS	1.15E-01	-0.0647	2.21E-01	NS
SKAP2	NS	1.15E-01	-0.0647	2.21E-01	NS
PTPN13	NS	1.15E-01	6.47E-02	2.21E-01	NS
DLX6	NS	1.15E-01	6.47E-02	2.21E-01	NS
CAMKK2	NS	1.15E-01	-0.0647	2.21E-01	NS
PRKRIP1	NS	1.15E-01	6.47E-02	2.21E-01	NS
PPFIA4	NS	1.15E-01	6.47E-02	2.21E-01	NS
GH1	NS	1.15E-01	-0.0647	2.21E-01	NS
TRIM72	NS	1.15E-01	-0.0647	2.21E-01	NS
SSR1	NS	1.15E-01	-0.0647	2.21E-01	NS
DCT	NS	1.15E-01	-0.0647	2.21E-01	NS

HUWE1	NS	1.15E-01	-0.0647	2.21E-01	NS
YTHDC2	NS	1.15E-01	-0.0647	2.21E-01	NS
TRIM50	NS	1.15E-01	6.47E-02	2.21E-01	NS
LGALS8	NS	1.15E-01	6.47E-02	2.21E-01	NS
SLC35D2	NS	1.15E-01	-0.0647	2.21E-01	NS
INPP5D	NS	1.15E-01	6.47E-02	2.21E-01	NS
GSN	NS	1.15E-01	-0.0647	2.21E-01	NS
SMIM31	NS	1.15E-01	-0.0647	2.21E-01	NS
C9orf24	NS	1.15E-01	-0.0646	2.21E-01	NS
KRTAP5-2	NS	1.15E-01	6.46E-02	2.21E-01	NS
MARVELD1	NS	1.15E-01	-0.0646	2.21E-01	NS
OR1L4	NS	1.15E-01	6.46E-02	2.21E-01	NS
RGS7	NS	1.15E-01	-0.0646	2.22E-01	NS
KPRP	NS	1.15E-01	6.46E-02	2.22E-01	NS
VDR	NS	1.16E-01	-0.0646	2.22E-01	NS
BTF3	NS	1.16E-01	-0.0646	2.22E-01	NS
COMP	NS	1.16E-01	-0.0646	2.22E-01	NS
DYNC2I1	NS	1.16E-01	-0.0646	2.22E-01	NS
ACAT2	NS	1.16E-01	6.46E-02	2.22E-01	NS
EFCAB14	NS	1.16E-01	-0.0646	2.22E-01	NS
VAT1	NS	1.16E-01	-0.0645	2.22E-01	NS
OPN5	NS	1.16E-01	-0.0645	2.22E-01	NS
GABRB2	NS	1.16E-01	-0.0645	2.22E-01	NS
TMED1	NS	1.16E-01	-0.0645	2.22E-01	NS
VSTM4	NS	1.16E-01	-0.0645	2.22E-01	NS
LSM3	NS	1.16E-01	-0.0645	2.22E-01	NS
KCNH6	NS	1.16E-01	6.45E-02	2.22E-01	NS
LAMTOR4	NS	1.16E-01	-0.0645	2.22E-01	NS
TNFRSF18	NS	1.16E-01	6.45E-02	2.22E-01	NS
CDHR4	NS	1.16E-01	-0.0645	2.22E-01	NS
ZBTB42	NS	1.16E-01	-0.0645	2.22E-01	NS
LRRC36	NS	1.16E-01	-0.0645	2.22E-01	NS
GBP3	NS	1.16E-01	-0.0645	2.22E-01	NS
NWD2	NS	1.16E-01	-0.0645	2.22E-01	NS
SLCO6A1	NS	1.16E-01	-0.0645	2.23E-01	NS
CCDC97	NS	1.16E-01	6.45E-02	2.23E-01	NS
SIGLEC14	NS	1.16E-01	-0.0645	2.23E-01	NS

ARID3B	NS	1.16E-01	6.45E-02	2.23E-01	NS
CRACD	NS	1.16E-01	-0.0644	2.23E-01	NS
ANKRD26	NS	1.16E-01	-0.0644	2.23E-01	NS
BANK1	NS	1.16E-01	-0.0644	2.23E-01	NS
RPL13	NS	1.16E-01	-0.0644	2.23E-01	NS
SCD5	NS	1.17E-01	-0.0644	2.23E-01	NS
ZNF233	NS	1.17E-01	-0.0644	2.23E-01	NS
UBQLN3	NS	1.17E-01	6.44E-02	2.23E-01	NS
FAM90A9P	NS	1.17E-01	-0.0644	2.23E-01	NS
UBL4A	NS	1.17E-01	6.44E-02	2.23E-01	NS
TAF6	NS	1.17E-01	6.43E-02	2.24E-01	NS
STXBP5	NS	1.17E-01	-0.0643	2.24E-01	NS
PTX4	NS	1.17E-01	6.43E-02	2.24E-01	NS
TRAPPC6A	NS	1.17E-01	-0.0643	2.24E-01	NS
GBX2	NS	1.17E-01	6.43E-02	2.24E-01	NS
SLC31A1	NS	1.17E-01	-0.0643	2.24E-01	NS
PHETA2	NS	1.17E-01	-0.0643	2.24E-01	NS
AAAS	NS	1.17E-01	-0.0643	2.24E-01	NS
SF3B1	NS	1.17E-01	-0.0643	2.24E-01	NS
ZNF595	NS	1.17E-01	-0.0642	2.24E-01	NS
LTBP2	NS	1.18E-01	-0.0642	2.25E-01	NS
HTR5A	NS	1.18E-01	6.42E-02	2.25E-01	NS
MEPCE	NS	1.18E-01	-0.0642	2.25E-01	NS
FGF13	NS	1.18E-01	-0.0642	2.25E-01	NS
TAMALIN	NS	1.18E-01	6.42E-02	2.25E-01	NS
SMPD2	NS	1.18E-01	6.42E-02	2.25E-01	NS
STK4	NS	1.18E-01	-0.0642	2.25E-01	NS
KRTAP20-3	NS	1.18E-01	-0.0642	2.25E-01	NS
MIB1	NS	1.18E-01	-0.0641	2.25E-01	NS
DEFB106B	NS	1.18E-01	-0.0641	2.25E-01	NS
CAPN5	NS	1.18E-01	-0.0641	2.25E-01	NS
BECN1	NS	1.18E-01	-0.0641	2.26E-01	NS
MVP	NS	1.18E-01	-0.0641	2.26E-01	NS
ABCG4	NS	1.18E-01	-0.0641	2.26E-01	NS
NEFM	NS	1.18E-01	-0.0641	2.26E-01	NS
KRTAP22-1	NS	1.18E-01	-0.0641	2.26E-01	NS
TM6SF2	NS	1.19E-01	6.40E-02	2.26E-01	NS

MT1E	NS	1.19E-01	-0.064	2.26E-01	NS
UHRF1BP1L	NS	1.19E-01	-0.064	2.26E-01	NS
MKKS	NS	1.19E-01	-0.064	2.26E-01	NS
KCNA3	NS	1.19E-01	6.40E-02	2.26E-01	NS
NIM1K	NS	1.19E-01	6.40E-02	2.26E-01	NS
TMX4	NS	1.19E-01	-0.064	2.27E-01	NS
CSNK1D	NS	1.19E-01	-0.064	2.27E-01	NS
FMOD	NS	1.19E-01	-0.064	2.27E-01	NS
ABCD3	NS	1.19E-01	6.40E-02	2.27E-01	NS
GCSAML	NS	1.19E-01	-0.0639	2.27E-01	NS
SOGA1	NS	1.19E-01	-0.0639	2.27E-01	NS
TECPR1	NS	1.19E-01	-0.0639	2.27E-01	NS
GPR135	NS	1.19E-01	6.39E-02	2.27E-01	NS
CXorf58	NS	1.19E-01	-0.0639	2.27E-01	NS
ATXN1	NS	1.19E-01	-0.0639	2.27E-01	NS
NANP	NS	1.20E-01	-0.0639	2.28E-01	NS
MTHFD2L	NS	1.20E-01	-0.0639	2.28E-01	NS
CCRL2	NS	1.20E-01	-0.0639	2.28E-01	NS
UBE2M	NS	1.20E-01	-0.0639	2.28E-01	NS
TRIM11	NS	1.20E-01	6.39E-02	2.28E-01	NS
ALKAL1	NS	1.20E-01	-0.0639	2.28E-01	NS
LRP2BP	NS	1.20E-01	-0.0639	2.28E-01	NS
ZFP28	NS	1.20E-01	-0.0639	2.28E-01	NS
PRELID3A	NS	1.20E-01	-0.0639	2.28E-01	NS
CALML5	NS	1.20E-01	-0.0639	2.28E-01	NS
ADO	NS	1.20E-01	-0.0638	2.28E-01	NS
SLC27A3	NS	1.20E-01	6.38E-02	2.28E-01	NS
TMEM102	NS	1.20E-01	-0.0638	2.28E-01	NS
ANKRD61	NS	1.20E-01	-0.0638	2.28E-01	NS
YLPM1	NS	1.20E-01	-0.0638	2.28E-01	NS
DHX40	NS	1.20E-01	-0.0638	2.28E-01	NS
ILF2	NS	1.20E-01	-0.0638	2.28E-01	NS
ECE2	NS	1.20E-01	-0.0638	2.28E-01	NS
CAMP	NS	1.20E-01	-0.0638	2.28E-01	NS
CHM	NS	1.20E-01	-0.0638	2.28E-01	NS
GPRASP1	NS	1.20E-01	-0.0638	2.28E-01	NS
LRRC24	NS	1.20E-01	-0.0638	2.28E-01	NS

EFNA2	NS	1.20E-01	6.38E-02	2.28E-01	NS
MAGEA1	NS	1.20E-01	-0.0638	2.28E-01	NS
RHOQ	NS	1.20E-01	-0.0638	2.28E-01	NS
POM121	NS	1.20E-01	6.38E-02	2.28E-01	NS
RAD54L	NS	1.20E-01	-0.0637	2.29E-01	NS
KMT5C	NS	1.20E-01	-0.0637	2.29E-01	NS
GJA9	NS	1.20E-01	-0.0637	2.29E-01	NS
TMEM181	NS	1.20E-01	-0.0637	2.29E-01	NS
DMRTA1	NS	1.21E-01	-0.0637	2.29E-01	NS
ACAD10	NS	1.21E-01	-0.0637	2.29E-01	NS
CLHC1	NS	1.21E-01	-0.0637	2.29E-01	NS
LYZL2	NS	1.21E-01	-0.0637	2.29E-01	NS
FSCN1	NS	1.21E-01	6.37E-02	2.29E-01	NS
HDAC10	NS	1.21E-01	-0.0637	2.29E-01	NS
MRRF	NS	1.21E-01	-0.0637	2.29E-01	NS
ZNF28	NS	1.21E-01	-0.0636	2.29E-01	NS
GMEB1	NS	1.21E-01	-0.0636	2.29E-01	NS
CGB2	NS	1.21E-01	6.36E-02	2.30E-01	NS
SCGB2A2	NS	1.21E-01	6.36E-02	2.30E-01	NS
CCDC47	NS	1.21E-01	-0.0636	2.30E-01	NS
DSC2	NS	1.21E-01	-0.0636	2.30E-01	NS
KBTBD12	NS	1.21E-01	-0.0636	2.30E-01	NS
NCOR1	NS	1.22E-01	-0.0635	2.30E-01	NS
BTNL9	NS	1.22E-01	6.35E-02	2.30E-01	NS
CCL4L2	NS	1.22E-01	6.35E-02	2.30E-01	NS
ASB10	NS	1.22E-01	6.35E-02	2.30E-01	NS
PSTPIP2	NS	1.22E-01	6.35E-02	2.30E-01	NS
TFEC	NS	1.22E-01	-0.0635	2.31E-01	NS
SLC12A4	NS	1.22E-01	-0.0635	2.31E-01	NS
TEPSIN	NS	1.22E-01	-0.0635	2.31E-01	NS
ZNF718	NS	1.22E-01	-0.0635	2.31E-01	NS
SLC35C2	NS	1.22E-01	-0.0635	2.31E-01	NS
SLC13A3	NS	1.22E-01	-0.0635	2.31E-01	NS
TBX21	NS	1.22E-01	-0.0635	2.31E-01	NS
ARHGEF38	NS	1.22E-01	6.35E-02	2.31E-01	NS
SDC1	NS	1.22E-01	6.35E-02	2.31E-01	NS
PPP1R14B	NS	1.22E-01	-0.0635	2.31E-01	NS

YTHDF1	NS	1.22E-01	-0.0634	2.31E-01	NS
MPHOSPH6	NS	1.22E-01	-0.0634	2.31E-01	NS
JHY	NS	1.22E-01	-0.0634	2.32E-01	NS
RHOBTB3	NS	1.22E-01	-0.0634	2.32E-01	NS
TEDC2	NS	1.22E-01	6.34E-02	2.32E-01	NS
PRAMEF13	NS	1.22E-01	-0.0634	2.32E-01	NS
SERINC4	NS	1.23E-01	-0.0634	2.32E-01	NS
RGS13	NS	1.23E-01	-0.0634	2.32E-01	NS
INSL4	NS	1.23E-01	-0.0633	2.32E-01	NS
DPPA4	NS	1.23E-01	6.33E-02	2.32E-01	NS
BLID	NS	1.23E-01	6.33E-02	2.32E-01	NS
FZD1	NS	1.23E-01	-0.0633	2.32E-01	NS
EVC2	NS	1.23E-01	-0.0633	2.32E-01	NS
PCYOX1L	NS	1.23E-01	-0.0633	2.32E-01	NS
RAD9B	NS	1.23E-01	-0.0633	2.32E-01	NS
TACC2	NS	1.23E-01	6.33E-02	2.32E-01	NS
TBL2	NS	1.23E-01	-0.0633	2.33E-01	NS
ECT2	NS	1.23E-01	-0.0633	2.33E-01	NS
NFIA	NS	1.23E-01	6.33E-02	2.33E-01	NS
PALD1	NS	1.23E-01	-0.0633	2.33E-01	NS
GPR88	NS	1.23E-01	6.33E-02	2.33E-01	NS
TSPYL1	NS	1.23E-01	-0.0633	2.33E-01	NS
TMPRSS12	NS	1.23E-01	-0.0633	2.33E-01	NS
SEPTIN14	NS	1.23E-01	6.32E-02	2.33E-01	NS
CMBL	NS	1.23E-01	-0.0632	2.33E-01	NS
ISCA2	NS	1.24E-01	-0.0632	2.33E-01	NS
SCARA5	NS	1.24E-01	-0.0632	2.33E-01	NS
IGFLR1	NS	1.24E-01	-0.0632	2.33E-01	NS
OR10Q1	NS	1.24E-01	6.32E-02	2.33E-01	NS
GPER1	NS	1.24E-01	-0.0632	2.33E-01	NS
TGFBRAP1	NS	1.24E-01	-0.0632	2.33E-01	NS
RAB21	NS	1.24E-01	-0.0631	2.34E-01	NS
ACTN4	NS	1.24E-01	6.31E-02	2.34E-01	NS
WDR61	NS	1.24E-01	6.31E-02	2.34E-01	NS
LONRF1	NS	1.24E-01	-0.0631	2.34E-01	NS
TAF11L9	NS	1.24E-01	-0.0631	2.34E-01	NS
ZNF583	NS	1.24E-01	6.31E-02	2.34E-01	NS

H2BC12	NS	1.24E-01	6.31E-02	2.34E-01	NS
ERVFRD-1	NS	1.24E-01	-0.0631	2.34E-01	NS
P2RX4	NS	1.24E-01	6.30E-02	2.35E-01	NS
MEP1B	NS	1.25E-01	-0.063	2.35E-01	NS
ZDHHC1	NS	1.25E-01	-0.063	2.35E-01	NS
ZNF709	NS	1.25E-01	-0.063	2.35E-01	NS
CD8B2	NS	1.25E-01	6.30E-02	2.35E-01	NS
PAGE2B	NS	1.25E-01	-0.063	2.35E-01	NS
CTHRC1	NS	1.25E-01	-0.063	2.35E-01	NS
ARMH1	NS	1.25E-01	-0.063	2.35E-01	NS
HOXD4	NS	1.25E-01	6.30E-02	2.35E-01	NS
PLEKHG3	NS	1.25E-01	6.30E-02	2.35E-01	NS
GAREM1	NS	1.25E-01	-0.063	2.35E-01	NS
ASRGL1	NS	1.25E-01	-0.063	2.35E-01	NS
CYP26A1	NS	1.25E-01	6.30E-02	2.35E-01	NS
NAA50	NS	1.25E-01	-0.063	2.35E-01	NS
E2F2	NS	1.25E-01	-0.0629	2.36E-01	NS
SNAPC1	NS	1.25E-01	-0.0629	2.36E-01	NS
LAMB3	NS	1.25E-01	-0.0629	2.36E-01	NS
DIRAS3	NS	1.25E-01	-0.0629	2.36E-01	NS
UBTFL1	NS	1.25E-01	-0.0629	2.36E-01	NS
TESK1	NS	1.25E-01	6.29E-02	2.36E-01	NS
AEBP2	NS	1.25E-01	-0.0629	2.36E-01	NS
LPCAT4	NS	1.25E-01	-0.0629	2.36E-01	NS
GALR2	NS	1.25E-01	-0.0629	2.36E-01	NS
EXOSC8	NS	1.26E-01	-0.0629	2.36E-01	NS
ATP11C	NS	1.26E-01	-0.0629	2.36E-01	NS
ZNF251	NS	1.26E-01	6.29E-02	2.36E-01	NS
SERTM1	NS	1.26E-01	-0.0629	2.36E-01	NS
C4BPB	NS	1.26E-01	-0.0629	2.36E-01	NS
M1AP	NS	1.26E-01	-0.0629	2.36E-01	NS
ZNF652	NS	1.26E-01	6.29E-02	2.36E-01	NS
GRIN2A	NS	1.26E-01	-0.0629	2.36E-01	NS
PIFO	NS	1.26E-01	6.28E-02	2.36E-01	NS
GOLIM4	NS	1.26E-01	-0.0628	2.36E-01	NS
PAGE5	NS	1.26E-01	-0.0628	2.36E-01	NS
ZNF33A	NS	1.26E-01	-0.0628	2.37E-01	NS

MRTO4	NS	1.26E-01	-0.0628	2.37E-01	NS
ADAM8	NS	1.26E-01	-0.0628	2.37E-01	NS
D2HGDH	NS	1.26E-01	6.28E-02	2.37E-01	NS
SEC62	NS	1.26E-01	-0.0628	2.37E-01	NS
PAFAH1B1	NS	1.26E-01	-0.0628	2.37E-01	NS
TOMM5	NS	1.26E-01	-0.0628	2.37E-01	NS
PSME3IP1	NS	1.26E-01	-0.0628	2.37E-01	NS
NCOR2	NS	1.26E-01	-0.0628	2.37E-01	NS
CNOT11	NS	1.26E-01	-0.0627	2.37E-01	NS
EPHB6	NS	1.26E-01	6.27E-02	2.37E-01	NS
RNF40	NS	1.26E-01	6.27E-02	2.37E-01	NS
WDR73	NS	1.26E-01	-0.0627	2.37E-01	NS
TLCD1	NS	1.27E-01	-0.0627	2.37E-01	NS
HELZ2	NS	1.27E-01	-0.0627	2.37E-01	NS
SCG2	NS	1.27E-01	-0.0627	2.37E-01	NS
C17orf97	NS	1.27E-01	-0.0627	2.37E-01	NS
EDNRA	NS	1.27E-01	-0.0627	2.38E-01	NS
NSFL1C	NS	1.27E-01	-0.0627	2.38E-01	NS
OCSTAMP	NS	1.27E-01	6.27E-02	2.38E-01	NS
OR5B17	NS	1.27E-01	6.27E-02	2.38E-01	NS
CILP2	NS	1.27E-01	6.26E-02	2.38E-01	NS
UBE2F-SCLY	NS	1.27E-01	6.26E-02	2.38E-01	NS
PHF20	NS	1.27E-01	6.26E-02	2.38E-01	NS
TEX15	NS	1.27E-01	-0.0626	2.38E-01	NS
PTRH2	NS	1.27E-01	-0.0626	2.38E-01	NS
GNA12	NS	1.27E-01	6.26E-02	2.38E-01	NS
PLCZ1	NS	1.27E-01	-0.0626	2.38E-01	NS
OR11A1	NS	1.27E-01	-0.0626	2.38E-01	NS
MSN	NS	1.27E-01	-0.0626	2.38E-01	NS
L3MBTL2	NS	1.27E-01	-0.0626	2.38E-01	NS
CPPED1	NS	1.27E-01	6.26E-02	2.38E-01	NS
SAYSD1	NS	1.27E-01	-0.0626	2.38E-01	NS
FAM174B	NS	1.27E-01	-0.0626	2.38E-01	NS
CCDC89	NS	1.27E-01	6.26E-02	2.39E-01	NS
PPM1B	NS	1.27E-01	-0.0626	2.39E-01	NS
SLC7A14	NS	1.27E-01	-0.0626	2.39E-01	NS
CIB4	NS	1.27E-01	-0.0626	2.39E-01	NS

RINL	NS	1.27E-01	-0.0626	2.39E-01	NS
SPDYE9	NS	1.27E-01	-0.0626	2.39E-01	NS
C1QTNF1	NS	1.28E-01	6.25E-02	2.39E-01	NS
CARTPT	NS	1.28E-01	-0.0625	2.39E-01	NS
CNDP1	NS	1.28E-01	-0.0625	2.39E-01	NS
ADGRG3	NS	1.28E-01	6.25E-02	2.39E-01	NS
PIGO	NS	1.28E-01	-0.0625	2.39E-01	NS
ITGAM	NS	1.28E-01	-0.0625	2.39E-01	NS
RPS19	NS	1.28E-01	-0.0625	2.39E-01	NS
KMT2C	NS	1.28E-01	-0.0625	2.39E-01	NS
PDZRN3	NS	1.28E-01	-0.0625	2.39E-01	NS
MAK	NS	1.28E-01	-0.0625	2.39E-01	NS
H2AZ1	NS	1.28E-01	-0.0625	2.39E-01	NS
FAM229A	NS	1.28E-01	-0.0625	2.39E-01	NS
SPANXN2	NS	1.28E-01	-0.0624	2.40E-01	NS
SSU72	NS	1.28E-01	-0.0624	2.40E-01	NS
VAV3	NS	1.28E-01	-0.0624	2.40E-01	NS
CLEC4D	NS	1.28E-01	-0.0624	2.40E-01	NS
KHDRBS1	NS	1.29E-01	-0.0624	2.40E-01	NS
FLNC	NS	1.29E-01	6.24E-02	2.40E-01	NS
NUP43	NS	1.29E-01	-0.0624	2.40E-01	NS
CMIP	NS	1.29E-01	-0.0624	2.40E-01	NS
MPZ	NS	1.29E-01	6.24E-02	2.40E-01	NS
CT47A9	NS	1.29E-01	6.23E-02	2.41E-01	NS
RMDN3	NS	1.29E-01	-0.0623	2.41E-01	NS
FBL	NS	1.29E-01	-0.0623	2.41E-01	NS
ABHD2	NS	1.29E-01	-0.0623	2.41E-01	NS
RNF44	NS	1.29E-01	6.23E-02	2.41E-01	NS
CNKSR2	NS	1.29E-01	-0.0623	2.41E-01	NS
OR1B1	NS	1.29E-01	-0.0623	2.41E-01	NS
MAN1A1	NS	1.29E-01	-0.0623	2.41E-01	NS
FBXO2	NS	1.29E-01	6.23E-02	2.41E-01	NS
DYDC2	NS	1.29E-01	-0.0623	2.41E-01	NS
FAT3	NS	1.29E-01	-0.0623	2.41E-01	NS
RTL10	NS	1.29E-01	-0.0623	2.41E-01	NS
MUC1	NS	1.29E-01	6.22E-02	2.41E-01	NS
C1QTNF12	NS	1.29E-01	6.22E-02	2.41E-01	NS

RPAP1	NS	1.30E-01	-0.0622	2.42E-01	NS
CDH23	NS	1.30E-01	-0.0622	2.42E-01	NS
TRIM24	NS	1.29E-01	-0.0622	2.42E-01	NS
SMIM42	NS	1.30E-01	6.22E-02	2.42E-01	NS
ITGA8	NS	1.30E-01	-0.0622	2.42E-01	NS
RNF141	NS	1.30E-01	-0.0622	2.42E-01	NS
ZNF814	NS	1.30E-01	6.22E-02	2.42E-01	NS
WTAP	NS	1.30E-01	-0.0622	2.42E-01	NS
MYBPHL	NS	1.30E-01	-0.0622	2.42E-01	NS
SGF29	NS	1.30E-01	-0.0622	2.42E-01	NS
SPDYE6	NS	1.30E-01	6.22E-02	2.42E-01	NS
ALOX5AP	NS	1.30E-01	6.22E-02	2.42E-01	NS
PTP4A2	NS	1.30E-01	6.21E-02	2.42E-01	NS
EDARADD	NS	1.30E-01	-0.0621	2.42E-01	NS
WDR45B	NS	1.30E-01	-0.0621	2.42E-01	NS
LPA	NS	1.30E-01	-0.0621	2.42E-01	NS
ATP1A1	NS	1.30E-01	-0.0621	2.42E-01	NS
JPT1	NS	1.30E-01	6.21E-02	2.42E-01	NS
RBMY1J	NS	1.30E-01	-0.0621	2.42E-01	NS
PANK2	NS	1.30E-01	-0.0621	2.42E-01	NS
UQCRH	NS	1.30E-01	-0.0621	2.42E-01	NS
FCGR3A	NS	1.30E-01	-0.0621	2.43E-01	NS
TMCC2	NS	1.30E-01	-0.0621	2.43E-01	NS
NOMO3	NS	1.31E-01	-0.062	2.43E-01	NS
ARSD	NS	1.31E-01	-0.062	2.43E-01	NS
PTGER2	NS	1.31E-01	6.20E-02	2.43E-01	NS
PAK2	NS	1.31E-01	-0.062	2.43E-01	NS
DNAH17	NS	1.31E-01	6.20E-02	2.43E-01	NS
RRAGB	NS	1.31E-01	-0.062	2.43E-01	NS
NFKBIZ	NS	1.31E-01	6.20E-02	2.43E-01	NS
PCDHGA8	NS	1.31E-01	-0.062	2.43E-01	NS
TRIM13	NS	1.31E-01	-0.062	2.44E-01	NS
BHLHE22	NS	1.31E-01	6.20E-02	2.44E-01	NS
IRAG1	NS	1.31E-01	-0.062	2.44E-01	NS
RHBG	NS	1.31E-01	-0.062	2.44E-01	NS
DROSHA	NS	1.31E-01	6.20E-02	2.44E-01	NS
RHBDL3	NS	1.31E-01	-0.062	2.44E-01	NS

TMEM132C	NS	1.31E-01	-0.0619	2.44E-01	NS
DAG1	NS	1.31E-01	6.19E-02	2.44E-01	NS
CT45A1	NS	1.31E-01	-0.0619	2.44E-01	NS
ZNF3	NS	1.31E-01	-0.0619	2.44E-01	NS
MMP26	NS	1.31E-01	6.19E-02	2.44E-01	NS
VTI1A	NS	1.31E-01	6.19E-02	2.44E-01	NS
ANTKMT	NS	1.31E-01	6.19E-02	2.44E-01	NS
WNT10A	NS	1.31E-01	6.19E-02	2.44E-01	NS
RSAD1	NS	1.32E-01	6.19E-02	2.45E-01	NS
USP40	NS	1.32E-01	-0.0619	2.45E-01	NS
MYO1B	NS	1.32E-01	-0.0619	2.45E-01	NS
POLE4	NS	1.32E-01	-0.0618	2.45E-01	NS
KRTAP4-12	NS	1.32E-01	6.18E-02	2.45E-01	NS
ACSS3	NS	1.32E-01	6.18E-02	2.45E-01	NS
FBXO44	NS	1.32E-01	-0.0618	2.45E-01	NS
UBTD1	NS	1.32E-01	6.18E-02	2.45E-01	NS
CKAP2	NS	1.32E-01	-0.0618	2.45E-01	NS
ANKRA2	NS	1.32E-01	-0.0618	2.45E-01	NS
PIGN	NS	1.32E-01	-0.0618	2.46E-01	NS
CHN1	NS	1.32E-01	-0.0618	2.46E-01	NS
NEK6	NS	1.33E-01	6.17E-02	2.46E-01	NS
TCF7L1	NS	1.33E-01	-0.0617	2.46E-01	NS
CTNND1	NS	1.33E-01	-0.0617	2.46E-01	NS
KIAA1210	NS	1.33E-01	6.17E-02	2.46E-01	NS
ARCN1	NS	1.33E-01	-0.0617	2.46E-01	NS
GGA3	NS	1.33E-01	-0.0617	2.46E-01	NS
TRAPPC8	NS	1.33E-01	-0.0617	2.46E-01	NS
EIF2S1	NS	1.33E-01	-0.0617	2.46E-01	NS
B4GALNT3	NS	1.33E-01	-0.0617	2.46E-01	NS
TAS2R1	NS	1.33E-01	-0.0617	2.46E-01	NS
PPL	NS	1.33E-01	-0.0617	2.46E-01	NS
PRMT7	NS	1.33E-01	-0.0617	2.46E-01	NS
SH3TC2	NS	1.33E-01	6.17E-02	2.46E-01	NS
TNIP2	NS	1.33E-01	6.17E-02	2.46E-01	NS
PABIR3	NS	1.33E-01	6.17E-02	2.47E-01	NS
YPEL3	NS	1.33E-01	6.16E-02	2.47E-01	NS
GGT6	NS	1.33E-01	6.16E-02	2.47E-01	NS

PCDH20	NS	1.33E-01	-0.0616	2.47E-01	NS
DPEP2	NS	1.33E-01	6.16E-02	2.47E-01	NS
SUSD2	NS	1.34E-01	6.16E-02	2.47E-01	NS
TAF11L10	NS	1.34E-01	-0.0616	2.47E-01	NS
WDR5B	NS	1.34E-01	-0.0616	2.47E-01	NS
WBP11	NS	1.34E-01	6.16E-02	2.47E-01	NS
RALY	NS	1.34E-01	6.15E-02	2.48E-01	NS
CC2D2A	NS	1.34E-01	-0.0615	2.48E-01	NS
PRKAA2	NS	1.34E-01	-0.0615	2.48E-01	NS
HOMER2	NS	1.34E-01	-0.0615	2.48E-01	NS
SLC12A1	NS	1.34E-01	-0.0615	2.48E-01	NS
ZNF697	NS	1.34E-01	-0.0615	2.48E-01	NS
C21orf58	NS	1.34E-01	-0.0615	2.48E-01	NS
FAM120C	NS	1.34E-01	-0.0615	2.48E-01	NS
NDUFAF5	NS	1.34E-01	-0.0615	2.48E-01	NS
A1CF	NS	1.34E-01	-0.0615	2.48E-01	NS
MSANTD2	NS	1.34E-01	6.15E-02	2.48E-01	NS
ADCYAP1	NS	1.34E-01	6.15E-02	2.48E-01	NS
IRF2	NS	1.34E-01	6.15E-02	2.48E-01	NS
PROSER1	NS	1.34E-01	6.15E-02	2.48E-01	NS
MISP	NS	1.34E-01	6.15E-02	2.48E-01	NS
WDR25	NS	1.34E-01	6.15E-02	2.48E-01	NS
CPT1B	NS	1.34E-01	6.15E-02	2.48E-01	NS
CPAMD8	NS	1.34E-01	-0.0614	2.48E-01	NS
C13orf46	NS	1.35E-01	-0.0614	2.49E-01	NS
LCE2C	NS	1.35E-01	-0.0614	2.49E-01	NS
HSPE1	NS	1.35E-01	-0.0614	2.49E-01	NS
GOPC	NS	1.35E-01	-0.0614	2.49E-01	NS
SSBP1	NS	1.35E-01	6.14E-02	2.49E-01	NS
TBC1D22A	NS	1.35E-01	6.14E-02	2.49E-01	NS
OR2D3	NS	1.35E-01	-0.0614	2.49E-01	NS
H3C2	NS	1.35E-01	-0.0614	2.49E-01	NS
SLC25A35	NS	1.35E-01	6.13E-02	2.49E-01	NS
TCF3	NS	1.35E-01	-0.0613	2.49E-01	NS
BAMBI	NS	1.35E-01	-0.0613	2.49E-01	NS
UBFD1	NS	1.35E-01	-0.0613	2.49E-01	NS
AIDA	NS	1.35E-01	-0.0613	2.49E-01	NS

TEAD4	NS	1.35E-01	-0.0613	2.49E-01	NS
TCAF2	NS	1.35E-01	-0.0613	2.50E-01	NS
MUC20	NS	1.35E-01	-0.0613	2.50E-01	NS
LRRC8E	NS	1.35E-01	6.13E-02	2.50E-01	NS
SLC35F1	NS	1.36E-01	-0.0613	2.50E-01	NS
SLC34A2	NS	1.36E-01	-0.0613	2.50E-01	NS
COL11A2	NS	1.36E-01	-0.0613	2.50E-01	NS
MFAP1	NS	1.36E-01	-0.0612	2.50E-01	NS
KRT26	NS	1.36E-01	-0.0612	2.50E-01	NS
ZFAND3	NS	1.36E-01	-0.0612	2.51E-01	NS
ANKRD16	NS	1.36E-01	-0.0612	2.51E-01	NS
HAUS6	NS	1.36E-01	-0.0612	2.51E-01	NS
SLC39A14	NS	1.36E-01	-0.0612	2.51E-01	NS
FAM193A	NS	1.36E-01	6.12E-02	2.51E-01	NS
CFAP100	NS	1.36E-01	-0.0612	2.51E-01	NS
AQP9	NS	1.36E-01	-0.0612	2.51E-01	NS
HNRNPCL3	NS	1.36E-01	6.12E-02	2.51E-01	NS
RBM27	NS	1.36E-01	6.12E-02	2.51E-01	NS
MYO6	NS	1.36E-01	-0.0612	2.51E-01	NS
PITPNM3	NS	1.36E-01	-0.0612	2.51E-01	NS
RPF1	NS	1.36E-01	-0.0611	2.51E-01	NS
RIPK3	NS	1.36E-01	-0.0611	2.51E-01	NS
APOBEC3A	NS	1.37E-01	-0.0611	2.51E-01	NS
ZDHHC3	NS	1.37E-01	6.11E-02	2.52E-01	NS
GRIK3	NS	1.37E-01	-0.0611	2.52E-01	NS
PNLIP	NS	1.37E-01	-0.0611	2.52E-01	NS
HSD17B12	NS	1.37E-01	-0.0611	2.52E-01	NS
TNIK	NS	1.37E-01	-0.0611	2.52E-01	NS
CALHM1	NS	1.37E-01	6.11E-02	2.52E-01	NS
PRSS45P	NS	1.37E-01	-0.0611	2.52E-01	NS
TM6SF1	NS	1.37E-01	6.11E-02	2.52E-01	NS
LUC7L3	NS	1.37E-01	6.11E-02	2.52E-01	NS
PRR14	NS	1.37E-01	6.10E-02	2.52E-01	NS
LASP1	NS	1.37E-01	-0.061	2.52E-01	NS
IPMK	NS	1.37E-01	6.10E-02	2.52E-01	NS
C1orf198	NS	1.37E-01	-0.061	2.52E-01	NS
MAML3	NS	1.37E-01	6.10E-02	2.52E-01	NS

LTN1	NS	1.37E-01	-0.061	2.52E-01	NS
PIK3R5	NS	1.37E-01	-0.061	2.52E-01	NS
GPR89B	NS	1.37E-01	6.10E-02	2.53E-01	NS
MTRF1	NS	1.37E-01	-0.061	2.53E-01	NS
AMT	NS	1.37E-01	-0.061	2.53E-01	NS
LTC4S	NS	1.38E-01	6.09E-02	2.53E-01	NS
F10	NS	1.38E-01	-0.0609	2.53E-01	NS
TRMT2B	NS	1.38E-01	-0.0609	2.53E-01	NS
EGR2	NS	1.38E-01	-0.0609	2.53E-01	NS
CYP1A2	NS	1.38E-01	-0.0609	2.53E-01	NS
RAD50	NS	1.38E-01	-0.0609	2.53E-01	NS
RAB41	NS	1.38E-01	-0.0609	2.53E-01	NS
P2RY8	NS	1.38E-01	6.09E-02	2.54E-01	NS
IFIT1B	NS	1.38E-01	-0.0609	2.54E-01	NS
RFPL4AL1	NS	1.38E-01	-0.0609	2.54E-01	NS
WASHC4	NS	1.38E-01	-0.0608	2.54E-01	NS
FLVCR1	NS	1.38E-01	-0.0608	2.54E-01	NS
HPCAL4	NS	1.38E-01	6.08E-02	2.54E-01	NS
SFTPA1	NS	1.38E-01	-0.0608	2.54E-01	NS
EML6	NS	1.39E-01	-0.0608	2.54E-01	NS
UBE2K	NS	1.39E-01	-0.0608	2.54E-01	NS
MS4A4E	NS	1.39E-01	6.08E-02	2.54E-01	NS
UHMK1	NS	1.39E-01	-0.0608	2.54E-01	NS
OR5B2	NS	1.39E-01	-0.0608	2.54E-01	NS
PDILT	NS	1.39E-01	6.08E-02	2.55E-01	NS
GNLY	NS	1.39E-01	-0.0607	2.55E-01	NS
NONO	NS	1.39E-01	-0.0607	2.55E-01	NS
PPP1R16B	NS	1.39E-01	-0.0607	2.55E-01	NS
LIPK	NS	1.39E-01	-0.0607	2.55E-01	NS
MYH1	NS	1.39E-01	-0.0607	2.55E-01	NS
SOAT2	NS	1.39E-01	6.07E-02	2.55E-01	NS
MCM9	NS	1.39E-01	6.07E-02	2.55E-01	NS
CYB561A3	NS	1.39E-01	-0.0607	2.55E-01	NS
ASB13	NS	1.39E-01	-0.0607	2.55E-01	NS
TIMM44	NS	1.40E-01	-0.0606	2.56E-01	NS
SNUPN	NS	1.39E-01	-0.0607	2.56E-01	NS
ZNF397	NS	1.40E-01	-0.0606	2.56E-01	NS

GPR31	NS	1.40E-01	-0.0606	2.56E-01	NS
AR	NS	1.40E-01	-0.0606	2.56E-01	NS
COL24A1	NS	1.40E-01	-0.0606	2.56E-01	NS
MTRF1L	NS	1.40E-01	-0.0606	2.56E-01	NS
ECHDC1	NS	1.40E-01	-0.0606	2.56E-01	NS
NPIPA7	NS	1.40E-01	6.06E-02	2.56E-01	NS
EME2	NS	1.40E-01	-0.0606	2.57E-01	NS
POU4F3	NS	1.40E-01	6.06E-02	2.57E-01	NS
KLF5	NS	1.40E-01	6.06E-02	2.57E-01	NS
PEX13	NS	1.40E-01	-0.0606	2.57E-01	NS
SRRM3	NS	1.40E-01	6.05E-02	2.57E-01	NS
BCOR	NS	1.40E-01	-0.0605	2.57E-01	NS
KRTAP23-1	NS	1.40E-01	-0.0605	2.57E-01	NS
FAH	NS	1.40E-01	-0.0605	2.57E-01	NS
ELMOD1	NS	1.40E-01	6.05E-02	2.57E-01	NS
RBM47	NS	1.40E-01	-0.0605	2.57E-01	NS
EPHX3	NS	1.40E-01	6.05E-02	2.57E-01	NS
SACS	NS	1.40E-01	-0.0605	2.57E-01	NS
IGFL1	NS	1.40E-01	6.05E-02	2.57E-01	NS
CLIC4	NS	1.40E-01	-0.0605	2.57E-01	NS
TGIF2LY	NS	1.41E-01	-0.0605	2.57E-01	NS
OR5AS1	NS	1.41E-01	6.05E-02	2.57E-01	NS
EFTUD2	NS	1.41E-01	-0.0605	2.57E-01	NS
SLC22A8	NS	1.41E-01	-0.0605	2.57E-01	NS
DISC1	NS	1.41E-01	-0.0605	2.57E-01	NS
PCNX3	NS	1.41E-01	-0.0605	2.57E-01	NS
TLR8	NS	1.41E-01	-0.0604	2.58E-01	NS
ZC3H11B	NS	1.41E-01	-0.0604	2.58E-01	NS
SPON1	NS	1.41E-01	-0.0604	2.58E-01	NS
ATP10B	NS	1.41E-01	-0.0604	2.58E-01	NS
MINDY4B	NS	1.41E-01	-0.0604	2.58E-01	NS
PCDHGA10	NS	1.41E-01	-0.0604	2.58E-01	NS
FOXJ3	NS	1.41E-01	6.04E-02	2.58E-01	NS
HPGDS	NS	1.41E-01	-0.0604	2.58E-01	NS
DTHD1	NS	1.41E-01	-0.0604	2.58E-01	NS
CRY2	NS	1.41E-01	6.04E-02	2.58E-01	NS
METTL25B	NS	1.41E-01	-0.0604	2.58E-01	NS

RSPO1	NS	1.41E-01	6.04E-02	2.58E-01	NS
RPS6KA6	NS	1.41E-01	6.04E-02	2.58E-01	NS
TONSL	NS	1.41E-01	-0.0603	2.58E-01	NS
ARIH2	NS	1.41E-01	-0.0603	2.58E-01	NS
FAM200A	NS	1.42E-01	-0.0603	2.58E-01	NS
ZC3H15	NS	1.42E-01	6.03E-02	2.58E-01	NS
EMC8	NS	1.42E-01	6.03E-02	2.58E-01	NS
DCTN3	NS	1.42E-01	6.03E-02	2.58E-01	NS
PSPH	NS	1.42E-01	-0.0603	2.58E-01	NS
DST	NS	1.42E-01	-0.0603	2.58E-01	NS
FIS1	NS	1.42E-01	-0.0603	2.59E-01	NS
NRCAM	NS	1.42E-01	-0.0603	2.59E-01	NS
MYO10	NS	1.42E-01	-0.0603	2.59E-01	NS
DPH5	NS	1.42E-01	-0.0603	2.59E-01	NS
TEX2	NS	1.42E-01	6.03E-02	2.59E-01	NS
ANPEP	NS	1.42E-01	-0.0603	2.59E-01	NS
RNF212	NS	1.42E-01	-0.0603	2.59E-01	NS
ZDBF2	NS	1.42E-01	6.03E-02	2.59E-01	NS
H2BC5	NS	1.42E-01	-0.0603	2.59E-01	NS
TTC38	NS	1.42E-01	-0.0603	2.59E-01	NS
OR4K5	NS	1.42E-01	6.03E-02	2.59E-01	NS
MYL6	NS	1.42E-01	-0.0603	2.59E-01	NS
RFWD3	NS	1.42E-01	-0.0603	2.59E-01	NS
UNC119B	NS	1.42E-01	-0.0603	2.59E-01	NS
STAP2	NS	1.42E-01	-0.0602	2.59E-01	NS
INSIG1	NS	1.42E-01	-0.0602	2.59E-01	NS
BAZ2B	NS	1.42E-01	-0.0602	2.59E-01	NS
KRTAP10-10	NS	1.42E-01	6.02E-02	2.59E-01	NS
PRR23D2	NS	1.42E-01	-0.0602	2.59E-01	NS
RHOD	NS	1.42E-01	-0.0602	2.59E-01	NS
RIPOR3	NS	1.42E-01	-0.0602	2.59E-01	NS
LRRC59	NS	1.42E-01	6.02E-02	2.59E-01	NS
HCFC2	NS	1.42E-01	-0.0602	2.59E-01	NS
APOC1	NS	1.42E-01	6.02E-02	2.59E-01	NS
ORMDL3	NS	1.43E-01	6.02E-02	2.59E-01	NS
SNX32	NS	1.43E-01	6.02E-02	2.59E-01	NS
BCLAF1	NS	1.43E-01	6.02E-02	2.60E-01	NS

LGSN	NS	1.43E-01	-0.0602	2.60E-01	NS
ZFP36	NS	1.43E-01	6.02E-02	2.60E-01	NS
KLHDC7B	NS	1.43E-01	6.01E-02	2.60E-01	NS
SCN10A	NS	1.43E-01	-0.0602	2.60E-01	NS
OR5M10	NS	1.43E-01	-0.0601	2.60E-01	NS
RAI1	NS	1.43E-01	6.01E-02	2.60E-01	NS
ATP5F1E	NS	1.43E-01	6.01E-02	2.60E-01	NS
ELAVL3	NS	1.43E-01	6.01E-02	2.60E-01	NS
HIF1A	NS	1.43E-01	-0.0601	2.60E-01	NS
CNOT2	NS	1.43E-01	-0.0601	2.60E-01	NS
UBIAD1	NS	1.43E-01	-0.0601	2.60E-01	NS
CDKN2B	NS	1.43E-01	-0.0601	2.60E-01	NS
TMEM250	NS	1.43E-01	6.01E-02	2.60E-01	NS
KIAA1755	NS	1.43E-01	-0.0601	2.60E-01	NS
GCK	NS	1.43E-01	-0.0601	2.60E-01	NS
FANCA	NS	1.43E-01	-0.0601	2.60E-01	NS
EZR	NS	1.43E-01	-0.0601	2.60E-01	NS
MIIP	NS	1.43E-01	6.01E-02	2.60E-01	NS
SPCS2	NS	1.43E-01	-0.0601	2.60E-01	NS
NSRP1	NS	1.43E-01	-0.0601	2.60E-01	NS
HACD1	NS	1.43E-01	6.01E-02	2.60E-01	NS
NXPE2	NS	1.43E-01	-0.0601	2.60E-01	NS
RPS4Y2	NS	1.43E-01	-0.0601	2.60E-01	NS
TACR2	NS	1.43E-01	-0.06	2.60E-01	NS
LRRC3B	NS	1.43E-01	-0.06	2.60E-01	NS
TRIM64	NS	1.43E-01	-0.06	2.60E-01	NS
CBLN4	NS	1.44E-01	-0.06	2.61E-01	NS
GAGE12F	NS	1.44E-01	-0.06	2.61E-01	NS
NASP	NS	1.44E-01	6.00E-02	2.61E-01	NS
PPP6R2	NS	1.44E-01	6.00E-02	2.61E-01	NS
HS6ST2	NS	1.44E-01	-0.06	2.61E-01	NS
ZNF324	NS	1.44E-01	-0.06	2.61E-01	NS
ZDHHC5	NS	1.44E-01	6.00E-02	2.61E-01	NS
PALM2AKAP2	NS	1.44E-01	-0.06	2.61E-01	NS
SCARA3	NS	1.44E-01	-0.06	2.61E-01	NS
PLXNB3	NS	1.44E-01	-0.06	2.61E-01	NS
ATP1A3	NS	1.44E-01	6.00E-02	2.61E-01	NS

MS4A6E	NS	1.44E-01	-0.0599	2.61E-01	NS
FAM50A	NS	1.44E-01	5.99E-02	2.62E-01	NS
CMAS	NS	1.44E-01	-0.0599	2.62E-01	NS
HAX1	NS	1.44E-01	-0.0599	2.62E-01	NS
TMEM209	NS	1.45E-01	-0.0599	2.62E-01	NS
HEYL	NS	1.45E-01	5.99E-02	2.62E-01	NS
KRTAP5-3	NS	1.45E-01	-0.0599	2.62E-01	NS
MTHFS	NS	1.45E-01	-0.0599	2.62E-01	NS
APOA5	NS	1.45E-01	5.99E-02	2.62E-01	NS
GOLGA8N	NS	1.45E-01	-0.0599	2.62E-01	NS
SEMA4F	NS	1.45E-01	-0.0599	2.62E-01	NS
TSPO	NS	1.45E-01	-0.0599	2.62E-01	NS
ATF6	NS	1.45E-01	-0.0598	2.62E-01	NS
CD69	NS	1.45E-01	-0.0598	2.62E-01	NS
DIPK1B	NS	1.45E-01	5.98E-02	2.62E-01	NS
SLC2A10	NS	1.45E-01	-0.0598	2.62E-01	NS
COQ5	NS	1.45E-01	5.98E-02	2.63E-01	NS
TMEM138	NS	1.45E-01	-0.0598	2.63E-01	NS
ZADH2	NS	1.45E-01	-0.0598	2.63E-01	NS
ZP4	NS	1.45E-01	-0.0598	2.63E-01	NS
KIF3B	NS	1.45E-01	-0.0598	2.63E-01	NS
TBC1D8B	NS	1.45E-01	-0.0598	2.63E-01	NS
IKZF2	NS	1.46E-01	-0.0597	2.63E-01	NS
DTD2	NS	1.46E-01	-0.0597	2.64E-01	NS
GSX2	NS	1.46E-01	5.97E-02	2.64E-01	NS
KIAA1614	NS	1.46E-01	-0.0597	2.64E-01	NS
EIF4E2	NS	1.46E-01	-0.0596	2.64E-01	NS
ANGPTL2	NS	1.46E-01	-0.0596	2.64E-01	NS
DBF4B	NS	1.46E-01	5.96E-02	2.64E-01	NS
NAA35	NS	1.46E-01	-0.0596	2.64E-01	NS
C8G	NS	1.46E-01	5.96E-02	2.64E-01	NS
TSPAN17	NS	1.46E-01	5.96E-02	2.65E-01	NS
SLC1A7	NS	1.46E-01	5.96E-02	2.65E-01	NS
PPP1R3B	NS	1.46E-01	-0.0596	2.65E-01	NS
MAPT	NS	1.46E-01	-0.0596	2.65E-01	NS
NPS	NS	1.47E-01	-0.0596	2.65E-01	NS
ATG14	NS	1.47E-01	5.96E-02	2.65E-01	NS

MYOM1	NS	1.47E-01	-0.0596	2.65E-01	NS
PARP6	NS	1.47E-01	5.96E-02	2.65E-01	NS
KLHL33	NS	1.47E-01	-0.0596	2.65E-01	NS
MAG	NS	1.47E-01	5.96E-02	2.65E-01	NS
PABIR2	NS	1.47E-01	5.96E-02	2.65E-01	NS
ATP5MF	NS	1.47E-01	-0.0596	2.65E-01	NS
BLACAT1	NS	1.47E-01	5.96E-02	2.65E-01	NS
SLC45A3	NS	1.47E-01	5.95E-02	2.65E-01	NS
MAGEA6	NS	1.47E-01	-0.0595	2.65E-01	NS
PPA2	NS	1.47E-01	-0.0595	2.65E-01	NS
DOCK9	NS	1.47E-01	-0.0595	2.65E-01	NS
LIPT2	NS	1.47E-01	-0.0595	2.65E-01	NS
POLB	NS	1.47E-01	5.95E-02	2.65E-01	NS
NALCN	NS	1.47E-01	5.95E-02	2.65E-01	NS
KLHL29	NS	1.47E-01	-0.0595	2.65E-01	NS
LCA5L	NS	1.47E-01	-0.0595	2.66E-01	NS
ACCS	NS	1.47E-01	-0.0595	2.66E-01	NS
TJAP1	NS	1.47E-01	-0.0595	2.66E-01	NS
SCML2	NS	1.47E-01	-0.0595	2.66E-01	NS
ZNF665	NS	1.47E-01	-0.0595	2.66E-01	NS
MBD3L4	NS	1.48E-01	5.94E-02	2.66E-01	NS
KIAA1549	NS	1.48E-01	-0.0594	2.66E-01	NS
UTP4	NS	1.48E-01	-0.0594	2.66E-01	NS
SSMEM1	NS	1.48E-01	-0.0594	2.66E-01	NS
SEMA4D	NS	1.48E-01	-0.0594	2.66E-01	NS
HHLA1	NS	1.48E-01	5.94E-02	2.66E-01	NS
RIIAD1	NS	1.48E-01	5.94E-02	2.67E-01	NS
KCNA4	NS	1.48E-01	-0.0594	2.67E-01	NS
INTS7	NS	1.48E-01	-0.0594	2.67E-01	NS
TM7SF2	NS	1.48E-01	-0.0594	2.67E-01	NS
SSX3	NS	1.48E-01	-0.0594	2.67E-01	NS
SLC19A2	NS	1.48E-01	5.94E-02	2.67E-01	NS
FUCA1	NS	1.48E-01	-0.0594	2.67E-01	NS
BTG1	NS	1.48E-01	5.93E-02	2.67E-01	NS
CROCC2	NS	1.48E-01	-0.0593	2.67E-01	NS
JAKMIP1	NS	1.48E-01	-0.0593	2.67E-01	NS
H2AP	NS	1.48E-01	5.93E-02	2.67E-01	NS

CHERP	NS	1.48E-01	5.93E-02	2.67E-01	NS
HAO1	NS	1.49E-01	-0.0593	2.67E-01	NS
CLC	NS	1.49E-01	-0.0593	2.67E-01	NS
C11orf98	NS	1.49E-01	-0.0593	2.67E-01	NS
IL22	NS	1.49E-01	5.93E-02	2.67E-01	NS
RPL35	NS	1.49E-01	-0.0593	2.68E-01	NS
FOXO4	NS	1.49E-01	-0.0593	2.68E-01	NS
MPZL1	NS	1.49E-01	5.93E-02	2.68E-01	NS
OR1J4	NS	1.49E-01	-0.0593	2.68E-01	NS
INO80B-WBP1	NS	1.49E-01	-0.0593	2.68E-01	NS
SEC61A1	NS	1.49E-01	-0.0592	2.68E-01	NS
TRAF5	NS	1.49E-01	-0.0592	2.68E-01	NS
TTC3	NS	1.49E-01	-0.0592	2.68E-01	NS
CTAGE8	NS	1.49E-01	-0.0592	2.68E-01	NS
ZFP57	NS	1.49E-01	-0.0592	2.68E-01	NS
TRH	NS	1.49E-01	-0.0592	2.68E-01	NS
KRT15	NS	1.49E-01	5.92E-02	2.68E-01	NS
TP53TG3B	NS	1.49E-01	-0.0592	2.68E-01	NS
ITPRID2	NS	1.49E-01	-0.0592	2.68E-01	NS
CRYAA	NS	1.49E-01	-0.0592	2.68E-01	NS
SLC2A7	NS	1.49E-01	-0.0592	2.68E-01	NS
IBSP	NS	1.49E-01	-0.0592	2.69E-01	NS
RNF19A	NS	1.49E-01	5.92E-02	2.69E-01	NS
OR10K1	NS	1.50E-01	-0.0591	2.69E-01	NS
ZC3H12B	NS	1.50E-01	-0.0591	2.69E-01	NS
ACSL1	NS	1.50E-01	-0.0591	2.69E-01	NS
EIF2AK4	NS	1.50E-01	5.91E-02	2.69E-01	NS
TRIM21	NS	1.50E-01	5.91E-02	2.69E-01	NS
CCDC18	NS	1.50E-01	-0.0591	2.69E-01	NS
CEACAM6	NS	1.50E-01	-0.0591	2.69E-01	NS
GPATCH2L	NS	1.50E-01	-0.0591	2.69E-01	NS
TANGO6	NS	1.50E-01	-0.0591	2.69E-01	NS
ISCA1	NS	1.50E-01	-0.0591	2.69E-01	NS
LCE1D	NS	1.50E-01	5.91E-02	2.70E-01	NS
OR6V1	NS	1.50E-01	5.91E-02	2.70E-01	NS
AFF1	NS	1.50E-01	5.91E-02	2.70E-01	NS
GYG2	NS	1.50E-01	-0.0591	2.70E-01	NS

SLC45A1	NS	1.50E-01	-0.059	2.70E-01	NS
ENO3	NS	1.51E-01	5.90E-02	2.70E-01	NS
ZNF655	NS	1.51E-01	-0.059	2.70E-01	NS
NPIPB11	NS	1.51E-01	-0.059	2.70E-01	NS
INSR	NS	1.51E-01	-0.059	2.70E-01	NS
A3GALT2	NS	1.51E-01	-0.059	2.70E-01	NS
MAN2A2	NS	1.51E-01	-0.059	2.70E-01	NS
TBC1D31	NS	1.51E-01	-0.059	2.70E-01	NS
ST3GAL4	NS	1.51E-01	-0.059	2.70E-01	NS
LY6H	NS	1.51E-01	5.90E-02	2.70E-01	NS
XPO6	NS	1.51E-01	5.90E-02	2.70E-01	NS
POLR2H	NS	1.51E-01	5.90E-02	2.70E-01	NS
DUSP7	NS	1.51E-01	5.89E-02	2.71E-01	NS
CRLF3	NS	1.51E-01	-0.0589	2.71E-01	NS
OR10H5	NS	1.51E-01	-0.0589	2.71E-01	NS
H3C10	NS	1.51E-01	-0.0589	2.71E-01	NS
ZNF329	NS	1.51E-01	-0.0589	2.71E-01	NS
CT47A10	NS	1.51E-01	5.89E-02	2.71E-01	NS
SORCS3	NS	1.51E-01	-0.0589	2.71E-01	NS
EXOSC3	NS	1.51E-01	-0.0589	2.71E-01	NS
SCN3A	NS	1.51E-01	-0.0589	2.71E-01	NS
EPB41L3	NS	1.51E-01	-0.0589	2.71E-01	NS
AAR2	NS	1.52E-01	-0.0589	2.71E-01	NS
TM4SF18	NS	1.52E-01	-0.0589	2.71E-01	NS
FHAD1	NS	1.52E-01	5.88E-02	2.72E-01	NS
ADGRL3	NS	1.52E-01	-0.0588	2.72E-01	NS
CNPY1	NS	1.52E-01	-0.0588	2.72E-01	NS
TMEM72	NS	1.52E-01	-0.0588	2.72E-01	NS
C2orf88	NS	1.52E-01	-0.0588	2.72E-01	NS
OR1E1	NS	1.52E-01	-0.0588	2.73E-01	NS
NELFB	NS	1.52E-01	-0.0588	2.73E-01	NS
SELENOS	NS	1.52E-01	-0.0587	2.73E-01	NS
ATP6V1B1	NS	1.52E-01	5.87E-02	2.73E-01	NS
MMADHC	NS	1.52E-01	-0.0587	2.73E-01	NS
ZNF469	NS	1.53E-01	-0.0587	2.73E-01	NS
C1orf56	NS	1.53E-01	-0.0587	2.73E-01	NS
RBIS	NS	1.53E-01	-0.0587	2.73E-01	NS

BTBD1	NS	1.53E-01	-0.0587	2.73E-01	NS
SMIM3	NS	1.53E-01	5.87E-02	2.73E-01	NS
ERFE	NS	1.53E-01	5.87E-02	2.73E-01	NS
ACAN	NS	1.53E-01	5.86E-02	2.74E-01	NS
FXYD1	NS	1.53E-01	5.86E-02	2.74E-01	NS
NANOGNB	NS	1.53E-01	5.86E-02	2.74E-01	NS
TRIO	NS	1.53E-01	5.86E-02	2.74E-01	NS
GLRX5	NS	1.53E-01	-0.0586	2.74E-01	NS
MAN1A2	NS	1.53E-01	-0.0586	2.74E-01	NS
WDR62	NS	1.53E-01	-0.0586	2.74E-01	NS
TINCR	NS	1.53E-01	5.86E-02	2.74E-01	NS
LRP2	NS	1.53E-01	5.86E-02	2.74E-01	NS
SCO2	NS	1.53E-01	5.86E-02	2.74E-01	NS
PCDHB9	NS	1.53E-01	5.86E-02	2.74E-01	NS
FAM186A	NS	1.53E-01	-0.0586	2.74E-01	NS
ZNF805	NS	1.53E-01	-0.0586	2.74E-01	NS
CEP128	NS	1.53E-01	-0.0586	2.74E-01	NS
UFC1	NS	1.53E-01	-0.0586	2.74E-01	NS
HACD4	NS	1.53E-01	-0.0586	2.74E-01	NS
TAS2R13	NS	1.53E-01	-0.0586	2.74E-01	NS
FAM89B	NS	1.54E-01	5.86E-02	2.74E-01	NS
DCAF12L2	NS	1.53E-01	5.86E-02	2.74E-01	NS
CHRNA3	NS	1.54E-01	-0.0586	2.74E-01	NS
CCDC33	NS	1.54E-01	-0.0586	2.74E-01	NS
TH	NS	1.54E-01	-0.0586	2.74E-01	NS
MITD1	NS	1.54E-01	5.86E-02	2.74E-01	NS
THOC7	NS	1.54E-01	-0.0585	2.74E-01	NS
HIBCH	NS	1.54E-01	-0.0585	2.74E-01	NS
SMPD3	NS	1.54E-01	-0.0585	2.74E-01	NS
TMC4	NS	1.54E-01	-0.0585	2.74E-01	NS
ACOT8	NS	1.54E-01	-0.0585	2.75E-01	NS
DCAF17	NS	1.54E-01	-0.0585	2.75E-01	NS
C3AR1	NS	1.54E-01	-0.0585	2.75E-01	NS
TAGLN	NS	1.54E-01	-0.0585	2.75E-01	NS
NAPG	NS	1.54E-01	-0.0585	2.75E-01	NS
CLPSL2	NS	1.54E-01	5.85E-02	2.75E-01	NS
OR4D11	NS	1.55E-01	5.84E-02	2.75E-01	NS

DPH2	NS	1.55E-01	-0.0584	2.75E-01	NS
PTPRCAP	NS	1.55E-01	-0.0584	2.75E-01	NS
POLR2B	NS	1.55E-01	-0.0584	2.75E-01	NS
ACAA1	NS	1.55E-01	-0.0584	2.76E-01	NS
CRELD1	NS	1.55E-01	-0.0584	2.76E-01	NS
SAXO2	NS	1.55E-01	5.84E-02	2.76E-01	NS
KCNC1	NS	1.55E-01	-0.0584	2.76E-01	NS
TEX38	NS	1.55E-01	-0.0584	2.76E-01	NS
ARMCX5-GPRASP2	NS	1.55E-01	-0.0584	2.76E-01	NS
PLEKHG1	NS	1.55E-01	-0.0584	2.76E-01	NS
ZIM3	NS	1.55E-01	-0.0584	2.76E-01	NS
CD300LB	NS	1.55E-01	-0.0584	2.76E-01	NS
NIBAN2	NS	1.55E-01	-0.0583	2.76E-01	NS
ST6GALNAC4	NS	1.55E-01	-0.0583	2.76E-01	NS
LRRTM2	NS	1.55E-01	-0.0583	2.76E-01	NS
CCDC80	NS	1.55E-01	-0.0583	2.76E-01	NS
KLF1	NS	1.55E-01	-0.0583	2.76E-01	NS
TAFA3	NS	1.55E-01	-0.0583	2.76E-01	NS
NAP1L1	NS	1.55E-01	-0.0583	2.76E-01	NS
SDK1	NS	1.55E-01	-0.0583	2.76E-01	NS
ZC3H12D	NS	1.55E-01	-0.0583	2.76E-01	NS
DIP2A	NS	1.55E-01	5.83E-02	2.76E-01	NS
TUBA4A	NS	1.56E-01	-0.0583	2.76E-01	NS
SPATA31A3	NS	1.56E-01	-0.0583	2.77E-01	NS
OTOGL	NS	1.56E-01	-0.0583	2.77E-01	NS
SPRYD3	NS	1.56E-01	-0.0583	2.77E-01	NS
TAF5L	NS	1.56E-01	-0.0583	2.77E-01	NS
PHF21A	NS	1.56E-01	5.83E-02	2.77E-01	NS
MMP7	NS	1.56E-01	-0.0582	2.77E-01	NS
CSF1R	NS	1.56E-01	-0.0582	2.77E-01	NS
TCF12	NS	1.56E-01	5.82E-02	2.77E-01	NS
HDGFL3	NS	1.56E-01	5.82E-02	2.77E-01	NS
TGIF2	NS	1.56E-01	-0.0582	2.77E-01	NS
GLT1D1	NS	1.56E-01	-0.0582	2.77E-01	NS
LRRC43	NS	1.56E-01	5.82E-02	2.77E-01	NS
ATP5F1C	NS	1.56E-01	-0.0582	2.77E-01	NS
ACP5	NS	1.56E-01	-0.0582	2.77E-01	NS

GJB4	NS	1.56E-01	-0.0582	2.78E-01	NS
TIGAR	NS	1.56E-01	-0.0582	2.78E-01	NS
SERPINB10	NS	1.56E-01	-0.0582	2.78E-01	NS
TIMM9	NS	1.57E-01	-0.0582	2.78E-01	NS
CLCNKB	NS	1.57E-01	-0.0582	2.78E-01	NS
SLC31A2	NS	1.57E-01	-0.0581	2.78E-01	NS
ANKZF1	NS	1.57E-01	-0.0581	2.78E-01	NS
PTTG1IP2	NS	1.57E-01	-0.0581	2.78E-01	NS
COL4A3	NS	1.57E-01	-0.0581	2.78E-01	NS
ARSB	NS	1.57E-01	5.81E-02	2.78E-01	NS
GPR146	NS	1.57E-01	-0.0581	2.78E-01	NS
NRDE2	NS	1.57E-01	-0.0581	2.78E-01	NS
CPEB3	NS	1.57E-01	5.81E-02	2.78E-01	NS
TWIST1	NS	1.57E-01	-0.0581	2.78E-01	NS
NT5DC4	NS	1.57E-01	-0.0581	2.78E-01	NS
OR4Q3	NS	1.57E-01	-0.0581	2.78E-01	NS
ZBED9	NS	1.57E-01	5.81E-02	2.78E-01	NS
PIGU	NS	1.57E-01	-0.0581	2.78E-01	NS
ANKRD13B	NS	1.57E-01	5.81E-02	2.79E-01	NS
GCSH	NS	1.57E-01	-0.058	2.79E-01	NS
FAM214B	NS	1.57E-01	5.80E-02	2.79E-01	NS
NT5C2	NS	1.57E-01	-0.058	2.79E-01	NS
LRRN4CL	NS	1.58E-01	5.80E-02	2.79E-01	NS
MEI1	NS	1.58E-01	-0.058	2.79E-01	NS
RANGAP1	NS	1.58E-01	-0.058	2.79E-01	NS
TUSC3	NS	1.58E-01	5.80E-02	2.79E-01	NS
TMPO	NS	1.58E-01	-0.058	2.79E-01	NS
A1BG	NS	1.58E-01	-0.058	2.79E-01	NS
CES5A	NS	1.58E-01	5.80E-02	2.79E-01	NS
CAPZB	NS	1.58E-01	5.80E-02	2.79E-01	NS
CPNE6	NS	1.58E-01	-0.058	2.79E-01	NS
BCAN	NS	1.58E-01	-0.058	2.79E-01	NS
PGD	NS	1.58E-01	-0.058	2.79E-01	NS
LAPTM5	NS	1.58E-01	-0.058	2.79E-01	NS
GSTT4	NS	1.58E-01	5.80E-02	2.79E-01	NS
IGLON5	NS	1.58E-01	5.80E-02	2.79E-01	NS
OR11G2	NS	1.58E-01	5.79E-02	2.80E-01	NS

NUBPL	NS	1.58E-01	-0.0579	2.80E-01	NS
ADGRA1	NS	1.58E-01	5.79E-02	2.80E-01	NS
PLSCR4	NS	1.58E-01	-0.0579	2.80E-01	NS
NPLOC4	NS	1.58E-01	-0.0579	2.80E-01	NS
SRP68	NS	1.58E-01	5.79E-02	2.80E-01	NS
ALKBH3	NS	1.59E-01	-0.0579	2.80E-01	NS
FAM227A	NS	1.59E-01	5.79E-02	2.80E-01	NS
COX7C	NS	1.59E-01	-0.0578	2.81E-01	NS
FANCI	NS	1.59E-01	-0.0578	2.81E-01	NS
OR8K1	NS	1.59E-01	-0.0578	2.81E-01	NS
LRRC27	NS	1.59E-01	-0.0578	2.81E-01	NS
MRPL12	NS	1.59E-01	-0.0578	2.81E-01	NS
GAA	NS	1.59E-01	-0.0578	2.82E-01	NS
FNDC9	NS	1.59E-01	-0.0578	2.82E-01	NS
PTEN	NS	1.59E-01	-0.0577	2.82E-01	NS
EIF3K	NS	1.60E-01	5.77E-02	2.82E-01	NS
PSMC6	NS	1.60E-01	-0.0577	2.82E-01	NS
WDR70	NS	1.60E-01	-0.0577	2.82E-01	NS
KLHL36	NS	1.60E-01	-0.0577	2.82E-01	NS
FOCAD	NS	1.60E-01	-0.0577	2.82E-01	NS
PLPBP	NS	1.60E-01	-0.0577	2.83E-01	NS
FZD5	NS	1.60E-01	5.77E-02	2.83E-01	NS
SERPINA10	NS	1.60E-01	-0.0577	2.83E-01	NS
TMEM163	NS	1.60E-01	-0.0576	2.83E-01	NS
FGA	NS	1.60E-01	-0.0576	2.83E-01	NS
SLC35A3	NS	1.60E-01	-0.0576	2.83E-01	NS
ZP2	NS	1.60E-01	-0.0576	2.83E-01	NS
CCDC38	NS	1.60E-01	-0.0576	2.83E-01	NS
RPL36A-HNRNPH2	NS	1.60E-01	-0.0576	2.83E-01	NS
HTATIP2	NS	1.60E-01	-0.0576	2.83E-01	NS
TDP2	NS	1.61E-01	5.76E-02	2.83E-01	NS
BUB1B-PAK6	NS	1.61E-01	5.76E-02	2.83E-01	NS
AGR2	NS	1.61E-01	-0.0576	2.83E-01	NS
MOCS3	NS	1.61E-01	-0.0576	2.83E-01	NS
MS4A2	NS	1.61E-01	5.76E-02	2.83E-01	NS
SPATA3	NS	1.61E-01	-0.0576	2.83E-01	NS
FUBP3	NS	1.61E-01	5.76E-02	2.84E-01	NS

ATP13A4	NS	1.61E-01	-0.0576	2.84E-01	NS
HABP2	NS	1.61E-01	-0.0576	2.84E-01	NS
WDR47	NS	1.61E-01	-0.0575	2.84E-01	NS
RPL22	NS	1.61E-01	-0.0575	2.84E-01	NS
CDK7	NS	1.61E-01	-0.0575	2.84E-01	NS
SPPL2A	NS	1.61E-01	-0.0575	2.84E-01	NS
GPX3	NS	1.61E-01	-0.0575	2.84E-01	NS
TOMM7	NS	1.61E-01	-0.0575	2.84E-01	NS
PRRT4	NS	1.61E-01	5.75E-02	2.84E-01	NS
POC1B	NS	1.61E-01	-0.0575	2.84E-01	NS
PDYN	NS	1.61E-01	-0.0575	2.84E-01	NS
RASGRP2	NS	1.62E-01	5.74E-02	2.85E-01	NS
ENAM	NS	1.62E-01	5.74E-02	2.85E-01	NS
SEC24C	NS	1.62E-01	5.74E-02	2.85E-01	NS
PER2	NS	1.62E-01	5.74E-02	2.85E-01	NS
OAZ2	NS	1.62E-01	-0.0574	2.85E-01	NS
LPAR2	NS	1.62E-01	-0.0574	2.85E-01	NS
CFAP52	NS	1.62E-01	-0.0574	2.85E-01	NS
BEX4	NS	1.62E-01	-0.0574	2.85E-01	NS
GGTA1	NS	1.62E-01	-0.0574	2.85E-01	NS
ZNF860	NS	1.62E-01	5.74E-02	2.85E-01	NS
SLC35E3	NS	1.62E-01	-0.0574	2.85E-01	NS
KLHDC1	NS	1.62E-01	-0.0574	2.86E-01	NS
KLHDC8B	NS	1.62E-01	-0.0574	2.86E-01	NS
TMTC1	NS	1.62E-01	5.73E-02	2.86E-01	NS
ANKRD7	NS	1.63E-01	-0.0573	2.86E-01	NS
TOPORS	NS	1.63E-01	-0.0573	2.86E-01	NS
H2AB1	NS	1.63E-01	5.73E-02	2.86E-01	NS
FN3K	NS	1.63E-01	5.73E-02	2.87E-01	NS
FAM20C	NS	1.63E-01	-0.0573	2.87E-01	NS
NOL6	NS	1.63E-01	-0.0573	2.87E-01	NS
OR4K17	NS	1.63E-01	5.73E-02	2.87E-01	NS
C1orf94	NS	1.63E-01	-0.0573	2.87E-01	NS
KDM8	NS	1.63E-01	-0.0573	2.87E-01	NS
LRIT3	NS	1.63E-01	5.73E-02	2.87E-01	NS
ELK4	NS	1.63E-01	-0.0573	2.87E-01	NS
CDR1	NS	1.63E-01	-0.0573	2.87E-01	NS

TAF11L4	NS	1.63E-01	5.72E-02	2.87E-01	NS
RTP2	NS	1.63E-01	-0.0572	2.87E-01	NS
TTL	NS	1.63E-01	-0.0572	2.87E-01	NS
EXOC8	NS	1.63E-01	-0.0572	2.87E-01	NS
VNN1	NS	1.63E-01	-0.0572	2.87E-01	NS
HP1BP3	NS	1.63E-01	-0.0572	2.87E-01	NS
RSC1A1	NS	1.63E-01	-0.0572	2.87E-01	NS
RBM38	NS	1.64E-01	5.72E-02	2.87E-01	NS
TXLNB	NS	1.64E-01	-0.0572	2.87E-01	NS
SLC66A3	NS	1.64E-01	-0.0572	2.87E-01	NS
ATXN3	NS	1.64E-01	5.72E-02	2.87E-01	NS
SEC14L5	NS	1.64E-01	-0.0572	2.87E-01	NS
SLC25A25	NS	1.64E-01	5.72E-02	2.87E-01	NS
ERG	NS	1.64E-01	5.72E-02	2.87E-01	NS
DCK	NS	1.64E-01	-0.0572	2.87E-01	NS
H2BC4	NS	1.64E-01	5.71E-02	2.87E-01	NS
PTPRH	NS	1.64E-01	-0.0571	2.88E-01	NS
TNFRSF10C	NS	1.64E-01	-0.0571	2.88E-01	NS
CLEC16A	NS	1.64E-01	-0.0571	2.88E-01	NS
PAX6	NS	1.64E-01	-0.0571	2.88E-01	NS
ANO9	NS	1.64E-01	5.71E-02	2.88E-01	NS
GSTP1	NS	1.64E-01	5.71E-02	2.88E-01	NS
ISY1-RAB43	NS	1.64E-01	-0.0571	2.88E-01	NS
TPT1	NS	1.64E-01	-0.0571	2.88E-01	NS
CARD18	NS	1.64E-01	-0.0571	2.88E-01	NS
NPM2	NS	1.64E-01	5.71E-02	2.88E-01	NS
FBLN2	NS	1.64E-01	-0.0571	2.88E-01	NS
MALSU1	NS	1.64E-01	-0.0571	2.88E-01	NS
ZNF784	NS	1.64E-01	-0.0571	2.88E-01	NS
CYP3A7	NS	1.64E-01	-0.0571	2.88E-01	NS
GUCA1A	NS	1.64E-01	5.71E-02	2.88E-01	NS
NMNAT1	NS	1.65E-01	-0.0571	2.88E-01	NS
ADAT1	NS	1.65E-01	-0.0571	2.88E-01	NS
UBASH3A	NS	1.65E-01	-0.057	2.89E-01	NS
QRICH2	NS	1.65E-01	5.70E-02	2.89E-01	NS
STX8	NS	1.65E-01	5.70E-02	2.89E-01	NS
PARS2	NS	1.65E-01	-0.057	2.89E-01	NS

MRPS10	NS	1.65E-01	-0.057	2.89E-01	NS
DPT	NS	1.65E-01	-0.057	2.89E-01	NS
TBC1D1	NS	1.65E-01	-0.057	2.89E-01	NS
EIF2D	NS	1.65E-01	5.70E-02	2.89E-01	NS
KDM6B	NS	1.65E-01	-0.057	2.89E-01	NS
CCNG2	NS	1.65E-01	5.70E-02	2.89E-01	NS
CNGA3	NS	1.65E-01	-0.057	2.89E-01	NS
DNTT	NS	1.65E-01	-0.057	2.89E-01	NS
ATP9B	NS	1.65E-01	-0.0569	2.90E-01	NS
PEDS1-UBE2V1	NS	1.65E-01	5.69E-02	2.90E-01	NS
TFF1	NS	1.65E-01	-0.0569	2.90E-01	NS
STX11	NS	1.66E-01	-0.0569	2.90E-01	NS
APOBEC3B	NS	1.66E-01	-0.0569	2.90E-01	NS
KLHL23	NS	1.66E-01	-0.0569	2.90E-01	NS
REEP1	NS	1.66E-01	-0.0569	2.90E-01	NS
TMEM131L	NS	1.66E-01	5.69E-02	2.90E-01	NS
ABCA5	NS	1.66E-01	-0.0569	2.90E-01	NS
VASH2	NS	1.66E-01	-0.0569	2.90E-01	NS
TOB2	NS	1.66E-01	-0.0569	2.90E-01	NS
FKBP1C	NS	1.66E-01	-0.0568	2.90E-01	NS
EDN1	NS	1.66E-01	-0.0568	2.91E-01	NS
UCKL1	NS	1.66E-01	5.68E-02	2.91E-01	NS
DEAF1	NS	1.66E-01	5.68E-02	2.91E-01	NS
RAC2	NS	1.66E-01	5.68E-02	2.91E-01	NS
ERCC2	NS	1.66E-01	-0.0568	2.91E-01	NS
KCNIP3	NS	1.67E-01	5.68E-02	2.91E-01	NS
CYP2C9	NS	1.67E-01	-0.0568	2.91E-01	NS
ARMC12	NS	1.66E-01	-0.0568	2.91E-01	NS
ATP5PF	NS	1.67E-01	-0.0568	2.91E-01	NS
TEX43	NS	1.67E-01	-0.0568	2.91E-01	NS
NID1	NS	1.67E-01	-0.0567	2.91E-01	NS
BEAN1	NS	1.67E-01	-0.0567	2.91E-01	NS
RAB24	NS	1.67E-01	-0.0567	2.92E-01	NS
PITPNM2	NS	1.67E-01	-0.0567	2.92E-01	NS
MTPAP	NS	1.67E-01	-0.0567	2.92E-01	NS
MYO3B	NS	1.67E-01	-0.0567	2.92E-01	NS
IDO1	NS	1.67E-01	-0.0567	2.92E-01	NS

NRXN2	NS	1.67E-01	5.67E-02	2.92E-01	NS
NXF2	NS	1.67E-01	-0.0567	2.92E-01	NS
KLK9	NS	1.67E-01	5.67E-02	2.92E-01	NS
DOC2A	NS	1.68E-01	5.67E-02	2.92E-01	NS
ZNF780B	NS	1.68E-01	-0.0567	2.92E-01	NS
L3MBTL3	NS	1.68E-01	-0.0566	2.92E-01	NS
RAB3GAP2	NS	1.68E-01	5.66E-02	2.92E-01	NS
REPIN1	NS	1.68E-01	5.66E-02	2.93E-01	NS
NFATC2	NS	1.68E-01	-0.0566	2.93E-01	NS
LMOD1	NS	1.68E-01	-0.0566	2.93E-01	NS
PLEKHM1	NS	1.68E-01	5.66E-02	2.93E-01	NS
GTSF1L	NS	1.68E-01	-0.0566	2.93E-01	NS
ITIH2	NS	1.68E-01	-0.0566	2.93E-01	NS
CSPG4	NS	1.68E-01	-0.0566	2.93E-01	NS
SERF1B	NS	1.68E-01	5.66E-02	2.93E-01	NS
IQCF6	NS	1.68E-01	5.66E-02	2.93E-01	NS
MTRNR2L11	NS	1.68E-01	5.66E-02	2.93E-01	NS
SULT1C2	NS	1.68E-01	-0.0566	2.93E-01	NS
SLURP1	NS	1.68E-01	5.66E-02	2.93E-01	NS
ETV5	NS	1.68E-01	-0.0566	2.93E-01	NS
SOD2	NS	1.68E-01	5.65E-02	2.93E-01	NS
GPR33	NS	1.68E-01	5.65E-02	2.93E-01	NS
FASTKD1	NS	1.68E-01	-0.0565	2.93E-01	NS
SH3BP5L	NS	1.69E-01	-0.0565	2.93E-01	NS
KDELR2	NS	1.69E-01	5.65E-02	2.94E-01	NS
TEX48	NS	1.69E-01	-0.0565	2.94E-01	NS
STX16	NS	1.69E-01	5.65E-02	2.94E-01	NS
IL12B	NS	1.69E-01	5.65E-02	2.94E-01	NS
CCDC187	NS	1.69E-01	5.65E-02	2.94E-01	NS
PAEP	NS	1.69E-01	5.65E-02	2.94E-01	NS
LIME1	NS	1.69E-01	5.65E-02	2.94E-01	NS
MAN2B2	NS	1.69E-01	5.65E-02	2.94E-01	NS
YTHDF2	NS	1.69E-01	-0.0565	2.94E-01	NS
PCDHB5	NS	1.69E-01	5.64E-02	2.94E-01	NS
SUMO4	NS	1.69E-01	-0.0564	2.94E-01	NS
SLC13A4	NS	1.69E-01	-0.0564	2.95E-01	NS
PRRT3	NS	1.69E-01	5.64E-02	2.95E-01	NS

GPATCH11	NS	1.69E-01	5.64E-02	2.95E-01	NS
PAGE2	NS	1.69E-01	-0.0564	2.95E-01	NS
BCL3	NS	1.70E-01	5.64E-02	2.95E-01	NS
SYF2	NS	1.70E-01	5.64E-02	2.95E-01	NS
ARHGEF16	NS	1.70E-01	-0.0564	2.95E-01	NS
SOCS5	NS	1.70E-01	-0.0564	2.95E-01	NS
ZNF217	NS	1.70E-01	-0.0564	2.95E-01	NS
SERBP1	NS	1.70E-01	-0.0564	2.95E-01	NS
AVPI1	NS	1.70E-01	-0.0563	2.95E-01	NS
MAD2L2	NS	1.70E-01	5.63E-02	2.95E-01	NS
USP20	NS	1.70E-01	-0.0563	2.95E-01	NS
MCTS1	NS	1.70E-01	-0.0563	2.95E-01	NS
MTMR2	NS	1.70E-01	-0.0563	2.95E-01	NS
IFI27	NS	1.70E-01	-0.0563	2.95E-01	NS
SMKR1	NS	1.70E-01	5.63E-02	2.95E-01	NS
MTG1	NS	1.70E-01	-0.0563	2.96E-01	NS
ATP6V0D1	NS	1.70E-01	5.63E-02	2.96E-01	NS
NBDY	NS	1.70E-01	-0.0563	2.96E-01	NS
RAMAC	NS	1.70E-01	-0.0563	2.96E-01	NS
FOXN3	NS	1.71E-01	-0.0563	2.96E-01	NS
HOXD9	NS	1.71E-01	5.63E-02	2.96E-01	NS
SMO	NS	1.71E-01	5.62E-02	2.96E-01	NS
IL6R	NS	1.71E-01	5.62E-02	2.96E-01	NS
DEFB103A	NS	1.71E-01	-0.0562	2.96E-01	NS
PXMP2	NS	1.71E-01	-0.0562	2.97E-01	NS
NPTXR	NS	1.71E-01	5.62E-02	2.97E-01	NS
DAPP1	NS	1.71E-01	-0.0562	2.97E-01	NS
TPD52	NS	1.71E-01	-0.0562	2.97E-01	NS
UBC	NS	1.71E-01	-0.0562	2.97E-01	NS
CCL18	NS	1.71E-01	-0.0562	2.97E-01	NS
KANTR	NS	1.71E-01	5.62E-02	2.97E-01	NS
FRMPD1	NS	1.71E-01	5.62E-02	2.97E-01	NS
MTRNR2L5	NS	1.71E-01	-0.0562	2.97E-01	NS
ZDHHC15	NS	1.71E-01	-0.0562	2.97E-01	NS
RUNX3	NS	1.72E-01	-0.0561	2.98E-01	NS
CAGE1	NS	1.72E-01	-0.0561	2.98E-01	NS
STPG2	NS	1.72E-01	-0.0561	2.98E-01	NS

APOD	NS	1.72E-01	-0.0561	2.98E-01	NS
QPCT	NS	1.72E-01	-0.0561	2.98E-01	NS
TFAP2C	NS	1.72E-01	-0.0561	2.98E-01	NS
TOP2B	NS	1.72E-01	-0.0561	2.98E-01	NS
RUFY2	NS	1.72E-01	5.61E-02	2.98E-01	NS
C4orf19	NS	1.72E-01	-0.0561	2.98E-01	NS
THBD	NS	1.72E-01	-0.0561	2.98E-01	NS
ADAM10	NS	1.72E-01	-0.0561	2.98E-01	NS
CMSS1	NS	1.72E-01	-0.056	2.98E-01	NS
SPATA31E1	NS	1.72E-01	5.60E-02	2.98E-01	NS
MICOS13	NS	1.72E-01	5.60E-02	2.98E-01	NS
ZNF679	NS	1.73E-01	-0.056	2.99E-01	NS
CUL9	NS	1.73E-01	-0.056	2.99E-01	NS
ISCU	NS	1.73E-01	-0.056	2.99E-01	NS
G6PC3	NS	1.73E-01	5.60E-02	2.99E-01	NS
ZNF146	NS	1.73E-01	-0.056	2.99E-01	NS
RANBP17	NS	1.73E-01	-0.056	2.99E-01	NS
LRP8	NS	1.73E-01	-0.056	2.99E-01	NS
POLR2J2	NS	1.73E-01	5.59E-02	2.99E-01	NS
XRN1	NS	1.73E-01	-0.0559	3.00E-01	NS
SPRED2	NS	1.73E-01	5.59E-02	3.00E-01	NS
FAM205A	NS	1.73E-01	5.59E-02	3.00E-01	NS
PKNOX1	NS	1.73E-01	5.59E-02	3.00E-01	NS
TMC1	NS	1.73E-01	-0.0559	3.00E-01	NS
APBB3	NS	1.73E-01	-0.0559	3.00E-01	NS
CYP2C18	NS	1.73E-01	-0.0559	3.00E-01	NS
SUV39H1	NS	1.73E-01	5.59E-02	3.00E-01	NS
KRT6C	NS	1.73E-01	-0.0559	3.00E-01	NS
CTRB1	NS	1.74E-01	5.59E-02	3.00E-01	NS
PHC2	NS	1.74E-01	5.59E-02	3.00E-01	NS
GALNT14	NS	1.74E-01	-0.0558	3.00E-01	NS
SPATA24	NS	1.74E-01	-0.0558	3.01E-01	NS
NXT2	NS	1.74E-01	-0.0558	3.01E-01	NS
C12orf43	NS	1.74E-01	-0.0558	3.01E-01	NS
WNT10B	NS	1.74E-01	5.58E-02	3.01E-01	NS
PODN	NS	1.74E-01	5.58E-02	3.01E-01	NS
MAGEB5	NS	1.74E-01	5.58E-02	3.01E-01	NS

OR2T10	NS	1.74E-01	5.58E-02	3.01E-01	NS
PROX1	NS	1.74E-01	5.58E-02	3.01E-01	NS
SATB2	NS	1.74E-01	-0.0558	3.01E-01	NS
OR2L13	NS	1.74E-01	-0.0558	3.01E-01	NS
KIF1B	NS	1.74E-01	-0.0557	3.01E-01	NS
DCX	NS	1.75E-01	-0.0557	3.02E-01	NS
ADAM2	NS	1.75E-01	-0.0557	3.02E-01	NS
DDX50	NS	1.75E-01	-0.0557	3.02E-01	NS
PLA2G2A	NS	1.75E-01	-0.0557	3.02E-01	NS
FAM171A1	NS	1.75E-01	5.57E-02	3.02E-01	NS
CT47A1	NS	1.75E-01	5.57E-02	3.02E-01	NS
UQCRFS1	NS	1.75E-01	-0.0557	3.02E-01	NS
PNPLA6	NS	1.75E-01	-0.0557	3.02E-01	NS
RBPMS2	NS	1.75E-01	-0.0557	3.02E-01	NS
YES1	NS	1.75E-01	-0.0557	3.02E-01	NS
BOLA2	NS	1.75E-01	5.57E-02	3.02E-01	NS
IGFBP1	NS	1.75E-01	-0.0557	3.02E-01	NS
HOOK1	NS	1.75E-01	5.57E-02	3.02E-01	NS
ZMYND15	NS	1.75E-01	5.57E-02	3.02E-01	NS
BEND7	NS	1.75E-01	-0.0557	3.02E-01	NS
MPP3	NS	1.75E-01	-0.0556	3.02E-01	NS
ZNF780A	NS	1.75E-01	-0.0556	3.02E-01	NS
H2AC20	NS	1.75E-01	-0.0556	3.02E-01	NS
AQP1	NS	1.75E-01	-0.0556	3.02E-01	NS
DNPEP	NS	1.75E-01	-0.0556	3.02E-01	NS
SLC25A26	NS	1.75E-01	-0.0556	3.02E-01	NS
SQSTM1	NS	1.75E-01	5.56E-02	3.02E-01	NS
MYO9B	NS	1.76E-01	5.56E-02	3.03E-01	NS
SLC35E4	NS	1.76E-01	5.56E-02	3.03E-01	NS
ME3	NS	1.76E-01	-0.0556	3.03E-01	NS
VWA2	NS	1.75E-01	-0.0556	3.03E-01	NS
KRT77	NS	1.76E-01	-0.0556	3.03E-01	NS
SELENOP	NS	1.76E-01	-0.0556	3.03E-01	NS
QSOX1	NS	1.76E-01	5.56E-02	3.03E-01	NS
ODC1	NS	1.76E-01	5.56E-02	3.03E-01	NS
UBN1	NS	1.76E-01	-0.0556	3.03E-01	NS
PORCN	NS	1.76E-01	-0.0556	3.03E-01	NS

MED28	NS	1.76E-01	-0.0556	3.03E-01	NS
GAR1	NS	1.76E-01	-0.0556	3.03E-01	NS
ARF3	NS	1.76E-01	-0.0556	3.03E-01	NS
ZNF106	NS	1.76E-01	-0.0555	3.03E-01	NS
FAM153A	NS	1.76E-01	-0.0555	3.03E-01	NS
AK6	NS	1.76E-01	-0.0555	3.03E-01	NS
CEP170	NS	1.76E-01	-0.0555	3.03E-01	NS
TIMM29	NS	1.76E-01	5.55E-02	3.03E-01	NS
OR2T11	NS	1.76E-01	5.55E-02	3.03E-01	NS
TRPT1	NS	1.76E-01	5.55E-02	3.03E-01	NS
CCDC102B	NS	1.76E-01	-0.0555	3.04E-01	NS
ELOVL5	NS	1.76E-01	-0.0555	3.04E-01	NS
MAX	NS	1.77E-01	5.55E-02	3.04E-01	NS
TNFAIP8L2	NS	1.77E-01	-0.0555	3.04E-01	NS
NOTCH1	NS	1.77E-01	5.55E-02	3.04E-01	NS
RNF113A	NS	1.77E-01	-0.0555	3.04E-01	NS
PROSER3	NS	1.77E-01	5.55E-02	3.04E-01	NS
PGLYRP1	NS	1.77E-01	-0.0555	3.04E-01	NS
TCF24	NS	1.77E-01	5.54E-02	3.04E-01	NS
USP2	NS	1.77E-01	5.54E-02	3.04E-01	NS
EEPD1	NS	1.77E-01	5.54E-02	3.04E-01	NS
PTPN1	NS	1.77E-01	-0.0554	3.04E-01	NS
FAM90A17P	NS	1.77E-01	5.54E-02	3.04E-01	NS
C17orf75	NS	1.77E-01	-0.0554	3.04E-01	NS
PIGX	NS	1.77E-01	5.54E-02	3.05E-01	NS
FBXW9	NS	1.77E-01	-0.0554	3.05E-01	NS
NOTCH2NLB	NS	1.77E-01	-0.0554	3.05E-01	NS
PTGR2	NS	1.78E-01	-0.0553	3.05E-01	NS
PCDH15	NS	1.78E-01	-0.0553	3.05E-01	NS
PDE9A	NS	1.78E-01	5.53E-02	3.05E-01	NS
MED31	NS	1.78E-01	-0.0553	3.06E-01	NS
NABP1	NS	1.78E-01	-0.0553	3.06E-01	NS
MMP24OS	NS	1.78E-01	5.53E-02	3.06E-01	NS
LIPH	NS	1.78E-01	-0.0553	3.06E-01	NS
RASIP1	NS	1.78E-01	-0.0553	3.06E-01	NS
CALCRL	NS	1.78E-01	-0.0553	3.06E-01	NS
FOXD4L5	NS	1.78E-01	5.53E-02	3.06E-01	NS

SPATA31A1	NS	1.78E-01	-0.0553	3.06E-01	NS
STK38	NS	1.78E-01	-0.0553	3.06E-01	NS
AADACL4	NS	1.78E-01	-0.0553	3.06E-01	NS
MYD88	NS	1.78E-01	-0.0552	3.06E-01	NS
OAS1	NS	1.79E-01	-0.0552	3.06E-01	NS
KPNA2	NS	1.79E-01	-0.0552	3.06E-01	NS
CSAG1	NS	1.79E-01	5.52E-02	3.06E-01	NS
CALCB	NS	1.79E-01	-0.0552	3.06E-01	NS
CEP89	NS	1.79E-01	5.52E-02	3.06E-01	NS
SH3BGRL3	NS	1.79E-01	5.52E-02	3.06E-01	NS
NNAT	NS	1.79E-01	-0.0552	3.07E-01	NS
TRPV3	NS	1.79E-01	5.52E-02	3.07E-01	NS
TIGD4	NS	1.79E-01	5.52E-02	3.07E-01	NS
PARP10	NS	1.79E-01	5.52E-02	3.07E-01	NS
STKLD1	NS	1.79E-01	-0.0551	3.07E-01	NS
SHISAL2A	NS	1.79E-01	5.51E-02	3.07E-01	NS
LIN52	NS	1.79E-01	-0.0551	3.07E-01	NS
RNFT2	NS	1.79E-01	-0.0551	3.07E-01	NS
UNC45A	NS	1.79E-01	-0.0551	3.07E-01	NS
HOXB9	NS	1.79E-01	5.51E-02	3.07E-01	NS
FAM76A	NS	1.79E-01	-0.0551	3.08E-01	NS
CCL22	NS	1.79E-01	-0.0551	3.08E-01	NS
PLEKHA8	NS	1.80E-01	5.51E-02	3.08E-01	NS
IKBKG	NS	1.80E-01	-0.0551	3.08E-01	NS
YBEY	NS	1.80E-01	-0.0551	3.08E-01	NS
ANKRD10	NS	1.80E-01	-0.0551	3.08E-01	NS
ASL	NS	1.80E-01	-0.0551	3.08E-01	NS
HUS1	NS	1.80E-01	-0.0551	3.08E-01	NS
CPNE1	NS	1.80E-01	-0.0551	3.08E-01	NS
PLCH1	NS	1.80E-01	-0.0551	3.08E-01	NS
GNAL	NS	1.80E-01	5.51E-02	3.08E-01	NS
HMGCL	NS	1.80E-01	5.50E-02	3.08E-01	NS
CTNND2	NS	1.80E-01	5.50E-02	3.08E-01	NS
FLACC1	NS	1.80E-01	-0.055	3.08E-01	NS
RETSAT	NS	1.80E-01	-0.055	3.08E-01	NS
GSS	NS	1.80E-01	5.50E-02	3.08E-01	NS
NMNAT2	NS	1.80E-01	-0.055	3.08E-01	NS

POLR3K	NS	1.80E-01	-0.055	3.08E-01	NS
UTS2B	NS	1.80E-01	-0.055	3.08E-01	NS
C6orf141	NS	1.80E-01	5.50E-02	3.08E-01	NS
PCNX2	NS	1.80E-01	-0.055	3.08E-01	NS
TBC1D3L	NS	1.80E-01	-0.055	3.08E-01	NS
EXOC4	NS	1.80E-01	-0.055	3.08E-01	NS
SCN1A	NS	1.80E-01	-0.055	3.08E-01	NS
LY6G6E	NS	1.80E-01	5.50E-02	3.08E-01	NS
CLTA	NS	1.80E-01	-0.055	3.08E-01	NS
TKTL2	NS	1.80E-01	-0.055	3.09E-01	NS
ASPG	NS	1.80E-01	5.50E-02	3.09E-01	NS
MTHFR	NS	1.80E-01	5.50E-02	3.09E-01	NS
CD24	NS	1.81E-01	-0.055	3.09E-01	NS
USP37	NS	1.81E-01	-0.0549	3.09E-01	NS
ROPN1B	NS	1.81E-01	5.49E-02	3.09E-01	NS
AXIN2	NS	1.81E-01	-0.0549	3.09E-01	NS
USP24	NS	1.81E-01	-0.0549	3.09E-01	NS
CIAO2A	NS	1.81E-01	-0.0549	3.10E-01	NS
SHROOM4	NS	1.81E-01	-0.0549	3.10E-01	NS
NBEA	NS	1.81E-01	5.49E-02	3.10E-01	NS
FCN1	NS	1.81E-01	-0.0549	3.10E-01	NS
BTBD9	NS	1.81E-01	5.49E-02	3.10E-01	NS
HSDL1	NS	1.82E-01	-0.0548	3.10E-01	NS
UNC13A	NS	1.82E-01	5.48E-02	3.10E-01	NS
USP17L26	NS	1.82E-01	-0.0548	3.10E-01	NS
DOCK4	NS	1.82E-01	-0.0548	3.10E-01	NS
CTSE	NS	1.82E-01	-0.0548	3.10E-01	NS
RABL6	NS	1.82E-01	-0.0548	3.11E-01	NS
ALPK2	NS	1.82E-01	-0.0548	3.11E-01	NS
EPRS1	NS	1.82E-01	5.48E-02	3.11E-01	NS
PLEK2	NS	1.82E-01	5.47E-02	3.11E-01	NS
CSTF1	NS	1.82E-01	-0.0547	3.11E-01	NS
KRT14	NS	1.82E-01	-0.0547	3.11E-01	NS
CDK11A	NS	1.82E-01	5.47E-02	3.11E-01	NS
SH3TC1	NS	1.82E-01	5.47E-02	3.11E-01	NS
C11orf86	NS	1.83E-01	-0.0547	3.11E-01	NS
MARCHF11	NS	1.83E-01	-0.0547	3.11E-01	NS

OR10G2	NS	1.83E-01	-0.0547	3.11E-01	NS
UBXN11	NS	1.83E-01	-0.0547	3.11E-01	NS
DNAAF10	NS	1.83E-01	-0.0547	3.12E-01	NS
LRIG3	NS	1.83E-01	-0.0547	3.12E-01	NS
TMEM235	NS	1.83E-01	5.47E-02	3.12E-01	NS
POM121C	NS	1.83E-01	5.47E-02	3.12E-01	NS
GJB1	NS	1.83E-01	-0.0547	3.12E-01	NS
ZNF622	NS	1.83E-01	-0.0547	3.12E-01	NS
S100A16	NS	1.83E-01	-0.0546	3.12E-01	NS
RASA1	NS	1.83E-01	-0.0546	3.12E-01	NS
GPSM2	NS	1.83E-01	-0.0546	3.12E-01	NS
MED12L	NS	1.83E-01	-0.0546	3.12E-01	NS
C19orf44	NS	1.83E-01	-0.0546	3.12E-01	NS
ELAPOR2	NS	1.83E-01	-0.0546	3.12E-01	NS
CALML4	NS	1.84E-01	-0.0546	3.13E-01	NS
KCNS3	NS	1.84E-01	5.46E-02	3.13E-01	NS
OGA	NS	1.84E-01	-0.0546	3.13E-01	NS
RIN2	NS	1.84E-01	5.46E-02	3.13E-01	NS
LARP1B	NS	1.84E-01	-0.0546	3.13E-01	NS
IL3RA	NS	1.84E-01	5.46E-02	3.13E-01	NS
MS4A15	NS	1.84E-01	-0.0546	3.13E-01	NS
FUZ	NS	1.84E-01	-0.0546	3.13E-01	NS
STAC3	NS	1.84E-01	5.45E-02	3.13E-01	NS
CCAR1	NS	1.84E-01	5.45E-02	3.13E-01	NS
NDN	NS	1.84E-01	5.45E-02	3.13E-01	NS
MRTFA	NS	1.84E-01	5.45E-02	3.13E-01	NS
SPTAN1	NS	1.84E-01	-0.0545	3.13E-01	NS
CELA3B	NS	1.84E-01	5.45E-02	3.13E-01	NS
SMG5	NS	1.84E-01	5.45E-02	3.14E-01	NS
WWC3	NS	1.84E-01	5.45E-02	3.14E-01	NS
SPANXD	NS	1.84E-01	-0.0545	3.14E-01	NS
AQR	NS	1.85E-01	-0.0545	3.14E-01	NS
KANK2	NS	1.85E-01	-0.0545	3.14E-01	NS
EXO5	NS	1.85E-01	-0.0545	3.14E-01	NS
CNNM2	NS	1.85E-01	-0.0545	3.14E-01	NS
SPINK7	NS	1.85E-01	5.44E-02	3.14E-01	NS
SLC13A2	NS	1.85E-01	-0.0544	3.14E-01	NS

LRIT1	NS	1.85E-01	-0.0544	3.15E-01	NS
ZFTA	NS	1.85E-01	-0.0544	3.15E-01	NS
KRTAP2-3	NS	1.85E-01	-0.0544	3.15E-01	NS
TNFRSF12A	NS	1.86E-01	-0.0543	3.15E-01	NS
TXLNG	NS	1.86E-01	-0.0543	3.15E-01	NS
HTR3E	NS	1.86E-01	-0.0543	3.15E-01	NS
PCGF1	NS	1.86E-01	-0.0543	3.15E-01	NS
GPR3	NS	1.86E-01	-0.0543	3.16E-01	NS
EFHC1	NS	1.86E-01	-0.0543	3.16E-01	NS
ARMC6	NS	1.86E-01	-0.0543	3.16E-01	NS
SAMD9L	NS	1.86E-01	-0.0543	3.16E-01	NS
RS1	NS	1.86E-01	-0.0543	3.16E-01	NS
ESRRB	NS	1.86E-01	-0.0543	3.16E-01	NS
TDRD9	NS	1.86E-01	-0.0543	3.16E-01	NS
APOF	NS	1.86E-01	5.43E-02	3.16E-01	NS
PRICKLE1	NS	1.86E-01	-0.0543	3.16E-01	NS
CHI3L1	NS	1.86E-01	-0.0543	3.16E-01	NS
MBD3L1	NS	1.86E-01	-0.0543	3.16E-01	NS
TRIM7	NS	1.87E-01	5.42E-02	3.17E-01	NS
SDCBP2	NS	1.87E-01	-0.0542	3.17E-01	NS
TIGD3	NS	1.87E-01	5.42E-02	3.17E-01	NS
SOCS7	NS	1.87E-01	-0.0542	3.17E-01	NS
KCTD8	NS	1.87E-01	-0.0542	3.17E-01	NS
STK17B	NS	1.87E-01	-0.0542	3.17E-01	NS
CAPN15	NS	1.87E-01	-0.0542	3.17E-01	NS
ASIC3	NS	1.87E-01	-0.0542	3.17E-01	NS
EMX1	NS	1.87E-01	-0.0542	3.17E-01	NS
LRRC32	NS	1.87E-01	-0.0542	3.17E-01	NS
KRT20	NS	1.87E-01	-0.0542	3.17E-01	NS
CT45A7	NS	1.87E-01	5.42E-02	3.17E-01	NS
PDK4	NS	1.87E-01	-0.0542	3.17E-01	NS
SYCE1	NS	1.87E-01	-0.0542	3.17E-01	NS
MAP2K1	NS	1.87E-01	5.42E-02	3.17E-01	NS
APOC3	NS	1.87E-01	5.41E-02	3.17E-01	NS
USP17L20	NS	1.87E-01	5.41E-02	3.17E-01	NS
FAM90A7P	NS	1.87E-01	-0.0541	3.17E-01	NS
SMCO2	NS	1.87E-01	-0.0541	3.17E-01	NS

EMX2	NS	1.87E-01	5.41E-02	3.17E-01	NS
TP53I3	NS	1.87E-01	-0.0541	3.17E-01	NS
MMGT1	NS	1.87E-01	-0.0541	3.17E-01	NS
CALHM5	NS	1.87E-01	-0.0541	3.17E-01	NS
LRBA	NS	1.87E-01	-0.0541	3.17E-01	NS
ZNF550	NS	1.87E-01	5.41E-02	3.17E-01	NS
RAB7B	NS	1.87E-01	-0.0541	3.17E-01	NS
ACVR1	NS	1.88E-01	5.41E-02	3.17E-01	NS
ANP32A	NS	1.88E-01	5.41E-02	3.17E-01	NS
TGM4	NS	1.88E-01	-0.0541	3.17E-01	NS
ZFP2	NS	1.88E-01	-0.0541	3.17E-01	NS
AP5Z1	NS	1.88E-01	-0.0541	3.17E-01	NS
AKAP7	NS	1.88E-01	-0.0541	3.17E-01	NS
PPP1R15A	NS	1.88E-01	-0.0541	3.17E-01	NS
MORC3	NS	1.88E-01	-0.0541	3.17E-01	NS
TADA2A	NS	1.88E-01	-0.0541	3.17E-01	NS
MFSD8	NS	1.88E-01	5.41E-02	3.17E-01	NS
OXNAD1	NS	1.88E-01	-0.0541	3.18E-01	NS
FTL	NS	1.88E-01	-0.0541	3.18E-01	NS
MFSD6L	NS	1.88E-01	5.41E-02	3.18E-01	NS
BDH1	NS	1.88E-01	-0.0541	3.18E-01	NS
ASIC2	NS	1.88E-01	-0.0541	3.18E-01	NS
ASAH1	NS	1.88E-01	-0.054	3.18E-01	NS
NUTM2F	NS	1.88E-01	-0.054	3.18E-01	NS
PPP1R21	NS	1.88E-01	5.40E-02	3.18E-01	NS
ARHGEF19	NS	1.88E-01	-0.054	3.18E-01	NS
ALK	NS	1.88E-01	5.40E-02	3.18E-01	NS
HSD11B2	NS	1.88E-01	-0.054	3.18E-01	NS
PRSS2	NS	1.89E-01	-0.054	3.19E-01	NS
H4C1	NS	1.89E-01	-0.054	3.19E-01	NS
YIF1B	NS	1.89E-01	-0.054	3.19E-01	NS
ACTL6B	NS	1.89E-01	5.39E-02	3.19E-01	NS
SMS	NS	1.89E-01	-0.0539	3.19E-01	NS
ADAMTS10	NS	1.89E-01	5.39E-02	3.19E-01	NS
TRIM63	NS	1.89E-01	-0.0539	3.19E-01	NS
FAM110C	NS	1.89E-01	-0.0539	3.19E-01	NS
FAHD1	NS	1.89E-01	-0.0539	3.19E-01	NS

C1S	NS	1.89E-01	-0.0539	3.19E-01	NS
BAX	NS	1.89E-01	5.39E-02	3.19E-01	NS
MRPL54	NS	1.89E-01	-0.0539	3.19E-01	NS
KDM5B	NS	1.89E-01	-0.0539	3.19E-01	NS
NTNG1	NS	1.89E-01	5.39E-02	3.19E-01	NS
SRSF4	NS	1.89E-01	-0.0539	3.19E-01	NS
TNPO1	NS	1.90E-01	5.39E-02	3.20E-01	NS
GDAP1	NS	1.89E-01	-0.0539	3.20E-01	NS
ZFR2	NS	1.89E-01	5.39E-02	3.20E-01	NS
ARL4A	NS	1.90E-01	5.39E-02	3.20E-01	NS
TCF25	NS	1.90E-01	5.39E-02	3.20E-01	NS
TIFA	NS	1.90E-01	-0.0539	3.20E-01	NS
GREB1	NS	1.90E-01	5.39E-02	3.20E-01	NS
STON1	NS	1.90E-01	-0.0539	3.20E-01	NS
TRNP1	NS	1.90E-01	-0.0539	3.20E-01	NS
FAM236B	NS	1.90E-01	-0.0539	3.20E-01	NS
OR4E1	NS	1.90E-01	-0.0539	3.20E-01	NS
CNGB1	NS	1.90E-01	-0.0538	3.20E-01	NS
ANKRD30BL	NS	1.90E-01	-0.0538	3.20E-01	NS
CLDN5	NS	1.90E-01	-0.0538	3.20E-01	NS
GJA1	NS	1.90E-01	-0.0538	3.20E-01	NS
LYPLA2	NS	1.90E-01	5.38E-02	3.20E-01	NS
ADGRB2	NS	1.90E-01	5.38E-02	3.20E-01	NS
GINS4	NS	1.90E-01	-0.0538	3.20E-01	NS
VRK2	NS	1.90E-01	-0.0538	3.20E-01	NS
PNKP	NS	1.90E-01	5.38E-02	3.20E-01	NS
NLGN4X	NS	1.90E-01	-0.0538	3.20E-01	NS
SLC15A2	NS	1.90E-01	-0.0538	3.20E-01	NS
MYMK	NS	1.90E-01	-0.0538	3.20E-01	NS
ARFGAP1	NS	1.90E-01	5.38E-02	3.20E-01	NS
CERS2	NS	1.90E-01	5.38E-02	3.20E-01	NS
ARPP19	NS	1.90E-01	-0.0538	3.20E-01	NS
ZNF671	NS	1.90E-01	-0.0538	3.20E-01	NS
NXPE4	NS	1.90E-01	-0.0538	3.21E-01	NS
VCX2	NS	1.90E-01	5.37E-02	3.21E-01	NS
R3HCC1	NS	1.91E-01	-0.0537	3.21E-01	NS
DDX46	NS	1.91E-01	-0.0537	3.21E-01	NS

HEXD	NS	1.91E-01	-0.0537	3.21E-01	NS
SHISAL1	NS	1.91E-01	5.37E-02	3.21E-01	NS
HIPK3	NS	1.91E-01	-0.0537	3.21E-01	NS
LRIF1	NS	1.91E-01	-0.0537	3.21E-01	NS
OR4C6	NS	1.91E-01	-0.0537	3.21E-01	NS
LGALS13	NS	1.91E-01	5.37E-02	3.21E-01	NS
SALL4	NS	1.91E-01	5.37E-02	3.21E-01	NS
SCIMP	NS	1.91E-01	-0.0537	3.21E-01	NS
CDC40	NS	1.91E-01	5.37E-02	3.21E-01	NS
TMPRSS7	NS	1.91E-01	-0.0537	3.21E-01	NS
KCTD12	NS	1.91E-01	5.37E-02	3.21E-01	NS
TMEM86B	NS	1.91E-01	-0.0537	3.21E-01	NS
TBL3	NS	1.91E-01	-0.0537	3.21E-01	NS
C12orf56	NS	1.91E-01	-0.0537	3.21E-01	NS
TRPM3	NS	1.91E-01	5.37E-02	3.21E-01	NS
TOGARAM1	NS	1.91E-01	-0.0537	3.21E-01	NS
NAA25	NS	1.91E-01	-0.0537	3.21E-01	NS
IGLL5	NS	1.91E-01	5.37E-02	3.21E-01	NS
NOTCH2NLA	NS	1.91E-01	-0.0537	3.21E-01	NS
RO60	NS	1.91E-01	-0.0536	3.21E-01	NS
MRS2	NS	1.91E-01	-0.0536	3.21E-01	NS
THNSL1	NS	1.91E-01	5.36E-02	3.21E-01	NS
LYNX1	NS	1.91E-01	-0.0536	3.21E-01	NS
OAZ1	NS	1.92E-01	-0.0536	3.22E-01	NS
POLE3	NS	1.92E-01	-0.0536	3.22E-01	NS
LDAH	NS	1.92E-01	-0.0536	3.22E-01	NS
CHD1L	NS	1.92E-01	-0.0536	3.22E-01	NS
KCTD15	NS	1.92E-01	5.36E-02	3.22E-01	NS
SLC2A6	NS	1.92E-01	-0.0536	3.22E-01	NS
SPIN2B	NS	1.92E-01	-0.0536	3.22E-01	NS
ZKSCAN7	NS	1.92E-01	-0.0536	3.22E-01	NS
PAQR3	NS	1.92E-01	-0.0535	3.22E-01	NS
PPP4R2	NS	1.92E-01	-0.0535	3.22E-01	NS
MANBA	NS	1.92E-01	-0.0535	3.23E-01	NS
PSMD6	NS	1.92E-01	5.35E-02	3.23E-01	NS
NIBAN1	NS	1.93E-01	-0.0535	3.23E-01	NS
PIWIL3	NS	1.93E-01	-0.0535	3.23E-01	NS

CHMP2A	NS	1.93E-01	5.35E-02	3.23E-01	NS
ARHGEF3	NS	1.93E-01	5.35E-02	3.23E-01	NS
MRPL21	NS	1.93E-01	-0.0535	3.23E-01	NS
RASL12	NS	1.93E-01	-0.0534	3.23E-01	NS
AIRE	NS	1.93E-01	5.34E-02	3.23E-01	NS
TYW1	NS	1.93E-01	-0.0534	3.23E-01	NS
TYSND1	NS	1.93E-01	-0.0534	3.24E-01	NS
CAPRIN2	NS	1.93E-01	-0.0534	3.24E-01	NS
BRI3BP	NS	1.93E-01	-0.0534	3.24E-01	NS
CD300LG	NS	1.93E-01	5.34E-02	3.24E-01	NS
NFAM1	NS	1.93E-01	-0.0534	3.24E-01	NS
HDAC7	NS	1.93E-01	5.34E-02	3.24E-01	NS
DEPDC5	NS	1.93E-01	5.34E-02	3.24E-01	NS
SIGLEC11	NS	1.93E-01	-0.0534	3.24E-01	NS
ZNF157	NS	1.93E-01	-0.0534	3.24E-01	NS
RSPO4	NS	1.94E-01	-0.0534	3.24E-01	NS
LIPC	NS	1.94E-01	-0.0534	3.24E-01	NS
MBD1	NS	1.94E-01	-0.0534	3.24E-01	NS
CCDC86	NS	1.94E-01	5.34E-02	3.24E-01	NS
BNC2	NS	1.94E-01	-0.0534	3.24E-01	NS
CDC23	NS	1.94E-01	-0.0533	3.25E-01	NS
CEP164	NS	1.94E-01	-0.0533	3.25E-01	NS
PHF3	NS	1.94E-01	5.33E-02	3.25E-01	NS
NTAQ1	NS	1.94E-01	-0.0533	3.25E-01	NS
RAB36	NS	1.94E-01	-0.0533	3.25E-01	NS
MUC3A	NS	1.94E-01	-0.0533	3.25E-01	NS
NAXD	NS	1.94E-01	5.33E-02	3.25E-01	NS
AMDHD2	NS	1.94E-01	5.33E-02	3.25E-01	NS
COPS7B	NS	1.94E-01	-0.0533	3.25E-01	NS
COMMD9	NS	1.94E-01	-0.0533	3.25E-01	NS
GRIN3A	NS	1.94E-01	5.33E-02	3.25E-01	NS
HTR1B	NS	1.94E-01	5.33E-02	3.25E-01	NS
LYPD5	NS	1.95E-01	5.32E-02	3.25E-01	NS
RFX2	NS	1.95E-01	5.32E-02	3.26E-01	NS
ACHE	NS	1.95E-01	5.32E-02	3.26E-01	NS
CNOT7	NS	1.95E-01	-0.0532	3.26E-01	NS
TCTE1	NS	1.95E-01	5.32E-02	3.26E-01	NS

TRNAU1AP	NS	1.95E-01	-0.0532	3.26E-01	NS
COG1	NS	1.95E-01	-0.0532	3.26E-01	NS
GOLPH3	NS	1.95E-01	5.32E-02	3.26E-01	NS
PTCD3	NS	1.95E-01	-0.0532	3.26E-01	NS
GTSF1	NS	1.95E-01	-0.0532	3.26E-01	NS
CANT1	NS	1.95E-01	-0.0532	3.26E-01	NS
DERL2	NS	1.95E-01	-0.0532	3.26E-01	NS
SBNO1	NS	1.95E-01	5.31E-02	3.26E-01	NS
GAB2	NS	1.96E-01	5.31E-02	3.26E-01	NS
ZNF629	NS	1.96E-01	-0.0531	3.26E-01	NS
TAF6L	NS	1.96E-01	5.31E-02	3.26E-01	NS
TACR3	NS	1.96E-01	-0.0531	3.26E-01	NS
CAPZA2	NS	1.96E-01	-0.0531	3.26E-01	NS
DEFB104A	NS	1.96E-01	-0.0531	3.26E-01	NS
DDX42	NS	1.96E-01	-0.0531	3.27E-01	NS
ATIC	NS	1.96E-01	-0.0531	3.27E-01	NS
USP17L3	NS	1.96E-01	-0.0531	3.27E-01	NS
UGT1A5	NS	1.96E-01	-0.0531	3.27E-01	NS
DPH1	NS	1.96E-01	-0.0531	3.27E-01	NS
SOX6	NS	1.96E-01	-0.0531	3.27E-01	NS
ZNF792	NS	1.96E-01	-0.0531	3.27E-01	NS
SASH3	NS	1.96E-01	-0.0531	3.27E-01	NS
SCN4B	NS	1.96E-01	5.31E-02	3.27E-01	NS
APOL1	NS	1.96E-01	5.31E-02	3.27E-01	NS
TMEM260	NS	1.96E-01	-0.0531	3.27E-01	NS
RYR3	NS	1.96E-01	-0.0531	3.27E-01	NS
SETD7	NS	1.96E-01	5.31E-02	3.27E-01	NS
PRMT1	NS	1.96E-01	-0.0531	3.27E-01	NS
MTMR10	NS	1.96E-01	5.30E-02	3.27E-01	NS
IL26	NS	1.96E-01	-0.053	3.27E-01	NS
VPS25	NS	1.97E-01	-0.053	3.28E-01	NS
TRAPPC1	NS	1.97E-01	-0.053	3.28E-01	NS
MFSD14B	NS	1.97E-01	-0.053	3.28E-01	NS
OR2L5	NS	1.97E-01	-0.053	3.28E-01	NS
TFG	NS	1.97E-01	5.30E-02	3.28E-01	NS
IFTAP	NS	1.97E-01	5.30E-02	3.28E-01	NS
HLA-C	NS	1.97E-01	-0.053	3.28E-01	NS

LRIT2	NS	1.97E-01	-0.053	3.28E-01	NS
CERS3	NS	1.97E-01	5.29E-02	3.28E-01	NS
RHBDD1	NS	1.97E-01	5.29E-02	3.28E-01	NS
BCL2L2	NS	1.97E-01	-0.0529	3.28E-01	NS
TMEM144	NS	1.97E-01	-0.0529	3.29E-01	NS
SERPINA9	NS	1.97E-01	-0.0529	3.29E-01	NS
CATSPER1	NS	1.97E-01	-0.0529	3.29E-01	NS
LRRTM4	NS	1.97E-01	-0.0529	3.29E-01	NS
PRL	NS	1.97E-01	-0.0529	3.29E-01	NS
OSBP	NS	1.98E-01	5.29E-02	3.29E-01	NS
TOLLIP	NS	1.98E-01	-0.0529	3.29E-01	NS
EIF5AL1	NS	1.98E-01	-0.0529	3.29E-01	NS
RCC1L	NS	1.98E-01	-0.0529	3.29E-01	NS
OR6A2	NS	1.98E-01	-0.0529	3.29E-01	NS
NEK8	NS	1.98E-01	-0.0529	3.29E-01	NS
GUSB	NS	1.98E-01	5.28E-02	3.29E-01	NS
DCPS	NS	1.98E-01	5.28E-02	3.30E-01	NS
C4orf50	NS	1.98E-01	-0.0528	3.30E-01	NS
PDCD6	NS	1.98E-01	-0.0528	3.30E-01	NS
GDA	NS	1.98E-01	-0.0528	3.30E-01	NS
BIRC7	NS	1.98E-01	5.28E-02	3.30E-01	NS
THYN1	NS	1.98E-01	5.28E-02	3.30E-01	NS
CEP250	NS	1.99E-01	-0.0528	3.30E-01	NS
ORM1	NS	1.99E-01	-0.0528	3.30E-01	NS
HEY2	NS	1.99E-01	5.28E-02	3.30E-01	NS
PAN3	NS	1.99E-01	5.27E-02	3.30E-01	NS
GALNT16	NS	1.99E-01	-0.0527	3.31E-01	NS
LY6K	NS	1.99E-01	5.27E-02	3.31E-01	NS
DOT1L	NS	1.99E-01	-0.0527	3.31E-01	NS
ATP5F1A	NS	1.99E-01	-0.0527	3.31E-01	NS
ACTL7A	NS	1.99E-01	-0.0527	3.31E-01	NS
SPOP	NS	1.99E-01	5.27E-02	3.31E-01	NS
ZNF653	NS	1.99E-01	-0.0527	3.31E-01	NS
MTCH1	NS	1.99E-01	5.27E-02	3.31E-01	NS
DNMT3L	NS	1.99E-01	5.27E-02	3.31E-01	NS
ARL6IP5	NS	1.99E-01	-0.0527	3.31E-01	NS
SMOC2	NS	1.99E-01	-0.0527	3.31E-01	NS

EFCC1	NS	2.00E-01	5.27E-02	3.31E-01	NS
BBS9	NS	2.00E-01	5.27E-02	3.31E-01	NS
WDR38	NS	2.00E-01	5.26E-02	3.32E-01	NS
PEMT	NS	2.00E-01	5.26E-02	3.32E-01	NS
ALDH1A1	NS	2.00E-01	-0.0526	3.32E-01	NS
AGMAT	NS	2.00E-01	5.26E-02	3.32E-01	NS
BIVM	NS	2.00E-01	-0.0526	3.32E-01	NS
IL31RA	NS	2.00E-01	5.26E-02	3.32E-01	NS
STXBP4	NS	2.00E-01	-0.0526	3.32E-01	NS
GRIK4	NS	2.00E-01	-0.0526	3.32E-01	NS
DDX43	NS	2.00E-01	-0.0526	3.32E-01	NS
GOLGA6C	NS	2.00E-01	-0.0526	3.32E-01	NS
OR1L1	NS	2.00E-01	-0.0526	3.32E-01	NS
HSD3B2	NS	2.00E-01	5.26E-02	3.32E-01	NS
IL27RA	NS	2.01E-01	5.26E-02	3.32E-01	NS
ADRM1	NS	2.01E-01	-0.0526	3.32E-01	NS
RCAN3	NS	2.01E-01	-0.0525	3.32E-01	NS
SCYL3	NS	2.01E-01	-0.0525	3.33E-01	NS
MOB4	NS	2.01E-01	-0.0525	3.33E-01	NS
VEGFA	NS	2.01E-01	5.25E-02	3.33E-01	NS
KSR1	NS	2.01E-01	5.25E-02	3.33E-01	NS
C1orf52	NS	2.01E-01	-0.0525	3.33E-01	NS
DIPK1C	NS	2.01E-01	-0.0525	3.33E-01	NS
NPTN	NS	2.01E-01	-0.0525	3.33E-01	NS
PFN3	NS	2.01E-01	5.25E-02	3.33E-01	NS
OR10S1	NS	2.01E-01	-0.0525	3.33E-01	NS
ZNF750	NS	2.01E-01	-0.0525	3.34E-01	NS
PCDH12	NS	2.01E-01	-0.0524	3.34E-01	NS
RADIL	NS	2.01E-01	-0.0524	3.34E-01	NS
OLFM1	NS	2.02E-01	-0.0524	3.34E-01	NS
HTR3A	NS	2.02E-01	-0.0524	3.34E-01	NS
TGIF2-RAB5IF	NS	2.02E-01	5.24E-02	3.34E-01	NS
HSPBP1	NS	2.02E-01	-0.0524	3.34E-01	NS
IFNA17	NS	2.02E-01	-0.0524	3.34E-01	NS
LGALS7B	NS	2.02E-01	-0.0524	3.34E-01	NS
GRWD1	NS	2.02E-01	-0.0524	3.34E-01	NS
MMP13	NS	2.02E-01	5.24E-02	3.34E-01	NS

MED17	NS	2.02E-01	-0.0524	3.34E-01	NS
PLB1	NS	2.02E-01	5.23E-02	3.35E-01	NS
PCYT1A	NS	2.03E-01	-0.0523	3.35E-01	NS
CDKAL1	NS	2.03E-01	5.23E-02	3.35E-01	NS
ZFPM2	NS	2.03E-01	-0.0523	3.35E-01	NS
MBLAC2	NS	2.03E-01	-0.0523	3.35E-01	NS
CA8	NS	2.03E-01	-0.0523	3.35E-01	NS
NTRK3	NS	2.03E-01	-0.0523	3.35E-01	NS
PDIA5	NS	2.03E-01	-0.0523	3.35E-01	NS
FZD3	NS	2.03E-01	-0.0523	3.35E-01	NS
TNFRSF17	NS	2.03E-01	-0.0523	3.35E-01	NS
DDX23	NS	2.03E-01	-0.0523	3.36E-01	NS
FRMD8	NS	2.03E-01	-0.0523	3.36E-01	NS
SFTPB	NS	2.03E-01	-0.0522	3.36E-01	NS
OR51A2	NS	2.03E-01	-0.0522	3.36E-01	NS
H2AC14	NS	2.03E-01	5.22E-02	3.36E-01	NS
DSTN	NS	2.03E-01	-0.0522	3.36E-01	NS
ZC3H10	NS	2.04E-01	-0.0522	3.36E-01	NS
CMC2	NS	2.04E-01	5.22E-02	3.36E-01	NS
NR1I3	NS	2.04E-01	-0.0522	3.36E-01	NS
UBE2T	NS	2.04E-01	-0.0522	3.36E-01	NS
CXCL1	NS	2.04E-01	-0.0522	3.36E-01	NS
WNT2B	NS	2.04E-01	-0.0522	3.37E-01	NS
KIF4B	NS	2.04E-01	5.22E-02	3.37E-01	NS
SAP30L	NS	2.04E-01	-0.0522	3.37E-01	NS
TEX22	NS	2.04E-01	5.21E-02	3.37E-01	NS
STT3A	NS	2.04E-01	5.21E-02	3.37E-01	NS
SLC9A5	NS	2.04E-01	5.21E-02	3.37E-01	NS
TPD52L2	NS	2.04E-01	-0.0521	3.37E-01	NS
FRS3	NS	2.05E-01	-0.0521	3.38E-01	NS
PIGT	NS	2.05E-01	-0.0521	3.38E-01	NS
DTX3L	NS	2.05E-01	-0.0521	3.38E-01	NS
HSP90B1	NS	2.05E-01	-0.0521	3.38E-01	NS
HBP1	NS	2.05E-01	5.21E-02	3.38E-01	NS
RPL7L1	NS	2.05E-01	-0.0521	3.38E-01	NS
ZNF444	NS	2.05E-01	5.21E-02	3.38E-01	NS
GNB4	NS	2.05E-01	-0.052	3.38E-01	NS

ABCF2-H2BE1	NS	2.05E-01	-0.052	3.38E-01	NS
FABP6	NS	2.05E-01	5.20E-02	3.38E-01	NS
AOAH	NS	2.05E-01	-0.052	3.38E-01	NS
CYGB	NS	2.05E-01	-0.052	3.38E-01	NS
BMERB1	NS	2.05E-01	-0.052	3.38E-01	NS
RHBDD2	NS	2.05E-01	5.20E-02	3.38E-01	NS
ATG12	NS	2.05E-01	5.20E-02	3.39E-01	NS
PAK1	NS	2.05E-01	5.20E-02	3.39E-01	NS
ABCF1	NS	2.05E-01	5.20E-02	3.39E-01	NS
STEAP1	NS	2.06E-01	-0.052	3.39E-01	NS
ADGRG1	NS	2.06E-01	-0.052	3.39E-01	NS
COPS7A	NS	2.06E-01	5.20E-02	3.39E-01	NS
NEIL1	NS	2.06E-01	-0.052	3.39E-01	NS
LCP1	NS	2.06E-01	-0.0519	3.39E-01	NS
DCAF7	NS	2.06E-01	-0.0519	3.39E-01	NS
INCENP	NS	2.06E-01	-0.0519	3.39E-01	NS
SPRR2G	NS	2.06E-01	-0.0519	3.39E-01	NS
USP6NL	NS	2.06E-01	5.19E-02	3.39E-01	NS
FEM1B	NS	2.06E-01	-0.0519	3.39E-01	NS
NAP1L5	NS	2.06E-01	-0.0519	3.40E-01	NS
DNAH2	NS	2.06E-01	-0.0519	3.40E-01	NS
ALG10	NS	2.06E-01	-0.0519	3.40E-01	NS
PSTK	NS	2.06E-01	5.19E-02	3.40E-01	NS
SLC2A9	NS	2.06E-01	5.19E-02	3.40E-01	NS
TMEM74	NS	2.06E-01	-0.0519	3.40E-01	NS
MRPL36	NS	2.06E-01	-0.0519	3.40E-01	NS
LIMCH1	NS	2.07E-01	-0.0519	3.40E-01	NS
H2AZ2	NS	2.07E-01	5.19E-02	3.40E-01	NS
PEX14	NS	2.07E-01	5.18E-02	3.40E-01	NS
SEC13	NS	2.07E-01	-0.0518	3.40E-01	NS
ZBTB8B	NS	2.07E-01	5.18E-02	3.40E-01	NS
TP53AIP1	NS	2.07E-01	-0.0518	3.40E-01	NS
INHA	NS	2.07E-01	5.18E-02	3.40E-01	NS
RPLP1	NS	2.07E-01	-0.0518	3.40E-01	NS
ZNF787	NS	2.07E-01	5.18E-02	3.40E-01	NS
ZNF587	NS	2.07E-01	-0.0518	3.41E-01	NS
KLF2	NS	2.07E-01	5.18E-02	3.41E-01	NS

DNAJA1	NS	2.07E-01	5.18E-02	3.41E-01	NS
MAP3K20	NS	2.07E-01	-0.0518	3.41E-01	NS
MYADM	NS	2.07E-01	5.18E-02	3.41E-01	NS
NECTIN4	NS	2.07E-01	-0.0518	3.41E-01	NS
LHFPL6	NS	2.07E-01	-0.0518	3.41E-01	NS
GAPVD1	NS	2.07E-01	-0.0518	3.41E-01	NS
FXYD5	NS	2.08E-01	5.17E-02	3.41E-01	NS
TMEM249	NS	2.08E-01	5.17E-02	3.41E-01	NS
PSMA6	NS	2.08E-01	-0.0517	3.41E-01	NS
NENF	NS	2.08E-01	5.17E-02	3.41E-01	NS
PKD1L1	NS	2.08E-01	5.17E-02	3.41E-01	NS
GTF2I	NS	2.08E-01	5.17E-02	3.41E-01	NS
PRAMEF27	NS	2.08E-01	-0.0517	3.41E-01	NS
GALM	NS	2.08E-01	-0.0517	3.42E-01	NS
GJD2	NS	2.08E-01	-0.0517	3.42E-01	NS
TAF1C	NS	2.08E-01	5.17E-02	3.42E-01	NS
ANXA11	NS	2.08E-01	5.17E-02	3.42E-01	NS
CNTNAP2	NS	2.08E-01	-0.0517	3.42E-01	NS
CHKB-CPT1B	NS	2.08E-01	-0.0517	3.42E-01	NS
SOX9	NS	2.08E-01	-0.0517	3.42E-01	NS
BZW1	NS	2.08E-01	-0.0517	3.42E-01	NS
GCKR	NS	2.08E-01	5.17E-02	3.42E-01	NS
ELOA3BP	NS	2.08E-01	-0.0517	3.42E-01	NS
PCLAF	NS	2.09E-01	-0.0516	3.42E-01	NS
SPINDOC	NS	2.09E-01	-0.0516	3.42E-01	NS
OR8U3	NS	2.09E-01	-0.0516	3.42E-01	NS
PARVG	NS	2.09E-01	5.16E-02	3.42E-01	NS
SPZ1	NS	2.09E-01	-0.0516	3.42E-01	NS
KRCC1	NS	2.09E-01	-0.0516	3.42E-01	NS
FMN1	NS	2.09E-01	5.16E-02	3.43E-01	NS
RPL24	NS	2.09E-01	-0.0516	3.43E-01	NS
ZCCHC3	NS	2.09E-01	-0.0516	3.43E-01	NS
TUNAR	NS	2.09E-01	-0.0516	3.43E-01	NS
SAP25	NS	2.09E-01	5.16E-02	3.43E-01	NS
CCNYL1	NS	2.09E-01	-0.0515	3.43E-01	NS
DNAH14	NS	2.09E-01	-0.0515	3.43E-01	NS
POLD1	NS	2.10E-01	-0.0515	3.43E-01	NS

ZNF761	NS	2.10E-01	-0.0515	3.43E-01	NS
CCDC138	NS	2.10E-01	-0.0515	3.43E-01	NS
CRAT	NS	2.10E-01	-0.0515	3.44E-01	NS
TTYH2	NS	2.10E-01	-0.0515	3.44E-01	NS
ABCB10	NS	2.10E-01	-0.0515	3.44E-01	NS
PXDNL	NS	2.10E-01	5.15E-02	3.44E-01	NS
BIRC6	NS	2.10E-01	-0.0515	3.44E-01	NS
NFASC	NS	2.10E-01	-0.0515	3.44E-01	NS
MOB3C	NS	2.10E-01	-0.0514	3.44E-01	NS
VPS29	NS	2.10E-01	-0.0514	3.45E-01	NS
SPEM2	NS	2.11E-01	5.14E-02	3.45E-01	NS
CCDC27	NS	2.11E-01	5.14E-02	3.45E-01	NS
SERTAD4	NS	2.11E-01	-0.0514	3.45E-01	NS
UNC13B	NS	2.11E-01	-0.0514	3.45E-01	NS
TBC1D9	NS	2.11E-01	-0.0514	3.45E-01	NS
TRABD2A	NS	2.11E-01	-0.0513	3.46E-01	NS
THG1L	NS	2.11E-01	-0.0513	3.46E-01	NS
HLA-DRA	NS	2.11E-01	-0.0513	3.46E-01	NS
EVI2A	NS	2.11E-01	-0.0513	3.46E-01	NS
ANGPTL4	NS	2.11E-01	5.13E-02	3.46E-01	NS
TRIM67	NS	2.11E-01	5.13E-02	3.46E-01	NS
KCNS1	NS	2.12E-01	-0.0513	3.46E-01	NS
SORBS2	NS	2.12E-01	-0.0513	3.46E-01	NS
TSNAXIP1	NS	2.12E-01	5.13E-02	3.46E-01	NS
MXI1	NS	2.12E-01	5.13E-02	3.46E-01	NS
NUAK2	NS	2.12E-01	5.13E-02	3.46E-01	NS
ZNF511	NS	2.12E-01	-0.0513	3.46E-01	NS
TRADD	NS	2.12E-01	5.13E-02	3.46E-01	NS
LRFN4	NS	2.12E-01	-0.0513	3.46E-01	NS
CPN2	NS	2.12E-01	5.12E-02	3.47E-01	NS
IMPG2	NS	2.12E-01	-0.0512	3.47E-01	NS
TRPC1	NS	2.12E-01	-0.0512	3.47E-01	NS
MAML1	NS	2.12E-01	-0.0512	3.47E-01	NS
PDE6G	NS	2.12E-01	-0.0512	3.47E-01	NS
EIF1B	NS	2.12E-01	5.12E-02	3.47E-01	NS
SMIM39	NS	2.12E-01	5.12E-02	3.47E-01	NS
RCE1	NS	2.12E-01	5.12E-02	3.47E-01	NS

RHOG	NS	2.12E-01	5.12E-02	3.47E-01	NS
BIN3	NS	2.13E-01	-0.0512	3.47E-01	NS
ANKRD60	NS	2.13E-01	5.12E-02	3.47E-01	NS
DDX49	NS	2.13E-01	-0.0512	3.47E-01	NS
AKAP10	NS	2.13E-01	-0.0512	3.47E-01	NS
OR4F4	NS	2.13E-01	-0.0512	3.47E-01	NS
BOD1	NS	2.13E-01	-0.0511	3.48E-01	NS
FAM153B	NS	2.13E-01	-0.0511	3.48E-01	NS
EPC1	NS	2.13E-01	5.11E-02	3.48E-01	NS
ERAP1	NS	2.13E-01	-0.0511	3.48E-01	NS
TSPY1	NS	2.13E-01	5.11E-02	3.48E-01	NS
ABCG5	NS	2.13E-01	5.11E-02	3.48E-01	NS
XRCC5	NS	2.13E-01	-0.0511	3.48E-01	NS
DGKB	NS	2.13E-01	5.11E-02	3.48E-01	NS
PSMD2	NS	2.13E-01	-0.0511	3.48E-01	NS
DDX60L	NS	2.13E-01	-0.0511	3.48E-01	NS
MAP2K7	NS	2.14E-01	-0.0511	3.49E-01	NS
DNAJB11	NS	2.14E-01	5.10E-02	3.49E-01	NS
MEP1A	NS	2.14E-01	-0.051	3.49E-01	NS
S100A11	NS	2.14E-01	5.10E-02	3.49E-01	NS
PIP5K1A	NS	2.14E-01	-0.051	3.49E-01	NS
HNRNPUL2-BSCL2	NS	2.14E-01	5.10E-02	3.49E-01	NS
RPL22L1	NS	2.14E-01	5.10E-02	3.49E-01	NS
MGAT4C	NS	2.14E-01	-0.051	3.49E-01	NS
DNAJC9	NS	2.14E-01	-0.051	3.49E-01	NS
STIM2	NS	2.14E-01	-0.051	3.49E-01	NS
CD40	NS	2.14E-01	-0.051	3.49E-01	NS
MFSD5	NS	2.14E-01	5.10E-02	3.49E-01	NS
ZNF320	NS	2.14E-01	-0.051	3.49E-01	NS
ENTPD7	NS	2.14E-01	-0.051	3.49E-01	NS
NGDN	NS	2.15E-01	-0.051	3.50E-01	NS
ARHGEF37	NS	2.15E-01	-0.051	3.50E-01	NS
PIAS4	NS	2.15E-01	5.09E-02	3.50E-01	NS
SFRP1	NS	2.15E-01	5.09E-02	3.50E-01	NS
GSTO1	NS	2.15E-01	-0.0509	3.50E-01	NS
CCDC106	NS	2.15E-01	-0.0509	3.51E-01	NS
BRD3	NS	2.15E-01	-0.0509	3.51E-01	NS

RPS21	NS	2.15E-01	-0.0509	3.51E-01	NS
KLHL34	NS	2.16E-01	5.08E-02	3.51E-01	NS
ST8SIA5	NS	2.16E-01	-0.0508	3.51E-01	NS
PROZ	NS	2.16E-01	5.08E-02	3.51E-01	NS
CA9	NS	2.16E-01	-0.0508	3.51E-01	NS
MREG	NS	2.16E-01	5.08E-02	3.51E-01	NS
RPL11	NS	2.16E-01	-0.0508	3.51E-01	NS
PSMD4	NS	2.16E-01	5.08E-02	3.51E-01	NS
TBC1D16	NS	2.16E-01	5.08E-02	3.51E-01	NS
CTSF	NS	2.16E-01	-0.0508	3.51E-01	NS
HK3	NS	2.16E-01	-0.0508	3.51E-01	NS
ERI2	NS	2.16E-01	-0.0508	3.51E-01	NS
SRSF10	NS	2.16E-01	-0.0508	3.51E-01	NS
AGPS	NS	2.16E-01	-0.0508	3.52E-01	NS
PDE3B	NS	2.16E-01	-0.0508	3.52E-01	NS
CLCN7	NS	2.16E-01	5.08E-02	3.52E-01	NS
ORC2	NS	2.16E-01	-0.0507	3.52E-01	NS
ATP1B4	NS	2.16E-01	5.07E-02	3.52E-01	NS
CPNE7	NS	2.16E-01	-0.0507	3.52E-01	NS
IFFO1	NS	2.17E-01	5.07E-02	3.52E-01	NS
APOBEC1	NS	2.17E-01	-0.0507	3.52E-01	NS
ZNF232	NS	2.17E-01	-0.0507	3.52E-01	NS
CCDC149	NS	2.17E-01	-0.0507	3.52E-01	NS
TNFAIP2	NS	2.17E-01	-0.0507	3.53E-01	NS
GABPB2	NS	2.17E-01	-0.0507	3.53E-01	NS
ZP3	NS	2.17E-01	-0.0507	3.53E-01	NS
STAU1	NS	2.17E-01	-0.0507	3.53E-01	NS
BICC1	NS	2.17E-01	5.07E-02	3.53E-01	NS
FCHSD1	NS	2.17E-01	-0.0506	3.53E-01	NS
CPLX1	NS	2.18E-01	5.06E-02	3.53E-01	NS
FAM107B	NS	2.18E-01	-0.0506	3.54E-01	NS
ARHGAP6	NS	2.18E-01	-0.0506	3.54E-01	NS
VEZF1	NS	2.18E-01	5.06E-02	3.54E-01	NS
SLITRK5	NS	2.18E-01	5.06E-02	3.54E-01	NS
AFF4	NS	2.18E-01	-0.0505	3.54E-01	NS
IMPDH1	NS	2.18E-01	-0.0505	3.54E-01	NS
TOP1	NS	2.18E-01	5.05E-02	3.54E-01	NS

BATF3	NS	2.19E-01	-0.0505	3.55E-01	NS
SGCE	NS	2.19E-01	-0.0505	3.55E-01	NS
GYPC	NS	2.19E-01	5.05E-02	3.55E-01	NS
LARP4	NS	2.19E-01	5.05E-02	3.55E-01	NS
SBF1	NS	2.19E-01	5.05E-02	3.55E-01	NS
PCNX4	NS	2.19E-01	-0.0505	3.55E-01	NS
ZNF439	NS	2.19E-01	-0.0505	3.55E-01	NS
ZFP91	NS	2.19E-01	5.05E-02	3.55E-01	NS
ZNF398	NS	2.19E-01	5.05E-02	3.55E-01	NS
ZNF18	NS	2.19E-01	-0.0505	3.55E-01	NS
NCEH1	NS	2.19E-01	-0.0505	3.55E-01	NS
SNRPB2	NS	2.19E-01	-0.0505	3.55E-01	NS
ALDH1B1	NS	2.19E-01	-0.0505	3.55E-01	NS
ZAN	NS	2.19E-01	-0.0505	3.55E-01	NS
TWIST2	NS	2.19E-01	-0.0505	3.55E-01	NS
PDGFRL	NS	2.19E-01	-0.0505	3.55E-01	NS
CALB2	NS	2.19E-01	-0.0505	3.55E-01	NS
POLE	NS	2.19E-01	-0.0505	3.55E-01	NS
SERPINB2	NS	2.19E-01	-0.0505	3.55E-01	NS
PACSIN2	NS	2.19E-01	-0.0505	3.55E-01	NS
SAMD10	NS	2.19E-01	5.04E-02	3.55E-01	NS
C9orf153	NS	2.19E-01	5.04E-02	3.55E-01	NS
FBXL8	NS	2.19E-01	-0.0504	3.55E-01	NS
OR8U1	NS	2.19E-01	-0.0504	3.55E-01	NS
ATG101	NS	2.19E-01	-0.0504	3.55E-01	NS
LBX1	NS	2.20E-01	5.04E-02	3.56E-01	NS
TNFSF11	NS	2.20E-01	5.04E-02	3.56E-01	NS
ZER1	NS	2.20E-01	-0.0504	3.56E-01	NS
UBXN2A	NS	2.20E-01	-0.0504	3.56E-01	NS
PPP1R14D	NS	2.20E-01	5.04E-02	3.56E-01	NS
CAPN1	NS	2.20E-01	5.04E-02	3.56E-01	NS
NHLRC1	NS	2.20E-01	-0.0504	3.56E-01	NS
DNAJB13	NS	2.20E-01	5.04E-02	3.56E-01	NS
TIFAB	NS	2.20E-01	-0.0504	3.56E-01	NS
TEP1	NS	2.20E-01	5.04E-02	3.56E-01	NS
SHISA4	NS	2.20E-01	5.04E-02	3.56E-01	NS
LCE3D	NS	2.20E-01	5.03E-02	3.56E-01	NS

ZNF716	NS	2.20E-01	-0.0503	3.56E-01	NS
PPT1	NS	2.20E-01	-0.0503	3.57E-01	NS
RSRC1	NS	2.20E-01	-0.0503	3.57E-01	NS
SET	NS	2.21E-01	5.03E-02	3.57E-01	NS
CSNK1E	NS	2.21E-01	-0.0503	3.57E-01	NS
KRT35	NS	2.21E-01	-0.0503	3.57E-01	NS
ASB1	NS	2.21E-01	-0.0503	3.57E-01	NS
DPP6	NS	2.21E-01	-0.0503	3.57E-01	NS
SENP5	NS	2.21E-01	5.03E-02	3.57E-01	NS
GLMP	NS	2.21E-01	5.03E-02	3.57E-01	NS
ANAPC5	NS	2.21E-01	-0.0502	3.57E-01	NS
ABLIM3	NS	2.21E-01	-0.0502	3.57E-01	NS
EML1	NS	2.21E-01	-0.0502	3.58E-01	NS
HLA-G	NS	2.21E-01	5.02E-02	3.58E-01	NS
AGER	NS	2.21E-01	5.02E-02	3.58E-01	NS
ANGPTL8	NS	2.21E-01	-0.0502	3.58E-01	NS
CAMK4	NS	2.21E-01	5.02E-02	3.58E-01	NS
ALG6	NS	2.22E-01	-0.0502	3.58E-01	NS
MPP1	NS	2.22E-01	5.02E-02	3.58E-01	NS
SUFU	NS	2.22E-01	5.02E-02	3.58E-01	NS
CLSTN2	NS	2.22E-01	-0.0502	3.58E-01	NS
PINX1	NS	2.22E-01	-0.0502	3.58E-01	NS
KCTD9	NS	2.22E-01	5.02E-02	3.58E-01	NS
BRCC3	NS	2.22E-01	-0.0502	3.58E-01	NS
ERF	NS	2.22E-01	5.02E-02	3.58E-01	NS
MALRD1	NS	2.22E-01	-0.0502	3.58E-01	NS
WDR91	NS	2.22E-01	5.01E-02	3.58E-01	NS
ENOX1	NS	2.22E-01	-0.0501	3.58E-01	NS
SUGP1	NS	2.22E-01	5.01E-02	3.58E-01	NS
PTBP2	NS	2.22E-01	-0.0501	3.58E-01	NS
LIMS3	NS	2.22E-01	-0.0501	3.58E-01	NS
SCAMP2	NS	2.22E-01	5.01E-02	3.59E-01	NS
MEGF8	NS	2.22E-01	5.01E-02	3.59E-01	NS
COL9A1	NS	2.22E-01	-0.0501	3.59E-01	NS
KLC1	NS	2.22E-01	5.01E-02	3.59E-01	NS
NOTCH4	NS	2.22E-01	5.01E-02	3.59E-01	NS
TLE2	NS	2.22E-01	5.01E-02	3.59E-01	NS

RAPGEF5	NS	2.22E-01	5.01E-02	3.59E-01	NS
PTP4A3	NS	2.22E-01	-0.0501	3.59E-01	NS
ADGRD2	NS	2.22E-01	5.01E-02	3.59E-01	NS
CRLF2	NS	2.22E-01	-0.0501	3.59E-01	NS
PCM1	NS	2.23E-01	-0.0501	3.59E-01	NS
QSOX2	NS	2.23E-01	-0.0501	3.59E-01	NS
PSG1	NS	2.23E-01	-0.0501	3.59E-01	NS
MLXIPL	NS	2.23E-01	5.01E-02	3.59E-01	NS
COMMD5	NS	2.23E-01	5.01E-02	3.59E-01	NS
RUSC2	NS	2.23E-01	5.01E-02	3.59E-01	NS
MCEE	NS	2.23E-01	-0.0501	3.59E-01	NS
CWF19L1	NS	2.23E-01	-0.05	3.59E-01	NS
ARHGAP12	NS	2.23E-01	-0.05	3.59E-01	NS
PIRT	NS	2.23E-01	-0.05	3.59E-01	NS
CCDC43	NS	2.23E-01	-0.05	3.59E-01	NS
LHX6	NS	2.23E-01	5.00E-02	3.60E-01	NS
CCNT1	NS	2.23E-01	5.00E-02	3.60E-01	NS
MIDN	NS	2.23E-01	5.00E-02	3.60E-01	NS
UBE2I	NS	2.23E-01	-0.05	3.60E-01	NS
RILPL2	NS	2.23E-01	-0.05	3.60E-01	NS
THAP6	NS	2.23E-01	5.00E-02	3.60E-01	NS
KRTAP3-3	NS	2.23E-01	-0.05	3.60E-01	NS
OSCAR	NS	2.24E-01	4.99E-02	3.60E-01	NS
H3-2	NS	2.24E-01	-0.0499	3.60E-01	NS
KCNK6	NS	2.24E-01	4.99E-02	3.61E-01	NS
FGF17	NS	2.24E-01	4.99E-02	3.61E-01	NS
ZNF124	NS	2.24E-01	-0.0499	3.61E-01	NS
XAB2	NS	2.24E-01	-0.0499	3.61E-01	NS
INTS2	NS	2.24E-01	-0.0499	3.61E-01	NS
SNW1	NS	2.24E-01	-0.0499	3.61E-01	NS
STK19	NS	2.24E-01	-0.0499	3.61E-01	NS
ARHGAP31	NS	2.24E-01	-0.0499	3.61E-01	NS
CD226	NS	2.25E-01	-0.0498	3.61E-01	NS
H1-5	NS	2.25E-01	-0.0498	3.61E-01	NS
TAF7L	NS	2.25E-01	-0.0498	3.62E-01	NS
EPHB3	NS	2.25E-01	4.98E-02	3.62E-01	NS
FKRP	NS	2.25E-01	-0.0498	3.62E-01	NS

NFKBIL1	NS	2.25E-01	-0.0498	3.62E-01	NS
POLR2J	NS	2.25E-01	4.98E-02	3.62E-01	NS
UTP20	NS	2.25E-01	-0.0498	3.62E-01	NS
LSR	NS	2.25E-01	4.98E-02	3.62E-01	NS
CD68	NS	2.25E-01	4.98E-02	3.62E-01	NS
NGLY1	NS	2.26E-01	-0.0497	3.63E-01	NS
RAB11FIP2	NS	2.26E-01	4.97E-02	3.63E-01	NS
BNIP3L	NS	2.26E-01	-0.0497	3.63E-01	NS
GPR78	NS	2.26E-01	4.97E-02	3.63E-01	NS
RLF	NS	2.26E-01	-0.0497	3.63E-01	NS
OR8J3	NS	2.26E-01	-0.0497	3.63E-01	NS
ZNF513	NS	2.26E-01	-0.0497	3.63E-01	NS
CMYA5	NS	2.26E-01	-0.0497	3.63E-01	NS
TNC	NS	2.26E-01	4.97E-02	3.63E-01	NS
SCP2D1	NS	2.26E-01	4.97E-02	3.63E-01	NS
COG8	NS	2.26E-01	4.97E-02	3.63E-01	NS
TRIM64C	NS	2.26E-01	4.97E-02	3.63E-01	NS
TGS1	NS	2.27E-01	-0.0496	3.64E-01	NS
RWDD4	NS	2.27E-01	-0.0496	3.64E-01	NS
CASQ1	NS	2.27E-01	-0.0496	3.64E-01	NS
SYN2	NS	2.27E-01	-0.0496	3.64E-01	NS
SHQ1	NS	2.27E-01	-0.0496	3.64E-01	NS
SEL1L	NS	2.27E-01	4.96E-02	3.64E-01	NS
CABYR	NS	2.27E-01	4.96E-02	3.64E-01	NS
DDB2	NS	2.27E-01	-0.0496	3.64E-01	NS
AGAP5	NS	2.27E-01	-0.0496	3.64E-01	NS
HLTF	NS	2.27E-01	4.96E-02	3.64E-01	NS
PRKCSH	NS	2.27E-01	4.96E-02	3.64E-01	NS
PPBP	NS	2.27E-01	4.96E-02	3.64E-01	NS
RAB31	NS	2.27E-01	-0.0496	3.64E-01	NS
HAMP	NS	2.27E-01	4.96E-02	3.64E-01	NS
COX7A2L	NS	2.27E-01	-0.0496	3.64E-01	NS
CALHM2	NS	2.27E-01	-0.0496	3.64E-01	NS
ABCB11	NS	2.27E-01	-0.0496	3.64E-01	NS
CNKSR3	NS	2.27E-01	4.96E-02	3.64E-01	NS
GDPD1	NS	2.27E-01	-0.0496	3.64E-01	NS
PHETA1	NS	2.27E-01	-0.0496	3.64E-01	NS

AVIL	NS	2.27E-01	-0.0496	3.65E-01	NS
SPO11	NS	2.28E-01	-0.0495	3.65E-01	NS
RBM14-RBM4	NS	2.28E-01	4.95E-02	3.65E-01	NS
NREP	NS	2.28E-01	-0.0495	3.65E-01	NS
PGAM4	NS	2.28E-01	-0.0495	3.65E-01	NS
BLVRA	NS	2.28E-01	-0.0495	3.65E-01	NS
PLA2G1B	NS	2.28E-01	4.95E-02	3.65E-01	NS
HPD	NS	2.28E-01	-0.0495	3.66E-01	NS
HMX2	NS	2.28E-01	4.95E-02	3.66E-01	NS
SPRY1	NS	2.28E-01	-0.0494	3.66E-01	NS
IGF1R	NS	2.29E-01	-0.0494	3.66E-01	NS
OR5AU1	NS	2.29E-01	4.94E-02	3.66E-01	NS
DDI1	NS	2.29E-01	-0.0494	3.66E-01	NS
MBOAT1	NS	2.29E-01	4.94E-02	3.66E-01	NS
ZNF551	NS	2.29E-01	4.94E-02	3.66E-01	NS
CD81	NS	2.29E-01	-0.0494	3.66E-01	NS
GABBR2	NS	2.29E-01	-0.0494	3.66E-01	NS
PDE2A	NS	2.29E-01	4.94E-02	3.66E-01	NS
HNRNPCL1	NS	2.29E-01	4.94E-02	3.66E-01	NS
GPR39	NS	2.29E-01	-0.0494	3.66E-01	NS
TRABD	NS	2.29E-01	-0.0494	3.67E-01	NS
PI16	NS	2.29E-01	4.94E-02	3.67E-01	NS
AKAP6	NS	2.29E-01	-0.0494	3.67E-01	NS
TBK1	NS	2.29E-01	4.94E-02	3.67E-01	NS
NDUFS2	NS	2.29E-01	-0.0494	3.67E-01	NS
CPSF3	NS	2.29E-01	-0.0493	3.67E-01	NS
NHSL2	NS	2.29E-01	-0.0493	3.67E-01	NS
LIFR	NS	2.30E-01	-0.0493	3.67E-01	NS
MT-ND6	NS	2.30E-01	4.93E-02	3.67E-01	NS
ERGIC3	NS	2.30E-01	4.93E-02	3.67E-01	NS
TRDMT1	NS	2.30E-01	-0.0493	3.67E-01	NS
PAN2	NS	2.30E-01	4.93E-02	3.67E-01	NS
AQP4	NS	2.30E-01	-0.0493	3.67E-01	NS
MAP3K15	NS	2.30E-01	-0.0493	3.67E-01	NS
MCCC1	NS	2.30E-01	4.93E-02	3.68E-01	NS
NIPBL	NS	2.30E-01	4.93E-02	3.68E-01	NS
CDK5RAP1	NS	2.30E-01	-0.0493	3.68E-01	NS

GUCA1B	NS	2.30E-01	-0.0493	3.68E-01	NS
PPP2R3B	NS	2.30E-01	4.93E-02	3.68E-01	NS
NUPR1	NS	2.30E-01	-0.0492	3.68E-01	NS
XRCC2	NS	2.31E-01	4.92E-02	3.68E-01	NS
CCDC63	NS	2.31E-01	-0.0492	3.68E-01	NS
CLPB	NS	2.31E-01	4.92E-02	3.68E-01	NS
COMMD1	NS	2.31E-01	-0.0492	3.69E-01	NS
SAG	NS	2.31E-01	4.92E-02	3.69E-01	NS
MEMO1	NS	2.31E-01	-0.0492	3.69E-01	NS
FERMT3	NS	2.31E-01	-0.0492	3.69E-01	NS
PHF23	NS	2.31E-01	-0.0492	3.69E-01	NS
GALNT12	NS	2.31E-01	-0.0492	3.69E-01	NS
SEPTIN7	NS	2.31E-01	-0.0491	3.69E-01	NS
OR9I1	NS	2.31E-01	-0.0491	3.69E-01	NS
MFSD11	NS	2.31E-01	-0.0491	3.69E-01	NS
OTOF	NS	2.32E-01	-0.0491	3.69E-01	NS
ELL2	NS	2.32E-01	4.91E-02	3.70E-01	NS
HNRNPCL4	NS	2.32E-01	-0.0491	3.70E-01	NS
NAP1L2	NS	2.32E-01	-0.0491	3.70E-01	NS
ERVV-1	NS	2.32E-01	4.91E-02	3.70E-01	NS
SGCD	NS	2.32E-01	-0.0491	3.70E-01	NS
GAPT	NS	2.32E-01	-0.0491	3.70E-01	NS
SLC9A2	NS	2.32E-01	-0.0491	3.70E-01	NS
ARPC5L	NS	2.32E-01	4.91E-02	3.70E-01	NS
ANGEL2	NS	2.32E-01	4.91E-02	3.70E-01	NS
SCYGR5	NS	2.32E-01	-0.0491	3.70E-01	NS
PLA2G4C	NS	2.32E-01	4.91E-02	3.70E-01	NS
MEX3C	NS	2.32E-01	-0.0491	3.70E-01	NS
GATAD2A	NS	2.32E-01	4.91E-02	3.70E-01	NS
MRGBP	NS	2.32E-01	4.90E-02	3.70E-01	NS
ID1	NS	2.32E-01	4.90E-02	3.70E-01	NS
FJX1	NS	2.32E-01	4.90E-02	3.70E-01	NS
LCK	NS	2.32E-01	-0.049	3.70E-01	NS
GGACT	NS	2.32E-01	4.90E-02	3.70E-01	NS
IFNL1	NS	2.33E-01	4.90E-02	3.70E-01	NS
CRMP1	NS	2.33E-01	-0.049	3.71E-01	NS
DNAJC30	NS	2.33E-01	-0.049	3.71E-01	NS

TPSB2	NS	2.33E-01	4.90E-02	3.71E-01	NS
OR2K2	NS	2.33E-01	-0.049	3.71E-01	NS
CRCT1	NS	2.33E-01	-0.049	3.71E-01	NS
OSBPL6	NS	2.33E-01	4.90E-02	3.71E-01	NS
USP35	NS	2.33E-01	4.90E-02	3.71E-01	NS
PDE1B	NS	2.33E-01	-0.049	3.71E-01	NS
PKDCC	NS	2.33E-01	4.90E-02	3.71E-01	NS
ZNF722P	NS	2.33E-01	4.90E-02	3.71E-01	NS
ECE1	NS	2.33E-01	-0.0489	3.71E-01	NS
ZNF593	NS	2.33E-01	4.89E-02	3.71E-01	NS
JOSD2	NS	2.33E-01	-0.049	3.71E-01	NS
CCDC14	NS	2.33E-01	-0.049	3.71E-01	NS
HEXIM1	NS	2.33E-01	-0.049	3.71E-01	NS
GH2	NS	2.33E-01	-0.0489	3.71E-01	NS
CCKAR	NS	2.33E-01	-0.0489	3.71E-01	NS
RNF7	NS	2.33E-01	4.89E-02	3.71E-01	NS
ZBTB48	NS	2.33E-01	-0.0489	3.71E-01	NS
MARVELD2	NS	2.33E-01	4.89E-02	3.71E-01	NS
NCK2	NS	2.34E-01	-0.0489	3.71E-01	NS
TNFSF14	NS	2.34E-01	-0.0489	3.71E-01	NS
SNED1	NS	2.34E-01	-0.0489	3.71E-01	NS
SLC44A3	NS	2.34E-01	4.89E-02	3.71E-01	NS
GDI2	NS	2.34E-01	4.89E-02	3.72E-01	NS
ETFBKMT	NS	2.34E-01	-0.0489	3.72E-01	NS
ETS2	NS	2.34E-01	-0.0489	3.72E-01	NS
LAMA1	NS	2.34E-01	-0.0489	3.72E-01	NS
BCL2L1	NS	2.34E-01	4.89E-02	3.72E-01	NS
MRPL41	NS	2.34E-01	-0.0489	3.72E-01	NS
COL1A1	NS	2.34E-01	-0.0488	3.72E-01	NS
SEC31A	NS	2.34E-01	4.89E-02	3.72E-01	NS
KCNA10	NS	2.34E-01	-0.0488	3.72E-01	NS
ACCSL	NS	2.34E-01	-0.0488	3.72E-01	NS
GIMD1	NS	2.34E-01	-0.0488	3.72E-01	NS
TBC1D3B	NS	2.34E-01	4.88E-02	3.72E-01	NS
ARL6	NS	2.34E-01	4.88E-02	3.72E-01	NS
ODF1	NS	2.34E-01	4.88E-02	3.72E-01	NS
RPL36	NS	2.34E-01	4.88E-02	3.72E-01	NS

AP2S1	NS	2.35E-01	-0.0488	3.72E-01	NS
BMP15	NS	2.35E-01	-0.0488	3.72E-01	NS
ALDH8A1	NS	2.35E-01	-0.0488	3.73E-01	NS
HOXB8	NS	2.35E-01	4.88E-02	3.73E-01	NS
CSN2	NS	2.35E-01	-0.0488	3.73E-01	NS
GJA5	NS	2.35E-01	-0.0488	3.73E-01	NS
SYS1-DBNDD2	NS	2.35E-01	4.88E-02	3.73E-01	NS
LZTS3	NS	2.35E-01	-0.0487	3.73E-01	NS
PLA2G4E	NS	2.35E-01	-0.0487	3.73E-01	NS
PTP4A1	NS	2.35E-01	4.87E-02	3.73E-01	NS
SLC6A5	NS	2.35E-01	-0.0487	3.73E-01	NS
PCDHA11	NS	2.35E-01	-0.0487	3.73E-01	NS
ODF3L1	NS	2.36E-01	4.87E-02	3.74E-01	NS
DNAJA4	NS	2.36E-01	-0.0487	3.74E-01	NS
ATP5MGL	NS	2.36E-01	-0.0487	3.74E-01	NS
MKX	NS	2.36E-01	-0.0487	3.74E-01	NS
RGS19	NS	2.36E-01	-0.0487	3.74E-01	NS
C4orf51	NS	2.36E-01	4.86E-02	3.74E-01	NS
ZFP90	NS	2.36E-01	-0.0486	3.74E-01	NS
BTBD19	NS	2.36E-01	4.86E-02	3.74E-01	NS
GK3P	NS	2.36E-01	4.86E-02	3.74E-01	NS
SMUG1	NS	2.36E-01	4.86E-02	3.74E-01	NS
MAP9	NS	2.36E-01	-0.0486	3.74E-01	NS
CHMP1B	NS	2.36E-01	4.86E-02	3.74E-01	NS
TP53TG5	NS	2.36E-01	-0.0486	3.75E-01	NS
PSMB9	NS	2.36E-01	4.86E-02	3.75E-01	NS
SLC19A1	NS	2.36E-01	-0.0486	3.75E-01	NS
U2SURP	NS	2.37E-01	-0.0486	3.75E-01	NS
PTGER4	NS	2.37E-01	-0.0486	3.75E-01	NS
TRIM45	NS	2.37E-01	-0.0486	3.75E-01	NS
FLYWCH1	NS	2.37E-01	4.86E-02	3.75E-01	NS
PSG8	NS	2.37E-01	-0.0486	3.75E-01	NS
SLIT3	NS	2.37E-01	-0.0486	3.75E-01	NS
ATP5PO	NS	2.37E-01	-0.0486	3.75E-01	NS
RTRAF	NS	2.37E-01	-0.0485	3.75E-01	NS
PDZK1	NS	2.37E-01	4.85E-02	3.76E-01	NS
ANKRD18B	NS	2.37E-01	-0.0485	3.76E-01	NS

GRIK5	NS	2.37E-01	4.85E-02	3.76E-01	NS
TBC1D8	NS	2.37E-01	-0.0485	3.76E-01	NS
SYNGR3	NS	2.37E-01	-0.0485	3.76E-01	NS
CFAP58	NS	2.37E-01	-0.0485	3.76E-01	NS
RASAL2	NS	2.38E-01	-0.0485	3.76E-01	NS
AJUBA	NS	2.38E-01	-0.0485	3.76E-01	NS
CA1	NS	2.38E-01	-0.0485	3.76E-01	NS
CPT2	NS	2.37E-01	4.85E-02	3.76E-01	NS
TMEM184A	NS	2.38E-01	4.85E-02	3.76E-01	NS
GPRC6A	NS	2.38E-01	-0.0485	3.76E-01	NS
LST1	NS	2.38E-01	-0.0485	3.76E-01	NS
ZNF891	NS	2.38E-01	-0.0485	3.76E-01	NS
BCAS4	NS	2.38E-01	-0.0485	3.76E-01	NS
ODR4	NS	2.38E-01	4.85E-02	3.76E-01	NS
RASSF10	NS	2.38E-01	4.85E-02	3.76E-01	NS
MTRFR	NS	2.38E-01	-0.0485	3.76E-01	NS
ABCB9	NS	2.38E-01	4.85E-02	3.76E-01	NS
SSTR2	NS	2.38E-01	-0.0485	3.76E-01	NS
MED13L	NS	2.38E-01	-0.0485	3.76E-01	NS
DHX8	NS	2.38E-01	-0.0484	3.76E-01	NS
AP1B1	NS	2.38E-01	-0.0485	3.76E-01	NS
NT5C3A	NS	2.38E-01	-0.0484	3.76E-01	NS
RTN3	NS	2.38E-01	4.84E-02	3.76E-01	NS
RBP5	NS	2.38E-01	-0.0484	3.76E-01	NS
STRC	NS	2.38E-01	-0.0484	3.76E-01	NS
LCE3A	NS	2.38E-01	4.84E-02	3.76E-01	NS
DEFB128	NS	2.38E-01	-0.0484	3.76E-01	NS
EFL1	NS	2.38E-01	4.84E-02	3.77E-01	NS
OR4X1	NS	2.38E-01	4.84E-02	3.77E-01	NS
CPXM2	NS	2.39E-01	4.84E-02	3.77E-01	NS
ADA	NS	2.39E-01	-0.0484	3.77E-01	NS
USP17L30	NS	2.39E-01	4.84E-02	3.77E-01	NS
SERPINA4	NS	2.39E-01	-0.0484	3.77E-01	NS
SLCO2B1	NS	2.39E-01	-0.0484	3.77E-01	NS
NECAP2	NS	2.39E-01	-0.0484	3.77E-01	NS
BCL9L	NS	2.39E-01	4.84E-02	3.77E-01	NS
PRR19	NS	2.39E-01	-0.0484	3.77E-01	NS

KIAA0040	NS	2.39E-01	-0.0484	3.77E-01	NS
ETV3L	NS	2.39E-01	4.83E-02	3.77E-01	NS
ATP9A	NS	2.39E-01	-0.0483	3.77E-01	NS
TSNAX	NS	2.39E-01	-0.0483	3.77E-01	NS
SLC30A5	NS	2.39E-01	4.83E-02	3.78E-01	NS
PSMG1	NS	2.39E-01	-0.0483	3.78E-01	NS
ANK2	NS	2.39E-01	-0.0483	3.78E-01	NS
USP4	NS	2.40E-01	4.83E-02	3.78E-01	NS
NCSTN	NS	2.40E-01	4.83E-02	3.78E-01	NS
RGS14	NS	2.40E-01	-0.0483	3.78E-01	NS
DPF1	NS	2.40E-01	4.83E-02	3.78E-01	NS
SLC6A9	NS	2.40E-01	4.83E-02	3.78E-01	NS
NCKAP5L	NS	2.40E-01	4.82E-02	3.78E-01	NS
MTAP	NS	2.40E-01	-0.0482	3.78E-01	NS
CDK19	NS	2.40E-01	-0.0482	3.78E-01	NS
CNNM1	NS	2.40E-01	-0.0482	3.79E-01	NS
CIB1	NS	2.40E-01	4.82E-02	3.79E-01	NS
FES	NS	2.40E-01	-0.0482	3.79E-01	NS
CIC	NS	2.40E-01	4.82E-02	3.79E-01	NS
PDE7B	NS	2.40E-01	-0.0482	3.79E-01	NS
OSR1	NS	2.41E-01	4.82E-02	3.79E-01	NS
MSL1	NS	2.41E-01	4.82E-02	3.79E-01	NS
TSPAN6	NS	2.41E-01	-0.0482	3.79E-01	NS
SMARCB1	NS	2.41E-01	4.82E-02	3.79E-01	NS
GPSM3	NS	2.41E-01	-0.0482	3.79E-01	NS
CDC14B	NS	2.41E-01	-0.0482	3.79E-01	NS
IFI6	NS	2.41E-01	-0.0481	3.79E-01	NS
GNG5	NS	2.41E-01	4.81E-02	3.79E-01	NS
SMIM22	NS	2.41E-01	-0.0481	3.79E-01	NS
RHCG	NS	2.41E-01	-0.0481	3.79E-01	NS
UNC79	NS	2.41E-01	4.81E-02	3.79E-01	NS
GPR83	NS	2.41E-01	-0.0481	3.79E-01	NS
PRDM16	NS	2.41E-01	4.81E-02	3.79E-01	NS
KCTD13	NS	2.41E-01	4.81E-02	3.79E-01	NS
SP100	NS	2.41E-01	4.81E-02	3.79E-01	NS
SLC6A3	NS	2.41E-01	4.81E-02	3.80E-01	NS
PAQR8	NS	2.41E-01	-0.0481	3.80E-01	NS

KIAA1217	NS	2.41E-01	-0.0481	3.80E-01	NS
IREB2	NS	2.42E-01	-0.0481	3.80E-01	NS
WDR13	NS	2.42E-01	-0.0481	3.80E-01	NS
MTRNR2L4	NS	2.42E-01	-0.0481	3.80E-01	NS
NOP16	NS	2.42E-01	-0.0481	3.80E-01	NS
DPM2	NS	2.42E-01	-0.0481	3.80E-01	NS
ZNF408	NS	2.42E-01	-0.0481	3.80E-01	NS
MAB21L2	NS	2.42E-01	-0.0481	3.80E-01	NS
NVL	NS	2.42E-01	4.81E-02	3.80E-01	NS
RANBP1	NS	2.42E-01	-0.048	3.80E-01	NS
CXCR5	NS	2.42E-01	4.80E-02	3.80E-01	NS
TERB2	NS	2.42E-01	4.80E-02	3.80E-01	NS
TEX13B	NS	2.42E-01	4.80E-02	3.80E-01	NS
SERINC3	NS	2.42E-01	-0.048	3.80E-01	NS
TMSB10	NS	2.42E-01	-0.048	3.80E-01	NS
C9orf131	NS	2.42E-01	-0.048	3.81E-01	NS
THEGL	NS	2.42E-01	-0.048	3.81E-01	NS
LCE5A	NS	2.42E-01	-0.048	3.81E-01	NS
RTL6	NS	2.42E-01	-0.048	3.81E-01	NS
GLOD4	NS	2.42E-01	4.80E-02	3.81E-01	NS
WDR37	NS	2.42E-01	-0.048	3.81E-01	NS
TPM4	NS	2.42E-01	-0.048	3.81E-01	NS
C1orf210	NS	2.43E-01	4.80E-02	3.81E-01	NS
UBE2B	NS	2.43E-01	-0.048	3.81E-01	NS
TRMT6	NS	2.43E-01	-0.048	3.81E-01	NS
CEP112	NS	2.43E-01	-0.048	3.81E-01	NS
NEK10	NS	2.43E-01	-0.048	3.81E-01	NS
CHP2	NS	2.43E-01	-0.048	3.81E-01	NS
NMUR1	NS	2.43E-01	-0.048	3.81E-01	NS
NPC2	NS	2.43E-01	-0.0479	3.81E-01	NS
B3GAT1	NS	2.43E-01	-0.0479	3.81E-01	NS
RIMKLA	NS	2.43E-01	-0.0479	3.81E-01	NS
WDR31	NS	2.43E-01	4.79E-02	3.81E-01	NS
IQGAP3	NS	2.43E-01	4.79E-02	3.81E-01	NS
SPN	NS	2.43E-01	4.79E-02	3.82E-01	NS
KRTAP12-3	NS	2.43E-01	4.79E-02	3.82E-01	NS
MGAT4B	NS	2.43E-01	4.79E-02	3.82E-01	NS

ABCA3	NS	2.43E-01	-0.0479	3.82E-01	NS
MYOD1	NS	2.44E-01	4.79E-02	3.82E-01	NS
SMCO1	NS	2.44E-01	-0.0479	3.82E-01	NS
HNRNPA1P48	NS	2.44E-01	-0.0479	3.82E-01	NS
DBX1	NS	2.44E-01	-0.0479	3.82E-01	NS
USP27X	NS	2.44E-01	-0.0479	3.82E-01	NS
ZNF34	NS	2.44E-01	-0.0479	3.82E-01	NS
LRRC3	NS	2.44E-01	4.79E-02	3.82E-01	NS
STK17A	NS	2.44E-01	-0.0478	3.82E-01	NS
PPARGC1A	NS	2.44E-01	-0.0478	3.83E-01	NS
XRRA1	NS	2.44E-01	-0.0478	3.83E-01	NS
IGHV3OR16-17	NS	2.44E-01	4.78E-02	3.83E-01	NS
CDH10	NS	2.44E-01	4.78E-02	3.83E-01	NS
DIS3	NS	2.44E-01	-0.0478	3.83E-01	NS
SNRPN	NS	2.44E-01	-0.0478	3.83E-01	NS
TSHZ2	NS	2.44E-01	-0.0478	3.83E-01	NS
SMDT1	NS	2.44E-01	4.78E-02	3.83E-01	NS
OCM	NS	2.44E-01	4.78E-02	3.83E-01	NS
CTSK	NS	2.44E-01	-0.0478	3.83E-01	NS
ANKS1B	NS	2.44E-01	4.78E-02	3.83E-01	NS
SCN4A	NS	2.45E-01	-0.0478	3.83E-01	NS
TRIP4	NS	2.45E-01	-0.0477	3.83E-01	NS
TLE7	NS	2.45E-01	4.77E-02	3.83E-01	NS
IGFBP3	NS	2.45E-01	-0.0477	3.83E-01	NS
ENY2	NS	2.45E-01	-0.0477	3.83E-01	NS
LRRC25	NS	2.45E-01	4.77E-02	3.84E-01	NS
TOMM40L	NS	2.45E-01	4.77E-02	3.84E-01	NS
SCAMP4	NS	2.45E-01	4.77E-02	3.84E-01	NS
SLAIN1	NS	2.45E-01	-0.0477	3.84E-01	NS
TTBK1	NS	2.45E-01	-0.0477	3.84E-01	NS
FBXO42	NS	2.46E-01	4.77E-02	3.84E-01	NS
DDIT4	NS	2.46E-01	-0.0477	3.84E-01	NS
BSPH1	NS	2.46E-01	4.77E-02	3.84E-01	NS
OLIG2	NS	2.46E-01	4.77E-02	3.84E-01	NS
DYNLT3	NS	2.46E-01	-0.0477	3.84E-01	NS
WNK4	NS	2.46E-01	4.76E-02	3.85E-01	NS
RSPO3	NS	2.46E-01	-0.0476	3.85E-01	NS

GBA	NS	2.46E-01	4.76E-02	3.85E-01	NS
AK4P3	NS	2.46E-01	-0.0476	3.85E-01	NS
PMM2	NS	2.46E-01	-0.0476	3.85E-01	NS
TNF	NS	2.46E-01	4.76E-02	3.85E-01	NS
SRRM4	NS	2.46E-01	4.76E-02	3.85E-01	NS
CENPO	NS	2.47E-01	4.76E-02	3.85E-01	NS
SOWAHB	NS	2.47E-01	4.76E-02	3.85E-01	NS
MYH6	NS	2.47E-01	-0.0476	3.85E-01	NS
CNTNAP3B	NS	2.47E-01	-0.0476	3.86E-01	NS
TMEM254	NS	2.47E-01	4.75E-02	3.86E-01	NS
CDC26	NS	2.47E-01	-0.0475	3.86E-01	NS
MRPL19	NS	2.47E-01	-0.0475	3.86E-01	NS
TENT4A	NS	2.47E-01	-0.0475	3.86E-01	NS
GSG1L2	NS	2.47E-01	4.75E-02	3.86E-01	NS
SPDYE11	NS	2.47E-01	-0.0475	3.86E-01	NS
ELSPBP1	NS	2.47E-01	4.75E-02	3.86E-01	NS
IFT20	NS	2.47E-01	4.75E-02	3.86E-01	NS
GNB1L	NS	2.48E-01	-0.0475	3.87E-01	NS
PCDH17	NS	2.48E-01	4.75E-02	3.87E-01	NS
RSPH3	NS	2.48E-01	-0.0475	3.87E-01	NS
SLC7A11	NS	2.48E-01	-0.0475	3.87E-01	NS
TMEM39A	NS	2.48E-01	-0.0475	3.87E-01	NS
CGB8	NS	2.48E-01	-0.0475	3.87E-01	NS
ALDH5A1	NS	2.48E-01	4.74E-02	3.87E-01	NS
PDGFB	NS	2.48E-01	-0.0474	3.87E-01	NS
MTMR6	NS	2.48E-01	-0.0474	3.87E-01	NS
PCSK6	NS	2.48E-01	-0.0474	3.87E-01	NS
IFI16	NS	2.48E-01	-0.0474	3.87E-01	NS
ZNF511-PRAP1	NS	2.48E-01	-0.0474	3.87E-01	NS
APOBEC3H	NS	2.48E-01	-0.0474	3.87E-01	NS
TRIM31	NS	2.48E-01	-0.0474	3.87E-01	NS
RREB1	NS	2.48E-01	-0.0474	3.87E-01	NS
PKHD1L1	NS	2.48E-01	-0.0474	3.87E-01	NS
KRT79	NS	2.48E-01	4.74E-02	3.88E-01	NS
GOLGA8R	NS	2.49E-01	-0.0474	3.88E-01	NS
PTGS1	NS	2.49E-01	4.74E-02	3.88E-01	NS
PRKX	NS	2.49E-01	-0.0474	3.88E-01	NS

ASPDH	NS	2.49E-01	-0.0474	3.88E-01	NS
NEURL1B	NS	2.49E-01	4.73E-02	3.88E-01	NS
ANKFY1	NS	2.49E-01	4.73E-02	3.88E-01	NS
B3GNT3	NS	2.49E-01	4.73E-02	3.88E-01	NS
LYNX1-SLURP2	NS	2.49E-01	4.73E-02	3.88E-01	NS
WDR82	NS	2.49E-01	-0.0473	3.88E-01	NS
CST9	NS	2.49E-01	-0.0473	3.88E-01	NS
EFCAB6	NS	2.49E-01	-0.0473	3.88E-01	NS
STRN	NS	2.50E-01	-0.0473	3.89E-01	NS
TMEM185A	NS	2.50E-01	4.72E-02	3.89E-01	NS
MPPED1	NS	2.50E-01	4.72E-02	3.89E-01	NS
NOM1	NS	2.50E-01	4.72E-02	3.89E-01	NS
FAM78B	NS	2.50E-01	4.72E-02	3.89E-01	NS
RNPS1	NS	2.50E-01	-0.0472	3.90E-01	NS
TBC1D3C	NS	2.50E-01	-0.0472	3.90E-01	NS
MVB12A	NS	2.50E-01	4.72E-02	3.90E-01	NS
HSPD1	NS	2.50E-01	-0.0472	3.90E-01	NS
SLC25A22	NS	2.50E-01	4.72E-02	3.90E-01	NS
ODF3	NS	2.50E-01	4.72E-02	3.90E-01	NS
GPR176	NS	2.51E-01	-0.0472	3.90E-01	NS
NOX4	NS	2.51E-01	4.72E-02	3.90E-01	NS
SLC12A5	NS	2.51E-01	4.72E-02	3.90E-01	NS
CLIC1	NS	2.51E-01	4.72E-02	3.90E-01	NS
ZNF616	NS	2.51E-01	4.72E-02	3.90E-01	NS
PTPN22	NS	2.51E-01	-0.0471	3.90E-01	NS
RNF123	NS	2.51E-01	-0.0471	3.90E-01	NS
WFDC3	NS	2.51E-01	-0.0471	3.90E-01	NS
DRC1	NS	2.51E-01	4.71E-02	3.91E-01	NS
MRC1	NS	2.51E-01	-0.0471	3.91E-01	NS
RIPOR2	NS	2.51E-01	-0.0471	3.91E-01	NS
ZNF257	NS	2.51E-01	-0.0471	3.91E-01	NS
PSMC3IP	NS	2.52E-01	-0.0471	3.91E-01	NS
ANKRD2	NS	2.52E-01	-0.0471	3.91E-01	NS
RGPD6	NS	2.52E-01	-0.0471	3.91E-01	NS
ARID2	NS	2.52E-01	-0.0471	3.91E-01	NS
TAF4B	NS	2.52E-01	4.71E-02	3.91E-01	NS
GALNT7	NS	2.52E-01	-0.0471	3.91E-01	NS

GPR55	NS	2.52E-01	-0.0471	3.91E-01	NS
PRR20D	NS	2.52E-01	-0.047	3.91E-01	NS
MCCD1	NS	2.52E-01	4.70E-02	3.92E-01	NS
CTNNBL1	NS	2.52E-01	-0.047	3.92E-01	NS
THBS3	NS	2.52E-01	4.70E-02	3.92E-01	NS
CRNN	NS	2.52E-01	-0.047	3.92E-01	NS
DCDC2B	NS	2.52E-01	-0.047	3.92E-01	NS
ZSWIM9	NS	2.52E-01	-0.047	3.92E-01	NS
STPG4	NS	2.53E-01	-0.047	3.92E-01	NS
CNTNAP1	NS	2.53E-01	4.70E-02	3.92E-01	NS
BSN	NS	2.53E-01	4.70E-02	3.92E-01	NS
CFAP65	NS	2.53E-01	-0.047	3.92E-01	NS
RPS10-NUDT3	NS	2.53E-01	-0.047	3.92E-01	NS
INTS9	NS	2.53E-01	-0.0469	3.93E-01	NS
SEMA6D	NS	2.53E-01	4.69E-02	3.93E-01	NS
SLC23A1	NS	2.53E-01	-0.0469	3.93E-01	NS
POP7	NS	2.53E-01	-0.0469	3.93E-01	NS
FPR2	NS	2.53E-01	-0.0469	3.93E-01	NS
HEBP1	NS	2.53E-01	-0.0469	3.93E-01	NS
INSIG2	NS	2.53E-01	-0.0469	3.93E-01	NS
RNPC3	NS	2.53E-01	-0.0469	3.93E-01	NS
SH3BP4	NS	2.53E-01	-0.0469	3.93E-01	NS
ALG5	NS	2.54E-01	-0.0469	3.93E-01	NS
AKAP17A	NS	2.54E-01	4.69E-02	3.93E-01	NS
RERE	NS	2.54E-01	4.69E-02	3.94E-01	NS
SLC6A11	NS	2.54E-01	-0.0469	3.94E-01	NS
BTF3L4	NS	2.54E-01	-0.0469	3.94E-01	NS
CXorf49	NS	2.54E-01	4.68E-02	3.94E-01	NS
KCNJ16	NS	2.54E-01	4.68E-02	3.94E-01	NS
TMEM126A	NS	2.54E-01	-0.0468	3.94E-01	NS
TSPAN9	NS	2.54E-01	-0.0468	3.94E-01	NS
TSHZ1	NS	2.54E-01	4.68E-02	3.94E-01	NS
C20orf144	NS	2.54E-01	4.68E-02	3.94E-01	NS
SUSD4	NS	2.54E-01	-0.0468	3.94E-01	NS
TOMM22	NS	2.54E-01	4.68E-02	3.94E-01	NS
SEPHS2	NS	2.54E-01	4.68E-02	3.94E-01	NS
TMCO6	NS	2.55E-01	-0.0468	3.95E-01	NS

RHOXF1	NS	2.55E-01	4.68E-02	3.95E-01	NS
OR2T12	NS	2.55E-01	4.68E-02	3.95E-01	NS
HDHD5	NS	2.55E-01	-0.0467	3.95E-01	NS
EIF5	NS	2.55E-01	-0.0467	3.95E-01	NS
ATP6V1F	NS	2.55E-01	4.67E-02	3.95E-01	NS
PRB1	NS	2.55E-01	-0.0467	3.95E-01	NS
DLGAP2	NS	2.55E-01	-0.0467	3.95E-01	NS
DRG1	NS	2.55E-01	4.67E-02	3.96E-01	NS
LUC7L	NS	2.55E-01	4.67E-02	3.96E-01	NS
PAFAH1B2	NS	2.55E-01	4.67E-02	3.96E-01	NS
BRIX1	NS	2.55E-01	-0.0467	3.96E-01	NS
EXOSC2	NS	2.56E-01	-0.0467	3.96E-01	NS
PIK3CG	NS	2.56E-01	4.67E-02	3.96E-01	NS
RNF103	NS	2.56E-01	-0.0467	3.96E-01	NS
TWF1	NS	2.56E-01	-0.0467	3.96E-01	NS
MDH2	NS	2.56E-01	4.66E-02	3.96E-01	NS
ADORA2B	NS	2.56E-01	4.66E-02	3.96E-01	NS
EGLN2	NS	2.56E-01	-0.0466	3.96E-01	NS
ELP1	NS	2.56E-01	-0.0466	3.97E-01	NS
MIF4GD	NS	2.56E-01	-0.0466	3.97E-01	NS
PARP3	NS	2.56E-01	-0.0466	3.97E-01	NS
CYP4A22	NS	2.56E-01	-0.0466	3.97E-01	NS
COL1A2	NS	2.56E-01	-0.0466	3.97E-01	NS
DCAKD	NS	2.57E-01	-0.0466	3.97E-01	NS
ADIPOQ	NS	2.57E-01	-0.0466	3.97E-01	NS
SOX13	NS	2.57E-01	-0.0466	3.97E-01	NS
ZNF639	NS	2.57E-01	4.66E-02	3.97E-01	NS
PPP2R2B	NS	2.57E-01	4.66E-02	3.97E-01	NS
RNF183	NS	2.57E-01	-0.0466	3.97E-01	NS
SLC22A5	NS	2.57E-01	-0.0465	3.97E-01	NS
CCM2	NS	2.57E-01	-0.0465	3.97E-01	NS
CYP3A43	NS	2.57E-01	-0.0465	3.97E-01	NS
EFCAB12	NS	2.57E-01	4.65E-02	3.97E-01	NS
PRELP	NS	2.57E-01	4.65E-02	3.97E-01	NS
KRT80	NS	2.58E-01	-0.0465	3.98E-01	NS
NRL	NS	2.58E-01	4.65E-02	3.98E-01	NS
FXYD7	NS	2.58E-01	-0.0465	3.98E-01	NS

ANKRD27	NS	2.58E-01	-0.0465	3.98E-01	NS
PTPN6	NS	2.58E-01	-0.0464	3.99E-01	NS
CDR2	NS	2.58E-01	-0.0464	3.99E-01	NS
CCDC191	NS	2.58E-01	-0.0464	3.99E-01	NS
SLC27A1	NS	2.58E-01	-0.0464	3.99E-01	NS
OGT	NS	2.58E-01	-0.0464	3.99E-01	NS
COPRS	NS	2.58E-01	-0.0464	3.99E-01	NS
PRM3	NS	2.58E-01	-0.0464	3.99E-01	NS
DPYD	NS	2.58E-01	-0.0464	3.99E-01	NS
NEDD8	NS	2.58E-01	-0.0464	3.99E-01	NS
TBC1D26	NS	2.58E-01	4.64E-02	3.99E-01	NS
TSPAN15	NS	2.58E-01	4.64E-02	3.99E-01	NS
GTF2H3	NS	2.58E-01	-0.0464	3.99E-01	NS
MATN2	NS	2.58E-01	-0.0464	3.99E-01	NS
DEGS2	NS	2.59E-01	-0.0464	3.99E-01	NS
ALDH6A1	NS	2.59E-01	-0.0464	3.99E-01	NS
SLC36A4	NS	2.59E-01	-0.0464	3.99E-01	NS
KRTAP9-4	NS	2.59E-01	-0.0464	3.99E-01	NS
IFNAR1	NS	2.59E-01	-0.0464	3.99E-01	NS
ADORA1	NS	2.59E-01	-0.0463	4.00E-01	NS
MS4A13	NS	2.59E-01	4.63E-02	4.00E-01	NS
ALDH1A3	NS	2.59E-01	4.63E-02	4.00E-01	NS
PEX26	NS	2.59E-01	4.63E-02	4.00E-01	NS
RNASE1	NS	2.59E-01	-0.0463	4.00E-01	NS
CRYGS	NS	2.59E-01	-0.0463	4.00E-01	NS
ENO4	NS	2.59E-01	-0.0463	4.00E-01	NS
LEXM	NS	2.59E-01	-0.0463	4.00E-01	NS
IFT172	NS	2.59E-01	-0.0463	4.00E-01	NS
IQCJ-SCHIP1	NS	2.60E-01	-0.0463	4.00E-01	NS
PURA	NS	2.60E-01	4.63E-02	4.00E-01	NS
AGRN	NS	2.60E-01	-0.0463	4.00E-01	NS
ARPC1B	NS	2.60E-01	-0.0463	4.00E-01	NS
NME5	NS	2.60E-01	-0.0463	4.01E-01	NS
OR5L2	NS	2.60E-01	-0.0463	4.01E-01	NS
ZNF701	NS	2.60E-01	-0.0462	4.01E-01	NS
NCDN	NS	2.60E-01	4.62E-02	4.01E-01	NS
IQGAP2	NS	2.60E-01	-0.0462	4.01E-01	NS

MAFK	NS	2.60E-01	4.62E-02	4.01E-01	NS
RNASE6	NS	2.60E-01	4.62E-02	4.01E-01	NS
HDAC4	NS	2.60E-01	-0.0462	4.01E-01	NS
ERLIN1	NS	2.61E-01	-0.0462	4.01E-01	NS
IFITM10	NS	2.61E-01	4.62E-02	4.01E-01	NS
CENPJ	NS	2.61E-01	-0.0462	4.02E-01	NS
OXT	NS	2.61E-01	-0.0461	4.02E-01	NS
ZC3H8	NS	2.61E-01	4.61E-02	4.02E-01	NS
POMZP3	NS	2.61E-01	4.61E-02	4.02E-01	NS
ASH2L	NS	2.61E-01	-0.0461	4.02E-01	NS
XKR4	NS	2.61E-01	-0.0461	4.02E-01	NS
PTS	NS	2.62E-01	-0.0461	4.03E-01	NS
NLRX1	NS	2.62E-01	4.61E-02	4.03E-01	NS
ZBTB14	NS	2.62E-01	-0.0461	4.03E-01	NS
TARS2	NS	2.62E-01	4.61E-02	4.03E-01	NS
GRIN2D	NS	2.62E-01	-0.0461	4.03E-01	NS
SLC20A2	NS	2.62E-01	4.61E-02	4.03E-01	NS
GTPBP2	NS	2.62E-01	-0.0461	4.03E-01	NS
UBAC1	NS	2.62E-01	4.61E-02	4.03E-01	NS
ZNF771	NS	2.62E-01	-0.0461	4.03E-01	NS
PHF2	NS	2.62E-01	4.61E-02	4.03E-01	NS
INPP5J	NS	2.62E-01	-0.046	4.03E-01	NS
TENT5B	NS	2.62E-01	-0.046	4.03E-01	NS
H2BC14	NS	2.62E-01	4.60E-02	4.04E-01	NS
ACOX2	NS	2.63E-01	4.60E-02	4.04E-01	NS
RALGAPB	NS	2.63E-01	-0.046	4.04E-01	NS
NDUFS7	NS	2.63E-01	4.60E-02	4.04E-01	NS
TFB1M	NS	2.63E-01	-0.046	4.04E-01	NS
TMEM258	NS	2.63E-01	-0.046	4.04E-01	NS
NOP2	NS	2.63E-01	-0.046	4.04E-01	NS
PRLH	NS	2.63E-01	4.59E-02	4.04E-01	NS
DNAI3	NS	2.63E-01	-0.0459	4.05E-01	NS
CX3CR1	NS	2.63E-01	-0.0459	4.05E-01	NS
SIN3A	NS	2.63E-01	4.59E-02	4.05E-01	NS
ABCE1	NS	2.63E-01	-0.0459	4.05E-01	NS
IL21R	NS	2.64E-01	-0.0459	4.05E-01	NS
TCTN3	NS	2.64E-01	4.59E-02	4.05E-01	NS

USE1	NS	2.64E-01	4.59E-02	4.05E-01	NS
CBX3	NS	2.64E-01	-0.0459	4.05E-01	NS
P4HA3	NS	2.64E-01	-0.0459	4.05E-01	NS
KRT40	NS	2.64E-01	4.58E-02	4.06E-01	NS
DLEC1	NS	2.64E-01	-0.0458	4.06E-01	NS
C17orf58	NS	2.64E-01	-0.0458	4.06E-01	NS
MED12	NS	2.64E-01	4.58E-02	4.06E-01	NS
ANKRD50	NS	2.64E-01	-0.0458	4.06E-01	NS
TRPC5OS	NS	2.64E-01	-0.0458	4.06E-01	NS
AHNAK2	NS	2.64E-01	-0.0458	4.06E-01	NS
ASPH	NS	2.65E-01	-0.0458	4.06E-01	NS
BTLA	NS	2.65E-01	-0.0458	4.06E-01	NS
UBE2S	NS	2.65E-01	4.58E-02	4.06E-01	NS
MTHFD1L	NS	2.65E-01	-0.0458	4.06E-01	NS
PGAM1	NS	2.65E-01	-0.0458	4.06E-01	NS
ADSS1	NS	2.65E-01	-0.0458	4.07E-01	NS
PPFIA3	NS	2.65E-01	4.58E-02	4.07E-01	NS
ITPRIPL1	NS	2.65E-01	-0.0458	4.07E-01	NS
MAGEB1	NS	2.65E-01	-0.0458	4.07E-01	NS
SLC7A6OS	NS	2.65E-01	-0.0457	4.07E-01	NS
GGT2	NS	2.65E-01	-0.0457	4.07E-01	NS
SMCR8	NS	2.65E-01	-0.0457	4.07E-01	NS
CDC42BPG	NS	2.66E-01	-0.0457	4.07E-01	NS
SUCNR1	NS	2.66E-01	-0.0457	4.07E-01	NS
HMGB1P1	NS	2.66E-01	4.57E-02	4.07E-01	NS
STPG3	NS	2.66E-01	4.57E-02	4.07E-01	NS
CBARP	NS	2.66E-01	4.57E-02	4.07E-01	NS
RAB40B	NS	2.66E-01	-0.0457	4.07E-01	NS
PRSS57	NS	2.66E-01	-0.0457	4.07E-01	NS
ASCL1	NS	2.66E-01	-0.0457	4.07E-01	NS
STX16-NPEPL1	NS	2.66E-01	-0.0457	4.07E-01	NS
KYAT1	NS	2.66E-01	-0.0457	4.08E-01	NS
MITF	NS	2.66E-01	4.57E-02	4.08E-01	NS
ZNF292	NS	2.66E-01	-0.0457	4.08E-01	NS
PLBD1	NS	2.66E-01	4.56E-02	4.08E-01	NS
KLHL6	NS	2.66E-01	-0.0456	4.08E-01	NS
GNPTAB	NS	2.66E-01	4.56E-02	4.08E-01	NS

FNTB	NS	2.67E-01	-0.0456	4.08E-01	NS
GCDH	NS	2.67E-01	-0.0456	4.08E-01	NS
GK2	NS	2.67E-01	-0.0456	4.09E-01	NS
FEZF2	NS	2.67E-01	4.56E-02	4.09E-01	NS
LLCFC1	NS	2.67E-01	4.56E-02	4.09E-01	NS
SMARCA1	NS	2.67E-01	-0.0456	4.09E-01	NS
ALDH1L1	NS	2.67E-01	4.56E-02	4.09E-01	NS
INTS10	NS	2.67E-01	4.56E-02	4.09E-01	NS
PDXP	NS	2.67E-01	-0.0456	4.09E-01	NS
PKD1	NS	2.67E-01	-0.0455	4.09E-01	NS
RNF24	NS	2.67E-01	4.56E-02	4.09E-01	NS
NOMO1	NS	2.67E-01	-0.0455	4.09E-01	NS
HOXA3	NS	2.67E-01	4.55E-02	4.09E-01	NS
PURB	NS	2.67E-01	-0.0455	4.09E-01	NS
SPDYE15	NS	2.67E-01	-0.0455	4.09E-01	NS
H2AC15	NS	2.67E-01	-0.0455	4.09E-01	NS
TEX50	NS	2.67E-01	-0.0455	4.09E-01	NS
ANK3	NS	2.68E-01	-0.0455	4.09E-01	NS
PIKFYVE	NS	2.68E-01	-0.0455	4.09E-01	NS
NMT1	NS	2.68E-01	-0.0455	4.09E-01	NS
ESCO1	NS	2.68E-01	-0.0455	4.09E-01	NS
CACHD1	NS	2.68E-01	-0.0455	4.09E-01	NS
MAGEA8	NS	2.68E-01	4.55E-02	4.09E-01	NS
TUBB2A	NS	2.68E-01	4.55E-02	4.09E-01	NS
LRRC61	NS	2.68E-01	4.55E-02	4.09E-01	NS
BCL6	NS	2.68E-01	4.55E-02	4.09E-01	NS
WIPI1	NS	2.68E-01	4.55E-02	4.09E-01	NS
NTRK2	NS	2.68E-01	-0.0455	4.10E-01	NS
AFAP1L1	NS	2.68E-01	4.55E-02	4.10E-01	NS
STEAP1B	NS	2.68E-01	-0.0455	4.10E-01	NS
PARP12	NS	2.68E-01	-0.0454	4.10E-01	NS
PLPPR2	NS	2.68E-01	-0.0454	4.10E-01	NS
TMEM88	NS	2.68E-01	-0.0454	4.10E-01	NS
MYT1	NS	2.68E-01	4.54E-02	4.10E-01	NS
BCL2L12	NS	2.68E-01	-0.0454	4.10E-01	NS
GOLGA6L6	NS	2.69E-01	-0.0454	4.10E-01	NS
SGK1	NS	2.69E-01	-0.0454	4.10E-01	NS

HSPA13	NS	2.69E-01	-0.0454	4.10E-01	NS
CYP4F11	NS	2.69E-01	4.54E-02	4.10E-01	NS
NDUFA13	NS	2.69E-01	-0.0454	4.10E-01	NS
WASHC1	NS	2.69E-01	4.54E-02	4.10E-01	NS
LYRM2	NS	2.69E-01	-0.0454	4.11E-01	NS
HSPA8	NS	2.69E-01	-0.0454	4.11E-01	NS
GHR	NS	2.70E-01	-0.0453	4.12E-01	NS
GTF2H2C	NS	2.70E-01	4.53E-02	4.12E-01	NS
FEV	NS	2.70E-01	-0.0453	4.12E-01	NS
FEZ1	NS	2.70E-01	4.53E-02	4.12E-01	NS
STMN4	NS	2.70E-01	4.53E-02	4.12E-01	NS
CASP2	NS	2.70E-01	4.53E-02	4.12E-01	NS
TARDBP	NS	2.70E-01	-0.0453	4.12E-01	NS
WNT9B	NS	2.70E-01	-0.0453	4.12E-01	NS
ANKMY2	NS	2.70E-01	-0.0453	4.12E-01	NS
RGPD5	NS	2.70E-01	-0.0453	4.12E-01	NS
GLI3	NS	2.70E-01	-0.0453	4.12E-01	NS
FAM181B	NS	2.70E-01	4.53E-02	4.12E-01	NS
HNRNPM	NS	2.71E-01	4.52E-02	4.12E-01	NS
RBPJL	NS	2.71E-01	4.52E-02	4.12E-01	NS
ARSL	NS	2.71E-01	4.52E-02	4.13E-01	NS
OR13A1	NS	2.71E-01	-0.0452	4.13E-01	NS
CLDN15	NS	2.71E-01	-0.0452	4.13E-01	NS
AKAP12	NS	2.71E-01	-0.0452	4.13E-01	NS
PYY	NS	2.71E-01	-0.0452	4.13E-01	NS
C1orf105	NS	2.71E-01	-0.0452	4.13E-01	NS
ARHGAP23	NS	2.71E-01	-0.0452	4.13E-01	NS
MOSPD2	NS	2.71E-01	4.52E-02	4.13E-01	NS
NUTF2	NS	2.71E-01	4.52E-02	4.13E-01	NS
WDR93	NS	2.71E-01	4.52E-02	4.13E-01	NS
SDC2	NS	2.71E-01	-0.0452	4.13E-01	NS
TEX261	NS	2.71E-01	-0.0452	4.13E-01	NS
RGCC	NS	2.71E-01	4.52E-02	4.13E-01	NS
MED25	NS	2.71E-01	-0.0452	4.13E-01	NS
NSDHL	NS	2.72E-01	4.51E-02	4.14E-01	NS
CT45A6	NS	2.72E-01	-0.0451	4.14E-01	NS
POTEF	NS	2.72E-01	4.51E-02	4.14E-01	NS

DDX21	NS	2.72E-01	4.51E-02	4.14E-01	NS
RPS26	NS	2.72E-01	-0.0451	4.14E-01	NS
PCDHA7	NS	2.72E-01	4.51E-02	4.14E-01	NS
UTP14A	NS	2.72E-01	-0.0451	4.14E-01	NS
IK	NS	2.72E-01	-0.0451	4.14E-01	NS
MAT1A	NS	2.72E-01	-0.0451	4.14E-01	NS
CYP4B1	NS	2.72E-01	4.51E-02	4.14E-01	NS
FBXW5	NS	2.72E-01	4.51E-02	4.14E-01	NS
RAB11B	NS	2.72E-01	4.51E-02	4.14E-01	NS
UBXN8	NS	2.72E-01	-0.0451	4.14E-01	NS
MAS1L	NS	2.72E-01	4.51E-02	4.14E-01	NS
NCR3LG1	NS	2.73E-01	-0.0451	4.14E-01	NS
SZT2	NS	2.73E-01	-0.0451	4.14E-01	NS
TMEM270	NS	2.73E-01	4.50E-02	4.15E-01	NS
FAM236C	NS	2.73E-01	4.50E-02	4.15E-01	NS
ARFRP1	NS	2.73E-01	-0.045	4.15E-01	NS
HLA-DQA1	NS	2.73E-01	-0.045	4.15E-01	NS
RPL7	NS	2.73E-01	-0.045	4.15E-01	NS
KCP	NS	2.73E-01	4.50E-02	4.15E-01	NS
CFAP298	NS	2.73E-01	-0.045	4.15E-01	NS
CYYR1	NS	2.73E-01	-0.045	4.15E-01	NS
ZXDB	NS	2.73E-01	-0.045	4.15E-01	NS
PTPN3	NS	2.74E-01	4.50E-02	4.16E-01	NS
CDKN1A	NS	2.74E-01	-0.045	4.16E-01	NS
ORC6	NS	2.74E-01	-0.0449	4.16E-01	NS
RXFP4	NS	2.74E-01	4.49E-02	4.16E-01	NS
HYI	NS	2.74E-01	4.49E-02	4.16E-01	NS
COX19	NS	2.74E-01	4.49E-02	4.16E-01	NS
ZNF670-ZNF695	NS	2.74E-01	-0.0449	4.16E-01	NS
WASF2	NS	2.74E-01	-0.0449	4.16E-01	NS
PXDC1	NS	2.74E-01	4.49E-02	4.16E-01	NS
OLFM2	NS	2.74E-01	-0.0449	4.16E-01	NS
CNIH1	NS	2.74E-01	-0.0449	4.16E-01	NS
CSF3R	NS	2.74E-01	4.49E-02	4.16E-01	NS
PLXNC1	NS	2.74E-01	-0.0449	4.16E-01	NS
COG5	NS	2.74E-01	4.49E-02	4.16E-01	NS
WASH6P	NS	2.74E-01	4.49E-02	4.16E-01	NS

PLCB4	NS	2.74E-01	-0.0449	4.16E-01	NS
C16orf89	NS	2.75E-01	4.49E-02	4.17E-01	NS
CLEC4G	NS	2.75E-01	-0.0449	4.17E-01	NS
CCNQ	NS	2.75E-01	-0.0449	4.17E-01	NS
FNBP4	NS	2.75E-01	-0.0449	4.17E-01	NS
HIRIP3	NS	2.75E-01	-0.0449	4.17E-01	NS
USP17L25	NS	2.75E-01	4.48E-02	4.17E-01	NS
C5AR2	NS	2.75E-01	-0.0448	4.17E-01	NS
STRADA	NS	2.75E-01	4.48E-02	4.17E-01	NS
ZDHHC8	NS	2.75E-01	-0.0448	4.17E-01	NS
KLC2	NS	2.75E-01	4.48E-02	4.17E-01	NS
DPYSL2	NS	2.75E-01	-0.0448	4.17E-01	NS
ATF1	NS	2.75E-01	-0.0448	4.18E-01	NS
ITGA3	NS	2.75E-01	4.48E-02	4.18E-01	NS
SMAD2	NS	2.76E-01	4.48E-02	4.18E-01	NS
SGCG	NS	2.76E-01	-0.0448	4.18E-01	NS
PCK1	NS	2.76E-01	-0.0448	4.18E-01	NS
GLIS2	NS	2.76E-01	-0.0448	4.18E-01	NS
SEC16A	NS	2.76E-01	4.48E-02	4.18E-01	NS
OR13F1	NS	2.76E-01	-0.0448	4.18E-01	NS
SETD9	NS	2.76E-01	-0.0447	4.18E-01	NS
NDRG4	NS	2.76E-01	4.47E-02	4.18E-01	NS
SEMA3G	NS	2.76E-01	-0.0447	4.18E-01	NS
UGT3A1	NS	2.76E-01	-0.0447	4.18E-01	NS
TRAIP	NS	2.76E-01	-0.0447	4.18E-01	NS
RPL27A	NS	2.77E-01	4.47E-02	4.19E-01	NS
ECSCR	NS	2.77E-01	4.47E-02	4.19E-01	NS
JUN	NS	2.77E-01	4.47E-02	4.19E-01	NS
MT-ND5	NS	2.77E-01	4.47E-02	4.19E-01	NS
TMEM268	NS	2.77E-01	-0.0446	4.20E-01	NS
IQGAP1	NS	2.77E-01	-0.0446	4.20E-01	NS
THAP3	NS	2.77E-01	4.46E-02	4.20E-01	NS
CHCHD3	NS	2.77E-01	-0.0446	4.20E-01	NS
HOMER3	NS	2.77E-01	-0.0446	4.20E-01	NS
MZF1	NS	2.77E-01	-0.0446	4.20E-01	NS
MBOAT7	NS	2.78E-01	4.46E-02	4.20E-01	NS
ATP6V1E2	NS	2.78E-01	-0.0446	4.20E-01	NS

DYNC1LI2	NS	2.78E-01	-0.0446	4.20E-01	NS
XPA	NS	2.78E-01	-0.0445	4.21E-01	NS
ZNF330	NS	2.78E-01	4.45E-02	4.21E-01	NS
PLD3	NS	2.78E-01	4.45E-02	4.21E-01	NS
MTURN	NS	2.78E-01	-0.0445	4.21E-01	NS
FCGR1B	NS	2.78E-01	-0.0445	4.21E-01	NS
SNX16	NS	2.78E-01	4.45E-02	4.21E-01	NS
PSPC1	NS	2.78E-01	4.45E-02	4.21E-01	NS
USP21	NS	2.78E-01	4.45E-02	4.21E-01	NS
HNRNPA3	NS	2.78E-01	-0.0445	4.21E-01	NS
ISG20L2	NS	2.78E-01	-0.0445	4.21E-01	NS
DIPK2A	NS	2.79E-01	-0.0445	4.21E-01	NS
SCYGR7	NS	2.79E-01	4.45E-02	4.21E-01	NS
CASZ1	NS	2.79E-01	-0.0445	4.21E-01	NS
LIN28A	NS	2.79E-01	-0.0445	4.21E-01	NS
FBXO43	NS	2.79E-01	-0.0445	4.21E-01	NS
ACRBP	NS	2.79E-01	4.45E-02	4.21E-01	NS
PEX6	NS	2.79E-01	-0.0444	4.22E-01	NS
CARD14	NS	2.79E-01	4.44E-02	4.22E-01	NS
SH3BGR	NS	2.79E-01	-0.0444	4.22E-01	NS
NUDCD3	NS	2.79E-01	-0.0444	4.22E-01	NS
KRT32	NS	2.79E-01	-0.0444	4.22E-01	NS
CTAG1B	NS	2.79E-01	-0.0444	4.22E-01	NS
JMJD1C	NS	2.79E-01	4.44E-02	4.22E-01	NS
FXYD4	NS	2.80E-01	-0.0444	4.23E-01	NS
CSH2	NS	2.80E-01	4.44E-02	4.23E-01	NS
B3GALT6	NS	2.80E-01	-0.0444	4.23E-01	NS
SERPINA11	NS	2.80E-01	-0.0444	4.23E-01	NS
EFCAB9	NS	2.80E-01	-0.0444	4.23E-01	NS
F7	NS	2.80E-01	-0.0443	4.23E-01	NS
ARPC3	NS	2.80E-01	-0.0443	4.23E-01	NS
VPS37A	NS	2.80E-01	-0.0443	4.23E-01	NS
B3GNT7	NS	2.80E-01	-0.0443	4.23E-01	NS
SHC1	NS	2.80E-01	-0.0443	4.23E-01	NS
CST1	NS	2.80E-01	4.43E-02	4.23E-01	NS
MYPOP	NS	2.81E-01	4.43E-02	4.23E-01	NS
CAPS	NS	2.81E-01	-0.0443	4.23E-01	NS

KCNK12	NS	2.81E-01	-0.0443	4.23E-01	NS
ALDH1A2	NS	2.81E-01	-0.0443	4.24E-01	NS
PSD4	NS	2.81E-01	-0.0443	4.24E-01	NS
TUBB6	NS	2.81E-01	-0.0443	4.24E-01	NS
METTL6	NS	2.81E-01	-0.0443	4.24E-01	NS
DBR1	NS	2.81E-01	4.42E-02	4.24E-01	NS
CEMP1	NS	2.81E-01	4.42E-02	4.24E-01	NS
CDK9	NS	2.81E-01	-0.0442	4.24E-01	NS
GMDS	NS	2.82E-01	-0.0442	4.25E-01	NS
ATF2	NS	2.82E-01	4.42E-02	4.25E-01	NS
COX15	NS	2.82E-01	-0.0442	4.25E-01	NS
CEP104	NS	2.82E-01	-0.0442	4.25E-01	NS
FBXL15	NS	2.82E-01	-0.0442	4.25E-01	NS
TESMIN	NS	2.82E-01	-0.0441	4.25E-01	NS
FCGRT	NS	2.82E-01	-0.0441	4.26E-01	NS
EHD2	NS	2.82E-01	-0.0441	4.26E-01	NS
CPZ	NS	2.82E-01	4.41E-02	4.26E-01	NS
DLG5	NS	2.83E-01	4.41E-02	4.26E-01	NS
PHKG2	NS	2.83E-01	4.41E-02	4.26E-01	NS
GNL2	NS	2.83E-01	-0.0441	4.26E-01	NS
ZCCHC2	NS	2.83E-01	-0.0441	4.26E-01	NS
STIL	NS	2.83E-01	-0.0441	4.26E-01	NS
CDY1	NS	2.83E-01	4.41E-02	4.26E-01	NS
EFEMP2	NS	2.83E-01	4.41E-02	4.26E-01	NS
LDLRAD3	NS	2.83E-01	-0.0441	4.26E-01	NS
DMD	NS	2.83E-01	-0.0441	4.26E-01	NS
MGLL	NS	2.83E-01	4.41E-02	4.26E-01	NS
SLC47A2	NS	2.83E-01	4.41E-02	4.26E-01	NS
RAB3D	NS	2.83E-01	-0.0441	4.26E-01	NS
ABHD11	NS	2.83E-01	4.41E-02	4.26E-01	NS
CLEC2B	NS	2.83E-01	-0.0441	4.26E-01	NS
C4orf36	NS	2.83E-01	4.41E-02	4.26E-01	NS
UTP15	NS	2.83E-01	4.41E-02	4.26E-01	NS
FBP1	NS	2.83E-01	4.40E-02	4.27E-01	NS
AGPAT1	NS	2.84E-01	-0.044	4.27E-01	NS
ZNF552	NS	2.84E-01	4.40E-02	4.27E-01	NS
SEC22B	NS	2.84E-01	-0.044	4.27E-01	NS

PTRHD1	NS	2.84E-01	4.40E-02	4.27E-01	NS
FAM98C	NS	2.84E-01	-0.044	4.27E-01	NS
HTR3B	NS	2.84E-01	-0.044	4.27E-01	NS
ZNF765	NS	2.84E-01	-0.044	4.27E-01	NS
SMIM10	NS	2.84E-01	-0.044	4.27E-01	NS
NXPH3	NS	2.84E-01	-0.044	4.27E-01	NS
LRP5	NS	2.84E-01	-0.044	4.28E-01	NS
UPK2	NS	2.85E-01	-0.0439	4.28E-01	NS
TECRL	NS	2.85E-01	-0.0439	4.28E-01	NS
GAREM2	NS	2.85E-01	-0.0439	4.28E-01	NS
SLC35G2	NS	2.85E-01	-0.0439	4.28E-01	NS
FAM228A	NS	2.85E-01	-0.0439	4.28E-01	NS
ATP5IF1	NS	2.85E-01	4.39E-02	4.28E-01	NS
ZNF414	NS	2.85E-01	4.39E-02	4.28E-01	NS
PCDHAC2	NS	2.85E-01	4.39E-02	4.28E-01	NS
CDV3	NS	2.85E-01	-0.0439	4.28E-01	NS
NRXN3	NS	2.85E-01	-0.0439	4.29E-01	NS
CAMTA2	NS	2.85E-01	4.39E-02	4.29E-01	NS
PRSS55	NS	2.86E-01	4.39E-02	4.29E-01	NS
TMEM179B	NS	2.86E-01	-0.0438	4.29E-01	NS
ANKRD65	NS	2.86E-01	4.38E-02	4.29E-01	NS
CFL2	NS	2.86E-01	-0.0438	4.29E-01	NS
DUSP29	NS	2.86E-01	4.38E-02	4.29E-01	NS
ABLIM1	NS	2.86E-01	-0.0438	4.29E-01	NS
SPINT4	NS	2.86E-01	4.38E-02	4.29E-01	NS
BSX	NS	2.86E-01	4.38E-02	4.29E-01	NS
THNSL2	NS	2.86E-01	4.38E-02	4.29E-01	NS
SF3A3	NS	2.86E-01	4.38E-02	4.29E-01	NS
RASGRP4	NS	2.86E-01	-0.0438	4.29E-01	NS
ZNF286A-TBC1D26	NS	2.86E-01	-0.0438	4.30E-01	NS
BLCAP	NS	2.86E-01	4.38E-02	4.30E-01	NS
SCPEP1	NS	2.86E-01	-0.0438	4.30E-01	NS
SNCB	NS	2.87E-01	4.38E-02	4.30E-01	NS
NOG	NS	2.87E-01	-0.0437	4.30E-01	NS
CIR1	NS	2.87E-01	-0.0437	4.30E-01	NS
HP	NS	2.87E-01	-0.0437	4.30E-01	NS
NAB2	NS	2.87E-01	-0.0437	4.30E-01	NS

REEP6	NS	2.87E-01	4.37E-02	4.31E-01	NS
IST1	NS	2.87E-01	4.37E-02	4.31E-01	NS
TMEM255B	NS	2.87E-01	-0.0437	4.31E-01	NS
ATXN7L3	NS	2.88E-01	-0.0437	4.31E-01	NS
MYOZ3	NS	2.88E-01	-0.0437	4.31E-01	NS
ZNF219	NS	2.88E-01	-0.0436	4.32E-01	NS
CCT2	NS	2.88E-01	4.36E-02	4.32E-01	NS
RGPD2	NS	2.88E-01	4.36E-02	4.32E-01	NS
SYTL3	NS	2.88E-01	-0.0436	4.32E-01	NS
LTB4R2	NS	2.88E-01	4.36E-02	4.32E-01	NS
PSEN1	NS	2.88E-01	-0.0436	4.32E-01	NS
SIPA1	NS	2.89E-01	4.36E-02	4.32E-01	NS
NUF2	NS	2.89E-01	-0.0436	4.32E-01	NS
PPDPFL	NS	2.89E-01	-0.0436	4.32E-01	NS
RPL31	NS	2.89E-01	-0.0436	4.32E-01	NS
KIAA2026	NS	2.89E-01	-0.0436	4.32E-01	NS
LCE1F	NS	2.89E-01	4.36E-02	4.32E-01	NS
CD1D	NS	2.89E-01	-0.0436	4.33E-01	NS
S100Z	NS	2.89E-01	-0.0435	4.33E-01	NS
CUX1	NS	2.89E-01	-0.0435	4.33E-01	NS
AGO3	NS	2.89E-01	-0.0435	4.33E-01	NS
KCTD6	NS	2.89E-01	-0.0435	4.33E-01	NS
CST6	NS	2.89E-01	-0.0435	4.33E-01	NS
HTRA2	NS	2.90E-01	-0.0435	4.33E-01	NS
CCDC59	NS	2.90E-01	4.35E-02	4.33E-01	NS
REX1BD	NS	2.90E-01	-0.0435	4.33E-01	NS
DCXR	NS	2.90E-01	-0.0435	4.33E-01	NS
SNCG	NS	2.90E-01	4.35E-02	4.34E-01	NS
AVP	NS	2.90E-01	4.35E-02	4.34E-01	NS
CD180	NS	2.90E-01	-0.0435	4.34E-01	NS
VTCN1	NS	2.90E-01	-0.0435	4.34E-01	NS
KBTBD2	NS	2.90E-01	4.34E-02	4.34E-01	NS
TOMM70	NS	2.90E-01	-0.0434	4.34E-01	NS
SH2D3A	NS	2.90E-01	4.34E-02	4.34E-01	NS
PADI3	NS	2.90E-01	-0.0434	4.34E-01	NS
GLB1	NS	2.90E-01	-0.0434	4.34E-01	NS
PROCA1	NS	2.90E-01	4.34E-02	4.34E-01	NS

NLRP11	NS	2.90E-01	-0.0434	4.34E-01	NS
CSH1	NS	2.90E-01	4.34E-02	4.34E-01	NS
METAP1	NS	2.90E-01	-0.0434	4.34E-01	NS
TRIM49D1	NS	2.90E-01	-0.0434	4.34E-01	NS
STMP1	NS	2.90E-01	-0.0434	4.34E-01	NS
NYX	NS	2.91E-01	4.34E-02	4.34E-01	NS
SLC35B1	NS	2.91E-01	4.34E-02	4.34E-01	NS
SLC12A7	NS	2.91E-01	-0.0433	4.35E-01	NS
TAS2R43	NS	2.91E-01	-0.0433	4.35E-01	NS
MTF1	NS	2.91E-01	-0.0433	4.35E-01	NS
AGAP1	NS	2.91E-01	-0.0433	4.35E-01	NS
B3GALNT1	NS	2.92E-01	-0.0433	4.36E-01	NS
CD300A	NS	2.92E-01	-0.0433	4.36E-01	NS
ZNF84	NS	2.92E-01	-0.0433	4.36E-01	NS
ACYP2	NS	2.92E-01	-0.0433	4.36E-01	NS
KIAA0319L	NS	2.92E-01	-0.0433	4.36E-01	NS
TUBA1A	NS	2.92E-01	-0.0433	4.36E-01	NS
CNGA4	NS	2.92E-01	4.33E-02	4.36E-01	NS
HCRT	NS	2.92E-01	4.33E-02	4.36E-01	NS
OR4A15	NS	2.92E-01	-0.0433	4.36E-01	NS
SEMA3F	NS	2.92E-01	-0.0433	4.36E-01	NS
TMEM43	NS	2.92E-01	4.32E-02	4.36E-01	NS
LRRC19	NS	2.92E-01	4.32E-02	4.36E-01	NS
EEF1E1-BLOC1S5	NS	2.92E-01	-0.0432	4.36E-01	NS
ARPC4-TTLL3	NS	2.92E-01	-0.0432	4.36E-01	NS
NACA2	NS	2.93E-01	-0.0432	4.37E-01	NS
IL18	NS	2.93E-01	4.32E-02	4.37E-01	NS
SPA17	NS	2.93E-01	4.32E-02	4.37E-01	NS
ALS2CL	NS	2.93E-01	-0.0432	4.37E-01	NS
H1-0	NS	2.93E-01	-0.0432	4.37E-01	NS
OR2T8	NS	2.93E-01	-0.0432	4.37E-01	NS
DHRS7C	NS	2.93E-01	-0.0432	4.37E-01	NS
KLHL3	NS	2.93E-01	4.32E-02	4.37E-01	NS
TNNT1	NS	2.93E-01	-0.0432	4.37E-01	NS
EOLA1	NS	2.93E-01	-0.0432	4.37E-01	NS
PPIL3	NS	2.93E-01	4.32E-02	4.37E-01	NS
RPS19BP1	NS	2.93E-01	-0.0432	4.37E-01	NS

ARPC4	NS	2.93E-01	-0.0432	4.37E-01	NS
ATP8A1	NS	2.93E-01	-0.0432	4.37E-01	NS
PAK3	NS	2.93E-01	-0.0432	4.37E-01	NS
OTUD4	NS	2.93E-01	-0.0432	4.37E-01	NS
KCNJ4	NS	2.93E-01	4.31E-02	4.37E-01	NS
NR5A2	NS	2.93E-01	-0.0431	4.37E-01	NS
CSRP2	NS	2.93E-01	-0.0431	4.37E-01	NS
ERMAP	NS	2.94E-01	-0.0431	4.38E-01	NS
OR51S1	NS	2.94E-01	-0.0431	4.38E-01	NS
OR51L1	NS	2.94E-01	-0.0431	4.38E-01	NS
PLA2G5	NS	2.94E-01	-0.0431	4.38E-01	NS
WBP1L	NS	2.94E-01	-0.0431	4.38E-01	NS
SPDYE18	NS	2.94E-01	4.31E-02	4.38E-01	NS
CCNT2	NS	2.94E-01	-0.0431	4.38E-01	NS
FRMPD3	NS	2.94E-01	-0.0431	4.38E-01	NS
ARSA	NS	2.94E-01	4.31E-02	4.38E-01	NS
PER3	NS	2.94E-01	-0.0431	4.38E-01	NS
ARHGAP9	NS	2.95E-01	4.30E-02	4.39E-01	NS
SYTL2	NS	2.95E-01	-0.043	4.39E-01	NS
FAM83A	NS	2.95E-01	4.30E-02	4.39E-01	NS
DYNLL1	NS	2.95E-01	-0.043	4.39E-01	NS
EBF1	NS	2.95E-01	-0.043	4.39E-01	NS
KIAA0513	NS	2.95E-01	-0.043	4.39E-01	NS
UQCR11	NS	2.95E-01	4.30E-02	4.39E-01	NS
ARC	NS	2.95E-01	4.30E-02	4.39E-01	NS
PRSS1	NS	2.95E-01	4.30E-02	4.39E-01	NS
RNF187	NS	2.95E-01	-0.043	4.39E-01	NS
BCORL1	NS	2.95E-01	4.30E-02	4.39E-01	NS
TASL	NS	2.95E-01	-0.043	4.39E-01	NS
SYNE3	NS	2.95E-01	-0.043	4.39E-01	NS
C1QTNF3	NS	2.95E-01	-0.043	4.39E-01	NS
PCDHA4	NS	2.95E-01	-0.043	4.39E-01	NS
DEGS1	NS	2.95E-01	-0.043	4.39E-01	NS
SETD3	NS	2.96E-01	-0.043	4.39E-01	NS
MSANTD1	NS	2.96E-01	-0.0429	4.40E-01	NS
FDXACB1	NS	2.96E-01	-0.0429	4.40E-01	NS
GUCY2C	NS	2.96E-01	-0.0429	4.40E-01	NS

RTP5	NS	2.96E-01	-0.0429	4.40E-01	NS
RPP38	NS	2.96E-01	-0.0429	4.40E-01	NS
EIF2B4	NS	2.96E-01	4.29E-02	4.40E-01	NS
SPDYA	NS	2.96E-01	-0.0429	4.40E-01	NS
FUT10	NS	2.96E-01	-0.0429	4.40E-01	NS
FBXO30	NS	2.96E-01	-0.0429	4.40E-01	NS
AKR1C2	NS	2.96E-01	-0.0429	4.40E-01	NS
MYO1E	NS	2.96E-01	-0.0429	4.40E-01	NS
VASN	NS	2.96E-01	4.29E-02	4.40E-01	NS
NDUFB10	NS	2.96E-01	-0.0429	4.40E-01	NS
VIRMA	NS	2.96E-01	-0.0429	4.40E-01	NS
TMEM242	NS	2.96E-01	-0.0429	4.40E-01	NS
TMCO4	NS	2.97E-01	-0.0429	4.40E-01	NS
HSPA14	NS	2.97E-01	-0.0429	4.40E-01	NS
RNF41	NS	2.97E-01	-0.0429	4.40E-01	NS
PGA4	NS	2.97E-01	-0.0429	4.40E-01	NS
TMBIM1	NS	2.97E-01	4.28E-02	4.41E-01	NS
TTLL11	NS	2.97E-01	4.28E-02	4.41E-01	NS
KCNMB3	NS	2.97E-01	-0.0428	4.41E-01	NS
VWA7	NS	2.97E-01	4.28E-02	4.41E-01	NS
PRAMEF14	NS	2.97E-01	-0.0428	4.41E-01	NS
ZNF518A	NS	2.98E-01	-0.0428	4.41E-01	NS
KIF1C	NS	2.98E-01	4.28E-02	4.42E-01	NS
RIPPLY1	NS	2.98E-01	-0.0428	4.42E-01	NS
CTDSP2	NS	2.98E-01	4.27E-02	4.42E-01	NS
PGA3	NS	2.98E-01	-0.0427	4.42E-01	NS
SCAPER	NS	2.98E-01	4.27E-02	4.42E-01	NS
GLS2	NS	2.98E-01	4.27E-02	4.42E-01	NS
AMELY	NS	2.98E-01	-0.0427	4.42E-01	NS
LYZ	NS	2.98E-01	-0.0427	4.42E-01	NS
PRDX6	NS	2.98E-01	-0.0427	4.43E-01	NS
CHST4	NS	2.99E-01	4.27E-02	4.43E-01	NS
OTX1	NS	2.99E-01	4.27E-02	4.43E-01	NS
PRR7	NS	2.99E-01	4.27E-02	4.43E-01	NS
ZNF335	NS	2.99E-01	4.27E-02	4.43E-01	NS
FAM98A	NS	2.99E-01	-0.0427	4.43E-01	NS
UBAP1	NS	2.99E-01	-0.0427	4.43E-01	NS

GEMIN8	NS	2.99E-01	-0.0426	4.43E-01	NS
RIOX1	NS	2.99E-01	-0.0427	4.43E-01	NS
TBC1D3E	NS	2.99E-01	-0.0426	4.43E-01	NS
ELAC2	NS	2.99E-01	4.26E-02	4.43E-01	NS
CTNNA3	NS	2.99E-01	-0.0426	4.43E-01	NS
DUT	NS	2.99E-01	-0.0426	4.43E-01	NS
UQCR10	NS	2.99E-01	4.26E-02	4.43E-01	NS
EIF4A3	NS	2.99E-01	-0.0426	4.43E-01	NS
AP5B1	NS	2.99E-01	4.26E-02	4.43E-01	NS
CCNK	NS	2.99E-01	4.26E-02	4.43E-01	NS
SLC43A3	NS	2.99E-01	-0.0426	4.43E-01	NS
APBA2	NS	3.00E-01	-0.0426	4.44E-01	NS
PIK3CD	NS	3.00E-01	4.26E-02	4.44E-01	NS
AP2A1	NS	3.00E-01	-0.0426	4.44E-01	NS
METTL7B	NS	3.00E-01	-0.0426	4.44E-01	NS
GPLD1	NS	3.00E-01	4.26E-02	4.44E-01	NS
MRE11	NS	3.00E-01	4.26E-02	4.44E-01	NS
COX18	NS	3.00E-01	-0.0426	4.44E-01	NS
CSRNP1	NS	3.00E-01	4.25E-02	4.44E-01	NS
FST	NS	3.00E-01	4.25E-02	4.45E-01	NS
AGTRAP	NS	3.01E-01	4.25E-02	4.45E-01	NS
DHX29	NS	3.01E-01	4.25E-02	4.45E-01	NS
TAF11L5	NS	3.01E-01	4.25E-02	4.45E-01	NS
MAPK6	NS	3.01E-01	-0.0425	4.45E-01	NS
SELENOT	NS	3.01E-01	-0.0425	4.45E-01	NS
CAPN9	NS	3.01E-01	-0.0425	4.45E-01	NS
ZNF727	NS	3.01E-01	4.25E-02	4.45E-01	NS
NIT2	NS	3.01E-01	4.25E-02	4.45E-01	NS
ARID4B	NS	3.01E-01	4.25E-02	4.45E-01	NS
PPT2-EGFL8	NS	3.01E-01	-0.0424	4.45E-01	NS
GLG1	NS	3.01E-01	-0.0424	4.46E-01	NS
OCEL1	NS	3.01E-01	-0.0424	4.46E-01	NS
TBR1	NS	3.01E-01	-0.0424	4.46E-01	NS
MTA2	NS	3.01E-01	-0.0424	4.46E-01	NS
BRAF	NS	3.01E-01	-0.0424	4.46E-01	NS
LONRF3	NS	3.01E-01	4.24E-02	4.46E-01	NS
BPIFB1	NS	3.02E-01	4.24E-02	4.46E-01	NS

GPR15	NS	3.02E-01	-0.0424	4.46E-01	NS
APOC4	NS	3.02E-01	4.24E-02	4.46E-01	NS
ZNF415	NS	3.02E-01	-0.0424	4.46E-01	NS
UBE2G1	NS	3.02E-01	4.24E-02	4.47E-01	NS
FAM151B	NS	3.03E-01	-0.0423	4.47E-01	NS
RBP7	NS	3.02E-01	-0.0423	4.47E-01	NS
CTXN2	NS	3.03E-01	-0.0423	4.47E-01	NS
PLEKHA6	NS	3.03E-01	-0.0423	4.47E-01	NS
TMEM200C	NS	3.03E-01	4.23E-02	4.47E-01	NS
RIMBP3B	NS	3.03E-01	4.23E-02	4.47E-01	NS
RPH3A	NS	3.03E-01	-0.0423	4.47E-01	NS
PCK2	NS	3.03E-01	-0.0423	4.47E-01	NS
TRIM65	NS	3.03E-01	-0.0423	4.48E-01	NS
PRX	NS	3.04E-01	-0.0422	4.48E-01	NS
CNDP2	NS	3.04E-01	-0.0422	4.48E-01	NS
PPP1R13L	NS	3.04E-01	4.22E-02	4.48E-01	NS
XRCC1	NS	3.04E-01	-0.0422	4.48E-01	NS
TP53I11	NS	3.04E-01	-0.0422	4.48E-01	NS
RPL26L1	NS	3.04E-01	-0.0422	4.49E-01	NS
RAB14	NS	3.04E-01	4.22E-02	4.49E-01	NS
VMAC	NS	3.04E-01	-0.0422	4.49E-01	NS
NPY5R	NS	3.04E-01	4.22E-02	4.49E-01	NS
GALR1	NS	3.04E-01	4.22E-02	4.49E-01	NS
CT47A5	NS	3.04E-01	4.22E-02	4.49E-01	NS
CTDNEP1	NS	3.04E-01	-0.0422	4.49E-01	NS
ITLN2	NS	3.05E-01	4.22E-02	4.49E-01	NS
ANKRD63	NS	3.05E-01	4.21E-02	4.49E-01	NS
FABP1	NS	3.05E-01	4.21E-02	4.49E-01	NS
CXCL9	NS	3.05E-01	4.21E-02	4.49E-01	NS
ANKH	NS	3.05E-01	-0.0421	4.50E-01	NS
GABRB3	NS	3.05E-01	-0.0421	4.50E-01	NS
AGAP2	NS	3.05E-01	4.21E-02	4.50E-01	NS
BLOC1S6	NS	3.05E-01	4.21E-02	4.50E-01	NS
CDH1	NS	3.05E-01	4.21E-02	4.50E-01	NS
AP3S1	NS	3.05E-01	-0.0421	4.50E-01	NS
CEACAM19	NS	3.05E-01	-0.0421	4.50E-01	NS
SRSF3	NS	3.05E-01	4.21E-02	4.50E-01	NS

UBE2D1	NS	3.05E-01	4.21E-02	4.50E-01	NS
SLC12A9	NS	3.06E-01	-0.0421	4.50E-01	NS
KIF18A	NS	3.06E-01	-0.0421	4.50E-01	NS
CHRDL2	NS	3.06E-01	-0.042	4.51E-01	NS
HDAC9	NS	3.06E-01	-0.042	4.51E-01	NS
TRIM49C	NS	3.06E-01	4.20E-02	4.51E-01	NS
ZNF689	NS	3.06E-01	4.20E-02	4.51E-01	NS
SLC10A3	NS	3.06E-01	4.20E-02	4.51E-01	NS
SIRT1	NS	3.07E-01	-0.042	4.51E-01	NS
RALGPS1	NS	3.07E-01	4.20E-02	4.51E-01	NS
NUDT7	NS	3.07E-01	-0.042	4.51E-01	NS
MYCBP	NS	3.07E-01	-0.042	4.51E-01	NS
TTC7B	NS	3.07E-01	-0.042	4.52E-01	NS
SCNN1G	NS	3.07E-01	4.20E-02	4.52E-01	NS
NIPA1	NS	3.07E-01	-0.042	4.52E-01	NS
RCSD1	NS	3.07E-01	4.20E-02	4.52E-01	NS
CRTC1	NS	3.07E-01	4.19E-02	4.52E-01	NS
TMEM87B	NS	3.07E-01	-0.0419	4.52E-01	NS
ELP3	NS	3.08E-01	-0.0419	4.52E-01	NS
DPH6	NS	3.08E-01	4.19E-02	4.53E-01	NS
AGBL1	NS	3.08E-01	-0.0419	4.53E-01	NS
TARBP1	NS	3.08E-01	4.19E-02	4.53E-01	NS
UPK1A	NS	3.08E-01	4.19E-02	4.53E-01	NS
ULK4	NS	3.08E-01	4.19E-02	4.53E-01	NS
CTXND2	NS	3.08E-01	-0.0418	4.53E-01	NS
WIPF1	NS	3.08E-01	-0.0418	4.53E-01	NS
TPI1	NS	3.08E-01	-0.0418	4.54E-01	NS
CENPVL1	NS	3.09E-01	4.18E-02	4.54E-01	NS
SLC13A5	NS	3.09E-01	-0.0418	4.54E-01	NS
PIK3AP1	NS	3.09E-01	-0.0418	4.54E-01	NS
LY6G5C	NS	3.09E-01	-0.0418	4.54E-01	NS
KLF11	NS	3.09E-01	4.18E-02	4.54E-01	NS
FECH	NS	3.09E-01	-0.0418	4.54E-01	NS
DIP2B	NS	3.09E-01	4.17E-02	4.55E-01	NS
SKP1	NS	3.09E-01	-0.0417	4.55E-01	NS
ZNF571	NS	3.10E-01	-0.0417	4.55E-01	NS
QSER1	NS	3.10E-01	4.17E-02	4.55E-01	NS

PIM3	NS	3.10E-01	4.17E-02	4.55E-01	NS
BID	NS	3.10E-01	-0.0417	4.55E-01	NS
INPP1	NS	3.10E-01	-0.0417	4.55E-01	NS
CD44	NS	3.10E-01	-0.0417	4.56E-01	NS
MCM8	NS	3.10E-01	-0.0417	4.56E-01	NS
CD80	NS	3.10E-01	-0.0417	4.56E-01	NS
SNX21	NS	3.10E-01	4.17E-02	4.56E-01	NS
LENG9	NS	3.10E-01	-0.0417	4.56E-01	NS
FLRT1	NS	3.11E-01	-0.0416	4.56E-01	NS
CACNA2D2	NS	3.11E-01	-0.0416	4.56E-01	NS
TPR	NS	3.11E-01	4.16E-02	4.56E-01	NS
IFNAR2	NS	3.11E-01	-0.0416	4.56E-01	NS
GASK1B	NS	3.11E-01	4.16E-02	4.56E-01	NS
ZKSCAN5	NS	3.11E-01	-0.0416	4.56E-01	NS
HMOX2	NS	3.11E-01	-0.0416	4.57E-01	NS
GPR108	NS	3.11E-01	-0.0416	4.57E-01	NS
THEMIS2	NS	3.11E-01	-0.0416	4.57E-01	NS
GATD3	NS	3.11E-01	-0.0416	4.57E-01	NS
LYZL1	NS	3.11E-01	-0.0416	4.57E-01	NS
STT3B	NS	3.11E-01	-0.0416	4.57E-01	NS
CLDN9	NS	3.12E-01	-0.0416	4.57E-01	NS
CHRDL1	NS	3.12E-01	4.15E-02	4.57E-01	NS
CHRNA6	NS	3.12E-01	4.15E-02	4.57E-01	NS
ZNF627	NS	3.12E-01	-0.0415	4.57E-01	NS
P2RX7	NS	3.12E-01	-0.0415	4.57E-01	NS
ORC3	NS	3.12E-01	-0.0415	4.57E-01	NS
SSC5D	NS	3.12E-01	4.15E-02	4.57E-01	NS
S100A13	NS	3.12E-01	-0.0415	4.57E-01	NS
ACSM3	NS	3.12E-01	-0.0415	4.57E-01	NS
MATR3	NS	3.12E-01	-0.0415	4.57E-01	NS
MS4A14	NS	3.12E-01	-0.0415	4.57E-01	NS
FMO4	NS	3.12E-01	-0.0415	4.57E-01	NS
SETD6	NS	3.12E-01	-0.0415	4.58E-01	NS
DDX56	NS	3.13E-01	-0.0415	4.58E-01	NS
SLC16A9	NS	3.13E-01	-0.0415	4.58E-01	NS
PSMD8	NS	3.13E-01	4.15E-02	4.58E-01	NS
NAA40	NS	3.13E-01	-0.0415	4.58E-01	NS

RDH11	NS	3.13E-01	-0.0414	4.58E-01	NS
INTS12	NS	3.13E-01	4.14E-02	4.58E-01	NS
IL17RE	NS	3.13E-01	-0.0414	4.59E-01	NS
KDM1B	NS	3.13E-01	-0.0414	4.59E-01	NS
DPF3	NS	3.13E-01	4.14E-02	4.59E-01	NS
GPR157	NS	3.13E-01	-0.0414	4.59E-01	NS
NPIPA9	NS	3.13E-01	4.14E-02	4.59E-01	NS
H2AC19	NS	3.13E-01	-0.0414	4.59E-01	NS
TBCC	NS	3.14E-01	4.14E-02	4.59E-01	NS
PDHA2	NS	3.14E-01	-0.0414	4.59E-01	NS
CXorf38	NS	3.14E-01	4.14E-02	4.59E-01	NS
HEY1	NS	3.14E-01	4.13E-02	4.60E-01	NS
OR2A14	NS	3.14E-01	4.13E-02	4.60E-01	NS
TMEM106B	NS	3.15E-01	-0.0413	4.61E-01	NS
AP1AR	NS	3.15E-01	-0.0413	4.61E-01	NS
ULBP2	NS	3.15E-01	4.13E-02	4.61E-01	NS
HTATSF1	NS	3.15E-01	-0.0413	4.61E-01	NS
DHDH	NS	3.15E-01	-0.0413	4.61E-01	NS
DEFB129	NS	3.15E-01	-0.0413	4.61E-01	NS
MRPL57	NS	3.15E-01	-0.0413	4.61E-01	NS
SPAST	NS	3.15E-01	-0.0413	4.61E-01	NS
ISL2	NS	3.15E-01	-0.0412	4.61E-01	NS
NYAP1	NS	3.15E-01	4.12E-02	4.61E-01	NS
NCOA1	NS	3.16E-01	-0.0412	4.61E-01	NS
RAB6A	NS	3.16E-01	4.12E-02	4.61E-01	NS
OR6P1	NS	3.16E-01	-0.0412	4.61E-01	NS
ACTR1B	NS	3.16E-01	-0.0412	4.62E-01	NS
KRT2	NS	3.16E-01	4.12E-02	4.62E-01	NS
DRD4	NS	3.16E-01	4.12E-02	4.62E-01	NS
CST9L	NS	3.16E-01	-0.0412	4.62E-01	NS
LARP6	NS	3.16E-01	4.12E-02	4.62E-01	NS
EZH1	NS	3.16E-01	-0.0412	4.62E-01	NS
GPR4	NS	3.16E-01	4.12E-02	4.62E-01	NS
STAT3	NS	3.16E-01	-0.0411	4.62E-01	NS
PRDM9	NS	3.16E-01	4.11E-02	4.62E-01	NS
RPL10	NS	3.17E-01	-0.0411	4.62E-01	NS
DENND4A	NS	3.17E-01	-0.0411	4.62E-01	NS

CPNE8	NS	3.17E-01	-0.0411	4.63E-01	NS
SMAD3	NS	3.17E-01	-0.0411	4.63E-01	NS
RSKR	NS	3.17E-01	-0.0411	4.63E-01	NS
ZNFX1	NS	3.17E-01	-0.0411	4.63E-01	NS
PTX3	NS	3.17E-01	4.11E-02	4.63E-01	NS
HPGD	NS	3.17E-01	-0.0411	4.63E-01	NS
FAM98B	NS	3.17E-01	-0.0411	4.63E-01	NS
C2orf80	NS	3.17E-01	-0.0411	4.63E-01	NS
SYDE2	NS	3.17E-01	-0.0411	4.63E-01	NS
NFE2L3	NS	3.18E-01	4.10E-02	4.64E-01	NS
CLEC5A	NS	3.18E-01	-0.041	4.64E-01	NS
PTK6	NS	3.18E-01	-0.041	4.64E-01	NS
CLDN34	NS	3.18E-01	4.10E-02	4.64E-01	NS
MAP3K14	NS	3.18E-01	4.10E-02	4.64E-01	NS
COX6B1	NS	3.18E-01	4.10E-02	4.64E-01	NS
PPP2R3C	NS	3.18E-01	-0.041	4.64E-01	NS
ANXA13	NS	3.18E-01	4.10E-02	4.64E-01	NS
ARHGAP17	NS	3.18E-01	4.10E-02	4.64E-01	NS
TTLL13P	NS	3.18E-01	-0.041	4.64E-01	NS
CHI3L2	NS	3.18E-01	-0.041	4.64E-01	NS
SCGB1D1	NS	3.18E-01	4.10E-02	4.64E-01	NS
IL13RA2	NS	3.19E-01	-0.041	4.65E-01	NS
VWCE	NS	3.19E-01	-0.0409	4.65E-01	NS
RNF26	NS	3.19E-01	-0.0409	4.65E-01	NS
PCBP3	NS	3.19E-01	-0.0409	4.65E-01	NS
PITPNB	NS	3.19E-01	-0.0409	4.65E-01	NS
YIPF4	NS	3.19E-01	-0.0409	4.65E-01	NS
FABP7	NS	3.19E-01	-0.0409	4.65E-01	NS
GJB6	NS	3.19E-01	-0.0409	4.66E-01	NS
OR52E8	NS	3.20E-01	-0.0409	4.66E-01	NS
MARCHF1	NS	3.20E-01	-0.0409	4.66E-01	NS
RBM7	NS	3.20E-01	-0.0409	4.66E-01	NS
CHAMP1	NS	3.20E-01	4.09E-02	4.66E-01	NS
SLC18A1	NS	3.20E-01	-0.0409	4.66E-01	NS
FBXO8	NS	3.20E-01	-0.0408	4.66E-01	NS
ACTG1	NS	3.20E-01	-0.0408	4.66E-01	NS
RNASEH2C	NS	3.20E-01	4.08E-02	4.66E-01	NS

SLC29A4	NS	3.20E-01	-0.0408	4.66E-01	NS
MC5R	NS	3.20E-01	-0.0408	4.66E-01	NS
GGCX	NS	3.21E-01	-0.0408	4.67E-01	NS
SGMS1	NS	3.21E-01	4.08E-02	4.67E-01	NS
C1QTNF9B	NS	3.21E-01	-0.0408	4.67E-01	NS
TEX14	NS	3.21E-01	-0.0408	4.67E-01	NS
SAR1B	NS	3.21E-01	4.07E-02	4.67E-01	NS
CFB	NS	3.21E-01	-0.0407	4.68E-01	NS
UHRF1BP1	NS	3.21E-01	4.07E-02	4.68E-01	NS
RPS15A	NS	3.21E-01	-0.0407	4.68E-01	NS
METTL15	NS	3.21E-01	4.07E-02	4.68E-01	NS
SCRIB	NS	3.21E-01	-0.0407	4.68E-01	NS
KCND2	NS	3.21E-01	4.07E-02	4.68E-01	NS
MBNL2	NS	3.21E-01	-0.0407	4.68E-01	NS
TMC8	NS	3.21E-01	4.07E-02	4.68E-01	NS
HSFX2	NS	3.22E-01	-0.0407	4.68E-01	NS
CHKA	NS	3.22E-01	4.07E-02	4.68E-01	NS
SLC39A8	NS	3.22E-01	-0.0407	4.68E-01	NS
PAQR7	NS	3.22E-01	-0.0407	4.69E-01	NS
PTGES3L-AARSD1	NS	3.22E-01	-0.0407	4.69E-01	NS
SHISA5	NS	3.22E-01	-0.0407	4.69E-01	NS
RAB4B-EGLN2	NS	3.22E-01	-0.0406	4.69E-01	NS
PNPLA7	NS	3.22E-01	-0.0406	4.69E-01	NS
SARNP	NS	3.23E-01	-0.0406	4.69E-01	NS
MRPS24	NS	3.23E-01	-0.0406	4.69E-01	NS
EIF4EBP3	NS	3.23E-01	-0.0406	4.69E-01	NS
ROM1	NS	3.23E-01	4.06E-02	4.69E-01	NS
RNF111	NS	3.23E-01	-0.0406	4.69E-01	NS
C3orf70	NS	3.23E-01	-0.0406	4.69E-01	NS
WDR86	NS	3.23E-01	-0.0406	4.69E-01	NS
SPAG16	NS	3.23E-01	4.06E-02	4.69E-01	NS
CITED1	NS	3.23E-01	-0.0406	4.69E-01	NS
PPP1R3D	NS	3.23E-01	-0.0406	4.69E-01	NS
URGCP-MRPS24	NS	3.23E-01	-0.0406	4.69E-01	NS
IGFBP5	NS	3.23E-01	-0.0406	4.69E-01	NS
FOXL2NB	NS	3.23E-01	-0.0406	4.69E-01	NS
TMEM25	NS	3.23E-01	-0.0406	4.69E-01	NS

DMGDH	NS	3.23E-01	-0.0406	4.70E-01	NS
CORO2A	NS	3.23E-01	4.06E-02	4.70E-01	NS
AQP6	NS	3.23E-01	4.06E-02	4.70E-01	NS
SRSF5	NS	3.23E-01	-0.0406	4.70E-01	NS
TEX44	NS	3.24E-01	4.05E-02	4.70E-01	NS
IRAG2	NS	3.24E-01	4.05E-02	4.70E-01	NS
ELOF1	NS	3.24E-01	-0.0405	4.70E-01	NS
YARS1	NS	3.24E-01	4.05E-02	4.70E-01	NS
CDCP2	NS	3.24E-01	-0.0405	4.70E-01	NS
TRIM66	NS	3.24E-01	-0.0405	4.70E-01	NS
WDR87	NS	3.24E-01	-0.0405	4.70E-01	NS
USP17L1	NS	3.24E-01	-0.0405	4.71E-01	NS
AEBP1	NS	3.24E-01	-0.0405	4.71E-01	NS
U2AF1	NS	3.24E-01	-0.0405	4.71E-01	NS
GAS7	NS	3.25E-01	-0.0405	4.71E-01	NS
ZIC5	NS	3.25E-01	4.04E-02	4.71E-01	NS
PKLR	NS	3.25E-01	-0.0404	4.71E-01	NS
PPIH	NS	3.25E-01	-0.0404	4.71E-01	NS
TIMP3	NS	3.25E-01	-0.0404	4.71E-01	NS
RPL18A	NS	3.25E-01	-0.0404	4.71E-01	NS
ZMIZ1	NS	3.25E-01	4.04E-02	4.71E-01	NS
H2BC1	NS	3.25E-01	-0.0404	4.71E-01	NS
PRDM4	NS	3.25E-01	-0.0404	4.71E-01	NS
NPIPA3	NS	3.25E-01	4.04E-02	4.72E-01	NS
SDHB	NS	3.25E-01	-0.0404	4.72E-01	NS
ARMC10	NS	3.25E-01	-0.0404	4.72E-01	NS
CLEC19A	NS	3.25E-01	-0.0404	4.72E-01	NS
WNT8B	NS	3.26E-01	-0.0404	4.72E-01	NS
SMARCD2	NS	3.26E-01	-0.0404	4.72E-01	NS
MESP2	NS	3.26E-01	4.04E-02	4.72E-01	NS
DIS3L	NS	3.26E-01	-0.0403	4.72E-01	NS
ZNF865	NS	3.26E-01	-0.0403	4.72E-01	NS
TSN	NS	3.26E-01	-0.0403	4.72E-01	NS
DNAAF5	NS	3.26E-01	-0.0403	4.72E-01	NS
MCM7	NS	3.26E-01	-0.0403	4.72E-01	NS
PPP1R14A	NS	3.26E-01	4.03E-02	4.73E-01	NS
LYG2	NS	3.26E-01	-0.0403	4.73E-01	NS

ARSH	NS	3.26E-01	-0.0403	4.73E-01	NS
FUT6	NS	3.26E-01	4.03E-02	4.73E-01	NS
PARVA	NS	3.26E-01	-0.0403	4.73E-01	NS
TBC1D2B	NS	3.26E-01	-0.0403	4.73E-01	NS
ZNF280A	NS	3.26E-01	-0.0403	4.73E-01	NS
ZNF710	NS	3.27E-01	-0.0403	4.73E-01	NS
SYT2	NS	3.27E-01	-0.0403	4.73E-01	NS
NCF2	NS	3.27E-01	4.03E-02	4.73E-01	NS
TET1	NS	3.27E-01	4.03E-02	4.73E-01	NS
NT5C	NS	3.27E-01	-0.0403	4.73E-01	NS
OR14I1	NS	3.27E-01	4.03E-02	4.73E-01	NS
SPP2	NS	3.27E-01	-0.0403	4.73E-01	NS
TRPV2	NS	3.27E-01	4.02E-02	4.73E-01	NS
SPSB4	NS	3.27E-01	4.02E-02	4.74E-01	NS
KIF19	NS	3.27E-01	4.02E-02	4.74E-01	NS
TAGLN3	NS	3.27E-01	-0.0402	4.74E-01	NS
LMNTD2	NS	3.27E-01	4.02E-02	4.74E-01	NS
CITED2	NS	3.28E-01	-0.0402	4.74E-01	NS
H2AC21	NS	3.28E-01	4.02E-02	4.74E-01	NS
HPS6	NS	3.28E-01	4.02E-02	4.74E-01	NS
CSDC2	NS	3.28E-01	-0.0402	4.74E-01	NS
HHATL	NS	3.28E-01	-0.0402	4.75E-01	NS
KAZALD1	NS	3.28E-01	-0.0402	4.75E-01	NS
CACNB1	NS	3.28E-01	4.01E-02	4.75E-01	NS
GPSM1	NS	3.28E-01	4.01E-02	4.75E-01	NS
AVEN	NS	3.28E-01	-0.0401	4.75E-01	NS
GINS2	NS	3.28E-01	-0.0401	4.75E-01	NS
NAT10	NS	3.28E-01	-0.0401	4.75E-01	NS
CCDC42	NS	3.28E-01	-0.0401	4.75E-01	NS
TDP1	NS	3.29E-01	-0.0401	4.75E-01	NS
CCDC51	NS	3.29E-01	-0.0401	4.75E-01	NS
COL9A3	NS	3.29E-01	4.01E-02	4.75E-01	NS
C19orf54	NS	3.29E-01	4.01E-02	4.75E-01	NS
LYPLAL1	NS	3.29E-01	-0.0401	4.75E-01	NS
OGFOD3	NS	3.29E-01	-0.0401	4.75E-01	NS
IL1RN	NS	3.29E-01	-0.0401	4.75E-01	NS
CGN	NS	3.29E-01	4.01E-02	4.75E-01	NS

PID1	NS	3.29E-01	-0.0401	4.75E-01	NS
UGT2B4	NS	3.30E-01	-0.04	4.76E-01	NS
TSC2	NS	3.30E-01	-0.04	4.76E-01	NS
PHGR1	NS	3.30E-01	4.00E-02	4.76E-01	NS
ATAD3B	NS	3.30E-01	4.00E-02	4.76E-01	NS
TNFRSF6B	NS	3.30E-01	4.00E-02	4.76E-01	NS
IGFBP2	NS	3.30E-01	4.00E-02	4.76E-01	NS
FAM189B	NS	3.30E-01	-0.04	4.76E-01	NS
ZNF512B	NS	3.30E-01	-0.04	4.76E-01	NS
USP42	NS	3.30E-01	4.00E-02	4.76E-01	NS
FKBP15	NS	3.30E-01	4.00E-02	4.76E-01	NS
ZNF7	NS	3.30E-01	-0.04	4.76E-01	NS
FAM189A1	NS	3.30E-01	4.00E-02	4.77E-01	NS
PABPN1L	NS	3.30E-01	-0.04	4.77E-01	NS
SLC1A5	NS	3.31E-01	-0.04	4.77E-01	NS
KREMEN1	NS	3.31E-01	-0.04	4.77E-01	NS
OR52E6	NS	3.31E-01	-0.0399	4.77E-01	NS
PFN1	NS	3.31E-01	3.99E-02	4.77E-01	NS
TDRD12	NS	3.31E-01	3.99E-02	4.78E-01	NS
NELFCD	NS	3.31E-01	-0.0399	4.78E-01	NS
ITPR2	NS	3.31E-01	-0.0399	4.78E-01	NS
EN2	NS	3.31E-01	3.99E-02	4.78E-01	NS
NKX6-3	NS	3.31E-01	-0.0399	4.78E-01	NS
PTDSS2	NS	3.31E-01	-0.0399	4.78E-01	NS
BPIFB4	NS	3.31E-01	-0.0399	4.78E-01	NS
SLC2A4RG	NS	3.32E-01	3.99E-02	4.78E-01	NS
ZNF236	NS	3.32E-01	3.99E-02	4.78E-01	NS
TAF13	NS	3.32E-01	-0.0399	4.78E-01	NS
NEK7	NS	3.32E-01	3.99E-02	4.78E-01	NS
KBTBD11	NS	3.32E-01	-0.0398	4.78E-01	NS
TBL1X	NS	3.32E-01	-0.0398	4.78E-01	NS
HDAC1	NS	3.32E-01	-0.0398	4.78E-01	NS
OR10P1	NS	3.32E-01	-0.0398	4.78E-01	NS
MTRNR2L10	NS	3.32E-01	3.98E-02	4.78E-01	NS
U2AF1L4	NS	3.32E-01	3.98E-02	4.79E-01	NS
KRTAP1-3	NS	3.32E-01	-0.0398	4.79E-01	NS
SLC7A8	NS	3.33E-01	-0.0398	4.79E-01	NS

GDF1	NS	3.33E-01	3.98E-02	4.79E-01	NS
TBC1D7	NS	3.33E-01	-0.0398	4.79E-01	NS
CDR2L	NS	3.33E-01	3.98E-02	4.79E-01	NS
PMAIP1	NS	3.33E-01	3.98E-02	4.79E-01	NS
RCBTB2	NS	3.33E-01	-0.0398	4.79E-01	NS
OVCA2	NS	3.33E-01	-0.0398	4.79E-01	NS
ADSL	NS	3.33E-01	-0.0398	4.79E-01	NS
RNASE8	NS	3.33E-01	-0.0397	4.80E-01	NS
LIMS2	NS	3.33E-01	-0.0397	4.80E-01	NS
CSRP1	NS	3.33E-01	-0.0397	4.80E-01	NS
OR1D2	NS	3.33E-01	-0.0397	4.80E-01	NS
WFIKKN1	NS	3.33E-01	3.97E-02	4.80E-01	NS
LCMT1	NS	3.33E-01	3.97E-02	4.80E-01	NS
TMEM9B	NS	3.33E-01	3.97E-02	4.80E-01	NS
HPS4	NS	3.34E-01	-0.0397	4.80E-01	NS
DEFA6	NS	3.34E-01	-0.0397	4.80E-01	NS
CHRM1	NS	3.34E-01	3.97E-02	4.80E-01	NS
RNF135	NS	3.34E-01	-0.0397	4.80E-01	NS
OVGP1	NS	3.34E-01	-0.0397	4.80E-01	NS
TNFAIP6	NS	3.34E-01	-0.0397	4.80E-01	NS
ECD	NS	3.34E-01	3.97E-02	4.80E-01	NS
MT1HL1	NS	3.34E-01	-0.0397	4.81E-01	NS
RPS3A	NS	3.34E-01	-0.0396	4.81E-01	NS
EIF2B1	NS	3.34E-01	-0.0396	4.81E-01	NS
PGPEP1L	NS	3.34E-01	3.96E-02	4.81E-01	NS
ADHFE1	NS	3.34E-01	-0.0396	4.81E-01	NS
PML	NS	3.35E-01	3.96E-02	4.81E-01	NS
COL20A1	NS	3.35E-01	3.96E-02	4.81E-01	NS
ZNF862	NS	3.35E-01	-0.0396	4.81E-01	NS
LYPD1	NS	3.35E-01	-0.0396	4.81E-01	NS
CD163	NS	3.35E-01	3.96E-02	4.82E-01	NS
RTL8C	NS	3.35E-01	-0.0396	4.82E-01	NS
TAB3	NS	3.36E-01	3.95E-02	4.82E-01	NS
RND1	NS	3.36E-01	-0.0395	4.83E-01	NS
SPPL2B	NS	3.36E-01	-0.0395	4.83E-01	NS
LONP2	NS	3.36E-01	-0.0395	4.83E-01	NS
GIP	NS	3.36E-01	3.95E-02	4.83E-01	NS

DPM1	NS	3.36E-01	-0.0395	4.83E-01	NS
NUDT19	NS	3.36E-01	-0.0395	4.83E-01	NS
IBA57	NS	3.36E-01	-0.0395	4.83E-01	NS
NPIPB4	NS	3.36E-01	3.95E-02	4.83E-01	NS
ENPP5	NS	3.37E-01	-0.0395	4.83E-01	NS
NUDT8	NS	3.37E-01	-0.0394	4.84E-01	NS
MACROH2A2	NS	3.37E-01	3.94E-02	4.84E-01	NS
FAM90A1	NS	3.37E-01	-0.0394	4.84E-01	NS
SMPDL3B	NS	3.37E-01	3.94E-02	4.84E-01	NS
RXFP3	NS	3.37E-01	-0.0394	4.84E-01	NS
VSTM5	NS	3.37E-01	-0.0394	4.84E-01	NS
RPS4X	NS	3.37E-01	-0.0394	4.84E-01	NS
SYNPO2L	NS	3.38E-01	3.94E-02	4.84E-01	NS
TICAM1	NS	3.38E-01	-0.0394	4.85E-01	NS
RABEPK	NS	3.38E-01	3.94E-02	4.85E-01	NS
RPS6KA3	NS	3.38E-01	-0.0394	4.85E-01	NS
EFCAB1	NS	3.38E-01	-0.0394	4.85E-01	NS
CNIH3	NS	3.38E-01	3.94E-02	4.85E-01	NS
RAB1A	NS	3.38E-01	-0.0393	4.85E-01	NS
VSX2	NS	3.38E-01	3.93E-02	4.85E-01	NS
FAIM2	NS	3.38E-01	3.93E-02	4.85E-01	NS
MAP6D1	NS	3.38E-01	-0.0393	4.85E-01	NS
C3orf20	NS	3.38E-01	-0.0393	4.85E-01	NS
CFTR	NS	3.38E-01	-0.0393	4.85E-01	NS
C3orf33	NS	3.38E-01	-0.0393	4.85E-01	NS
PRAMEF26	NS	3.38E-01	-0.0393	4.85E-01	NS
OR2C1	NS	3.39E-01	3.93E-02	4.85E-01	NS
SLC52A1	NS	3.39E-01	3.93E-02	4.85E-01	NS
TMEM97	NS	3.39E-01	-0.0393	4.85E-01	NS
SPTLC1	NS	3.39E-01	3.93E-02	4.85E-01	NS
AMER3	NS	3.39E-01	3.93E-02	4.85E-01	NS
HLA-E	NS	3.39E-01	-0.0393	4.85E-01	NS
POU3F2	NS	3.39E-01	-0.0393	4.85E-01	NS
MCM5	NS	3.39E-01	3.93E-02	4.85E-01	NS
RAD51B	NS	3.39E-01	-0.0393	4.85E-01	NS
UPRT	NS	3.39E-01	-0.0393	4.86E-01	NS
XKRX	NS	3.39E-01	-0.0393	4.86E-01	NS

NXF2B	NS	3.39E-01	-0.0393	4.86E-01	NS
SMARCA2	NS	3.39E-01	3.93E-02	4.86E-01	NS
PKIA	NS	3.39E-01	-0.0392	4.86E-01	NS
KPNA5	NS	3.39E-01	-0.0392	4.86E-01	NS
ASNSD1	NS	3.40E-01	3.92E-02	4.86E-01	NS
LTF	NS	3.40E-01	-0.0392	4.86E-01	NS
ZNF319	NS	3.40E-01	3.92E-02	4.86E-01	NS
PMM1	NS	3.40E-01	-0.0392	4.86E-01	NS
OR51V1	NS	3.40E-01	-0.0392	4.86E-01	NS
REM1	NS	3.40E-01	-0.0392	4.87E-01	NS
LOX	NS	3.40E-01	-0.0392	4.87E-01	NS
SPATA32	NS	3.40E-01	3.92E-02	4.87E-01	NS
MSH2	NS	3.40E-01	-0.0392	4.87E-01	NS
HMGB2	NS	3.40E-01	3.92E-02	4.87E-01	NS
BOLA2-SMG1P6	NS	3.40E-01	3.92E-02	4.87E-01	NS
RBMY1F	NS	3.40E-01	-0.0392	4.87E-01	NS
OR14C36	NS	3.40E-01	-0.0392	4.87E-01	NS
FAM222A	NS	3.40E-01	3.92E-02	4.87E-01	NS
ANKMY1	NS	3.40E-01	-0.0391	4.87E-01	NS
DCP1B	NS	3.41E-01	-0.0391	4.87E-01	NS
TEKT5	NS	3.40E-01	3.91E-02	4.87E-01	NS
R3HDM4	NS	3.40E-01	3.91E-02	4.87E-01	NS
FUBP1	NS	3.41E-01	-0.0391	4.87E-01	NS
MRPS26	NS	3.41E-01	-0.0391	4.87E-01	NS
KRTAP19-5	NS	3.41E-01	-0.0391	4.87E-01	NS
BCKDK	NS	3.41E-01	-0.0391	4.87E-01	NS
LAMTOR1	NS	3.41E-01	-0.0391	4.87E-01	NS
RNF4	NS	3.41E-01	-0.0391	4.87E-01	NS
ZNF80	NS	3.41E-01	-0.0391	4.87E-01	NS
PPP2R1A	NS	3.41E-01	-0.0391	4.88E-01	NS
NT5DC2	NS	3.41E-01	-0.0391	4.88E-01	NS
POTEB3	NS	3.41E-01	-0.0391	4.88E-01	NS
BAG1	NS	3.41E-01	3.91E-02	4.88E-01	NS
EEA1	NS	3.42E-01	-0.0391	4.88E-01	NS
TRPM4	NS	3.41E-01	3.91E-02	4.88E-01	NS
TK2	NS	3.42E-01	-0.0391	4.88E-01	NS
ZNF428	NS	3.42E-01	-0.0391	4.88E-01	NS

CCDC113	NS	3.42E-01	-0.039	4.88E-01	NS
LTBP4	NS	3.42E-01	-0.039	4.88E-01	NS
DUOX2	NS	3.42E-01	-0.039	4.88E-01	NS
KRT33B	NS	3.42E-01	-0.039	4.88E-01	NS
KPTN	NS	3.42E-01	-0.039	4.88E-01	NS
ACVRL1	NS	3.42E-01	3.90E-02	4.88E-01	NS
C8orf74	NS	3.42E-01	-0.039	4.88E-01	NS
LAX1	NS	3.42E-01	-0.039	4.89E-01	NS
S100A10	NS	3.42E-01	-0.039	4.89E-01	NS
OR2F2	NS	3.42E-01	-0.039	4.89E-01	NS
CSE1L	NS	3.43E-01	-0.039	4.89E-01	NS
MFF	NS	3.43E-01	-0.039	4.89E-01	NS
FITM2	NS	3.43E-01	-0.0389	4.89E-01	NS
SOX4	NS	3.43E-01	3.89E-02	4.89E-01	NS
MORF4L1	NS	3.43E-01	3.89E-02	4.90E-01	NS
OOSP4B	NS	3.43E-01	-0.0389	4.90E-01	NS
KNDC1	NS	3.43E-01	-0.0389	4.90E-01	NS
KMT2B	NS	3.43E-01	-0.0389	4.90E-01	NS
HMGXB4	NS	3.43E-01	-0.0389	4.90E-01	NS
OSBPL11	NS	3.43E-01	3.89E-02	4.90E-01	NS
ZNF407	NS	3.44E-01	-0.0389	4.90E-01	NS
TRIM58	NS	3.44E-01	3.89E-02	4.90E-01	NS
HYAL3	NS	3.44E-01	-0.0389	4.90E-01	NS
LAT	NS	3.44E-01	-0.0389	4.90E-01	NS
POF1B	NS	3.44E-01	-0.0388	4.91E-01	NS
RFX1	NS	3.44E-01	3.88E-02	4.91E-01	NS
PF4V1	NS	3.44E-01	3.88E-02	4.91E-01	NS
PAIP2	NS	3.44E-01	-0.0388	4.91E-01	NS
TRMT9B	NS	3.44E-01	-0.0388	4.91E-01	NS
SLC22A31	NS	3.44E-01	3.88E-02	4.91E-01	NS
TAS2R39	NS	3.44E-01	-0.0388	4.91E-01	NS
MARCHF4	NS	3.45E-01	-0.0388	4.91E-01	NS
MYOZ2	NS	3.45E-01	-0.0388	4.91E-01	NS
DMBX1	NS	3.45E-01	3.88E-02	4.91E-01	NS
ADD2	NS	3.45E-01	-0.0388	4.91E-01	NS
C19orf67	NS	3.45E-01	3.88E-02	4.91E-01	NS
OR6C65	NS	3.45E-01	3.88E-02	4.91E-01	NS

EFHD1	NS	3.45E-01	3.88E-02	4.91E-01	NS
KLC4	NS	3.45E-01	3.88E-02	4.91E-01	NS
CMTM3	NS	3.45E-01	3.88E-02	4.92E-01	NS
PRLR	NS	3.45E-01	-0.0388	4.92E-01	NS
SLC6A16	NS	3.45E-01	-0.0388	4.92E-01	NS
NEUROG3	NS	3.45E-01	3.87E-02	4.92E-01	NS
ALG1	NS	3.46E-01	3.87E-02	4.92E-01	NS
STEAP2	NS	3.46E-01	3.87E-02	4.92E-01	NS
B4GALNT1	NS	3.46E-01	-0.0387	4.92E-01	NS
H2AC12	NS	3.46E-01	3.87E-02	4.92E-01	NS
DSCC1	NS	3.46E-01	-0.0387	4.92E-01	NS
CYP11A1	NS	3.46E-01	-0.0387	4.92E-01	NS
AFG3L2	NS	3.46E-01	3.87E-02	4.92E-01	NS
PYCR2	NS	3.46E-01	-0.0387	4.92E-01	NS
CYP27C1	NS	3.46E-01	-0.0387	4.92E-01	NS
TSR1	NS	3.46E-01	-0.0387	4.92E-01	NS
P2RY13	NS	3.46E-01	3.87E-02	4.92E-01	NS
CLN3	NS	3.46E-01	3.87E-02	4.92E-01	NS
GFY	NS	3.46E-01	-0.0387	4.92E-01	NS
RWDD1	NS	3.46E-01	-0.0387	4.93E-01	NS
CALCOCO1	NS	3.46E-01	-0.0387	4.93E-01	NS
LLPH	NS	3.46E-01	-0.0387	4.93E-01	NS
ASAP3	NS	3.47E-01	-0.0387	4.93E-01	NS
NIPAL4	NS	3.46E-01	3.87E-02	4.93E-01	NS
PPP1CC	NS	3.47E-01	3.86E-02	4.93E-01	NS
CRADD	NS	3.47E-01	3.86E-02	4.93E-01	NS
EXO1	NS	3.47E-01	-0.0386	4.93E-01	NS
DNM1L	NS	3.47E-01	-0.0386	4.93E-01	NS
HSPB3	NS	3.47E-01	3.86E-02	4.93E-01	NS
PPIL2	NS	3.47E-01	-0.0386	4.93E-01	NS
USP10	NS	3.47E-01	3.86E-02	4.93E-01	NS
HERC1	NS	3.47E-01	-0.0386	4.93E-01	NS
RAB27A	NS	3.47E-01	-0.0386	4.93E-01	NS
FAM199X	NS	3.47E-01	3.86E-02	4.93E-01	NS
MIEN1	NS	3.47E-01	-0.0386	4.93E-01	NS
GALC	NS	3.47E-01	-0.0386	4.93E-01	NS
RNF144B	NS	3.47E-01	-0.0386	4.94E-01	NS

TMIGD2	NS	3.48E-01	-0.0386	4.94E-01	NS
SLC26A6	NS	3.48E-01	-0.0386	4.94E-01	NS
RAB33B	NS	3.48E-01	-0.0386	4.94E-01	NS
SMIM28	NS	3.48E-01	-0.0386	4.94E-01	NS
CYP2B6	NS	3.48E-01	-0.0385	4.94E-01	NS
MRLN	NS	3.48E-01	3.85E-02	4.94E-01	NS
TPP1	NS	3.48E-01	3.85E-02	4.94E-01	NS
PFDN5	NS	3.49E-01	-0.0385	4.95E-01	NS
PTPN11	NS	3.49E-01	-0.0385	4.95E-01	NS
SPATA13	NS	3.49E-01	3.85E-02	4.95E-01	NS
CCL1	NS	3.49E-01	3.85E-02	4.95E-01	NS
ODAD4	NS	3.49E-01	-0.0385	4.95E-01	NS
PPME1	NS	3.49E-01	-0.0385	4.95E-01	NS
ZC3H4	NS	3.49E-01	3.85E-02	4.95E-01	NS
COL8A1	NS	3.49E-01	-0.0385	4.95E-01	NS
NAXE	NS	3.49E-01	3.84E-02	4.95E-01	NS
RFC4	NS	3.49E-01	-0.0384	4.96E-01	NS
GADD45G	NS	3.50E-01	3.84E-02	4.96E-01	NS
PBLD	NS	3.50E-01	-0.0384	4.96E-01	NS
KIAA2012	NS	3.50E-01	3.84E-02	4.96E-01	NS
WAC	NS	3.50E-01	3.84E-02	4.96E-01	NS
PLA2G2D	NS	3.50E-01	3.84E-02	4.96E-01	NS
SLC5A10	NS	3.50E-01	3.84E-02	4.96E-01	NS
H3C1	NS	3.50E-01	3.84E-02	4.96E-01	NS
EMID1	NS	3.50E-01	-0.0384	4.96E-01	NS
C12orf42	NS	3.50E-01	-0.0384	4.96E-01	NS
MCL1	NS	3.50E-01	-0.0384	4.96E-01	NS
NCLN	NS	3.50E-01	-0.0384	4.96E-01	NS
BBX	NS	3.50E-01	-0.0384	4.96E-01	NS
NRG1	NS	3.50E-01	-0.0383	4.97E-01	NS
OGG1	NS	3.50E-01	-0.0383	4.97E-01	NS
GAS2L1	NS	3.51E-01	3.83E-02	4.97E-01	NS
GCNA	NS	3.51E-01	-0.0383	4.97E-01	NS
SNAPC3	NS	3.51E-01	-0.0383	4.97E-01	NS
CSTA	NS	3.51E-01	-0.0383	4.97E-01	NS
TNRC6A	NS	3.51E-01	-0.0383	4.97E-01	NS
SMIM1	NS	3.51E-01	3.83E-02	4.97E-01	NS

COLEC11	NS	3.51E-01	-0.0383	4.97E-01	NS
CLBA1	NS	3.51E-01	3.83E-02	4.98E-01	NS
BMI1	NS	3.51E-01	-0.0383	4.98E-01	NS
ARHGEF40	NS	3.52E-01	-0.0383	4.98E-01	NS
SAMD13	NS	3.52E-01	3.82E-02	4.98E-01	NS
AGBL5	NS	3.52E-01	-0.0382	4.98E-01	NS
ZNF254	NS	3.52E-01	-0.0382	4.98E-01	NS
H4C7	NS	3.52E-01	3.82E-02	4.98E-01	NS
CCP110	NS	3.52E-01	-0.0382	4.98E-01	NS
EDEM3	NS	3.52E-01	3.82E-02	4.98E-01	NS
PTPN2	NS	3.52E-01	-0.0382	4.98E-01	NS
MPND	NS	3.53E-01	-0.0382	4.99E-01	NS
MT1A	NS	3.53E-01	-0.0382	4.99E-01	NS
SLC35A5	NS	3.53E-01	-0.0382	4.99E-01	NS
CISH	NS	3.53E-01	3.82E-02	4.99E-01	NS
THUMPD2	NS	3.53E-01	-0.0381	4.99E-01	NS
FAXC	NS	3.53E-01	-0.0381	4.99E-01	NS
C10orf62	NS	3.53E-01	-0.0381	5.00E-01	NS
MADD	NS	3.53E-01	-0.0381	5.00E-01	NS
FAM104A	NS	3.53E-01	-0.0381	5.00E-01	NS
RPL37	NS	3.53E-01	-0.0381	5.00E-01	NS
TUBB3	NS	3.53E-01	-0.0381	5.00E-01	NS
PRSS21	NS	3.54E-01	3.81E-02	5.00E-01	NS
VCPKMT	NS	3.54E-01	-0.0381	5.00E-01	NS
SLX4IP	NS	3.54E-01	-0.0381	5.00E-01	NS
TRAF3IP1	NS	3.54E-01	-0.0381	5.00E-01	NS
SLC37A2	NS	3.54E-01	3.81E-02	5.00E-01	NS
TAFA4	NS	3.54E-01	-0.0381	5.00E-01	NS
UCP2	NS	3.54E-01	-0.0381	5.00E-01	NS
SPINK8	NS	3.54E-01	-0.0381	5.00E-01	NS
CATSPERB	NS	3.54E-01	-0.0381	5.00E-01	NS
MMP24	NS	3.54E-01	-0.038	5.01E-01	NS
FERMT1	NS	3.54E-01	-0.038	5.01E-01	NS
FBXO3	NS	3.54E-01	-0.038	5.01E-01	NS
NPHS1	NS	3.54E-01	3.80E-02	5.01E-01	NS
ANKRD40CL	NS	3.54E-01	-0.038	5.01E-01	NS
SLC3A1	NS	3.54E-01	-0.038	5.01E-01	NS

IPO5	NS	3.54E-01	-0.038	5.01E-01	NS
CENPT	NS	3.54E-01	3.80E-02	5.01E-01	NS
MAPRE2	NS	3.55E-01	3.80E-02	5.01E-01	NS
ZNF337	NS	3.55E-01	-0.038	5.01E-01	NS
MSMB	NS	3.55E-01	-0.038	5.01E-01	NS
TMED7	NS	3.55E-01	-0.038	5.01E-01	NS
TOR3A	NS	3.55E-01	3.80E-02	5.01E-01	NS
CARMIL3	NS	3.55E-01	3.80E-02	5.01E-01	NS
YKT6	NS	3.55E-01	3.80E-02	5.01E-01	NS
KCNN1	NS	3.55E-01	3.80E-02	5.01E-01	NS
ADI1	NS	3.55E-01	3.80E-02	5.01E-01	NS
ZNF212	NS	3.56E-01	-0.0379	5.02E-01	NS
TRIM10	NS	3.56E-01	-0.0379	5.02E-01	NS
MAPK1	NS	3.56E-01	-0.0379	5.02E-01	NS
ANKRD29	NS	3.56E-01	-0.0379	5.02E-01	NS
ITGA2	NS	3.56E-01	-0.0379	5.02E-01	NS
TCTN1	NS	3.56E-01	3.79E-02	5.02E-01	NS
CFH	NS	3.56E-01	-0.0379	5.02E-01	NS
GP1BA	NS	3.56E-01	3.79E-02	5.02E-01	NS
RBL2	NS	3.56E-01	-0.0379	5.02E-01	NS
CFI	NS	3.56E-01	-0.0379	5.02E-01	NS
CLEC4M	NS	3.56E-01	-0.0379	5.02E-01	NS
AHRR	NS	3.56E-01	-0.0379	5.03E-01	NS
POLN	NS	3.56E-01	3.79E-02	5.03E-01	NS
ZBTB17	NS	3.57E-01	-0.0379	5.03E-01	NS
LINC02203	NS	3.57E-01	-0.0378	5.03E-01	NS
TESC	NS	3.57E-01	3.78E-02	5.03E-01	NS
SLC5A6	NS	3.57E-01	3.78E-02	5.03E-01	NS
SPAG1	NS	3.57E-01	-0.0378	5.03E-01	NS
BUB1B	NS	3.57E-01	3.78E-02	5.04E-01	NS
RMI2	NS	3.58E-01	3.78E-02	5.04E-01	NS
PLD4	NS	3.58E-01	-0.0378	5.04E-01	NS
MIS18BP1	NS	3.58E-01	3.78E-02	5.04E-01	NS
TMEM139	NS	3.58E-01	-0.0378	5.04E-01	NS
CIB2	NS	3.58E-01	-0.0377	5.04E-01	NS
SPRY4	NS	3.58E-01	3.77E-02	5.04E-01	NS
FIG4	NS	3.58E-01	3.77E-02	5.05E-01	NS

S100PBP	NS	3.58E-01	3.77E-02	5.05E-01	NS
CCDC91	NS	3.58E-01	3.77E-02	5.05E-01	NS
HAVCR2	NS	3.58E-01	-0.0377	5.05E-01	NS
TBX15	NS	3.58E-01	-0.0377	5.05E-01	NS
FOXA2	NS	3.58E-01	3.77E-02	5.05E-01	NS
SUN5	NS	3.59E-01	-0.0377	5.05E-01	NS
SUGP2	NS	3.59E-01	-0.0377	5.05E-01	NS
ADAP1	NS	3.59E-01	-0.0377	5.05E-01	NS
IZUMO1R	NS	3.59E-01	-0.0377	5.05E-01	NS
SIRPG	NS	3.59E-01	3.77E-02	5.05E-01	NS
CAMKK1	NS	3.59E-01	-0.0377	5.05E-01	NS
NFKBIB	NS	3.59E-01	3.77E-02	5.05E-01	NS
ARFGAP2	NS	3.59E-01	-0.0377	5.05E-01	NS
TBPL2	NS	3.59E-01	-0.0377	5.05E-01	NS
RASSF8	NS	3.59E-01	3.77E-02	5.05E-01	NS
PTDSS1	NS	3.59E-01	-0.0376	5.06E-01	NS
PSRC1	NS	3.59E-01	3.76E-02	5.06E-01	NS
HIPK2	NS	3.59E-01	-0.0376	5.06E-01	NS
SLC4A1	NS	3.60E-01	3.76E-02	5.06E-01	NS
ECI2	NS	3.60E-01	-0.0376	5.06E-01	NS
PLK2	NS	3.60E-01	3.76E-02	5.06E-01	NS
ZAR1L	NS	3.60E-01	3.76E-02	5.06E-01	NS
TAFA2	NS	3.60E-01	3.76E-02	5.06E-01	NS
CHURC1	NS	3.60E-01	-0.0376	5.06E-01	NS
GUCA2B	NS	3.60E-01	3.76E-02	5.06E-01	NS
SCCPDH	NS	3.60E-01	-0.0376	5.06E-01	NS
CDH18	NS	3.60E-01	3.76E-02	5.06E-01	NS
SMIM35	NS	3.60E-01	-0.0376	5.06E-01	NS
VSIG2	NS	3.60E-01	-0.0376	5.06E-01	NS
ARHGEF10L	NS	3.60E-01	-0.0376	5.06E-01	NS
SLC38A7	NS	3.60E-01	-0.0376	5.06E-01	NS
CHST3	NS	3.60E-01	-0.0376	5.06E-01	NS
ZRANB2	NS	3.60E-01	-0.0376	5.06E-01	NS
TTI1	NS	3.60E-01	-0.0376	5.06E-01	NS
CFAP95	NS	3.61E-01	-0.0375	5.07E-01	NS
LARGE1	NS	3.61E-01	-0.0375	5.07E-01	NS
RPL30	NS	3.61E-01	-0.0375	5.07E-01	NS

C10orf143	NS	3.61E-01	-0.0375	5.07E-01	NS
CNN2	NS	3.61E-01	-0.0375	5.07E-01	NS
FKBP9	NS	3.61E-01	-0.0375	5.07E-01	NS
GTF2H2	NS	3.61E-01	-0.0375	5.07E-01	NS
HMGN1	NS	3.61E-01	-0.0375	5.07E-01	NS
NME1-NME2	NS	3.61E-01	-0.0375	5.07E-01	NS
ATP5MC2	NS	3.62E-01	-0.0375	5.08E-01	NS
TRAF4	NS	3.62E-01	-0.0375	5.08E-01	NS
OR4P4	NS	3.62E-01	3.75E-02	5.08E-01	NS
FAM90A10P	NS	3.62E-01	-0.0374	5.08E-01	NS
ST8SIA2	NS	3.62E-01	-0.0374	5.08E-01	NS
LIPT1	NS	3.62E-01	3.74E-02	5.08E-01	NS
ANAPC10	NS	3.62E-01	3.74E-02	5.08E-01	NS
BPI	NS	3.62E-01	3.74E-02	5.08E-01	NS
FAM13B	NS	3.63E-01	-0.0374	5.09E-01	NS
H2BU1	NS	3.63E-01	-0.0374	5.09E-01	NS
REXO1	NS	3.63E-01	-0.0373	5.09E-01	NS
NECAP1	NS	3.63E-01	3.73E-02	5.09E-01	NS
HMGXB3	NS	3.63E-01	-0.0373	5.09E-01	NS
STRA6	NS	3.63E-01	3.73E-02	5.09E-01	NS
NICN1	NS	3.63E-01	-0.0373	5.09E-01	NS
MCHR2	NS	3.63E-01	-0.0373	5.09E-01	NS
FAM111B	NS	3.63E-01	3.73E-02	5.09E-01	NS
SELENOH	NS	3.63E-01	-0.0373	5.09E-01	NS
PDE7A	NS	3.63E-01	3.73E-02	5.09E-01	NS
NPC1	NS	3.64E-01	-0.0373	5.09E-01	NS
FNDC10	NS	3.64E-01	-0.0373	5.10E-01	NS
ARHGAP40	NS	3.64E-01	-0.0373	5.10E-01	NS
LRTOMT	NS	3.64E-01	-0.0373	5.10E-01	NS
SPG21	NS	3.64E-01	-0.0373	5.10E-01	NS
ZNF20	NS	3.64E-01	-0.0373	5.10E-01	NS
TBRG4	NS	3.64E-01	-0.0373	5.10E-01	NS
TENM2	NS	3.64E-01	3.73E-02	5.10E-01	NS
HTR7	NS	3.64E-01	-0.0373	5.10E-01	NS
CCPG1	NS	3.64E-01	-0.0373	5.10E-01	NS
OR1S1	NS	3.64E-01	3.73E-02	5.10E-01	NS
BPIFA3	NS	3.64E-01	-0.0373	5.10E-01	NS

TMEM253	NS	3.64E-01	-0.0373	5.10E-01	NS
KLHL42	NS	3.64E-01	-0.0373	5.10E-01	NS
PRPF38B	NS	3.64E-01	3.72E-02	5.10E-01	NS
TAS1R1	NS	3.64E-01	3.72E-02	5.10E-01	NS
AARSD1	NS	3.64E-01	-0.0372	5.10E-01	NS
NPR2	NS	3.65E-01	3.72E-02	5.10E-01	NS
RTCA	NS	3.65E-01	-0.0372	5.10E-01	NS
OR2M5	NS	3.65E-01	-0.0372	5.10E-01	NS
BPIFC	NS	3.65E-01	3.72E-02	5.10E-01	NS
TMA7	NS	3.65E-01	3.72E-02	5.10E-01	NS
PPP1R32	NS	3.65E-01	-0.0372	5.11E-01	NS
FASTKD5	NS	3.65E-01	-0.0372	5.11E-01	NS
STIMATE-MUSTN1	NS	3.65E-01	3.72E-02	5.11E-01	NS
ATP2A3	NS	3.65E-01	3.72E-02	5.11E-01	NS
ZBED6	NS	3.65E-01	-0.0372	5.11E-01	NS
FAM149B1	NS	3.65E-01	-0.0372	5.11E-01	NS
NDC1	NS	3.65E-01	-0.0372	5.11E-01	NS
EIPR1	NS	3.66E-01	-0.0372	5.11E-01	NS
RAB28	NS	3.66E-01	-0.0372	5.11E-01	NS
TRAF3	NS	3.66E-01	3.72E-02	5.11E-01	NS
ZNF624	NS	3.66E-01	-0.0371	5.11E-01	NS
MAGEB6	NS	3.66E-01	3.71E-02	5.12E-01	NS
RNF5	NS	3.66E-01	3.71E-02	5.12E-01	NS
USP17L17	NS	3.66E-01	3.71E-02	5.12E-01	NS
USP54	NS	3.66E-01	3.71E-02	5.12E-01	NS
MTMR4	NS	3.67E-01	-0.0371	5.12E-01	NS
TEX13C	NS	3.67E-01	-0.0371	5.12E-01	NS
GLCE	NS	3.67E-01	-0.0371	5.12E-01	NS
PIP4K2B	NS	3.67E-01	-0.0371	5.13E-01	NS
TPTE	NS	3.67E-01	3.70E-02	5.13E-01	NS
CCDC93	NS	3.67E-01	3.70E-02	5.13E-01	NS
DEK	NS	3.67E-01	-0.037	5.13E-01	NS
EVA1B	NS	3.67E-01	-0.037	5.13E-01	NS
MCCC2	NS	3.68E-01	-0.037	5.13E-01	NS
AUH	NS	3.68E-01	3.70E-02	5.13E-01	NS
PDPN	NS	3.68E-01	3.70E-02	5.13E-01	NS
EIF4H	NS	3.68E-01	-0.037	5.14E-01	NS

PRXL2B	NS	3.68E-01	-0.037	5.14E-01	NS
YWHAG	NS	3.68E-01	-0.037	5.14E-01	NS
IL27	NS	3.68E-01	-0.037	5.14E-01	NS
RAP1GAP	NS	3.68E-01	3.69E-02	5.14E-01	NS
C1orf141	NS	3.68E-01	-0.0369	5.14E-01	NS
SDC4	NS	3.68E-01	-0.0369	5.14E-01	NS
KIR2DL1	NS	3.69E-01	-0.0369	5.14E-01	NS
GJA4	NS	3.69E-01	3.69E-02	5.14E-01	NS
CENPW	NS	3.69E-01	-0.0369	5.14E-01	NS
PCDHA10	NS	3.69E-01	3.69E-02	5.14E-01	NS
REG4	NS	3.69E-01	-0.0369	5.14E-01	NS
KRTAP9-2	NS	3.69E-01	3.69E-02	5.14E-01	NS
TMEM59L	NS	3.69E-01	3.69E-02	5.15E-01	NS
RNF38	NS	3.69E-01	3.69E-02	5.15E-01	NS
DPP7	NS	3.69E-01	3.69E-02	5.15E-01	NS
CAV2	NS	3.69E-01	3.69E-02	5.15E-01	NS
TRPM7	NS	3.69E-01	3.69E-02	5.15E-01	NS
TRAPPC2L	NS	3.69E-01	-0.0369	5.15E-01	NS
USP17L13	NS	3.70E-01	3.68E-02	5.16E-01	NS
CST3	NS	3.70E-01	3.68E-02	5.16E-01	NS
DPYSL3	NS	3.70E-01	-0.0368	5.16E-01	NS
PVALB	NS	3.70E-01	-0.0368	5.16E-01	NS
HSPA5	NS	3.70E-01	3.68E-02	5.16E-01	NS
SPDYE5	NS	3.70E-01	3.68E-02	5.16E-01	NS
EMC6	NS	3.70E-01	-0.0368	5.16E-01	NS
PFKFB2	NS	3.70E-01	-0.0368	5.16E-01	NS
BVES	NS	3.71E-01	-0.0368	5.17E-01	NS
MIP	NS	3.71E-01	-0.0367	5.17E-01	NS
AK4	NS	3.71E-01	-0.0367	5.17E-01	NS
OR2T3	NS	3.71E-01	-0.0367	5.17E-01	NS
DDHD1	NS	3.71E-01	-0.0367	5.17E-01	NS
HOXB4	NS	3.71E-01	3.67E-02	5.17E-01	NS
ARL6IP4	NS	3.71E-01	-0.0367	5.17E-01	NS
YWHAH	NS	3.72E-01	-0.0367	5.18E-01	NS
TMEM132A	NS	3.72E-01	3.66E-02	5.18E-01	NS
DGKD	NS	3.72E-01	3.66E-02	5.18E-01	NS
CNTNAP4	NS	3.72E-01	3.66E-02	5.18E-01	NS

INKA2	NS	3.72E-01	-0.0366	5.18E-01	NS
ENPP1	NS	3.72E-01	-0.0366	5.18E-01	NS
AGBL3	NS	3.72E-01	-0.0366	5.18E-01	NS
NCKIPSD	NS	3.72E-01	3.66E-02	5.18E-01	NS
ADGRG2	NS	3.73E-01	-0.0366	5.19E-01	NS
SRSF11	NS	3.73E-01	-0.0366	5.19E-01	NS
KIAA1109	NS	3.73E-01	-0.0366	5.19E-01	NS
ZMYM4	NS	3.73E-01	3.66E-02	5.19E-01	NS
LSM12	NS	3.73E-01	-0.0366	5.19E-01	NS
COX6C	NS	3.73E-01	-0.0366	5.19E-01	NS
SULT1A4	NS	3.73E-01	3.66E-02	5.19E-01	NS
PGAP2	NS	3.73E-01	-0.0366	5.19E-01	NS
CFAP97D2	NS	3.73E-01	-0.0366	5.19E-01	NS
RNF157	NS	3.73E-01	3.66E-02	5.19E-01	NS
PLEKHM2	NS	3.74E-01	-0.0365	5.20E-01	NS
TAF4	NS	3.74E-01	-0.0365	5.20E-01	NS
ANGPT2	NS	3.74E-01	-0.0365	5.20E-01	NS
DCAF4L2	NS	3.74E-01	-0.0365	5.20E-01	NS
ACTL7B	NS	3.74E-01	3.65E-02	5.20E-01	NS
CGB5	NS	3.74E-01	3.65E-02	5.20E-01	NS
SGK2	NS	3.74E-01	-0.0365	5.20E-01	NS
MTM1	NS	3.74E-01	-0.0365	5.20E-01	NS
NCOA4	NS	3.74E-01	-0.0365	5.20E-01	NS
CD302	NS	3.74E-01	-0.0365	5.21E-01	NS
INTS1	NS	3.75E-01	-0.0365	5.21E-01	NS
TLE6	NS	3.75E-01	-0.0365	5.21E-01	NS
MOB2	NS	3.75E-01	-0.0365	5.21E-01	NS
PRDM11	NS	3.75E-01	-0.0365	5.21E-01	NS
CABLES2	NS	3.75E-01	-0.0364	5.21E-01	NS
TENM3	NS	3.75E-01	3.64E-02	5.21E-01	NS
CTCF	NS	3.75E-01	-0.0364	5.21E-01	NS
RNASE7	NS	3.75E-01	3.64E-02	5.21E-01	NS
SMIM38	NS	3.75E-01	3.64E-02	5.21E-01	NS
HNRNPC	NS	3.75E-01	3.64E-02	5.22E-01	NS
NECAB3	NS	3.76E-01	3.64E-02	5.22E-01	NS
PDLIM5	NS	3.76E-01	-0.0364	5.22E-01	NS
HSFX1	NS	3.76E-01	-0.0364	5.22E-01	NS

SLC22A18AS	NS	3.76E-01	3.64E-02	5.22E-01	NS
TSSK2	NS	3.76E-01	3.64E-02	5.22E-01	NS
TLCD5	NS	3.76E-01	-0.0364	5.22E-01	NS
STAB1	NS	3.76E-01	3.63E-02	5.22E-01	NS
MIER2	NS	3.76E-01	3.63E-02	5.22E-01	NS
DUSP4	NS	3.76E-01	3.63E-02	5.22E-01	NS
MAPK1IP1L	NS	3.76E-01	-0.0363	5.22E-01	NS
RPH3AL	NS	3.76E-01	-0.0363	5.22E-01	NS
ATAD5	NS	3.76E-01	-0.0363	5.22E-01	NS
SERPINB11	NS	3.77E-01	3.63E-02	5.23E-01	NS
RAB11FIP4	NS	3.77E-01	-0.0363	5.23E-01	NS
ZNF562	NS	3.77E-01	-0.0363	5.23E-01	NS
TP63	NS	3.77E-01	3.63E-02	5.23E-01	NS
TBC1D4	NS	3.77E-01	-0.0363	5.23E-01	NS
GATC	NS	3.77E-01	-0.0363	5.23E-01	NS
ZNF766	NS	3.77E-01	-0.0363	5.23E-01	NS
E2F5	NS	3.77E-01	-0.0363	5.23E-01	NS
RAB3IP	NS	3.77E-01	-0.0363	5.23E-01	NS
KRI1	NS	3.77E-01	-0.0363	5.23E-01	NS
RPS9	NS	3.77E-01	3.63E-02	5.23E-01	NS
USP17L2	NS	3.77E-01	-0.0363	5.23E-01	NS
APOA4	NS	3.77E-01	3.63E-02	5.23E-01	NS
HBG1	NS	3.77E-01	3.63E-02	5.23E-01	NS
TSPAN8	NS	3.77E-01	-0.0363	5.23E-01	NS
MAN1B1	NS	3.77E-01	-0.0363	5.23E-01	NS
GNAQ	NS	3.78E-01	-0.0362	5.23E-01	NS
IGBP1P2	NS	3.78E-01	3.62E-02	5.23E-01	NS
PRKAG3	NS	3.78E-01	-0.0362	5.23E-01	NS
LTK	NS	3.78E-01	3.62E-02	5.23E-01	NS
LCE3E	NS	3.78E-01	3.62E-02	5.23E-01	NS
LCE1E	NS	3.78E-01	-0.0362	5.23E-01	NS
SERINC5	NS	3.78E-01	-0.0362	5.23E-01	NS
INVS	NS	3.78E-01	3.62E-02	5.24E-01	NS
NFRKB	NS	3.78E-01	-0.0362	5.24E-01	NS
PPP2R2A	NS	3.78E-01	-0.0362	5.24E-01	NS
SLC35D1	NS	3.78E-01	-0.0362	5.24E-01	NS
ELOVL7	NS	3.78E-01	-0.0362	5.24E-01	NS

FAM219A	NS	3.78E-01	3.62E-02	5.24E-01	NS
L1TD1	NS	3.79E-01	-0.0362	5.24E-01	NS
ROBO1	NS	3.79E-01	-0.0362	5.24E-01	NS
CLRN3	NS	3.79E-01	-0.0361	5.25E-01	NS
KLRG1	NS	3.79E-01	-0.0361	5.25E-01	NS
CCL24	NS	3.79E-01	-0.0361	5.25E-01	NS
CAPN3	NS	3.79E-01	3.61E-02	5.25E-01	NS
NFIX	NS	3.79E-01	3.61E-02	5.25E-01	NS
ZFYVE27	NS	3.80E-01	-0.0361	5.25E-01	NS
RBKS	NS	3.80E-01	-0.0361	5.26E-01	NS
USP33	NS	3.80E-01	-0.0361	5.26E-01	NS
NFYC	NS	3.80E-01	3.61E-02	5.26E-01	NS
CYTH1	NS	3.80E-01	3.61E-02	5.26E-01	NS
PPP1R2	NS	3.80E-01	3.61E-02	5.26E-01	NS
MGP	NS	3.80E-01	-0.036	5.26E-01	NS
ATP5MJ	NS	3.80E-01	3.60E-02	5.26E-01	NS
RGN	NS	3.80E-01	3.60E-02	5.26E-01	NS
GLRX	NS	3.80E-01	-0.036	5.26E-01	NS
KCNV1	NS	3.81E-01	-0.036	5.27E-01	NS
LFNG	NS	3.81E-01	-0.036	5.27E-01	NS
SNIP1	NS	3.81E-01	-0.036	5.27E-01	NS
SBK3	NS	3.81E-01	3.60E-02	5.27E-01	NS
PKP1	NS	3.81E-01	-0.036	5.27E-01	NS
PREX2	NS	3.81E-01	-0.036	5.27E-01	NS
CD55	NS	3.81E-01	-0.0359	5.27E-01	NS
ZNF438	NS	3.81E-01	-0.0359	5.27E-01	NS
FAM229B	NS	3.82E-01	-0.0359	5.28E-01	NS
N4BP3	NS	3.82E-01	3.59E-02	5.28E-01	NS
TOR1A	NS	3.82E-01	3.59E-02	5.28E-01	NS
IER2	NS	3.82E-01	3.59E-02	5.28E-01	NS
ATP1A2	NS	3.82E-01	3.59E-02	5.28E-01	NS
HJURP	NS	3.82E-01	3.59E-02	5.28E-01	NS
COX4I2	NS	3.82E-01	3.59E-02	5.28E-01	NS
GPALPP1	NS	3.82E-01	-0.0359	5.28E-01	NS
MYRIP	NS	3.82E-01	-0.0359	5.28E-01	NS
ACP7	NS	3.83E-01	3.59E-02	5.28E-01	NS
CYB5D1	NS	3.83E-01	-0.0359	5.29E-01	NS

SEMA3C	NS	3.83E-01	-0.0359	5.29E-01	NS
CDKN1B	NS	3.83E-01	3.58E-02	5.29E-01	NS
RGPD8	NS	3.83E-01	-0.0358	5.29E-01	NS
DGUOK	NS	3.83E-01	-0.0358	5.29E-01	NS
GLB1L2	NS	3.83E-01	3.58E-02	5.29E-01	NS
SH2D2A	NS	3.83E-01	-0.0358	5.29E-01	NS
SEC11A	NS	3.83E-01	3.58E-02	5.29E-01	NS
UROD	NS	3.83E-01	3.58E-02	5.29E-01	NS
TRIM2	NS	3.83E-01	-0.0358	5.29E-01	NS
HDHD2	NS	3.83E-01	-0.0358	5.29E-01	NS
H2BW2	NS	3.83E-01	-0.0358	5.29E-01	NS
PRIM2	NS	3.83E-01	-0.0358	5.29E-01	NS
GID8	NS	3.84E-01	-0.0358	5.29E-01	NS
GPR50	NS	3.84E-01	3.58E-02	5.29E-01	NS
PRR20B	NS	3.84E-01	-0.0358	5.29E-01	NS
PARP2	NS	3.84E-01	-0.0358	5.30E-01	NS
ZC3H13	NS	3.84E-01	-0.0358	5.30E-01	NS
NCS1	NS	3.84E-01	-0.0357	5.30E-01	NS
NRF1	NS	3.84E-01	-0.0357	5.30E-01	NS
C20orf203	NS	3.84E-01	3.57E-02	5.30E-01	NS
MPHOSPH9	NS	3.84E-01	-0.0357	5.30E-01	NS
RCHY1	NS	3.84E-01	-0.0357	5.30E-01	NS
APOBR	NS	3.85E-01	3.57E-02	5.30E-01	NS
MBOAT2	NS	3.85E-01	3.57E-02	5.30E-01	NS
KNG1	NS	3.85E-01	-0.0357	5.30E-01	NS
OARD1	NS	3.85E-01	3.57E-02	5.30E-01	NS
NAGPA	NS	3.85E-01	-0.0357	5.31E-01	NS
SULT2B1	NS	3.85E-01	3.57E-02	5.31E-01	NS
PROCR	NS	3.85E-01	-0.0357	5.31E-01	NS
POMT2	NS	3.85E-01	-0.0357	5.31E-01	NS
ARNT2	NS	3.85E-01	3.57E-02	5.31E-01	NS
OCIAD1	NS	3.85E-01	-0.0357	5.31E-01	NS
CTBP2	NS	3.85E-01	-0.0356	5.31E-01	NS
LYRM4	NS	3.85E-01	-0.0357	5.31E-01	NS
MYOM3	NS	3.86E-01	-0.0356	5.31E-01	NS
OR2L3	NS	3.86E-01	-0.0356	5.31E-01	NS
GUCD1	NS	3.86E-01	3.56E-02	5.32E-01	NS

UTP18	NS	3.86E-01	3.56E-02	5.32E-01	NS
GPD1	NS	3.86E-01	3.56E-02	5.32E-01	NS
OR6F1	NS	3.86E-01	-0.0356	5.32E-01	NS
KCNAB3	NS	3.86E-01	-0.0356	5.32E-01	NS
GOLGA6L10	NS	3.86E-01	3.56E-02	5.32E-01	NS
OR2L2	NS	3.86E-01	-0.0356	5.32E-01	NS
SFT2D2	NS	3.87E-01	-0.0355	5.33E-01	NS
AKIP1	NS	3.87E-01	3.55E-02	5.33E-01	NS
MCTP2	NS	3.87E-01	-0.0355	5.33E-01	NS
ESM1	NS	3.88E-01	-0.0355	5.33E-01	NS
GNG7	NS	3.88E-01	-0.0355	5.33E-01	NS
MARCKS	NS	3.88E-01	-0.0355	5.33E-01	NS
ODAPH	NS	3.88E-01	3.55E-02	5.33E-01	NS
CXCR2	NS	3.88E-01	-0.0355	5.33E-01	NS
DNAJC3	NS	3.88E-01	-0.0355	5.34E-01	NS
TFDP3	NS	3.88E-01	3.55E-02	5.34E-01	NS
PRKCG	NS	3.88E-01	3.55E-02	5.34E-01	NS
GTF2E2	NS	3.88E-01	3.55E-02	5.34E-01	NS
AP1S2	NS	3.88E-01	-0.0354	5.34E-01	NS
IRS4	NS	3.89E-01	-0.0354	5.34E-01	NS
DIP2C	NS	3.89E-01	-0.0354	5.34E-01	NS
ZMIZ2	NS	3.89E-01	3.54E-02	5.35E-01	NS
COMMD3-BMI1	NS	3.89E-01	-0.0354	5.35E-01	NS
TBC1D28	NS	3.89E-01	-0.0354	5.35E-01	NS
ANKRD39	NS	3.89E-01	3.54E-02	5.35E-01	NS
CEP44	NS	3.89E-01	-0.0354	5.35E-01	NS
HELLS	NS	3.89E-01	-0.0354	5.35E-01	NS
SHROOM3	NS	3.90E-01	-0.0353	5.35E-01	NS
ATP6V1B2	NS	3.90E-01	-0.0353	5.35E-01	NS
NUP35	NS	3.90E-01	-0.0353	5.35E-01	NS
OR1C1	NS	3.90E-01	-0.0353	5.36E-01	NS
DUS2	NS	3.90E-01	-0.0353	5.36E-01	NS
SSX4	NS	3.90E-01	3.53E-02	5.36E-01	NS
PTBP3	NS	3.90E-01	3.53E-02	5.36E-01	NS
PRAMEF25	NS	3.90E-01	-0.0353	5.36E-01	NS
PHC1	NS	3.90E-01	-0.0353	5.36E-01	NS
ARAP3	NS	3.90E-01	-0.0353	5.36E-01	NS

GDF5	NS	3.90E-01	-0.0353	5.36E-01	NS
COPG2	NS	3.90E-01	-0.0353	5.36E-01	NS
KRTAP5-1	NS	3.90E-01	3.53E-02	5.36E-01	NS
TSPAN3	NS	3.90E-01	-0.0353	5.36E-01	NS
ASB18	NS	3.91E-01	3.53E-02	5.37E-01	NS
LYRM7	NS	3.91E-01	-0.0352	5.37E-01	NS
HES6	NS	3.91E-01	3.52E-02	5.37E-01	NS
DBH	NS	3.91E-01	-0.0352	5.37E-01	NS
BCL10	NS	3.91E-01	-0.0352	5.37E-01	NS
BET1L	NS	3.91E-01	-0.0352	5.37E-01	NS
CHST5	NS	3.91E-01	-0.0352	5.37E-01	NS
AFAP1L2	NS	3.91E-01	-0.0352	5.37E-01	NS
SLIRP	NS	3.91E-01	-0.0352	5.37E-01	NS
HAP1	NS	3.91E-01	3.52E-02	5.37E-01	NS
COX20	NS	3.91E-01	3.52E-02	5.37E-01	NS
COQ8B	NS	3.91E-01	-0.0352	5.37E-01	NS
KCTD10	NS	3.92E-01	-0.0352	5.37E-01	NS
ACTG2	NS	3.92E-01	-0.0352	5.37E-01	NS
RNF186	NS	3.92E-01	3.52E-02	5.37E-01	NS
CIDEC	NS	3.92E-01	3.52E-02	5.37E-01	NS
PABPC3	NS	3.92E-01	-0.0352	5.37E-01	NS
PAX4	NS	3.92E-01	-0.0351	5.38E-01	NS
MAGEA4	NS	3.92E-01	3.51E-02	5.38E-01	NS
H2BC10	NS	3.92E-01	3.51E-02	5.38E-01	NS
DPF2	NS	3.92E-01	3.51E-02	5.38E-01	NS
LAMB2	NS	3.92E-01	-0.0351	5.38E-01	NS
TMEM236	NS	3.92E-01	3.51E-02	5.38E-01	NS
PGM3	NS	3.92E-01	-0.0351	5.38E-01	NS
SHISA9	NS	3.92E-01	3.51E-02	5.38E-01	NS
LRTM2	NS	3.93E-01	-0.0351	5.38E-01	NS
AASS	NS	3.93E-01	-0.0351	5.39E-01	NS
IGSF6	NS	3.93E-01	3.51E-02	5.39E-01	NS
BANP	NS	3.93E-01	3.51E-02	5.39E-01	NS
ZNF467	NS	3.93E-01	-0.0351	5.39E-01	NS
OR5AP2	NS	3.93E-01	-0.0351	5.39E-01	NS
PPIP5K2	NS	3.93E-01	-0.0351	5.39E-01	NS
ZNF454	NS	3.93E-01	3.51E-02	5.39E-01	NS

ATF7-NPFF	NS	3.93E-01	3.51E-02	5.39E-01	NS
HECTD3	NS	3.93E-01	3.51E-02	5.39E-01	NS
TMEM115	NS	3.93E-01	3.50E-02	5.39E-01	NS
NDUFS5	NS	3.93E-01	3.50E-02	5.39E-01	NS
ZSWIM7	NS	3.93E-01	-0.0351	5.39E-01	NS
ABCC2	NS	3.94E-01	-0.035	5.39E-01	NS
COPA	NS	3.94E-01	-0.035	5.39E-01	NS
RGS16	NS	3.94E-01	3.50E-02	5.39E-01	NS
ZKSCAN8	NS	3.94E-01	3.50E-02	5.39E-01	NS
MAGEB10	NS	3.94E-01	3.50E-02	5.39E-01	NS
HTRA4	NS	3.94E-01	-0.035	5.39E-01	NS
GPR151	NS	3.94E-01	-0.035	5.39E-01	NS
CHRNB2	NS	3.94E-01	-0.035	5.40E-01	NS
GOLGA4	NS	3.94E-01	-0.035	5.40E-01	NS
CYP27A1	NS	3.95E-01	-0.035	5.40E-01	NS
RNF25	NS	3.95E-01	-0.035	5.40E-01	NS
DUSP28	NS	3.95E-01	3.49E-02	5.40E-01	NS
ZNF200	NS	3.95E-01	-0.0349	5.41E-01	NS
GNA15	NS	3.95E-01	3.49E-02	5.41E-01	NS
TCTN2	NS	3.95E-01	-0.0349	5.41E-01	NS
KRT31	NS	3.95E-01	3.49E-02	5.41E-01	NS
OR8D4	NS	3.95E-01	-0.0349	5.41E-01	NS
LGI3	NS	3.95E-01	3.49E-02	5.41E-01	NS
DGKH	NS	3.96E-01	3.49E-02	5.41E-01	NS
CDIP1	NS	3.96E-01	-0.0349	5.41E-01	NS
EHD1	NS	3.96E-01	-0.0349	5.42E-01	NS
C6orf120	NS	3.96E-01	3.49E-02	5.42E-01	NS
EXTL1	NS	3.96E-01	3.49E-02	5.42E-01	NS
TMEM120A	NS	3.96E-01	-0.0349	5.42E-01	NS
CEP350	NS	3.96E-01	-0.0349	5.42E-01	NS
SPOCK1	NS	3.96E-01	-0.0348	5.42E-01	NS
SMG6	NS	3.97E-01	3.48E-02	5.42E-01	NS
NDUFA9	NS	3.97E-01	3.48E-02	5.42E-01	NS
TERF2IP	NS	3.97E-01	3.48E-02	5.42E-01	NS
NSG2	NS	3.97E-01	-0.0348	5.42E-01	NS
PPEF2	NS	3.97E-01	3.48E-02	5.42E-01	NS
OXR1	NS	3.97E-01	3.48E-02	5.42E-01	NS

DACT1	NS	3.97E-01	3.48E-02	5.42E-01	NS
CDHR3	NS	3.97E-01	3.48E-02	5.43E-01	NS
OR52D1	NS	3.97E-01	3.48E-02	5.43E-01	NS
TLE5	NS	3.97E-01	3.48E-02	5.43E-01	NS
NDUFAF3	NS	3.97E-01	-0.0348	5.43E-01	NS
CYP2F1	NS	3.97E-01	3.48E-02	5.43E-01	NS
KERA	NS	3.97E-01	-0.0348	5.43E-01	NS
SCD	NS	3.97E-01	3.47E-02	5.43E-01	NS
ADAMTS17	NS	3.97E-01	-0.0348	5.43E-01	NS
HEATR6	NS	3.98E-01	-0.0347	5.43E-01	NS
DSC1	NS	3.98E-01	-0.0347	5.43E-01	NS
CFAP44	NS	3.98E-01	-0.0347	5.43E-01	NS
DNAJC13	NS	3.98E-01	-0.0347	5.43E-01	NS
GCNT1	NS	3.98E-01	-0.0347	5.43E-01	NS
IMP4	NS	3.98E-01	3.47E-02	5.43E-01	NS
PCNX1	NS	3.98E-01	3.47E-02	5.43E-01	NS
GFER	NS	3.98E-01	3.47E-02	5.43E-01	NS
OR51D1	NS	3.98E-01	3.47E-02	5.44E-01	NS
EIF4EBP2	NS	3.99E-01	-0.0347	5.44E-01	NS
NBEAL2	NS	3.99E-01	3.47E-02	5.44E-01	NS
ZFYVE1	NS	3.99E-01	-0.0347	5.44E-01	NS
CATSPER2	NS	3.98E-01	-0.0347	5.44E-01	NS
TMEM11	NS	3.98E-01	-0.0347	5.44E-01	NS
FOXI3	NS	3.99E-01	3.47E-02	5.44E-01	NS
A2ML1	NS	3.99E-01	3.47E-02	5.44E-01	NS
KCNK10	NS	3.99E-01	-0.0347	5.44E-01	NS
PLEKHA3	NS	3.99E-01	-0.0347	5.44E-01	NS
SNCAIP	NS	3.99E-01	3.46E-02	5.44E-01	NS
CCL19	NS	3.99E-01	3.46E-02	5.44E-01	NS
GET1-SH3BGR	NS	3.99E-01	-0.0346	5.44E-01	NS
NUDT16L1	NS	3.99E-01	-0.0346	5.44E-01	NS
SH3BP2	NS	3.99E-01	-0.0346	5.44E-01	NS
TAF11L3	NS	3.99E-01	-0.0346	5.44E-01	NS
MAPKAPK5	NS	3.99E-01	3.46E-02	5.44E-01	NS
CABS1	NS	4.00E-01	3.46E-02	5.45E-01	NS
RPL36A	NS	4.00E-01	-0.0346	5.45E-01	NS
EDA	NS	4.00E-01	3.46E-02	5.45E-01	NS

DACT2	NS	4.00E-01	-0.0346	5.45E-01	NS
HOOK3	NS	4.00E-01	3.46E-02	5.45E-01	NS
C5orf24	NS	4.00E-01	-0.0346	5.45E-01	NS
GADD45A	NS	4.00E-01	3.46E-02	5.45E-01	NS
SURF4	NS	4.00E-01	-0.0345	5.45E-01	NS
IGBP1	NS	4.00E-01	-0.0345	5.45E-01	NS
UNC93B1	NS	4.00E-01	3.45E-02	5.45E-01	NS
FYB1	NS	4.01E-01	3.45E-02	5.46E-01	NS
MTBP	NS	4.01E-01	-0.0345	5.46E-01	NS
IPO7	NS	4.01E-01	-0.0345	5.46E-01	NS
STIP1	NS	4.01E-01	-0.0345	5.46E-01	NS
CEBPA	NS	4.01E-01	3.45E-02	5.46E-01	NS
CEACAM16	NS	4.01E-01	3.45E-02	5.46E-01	NS
GXYLT1	NS	4.01E-01	-0.0345	5.46E-01	NS
NKRF	NS	4.01E-01	-0.0345	5.46E-01	NS
HPCA	NS	4.01E-01	-0.0345	5.46E-01	NS
VPS4B	NS	4.02E-01	3.44E-02	5.47E-01	NS
ANKRD36B	NS	4.02E-01	-0.0344	5.47E-01	NS
RASSF2	NS	4.02E-01	-0.0344	5.47E-01	NS
TRIM74	NS	4.02E-01	3.44E-02	5.47E-01	NS
HVCN1	NS	4.02E-01	3.44E-02	5.47E-01	NS
HSPB1	NS	4.02E-01	3.44E-02	5.47E-01	NS
TMEM179	NS	4.02E-01	-0.0344	5.47E-01	NS
TMEM74B	NS	4.02E-01	-0.0344	5.47E-01	NS
CD101	NS	4.02E-01	-0.0344	5.48E-01	NS
IGHMBP2	NS	4.03E-01	-0.0344	5.48E-01	NS
PLPP5	NS	4.03E-01	-0.0344	5.48E-01	NS
CYSLTR2	NS	4.03E-01	-0.0344	5.48E-01	NS
TMEM202	NS	4.03E-01	3.44E-02	5.48E-01	NS
SPATA25	NS	4.03E-01	-0.0344	5.48E-01	NS
CARF	NS	4.03E-01	-0.0344	5.48E-01	NS
C5orf58	NS	4.03E-01	-0.0344	5.48E-01	NS
HSF2	NS	4.03E-01	3.44E-02	5.48E-01	NS
OR4B1	NS	4.03E-01	3.44E-02	5.48E-01	NS
RNF126	NS	4.03E-01	3.43E-02	5.48E-01	NS
ABCC1	NS	4.03E-01	-0.0343	5.48E-01	NS
SLC10A4	NS	4.03E-01	3.43E-02	5.48E-01	NS

CNOT8	NS	4.03E-01	3.43E-02	5.48E-01	NS
SHANK2	NS	4.03E-01	-0.0343	5.48E-01	NS
ACTBL2	NS	4.03E-01	-0.0343	5.48E-01	NS
PWP1	NS	4.03E-01	-0.0343	5.48E-01	NS
SLC25A30	NS	4.03E-01	-0.0343	5.48E-01	NS
ZNF570	NS	4.04E-01	3.43E-02	5.48E-01	NS
UVSSA	NS	4.04E-01	-0.0343	5.48E-01	NS
CLPX	NS	4.04E-01	-0.0343	5.48E-01	NS
JUP	NS	4.04E-01	3.43E-02	5.48E-01	NS
LCLAT1	NS	4.04E-01	-0.0343	5.48E-01	NS
VRK3	NS	4.04E-01	-0.0343	5.49E-01	NS
VGLL3	NS	4.04E-01	-0.0343	5.49E-01	NS
TBC1D3D	NS	4.04E-01	-0.0343	5.49E-01	NS
NDUFB9	NS	4.04E-01	-0.0342	5.49E-01	NS
FOXC1	NS	4.04E-01	-0.0342	5.49E-01	NS
NPRL3	NS	4.05E-01	-0.0342	5.49E-01	NS
ADAM18	NS	4.05E-01	-0.0342	5.49E-01	NS
UBA5	NS	4.05E-01	3.42E-02	5.49E-01	NS
SKP2	NS	4.05E-01	-0.0342	5.49E-01	NS
LRWD1	NS	4.05E-01	3.42E-02	5.49E-01	NS
APAF1	NS	4.05E-01	-0.0342	5.49E-01	NS
LBHD1	NS	4.05E-01	-0.0342	5.49E-01	NS
SPIC	NS	4.05E-01	-0.0342	5.50E-01	NS
FARP2	NS	4.05E-01	-0.0342	5.50E-01	NS
DDIT4L	NS	4.05E-01	3.42E-02	5.50E-01	NS
CCDC103	NS	4.05E-01	-0.0342	5.50E-01	NS
ANKRD33B	NS	4.05E-01	-0.0342	5.50E-01	NS
MCAT	NS	4.05E-01	3.42E-02	5.50E-01	NS
ABHD15	NS	4.05E-01	-0.0342	5.50E-01	NS
KIF22	NS	4.05E-01	-0.0342	5.50E-01	NS
SMARCA5	NS	4.05E-01	3.42E-02	5.50E-01	NS
ABTB1	NS	4.06E-01	3.42E-02	5.50E-01	NS
ZNF609	NS	4.06E-01	3.41E-02	5.50E-01	NS
ZNF333	NS	4.06E-01	-0.0341	5.51E-01	NS
SENP3	NS	4.06E-01	3.41E-02	5.51E-01	NS
EYA4	NS	4.06E-01	-0.0341	5.51E-01	NS
EMILIN1	NS	4.06E-01	3.41E-02	5.51E-01	NS

SPATC1L	NS	4.06E-01	-0.0341	5.51E-01	NS
ACTL10	NS	4.06E-01	3.41E-02	5.51E-01	NS
ZNF69	NS	4.07E-01	-0.0341	5.51E-01	NS
ST6GALNAC2	NS	4.07E-01	3.41E-02	5.51E-01	NS
CLDN6	NS	4.07E-01	3.41E-02	5.51E-01	NS
FRZB	NS	4.07E-01	-0.0341	5.52E-01	NS
GALR3	NS	4.07E-01	-0.034	5.52E-01	NS
PCDHA2	NS	4.07E-01	-0.034	5.52E-01	NS
KATNAL1	NS	4.07E-01	3.40E-02	5.52E-01	NS
KAT5	NS	4.07E-01	3.40E-02	5.52E-01	NS
SRGAP1	NS	4.08E-01	-0.034	5.52E-01	NS
KRT10	NS	4.08E-01	3.40E-02	5.52E-01	NS
AGXT2	NS	4.08E-01	3.40E-02	5.52E-01	NS
HSPA2	NS	4.08E-01	-0.034	5.52E-01	NS
RHPN1	NS	4.08E-01	-0.034	5.52E-01	NS
PPP3CA	NS	4.08E-01	-0.034	5.53E-01	NS
CCL14	NS	4.08E-01	3.40E-02	5.53E-01	NS
PRKAB2	NS	4.08E-01	3.40E-02	5.53E-01	NS
CLUL1	NS	4.08E-01	3.39E-02	5.53E-01	NS
OR1J1	NS	4.09E-01	3.39E-02	5.53E-01	NS
PIBF1	NS	4.09E-01	-0.0339	5.53E-01	NS
ZNF143	NS	4.09E-01	-0.0339	5.53E-01	NS
OR1Q1	NS	4.09E-01	3.39E-02	5.53E-01	NS
POLG2	NS	4.09E-01	-0.0339	5.53E-01	NS
BARHL2	NS	4.09E-01	3.39E-02	5.54E-01	NS
FBLN7	NS	4.09E-01	3.39E-02	5.54E-01	NS
ZBTB11	NS	4.09E-01	3.39E-02	5.54E-01	NS
TEX36	NS	4.10E-01	3.39E-02	5.54E-01	NS
COG7	NS	4.10E-01	-0.0339	5.54E-01	NS
MYF5	NS	4.10E-01	-0.0339	5.54E-01	NS
AMOTL1	NS	4.10E-01	-0.0339	5.54E-01	NS
EPHA10	NS	4.10E-01	3.38E-02	5.54E-01	NS
PCP2	NS	4.10E-01	-0.0338	5.55E-01	NS
RAD17	NS	4.10E-01	-0.0338	5.55E-01	NS
GNE	NS	4.10E-01	-0.0338	5.55E-01	NS
CENPH	NS	4.10E-01	-0.0338	5.55E-01	NS
COL23A1	NS	4.10E-01	3.38E-02	5.55E-01	NS

CLIP2	NS	4.10E-01	-0.0338	5.55E-01	NS
KIAA0232	NS	4.11E-01	-0.0338	5.55E-01	NS
MT1H	NS	4.11E-01	3.38E-02	5.55E-01	NS
REEP2	NS	4.11E-01	-0.0338	5.55E-01	NS
TAC3	NS	4.11E-01	-0.0338	5.55E-01	NS
FAM102A	NS	4.11E-01	-0.0337	5.56E-01	NS
HNRNPUL1	NS	4.12E-01	3.37E-02	5.56E-01	NS
SPATA1	NS	4.12E-01	3.37E-02	5.56E-01	NS
C17orf100	NS	4.12E-01	-0.0337	5.56E-01	NS
PTPN12	NS	4.12E-01	-0.0337	5.57E-01	NS
ZC3H11A	NS	4.12E-01	-0.0337	5.57E-01	NS
TIAM1	NS	4.12E-01	3.37E-02	5.57E-01	NS
IL4	NS	4.12E-01	3.37E-02	5.57E-01	NS
LGI4	NS	4.12E-01	-0.0337	5.57E-01	NS
ITPRIP	NS	4.13E-01	3.36E-02	5.57E-01	NS
DDO	NS	4.13E-01	-0.0336	5.58E-01	NS
ZAP70	NS	4.13E-01	-0.0336	5.58E-01	NS
TUFT1	NS	4.13E-01	3.36E-02	5.58E-01	NS
IGSF23	NS	4.13E-01	3.36E-02	5.58E-01	NS
PDS5B	NS	4.13E-01	-0.0336	5.58E-01	NS
BBOF1	NS	4.13E-01	-0.0336	5.58E-01	NS
WDFY3	NS	4.13E-01	-0.0336	5.58E-01	NS
KLK6	NS	4.13E-01	-0.0336	5.58E-01	NS
PRDM15	NS	4.14E-01	-0.0336	5.58E-01	NS
RBM24	NS	4.14E-01	-0.0335	5.59E-01	NS
COA4	NS	4.14E-01	3.35E-02	5.59E-01	NS
HAPLN3	NS	4.14E-01	-0.0335	5.59E-01	NS
C1orf174	NS	4.15E-01	-0.0335	5.60E-01	NS
THOC6	NS	4.15E-01	-0.0335	5.60E-01	NS
CD200	NS	4.15E-01	3.35E-02	5.60E-01	NS
FAM71F1	NS	4.15E-01	-0.0335	5.60E-01	NS
TRIM69	NS	4.15E-01	3.35E-02	5.60E-01	NS
GPR89A	NS	4.15E-01	3.35E-02	5.60E-01	NS
LAMP2	NS	4.15E-01	-0.0335	5.60E-01	NS
ERBB2	NS	4.15E-01	-0.0335	5.60E-01	NS
HTR2C	NS	4.15E-01	3.35E-02	5.60E-01	NS
RIMS1	NS	4.16E-01	3.34E-02	5.60E-01	NS

ZBTB32	NS	4.16E-01	-0.0334	5.61E-01	NS
NFILZ	NS	4.16E-01	3.34E-02	5.61E-01	NS
ZNF169	NS	4.16E-01	3.34E-02	5.61E-01	NS
CAV1	NS	4.16E-01	3.34E-02	5.61E-01	NS
FYN	NS	4.16E-01	3.34E-02	5.61E-01	NS
LAPTM4A	NS	4.16E-01	-0.0334	5.61E-01	NS
ERGIC2	NS	4.16E-01	-0.0334	5.61E-01	NS
OR10G8	NS	4.16E-01	3.34E-02	5.61E-01	NS
ACP1	NS	4.16E-01	-0.0334	5.61E-01	NS
CPA5	NS	4.17E-01	3.34E-02	5.61E-01	NS
CMKLR2	NS	4.17E-01	3.34E-02	5.61E-01	NS
TRMT1L	NS	4.17E-01	3.33E-02	5.62E-01	NS
BPY2	NS	4.17E-01	-0.0333	5.62E-01	NS
BPY2B	NS	4.17E-01	-0.0333	5.62E-01	NS
BPY2C	NS	4.17E-01	-0.0333	5.62E-01	NS
RAP2A	NS	4.17E-01	-0.0333	5.62E-01	NS
MPC1L	NS	4.17E-01	-0.0333	5.62E-01	NS
ZNF560	NS	4.17E-01	3.33E-02	5.62E-01	NS
SPACA6	NS	4.17E-01	-0.0333	5.62E-01	NS
SPTLC2	NS	4.17E-01	3.33E-02	5.62E-01	NS
NLRP3	NS	4.17E-01	-0.0333	5.62E-01	NS
PHF6	NS	4.18E-01	-0.0333	5.62E-01	NS
COMMD10	NS	4.18E-01	-0.0333	5.62E-01	NS
NSG1	NS	4.18E-01	-0.0333	5.62E-01	NS
ANKS4B	NS	4.18E-01	-0.0333	5.62E-01	NS
TEDDM1	NS	4.18E-01	-0.0333	5.62E-01	NS
VWF	NS	4.18E-01	3.33E-02	5.62E-01	NS
ARL6IP1	NS	4.18E-01	-0.0333	5.63E-01	NS
MGST3	NS	4.18E-01	-0.0333	5.63E-01	NS
FMR1	NS	4.18E-01	3.32E-02	5.63E-01	NS
CCDC112	NS	4.18E-01	-0.0332	5.63E-01	NS
OTUD7A	NS	4.18E-01	-0.0333	5.63E-01	NS
MOB1B	NS	4.18E-01	3.32E-02	5.63E-01	NS
ERO1A	NS	4.19E-01	-0.0332	5.63E-01	NS
LIMS4	NS	4.19E-01	-0.0332	5.63E-01	NS
C2orf69	NS	4.19E-01	-0.0332	5.63E-01	NS
DNAH12	NS	4.19E-01	3.32E-02	5.63E-01	NS

TUG1	NS	4.19E-01	-0.0332	5.63E-01	NS
LDB1	NS	4.19E-01	3.32E-02	5.63E-01	NS
OR6J1	NS	4.19E-01	3.32E-02	5.63E-01	NS
UBXN2B	NS	4.19E-01	-0.0332	5.64E-01	NS
ZNF620	NS	4.19E-01	3.32E-02	5.64E-01	NS
ABTB2	NS	4.19E-01	-0.0332	5.64E-01	NS
ENC1	NS	4.19E-01	3.32E-02	5.64E-01	NS
WDR81	NS	4.20E-01	3.32E-02	5.64E-01	NS
ZNF845	NS	4.20E-01	-0.0332	5.64E-01	NS
REV3L	NS	4.20E-01	-0.0331	5.64E-01	NS
SYTL4	NS	4.20E-01	3.31E-02	5.64E-01	NS
GRAMD1B	NS	4.20E-01	3.31E-02	5.64E-01	NS
CRYAB	NS	4.20E-01	3.31E-02	5.64E-01	NS
FBXW10	NS	4.20E-01	3.31E-02	5.64E-01	NS
DPEP2NB	NS	4.20E-01	-0.0331	5.64E-01	NS
NR2E3	NS	4.20E-01	3.31E-02	5.64E-01	NS
SFRP4	NS	4.20E-01	-0.0331	5.64E-01	NS
EGFR	NS	4.20E-01	-0.0331	5.64E-01	NS
SMG8	NS	4.20E-01	-0.0331	5.64E-01	NS
GPR82	NS	4.21E-01	-0.0331	5.65E-01	NS
FAM53A	NS	4.21E-01	-0.0331	5.65E-01	NS
CRYZL1	NS	4.21E-01	-0.0331	5.65E-01	NS
XKR6	NS	4.21E-01	-0.0331	5.65E-01	NS
SMCO4	NS	4.21E-01	-0.0331	5.65E-01	NS
USP17L15	NS	4.21E-01	-0.0331	5.65E-01	NS
TRIM9	NS	4.21E-01	-0.033	5.65E-01	NS
EYA2	NS	4.21E-01	-0.033	5.65E-01	NS
JPH1	NS	4.21E-01	-0.033	5.65E-01	NS
FBXO6	NS	4.21E-01	-0.033	5.65E-01	NS
GABARAPL1	NS	4.21E-01	-0.033	5.66E-01	NS
H2BC3	NS	4.22E-01	-0.033	5.66E-01	NS
PLEKHG4B	NS	4.22E-01	-0.033	5.66E-01	NS
OPRPN	NS	4.22E-01	-0.033	5.66E-01	NS
SCAI	NS	4.22E-01	-0.033	5.66E-01	NS
RPL14	NS	4.22E-01	-0.033	5.66E-01	NS
NPIPB8	NS	4.22E-01	3.30E-02	5.66E-01	NS
STON2	NS	4.22E-01	3.30E-02	5.66E-01	NS

KRT74	NS	4.22E-01	3.30E-02	5.66E-01	NS
NEXN	NS	4.22E-01	-0.033	5.66E-01	NS
MOGAT2	NS	4.22E-01	-0.033	5.66E-01	NS
KRTAP10-2	NS	4.22E-01	3.30E-02	5.66E-01	NS
ZNF730	NS	4.22E-01	-0.033	5.66E-01	NS
PPIE	NS	4.22E-01	-0.033	5.66E-01	NS
ZMYND10	NS	4.22E-01	-0.0329	5.66E-01	NS
MYDGF	NS	4.22E-01	-0.0329	5.66E-01	NS
FOSB	NS	4.22E-01	3.30E-02	5.66E-01	NS
SERF2	NS	4.23E-01	3.29E-02	5.67E-01	NS
PDE10A	NS	4.23E-01	-0.0329	5.67E-01	NS
NPFFR1	NS	4.23E-01	-0.0329	5.67E-01	NS
CBX2	NS	4.23E-01	3.29E-02	5.67E-01	NS
FAM9B	NS	4.23E-01	-0.0329	5.67E-01	NS
SPEG	NS	4.23E-01	3.29E-02	5.67E-01	NS
ADCY4	NS	4.23E-01	-0.0329	5.67E-01	NS
NIPSNAP2	NS	4.24E-01	3.29E-02	5.67E-01	NS
ZSCAN2	NS	4.24E-01	-0.0329	5.67E-01	NS
OASL	NS	4.24E-01	-0.0329	5.68E-01	NS
IZUMO1	NS	4.24E-01	3.28E-02	5.68E-01	NS
CYBC1	NS	4.24E-01	-0.0328	5.68E-01	NS
SPG11	NS	4.24E-01	-0.0328	5.68E-01	NS
ANKRD36C	NS	4.24E-01	-0.0328	5.68E-01	NS
SKA3	NS	4.24E-01	3.28E-02	5.68E-01	NS
MOGAT1	NS	4.24E-01	-0.0328	5.68E-01	NS
GGT7	NS	4.24E-01	-0.0328	5.68E-01	NS
GJE1	NS	4.24E-01	-0.0328	5.68E-01	NS
RNF216	NS	4.24E-01	3.28E-02	5.68E-01	NS
EIF3A	NS	4.25E-01	3.28E-02	5.68E-01	NS
DRD3	NS	4.25E-01	-0.0328	5.68E-01	NS
PSMC2	NS	4.25E-01	-0.0328	5.68E-01	NS
PLGLB1	NS	4.25E-01	-0.0328	5.68E-01	NS
SCP2	NS	4.25E-01	-0.0328	5.69E-01	NS
OLFML2B	NS	4.25E-01	-0.0328	5.69E-01	NS
PCDH9	NS	4.25E-01	-0.0328	5.69E-01	NS
KXD1	NS	4.25E-01	3.28E-02	5.69E-01	NS
CIITA	NS	4.25E-01	-0.0327	5.69E-01	NS

PLCB1	NS	4.25E-01	-0.0327	5.69E-01	NS
CRY1	NS	4.26E-01	-0.0327	5.69E-01	NS
CYC1	NS	4.26E-01	-0.0327	5.69E-01	NS
WNK1	NS	4.26E-01	-0.0327	5.69E-01	NS
REG1A	NS	4.26E-01	3.27E-02	5.69E-01	NS
CENPF	NS	4.26E-01	3.27E-02	5.69E-01	NS
CZIB	NS	4.26E-01	-0.0327	5.69E-01	NS
PIP4P1	NS	4.26E-01	3.27E-02	5.69E-01	NS
HSPA12A	NS	4.26E-01	3.27E-02	5.69E-01	NS
NFU1	NS	4.26E-01	-0.0327	5.69E-01	NS
CRYBA4	NS	4.26E-01	-0.0327	5.69E-01	NS
DYNC2LI1	NS	4.26E-01	3.27E-02	5.70E-01	NS
CLK1	NS	4.26E-01	3.27E-02	5.70E-01	NS
CCDC24	NS	4.26E-01	-0.0327	5.70E-01	NS
KRTAP5-8	NS	4.26E-01	-0.0327	5.70E-01	NS
SH3RF1	NS	4.27E-01	-0.0327	5.70E-01	NS
CHST11	NS	4.27E-01	-0.0327	5.70E-01	NS
ZFAND4	NS	4.27E-01	-0.0327	5.70E-01	NS
CNOT6	NS	4.27E-01	-0.0326	5.70E-01	NS
DLGAP4	NS	4.27E-01	3.26E-02	5.70E-01	NS
INHBC	NS	4.27E-01	-0.0326	5.71E-01	NS
OR4K14	NS	4.28E-01	-0.0326	5.71E-01	NS
PHB2	NS	4.27E-01	3.26E-02	5.71E-01	NS
SNX13	NS	4.28E-01	-0.0325	5.72E-01	NS
TSPAN2	NS	4.28E-01	3.25E-02	5.72E-01	NS
TTC8	NS	4.28E-01	-0.0325	5.72E-01	NS
ZNF567	NS	4.28E-01	-0.0325	5.72E-01	NS
NBPF4	NS	4.28E-01	-0.0325	5.72E-01	NS
RUNDC3A	NS	4.29E-01	3.25E-02	5.72E-01	NS
APOL2	NS	4.29E-01	3.25E-02	5.72E-01	NS
PDLIM1	NS	4.29E-01	-0.0325	5.73E-01	NS
AREL1	NS	4.29E-01	-0.0325	5.73E-01	NS
DENND2D	NS	4.29E-01	-0.0325	5.73E-01	NS
ZNF835	NS	4.29E-01	3.25E-02	5.73E-01	NS
TP53TG3C	NS	4.29E-01	-0.0325	5.73E-01	NS
PLAGL2	NS	4.30E-01	-0.0324	5.73E-01	NS
SAE1	NS	4.30E-01	-0.0324	5.73E-01	NS

SPRING1	NS	4.30E-01	-0.0324	5.73E-01	NS
KLK8	NS	4.30E-01	-0.0324	5.74E-01	NS
ULK2	NS	4.30E-01	-0.0324	5.74E-01	NS
FGF8	NS	4.30E-01	3.24E-02	5.74E-01	NS
CSMD3	NS	4.30E-01	-0.0324	5.74E-01	NS
TEKT1	NS	4.30E-01	-0.0324	5.74E-01	NS
POLDIP3	NS	4.30E-01	3.24E-02	5.74E-01	NS
SEC61A2	NS	4.30E-01	3.24E-02	5.74E-01	NS
ABCA1	NS	4.30E-01	-0.0324	5.74E-01	NS
PCDHAC1	NS	4.30E-01	3.24E-02	5.74E-01	NS
KLF4	NS	4.31E-01	3.24E-02	5.74E-01	NS
ATP6V1G1	NS	4.31E-01	-0.0324	5.74E-01	NS
CACNA1G	NS	4.31E-01	3.24E-02	5.74E-01	NS
DUSP12	NS	4.31E-01	-0.0324	5.74E-01	NS
SULT1A3	NS	4.31E-01	3.23E-02	5.74E-01	NS
ZBTB7B	NS	4.31E-01	3.23E-02	5.74E-01	NS
KRTAP5-9	NS	4.31E-01	-0.0323	5.75E-01	NS
AAMDC	NS	4.31E-01	-0.0323	5.75E-01	NS
PHF1	NS	4.32E-01	-0.0323	5.75E-01	NS
TPP2	NS	4.32E-01	-0.0323	5.75E-01	NS
NSA2	NS	4.32E-01	-0.0323	5.75E-01	NS
FIGLA	NS	4.32E-01	-0.0323	5.75E-01	NS
HYPK	NS	4.32E-01	-0.0323	5.76E-01	NS
DPAGT1	NS	4.32E-01	-0.0323	5.76E-01	NS
CASP14	NS	4.32E-01	3.22E-02	5.76E-01	NS
ZNF385B	NS	4.32E-01	-0.0322	5.76E-01	NS
GPHN	NS	4.32E-01	3.22E-02	5.76E-01	NS
CLDN4	NS	4.32E-01	3.22E-02	5.76E-01	NS
PCDHGC5	NS	4.33E-01	-0.0322	5.76E-01	NS
MR1	NS	4.33E-01	3.22E-02	5.76E-01	NS
ALOX15B	NS	4.33E-01	-0.0322	5.76E-01	NS
FADS1	NS	4.33E-01	-0.0322	5.76E-01	NS
MAP3K2	NS	4.33E-01	-0.0322	5.76E-01	NS
ZNF263	NS	4.33E-01	3.22E-02	5.76E-01	NS
ERVK3-1	NS	4.33E-01	3.22E-02	5.76E-01	NS
ZDHHC12	NS	4.33E-01	-0.0322	5.76E-01	NS
DLX2	NS	4.33E-01	3.22E-02	5.76E-01	NS

GGTLC1	NS	4.33E-01	-0.0322	5.76E-01	NS
ZNF334	NS	4.33E-01	-0.0322	5.76E-01	NS
PTPRN2	NS	4.33E-01	3.22E-02	5.76E-01	NS
DTNB	NS	4.33E-01	3.22E-02	5.77E-01	NS
GPD2	NS	4.34E-01	-0.0322	5.77E-01	NS
MTHFSD	NS	4.34E-01	-0.0322	5.77E-01	NS
METTL8	NS	4.34E-01	-0.0322	5.77E-01	NS
RORC	NS	4.34E-01	3.22E-02	5.77E-01	NS
EMP2	NS	4.34E-01	-0.0322	5.77E-01	NS
MED21	NS	4.34E-01	3.22E-02	5.77E-01	NS
FANCC	NS	4.34E-01	-0.0321	5.77E-01	NS
CEBPE	NS	4.34E-01	-0.0321	5.77E-01	NS
GCM1	NS	4.34E-01	-0.0321	5.77E-01	NS
SYT12	NS	4.34E-01	-0.0321	5.78E-01	NS
KCNH1	NS	4.35E-01	-0.0321	5.78E-01	NS
TCTA	NS	4.35E-01	-0.0321	5.78E-01	NS
WWP2	NS	4.35E-01	-0.0321	5.78E-01	NS
ZNF121	NS	4.35E-01	-0.0321	5.78E-01	NS
ZCCHC14	NS	4.35E-01	-0.0321	5.78E-01	NS
ANKRD45	NS	4.35E-01	3.21E-02	5.78E-01	NS
MLN	NS	4.35E-01	3.20E-02	5.78E-01	NS
SZRD1	NS	4.35E-01	-0.032	5.79E-01	NS
CDC16	NS	4.36E-01	-0.032	5.79E-01	NS
BARX2	NS	4.36E-01	-0.032	5.79E-01	NS
MST1	NS	4.36E-01	-0.032	5.79E-01	NS
LUC7L2	NS	4.36E-01	-0.032	5.79E-01	NS
TMOD1	NS	4.36E-01	-0.032	5.79E-01	NS
SKIV2L	NS	4.36E-01	3.20E-02	5.79E-01	NS
CCDC134	NS	4.36E-01	3.20E-02	5.79E-01	NS
IKBKB	NS	4.36E-01	-0.032	5.79E-01	NS
SLC16A7	NS	4.36E-01	-0.032	5.80E-01	NS
NEGR1	NS	4.36E-01	-0.032	5.80E-01	NS
PPP2R5B	NS	4.37E-01	3.19E-02	5.80E-01	NS
SYNDIG1	NS	4.37E-01	-0.0319	5.80E-01	NS
SPDYC	NS	4.37E-01	-0.0319	5.80E-01	NS
NATD1	NS	4.37E-01	-0.0319	5.80E-01	NS
POLA1	NS	4.37E-01	-0.0319	5.80E-01	NS

HSH2D	NS	4.37E-01	-0.0319	5.80E-01	NS
COLCA2	NS	4.37E-01	-0.0319	5.80E-01	NS
LGALS9	NS	4.37E-01	3.19E-02	5.80E-01	NS
NUDT9	NS	4.37E-01	-0.0319	5.80E-01	NS
STAMBP	NS	4.37E-01	-0.0319	5.80E-01	NS
KCNJ9	NS	4.37E-01	3.19E-02	5.80E-01	NS
DRGX	NS	4.37E-01	-0.0319	5.80E-01	NS
BTK	NS	4.37E-01	3.19E-02	5.80E-01	NS
PLEKHO2	NS	4.38E-01	3.19E-02	5.81E-01	NS
DAPK3	NS	4.38E-01	-0.0319	5.81E-01	NS
LIMK2	NS	4.38E-01	-0.0319	5.81E-01	NS
CRYL1	NS	4.38E-01	3.18E-02	5.81E-01	NS
STX10	NS	4.38E-01	3.18E-02	5.81E-01	NS
ZBTB10	NS	4.38E-01	3.18E-02	5.81E-01	NS
CNBP	NS	4.38E-01	3.18E-02	5.81E-01	NS
DOCK5	NS	4.39E-01	-0.0318	5.82E-01	NS
CRHR2	NS	4.39E-01	3.18E-02	5.82E-01	NS
PUS10	NS	4.39E-01	-0.0318	5.82E-01	NS
ADGRD1	NS	4.39E-01	3.18E-02	5.82E-01	NS
RINT1	NS	4.39E-01	-0.0318	5.82E-01	NS
FUT8	NS	4.39E-01	-0.0318	5.82E-01	NS
MCUR1	NS	4.39E-01	-0.0318	5.82E-01	NS
EBPL	NS	4.39E-01	-0.0318	5.82E-01	NS
ELN	NS	4.40E-01	3.17E-02	5.83E-01	NS
TBCCD1	NS	4.40E-01	-0.0317	5.83E-01	NS
ABCA13	NS	4.40E-01	-0.0317	5.83E-01	NS
MCRS1	NS	4.40E-01	-0.0317	5.83E-01	NS
POU6F2	NS	4.40E-01	-0.0317	5.83E-01	NS
FN1	NS	4.40E-01	3.17E-02	5.83E-01	NS
KIF2B	NS	4.40E-01	3.17E-02	5.83E-01	NS
FABP4	NS	4.40E-01	-0.0317	5.83E-01	NS
COPG1	NS	4.40E-01	3.17E-02	5.83E-01	NS
TAF11L14	NS	4.40E-01	3.17E-02	5.83E-01	NS
OR2M3	NS	4.41E-01	3.17E-02	5.83E-01	NS
TMED3	NS	4.41E-01	-0.0317	5.83E-01	NS
MAP6	NS	4.41E-01	3.17E-02	5.83E-01	NS
SOS1	NS	4.41E-01	-0.0317	5.84E-01	NS

SMYD1	NS	4.41E-01	-0.0317	5.84E-01	NS
COPS5	NS	4.41E-01	-0.0316	5.84E-01	NS
NAPB	NS	4.41E-01	3.16E-02	5.84E-01	NS
NSUN4	NS	4.41E-01	3.16E-02	5.84E-01	NS
STIMATE	NS	4.41E-01	3.16E-02	5.84E-01	NS
TMLHE	NS	4.41E-01	3.16E-02	5.84E-01	NS
TOR4A	NS	4.42E-01	-0.0316	5.84E-01	NS
INO80C	NS	4.42E-01	-0.0316	5.84E-01	NS
GTSE1	NS	4.42E-01	3.16E-02	5.85E-01	NS
SMIM44	NS	4.42E-01	3.16E-02	5.85E-01	NS
ROGDI	NS	4.42E-01	3.16E-02	5.85E-01	NS
CHGB	NS	4.42E-01	-0.0316	5.85E-01	NS
VRTN	NS	4.42E-01	3.16E-02	5.85E-01	NS
GOLGA1	NS	4.43E-01	-0.0315	5.85E-01	NS
ZBED1	NS	4.43E-01	3.15E-02	5.85E-01	NS
TRIM51GP	NS	4.43E-01	-0.0315	5.85E-01	NS
EMD	NS	4.43E-01	3.15E-02	5.86E-01	NS
HPS1	NS	4.43E-01	-0.0315	5.86E-01	NS
DUSP11	NS	4.43E-01	-0.0315	5.86E-01	NS
ENTR1	NS	4.43E-01	3.15E-02	5.86E-01	NS
DNAJC12	NS	4.43E-01	-0.0315	5.86E-01	NS
BRPF1	NS	4.43E-01	3.15E-02	5.86E-01	NS
ASPHD1	NS	4.43E-01	3.15E-02	5.86E-01	NS
RASSF1	NS	4.43E-01	-0.0315	5.86E-01	NS
SHE	NS	4.43E-01	-0.0315	5.86E-01	NS
VPS53	NS	4.44E-01	3.15E-02	5.86E-01	NS
OPRL1	NS	4.44E-01	-0.0315	5.86E-01	NS
ERC1	NS	4.44E-01	3.15E-02	5.86E-01	NS
COQ8A	NS	4.44E-01	3.14E-02	5.86E-01	NS
PRAMEF10	NS	4.44E-01	3.14E-02	5.86E-01	NS
TMEM184B	NS	4.44E-01	-0.0314	5.87E-01	NS
YAF2	NS	4.44E-01	-0.0314	5.87E-01	NS
POC1B-GALNT4	NS	4.44E-01	-0.0314	5.87E-01	NS
PLEKHG2	NS	4.44E-01	-0.0314	5.87E-01	NS
TTLL12	NS	4.44E-01	-0.0314	5.87E-01	NS
ITGA10	NS	4.45E-01	-0.0314	5.87E-01	NS
LRPAP1	NS	4.45E-01	-0.0314	5.87E-01	NS

OGDHL	NS	4.45E-01	3.14E-02	5.88E-01	NS
SCNM1	NS	4.45E-01	-0.0314	5.88E-01	NS
FOXR1	NS	4.45E-01	3.14E-02	5.88E-01	NS
PDXK	NS	4.45E-01	-0.0314	5.88E-01	NS
NAA38	NS	4.46E-01	3.13E-02	5.88E-01	NS
C16orf78	NS	4.46E-01	-0.0313	5.88E-01	NS
AMHR2	NS	4.46E-01	3.13E-02	5.89E-01	NS
KCNE4	NS	4.46E-01	-0.0313	5.89E-01	NS
RBM4	NS	4.46E-01	3.13E-02	5.89E-01	NS
MROH7-TTC4	NS	4.46E-01	-0.0313	5.89E-01	NS
ATP5MC1	NS	4.46E-01	-0.0313	5.89E-01	NS
PLN	NS	4.46E-01	-0.0313	5.89E-01	NS
RASGRF1	NS	4.46E-01	-0.0313	5.89E-01	NS
ALOX12	NS	4.46E-01	-0.0313	5.89E-01	NS
TUSC1	NS	4.47E-01	-0.0313	5.89E-01	NS
MRI1	NS	4.47E-01	3.13E-02	5.89E-01	NS
PRKACA	NS	4.47E-01	-0.0313	5.89E-01	NS
CDC37L1	NS	4.47E-01	-0.0312	5.89E-01	NS
MYO15A	NS	4.47E-01	3.12E-02	5.89E-01	NS
MYOC	NS	4.47E-01	-0.0312	5.89E-01	NS
LINC02218	NS	4.47E-01	-0.0312	5.90E-01	NS
OOSP3	NS	4.47E-01	-0.0312	5.90E-01	NS
CORO7	NS	4.47E-01	3.12E-02	5.90E-01	NS
SPATS2	NS	4.48E-01	3.12E-02	5.90E-01	NS
ZNF578	NS	4.48E-01	-0.0312	5.90E-01	NS
CLIP4	NS	4.48E-01	3.12E-02	5.90E-01	NS
CEP41	NS	4.48E-01	3.12E-02	5.90E-01	NS
ARMT1	NS	4.48E-01	-0.0312	5.90E-01	NS
PRAME	NS	4.48E-01	3.12E-02	5.90E-01	NS
SAA2	NS	4.48E-01	-0.0312	5.90E-01	NS
ETNK2	NS	4.48E-01	-0.0312	5.90E-01	NS
PIP4K2A	NS	4.48E-01	-0.0312	5.90E-01	NS
GOLGA7B	NS	4.48E-01	3.12E-02	5.90E-01	NS
SYNPR	NS	4.48E-01	-0.0312	5.90E-01	NS
FADS2	NS	4.48E-01	3.12E-02	5.90E-01	NS
ULBP1	NS	4.48E-01	3.11E-02	5.90E-01	NS
MCF2L	NS	4.48E-01	3.11E-02	5.90E-01	NS

KCTD3	NS	4.48E-01	-0.0311	5.91E-01	NS
FHIT	NS	4.49E-01	-0.0311	5.91E-01	NS
AP3M2	NS	4.49E-01	-0.0311	5.92E-01	NS
ATP6AP1	NS	4.49E-01	3.11E-02	5.92E-01	NS
TTC9C	NS	4.49E-01	-0.0311	5.92E-01	NS
RPS10	NS	4.49E-01	-0.0311	5.92E-01	NS
ACSS2	NS	4.49E-01	-0.0311	5.92E-01	NS
NDRG1	NS	4.50E-01	3.10E-02	5.92E-01	NS
BOLL	NS	4.50E-01	-0.031	5.92E-01	NS
SERPINA12	NS	4.50E-01	3.10E-02	5.92E-01	NS
ZPBP	NS	4.50E-01	-0.031	5.92E-01	NS
FAM91A1	NS	4.50E-01	3.10E-02	5.92E-01	NS
GSG1	NS	4.50E-01	-0.031	5.92E-01	NS
SCIN	NS	4.50E-01	-0.031	5.92E-01	NS
TCOF1	NS	4.51E-01	-0.031	5.92E-01	NS
MKNK1	NS	4.50E-01	3.10E-02	5.92E-01	NS
XYLT1	NS	4.50E-01	-0.031	5.92E-01	NS
MXD4	NS	4.51E-01	-0.031	5.92E-01	NS
BTN1A1	NS	4.51E-01	-0.031	5.92E-01	NS
RAB2B	NS	4.50E-01	-0.031	5.92E-01	NS
CDK8	NS	4.50E-01	3.10E-02	5.92E-01	NS
ITGB7	NS	4.51E-01	-0.031	5.92E-01	NS
ARF1	NS	4.51E-01	3.10E-02	5.92E-01	NS
MFSD6	NS	4.51E-01	3.10E-02	5.92E-01	NS
MAGED4	NS	4.51E-01	3.10E-02	5.92E-01	NS
PLPP3	NS	4.51E-01	-0.031	5.92E-01	NS
SIAH2	NS	4.50E-01	-0.031	5.92E-01	NS
GBP6	NS	4.51E-01	-0.031	5.92E-01	NS
ERC2	NS	4.50E-01	-0.031	5.92E-01	NS
CALM1	NS	4.51E-01	-0.031	5.92E-01	NS
GIMAP1-GIMAP5	NS	4.51E-01	3.10E-02	5.92E-01	NS
DUS4L-BCAP29	NS	4.50E-01	-0.031	5.92E-01	NS
CDK2AP2	NS	4.51E-01	3.10E-02	5.93E-01	NS
PPP2R5C	NS	4.51E-01	-0.0309	5.93E-01	NS
MGAT5B	NS	4.51E-01	-0.0309	5.93E-01	NS
RBSN	NS	4.51E-01	-0.0309	5.93E-01	NS
PPIG	NS	4.51E-01	-0.0309	5.93E-01	NS

P2RX2	NS	4.51E-01	3.09E-02	5.93E-01	NS
TUBA3D	NS	4.52E-01	-0.0309	5.93E-01	NS
DBNL	NS	4.52E-01	-0.0309	5.93E-01	NS
CACNG8	NS	4.52E-01	-0.0309	5.93E-01	NS
SLC20A1	NS	4.52E-01	3.09E-02	5.93E-01	NS
SLC8B1	NS	4.52E-01	-0.0309	5.93E-01	NS
ATP5MG	NS	4.52E-01	3.09E-02	5.93E-01	NS
FSCN2	NS	4.52E-01	3.09E-02	5.93E-01	NS
SLC35F6	NS	4.52E-01	-0.0309	5.93E-01	NS
COL13A1	NS	4.52E-01	-0.0309	5.93E-01	NS
LRRN4	NS	4.52E-01	3.09E-02	5.94E-01	NS
BSDC1	NS	4.52E-01	3.09E-02	5.94E-01	NS
MGAT4A	NS	4.52E-01	3.09E-02	5.94E-01	NS
ABHD1	NS	4.52E-01	-0.0309	5.94E-01	NS
TMPRSS15	NS	4.53E-01	3.08E-02	5.94E-01	NS
CACNB4	NS	4.53E-01	-0.0308	5.94E-01	NS
UBA7	NS	4.53E-01	-0.0308	5.94E-01	NS
IL13RA1	NS	4.53E-01	3.08E-02	5.95E-01	NS
POLG	NS	4.53E-01	-0.0308	5.95E-01	NS
OTOP2	NS	4.53E-01	-0.0308	5.95E-01	NS
CD22	NS	4.53E-01	3.08E-02	5.95E-01	NS
ARAP2	NS	4.54E-01	3.08E-02	5.95E-01	NS
SEMA4C	NS	4.53E-01	-0.0308	5.95E-01	NS
TRIP10	NS	4.54E-01	-0.0308	5.95E-01	NS
RIBC1	NS	4.54E-01	-0.0308	5.95E-01	NS
SAP18	NS	4.54E-01	3.08E-02	5.95E-01	NS
RSPH10B	NS	4.54E-01	-0.0308	5.95E-01	NS
ACOX3	NS	4.54E-01	-0.0308	5.95E-01	NS
EOGT	NS	4.54E-01	-0.0308	5.95E-01	NS
DIRAS2	NS	4.54E-01	-0.0308	5.95E-01	NS
NMD3	NS	4.54E-01	-0.0308	5.95E-01	NS
OR8I2	NS	4.54E-01	-0.0308	5.95E-01	NS
UBQLN2	NS	4.54E-01	3.08E-02	5.95E-01	NS
INF2	NS	4.54E-01	-0.0307	5.95E-01	NS
ST3GAL5	NS	4.54E-01	-0.0307	5.95E-01	NS
GATB	NS	4.54E-01	-0.0307	5.95E-01	NS
DOK7	NS	4.54E-01	3.07E-02	5.95E-01	NS

TEAD3	NS	4.55E-01	3.07E-02	5.96E-01	NS
PAFAH2	NS	4.55E-01	-0.0307	5.96E-01	NS
TWNK	NS	4.55E-01	-0.0307	5.96E-01	NS
GSC	NS	4.55E-01	3.07E-02	5.96E-01	NS
MINAR2	NS	4.55E-01	-0.0307	5.96E-01	NS
SELPLG	NS	4.55E-01	3.07E-02	5.96E-01	NS
PABPC4L	NS	4.55E-01	3.07E-02	5.96E-01	NS
METTL17	NS	4.55E-01	3.07E-02	5.96E-01	NS
ROBO4	NS	4.56E-01	-0.0306	5.97E-01	NS
LARS2	NS	4.56E-01	-0.0306	5.97E-01	NS
HDAC5	NS	4.56E-01	3.06E-02	5.97E-01	NS
VCX	NS	4.56E-01	3.06E-02	5.97E-01	NS
R3HCC1L	NS	4.56E-01	3.06E-02	5.97E-01	NS
DRAM2	NS	4.57E-01	-0.0306	5.98E-01	NS
AMOTL2	NS	4.57E-01	-0.0306	5.98E-01	NS
SLCO2A1	NS	4.57E-01	3.06E-02	5.98E-01	NS
SIGLEC5	NS	4.57E-01	-0.0305	5.98E-01	NS
NR6A1	NS	4.57E-01	3.05E-02	5.98E-01	NS
GRIN2C	NS	4.57E-01	-0.0305	5.98E-01	NS
CGAS	NS	4.57E-01	-0.0305	5.98E-01	NS
CYCS	NS	4.57E-01	-0.0305	5.98E-01	NS
CNEP1R1	NS	4.57E-01	-0.0305	5.98E-01	NS
KRTAP4-11	NS	4.58E-01	-0.0305	5.98E-01	NS
CECR2	NS	4.58E-01	3.05E-02	5.99E-01	NS
UBQLNL	NS	4.58E-01	3.05E-02	5.99E-01	NS
UNC5D	NS	4.58E-01	-0.0305	5.99E-01	NS
CCR3	NS	4.58E-01	-0.0305	5.99E-01	NS
HLA-DQA2	NS	4.58E-01	3.05E-02	5.99E-01	NS
DGCR6	NS	4.58E-01	-0.0305	5.99E-01	NS
DKK3	NS	4.58E-01	-0.0305	5.99E-01	NS
CRAMP1	NS	4.58E-01	-0.0305	5.99E-01	NS
OR2M2	NS	4.58E-01	-0.0305	5.99E-01	NS
GAGE1	NS	4.58E-01	-0.0305	5.99E-01	NS
RBMX	NS	4.58E-01	3.05E-02	5.99E-01	NS
TSPAN16	NS	4.59E-01	-0.0304	5.99E-01	NS
KCNT1	NS	4.59E-01	-0.0304	6.00E-01	NS
UBE3B	NS	4.59E-01	3.04E-02	6.00E-01	NS

OTULINL	NS	4.59E-01	3.04E-02	6.00E-01	NS
ARRDC4	NS	4.59E-01	-0.0304	6.00E-01	NS
DAZ3	NS	4.59E-01	3.04E-02	6.00E-01	NS
MAP3K12	NS	4.59E-01	3.04E-02	6.00E-01	NS
CORO7-PAM16	NS	4.60E-01	-0.0304	6.00E-01	NS
GNAI1	NS	4.60E-01	-0.0304	6.00E-01	NS
EMP1	NS	4.60E-01	-0.0304	6.00E-01	NS
MAGEC1	NS	4.60E-01	-0.0304	6.00E-01	NS
TLR3	NS	4.60E-01	-0.0304	6.00E-01	NS
TPSAB1	NS	4.60E-01	3.04E-02	6.00E-01	NS
IL17RC	NS	4.60E-01	-0.0304	6.00E-01	NS
ZDHHC16	NS	4.60E-01	3.04E-02	6.00E-01	NS
OR9G4	NS	4.60E-01	3.04E-02	6.01E-01	NS
AARS2	NS	4.60E-01	-0.0303	6.01E-01	NS
TSSC4	NS	4.60E-01	-0.0303	6.01E-01	NS
MTA3	NS	4.60E-01	3.03E-02	6.01E-01	NS
TRIM34	NS	4.60E-01	-0.0303	6.01E-01	NS
BTBD18	NS	4.61E-01	3.03E-02	6.01E-01	NS
RHEX	NS	4.61E-01	-0.0303	6.01E-01	NS
RIMBP3C	NS	4.61E-01	3.03E-02	6.01E-01	NS
SNX7	NS	4.61E-01	-0.0303	6.01E-01	NS
HCFC1	NS	4.61E-01	3.03E-02	6.01E-01	NS
IFNA2	NS	4.61E-01	-0.0303	6.02E-01	NS
TVP23C	NS	4.61E-01	-0.0303	6.02E-01	NS
USH1G	NS	4.61E-01	3.03E-02	6.02E-01	NS
RPF2	NS	4.61E-01	-0.0303	6.02E-01	NS
RANBP3	NS	4.61E-01	-0.0303	6.02E-01	NS
OR1I1	NS	4.62E-01	3.02E-02	6.02E-01	NS
SIGLEC9	NS	4.62E-01	-0.0302	6.02E-01	NS
RBFOX3	NS	4.62E-01	-0.0302	6.02E-01	NS
MID1IP1	NS	4.62E-01	3.02E-02	6.03E-01	NS
CRABP1	NS	4.62E-01	-0.0302	6.03E-01	NS
PHLDA2	NS	4.62E-01	3.02E-02	6.03E-01	NS
ATG4D	NS	4.62E-01	-0.0302	6.03E-01	NS
CPM	NS	4.63E-01	3.02E-02	6.03E-01	NS
CALU	NS	4.63E-01	-0.0302	6.03E-01	NS
HLA-DQB1	NS	4.63E-01	-0.0302	6.03E-01	NS

EPG5	NS	4.63E-01	3.02E-02	6.03E-01	NS
TFR2	NS	4.63E-01	3.01E-02	6.04E-01	NS
C3orf22	NS	4.63E-01	-0.0301	6.04E-01	NS
SNX1	NS	4.63E-01	3.01E-02	6.04E-01	NS
RSPH9	NS	4.63E-01	-0.0301	6.04E-01	NS
OSBPL10	NS	4.63E-01	-0.0301	6.04E-01	NS
TSEN2	NS	4.64E-01	-0.0301	6.04E-01	NS
ATRIP	NS	4.64E-01	-0.0301	6.04E-01	NS
COPZ1	NS	4.64E-01	-0.0301	6.04E-01	NS
KLHL35	NS	4.64E-01	-0.0301	6.04E-01	NS
RASA3	NS	4.64E-01	-0.0301	6.04E-01	NS
ZRANB1	NS	4.64E-01	3.01E-02	6.05E-01	NS
GPR17	NS	4.64E-01	3.01E-02	6.05E-01	NS
EML3	NS	4.64E-01	3.01E-02	6.05E-01	NS
ETDC	NS	4.64E-01	-0.0301	6.05E-01	NS
ZFR	NS	4.64E-01	3.01E-02	6.05E-01	NS
SMARCAL1	NS	4.64E-01	3.00E-02	6.05E-01	NS
MOB3A	NS	4.65E-01	-0.03	6.05E-01	NS
SPDYE21	NS	4.65E-01	3.00E-02	6.05E-01	NS
BLK	NS	4.65E-01	3.00E-02	6.05E-01	NS
SYT13	NS	4.65E-01	-0.03	6.05E-01	NS
EPO	NS	4.65E-01	3.00E-02	6.05E-01	NS
AGTPBP1	NS	4.65E-01	-0.03	6.05E-01	NS
PSMG3	NS	4.65E-01	-0.03	6.05E-01	NS
PACS1	NS	4.65E-01	3.00E-02	6.05E-01	NS
CD63	NS	4.65E-01	-0.03	6.05E-01	NS
OLIG3	NS	4.65E-01	3.00E-02	6.05E-01	NS
LMF1	NS	4.66E-01	-0.03	6.06E-01	NS
GRP	NS	4.65E-01	-0.03	6.06E-01	NS
PRAMEF15	NS	4.66E-01	-0.03	6.06E-01	NS
SYP	NS	4.66E-01	-0.03	6.06E-01	NS
MAP1LC3B	NS	4.66E-01	3.00E-02	6.06E-01	NS
LRRC45	NS	4.66E-01	-0.03	6.06E-01	NS
PIK3C3	NS	4.66E-01	-0.03	6.06E-01	NS
NHEJ1	NS	4.66E-01	-0.03	6.06E-01	NS
AJAP1	NS	4.66E-01	-0.0299	6.06E-01	NS
CERS1	NS	4.66E-01	-0.0299	6.06E-01	NS

PRR29	NS	4.66E-01	-0.0299	6.06E-01	NS
GOT2	NS	4.66E-01	2.99E-02	6.06E-01	NS
ZNF207	NS	4.66E-01	-0.0299	6.06E-01	NS
CLDN23	NS	4.66E-01	-0.0299	6.06E-01	NS
INSRR	NS	4.66E-01	-0.0299	6.06E-01	NS
ZNF394	NS	4.66E-01	-0.0299	6.06E-01	NS
SMAD7	NS	4.67E-01	2.99E-02	6.06E-01	NS
ANKS6	NS	4.67E-01	2.99E-02	6.07E-01	NS
C19orf47	NS	4.67E-01	2.99E-02	6.07E-01	NS
ELOA2	NS	4.67E-01	-0.0299	6.07E-01	NS
PHYKPL	NS	4.68E-01	2.98E-02	6.08E-01	NS
ARL14	NS	4.68E-01	2.98E-02	6.08E-01	NS
BCL2L13	NS	4.68E-01	2.98E-02	6.08E-01	NS
RBFA	NS	4.68E-01	-0.0298	6.08E-01	NS
SCN2B	NS	4.68E-01	2.98E-02	6.08E-01	NS
MARK4	NS	4.68E-01	2.98E-02	6.08E-01	NS
CRACR2A	NS	4.68E-01	2.98E-02	6.08E-01	NS
C12orf71	NS	4.68E-01	-0.0298	6.08E-01	NS
LRRC52	NS	4.68E-01	-0.0298	6.08E-01	NS
TMEM17	NS	4.68E-01	2.98E-02	6.08E-01	NS
HOXA13	NS	4.69E-01	-0.0298	6.08E-01	NS
LAIR2	NS	4.69E-01	-0.0298	6.09E-01	NS
LHFPL3	NS	4.69E-01	-0.0298	6.09E-01	NS
YIPF1	NS	4.69E-01	2.97E-02	6.09E-01	NS
MCM4	NS	4.69E-01	-0.0297	6.09E-01	NS
CCND2	NS	4.69E-01	-0.0297	6.09E-01	NS
UTY	NS	4.69E-01	2.97E-02	6.09E-01	NS
SLITRK3	NS	4.69E-01	2.97E-02	6.09E-01	NS
NUDT10	NS	4.69E-01	-0.0297	6.09E-01	NS
STARD9	NS	4.69E-01	-0.0297	6.09E-01	NS
CBLN3	NS	4.69E-01	-0.0297	6.09E-01	NS
DNAJC21	NS	4.70E-01	-0.0297	6.09E-01	NS
LRRC41	NS	4.70E-01	2.97E-02	6.10E-01	NS
GLYATL2	NS	4.70E-01	-0.0297	6.10E-01	NS
RUNX1	NS	4.70E-01	-0.0297	6.10E-01	NS
DEFB1	NS	4.70E-01	2.97E-02	6.10E-01	NS
EXD3	NS	4.70E-01	-0.0297	6.10E-01	NS

EHD3	NS	4.70E-01	-0.0296	6.10E-01	NS
CHN2	NS	4.70E-01	-0.0296	6.10E-01	NS
FBXL18	NS	4.70E-01	-0.0296	6.10E-01	NS
TUBA1C	NS	4.71E-01	-0.0296	6.10E-01	NS
CHURC1-FNTB	NS	4.71E-01	-0.0296	6.10E-01	NS
MZT2B	NS	4.71E-01	-0.0296	6.10E-01	NS
TAF15	NS	4.71E-01	2.96E-02	6.10E-01	NS
EVI5	NS	4.71E-01	-0.0296	6.10E-01	NS
FXYD3	NS	4.71E-01	2.96E-02	6.10E-01	NS
TNFSF12	NS	4.71E-01	-0.0296	6.10E-01	NS
CD37	NS	4.71E-01	2.96E-02	6.10E-01	NS
KAT2B	NS	4.71E-01	-0.0296	6.10E-01	NS
OR11H6	NS	4.71E-01	2.96E-02	6.11E-01	NS
CRBN	NS	4.71E-01	-0.0296	6.11E-01	NS
PRPF40A	NS	4.71E-01	-0.0296	6.11E-01	NS
SCNN1B	NS	4.71E-01	2.96E-02	6.11E-01	NS
LRRC23	NS	4.71E-01	2.96E-02	6.11E-01	NS
PRRX2	NS	4.71E-01	2.96E-02	6.11E-01	NS
SLC12A3	NS	4.72E-01	2.96E-02	6.11E-01	NS
TAF11L6	NS	4.72E-01	-0.0296	6.11E-01	NS
TBC1D9B	NS	4.72E-01	-0.0295	6.11E-01	NS
FTCDNL1	NS	4.72E-01	-0.0295	6.12E-01	NS
BHLHA15	NS	4.72E-01	-0.0295	6.12E-01	NS
RBM5	NS	4.73E-01	-0.0295	6.12E-01	NS
CD274	NS	4.73E-01	-0.0295	6.12E-01	NS
MBP	NS	4.73E-01	-0.0295	6.12E-01	NS
ZSCAN31	NS	4.73E-01	-0.0295	6.12E-01	NS
ISY1	NS	4.73E-01	-0.0295	6.12E-01	NS
TASOR	NS	4.73E-01	2.95E-02	6.12E-01	NS
ECEL1	NS	4.73E-01	2.95E-02	6.12E-01	NS
PLEKHJ1	NS	4.73E-01	-0.0295	6.12E-01	NS
C15orf65	NS	4.73E-01	-0.0295	6.12E-01	NS
GBA2	NS	4.73E-01	-0.0295	6.12E-01	NS
GPR119	NS	4.73E-01	2.95E-02	6.12E-01	NS
SSR4	NS	4.73E-01	-0.0295	6.12E-01	NS
ADRA2B	NS	4.73E-01	-0.0295	6.12E-01	NS
GBF1	NS	4.74E-01	2.94E-02	6.13E-01	NS

CFDP1	NS	4.74E-01	2.94E-02	6.13E-01	NS
SETDB1	NS	4.74E-01	-0.0294	6.13E-01	NS
TMEM51	NS	4.74E-01	-0.0294	6.13E-01	NS
KCNH2	NS	4.74E-01	2.94E-02	6.13E-01	NS
SLC6A8	NS	4.74E-01	-0.0294	6.13E-01	NS
TIAL1	NS	4.74E-01	-0.0294	6.14E-01	NS
NRBP1	NS	4.74E-01	-0.0294	6.14E-01	NS
PAGR1	NS	4.75E-01	2.94E-02	6.14E-01	NS
AKAP8	NS	4.75E-01	2.94E-02	6.14E-01	NS
LRRC4B	NS	4.75E-01	-0.0294	6.14E-01	NS
GALK2	NS	4.75E-01	2.93E-02	6.14E-01	NS
TMED10	NS	4.75E-01	-0.0294	6.14E-01	NS
MAGEA12	NS	4.75E-01	2.93E-02	6.14E-01	NS
CNNM3	NS	4.75E-01	-0.0293	6.14E-01	NS
CCDC198	NS	4.75E-01	2.93E-02	6.14E-01	NS
RFPL2	NS	4.75E-01	2.93E-02	6.14E-01	NS
NUTM1	NS	4.75E-01	-0.0293	6.14E-01	NS
UPK3BL1	NS	4.75E-01	-0.0293	6.14E-01	NS
ABI1	NS	4.76E-01	2.93E-02	6.14E-01	NS
MPV17	NS	4.76E-01	-0.0293	6.15E-01	NS
FLG2	NS	4.76E-01	-0.0293	6.15E-01	NS
OTUD5	NS	4.76E-01	2.93E-02	6.15E-01	NS
FAM237B	NS	4.76E-01	2.93E-02	6.15E-01	NS
SLC25A28	NS	4.76E-01	-0.0293	6.15E-01	NS
MAGEC3	NS	4.76E-01	2.93E-02	6.15E-01	NS
OR52M1	NS	4.76E-01	-0.0293	6.15E-01	NS
ZFP82	NS	4.76E-01	-0.0293	6.15E-01	NS
WDR6	NS	4.76E-01	-0.0293	6.15E-01	NS
USP15	NS	4.76E-01	2.92E-02	6.15E-01	NS
COL25A1	NS	4.77E-01	-0.0292	6.15E-01	NS
TNIP3	NS	4.77E-01	-0.0292	6.15E-01	NS
NLK	NS	4.77E-01	-0.0292	6.15E-01	NS
GPATCH2	NS	4.77E-01	2.92E-02	6.15E-01	NS
DRG2	NS	4.77E-01	2.92E-02	6.15E-01	NS
SEPTIN6	NS	4.77E-01	-0.0292	6.15E-01	NS
PSMB8	NS	4.77E-01	2.92E-02	6.15E-01	NS
NARF	NS	4.77E-01	-0.0292	6.15E-01	NS

CRTAP	NS	4.77E-01	2.92E-02	6.15E-01	NS
CD74	NS	4.77E-01	2.92E-02	6.15E-01	NS
RYR1	NS	4.77E-01	-0.0292	6.15E-01	NS
REEP3	NS	4.77E-01	-0.0292	6.16E-01	NS
SMIM14	NS	4.77E-01	-0.0292	6.16E-01	NS
USP48	NS	4.77E-01	2.92E-02	6.16E-01	NS
LRP1	NS	4.77E-01	-0.0292	6.16E-01	NS
C17orf113	NS	4.77E-01	-0.0292	6.16E-01	NS
ALPL	NS	4.77E-01	2.92E-02	6.16E-01	NS
HSPB8	NS	4.78E-01	-0.0292	6.16E-01	NS
FBRSL1	NS	4.78E-01	2.92E-02	6.16E-01	NS
WEE1	NS	4.78E-01	2.92E-02	6.16E-01	NS
LONP1	NS	4.78E-01	-0.0292	6.16E-01	NS
DGLUCY	NS	4.78E-01	-0.0292	6.16E-01	NS
ZXDA	NS	4.78E-01	2.92E-02	6.16E-01	NS
MAFB	NS	4.78E-01	-0.0292	6.16E-01	NS
NUP50	NS	4.78E-01	-0.0292	6.16E-01	NS
RNASE2	NS	4.78E-01	2.91E-02	6.16E-01	NS
ADAM21	NS	4.78E-01	2.91E-02	6.16E-01	NS
C12orf50	NS	4.78E-01	2.91E-02	6.16E-01	NS
GLB1L	NS	4.78E-01	-0.0291	6.16E-01	NS
MED10	NS	4.78E-01	2.91E-02	6.16E-01	NS
IL21	NS	4.78E-01	-0.0291	6.16E-01	NS
PFKFB3	NS	4.79E-01	-0.0291	6.17E-01	NS
CHRM5	NS	4.79E-01	-0.0291	6.17E-01	NS
SYT15	NS	4.79E-01	2.91E-02	6.17E-01	NS
B3GALT5	NS	4.79E-01	2.91E-02	6.17E-01	NS
KRTAP21-2	NS	4.79E-01	2.91E-02	6.17E-01	NS
SHOX	NS	4.79E-01	-0.0291	6.17E-01	NS
CRNKL1	NS	4.79E-01	-0.0291	6.17E-01	NS
HHEX	NS	4.79E-01	2.91E-02	6.17E-01	NS
UBLCP1	NS	4.80E-01	2.90E-02	6.18E-01	NS
ARID3A	NS	4.80E-01	-0.029	6.18E-01	NS
TSGA10	NS	4.80E-01	2.90E-02	6.18E-01	NS
TIMM23	NS	4.80E-01	2.90E-02	6.18E-01	NS
SNRPB	NS	4.80E-01	-0.029	6.18E-01	NS
MGST1	NS	4.80E-01	2.90E-02	6.18E-01	NS

TNXB	NS	4.80E-01	2.90E-02	6.18E-01	NS
OGDH	NS	4.80E-01	-0.029	6.18E-01	NS
NUSAP1	NS	4.80E-01	2.90E-02	6.18E-01	NS
MAGI1	NS	4.81E-01	2.90E-02	6.19E-01	NS
ACIN1	NS	4.81E-01	-0.029	6.19E-01	NS
PPARD	NS	4.81E-01	2.90E-02	6.19E-01	NS
CEL	NS	4.81E-01	2.90E-02	6.19E-01	NS
AMZ1	NS	4.81E-01	-0.029	6.19E-01	NS
IRAK2	NS	4.81E-01	-0.029	6.19E-01	NS
CCT3	NS	4.81E-01	-0.0289	6.19E-01	NS
RARG	NS	4.81E-01	-0.0289	6.19E-01	NS
LATS1	NS	4.81E-01	2.89E-02	6.19E-01	NS
MAPK3	NS	4.81E-01	2.89E-02	6.19E-01	NS
GUCY1A2	NS	4.81E-01	2.89E-02	6.19E-01	NS
GFAP	NS	4.81E-01	-0.0289	6.19E-01	NS
GNAI2	NS	4.82E-01	2.89E-02	6.19E-01	NS
TLR6	NS	4.82E-01	-0.0289	6.19E-01	NS
OR2C3	NS	4.82E-01	2.89E-02	6.19E-01	NS
GPBP1L1	NS	4.82E-01	2.89E-02	6.19E-01	NS
MIDEAS	NS	4.82E-01	2.89E-02	6.20E-01	NS
KIFC3	NS	4.82E-01	-0.0289	6.20E-01	NS
SLC7A9	NS	4.82E-01	-0.0289	6.20E-01	NS
SART3	NS	4.82E-01	2.89E-02	6.20E-01	NS
IFNA21	NS	4.82E-01	-0.0289	6.20E-01	NS
TMEM196	NS	4.82E-01	-0.0289	6.20E-01	NS
ZNF772	NS	4.82E-01	2.89E-02	6.20E-01	NS
GIPR	NS	4.82E-01	2.88E-02	6.20E-01	NS
UBE2D3	NS	4.83E-01	2.88E-02	6.20E-01	NS
PPP1R7	NS	4.83E-01	-0.0288	6.20E-01	NS
HAUS4	NS	4.83E-01	-0.0288	6.20E-01	NS
MTDH	NS	4.83E-01	2.88E-02	6.20E-01	NS
SPATA21	NS	4.83E-01	-0.0288	6.20E-01	NS
ZNF800	NS	4.83E-01	2.88E-02	6.20E-01	NS
USP13	NS	4.83E-01	-0.0288	6.20E-01	NS
DBP	NS	4.83E-01	2.88E-02	6.20E-01	NS
ARRDC1	NS	4.83E-01	2.88E-02	6.20E-01	NS
TMEM211	NS	4.83E-01	-0.0288	6.20E-01	NS

NTHL1	NS	4.83E-01	-0.0288	6.20E-01	NS
HSD17B10	NS	4.83E-01	2.88E-02	6.20E-01	NS
FAM71E1	NS	4.83E-01	-0.0288	6.20E-01	NS
RGPD3	NS	4.83E-01	-0.0288	6.21E-01	NS
COL2A1	NS	4.83E-01	-0.0288	6.21E-01	NS
CEACAM20	NS	4.84E-01	2.88E-02	6.21E-01	NS
H4C12	NS	4.84E-01	-0.0287	6.21E-01	NS
ABCA9	NS	4.84E-01	2.87E-02	6.21E-01	NS
CCL28	NS	4.84E-01	2.87E-02	6.21E-01	NS
DNAJC25	NS	4.84E-01	2.87E-02	6.22E-01	NS
MMP19	NS	4.84E-01	2.87E-02	6.22E-01	NS
PBX2	NS	4.84E-01	2.87E-02	6.22E-01	NS
IRGM	NS	4.85E-01	-0.0287	6.22E-01	NS
VSTM2A	NS	4.85E-01	2.87E-02	6.22E-01	NS
VPS26C	NS	4.85E-01	2.87E-02	6.22E-01	NS
MUC16	NS	4.85E-01	-0.0287	6.22E-01	NS
YTHDC1	NS	4.85E-01	2.87E-02	6.22E-01	NS
RBL1	NS	4.85E-01	-0.0287	6.22E-01	NS
PNRC1	NS	4.86E-01	2.86E-02	6.23E-01	NS
LCP2	NS	4.86E-01	-0.0286	6.23E-01	NS
ARAP1	NS	4.86E-01	2.86E-02	6.23E-01	NS
PCDHB14	NS	4.86E-01	-0.0286	6.23E-01	NS
OR5M11	NS	4.86E-01	2.86E-02	6.23E-01	NS
ARL5B	NS	4.86E-01	-0.0286	6.23E-01	NS
TRIM56	NS	4.86E-01	-0.0286	6.23E-01	NS
ASDURF	NS	4.86E-01	2.86E-02	6.23E-01	NS
MINK1	NS	4.86E-01	2.86E-02	6.24E-01	NS
GRK2	NS	4.87E-01	2.86E-02	6.24E-01	NS
MGME1	NS	4.87E-01	-0.0286	6.24E-01	NS
PIP4K2C	NS	4.87E-01	-0.0286	6.24E-01	NS
ADCY3	NS	4.87E-01	-0.0286	6.24E-01	NS
SPRR4	NS	4.87E-01	2.86E-02	6.24E-01	NS
SNAI3	NS	4.87E-01	-0.0286	6.24E-01	NS
TMEM212	NS	4.87E-01	-0.0286	6.24E-01	NS
NUP54	NS	4.87E-01	2.85E-02	6.24E-01	NS
POGLUT3	NS	4.87E-01	-0.0285	6.24E-01	NS
NETO1	NS	4.87E-01	-0.0285	6.24E-01	NS

VCX3A	NS	4.87E-01	2.85E-02	6.24E-01	NS
SP110	NS	4.88E-01	-0.0285	6.24E-01	NS
NDUFA11	NS	4.88E-01	-0.0285	6.24E-01	NS
ZFP92	NS	4.88E-01	2.85E-02	6.25E-01	NS
LMNA	NS	4.88E-01	2.85E-02	6.25E-01	NS
NGF	NS	4.88E-01	-0.0285	6.25E-01	NS
HK1	NS	4.88E-01	-0.0285	6.25E-01	NS
FBRS	NS	4.88E-01	2.85E-02	6.25E-01	NS
SIAE	NS	4.88E-01	-0.0285	6.25E-01	NS
SGPP1	NS	4.88E-01	2.85E-02	6.25E-01	NS
ESYT1	NS	4.88E-01	-0.0285	6.25E-01	NS
CDH22	NS	4.88E-01	2.85E-02	6.25E-01	NS
TLX2	NS	4.89E-01	-0.0284	6.25E-01	NS
PTRH1	NS	4.89E-01	-0.0284	6.25E-01	NS
DCP1A	NS	4.89E-01	2.84E-02	6.25E-01	NS
NOP10	NS	4.89E-01	2.84E-02	6.26E-01	NS
KCNMB1	NS	4.89E-01	-0.0284	6.26E-01	NS
TEX13A	NS	4.89E-01	2.84E-02	6.26E-01	NS
PLCL1	NS	4.89E-01	-0.0284	6.26E-01	NS
ERCC1	NS	4.89E-01	-0.0284	6.26E-01	NS
OR7A17	NS	4.89E-01	-0.0284	6.26E-01	NS
METTL22	NS	4.89E-01	-0.0284	6.26E-01	NS
CD109	NS	4.89E-01	-0.0284	6.26E-01	NS
VWC2	NS	4.89E-01	2.84E-02	6.26E-01	NS
ITPKB	NS	4.90E-01	2.84E-02	6.26E-01	NS
ANO10	NS	4.90E-01	2.84E-02	6.26E-01	NS
SERTAD1	NS	4.90E-01	-0.0284	6.26E-01	NS
NKAPD1	NS	4.90E-01	2.84E-02	6.27E-01	NS
SMR3B	NS	4.90E-01	2.84E-02	6.27E-01	NS
TMPPE	NS	4.90E-01	-0.0283	6.27E-01	NS
MIEF1	NS	4.90E-01	-0.0283	6.27E-01	NS
MRGPRF	NS	4.90E-01	-0.0283	6.27E-01	NS
ZBTB24	NS	4.91E-01	2.83E-02	6.27E-01	NS
UFM1	NS	4.91E-01	-0.0283	6.27E-01	NS
CHPF	NS	4.91E-01	2.83E-02	6.27E-01	NS
FAM209A	NS	4.91E-01	-0.0283	6.27E-01	NS
GAB1	NS	4.91E-01	-0.0283	6.27E-01	NS

JAML	NS	4.91E-01	-0.0283	6.27E-01	NS
SIM1	NS	4.91E-01	2.83E-02	6.27E-01	NS
HMCN1	NS	4.91E-01	-0.0283	6.27E-01	NS
ZSWIM8	NS	4.91E-01	-0.0283	6.27E-01	NS
SLC17A5	NS	4.91E-01	2.83E-02	6.27E-01	NS
SH3BGRL	NS	4.91E-01	2.83E-02	6.27E-01	NS
CCDC180	NS	4.91E-01	-0.0283	6.27E-01	NS
RNF208	NS	4.91E-01	2.83E-02	6.27E-01	NS
UHRF1	NS	4.91E-01	2.83E-02	6.27E-01	NS
DGCR2	NS	4.91E-01	2.83E-02	6.28E-01	NS
VILL	NS	4.91E-01	2.83E-02	6.28E-01	NS
VPS45	NS	4.91E-01	2.83E-02	6.28E-01	NS
EPHB2	NS	4.92E-01	-0.0283	6.28E-01	NS
DCTN4	NS	4.92E-01	-0.0282	6.28E-01	NS
OR51F1	NS	4.92E-01	-0.0282	6.28E-01	NS
CDCA5	NS	4.92E-01	-0.0282	6.28E-01	NS
CSNK2B	NS	4.92E-01	-0.0282	6.28E-01	NS
PACSIN3	NS	4.92E-01	2.82E-02	6.28E-01	NS
ZNF775	NS	4.92E-01	-0.0282	6.28E-01	NS
CAPN7	NS	4.92E-01	-0.0282	6.28E-01	NS
ELF1	NS	4.92E-01	-0.0282	6.28E-01	NS
EFCAB2	NS	4.92E-01	2.82E-02	6.28E-01	NS
SV2A	NS	4.93E-01	2.82E-02	6.28E-01	NS
CRYBB3	NS	4.93E-01	2.82E-02	6.29E-01	NS
SMAD1	NS	4.93E-01	-0.0282	6.29E-01	NS
SLC7A5	NS	4.93E-01	2.81E-02	6.29E-01	NS
SLC25A15	NS	4.93E-01	2.81E-02	6.29E-01	NS
ZNF57	NS	4.93E-01	-0.0281	6.29E-01	NS
ALOX5	NS	4.93E-01	-0.0281	6.29E-01	NS
NSUN2	NS	4.93E-01	-0.0281	6.29E-01	NS
BRAP	NS	4.94E-01	2.81E-02	6.29E-01	NS
WDR7	NS	4.94E-01	-0.0281	6.29E-01	NS
RAD23A	NS	4.94E-01	-0.0281	6.29E-01	NS
USP17L10	NS	4.93E-01	2.81E-02	6.29E-01	NS
PYGM	NS	4.94E-01	-0.0281	6.29E-01	NS
UMAD1	NS	4.94E-01	-0.0281	6.29E-01	NS
PDSS1	NS	4.94E-01	2.81E-02	6.29E-01	NS

MCFD2	NS	4.94E-01	2.81E-02	6.29E-01	NS
LRRC37B	NS	4.94E-01	-0.0281	6.30E-01	NS
DOHH	NS	4.94E-01	2.81E-02	6.30E-01	NS
NELFA	NS	4.94E-01	-0.0281	6.30E-01	NS
STX7	NS	4.94E-01	-0.0281	6.30E-01	NS
SRPX2	NS	4.95E-01	2.81E-02	6.30E-01	NS
CYP7A1	NS	4.95E-01	-0.0281	6.30E-01	NS
ADAMTS1	NS	4.95E-01	2.80E-02	6.30E-01	NS
CPA4	NS	4.95E-01	2.80E-02	6.30E-01	NS
C17orf114	NS	4.95E-01	-0.028	6.30E-01	NS
FEM1C	NS	4.95E-01	-0.028	6.30E-01	NS
APPBP2	NS	4.95E-01	2.80E-02	6.31E-01	NS
APPL1	NS	4.95E-01	2.80E-02	6.31E-01	NS
SMIM13	NS	4.95E-01	2.80E-02	6.31E-01	NS
CROCC	NS	4.96E-01	2.80E-02	6.31E-01	NS
C18orf21	NS	4.96E-01	-0.028	6.31E-01	NS
TMEM239	NS	4.96E-01	-0.028	6.31E-01	NS
IFI30	NS	4.96E-01	2.80E-02	6.31E-01	NS
BRINP3	NS	4.96E-01	-0.028	6.31E-01	NS
MASTL	NS	4.96E-01	-0.028	6.31E-01	NS
HMSD	NS	4.96E-01	-0.028	6.31E-01	NS
CAPZA1	NS	4.96E-01	-0.028	6.32E-01	NS
GPAT4	NS	4.96E-01	2.79E-02	6.32E-01	NS
COX14	NS	4.96E-01	-0.0279	6.32E-01	NS
CEP57	NS	4.97E-01	2.79E-02	6.32E-01	NS
MAP2K6	NS	4.97E-01	-0.0279	6.32E-01	NS
ZNF586	NS	4.97E-01	-0.0279	6.33E-01	NS
TXNRD3	NS	4.98E-01	-0.0279	6.33E-01	NS
TNFRSF10D	NS	4.98E-01	2.79E-02	6.33E-01	NS
CXCL6	NS	4.98E-01	-0.0278	6.33E-01	NS
FCSK	NS	4.98E-01	-0.0278	6.34E-01	NS
PCDHB15	NS	4.98E-01	2.78E-02	6.34E-01	NS
TRIM15	NS	4.98E-01	-0.0278	6.34E-01	NS
NRN1	NS	4.98E-01	-0.0278	6.34E-01	NS
SACM1L	NS	4.98E-01	-0.0278	6.34E-01	NS
MYMX	NS	4.99E-01	2.78E-02	6.34E-01	NS
SMIM23	NS	4.99E-01	2.78E-02	6.34E-01	NS

NOL4L	NS	4.99E-01	2.78E-02	6.34E-01	NS
NPIPB13	NS	4.99E-01	2.78E-02	6.34E-01	NS
CACNG2	NS	4.99E-01	-0.0278	6.35E-01	NS
WIPF3	NS	4.99E-01	2.78E-02	6.35E-01	NS
CLCA1	NS	4.99E-01	-0.0277	6.35E-01	NS
IKZF1	NS	5.00E-01	-0.0277	6.36E-01	NS
POLR1E	NS	5.00E-01	-0.0277	6.36E-01	NS
CSRNP2	NS	5.00E-01	2.77E-02	6.36E-01	NS
FNDC7	NS	5.00E-01	-0.0277	6.36E-01	NS
H4-16	NS	5.00E-01	2.77E-02	6.36E-01	NS
SLC30A8	NS	5.01E-01	2.77E-02	6.36E-01	NS
KRT82	NS	5.01E-01	2.77E-02	6.36E-01	NS
ANKLE2	NS	5.01E-01	-0.0277	6.36E-01	NS
OR10H4	NS	5.01E-01	-0.0276	6.36E-01	NS
SLC36A3	NS	5.01E-01	-0.0276	6.36E-01	NS
CCDC196	NS	5.01E-01	-0.0276	6.36E-01	NS
DNAAF6	NS	5.01E-01	2.76E-02	6.37E-01	NS
YWHAE	NS	5.01E-01	2.76E-02	6.37E-01	NS
TGM3	NS	5.01E-01	-0.0276	6.37E-01	NS
OR1L6	NS	5.01E-01	-0.0276	6.37E-01	NS
CRYM	NS	5.02E-01	2.76E-02	6.37E-01	NS
AK2	NS	5.02E-01	2.76E-02	6.37E-01	NS
MAMLD1	NS	5.02E-01	-0.0276	6.37E-01	NS
PPIF	NS	5.02E-01	-0.0276	6.37E-01	NS
EZHIP	NS	5.02E-01	2.76E-02	6.37E-01	NS
KRT6A	NS	5.02E-01	2.76E-02	6.37E-01	NS
ESAM	NS	5.02E-01	-0.0276	6.37E-01	NS
TPCN2	NS	5.02E-01	-0.0276	6.37E-01	NS
TMEM63A	NS	5.03E-01	-0.0275	6.38E-01	NS
H2AC4	NS	5.03E-01	-0.0275	6.38E-01	NS
DNMBP	NS	5.03E-01	2.75E-02	6.38E-01	NS
EIF6	NS	5.03E-01	-0.0275	6.38E-01	NS
RBAK-RBAKDN	NS	5.03E-01	-0.0275	6.39E-01	NS
BOD1L1	NS	5.04E-01	-0.0275	6.39E-01	NS
TOP3A	NS	5.04E-01	2.75E-02	6.39E-01	NS
C8orf58	NS	5.04E-01	2.75E-02	6.39E-01	NS
HCCS	NS	5.04E-01	-0.0274	6.39E-01	NS

UBE2A	NS	5.04E-01	2.74E-02	6.39E-01	NS
CERT1	NS	5.04E-01	2.74E-02	6.39E-01	NS
TARS3	NS	5.04E-01	-0.0275	6.39E-01	NS
HELZ	NS	5.04E-01	-0.0274	6.39E-01	NS
RAD51	NS	5.04E-01	-0.0274	6.39E-01	NS
CEP68	NS	5.04E-01	2.74E-02	6.39E-01	NS
C2orf49	NS	5.04E-01	-0.0274	6.39E-01	NS
TBC1D3H	NS	5.04E-01	2.74E-02	6.39E-01	NS
OR6C74	NS	5.04E-01	-0.0274	6.39E-01	NS
MEIOC	NS	5.05E-01	2.74E-02	6.40E-01	NS
PNMA6F	NS	5.05E-01	-0.0274	6.40E-01	NS
ALPK1	NS	5.05E-01	2.74E-02	6.40E-01	NS
FANCM	NS	5.05E-01	-0.0274	6.40E-01	NS
KCNQ2	NS	5.05E-01	2.74E-02	6.40E-01	NS
KDM3B	NS	5.05E-01	2.74E-02	6.40E-01	NS
OR52B6	NS	5.05E-01	-0.0274	6.40E-01	NS
MYNN	NS	5.05E-01	-0.0274	6.40E-01	NS
TTC31	NS	5.05E-01	-0.0274	6.40E-01	NS
FGF10	NS	5.05E-01	-0.0274	6.40E-01	NS
RNF19B	NS	5.05E-01	-0.0274	6.40E-01	NS
PRKAG2	NS	5.06E-01	-0.0273	6.40E-01	NS
ARHGAP22	NS	5.06E-01	2.73E-02	6.40E-01	NS
SUPT20HL1	NS	5.06E-01	-0.0273	6.40E-01	NS
CALN1	NS	5.06E-01	2.73E-02	6.41E-01	NS
FOXD3	NS	5.06E-01	2.73E-02	6.41E-01	NS
SDHA	NS	5.06E-01	-0.0273	6.41E-01	NS
PEPD	NS	5.06E-01	-0.0273	6.41E-01	NS
SLC25A13	NS	5.06E-01	2.73E-02	6.41E-01	NS
ARL2	NS	5.06E-01	-0.0273	6.41E-01	NS
LAPTM4B	NS	5.06E-01	-0.0273	6.41E-01	NS
FAR1	NS	5.07E-01	2.73E-02	6.41E-01	NS
CDK15	NS	5.07E-01	-0.0273	6.41E-01	NS
TMEM273	NS	5.07E-01	-0.0273	6.41E-01	NS
DDR1	NS	5.07E-01	-0.0273	6.41E-01	NS
KRTAP4-7	NS	5.07E-01	2.73E-02	6.41E-01	NS
NDUFC2-KCTD14	NS	5.07E-01	2.73E-02	6.41E-01	NS
OR8K5	NS	5.07E-01	-0.0272	6.42E-01	NS

SMCHD1	NS	5.07E-01	-0.0272	6.42E-01	NS
SAFB	NS	5.07E-01	-0.0272	6.42E-01	NS
BORCS6	NS	5.07E-01	-0.0272	6.42E-01	NS
TMEM130	NS	5.07E-01	-0.0272	6.42E-01	NS
POLA2	NS	5.08E-01	-0.0272	6.42E-01	NS
ABCC3	NS	5.08E-01	2.72E-02	6.42E-01	NS
LPIN1	NS	5.08E-01	2.72E-02	6.42E-01	NS
ZNF410	NS	5.08E-01	-0.0272	6.42E-01	NS
CDS1	NS	5.08E-01	-0.0272	6.42E-01	NS
FOXP1	NS	5.08E-01	-0.0272	6.42E-01	NS
GEM	NS	5.09E-01	-0.0272	6.43E-01	NS
KCNK9	NS	5.08E-01	2.72E-02	6.43E-01	NS
ANHX	NS	5.09E-01	2.72E-02	6.43E-01	NS
KIAA0586	NS	5.09E-01	2.72E-02	6.43E-01	NS
CLCNKA	NS	5.09E-01	-0.0271	6.43E-01	NS
OR5V1	NS	5.09E-01	-0.0271	6.43E-01	NS
RPS25	NS	5.09E-01	2.71E-02	6.43E-01	NS
DNAAF9	NS	5.09E-01	-0.0271	6.43E-01	NS
CFHR3	NS	5.09E-01	-0.0271	6.43E-01	NS
DNAAF4	NS	5.10E-01	2.71E-02	6.44E-01	NS
PIWIL4	NS	5.10E-01	-0.0271	6.44E-01	NS
ABCC11	NS	5.10E-01	2.71E-02	6.44E-01	NS
MRPL37	NS	5.10E-01	2.71E-02	6.44E-01	NS
ZNF841	NS	5.10E-01	-0.027	6.45E-01	NS
TNFRSF1A	NS	5.11E-01	-0.027	6.45E-01	NS
TIMM23B	NS	5.11E-01	-0.027	6.45E-01	NS
EBLN1	NS	5.11E-01	-0.027	6.45E-01	NS
CDH7	NS	5.11E-01	-0.027	6.45E-01	NS
EFEMP1	NS	5.12E-01	-0.027	6.46E-01	NS
CALD1	NS	5.12E-01	-0.027	6.46E-01	NS
EFS	NS	5.12E-01	-0.0269	6.46E-01	NS
ZNF205	NS	5.12E-01	-0.0269	6.46E-01	NS
PRKACB	NS	5.12E-01	2.69E-02	6.46E-01	NS
TNFRSF19	NS	5.12E-01	-0.0269	6.46E-01	NS
NLRP2B	NS	5.12E-01	2.69E-02	6.47E-01	NS
PPWD1	NS	5.12E-01	2.69E-02	6.47E-01	NS
DHCR24	NS	5.12E-01	2.69E-02	6.47E-01	NS

CDCP1	NS	5.13E-01	-0.0269	6.47E-01	NS
SIGLECL1	NS	5.13E-01	-0.0269	6.47E-01	NS
MLLT6	NS	5.12E-01	-0.0269	6.47E-01	NS
UNC5A	NS	5.13E-01	-0.0269	6.47E-01	NS
DYNLL2	NS	5.13E-01	2.69E-02	6.47E-01	NS
LIF	NS	5.13E-01	-0.0269	6.47E-01	NS
RAMP2	NS	5.13E-01	2.69E-02	6.47E-01	NS
EP300	NS	5.13E-01	2.69E-02	6.47E-01	NS
PIK3R1	NS	5.13E-01	2.69E-02	6.47E-01	NS
KRT75	NS	5.13E-01	-0.0269	6.47E-01	NS
BMP7	NS	5.13E-01	2.69E-02	6.47E-01	NS
RASAL3	NS	5.13E-01	2.69E-02	6.47E-01	NS
ADAMTS14	NS	5.13E-01	-0.0269	6.47E-01	NS
SFXN5	NS	5.13E-01	2.69E-02	6.47E-01	NS
CEP72	NS	5.13E-01	-0.0268	6.47E-01	NS
FAM53B	NS	5.13E-01	2.68E-02	6.47E-01	NS
CDH3	NS	5.13E-01	-0.0268	6.47E-01	NS
ATG4B	NS	5.14E-01	2.68E-02	6.47E-01	NS
TTC22	NS	5.14E-01	2.68E-02	6.48E-01	NS
TMEM248	NS	5.14E-01	-0.0268	6.48E-01	NS
CENPV	NS	5.14E-01	-0.0268	6.48E-01	NS
NAT2	NS	5.14E-01	2.68E-02	6.48E-01	NS
TMPRSS3	NS	5.14E-01	-0.0268	6.48E-01	NS
CRIP1	NS	5.14E-01	2.68E-02	6.48E-01	NS
ZNF260	NS	5.15E-01	-0.0268	6.48E-01	NS
GPATCH8	NS	5.15E-01	-0.0268	6.48E-01	NS
UBXN7	NS	5.15E-01	-0.0268	6.49E-01	NS
STAG3	NS	5.15E-01	2.68E-02	6.49E-01	NS
HMGCS1	NS	5.15E-01	-0.0268	6.49E-01	NS
COL26A1	NS	5.15E-01	2.67E-02	6.49E-01	NS
RNF169	NS	5.15E-01	-0.0267	6.49E-01	NS
VPS51	NS	5.15E-01	-0.0267	6.49E-01	NS
RPIA	NS	5.15E-01	-0.0267	6.49E-01	NS
MINDY2	NS	5.15E-01	-0.0267	6.49E-01	NS
NINJ1	NS	5.15E-01	-0.0267	6.49E-01	NS
GPM6A	NS	5.15E-01	-0.0267	6.49E-01	NS
FBXO16	NS	5.15E-01	-0.0267	6.49E-01	NS

PAX9	NS	5.15E-01	-0.0267	6.49E-01	NS
FAM83H	NS	5.15E-01	-0.0267	6.49E-01	NS
AKR1B1	NS	5.16E-01	-0.0267	6.49E-01	NS
SUPT5H	NS	5.16E-01	2.67E-02	6.49E-01	NS
ZC3H7A	NS	5.16E-01	-0.0267	6.49E-01	NS
ZMYM5	NS	5.16E-01	-0.0267	6.50E-01	NS
MAP2K3	NS	5.16E-01	-0.0267	6.50E-01	NS
PKN1	NS	5.16E-01	2.67E-02	6.50E-01	NS
THSD4	NS	5.16E-01	2.67E-02	6.50E-01	NS
HBA2	NS	5.16E-01	2.66E-02	6.50E-01	NS
DUOXA2	NS	5.17E-01	-0.0266	6.50E-01	NS
GATM	NS	5.17E-01	2.66E-02	6.50E-01	NS
FBXO46	NS	5.17E-01	-0.0266	6.50E-01	NS
CKMT1A	NS	5.17E-01	-0.0266	6.50E-01	NS
OR56A3	NS	5.17E-01	-0.0266	6.50E-01	NS
GRAMD1A	NS	5.17E-01	2.66E-02	6.50E-01	NS
DCLK2	NS	5.17E-01	-0.0266	6.51E-01	NS
SFPQ	NS	5.18E-01	2.66E-02	6.51E-01	NS
C20orf141	NS	5.18E-01	-0.0265	6.52E-01	NS
ANKRD34C	NS	5.18E-01	2.65E-02	6.52E-01	NS
HLA-A	NS	5.18E-01	2.65E-02	6.52E-01	NS
ZNF275	NS	5.19E-01	2.65E-02	6.52E-01	NS
ADAM30	NS	5.19E-01	-0.0265	6.52E-01	NS
KIRREL3	NS	5.19E-01	2.65E-02	6.52E-01	NS
IL19	NS	5.19E-01	-0.0265	6.52E-01	NS
SOSTDC1	NS	5.19E-01	-0.0265	6.52E-01	NS
KCNJ14	NS	5.19E-01	-0.0265	6.52E-01	NS
CSNK2A1	NS	5.19E-01	2.65E-02	6.52E-01	NS
ZNF638	NS	5.19E-01	2.65E-02	6.52E-01	NS
ANKRD9	NS	5.19E-01	2.65E-02	6.52E-01	NS
NPIPB3	NS	5.19E-01	2.65E-02	6.52E-01	NS
LRTM1	NS	5.19E-01	-0.0265	6.53E-01	NS
SLC5A12	NS	5.20E-01	-0.0264	6.53E-01	NS
HOXA5	NS	5.20E-01	2.64E-02	6.53E-01	NS
PLEKHG5	NS	5.20E-01	2.64E-02	6.54E-01	NS
GRK6	NS	5.20E-01	2.64E-02	6.54E-01	NS
KRTAP5-11	NS	5.20E-01	2.64E-02	6.54E-01	NS

SEC23IP	NS	5.20E-01	-0.0264	6.54E-01	NS
FAM9A	NS	5.20E-01	2.64E-02	6.54E-01	NS
SCN3B	NS	5.21E-01	-0.0264	6.54E-01	NS
CISD3	NS	5.21E-01	-0.0263	6.55E-01	NS
ACSL3	NS	5.21E-01	2.63E-02	6.55E-01	NS
OTUD1	NS	5.21E-01	2.63E-02	6.55E-01	NS
ERRFI1	NS	5.22E-01	2.63E-02	6.55E-01	NS
TSHZ3	NS	5.22E-01	2.63E-02	6.55E-01	NS
INA	NS	5.22E-01	2.63E-02	6.55E-01	NS
LAS1L	NS	5.22E-01	2.63E-02	6.55E-01	NS
PRKRA	NS	5.22E-01	-0.0263	6.55E-01	NS
STOM	NS	5.22E-01	2.63E-02	6.55E-01	NS
ZNF432	NS	5.22E-01	2.63E-02	6.56E-01	NS
TLDC2	NS	5.23E-01	-0.0263	6.56E-01	NS
C1QTNF9	NS	5.23E-01	2.62E-02	6.56E-01	NS
PSMB2	NS	5.23E-01	-0.0262	6.56E-01	NS
ING3	NS	5.23E-01	-0.0262	6.56E-01	NS
GPRIN2	NS	5.23E-01	2.62E-02	6.56E-01	NS
EXOSC6	NS	5.23E-01	-0.0262	6.56E-01	NS
PCARE	NS	5.23E-01	-0.0262	6.56E-01	NS
PLCD1	NS	5.23E-01	-0.0262	6.56E-01	NS
KLHL2	NS	5.23E-01	-0.0262	6.56E-01	NS
DYNLT2B	NS	5.23E-01	-0.0262	6.56E-01	NS
PRR11	NS	5.24E-01	2.62E-02	6.57E-01	NS
TDRKH	NS	5.24E-01	-0.0262	6.57E-01	NS
OVOL3	NS	5.24E-01	2.62E-02	6.57E-01	NS
PLEK	NS	5.24E-01	2.62E-02	6.57E-01	NS
KRTAP4-4	NS	5.24E-01	2.62E-02	6.57E-01	NS
LVRN	NS	5.24E-01	-0.0262	6.57E-01	NS
ADAP2	NS	5.24E-01	2.62E-02	6.57E-01	NS
XKR7	NS	5.24E-01	2.62E-02	6.57E-01	NS
PTPRA	NS	5.24E-01	2.62E-02	6.57E-01	NS
DUSP22	NS	5.24E-01	2.61E-02	6.57E-01	NS
CBY2	NS	5.25E-01	-0.0261	6.57E-01	NS
RAB13	NS	5.25E-01	-0.0261	6.58E-01	NS
SLC8A1	NS	5.25E-01	-0.0261	6.58E-01	NS
ZNF790	NS	5.25E-01	2.61E-02	6.58E-01	NS

ERBIN	NS	5.25E-01	-0.0261	6.58E-01	NS
SIX4	NS	5.25E-01	2.61E-02	6.58E-01	NS
ITGAL	NS	5.25E-01	-0.0261	6.58E-01	NS
KIRREL2	NS	5.25E-01	2.61E-02	6.58E-01	NS
CARD19	NS	5.25E-01	2.61E-02	6.58E-01	NS
PTCHD1	NS	5.25E-01	2.61E-02	6.58E-01	NS
RXRB	NS	5.25E-01	-0.0261	6.58E-01	NS
TNRC6C	NS	5.26E-01	2.61E-02	6.58E-01	NS
KRTAP5-6	NS	5.26E-01	-0.0261	6.58E-01	NS
SPINT1	NS	5.26E-01	2.61E-02	6.58E-01	NS
AWAT1	NS	5.26E-01	2.61E-02	6.58E-01	NS
SPATA6L	NS	5.26E-01	-0.026	6.59E-01	NS
FER	NS	5.26E-01	-0.026	6.59E-01	NS
S100A14	NS	5.26E-01	-0.026	6.59E-01	NS
RPL12	NS	5.26E-01	-0.026	6.59E-01	NS
SOWAHA	NS	5.26E-01	-0.026	6.59E-01	NS
IQSEC2	NS	5.26E-01	-0.026	6.59E-01	NS
MRPS21	NS	5.26E-01	-0.026	6.59E-01	NS
CDON	NS	5.27E-01	-0.026	6.59E-01	NS
MIGA2	NS	5.27E-01	-0.026	6.59E-01	NS
SBDS	NS	5.27E-01	2.60E-02	6.59E-01	NS
SLFN12	NS	5.27E-01	-0.026	6.59E-01	NS
KRTAP22-2	NS	5.27E-01	-0.026	6.59E-01	NS
USP17L28	NS	5.27E-01	2.60E-02	6.59E-01	NS
CFAP97	NS	5.27E-01	-0.026	6.59E-01	NS
C1orf87	NS	5.27E-01	2.60E-02	6.59E-01	NS
FBXO28	NS	5.27E-01	-0.026	6.59E-01	NS
PCDHB11	NS	5.27E-01	-0.026	6.59E-01	NS
PET100	NS	5.27E-01	-0.026	6.59E-01	NS
OR51B6	NS	5.27E-01	2.60E-02	6.59E-01	NS
FARSB	NS	5.27E-01	-0.026	6.59E-01	NS
VPS37C	NS	5.27E-01	2.60E-02	6.59E-01	NS
TBXT	NS	5.27E-01	-0.026	6.59E-01	NS
ILDR2	NS	5.28E-01	-0.0259	6.60E-01	NS
PRDM14	NS	5.28E-01	2.59E-02	6.60E-01	NS
RHD	NS	5.28E-01	2.59E-02	6.60E-01	NS
NLE1	NS	5.28E-01	2.59E-02	6.60E-01	NS

CACNG1	NS	5.28E-01	-0.0259	6.60E-01	NS
MAP3K19	NS	5.28E-01	-0.0259	6.60E-01	NS
NAF1	NS	5.28E-01	2.59E-02	6.60E-01	NS
TM7SF3	NS	5.28E-01	-0.0259	6.60E-01	NS
TADA2B	NS	5.28E-01	2.59E-02	6.60E-01	NS
USP17L18	NS	5.29E-01	2.59E-02	6.60E-01	NS
ZNF431	NS	5.29E-01	-0.0259	6.60E-01	NS
POTEG	NS	5.29E-01	2.59E-02	6.60E-01	NS
STK32B	NS	5.29E-01	-0.0259	6.61E-01	NS
NCF4	NS	5.29E-01	-0.0259	6.61E-01	NS
STX1B	NS	5.29E-01	2.58E-02	6.61E-01	NS
TOE1	NS	5.29E-01	-0.0258	6.61E-01	NS
MTA1	NS	5.29E-01	-0.0258	6.61E-01	NS
UBE2E2	NS	5.30E-01	2.58E-02	6.61E-01	NS
TBC1D15	NS	5.30E-01	2.58E-02	6.62E-01	NS
PHLDB3	NS	5.30E-01	2.58E-02	6.62E-01	NS
COL12A1	NS	5.30E-01	-0.0258	6.62E-01	NS
TMED8	NS	5.30E-01	-0.0258	6.62E-01	NS
XCR1	NS	5.30E-01	2.58E-02	6.62E-01	NS
RTN4	NS	5.31E-01	2.57E-02	6.63E-01	NS
PLPP2	NS	5.31E-01	-0.0257	6.63E-01	NS
PMPCA	NS	5.31E-01	-0.0257	6.63E-01	NS
MFAP4	NS	5.31E-01	-0.0257	6.63E-01	NS
REV1	NS	5.31E-01	-0.0257	6.63E-01	NS
WWOX	NS	5.31E-01	-0.0257	6.63E-01	NS
DNASE1	NS	5.31E-01	-0.0257	6.63E-01	NS
TNFRSF25	NS	5.31E-01	-0.0257	6.63E-01	NS
FUS	NS	5.31E-01	2.57E-02	6.63E-01	NS
STX2	NS	5.31E-01	2.57E-02	6.63E-01	NS
PLVAP	NS	5.31E-01	2.57E-02	6.63E-01	NS
GNGT1	NS	5.31E-01	-0.0257	6.63E-01	NS
SPATA31A7	NS	5.32E-01	-0.0257	6.63E-01	NS
DDHD2	NS	5.32E-01	2.57E-02	6.63E-01	NS
CEP290	NS	5.32E-01	2.57E-02	6.63E-01	NS
COX5B	NS	5.32E-01	-0.0257	6.63E-01	NS
MAGEA10	NS	5.32E-01	2.57E-02	6.64E-01	NS
HDGF	NS	5.32E-01	-0.0257	6.64E-01	NS

DOK2	NS	5.32E-01	-0.0257	6.64E-01	NS
RPRD2	NS	5.32E-01	-0.0257	6.64E-01	NS
ESR2	NS	5.32E-01	2.56E-02	6.64E-01	NS
HSF5	NS	5.32E-01	-0.0257	6.64E-01	NS
DDX3Y	NS	5.32E-01	2.56E-02	6.64E-01	NS
UNC5B	NS	5.32E-01	-0.0256	6.64E-01	NS
ACOT9	NS	5.32E-01	2.56E-02	6.64E-01	NS
SREBF2	NS	5.33E-01	-0.0256	6.64E-01	NS
FCGR1A	NS	5.33E-01	2.56E-02	6.64E-01	NS
SLC1A4	NS	5.33E-01	-0.0256	6.64E-01	NS
WDR41	NS	5.33E-01	2.56E-02	6.64E-01	NS
ZNF618	NS	5.33E-01	2.56E-02	6.64E-01	NS
ABR	NS	5.33E-01	-0.0256	6.64E-01	NS
PRDX3	NS	5.33E-01	-0.0256	6.64E-01	NS
MAPK10	NS	5.34E-01	-0.0256	6.65E-01	NS
FBXL5	NS	5.34E-01	-0.0256	6.65E-01	NS
TTC36	NS	5.34E-01	-0.0256	6.65E-01	NS
GNRHR	NS	5.34E-01	2.55E-02	6.65E-01	NS
CCL27	NS	5.34E-01	2.55E-02	6.65E-01	NS
COL5A1	NS	5.34E-01	-0.0255	6.65E-01	NS
RPL7A	NS	5.34E-01	-0.0255	6.65E-01	NS
PIEZO1	NS	5.34E-01	-0.0255	6.65E-01	NS
EPS8L1	NS	5.34E-01	2.55E-02	6.65E-01	NS
PANK4	NS	5.34E-01	2.55E-02	6.65E-01	NS
FARSA	NS	5.35E-01	-0.0255	6.65E-01	NS
PDCD6-AHRR	NS	5.35E-01	2.55E-02	6.65E-01	NS
GUCA1ANB	NS	5.35E-01	2.55E-02	6.66E-01	NS
CMA1	NS	5.35E-01	-0.0255	6.66E-01	NS
SRSF1	NS	5.35E-01	2.55E-02	6.66E-01	NS
GPR182	NS	5.35E-01	2.55E-02	6.66E-01	NS
WDPCP	NS	5.35E-01	-0.0255	6.66E-01	NS
ZNF837	NS	5.35E-01	-0.0255	6.66E-01	NS
UBE2H	NS	5.36E-01	-0.0254	6.66E-01	NS
ZNF565	NS	5.36E-01	2.54E-02	6.67E-01	NS
GPHA2	NS	5.37E-01	2.54E-02	6.68E-01	NS
LGALS9C	NS	5.37E-01	2.54E-02	6.68E-01	NS
CFHR1	NS	5.37E-01	2.54E-02	6.68E-01	NS

MCHR1	NS	5.37E-01	2.54E-02	6.68E-01	NS
MS4A10	NS	5.37E-01	2.54E-02	6.68E-01	NS
FKBP1A	NS	5.37E-01	-0.0254	6.68E-01	NS
TTLL9	NS	5.37E-01	-0.0253	6.68E-01	NS
OR2M4	NS	5.37E-01	-0.0253	6.68E-01	NS
CLIC3	NS	5.37E-01	-0.0253	6.68E-01	NS
PIP5K1C	NS	5.38E-01	2.53E-02	6.69E-01	NS
TRIB1	NS	5.38E-01	2.53E-02	6.69E-01	NS
TMEM234	NS	5.38E-01	-0.0253	6.69E-01	NS
YPEL5	NS	5.38E-01	2.53E-02	6.69E-01	NS
KIF3C	NS	5.38E-01	-0.0253	6.69E-01	NS
CCNJL	NS	5.38E-01	2.53E-02	6.69E-01	NS
KRTAP21-1	NS	5.38E-01	-0.0253	6.69E-01	NS
MS4A6A	NS	5.39E-01	2.53E-02	6.69E-01	NS
RNASEH2B	NS	5.39E-01	2.52E-02	6.69E-01	NS
CUZD1	NS	5.39E-01	2.52E-02	6.69E-01	NS
FGD2	NS	5.39E-01	-0.0252	6.69E-01	NS
ANP32D	NS	5.39E-01	-0.0252	6.70E-01	NS
EWSR1	NS	5.39E-01	-0.0252	6.70E-01	NS
SETBP1	NS	5.39E-01	2.52E-02	6.70E-01	NS
ARID5B	NS	5.39E-01	-0.0252	6.70E-01	NS
CCNE1	NS	5.39E-01	-0.0252	6.70E-01	NS
FOXJ1	NS	5.39E-01	2.52E-02	6.70E-01	NS
ERLIN2	NS	5.39E-01	2.52E-02	6.70E-01	NS
PAK1IP1	NS	5.40E-01	-0.0252	6.70E-01	NS
CCDC69	NS	5.40E-01	-0.0252	6.70E-01	NS
SELENON	NS	5.40E-01	-0.0252	6.70E-01	NS
ZDHHC4	NS	5.40E-01	-0.0252	6.70E-01	NS
EFCAB11	NS	5.40E-01	-0.0252	6.70E-01	NS
ABHD14A	NS	5.40E-01	2.52E-02	6.71E-01	NS
ID2	NS	5.40E-01	2.52E-02	6.71E-01	NS
MYB	NS	5.40E-01	-0.0252	6.71E-01	NS
FCER1G	NS	5.40E-01	2.52E-02	6.71E-01	NS
GART	NS	5.40E-01	-0.0252	6.71E-01	NS
ZNF641	NS	5.40E-01	2.52E-02	6.71E-01	NS
KRTAP19-6	NS	5.40E-01	2.52E-02	6.71E-01	NS
MIA2	NS	5.40E-01	2.52E-02	6.71E-01	NS

APOBEC3F	NS	5.41E-01	2.51E-02	6.71E-01	NS
KLC3	NS	5.41E-01	-0.0251	6.71E-01	NS
DNAJC28	NS	5.41E-01	2.51E-02	6.71E-01	NS
ADCY2	NS	5.41E-01	-0.0251	6.71E-01	NS
YIPF3	NS	5.41E-01	2.51E-02	6.71E-01	NS
POLR2M	NS	5.41E-01	2.51E-02	6.71E-01	NS
PPM1K	NS	5.41E-01	2.51E-02	6.71E-01	NS
WDR26	NS	5.41E-01	-0.0251	6.71E-01	NS
ASXL3	NS	5.41E-01	2.51E-02	6.71E-01	NS
MLST8	NS	5.41E-01	2.51E-02	6.71E-01	NS
ATG16L2	NS	5.41E-01	-0.0251	6.71E-01	NS
TSNARE1	NS	5.41E-01	-0.0251	6.71E-01	NS
DLGAP3	NS	5.42E-01	2.51E-02	6.72E-01	NS
GTF3C4	NS	5.42E-01	2.51E-02	6.72E-01	NS
DCTN5	NS	5.42E-01	2.51E-02	6.72E-01	NS
PRTFDC1	NS	5.42E-01	-0.0251	6.72E-01	NS
HM13	NS	5.42E-01	2.50E-02	6.72E-01	NS
SRP9	NS	5.42E-01	2.51E-02	6.72E-01	NS
RPS6KC1	NS	5.42E-01	-0.025	6.72E-01	NS
YEATS2	NS	5.42E-01	-0.025	6.72E-01	NS
CCDC121	NS	5.43E-01	2.50E-02	6.72E-01	NS
MBD4	NS	5.43E-01	-0.025	6.72E-01	NS
RAB40A	NS	5.43E-01	-0.025	6.72E-01	NS
SLC12A8	NS	5.43E-01	-0.025	6.73E-01	NS
NABP2	NS	5.43E-01	-0.025	6.73E-01	NS
HCN3	NS	5.43E-01	-0.025	6.73E-01	NS
CALHM3	NS	5.43E-01	2.50E-02	6.73E-01	NS
DMAC2	NS	5.43E-01	-0.025	6.73E-01	NS
COMT	NS	5.43E-01	-0.025	6.73E-01	NS
GLE1	NS	5.43E-01	-0.025	6.73E-01	NS
FAM187B	NS	5.43E-01	2.50E-02	6.73E-01	NS
TERF2	NS	5.43E-01	-0.025	6.73E-01	NS
FGL1	NS	5.44E-01	2.49E-02	6.73E-01	NS
LIN54	NS	5.44E-01	2.49E-02	6.73E-01	NS
KIF2A	NS	5.44E-01	2.49E-02	6.73E-01	NS
RPS12	NS	5.44E-01	-0.0249	6.73E-01	NS
MYL3	NS	5.44E-01	2.49E-02	6.73E-01	NS

DHX57	NS	5.44E-01	-0.0249	6.73E-01	NS
GREM1	NS	5.44E-01	-0.0249	6.73E-01	NS
CCDC136	NS	5.44E-01	2.49E-02	6.73E-01	NS
NFATC1	NS	5.44E-01	-0.0249	6.73E-01	NS
C17orf98	NS	5.44E-01	-0.0249	6.73E-01	NS
WDR72	NS	5.44E-01	2.49E-02	6.74E-01	NS
OTULIN	NS	5.45E-01	-0.0249	6.74E-01	NS
NKPD1	NS	5.45E-01	2.49E-02	6.74E-01	NS
LRAT	NS	5.45E-01	-0.0249	6.74E-01	NS
BMP4	NS	5.45E-01	-0.0249	6.74E-01	NS
ATPAF1	NS	5.45E-01	-0.0248	6.74E-01	NS
PSAP	NS	5.45E-01	-0.0248	6.74E-01	NS
RBMY1D	NS	5.45E-01	2.48E-02	6.75E-01	NS
LHFPL5	NS	5.46E-01	-0.0248	6.75E-01	NS
MTCH2	NS	5.46E-01	2.48E-02	6.75E-01	NS
DMRT1	NS	5.46E-01	2.48E-02	6.75E-01	NS
CEP78	NS	5.46E-01	-0.0248	6.75E-01	NS
MYO18A	NS	5.46E-01	-0.0248	6.75E-01	NS
OR6X1	NS	5.47E-01	-0.0248	6.76E-01	NS
C2CD5	NS	5.47E-01	2.48E-02	6.76E-01	NS
OR4N4C	NS	5.47E-01	-0.0248	6.76E-01	NS
TUBB1	NS	5.47E-01	2.47E-02	6.76E-01	NS
TLX1	NS	5.47E-01	2.47E-02	6.76E-01	NS
TRAPPC5	NS	5.47E-01	2.47E-02	6.76E-01	NS
ZYG11A	NS	5.47E-01	2.47E-02	6.76E-01	NS
TSPAN7	NS	5.47E-01	-0.0247	6.76E-01	NS
KLHL9	NS	5.47E-01	2.47E-02	6.76E-01	NS
KCND3	NS	5.47E-01	-0.0247	6.76E-01	NS
ZMYM3	NS	5.47E-01	2.47E-02	6.76E-01	NS
ITGB6	NS	5.47E-01	2.47E-02	6.76E-01	NS
ADGRG4	NS	5.47E-01	-0.0247	6.76E-01	NS
YBX1	NS	5.48E-01	2.47E-02	6.76E-01	NS
PBX4	NS	5.48E-01	-0.0247	6.76E-01	NS
CCKBR	NS	5.48E-01	2.47E-02	6.76E-01	NS
MRPS15	NS	5.48E-01	2.47E-02	6.76E-01	NS
BCL2A1	NS	5.48E-01	-0.0247	6.76E-01	NS
RPS8	NS	5.48E-01	-0.0247	6.76E-01	NS

ADRA1B	NS	5.48E-01	-0.0247	6.76E-01	NS
HOXC12	NS	5.48E-01	2.47E-02	6.76E-01	NS
RGS4	NS	5.48E-01	2.47E-02	6.76E-01	NS
PIK3R6	NS	5.48E-01	-0.0247	6.77E-01	NS
NKAIN2	NS	5.48E-01	-0.0247	6.77E-01	NS
SRC	NS	5.48E-01	-0.0247	6.77E-01	NS
WASL	NS	5.49E-01	-0.0246	6.77E-01	NS
KRTAP1-4	NS	5.49E-01	2.46E-02	6.77E-01	NS
RTEL1-TNFRSF6B	NS	5.49E-01	2.46E-02	6.77E-01	NS
TNFRSF1B	NS	5.49E-01	-0.0246	6.77E-01	NS
TSPAN32	NS	5.49E-01	2.46E-02	6.78E-01	NS
ZNF211	NS	5.49E-01	2.46E-02	6.78E-01	NS
GPN2	NS	5.49E-01	-0.0246	6.78E-01	NS
LY6E	NS	5.49E-01	-0.0246	6.78E-01	NS
CASTOR1	NS	5.49E-01	-0.0246	6.78E-01	NS
GPR137C	NS	5.50E-01	2.46E-02	6.78E-01	NS
YPEL2	NS	5.50E-01	2.46E-02	6.78E-01	NS
GJC1	NS	5.50E-01	-0.0246	6.78E-01	NS
CENPN	NS	5.50E-01	2.46E-02	6.78E-01	NS
PGC	NS	5.50E-01	2.45E-02	6.78E-01	NS
KIAA0100	NS	5.50E-01	-0.0245	6.78E-01	NS
VN1R4	NS	5.50E-01	2.45E-02	6.78E-01	NS
RBMY1B	NS	5.50E-01	2.45E-02	6.78E-01	NS
VMP1	NS	5.50E-01	-0.0245	6.78E-01	NS
ZDHHC17	NS	5.50E-01	-0.0245	6.78E-01	NS
STX5	NS	5.50E-01	2.45E-02	6.79E-01	NS
POGK	NS	5.51E-01	2.45E-02	6.79E-01	NS
TBC1D3I	NS	5.51E-01	-0.0245	6.79E-01	NS
SV2B	NS	5.51E-01	-0.0245	6.79E-01	NS
TOX4	NS	5.51E-01	2.45E-02	6.79E-01	NS
HOXA6	NS	5.51E-01	-0.0245	6.79E-01	NS
CELSR3	NS	5.51E-01	-0.0245	6.79E-01	NS
SP4	NS	5.51E-01	-0.0245	6.79E-01	NS
EBF2	NS	5.52E-01	2.45E-02	6.79E-01	NS
GUCA2A	NS	5.52E-01	2.45E-02	6.80E-01	NS
DPCD	NS	5.52E-01	-0.0245	6.80E-01	NS
SEMA5A	NS	5.52E-01	-0.0244	6.80E-01	NS

TP73	NS	5.52E-01	2.44E-02	6.80E-01	NS
LZTS2	NS	5.52E-01	2.44E-02	6.80E-01	NS
MAP7	NS	5.52E-01	-0.0244	6.80E-01	NS
COP1	NS	5.52E-01	2.44E-02	6.80E-01	NS
HIGD2B	NS	5.52E-01	-0.0244	6.80E-01	NS
VSTM1	NS	5.52E-01	2.44E-02	6.80E-01	NS
EGLN3	NS	5.52E-01	-0.0244	6.80E-01	NS
CD177	NS	5.52E-01	-0.0244	6.80E-01	NS
ABCC8	NS	5.53E-01	-0.0244	6.80E-01	NS
CYP46A1	NS	5.53E-01	-0.0244	6.80E-01	NS
UNC13D	NS	5.53E-01	-0.0244	6.80E-01	NS
ITGB4	NS	5.53E-01	2.44E-02	6.80E-01	NS
FBXO36	NS	5.53E-01	-0.0244	6.80E-01	NS
TMEM52B	NS	5.53E-01	2.44E-02	6.80E-01	NS
POLR3D	NS	5.53E-01	2.44E-02	6.80E-01	NS
ATOX1	NS	5.53E-01	2.44E-02	6.80E-01	NS
TUBA3C	NS	5.53E-01	2.44E-02	6.80E-01	NS
KRTAP5-10	NS	5.53E-01	-0.0244	6.80E-01	NS
LRRC3C	NS	5.53E-01	-0.0244	6.80E-01	NS
GATA4	NS	5.53E-01	-0.0244	6.80E-01	NS
CRYBG2	NS	5.53E-01	2.44E-02	6.80E-01	NS
CYP21A2	NS	5.53E-01	2.44E-02	6.80E-01	NS
CEP162	NS	5.53E-01	-0.0244	6.80E-01	NS
CEP97	NS	5.53E-01	2.43E-02	6.81E-01	NS
GLT6D1	NS	5.53E-01	2.43E-02	6.81E-01	NS
TUFM	NS	5.54E-01	-0.0243	6.81E-01	NS
MRPS31	NS	5.54E-01	-0.0243	6.82E-01	NS
YPEL4	NS	5.55E-01	2.43E-02	6.82E-01	NS
SRY	NS	5.55E-01	2.43E-02	6.82E-01	NS
H2BS1	NS	5.54E-01	2.43E-02	6.82E-01	NS
BHMT2	NS	5.55E-01	2.43E-02	6.82E-01	NS
ARL5C	NS	5.55E-01	2.42E-02	6.82E-01	NS
OR2I1P	NS	5.55E-01	2.43E-02	6.82E-01	NS
MUC13	NS	5.55E-01	-0.0242	6.82E-01	NS
CNP	NS	5.55E-01	-0.0242	6.82E-01	NS
ZNF135	NS	5.55E-01	-0.0242	6.82E-01	NS
ZMYND19	NS	5.56E-01	2.42E-02	6.83E-01	NS

AP2A2	NS	5.56E-01	-0.0242	6.83E-01	NS
STXBP3	NS	5.56E-01	-0.0242	6.83E-01	NS
SYCP2	NS	5.56E-01	-0.0242	6.83E-01	NS
ZSCAN5B	NS	5.56E-01	2.42E-02	6.83E-01	NS
CCDC3	NS	5.56E-01	2.42E-02	6.83E-01	NS
CDKL3	NS	5.56E-01	-0.0242	6.83E-01	NS
RIPK2	NS	5.56E-01	2.42E-02	6.83E-01	NS
CFAP299	NS	5.56E-01	2.42E-02	6.83E-01	NS
SAA2-SAA4	NS	5.57E-01	-0.0242	6.83E-01	NS
C11orf21	NS	5.57E-01	2.41E-02	6.84E-01	NS
HECTD4	NS	5.57E-01	-0.0241	6.84E-01	NS
SLC34A1	NS	5.57E-01	2.41E-02	6.84E-01	NS
C16orf54	NS	5.57E-01	2.41E-02	6.84E-01	NS
NDUFA2	NS	5.57E-01	2.41E-02	6.84E-01	NS
AZIN1	NS	5.57E-01	2.41E-02	6.84E-01	NS
GALNT17	NS	5.57E-01	-0.0241	6.84E-01	NS
ZNF713	NS	5.57E-01	2.41E-02	6.84E-01	NS
XYLB	NS	5.57E-01	-0.0241	6.84E-01	NS
LCE2A	NS	5.57E-01	-0.0241	6.84E-01	NS
PALB2	NS	5.58E-01	-0.0241	6.84E-01	NS
MTMR11	NS	5.58E-01	2.41E-02	6.84E-01	NS
C11orf97	NS	5.58E-01	2.41E-02	6.84E-01	NS
TSPAN33	NS	5.58E-01	-0.0241	6.84E-01	NS
TNFRSF14	NS	5.58E-01	2.41E-02	6.84E-01	NS
NUTM2D	NS	5.58E-01	-0.0241	6.85E-01	NS
GATA1	NS	5.58E-01	-0.024	6.85E-01	NS
SAMD1	NS	5.58E-01	-0.024	6.85E-01	NS
OLFML2A	NS	5.58E-01	-0.024	6.85E-01	NS
CYBA	NS	5.58E-01	-0.024	6.85E-01	NS
GNG14	NS	5.59E-01	-0.024	6.85E-01	NS
NCL	NS	5.59E-01	-0.024	6.85E-01	NS
MOAP1	NS	5.59E-01	-0.024	6.85E-01	NS
SLC1A6	NS	5.59E-01	2.40E-02	6.85E-01	NS
TPPP	NS	5.59E-01	-0.024	6.85E-01	NS
RHPN2	NS	5.59E-01	2.40E-02	6.85E-01	NS
LGALS3	NS	5.59E-01	-0.024	6.85E-01	NS
CDC42EP1	NS	5.59E-01	2.40E-02	6.85E-01	NS

SRARP	NS	5.59E-01	-0.024	6.86E-01	NS
CACNA1S	NS	5.60E-01	-0.024	6.86E-01	NS
OBP2A	NS	5.60E-01	2.40E-02	6.86E-01	NS
SFI1	NS	5.60E-01	2.40E-02	6.86E-01	NS
PCED1B	NS	5.60E-01	-0.024	6.86E-01	NS
SLC7A3	NS	5.60E-01	-0.024	6.86E-01	NS
IER5	NS	5.60E-01	2.39E-02	6.86E-01	NS
TGM5	NS	5.60E-01	2.39E-02	6.86E-01	NS
DUSP3	NS	5.60E-01	-0.0239	6.86E-01	NS
OR1S2	NS	5.61E-01	2.39E-02	6.87E-01	NS
GLUD2	NS	5.61E-01	-0.0239	6.87E-01	NS
RTL8A	NS	5.61E-01	2.39E-02	6.87E-01	NS
ZNF25	NS	5.61E-01	-0.0239	6.87E-01	NS
SPTY2D1	NS	5.61E-01	-0.0239	6.87E-01	NS
FMNL1	NS	5.61E-01	2.39E-02	6.87E-01	NS
CLCN6	NS	5.62E-01	2.38E-02	6.88E-01	NS
CCNY	NS	5.62E-01	2.38E-02	6.88E-01	NS
HOXC13	NS	5.62E-01	-0.0238	6.88E-01	NS
TGFB1I1	NS	5.62E-01	2.38E-02	6.88E-01	NS
DRAM1	NS	5.62E-01	2.38E-02	6.88E-01	NS
CPLANE2	NS	5.62E-01	-0.0238	6.88E-01	NS
OR51T1	NS	5.62E-01	2.38E-02	6.88E-01	NS
H2AC6	NS	5.62E-01	-0.0238	6.88E-01	NS
MAGEA3	NS	5.62E-01	2.38E-02	6.88E-01	NS
EMB	NS	5.62E-01	2.38E-02	6.88E-01	NS
NOP9	NS	5.63E-01	2.38E-02	6.88E-01	NS
GCFC2	NS	5.63E-01	2.38E-02	6.89E-01	NS
KRTAP4-9	NS	5.63E-01	2.38E-02	6.89E-01	NS
TCF7L2	NS	5.63E-01	2.38E-02	6.89E-01	NS
MRPL10	NS	5.63E-01	-0.0238	6.89E-01	NS
PILRB	NS	5.63E-01	-0.0238	6.89E-01	NS
TEDC1	NS	5.63E-01	-0.0237	6.89E-01	NS
TBCB	NS	5.64E-01	-0.0237	6.89E-01	NS
GFPT1	NS	5.64E-01	-0.0237	6.89E-01	NS
PLCG1	NS	5.64E-01	-0.0237	6.89E-01	NS
RNF225	NS	5.64E-01	2.37E-02	6.89E-01	NS
POTED	NS	5.64E-01	-0.0237	6.89E-01	NS

CRKL	NS	5.64E-01	2.37E-02	6.89E-01	NS
ADGRG5	NS	5.64E-01	-0.0237	6.89E-01	NS
HAUS5	NS	5.64E-01	-0.0237	6.90E-01	NS
CRP	NS	5.64E-01	-0.0237	6.90E-01	NS
ZSCAN25	NS	5.64E-01	2.37E-02	6.90E-01	NS
UBE3A	NS	5.64E-01	-0.0237	6.90E-01	NS
TAF1	NS	5.64E-01	-0.0237	6.90E-01	NS
CDS2	NS	5.65E-01	-0.0237	6.90E-01	NS
ZNF2	NS	5.65E-01	2.37E-02	6.90E-01	NS
POLR3E	NS	5.65E-01	2.36E-02	6.90E-01	NS
SEMA4G	NS	5.65E-01	-0.0236	6.90E-01	NS
ZMYM2	NS	5.65E-01	-0.0236	6.90E-01	NS
ABCC6	NS	5.65E-01	2.36E-02	6.90E-01	NS
ZNF362	NS	5.65E-01	2.36E-02	6.91E-01	NS
MRPL43	NS	5.65E-01	2.36E-02	6.91E-01	NS
CNOT3	NS	5.65E-01	2.36E-02	6.91E-01	NS
ACAD8	NS	5.65E-01	-0.0236	6.91E-01	NS
SP1	NS	5.65E-01	-0.0236	6.91E-01	NS
ZDHHC14	NS	5.66E-01	-0.0236	6.91E-01	NS
NOVA1	NS	5.66E-01	-0.0236	6.91E-01	NS
MGARP	NS	5.66E-01	2.36E-02	6.92E-01	NS
SLPI	NS	5.67E-01	-0.0235	6.92E-01	NS
CDH13	NS	5.67E-01	-0.0235	6.92E-01	NS
SERHL2	NS	5.67E-01	2.35E-02	6.92E-01	NS
IGFALS	NS	5.67E-01	2.35E-02	6.92E-01	NS
KCNN2	NS	5.67E-01	2.35E-02	6.92E-01	NS
BIRC5	NS	5.67E-01	2.35E-02	6.92E-01	NS
H2BC8	NS	5.67E-01	-0.0235	6.92E-01	NS
KLF18	NS	5.67E-01	-0.0235	6.92E-01	NS
SIRT2	NS	5.67E-01	-0.0235	6.92E-01	NS
MYCBPAP	NS	5.67E-01	-0.0235	6.92E-01	NS
PRG3	NS	5.67E-01	-0.0235	6.92E-01	NS
TSR3	NS	5.67E-01	2.35E-02	6.92E-01	NS
HMX3	NS	5.67E-01	2.35E-02	6.92E-01	NS
UQCC3	NS	5.67E-01	2.35E-02	6.92E-01	NS
DUSP13	NS	5.68E-01	2.35E-02	6.92E-01	NS
SHISA7	NS	5.68E-01	2.35E-02	6.93E-01	NS

MIXL1	NS	5.68E-01	2.35E-02	6.93E-01	NS
SLC6A4	NS	5.68E-01	2.35E-02	6.93E-01	NS
CLPSL1	NS	5.68E-01	-0.0235	6.93E-01	NS
UQCC1	NS	5.68E-01	-0.0234	6.93E-01	NS
SH2B2	NS	5.68E-01	-0.0234	6.93E-01	NS
FCHO1	NS	5.69E-01	2.34E-02	6.93E-01	NS
PRR5L	NS	5.69E-01	2.34E-02	6.94E-01	NS
CXorf66	NS	5.69E-01	2.34E-02	6.94E-01	NS
BMS1	NS	5.69E-01	-0.0234	6.94E-01	NS
GTF3A	NS	5.69E-01	-0.0234	6.94E-01	NS
RPL29	NS	5.69E-01	-0.0234	6.94E-01	NS
SAC3D1	NS	5.69E-01	-0.0234	6.94E-01	NS
NUTM2A	NS	5.69E-01	-0.0234	6.94E-01	NS
HESX1	NS	5.69E-01	-0.0234	6.94E-01	NS
DBNDD2	NS	5.70E-01	-0.0234	6.94E-01	NS
PAK6	NS	5.70E-01	2.34E-02	6.94E-01	NS
YARS2	NS	5.70E-01	-0.0233	6.94E-01	NS
TMEM229A	NS	5.70E-01	-0.0233	6.95E-01	NS
ENTHD1	NS	5.70E-01	-0.0233	6.95E-01	NS
MRPL55	NS	5.70E-01	-0.0233	6.95E-01	NS
SUCLG2	NS	5.70E-01	-0.0233	6.95E-01	NS
TMEM262	NS	5.70E-01	2.33E-02	6.95E-01	NS
MMS19	NS	5.71E-01	-0.0233	6.95E-01	NS
IFFO2	NS	5.71E-01	-0.0233	6.96E-01	NS
JMJD6	NS	5.71E-01	2.33E-02	6.96E-01	NS
PF4	NS	5.72E-01	-0.0232	6.96E-01	NS
MYEOV	NS	5.72E-01	2.32E-02	6.96E-01	NS
AP1M1	NS	5.72E-01	-0.0232	6.96E-01	NS
NTN5	NS	5.72E-01	2.32E-02	6.96E-01	NS
MAGEE1	NS	5.72E-01	2.32E-02	6.97E-01	NS
TMEM121B	NS	5.72E-01	2.32E-02	6.97E-01	NS
MED9	NS	5.72E-01	-0.0232	6.97E-01	NS
CDHR1	NS	5.72E-01	-0.0232	6.97E-01	NS
LSM14A	NS	5.73E-01	-0.0232	6.97E-01	NS
C19orf25	NS	5.73E-01	2.32E-02	6.97E-01	NS
NEURL4	NS	5.73E-01	-0.0232	6.97E-01	NS
C1orf127	NS	5.73E-01	-0.0231	6.98E-01	NS

ARL3	NS	5.73E-01	-0.0231	6.98E-01	NS
ATMIN	NS	5.73E-01	-0.0231	6.98E-01	NS
ZNF493	NS	5.73E-01	2.31E-02	6.98E-01	NS
TMEM87A	NS	5.73E-01	2.31E-02	6.98E-01	NS
EIF1AD	NS	5.74E-01	2.31E-02	6.98E-01	NS
USP17L5	NS	5.74E-01	2.31E-02	6.98E-01	NS
OSGIN1	NS	5.74E-01	2.31E-02	6.98E-01	NS
ARHGAP28	NS	5.74E-01	2.31E-02	6.98E-01	NS
ARHGAP33	NS	5.74E-01	2.31E-02	6.98E-01	NS
MRPS35	NS	5.74E-01	2.31E-02	6.98E-01	NS
RASGEF1C	NS	5.74E-01	2.31E-02	6.98E-01	NS
CA4	NS	5.74E-01	-0.0231	6.98E-01	NS
LDOC1	NS	5.74E-01	-0.0231	6.98E-01	NS
QTRT2	NS	5.74E-01	-0.0231	6.98E-01	NS
TIE1	NS	5.74E-01	-0.0231	6.98E-01	NS
ALMS1	NS	5.75E-01	-0.0231	6.99E-01	NS
ANKS1A	NS	5.75E-01	2.30E-02	6.99E-01	NS
KIF27	NS	5.75E-01	2.30E-02	6.99E-01	NS
SETDB2	NS	5.75E-01	-0.023	6.99E-01	NS
CACNA1I	NS	5.75E-01	2.30E-02	6.99E-01	NS
UXT	NS	5.75E-01	-0.023	6.99E-01	NS
CILK1	NS	5.75E-01	2.30E-02	6.99E-01	NS
GCGR	NS	5.75E-01	2.30E-02	6.99E-01	NS
AKIRIN1	NS	5.75E-01	2.30E-02	6.99E-01	NS
RBMS1	NS	5.75E-01	-0.023	6.99E-01	NS
CYP4F8	NS	5.75E-01	2.30E-02	6.99E-01	NS
LCORL	NS	5.76E-01	2.30E-02	6.99E-01	NS
RABGGTA	NS	5.76E-01	2.30E-02	6.99E-01	NS
LCE2B	NS	5.76E-01	-0.023	6.99E-01	NS
BRD7	NS	5.76E-01	-0.023	6.99E-01	NS
CDIN1	NS	5.76E-01	2.30E-02	6.99E-01	NS
RAN	NS	5.76E-01	-0.023	7.00E-01	NS
RER1	NS	5.76E-01	-0.023	7.00E-01	NS
WNT11	NS	5.76E-01	2.29E-02	7.00E-01	NS
RBPMS	NS	5.77E-01	-0.0229	7.00E-01	NS
PLXNA1	NS	5.77E-01	2.29E-02	7.00E-01	NS
CUL4B	NS	5.77E-01	2.29E-02	7.00E-01	NS

ABHD16B	NS	5.77E-01	2.29E-02	7.01E-01	NS
PGA5	NS	5.77E-01	-0.0229	7.01E-01	NS
ISYNA1	NS	5.77E-01	2.29E-02	7.01E-01	NS
HERPUD1	NS	5.78E-01	-0.0229	7.01E-01	NS
NUP153	NS	5.78E-01	2.29E-02	7.01E-01	NS
GALNT8	NS	5.78E-01	-0.0229	7.01E-01	NS
TMEM8B	NS	5.78E-01	2.29E-02	7.01E-01	NS
SPDYE17	NS	5.78E-01	-0.0229	7.01E-01	NS
STX4	NS	5.78E-01	2.29E-02	7.01E-01	NS
SH2B3	NS	5.78E-01	2.29E-02	7.01E-01	NS
C1QTNF5	NS	5.78E-01	2.29E-02	7.01E-01	NS
ELOA3DP	NS	5.78E-01	2.29E-02	7.01E-01	NS
NOL11	NS	5.78E-01	-0.0228	7.01E-01	NS
AQP12A	NS	5.78E-01	2.28E-02	7.01E-01	NS
BEGAIN	NS	5.78E-01	-0.0228	7.02E-01	NS
TOGARAM2	NS	5.78E-01	-0.0228	7.02E-01	NS
MFN2	NS	5.79E-01	2.28E-02	7.02E-01	NS
CTSB	NS	5.79E-01	-0.0228	7.02E-01	NS
PURG	NS	5.79E-01	2.28E-02	7.02E-01	NS
ANKRD28	NS	5.79E-01	-0.0228	7.02E-01	NS
SMYD5	NS	5.79E-01	-0.0228	7.02E-01	NS
TADA3	NS	5.79E-01	-0.0228	7.03E-01	NS
TMEM182	NS	5.79E-01	-0.0228	7.03E-01	NS
ADCYAP1R1	NS	5.80E-01	-0.0227	7.03E-01	NS
HSP90AA1	NS	5.80E-01	2.27E-02	7.03E-01	NS
LPCAT2	NS	5.80E-01	-0.0227	7.03E-01	NS
FBP2	NS	5.80E-01	-0.0227	7.03E-01	NS
JMY	NS	5.80E-01	-0.0228	7.03E-01	NS
LY96	NS	5.80E-01	2.28E-02	7.03E-01	NS
CDK12	NS	5.80E-01	-0.0227	7.03E-01	NS
OR7D4	NS	5.80E-01	2.27E-02	7.03E-01	NS
CAPS2	NS	5.80E-01	2.27E-02	7.03E-01	NS
SMOC1	NS	5.80E-01	-0.0227	7.03E-01	NS
NAP1L3	NS	5.80E-01	2.27E-02	7.03E-01	NS
THRAP3	NS	5.80E-01	2.27E-02	7.03E-01	NS
MBNL1	NS	5.80E-01	-0.0227	7.03E-01	NS
ZBTB8A	NS	5.80E-01	-0.0227	7.03E-01	NS

GBGT1	NS	5.81E-01	-0.0227	7.03E-01	NS
ADAM29	NS	5.81E-01	2.27E-02	7.04E-01	NS
KRTAP1-5	NS	5.81E-01	2.27E-02	7.04E-01	NS
IFNB1	NS	5.81E-01	-0.0227	7.04E-01	NS
DAO	NS	5.81E-01	-0.0227	7.04E-01	NS
GRIK2	NS	5.81E-01	-0.0227	7.04E-01	NS
GORASP2	NS	5.81E-01	2.26E-02	7.04E-01	NS
DHRSX	NS	5.82E-01	2.26E-02	7.04E-01	NS
BCR	NS	5.82E-01	-0.0226	7.04E-01	NS
AKNA	NS	5.82E-01	2.26E-02	7.04E-01	NS
TARS1	NS	5.82E-01	2.26E-02	7.04E-01	NS
ATP2A2	NS	5.82E-01	-0.0226	7.04E-01	NS
UNC5CL	NS	5.82E-01	2.26E-02	7.04E-01	NS
DNHD1	NS	5.82E-01	2.26E-02	7.04E-01	NS
SMC4	NS	5.82E-01	-0.0226	7.05E-01	NS
KRT12	NS	5.82E-01	-0.0226	7.05E-01	NS
ANKRD55	NS	5.82E-01	2.26E-02	7.05E-01	NS
POLDIP2	NS	5.83E-01	2.26E-02	7.05E-01	NS
FITM1	NS	5.83E-01	2.26E-02	7.05E-01	NS
BPIFB3	NS	5.83E-01	2.26E-02	7.05E-01	NS
DNM1	NS	5.83E-01	2.26E-02	7.05E-01	NS
SLCO1A2	NS	5.83E-01	2.26E-02	7.05E-01	NS
RAF1	NS	5.83E-01	-0.0226	7.05E-01	NS
GABARAP	NS	5.83E-01	-0.0226	7.05E-01	NS
BMPR1B	NS	5.83E-01	-0.0225	7.05E-01	NS
KIR2DL3	NS	5.83E-01	2.25E-02	7.05E-01	NS
GALNT2	NS	5.83E-01	-0.0225	7.05E-01	NS
SRSF2	NS	5.83E-01	-0.0225	7.05E-01	NS
AGPAT4	NS	5.83E-01	-0.0225	7.05E-01	NS
DNAJB9	NS	5.83E-01	2.25E-02	7.05E-01	NS
CCDC157	NS	5.83E-01	-0.0225	7.05E-01	NS
ZNF791	NS	5.84E-01	2.25E-02	7.06E-01	NS
MTPN	NS	5.84E-01	-0.0225	7.06E-01	NS
ARF6	NS	5.84E-01	2.25E-02	7.06E-01	NS
NUTM2B	NS	5.84E-01	-0.0225	7.06E-01	NS
HBE1	NS	5.84E-01	-0.0225	7.06E-01	NS
NSD2	NS	5.84E-01	2.25E-02	7.06E-01	NS

KCTD19	NS	5.84E-01	-0.0225	7.06E-01	NS
EPHX4	NS	5.84E-01	2.25E-02	7.06E-01	NS
ATP6V0A1	NS	5.84E-01	-0.0225	7.06E-01	NS
SLC5A8	NS	5.84E-01	-0.0225	7.06E-01	NS
RBM28	NS	5.85E-01	2.25E-02	7.06E-01	NS
TNIP1	NS	5.85E-01	-0.0224	7.07E-01	NS
MUC5B	NS	5.85E-01	-0.0224	7.07E-01	NS
PIGW	NS	5.85E-01	-0.0224	7.07E-01	NS
LECT2	NS	5.85E-01	-0.0224	7.07E-01	NS
SHISA2	NS	5.85E-01	2.24E-02	7.07E-01	NS
CACNA1D	NS	5.86E-01	-0.0224	7.07E-01	NS
COLEC12	NS	5.86E-01	-0.0224	7.07E-01	NS
KRTAP19-1	NS	5.85E-01	-0.0224	7.07E-01	NS
TAS2R7	NS	5.86E-01	2.24E-02	7.07E-01	NS
NME4	NS	5.86E-01	2.24E-02	7.07E-01	NS
VKORC1L1	NS	5.86E-01	-0.0224	7.07E-01	NS
BRS3	NS	5.86E-01	2.24E-02	7.07E-01	NS
HOXC9	NS	5.86E-01	2.24E-02	7.08E-01	NS
SMC3	NS	5.86E-01	-0.0224	7.08E-01	NS
PRR13	NS	5.87E-01	-0.0223	7.08E-01	NS
USP17L21	NS	5.87E-01	2.23E-02	7.09E-01	NS
IFITM5	NS	5.87E-01	2.23E-02	7.09E-01	NS
NOX5	NS	5.88E-01	-0.0223	7.09E-01	NS
SFTA2	NS	5.88E-01	-0.0223	7.09E-01	NS
TESK2	NS	5.88E-01	-0.0223	7.09E-01	NS
ADRA1A	NS	5.88E-01	2.23E-02	7.09E-01	NS
RTL1	NS	5.88E-01	-0.0223	7.09E-01	NS
TPTEP2-CSNK1E	NS	5.88E-01	2.23E-02	7.09E-01	NS
GGCT	NS	5.88E-01	2.22E-02	7.10E-01	NS
RABL2A	NS	5.88E-01	2.22E-02	7.10E-01	NS
HNF4A	NS	5.88E-01	2.22E-02	7.10E-01	NS
WTIP	NS	5.88E-01	2.22E-02	7.10E-01	NS
TAF8	NS	5.88E-01	-0.0222	7.10E-01	NS
AVPR1A	NS	5.89E-01	-0.0222	7.10E-01	NS
RALB	NS	5.89E-01	2.22E-02	7.10E-01	NS
PTMA	NS	5.89E-01	2.22E-02	7.10E-01	NS
FNTA	NS	5.89E-01	2.22E-02	7.10E-01	NS

OR5M3	NS	5.89E-01	-0.0222	7.10E-01	NS
ZBTB6	NS	5.89E-01	2.22E-02	7.10E-01	NS
CLPS	NS	5.89E-01	-0.0222	7.10E-01	NS
DENND1A	NS	5.89E-01	-0.0222	7.11E-01	NS
RCBTB1	NS	5.90E-01	-0.0221	7.11E-01	NS
FCF1	NS	5.90E-01	-0.0221	7.11E-01	NS
VWA5A	NS	5.90E-01	-0.0221	7.11E-01	NS
GXYLT2	NS	5.90E-01	2.21E-02	7.11E-01	NS
LCE4A	NS	5.90E-01	-0.0221	7.12E-01	NS
LORICRIN	NS	5.90E-01	-0.0221	7.12E-01	NS
STXBP6	NS	5.90E-01	-0.0221	7.12E-01	NS
ZNF367	NS	5.91E-01	2.21E-02	7.12E-01	NS
PLEC	NS	5.91E-01	-0.0221	7.12E-01	NS
NUFIP1	NS	5.91E-01	2.21E-02	7.12E-01	NS
PRAM1	NS	5.91E-01	2.21E-02	7.12E-01	NS
CIDEB	NS	5.91E-01	2.21E-02	7.12E-01	NS
RPEL1	NS	5.91E-01	-0.0221	7.12E-01	NS
WNK2	NS	5.91E-01	-0.0221	7.12E-01	NS
MTERF4	NS	5.91E-01	-0.0221	7.12E-01	NS
SLC2A1	NS	5.92E-01	2.20E-02	7.12E-01	NS
TMEM109	NS	5.92E-01	2.20E-02	7.13E-01	NS
TAS2R4	NS	5.92E-01	-0.022	7.13E-01	NS
CDH4	NS	5.92E-01	2.20E-02	7.13E-01	NS
TTC12	NS	5.92E-01	-0.022	7.13E-01	NS
TSPEAR	NS	5.92E-01	2.20E-02	7.13E-01	NS
SMIM10L2B	NS	5.92E-01	-0.022	7.13E-01	NS
SGTB	NS	5.92E-01	-0.022	7.13E-01	NS
THUMPD3	NS	5.93E-01	2.20E-02	7.14E-01	NS
AMPD1	NS	5.93E-01	2.20E-02	7.14E-01	NS
RUVBL1	NS	5.93E-01	2.19E-02	7.14E-01	NS
ATAD3A	NS	5.93E-01	2.19E-02	7.14E-01	NS
SERPINB1	NS	5.93E-01	-0.0219	7.14E-01	NS
PPM1D	NS	5.93E-01	-0.0219	7.14E-01	NS
S100A7A	NS	5.93E-01	-0.0219	7.14E-01	NS
C14orf132	NS	5.93E-01	-0.0219	7.14E-01	NS
EDDM13	NS	5.93E-01	-0.0219	7.14E-01	NS
CCDC13	NS	5.94E-01	2.19E-02	7.14E-01	NS

A4GNT	NS	5.94E-01	2.19E-02	7.14E-01	NS
H1-1	NS	5.94E-01	2.19E-02	7.14E-01	NS
EML2	NS	5.94E-01	2.19E-02	7.15E-01	NS
MFSD13A	NS	5.94E-01	-0.0219	7.15E-01	NS
RBM33	NS	5.94E-01	2.19E-02	7.15E-01	NS
OR5T1	NS	5.94E-01	-0.0219	7.15E-01	NS
FAM124A	NS	5.94E-01	2.19E-02	7.15E-01	NS
SH2D4A	NS	5.95E-01	2.19E-02	7.15E-01	NS
JARID2	NS	5.95E-01	2.19E-02	7.15E-01	NS
DNAJC2	NS	5.95E-01	-0.0219	7.15E-01	NS
GPR35	NS	5.95E-01	-0.0218	7.15E-01	NS
TERB1	NS	5.95E-01	2.18E-02	7.15E-01	NS
PCSK7	NS	5.95E-01	-0.0218	7.15E-01	NS
C1orf100	NS	5.95E-01	-0.0218	7.16E-01	NS
LMX1B	NS	5.95E-01	2.18E-02	7.16E-01	NS
PDLIM3	NS	5.95E-01	-0.0218	7.16E-01	NS
HIKESHI	NS	5.95E-01	-0.0218	7.16E-01	NS
C9orf43	NS	5.96E-01	-0.0218	7.16E-01	NS
MCRIP2	NS	5.96E-01	2.18E-02	7.16E-01	NS
DNAJC11	NS	5.96E-01	-0.0218	7.16E-01	NS
EIF2AK1	NS	5.96E-01	2.18E-02	7.16E-01	NS
PRAMEF12	NS	5.96E-01	-0.0218	7.16E-01	NS
ZNF573	NS	5.96E-01	-0.0218	7.16E-01	NS
EEF1AKMT3	NS	5.96E-01	2.18E-02	7.16E-01	NS
SLC35G1	NS	5.96E-01	2.18E-02	7.16E-01	NS
NDUFA6	NS	5.96E-01	-0.0218	7.16E-01	NS
ZC3H12C	NS	5.96E-01	-0.0218	7.16E-01	NS
SPAG5	NS	5.97E-01	-0.0217	7.17E-01	NS
SLC43A1	NS	5.97E-01	2.17E-02	7.17E-01	NS
DSEL	NS	5.97E-01	-0.0217	7.17E-01	NS
MAP4K2	NS	5.97E-01	-0.0217	7.17E-01	NS
ZSCAN22	NS	5.97E-01	-0.0217	7.17E-01	NS
PGM1	NS	5.97E-01	2.17E-02	7.17E-01	NS
ENTPD1	NS	5.97E-01	-0.0217	7.17E-01	NS
LENEP	NS	5.97E-01	2.17E-02	7.17E-01	NS
PTTG1IP	NS	5.97E-01	2.17E-02	7.17E-01	NS
SIPA1L1	NS	5.97E-01	2.17E-02	7.17E-01	NS

CRLS1	NS	5.98E-01	2.17E-02	7.17E-01	NS
UBA3	NS	5.98E-01	-0.0217	7.17E-01	NS
DLG4	NS	5.98E-01	-0.0217	7.17E-01	NS
HMGB3	NS	5.98E-01	2.17E-02	7.17E-01	NS
S1PR4	NS	5.98E-01	2.17E-02	7.17E-01	NS
GAL3ST2	NS	5.98E-01	2.17E-02	7.17E-01	NS
ABCD1	NS	5.98E-01	-0.0217	7.17E-01	NS
TPO	NS	5.98E-01	2.17E-02	7.17E-01	NS
TMEM116	NS	5.98E-01	-0.0217	7.18E-01	NS
MVK	NS	5.98E-01	-0.0216	7.18E-01	NS
TSPAN4	NS	5.98E-01	-0.0216	7.18E-01	NS
AP4M1	NS	5.98E-01	-0.0216	7.18E-01	NS
TOM1	NS	5.99E-01	2.16E-02	7.18E-01	NS
MFSD1	NS	5.99E-01	2.16E-02	7.18E-01	NS
CREB3L2	NS	5.99E-01	2.16E-02	7.18E-01	NS
KATNB1	NS	5.99E-01	-0.0216	7.18E-01	NS
WHAMM	NS	5.99E-01	2.16E-02	7.18E-01	NS
BEST3	NS	5.99E-01	-0.0216	7.18E-01	NS
USO1	NS	5.99E-01	-0.0216	7.18E-01	NS
DFFB	NS	5.99E-01	-0.0216	7.18E-01	NS
MARF1	NS	5.99E-01	-0.0216	7.18E-01	NS
BLM	NS	5.99E-01	2.16E-02	7.18E-01	NS
SIX2	NS	5.99E-01	2.16E-02	7.18E-01	NS
IL2RG	NS	6.00E-01	-0.0216	7.19E-01	NS
PPFIBP2	NS	6.00E-01	2.16E-02	7.19E-01	NS
PRPS1	NS	6.00E-01	-0.0215	7.19E-01	NS
ELOA3CP	NS	6.00E-01	2.15E-02	7.19E-01	NS
HINT3	NS	6.00E-01	-0.0215	7.19E-01	NS
SLC4A9	NS	6.00E-01	-0.0215	7.19E-01	NS
SAMD4B	NS	6.00E-01	-0.0215	7.19E-01	NS
INPP5K	NS	6.00E-01	2.15E-02	7.19E-01	NS
ZNF692	NS	6.00E-01	2.15E-02	7.19E-01	NS
LDLRAD1	NS	6.00E-01	2.15E-02	7.19E-01	NS
CA3	NS	6.01E-01	-0.0215	7.19E-01	NS
FLVCR2	NS	6.01E-01	-0.0215	7.20E-01	NS
TERF1	NS	6.01E-01	2.15E-02	7.20E-01	NS
ALG12	NS	6.01E-01	-0.0215	7.20E-01	NS

PRSS51	NS	6.01E-01	-0.0215	7.20E-01	NS
SLCO5A1	NS	6.01E-01	-0.0215	7.20E-01	NS
RBM8A	NS	6.01E-01	-0.0215	7.20E-01	NS
VAMP3	NS	6.02E-01	-0.0215	7.20E-01	NS
FLT4	NS	6.02E-01	2.14E-02	7.20E-01	NS
UQCRC1	NS	6.02E-01	2.14E-02	7.20E-01	NS
CLCA2	NS	6.02E-01	-0.0214	7.20E-01	NS
CFAP74	NS	6.02E-01	2.14E-02	7.20E-01	NS
CHCHD10	NS	6.02E-01	2.14E-02	7.20E-01	NS
FGF5	NS	6.02E-01	2.14E-02	7.20E-01	NS
SMIM40	NS	6.02E-01	-0.0214	7.20E-01	NS
FAM131B	NS	6.02E-01	-0.0214	7.21E-01	NS
NUP42	NS	6.02E-01	-0.0214	7.21E-01	NS
ZNF483	NS	6.02E-01	2.14E-02	7.21E-01	NS
TMPRSS4	NS	6.03E-01	2.14E-02	7.21E-01	NS
GTF2IRD2	NS	6.03E-01	2.14E-02	7.21E-01	NS
SCARB2	NS	6.03E-01	2.14E-02	7.21E-01	NS
GET3	NS	6.03E-01	-0.0214	7.21E-01	NS
MED23	NS	6.03E-01	-0.0214	7.21E-01	NS
DUOXA1	NS	6.03E-01	-0.0214	7.21E-01	NS
PEX11G	NS	6.03E-01	-0.0213	7.21E-01	NS
CGNL1	NS	6.03E-01	-0.0213	7.22E-01	NS
ZNF33B	NS	6.03E-01	2.13E-02	7.22E-01	NS
SMG7	NS	6.04E-01	-0.0213	7.22E-01	NS
LGALS2	NS	6.04E-01	2.13E-02	7.22E-01	NS
PCDHA3	NS	6.04E-01	2.13E-02	7.22E-01	NS
ETFA	NS	6.04E-01	-0.0213	7.22E-01	NS
NDUFA10	NS	6.04E-01	-0.0213	7.22E-01	NS
CPT1C	NS	6.04E-01	2.13E-02	7.22E-01	NS
SELL	NS	6.04E-01	2.13E-02	7.22E-01	NS
ILK	NS	6.05E-01	-0.0213	7.23E-01	NS
PRAMEF18	NS	6.05E-01	-0.0213	7.23E-01	NS
CLVS1	NS	6.05E-01	-0.0213	7.23E-01	NS
SLC24A2	NS	6.05E-01	-0.0213	7.23E-01	NS
ZFYVE19	NS	6.05E-01	-0.0212	7.23E-01	NS
RRP1B	NS	6.05E-01	-0.0212	7.23E-01	NS
MBNL3	NS	6.05E-01	2.12E-02	7.23E-01	NS

MAU2	NS	6.05E-01	-0.0212	7.23E-01	NS
TIMP2	NS	6.05E-01	-0.0212	7.23E-01	NS
AAGAB	NS	6.05E-01	2.12E-02	7.23E-01	NS
SVOPL	NS	6.06E-01	-0.0212	7.23E-01	NS
RGPD4	NS	6.06E-01	-0.0212	7.23E-01	NS
KRT19	NS	6.06E-01	-0.0212	7.23E-01	NS
RHOJ	NS	6.06E-01	-0.0212	7.24E-01	NS
ZNF736	NS	6.06E-01	-0.0212	7.24E-01	NS
CPXM1	NS	6.06E-01	-0.0212	7.24E-01	NS
CSTB	NS	6.06E-01	2.12E-02	7.24E-01	NS
OR6C75	NS	6.07E-01	-0.0212	7.24E-01	NS
CTXND1	NS	6.06E-01	-0.0212	7.24E-01	NS
STIM1	NS	6.07E-01	2.12E-02	7.24E-01	NS
CFAP157	NS	6.07E-01	-0.0211	7.24E-01	NS
TMEM147	NS	6.07E-01	-0.0211	7.24E-01	NS
SYNE4	NS	6.07E-01	2.11E-02	7.24E-01	NS
MX1	NS	6.07E-01	2.11E-02	7.24E-01	NS
MON1B	NS	6.07E-01	-0.0211	7.25E-01	NS
ELOA	NS	6.07E-01	2.11E-02	7.25E-01	NS
FAM32A	NS	6.08E-01	-0.0211	7.25E-01	NS
PHYHIP	NS	6.08E-01	2.11E-02	7.25E-01	NS
MAGI2	NS	6.08E-01	-0.0211	7.25E-01	NS
TNFSF12-TNFSF13	NS	6.08E-01	-0.0211	7.25E-01	NS
CCNI	NS	6.08E-01	2.11E-02	7.25E-01	NS
PDZD8	NS	6.08E-01	2.11E-02	7.25E-01	NS
RAB6B	NS	6.08E-01	-0.0211	7.25E-01	NS
RIMBP3	NS	6.08E-01	2.11E-02	7.25E-01	NS
SYCE2	NS	6.09E-01	-0.021	7.26E-01	NS
ZNF623	NS	6.09E-01	-0.021	7.26E-01	NS
FBXO7	NS	6.09E-01	-0.021	7.26E-01	NS
CASKIN1	NS	6.09E-01	-0.021	7.26E-01	NS
PPM1A	NS	6.09E-01	-0.021	7.26E-01	NS
GLUL	NS	6.09E-01	2.10E-02	7.26E-01	NS
APEH	NS	6.09E-01	-0.021	7.26E-01	NS
PHF10	NS	6.09E-01	-0.021	7.26E-01	NS
PPTC7	NS	6.09E-01	2.10E-02	7.26E-01	NS
RCL1	NS	6.09E-01	2.10E-02	7.26E-01	NS

AFF3	NS	6.10E-01	-0.021	7.26E-01	NS
LITAFD	NS	6.09E-01	2.10E-02	7.26E-01	NS
REST	NS	6.10E-01	2.10E-02	7.26E-01	NS
WASHC2A	NS	6.10E-01	-0.021	7.26E-01	NS
BRD1	NS	6.10E-01	2.10E-02	7.26E-01	NS
CDH6	NS	6.10E-01	2.10E-02	7.26E-01	NS
DNMT1	NS	6.10E-01	2.10E-02	7.26E-01	NS
SLC25A44	NS	6.10E-01	-0.021	7.26E-01	NS
MAPK12	NS	6.10E-01	-0.021	7.26E-01	NS
HS3ST6	NS	6.10E-01	2.10E-02	7.27E-01	NS
DEDD	NS	6.10E-01	2.09E-02	7.27E-01	NS
PTPRE	NS	6.11E-01	-0.0209	7.27E-01	NS
USP1	NS	6.11E-01	-0.0209	7.27E-01	NS
C1orf109	NS	6.11E-01	-0.0209	7.27E-01	NS
SH2D7	NS	6.11E-01	-0.0209	7.27E-01	NS
RTN4RL2	NS	6.11E-01	-0.0209	7.28E-01	NS
SPINK1	NS	6.11E-01	-0.0209	7.28E-01	NS
RPL9	NS	6.12E-01	2.09E-02	7.28E-01	NS
KIAA1191	NS	6.12E-01	2.09E-02	7.28E-01	NS
IL1RAP	NS	6.12E-01	-0.0209	7.28E-01	NS
BTBD17	NS	6.12E-01	2.09E-02	7.28E-01	NS
KCNK17	NS	6.12E-01	2.08E-02	7.28E-01	NS
TIMM17B	NS	6.12E-01	2.08E-02	7.28E-01	NS
PTCH1	NS	6.12E-01	-0.0208	7.28E-01	NS
NT5DC3	NS	6.12E-01	2.08E-02	7.29E-01	NS
POU2F2	NS	6.13E-01	2.08E-02	7.29E-01	NS
TPM3	NS	6.13E-01	2.08E-02	7.29E-01	NS
GABRA6	NS	6.13E-01	2.08E-02	7.29E-01	NS
GPR84	NS	6.13E-01	2.08E-02	7.29E-01	NS
WNT7A	NS	6.13E-01	-0.0208	7.29E-01	NS
CBX5	NS	6.13E-01	2.08E-02	7.30E-01	NS
PNP	NS	6.13E-01	2.08E-02	7.30E-01	NS
IHH	NS	6.14E-01	2.07E-02	7.30E-01	NS
CYLC2	NS	6.14E-01	2.07E-02	7.30E-01	NS
VN1R2	NS	6.14E-01	-0.0207	7.30E-01	NS
KIAA1522	NS	6.14E-01	-0.0207	7.30E-01	NS
USP38	NS	6.14E-01	-0.0207	7.30E-01	NS

TNFRSF9	NS	6.14E-01	-0.0207	7.30E-01	NS
PRR20A	NS	6.14E-01	2.07E-02	7.30E-01	NS
HTT	NS	6.14E-01	2.07E-02	7.31E-01	NS
ALG9	NS	6.15E-01	2.07E-02	7.31E-01	NS
LDB3	NS	6.15E-01	2.07E-02	7.31E-01	NS
UFSP1	NS	6.15E-01	-0.0207	7.31E-01	NS
OR56A4	NS	6.15E-01	-0.0207	7.31E-01	NS
RAB3B	NS	6.15E-01	-0.0207	7.31E-01	NS
APOC2	NS	6.15E-01	2.07E-02	7.31E-01	NS
AGAP3	NS	6.15E-01	2.06E-02	7.31E-01	NS
KIAA1549L	NS	6.15E-01	-0.0206	7.31E-01	NS
ANP32B	NS	6.16E-01	-0.0206	7.31E-01	NS
FRAS1	NS	6.16E-01	2.06E-02	7.31E-01	NS
IQCF1	NS	6.16E-01	2.06E-02	7.32E-01	NS
TANGO2	NS	6.16E-01	-0.0206	7.32E-01	NS
OXSR1	NS	6.16E-01	2.06E-02	7.32E-01	NS
MYRF	NS	6.16E-01	2.06E-02	7.32E-01	NS
LRP10	NS	6.17E-01	2.06E-02	7.32E-01	NS
UBE2QL1	NS	6.17E-01	-0.0206	7.32E-01	NS
ERVMER34-1	NS	6.16E-01	2.06E-02	7.32E-01	NS
PISD	NS	6.16E-01	2.06E-02	7.32E-01	NS
JADE2	NS	6.17E-01	-0.0206	7.32E-01	NS
CLDN7	NS	6.17E-01	-0.0206	7.32E-01	NS
MRFAP1	NS	6.17E-01	-0.0206	7.32E-01	NS
ALDH7A1	NS	6.17E-01	2.06E-02	7.32E-01	NS
CD19	NS	6.17E-01	2.05E-02	7.32E-01	NS
KRTAP29-1	NS	6.17E-01	-0.0205	7.33E-01	NS
RAB34	NS	6.17E-01	-0.0205	7.33E-01	NS
BICDL1	NS	6.17E-01	2.05E-02	7.33E-01	NS
CTCFL	NS	6.17E-01	-0.0205	7.33E-01	NS
LOXHD1	NS	6.17E-01	-0.0205	7.33E-01	NS
OR9Q2	NS	6.18E-01	-0.0205	7.33E-01	NS
FBF1	NS	6.18E-01	-0.0205	7.33E-01	NS
SLC25A29	NS	6.18E-01	-0.0205	7.33E-01	NS
KDM6A	NS	6.18E-01	-0.0205	7.33E-01	NS
NCK1	NS	6.18E-01	2.05E-02	7.33E-01	NS
PSMB4	NS	6.18E-01	-0.0205	7.33E-01	NS

ASB3	NS	6.18E-01	-0.0205	7.33E-01	NS
DDX27	NS	6.18E-01	2.05E-02	7.33E-01	NS
IGSF9	NS	6.19E-01	-0.0205	7.34E-01	NS
BLVRB	NS	6.19E-01	-0.0205	7.34E-01	NS
PTGDR2	NS	6.18E-01	2.05E-02	7.34E-01	NS
POM121L12	NS	6.18E-01	2.05E-02	7.34E-01	NS
USP12	NS	6.19E-01	-0.0204	7.34E-01	NS
RENBP	NS	6.19E-01	2.04E-02	7.34E-01	NS
ARFIP1	NS	6.19E-01	-0.0204	7.34E-01	NS
TMEM215	NS	6.19E-01	2.04E-02	7.34E-01	NS
B4GALT3	NS	6.19E-01	-0.0204	7.34E-01	NS
CTSC	NS	6.19E-01	-0.0204	7.34E-01	NS
SNTB2	NS	6.19E-01	2.04E-02	7.34E-01	NS
TXNDC15	NS	6.20E-01	-0.0204	7.34E-01	NS
OR2G3	NS	6.20E-01	2.04E-02	7.34E-01	NS
TEX45	NS	6.20E-01	-0.0204	7.35E-01	NS
RELT	NS	6.20E-01	2.04E-02	7.35E-01	NS
CREG2	NS	6.20E-01	2.03E-02	7.35E-01	NS
GMIP	NS	6.20E-01	-0.0203	7.35E-01	NS
B3GALT9	NS	6.21E-01	-0.0203	7.35E-01	NS
FSHR	NS	6.21E-01	-0.0203	7.35E-01	NS
CELF1	NS	6.21E-01	2.03E-02	7.35E-01	NS
TMEM233	NS	6.21E-01	2.03E-02	7.35E-01	NS
VEZT	NS	6.21E-01	-0.0203	7.35E-01	NS
DPP3	NS	6.21E-01	2.03E-02	7.36E-01	NS
CCDC9	NS	6.21E-01	2.03E-02	7.36E-01	NS
GPRC5C	NS	6.21E-01	-0.0203	7.36E-01	NS
DNAAF1	NS	6.21E-01	-0.0203	7.36E-01	NS
BDNF	NS	6.21E-01	2.03E-02	7.36E-01	NS
PI4KA	NS	6.21E-01	2.03E-02	7.36E-01	NS
OR10V1	NS	6.22E-01	-0.0203	7.36E-01	NS
IFT22	NS	6.22E-01	2.03E-02	7.36E-01	NS
SYT17	NS	6.22E-01	-0.0202	7.36E-01	NS
S100A8	NS	6.22E-01	-0.0202	7.36E-01	NS
ADD3	NS	6.22E-01	2.02E-02	7.36E-01	NS
RDH14	NS	6.22E-01	-0.0202	7.36E-01	NS
FER1L6	NS	6.22E-01	-0.0202	7.36E-01	NS

ACER3	NS	6.23E-01	2.02E-02	7.37E-01	NS
LRRC42	NS	6.23E-01	-0.0202	7.37E-01	NS
LRP11	NS	6.23E-01	2.02E-02	7.37E-01	NS
ME2	NS	6.23E-01	-0.0202	7.37E-01	NS
C2CD2	NS	6.23E-01	-0.0202	7.37E-01	NS
RNASE3	NS	6.23E-01	-0.0202	7.37E-01	NS
RRP7A	NS	6.23E-01	-0.0202	7.37E-01	NS
FDXR	NS	6.23E-01	-0.0202	7.37E-01	NS
ZNF592	NS	6.23E-01	-0.0202	7.37E-01	NS
BRF1	NS	6.24E-01	-0.0202	7.38E-01	NS
AKT3	NS	6.24E-01	-0.0201	7.38E-01	NS
UFSP2	NS	6.24E-01	-0.0201	7.38E-01	NS
PHACTR1	NS	6.24E-01	-0.0201	7.38E-01	NS
CHMP1A	NS	6.24E-01	-0.0201	7.38E-01	NS
BRD4	NS	6.24E-01	-0.0201	7.38E-01	NS
REP15	NS	6.24E-01	2.01E-02	7.38E-01	NS
MUC22	NS	6.24E-01	2.01E-02	7.38E-01	NS
NUB1	NS	6.25E-01	2.01E-02	7.38E-01	NS
OGFOD1	NS	6.25E-01	-0.0201	7.38E-01	NS
COLEC10	NS	6.25E-01	-0.0201	7.38E-01	NS
NCOA2	NS	6.25E-01	2.01E-02	7.38E-01	NS
DNAJC15	NS	6.25E-01	-0.0201	7.38E-01	NS
PDE4A	NS	6.25E-01	-0.0201	7.38E-01	NS
SHLD2	NS	6.25E-01	-0.0201	7.38E-01	NS
ZNF12	NS	6.25E-01	-0.0201	7.38E-01	NS
LGALS16	NS	6.25E-01	2.01E-02	7.38E-01	NS
JAM3	NS	6.25E-01	-0.0201	7.38E-01	NS
FADS6	NS	6.25E-01	2.01E-02	7.38E-01	NS
DIO1	NS	6.25E-01	-0.0201	7.38E-01	NS
USF3	NS	6.25E-01	-0.0201	7.38E-01	NS
LRP1B	NS	6.25E-01	2.01E-02	7.38E-01	NS
FKBP5	NS	6.25E-01	-0.0201	7.39E-01	NS
SEC31B	NS	6.25E-01	-0.0201	7.39E-01	NS
E4F1	NS	6.26E-01	-0.02	7.39E-01	NS
APOL4	NS	6.26E-01	-0.02	7.39E-01	NS
MARK3	NS	6.26E-01	-0.02	7.39E-01	NS
RP1L1	NS	6.26E-01	-0.02	7.39E-01	NS

MAPKBP1	NS	6.26E-01	2.00E-02	7.39E-01	NS
TTYH1	NS	6.26E-01	-0.02	7.39E-01	NS
MYO1A	NS	6.26E-01	-0.02	7.39E-01	NS
MMP9	NS	6.26E-01	-0.02	7.39E-01	NS
PDGFRB	NS	6.26E-01	2.00E-02	7.40E-01	NS
KAT6A	NS	6.27E-01	2.00E-02	7.40E-01	NS
FAAH	NS	6.27E-01	-0.02	7.40E-01	NS
TRIM46	NS	6.27E-01	-0.02	7.40E-01	NS
CD38	NS	6.27E-01	2.00E-02	7.40E-01	NS
KCNK16	NS	6.27E-01	1.99E-02	7.40E-01	NS
TRMT2A	NS	6.27E-01	-0.0199	7.40E-01	NS
COL10A1	NS	6.27E-01	-0.0199	7.40E-01	NS
MRPS34	NS	6.27E-01	-0.0199	7.40E-01	NS
GFI1B	NS	6.28E-01	1.99E-02	7.40E-01	NS
SPATA31A6	NS	6.28E-01	-0.0199	7.40E-01	NS
ROBO3	NS	6.28E-01	1.99E-02	7.40E-01	NS
CUL4A	NS	6.28E-01	-0.0199	7.41E-01	NS
KHDC3L	NS	6.28E-01	-0.0199	7.41E-01	NS
MUS81	NS	6.28E-01	-0.0199	7.41E-01	NS
RP1	NS	6.28E-01	-0.0199	7.41E-01	NS
OPN1MW2	NS	6.28E-01	-0.0199	7.41E-01	NS
RAPGEFL1	NS	6.28E-01	-0.0199	7.41E-01	NS
CHUK	NS	6.28E-01	1.99E-02	7.41E-01	NS
FAM118A	NS	6.29E-01	-0.0199	7.41E-01	NS
BARHL1	NS	6.29E-01	-0.0199	7.41E-01	NS
PPP1R27	NS	6.29E-01	1.99E-02	7.41E-01	NS
PRAMEF2	NS	6.29E-01	1.98E-02	7.42E-01	NS
FBXO32	NS	6.29E-01	-0.0198	7.42E-01	NS
IFNA16	NS	6.29E-01	-0.0198	7.42E-01	NS
HERC4	NS	6.30E-01	1.98E-02	7.42E-01	NS
ADTRP	NS	6.30E-01	1.98E-02	7.42E-01	NS
LARP7	NS	6.30E-01	-0.0198	7.42E-01	NS
ZAR1	NS	6.30E-01	1.98E-02	7.42E-01	NS
CIT	NS	6.30E-01	-0.0198	7.42E-01	NS
CORO1C	NS	6.30E-01	-0.0198	7.42E-01	NS
CTNNB1	NS	6.30E-01	1.98E-02	7.42E-01	NS
TMUB2	NS	6.30E-01	1.98E-02	7.42E-01	NS

CACYBP	NS	6.31E-01	1.98E-02	7.43E-01	NS
CXCR1	NS	6.31E-01	-0.0198	7.43E-01	NS
THAP4	NS	6.30E-01	-0.0198	7.43E-01	NS
RHBDF1	NS	6.31E-01	1.98E-02	7.43E-01	NS
MCPH1	NS	6.31E-01	-0.0197	7.43E-01	NS
OR4D9	NS	6.31E-01	-0.0197	7.43E-01	NS
POGZ	NS	6.31E-01	1.97E-02	7.43E-01	NS
FCN3	NS	6.31E-01	1.97E-02	7.43E-01	NS
PRRC2C	NS	6.31E-01	-0.0197	7.43E-01	NS
TSPAN31	NS	6.31E-01	1.97E-02	7.43E-01	NS
RPS29	NS	6.31E-01	-0.0197	7.43E-01	NS
PDCD7	NS	6.32E-01	-0.0197	7.43E-01	NS
KTN1	NS	6.32E-01	1.97E-02	7.43E-01	NS
LEMD3	NS	6.32E-01	-0.0197	7.43E-01	NS
PRR14L	NS	6.32E-01	-0.0197	7.43E-01	NS
FAM47E	NS	6.32E-01	-0.0197	7.43E-01	NS
AKR1B10	NS	6.32E-01	1.97E-02	7.43E-01	NS
ELAVL1	NS	6.32E-01	1.97E-02	7.44E-01	NS
TRAPPC11	NS	6.32E-01	-0.0197	7.44E-01	NS
SFRP2	NS	6.32E-01	-0.0197	7.44E-01	NS
NDC80	NS	6.33E-01	-0.0196	7.44E-01	NS
EDAR	NS	6.33E-01	1.96E-02	7.44E-01	NS
CKMT1B	NS	6.33E-01	1.96E-02	7.44E-01	NS
INMT	NS	6.33E-01	-0.0196	7.44E-01	NS
SNX10	NS	6.33E-01	1.96E-02	7.45E-01	NS
GFUS	NS	6.33E-01	-0.0196	7.45E-01	NS
NXF3	NS	6.33E-01	1.96E-02	7.45E-01	NS
RPRD1B	NS	6.34E-01	1.96E-02	7.45E-01	NS
YIPF2	NS	6.34E-01	-0.0196	7.45E-01	NS
SEMA3B	NS	6.34E-01	1.96E-02	7.45E-01	NS
IFNL2	NS	6.34E-01	1.96E-02	7.45E-01	NS
DPPA5	NS	6.34E-01	1.96E-02	7.45E-01	NS
EYS	NS	6.34E-01	-0.0196	7.45E-01	NS
CYB5R4	NS	6.34E-01	-0.0196	7.45E-01	NS
MCMDC2	NS	6.34E-01	1.96E-02	7.45E-01	NS
MNT	NS	6.34E-01	-0.0195	7.46E-01	NS
RABL2B	NS	6.34E-01	-0.0195	7.46E-01	NS

EVI2B	NS	6.34E-01	1.95E-02	7.46E-01	NS
NBPF6	NS	6.34E-01	1.95E-02	7.46E-01	NS
PRICKLE4	NS	6.34E-01	1.95E-02	7.46E-01	NS
SEZ6L2	NS	6.35E-01	-0.0195	7.46E-01	NS
ATP13A1	NS	6.35E-01	-0.0195	7.46E-01	NS
LARGE2	NS	6.35E-01	1.95E-02	7.46E-01	NS
FTCD	NS	6.35E-01	-0.0195	7.46E-01	NS
ACTRT3	NS	6.35E-01	-0.0195	7.46E-01	NS
FGFBP1	NS	6.35E-01	1.95E-02	7.46E-01	NS
ZNF91	NS	6.35E-01	-0.0195	7.46E-01	NS
GLIS3	NS	6.36E-01	1.95E-02	7.47E-01	NS
NLGN2	NS	6.36E-01	1.95E-02	7.47E-01	NS
ADM	NS	6.36E-01	-0.0195	7.47E-01	NS
HBD	NS	6.36E-01	-0.0195	7.47E-01	NS
GSTO2	NS	6.36E-01	-0.0194	7.47E-01	NS
CASP1	NS	6.36E-01	-0.0194	7.47E-01	NS
OR1F1	NS	6.36E-01	-0.0194	7.47E-01	NS
CCNP	NS	6.36E-01	-0.0194	7.47E-01	NS
BRSK1	NS	6.36E-01	-0.0194	7.47E-01	NS
NAP1L4	NS	6.37E-01	1.94E-02	7.47E-01	NS
PPDPF	NS	6.37E-01	-0.0194	7.48E-01	NS
HSD17B14	NS	6.37E-01	-0.0194	7.48E-01	NS
RALGPS2	NS	6.37E-01	-0.0194	7.48E-01	NS
ADAMTSL1	NS	6.37E-01	1.94E-02	7.48E-01	NS
SIRT7	NS	6.38E-01	1.93E-02	7.48E-01	NS
CARD9	NS	6.38E-01	-0.0193	7.48E-01	NS
KDM4E	NS	6.38E-01	-0.0194	7.48E-01	NS
SLC35G6	NS	6.38E-01	1.94E-02	7.48E-01	NS
ZNF683	NS	6.38E-01	1.93E-02	7.48E-01	NS
HCFC1R1	NS	6.38E-01	1.93E-02	7.48E-01	NS
RAD18	NS	6.38E-01	-0.0193	7.48E-01	NS
OVOL2	NS	6.38E-01	1.93E-02	7.48E-01	NS
PAWR	NS	6.38E-01	1.93E-02	7.49E-01	NS
H3C15	NS	6.38E-01	-0.0193	7.49E-01	NS
PPP1R37	NS	6.39E-01	1.93E-02	7.49E-01	NS
TANK	NS	6.39E-01	1.93E-02	7.49E-01	NS
GABRE	NS	6.39E-01	-0.0193	7.49E-01	NS

FOLR1	NS	6.39E-01	1.93E-02	7.49E-01	NS
SSC4D	NS	6.39E-01	1.93E-02	7.49E-01	NS
LCN2	NS	6.39E-01	-0.0193	7.49E-01	NS
ING5	NS	6.39E-01	-0.0193	7.49E-01	NS
P4HB	NS	6.39E-01	1.93E-02	7.49E-01	NS
FAM200B	NS	6.39E-01	-0.0193	7.49E-01	NS
RNF20	NS	6.39E-01	-0.0193	7.49E-01	NS
COPE	NS	6.39E-01	-0.0193	7.49E-01	NS
FUT7	NS	6.40E-01	-0.0192	7.49E-01	NS
ICAM1	NS	6.40E-01	1.92E-02	7.50E-01	NS
SIM2	NS	6.40E-01	-0.0192	7.50E-01	NS
PITPNC1	NS	6.40E-01	1.92E-02	7.50E-01	NS
SLC35E2B	NS	6.40E-01	-0.0192	7.50E-01	NS
OR4C46	NS	6.40E-01	1.92E-02	7.50E-01	NS
STAT1	NS	6.40E-01	-0.0192	7.50E-01	NS
SPRR1B	NS	6.41E-01	1.92E-02	7.50E-01	NS
USP14	NS	6.41E-01	1.92E-02	7.51E-01	NS
OR10A6	NS	6.41E-01	-0.0191	7.51E-01	NS
WNT8A	NS	6.41E-01	-0.0191	7.51E-01	NS
ARMCX3	NS	6.41E-01	-0.0191	7.51E-01	NS
PADI1	NS	6.41E-01	-0.0191	7.51E-01	NS
SLC66A1	NS	6.42E-01	1.91E-02	7.52E-01	NS
FAM172A	NS	6.42E-01	1.91E-02	7.52E-01	NS
BST1	NS	6.42E-01	1.91E-02	7.52E-01	NS
SCAMP3	NS	6.42E-01	1.91E-02	7.52E-01	NS
MOK	NS	6.42E-01	1.91E-02	7.52E-01	NS
NCOA3	NS	6.42E-01	-0.0191	7.52E-01	NS
PSMB6	NS	6.43E-01	1.91E-02	7.52E-01	NS
HECTD1	NS	6.43E-01	1.91E-02	7.52E-01	NS
PRKAB1	NS	6.43E-01	-0.0191	7.52E-01	NS
FCRL2	NS	6.43E-01	-0.0191	7.52E-01	NS
POLR2J3	NS	6.43E-01	1.90E-02	7.52E-01	NS
CSAG2	NS	6.43E-01	-0.0191	7.52E-01	NS
H2AC13	NS	6.43E-01	1.90E-02	7.52E-01	NS
NAGK	NS	6.43E-01	1.90E-02	7.53E-01	NS
ORAI2	NS	6.43E-01	1.90E-02	7.53E-01	NS
CRX	NS	6.43E-01	1.90E-02	7.53E-01	NS

FAM193B	NS	6.43E-01	-0.019	7.53E-01	NS
ZNF160	NS	6.43E-01	-0.019	7.53E-01	NS
IDH3B	NS	6.44E-01	-0.019	7.53E-01	NS
PSMA3	NS	6.44E-01	-0.019	7.53E-01	NS
VIPAS39	NS	6.44E-01	1.90E-02	7.53E-01	NS
IL1F10	NS	6.44E-01	1.90E-02	7.53E-01	NS
MED24	NS	6.44E-01	1.90E-02	7.53E-01	NS
FAM162B	NS	6.44E-01	1.90E-02	7.53E-01	NS
VTA1	NS	6.44E-01	-0.019	7.53E-01	NS
REPS1	NS	6.44E-01	-0.019	7.53E-01	NS
PNMA6E	NS	6.44E-01	1.90E-02	7.53E-01	NS
SETD1B	NS	6.45E-01	1.90E-02	7.53E-01	NS
KIF15	NS	6.45E-01	-0.019	7.53E-01	NS
DHRS9	NS	6.45E-01	1.89E-02	7.54E-01	NS
CBX7	NS	6.45E-01	-0.0189	7.54E-01	NS
DNAI4	NS	6.45E-01	1.89E-02	7.54E-01	NS
POU5F2	NS	6.45E-01	1.89E-02	7.54E-01	NS
DTX3	NS	6.45E-01	1.89E-02	7.54E-01	NS
DCTD	NS	6.46E-01	-0.0189	7.54E-01	NS
VANGL2	NS	6.46E-01	-0.0189	7.54E-01	NS
GCM2	NS	6.46E-01	-0.0189	7.55E-01	NS
KASH5	NS	6.46E-01	-0.0189	7.55E-01	NS
STARD10	NS	6.46E-01	1.89E-02	7.55E-01	NS
RAB3C	NS	6.46E-01	-0.0189	7.55E-01	NS
MLC1	NS	6.46E-01	-0.0188	7.55E-01	NS
VAX2	NS	6.46E-01	1.88E-02	7.55E-01	NS
CENPC	NS	6.46E-01	1.89E-02	7.55E-01	NS
GSTA5	NS	6.46E-01	-0.0188	7.55E-01	NS
RPL18	NS	6.47E-01	-0.0188	7.55E-01	NS
ERN1	NS	6.47E-01	1.88E-02	7.55E-01	NS
VCP	NS	6.47E-01	-0.0188	7.55E-01	NS
DHRS4	NS	6.47E-01	1.88E-02	7.55E-01	NS
ZNF676	NS	6.47E-01	-0.0188	7.55E-01	NS
MRPS18A	NS	6.47E-01	1.88E-02	7.56E-01	NS
PCDH18	NS	6.47E-01	1.88E-02	7.56E-01	NS
OR52E2	NS	6.48E-01	-0.0188	7.56E-01	NS
HCK	NS	6.48E-01	-0.0187	7.57E-01	NS

SRFBP1	NS	6.48E-01	-0.0187	7.57E-01	NS
NFKB1	NS	6.48E-01	-0.0187	7.57E-01	NS
MTR	NS	6.49E-01	-0.0187	7.57E-01	NS
FOXD4L3	NS	6.49E-01	1.87E-02	7.57E-01	NS
HARBI1	NS	6.49E-01	1.87E-02	7.58E-01	NS
ZNF846	NS	6.49E-01	-0.0187	7.58E-01	NS
IFNA6	NS	6.49E-01	-0.0187	7.58E-01	NS
SMIM33	NS	6.50E-01	1.87E-02	7.58E-01	NS
LRRC72	NS	6.50E-01	1.87E-02	7.58E-01	NS
DOCK3	NS	6.50E-01	-0.0187	7.58E-01	NS
POU6F1	NS	6.50E-01	-0.0187	7.58E-01	NS
C1orf21	NS	6.50E-01	1.87E-02	7.58E-01	NS
DAP3	NS	6.50E-01	-0.0187	7.58E-01	NS
ISM1	NS	6.50E-01	-0.0186	7.58E-01	NS
PPP3CB	NS	6.50E-01	-0.0186	7.58E-01	NS
FAM168B	NS	6.50E-01	1.86E-02	7.58E-01	NS
IL13	NS	6.50E-01	1.86E-02	7.58E-01	NS
BBLN	NS	6.50E-01	1.86E-02	7.58E-01	NS
REN	NS	6.50E-01	-0.0186	7.58E-01	NS
TTC13	NS	6.50E-01	-0.0186	7.58E-01	NS
POLRMT	NS	6.50E-01	1.86E-02	7.58E-01	NS
C5orf22	NS	6.51E-01	-0.0186	7.58E-01	NS
FAN1	NS	6.51E-01	1.86E-02	7.58E-01	NS
CD207	NS	6.51E-01	1.86E-02	7.59E-01	NS
PIANP	NS	6.51E-01	1.86E-02	7.59E-01	NS
GSDMB	NS	6.51E-01	-0.0186	7.59E-01	NS
FFAR1	NS	6.51E-01	1.86E-02	7.59E-01	NS
FXR1	NS	6.51E-01	-0.0186	7.59E-01	NS
DOK6	NS	6.51E-01	-0.0186	7.59E-01	NS
VIPR1	NS	6.51E-01	1.86E-02	7.59E-01	NS
EIF4G3	NS	6.51E-01	-0.0186	7.59E-01	NS
TYROBP	NS	6.52E-01	-0.0185	7.59E-01	NS
C16orf95	NS	6.52E-01	1.85E-02	7.59E-01	NS
CS	NS	6.52E-01	1.85E-02	7.59E-01	NS
TMTC2	NS	6.52E-01	-0.0185	7.60E-01	NS
CCDC160	NS	6.52E-01	-0.0185	7.60E-01	NS
GALT	NS	6.52E-01	-0.0185	7.60E-01	NS

CLASP1	NS	6.53E-01	1.85E-02	7.60E-01	NS
IRAK3	NS	6.53E-01	-0.0185	7.60E-01	NS
OR4A5	NS	6.53E-01	-0.0185	7.60E-01	NS
MYLK3	NS	6.53E-01	-0.0185	7.60E-01	NS
ZNF557	NS	6.53E-01	-0.0185	7.60E-01	NS
USP17L12	NS	6.53E-01	1.85E-02	7.60E-01	NS
KCNA1	NS	6.53E-01	-0.0185	7.60E-01	NS
DTX1	NS	6.53E-01	-0.0185	7.60E-01	NS
LUZP2	NS	6.53E-01	-0.0185	7.60E-01	NS
KDM4F	NS	6.53E-01	1.85E-02	7.60E-01	NS
KANK4	NS	6.53E-01	1.85E-02	7.60E-01	NS
ZNF354A	NS	6.53E-01	-0.0185	7.60E-01	NS
GATAD1	NS	6.53E-01	1.84E-02	7.60E-01	NS
WAS	NS	6.53E-01	1.84E-02	7.60E-01	NS
ZCCHC8	NS	6.54E-01	1.84E-02	7.60E-01	NS
GBE1	NS	6.54E-01	-0.0184	7.60E-01	NS
PNPLA5	NS	6.54E-01	1.84E-02	7.61E-01	NS
PGLYRP3	NS	6.54E-01	-0.0184	7.61E-01	NS
ODF2	NS	6.54E-01	1.84E-02	7.61E-01	NS
CLPP	NS	6.54E-01	-0.0184	7.61E-01	NS
RPL34	NS	6.54E-01	-0.0184	7.61E-01	NS
CD83	NS	6.54E-01	-0.0184	7.61E-01	NS
ZNF541	NS	6.54E-01	1.84E-02	7.61E-01	NS
EPHA1	NS	6.55E-01	-0.0184	7.61E-01	NS
IRX3	NS	6.55E-01	1.84E-02	7.61E-01	NS
RNF167	NS	6.55E-01	-0.0184	7.61E-01	NS
LBR	NS	6.55E-01	-0.0184	7.61E-01	NS
CNPY4	NS	6.55E-01	-0.0184	7.61E-01	NS
SFTPC	NS	6.55E-01	1.84E-02	7.61E-01	NS
OR51E1	NS	6.55E-01	-0.0184	7.61E-01	NS
B4GALT6	NS	6.55E-01	-0.0183	7.62E-01	NS
HSPG2	NS	6.55E-01	-0.0183	7.62E-01	NS
TBC1D17	NS	6.55E-01	1.83E-02	7.62E-01	NS
COX8A	NS	6.55E-01	1.83E-02	7.62E-01	NS
SLC7A10	NS	6.56E-01	-0.0183	7.62E-01	NS
RB1	NS	6.56E-01	-0.0183	7.62E-01	NS
PEX7	NS	6.56E-01	-0.0183	7.62E-01	NS

XBP1	NS	6.56E-01	1.83E-02	7.63E-01	NS
LRRC47	NS	6.57E-01	1.83E-02	7.63E-01	NS
PXMP4	NS	6.57E-01	-0.0183	7.63E-01	NS
OR2AJ1	NS	6.57E-01	-0.0183	7.63E-01	NS
EFNB2	NS	6.57E-01	-0.0182	7.63E-01	NS
C1orf159	NS	6.57E-01	1.82E-02	7.63E-01	NS
HEATR1	NS	6.57E-01	1.82E-02	7.63E-01	NS
TSKS	NS	6.57E-01	-0.0182	7.63E-01	NS
ZFP14	NS	6.57E-01	-0.0182	7.63E-01	NS
USB1	NS	6.57E-01	1.82E-02	7.63E-01	NS
RTEL1	NS	6.57E-01	-0.0182	7.63E-01	NS
BCAS3	NS	6.58E-01	1.82E-02	7.63E-01	NS
IGF2BP2	NS	6.58E-01	1.82E-02	7.64E-01	NS
PSAPL1	NS	6.58E-01	1.82E-02	7.64E-01	NS
LRRK2	NS	6.58E-01	-0.0182	7.64E-01	NS
SPACA1	NS	6.58E-01	-0.0182	7.64E-01	NS
C2CD6	NS	6.58E-01	-0.0182	7.64E-01	NS
PRICKLE3	NS	6.59E-01	-0.0182	7.64E-01	NS
POLI	NS	6.59E-01	-0.0181	7.64E-01	NS
KRT38	NS	6.59E-01	-0.0181	7.64E-01	NS
MUC2	NS	6.59E-01	1.81E-02	7.64E-01	NS
TAF9	NS	6.59E-01	-0.0181	7.65E-01	NS
TDRD10	NS	6.59E-01	-0.0181	7.65E-01	NS
SLC25A34	NS	6.59E-01	-0.0181	7.65E-01	NS
HEATR5A	NS	6.59E-01	-0.0181	7.65E-01	NS
BCAR3	NS	6.60E-01	1.81E-02	7.65E-01	NS
CCIN	NS	6.60E-01	1.81E-02	7.65E-01	NS
SMTN	NS	6.60E-01	-0.0181	7.65E-01	NS
RBMY1A1	NS	6.60E-01	-0.0181	7.65E-01	NS
TEX19	NS	6.60E-01	1.81E-02	7.65E-01	NS
KIRREL1	NS	6.60E-01	-0.0181	7.65E-01	NS
PHYH	NS	6.60E-01	-0.0181	7.65E-01	NS
HBEGF	NS	6.60E-01	1.81E-02	7.65E-01	NS
WDR33	NS	6.60E-01	-0.0181	7.65E-01	NS
TREX1	NS	6.60E-01	1.81E-02	7.65E-01	NS
FAM78A	NS	6.60E-01	-0.0181	7.65E-01	NS
METTL26	NS	6.60E-01	-0.0181	7.65E-01	NS

TET3	NS	6.60E-01	-0.0181	7.65E-01	NS
SLC6A15	NS	6.61E-01	-0.018	7.66E-01	NS
CD9	NS	6.61E-01	-0.018	7.66E-01	NS
XKR3	NS	6.61E-01	-0.018	7.66E-01	NS
PCTP	NS	6.61E-01	1.80E-02	7.66E-01	NS
BTAF1	NS	6.61E-01	1.80E-02	7.66E-01	NS
NR2C1	NS	6.61E-01	-0.018	7.66E-01	NS
LAT2	NS	6.61E-01	-0.018	7.66E-01	NS
TSPYL4	NS	6.62E-01	1.80E-02	7.67E-01	NS
TAX1BP1	NS	6.62E-01	1.80E-02	7.67E-01	NS
IL23A	NS	6.62E-01	-0.018	7.67E-01	NS
TNFRSF10A	NS	6.62E-01	-0.018	7.67E-01	NS
LPP	NS	6.62E-01	-0.0179	7.67E-01	NS
KRTAP19-4	NS	6.62E-01	1.79E-02	7.67E-01	NS
RDH5	NS	6.63E-01	-0.0179	7.67E-01	NS
RAB1B	NS	6.63E-01	1.79E-02	7.67E-01	NS
SKOR1	NS	6.63E-01	-0.0179	7.67E-01	NS
NFATC4	NS	6.63E-01	-0.0179	7.68E-01	NS
NMU	NS	6.63E-01	1.79E-02	7.68E-01	NS
ITGA4	NS	6.63E-01	-0.0179	7.68E-01	NS
LGALSL	NS	6.63E-01	-0.0179	7.68E-01	NS
SLC39A6	NS	6.63E-01	-0.0179	7.68E-01	NS
HNRNPLL	NS	6.63E-01	1.79E-02	7.68E-01	NS
P2RY1	NS	6.63E-01	-0.0179	7.68E-01	NS
ZNF417	NS	6.63E-01	-0.0179	7.68E-01	NS
ZNF75D	NS	6.63E-01	-0.0179	7.68E-01	NS
SUMO2	NS	6.63E-01	1.79E-02	7.68E-01	NS
PDLIM7	NS	6.63E-01	1.79E-02	7.68E-01	NS
HBA1	NS	6.63E-01	1.79E-02	7.68E-01	NS
MCIDAS	NS	6.63E-01	-0.0179	7.68E-01	NS
RALA	NS	6.64E-01	-0.0179	7.68E-01	NS
MAP1S	NS	6.64E-01	1.79E-02	7.68E-01	NS
SNRNP25	NS	6.64E-01	-0.0179	7.68E-01	NS
FAM131A	NS	6.64E-01	1.79E-02	7.68E-01	NS
RBMY1E	NS	6.64E-01	-0.0179	7.68E-01	NS
TXNIP	NS	6.64E-01	1.79E-02	7.68E-01	NS
OAT	NS	6.64E-01	-0.0178	7.68E-01	NS

MAPK8	NS	6.64E-01	1.78E-02	7.68E-01	NS
MTX1	NS	6.64E-01	1.78E-02	7.68E-01	NS
C11orf53	NS	6.64E-01	1.78E-02	7.68E-01	NS
PNMA2	NS	6.64E-01	1.78E-02	7.68E-01	NS
GSDME	NS	6.64E-01	-0.0178	7.68E-01	NS
TFPI	NS	6.65E-01	-0.0178	7.68E-01	NS
GATA3	NS	6.65E-01	-0.0178	7.68E-01	NS
SLC1A2	NS	6.65E-01	-0.0178	7.68E-01	NS
C11orf58	NS	6.65E-01	-0.0178	7.69E-01	NS
PRRG2	NS	6.65E-01	1.78E-02	7.69E-01	NS
HADHB	NS	6.65E-01	1.78E-02	7.69E-01	NS
TAF10	NS	6.65E-01	-0.0178	7.69E-01	NS
OR4S1	NS	6.65E-01	-0.0178	7.69E-01	NS
DDX51	NS	6.65E-01	-0.0178	7.69E-01	NS
ZDHHC6	NS	6.65E-01	-0.0178	7.69E-01	NS
CHAT	NS	6.65E-01	1.78E-02	7.69E-01	NS
ALKBH7	NS	6.65E-01	-0.0178	7.69E-01	NS
SPDYE3	NS	6.65E-01	1.78E-02	7.69E-01	NS
ATL2	NS	6.65E-01	1.78E-02	7.69E-01	NS
TSEN15	NS	6.65E-01	1.78E-02	7.69E-01	NS
YTHDF3	NS	6.66E-01	1.78E-02	7.69E-01	NS
IPO13	NS	6.66E-01	1.77E-02	7.69E-01	NS
MT2A	NS	6.66E-01	-0.0177	7.69E-01	NS
RGS5	NS	6.66E-01	1.77E-02	7.69E-01	NS
C4BPA	NS	6.66E-01	-0.0177	7.70E-01	NS
STXBP1	NS	6.67E-01	1.77E-02	7.70E-01	NS
C1QA	NS	6.67E-01	-0.0177	7.70E-01	NS
SLC17A7	NS	6.67E-01	1.77E-02	7.70E-01	NS
NDEL1	NS	6.67E-01	1.77E-02	7.70E-01	NS
CDC42EP4	NS	6.68E-01	-0.0176	7.71E-01	NS
NDUFB4	NS	6.68E-01	-0.0176	7.71E-01	NS
UACA	NS	6.68E-01	-0.0176	7.71E-01	NS
CAB39	NS	6.68E-01	-0.0176	7.71E-01	NS
KNSTRN	NS	6.68E-01	1.76E-02	7.71E-01	NS
SDF2	NS	6.68E-01	-0.0176	7.71E-01	NS
KCNJ5	NS	6.68E-01	1.76E-02	7.71E-01	NS
MRPL1	NS	6.68E-01	1.76E-02	7.71E-01	NS

MYH9	NS	6.69E-01	1.76E-02	7.72E-01	NS
AKAP9	NS	6.69E-01	-0.0176	7.72E-01	NS
GSPT1	NS	6.69E-01	-0.0176	7.72E-01	NS
KCMF1	NS	6.69E-01	1.76E-02	7.72E-01	NS
ZNF708	NS	6.69E-01	-0.0175	7.72E-01	NS
KIF14	NS	6.69E-01	-0.0175	7.72E-01	NS
NUP210	NS	6.69E-01	1.75E-02	7.72E-01	NS
SLC25A10	NS	6.69E-01	-0.0175	7.72E-01	NS
EIF4A2	NS	6.70E-01	-0.0175	7.72E-01	NS
FRMPD2	NS	6.70E-01	-0.0175	7.72E-01	NS
GTPBP3	NS	6.70E-01	-0.0175	7.73E-01	NS
PGLS	NS	6.70E-01	1.75E-02	7.73E-01	NS
SUPT3H	NS	6.70E-01	1.75E-02	7.73E-01	NS
STARD4	NS	6.70E-01	1.75E-02	7.73E-01	NS
NOB1	NS	6.70E-01	-0.0175	7.73E-01	NS
NOTCH2NLR	NS	6.70E-01	-0.0175	7.73E-01	NS
ZNF250	NS	6.70E-01	-0.0175	7.73E-01	NS
TMEM30A	NS	6.71E-01	-0.0175	7.73E-01	NS
USF1	NS	6.71E-01	-0.0174	7.74E-01	NS
CTBP1	NS	6.71E-01	-0.0174	7.74E-01	NS
ZNF514	NS	6.72E-01	-0.0174	7.74E-01	NS
TBX1	NS	6.72E-01	1.74E-02	7.74E-01	NS
ZNF735	NS	6.72E-01	-0.0174	7.74E-01	NS
MSLN	NS	6.72E-01	1.74E-02	7.74E-01	NS
B3GALT1	NS	6.72E-01	1.74E-02	7.74E-01	NS
SF3A1	NS	6.72E-01	1.74E-02	7.74E-01	NS
MAN1C1	NS	6.72E-01	-0.0174	7.74E-01	NS
C2orf50	NS	6.72E-01	-0.0174	7.74E-01	NS
CXCL16	NS	6.72E-01	-0.0174	7.74E-01	NS
OR7G2	NS	6.72E-01	-0.0174	7.74E-01	NS
GADD45GIP1	NS	6.72E-01	1.74E-02	7.74E-01	NS
OR4C15	NS	6.72E-01	1.74E-02	7.74E-01	NS
MZT1	NS	6.72E-01	-0.0174	7.74E-01	NS
MARCHF5	NS	6.72E-01	-0.0174	7.74E-01	NS
CDC6	NS	6.73E-01	1.73E-02	7.75E-01	NS
DGAT1	NS	6.73E-01	-0.0173	7.75E-01	NS
ATP6V1G2-DDX39B	NS	6.73E-01	-0.0173	7.75E-01	NS

ATP6V1FNB	NS	6.73E-01	1.73E-02	7.75E-01	NS
UCK2	NS	6.73E-01	1.73E-02	7.75E-01	NS
GJA8	NS	6.73E-01	1.73E-02	7.75E-01	NS
ISOC2	NS	6.73E-01	1.73E-02	7.75E-01	NS
RAMP3	NS	6.73E-01	1.73E-02	7.75E-01	NS
JAG1	NS	6.74E-01	1.73E-02	7.75E-01	NS
CHD4	NS	6.74E-01	-0.0173	7.75E-01	NS
PADI2	NS	6.74E-01	-0.0173	7.75E-01	NS
SCYL2	NS	6.74E-01	1.73E-02	7.75E-01	NS
AZIN2	NS	6.74E-01	-0.0173	7.75E-01	NS
NDNF	NS	6.74E-01	1.73E-02	7.75E-01	NS
PLEKHD1	NS	6.74E-01	-0.0173	7.75E-01	NS
FAM217B	NS	6.74E-01	1.73E-02	7.75E-01	NS
LMAN2	NS	6.74E-01	-0.0173	7.76E-01	NS
BRSK2	NS	6.74E-01	1.73E-02	7.76E-01	NS
NFIB	NS	6.75E-01	-0.0172	7.76E-01	NS
DEPDC4	NS	6.75E-01	1.72E-02	7.76E-01	NS
IL17B	NS	6.75E-01	-0.0172	7.76E-01	NS
ARMS2	NS	6.75E-01	1.72E-02	7.76E-01	NS
SLC5A2	NS	6.75E-01	1.72E-02	7.76E-01	NS
SPATA46	NS	6.75E-01	-0.0172	7.76E-01	NS
TBCD	NS	6.75E-01	1.72E-02	7.76E-01	NS
TRIM22	NS	6.75E-01	-0.0172	7.76E-01	NS
STARD5	NS	6.75E-01	1.72E-02	7.76E-01	NS
STAT6	NS	6.75E-01	-0.0172	7.77E-01	NS
FAM189A2	NS	6.75E-01	-0.0172	7.77E-01	NS
SAMD8	NS	6.75E-01	-0.0172	7.77E-01	NS
JPT2	NS	6.76E-01	-0.0172	7.77E-01	NS
LYPD2	NS	6.76E-01	1.72E-02	7.77E-01	NS
OR4D2	NS	6.76E-01	-0.0172	7.77E-01	NS
RGS17	NS	6.76E-01	1.72E-02	7.77E-01	NS
KATNIP	NS	6.76E-01	1.71E-02	7.77E-01	NS
PYCARD	NS	6.76E-01	-0.0171	7.77E-01	NS
CLPTM1L	NS	6.77E-01	-0.0171	7.78E-01	NS
PLEKHH1	NS	6.77E-01	-0.0171	7.78E-01	NS
ELMO2	NS	6.77E-01	1.71E-02	7.78E-01	NS
GPBP1	NS	6.77E-01	1.71E-02	7.78E-01	NS

UBE2D4	NS	6.78E-01	-0.0171	7.78E-01	NS
GNAS	NS	6.78E-01	-0.0171	7.78E-01	NS
NECTIN2	NS	6.78E-01	1.71E-02	7.78E-01	NS
IRX4	NS	6.78E-01	1.71E-02	7.79E-01	NS
C1orf232	NS	6.78E-01	1.71E-02	7.79E-01	NS
AIF1	NS	6.78E-01	1.71E-02	7.79E-01	NS
ZFAND2A	NS	6.78E-01	1.71E-02	7.79E-01	NS
NHSL1	NS	6.78E-01	1.70E-02	7.79E-01	NS
USP8	NS	6.78E-01	-0.017	7.79E-01	NS
DENND2C	NS	6.78E-01	1.70E-02	7.79E-01	NS
B3GNT4	NS	6.78E-01	-0.017	7.79E-01	NS
KCNJ3	NS	6.78E-01	-0.017	7.79E-01	NS
KNTC1	NS	6.78E-01	-0.017	7.79E-01	NS
HOGA1	NS	6.79E-01	1.70E-02	7.79E-01	NS
GTF2IRD1	NS	6.79E-01	1.70E-02	7.79E-01	NS
ANXA1	NS	6.79E-01	-0.017	7.79E-01	NS
TMEM104	NS	6.79E-01	-0.017	7.79E-01	NS
SGPP2	NS	6.79E-01	-0.017	7.79E-01	NS
CCNDBP1	NS	6.79E-01	-0.017	7.79E-01	NS
PSMB11	NS	6.79E-01	-0.017	7.80E-01	NS
DHX32	NS	6.79E-01	1.70E-02	7.80E-01	NS
CAPG	NS	6.80E-01	-0.017	7.80E-01	NS
PHYHD1	NS	6.80E-01	1.70E-02	7.80E-01	NS
NBEAL1	NS	6.80E-01	1.69E-02	7.80E-01	NS
FOXS1	NS	6.80E-01	-0.0169	7.80E-01	NS
ARHGEF15	NS	6.80E-01	-0.0169	7.80E-01	NS
CLCC1	NS	6.80E-01	-0.0169	7.80E-01	NS
C11orf71	NS	6.80E-01	-0.0169	7.80E-01	NS
C8orf48	NS	6.81E-01	-0.0169	7.81E-01	NS
CHST8	NS	6.81E-01	-0.0169	7.81E-01	NS
ZFAND1	NS	6.81E-01	-0.0169	7.81E-01	NS
ZNF519	NS	6.81E-01	-0.0169	7.81E-01	NS
AKIRIN2	NS	6.81E-01	-0.0169	7.81E-01	NS
DNTTIP1	NS	6.82E-01	1.69E-02	7.81E-01	NS
RBM17	NS	6.82E-01	-0.0169	7.81E-01	NS
XPC	NS	6.82E-01	-0.0169	7.81E-01	NS
GPX5	NS	6.82E-01	-0.0169	7.81E-01	NS

DZIP1	NS	6.82E-01	-0.0168	7.82E-01	NS
NDUFA12	NS	6.82E-01	1.68E-02	7.82E-01	NS
ACTB	NS	6.82E-01	1.68E-02	7.82E-01	NS
ARHGDIA	NS	6.82E-01	1.68E-02	7.82E-01	NS
KCNAB2	NS	6.82E-01	-0.0168	7.82E-01	NS
PFKP	NS	6.83E-01	1.68E-02	7.82E-01	NS
PSMC5	NS	6.83E-01	-0.0168	7.82E-01	NS
PYGL	NS	6.83E-01	1.68E-02	7.82E-01	NS
ATN1	NS	6.83E-01	1.68E-02	7.82E-01	NS
PTGES2	NS	6.83E-01	1.68E-02	7.82E-01	NS
ATP2B2	NS	6.83E-01	1.68E-02	7.82E-01	NS
PLK5	NS	6.83E-01	1.68E-02	7.82E-01	NS
KCNN4	NS	6.83E-01	1.68E-02	7.82E-01	NS
SMIM8	NS	6.83E-01	-0.0168	7.82E-01	NS
TINAGL1	NS	6.83E-01	1.68E-02	7.82E-01	NS
CCND1	NS	6.83E-01	1.68E-02	7.82E-01	NS
CCZ1	NS	6.83E-01	-0.0168	7.82E-01	NS
ACTR3B	NS	6.83E-01	-0.0168	7.82E-01	NS
PLEKHA4	NS	6.83E-01	1.68E-02	7.83E-01	NS
KLF16	NS	6.83E-01	-0.0168	7.83E-01	NS
FAM117B	NS	6.83E-01	1.68E-02	7.83E-01	NS
DCDC1	NS	6.84E-01	-0.0167	7.83E-01	NS
NOVA2	NS	6.84E-01	1.67E-02	7.83E-01	NS
LIAS	NS	6.84E-01	-0.0167	7.83E-01	NS
LTBR	NS	6.85E-01	-0.0167	7.84E-01	NS
CORO1B	NS	6.85E-01	-0.0167	7.84E-01	NS
MT3	NS	6.85E-01	-0.0167	7.84E-01	NS
CARNS1	NS	6.85E-01	-0.0167	7.84E-01	NS
PRDM5	NS	6.85E-01	1.67E-02	7.84E-01	NS
H4C9	NS	6.85E-01	1.67E-02	7.84E-01	NS
CXADR	NS	6.85E-01	-0.0167	7.84E-01	NS
DCLRE1C	NS	6.85E-01	-0.0166	7.84E-01	NS
FGF20	NS	6.86E-01	1.66E-02	7.85E-01	NS
BIN1	NS	6.86E-01	-0.0166	7.85E-01	NS
CEACAM8	NS	6.86E-01	-0.0166	7.85E-01	NS
ENTPD5	NS	6.86E-01	1.66E-02	7.85E-01	NS
UBE2L5	NS	6.86E-01	-0.0166	7.85E-01	NS

RBM10	NS	6.86E-01	-0.0166	7.85E-01	NS
IGDCC3	NS	6.86E-01	1.66E-02	7.85E-01	NS
COL18A1	NS	6.86E-01	-0.0166	7.85E-01	NS
RUSC1	NS	6.87E-01	-0.0166	7.85E-01	NS
NFYA	NS	6.87E-01	-0.0166	7.85E-01	NS
SNRNP70	NS	6.87E-01	1.66E-02	7.85E-01	NS
ATP6V0A4	NS	6.87E-01	-0.0166	7.85E-01	NS
AMY1B	NS	6.87E-01	-0.0166	7.85E-01	NS
NR1H3	NS	6.87E-01	-0.0166	7.85E-01	NS
KAT2A	NS	6.87E-01	-0.0165	7.85E-01	NS
GPBAR1	NS	6.87E-01	-0.0165	7.85E-01	NS
AP3M1	NS	6.87E-01	1.65E-02	7.85E-01	NS
RASSF9	NS	6.87E-01	1.65E-02	7.85E-01	NS
OR51G1	NS	6.87E-01	-0.0165	7.85E-01	NS
PPP1R3G	NS	6.87E-01	1.65E-02	7.86E-01	NS
FAM161B	NS	6.87E-01	1.65E-02	7.86E-01	NS
PRCP	NS	6.88E-01	-0.0165	7.86E-01	NS
GIT2	NS	6.88E-01	-0.0165	7.86E-01	NS
BOC	NS	6.88E-01	-0.0165	7.86E-01	NS
GLI2	NS	6.88E-01	1.65E-02	7.86E-01	NS
CAND1	NS	6.88E-01	1.65E-02	7.86E-01	NS
ULK1	NS	6.88E-01	1.65E-02	7.86E-01	NS
GANAB	NS	6.88E-01	-0.0165	7.86E-01	NS
WDR74	NS	6.88E-01	-0.0165	7.86E-01	NS
KIDINS220	NS	6.88E-01	-0.0165	7.86E-01	NS
DYSF	NS	6.88E-01	1.65E-02	7.86E-01	NS
GPD1L	NS	6.89E-01	-0.0165	7.86E-01	NS
USPL1	NS	6.89E-01	-0.0165	7.86E-01	NS
TRIP11	NS	6.89E-01	-0.0165	7.86E-01	NS
EXOSC10	NS	6.89E-01	-0.0165	7.86E-01	NS
DACT3	NS	6.89E-01	1.64E-02	7.87E-01	NS
CRYBB2	NS	6.89E-01	1.64E-02	7.87E-01	NS
KDM5C	NS	6.89E-01	1.64E-02	7.87E-01	NS
PNISR	NS	6.89E-01	-0.0164	7.87E-01	NS
TXNDC2	NS	6.89E-01	1.64E-02	7.87E-01	NS
ZNF185	NS	6.89E-01	-0.0164	7.87E-01	NS
RRP1	NS	6.90E-01	1.64E-02	7.87E-01	NS

TRIM39	NS	6.90E-01	-0.0164	7.87E-01	NS
APBB2	NS	6.90E-01	-0.0164	7.87E-01	NS
VDAC2	NS	6.90E-01	-0.0164	7.87E-01	NS
PALM3	NS	6.90E-01	1.64E-02	7.87E-01	NS
SMYD2	NS	6.90E-01	1.64E-02	7.87E-01	NS
BORCS8-MEF2B	NS	6.90E-01	-0.0164	7.87E-01	NS
SPTBN5	NS	6.90E-01	1.64E-02	7.87E-01	NS
SLC38A3	NS	6.90E-01	-0.0164	7.87E-01	NS
RUVBL2	NS	6.90E-01	-0.0164	7.88E-01	NS
ALOXE3	NS	6.91E-01	1.63E-02	7.88E-01	NS
IFNA1	NS	6.91E-01	-0.0163	7.88E-01	NS
RELB	NS	6.91E-01	-0.0163	7.88E-01	NS
OR9G1	NS	6.91E-01	-0.0163	7.88E-01	NS
NPAP1	NS	6.91E-01	-0.0163	7.88E-01	NS
ALB	NS	6.91E-01	-0.0163	7.88E-01	NS
SLC10A7	NS	6.91E-01	1.63E-02	7.88E-01	NS
DDX19B	NS	6.91E-01	1.63E-02	7.88E-01	NS
ZBTB41	NS	6.91E-01	-0.0163	7.88E-01	NS
FAM222B	NS	6.92E-01	-0.0163	7.88E-01	NS
WFDC2	NS	6.92E-01	-0.0163	7.88E-01	NS
MAD2L1BP	NS	6.92E-01	1.63E-02	7.88E-01	NS
ZNF274	NS	6.92E-01	1.63E-02	7.89E-01	NS
SNX9	NS	6.92E-01	-0.0163	7.89E-01	NS
MEA1	NS	6.92E-01	-0.0163	7.89E-01	NS
JAZF1	NS	6.93E-01	1.62E-02	7.89E-01	NS
DYRK1A	NS	6.93E-01	-0.0162	7.89E-01	NS
ALG1L2	NS	6.93E-01	-0.0162	7.90E-01	NS
COL27A1	NS	6.93E-01	-0.0162	7.90E-01	NS
TEX37	NS	6.94E-01	1.62E-02	7.90E-01	NS
PARP8	NS	6.94E-01	-0.0162	7.90E-01	NS
ZNF488	NS	6.94E-01	1.62E-02	7.91E-01	NS
SIN3B	NS	6.94E-01	-0.0162	7.91E-01	NS
SPG7	NS	6.94E-01	-0.0161	7.91E-01	NS
KHDC1	NS	6.95E-01	1.61E-02	7.91E-01	NS
IFNGR2	NS	6.95E-01	1.61E-02	7.91E-01	NS
MAB21L4	NS	6.95E-01	1.61E-02	7.91E-01	NS
KATNA1	NS	6.95E-01	1.61E-02	7.91E-01	NS

PHF20L1	NS	6.95E-01	-0.0161	7.91E-01	NS
BMP1	NS	6.95E-01	-0.0161	7.91E-01	NS
NRTN	NS	6.95E-01	-0.0161	7.91E-01	NS
OR14A16	NS	6.95E-01	1.61E-02	7.91E-01	NS
ARHGAP32	NS	6.95E-01	-0.0161	7.91E-01	NS
LOXL2	NS	6.95E-01	-0.0161	7.91E-01	NS
SLC30A10	NS	6.95E-01	-0.0161	7.92E-01	NS
H2BC18	NS	6.96E-01	1.61E-02	7.92E-01	NS
ZSCAN18	NS	6.96E-01	1.61E-02	7.92E-01	NS
CLN6	NS	6.96E-01	1.61E-02	7.92E-01	NS
SLC25A45	NS	6.96E-01	-0.0161	7.92E-01	NS
GPX6	NS	6.96E-01	-0.0161	7.92E-01	NS
CD72	NS	6.96E-01	1.61E-02	7.92E-01	NS
TRIML2	NS	6.96E-01	-0.016	7.92E-01	NS
FAM90A14P	NS	6.96E-01	-0.016	7.92E-01	NS
ZNF490	NS	6.97E-01	-0.016	7.92E-01	NS
PLEKHF1	NS	6.97E-01	-0.016	7.92E-01	NS
MPLKIP	NS	6.97E-01	1.60E-02	7.93E-01	NS
SNN	NS	6.97E-01	1.60E-02	7.93E-01	NS
SREK1	NS	6.97E-01	-0.016	7.93E-01	NS
RNF39	NS	6.97E-01	-0.016	7.93E-01	NS
ADCK1	NS	6.97E-01	1.60E-02	7.93E-01	NS
LILRA2	NS	6.98E-01	1.60E-02	7.93E-01	NS
MEIOSIN	NS	6.98E-01	-0.016	7.93E-01	NS
HDAC6	NS	6.98E-01	1.59E-02	7.94E-01	NS
ACKR3	NS	6.98E-01	-0.0159	7.94E-01	NS
TNP2	NS	6.98E-01	1.59E-02	7.94E-01	NS
EPCAM	NS	6.98E-01	1.59E-02	7.94E-01	NS
RBM19	NS	6.98E-01	1.59E-02	7.94E-01	NS
ANGPTL3	NS	6.98E-01	-0.0159	7.94E-01	NS
ZNF687	NS	6.98E-01	-0.0159	7.94E-01	NS
CHMP4C	NS	6.98E-01	-0.0159	7.94E-01	NS
TMEM150A	NS	6.98E-01	1.59E-02	7.94E-01	NS
COBL	NS	6.98E-01	1.59E-02	7.94E-01	NS
SLC9A3	NS	6.99E-01	-0.0159	7.94E-01	NS
CDK20	NS	6.99E-01	1.59E-02	7.94E-01	NS
CSGALNACT2	NS	6.99E-01	-0.0159	7.94E-01	NS

PARL	NS	6.99E-01	1.59E-02	7.94E-01	NS
CARMIL1	NS	6.99E-01	1.59E-02	7.94E-01	NS
HNRNPK	NS	6.99E-01	-0.0159	7.95E-01	NS
ZNF581	NS	6.99E-01	1.59E-02	7.95E-01	NS
FMC1-LUC7L2	NS	7.00E-01	-0.0159	7.95E-01	NS
LRRC66	NS	7.00E-01	1.58E-02	7.95E-01	NS
ABHD17A	NS	7.00E-01	-0.0158	7.95E-01	NS
IRF2BP1	NS	7.00E-01	1.58E-02	7.95E-01	NS
GTPBP4	NS	7.00E-01	1.58E-02	7.95E-01	NS
MCM3AP	NS	7.00E-01	1.58E-02	7.95E-01	NS
RPAP3	NS	7.01E-01	-0.0158	7.96E-01	NS
DAPK1	NS	7.01E-01	1.58E-02	7.96E-01	NS
RPL17-C18orf32	NS	7.01E-01	1.58E-02	7.96E-01	NS
KCNC4	NS	7.01E-01	-0.0158	7.96E-01	NS
NHP2	NS	7.01E-01	-0.0158	7.96E-01	NS
INSM2	NS	7.01E-01	1.58E-02	7.96E-01	NS
PPM1E	NS	7.01E-01	-0.0158	7.96E-01	NS
CEP57L1	NS	7.01E-01	1.58E-02	7.96E-01	NS
ARHGEF9	NS	7.01E-01	1.58E-02	7.96E-01	NS
C9orf78	NS	7.01E-01	1.58E-02	7.96E-01	NS
NIBAN3	NS	7.01E-01	-0.0158	7.96E-01	NS
SLC22A11	NS	7.01E-01	1.58E-02	7.96E-01	NS
BGN	NS	7.01E-01	1.58E-02	7.96E-01	NS
CDCA2	NS	7.01E-01	-0.0158	7.96E-01	NS
ARMC1	NS	7.02E-01	-0.0157	7.96E-01	NS
PLEKHB2	NS	7.02E-01	1.57E-02	7.96E-01	NS
CRYGN	NS	7.02E-01	-0.0157	7.96E-01	NS
CLEC4A	NS	7.02E-01	1.57E-02	7.96E-01	NS
VOPP1	NS	7.02E-01	-0.0157	7.96E-01	NS
GTF2A2	NS	7.02E-01	-0.0157	7.97E-01	NS
GRIN3B	NS	7.03E-01	-0.0157	7.97E-01	NS
OR2B2	NS	7.03E-01	1.57E-02	7.97E-01	NS
SLC25A36	NS	7.03E-01	1.57E-02	7.97E-01	NS
PPARGC1B	NS	7.03E-01	1.57E-02	7.97E-01	NS
ALG8	NS	7.03E-01	1.57E-02	7.97E-01	NS
H4C8	NS	7.03E-01	-0.0157	7.97E-01	NS
RBM6	NS	7.03E-01	-0.0156	7.97E-01	NS

MORN5	NS	7.04E-01	1.56E-02	7.98E-01	NS
CTNS	NS	7.04E-01	1.56E-02	7.99E-01	NS
SMLR1	NS	7.05E-01	1.56E-02	7.99E-01	NS
PLXNA4	NS	7.05E-01	1.56E-02	7.99E-01	NS
ZNF391	NS	7.05E-01	-0.0156	7.99E-01	NS
OBSL1	NS	7.05E-01	1.55E-02	7.99E-01	NS
PEA15	NS	7.06E-01	-0.0155	8.00E-01	NS
DENND4B	NS	7.06E-01	-0.0155	8.00E-01	NS
DVL2	NS	7.06E-01	1.55E-02	8.00E-01	NS
SMOX	NS	7.06E-01	-0.0155	8.00E-01	NS
FABP9	NS	7.06E-01	-0.0155	8.00E-01	NS
MED18	NS	7.06E-01	-0.0155	8.00E-01	NS
CNIH2	NS	7.06E-01	-0.0155	8.00E-01	NS
KCNMB4	NS	7.06E-01	1.55E-02	8.00E-01	NS
AAK1	NS	7.06E-01	1.55E-02	8.00E-01	NS
DIAPH1	NS	7.06E-01	-0.0155	8.00E-01	NS
PHF8	NS	7.06E-01	-0.0155	8.00E-01	NS
CADM2	NS	7.07E-01	-0.0155	8.00E-01	NS
FADS3	NS	7.07E-01	-0.0155	8.00E-01	NS
UPK3BL2	NS	7.07E-01	-0.0155	8.00E-01	NS
CENATAC	NS	7.07E-01	1.54E-02	8.00E-01	NS
PARD3B	NS	7.07E-01	-0.0154	8.01E-01	NS
ENKD1	NS	7.07E-01	-0.0154	8.01E-01	NS
OR8B2	NS	7.07E-01	-0.0154	8.01E-01	NS
ATP8B2	NS	7.07E-01	-0.0154	8.01E-01	NS
KRBOX1	NS	7.07E-01	-0.0154	8.01E-01	NS
MRM1	NS	7.08E-01	1.54E-02	8.01E-01	NS
RHO	NS	7.08E-01	-0.0154	8.01E-01	NS
ARHGAP19-SLIT1	NS	7.08E-01	1.54E-02	8.01E-01	NS
RABGEF1	NS	7.08E-01	-0.0154	8.02E-01	NS
TOP1MT	NS	7.08E-01	-0.0154	8.02E-01	NS
SKI	NS	7.08E-01	-0.0154	8.02E-01	NS
SLIT2	NS	7.09E-01	1.54E-02	8.02E-01	NS
SKIDA1	NS	7.09E-01	1.53E-02	8.02E-01	NS
SH3GL1	NS	7.09E-01	1.53E-02	8.02E-01	NS
PLXND1	NS	7.09E-01	-0.0153	8.02E-01	NS
PDCD2	NS	7.09E-01	1.53E-02	8.02E-01	NS

MIOX	NS	7.09E-01	1.53E-02	8.02E-01	NS
KRTAP13-4	NS	7.09E-01	-0.0153	8.02E-01	NS
PHLDA3	NS	7.09E-01	-0.0153	8.02E-01	NS
PPEF1	NS	7.10E-01	1.53E-02	8.03E-01	NS
OR6K2	NS	7.10E-01	1.53E-02	8.03E-01	NS
H3C3	NS	7.10E-01	1.53E-02	8.03E-01	NS
CAPN2	NS	7.10E-01	-0.0153	8.03E-01	NS
CNOT4	NS	7.10E-01	-0.0153	8.03E-01	NS
NXPE3	NS	7.10E-01	-0.0153	8.03E-01	NS
PCDHA1	NS	7.10E-01	1.53E-02	8.03E-01	NS
COQ4	NS	7.10E-01	1.53E-02	8.03E-01	NS
LILRB4	NS	7.10E-01	-0.0153	8.03E-01	NS
BCL7C	NS	7.11E-01	1.52E-02	8.03E-01	NS
F12	NS	7.11E-01	1.52E-02	8.03E-01	NS
FATE1	NS	7.11E-01	1.52E-02	8.03E-01	NS
CFAP276	NS	7.11E-01	-0.0152	8.03E-01	NS
HAS3	NS	7.11E-01	1.52E-02	8.03E-01	NS
PSMD7	NS	7.11E-01	1.52E-02	8.04E-01	NS
DLG1	NS	7.12E-01	-0.0152	8.04E-01	NS
UBTF	NS	7.12E-01	1.52E-02	8.04E-01	NS
DEFB135	NS	7.12E-01	-0.0152	8.04E-01	NS
MXD1	NS	7.12E-01	-0.0152	8.05E-01	NS
ENO2	NS	7.12E-01	1.52E-02	8.05E-01	NS
JPH3	NS	7.12E-01	-0.0152	8.05E-01	NS
LHFPL2	NS	7.13E-01	-0.0151	8.05E-01	NS
RNF149	NS	7.13E-01	1.51E-02	8.05E-01	NS
KCNJ12	NS	7.13E-01	-0.0151	8.05E-01	NS
MED27	NS	7.13E-01	-0.0151	8.05E-01	NS
QPRT	NS	7.13E-01	-0.0151	8.05E-01	NS
OSBP2	NS	7.13E-01	-0.0151	8.06E-01	NS
MMP14	NS	7.14E-01	1.51E-02	8.06E-01	NS
EPHX2	NS	7.14E-01	1.50E-02	8.06E-01	NS
CFAP45	NS	7.14E-01	1.50E-02	8.06E-01	NS
KCNS2	NS	7.14E-01	1.50E-02	8.06E-01	NS
FBXL19	NS	7.14E-01	1.50E-02	8.06E-01	NS
TULP4	NS	7.14E-01	1.50E-02	8.06E-01	NS
C2orf68	NS	7.14E-01	1.50E-02	8.06E-01	NS

RPL32	NS	7.15E-01	-0.015	8.07E-01	NS
WRAP53	NS	7.15E-01	1.50E-02	8.07E-01	NS
OPN3	NS	7.15E-01	-0.015	8.07E-01	NS
AP3B2	NS	7.15E-01	-0.015	8.07E-01	NS
SAMD14	NS	7.15E-01	1.50E-02	8.07E-01	NS
SLC39A3	NS	7.16E-01	-0.015	8.07E-01	NS
SCN1B	NS	7.16E-01	-0.015	8.07E-01	NS
SLC30A3	NS	7.16E-01	1.50E-02	8.07E-01	NS
ALDH3B2	NS	7.16E-01	-0.015	8.07E-01	NS
CLDN18	NS	7.16E-01	-0.0149	8.08E-01	NS
SYT5	NS	7.16E-01	1.49E-02	8.08E-01	NS
GALNT15	NS	7.16E-01	1.49E-02	8.08E-01	NS
PNLIPRP1	NS	7.16E-01	1.49E-02	8.08E-01	NS
MANSC4	NS	7.16E-01	1.49E-02	8.08E-01	NS
PRR20C	NS	7.16E-01	-0.0149	8.08E-01	NS
ERLEC1	NS	7.16E-01	-0.0149	8.08E-01	NS
TMEM61	NS	7.17E-01	1.49E-02	8.08E-01	NS
CD84	NS	7.17E-01	-0.0149	8.08E-01	NS
MYO9A	NS	7.17E-01	-0.0149	8.09E-01	NS
RHNO1	NS	7.17E-01	-0.0149	8.09E-01	NS
SLC25A52	NS	7.18E-01	-0.0149	8.09E-01	NS
SMAD5	NS	7.18E-01	-0.0148	8.09E-01	NS
DNAJC4	NS	7.18E-01	-0.0148	8.09E-01	NS
GATA2	NS	7.18E-01	1.48E-02	8.09E-01	NS
STK10	NS	7.18E-01	1.48E-02	8.09E-01	NS
KRTAP19-2	NS	7.18E-01	1.48E-02	8.09E-01	NS
PWWP3B	NS	7.18E-01	1.48E-02	8.09E-01	NS
YBX3	NS	7.19E-01	-0.0148	8.10E-01	NS
TYK2	NS	7.19E-01	1.48E-02	8.10E-01	NS
PPP2R5A	NS	7.19E-01	-0.0148	8.10E-01	NS
ICMT	NS	7.19E-01	-0.0148	8.10E-01	NS
SLC9B1	NS	7.19E-01	-0.0148	8.10E-01	NS
FLAD1	NS	7.19E-01	-0.0148	8.10E-01	NS
IVL	NS	7.19E-01	1.48E-02	8.10E-01	NS
SLC28A1	NS	7.19E-01	1.48E-02	8.10E-01	NS
GCN1	NS	7.19E-01	-0.0148	8.10E-01	NS
SLC35G4	NS	7.19E-01	-0.0148	8.10E-01	NS

CNN3	NS	7.19E-01	-0.0148	8.10E-01	NS
SDS	NS	7.19E-01	-0.0148	8.10E-01	NS
NACC2	NS	7.19E-01	1.48E-02	8.10E-01	NS
PJA2	NS	7.19E-01	-0.0148	8.10E-01	NS
TWF2	NS	7.19E-01	-0.0148	8.10E-01	NS
TAP1	NS	7.20E-01	1.47E-02	8.10E-01	NS
INTS6	NS	7.20E-01	1.47E-02	8.10E-01	NS
P3H1	NS	7.20E-01	-0.0147	8.10E-01	NS
SLC6A1	NS	7.20E-01	-0.0147	8.10E-01	NS
MPI	NS	7.20E-01	-0.0147	8.10E-01	NS
ADAMDEC1	NS	7.20E-01	-0.0147	8.10E-01	NS
ELOVL1	NS	7.20E-01	1.47E-02	8.10E-01	NS
SBSN	NS	7.20E-01	-0.0147	8.10E-01	NS
LILRA1	NS	7.20E-01	1.47E-02	8.11E-01	NS
LIMS1	NS	7.21E-01	-0.0147	8.11E-01	NS
WDR54	NS	7.21E-01	-0.0147	8.11E-01	NS
IL32	NS	7.21E-01	-0.0147	8.11E-01	NS
PILRA	NS	7.21E-01	-0.0147	8.11E-01	NS
PRXL2A	NS	7.21E-01	1.47E-02	8.11E-01	NS
ING2	NS	7.21E-01	-0.0147	8.11E-01	NS
DIMT1	NS	7.21E-01	-0.0147	8.11E-01	NS
CCDC50	NS	7.21E-01	-0.0147	8.11E-01	NS
FRRS1L	NS	7.22E-01	1.46E-02	8.11E-01	NS
GIGYF1	NS	7.22E-01	-0.0146	8.12E-01	NS
XKR5	NS	7.22E-01	1.46E-02	8.12E-01	NS
CDKN2AIP	NS	7.22E-01	-0.0146	8.12E-01	NS
TRAPPC3	NS	7.22E-01	1.46E-02	8.12E-01	NS
VASH1	NS	7.22E-01	-0.0146	8.12E-01	NS
TFAP2E	NS	7.22E-01	-0.0146	8.12E-01	NS
GTF2IRD2B	NS	7.23E-01	1.46E-02	8.13E-01	NS
KAT7	NS	7.23E-01	1.46E-02	8.13E-01	NS
OR52N2	NS	7.23E-01	-0.0145	8.13E-01	NS
SPDYE13	NS	7.24E-01	-0.0145	8.13E-01	NS
SENP7	NS	7.24E-01	-0.0145	8.13E-01	NS
TMEM132E	NS	7.24E-01	-0.0145	8.13E-01	NS
RPL26	NS	7.24E-01	-0.0145	8.13E-01	NS
TSPAN11	NS	7.24E-01	1.45E-02	8.13E-01	NS

LNP1	NS	7.24E-01	-0.0145	8.13E-01	NS
CHGA	NS	7.24E-01	-0.0145	8.14E-01	NS
CSF2RA	NS	7.24E-01	-0.0145	8.14E-01	NS
ZNF324B	NS	7.25E-01	1.45E-02	8.14E-01	NS
TMEM150B	NS	7.25E-01	-0.0145	8.14E-01	NS
TRAFD1	NS	7.25E-01	-0.0145	8.14E-01	NS
RIOX2	NS	7.25E-01	-0.0145	8.14E-01	NS
ITGA5	NS	7.25E-01	1.44E-02	8.14E-01	NS
CYRIB	NS	7.25E-01	1.44E-02	8.14E-01	NS
UBE2R2	NS	7.25E-01	-0.0144	8.14E-01	NS
C11orf65	NS	7.26E-01	1.44E-02	8.15E-01	NS
RBCK1	NS	7.26E-01	-0.0144	8.15E-01	NS
DHCR7	NS	7.26E-01	-0.0144	8.15E-01	NS
EDEM1	NS	7.26E-01	-0.0144	8.15E-01	NS
DEDD2	NS	7.26E-01	1.44E-02	8.15E-01	NS
TKT	NS	7.26E-01	-0.0144	8.15E-01	NS
FNDC3B	NS	7.27E-01	1.44E-02	8.15E-01	NS
C17orf80	NS	7.27E-01	1.44E-02	8.15E-01	NS
DIPK2B	NS	7.27E-01	-0.0144	8.15E-01	NS
EEF2	NS	7.27E-01	-0.0144	8.15E-01	NS
IRF2BP2	NS	7.27E-01	-0.0144	8.15E-01	NS
FSTL4	NS	7.27E-01	-0.0143	8.15E-01	NS
GPR75	NS	7.27E-01	-0.0144	8.15E-01	NS
MYL9	NS	7.27E-01	-0.0143	8.16E-01	NS
TMEM71	NS	7.27E-01	-0.0143	8.16E-01	NS
KCNK4	NS	7.27E-01	1.43E-02	8.16E-01	NS
SPRR5	NS	7.27E-01	-0.0143	8.16E-01	NS
MMP23B	NS	7.27E-01	1.43E-02	8.16E-01	NS
MCU	NS	7.28E-01	-0.0143	8.16E-01	NS
PDZD3	NS	7.28E-01	1.43E-02	8.16E-01	NS
SUCLG1	NS	7.28E-01	1.43E-02	8.16E-01	NS
EGF	NS	7.28E-01	-0.0143	8.16E-01	NS
ZNF131	NS	7.28E-01	-0.0143	8.16E-01	NS
KRTAP20-2	NS	7.28E-01	1.43E-02	8.17E-01	NS
MAVS	NS	7.29E-01	-0.0142	8.17E-01	NS
FGFR1OP2	NS	7.29E-01	1.42E-02	8.17E-01	NS
GCH1	NS	7.29E-01	-0.0142	8.17E-01	NS

RAB15	NS	7.29E-01	-0.0142	8.17E-01	NS
DDT	NS	7.29E-01	1.42E-02	8.18E-01	NS
MAST3	NS	7.30E-01	-0.0142	8.18E-01	NS
ATF7	NS	7.30E-01	1.42E-02	8.18E-01	NS
COX4I1	NS	7.30E-01	-0.0142	8.18E-01	NS
ST7L	NS	7.30E-01	1.42E-02	8.18E-01	NS
HASPIN	NS	7.30E-01	1.42E-02	8.18E-01	NS
SLC11A1	NS	7.30E-01	1.42E-02	8.18E-01	NS
VANGL1	NS	7.30E-01	-0.0142	8.18E-01	NS
GABRR2	NS	7.31E-01	-0.0141	8.18E-01	NS
CHMP4B	NS	7.31E-01	-0.0141	8.19E-01	NS
MPDU1	NS	7.31E-01	-0.0141	8.19E-01	NS
RAB11FIP5	NS	7.31E-01	1.41E-02	8.19E-01	NS
SMG1	NS	7.31E-01	-0.0141	8.19E-01	NS
ZNF253	NS	7.31E-01	-0.0141	8.19E-01	NS
RAB40C	NS	7.31E-01	1.41E-02	8.19E-01	NS
PMS2	NS	7.31E-01	-0.0141	8.19E-01	NS
LCE3C	NS	7.31E-01	-0.0141	8.19E-01	NS
APBB1IP	NS	7.32E-01	1.41E-02	8.19E-01	NS
NPY	NS	7.32E-01	1.41E-02	8.19E-01	NS
ZDHHC20	NS	7.32E-01	-0.0141	8.20E-01	NS
ZNF626	NS	7.32E-01	-0.0141	8.20E-01	NS
DLL4	NS	7.32E-01	-0.0141	8.20E-01	NS
DHFR	NS	7.32E-01	1.41E-02	8.20E-01	NS
DBI	NS	7.32E-01	1.41E-02	8.20E-01	NS
SLC15A4	NS	7.32E-01	1.40E-02	8.20E-01	NS
GPKOW	NS	7.33E-01	-0.014	8.20E-01	NS
MAPK4	NS	7.33E-01	-0.014	8.20E-01	NS
ENG	NS	7.33E-01	1.40E-02	8.20E-01	NS
TRAF7	NS	7.33E-01	1.40E-02	8.20E-01	NS
C5	NS	7.33E-01	-0.014	8.20E-01	NS
HSD17B2	NS	7.33E-01	-0.014	8.20E-01	NS
HDC	NS	7.34E-01	-0.014	8.21E-01	NS
CKM	NS	7.34E-01	-0.014	8.21E-01	NS
FOXO3	NS	7.34E-01	-0.014	8.21E-01	NS
OPN4	NS	7.34E-01	1.40E-02	8.21E-01	NS
LEF1	NS	7.34E-01	-0.0139	8.21E-01	NS

EIF3I	NS	7.35E-01	1.39E-02	8.22E-01	NS
NOA1	NS	7.35E-01	-0.0139	8.22E-01	NS
OCM2	NS	7.35E-01	1.39E-02	8.22E-01	NS
BRWD3	NS	7.35E-01	1.39E-02	8.22E-01	NS
CD70	NS	7.35E-01	1.39E-02	8.22E-01	NS
RBP4	NS	7.36E-01	1.39E-02	8.23E-01	NS
LIMD2	NS	7.36E-01	-0.0139	8.23E-01	NS
SFTPA2	NS	7.36E-01	-0.0138	8.23E-01	NS
SP6	NS	7.36E-01	-0.0138	8.23E-01	NS
LRRC10	NS	7.37E-01	-0.0138	8.23E-01	NS
SPATA22	NS	7.37E-01	-0.0138	8.23E-01	NS
DCBLD1	NS	7.37E-01	-0.0138	8.23E-01	NS
PRMT8	NS	7.37E-01	1.38E-02	8.24E-01	NS
MUC6	NS	7.37E-01	1.38E-02	8.24E-01	NS
PNMA8C	NS	7.37E-01	1.38E-02	8.24E-01	NS
UFL1	NS	7.38E-01	-0.0138	8.24E-01	NS
SLC23A2	NS	7.38E-01	-0.0138	8.24E-01	NS
CRIPT	NS	7.38E-01	1.38E-02	8.24E-01	NS
MAL	NS	7.38E-01	-0.0138	8.24E-01	NS
PSG3	NS	7.38E-01	-0.0138	8.24E-01	NS
RBM42	NS	7.38E-01	-0.0137	8.24E-01	NS
SLC4A2	NS	7.38E-01	1.37E-02	8.24E-01	NS
FRS2	NS	7.38E-01	-0.0137	8.24E-01	NS
LRRC28	NS	7.38E-01	1.37E-02	8.24E-01	NS
SYNDIG1L	NS	7.38E-01	-0.0137	8.24E-01	NS
HDDC3	NS	7.38E-01	-0.0137	8.24E-01	NS
SLC1A3	NS	7.38E-01	-0.0137	8.24E-01	NS
TAF3	NS	7.38E-01	1.37E-02	8.24E-01	NS
TEX49	NS	7.39E-01	1.37E-02	8.25E-01	NS
SPDYE14	NS	7.39E-01	-0.0137	8.25E-01	NS
SURF1	NS	7.39E-01	-0.0137	8.25E-01	NS
IFNA5	NS	7.39E-01	-0.0137	8.25E-01	NS
SLC25A14	NS	7.39E-01	1.37E-02	8.25E-01	NS
PTGES3	NS	7.39E-01	-0.0137	8.25E-01	NS
YWHAQ	NS	7.39E-01	1.37E-02	8.25E-01	NS
SNTB1	NS	7.39E-01	1.37E-02	8.25E-01	NS
SYTL1	NS	7.39E-01	1.37E-02	8.25E-01	NS

TALDO1	NS	7.40E-01	1.37E-02	8.25E-01	NS
BRAT1	NS	7.40E-01	1.37E-02	8.25E-01	NS
NTNG2	NS	7.40E-01	1.36E-02	8.26E-01	NS
ALDH16A1	NS	7.40E-01	-0.0136	8.26E-01	NS
SEC22A	NS	7.40E-01	-0.0136	8.26E-01	NS
SEMA6C	NS	7.40E-01	-0.0136	8.26E-01	NS
PHF19	NS	7.41E-01	-0.0136	8.26E-01	NS
GPR61	NS	7.41E-01	-0.0136	8.26E-01	NS
JOSD1	NS	7.41E-01	1.36E-02	8.26E-01	NS
TNFRSF13C	NS	7.41E-01	-0.0136	8.26E-01	NS
ARL4C	NS	7.41E-01	1.36E-02	8.27E-01	NS
DNAJC8	NS	7.42E-01	-0.0135	8.27E-01	NS
TAPBPL	NS	7.42E-01	-0.0135	8.27E-01	NS
PLCXD1	NS	7.42E-01	-0.0135	8.27E-01	NS
CD36	NS	7.42E-01	-0.0135	8.28E-01	NS
NPIPA1	NS	7.42E-01	-0.0135	8.28E-01	NS
SPINK2	NS	7.42E-01	1.35E-02	8.28E-01	NS
NOD2	NS	7.43E-01	-0.0135	8.28E-01	NS
OR6Y1	NS	7.43E-01	1.35E-02	8.28E-01	NS
DLGAP1	NS	7.43E-01	-0.0135	8.28E-01	NS
ZNF605	NS	7.43E-01	-0.0134	8.29E-01	NS
MAGEB6B	NS	7.43E-01	-0.0134	8.29E-01	NS
RBM4B	NS	7.43E-01	1.34E-02	8.29E-01	NS
SCAP	NS	7.44E-01	1.34E-02	8.29E-01	NS
ZNF705A	NS	7.44E-01	-0.0134	8.29E-01	NS
LAYN	NS	7.44E-01	1.34E-02	8.29E-01	NS
NFATC3	NS	7.44E-01	1.34E-02	8.29E-01	NS
UPP1	NS	7.44E-01	1.34E-02	8.29E-01	NS
ARID4A	NS	7.45E-01	1.34E-02	8.30E-01	NS
POLR2C	NS	7.45E-01	1.34E-02	8.30E-01	NS
FOXD4L4	NS	7.45E-01	-0.0134	8.30E-01	NS
WDR17	NS	7.45E-01	1.34E-02	8.30E-01	NS
TRIM73	NS	7.45E-01	1.34E-02	8.30E-01	NS
MSH3	NS	7.45E-01	1.34E-02	8.30E-01	NS
MICAL2	NS	7.45E-01	1.34E-02	8.30E-01	NS
TIGD5	NS	7.45E-01	-0.0134	8.30E-01	NS
FREM1	NS	7.45E-01	-0.0133	8.30E-01	NS

RALGDS	NS	7.45E-01	1.33E-02	8.30E-01	NS
CCDC171	NS	7.45E-01	-0.0133	8.30E-01	NS
RPL37A	NS	7.46E-01	-0.0133	8.30E-01	NS
NLGN3	NS	7.46E-01	-0.0133	8.30E-01	NS
SCUBE2	NS	7.46E-01	1.33E-02	8.30E-01	NS
PLOD3	NS	7.46E-01	1.33E-02	8.30E-01	NS
NOL3	NS	7.46E-01	-0.0133	8.30E-01	NS
WFDC8	NS	7.46E-01	-0.0133	8.30E-01	NS
RAB43	NS	7.46E-01	1.33E-02	8.30E-01	NS
MET	NS	7.46E-01	-0.0133	8.31E-01	NS
AHR	NS	7.46E-01	-0.0133	8.31E-01	NS
LCT	NS	7.47E-01	1.33E-02	8.31E-01	NS
MLX	NS	7.47E-01	1.33E-02	8.31E-01	NS
NDUFV3	NS	7.47E-01	-0.0133	8.31E-01	NS
AHI1	NS	7.47E-01	-0.0133	8.31E-01	NS
APMAP	NS	7.47E-01	-0.0133	8.31E-01	NS
DPPA2	NS	7.47E-01	-0.0132	8.31E-01	NS
NPPB	NS	7.48E-01	1.32E-02	8.32E-01	NS
RPL8	NS	7.48E-01	-0.0132	8.32E-01	NS
DAGLB	NS	7.48E-01	-0.0132	8.32E-01	NS
ZIM2	NS	7.48E-01	1.32E-02	8.32E-01	NS
CCL15-CCL14	NS	7.48E-01	1.32E-02	8.32E-01	NS
TRIM40	NS	7.48E-01	1.32E-02	8.32E-01	NS
TMEM156	NS	7.49E-01	1.32E-02	8.33E-01	NS
ZNF354B	NS	7.49E-01	1.32E-02	8.33E-01	NS
SULF2	NS	7.49E-01	1.32E-02	8.33E-01	NS
ENTPD6	NS	7.49E-01	-0.0132	8.33E-01	NS
OSTC	NS	7.49E-01	-0.0132	8.33E-01	NS
ERCC6	NS	7.49E-01	-0.0132	8.33E-01	NS
TBC1D3K	NS	7.49E-01	-0.0132	8.33E-01	NS
H3-3B	NS	7.49E-01	1.31E-02	8.33E-01	NS
SPIN2A	NS	7.49E-01	-0.0131	8.33E-01	NS
COL6A2	NS	7.49E-01	1.31E-02	8.33E-01	NS
CENPI	NS	7.50E-01	-0.0131	8.33E-01	NS
ARHGEF7	NS	7.50E-01	-0.0131	8.33E-01	NS
PARP9	NS	7.50E-01	-0.0131	8.33E-01	NS
USP17L22	NS	7.50E-01	1.31E-02	8.33E-01	NS

WDFY1	NS	7.50E-01	1.31E-02	8.33E-01	NS
B3GLCT	NS	7.50E-01	-0.0131	8.33E-01	NS
MLLT10	NS	7.50E-01	1.31E-02	8.33E-01	NS
AGFG2	NS	7.50E-01	1.31E-02	8.33E-01	NS
PHF7	NS	7.50E-01	1.31E-02	8.33E-01	NS
C2orf81	NS	7.50E-01	-0.0131	8.34E-01	NS
FAM117A	NS	7.50E-01	1.31E-02	8.34E-01	NS
TP53	NS	7.50E-01	-0.0131	8.34E-01	NS
DNM2	NS	7.51E-01	1.31E-02	8.34E-01	NS
CDCA4	NS	7.51E-01	1.31E-02	8.34E-01	NS
LRRC31	NS	7.51E-01	1.30E-02	8.34E-01	NS
CD52	NS	7.51E-01	-0.013	8.34E-01	NS
NQO2	NS	7.51E-01	1.30E-02	8.34E-01	NS
VPS36	NS	7.51E-01	1.30E-02	8.34E-01	NS
COMMD4	NS	7.51E-01	1.30E-02	8.34E-01	NS
IL31	NS	7.52E-01	1.30E-02	8.35E-01	NS
CPSF6	NS	7.52E-01	-0.013	8.35E-01	NS
RPS5	NS	7.52E-01	1.30E-02	8.35E-01	NS
FBXW2	NS	7.52E-01	1.30E-02	8.35E-01	NS
BNIP2	NS	7.52E-01	1.30E-02	8.35E-01	NS
RYK	NS	7.52E-01	-0.013	8.35E-01	NS
TBC1D3G	NS	7.52E-01	-0.013	8.35E-01	NS
LRRFIP2	NS	7.53E-01	1.29E-02	8.35E-01	NS
TRAK2	NS	7.53E-01	-0.013	8.35E-01	NS
GRID2IP	NS	7.53E-01	1.29E-02	8.35E-01	NS
CPB1	NS	7.53E-01	1.29E-02	8.36E-01	NS
CDC37	NS	7.53E-01	1.29E-02	8.36E-01	NS
ZNF221	NS	7.53E-01	1.29E-02	8.36E-01	NS
PPM1M	NS	7.53E-01	1.29E-02	8.36E-01	NS
MARCO	NS	7.54E-01	-0.0129	8.36E-01	NS
SMAP2	NS	7.54E-01	1.29E-02	8.36E-01	NS
PCP4	NS	7.54E-01	1.29E-02	8.36E-01	NS
RAI2	NS	7.54E-01	1.29E-02	8.36E-01	NS
MCMBP	NS	7.54E-01	-0.0129	8.37E-01	NS
SPTLC3	NS	7.54E-01	-0.0129	8.37E-01	NS
CHCHD4	NS	7.55E-01	-0.0128	8.37E-01	NS
CASP8AP2	NS	7.55E-01	-0.0128	8.37E-01	NS

ABCC5	NS	7.55E-01	1.28E-02	8.37E-01	NS
ADGRB1	NS	7.55E-01	-0.0128	8.37E-01	NS
ABL1	NS	7.55E-01	-0.0128	8.37E-01	NS
COLQ	NS	7.56E-01	-0.0128	8.38E-01	NS
CDH26	NS	7.56E-01	-0.0128	8.38E-01	NS
ANKRD44	NS	7.56E-01	-0.0128	8.38E-01	NS
OR56A5	NS	7.56E-01	1.28E-02	8.38E-01	NS
EGR1	NS	7.56E-01	1.28E-02	8.38E-01	NS
CDKN1C	NS	7.56E-01	-0.0127	8.38E-01	NS
FRY	NS	7.57E-01	-0.0127	8.38E-01	NS
MAN2B1	NS	7.57E-01	1.27E-02	8.38E-01	NS
ZNRF1	NS	7.57E-01	1.27E-02	8.38E-01	NS
HTR3C	NS	7.57E-01	1.27E-02	8.39E-01	NS
CCDC177	NS	7.57E-01	-0.0127	8.39E-01	NS
ZFP64	NS	7.57E-01	1.27E-02	8.39E-01	NS
NRBF2	NS	7.57E-01	1.27E-02	8.39E-01	NS
SARS1	NS	7.57E-01	1.27E-02	8.39E-01	NS
GIMAP1	NS	7.57E-01	1.27E-02	8.39E-01	NS
TPRN	NS	7.58E-01	-0.0127	8.39E-01	NS
SARM1	NS	7.58E-01	-0.0127	8.39E-01	NS
CTXN3	NS	7.58E-01	1.27E-02	8.39E-01	NS
VWA3A	NS	7.58E-01	-0.0127	8.39E-01	NS
CYP26B1	NS	7.58E-01	1.27E-02	8.39E-01	NS
GNAI3	NS	7.58E-01	-0.0127	8.39E-01	NS
BMPR1A	NS	7.58E-01	1.27E-02	8.39E-01	NS
NUP98	NS	7.58E-01	1.27E-02	8.39E-01	NS
CHPT1	NS	7.58E-01	-0.0127	8.39E-01	NS
ZCWPW2	NS	7.58E-01	1.27E-02	8.39E-01	NS
SCART1	NS	7.58E-01	-0.0127	8.39E-01	NS
RPS6KB2	NS	7.58E-01	1.26E-02	8.39E-01	NS
ARL5A	NS	7.58E-01	-0.0126	8.39E-01	NS
RAB11FIP3	NS	7.59E-01	-0.0126	8.39E-01	NS
CHRNA4	NS	7.59E-01	-0.0126	8.39E-01	NS
HIPK1	NS	7.58E-01	-0.0126	8.39E-01	NS
MACO1	NS	7.59E-01	-0.0126	8.39E-01	NS
SLITRK2	NS	7.59E-01	-0.0126	8.39E-01	NS
MT-ND3	NS	7.59E-01	-0.0126	8.39E-01	NS

STAT5B	NS	7.59E-01	-0.0126	8.40E-01	NS
C3orf62	NS	7.60E-01	-0.0126	8.40E-01	NS
TNNC2	NS	7.60E-01	1.26E-02	8.40E-01	NS
WIPF2	NS	7.60E-01	1.26E-02	8.40E-01	NS
PTPN23	NS	7.60E-01	-0.0125	8.40E-01	NS
PIGM	NS	7.60E-01	-0.0125	8.40E-01	NS
ACOXL	NS	7.60E-01	-0.0126	8.40E-01	NS
COA8	NS	7.60E-01	-0.0126	8.40E-01	NS
KLHL17	NS	7.60E-01	1.25E-02	8.41E-01	NS
RECQL5	NS	7.61E-01	1.25E-02	8.41E-01	NS
IMPA2	NS	7.61E-01	-0.0125	8.41E-01	NS
KISS1	NS	7.61E-01	-0.0125	8.41E-01	NS
SIRPA	NS	7.61E-01	-0.0125	8.41E-01	NS
TBXAS1	NS	7.61E-01	1.25E-02	8.41E-01	NS
ST8SIA6	NS	7.61E-01	-0.0125	8.41E-01	NS
SPRR3	NS	7.61E-01	1.25E-02	8.41E-01	NS
VPS9D1	NS	7.61E-01	1.25E-02	8.41E-01	NS
IFT46	NS	7.61E-01	1.25E-02	8.41E-01	NS
PTH1R	NS	7.61E-01	-0.0125	8.41E-01	NS
FOXP4	NS	7.61E-01	1.25E-02	8.41E-01	NS
WNT1	NS	7.62E-01	1.25E-02	8.42E-01	NS
TACO1	NS	7.62E-01	1.25E-02	8.42E-01	NS
GTF2H5	NS	7.62E-01	-0.0124	8.42E-01	NS
SCGB1D4	NS	7.62E-01	-0.0124	8.42E-01	NS
SYMPK	NS	7.62E-01	-0.0124	8.42E-01	NS
ZDHHC9	NS	7.62E-01	1.24E-02	8.42E-01	NS
GDNF	NS	7.62E-01	-0.0124	8.42E-01	NS
P3R3URF	NS	7.62E-01	-0.0124	8.42E-01	NS
DOCK11	NS	7.62E-01	1.24E-02	8.42E-01	NS
IL16	NS	7.62E-01	1.24E-02	8.42E-01	NS
SSX4B	NS	7.62E-01	1.24E-02	8.42E-01	NS
SPRYD4	NS	7.63E-01	1.24E-02	8.42E-01	NS
RGSL1	NS	7.63E-01	1.24E-02	8.42E-01	NS
TIGD6	NS	7.63E-01	1.24E-02	8.43E-01	NS
SFXN4	NS	7.63E-01	-0.0124	8.43E-01	NS
HOXB13	NS	7.63E-01	-0.0124	8.43E-01	NS
MCOLN3	NS	7.63E-01	-0.0124	8.43E-01	NS

SCFD1	NS	7.64E-01	-0.0124	8.43E-01	NS
VPS4A	NS	7.64E-01	1.24E-02	8.43E-01	NS
ANKRD42	NS	7.64E-01	1.23E-02	8.43E-01	NS
FZD7	NS	7.64E-01	1.23E-02	8.43E-01	NS
MRPL16	NS	7.64E-01	-0.0123	8.43E-01	NS
CERS5	NS	7.64E-01	-0.0123	8.43E-01	NS
ORAI1	NS	7.64E-01	-0.0123	8.43E-01	NS
OXCT2	NS	7.64E-01	-0.0123	8.43E-01	NS
FER1L5	NS	7.64E-01	-0.0123	8.43E-01	NS
HGSNAT	NS	7.64E-01	1.23E-02	8.43E-01	NS
AICDA	NS	7.65E-01	1.23E-02	8.44E-01	NS
PCDH8	NS	7.65E-01	-0.0123	8.44E-01	NS
H4C6	NS	7.65E-01	1.23E-02	8.44E-01	NS
SYN1	NS	7.65E-01	1.23E-02	8.44E-01	NS
SIK3	NS	7.65E-01	-0.0123	8.44E-01	NS
ZBTB21	NS	7.65E-01	1.23E-02	8.44E-01	NS
KBTBD8	NS	7.65E-01	-0.0123	8.44E-01	NS
IGLL1	NS	7.65E-01	1.23E-02	8.44E-01	NS
MACROH2A1	NS	7.66E-01	1.22E-02	8.44E-01	NS
SLC12A6	NS	7.66E-01	1.22E-02	8.44E-01	NS
HBS1L	NS	7.66E-01	-0.0122	8.44E-01	NS
DOK1	NS	7.66E-01	1.22E-02	8.45E-01	NS
SNRNP40	NS	7.66E-01	-0.0122	8.45E-01	NS
TIPARP	NS	7.66E-01	-0.0122	8.45E-01	NS
FRMD4B	NS	7.66E-01	-0.0122	8.45E-01	NS
RHBDL1	NS	7.67E-01	-0.0122	8.45E-01	NS
MAL2	NS	7.67E-01	-0.0122	8.45E-01	NS
RAB4B	NS	7.67E-01	-0.0122	8.45E-01	NS
SDHD	NS	7.67E-01	1.22E-02	8.45E-01	NS
ART3	NS	7.67E-01	-0.0122	8.45E-01	NS
GTDC1	NS	7.68E-01	1.21E-02	8.46E-01	NS
EPC2	NS	7.68E-01	-0.0121	8.46E-01	NS
CHML	NS	7.67E-01	-0.0121	8.46E-01	NS
CNTNAP3	NS	7.68E-01	-0.0121	8.46E-01	NS
TAS2R8	NS	7.68E-01	1.21E-02	8.46E-01	NS
TES	NS	7.68E-01	-0.0121	8.46E-01	NS
MRPL44	NS	7.68E-01	-0.0121	8.46E-01	NS

AGFG1	NS	7.68E-01	-0.0121	8.46E-01	NS
H2AX	NS	7.68E-01	-0.0121	8.46E-01	NS
FAM13A	NS	7.68E-01	-0.0121	8.46E-01	NS
CALR3	NS	7.68E-01	1.21E-02	8.46E-01	NS
DBF4	NS	7.69E-01	-0.0121	8.46E-01	NS
GLIS1	NS	7.69E-01	1.21E-02	8.46E-01	NS
ATG13	NS	7.69E-01	-0.0121	8.46E-01	NS
ZNF793	NS	7.69E-01	-0.0121	8.47E-01	NS
PIGZ	NS	7.69E-01	-0.0121	8.47E-01	NS
ARSK	NS	7.69E-01	1.20E-02	8.47E-01	NS
KRTAP1-1	NS	7.70E-01	-0.012	8.47E-01	NS
TECR	NS	7.70E-01	1.20E-02	8.47E-01	NS
WRAP73	NS	7.70E-01	-0.012	8.47E-01	NS
MORN3	NS	7.70E-01	1.20E-02	8.48E-01	NS
ATG3	NS	7.70E-01	-0.012	8.48E-01	NS
PRSS16	NS	7.70E-01	-0.012	8.48E-01	NS
HLX	NS	7.70E-01	1.20E-02	8.48E-01	NS
PRPF4	NS	7.70E-01	1.20E-02	8.48E-01	NS
SDCBP	NS	7.70E-01	1.20E-02	8.48E-01	NS
PRDX5	NS	7.71E-01	1.20E-02	8.48E-01	NS
IFT74	NS	7.71E-01	1.20E-02	8.48E-01	NS
ZFP1	NS	7.71E-01	-0.012	8.48E-01	NS
DDX11	NS	7.71E-01	-0.012	8.48E-01	NS
TTLL5	NS	7.71E-01	1.19E-02	8.48E-01	NS
DUSP18	NS	7.71E-01	-0.0119	8.48E-01	NS
C2CD2L	NS	7.71E-01	1.19E-02	8.48E-01	NS
S100A9	NS	7.72E-01	1.19E-02	8.49E-01	NS
PAX7	NS	7.72E-01	-0.0119	8.49E-01	NS
TAS2R5	NS	7.72E-01	-0.0119	8.49E-01	NS
CTF1	NS	7.72E-01	1.19E-02	8.49E-01	NS
PCBP2	NS	7.72E-01	1.19E-02	8.49E-01	NS
SOBP	NS	7.72E-01	-0.0119	8.49E-01	NS
LCN15	NS	7.72E-01	-0.0119	8.49E-01	NS
SMIM43	NS	7.73E-01	1.19E-02	8.49E-01	NS
RWDD2A	NS	7.73E-01	1.19E-02	8.49E-01	NS
SLC2A3	NS	7.73E-01	1.19E-02	8.49E-01	NS
COASY	NS	7.73E-01	-0.0119	8.49E-01	NS

GTF3C3	NS	7.73E-01	-0.0119	8.49E-01	NS
PDE6B	NS	7.73E-01	1.19E-02	8.49E-01	NS
TMEM50A	NS	7.73E-01	-0.0119	8.49E-01	NS
SDHAF2	NS	7.73E-01	-0.0118	8.50E-01	NS
F5	NS	7.73E-01	1.18E-02	8.50E-01	NS
BSG	NS	7.73E-01	-0.0118	8.50E-01	NS
VWA5B1	NS	7.73E-01	1.18E-02	8.50E-01	NS
AHCTF1	NS	7.73E-01	1.18E-02	8.50E-01	NS
PLOD1	NS	7.74E-01	-0.0118	8.50E-01	NS
RNF130	NS	7.74E-01	1.18E-02	8.50E-01	NS
CEBPG	NS	7.74E-01	-0.0118	8.50E-01	NS
CDC42	NS	7.74E-01	-0.0118	8.50E-01	NS
STARD8	NS	7.74E-01	1.18E-02	8.50E-01	NS
ZNF768	NS	7.74E-01	-0.0118	8.50E-01	NS
DNAJC25-GNG10	NS	7.74E-01	-0.0118	8.50E-01	NS
ZC3H18	NS	7.74E-01	-0.0118	8.50E-01	NS
DCHS1	NS	7.75E-01	-0.0118	8.50E-01	NS
SLC6A19	NS	7.75E-01	-0.0118	8.50E-01	NS
TREM1	NS	7.75E-01	1.17E-02	8.51E-01	NS
CCK	NS	7.75E-01	-0.0117	8.51E-01	NS
RTF2	NS	7.75E-01	-0.0117	8.51E-01	NS
POLD4	NS	7.75E-01	1.17E-02	8.51E-01	NS
TPD52L3	NS	7.76E-01	-0.0117	8.51E-01	NS
NDUFA3	NS	7.76E-01	1.17E-02	8.51E-01	NS
SAMD5	NS	7.76E-01	-0.0117	8.51E-01	NS
LETM1	NS	7.76E-01	1.17E-02	8.52E-01	NS
LY75-CD302	NS	7.76E-01	-0.0117	8.52E-01	NS
SPAG6	NS	7.76E-01	1.17E-02	8.52E-01	NS
ATP1A4	NS	7.76E-01	1.17E-02	8.52E-01	NS
AP2M1	NS	7.77E-01	1.17E-02	8.52E-01	NS
CCDC110	NS	7.76E-01	1.17E-02	8.52E-01	NS
ZBED8	NS	7.77E-01	1.17E-02	8.52E-01	NS
FAM8A1	NS	7.77E-01	-0.0117	8.52E-01	NS
WDFY2	NS	7.77E-01	1.16E-02	8.52E-01	NS
CRYGC	NS	7.77E-01	1.16E-02	8.52E-01	NS
NOS1AP	NS	7.77E-01	1.17E-02	8.52E-01	NS
TTYH3	NS	7.77E-01	-0.0116	8.52E-01	NS

PAQR9	NS	7.77E-01	1.16E-02	8.53E-01	NS
TBC1D3F	NS	7.78E-01	1.16E-02	8.53E-01	NS
CNFN	NS	7.78E-01	-0.0116	8.53E-01	NS
PTGIS	NS	7.78E-01	1.16E-02	8.53E-01	NS
TPM1	NS	7.78E-01	-0.0116	8.53E-01	NS
C10orf82	NS	7.78E-01	1.16E-02	8.53E-01	NS
SLCO3A1	NS	7.78E-01	-0.0116	8.53E-01	NS
CCDC154	NS	7.78E-01	-0.0116	8.53E-01	NS
PMS1	NS	7.78E-01	-0.0116	8.53E-01	NS
RPS15	NS	7.79E-01	-0.0116	8.53E-01	NS
ASNS	NS	7.79E-01	1.15E-02	8.54E-01	NS
XRCC3	NS	7.79E-01	1.15E-02	8.54E-01	NS
CPNE2	NS	7.79E-01	-0.0115	8.54E-01	NS
WFIKKN2	NS	7.79E-01	1.15E-02	8.54E-01	NS
SUZ12	NS	7.79E-01	-0.0115	8.54E-01	NS
EXTL3	NS	7.79E-01	-0.0115	8.54E-01	NS
MIOS	NS	7.79E-01	1.15E-02	8.54E-01	NS
GHRHR	NS	7.79E-01	1.15E-02	8.54E-01	NS
SLC25A6	NS	7.79E-01	1.15E-02	8.54E-01	NS
GRIPAP1	NS	7.80E-01	-0.0115	8.54E-01	NS
RBM39	NS	7.80E-01	-0.0115	8.54E-01	NS
SLTM	NS	7.80E-01	1.15E-02	8.54E-01	NS
FUT4	NS	7.80E-01	-0.0115	8.54E-01	NS
PARP15	NS	7.80E-01	-0.0115	8.54E-01	NS
CYB5RL	NS	7.80E-01	1.15E-02	8.55E-01	NS
PAXBP1	NS	7.81E-01	-0.0114	8.55E-01	NS
MRPL14	NS	7.81E-01	1.14E-02	8.55E-01	NS
TMEM164	NS	7.81E-01	-0.0114	8.55E-01	NS
PRC1	NS	7.81E-01	-0.0114	8.55E-01	NS
RFC2	NS	7.81E-01	1.14E-02	8.55E-01	NS
CWC15	NS	7.81E-01	-0.0114	8.55E-01	NS
KRTAP10-5	NS	7.81E-01	1.14E-02	8.55E-01	NS
ACTR3	NS	7.81E-01	-0.0114	8.55E-01	NS
SUPV3L1	NS	7.82E-01	1.14E-02	8.55E-01	NS
FAIM	NS	7.82E-01	-0.0114	8.55E-01	NS
NTRK1	NS	7.82E-01	-0.0114	8.56E-01	NS
H2BC21	NS	7.82E-01	-0.0114	8.56E-01	NS

KIFBP	NS	7.82E-01	-0.0114	8.56E-01	NS
KBTBD4	NS	7.82E-01	-0.0114	8.56E-01	NS
OLA1	NS	7.82E-01	1.14E-02	8.56E-01	NS
PIGH	NS	7.82E-01	1.13E-02	8.56E-01	NS
C22orf31	NS	7.83E-01	-0.0113	8.56E-01	NS
CRISPLD2	NS	7.83E-01	1.13E-02	8.56E-01	NS
EXOC5	NS	7.83E-01	-0.0113	8.56E-01	NS
CD33	NS	7.83E-01	-0.0113	8.56E-01	NS
ERP29	NS	7.83E-01	1.13E-02	8.56E-01	NS
TSPAN18	NS	7.84E-01	1.13E-02	8.57E-01	NS
SPNS2	NS	7.84E-01	-0.0113	8.57E-01	NS
ANKRD49	NS	7.84E-01	-0.0113	8.57E-01	NS
STARD6	NS	7.84E-01	1.13E-02	8.57E-01	NS
FOXB2	NS	7.84E-01	1.13E-02	8.57E-01	NS
FURIN	NS	7.84E-01	-0.0113	8.57E-01	NS
LRRC49	NS	7.84E-01	-0.0113	8.57E-01	NS
ERICH3	NS	7.84E-01	-0.0113	8.57E-01	NS
RPL17	NS	7.84E-01	-0.0113	8.57E-01	NS
CSK	NS	7.85E-01	1.12E-02	8.58E-01	NS
PUM2	NS	7.85E-01	1.12E-02	8.58E-01	NS
CFAP54	NS	7.85E-01	-0.0112	8.58E-01	NS
MLLT1	NS	7.86E-01	-0.0112	8.59E-01	NS
ITLN1	NS	7.86E-01	-0.0112	8.59E-01	NS
HNRNPUL2	NS	7.86E-01	1.12E-02	8.59E-01	NS
CPLX3	NS	7.86E-01	-0.0112	8.59E-01	NS
TFE3	NS	7.86E-01	1.12E-02	8.59E-01	NS
ASAP2	NS	7.86E-01	-0.0112	8.59E-01	NS
TMED4	NS	7.86E-01	-0.0111	8.59E-01	NS
NDRG2	NS	7.86E-01	1.11E-02	8.59E-01	NS
PI3	NS	7.87E-01	-0.0111	8.59E-01	NS
KRTCAP2	NS	7.87E-01	1.11E-02	8.59E-01	NS
GPIHBP1	NS	7.87E-01	1.11E-02	8.59E-01	NS
MYL4	NS	7.87E-01	-0.0111	8.59E-01	NS
EIF4A1	NS	7.87E-01	1.11E-02	8.60E-01	NS
CCL4	NS	7.87E-01	1.11E-02	8.60E-01	NS
PSMD14	NS	7.87E-01	-0.0111	8.60E-01	NS
GDPD5	NS	7.87E-01	1.11E-02	8.60E-01	NS

TMEM222	NS	7.87E-01	-0.0111	8.60E-01	NS
SLC10A6	NS	7.87E-01	-0.0111	8.60E-01	NS
DEFB112	NS	7.88E-01	-0.0111	8.60E-01	NS
GPR141	NS	7.88E-01	-0.0111	8.60E-01	NS
PNMA8B	NS	7.88E-01	1.11E-02	8.60E-01	NS
ZNF423	NS	7.89E-01	1.10E-02	8.61E-01	NS
GLT8D1	NS	7.89E-01	-0.011	8.61E-01	NS
PATL2	NS	7.89E-01	1.10E-02	8.61E-01	NS
MPST	NS	7.89E-01	-0.011	8.61E-01	NS
ZSCAN10	NS	7.89E-01	-0.011	8.61E-01	NS
LILRB2	NS	7.89E-01	1.10E-02	8.62E-01	NS
TUBA1B	NS	7.90E-01	-0.011	8.62E-01	NS
SLN	NS	7.90E-01	-0.011	8.62E-01	NS
NRIP1	NS	7.90E-01	1.10E-02	8.62E-01	NS
NPY4R2	NS	7.90E-01	1.09E-02	8.62E-01	NS
ELOB	NS	7.90E-01	-0.0109	8.62E-01	NS
UCHL3	NS	7.90E-01	-0.0109	8.62E-01	NS
ORMDL1	NS	7.91E-01	-0.0109	8.62E-01	NS
GNG5P2	NS	7.90E-01	-0.0109	8.62E-01	NS
ZFP36L2	NS	7.91E-01	-0.0109	8.62E-01	NS
PRSS50	NS	7.91E-01	1.09E-02	8.62E-01	NS
CHCHD5	NS	7.91E-01	1.09E-02	8.63E-01	NS
GOLGA6L9	NS	7.91E-01	1.09E-02	8.63E-01	NS
NAAA	NS	7.92E-01	-0.0109	8.63E-01	NS
MOB3B	NS	7.92E-01	1.08E-02	8.63E-01	NS
IL22RA1	NS	7.92E-01	1.08E-02	8.64E-01	NS
TBX20	NS	7.92E-01	-0.0108	8.64E-01	NS
PLA2G12B	NS	7.92E-01	1.08E-02	8.64E-01	NS
LMBRD2	NS	7.92E-01	1.08E-02	8.64E-01	NS
CA2	NS	7.93E-01	1.08E-02	8.64E-01	NS
THAP11	NS	7.93E-01	1.08E-02	8.64E-01	NS
WWP1	NS	7.93E-01	1.08E-02	8.64E-01	NS
SFXN2	NS	7.93E-01	1.08E-02	8.64E-01	NS
SALL2	NS	7.93E-01	-0.0108	8.64E-01	NS
OR2T6	NS	7.93E-01	-0.0108	8.65E-01	NS
ITPR1	NS	7.93E-01	1.08E-02	8.65E-01	NS
SLC6A13	NS	7.94E-01	-0.0107	8.65E-01	NS

KRR1	NS	7.94E-01	1.07E-02	8.65E-01	NS
DEFB118	NS	7.94E-01	-0.0107	8.66E-01	NS
DOCK8	NS	7.95E-01	-0.0107	8.66E-01	NS
PIGF	NS	7.95E-01	-0.0107	8.66E-01	NS
RBBP5	NS	7.95E-01	1.07E-02	8.66E-01	NS
CARS2	NS	7.95E-01	-0.0107	8.66E-01	NS
CAPSL	NS	7.95E-01	-0.0106	8.66E-01	NS
TMEM143	NS	7.95E-01	1.07E-02	8.66E-01	NS
CKAP5	NS	7.95E-01	1.06E-02	8.66E-01	NS
FBXL7	NS	7.95E-01	1.07E-02	8.66E-01	NS
EVX2	NS	7.96E-01	-0.0106	8.66E-01	NS
SMC5	NS	7.96E-01	-0.0106	8.66E-01	NS
NUP85	NS	7.96E-01	1.06E-02	8.67E-01	NS
SRP14	NS	7.96E-01	-0.0106	8.67E-01	NS
PIH1D2	NS	7.96E-01	-0.0106	8.67E-01	NS
PPP1R1B	NS	7.96E-01	-0.0106	8.67E-01	NS
GIMAP5	NS	7.96E-01	1.06E-02	8.67E-01	NS
LHX1	NS	7.96E-01	-0.0106	8.67E-01	NS
TMEM41A	NS	7.96E-01	1.06E-02	8.67E-01	NS
FOXD4L6	NS	7.96E-01	-0.0106	8.67E-01	NS
KAAG1	NS	7.97E-01	-0.0106	8.67E-01	NS
AP5M1	NS	7.97E-01	-0.0106	8.67E-01	NS
TMEM98	NS	7.97E-01	1.06E-02	8.67E-01	NS
TAS1R3	NS	7.97E-01	-0.0106	8.68E-01	NS
MSGN1	NS	7.97E-01	1.05E-02	8.68E-01	NS
OR13J1	NS	7.97E-01	1.06E-02	8.68E-01	NS
H1-7	NS	7.97E-01	1.05E-02	8.68E-01	NS
SENP8	NS	7.97E-01	1.05E-02	8.68E-01	NS
HAUS3	NS	7.98E-01	1.05E-02	8.68E-01	NS
DCAF13	NS	7.98E-01	-0.0105	8.68E-01	NS
OR4K2	NS	7.98E-01	-0.0105	8.68E-01	NS
MLXIP	NS	7.98E-01	1.05E-02	8.68E-01	NS
REEP4	NS	7.98E-01	1.05E-02	8.68E-01	NS
OR2W1	NS	7.98E-01	-0.0105	8.68E-01	NS
CNOT1	NS	7.99E-01	1.05E-02	8.69E-01	NS
TSPY2	NS	7.99E-01	1.05E-02	8.69E-01	NS
FAM83F	NS	7.99E-01	-0.0105	8.69E-01	NS

G3BP2	NS	7.99E-01	-0.0105	8.69E-01	NS
ZNF644	NS	7.99E-01	1.05E-02	8.69E-01	NS
ALOX12B	NS	7.99E-01	-0.0104	8.69E-01	NS
PSMG2	NS	8.00E-01	1.04E-02	8.69E-01	NS
TSSK1B	NS	8.00E-01	-0.0104	8.69E-01	NS
SUPT20H	NS	8.00E-01	1.04E-02	8.69E-01	NS
ZFAND2B	NS	8.00E-01	1.04E-02	8.70E-01	NS
MRPL2	NS	8.00E-01	-0.0104	8.70E-01	NS
BATF2	NS	8.00E-01	-0.0104	8.70E-01	NS
OR1R1P	NS	8.00E-01	1.04E-02	8.70E-01	NS
NALF1	NS	8.01E-01	1.04E-02	8.70E-01	NS
HSPH1	NS	8.01E-01	1.04E-02	8.70E-01	NS
OSMR	NS	8.01E-01	1.04E-02	8.70E-01	NS
MERTK	NS	8.01E-01	1.04E-02	8.70E-01	NS
CDKL2	NS	8.01E-01	-0.0103	8.71E-01	NS
C6orf136	NS	8.01E-01	1.03E-02	8.71E-01	NS
PRUNE1	NS	8.02E-01	-0.0103	8.71E-01	NS
SLC5A4	NS	8.02E-01	-0.0103	8.71E-01	NS
ANKRD30B	NS	8.02E-01	-0.0103	8.71E-01	NS
DGKA	NS	8.02E-01	-0.0103	8.71E-01	NS
ATP8B3	NS	8.02E-01	-0.0103	8.71E-01	NS
NSD3	NS	8.02E-01	-0.0103	8.71E-01	NS
MIEF2	NS	8.02E-01	-0.0103	8.71E-01	NS
ANKRD53	NS	8.02E-01	1.03E-02	8.71E-01	NS
L1CAM	NS	8.02E-01	-0.0103	8.71E-01	NS
ARGLU1	NS	8.03E-01	-0.0103	8.72E-01	NS
QDPR	NS	8.03E-01	1.03E-02	8.72E-01	NS
TPM2	NS	8.03E-01	1.03E-02	8.72E-01	NS
TMEM101	NS	8.03E-01	-0.0102	8.72E-01	NS
CDH24	NS	8.03E-01	-0.0102	8.72E-01	NS
GSTA2	NS	8.03E-01	-0.0102	8.72E-01	NS
CHMP7	NS	8.03E-01	-0.0102	8.72E-01	NS
MICU1	NS	8.04E-01	-0.0102	8.72E-01	NS
SMIM10L2A	NS	8.04E-01	-0.0102	8.72E-01	NS
OR4N4	NS	8.04E-01	-0.0102	8.72E-01	NS
FAM43A	NS	8.04E-01	-0.0102	8.72E-01	NS
CCDC61	NS	8.04E-01	1.02E-02	8.72E-01	NS

SSX1	NS	8.04E-01	-0.0102	8.72E-01	NS
MRPL27	NS	8.04E-01	-0.0102	8.73E-01	NS
KRT76	NS	8.05E-01	-0.0102	8.73E-01	NS
LRRC8C	NS	8.05E-01	-0.0102	8.73E-01	NS
TOX2	NS	8.05E-01	1.01E-02	8.73E-01	NS
ZNF331	NS	8.05E-01	-0.0101	8.73E-01	NS
BMP2K	NS	8.05E-01	1.01E-02	8.73E-01	NS
ZNF888	NS	8.05E-01	1.01E-02	8.73E-01	NS
RLN1	NS	8.06E-01	-0.0101	8.74E-01	NS
EGFL7	NS	8.06E-01	-0.0101	8.74E-01	NS
RIMS4	NS	8.06E-01	-0.0101	8.74E-01	NS
INTS8	NS	8.07E-01	-0.0101	8.75E-01	NS
PWP2	NS	8.07E-01	1.01E-02	8.75E-01	NS
OR1G1	NS	8.07E-01	1.01E-02	8.75E-01	NS
ACTA1	NS	8.07E-01	1.00E-02	8.75E-01	NS
ATP1B2	NS	8.07E-01	-0.01	8.75E-01	NS
NAA80	NS	8.07E-01	1.00E-02	8.75E-01	NS
ATP5F1B	NS	8.07E-01	1.00E-02	8.75E-01	NS
TPH1	NS	8.07E-01	-0.01	8.75E-01	NS
MYO1F	NS	8.08E-01	1.00E-02	8.75E-01	NS
RP9	NS	8.08E-01	1.00E-02	8.75E-01	NS
UBE2L6	NS	8.08E-01	9.98E-03	8.76E-01	NS
GIMAP2	NS	8.08E-01	9.97E-03	8.76E-01	NS
ATP8B4	NS	8.08E-01	-0.00996	8.76E-01	NS
FANCD2	NS	8.08E-01	9.96E-03	8.76E-01	NS
HOPX	NS	8.09E-01	9.96E-03	8.76E-01	NS
ESYT2	NS	8.09E-01	-0.00994	8.76E-01	NS
SOAT1	NS	8.09E-01	-0.00993	8.76E-01	NS
DAGLA	NS	8.09E-01	9.92E-03	8.77E-01	NS
SIK2	NS	8.09E-01	-0.00992	8.77E-01	NS
TACC3	NS	8.09E-01	-0.00991	8.77E-01	NS
COL6A3	NS	8.09E-01	9.91E-03	8.77E-01	NS
ITGB1	NS	8.09E-01	-0.00991	8.77E-01	NS
PLEKHA5	NS	8.10E-01	9.89E-03	8.77E-01	NS
PDIA4	NS	8.10E-01	9.89E-03	8.77E-01	NS
CLCF1	NS	8.10E-01	9.88E-03	8.77E-01	NS
KIF18B	NS	8.10E-01	9.88E-03	8.77E-01	NS

OR5C1	NS	8.10E-01	-0.00986	8.77E-01	NS
MAP4K5	NS	8.10E-01	9.85E-03	8.78E-01	NS
PDGFRA	NS	8.11E-01	-0.00985	8.78E-01	NS
JTB	NS	8.11E-01	9.82E-03	8.78E-01	NS
OR7D2	NS	8.11E-01	-0.00982	8.78E-01	NS
C8orf76	NS	8.11E-01	9.83E-03	8.78E-01	NS
DAXX	NS	8.11E-01	-0.00982	8.78E-01	NS
NEU1	NS	8.11E-01	-0.00981	8.78E-01	NS
MAST2	NS	8.11E-01	-0.00981	8.78E-01	NS
ATE1	NS	8.11E-01	-0.00981	8.78E-01	NS
USP7	NS	8.12E-01	9.79E-03	8.78E-01	NS
H3C8	NS	8.12E-01	-0.00979	8.78E-01	NS
NAT14	NS	8.12E-01	9.78E-03	8.78E-01	NS
MTX3	NS	8.12E-01	9.78E-03	8.78E-01	NS
ZNF619	NS	8.12E-01	-0.00978	8.78E-01	NS
NTN1	NS	8.12E-01	-0.00977	8.79E-01	NS
GTPBP8	NS	8.12E-01	-0.00976	8.79E-01	NS
LDLRAD2	NS	8.12E-01	-0.00977	8.79E-01	NS
NTN4	NS	8.12E-01	-0.00976	8.79E-01	NS
SPACA9	NS	8.12E-01	-0.00976	8.79E-01	NS
CCDC78	NS	8.12E-01	9.75E-03	8.79E-01	NS
POU5F1B	NS	8.12E-01	9.75E-03	8.79E-01	NS
KCNJ8	NS	8.13E-01	-0.00973	8.79E-01	NS
LOXL4	NS	8.13E-01	-0.00973	8.79E-01	NS
NIPAL2	NS	8.13E-01	-0.00973	8.79E-01	NS
VIPR2	NS	8.13E-01	-0.00973	8.79E-01	NS
OOSP2	NS	8.14E-01	9.68E-03	8.80E-01	NS
C9orf116	NS	8.14E-01	-0.00967	8.80E-01	NS
AKNAD1	NS	8.14E-01	-0.00967	8.80E-01	NS
LRRC15	NS	8.14E-01	9.68E-03	8.80E-01	NS
NPEPL1	NS	8.14E-01	-0.00967	8.80E-01	NS
GPR162	NS	8.14E-01	-0.00967	8.80E-01	NS
EML4	NS	8.14E-01	-0.00966	8.80E-01	NS
OPRD1	NS	8.14E-01	9.65E-03	8.80E-01	NS
S100A12	NS	8.14E-01	-0.00965	8.80E-01	NS
CLDN25	NS	8.14E-01	9.65E-03	8.80E-01	NS
RGPD1	NS	8.14E-01	9.64E-03	8.80E-01	NS

ADAM15	NS	8.15E-01	-0.00964	8.80E-01	NS
DMP1	NS	8.15E-01	-0.00963	8.80E-01	NS
GALNT3	NS	8.15E-01	-0.00963	8.80E-01	NS
PPRC1	NS	8.15E-01	-0.00963	8.80E-01	NS
FANCL	NS	8.15E-01	9.62E-03	8.80E-01	NS
CAMK2G	NS	8.15E-01	9.62E-03	8.80E-01	NS
MRPL13	NS	8.15E-01	9.62E-03	8.80E-01	NS
OR2A5	NS	8.15E-01	-0.00962	8.80E-01	NS
ESD	NS	8.15E-01	9.61E-03	8.80E-01	NS
IGFN1	NS	8.15E-01	-0.0096	8.81E-01	NS
H2AC18	NS	8.15E-01	9.59E-03	8.81E-01	NS
DNAAF11	NS	8.16E-01	-0.00958	8.81E-01	NS
PPP4R3B	NS	8.16E-01	9.58E-03	8.81E-01	NS
CCHCR1	NS	8.16E-01	-0.00958	8.81E-01	NS
TTC7A	NS	8.16E-01	9.57E-03	8.81E-01	NS
SRPK3	NS	8.16E-01	-0.00957	8.81E-01	NS
ADAMTS8	NS	8.16E-01	9.55E-03	8.81E-01	NS
IMPDH2	NS	8.16E-01	9.55E-03	8.81E-01	NS
RNF227	NS	8.16E-01	-0.00954	8.81E-01	NS
TNFSF13B	NS	8.16E-01	-0.00954	8.81E-01	NS
TCF4	NS	8.17E-01	9.53E-03	8.81E-01	NS
DDX39B	NS	8.17E-01	-0.00952	8.82E-01	NS
CBFA2T2	NS	8.17E-01	-0.0095	8.82E-01	NS
KHDRBS3	NS	8.17E-01	9.49E-03	8.82E-01	NS
CADM4	NS	8.18E-01	-0.00947	8.82E-01	NS
SRSF6	NS	8.18E-01	9.47E-03	8.82E-01	NS
SATB1	NS	8.18E-01	9.47E-03	8.82E-01	NS
PLG	NS	8.18E-01	9.46E-03	8.82E-01	NS
ARHGEF11	NS	8.18E-01	9.46E-03	8.82E-01	NS
ARHGAP19	NS	8.18E-01	9.46E-03	8.82E-01	NS
DENND6A	NS	8.18E-01	9.45E-03	8.82E-01	NS
SIRT6	NS	8.18E-01	9.43E-03	8.83E-01	NS
SYNE1	NS	8.19E-01	-0.00942	8.83E-01	NS
SNAP91	NS	8.19E-01	9.42E-03	8.83E-01	NS
PCDHB3	NS	8.19E-01	9.39E-03	8.83E-01	NS
NUMA1	NS	8.20E-01	9.37E-03	8.84E-01	NS
RGS12	NS	8.20E-01	-0.00936	8.84E-01	NS

HIBADH	NS	8.20E-01	9.35E-03	8.84E-01	NS
LRRC8D	NS	8.20E-01	-0.00935	8.84E-01	NS
ZNF468	NS	8.20E-01	-0.00933	8.84E-01	NS
CREB3	NS	8.21E-01	9.31E-03	8.85E-01	NS
GDF7	NS	8.21E-01	-0.00931	8.85E-01	NS
CTRC	NS	8.21E-01	-0.00931	8.85E-01	NS
PRKCE	NS	8.21E-01	-0.00931	8.85E-01	NS
MMP1	NS	8.21E-01	-0.00932	8.85E-01	NS
SDHC	NS	8.21E-01	-0.00928	8.85E-01	NS
TREX2	NS	8.21E-01	-0.00927	8.85E-01	NS
BECN2	NS	8.21E-01	9.27E-03	8.85E-01	NS
KRTAP4-2	NS	8.22E-01	9.26E-03	8.85E-01	NS
CPXCR1	NS	8.22E-01	9.25E-03	8.85E-01	NS
FRG1	NS	8.22E-01	9.25E-03	8.85E-01	NS
HEXIM2	NS	8.22E-01	9.25E-03	8.85E-01	NS
CBLB	NS	8.22E-01	9.24E-03	8.85E-01	NS
ASCL2	NS	8.22E-01	9.24E-03	8.85E-01	NS
PABPC4	NS	8.22E-01	-0.00924	8.85E-01	NS
LARP4B	NS	8.22E-01	-0.00924	8.85E-01	NS
CDC5L	NS	8.22E-01	9.22E-03	8.86E-01	NS
MAPKAP1	NS	8.23E-01	-0.00921	8.86E-01	NS
USP17L8	NS	8.23E-01	-0.00921	8.86E-01	NS
ARMH3	NS	8.23E-01	-0.0092	8.86E-01	NS
IKBKE	NS	8.23E-01	-0.0092	8.86E-01	NS
CHDH	NS	8.23E-01	-0.0092	8.86E-01	NS
DIS3L2	NS	8.23E-01	-0.00919	8.86E-01	NS
DDX3X	NS	8.23E-01	-0.00919	8.86E-01	NS
WASHC5	NS	8.23E-01	9.19E-03	8.86E-01	NS
ERMARD	NS	8.23E-01	-0.00918	8.86E-01	NS
SH3BP5	NS	8.23E-01	-0.00918	8.86E-01	NS
GNG12	NS	8.23E-01	-0.00917	8.86E-01	NS
AGO4	NS	8.23E-01	-0.00916	8.86E-01	NS
PRIM1	NS	8.24E-01	-0.00914	8.87E-01	NS
G6PD	NS	8.24E-01	-0.00912	8.87E-01	NS
SMIM41	NS	8.24E-01	-0.00912	8.87E-01	NS
TENM4	NS	8.25E-01	9.11E-03	8.87E-01	NS
TFF2	NS	8.25E-01	9.10E-03	8.87E-01	NS

TMEM171	NS	8.25E-01	9.10E-03	8.87E-01	NS
OOSP1	NS	8.25E-01	9.10E-03	8.87E-01	NS
NDUFAB1	NS	8.25E-01	-0.00908	8.88E-01	NS
SEPTIN9	NS	8.25E-01	9.06E-03	8.88E-01	NS
CBY3	NS	8.25E-01	-0.00906	8.88E-01	NS
CD164	NS	8.26E-01	9.06E-03	8.88E-01	NS
LILRB3	NS	8.26E-01	-0.00905	8.88E-01	NS
DENND10	NS	8.26E-01	-0.00904	8.88E-01	NS
IGSF21	NS	8.26E-01	-0.00904	8.88E-01	NS
GPR160	NS	8.26E-01	-0.00903	8.88E-01	NS
ZNF285	NS	8.26E-01	-0.00903	8.88E-01	NS
KCTD5	NS	8.26E-01	9.01E-03	8.89E-01	NS
RNF170	NS	8.27E-01	8.99E-03	8.89E-01	NS
KRT78	NS	8.27E-01	8.98E-03	8.89E-01	NS
ACTN3	NS	8.27E-01	-0.00897	8.89E-01	NS
OR10A3	NS	8.27E-01	-0.00896	8.89E-01	NS
RRM2B	NS	8.28E-01	-0.00895	8.89E-01	NS
GHITM	NS	8.28E-01	-0.00895	8.89E-01	NS
TMEM38A	NS	8.28E-01	8.94E-03	8.89E-01	NS
KRT85	NS	8.28E-01	8.94E-03	8.89E-01	NS
TXNL4B	NS	8.28E-01	8.93E-03	8.90E-01	NS
IHO1	NS	8.28E-01	-0.00892	8.90E-01	NS
ARHGAP21	NS	8.28E-01	-0.00892	8.90E-01	NS
CAPNS1	NS	8.28E-01	-0.00892	8.90E-01	NS
SCLY	NS	8.28E-01	-0.00891	8.90E-01	NS
C1GALT1C1L	NS	8.29E-01	8.89E-03	8.90E-01	NS
FXYD6-FXYD2	NS	8.29E-01	8.87E-03	8.91E-01	NS
COL7A1	NS	8.29E-01	8.86E-03	8.91E-01	NS
SEMA5B	NS	8.29E-01	-0.00885	8.91E-01	NS
OSBPL2	NS	8.30E-01	8.82E-03	8.91E-01	NS
BLOC1S3	NS	8.30E-01	-0.00883	8.91E-01	NS
BAP1	NS	8.30E-01	-0.00882	8.91E-01	NS
TOR1AIP2	NS	8.30E-01	8.81E-03	8.92E-01	NS
DGKG	NS	8.30E-01	8.80E-03	8.92E-01	NS
ASCC2	NS	8.31E-01	-0.00879	8.92E-01	NS
TNKS1BP1	NS	8.31E-01	-0.00879	8.92E-01	NS
CFAP418	NS	8.31E-01	8.79E-03	8.92E-01	NS

PITX2	NS	8.30E-01	-0.0088	8.92E-01	NS
UBE2V1	NS	8.30E-01	-0.0088	8.92E-01	NS
KMT2D	NS	8.31E-01	-0.00878	8.92E-01	NS
RFNG	NS	8.31E-01	8.78E-03	8.92E-01	NS
H2BC13	NS	8.31E-01	-0.00878	8.92E-01	NS
WNT5B	NS	8.31E-01	-0.00876	8.92E-01	NS
CTH	NS	8.31E-01	8.75E-03	8.92E-01	NS
TRPM2	NS	8.31E-01	8.75E-03	8.92E-01	NS
FOXO1	NS	8.31E-01	-0.00875	8.92E-01	NS
RCC1	NS	8.31E-01	-0.00876	8.92E-01	NS
OR5P3	NS	8.31E-01	-0.00876	8.92E-01	NS
ZNF563	NS	8.31E-01	-0.00877	8.92E-01	NS
CCL5	NS	8.31E-01	-0.00875	8.92E-01	NS
RNF222	NS	8.31E-01	8.75E-03	8.92E-01	NS
SYNGR2	NS	8.31E-01	-0.00874	8.92E-01	NS
PUF60	NS	8.32E-01	-0.00873	8.92E-01	NS
BICDL2	NS	8.32E-01	-0.00873	8.92E-01	NS
MOCOS	NS	8.32E-01	8.72E-03	8.92E-01	NS
RPLP2	NS	8.32E-01	-0.00872	8.92E-01	NS
TRIM25	NS	8.32E-01	8.71E-03	8.92E-01	NS
SDAD1	NS	8.33E-01	-0.00868	8.93E-01	NS
PSME4	NS	8.33E-01	-0.00867	8.93E-01	NS
TMEM132D	NS	8.33E-01	-0.00867	8.93E-01	NS
SLC16A14	NS	8.33E-01	8.65E-03	8.93E-01	NS
NPIPB12	NS	8.33E-01	8.66E-03	8.93E-01	NS
RAB25	NS	8.33E-01	-0.00864	8.93E-01	NS
TF	NS	8.34E-01	-0.00864	8.93E-01	NS
LLGL1	NS	8.33E-01	-0.00864	8.93E-01	NS
FGF23	NS	8.34E-01	-0.00862	8.94E-01	NS
HNRNPA0	NS	8.34E-01	-0.00862	8.94E-01	NS
APLNR	NS	8.34E-01	-0.00862	8.94E-01	NS
CLNS1A	NS	8.34E-01	8.60E-03	8.94E-01	NS
MPP2	NS	8.34E-01	-0.00859	8.94E-01	NS
ST6GALNAC6	NS	8.34E-01	8.59E-03	8.94E-01	NS
ADGRG7	NS	8.35E-01	8.58E-03	8.94E-01	NS
ACD	NS	8.35E-01	8.57E-03	8.94E-01	NS
RPS6KA1	NS	8.35E-01	-0.00857	8.94E-01	NS

RPL23	NS	8.35E-01	-0.00857	8.94E-01	NS
NDUFS8	NS	8.35E-01	-0.00856	8.94E-01	NS
FMR1NB	NS	8.35E-01	8.55E-03	8.95E-01	NS
ASAH2B	NS	8.35E-01	8.54E-03	8.95E-01	NS
ZC3H3	NS	8.35E-01	8.54E-03	8.95E-01	NS
FOSL2	NS	8.36E-01	-0.00853	8.95E-01	NS
ZNF461	NS	8.36E-01	8.52E-03	8.95E-01	NS
PDAP1	NS	8.36E-01	-0.00849	8.96E-01	NS
LYPLA1	NS	8.36E-01	8.49E-03	8.96E-01	NS
EXT1	NS	8.36E-01	-0.00849	8.96E-01	NS
FOXD2	NS	8.37E-01	-0.00847	8.96E-01	NS
NAA60	NS	8.37E-01	-0.00847	8.96E-01	NS
SRRM1	NS	8.37E-01	8.47E-03	8.96E-01	NS
OR8H2	NS	8.37E-01	-0.00846	8.96E-01	NS
RBBP6	NS	8.37E-01	8.45E-03	8.96E-01	NS
POU4F2	NS	8.37E-01	8.44E-03	8.96E-01	NS
TTPAL	NS	8.37E-01	-0.00843	8.96E-01	NS
OSR2	NS	8.37E-01	8.43E-03	8.96E-01	NS
DEFB134	NS	8.37E-01	-0.00843	8.96E-01	NS
TBC1D24	NS	8.38E-01	-0.00842	8.96E-01	NS
ARHGEF4	NS	8.38E-01	8.41E-03	8.96E-01	NS
NEUROD6	NS	8.38E-01	-0.00841	8.96E-01	NS
RBM48	NS	8.38E-01	8.41E-03	8.97E-01	NS
NUP88	NS	8.38E-01	-0.00839	8.97E-01	NS
UCHL1	NS	8.38E-01	-0.00839	8.97E-01	NS
SPEN	NS	8.39E-01	-0.00837	8.97E-01	NS
MCM6	NS	8.39E-01	-0.00837	8.97E-01	NS
A4GALT	NS	8.39E-01	8.37E-03	8.97E-01	NS
ATG7	NS	8.39E-01	-0.00837	8.97E-01	NS
TXNDC8	NS	8.39E-01	-0.00837	8.97E-01	NS
NTSR1	NS	8.39E-01	-0.00836	8.97E-01	NS
UBE4A	NS	8.39E-01	-0.00836	8.97E-01	NS
CHMP4A	NS	8.39E-01	8.36E-03	8.97E-01	NS
KIF4A	NS	8.39E-01	-0.00835	8.97E-01	NS
RFPL1	NS	8.39E-01	8.34E-03	8.97E-01	NS
ZBTB18	NS	8.39E-01	8.33E-03	8.97E-01	NS
TP53TG3F	NS	8.39E-01	-0.00833	8.97E-01	NS

CCR2	NS	8.39E-01	-0.00832	8.97E-01	NS
ZNF781	NS	8.40E-01	8.32E-03	8.98E-01	NS
KPNA3	NS	8.40E-01	-0.00829	8.98E-01	NS
DMTF1	NS	8.40E-01	-0.0083	8.98E-01	NS
OTOP1	NS	8.40E-01	-0.00829	8.98E-01	NS
DNAAF8	NS	8.40E-01	-0.0083	8.98E-01	NS
WDR97	NS	8.40E-01	8.29E-03	8.98E-01	NS
ANKRD52	NS	8.40E-01	-0.00828	8.98E-01	NS
CD82	NS	8.41E-01	8.27E-03	8.98E-01	NS
HYKK	NS	8.41E-01	-0.00826	8.98E-01	NS
RUNX2	NS	8.41E-01	-0.00826	8.98E-01	NS
BTNL8	NS	8.41E-01	-0.00824	8.98E-01	NS
SOX14	NS	8.41E-01	8.25E-03	8.98E-01	NS
USP47	NS	8.41E-01	-0.00825	8.98E-01	NS
MSH5-SAPCD1	NS	8.41E-01	8.24E-03	8.98E-01	NS
ST6GALNAC1	NS	8.41E-01	-0.00823	8.98E-01	NS
SUPT16H	NS	8.41E-01	8.23E-03	8.98E-01	NS
BCL7B	NS	8.41E-01	-0.00823	8.98E-01	NS
MICAL1	NS	8.41E-01	-0.00823	8.98E-01	NS
FAM104B	NS	8.41E-01	-0.00823	8.98E-01	NS
B4GALT5	NS	8.41E-01	-0.00822	8.99E-01	NS
FMO3	NS	8.41E-01	8.22E-03	8.99E-01	NS
OR1L3	NS	8.41E-01	-0.00822	8.99E-01	NS
VASP	NS	8.42E-01	8.21E-03	8.99E-01	NS
SMC1A	NS	8.42E-01	-0.00818	8.99E-01	NS
MLEC	NS	8.42E-01	-0.00819	8.99E-01	NS
PCDHB6	NS	8.42E-01	-0.00817	8.99E-01	NS
FRAT1	NS	8.42E-01	-0.00818	8.99E-01	NS
RAB8A	NS	8.42E-01	-0.00818	8.99E-01	NS
SERPINA1	NS	8.42E-01	-0.00817	8.99E-01	NS
PPP1R11	NS	8.42E-01	8.17E-03	8.99E-01	NS
PPP1R36	NS	8.42E-01	8.16E-03	8.99E-01	NS
ATF6B	NS	8.42E-01	-0.00817	8.99E-01	NS
CASQ2	NS	8.43E-01	-0.00815	8.99E-01	NS
MEGF9	NS	8.43E-01	8.14E-03	8.99E-01	NS
NME3	NS	8.43E-01	-0.00814	8.99E-01	NS
ANO3	NS	8.43E-01	8.14E-03	8.99E-01	NS

GLIPR2	NS	8.43E-01	-0.00812	9.00E-01	NS
PACSIN1	NS	8.43E-01	-0.00812	9.00E-01	NS
TMEM151B	NS	8.43E-01	8.12E-03	9.00E-01	NS
CLEC4C	NS	8.43E-01	-0.00812	9.00E-01	NS
STX1A	NS	8.44E-01	-0.00811	9.00E-01	NS
PRKAG1	NS	8.44E-01	8.11E-03	9.00E-01	NS
TMEM175	NS	8.44E-01	-0.00809	9.00E-01	NS
VPS52	NS	8.44E-01	-0.00807	9.00E-01	NS
ERAS	NS	8.44E-01	-0.00806	9.00E-01	NS
SMIM19	NS	8.45E-01	8.06E-03	9.01E-01	NS
OR4E2	NS	8.45E-01	8.05E-03	9.01E-01	NS
S1PR3	NS	8.45E-01	-0.00804	9.01E-01	NS
TMC2	NS	8.45E-01	8.04E-03	9.01E-01	NS
FAM166C	NS	8.45E-01	8.04E-03	9.01E-01	NS
CXorf49B	NS	8.45E-01	-0.00803	9.01E-01	NS
ATCAY	NS	8.45E-01	-0.00803	9.01E-01	NS
LOXL1	NS	8.45E-01	-0.00802	9.01E-01	NS
STX6	NS	8.46E-01	-0.008	9.01E-01	NS
SP140L	NS	8.46E-01	-0.008	9.01E-01	NS
CRYBA2	NS	8.46E-01	7.99E-03	9.01E-01	NS
SLC7A6	NS	8.46E-01	7.98E-03	9.01E-01	NS
SWI5	NS	8.46E-01	7.98E-03	9.01E-01	NS
UBXN4	NS	8.46E-01	-0.00796	9.02E-01	NS
IRAK1	NS	8.46E-01	-0.00796	9.02E-01	NS
CAPN11	NS	8.46E-01	7.95E-03	9.02E-01	NS
NEK3	NS	8.47E-01	7.94E-03	9.02E-01	NS
RHEB	NS	8.47E-01	-0.00793	9.02E-01	NS
ZNF280D	NS	8.47E-01	7.92E-03	9.02E-01	NS
WBP1	NS	8.47E-01	-0.00792	9.02E-01	NS
ARSF	NS	8.47E-01	7.91E-03	9.02E-01	NS
SEM1	NS	8.47E-01	-0.00791	9.02E-01	NS
FBXO33	NS	8.47E-01	-0.00791	9.02E-01	NS
VAV2	NS	8.47E-01	7.90E-03	9.02E-01	NS
MGAT4D	NS	8.48E-01	-0.00789	9.03E-01	NS
SGCA	NS	8.48E-01	-0.00786	9.03E-01	NS
LRRC75A	NS	8.48E-01	-0.00786	9.03E-01	NS
SKOR2	NS	8.49E-01	7.85E-03	9.03E-01	NS

SLC26A11	NS	8.49E-01	7.83E-03	9.04E-01	NS
UAP1L1	NS	8.49E-01	7.82E-03	9.04E-01	NS
DHX15	NS	8.49E-01	-0.0078	9.04E-01	NS
AKAP13	NS	8.50E-01	-0.00778	9.05E-01	NS
RAD54L2	NS	8.50E-01	-0.00778	9.05E-01	NS
KPNA6	NS	8.50E-01	-0.00777	9.05E-01	NS
MSANTD4	NS	8.50E-01	-0.00777	9.05E-01	NS
CDC20	NS	8.51E-01	-0.00774	9.05E-01	NS
THOC2	NS	8.50E-01	-0.00775	9.05E-01	NS
NPAT	NS	8.50E-01	7.75E-03	9.05E-01	NS
TMEM192	NS	8.51E-01	-0.00774	9.05E-01	NS
XXYLT1	NS	8.50E-01	-0.00775	9.05E-01	NS
ADARB2	NS	8.51E-01	-0.00774	9.05E-01	NS
CNR2	NS	8.50E-01	-0.00776	9.05E-01	NS
MFRP	NS	8.50E-01	-0.00775	9.05E-01	NS
OGN	NS	8.51E-01	7.72E-03	9.05E-01	NS
PCDH11X	NS	8.51E-01	7.71E-03	9.05E-01	NS
BANF1	NS	8.51E-01	-0.0077	9.05E-01	NS
EPN2	NS	8.51E-01	7.70E-03	9.05E-01	NS
TIPIN	NS	8.52E-01	7.69E-03	9.06E-01	NS
AFMID	NS	8.52E-01	-0.00769	9.06E-01	NS
REPS2	NS	8.52E-01	7.66E-03	9.06E-01	NS
YJU2B	NS	8.52E-01	7.65E-03	9.06E-01	NS
MYH3	NS	8.52E-01	-0.00765	9.06E-01	NS
ARMC8	NS	8.52E-01	7.65E-03	9.06E-01	NS
CYFIP1	NS	8.52E-01	7.65E-03	9.06E-01	NS
GSG1L	NS	8.53E-01	7.64E-03	9.06E-01	NS
RPLP0	NS	8.53E-01	-0.00763	9.06E-01	NS
MYLK	NS	8.53E-01	-0.00762	9.06E-01	NS
ACTR8	NS	8.53E-01	-0.00762	9.06E-01	NS
GHRH	NS	8.53E-01	7.61E-03	9.06E-01	NS
XAGE3	NS	8.53E-01	-0.00762	9.06E-01	NS
METTL7A	NS	8.54E-01	-0.00757	9.07E-01	NS
SLC39A11	NS	8.54E-01	-0.00756	9.07E-01	NS
ST6GALNAC3	NS	8.54E-01	7.57E-03	9.07E-01	NS
MIA-RAB4B	NS	8.54E-01	-0.00756	9.07E-01	NS
COX10	NS	8.54E-01	7.55E-03	9.08E-01	NS

FGF6	NS	8.54E-01	7.54E-03	9.08E-01	NS
USP49	NS	8.55E-01	7.53E-03	9.08E-01	NS
MKRN2	NS	8.55E-01	7.53E-03	9.08E-01	NS
ZMYND11	NS	8.55E-01	-0.00752	9.08E-01	NS
PRKD3	NS	8.55E-01	7.51E-03	9.08E-01	NS
RSU1	NS	8.55E-01	7.50E-03	9.08E-01	NS
BPGM	NS	8.55E-01	-0.0075	9.08E-01	NS
GGTLC3	NS	8.55E-01	-0.0075	9.08E-01	NS
FKBP7	NS	8.55E-01	-0.00749	9.08E-01	NS
HRH4	NS	8.55E-01	-0.00749	9.08E-01	NS
PDCD6IP	NS	8.56E-01	-0.00747	9.09E-01	NS
GPR161	NS	8.56E-01	7.47E-03	9.09E-01	NS
FAM187A	NS	8.56E-01	-0.00747	9.09E-01	NS
SPON2	NS	8.56E-01	7.45E-03	9.09E-01	NS
AMY2A	NS	8.56E-01	7.45E-03	9.09E-01	NS
FMNL2	NS	8.56E-01	7.44E-03	9.09E-01	NS
OR6K6	NS	8.56E-01	-0.00744	9.09E-01	NS
BFSP1	NS	8.56E-01	7.43E-03	9.09E-01	NS
KRT71	NS	8.57E-01	-0.00743	9.09E-01	NS
ACTR1A	NS	8.57E-01	-0.00741	9.09E-01	NS
MSRB3	NS	8.57E-01	7.40E-03	9.09E-01	NS
SYNPO2	NS	8.57E-01	7.39E-03	9.10E-01	NS
FAM219B	NS	8.57E-01	7.39E-03	9.10E-01	NS
GLIPR1	NS	8.57E-01	-0.00739	9.10E-01	NS
TTLL7	NS	8.57E-01	-0.00738	9.10E-01	NS
SYS1	NS	8.57E-01	7.38E-03	9.10E-01	NS
PSAT1	NS	8.58E-01	-0.00737	9.10E-01	NS
CRACDL	NS	8.58E-01	-0.00737	9.10E-01	NS
CARD8	NS	8.58E-01	7.36E-03	9.10E-01	NS
PHF11	NS	8.58E-01	7.36E-03	9.10E-01	NS
SEC24B	NS	8.58E-01	7.36E-03	9.10E-01	NS
PC	NS	8.58E-01	7.36E-03	9.10E-01	NS
CYP2A7	NS	8.58E-01	-0.00736	9.10E-01	NS
SMPD1	NS	8.58E-01	7.34E-03	9.10E-01	NS
BCAS2	NS	8.58E-01	-0.00733	9.10E-01	NS
GATAD2B	NS	8.59E-01	7.32E-03	9.10E-01	NS
SLC16A10	NS	8.59E-01	-0.00731	9.10E-01	NS

SLC3A2	NS	8.59E-01	7.31E-03	9.10E-01	NS
PGPEP1	NS	8.59E-01	-0.00731	9.10E-01	NS
CORO1A	NS	8.59E-01	7.29E-03	9.11E-01	NS
OPRM1	NS	8.59E-01	7.29E-03	9.11E-01	NS
NIFK	NS	8.59E-01	7.28E-03	9.11E-01	NS
SERPINB6	NS	8.59E-01	-0.00728	9.11E-01	NS
ZBTB39	NS	8.59E-01	7.28E-03	9.11E-01	NS
FAM47B	NS	8.59E-01	7.28E-03	9.11E-01	NS
CUEDC1	NS	8.59E-01	-0.00727	9.11E-01	NS
PPP1R9B	NS	8.60E-01	7.27E-03	9.11E-01	NS
PADI4	NS	8.60E-01	7.27E-03	9.11E-01	NS
NEK5	NS	8.60E-01	-0.00726	9.11E-01	NS
CYBRD1	NS	8.60E-01	-0.00726	9.11E-01	NS
PBXIP1	NS	8.60E-01	7.26E-03	9.11E-01	NS
CIPC	NS	8.60E-01	-0.00725	9.11E-01	NS
SYNPO	NS	8.60E-01	-0.00725	9.11E-01	NS
PIN1	NS	8.60E-01	7.23E-03	9.11E-01	NS
JPH2	NS	8.60E-01	-0.00724	9.11E-01	NS
APBB1	NS	8.60E-01	7.24E-03	9.11E-01	NS
PGBD5	NS	8.60E-01	7.24E-03	9.11E-01	NS
CXXC5	NS	8.60E-01	7.23E-03	9.11E-01	NS
ST6GAL1	NS	8.60E-01	7.23E-03	9.11E-01	NS
SNX29	NS	8.61E-01	7.21E-03	9.11E-01	NS
NANOS2	NS	8.61E-01	7.21E-03	9.11E-01	NS
PDE6A	NS	8.61E-01	7.21E-03	9.11E-01	NS
MTFR1	NS	8.61E-01	7.20E-03	9.11E-01	NS
PRPF3	NS	8.61E-01	-0.0072	9.11E-01	NS
AQP10	NS	8.61E-01	-0.00718	9.12E-01	NS
SVBP	NS	8.61E-01	7.18E-03	9.12E-01	NS
FOXRED2	NS	8.61E-01	-0.00718	9.12E-01	NS
EAF1	NS	8.61E-01	7.17E-03	9.12E-01	NS
PRPF4B	NS	8.62E-01	7.16E-03	9.12E-01	NS
TIMM10B	NS	8.62E-01	-0.00715	9.12E-01	NS
HAUS7	NS	8.62E-01	-0.00715	9.12E-01	NS
GPR37L1	NS	8.62E-01	7.14E-03	9.12E-01	NS
STRADB	NS	8.62E-01	7.13E-03	9.12E-01	NS
OR2V2	NS	8.62E-01	7.13E-03	9.12E-01	NS

FAM81A	NS	8.62E-01	7.12E-03	9.12E-01	NS
RPN1	NS	8.62E-01	-0.00712	9.12E-01	NS
LILRB1	NS	8.63E-01	7.10E-03	9.13E-01	NS
CLEC3A	NS	8.63E-01	-0.0071	9.13E-01	NS
FSIP2	NS	8.63E-01	-0.0071	9.13E-01	NS
TRIP13	NS	8.64E-01	7.05E-03	9.14E-01	NS
PAF1	NS	8.64E-01	7.03E-03	9.14E-01	NS
TRPM5	NS	8.64E-01	7.02E-03	9.14E-01	NS
RHOC	NS	8.64E-01	7.02E-03	9.14E-01	NS
KNOP1	NS	8.65E-01	-0.00699	9.14E-01	NS
TTK	NS	8.65E-01	6.99E-03	9.14E-01	NS
ZSWIM4	NS	8.65E-01	7.00E-03	9.14E-01	NS
SECISBP2L	NS	8.65E-01	7.00E-03	9.14E-01	NS
IFNA14	NS	8.65E-01	-0.00699	9.14E-01	NS
MAP3K8	NS	8.65E-01	-0.00699	9.14E-01	NS
BLMH	NS	8.65E-01	6.98E-03	9.14E-01	NS
GAPDH	NS	8.65E-01	6.98E-03	9.14E-01	NS
ELOVL6	NS	8.65E-01	6.98E-03	9.14E-01	NS
IDH3G	NS	8.65E-01	-0.00697	9.15E-01	NS
ATAD2	NS	8.65E-01	6.97E-03	9.15E-01	NS
DDTL	NS	8.65E-01	6.96E-03	9.15E-01	NS
CNOT9	NS	8.66E-01	6.95E-03	9.15E-01	NS
NINJ2	NS	8.66E-01	6.95E-03	9.15E-01	NS
H2BC11	NS	8.66E-01	6.94E-03	9.15E-01	NS
EIF4E	NS	8.66E-01	6.94E-03	9.15E-01	NS
NAIP	NS	8.66E-01	6.94E-03	9.15E-01	NS
CLK2	NS	8.66E-01	-0.00693	9.15E-01	NS
DLG3	NS	8.66E-01	6.91E-03	9.15E-01	NS
IER3	NS	8.66E-01	-0.00691	9.15E-01	NS
PDGFA	NS	8.66E-01	6.91E-03	9.15E-01	NS
VSIG10L2	NS	8.67E-01	6.90E-03	9.15E-01	NS
CCT4	NS	8.67E-01	-0.00688	9.16E-01	NS
NGRN	NS	8.67E-01	6.87E-03	9.16E-01	NS
NANS	NS	8.67E-01	6.86E-03	9.16E-01	NS
BAIAP2L1	NS	8.68E-01	6.85E-03	9.16E-01	NS
CCNF	NS	8.68E-01	-0.00685	9.16E-01	NS
HERPUD2	NS	8.68E-01	6.84E-03	9.16E-01	NS

UCP1	NS	8.68E-01	6.84E-03	9.16E-01	NS
CAP2	NS	8.68E-01	6.83E-03	9.16E-01	NS
FBXO15	NS	8.68E-01	6.83E-03	9.16E-01	NS
RAB11A	NS	8.68E-01	-0.00682	9.17E-01	NS
TMOD3	NS	8.69E-01	6.79E-03	9.17E-01	NS
GALNTL5	NS	8.69E-01	6.77E-03	9.17E-01	NS
SLC39A4	NS	8.69E-01	6.77E-03	9.17E-01	NS
ELAVL2	NS	8.69E-01	-0.00676	9.17E-01	NS
ELP5	NS	8.69E-01	-0.00675	9.18E-01	NS
ZNF648	NS	8.70E-01	6.75E-03	9.18E-01	NS
LEFTY1	NS	8.70E-01	-0.00674	9.18E-01	NS
TFAP4	NS	8.70E-01	6.72E-03	9.18E-01	NS
MAP4	NS	8.70E-01	6.71E-03	9.18E-01	NS
DNASE1L1	NS	8.71E-01	6.69E-03	9.19E-01	NS
COL4A1	NS	8.71E-01	6.67E-03	9.19E-01	NS
NUP93	NS	8.71E-01	6.67E-03	9.19E-01	NS
HEBP2	NS	8.71E-01	6.65E-03	9.19E-01	NS
CREM	NS	8.71E-01	6.65E-03	9.19E-01	NS
SUSD5	NS	8.71E-01	-0.00665	9.19E-01	NS
ZBTB43	NS	8.72E-01	6.64E-03	9.19E-01	NS
PYDC2	NS	8.72E-01	6.64E-03	9.19E-01	NS
C11orf96	NS	8.72E-01	6.64E-03	9.19E-01	NS
USP32	NS	8.72E-01	-0.00663	9.19E-01	NS
ASB8	NS	8.72E-01	6.63E-03	9.19E-01	NS
GALNT9	NS	8.72E-01	6.62E-03	9.19E-01	NS
ANO7	NS	8.72E-01	6.61E-03	9.20E-01	NS
LGALS12	NS	8.72E-01	-0.0066	9.20E-01	NS
EMC3	NS	8.72E-01	-0.0066	9.20E-01	NS
SNRPD2	NS	8.73E-01	-0.00658	9.20E-01	NS
ISM2	NS	8.73E-01	-0.00658	9.20E-01	NS
WDR27	NS	8.73E-01	-0.00657	9.20E-01	NS
PPP1R3C	NS	8.73E-01	-0.00656	9.20E-01	NS
FKBP3	NS	8.73E-01	-0.00656	9.20E-01	NS
OR2AG2	NS	8.73E-01	6.55E-03	9.20E-01	NS
ZNF534	NS	8.73E-01	6.55E-03	9.20E-01	NS
FOXN2	NS	8.74E-01	-0.00652	9.21E-01	NS
ENTPD4	NS	8.74E-01	6.51E-03	9.21E-01	NS

HAT1	NS	8.74E-01	6.50E-03	9.21E-01	NS
TUT4	NS	8.75E-01	-0.00649	9.21E-01	NS
VPS11	NS	8.75E-01	-0.00648	9.22E-01	NS
RHOT2	NS	8.75E-01	6.47E-03	9.22E-01	NS
TMEM119	NS	8.75E-01	-0.00647	9.22E-01	NS
TRIM55	NS	8.75E-01	-0.00646	9.22E-01	NS
ESRP1	NS	8.75E-01	6.45E-03	9.22E-01	NS
PUS7L	NS	8.75E-01	6.45E-03	9.22E-01	NS
BRD3OS	NS	8.75E-01	6.46E-03	9.22E-01	NS
PKP2	NS	8.75E-01	6.44E-03	9.22E-01	NS
CNTRL	NS	8.76E-01	6.44E-03	9.22E-01	NS
RPUSD3	NS	8.76E-01	-0.00643	9.22E-01	NS
BCO2	NS	8.76E-01	6.44E-03	9.22E-01	NS
HSBP1	NS	8.76E-01	-0.00644	9.22E-01	NS
GYPB	NS	8.75E-01	6.44E-03	9.22E-01	NS
PSTPIP1	NS	8.76E-01	6.42E-03	9.22E-01	NS
UPK1B	NS	8.76E-01	-0.0064	9.22E-01	NS
MROH7	NS	8.76E-01	-0.0064	9.22E-01	NS
SELENOO	NS	8.76E-01	6.40E-03	9.22E-01	NS
ZPBP2	NS	8.76E-01	6.39E-03	9.22E-01	NS
AAMP	NS	8.76E-01	6.39E-03	9.22E-01	NS
ARHGAP26	NS	8.76E-01	6.39E-03	9.22E-01	NS
NDUFS3	NS	8.77E-01	6.38E-03	9.22E-01	NS
NUFIP2	NS	8.77E-01	6.37E-03	9.23E-01	NS
STAT2	NS	8.77E-01	6.36E-03	9.23E-01	NS
NAA30	NS	8.77E-01	-0.00635	9.23E-01	NS
FGD3	NS	8.78E-01	6.33E-03	9.23E-01	NS
CCDC167	NS	8.78E-01	-0.00633	9.23E-01	NS
POU3F4	NS	8.78E-01	6.32E-03	9.23E-01	NS
LATS2	NS	8.78E-01	6.32E-03	9.23E-01	NS
CFAP221	NS	8.78E-01	6.30E-03	9.24E-01	NS
UBAP1L	NS	8.78E-01	-0.00629	9.24E-01	NS
H3C13	NS	8.78E-01	6.29E-03	9.24E-01	NS
OAS2	NS	8.78E-01	-0.00628	9.24E-01	NS
OR2A25	NS	8.78E-01	-0.00629	9.24E-01	NS
KAZN	NS	8.79E-01	6.27E-03	9.24E-01	NS
JKAMP	NS	8.79E-01	6.27E-03	9.24E-01	NS

NPIPA2	NS	8.79E-01	6.26E-03	9.24E-01	NS
PLEKHA1	NS	8.79E-01	6.25E-03	9.24E-01	NS
CCDC183	NS	8.79E-01	-0.00625	9.24E-01	NS
CST4	NS	8.79E-01	6.23E-03	9.24E-01	NS
DVL1	NS	8.79E-01	-0.00623	9.24E-01	NS
OR5AK2	NS	8.79E-01	-0.00623	9.24E-01	NS
POLR1D	NS	8.80E-01	-0.00622	9.25E-01	NS
CCDC201	NS	8.80E-01	6.22E-03	9.25E-01	NS
XAGE2	NS	8.80E-01	-0.00621	9.25E-01	NS
TMEM198	NS	8.80E-01	6.21E-03	9.25E-01	NS
APOA2	NS	8.80E-01	6.20E-03	9.25E-01	NS
YOD1	NS	8.80E-01	6.19E-03	9.25E-01	NS
DTNA	NS	8.80E-01	6.19E-03	9.25E-01	NS
CCDC87	NS	8.80E-01	6.19E-03	9.25E-01	NS
KCNK18	NS	8.80E-01	-0.00618	9.25E-01	NS
SOX12	NS	8.81E-01	-0.00618	9.25E-01	NS
PEG10	NS	8.81E-01	6.17E-03	9.25E-01	NS
SERP1	NS	8.81E-01	-0.00616	9.25E-01	NS
SLC22A24	NS	8.81E-01	6.16E-03	9.25E-01	NS
GRM7	NS	8.81E-01	-0.00615	9.25E-01	NS
GABRD	NS	8.81E-01	6.14E-03	9.25E-01	NS
RPS3	NS	8.81E-01	-0.00613	9.26E-01	NS
GRK5	NS	8.81E-01	-0.00613	9.26E-01	NS
NLRP2	NS	8.82E-01	6.11E-03	9.26E-01	NS
MRPS16	NS	8.82E-01	-0.0061	9.26E-01	NS
ZNF836	NS	8.82E-01	6.10E-03	9.26E-01	NS
CCL20	NS	8.82E-01	-0.00609	9.26E-01	NS
CDH15	NS	8.82E-01	-0.00608	9.26E-01	NS
ARTN	NS	8.82E-01	6.08E-03	9.26E-01	NS
OR10R2	NS	8.83E-01	-0.00606	9.27E-01	NS
PLIN3	NS	8.83E-01	6.05E-03	9.27E-01	NS
USP25	NS	8.83E-01	-0.00605	9.27E-01	NS
GOLGA6L7	NS	8.83E-01	6.03E-03	9.27E-01	NS
KLHL18	NS	8.83E-01	6.03E-03	9.27E-01	NS
IRF2BPL	NS	8.84E-01	-0.00602	9.27E-01	NS
STAT5A	NS	8.83E-01	-0.00602	9.27E-01	NS
AKR1A1	NS	8.84E-01	-0.00601	9.27E-01	NS

LDLR	NS	8.84E-01	5.99E-03	9.27E-01	NS
ZNF654	NS	8.84E-01	-0.006	9.27E-01	NS
NACA	NS	8.84E-01	5.99E-03	9.27E-01	NS
MAGEA2	NS	8.84E-01	5.99E-03	9.27E-01	NS
P3H2	NS	8.84E-01	5.99E-03	9.27E-01	NS
GGA1	NS	8.84E-01	-0.00598	9.28E-01	NS
MYBPH	NS	8.84E-01	5.97E-03	9.28E-01	NS
MYG1	NS	8.85E-01	-0.00595	9.28E-01	NS
FOXO3B	NS	8.85E-01	-0.00595	9.28E-01	NS
CTDSPL2	NS	8.85E-01	-0.00593	9.28E-01	NS
SMIM4	NS	8.85E-01	5.92E-03	9.28E-01	NS
DEFB130A	NS	8.86E-01	-0.00591	9.29E-01	NS
ITGB3BP	NS	8.86E-01	5.90E-03	9.29E-01	NS
SULT1B1	NS	8.86E-01	-0.00589	9.29E-01	NS
WIZ	NS	8.86E-01	5.88E-03	9.29E-01	NS
TUBB8	NS	8.86E-01	5.88E-03	9.29E-01	NS
OR5AR1	NS	8.86E-01	5.87E-03	9.29E-01	NS
BAZ1A	NS	8.87E-01	5.86E-03	9.29E-01	NS
NDUFC2	NS	8.87E-01	5.85E-03	9.29E-01	NS
TXLNA	NS	8.87E-01	5.83E-03	9.30E-01	NS
GOLGA8A	NS	8.87E-01	5.83E-03	9.30E-01	NS
MYBL1	NS	8.87E-01	-0.00583	9.30E-01	NS
SRSF8	NS	8.87E-01	-0.00583	9.30E-01	NS
TRAK1	NS	8.87E-01	5.82E-03	9.30E-01	NS
PRKD2	NS	8.88E-01	5.80E-03	9.30E-01	NS
RPS13	NS	8.88E-01	-0.0058	9.30E-01	NS
NDOR1	NS	8.88E-01	-0.00581	9.30E-01	NS
ATAD2B	NS	8.88E-01	5.79E-03	9.30E-01	NS
PRAP1	NS	8.88E-01	-0.00579	9.30E-01	NS
SNAI1	NS	8.88E-01	-0.00578	9.30E-01	NS
TMOD2	NS	8.88E-01	5.78E-03	9.30E-01	NS
SLC46A1	NS	8.88E-01	-0.00577	9.30E-01	NS
HES1	NS	8.89E-01	5.76E-03	9.31E-01	NS
DLK2	NS	8.88E-01	-0.00576	9.31E-01	NS
ADIPOR1	NS	8.89E-01	5.74E-03	9.31E-01	NS
CNKSR1	NS	8.89E-01	5.72E-03	9.31E-01	NS
PWWP4	NS	8.89E-01	5.72E-03	9.31E-01	NS

FBLIM1	NS	8.89E-01	-0.00572	9.31E-01	NS
ACP6	NS	8.89E-01	-0.00572	9.31E-01	NS
STRIP1	NS	8.89E-01	-0.00571	9.31E-01	NS
WNT2	NS	8.90E-01	-0.0057	9.31E-01	NS
ZNF462	NS	8.90E-01	-0.0057	9.31E-01	NS
CCNH	NS	8.90E-01	-0.00569	9.31E-01	NS
FAM170A	NS	8.90E-01	-0.0057	9.31E-01	NS
RNF138	NS	8.90E-01	-0.00567	9.32E-01	NS
CASP8	NS	8.91E-01	5.65E-03	9.32E-01	NS
FH	NS	8.91E-01	-0.00565	9.32E-01	NS
PAIP1	NS	8.91E-01	5.64E-03	9.32E-01	NS
KRTAP9-3	NS	8.92E-01	5.60E-03	9.33E-01	NS
KCNA5	NS	8.92E-01	5.57E-03	9.34E-01	NS
FGFR1	NS	8.93E-01	-0.00552	9.34E-01	NS
RPA1	NS	8.93E-01	5.51E-03	9.35E-01	NS
FAHD2B	NS	8.93E-01	5.51E-03	9.35E-01	NS
BNIPL	NS	8.93E-01	-0.00551	9.35E-01	NS
TRMT61B	NS	8.94E-01	-0.0055	9.35E-01	NS
MYH4	NS	8.94E-01	-0.0055	9.35E-01	NS
RAB19	NS	8.94E-01	5.48E-03	9.35E-01	NS
CBR1	NS	8.94E-01	5.48E-03	9.35E-01	NS
CBX6	NS	8.94E-01	-0.00548	9.35E-01	NS
F13A1	NS	8.94E-01	-0.00547	9.35E-01	NS
RIMS2	NS	8.94E-01	-0.00547	9.35E-01	NS
OR4F6	NS	8.94E-01	5.47E-03	9.35E-01	NS
IZUMO4	NS	8.95E-01	-0.00544	9.35E-01	NS
ARFGEF3	NS	8.95E-01	5.44E-03	9.35E-01	NS
RBP1	NS	8.95E-01	5.44E-03	9.35E-01	NS
AP4B1	NS	8.95E-01	-0.00545	9.35E-01	NS
NOLC1	NS	8.95E-01	5.44E-03	9.35E-01	NS
ATOH1	NS	8.94E-01	5.45E-03	9.35E-01	NS
ANKUB1	NS	8.95E-01	5.44E-03	9.35E-01	NS
RRAGD	NS	8.95E-01	-0.00543	9.35E-01	NS
ZC3H14	NS	8.95E-01	5.43E-03	9.35E-01	NS
OR4D1	NS	8.95E-01	-0.00543	9.35E-01	NS
TFEB	NS	8.95E-01	5.42E-03	9.35E-01	NS
TAPBP	NS	8.95E-01	-0.00541	9.35E-01	NS

SYNRG	NS	8.95E-01	-0.0054	9.36E-01	NS
PMP22	NS	8.96E-01	5.38E-03	9.36E-01	NS
HR	NS	8.96E-01	-0.00538	9.36E-01	NS
NEK4	NS	8.96E-01	-0.00537	9.36E-01	NS
PTK2	NS	8.96E-01	-0.00537	9.36E-01	NS
UBP1	NS	8.96E-01	-0.00536	9.36E-01	NS
ARFGEF2	NS	8.96E-01	5.35E-03	9.36E-01	NS
EVPLL	NS	8.97E-01	5.34E-03	9.36E-01	NS
POLE2	NS	8.97E-01	-0.00533	9.37E-01	NS
GRAMD4	NS	8.97E-01	-0.00532	9.37E-01	NS
YY1	NS	8.97E-01	5.32E-03	9.37E-01	NS
PLEKHA7	NS	8.97E-01	-0.00532	9.37E-01	NS
PLP2	NS	8.97E-01	5.31E-03	9.37E-01	NS
GPR45	NS	8.97E-01	5.31E-03	9.37E-01	NS
EXD2	NS	8.97E-01	-0.0053	9.37E-01	NS
TRAF3IP2	NS	8.97E-01	5.30E-03	9.37E-01	NS
SMARCA4	NS	8.98E-01	5.29E-03	9.37E-01	NS
TAAR9	NS	8.98E-01	5.28E-03	9.37E-01	NS
S100A1	NS	8.98E-01	-0.00527	9.37E-01	NS
FAM83B	NS	8.98E-01	5.27E-03	9.37E-01	NS
USP17L11	NS	8.98E-01	-0.00527	9.37E-01	NS
DNAJB14	NS	8.98E-01	-0.00526	9.37E-01	NS
PVRIG	NS	8.98E-01	-0.00525	9.37E-01	NS
ISL1	NS	8.98E-01	5.24E-03	9.38E-01	NS
NXF1	NS	8.99E-01	5.24E-03	9.38E-01	NS
PLXNB2	NS	8.99E-01	-0.00524	9.38E-01	NS
TRIM61	NS	8.99E-01	-0.00523	9.38E-01	NS
TCHP	NS	8.99E-01	5.23E-03	9.38E-01	NS
ARL6IP6	NS	8.99E-01	-0.00522	9.38E-01	NS
DIAPH3	NS	8.99E-01	-0.00521	9.38E-01	NS
PNPLA8	NS	8.99E-01	5.20E-03	9.38E-01	NS
CD46	NS	8.99E-01	-0.00519	9.38E-01	NS
DYRK3	NS	8.99E-01	5.19E-03	9.38E-01	NS
SLC2A14	NS	8.99E-01	5.19E-03	9.38E-01	NS
CYP4X1	NS	8.99E-01	-0.0052	9.38E-01	NS
E2F4	NS	9.00E-01	-0.00519	9.38E-01	NS
SMARCE1	NS	9.00E-01	5.18E-03	9.38E-01	NS

OR6C4	NS	9.00E-01	-0.00518	9.38E-01	NS
PSMB3	NS	9.00E-01	5.18E-03	9.38E-01	NS
ERP44	NS	9.00E-01	5.16E-03	9.38E-01	NS
ACSL5	NS	9.00E-01	5.15E-03	9.39E-01	NS
MPPED2	NS	9.00E-01	5.14E-03	9.39E-01	NS
PCBD1	NS	9.00E-01	5.14E-03	9.39E-01	NS
KIAA0753	NS	9.00E-01	-0.00514	9.39E-01	NS
USP17L27	NS	9.00E-01	5.14E-03	9.39E-01	NS
RITA1	NS	9.01E-01	-0.00513	9.39E-01	NS
NUTM2G	NS	9.01E-01	5.13E-03	9.39E-01	NS
ZNF442	NS	9.01E-01	-0.00513	9.39E-01	NS
R3HDM1	NS	9.01E-01	5.12E-03	9.39E-01	NS
DNAJC27	NS	9.01E-01	-0.00511	9.39E-01	NS
ZNF558	NS	9.01E-01	-0.0051	9.39E-01	NS
SYNC	NS	9.02E-01	5.08E-03	9.39E-01	NS
CACNG5	NS	9.02E-01	5.08E-03	9.39E-01	NS
MAS1	NS	9.02E-01	-0.00506	9.40E-01	NS
LIMK1	NS	9.02E-01	-0.00506	9.40E-01	NS
MAGEA2B	NS	9.02E-01	-0.00506	9.40E-01	NS
ATP4A	NS	9.02E-01	-0.00505	9.40E-01	NS
SPDYE1	NS	9.02E-01	5.04E-03	9.40E-01	NS
MEF2B	NS	9.02E-01	-0.00504	9.40E-01	NS
CA6	NS	9.03E-01	5.03E-03	9.40E-01	NS
S100A7L2	NS	9.03E-01	5.02E-03	9.40E-01	NS
CSHL1	NS	9.03E-01	-0.00503	9.40E-01	NS
OR52R1	NS	9.03E-01	-0.00502	9.40E-01	NS
STARD7	NS	9.03E-01	-0.00502	9.40E-01	NS
DDX5	NS	9.03E-01	-0.00501	9.40E-01	NS
MAP3K1	NS	9.03E-01	5.01E-03	9.40E-01	NS
COL4A4	NS	9.03E-01	-0.00499	9.40E-01	NS
KIF9	NS	9.03E-01	-0.00499	9.40E-01	NS
KLK14	NS	9.03E-01	5.00E-03	9.40E-01	NS
ETV6	NS	9.03E-01	4.99E-03	9.40E-01	NS
TRAM1L1	NS	9.03E-01	4.99E-03	9.40E-01	NS
ZNF844	NS	9.03E-01	4.99E-03	9.40E-01	NS
NUP205	NS	9.03E-01	-0.00498	9.40E-01	NS
CYTL1	NS	9.04E-01	4.97E-03	9.40E-01	NS

MYL6B	NS	9.04E-01	-0.00497	9.40E-01	NS
GDF15	NS	9.04E-01	4.96E-03	9.41E-01	NS
MRAP2	NS	9.04E-01	4.95E-03	9.41E-01	NS
RBM26	NS	9.04E-01	4.95E-03	9.41E-01	NS
NEMP1	NS	9.04E-01	-0.00495	9.41E-01	NS
ARHGEF33	NS	9.04E-01	4.94E-03	9.41E-01	NS
RBBP4	NS	9.04E-01	4.93E-03	9.41E-01	NS
BCL2L15	NS	9.05E-01	4.93E-03	9.41E-01	NS
CCDC30	NS	9.05E-01	4.91E-03	9.41E-01	NS
CREB3L4	NS	9.05E-01	4.90E-03	9.41E-01	NS
C1orf50	NS	9.05E-01	4.90E-03	9.41E-01	NS
COG2	NS	9.05E-01	-0.0049	9.41E-01	NS
TCFL5	NS	9.05E-01	-0.00489	9.41E-01	NS
SH3D21	NS	9.05E-01	4.90E-03	9.41E-01	NS
MOB1A	NS	9.05E-01	4.88E-03	9.41E-01	NS
PRR9	NS	9.05E-01	-0.00488	9.41E-01	NS
C7orf26	NS	9.06E-01	4.87E-03	9.42E-01	NS
MAEL	NS	9.06E-01	-0.00486	9.42E-01	NS
MZT2A	NS	9.06E-01	-0.00486	9.42E-01	NS
KRTAP2-1	NS	9.06E-01	4.85E-03	9.42E-01	NS
DDX17	NS	9.06E-01	4.84E-03	9.42E-01	NS
PRSS35	NS	9.07E-01	4.82E-03	9.42E-01	NS
POLR1H	NS	9.07E-01	-0.00482	9.42E-01	NS
LLGL2	NS	9.07E-01	4.81E-03	9.42E-01	NS
DAAM1	NS	9.07E-01	-0.00481	9.42E-01	NS
ASB6	NS	9.07E-01	-0.00478	9.43E-01	NS
SPATA12	NS	9.07E-01	4.78E-03	9.43E-01	NS
DELE1	NS	9.08E-01	4.77E-03	9.43E-01	NS
OR5W2	NS	9.08E-01	-0.00476	9.43E-01	NS
CT47A2	NS	9.08E-01	4.76E-03	9.43E-01	NS
VPS35	NS	9.08E-01	-0.00475	9.43E-01	NS
RNF13	NS	9.08E-01	-0.00474	9.43E-01	NS
TENT4B	NS	9.08E-01	4.74E-03	9.43E-01	NS
TAOK3	NS	9.08E-01	4.74E-03	9.43E-01	NS
AURKA	NS	9.08E-01	4.73E-03	9.43E-01	NS
DAZ2	NS	9.08E-01	-0.00473	9.43E-01	NS
ASGR2	NS	9.09E-01	-0.00471	9.44E-01	NS

OLR1	NS	9.09E-01	4.70E-03	9.44E-01	NS
MYBL2	NS	9.09E-01	4.69E-03	9.44E-01	NS
ZNF142	NS	9.09E-01	4.69E-03	9.44E-01	NS
SPEM1	NS	9.09E-01	-0.00469	9.44E-01	NS
NPIPA5	NS	9.09E-01	4.68E-03	9.44E-01	NS
ZFPL1	NS	9.10E-01	-0.00467	9.44E-01	NS
BCAP31	NS	9.09E-01	4.67E-03	9.44E-01	NS
LY6G5B	NS	9.10E-01	4.67E-03	9.44E-01	NS
MCEMP1	NS	9.10E-01	4.67E-03	9.44E-01	NS
ARID1A	NS	9.10E-01	-0.00465	9.44E-01	NS
KLB	NS	9.10E-01	4.65E-03	9.44E-01	NS
APP	NS	9.10E-01	4.64E-03	9.45E-01	NS
B4GAT1	NS	9.10E-01	4.63E-03	9.45E-01	NS
RFX7	NS	9.10E-01	4.63E-03	9.45E-01	NS
CRLF1	NS	9.11E-01	4.62E-03	9.45E-01	NS
PALS1	NS	9.11E-01	-0.00461	9.45E-01	NS
EDF1	NS	9.11E-01	4.61E-03	9.45E-01	NS
SNX4	NS	9.11E-01	-0.00461	9.45E-01	NS
DUSP23	NS	9.11E-01	-0.00461	9.45E-01	NS
TMEM120B	NS	9.11E-01	4.62E-03	9.45E-01	NS
APOC4-APOC2	NS	9.10E-01	4.62E-03	9.45E-01	NS
NOL12	NS	9.10E-01	4.62E-03	9.45E-01	NS
FLII	NS	9.11E-01	-0.00459	9.45E-01	NS
STH	NS	9.11E-01	4.59E-03	9.45E-01	NS
BUB3	NS	9.11E-01	4.59E-03	9.45E-01	NS
ZNF587B	NS	9.11E-01	-0.00458	9.45E-01	NS
CD244	NS	9.11E-01	4.58E-03	9.45E-01	NS
PANX1	NS	9.12E-01	-0.00456	9.45E-01	NS
RIF1	NS	9.12E-01	-0.00455	9.45E-01	NS
TULP1	NS	9.12E-01	-0.00456	9.45E-01	NS
ZNF525	NS	9.12E-01	-0.00456	9.45E-01	NS
NDUFA1	NS	9.12E-01	4.55E-03	9.45E-01	NS
UBE2J1	NS	9.12E-01	-0.00454	9.45E-01	NS
DUSP15	NS	9.12E-01	-0.00452	9.46E-01	NS
TSPAN5	NS	9.12E-01	4.52E-03	9.46E-01	NS
TUBB4B	NS	9.12E-01	4.52E-03	9.46E-01	NS
ENTPD8	NS	9.12E-01	4.52E-03	9.46E-01	NS

DHX16	NS	9.12E-01	-0.00452	9.46E-01	NS
PTPN9	NS	9.13E-01	4.48E-03	9.46E-01	NS
C11orf16	NS	9.13E-01	-0.00448	9.46E-01	NS
TMEM123	NS	9.13E-01	-0.00448	9.46E-01	NS
RAB5B	NS	9.13E-01	-0.00447	9.46E-01	NS
SMNDC1	NS	9.13E-01	-0.00446	9.46E-01	NS
ANAPC1	NS	9.13E-01	4.46E-03	9.46E-01	NS
ACAP2	NS	9.14E-01	4.46E-03	9.46E-01	NS
GRM6	NS	9.14E-01	4.44E-03	9.47E-01	NS
UBE2O	NS	9.14E-01	-0.00444	9.47E-01	NS
DNAH3	NS	9.14E-01	-0.00444	9.47E-01	NS
BTBD6	NS	9.14E-01	4.44E-03	9.47E-01	NS
MTMR1	NS	9.14E-01	4.43E-03	9.47E-01	NS
KRT84	NS	9.14E-01	-0.00442	9.47E-01	NS
CPSF1	NS	9.15E-01	-0.00441	9.47E-01	NS
ERBB3	NS	9.15E-01	4.40E-03	9.47E-01	NS
NDST1	NS	9.15E-01	4.39E-03	9.47E-01	NS
C1QTNF8	NS	9.15E-01	4.39E-03	9.47E-01	NS
TPCN1	NS	9.15E-01	4.39E-03	9.47E-01	NS
MC2R	NS	9.15E-01	4.37E-03	9.47E-01	NS
BLOC1S5	NS	9.15E-01	4.37E-03	9.47E-01	NS
PTCD1	NS	9.16E-01	-0.00436	9.48E-01	NS
SCNN1A	NS	9.16E-01	4.36E-03	9.48E-01	NS
RELL1	NS	9.16E-01	-0.00435	9.48E-01	NS
RBM14	NS	9.16E-01	4.35E-03	9.48E-01	NS
CACNA2D4	NS	9.16E-01	4.33E-03	9.48E-01	NS
CCL15	NS	9.16E-01	-0.00432	9.48E-01	NS
SELENOF	NS	9.17E-01	-0.0043	9.49E-01	NS
COX6A1	NS	9.17E-01	4.29E-03	9.49E-01	NS
EDDM3A	NS	9.17E-01	4.29E-03	9.49E-01	NS
PRAF2	NS	9.17E-01	4.27E-03	9.49E-01	NS
NACC1	NS	9.17E-01	4.26E-03	9.49E-01	NS
VDAC3	NS	9.18E-01	-0.00424	9.50E-01	NS
AMER2	NS	9.18E-01	-0.00423	9.50E-01	NS
TLE3	NS	9.18E-01	4.22E-03	9.50E-01	NS
HSPA1B	NS	9.18E-01	4.22E-03	9.50E-01	NS
KRTAP2-2	NS	9.18E-01	4.22E-03	9.50E-01	NS

INPPL1	NS	9.18E-01	4.21E-03	9.50E-01	NS
C4orf33	NS	9.19E-01	-0.00419	9.50E-01	NS
KLHDC9	NS	9.19E-01	4.19E-03	9.50E-01	NS
MS4A3	NS	9.19E-01	-0.00418	9.50E-01	NS
NUDT5	NS	9.19E-01	4.18E-03	9.50E-01	NS
ZIC4	NS	9.19E-01	4.18E-03	9.50E-01	NS
MRPL45	NS	9.19E-01	4.16E-03	9.51E-01	NS
UNC80	NS	9.20E-01	-0.00415	9.51E-01	NS
FIZ1	NS	9.20E-01	-0.00415	9.51E-01	NS
PIP	NS	9.20E-01	-0.00414	9.51E-01	NS
IDO2	NS	9.20E-01	4.15E-03	9.51E-01	NS
TULP3	NS	9.20E-01	-0.00414	9.51E-01	NS
GUCA1C	NS	9.20E-01	4.13E-03	9.51E-01	NS
SLC43A2	NS	9.20E-01	4.13E-03	9.51E-01	NS
ADCY5	NS	9.20E-01	4.14E-03	9.51E-01	NS
MFAP2	NS	9.20E-01	-0.00413	9.51E-01	NS
AUP1	NS	9.20E-01	-0.00412	9.51E-01	NS
ITPRID1	NS	9.20E-01	4.12E-03	9.51E-01	NS
CLCN2	NS	9.21E-01	-0.0041	9.51E-01	NS
PPP6C	NS	9.20E-01	4.10E-03	9.51E-01	NS
TXN	NS	9.20E-01	4.11E-03	9.51E-01	NS
PWWP2B	NS	9.21E-01	4.09E-03	9.51E-01	NS
PACS2	NS	9.20E-01	-0.0041	9.51E-01	NS
RPRML	NS	9.20E-01	-0.00411	9.51E-01	NS
ZNF70	NS	9.20E-01	4.11E-03	9.51E-01	NS
PRAMEF20	NS	9.21E-01	4.09E-03	9.51E-01	NS
PARG	NS	9.21E-01	-0.00409	9.51E-01	NS
MILR1	NS	9.21E-01	-0.00409	9.51E-01	NS
ABCB5	NS	9.21E-01	4.08E-03	9.51E-01	NS
ITIH5	NS	9.22E-01	-0.00405	9.52E-01	NS
ARMC7	NS	9.22E-01	-0.00405	9.52E-01	NS
TSGA13	NS	9.22E-01	-0.00404	9.52E-01	NS
CREBL2	NS	9.22E-01	4.03E-03	9.52E-01	NS
TMEM167B	NS	9.22E-01	-0.00403	9.52E-01	NS
ATAT1	NS	9.22E-01	4.03E-03	9.52E-01	NS
DSP	NS	9.22E-01	4.02E-03	9.52E-01	NS
HACE1	NS	9.22E-01	4.00E-03	9.52E-01	NS

VPS28	NS	9.22E-01	-0.004	9.52E-01	NS
ARNTL	NS	9.23E-01	-0.00399	9.52E-01	NS
AZGP1	NS	9.23E-01	-0.00398	9.52E-01	NS
CHPF2	NS	9.23E-01	3.97E-03	9.52E-01	NS
BTN3A3	NS	9.23E-01	-0.00395	9.53E-01	NS
RICTOR	NS	9.24E-01	3.93E-03	9.53E-01	NS
INMT-MINDY4	NS	9.24E-01	3.92E-03	9.53E-01	NS
CYTIP	NS	9.24E-01	3.92E-03	9.53E-01	NS
FGF1	NS	9.24E-01	-0.00391	9.53E-01	NS
OIT3	NS	9.24E-01	-0.0039	9.53E-01	NS
MMP17	NS	9.24E-01	3.90E-03	9.53E-01	NS
CAMSAP2	NS	9.24E-01	-0.0039	9.53E-01	NS
TM2D3	NS	9.25E-01	-0.00389	9.54E-01	NS
PLA2G4D	NS	9.25E-01	-0.00388	9.54E-01	NS
GPX1	NS	9.25E-01	-0.00388	9.54E-01	NS
FBXW8	NS	9.25E-01	3.87E-03	9.54E-01	NS
RSBN1	NS	9.25E-01	-0.00387	9.54E-01	NS
MSRB1	NS	9.25E-01	-0.00386	9.54E-01	NS
SELENOW	NS	9.25E-01	-0.00386	9.54E-01	NS
RPS27	NS	9.25E-01	-0.00385	9.54E-01	NS
TM4SF5	NS	9.26E-01	3.81E-03	9.55E-01	NS
RYR2	NS	9.26E-01	-0.00381	9.55E-01	NS
ROCK1	NS	9.27E-01	-0.00378	9.55E-01	NS
OR2T1	NS	9.27E-01	3.78E-03	9.55E-01	NS
CAPN12	NS	9.27E-01	-0.00378	9.55E-01	NS
C15orf39	NS	9.27E-01	-0.00375	9.56E-01	NS
DCAF11	NS	9.28E-01	-0.00371	9.56E-01	NS
TXNRD1	NS	9.28E-01	-0.00371	9.56E-01	NS
GRXCR2	NS	9.28E-01	-0.00371	9.56E-01	NS
CDAN1	NS	9.28E-01	3.70E-03	9.56E-01	NS
CALML3	NS	9.28E-01	3.70E-03	9.56E-01	NS
CATSPER4	NS	9.28E-01	3.70E-03	9.56E-01	NS
AS3MT	NS	9.28E-01	-0.00369	9.56E-01	NS
TNK2	NS	9.29E-01	-0.00368	9.56E-01	NS
RRP8	NS	9.29E-01	3.69E-03	9.56E-01	NS
RNF17	NS	9.29E-01	3.68E-03	9.56E-01	NS
OR5D18	NS	9.28E-01	-0.00369	9.56E-01	NS

HNRNPAB	NS	9.29E-01	-0.00367	9.57E-01	NS
SAT2	NS	9.29E-01	3.66E-03	9.57E-01	NS
C10orf67	NS	9.29E-01	-0.00365	9.57E-01	NS
TTC17	NS	9.30E-01	3.62E-03	9.57E-01	NS
DNAJA3	NS	9.30E-01	3.62E-03	9.57E-01	NS
RPS6KA4	NS	9.30E-01	-0.00362	9.57E-01	NS
C19orf84	NS	9.30E-01	-0.00362	9.57E-01	NS
RASSF5	NS	9.30E-01	-0.00362	9.57E-01	NS
TRAPPC12	NS	9.30E-01	-0.00361	9.57E-01	NS
SSH2	NS	9.30E-01	-0.00358	9.58E-01	NS
NECAB2	NS	9.31E-01	-0.00357	9.58E-01	NS
C10orf71	NS	9.31E-01	3.57E-03	9.58E-01	NS
CPLX2	NS	9.31E-01	3.56E-03	9.58E-01	NS
IGFBP6	NS	9.31E-01	-0.00356	9.58E-01	NS
RANBP3L	NS	9.31E-01	-0.00355	9.58E-01	NS
NEUROG2	NS	9.31E-01	3.55E-03	9.58E-01	NS
LMBR1L	NS	9.31E-01	-0.00354	9.58E-01	NS
DHRS1	NS	9.31E-01	3.54E-03	9.58E-01	NS
C11orf68	NS	9.31E-01	3.54E-03	9.58E-01	NS
DNAJB4	NS	9.32E-01	3.52E-03	9.59E-01	NS
PRSS27	NS	9.32E-01	-0.00352	9.59E-01	NS
ZNF396	NS	9.32E-01	-0.00352	9.59E-01	NS
EDEM2	NS	9.32E-01	3.51E-03	9.59E-01	NS
CYP24A1	NS	9.32E-01	-0.0035	9.59E-01	NS
PRR3	NS	9.32E-01	-0.0035	9.59E-01	NS
IGSF3	NS	9.32E-01	3.50E-03	9.59E-01	NS
VHL	NS	9.32E-01	-0.00349	9.59E-01	NS
INAVA	NS	9.33E-01	-0.00348	9.59E-01	NS
BMP2	NS	9.33E-01	3.47E-03	9.59E-01	NS
FBXL20	NS	9.33E-01	3.46E-03	9.59E-01	NS
FAM220A	NS	9.33E-01	3.46E-03	9.59E-01	NS
ZNF684	NS	9.33E-01	3.46E-03	9.59E-01	NS
DMRTA2	NS	9.33E-01	3.45E-03	9.59E-01	NS
ANTXRL	NS	9.33E-01	-0.00345	9.59E-01	NS
RPUSD1	NS	9.33E-01	-0.00344	9.60E-01	NS
TMEM50B	NS	9.33E-01	3.44E-03	9.60E-01	NS
EAPP	NS	9.34E-01	-0.00342	9.60E-01	NS

TCEANC2	NS	9.34E-01	3.41E-03	9.60E-01	NS
KIAA2013	NS	9.34E-01	3.41E-03	9.60E-01	NS
IL37	NS	9.34E-01	-0.00341	9.60E-01	NS
TMEM219	NS	9.34E-01	-0.00342	9.60E-01	NS
OR2T27	NS	9.34E-01	3.41E-03	9.60E-01	NS
EI24	NS	9.34E-01	3.40E-03	9.60E-01	NS
DLX3	NS	9.34E-01	-0.00338	9.60E-01	NS
PCIF1	NS	9.34E-01	-0.00339	9.60E-01	NS
FGF7	NS	9.34E-01	3.38E-03	9.60E-01	NS
SNX22	NS	9.34E-01	-0.0034	9.60E-01	NS
TNFSF13	NS	9.34E-01	3.39E-03	9.60E-01	NS
TEX26	NS	9.34E-01	3.38E-03	9.60E-01	NS
ATP5MF-PTCD1	NS	9.34E-01	-0.00339	9.60E-01	NS
NGEF	NS	9.35E-01	3.38E-03	9.60E-01	NS
NR3C2	NS	9.35E-01	-0.00335	9.60E-01	NS
SH3BP1	NS	9.35E-01	-0.00334	9.60E-01	NS
TAF12	NS	9.35E-01	3.34E-03	9.60E-01	NS
SVOP	NS	9.35E-01	-0.00334	9.60E-01	NS
HOXB2	NS	9.35E-01	3.35E-03	9.60E-01	NS
SLC22A25	NS	9.35E-01	-0.00333	9.60E-01	NS
BHLHE41	NS	9.35E-01	3.33E-03	9.60E-01	NS
PTGR1	NS	9.36E-01	3.32E-03	9.61E-01	NS
BBS5	NS	9.36E-01	3.32E-03	9.61E-01	NS
CEP43	NS	9.36E-01	3.32E-03	9.61E-01	NS
PIGB	NS	9.36E-01	-0.00331	9.61E-01	NS
KCNQ3	NS	9.36E-01	-0.00331	9.61E-01	NS
SPATA6	NS	9.36E-01	3.30E-03	9.61E-01	NS
RLIM	NS	9.36E-01	3.30E-03	9.61E-01	NS
TMEM178A	NS	9.36E-01	-0.0033	9.61E-01	NS
LTA	NS	9.36E-01	-0.00328	9.61E-01	NS
PRR20G	NS	9.36E-01	3.28E-03	9.61E-01	NS
MAP4K1	NS	9.36E-01	3.28E-03	9.61E-01	NS
LYRM9	NS	9.37E-01	-0.00326	9.61E-01	NS
RIMS3	NS	9.37E-01	-0.00325	9.61E-01	NS
OR4C13	NS	9.37E-01	3.26E-03	9.61E-01	NS
HDLBP	NS	9.37E-01	-0.00324	9.61E-01	NS
MED14	NS	9.37E-01	-0.00323	9.62E-01	NS

FAM47C	NS	9.37E-01	3.23E-03	9.62E-01	NS
KCNIP2	NS	9.38E-01	-0.00322	9.62E-01	NS
SLC22A16	NS	9.38E-01	-0.00322	9.62E-01	NS
PPAN	NS	9.38E-01	-0.00321	9.62E-01	NS
ZNF273	NS	9.38E-01	3.20E-03	9.62E-01	NS
MKRN2OS	NS	9.38E-01	-0.0032	9.62E-01	NS
OR10G4	NS	9.38E-01	3.17E-03	9.62E-01	NS
PRAMEF19	NS	9.39E-01	3.15E-03	9.63E-01	NS
ICAM4	NS	9.39E-01	-0.00315	9.63E-01	NS
POU1F1	NS	9.39E-01	-0.00314	9.63E-01	NS
EPB41L4B	NS	9.39E-01	3.12E-03	9.63E-01	NS
FOSL1	NS	9.40E-01	-0.00311	9.63E-01	NS
UPF3B	NS	9.40E-01	-0.0031	9.63E-01	NS
LRFN5	NS	9.40E-01	-0.0031	9.63E-01	NS
STARD3NL	NS	9.40E-01	-0.00309	9.64E-01	NS
SPPL2C	NS	9.40E-01	-0.00307	9.64E-01	NS
PRKCH	NS	9.40E-01	-0.00307	9.64E-01	NS
SRCAP	NS	9.41E-01	-0.00306	9.64E-01	NS
MTMR3	NS	9.41E-01	-0.00304	9.64E-01	NS
PDHX	NS	9.41E-01	3.06E-03	9.64E-01	NS
C11orf49	NS	9.41E-01	-0.00306	9.64E-01	NS
NR4A2	NS	9.41E-01	-0.00305	9.64E-01	NS
CDH12	NS	9.41E-01	-0.00304	9.64E-01	NS
CYP2C19	NS	9.41E-01	3.05E-03	9.64E-01	NS
RPL38	NS	9.41E-01	-0.00305	9.64E-01	NS
TLR1	NS	9.41E-01	-0.00305	9.64E-01	NS
WFDC10A	NS	9.41E-01	-0.00305	9.64E-01	NS
MBD3L3	NS	9.41E-01	-0.00304	9.64E-01	NS
ZBTB7C	NS	9.41E-01	3.04E-03	9.64E-01	NS
PCYT2	NS	9.41E-01	-0.00305	9.64E-01	NS
EQTN	NS	9.41E-01	3.03E-03	9.64E-01	NS
DOCK7	NS	9.41E-01	3.02E-03	9.64E-01	NS
PGRMC1	NS	9.41E-01	-0.00302	9.64E-01	NS
ASCC1	NS	9.42E-01	3.01E-03	9.64E-01	NS
TMEM160	NS	9.42E-01	-0.00299	9.65E-01	NS
GPRC5A	NS	9.42E-01	-0.00299	9.65E-01	NS
LAMC2	NS	9.42E-01	-0.00298	9.65E-01	NS

JMJD4	NS	9.42E-01	2.97E-03	9.65E-01	NS
OR6C2	NS	9.42E-01	2.97E-03	9.65E-01	NS
H3-5	NS	9.42E-01	2.98E-03	9.65E-01	NS
SMURF1	NS	9.42E-01	2.98E-03	9.65E-01	NS
CCDC200	NS	9.42E-01	2.97E-03	9.65E-01	NS
NPR1	NS	9.42E-01	-0.00296	9.65E-01	NS
POLR2D	NS	9.43E-01	2.95E-03	9.65E-01	NS
GCNT7	NS	9.43E-01	2.94E-03	9.65E-01	NS
GAMT	NS	9.43E-01	-0.00293	9.65E-01	NS
KRBA2	NS	9.44E-01	-0.00291	9.66E-01	NS
ATG16L1	NS	9.44E-01	2.89E-03	9.66E-01	NS
KCNG4	NS	9.44E-01	2.88E-03	9.66E-01	NS
MOXD1	NS	9.44E-01	2.87E-03	9.66E-01	NS
LDHAL6B	NS	9.44E-01	-0.00286	9.66E-01	NS
LRRC57	NS	9.45E-01	-0.00285	9.66E-01	NS
IRF9	NS	9.45E-01	-0.00285	9.66E-01	NS
GDF11	NS	9.45E-01	-0.00284	9.66E-01	NS
ARHGEF39	NS	9.45E-01	2.84E-03	9.66E-01	NS
RPP25	NS	9.45E-01	2.84E-03	9.66E-01	NS
TXNRD2	NS	9.45E-01	2.83E-03	9.66E-01	NS
NUGGC	NS	9.45E-01	-0.00283	9.66E-01	NS
DGKZ	NS	9.45E-01	2.83E-03	9.67E-01	NS
DNAL1	NS	9.45E-01	-0.00282	9.67E-01	NS
PPP1R12B	NS	9.45E-01	-0.00281	9.67E-01	NS
PODXL2	NS	9.45E-01	2.82E-03	9.67E-01	NS
NTS	NS	9.45E-01	2.81E-03	9.67E-01	NS
PKP4	NS	9.45E-01	2.82E-03	9.67E-01	NS
ATP10D	NS	9.45E-01	-0.00281	9.67E-01	NS
USP17L23	NS	9.46E-01	2.80E-03	9.67E-01	NS
OR4X2	NS	9.46E-01	2.80E-03	9.67E-01	NS
PAXIP1	NS	9.46E-01	-0.00277	9.67E-01	NS
C1orf54	NS	9.47E-01	-0.00275	9.68E-01	NS
MBLAC1	NS	9.47E-01	-0.00274	9.68E-01	NS
GOLGA8B	NS	9.47E-01	2.74E-03	9.68E-01	NS
IAPP	NS	9.47E-01	2.73E-03	9.68E-01	NS
TRIM4	NS	9.47E-01	2.72E-03	9.68E-01	NS
KRT16	NS	9.47E-01	2.72E-03	9.68E-01	NS

PHF13	NS	9.47E-01	-0.00272	9.68E-01	NS
CSNK2A2	NS	9.47E-01	-0.00271	9.68E-01	NS
VAV1	NS	9.48E-01	2.69E-03	9.68E-01	NS
DMRTC2	NS	9.48E-01	2.68E-03	9.68E-01	NS
FAM241A	NS	9.48E-01	-0.00268	9.68E-01	NS
FUT2	NS	9.48E-01	-0.00269	9.68E-01	NS
KCNE1	NS	9.48E-01	2.68E-03	9.68E-01	NS
TCN2	NS	9.48E-01	2.69E-03	9.68E-01	NS
CD300C	NS	9.48E-01	-0.00268	9.68E-01	NS
STPG1	NS	9.48E-01	-0.00267	9.68E-01	NS
MEOX1	NS	9.48E-01	2.67E-03	9.68E-01	NS
PCYT1B	NS	9.48E-01	2.67E-03	9.68E-01	NS
CLDN11	NS	9.48E-01	2.66E-03	9.68E-01	NS
PRRC2B	NS	9.49E-01	2.65E-03	9.68E-01	NS
TROAP	NS	9.49E-01	2.65E-03	9.68E-01	NS
MBD3L2B	NS	9.49E-01	-0.00264	9.69E-01	NS
KBTBD7	NS	9.49E-01	-0.00263	9.69E-01	NS
NCKAP1L	NS	9.49E-01	-0.00264	9.69E-01	NS
XG	NS	9.49E-01	2.62E-03	9.69E-01	NS
CT45A8	NS	9.49E-01	-0.00262	9.69E-01	NS
KLF9	NS	9.50E-01	2.60E-03	9.69E-01	NS
TRAPPC10	NS	9.49E-01	-0.0026	9.69E-01	NS
IL36RN	NS	9.50E-01	2.59E-03	9.69E-01	NS
CT45A3	NS	9.50E-01	-0.00259	9.69E-01	NS
UBAP2L	NS	9.50E-01	-0.00257	9.69E-01	NS
TSKU	NS	9.50E-01	-0.00256	9.70E-01	NS
TUBB8B	NS	9.50E-01	-0.00256	9.70E-01	NS
ZNF540	NS	9.50E-01	-0.00256	9.70E-01	NS
SASS6	NS	9.50E-01	2.55E-03	9.70E-01	NS
MARCHF10	NS	9.51E-01	2.55E-03	9.70E-01	NS
GLDC	NS	9.51E-01	2.53E-03	9.70E-01	NS
SLC6A17	NS	9.51E-01	-0.00253	9.70E-01	NS
RAB6C	NS	9.51E-01	2.54E-03	9.70E-01	NS
TDGF1P3	NS	9.51E-01	2.54E-03	9.70E-01	NS
GDE1	NS	9.51E-01	2.52E-03	9.70E-01	NS
MED1	NS	9.51E-01	-0.00252	9.70E-01	NS
VAMP1	NS	9.51E-01	2.52E-03	9.70E-01	NS

RIN1	NS	9.51E-01	2.52E-03	9.70E-01	NS
FAM166A	NS	9.51E-01	-0.00252	9.70E-01	NS
STK32A	NS	9.51E-01	-0.0025	9.70E-01	NS
S1PR5	NS	9.52E-01	-0.0025	9.70E-01	NS
HOXA1	NS	9.52E-01	2.48E-03	9.70E-01	NS
ZNF473	NS	9.52E-01	2.48E-03	9.70E-01	NS
NEMF	NS	9.52E-01	-0.00248	9.70E-01	NS
TBC1D25	NS	9.52E-01	-0.00246	9.71E-01	NS
DYNC1I2	NS	9.52E-01	-0.00245	9.71E-01	NS
CNST	NS	9.53E-01	-0.00244	9.71E-01	NS
NSMCE1	NS	9.53E-01	2.44E-03	9.71E-01	NS
GGT5	NS	9.53E-01	-0.00243	9.71E-01	NS
C16orf87	NS	9.53E-01	-0.00243	9.71E-01	NS
PIK3C2A	NS	9.53E-01	2.41E-03	9.71E-01	NS
PDE4D	NS	9.53E-01	2.41E-03	9.71E-01	NS
RABGAP1L	NS	9.53E-01	-0.00241	9.71E-01	NS
KRT9	NS	9.53E-01	-0.00241	9.71E-01	NS
SRXN1	NS	9.53E-01	-0.00242	9.71E-01	NS
MAGED4B	NS	9.53E-01	-0.0024	9.71E-01	NS
CARD16	NS	9.53E-01	2.40E-03	9.71E-01	NS
SCTR	NS	9.54E-01	2.38E-03	9.71E-01	NS
HNRNPA1L2	NS	9.54E-01	-0.00239	9.71E-01	NS
MLKL	NS	9.54E-01	-0.00239	9.71E-01	NS
CCDC116	NS	9.54E-01	2.37E-03	9.71E-01	NS
PPP4R3A	NS	9.54E-01	2.37E-03	9.71E-01	NS
NFKBID	NS	9.54E-01	-0.00237	9.71E-01	NS
DCTN2	NS	9.54E-01	-0.00235	9.72E-01	NS
BRMS1	NS	9.54E-01	-0.00235	9.72E-01	NS
C3orf84	NS	9.55E-01	-0.00233	9.72E-01	NS
CDC7	NS	9.55E-01	-0.00231	9.72E-01	NS
PCDHB8	NS	9.55E-01	-0.00231	9.72E-01	NS
HAL	NS	9.55E-01	-0.0023	9.72E-01	NS
OR7C1	NS	9.55E-01	-0.0023	9.72E-01	NS
ZC3HAV1L	NS	9.56E-01	-0.00229	9.73E-01	NS
NAT9	NS	9.56E-01	2.27E-03	9.73E-01	NS
PGAP6	NS	9.56E-01	2.26E-03	9.73E-01	NS
TOR2A	NS	9.56E-01	-0.00227	9.73E-01	NS

FBXL12	NS	9.56E-01	2.26E-03	9.73E-01	NS
ASF1A	NS	9.56E-01	2.25E-03	9.73E-01	NS
SFR1	NS	9.56E-01	-0.00224	9.73E-01	NS
MT1G	NS	9.57E-01	-0.00223	9.73E-01	NS
KRTAP19-8	NS	9.57E-01	2.22E-03	9.74E-01	NS
DUS4L	NS	9.57E-01	-0.00221	9.74E-01	NS
ZBTB20	NS	9.57E-01	2.20E-03	9.74E-01	NS
CT47A4	NS	9.57E-01	2.20E-03	9.74E-01	NS
ZSWIM5	NS	9.57E-01	-0.00219	9.74E-01	NS
NEDD8-MDP1	NS	9.57E-01	2.19E-03	9.74E-01	NS
MATN1	NS	9.58E-01	-0.00218	9.74E-01	NS
FGF19	NS	9.58E-01	2.17E-03	9.74E-01	NS
TSSK3	NS	9.58E-01	2.17E-03	9.74E-01	NS
CRTC2	NS	9.58E-01	-0.00216	9.74E-01	NS
BMPR2	NS	9.58E-01	-0.00215	9.74E-01	NS
SNX5	NS	9.59E-01	-0.00214	9.75E-01	NS
APEX2	NS	9.59E-01	-0.00213	9.75E-01	NS
WNK3	NS	9.59E-01	2.13E-03	9.75E-01	NS
TUT7	NS	9.59E-01	2.11E-03	9.75E-01	NS
EBF3	NS	9.59E-01	2.11E-03	9.75E-01	NS
GNPAT	NS	9.59E-01	-0.00211	9.75E-01	NS
OR5H6	NS	9.59E-01	2.11E-03	9.75E-01	NS
CATSPERD	NS	9.59E-01	-0.0021	9.75E-01	NS
ZNF747	NS	9.59E-01	2.09E-03	9.75E-01	NS
PIK3CB	NS	9.60E-01	2.06E-03	9.75E-01	NS
PTPN18	NS	9.60E-01	-0.00206	9.75E-01	NS
P4HA2	NS	9.60E-01	2.08E-03	9.75E-01	NS
TEKT2	NS	9.60E-01	2.07E-03	9.75E-01	NS
DNPH1	NS	9.60E-01	-0.00206	9.75E-01	NS
SPECC1	NS	9.60E-01	2.06E-03	9.75E-01	NS
DENND4C	NS	9.60E-01	-0.00207	9.75E-01	NS
EYA3	NS	9.60E-01	2.06E-03	9.75E-01	NS
SSTR3	NS	9.60E-01	2.06E-03	9.75E-01	NS
NCBP1	NS	9.60E-01	-0.00205	9.75E-01	NS
CCR4	NS	9.60E-01	-0.00204	9.76E-01	NS
KARS1	NS	9.61E-01	-0.00203	9.76E-01	NS
MUC21	NS	9.61E-01	2.02E-03	9.76E-01	NS

ARHGEF35	NS	9.61E-01	-0.00201	9.76E-01	NS
FRMD4A	NS	9.61E-01	-0.00198	9.76E-01	NS
STK11	NS	9.62E-01	1.97E-03	9.77E-01	NS
MYOZ1	NS	9.62E-01	-0.00197	9.77E-01	NS
ZNF107	NS	9.62E-01	1.97E-03	9.77E-01	NS
DHRS3	NS	9.62E-01	-0.00195	9.77E-01	NS
PAM16	NS	9.62E-01	-0.00195	9.77E-01	NS
NAA16	NS	9.62E-01	-0.00194	9.77E-01	NS
IGF2BP3	NS	9.63E-01	1.93E-03	9.77E-01	NS
PLCH2	NS	9.63E-01	1.92E-03	9.77E-01	NS
ATAD3C	NS	9.63E-01	-0.00192	9.77E-01	NS
LINS1	NS	9.63E-01	-0.0019	9.77E-01	NS
FBXL17	NS	9.63E-01	1.90E-03	9.77E-01	NS
VLDLR	NS	9.63E-01	-0.0019	9.77E-01	NS
PLCG2	NS	9.63E-01	1.89E-03	9.78E-01	NS
CPA1	NS	9.63E-01	1.88E-03	9.78E-01	NS
NRSN1	NS	9.64E-01	-0.00188	9.78E-01	NS
C9orf57	NS	9.64E-01	-0.00186	9.78E-01	NS
RGL2	NS	9.64E-01	1.86E-03	9.78E-01	NS
NSMAF	NS	9.64E-01	-0.00185	9.78E-01	NS
MROH1	NS	9.64E-01	1.85E-03	9.78E-01	NS
MFAP5	NS	9.64E-01	-0.00184	9.78E-01	NS
CNTFR	NS	9.64E-01	-0.00184	9.78E-01	NS
HSPA1A	NS	9.65E-01	1.82E-03	9.78E-01	NS
TRAF3IP3	NS	9.65E-01	1.81E-03	9.79E-01	NS
PLAUR	NS	9.65E-01	-0.00181	9.79E-01	NS
ZDHHC21	NS	9.65E-01	-0.0018	9.79E-01	NS
SPATA31A5	NS	9.65E-01	1.80E-03	9.79E-01	NS
RBBP8	NS	9.65E-01	1.79E-03	9.79E-01	NS
RBM25	NS	9.65E-01	1.79E-03	9.79E-01	NS
JSRP1	NS	9.65E-01	1.78E-03	9.79E-01	NS
SEPTIN11	NS	9.66E-01	1.78E-03	9.79E-01	NS
SRSF9	NS	9.66E-01	1.77E-03	9.79E-01	NS
CLDN19	NS	9.66E-01	-0.00177	9.79E-01	NS
CCDC28B	NS	9.66E-01	1.77E-03	9.79E-01	NS
MMP25	NS	9.66E-01	1.76E-03	9.79E-01	NS
TERT	NS	9.66E-01	-0.00175	9.79E-01	NS

ZBTB25	NS	9.66E-01	-0.00174	9.79E-01	NS
CPSF4	NS	9.66E-01	1.74E-03	9.79E-01	NS
PRKN	NS	9.66E-01	1.73E-03	9.79E-01	NS
SDR9C7	NS	9.66E-01	1.73E-03	9.79E-01	NS
IRF1	NS	9.67E-01	-0.00171	9.80E-01	NS
CRELD2	NS	9.67E-01	1.71E-03	9.80E-01	NS
TEX33	NS	9.67E-01	1.71E-03	9.80E-01	NS
RSF1	NS	9.67E-01	1.70E-03	9.80E-01	NS
PXN	NS	9.67E-01	1.70E-03	9.80E-01	NS
SNAPC5	NS	9.67E-01	-0.0017	9.80E-01	NS
TMEM70	NS	9.67E-01	1.70E-03	9.80E-01	NS
OR51F2	NS	9.67E-01	-0.00169	9.80E-01	NS
SFRP5	NS	9.67E-01	-0.00169	9.80E-01	NS
C20orf85	NS	9.67E-01	-0.00168	9.80E-01	NS
CAMKV	NS	9.67E-01	1.68E-03	9.80E-01	NS
CHD5	NS	9.68E-01	-0.00167	9.80E-01	NS
CKB	NS	9.68E-01	1.67E-03	9.80E-01	NS
EXOC7	NS	9.68E-01	-0.00167	9.80E-01	NS
CEP126	NS	9.68E-01	-0.00166	9.80E-01	NS
GPR12	NS	9.68E-01	-0.00165	9.80E-01	NS
TMUB1	NS	9.68E-01	1.64E-03	9.80E-01	NS
CGB3	NS	9.68E-01	1.64E-03	9.80E-01	NS
C16orf74	NS	9.68E-01	1.63E-03	9.80E-01	NS
HSF2BP	NS	9.68E-01	1.64E-03	9.80E-01	NS
OR10H2	NS	9.68E-01	-0.00164	9.80E-01	NS
KRTAP13-1	NS	9.68E-01	1.63E-03	9.80E-01	NS
PDPK1	NS	9.68E-01	1.63E-03	9.80E-01	NS
MROH8	NS	9.69E-01	-0.00162	9.80E-01	NS
CST2	NS	9.69E-01	1.62E-03	9.80E-01	NS
CCDC124	NS	9.69E-01	1.61E-03	9.80E-01	NS
TIMM13	NS	9.69E-01	1.61E-03	9.80E-01	NS
BLOC1S1	NS	9.69E-01	1.57E-03	9.81E-01	NS
SWSAP1	NS	9.70E-01	1.57E-03	9.81E-01	NS
ZNF230	NS	9.70E-01	1.55E-03	9.81E-01	NS
ZNF778	NS	9.70E-01	-0.00155	9.81E-01	NS
MYBPC1	NS	9.70E-01	-0.00155	9.81E-01	NS
NTPCR	NS	9.70E-01	-0.00154	9.81E-01	NS

E2F8	NS	9.70E-01	-0.00153	9.82E-01	NS
CCDC15	NS	9.70E-01	1.53E-03	9.82E-01	NS
EVI5L	NS	9.70E-01	-0.00152	9.82E-01	NS
TSBP1	NS	9.71E-01	-0.00152	9.82E-01	NS
NAGA	NS	9.71E-01	-0.00151	9.82E-01	NS
NDUFA4L2	NS	9.71E-01	1.50E-03	9.82E-01	NS
ZBTB8OS	NS	9.71E-01	-0.0015	9.82E-01	NS
DEFB130B	NS	9.71E-01	1.49E-03	9.82E-01	NS
APOL3	NS	9.71E-01	1.48E-03	9.82E-01	NS
OR1N1	NS	9.71E-01	1.48E-03	9.82E-01	NS
MTFR1L	NS	9.71E-01	1.47E-03	9.82E-01	NS
OR6B2	NS	9.72E-01	-0.00146	9.82E-01	NS
NYAP2	NS	9.72E-01	-0.00145	9.83E-01	NS
CSF1	NS	9.72E-01	-0.00143	9.83E-01	NS
DEFA4	NS	9.72E-01	1.42E-03	9.83E-01	NS
FLOT2	NS	9.73E-01	-0.00141	9.83E-01	NS
FGR	NS	9.73E-01	1.40E-03	9.83E-01	NS
TRIM38	NS	9.73E-01	-0.00139	9.83E-01	NS
KDM4C	NS	9.73E-01	1.37E-03	9.84E-01	NS
AK3	NS	9.73E-01	-0.00137	9.84E-01	NS
NPIPB5	NS	9.73E-01	-0.00138	9.84E-01	NS
PRODH2	NS	9.73E-01	-0.00137	9.84E-01	NS
CCDC90B	NS	9.74E-01	-0.00135	9.84E-01	NS
XAGE5	NS	9.74E-01	1.35E-03	9.84E-01	NS
RUFY1	NS	9.74E-01	1.35E-03	9.84E-01	NS
TMEM187	NS	9.74E-01	-0.00135	9.84E-01	NS
DEFB116	NS	9.74E-01	-0.00133	9.84E-01	NS
ATXN7	NS	9.74E-01	1.33E-03	9.84E-01	NS
HLF	NS	9.74E-01	1.31E-03	9.84E-01	NS
RHAG	NS	9.74E-01	-0.00131	9.84E-01	NS
ECI1	NS	9.74E-01	1.32E-03	9.84E-01	NS
RECQL4	NS	9.75E-01	-0.00131	9.84E-01	NS
FCHSD2	NS	9.75E-01	-0.00128	9.85E-01	NS
RASL10A	NS	9.75E-01	-0.00127	9.85E-01	NS
ARHGEF18	NS	9.75E-01	-0.00127	9.85E-01	NS
PIEZO2	NS	9.76E-01	1.26E-03	9.85E-01	NS
THOC1	NS	9.76E-01	1.25E-03	9.85E-01	NS

DAD1	NS	9.76E-01	1.25E-03	9.85E-01	NS
TMEM14B	NS	9.76E-01	1.25E-03	9.85E-01	NS
ZNF496	NS	9.76E-01	-0.00125	9.85E-01	NS
SLC6A20	NS	9.76E-01	-0.00124	9.85E-01	NS
B4GALNT2	NS	9.76E-01	-0.00124	9.85E-01	NS
SAXO1	NS	9.76E-01	1.23E-03	9.85E-01	NS
MEIOB	NS	9.76E-01	-0.00123	9.85E-01	NS
PLIN2	NS	9.76E-01	1.22E-03	9.85E-01	NS
UPB1	NS	9.76E-01	-0.00121	9.85E-01	NS
ADAMTS13	NS	9.76E-01	1.21E-03	9.85E-01	NS
HIP1R	NS	9.77E-01	-0.00119	9.86E-01	NS
ADAMTS4	NS	9.77E-01	1.18E-03	9.86E-01	NS
ARHGAP27	NS	9.77E-01	1.18E-03	9.86E-01	NS
EPOR	NS	9.77E-01	1.18E-03	9.86E-01	NS
POMGNT1	NS	9.78E-01	1.16E-03	9.86E-01	NS
CBS	NS	9.77E-01	1.16E-03	9.86E-01	NS
ZNF148	NS	9.77E-01	-0.00116	9.86E-01	NS
TMEM37	NS	9.78E-01	-0.00116	9.86E-01	NS
KIR3DL2	NS	9.77E-01	1.16E-03	9.86E-01	NS
SLK	NS	9.78E-01	-0.00115	9.86E-01	NS
PLAG1	NS	9.78E-01	-0.00115	9.86E-01	NS
CPLANE1	NS	9.78E-01	-0.00115	9.86E-01	NS
TP53INP1	NS	9.78E-01	1.14E-03	9.86E-01	NS
FARP1	NS	9.78E-01	-0.00114	9.86E-01	NS
COG4	NS	9.78E-01	1.13E-03	9.86E-01	NS
STK24	NS	9.78E-01	1.13E-03	9.86E-01	NS
KLHL11	NS	9.78E-01	1.12E-03	9.86E-01	NS
ZBTB1	NS	9.78E-01	-0.00112	9.86E-01	NS
C8orf34	NS	9.78E-01	1.11E-03	9.86E-01	NS
PXT1	NS	9.79E-01	1.10E-03	9.87E-01	NS
MPV17L	NS	9.79E-01	-0.00109	9.87E-01	NS
TCHH	NS	9.79E-01	1.08E-03	9.87E-01	NS
ABAT	NS	9.79E-01	-0.00108	9.87E-01	NS
INCA1	NS	9.79E-01	-0.00109	9.87E-01	NS
TMEM178B	NS	9.79E-01	1.09E-03	9.87E-01	NS
ABHD17B	NS	9.79E-01	-0.00107	9.87E-01	NS
CRIP3	NS	9.79E-01	-0.00108	9.87E-01	NS

SHPK	NS	9.79E-01	-0.00107	9.87E-01	NS
NKX1-2	NS	9.79E-01	1.07E-03	9.87E-01	NS
MYT1L	NS	9.79E-01	-0.00106	9.87E-01	NS
MINDY1	NS	9.80E-01	-0.00106	9.87E-01	NS
KIAA0895	NS	9.80E-01	-0.00104	9.87E-01	NS
RNPEP	NS	9.80E-01	1.04E-03	9.87E-01	NS
MEST	NS	9.80E-01	-0.00103	9.87E-01	NS
RPN2	NS	9.80E-01	-0.00101	9.87E-01	NS
TSPAN10	NS	9.80E-01	1.01E-03	9.87E-01	NS
GDPGP1	NS	9.80E-01	-0.00102	9.87E-01	NS
SENP3-EIF4A1	NS	9.80E-01	-0.00101	9.87E-01	NS
MINDY4	NS	9.80E-01	-0.00101	9.87E-01	NS
SCUBE1	NS	9.81E-01	9.98E-04	9.87E-01	NS
C12orf76	NS	9.81E-01	-0.000998	9.87E-01	NS
WDR18	NS	9.81E-01	9.84E-04	9.88E-01	NS
POTEM	NS	9.82E-01	-0.000936	9.89E-01	NS
KRTAP12-2	NS	9.82E-01	-0.000932	9.89E-01	NS
GAP43	NS	9.82E-01	-0.000915	9.89E-01	NS
NFATC2IP	NS	9.82E-01	9.16E-04	9.89E-01	NS
ARHGDIB	NS	9.83E-01	8.97E-04	9.89E-01	NS
C1orf185	NS	9.83E-01	8.67E-04	9.90E-01	NS
NPY4R	NS	9.83E-01	8.62E-04	9.90E-01	NS
SULT2A1	NS	9.83E-01	-0.000859	9.90E-01	NS
SHANK3	NS	9.84E-01	-0.000848	9.90E-01	NS
MYLPF	NS	9.84E-01	8.42E-04	9.90E-01	NS
PNMA3	NS	9.84E-01	-0.000838	9.90E-01	NS
BTBD8	NS	9.84E-01	-0.00084	9.90E-01	NS
SLC25A1	NS	9.84E-01	8.28E-04	9.90E-01	NS
PODNL1	NS	9.84E-01	8.27E-04	9.90E-01	NS
CLSPN	NS	9.84E-01	8.15E-04	9.90E-01	NS
SNRPC	NS	9.84E-01	-0.000811	9.90E-01	NS
SRPK2	NS	9.84E-01	8.10E-04	9.90E-01	NS
HLA-DQB2	NS	9.85E-01	7.95E-04	9.90E-01	NS
USP17L4	NS	9.85E-01	-0.00079	9.90E-01	NS
RAB22A	NS	9.85E-01	-0.000784	9.91E-01	NS
RARS2	NS	9.85E-01	7.77E-04	9.91E-01	NS
R3HDML	NS	9.85E-01	7.72E-04	9.91E-01	NS

RPS2	NS	9.85E-01	-0.000768	9.91E-01	NS
TMEM63B	NS	9.85E-01	-0.00076	9.91E-01	NS
MBTPS2	NS	9.86E-01	-0.000728	9.91E-01	NS
CGB7	NS	9.86E-01	7.27E-04	9.91E-01	NS
TPPP2	NS	9.86E-01	7.20E-04	9.91E-01	NS
ABHD13	NS	9.86E-01	7.14E-04	9.91E-01	NS
SNX20	NS	9.86E-01	-0.000703	9.92E-01	NS
TMC3	NS	9.86E-01	-0.000705	9.92E-01	NS
RAB5C	NS	9.86E-01	6.97E-04	9.92E-01	NS
NOBOX	NS	9.87E-01	-0.000686	9.92E-01	NS
UGGT1	NS	9.87E-01	-0.000677	9.92E-01	NS
TLN2	NS	9.87E-01	-0.000677	9.92E-01	NS
VPS35L	NS	9.87E-01	6.71E-04	9.92E-01	NS
TSPAN1	NS	9.87E-01	-0.000664	9.92E-01	NS
PEX11A	NS	9.87E-01	6.62E-04	9.92E-01	NS
C11orf80	NS	9.87E-01	-0.000669	9.92E-01	NS
WT1	NS	9.87E-01	6.66E-04	9.92E-01	NS
CIDEA	NS	9.87E-01	6.59E-04	9.92E-01	NS
ZFC3H1	NS	9.88E-01	6.43E-04	9.92E-01	NS
CCDC107	NS	9.88E-01	6.38E-04	9.92E-01	NS
SOGA3	NS	9.88E-01	6.32E-04	9.92E-01	NS
NOCT	NS	9.88E-01	-0.000627	9.92E-01	NS
LGALS7	NS	9.88E-01	6.13E-04	9.93E-01	NS
TMEM174	NS	9.88E-01	6.07E-04	9.93E-01	NS
RPL5	NS	9.88E-01	-0.000599	9.93E-01	NS
MSH5	NS	9.88E-01	5.93E-04	9.93E-01	NS
SLC2A4	NS	9.89E-01	-0.00059	9.93E-01	NS
PTPMT1	NS	9.89E-01	-0.000578	9.93E-01	NS
IL18BP	NS	9.89E-01	-0.000576	9.93E-01	NS
HAAO	NS	9.89E-01	-0.000578	9.93E-01	NS
CAT	NS	9.89E-01	-0.000567	9.93E-01	NS
EIF4EBP1	NS	9.89E-01	5.58E-04	9.93E-01	NS
ACSM4	NS	9.89E-01	-0.000555	9.93E-01	NS
ARNT	NS	9.89E-01	5.46E-04	9.93E-01	NS
NDST2	NS	9.90E-01	5.39E-04	9.93E-01	NS
TMEM134	NS	9.90E-01	-0.000537	9.93E-01	NS
HES2	NS	9.90E-01	5.11E-04	9.94E-01	NS

HIGD2A	NS	9.90E-01	4.98E-04	9.94E-01	NS
DNAI1	NS	9.91E-01	-0.000484	9.94E-01	NS
HLA-F	NS	9.91E-01	4.85E-04	9.94E-01	NS
UBE2F	NS	9.91E-01	4.79E-04	9.94E-01	NS
CERCAM	NS	9.91E-01	-0.000475	9.94E-01	NS
WSCD1	NS	9.91E-01	4.59E-04	9.94E-01	NS
AXDND1	NS	9.91E-01	-0.000456	9.94E-01	NS
VSX1	NS	9.91E-01	4.46E-04	9.95E-01	NS
RPE65	NS	9.91E-01	4.47E-04	9.95E-01	NS
AK8	NS	9.91E-01	-0.00044	9.95E-01	NS
H3C14	NS	9.91E-01	4.42E-04	9.95E-01	NS
PRAMEF5	NS	9.91E-01	4.42E-04	9.95E-01	NS
LEMD2	NS	9.92E-01	-0.000432	9.95E-01	NS
CBLL2	NS	9.92E-01	4.35E-04	9.95E-01	NS
GPC2	NS	9.92E-01	-0.000421	9.95E-01	NS
NFKB2	NS	9.92E-01	-0.000417	9.95E-01	NS
MUC4	NS	9.92E-01	-0.000405	9.95E-01	NS
ADAMTS19	NS	9.92E-01	3.99E-04	9.95E-01	NS
IGF2	NS	9.93E-01	-0.000383	9.95E-01	NS
HACD2	NS	9.93E-01	3.85E-04	9.95E-01	NS
SANBR	NS	9.93E-01	-0.000378	9.95E-01	NS
NUBP2	NS	9.93E-01	3.61E-04	9.95E-01	NS
GADD45B	NS	9.93E-01	3.64E-04	9.95E-01	NS
BAALC	NS	9.93E-01	-0.000357	9.95E-01	NS
WIF1	NS	9.93E-01	-0.00035	9.96E-01	NS
KCNA6	NS	9.93E-01	3.46E-04	9.96E-01	NS
SRA1	NS	9.94E-01	-0.00033	9.96E-01	NS
AP3D1	NS	9.94E-01	3.27E-04	9.96E-01	NS
SLC35A1	NS	9.94E-01	3.19E-04	9.96E-01	NS
SLFN11	NS	9.94E-01	-0.000315	9.96E-01	NS
TFAP2B	NS	9.94E-01	-0.00029	9.96E-01	NS
PLXNA3	NS	9.94E-01	-0.000294	9.96E-01	NS
DNAJB5	NS	9.94E-01	2.95E-04	9.96E-01	NS
COMMD3	NS	9.94E-01	2.98E-04	9.96E-01	NS
MPPE1	NS	9.94E-01	3.00E-04	9.96E-01	NS
ODAD3	NS	9.94E-01	2.92E-04	9.96E-01	NS
SYCP1	NS	9.94E-01	-0.000304	9.96E-01	NS

TCEA2	NS	9.94E-01	-0.000284	9.96E-01	NS
CMKLR1	NS	9.95E-01	2.75E-04	9.96E-01	NS
NLRP1	NS	9.95E-01	2.71E-04	9.96E-01	NS
ENPP7	NS	9.95E-01	2.67E-04	9.96E-01	NS
TUBGCP2	NS	9.95E-01	-0.000255	9.96E-01	NS
PIP5K1B	NS	9.95E-01	-0.000248	9.97E-01	NS
FBXW4	NS	9.95E-01	-0.000243	9.97E-01	NS
IL17A	NS	9.96E-01	-0.000219	9.97E-01	NS
SLC25A51	NS	9.96E-01	2.10E-04	9.97E-01	NS
HSPA9	NS	9.96E-01	-0.000204	9.97E-01	NS
WBP2	NS	9.96E-01	-0.000184	9.97E-01	NS
RIC8A	NS	9.96E-01	1.86E-04	9.97E-01	NS
TTC1	NS	9.97E-01	-0.000173	9.98E-01	NS
TMEM45A	NS	9.97E-01	-0.00015	9.98E-01	NS
AMIGO1	NS	9.97E-01	1.40E-04	9.98E-01	NS
SHLD1	NS	9.97E-01	-0.000136	9.98E-01	NS
PSG2	NS	9.98E-01	1.15E-04	9.99E-01	NS
EFCAB5	NS	9.98E-01	-0.00011	9.99E-01	NS
SLC38A9	NS	9.98E-01	1.11E-04	9.99E-01	NS
ARF5	NS	9.98E-01	-0.0000908	9.99E-01	NS
TNK1	NS	9.98E-01	9.51E-05	9.99E-01	NS
C4orf47	NS	9.98E-01	9.31E-05	9.99E-01	NS
VARS2	NS	9.98E-01	8.44E-05	9.99E-01	NS
ELAPOR1	NS	9.98E-01	-7.85E-05	9.99E-01	NS
TP53TG3E	NS	9.99E-01	-7.35E-05	9.99E-01	NS
CXCL12	NS	9.99E-01	-3.02E-05	1.00E+00	NS
MAP3K6	NS	1.00E+00	-2.30E-05	1.00E+00	NS
ZZEF1	NS	1.00E+00	-1.22E-06	1.00E+00	NS
FHIP2B	NS	1.00E+00	4.33E-06	1.00E+00	NS
SPIB	NS	1.00E+00	-8.73E-07	1.00E+00	NS
