# Supplementary material for: RBAD: The first database dedicated alterations of blood RNA in individuals with Alzheimer’s disease and their clinical relevance
Source: Neural Regen Res. 2025 Mar 25;21(6):2553–62. doi: 10.4103/NRR.NRR-D-24-01165 (PMC13211806; doi:10.4103/NRR.NRR-D-24-01165)
Supplement: Supplementary file 24 [file NRR-21-2553_Suppl2.pdf]

Additional Table 5. Disease-associated pathways enriched by disease-associated DEGs detected in RBAD

ID	ONTOLOGY	Description	Group (data type, dataset, comparison)	Strength of evidence
BP	GO:0006641	triglyceride metabolic process	bulk RNA-seq,ACOM,AD.vs.control; bulk RNA-seq,Homo_723,AD.vs.MCI; bulk RNA-seq,Homo_723,MCI.vs.control; bulk RNA-seq,Homo_714,AD.vs.control; bulk RNA-seq,Homo_714,AD.vs.MCI; bulk RNA-seq,Homo_714,MCI.vs.control; bulk RNA-seq,Homo_633,AD.vs.control; bulk RNA-seq,Homo_633,AD.vs.MCI	8
CC	GO:0062023	collagen-containing extracellular matrix	bulk RNA-seq,ACOM,AD.vs.control; bulk RNA-seq,Homo_723,AD.vs.control; bulk RNA-seq,Homo_723,AD.vs.MCI; bulk RNA-seq,Homo_714,AD.vs.control; bulk RNA-seq,Homo_714,AD.vs.MCI; bulk RNA-seq,Homo_633,AD.vs.control; bulk RNA-seq,Homo_633,AD.vs.MCI; bulk RNA-seq,ROSMAP,AD.vs.control; bulk RNA-seq,ROSMAP,MCI.vs.control; bulk RNA-seq,SRP223445,AD.vs.control	10
BP	GO:0034367	protein-containing complex remodeling	bulk RNA-seq,ACOM,AD.vs.control; bulk RNA-seq,Homo_723,AD.vs.control; bulk RNA-seq,Homo_723,AD.vs.MCI; bulk RNA-seq,Homo_723,MCI.vs.control; bulk RNA-seq,Homo_714,AD.vs.control; bulk RNA-seq,Homo_714,AD.vs.MCI; bulk RNA-seq,Homo_714,MCI.vs.control	7
BP	GO:0071827	plasma lipoprotein particle organization	bulk RNA-seq,ACOM,AD.vs.control; bulk RNA-seq,Homo_723,AD.vs.MCI; bulk RNA-seq,Homo_723,MCI.vs.control; bulk RNA-seq,Homo_714,AD.vs.MCI; bulk RNA-seq,Homo_714,MCI.vs.control; bulk RNA-seq,Homo_633,AD.vs.MCI	6
BP	GO:0071825	protein-lipid complex subunit organization	bulk RNA-seq,ACOM,AD.vs.control; bulk RNA-seq,Homo_723,AD.vs.MCI; bulk RNA-seq,Homo_723,MCI.vs.control; bulk RNA-seq,Homo_714,AD.vs.MCI; bulk RNA-seq,Homo_714,MCI.vs.control; bulk RNA-seq,Homo_633,AD.vs.MCI	6
BP	GO:0034368	protein-lipid complex remodeling	bulk RNA-seq,ACOM,AD.vs.control; bulk RNA-seq,Homo_723,AD.vs.control; bulk RNA-seq,Homo_723,AD.vs.MCI; bulk RNA-seq,Homo_723,MCI.vs.control; bulk RNA-seq,Homo_714,AD.vs.control; bulk RNA-seq,Homo_714,AD.vs.MCI; bulk RNA-seq,Homo_714,MCI.vs.control	7
BP	GO:0034369	plasma lipoprotein particle remodeling	bulk RNA-seq,ACOM,AD.vs.control; bulk RNA-seq,Homo_723,AD.vs.control; bulk RNA-seq,Homo_723,AD.vs.MCI; bulk RNA-seq,Homo_723,MCI.vs.control; bulk RNA-seq,Homo_714,AD.vs.control; bulk RNA-seq,Homo_714,AD.vs.MCI; bulk RNA-seq,Homo_714,MCI.vs.control	7
BP	GO:0006638	neutral lipid metabolic process	bulk RNA-seq,ACOM,AD.vs.control; bulk RNA-seq,Homo_723,AD.vs.MCI; bulk RNA-seq,Homo_723,MCI.vs.control; bulk RNA-seq,Homo_714,AD.vs.control; bulk RNA-seq,Homo_714,AD.vs.MCI; bulk RNA-seq,Homo_633,AD.vs.control; bulk RNA-seq,Homo_633,AD.vs.MCI	7
BP	GO:0006639	acylglycerol metabolic process	bulk RNA-seq,ACOM,AD.vs.control; bulk RNA-seq,Homo_723,AD.vs.MCI; bulk RNA-seq,Homo_723,MCI.vs.control; bulk RNA-seq,Homo_714,AD.vs.control; bulk RNA-seq,Homo_714,AD.vs.MCI; bulk RNA-seq,Homo_633,AD.vs.control; bulk RNA-seq,Homo_633,AD.vs.MCI	7
CC	GO:0005788	endoplasmic reticulum lumen	bulk RNA-seq,ACOM,AD.vs.control; bulk RNA-seq,Emory,MCI.vs.control; bulk RNA-seq,Homo_723,AD.vs.control; bulk RNA-seq,Homo_723,AD.vs.MCI; bulk RNA-seq,Homo_723,MCI.vs.control; bulk RNA-seq,Homo_714,AD.vs.control; bulk RNA-seq,Homo_714,AD.vs.MCI; bulk RNA-seq,Homo_633,AD.vs.control; bulk RNA-seq,Homo_633,AD.vs.MCI	9
BP	GO:0031667	response to nutrient levels	bulk RNA-seq,ACOM,AD.vs.control; bulk RNA-seq,Homo_723,AD.vs.control; bulk RNA-seq,Homo_723,AD.vs.MCI; bulk RNA-seq,Homo_714,AD.vs.control; bulk RNA-seq,Homo_714,AD.vs.MCI; bulk RNA-seq,Homo_633,AD.vs.control; bulk RNA-seq,Homo_633,AD.vs.MCI	7
BP	GO:0015850	organic hydroxy compound transport	bulk RNA-seq,ACOM,AD.vs.control; bulk RNA-seq,Homo_723,AD.vs.control; bulk RNA-seq,Homo_723,AD.vs.MCI; bulk RNA-seq,Homo_723,MCI.vs.control; bulk RNA-seq,Homo_714,AD.vs.control; bulk RNA-seq,Homo_714,AD.vs.MCI; bulk RNA-seq,Homo_633,AD.vs.control; bulk RNA-seq,Homo_633,AD.vs.MCI; bulk RNA-seq,ROSMAP,MCI.vs.control; bulk RNA-seq,SRP325058,AD.vs.control	10
CC	GO:0034361	very-low-density lipoprotein particle	bulk RNA-seq,ACOM,AD.vs.control; bulk RNA-seq,Homo_723,AD.vs.MCI; bulk RNA-seq,Homo_723,MCI.vs.control; bulk RNA-seq,Homo_714,AD.vs.MCI; bulk RNA-seq,Homo_714,MCI.vs.control	5
CC	GO:0034385	triglyceride-rich plasma lipoprotein particle	bulk RNA-seq,ACOM,AD.vs.control; bulk RNA-seq,Homo_723,AD.vs.MCI; bulk RNA-seq,Homo_723,MCI.vs.control; bulk RNA-seq,Homo_714,AD.vs.MCI; bulk RNA-seq,Homo_714,MCI.vs.control	5
BP	GO:0097006	regulation of plasma lipoprotein particle levels	bulk RNA-seq,ACOM,AD.vs.control; bulk RNA-seq,Homo_723,AD.vs.MCI; bulk RNA-seq,Homo_723,MCI.vs.control; bulk RNA-seq,Homo_714,AD.vs.control; bulk RNA-seq,Homo_714,AD.vs.MCI; bulk RNA-seq,Homo_714,MCI.vs.control; bulk RNA-seq,Homo_633,AD.vs.control; bulk RNA-seq,Homo_633,AD.vs.MCI	8
BP	GO:0050878	regulation of body fluid levels	bulk RNA-seq,ACOM,AD.vs.control; bulk RNA-seq,Emory,MCI.vs.control; bulk RNA-seq,Homo_723,AD.vs.control; bulk RNA-seq,Homo_723,AD.vs.MCI; bulk RNA-seq,Homo_714,AD.vs.control; bulk RNA-seq,Homo_714,AD.vs.MCI; bulk RNA-seq,Homo_633,AD.vs.control; bulk RNA-seq,Homo_633,AD.vs.MCI	8
BP	GO:0009991	response to extracellular stimulus	bulk RNA-seq,ACOM,AD.vs.control; bulk RNA-seq,Homo_633,AD.vs.control; bulk RNA-seq,Homo_633,AD.vs.MCI	3
BP	GO:0055090	acylglycerol homeostasis	bulk RNA-seq,ACOM,AD.vs.control; bulk RNA-seq,Homo_723,AD.vs.MCI; bulk RNA-seq,Homo_723,MCI.vs.control; bulk RNA-seq,Homo_714,AD.vs.control; bulk RNA-seq,Homo_714,AD.vs.MCI; bulk RNA-seq,Homo_714,MCI.vs.control	6
BP	GO:0070328	triglyceride homeostasis	bulk RNA-seq,ACOM,AD.vs.control; bulk RNA-seq,Homo_723,AD.vs.MCI; bulk RNA-seq,Homo_723,MCI.vs.control; bulk RNA-seq,Homo_714,AD.vs.control; bulk RNA-seq,Homo_714,AD.vs.MCI; bulk RNA-seq,Homo_714,MCI.vs.control	6
CC	GO:0042627	chylomicron	bulk RNA-seq,ACOM,AD.vs.control; bulk RNA-seq,Homo_723,AD.vs.MCI; bulk RNA-seq,Homo_723,MCI.vs.control; bulk RNA-seq,Homo_714,AD.vs.control; bulk RNA-seq,Homo_714,AD.vs.MCI; bulk RNA-seq,Homo_714,MCI.vs.control	6
BP	GO:0042157	lipoprotein metabolic process	bulk RNA-seq,ACOM,AD.vs.control; bulk RNA-seq,Homo_723,MCI.vs.control; bulk RNA-seq,Homo_633,AD.vs.control; bulk RNA-seq,Homo_633,AD.vs.MCI	4
BP	GO:0046503	glycerolipid catabolic process	bulk RNA-seq,ACOM,AD.vs.control; bulk RNA-seq,Homo_723,AD.vs.MCI; bulk RNA-seq,Homo_723,MCI.vs.control; bulk RNA-seq,Homo_714,AD.vs.MCI; bulk RNA-seq,Homo_714,MCI.vs.control; bulk RNA-seq,Homo_633,AD.vs.control; bulk RNA-seq,Homo_633,AD.vs.MCI	7
BP	GO:0055088	lipid homeostasis	bulk RNA-seq,ACOM,AD.vs.control; bulk RNA-seq,Homo_723,AD.vs.MCI; bulk RNA-seq,Homo_723,MCI.vs.control; bulk RNA-seq,Homo_714,AD.vs.control; bulk RNA-seq,Homo_714,AD.vs.MCI; bulk RNA-seq,Homo_633,AD.vs.control; bulk RNA-seq,Homo_633,AD.vs.MCI	7
BP	GO:0070371	ERK1 and ERK2 cascade	bulk RNA-seq,ACOM,AD.vs.control; bulk RNA-seq,Homo_723,AD.vs.control; bulk RNA-seq,Homo_723,AD.vs.MCI; bulk RNA-seq,Homo_714,AD.vs.control; bulk RNA-seq,Homo_714,AD.vs.MCI; bulk RNA-seq,Homo_633,AD.vs.control; bulk RNA-seq,Homo_633,AD.vs.MCI	7
BP	GO:0060191	regulation of lipase activity	bulk RNA-seq,ACOM,AD.vs.control; bulk RNA-seq,Homo_723,AD.vs.control; bulk RNA-seq,Homo_723,AD.vs.MCI; bulk RNA-seq,Homo_723,MCI.vs.control; bulk RNA-seq,Homo_714,AD.vs.control; bulk RNA-seq,Homo_714,AD.vs.MCI; bulk RNA-seq,Homo_714,MCI.vs.control; bulk RNA-seq,Homo_633,AD.vs.control; bulk RNA-seq,Homo_633,AD.vs.MCI	9
BP	GO:0055092	sterol homeostasis	bulk RNA-seq,ACOM,AD.vs.control; bulk RNA-seq,Homo_723,AD.vs.control; bulk RNA-seq,Homo_723,AD.vs.MCI; bulk RNA-seq,Homo_723,MCI.vs.control; bulk RNA-seq,Homo_714,AD.vs.MCI; bulk RNA-seq,Homo_714,MCI.vs.control; bulk RNA-seq,Homo_633,AD.vs.control; bulk RNA-seq,Homo_633,AD.vs.MCI	8
BP	GO:0034219	carbohydrate transmembrane transport	bulk RNA-seq,ACOM,AD.vs.control; bulk RNA-seq,Homo_723,AD.vs.MCI; bulk RNA-seq,Homo_723,MCI.vs.control; bulk RNA-seq,Homo_714,AD.vs.MCI; bulk RNA-seq,Homo_633,AD.vs.control; bulk RNA-seq,Homo_633,AD.vs.MCI	6
BP	GO:0043410	positive regulation of MAPK cascade	bulk RNA-seq,ACOM,AD.vs.control; bulk RNA-seq,Homo_723,AD.vs.control; bulk RNA-seq,Homo_723,AD.vs.MCI; bulk RNA-seq,Homo_714,AD.vs.control; bulk RNA-seq,Homo_714,AD.vs.MCI; bulk RNA-seq,Homo_633,AD.vs.control; bulk RNA-seq,Homo_633,AD.vs.MCI	7

CC	GO:0043025	neuronal cell body	bulk RNA-seq,ACOM,AD.vs.control; bulk RNA-seq,Emory,MCI.vs.control; bulk RNA-seq,Homo_723,AD.vs.control; bulk RNA-seq,Homo_723,AD.vs.MCI; bulk RNA-seq,Homo_714,AD.vs.control; bulk RNA-seq,Homo_714,AD.vs.MCI; bulk RNA-seq,Homo_633,AD.vs.control; bulk RNA-seq,Homo_633,AD.vs.MCI; bulk RNA-seq,ROSMAP,AD.vs.control; bulk RNA-seq,ROSMAP,MCI.vs.control; bulk RNA-seq,SRP223445,AD.vs.control	11
BP	GO:0046461	neutral lipid catabolic process	bulk RNA-seq,ACOM,AD.vs.control; bulk RNA-seq,Homo_723,AD.vs.MCI; bulk RNA-seq,Homo_723,MCI.vs.control; bulk RNA-seq,Homo_714,AD.vs.MCI; bulk RNA-seq,Homo_714,MCI.vs.control	5
BP	GO:0046464	acylglycerol catabolic process	bulk RNA-seq,ACOM,AD.vs.control; bulk RNA-seq,Homo_723,AD.vs.MCI; bulk RNA-seq,Homo_723,MCI.vs.control; bulk RNA-seq,Homo_714,AD.vs.MCI; bulk RNA-seq,Homo_714,MCI.vs.control	5
BP	GO:0034370	triglyceride-rich lipoprotein particle remodeling	bulk RNA-seq,ACOM,AD.vs.control; bulk RNA-seq,Homo_723,AD.vs.MCI; bulk RNA-seq,Homo_723,MCI.vs.control; bulk RNA-seq,Homo_714,AD.vs.control; bulk RNA-seq,Homo_714,AD.vs.MCI; bulk RNA-seq,Homo_714,MCI.vs.control	6
BP	GO:0034372	very-low-density lipoprotein particle remodeling	bulk RNA-seq,ACOM,AD.vs.control; bulk RNA-seq,Homo_723,AD.vs.MCI; bulk RNA-seq,Homo_723,MCI.vs.control; bulk RNA-seq,Homo_714,AD.vs.control; bulk RNA-seq,Homo_714,AD.vs.MCI; bulk RNA-seq,Homo_714,MCI.vs.control	6
BP	GO:0050817	coagulation	bulk RNA-seq,ACOM,AD.vs.control; bulk RNA-seq,Homo_723,AD.vs.control; bulk RNA-seq,Homo_723,AD.vs.MCI; bulk RNA-seq,Homo_723,MCI.vs.control; bulk RNA-seq,Homo_714,AD.vs.control; bulk RNA-seq,Homo_714,AD.vs.MCI; bulk RNA-seq,Homo_633,AD.vs.control; bulk RNA-seq,Homo_633,AD.vs.MCI	8
BP	GO:0042632	cholesterol homeostasis	bulk RNA-seq,ACOM,AD.vs.control; bulk RNA-seq,Homo_723,AD.vs.MCI; bulk RNA-seq,Homo_723,MCI.vs.control; bulk RNA-seq,Homo_714,AD.vs.MCI; bulk RNA-seq,Homo_714,MCI.vs.control; bulk RNA-seq,Homo_633,AD.vs.control; bulk RNA-seq,Homo_633,AD.vs.MCI	7
BP	GO:0008643	carbohydrate transport	bulk RNA-seq,ACOM,AD.vs.control; bulk RNA-seq,Homo_723,MCI.vs.control; bulk RNA-seq,Homo_714,AD.vs.MCI; bulk RNA-seq,Homo_633,AD.vs.control; bulk RNA-seq,Homo_633,AD.vs.MCI	5
BP	GO:0015748	organophosphate ester transport	bulk RNA-seq,ACOM,AD.vs.control; bulk RNA-seq,Homo_723,AD.vs.MCI; bulk RNA-seq,Homo_723,MCI.vs.control; bulk RNA-seq,Homo_714,AD.vs.MCI; bulk RNA-seq,Homo_633,AD.vs.control; bulk RNA-seq,Homo_633,AD.vs.MCI; scRNA-seq,SRP330776,Naive CD8+ T cell_2-AD.vs.control	7
BP	GO:0003018	vascular process in circulatory system	bulk RNA-seq,ACOM,AD.vs.control; bulk RNA-seq,Homo_723,AD.vs.control; bulk RNA-seq,Homo_723,AD.vs.MCI; bulk RNA-seq,Homo_723,MCI.vs.control; bulk RNA-seq,Homo_714,AD.vs.control; bulk RNA-seq,Homo_714,AD.vs.MCI; bulk RNA-seq,Homo_633,AD.vs.control; bulk RNA-seq,Homo_633,AD.vs.MCI	8
BP	GO:0007599	hemostasis	bulk RNA-seq,ACOM,AD.vs.control; bulk RNA-seq,Homo_723,AD.vs.control; bulk RNA-seq,Homo_723,AD.vs.MCI; bulk RNA-seq,Homo_723,MCI.vs.control; bulk RNA-seq,Homo_714,AD.vs.control; bulk RNA-seq,Homo_714,AD.vs.MCI; bulk RNA-seq,Homo_633,AD.vs.control; bulk RNA-seq,Homo_633,AD.vs.MCI	8
BP	GO:0031331	positive regulation of cellular catabolic process	bulk RNA-seq,ACOM,AD.vs.control; bulk RNA-seq,Homo_723,AD.vs.MCI; bulk RNA-seq,Homo_714,AD.vs.control; bulk RNA-seq,Homo_714,AD.vs.MCI; bulk RNA-seq,Homo_633,AD.vs.control; bulk RNA-seq,Homo_633,AD.vs.MCI	6
BP	GO:0007596	blood coagulation	bulk RNA-seq,ACOM,AD.vs.control; bulk RNA-seq,Homo_723,AD.vs.control; bulk RNA-seq,Homo_723,AD.vs.MCI; bulk RNA-seq,Homo_723,MCI.vs.control; bulk RNA-seq,Homo_714,AD.vs.control; bulk RNA-seq,Homo_714,AD.vs.MCI; bulk RNA-seq,Homo_633,AD.vs.control; bulk RNA-seq,Homo_633,AD.vs.MCI	8
BP	GO:0051346	negative regulation of hydrolase activity	bulk RNA-seq,ACOM,AD.vs.control; bulk RNA-seq,Homo_723,AD.vs.control; bulk RNA-seq,Homo_723,AD.vs.MCI; bulk RNA-seq,Homo_714,AD.vs.control; bulk RNA-seq,Homo_714,AD.vs.MCI; bulk RNA-seq,Homo_633,AD.vs.control; bulk RNA-seq,Homo_633,AD.vs.MCI	7
BP	GO:0032355	response to estradiol	bulk RNA-seq,ACOM,AD.vs.control; bulk RNA-seq,Homo_723,AD.vs.control; bulk RNA-seq,Homo_723,AD.vs.MCI; bulk RNA-seq,Homo_723,MCI.vs.control; bulk RNA-seq,Homo_714,AD.vs.MCI; bulk RNA-seq,Homo_714,MCI.vs.control; bulk RNA-seq,Homo_633,AD.vs.control; bulk RNA-seq,Homo_633,AD.vs.MCI	8
BP	GO:0046486	glycerolipid metabolic process	bulk RNA-seq,ACOM,AD.vs.control; bulk RNA-seq,Homo_723,AD.vs.MCI; bulk RNA-seq,Homo_714,AD.vs.MCI; bulk RNA-seq,Homo_633,AD.vs.control; bulk RNA-seq,Homo_633,AD.vs.MCI; scRNA-seq,SRP330776,CD8+ T cell_1-AD.vs.control; scRNA-seq,SRP330776,CD8+ T cell_2-AD.vs.control; scRNA-seq,SRP330776,Naive CD8+ T cell_2-AD.vs.control	8
CC	GO:0072562	blood microparticle	bulk RNA-seq,ACOM,AD.vs.control; bulk RNA-seq,Homo_723,AD.vs.control; bulk RNA-seq,Homo_723,AD.vs.MCI; bulk RNA-seq,Homo_723,MCI.vs.control; bulk RNA-seq,Homo_714,AD.vs.MCI; bulk RNA-seq,Homo_633,AD.vs.control; bulk RNA-seq,Homo_633,AD.vs.MCI; bulk RNA-seq,SRP223445,AD.vs.control; scRNA-seq,SRP330776,Naive CD8+ T cell_1-AD.vs.control; scRNA-seq,SRP330776,Naive CD8+ T cell_2-AD.vs.control	10
BP	GO:0050892	intestinal absorption	bulk RNA-seq,ACOM,AD.vs.control; bulk RNA-seq,Homo_723,AD.vs.MCI; bulk RNA-seq,Homo_723,MCI.vs.control; bulk RNA-seq,Homo_714,AD.vs.MCI; bulk RNA-seq,Homo_714,MCI.vs.control; bulk RNA-seq,Homo_633,AD.vs.control; bulk RNA-seq,Homo_633,AD.vs.MCI	7
BP	GO:0034381	plasma lipoprotein particle clearance	bulk RNA-seq,ACOM,AD.vs.control; bulk RNA-seq,Homo_723,AD.vs.control; bulk RNA-seq,Homo_723,AD.vs.MCI; bulk RNA-seq,Homo_723,MCI.vs.control; bulk RNA-seq,Homo_714,AD.vs.control; bulk RNA-seq,Homo_714,AD.vs.MCI; bulk RNA-seq,Homo_714,MCI.vs.control	7
BP	GO:0034377	plasma lipoprotein particle assembly	bulk RNA-seq,ACOM,AD.vs.control; bulk RNA-seq,Homo_723,AD.vs.MCI; bulk RNA-seq,Homo_723,MCI.vs.control; bulk RNA-seq,Homo_714,AD.vs.MCI; bulk RNA-seq,Homo_714,MCI.vs.control	5
CC	GO:0097060	synaptic membrane	bulk RNA-seq,ACOM,AD.vs.control; bulk RNA-seq,Emory,MCI.vs.control; bulk RNA-seq,Homo_723,AD.vs.control; bulk RNA-seq,Homo_723,AD.vs.MCI; bulk RNA-seq,Homo_714,AD.vs.control; bulk RNA-seq,Homo_714,AD.vs.MCI; bulk RNA-seq,Homo_633,AD.vs.control; bulk RNA-seq,Homo_633,AD.vs.MCI; bulk RNA-seq,Homo_633,MCI.vs.control; bulk RNA-seq,ROSMAP,AD.vs.control; bulk RNA-seq,ROSMAP,MCI.vs.control; bulk RNA-seq,SRP223445,AD.vs.control	12
BP	GO:1903522	regulation of blood circulation	bulk RNA-seq,ACOM,AD.vs.control; bulk RNA-seq,Emory,MCI.vs.control; bulk RNA-seq,Homo_723,AD.vs.control; bulk RNA-seq,Homo_723,AD.vs.MCI; bulk RNA-seq,Homo_723,MCI.vs.control; bulk RNA-seq,Homo_714,AD.vs.control; bulk RNA-seq,Homo_714,AD.vs.MCI; bulk RNA-seq,Homo_633,AD.vs.control	8
BP	GO:0044058	regulation of digestive system process	bulk RNA-seq,ACOM,AD.vs.control; bulk RNA-seq,Homo_723,MCI.vs.control	2
BP	GO:0015918	sterol transport	bulk RNA-seq,ACOM,AD.vs.control; bulk RNA-seq,Homo_723,AD.vs.MCI; bulk RNA-seq,Homo_723,MCI.vs.control; bulk RNA-seq,Homo_714,AD.vs.MCI; bulk RNA-seq,Homo_633,AD.vs.control; bulk RNA-seq,Homo_633,AD.vs.MCI	6
BP	GO:0019433	triglyceride catabolic process	bulk RNA-seq,ACOM,AD.vs.control; bulk RNA-seq,Homo_723,AD.vs.MCI; bulk RNA-seq,Homo_723,MCI.vs.control; bulk RNA-seq,Homo_714,AD.vs.MCI; bulk RNA-seq,Homo_714,MCI.vs.control	5
BP	GO:0050996	positive regulation of lipid catabolic process	bulk RNA-seq,ACOM,AD.vs.control; bulk RNA-seq,Homo_723,AD.vs.MCI; bulk RNA-seq,Homo_723,MCI.vs.control; bulk RNA-seq,Homo_714,AD.vs.MCI; bulk RNA-seq,Homo_714,MCI.vs.control; bulk RNA-seq,Homo_633,AD.vs.control; bulk RNA-seq,Homo_633,AD.vs.MCI	7
BP	GO:0015749	monosaccharide transmembrane transport	bulk RNA-seq,ACOM,AD.vs.control; bulk RNA-seq,Homo_723,AD.vs.MCI; bulk RNA-seq,Homo_723,MCI.vs.control; bulk RNA-seq,Homo_714,AD.vs.MCI; bulk RNA-seq,Homo_714,MCI.vs.control; bulk RNA-seq,Homo_633,AD.vs.control; bulk RNA-seq,Homo_633,AD.vs.MCI	7
BP	GO:0055065	metal ion homeostasis	bulk RNA-seq,ACOM,AD.vs.control; scRNA-seq,SRP330776,Naive CD8+ T cell_2-AD.vs.control	2
BP	GO:0030301	cholesterol transport	bulk RNA-seq,ACOM,AD.vs.control; bulk RNA-seq,Homo_723,AD.vs.MCI; bulk RNA-seq,Homo_723,MCI.vs.control; bulk RNA-seq,Homo_714,AD.vs.MCI; bulk RNA-seq,Homo_714,MCI.vs.control; bulk RNA-seq,Homo_633,AD.vs.control; bulk RNA-seq,Homo_633,AD.vs.MCI	7
BP	GO:0007586	digestion	bulk RNA-seq,ACOM,AD.vs.control; bulk RNA-seq,Emory,MCI.vs.control; bulk RNA-seq,Homo_723,AD.vs.control; bulk RNA-seq,Homo_723,AD.vs.MCI; bulk RNA-seq,Homo_723,MCI.vs.control; bulk RNA-seq,Homo_714,AD.vs.control; bulk RNA-seq,Homo_714,AD.vs.MCI; bulk RNA-seq,SRP223445,AD.vs.control	8
BP	GO:0035264	multicellular organism growth	bulk RNA-seq,ACOM,AD.vs.control; bulk RNA-seq,Homo_723,AD.vs.MCI; bulk RNA-seq,Homo_723,MCI.vs.control; bulk RNA-seq,Homo_714,AD.vs.MCI; bulk RNA-seq,Homo_633,AD.vs.control; bulk RNA-seq,Homo_633,AD.vs.MCI	6

BP	GO:0032103	positive regulation of response to external stimulus	bulk RNA-seq,ACOM,AD.vs.control; bulk RNA-seq,Homo_723,AD.vs.MCI; bulk RNA-seq,Homo_714,AD.vs.MCI; bulk RNA-seq,Homo_633,AD.vs.control; bulk RNA-seq,Homo_633,AD.vs.MCI; scRNA-seq,SRP330776,Naive CD8+ T cell_2-AD.vs.control	6
BP	GO:0003012	muscle system process	bulk RNA-seq,ACOM,AD.vs.control; bulk RNA-seq,Homo_723,AD.vs.control; bulk RNA-seq,Homo_723,AD.vs.MCI; bulk RNA-seq,Homo_714,AD.vs.control; bulk RNA-seq,Homo_714,AD.vs.MCI; bulk RNA-seq,Homo_633,AD.vs.control; bulk RNA-seq,Homo_633,AD.vs.MCI	7
BP	GO:1905918	regulation of CoA-transferase activity	bulk RNA-seq,ACOM,AD.vs.control; bulk RNA-seq,Homo_723,AD.vs.MCI; bulk RNA-seq,Homo_723,MCI.vs.control; bulk RNA-seq,Homo_714,AD.vs.control; bulk RNA-seq,Homo_714,AD.vs.MCI; bulk RNA-seq,Homo_714,MCI.vs.control	6
BP	GO:0043171	peptide catabolic process	bulk RNA-seq,ACOM,AD.vs.control; bulk RNA-seq,Homo_723,MCI.vs.control; bulk RNA-seq,Homo_714,MCI.vs.control; bulk RNA-seq,Homo_633,AD.vs.control; bulk RNA-seq,Homo_633,AD.vs.MCI	5
BP	GO:0045765	regulation of angiogenesis	bulk RNA-seq,ACOM,AD.vs.control; bulk RNA-seq,Homo_723,AD.vs.control; bulk RNA-seq,Homo_723,AD.vs.MCI; bulk RNA-seq,Homo_714,AD.vs.control; bulk RNA-seq,Homo_714,AD.vs.MCI; bulk RNA-seq,Homo_633,AD.vs.control; bulk RNA-seq,Homo_633,AD.vs.MCI	7
BP	GO:0033700	phospholipid efflux	bulk RNA-seq,ACOM,AD.vs.control; bulk RNA-seq,Homo_723,AD.vs.MCI; bulk RNA-seq,Homo_723,MCI.vs.control; bulk RNA-seq,Homo_714,AD.vs.control; bulk RNA-seq,Homo_714,AD.vs.MCI; bulk RNA-seq,Homo_714,MCI.vs.control	6
BP	GO:1901342	regulation of vasculature development	bulk RNA-seq,ACOM,AD.vs.control; bulk RNA-seq,Homo_723,AD.vs.control; bulk RNA-seq,Homo_723,AD.vs.MCI; bulk RNA-seq,Homo_714,AD.vs.control; bulk RNA-seq,Homo_714,AD.vs.MCI; bulk RNA-seq,Homo_633,AD.vs.control; bulk RNA-seq,Homo_633,AD.vs.MCI	7
BP	GO:0042060	wound healing	bulk RNA-seq,ACOM,AD.vs.control; bulk RNA-seq,Homo_723,AD.vs.control; bulk RNA-seq,Homo_723,AD.vs.MCI; bulk RNA-seq,Homo_714,AD.vs.control; bulk RNA-seq,Homo_714,AD.vs.MCI; bulk RNA-seq,Homo_633,AD.vs.control; bulk RNA-seq,Homo_633,AD.vs.MCI	7
CC	GO:0043235	receptor complex	bulk RNA-seq,ACOM,AD.vs.control; bulk RNA-seq,ROSMAP,AD.vs.control; bulk RNA-seq,ROSMAP,MCI.vs.control; scRNA-seq,SRP330776,Natural killer cell_1-AD.vs.control; scRNA-seq,SRP215507,CD8+ T cell_1-AD.vs.control; scRNA-seq,SRP215507,CD8+ T cell_1-MCI.vs.control; scRNA-	7
BP	GO:0008202	steroid metabolic process	bulk RNA-seq,ACOM,AD.vs.control; bulk RNA-seq,Homo_723,AD.vs.control; bulk RNA-seq,Homo_723,AD.vs.MCI; bulk RNA-seq,Homo_723,MCI.vs.control; bulk RNA-seq,Homo_714,AD.vs.control; bulk RNA-seq,Homo_714,AD.vs.MCI; bulk RNA-seq,Homo_633,AD.vs.control; bulk RNA-seq,Homo_633,AD.vs.MCI	8
BP	GO:0006869	lipid transport	bulk RNA-seq,ACOM,AD.vs.control; bulk RNA-seq,Homo_723,AD.vs.control; bulk RNA-seq,Homo_723,AD.vs.MCI; bulk RNA-seq,Homo_714,AD.vs.control; bulk RNA-seq,Homo_714,AD.vs.MCI; bulk RNA-seq,Homo_633,AD.vs.control; bulk RNA-seq,Homo_633,AD.vs.MCI	7
BP	GO:0050818	regulation of coagulation	bulk RNA-seq,ACOM,AD.vs.control; bulk RNA-seq,Homo_723,AD.vs.control; bulk RNA-seq,Homo_723,AD.vs.MCI; bulk RNA-seq,Homo_723,MCI.vs.control; bulk RNA-seq,Homo_714,AD.vs.control; bulk RNA-seq,Homo_714,AD.vs.MCI; bulk RNA-seq,Homo_714,MCI.vs.control; bulk RNA-seq,Homo_633,AD.vs.control; bulk RNA-seq,Homo_633,AD.vs.MCI	9
BP	GO:0065005	protein-lipid complex assembly	bulk RNA-seq,ACOM,AD.vs.control; bulk RNA-seq,Homo_723,AD.vs.MCI; bulk RNA-seq,Homo_723,MCI.vs.control; bulk RNA-seq,Homo_714,AD.vs.MCI; bulk RNA-seq,Homo_714,MCI.vs.control; bulk RNA-seq,Homo_633,AD.vs.MCI	6
BP	GO:0019216	regulation of lipid metabolic process	bulk RNA-seq,ACOM,AD.vs.control; bulk RNA-seq,Homo_723,AD.vs.control; bulk RNA-seq,Homo_723,AD.vs.MCI; bulk RNA-seq,Homo_714,AD.vs.control; bulk RNA-seq,Homo_714,AD.vs.MCI; bulk RNA-seq,Homo_633,AD.vs.control; bulk RNA-seq,Homo_633,AD.vs.MCI	7
BP	GO:0023061	signal release	bulk RNA-seq,ACOM,AD.vs.control; bulk RNA-seq,Emory,MCI.vs.control; bulk RNA-seq,Homo_723,AD.vs.MCI; bulk RNA-seq,Homo_714,AD.vs.control; bulk RNA-seq,Homo_714,AD.vs.MCI; bulk RNA-seq,Homo_633,AD.vs.control; bulk RNA-seq,Homo_633,AD.vs.MCI	8
BP	GO:0045834	positive regulation of lipid metabolic process	bulk RNA-seq,ACOM,AD.vs.control; bulk RNA-seq,Homo_723,AD.vs.MCI; bulk RNA-seq,Homo_723,MCI.vs.control; bulk RNA-seq,Homo_714,AD.vs.MCI; bulk RNA-seq,Homo_633,AD.vs.control; bulk RNA-seq,Homo_633,AD.vs.MCI	6
BP	GO:0031100	animal organ regeneration	bulk RNA-seq,ACOM,AD.vs.control; bulk RNA-seq,Homo_723,AD.vs.MCI; bulk RNA-seq,Homo_723,MCI.vs.control; bulk RNA-seq,Homo_714,AD.vs.MCI; bulk RNA-seq,Homo_714,MCI.vs.control; bulk RNA-seq,Homo_633,AD.vs.control; bulk RNA-seq,Homo_633,AD.vs.MCI	7
BP	GO:0008645	hexose transmembrane transport	bulk RNA-seq,ACOM,AD.vs.control; bulk RNA-seq,Homo_723,AD.vs.MCI; bulk RNA-seq,Homo_723,MCI.vs.control; bulk RNA-seq,Homo_714,AD.vs.MCI; bulk RNA-seq,Homo_714,MCI.vs.control; bulk RNA-seq,Homo_633,AD.vs.control; bulk RNA-seq,Homo_633,AD.vs.MCI	7
BP	GO:1904659	glucose transmembrane transport	bulk RNA-seq,ACOM,AD.vs.control; bulk RNA-seq,Homo_723,AD.vs.MCI; bulk RNA-seq,Homo_723,MCI.vs.control; bulk RNA-seq,Homo_714,AD.vs.MCI; bulk RNA-seq,Homo_714,MCI.vs.control; bulk RNA-seq,Homo_633,AD.vs.control; bulk RNA-seq,Homo_633,AD.vs.MCI	7
BP	GO:0010817	regulation of hormone levels	bulk RNA-seq,ACOM,AD.vs.control	1
BP	GO:0070372	regulation of ERK1 and ERK2 cascade	bulk RNA-seq,ACOM,AD.vs.control; bulk RNA-seq,Homo_723,AD.vs.control; bulk RNA-seq,Homo_723,AD.vs.MCI; bulk RNA-seq,Homo_723,MCI.vs.control; bulk RNA-seq,Homo_714,AD.vs.control; bulk RNA-seq,Homo_714,AD.vs.MCI; bulk RNA-seq,Homo_633,AD.vs.control; bulk RNA-seq,Homo_633,AD.vs.MCI	8
BP	GO:0016042	lipid catabolic process	bulk RNA-seq,ACOM,AD.vs.control; bulk RNA-seq,Homo_723,AD.vs.MCI; bulk RNA-seq,Homo_714,AD.vs.MCI; bulk RNA-seq,Homo_633,AD.vs.control; bulk RNA-seq,Homo_633,AD.vs.MCI	5
CC	GO:0031091	platelet alpha granule	bulk RNA-seq,ACOM,AD.vs.control; bulk RNA-seq,Homo_723,AD.vs.control; bulk RNA-seq,Homo_723,AD.vs.MCI; bulk RNA-seq,Homo_723,MCI.vs.control; bulk RNA-seq,Homo_714,AD.vs.control; bulk RNA-seq,Homo_714,AD.vs.MCI; bulk RNA-seq,Homo_714,MCI.vs.control; bulk RNA-seq,Homo_633,AD.vs.control; bulk RNA-seq,Homo_633,AD.vs.MCI; scRNA-seq,SRP330776,Naive CD8+ T cell_2-AD.vs.control; scRNA-seq,SRP215507,CD8+	11
BP	GO:0061045	negative regulation of wound healing	bulk RNA-seq,ACOM,AD.vs.control; bulk RNA-seq,Homo_723,AD.vs.control; bulk RNA-seq,Homo_723,AD.vs.MCI; bulk RNA-seq,Homo_723,MCI.vs.control; bulk RNA-seq,Homo_714,AD.vs.control; bulk RNA-seq,Homo_714,AD.vs.MCI; bulk RNA-seq,Homo_714,MCI.vs.control	7
BP	GO:0010876	lipid localization	bulk RNA-seq,ACOM,AD.vs.control; bulk RNA-seq,Homo_723,AD.vs.control; bulk RNA-seq,Homo_723,AD.vs.MCI; bulk RNA-seq,Homo_714,AD.vs.control; bulk RNA-seq,Homo_714,AD.vs.MCI; bulk RNA-seq,Homo_633,AD.vs.control; bulk RNA-seq,Homo_633,AD.vs.MCI	7
CC	GO:0030133	transport vesicle	bulk RNA-seq,ACOM,AD.vs.control; bulk RNA-seq,Homo_723,AD.vs.control; bulk RNA-seq,Homo_723,AD.vs.MCI; bulk RNA-seq,Homo_714,AD.vs.control; bulk RNA-seq,Homo_714,AD.vs.MCI; bulk RNA-seq,Homo_633,AD.vs.control; bulk RNA-seq,Homo_633,AD.vs.MCI	7
BP	GO:0016441	post-transcriptional gene silencing	bulk RNA-seq,ACOM,AD.vs.control	1
BP	GO:0035194	post-transcriptional gene silencing by RNA	bulk RNA-seq,ACOM,AD.vs.control	1
BP	GO:0015914	phospholipid transport	bulk RNA-seq,ACOM,AD.vs.control; bulk RNA-seq,Homo_723,AD.vs.MCI; bulk RNA-seq,Homo_723,MCI.vs.control; bulk RNA-seq,Homo_714,AD.vs.MCI; bulk RNA-seq,Homo_714,MCI.vs.control; bulk RNA-seq,Homo_633,AD.vs.control; bulk RNA-seq,Homo_633,AD.vs.MCI	7
BP	GO:0010959	regulation of metal ion transport	bulk RNA-seq,ACOM,AD.vs.control; bulk RNA-seq,Homo_723,AD.vs.control; bulk RNA-seq,Homo_723,AD.vs.MCI; bulk RNA-seq,Homo_714,AD.vs.control; bulk RNA-seq,Homo_714,AD.vs.MCI; bulk RNA-seq,Homo_633,AD.vs.control; bulk RNA-seq,Homo_633,AD.vs.MCI	7
MF	GO:0120015	sterol transfer activity	bulk RNA-seq,ACOM,AD.vs.control; bulk RNA-seq,Homo_723,AD.vs.MCI; bulk RNA-seq,Homo_723,MCI.vs.control; bulk RNA-seq,Homo_714,AD.vs.MCI; bulk RNA-seq,Homo_714,MCI.vs.control; bulk RNA-seq,Homo_633,AD.vs.control	6
BP	GO:0030193	regulation of blood coagulation	bulk RNA-seq,ACOM,AD.vs.control; bulk RNA-seq,Homo_723,AD.vs.control; bulk RNA-seq,Homo_723,AD.vs.MCI; bulk RNA-seq,Homo_723,MCI.vs.control; bulk RNA-seq,Homo_714,AD.vs.control; bulk RNA-seq,Homo_714,AD.vs.MCI; bulk RNA-seq,Homo_714,MCI.vs.control; bulk RNA-seq,Homo_633,AD.vs.control; bulk RNA-seq,Homo_633,AD.vs.MCI	9

BP	GO:0014065	phosphatidylinositol 3-kinase signaling	bulk RNA-seq,ACOM,AD.vs.control; bulk RNA-seq,Homo_723,AD.vs.control; bulk RNA-seq,Homo_723,AD.vs.MCI; bulk RNA-seq,Homo_723,MCI.vs.control; bulk RNA-seq,Homo_714,AD.vs.MCI; bulk RNA-seq,Homo_633,AD.vs.control; bulk RNA-seq,Homo_633,AD.vs.MCI; bulk RNA-seq,ROSMAP,AD.vs.control; bulk RNA-seq,ROSMAP,MCI.vs.control	9
BP	GO:0001889	liver development	bulk RNA-seq,ACOM,AD.vs.control; bulk RNA-seq,Homo_723,AD.vs.control; bulk RNA-seq,Homo_723,AD.vs.MCI; bulk RNA-seq,Homo_723,MCI.vs.control; bulk RNA-seq,Homo_714,AD.vs.control; bulk RNA-seq,Homo_714,AD.vs.MCI; bulk RNA-seq,Homo_633,AD.vs.control; bulk RNA-seq,Homo_633,AD.vs.MCI	8
BP	GO:0005996	monosaccharide metabolic process	bulk RNA-seq,ACOM,AD.vs.control; bulk RNA-seq,Homo_723,MCI.vs.control; bulk RNA-seq,Homo_714,AD.vs.MCI; bulk RNA-seq,Homo_633,AD.vs.control; bulk RNA-seq,Homo_633,AD.vs.MCI	5
BP	GO:0001819	positive regulation of cytokine production	bulk RNA-seq,ACOM,AD.vs.control; bulk RNA-seq,Homo_723,AD.vs.MCI; bulk RNA-seq,Homo_714,AD.vs.MCI; bulk RNA-seq,Homo_633,AD.vs.control; bulk RNA-seq,Homo_633,AD.vs.MCI	5
MF	GO:0043177	organic acid binding	bulk RNA-seq,ACOM,AD.vs.control; bulk RNA-seq,Homo_723,AD.vs.control; bulk RNA-seq,Homo_723,AD.vs.MCI; bulk RNA-seq,Homo_723,MCI.vs.control; bulk RNA-seq,Homo_714,AD.vs.MCI; bulk RNA-seq,Homo_633,AD.vs.control; bulk RNA-seq,Homo_633,AD.vs.MCI	7
BP	GO:0051604	protein maturation	bulk RNA-seq,ACOM,AD.vs.control; bulk RNA-seq,Homo_723,AD.vs.control; bulk RNA-seq,Homo_723,AD.vs.MCI; bulk RNA-seq,Homo_714,AD.vs.MCI; bulk RNA-seq,Homo_633,AD.vs.control; bulk RNA-seq,Homo_633,AD.vs.MCI	6
BP	GO:0014066	regulation of phosphatidylinositol 3-kinase signaling	bulk RNA-seq,ACOM,AD.vs.control; bulk RNA-seq,Homo_723,AD.vs.control; bulk RNA-seq,Homo_723,AD.vs.MCI; bulk RNA-seq,Homo_723,MCI.vs.control; bulk RNA-seq,Homo_714,AD.vs.control; bulk RNA-seq,Homo_714,AD.vs.MCI; bulk RNA-seq,Homo_714,MCI.vs.control; bulk RNA-seq,Homo_633,AD.vs.control; bulk RNA-seq,Homo_633,AD.vs.MCI	9
BP	GO:0006836	neurotransmitter transport	bulk RNA-seq,ACOM,AD.vs.control; bulk RNA-seq,Homo_723,AD.vs.control; bulk RNA-seq,Homo_723,AD.vs.MCI; bulk RNA-seq,Homo_723,MCI.vs.control; bulk RNA-seq,Homo_714,AD.vs.control; bulk RNA-seq,Homo_714,AD.vs.MCI; bulk RNA-seq,Homo_633,AD.vs.control; bulk RNA-seq,Homo_633,AD.vs.MCI; bulk RNA-seq,ROSMAP,AD.vs.control; bulk RNA-seq,ROSMAP,MCI.vs.control	10
BP	GO:0034374	low-density lipoprotein particle remodeling	bulk RNA-seq,ACOM,AD.vs.control; bulk RNA-seq,Homo_723,AD.vs.control; bulk RNA-seq,Homo_723,AD.vs.MCI; bulk RNA-seq,Homo_723,MCI.vs.control; bulk RNA-seq,Homo_714,AD.vs.control; bulk RNA-seq,Homo_714,AD.vs.MCI; bulk RNA-seq,Homo_714,MCI.vs.control	7
BP	GO:0010466	negative regulation of peptidase activity	bulk RNA-seq,ACOM,AD.vs.control; bulk RNA-seq,Homo_723,AD.vs.control; bulk RNA-seq,Homo_723,AD.vs.MCI; bulk RNA-seq,Homo_723,MCI.vs.control; bulk RNA-seq,Homo_714,AD.vs.control; bulk RNA-seq,Homo_714,AD.vs.MCI; bulk RNA-seq,Homo_633,AD.vs.control; bulk RNA-seq,Homo_633,AD.vs.MCI	8
CC	GO:0034358	plasma lipoprotein particle	bulk RNA-seq,ACOM,AD.vs.control; bulk RNA-seq,Homo_723,AD.vs.MCI; bulk RNA-seq,Homo_723,MCI.vs.control; bulk RNA-seq,Homo_714,AD.vs.MCI; bulk RNA-seq,Homo_714,MCI.vs.control; bulk RNA-seq,Homo_633,AD.vs.control; bulk RNA-seq,Homo_633,AD.vs.MCI	7
CC	GO:1990777	lipoprotein particle	bulk RNA-seq,ACOM,AD.vs.control; bulk RNA-seq,Homo_723,AD.vs.MCI; bulk RNA-seq,Homo_723,MCI.vs.control; bulk RNA-seq,Homo_714,AD.vs.MCI; bulk RNA-seq,Homo_714,MCI.vs.control; bulk RNA-seq,Homo_633,AD.vs.control; bulk RNA-seq,Homo_633,AD.vs.MCI	7
BP	GO:0034375	high-density lipoprotein particle remodeling	bulk RNA-seq,ACOM,AD.vs.control; bulk RNA-seq,Homo_723,AD.vs.control; bulk RNA-seq,Homo_723,AD.vs.MCI; bulk RNA-seq,Homo_723,MCI.vs.control; bulk RNA-seq,Homo_714,AD.vs.control; bulk RNA-seq,Homo_714,AD.vs.MCI; bulk RNA-seq,Homo_714,MCI.vs.control	7
BP	GO:0061041	regulation of wound healing	bulk RNA-seq,ACOM,AD.vs.control; bulk RNA-seq,Homo_723,AD.vs.control; bulk RNA-seq,Homo_723,AD.vs.MCI; bulk RNA-seq,Homo_723,MCI.vs.control; bulk RNA-seq,Homo_714,AD.vs.control; bulk RNA-seq,Homo_714,AD.vs.MCI; bulk RNA-seq,Homo_633,AD.vs.control; bulk RNA-seq,Homo_633,AD.vs.MCI	8
BP	GO:0019318	hexose metabolic process	bulk RNA-seq,ACOM,AD.vs.control; bulk RNA-seq,Homo_723,MCI.vs.control; bulk RNA-seq,Homo_714,AD.vs.MCI; bulk RNA-seq,Homo_633,AD.vs.control; bulk RNA-seq,Homo_633,AD.vs.MCI	5
BP	GO:0098657	import into cell	bulk RNA-seq,ACOM,AD.vs.control; bulk RNA-seq,Homo_723,AD.vs.control; bulk RNA-seq,Homo_723,AD.vs.MCI; bulk RNA-seq,Homo_723,MCI.vs.control; bulk RNA-seq,Homo_714,AD.vs.MCI; bulk RNA-seq,Homo_633,AD.vs.control; bulk RNA-seq,Homo_633,AD.vs.MCI; bulk RNA-seq,ROSMAP,AD.vs.control; bulk RNA-seq,SRP223445,AD.vs.control	9
CC	GO:0016442	RISC complex	bulk RNA-seq,ACOM,AD.vs.control; bulk RNA-seq,Homo_723,AD.vs.control; bulk RNA-seq,Homo_723,AD.vs.MCI; bulk RNA-seq,Homo_723,MCI.vs.control; bulk RNA-seq,Homo_714,AD.vs.control; bulk RNA-seq,Homo_714,AD.vs.MCI; bulk RNA-seq,Homo_714,MCI.vs.control; bulk RNA-seq,Homo_633,MCI.vs.control; bulk RNA-seq,SRP223445,AD.vs.control	9
CC	GO:0031332	RNAi effector complex	bulk RNA-seq,ACOM,AD.vs.control; bulk RNA-seq,Homo_723,AD.vs.control; bulk RNA-seq,Homo_723,AD.vs.MCI; bulk RNA-seq,Homo_723,MCI.vs.control; bulk RNA-seq,Homo_714,AD.vs.control; bulk RNA-seq,Homo_714,AD.vs.MCI; bulk RNA-seq,Homo_714,MCI.vs.control; bulk RNA-seq,Homo_633,MCI.vs.control; bulk RNA-seq,SRP223445,AD.vs.control	9
MF	GO:0015248	sterol transporter activity	bulk RNA-seq,ACOM,AD.vs.control; bulk RNA-seq,Homo_723,AD.vs.MCI; bulk RNA-seq,Homo_723,MCI.vs.control; bulk RNA-seq,Homo_714,AD.vs.MCI; bulk RNA-seq,Homo_714,MCI.vs.control; bulk RNA-seq,Homo_633,AD.vs.control; bulk RNA-seq,Homo_633,AD.vs.MCI	7
BP	GO:0072378	blood coagulation, fibrin clot formation	bulk RNA-seq,ACOM,AD.vs.control; bulk RNA-seq,Homo_723,AD.vs.control; bulk RNA-seq,Homo_723,AD.vs.MCI; bulk RNA-seq,Homo_723,MCI.vs.control; bulk RNA-seq,Homo_714,AD.vs.control; bulk RNA-seq,Homo_714,AD.vs.MCI; bulk RNA-seq,Homo_714,MCI.vs.control	7
CC	GO:0032994	protein-lipid complex	bulk RNA-seq,ACOM,AD.vs.control; bulk RNA-seq,Homo_723,AD.vs.MCI; bulk RNA-seq,Homo_723,MCI.vs.control; bulk RNA-seq,Homo_714,AD.vs.MCI; bulk RNA-seq,Homo_714,MCI.vs.control; bulk RNA-seq,Homo_633,AD.vs.control; bulk RNA-seq,Homo_633,AD.vs.MCI	7
BP	GO:0006006	glucose metabolic process	bulk RNA-seq,ACOM,AD.vs.control; bulk RNA-seq,Homo_723,MCI.vs.control; bulk RNA-seq,Homo_633,AD.vs.control; bulk RNA-seq,Homo_633,AD.vs.MCI	4
MF	GO:1901681	sulfur compound binding	bulk RNA-seq,ACOM,AD.vs.control; bulk RNA-seq,Homo_723,AD.vs.control; bulk RNA-seq,Homo_723,AD.vs.MCI; bulk RNA-seq,Homo_723,MCI.vs.control; bulk RNA-seq,Homo_714,AD.vs.control; bulk RNA-seq,Homo_714,AD.vs.MCI; bulk RNA-seq,Homo_633,AD.vs.control; bulk RNA-seq,Homo_633,AD.vs.MCI	8
BP	GO:0061008	hepaticobiliary system development	bulk RNA-seq,ACOM,AD.vs.control; bulk RNA-seq,Homo_723,AD.vs.control; bulk RNA-seq,Homo_723,AD.vs.MCI; bulk RNA-seq,Homo_723,MCI.vs.control; bulk RNA-seq,Homo_714,AD.vs.control; bulk RNA-seq,Homo_714,AD.vs.MCI; bulk RNA-seq,Homo_633,AD.vs.control; bulk RNA-seq,Homo_633,AD.vs.MCI	8
BP	GO:0009636	response to toxic substance	bulk RNA-seq,ACOM,AD.vs.control; bulk RNA-seq,Homo_723,AD.vs.MCI; bulk RNA-seq,Homo_723,MCI.vs.control; bulk RNA-seq,Homo_714,AD.vs.MCI; bulk RNA-seq,Homo_633,AD.vs.control; bulk RNA-seq,Homo_633,AD.vs.MCI	6
BP	GO:0072507	divalent inorganic cation homeostasis	bulk RNA-seq,ACOM,AD.vs.control; bulk RNA-seq,Homo_723,AD.vs.control; bulk RNA-seq,Homo_723,AD.vs.MCI; bulk RNA-seq,Homo_714,AD.vs.control; bulk RNA-seq,Homo_714,AD.vs.MCI; bulk RNA-seq,Homo_633,AD.vs.control; bulk RNA-seq,Homo_633,AD.vs.MCI	7
BP	GO:0019229	regulation of vasoconstriction	bulk RNA-seq,ACOM,AD.vs.control; bulk RNA-seq,Homo_723,AD.vs.control; bulk RNA-seq,Homo_723,AD.vs.MCI; bulk RNA-seq,Homo_723,MCI.vs.control; bulk RNA-seq,Homo_714,AD.vs.control; bulk RNA-seq,Homo_714,AD.vs.MCI; bulk RNA-seq,Homo_714,MCI.vs.control	7
BP	GO:0043651	linoleic acid metabolic process	bulk RNA-seq,ACOM,AD.vs.control; bulk RNA-seq,Homo_723,MCI.vs.control; bulk RNA-seq,Homo_714,MCI.vs.control	3
MF	GO:0120020	cholesterol transfer activity	bulk RNA-seq,ACOM,AD.vs.control; bulk RNA-seq,Homo_723,AD.vs.MCI; bulk RNA-seq,Homo_723,MCI.vs.control; bulk RNA-seq,Homo_714,AD.vs.MCI; bulk RNA-seq,Homo_714,MCI.vs.control	5
BP	GO:0045861	negative regulation of proteolysis	bulk RNA-seq,ACOM,AD.vs.control; bulk RNA-seq,Homo_723,AD.vs.control; bulk RNA-seq,Homo_723,AD.vs.MCI; bulk RNA-seq,Homo_714,AD.vs.control; bulk RNA-seq,Homo_714,AD.vs.MCI; bulk RNA-seq,Homo_633,AD.vs.control; bulk RNA-seq,Homo_633,AD.vs.MCI	7

BP	GO:0035592	establishment of protein localization to extracellular region	bulk RNA-seq,ACOM,AD.vs.control; bulk RNA-seq,Homo_723,AD.vs.control; bulk RNA-seq,Homo_723,AD.vs.MCI; bulk RNA-seq,Homo_714,AD.vs.MCI; bulk RNA-seq,Homo_633,AD.vs.control; bulk RNA-seq,Homo_633,AD.vs.MCI	6
BP	GO:0062012	regulation of small molecule metabolic process	bulk RNA-seq,ACOM,AD.vs.control; bulk RNA-seq,Homo_723,AD.vs.MCI; bulk RNA-seq,Homo_714,AD.vs.MCI; bulk RNA-seq,Homo_633,AD.vs.control; bulk RNA-seq,Homo_633,AD.vs.MCI	5
BP	GO:0035195	miRNA-mediated gene silencing	bulk RNA-seq,ACOM,AD.vs.control	1
BP	GO:0010951	negative regulation of endopeptidase activity	bulk RNA-seq,ACOM,AD.vs.control; bulk RNA-seq,Homo_723,AD.vs.control; bulk RNA-seq,Homo_723,AD.vs.MCI; bulk RNA-seq,Homo_723,MCI.vs.control; bulk RNA-seq,Homo_714,AD.vs.control; bulk RNA-seq,Homo_714,AD.vs.MCI; bulk RNA-seq,Homo_633,AD.vs.control; bulk RNA-seq,Homo_633,AD.vs.MCI	8
BP	GO:0043691	reverse cholesterol transport	bulk RNA-seq,ACOM,AD.vs.control; bulk RNA-seq,Homo_723,AD.vs.MCI; bulk RNA-seq,Homo_723,MCI.vs.control; bulk RNA-seq,Homo_714,AD.vs.MCI; bulk RNA-seq,Homo_714,MCI.vs.control; bulk RNA-seq,Homo_633,AD.vs.control; bulk RNA-seq,Homo_633,AD.vs.MCI	7
CC	GO:0031093	platelet alpha granule lumen	bulk RNA-seq,ACOM,AD.vs.control; bulk RNA-seq,Homo_723,AD.vs.control; bulk RNA-seq,Homo_723,AD.vs.MCI; bulk RNA-seq,Homo_723,MCI.vs.control; bulk RNA-seq,Homo_714,AD.vs.control; bulk RNA-seq,Homo_714,AD.vs.MCI; bulk RNA-seq,Homo_714,MCI.vs.control; bulk RNA-seq,Homo_633,AD.vs.control; bulk RNA-seq,Homo_633,AD.vs.MCI; scRNA-seq,SRP215507,CD8+ T cell_3-AD.vs.control	10
BP	GO:0051051	negative regulation of transport	bulk RNA-seq,ACOM,AD.vs.control; bulk RNA-seq,Homo_723,AD.vs.control; bulk RNA-seq,Homo_723,AD.vs.MCI; bulk RNA-seq,Homo_714,AD.vs.control; bulk RNA-seq,Homo_714,AD.vs.MCI; bulk RNA-seq,Homo_633,AD.vs.control; bulk RNA-seq,Homo_633,AD.vs.MCI	7
BP	GO:0051004	regulation of lipoprotein lipase activity	bulk RNA-seq,ACOM,AD.vs.control; bulk RNA-seq,Homo_723,AD.vs.MCI; bulk RNA-seq,Homo_723,MCI.vs.control; bulk RNA-seq,Homo_714,AD.vs.MCI; bulk RNA-seq,Homo_714,MCI.vs.control; bulk RNA-seq,ROSMAP,AD.vs.control	6
BP	GO:1905952	regulation of lipid localization	bulk RNA-seq,ACOM,AD.vs.control; bulk RNA-seq,Homo_723,AD.vs.MCI; bulk RNA-seq,Homo_723,MCI.vs.control; bulk RNA-seq,Homo_714,AD.vs.MCI; bulk RNA-seq,Homo_633,AD.vs.control; bulk RNA-seq,Homo_633,AD.vs.MCI	6
MF	GO:0004806	triglyceride lipase activity	bulk RNA-seq,ACOM,AD.vs.control	1
BP	GO:0048015	phosphatidylinositol-mediated signaling	bulk RNA-seq,ACOM,AD.vs.control; bulk RNA-seq,Homo_723,AD.vs.control; bulk RNA-seq,Homo_723,AD.vs.MCI; bulk RNA-seq,Homo_723,MCI.vs.control; bulk RNA-seq,Homo_714,AD.vs.MCI; bulk RNA-seq,Homo_633,AD.vs.control; bulk RNA-seq,Homo_633,AD.vs.MCI; bulk RNA-seq,ROSMAP,AD.vs.control	8
BP	GO:0001935	endothelial cell proliferation	bulk RNA-seq,ACOM,AD.vs.control; bulk RNA-seq,Homo_723,AD.vs.control; bulk RNA-seq,Homo_723,AD.vs.MCI; bulk RNA-seq,Homo_723,MCI.vs.control; bulk RNA-seq,Homo_714,AD.vs.control; bulk RNA-seq,Homo_714,AD.vs.MCI; bulk RNA-seq,Homo_633,AD.vs.control; bulk RNA-seq,Homo_633,AD.vs.MCI	8
BP	GO:0071692	protein localization to extracellular region	bulk RNA-seq,ACOM,AD.vs.control; bulk RNA-seq,Homo_723,AD.vs.control; bulk RNA-seq,Homo_723,AD.vs.MCI; bulk RNA-seq,Homo_714,AD.vs.MCI; bulk RNA-seq,Homo_633,AD.vs.control; bulk RNA-seq,Homo_633,AD.vs.MCI	6
BP	GO:0016125	sterol metabolic process	bulk RNA-seq,ACOM,AD.vs.control; bulk RNA-seq,Homo_723,AD.vs.MCI; bulk RNA-seq,Homo_723,MCI.vs.control; bulk RNA-seq,Homo_714,AD.vs.MCI; bulk RNA-seq,Homo_633,AD.vs.control; bulk RNA-seq,Homo_633,AD.vs.MCI	6
BP	GO:0070374	positive regulation of ERK1 and ERK2 cascade	bulk RNA-seq,ACOM,AD.vs.control; bulk RNA-seq,Homo_723,AD.vs.control; bulk RNA-seq,Homo_723,AD.vs.MCI; bulk RNA-seq,Homo_723,MCI.vs.control; bulk RNA-seq,Homo_714,AD.vs.control; bulk RNA-seq,Homo_714,AD.vs.MCI; bulk RNA-seq,Homo_633,AD.vs.control; bulk RNA-seq,Homo_633,AD.vs.MCI	8
MF	GO:0043178	alcohol binding	bulk RNA-seq,ACOM,AD.vs.control; bulk RNA-seq,Homo_723,AD.vs.MCI; bulk RNA-seq,Homo_723,MCI.vs.control; bulk RNA-seq,Homo_714,AD.vs.MCI; bulk RNA-seq,Homo_714,MCI.vs.control; bulk RNA-seq,Homo_633,AD.vs.control; bulk RNA-seq,Homo_633,AD.vs.MCI	7
BP	GO:0043568	positive regulation of insulin-like growth factor receptor signaling pathway	bulk RNA-seq,ACOM,AD.vs.control; bulk RNA-seq,Homo_723,AD.vs.MCI; bulk RNA-seq,Homo_723,MCI.vs.control; bulk RNA-seq,Homo_714,AD.vs.MCI; bulk RNA-seq,Homo_714,MCI.vs.control	5
BP	GO:0043567	regulation of insulin-like growth factor receptor signaling pathway	bulk RNA-seq,ACOM,AD.vs.control; bulk RNA-seq,Homo_723,AD.vs.MCI; bulk RNA-seq,Homo_723,MCI.vs.control; bulk RNA-seq,Homo_714,AD.vs.MCI; bulk RNA-seq,Homo_714,MCI.vs.control	5
MF	GO:0140104	molecular carrier activity	bulk RNA-seq,ACOM,AD.vs.control; bulk RNA-seq,Homo_723,AD.vs.control; bulk RNA-seq,Homo_723,MCI.vs.control; bulk RNA-seq,Homo_714,AD.vs.control; bulk RNA-seq,Homo_714,MCI.vs.control; bulk RNA-seq,Homo_633,AD.vs.control; bulk RNA-seq,Homo_633,AD.vs.MCI	7
BP	GO:0002526	acute inflammatory response	bulk RNA-seq,ACOM,AD.vs.control; bulk RNA-seq,Homo_723,AD.vs.MCI; bulk RNA-seq,Homo_723,MCI.vs.control; bulk RNA-seq,Homo_714,AD.vs.MCI; bulk RNA-seq,Homo_714,MCI.vs.control; bulk RNA-seq,Homo_633,AD.vs.control; bulk RNA-seq,Homo_633,AD.vs.MCI; bulk RNA-	8
BP	GO:0001667	ameboidal-type cell migration	bulk RNA-seq,ACOM,AD.vs.control; bulk RNA-seq,Homo_723,AD.vs.control; bulk RNA-seq,Homo_723,AD.vs.MCI; bulk RNA-seq,Homo_714,AD.vs.control; bulk RNA-seq,Homo_714,AD.vs.MCI; bulk RNA-seq,Homo_633,AD.vs.control; bulk RNA-seq,Homo_633,AD.vs.MCI; scRNA-seq,SRP330776,Naive CD8+ T	8
BP	GO:1901535	regulation of DNA demethylation	bulk RNA-seq,ACOM,AD.vs.control	1
BP	GO:0015837	amine transport	bulk RNA-seq,ACOM,AD.vs.control; bulk RNA-seq,Homo_723,AD.vs.control; bulk RNA-seq,Homo_723,AD.vs.MCI; bulk RNA-seq,Homo_723,MCI.vs.control; bulk RNA-seq,Homo_714,AD.vs.control; bulk RNA-seq,Homo_714,AD.vs.MCI; bulk RNA-seq,Homo_633,MCI.vs.control; bulk RNA-	8
BP	GO:0006066	alcohol metabolic process	bulk RNA-seq,ACOM,AD.vs.control; bulk RNA-seq,Homo_723,AD.vs.MCI; bulk RNA-seq,Homo_714,AD.vs.MCI; bulk RNA-seq,Homo_633,AD.vs.control; bulk RNA-seq,Homo_633,AD.vs.MCI	5
BP	GO:0046427	positive regulation of receptor signaling pathway via JAK-	bulk RNA-seq,ACOM,AD.vs.control; bulk RNA-seq,Homo_714,AD.vs.MCI	2
BP	GO:0050819	negative regulation of coagulation	bulk RNA-seq,ACOM,AD.vs.control; bulk RNA-seq,Homo_723,AD.vs.control; bulk RNA-seq,Homo_723,AD.vs.MCI; bulk RNA-seq,Homo_723,MCI.vs.control; bulk RNA-seq,Homo_714,AD.vs.control; bulk RNA-seq,Homo_714,AD.vs.MCI; bulk RNA-seq,Homo_714,MCI.vs.control; bulk RNA-seq,Homo_633,AD.vs.control; bulk RNA-seq,Homo_633,AD.vs.MCI	9
BP	GO:0033344	cholesterol efflux	bulk RNA-seq,ACOM,AD.vs.control; bulk RNA-seq,Homo_723,AD.vs.MCI; bulk RNA-seq,Homo_723,MCI.vs.control; bulk RNA-seq,Homo_714,AD.vs.MCI; bulk RNA-seq,Homo_714,MCI.vs.control	5
BP	GO:0008203	cholesterol metabolic process	bulk RNA-seq,ACOM,AD.vs.control; bulk RNA-seq,Homo_723,AD.vs.MCI; bulk RNA-seq,Homo_723,MCI.vs.control; bulk RNA-seq,Homo_714,AD.vs.MCI; bulk RNA-seq,Homo_714,MCI.vs.control; bulk RNA-seq,Homo_633,AD.vs.control; bulk RNA-seq,Homo_633,AD.vs.MCI	7
BP	GO:0040018	positive regulation of multicellular organism growth	bulk RNA-seq,ACOM,AD.vs.control; bulk RNA-seq,Homo_723,MCI.vs.control; bulk RNA-seq,Homo_714,MCI.vs.control; bulk RNA-seq,Homo_633,AD.vs.control; bulk RNA-seq,Homo_633,AD.vs.MCI	5
BP	GO:0032602	chemokine production	bulk RNA-seq,ACOM,AD.vs.control; bulk RNA-seq,Homo_723,AD.vs.MCI; bulk RNA-seq,Homo_723,MCI.vs.control; bulk RNA-seq,Homo_714,AD.vs.MCI; bulk RNA-seq,Homo_714,MCI.vs.control; bulk RNA-seq,Homo_633,AD.vs.control; bulk RNA-seq,Homo_633,AD.vs.MCI	7
BP	GO:0040014	regulation of multicellular organism growth	bulk RNA-seq,ACOM,AD.vs.control; bulk RNA-seq,Homo_723,AD.vs.MCI; bulk RNA-seq,Homo_723,MCI.vs.control; bulk RNA-seq,Homo_714,AD.vs.MCI; bulk RNA-seq,Homo_714,MCI.vs.control; bulk RNA-seq,Homo_633,AD.vs.control; bulk RNA-seq,Homo_633,AD.vs.MCI	7
BP	GO:1902652	secondary alcohol metabolic process	bulk RNA-seq,ACOM,AD.vs.control; bulk RNA-seq,Homo_723,AD.vs.MCI; bulk RNA-seq,Homo_723,MCI.vs.control; bulk RNA-seq,Homo_714,AD.vs.MCI; bulk RNA-seq,Homo_633,AD.vs.control; bulk RNA-seq,Homo_633,AD.vs.MCI	6
BP	GO:0042976	activation of Janus kinase activity	bulk RNA-seq,ACOM,AD.vs.control	1
BP	GO:0042044	fluid transport	bulk RNA-seq,ACOM,AD.vs.control; bulk RNA-seq,Homo_723,AD.vs.MCI; bulk RNA-seq,Homo_723,MCI.vs.control; bulk RNA-seq,Homo_714,AD.vs.MCI; bulk RNA-seq,Homo_714,MCI.vs.control; bulk RNA-seq,Homo_633,AD.vs.control; bulk RNA-seq,Homo_633,AD.vs.MCI	7

BP	GO:0032368	regulation of lipid transport	bulk RNA-seq,ACOM,AD.vs.control; bulk RNA-seq,Homo_723,AD.vs.MCI; bulk RNA-seq,Homo_723,MCI.vs.control; bulk RNA-seq,Homo_714,AD.vs.MCI; bulk RNA-seq,Homo_633,AD.vs.control; bulk RNA-seq,Homo_633,AD.vs.MCI	6
BP	GO:0055074	calcium ion homeostasis	bulk RNA-seq,ACOM,AD.vs.control; bulk RNA-seq,Homo_723,AD.vs.control; bulk RNA-seq,Homo_723,AD.vs.MCI; bulk RNA-seq,Homo_723,MCI.vs.control; bulk RNA-seq,Homo_714,AD.vs.control; bulk RNA-seq,Homo_714,AD.vs.MCI; bulk RNA-seq,Homo_633,AD.vs.control; bulk RNA-seq,Homo_633,AD.vs.MCI	8
BP	GO:0120009	intermembrane lipid transfer	bulk RNA-seq,ACOM,AD.vs.control; bulk RNA-seq,Homo_723,AD.vs.MCI; bulk RNA-seq,Homo_723,MCI.vs.control; bulk RNA-seq,Homo_714,AD.vs.MCI; bulk RNA-seq,Homo_714,MCI.vs.control; bulk RNA-seq,Homo_633,AD.vs.control; bulk RNA-seq,Homo_633,AD.vs.MCI	7
MF	GO:0120013	lipid transfer activity	bulk RNA-seq,ACOM,AD.vs.control; bulk RNA-seq,Homo_723,AD.vs.MCI; bulk RNA-seq,Homo_723,MCI.vs.control; bulk RNA-seq,Homo_714,AD.vs.MCI; bulk RNA-seq,Homo_714,MCI.vs.control; bulk RNA-seq,Homo_633,AD.vs.control; bulk RNA-seq,Homo_633,AD.vs.MCI	7
BP	GO:0090130	tissue migration	bulk RNA-seq,ACOM,AD.vs.control; bulk RNA-seq,Homo_723,AD.vs.control; bulk RNA-seq,Homo_723,AD.vs.MCI; bulk RNA-seq,Homo_714,AD.vs.control; bulk RNA-seq,Homo_714,AD.vs.MCI; bulk RNA-seq,Homo_633,AD.vs.control; bulk RNA-seq,Homo_633,AD.vs.MCI; scRNA-seq,SRP330776,Naive CD8+ T	8
BP	GO:0090207	regulation of triglyceride metabolic process	bulk RNA-seq,ACOM,AD.vs.control; bulk RNA-seq,Homo_723,AD.vs.MCI; bulk RNA-seq,Homo_723,MCI.vs.control; bulk RNA-seq,Homo_714,AD.vs.control; bulk RNA-seq,Homo_714,AD.vs.MCI; bulk RNA-seq,Homo_714,MCI.vs.control; bulk RNA-seq,Homo_633,AD.vs.control; bulk RNA-seq,Homo_633,AD.vs.MCI	8
BP	GO:0009064	glutamine family amino acid metabolic process	bulk RNA-seq,ACOM,AD.vs.control; bulk RNA-seq,Homo_723,AD.vs.MCI; bulk RNA-seq,Homo_723,MCI.vs.control; bulk RNA-seq,Homo_714,AD.vs.MCI; bulk RNA-seq,Homo_714,MCI.vs.control; bulk RNA-seq,Homo_633,AD.vs.control; bulk RNA-seq,Homo_633,AD.vs.MCI	7
BP	GO:0035150	regulation of tube size	bulk RNA-seq,ACOM,AD.vs.control; bulk RNA-seq,Homo_723,AD.vs.control; bulk RNA-seq,Homo_723,AD.vs.MCI; bulk RNA-seq,Homo_723,MCI.vs.control; bulk RNA-seq,Homo_714,AD.vs.control; bulk RNA-seq,Homo_714,AD.vs.MCI; bulk RNA-seq,Homo_633,AD.vs.control; bulk RNA-seq,Homo_633,AD.vs.MCI	8
BP	GO:0048017	inositol lipid-mediated signaling	bulk RNA-seq,ACOM,AD.vs.control; bulk RNA-seq,Homo_723,AD.vs.control; bulk RNA-seq,Homo_723,AD.vs.MCI; bulk RNA-seq,Homo_723,MCI.vs.control; bulk RNA-seq,Homo_714,AD.vs.MCI; bulk RNA-seq,Homo_633,AD.vs.control; bulk RNA-seq,Homo_633,AD.vs.MCI; bulk RNA-seq,ROSMAP,AD.vs.control	8
CC	GO:0045211	postsynaptic membrane	bulk RNA-seq,ACOM,AD.vs.control; bulk RNA-seq,Emory,MCI.vs.control; bulk RNA-seq,Homo_723,AD.vs.control; bulk RNA-seq,Homo_723,AD.vs.MCI; bulk RNA-seq,Homo_723,MCI.vs.control; bulk RNA-seq,Homo_714,AD.vs.control; bulk RNA-seq,Homo_714,AD.vs.MCI; bulk RNA-seq,Homo_633,AD.vs.control; bulk RNA-seq,Homo_633,AD.vs.MCI; bulk RNA-seq,Homo_633,MCI.vs.control; bulk RNA-seq,ROSMAP,AD.vs.control; bulk RNA-seq,ROSMAP,MCI.vs.control; bulk RNA-seq,SRP223445,AD.vs.control	13
BP	GO:0006874	cellular calcium ion homeostasis	bulk RNA-seq,ACOM,AD.vs.control; bulk RNA-seq,Homo_723,AD.vs.control; bulk RNA-seq,Homo_723,AD.vs.MCI; bulk RNA-seq,Homo_723,MCI.vs.control; bulk RNA-seq,Homo_714,AD.vs.control; bulk RNA-seq,Homo_714,AD.vs.MCI; bulk RNA-seq,Homo_633,AD.vs.control; bulk RNA-seq,Homo_633,AD.vs.MCI	8
MF	GO:0015144	carbohydrate transmembrane transporter activity	bulk RNA-seq,ACOM,AD.vs.control; bulk RNA-seq,Homo_723,MCI.vs.control; bulk RNA-seq,Homo_714,MCI.vs.control; bulk RNA-seq,Homo_633,AD.vs.control; bulk RNA-seq,Homo_633,AD.vs.MCI	5
MF	GO:0015485	cholesterol binding	bulk RNA-seq,ACOM,AD.vs.control; bulk RNA-seq,Homo_723,AD.vs.MCI; bulk RNA-seq,Homo_723,MCI.vs.control; bulk RNA-seq,Homo_714,AD.vs.MCI; bulk RNA-seq,Homo_714,MCI.vs.control; bulk RNA-seq,Homo_633,AD.vs.control; bulk RNA-seq,Homo_633,AD.vs.MCI	7
BP	GO:0060193	positive regulation of lipase activity	bulk RNA-seq,ACOM,AD.vs.control; bulk RNA-seq,Homo_723,AD.vs.control; bulk RNA-seq,Homo_723,AD.vs.MCI; bulk RNA-seq,Homo_723,MCI.vs.control; bulk RNA-seq,Homo_714,AD.vs.MCI; bulk RNA-seq,Homo_714,MCI.vs.control; bulk RNA-seq,Homo_633,AD.vs.control; bulk RNA-seq,Homo_633,AD.vs.MCI	8
BP	GO:0006939	smooth muscle contraction	bulk RNA-seq,ACOM,AD.vs.control; bulk RNA-seq,Homo_723,AD.vs.control; bulk RNA-seq,Homo_723,AD.vs.MCI; bulk RNA-seq,Homo_723,MCI.vs.control; bulk RNA-seq,Homo_714,AD.vs.control; bulk RNA-seq,Homo_714,AD.vs.MCI; bulk RNA-seq,Homo_714,MCI.vs.control; bulk RNA-seq,Homo_633,AD.vs.control; bulk RNA-seq,Homo_633,AD.vs.MCI	9
MF	GO:0005496	steroid binding	bulk RNA-seq,ACOM,AD.vs.control; bulk RNA-seq,Homo_723,AD.vs.MCI; bulk RNA-seq,Homo_723,MCI.vs.control; bulk RNA-seq,Homo_714,AD.vs.MCI; bulk RNA-seq,Homo_714,MCI.vs.control; bulk RNA-seq,Homo_633,AD.vs.control; bulk RNA-seq,Homo_633,AD.vs.MCI	7
BP	GO:0001936	regulation of endothelial cell proliferation	bulk RNA-seq,ACOM,AD.vs.control; bulk RNA-seq,Homo_723,AD.vs.control; bulk RNA-seq,Homo_723,AD.vs.MCI; bulk RNA-seq,Homo_723,MCI.vs.control; bulk RNA-seq,Homo_714,AD.vs.control; bulk RNA-seq,Homo_714,AD.vs.MCI; bulk RNA-seq,Homo_633,AD.vs.control	7
BP	GO:0001505	regulation of neurotransmitter levels	bulk RNA-seq,ACOM,AD.vs.control; bulk RNA-seq,Homo_723,AD.vs.control; bulk RNA-seq,Homo_723,AD.vs.MCI; bulk RNA-seq,Homo_723,MCI.vs.control; bulk RNA-seq,Homo_714,AD.vs.control; bulk RNA-seq,Homo_714,AD.vs.MCI; bulk RNA-seq,Homo_633,AD.vs.control; bulk RNA-seq,Homo_633,AD.vs.MCI; bulk RNA-seq,ROSMAP,AD.vs.control; bulk RNA-seq,ROSMAP,MCI.vs.control	10
MF	GO:0004857	enzyme inhibitor activity	bulk RNA-seq,ACOM,AD.vs.control; bulk RNA-seq,Homo_723,AD.vs.control; bulk RNA-seq,Homo_723,AD.vs.MCI; bulk RNA-seq,Homo_714,AD.vs.control; bulk RNA-seq,Homo_714,AD.vs.MCI; bulk RNA-seq,Homo_633,AD.vs.control; bulk RNA-seq,Homo_633,AD.vs.MCI	7
BP	GO:0050900	leukocyte migration	bulk RNA-seq,ACOM,AD.vs.control; bulk RNA-seq,Homo_723,AD.vs.MCI; bulk RNA-seq,Homo_714,AD.vs.MCI; bulk RNA-seq,Homo_633,AD.vs.control; bulk RNA-seq,Homo_633,AD.vs.MCI; bulk RNA-seq,SRP223445,AD.vs.control	6
BP	GO:0007631	feeding behavior	bulk RNA-seq,ACOM,AD.vs.control; bulk RNA-seq,Homo_723,AD.vs.control; bulk RNA-seq,Homo_723,AD.vs.MCI; bulk RNA-seq,Homo_723,MCI.vs.control; bulk RNA-seq,Homo_714,AD.vs.control; bulk RNA-seq,Homo_714,AD.vs.MCI	6
BP	GO:0001666	response to hypoxia	bulk RNA-seq,ACOM,AD.vs.control; bulk RNA-seq,Homo_723,AD.vs.MCI; bulk RNA-seq,Homo_723,MCI.vs.control; bulk RNA-seq,Homo_714,AD.vs.MCI; bulk RNA-seq,Homo_633,AD.vs.control; bulk RNA-seq,Homo_633,AD.vs.MCI	6
BP	GO:0060192	negative regulation of lipase activity	bulk RNA-seq,ACOM,AD.vs.control; bulk RNA-seq,Homo_723,AD.vs.MCI; bulk RNA-seq,Homo_723,MCI.vs.control; bulk RNA-seq,Homo_714,AD.vs.MCI; bulk RNA-seq,Homo_714,MCI.vs.control; bulk RNA-seq,Homo_633,AD.vs.control; bulk RNA-seq,Homo_633,AD.vs.MCI	7
BP	GO:0072503	cellular divalent inorganic cation homeostasis	bulk RNA-seq,ACOM,AD.vs.control; bulk RNA-seq,Homo_723,AD.vs.control; bulk RNA-seq,Homo_723,AD.vs.MCI; bulk RNA-seq,Homo_723,MCI.vs.control; bulk RNA-seq,Homo_714,AD.vs.control; bulk RNA-seq,Homo_714,AD.vs.MCI; bulk RNA-seq,Homo_633,AD.vs.control; bulk RNA-seq,Homo_633,AD.vs.MCI	8
BP	GO:1903053	regulation of extracellular matrix organization	bulk RNA-seq,ACOM,AD.vs.control; bulk RNA-seq,Homo_723,AD.vs.MCI; bulk RNA-seq,Homo_723,MCI.vs.control; bulk RNA-seq,Homo_714,AD.vs.MCI; bulk RNA-seq,Homo_633,AD.vs.control; bulk RNA-seq,Homo_633,AD.vs.MCI	6
BP	GO:0009306	protein secretion	bulk RNA-seq,ACOM,AD.vs.control; bulk RNA-seq,Homo_723,AD.vs.control; bulk RNA-seq,Homo_723,AD.vs.MCI; bulk RNA-seq,Homo_714,AD.vs.MCI; bulk RNA-seq,Homo_633,AD.vs.control; bulk RNA-seq,Homo_633,AD.vs.MCI	6
MF	GO:0061134	peptidase regulator activity	bulk RNA-seq,ACOM,AD.vs.control; bulk RNA-seq,Homo_723,AD.vs.control; bulk RNA-seq,Homo_723,AD.vs.MCI; bulk RNA-seq,Homo_723,MCI.vs.control; bulk RNA-seq,Homo_714,AD.vs.control; bulk RNA-seq,Homo_714,AD.vs.MCI; bulk RNA-seq,Homo_633,AD.vs.control; bulk RNA-seq,Homo_633,AD.vs.MCI	8
BP	GO:1900046	regulation of hemostasis	bulk RNA-seq,ACOM,AD.vs.control; bulk RNA-seq,Homo_723,AD.vs.control; bulk RNA-seq,Homo_723,AD.vs.MCI; bulk RNA-seq,Homo_723,MCI.vs.control; bulk RNA-seq,Homo_714,AD.vs.control; bulk RNA-seq,Homo_714,AD.vs.MCI; bulk RNA-seq,Homo_714,MCI.vs.control; bulk RNA-seq,Homo_633,AD.vs.control; bulk RNA-seq,Homo_633,AD.vs.MCI	9
BP	GO:0010038	response to metal ion	bulk RNA-seq,ACOM,AD.vs.control; bulk RNA-seq,Emory,MCI.vs.control; bulk RNA-seq,Homo_723,AD.vs.control; bulk RNA-seq,Homo_723,AD.vs.MCI; bulk RNA-seq,Homo_714,AD.vs.control; bulk RNA-seq,Homo_714,AD.vs.MCI; bulk RNA-seq,Homo_633,AD.vs.control; bulk RNA-seq,Homo_633,AD.vs.MCI	8
BP	GO:0090132	epithelium migration	bulk RNA-seq,ACOM,AD.vs.control; bulk RNA-seq,Homo_723,AD.vs.control; bulk RNA-seq,Homo_723,AD.vs.MCI; bulk RNA-seq,Homo_714,AD.vs.control; bulk RNA-seq,Homo_714,AD.vs.MCI; bulk RNA-seq,Homo_633,AD.vs.control; bulk RNA-seq,Homo_633,AD.vs.MCI; scRNA-seq,SRP330776,Naive CD8+ T	8

BP	GO:0051047	positive regulation of secretion	bulk RNA-seq,ACOM,AD.vs.control; bulk RNA-seq,Homo_723,AD.vs.control; bulk RNA-seq,Homo_723,AD.vs.MCI; bulk RNA-seq,Homo_723,MCI.vs.control; bulk RNA-seq,Homo_714,AD.vs.control; bulk RNA-seq,Homo_714,AD.vs.MCI; bulk RNA-seq,Homo_633,AD.vs.control; bulk RNA-seq,Homo_633,AD.vs.MCI	8
MF	GO:0032934	sterol binding	bulk RNA-seq,ACOM,AD.vs.control; bulk RNA-seq,Homo_723,AD.vs.MCI; bulk RNA-seq,Homo_723,MCI.vs.control; bulk RNA-seq,Homo_714,MCI.vs.control; bulk RNA-seq,Homo_633,AD.vs.control; bulk RNA-seq,Homo_633,AD.vs.MCI	6
BP	GO:0001885	endothelial cell development	bulk RNA-seq,ACOM,AD.vs.control; bulk RNA-seq,Homo_723,AD.vs.control; bulk RNA-seq,Homo_723,AD.vs.MCI; bulk RNA-seq,Homo_723,MCI.vs.control; bulk RNA-seq,Homo_714,AD.vs.MCI; bulk RNA-seq,Homo_714,MCI.vs.control; bulk RNA-seq,Homo_633,AD.vs.control; bulk RNA-seq,Homo_633,AD.vs.MCI	8
CC	GO:0034364	high-density lipoprotein particle	bulk RNA-seq,ACOM,AD.vs.control; bulk RNA-seq,Homo_723,AD.vs.MCI; bulk RNA-seq,Homo_723,MCI.vs.control; bulk RNA-seq,Homo_714,AD.vs.control; bulk RNA-seq,Homo_714,AD.vs.MCI; bulk RNA-seq,Homo_714,MCI.vs.control	6
BP	GO:0060396	growth hormone receptor signaling pathway	bulk RNA-seq,ACOM,AD.vs.control; bulk RNA-seq,Homo_714,AD.vs.control	2
BP	GO:0071378	cellular response to growth hormone stimulus	bulk RNA-seq,ACOM,AD.vs.control; bulk RNA-seq,Homo_714,AD.vs.control	2
BP	GO:0014068	positive regulation of phosphatidylinositol 3-kinase signaling	bulk RNA-seq,ACOM,AD.vs.control; bulk RNA-seq,Homo_723,AD.vs.control; bulk RNA-seq,Homo_723,AD.vs.MCI; bulk RNA-seq,Homo_723,MCI.vs.control; bulk RNA-seq,Homo_714,AD.vs.control; bulk RNA-seq,Homo_714,AD.vs.MCI; bulk RNA-seq,Homo_714,MCI.vs.control; bulk RNA-seq,Homo_633,AD.vs.control; bulk RNA-seq,ROSMAP,MCI.vs.control	9
MF	GO:0099094	ligand-gated cation channel activity	bulk RNA-seq,ACOM,AD.vs.control; bulk RNA-seq,Homo_723,AD.vs.control; bulk RNA-seq,Homo_723,AD.vs.MCI; bulk RNA-seq,Homo_723,MCI.vs.control; bulk RNA-seq,Homo_714,AD.vs.control; bulk RNA-seq,Homo_714,AD.vs.MCI; bulk RNA-seq,Homo_714,MCI.vs.control; bulk RNA-seq,Homo_633,AD.vs.control; bulk RNA-seq,Homo_633,AD.vs.MCI; bulk RNA-seq,ROSMAP,AD.vs.control; bulk RNA-seq,SRP223445,AD.vs.control	11
BP	GO:0022600	digestive system process	bulk RNA-seq,ACOM,AD.vs.control; bulk RNA-seq,Homo_723,AD.vs.control; bulk RNA-seq,Homo_723,AD.vs.MCI; bulk RNA-seq,Homo_723,MCI.vs.control; bulk RNA-seq,Homo_714,AD.vs.MCI; bulk RNA-seq,Homo_714,MCI.vs.control; bulk RNA-seq,Homo_633,AD.vs.control	7
BP	GO:0033674	positive regulation of kinase activity	bulk RNA-seq,ACOM,AD.vs.control; bulk RNA-seq,Homo_723,AD.vs.control; bulk RNA-seq,Homo_723,AD.vs.MCI; bulk RNA-seq,Homo_714,AD.vs.MCI; bulk RNA-seq,Homo_633,AD.vs.control; bulk RNA-seq,Homo_633,AD.vs.MCI	6
CC	GO:0030658	transport vesicle membrane	bulk RNA-seq,ACOM,AD.vs.control; bulk RNA-seq,Homo_723,AD.vs.control; bulk RNA-seq,Homo_723,AD.vs.MCI; bulk RNA-seq,Homo_723,MCI.vs.control; bulk RNA-seq,Homo_714,AD.vs.MCI; bulk RNA-seq,Homo_633,AD.vs.control; bulk RNA-seq,Homo_633,AD.vs.MCI	7
BP	GO:0098754	detoxification	bulk RNA-seq,ACOM,AD.vs.control; bulk RNA-seq,Homo_723,MCI.vs.control; bulk RNA-seq,Homo_714,AD.vs.MCI; bulk RNA-seq,Homo_633,AD.vs.control; bulk RNA-seq,Homo_633,AD.vs.MCI; bulk RNA-seq,ROSMAP,MCI.vs.control	6
BP	GO:0044242	cellular lipid catabolic process	bulk RNA-seq,ACOM,AD.vs.control; bulk RNA-seq,Homo_723,AD.vs.MCI; bulk RNA-seq,Homo_723,MCI.vs.control; bulk RNA-seq,Homo_714,AD.vs.control; bulk RNA-seq,Homo_714,AD.vs.MCI; bulk RNA-seq,Homo_633,AD.vs.control; bulk RNA-seq,Homo_633,AD.vs.MCI	7
BP	GO:0009914	hormone transport	bulk RNA-seq,ACOM,AD.vs.control; bulk RNA-seq,Emory,MCI.vs.control; bulk RNA-seq,Homo_723,AD.vs.control; bulk RNA-seq,Homo_723,AD.vs.MCI; bulk RNA-seq,Homo_723,MCI.vs.control; bulk RNA-seq,Homo_714,AD.vs.control; bulk RNA-seq,Homo_714,AD.vs.MCI; bulk RNA-seq,Homo_633,AD.vs.control; bulk RNA-seq,Homo_633,AD.vs.MCI	9
BP	GO:0032094	response to food	bulk RNA-seq,ACOM,AD.vs.control; bulk RNA-seq,Homo_723,AD.vs.MCI; bulk RNA-seq,Homo_723,MCI.vs.control	3
MF	GO:0015145	monosaccharide transmembrane transporter activity	bulk RNA-seq,ACOM,AD.vs.control; bulk RNA-seq,Homo_723,MCI.vs.control; bulk RNA-seq,Homo_633,AD.vs.control	3
BP	GO:0009395	phospholipid catabolic process	bulk RNA-seq,ACOM,AD.vs.control; bulk RNA-seq,Homo_723,MCI.vs.control; bulk RNA-seq,Homo_714,MCI.vs.control; bulk RNA-seq,Homo_633,AD.vs.control; bulk RNA-seq,Homo_633,AD.vs.MCI	5
BP	GO:0043550	regulation of lipid kinase activity	bulk RNA-seq,ACOM,AD.vs.control; bulk RNA-seq,Homo_723,AD.vs.MCI; bulk RNA-seq,Homo_723,MCI.vs.control; bulk RNA-seq,Homo_714,AD.vs.MCI; bulk RNA-seq,Homo_714,MCI.vs.control; bulk RNA-seq,Homo_633,AD.vs.control; bulk RNA-seq,Homo_633,AD.vs.MCI	7
BP	GO:0035296	regulation of tube diameter	bulk RNA-seq,ACOM,AD.vs.control; bulk RNA-seq,Homo_723,AD.vs.control; bulk RNA-seq,Homo_723,AD.vs.MCI; bulk RNA-seq,Homo_723,MCI.vs.control; bulk RNA-seq,Homo_714,AD.vs.control; bulk RNA-seq,Homo_714,AD.vs.MCI; bulk RNA-seq,Homo_633,AD.vs.control; bulk RNA-seq,Homo_633,AD.vs.MCI	8
BP	GO:0097746	blood vessel diameter maintenance	bulk RNA-seq,ACOM,AD.vs.control; bulk RNA-seq,Homo_723,AD.vs.control; bulk RNA-seq,Homo_723,AD.vs.MCI; bulk RNA-seq,Homo_723,MCI.vs.control; bulk RNA-seq,Homo_714,AD.vs.control; bulk RNA-seq,Homo_714,AD.vs.MCI; bulk RNA-seq,Homo_633,AD.vs.control; bulk RNA-seq,Homo_633,AD.vs.MCI	8
MF	GO:0020037	heme binding	bulk RNA-seq,ACOM,AD.vs.control; bulk RNA-seq,Homo_723,AD.vs.MCI; bulk RNA-seq,Homo_723,MCI.vs.control; bulk RNA-seq,Homo_714,AD.vs.MCI; bulk RNA-seq,Homo_633,AD.vs.control; bulk RNA-seq,Homo_633,AD.vs.MCI; bulk RNA-seq,SRP223445,AD.vs.control	7
BP	GO:0090208	positive regulation of triglyceride metabolic process	bulk RNA-seq,ACOM,AD.vs.control; bulk RNA-seq,Homo_723,MCI.vs.control; bulk RNA-seq,Homo_714,AD.vs.MCI; bulk RNA-seq,Homo_714,MCI.vs.control	4
MF	GO:0060229	lipase activator activity	bulk RNA-seq,ACOM,AD.vs.control; bulk RNA-seq,Homo_723,AD.vs.MCI; bulk RNA-seq,Homo_723,MCI.vs.control; bulk RNA-seq,Homo_714,AD.vs.control; bulk RNA-seq,Homo_714,AD.vs.MCI; bulk RNA-seq,Homo_714,MCI.vs.control; bulk RNA-seq,Homo_633,AD.vs.control; bulk RNA-seq,Homo_633,AD.vs.MCI	8
BP	GO:0030195	negative regulation of blood coagulation	bulk RNA-seq,ACOM,AD.vs.control; bulk RNA-seq,Homo_723,AD.vs.control; bulk RNA-seq,Homo_723,AD.vs.MCI; bulk RNA-seq,Homo_723,MCI.vs.control; bulk RNA-seq,Homo_714,AD.vs.control; bulk RNA-seq,Homo_714,AD.vs.MCI; bulk RNA-seq,Homo_714,MCI.vs.control	7
BP	GO:0046434	organophosphate catabolic process	bulk RNA-seq,ACOM,AD.vs.control; bulk RNA-seq,Homo_723,AD.vs.control; bulk RNA-seq,Homo_723,MCI.vs.control; bulk RNA-seq,Homo_714,AD.vs.control; bulk RNA-seq,Homo_633,AD.vs.control; bulk RNA-seq,Homo_633,AD.vs.MCI; scRNA-seq,SRP330776,B cell_1-AD.vs.control;	8
MF	GO:0005539	glycosaminoglycan binding	bulk RNA-seq,ACOM,AD.vs.control; bulk RNA-seq,Homo_723,AD.vs.control; bulk RNA-seq,Homo_723,AD.vs.MCI; bulk RNA-seq,Homo_723,MCI.vs.control; bulk RNA-seq,Homo_714,AD.vs.control; bulk RNA-seq,Homo_714,AD.vs.MCI; bulk RNA-seq,Homo_633,AD.vs.control; bulk RNA-seq,Homo_633,AD.vs.MCI; bulk RNA-seq,ROSMAP,MCI.vs.control	9
BP	GO:0018108	peptidyl-tyrosine phosphorylation	bulk RNA-seq,ACOM,AD.vs.control; bulk RNA-seq,Homo_723,AD.vs.control; bulk RNA-seq,Homo_723,AD.vs.MCI; bulk RNA-seq,Homo_714,AD.vs.MCI; bulk RNA-seq,Homo_633,AD.vs.control; bulk RNA-seq,Homo_633,AD.vs.MCI	6
BP	GO:0034379	very-low-density lipoprotein particle assembly	bulk RNA-seq,ACOM,AD.vs.control; bulk RNA-seq,Homo_723,MCI.vs.control; bulk RNA-seq,Homo_714,MCI.vs.control	3
CC	GO:0009897	external side of plasma membrane	bulk RNA-seq,ACOM,AD.vs.control; bulk RNA-seq,Homo_723,AD.vs.MCI; bulk RNA-seq,Homo_714,AD.vs.MCI; bulk RNA-seq,Homo_633,AD.vs.control; bulk RNA-seq,Homo_633,AD.vs.MCI; scRNA-seq,SRP330776,Monocyte_2-AD.vs.control; scRNA-seq,SRP330776,Naive CD8+ T cell_2-AD.vs.control; scRNA-seq,SRP309935,B cell_2-AD.vs.control; scRNA-seq,SRP309935,Monocyte_2-AD.vs.control; scRNA-seq,SRP215507,CD8+ T cell_3-AD.vs.control; scRNA-	11
BP	GO:0008217	regulation of blood pressure	bulk RNA-seq,ACOM,AD.vs.control; bulk RNA-seq,Homo_723,AD.vs.control; bulk RNA-seq,Homo_723,AD.vs.MCI; bulk RNA-seq,Homo_723,MCI.vs.control; bulk RNA-seq,Homo_714,AD.vs.control; bulk RNA-seq,Homo_714,AD.vs.MCI; bulk RNA-seq,Homo_633,AD.vs.control; bulk RNA-seq,Homo_633,AD.vs.MCI	8
CC	GO:0008021	synaptic vesicle	bulk RNA-seq,ACOM,AD.vs.control; bulk RNA-seq,Homo_723,AD.vs.control; bulk RNA-seq,Homo_723,AD.vs.MCI; bulk RNA-seq,Homo_723,MCI.vs.control; bulk RNA-seq,Homo_714,AD.vs.control; bulk RNA-seq,Homo_714,AD.vs.MCI; bulk RNA-seq,Homo_633,AD.vs.control; bulk RNA-seq,Homo_633,AD.vs.MCI	8
BP	GO:0070076	histone lysine demethylation	bulk RNA-seq,ACOM,AD.vs.control; bulk RNA-seq,Homo_723,MCI.vs.control; bulk RNA-seq,Homo_714,AD.vs.MCI; bulk RNA-seq,Homo_714,MCI.vs.control; bulk RNA-seq,Homo_633,AD.vs.control; bulk RNA-seq,Homo_633,AD.vs.MCI; bulk RNA-seq,Homo_633,MCI.vs.control	7
BP	GO:0010631	epithelial cell migration	bulk RNA-seq,ACOM,AD.vs.control; bulk RNA-seq,Homo_723,AD.vs.control; bulk RNA-seq,Homo_723,AD.vs.MCI; bulk RNA-seq,Homo_714,AD.vs.control; bulk RNA-seq,Homo_714,AD.vs.MCI; bulk RNA-seq,Homo_633,AD.vs.control; bulk RNA-seq,Homo_633,AD.vs.MCI; scRNA-seq,SRP330776,Naive CD8+ T	8

BP	GO:0050820	positive regulation of coagulation	bulk RNA-seq,ACOM,AD.vs.control; bulk RNA-seq,Homo_723,AD.vs.MCI; bulk RNA-seq,Homo_723,MCI.vs.control; bulk RNA-seq,Homo_714,AD.vs.MCI; bulk RNA-seq,Homo_714,MCI.vs.control; bulk RNA-seq,Homo_633,AD.vs.control; bulk RNA-seq,Homo_633,AD.vs.MCI	7
BP	GO:0006813	potassium ion transport	bulk RNA-seq,ACOM,AD.vs.control; bulk RNA-seq,Homo_723,AD.vs.control; bulk RNA-seq,Homo_723,AD.vs.MCI; bulk RNA-seq,Homo_723,MCI.vs.control; bulk RNA-seq,Homo_714,AD.vs.control; bulk RNA-seq,Homo_714,AD.vs.MCI; bulk RNA-seq,Homo_633,AD.vs.control; bulk RNA-seq,Homo_633,AD.vs.MCI; bulk RNA-seq,SRP223445,AD.vs.control	9
MF	GO:0043024	ribosomal small subunit binding	bulk RNA-seq,ACOM,AD.vs.control; bulk RNA-seq,Homo_723,MCI.vs.control; bulk RNA-seq,Homo_714,MCI.vs.control; bulk RNA-seq,Homo_633,AD.vs.control; bulk RNA-seq,Homo_633,AD.vs.MCI	5
BP	GO:0006644	phospholipid metabolic process	bulk RNA-seq,ACOM,AD.vs.control; bulk RNA-seq,Homo_723,AD.vs.MCI; bulk RNA-seq,Homo_714,AD.vs.control; bulk RNA-seq,Homo_714,AD.vs.MCI; bulk RNA-seq,Homo_633,AD.vs.control; bulk RNA-seq,Homo_633,AD.vs.MCI; scRNA-seq,SRP330776,CD8+ T cell_2-AD.vs.control	7
BP	GO:0060416	response to growth hormone	bulk RNA-seq,ACOM,AD.vs.control; bulk RNA-seq,Homo_723,AD.vs.MCI; bulk RNA-seq,Homo_723,MCI.vs.control; bulk RNA-seq,Homo_714,AD.vs.MCI; bulk RNA-seq,Homo_714,MCI.vs.control	5
BP	GO:0006875	cellular metal ion homeostasis	bulk RNA-seq,ACOM,AD.vs.control; bulk RNA-seq,Homo_723,AD.vs.control; bulk RNA-seq,Homo_723,AD.vs.MCI; bulk RNA-seq,Homo_714,AD.vs.control; bulk RNA-seq,Homo_714,AD.vs.MCI; bulk RNA-seq,Homo_633,AD.vs.control; bulk RNA-seq,Homo_633,AD.vs.MCI; scRNA-seq,SRP330776,Naive CD8+ T	8
BP	GO:0098856	intestinal lipid absorption	bulk RNA-seq,ACOM,AD.vs.control; bulk RNA-seq,Homo_723,AD.vs.MCI; bulk RNA-seq,Homo_723,MCI.vs.control; bulk RNA-seq,Homo_714,AD.vs.MCI; bulk RNA-seq,Homo_714,MCI.vs.control	5
BP	GO:0045766	positive regulation of angiogenesis	bulk RNA-seq,ACOM,AD.vs.control; bulk RNA-seq,Homo_723,AD.vs.control; bulk RNA-seq,Homo_723,AD.vs.MCI; bulk RNA-seq,Homo_723,MCI.vs.control; bulk RNA-seq,Homo_714,AD.vs.control; bulk RNA-seq,Homo_714,AD.vs.MCI; bulk RNA-seq,Homo_633,AD.vs.control; bulk RNA-seq,Homo_633,AD.vs.MCI	8
BP	GO:1904018	positive regulation of vasculature development	bulk RNA-seq,ACOM,AD.vs.control; bulk RNA-seq,Homo_723,AD.vs.control; bulk RNA-seq,Homo_723,AD.vs.MCI; bulk RNA-seq,Homo_723,MCI.vs.control; bulk RNA-seq,Homo_714,AD.vs.control; bulk RNA-seq,Homo_714,AD.vs.MCI; bulk RNA-seq,Homo_633,AD.vs.control; bulk RNA-seq,Homo_633,AD.vs.MCI	8
CC	GO:0070382	exocytic vesicle	bulk RNA-seq,ACOM,AD.vs.control; bulk RNA-seq,Homo_723,AD.vs.control; bulk RNA-seq,Homo_723,AD.vs.MCI; bulk RNA-seq,Homo_723,MCI.vs.control; bulk RNA-seq,Homo_714,AD.vs.control; bulk RNA-seq,Homo_714,AD.vs.MCI; bulk RNA-seq,Homo_633,AD.vs.control; bulk RNA-seq,Homo_633,AD.vs.MCI	8
MF	GO:0046873	metal ion transmembrane transporter activity	bulk RNA-seq,ACOM,AD.vs.control; bulk RNA-seq,Emory,MCI.vs.control; bulk RNA-seq,Homo_723,AD.vs.control; bulk RNA-seq,Homo_723,AD.vs.MCI; bulk RNA-seq,Homo_714,AD.vs.control; bulk RNA-seq,Homo_714,AD.vs.MCI; bulk RNA-seq,Homo_633,AD.vs.control; bulk RNA-seq,Homo_633,AD.vs.MCI; bulk RNA-seq,ROSMAP,AD.vs.control; bulk RNA-seq,ROSMAP,MCI.vs.control; bulk RNA-seq,SRP223445,AD.vs.control	11
BP	GO:0043434	response to peptide hormone	bulk RNA-seq,ACOM,AD.vs.control; bulk RNA-seq,Homo_723,AD.vs.control; bulk RNA-seq,Homo_723,AD.vs.MCI; bulk RNA-seq,Homo_714,AD.vs.control; bulk RNA-seq,Homo_714,AD.vs.MCI; bulk RNA-seq,Homo_633,AD.vs.control; bulk RNA-seq,Homo_633,AD.vs.MCI	7
BP	GO:0034764	positive regulation of transmembrane transport	bulk RNA-seq,ACOM,AD.vs.control; bulk RNA-seq,Homo_723,AD.vs.control; bulk RNA-seq,Homo_723,AD.vs.MCI; bulk RNA-seq,Homo_723,MCI.vs.control; bulk RNA-seq,Homo_714,AD.vs.control; bulk RNA-seq,Homo_714,AD.vs.MCI; bulk RNA-seq,Homo_633,AD.vs.control; bulk RNA-seq,Homo_633,AD.vs.MCI	8
BP	GO:1903035	negative regulation of response to wounding	bulk RNA-seq,ACOM,AD.vs.control; bulk RNA-seq,Homo_723,AD.vs.control; bulk RNA-seq,Homo_723,AD.vs.MCI; bulk RNA-seq,Homo_723,MCI.vs.control; bulk RNA-seq,Homo_714,AD.vs.control; bulk RNA-seq,Homo_714,AD.vs.MCI; bulk RNA-seq,Homo_714,MCI.vs.control; bulk RNA-seq,Homo_633,AD.vs.control; bulk RNA-seq,Homo_633,AD.vs.MCI	9
CC	GO:0043197	dendritic spine	bulk RNA-seq,ACOM,AD.vs.control; bulk RNA-seq,Emory,MCI.vs.control; bulk RNA-seq,Homo_723,AD.vs.control; bulk RNA-seq,Homo_723,AD.vs.MCI; bulk RNA-seq,Homo_723,MCI.vs.control; bulk RNA-seq,Homo_714,AD.vs.MCI; bulk RNA-seq,Homo_633,AD.vs.control; bulk RNA-seq,Homo_633,AD.vs.MCI	8
BP	GO:0046879	hormone secretion	bulk RNA-seq,ACOM,AD.vs.control; bulk RNA-seq,Emory,MCI.vs.control; bulk RNA-seq,Homo_723,AD.vs.control; bulk RNA-seq,Homo_723,AD.vs.MCI; bulk RNA-seq,Homo_723,MCI.vs.control; bulk RNA-seq,Homo_714,AD.vs.control; bulk RNA-seq,Homo_714,AD.vs.MCI; bulk RNA-seq,Homo_633,AD.vs.control; bulk RNA-seq,Homo_633,AD.vs.MCI	9
BP	GO:0042445	hormone metabolic process	bulk RNA-seq,ACOM,AD.vs.control; bulk RNA-seq,Homo_723,AD.vs.control; bulk RNA-seq,Homo_723,AD.vs.MCI; bulk RNA-seq,Homo_723,MCI.vs.control; bulk RNA-seq,Homo_714,AD.vs.control; bulk RNA-seq,Homo_714,AD.vs.MCI; bulk RNA-seq,Homo_633,AD.vs.control; bulk RNA-seq,Homo_633,AD.vs.MCI	8
BP	GO:0033002	muscle cell proliferation	bulk RNA-seq,ACOM,AD.vs.control; bulk RNA-seq,Homo_723,AD.vs.control; bulk RNA-seq,Homo_723,AD.vs.MCI; bulk RNA-seq,Homo_723,MCI.vs.control; bulk RNA-seq,Homo_714,AD.vs.control; bulk RNA-seq,Homo_714,AD.vs.MCI; bulk RNA-seq,Homo_633,AD.vs.control; bulk RNA-seq,Homo_633,AD.vs.MCI	8
BP	GO:0044089	positive regulation of cellular component biogenesis	bulk RNA-seq,ACOM,AD.vs.control; bulk RNA-seq,Homo_723,AD.vs.control; bulk RNA-seq,Homo_723,AD.vs.MCI; bulk RNA-seq,Homo_714,AD.vs.MCI; bulk RNA-seq,Homo_633,AD.vs.control; bulk RNA-seq,Homo_633,AD.vs.MCI	6
BP	GO:0030194	positive regulation of blood coagulation	bulk RNA-seq,ACOM,AD.vs.control; bulk RNA-seq,Homo_723,AD.vs.MCI; bulk RNA-seq,Homo_723,MCI.vs.control; bulk RNA-seq,Homo_714,AD.vs.MCI; bulk RNA-seq,Homo_714,MCI.vs.control; bulk RNA-seq,Homo_633,AD.vs.control; bulk RNA-seq,Homo_633,AD.vs.MCI	7
BP	GO:1900048	positive regulation of hemostasis	bulk RNA-seq,ACOM,AD.vs.control; bulk RNA-seq,Homo_723,AD.vs.MCI; bulk RNA-seq,Homo_723,MCI.vs.control; bulk RNA-seq,Homo_714,AD.vs.MCI; bulk RNA-seq,Homo_714,MCI.vs.control; bulk RNA-seq,Homo_633,AD.vs.control; bulk RNA-seq,Homo_633,AD.vs.MCI	7
BP	GO:1903532	positive regulation of secretion by cell	bulk RNA-seq,ACOM,AD.vs.control; bulk RNA-seq,Homo_723,AD.vs.control; bulk RNA-seq,Homo_723,AD.vs.MCI; bulk RNA-seq,Homo_723,MCI.vs.control; bulk RNA-seq,Homo_714,AD.vs.MCI; bulk RNA-seq,Homo_633,AD.vs.control; bulk RNA-seq,Homo_633,AD.vs.MCI	7
MF	GO:0051119	sugar transmembrane transporter activity	bulk RNA-seq,ACOM,AD.vs.control; bulk RNA-seq,Homo_723,MCI.vs.control; bulk RNA-seq,Homo_714,MCI.vs.control; bulk RNA-seq,Homo_633,AD.vs.control; bulk RNA-seq,Homo_633,AD.vs.MCI	5
BP	GO:0042744	hydrogen peroxide catabolic process	bulk RNA-seq,ACOM,AD.vs.control; scRNA-seq,SRP309935,Erythroid cell-AD.vs.control	2
BP	GO:0015844	monoamine transport	bulk RNA-seq,ACOM,AD.vs.control; bulk RNA-seq,Homo_723,AD.vs.control; bulk RNA-seq,Homo_723,AD.vs.MCI; bulk RNA-seq,Homo_723,MCI.vs.control; bulk RNA-seq,Homo_714,AD.vs.control; bulk RNA-seq,Homo_714,AD.vs.MCI; bulk RNA-seq,Homo_633,MCI.vs.control	7
BP	GO:0002697	regulation of immune effector process	bulk RNA-seq,ACOM,AD.vs.control; bulk RNA-seq,Homo_714,AD.vs.MCI; bulk RNA-seq,Homo_633,AD.vs.control; bulk RNA-seq,Homo_633,AD.vs.MCI; scRNA-seq,SRP330776,Naive CD8+ T cell_2-AD.vs.control; scRNA-seq,SRP215507,CD8+ T cell_3-AD.vs.control	6
BP	GO:0006816	calcium ion transport	bulk RNA-seq,ACOM,AD.vs.control; bulk RNA-seq,Homo_723,AD.vs.control; bulk RNA-seq,Homo_723,AD.vs.MCI; bulk RNA-seq,Homo_714,AD.vs.control; bulk RNA-seq,Homo_714,AD.vs.MCI; bulk RNA-seq,Homo_633,AD.vs.control; bulk RNA-seq,Homo_633,AD.vs.MCI	7
BP	GO:0051937	catecholamine transport	bulk RNA-seq,ACOM,AD.vs.control; bulk RNA-seq,Homo_723,AD.vs.control; bulk RNA-seq,Homo_723,AD.vs.MCI; bulk RNA-seq,Homo_723,MCI.vs.control; bulk RNA-seq,Homo_714,AD.vs.control; bulk RNA-seq,Homo_714,AD.vs.MCI; bulk RNA-seq,SRP325058,AD.vs.control	7
BP	GO:0097237	cellular response to toxic substance	bulk RNA-seq,ACOM,AD.vs.control; bulk RNA-seq,Homo_723,AD.vs.MCI; bulk RNA-seq,Homo_723,MCI.vs.control; bulk RNA-seq,Homo_714,AD.vs.MCI; bulk RNA-seq,Homo_714,MCI.vs.control; bulk RNA-seq,Homo_633,AD.vs.control; bulk RNA-seq,Homo_633,AD.vs.MCI; bulk RNA-	8
BP	GO:1900047	negative regulation of hemostasis	bulk RNA-seq,ACOM,AD.vs.control; bulk RNA-seq,Homo_723,AD.vs.control; bulk RNA-seq,Homo_723,AD.vs.MCI; bulk RNA-seq,Homo_723,MCI.vs.control; bulk RNA-seq,Homo_714,AD.vs.control; bulk RNA-seq,Homo_714,AD.vs.MCI; bulk RNA-seq,Homo_714,MCI.vs.control	7
BP	GO:0003300	cardiac muscle hypertrophy	bulk RNA-seq,ACOM,AD.vs.control; bulk RNA-seq,Homo_723,AD.vs.control; bulk RNA-seq,Homo_723,AD.vs.MCI; bulk RNA-seq,Homo_723,MCI.vs.control; bulk RNA-seq,Homo_714,AD.vs.control; bulk RNA-seq,Homo_714,AD.vs.MCI; bulk RNA-seq,Homo_714,MCI.vs.control; bulk RNA-	8

BP	GO:0009308	amine metabolic process	bulk RNA-seq,ACOM,AD.vs.control; bulk RNA-seq,Homo_723,AD.vs.control; bulk RNA-seq,Homo_723,AD.vs.MCI; bulk RNA-seq,Homo_723,MCI.vs.control; bulk RNA-seq,Homo_714,AD.vs.control; bulk RNA-seq,Homo_714,AD.vs.MCI; bulk RNA-seq,Homo_714,MCI.vs.control; bulk RNA-	8
BP	GO:0071375	cellular response to peptide hormone stimulus	bulk RNA-seq,ACOM,AD.vs.control; bulk RNA-seq,Homo_723,AD.vs.MCI; bulk RNA-seq,Homo_723,MCI.vs.control; bulk RNA-seq,Homo_714,AD.vs.control; bulk RNA-seq,Homo_714,AD.vs.MCI; bulk RNA-seq,Homo_633,AD.vs.control; bulk RNA-seq,Homo_633,AD.vs.MCI	7
BP	GO:0036293	response to decreased oxygen levels	bulk RNA-seq,ACOM,AD.vs.control; bulk RNA-seq,Homo_723,AD.vs.MCI; bulk RNA-seq,Homo_723,MCI.vs.control; bulk RNA-seq,Homo_714,AD.vs.MCI; bulk RNA-seq,Homo_633,AD.vs.control; bulk RNA-seq,Homo_633,AD.vs.MCI	6
BP	GO:0044106	cellular amine metabolic process	bulk RNA-seq,ACOM,AD.vs.control; bulk RNA-seq,Homo_723,AD.vs.control; bulk RNA-seq,Homo_723,AD.vs.MCI; bulk RNA-seq,Homo_723,MCI.vs.control; bulk RNA-seq,Homo_714,AD.vs.control; bulk RNA-seq,Homo_714,AD.vs.MCI; bulk RNA-seq,Homo_714,MCI.vs.control; bulk RNA-	8
BP	GO:0042311	vasodilation	bulk RNA-seq,ACOM,AD.vs.control; bulk RNA-seq,Homo_723,AD.vs.control; bulk RNA-seq,Homo_723,AD.vs.MCI; bulk RNA-seq,Homo_723,MCI.vs.control; bulk RNA-seq,Homo_714,AD.vs.MCI; bulk RNA-seq,Homo_714,MCI.vs.control; bulk RNA-seq,Homo_633,AD.vs.control; bulk RNA-seq,Homo_633,AD.vs.MCI	8
BP	GO:0071731	response to nitric oxide	bulk RNA-seq,ACOM,AD.vs.control; bulk RNA-seq,Homo_723,MCI.vs.control; bulk RNA-seq,Homo_633,AD.vs.control	3
BP	GO:0043551	regulation of phosphatidylinositol 3-kinase activity	bulk RNA-seq,ACOM,AD.vs.control; bulk RNA-seq,Homo_723,AD.vs.MCI; bulk RNA-seq,Homo_723,MCI.vs.control; bulk RNA-seq,Homo_714,AD.vs.MCI; bulk RNA-seq,Homo_714,MCI.vs.control; bulk RNA-seq,Homo_633,AD.vs.control; bulk RNA-seq,Homo_633,AD.vs.MCI	7
BP	GO:0032642	regulation of chemokine production	bulk RNA-seq,ACOM,AD.vs.control; bulk RNA-seq,Homo_723,AD.vs.MCI; bulk RNA-seq,Homo_723,MCI.vs.control; bulk RNA-seq,Homo_714,AD.vs.MCI; bulk RNA-seq,Homo_714,MCI.vs.control; bulk RNA-seq,Homo_633,AD.vs.control; bulk RNA-seq,Homo_633,AD.vs.MCI	7
MF	GO:0008238	exopeptidase activity	bulk RNA-seq,ACOM,AD.vs.control; bulk RNA-seq,Homo_723,AD.vs.MCI; bulk RNA-seq,Homo_723,MCI.vs.control; bulk RNA-seq,Homo_714,AD.vs.MCI; bulk RNA-seq,Homo_714,MCI.vs.control; bulk RNA-seq,Homo_633,AD.vs.control; bulk RNA-seq,Homo_633,AD.vs.MCI	7
CC	GO:0044309	neuron spine	bulk RNA-seq,ACOM,AD.vs.control; bulk RNA-seq,Emory,MCI.vs.control; bulk RNA-seq,Homo_723,AD.vs.control; bulk RNA-seq,Homo_723,AD.vs.MCI; bulk RNA-seq,Homo_723,MCI.vs.control; bulk RNA-seq,Homo_714,AD.vs.MCI; bulk RNA-seq,Homo_633,AD.vs.control; bulk RNA-seq,Homo_633,AD.vs.MCI	8
BP	GO:0002685	regulation of leukocyte migration	bulk RNA-seq,ACOM,AD.vs.control; bulk RNA-seq,Homo_723,AD.vs.MCI; bulk RNA-seq,Homo_723,MCI.vs.control; bulk RNA-seq,Homo_714,AD.vs.MCI; bulk RNA-seq,Homo_633,AD.vs.control; bulk RNA-seq,Homo_633,AD.vs.MCI	6
BP	GO:0032488	Cdc42 protein signal transduction	bulk RNA-seq,ACOM,AD.vs.control; bulk RNA-seq,Homo_723,AD.vs.control; bulk RNA-seq,Homo_723,AD.vs.MCI; bulk RNA-seq,Homo_723,MCI.vs.control; bulk RNA-seq,Homo_714,AD.vs.MCI; bulk RNA-seq,Homo_714,MCI.vs.control; bulk RNA-seq,Homo_633,AD.vs.control; bulk RNA-seq,Homo_633,AD.vs.MCI	8
MF	GO:0016597	amino acid binding	bulk RNA-seq,ACOM,AD.vs.control; bulk RNA-seq,Homo_723,AD.vs.control; bulk RNA-seq,Homo_723,AD.vs.MCI; bulk RNA-seq,Homo_723,MCI.vs.control; bulk RNA-seq,Homo_714,AD.vs.MCI; bulk RNA-seq,Homo_714,MCI.vs.control; bulk RNA-seq,Homo_633,AD.vs.control	7
MF	GO:0015276	ligand-gated ion channel activity	bulk RNA-seq,ACOM,AD.vs.control; bulk RNA-seq,Homo_723,AD.vs.control; bulk RNA-seq,Homo_723,AD.vs.MCI; bulk RNA-seq,Homo_723,MCI.vs.control; bulk RNA-seq,Homo_714,AD.vs.control; bulk RNA-seq,Homo_714,AD.vs.MCI; bulk RNA-seq,Homo_633,AD.vs.control; bulk RNA-seq,Homo_633,AD.vs.MCI; bulk RNA-seq,ROSMAP,AD.vs.control; bulk RNA-seq,SRP223445,AD.vs.control	10
MF	GO:0022834	ligand-gated channel activity	bulk RNA-seq,ACOM,AD.vs.control; bulk RNA-seq,Homo_723,AD.vs.control; bulk RNA-seq,Homo_723,AD.vs.MCI; bulk RNA-seq,Homo_723,MCI.vs.control; bulk RNA-seq,Homo_714,AD.vs.control; bulk RNA-seq,Homo_714,AD.vs.MCI; bulk RNA-seq,Homo_633,AD.vs.control; bulk RNA-seq,Homo_633,AD.vs.MCI; bulk RNA-seq,ROSMAP,AD.vs.control; bulk RNA-seq,SRP223445,AD.vs.control	10
BP	GO:0032956	regulation of actin cytoskeleton organization	bulk RNA-seq,ACOM,AD.vs.control; bulk RNA-seq,Homo_723,AD.vs.control; bulk RNA-seq,Homo_723,AD.vs.MCI; bulk RNA-seq,Homo_714,AD.vs.MCI; bulk RNA-seq,Homo_633,AD.vs.control; bulk RNA-seq,Homo_633,AD.vs.MCI	6
BP	GO:1904894	positive regulation of receptor signaling pathway via STAT	bulk RNA-seq,ACOM,AD.vs.control; bulk RNA-seq,Homo_714,AD.vs.MCI	2
BP	GO:0032371	regulation of sterol transport	bulk RNA-seq,ACOM,AD.vs.control; bulk RNA-seq,Homo_723,AD.vs.MCI; bulk RNA-seq,Homo_723,MCI.vs.control; bulk RNA-seq,Homo_714,AD.vs.MCI; bulk RNA-seq,Homo_714,MCI.vs.control; bulk RNA-seq,Homo_633,AD.vs.control; bulk RNA-seq,Homo_633,AD.vs.MCI	7
BP	GO:0032374	regulation of cholesterol transport	bulk RNA-seq,ACOM,AD.vs.control; bulk RNA-seq,Homo_723,AD.vs.MCI; bulk RNA-seq,Homo_723,MCI.vs.control; bulk RNA-seq,Homo_714,AD.vs.MCI; bulk RNA-seq,Homo_714,MCI.vs.control; bulk RNA-seq,Homo_633,AD.vs.control; bulk RNA-seq,Homo_633,AD.vs.MCI	7
BP	GO:0002532	production of molecular mediator involved in inflammatory	bulk RNA-seq,ACOM,AD.vs.control; bulk RNA-seq,Homo_714,AD.vs.MCI; bulk RNA-seq,Homo_633,AD.vs.control; bulk RNA-seq,Homo_633,AD.vs.MCI	4
MF	GO:0005201	extracellular matrix structural constituent	bulk RNA-seq,ACOM,AD.vs.control; bulk RNA-seq,Emory,MCI.vs.control; bulk RNA-seq,Homo_723,AD.vs.control; bulk RNA-seq,Homo_723,AD.vs.MCI; bulk RNA-seq,Homo_723,MCI.vs.control; bulk RNA-seq,Homo_714,AD.vs.control; bulk RNA-seq,Homo_714,AD.vs.MCI; bulk RNA-seq,ROSMAP,AD.vs.control; bulk RNA-seq,ROSMAP,MCI.vs.control; bulk RNA-seq,SRP223445,AD.vs.control; scRNA-seq,SRP330776,Monocyte_1-AD.vs.control	11
BP	GO:2001237	negative regulation of extrinsic apoptotic signaling pathway	bulk RNA-seq,ACOM,AD.vs.control; bulk RNA-seq,Homo_723,AD.vs.control; bulk RNA-seq,Homo_723,AD.vs.MCI; bulk RNA-seq,Homo_723,MCI.vs.control; bulk RNA-seq,Homo_714,AD.vs.MCI; bulk RNA-seq,Homo_714,MCI.vs.control; bulk RNA-seq,Homo_633,AD.vs.control; bulk RNA-seq,Homo_633,AD.vs.MCI	8
BP	GO:0044241	lipid digestion	bulk RNA-seq,ACOM,AD.vs.control; bulk RNA-seq,Homo_723,AD.vs.MCI; bulk RNA-seq,Homo_723,MCI.vs.control; bulk RNA-seq,Homo_714,AD.vs.MCI; bulk RNA-seq,Homo_714,MCI.vs.control	5
MF	GO:0019825	oxygen binding	bulk RNA-seq,ACOM,AD.vs.control; bulk RNA-seq,Homo_723,AD.vs.MCI; bulk RNA-seq,Homo_723,MCI.vs.control; bulk RNA-seq,Homo_714,AD.vs.MCI; bulk RNA-seq,Homo_714,MCI.vs.control; bulk RNA-seq,SRP223445,AD.vs.control	6
BP	GO:0018212	peptidyl-tyrosine modification	bulk RNA-seq,ACOM,AD.vs.control; bulk RNA-seq,Homo_723,AD.vs.control; bulk RNA-seq,Homo_723,AD.vs.MCI; bulk RNA-seq,Homo_714,AD.vs.MCI; bulk RNA-seq,Homo_633,AD.vs.control; bulk RNA-seq,Homo_633,AD.vs.MCI	6
BP	GO:0022604	regulation of cell morphogenesis	bulk RNA-seq,ACOM,AD.vs.control; bulk RNA-seq,Homo_723,AD.vs.control; bulk RNA-seq,Homo_723,AD.vs.MCI; bulk RNA-seq,Homo_723,MCI.vs.control; bulk RNA-seq,Homo_714,AD.vs.MCI; bulk RNA-seq,Homo_633,AD.vs.control; bulk RNA-seq,Homo_633,AD.vs.MCI	7
CC	GO:0031983	vesicle lumen	bulk RNA-seq,ACOM,AD.vs.control; bulk RNA-seq,Homo_723,AD.vs.control; bulk RNA-seq,Homo_723,AD.vs.MCI; bulk RNA-seq,Homo_723,MCI.vs.control; bulk RNA-seq,Homo_714,AD.vs.control; bulk RNA-seq,Homo_714,AD.vs.MCI; bulk RNA-seq,Homo_633,AD.vs.control; bulk RNA-seq,Homo_633,AD.vs.MCI; bulk RNA-seq,MCSA,MCI.vs.control; bulk RNA-seq,SRP223445,AD.vs.control	10
BP	GO:0001654	eye development	bulk RNA-seq,Emory,MCI.vs.control; bulk RNA-seq,Homo_723,AD.vs.control; bulk RNA-seq,Homo_723,AD.vs.MCI; bulk RNA-seq,Homo_714,AD.vs.control; bulk RNA-seq,Homo_714,AD.vs.MCI; bulk RNA-seq,Homo_633,AD.vs.control; bulk RNA-seq,Homo_633,AD.vs.MCI; bulk RNA-seq,ROSMAP,AD.vs.control; bulk RNA-seq,SRP223445,AD.vs.control	9
CC	GO:0099240	intrinsic component of synaptic membrane	bulk RNA-seq,Emory,MCI.vs.control; bulk RNA-seq,Homo_723,AD.vs.control; bulk RNA-seq,Homo_723,AD.vs.MCI; bulk RNA-seq,Homo_723,MCI.vs.control; bulk RNA-seq,Homo_714,AD.vs.control; bulk RNA-seq,Homo_714,AD.vs.MCI; bulk RNA-seq,Homo_633,AD.vs.MCI; bulk RNA-seq,Homo_633,MCI.vs.control; bulk RNA-seq,ROSMAP,AD.vs.control; bulk RNA-seq,ROSMAP,MCI.vs.control; bulk RNA-seq,SRP223445,AD.vs.control	11
BP	GO:0048880	sensory system development	bulk RNA-seq,Emory,MCI.vs.control; bulk RNA-seq,Homo_723,AD.vs.control; bulk RNA-seq,Homo_723,AD.vs.MCI; bulk RNA-seq,Homo_714,AD.vs.control; bulk RNA-seq,Homo_714,AD.vs.MCI; bulk RNA-seq,Homo_633,AD.vs.control; bulk RNA-seq,Homo_633,AD.vs.MCI; bulk RNA-seq,ROSMAP,AD.vs.control; bulk RNA-seq,SRP223445,AD.vs.control	9

BP	GO:0050808	synapse organization	bulk RNA-seq,Emory,MCI.vs.control; bulk RNA-seq,Homo_723,AD.vs.control; bulk RNA-seq,Homo_723,AD.vs.MCI; bulk RNA-seq,Homo_714,AD.vs.control; bulk RNA-seq,Homo_714,AD.vs.MCI; bulk RNA-seq,Homo_633,AD.vs.control; bulk RNA-seq,Homo_633,AD.vs.MCI; bulk RNA-seq,ROSMAP,AD.vs.control; bulk RNA-seq,ROSMAP,MCI.vs.control; bulk RNA-seq,SRP223445,AD.vs.control	10
BP	GO:0150063	visual system development	bulk RNA-seq,Emory,MCI.vs.control; bulk RNA-seq,Homo_723,AD.vs.control; bulk RNA-seq,Homo_723,AD.vs.MCI; bulk RNA-seq,Homo_714,AD.vs.control; bulk RNA-seq,Homo_714,AD.vs.MCI; bulk RNA-seq,Homo_633,AD.vs.control; bulk RNA-seq,Homo_633,AD.vs.MCI; bulk RNA-seq,ROSMAP,AD.vs.control; bulk RNA-seq,SRP223445,AD.vs.control	9
BP	GO:0043010	camera-type eye development	bulk RNA-seq,Emory,MCI.vs.control; bulk RNA-seq,Homo_723,AD.vs.control; bulk RNA-seq,Homo_723,AD.vs.MCI; bulk RNA-seq,Homo_714,AD.vs.control; bulk RNA-seq,Homo_714,AD.vs.MCI; bulk RNA-seq,Homo_633,AD.vs.control; bulk RNA-seq,Homo_633,AD.vs.MCI; bulk RNA-seq,ROSMAP,AD.vs.control; bulk RNA-seq,SRP223445,AD.vs.control	9
BP	GO:0007416	synapse assembly	bulk RNA-seq,Emory,MCI.vs.control; bulk RNA-seq,Homo_723,AD.vs.control; bulk RNA-seq,Homo_723,AD.vs.MCI; bulk RNA-seq,Homo_723,MCI.vs.control; bulk RNA-seq,Homo_714,AD.vs.control; bulk RNA-seq,Homo_714,AD.vs.MCI; bulk RNA-seq,Homo_633,AD.vs.MCI; bulk RNA-seq,Homo_633,MCI.vs.control; bulk RNA-seq,ROSMAP,AD.vs.control; bulk RNA-seq,ROSMAP,MCI.vs.control; bulk RNA-seq,SRP223445,AD.vs.control	11
BP	GO:0060541	respiratory system development	bulk RNA-seq,Emory,MCI.vs.control; bulk RNA-seq,Homo_723,AD.vs.control; bulk RNA-seq,Homo_723,AD.vs.MCI; bulk RNA-seq,Homo_723,MCI.vs.control; bulk RNA-seq,Homo_714,AD.vs.control; bulk RNA-seq,Homo_714,AD.vs.MCI; bulk RNA-seq,Homo_633,AD.vs.control; bulk RNA-seq,Homo_633,AD.vs.MCI	8
CC	GO:0042734	presynaptic membrane	bulk RNA-seq,Emory,MCI.vs.control; bulk RNA-seq,Homo_723,AD.vs.control; bulk RNA-seq,Homo_723,AD.vs.MCI; bulk RNA-seq,Homo_723,MCI.vs.control; bulk RNA-seq,Homo_714,AD.vs.control; bulk RNA-seq,Homo_714,AD.vs.MCI; bulk RNA-seq,Homo_633,AD.vs.control; bulk RNA-seq,Homo_633,AD.vs.MCI; bulk RNA-seq,ROSMAP,AD.vs.control; bulk RNA-seq,ROSMAP,MCI.vs.control	10
BP	GO:0048562	embryonic organ morphogenesis	bulk RNA-seq,Emory,MCI.vs.control; bulk RNA-seq,Homo_723,AD.vs.control; bulk RNA-seq,Homo_723,AD.vs.MCI; bulk RNA-seq,Homo_723,MCI.vs.control; bulk RNA-seq,Homo_714,AD.vs.control; bulk RNA-seq,Homo_714,AD.vs.MCI; bulk RNA-seq,Homo_633,AD.vs.control; bulk RNA-seq,Homo_633,AD.vs.MCI; bulk RNA-seq,Homo_633,MCI.vs.control; bulk RNA-seq,SRP223445,AD.vs.control	10
CC	GO:0099699	integral component of synaptic membrane	bulk RNA-seq,Emory,MCI.vs.control; bulk RNA-seq,Homo_723,AD.vs.control; bulk RNA-seq,Homo_723,AD.vs.MCI; bulk RNA-seq,Homo_723,MCI.vs.control; bulk RNA-seq,Homo_714,AD.vs.control; bulk RNA-seq,Homo_714,AD.vs.MCI; bulk RNA-seq,Homo_633,MCI.vs.control; bulk RNA-seq,ROSMAP,AD.vs.control; bulk RNA-seq,ROSMAP,MCI.vs.control; bulk RNA-seq,SRP223445,AD.vs.control	10
CC	GO:0099056	integral component of presynaptic membrane	bulk RNA-seq,Emory,MCI.vs.control; bulk RNA-seq,Homo_723,AD.vs.control; bulk RNA-seq,Homo_723,AD.vs.MCI; bulk RNA-seq,Homo_723,MCI.vs.control; bulk RNA-seq,Homo_714,AD.vs.control; bulk RNA-seq,Homo_714,AD.vs.MCI; bulk RNA-seq,Homo_714,MCI.vs.control; bulk RNA-seq,Homo_633,MCI.vs.control; bulk RNA-seq,SRP223445,AD.vs.control	9
BP	GO:0043583	ear development	bulk RNA-seq,Emory,MCI.vs.control; bulk RNA-seq,Homo_723,AD.vs.control; bulk RNA-seq,Homo_723,AD.vs.MCI; bulk RNA-seq,Homo_723,MCI.vs.control; bulk RNA-seq,Homo_714,AD.vs.control; bulk RNA-seq,Homo_714,AD.vs.MCI; bulk RNA-seq,Homo_633,AD.vs.control; bulk RNA-seq,Homo_633,AD.vs.MCI; bulk RNA-seq,Homo_633,MCI.vs.control; bulk RNA-seq,SRP223445,AD.vs.control	10
MF	GO:0030594	neurotransmitter receptor activity	bulk RNA-seq,Emory,MCI.vs.control; bulk RNA-seq,Homo_723,AD.vs.control; bulk RNA-seq,Homo_723,AD.vs.MCI; bulk RNA-seq,Homo_723,MCI.vs.control; bulk RNA-seq,Homo_714,AD.vs.control; bulk RNA-seq,Homo_714,AD.vs.MCI; bulk RNA-seq,Homo_633,MCI.vs.control; bulk RNA-	8
CC	GO:0015934	large ribosomal subunit	bulk RNA-seq,Emory,MCI.vs.control; bulk RNA-seq,Homo_723,AD.vs.control; bulk RNA-seq,Homo_723,AD.vs.MCI; bulk RNA-seq,Homo_723,MCI.vs.control; bulk RNA-seq,Homo_714,AD.vs.control; bulk RNA-seq,Homo_714,AD.vs.MCI; bulk RNA-seq,Homo_714,MCI.vs.control; bulk RNA-seq,Homo_633,AD.vs.control; bulk RNA-seq,Homo_633,AD.vs.MCI; bulk RNA-seq,SRP223445,AD.vs.control; scRNA-seq,SRP330776,Megakaryocyte_2-	11
BP	GO:0060562	epithelial tube morphogenesis	bulk RNA-seq,Emory,MCI.vs.control; bulk RNA-seq,Homo_723,AD.vs.control; bulk RNA-seq,Homo_723,AD.vs.MCI; bulk RNA-seq,Homo_714,AD.vs.control; bulk RNA-seq,Homo_714,AD.vs.MCI; bulk RNA-seq,Homo_633,AD.vs.control; bulk RNA-seq,Homo_633,AD.vs.MCI; bulk RNA-seq,ROSMAP,AD.vs.control; bulk RNA-seq,SRP223445,AD.vs.control	9
MF	GO:0022836	gated channel activity	bulk RNA-seq,Emory,MCI.vs.control; bulk RNA-seq,Homo_723,AD.vs.control; bulk RNA-seq,Homo_723,AD.vs.MCI; bulk RNA-seq,Homo_714,AD.vs.control; bulk RNA-seq,Homo_714,AD.vs.MCI; bulk RNA-seq,Homo_633,AD.vs.control; bulk RNA-seq,Homo_633,AD.vs.MCI; bulk RNA-seq,ROSMAP,AD.vs.control; bulk RNA-seq,ROSMAP,MCI.vs.control; bulk RNA-seq,SRP223445,AD.vs.control	10
BP	GO:0007389	pattern specification process	bulk RNA-seq,Emory,MCI.vs.control; bulk RNA-seq,Homo_723,AD.vs.control; bulk RNA-seq,Homo_723,AD.vs.MCI; bulk RNA-seq,Homo_714,AD.vs.control; bulk RNA-seq,Homo_714,AD.vs.MCI; bulk RNA-seq,Homo_633,AD.vs.control; bulk RNA-seq,Homo_633,AD.vs.MCI; bulk RNA-seq,Homo_633,MCI.vs.control; bulk RNA-seq,ROSMAP,AD.vs.control; bulk RNA-seq,SRP223445,AD.vs.control	10
BP	GO:0042733	embryonic digit morphogenesis	bulk RNA-seq,Emory,MCI.vs.control; bulk RNA-seq,Homo_723,AD.vs.control; bulk RNA-seq,Homo_723,AD.vs.MCI; bulk RNA-seq,Homo_723,MCI.vs.control; bulk RNA-seq,Homo_714,AD.vs.MCI; bulk RNA-seq,Homo_633,AD.vs.control; bulk RNA-seq,Homo_633,AD.vs.MCI	7
CC	GO:0098889	intrinsic component of presynaptic membrane	bulk RNA-seq,Emory,MCI.vs.control; bulk RNA-seq,Homo_723,AD.vs.control; bulk RNA-seq,Homo_723,AD.vs.MCI; bulk RNA-seq,Homo_723,MCI.vs.control; bulk RNA-seq,Homo_714,AD.vs.control; bulk RNA-seq,Homo_714,AD.vs.MCI; bulk RNA-seq,Homo_714,MCI.vs.control; bulk RNA-seq,Homo_633,MCI.vs.control; bulk RNA-seq,SRP223445,AD.vs.control	9
MF	GO:0015267	channel activity	bulk RNA-seq,Emory,MCI.vs.control; bulk RNA-seq,Homo_723,AD.vs.control; bulk RNA-seq,Homo_723,AD.vs.MCI; bulk RNA-seq,Homo_714,AD.vs.control; bulk RNA-seq,Homo_714,AD.vs.MCI; bulk RNA-seq,Homo_633,AD.vs.control; bulk RNA-seq,Homo_633,AD.vs.MCI; bulk RNA-seq,ROSMAP,AD.vs.control; bulk RNA-seq,ROSMAP,MCI.vs.control; bulk RNA-seq,SRP223445,AD.vs.control	10
BP	GO:0097485	neuron projection guidance	bulk RNA-seq,Emory,MCI.vs.control; bulk RNA-seq,Homo_723,AD.vs.control; bulk RNA-seq,Homo_723,AD.vs.MCI; bulk RNA-seq,Homo_723,MCI.vs.control; bulk RNA-seq,Homo_714,AD.vs.control; bulk RNA-seq,Homo_714,AD.vs.MCI; bulk RNA-seq,Homo_633,AD.vs.control; bulk RNA-seq,Homo_633,AD.vs.MCI; bulk RNA-seq,Homo_633,MCI.vs.control; bulk RNA-seq,ROSMAP,AD.vs.control; bulk RNA-seq,SRP223445,AD.vs.control	11
MF	GO:0005216	ion channel activity	bulk RNA-seq,Emory,MCI.vs.control; bulk RNA-seq,Homo_723,AD.vs.control; bulk RNA-seq,Homo_723,AD.vs.MCI; bulk RNA-seq,Homo_714,AD.vs.control; bulk RNA-seq,Homo_714,AD.vs.MCI; bulk RNA-seq,Homo_633,AD.vs.control; bulk RNA-seq,Homo_633,AD.vs.MCI; bulk RNA-seq,ROSMAP,AD.vs.control; bulk RNA-seq,ROSMAP,MCI.vs.control; bulk RNA-seq,SRP223445,AD.vs.control	10
BP	GO:0001763	morphogenesis of a branching structure	bulk RNA-seq,Emory,MCI.vs.control; bulk RNA-seq,Homo_723,AD.vs.control; bulk RNA-seq,Homo_723,AD.vs.MCI; bulk RNA-seq,Homo_723,MCI.vs.control; bulk RNA-seq,Homo_714,AD.vs.control; bulk RNA-seq,Homo_714,AD.vs.MCI; bulk RNA-seq,Homo_633,AD.vs.control; bulk RNA-seq,Homo_633,AD.vs.MCI; bulk RNA-seq,SRP223445,AD.vs.control	9
CC	GO:0098978	glutamatergic synapse	bulk RNA-seq,Emory,MCI.vs.control; bulk RNA-seq,Homo_723,AD.vs.control; bulk RNA-seq,Homo_723,AD.vs.MCI; bulk RNA-seq,Homo_723,MCI.vs.control; bulk RNA-seq,Homo_714,AD.vs.control; bulk RNA-seq,Homo_714,AD.vs.MCI; bulk RNA-seq,Homo_633,AD.vs.control; bulk RNA-seq,Homo_633,AD.vs.MCI; bulk RNA-seq,ROSMAP,AD.vs.control; bulk RNA-seq,ROSMAP,MCI.vs.control; bulk RNA-seq,SRP223445,AD.vs.control	11
BP	GO:0061564	axon development	bulk RNA-seq,Emory,MCI.vs.control; bulk RNA-seq,Homo_723,AD.vs.control; bulk RNA-seq,Homo_723,AD.vs.MCI; bulk RNA-seq,Homo_714,AD.vs.control; bulk RNA-seq,Homo_714,AD.vs.MCI; bulk RNA-seq,Homo_633,AD.vs.control; bulk RNA-seq,Homo_633,AD.vs.MCI; bulk RNA-seq,ROSMAP,AD.vs.control; bulk RNA-seq,ROSMAP,MCI.vs.control; bulk RNA-seq,SRP223445,AD.vs.control	10

BP	GO:0042391	regulation of membrane potential	bulk RNA-seq,Emory,MCI.vs.control; bulk RNA-seq,Homo_723,AD.vs.control; bulk RNA-seq,Homo_723,AD.vs.MCI; bulk RNA-seq,Homo_714,AD.vs.control; bulk RNA-seq,Homo_714,AD.vs.MCI; bulk RNA-seq,Homo_633,AD.vs.control; bulk RNA-seq,Homo_633,AD.vs.MCI; bulk RNA-seq,ROSMAP,AD.vs.control; bulk RNA-seq,ROSMAP,MCI.vs.control; bulk RNA-seq,SRP223445,AD.vs.control; scRNA-seq,SRP330776,Naive CD8+ T cell_2-AD.vs.control	11
BP	GO:0007411	axon guidance	bulk RNA-seq,Emory,MCI.vs.control; bulk RNA-seq,Homo_723,AD.vs.control; bulk RNA-seq,Homo_723,AD.vs.MCI; bulk RNA-seq,Homo_723,MCI.vs.control; bulk RNA-seq,Homo_714,AD.vs.control; bulk RNA-seq,Homo_714,AD.vs.MCI; bulk RNA-seq,Homo_633,AD.vs.control; bulk RNA-seq,Homo_633,AD.vs.MCI; bulk RNA-seq,Homo_633,MCI.vs.control; bulk RNA-seq,ROSMAP,AD.vs.control; bulk RNA-seq,SRP223445,AD.vs.control	11
BP	GO:0048839	inner ear development	bulk RNA-seq,Emory,MCI.vs.control; bulk RNA-seq,Homo_723,AD.vs.control; bulk RNA-seq,Homo_723,AD.vs.MCI; bulk RNA-seq,Homo_723,MCI.vs.control; bulk RNA-seq,Homo_714,AD.vs.control; bulk RNA-seq,Homo_714,AD.vs.MCI; bulk RNA-seq,Homo_633,AD.vs.control; bulk RNA-seq,Homo_633,AD.vs.MCI; bulk RNA-seq,Homo_633,MCI.vs.control; bulk RNA-seq,SRP223445,AD.vs.control	10
MF	GO:0022803	passive transmembrane transporter activity	bulk RNA-seq,Emory,MCI.vs.control; bulk RNA-seq,Homo_723,AD.vs.control; bulk RNA-seq,Homo_723,AD.vs.MCI; bulk RNA-seq,Homo_714,AD.vs.control; bulk RNA-seq,Homo_714,AD.vs.MCI; bulk RNA-seq,Homo_633,AD.vs.control; bulk RNA-seq,Homo_633,AD.vs.MCI; bulk RNA-seq,ROSMAP,AD.vs.control; bulk RNA-seq,ROSMAP,MCI.vs.control; bulk RNA-seq,SRP223445,AD.vs.control	10
BP	GO:0007188	adenylate cyclase-modulating G protein-coupled receptor signaling pathway	bulk RNA-seq,Emory,MCI.vs.control; bulk RNA-seq,Homo_723,AD.vs.control; bulk RNA-seq,Homo_723,AD.vs.MCI; bulk RNA-seq,Homo_723,MCI.vs.control; bulk RNA-seq,Homo_714,AD.vs.control; bulk RNA-seq,Homo_714,AD.vs.MCI; bulk RNA-seq,Homo_633,MCI.vs.control	7
BP	GO:0060537	muscle tissue development	bulk RNA-seq,Emory,MCI.vs.control; bulk RNA-seq,Homo_723,AD.vs.control; bulk RNA-seq,Homo_723,AD.vs.MCI; bulk RNA-seq,Homo_714,AD.vs.control; bulk RNA-seq,Homo_714,AD.vs.MCI; bulk RNA-seq,Homo_633,AD.vs.control; bulk RNA-seq,Homo_633,AD.vs.MCI; bulk RNA-seq,SRP223445,AD.vs.control	8
BP	GO:0002791	regulation of peptide secretion	bulk RNA-seq,Emory,MCI.vs.control; bulk RNA-seq,Homo_723,AD.vs.control; bulk RNA-seq,Homo_723,AD.vs.MCI; bulk RNA-seq,Homo_723,MCI.vs.control; bulk RNA-seq,Homo_714,AD.vs.MCI; bulk RNA-seq,Homo_633,AD.vs.control; bulk RNA-seq,Homo_633,AD.vs.MCI; bulk RNA-seq,SRP325058,AD.vs.control	8
BP	GO:0048754	branching morphogenesis of an epithelial tube	bulk RNA-seq,Emory,MCI.vs.control; bulk RNA-seq,Homo_723,AD.vs.control; bulk RNA-seq,Homo_723,AD.vs.MCI; bulk RNA-seq,Homo_723,MCI.vs.control; bulk RNA-seq,Homo_714,AD.vs.control; bulk RNA-seq,Homo_714,AD.vs.MCI; bulk RNA-seq,Homo_633,AD.vs.control; bulk RNA-seq,Homo_633,AD.vs.MCI; bulk RNA-seq,SRP223445,AD.vs.control	9
CC	GO:0034702	ion channel complex	bulk RNA-seq,Emory,MCI.vs.control; bulk RNA-seq,Homo_723,AD.vs.control; bulk RNA-seq,Homo_723,AD.vs.MCI; bulk RNA-seq,Homo_723,MCI.vs.control; bulk RNA-seq,Homo_714,AD.vs.control; bulk RNA-seq,Homo_714,AD.vs.MCI; bulk RNA-seq,Homo_633,AD.vs.MCI; bulk RNA-seq,Homo_633,MCI.vs.control; bulk RNA-seq,ROSMAP,AD.vs.control; bulk RNA-seq,ROSMAP,MCI.vs.control; bulk RNA-seq,SRP223445,AD.vs.control; bulk RNA-	12
BP	GO:0090596	sensory organ morphogenesis	bulk RNA-seq,Emory,MCI.vs.control; bulk RNA-seq,Homo_723,AD.vs.control; bulk RNA-seq,Homo_723,AD.vs.MCI; bulk RNA-seq,Homo_723,MCI.vs.control; bulk RNA-seq,Homo_714,AD.vs.control; bulk RNA-seq,Homo_714,AD.vs.MCI; bulk RNA-seq,Homo_633,AD.vs.control; bulk RNA-seq,Homo_633,AD.vs.MCI; bulk RNA-seq,SRP223445,AD.vs.control	9
BP	GO:0030323	respiratory tube development	bulk RNA-seq,Emory,MCI.vs.control; bulk RNA-seq,Homo_723,AD.vs.control; bulk RNA-seq,Homo_723,AD.vs.MCI; bulk RNA-seq,Homo_723,MCI.vs.control; bulk RNA-seq,Homo_714,AD.vs.control; bulk RNA-seq,Homo_714,AD.vs.MCI; bulk RNA-seq,Homo_633,AD.vs.control; bulk RNA-seq,Homo_633,AD.vs.MCI; bulk RNA-seq,ROSMAP,AD.vs.control	9
BP	GO:0090276	regulation of peptide hormone secretion	bulk RNA-seq,Emory,MCI.vs.control; bulk RNA-seq,Homo_723,AD.vs.control; bulk RNA-seq,Homo_723,AD.vs.MCI; bulk RNA-seq,Homo_723,MCI.vs.control; bulk RNA-seq,Homo_714,AD.vs.MCI; bulk RNA-seq,Homo_633,AD.vs.control; bulk RNA-seq,Homo_633,AD.vs.MCI	7
BP	GO:0030324	lung development	bulk RNA-seq,Emory,MCI.vs.control; bulk RNA-seq,Homo_723,AD.vs.control; bulk RNA-seq,Homo_723,AD.vs.MCI; bulk RNA-seq,Homo_723,MCI.vs.control; bulk RNA-seq,Homo_714,AD.vs.control; bulk RNA-seq,Homo_714,AD.vs.MCI; bulk RNA-seq,Homo_633,AD.vs.control; bulk RNA-seq,Homo_633,AD.vs.MCI	8
BP	GO:0034329	cell junction assembly	bulk RNA-seq,Emory,MCI.vs.control; bulk RNA-seq,Homo_723,AD.vs.control; bulk RNA-seq,Homo_723,AD.vs.MCI; bulk RNA-seq,Homo_714,AD.vs.control; bulk RNA-seq,Homo_714,AD.vs.MCI; bulk RNA-seq,Homo_633,AD.vs.control; bulk RNA-seq,Homo_633,AD.vs.MCI; bulk RNA-seq,ROSMAP,AD.vs.control; bulk RNA-seq,ROSMAP,MCI.vs.control; bulk RNA-seq,SRP223445,AD.vs.control	10
CC	GO:1902495	transmembrane transporter complex	bulk RNA-seq,Emory,MCI.vs.control; bulk RNA-seq,Homo_723,AD.vs.control; bulk RNA-seq,Homo_723,AD.vs.MCI; bulk RNA-seq,Homo_714,AD.vs.control; bulk RNA-seq,Homo_714,AD.vs.MCI; bulk RNA-seq,Homo_633,AD.vs.control; bulk RNA-seq,Homo_633,AD.vs.MCI; bulk RNA-seq,ROSMAP,AD.vs.control; bulk RNA-seq,ROSMAP,MCI.vs.control; bulk RNA-seq,SRP223445,AD.vs.control	10
BP	GO:0090087	regulation of peptide transport	bulk RNA-seq,Emory,MCI.vs.control; bulk RNA-seq,Homo_723,AD.vs.control; bulk RNA-seq,Homo_723,AD.vs.MCI; bulk RNA-seq,Homo_723,MCI.vs.control; bulk RNA-seq,Homo_714,AD.vs.MCI; bulk RNA-seq,Homo_633,AD.vs.control; bulk RNA-seq,Homo_633,AD.vs.MCI	7
BP	GO:0048568	embryonic organ development	bulk RNA-seq,Emory,MCI.vs.control; bulk RNA-seq,Homo_723,AD.vs.control; bulk RNA-seq,Homo_723,AD.vs.MCI; bulk RNA-seq,Homo_714,AD.vs.control; bulk RNA-seq,Homo_714,AD.vs.MCI; bulk RNA-seq,Homo_633,AD.vs.control; bulk RNA-seq,Homo_633,AD.vs.MCI; bulk RNA-seq,SRP223445,AD.vs.control	8
BP	GO:0007409	axonogenesis	bulk RNA-seq,Emory,MCI.vs.control; bulk RNA-seq,Homo_723,AD.vs.control; bulk RNA-seq,Homo_723,AD.vs.MCI; bulk RNA-seq,Homo_714,AD.vs.control; bulk RNA-seq,Homo_714,AD.vs.MCI; bulk RNA-seq,Homo_633,AD.vs.control; bulk RNA-seq,Homo_633,AD.vs.MCI; bulk RNA-seq,ROSMAP,AD.vs.control; bulk RNA-seq,ROSMAP,MCI.vs.control; bulk RNA-seq,SRP223445,AD.vs.control	10
BP	GO:0002181	cytoplasmic translation	bulk RNA-seq,Emory,MCI.vs.control; bulk RNA-seq,Homo_723,AD.vs.control; bulk RNA-seq,Homo_723,AD.vs.MCI; bulk RNA-seq,Homo_723,MCI.vs.control; bulk RNA-seq,Homo_714,AD.vs.control; bulk RNA-seq,Homo_714,AD.vs.MCI; bulk RNA-seq,Homo_633,AD.vs.control; bulk RNA-seq,Homo_633,AD.vs.MCI; bulk RNA-seq,SRP223445,AD.vs.control; scRNA-seq,SRP330776,Megakaryocyte_2-AD.vs.control	10
BP	GO:0045165	cell fate commitment	bulk RNA-seq,Emory,MCI.vs.control; bulk RNA-seq,Homo_723,AD.vs.control; bulk RNA-seq,Homo_723,AD.vs.MCI; bulk RNA-seq,Homo_723,MCI.vs.control; bulk RNA-seq,Homo_714,AD.vs.control; bulk RNA-seq,Homo_714,AD.vs.MCI; bulk RNA-seq,Homo_633,MCI.vs.control; bulk RNA-	8
CC	GO:1990351	transporter complex	bulk RNA-seq,Emory,MCI.vs.control; bulk RNA-seq,Homo_723,AD.vs.control; bulk RNA-seq,Homo_723,AD.vs.MCI; bulk RNA-seq,Homo_714,AD.vs.control; bulk RNA-seq,Homo_714,AD.vs.MCI; bulk RNA-seq,Homo_633,AD.vs.control; bulk RNA-seq,Homo_633,AD.vs.MCI; bulk RNA-seq,ROSMAP,AD.vs.control; bulk RNA-seq,SRP223445,AD.vs.control	9
BP	GO:0060438	trachea development	bulk RNA-seq,Emory,MCI.vs.control; bulk RNA-seq,Homo_723,AD.vs.control; bulk RNA-seq,Homo_723,AD.vs.MCI; bulk RNA-seq,Homo_723,MCI.vs.control; bulk RNA-seq,Homo_714,AD.vs.MCI	5
BP	GO:0001764	neuron migration	bulk RNA-seq,Emory,MCI.vs.control; bulk RNA-seq,Homo_723,AD.vs.control; bulk RNA-seq,Homo_723,AD.vs.MCI; bulk RNA-seq,Homo_723,MCI.vs.control; bulk RNA-seq,Homo_714,AD.vs.control; bulk RNA-seq,Homo_714,AD.vs.MCI; bulk RNA-seq,Homo_633,AD.vs.control; bulk RNA-seq,ROSMAP,AD.vs.control	8
BP	GO:0035136	forelimb morphogenesis	bulk RNA-seq,Emory,MCI.vs.control; bulk RNA-seq,Homo_723,AD.vs.control; bulk RNA-seq,Homo_723,AD.vs.MCI; bulk RNA-seq,Homo_723,MCI.vs.control; bulk RNA-seq,Homo_714,AD.vs.control; bulk RNA-seq,Homo_714,AD.vs.MCI; bulk RNA-seq,Homo_714,MCI.vs.control; bulk RNA-	8
MF	GO:0001228	DNA-binding transcription activator activity, RNA polymerase II-specific	bulk RNA-seq,Emory,MCI.vs.control; bulk RNA-seq,Homo_723,AD.vs.control; bulk RNA-seq,Homo_723,AD.vs.MCI; bulk RNA-seq,Homo_714,AD.vs.control; bulk RNA-seq,Homo_714,AD.vs.MCI; bulk RNA-seq,Homo_633,AD.vs.control; bulk RNA-seq,Homo_633,AD.vs.MCI; bulk RNA-seq,SRP223445,AD.vs.control; scRNA-seq,SRP330776,Naive CD8+ T cell_2-AD.vs.control	9
MF	GO:0050681	nuclear androgen receptor binding	bulk RNA-seq,Emory,MCI.vs.control; bulk RNA-seq,Homo_723,MCI.vs.control; bulk RNA-seq,Homo_714,MCI.vs.control; bulk RNA-seq,Homo_633,AD.vs.control; bulk RNA-seq,Homo_633,AD.vs.MCI; bulk RNA-seq,Homo_633,MCI.vs.control	6

BP	GO:0007281	germ cell development	bulk RNA-seq,Emory,MCI.vs.control; bulk RNA-seq,Homo_723,AD.vs.control; bulk RNA-seq,Homo_723,AD.vs.MCI; bulk RNA-seq,Homo_723,MCI.vs.control; bulk RNA-seq,Homo_714,AD.vs.control; bulk RNA-seq,Homo_714,AD.vs.MCI; bulk RNA-seq,Homo_633,AD.vs.control; bulk RNA-seq,Homo_633,AD.vs.MCI	8
BP	GO:0048706	embryonic skeletal system development	bulk RNA-seq,Emory,MCI.vs.control; bulk RNA-seq,Homo_723,AD.vs.control; bulk RNA-seq,Homo_723,AD.vs.MCI; bulk RNA-seq,Homo_723,MCI.vs.control; bulk RNA-seq,Homo_714,AD.vs.control; bulk RNA-seq,Homo_714,AD.vs.MCI; bulk RNA-seq,Homo_714,MCI.vs.control; bulk RNA-seq,ROSMAP,AD.vs.control; bulk RNA-seq,SRP223445,AD.vs.control	9
MF	GO:0001216	DNA-binding transcription activator activity	bulk RNA-seq,Emory,MCI.vs.control; bulk RNA-seq,Homo_723,AD.vs.control; bulk RNA-seq,Homo_723,AD.vs.MCI; bulk RNA-seq,Homo_714,AD.vs.control; bulk RNA-seq,Homo_714,AD.vs.MCI; bulk RNA-seq,Homo_633,AD.vs.control; bulk RNA-seq,Homo_633,AD.vs.MCI; bulk RNA-seq,Homo_633,MCI.vs.control; bulk RNA-seq,SRP223445,AD.vs.control; scRNA-seq,SRP330776,Naive CD8+ T cell_2-AD.vs.control	10
BP	GO:0007215	glutamate receptor signaling pathway	bulk RNA-seq,Emory,MCI.vs.control; bulk RNA-seq,Homo_723,AD.vs.control; bulk RNA-seq,Homo_723,AD.vs.MCI; bulk RNA-seq,Homo_723,MCI.vs.control; bulk RNA-seq,Homo_714,AD.vs.MCI; bulk RNA-seq,Homo_714,MCI.vs.control; bulk RNA-seq,Homo_633,AD.vs.control; bulk RNA-seq,Homo_633,AD.vs.MCI	8
BP	GO:0051932	synaptic transmission, GABAergic	bulk RNA-seq,Emory,MCI.vs.control; bulk RNA-seq,Homo_723,AD.vs.control; bulk RNA-seq,Homo_723,AD.vs.MCI; bulk RNA-seq,Homo_723,MCI.vs.control; bulk RNA-seq,Homo_714,AD.vs.control; bulk RNA-seq,Homo_714,AD.vs.MCI; bulk RNA-seq,Homo_633,MCI.vs.control	7
MF	GO:0005261	cation channel activity	bulk RNA-seq,Emory,MCI.vs.control; bulk RNA-seq,Homo_723,AD.vs.control; bulk RNA-seq,Homo_723,AD.vs.MCI; bulk RNA-seq,Homo_714,AD.vs.control; bulk RNA-seq,Homo_714,AD.vs.MCI; bulk RNA-seq,Homo_633,AD.vs.control; bulk RNA-seq,Homo_633,AD.vs.MCI; bulk RNA-seq,ROSMAP,AD.vs.control; bulk RNA-seq,ROSMAP,MCI.vs.control; bulk RNA-seq,SRP223445,AD.vs.control	10
BP	GO:0035137	hindlimb morphogenesis	bulk RNA-seq,Emory,MCI.vs.control; bulk RNA-seq,Homo_723,AD.vs.control; bulk RNA-seq,Homo_723,AD.vs.MCI; bulk RNA-seq,Homo_723,MCI.vs.control; bulk RNA-seq,Homo_714,AD.vs.MCI; bulk RNA-seq,Homo_714,MCI.vs.control	6
BP	GO:0007193	adenylate cyclase-inhibiting G protein-coupled receptor signaling pathway	bulk RNA-seq,Emory,MCI.vs.control; bulk RNA-seq,Homo_723,AD.vs.control; bulk RNA-seq,Homo_723,AD.vs.MCI; bulk RNA-seq,Homo_714,AD.vs.control; bulk RNA-seq,Homo_714,AD.vs.MCI; bulk RNA-seq,Homo_633,MCI.vs.control	6
BP	GO:0003002	regionalization	bulk RNA-seq,Emory,MCI.vs.control; bulk RNA-seq,Homo_723,AD.vs.control; bulk RNA-seq,Homo_723,AD.vs.MCI; bulk RNA-seq,Homo_714,AD.vs.control; bulk RNA-seq,Homo_714,AD.vs.MCI; bulk RNA-seq,Homo_633,AD.vs.control; bulk RNA-seq,Homo_633,AD.vs.MCI; bulk RNA-seq,Homo_633,MCI.vs.control; bulk RNA-seq,ROSMAP,AD.vs.control; bulk RNA-seq,SRP223445,AD.vs.control	10
BP	GO:0099563	modification of synaptic structure	bulk RNA-seq,Emory,MCI.vs.control; bulk RNA-seq,Homo_723,AD.vs.control; bulk RNA-seq,Homo_723,AD.vs.MCI; bulk RNA-seq,Homo_723,MCI.vs.control; bulk RNA-seq,Homo_714,AD.vs.MCI; bulk RNA-seq,Homo_714,MCI.vs.control; bulk RNA-seq,Homo_633,AD.vs.control; bulk RNA-seq,Homo_633,AD.vs.MCI	8
BP	GO:0048511	rhythmic process	bulk RNA-seq,Emory,MCI.vs.control; bulk RNA-seq,Homo_723,AD.vs.control; bulk RNA-seq,Homo_723,AD.vs.MCI; bulk RNA-seq,Homo_723,MCI.vs.control; bulk RNA-seq,Homo_714,AD.vs.control; bulk RNA-seq,Homo_714,AD.vs.MCI; bulk RNA-seq,Homo_633,AD.vs.control; bulk RNA-seq,Homo_633,AD.vs.MCI; bulk RNA-seq,SRP223445,AD.vs.control	9
BP	GO:0021537	telencephalon development	bulk RNA-seq,Emory,MCI.vs.control; bulk RNA-seq,Homo_723,AD.vs.control; bulk RNA-seq,Homo_723,AD.vs.MCI; bulk RNA-seq,Homo_723,MCI.vs.control; bulk RNA-seq,Homo_714,AD.vs.control; bulk RNA-seq,Homo_714,AD.vs.MCI; bulk RNA-seq,Homo_633,AD.vs.control; bulk RNA-seq,Homo_633,AD.vs.MCI; bulk RNA-seq,ROSMAP,AD.vs.control; bulk RNA-seq,SRP223445,AD.vs.control	10
CC	GO:0031253	cell projection membrane	bulk RNA-seq,Emory,MCI.vs.control; bulk RNA-seq,Homo_723,AD.vs.control; bulk RNA-seq,Homo_723,AD.vs.MCI; bulk RNA-seq,Homo_714,AD.vs.control; bulk RNA-seq,Homo_714,AD.vs.MCI; bulk RNA-seq,Homo_633,AD.vs.control; bulk RNA-seq,Homo_633,AD.vs.MCI; bulk RNA-seq,ROSMAP,AD.vs.control	8
BP	GO:0042274	ribosomal small subunit biogenesis	bulk RNA-seq,Emory,MCI.vs.control; bulk RNA-seq,Homo_723,AD.vs.control; bulk RNA-seq,Homo_723,AD.vs.MCI; bulk RNA-seq,Homo_723,MCI.vs.control; bulk RNA-seq,Homo_714,AD.vs.control; bulk RNA-seq,Homo_714,AD.vs.MCI; bulk RNA-seq,Homo_714,MCI.vs.control; bulk RNA-seq,Homo_633,AD.vs.control; bulk RNA-seq,Homo_633,AD.vs.MCI; bulk RNA-seq,SRP223445,AD.vs.control	10
BP	GO:0060765	regulation of androgen receptor signaling pathway	bulk RNA-seq,Emory,MCI.vs.control; bulk RNA-seq,Homo_723,MCI.vs.control; bulk RNA-seq,Homo_714,MCI.vs.control; bulk RNA-seq,Homo_633,AD.vs.control; bulk RNA-seq,Homo_633,AD.vs.MCI	5
MF	GO:0140359	ABC-type transporter activity	bulk RNA-seq,Emory,MCI.vs.control; bulk RNA-seq,Homo_723,AD.vs.MCI; bulk RNA-seq,Homo_723,MCI.vs.control; bulk RNA-seq,Homo_714,AD.vs.MCI; bulk RNA-seq,Homo_714,MCI.vs.control; bulk RNA-seq,Homo_633,AD.vs.control; bulk RNA-seq,Homo_633,AD.vs.MCI	7
CC	GO:0098936	intrinsic component of postsynaptic membrane	bulk RNA-seq,Emory,MCI.vs.control; bulk RNA-seq,Homo_723,AD.vs.control; bulk RNA-seq,Homo_723,AD.vs.MCI; bulk RNA-seq,Homo_723,MCI.vs.control; bulk RNA-seq,Homo_714,AD.vs.control; bulk RNA-seq,Homo_714,AD.vs.MCI; bulk RNA-seq,Homo_714,MCI.vs.control; bulk RNA-seq,Homo_633,MCI.vs.control; bulk RNA-seq,ROSMAP,AD.vs.control; bulk RNA-seq,ROSMAP,MCI.vs.control; bulk RNA-seq,SRP223445,AD.vs.control	11
BP	GO:0035107	appendage morphogenesis	bulk RNA-seq,Emory,MCI.vs.control; bulk RNA-seq,Homo_723,AD.vs.control; bulk RNA-seq,Homo_723,AD.vs.MCI; bulk RNA-seq,Homo_723,MCI.vs.control; bulk RNA-seq,Homo_714,AD.vs.control; bulk RNA-seq,Homo_714,AD.vs.MCI; bulk RNA-seq,Homo_633,AD.vs.control; bulk RNA-seq,Homo_633,AD.vs.MCI; bulk RNA-seq,Homo_633,MCI.vs.control; bulk RNA-seq,SRP223445,AD.vs.control	10
BP	GO:0035108	limb morphogenesis	bulk RNA-seq,Emory,MCI.vs.control; bulk RNA-seq,Homo_723,AD.vs.control; bulk RNA-seq,Homo_723,AD.vs.MCI; bulk RNA-seq,Homo_723,MCI.vs.control; bulk RNA-seq,Homo_714,AD.vs.control; bulk RNA-seq,Homo_714,AD.vs.MCI; bulk RNA-seq,Homo_633,AD.vs.control; bulk RNA-seq,Homo_633,AD.vs.MCI; bulk RNA-seq,Homo_633,MCI.vs.control; bulk RNA-seq,SRP223445,AD.vs.control	10
BP	GO:0140053	mitochondrial gene expression	bulk RNA-seq,Emory,MCI.vs.control; bulk RNA-seq,Homo_723,AD.vs.control; bulk RNA-seq,Homo_723,AD.vs.MCI; bulk RNA-seq,Homo_723,MCI.vs.control; bulk RNA-seq,Homo_714,AD.vs.control; bulk RNA-seq,Homo_714,AD.vs.MCI; bulk RNA-seq,Homo_633,AD.vs.control; bulk RNA-seq,Homo_633,AD.vs.MCI; bulk RNA-seq,SRP223445,AD.vs.control	9
BP	GO:0030072	peptide hormone secretion	bulk RNA-seq,Emory,MCI.vs.control; bulk RNA-seq,Homo_723,AD.vs.control; bulk RNA-seq,Homo_723,AD.vs.MCI; bulk RNA-seq,Homo_723,MCI.vs.control; bulk RNA-seq,Homo_714,AD.vs.control; bulk RNA-seq,Homo_714,AD.vs.MCI; bulk RNA-seq,Homo_633,AD.vs.control; bulk RNA-seq,Homo_633,AD.vs.MCI	8
CC	GO:0099572	postsynaptic specialization	bulk RNA-seq,Emory,MCI.vs.control; bulk RNA-seq,Homo_723,AD.vs.control; bulk RNA-seq,Homo_723,AD.vs.MCI; bulk RNA-seq,Homo_714,AD.vs.control; bulk RNA-seq,Homo_714,AD.vs.MCI; bulk RNA-seq,Homo_633,AD.vs.control; bulk RNA-seq,Homo_633,AD.vs.MCI; bulk RNA-seq,ROSMAP,AD.vs.control; bulk RNA-seq,SRP223445,AD.vs.control	9
BP	GO:0001656	metanephros development	bulk RNA-seq,Emory,MCI.vs.control; bulk RNA-seq,Homo_723,AD.vs.control; bulk RNA-seq,Homo_723,AD.vs.MCI; bulk RNA-seq,Homo_723,MCI.vs.control; bulk RNA-seq,Homo_714,AD.vs.control; bulk RNA-seq,Homo_714,AD.vs.MCI; bulk RNA-seq,Homo_714,MCI.vs.control; bulk RNA-seq,Homo_633,AD.vs.MCI; bulk RNA-seq,Homo_633,MCI.vs.control; bulk RNA-seq,SRP223445,AD.vs.control	10
CC	GO:0031256	leading edge membrane	bulk RNA-seq,Emory,MCI.vs.control; bulk RNA-seq,Homo_723,AD.vs.control; bulk RNA-seq,Homo_723,AD.vs.MCI; bulk RNA-seq,Homo_723,MCI.vs.control; bulk RNA-seq,Homo_714,AD.vs.control; bulk RNA-seq,Homo_714,AD.vs.MCI; bulk RNA-seq,Homo_633,AD.vs.control; bulk RNA-seq,Homo_633,AD.vs.MCI; bulk RNA-seq,SRP223445,AD.vs.control	9
BP	GO:0009952	anterior/posterior pattern specification	bulk RNA-seq,Emory,MCI.vs.control; bulk RNA-seq,Homo_723,AD.vs.control; bulk RNA-seq,Homo_723,AD.vs.MCI; bulk RNA-seq,Homo_723,MCI.vs.control; bulk RNA-seq,Homo_714,AD.vs.control; bulk RNA-seq,Homo_714,AD.vs.MCI; bulk RNA-seq,Homo_633,AD.vs.control; bulk RNA-seq,Homo_633,AD.vs.MCI; bulk RNA-seq,ROSMAP,AD.vs.control; bulk RNA-seq,SRP223445,AD.vs.control	10

MF	GO:0008227	G protein-coupled amine receptor activity	bulk RNA-seq,Emory,MCI.vs.control; bulk RNA-seq,Homo_723,AD.vs.control; bulk RNA-seq,Homo_723,AD.vs.MCI; bulk RNA-seq,Homo_714,AD.vs.control; bulk RNA-seq,Homo_714,AD.vs.MCI; bulk RNA-seq,Homo_633,MCI.vs.control	6
BP	GO:0061138	morphogenesis of a branching epithelium	bulk RNA-seq,Emory,MCI.vs.control; bulk RNA-seq,Homo_723,AD.vs.control; bulk RNA-seq,Homo_723,AD.vs.MCI; bulk RNA-seq,Homo_723,MCI.vs.control; bulk RNA-seq,Homo_714,AD.vs.control; bulk RNA-seq,Homo_714,AD.vs.MCI; bulk RNA-seq,Homo_633,AD.vs.control; bulk RNA-seq,Homo_633,AD.vs.MCI; bulk RNA-seq,SRP223445,AD.vs.control	9
BP	GO:0050807	regulation of synapse organization	bulk RNA-seq,Emory,MCI.vs.control; bulk RNA-seq,Homo_723,AD.vs.control; bulk RNA-seq,Homo_723,AD.vs.MCI; bulk RNA-seq,Homo_723,MCI.vs.control; bulk RNA-seq,Homo_714,AD.vs.control; bulk RNA-seq,Homo_714,AD.vs.MCI; bulk RNA-seq,Homo_633,AD.vs.control; bulk RNA-seq,Homo_633,AD.vs.MCI; bulk RNA-seq,ROSMAP,AD.vs.control; bulk RNA-seq,ROSMAP,MCI.vs.control	10
BP	GO:0031016	pancreas development	bulk RNA-seq,Emory,MCI.vs.control; bulk RNA-seq,Homo_723,AD.vs.control; bulk RNA-seq,Homo_723,AD.vs.MCI; bulk RNA-seq,Homo_723,MCI.vs.control; bulk RNA-seq,Homo_714,AD.vs.control; bulk RNA-seq,Homo_714,AD.vs.MCI; bulk RNA-seq,Homo_714,MCI.vs.control; bulk RNA-seq,Homo_633,MCI.vs.control; bulk RNA-seq,SRP223445,AD.vs.control	9
BP	GO:0060078	regulation of postsynaptic membrane potential	bulk RNA-seq,Emory,MCI.vs.control; bulk RNA-seq,Homo_723,AD.vs.control; bulk RNA-seq,Homo_723,AD.vs.MCI; bulk RNA-seq,Homo_723,MCI.vs.control; bulk RNA-seq,Homo_714,AD.vs.control; bulk RNA-seq,Homo_714,AD.vs.MCI; bulk RNA-seq,ROSMAP,AD.vs.control; bulk RNA-seq,ROSMAP,MCI.vs.control; bulk RNA-seq,SRP223445,AD.vs.control	9
BP	GO:0002790	peptide secretion	bulk RNA-seq,Emory,MCI.vs.control; bulk RNA-seq,Homo_723,AD.vs.control; bulk RNA-seq,Homo_723,AD.vs.MCI; bulk RNA-seq,Homo_723,MCI.vs.control; bulk RNA-seq,Homo_714,AD.vs.control; bulk RNA-seq,Homo_714,AD.vs.MCI; bulk RNA-seq,Homo_633,AD.vs.control; bulk RNA-seq,Homo_633,AD.vs.MCI	8
BP	GO:0050796	regulation of insulin secretion	bulk RNA-seq,Emory,MCI.vs.control; bulk RNA-seq,Homo_723,AD.vs.control; bulk RNA-seq,Homo_723,AD.vs.MCI; bulk RNA-seq,Homo_723,MCI.vs.control; bulk RNA-seq,Homo_714,AD.vs.MCI; bulk RNA-seq,Homo_633,AD.vs.control; bulk RNA-seq,Homo_633,AD.vs.MCI; bulk RNA-seq,SRP325058,AD.vs.control	8
BP	GO:0071300	cellular response to retinoic acid	bulk RNA-seq,Emory,MCI.vs.control; bulk RNA-seq,Homo_723,AD.vs.control; bulk RNA-seq,Homo_723,AD.vs.MCI; bulk RNA-seq,Homo_723,MCI.vs.control; bulk RNA-seq,Homo_714,AD.vs.MCI; bulk RNA-seq,Homo_714,MCI.vs.control; bulk RNA-seq,Homo_633,AD.vs.control; bulk RNA-seq,Homo_633,AD.vs.MCI; bulk RNA-seq,SRP223445,AD.vs.control	9
MF	GO:0098960	postsynaptic neurotransmitter receptor activity	bulk RNA-seq,Emory,MCI.vs.control; bulk RNA-seq,Homo_723,AD.vs.control; bulk RNA-seq,Homo_723,AD.vs.MCI; bulk RNA-seq,Homo_723,MCI.vs.control; bulk RNA-seq,Homo_714,AD.vs.control; bulk RNA-seq,Homo_714,AD.vs.MCI; bulk RNA-seq,Homo_633,MCI.vs.control; bulk RNA-	8
BP	GO:0030326	embryonic limb morphogenesis	bulk RNA-seq,Emory,MCI.vs.control; bulk RNA-seq,Homo_723,AD.vs.control; bulk RNA-seq,Homo_723,AD.vs.MCI; bulk RNA-seq,Homo_723,MCI.vs.control; bulk RNA-seq,Homo_714,AD.vs.control; bulk RNA-seq,Homo_714,AD.vs.MCI; bulk RNA-seq,Homo_714,MCI.vs.control; bulk RNA-seq,Homo_633,AD.vs.control; bulk RNA-seq,Homo_633,AD.vs.MCI; bulk RNA-seq,Homo_633,MCI.vs.control; bulk RNA-seq,SRP223445,AD.vs.control	11
BP	GO:0035113	embryonic appendage morphogenesis	bulk RNA-seq,Emory,MCI.vs.control; bulk RNA-seq,Homo_723,AD.vs.control; bulk RNA-seq,Homo_723,AD.vs.MCI; bulk RNA-seq,Homo_723,MCI.vs.control; bulk RNA-seq,Homo_714,AD.vs.control; bulk RNA-seq,Homo_714,AD.vs.MCI; bulk RNA-seq,Homo_714,MCI.vs.control; bulk RNA-seq,Homo_633,AD.vs.control; bulk RNA-seq,Homo_633,AD.vs.MCI; bulk RNA-seq,Homo_633,MCI.vs.control; bulk RNA-seq,SRP223445,AD.vs.control	11
BP	GO:0042471	ear morphogenesis	bulk RNA-seq,Emory,MCI.vs.control; bulk RNA-seq,Homo_723,AD.vs.control; bulk RNA-seq,Homo_723,AD.vs.MCI; bulk RNA-seq,Homo_723,MCI.vs.control; bulk RNA-seq,Homo_714,AD.vs.control; bulk RNA-seq,Homo_714,AD.vs.MCI; bulk RNA-seq,Homo_714,MCI.vs.control; bulk RNA-seq,Homo_633,MCI.vs.control; bulk RNA-seq,SRP223445,AD.vs.control	9
BP	GO:0001655	urogenital system development	bulk RNA-seq,Emory,MCI.vs.control; bulk RNA-seq,Homo_723,AD.vs.control; bulk RNA-seq,Homo_723,AD.vs.MCI; bulk RNA-seq,Homo_714,AD.vs.control; bulk RNA-seq,Homo_714,AD.vs.MCI; bulk RNA-seq,Homo_633,AD.vs.control; bulk RNA-seq,Homo_633,AD.vs.MCI; bulk RNA-seq,ROSMAP,AD.vs.control; bulk RNA-seq,ROSMAP,MCI.vs.control; bulk RNA-seq,SRP223445,AD.vs.control	10
BP	GO:0042461	photoreceptor cell development	bulk RNA-seq,Emory,MCI.vs.control; bulk RNA-seq,Homo_723,AD.vs.control; bulk RNA-seq,Homo_723,AD.vs.MCI; bulk RNA-seq,Homo_723,MCI.vs.control; bulk RNA-seq,Homo_714,AD.vs.MCI; bulk RNA-seq,Homo_633,AD.vs.control; bulk RNA-seq,Homo_633,AD.vs.MCI	7
CC	GO:0099055	integral component of postsynaptic membrane	bulk RNA-seq,Emory,MCI.vs.control; bulk RNA-seq,Homo_723,AD.vs.control; bulk RNA-seq,Homo_723,AD.vs.MCI; bulk RNA-seq,Homo_723,MCI.vs.control; bulk RNA-seq,Homo_714,AD.vs.control; bulk RNA-seq,Homo_714,AD.vs.MCI; bulk RNA-seq,Homo_714,MCI.vs.control; bulk RNA-seq,Homo_633,MCI.vs.control; bulk RNA-seq,SRP223445,AD.vs.control	9
BP	GO:0050953	sensory perception of light stimulus	bulk RNA-seq,Emory,MCI.vs.control; bulk RNA-seq,Homo_723,AD.vs.control; bulk RNA-seq,Homo_723,AD.vs.MCI; bulk RNA-seq,Homo_723,MCI.vs.control; bulk RNA-seq,Homo_714,AD.vs.control; bulk RNA-seq,Homo_714,AD.vs.MCI; bulk RNA-seq,Homo_633,MCI.vs.control; bulk RNA-seq,ROSMAP,AD.vs.control; bulk RNA-seq,SRP223445,AD.vs.control	9
CC	GO:0150034	distal axon	bulk RNA-seq,Emory,MCI.vs.control; bulk RNA-seq,Homo_723,AD.vs.control; bulk RNA-seq,Homo_723,AD.vs.MCI; bulk RNA-seq,Homo_723,MCI.vs.control; bulk RNA-seq,Homo_714,AD.vs.MCI; bulk RNA-seq,Homo_633,AD.vs.control; bulk RNA-seq,Homo_633,AD.vs.MCI; bulk RNA-seq,ROSMAP,AD.vs.control; bulk RNA-seq,ROSMAP,MCI.vs.control; bulk RNA-seq,SRP223445,AD.vs.control	10
CC	GO:0098984	neuron to neuron synapse	bulk RNA-seq,Emory,MCI.vs.control; bulk RNA-seq,Homo_723,AD.vs.control; bulk RNA-seq,Homo_723,AD.vs.MCI; bulk RNA-seq,Homo_714,AD.vs.control; bulk RNA-seq,Homo_714,AD.vs.MCI; bulk RNA-seq,Homo_633,AD.vs.control; bulk RNA-seq,Homo_633,AD.vs.MCI; bulk RNA-seq,ROSMAP,AD.vs.control; bulk RNA-seq,SRP223445,AD.vs.control	9
CC	GO:0032590	dendrite membrane	bulk RNA-seq,Emory,MCI.vs.control; bulk RNA-seq,Homo_723,AD.vs.control; bulk RNA-seq,Homo_723,AD.vs.MCI; bulk RNA-seq,Homo_723,MCI.vs.control; bulk RNA-seq,Homo_714,AD.vs.control; bulk RNA-seq,Homo_714,AD.vs.MCI; bulk RNA-seq,Homo_633,MCI.vs.control	7
BP	GO:0120316	sperm flagellum assembly	bulk RNA-seq,Emory,MCI.vs.control; bulk RNA-seq,Homo_723,AD.vs.control; bulk RNA-seq,Homo_723,AD.vs.MCI; bulk RNA-seq,Homo_723,MCI.vs.control	4
BP	GO:0048645	animal organ formation	bulk RNA-seq,Emory,MCI.vs.control; bulk RNA-seq,Homo_723,AD.vs.control; bulk RNA-seq,Homo_723,AD.vs.MCI; bulk RNA-seq,Homo_723,MCI.vs.control; bulk RNA-seq,Homo_714,AD.vs.control; bulk RNA-seq,Homo_714,AD.vs.MCI; bulk RNA-seq,Homo_714,MCI.vs.control; bulk RNA-seq,Homo_633,MCI.vs.control; bulk RNA-seq,ROSMAP,MCI.vs.control; bulk RNA-seq,SRP223445,AD.vs.control; scRNA-seq,SRP330776,Naive CD8+ T cell_1-	11
BP	GO:0002066	columnar/cuboidal epithelial cell development	bulk RNA-seq,Emory,MCI.vs.control; bulk RNA-seq,Homo_723,AD.vs.control; bulk RNA-seq,Homo_723,AD.vs.MCI; bulk RNA-seq,Homo_723,MCI.vs.control; bulk RNA-seq,Homo_714,AD.vs.MCI	5
BP	GO:0042462	eye photoreceptor cell development	bulk RNA-seq,Emory,MCI.vs.control; bulk RNA-seq,Homo_723,AD.vs.control; bulk RNA-seq,Homo_723,AD.vs.MCI; bulk RNA-seq,Homo_723,MCI.vs.control; bulk RNA-seq,Homo_714,AD.vs.MCI	5
BP	GO:0060441	epithelial tube branching involved in lung morphogenesis	bulk RNA-seq,Emory,MCI.vs.control; bulk RNA-seq,Homo_723,AD.vs.control; bulk RNA-seq,Homo_723,AD.vs.MCI; bulk RNA-seq,Homo_723,MCI.vs.control; bulk RNA-seq,Homo_714,AD.vs.MCI; bulk RNA-seq,Homo_714,MCI.vs.control; bulk RNA-seq,Homo_633,MCI.vs.control	7
BP	GO:0030900	forebrain development	bulk RNA-seq,Emory,MCI.vs.control; bulk RNA-seq,Homo_723,AD.vs.control; bulk RNA-seq,Homo_723,AD.vs.MCI; bulk RNA-seq,Homo_714,AD.vs.control; bulk RNA-seq,Homo_714,AD.vs.MCI; bulk RNA-seq,Homo_633,AD.vs.control; bulk RNA-seq,Homo_633,AD.vs.MCI; bulk RNA-seq,ROSMAP,AD.vs.control; bulk RNA-seq,SRP223445,AD.vs.control	9
BP	GO:0015833	peptide transport	bulk RNA-seq,Emory,MCI.vs.control; bulk RNA-seq,Homo_723,AD.vs.control; bulk RNA-seq,Homo_723,AD.vs.MCI; bulk RNA-seq,Homo_723,MCI.vs.control; bulk RNA-seq,Homo_714,AD.vs.control; bulk RNA-seq,Homo_714,AD.vs.MCI; bulk RNA-seq,Homo_633,AD.vs.control; bulk RNA-seq,Homo_633,AD.vs.MCI	8

BP	GO:0032526	response to retinoic acid	bulk RNA-seq,Emory,MCI.vs.control; bulk RNA-seq,Homo_723,AD.vs.control; bulk RNA-seq,Homo_723,AD.vs.MCI; bulk RNA-seq,Homo_723,MCI.vs.control; bulk RNA-seq,Homo_714,AD.vs.MCI; bulk RNA-seq,Homo_714,MCI.vs.control; bulk RNA-seq,Homo_633,AD.vs.control; bulk RNA-seq,Homo_633,AD.vs.MCI; bulk RNA-seq,SRP223445,AD.vs.control	9
BP	GO:0030073	insulin secretion	bulk RNA-seq,Emory,MCI.vs.control; bulk RNA-seq,Homo_723,AD.vs.control; bulk RNA-seq,Homo_723,AD.vs.MCI; bulk RNA-seq,Homo_723,MCI.vs.control; bulk RNA-seq,Homo_714,AD.vs.MCI; bulk RNA-seq,Homo_633,AD.vs.control; bulk RNA-seq,Homo_633,AD.vs.MCI	7
BP	GO:0007156	homophilic cell adhesion via plasma membrane adhesion molecules	bulk RNA-seq,Emory,MCI.vs.control; bulk RNA-seq,Homo_723,AD.vs.control; bulk RNA-seq,Homo_723,AD.vs.MCI; bulk RNA-seq,Homo_723,MCI.vs.control; bulk RNA-seq,Homo_714,AD.vs.control; bulk RNA-seq,Homo_714,AD.vs.MCI; bulk RNA-seq,Homo_633,MCI.vs.control; bulk RNA-seq,ROSMAP,AD.vs.control; bulk RNA-seq,ROSMAP,MCI.vs.control; bulk RNA-seq,SRP223445,AD.vs.control; scRNA-seq,SRP215507,CD8+ T cell_1-	11
BP	GO:0009642	response to light intensity	bulk RNA-seq,Emory,MCI.vs.control	1
BP	GO:0006821	chloride transport	bulk RNA-seq,Emory,MCI.vs.control; bulk RNA-seq,Homo_723,AD.vs.control; bulk RNA-seq,Homo_723,AD.vs.MCI; bulk RNA-seq,Homo_723,MCI.vs.control; bulk RNA-seq,Homo_714,AD.vs.control; bulk RNA-seq,Homo_714,AD.vs.MCI; bulk RNA-seq,Homo_714,MCI.vs.control; bulk RNA-	8
BP	GO:2000241	regulation of reproductive process	bulk RNA-seq,Emory,MCI.vs.control; bulk RNA-seq,Homo_723,AD.vs.control; bulk RNA-seq,Homo_723,AD.vs.MCI; bulk RNA-seq,Homo_723,MCI.vs.control; bulk RNA-seq,Homo_714,AD.vs.control; bulk RNA-seq,Homo_714,AD.vs.MCI; bulk RNA-seq,Homo_633,AD.vs.control; bulk RNA-seq,Homo_633,AD.vs.MCI	8
BP	GO:0046883	regulation of hormone secretion	bulk RNA-seq,Emory,MCI.vs.control; bulk RNA-seq,Homo_723,AD.vs.control; bulk RNA-seq,Homo_723,AD.vs.MCI; bulk RNA-seq,Homo_723,MCI.vs.control; bulk RNA-seq,Homo_714,AD.vs.control; bulk RNA-seq,Homo_714,AD.vs.MCI; bulk RNA-seq,Homo_633,AD.vs.control; bulk RNA-seq,Homo_633,AD.vs.MCI	8
BP	GO:0098742	cell-cell adhesion via plasma-membrane adhesion molecules	bulk RNA-seq,Emory,MCI.vs.control; bulk RNA-seq,Homo_723,AD.vs.control; bulk RNA-seq,Homo_723,AD.vs.MCI; bulk RNA-seq,Homo_723,MCI.vs.control; bulk RNA-seq,Homo_714,AD.vs.control; bulk RNA-seq,Homo_714,AD.vs.MCI; bulk RNA-seq,Homo_633,MCI.vs.control; bulk RNA-seq,ROSMAP,AD.vs.control; bulk RNA-seq,ROSMAP,MCI.vs.control; bulk RNA-seq,SRP223445,AD.vs.control	10
BP	GO:0071498	cellular response to fluid shear stress	bulk RNA-seq,Emory,MCI.vs.control; bulk RNA-seq,Homo_723,AD.vs.MCI; bulk RNA-seq,Homo_723,MCI.vs.control; bulk RNA-seq,Homo_714,AD.vs.MCI; bulk RNA-seq,Homo_714,MCI.vs.control; bulk RNA-seq,Homo_633,AD.vs.control; bulk RNA-seq,Homo_633,AD.vs.MCI	7
BP	GO:0030879	mammary gland development	bulk RNA-seq,Emory,MCI.vs.control; bulk RNA-seq,Homo_723,AD.vs.control; bulk RNA-seq,Homo_723,AD.vs.MCI; bulk RNA-seq,Homo_723,MCI.vs.control; bulk RNA-seq,Homo_714,AD.vs.control; bulk RNA-seq,Homo_714,AD.vs.MCI; bulk RNA-seq,Homo_633,AD.vs.control	7
BP	GO:0072001	renal system development	bulk RNA-seq,Emory,MCI.vs.control; bulk RNA-seq,Homo_723,AD.vs.control; bulk RNA-seq,Homo_723,AD.vs.MCI; bulk RNA-seq,Homo_714,AD.vs.control; bulk RNA-seq,Homo_714,AD.vs.MCI; bulk RNA-seq,Homo_633,AD.vs.control; bulk RNA-seq,Homo_633,AD.vs.MCI; bulk RNA-seq,ROSMAP,AD.vs.control; bulk RNA-seq,ROSMAP,MCI.vs.control; bulk RNA-seq,SRP223445,AD.vs.control	10
MF	GO:0005516	calmodulin binding	bulk RNA-seq,Emory,MCI.vs.control; bulk RNA-seq,Homo_723,AD.vs.control; bulk RNA-seq,Homo_723,AD.vs.MCI; bulk RNA-seq,Homo_723,MCI.vs.control; bulk RNA-seq,Homo_714,AD.vs.MCI; bulk RNA-seq,Homo_633,AD.vs.control; bulk RNA-seq,ROSMAP,AD.vs.control	7
BP	GO:0007200	phospholipase C-activating G protein-coupled receptor signaling pathway	bulk RNA-seq,Emory,MCI.vs.control; bulk RNA-seq,Homo_723,AD.vs.control; bulk RNA-seq,Homo_723,AD.vs.MCI; bulk RNA-seq,Homo_723,MCI.vs.control; bulk RNA-seq,Homo_714,AD.vs.control; bulk RNA-seq,Homo_714,AD.vs.MCI; bulk RNA-seq,Homo_633,MCI.vs.control	7
BP	GO:0001570	vasculogenesis	bulk RNA-seq,Emory,MCI.vs.control; bulk RNA-seq,Homo_723,AD.vs.control; bulk RNA-seq,Homo_723,AD.vs.MCI; bulk RNA-seq,Homo_723,MCI.vs.control; bulk RNA-seq,Homo_714,AD.vs.control; bulk RNA-seq,Homo_714,AD.vs.MCI; bulk RNA-seq,Homo_714,MCI.vs.control; bulk RNA-seq,Homo_633,AD.vs.control; bulk RNA-seq,Homo_633,AD.vs.MCI	9
BP	GO:0042886	amide transport	bulk RNA-seq,Emory,MCI.vs.control; bulk RNA-seq,Homo_723,AD.vs.control; bulk RNA-seq,Homo_723,AD.vs.MCI; bulk RNA-seq,Homo_723,MCI.vs.control; bulk RNA-seq,Homo_714,AD.vs.control; bulk RNA-seq,Homo_714,AD.vs.MCI; bulk RNA-seq,Homo_633,AD.vs.control; bulk RNA-seq,Homo_633,AD.vs.MCI	8
BP	GO:0048863	stem cell differentiation	bulk RNA-seq,Emory,MCI.vs.control; bulk RNA-seq,Homo_723,AD.vs.control; bulk RNA-seq,Homo_723,AD.vs.MCI; bulk RNA-seq,Homo_723,MCI.vs.control; bulk RNA-seq,Homo_714,AD.vs.control; bulk RNA-seq,Homo_714,AD.vs.MCI; bulk RNA-seq,Homo_633,AD.vs.control; bulk RNA-seq,Homo_633,AD.vs.MCI; bulk RNA-seq,ROSMAP,AD.vs.control; bulk RNA-seq,SRP223445,AD.vs.control	10
MF	GO:0035035	histone acetyltransferase binding	bulk RNA-seq,Emory,MCI.vs.control; bulk RNA-seq,Homo_723,MCI.vs.control; bulk RNA-seq,Homo_633,AD.vs.control; bulk RNA-seq,Homo_633,AD.vs.MCI	4
BP	GO:0099010	modification of postsynaptic structure	bulk RNA-seq,Emory,MCI.vs.control; bulk RNA-seq,Homo_723,AD.vs.control; bulk RNA-seq,Homo_723,AD.vs.MCI; bulk RNA-seq,Homo_723,MCI.vs.control; bulk RNA-seq,Homo_714,AD.vs.MCI; bulk RNA-seq,Homo_714,MCI.vs.control	6
BP	GO:0035115	embryonic forelimb morphogenesis	bulk RNA-seq,Emory,MCI.vs.control; bulk RNA-seq,Homo_723,AD.vs.control; bulk RNA-seq,Homo_723,AD.vs.MCI; bulk RNA-seq,Homo_723,MCI.vs.control; bulk RNA-seq,Homo_714,AD.vs.MCI; bulk RNA-seq,Homo_714,MCI.vs.control; bulk RNA-seq,SRP223445,AD.vs.control	7
BP	GO:0045664	regulation of neuron differentiation	bulk RNA-seq,Emory,MCI.vs.control; bulk RNA-seq,Homo_723,AD.vs.control; bulk RNA-seq,Homo_723,AD.vs.MCI; bulk RNA-seq,Homo_723,MCI.vs.control; bulk RNA-seq,Homo_714,AD.vs.control; bulk RNA-seq,Homo_714,AD.vs.MCI; bulk RNA-seq,Homo_633,AD.vs.control; bulk RNA-seq,Homo_633,AD.vs.MCI; bulk RNA-seq,ROSMAP,AD.vs.control; bulk RNA-seq,SRP223445,AD.vs.control	10
BP	GO:0048732	gland development	bulk RNA-seq,Emory,MCI.vs.control; bulk RNA-seq,Homo_723,AD.vs.control; bulk RNA-seq,Homo_723,AD.vs.MCI; bulk RNA-seq,Homo_714,AD.vs.control; bulk RNA-seq,Homo_714,AD.vs.MCI; bulk RNA-seq,Homo_633,AD.vs.control; bulk RNA-seq,Homo_633,AD.vs.MCI; bulk RNA-seq,SRP223445,AD.vs.control; scRNA-seq,SRP330776,Naive CD8+ T cell_3-AD.vs.control; scRNA-seq,SRP330776,Natural killer cell_1-AD.vs.control	10
BP	GO:0035116	embryonic hindlimb morphogenesis	bulk RNA-seq,Emory,MCI.vs.control; bulk RNA-seq,Homo_723,AD.vs.control; bulk RNA-seq,Homo_723,AD.vs.MCI; bulk RNA-seq,Homo_723,MCI.vs.control; bulk RNA-seq,Homo_714,AD.vs.MCI; bulk RNA-seq,Homo_714,MCI.vs.control	6
CC	GO:0043204	perikaryon	bulk RNA-seq,Emory,MCI.vs.control; bulk RNA-seq,Homo_723,AD.vs.control; bulk RNA-seq,Homo_723,AD.vs.MCI; bulk RNA-seq,Homo_723,MCI.vs.control; bulk RNA-seq,Homo_714,AD.vs.control; bulk RNA-seq,Homo_714,AD.vs.MCI; bulk RNA-seq,Homo_633,AD.vs.control; bulk RNA-seq,Homo_633,AD.vs.MCI; bulk RNA-seq,SRP223445,AD.vs.control	9
MF	GO:0000146	microfilament motor activity	bulk RNA-seq,Emory,MCI.vs.control; bulk RNA-seq,Homo_723,AD.vs.control; bulk RNA-seq,Homo_723,AD.vs.MCI; bulk RNA-seq,Homo_723,MCI.vs.control; bulk RNA-seq,Homo_714,AD.vs.MCI; bulk RNA-seq,Homo_714,MCI.vs.control	6
BP	GO:0007623	circadian rhythm	bulk RNA-seq,Emory,MCI.vs.control; bulk RNA-seq,Homo_723,AD.vs.control; bulk RNA-seq,Homo_723,AD.vs.MCI; bulk RNA-seq,Homo_723,MCI.vs.control; bulk RNA-seq,Homo_714,AD.vs.control; bulk RNA-seq,Homo_714,AD.vs.MCI; bulk RNA-seq,Homo_633,AD.vs.control; bulk RNA-seq,Homo_633,AD.vs.MCI	8
BP	GO:0048705	skeletal system morphogenesis	bulk RNA-seq,Emory,MCI.vs.control; bulk RNA-seq,Homo_723,AD.vs.control; bulk RNA-seq,Homo_723,AD.vs.MCI; bulk RNA-seq,Homo_723,MCI.vs.control; bulk RNA-seq,Homo_714,AD.vs.control; bulk RNA-seq,Homo_714,AD.vs.MCI; bulk RNA-seq,Homo_633,AD.vs.control; bulk RNA-seq,Homo_633,AD.vs.MCI; bulk RNA-seq,ROSMAP,AD.vs.control; bulk RNA-seq,SRP223445,AD.vs.control	10
BP	GO:0060294	cilium movement involved in cell motility	bulk RNA-seq,Emory,MCI.vs.control; bulk RNA-seq,Homo_723,AD.vs.control; bulk RNA-seq,Homo_723,AD.vs.MCI; bulk RNA-seq,Homo_723,MCI.vs.control; bulk RNA-seq,Homo_714,AD.vs.control; bulk RNA-seq,Homo_714,AD.vs.MCI; bulk RNA-seq,Homo_633,AD.vs.control; bulk RNA-seq,Homo_633,AD.vs.MCI	8
CC	GO:0022625	cytosolic large ribosomal subunit	bulk RNA-seq,Emory,MCI.vs.control; bulk RNA-seq,Homo_723,AD.vs.control; bulk RNA-seq,Homo_723,MCI.vs.control; bulk RNA-seq,Homo_714,AD.vs.control; bulk RNA-seq,Homo_714,MCI.vs.control; bulk RNA-seq,Homo_633,AD.vs.control; bulk RNA-seq,Homo_633,AD.vs.MCI; bulk RNA-seq,ROSMAP,AD.vs.control; bulk RNA-seq,ROSMAP,MCI.vs.control; scRNA-seq,SRP330776,Megakaryocyte_2-AD.vs.control; scRNA-	11

BP	GO:0051653	spindle localization	bulk RNA-seq,Emory,MCI.vs.control; bulk RNA-seq,Homo_723,AD.vs.MCI; bulk RNA-seq,Homo_723,MCI.vs.control; bulk RNA-seq,Homo_714,MCI.vs.control; bulk RNA-seq,Homo_633,AD.vs.control; bulk RNA-seq,Homo_633,AD.vs.MCI	6
BP	GO:0042472	inner ear morphogenesis	bulk RNA-seq,Emory,MCI.vs.control; bulk RNA-seq,Homo_723,AD.vs.control; bulk RNA-seq,Homo_723,AD.vs.MCI; bulk RNA-seq,Homo_723,MCI.vs.control; bulk RNA-seq,Homo_714,AD.vs.control; bulk RNA-seq,Homo_714,AD.vs.MCI; bulk RNA-seq,Homo_714,MCI.vs.control; bulk RNA-	8
BP	GO:0048592	eye morphogenesis	bulk RNA-seq,Emory,MCI.vs.control; bulk RNA-seq,Homo_723,AD.vs.control; bulk RNA-seq,Homo_723,AD.vs.MCI; bulk RNA-seq,Homo_723,MCI.vs.control; bulk RNA-seq,Homo_714,AD.vs.control; bulk RNA-seq,Homo_714,AD.vs.MCI; bulk RNA-seq,Homo_633,AD.vs.control; bulk RNA-seq,Homo_633,AD.vs.MCI; bulk RNA-seq,ROSMAP,AD.vs.control; bulk RNA-seq,SRP223445,AD.vs.control	10
CC	GO:0032279	asymmetric synapse	bulk RNA-seq,Emory,MCI.vs.control; bulk RNA-seq,Homo_723,AD.vs.control; bulk RNA-seq,Homo_723,AD.vs.MCI; bulk RNA-seq,Homo_723,MCI.vs.control; bulk RNA-seq,Homo_714,AD.vs.MCI; bulk RNA-seq,Homo_633,AD.vs.control; bulk RNA-seq,Homo_633,AD.vs.MCI; bulk RNA-seq,ROSMAP,AD.vs.control; bulk RNA-seq,SRP223445,AD.vs.control	9
BP	GO:0060041	retina development in camera-type eye	bulk RNA-seq,Emory,MCI.vs.control; bulk RNA-seq,Homo_723,AD.vs.control; bulk RNA-seq,Homo_723,AD.vs.MCI; bulk RNA-seq,Homo_723,MCI.vs.control; bulk RNA-seq,Homo_714,AD.vs.control; bulk RNA-seq,Homo_714,AD.vs.MCI; bulk RNA-seq,Homo_633,AD.vs.control; bulk RNA-seq,Homo_633,AD.vs.MCI; bulk RNA-seq,SRP223445,AD.vs.control	9
CC	GO:0098982	GABA-ergic synapse	bulk RNA-seq,Emory,MCI.vs.control; bulk RNA-seq,Homo_723,AD.vs.control; bulk RNA-seq,Homo_723,AD.vs.MCI; bulk RNA-seq,Homo_723,MCI.vs.control; bulk RNA-seq,Homo_714,AD.vs.control; bulk RNA-seq,Homo_714,AD.vs.MCI; bulk RNA-seq,Homo_714,MCI.vs.control; bulk RNA-seq,Homo_633,MCI.vs.control; bulk RNA-seq,SRP223445,AD.vs.control	9
BP	GO:0048593	camera-type eye morphogenesis	bulk RNA-seq,Emory,MCI.vs.control; bulk RNA-seq,Homo_723,AD.vs.control; bulk RNA-seq,Homo_723,AD.vs.MCI; bulk RNA-seq,Homo_723,MCI.vs.control; bulk RNA-seq,Homo_714,AD.vs.control; bulk RNA-seq,Homo_714,AD.vs.MCI; bulk RNA-seq,Homo_633,AD.vs.control; bulk RNA-seq,Homo_633,AD.vs.MCI; bulk RNA-seq,ROSMAP,AD.vs.control; bulk RNA-seq,SRP223445,AD.vs.control	10
CC	GO:0098948	intrinsic component of postsynaptic specialization membrane	bulk RNA-seq,Emory,MCI.vs.control; bulk RNA-seq,Homo_723,AD.vs.control; bulk RNA-seq,Homo_723,AD.vs.MCI; bulk RNA-seq,Homo_723,MCI.vs.control; bulk RNA-seq,Homo_714,AD.vs.control; bulk RNA-seq,Homo_714,AD.vs.MCI; bulk RNA-seq,Homo_633,MCI.vs.control; bulk RNA-	8
BP	GO:0030490	maturation of SSU-rRNA	bulk RNA-seq,Emory,MCI.vs.control; bulk RNA-seq,Homo_723,AD.vs.control; bulk RNA-seq,Homo_723,AD.vs.MCI; bulk RNA-seq,Homo_723,MCI.vs.control; bulk RNA-seq,Homo_714,AD.vs.control; bulk RNA-seq,Homo_714,AD.vs.MCI; bulk RNA-seq,Homo_714,MCI.vs.control; bulk RNA-seq,Homo_633,AD.vs.control; bulk RNA-seq,Homo_633,AD.vs.MCI	9
BP	GO:0060914	heart formation	bulk RNA-seq,Emory,MCI.vs.control; bulk RNA-seq,Homo_723,AD.vs.control; bulk RNA-seq,Homo_723,AD.vs.MCI; bulk RNA-seq,Homo_723,MCI.vs.control; bulk RNA-seq,Homo_714,AD.vs.MCI; bulk RNA-seq,Homo_714,MCI.vs.control	6
BP	GO:0051339	regulation of lyase activity	bulk RNA-seq,Emory,MCI.vs.control; bulk RNA-seq,Homo_723,AD.vs.control; bulk RNA-seq,Homo_723,AD.vs.MCI; bulk RNA-seq,Homo_723,MCI.vs.control; bulk RNA-seq,Homo_714,AD.vs.control; bulk RNA-seq,Homo_714,AD.vs.MCI; bulk RNA-seq,SRP223445,AD.vs.control	7
BP	GO:0002064	epithelial cell development	bulk RNA-seq,Emory,MCI.vs.control; bulk RNA-seq,Homo_723,AD.vs.control; bulk RNA-seq,Homo_723,AD.vs.MCI; bulk RNA-seq,Homo_723,MCI.vs.control; bulk RNA-seq,Homo_714,AD.vs.control; bulk RNA-seq,Homo_714,AD.vs.MCI; bulk RNA-seq,Homo_633,AD.vs.control; bulk RNA-seq,Homo_633,AD.vs.MCI	8
BP	GO:0007601	visual perception	bulk RNA-seq,Emory,MCI.vs.control; bulk RNA-seq,Homo_723,AD.vs.control; bulk RNA-seq,Homo_723,AD.vs.MCI; bulk RNA-seq,Homo_723,MCI.vs.control; bulk RNA-seq,Homo_714,AD.vs.control; bulk RNA-seq,Homo_714,AD.vs.MCI; bulk RNA-seq,ROSMAP,AD.vs.control; bulk RNA-seq,SRP223445,AD.vs.control	8
CC	GO:0034703	cation channel complex	bulk RNA-seq,Emory,MCI.vs.control; bulk RNA-seq,Homo_723,AD.vs.control; bulk RNA-seq,Homo_723,AD.vs.MCI; bulk RNA-seq,Homo_723,MCI.vs.control; bulk RNA-seq,Homo_714,AD.vs.control; bulk RNA-seq,Homo_714,AD.vs.MCI; bulk RNA-seq,ROSMAP,AD.vs.control; bulk RNA-seq,ROSMAP,MCI.vs.control; bulk RNA-seq,SRP223445,AD.vs.control	9
CC	GO:0016460	myosin II complex	bulk RNA-seq,Emory,MCI.vs.control; bulk RNA-seq,Homo_723,AD.vs.control; bulk RNA-seq,Homo_723,AD.vs.MCI; bulk RNA-seq,Homo_714,AD.vs.control; bulk RNA-seq,Homo_714,AD.vs.MCI	5
BP	GO:0042273	ribosomal large subunit biogenesis	bulk RNA-seq,Emory,MCI.vs.control; bulk RNA-seq,Homo_723,AD.vs.control; bulk RNA-seq,Homo_723,AD.vs.MCI; bulk RNA-seq,Homo_723,MCI.vs.control; bulk RNA-seq,Homo_714,AD.vs.control; bulk RNA-seq,Homo_714,AD.vs.MCI; bulk RNA-seq,Homo_714,MCI.vs.control; bulk RNA-seq,Homo_633,AD.vs.control; bulk RNA-seq,Homo_633,AD.vs.MCI; bulk RNA-seq,SRP223445,AD.vs.control	10
BP	GO:0001704	formation of primary germ layer	bulk RNA-seq,Emory,MCI.vs.control; bulk RNA-seq,Homo_723,AD.vs.control; bulk RNA-seq,Homo_723,AD.vs.MCI; bulk RNA-seq,Homo_723,MCI.vs.control; bulk RNA-seq,Homo_714,AD.vs.control; bulk RNA-seq,Homo_714,AD.vs.MCI; bulk RNA-seq,Homo_714,MCI.vs.control; bulk RNA-	8
CC	GO:0032589	neuron projection membrane	bulk RNA-seq,Emory,MCI.vs.control; bulk RNA-seq,Homo_723,AD.vs.control; bulk RNA-seq,Homo_723,AD.vs.MCI; bulk RNA-seq,Homo_723,MCI.vs.control; bulk RNA-seq,Homo_714,AD.vs.control; bulk RNA-seq,Homo_714,AD.vs.MCI	6
BP	GO:0045666	positive regulation of neuron differentiation	bulk RNA-seq,Emory,MCI.vs.control; bulk RNA-seq,Homo_723,AD.vs.control; bulk RNA-seq,Homo_723,AD.vs.MCI; bulk RNA-seq,Homo_723,MCI.vs.control; bulk RNA-seq,Homo_714,AD.vs.control; bulk RNA-seq,Homo_714,AD.vs.MCI; bulk RNA-seq,Homo_714,MCI.vs.control; bulk RNA-seq,Homo_633,AD.vs.control; bulk RNA-seq,Homo_633,AD.vs.MCI; bulk RNA-seq,Homo_633,MCI.vs.control; bulk RNA-seq,SRP223445,AD.vs.control	11
BP	GO:0016331	morphogenesis of embryonic epithelium	bulk RNA-seq,Emory,MCI.vs.control; bulk RNA-seq,Homo_723,AD.vs.control; bulk RNA-seq,Homo_723,AD.vs.MCI; bulk RNA-seq,Homo_723,MCI.vs.control; bulk RNA-seq,Homo_714,AD.vs.MCI; bulk RNA-seq,Homo_633,AD.vs.control; bulk RNA-seq,Homo_633,AD.vs.MCI; bulk RNA-seq,SRP223445,AD.vs.control	8
BP	GO:0044458	motile cilium assembly	bulk RNA-seq,Emory,MCI.vs.control; bulk RNA-seq,Homo_723,AD.vs.control; bulk RNA-seq,Homo_723,AD.vs.MCI; bulk RNA-seq,Homo_723,MCI.vs.control; bulk RNA-seq,Homo_633,AD.vs.control; bulk RNA-seq,Homo_633,AD.vs.MCI	6
BP	GO:0001539	cilium or flagellum-dependent cell motility	bulk RNA-seq,Emory,MCI.vs.control; bulk RNA-seq,Homo_723,AD.vs.control; bulk RNA-seq,Homo_723,AD.vs.MCI; bulk RNA-seq,Homo_723,MCI.vs.control; bulk RNA-seq,Homo_714,AD.vs.control; bulk RNA-seq,Homo_714,AD.vs.MCI; bulk RNA-seq,Homo_633,AD.vs.control; bulk RNA-seq,Homo_633,AD.vs.MCI	8
BP	GO:0060285	cilium-dependent cell motility	bulk RNA-seq,Emory,MCI.vs.control; bulk RNA-seq,Homo_723,AD.vs.control; bulk RNA-seq,Homo_723,AD.vs.MCI; bulk RNA-seq,Homo_723,MCI.vs.control; bulk RNA-seq,Homo_714,AD.vs.control; bulk RNA-seq,Homo_714,AD.vs.MCI; bulk RNA-seq,Homo_633,AD.vs.control; bulk RNA-seq,Homo_633,AD.vs.MCI	8
BP	GO:0007517	muscle organ development	bulk RNA-seq,Emory,MCI.vs.control; bulk RNA-seq,Homo_723,AD.vs.control; bulk RNA-seq,Homo_723,AD.vs.MCI; bulk RNA-seq,Homo_714,AD.vs.control; bulk RNA-seq,Homo_714,AD.vs.MCI; bulk RNA-seq,Homo_633,AD.vs.control; bulk RNA-seq,Homo_633,AD.vs.MCI; bulk RNA-seq,SRP223445,AD.vs.control	8
BP	GO:0019932	second-messenger-mediated signaling	bulk RNA-seq,Emory,MCI.vs.control; bulk RNA-seq,Homo_723,AD.vs.control; bulk RNA-seq,Homo_723,AD.vs.MCI; bulk RNA-seq,Homo_723,MCI.vs.control; bulk RNA-seq,Homo_714,AD.vs.control; bulk RNA-seq,Homo_714,AD.vs.MCI; bulk RNA-seq,Homo_633,AD.vs.control; bulk RNA-seq,Homo_633,AD.vs.MCI	8
BP	GO:0048704	embryonic skeletal system morphogenesis	bulk RNA-seq,Emory,MCI.vs.control; bulk RNA-seq,Homo_723,AD.vs.control; bulk RNA-seq,Homo_723,AD.vs.MCI; bulk RNA-seq,Homo_723,MCI.vs.control; bulk RNA-seq,Homo_714,AD.vs.control; bulk RNA-seq,Homo_714,AD.vs.MCI; bulk RNA-seq,Homo_714,MCI.vs.control; bulk RNA-	8
BP	GO:0001708	cell fate specification	bulk RNA-seq,Emory,MCI.vs.control; bulk RNA-seq,Homo_723,AD.vs.control; bulk RNA-seq,Homo_723,AD.vs.MCI; bulk RNA-seq,Homo_723,MCI.vs.control; bulk RNA-seq,Homo_714,AD.vs.control; bulk RNA-seq,Homo_714,AD.vs.MCI; bulk RNA-seq,Homo_714,MCI.vs.control; bulk RNA-seq,Homo_633,MCI.vs.control; bulk RNA-seq,SRP223445,AD.vs.control	9
MF	GO:0005230	extracellular ligand-gated ion channel activity	bulk RNA-seq,Emory,MCI.vs.control; bulk RNA-seq,Homo_723,AD.vs.control; bulk RNA-seq,Homo_723,AD.vs.MCI; bulk RNA-seq,Homo_723,MCI.vs.control; bulk RNA-seq,Homo_714,AD.vs.control; bulk RNA-seq,Homo_714,AD.vs.MCI; bulk RNA-seq,Homo_633,MCI.vs.control; bulk RNA-	8

CC	GO:0014069	postsynaptic density	bulk RNA-seq,Emory,MCI.vs.control; bulk RNA-seq,Homo_723,AD.vs.control; bulk RNA-seq,Homo_723,AD.vs.MCI; bulk RNA-seq,Homo_723,MCI.vs.control; bulk RNA-seq,Homo_714,AD.vs.MCI; bulk RNA-seq,Homo_633,AD.vs.control; bulk RNA-seq,Homo_633,AD.vs.MCI; bulk RNA-seq,ROSMAP,AD.vs.control; bulk RNA-seq,SRP223445,AD.vs.control	9
BP	GO:2000677	regulation of transcription regulatory region DNA binding	bulk RNA-seq,Emory,MCI.vs.control; bulk RNA-seq,Homo_723,AD.vs.control; bulk RNA-seq,Homo_723,AD.vs.MCI; bulk RNA-seq,Homo_723,MCI.vs.control; bulk RNA-seq,Homo_714,AD.vs.MCI; bulk RNA-seq,Homo_633,AD.vs.control; bulk RNA-seq,Homo_633,AD.vs.MCI	7
BP	GO:0000462	maturation of SSU-rRNA from tricistronic rRNA transcript (SSU-rRNA, 5.8S rRNA, LSU-rRNA)	bulk RNA-seq,Emory,MCI.vs.control; bulk RNA-seq,Homo_723,AD.vs.control; bulk RNA-seq,Homo_723,AD.vs.MCI; bulk RNA-seq,Homo_714,AD.vs.control; bulk RNA-seq,Homo_714,MCI.vs.control; bulk RNA-seq,Homo_633,AD.vs.control; bulk RNA-seq,Homo_633,AD.vs.MCI	7
BP	GO:0030317	flagellated sperm motility	bulk RNA-seq,Emory,MCI.vs.control; bulk RNA-seq,Homo_723,AD.vs.control; bulk RNA-seq,Homo_723,AD.vs.MCI; bulk RNA-seq,Homo_723,MCI.vs.control; bulk RNA-seq,Homo_714,AD.vs.control; bulk RNA-seq,Homo_714,AD.vs.MCI; bulk RNA-seq,Homo_633,AD.vs.control; bulk RNA-seq,Homo_633,AD.vs.MCI	8
BP	GO:0097722	sperm motility	bulk RNA-seq,Emory,MCI.vs.control; bulk RNA-seq,Homo_723,AD.vs.control; bulk RNA-seq,Homo_723,AD.vs.MCI; bulk RNA-seq,Homo_723,MCI.vs.control; bulk RNA-seq,Homo_714,AD.vs.control; bulk RNA-seq,Homo_714,AD.vs.MCI; bulk RNA-seq,Homo_633,AD.vs.control; bulk RNA-seq,Homo_633,AD.vs.MCI	8
BP	GO:0003007	heart morphogenesis	bulk RNA-seq,Emory,MCI.vs.control; bulk RNA-seq,Homo_723,AD.vs.control; bulk RNA-seq,Homo_723,AD.vs.MCI; bulk RNA-seq,Homo_723,MCI.vs.control; bulk RNA-seq,Homo_714,AD.vs.control; bulk RNA-seq,Homo_714,AD.vs.MCI; bulk RNA-seq,Homo_633,AD.vs.control; bulk RNA-seq,Homo_633,AD.vs.MCI; bulk RNA-seq,SRP223445,AD.vs.control	9
BP	GO:0001706	endoderm formation	bulk RNA-seq,Emory,MCI.vs.control; bulk RNA-seq,Homo_723,AD.vs.control; bulk RNA-seq,Homo_723,AD.vs.MCI; bulk RNA-seq,Homo_723,MCI.vs.control; bulk RNA-seq,Homo_714,AD.vs.MCI; bulk RNA-seq,Homo_714,MCI.vs.control	6
BP	GO:0003231	cardiac ventricle development	bulk RNA-seq,Emory,MCI.vs.control; bulk RNA-seq,Homo_723,AD.vs.control; bulk RNA-seq,Homo_723,AD.vs.MCI; bulk RNA-seq,Homo_723,MCI.vs.control; bulk RNA-seq,Homo_714,AD.vs.control; bulk RNA-seq,Homo_714,AD.vs.MCI; bulk RNA-seq,Homo_714,MCI.vs.control; bulk RNA-seq,Homo_633,AD.vs.control; bulk RNA-seq,SRP223445,AD.vs.control	9
BP	GO:0050803	regulation of synapse structure or activity	bulk RNA-seq,Emory,MCI.vs.control; bulk RNA-seq,Homo_723,AD.vs.control; bulk RNA-seq,Homo_723,AD.vs.MCI; bulk RNA-seq,Homo_723,MCI.vs.control; bulk RNA-seq,Homo_714,AD.vs.control; bulk RNA-seq,Homo_714,AD.vs.MCI; bulk RNA-seq,Homo_633,AD.vs.control; bulk RNA-seq,Homo_633,AD.vs.MCI; bulk RNA-seq,ROSMAP,AD.vs.control; bulk RNA-seq,ROSMAP,MCI.vs.control	10
BP	GO:2001222	regulation of neuron migration	bulk RNA-seq,Emory,MCI.vs.control; bulk RNA-seq,Homo_723,AD.vs.control; bulk RNA-seq,Homo_723,AD.vs.MCI; bulk RNA-seq,Homo_723,MCI.vs.control; bulk RNA-seq,Homo_714,AD.vs.MCI; bulk RNA-seq,Homo_714,MCI.vs.control; bulk RNA-seq,Homo_633,AD.vs.control; bulk RNA-seq,Homo_633,MCI.vs.control; bulk RNA-seq,ROSMAP,MCI.vs.control	9
BP	GO:0060325	face morphogenesis	bulk RNA-seq,Emory,MCI.vs.control; bulk RNA-seq,Homo_723,AD.vs.control; bulk RNA-seq,Homo_723,AD.vs.MCI; bulk RNA-seq,Homo_723,MCI.vs.control; bulk RNA-seq,Homo_714,AD.vs.MCI; bulk RNA-seq,Homo_714,MCI.vs.control	6
BP	GO:0060079	excitatory postsynaptic potential	bulk RNA-seq,Emory,MCI.vs.control; bulk RNA-seq,Homo_723,AD.vs.control; bulk RNA-seq,Homo_723,AD.vs.MCI; bulk RNA-seq,Homo_723,MCI.vs.control; bulk RNA-seq,Homo_714,AD.vs.control; bulk RNA-seq,Homo_714,AD.vs.MCI; bulk RNA-seq,Homo_714,MCI.vs.control; bulk RNA-seq,ROSMAP,AD.vs.control; bulk RNA-seq,ROSMAP,MCI.vs.control	9
BP	GO:0071364	cellular response to epidermal growth factor stimulus	bulk RNA-seq,Emory,MCI.vs.control; bulk RNA-seq,Homo_723,AD.vs.control; bulk RNA-seq,Homo_723,AD.vs.MCI; bulk RNA-seq,Homo_723,MCI.vs.control; bulk RNA-seq,Homo_714,AD.vs.MCI; bulk RNA-seq,Homo_714,MCI.vs.control; bulk RNA-seq,Homo_633,AD.vs.control; bulk RNA-seq,Homo_633,AD.vs.MCI	8
MF	GO:0005237	inhibitory extracellular ligand-gated ion channel activity	bulk RNA-seq,Emory,MCI.vs.control; bulk RNA-seq,Homo_723,AD.vs.control; bulk RNA-seq,Homo_723,AD.vs.MCI; bulk RNA-seq,Homo_714,AD.vs.control; bulk RNA-seq,Homo_714,AD.vs.MCI; bulk RNA-seq,Homo_633,AD.vs.control	6
BP	GO:2000242	negative regulation of reproductive process	bulk RNA-seq,Emory,MCI.vs.control; bulk RNA-seq,Homo_723,AD.vs.control; bulk RNA-seq,Homo_723,AD.vs.MCI; bulk RNA-seq,Homo_723,MCI.vs.control; bulk RNA-seq,Homo_714,AD.vs.MCI; bulk RNA-seq,Homo_714,MCI.vs.control	6
CC	GO:0034705	potassium channel complex	bulk RNA-seq,Emory,MCI.vs.control; bulk RNA-seq,Homo_723,AD.vs.control; bulk RNA-seq,Homo_723,AD.vs.MCI; bulk RNA-seq,Homo_723,MCI.vs.control; bulk RNA-seq,Homo_714,AD.vs.control; bulk RNA-seq,Homo_714,AD.vs.MCI; bulk RNA-seq,Homo_633,MCI.vs.control; bulk RNA-	8
BP	GO:0061371	determination of heart left/right asymmetry	bulk RNA-seq,Emory,MCI.vs.control; bulk RNA-seq,Homo_723,AD.vs.control; bulk RNA-seq,Homo_723,AD.vs.MCI; bulk RNA-seq,Homo_723,MCI.vs.control; bulk RNA-seq,Homo_714,AD.vs.control; bulk RNA-seq,Homo_714,AD.vs.MCI; bulk RNA-seq,Homo_714,MCI.vs.control	7
BP	GO:0046660	female sex differentiation	bulk RNA-seq,Emory,MCI.vs.control; bulk RNA-seq,Homo_723,AD.vs.control; bulk RNA-seq,Homo_723,AD.vs.MCI; bulk RNA-seq,Homo_723,MCI.vs.control; bulk RNA-seq,Homo_714,AD.vs.control; bulk RNA-seq,Homo_714,AD.vs.MCI; bulk RNA-seq,Homo_714,MCI.vs.control; bulk RNA-seq,Homo_633,AD.vs.control; bulk RNA-seq,SRP223445,AD.vs.control	9
MF	GO:0019843	rRNA binding	bulk RNA-seq,Emory,MCI.vs.control; bulk RNA-seq,Homo_723,AD.vs.control; bulk RNA-seq,Homo_723,AD.vs.MCI; bulk RNA-seq,Homo_723,MCI.vs.control; bulk RNA-seq,Homo_714,AD.vs.control; bulk RNA-seq,Homo_714,AD.vs.MCI; bulk RNA-seq,Homo_714,MCI.vs.control; bulk RNA-seq,Homo_633,AD.vs.control; bulk RNA-seq,Homo_633,AD.vs.MCI	9
BP	GO:0007189	adenylate cyclase-activating G protein-coupled receptor signaling pathway	bulk RNA-seq,Emory,MCI.vs.control; bulk RNA-seq,Homo_723,AD.vs.control; bulk RNA-seq,Homo_723,AD.vs.MCI; bulk RNA-seq,Homo_723,MCI.vs.control; bulk RNA-seq,Homo_714,AD.vs.control; bulk RNA-seq,Homo_714,AD.vs.MCI	6
BP	GO:0034204	lipid translocation	bulk RNA-seq,Emory,MCI.vs.control; bulk RNA-seq,Homo_723,AD.vs.MCI; bulk RNA-seq,Homo_723,MCI.vs.control; bulk RNA-seq,Homo_714,AD.vs.MCI; bulk RNA-seq,Homo_714,MCI.vs.control; bulk RNA-seq,Homo_633,AD.vs.control; bulk RNA-seq,Homo_633,AD.vs.MCI	7
BP	GO:0031280	negative regulation of cyclase activity	bulk RNA-seq,Emory,MCI.vs.control; bulk RNA-seq,Homo_723,AD.vs.control; bulk RNA-seq,Homo_723,AD.vs.MCI; bulk RNA-seq,Homo_723,MCI.vs.control	4
MF	GO:0015108	chloride transmembrane transporter activity	bulk RNA-seq,Emory,MCI.vs.control; bulk RNA-seq,Homo_723,AD.vs.control; bulk RNA-seq,Homo_723,AD.vs.MCI; bulk RNA-seq,Homo_723,MCI.vs.control; bulk RNA-seq,Homo_714,AD.vs.control; bulk RNA-seq,Homo_714,AD.vs.MCI; bulk RNA-seq,Homo_714,MCI.vs.control; bulk RNA-	8
CC	GO:0044304	main axon	bulk RNA-seq,Emory,MCI.vs.control; bulk RNA-seq,Homo_723,AD.vs.control; bulk RNA-seq,Homo_723,AD.vs.MCI; bulk RNA-seq,Homo_723,MCI.vs.control; bulk RNA-seq,Homo_714,AD.vs.MCI; bulk RNA-seq,Homo_714,MCI.vs.control; bulk RNA-seq,Homo_633,AD.vs.control	7
BP	GO:0007519	skeletal muscle tissue development	bulk RNA-seq,Emory,MCI.vs.control; bulk RNA-seq,Homo_723,AD.vs.control; bulk RNA-seq,Homo_723,AD.vs.MCI; bulk RNA-seq,Homo_723,MCI.vs.control; bulk RNA-seq,Homo_714,AD.vs.control; bulk RNA-seq,Homo_714,AD.vs.MCI; bulk RNA-seq,Homo_633,AD.vs.control; bulk RNA-seq,Homo_633,AD.vs.MCI	8
BP	GO:0051966	regulation of synaptic transmission, glutamatergic	bulk RNA-seq,Emory,MCI.vs.control; bulk RNA-seq,Homo_723,AD.vs.control; bulk RNA-seq,Homo_723,AD.vs.MCI; bulk RNA-seq,Homo_723,MCI.vs.control; bulk RNA-seq,Homo_714,AD.vs.control; bulk RNA-seq,Homo_714,AD.vs.MCI; bulk RNA-seq,Homo_714,MCI.vs.control	7
CC	GO:0098686	hippocampal mossy fiber to CA3 synapse	bulk RNA-seq,Emory,MCI.vs.control; bulk RNA-seq,Homo_723,AD.vs.control; bulk RNA-seq,Homo_723,AD.vs.MCI; bulk RNA-seq,Homo_723,MCI.vs.control; bulk RNA-seq,Homo_714,AD.vs.MCI; bulk RNA-seq,Homo_633,MCI.vs.control	6
BP	GO:0050954	sensory perception of mechanical stimulus	bulk RNA-seq,Emory,MCI.vs.control; bulk RNA-seq,Homo_723,AD.vs.control; bulk RNA-seq,Homo_723,AD.vs.MCI; bulk RNA-seq,Homo_723,MCI.vs.control; bulk RNA-seq,Homo_714,AD.vs.control; bulk RNA-seq,Homo_714,AD.vs.MCI; bulk RNA-seq,Homo_633,AD.vs.control; bulk RNA-seq,Homo_633,AD.vs.MCI; bulk RNA-seq,ROSMAP,AD.vs.control; bulk RNA-seq,ROSMAP,MCI.vs.control; bulk RNA-seq,SRP223445,AD.vs.control	11
BP	GO:0019228	neuronal action potential	bulk RNA-seq,Emory,MCI.vs.control; bulk RNA-seq,Homo_723,AD.vs.control; bulk RNA-seq,Homo_723,AD.vs.MCI; bulk RNA-seq,Homo_723,MCI.vs.control; bulk RNA-seq,Homo_714,AD.vs.MCI; bulk RNA-seq,Homo_633,MCI.vs.control; bulk RNA-seq,SRP223445,AD.vs.control	7

BP	GO:0098661	inorganic anion transmembrane transport	bulk RNA-seq,Emory,MCI.vs.control; bulk RNA-seq,Homo_723,AD.vs.control; bulk RNA-seq,Homo_723,AD.vs.MCI; bulk RNA-seq,Homo_723,MCI.vs.control; bulk RNA-seq,Homo_714,AD.vs.control; bulk RNA-seq,Homo_714,AD.vs.MCI; bulk RNA-seq,Homo_714,MCI.vs.control; bulk RNA-seq,Homo_633,AD.vs.control; bulk RNA-seq,ROSMAP,MCI.vs.control	9
BP	GO:0060439	trachea morphogenesis	bulk RNA-seq,Emory,MCI.vs.control; bulk RNA-seq,Homo_723,AD.vs.control; bulk RNA-seq,Homo_723,AD.vs.MCI; bulk RNA-seq,Homo_723,MCI.vs.control	4
BP	GO:0046500	S-adenosylmethionine metabolic process	bulk RNA-seq,Emory,MCI.vs.control; bulk RNA-seq,Homo_723,AD.vs.control; bulk RNA-seq,Homo_723,MCI.vs.control; bulk RNA-seq,Homo_714,MCI.vs.control; bulk RNA-seq,Homo_633,AD.vs.control; bulk RNA-seq,Homo_633,AD.vs.MCI	6
BP	GO:0098664	G protein-coupled serotonin receptor signaling pathway	bulk RNA-seq,Emory,MCI.vs.control; bulk RNA-seq,Homo_723,AD.vs.control; bulk RNA-seq,Homo_723,AD.vs.MCI; bulk RNA-seq,Homo_714,AD.vs.control; bulk RNA-seq,Homo_714,AD.vs.MCI; bulk RNA-seq,Homo_633,MCI.vs.control	6
BP	GO:0045761	regulation of adenylate cyclase activity	bulk RNA-seq,Emory,MCI.vs.control; bulk RNA-seq,Homo_723,AD.vs.control; bulk RNA-seq,Homo_723,AD.vs.MCI; bulk RNA-seq,Homo_723,MCI.vs.control; bulk RNA-seq,Homo_714,AD.vs.control; bulk RNA-seq,Homo_714,AD.vs.MCI	6
CC	GO:0022626	cytosolic ribosome	bulk RNA-seq,Emory,MCI.vs.control; bulk RNA-seq,Homo_723,AD.vs.control; bulk RNA-seq,Homo_723,MCI.vs.control; bulk RNA-seq,Homo_714,AD.vs.control; bulk RNA-seq,Homo_714,MCI.vs.control; bulk RNA-seq,Homo_633,AD.vs.control; bulk RNA-seq,Homo_633,AD.vs.MCI; bulk RNA-seq,SRP325058,AD.vs.control; scRNA-seq,SRP330776,Megakaryocyte_2-AD.vs.control; scRNA-seq,SRP215507,CD8+ T cell_4-AD.vs.control	10
MF	GO:1904315	transmitter-gated ion channel activity involved in regulation of postsynaptic membrane potential	bulk RNA-seq,Emory,MCI.vs.control; bulk RNA-seq,Homo_723,AD.vs.control; bulk RNA-seq,Homo_723,AD.vs.MCI; bulk RNA-seq,Homo_723,MCI.vs.control; bulk RNA-seq,Homo_714,AD.vs.control; bulk RNA-seq,Homo_714,AD.vs.MCI	6
BP	GO:0003014	renal system process	bulk RNA-seq,Emory,MCI.vs.control; bulk RNA-seq,Homo_723,AD.vs.control; bulk RNA-seq,Homo_723,AD.vs.MCI; bulk RNA-seq,Homo_723,MCI.vs.control; bulk RNA-seq,Homo_714,AD.vs.control; bulk RNA-seq,Homo_714,AD.vs.MCI; bulk RNA-seq,Homo_714,MCI.vs.control; bulk RNA-seq,Homo_633,AD.vs.control; bulk RNA-seq,Homo_633,AD.vs.MCI; bulk RNA-seq,SRP223445,AD.vs.control	10
BP	GO:0015698	inorganic anion transport	bulk RNA-seq,Emory,MCI.vs.control; bulk RNA-seq,Homo_723,AD.vs.control; bulk RNA-seq,Homo_723,AD.vs.MCI; bulk RNA-seq,Homo_723,MCI.vs.control; bulk RNA-seq,Homo_714,AD.vs.control; bulk RNA-seq,Homo_714,AD.vs.MCI; bulk RNA-seq,Homo_633,AD.vs.control; bulk RNA-seq,Homo_633,AD.vs.MCI	8
MF	GO:0022824	transmitter-gated ion channel activity	bulk RNA-seq,Emory,MCI.vs.control; bulk RNA-seq,Homo_723,AD.vs.control; bulk RNA-seq,Homo_723,AD.vs.MCI; bulk RNA-seq,Homo_723,MCI.vs.control; bulk RNA-seq,Homo_714,AD.vs.control; bulk RNA-seq,Homo_714,AD.vs.MCI; bulk RNA-seq,Homo_633,MCI.vs.control	7
MF	GO:0022835	transmitter-gated channel activity	bulk RNA-seq,Emory,MCI.vs.control; bulk RNA-seq,Homo_723,AD.vs.control; bulk RNA-seq,Homo_723,AD.vs.MCI; bulk RNA-seq,Homo_723,MCI.vs.control; bulk RNA-seq,Homo_714,AD.vs.control; bulk RNA-seq,Homo_714,AD.vs.MCI; bulk RNA-seq,Homo_633,MCI.vs.control	7
BP	GO:0031589	cell-substrate adhesion	bulk RNA-seq,Emory,MCI.vs.control; bulk RNA-seq,Homo_723,AD.vs.control; bulk RNA-seq,Homo_723,AD.vs.MCI; bulk RNA-seq,Homo_714,AD.vs.control; bulk RNA-seq,Homo_714,AD.vs.MCI; bulk RNA-seq,Homo_633,AD.vs.control; bulk RNA-seq,Homo_633,AD.vs.MCI; bulk RNA-seq,ROSMAP,AD.vs.control; bulk RNA-seq,SRP223445,AD.vs.control	9
CC	GO:0000407	phagophore assembly site	bulk RNA-seq,Emory,MCI.vs.control; bulk RNA-seq,Homo_723,AD.vs.control; bulk RNA-seq,Homo_723,AD.vs.MCI; bulk RNA-seq,Homo_714,AD.vs.control; bulk RNA-seq,Homo_633,AD.vs.control; bulk RNA-seq,Homo_633,AD.vs.MCI	6
MF	GO:0099529	neurotransmitter receptor activity involved in regulation of postsynaptic membrane potential	bulk RNA-seq,Emory,MCI.vs.control; bulk RNA-seq,Homo_723,AD.vs.control; bulk RNA-seq,Homo_723,AD.vs.MCI; bulk RNA-seq,Homo_723,MCI.vs.control; bulk RNA-seq,Homo_714,AD.vs.control; bulk RNA-seq,Homo_714,AD.vs.MCI	6
CC	GO:0099634	postsynaptic specialization membrane	bulk RNA-seq,Emory,MCI.vs.control; bulk RNA-seq,Homo_723,AD.vs.control; bulk RNA-seq,Homo_723,AD.vs.MCI; bulk RNA-seq,Homo_723,MCI.vs.control; bulk RNA-seq,Homo_714,AD.vs.control; bulk RNA-seq,Homo_714,AD.vs.MCI; bulk RNA-seq,Homo_633,AD.vs.MCI; bulk RNA-seq,ROSMAP,AD.vs.control; bulk RNA-seq,SRP223445,AD.vs.control	9
BP	GO:0043038	amino acid activation	bulk RNA-seq,Emory,MCI.vs.control; bulk RNA-seq,Homo_723,AD.vs.control; bulk RNA-seq,Homo_723,MCI.vs.control; bulk RNA-seq,Homo_714,AD.vs.control; bulk RNA-seq,Homo_714,AD.vs.MCI; bulk RNA-seq,Homo_714,MCI.vs.control; bulk RNA-seq,Homo_633,AD.vs.control; bulk	8
MF	GO:0140303	intramembrane lipid transporter activity	bulk RNA-seq,Emory,MCI.vs.control; bulk RNA-seq,Homo_723,AD.vs.MCI; bulk RNA-seq,Homo_723,MCI.vs.control; bulk RNA-seq,Homo_714,AD.vs.MCI; bulk RNA-seq,Homo_714,MCI.vs.control; bulk RNA-seq,Homo_633,AD.vs.control; bulk RNA-seq,Homo_633,AD.vs.MCI	7
MF	GO:0005178	integrin binding	bulk RNA-seq,Emory,MCI.vs.control; bulk RNA-seq,Homo_723,AD.vs.control; bulk RNA-seq,Homo_723,AD.vs.MCI; bulk RNA-seq,Homo_723,MCI.vs.control; bulk RNA-seq,Homo_714,AD.vs.MCI; bulk RNA-seq,Homo_633,AD.vs.control; bulk RNA-seq,Homo_633,AD.vs.MCI; bulk RNA-seq,ROSMAP,AD.vs.control	8
BP	GO:0050708	regulation of protein secretion	bulk RNA-seq,Emory,MCI.vs.control; bulk RNA-seq,Homo_723,AD.vs.control; bulk RNA-seq,Homo_723,AD.vs.MCI; bulk RNA-seq,Homo_723,MCI.vs.control; bulk RNA-seq,Homo_714,AD.vs.MCI; bulk RNA-seq,Homo_633,AD.vs.control; bulk RNA-seq,Homo_633,AD.vs.MCI; bulk RNA-seq,SRP325058,AD.vs.control	8
CC	GO:0016459	myosin complex	bulk RNA-seq,Emory,MCI.vs.control; bulk RNA-seq,Homo_723,AD.vs.control; bulk RNA-seq,Homo_723,AD.vs.MCI; bulk RNA-seq,Homo_714,AD.vs.control; bulk RNA-seq,Homo_714,AD.vs.MCI	5
BP	GO:0070525	tRNA threonylcarbamoyladenosine metabolic process	bulk RNA-seq,Emory,MCI.vs.control; bulk RNA-seq,Homo_723,AD.vs.control; bulk RNA-seq,Homo_714,AD.vs.control; bulk RNA-seq,Homo_633,AD.vs.control; bulk RNA-seq,Homo_633,AD.vs.MCI	5
BP	GO:0048736	appendage development	bulk RNA-seq,Emory,MCI.vs.control; bulk RNA-seq,Homo_723,AD.vs.control; bulk RNA-seq,Homo_723,AD.vs.MCI; bulk RNA-seq,Homo_723,MCI.vs.control; bulk RNA-seq,Homo_714,AD.vs.control; bulk RNA-seq,Homo_714,AD.vs.MCI; bulk RNA-seq,Homo_633,AD.vs.control; bulk RNA-seq,Homo_633,AD.vs.MCI; bulk RNA-seq,Homo_633,MCI.vs.control; bulk RNA-seq,SRP223445,AD.vs.control	10
BP	GO:0060173	limb development	bulk RNA-seq,Emory,MCI.vs.control; bulk RNA-seq,Homo_723,AD.vs.control; bulk RNA-seq,Homo_723,AD.vs.MCI; bulk RNA-seq,Homo_723,MCI.vs.control; bulk RNA-seq,Homo_714,AD.vs.control; bulk RNA-seq,Homo_714,AD.vs.MCI; bulk RNA-seq,Homo_633,AD.vs.control; bulk RNA-seq,Homo_633,AD.vs.MCI; bulk RNA-seq,Homo_633,MCI.vs.control; bulk RNA-seq,SRP223445,AD.vs.control	10
BP	GO:0050767	regulation of neurogenesis	bulk RNA-seq,Emory,MCI.vs.control; bulk RNA-seq,Homo_723,AD.vs.control; bulk RNA-seq,Homo_723,AD.vs.MCI; bulk RNA-seq,Homo_714,AD.vs.control; bulk RNA-seq,Homo_714,AD.vs.MCI; bulk RNA-seq,Homo_633,AD.vs.control; bulk RNA-seq,Homo_633,AD.vs.MCI; bulk RNA-seq,ROSMAP,AD.vs.control; bulk RNA-seq,SRP223445,AD.vs.control	9
BP	GO:0072132	mesenchyme morphogenesis	bulk RNA-seq,Emory,MCI.vs.control; bulk RNA-seq,Homo_723,AD.vs.control; bulk RNA-seq,Homo_723,AD.vs.MCI; bulk RNA-seq,Homo_723,MCI.vs.control; bulk RNA-seq,Homo_714,AD.vs.control; bulk RNA-seq,Homo_714,AD.vs.MCI; bulk RNA-seq,Homo_714,MCI.vs.control	7
BP	GO:0003143	embryonic heart tube morphogenesis	bulk RNA-seq,Emory,MCI.vs.control; bulk RNA-seq,Homo_723,AD.vs.control; bulk RNA-seq,Homo_723,AD.vs.MCI; bulk RNA-seq,Homo_723,MCI.vs.control; bulk RNA-seq,Homo_714,AD.vs.control; bulk RNA-seq,Homo_714,AD.vs.MCI; bulk RNA-seq,Homo_714,MCI.vs.control; bulk RNA-seq,Homo_633,AD.vs.control; bulk RNA-seq,SRP223445,AD.vs.control	9
MF	GO:0005244	voltage-gated ion channel activity	bulk RNA-seq,Emory,MCI.vs.control; bulk RNA-seq,Homo_723,AD.vs.control; bulk RNA-seq,Homo_723,AD.vs.MCI; bulk RNA-seq,Homo_723,MCI.vs.control; bulk RNA-seq,Homo_714,AD.vs.control; bulk RNA-seq,Homo_714,AD.vs.MCI; bulk RNA-seq,Homo_633,MCI.vs.control; bulk RNA-seq,ROSMAP,AD.vs.control; bulk RNA-seq,SRP223445,AD.vs.control	9
MF	GO:0022832	voltage-gated channel activity	bulk RNA-seq,Emory,MCI.vs.control; bulk RNA-seq,Homo_723,AD.vs.control; bulk RNA-seq,Homo_723,AD.vs.MCI; bulk RNA-seq,Homo_723,MCI.vs.control; bulk RNA-seq,Homo_714,AD.vs.control; bulk RNA-seq,Homo_714,AD.vs.MCI; bulk RNA-seq,Homo_633,MCI.vs.control; bulk RNA-seq,ROSMAP,AD.vs.control; bulk RNA-seq,SRP223445,AD.vs.control	9

BP	GO:0043062	extracellular structure organization	bulk RNA-seq,Emory,MCI.vs.control; bulk RNA-seq,Homo_723,AD.vs.control; bulk RNA-seq,Homo_723,AD.vs.MCI; bulk RNA-seq,Homo_723,MCI.vs.control; bulk RNA-seq,Homo_714,AD.vs.control; bulk RNA-seq,Homo_714,AD.vs.MCI; bulk RNA-seq,Homo_633,AD.vs.control; bulk RNA-seq,Homo_633,AD.vs.MCI; bulk RNA-seq,ROSMAP,AD.vs.control; bulk RNA-seq,ROSMAP,MCI.vs.control; bulk RNA-seq,SRP223445,AD.vs.control	11
BP	GO:0036498	IRE1-mediated unfolded protein response	bulk RNA-seq,Emory,MCI.vs.control; bulk RNA-seq,Homo_633,AD.vs.control	2
BP	GO:0031279	regulation of cyclase activity	bulk RNA-seq,Emory,MCI.vs.control; bulk RNA-seq,Homo_723,AD.vs.control; bulk RNA-seq,Homo_723,AD.vs.MCI; bulk RNA-seq,Homo_723,MCI.vs.control; bulk RNA-seq,Homo_714,AD.vs.control; bulk RNA-seq,Homo_714,AD.vs.MCI; bulk RNA-seq,SRP223445,AD.vs.control	7
BP	GO:0060113	inner ear receptor cell differentiation	bulk RNA-seq,Emory,MCI.vs.control; bulk RNA-seq,Homo_723,AD.vs.control; bulk RNA-seq,Homo_723,AD.vs.MCI; bulk RNA-seq,Homo_723,MCI.vs.control; bulk RNA-seq,Homo_714,AD.vs.MCI; bulk RNA-seq,Homo_714,MCI.vs.control	6
CC	GO:0098858	actin-based cell projection	bulk RNA-seq,Emory,MCI.vs.control; bulk RNA-seq,Homo_723,AD.vs.control; bulk RNA-seq,Homo_723,AD.vs.MCI; bulk RNA-seq,Homo_723,MCI.vs.control; bulk RNA-seq,Homo_714,AD.vs.control; bulk RNA-seq,Homo_714,AD.vs.MCI; bulk RNA-seq,Homo_633,AD.vs.control; bulk RNA-seq,Homo_633,AD.vs.MCI; bulk RNA-seq,ROSMAP,AD.vs.control; bulk RNA-seq,SRP223445,AD.vs.control	10
BP	GO:1901142	insulin metabolic process	bulk RNA-seq,Emory,MCI.vs.control	1
CC	GO:0015935	small ribosomal subunit	bulk RNA-seq,Emory,MCI.vs.control; bulk RNA-seq,Homo_723,AD.vs.control; bulk RNA-seq,Homo_723,AD.vs.MCI; bulk RNA-seq,Homo_723,MCI.vs.control; bulk RNA-seq,Homo_714,AD.vs.control; bulk RNA-seq,Homo_714,MCI.vs.control; bulk RNA-seq,Homo_633,AD.vs.control; bulk RNA-	8
BP	GO:0048013	ephrin receptor signaling pathway	bulk RNA-seq,Emory,MCI.vs.control; bulk RNA-seq,Homo_723,AD.vs.control; bulk RNA-seq,Homo_723,AD.vs.MCI; bulk RNA-seq,Homo_723,MCI.vs.control; bulk RNA-seq,Homo_714,AD.vs.MCI; bulk RNA-seq,Homo_714,MCI.vs.control; bulk RNA-seq,Homo_633,AD.vs.control; bulk RNA-seq,Homo_633,AD.vs.MCI	8
BP	GO:0007605	sensory perception of sound	bulk RNA-seq,Emory,MCI.vs.control; bulk RNA-seq,Homo_723,AD.vs.control; bulk RNA-seq,Homo_723,AD.vs.MCI; bulk RNA-seq,Homo_723,MCI.vs.control; bulk RNA-seq,Homo_714,AD.vs.control; bulk RNA-seq,Homo_714,AD.vs.MCI; bulk RNA-seq,Homo_633,AD.vs.control; bulk RNA-seq,Homo_633,AD.vs.MCI; bulk RNA-seq,ROSMAP,AD.vs.control; bulk RNA-seq,ROSMAP,MCI.vs.control; bulk RNA-seq,SRP223445,AD.vs.control	11
BP	GO:0003341	cilium movement	bulk RNA-seq,Emory,MCI.vs.control; bulk RNA-seq,Homo_723,AD.vs.control; bulk RNA-seq,Homo_723,AD.vs.MCI; bulk RNA-seq,Homo_723,MCI.vs.control; bulk RNA-seq,Homo_714,AD.vs.control; bulk RNA-seq,Homo_714,AD.vs.MCI; bulk RNA-seq,Homo_633,AD.vs.control; bulk RNA-seq,Homo_633,AD.vs.MCI	8
BP	GO:0001822	kidney development	bulk RNA-seq,Emory,MCI.vs.control; bulk RNA-seq,Homo_723,AD.vs.control; bulk RNA-seq,Homo_723,AD.vs.MCI; bulk RNA-seq,Homo_723,MCI.vs.control; bulk RNA-seq,Homo_714,AD.vs.control; bulk RNA-seq,Homo_714,AD.vs.MCI; bulk RNA-seq,Homo_633,AD.vs.control; bulk RNA-seq,Homo_633,AD.vs.MCI; bulk RNA-seq,ROSMAP,AD.vs.control; bulk RNA-seq,ROSMAP,MCI.vs.control; bulk RNA-seq,SRP223445,AD.vs.control	11
BP	GO:0043039	tRNA aminoacylation	bulk RNA-seq,Emory,MCI.vs.control; bulk RNA-seq,Homo_723,AD.vs.control; bulk RNA-seq,Homo_723,MCI.vs.control; bulk RNA-seq,Homo_714,AD.vs.control; bulk RNA-seq,Homo_714,AD.vs.MCI; bulk RNA-seq,Homo_714,MCI.vs.control; bulk RNA-seq,Homo_633,AD.vs.control; bulk	8
CC	GO:0000313	organellar ribosome	bulk RNA-seq,Emory,MCI.vs.control; bulk RNA-seq,Homo_723,AD.vs.control; bulk RNA-seq,Homo_723,AD.vs.MCI; bulk RNA-seq,Homo_723,MCI.vs.control; bulk RNA-seq,Homo_714,AD.vs.control; bulk RNA-seq,Homo_714,AD.vs.MCI; bulk RNA-seq,Homo_714,MCI.vs.control; bulk RNA-seq,Homo_633,AD.vs.control; bulk RNA-seq,Homo_633,AD.vs.MCI; bulk RNA-seq,SRP223445,AD.vs.control	10
CC	GO:0005761	mitochondrial ribosome	bulk RNA-seq,Emory,MCI.vs.control; bulk RNA-seq,Homo_723,AD.vs.control; bulk RNA-seq,Homo_723,AD.vs.MCI; bulk RNA-seq,Homo_723,MCI.vs.control; bulk RNA-seq,Homo_714,AD.vs.control; bulk RNA-seq,Homo_714,AD.vs.MCI; bulk RNA-seq,Homo_714,MCI.vs.control; bulk RNA-seq,Homo_633,AD.vs.control; bulk RNA-seq,Homo_633,AD.vs.MCI; bulk RNA-seq,SRP223445,AD.vs.control	10
BP	GO:0061448	connective tissue development	bulk RNA-seq,Emory,MCI.vs.control; bulk RNA-seq,Homo_723,AD.vs.control; bulk RNA-seq,Homo_723,AD.vs.MCI; bulk RNA-seq,Homo_723,MCI.vs.control; bulk RNA-seq,Homo_714,AD.vs.control; bulk RNA-seq,Homo_714,AD.vs.MCI; bulk RNA-seq,Homo_633,AD.vs.control; bulk RNA-seq,Homo_633,AD.vs.MCI; bulk RNA-seq,SRP223445,AD.vs.control; scRNA-seq,SRP330776,Naive CD8+ T cell_2-AD.vs.control	10
BP	GO:0033108	mitochondrial respiratory chain complex assembly	bulk RNA-seq,Emory,MCI.vs.control; bulk RNA-seq,Homo_723,AD.vs.control; bulk RNA-seq,Homo_723,AD.vs.MCI; bulk RNA-seq,Homo_723,MCI.vs.control; bulk RNA-seq,Homo_714,AD.vs.control; bulk RNA-seq,Homo_714,AD.vs.MCI; bulk RNA-seq,Homo_714,MCI.vs.control; bulk RNA-seq,Homo_633,AD.vs.control; bulk RNA-seq,Homo_633,AD.vs.MCI	9
BP	GO:0010642	negative regulation of platelet-derived growth factor receptor signaling pathway	bulk RNA-seq,Emory,MCI.vs.control; bulk RNA-seq,Homo_723,MCI.vs.control; bulk RNA-seq,Homo_633,AD.vs.control; bulk RNA-seq,Homo_633,AD.vs.MCI	4
MF	GO:0008066	glutamate receptor activity	bulk RNA-seq,Emory,MCI.vs.control; bulk RNA-seq,Homo_723,AD.vs.control; bulk RNA-seq,Homo_723,AD.vs.MCI; bulk RNA-seq,Homo_714,AD.vs.MCI; bulk RNA-seq,Homo_633,MCI.vs.control	5
BP	GO:0048745	smooth muscle tissue development	bulk RNA-seq,Emory,MCI.vs.control; bulk RNA-seq,Homo_723,AD.vs.MCI; bulk RNA-seq,Homo_723,MCI.vs.control; bulk RNA-seq,Homo_714,AD.vs.MCI; bulk RNA-seq,Homo_714,MCI.vs.control; bulk RNA-seq,SRP223445,AD.vs.control	6
MF	GO:0099095	ligand-gated anion channel activity	bulk RNA-seq,Emory,MCI.vs.control; bulk RNA-seq,Homo_723,AD.vs.control; bulk RNA-seq,Homo_723,AD.vs.MCI; bulk RNA-seq,Homo_723,MCI.vs.control; bulk RNA-seq,Homo_714,AD.vs.control; bulk RNA-seq,Homo_714,AD.vs.MCI	6
BP	GO:0046320	regulation of fatty acid oxidation	bulk RNA-seq,Emory,MCI.vs.control; bulk RNA-seq,Homo_723,MCI.vs.control; bulk RNA-seq,Homo_714,MCI.vs.control; bulk RNA-seq,Homo_633,AD.vs.control; bulk RNA-seq,Homo_633,AD.vs.MCI	5
CC	GO:0030684	preribosome	bulk RNA-seq,Emory,MCI.vs.control; bulk RNA-seq,Homo_723,AD.vs.control; bulk RNA-seq,Homo_723,AD.vs.MCI; bulk RNA-seq,Homo_723,MCI.vs.control; bulk RNA-seq,Homo_714,AD.vs.control; bulk RNA-seq,Homo_714,AD.vs.MCI; bulk RNA-seq,Homo_714,MCI.vs.control; bulk RNA-seq,Homo_633,AD.vs.control; bulk RNA-seq,Homo_633,AD.vs.MCI; bulk RNA-seq,SRP223445,AD.vs.control	10
BP	GO:0060021	roof of mouth development	bulk RNA-seq,Emory,MCI.vs.control; bulk RNA-seq,Homo_723,AD.vs.control; bulk RNA-seq,Homo_723,AD.vs.MCI; bulk RNA-seq,Homo_723,MCI.vs.control; bulk RNA-seq,Homo_714,AD.vs.control; bulk RNA-seq,Homo_714,AD.vs.MCI; bulk RNA-seq,Homo_714,MCI.vs.control; bulk RNA-	8
BP	GO:0009948	anterior/posterior axis specification	bulk RNA-seq,Emory,MCI.vs.control; bulk RNA-seq,Homo_723,AD.vs.control; bulk RNA-seq,Homo_723,AD.vs.MCI; bulk RNA-seq,Homo_723,MCI.vs.control; bulk RNA-seq,Homo_714,AD.vs.control; bulk RNA-seq,Homo_714,AD.vs.MCI	6
MF	GO:0015103	inorganic anion transmembrane transporter activity	bulk RNA-seq,Emory,MCI.vs.control; bulk RNA-seq,Homo_723,AD.vs.control; bulk RNA-seq,Homo_723,AD.vs.MCI; bulk RNA-seq,Homo_723,MCI.vs.control; bulk RNA-seq,Homo_714,AD.vs.control; bulk RNA-seq,Homo_714,AD.vs.MCI; bulk RNA-seq,Homo_633,AD.vs.control; bulk RNA-seq,Homo_633,AD.vs.MCI; bulk RNA-seq,ROSMAP,MCI.vs.control	9
BP	GO:0060349	bone morphogenesis	bulk RNA-seq,Emory,MCI.vs.control; bulk RNA-seq,Homo_723,AD.vs.control; bulk RNA-seq,Homo_723,AD.vs.MCI; bulk RNA-seq,Homo_723,MCI.vs.control; bulk RNA-seq,Homo_714,AD.vs.MCI; bulk RNA-seq,Homo_714,MCI.vs.control; bulk RNA-seq,Homo_633,AD.vs.control; bulk RNA-seq,Homo_633,AD.vs.MCI; bulk RNA-seq,SRP223445,AD.vs.control	9
BP	GO:0046530	photoreceptor cell differentiation	bulk RNA-seq,Emory,MCI.vs.control; bulk RNA-seq,Homo_723,AD.vs.control; bulk RNA-seq,Homo_723,AD.vs.MCI; bulk RNA-seq,Homo_723,MCI.vs.control; bulk RNA-seq,Homo_714,AD.vs.control; bulk RNA-seq,Homo_714,AD.vs.MCI; bulk RNA-seq,Homo_633,AD.vs.control; bulk RNA-seq,Homo_633,AD.vs.MCI	8
BP	GO:2000679	positive regulation of transcription regulatory region DNA binding	bulk RNA-seq,Emory,MCI.vs.control; bulk RNA-seq,Homo_723,AD.vs.control; bulk RNA-seq,Homo_723,AD.vs.MCI; bulk RNA-seq,Homo_723,MCI.vs.control; bulk RNA-seq,Homo_633,AD.vs.control	5

MF	GO:0051015	actin filament binding	bulk RNA-seq,Emory,MCI.vs.control; bulk RNA-seq,Homo_723,AD.vs.MCI; bulk RNA-seq,Homo_723,MCI.vs.control; bulk RNA-seq,Homo_714,AD.vs.MCI; bulk RNA-seq,Homo_633,AD.vs.control; bulk RNA-seq,Homo_633,AD.vs.MCI; bulk RNA-seq,ROSMAP,AD.vs.control	7
BP	GO:0099565	chemical synaptic transmission, postsynaptic	bulk RNA-seq,Emory,MCI.vs.control; bulk RNA-seq,Homo_723,AD.vs.control; bulk RNA-seq,Homo_723,AD.vs.MCI; bulk RNA-seq,Homo_723,MCI.vs.control; bulk RNA-seq,Homo_714,AD.vs.control; bulk RNA-seq,Homo_714,AD.vs.MCI; bulk RNA-seq,ROSMAP,AD.vs.control; bulk RNA-seq,ROSMAP,MCI.vs.control; bulk RNA-seq,SRP223445,AD.vs.control	9
BP	GO:0098781	ncRNA transcription	bulk RNA-seq,Emory,MCI.vs.control; bulk RNA-seq,Homo_723,AD.vs.control; bulk RNA-seq,Homo_723,MCI.vs.control; bulk RNA-seq,Homo_714,AD.vs.control; bulk RNA-seq,Homo_714,MCI.vs.control; bulk RNA-seq,Homo_633,AD.vs.control; bulk RNA-seq,Homo_633,AD.vs.MCI	7
CC	GO:0030175	filopodium	bulk RNA-seq,Emory,MCI.vs.control; bulk RNA-seq,Homo_723,AD.vs.control; bulk RNA-seq,Homo_723,AD.vs.MCI; bulk RNA-seq,Homo_723,MCI.vs.control; bulk RNA-seq,Homo_714,AD.vs.MCI; bulk RNA-seq,Homo_714,MCI.vs.control; bulk RNA-seq,Homo_633,AD.vs.control; bulk RNA-seq,Homo_633,AD.vs.MCI; bulk RNA-seq,ROSMAP,AD.vs.control	9
BP	GO:0019532	oxalate transport	bulk RNA-seq,Emory,MCI.vs.control; bulk RNA-seq,ROSMAP,AD.vs.control	2
BP	GO:0009581	detection of external stimulus	bulk RNA-seq,Emory,MCI.vs.control; bulk RNA-seq,Homo_723,AD.vs.control; bulk RNA-seq,Homo_723,AD.vs.MCI; bulk RNA-seq,Homo_723,MCI.vs.control; bulk RNA-seq,Homo_714,AD.vs.control; bulk RNA-seq,Homo_714,AD.vs.MCI; bulk RNA-seq,Homo_633,AD.vs.MCI; bulk RNA-seq,SRP223445,AD.vs.control	8
BP	GO:0009582	detection of abiotic stimulus	bulk RNA-seq,Emory,MCI.vs.control; bulk RNA-seq,Homo_723,AD.vs.control; bulk RNA-seq,Homo_723,AD.vs.MCI; bulk RNA-seq,Homo_723,MCI.vs.control; bulk RNA-seq,Homo_714,AD.vs.control; bulk RNA-seq,Homo_714,AD.vs.MCI; bulk RNA-seq,Homo_633,AD.vs.MCI; bulk RNA-seq,SRP223445,AD.vs.control	8
BP	GO:0060119	inner ear receptor cell development	bulk RNA-seq,Emory,MCI.vs.control; bulk RNA-seq,Homo_723,AD.vs.control; bulk RNA-seq,Homo_723,AD.vs.MCI; bulk RNA-seq,Homo_723,MCI.vs.control; bulk RNA-seq,Homo_714,AD.vs.MCI	5
BP	GO:0048813	dendrite morphogenesis	bulk RNA-seq,Emory,MCI.vs.control; bulk RNA-seq,Homo_723,AD.vs.control; bulk RNA-seq,Homo_723,AD.vs.MCI; bulk RNA-seq,Homo_723,MCI.vs.control; bulk RNA-seq,Homo_714,AD.vs.MCI; bulk RNA-seq,Homo_633,AD.vs.control; bulk RNA-seq,Homo_633,AD.vs.MCI; bulk RNA-seq,ROSMAP,AD.vs.control; bulk RNA-seq,ROSMAP,MCI.vs.control	9
BP	GO:0021915	neural tube development	bulk RNA-seq,Emory,MCI.vs.control; bulk RNA-seq,Homo_723,AD.vs.control; bulk RNA-seq,Homo_723,AD.vs.MCI; bulk RNA-seq,Homo_723,MCI.vs.control; bulk RNA-seq,Homo_714,AD.vs.MCI; bulk RNA-seq,Homo_633,AD.vs.control; bulk RNA-seq,Homo_633,AD.vs.MCI	7
BP	GO:0048515	spermatid differentiation	bulk RNA-seq,Emory,MCI.vs.control; bulk RNA-seq,Homo_723,AD.vs.control; bulk RNA-seq,Homo_723,AD.vs.MCI; bulk RNA-seq,Homo_714,AD.vs.control; bulk RNA-seq,Homo_714,AD.vs.MCI; bulk RNA-seq,Homo_633,AD.vs.control; bulk RNA-seq,Homo_633,AD.vs.MCI	7
BP	GO:0051293	establishment of spindle localization	bulk RNA-seq,Emory,MCI.vs.control; bulk RNA-seq,Homo_723,AD.vs.MCI; bulk RNA-seq,Homo_723,MCI.vs.control; bulk RNA-seq,Homo_714,MCI.vs.control; bulk RNA-seq,Homo_633,AD.vs.control; bulk RNA-seq,Homo_633,AD.vs.MCI	6
CC	GO:0030016	myofibril	bulk RNA-seq,Emory,MCI.vs.control; bulk RNA-seq,Homo_723,AD.vs.control; bulk RNA-seq,Homo_723,AD.vs.MCI; bulk RNA-seq,Homo_723,MCI.vs.control; bulk RNA-seq,Homo_714,AD.vs.control; bulk RNA-seq,Homo_714,AD.vs.MCI; bulk RNA-seq,Homo_633,AD.vs.control; bulk RNA-seq,Homo_633,AD.vs.MCI	8
BP	GO:0007528	neuromuscular junction development	bulk RNA-seq,Emory,MCI.vs.control; bulk RNA-seq,Homo_723,AD.vs.control; bulk RNA-seq,Homo_723,AD.vs.MCI; bulk RNA-seq,Homo_723,MCI.vs.control; bulk RNA-seq,Homo_714,AD.vs.MCI; bulk RNA-seq,Homo_714,MCI.vs.control; bulk RNA-seq,Homo_633,AD.vs.control; bulk RNA-seq,Homo_633,AD.vs.MCI	8
BP	GO:0048536	spleen development	bulk RNA-seq,Emory,MCI.vs.control; bulk RNA-seq,Homo_633,AD.vs.control; bulk RNA-seq,Homo_633,AD.vs.MCI	3
BP	GO:0021885	forebrain cell migration	bulk RNA-seq,Emory,MCI.vs.control; bulk RNA-seq,Homo_723,AD.vs.control; bulk RNA-seq,Homo_723,AD.vs.MCI; bulk RNA-seq,Homo_723,MCI.vs.control; bulk RNA-seq,Homo_714,AD.vs.MCI; bulk RNA-seq,Homo_714,MCI.vs.control; bulk RNA-seq,Homo_633,AD.vs.control	7
BP	GO:0030198	extracellular matrix organization	bulk RNA-seq,Emory,MCI.vs.control; bulk RNA-seq,Homo_723,AD.vs.control; bulk RNA-seq,Homo_723,AD.vs.MCI; bulk RNA-seq,Homo_723,MCI.vs.control; bulk RNA-seq,Homo_714,AD.vs.control; bulk RNA-seq,Homo_714,AD.vs.MCI; bulk RNA-seq,Homo_633,AD.vs.control; bulk RNA-seq,Homo_633,AD.vs.MCI; bulk RNA-seq,ROSMAP,AD.vs.control; bulk RNA-seq,ROSMAP,MCI.vs.control; bulk RNA-seq,SRP223445,AD.vs.control	11
MF	GO:0004812	aminoacyl-tRNA ligase activity	bulk RNA-seq,Emory,MCI.vs.control; bulk RNA-seq,Homo_723,AD.vs.control; bulk RNA-seq,Homo_723,MCI.vs.control; bulk RNA-seq,Homo_714,AD.vs.control; bulk RNA-seq,Homo_714,AD.vs.MCI; bulk RNA-seq,Homo_714,MCI.vs.control; bulk RNA-seq,Homo_633,AD.vs.control; bulk	8
BP	GO:0006418	tRNA aminoacylation for protein translation	bulk RNA-seq,Emory,MCI.vs.control; bulk RNA-seq,Homo_723,AD.vs.control; bulk RNA-seq,Homo_723,MCI.vs.control; bulk RNA-seq,Homo_714,AD.vs.control; bulk RNA-seq,Homo_714,AD.vs.MCI; bulk RNA-seq,Homo_714,MCI.vs.control; bulk RNA-seq,Homo_633,AD.vs.control; bulk	8
MF	GO:0016875	ligase activity, forming carbon-oxygen bonds	bulk RNA-seq,Emory,MCI.vs.control; bulk RNA-seq,Homo_723,AD.vs.control; bulk RNA-seq,Homo_723,MCI.vs.control; bulk RNA-seq,Homo_714,AD.vs.control; bulk RNA-seq,Homo_714,AD.vs.MCI; bulk RNA-seq,Homo_714,MCI.vs.control; bulk RNA-seq,Homo_633,AD.vs.control; bulk	8
MF	GO:0005283	amino acid:sodium symporter activity	bulk RNA-seq,Emory,MCI.vs.control; bulk RNA-seq,Homo_723,AD.vs.control; bulk RNA-seq,Homo_723,AD.vs.MCI; bulk RNA-seq,Homo_714,AD.vs.MCI; bulk RNA-seq,Homo_633,MCI.vs.control; bulk RNA-seq,ROSMAP,MCI.vs.control	6
BP	GO:0060425	lung morphogenesis	bulk RNA-seq,Emory,MCI.vs.control; bulk RNA-seq,Homo_723,AD.vs.control; bulk RNA-seq,Homo_723,AD.vs.MCI; bulk RNA-seq,Homo_723,MCI.vs.control; bulk RNA-seq,Homo_714,AD.vs.control; bulk RNA-seq,Homo_714,AD.vs.MCI; bulk RNA-seq,Homo_714,MCI.vs.control; bulk RNA-	8
BP	GO:0021987	cerebral cortex development	bulk RNA-seq,Emory,MCI.vs.control; bulk RNA-seq,Homo_723,AD.vs.control; bulk RNA-seq,Homo_723,AD.vs.MCI; bulk RNA-seq,Homo_723,MCI.vs.control; bulk RNA-seq,Homo_714,AD.vs.MCI; bulk RNA-seq,Homo_714,MCI.vs.control; bulk RNA-seq,Homo_633,AD.vs.control; bulk RNA-seq,Homo_633,AD.vs.MCI; bulk RNA-seq,ROSMAP,AD.vs.control	9
BP	GO:0042417	dopamine metabolic process	bulk RNA-seq,Emory,MCI.vs.control; bulk RNA-seq,Homo_723,AD.vs.control; bulk RNA-seq,Homo_723,AD.vs.MCI; bulk RNA-seq,Homo_723,MCI.vs.control; bulk RNA-seq,Homo_714,AD.vs.control; bulk RNA-seq,Homo_714,AD.vs.MCI; bulk RNA-seq,Homo_633,MCI.vs.control	7
BP	GO:0071312	cellular response to alkaloid	bulk RNA-seq,Emory,MCI.vs.control; bulk RNA-seq,Homo_633,AD.vs.control	2
BP	GO:0051101	regulation of DNA binding	bulk RNA-seq,Emory,MCI.vs.control; bulk RNA-seq,Homo_723,AD.vs.control; bulk RNA-seq,Homo_723,AD.vs.MCI; bulk RNA-seq,Homo_723,MCI.vs.control; bulk RNA-seq,Homo_714,AD.vs.MCI; bulk RNA-seq,Homo_714,MCI.vs.control; bulk RNA-seq,Homo_633,AD.vs.control; bulk RNA-seq,Homo_633,AD.vs.MCI	8
BP	GO:0072189	ureter development	bulk RNA-seq,Emory,MCI.vs.control; bulk RNA-seq,Homo_723,AD.vs.control; bulk RNA-seq,Homo_723,AD.vs.MCI; bulk RNA-seq,Homo_723,MCI.vs.control; bulk RNA-seq,Homo_714,AD.vs.MCI; bulk RNA-seq,Homo_714,MCI.vs.control	6
CC	GO:0099060	integral component of postsynaptic specialization membrane	bulk RNA-seq,Emory,MCI.vs.control; bulk RNA-seq,Homo_723,AD.vs.control; bulk RNA-seq,Homo_723,AD.vs.MCI; bulk RNA-seq,Homo_723,MCI.vs.control; bulk RNA-seq,Homo_714,AD.vs.control; bulk RNA-seq,Homo_714,AD.vs.MCI; bulk RNA-seq,Homo_714,MCI.vs.control; bulk RNA-seq,Homo_633,MCI.vs.control; bulk RNA-seq,SRP223445,AD.vs.control	9
BP	GO:0032793	positive regulation of CREB transcription factor activity	bulk RNA-seq,Emory,MCI.vs.control; bulk RNA-seq,Homo_723,AD.vs.control; bulk RNA-seq,Homo_723,AD.vs.MCI; bulk RNA-seq,Homo_723,MCI.vs.control; bulk RNA-seq,Homo_714,AD.vs.MCI; bulk RNA-seq,Homo_714,MCI.vs.control	6
BP	GO:0022612	gland morphogenesis	bulk RNA-seq,Emory,MCI.vs.control; bulk RNA-seq,Homo_723,AD.vs.control; bulk RNA-seq,Homo_723,AD.vs.MCI; bulk RNA-seq,Homo_723,MCI.vs.control; bulk RNA-seq,Homo_714,AD.vs.control; bulk RNA-seq,Homo_714,AD.vs.MCI; bulk RNA-seq,Homo_714,MCI.vs.control; bulk RNA-seq,Homo_633,AD.vs.control; bulk RNA-seq,Homo_633,AD.vs.MCI	9

BP	GO:0060323	head morphogenesis	bulk RNA-seq,Emory,MCI.vs.control; bulk RNA-seq,Homo_723,AD.vs.control; bulk RNA-seq,Homo_723,AD.vs.MCI; bulk RNA-seq,Homo_723,MCI.vs.control; bulk RNA-seq,Homo_714,AD.vs.MCI; bulk RNA-seq,Homo_714,MCI.vs.control; bulk RNA-seq,Homo_633,AD.vs.control; bulk RNA-seq,Homo_633,AD.vs.MCI	8
BP	GO:0060538	skeletal muscle organ development	bulk RNA-seq,Emory,MCI.vs.control; bulk RNA-seq,Homo_723,AD.vs.control; bulk RNA-seq,Homo_723,AD.vs.MCI; bulk RNA-seq,Homo_723,MCI.vs.control; bulk RNA-seq,Homo_714,AD.vs.control; bulk RNA-seq,Homo_714,AD.vs.MCI; bulk RNA-seq,Homo_633,AD.vs.control; bulk RNA-seq,Homo_633,AD.vs.MCI	8
BP	GO:2000136	regulation of cell proliferation involved in heart morphogenesis	bulk RNA-seq,Emory,MCI.vs.control; bulk RNA-seq,Homo_723,AD.vs.control; bulk RNA-seq,Homo_723,AD.vs.MCI; bulk RNA-seq,Homo_723,MCI.vs.control; bulk RNA-seq,Homo_714,AD.vs.MCI; bulk RNA-seq,Homo_714,MCI.vs.control	6
BP	GO:0001754	eye photoreceptor cell differentiation	bulk RNA-seq,Emory,MCI.vs.control; bulk RNA-seq,Homo_723,AD.vs.control; bulk RNA-seq,Homo_723,AD.vs.MCI; bulk RNA-seq,Homo_723,MCI.vs.control; bulk RNA-seq,Homo_714,AD.vs.MCI	5
BP	GO:0007214	gamma-aminobutyric acid signaling pathway	bulk RNA-seq,Emory,MCI.vs.control; bulk RNA-seq,Homo_723,AD.vs.control; bulk RNA-seq,Homo_723,AD.vs.MCI; bulk RNA-seq,Homo_714,AD.vs.control; bulk RNA-seq,Homo_714,AD.vs.MCI	5
BP	GO:0042752	regulation of circadian rhythm	bulk RNA-seq,Emory,MCI.vs.control; bulk RNA-seq,Homo_723,AD.vs.control; bulk RNA-seq,Homo_723,AD.vs.MCI; bulk RNA-seq,Homo_723,MCI.vs.control; bulk RNA-seq,Homo_714,AD.vs.MCI; bulk RNA-seq,Homo_714,MCI.vs.control; bulk RNA-seq,Homo_633,AD.vs.control; bulk RNA-seq,Homo_633,AD.vs.MCI	8
BP	GO:0060068	vagina development	bulk RNA-seq,Emory,MCI.vs.control; bulk RNA-seq,Homo_723,AD.vs.control; bulk RNA-seq,Homo_723,AD.vs.MCI; bulk RNA-seq,Homo_723,MCI.vs.control	4
BP	GO:0051294	establishment of spindle orientation	bulk RNA-seq,Emory,MCI.vs.control; bulk RNA-seq,Homo_633,AD.vs.control; bulk RNA-seq,Homo_633,AD.vs.MCI	3
BP	GO:1901160	primary amino compound metabolic process	bulk RNA-seq,Emory,MCI.vs.control; bulk RNA-seq,Homo_723,AD.vs.control; bulk RNA-seq,Homo_723,AD.vs.MCI; bulk RNA-seq,Homo_723,MCI.vs.control	4
BP	GO:0043279	response to alkaloid	bulk RNA-seq,Emory,MCI.vs.control; bulk RNA-seq,Homo_723,AD.vs.control; bulk RNA-seq,Homo_723,AD.vs.MCI; bulk RNA-seq,Homo_723,MCI.vs.control; bulk RNA-seq,Homo_714,AD.vs.control; bulk RNA-seq,Homo_714,AD.vs.MCI; bulk RNA-seq,Homo_633,AD.vs.control; bulk RNA-seq,Homo_633,AD.vs.MCI	8
BP	GO:0006814	sodium ion transport	bulk RNA-seq,Emory,MCI.vs.control; bulk RNA-seq,Homo_723,AD.vs.control; bulk RNA-seq,Homo_723,AD.vs.MCI; bulk RNA-seq,Homo_723,MCI.vs.control; bulk RNA-seq,Homo_714,AD.vs.control; bulk RNA-seq,Homo_714,AD.vs.MCI; bulk RNA-seq,Homo_633,AD.vs.control; bulk RNA-seq,Homo_633,AD.vs.MCI; bulk RNA-seq,ROSMAP,AD.vs.control; bulk RNA-seq,ROSMAP,MCI.vs.control; bulk RNA-seq,SRP223445,AD.vs.control	11
BP	GO:0042753	positive regulation of circadian rhythm	bulk RNA-seq,Emory,MCI.vs.control; bulk RNA-seq,Homo_714,AD.vs.control; bulk RNA-seq,Homo_633,AD.vs.control; bulk RNA-seq,Homo_633,AD.vs.MCI	4
MF	GO:0070742	C2H2 zinc finger domain binding	bulk RNA-seq,Emory,MCI.vs.control; bulk RNA-seq,Homo_723,MCI.vs.control	2
CC	GO:0089717	spanning component of membrane	bulk RNA-seq,Emory,MCI.vs.control; bulk RNA-seq,Homo_723,AD.vs.control; bulk RNA-seq,Homo_723,AD.vs.MCI; bulk RNA-seq,Homo_723,MCI.vs.control; bulk RNA-seq,Homo_714,AD.vs.MCI; bulk RNA-seq,Homo_714,MCI.vs.control	6
BP	GO:1902476	chloride transmembrane transport	bulk RNA-seq,Emory,MCI.vs.control; bulk RNA-seq,Homo_723,AD.vs.control; bulk RNA-seq,Homo_723,AD.vs.MCI; bulk RNA-seq,Homo_723,MCI.vs.control; bulk RNA-seq,Homo_714,AD.vs.control; bulk RNA-seq,Homo_714,AD.vs.MCI; bulk RNA-seq,Homo_714,MCI.vs.control; bulk RNA-	8
BP	GO:1901861	regulation of muscle tissue development	bulk RNA-seq,Emory,MCI.vs.control; bulk RNA-seq,Homo_723,AD.vs.control; bulk RNA-seq,Homo_723,AD.vs.MCI; bulk RNA-seq,Homo_723,MCI.vs.control; bulk RNA-seq,Homo_714,AD.vs.MCI; bulk RNA-seq,Homo_714,MCI.vs.control; bulk RNA-seq,Homo_633,AD.vs.control; bulk RNA-	8
BP	GO:0007286	spermatid development	bulk RNA-seq,Emory,MCI.vs.control; bulk RNA-seq,Homo_723,AD.vs.control; bulk RNA-seq,Homo_723,AD.vs.MCI; bulk RNA-seq,Homo_714,AD.vs.control; bulk RNA-seq,Homo_714,AD.vs.MCI; bulk RNA-seq,Homo_633,AD.vs.control; bulk RNA-seq,Homo_633,AD.vs.MCI	7
MF	GO:0004497	monooxygenase activity	bulk RNA-seq,Emory,MCI.vs.control; bulk RNA-seq,Homo_723,AD.vs.control; bulk RNA-seq,Homo_723,AD.vs.MCI; bulk RNA-seq,Homo_723,MCI.vs.control; bulk RNA-seq,Homo_714,AD.vs.MCI; bulk RNA-seq,Homo_714,MCI.vs.control; bulk RNA-seq,Homo_633,AD.vs.control; bulk RNA-	8
BP	GO:0099177	regulation of trans-synaptic signaling	bulk RNA-seq,Emory,MCI.vs.control; bulk RNA-seq,Homo_723,AD.vs.control; bulk RNA-seq,Homo_723,AD.vs.MCI; bulk RNA-seq,Homo_714,AD.vs.control; bulk RNA-seq,Homo_714,AD.vs.MCI; bulk RNA-seq,Homo_633,AD.vs.control; bulk RNA-seq,Homo_633,AD.vs.MCI; bulk RNA-seq,ROSMAP,AD.vs.control; bulk RNA-seq,ROSMAP,MCI.vs.control; bulk RNA-seq,SRP223445,AD.vs.control; scRNA-seq,SRP330776,Naive CD8+ T cell_3-AD.vs.control	11
MF	GO:0017022	myosin binding	bulk RNA-seq,Emory,MCI.vs.control; bulk RNA-seq,Homo_723,AD.vs.control; bulk RNA-seq,Homo_723,AD.vs.MCI; bulk RNA-seq,Homo_723,MCI.vs.control; bulk RNA-seq,Homo_714,AD.vs.control; bulk RNA-seq,Homo_714,AD.vs.MCI; bulk RNA-seq,Homo_714,MCI.vs.control; bulk RNA-	8
BP	GO:0030534	adult behavior	bulk RNA-seq,Emory,MCI.vs.control; bulk RNA-seq,Homo_723,AD.vs.control; bulk RNA-seq,Homo_723,AD.vs.MCI; bulk RNA-seq,Homo_723,MCI.vs.control; bulk RNA-seq,Homo_714,AD.vs.control; bulk RNA-seq,Homo_714,AD.vs.MCI; bulk RNA-seq,Homo_633,AD.vs.control; bulk RNA-seq,Homo_633,AD.vs.MCI; bulk RNA-seq,SRP325058,AD.vs.control	9
BP	GO:0010875	positive regulation of cholesterol efflux	bulk RNA-seq,Emory,MCI.vs.control; bulk RNA-seq,Homo_723,AD.vs.MCI; bulk RNA-seq,Homo_723,MCI.vs.control; bulk RNA-seq,Homo_714,AD.vs.MCI; bulk RNA-seq,Homo_714,MCI.vs.control	5
BP	GO:0060428	lung epithelium development	bulk RNA-seq,Emory,MCI.vs.control; bulk RNA-seq,Homo_723,AD.vs.control; bulk RNA-seq,Homo_723,AD.vs.MCI; bulk RNA-seq,Homo_723,MCI.vs.control; bulk RNA-seq,Homo_714,AD.vs.MCI; bulk RNA-seq,Homo_714,MCI.vs.control	6
BP	GO:0061298	retina vasculature development in camera-type eye	bulk RNA-seq,Emory,MCI.vs.control; bulk RNA-seq,Homo_723,AD.vs.control; bulk RNA-seq,Homo_723,AD.vs.MCI; bulk RNA-seq,Homo_723,MCI.vs.control; bulk RNA-seq,Homo_714,AD.vs.MCI; bulk RNA-seq,Homo_714,MCI.vs.control; bulk RNA-seq,Homo_633,AD.vs.control	7
MF	GO:0005324	long-chain fatty acid transporter activity	bulk RNA-seq,Emory,MCI.vs.control; bulk RNA-seq,Homo_723,AD.vs.MCI; bulk RNA-seq,Homo_723,MCI.vs.control; bulk RNA-seq,Homo_714,AD.vs.MCI; bulk RNA-seq,Homo_714,MCI.vs.control; bulk RNA-seq,Homo_633,AD.vs.control; bulk RNA-seq,Homo_633,AD.vs.MCI	7
BP	GO:0070849	response to epidermal growth factor	bulk RNA-seq,Emory,MCI.vs.control; bulk RNA-seq,Homo_723,AD.vs.control; bulk RNA-seq,Homo_723,AD.vs.MCI; bulk RNA-seq,Homo_723,MCI.vs.control; bulk RNA-seq,Homo_714,AD.vs.MCI; bulk RNA-seq,Homo_714,MCI.vs.control; bulk RNA-seq,Homo_633,AD.vs.control; bulk RNA-seq,Homo_633,AD.vs.MCI	8
BP	GO:0042428	serotonin metabolic process	bulk RNA-seq,Emory,MCI.vs.control; bulk RNA-seq,Homo_723,AD.vs.control; bulk RNA-seq,Homo_723,AD.vs.MCI; bulk RNA-seq,Homo_723,MCI.vs.control; bulk RNA-seq,Homo_714,AD.vs.MCI; bulk RNA-seq,Homo_633,MCI.vs.control	6
MF	GO:0004115	3',5'-cyclic-AMP phosphodiesterase activity	bulk RNA-seq,Emory,MCI.vs.control; bulk RNA-seq,Homo_723,AD.vs.MCI; bulk RNA-seq,Homo_723,MCI.vs.control; bulk RNA-seq,Homo_714,AD.vs.MCI; bulk RNA-seq,Homo_714,MCI.vs.control; bulk RNA-seq,Homo_633,AD.vs.control; bulk RNA-seq,ROSMAP,AD.vs.control	7
BP	GO:0061180	mammary gland epithelium development	bulk RNA-seq,Emory,MCI.vs.control; bulk RNA-seq,Homo_723,AD.vs.control; bulk RNA-seq,Homo_723,AD.vs.MCI; bulk RNA-seq,Homo_723,MCI.vs.control; bulk RNA-seq,Homo_714,AD.vs.MCI; bulk RNA-seq,Homo_714,MCI.vs.control; bulk RNA-seq,Homo_633,AD.vs.control	7
MF	GO:0003779	actin binding	bulk RNA-seq,Emory,MCI.vs.control; bulk RNA-seq,Homo_723,AD.vs.control; bulk RNA-seq,Homo_723,AD.vs.MCI; bulk RNA-seq,Homo_714,AD.vs.control; bulk RNA-seq,Homo_714,AD.vs.MCI; bulk RNA-seq,Homo_633,AD.vs.control; bulk RNA-seq,Homo_633,AD.vs.MCI; bulk RNA-seq,SRP223445,AD.vs.control	8
CC	GO:0005684	U2-type spliceosomal complex	bulk RNA-seq,Emory,MCI.vs.control; bulk RNA-seq,Homo_723,AD.vs.control; bulk RNA-seq,Homo_723,AD.vs.MCI; bulk RNA-seq,Homo_723,MCI.vs.control; bulk RNA-seq,Homo_714,AD.vs.control; bulk RNA-seq,Homo_714,AD.vs.MCI; bulk RNA-seq,Homo_714,MCI.vs.control; bulk RNA-seq,Homo_633,AD.vs.control; bulk RNA-seq,Homo_633,AD.vs.MCI; scRNA-seq,SRP309935,Megakaryocyte_2-AD.vs.control	10
BP	GO:0060019	radial glial cell differentiation	bulk RNA-seq,Emory,MCI.vs.control; bulk RNA-seq,Homo_723,AD.vs.control; bulk RNA-seq,Homo_723,AD.vs.MCI; bulk RNA-seq,Homo_723,MCI.vs.control; bulk RNA-seq,Homo_714,AD.vs.MCI	5
BP	GO:0050482	arachidonic acid secretion	bulk RNA-seq,Emory,MCI.vs.control; bulk RNA-seq,Homo_723,AD.vs.MCI; bulk RNA-seq,Homo_714,AD.vs.control; bulk RNA-seq,Homo_714,AD.vs.MCI	4

BP	GO:1903963	arachidonate transport	bulk RNA-seq,Emory,MCI.vs.control; bulk RNA-seq,Homo_723,AD.vs.MCI; bulk RNA-seq,Homo_714,AD.vs.control; bulk RNA-seq,Homo_714,AD.vs.MCI	4
BP	GO:2000049	positive regulation of cell-cell adhesion mediated by cadherin	bulk RNA-seq,Emory,MCI.vs.control; bulk RNA-seq,Homo_723,AD.vs.control; bulk RNA-seq,Homo_723,AD.vs.MCI; bulk RNA-seq,Homo_723,MCI.vs.control; bulk RNA-seq,Homo_714,AD.vs.MCI	5
BP	GO:0006936	muscle contraction	bulk RNA-seq,Emory,MCI.vs.control; bulk RNA-seq,Homo_723,AD.vs.control; bulk RNA-seq,Homo_723,AD.vs.MCI; bulk RNA-seq,Homo_714,AD.vs.control; bulk RNA-seq,Homo_714,AD.vs.MCI; bulk RNA-seq,Homo_633,AD.vs.control; bulk RNA-seq,Homo_633,AD.vs.MCI	7
BP	GO:0043388	positive regulation of DNA binding	bulk RNA-seq,Emory,MCI.vs.control; bulk RNA-seq,Homo_723,AD.vs.control; bulk RNA-seq,Homo_723,AD.vs.MCI; bulk RNA-seq,Homo_723,MCI.vs.control; bulk RNA-seq,Homo_714,MCI.vs.control; bulk RNA-seq,Homo_633,AD.vs.control; bulk RNA-seq,Homo_633,AD.vs.MCI	7
BP	GO:0072178	nephric duct morphogenesis	bulk RNA-seq,Emory,MCI.vs.control; bulk RNA-seq,Homo_723,AD.vs.MCI; bulk RNA-seq,Homo_723,MCI.vs.control; bulk RNA-seq,Homo_714,AD.vs.MCI; bulk RNA-seq,Homo_714,MCI.vs.control	5
BP	GO:0032543	mitochondrial translation	bulk RNA-seq,Emory,MCI.vs.control; bulk RNA-seq,Homo_723,AD.vs.control; bulk RNA-seq,Homo_723,AD.vs.MCI; bulk RNA-seq,Homo_723,MCI.vs.control; bulk RNA-seq,Homo_714,AD.vs.control; bulk RNA-seq,Homo_714,AD.vs.MCI; bulk RNA-seq,Homo_633,AD.vs.control; bulk RNA-seq,Homo_633,AD.vs.MCI; bulk RNA-seq,SRP223445,AD.vs.control	9
BP	GO:0045229	external encapsulating structure organization	bulk RNA-seq,Emory,MCI.vs.control; bulk RNA-seq,Homo_723,AD.vs.control; bulk RNA-seq,Homo_723,AD.vs.MCI; bulk RNA-seq,Homo_723,MCI.vs.control; bulk RNA-seq,Homo_714,AD.vs.control; bulk RNA-seq,Homo_714,AD.vs.MCI; bulk RNA-seq,Homo_633,AD.vs.control; bulk RNA-seq,Homo_633,AD.vs.MCI; bulk RNA-seq,ROSMAP,AD.vs.control; bulk RNA-seq,ROSMAP,MCI.vs.control; bulk RNA-seq,SRP223445,AD.vs.control	11
BP	GO:0042693	muscle cell fate commitment	bulk RNA-seq,Emory,MCI.vs.control; bulk RNA-seq,Homo_723,AD.vs.control; bulk RNA-seq,Homo_723,AD.vs.MCI; bulk RNA-seq,Homo_723,MCI.vs.control; bulk RNA-seq,Homo_714,AD.vs.control; bulk RNA-seq,Homo_714,AD.vs.MCI; bulk RNA-seq,Homo_714,MCI.vs.control; bulk RNA-	8
CC	GO:0030686	90S preribosome	bulk RNA-seq,Emory,MCI.vs.control; bulk RNA-seq,Homo_723,AD.vs.control; bulk RNA-seq,Homo_723,MCI.vs.control; bulk RNA-seq,Homo_714,AD.vs.control; bulk RNA-seq,Homo_714,MCI.vs.control; bulk RNA-seq,Homo_633,AD.vs.control; bulk RNA-seq,Homo_633,AD.vs.MCI; bulk	8
BP	GO:0014012	peripheral nervous system axon regeneration	bulk RNA-seq,Emory,MCI.vs.control; bulk RNA-seq,Homo_723,AD.vs.MCI; bulk RNA-seq,Homo_723,MCI.vs.control; bulk RNA-seq,Homo_714,AD.vs.MCI; bulk RNA-seq,Homo_714,MCI.vs.control	5
BP	GO:0050804	modulation of chemical synaptic transmission	bulk RNA-seq,Emory,MCI.vs.control; bulk RNA-seq,Homo_723,AD.vs.control; bulk RNA-seq,Homo_723,AD.vs.MCI; bulk RNA-seq,Homo_714,AD.vs.control; bulk RNA-seq,Homo_714,AD.vs.MCI; bulk RNA-seq,Homo_633,AD.vs.control; bulk RNA-seq,Homo_633,AD.vs.MCI; bulk RNA-seq,ROSMAP,AD.vs.control; bulk RNA-seq,ROSMAP,MCI.vs.control; bulk RNA-seq,SRP223445,AD.vs.control; scRNA-seq,SRP330776,Naive CD8+ T cell_3-AD.vs.control	11
BP	GO:0072176	nephric duct development	bulk RNA-seq,Emory,MCI.vs.control; bulk RNA-seq,Homo_723,AD.vs.MCI; bulk RNA-seq,Homo_723,MCI.vs.control; bulk RNA-seq,Homo_714,AD.vs.MCI; bulk RNA-seq,Homo_714,MCI.vs.control	5
BP	GO:0007351	tripartite regional subdivision	bulk RNA-seq,Emory,MCI.vs.control; bulk RNA-seq,Homo_723,AD.vs.control; bulk RNA-seq,Homo_723,AD.vs.MCI; bulk RNA-seq,Homo_723,MCI.vs.control; bulk RNA-seq,Homo_714,AD.vs.control; bulk RNA-seq,Homo_714,AD.vs.MCI; bulk RNA-seq,Homo_633,MCI.vs.control	7
BP	GO:0008595	anterior/posterior axis specification, embryo	bulk RNA-seq,Emory,MCI.vs.control; bulk RNA-seq,Homo_723,AD.vs.control; bulk RNA-seq,Homo_723,AD.vs.MCI; bulk RNA-seq,Homo_723,MCI.vs.control; bulk RNA-seq,Homo_714,AD.vs.control; bulk RNA-seq,Homo_714,AD.vs.MCI; bulk RNA-seq,Homo_633,MCI.vs.control	7
BP	GO:0003151	outflow tract morphogenesis	bulk RNA-seq,Emory,MCI.vs.control; bulk RNA-seq,Homo_723,AD.vs.control; bulk RNA-seq,Homo_723,AD.vs.MCI; bulk RNA-seq,Homo_723,MCI.vs.control; bulk RNA-seq,Homo_714,AD.vs.control; bulk RNA-seq,Homo_714,AD.vs.MCI; bulk RNA-seq,Homo_714,MCI.vs.control; bulk RNA-	8
BP	GO:0014706	striated muscle tissue development	bulk RNA-seq,Emory,MCI.vs.control; bulk RNA-seq,Homo_723,AD.vs.control; bulk RNA-seq,Homo_723,AD.vs.MCI; bulk RNA-seq,Homo_723,MCI.vs.control; bulk RNA-seq,Homo_714,AD.vs.control; bulk RNA-seq,Homo_714,AD.vs.MCI; bulk RNA-seq,Homo_633,AD.vs.control; bulk RNA-	8
BP	GO:0060560	developmental growth involved in morphogenesis	bulk RNA-seq,Emory,MCI.vs.control; bulk RNA-seq,Homo_723,AD.vs.control; bulk RNA-seq,Homo_723,AD.vs.MCI; bulk RNA-seq,Homo_723,MCI.vs.control; bulk RNA-seq,Homo_714,AD.vs.control; bulk RNA-seq,Homo_714,AD.vs.MCI; bulk RNA-seq,Homo_633,AD.vs.control; bulk RNA-seq,Homo_633,AD.vs.MCI; bulk RNA-seq,SRP223445,AD.vs.control	9
BP	GO:0000959	mitochondrial RNA metabolic process	bulk RNA-seq,Emory,MCI.vs.control; bulk RNA-seq,Homo_723,AD.vs.control; bulk RNA-seq,Homo_723,AD.vs.MCI; bulk RNA-seq,Homo_723,MCI.vs.control; bulk RNA-seq,Homo_714,AD.vs.control; bulk RNA-seq,Homo_714,AD.vs.MCI; bulk RNA-seq,Homo_714,MCI.vs.control; bulk RNA-seq,Homo_633,AD.vs.control; bulk RNA-seq,Homo_633,AD.vs.MCI	9
BP	GO:0009214	cyclic nucleotide catabolic process	bulk RNA-seq,Emory,MCI.vs.control; bulk RNA-seq,Homo_723,MCI.vs.control; bulk RNA-seq,Homo_714,AD.vs.MCI; bulk RNA-seq,Homo_714,MCI.vs.control	4
BP	GO:0048286	lung alveolus development	bulk RNA-seq,Emory,MCI.vs.control; bulk RNA-seq,Homo_723,AD.vs.MCI; bulk RNA-seq,Homo_723,MCI.vs.control; bulk RNA-seq,Homo_714,AD.vs.MCI; bulk RNA-seq,Homo_714,MCI.vs.control; bulk RNA-seq,Homo_633,AD.vs.control; bulk RNA-seq,Homo_633,AD.vs.MCI	7
BP	GO:0019896	axonal transport of mitochondrion	bulk RNA-seq,Emory,MCI.vs.control; bulk RNA-seq,Homo_723,MCI.vs.control; bulk RNA-seq,Homo_633,AD.vs.control	3
BP	GO:0021871	forebrain regionalization	bulk RNA-seq,Emory,MCI.vs.control; bulk RNA-seq,Homo_723,AD.vs.control; bulk RNA-seq,Homo_723,AD.vs.MCI; bulk RNA-seq,Homo_723,MCI.vs.control; bulk RNA-seq,Homo_714,AD.vs.MCI	5
BP	GO:2000047	regulation of cell-cell adhesion mediated by cadherin	bulk RNA-seq,Emory,MCI.vs.control; bulk RNA-seq,Homo_723,AD.vs.control; bulk RNA-seq,Homo_723,AD.vs.MCI; bulk RNA-seq,Homo_723,MCI.vs.control; bulk RNA-seq,Homo_714,AD.vs.MCI	5
MF	GO:0016303	1-phosphatidylinositol-3-kinase activity	bulk RNA-seq,Emory,MCI.vs.control; bulk RNA-seq,Homo_723,MCI.vs.control; bulk RNA-seq,Homo_714,MCI.vs.control; bulk RNA-seq,Homo_633,AD.vs.control; bulk RNA-seq,Homo_633,AD.vs.MCI	5
BP	GO:0046847	filopodium assembly	bulk RNA-seq,Emory,MCI.vs.control; bulk RNA-seq,Homo_723,AD.vs.MCI; bulk RNA-seq,Homo_723,MCI.vs.control; bulk RNA-seq,Homo_714,AD.vs.MCI; bulk RNA-seq,Homo_714,MCI.vs.control; bulk RNA-seq,Homo_633,AD.vs.control; bulk RNA-seq,Homo_633,AD.vs.MCI; bulk RNA-	8
MF	GO:0005319	lipid transporter activity	bulk RNA-seq,Emory,MCI.vs.control; bulk RNA-seq,Homo_723,AD.vs.control; bulk RNA-seq,Homo_723,AD.vs.MCI; bulk RNA-seq,Homo_723,MCI.vs.control; bulk RNA-seq,Homo_714,AD.vs.control; bulk RNA-seq,Homo_714,AD.vs.MCI; bulk RNA-seq,Homo_633,AD.vs.control; bulk RNA-seq,Homo_633,AD.vs.MCI	8
CC	GO:0030017	sarcomere	bulk RNA-seq,Emory,MCI.vs.control; bulk RNA-seq,Homo_723,AD.vs.control; bulk RNA-seq,Homo_723,AD.vs.MCI; bulk RNA-seq,Homo_723,MCI.vs.control; bulk RNA-seq,Homo_714,AD.vs.control; bulk RNA-seq,Homo_714,AD.vs.MCI; bulk RNA-seq,Homo_633,AD.vs.control; bulk RNA-seq,Homo_633,AD.vs.MCI	8
BP	GO:0042220	response to cocaine	bulk RNA-seq,Emory,MCI.vs.control; bulk RNA-seq,Homo_723,AD.vs.control; bulk RNA-seq,Homo_723,AD.vs.MCI; bulk RNA-seq,Homo_723,MCI.vs.control; bulk RNA-seq,Homo_714,AD.vs.control; bulk RNA-seq,Homo_714,AD.vs.MCI; bulk RNA-seq,Homo_633,MCI.vs.control	7
BP	GO:0009880	embryonic pattern specification	bulk RNA-seq,Emory,MCI.vs.control; bulk RNA-seq,Homo_723,AD.vs.control; bulk RNA-seq,Homo_723,AD.vs.MCI; bulk RNA-seq,Homo_723,MCI.vs.control; bulk RNA-seq,Homo_714,AD.vs.control; bulk RNA-seq,Homo_714,AD.vs.MCI; bulk RNA-seq,Homo_714,MCI.vs.control	7
BP	GO:0007492	endoderm development	bulk RNA-seq,Emory,MCI.vs.control; bulk RNA-seq,Homo_723,AD.vs.control; bulk RNA-seq,Homo_723,AD.vs.MCI; bulk RNA-seq,Homo_723,MCI.vs.control; bulk RNA-seq,Homo_714,AD.vs.MCI; bulk RNA-seq,Homo_714,MCI.vs.control	6
BP	GO:0007216	G protein-coupled glutamate receptor signaling pathway	bulk RNA-seq,Emory,MCI.vs.control; bulk RNA-seq,Homo_723,AD.vs.control; bulk RNA-seq,Homo_723,AD.vs.MCI; bulk RNA-seq,Homo_714,AD.vs.MCI	4
BP	GO:0042659	regulation of cell fate specification	bulk RNA-seq,Emory,MCI.vs.control; bulk RNA-seq,Homo_723,AD.vs.control; bulk RNA-seq,Homo_723,AD.vs.MCI; bulk RNA-seq,Homo_723,MCI.vs.control; bulk RNA-seq,Homo_714,AD.vs.MCI; bulk RNA-seq,Homo_714,MCI.vs.control	6

BP	GO:0032228	regulation of synaptic transmission, GABAergic	bulk RNA-seq,Emory,MCI.vs.control; bulk RNA-seq,Homo_723,AD.vs.control; bulk RNA-seq,Homo_723,AD.vs.MCI; bulk RNA-seq,Homo_714,AD.vs.MCI	4
CC	GO:0008076	voltage-gated potassium channel complex	bulk RNA-seq,Emory,MCI.vs.control; bulk RNA-seq,Homo_723,AD.vs.control; bulk RNA-seq,Homo_723,AD.vs.MCI; bulk RNA-seq,Homo_723,MCI.vs.control; bulk RNA-seq,Homo_714,AD.vs.control; bulk RNA-seq,Homo_714,AD.vs.MCI; bulk RNA-seq,Homo_633,MCI.vs.control; bulk RNA-	8
BP	GO:0019370	leukotriene biosynthetic process	bulk RNA-seq,Emory,MCI.vs.control; bulk RNA-seq,Homo_633,AD.vs.control; bulk RNA-seq,Homo_633,AD.vs.MCI	3
BP	GO:0010257	NADH dehydrogenase complex assembly	bulk RNA-seq,Emory,MCI.vs.control; bulk RNA-seq,Homo_723,AD.vs.control; bulk RNA-seq,Homo_723,AD.vs.MCI; bulk RNA-seq,Homo_723,MCI.vs.control; bulk RNA-seq,Homo_714,AD.vs.control; bulk RNA-seq,Homo_714,AD.vs.MCI; bulk RNA-seq,Homo_714,MCI.vs.control; bulk RNA-seq,Homo_633,AD.vs.control; bulk RNA-seq,Homo_633,AD.vs.MCI	9
BP	GO:0032981	mitochondrial respiratory chain complex I assembly	bulk RNA-seq,Emory,MCI.vs.control; bulk RNA-seq,Homo_723,AD.vs.control; bulk RNA-seq,Homo_723,AD.vs.MCI; bulk RNA-seq,Homo_723,MCI.vs.control; bulk RNA-seq,Homo_714,AD.vs.control; bulk RNA-seq,Homo_714,AD.vs.MCI; bulk RNA-seq,Homo_714,MCI.vs.control; bulk RNA-seq,Homo_633,AD.vs.control; bulk RNA-seq,Homo_633,AD.vs.MCI	9
BP	GO:0050982	detection of mechanical stimulus	bulk RNA-seq,Emory,MCI.vs.control; bulk RNA-seq,Homo_723,AD.vs.control; bulk RNA-seq,Homo_723,AD.vs.MCI; bulk RNA-seq,Homo_723,MCI.vs.control; bulk RNA-seq,Homo_714,AD.vs.control; bulk RNA-seq,Homo_714,AD.vs.MCI	6
BP	GO:0097035	regulation of membrane lipid distribution	bulk RNA-seq,Emory,MCI.vs.control; bulk RNA-seq,Homo_723,MCI.vs.control; bulk RNA-seq,Homo_714,AD.vs.MCI; bulk RNA-seq,Homo_714,MCI.vs.control; bulk RNA-seq,Homo_633,AD.vs.control; bulk RNA-seq,Homo_633,AD.vs.MCI	6
BP	GO:0035747	natural killer cell chemotaxis	bulk RNA-seq,Emory,MCI.vs.control	1
BP	GO:0008272	sulfate transport	bulk RNA-seq,Emory,MCI.vs.control; bulk RNA-seq,Homo_633,AD.vs.control; bulk RNA-seq,Homo_633,AD.vs.MCI	3
BP	GO:0007288	sperm axoneme assembly	bulk RNA-seq,Emory,MCI.vs.control; bulk RNA-seq,Homo_723,AD.vs.control; bulk RNA-seq,Homo_723,AD.vs.MCI; bulk RNA-seq,Homo_723,MCI.vs.control; bulk RNA-seq,Homo_633,AD.vs.control	5
BP	GO:0035987	endodermal cell differentiation	bulk RNA-seq,Emory,MCI.vs.control; bulk RNA-seq,Homo_723,AD.vs.control; bulk RNA-seq,Homo_723,AD.vs.MCI; bulk RNA-seq,Homo_723,MCI.vs.control; bulk RNA-seq,Homo_714,AD.vs.MCI; bulk RNA-seq,Homo_714,MCI.vs.control	6
BP	GO:0072348	sulfur compound transport	bulk RNA-seq,Emory,MCI.vs.control; bulk RNA-seq,Homo_723,AD.vs.MCI; bulk RNA-seq,Homo_723,MCI.vs.control; bulk RNA-seq,Homo_714,MCI.vs.control; bulk RNA-seq,Homo_633,AD.vs.control; bulk RNA-seq,Homo_633,AD.vs.MCI	6
CC	GO:0042101	T cell receptor complex	bulk RNA-seq,Homo_723,AD.vs.control; bulk RNA-seq,Homo_723,AD.vs.MCI; bulk RNA-seq,Homo_723,MCI.vs.control; bulk RNA-seq,Homo_714,AD.vs.control; bulk RNA-seq,Homo_714,AD.vs.MCI; bulk RNA-seq,Homo_633,AD.vs.control; bulk RNA-seq,Homo_633,AD.vs.MCI; scRNA-seq,SRP330776,B cell_2-AD.vs.control; scRNA-seq,SRP330776,CD8+ T cell_2-AD.vs.control; scRNA-seq,SRP330776,Natural killer cell_1-AD.vs.control; scRNA-seq,SRP309935,B cell_1-AD.vs.control; scRNA-seq,SRP309935,Megakaryocyte_2-AD.vs.control; scRNA-seq,SRP215507,CD8+ T cell_1-AD.vs.control; scRNA-seq,SRP215507,CD8+ T cell_1-MCI.vs.control; scRNA-seq,SRP215507,CD8+ T cell_4-AD.vs.MCI	15
CC	GO:0019814	immunoglobulin complex	bulk RNA-seq,Homo_723,AD.vs.control; bulk RNA-seq,Homo_723,AD.vs.MCI; bulk RNA-seq,Homo_714,AD.vs.control; bulk RNA-seq,Homo_714,AD.vs.MCI; bulk RNA-seq,Homo_633,AD.vs.control; bulk RNA-seq,Homo_633,AD.vs.MCI; bulk RNA-seq,MCSA,MCI.vs.control; bulk RNA-seq,SRP223445,AD.vs.control; scRNA-seq,SRP330776,CD8+ T cell_1-AD.vs.control; scRNA-seq,SRP330776,Memory T cell_2-AD.vs.control; scRNA-seq,SRP330776,Naive CD8+ T cell_1-AD.vs.control; scRNA-seq,SRP330776,Naive CD8+ T cell_2-AD.vs.control; scRNA-seq,SRP309935,Dendritic cell-AD.vs.control; scRNA-seq,SRP309935,Monocyte_2-AD.vs.control; scRNA-seq,SRP309935,Naive CD8+ T cell_1-AD.vs.control	15
BP	GO:0002377	immunoglobulin production	bulk RNA-seq,Homo_723,AD.vs.control; bulk RNA-seq,Homo_723,AD.vs.MCI; bulk RNA-seq,Homo_723,MCI.vs.control; bulk RNA-seq,Homo_714,AD.vs.control; bulk RNA-seq,Homo_714,AD.vs.MCI; bulk RNA-seq,Homo_633,AD.vs.control; bulk RNA-seq,Homo_633,AD.vs.MCI; bulk RNA-seq,MCSA,MCI.vs.control; scRNA-seq,SRP330776,Naive CD8+ T cell_2-AD.vs.control; scRNA-seq,SRP215507,CD8+ T cell_1-MCI.vs.control; scRNA-seq,SRP215507,CD8+ T cell_3-AD.vs.control; scRNA-seq,SRP215507,CD8+ T cell_3-MCI.vs.control	12
MF	GO:0003823	antigen binding	bulk RNA-seq,Homo_723,AD.vs.control; bulk RNA-seq,Homo_723,AD.vs.MCI; bulk RNA-seq,Homo_714,AD.vs.control; bulk RNA-seq,Homo_714,AD.vs.MCI; bulk RNA-seq,Homo_633,AD.vs.control; bulk RNA-seq,Homo_633,AD.vs.MCI; bulk RNA-seq,MCSA,MCI.vs.control; scRNA-seq,SRP330776,Naive CD8+ T cell_1-AD.vs.control; scRNA-seq,SRP330776,Naive CD8+ T cell_2-AD.vs.control; scRNA-seq,SRP330776,Natural killer cell_1-AD.vs.control; scRNA-seq,SRP309935,Dendritic cell-AD.vs.control; scRNA-seq,SRP309935,Monocyte_2-AD.vs.control; scRNA-seq,SRP215507,CD8+ T cell_3-AD.vs.control; scRNA-	14
BP	GO:0006399	tRNA metabolic process	bulk RNA-seq,Homo_723,AD.vs.control; bulk RNA-seq,Homo_723,AD.vs.MCI; bulk RNA-seq,Homo_723,MCI.vs.control; bulk RNA-seq,Homo_714,AD.vs.control; bulk RNA-seq,Homo_714,AD.vs.MCI; bulk RNA-seq,Homo_633,AD.vs.control; bulk RNA-seq,Homo_633,AD.vs.MCI; bulk RNA-	8
BP	GO:0006364	rRNA processing	bulk RNA-seq,Homo_723,AD.vs.control; bulk RNA-seq,Homo_723,AD.vs.MCI; bulk RNA-seq,Homo_723,MCI.vs.control; bulk RNA-seq,Homo_714,AD.vs.control; bulk RNA-seq,Homo_714,AD.vs.MCI; bulk RNA-seq,Homo_633,AD.vs.control; bulk RNA-seq,Homo_633,AD.vs.MCI; bulk RNA-seq,SRP223445,AD.vs.control; scRNA-seq,SRP309935,Megakaryocyte_2-AD.vs.control	9
BP	GO:0042254	ribosome biogenesis	bulk RNA-seq,Homo_723,AD.vs.control; bulk RNA-seq,Homo_723,AD.vs.MCI; bulk RNA-seq,Homo_723,MCI.vs.control; bulk RNA-seq,Homo_714,AD.vs.control; bulk RNA-seq,Homo_714,AD.vs.MCI; bulk RNA-seq,Homo_633,AD.vs.control; bulk RNA-seq,Homo_633,AD.vs.MCI; bulk RNA-	8
BP	GO:0016072	rRNA metabolic process	bulk RNA-seq,Homo_723,AD.vs.control; bulk RNA-seq,Homo_723,AD.vs.MCI; bulk RNA-seq,Homo_723,MCI.vs.control; bulk RNA-seq,Homo_714,AD.vs.control; bulk RNA-seq,Homo_714,AD.vs.MCI; bulk RNA-seq,Homo_633,AD.vs.control; bulk RNA-seq,Homo_633,AD.vs.MCI; bulk RNA-	8
BP	GO:0008033	tRNA processing	bulk RNA-seq,Homo_723,AD.vs.control; bulk RNA-seq,Homo_723,AD.vs.MCI; bulk RNA-seq,Homo_723,MCI.vs.control; bulk RNA-seq,Homo_714,AD.vs.control; bulk RNA-seq,Homo_714,AD.vs.MCI; bulk RNA-seq,Homo_633,AD.vs.control; bulk RNA-seq,Homo_633,AD.vs.MCI	7
MF	GO:0034987	immunoglobulin receptor binding	bulk RNA-seq,Homo_723,AD.vs.control; bulk RNA-seq,Homo_723,AD.vs.MCI; bulk RNA-seq,Homo_714,AD.vs.control; bulk RNA-seq,Homo_714,AD.vs.MCI; bulk RNA-seq,Homo_633,AD.vs.control; bulk RNA-seq,Homo_633,AD.vs.MCI; bulk RNA-seq,MCSA,MCI.vs.control; scRNA-seq,SRP330776,Memory T cell_2-AD.vs.control; scRNA-seq,SRP330776,Naive CD8+ T cell_2-AD.vs.control; scRNA-seq,SRP309935,Dendritic cell-AD.vs.control; scRNA-seq,SRP309935,Megakaryocyte_1-AD.vs.control; scRNA-seq,SRP309935,Monocyte_2-AD.vs.control; scRNA-seq,SRP309935,Naive CD8+ T cell_1-AD.vs.control	13
CC	GO:0044391	ribosomal subunit	bulk RNA-seq,Homo_723,AD.vs.control; bulk RNA-seq,Homo_723,AD.vs.MCI; bulk RNA-seq,Homo_723,MCI.vs.control; bulk RNA-seq,Homo_714,AD.vs.control; bulk RNA-seq,Homo_714,AD.vs.MCI; bulk RNA-seq,Homo_633,AD.vs.control; bulk RNA-seq,Homo_633,AD.vs.MCI; bulk RNA-seq,SRP223445,AD.vs.control; bulk RNA-seq,SRP325058,AD.vs.control; scRNA-seq,SRP330776,B cell_4-AD.vs.control; scRNA-	11
CC	GO:0042571	immunoglobulin complex, circulating	bulk RNA-seq,Homo_723,AD.vs.control; bulk RNA-seq,Homo_723,AD.vs.MCI; bulk RNA-seq,Homo_714,AD.vs.control; bulk RNA-seq,Homo_714,AD.vs.MCI; bulk RNA-seq,Homo_633,AD.vs.control; bulk RNA-seq,Homo_633,AD.vs.MCI; bulk RNA-seq,MCSA,MCI.vs.control; bulk RNA-seq,SRP223445,AD.vs.control; scRNA-seq,SRP330776,CD8+ T cell_1-AD.vs.control; scRNA-seq,SRP330776,Naive CD8+ T cell_1-AD.vs.control; scRNA-seq,SRP330776,Naive CD8+ T cell_2-AD.vs.control; scRNA-seq,SRP309935,Dendritic cell-AD.vs.control; scRNA-seq,SRP309935,Megakaryocyte_1-AD.vs.control; scRNA-seq,SRP309935,Monocyte_2-AD.vs.control; scRNA-seq,SRP309935,Naive CD8+ T cell_1-AD.vs.control; scRNA-seq,SRP309935,Natural killer cell_1-	16

BP	GO:0050871	positive regulation of B cell activation	bulk RNA-seq,Homo_723,AD.vs.control; bulk RNA-seq,Homo_723,AD.vs.MCI; bulk RNA-seq,Homo_714,AD.vs.control; bulk RNA-seq,Homo_714,AD.vs.MCI; bulk RNA-seq,Homo_633,AD.vs.control; bulk RNA-seq,Homo_633,AD.vs.MCI; bulk RNA-seq,MCSA,MCI.vs.control; scRNA-seq,SRP330776,Naive CD8+ T cell_2-AD.vs.control; scRNA-seq,SRP309935,Monocyte_2-AD.vs.control	9
CC	GO:0071013	catalytic step 2 spliceosome	bulk RNA-seq,Homo_723,AD.vs.control; bulk RNA-seq,Homo_723,AD.vs.MCI; bulk RNA-seq,Homo_723,MCI.vs.control; bulk RNA-seq,Homo_714,AD.vs.control; bulk RNA-seq,Homo_714,AD.vs.MCI; bulk RNA-seq,Homo_714,MCI.vs.control; bulk RNA-seq,Homo_633,AD.vs.control; bulk	8
MF	GO:0140101	catalytic activity, acting on a tRNA	bulk RNA-seq,Homo_723,AD.vs.control; bulk RNA-seq,Homo_723,AD.vs.MCI; bulk RNA-seq,Homo_723,MCI.vs.control; bulk RNA-seq,Homo_714,AD.vs.control; bulk RNA-seq,Homo_714,AD.vs.MCI; bulk RNA-seq,Homo_714,MCI.vs.control; bulk RNA-seq,Homo_633,AD.vs.control; bulk	8
CC	GO:0000315	organellar large ribosomal subunit	bulk RNA-seq,Homo_723,AD.vs.control; bulk RNA-seq,Homo_723,AD.vs.MCI; bulk RNA-seq,Homo_723,MCI.vs.control; bulk RNA-seq,Homo_714,AD.vs.control; bulk RNA-seq,Homo_714,AD.vs.MCI; bulk RNA-seq,Homo_714,MCI.vs.control; bulk RNA-seq,Homo_633,AD.vs.control; bulk	8
CC	GO:0005762	mitochondrial large ribosomal subunit	bulk RNA-seq,Homo_723,AD.vs.control; bulk RNA-seq,Homo_723,AD.vs.MCI; bulk RNA-seq,Homo_723,MCI.vs.control; bulk RNA-seq,Homo_714,AD.vs.control; bulk RNA-seq,Homo_714,AD.vs.MCI; bulk RNA-seq,Homo_714,MCI.vs.control; bulk RNA-seq,Homo_633,AD.vs.control; bulk	8
BP	GO:0050853	B cell receptor signaling pathway	bulk RNA-seq,Homo_723,AD.vs.control; bulk RNA-seq,Homo_723,AD.vs.MCI; bulk RNA-seq,Homo_714,AD.vs.control; bulk RNA-seq,Homo_714,AD.vs.MCI; bulk RNA-seq,Homo_633,AD.vs.control; bulk RNA-seq,Homo_633,AD.vs.MCI; bulk RNA-seq,MCSA,MCI.vs.control; scRNA-seq,SRP330776,Naive CD8+ T cell_2-AD.vs.control; scRNA-seq,SRP309935,Dendritic cell-AD.vs.control; scRNA-seq,SRP309935,Monocyte_2-AD.vs.control; scRNA-seq,SRP215507,CD8+ T	11
CC	GO:0098798	mitochondrial protein-containing complex	bulk RNA-seq,Homo_723,AD.vs.control; bulk RNA-seq,Homo_723,AD.vs.MCI; bulk RNA-seq,Homo_723,MCI.vs.control; bulk RNA-seq,Homo_714,AD.vs.control; bulk RNA-seq,Homo_714,AD.vs.MCI; bulk RNA-seq,Homo_633,AD.vs.control; bulk RNA-seq,Homo_633,AD.vs.MCI; scRNA-seq,SRP215507,CD8+ T cell_4-MCI.vs.control; scRNA-seq,SRP215507,CD8+ T cell_8-MCI.vs.control	9
BP	GO:0002440	production of molecular mediator of immune response	bulk RNA-seq,Homo_723,AD.vs.control; bulk RNA-seq,Homo_723,AD.vs.MCI; bulk RNA-seq,Homo_714,AD.vs.control; bulk RNA-seq,Homo_714,AD.vs.MCI; bulk RNA-seq,Homo_633,AD.vs.control; bulk RNA-seq,Homo_633,AD.vs.MCI; bulk RNA-seq,MCSA,MCI.vs.control; scRNA-seq,SRP330776,Naive CD8+ T cell_2-AD.vs.control; scRNA-seq,SRP215507,CD8+ T cell_3-AD.vs.control	9
BP	GO:0009451	RNA modification	bulk RNA-seq,Homo_723,AD.vs.control; bulk RNA-seq,Homo_723,AD.vs.MCI; bulk RNA-seq,Homo_723,MCI.vs.control; bulk RNA-seq,Homo_714,AD.vs.control; bulk RNA-seq,Homo_714,AD.vs.MCI; bulk RNA-seq,Homo_633,AD.vs.control; bulk RNA-seq,Homo_633,AD.vs.MCI	7
MF	GO:0003735	structural constituent of ribosome	bulk RNA-seq,Homo_723,AD.vs.control; bulk RNA-seq,Homo_723,AD.vs.MCI; bulk RNA-seq,Homo_723,MCI.vs.control; bulk RNA-seq,Homo_714,AD.vs.control; bulk RNA-seq,Homo_714,AD.vs.MCI; bulk RNA-seq,Homo_633,AD.vs.control; bulk RNA-seq,Homo_633,AD.vs.MCI; bulk RNA-seq,ROSMAP,MCI.vs.control; bulk RNA-seq,SRP325058,AD.vs.control; scRNA-seq,SRP330776,Megakaryocyte_2-AD.vs.control; scRNA-seq,SRP215507,CD8+	11
CC	GO:0071011	precatalytic spliceosome	bulk RNA-seq,Homo_723,AD.vs.control; bulk RNA-seq,Homo_723,AD.vs.MCI; bulk RNA-seq,Homo_723,MCI.vs.control; bulk RNA-seq,Homo_714,AD.vs.control; bulk RNA-seq,Homo_714,AD.vs.MCI; bulk RNA-seq,Homo_714,MCI.vs.control; bulk RNA-seq,Homo_633,AD.vs.control; bulk	8
CC	GO:0098802	plasma membrane signaling receptor complex	bulk RNA-seq,Homo_723,AD.vs.control; bulk RNA-seq,Homo_723,AD.vs.MCI; bulk RNA-seq,Homo_714,AD.vs.control; bulk RNA-seq,Homo_714,AD.vs.MCI; bulk RNA-seq,Homo_633,AD.vs.control; bulk RNA-seq,Homo_633,AD.vs.MCI; scRNA-seq,SRP330776,Natural killer cell_1-AD.vs.control; scRNA-seq,SRP215507,CD8+ T cell_1-AD.vs.control; scRNA-seq,SRP215507,CD8+ T cell_1-MCI.vs.control; scRNA-seq,SRP215507,CD8+ T cell_4-AD.vs.MCI	10
BP	GO:0050864	regulation of B cell activation	bulk RNA-seq,Homo_723,AD.vs.control; bulk RNA-seq,Homo_723,AD.vs.MCI; bulk RNA-seq,Homo_714,AD.vs.control; bulk RNA-seq,Homo_714,AD.vs.MCI; bulk RNA-seq,Homo_633,AD.vs.control; bulk RNA-seq,Homo_633,AD.vs.MCI; bulk RNA-seq,MCSA,MCI.vs.control; scRNA-seq,SRP330776,Naive CD8+ T cell_2-AD.vs.control; scRNA-seq,SRP309935,Monocyte_2-AD.vs.control	9
CC	GO:0005681	spliceosomal complex	bulk RNA-seq,Homo_723,AD.vs.control; bulk RNA-seq,Homo_723,AD.vs.MCI; bulk RNA-seq,Homo_723,MCI.vs.control; bulk RNA-seq,Homo_714,AD.vs.control; bulk RNA-seq,Homo_714,AD.vs.MCI; bulk RNA-seq,Homo_633,AD.vs.control; bulk RNA-seq,Homo_633,AD.vs.MCI; scRNA-	8
BP	GO:0050851	antigen receptor-mediated signaling pathway	bulk RNA-seq,Homo_723,AD.vs.control; bulk RNA-seq,Homo_723,AD.vs.MCI; bulk RNA-seq,Homo_723,MCI.vs.control; bulk RNA-seq,Homo_714,AD.vs.control; bulk RNA-seq,Homo_714,AD.vs.MCI; bulk RNA-seq,Homo_633,AD.vs.control; bulk RNA-seq,Homo_633,AD.vs.MCI; bulk RNA-seq,MCSA,MCI.vs.control; scRNA-seq,SRP330776,Naive CD8+ T cell_2-AD.vs.control; scRNA-seq,SRP309935,Monocyte_2-AD.vs.control; scRNA-	11
CC	GO:0071005	U2-type precatalytic spliceosome	bulk RNA-seq,Homo_723,AD.vs.control; bulk RNA-seq,Homo_723,AD.vs.MCI; bulk RNA-seq,Homo_723,MCI.vs.control; bulk RNA-seq,Homo_714,AD.vs.control; bulk RNA-seq,Homo_714,AD.vs.MCI; bulk RNA-seq,Homo_714,MCI.vs.control; bulk RNA-seq,Homo_633,AD.vs.control; bulk	8
BP	GO:0016064	immunoglobulin mediated immune response	bulk RNA-seq,Homo_723,AD.vs.control; bulk RNA-seq,Homo_723,AD.vs.MCI; bulk RNA-seq,Homo_723,MCI.vs.control; bulk RNA-seq,Homo_714,AD.vs.control; bulk RNA-seq,Homo_714,AD.vs.MCI; bulk RNA-seq,Homo_633,AD.vs.control; bulk RNA-seq,Homo_633,AD.vs.MCI; scRNA-seq,SRP330776,Naive CD8+ T cell_2-AD.vs.control; scRNA-seq,SRP309935,Monocyte_2-AD.vs.control; scRNA-seq,SRP215507,CD8+ T cell_3-AD.vs.control;	11
BP	GO:0019724	B cell mediated immunity	bulk RNA-seq,Homo_723,AD.vs.control; bulk RNA-seq,Homo_723,AD.vs.MCI; bulk RNA-seq,Homo_723,MCI.vs.control; bulk RNA-seq,Homo_714,AD.vs.control; bulk RNA-seq,Homo_714,AD.vs.MCI; bulk RNA-seq,Homo_633,AD.vs.control; bulk RNA-seq,Homo_633,AD.vs.MCI; scRNA-seq,SRP330776,Naive CD8+ T cell_2-AD.vs.control; scRNA-seq,SRP309935,Monocyte_2-AD.vs.control; scRNA-seq,SRP215507,CD8+ T cell_3-AD.vs.control	10
BP	GO:0006400	tRNA modification	bulk RNA-seq,Homo_723,AD.vs.control; bulk RNA-seq,Homo_723,AD.vs.MCI; bulk RNA-seq,Homo_723,MCI.vs.control; bulk RNA-seq,Homo_714,AD.vs.control; bulk RNA-seq,Homo_714,AD.vs.MCI; bulk RNA-seq,Homo_714,MCI.vs.control; bulk RNA-seq,Homo_633,AD.vs.control; bulk	8
BP	GO:0002449	lymphocyte mediated immunity	bulk RNA-seq,Homo_723,AD.vs.control; bulk RNA-seq,Homo_723,AD.vs.MCI; bulk RNA-seq,Homo_714,AD.vs.control; bulk RNA-seq,Homo_714,AD.vs.MCI; bulk RNA-seq,Homo_633,AD.vs.control; bulk RNA-seq,Homo_633,AD.vs.MCI; scRNA-seq,SRP330776,Naive CD8+ T cell_2-AD.vs.control; scRNA-seq,SRP309935,Monocyte_2-AD.vs.control; scRNA-seq,SRP215507,CD8+ T cell_3-AD.vs.control	9
BP	GO:0001510	RNA methylation	bulk RNA-seq,Homo_723,AD.vs.control; bulk RNA-seq,Homo_723,AD.vs.MCI; bulk RNA-seq,Homo_723,MCI.vs.control; bulk RNA-seq,Homo_714,AD.vs.control; bulk RNA-seq,Homo_714,AD.vs.MCI; bulk RNA-seq,Homo_714,MCI.vs.control; bulk RNA-seq,Homo_633,AD.vs.control; bulk	8
BP	GO:0006368	transcription elongation by RNA polymerase II promoter	bulk RNA-seq,Homo_723,AD.vs.control; bulk RNA-seq,Homo_723,AD.vs.MCI; bulk RNA-seq,Homo_723,MCI.vs.control; bulk RNA-seq,Homo_714,AD.vs.control; bulk RNA-seq,Homo_714,MCI.vs.control; bulk RNA-seq,Homo_633,AD.vs.control; bulk RNA-seq,Homo_633,AD.vs.MCI	7
BP	GO:0048663	neuron fate commitment	bulk RNA-seq,Homo_723,AD.vs.control; bulk RNA-seq,Homo_723,AD.vs.MCI; bulk RNA-seq,Homo_723,MCI.vs.control; bulk RNA-seq,Homo_714,AD.vs.control; bulk RNA-seq,Homo_714,AD.vs.MCI; bulk RNA-seq,Homo_633,MCI.vs.control; bulk RNA-seq,SRP223445,AD.vs.control	7
BP	GO:0050911	detection of chemical stimulus involved in sensory perception of smell	bulk RNA-seq,Homo_723,AD.vs.control; bulk RNA-seq,Homo_723,AD.vs.MCI; bulk RNA-seq,Homo_723,MCI.vs.control; bulk RNA-seq,Homo_714,AD.vs.control; bulk RNA-seq,Homo_714,AD.vs.MCI; bulk RNA-seq,Homo_714,MCI.vs.control; bulk RNA-seq,Homo_633,MCI.vs.control; bulk RNA-seq,ROSMAP,AD.vs.MCI; bulk RNA-seq,SRP223445,AD.vs.control	9
MF	GO:0004984	olfactory receptor activity	bulk RNA-seq,Homo_723,AD.vs.control; bulk RNA-seq,Homo_723,AD.vs.MCI; bulk RNA-seq,Homo_723,MCI.vs.control; bulk RNA-seq,Homo_714,AD.vs.control; bulk RNA-seq,Homo_714,AD.vs.MCI; bulk RNA-seq,Homo_714,MCI.vs.control; bulk RNA-seq,Homo_633,MCI.vs.control; bulk RNA-seq,ROSMAP,AD.vs.MCI; bulk RNA-seq,SRP223445,AD.vs.control	9
MF	GO:0005549	odorant binding	bulk RNA-seq,Homo_723,AD.vs.control; bulk RNA-seq,Homo_723,AD.vs.MCI; bulk RNA-seq,Homo_723,MCI.vs.control; bulk RNA-seq,Homo_714,AD.vs.control; bulk RNA-seq,Homo_714,AD.vs.MCI; bulk RNA-seq,Homo_714,MCI.vs.control; bulk RNA-seq,Homo_633,MCI.vs.control; bulk RNA-seq,ROSMAP,AD.vs.control; bulk RNA-seq,ROSMAP,AD.vs.MCI; bulk RNA-seq,SRP223445,AD.vs.control	10

BP	GO:0007608	sensory perception of smell	bulk RNA-seq,Homo_723,AD.vs.control; bulk RNA-seq,Homo_723,AD.vs.MCI; bulk RNA-seq,Homo_723,MCI.vs.control; bulk RNA-seq,Homo_714,AD.vs.control; bulk RNA-seq,Homo_714,AD.vs.MCI; bulk RNA-seq,Homo_714,MCI.vs.control; bulk RNA-seq,Homo_633,MCI.vs.control; bulk RNA-seq,ROSMAP,AD.vs.MCI; bulk RNA-seq,SRP223445,AD.vs.control	9
CC	GO:0099061	integral component of postsynaptic density membrane	bulk RNA-seq,Homo_723,AD.vs.control; bulk RNA-seq,Homo_723,AD.vs.MCI; bulk RNA-seq,Homo_723,MCI.vs.control; bulk RNA-seq,Homo_714,AD.vs.control; bulk RNA-seq,Homo_714,AD.vs.MCI; bulk RNA-seq,Homo_633,MCI.vs.control	6
CC	GO:0005840	ribosome	bulk RNA-seq,Homo_723,AD.vs.control; bulk RNA-seq,Homo_723,AD.vs.MCI; bulk RNA-seq,Homo_723,MCI.vs.control; bulk RNA-seq,Homo_714,AD.vs.control; bulk RNA-seq,Homo_714,AD.vs.MCI; bulk RNA-seq,Homo_633,AD.vs.control; bulk RNA-seq,Homo_633,AD.vs.MCI; bulk RNA-seq,SRP325058,AD.vs.control; scRNA-seq,SRP330776,Megakaryocyte_2-AD.vs.control	9
BP	GO:0002381	immunoglobulin production involved in immunoglobulin-mediated immune response	bulk RNA-seq,Homo_723,AD.vs.control; bulk RNA-seq,Homo_723,AD.vs.MCI; bulk RNA-seq,Homo_723,MCI.vs.control; bulk RNA-seq,Homo_714,AD.vs.control; bulk RNA-seq,Homo_714,MCI.vs.control; bulk RNA-seq,Homo_633,AD.vs.control; bulk RNA-seq,Homo_633,AD.vs.MCI; scRNA-seq,SRP215507,CD8+ T cell_3-AD.vs.control; scRNA-seq,SRP215507,CD8+ T cell_3-MCI.vs.control	9
CC	GO:0060076	excitatory synapse	bulk RNA-seq,Homo_723,AD.vs.control; bulk RNA-seq,Homo_723,AD.vs.MCI; bulk RNA-seq,Homo_723,MCI.vs.control; bulk RNA-seq,Homo_714,AD.vs.control; bulk RNA-seq,Homo_714,AD.vs.MCI; bulk RNA-seq,SRP223445,AD.vs.control	6
BP	GO:0050907	detection of chemical stimulus involved in sensory perception	bulk RNA-seq,Homo_723,AD.vs.control; bulk RNA-seq,Homo_723,AD.vs.MCI; bulk RNA-seq,Homo_723,MCI.vs.control; bulk RNA-seq,Homo_714,AD.vs.control; bulk RNA-seq,Homo_714,AD.vs.MCI; bulk RNA-seq,Homo_714,MCI.vs.control; bulk RNA-seq,Homo_633,MCI.vs.control; bulk RNA-seq,ROSMAP,AD.vs.MCI; bulk RNA-seq,SRP223445,AD.vs.control	9
CC	GO:0099146	intrinsic component of postsynaptic density membrane	bulk RNA-seq,Homo_723,AD.vs.control; bulk RNA-seq,Homo_723,AD.vs.MCI; bulk RNA-seq,Homo_723,MCI.vs.control; bulk RNA-seq,Homo_714,AD.vs.control; bulk RNA-seq,Homo_714,AD.vs.MCI; bulk RNA-seq,Homo_633,MCI.vs.control	6
BP	GO:0048665	neuron fate specification	bulk RNA-seq,Homo_723,AD.vs.control; bulk RNA-seq,Homo_723,AD.vs.MCI; bulk RNA-seq,Homo_723,MCI.vs.control; bulk RNA-seq,Homo_714,AD.vs.control; bulk RNA-seq,Homo_714,AD.vs.MCI	5
BP	GO:0009593	detection of chemical stimulus	bulk RNA-seq,Homo_723,AD.vs.control; bulk RNA-seq,Homo_723,AD.vs.MCI; bulk RNA-seq,Homo_723,MCI.vs.control; bulk RNA-seq,Homo_714,AD.vs.control; bulk RNA-seq,Homo_714,AD.vs.MCI; bulk RNA-seq,Homo_714,MCI.vs.control; bulk RNA-seq,Homo_633,MCI.vs.control; bulk RNA-seq,ROSMAP,AD.vs.MCI; bulk RNA-seq,SRP223445,AD.vs.control	9
BP	GO:0021536	diencephalon development	bulk RNA-seq,Homo_723,AD.vs.control; bulk RNA-seq,Homo_723,AD.vs.MCI; bulk RNA-seq,Homo_723,MCI.vs.control; bulk RNA-seq,Homo_714,AD.vs.control; bulk RNA-seq,Homo_714,AD.vs.MCI; bulk RNA-seq,Homo_633,MCI.vs.control	6
BP	GO:0021761	limbic system development	bulk RNA-seq,Homo_723,AD.vs.control; bulk RNA-seq,Homo_723,AD.vs.MCI; bulk RNA-seq,Homo_723,MCI.vs.control; bulk RNA-seq,Homo_714,AD.vs.control; bulk RNA-seq,Homo_714,AD.vs.MCI; bulk RNA-seq,Homo_714,MCI.vs.control; bulk RNA-seq,Homo_633,AD.vs.control; bulk RNA-seq,Homo_633,AD.vs.MCI; bulk RNA-seq,Homo_633,MCI.vs.control	9
CC	GO:1904813	ficolin-1-rich granule lumen	bulk RNA-seq,Homo_723,AD.vs.control; bulk RNA-seq,Homo_723,AD.vs.MCI; bulk RNA-seq,Homo_714,AD.vs.control; bulk RNA-seq,Homo_714,AD.vs.MCI; bulk RNA-seq,Homo_633,AD.vs.control; bulk RNA-seq,Homo_633,AD.vs.MCI; bulk RNA-seq,SRP223445,AD.vs.control	7
BP	GO:0099068	postsynapse assembly	bulk RNA-seq,Homo_723,AD.vs.control; bulk RNA-seq,Homo_723,AD.vs.MCI; bulk RNA-seq,Homo_723,MCI.vs.control; bulk RNA-seq,Homo_714,AD.vs.MCI; bulk RNA-seq,Homo_714,MCI.vs.control	5
BP	GO:2000179	positive regulation of neural precursor cell proliferation	bulk RNA-seq,Homo_723,AD.vs.control; bulk RNA-seq,Homo_723,AD.vs.MCI; bulk RNA-seq,Homo_723,MCI.vs.control; bulk RNA-seq,Homo_714,AD.vs.control; bulk RNA-seq,Homo_714,AD.vs.MCI; bulk RNA-seq,Homo_714,MCI.vs.control	6
BP	GO:0002456	T cell mediated immunity	bulk RNA-seq,Homo_723,AD.vs.control; bulk RNA-seq,Homo_723,AD.vs.MCI; bulk RNA-seq,Homo_723,MCI.vs.control; bulk RNA-seq,Homo_714,AD.vs.control; bulk RNA-seq,Homo_714,MCI.vs.control; bulk RNA-seq,Homo_633,AD.vs.control; bulk RNA-seq,Homo_633,AD.vs.MCI	7
BP	GO:0021953	central nervous system neuron differentiation	bulk RNA-seq,Homo_723,AD.vs.control; bulk RNA-seq,Homo_723,AD.vs.MCI; bulk RNA-seq,Homo_723,MCI.vs.control; bulk RNA-seq,Homo_714,AD.vs.control; bulk RNA-seq,Homo_714,AD.vs.MCI; bulk RNA-seq,Homo_633,AD.vs.control; bulk RNA-seq,Homo_633,AD.vs.MCI; bulk RNA-seq,ROSMAP,AD.vs.control; bulk RNA-seq,SRP223445,AD.vs.control	9
BP	GO:0099084	postsynaptic specialization organization	bulk RNA-seq,Homo_723,AD.vs.control; bulk RNA-seq,Homo_723,AD.vs.MCI; bulk RNA-seq,Homo_723,MCI.vs.control; bulk RNA-seq,Homo_714,AD.vs.control; bulk RNA-seq,Homo_714,AD.vs.MCI; bulk RNA-seq,Homo_714,MCI.vs.control	6
BP	GO:2000027	regulation of animal organ morphogenesis	bulk RNA-seq,Homo_723,AD.vs.control; bulk RNA-seq,Homo_723,AD.vs.MCI; bulk RNA-seq,Homo_723,MCI.vs.control; bulk RNA-seq,Homo_714,AD.vs.control; bulk RNA-seq,Homo_714,AD.vs.MCI; bulk RNA-seq,SRP223445,AD.vs.control	6
CC	GO:0098878	neurotransmitter receptor complex	bulk RNA-seq,Homo_723,AD.vs.control; bulk RNA-seq,Homo_723,AD.vs.MCI; bulk RNA-seq,Homo_723,MCI.vs.control; bulk RNA-seq,Homo_714,AD.vs.control; bulk RNA-seq,Homo_714,AD.vs.MCI	5
BP	GO:0002052	positive regulation of neuroblast proliferation	bulk RNA-seq,Homo_723,AD.vs.control; bulk RNA-seq,Homo_723,AD.vs.MCI; bulk RNA-seq,Homo_723,MCI.vs.control; bulk RNA-seq,Homo_714,AD.vs.control; bulk RNA-seq,Homo_714,AD.vs.MCI; bulk RNA-seq,Homo_714,MCI.vs.control	6
BP	GO:0000956	nuclear-transcribed mRNA catabolic process	bulk RNA-seq,Homo_723,AD.vs.control; bulk RNA-seq,Homo_723,AD.vs.MCI; bulk RNA-seq,Homo_723,MCI.vs.control; bulk RNA-seq,Homo_714,AD.vs.control; bulk RNA-seq,Homo_714,AD.vs.MCI; bulk RNA-seq,Homo_714,MCI.vs.control; bulk RNA-seq,Homo_633,AD.vs.control; bulk	8
BP	GO:0006958	complement activation, classical pathway	bulk RNA-seq,Homo_723,AD.vs.control; bulk RNA-seq,Homo_723,AD.vs.MCI; bulk RNA-seq,Homo_714,AD.vs.control; bulk RNA-seq,Homo_714,AD.vs.MCI; bulk RNA-seq,Homo_633,AD.vs.control; bulk RNA-seq,Homo_633,AD.vs.MCI; scRNA-seq,SRP330776,Naive CD8+ T cell_1-AD.vs.control; scRNA-seq,SRP330776,Naive CD8+ T cell_2-AD.vs.control; scRNA-seq,SRP309935,Monocyte_2-AD.vs.control; scRNA-seq,SRP309935,Naive CD8+ T cell_1-	10
BP	GO:0097106	postsynaptic density organization	bulk RNA-seq,Homo_723,AD.vs.control; bulk RNA-seq,Homo_723,AD.vs.MCI; bulk RNA-seq,Homo_723,MCI.vs.control; bulk RNA-seq,Homo_714,AD.vs.control; bulk RNA-seq,Homo_714,AD.vs.MCI; bulk RNA-seq,Homo_714,MCI.vs.control	6
BP	GO:0071542	dopaminergic neuron differentiation	bulk RNA-seq,Homo_723,AD.vs.control; bulk RNA-seq,Homo_723,AD.vs.MCI; bulk RNA-seq,Homo_723,MCI.vs.control; bulk RNA-seq,Homo_714,AD.vs.control; bulk RNA-seq,Homo_714,AD.vs.MCI; bulk RNA-seq,Homo_633,MCI.vs.control; bulk RNA-seq,SRP223445,AD.vs.control	7
BP	GO:0021872	forebrain generation of neurons	bulk RNA-seq,Homo_723,AD.vs.control; bulk RNA-seq,Homo_723,AD.vs.MCI; bulk RNA-seq,Homo_723,MCI.vs.control; bulk RNA-seq,Homo_714,AD.vs.control; bulk RNA-seq,Homo_714,AD.vs.MCI; bulk RNA-seq,Homo_714,MCI.vs.control; bulk RNA-seq,Homo_633,MCI.vs.control; bulk	8
BP	GO:2000177	regulation of neural precursor cell proliferation	bulk RNA-seq,Homo_723,AD.vs.control; bulk RNA-seq,Homo_723,AD.vs.MCI; bulk RNA-seq,Homo_723,MCI.vs.control; bulk RNA-seq,Homo_714,AD.vs.control; bulk RNA-seq,Homo_714,AD.vs.MCI; bulk RNA-seq,Homo_714,MCI.vs.control; bulk RNA-seq,Homo_633,AD.vs.control; bulk	8
BP	GO:0099054	presynapse assembly	bulk RNA-seq,Homo_723,AD.vs.control; bulk RNA-seq,Homo_723,AD.vs.MCI; bulk RNA-seq,Homo_723,MCI.vs.control; bulk RNA-seq,Homo_714,AD.vs.control; bulk RNA-seq,Homo_714,AD.vs.MCI; bulk RNA-seq,Homo_714,MCI.vs.control; bulk RNA-seq,ROSMAP,MCI.vs.control	7
BP	GO:0002455	humoral immune response mediated by circulating immunoglobulin	bulk RNA-seq,Homo_723,AD.vs.control; bulk RNA-seq,Homo_723,AD.vs.MCI; bulk RNA-seq,Homo_714,AD.vs.control; bulk RNA-seq,Homo_714,AD.vs.MCI; bulk RNA-seq,Homo_633,AD.vs.control; bulk RNA-seq,Homo_633,AD.vs.MCI; scRNA-seq,SRP330776,Naive CD8+ T cell_1-AD.vs.control; scRNA-	8

BP	GO:0035249	synaptic transmission, glutamatergic	bulk RNA-seq,Homo_723,AD.vs.control; bulk RNA-seq,Homo_723,AD.vs.MCI; bulk RNA-seq,Homo_723,MCI.vs.control; bulk RNA-seq,Homo_714,AD.vs.control; bulk RNA-seq,Homo_714,AD.vs.MCI; bulk RNA-seq,Homo_714,MCI.vs.control; bulk RNA-seq,Homo_633,AD.vs.control; bulk RNA-seq,Homo_633,AD.vs.MCI; bulk RNA-seq,ROSMAP,MCI.vs.control	9
CC	GO:0008328	ionotropic glutamate receptor complex	bulk RNA-seq,Homo_723,AD.vs.control; bulk RNA-seq,Homo_723,AD.vs.MCI; bulk RNA-seq,Homo_723,MCI.vs.control; bulk RNA-seq,Homo_714,AD.vs.control; bulk RNA-seq,Homo_714,AD.vs.MCI	5
BP	GO:0002824	positive regulation of adaptive immune response based on somatic recombination of immune receptors built from immunoglobulin superfamily domains	bulk RNA-seq,Homo_723,AD.vs.control; bulk RNA-seq,Homo_723,AD.vs.MCI; bulk RNA-seq,Homo_723,MCI.vs.control; bulk RNA-seq,Homo_714,AD.vs.control; bulk RNA-seq,Homo_714,MCI.vs.control; bulk RNA-seq,Homo_633,AD.vs.control; bulk RNA-seq,Homo_633,AD.vs.MCI	7
BP	GO:0001823	mesonephros development	bulk RNA-seq,Homo_723,AD.vs.control; bulk RNA-seq,Homo_723,AD.vs.MCI; bulk RNA-seq,Homo_723,MCI.vs.control; bulk RNA-seq,Homo_714,AD.vs.control; bulk RNA-seq,Homo_714,AD.vs.MCI; bulk RNA-seq,Homo_714,MCI.vs.control; bulk RNA-seq,Homo_633,AD.vs.MCI; bulk RNA-	8
BP	GO:0002429	immune response-activating cell surface receptor signaling pathway	bulk RNA-seq,Homo_723,AD.vs.control; bulk RNA-seq,Homo_723,AD.vs.MCI; bulk RNA-seq,Homo_723,MCI.vs.control; bulk RNA-seq,Homo_714,AD.vs.control; bulk RNA-seq,Homo_633,AD.vs.control; bulk RNA-seq,Homo_633,AD.vs.MCI; scRNA-seq,SRP330776,Naive CD8+ T cell_2-	8
BP	GO:0002757	immune response-activating signal transduction	bulk RNA-seq,Homo_723,AD.vs.control; bulk RNA-seq,Homo_723,AD.vs.MCI; bulk RNA-seq,Homo_723,MCI.vs.control; bulk RNA-seq,Homo_714,AD.vs.control; bulk RNA-seq,Homo_633,AD.vs.control; bulk RNA-seq,Homo_633,AD.vs.MCI; scRNA-seq,SRP330776,Naive CD8+ T cell_2-	8
BP	GO:0090497	mesenchymal cell migration	bulk RNA-seq,Homo_723,AD.vs.control; bulk RNA-seq,Homo_723,AD.vs.MCI; bulk RNA-seq,Homo_723,MCI.vs.control; bulk RNA-seq,Homo_714,AD.vs.control; bulk RNA-seq,Homo_714,AD.vs.MCI; bulk RNA-seq,Homo_714,MCI.vs.control	6
BP	GO:0072163	mesonephric epithelium development	bulk RNA-seq,Homo_723,AD.vs.control; bulk RNA-seq,Homo_723,AD.vs.MCI; bulk RNA-seq,Homo_723,MCI.vs.control; bulk RNA-seq,Homo_714,AD.vs.control; bulk RNA-seq,Homo_714,AD.vs.MCI; bulk RNA-seq,Homo_714,MCI.vs.control; bulk RNA-seq,SRP223445,AD.vs.control	7
BP	GO:0072164	mesonephric tubule development	bulk RNA-seq,Homo_723,AD.vs.control; bulk RNA-seq,Homo_723,AD.vs.MCI; bulk RNA-seq,Homo_723,MCI.vs.control; bulk RNA-seq,Homo_714,AD.vs.control; bulk RNA-seq,Homo_714,AD.vs.MCI; bulk RNA-seq,Homo_714,MCI.vs.control; bulk RNA-seq,SRP223445,AD.vs.control	7
BP	GO:0072073	kidney epithelium development	bulk RNA-seq,Homo_723,AD.vs.control; bulk RNA-seq,Homo_723,AD.vs.MCI; bulk RNA-seq,Homo_723,MCI.vs.control; bulk RNA-seq,Homo_714,AD.vs.control; bulk RNA-seq,Homo_714,AD.vs.MCI; bulk RNA-seq,Homo_633,AD.vs.control; bulk RNA-seq,Homo_633,AD.vs.MCI; bulk RNA-seq,ROSMAP,AD.vs.control; bulk RNA-seq,SRP223445,AD.vs.control	9
CC	GO:0098800	inner mitochondrial membrane protein complex	bulk RNA-seq,Homo_723,AD.vs.control; bulk RNA-seq,Homo_723,AD.vs.MCI; bulk RNA-seq,Homo_723,MCI.vs.control; bulk RNA-seq,Homo_714,AD.vs.control; bulk RNA-seq,Homo_714,AD.vs.MCI; bulk RNA-seq,Homo_633,AD.vs.control; bulk RNA-seq,Homo_633,AD.vs.MCI	7
BP	GO:0001657	ureteric bud development	bulk RNA-seq,Homo_723,AD.vs.control; bulk RNA-seq,Homo_723,AD.vs.MCI; bulk RNA-seq,Homo_723,MCI.vs.control; bulk RNA-seq,Homo_714,AD.vs.control; bulk RNA-seq,Homo_714,AD.vs.MCI; bulk RNA-seq,Homo_714,MCI.vs.control; bulk RNA-seq,ROSMAP,AD.vs.control; bulk	8
BP	GO:0050919	negative chemotaxis	bulk RNA-seq,Homo_723,AD.vs.control; bulk RNA-seq,Homo_723,AD.vs.MCI; bulk RNA-seq,Homo_723,MCI.vs.control; bulk RNA-seq,Homo_714,AD.vs.control; bulk RNA-seq,Homo_714,AD.vs.MCI; bulk RNA-seq,Homo_714,MCI.vs.control	6
BP	GO:0051965	positive regulation of synapse assembly	bulk RNA-seq,Homo_723,AD.vs.control; bulk RNA-seq,Homo_723,AD.vs.MCI; bulk RNA-seq,Homo_723,MCI.vs.control; bulk RNA-seq,Homo_714,AD.vs.MCI	4
BP	GO:0042596	fear response	bulk RNA-seq,Homo_723,AD.vs.control; bulk RNA-seq,Homo_723,AD.vs.MCI; bulk RNA-seq,Homo_723,MCI.vs.control; bulk RNA-seq,Homo_714,AD.vs.control; bulk RNA-seq,Homo_714,AD.vs.MCI; bulk RNA-seq,Homo_714,MCI.vs.control	6
BP	GO:0051963	regulation of synapse assembly	bulk RNA-seq,Homo_723,AD.vs.control; bulk RNA-seq,Homo_723,AD.vs.MCI; bulk RNA-seq,Homo_723,MCI.vs.control; bulk RNA-seq,Homo_714,AD.vs.control; bulk RNA-seq,Homo_714,AD.vs.MCI; bulk RNA-seq,Homo_714,MCI.vs.control; bulk RNA-seq,Homo_633,AD.vs.MCI; bulk RNA-seq,ROSMAP,AD.vs.control; bulk RNA-seq,ROSMAP,MCI.vs.control	9
BP	GO:0033555	multicellular organismal response to stress	bulk RNA-seq,Homo_723,AD.vs.control; bulk RNA-seq,Homo_723,AD.vs.MCI; bulk RNA-seq,Homo_723,MCI.vs.control; bulk RNA-seq,Homo_714,AD.vs.control; bulk RNA-seq,Homo_714,AD.vs.MCI; bulk RNA-seq,Homo_714,MCI.vs.control; bulk RNA-seq,Homo_633,AD.vs.control; bulk	8
BP	GO:0002708	positive regulation of lymphocyte mediated immunity	bulk RNA-seq,Homo_723,AD.vs.control; bulk RNA-seq,Homo_723,AD.vs.MCI; bulk RNA-seq,Homo_723,MCI.vs.control; bulk RNA-seq,Homo_714,AD.vs.control; bulk RNA-seq,Homo_714,MCI.vs.control; bulk RNA-seq,Homo_633,AD.vs.control; bulk RNA-seq,Homo_633,AD.vs.MCI	7
MF	GO:0043021	ribonucleoprotein complex binding	bulk RNA-seq,Homo_723,AD.vs.control; bulk RNA-seq,Homo_723,MCI.vs.control; bulk RNA-seq,Homo_714,AD.vs.control; bulk RNA-seq,Homo_633,AD.vs.control; bulk RNA-seq,Homo_633,AD.vs.MCI	5
BP	GO:0002706	regulation of lymphocyte mediated immunity	bulk RNA-seq,Homo_723,AD.vs.control; bulk RNA-seq,Homo_723,AD.vs.MCI; bulk RNA-seq,Homo_723,MCI.vs.control; bulk RNA-seq,Homo_714,AD.vs.control; bulk RNA-seq,Homo_633,AD.vs.control; bulk RNA-seq,Homo_633,AD.vs.MCI	6
BP	GO:0007157	heterophilic cell-cell adhesion via plasma membrane cell adhesion molecules	bulk RNA-seq,Homo_723,AD.vs.control; bulk RNA-seq,Homo_723,AD.vs.MCI; bulk RNA-seq,Homo_723,MCI.vs.control; bulk RNA-seq,Homo_714,AD.vs.control; bulk RNA-seq,Homo_714,AD.vs.MCI	5
BP	GO:0021879	forebrain neuron differentiation	bulk RNA-seq,Homo_723,AD.vs.control; bulk RNA-seq,Homo_723,AD.vs.MCI; bulk RNA-seq,Homo_723,MCI.vs.control; bulk RNA-seq,Homo_714,AD.vs.control; bulk RNA-seq,Homo_714,AD.vs.MCI; bulk RNA-seq,Homo_714,MCI.vs.control; bulk RNA-seq,Homo_633,MCI.vs.control	7
BP	GO:0002768	immune response-regulating cell surface receptor signaling pathway	bulk RNA-seq,Homo_723,AD.vs.control; bulk RNA-seq,Homo_723,AD.vs.MCI; bulk RNA-seq,Homo_723,MCI.vs.control; bulk RNA-seq,Homo_714,AD.vs.control; bulk RNA-seq,Homo_633,AD.vs.control; bulk RNA-seq,Homo_633,AD.vs.MCI; scRNA-seq,SRP330776,Naive CD8+ T cell_2-	8
BP	GO:0006354	DNA-templated transcription elongation	bulk RNA-seq,Homo_723,AD.vs.control; bulk RNA-seq,Homo_723,MCI.vs.control; bulk RNA-seq,Homo_714,AD.vs.control; bulk RNA-seq,Homo_633,AD.vs.control; bulk RNA-seq,Homo_633,AD.vs.MCI; scRNA-seq,SRP309935,Megakaryocyte_2-AD.vs.control	6
BP	GO:0072009	nephron epithelium development	bulk RNA-seq,Homo_723,AD.vs.control; bulk RNA-seq,Homo_723,AD.vs.MCI; bulk RNA-seq,Homo_723,MCI.vs.control; bulk RNA-seq,Homo_714,AD.vs.control; bulk RNA-seq,Homo_714,AD.vs.MCI; bulk RNA-seq,Homo_714,MCI.vs.control; bulk RNA-seq,SRP223445,AD.vs.control	7
BP	GO:0001755	neural crest cell migration	bulk RNA-seq,Homo_723,AD.vs.control; bulk RNA-seq,Homo_723,AD.vs.MCI; bulk RNA-seq,Homo_723,MCI.vs.control; bulk RNA-seq,Homo_714,AD.vs.control; bulk RNA-seq,Homo_714,AD.vs.MCI; bulk RNA-seq,Homo_714,MCI.vs.control	6
BP	GO:0001662	behavioral fear response	bulk RNA-seq,Homo_723,AD.vs.control; bulk RNA-seq,Homo_723,AD.vs.MCI; bulk RNA-seq,Homo_723,MCI.vs.control; bulk RNA-seq,Homo_714,AD.vs.control; bulk RNA-seq,Homo_714,AD.vs.MCI; bulk RNA-seq,Homo_714,MCI.vs.control	6
BP	GO:0072655	establishment of protein localization to mitochondrion	bulk RNA-seq,Homo_723,AD.vs.control; bulk RNA-seq,Homo_723,MCI.vs.control; bulk RNA-seq,Homo_714,AD.vs.control; bulk RNA-seq,Homo_714,MCI.vs.control; bulk RNA-seq,Homo_633,AD.vs.control; bulk RNA-seq,Homo_633,AD.vs.MCI	6
BP	GO:0035637	multicellular organismal signaling	bulk RNA-seq,Homo_723,AD.vs.control; bulk RNA-seq,Homo_723,AD.vs.MCI; bulk RNA-seq,Homo_723,MCI.vs.control; bulk RNA-seq,Homo_714,AD.vs.control; bulk RNA-seq,Homo_714,AD.vs.MCI; bulk RNA-seq,Homo_633,AD.vs.control; bulk RNA-seq,ROSMAP,MCI.vs.control; bulk	8
BP	GO:0014032	neural crest cell development	bulk RNA-seq,Homo_723,AD.vs.control; bulk RNA-seq,Homo_723,AD.vs.MCI; bulk RNA-seq,Homo_723,MCI.vs.control; bulk RNA-seq,Homo_714,AD.vs.control; bulk RNA-seq,Homo_714,AD.vs.MCI; bulk RNA-seq,Homo_714,MCI.vs.control	6
BP	GO:0099172	presynapse organization	bulk RNA-seq,Homo_723,AD.vs.control; bulk RNA-seq,Homo_723,AD.vs.MCI; bulk RNA-seq,Homo_723,MCI.vs.control; bulk RNA-seq,Homo_714,AD.vs.control; bulk RNA-seq,Homo_714,AD.vs.MCI; bulk RNA-seq,Homo_714,MCI.vs.control; bulk RNA-seq,ROSMAP,MCI.vs.control	7

BP	GO:0048565	digestive tract development	bulk RNA-seq,Homo_723,AD.vs.control; bulk RNA-seq,Homo_723,AD.vs.MCI; bulk RNA-seq,Homo_723,MCI.vs.control; bulk RNA-seq,Homo_714,AD.vs.control; bulk RNA-seq,Homo_714,AD.vs.MCI; bulk RNA-seq,SRP223445,AD.vs.control	6
BP	GO:0090501	RNA phosphodiester bond hydrolysis	bulk RNA-seq,Homo_723,AD.vs.control; bulk RNA-seq,Homo_723,AD.vs.MCI; bulk RNA-seq,Homo_723,MCI.vs.control; bulk RNA-seq,Homo_714,AD.vs.control; bulk RNA-seq,Homo_714,AD.vs.MCI; bulk RNA-seq,Homo_633,AD.vs.control; bulk RNA-seq,Homo_633,AD.vs.MCI	7
MF	GO:0005254	chloride channel activity	bulk RNA-seq,Homo_723,AD.vs.control; bulk RNA-seq,Homo_723,AD.vs.MCI; bulk RNA-seq,Homo_723,MCI.vs.control; bulk RNA-seq,Homo_714,AD.vs.control; bulk RNA-seq,Homo_714,AD.vs.MCI; bulk RNA-seq,Homo_714,MCI.vs.control; bulk RNA-seq,ROSMAP,MCI.vs.control	7
BP	GO:0007405	neuroblast proliferation	bulk RNA-seq,Homo_723,AD.vs.control; bulk RNA-seq,Homo_723,AD.vs.MCI; bulk RNA-seq,Homo_723,MCI.vs.control; bulk RNA-seq,Homo_714,AD.vs.control; bulk RNA-seq,Homo_714,AD.vs.MCI; bulk RNA-seq,Homo_714,MCI.vs.control; bulk RNA-seq,Homo_633,AD.vs.MCI	7
BP	GO:0060675	ureteric bud morphogenesis	bulk RNA-seq,Homo_723,AD.vs.control; bulk RNA-seq,Homo_723,AD.vs.MCI; bulk RNA-seq,Homo_723,MCI.vs.control; bulk RNA-seq,Homo_714,AD.vs.control; bulk RNA-seq,Homo_714,AD.vs.MCI; bulk RNA-seq,Homo_714,MCI.vs.control; bulk RNA-seq,SRP223445,AD.vs.control	7
BP	GO:0061351	neural precursor cell proliferation	bulk RNA-seq,Homo_723,AD.vs.control; bulk RNA-seq,Homo_723,AD.vs.MCI; bulk RNA-seq,Homo_723,MCI.vs.control; bulk RNA-seq,Homo_714,AD.vs.control; bulk RNA-seq,Homo_714,AD.vs.MCI; bulk RNA-seq,Homo_633,AD.vs.control; bulk RNA-seq,Homo_633,AD.vs.MCI; bulk RNA-	8
BP	GO:0007218	neuropeptide signaling pathway	bulk RNA-seq,Homo_723,AD.vs.control; bulk RNA-seq,Homo_723,AD.vs.MCI; bulk RNA-seq,Homo_723,MCI.vs.control; bulk RNA-seq,Homo_714,AD.vs.control; bulk RNA-seq,Homo_714,AD.vs.MCI; bulk RNA-seq,Homo_633,MCI.vs.control; bulk RNA-seq,SRP223445,AD.vs.control	7
CC	GO:0098839	postsynaptic density membrane	bulk RNA-seq,Homo_723,AD.vs.control; bulk RNA-seq,Homo_723,AD.vs.MCI; bulk RNA-seq,Homo_723,MCI.vs.control; bulk RNA-seq,Homo_714,AD.vs.control; bulk RNA-seq,Homo_714,AD.vs.MCI; bulk RNA-seq,Homo_633,AD.vs.control; bulk RNA-seq,Homo_633,AD.vs.MCI; bulk RNA-	8
BP	GO:0099504	synaptic vesicle cycle	bulk RNA-seq,Homo_723,AD.vs.control; bulk RNA-seq,Homo_723,AD.vs.MCI; bulk RNA-seq,Homo_723,MCI.vs.control; bulk RNA-seq,Homo_714,AD.vs.control; bulk RNA-seq,Homo_714,AD.vs.MCI; bulk RNA-seq,Homo_633,AD.vs.control; bulk RNA-seq,Homo_633,AD.vs.MCI; bulk RNA-seq,ROSMAP,AD.vs.control; bulk RNA-seq,ROSMAP,MCI.vs.control	9
BP	GO:0072078	nephron tubule morphogenesis	bulk RNA-seq,Homo_723,AD.vs.control; bulk RNA-seq,Homo_723,AD.vs.MCI; bulk RNA-seq,Homo_723,MCI.vs.control; bulk RNA-seq,Homo_714,AD.vs.control; bulk RNA-seq,Homo_714,AD.vs.MCI; bulk RNA-seq,Homo_714,MCI.vs.control; bulk RNA-seq,SRP223445,AD.vs.control	7
BP	GO:0051952	regulation of amine transport	bulk RNA-seq,Homo_723,AD.vs.control; bulk RNA-seq,Homo_723,AD.vs.MCI; bulk RNA-seq,Homo_723,MCI.vs.control; bulk RNA-seq,Homo_714,AD.vs.control; bulk RNA-seq,Homo_714,AD.vs.MCI; bulk RNA-seq,Homo_633,MCI.vs.control	6
BP	GO:0021675	nerve development	bulk RNA-seq,Homo_723,AD.vs.control; bulk RNA-seq,Homo_723,AD.vs.MCI; bulk RNA-seq,Homo_723,MCI.vs.control; bulk RNA-seq,Homo_714,AD.vs.control; bulk RNA-seq,Homo_714,AD.vs.MCI; bulk RNA-seq,Homo_714,MCI.vs.control; bulk RNA-seq,Homo_633,AD.vs.MCI	7
BP	GO:0060688	regulation of morphogenesis of a branching structure	bulk RNA-seq,Homo_723,AD.vs.control; bulk RNA-seq,Homo_723,AD.vs.MCI; bulk RNA-seq,Homo_723,MCI.vs.control; bulk RNA-seq,Homo_714,AD.vs.control; bulk RNA-seq,Homo_714,AD.vs.MCI; bulk RNA-seq,Homo_714,MCI.vs.control; bulk RNA-seq,SRP223445,AD.vs.control	7
BP	GO:0072028	nephron morphogenesis	bulk RNA-seq,Homo_723,AD.vs.control; bulk RNA-seq,Homo_723,AD.vs.MCI; bulk RNA-seq,Homo_723,MCI.vs.control; bulk RNA-seq,Homo_714,AD.vs.control; bulk RNA-seq,Homo_714,AD.vs.MCI; bulk RNA-seq,Homo_714,MCI.vs.control; bulk RNA-seq,SRP223445,AD.vs.control	7
BP	GO:0009953	dorsal/ventral pattern formation	bulk RNA-seq,Homo_723,AD.vs.control; bulk RNA-seq,Homo_723,AD.vs.MCI; bulk RNA-seq,Homo_723,MCI.vs.control; bulk RNA-seq,Homo_714,AD.vs.control; bulk RNA-seq,Homo_714,AD.vs.MCI; bulk RNA-seq,Homo_714,MCI.vs.control; bulk RNA-seq,Homo_633,AD.vs.control; bulk	8
BP	GO:0019226	transmission of nerve impulse	bulk RNA-seq,Homo_723,AD.vs.control; bulk RNA-seq,Homo_723,AD.vs.MCI; bulk RNA-seq,Homo_723,MCI.vs.control; bulk RNA-seq,Homo_714,AD.vs.control; bulk RNA-seq,Homo_714,AD.vs.MCI; bulk RNA-seq,Homo_633,MCI.vs.control; bulk RNA-seq,ROSMAP,MCI.vs.control; bulk	8
BP	GO:0060993	kidney morphogenesis	bulk RNA-seq,Homo_723,AD.vs.control; bulk RNA-seq,Homo_723,AD.vs.MCI; bulk RNA-seq,Homo_723,MCI.vs.control; bulk RNA-seq,Homo_714,AD.vs.control; bulk RNA-seq,Homo_714,AD.vs.MCI; bulk RNA-seq,Homo_714,MCI.vs.control; bulk RNA-seq,SRP223445,AD.vs.control	7
BP	GO:1901890	positive regulation of cell junction assembly	bulk RNA-seq,Homo_723,AD.vs.control; bulk RNA-seq,Homo_723,AD.vs.MCI; bulk RNA-seq,Homo_723,MCI.vs.control; bulk RNA-seq,Homo_714,AD.vs.control; bulk RNA-seq,Homo_714,AD.vs.MCI; bulk RNA-seq,Homo_714,MCI.vs.control; bulk RNA-seq,Homo_633,AD.vs.control; bulk RNA-seq,Homo_633,AD.vs.MCI; bulk RNA-seq,ROSMAP,AD.vs.control	9
BP	GO:0001658	branching involved in ureteric bud morphogenesis	bulk RNA-seq,Homo_723,AD.vs.control; bulk RNA-seq,Homo_723,AD.vs.MCI; bulk RNA-seq,Homo_723,MCI.vs.control; bulk RNA-seq,Homo_714,AD.vs.control; bulk RNA-seq,Homo_714,AD.vs.MCI; bulk RNA-seq,Homo_714,MCI.vs.control; bulk RNA-seq,SRP223445,AD.vs.control	7
BP	GO:0072171	mesonephric tubule morphogenesis	bulk RNA-seq,Homo_723,AD.vs.control; bulk RNA-seq,Homo_723,AD.vs.MCI; bulk RNA-seq,Homo_723,MCI.vs.control; bulk RNA-seq,Homo_714,AD.vs.control; bulk RNA-seq,Homo_714,AD.vs.MCI; bulk RNA-seq,Homo_714,MCI.vs.control; bulk RNA-seq,SRP223445,AD.vs.control	7
BP	GO:0050770	regulation of axonogenesis	bulk RNA-seq,Homo_723,AD.vs.control; bulk RNA-seq,Homo_723,AD.vs.MCI; bulk RNA-seq,Homo_723,MCI.vs.control; bulk RNA-seq,Homo_714,AD.vs.control; bulk RNA-seq,Homo_714,AD.vs.MCI; bulk RNA-seq,Homo_633,AD.vs.control; bulk RNA-seq,Homo_633,AD.vs.MCI; bulk RNA-	8
BP	GO:0061333	renal tubule morphogenesis	bulk RNA-seq,Homo_723,AD.vs.control; bulk RNA-seq,Homo_723,AD.vs.MCI; bulk RNA-seq,Homo_723,MCI.vs.control; bulk RNA-seq,Homo_714,AD.vs.control; bulk RNA-seq,Homo_714,AD.vs.MCI; bulk RNA-seq,Homo_714,MCI.vs.control; bulk RNA-seq,SRP223445,AD.vs.control	7
BP	GO:0072088	nephron epithelium morphogenesis	bulk RNA-seq,Homo_723,AD.vs.control; bulk RNA-seq,Homo_723,AD.vs.MCI; bulk RNA-seq,Homo_723,MCI.vs.control; bulk RNA-seq,Homo_714,AD.vs.control; bulk RNA-seq,Homo_714,AD.vs.MCI; bulk RNA-seq,Homo_714,MCI.vs.control; bulk RNA-seq,SRP223445,AD.vs.control	7
BP	GO:0048864	stem cell development	bulk RNA-seq,Homo_723,AD.vs.control; bulk RNA-seq,Homo_723,AD.vs.MCI; bulk RNA-seq,Homo_723,MCI.vs.control; bulk RNA-seq,Homo_714,AD.vs.control; bulk RNA-seq,Homo_714,AD.vs.MCI; bulk RNA-seq,Homo_714,MCI.vs.control	6
BP	GO:0099003	vesicle-mediated transport in synapse	bulk RNA-seq,Homo_723,AD.vs.control; bulk RNA-seq,Homo_723,AD.vs.MCI; bulk RNA-seq,Homo_723,MCI.vs.control; bulk RNA-seq,Homo_714,AD.vs.control; bulk RNA-seq,Homo_714,AD.vs.MCI; bulk RNA-seq,Homo_633,AD.vs.control; bulk RNA-seq,Homo_633,AD.vs.MCI; bulk RNA-seq,ROSMAP,AD.vs.control; bulk RNA-seq,ROSMAP,MCI.vs.control	9
BP	GO:0048483	autonomic nervous system development	bulk RNA-seq,Homo_723,AD.vs.control; bulk RNA-seq,Homo_723,AD.vs.MCI; bulk RNA-seq,Homo_723,MCI.vs.control; bulk RNA-seq,Homo_714,AD.vs.control; bulk RNA-seq,Homo_714,AD.vs.MCI; bulk RNA-seq,Homo_714,MCI.vs.control	6
BP	GO:0090305	nucleic acid phosphodiester bond hydrolysis	bulk RNA-seq,Homo_723,AD.vs.control; bulk RNA-seq,Homo_723,AD.vs.MCI; bulk RNA-seq,Homo_723,MCI.vs.control; bulk RNA-seq,Homo_714,AD.vs.control; bulk RNA-seq,Homo_633,AD.vs.control; bulk RNA-seq,Homo_633,AD.vs.MCI	6
MF	GO:0005253	anion channel activity	bulk RNA-seq,Homo_723,AD.vs.control; bulk RNA-seq,Homo_723,AD.vs.MCI; bulk RNA-seq,Homo_723,MCI.vs.control; bulk RNA-seq,Homo_714,AD.vs.control; bulk RNA-seq,Homo_714,AD.vs.MCI; bulk RNA-seq,Homo_714,MCI.vs.control	6
BP	GO:0061326	renal tubule development	bulk RNA-seq,Homo_723,AD.vs.control; bulk RNA-seq,Homo_723,AD.vs.MCI; bulk RNA-seq,Homo_723,MCI.vs.control; bulk RNA-seq,Homo_714,AD.vs.control; bulk RNA-seq,Homo_714,AD.vs.MCI; bulk RNA-seq,Homo_714,MCI.vs.control; bulk RNA-seq,SRP223445,AD.vs.control	7
BP	GO:0099173	postsynapse organization	bulk RNA-seq,Homo_723,AD.vs.control; bulk RNA-seq,Homo_723,AD.vs.MCI; bulk RNA-seq,Homo_723,MCI.vs.control; bulk RNA-seq,Homo_714,AD.vs.control; bulk RNA-seq,Homo_714,AD.vs.MCI; bulk RNA-seq,Homo_633,AD.vs.control; bulk RNA-seq,Homo_633,AD.vs.MCI; scRNA-	8
CC	GO:0044306	neuron projection terminus	bulk RNA-seq,Homo_723,AD.vs.control; bulk RNA-seq,Homo_723,AD.vs.MCI; bulk RNA-seq,Homo_723,MCI.vs.control; bulk RNA-seq,Homo_714,AD.vs.control; bulk RNA-seq,Homo_714,AD.vs.MCI; bulk RNA-seq,Homo_633,AD.vs.control; bulk RNA-seq,Homo_633,AD.vs.MCI; bulk RNA-	8

CC	GO:0005882	intermediate filament	bulk RNA-seq,Homo_723,AD.vs.control; bulk RNA-seq,Homo_723,AD.vs.MCI; bulk RNA-seq,Homo_714,AD.vs.control; bulk RNA-seq,Homo_714,AD.vs.MCI; bulk RNA-seq,Homo_633,MCI.vs.control; bulk RNA-seq,SRP223445,AD.vs.control	6
BP	GO:1905330	regulation of morphogenesis of an epithelium	bulk RNA-seq,Homo_723,AD.vs.control; bulk RNA-seq,Homo_723,AD.vs.MCI; bulk RNA-seq,Homo_723,MCI.vs.control; bulk RNA-seq,Homo_714,AD.vs.control; bulk RNA-seq,Homo_714,AD.vs.MCI; bulk RNA-seq,Homo_714,MCI.vs.control; bulk RNA-seq,SRP223445,AD.vs.control	7
BP	GO:0031644	regulation of nervous system process	bulk RNA-seq,Homo_723,AD.vs.control; bulk RNA-seq,Homo_723,AD.vs.MCI; bulk RNA-seq,Homo_723,MCI.vs.control; bulk RNA-seq,Homo_714,AD.vs.control; bulk RNA-seq,Homo_714,AD.vs.MCI; bulk RNA-seq,Homo_633,AD.vs.control; bulk RNA-seq,Homo_633,AD.vs.MCI	7
BP	GO:0072006	nephron development	bulk RNA-seq,Homo_723,AD.vs.control; bulk RNA-seq,Homo_723,AD.vs.MCI; bulk RNA-seq,Homo_723,MCI.vs.control; bulk RNA-seq,Homo_714,AD.vs.control; bulk RNA-seq,Homo_714,AD.vs.MCI; bulk RNA-seq,Homo_633,AD.vs.control; bulk RNA-seq,Homo_633,AD.vs.MCI; bulk RNA-seq,ROSMAP,AD.vs.control; bulk RNA-seq,SRP223445,AD.vs.control	9
BP	GO:0072080	nephron tubule development	bulk RNA-seq,Homo_723,AD.vs.control; bulk RNA-seq,Homo_723,AD.vs.MCI; bulk RNA-seq,Homo_723,MCI.vs.control; bulk RNA-seq,Homo_714,AD.vs.control; bulk RNA-seq,Homo_714,AD.vs.MCI; bulk RNA-seq,Homo_714,MCI.vs.control; bulk RNA-seq,SRP223445,AD.vs.control	7
MF	GO:0022843	voltage-gated cation channel activity	bulk RNA-seq,Homo_723,AD.vs.control; bulk RNA-seq,Homo_723,AD.vs.MCI; bulk RNA-seq,Homo_723,MCI.vs.control; bulk RNA-seq,Homo_714,AD.vs.control; bulk RNA-seq,Homo_714,AD.vs.MCI; bulk RNA-seq,Homo_633,MCI.vs.control; bulk RNA-seq,ROSMAP,AD.vs.control; bulk	8
BP	GO:0021543	pallium development	bulk RNA-seq,Homo_723,AD.vs.control; bulk RNA-seq,Homo_723,AD.vs.MCI; bulk RNA-seq,Homo_723,MCI.vs.control; bulk RNA-seq,Homo_714,AD.vs.control; bulk RNA-seq,Homo_714,AD.vs.MCI; bulk RNA-seq,Homo_633,AD.vs.control; bulk RNA-seq,Homo_633,AD.vs.MCI; bulk RNA-	8
CC	GO:0043679	axon terminus	bulk RNA-seq,Homo_723,AD.vs.control; bulk RNA-seq,Homo_723,AD.vs.MCI; bulk RNA-seq,Homo_723,MCI.vs.control; bulk RNA-seq,Homo_714,AD.vs.control; bulk RNA-seq,Homo_714,AD.vs.MCI; bulk RNA-seq,Homo_714,MCI.vs.control; bulk RNA-seq,ROSMAP,MCI.vs.control	7
BP	GO:0021954	central nervous system neuron development	bulk RNA-seq,Homo_723,AD.vs.control; bulk RNA-seq,Homo_723,AD.vs.MCI; bulk RNA-seq,Homo_723,MCI.vs.control; bulk RNA-seq,Homo_714,AD.vs.control; bulk RNA-seq,Homo_714,AD.vs.MCI; bulk RNA-seq,Homo_714,MCI.vs.control; bulk RNA-seq,Homo_633,AD.vs.control; bulk	8
BP	GO:0007611	learning or memory	bulk RNA-seq,Homo_723,AD.vs.control; bulk RNA-seq,Homo_723,AD.vs.MCI; bulk RNA-seq,Homo_723,MCI.vs.control; bulk RNA-seq,Homo_714,AD.vs.control; bulk RNA-seq,Homo_714,AD.vs.MCI; bulk RNA-seq,Homo_633,AD.vs.control; bulk RNA-seq,Homo_633,AD.vs.MCI; bulk RNA-seq,ROSMAP,AD.vs.control; bulk RNA-seq,SRP223445,AD.vs.control	9
BP	GO:0021766	hippocampus development	bulk RNA-seq,Homo_723,AD.vs.control; bulk RNA-seq,Homo_723,AD.vs.MCI; bulk RNA-seq,Homo_723,MCI.vs.control; bulk RNA-seq,Homo_714,AD.vs.control; bulk RNA-seq,Homo_714,AD.vs.MCI; bulk RNA-seq,Homo_714,MCI.vs.control; bulk RNA-seq,Homo_633,AD.vs.control; bulk	8
CC	GO:0030672	synaptic vesicle membrane	bulk RNA-seq,Homo_723,AD.vs.control; bulk RNA-seq,Homo_723,AD.vs.MCI; bulk RNA-seq,Homo_723,MCI.vs.control; bulk RNA-seq,Homo_714,AD.vs.MCI; bulk RNA-seq,Homo_633,AD.vs.control; bulk RNA-seq,Homo_633,AD.vs.MCI; bulk RNA-seq,SRP223445,AD.vs.control	7
CC	GO:0099501	exocytic vesicle membrane	bulk RNA-seq,Homo_723,AD.vs.control; bulk RNA-seq,Homo_723,AD.vs.MCI; bulk RNA-seq,Homo_723,MCI.vs.control; bulk RNA-seq,Homo_714,AD.vs.MCI; bulk RNA-seq,Homo_633,AD.vs.control; bulk RNA-seq,Homo_633,AD.vs.MCI; bulk RNA-seq,SRP223445,AD.vs.control	7
BP	GO:0035050	embryonic heart tube development	bulk RNA-seq,Homo_723,AD.vs.control; bulk RNA-seq,Homo_723,AD.vs.MCI; bulk RNA-seq,Homo_723,MCI.vs.control; bulk RNA-seq,Homo_714,AD.vs.control; bulk RNA-seq,Homo_714,AD.vs.MCI; bulk RNA-seq,Homo_714,MCI.vs.control	6
BP	GO:0014033	neural crest cell differentiation	bulk RNA-seq,Homo_723,AD.vs.control; bulk RNA-seq,Homo_723,AD.vs.MCI; bulk RNA-seq,Homo_723,MCI.vs.control; bulk RNA-seq,Homo_714,AD.vs.control; bulk RNA-seq,Homo_714,AD.vs.MCI; bulk RNA-seq,Homo_714,MCI.vs.control; bulk RNA-seq,Homo_633,AD.vs.control; bulk	8
BP	GO:0006839	mitochondrial transport	bulk RNA-seq,Homo_723,AD.vs.control; bulk RNA-seq,Homo_723,MCI.vs.control; bulk RNA-seq,Homo_714,AD.vs.control; bulk RNA-seq,Homo_633,AD.vs.control; bulk RNA-seq,Homo_633,AD.vs.MCI	5
BP	GO:0060485	mesenchyme development	bulk RNA-seq,Homo_723,AD.vs.control; bulk RNA-seq,Homo_723,AD.vs.MCI; bulk RNA-seq,Homo_723,MCI.vs.control; bulk RNA-seq,Homo_714,AD.vs.control; bulk RNA-seq,Homo_714,AD.vs.MCI; bulk RNA-seq,Homo_633,AD.vs.control; bulk RNA-seq,Homo_633,AD.vs.MCI; bulk RNA-seq,ROSMAP,AD.vs.control; bulk RNA-seq,SRP223445,AD.vs.control	9
BP	GO:0051962	positive regulation of nervous system development	bulk RNA-seq,Homo_723,AD.vs.control; bulk RNA-seq,Homo_723,AD.vs.MCI; bulk RNA-seq,Homo_723,MCI.vs.control; bulk RNA-seq,Homo_714,AD.vs.control; bulk RNA-seq,Homo_714,AD.vs.MCI; bulk RNA-seq,Homo_633,AD.vs.control; bulk RNA-seq,Homo_633,AD.vs.MCI; bulk RNA-	8
BP	GO:0007626	locomotory behavior	bulk RNA-seq,Homo_723,AD.vs.control; bulk RNA-seq,Homo_723,AD.vs.MCI; bulk RNA-seq,Homo_723,MCI.vs.control; bulk RNA-seq,Homo_714,AD.vs.control; bulk RNA-seq,Homo_714,AD.vs.MCI; bulk RNA-seq,Homo_633,AD.vs.control; bulk RNA-seq,Homo_633,AD.vs.MCI; bulk RNA-	8
BP	GO:0055123	digestive system development	bulk RNA-seq,Homo_723,AD.vs.control; bulk RNA-seq,Homo_723,AD.vs.MCI; bulk RNA-seq,Homo_723,MCI.vs.control; bulk RNA-seq,Homo_714,AD.vs.control; bulk RNA-seq,Homo_714,AD.vs.MCI; bulk RNA-seq,SRP223445,AD.vs.control	6
MF	GO:0005249	voltage-gated potassium channel activity	bulk RNA-seq,Homo_723,AD.vs.control; bulk RNA-seq,Homo_723,AD.vs.MCI; bulk RNA-seq,Homo_723,MCI.vs.control; bulk RNA-seq,Homo_714,AD.vs.control; bulk RNA-seq,Homo_714,AD.vs.MCI; bulk RNA-seq,Homo_633,MCI.vs.control; bulk RNA-seq,SRP223445,AD.vs.control	7
BP	GO:0051961	negative regulation of nervous system development	bulk RNA-seq,Homo_723,AD.vs.control; bulk RNA-seq,Homo_723,AD.vs.MCI; bulk RNA-seq,Homo_723,MCI.vs.control; bulk RNA-seq,Homo_714,AD.vs.control; bulk RNA-seq,Homo_714,AD.vs.MCI; bulk RNA-seq,Homo_633,AD.vs.control; bulk RNA-seq,Homo_633,AD.vs.MCI	7
BP	GO:0007613	memory	bulk RNA-seq,Homo_723,AD.vs.control; bulk RNA-seq,Homo_723,AD.vs.MCI; bulk RNA-seq,Homo_723,MCI.vs.control; bulk RNA-seq,Homo_714,AD.vs.control; bulk RNA-seq,Homo_714,AD.vs.MCI; bulk RNA-seq,Homo_714,MCI.vs.control	6
BP	GO:0002819	regulation of adaptive immune response	bulk RNA-seq,Homo_723,AD.vs.control; bulk RNA-seq,Homo_723,MCI.vs.control; bulk RNA-seq,Homo_714,AD.vs.control; bulk RNA-seq,Homo_633,AD.vs.control; bulk RNA-seq,Homo_633,AD.vs.MCI	5
BP	GO:0051216	cartilage development	bulk RNA-seq,Homo_723,AD.vs.control; bulk RNA-seq,Homo_723,AD.vs.MCI; bulk RNA-seq,Homo_723,MCI.vs.control; bulk RNA-seq,Homo_714,AD.vs.control; bulk RNA-seq,Homo_714,AD.vs.MCI; bulk RNA-seq,Homo_633,AD.vs.control; bulk RNA-seq,Homo_633,AD.vs.MCI; bulk RNA-seq,SRP223445,AD.vs.control; scRNA-seq,SRP330776,Naive CD8+ T cell_2-AD.vs.control	9
CC	GO:0101002	ficolin-1-rich granule	bulk RNA-seq,Homo_723,AD.vs.control; bulk RNA-seq,Homo_723,AD.vs.MCI; bulk RNA-seq,Homo_714,AD.vs.control; bulk RNA-seq,Homo_633,AD.vs.control; bulk RNA-seq,Homo_633,AD.vs.MCI; bulk RNA-seq,SRP223445,AD.vs.control	6
BP	GO:0002065	columnar/cuboidal epithelial cell differentiation	bulk RNA-seq,Homo_723,AD.vs.control; bulk RNA-seq,Homo_723,AD.vs.MCI; bulk RNA-seq,Homo_723,MCI.vs.control; bulk RNA-seq,Homo_714,AD.vs.control; bulk RNA-seq,Homo_714,AD.vs.MCI; bulk RNA-seq,Homo_714,MCI.vs.control; bulk RNA-seq,SRP223445,AD.vs.control	7
BP	GO:0008344	adult locomotory behavior	bulk RNA-seq,Homo_723,AD.vs.control; bulk RNA-seq,Homo_723,AD.vs.MCI; bulk RNA-seq,Homo_723,MCI.vs.control; bulk RNA-seq,Homo_714,AD.vs.control; bulk RNA-seq,Homo_714,AD.vs.MCI; bulk RNA-seq,Homo_714,MCI.vs.control; bulk RNA-seq,Homo_633,AD.vs.control	7
BP	GO:0048762	mesenchymal cell differentiation	bulk RNA-seq,Homo_723,AD.vs.control; bulk RNA-seq,Homo_723,AD.vs.MCI; bulk RNA-seq,Homo_723,MCI.vs.control; bulk RNA-seq,Homo_714,AD.vs.control; bulk RNA-seq,Homo_714,AD.vs.MCI; bulk RNA-seq,Homo_633,AD.vs.control; bulk RNA-seq,Homo_633,AD.vs.MCI; bulk RNA-	8
BP	GO:0010721	negative regulation of cell development	bulk RNA-seq,Homo_723,AD.vs.control; bulk RNA-seq,Homo_723,AD.vs.MCI; bulk RNA-seq,Homo_723,MCI.vs.control; bulk RNA-seq,Homo_714,AD.vs.control; bulk RNA-seq,Homo_714,AD.vs.MCI; bulk RNA-seq,Homo_633,AD.vs.control; bulk RNA-seq,Homo_633,AD.vs.MCI; bulk RNA-	8

BP	GO:0035270	endocrine system development	bulk RNA-seq,Homo_723,AD.vs.control; bulk RNA-seq,Homo_723,AD.vs.MCI; bulk RNA-seq,Homo_723,MCI.vs.control; bulk RNA-seq,Homo_714,AD.vs.control; bulk RNA-seq,Homo_714,AD.vs.MCI; bulk RNA-seq,Homo_633,AD.vs.control; bulk RNA-seq,Homo_633,MCI.vs.control; bulk RNA-seq,SRP223445,AD.vs.control; scRNA-seq,SRP330776,Natural killer cell_1-AD.vs.control	9
BP	GO:0051960	regulation of nervous system development	bulk RNA-seq,Homo_723,AD.vs.control; bulk RNA-seq,Homo_723,AD.vs.MCI; bulk RNA-seq,Homo_714,AD.vs.control; bulk RNA-seq,Homo_714,AD.vs.MCI; bulk RNA-seq,Homo_633,AD.vs.control; bulk RNA-seq,Homo_633,AD.vs.MCI; bulk RNA-seq,ROSMAP,AD.vs.control; bulk RNA-seq,SRP223445,AD.vs.control	8
BP	GO:0050890	cognition	bulk RNA-seq,Homo_723,AD.vs.control; bulk RNA-seq,Homo_723,AD.vs.MCI; bulk RNA-seq,Homo_723,MCI.vs.control; bulk RNA-seq,Homo_714,AD.vs.control; bulk RNA-seq,Homo_714,AD.vs.MCI; bulk RNA-seq,Homo_633,AD.vs.control; bulk RNA-seq,Homo_633,AD.vs.MCI; bulk RNA-	8
BP	GO:0050768	negative regulation of neurogenesis	bulk RNA-seq,Homo_723,AD.vs.control; bulk RNA-seq,Homo_723,AD.vs.MCI; bulk RNA-seq,Homo_723,MCI.vs.control; bulk RNA-seq,Homo_714,AD.vs.control; bulk RNA-seq,Homo_714,AD.vs.MCI; bulk RNA-seq,Homo_633,AD.vs.control; bulk RNA-seq,Homo_633,AD.vs.MCI	7
BP	GO:0071774	response to fibroblast growth factor	bulk RNA-seq,Homo_723,AD.vs.control; bulk RNA-seq,Homo_723,AD.vs.MCI; bulk RNA-seq,Homo_723,MCI.vs.control; bulk RNA-seq,Homo_714,AD.vs.control; bulk RNA-seq,Homo_714,AD.vs.MCI; bulk RNA-seq,Homo_714,MCI.vs.control; bulk RNA-seq,Homo_633,AD.vs.control; bulk	8
BP	GO:0043524	negative regulation of neuron apoptotic process	bulk RNA-seq,Homo_723,AD.vs.control; bulk RNA-seq,Homo_723,AD.vs.MCI; bulk RNA-seq,Homo_723,MCI.vs.control; bulk RNA-seq,Homo_714,AD.vs.control; bulk RNA-seq,Homo_714,AD.vs.MCI; bulk RNA-seq,Homo_633,AD.vs.control; bulk RNA-seq,Homo_633,AD.vs.MCI	7
BP	GO:0044344	cellular response to fibroblast growth factor stimulus	bulk RNA-seq,Homo_723,AD.vs.control; bulk RNA-seq,Homo_723,AD.vs.MCI; bulk RNA-seq,Homo_723,MCI.vs.control; bulk RNA-seq,Homo_714,AD.vs.control; bulk RNA-seq,Homo_714,AD.vs.MCI; bulk RNA-seq,Homo_714,MCI.vs.control; bulk RNA-seq,Homo_633,AD.vs.control	7
BP	GO:0007612	learning	bulk RNA-seq,Homo_723,AD.vs.control; bulk RNA-seq,Homo_723,AD.vs.MCI; bulk RNA-seq,Homo_723,MCI.vs.control; bulk RNA-seq,Homo_714,AD.vs.control; bulk RNA-seq,Homo_714,AD.vs.MCI; bulk RNA-seq,Homo_633,AD.vs.control	6
BP	GO:0071826	ribonucleoprotein complex subunit organization	bulk RNA-seq,Homo_723,AD.vs.control; bulk RNA-seq,Homo_723,MCI.vs.control; bulk RNA-seq,Homo_714,AD.vs.control; bulk RNA-seq,Homo_714,AD.vs.MCI; bulk RNA-seq,Homo_633,AD.vs.control; bulk RNA-seq,Homo_633,AD.vs.MCI	6
BP	GO:0051588	regulation of neurotransmitter transport	bulk RNA-seq,Homo_723,AD.vs.control; bulk RNA-seq,Homo_723,AD.vs.MCI; bulk RNA-seq,Homo_723,MCI.vs.control; bulk RNA-seq,Homo_714,AD.vs.MCI; bulk RNA-seq,Homo_714,MCI.vs.control; bulk RNA-seq,Homo_633,AD.vs.control; bulk RNA-seq,Homo_633,AD.vs.MCI	7
BP	GO:0008543	fibroblast growth factor receptor signaling pathway	bulk RNA-seq,Homo_723,AD.vs.control; bulk RNA-seq,Homo_723,AD.vs.MCI; bulk RNA-seq,Homo_723,MCI.vs.control; bulk RNA-seq,Homo_714,AD.vs.control; bulk RNA-seq,Homo_714,AD.vs.MCI; bulk RNA-seq,Homo_714,MCI.vs.control	6
BP	GO:0007269	neurotransmitter secretion	bulk RNA-seq,Homo_723,AD.vs.control; bulk RNA-seq,Homo_723,AD.vs.MCI; bulk RNA-seq,Homo_723,MCI.vs.control; bulk RNA-seq,Homo_714,AD.vs.MCI; bulk RNA-seq,Homo_633,AD.vs.control; bulk RNA-seq,Homo_633,AD.vs.MCI	6
BP	GO:0099643	signal release from synapse	bulk RNA-seq,Homo_723,AD.vs.control; bulk RNA-seq,Homo_723,AD.vs.MCI; bulk RNA-seq,Homo_723,MCI.vs.control; bulk RNA-seq,Homo_714,AD.vs.MCI; bulk RNA-seq,Homo_633,AD.vs.control; bulk RNA-seq,Homo_633,AD.vs.MCI	6
BP	GO:0001508	action potential	bulk RNA-seq,Homo_723,AD.vs.control; bulk RNA-seq,Homo_723,AD.vs.MCI; bulk RNA-seq,Homo_723,MCI.vs.control; bulk RNA-seq,Homo_714,AD.vs.control; bulk RNA-seq,Homo_714,AD.vs.MCI; bulk RNA-seq,Homo_633,AD.vs.control; bulk RNA-seq,Homo_633,AD.vs.MCI	7
BP	GO:0007498	mesoderm development	bulk RNA-seq,Homo_723,AD.vs.control; bulk RNA-seq,Homo_723,AD.vs.MCI; bulk RNA-seq,Homo_723,MCI.vs.control; bulk RNA-seq,Homo_714,AD.vs.control; bulk RNA-seq,Homo_714,AD.vs.MCI; bulk RNA-seq,ROSMAP,AD.vs.control; bulk RNA-seq,SRP223445,AD.vs.control	7
BP	GO:1901888	regulation of cell junction assembly	bulk RNA-seq,Homo_723,AD.vs.control; bulk RNA-seq,Homo_723,AD.vs.MCI; bulk RNA-seq,Homo_723,MCI.vs.control; bulk RNA-seq,Homo_714,AD.vs.control; bulk RNA-seq,Homo_714,AD.vs.MCI; bulk RNA-seq,Homo_633,AD.vs.control; bulk RNA-seq,Homo_633,AD.vs.MCI; bulk RNA-seq,ROSMAP,AD.vs.control; bulk RNA-seq,ROSMAP,MCI.vs.control	9
BP	GO:0016079	synaptic vesicle exocytosis	bulk RNA-seq,Homo_723,AD.vs.control; bulk RNA-seq,Homo_723,AD.vs.MCI; bulk RNA-seq,Homo_723,MCI.vs.control; bulk RNA-seq,Homo_714,AD.vs.MCI; bulk RNA-seq,Homo_714,MCI.vs.control; bulk RNA-seq,Homo_633,AD.vs.control; bulk RNA-seq,Homo_633,AD.vs.MCI	7
MF	GO:0008083	growth factor activity	bulk RNA-seq,Homo_723,AD.vs.control; bulk RNA-seq,Homo_723,AD.vs.MCI; bulk RNA-seq,Homo_723,MCI.vs.control; bulk RNA-seq,Homo_714,AD.vs.control; bulk RNA-seq,Homo_714,AD.vs.MCI; bulk RNA-seq,SRP223445,AD.vs.control	6
BP	GO:0021510	spinal cord development	bulk RNA-seq,Homo_723,AD.vs.control; bulk RNA-seq,Homo_723,AD.vs.MCI; bulk RNA-seq,Homo_723,MCI.vs.control; bulk RNA-seq,Homo_714,AD.vs.control; bulk RNA-seq,Homo_714,AD.vs.MCI; bulk RNA-seq,Homo_714,MCI.vs.control; bulk RNA-seq,ROSMAP,AD.vs.control; bulk	8
BP	GO:0050806	positive regulation of synaptic transmission	bulk RNA-seq,Homo_723,AD.vs.control; bulk RNA-seq,Homo_723,AD.vs.MCI; bulk RNA-seq,Homo_723,MCI.vs.control; bulk RNA-seq,Homo_714,AD.vs.control; bulk RNA-seq,Homo_714,AD.vs.MCI; bulk RNA-seq,Homo_633,AD.vs.control; bulk RNA-seq,Homo_633,AD.vs.MCI	7
BP	GO:0048167	regulation of synaptic plasticity	bulk RNA-seq,Homo_723,AD.vs.control; bulk RNA-seq,Homo_723,AD.vs.MCI; bulk RNA-seq,Homo_723,MCI.vs.control; bulk RNA-seq,Homo_714,AD.vs.control; bulk RNA-seq,Homo_714,AD.vs.MCI; bulk RNA-seq,Homo_633,AD.vs.control; bulk RNA-seq,Homo_633,AD.vs.MCI; bulk RNA-seq,ROSMAP,AD.vs.control; scRNA-seq,SRP330776,Naive CD8+ T cell_3-AD.vs.control	9
BP	GO:0051592	response to calcium ion	bulk RNA-seq,Homo_723,AD.vs.control; bulk RNA-seq,Homo_723,AD.vs.MCI; bulk RNA-seq,Homo_723,MCI.vs.control; bulk RNA-seq,Homo_714,AD.vs.control; bulk RNA-seq,Homo_714,AD.vs.MCI; bulk RNA-seq,Homo_633,AD.vs.control; bulk RNA-seq,Homo_633,AD.vs.MCI	7
CC	GO:0045111	intermediate filament cytoskeleton	bulk RNA-seq,Homo_723,AD.vs.control; bulk RNA-seq,Homo_723,AD.vs.MCI; bulk RNA-seq,Homo_714,AD.vs.control; bulk RNA-seq,Homo_714,AD.vs.MCI; bulk RNA-seq,SRP223445,AD.vs.control	5
BP	GO:0061387	regulation of extent of cell growth	bulk RNA-seq,Homo_723,AD.vs.control; bulk RNA-seq,Homo_723,AD.vs.MCI; bulk RNA-seq,Homo_723,MCI.vs.control; bulk RNA-seq,Homo_714,AD.vs.control; bulk RNA-seq,Homo_714,AD.vs.MCI; bulk RNA-seq,Homo_714,MCI.vs.control; bulk RNA-seq,Homo_633,AD.vs.control; bulk	8
CC	GO:0005604	basement membrane	bulk RNA-seq,Homo_723,AD.vs.control; bulk RNA-seq,Homo_723,AD.vs.MCI; bulk RNA-seq,Homo_723,MCI.vs.control; bulk RNA-seq,Homo_714,AD.vs.control; bulk RNA-seq,Homo_714,AD.vs.MCI; bulk RNA-seq,Homo_714,MCI.vs.control; bulk RNA-seq,Homo_633,AD.vs.control; bulk RNA-seq,Homo_633,AD.vs.MCI; bulk RNA-seq,ROSMAP,AD.vs.control; bulk RNA-seq,ROSMAP,MCI.vs.control	10
BP	GO:0010975	regulation of neuron projection development	bulk RNA-seq,Homo_723,AD.vs.control; bulk RNA-seq,Homo_723,AD.vs.MCI; bulk RNA-seq,Homo_714,AD.vs.control; bulk RNA-seq,Homo_714,AD.vs.MCI; bulk RNA-seq,Homo_633,AD.vs.control; bulk RNA-seq,Homo_633,AD.vs.MCI; bulk RNA-seq,ROSMAP,AD.vs.control; bulk RNA-seq,ROSMAP,MCI.vs.control; bulk RNA-seq,SRP223445,AD.vs.control; scRNA-seq,SRP330776,Naive CD8+ T cell_2-AD.vs.control	10
BP	GO:0016358	dendrite development	bulk RNA-seq,Homo_723,AD.vs.control; bulk RNA-seq,Homo_723,AD.vs.MCI; bulk RNA-seq,Homo_723,MCI.vs.control; bulk RNA-seq,Homo_714,AD.vs.MCI; bulk RNA-seq,Homo_633,AD.vs.control; bulk RNA-seq,Homo_633,AD.vs.MCI; bulk RNA-seq,ROSMAP,MCI.vs.control	7
BP	GO:0022618	ribonucleoprotein complex assembly	bulk RNA-seq,Homo_723,AD.vs.control; bulk RNA-seq,Homo_723,MCI.vs.control; bulk RNA-seq,Homo_714,AD.vs.control; bulk RNA-seq,Homo_633,AD.vs.control; bulk RNA-seq,Homo_633,AD.vs.MCI	5
BP	GO:0003015	heart process	bulk RNA-seq,Homo_723,AD.vs.control; bulk RNA-seq,Homo_723,AD.vs.MCI; bulk RNA-seq,Homo_723,MCI.vs.control; bulk RNA-seq,Homo_714,AD.vs.control; bulk RNA-seq,Homo_714,AD.vs.MCI; bulk RNA-seq,Homo_633,AD.vs.control; bulk RNA-seq,Homo_633,AD.vs.MCI	7
BP	GO:0050905	neuromuscular process	bulk RNA-seq,Homo_723,AD.vs.control; bulk RNA-seq,Homo_723,AD.vs.MCI; bulk RNA-seq,Homo_723,MCI.vs.control; bulk RNA-seq,Homo_714,AD.vs.control; bulk RNA-seq,Homo_714,AD.vs.MCI; bulk RNA-seq,Homo_633,AD.vs.control; bulk RNA-seq,Homo_633,AD.vs.MCI; bulk RNA-	8

BP	GO:0003206	cardiac chamber morphogenesis	bulk RNA-seq,Homo_723,AD.vs.control; bulk RNA-seq,Homo_723,AD.vs.MCI; bulk RNA-seq,Homo_723,MCI.vs.control; bulk RNA-seq,Homo_714,AD.vs.control; bulk RNA-seq,Homo_714,AD.vs.MCI; bulk RNA-seq,Homo_714,MCI.vs.control; bulk RNA-seq,Homo_633,AD.vs.control; bulk	8
BP	GO:0008016	regulation of heart contraction	bulk RNA-seq,Homo_723,AD.vs.control; bulk RNA-seq,Homo_723,AD.vs.MCI; bulk RNA-seq,Homo_723,MCI.vs.control; bulk RNA-seq,Homo_714,AD.vs.control; bulk RNA-seq,Homo_714,AD.vs.MCI	5
BP	GO:0045088	regulation of innate immune response	bulk RNA-seq,Homo_723,AD.vs.control; bulk RNA-seq,Homo_723,MCI.vs.control; bulk RNA-seq,Homo_714,AD.vs.control; bulk RNA-seq,Homo_633,AD.vs.control; bulk RNA-seq,Homo_633,AD.vs.MCI; scRNA-seq,SRP309935,Megakaryocyte_2-AD.vs.control	6
BP	GO:0060047	heart contraction	bulk RNA-seq,Homo_723,AD.vs.control; bulk RNA-seq,Homo_723,AD.vs.MCI; bulk RNA-seq,Homo_723,MCI.vs.control; bulk RNA-seq,Homo_714,AD.vs.control; bulk RNA-seq,Homo_714,AD.vs.MCI; bulk RNA-seq,Homo_633,AD.vs.control	6
BP	GO:0030902	hindbrain development	bulk RNA-seq,Homo_723,AD.vs.control; bulk RNA-seq,Homo_723,AD.vs.MCI; bulk RNA-seq,Homo_723,MCI.vs.control; bulk RNA-seq,Homo_714,AD.vs.control; bulk RNA-seq,Homo_714,AD.vs.MCI; bulk RNA-seq,Homo_633,AD.vs.control; bulk RNA-seq,Homo_633,AD.vs.MCI; bulk RNA-	8
BP	GO:0048738	cardiac muscle tissue development	bulk RNA-seq,Homo_723,AD.vs.control; bulk RNA-seq,Homo_723,AD.vs.MCI; bulk RNA-seq,Homo_723,MCI.vs.control; bulk RNA-seq,Homo_714,AD.vs.control; bulk RNA-seq,Homo_714,AD.vs.MCI; bulk RNA-seq,SRP223445,AD.vs.control	6
MF	GO:0015079	potassium ion transmembrane transporter activity	bulk RNA-seq,Homo_723,AD.vs.control; bulk RNA-seq,Homo_723,AD.vs.MCI; bulk RNA-seq,Homo_723,MCI.vs.control; bulk RNA-seq,Homo_714,AD.vs.control; bulk RNA-seq,Homo_714,AD.vs.MCI; bulk RNA-seq,SRP223445,AD.vs.control	6
BP	GO:0043588	skin development	bulk RNA-seq,Homo_723,AD.vs.control; bulk RNA-seq,Homo_723,AD.vs.MCI; bulk RNA-seq,Homo_723,MCI.vs.control; bulk RNA-seq,Homo_714,AD.vs.control; bulk RNA-seq,Homo_714,AD.vs.MCI; bulk RNA-seq,Homo_633,AD.vs.control; bulk RNA-seq,Homo_633,AD.vs.MCI; bulk RNA-	8
BP	GO:0003205	cardiac chamber development	bulk RNA-seq,Homo_723,AD.vs.control; bulk RNA-seq,Homo_723,AD.vs.MCI; bulk RNA-seq,Homo_723,MCI.vs.control; bulk RNA-seq,Homo_714,AD.vs.control; bulk RNA-seq,Homo_714,AD.vs.MCI; bulk RNA-seq,Homo_633,AD.vs.control; bulk RNA-seq,SRP223445,AD.vs.control	7
BP	GO:0042476	odontogenesis	bulk RNA-seq,Homo_723,AD.vs.control; bulk RNA-seq,Homo_723,AD.vs.MCI; bulk RNA-seq,Homo_723,MCI.vs.control; bulk RNA-seq,Homo_714,AD.vs.control; bulk RNA-seq,Homo_714,AD.vs.MCI; bulk RNA-seq,SRP223445,AD.vs.control	6
BP	GO:0006401	RNA catabolic process	bulk RNA-seq,Homo_723,AD.vs.control; bulk RNA-seq,Homo_723,AD.vs.MCI; bulk RNA-seq,Homo_723,MCI.vs.control; bulk RNA-seq,Homo_714,AD.vs.control; bulk RNA-seq,Homo_633,AD.vs.control; bulk RNA-seq,Homo_633,AD.vs.MCI	6
BP	GO:1990138	neuron projection extension	bulk RNA-seq,Homo_723,AD.vs.control; bulk RNA-seq,Homo_723,AD.vs.MCI; bulk RNA-seq,Homo_723,MCI.vs.control; bulk RNA-seq,Homo_714,AD.vs.MCI; bulk RNA-seq,Homo_633,AD.vs.control; bulk RNA-seq,Homo_633,AD.vs.MCI	6
MF	GO:0048018	receptor ligand activity	bulk RNA-seq,Homo_723,AD.vs.control; bulk RNA-seq,Homo_723,AD.vs.MCI; bulk RNA-seq,Homo_714,AD.vs.control; bulk RNA-seq,Homo_714,AD.vs.MCI; bulk RNA-seq,ROSMAP,AD.vs.MCI; bulk RNA-seq,SRP223445,AD.vs.control	6
BP	GO:0071805	potassium ion transmembrane transport	bulk RNA-seq,Homo_723,AD.vs.control; bulk RNA-seq,Homo_723,AD.vs.MCI; bulk RNA-seq,Homo_723,MCI.vs.control; bulk RNA-seq,Homo_714,AD.vs.control; bulk RNA-seq,Homo_714,AD.vs.MCI; bulk RNA-seq,Homo_633,AD.vs.control; bulk RNA-seq,SRP223445,AD.vs.control	7
BP	GO:0007548	sex differentiation	bulk RNA-seq,Homo_723,AD.vs.control; bulk RNA-seq,Homo_723,AD.vs.MCI; bulk RNA-seq,Homo_723,MCI.vs.control; bulk RNA-seq,Homo_714,AD.vs.control; bulk RNA-seq,Homo_714,AD.vs.MCI; bulk RNA-seq,Homo_633,AD.vs.control; bulk RNA-seq,Homo_633,AD.vs.MCI; bulk RNA-seq,ROSMAP,AD.vs.control; bulk RNA-seq,SRP223445,AD.vs.control	9
BP	GO:1903305	regulation of regulated secretory pathway	bulk RNA-seq,Homo_723,AD.vs.control; bulk RNA-seq,Homo_723,AD.vs.MCI; bulk RNA-seq,Homo_723,MCI.vs.control; bulk RNA-seq,Homo_714,AD.vs.MCI; bulk RNA-seq,Homo_633,AD.vs.control; bulk RNA-seq,Homo_633,AD.vs.MCI	6
MF	GO:0030546	signaling receptor activator activity	bulk RNA-seq,Homo_723,AD.vs.control; bulk RNA-seq,Homo_723,AD.vs.MCI; bulk RNA-seq,Homo_714,AD.vs.control; bulk RNA-seq,Homo_714,AD.vs.MCI; bulk RNA-seq,ROSMAP,AD.vs.MCI; bulk RNA-seq,SRP223445,AD.vs.control	6
BP	GO:0061458	reproductive system development	bulk RNA-seq,Homo_723,AD.vs.control; bulk RNA-seq,Homo_723,AD.vs.MCI; bulk RNA-seq,Homo_723,MCI.vs.control; bulk RNA-seq,Homo_714,AD.vs.control; bulk RNA-seq,Homo_714,AD.vs.MCI; bulk RNA-seq,Homo_633,AD.vs.control; bulk RNA-seq,Homo_633,AD.vs.MCI; bulk RNA-seq,ROSMAP,AD.vs.control; bulk RNA-seq,ROSMAP,MCI.vs.control; bulk RNA-seq,SRP223445,AD.vs.control	10
BP	GO:0050769	positive regulation of neurogenesis	bulk RNA-seq,Homo_723,AD.vs.control; bulk RNA-seq,Homo_723,AD.vs.MCI; bulk RNA-seq,Homo_723,MCI.vs.control; bulk RNA-seq,Homo_714,AD.vs.control; bulk RNA-seq,Homo_714,AD.vs.MCI; bulk RNA-seq,Homo_633,AD.vs.control; bulk RNA-seq,Homo_633,AD.vs.MCI; bulk RNA-	8
BP	GO:0048608	reproductive structure development	bulk RNA-seq,Homo_723,AD.vs.control; bulk RNA-seq,Homo_723,AD.vs.MCI; bulk RNA-seq,Homo_723,MCI.vs.control; bulk RNA-seq,Homo_714,AD.vs.control; bulk RNA-seq,Homo_714,AD.vs.MCI; bulk RNA-seq,Homo_633,AD.vs.control; bulk RNA-seq,Homo_633,AD.vs.MCI; bulk RNA-seq,ROSMAP,AD.vs.control; bulk RNA-seq,ROSMAP,MCI.vs.control; bulk RNA-seq,SRP223445,AD.vs.control	10
BP	GO:0008544	epidermis development	bulk RNA-seq,Homo_723,AD.vs.control; bulk RNA-seq,Homo_723,AD.vs.MCI; bulk RNA-seq,Homo_714,AD.vs.control; bulk RNA-seq,Homo_714,AD.vs.MCI; bulk RNA-seq,Homo_633,AD.vs.control; bulk RNA-seq,Homo_633,AD.vs.MCI; bulk RNA-seq,SRP223445,AD.vs.control	7
BP	GO:0048588	developmental cell growth	bulk RNA-seq,Homo_723,AD.vs.control; bulk RNA-seq,Homo_723,AD.vs.MCI; bulk RNA-seq,Homo_723,MCI.vs.control; bulk RNA-seq,Homo_714,AD.vs.control; bulk RNA-seq,Homo_714,AD.vs.MCI; bulk RNA-seq,Homo_633,AD.vs.control; bulk RNA-seq,Homo_633,AD.vs.MCI; bulk RNA-	8
BP	GO:0017157	regulation of exocytosis	bulk RNA-seq,Homo_723,AD.vs.control; bulk RNA-seq,Homo_723,AD.vs.MCI; bulk RNA-seq,Homo_723,MCI.vs.control; bulk RNA-seq,Homo_714,AD.vs.MCI; bulk RNA-seq,Homo_633,AD.vs.control; bulk RNA-seq,Homo_633,AD.vs.MCI	6
BP	GO:0001837	epithelial to mesenchymal transition	bulk RNA-seq,Homo_723,AD.vs.control; bulk RNA-seq,Homo_723,AD.vs.MCI; bulk RNA-seq,Homo_723,MCI.vs.control; bulk RNA-seq,Homo_714,AD.vs.control; bulk RNA-seq,Homo_714,AD.vs.MCI; bulk RNA-seq,Homo_633,AD.vs.control; bulk RNA-seq,Homo_633,AD.vs.MCI	7
CC	GO:0031225	anchored component of membrane	bulk RNA-seq,Homo_723,AD.vs.control; bulk RNA-seq,Homo_723,AD.vs.MCI; bulk RNA-seq,Homo_723,MCI.vs.control; bulk RNA-seq,Homo_714,AD.vs.control; bulk RNA-seq,Homo_714,AD.vs.MCI	5
BP	GO:0007369	gastrulation	bulk RNA-seq,Homo_723,AD.vs.control; bulk RNA-seq,Homo_723,AD.vs.MCI; bulk RNA-seq,Homo_723,MCI.vs.control; bulk RNA-seq,Homo_714,AD.vs.control; bulk RNA-seq,Homo_714,AD.vs.MCI; bulk RNA-seq,Homo_633,AD.vs.control; bulk RNA-seq,Homo_633,AD.vs.MCI	7
BP	GO:0045137	development of primary sexual characteristics	bulk RNA-seq,Homo_723,AD.vs.control; bulk RNA-seq,Homo_723,AD.vs.MCI; bulk RNA-seq,Homo_723,MCI.vs.control; bulk RNA-seq,Homo_714,AD.vs.control; bulk RNA-seq,Homo_714,AD.vs.MCI; bulk RNA-seq,Homo_633,AD.vs.control; bulk RNA-seq,Homo_633,AD.vs.MCI; bulk RNA-	8
BP	GO:0043523	regulation of neuron apoptotic process	bulk RNA-seq,Homo_723,AD.vs.control; bulk RNA-seq,Homo_723,AD.vs.MCI; bulk RNA-seq,Homo_723,MCI.vs.control; bulk RNA-seq,Homo_714,AD.vs.control; bulk RNA-seq,Homo_714,AD.vs.MCI; bulk RNA-seq,Homo_633,AD.vs.control; bulk RNA-seq,Homo_633,AD.vs.MCI	7
MF	GO:0008201	heparin binding	bulk RNA-seq,Homo_723,AD.vs.control; bulk RNA-seq,Homo_723,AD.vs.MCI; bulk RNA-seq,Homo_723,MCI.vs.control; bulk RNA-seq,Homo_714,AD.vs.control; bulk RNA-seq,Homo_714,AD.vs.MCI; bulk RNA-seq,Homo_633,AD.vs.control; bulk RNA-seq,Homo_633,AD.vs.MCI	7
BP	GO:0060070	canonical Wnt signaling pathway	bulk RNA-seq,Homo_723,AD.vs.control; bulk RNA-seq,Homo_723,AD.vs.MCI; bulk RNA-seq,Homo_723,MCI.vs.control; bulk RNA-seq,Homo_714,AD.vs.control; bulk RNA-seq,Homo_714,AD.vs.MCI; bulk RNA-seq,Homo_633,AD.vs.control; bulk RNA-seq,Homo_633,AD.vs.MCI; bulk RNA-	8
BP	GO:0009913	epidermal cell differentiation	bulk RNA-seq,Homo_723,AD.vs.control; bulk RNA-seq,Homo_723,AD.vs.MCI; bulk RNA-seq,Homo_723,MCI.vs.control; bulk RNA-seq,Homo_714,AD.vs.control; bulk RNA-seq,Homo_714,AD.vs.MCI; bulk RNA-seq,Homo_633,AD.vs.control; bulk RNA-seq,Homo_633,AD.vs.MCI; bulk RNA-	8

BP	GO:0008406	gonad development	bulk RNA-seq,Homo_723,AD.vs.control; bulk RNA-seq,Homo_723,AD.vs.MCI; bulk RNA-seq,Homo_723,MCI.vs.control; bulk RNA-seq,Homo_714,AD.vs.control; bulk RNA-seq,Homo_714,AD.vs.MCI; bulk RNA-seq,Homo_633,AD.vs.control; bulk RNA-seq,Homo_633,AD.vs.MCI; bulk RNA-	8
BP	GO:0060828	regulation of canonical Wnt signaling pathway	bulk RNA-seq,Homo_723,AD.vs.control; bulk RNA-seq,Homo_723,AD.vs.MCI; bulk RNA-seq,Homo_723,MCI.vs.control; bulk RNA-seq,Homo_714,AD.vs.control; bulk RNA-seq,Homo_714,AD.vs.MCI; bulk RNA-seq,Homo_633,AD.vs.control; bulk RNA-seq,Homo_633,AD.vs.MCI	7
BP	GO:0010720	positive regulation of cell development	bulk RNA-seq,Homo_723,AD.vs.control; bulk RNA-seq,Homo_723,AD.vs.MCI; bulk RNA-seq,Homo_723,MCI.vs.control; bulk RNA-seq,Homo_714,AD.vs.control; bulk RNA-seq,Homo_714,AD.vs.MCI; bulk RNA-seq,Homo_633,AD.vs.control; bulk RNA-seq,Homo_633,AD.vs.MCI; bulk RNA-	8
BP	GO:0016055	Wnt signaling pathway	bulk RNA-seq,Homo_723,AD.vs.control; bulk RNA-seq,Homo_723,AD.vs.MCI; bulk RNA-seq,Homo_714,AD.vs.control; bulk RNA-seq,Homo_714,AD.vs.MCI; bulk RNA-seq,Homo_633,AD.vs.control; bulk RNA-seq,Homo_633,AD.vs.MCI; bulk RNA-seq,ROSMAP,AD.vs.control; bulk RNA-seq,SRP223445,AD.vs.control	8
BP	GO:0051402	neuron apoptotic process	bulk RNA-seq,Homo_723,AD.vs.control; bulk RNA-seq,Homo_723,AD.vs.MCI; bulk RNA-seq,Homo_723,MCI.vs.control; bulk RNA-seq,Homo_714,AD.vs.control; bulk RNA-seq,Homo_714,AD.vs.MCI; bulk RNA-seq,Homo_633,AD.vs.control; bulk RNA-seq,Homo_633,AD.vs.MCI	7
BP	GO:0030111	regulation of Wnt signaling pathway	bulk RNA-seq,Homo_723,AD.vs.control; bulk RNA-seq,Homo_723,AD.vs.MCI; bulk RNA-seq,Homo_714,AD.vs.control; bulk RNA-seq,Homo_714,AD.vs.MCI; bulk RNA-seq,Homo_633,AD.vs.control; bulk RNA-seq,Homo_633,AD.vs.MCI	6
BP	GO:0048193	Golgi vesicle transport	bulk RNA-seq,Homo_723,AD.vs.control; bulk RNA-seq,Homo_723,MCI.vs.control; bulk RNA-seq,Homo_714,AD.vs.control; bulk RNA-seq,Homo_633,AD.vs.control; bulk RNA-seq,Homo_633,AD.vs.MCI	5
BP	GO:0045055	regulated exocytosis	bulk RNA-seq,Homo_723,AD.vs.control; bulk RNA-seq,Homo_723,AD.vs.MCI; bulk RNA-seq,Homo_723,MCI.vs.control; bulk RNA-seq,Homo_714,AD.vs.MCI; bulk RNA-seq,Homo_633,AD.vs.control; bulk RNA-seq,Homo_633,AD.vs.MCI	6
BP	GO:0198738	cell-cell signaling by wnt	bulk RNA-seq,Homo_723,AD.vs.control; bulk RNA-seq,Homo_723,AD.vs.MCI; bulk RNA-seq,Homo_714,AD.vs.control; bulk RNA-seq,Homo_714,AD.vs.MCI; bulk RNA-seq,Homo_633,AD.vs.control; bulk RNA-seq,Homo_633,AD.vs.MCI; bulk RNA-seq,ROSMAP,AD.vs.control; bulk RNA-seq,SRP223445,AD.vs.control	8
MF	GO:1903231	mRNA base-pairing post-transcriptional repressor activity	bulk RNA-seq,Homo_723,AD.vs.control; bulk RNA-seq,Homo_723,MCI.vs.control; bulk RNA-seq,Homo_714,AD.vs.control; bulk RNA-seq,Homo_714,AD.vs.MCI; bulk RNA-seq,Homo_714,MCI.vs.control; bulk RNA-seq,Homo_633,MCI.vs.control; bulk RNA-seq,SRP223445,AD.vs.control	7
MF	GO:0008509	anion transmembrane transporter activity	bulk RNA-seq,Homo_723,AD.vs.control; bulk RNA-seq,Homo_723,AD.vs.MCI; bulk RNA-seq,Homo_723,MCI.vs.control; bulk RNA-seq,Homo_714,AD.vs.control; bulk RNA-seq,Homo_714,AD.vs.MCI; bulk RNA-seq,Homo_633,AD.vs.control; bulk RNA-seq,Homo_633,AD.vs.MCI	7
BP	GO:0032412	regulation of ion transmembrane transporter activity	bulk RNA-seq,Homo_723,AD.vs.control; bulk RNA-seq,Homo_723,AD.vs.MCI; bulk RNA-seq,Homo_723,MCI.vs.control; bulk RNA-seq,Homo_714,AD.vs.control; bulk RNA-seq,Homo_714,AD.vs.MCI; bulk RNA-seq,Homo_633,AD.vs.control; bulk RNA-seq,Homo_633,AD.vs.MCI	7
CC	GO:0031514	motile cilium	bulk RNA-seq,Homo_723,AD.vs.control; bulk RNA-seq,Homo_723,AD.vs.MCI; bulk RNA-seq,Homo_723,MCI.vs.control; bulk RNA-seq,Homo_714,AD.vs.control; bulk RNA-seq,Homo_714,AD.vs.MCI; bulk RNA-seq,Homo_633,AD.vs.control; bulk RNA-seq,Homo_633,AD.vs.MCI	7
BP	GO:0090257	regulation of muscle system process	bulk RNA-seq,Homo_723,AD.vs.control; bulk RNA-seq,Homo_723,AD.vs.MCI; bulk RNA-seq,Homo_723,MCI.vs.control; bulk RNA-seq,Homo_714,AD.vs.control; bulk RNA-seq,Homo_714,AD.vs.MCI; bulk RNA-seq,Homo_633,AD.vs.control; bulk RNA-seq,Homo_633,AD.vs.MCI	7
BP	GO:0022898	regulation of transmembrane transporter activity	bulk RNA-seq,Homo_723,AD.vs.control; bulk RNA-seq,Homo_723,AD.vs.MCI; bulk RNA-seq,Homo_723,MCI.vs.control; bulk RNA-seq,Homo_714,AD.vs.control; bulk RNA-seq,Homo_714,AD.vs.MCI; bulk RNA-seq,Homo_633,AD.vs.control; bulk RNA-seq,Homo_633,AD.vs.MCI	7
BP	GO:0042063	gliogenesis	bulk RNA-seq,Homo_723,AD.vs.control; bulk RNA-seq,Homo_723,AD.vs.MCI; bulk RNA-seq,Homo_723,MCI.vs.control; bulk RNA-seq,Homo_714,AD.vs.control; bulk RNA-seq,Homo_714,AD.vs.MCI; bulk RNA-seq,Homo_633,AD.vs.control; bulk RNA-seq,Homo_633,AD.vs.MCI; bulk RNA-	8
BP	GO:0098656	anion transmembrane transport	bulk RNA-seq,Homo_723,AD.vs.control; bulk RNA-seq,Homo_723,AD.vs.MCI; bulk RNA-seq,Homo_723,MCI.vs.control; bulk RNA-seq,Homo_714,AD.vs.control; bulk RNA-seq,Homo_714,AD.vs.MCI; bulk RNA-seq,Homo_633,AD.vs.control; bulk RNA-seq,Homo_633,AD.vs.MCI	7
BP	GO:0000377	RNA splicing, via transesterification reactions with bulged adenosine as nucleophile	bulk RNA-seq,Homo_723,AD.vs.control; bulk RNA-seq,Homo_723,MCI.vs.control; bulk RNA-seq,Homo_714,AD.vs.control; bulk RNA-seq,Homo_633,AD.vs.control; bulk RNA-seq,Homo_633,AD.vs.MCI; scRNA-seq,SRP309935,Megakaryocyte_2-AD.vs.control	6
BP	GO:0000398	mRNA splicing, via spliceosome	bulk RNA-seq,Homo_723,AD.vs.control; bulk RNA-seq,Homo_723,MCI.vs.control; bulk RNA-seq,Homo_714,AD.vs.control; bulk RNA-seq,Homo_633,AD.vs.control; bulk RNA-seq,Homo_633,AD.vs.MCI; scRNA-seq,SRP309935,Megakaryocyte_2-AD.vs.control	6
BP	GO:0001503	ossification	bulk RNA-seq,Homo_723,AD.vs.control; bulk RNA-seq,Homo_723,AD.vs.MCI; bulk RNA-seq,Homo_714,AD.vs.control; bulk RNA-seq,Homo_714,AD.vs.MCI; bulk RNA-seq,Homo_633,AD.vs.control; bulk RNA-seq,Homo_633,AD.vs.MCI; bulk RNA-seq,SRP223445,AD.vs.control	7
BP	GO:0048638	regulation of developmental growth	bulk RNA-seq,Homo_723,AD.vs.control; bulk RNA-seq,Homo_723,AD.vs.MCI; bulk RNA-seq,Homo_723,MCI.vs.control; bulk RNA-seq,Homo_714,AD.vs.control; bulk RNA-seq,Homo_714,AD.vs.MCI; bulk RNA-seq,Homo_633,AD.vs.control; bulk RNA-seq,Homo_633,AD.vs.MCI; bulk RNA-	8
BP	GO:0021700	developmental maturation	bulk RNA-seq,Homo_723,AD.vs.control; bulk RNA-seq,Homo_723,AD.vs.MCI; bulk RNA-seq,Homo_723,MCI.vs.control; bulk RNA-seq,Homo_714,AD.vs.control; bulk RNA-seq,Homo_714,AD.vs.MCI; bulk RNA-seq,Homo_633,AD.vs.control; bulk RNA-seq,Homo_633,AD.vs.MCI; bulk RNA-	8
BP	GO:0051146	striated muscle cell differentiation	bulk RNA-seq,Homo_723,AD.vs.control; bulk RNA-seq,Homo_723,AD.vs.MCI; bulk RNA-seq,Homo_723,MCI.vs.control; bulk RNA-seq,Homo_714,AD.vs.control; bulk RNA-seq,Homo_714,AD.vs.MCI; bulk RNA-seq,Homo_633,AD.vs.control; bulk RNA-seq,Homo_633,AD.vs.MCI	7
BP	GO:1904062	regulation of cation transmembrane transport	bulk RNA-seq,Homo_723,AD.vs.control; bulk RNA-seq,Homo_723,AD.vs.MCI; bulk RNA-seq,Homo_714,AD.vs.control; bulk RNA-seq,Homo_714,AD.vs.MCI; bulk RNA-seq,Homo_633,AD.vs.control; bulk RNA-seq,Homo_633,AD.vs.MCI	6
BP	GO:0090287	regulation of cellular response to growth factor stimulus	bulk RNA-seq,Homo_723,AD.vs.control; bulk RNA-seq,Homo_723,AD.vs.MCI; bulk RNA-seq,Homo_714,AD.vs.control; bulk RNA-seq,Homo_714,AD.vs.MCI; bulk RNA-seq,Homo_633,AD.vs.control; bulk RNA-seq,Homo_633,AD.vs.MCI; bulk RNA-seq,SRP223445,AD.vs.control	7
BP	GO:0032409	regulation of transporter activity	bulk RNA-seq,Homo_723,AD.vs.control; bulk RNA-seq,Homo_723,AD.vs.MCI; bulk RNA-seq,Homo_723,MCI.vs.control; bulk RNA-seq,Homo_714,AD.vs.control; bulk RNA-seq,Homo_714,AD.vs.MCI; bulk RNA-seq,Homo_633,AD.vs.control; bulk RNA-seq,Homo_633,AD.vs.MCI	7
BP	GO:0042692	muscle cell differentiation	bulk RNA-seq,Homo_723,AD.vs.control; bulk RNA-seq,Homo_723,AD.vs.MCI; bulk RNA-seq,Homo_714,AD.vs.control; bulk RNA-seq,Homo_714,AD.vs.MCI; bulk RNA-seq,Homo_633,AD.vs.control; bulk RNA-seq,Homo_633,AD.vs.MCI	6
BP	GO:0009410	response to xenobiotic stimulus	bulk RNA-seq,Homo_723,AD.vs.control; bulk RNA-seq,Homo_723,AD.vs.MCI; bulk RNA-seq,Homo_714,AD.vs.control; bulk RNA-seq,Homo_714,AD.vs.MCI; bulk RNA-seq,Homo_633,AD.vs.control; bulk RNA-seq,Homo_633,AD.vs.MCI	6
BP	GO:0050678	regulation of epithelial cell proliferation	bulk RNA-seq,Homo_723,AD.vs.control; bulk RNA-seq,Homo_723,AD.vs.MCI; bulk RNA-seq,Homo_714,AD.vs.control; bulk RNA-seq,Homo_714,AD.vs.MCI; bulk RNA-seq,Homo_633,AD.vs.control; bulk RNA-seq,Homo_633,AD.vs.MCI	6
BP	GO:0050673	epithelial cell proliferation	bulk RNA-seq,Homo_723,AD.vs.control; bulk RNA-seq,Homo_723,AD.vs.MCI; bulk RNA-seq,Homo_714,AD.vs.control; bulk RNA-seq,Homo_714,AD.vs.MCI; bulk RNA-seq,Homo_633,AD.vs.control; bulk RNA-seq,Homo_633,AD.vs.MCI	6
BP	GO:0016049	cell growth	bulk RNA-seq,Homo_723,AD.vs.control; bulk RNA-seq,Homo_723,AD.vs.MCI; bulk RNA-seq,Homo_714,AD.vs.control; bulk RNA-seq,Homo_714,AD.vs.MCI; bulk RNA-seq,Homo_633,AD.vs.control; bulk RNA-seq,Homo_633,AD.vs.MCI	6
CC	GO:0015629	actin cytoskeleton	bulk RNA-seq,Homo_723,AD.vs.control; bulk RNA-seq,Homo_723,AD.vs.MCI; bulk RNA-seq,Homo_714,AD.vs.control; bulk RNA-seq,Homo_714,AD.vs.MCI; bulk RNA-seq,Homo_633,AD.vs.control; bulk RNA-seq,Homo_633,AD.vs.MCI; bulk RNA-seq,ROSMAP,AD.vs.control; bulk RNA-seq,SRP223445,AD.vs.control	8
CC	GO:0031252	cell leading edge	bulk RNA-seq,Homo_723,AD.vs.control; bulk RNA-seq,Homo_723,AD.vs.MCI; bulk RNA-seq,Homo_714,AD.vs.control; bulk RNA-seq,Homo_714,AD.vs.MCI; bulk RNA-seq,Homo_633,AD.vs.control; bulk RNA-seq,Homo_633,AD.vs.MCI	6

BP	GO:0031346	positive regulation of cell projection organization	bulk RNA-seq,Homo_723,AD.vs.control; bulk RNA-seq,Homo_723,AD.vs.MCI; bulk RNA-seq,Homo_714,AD.vs.MCI; bulk RNA-seq,Homo_633,AD.vs.control; bulk RNA-seq,Homo_633,AD.vs.MCI; bulk RNA-seq,ROSMAP,AD.vs.control; bulk RNA-seq,ROSMAP,MCI.vs.control; bulk RNA-seq,SRP223445,AD.vs.control	8
BP	GO:0009954	proximal/distal pattern formation	bulk RNA-seq,Homo_723,AD.vs.control; bulk RNA-seq,Homo_723,AD.vs.MCI; bulk RNA-seq,Homo_723,MCI.vs.control; bulk RNA-seq,Homo_714,AD.vs.control; bulk RNA-seq,Homo_714,AD.vs.MCI; bulk RNA-seq,Homo_714,MCI.vs.control; bulk RNA-seq,Homo_633,MCI.vs.control; bulk	8
BP	GO:0002209	behavioral defense response	bulk RNA-seq,Homo_723,AD.vs.control; bulk RNA-seq,Homo_723,AD.vs.MCI; bulk RNA-seq,Homo_723,MCI.vs.control; bulk RNA-seq,Homo_714,AD.vs.control; bulk RNA-seq,Homo_714,AD.vs.MCI; bulk RNA-seq,Homo_714,MCI.vs.control	6
BP	GO:0032784	regulation of DNA-templated transcription elongation	bulk RNA-seq,Homo_723,AD.vs.control; bulk RNA-seq,Homo_723,AD.vs.MCI; bulk RNA-seq,Homo_723,MCI.vs.control; bulk RNA-seq,Homo_714,AD.vs.control; bulk RNA-seq,Homo_714,MCI.vs.control; bulk RNA-seq,Homo_633,AD.vs.control; bulk RNA-seq,Homo_633,AD.vs.MCI	7
BP	GO:0006865	amino acid transport	bulk RNA-seq,Homo_723,AD.vs.control; bulk RNA-seq,Homo_723,AD.vs.MCI; bulk RNA-seq,Homo_723,MCI.vs.control; bulk RNA-seq,Homo_714,AD.vs.control; bulk RNA-seq,Homo_714,AD.vs.MCI; bulk RNA-seq,Homo_633,AD.vs.control; bulk RNA-seq,Homo_633,AD.vs.MCI	7
CC	GO:0000502	proteasome complex	bulk RNA-seq,Homo_723,AD.vs.control; bulk RNA-seq,Homo_723,AD.vs.MCI; bulk RNA-seq,Homo_714,AD.vs.control; bulk RNA-seq,Homo_714,AD.vs.MCI; bulk RNA-seq,Homo_633,AD.vs.control; bulk RNA-seq,Homo_633,AD.vs.MCI	6
BP	GO:0017156	calcium-ion regulated exocytosis	bulk RNA-seq,Homo_723,AD.vs.control; bulk RNA-seq,Homo_723,AD.vs.MCI; bulk RNA-seq,Homo_723,MCI.vs.control; bulk RNA-seq,Homo_714,AD.vs.MCI; bulk RNA-seq,Homo_714,MCI.vs.control; bulk RNA-seq,Homo_633,AD.vs.control	6
BP	GO:0000375	RNA splicing, via transesterification reactions	bulk RNA-seq,Homo_723,AD.vs.control; bulk RNA-seq,Homo_714,AD.vs.control; bulk RNA-seq,Homo_633,AD.vs.control; bulk RNA-seq,Homo_633,AD.vs.MCI; scRNA-seq,SRP309935,Megakaryocyte_2-AD.vs.control	5
BP	GO:0006887	exocytosis	bulk RNA-seq,Homo_723,AD.vs.control; bulk RNA-seq,Homo_723,AD.vs.MCI; bulk RNA-seq,Homo_714,AD.vs.MCI; bulk RNA-seq,Homo_633,AD.vs.control; bulk RNA-seq,Homo_633,AD.vs.MCI; scRNA-seq,SRP330776,Naive CD8+ T cell_2-AD.vs.control	6
MF	GO:0005267	potassium channel activity	bulk RNA-seq,Homo_723,AD.vs.control; bulk RNA-seq,Homo_723,AD.vs.MCI; bulk RNA-seq,Homo_723,MCI.vs.control; bulk RNA-seq,Homo_714,AD.vs.control; bulk RNA-seq,Homo_714,AD.vs.MCI; bulk RNA-seq,SRP223445,AD.vs.control	6
BP	GO:0010001	glial cell differentiation	bulk RNA-seq,Homo_723,AD.vs.control; bulk RNA-seq,Homo_723,AD.vs.MCI; bulk RNA-seq,Homo_723,MCI.vs.control; bulk RNA-seq,Homo_714,AD.vs.MCI; bulk RNA-seq,Homo_633,AD.vs.control; bulk RNA-seq,Homo_633,AD.vs.MCI	6
CC	GO:0034707	chloride channel complex	bulk RNA-seq,Homo_723,AD.vs.control; bulk RNA-seq,Homo_723,AD.vs.MCI; bulk RNA-seq,Homo_723,MCI.vs.control; bulk RNA-seq,Homo_714,AD.vs.control; bulk RNA-seq,Homo_714,AD.vs.MCI	5
BP	GO:0002822	regulation of adaptive immune response based on somatic recombination of immune receptors built from immunoglobulin superfamily domains	bulk RNA-seq,Homo_723,AD.vs.control; bulk RNA-seq,Homo_723,MCI.vs.control; bulk RNA-seq,Homo_714,AD.vs.control; bulk RNA-seq,Homo_633,AD.vs.control; bulk RNA-seq,Homo_633,AD.vs.MCI	5
CC	GO:0097729	9+2 motile cilium	bulk RNA-seq,Homo_723,AD.vs.control; bulk RNA-seq,Homo_723,AD.vs.MCI; bulk RNA-seq,Homo_723,MCI.vs.control; bulk RNA-seq,Homo_714,AD.vs.control; bulk RNA-seq,Homo_714,AD.vs.MCI; bulk RNA-seq,Homo_633,AD.vs.control; bulk RNA-seq,Homo_633,AD.vs.MCI	7
BP	GO:0002705	positive regulation of leukocyte mediated immunity	bulk RNA-seq,Homo_723,AD.vs.control; bulk RNA-seq,Homo_723,AD.vs.MCI; bulk RNA-seq,Homo_723,MCI.vs.control; bulk RNA-seq,Homo_714,AD.vs.control; bulk RNA-seq,Homo_633,AD.vs.control; bulk RNA-seq,Homo_633,AD.vs.MCI	6
MF	GO:0000049	tRNA binding	bulk RNA-seq,Homo_723,AD.vs.control; bulk RNA-seq,Homo_723,AD.vs.MCI; bulk RNA-seq,Homo_723,MCI.vs.control; bulk RNA-seq,Homo_714,AD.vs.control; bulk RNA-seq,Homo_714,AD.vs.MCI; bulk RNA-seq,Homo_714,MCI.vs.control; bulk RNA-seq,Homo_633,AD.vs.control; bulk	8
BP	GO:0060249	anatomical structure homeostasis	bulk RNA-seq,Homo_723,AD.vs.control; bulk RNA-seq,Homo_723,AD.vs.MCI; bulk RNA-seq,Homo_723,MCI.vs.control; bulk RNA-seq,Homo_714,AD.vs.control; bulk RNA-seq,Homo_714,AD.vs.MCI; bulk RNA-seq,Homo_633,AD.vs.control; bulk RNA-seq,Homo_633,AD.vs.MCI	7
BP	GO:0045333	cellular respiration	bulk RNA-seq,Homo_723,AD.vs.control; bulk RNA-seq,Homo_723,MCI.vs.control; bulk RNA-seq,Homo_714,AD.vs.control; bulk RNA-seq,Homo_633,AD.vs.control; bulk RNA-seq,Homo_633,AD.vs.MCI	5
BP	GO:0008038	neuron recognition	bulk RNA-seq,Homo_723,AD.vs.control; bulk RNA-seq,Homo_723,AD.vs.MCI; bulk RNA-seq,Homo_723,MCI.vs.control; bulk RNA-seq,Homo_714,AD.vs.MCI; bulk RNA-seq,Homo_714,MCI.vs.control; bulk RNA-seq,Homo_633,AD.vs.control; bulk RNA-seq,Homo_633,AD.vs.MCI	7
CC	GO:0043292	contractile fiber	bulk RNA-seq,Homo_723,AD.vs.control; bulk RNA-seq,Homo_723,AD.vs.MCI; bulk RNA-seq,Homo_723,MCI.vs.control; bulk RNA-seq,Homo_714,AD.vs.control; bulk RNA-seq,Homo_714,AD.vs.MCI; bulk RNA-seq,Homo_633,AD.vs.control; bulk RNA-seq,Homo_633,AD.vs.MCI	7
BP	GO:2001257	regulation of cation channel activity	bulk RNA-seq,Homo_723,AD.vs.control; bulk RNA-seq,Homo_723,AD.vs.MCI; bulk RNA-seq,Homo_723,MCI.vs.control; bulk RNA-seq,Homo_714,AD.vs.control; bulk RNA-seq,Homo_714,AD.vs.MCI; bulk RNA-seq,Homo_633,AD.vs.control	6
CC	GO:0005581	collagen trimer	bulk RNA-seq,Homo_723,AD.vs.control; bulk RNA-seq,Homo_723,AD.vs.MCI; bulk RNA-seq,Homo_723,MCI.vs.control; bulk RNA-seq,Homo_714,AD.vs.control; bulk RNA-seq,Homo_714,AD.vs.MCI; bulk RNA-seq,Homo_714,MCI.vs.control; bulk RNA-seq,ROSMAP,AD.vs.control; bulk	8
BP	GO:0006402	mRNA catabolic process	bulk RNA-seq,Homo_723,AD.vs.control; bulk RNA-seq,Homo_723,AD.vs.MCI; bulk RNA-seq,Homo_723,MCI.vs.control; bulk RNA-seq,Homo_714,AD.vs.control; bulk RNA-seq,Homo_633,AD.vs.control; bulk RNA-seq,Homo_633,AD.vs.MCI	6
BP	GO:1902692	regulation of neuroblast proliferation	bulk RNA-seq,Homo_723,AD.vs.control; bulk RNA-seq,Homo_723,AD.vs.MCI; bulk RNA-seq,Homo_723,MCI.vs.control; bulk RNA-seq,Homo_714,AD.vs.control; bulk RNA-seq,Homo_714,AD.vs.MCI; bulk RNA-seq,Homo_714,MCI.vs.control	6
BP	GO:0006937	regulation of muscle contraction	bulk RNA-seq,Homo_723,AD.vs.control; bulk RNA-seq,Homo_723,AD.vs.MCI; bulk RNA-seq,Homo_723,MCI.vs.control; bulk RNA-seq,Homo_714,AD.vs.control; bulk RNA-seq,Homo_714,AD.vs.MCI; bulk RNA-seq,Homo_633,AD.vs.control	6
BP	GO:0010324	membrane invagination	bulk RNA-seq,Homo_723,AD.vs.control; bulk RNA-seq,Homo_723,AD.vs.MCI; bulk RNA-seq,Homo_714,AD.vs.control; bulk RNA-seq,Homo_714,AD.vs.MCI; bulk RNA-seq,Homo_633,AD.vs.control; bulk RNA-seq,Homo_633,AD.vs.MCI; bulk RNA-seq,MCSA,MCI.vs.control; scRNA-seq,SRP330776,Naive CD8+ T cell_2-AD.vs.control; scRNA-seq,SRP309935,Monocyte_2-AD.vs.control	9
MF	GO:0030414	peptidase inhibitor activity	bulk RNA-seq,Homo_723,AD.vs.control; bulk RNA-seq,Homo_723,AD.vs.MCI; bulk RNA-seq,Homo_723,MCI.vs.control; bulk RNA-seq,Homo_714,AD.vs.control; bulk RNA-seq,Homo_714,AD.vs.MCI; bulk RNA-seq,Homo_633,AD.vs.MCI	6
MF	GO:0019838	growth factor binding	bulk RNA-seq,Homo_723,AD.vs.control; bulk RNA-seq,Homo_723,AD.vs.MCI; bulk RNA-seq,Homo_723,MCI.vs.control; bulk RNA-seq,Homo_714,AD.vs.control; bulk RNA-seq,Homo_714,AD.vs.MCI; bulk RNA-seq,Homo_633,AD.vs.control; bulk RNA-seq,Homo_633,AD.vs.MCI; bulk RNA-seq,ROSMAP,AD.vs.control; bulk RNA-seq,ROSMAP,MCI.vs.control	9
BP	GO:0002703	regulation of leukocyte mediated immunity	bulk RNA-seq,Homo_723,AD.vs.control; bulk RNA-seq,Homo_723,MCI.vs.control; bulk RNA-seq,Homo_714,AD.vs.control; bulk RNA-seq,Homo_633,AD.vs.control; bulk RNA-seq,Homo_633,AD.vs.MCI	5
CC	GO:0045095	keratin filament	bulk RNA-seq,Homo_723,AD.vs.control; bulk RNA-seq,Homo_723,AD.vs.MCI; bulk RNA-seq,Homo_714,AD.vs.control; bulk RNA-seq,Homo_714,AD.vs.MCI; bulk RNA-seq,Homo_633,MCI.vs.control; bulk RNA-seq,SRP223445,AD.vs.control	6
BP	GO:0002183	cytoplasmic translational initiation	bulk RNA-seq,Homo_723,AD.vs.control; bulk RNA-seq,Homo_723,AD.vs.MCI; bulk RNA-seq,Homo_723,MCI.vs.control; bulk RNA-seq,Homo_714,AD.vs.control; bulk RNA-seq,Homo_714,AD.vs.MCI; bulk RNA-seq,Homo_714,MCI.vs.control; bulk RNA-seq,Homo_633,AD.vs.control; bulk	8

MF	GO:0030371	translation repressor activity	bulk RNA-seq,Homo_723,AD.vs.control; bulk RNA-seq,Homo_723,MCI.vs.control; bulk RNA-seq,Homo_714,AD.vs.control; bulk RNA-seq,Homo_714,AD.vs.MCI; bulk RNA-seq,Homo_714,MCI.vs.control; bulk RNA-seq,Homo_633,MCI.vs.control; bulk RNA-seq,SRP223445,AD.vs.control	7
MF	GO:0001664	G protein-coupled receptor binding	bulk RNA-seq,Homo_723,AD.vs.control; bulk RNA-seq,Homo_723,AD.vs.MCI; bulk RNA-seq,Homo_723,MCI.vs.control; bulk RNA-seq,Homo_714,AD.vs.control; bulk RNA-seq,Homo_714,AD.vs.MCI	5
BP	GO:0009566	fertilization	bulk RNA-seq,Homo_723,AD.vs.control; bulk RNA-seq,Homo_723,AD.vs.MCI; bulk RNA-seq,Homo_714,AD.vs.control; bulk RNA-seq,Homo_714,AD.vs.MCI; bulk RNA-seq,Homo_633,AD.vs.control; bulk RNA-seq,Homo_633,AD.vs.MCI; bulk RNA-seq,SRP223445,AD.vs.control	7
BP	GO:0001894	tissue homeostasis	bulk RNA-seq,Homo_723,AD.vs.control; bulk RNA-seq,Homo_723,AD.vs.MCI; bulk RNA-seq,Homo_723,MCI.vs.control; bulk RNA-seq,Homo_714,AD.vs.control; bulk RNA-seq,Homo_714,AD.vs.MCI; bulk RNA-seq,Homo_633,AD.vs.control; bulk RNA-seq,Homo_633,AD.vs.MCI	7
BP	GO:0015849	organic acid transport	bulk RNA-seq,Homo_723,AD.vs.control; bulk RNA-seq,Homo_723,AD.vs.MCI; bulk RNA-seq,Homo_723,MCI.vs.control; bulk RNA-seq,Homo_714,AD.vs.control; bulk RNA-seq,Homo_714,AD.vs.MCI; bulk RNA-seq,Homo_633,AD.vs.control; bulk RNA-seq,Homo_633,AD.vs.MCI	7
BP	GO:0015986	proton motive force-driven ATP synthesis	bulk RNA-seq,Homo_723,AD.vs.control; bulk RNA-seq,Homo_723,AD.vs.MCI; bulk RNA-seq,Homo_723,MCI.vs.control; bulk RNA-seq,Homo_714,AD.vs.control; bulk RNA-seq,Homo_714,AD.vs.MCI; bulk RNA-seq,Homo_714,MCI.vs.control; bulk RNA-seq,Homo_633,AD.vs.control; bulk	8
BP	GO:0001558	regulation of cell growth	bulk RNA-seq,Homo_723,AD.vs.control; bulk RNA-seq,Homo_723,AD.vs.MCI; bulk RNA-seq,Homo_714,AD.vs.control; bulk RNA-seq,Homo_714,AD.vs.MCI; bulk RNA-seq,Homo_633,AD.vs.control; bulk RNA-seq,Homo_633,AD.vs.MCI	6
CC	GO:0045177	apical part of cell	bulk RNA-seq,Homo_723,AD.vs.control; bulk RNA-seq,Homo_723,AD.vs.MCI; bulk RNA-seq,Homo_714,AD.vs.control; bulk RNA-seq,Homo_714,AD.vs.MCI; bulk RNA-seq,Homo_633,AD.vs.control; bulk RNA-seq,Homo_633,AD.vs.MCI; bulk RNA-seq,ROSMAP,AD.vs.control; bulk RNA-seq,ROSMAP,MCI.vs.control; bulk RNA-seq,SRP223445,AD.vs.control	9
BP	GO:0099175	regulation of postsynapse organization	bulk RNA-seq,Homo_723,AD.vs.control; bulk RNA-seq,Homo_723,AD.vs.MCI; bulk RNA-seq,Homo_723,MCI.vs.control; bulk RNA-seq,Homo_714,AD.vs.MCI; bulk RNA-seq,Homo_714,MCI.vs.control; bulk RNA-seq,Homo_633,AD.vs.control; bulk RNA-seq,Homo_633,AD.vs.MCI; bulk RNA-	8
BP	GO:0002821	positive regulation of adaptive immune response	bulk RNA-seq,Homo_723,AD.vs.control; bulk RNA-seq,Homo_723,AD.vs.MCI; bulk RNA-seq,Homo_723,MCI.vs.control; bulk RNA-seq,Homo_714,AD.vs.control; bulk RNA-seq,Homo_714,MCI.vs.control; bulk RNA-seq,Homo_633,AD.vs.control; bulk RNA-seq,Homo_633,AD.vs.MCI	7
BP	GO:0006941	striated muscle contraction	bulk RNA-seq,Homo_723,AD.vs.control; bulk RNA-seq,Homo_723,AD.vs.MCI; bulk RNA-seq,Homo_723,MCI.vs.control; bulk RNA-seq,Homo_714,AD.vs.control; bulk RNA-seq,Homo_714,AD.vs.MCI; bulk RNA-seq,Homo_633,AD.vs.control	6
CC	GO:0000932	P-body	bulk RNA-seq,Homo_723,AD.vs.control; bulk RNA-seq,Homo_723,AD.vs.MCI; bulk RNA-seq,Homo_723,MCI.vs.control; bulk RNA-seq,Homo_714,AD.vs.control; bulk RNA-seq,Homo_714,AD.vs.MCI; bulk RNA-seq,Homo_714,MCI.vs.control; bulk RNA-seq,Homo_633,AD.vs.control; bulk	8
BP	GO:0140546	defense response to symbiont	bulk RNA-seq,Homo_723,AD.vs.control; bulk RNA-seq,Homo_723,MCI.vs.control; bulk RNA-seq,Homo_714,AD.vs.control; bulk RNA-seq,Homo_633,AD.vs.control; bulk RNA-seq,Homo_633,AD.vs.MCI; scRNA-seq,SRP215507,CD8+ T cell_3-AD.vs.control	6
BP	GO:0043270	positive regulation of ion transport	bulk RNA-seq,Homo_723,AD.vs.control; bulk RNA-seq,Homo_723,AD.vs.MCI; bulk RNA-seq,Homo_723,MCI.vs.control; bulk RNA-seq,Homo_714,AD.vs.control; bulk RNA-seq,Homo_714,AD.vs.MCI; bulk RNA-seq,Homo_633,AD.vs.control; bulk RNA-seq,Homo_633,AD.vs.MCI	7
BP	GO:0021517	ventral spinal cord development	bulk RNA-seq,Homo_723,AD.vs.control; bulk RNA-seq,Homo_723,AD.vs.MCI; bulk RNA-seq,Homo_723,MCI.vs.control; bulk RNA-seq,Homo_714,AD.vs.control; bulk RNA-seq,Homo_714,AD.vs.MCI; bulk RNA-seq,SRP223445,AD.vs.control	6
MF	GO:0005179	hormone activity	bulk RNA-seq,Homo_723,AD.vs.control; bulk RNA-seq,Homo_723,AD.vs.MCI; bulk RNA-seq,Homo_723,MCI.vs.control; bulk RNA-seq,Homo_714,AD.vs.control; bulk RNA-seq,Homo_714,AD.vs.MCI; bulk RNA-seq,SRP223445,AD.vs.control	6
CC	GO:0048786	presynaptic active zone	bulk RNA-seq,Homo_723,AD.vs.control; bulk RNA-seq,Homo_723,AD.vs.MCI; bulk RNA-seq,Homo_723,MCI.vs.control; bulk RNA-seq,Homo_714,AD.vs.MCI; bulk RNA-seq,Homo_714,MCI.vs.control; bulk RNA-seq,Homo_633,AD.vs.control; bulk RNA-seq,Homo_633,AD.vs.MCI	7
BP	GO:0055001	muscle cell development	bulk RNA-seq,Homo_723,AD.vs.control; bulk RNA-seq,Homo_723,AD.vs.MCI; bulk RNA-seq,Homo_723,MCI.vs.control; bulk RNA-seq,Homo_714,AD.vs.control; bulk RNA-seq,Homo_714,AD.vs.MCI; bulk RNA-seq,Homo_633,AD.vs.control; bulk RNA-seq,Homo_633,AD.vs.MCI	7
BP	GO:0034243	regulation of transcription elongation by RNA polymerase II	bulk RNA-seq,Homo_723,AD.vs.control; bulk RNA-seq,Homo_723,AD.vs.MCI; bulk RNA-seq,Homo_723,MCI.vs.control; bulk RNA-seq,Homo_714,AD.vs.control; bulk RNA-seq,Homo_714,MCI.vs.control; bulk RNA-seq,Homo_633,AD.vs.control; bulk RNA-seq,Homo_633,AD.vs.MCI	7
BP	GO:0030216	keratinocyte differentiation	bulk RNA-seq,Homo_723,AD.vs.control; bulk RNA-seq,Homo_723,AD.vs.MCI; bulk RNA-seq,Homo_723,MCI.vs.control; bulk RNA-seq,Homo_714,AD.vs.control; bulk RNA-seq,Homo_714,AD.vs.MCI; bulk RNA-seq,Homo_633,AD.vs.control; bulk RNA-seq,Homo_633,AD.vs.MCI; bulk RNA-	8
BP	GO:0099024	plasma membrane invagination	bulk RNA-seq,Homo_723,AD.vs.control; bulk RNA-seq,Homo_723,AD.vs.MCI; bulk RNA-seq,Homo_714,AD.vs.control; bulk RNA-seq,Homo_714,AD.vs.MCI; bulk RNA-seq,Homo_633,AD.vs.control; bulk RNA-seq,Homo_633,AD.vs.MCI; bulk RNA-seq,MCSA,MCI.vs.control; scRNA-seq,SRP330776,Naive CD8+ T cell_2-AD.vs.control; scRNA-seq,SRP309935,Monocyte_2-AD.vs.control	9
BP	GO:0061337	cardiac conduction	bulk RNA-seq,Homo_723,AD.vs.control; bulk RNA-seq,Homo_723,AD.vs.MCI; bulk RNA-seq,Homo_723,MCI.vs.control; bulk RNA-seq,Homo_714,AD.vs.control; bulk RNA-seq,Homo_714,AD.vs.MCI; bulk RNA-seq,Homo_714,MCI.vs.control	6
BP	GO:0070997	neuron death	bulk RNA-seq,Homo_723,AD.vs.control; bulk RNA-seq,Homo_723,AD.vs.MCI; bulk RNA-seq,Homo_714,AD.vs.control; bulk RNA-seq,Homo_714,AD.vs.MCI; bulk RNA-seq,Homo_633,AD.vs.control; bulk RNA-seq,Homo_633,AD.vs.MCI	6
BP	GO:0051607	defense response to virus	bulk RNA-seq,Homo_723,AD.vs.control; bulk RNA-seq,Homo_723,MCI.vs.control; bulk RNA-seq,Homo_714,AD.vs.control; bulk RNA-seq,Homo_633,AD.vs.control; bulk RNA-seq,Homo_633,AD.vs.MCI; scRNA-seq,SRP215507,CD8+ T cell_3-AD.vs.control	6
BP	GO:0035176	social behavior	bulk RNA-seq,Homo_723,AD.vs.control; bulk RNA-seq,Homo_723,AD.vs.MCI; bulk RNA-seq,Homo_723,MCI.vs.control; bulk RNA-seq,Homo_714,AD.vs.control; bulk RNA-seq,Homo_714,AD.vs.MCI; bulk RNA-seq,Homo_714,MCI.vs.control	6
BP	GO:0035051	cardiocyte differentiation	bulk RNA-seq,Homo_723,AD.vs.control; bulk RNA-seq,Homo_723,AD.vs.MCI; bulk RNA-seq,Homo_723,MCI.vs.control; bulk RNA-seq,Homo_714,AD.vs.control; bulk RNA-seq,Homo_714,AD.vs.MCI	5
CC	GO:0032809	neuronal cell body membrane	bulk RNA-seq,Homo_723,AD.vs.control; bulk RNA-seq,Homo_723,AD.vs.MCI; bulk RNA-seq,Homo_723,MCI.vs.control; bulk RNA-seq,Homo_714,AD.vs.MCI	4
BP	GO:0031424	keratinization	bulk RNA-seq,Homo_723,AD.vs.control; bulk RNA-seq,Homo_723,AD.vs.MCI; bulk RNA-seq,Homo_714,AD.vs.control; bulk RNA-seq,Homo_714,AD.vs.MCI; bulk RNA-seq,Homo_633,AD.vs.control; bulk RNA-seq,Homo_633,MCI.vs.control; bulk RNA-seq,SRP223445,AD.vs.control	7
MF	GO:0008173	RNA methyltransferase activity	bulk RNA-seq,Homo_723,AD.vs.control; bulk RNA-seq,Homo_723,AD.vs.MCI; bulk RNA-seq,Homo_723,MCI.vs.control; bulk RNA-seq,Homo_714,AD.vs.control; bulk RNA-seq,Homo_714,MCI.vs.control; bulk RNA-seq,Homo_633,AD.vs.control; bulk RNA-seq,Homo_633,AD.vs.MCI	7
BP	GO:0050679	positive regulation of epithelial cell proliferation	bulk RNA-seq,Homo_723,AD.vs.control; bulk RNA-seq,Homo_723,AD.vs.MCI; bulk RNA-seq,Homo_723,MCI.vs.control; bulk RNA-seq,Homo_714,AD.vs.control; bulk RNA-seq,Homo_714,AD.vs.MCI; bulk RNA-seq,Homo_633,AD.vs.control; bulk RNA-seq,Homo_633,AD.vs.MCI	7
BP	GO:0021983	pituitary gland development	bulk RNA-seq,Homo_723,AD.vs.control; bulk RNA-seq,Homo_723,AD.vs.MCI; bulk RNA-seq,Homo_723,MCI.vs.control; bulk RNA-seq,Homo_714,AD.vs.control; bulk RNA-seq,Homo_714,AD.vs.MCI; bulk RNA-seq,Homo_633,MCI.vs.control	6
BP	GO:0030516	regulation of axon extension	bulk RNA-seq,Homo_723,AD.vs.control; bulk RNA-seq,Homo_723,AD.vs.MCI; bulk RNA-seq,Homo_723,MCI.vs.control; bulk RNA-seq,Homo_714,AD.vs.control; bulk RNA-seq,Homo_714,AD.vs.MCI; bulk RNA-seq,Homo_714,MCI.vs.control; bulk RNA-seq,Homo_633,AD.vs.control; bulk	8

BP	GO:0070585	protein localization to mitochondrion	bulk RNA-seq,Homo_723,AD.vs.control; bulk RNA-seq,Homo_723,MCI.vs.control; bulk RNA-seq,Homo_714,AD.vs.control; bulk RNA-seq,Homo_714,MCI.vs.control; bulk RNA-seq,Homo_633,AD.vs.control; bulk RNA-seq,Homo_633,AD.vs.MCI	6
BP	GO:0051703	biological process involved in intraspecies interaction between organisms	bulk RNA-seq,Homo_723,AD.vs.control; bulk RNA-seq,Homo_723,AD.vs.MCI; bulk RNA-seq,Homo_723,MCI.vs.control; bulk RNA-seq,Homo_714,AD.vs.control; bulk RNA-seq,Homo_714,AD.vs.MCI; bulk RNA-seq,Homo_714,MCI.vs.control	6
BP	GO:1901215	negative regulation of neuron death	bulk RNA-seq,Homo_723,AD.vs.control; bulk RNA-seq,Homo_723,AD.vs.MCI; bulk RNA-seq,Homo_723,MCI.vs.control; bulk RNA-seq,Homo_714,AD.vs.control; bulk RNA-seq,Homo_714,AD.vs.MCI; bulk RNA-seq,Homo_633,AD.vs.control; bulk RNA-seq,Homo_633,AD.vs.MCI	7
BP	GO:1901214	regulation of neuron death	bulk RNA-seq,Homo_723,AD.vs.control; bulk RNA-seq,Homo_723,AD.vs.MCI; bulk RNA-seq,Homo_723,MCI.vs.control; bulk RNA-seq,Homo_714,AD.vs.control; bulk RNA-seq,Homo_714,AD.vs.MCI; bulk RNA-seq,Homo_633,AD.vs.control; bulk RNA-seq,Homo_633,AD.vs.MCI	7
BP	GO:0046661	male sex differentiation	bulk RNA-seq,Homo_723,AD.vs.control; bulk RNA-seq,Homo_723,AD.vs.MCI; bulk RNA-seq,Homo_723,MCI.vs.control; bulk RNA-seq,Homo_714,AD.vs.control; bulk RNA-seq,Homo_714,AD.vs.MCI; bulk RNA-seq,Homo_633,AD.vs.control; bulk RNA-seq,Homo_633,AD.vs.MCI	7
BP	GO:0021515	cell differentiation in spinal cord	bulk RNA-seq,Homo_723,AD.vs.control; bulk RNA-seq,Homo_723,AD.vs.MCI; bulk RNA-seq,Homo_723,MCI.vs.control; bulk RNA-seq,Homo_714,AD.vs.control; bulk RNA-seq,Homo_714,AD.vs.MCI; bulk RNA-seq,SRP223445,AD.vs.control	6
MF	GO:0001540	amyloid-beta binding	bulk RNA-seq,Homo_723,AD.vs.control; bulk RNA-seq,Homo_723,AD.vs.MCI; bulk RNA-seq,Homo_723,MCI.vs.control; bulk RNA-seq,Homo_714,AD.vs.MCI; bulk RNA-seq,Homo_714,MCI.vs.control; bulk RNA-seq,Homo_633,AD.vs.control; bulk RNA-seq,Homo_633,AD.vs.MCI	7
BP	GO:0010469	regulation of signaling receptor activity	bulk RNA-seq,Homo_723,AD.vs.control; bulk RNA-seq,Homo_723,AD.vs.MCI; bulk RNA-seq,Homo_723,MCI.vs.control; bulk RNA-seq,Homo_714,AD.vs.control; bulk RNA-seq,Homo_714,AD.vs.MCI; bulk RNA-seq,Homo_633,AD.vs.control	6
BP	GO:0090102	cochlea development	bulk RNA-seq,Homo_723,AD.vs.control; bulk RNA-seq,Homo_723,AD.vs.MCI; bulk RNA-seq,Homo_723,MCI.vs.control; bulk RNA-seq,Homo_714,AD.vs.control; bulk RNA-seq,Homo_714,AD.vs.MCI; bulk RNA-seq,Homo_714,MCI.vs.control	6
CC	GO:0032281	AMPA glutamate receptor complex	bulk RNA-seq,Homo_723,AD.vs.control; bulk RNA-seq,Homo_723,AD.vs.MCI; bulk RNA-seq,Homo_723,MCI.vs.control; bulk RNA-seq,Homo_714,AD.vs.MCI	4
BP	GO:0000154	rRNA modification	bulk RNA-seq,Homo_723,AD.vs.control; bulk RNA-seq,Homo_723,AD.vs.MCI; bulk RNA-seq,Homo_723,MCI.vs.control; bulk RNA-seq,Homo_714,AD.vs.control; bulk RNA-seq,Homo_714,AD.vs.MCI; bulk RNA-seq,Homo_714,MCI.vs.control; bulk RNA-seq,Homo_633,AD.vs.control; bulk	8
BP	GO:0006888	endoplasmic reticulum to Golgi vesicle-mediated transport	bulk RNA-seq,Homo_723,AD.vs.control; bulk RNA-seq,Homo_723,MCI.vs.control; bulk RNA-seq,Homo_714,AD.vs.control; bulk RNA-seq,Homo_633,AD.vs.control; bulk RNA-seq,Homo_633,AD.vs.MCI	5
BP	GO:0021545	cranial nerve development	bulk RNA-seq,Homo_723,AD.vs.control; bulk RNA-seq,Homo_723,AD.vs.MCI; bulk RNA-seq,Homo_723,MCI.vs.control; bulk RNA-seq,Homo_714,AD.vs.control; bulk RNA-seq,Homo_714,AD.vs.MCI; bulk RNA-seq,Homo_714,MCI.vs.control	6
BP	GO:0030856	regulation of epithelial cell differentiation	bulk RNA-seq,Homo_723,AD.vs.control; bulk RNA-seq,Homo_723,AD.vs.MCI; bulk RNA-seq,Homo_723,MCI.vs.control; bulk RNA-seq,Homo_714,AD.vs.control; bulk RNA-seq,Homo_714,AD.vs.MCI; bulk RNA-seq,Homo_633,AD.vs.control; bulk RNA-seq,Homo_633,AD.vs.MCI	7
BP	GO:0001738	morphogenesis of a polarized epithelium	bulk RNA-seq,Homo_723,AD.vs.control; bulk RNA-seq,Homo_723,AD.vs.MCI; bulk RNA-seq,Homo_723,MCI.vs.control; bulk RNA-seq,Homo_714,AD.vs.MCI; bulk RNA-seq,Homo_714,MCI.vs.control; bulk RNA-seq,Homo_633,AD.vs.control; bulk RNA-seq,Homo_633,AD.vs.MCI; bulk RNA-	8
BP	GO:0030217	T cell differentiation	bulk RNA-seq,Homo_723,AD.vs.control; bulk RNA-seq,Homo_723,MCI.vs.control; bulk RNA-seq,Homo_714,AD.vs.control; bulk RNA-seq,Homo_633,AD.vs.control; bulk RNA-seq,Homo_633,AD.vs.MCI	5
BP	GO:0002062	chondrocyte differentiation	bulk RNA-seq,Homo_723,AD.vs.control; bulk RNA-seq,Homo_723,AD.vs.MCI; bulk RNA-seq,Homo_723,MCI.vs.control; bulk RNA-seq,Homo_714,AD.vs.MCI; bulk RNA-seq,Homo_714,MCI.vs.control; bulk RNA-seq,Homo_633,AD.vs.control; bulk RNA-seq,Homo_633,AD.vs.MCI; scRNA-seq,SRP330776,Naive CD8+ T	8
BP	GO:0042303	molting cycle	bulk RNA-seq,Homo_723,AD.vs.control; bulk RNA-seq,Homo_723,AD.vs.MCI; bulk RNA-seq,Homo_723,MCI.vs.control; bulk RNA-seq,Homo_714,AD.vs.control; bulk RNA-seq,Homo_714,AD.vs.MCI; bulk RNA-seq,Homo_714,MCI.vs.control; bulk RNA-seq,Homo_633,AD.vs.control; bulk RNA-seq,Homo_633,AD.vs.MCI; bulk RNA-seq,SRP223445,AD.vs.control	9
BP	GO:0042633	hair cycle	bulk RNA-seq,Homo_723,AD.vs.control; bulk RNA-seq,Homo_723,AD.vs.MCI; bulk RNA-seq,Homo_723,MCI.vs.control; bulk RNA-seq,Homo_714,AD.vs.control; bulk RNA-seq,Homo_714,AD.vs.MCI; bulk RNA-seq,Homo_714,MCI.vs.control; bulk RNA-seq,Homo_633,AD.vs.control; bulk RNA-seq,Homo_633,AD.vs.MCI; bulk RNA-seq,SRP223445,AD.vs.control	9
BP	GO:0003338	metanephros morphogenesis	bulk RNA-seq,Homo_723,AD.vs.control; bulk RNA-seq,Homo_723,AD.vs.MCI; bulk RNA-seq,Homo_723,MCI.vs.control; bulk RNA-seq,Homo_714,AD.vs.control; bulk RNA-seq,Homo_714,AD.vs.MCI; bulk RNA-seq,Homo_714,MCI.vs.control; bulk RNA-seq,Homo_633,MCI.vs.control	7
BP	GO:0060348	bone development	bulk RNA-seq,Homo_723,AD.vs.control; bulk RNA-seq,Homo_723,AD.vs.MCI; bulk RNA-seq,Homo_723,MCI.vs.control; bulk RNA-seq,Homo_714,AD.vs.control; bulk RNA-seq,Homo_714,AD.vs.MCI; bulk RNA-seq,Homo_633,AD.vs.control; bulk RNA-seq,Homo_633,AD.vs.MCI	7
BP	GO:0007422	peripheral nervous system development	bulk RNA-seq,Homo_723,AD.vs.control; bulk RNA-seq,Homo_723,AD.vs.MCI; bulk RNA-seq,Homo_723,MCI.vs.control; bulk RNA-seq,Homo_714,AD.vs.control; bulk RNA-seq,Homo_714,AD.vs.MCI; bulk RNA-seq,Homo_714,MCI.vs.control	6
BP	GO:0090503	RNA phosphodiester bond hydrolysis, exonucleolytic	bulk RNA-seq,Homo_723,AD.vs.control; bulk RNA-seq,Homo_723,AD.vs.MCI; bulk RNA-seq,Homo_714,AD.vs.control; bulk RNA-seq,Homo_714,AD.vs.MCI; bulk RNA-seq,Homo_633,AD.vs.control; bulk RNA-seq,Homo_633,AD.vs.MCI	6
MF	GO:0004518	nuclease activity	bulk RNA-seq,Homo_723,AD.vs.control; bulk RNA-seq,Homo_723,MCI.vs.control; bulk RNA-seq,Homo_714,AD.vs.control; bulk RNA-seq,Homo_633,AD.vs.control; bulk RNA-seq,Homo_633,AD.vs.MCI	5
BP	GO:0009798	axis specification	bulk RNA-seq,Homo_723,AD.vs.control; bulk RNA-seq,Homo_723,AD.vs.MCI; bulk RNA-seq,Homo_723,MCI.vs.control; bulk RNA-seq,Homo_714,AD.vs.control; bulk RNA-seq,Homo_714,AD.vs.MCI; bulk RNA-seq,Homo_714,MCI.vs.control	6
BP	GO:0002067	glandular epithelial cell differentiation	bulk RNA-seq,Homo_723,AD.vs.control; bulk RNA-seq,Homo_723,AD.vs.MCI; bulk RNA-seq,Homo_723,MCI.vs.control; bulk RNA-seq,Homo_714,AD.vs.control; bulk RNA-seq,Homo_714,AD.vs.MCI; bulk RNA-seq,Homo_714,MCI.vs.control	6
BP	GO:0006911	phagocytosis, engulfment	bulk RNA-seq,Homo_723,AD.vs.control; bulk RNA-seq,Homo_723,AD.vs.MCI; bulk RNA-seq,Homo_714,AD.vs.control; bulk RNA-seq,Homo_714,AD.vs.MCI; bulk RNA-seq,Homo_633,AD.vs.control; bulk RNA-seq,Homo_633,AD.vs.MCI; bulk RNA-seq,MCSA,MCI.vs.control; scRNA-seq,SRP330776,Naive CD8+ T cell_2-AD.vs.control; scRNA-seq,SRP309935,Monocyte_2-AD.vs.control; scRNA-seq,SRP309935,Plasmacytoid dendritic cell_1-AD.vs.control	10
BP	GO:0098698	postsynaptic specialization assembly	bulk RNA-seq,Homo_723,AD.vs.control; bulk RNA-seq,Homo_723,AD.vs.MCI; bulk RNA-seq,Homo_723,MCI.vs.control; bulk RNA-seq,Homo_714,AD.vs.MCI; bulk RNA-seq,Homo_714,MCI.vs.control	5
BP	GO:0002709	regulation of T cell mediated immunity	bulk RNA-seq,Homo_723,AD.vs.control; bulk RNA-seq,Homo_723,AD.vs.MCI; bulk RNA-seq,Homo_723,MCI.vs.control; bulk RNA-seq,Homo_714,AD.vs.control; bulk RNA-seq,Homo_714,MCI.vs.control; bulk RNA-seq,Homo_633,AD.vs.control; bulk RNA-seq,Homo_633,AD.vs.MCI	7
BP	GO:0051954	positive regulation of amine transport	bulk RNA-seq,Homo_723,AD.vs.control; bulk RNA-seq,Homo_723,AD.vs.MCI; bulk RNA-seq,Homo_723,MCI.vs.control; bulk RNA-seq,Homo_714,AD.vs.control; bulk RNA-seq,Homo_714,AD.vs.MCI; bulk RNA-seq,SRP325058,AD.vs.control	6
MF	GO:0016796	exonuclease activity, active with either ribo- or deoxyribonucleic acids and producing 5'-phosphomonoesters	bulk RNA-seq,Homo_723,AD.vs.control; bulk RNA-seq,Homo_723,AD.vs.MCI; bulk RNA-seq,Homo_714,AD.vs.control; bulk RNA-seq,Homo_714,AD.vs.MCI; bulk RNA-seq,Homo_714,MCI.vs.control; bulk RNA-seq,Homo_633,AD.vs.control; bulk RNA-seq,Homo_633,AD.vs.MCI	7
BP	GO:0050772	positive regulation of axonogenesis	bulk RNA-seq,Homo_723,AD.vs.control; bulk RNA-seq,Homo_723,AD.vs.MCI; bulk RNA-seq,Homo_723,MCI.vs.control; bulk RNA-seq,Homo_714,AD.vs.control; bulk RNA-seq,Homo_714,AD.vs.MCI; bulk RNA-seq,Homo_714,MCI.vs.control; bulk RNA-seq,Homo_633,AD.vs.control; bulk	8

MF	GO:0004866	endopeptidase inhibitor activity	bulk RNA-seq,Homo_723,AD.vs.control; bulk RNA-seq,Homo_723,AD.vs.MCI; bulk RNA-seq,Homo_723,MCI.vs.control; bulk RNA-seq,Homo_714,AD.vs.control; bulk RNA-seq,Homo_714,AD.vs.MCI; bulk RNA-seq,Homo_633,AD.vs.control; bulk RNA-seq,Homo_633,AD.vs.MCI	7
BP	GO:0030177	positive regulation of Wnt signaling pathway	bulk RNA-seq,Homo_723,AD.vs.control; bulk RNA-seq,Homo_723,AD.vs.MCI; bulk RNA-seq,Homo_723,MCI.vs.control; bulk RNA-seq,Homo_714,AD.vs.control; bulk RNA-seq,Homo_714,AD.vs.MCI; bulk RNA-seq,Homo_633,AD.vs.control; bulk RNA-seq,Homo_633,AD.vs.MCI	7
CC	GO:0016324	apical plasma membrane	bulk RNA-seq,Homo_723,AD.vs.control; bulk RNA-seq,Homo_723,AD.vs.MCI; bulk RNA-seq,Homo_714,AD.vs.control; bulk RNA-seq,Homo_714,AD.vs.MCI; bulk RNA-seq,Homo_633,AD.vs.control; bulk RNA-seq,Homo_633,AD.vs.MCI; bulk RNA-seq,ROSMAP,AD.vs.control; bulk RNA-seq,SRP223445,AD.vs.control	8
BP	GO:0042475	odontogenesis of dentin-containing tooth	bulk RNA-seq,Homo_723,AD.vs.control; bulk RNA-seq,Homo_723,AD.vs.MCI; bulk RNA-seq,Homo_723,MCI.vs.control; bulk RNA-seq,Homo_714,AD.vs.control; bulk RNA-seq,Homo_714,AD.vs.MCI; bulk RNA-seq,Homo_714,MCI.vs.control; bulk RNA-seq,SRP223445,AD.vs.control	7
BP	GO:0003156	regulation of animal organ formation	bulk RNA-seq,Homo_723,AD.vs.control; bulk RNA-seq,Homo_723,AD.vs.MCI; bulk RNA-seq,Homo_723,MCI.vs.control; bulk RNA-seq,Homo_714,AD.vs.control; bulk RNA-seq,Homo_714,AD.vs.MCI; bulk RNA-seq,Homo_714,MCI.vs.control; bulk RNA-seq,Homo_633,MCI.vs.control	7
MF	GO:0051536	iron-sulfur cluster binding	bulk RNA-seq,Homo_723,AD.vs.control; bulk RNA-seq,Homo_723,MCI.vs.control; bulk RNA-seq,Homo_714,AD.vs.control; bulk RNA-seq,Homo_714,MCI.vs.control; bulk RNA-seq,Homo_633,AD.vs.control; bulk RNA-seq,Homo_633,AD.vs.MCI	6
MF	GO:0051540	metal cluster binding	bulk RNA-seq,Homo_723,AD.vs.control; bulk RNA-seq,Homo_723,MCI.vs.control; bulk RNA-seq,Homo_714,AD.vs.control; bulk RNA-seq,Homo_714,MCI.vs.control; bulk RNA-seq,Homo_633,AD.vs.control; bulk RNA-seq,Homo_633,AD.vs.MCI	6
BP	GO:0046928	regulation of neurotransmitter secretion	bulk RNA-seq,Homo_723,AD.vs.control; bulk RNA-seq,Homo_723,AD.vs.MCI; bulk RNA-seq,Homo_723,MCI.vs.control; bulk RNA-seq,Homo_714,AD.vs.MCI; bulk RNA-seq,Homo_714,MCI.vs.control; bulk RNA-seq,Homo_633,AD.vs.control; bulk RNA-seq,Homo_633,AD.vs.MCI	7
BP	GO:0031214	biomineral tissue development	bulk RNA-seq,Homo_723,AD.vs.control; bulk RNA-seq,Homo_723,AD.vs.MCI; bulk RNA-seq,Homo_723,MCI.vs.control; bulk RNA-seq,Homo_714,AD.vs.control; bulk RNA-seq,Homo_714,AD.vs.MCI; bulk RNA-seq,Homo_633,AD.vs.control; bulk RNA-seq,Homo_633,AD.vs.MCI; bulk RNA-	8
BP	GO:0000723	telomere maintenance	bulk RNA-seq,Homo_723,AD.vs.control; bulk RNA-seq,Homo_723,MCI.vs.control; bulk RNA-seq,Homo_714,AD.vs.control; bulk RNA-seq,Homo_633,AD.vs.control; bulk RNA-seq,Homo_633,AD.vs.MCI	5
MF	GO:0004714	transmembrane receptor protein tyrosine kinase activity	bulk RNA-seq,Homo_723,AD.vs.control; bulk RNA-seq,Homo_723,AD.vs.MCI; bulk RNA-seq,Homo_723,MCI.vs.control; bulk RNA-seq,Homo_714,AD.vs.MCI; bulk RNA-seq,Homo_714,MCI.vs.control; bulk RNA-seq,Homo_633,AD.vs.control; bulk RNA-seq,Homo_633,AD.vs.MCI; bulk RNA-seq,ROSMAP,AD.vs.control; bulk RNA-seq,ROSMAP,MCI.vs.control	9
BP	GO:0021542	dentate gyrus development	bulk RNA-seq,Homo_723,AD.vs.control; bulk RNA-seq,Homo_723,AD.vs.MCI; bulk RNA-seq,Homo_723,MCI.vs.control; bulk RNA-seq,Homo_714,AD.vs.control; bulk RNA-seq,Homo_714,AD.vs.MCI	5
BP	GO:0031128	developmental induction	bulk RNA-seq,Homo_723,AD.vs.control; bulk RNA-seq,Homo_723,AD.vs.MCI; bulk RNA-seq,Homo_723,MCI.vs.control; bulk RNA-seq,Homo_714,AD.vs.control; bulk RNA-seq,Homo_714,AD.vs.MCI; bulk RNA-seq,Homo_714,MCI.vs.control; bulk RNA-seq,Homo_633,MCI.vs.control; bulk	8
BP	GO:0034754	cellular hormone metabolic process	bulk RNA-seq,Homo_723,AD.vs.control; bulk RNA-seq,Homo_723,AD.vs.MCI; bulk RNA-seq,Homo_723,MCI.vs.control; bulk RNA-seq,Homo_714,AD.vs.control; bulk RNA-seq,Homo_714,AD.vs.MCI	5
CC	GO:0098563	intrinsic component of synaptic vesicle membrane	bulk RNA-seq,Homo_723,AD.vs.control; bulk RNA-seq,Homo_723,AD.vs.MCI; bulk RNA-seq,Homo_723,MCI.vs.control; bulk RNA-seq,Homo_714,AD.vs.MCI	4
MF	GO:0004175	endopeptidase activity	bulk RNA-seq,Homo_723,AD.vs.control; bulk RNA-seq,Homo_723,AD.vs.MCI; bulk RNA-seq,Homo_714,AD.vs.control; bulk RNA-seq,Homo_714,AD.vs.MCI; bulk RNA-seq,Homo_633,AD.vs.control; bulk RNA-seq,Homo_633,AD.vs.MCI; bulk RNA-seq,SRP223445,AD.vs.control	7
BP	GO:1904861	excitatory synapse assembly	bulk RNA-seq,Homo_723,AD.vs.control; bulk RNA-seq,Homo_723,AD.vs.MCI; bulk RNA-seq,Homo_723,MCI.vs.control; bulk RNA-seq,Homo_714,AD.vs.MCI; bulk RNA-seq,Homo_714,MCI.vs.control	5
CC	GO:0044298	cell body membrane	bulk RNA-seq,Homo_723,AD.vs.control; bulk RNA-seq,Homo_723,AD.vs.MCI; bulk RNA-seq,Homo_723,MCI.vs.control; bulk RNA-seq,Homo_714,AD.vs.MCI	4
BP	GO:1902742	apoptotic process involved in development	bulk RNA-seq,Homo_723,AD.vs.control; bulk RNA-seq,Homo_723,AD.vs.MCI; bulk RNA-seq,Homo_723,MCI.vs.control; bulk RNA-seq,Homo_714,AD.vs.control; bulk RNA-seq,Homo_714,AD.vs.MCI; bulk RNA-seq,Homo_633,MCI.vs.control	6
MF	GO:0005231	excitatory extracellular ligand-gated ion channel activity	bulk RNA-seq,Homo_723,AD.vs.control; bulk RNA-seq,Homo_723,AD.vs.MCI; bulk RNA-seq,Homo_723,MCI.vs.control; bulk RNA-seq,Homo_714,AD.vs.control; bulk RNA-seq,Homo_714,AD.vs.MCI	5
BP	GO:0032786	positive regulation of DNA-templated transcription, elongation	bulk RNA-seq,Homo_723,AD.vs.control; bulk RNA-seq,Homo_723,AD.vs.MCI; bulk RNA-seq,Homo_723,MCI.vs.control; bulk RNA-seq,Homo_714,AD.vs.control; bulk RNA-seq,Homo_714,MCI.vs.control; bulk RNA-seq,Homo_633,AD.vs.control; bulk RNA-seq,Homo_633,AD.vs.MCI	7
BP	GO:0015872	dopamine transport	bulk RNA-seq,Homo_723,AD.vs.control; bulk RNA-seq,Homo_723,AD.vs.MCI; bulk RNA-seq,Homo_723,MCI.vs.control; bulk RNA-seq,Homo_714,AD.vs.control; bulk RNA-seq,Homo_714,AD.vs.MCI	5
BP	GO:0050771	negative regulation of axonogenesis	bulk RNA-seq,Homo_723,AD.vs.control; bulk RNA-seq,Homo_723,AD.vs.MCI; bulk RNA-seq,Homo_723,MCI.vs.control; bulk RNA-seq,Homo_714,AD.vs.MCI; bulk RNA-seq,Homo_714,MCI.vs.control; bulk RNA-seq,Homo_633,AD.vs.control; bulk RNA-seq,ROSMAP,AD.vs.control	7
BP	GO:0019080	viral gene expression	bulk RNA-seq,Homo_723,AD.vs.control; bulk RNA-seq,Homo_723,AD.vs.MCI; bulk RNA-seq,Homo_723,MCI.vs.control; bulk RNA-seq,Homo_714,AD.vs.control; bulk RNA-seq,Homo_714,MCI.vs.control; bulk RNA-seq,Homo_633,AD.vs.control; bulk RNA-seq,Homo_633,AD.vs.MCI	7
BP	GO:0021988	olfactory lobe development	bulk RNA-seq,Homo_723,AD.vs.control; bulk RNA-seq,Homo_723,AD.vs.MCI; bulk RNA-seq,Homo_723,MCI.vs.control; bulk RNA-seq,Homo_714,AD.vs.control; bulk RNA-seq,Homo_714,AD.vs.MCI; bulk RNA-seq,Homo_633,MCI.vs.control	6
BP	GO:0042255	ribosome assembly	bulk RNA-seq,Homo_723,AD.vs.control; bulk RNA-seq,Homo_723,AD.vs.MCI; bulk RNA-seq,Homo_723,MCI.vs.control; bulk RNA-seq,Homo_714,AD.vs.control; bulk RNA-seq,Homo_714,MCI.vs.control; bulk RNA-seq,Homo_633,AD.vs.control; bulk RNA-seq,Homo_633,AD.vs.MCI	7
BP	GO:0035265	organ growth	bulk RNA-seq,Homo_723,AD.vs.control; bulk RNA-seq,Homo_723,AD.vs.MCI; bulk RNA-seq,Homo_723,MCI.vs.control; bulk RNA-seq,Homo_714,AD.vs.control; bulk RNA-seq,Homo_714,AD.vs.MCI; bulk RNA-seq,Homo_633,AD.vs.control; bulk RNA-seq,Homo_633,AD.vs.MCI; bulk RNA-	8
BP	GO:0001941	postsynaptic membrane organization	bulk RNA-seq,Homo_723,AD.vs.control; bulk RNA-seq,Homo_723,AD.vs.MCI; bulk RNA-seq,Homo_723,MCI.vs.control; bulk RNA-seq,Homo_714,AD.vs.MCI; bulk RNA-seq,Homo_714,MCI.vs.control	5
MF	GO:0030515	snoRNA binding	bulk RNA-seq,Homo_723,AD.vs.control; bulk RNA-seq,Homo_723,AD.vs.MCI; bulk RNA-seq,Homo_723,MCI.vs.control; bulk RNA-seq,Homo_714,AD.vs.control; bulk RNA-seq,Homo_714,AD.vs.MCI; bulk RNA-seq,Homo_714,MCI.vs.control; bulk RNA-seq,Homo_633,AD.vs.control; bulk RNA-seq,Homo_633,AD.vs.MCI; bulk RNA-seq,Homo_633,MCI.vs.control	9
BP	GO:0045216	cell-cell junction organization	bulk RNA-seq,Homo_723,AD.vs.control; bulk RNA-seq,Homo_723,AD.vs.MCI; bulk RNA-seq,Homo_723,MCI.vs.control; bulk RNA-seq,Homo_714,AD.vs.control; bulk RNA-seq,Homo_714,AD.vs.MCI; bulk RNA-seq,Homo_633,AD.vs.control; bulk RNA-seq,Homo_633,AD.vs.MCI; bulk RNA-	8
BP	GO:0072210	metanephric nephron development	bulk RNA-seq,Homo_723,AD.vs.control; bulk RNA-seq,Homo_723,AD.vs.MCI; bulk RNA-seq,Homo_723,MCI.vs.control; bulk RNA-seq,Homo_714,AD.vs.control; bulk RNA-seq,Homo_714,AD.vs.MCI; bulk RNA-seq,Homo_714,MCI.vs.control; bulk RNA-seq,Homo_633,MCI.vs.control	7
BP	GO:0046942	carboxylic acid transport	bulk RNA-seq,Homo_723,AD.vs.control; bulk RNA-seq,Homo_723,AD.vs.MCI; bulk RNA-seq,Homo_723,MCI.vs.control; bulk RNA-seq,Homo_714,AD.vs.control; bulk RNA-seq,Homo_714,AD.vs.MCI; bulk RNA-seq,Homo_633,AD.vs.control; bulk RNA-seq,Homo_633,AD.vs.MCI; bulk RNA-seq,ROSMAP,AD.vs.control; bulk RNA-seq,ROSMAP,MCI.vs.control	9

BP	GO:0060291	long-term synaptic potentiation	bulk RNA-seq,Homo_723,AD.vs.control; bulk RNA-seq,Homo_723,AD.vs.MCI; bulk RNA-seq,Homo_723,MCI.vs.control; bulk RNA-seq,Homo_714,AD.vs.control; bulk RNA-seq,Homo_714,AD.vs.MCI; bulk RNA-seq,Homo_714,MCI.vs.control	6
BP	GO:0110148	biomineralization	bulk RNA-seq,Homo_723,AD.vs.control; bulk RNA-seq,Homo_723,AD.vs.MCI; bulk RNA-seq,Homo_723,MCI.vs.control; bulk RNA-seq,Homo_714,AD.vs.control; bulk RNA-seq,Homo_714,AD.vs.MCI; bulk RNA-seq,Homo_633,AD.vs.control; bulk RNA-seq,Homo_633,AD.vs.MCI; bulk RNA-	8
BP	GO:0002027	regulation of heart rate	bulk RNA-seq,Homo_723,AD.vs.control; bulk RNA-seq,Homo_723,AD.vs.MCI; bulk RNA-seq,Homo_723,MCI.vs.control; bulk RNA-seq,Homo_714,AD.vs.control; bulk RNA-seq,Homo_714,AD.vs.MCI; bulk RNA-seq,Homo_714,MCI.vs.control	6
BP	GO:0006910	phagocytosis, recognition	bulk RNA-seq,Homo_723,AD.vs.control; bulk RNA-seq,Homo_723,AD.vs.MCI; bulk RNA-seq,Homo_714,AD.vs.control; bulk RNA-seq,Homo_714,AD.vs.MCI; bulk RNA-seq,Homo_633,AD.vs.control; bulk RNA-seq,Homo_633,AD.vs.MCI; scRNA-seq,SRP330776,Naive CD8+ T cell_1-AD.vs.control; scRNA-	8
BP	GO:0010977	negative regulation of neuron projection development	bulk RNA-seq,Homo_723,AD.vs.control; bulk RNA-seq,Homo_723,AD.vs.MCI; bulk RNA-seq,Homo_723,MCI.vs.control; bulk RNA-seq,Homo_714,AD.vs.MCI; bulk RNA-seq,Homo_633,AD.vs.control; bulk RNA-seq,Homo_633,AD.vs.MCI; bulk RNA-seq,ROSMAP,AD.vs.control; scRNA-seq,SRP330776,Naive CD8+ T	8
BP	GO:0048675	axon extension	bulk RNA-seq,Homo_723,AD.vs.control; bulk RNA-seq,Homo_723,AD.vs.MCI; bulk RNA-seq,Homo_723,MCI.vs.control; bulk RNA-seq,Homo_714,AD.vs.MCI; bulk RNA-seq,Homo_714,MCI.vs.control; bulk RNA-seq,Homo_633,AD.vs.control; bulk RNA-seq,Homo_633,AD.vs.MCI	7
BP	GO:0050920	regulation of chemotaxis	bulk RNA-seq,Homo_723,AD.vs.control; bulk RNA-seq,Homo_723,AD.vs.MCI; bulk RNA-seq,Homo_723,MCI.vs.control; bulk RNA-seq,Homo_714,AD.vs.control; bulk RNA-seq,Homo_714,AD.vs.MCI; bulk RNA-seq,Homo_633,AD.vs.control; bulk RNA-seq,Homo_633,AD.vs.MCI; scRNA-	8
BP	GO:0032968	positive regulation of transcription elongation by RNA polymerase II	bulk RNA-seq,Homo_723,AD.vs.control; bulk RNA-seq,Homo_723,AD.vs.MCI; bulk RNA-seq,Homo_723,MCI.vs.control; bulk RNA-seq,Homo_714,AD.vs.control; bulk RNA-seq,Homo_714,MCI.vs.control; bulk RNA-seq,Homo_633,AD.vs.control; bulk RNA-seq,Homo_633,AD.vs.MCI	7
BP	GO:0015711	organic anion transport	bulk RNA-seq,Homo_723,AD.vs.control; bulk RNA-seq,Homo_723,AD.vs.MCI; bulk RNA-seq,Homo_714,AD.vs.control; bulk RNA-seq,Homo_714,AD.vs.MCI; bulk RNA-seq,Homo_633,AD.vs.control; bulk RNA-seq,Homo_633,AD.vs.MCI; bulk RNA-seq,ROSMAP,AD.vs.control	7
BP	GO:0010092	specification of animal organ identity	bulk RNA-seq,Homo_723,AD.vs.control; bulk RNA-seq,Homo_723,AD.vs.MCI; bulk RNA-seq,Homo_723,MCI.vs.control; bulk RNA-seq,Homo_714,AD.vs.control; bulk RNA-seq,Homo_714,AD.vs.MCI; bulk RNA-seq,Homo_714,MCI.vs.control; bulk RNA-seq,Homo_633,MCI.vs.control	7
BP	GO:0003407	neural retina development	bulk RNA-seq,Homo_723,AD.vs.control; bulk RNA-seq,Homo_723,AD.vs.MCI; bulk RNA-seq,Homo_723,MCI.vs.control; bulk RNA-seq,Homo_714,AD.vs.MCI; bulk RNA-seq,Homo_714,MCI.vs.control; bulk RNA-seq,Homo_633,AD.vs.control; bulk RNA-seq,Homo_633,AD.vs.MCI; bulk RNA-	8
BP	GO:0007368	determination of left/right symmetry	bulk RNA-seq,Homo_723,AD.vs.control; bulk RNA-seq,Homo_723,AD.vs.MCI; bulk RNA-seq,Homo_723,MCI.vs.control; bulk RNA-seq,Homo_714,AD.vs.control; bulk RNA-seq,Homo_714,AD.vs.MCI; bulk RNA-seq,Homo_714,MCI.vs.control; bulk RNA-seq,Homo_633,AD.vs.control; bulk	8
BP	GO:0035725	sodium ion transmembrane transport	bulk RNA-seq,Homo_723,AD.vs.control; bulk RNA-seq,Homo_723,AD.vs.MCI; bulk RNA-seq,Homo_723,MCI.vs.control; bulk RNA-seq,Homo_714,AD.vs.control; bulk RNA-seq,Homo_714,AD.vs.MCI; bulk RNA-seq,Homo_633,AD.vs.control; bulk RNA-seq,Homo_633,AD.vs.MCI; bulk RNA-seq,ROSMAP,AD.vs.control; bulk RNA-seq,ROSMAP,MCI.vs.control; bulk RNA-seq,SRP223445,AD.vs.control	10
BP	GO:0016445	somatic diversification of immunoglobulins	bulk RNA-seq,Homo_723,AD.vs.control; bulk RNA-seq,Homo_723,MCI.vs.control; bulk RNA-seq,Homo_714,AD.vs.control; bulk RNA-seq,Homo_714,MCI.vs.control; bulk RNA-seq,Homo_633,AD.vs.control; bulk RNA-seq,Homo_633,AD.vs.MCI	6
BP	GO:0008306	associative learning	bulk RNA-seq,Homo_723,AD.vs.control; bulk RNA-seq,Homo_723,AD.vs.MCI; bulk RNA-seq,Homo_723,MCI.vs.control; bulk RNA-seq,Homo_714,AD.vs.control; bulk RNA-seq,Homo_714,AD.vs.MCI; bulk RNA-seq,Homo_714,MCI.vs.control; bulk RNA-seq,Homo_633,AD.vs.control	7
BP	GO:2000311	regulation of AMPA receptor activity	bulk RNA-seq,Homo_723,AD.vs.control; bulk RNA-seq,Homo_723,AD.vs.MCI; bulk RNA-seq,Homo_723,MCI.vs.control; bulk RNA-seq,Homo_714,AD.vs.MCI; bulk RNA-seq,Homo_714,MCI.vs.control	5
BP	GO:0060074	synapse maturation	bulk RNA-seq,Homo_723,AD.vs.control; bulk RNA-seq,Homo_723,AD.vs.MCI; bulk RNA-seq,Homo_723,MCI.vs.control; bulk RNA-seq,Homo_714,AD.vs.control; bulk RNA-seq,Homo_714,AD.vs.MCI; bulk RNA-seq,Homo_714,MCI.vs.control	6
BP	GO:0031032	actomyosin structure organization	bulk RNA-seq,Homo_723,AD.vs.control; bulk RNA-seq,Homo_723,AD.vs.MCI; bulk RNA-seq,Homo_723,MCI.vs.control; bulk RNA-seq,Homo_714,AD.vs.control; bulk RNA-seq,Homo_714,AD.vs.MCI; bulk RNA-seq,Homo_633,AD.vs.control; bulk RNA-seq,Homo_633,AD.vs.MCI	7
BP	GO:0007628	adult walking behavior	bulk RNA-seq,Homo_723,AD.vs.control; bulk RNA-seq,Homo_723,AD.vs.MCI; bulk RNA-seq,Homo_723,MCI.vs.control; bulk RNA-seq,Homo_714,AD.vs.control; bulk RNA-seq,Homo_714,AD.vs.MCI; bulk RNA-seq,Homo_714,MCI.vs.control; bulk RNA-seq,Homo_633,AD.vs.control; bulk	8
MF	GO:0019199	transmembrane receptor protein kinase activity	bulk RNA-seq,Homo_723,AD.vs.control; bulk RNA-seq,Homo_723,AD.vs.MCI; bulk RNA-seq,Homo_723,MCI.vs.control; bulk RNA-seq,Homo_714,AD.vs.control; bulk RNA-seq,Homo_714,AD.vs.MCI; bulk RNA-seq,Homo_714,MCI.vs.control; bulk RNA-seq,Homo_633,AD.vs.control; bulk RNA-seq,Homo_633,AD.vs.MCI; bulk RNA-seq,ROSMAP,AD.vs.control; bulk RNA-seq,ROSMAP,MCI.vs.control	10
BP	GO:0050433	regulation of catecholamine secretion	bulk RNA-seq,Homo_723,AD.vs.control; bulk RNA-seq,Homo_723,AD.vs.MCI; bulk RNA-seq,Homo_723,MCI.vs.control; bulk RNA-seq,Homo_714,AD.vs.control; bulk RNA-seq,Homo_714,AD.vs.MCI; bulk RNA-seq,SRP325058,AD.vs.control	6
BP	GO:0045665	negative regulation of neuron differentiation	bulk RNA-seq,Homo_723,AD.vs.control; bulk RNA-seq,Homo_723,AD.vs.MCI; bulk RNA-seq,Homo_723,MCI.vs.control; bulk RNA-seq,Homo_714,AD.vs.MCI; bulk RNA-seq,Homo_714,MCI.vs.control; bulk RNA-seq,Homo_633,AD.vs.control; bulk RNA-seq,Homo_633,AD.vs.MCI; bulk RNA-	8
BP	GO:0097107	postsynaptic density assembly	bulk RNA-seq,Homo_723,AD.vs.control; bulk RNA-seq,Homo_723,AD.vs.MCI; bulk RNA-seq,Homo_723,MCI.vs.control; bulk RNA-seq,Homo_714,AD.vs.MCI; bulk RNA-seq,Homo_714,MCI.vs.control	5
MF	GO:0005501	retinoid binding	bulk RNA-seq,Homo_723,AD.vs.control; bulk RNA-seq,Homo_723,AD.vs.MCI; bulk RNA-seq,Homo_723,MCI.vs.control; bulk RNA-seq,Homo_714,AD.vs.control; bulk RNA-seq,Homo_714,AD.vs.MCI; bulk RNA-seq,Homo_633,MCI.vs.control	6
MF	GO:0019840	isoprenoid binding	bulk RNA-seq,Homo_723,AD.vs.control; bulk RNA-seq,Homo_723,AD.vs.MCI; bulk RNA-seq,Homo_723,MCI.vs.control; bulk RNA-seq,Homo_714,AD.vs.control; bulk RNA-seq,Homo_714,AD.vs.MCI; bulk RNA-seq,Homo_633,MCI.vs.control	6
MF	GO:0016896	exoribonuclease activity, producing 5'-phosphomonoesters	bulk RNA-seq,Homo_723,AD.vs.control; bulk RNA-seq,Homo_723,AD.vs.MCI; bulk RNA-seq,Homo_714,AD.vs.control; bulk RNA-seq,Homo_714,AD.vs.MCI; bulk RNA-seq,Homo_633,AD.vs.control; bulk RNA-seq,Homo_633,AD.vs.MCI	6
CC	GO:0060077	inhibitory synapse	bulk RNA-seq,Homo_723,AD.vs.control; bulk RNA-seq,Homo_723,AD.vs.MCI; bulk RNA-seq,Homo_723,MCI.vs.control; bulk RNA-seq,Homo_714,AD.vs.control; bulk RNA-seq,Homo_714,AD.vs.MCI; bulk RNA-seq,Homo_714,MCI.vs.control	6
BP	GO:0030178	negative regulation of Wnt signaling pathway	bulk RNA-seq,Homo_723,AD.vs.control; bulk RNA-seq,Homo_723,AD.vs.MCI; bulk RNA-seq,Homo_723,MCI.vs.control; bulk RNA-seq,Homo_714,AD.vs.MCI; bulk RNA-seq,Homo_633,AD.vs.control	5
BP	GO:0009799	specification of symmetry	bulk RNA-seq,Homo_723,AD.vs.control; bulk RNA-seq,Homo_723,AD.vs.MCI; bulk RNA-seq,Homo_723,MCI.vs.control; bulk RNA-seq,Homo_714,AD.vs.control; bulk RNA-seq,Homo_714,AD.vs.MCI; bulk RNA-seq,Homo_633,AD.vs.control; bulk RNA-seq,Homo_633,AD.vs.MCI	7
MF	GO:0051539	4 iron, 4 sulfur cluster binding	bulk RNA-seq,Homo_723,AD.vs.control; bulk RNA-seq,Homo_723,MCI.vs.control; bulk RNA-seq,Homo_714,AD.vs.control; bulk RNA-seq,Homo_714,MCI.vs.control; bulk RNA-seq,Homo_633,AD.vs.control; bulk RNA-seq,Homo_633,AD.vs.MCI	6
CC	GO:0016342	catenin complex	bulk RNA-seq,Homo_723,AD.vs.control; bulk RNA-seq,Homo_723,AD.vs.MCI; bulk RNA-seq,Homo_723,MCI.vs.control; bulk RNA-seq,Homo_714,AD.vs.MCI; bulk RNA-seq,Homo_714,MCI.vs.control; bulk RNA-seq,Homo_633,MCI.vs.control	6
BP	GO:0031960	response to corticosteroid	bulk RNA-seq,Homo_723,AD.vs.control; bulk RNA-seq,Homo_723,AD.vs.MCI; bulk RNA-seq,Homo_723,MCI.vs.control; bulk RNA-seq,Homo_714,AD.vs.control; bulk RNA-seq,Homo_714,AD.vs.MCI; bulk RNA-seq,Homo_633,AD.vs.control; bulk RNA-seq,Homo_633,AD.vs.MCI	7

BP	GO:0050432	catecholamine secretion	bulk RNA-seq,Homo_723,AD.vs.control; bulk RNA-seq,Homo_723,AD.vs.MCI; bulk RNA-seq,Homo_723,MCI.vs.control; bulk RNA-seq,Homo_714,AD.vs.control; bulk RNA-seq,Homo_714,AD.vs.MCI; bulk RNA-seq,SRP325058,AD.vs.control	6
BP	GO:0007338	single fertilization	bulk RNA-seq,Homo_723,AD.vs.control; bulk RNA-seq,Homo_723,AD.vs.MCI; bulk RNA-seq,Homo_714,AD.vs.control; bulk RNA-seq,Homo_714,AD.vs.MCI	4
BP	GO:0021854	hypothalamus development	bulk RNA-seq,Homo_723,AD.vs.control; bulk RNA-seq,Homo_723,AD.vs.MCI; bulk RNA-seq,Homo_714,AD.vs.control; bulk RNA-seq,Homo_714,AD.vs.MCI; bulk RNA-seq,Homo_633,MCI.vs.control	5
BP	GO:0046545	development of primary female sexual characteristics	bulk RNA-seq,Homo_723,AD.vs.control; bulk RNA-seq,Homo_723,AD.vs.MCI; bulk RNA-seq,Homo_723,MCI.vs.control; bulk RNA-seq,Homo_714,AD.vs.control; bulk RNA-seq,Homo_714,AD.vs.MCI; bulk RNA-seq,Homo_714,MCI.vs.control; bulk RNA-seq,Homo_633,AD.vs.control	7
BP	GO:0051384	response to glucocorticoid	bulk RNA-seq,Homo_723,AD.vs.control; bulk RNA-seq,Homo_723,AD.vs.MCI; bulk RNA-seq,Homo_723,MCI.vs.control; bulk RNA-seq,Homo_714,AD.vs.control; bulk RNA-seq,Homo_714,AD.vs.MCI; bulk RNA-seq,Homo_633,AD.vs.control; bulk RNA-seq,Homo_633,AD.vs.MCI	7
BP	GO:0007018	microtubule-based movement	bulk RNA-seq,Homo_723,AD.vs.control; bulk RNA-seq,Homo_723,AD.vs.MCI; bulk RNA-seq,Homo_714,AD.vs.control; bulk RNA-seq,Homo_714,AD.vs.MCI; bulk RNA-seq,Homo_633,AD.vs.control; bulk RNA-seq,Homo_633,AD.vs.MCI; bulk RNA-seq,ROSMAP,AD.vs.control; bulk RNA-seq,ROSMAP,MCI.vs.control	8
BP	GO:0016339	calcium-dependent cell-cell adhesion via plasma membrane cell adhesion molecules	bulk RNA-seq,Homo_723,AD.vs.control; bulk RNA-seq,Homo_723,AD.vs.MCI; bulk RNA-seq,Homo_723,MCI.vs.control; bulk RNA-seq,Homo_714,AD.vs.control; bulk RNA-seq,Homo_714,AD.vs.MCI	5
BP	GO:0007187	G protein-coupled receptor signaling pathway, coupled to cyclic nucleotide second messenger	bulk RNA-seq,Homo_723,AD.vs.control; bulk RNA-seq,Homo_723,AD.vs.MCI; bulk RNA-seq,Homo_714,AD.vs.control; bulk RNA-seq,Homo_714,AD.vs.MCI; bulk RNA-seq,Homo_633,MCI.vs.control; bulk RNA-seq,SRP223445,AD.vs.control	6
BP	GO:0032970	regulation of actin filament-based process	bulk RNA-seq,Homo_723,AD.vs.control; bulk RNA-seq,Homo_723,AD.vs.MCI; bulk RNA-seq,Homo_714,AD.vs.control; bulk RNA-seq,Homo_714,AD.vs.MCI; bulk RNA-seq,Homo_633,AD.vs.control; bulk RNA-seq,Homo_633,AD.vs.MCI	6
BP	GO:0017158	regulation of calcium ion-dependent exocytosis	bulk RNA-seq,Homo_723,AD.vs.control; bulk RNA-seq,Homo_723,AD.vs.MCI; bulk RNA-seq,Homo_723,MCI.vs.control; bulk RNA-seq,Homo_714,AD.vs.MCI	4
CC	GO:0071007	U2-type catalytic step 2 spliceosome	bulk RNA-seq,Homo_723,AD.vs.control; bulk RNA-seq,Homo_723,AD.vs.MCI; bulk RNA-seq,Homo_723,MCI.vs.control; bulk RNA-seq,Homo_714,AD.vs.control; bulk RNA-seq,Homo_714,AD.vs.MCI; bulk RNA-seq,Homo_714,MCI.vs.control; bulk RNA-seq,Homo_633,AD.vs.control; bulk RNA-seq,Homo_633,AD.vs.MCI; bulk RNA-seq,Homo_633,MCI.vs.control	9
BP	GO:0060048	cardiac muscle contraction	bulk RNA-seq,Homo_723,AD.vs.control; bulk RNA-seq,Homo_723,AD.vs.MCI; bulk RNA-seq,Homo_723,MCI.vs.control; bulk RNA-seq,Homo_714,AD.vs.control; bulk RNA-seq,Homo_714,AD.vs.MCI; bulk RNA-seq,Homo_633,AD.vs.control	6
BP	GO:0009142	nucleoside triphosphate biosynthetic process	bulk RNA-seq,Homo_723,AD.vs.control; bulk RNA-seq,Homo_723,MCI.vs.control; bulk RNA-seq,Homo_714,AD.vs.control; bulk RNA-seq,Homo_633,AD.vs.control; bulk RNA-seq,Homo_633,AD.vs.MCI	5
MF	GO:0008194	UDP-glycosyltransferase activity	bulk RNA-seq,Homo_723,AD.vs.control; bulk RNA-seq,Homo_723,AD.vs.MCI; bulk RNA-seq,Homo_723,MCI.vs.control; bulk RNA-seq,Homo_714,AD.vs.control; bulk RNA-seq,Homo_714,AD.vs.MCI; bulk RNA-seq,Homo_633,AD.vs.control; bulk RNA-seq,Homo_633,AD.vs.MCI	7
BP	GO:0006754	ATP biosynthetic process	bulk RNA-seq,Homo_723,AD.vs.control; bulk RNA-seq,Homo_723,MCI.vs.control; bulk RNA-seq,Homo_714,AD.vs.control; bulk RNA-seq,Homo_714,MCI.vs.control; bulk RNA-seq,Homo_633,AD.vs.control; bulk RNA-seq,Homo_633,AD.vs.MCI	6
BP	GO:0090659	walking behavior	bulk RNA-seq,Homo_723,AD.vs.control; bulk RNA-seq,Homo_723,AD.vs.MCI; bulk RNA-seq,Homo_723,MCI.vs.control; bulk RNA-seq,Homo_714,AD.vs.control; bulk RNA-seq,Homo_714,AD.vs.MCI; bulk RNA-seq,Homo_633,AD.vs.control; bulk RNA-seq,Homo_633,AD.vs.MCI	7
BP	GO:0002200	somatic diversification of immune receptors	bulk RNA-seq,Homo_723,AD.vs.control; bulk RNA-seq,Homo_723,MCI.vs.control; bulk RNA-seq,Homo_714,AD.vs.control; bulk RNA-seq,Homo_714,MCI.vs.control; bulk RNA-seq,Homo_633,AD.vs.control; bulk RNA-seq,Homo_633,AD.vs.MCI	6
BP	GO:0110110	positive regulation of animal organ morphogenesis	bulk RNA-seq,Homo_723,AD.vs.control; bulk RNA-seq,Homo_723,AD.vs.MCI; bulk RNA-seq,Homo_723,MCI.vs.control; bulk RNA-seq,Homo_714,AD.vs.control; bulk RNA-seq,Homo_714,AD.vs.MCI; bulk RNA-seq,Homo_714,MCI.vs.control; bulk RNA-seq,Homo_633,MCI.vs.control	7
BP	GO:0098773	skin epidermis development	bulk RNA-seq,Homo_723,AD.vs.control; bulk RNA-seq,Homo_723,AD.vs.MCI; bulk RNA-seq,Homo_723,MCI.vs.control; bulk RNA-seq,Homo_714,AD.vs.control; bulk RNA-seq,Homo_714,AD.vs.MCI; bulk RNA-seq,Homo_714,MCI.vs.control; bulk RNA-seq,Homo_633,AD.vs.control; bulk	8
BP	GO:0009855	determination of bilateral symmetry	bulk RNA-seq,Homo_723,AD.vs.control; bulk RNA-seq,Homo_723,AD.vs.MCI; bulk RNA-seq,Homo_723,MCI.vs.control; bulk RNA-seq,Homo_714,AD.vs.control; bulk RNA-seq,Homo_714,AD.vs.MCI; bulk RNA-seq,Homo_714,MCI.vs.control; bulk RNA-seq,Homo_633,AD.vs.control; bulk	8
BP	GO:0022412	cellular process involved in reproduction in multicellular organism	bulk RNA-seq,Homo_723,AD.vs.control; bulk RNA-seq,Homo_723,AD.vs.MCI; bulk RNA-seq,Homo_714,AD.vs.control; bulk RNA-seq,Homo_714,AD.vs.MCI; bulk RNA-seq,Homo_633,AD.vs.control; bulk RNA-seq,Homo_633,AD.vs.MCI	6
BP	GO:0006403	RNA localization	bulk RNA-seq,Homo_723,AD.vs.control; bulk RNA-seq,Homo_723,MCI.vs.control; bulk RNA-seq,Homo_714,AD.vs.control; bulk RNA-seq,Homo_633,AD.vs.control; bulk RNA-seq,Homo_633,AD.vs.MCI	5
CC	GO:0031305	integral component of mitochondrial inner membrane	bulk RNA-seq,Homo_723,AD.vs.control; bulk RNA-seq,Homo_723,AD.vs.MCI; bulk RNA-seq,Homo_723,MCI.vs.control; bulk RNA-seq,Homo_714,AD.vs.control; bulk RNA-seq,Homo_714,AD.vs.MCI; bulk RNA-seq,Homo_714,MCI.vs.control; bulk RNA-seq,Homo_633,AD.vs.control; bulk RNA-seq,Homo_633,AD.vs.MCI; bulk RNA-seq,SRP325058,AD.vs.control	9
CC	GO:0036126	sperm flagellum	bulk RNA-seq,Homo_723,AD.vs.control; bulk RNA-seq,Homo_723,AD.vs.MCI; bulk RNA-seq,Homo_723,MCI.vs.control; bulk RNA-seq,Homo_714,AD.vs.control; bulk RNA-seq,Homo_714,AD.vs.MCI; bulk RNA-seq,Homo_633,AD.vs.control; bulk RNA-seq,Homo_633,AD.vs.MCI	7
MF	GO:0048019	receptor antagonist activity	bulk RNA-seq,Homo_723,AD.vs.control; bulk RNA-seq,Homo_723,AD.vs.MCI; bulk RNA-seq,Homo_714,AD.vs.control; bulk RNA-seq,Homo_714,AD.vs.MCI	4
MF	GO:0015081	sodium ion transmembrane transporter activity	bulk RNA-seq,Homo_723,AD.vs.control; bulk RNA-seq,Homo_723,AD.vs.MCI; bulk RNA-seq,Homo_723,MCI.vs.control; bulk RNA-seq,Homo_714,AD.vs.control; bulk RNA-seq,Homo_714,AD.vs.MCI; bulk RNA-seq,ROSMAP,AD.vs.control; bulk RNA-seq,ROSMAP,MCI.vs.control	7
CC	GO:0099568	cytoplasmic region	bulk RNA-seq,Homo_723,AD.vs.control; bulk RNA-seq,Homo_723,AD.vs.MCI; bulk RNA-seq,Homo_723,MCI.vs.control; bulk RNA-seq,Homo_714,AD.vs.MCI; bulk RNA-seq,Homo_633,AD.vs.control; bulk RNA-seq,Homo_633,AD.vs.MCI; bulk RNA-seq,ROSMAP,AD.vs.control; bulk RNA-seq,ROSMAP,MCI.vs.control	8
BP	GO:0021513	spinal cord dorsal/ventral patterning	bulk RNA-seq,Homo_723,AD.vs.control; bulk RNA-seq,Homo_723,AD.vs.MCI; bulk RNA-seq,Homo_723,MCI.vs.control; bulk RNA-seq,Homo_714,AD.vs.control; bulk RNA-seq,Homo_714,AD.vs.MCI	5
BP	GO:0042776	proton motive force-driven mitochondrial ATP synthesis	bulk RNA-seq,Homo_723,AD.vs.control; bulk RNA-seq,Homo_723,AD.vs.MCI; bulk RNA-seq,Homo_723,MCI.vs.control; bulk RNA-seq,Homo_714,AD.vs.control; bulk RNA-seq,Homo_714,AD.vs.MCI; bulk RNA-seq,Homo_714,MCI.vs.control; bulk RNA-seq,Homo_633,AD.vs.control; bulk	8
BP	GO:0030003	cellular cation homeostasis	bulk RNA-seq,Homo_723,AD.vs.control; bulk RNA-seq,Homo_723,AD.vs.MCI; bulk RNA-seq,Homo_714,AD.vs.control; bulk RNA-seq,Homo_714,AD.vs.MCI; bulk RNA-seq,Homo_633,AD.vs.control; bulk RNA-seq,Homo_633,AD.vs.MCI; scRNA-seq,SRP330776,Naive CD8+ T cell_2-AD.vs.control	7
CC	GO:0035580	specific granule lumen	bulk RNA-seq,Homo_723,AD.vs.control; bulk RNA-seq,Homo_723,AD.vs.MCI; bulk RNA-seq,Homo_714,AD.vs.control; bulk RNA-seq,Homo_714,MCI.vs.control; bulk RNA-seq,Homo_633,AD.vs.control; bulk RNA-seq,Homo_633,AD.vs.MCI; bulk RNA-seq,SRP223445,AD.vs.control	7
BP	GO:0021511	spinal cord patterning	bulk RNA-seq,Homo_723,AD.vs.control; bulk RNA-seq,Homo_723,AD.vs.MCI; bulk RNA-seq,Homo_723,MCI.vs.control; bulk RNA-seq,Homo_714,AD.vs.control; bulk RNA-seq,Homo_714,AD.vs.MCI	5
BP	GO:0000460	maturation of 5.8S rRNA	bulk RNA-seq,Homo_723,AD.vs.control; bulk RNA-seq,Homo_723,AD.vs.MCI; bulk RNA-seq,Homo_723,MCI.vs.control; bulk RNA-seq,Homo_714,AD.vs.control; bulk RNA-seq,Homo_714,AD.vs.MCI; bulk RNA-seq,Homo_714,MCI.vs.control; bulk RNA-seq,Homo_633,AD.vs.control; bulk	8

BP	GO:0002711	positive regulation of T cell mediated immunity	bulk RNA-seq,Homo_723,AD.vs.control; bulk RNA-seq,Homo_723,AD.vs.MCI; bulk RNA-seq,Homo_723,MCI.vs.control; bulk RNA-seq,Homo_714,AD.vs.control; bulk RNA-seq,Homo_714,MCI.vs.control; bulk RNA-seq,Homo_633,AD.vs.control; bulk RNA-seq,Homo_633,AD.vs.MCI; scRNA-	8
BP	GO:0033044	regulation of chromosome organization	bulk RNA-seq,Homo_723,AD.vs.control; bulk RNA-seq,Homo_723,MCI.vs.control; bulk RNA-seq,Homo_714,AD.vs.control; bulk RNA-seq,Homo_633,AD.vs.control; bulk RNA-seq,Homo_633,AD.vs.MCI	5
BP	GO:0001947	heart looping	bulk RNA-seq,Homo_723,AD.vs.control; bulk RNA-seq,Homo_723,AD.vs.MCI; bulk RNA-seq,Homo_723,MCI.vs.control; bulk RNA-seq,Homo_714,AD.vs.control; bulk RNA-seq,Homo_714,AD.vs.MCI; bulk RNA-seq,Homo_714,MCI.vs.control; bulk RNA-seq,SRP223445,AD.vs.control	7
BP	GO:0010463	mesenchymal cell proliferation	bulk RNA-seq,Homo_723,AD.vs.control; bulk RNA-seq,Homo_723,AD.vs.MCI; bulk RNA-seq,Homo_723,MCI.vs.control; bulk RNA-seq,Homo_714,AD.vs.control; bulk RNA-seq,Homo_714,AD.vs.MCI; bulk RNA-seq,Homo_714,MCI.vs.control; bulk RNA-seq,Homo_633,MCI.vs.control; bulk	8
MF	GO:0004532	exoribonuclease activity	bulk RNA-seq,Homo_723,AD.vs.control; bulk RNA-seq,Homo_723,AD.vs.MCI; bulk RNA-seq,Homo_714,AD.vs.control; bulk RNA-seq,Homo_714,AD.vs.MCI; bulk RNA-seq,Homo_633,AD.vs.control; bulk RNA-seq,Homo_633,AD.vs.MCI	6
BP	GO:0006413	translational initiation	bulk RNA-seq,Homo_723,AD.vs.control; bulk RNA-seq,Homo_723,AD.vs.MCI; bulk RNA-seq,Homo_723,MCI.vs.control; bulk RNA-seq,Homo_714,AD.vs.control; bulk RNA-seq,Homo_714,AD.vs.MCI; bulk RNA-seq,Homo_714,MCI.vs.control; bulk RNA-seq,Homo_633,AD.vs.control; bulk	8
BP	GO:0001502	cartilage condensation	bulk RNA-seq,Homo_723,AD.vs.control; bulk RNA-seq,Homo_723,AD.vs.MCI; bulk RNA-seq,Homo_723,MCI.vs.control; bulk RNA-seq,Homo_714,AD.vs.control; bulk RNA-seq,Homo_714,AD.vs.MCI	5
BP	GO:0070588	calcium ion transmembrane transport	bulk RNA-seq,Homo_723,AD.vs.control; bulk RNA-seq,Homo_723,AD.vs.MCI; bulk RNA-seq,Homo_714,AD.vs.control; bulk RNA-seq,Homo_714,AD.vs.MCI; bulk RNA-seq,Homo_633,AD.vs.control; bulk RNA-seq,Homo_633,AD.vs.MCI; bulk RNA-seq,ROSMAP,AD.vs.control	7
MF	GO:0004725	protein tyrosine phosphatase activity	bulk RNA-seq,Homo_723,AD.vs.control; bulk RNA-seq,Homo_723,AD.vs.MCI; bulk RNA-seq,Homo_723,MCI.vs.control; bulk RNA-seq,Homo_714,AD.vs.MCI; bulk RNA-seq,Homo_714,MCI.vs.control; bulk RNA-seq,Homo_633,AD.vs.control; bulk RNA-seq,Homo_633,AD.vs.MCI	7
BP	GO:0048846	axon extension involved in axon guidance	bulk RNA-seq,Homo_723,AD.vs.control; bulk RNA-seq,Homo_723,AD.vs.MCI; bulk RNA-seq,Homo_723,MCI.vs.control; bulk RNA-seq,Homo_714,AD.vs.MCI; bulk RNA-seq,Homo_714,MCI.vs.control; bulk RNA-seq,Homo_633,AD.vs.control	6
BP	GO:1902284	neuron projection extension involved in neuron projection guidance	bulk RNA-seq,Homo_723,AD.vs.control; bulk RNA-seq,Homo_723,AD.vs.MCI; bulk RNA-seq,Homo_723,MCI.vs.control; bulk RNA-seq,Homo_714,AD.vs.MCI; bulk RNA-seq,Homo_714,MCI.vs.control; bulk RNA-seq,Homo_633,AD.vs.control	6
BP	GO:1905874	regulation of postsynaptic density organization	bulk RNA-seq,Homo_723,AD.vs.control; bulk RNA-seq,Homo_723,AD.vs.MCI; bulk RNA-seq,Homo_723,MCI.vs.control; bulk RNA-seq,Homo_714,AD.vs.MCI	4
BP	GO:0010976	positive regulation of neuron projection development	bulk RNA-seq,Homo_723,AD.vs.control; bulk RNA-seq,Homo_723,AD.vs.MCI; bulk RNA-seq,Homo_723,MCI.vs.control; bulk RNA-seq,Homo_714,AD.vs.MCI; bulk RNA-seq,Homo_633,AD.vs.control; bulk RNA-seq,Homo_633,AD.vs.MCI	6
BP	GO:0006626	protein targeting to mitochondrion	bulk RNA-seq,Homo_723,AD.vs.control; bulk RNA-seq,Homo_723,MCI.vs.control; bulk RNA-seq,Homo_714,AD.vs.control; bulk RNA-seq,Homo_714,MCI.vs.control; bulk RNA-seq,Homo_633,AD.vs.control; bulk RNA-seq,Homo_633,AD.vs.MCI	6
BP	GO:0001709	cell fate determination	bulk RNA-seq,Homo_723,AD.vs.control; bulk RNA-seq,Homo_723,AD.vs.MCI; bulk RNA-seq,Homo_723,MCI.vs.control; bulk RNA-seq,Homo_714,AD.vs.control; bulk RNA-seq,Homo_714,AD.vs.MCI; bulk RNA-seq,Homo_714,MCI.vs.control; bulk RNA-seq,Homo_633,MCI.vs.control; bulk	8
BP	GO:0060071	Wnt signaling pathway, planar cell polarity pathway	bulk RNA-seq,Homo_723,AD.vs.control; bulk RNA-seq,Homo_723,AD.vs.MCI; bulk RNA-seq,Homo_723,MCI.vs.control; bulk RNA-seq,Homo_714,AD.vs.MCI; bulk RNA-seq,Homo_714,MCI.vs.control; bulk RNA-seq,Homo_633,AD.vs.control	6
BP	GO:0001736	establishment of planar polarity	bulk RNA-seq,Homo_723,AD.vs.control; bulk RNA-seq,Homo_723,AD.vs.MCI; bulk RNA-seq,Homo_723,MCI.vs.control; bulk RNA-seq,Homo_714,AD.vs.MCI; bulk RNA-seq,Homo_714,MCI.vs.control; bulk RNA-seq,Homo_633,AD.vs.control; bulk RNA-seq,Homo_633,AD.vs.MCI	7
BP	GO:0007164	establishment of tissue polarity	bulk RNA-seq,Homo_723,AD.vs.control; bulk RNA-seq,Homo_723,AD.vs.MCI; bulk RNA-seq,Homo_723,MCI.vs.control; bulk RNA-seq,Homo_714,AD.vs.MCI; bulk RNA-seq,Homo_714,MCI.vs.control; bulk RNA-seq,Homo_633,AD.vs.control; bulk RNA-seq,Homo_633,AD.vs.MCI	7
MF	GO:0017171	serine hydrolase activity	bulk RNA-seq,Homo_723,AD.vs.control; bulk RNA-seq,Homo_723,AD.vs.MCI; bulk RNA-seq,Homo_723,MCI.vs.control; bulk RNA-seq,Homo_714,AD.vs.control; bulk RNA-seq,Homo_714,AD.vs.MCI; bulk RNA-seq,Homo_633,AD.vs.control; bulk RNA-seq,Homo_633,AD.vs.MCI; bulk RNA-	8
MF	GO:0061135	endopeptidase regulator activity	bulk RNA-seq,Homo_723,AD.vs.control; bulk RNA-seq,Homo_723,AD.vs.MCI; bulk RNA-seq,Homo_723,MCI.vs.control; bulk RNA-seq,Homo_714,AD.vs.control; bulk RNA-seq,Homo_714,AD.vs.MCI; bulk RNA-seq,Homo_633,AD.vs.control; bulk RNA-seq,Homo_633,AD.vs.MCI	7
BP	GO:0048048	embryonic eye morphogenesis	bulk RNA-seq,Homo_723,AD.vs.control; bulk RNA-seq,Homo_723,AD.vs.MCI; bulk RNA-seq,Homo_723,MCI.vs.control; bulk RNA-seq,Homo_714,AD.vs.MCI; bulk RNA-seq,Homo_714,MCI.vs.control	5
BP	GO:0060512	prostate gland morphogenesis	bulk RNA-seq,Homo_723,AD.vs.control; bulk RNA-seq,Homo_723,AD.vs.MCI; bulk RNA-seq,Homo_723,MCI.vs.control; bulk RNA-seq,Homo_714,AD.vs.control; bulk RNA-seq,Homo_714,AD.vs.MCI; bulk RNA-seq,Homo_714,MCI.vs.control; bulk RNA-seq,Homo_633,MCI.vs.control	7
MF	GO:0016247	channel regulator activity	bulk RNA-seq,Homo_723,AD.vs.control; bulk RNA-seq,Homo_723,AD.vs.MCI; bulk RNA-seq,Homo_723,MCI.vs.control; bulk RNA-seq,Homo_714,AD.vs.MCI; bulk RNA-seq,Homo_633,AD.vs.control; bulk RNA-seq,Homo_633,AD.vs.MCI	6
CC	GO:0031304	intrinsic component of mitochondrial inner membrane	bulk RNA-seq,Homo_723,AD.vs.control; bulk RNA-seq,Homo_723,AD.vs.MCI; bulk RNA-seq,Homo_723,MCI.vs.control; bulk RNA-seq,Homo_714,AD.vs.control; bulk RNA-seq,Homo_714,AD.vs.MCI; bulk RNA-seq,Homo_714,MCI.vs.control; bulk RNA-seq,Homo_633,AD.vs.control; bulk RNA-seq,Homo_633,AD.vs.MCI; bulk RNA-seq,SRP325058,AD.vs.control	9
BP	GO:0031345	negative regulation of cell projection organization	bulk RNA-seq,Homo_723,AD.vs.control; bulk RNA-seq,Homo_723,AD.vs.MCI; bulk RNA-seq,Homo_723,MCI.vs.control; bulk RNA-seq,Homo_714,AD.vs.MCI; bulk RNA-seq,Homo_633,AD.vs.control; bulk RNA-seq,Homo_633,AD.vs.MCI; bulk RNA-seq,ROSMAP,AD.vs.control; scRNA-seq,SRP330776,Naive CD8+ T	8
CC	GO:0035770	ribonucleoprotein granule	bulk RNA-seq,Homo_723,AD.vs.control; bulk RNA-seq,Homo_723,MCI.vs.control; bulk RNA-seq,Homo_714,AD.vs.control; bulk RNA-seq,Homo_633,AD.vs.control; bulk RNA-seq,Homo_633,AD.vs.MCI	5
MF	GO:0008236	serine-type peptidase activity	bulk RNA-seq,Homo_723,AD.vs.control; bulk RNA-seq,Homo_723,AD.vs.MCI; bulk RNA-seq,Homo_723,MCI.vs.control; bulk RNA-seq,Homo_714,AD.vs.control; bulk RNA-seq,Homo_714,AD.vs.MCI; bulk RNA-seq,Homo_633,AD.vs.control; bulk RNA-seq,Homo_633,AD.vs.MCI; bulk RNA-	8
BP	GO:0017004	cytochrome complex assembly	bulk RNA-seq,Homo_723,AD.vs.control; bulk RNA-seq,Homo_723,AD.vs.MCI; bulk RNA-seq,Homo_723,MCI.vs.control; bulk RNA-seq,Homo_714,AD.vs.control; bulk RNA-seq,Homo_714,MCI.vs.control; bulk RNA-seq,Homo_633,AD.vs.control; bulk RNA-seq,Homo_633,AD.vs.MCI	7
CC	GO:0098644	complex of collagen trimers	bulk RNA-seq,Homo_723,AD.vs.control; bulk RNA-seq,Homo_723,AD.vs.MCI; bulk RNA-seq,Homo_723,MCI.vs.control; bulk RNA-seq,Homo_714,AD.vs.MCI; bulk RNA-seq,ROSMAP,AD.vs.control; bulk RNA-seq,SRP223445,AD.vs.control	6
BP	GO:0048678	response to axon injury	bulk RNA-seq,Homo_723,AD.vs.control; bulk RNA-seq,Homo_723,AD.vs.MCI; bulk RNA-seq,Homo_723,MCI.vs.control; bulk RNA-seq,Homo_714,AD.vs.MCI; bulk RNA-seq,Homo_714,MCI.vs.control; bulk RNA-seq,Homo_633,AD.vs.control; bulk RNA-seq,Homo_633,AD.vs.MCI	7
BP	GO:0051968	positive regulation of synaptic transmission, glutamatergic	bulk RNA-seq,Homo_723,AD.vs.control; bulk RNA-seq,Homo_723,AD.vs.MCI; bulk RNA-seq,Homo_723,MCI.vs.control; bulk RNA-seq,Homo_714,AD.vs.control; bulk RNA-seq,Homo_714,AD.vs.MCI; bulk RNA-seq,Homo_714,MCI.vs.control	6
BP	GO:0009612	response to mechanical stimulus	bulk RNA-seq,Homo_723,AD.vs.control; bulk RNA-seq,Homo_723,AD.vs.MCI; bulk RNA-seq,Homo_723,MCI.vs.control; bulk RNA-seq,Homo_714,AD.vs.control; bulk RNA-seq,Homo_714,AD.vs.MCI; bulk RNA-seq,Homo_633,AD.vs.control; bulk RNA-seq,Homo_633,AD.vs.MCI	7

BP	GO:0006956	complement activation	bulk RNA-seq,Homo_723,AD.vs.control; bulk RNA-seq,Homo_723,AD.vs.MCI; bulk RNA-seq,Homo_714,AD.vs.control; bulk RNA-seq,Homo_714,MCI.vs.control; bulk RNA-seq,Homo_633,AD.vs.control; bulk RNA-seq,Homo_633,AD.vs.MCI; scRNA-seq,SRP330776,Naive CD8+ T cell_1-AD.vs.control; scRNA-seq,SRP330776,Naive CD8+ T cell_2-AD.vs.control; scRNA-seq,SRP309935,Monocyte_2-AD.vs.control; scRNA-seq,SRP309935,Naive	10
BP	GO:0008585	female gonad development	bulk RNA-seq,Homo_723,AD.vs.control; bulk RNA-seq,Homo_723,MCI.vs.control; bulk RNA-seq,Homo_714,AD.vs.control; bulk RNA-seq,Homo_714,AD.vs.MCI; bulk RNA-seq,Homo_714,MCI.vs.control; bulk RNA-seq,Homo_633,AD.vs.control	7
BP	GO:0006457	protein folding	bulk RNA-seq,Homo_723,AD.vs.control; bulk RNA-seq,Homo_723,MCI.vs.control; bulk RNA-seq,Homo_714,AD.vs.control; bulk RNA-seq,Homo_633,AD.vs.control; bulk RNA-seq,Homo_633,AD.vs.MCI	5
BP	GO:0098743	cell aggregation	bulk RNA-seq,Homo_723,AD.vs.control; bulk RNA-seq,Homo_723,AD.vs.MCI; bulk RNA-seq,Homo_723,MCI.vs.control; bulk RNA-seq,Homo_714,AD.vs.control; bulk RNA-seq,Homo_714,AD.vs.MCI	5
BP	GO:0009201	ribonucleoside triphosphate biosynthetic process	bulk RNA-seq,Homo_723,AD.vs.control; bulk RNA-seq,Homo_723,MCI.vs.control; bulk RNA-seq,Homo_714,AD.vs.control; bulk RNA-seq,Homo_714,MCI.vs.control; bulk RNA-seq,Homo_633,AD.vs.control; bulk RNA-seq,Homo_633,AD.vs.MCI	6
BP	GO:0090175	regulation of establishment of planar polarity	bulk RNA-seq,Homo_723,AD.vs.control; bulk RNA-seq,Homo_723,AD.vs.MCI; bulk RNA-seq,Homo_723,MCI.vs.control; bulk RNA-seq,Homo_714,AD.vs.MCI; bulk RNA-seq,Homo_714,MCI.vs.control; bulk RNA-seq,Homo_633,AD.vs.control; bulk RNA-seq,Homo_633,AD.vs.MCI	7
BP	GO:0001649	osteoblast differentiation	bulk RNA-seq,Homo_723,AD.vs.control; bulk RNA-seq,Homo_723,AD.vs.MCI; bulk RNA-seq,Homo_723,MCI.vs.control; bulk RNA-seq,Homo_714,AD.vs.control; bulk RNA-seq,Homo_714,AD.vs.MCI; bulk RNA-seq,Homo_633,AD.vs.control; bulk RNA-seq,Homo_633,AD.vs.MCI; scRNA-	8
MF	GO:0030547	signaling receptor inhibitor activity	bulk RNA-seq,Homo_723,AD.vs.control; bulk RNA-seq,Homo_723,AD.vs.MCI; bulk RNA-seq,Homo_723,MCI.vs.control; bulk RNA-seq,Homo_714,AD.vs.control; bulk RNA-seq,Homo_714,AD.vs.MCI	5
BP	GO:0055007	cardiac muscle cell differentiation	bulk RNA-seq,Homo_723,AD.vs.control; bulk RNA-seq,Homo_723,AD.vs.MCI; bulk RNA-seq,Homo_723,MCI.vs.control; bulk RNA-seq,Homo_714,AD.vs.control; bulk RNA-seq,Homo_714,AD.vs.MCI; bulk RNA-seq,Homo_714,MCI.vs.control	6
BP	GO:0045956	positive regulation of calcium ion-dependent exocytosis	bulk RNA-seq,Homo_723,AD.vs.control; bulk RNA-seq,Homo_723,AD.vs.MCI; bulk RNA-seq,Homo_723,MCI.vs.control; bulk RNA-seq,Homo_714,AD.vs.MCI	4
BP	GO:0060411	cardiac septum morphogenesis	bulk RNA-seq,Homo_723,AD.vs.control; bulk RNA-seq,Homo_723,AD.vs.MCI; bulk RNA-seq,Homo_723,MCI.vs.control; bulk RNA-seq,Homo_714,AD.vs.control; bulk RNA-seq,Homo_714,AD.vs.MCI; bulk RNA-seq,Homo_714,MCI.vs.control; bulk RNA-seq,Homo_633,AD.vs.control; bulk	8
BP	GO:0021772	olfactory bulb development	bulk RNA-seq,Homo_723,AD.vs.control; bulk RNA-seq,Homo_723,AD.vs.MCI; bulk RNA-seq,Homo_723,MCI.vs.control; bulk RNA-seq,Homo_714,AD.vs.MCI; bulk RNA-seq,Homo_633,MCI.vs.control	5
BP	GO:0070252	actin-mediated cell contraction	bulk RNA-seq,Homo_723,AD.vs.control; bulk RNA-seq,Homo_723,AD.vs.MCI; bulk RNA-seq,Homo_723,MCI.vs.control; bulk RNA-seq,Homo_714,AD.vs.control; bulk RNA-seq,Homo_714,AD.vs.MCI; bulk RNA-seq,Homo_714,MCI.vs.control; bulk RNA-seq,Homo_633,AD.vs.control	7
BP	GO:0035282	segmentation	bulk RNA-seq,Homo_723,AD.vs.control; bulk RNA-seq,Homo_723,AD.vs.MCI; bulk RNA-seq,Homo_723,MCI.vs.control; bulk RNA-seq,Homo_714,AD.vs.MCI; bulk RNA-seq,Homo_714,MCI.vs.control; bulk RNA-seq,Homo_633,AD.vs.control; bulk RNA-seq,SRP223445,AD.vs.control	7
BP	GO:0021889	olfactory bulb interneuron differentiation	bulk RNA-seq,Homo_723,AD.vs.control; bulk RNA-seq,Homo_723,AD.vs.MCI; bulk RNA-seq,Homo_723,MCI.vs.control; bulk RNA-seq,Homo_714,AD.vs.control; bulk RNA-seq,Homo_714,AD.vs.MCI; bulk RNA-seq,Homo_633,MCI.vs.control	6
CC	GO:0005938	cell cortex	bulk RNA-seq,Homo_723,AD.vs.control; bulk RNA-seq,Homo_723,AD.vs.MCI; bulk RNA-seq,Homo_723,MCI.vs.control; bulk RNA-seq,Homo_714,AD.vs.control; bulk RNA-seq,Homo_714,AD.vs.MCI; bulk RNA-seq,Homo_633,AD.vs.control; bulk RNA-seq,Homo_633,AD.vs.MCI; bulk RNA-seq,ROSMAP,AD.vs.control; bulk RNA-seq,ROSMAP,MCI.vs.control	9
MF	GO:0004527	exonuclease activity	bulk RNA-seq,Homo_723,AD.vs.control; bulk RNA-seq,Homo_723,AD.vs.MCI; bulk RNA-seq,Homo_723,MCI.vs.control; bulk RNA-seq,Homo_714,AD.vs.control; bulk RNA-seq,Homo_714,MCI.vs.control; bulk RNA-seq,Homo_633,AD.vs.control; bulk RNA-seq,Homo_633,AD.vs.MCI	7
MF	GO:0008528	G protein-coupled peptide receptor activity	bulk RNA-seq,Homo_723,AD.vs.control; bulk RNA-seq,Homo_723,AD.vs.MCI; bulk RNA-seq,Homo_723,MCI.vs.control; bulk RNA-seq,Homo_714,AD.vs.control; bulk RNA-seq,Homo_714,AD.vs.MCI; bulk RNA-seq,Homo_633,MCI.vs.control; bulk RNA-seq,SRP223445,AD.vs.control	7
BP	GO:0098739	import across plasma membrane	bulk RNA-seq,Homo_723,AD.vs.control; bulk RNA-seq,Homo_723,AD.vs.MCI; bulk RNA-seq,Homo_723,MCI.vs.control; bulk RNA-seq,Homo_714,AD.vs.MCI; bulk RNA-seq,Homo_633,AD.vs.control; bulk RNA-seq,ROSMAP,AD.vs.control	6
CC	GO:0000314	organellar small ribosomal subunit	bulk RNA-seq,Homo_723,AD.vs.control; bulk RNA-seq,Homo_723,AD.vs.MCI; bulk RNA-seq,Homo_723,MCI.vs.control; bulk RNA-seq,Homo_714,AD.vs.control; bulk RNA-seq,Homo_714,MCI.vs.control; bulk RNA-seq,Homo_633,AD.vs.control; bulk RNA-seq,Homo_633,AD.vs.MCI; bulk	8
CC	GO:0005763	mitochondrial small ribosomal subunit	bulk RNA-seq,Homo_723,AD.vs.control; bulk RNA-seq,Homo_723,AD.vs.MCI; bulk RNA-seq,Homo_723,MCI.vs.control; bulk RNA-seq,Homo_714,AD.vs.control; bulk RNA-seq,Homo_714,MCI.vs.control; bulk RNA-seq,Homo_633,AD.vs.control; bulk RNA-seq,Homo_633,AD.vs.MCI; bulk	8
BP	GO:0001942	hair follicle development	bulk RNA-seq,Homo_723,AD.vs.control; bulk RNA-seq,Homo_723,AD.vs.MCI; bulk RNA-seq,Homo_723,MCI.vs.control; bulk RNA-seq,Homo_714,AD.vs.control; bulk RNA-seq,Homo_714,AD.vs.MCI; bulk RNA-seq,Homo_714,MCI.vs.control; bulk RNA-seq,Homo_633,AD.vs.control; bulk	8
BP	GO:0060740	prostate gland epithelium morphogenesis	bulk RNA-seq,Homo_723,AD.vs.control; bulk RNA-seq,Homo_723,AD.vs.MCI; bulk RNA-seq,Homo_723,MCI.vs.control; bulk RNA-seq,Homo_714,AD.vs.control; bulk RNA-seq,Homo_714,AD.vs.MCI; bulk RNA-seq,Homo_714,MCI.vs.control; bulk RNA-seq,Homo_633,MCI.vs.control	7
BP	GO:0007224	smoothened signaling pathway	bulk RNA-seq,Homo_723,AD.vs.control; bulk RNA-seq,Homo_723,AD.vs.MCI; bulk RNA-seq,Homo_723,MCI.vs.control; bulk RNA-seq,Homo_714,AD.vs.MCI; bulk RNA-seq,Homo_633,AD.vs.control; bulk RNA-seq,Homo_633,AD.vs.MCI; bulk RNA-seq,SRP223445,AD.vs.control	7
CC	GO:0043195	terminal bouton	bulk RNA-seq,Homo_723,AD.vs.control; bulk RNA-seq,Homo_723,AD.vs.MCI; bulk RNA-seq,Homo_723,MCI.vs.control; bulk RNA-seq,Homo_714,AD.vs.MCI	4
BP	GO:0035567	non-canonical Wnt signaling pathway	bulk RNA-seq,Homo_723,AD.vs.control; bulk RNA-seq,Homo_723,AD.vs.MCI; bulk RNA-seq,Homo_723,MCI.vs.control; bulk RNA-seq,Homo_714,AD.vs.MCI; bulk RNA-seq,Homo_714,MCI.vs.control; bulk RNA-seq,Homo_633,AD.vs.control; bulk RNA-seq,Homo_633,AD.vs.MCI	7
MF	GO:0000175	3'-5'-exoribonuclease activity	bulk RNA-seq,Homo_723,AD.vs.control; bulk RNA-seq,Homo_723,AD.vs.MCI; bulk RNA-seq,Homo_714,AD.vs.control; bulk RNA-seq,Homo_714,AD.vs.MCI; bulk RNA-seq,Homo_633,AD.vs.control; bulk RNA-seq,Homo_633,AD.vs.MCI	6
MF	GO:0023023	MHC protein complex binding	bulk RNA-seq,Homo_723,AD.vs.control; bulk RNA-seq,Homo_723,AD.vs.MCI; bulk RNA-seq,Homo_714,AD.vs.control; bulk RNA-seq,Homo_714,MCI.vs.control; bulk RNA-seq,Homo_633,AD.vs.control; bulk RNA-seq,Homo_633,AD.vs.MCI; scRNA-seq,SRP215507,CD8+ T cell_3-	8
MF	GO:0042287	MHC protein binding	bulk RNA-seq,Homo_723,AD.vs.control; bulk RNA-seq,Homo_723,AD.vs.MCI; bulk RNA-seq,Homo_714,AD.vs.control; bulk RNA-seq,Homo_714,AD.vs.MCI; bulk RNA-seq,Homo_633,AD.vs.control; bulk RNA-seq,Homo_633,AD.vs.MCI; scRNA-seq,SRP330776,Natural killer cell_1-AD.vs.control; scRNA-	8
BP	GO:0003279	cardiac septum development	bulk RNA-seq,Homo_723,AD.vs.control; bulk RNA-seq,Homo_723,AD.vs.MCI; bulk RNA-seq,Homo_723,MCI.vs.control; bulk RNA-seq,Homo_714,AD.vs.control; bulk RNA-seq,Homo_714,AD.vs.MCI; bulk RNA-seq,Homo_714,MCI.vs.control; bulk RNA-seq,Homo_633,AD.vs.control	7
BP	GO:0062125	regulation of mitochondrial gene expression	bulk RNA-seq,Homo_723,AD.vs.control; bulk RNA-seq,Homo_723,AD.vs.MCI; bulk RNA-seq,Homo_723,MCI.vs.control; bulk RNA-seq,Homo_714,AD.vs.control; bulk RNA-seq,Homo_714,AD.vs.MCI; bulk RNA-seq,Homo_714,MCI.vs.control; bulk RNA-seq,Homo_633,AD.vs.control; bulk RNA-seq,Homo_633,AD.vs.MCI; bulk RNA-seq,Homo_633,MCI.vs.control	9

BP	GO:0007178	transmembrane receptor protein serine/threonine kinase signaling pathway	bulk RNA-seq,Homo_723,AD.vs.control; bulk RNA-seq,Homo_723,AD.vs.MCI; bulk RNA-seq,Homo_714,AD.vs.control; bulk RNA-seq,Homo_714,AD.vs.MCI; bulk RNA-seq,Homo_633,AD.vs.control; bulk RNA-seq,Homo_633,AD.vs.MCI; bulk RNA-seq,SRP223445,AD.vs.control; scRNA-seq,SRP330776,B cell_1-AD.vs.control; scRNA-seq,SRP330776,CD8+ T cell_1-AD.vs.control; scRNA-seq,SRP330776,CD8+ T cell_2-AD.vs.control; scRNA-seq,SRP330776,Memory T cell_2-AD.vs.control; scRNA-seq,SRP330776,Naive CD8+ T cell_1-AD.vs.control; scRNA-seq,SRP330776,Natural killer T cell-AD.vs.control	13
BP	GO:0009145	purine nucleoside triphosphate biosynthetic process	bulk RNA-seq,Homo_723,AD.vs.control; bulk RNA-seq,Homo_723,MCI.vs.control; bulk RNA-seq,Homo_714,AD.vs.control; bulk RNA-seq,Homo_714,MCI.vs.control; bulk RNA-seq,Homo_633,AD.vs.control; bulk RNA-seq,Homo_633,AD.vs.MCI	6
BP	GO:0050688	regulation of defense response to virus	bulk RNA-seq,Homo_723,AD.vs.control; bulk RNA-seq,Homo_723,AD.vs.MCI; bulk RNA-seq,Homo_723,MCI.vs.control; bulk RNA-seq,Homo_714,AD.vs.control; bulk RNA-seq,Homo_714,MCI.vs.control; bulk RNA-seq,Homo_633,AD.vs.control; bulk RNA-seq,Homo_633,AD.vs.MCI	7
BP	GO:0090092	regulation of transmembrane receptor protein serine/threonine kinase signaling pathway	bulk RNA-seq,Homo_723,AD.vs.control; bulk RNA-seq,Homo_723,AD.vs.MCI; bulk RNA-seq,Homo_723,MCI.vs.control; bulk RNA-seq,Homo_714,AD.vs.control; bulk RNA-seq,Homo_714,AD.vs.MCI; bulk RNA-seq,Homo_633,AD.vs.control; bulk RNA-seq,Homo_633,AD.vs.MCI; bulk RNA-seq,SRP223445,AD.vs.control; scRNA-seq,SRP330776,Naive CD8+ T cell_1-AD.vs.control	9
CC	GO:0009925	basal plasma membrane	bulk RNA-seq,Homo_723,AD.vs.control; bulk RNA-seq,Homo_723,AD.vs.MCI; bulk RNA-seq,Homo_723,MCI.vs.control; bulk RNA-seq,Homo_714,AD.vs.control; bulk RNA-seq,Homo_714,AD.vs.MCI; bulk RNA-seq,Homo_633,AD.vs.control; bulk RNA-seq,Homo_633,AD.vs.MCI; bulk RNA-seq,ROSMAP,AD.vs.control; bulk RNA-seq,ROSMAP,MCI.vs.control; bulk RNA-seq,SRP223445,AD.vs.control	10
BP	GO:0030307	positive regulation of cell growth	bulk RNA-seq,Homo_723,AD.vs.control; bulk RNA-seq,Homo_723,AD.vs.MCI; bulk RNA-seq,Homo_723,MCI.vs.control; bulk RNA-seq,Homo_714,AD.vs.control; bulk RNA-seq,Homo_714,AD.vs.MCI; bulk RNA-seq,Homo_633,AD.vs.control; bulk RNA-seq,Homo_633,AD.vs.MCI	7
BP	GO:0030850	prostate gland development	bulk RNA-seq,Homo_723,AD.vs.control; bulk RNA-seq,Homo_723,AD.vs.MCI; bulk RNA-seq,Homo_723,MCI.vs.control; bulk RNA-seq,Homo_714,AD.vs.control; bulk RNA-seq,Homo_714,AD.vs.MCI; bulk RNA-seq,Homo_714,MCI.vs.control; bulk RNA-seq,Homo_633,AD.vs.control; bulk RNA-seq,Homo_633,AD.vs.MCI; bulk RNA-seq,Homo_633,MCI.vs.control	9
BP	GO:0007340	acrosome reaction	bulk RNA-seq,Homo_723,AD.vs.control; bulk RNA-seq,Homo_723,AD.vs.MCI; bulk RNA-seq,Homo_714,AD.vs.control; bulk RNA-seq,Homo_714,AD.vs.MCI	4
MF	GO:0042605	peptide antigen binding	bulk RNA-seq,Homo_723,AD.vs.control; bulk RNA-seq,Homo_723,AD.vs.MCI; bulk RNA-seq,Homo_723,MCI.vs.control; bulk RNA-seq,Homo_714,AD.vs.control; bulk RNA-seq,Homo_714,MCI.vs.control; bulk RNA-seq,Homo_633,AD.vs.control; bulk RNA-seq,Homo_633,AD.vs.MCI; scRNA-seq,SRP330776,Natural killer cell_1-AD.vs.control; scRNA-seq,SRP215507,CD8+ T cell_3-MCI.vs.control	9
BP	GO:0006302	double-strand break repair	bulk RNA-seq,Homo_723,AD.vs.control; bulk RNA-seq,Homo_723,MCI.vs.control; bulk RNA-seq,Homo_714,AD.vs.control; bulk RNA-seq,Homo_633,AD.vs.control; bulk RNA-seq,Homo_633,AD.vs.MCI	5
BP	GO:0071772	response to BMP	bulk RNA-seq,Homo_723,AD.vs.control; bulk RNA-seq,Homo_723,AD.vs.MCI; bulk RNA-seq,Homo_723,MCI.vs.control; bulk RNA-seq,Homo_714,AD.vs.control; bulk RNA-seq,Homo_714,AD.vs.MCI; bulk RNA-seq,SRP223445,AD.vs.control	6
BP	GO:0071773	cellular response to BMP stimulus	bulk RNA-seq,Homo_723,AD.vs.control; bulk RNA-seq,Homo_723,AD.vs.MCI; bulk RNA-seq,Homo_723,MCI.vs.control; bulk RNA-seq,Homo_714,AD.vs.control; bulk RNA-seq,Homo_714,AD.vs.MCI; bulk RNA-seq,SRP223445,AD.vs.control	6
MF	GO:0001217	DNA-binding transcription repressor activity	bulk RNA-seq,Homo_723,AD.vs.control; bulk RNA-seq,Homo_723,AD.vs.MCI; bulk RNA-seq,Homo_723,MCI.vs.control; bulk RNA-seq,Homo_714,AD.vs.MCI; bulk RNA-seq,Homo_633,AD.vs.control; bulk RNA-seq,Homo_633,AD.vs.MCI; bulk RNA-seq,SRP223445,AD.vs.control	7
BP	GO:0090263	positive regulation of canonical Wnt signaling pathway	bulk RNA-seq,Homo_723,AD.vs.control; bulk RNA-seq,Homo_723,AD.vs.MCI; bulk RNA-seq,Homo_723,MCI.vs.control; bulk RNA-seq,Homo_714,AD.vs.control; bulk RNA-seq,Homo_714,AD.vs.MCI; bulk RNA-seq,Homo_714,MCI.vs.control; bulk RNA-seq,Homo_633,AD.vs.control; bulk	8
MF	GO:0016917	GABA receptor activity	bulk RNA-seq,Homo_723,AD.vs.control; bulk RNA-seq,Homo_723,AD.vs.MCI; bulk RNA-seq,Homo_723,MCI.vs.control; bulk RNA-seq,Homo_714,AD.vs.control; bulk RNA-seq,Homo_714,AD.vs.MCI; bulk RNA-seq,Homo_633,MCI.vs.control	6
BP	GO:0006353	DNA-templated transcription termination	bulk RNA-seq,Homo_723,AD.vs.control; bulk RNA-seq,Homo_723,AD.vs.MCI; bulk RNA-seq,Homo_723,MCI.vs.control; bulk RNA-seq,Homo_714,AD.vs.control; bulk RNA-seq,Homo_714,AD.vs.MCI; bulk RNA-seq,Homo_714,MCI.vs.control; bulk RNA-seq,Homo_633,AD.vs.control; bulk RNA-seq,Homo_633,AD.vs.MCI; bulk RNA-seq,Homo_633,MCI.vs.control	9
CC	GO:0098573	intrinsic component of mitochondrial membrane	bulk RNA-seq,Homo_723,AD.vs.control; bulk RNA-seq,Homo_723,AD.vs.MCI; bulk RNA-seq,Homo_723,MCI.vs.control; bulk RNA-seq,Homo_714,AD.vs.control; bulk RNA-seq,Homo_714,MCI.vs.control; bulk RNA-seq,Homo_633,AD.vs.control; bulk RNA-seq,Homo_633,AD.vs.MCI	7
BP	GO:0021696	cerebellar cortex morphogenesis	bulk RNA-seq,Homo_723,AD.vs.control; bulk RNA-seq,Homo_723,AD.vs.MCI; bulk RNA-seq,Homo_723,MCI.vs.control; bulk RNA-seq,Homo_714,AD.vs.MCI; bulk RNA-seq,Homo_714,MCI.vs.control; bulk RNA-seq,Homo_633,AD.vs.control; bulk RNA-seq,Homo_633,AD.vs.MCI	7
BP	GO:2001020	regulation of response to DNA damage stimulus	bulk RNA-seq,Homo_723,AD.vs.control; bulk RNA-seq,Homo_723,MCI.vs.control; bulk RNA-seq,Homo_714,AD.vs.control; bulk RNA-seq,Homo_633,AD.vs.control; bulk RNA-seq,Homo_633,AD.vs.MCI	5
BP	GO:0032200	telomere organization	bulk RNA-seq,Homo_723,AD.vs.control; bulk RNA-seq,Homo_723,MCI.vs.control; bulk RNA-seq,Homo_714,AD.vs.control; bulk RNA-seq,Homo_633,AD.vs.control; bulk RNA-seq,Homo_633,AD.vs.MCI	5
BP	GO:0099174	regulation of presynapse organization	bulk RNA-seq,Homo_723,AD.vs.control; bulk RNA-seq,Homo_723,AD.vs.MCI; bulk RNA-seq,Homo_723,MCI.vs.control; bulk RNA-seq,Homo_714,AD.vs.control; bulk RNA-seq,Homo_714,AD.vs.MCI	5
BP	GO:1905606	regulation of presynapse assembly	bulk RNA-seq,Homo_723,AD.vs.control; bulk RNA-seq,Homo_723,AD.vs.MCI; bulk RNA-seq,Homo_723,MCI.vs.control; bulk RNA-seq,Homo_714,AD.vs.control; bulk RNA-seq,Homo_714,AD.vs.MCI	5
BP	GO:0031623	receptor internalization	bulk RNA-seq,Homo_723,AD.vs.control; bulk RNA-seq,Homo_723,AD.vs.MCI; bulk RNA-seq,Homo_723,MCI.vs.control; bulk RNA-seq,Homo_714,AD.vs.control; bulk RNA-seq,Homo_714,AD.vs.MCI; bulk RNA-seq,Homo_714,MCI.vs.control; bulk RNA-seq,Homo_633,AD.vs.control; bulk	8
MF	GO:0001227	DNA-binding transcription repressor activity, RNA polymerase II-specific	bulk RNA-seq,Homo_723,AD.vs.control; bulk RNA-seq,Homo_723,AD.vs.MCI; bulk RNA-seq,Homo_723,MCI.vs.control; bulk RNA-seq,Homo_714,AD.vs.MCI; bulk RNA-seq,Homo_633,AD.vs.control; bulk RNA-seq,Homo_633,AD.vs.MCI; bulk RNA-seq,SRP223445,AD.vs.control	7
BP	GO:0009060	aerobic respiration	bulk RNA-seq,Homo_723,AD.vs.control; bulk RNA-seq,Homo_723,MCI.vs.control; bulk RNA-seq,Homo_714,AD.vs.control; bulk RNA-seq,Homo_633,AD.vs.control; bulk RNA-seq,Homo_633,AD.vs.MCI	5
CC	GO:0032040	small-subunit processome	bulk RNA-seq,Homo_723,AD.vs.control; bulk RNA-seq,Homo_723,MCI.vs.control; bulk RNA-seq,Homo_714,AD.vs.control; bulk RNA-seq,Homo_714,MCI.vs.control; bulk RNA-seq,Homo_633,AD.vs.control; bulk RNA-seq,Homo_633,AD.vs.MCI	6
MF	GO:0008408	3'-5' exonuclease activity	bulk RNA-seq,Homo_723,AD.vs.control; bulk RNA-seq,Homo_723,AD.vs.MCI; bulk RNA-seq,Homo_723,MCI.vs.control; bulk RNA-seq,Homo_714,AD.vs.control; bulk RNA-seq,Homo_714,AD.vs.MCI; bulk RNA-seq,Homo_714,MCI.vs.control; bulk RNA-seq,Homo_633,AD.vs.control; bulk	8
MF	GO:0140030	modification-dependent protein binding	bulk RNA-seq,Homo_723,AD.vs.control; bulk RNA-seq,Homo_723,MCI.vs.control; bulk RNA-seq,Homo_714,AD.vs.control; bulk RNA-seq,Homo_633,AD.vs.control; bulk RNA-seq,Homo_633,AD.vs.MCI	5
BP	GO:0086065	cell communication involved in cardiac conduction	bulk RNA-seq,Homo_723,AD.vs.control; bulk RNA-seq,Homo_723,AD.vs.MCI; bulk RNA-seq,Homo_723,MCI.vs.control; bulk RNA-seq,Homo_714,AD.vs.control; bulk RNA-seq,Homo_714,AD.vs.MCI; bulk RNA-seq,Homo_714,MCI.vs.control	6
MF	GO:0008757	S-adenosylmethionine-dependent methyltransferase activity	bulk RNA-seq,Homo_723,AD.vs.control; bulk RNA-seq,Homo_723,MCI.vs.control; bulk RNA-seq,Homo_714,AD.vs.control; bulk RNA-seq,Homo_633,AD.vs.control; bulk RNA-seq,Homo_633,AD.vs.MCI	5

BP	GO:0072091	regulation of stem cell proliferation	bulk RNA-seq,Homo_723,AD.vs.control; bulk RNA-seq,Homo_723,AD.vs.MCI; bulk RNA-seq,Homo_723,MCI.vs.control; bulk RNA-seq,Homo_714,AD.vs.control; bulk RNA-seq,Homo_714,AD.vs.MCI; bulk RNA-seq,Homo_714,MCI.vs.control	6
CC	GO:0031674	I band	bulk RNA-seq,Homo_723,AD.vs.control; bulk RNA-seq,Homo_723,AD.vs.MCI; bulk RNA-seq,Homo_723,MCI.vs.control; bulk RNA-seq,Homo_714,AD.vs.control; bulk RNA-seq,Homo_714,AD.vs.MCI; bulk RNA-seq,Homo_633,AD.vs.control; bulk RNA-seq,Homo_633,AD.vs.MCI	7
MF	GO:0003774	cytoskeletal motor activity	bulk RNA-seq,Homo_723,AD.vs.control; bulk RNA-seq,Homo_723,AD.vs.MCI; bulk RNA-seq,Homo_723,MCI.vs.control; bulk RNA-seq,Homo_714,AD.vs.MCI; bulk RNA-seq,Homo_714,MCI.vs.control; bulk RNA-seq,Homo_633,AD.vs.control; bulk RNA-seq,Homo_633,AD.vs.MCI; bulk RNA-	8
BP	GO:2000232	regulation of rRNA processing	bulk RNA-seq,Homo_723,AD.vs.control; bulk RNA-seq,Homo_723,AD.vs.MCI; bulk RNA-seq,Homo_714,AD.vs.control; bulk RNA-seq,Homo_714,AD.vs.MCI; bulk RNA-seq,Homo_633,AD.vs.control; bulk RNA-seq,Homo_633,AD.vs.MCI	6
BP	GO:0099590	neurotransmitter receptor internalization	bulk RNA-seq,Homo_723,AD.vs.control; bulk RNA-seq,Homo_723,AD.vs.MCI; bulk RNA-seq,Homo_723,MCI.vs.control; bulk RNA-seq,Homo_714,AD.vs.MCI; bulk RNA-seq,Homo_714,MCI.vs.control	5
BP	GO:0007616	long-term memory	bulk RNA-seq,Homo_723,AD.vs.control; bulk RNA-seq,Homo_723,AD.vs.MCI; bulk RNA-seq,Homo_723,MCI.vs.control; bulk RNA-seq,Homo_714,AD.vs.MCI	4
BP	GO:0050852	T cell receptor signaling pathway	bulk RNA-seq,Homo_723,AD.vs.control; bulk RNA-seq,Homo_723,MCI.vs.control; bulk RNA-seq,Homo_714,AD.vs.control; bulk RNA-seq,Homo_714,MCI.vs.control; bulk RNA-seq,Homo_633,AD.vs.control; bulk RNA-seq,Homo_633,AD.vs.MCI	6
CC	GO:0030285	integral component of synaptic vesicle membrane	bulk RNA-seq,Homo_723,AD.vs.control; bulk RNA-seq,Homo_723,AD.vs.MCI; bulk RNA-seq,Homo_723,MCI.vs.control; bulk RNA-seq,Homo_714,AD.vs.MCI	4
CC	GO:0005747	mitochondrial respiratory chain complex I	bulk RNA-seq,Homo_723,AD.vs.control; bulk RNA-seq,Homo_723,AD.vs.MCI; bulk RNA-seq,Homo_723,MCI.vs.control; bulk RNA-seq,Homo_714,AD.vs.control; bulk RNA-seq,Homo_714,AD.vs.MCI; bulk RNA-seq,Homo_714,MCI.vs.control; bulk RNA-seq,Homo_633,AD.vs.control; bulk	8
CC	GO:0030964	NADH dehydrogenase complex	bulk RNA-seq,Homo_723,AD.vs.control; bulk RNA-seq,Homo_723,AD.vs.MCI; bulk RNA-seq,Homo_723,MCI.vs.control; bulk RNA-seq,Homo_714,AD.vs.control; bulk RNA-seq,Homo_714,AD.vs.MCI; bulk RNA-seq,Homo_714,MCI.vs.control; bulk RNA-seq,Homo_633,AD.vs.control; bulk	8
CC	GO:0045271	respiratory chain complex I	bulk RNA-seq,Homo_723,AD.vs.control; bulk RNA-seq,Homo_723,AD.vs.MCI; bulk RNA-seq,Homo_723,MCI.vs.control; bulk RNA-seq,Homo_714,AD.vs.control; bulk RNA-seq,Homo_714,AD.vs.MCI; bulk RNA-seq,Homo_714,MCI.vs.control; bulk RNA-seq,Homo_633,AD.vs.control; bulk	8
MF	GO:0004970	ionotropic glutamate receptor activity	bulk RNA-seq,Homo_723,AD.vs.control; bulk RNA-seq,Homo_723,AD.vs.MCI; bulk RNA-seq,Homo_723,MCI.vs.control; bulk RNA-seq,Homo_714,AD.vs.MCI	4
MF	GO:0001653	peptide receptor activity	bulk RNA-seq,Homo_723,AD.vs.control; bulk RNA-seq,Homo_723,AD.vs.MCI; bulk RNA-seq,Homo_723,MCI.vs.control; bulk RNA-seq,Homo_714,AD.vs.control; bulk RNA-seq,Homo_714,AD.vs.MCI; bulk RNA-seq,Homo_633,MCI.vs.control; bulk RNA-seq,SRP223445,AD.vs.control	7
MF	GO:0004386	helicase activity	bulk RNA-seq,Homo_723,AD.vs.control; bulk RNA-seq,Homo_723,MCI.vs.control; bulk RNA-seq,Homo_714,AD.vs.control; bulk RNA-seq,Homo_633,AD.vs.control; bulk RNA-seq,Homo_633,AD.vs.MCI	5
CC	GO:1990204	oxidoreductase complex	bulk RNA-seq,Homo_723,AD.vs.control; bulk RNA-seq,Homo_723,MCI.vs.control; bulk RNA-seq,Homo_714,AD.vs.control; bulk RNA-seq,Homo_714,MCI.vs.control; bulk RNA-seq,Homo_633,AD.vs.control; bulk RNA-seq,Homo_633,AD.vs.MCI	6
BP	GO:0031124	mRNA 3'-end processing	bulk RNA-seq,Homo_723,AD.vs.control; bulk RNA-seq,Homo_723,AD.vs.MCI; bulk RNA-seq,Homo_723,MCI.vs.control; bulk RNA-seq,Homo_714,AD.vs.control; bulk RNA-seq,Homo_714,MCI.vs.control; bulk RNA-seq,Homo_633,AD.vs.control; bulk RNA-seq,Homo_633,AD.vs.MCI	7
BP	GO:0009206	purine ribonucleoside triphosphate biosynthetic process	bulk RNA-seq,Homo_723,AD.vs.control; bulk RNA-seq,Homo_723,MCI.vs.control; bulk RNA-seq,Homo_714,AD.vs.control; bulk RNA-seq,Homo_714,MCI.vs.control; bulk RNA-seq,Homo_633,AD.vs.control; bulk RNA-seq,Homo_633,AD.vs.MCI	6
BP	GO:0007043	cell-cell junction assembly	bulk RNA-seq,Homo_723,AD.vs.control; bulk RNA-seq,Homo_723,AD.vs.MCI; bulk RNA-seq,Homo_723,MCI.vs.control; bulk RNA-seq,Homo_714,AD.vs.control; bulk RNA-seq,Homo_714,AD.vs.MCI; bulk RNA-seq,Homo_633,AD.vs.control; bulk RNA-seq,SRP223445,AD.vs.control	7
BP	GO:0022404	molting cycle process	bulk RNA-seq,Homo_723,AD.vs.control; bulk RNA-seq,Homo_723,AD.vs.MCI; bulk RNA-seq,Homo_723,MCI.vs.control; bulk RNA-seq,Homo_714,AD.vs.control; bulk RNA-seq,Homo_714,AD.vs.MCI; bulk RNA-seq,Homo_714,MCI.vs.control; bulk RNA-seq,Homo_633,AD.vs.control; bulk	8
BP	GO:0022405	hair cycle process	bulk RNA-seq,Homo_723,AD.vs.control; bulk RNA-seq,Homo_723,AD.vs.MCI; bulk RNA-seq,Homo_723,MCI.vs.control; bulk RNA-seq,Homo_714,AD.vs.control; bulk RNA-seq,Homo_714,AD.vs.MCI; bulk RNA-seq,Homo_714,MCI.vs.control; bulk RNA-seq,Homo_633,AD.vs.control; bulk	8
BP	GO:0034767	positive regulation of ion transmembrane transport	bulk RNA-seq,Homo_723,AD.vs.control; bulk RNA-seq,Homo_723,AD.vs.MCI; bulk RNA-seq,Homo_723,MCI.vs.control; bulk RNA-seq,Homo_714,AD.vs.control; bulk RNA-seq,Homo_714,AD.vs.MCI; bulk RNA-seq,Homo_633,AD.vs.control	6
BP	GO:0048709	oligodendrocyte differentiation	bulk RNA-seq,Homo_723,AD.vs.control; bulk RNA-seq,Homo_723,AD.vs.MCI; bulk RNA-seq,Homo_723,MCI.vs.control; bulk RNA-seq,Homo_714,AD.vs.MCI; bulk RNA-seq,Homo_714,MCI.vs.control; bulk RNA-seq,Homo_633,AD.vs.control; bulk RNA-seq,Homo_633,AD.vs.MCI	7
BP	GO:0003229	ventricular cardiac muscle tissue development	bulk RNA-seq,Homo_723,AD.vs.control; bulk RNA-seq,Homo_723,AD.vs.MCI; bulk RNA-seq,Homo_723,MCI.vs.control; bulk RNA-seq,Homo_714,AD.vs.control; bulk RNA-seq,Homo_714,AD.vs.MCI; bulk RNA-seq,Homo_714,MCI.vs.control; scRNA-seq,SRP330776,Naive CD8+ T cell_2-	7
MF	GO:0004252	serine-type endopeptidase activity	bulk RNA-seq,Homo_723,AD.vs.control; bulk RNA-seq,Homo_723,AD.vs.MCI; bulk RNA-seq,Homo_723,MCI.vs.control; bulk RNA-seq,Homo_714,AD.vs.control; bulk RNA-seq,Homo_714,AD.vs.MCI; bulk RNA-seq,SRP223445,AD.vs.control	6
CC	GO:0045178	basal part of cell	bulk RNA-seq,Homo_723,AD.vs.control; bulk RNA-seq,Homo_723,AD.vs.MCI; bulk RNA-seq,Homo_723,MCI.vs.control; bulk RNA-seq,Homo_714,AD.vs.control; bulk RNA-seq,Homo_714,AD.vs.MCI; bulk RNA-seq,Homo_633,AD.vs.control; bulk RNA-seq,Homo_633,AD.vs.MCI; bulk RNA-seq,ROSMAP,AD.vs.control; bulk RNA-seq,ROSMAP,MCI.vs.control; bulk RNA-seq,SRP223445,AD.vs.control	10
BP	GO:0019233	sensory perception of pain	bulk RNA-seq,Homo_723,AD.vs.control; bulk RNA-seq,Homo_723,AD.vs.MCI; bulk RNA-seq,Homo_723,MCI.vs.control; bulk RNA-seq,Homo_714,AD.vs.control; bulk RNA-seq,Homo_714,AD.vs.MCI; bulk RNA-seq,Homo_714,MCI.vs.control	6
BP	GO:0060840	artery development	bulk RNA-seq,Homo_723,AD.vs.control; bulk RNA-seq,Homo_723,AD.vs.MCI; bulk RNA-seq,Homo_723,MCI.vs.control; bulk RNA-seq,Homo_714,AD.vs.control; bulk RNA-seq,Homo_714,AD.vs.MCI; bulk RNA-seq,Homo_714,MCI.vs.control; bulk RNA-seq,SRP223445,AD.vs.control	7
MF	GO:0099106	ion channel regulator activity	bulk RNA-seq,Homo_723,AD.vs.control; bulk RNA-seq,Homo_723,AD.vs.MCI; bulk RNA-seq,Homo_723,MCI.vs.control; bulk RNA-seq,Homo_714,AD.vs.MCI; bulk RNA-seq,Homo_714,MCI.vs.control; bulk RNA-seq,Homo_633,AD.vs.control; bulk RNA-seq,Homo_633,AD.vs.MCI	7
BP	GO:0007015	actin filament organization	bulk RNA-seq,Homo_723,AD.vs.control; bulk RNA-seq,Homo_723,AD.vs.MCI; bulk RNA-seq,Homo_714,AD.vs.control; bulk RNA-seq,Homo_714,AD.vs.MCI; bulk RNA-seq,Homo_633,AD.vs.control; bulk RNA-seq,Homo_633,AD.vs.MCI	6
BP	GO:0050918	positive chemotaxis	bulk RNA-seq,Homo_723,AD.vs.control; bulk RNA-seq,Homo_723,AD.vs.MCI; bulk RNA-seq,Homo_723,MCI.vs.control; bulk RNA-seq,Homo_714,AD.vs.control; bulk RNA-seq,Homo_714,AD.vs.MCI; bulk RNA-seq,Homo_714,MCI.vs.control	6
BP	GO:0071277	cellular response to calcium ion	bulk RNA-seq,Homo_723,AD.vs.control; bulk RNA-seq,Homo_723,AD.vs.MCI; bulk RNA-seq,Homo_723,MCI.vs.control; bulk RNA-seq,Homo_714,AD.vs.MCI; bulk RNA-seq,Homo_714,MCI.vs.control; bulk RNA-seq,Homo_633,AD.vs.control	6
BP	GO:0010717	regulation of epithelial to mesenchymal transition	bulk RNA-seq,Homo_723,AD.vs.control; bulk RNA-seq,Homo_723,AD.vs.MCI; bulk RNA-seq,Homo_723,MCI.vs.control; bulk RNA-seq,Homo_714,AD.vs.control; bulk RNA-seq,Homo_714,AD.vs.MCI; bulk RNA-seq,Homo_714,MCI.vs.control; bulk RNA-seq,Homo_633,AD.vs.control; bulk	8
BP	GO:0034446	substrate adhesion-dependent cell spreading	bulk RNA-seq,Homo_723,AD.vs.control; bulk RNA-seq,Homo_723,AD.vs.MCI; bulk RNA-seq,Homo_723,MCI.vs.control; bulk RNA-seq,Homo_714,AD.vs.control; bulk RNA-seq,Homo_714,AD.vs.MCI; bulk RNA-seq,Homo_714,MCI.vs.control; bulk RNA-seq,Homo_633,AD.vs.control; bulk	8

BP	GO:0050805	negative regulation of synaptic transmission	bulk RNA-seq,Homo_723,AD.vs.control; bulk RNA-seq,Homo_723,AD.vs.MCI; bulk RNA-seq,Homo_723,MCI.vs.control; bulk RNA-seq,Homo_714,AD.vs.control; bulk RNA-seq,Homo_714,AD.vs.MCI; bulk RNA-seq,Homo_714,MCI.vs.control; bulk RNA-seq,Homo_633,AD.vs.control	7
BP	GO:0099022	vesicle tethering	bulk RNA-seq,Homo_723,AD.vs.control; bulk RNA-seq,Homo_723,AD.vs.MCI; bulk RNA-seq,Homo_723,MCI.vs.control; bulk RNA-seq,Homo_714,AD.vs.control; bulk RNA-seq,Homo_714,AD.vs.MCI; bulk RNA-seq,Homo_714,MCI.vs.control; bulk RNA-seq,Homo_633,AD.vs.control; bulk RNA-seq,Homo_633,AD.vs.MCI; bulk RNA-seq,Homo_633,MCI.vs.control	9
BP	GO:0045824	negative regulation of innate immune response	bulk RNA-seq,Homo_723,AD.vs.control; bulk RNA-seq,Homo_723,AD.vs.MCI; bulk RNA-seq,Homo_723,MCI.vs.control; bulk RNA-seq,Homo_714,AD.vs.control; bulk RNA-seq,Homo_714,MCI.vs.control; bulk RNA-seq,Homo_633,AD.vs.control; bulk RNA-seq,Homo_633,AD.vs.MCI	7
CC	GO:0030880	RNA polymerase complex	bulk RNA-seq,Homo_723,AD.vs.control; bulk RNA-seq,Homo_723,MCI.vs.control; bulk RNA-seq,Homo_714,AD.vs.control; bulk RNA-seq,Homo_714,MCI.vs.control; bulk RNA-seq,Homo_633,AD.vs.control; bulk RNA-seq,Homo_633,AD.vs.MCI	6
BP	GO:0090090	negative regulation of canonical Wnt signaling pathway	bulk RNA-seq,Homo_723,AD.vs.control; bulk RNA-seq,Homo_723,AD.vs.MCI; bulk RNA-seq,Homo_723,MCI.vs.control; bulk RNA-seq,Homo_714,AD.vs.MCI; bulk RNA-seq,Homo_633,AD.vs.control	5
BP	GO:0019935	cyclic-nucleotide-mediated signaling	bulk RNA-seq,Homo_723,AD.vs.control; bulk RNA-seq,Homo_723,AD.vs.MCI; bulk RNA-seq,Homo_723,MCI.vs.control; bulk RNA-seq,Homo_714,AD.vs.control; bulk RNA-seq,Homo_714,AD.vs.MCI; bulk RNA-seq,Homo_714,MCI.vs.control; bulk RNA-seq,Homo_633,AD.vs.control; bulk	8
BP	GO:0007160	cell-matrix adhesion	bulk RNA-seq,Homo_723,AD.vs.control; bulk RNA-seq,Homo_723,AD.vs.MCI; bulk RNA-seq,Homo_723,MCI.vs.control; bulk RNA-seq,Homo_714,AD.vs.control; bulk RNA-seq,Homo_714,AD.vs.MCI; bulk RNA-seq,Homo_633,AD.vs.control; bulk RNA-seq,Homo_633,AD.vs.MCI	7
BP	GO:0006909	phagocytosis	bulk RNA-seq,Homo_723,AD.vs.control; bulk RNA-seq,Homo_723,AD.vs.MCI; bulk RNA-seq,Homo_723,MCI.vs.control; bulk RNA-seq,Homo_633,AD.vs.control; bulk RNA-seq,Homo_633,AD.vs.MCI; scRNA-seq,SRP330776,Naive CD8+ T cell_2-AD.vs.control; scRNA-seq,SRP215507,CD8+	7
BP	GO:0048566	embryonic digestive tract development	bulk RNA-seq,Homo_723,AD.vs.control; bulk RNA-seq,Homo_723,AD.vs.MCI; bulk RNA-seq,Homo_723,MCI.vs.control; bulk RNA-seq,Homo_714,AD.vs.control; bulk RNA-seq,Homo_714,AD.vs.MCI; bulk RNA-seq,Homo_714,MCI.vs.control	6
BP	GO:1905332	positive regulation of morphogenesis of an epithelium	bulk RNA-seq,Homo_723,AD.vs.control; bulk RNA-seq,Homo_723,AD.vs.MCI; bulk RNA-seq,Homo_723,MCI.vs.control; bulk RNA-seq,Homo_714,AD.vs.MCI; bulk RNA-seq,Homo_714,MCI.vs.control	5
CC	GO:0032592	integral component of mitochondrial membrane	bulk RNA-seq,Homo_723,AD.vs.control; bulk RNA-seq,Homo_723,MCI.vs.control; bulk RNA-seq,Homo_714,AD.vs.control; bulk RNA-seq,Homo_714,MCI.vs.control; bulk RNA-seq,Homo_633,AD.vs.control; bulk RNA-seq,Homo_633,AD.vs.MCI	6
BP	GO:0000469	cleavage involved in rRNA processing	bulk RNA-seq,Homo_723,AD.vs.control; bulk RNA-seq,Homo_723,AD.vs.MCI; bulk RNA-seq,Homo_714,AD.vs.control; bulk RNA-seq,Homo_714,AD.vs.MCI; bulk RNA-seq,Homo_633,AD.vs.control; bulk RNA-seq,Homo_633,AD.vs.MCI; bulk RNA-seq,Homo_633,MCI.vs.control	7
BP	GO:1904064	positive regulation of cation transmembrane transport	bulk RNA-seq,Homo_723,AD.vs.control; bulk RNA-seq,Homo_723,AD.vs.MCI; bulk RNA-seq,Homo_723,MCI.vs.control; bulk RNA-seq,Homo_714,AD.vs.MCI; bulk RNA-seq,Homo_633,AD.vs.control; bulk RNA-seq,Homo_633,AD.vs.MCI	6
CC	GO:0070469	respirasome	bulk RNA-seq,Homo_723,AD.vs.control; bulk RNA-seq,Homo_723,AD.vs.MCI; bulk RNA-seq,Homo_723,MCI.vs.control; bulk RNA-seq,Homo_714,AD.vs.control; bulk RNA-seq,Homo_714,MCI.vs.control; bulk RNA-seq,Homo_633,AD.vs.control; bulk RNA-seq,Homo_633,AD.vs.MCI	7
BP	GO:0002369	T cell cytokine production	bulk RNA-seq,Homo_723,AD.vs.control; bulk RNA-seq,Homo_723,AD.vs.MCI; bulk RNA-seq,Homo_723,MCI.vs.control; bulk RNA-seq,Homo_714,AD.vs.control; bulk RNA-seq,Homo_714,MCI.vs.control; bulk RNA-seq,Homo_633,AD.vs.control; bulk RNA-seq,Homo_633,AD.vs.MCI; scRNA-	8
BP	GO:0002724	regulation of T cell cytokine production	bulk RNA-seq,Homo_723,AD.vs.control; bulk RNA-seq,Homo_723,AD.vs.MCI; bulk RNA-seq,Homo_723,MCI.vs.control; bulk RNA-seq,Homo_714,AD.vs.control; bulk RNA-seq,Homo_714,MCI.vs.control; bulk RNA-seq,Homo_633,AD.vs.control; bulk RNA-seq,Homo_633,AD.vs.MCI; scRNA-	8
BP	GO:0007006	mitochondrial membrane organization	bulk RNA-seq,Homo_723,AD.vs.control; bulk RNA-seq,Homo_723,MCI.vs.control; bulk RNA-seq,Homo_714,AD.vs.control; bulk RNA-seq,Homo_714,MCI.vs.control; bulk RNA-seq,Homo_633,AD.vs.control; bulk RNA-seq,Homo_633,AD.vs.MCI	6
CC	GO:0055029	nuclear DNA-directed RNA polymerase complex	bulk RNA-seq,Homo_723,AD.vs.control; bulk RNA-seq,Homo_723,MCI.vs.control; bulk RNA-seq,Homo_714,AD.vs.control; bulk RNA-seq,Homo_714,MCI.vs.control; bulk RNA-seq,Homo_633,AD.vs.control; bulk RNA-seq,Homo_633,AD.vs.MCI	6
BP	GO:0002292	T cell differentiation involved in immune response	bulk RNA-seq,Homo_723,AD.vs.control; bulk RNA-seq,Homo_723,MCI.vs.control; bulk RNA-seq,Homo_714,AD.vs.control; bulk RNA-seq,Homo_714,MCI.vs.control; bulk RNA-seq,Homo_633,AD.vs.control; bulk RNA-seq,Homo_633,AD.vs.MCI	6
BP	GO:0090183	regulation of kidney development	bulk RNA-seq,Homo_723,AD.vs.control; bulk RNA-seq,Homo_723,AD.vs.MCI; bulk RNA-seq,Homo_723,MCI.vs.control; bulk RNA-seq,Homo_714,AD.vs.control; bulk RNA-seq,Homo_714,AD.vs.MCI	5
BP	GO:0048713	regulation of oligodendrocyte differentiation	bulk RNA-seq,Homo_723,AD.vs.control; bulk RNA-seq,Homo_723,AD.vs.MCI; bulk RNA-seq,Homo_723,MCI.vs.control; bulk RNA-seq,Homo_714,AD.vs.MCI; bulk RNA-seq,Homo_714,MCI.vs.control	5
BP	GO:0048806	genitalia development	bulk RNA-seq,Homo_723,AD.vs.control; bulk RNA-seq,Homo_723,AD.vs.MCI; bulk RNA-seq,Homo_723,MCI.vs.control; bulk RNA-seq,Homo_714,AD.vs.control; bulk RNA-seq,Homo_714,AD.vs.MCI; bulk RNA-seq,Homo_714,MCI.vs.control; bulk RNA-seq,Homo_633,AD.vs.control; bulk	8
BP	GO:1903034	regulation of response to wounding	bulk RNA-seq,Homo_723,AD.vs.control; bulk RNA-seq,Homo_723,AD.vs.MCI; bulk RNA-seq,Homo_723,MCI.vs.control; bulk RNA-seq,Homo_714,AD.vs.control; bulk RNA-seq,Homo_714,AD.vs.MCI; bulk RNA-seq,Homo_633,AD.vs.control; bulk RNA-seq,Homo_633,AD.vs.MCI	7
BP	GO:0022037	metencephalon development	bulk RNA-seq,Homo_723,AD.vs.control; bulk RNA-seq,Homo_723,AD.vs.MCI; bulk RNA-seq,Homo_723,MCI.vs.control; bulk RNA-seq,Homo_714,AD.vs.MCI; bulk RNA-seq,Homo_714,MCI.vs.control; bulk RNA-seq,Homo_633,AD.vs.control; bulk RNA-seq,Homo_633,AD.vs.MCI	7
BP	GO:1990542	mitochondrial transmembrane transport	bulk RNA-seq,Homo_723,AD.vs.control; bulk RNA-seq,Homo_723,MCI.vs.control; bulk RNA-seq,Homo_714,AD.vs.control; bulk RNA-seq,Homo_714,MCI.vs.control; bulk RNA-seq,Homo_633,AD.vs.control; bulk RNA-seq,Homo_633,AD.vs.MCI	6
BP	GO:1903008	organelle disassembly	bulk RNA-seq,Homo_723,AD.vs.control; bulk RNA-seq,Homo_723,MCI.vs.control; bulk RNA-seq,Homo_714,AD.vs.control; bulk RNA-seq,Homo_633,AD.vs.control; bulk RNA-seq,Homo_633,AD.vs.MCI	5
BP	GO:0008584	male gonad development	bulk RNA-seq,Homo_723,AD.vs.control; bulk RNA-seq,Homo_723,AD.vs.MCI; bulk RNA-seq,Homo_723,MCI.vs.control; bulk RNA-seq,Homo_714,AD.vs.control; bulk RNA-seq,Homo_714,AD.vs.MCI; bulk RNA-seq,Homo_633,AD.vs.control; bulk RNA-seq,Homo_633,AD.vs.MCI	7
CC	GO:0030120	vesicle coat	bulk RNA-seq,Homo_723,AD.vs.control; bulk RNA-seq,Homo_723,MCI.vs.control; bulk RNA-seq,Homo_714,AD.vs.control; bulk RNA-seq,Homo_714,MCI.vs.control; bulk RNA-seq,Homo_633,AD.vs.control	5
CC	GO:0000178	exosome (RNase complex)	bulk RNA-seq,Homo_723,AD.vs.control; bulk RNA-seq,Homo_723,AD.vs.MCI; bulk RNA-seq,Homo_723,MCI.vs.control; bulk RNA-seq,Homo_714,AD.vs.control; bulk RNA-seq,Homo_714,AD.vs.MCI; bulk RNA-seq,Homo_633,AD.vs.control; bulk RNA-seq,Homo_633,AD.vs.MCI	7
MF	GO:0004867	serine-type endopeptidase inhibitor activity	bulk RNA-seq,Homo_723,AD.vs.control; bulk RNA-seq,Homo_723,AD.vs.MCI; bulk RNA-seq,Homo_723,MCI.vs.control; bulk RNA-seq,Homo_714,AD.vs.control; bulk RNA-seq,Homo_714,AD.vs.MCI; bulk RNA-seq,Homo_714,MCI.vs.control	6
MF	GO:0003955	NAD(P)H dehydrogenase (quinone) activity	bulk RNA-seq,Homo_723,AD.vs.control; bulk RNA-seq,Homo_723,AD.vs.MCI; bulk RNA-seq,Homo_723,MCI.vs.control; bulk RNA-seq,Homo_714,AD.vs.control; bulk RNA-seq,Homo_714,MCI.vs.control; bulk RNA-seq,Homo_633,AD.vs.control; bulk RNA-seq,Homo_633,AD.vs.MCI	7
BP	GO:0043200	response to amino acid	bulk RNA-seq,Homo_723,AD.vs.control; bulk RNA-seq,Homo_723,AD.vs.MCI; bulk RNA-seq,Homo_723,MCI.vs.control; bulk RNA-seq,Homo_714,AD.vs.MCI; bulk RNA-seq,Homo_714,MCI.vs.control; bulk RNA-seq,Homo_633,AD.vs.control; bulk RNA-seq,Homo_633,AD.vs.MCI; bulk RNA-	8

BP	GO:0019098	reproductive behavior	bulk RNA-seq,Homo_723,AD.vs.control; bulk RNA-seq,Homo_723,AD.vs.MCI; bulk RNA-seq,Homo_714,AD.vs.control; bulk RNA-seq,Homo_714,AD.vs.MCI; bulk RNA-seq,Homo_633,AD.vs.control	5
BP	GO:0035082	axoneme assembly	bulk RNA-seq,Homo_723,AD.vs.control; bulk RNA-seq,Homo_723,AD.vs.MCI; bulk RNA-seq,Homo_723,MCI.vs.control; bulk RNA-seq,Homo_714,AD.vs.MCI; bulk RNA-seq,Homo_714,MCI.vs.control; bulk RNA-seq,Homo_633,AD.vs.control; bulk RNA-seq,Homo_633,AD.vs.MCI	7
BP	GO:0045927	positive regulation of growth	bulk RNA-seq,Homo_723,AD.vs.control; bulk RNA-seq,Homo_723,AD.vs.MCI; bulk RNA-seq,Homo_723,MCI.vs.control; bulk RNA-seq,Homo_714,AD.vs.control; bulk RNA-seq,Homo_714,AD.vs.MCI; bulk RNA-seq,Homo_633,AD.vs.control; bulk RNA-seq,Homo_633,AD.vs.MCI	7
BP	GO:0048168	regulation of neuronal synaptic plasticity	bulk RNA-seq,Homo_723,AD.vs.control; bulk RNA-seq,Homo_723,AD.vs.MCI; bulk RNA-seq,Homo_723,MCI.vs.control; bulk RNA-seq,Homo_714,AD.vs.MCI; bulk RNA-seq,Homo_714,MCI.vs.control; bulk RNA-seq,Homo_633,AD.vs.control	6
BP	GO:0001759	organ induction	bulk RNA-seq,Homo_723,AD.vs.control; bulk RNA-seq,Homo_723,AD.vs.MCI; bulk RNA-seq,Homo_723,MCI.vs.control; bulk RNA-seq,Homo_714,AD.vs.control; bulk RNA-seq,Homo_714,AD.vs.MCI; bulk RNA-seq,Homo_714,MCI.vs.control; bulk RNA-seq,Homo_633,MCI.vs.control	7
CC	GO:0097014	ciliary plasm	bulk RNA-seq,Homo_723,AD.vs.control; bulk RNA-seq,Homo_723,AD.vs.MCI; bulk RNA-seq,Homo_723,MCI.vs.control; bulk RNA-seq,Homo_714,AD.vs.MCI; bulk RNA-seq,Homo_633,AD.vs.control; bulk RNA-seq,Homo_633,AD.vs.MCI; bulk RNA-seq,ROSMAP,MCI.vs.control	7
BP	GO:0000963	mitochondrial RNA processing	bulk RNA-seq,Homo_723,AD.vs.control; bulk RNA-seq,Homo_723,AD.vs.MCI; bulk RNA-seq,Homo_723,MCI.vs.control; bulk RNA-seq,Homo_714,AD.vs.control; bulk RNA-seq,Homo_714,AD.vs.MCI; bulk RNA-seq,Homo_714,MCI.vs.control; bulk RNA-seq,Homo_633,AD.vs.control; bulk	8
BP	GO:0021602	cranial nerve morphogenesis	bulk RNA-seq,Homo_723,AD.vs.control; bulk RNA-seq,Homo_723,AD.vs.MCI; bulk RNA-seq,Homo_723,MCI.vs.control; bulk RNA-seq,Homo_714,AD.vs.control; bulk RNA-seq,Homo_714,AD.vs.MCI	5
BP	GO:0014046	dopamine secretion	bulk RNA-seq,Homo_723,AD.vs.control; bulk RNA-seq,Homo_723,AD.vs.MCI; bulk RNA-seq,Homo_723,MCI.vs.control; bulk RNA-seq,Homo_714,AD.vs.MCI	4
BP	GO:0014059	regulation of dopamine secretion	bulk RNA-seq,Homo_723,AD.vs.control; bulk RNA-seq,Homo_723,AD.vs.MCI; bulk RNA-seq,Homo_723,MCI.vs.control; bulk RNA-seq,Homo_714,AD.vs.MCI	4
BP	GO:0051897	positive regulation of protein kinase B signaling	bulk RNA-seq,Homo_723,AD.vs.control; bulk RNA-seq,Homo_723,AD.vs.MCI; bulk RNA-seq,Homo_723,MCI.vs.control; bulk RNA-seq,Homo_714,AD.vs.control; bulk RNA-seq,Homo_714,AD.vs.MCI; bulk RNA-seq,Homo_714,MCI.vs.control; bulk RNA-seq,Homo_633,AD.vs.control; bulk	8
BP	GO:0048596	embryonic camera-type eye morphogenesis	bulk RNA-seq,Homo_723,AD.vs.control; bulk RNA-seq,Homo_723,AD.vs.MCI; bulk RNA-seq,Homo_723,MCI.vs.control; bulk RNA-seq,Homo_714,AD.vs.MCI; bulk RNA-seq,Homo_714,MCI.vs.control; bulk RNA-seq,Homo_633,MCI.vs.control	6
MF	GO:0045499	chemorepellent activity	bulk RNA-seq,Homo_723,AD.vs.control; bulk RNA-seq,Homo_723,AD.vs.MCI; bulk RNA-seq,Homo_723,MCI.vs.control; bulk RNA-seq,Homo_714,AD.vs.MCI; bulk RNA-seq,Homo_714,MCI.vs.control	5
BP	GO:0038179	neurotrophin signaling pathway	bulk RNA-seq,Homo_723,AD.vs.control; bulk RNA-seq,Homo_723,AD.vs.MCI; bulk RNA-seq,Homo_723,MCI.vs.control; bulk RNA-seq,Homo_714,AD.vs.control; bulk RNA-seq,Homo_714,AD.vs.MCI; bulk RNA-seq,Homo_633,AD.vs.control	6
BP	GO:0016447	somatic recombination of immunoglobulin gene segments	bulk RNA-seq,Homo_723,AD.vs.control; bulk RNA-seq,Homo_723,MCI.vs.control; bulk RNA-seq,Homo_714,AD.vs.control; bulk RNA-seq,Homo_714,MCI.vs.control; bulk RNA-seq,Homo_633,AD.vs.control; bulk RNA-seq,Homo_633,AD.vs.MCI	6
BP	GO:0021575	hindbrain morphogenesis	bulk RNA-seq,Homo_723,AD.vs.control; bulk RNA-seq,Homo_723,AD.vs.MCI; bulk RNA-seq,Homo_723,MCI.vs.control; bulk RNA-seq,Homo_714,AD.vs.MCI; bulk RNA-seq,Homo_714,MCI.vs.control; bulk RNA-seq,Homo_633,AD.vs.control; bulk RNA-seq,Homo_633,AD.vs.MCI	7
CC	GO:1905354	exoribonuclease complex	bulk RNA-seq,Homo_723,AD.vs.control; bulk RNA-seq,Homo_723,AD.vs.MCI; bulk RNA-seq,Homo_723,MCI.vs.control; bulk RNA-seq,Homo_714,AD.vs.control; bulk RNA-seq,Homo_714,AD.vs.MCI; bulk RNA-seq,Homo_714,MCI.vs.control; bulk RNA-seq,Homo_633,AD.vs.control; bulk	8
BP	GO:0007568	aging	bulk RNA-seq,Homo_723,AD.vs.control; bulk RNA-seq,Homo_723,AD.vs.MCI; bulk RNA-seq,Homo_723,MCI.vs.control; bulk RNA-seq,Homo_714,AD.vs.control; bulk RNA-seq,Homo_714,AD.vs.MCI; bulk RNA-seq,Homo_633,AD.vs.control; bulk RNA-seq,Homo_633,AD.vs.MCI	7
BP	GO:0072273	metanephric nephron morphogenesis	bulk RNA-seq,Homo_723,AD.vs.control; bulk RNA-seq,Homo_723,AD.vs.MCI; bulk RNA-seq,Homo_723,MCI.vs.control; bulk RNA-seq,Homo_714,AD.vs.MCI; bulk RNA-seq,Homo_714,MCI.vs.control; bulk RNA-seq,Homo_633,MCI.vs.control	6
BP	GO:0006360	transcription by RNA polymerase I	bulk RNA-seq,Homo_723,AD.vs.control; bulk RNA-seq,Homo_723,AD.vs.MCI; bulk RNA-seq,Homo_723,MCI.vs.control; bulk RNA-seq,Homo_714,AD.vs.control; bulk RNA-seq,Homo_714,MCI.vs.control; bulk RNA-seq,Homo_633,AD.vs.control; bulk RNA-seq,Homo_633,AD.vs.MCI	7
CC	GO:0000428	DNA-directed RNA polymerase complex	bulk RNA-seq,Homo_723,AD.vs.control; bulk RNA-seq,Homo_723,MCI.vs.control; bulk RNA-seq,Homo_714,AD.vs.control; bulk RNA-seq,Homo_714,MCI.vs.control; bulk RNA-seq,Homo_633,AD.vs.control; bulk RNA-seq,Homo_633,AD.vs.MCI	6
MF	GO:0070851	growth factor receptor binding	bulk RNA-seq,Homo_723,AD.vs.control; bulk RNA-seq,Homo_723,AD.vs.MCI; bulk RNA-seq,Homo_723,MCI.vs.control; bulk RNA-seq,Homo_714,AD.vs.control; bulk RNA-seq,Homo_714,AD.vs.MCI; bulk RNA-seq,Homo_633,AD.vs.control	6
MF	GO:0031406	carboxylic acid binding	bulk RNA-seq,Homo_723,AD.vs.control; bulk RNA-seq,Homo_723,AD.vs.MCI; bulk RNA-seq,Homo_723,MCI.vs.control; bulk RNA-seq,Homo_714,AD.vs.control; bulk RNA-seq,Homo_714,AD.vs.MCI; bulk RNA-seq,Homo_633,AD.vs.control; bulk RNA-seq,Homo_633,AD.vs.MCI	7
BP	GO:0072175	epithelial tube formation	bulk RNA-seq,Homo_723,AD.vs.control; bulk RNA-seq,Homo_723,AD.vs.MCI; bulk RNA-seq,Homo_723,MCI.vs.control; bulk RNA-seq,Homo_714,AD.vs.MCI; bulk RNA-seq,Homo_633,AD.vs.control; bulk RNA-seq,Homo_633,AD.vs.MCI	6
BP	GO:0035456	response to interferon-beta	bulk RNA-seq,Homo_723,AD.vs.control; bulk RNA-seq,Homo_723,AD.vs.MCI; bulk RNA-seq,Homo_723,MCI.vs.control; bulk RNA-seq,Homo_714,AD.vs.control; bulk RNA-seq,Homo_714,MCI.vs.control; bulk RNA-seq,Homo_633,AD.vs.control; bulk RNA-seq,Homo_633,AD.vs.MCI; bulk	8
BP	GO:1990089	response to nerve growth factor	bulk RNA-seq,Homo_723,AD.vs.control; bulk RNA-seq,Homo_723,AD.vs.MCI; bulk RNA-seq,Homo_723,MCI.vs.control; bulk RNA-seq,Homo_714,AD.vs.MCI	4
BP	GO:1902414	protein localization to cell junction	bulk RNA-seq,Homo_723,AD.vs.control; bulk RNA-seq,Homo_723,AD.vs.MCI; bulk RNA-seq,Homo_723,MCI.vs.control; bulk RNA-seq,Homo_714,AD.vs.control; bulk RNA-seq,Homo_714,AD.vs.MCI; bulk RNA-seq,Homo_714,MCI.vs.control; bulk RNA-seq,Homo_633,AD.vs.control; bulk	8
BP	GO:0070129	regulation of mitochondrial translation	bulk RNA-seq,Homo_723,AD.vs.control; bulk RNA-seq,Homo_723,AD.vs.MCI; bulk RNA-seq,Homo_714,AD.vs.control; bulk RNA-seq,Homo_714,AD.vs.MCI; bulk RNA-seq,Homo_714,MCI.vs.control; bulk RNA-seq,Homo_633,AD.vs.control; bulk RNA-seq,Homo_633,AD.vs.MCI; bulk RNA-	8
BP	GO:0045109	intermediate filament organization	bulk RNA-seq,Homo_723,AD.vs.control; bulk RNA-seq,Homo_723,AD.vs.MCI; bulk RNA-seq,Homo_714,AD.vs.control; bulk RNA-seq,Homo_714,AD.vs.MCI; bulk RNA-seq,SRP223445,AD.vs.control	5
CC	GO:0005930	axoneme	bulk RNA-seq,Homo_723,AD.vs.control; bulk RNA-seq,Homo_723,AD.vs.MCI; bulk RNA-seq,Homo_723,MCI.vs.control; bulk RNA-seq,Homo_714,AD.vs.MCI; bulk RNA-seq,Homo_633,AD.vs.control; bulk RNA-seq,Homo_633,AD.vs.MCI; bulk RNA-seq,ROSMAP,AD.vs.control; bulk RNA-seq,ROSMAP,MCI.vs.control	8
BP	GO:0051123	RNA polymerase II preinitiation complex assembly	bulk RNA-seq,Homo_723,AD.vs.control; bulk RNA-seq,Homo_723,MCI.vs.control; bulk RNA-seq,Homo_714,AD.vs.control; bulk RNA-seq,Homo_714,MCI.vs.control; bulk RNA-seq,Homo_633,AD.vs.control; bulk RNA-seq,Homo_633,AD.vs.MCI	6
BP	GO:0007638	mechanosensory behavior	bulk RNA-seq,Homo_723,AD.vs.control; bulk RNA-seq,Homo_723,AD.vs.MCI; bulk RNA-seq,Homo_723,MCI.vs.control; bulk RNA-seq,Homo_714,AD.vs.control; bulk RNA-seq,Homo_714,AD.vs.MCI	5
BP	GO:0030048	actin filament-based movement	bulk RNA-seq,Homo_723,AD.vs.control; bulk RNA-seq,Homo_723,AD.vs.MCI; bulk RNA-seq,Homo_723,MCI.vs.control; bulk RNA-seq,Homo_714,AD.vs.control; bulk RNA-seq,Homo_714,AD.vs.MCI; bulk RNA-seq,Homo_633,AD.vs.control	6
BP	GO:0048841	regulation of axon extension involved in axon guidance	bulk RNA-seq,Homo_723,AD.vs.control; bulk RNA-seq,Homo_723,AD.vs.MCI; bulk RNA-seq,Homo_723,MCI.vs.control; bulk RNA-seq,Homo_714,AD.vs.MCI; bulk RNA-seq,Homo_714,MCI.vs.control	5

CC	GO:0022627	cytosolic small ribosomal subunit	bulk RNA-seq,Homo_723,AD.vs.control; bulk RNA-seq,Homo_723,MCI.vs.control; bulk RNA-seq,Homo_714,AD.vs.control; bulk RNA-seq,Homo_714,MCI.vs.control; bulk RNA-seq,Homo_633,AD.vs.control; bulk RNA-seq,Homo_633,AD.vs.MCI	6
BP	GO:0007263	nitric oxide mediated signal transduction	bulk RNA-seq,Homo_723,AD.vs.control; bulk RNA-seq,Homo_723,AD.vs.MCI; bulk RNA-seq,Homo_723,MCI.vs.control; bulk RNA-seq,Homo_714,AD.vs.control; bulk RNA-seq,Homo_714,AD.vs.MCI	5
BP	GO:0060443	mammary gland morphogenesis	bulk RNA-seq,Homo_723,AD.vs.control; bulk RNA-seq,Homo_723,AD.vs.MCI; bulk RNA-seq,Homo_723,MCI.vs.control; bulk RNA-seq,Homo_714,AD.vs.control; bulk RNA-seq,Homo_714,AD.vs.MCI; bulk RNA-seq,Homo_714,MCI.vs.control; bulk RNA-seq,Homo_633,AD.vs.control; bulk	8
BP	GO:0045494	photoreceptor cell maintenance	bulk RNA-seq,Homo_723,AD.vs.control; bulk RNA-seq,Homo_723,AD.vs.MCI; bulk RNA-seq,Homo_723,MCI.vs.control; bulk RNA-seq,Homo_714,AD.vs.control; bulk RNA-seq,Homo_714,AD.vs.MCI; bulk RNA-seq,Homo_633,AD.vs.control	6
BP	GO:0035148	tube formation	bulk RNA-seq,Homo_723,AD.vs.control; bulk RNA-seq,Homo_723,AD.vs.MCI; bulk RNA-seq,Homo_723,MCI.vs.control; bulk RNA-seq,Homo_714,AD.vs.MCI; bulk RNA-seq,Homo_633,AD.vs.control; bulk RNA-seq,Homo_633,AD.vs.MCI	6
BP	GO:0038065	collagen-activated signaling pathway	bulk RNA-seq,Homo_723,AD.vs.control; bulk RNA-seq,Homo_723,AD.vs.MCI; bulk RNA-seq,Homo_723,MCI.vs.control; bulk RNA-seq,Homo_714,AD.vs.control; bulk RNA-seq,Homo_714,AD.vs.MCI; bulk RNA-seq,Homo_714,MCI.vs.control	6
BP	GO:0048844	artery morphogenesis	bulk RNA-seq,Homo_723,AD.vs.control; bulk RNA-seq,Homo_723,AD.vs.MCI; bulk RNA-seq,Homo_723,MCI.vs.control; bulk RNA-seq,Homo_714,AD.vs.control; bulk RNA-seq,Homo_714,AD.vs.MCI; bulk RNA-seq,Homo_714,MCI.vs.control	6
MF	GO:0016741	transferase activity, transferring one-carbon groups	bulk RNA-seq,Homo_723,AD.vs.control; bulk RNA-seq,Homo_723,MCI.vs.control; bulk RNA-seq,Homo_714,AD.vs.control; bulk RNA-seq,Homo_633,AD.vs.control; bulk RNA-seq,Homo_633,AD.vs.MCI	5
BP	GO:0014075	response to amine	bulk RNA-seq,Homo_723,AD.vs.control; bulk RNA-seq,Homo_723,AD.vs.MCI; bulk RNA-seq,Homo_723,MCI.vs.control; bulk RNA-seq,Homo_714,AD.vs.control; bulk RNA-seq,Homo_714,AD.vs.MCI	5
BP	GO:0002726	positive regulation of T cell cytokine production	bulk RNA-seq,Homo_723,AD.vs.control; bulk RNA-seq,Homo_723,AD.vs.MCI; bulk RNA-seq,Homo_723,MCI.vs.control; bulk RNA-seq,Homo_714,AD.vs.control; bulk RNA-seq,Homo_714,MCI.vs.control; bulk RNA-seq,Homo_633,AD.vs.control; bulk RNA-seq,Homo_633,AD.vs.MCI; bulk RNA-seq,Homo_633,MCI.vs.control; scRNA-seq,SRP330776,CD8+ T cell_2-AD.vs.control	9
MF	GO:0016779	nucleotidyltransferase activity	bulk RNA-seq,Homo_723,AD.vs.control; bulk RNA-seq,Homo_723,MCI.vs.control; bulk RNA-seq,Homo_714,AD.vs.control; bulk RNA-seq,Homo_714,MCI.vs.control; bulk RNA-seq,Homo_633,AD.vs.control; bulk RNA-seq,Homo_633,AD.vs.MCI	6
BP	GO:0048546	digestive tract morphogenesis	bulk RNA-seq,Homo_723,AD.vs.control; bulk RNA-seq,Homo_723,AD.vs.MCI; bulk RNA-seq,Homo_723,MCI.vs.control; bulk RNA-seq,Homo_714,AD.vs.control; bulk RNA-seq,Homo_714,AD.vs.MCI	5
BP	GO:1904862	inhibitory synapse assembly	bulk RNA-seq,Homo_723,AD.vs.control; bulk RNA-seq,Homo_723,AD.vs.MCI; bulk RNA-seq,Homo_723,MCI.vs.control; bulk RNA-seq,Homo_714,AD.vs.MCI; bulk RNA-seq,Homo_633,MCI.vs.control	5
CC	GO:1902710	GABA receptor complex	bulk RNA-seq,Homo_723,AD.vs.control; bulk RNA-seq,Homo_723,AD.vs.MCI; bulk RNA-seq,Homo_714,AD.vs.control; bulk RNA-seq,Homo_714,AD.vs.MCI; bulk RNA-seq,Homo_633,MCI.vs.control	5
BP	GO:0099150	regulation of postsynaptic specialization assembly	bulk RNA-seq,Homo_723,AD.vs.control; bulk RNA-seq,Homo_723,AD.vs.MCI; bulk RNA-seq,Homo_723,MCI.vs.control; bulk RNA-seq,Homo_714,AD.vs.MCI	4
BP	GO:0061005	cell differentiation involved in kidney development	bulk RNA-seq,Homo_723,AD.vs.control; bulk RNA-seq,Homo_723,AD.vs.MCI; bulk RNA-seq,Homo_723,MCI.vs.control; bulk RNA-seq,Homo_714,AD.vs.MCI; bulk RNA-seq,Homo_714,MCI.vs.control	5
BP	GO:0050777	negative regulation of immune response	bulk RNA-seq,Homo_723,AD.vs.control; bulk RNA-seq,Homo_723,MCI.vs.control; bulk RNA-seq,Homo_714,AD.vs.control; bulk RNA-seq,Homo_633,AD.vs.control; bulk RNA-seq,Homo_633,AD.vs.MCI	5
BP	GO:0099601	regulation of neurotransmitter receptor activity	bulk RNA-seq,Homo_723,AD.vs.control; bulk RNA-seq,Homo_723,AD.vs.MCI; bulk RNA-seq,Homo_723,MCI.vs.control; bulk RNA-seq,Homo_714,AD.vs.MCI; bulk RNA-seq,Homo_714,MCI.vs.control; bulk RNA-seq,Homo_633,AD.vs.control; bulk RNA-seq,SRP223445,AD.vs.control	7
BP	GO:0002396	MHC protein complex assembly	bulk RNA-seq,Homo_723,AD.vs.control; bulk RNA-seq,Homo_723,AD.vs.MCI; bulk RNA-seq,Homo_723,MCI.vs.control; bulk RNA-seq,Homo_714,AD.vs.control; bulk RNA-seq,Homo_714,AD.vs.MCI; bulk RNA-seq,Homo_714,MCI.vs.control; bulk RNA-seq,Homo_633,AD.vs.control; bulk RNA-seq,Homo_633,AD.vs.MCI; bulk RNA-seq,Homo_633,MCI.vs.control; scRNA-seq,SRP215507,CD8+ T cell_1-MCI.vs.control; scRNA-seq,SRP215507,CD8+ T cell_3-AD.vs.control; scRNA-seq,SRP215507,CD8+ T cell_3-MCI.vs.control	12
BP	GO:0002501	peptide antigen assembly with MHC protein complex	bulk RNA-seq,Homo_723,AD.vs.control; bulk RNA-seq,Homo_723,AD.vs.MCI; bulk RNA-seq,Homo_723,MCI.vs.control; bulk RNA-seq,Homo_714,AD.vs.control; bulk RNA-seq,Homo_714,AD.vs.MCI; bulk RNA-seq,Homo_714,MCI.vs.control; bulk RNA-seq,Homo_633,AD.vs.control; bulk RNA-seq,Homo_633,AD.vs.MCI; bulk RNA-seq,Homo_633,MCI.vs.control; scRNA-seq,SRP215507,CD8+ T cell_1-MCI.vs.control; scRNA-seq,SRP215507,CD8+ T cell_3-AD.vs.control; scRNA-seq,SRP215507,CD8+ T cell_3-MCI.vs.control	12
BP	GO:0045471	response to ethanol	bulk RNA-seq,Homo_723,AD.vs.control; bulk RNA-seq,Homo_723,AD.vs.MCI; bulk RNA-seq,Homo_723,MCI.vs.control; bulk RNA-seq,Homo_714,AD.vs.control; bulk RNA-seq,Homo_714,AD.vs.MCI; bulk RNA-seq,Homo_714,MCI.vs.control; bulk RNA-seq,Homo_633,AD.vs.control; bulk	8
BP	GO:0071241	cellular response to inorganic substance	bulk RNA-seq,Homo_723,AD.vs.control; bulk RNA-seq,Homo_723,AD.vs.MCI; bulk RNA-seq,Homo_723,MCI.vs.control; bulk RNA-seq,Homo_714,AD.vs.control; bulk RNA-seq,Homo_714,AD.vs.MCI; bulk RNA-seq,Homo_633,AD.vs.control; bulk RNA-seq,Homo_633,AD.vs.MCI	7
BP	GO:0006378	mRNA polyadenylation	bulk RNA-seq,Homo_723,AD.vs.control; bulk RNA-seq,Homo_723,AD.vs.MCI; bulk RNA-seq,Homo_723,MCI.vs.control; bulk RNA-seq,Homo_714,AD.vs.control; bulk RNA-seq,Homo_714,MCI.vs.control; bulk RNA-seq,Homo_633,AD.vs.control; bulk RNA-seq,Homo_633,AD.vs.MCI	7
BP	GO:0035418	protein localization to synapse	bulk RNA-seq,Homo_723,AD.vs.control; bulk RNA-seq,Homo_723,AD.vs.MCI; bulk RNA-seq,Homo_723,MCI.vs.control; bulk RNA-seq,Homo_714,AD.vs.control; bulk RNA-seq,Homo_714,AD.vs.MCI; bulk RNA-seq,Homo_714,MCI.vs.control; bulk RNA-seq,Homo_633,AD.vs.control; bulk	8
CC	GO:0022624	proteasome accessory complex	bulk RNA-seq,Homo_723,AD.vs.control; bulk RNA-seq,Homo_723,AD.vs.MCI; bulk RNA-seq,Homo_714,AD.vs.control; bulk RNA-seq,Homo_714,AD.vs.MCI; bulk RNA-seq,Homo_633,AD.vs.control; bulk RNA-seq,Homo_633,AD.vs.MCI	6
BP	GO:0009311	oligosaccharide metabolic process	bulk RNA-seq,Homo_723,AD.vs.control; bulk RNA-seq,Homo_723,AD.vs.MCI; bulk RNA-seq,Homo_723,MCI.vs.control; bulk RNA-seq,Homo_714,AD.vs.MCI; bulk RNA-seq,Homo_633,AD.vs.control; bulk RNA-seq,Homo_633,AD.vs.MCI	6
BP	GO:0032330	regulation of chondrocyte differentiation	bulk RNA-seq,Homo_723,AD.vs.control; bulk RNA-seq,Homo_723,AD.vs.MCI; bulk RNA-seq,Homo_723,MCI.vs.control; bulk RNA-seq,Homo_714,AD.vs.MCI; bulk RNA-seq,Homo_714,MCI.vs.control; bulk RNA-seq,Homo_633,AD.vs.control	6
CC	GO:0090734	site of DNA damage	bulk RNA-seq,Homo_723,AD.vs.control; bulk RNA-seq,Homo_723,MCI.vs.control; bulk RNA-seq,Homo_714,AD.vs.control; bulk RNA-seq,Homo_714,MCI.vs.control; bulk RNA-seq,Homo_633,AD.vs.control; bulk RNA-seq,Homo_633,AD.vs.MCI	6
BP	GO:0050885	neuromuscular process controlling balance	bulk RNA-seq,Homo_723,AD.vs.control; bulk RNA-seq,Homo_723,AD.vs.MCI; bulk RNA-seq,Homo_723,MCI.vs.control; bulk RNA-seq,Homo_714,AD.vs.control; bulk RNA-seq,Homo_714,AD.vs.MCI; bulk RNA-seq,Homo_714,MCI.vs.control	6
BP	GO:0003197	endocardial cushion development	bulk RNA-seq,Homo_723,AD.vs.control; bulk RNA-seq,Homo_723,AD.vs.MCI; bulk RNA-seq,Homo_723,MCI.vs.control; bulk RNA-seq,Homo_714,AD.vs.control; bulk RNA-seq,Homo_714,AD.vs.MCI; bulk RNA-seq,Homo_714,MCI.vs.control; bulk RNA-seq,SRP223445,AD.vs.control	7
CC	GO:0034708	methyltransferase complex	bulk RNA-seq,Homo_723,AD.vs.control; bulk RNA-seq,Homo_723,MCI.vs.control; bulk RNA-seq,Homo_714,AD.vs.control; bulk RNA-seq,Homo_714,MCI.vs.control; bulk RNA-seq,Homo_633,AD.vs.control; bulk RNA-seq,Homo_633,AD.vs.MCI; scRNA-seq,SRP309935,Megakaryocyte_2-	7

BP	GO:0046546	development of primary male sexual characteristics	bulk RNA-seq,Homo_723,AD.vs.control; bulk RNA-seq,Homo_723,AD.vs.MCI; bulk RNA-seq,Homo_723,MCI.vs.control; bulk RNA-seq,Homo_714,AD.vs.control; bulk RNA-seq,Homo_714,AD.vs.MCI; bulk RNA-seq,Homo_633,AD.vs.control; bulk RNA-seq,Homo_633,AD.vs.MCI	7
BP	GO:0051896	regulation of protein kinase B signaling	bulk RNA-seq,Homo_723,AD.vs.control; bulk RNA-seq,Homo_723,AD.vs.MCI; bulk RNA-seq,Homo_723,MCI.vs.control; bulk RNA-seq,Homo_714,AD.vs.control; bulk RNA-seq,Homo_714,AD.vs.MCI; bulk RNA-seq,Homo_633,AD.vs.control; bulk RNA-seq,Homo_633,AD.vs.MCI	7
CC	GO:0016323	basolateral plasma membrane	bulk RNA-seq,Homo_723,AD.vs.control; bulk RNA-seq,Homo_723,AD.vs.MCI; bulk RNA-seq,Homo_723,MCI.vs.control; bulk RNA-seq,Homo_714,AD.vs.control; bulk RNA-seq,Homo_714,AD.vs.MCI; bulk RNA-seq,Homo_633,AD.vs.control; bulk RNA-seq,Homo_633,AD.vs.MCI; bulk RNA-seq,ROSMAP,AD.vs.control; bulk RNA-seq,SRP223445,AD.vs.control	9
MF	GO:0017147	Wnt-protein binding	bulk RNA-seq,Homo_723,AD.vs.control; bulk RNA-seq,Homo_723,AD.vs.MCI; bulk RNA-seq,Homo_723,MCI.vs.control; bulk RNA-seq,Homo_714,AD.vs.MCI; bulk RNA-seq,SRP223445,AD.vs.control	5
BP	GO:0071248	cellular response to metal ion	bulk RNA-seq,Homo_723,AD.vs.control; bulk RNA-seq,Homo_723,AD.vs.MCI; bulk RNA-seq,Homo_723,MCI.vs.control; bulk RNA-seq,Homo_714,AD.vs.control; bulk RNA-seq,Homo_714,AD.vs.MCI; bulk RNA-seq,Homo_633,AD.vs.control; bulk RNA-seq,Homo_633,AD.vs.MCI	7
BP	GO:0002562	somatic diversification of immune receptors via germline recombination within a single locus	bulk RNA-seq,Homo_723,AD.vs.control; bulk RNA-seq,Homo_723,MCI.vs.control; bulk RNA-seq,Homo_714,AD.vs.control; bulk RNA-seq,Homo_714,MCI.vs.control; bulk RNA-seq,Homo_633,AD.vs.control; bulk RNA-seq,Homo_633,AD.vs.MCI	6
BP	GO:0016444	somatic cell DNA recombination	bulk RNA-seq,Homo_723,AD.vs.control; bulk RNA-seq,Homo_723,MCI.vs.control; bulk RNA-seq,Homo_714,AD.vs.control; bulk RNA-seq,Homo_714,MCI.vs.control; bulk RNA-seq,Homo_633,AD.vs.control; bulk RNA-seq,Homo_633,AD.vs.MCI	6
BP	GO:0015800	acidic amino acid transport	bulk RNA-seq,Homo_723,AD.vs.control; bulk RNA-seq,Homo_723,AD.vs.MCI; bulk RNA-seq,Homo_723,MCI.vs.control; bulk RNA-seq,Homo_714,AD.vs.MCI; bulk RNA-seq,Homo_633,AD.vs.control; bulk RNA-seq,Homo_633,AD.vs.MCI	6
CC	GO:0036464	cytoplasmic ribonucleoprotein granule	bulk RNA-seq,Homo_723,AD.vs.control; bulk RNA-seq,Homo_723,MCI.vs.control; bulk RNA-seq,Homo_714,AD.vs.control; bulk RNA-seq,Homo_633,AD.vs.control; bulk RNA-seq,Homo_633,AD.vs.MCI	5
BP	GO:0016236	macroautophagy	bulk RNA-seq,Homo_723,AD.vs.control; bulk RNA-seq,Homo_723,MCI.vs.control; bulk RNA-seq,Homo_714,AD.vs.control; bulk RNA-seq,Homo_633,AD.vs.control; bulk RNA-seq,Homo_633,AD.vs.MCI	5
BP	GO:0006120	mitochondrial electron transport, NADH to ubiquinone	bulk RNA-seq,Homo_723,AD.vs.control; bulk RNA-seq,Homo_723,AD.vs.MCI; bulk RNA-seq,Homo_723,MCI.vs.control; bulk RNA-seq,Homo_714,AD.vs.control; bulk RNA-seq,Homo_714,MCI.vs.control; bulk RNA-seq,Homo_633,AD.vs.control; bulk RNA-seq,Homo_633,AD.vs.MCI	7
BP	GO:0006310	DNA recombination	bulk RNA-seq,Homo_723,AD.vs.control; bulk RNA-seq,Homo_723,MCI.vs.control; bulk RNA-seq,Homo_714,AD.vs.control; bulk RNA-seq,Homo_633,AD.vs.control; bulk RNA-seq,Homo_633,AD.vs.MCI	5
MF	GO:0140097	catalytic activity, acting on DNA	bulk RNA-seq,Homo_723,AD.vs.control; bulk RNA-seq,Homo_723,MCI.vs.control; bulk RNA-seq,Homo_714,AD.vs.control; bulk RNA-seq,Homo_633,AD.vs.control; bulk RNA-seq,Homo_633,AD.vs.MCI	5
BP	GO:0001578	microtubule bundle formation	bulk RNA-seq,Homo_723,AD.vs.control; bulk RNA-seq,Homo_723,AD.vs.MCI; bulk RNA-seq,Homo_723,MCI.vs.control; bulk RNA-seq,Homo_714,AD.vs.MCI; bulk RNA-seq,Homo_714,MCI.vs.control; bulk RNA-seq,Homo_633,AD.vs.control; bulk RNA-seq,Homo_633,AD.vs.MCI	7
MF	GO:0008188	neuropeptide receptor activity	bulk RNA-seq,Homo_723,AD.vs.control; bulk RNA-seq,Homo_723,AD.vs.MCI; bulk RNA-seq,Homo_714,AD.vs.control; bulk RNA-seq,Homo_714,AD.vs.MCI; bulk RNA-seq,Homo_633,MCI.vs.control; bulk RNA-seq,SRP223445,AD.vs.control	6
BP	GO:0072089	stem cell proliferation	bulk RNA-seq,Homo_723,AD.vs.control; bulk RNA-seq,Homo_723,AD.vs.MCI; bulk RNA-seq,Homo_723,MCI.vs.control; bulk RNA-seq,Homo_714,AD.vs.control; bulk RNA-seq,Homo_714,AD.vs.MCI; bulk RNA-seq,Homo_714,MCI.vs.control; bulk RNA-seq,Homo_633,AD.vs.control	7
BP	GO:0031646	positive regulation of nervous system process	bulk RNA-seq,Homo_723,AD.vs.control; bulk RNA-seq,Homo_723,AD.vs.MCI; bulk RNA-seq,Homo_723,MCI.vs.control; bulk RNA-seq,Homo_714,AD.vs.control; bulk RNA-seq,Homo_714,AD.vs.MCI; bulk RNA-seq,Homo_633,AD.vs.MCI; bulk RNA-seq,SRP325058,AD.vs.control	7
BP	GO:0022904	respiratory electron transport chain	bulk RNA-seq,Homo_723,AD.vs.control; bulk RNA-seq,Homo_723,MCI.vs.control; bulk RNA-seq,Homo_714,AD.vs.control; bulk RNA-seq,Homo_714,MCI.vs.control; bulk RNA-seq,Homo_633,AD.vs.control; bulk RNA-seq,Homo_633,AD.vs.MCI	6
MF	GO:0008137	NADH dehydrogenase (ubiquinone) activity	bulk RNA-seq,Homo_723,AD.vs.control; bulk RNA-seq,Homo_723,AD.vs.MCI; bulk RNA-seq,Homo_723,MCI.vs.control; bulk RNA-seq,Homo_714,AD.vs.control; bulk RNA-seq,Homo_714,MCI.vs.control; bulk RNA-seq,Homo_633,AD.vs.control; bulk RNA-seq,Homo_633,AD.vs.MCI	7
BP	GO:0042490	mechanoreceptor differentiation	bulk RNA-seq,Homo_723,AD.vs.control; bulk RNA-seq,Homo_723,AD.vs.MCI; bulk RNA-seq,Homo_723,MCI.vs.control; bulk RNA-seq,Homo_714,AD.vs.control; bulk RNA-seq,Homo_714,AD.vs.MCI; bulk RNA-seq,Homo_714,MCI.vs.control	6
BP	GO:0061042	vascular wound healing	bulk RNA-seq,Homo_723,AD.vs.control; bulk RNA-seq,Homo_723,AD.vs.MCI; bulk RNA-seq,Homo_723,MCI.vs.control; bulk RNA-seq,Homo_714,AD.vs.control; bulk RNA-seq,Homo_714,AD.vs.MCI; bulk RNA-seq,Homo_714,MCI.vs.control	6
MF	GO:0003684	damaged DNA binding	bulk RNA-seq,Homo_723,AD.vs.control; bulk RNA-seq,Homo_723,MCI.vs.control; bulk RNA-seq,Homo_714,AD.vs.control; bulk RNA-seq,Homo_714,MCI.vs.control; bulk RNA-seq,Homo_633,AD.vs.control; bulk RNA-seq,Homo_633,AD.vs.MCI	6
MF	GO:0030020	extracellular matrix structural constituent conferring tensile strength	bulk RNA-seq,Homo_723,AD.vs.control; bulk RNA-seq,Homo_723,AD.vs.MCI; bulk RNA-seq,Homo_723,MCI.vs.control; bulk RNA-seq,Homo_714,AD.vs.MCI; bulk RNA-seq,Homo_714,MCI.vs.control; bulk RNA-seq,ROSMAP,AD.vs.control	6
BP	GO:0006278	RNA-templated DNA biosynthetic process	bulk RNA-seq,Homo_723,AD.vs.control; bulk RNA-seq,Homo_723,MCI.vs.control; bulk RNA-seq,Homo_714,AD.vs.control; bulk RNA-seq,Homo_714,MCI.vs.control; bulk RNA-seq,Homo_633,AD.vs.control; bulk RNA-seq,Homo_633,AD.vs.MCI	6
BP	GO:0010669	epithelial structure maintenance	bulk RNA-seq,Homo_723,AD.vs.control; bulk RNA-seq,Homo_723,AD.vs.MCI; bulk RNA-seq,Homo_723,MCI.vs.control; bulk RNA-seq,Homo_714,AD.vs.control; bulk RNA-seq,Homo_714,AD.vs.MCI; bulk RNA-seq,Homo_714,MCI.vs.control	6
BP	GO:0007584	response to nutrient	bulk RNA-seq,Homo_723,AD.vs.control; bulk RNA-seq,Homo_723,AD.vs.MCI; bulk RNA-seq,Homo_723,MCI.vs.control; bulk RNA-seq,Homo_714,AD.vs.control; bulk RNA-seq,Homo_714,AD.vs.MCI; bulk RNA-seq,Homo_633,AD.vs.control; bulk RNA-seq,Homo_633,AD.vs.MCI	7
BP	GO:0002832	negative regulation of response to biotic stimulus	bulk RNA-seq,Homo_723,AD.vs.control; bulk RNA-seq,Homo_723,MCI.vs.control; bulk RNA-seq,Homo_714,AD.vs.control; bulk RNA-seq,Homo_714,MCI.vs.control; bulk RNA-seq,Homo_633,AD.vs.control; bulk RNA-seq,Homo_633,AD.vs.MCI	6
BP	GO:0086019	cell-cell signaling involved in cardiac conduction	bulk RNA-seq,Homo_723,AD.vs.control; bulk RNA-seq,Homo_723,AD.vs.MCI; bulk RNA-seq,Homo_723,MCI.vs.control; bulk RNA-seq,Homo_714,AD.vs.control; bulk RNA-seq,Homo_714,AD.vs.MCI; bulk RNA-seq,Homo_714,MCI.vs.control	6
CC	GO:0030117	membrane coat	bulk RNA-seq,Homo_723,AD.vs.control; bulk RNA-seq,Homo_723,MCI.vs.control; bulk RNA-seq,Homo_714,AD.vs.control; bulk RNA-seq,Homo_714,MCI.vs.control; bulk RNA-seq,Homo_633,AD.vs.control; bulk RNA-seq,Homo_633,AD.vs.MCI	6
CC	GO:0048475	coated membrane	bulk RNA-seq,Homo_723,AD.vs.control; bulk RNA-seq,Homo_723,MCI.vs.control; bulk RNA-seq,Homo_714,AD.vs.control; bulk RNA-seq,Homo_714,MCI.vs.control; bulk RNA-seq,Homo_633,AD.vs.control; bulk RNA-seq,Homo_633,AD.vs.MCI	6
BP	GO:0021549	cerebellum development	bulk RNA-seq,Homo_723,AD.vs.control; bulk RNA-seq,Homo_723,AD.vs.MCI; bulk RNA-seq,Homo_723,MCI.vs.control; bulk RNA-seq,Homo_714,AD.vs.MCI; bulk RNA-seq,Homo_714,MCI.vs.control; bulk RNA-seq,Homo_633,AD.vs.control; bulk RNA-seq,Homo_633,AD.vs.MCI	7
CC	GO:0005746	mitochondrial respirasome	bulk RNA-seq,Homo_723,AD.vs.control; bulk RNA-seq,Homo_723,MCI.vs.control; bulk RNA-seq,Homo_714,AD.vs.control; bulk RNA-seq,Homo_714,MCI.vs.control; bulk RNA-seq,Homo_633,AD.vs.control; bulk RNA-seq,Homo_633,AD.vs.MCI	6

BP	GO:0010833	telomere maintenance via telomere lengthening	bulk RNA-seq,Homo_723,AD.vs.control; bulk RNA-seq,Homo_723,MCI.vs.control; bulk RNA-seq,Homo_714,AD.vs.control; bulk RNA-seq,Homo_714,MCI.vs.control; bulk RNA-seq,Homo_633,AD.vs.control; bulk RNA-seq,Homo_633,AD.vs.MCI	6
MF	GO:0050136	NADH dehydrogenase (quinone) activity	bulk RNA-seq,Homo_723,AD.vs.control; bulk RNA-seq,Homo_723,MCI.vs.control; bulk RNA-seq,Homo_714,AD.vs.control; bulk RNA-seq,Homo_714,MCI.vs.control; bulk RNA-seq,Homo_633,AD.vs.control; bulk RNA-seq,Homo_633,AD.vs.MCI	6
BP	GO:0060042	retina morphogenesis in camera-type eye	bulk RNA-seq,Homo_723,AD.vs.control; bulk RNA-seq,Homo_723,AD.vs.MCI; bulk RNA-seq,Homo_723,MCI.vs.control; bulk RNA-seq,Homo_714,AD.vs.MCI; bulk RNA-seq,Homo_714,MCI.vs.control; bulk RNA-seq,Homo_633,AD.vs.control; bulk RNA-seq,Homo_633,AD.vs.MCI; bulk RNA-seq,ROSMAP,AD.vs.control; bulk RNA-seq,SRP223445,AD.vs.control	9
BP	GO:0001101	response to acid chemical	bulk RNA-seq,Homo_723,AD.vs.control; bulk RNA-seq,Homo_723,AD.vs.MCI; bulk RNA-seq,Homo_723,MCI.vs.control; bulk RNA-seq,Homo_714,AD.vs.MCI; bulk RNA-seq,Homo_633,AD.vs.control; bulk RNA-seq,Homo_633,AD.vs.MCI; scRNA-seq,SRP330776,CD8+ T cell_1-AD.vs.control; scRNA-seq,SRP330776,Naive CD8+ T cell_1-AD.vs.control	8
BP	GO:0035335	peptidyl-tyrosine dephosphorylation	bulk RNA-seq,Homo_723,AD.vs.control; bulk RNA-seq,Homo_723,AD.vs.MCI; bulk RNA-seq,Homo_723,MCI.vs.control; bulk RNA-seq,Homo_714,AD.vs.MCI; bulk RNA-seq,Homo_714,MCI.vs.control; bulk RNA-seq,Homo_633,AD.vs.control	6
BP	GO:0006721	terpenoid metabolic process	bulk RNA-seq,Homo_723,AD.vs.control; bulk RNA-seq,Homo_723,AD.vs.MCI; bulk RNA-seq,Homo_723,MCI.vs.control; bulk RNA-seq,Homo_714,AD.vs.control; bulk RNA-seq,Homo_714,AD.vs.MCI; bulk RNA-seq,Homo_714,MCI.vs.control; bulk RNA-seq,Homo_633,AD.vs.control; bulk	8
BP	GO:0007004	telomere maintenance via telomerase	bulk RNA-seq,Homo_723,AD.vs.control; bulk RNA-seq,Homo_723,MCI.vs.control; bulk RNA-seq,Homo_714,AD.vs.control; bulk RNA-seq,Homo_714,MCI.vs.control; bulk RNA-seq,Homo_633,AD.vs.control; bulk RNA-seq,Homo_633,AD.vs.MCI	6
BP	GO:0031167	rRNA methylation	bulk RNA-seq,Homo_723,AD.vs.control; bulk RNA-seq,Homo_723,AD.vs.MCI; bulk RNA-seq,Homo_723,MCI.vs.control; bulk RNA-seq,Homo_714,AD.vs.control; bulk RNA-seq,Homo_714,MCI.vs.control; bulk RNA-seq,Homo_633,AD.vs.control; bulk RNA-seq,Homo_633,AD.vs.MCI; bulk	8
BP	GO:0034661	ncRNA catabolic process	bulk RNA-seq,Homo_723,AD.vs.control; bulk RNA-seq,Homo_723,AD.vs.MCI; bulk RNA-seq,Homo_714,AD.vs.control; bulk RNA-seq,Homo_714,AD.vs.MCI; bulk RNA-seq,Homo_633,AD.vs.control; bulk RNA-seq,Homo_633,AD.vs.MCI	6
MF	GO:0008168	methyltransferase activity	bulk RNA-seq,Homo_723,AD.vs.control; bulk RNA-seq,Homo_723,MCI.vs.control; bulk RNA-seq,Homo_714,AD.vs.control; bulk RNA-seq,Homo_633,AD.vs.control; bulk RNA-seq,Homo_633,AD.vs.MCI	5
BP	GO:0048791	calcium ion-regulated exocytosis of neurotransmitter	bulk RNA-seq,Homo_723,AD.vs.control; bulk RNA-seq,Homo_723,AD.vs.MCI; bulk RNA-seq,Homo_723,MCI.vs.control; bulk RNA-seq,Homo_714,AD.vs.control; bulk RNA-seq,Homo_714,AD.vs.MCI	5
BP	GO:0090151	establishment of protein localization to mitochondrial membrane	bulk RNA-seq,Homo_723,AD.vs.control; bulk RNA-seq,Homo_723,MCI.vs.control; bulk RNA-seq,Homo_714,AD.vs.control; bulk RNA-seq,Homo_714,MCI.vs.control; bulk RNA-seq,Homo_633,AD.vs.control; bulk RNA-seq,Homo_633,AD.vs.MCI	6
BP	GO:0000018	regulation of DNA recombination	bulk RNA-seq,Homo_723,AD.vs.control; bulk RNA-seq,Homo_723,MCI.vs.control; bulk RNA-seq,Homo_714,AD.vs.control; bulk RNA-seq,Homo_633,AD.vs.control; bulk RNA-seq,Homo_633,AD.vs.MCI	5
CC	GO:1905348	endonuclease complex	bulk RNA-seq,Homo_723,AD.vs.control; bulk RNA-seq,Homo_723,AD.vs.MCI; bulk RNA-seq,Homo_714,AD.vs.control; bulk RNA-seq,Homo_714,MCI.vs.control; bulk RNA-seq,Homo_633,AD.vs.control; bulk RNA-seq,Homo_633,AD.vs.MCI	6
BP	GO:0090189	regulation of branching involved in ureteric bud	bulk RNA-seq,Homo_723,AD.vs.control; bulk RNA-seq,Homo_723,AD.vs.MCI; bulk RNA-seq,Homo_723,MCI.vs.control; bulk RNA-seq,Homo_714,AD.vs.MCI	4
BP	GO:0007494	midgut development	bulk RNA-seq,Homo_723,AD.vs.control; bulk RNA-seq,Homo_723,AD.vs.MCI; bulk RNA-seq,Homo_723,MCI.vs.control; bulk RNA-seq,Homo_714,AD.vs.control; bulk RNA-seq,Homo_714,AD.vs.MCI; bulk RNA-seq,Homo_714,MCI.vs.control	6
BP	GO:0035235	ionotropic glutamate receptor signaling pathway	bulk RNA-seq,Homo_723,AD.vs.control; bulk RNA-seq,Homo_723,AD.vs.MCI; bulk RNA-seq,Homo_723,MCI.vs.control; bulk RNA-seq,Homo_714,AD.vs.MCI; bulk RNA-seq,Homo_633,AD.vs.control	5
BP	GO:1990806	ligand-gated ion channel signaling pathway	bulk RNA-seq,Homo_723,AD.vs.control; bulk RNA-seq,Homo_723,AD.vs.MCI; bulk RNA-seq,Homo_723,MCI.vs.control; bulk RNA-seq,Homo_714,AD.vs.MCI; bulk RNA-seq,Homo_633,AD.vs.control	5
MF	GO:0001098	basal transcription machinery binding	bulk RNA-seq,Homo_723,AD.vs.control; bulk RNA-seq,Homo_723,MCI.vs.control; bulk RNA-seq,Homo_714,AD.vs.control; bulk RNA-seq,Homo_714,MCI.vs.control; bulk RNA-seq,Homo_633,AD.vs.control; bulk RNA-seq,Homo_633,AD.vs.MCI	6
MF	GO:0001099	basal RNA polymerase II transcription machinery binding	bulk RNA-seq,Homo_723,AD.vs.control; bulk RNA-seq,Homo_723,MCI.vs.control; bulk RNA-seq,Homo_714,AD.vs.control; bulk RNA-seq,Homo_714,MCI.vs.control; bulk RNA-seq,Homo_633,AD.vs.control; bulk RNA-seq,Homo_633,AD.vs.MCI	6
BP	GO:2001028	positive regulation of endothelial cell chemotaxis	bulk RNA-seq,Homo_723,AD.vs.control; bulk RNA-seq,Homo_723,AD.vs.MCI; bulk RNA-seq,Homo_723,MCI.vs.control; bulk RNA-seq,Homo_714,AD.vs.control; bulk RNA-seq,Homo_714,AD.vs.MCI; bulk RNA-seq,Homo_714,MCI.vs.control	6
BP	GO:0001523	retinoid metabolic process	bulk RNA-seq,Homo_723,AD.vs.control; bulk RNA-seq,Homo_723,AD.vs.MCI; bulk RNA-seq,Homo_723,MCI.vs.control; bulk RNA-seq,Homo_714,AD.vs.control; bulk RNA-seq,Homo_714,AD.vs.MCI; bulk RNA-seq,Homo_714,MCI.vs.control; bulk RNA-seq,Homo_633,AD.vs.MCI	7
BP	GO:0030857	negative regulation of epithelial cell differentiation	bulk RNA-seq,Homo_723,AD.vs.control; bulk RNA-seq,Homo_723,AD.vs.MCI; bulk RNA-seq,Homo_723,MCI.vs.control; bulk RNA-seq,Homo_714,AD.vs.control; bulk RNA-seq,Homo_714,AD.vs.MCI; bulk RNA-seq,Homo_714,MCI.vs.control	6
BP	GO:0090288	negative regulation of cellular response to growth factor stimulus	bulk RNA-seq,Homo_723,AD.vs.control; bulk RNA-seq,Homo_723,AD.vs.MCI; bulk RNA-seq,Homo_723,MCI.vs.control; bulk RNA-seq,Homo_714,AD.vs.MCI; bulk RNA-seq,Homo_714,MCI.vs.control; bulk RNA-seq,SRP223445,AD.vs.control	6
MF	GO:0005125	cytokine activity	bulk RNA-seq,Homo_723,AD.vs.control; bulk RNA-seq,Homo_723,AD.vs.MCI; bulk RNA-seq,Homo_723,MCI.vs.control; bulk RNA-seq,Homo_714,AD.vs.control; bulk RNA-seq,Homo_714,AD.vs.MCI	5
BP	GO:0051955	regulation of amino acid transport	bulk RNA-seq,Homo_723,AD.vs.control; bulk RNA-seq,Homo_723,AD.vs.MCI; bulk RNA-seq,Homo_723,MCI.vs.control; bulk RNA-seq,Homo_714,AD.vs.control; bulk RNA-seq,Homo_714,AD.vs.MCI	5
BP	GO:0021884	forebrain neuron development	bulk RNA-seq,Homo_723,AD.vs.control; bulk RNA-seq,Homo_723,AD.vs.MCI; bulk RNA-seq,Homo_723,MCI.vs.control; bulk RNA-seq,Homo_714,AD.vs.MCI; bulk RNA-seq,Homo_633,AD.vs.control	5
BP	GO:0001702	gastrulation with mouth forming second	bulk RNA-seq,Homo_723,AD.vs.control; bulk RNA-seq,Homo_723,AD.vs.MCI; bulk RNA-seq,Homo_723,MCI.vs.control; bulk RNA-seq,Homo_714,AD.vs.MCI; bulk RNA-seq,Homo_633,AD.vs.control; bulk RNA-seq,Homo_633,AD.vs.MCI	6
BP	GO:0030517	negative regulation of axon extension	bulk RNA-seq,Homo_723,AD.vs.control; bulk RNA-seq,Homo_723,AD.vs.MCI; bulk RNA-seq,Homo_723,MCI.vs.control; bulk RNA-seq,Homo_714,AD.vs.MCI; bulk RNA-seq,Homo_714,MCI.vs.control; bulk RNA-seq,Homo_633,AD.vs.control	6
BP	GO:0010810	regulation of cell-substrate adhesion	bulk RNA-seq,Homo_723,AD.vs.control; bulk RNA-seq,Homo_723,AD.vs.MCI; bulk RNA-seq,Homo_723,MCI.vs.control; bulk RNA-seq,Homo_714,AD.vs.control; bulk RNA-seq,Homo_714,AD.vs.MCI; bulk RNA-seq,Homo_633,AD.vs.control; bulk RNA-seq,Homo_633,AD.vs.MCI; bulk RNA-	8
MF	GO:0023026	MHC class II protein complex binding	bulk RNA-seq,Homo_723,AD.vs.control; bulk RNA-seq,Homo_723,AD.vs.MCI; bulk RNA-seq,Homo_723,MCI.vs.control; bulk RNA-seq,Homo_714,AD.vs.control; bulk RNA-seq,Homo_714,MCI.vs.control; bulk RNA-seq,Homo_633,AD.vs.control; bulk RNA-seq,Homo_633,AD.vs.MCI; bulk RNA-seq,Homo_633,MCI.vs.control; scRNA-seq,SRP215507,CD8+ T cell_3-AD.vs.control; scRNA-seq,SRP215507,CD8+ T cell_3-MCI.vs.control	10
BP	GO:0000966	RNA 5'-end processing	bulk RNA-seq,Homo_723,AD.vs.control; bulk RNA-seq,Homo_723,AD.vs.MCI; bulk RNA-seq,Homo_714,AD.vs.control; bulk RNA-seq,Homo_714,AD.vs.MCI; bulk RNA-seq,Homo_633,AD.vs.control; bulk RNA-seq,Homo_633,AD.vs.MCI	6

BP	GO:0008589	regulation of smoothened signaling pathway	bulk RNA-seq,Homo_723,AD.vs.control; bulk RNA-seq,Homo_723,AD.vs.MCI; bulk RNA-seq,Homo_723,MCI.vs.control; bulk RNA-seq,Homo_714,AD.vs.MCI; bulk RNA-seq,Homo_714,MCI.vs.control; bulk RNA-seq,Homo_633,AD.vs.control; bulk RNA-seq,Homo_633,AD.vs.MCI; bulk RNA-	8
CC	GO:0030666	endocytic vesicle membrane	bulk RNA-seq,Homo_723,AD.vs.control; bulk RNA-seq,Homo_723,AD.vs.MCI; bulk RNA-seq,Homo_723,MCI.vs.control; bulk RNA-seq,Homo_714,AD.vs.MCI; bulk RNA-seq,Homo_633,AD.vs.control; bulk RNA-seq,Homo_633,AD.vs.MCI	6
BP	GO:0045685	regulation of glial cell differentiation	bulk RNA-seq,Homo_723,AD.vs.control; bulk RNA-seq,Homo_723,AD.vs.MCI; bulk RNA-seq,Homo_723,MCI.vs.control; bulk RNA-seq,Homo_714,AD.vs.MCI; bulk RNA-seq,Homo_714,MCI.vs.control	5
CC	GO:0001772	immunological synapse	bulk RNA-seq,Homo_723,AD.vs.control; bulk RNA-seq,Homo_723,AD.vs.MCI; bulk RNA-seq,Homo_723,MCI.vs.control; bulk RNA-seq,Homo_714,AD.vs.control; bulk RNA-seq,Homo_714,AD.vs.MCI; bulk RNA-seq,Homo_714,MCI.vs.control; bulk RNA-seq,Homo_633,AD.vs.control; bulk	8
BP	GO:0071695	anatomical structure maturation	bulk RNA-seq,Homo_723,AD.vs.control; bulk RNA-seq,Homo_723,AD.vs.MCI; bulk RNA-seq,Homo_723,MCI.vs.control; bulk RNA-seq,Homo_714,AD.vs.MCI; bulk RNA-seq,Homo_633,AD.vs.control; bulk RNA-seq,Homo_633,AD.vs.MCI	6
BP	GO:0043631	RNA polyadenylation	bulk RNA-seq,Homo_723,AD.vs.control; bulk RNA-seq,Homo_723,AD.vs.MCI; bulk RNA-seq,Homo_723,MCI.vs.control; bulk RNA-seq,Homo_714,AD.vs.control; bulk RNA-seq,Homo_714,AD.vs.MCI; bulk RNA-seq,Homo_714,MCI.vs.control; bulk RNA-seq,Homo_633,AD.vs.control; bulk	8
MF	GO:0042562	hormone binding	bulk RNA-seq,Homo_723,AD.vs.control; bulk RNA-seq,Homo_723,AD.vs.MCI; bulk RNA-seq,Homo_723,MCI.vs.control; bulk RNA-seq,Homo_714,AD.vs.control; bulk RNA-seq,Homo_714,AD.vs.MCI; bulk RNA-seq,Homo_714,MCI.vs.control	6
BP	GO:0060563	neuroepithelial cell differentiation	bulk RNA-seq,Homo_723,AD.vs.control; bulk RNA-seq,Homo_723,AD.vs.MCI; bulk RNA-seq,Homo_714,AD.vs.control; bulk RNA-seq,Homo_714,AD.vs.MCI	4
BP	GO:0044782	cilium organization	bulk RNA-seq,Homo_723,AD.vs.control; bulk RNA-seq,Homo_723,AD.vs.MCI; bulk RNA-seq,Homo_714,AD.vs.MCI; bulk RNA-seq,Homo_633,AD.vs.control; bulk RNA-seq,Homo_633,AD.vs.MCI; bulk RNA-seq,ROSMAP,AD.vs.control; bulk RNA-seq,ROSMAP,MCI.vs.control	7
MF	GO:0008503	benzodiazepine receptor activity	bulk RNA-seq,Homo_723,AD.vs.control; bulk RNA-seq,Homo_723,AD.vs.MCI; bulk RNA-seq,Homo_714,AD.vs.control; bulk RNA-seq,Homo_714,AD.vs.MCI	4
CC	GO:0001669	acrosomal vesicle	bulk RNA-seq,Homo_723,AD.vs.control; bulk RNA-seq,Homo_723,AD.vs.MCI; bulk RNA-seq,Homo_714,AD.vs.control; bulk RNA-seq,Homo_714,AD.vs.MCI	4
CC	GO:0042611	MHC protein complex	bulk RNA-seq,Homo_723,AD.vs.control; bulk RNA-seq,Homo_723,AD.vs.MCI; bulk RNA-seq,Homo_723,MCI.vs.control; bulk RNA-seq,Homo_714,AD.vs.control; bulk RNA-seq,Homo_714,MCI.vs.control; bulk RNA-seq,Homo_633,AD.vs.control; bulk RNA-seq,Homo_633,MCI.vs.control; scRNA-seq,SRP215507,CD8+ T cell_1-MCI.vs.control; scRNA-seq,SRP215507,CD8+ T cell_3-AD.vs.control; scRNA-seq,SRP215507,CD8+ T cell_3-	10
MF	GO:0140102	catalytic activity, acting on a rRNA	bulk RNA-seq,Homo_723,AD.vs.control; bulk RNA-seq,Homo_723,AD.vs.MCI; bulk RNA-seq,Homo_723,MCI.vs.control; bulk RNA-seq,Homo_714,AD.vs.control; bulk RNA-seq,Homo_714,MCI.vs.control; bulk RNA-seq,Homo_633,AD.vs.control; bulk RNA-seq,Homo_633,AD.vs.MCI; bulk	8
BP	GO:0009199	ribonucleoside triphosphate metabolic process	bulk RNA-seq,Homo_723,AD.vs.control; bulk RNA-seq,Homo_723,MCI.vs.control; bulk RNA-seq,Homo_714,AD.vs.control; bulk RNA-seq,Homo_633,AD.vs.control; bulk RNA-seq,Homo_633,AD.vs.MCI	5
BP	GO:0048644	muscle organ morphogenesis	bulk RNA-seq,Homo_723,AD.vs.control; bulk RNA-seq,Homo_723,AD.vs.MCI; bulk RNA-seq,Homo_723,MCI.vs.control; bulk RNA-seq,Homo_714,AD.vs.control; bulk RNA-seq,Homo_714,AD.vs.MCI; bulk RNA-seq,Homo_714,MCI.vs.control; bulk RNA-seq,SRP223445,AD.vs.control	7
BP	GO:0099560	synaptic membrane adhesion	bulk RNA-seq,Homo_723,AD.vs.control; bulk RNA-seq,Homo_723,AD.vs.MCI; bulk RNA-seq,Homo_723,MCI.vs.control; bulk RNA-seq,Homo_714,AD.vs.control; bulk RNA-seq,Homo_714,AD.vs.MCI; bulk RNA-seq,Homo_714,MCI.vs.control	6
BP	GO:0003333	amino acid transmembrane transport	bulk RNA-seq,Homo_723,AD.vs.control; bulk RNA-seq,Homo_723,AD.vs.MCI; bulk RNA-seq,Homo_723,MCI.vs.control; bulk RNA-seq,Homo_714,AD.vs.MCI; bulk RNA-seq,Homo_633,AD.vs.control; bulk RNA-seq,Homo_633,AD.vs.MCI	6
CC	GO:0005796	Golgi lumen	bulk RNA-seq,Homo_723,AD.vs.control; bulk RNA-seq,Homo_723,AD.vs.MCI; bulk RNA-seq,Homo_723,MCI.vs.control; bulk RNA-seq,Homo_714,AD.vs.control; bulk RNA-seq,Homo_714,AD.vs.MCI; bulk RNA-seq,Homo_714,MCI.vs.control	6
CC	GO:0098685	Schaffer collateral - CA1 synapse	bulk RNA-seq,Homo_723,AD.vs.control; bulk RNA-seq,Homo_723,AD.vs.MCI; bulk RNA-seq,Homo_723,MCI.vs.control; bulk RNA-seq,Homo_714,AD.vs.MCI; bulk RNA-seq,Homo_714,MCI.vs.control; bulk RNA-seq,Homo_633,AD.vs.control; bulk RNA-seq,Homo_633,AD.vs.MCI	7
BP	GO:0016101	diterpenoid metabolic process	bulk RNA-seq,Homo_723,AD.vs.control; bulk RNA-seq,Homo_723,AD.vs.MCI; bulk RNA-seq,Homo_723,MCI.vs.control; bulk RNA-seq,Homo_714,AD.vs.control; bulk RNA-seq,Homo_714,AD.vs.MCI; bulk RNA-seq,Homo_714,MCI.vs.control; bulk RNA-seq,Homo_633,AD.vs.control; bulk	8
BP	GO:0031647	regulation of protein stability	bulk RNA-seq,Homo_723,AD.vs.control; bulk RNA-seq,Homo_723,MCI.vs.control; bulk RNA-seq,Homo_714,AD.vs.control; bulk RNA-seq,Homo_633,AD.vs.control; bulk RNA-seq,Homo_633,AD.vs.MCI	5
CC	GO:0005657	replication fork	bulk RNA-seq,Homo_723,AD.vs.control; bulk RNA-seq,Homo_723,MCI.vs.control; bulk RNA-seq,Homo_714,AD.vs.control; bulk RNA-seq,Homo_714,MCI.vs.control; bulk RNA-seq,Homo_633,AD.vs.control; bulk RNA-seq,Homo_633,AD.vs.MCI	6
BP	GO:0038202	TORC1 signaling	bulk RNA-seq,Homo_723,AD.vs.control; bulk RNA-seq,Homo_723,MCI.vs.control; bulk RNA-seq,Homo_714,AD.vs.control; bulk RNA-seq,Homo_714,MCI.vs.control; bulk RNA-seq,Homo_633,AD.vs.control; bulk RNA-seq,Homo_633,AD.vs.MCI	6
MF	GO:0003725	double-stranded RNA binding	bulk RNA-seq,Homo_723,AD.vs.control; bulk RNA-seq,Homo_723,MCI.vs.control; bulk RNA-seq,Homo_714,AD.vs.control; bulk RNA-seq,Homo_714,MCI.vs.control; bulk RNA-seq,Homo_633,AD.vs.control; bulk RNA-seq,Homo_633,AD.vs.MCI	6
MF	GO:0004222	metalloendopeptidase activity	bulk RNA-seq,Homo_723,AD.vs.control; bulk RNA-seq,Homo_723,AD.vs.MCI; bulk RNA-seq,Homo_723,MCI.vs.control; bulk RNA-seq,Homo_714,AD.vs.control; bulk RNA-seq,Homo_714,AD.vs.MCI; bulk RNA-seq,Homo_714,MCI.vs.control; bulk RNA-seq,Homo_633,AD.vs.control; bulk	8
BP	GO:0036260	RNA capping	bulk RNA-seq,Homo_723,AD.vs.control; bulk RNA-seq,Homo_723,MCI.vs.control; bulk RNA-seq,Homo_714,AD.vs.control; bulk RNA-seq,Homo_714,MCI.vs.control; bulk RNA-seq,Homo_633,AD.vs.control; bulk RNA-seq,Homo_633,AD.vs.MCI; bulk RNA-seq,Homo_633,MCI.vs.control	7
CC	GO:0005859	muscle myosin complex	bulk RNA-seq,Homo_723,AD.vs.control; bulk RNA-seq,Homo_723,AD.vs.MCI; bulk RNA-seq,Homo_714,AD.vs.control; bulk RNA-seq,Homo_714,AD.vs.MCI	4
BP	GO:0060004	reflex	bulk RNA-seq,Homo_723,AD.vs.control; bulk RNA-seq,Homo_723,AD.vs.MCI; bulk RNA-seq,Homo_723,MCI.vs.control; bulk RNA-seq,Homo_714,AD.vs.control; bulk RNA-seq,Homo_714,AD.vs.MCI	5
BP	GO:0098659	inorganic cation import across plasma membrane	bulk RNA-seq,Homo_723,AD.vs.control; bulk RNA-seq,Homo_723,AD.vs.MCI; bulk RNA-seq,Homo_723,MCI.vs.control; bulk RNA-seq,Homo_714,AD.vs.MCI; bulk RNA-seq,Homo_714,MCI.vs.control; bulk RNA-seq,SRP223445,AD.vs.control	6
BP	GO:0099587	inorganic ion import across plasma membrane	bulk RNA-seq,Homo_723,AD.vs.control; bulk RNA-seq,Homo_723,AD.vs.MCI; bulk RNA-seq,Homo_723,MCI.vs.control; bulk RNA-seq,Homo_714,AD.vs.MCI; bulk RNA-seq,Homo_714,MCI.vs.control; bulk RNA-seq,SRP223445,AD.vs.control	6
BP	GO:0060415	muscle tissue morphogenesis	bulk RNA-seq,Homo_723,AD.vs.control; bulk RNA-seq,Homo_723,AD.vs.MCI; bulk RNA-seq,Homo_723,MCI.vs.control; bulk RNA-seq,Homo_714,AD.vs.control; bulk RNA-seq,Homo_714,AD.vs.MCI; bulk RNA-seq,Homo_714,MCI.vs.control	6
BP	GO:0071526	semaphorin-plexin signaling pathway	bulk RNA-seq,Homo_723,AD.vs.control; bulk RNA-seq,Homo_723,AD.vs.MCI; bulk RNA-seq,Homo_723,MCI.vs.control; bulk RNA-seq,Homo_714,AD.vs.MCI; bulk RNA-seq,Homo_714,MCI.vs.control; bulk RNA-seq,Homo_633,AD.vs.control; bulk RNA-seq,Homo_633,AD.vs.MCI	7
BP	GO:0090670	RNA localization to Cajal body	bulk RNA-seq,Homo_723,AD.vs.control; bulk RNA-seq,Homo_723,AD.vs.MCI; bulk RNA-seq,Homo_723,MCI.vs.control; bulk RNA-seq,Homo_714,AD.vs.control; bulk RNA-seq,Homo_714,AD.vs.MCI; bulk RNA-seq,Homo_714,MCI.vs.control; bulk RNA-seq,Homo_633,AD.vs.control; bulk RNA-seq,Homo_633,AD.vs.MCI; bulk RNA-seq,Homo_633,MCI.vs.control	9

BP	GO:0090671	telomerase RNA localization to Cajal body	bulk RNA-seq,Homo_723,AD.vs.control; bulk RNA-seq,Homo_723,AD.vs.MCI; bulk RNA-seq,Homo_723,MCI.vs.control; bulk RNA-seq,Homo_714,AD.vs.control; bulk RNA-seq,Homo_714,AD.vs.MCI; bulk RNA-seq,Homo_714,MCI.vs.control; bulk RNA-seq,Homo_633,AD.vs.control; bulk RNA-seq,Homo_633,AD.vs.MCI; bulk RNA-seq,Homo_633,MCI.vs.control	9
BP	GO:0090672	telomerase RNA localization	bulk RNA-seq,Homo_723,AD.vs.control; bulk RNA-seq,Homo_723,AD.vs.MCI; bulk RNA-seq,Homo_723,MCI.vs.control; bulk RNA-seq,Homo_714,AD.vs.control; bulk RNA-seq,Homo_714,AD.vs.MCI; bulk RNA-seq,Homo_714,MCI.vs.control; bulk RNA-seq,Homo_633,AD.vs.control; bulk RNA-seq,Homo_633,AD.vs.MCI; bulk RNA-seq,Homo_633,MCI.vs.control	9
BP	GO:0090685	RNA localization to nucleus	bulk RNA-seq,Homo_723,AD.vs.control; bulk RNA-seq,Homo_723,AD.vs.MCI; bulk RNA-seq,Homo_723,MCI.vs.control; bulk RNA-seq,Homo_714,AD.vs.control; bulk RNA-seq,Homo_714,AD.vs.MCI; bulk RNA-seq,Homo_714,MCI.vs.control; bulk RNA-seq,Homo_633,AD.vs.control; bulk RNA-seq,Homo_633,AD.vs.MCI; bulk RNA-seq,Homo_633,MCI.vs.control	9
BP	GO:0097091	synaptic vesicle clustering	bulk RNA-seq,Homo_723,AD.vs.control; bulk RNA-seq,Homo_723,AD.vs.MCI; bulk RNA-seq,Homo_723,MCI.vs.control; bulk RNA-seq,Homo_714,AD.vs.MCI	4
BP	GO:0090103	cochlea morphogenesis	bulk RNA-seq,Homo_723,AD.vs.control; bulk RNA-seq,Homo_723,AD.vs.MCI; bulk RNA-seq,Homo_723,MCI.vs.control; bulk RNA-seq,Homo_714,AD.vs.control; bulk RNA-seq,Homo_714,AD.vs.MCI	5
BP	GO:1903307	positive regulation of regulated secretory pathway	bulk RNA-seq,Homo_723,AD.vs.control; bulk RNA-seq,Homo_723,AD.vs.MCI; bulk RNA-seq,Homo_723,MCI.vs.control; bulk RNA-seq,Homo_714,AD.vs.MCI	4
CC	GO:0061695	transferase complex, transferring phosphorus-containing groups	bulk RNA-seq,Homo_723,AD.vs.control; bulk RNA-seq,Homo_723,MCI.vs.control; bulk RNA-seq,Homo_714,AD.vs.control; bulk RNA-seq,Homo_633,AD.vs.control; bulk RNA-seq,Homo_633,AD.vs.MCI	5
BP	GO:0031929	TOR signaling	bulk RNA-seq,Homo_723,AD.vs.control; bulk RNA-seq,Homo_723,MCI.vs.control; bulk RNA-seq,Homo_714,AD.vs.control; bulk RNA-seq,Homo_714,MCI.vs.control; bulk RNA-seq,Homo_633,AD.vs.control; bulk RNA-seq,Homo_633,AD.vs.MCI	6
BP	GO:0006805	xenobiotic metabolic process	bulk RNA-seq,Homo_723,AD.vs.control; bulk RNA-seq,Homo_723,AD.vs.MCI; bulk RNA-seq,Homo_723,MCI.vs.control; bulk RNA-seq,Homo_714,AD.vs.control; bulk RNA-seq,Homo_714,AD.vs.MCI; bulk RNA-seq,Homo_714,MCI.vs.control; bulk RNA-seq,Homo_633,AD.vs.control	7
BP	GO:0048639	positive regulation of developmental growth	bulk RNA-seq,Homo_723,AD.vs.control; bulk RNA-seq,Homo_723,AD.vs.MCI; bulk RNA-seq,Homo_723,MCI.vs.control; bulk RNA-seq,Homo_714,AD.vs.MCI; bulk RNA-seq,Homo_633,AD.vs.control; bulk RNA-seq,Homo_633,AD.vs.MCI; bulk RNA-seq,SRP223445,AD.vs.control	7
BP	GO:0006635	fatty acid beta-oxidation	bulk RNA-seq,Homo_723,AD.vs.control; bulk RNA-seq,Homo_723,MCI.vs.control; bulk RNA-seq,Homo_714,AD.vs.control; bulk RNA-seq,Homo_714,MCI.vs.control; bulk RNA-seq,Homo_633,AD.vs.control; bulk RNA-seq,Homo_633,AD.vs.MCI	6
CC	GO:0005838	proteasome regulatory particle	bulk RNA-seq,Homo_723,AD.vs.control; bulk RNA-seq,Homo_723,AD.vs.MCI; bulk RNA-seq,Homo_714,AD.vs.control; bulk RNA-seq,Homo_714,AD.vs.MCI; bulk RNA-seq,Homo_633,AD.vs.control; bulk RNA-seq,Homo_633,AD.vs.MCI	6
BP	GO:0043491	protein kinase B signaling	bulk RNA-seq,Homo_723,AD.vs.control; bulk RNA-seq,Homo_723,AD.vs.MCI; bulk RNA-seq,Homo_723,MCI.vs.control; bulk RNA-seq,Homo_714,AD.vs.control; bulk RNA-seq,Homo_714,AD.vs.MCI; bulk RNA-seq,Homo_633,AD.vs.control; bulk RNA-seq,Homo_633,AD.vs.MCI	7
MF	GO:0005164	tumor necrosis factor receptor binding	bulk RNA-seq,Homo_723,AD.vs.control; bulk RNA-seq,Homo_723,AD.vs.MCI; bulk RNA-seq,Homo_714,AD.vs.control; bulk RNA-seq,Homo_714,AD.vs.MCI; bulk RNA-seq,Homo_633,AD.vs.control	5
BP	GO:0098815	modulation of excitatory postsynaptic potential	bulk RNA-seq,Homo_723,AD.vs.control; bulk RNA-seq,Homo_723,AD.vs.MCI; bulk RNA-seq,Homo_723,MCI.vs.control; bulk RNA-seq,Homo_714,AD.vs.control; bulk RNA-seq,Homo_714,AD.vs.MCI; bulk RNA-seq,Homo_714,MCI.vs.control; bulk RNA-seq,ROSMAP,AD.vs.control	7
BP	GO:0050870	positive regulation of T cell activation	bulk RNA-seq,Homo_723,AD.vs.control; bulk RNA-seq,Homo_723,MCI.vs.control; bulk RNA-seq,Homo_714,AD.vs.control; bulk RNA-seq,Homo_633,AD.vs.control; bulk RNA-seq,Homo_633,AD.vs.MCI; scRNA-seq,SRP215507,CD8+ T cell_1-MCI.vs.control; scRNA-seq,SRP215507,CD8+ T cell_3-AD.vs.control; scRNA-seq,SRP215507,CD8+ T cell_3-MCI.vs.control	8
BP	GO:0048485	sympathetic nervous system development	bulk RNA-seq,Homo_723,AD.vs.control; bulk RNA-seq,Homo_723,AD.vs.MCI; bulk RNA-seq,Homo_723,MCI.vs.control; bulk RNA-seq,Homo_714,AD.vs.MCI	4
BP	GO:0086003	cardiac muscle cell contraction	bulk RNA-seq,Homo_723,AD.vs.control; bulk RNA-seq,Homo_723,AD.vs.MCI; bulk RNA-seq,Homo_723,MCI.vs.control; bulk RNA-seq,Homo_714,AD.vs.control; bulk RNA-seq,Homo_714,AD.vs.MCI; bulk RNA-seq,Homo_714,MCI.vs.control	6
BP	GO:0019934	cGMP-mediated signaling	bulk RNA-seq,Homo_723,AD.vs.control; bulk RNA-seq,Homo_723,AD.vs.MCI; bulk RNA-seq,Homo_723,MCI.vs.control; bulk RNA-seq,Homo_714,AD.vs.control; bulk RNA-seq,Homo_714,AD.vs.MCI; bulk RNA-seq,Homo_714,MCI.vs.control; bulk RNA-seq,Homo_633,AD.vs.control; bulk	8
BP	GO:0009205	purine ribonucleoside triphosphate metabolic process	bulk RNA-seq,Homo_723,AD.vs.control; bulk RNA-seq,Homo_723,MCI.vs.control; bulk RNA-seq,Homo_714,AD.vs.control; bulk RNA-seq,Homo_633,AD.vs.control; bulk RNA-seq,Homo_633,AD.vs.MCI	5
BP	GO:0097553	calcium ion transmembrane import into cytosol	bulk RNA-seq,Homo_723,AD.vs.control; bulk RNA-seq,Homo_723,AD.vs.MCI; bulk RNA-seq,Homo_723,MCI.vs.control; bulk RNA-seq,Homo_714,AD.vs.control; bulk RNA-seq,Homo_714,AD.vs.MCI; bulk RNA-seq,Homo_633,AD.vs.control; bulk RNA-seq,Homo_633,AD.vs.MCI; bulk RNA-	8
BP	GO:0010232	vascular transport	bulk RNA-seq,Homo_723,AD.vs.control; bulk RNA-seq,Homo_723,AD.vs.MCI; bulk RNA-seq,Homo_723,MCI.vs.control; bulk RNA-seq,Homo_714,AD.vs.MCI; bulk RNA-seq,Homo_714,MCI.vs.control; bulk RNA-seq,Homo_633,AD.vs.control	6
BP	GO:0150104	transport across blood-brain barrier	bulk RNA-seq,Homo_723,AD.vs.control; bulk RNA-seq,Homo_723,AD.vs.MCI; bulk RNA-seq,Homo_723,MCI.vs.control; bulk RNA-seq,Homo_714,AD.vs.MCI; bulk RNA-seq,Homo_714,MCI.vs.control; bulk RNA-seq,Homo_633,AD.vs.control	6
BP	GO:0003401	axis elongation	bulk RNA-seq,Homo_723,AD.vs.control; bulk RNA-seq,Homo_723,AD.vs.MCI; bulk RNA-seq,Homo_723,MCI.vs.control; bulk RNA-seq,Homo_714,AD.vs.MCI	4
BP	GO:0008535	respiratory chain complex IV assembly	bulk RNA-seq,Homo_723,AD.vs.control; bulk RNA-seq,Homo_723,AD.vs.MCI; bulk RNA-seq,Homo_723,MCI.vs.control; bulk RNA-seq,Homo_714,AD.vs.control; bulk RNA-seq,Homo_714,MCI.vs.control; bulk RNA-seq,Homo_633,AD.vs.control; bulk RNA-seq,Homo_633,AD.vs.MCI; bulk	8
BP	GO:0009144	purine nucleoside triphosphate metabolic process	bulk RNA-seq,Homo_723,AD.vs.control; bulk RNA-seq,Homo_723,MCI.vs.control; bulk RNA-seq,Homo_714,AD.vs.control; bulk RNA-seq,Homo_633,AD.vs.control; bulk RNA-seq,Homo_633,AD.vs.MCI	5
BP	GO:0022900	electron transport chain	bulk RNA-seq,Homo_723,AD.vs.control; bulk RNA-seq,Homo_723,MCI.vs.control; bulk RNA-seq,Homo_714,AD.vs.control; bulk RNA-seq,Homo_633,AD.vs.control; bulk RNA-seq,Homo_633,AD.vs.MCI	5
BP	GO:0046390	ribose phosphate biosynthetic process	bulk RNA-seq,Homo_723,AD.vs.control; bulk RNA-seq,Homo_723,MCI.vs.control; bulk RNA-seq,Homo_714,AD.vs.control; bulk RNA-seq,Homo_633,AD.vs.control; bulk RNA-seq,Homo_633,AD.vs.MCI	5
BP	GO:0099151	regulation of postsynaptic density assembly	bulk RNA-seq,Homo_723,AD.vs.control; bulk RNA-seq,Homo_723,AD.vs.MCI; bulk RNA-seq,Homo_723,MCI.vs.control	3
BP	GO:2000272	negative regulation of signaling receptor activity	bulk RNA-seq,Homo_723,AD.vs.control; bulk RNA-seq,Homo_723,AD.vs.MCI; bulk RNA-seq,Homo_723,MCI.vs.control; bulk RNA-seq,Homo_714,AD.vs.MCI	4
BP	GO:0016048	detection of temperature stimulus	bulk RNA-seq,Homo_723,AD.vs.control; bulk RNA-seq,Homo_723,AD.vs.MCI; bulk RNA-seq,Homo_723,MCI.vs.control; bulk RNA-seq,Homo_714,AD.vs.control; bulk RNA-seq,Homo_714,AD.vs.MCI	5
CC	GO:0000177	cytoplasmic exosome (RNase complex)	bulk RNA-seq,Homo_723,AD.vs.control; bulk RNA-seq,Homo_723,AD.vs.MCI; bulk RNA-seq,Homo_714,AD.vs.control; bulk RNA-seq,Homo_714,AD.vs.MCI; bulk RNA-seq,Homo_633,AD.vs.control; bulk RNA-seq,Homo_633,AD.vs.MCI	6
MF	GO:0042393	histone binding	bulk RNA-seq,Homo_723,AD.vs.control; bulk RNA-seq,Homo_723,MCI.vs.control; bulk RNA-seq,Homo_714,AD.vs.control; bulk RNA-seq,Homo_633,AD.vs.control; bulk RNA-seq,Homo_633,AD.vs.MCI	5

BP	GO:0003158	endothelium development	bulk RNA-seq,Homo_723,AD.vs.control; bulk RNA-seq,Homo_723,AD.vs.MCI; bulk RNA-seq,Homo_723,MCI.vs.control; bulk RNA-seq,Homo_714,AD.vs.control; bulk RNA-seq,Homo_714,AD.vs.MCI; bulk RNA-seq,Homo_633,AD.vs.control; bulk RNA-seq,Homo_633,AD.vs.MCI	7
MF	GO:0015453	oxidoreduction-driven active transmembrane transporter activity	bulk RNA-seq,Homo_723,AD.vs.control; bulk RNA-seq,Homo_723,AD.vs.MCI; bulk RNA-seq,Homo_723,MCI.vs.control; bulk RNA-seq,Homo_714,AD.vs.control; bulk RNA-seq,Homo_714,MCI.vs.control; bulk RNA-seq,Homo_633,AD.vs.control; bulk RNA-seq,Homo_633,AD.vs.MCI	7
BP	GO:0009303	rRNA transcription	bulk RNA-seq,Homo_723,AD.vs.control; bulk RNA-seq,Homo_723,MCI.vs.control; bulk RNA-seq,Homo_714,AD.vs.control; bulk RNA-seq,Homo_714,MCI.vs.control; bulk RNA-seq,Homo_633,AD.vs.control; bulk RNA-seq,Homo_633,AD.vs.MCI	6
BP	GO:0045104	intermediate filament cytoskeleton organization	bulk RNA-seq,Homo_723,AD.vs.control; bulk RNA-seq,Homo_723,AD.vs.MCI; bulk RNA-seq,Homo_723,MCI.vs.control; bulk RNA-seq,Homo_714,AD.vs.control; bulk RNA-seq,Homo_714,AD.vs.MCI; bulk RNA-seq,Homo_714,MCI.vs.control; bulk RNA-seq,SRP223445,AD.vs.control	7
BP	GO:0009141	nucleoside triphosphate metabolic process	bulk RNA-seq,Homo_723,AD.vs.control; bulk RNA-seq,Homo_723,MCI.vs.control; bulk RNA-seq,Homo_714,AD.vs.control; bulk RNA-seq,Homo_633,AD.vs.control; bulk RNA-seq,Homo_633,AD.vs.MCI	5
BP	GO:0048854	brain morphogenesis	bulk RNA-seq,Homo_723,AD.vs.control; bulk RNA-seq,Homo_723,AD.vs.MCI; bulk RNA-seq,Homo_723,MCI.vs.control; bulk RNA-seq,Homo_714,AD.vs.MCI; bulk RNA-seq,Homo_714,MCI.vs.control; bulk RNA-seq,Homo_633,AD.vs.control; bulk RNA-seq,Homo_633,AD.vs.MCI	7
MF	GO:0046966	nuclear thyroid hormone receptor binding	bulk RNA-seq,Homo_723,AD.vs.control; bulk RNA-seq,Homo_723,MCI.vs.control; bulk RNA-seq,Homo_714,AD.vs.control; bulk RNA-seq,Homo_714,MCI.vs.control; bulk RNA-seq,Homo_633,AD.vs.control; bulk RNA-seq,Homo_633,AD.vs.MCI; bulk RNA-seq,Homo_633,MCI.vs.control	7
BP	GO:0007617	mating behavior	bulk RNA-seq,Homo_723,AD.vs.control; bulk RNA-seq,Homo_723,AD.vs.MCI; bulk RNA-seq,Homo_714,AD.vs.control; bulk RNA-seq,Homo_714,AD.vs.MCI; bulk RNA-seq,Homo_633,AD.vs.control	5
CC	GO:0031902	late endosome membrane	bulk RNA-seq,Homo_723,AD.vs.control; bulk RNA-seq,Homo_723,MCI.vs.control; bulk RNA-seq,Homo_714,AD.vs.control; bulk RNA-seq,Homo_633,AD.vs.control; bulk RNA-seq,Homo_633,AD.vs.MCI	5
BP	GO:0071025	RNA surveillance	bulk RNA-seq,Homo_723,AD.vs.control; bulk RNA-seq,Homo_723,AD.vs.MCI; bulk RNA-seq,Homo_714,AD.vs.control; bulk RNA-seq,Homo_714,AD.vs.MCI; bulk RNA-seq,Homo_633,AD.vs.control	5
BP	GO:0060579	ventral spinal cord interneuron fate commitment	bulk RNA-seq,Homo_723,AD.vs.control; bulk RNA-seq,Homo_723,AD.vs.MCI; bulk RNA-seq,Homo_723,MCI.vs.control; bulk RNA-seq,Homo_714,AD.vs.control; bulk RNA-seq,Homo_714,AD.vs.MCI	5
BP	GO:0060581	cell fate commitment involved in pattern specification	bulk RNA-seq,Homo_723,AD.vs.control; bulk RNA-seq,Homo_723,AD.vs.MCI; bulk RNA-seq,Homo_723,MCI.vs.control; bulk RNA-seq,Homo_714,AD.vs.control; bulk RNA-seq,Homo_714,AD.vs.MCI	5
BP	GO:0086002	cardiac muscle cell action potential involved in contraction	bulk RNA-seq,Homo_723,AD.vs.control; bulk RNA-seq,Homo_723,AD.vs.MCI; bulk RNA-seq,Homo_723,MCI.vs.control; bulk RNA-seq,Homo_714,AD.vs.control; bulk RNA-seq,Homo_714,AD.vs.MCI; bulk RNA-seq,Homo_714,MCI.vs.control	6
BP	GO:0032204	regulation of telomere maintenance	bulk RNA-seq,Homo_723,AD.vs.control; bulk RNA-seq,Homo_723,MCI.vs.control; bulk RNA-seq,Homo_714,AD.vs.control; bulk RNA-seq,Homo_714,MCI.vs.control; bulk RNA-seq,Homo_633,AD.vs.control; bulk RNA-seq,Homo_633,AD.vs.MCI	6
BP	GO:0048557	embryonic digestive tract morphogenesis	bulk RNA-seq,Homo_723,AD.vs.control; bulk RNA-seq,Homo_723,AD.vs.MCI; bulk RNA-seq,Homo_723,MCI.vs.control; bulk RNA-seq,Homo_714,AD.vs.control; bulk RNA-seq,Homo_714,AD.vs.MCI	5
CC	GO:0098803	respiratory chain complex	bulk RNA-seq,Homo_723,AD.vs.control; bulk RNA-seq,Homo_723,MCI.vs.control; bulk RNA-seq,Homo_714,AD.vs.control; bulk RNA-seq,Homo_714,MCI.vs.control; bulk RNA-seq,Homo_633,AD.vs.control; bulk RNA-seq,Homo_633,AD.vs.MCI	6
BP	GO:0046631	alpha-beta T cell activation	bulk RNA-seq,Homo_723,AD.vs.control; bulk RNA-seq,Homo_723,MCI.vs.control; bulk RNA-seq,Homo_714,AD.vs.control; bulk RNA-seq,Homo_633,AD.vs.control; bulk RNA-seq,Homo_633,AD.vs.MCI	5
BP	GO:1903825	organic acid transmembrane transport	bulk RNA-seq,Homo_723,AD.vs.control; bulk RNA-seq,Homo_723,AD.vs.MCI; bulk RNA-seq,Homo_723,MCI.vs.control; bulk RNA-seq,Homo_714,AD.vs.MCI; bulk RNA-seq,Homo_633,AD.vs.control; bulk RNA-seq,Homo_633,AD.vs.MCI	6
CC	GO:1902555	endoribonuclease complex	bulk RNA-seq,Homo_723,AD.vs.control; bulk RNA-seq,Homo_723,AD.vs.MCI; bulk RNA-seq,Homo_714,AD.vs.control; bulk RNA-seq,Homo_633,AD.vs.control; bulk RNA-seq,Homo_633,AD.vs.MCI	5
BP	GO:0002090	regulation of receptor internalization	bulk RNA-seq,Homo_723,AD.vs.control; bulk RNA-seq,Homo_723,AD.vs.MCI; bulk RNA-seq,Homo_723,MCI.vs.control; bulk RNA-seq,Homo_714,AD.vs.MCI; bulk RNA-seq,Homo_714,MCI.vs.control; bulk RNA-seq,Homo_633,AD.vs.control; bulk RNA-seq,Homo_633,AD.vs.MCI	7
BP	GO:0007158	neuron cell-cell adhesion	bulk RNA-seq,Homo_723,AD.vs.control; bulk RNA-seq,Homo_723,AD.vs.MCI; bulk RNA-seq,Homo_723,MCI.vs.control; bulk RNA-seq,Homo_714,AD.vs.MCI; bulk RNA-seq,Homo_714,MCI.vs.control; bulk RNA-seq,SRP223445,AD.vs.control	6
BP	GO:0007044	cell-substrate junction assembly	bulk RNA-seq,Homo_723,AD.vs.control; bulk RNA-seq,Homo_723,AD.vs.MCI; bulk RNA-seq,Homo_723,MCI.vs.control; bulk RNA-seq,Homo_714,AD.vs.control; bulk RNA-seq,Homo_714,AD.vs.MCI; bulk RNA-seq,Homo_714,MCI.vs.control; bulk RNA-seq,Homo_633,AD.vs.control; bulk	8
BP	GO:0097475	motor neuron migration	bulk RNA-seq,Homo_723,AD.vs.control; bulk RNA-seq,Homo_723,AD.vs.MCI; bulk RNA-seq,Homo_723,MCI.vs.control; bulk RNA-seq,Homo_714,AD.vs.MCI	4
CC	GO:0031594	neuromuscular junction	bulk RNA-seq,Homo_723,AD.vs.control; bulk RNA-seq,Homo_723,AD.vs.MCI; bulk RNA-seq,Homo_723,MCI.vs.control; bulk RNA-seq,Homo_714,AD.vs.control; bulk RNA-seq,Homo_714,AD.vs.MCI; bulk RNA-seq,Homo_714,MCI.vs.control; bulk RNA-seq,Homo_633,AD.vs.control; bulk	8
BP	GO:0048934	peripheral nervous system neuron differentiation	bulk RNA-seq,Homo_723,AD.vs.control; bulk RNA-seq,Homo_723,AD.vs.MCI; bulk RNA-seq,Homo_723,MCI.vs.control; bulk RNA-seq,Homo_714,AD.vs.control; bulk RNA-seq,Homo_714,AD.vs.MCI	5
BP	GO:0048935	peripheral nervous system neuron development	bulk RNA-seq,Homo_723,AD.vs.control; bulk RNA-seq,Homo_723,AD.vs.MCI; bulk RNA-seq,Homo_723,MCI.vs.control; bulk RNA-seq,Homo_714,AD.vs.control; bulk RNA-seq,Homo_714,AD.vs.MCI	5
BP	GO:0030277	maintenance of gastrointestinal epithelium	bulk RNA-seq,Homo_723,AD.vs.control; bulk RNA-seq,Homo_723,AD.vs.MCI; bulk RNA-seq,Homo_723,MCI.vs.control; bulk RNA-seq,Homo_714,AD.vs.control; bulk RNA-seq,Homo_714,AD.vs.MCI; bulk RNA-seq,Homo_714,MCI.vs.control	6
BP	GO:0045089	positive regulation of innate immune response	bulk RNA-seq,Homo_723,AD.vs.control; bulk RNA-seq,Homo_723,MCI.vs.control; bulk RNA-seq,Homo_714,AD.vs.control; bulk RNA-seq,Homo_633,AD.vs.control; bulk RNA-seq,Homo_633,AD.vs.MCI	5
BP	GO:0008361	regulation of cell size	bulk RNA-seq,Homo_723,AD.vs.control; bulk RNA-seq,Homo_723,AD.vs.MCI; bulk RNA-seq,Homo_723,MCI.vs.control; bulk RNA-seq,Homo_714,AD.vs.MCI; bulk RNA-seq,Homo_633,AD.vs.control; bulk RNA-seq,Homo_633,AD.vs.MCI	6
CC	GO:0042613	MHC class II protein complex	bulk RNA-seq,Homo_723,AD.vs.control; bulk RNA-seq,Homo_723,AD.vs.MCI; bulk RNA-seq,Homo_723,MCI.vs.control; bulk RNA-seq,Homo_714,AD.vs.control; bulk RNA-seq,Homo_714,MCI.vs.control; bulk RNA-seq,Homo_633,AD.vs.control; bulk RNA-seq,Homo_633,MCI.vs.control; scRNA-seq,SRP215507,CD8+ T cell_1-MCI.vs.control; scRNA-seq,SRP215507,CD8+ T cell_3-AD.vs.control; scRNA-seq,SRP215507,CD8+ T cell_3-	10
CC	GO:0046658	anchored component of plasma membrane	bulk RNA-seq,Homo_723,AD.vs.control; bulk RNA-seq,Homo_723,AD.vs.MCI; bulk RNA-seq,Homo_723,MCI.vs.control; bulk RNA-seq,Homo_714,AD.vs.MCI	4
BP	GO:0050691	regulation of defense response to virus by host	bulk RNA-seq,Homo_723,AD.vs.control; bulk RNA-seq,Homo_723,AD.vs.MCI; bulk RNA-seq,Homo_723,MCI.vs.control; bulk RNA-seq,Homo_714,AD.vs.control; bulk RNA-seq,Homo_714,MCI.vs.control; bulk RNA-seq,Homo_633,AD.vs.control; bulk RNA-seq,Homo_633,AD.vs.MCI	7
BP	GO:0048486	parasympathetic nervous system development	bulk RNA-seq,Homo_723,AD.vs.control; bulk RNA-seq,Homo_723,AD.vs.MCI; bulk RNA-seq,Homo_723,MCI.vs.control; bulk RNA-seq,Homo_714,AD.vs.control; bulk RNA-seq,Homo_714,AD.vs.MCI	5

BP	GO:0031076	embryonic camera-type eye development	bulk RNA-seq,Homo_723,AD.vs.control; bulk RNA-seq,Homo_723,AD.vs.MCI; bulk RNA-seq,Homo_723,MCI.vs.control; bulk RNA-seq,Homo_714,AD.vs.control; bulk RNA-seq,Homo_714,AD.vs.MCI; bulk RNA-seq,Homo_714,MCI.vs.control	6
BP	GO:0048545	response to steroid hormone	bulk RNA-seq,Homo_723,AD.vs.control; bulk RNA-seq,Homo_723,AD.vs.MCI; bulk RNA-seq,Homo_714,AD.vs.control; bulk RNA-seq,Homo_714,AD.vs.MCI; bulk RNA-seq,Homo_633,AD.vs.control; bulk RNA-seq,Homo_633,AD.vs.MCI	6
MF	GO:0001786	phosphatidylserine binding	bulk RNA-seq,Homo_723,AD.vs.control; bulk RNA-seq,Homo_723,AD.vs.MCI; bulk RNA-seq,Homo_723,MCI.vs.control; bulk RNA-seq,Homo_714,AD.vs.MCI; bulk RNA-seq,Homo_633,AD.vs.control; bulk RNA-seq,Homo_633,AD.vs.MCI	6
BP	GO:0097305	response to alcohol	bulk RNA-seq,Homo_723,AD.vs.control; bulk RNA-seq,Homo_723,AD.vs.MCI; bulk RNA-seq,Homo_723,MCI.vs.control; bulk RNA-seq,Homo_714,AD.vs.control; bulk RNA-seq,Homo_714,AD.vs.MCI; bulk RNA-seq,Homo_633,AD.vs.control; bulk RNA-seq,Homo_633,AD.vs.MCI; scRNA-	8
CC	GO:0030018	Z disc	bulk RNA-seq,Homo_723,AD.vs.control; bulk RNA-seq,Homo_723,AD.vs.MCI; bulk RNA-seq,Homo_723,MCI.vs.control; bulk RNA-seq,Homo_714,AD.vs.control; bulk RNA-seq,Homo_714,AD.vs.MCI; bulk RNA-seq,Homo_633,AD.vs.control; bulk RNA-seq,Homo_633,AD.vs.MCI	7
BP	GO:0055013	cardiac muscle cell development	bulk RNA-seq,Homo_723,AD.vs.control; bulk RNA-seq,Homo_723,AD.vs.MCI; bulk RNA-seq,Homo_723,MCI.vs.control; bulk RNA-seq,Homo_714,AD.vs.control; bulk RNA-seq,Homo_714,AD.vs.MCI; bulk RNA-seq,Homo_714,MCI.vs.control	6
BP	GO:0022029	telencephalon cell migration	bulk RNA-seq,Homo_723,AD.vs.control; bulk RNA-seq,Homo_723,AD.vs.MCI; bulk RNA-seq,Homo_723,MCI.vs.control; bulk RNA-seq,Homo_714,AD.vs.MCI; bulk RNA-seq,Homo_714,MCI.vs.control; bulk RNA-seq,Homo_633,AD.vs.control	6
BP	GO:0048332	mesoderm morphogenesis	bulk RNA-seq,Homo_723,AD.vs.control; bulk RNA-seq,Homo_723,AD.vs.MCI; bulk RNA-seq,Homo_723,MCI.vs.control; bulk RNA-seq,Homo_714,AD.vs.control; bulk RNA-seq,Homo_714,AD.vs.MCI; bulk RNA-seq,Homo_714,MCI.vs.control	6
BP	GO:1904936	interneuron migration	bulk RNA-seq,Homo_723,AD.vs.control; bulk RNA-seq,Homo_723,AD.vs.MCI; bulk RNA-seq,Homo_723,MCI.vs.control; bulk RNA-seq,Homo_714,AD.vs.control; bulk RNA-seq,Homo_714,AD.vs.MCI; bulk RNA-seq,Homo_633,MCI.vs.control	6
BP	GO:0044706	multi-multicellular organism process	bulk RNA-seq,Homo_723,AD.vs.control; bulk RNA-seq,Homo_723,AD.vs.MCI; bulk RNA-seq,Homo_723,MCI.vs.control; bulk RNA-seq,Homo_714,AD.vs.control; bulk RNA-seq,Homo_714,AD.vs.MCI; bulk RNA-seq,Homo_633,AD.vs.control; bulk RNA-seq,Homo_633,AD.vs.MCI	7
BP	GO:0002063	chondrocyte development	bulk RNA-seq,Homo_723,AD.vs.control; bulk RNA-seq,Homo_723,AD.vs.MCI; bulk RNA-seq,Homo_723,MCI.vs.control; bulk RNA-seq,Homo_714,AD.vs.MCI; bulk RNA-seq,Homo_714,MCI.vs.control; bulk RNA-seq,Homo_633,AD.vs.control; bulk RNA-seq,Homo_633,AD.vs.MCI	7
MF	GO:0098631	cell adhesion mediator activity	bulk RNA-seq,Homo_723,AD.vs.control; bulk RNA-seq,Homo_723,AD.vs.MCI; bulk RNA-seq,Homo_723,MCI.vs.control; bulk RNA-seq,Homo_714,AD.vs.MCI; bulk RNA-seq,Homo_714,MCI.vs.control; bulk RNA-seq,Homo_633,AD.vs.control	6
BP	GO:0036465	synaptic vesicle recycling	bulk RNA-seq,Homo_723,AD.vs.control; bulk RNA-seq,Homo_723,AD.vs.MCI; bulk RNA-seq,Homo_723,MCI.vs.control; bulk RNA-seq,Homo_714,AD.vs.MCI; bulk RNA-seq,Homo_714,MCI.vs.control; bulk RNA-seq,Homo_633,AD.vs.control; bulk RNA-seq,Homo_633,AD.vs.MCI	7
MF	GO:0008649	rRNA methyltransferase activity	bulk RNA-seq,Homo_723,AD.vs.control; bulk RNA-seq,Homo_723,AD.vs.MCI; bulk RNA-seq,Homo_723,MCI.vs.control; bulk RNA-seq,Homo_714,AD.vs.control; bulk RNA-seq,Homo_714,MCI.vs.control; bulk RNA-seq,Homo_633,AD.vs.control; bulk RNA-seq,Homo_633,AD.vs.MCI; bulk	8
BP	GO:0030282	bone mineralization	bulk RNA-seq,Homo_723,AD.vs.control; bulk RNA-seq,Homo_723,AD.vs.MCI; bulk RNA-seq,Homo_723,MCI.vs.control; bulk RNA-seq,Homo_714,AD.vs.MCI; bulk RNA-seq,Homo_714,MCI.vs.control; bulk RNA-seq,Homo_633,AD.vs.control; bulk RNA-seq,Homo_633,AD.vs.MCI	7
BP	GO:0031123	RNA 3'-end processing	bulk RNA-seq,Homo_723,AD.vs.control; bulk RNA-seq,Homo_723,AD.vs.MCI; bulk RNA-seq,Homo_723,MCI.vs.control; bulk RNA-seq,Homo_714,AD.vs.control; bulk RNA-seq,Homo_714,MCI.vs.control; bulk RNA-seq,Homo_633,AD.vs.control; bulk RNA-seq,Homo_633,AD.vs.MCI	7
MF	GO:0004890	GABA-A receptor activity	bulk RNA-seq,Homo_723,AD.vs.control; bulk RNA-seq,Homo_723,AD.vs.MCI; bulk RNA-seq,Homo_723,MCI.vs.control; bulk RNA-seq,Homo_714,AD.vs.control; bulk RNA-seq,Homo_714,AD.vs.MCI	5
CC	GO:1902711	GABA-A receptor complex	bulk RNA-seq,Homo_723,AD.vs.control; bulk RNA-seq,Homo_723,AD.vs.MCI; bulk RNA-seq,Homo_723,MCI.vs.control; bulk RNA-seq,Homo_714,AD.vs.control; bulk RNA-seq,Homo_714,AD.vs.MCI	5
BP	GO:0086001	cardiac muscle cell action potential	bulk RNA-seq,Homo_723,AD.vs.control; bulk RNA-seq,Homo_723,AD.vs.MCI; bulk RNA-seq,Homo_723,MCI.vs.control; bulk RNA-seq,Homo_714,AD.vs.control; bulk RNA-seq,Homo_714,AD.vs.MCI; bulk RNA-seq,Homo_714,MCI.vs.control; bulk RNA-seq,Homo_633,AD.vs.control	7
BP	GO:2000243	positive regulation of reproductive process	bulk RNA-seq,Homo_723,AD.vs.control; bulk RNA-seq,Homo_723,AD.vs.MCI; bulk RNA-seq,Homo_723,MCI.vs.control; bulk RNA-seq,Homo_714,AD.vs.control; bulk RNA-seq,Homo_714,AD.vs.MCI; bulk RNA-seq,Homo_714,MCI.vs.control	6
BP	GO:0010842	retina layer formation	bulk RNA-seq,Homo_723,AD.vs.control; bulk RNA-seq,Homo_723,AD.vs.MCI; bulk RNA-seq,Homo_723,MCI.vs.control; bulk RNA-seq,Homo_714,AD.vs.MCI; bulk RNA-seq,SRP223445,AD.vs.control	5
MF	GO:0015171	amino acid transmembrane transporter activity	bulk RNA-seq,Homo_723,AD.vs.control; bulk RNA-seq,Homo_723,AD.vs.MCI; bulk RNA-seq,Homo_723,MCI.vs.control; bulk RNA-seq,Homo_714,AD.vs.MCI; bulk RNA-seq,Homo_714,MCI.vs.control; bulk RNA-seq,Homo_633,AD.vs.control; bulk RNA-seq,Homo_633,AD.vs.MCI	7
BP	GO:1904872	regulation of telomerase RNA localization to Cajal body	bulk RNA-seq,Homo_723,AD.vs.control; bulk RNA-seq,Homo_723,AD.vs.MCI; bulk RNA-seq,Homo_723,MCI.vs.control; bulk RNA-seq,Homo_714,AD.vs.control; bulk RNA-seq,Homo_714,AD.vs.MCI; bulk RNA-seq,Homo_714,MCI.vs.control; bulk RNA-seq,Homo_633,AD.vs.control; bulk RNA-seq,Homo_633,AD.vs.MCI; bulk RNA-seq,Homo_633,MCI.vs.control	9
BP	GO:0031103	axon regeneration	bulk RNA-seq,Homo_723,AD.vs.control; bulk RNA-seq,Homo_723,AD.vs.MCI; bulk RNA-seq,Homo_723,MCI.vs.control; bulk RNA-seq,Homo_714,AD.vs.MCI; bulk RNA-seq,Homo_714,MCI.vs.control; bulk RNA-seq,Homo_633,AD.vs.control	6
BP	GO:0000466	maturation of 5.8S rRNA from tricistronic rRNA transcript (SSU-rRNA, 5.8S rRNA, LSU-rRNA)	bulk RNA-seq,Homo_723,AD.vs.control; bulk RNA-seq,Homo_723,AD.vs.MCI; bulk RNA-seq,Homo_714,AD.vs.control; bulk RNA-seq,Homo_714,AD.vs.MCI; bulk RNA-seq,Homo_633,AD.vs.control; bulk RNA-seq,Homo_633,AD.vs.MCI; bulk RNA-seq,Homo_633,MCI.vs.control	7
BP	GO:0052695	cellular glucuronidation	bulk RNA-seq,Homo_723,AD.vs.control; bulk RNA-seq,Homo_723,AD.vs.MCI; bulk RNA-seq,Homo_714,AD.vs.control; bulk RNA-seq,Homo_714,AD.vs.MCI	4
BP	GO:0045103	intermediate filament-based process	bulk RNA-seq,Homo_723,AD.vs.control; bulk RNA-seq,Homo_723,AD.vs.MCI; bulk RNA-seq,Homo_723,MCI.vs.control; bulk RNA-seq,Homo_714,AD.vs.control; bulk RNA-seq,Homo_714,AD.vs.MCI; bulk RNA-seq,Homo_714,MCI.vs.control; bulk RNA-seq,SRP223445,AD.vs.control	7
CC	GO:0042581	specific granule	bulk RNA-seq,Homo_723,AD.vs.control; bulk RNA-seq,Homo_723,MCI.vs.control; bulk RNA-seq,Homo_714,AD.vs.control; bulk RNA-seq,Homo_633,AD.vs.control; bulk RNA-seq,Homo_633,AD.vs.MCI; bulk RNA-seq,MCSA,MCI.vs.control; bulk RNA-seq,SRP223445,AD.vs.control	7
BP	GO:0002287	alpha-beta T cell activation involved in immune response	bulk RNA-seq,Homo_723,AD.vs.control; bulk RNA-seq,Homo_723,MCI.vs.control; bulk RNA-seq,Homo_714,AD.vs.control; bulk RNA-seq,Homo_714,MCI.vs.control; bulk RNA-seq,Homo_633,AD.vs.control; bulk RNA-seq,Homo_633,AD.vs.MCI	6
BP	GO:0002293	alpha-beta T cell differentiation involved in immune response	bulk RNA-seq,Homo_723,AD.vs.control; bulk RNA-seq,Homo_723,MCI.vs.control; bulk RNA-seq,Homo_714,AD.vs.control; bulk RNA-seq,Homo_714,MCI.vs.control; bulk RNA-seq,Homo_633,AD.vs.control; bulk RNA-seq,Homo_633,AD.vs.MCI	6
BP	GO:0022616	DNA strand elongation	bulk RNA-seq,Homo_723,AD.vs.control; bulk RNA-seq,Homo_723,MCI.vs.control; bulk RNA-seq,Homo_714,AD.vs.control; bulk RNA-seq,Homo_714,MCI.vs.control; bulk RNA-seq,Homo_633,AD.vs.control; bulk RNA-seq,Homo_633,AD.vs.MCI	6
BP	GO:0060602	branch elongation of an epithelium	bulk RNA-seq,Homo_723,AD.vs.control; bulk RNA-seq,Homo_723,AD.vs.MCI; bulk RNA-seq,Homo_723,MCI.vs.control; bulk RNA-seq,Homo_714,AD.vs.MCI; bulk RNA-seq,Homo_633,MCI.vs.control	5
BP	GO:0030903	notochord development	bulk RNA-seq,Homo_723,AD.vs.control; bulk RNA-seq,Homo_723,AD.vs.MCI; bulk RNA-seq,Homo_723,MCI.vs.control; bulk RNA-seq,Homo_714,AD.vs.MCI	4

BP	GO:0007210	serotonin receptor signaling pathway	bulk RNA-seq,Homo_723,AD.vs.control; bulk RNA-seq,Homo_723,AD.vs.MCI; bulk RNA-seq,Homo_714,AD.vs.control; bulk RNA-seq,Homo_714,AD.vs.MCI	4
MF	GO:0022839	ion gated channel activity	bulk RNA-seq,Homo_723,AD.vs.control; bulk RNA-seq,Homo_723,AD.vs.MCI; bulk RNA-seq,Homo_723,MCI.vs.control; bulk RNA-seq,Homo_714,AD.vs.control; bulk RNA-seq,Homo_714,AD.vs.MCI; bulk RNA-seq,Homo_714,MCI.vs.control	6
BP	GO:1903241	U2-type prespliceosome assembly	bulk RNA-seq,Homo_723,AD.vs.control; bulk RNA-seq,Homo_723,AD.vs.MCI; bulk RNA-seq,Homo_723,MCI.vs.control; bulk RNA-seq,Homo_714,AD.vs.control; bulk RNA-seq,Homo_714,MCI.vs.control; bulk RNA-seq,Homo_633,AD.vs.control; bulk RNA-seq,Homo_633,AD.vs.MCI; bulk	8
BP	GO:0014072	response to isoquinoline alkaloid	bulk RNA-seq,Homo_723,AD.vs.control; bulk RNA-seq,Homo_723,AD.vs.MCI; bulk RNA-seq,Homo_723,MCI.vs.control; bulk RNA-seq,Homo_714,AD.vs.control; bulk RNA-seq,Homo_714,AD.vs.MCI	5
BP	GO:0043278	response to morphine	bulk RNA-seq,Homo_723,AD.vs.control; bulk RNA-seq,Homo_723,AD.vs.MCI; bulk RNA-seq,Homo_723,MCI.vs.control; bulk RNA-seq,Homo_714,AD.vs.control; bulk RNA-seq,Homo_714,AD.vs.MCI	5
BP	GO:0002399	MHC class II protein complex assembly	bulk RNA-seq,Homo_723,AD.vs.control; bulk RNA-seq,Homo_723,AD.vs.MCI; bulk RNA-seq,Homo_723,MCI.vs.control; bulk RNA-seq,Homo_714,AD.vs.control; bulk RNA-seq,Homo_714,MCI.vs.control; bulk RNA-seq,Homo_633,AD.vs.control; bulk RNA-seq,Homo_633,MCI.vs.control; scRNA-seq,SRP215507,CD8+ T cell_1-MCI.vs.control; scRNA-seq,SRP215507,CD8+ T cell_3-AD.vs.control; scRNA-seq,SRP215507,CD8+ T cell_3-	10
BP	GO:0002503	peptide antigen assembly with MHC class II protein complex	bulk RNA-seq,Homo_723,AD.vs.control; bulk RNA-seq,Homo_723,AD.vs.MCI; bulk RNA-seq,Homo_723,MCI.vs.control; bulk RNA-seq,Homo_714,AD.vs.control; bulk RNA-seq,Homo_714,MCI.vs.control; bulk RNA-seq,Homo_633,AD.vs.control; bulk RNA-seq,Homo_633,MCI.vs.control; scRNA-seq,SRP215507,CD8+ T cell_1-MCI.vs.control; scRNA-seq,SRP215507,CD8+ T cell_3-AD.vs.control; scRNA-seq,SRP215507,CD8+ T cell_3-	10
BP	GO:0021697	cerebellar cortex formation	bulk RNA-seq,Homo_723,AD.vs.control; bulk RNA-seq,Homo_723,AD.vs.MCI; bulk RNA-seq,Homo_723,MCI.vs.control; bulk RNA-seq,Homo_714,AD.vs.MCI; bulk RNA-seq,Homo_633,AD.vs.control; bulk RNA-seq,Homo_633,AD.vs.MCI	6
BP	GO:0043266	regulation of potassium ion transport	bulk RNA-seq,Homo_723,AD.vs.control; bulk RNA-seq,Homo_723,AD.vs.MCI; bulk RNA-seq,Homo_723,MCI.vs.control; bulk RNA-seq,Homo_714,AD.vs.control; bulk RNA-seq,Homo_714,AD.vs.MCI	5
BP	GO:0006063	uronic acid metabolic process	bulk RNA-seq,Homo_723,AD.vs.control; bulk RNA-seq,Homo_723,AD.vs.MCI; bulk RNA-seq,Homo_714,AD.vs.control; bulk RNA-seq,Homo_714,AD.vs.MCI	4
BP	GO:0019585	glucuronate metabolic process	bulk RNA-seq,Homo_723,AD.vs.control; bulk RNA-seq,Homo_723,AD.vs.MCI; bulk RNA-seq,Homo_714,AD.vs.control; bulk RNA-seq,Homo_714,AD.vs.MCI	4
BP	GO:0030100	regulation of endocytosis	bulk RNA-seq,Homo_723,AD.vs.control; bulk RNA-seq,Homo_723,AD.vs.MCI; bulk RNA-seq,Homo_723,MCI.vs.control; bulk RNA-seq,Homo_714,AD.vs.control; bulk RNA-seq,Homo_714,AD.vs.MCI; bulk RNA-seq,Homo_633,AD.vs.control; bulk RNA-seq,Homo_633,AD.vs.MCI	7
CC	GO:0032838	plasma membrane bounded cell projection cytoplasm	bulk RNA-seq,Homo_723,AD.vs.control; bulk RNA-seq,Homo_723,AD.vs.MCI; bulk RNA-seq,Homo_723,MCI.vs.control; bulk RNA-seq,Homo_714,AD.vs.MCI; bulk RNA-seq,Homo_633,AD.vs.control; bulk RNA-seq,Homo_633,AD.vs.MCI; bulk RNA-seq,ROSMAP,AD.vs.control; bulk RNA-seq,ROSMAP,MCI.vs.control	8
BP	GO:0097104	postsynaptic membrane assembly	bulk RNA-seq,Homo_723,AD.vs.control; bulk RNA-seq,Homo_723,AD.vs.MCI; bulk RNA-seq,Homo_723,MCI.vs.control; bulk RNA-seq,Homo_714,AD.vs.MCI; bulk RNA-seq,Homo_714,MCI.vs.control; bulk RNA-seq,ROSMAP,AD.vs.control; bulk RNA-seq,ROSMAP,MCI.vs.control	7
BP	GO:0032535	regulation of cellular component size	bulk RNA-seq,Homo_723,AD.vs.control; bulk RNA-seq,Homo_723,AD.vs.MCI; bulk RNA-seq,Homo_714,AD.vs.MCI; bulk RNA-seq,Homo_633,AD.vs.control; bulk RNA-seq,Homo_633,AD.vs.MCI	5
CC	GO:0043194	axon initial segment	bulk RNA-seq,Homo_723,AD.vs.control; bulk RNA-seq,Homo_723,AD.vs.MCI; bulk RNA-seq,Homo_723,MCI.vs.control; bulk RNA-seq,Homo_714,AD.vs.MCI	4
BP	GO:0001707	mesoderm formation	bulk RNA-seq,Homo_723,AD.vs.control; bulk RNA-seq,Homo_723,AD.vs.MCI; bulk RNA-seq,Homo_723,MCI.vs.control; bulk RNA-seq,Homo_714,AD.vs.control; bulk RNA-seq,Homo_714,AD.vs.MCI; bulk RNA-seq,Homo_714,MCI.vs.control	6
BP	GO:0021514	ventral spinal cord interneuron differentiation	bulk RNA-seq,Homo_723,AD.vs.control; bulk RNA-seq,Homo_723,AD.vs.MCI; bulk RNA-seq,Homo_723,MCI.vs.control; bulk RNA-seq,Homo_714,AD.vs.control; bulk RNA-seq,Homo_714,AD.vs.MCI	5
BP	GO:0072578	neurotransmitter-gated ion channel clustering	bulk RNA-seq,Homo_723,AD.vs.control; bulk RNA-seq,Homo_723,AD.vs.MCI; bulk RNA-seq,Homo_723,MCI.vs.control; bulk RNA-seq,Homo_714,AD.vs.MCI; bulk RNA-seq,Homo_714,MCI.vs.control	5
CC	GO:1905369	endopeptidase complex	bulk RNA-seq,Homo_723,AD.vs.control; bulk RNA-seq,Homo_723,MCI.vs.control; bulk RNA-seq,Homo_714,AD.vs.control; bulk RNA-seq,Homo_714,MCI.vs.control; bulk RNA-seq,Homo_633,AD.vs.control; bulk RNA-seq,Homo_633,AD.vs.MCI	6
BP	GO:0043576	regulation of respiratory gaseous exchange	bulk RNA-seq,Homo_723,AD.vs.control; bulk RNA-seq,Homo_723,AD.vs.MCI; bulk RNA-seq,Homo_723,MCI.vs.control; bulk RNA-seq,Homo_714,AD.vs.control; bulk RNA-seq,Homo_714,AD.vs.MCI	5
BP	GO:0009225	nucleotide-sugar metabolic process	bulk RNA-seq,Homo_723,AD.vs.control; bulk RNA-seq,Homo_723,AD.vs.MCI; bulk RNA-seq,Homo_714,AD.vs.control; bulk RNA-seq,Homo_714,AD.vs.MCI; bulk RNA-seq,Homo_633,AD.vs.control; bulk RNA-seq,Homo_633,AD.vs.MCI	6
CC	GO:1905368	peptidase complex	bulk RNA-seq,Homo_723,AD.vs.control; bulk RNA-seq,Homo_723,MCI.vs.control; bulk RNA-seq,Homo_714,AD.vs.control; bulk RNA-seq,Homo_714,MCI.vs.control; bulk RNA-seq,Homo_633,AD.vs.control; bulk RNA-seq,Homo_633,AD.vs.MCI	6
MF	GO:0005005	transmembrane-ephrin receptor activity	bulk RNA-seq,Homo_723,AD.vs.control; bulk RNA-seq,Homo_723,AD.vs.MCI; bulk RNA-seq,Homo_723,MCI.vs.control; bulk RNA-seq,Homo_714,AD.vs.MCI; bulk RNA-seq,Homo_714,MCI.vs.control; bulk RNA-seq,ROSMAP,AD.vs.control; bulk RNA-seq,ROSMAP,MCI.vs.control	7
BP	GO:0051924	regulation of calcium ion transport	bulk RNA-seq,Homo_723,AD.vs.control; bulk RNA-seq,Homo_723,AD.vs.MCI; bulk RNA-seq,Homo_723,MCI.vs.control; bulk RNA-seq,Homo_714,AD.vs.MCI; bulk RNA-seq,Homo_633,AD.vs.control; bulk RNA-seq,Homo_633,AD.vs.MCI	6
MF	GO:0004540	ribonuclease activity	bulk RNA-seq,Homo_723,AD.vs.control; bulk RNA-seq,Homo_723,AD.vs.MCI; bulk RNA-seq,Homo_714,AD.vs.control; bulk RNA-seq,Homo_633,AD.vs.control; bulk RNA-seq,Homo_633,AD.vs.MCI	5
BP	GO:0006303	double-strand break repair via nonhomologous end joining	bulk RNA-seq,Homo_723,AD.vs.control; bulk RNA-seq,Homo_723,MCI.vs.control; bulk RNA-seq,Homo_714,AD.vs.control; bulk RNA-seq,Homo_714,MCI.vs.control; bulk RNA-seq,Homo_633,AD.vs.control; bulk RNA-seq,Homo_633,AD.vs.MCI	6
BP	GO:0016226	iron-sulfur cluster assembly	bulk RNA-seq,Homo_723,AD.vs.control; bulk RNA-seq,Homo_723,AD.vs.MCI; bulk RNA-seq,Homo_723,MCI.vs.control; bulk RNA-seq,Homo_714,AD.vs.control; bulk RNA-seq,Homo_714,MCI.vs.control; bulk RNA-seq,Homo_633,AD.vs.control; bulk RNA-seq,Homo_633,AD.vs.MCI; bulk	8
BP	GO:0031163	metallo-sulfur cluster assembly	bulk RNA-seq,Homo_723,AD.vs.control; bulk RNA-seq,Homo_723,AD.vs.MCI; bulk RNA-seq,Homo_723,MCI.vs.control; bulk RNA-seq,Homo_714,AD.vs.control; bulk RNA-seq,Homo_714,MCI.vs.control; bulk RNA-seq,Homo_633,AD.vs.control; bulk RNA-seq,Homo_633,AD.vs.MCI; bulk	8
MF	GO:0070034	telomerase RNA binding	bulk RNA-seq,Homo_723,AD.vs.control; bulk RNA-seq,Homo_723,AD.vs.MCI; bulk RNA-seq,Homo_723,MCI.vs.control; bulk RNA-seq,Homo_714,AD.vs.control; bulk RNA-seq,Homo_714,MCI.vs.control; bulk RNA-seq,Homo_633,AD.vs.control; bulk RNA-seq,Homo_633,AD.vs.MCI; bulk	8
BP	GO:0070293	renal absorption	bulk RNA-seq,Homo_723,AD.vs.control; bulk RNA-seq,Homo_723,AD.vs.MCI; bulk RNA-seq,Homo_723,MCI.vs.control; bulk RNA-seq,Homo_714,AD.vs.control; bulk RNA-seq,Homo_714,AD.vs.MCI; bulk RNA-seq,Homo_714,MCI.vs.control; bulk RNA-seq,Homo_633,AD.vs.control; bulk	8
BP	GO:0002285	lymphocyte activation involved in immune response	bulk RNA-seq,Homo_723,AD.vs.control; bulk RNA-seq,Homo_723,MCI.vs.control; bulk RNA-seq,Homo_714,AD.vs.control; bulk RNA-seq,Homo_633,AD.vs.control; bulk RNA-seq,Homo_633,AD.vs.MCI	5
BP	GO:0009260	ribonucleotide biosynthetic process	bulk RNA-seq,Homo_723,AD.vs.control; bulk RNA-seq,Homo_723,MCI.vs.control; bulk RNA-seq,Homo_714,AD.vs.control; bulk RNA-seq,Homo_633,AD.vs.control; bulk RNA-seq,Homo_633,AD.vs.MCI	5

BP	GO:0071711	basement membrane organization	bulk RNA-seq,Homo_723,AD.vs.control; bulk RNA-seq,Homo_723,AD.vs.MCI; bulk RNA-seq,Homo_723,MCI.vs.control; bulk RNA-seq,Homo_714,AD.vs.control; bulk RNA-seq,Homo_714,AD.vs.MCI; bulk RNA-seq,Homo_714,MCI.vs.control; bulk RNA-seq,Homo_633,AD.vs.control; bulk	8
BP	GO:0048333	mesodermal cell differentiation	bulk RNA-seq,Homo_723,AD.vs.control; bulk RNA-seq,Homo_723,AD.vs.MCI; bulk RNA-seq,Homo_723,MCI.vs.control; bulk RNA-seq,Homo_714,AD.vs.control; bulk RNA-seq,Homo_714,AD.vs.MCI	5
BP	GO:0052547	regulation of peptidase activity	bulk RNA-seq,Homo_723,AD.vs.control; bulk RNA-seq,Homo_723,AD.vs.MCI; bulk RNA-seq,Homo_714,AD.vs.control; bulk RNA-seq,Homo_714,AD.vs.MCI; bulk RNA-seq,Homo_633,AD.vs.control; bulk RNA-seq,Homo_633,AD.vs.MCI; bulk RNA-seq,SRP223445,AD.vs.control	7
BP	GO:0007229	integrin-mediated signaling pathway	bulk RNA-seq,Homo_723,AD.vs.control; bulk RNA-seq,Homo_723,AD.vs.MCI; bulk RNA-seq,Homo_723,MCI.vs.control; bulk RNA-seq,Homo_714,AD.vs.MCI; bulk RNA-seq,Homo_714,MCI.vs.control; bulk RNA-seq,Homo_633,AD.vs.control; bulk RNA-seq,Homo_633,AD.vs.MCI	7
BP	GO:1902475	L-alpha-amino acid transmembrane transport	bulk RNA-seq,Homo_723,AD.vs.control; bulk RNA-seq,Homo_723,AD.vs.MCI; bulk RNA-seq,Homo_723,MCI.vs.control; bulk RNA-seq,Homo_714,AD.vs.MCI; bulk RNA-seq,Homo_633,AD.vs.control; bulk RNA-seq,Homo_633,AD.vs.MCI	6
BP	GO:0003208	cardiac ventricle morphogenesis	bulk RNA-seq,Homo_723,AD.vs.control; bulk RNA-seq,Homo_723,AD.vs.MCI; bulk RNA-seq,Homo_723,MCI.vs.control; bulk RNA-seq,Homo_714,AD.vs.control; bulk RNA-seq,Homo_714,AD.vs.MCI; bulk RNA-seq,Homo_714,MCI.vs.control; bulk RNA-seq,Homo_633,AD.vs.control; bulk	8
BP	GO:0009062	fatty acid catabolic process	bulk RNA-seq,Homo_723,AD.vs.control; bulk RNA-seq,Homo_723,MCI.vs.control; bulk RNA-seq,Homo_714,AD.vs.control; bulk RNA-seq,Homo_714,MCI.vs.control; bulk RNA-seq,Homo_633,AD.vs.control; bulk RNA-seq,Homo_633,AD.vs.MCI	6
MF	GO:0005242	inward rectifier potassium channel activity	bulk RNA-seq,Homo_723,AD.vs.control; bulk RNA-seq,Homo_723,AD.vs.MCI; bulk RNA-seq,Homo_723,MCI.vs.control; bulk RNA-seq,Homo_714,AD.vs.MCI	4
BP	GO:0150115	cell-substrate junction organization	bulk RNA-seq,Homo_723,AD.vs.control; bulk RNA-seq,Homo_723,AD.vs.MCI; bulk RNA-seq,Homo_723,MCI.vs.control; bulk RNA-seq,Homo_714,AD.vs.control; bulk RNA-seq,Homo_714,AD.vs.MCI; bulk RNA-seq,Homo_714,MCI.vs.control; bulk RNA-seq,Homo_633,AD.vs.control; bulk	8
BP	GO:0071625	vocalization behavior	bulk RNA-seq,Homo_723,AD.vs.control; bulk RNA-seq,Homo_723,AD.vs.MCI; bulk RNA-seq,Homo_723,MCI.vs.control; bulk RNA-seq,Homo_714,AD.vs.MCI; bulk RNA-seq,Homo_714,MCI.vs.control; bulk RNA-seq,ROSMAP,AD.vs.control; bulk RNA-seq,SRP223445,AD.vs.control	7
BP	GO:0006029	proteoglycan metabolic process	bulk RNA-seq,Homo_723,AD.vs.control; bulk RNA-seq,Homo_723,AD.vs.MCI; bulk RNA-seq,Homo_723,MCI.vs.control; bulk RNA-seq,Homo_714,AD.vs.MCI; bulk RNA-seq,Homo_714,MCI.vs.control; bulk RNA-seq,Homo_633,AD.vs.control; bulk RNA-seq,Homo_633,AD.vs.MCI	7
BP	GO:0072077	renal vesicle morphogenesis	bulk RNA-seq,Homo_723,AD.vs.control; bulk RNA-seq,Homo_723,AD.vs.MCI; bulk RNA-seq,Homo_723,MCI.vs.control; bulk RNA-seq,Homo_714,AD.vs.MCI; bulk RNA-seq,Homo_714,MCI.vs.control; bulk RNA-seq,Homo_633,MCI.vs.control	6
CC	GO:0098688	parallel fiber to Purkinje cell synapse	bulk RNA-seq,Homo_723,AD.vs.control; bulk RNA-seq,Homo_723,AD.vs.MCI; bulk RNA-seq,Homo_723,MCI.vs.control	3
MF	GO:0005003	ephrin receptor activity	bulk RNA-seq,Homo_723,AD.vs.control; bulk RNA-seq,Homo_723,AD.vs.MCI; bulk RNA-seq,Homo_723,MCI.vs.control; bulk RNA-seq,Homo_714,AD.vs.MCI; bulk RNA-seq,Homo_714,MCI.vs.control; bulk RNA-seq,ROSMAP,AD.vs.control; bulk RNA-seq,ROSMAP,MCI.vs.control	7
BP	GO:0002204	somatic recombination of immunoglobulin genes involved in immune response	bulk RNA-seq,Homo_723,AD.vs.control; bulk RNA-seq,Homo_723,MCI.vs.control; bulk RNA-seq,Homo_714,AD.vs.control; bulk RNA-seq,Homo_714,MCI.vs.control; bulk RNA-seq,Homo_633,AD.vs.control; bulk RNA-seq,Homo_633,AD.vs.MCI	6
BP	GO:0002208	somatic diversification of immunoglobulins involved in immune response	bulk RNA-seq,Homo_723,AD.vs.control; bulk RNA-seq,Homo_723,MCI.vs.control; bulk RNA-seq,Homo_714,AD.vs.control; bulk RNA-seq,Homo_714,MCI.vs.control; bulk RNA-seq,Homo_633,AD.vs.control; bulk RNA-seq,Homo_633,AD.vs.MCI	6
BP	GO:0045190	isotype switching	bulk RNA-seq,Homo_723,AD.vs.control; bulk RNA-seq,Homo_723,MCI.vs.control; bulk RNA-seq,Homo_714,AD.vs.control; bulk RNA-seq,Homo_714,MCI.vs.control; bulk RNA-seq,Homo_633,AD.vs.control; bulk RNA-seq,Homo_633,AD.vs.MCI	6
BP	GO:1903311	regulation of mRNA metabolic process	bulk RNA-seq,Homo_723,AD.vs.control; bulk RNA-seq,Homo_723,MCI.vs.control; bulk RNA-seq,Homo_714,AD.vs.control; bulk RNA-seq,Homo_633,AD.vs.control; bulk RNA-seq,Homo_633,AD.vs.MCI	5
BP	GO:0006940	regulation of smooth muscle contraction	bulk RNA-seq,Homo_723,AD.vs.control; bulk RNA-seq,Homo_723,AD.vs.MCI; bulk RNA-seq,Homo_723,MCI.vs.control; bulk RNA-seq,Homo_714,AD.vs.control; bulk RNA-seq,Homo_714,AD.vs.MCI; bulk RNA-seq,Homo_714,MCI.vs.control	6
CC	GO:0045121	membrane raft	bulk RNA-seq,Homo_723,AD.vs.control; bulk RNA-seq,Homo_723,AD.vs.MCI; bulk RNA-seq,Homo_723,MCI.vs.control; bulk RNA-seq,Homo_714,AD.vs.MCI; bulk RNA-seq,Homo_633,AD.vs.control; bulk RNA-seq,Homo_633,AD.vs.MCI	6
CC	GO:0016580	Sin3 complex	bulk RNA-seq,Homo_723,AD.vs.control; bulk RNA-seq,Homo_723,AD.vs.MCI; bulk RNA-seq,Homo_723,MCI.vs.control; bulk RNA-seq,Homo_714,AD.vs.control; bulk RNA-seq,Homo_714,AD.vs.MCI; bulk RNA-seq,Homo_714,MCI.vs.control; bulk RNA-seq,Homo_633,AD.vs.control; bulk RNA-seq,Homo_633,AD.vs.MCI; bulk RNA-seq,Homo_633,MCI.vs.control	9
CC	GO:0070822	Sin3-type complex	bulk RNA-seq,Homo_723,AD.vs.control; bulk RNA-seq,Homo_723,AD.vs.MCI; bulk RNA-seq,Homo_723,MCI.vs.control; bulk RNA-seq,Homo_714,AD.vs.control; bulk RNA-seq,Homo_714,AD.vs.MCI; bulk RNA-seq,Homo_714,MCI.vs.control; bulk RNA-seq,Homo_633,AD.vs.control; bulk RNA-seq,Homo_633,AD.vs.MCI; bulk RNA-seq,Homo_633,MCI.vs.control	9
BP	GO:0051930	regulation of sensory perception of pain	bulk RNA-seq,Homo_723,AD.vs.control; bulk RNA-seq,Homo_723,AD.vs.MCI; bulk RNA-seq,Homo_723,MCI.vs.control; bulk RNA-seq,Homo_714,AD.vs.control; bulk RNA-seq,Homo_714,AD.vs.MCI; bulk RNA-seq,Homo_714,MCI.vs.control	6
BP	GO:0051931	regulation of sensory perception	bulk RNA-seq,Homo_723,AD.vs.control; bulk RNA-seq,Homo_723,AD.vs.MCI; bulk RNA-seq,Homo_723,MCI.vs.control; bulk RNA-seq,Homo_714,AD.vs.control; bulk RNA-seq,Homo_714,AD.vs.MCI; bulk RNA-seq,Homo_714,MCI.vs.control	6
BP	GO:0050927	positive regulation of positive chemotaxis	bulk RNA-seq,Homo_723,AD.vs.control; bulk RNA-seq,Homo_723,AD.vs.MCI; bulk RNA-seq,Homo_723,MCI.vs.control; bulk RNA-seq,Homo_714,AD.vs.control; bulk RNA-seq,Homo_714,AD.vs.MCI; bulk RNA-seq,Homo_714,MCI.vs.control	6
BP	GO:0042559	pteridine-containing compound biosynthetic process	bulk RNA-seq,Homo_723,AD.vs.control; bulk RNA-seq,Homo_723,AD.vs.MCI; bulk RNA-seq,Homo_723,MCI.vs.control; bulk RNA-seq,Homo_714,AD.vs.control; bulk RNA-seq,Homo_714,MCI.vs.control; bulk RNA-seq,Homo_633,AD.vs.control; bulk RNA-seq,Homo_633,AD.vs.MCI; bulk	8
BP	GO:0030488	tRNA methylation	bulk RNA-seq,Homo_723,AD.vs.control; bulk RNA-seq,Homo_723,MCI.vs.control; bulk RNA-seq,Homo_714,AD.vs.control; bulk RNA-seq,Homo_714,MCI.vs.control; bulk RNA-seq,Homo_633,AD.vs.control; bulk RNA-seq,Homo_633,AD.vs.MCI	6
BP	GO:0061548	ganglion development	bulk RNA-seq,Homo_723,AD.vs.control; bulk RNA-seq,Homo_723,AD.vs.MCI; bulk RNA-seq,Homo_723,MCI.vs.control; bulk RNA-seq,Homo_714,AD.vs.MCI	4
MF	GO:0030165	PDZ domain binding	bulk RNA-seq,Homo_723,AD.vs.control; bulk RNA-seq,Homo_723,AD.vs.MCI; bulk RNA-seq,Homo_723,MCI.vs.control; bulk RNA-seq,Homo_714,AD.vs.MCI; bulk RNA-seq,Homo_714,MCI.vs.control; bulk RNA-seq,Homo_633,AD.vs.control	6
BP	GO:0032835	glomerulus development	bulk RNA-seq,Homo_723,AD.vs.control; bulk RNA-seq,Homo_723,AD.vs.MCI; bulk RNA-seq,Homo_723,MCI.vs.control; bulk RNA-seq,Homo_714,AD.vs.MCI; bulk RNA-seq,Homo_714,MCI.vs.control; bulk RNA-seq,Homo_633,AD.vs.control	6
BP	GO:0060080	inhibitory postsynaptic potential	bulk RNA-seq,Homo_723,AD.vs.control; bulk RNA-seq,Homo_723,AD.vs.MCI; bulk RNA-seq,Homo_723,MCI.vs.control; bulk RNA-seq,Homo_714,AD.vs.MCI	4
BP	GO:0045921	positive regulation of exocytosis	bulk RNA-seq,Homo_723,AD.vs.control; bulk RNA-seq,Homo_723,AD.vs.MCI; bulk RNA-seq,Homo_723,MCI.vs.control; bulk RNA-seq,Homo_714,AD.vs.MCI; bulk RNA-seq,Homo_714,MCI.vs.control; bulk RNA-seq,Homo_633,AD.vs.control; bulk RNA-seq,Homo_633,AD.vs.MCI	7
BP	GO:0015980	energy derivation by oxidation of organic compounds	bulk RNA-seq,Homo_723,AD.vs.control; bulk RNA-seq,Homo_714,AD.vs.control; bulk RNA-seq,Homo_633,AD.vs.control; bulk RNA-seq,Homo_633,AD.vs.MCI	4
BP	GO:2000300	regulation of synaptic vesicle exocytosis	bulk RNA-seq,Homo_723,AD.vs.control; bulk RNA-seq,Homo_723,AD.vs.MCI; bulk RNA-seq,Homo_723,MCI.vs.control; bulk RNA-seq,Homo_714,AD.vs.MCI; bulk RNA-seq,Homo_714,MCI.vs.control; bulk RNA-seq,Homo_633,AD.vs.control; bulk RNA-seq,Homo_633,AD.vs.MCI	7

BP	GO:0048843	negative regulation of axon extension involved in axon guidance	bulk RNA-seq,Homo_723,AD.vs.control; bulk RNA-seq,Homo_723,AD.vs.MCI; bulk RNA-seq,Homo_723,MCI.vs.control; bulk RNA-seq,Homo_714,AD.vs.MCI; bulk RNA-seq,Homo_714,MCI.vs.control	5
BP	GO:0055002	striated muscle cell development	bulk RNA-seq,Homo_723,AD.vs.control; bulk RNA-seq,Homo_723,AD.vs.MCI; bulk RNA-seq,Homo_723,MCI.vs.control; bulk RNA-seq,Homo_714,AD.vs.MCI; bulk RNA-seq,Homo_714,MCI.vs.control; bulk RNA-seq,Homo_633,AD.vs.control	6
MF	GO:0033293	monocarboxylic acid binding	bulk RNA-seq,Homo_723,AD.vs.control; bulk RNA-seq,Homo_723,AD.vs.MCI; bulk RNA-seq,Homo_723,MCI.vs.control; bulk RNA-seq,Homo_714,AD.vs.control; bulk RNA-seq,Homo_714,AD.vs.MCI; bulk RNA-seq,Homo_714,MCI.vs.control	6
BP	GO:0007350	blastoderm segmentation	bulk RNA-seq,Homo_723,AD.vs.control; bulk RNA-seq,Homo_723,AD.vs.MCI; bulk RNA-seq,Homo_723,MCI.vs.control; bulk RNA-seq,Homo_714,AD.vs.control; bulk RNA-seq,Homo_714,AD.vs.MCI; bulk RNA-seq,Homo_714,MCI.vs.control	6
BP	GO:0043652	engulfment of apoptotic cell	bulk RNA-seq,Homo_723,AD.vs.control; bulk RNA-seq,Homo_723,AD.vs.MCI; bulk RNA-seq,Homo_723,MCI.vs.control; bulk RNA-seq,Homo_714,AD.vs.MCI; bulk RNA-seq,Homo_633,AD.vs.control	5
BP	GO:0021534	cell proliferation in hindbrain	bulk RNA-seq,Homo_723,AD.vs.control; bulk RNA-seq,Homo_723,AD.vs.MCI; bulk RNA-seq,Homo_723,MCI.vs.control; bulk RNA-seq,Homo_714,AD.vs.MCI	4
BP	GO:0021924	cell proliferation in external granule layer	bulk RNA-seq,Homo_723,AD.vs.control; bulk RNA-seq,Homo_723,AD.vs.MCI; bulk RNA-seq,Homo_723,MCI.vs.control; bulk RNA-seq,Homo_714,AD.vs.MCI	4
BP	GO:0021930	cerebellar granule cell precursor proliferation	bulk RNA-seq,Homo_723,AD.vs.control; bulk RNA-seq,Homo_723,AD.vs.MCI; bulk RNA-seq,Homo_723,MCI.vs.control; bulk RNA-seq,Homo_714,AD.vs.MCI	4
MF	GO:0015020	glucuronosyltransferase activity	bulk RNA-seq,Homo_723,AD.vs.control; bulk RNA-seq,Homo_723,AD.vs.MCI; bulk RNA-seq,Homo_714,AD.vs.control; bulk RNA-seq,Homo_714,AD.vs.MCI	4
BP	GO:0019079	viral genome replication	bulk RNA-seq,Homo_723,AD.vs.control; bulk RNA-seq,Homo_723,MCI.vs.control; bulk RNA-seq,Homo_714,AD.vs.control; bulk RNA-seq,Homo_633,AD.vs.control; bulk RNA-seq,Homo_633,AD.vs.MCI	5
BP	GO:0006289	nucleotide-excision repair	bulk RNA-seq,Homo_723,AD.vs.control; bulk RNA-seq,Homo_723,MCI.vs.control; bulk RNA-seq,Homo_714,AD.vs.control; bulk RNA-seq,Homo_714,MCI.vs.control; bulk RNA-seq,Homo_633,AD.vs.control; bulk RNA-seq,Homo_633,AD.vs.MCI	6
BP	GO:0032331	negative regulation of chondrocyte differentiation	bulk RNA-seq,Homo_723,AD.vs.control; bulk RNA-seq,Homo_723,AD.vs.MCI; bulk RNA-seq,Homo_723,MCI.vs.control; bulk RNA-seq,Homo_714,AD.vs.MCI; bulk RNA-seq,Homo_714,MCI.vs.control	5
BP	GO:0043113	receptor clustering	bulk RNA-seq,Homo_723,AD.vs.control; bulk RNA-seq,Homo_723,AD.vs.MCI; bulk RNA-seq,Homo_723,MCI.vs.control; bulk RNA-seq,Homo_714,AD.vs.MCI; bulk RNA-seq,Homo_714,MCI.vs.control; bulk RNA-seq,Homo_633,AD.vs.control; bulk RNA-seq,Homo_633,AD.vs.MCI	7
BP	GO:0016075	rRNA catabolic process	bulk RNA-seq,Homo_723,AD.vs.control; bulk RNA-seq,Homo_723,AD.vs.MCI; bulk RNA-seq,Homo_714,AD.vs.control; bulk RNA-seq,Homo_714,AD.vs.MCI; bulk RNA-seq,Homo_633,AD.vs.control; bulk RNA-seq,Homo_633,AD.vs.MCI	6
BP	GO:0051168	nuclear export	bulk RNA-seq,Homo_723,AD.vs.control; bulk RNA-seq,Homo_723,MCI.vs.control; bulk RNA-seq,Homo_714,AD.vs.control; bulk RNA-seq,Homo_633,AD.vs.control; bulk RNA-seq,Homo_633,AD.vs.MCI	5
BP	GO:0045621	positive regulation of lymphocyte differentiation	bulk RNA-seq,Homo_723,AD.vs.control; bulk RNA-seq,Homo_723,MCI.vs.control; bulk RNA-seq,Homo_714,AD.vs.control; bulk RNA-seq,Homo_633,AD.vs.control; bulk RNA-seq,Homo_633,AD.vs.MCI	5
BP	GO:0021895	cerebral cortex neuron differentiation	bulk RNA-seq,Homo_723,AD.vs.control; bulk RNA-seq,Homo_723,AD.vs.MCI; bulk RNA-seq,Homo_723,MCI.vs.control; bulk RNA-seq,Homo_714,AD.vs.MCI; bulk RNA-seq,Homo_714,MCI.vs.control	5
BP	GO:0086070	SA node cell to atrial cardiac muscle cell communication	bulk RNA-seq,Homo_723,AD.vs.control; bulk RNA-seq,Homo_723,AD.vs.MCI; bulk RNA-seq,Homo_723,MCI.vs.control; bulk RNA-seq,Homo_714,AD.vs.control; bulk RNA-seq,Homo_714,AD.vs.MCI; bulk RNA-seq,Homo_714,MCI.vs.control	6
MF	GO:0001972	retinoic acid binding	bulk RNA-seq,Homo_723,AD.vs.control; bulk RNA-seq,Homo_723,AD.vs.MCI; bulk RNA-seq,Homo_723,MCI.vs.control; bulk RNA-seq,Homo_714,AD.vs.control; bulk RNA-seq,Homo_714,AD.vs.MCI; bulk RNA-seq,Homo_633,MCI.vs.control	6
BP	GO:2001026	regulation of endothelial cell chemotaxis	bulk RNA-seq,Homo_723,AD.vs.control; bulk RNA-seq,Homo_723,AD.vs.MCI; bulk RNA-seq,Homo_723,MCI.vs.control; bulk RNA-seq,Homo_714,AD.vs.control; bulk RNA-seq,Homo_714,AD.vs.MCI; bulk RNA-seq,Homo_714,MCI.vs.control	6
CC	GO:0005901	caveola	bulk RNA-seq,Homo_723,AD.vs.control; bulk RNA-seq,Homo_723,AD.vs.MCI; bulk RNA-seq,Homo_723,MCI.vs.control; bulk RNA-seq,Homo_714,AD.vs.control; bulk RNA-seq,Homo_714,AD.vs.MCI; bulk RNA-seq,Homo_714,MCI.vs.control; bulk RNA-seq,Homo_633,AD.vs.control	7
BP	GO:0050921	positive regulation of chemotaxis	bulk RNA-seq,Homo_723,AD.vs.control; bulk RNA-seq,Homo_723,AD.vs.MCI; bulk RNA-seq,Homo_723,MCI.vs.control; bulk RNA-seq,Homo_714,AD.vs.control; bulk RNA-seq,Homo_714,AD.vs.MCI; bulk RNA-seq,Homo_633,AD.vs.control; scRNA-seq,SRP330776,Memory T cell_2-AD.vs.control; scRNA-seq,SRP330776,Naive CD8+ T cell_2-AD.vs.control	8
BP	GO:1990173	protein localization to nucleoplasm	bulk RNA-seq,Homo_723,AD.vs.control; bulk RNA-seq,Homo_723,MCI.vs.control; bulk RNA-seq,Homo_714,AD.vs.control; bulk RNA-seq,Homo_714,MCI.vs.control; bulk RNA-seq,Homo_633,AD.vs.control; bulk RNA-seq,Homo_633,AD.vs.MCI; bulk RNA-seq,Homo_633,MCI.vs.control	7
BP	GO:0033059	cellular pigmentation	bulk RNA-seq,Homo_723,AD.vs.control; bulk RNA-seq,Homo_723,MCI.vs.control; bulk RNA-seq,Homo_714,AD.vs.control; bulk RNA-seq,Homo_714,MCI.vs.control; bulk RNA-seq,Homo_633,AD.vs.control; bulk RNA-seq,Homo_633,AD.vs.MCI	6
BP	GO:1905809	negative regulation of synapse organization	bulk RNA-seq,Homo_723,AD.vs.control; bulk RNA-seq,Homo_723,AD.vs.MCI; bulk RNA-seq,Homo_723,MCI.vs.control; bulk RNA-seq,Homo_714,AD.vs.control; bulk RNA-seq,Homo_714,AD.vs.MCI; bulk RNA-seq,Homo_714,MCI.vs.control	6
BP	GO:0070536	protein K63-linked deubiquitination	bulk RNA-seq,Homo_723,AD.vs.control; bulk RNA-seq,Homo_723,MCI.vs.control; bulk RNA-seq,Homo_714,AD.vs.control; bulk RNA-seq,Homo_714,MCI.vs.control; bulk RNA-seq,Homo_633,AD.vs.control; bulk RNA-seq,Homo_633,AD.vs.MCI	6
BP	GO:0043409	negative regulation of MAPK cascade	bulk RNA-seq,Homo_723,AD.vs.control; bulk RNA-seq,Homo_723,AD.vs.MCI; bulk RNA-seq,Homo_723,MCI.vs.control; bulk RNA-seq,Homo_714,AD.vs.MCI; bulk RNA-seq,Homo_633,AD.vs.control; bulk RNA-seq,Homo_633,AD.vs.MCI	6
BP	GO:1903432	regulation of TORC1 signaling	bulk RNA-seq,Homo_723,AD.vs.control; bulk RNA-seq,Homo_723,MCI.vs.control; bulk RNA-seq,Homo_714,AD.vs.control; bulk RNA-seq,Homo_714,MCI.vs.control; bulk RNA-seq,Homo_633,AD.vs.control; bulk RNA-seq,Homo_633,AD.vs.MCI	6
MF	GO:0005342	organic acid transmembrane transporter activity	bulk RNA-seq,Homo_723,AD.vs.control; bulk RNA-seq,Homo_723,AD.vs.MCI; bulk RNA-seq,Homo_723,MCI.vs.control; bulk RNA-seq,Homo_714,AD.vs.control; bulk RNA-seq,Homo_714,AD.vs.MCI; bulk RNA-seq,Homo_633,AD.vs.control; bulk RNA-seq,Homo_633,AD.vs.MCI	7
BP	GO:0001895	retina homeostasis	bulk RNA-seq,Homo_723,AD.vs.control; bulk RNA-seq,Homo_723,AD.vs.MCI; bulk RNA-seq,Homo_723,MCI.vs.control; bulk RNA-seq,Homo_714,AD.vs.control; bulk RNA-seq,Homo_714,AD.vs.MCI; bulk RNA-seq,Homo_633,AD.vs.control; bulk RNA-seq,Homo_633,AD.vs.MCI	7
BP	GO:0070897	transcription preinitiation complex assembly	bulk RNA-seq,Homo_723,AD.vs.control; bulk RNA-seq,Homo_723,MCI.vs.control; bulk RNA-seq,Homo_714,AD.vs.control; bulk RNA-seq,Homo_714,MCI.vs.control; bulk RNA-seq,Homo_633,AD.vs.control; bulk RNA-seq,Homo_633,AD.vs.MCI	6
MF	GO:0008186	ATP-dependent activity, acting on RNA	bulk RNA-seq,Homo_723,AD.vs.control; bulk RNA-seq,Homo_723,MCI.vs.control; bulk RNA-seq,Homo_714,AD.vs.control; bulk RNA-seq,Homo_714,MCI.vs.control; bulk RNA-seq,Homo_633,AD.vs.control; bulk RNA-seq,Homo_633,AD.vs.MCI	6
MF	GO:0016655	oxidoreductase activity, acting on NAD(P)H, quinone or similar compound as acceptor	bulk RNA-seq,Homo_723,AD.vs.control; bulk RNA-seq,Homo_723,MCI.vs.control; bulk RNA-seq,Homo_714,AD.vs.control; bulk RNA-seq,Homo_714,MCI.vs.control; bulk RNA-seq,Homo_633,AD.vs.control; bulk RNA-seq,Homo_633,AD.vs.MCI	6
BP	GO:0042573	retinoic acid metabolic process	bulk RNA-seq,Homo_723,AD.vs.control; bulk RNA-seq,Homo_723,AD.vs.MCI; bulk RNA-seq,Homo_723,MCI.vs.control; bulk RNA-seq,Homo_714,AD.vs.control; bulk RNA-seq,Homo_714,AD.vs.MCI; bulk RNA-seq,Homo_714,MCI.vs.control	6

BP	GO:0019646	aerobic electron transport chain	bulk RNA-seq,Homo_723,AD.vs.control; bulk RNA-seq,Homo_723,MCI.vs.control; bulk RNA-seq,Homo_714,AD.vs.control; bulk RNA-seq,Homo_714,MCI.vs.control; bulk RNA-seq,Homo_633,AD.vs.control; bulk RNA-seq,Homo_633,AD.vs.MCI	6
BP	GO:0033962	P-body assembly	bulk RNA-seq,Homo_723,AD.vs.control; bulk RNA-seq,Homo_723,AD.vs.MCI; bulk RNA-seq,Homo_723,MCI.vs.control; bulk RNA-seq,Homo_714,AD.vs.control; bulk RNA-seq,Homo_714,AD.vs.MCI; bulk RNA-seq,Homo_714,MCI.vs.control; bulk RNA-seq,Homo_633,AD.vs.control; bulk RNA-seq,Homo_633,AD.vs.MCI; bulk RNA-seq,Homo_633,MCI.vs.control	9
BP	GO:0060350	endochondral bone morphogenesis	bulk RNA-seq,Homo_723,AD.vs.control; bulk RNA-seq,Homo_723,AD.vs.MCI; bulk RNA-seq,Homo_723,MCI.vs.control; bulk RNA-seq,Homo_714,AD.vs.MCI; bulk RNA-seq,Homo_714,MCI.vs.control; bulk RNA-seq,Homo_633,AD.vs.control; bulk RNA-seq,Homo_633,AD.vs.MCI	7
CC	GO:0043296	apical junction complex	bulk RNA-seq,Homo_723,AD.vs.control; bulk RNA-seq,Homo_723,AD.vs.MCI; bulk RNA-seq,Homo_723,MCI.vs.control; bulk RNA-seq,Homo_714,AD.vs.control; bulk RNA-seq,Homo_714,AD.vs.MCI; bulk RNA-seq,Homo_633,AD.vs.control; bulk RNA-seq,Homo_633,AD.vs.MCI	7
BP	GO:0002312	B cell activation involved in immune response	bulk RNA-seq,Homo_723,AD.vs.control; bulk RNA-seq,Homo_723,MCI.vs.control; bulk RNA-seq,Homo_714,AD.vs.control; bulk RNA-seq,Homo_714,MCI.vs.control; bulk RNA-seq,Homo_633,AD.vs.control; bulk RNA-seq,Homo_633,AD.vs.MCI	6
BP	GO:0043954	cellular component maintenance	bulk RNA-seq,Homo_723,AD.vs.control; bulk RNA-seq,Homo_723,AD.vs.MCI; bulk RNA-seq,Homo_723,MCI.vs.control; bulk RNA-seq,Homo_714,AD.vs.MCI; bulk RNA-seq,Homo_714,MCI.vs.control; bulk RNA-seq,Homo_633,AD.vs.control; bulk RNA-seq,Homo_633,AD.vs.MCI; bulk RNA-seq,ROSMAP,AD.vs.control; scRNA-seq,SRP330776,Natural killer cell_1-AD.vs.control	9
CC	GO:0001917	photoreceptor inner segment	bulk RNA-seq,Homo_723,AD.vs.control; bulk RNA-seq,Homo_723,AD.vs.MCI; bulk RNA-seq,Homo_723,MCI.vs.control; bulk RNA-seq,Homo_714,AD.vs.control; bulk RNA-seq,Homo_714,AD.vs.MCI; bulk RNA-seq,Homo_714,MCI.vs.control	6
MF	GO:0003724	RNA helicase activity	bulk RNA-seq,Homo_723,AD.vs.control; bulk RNA-seq,Homo_723,MCI.vs.control; bulk RNA-seq,Homo_714,AD.vs.control; bulk RNA-seq,Homo_714,MCI.vs.control; bulk RNA-seq,Homo_633,AD.vs.control; bulk RNA-seq,Homo_633,AD.vs.MCI	6
BP	GO:0032414	positive regulation of ion transmembrane transporter activity	bulk RNA-seq,Homo_723,AD.vs.control; bulk RNA-seq,Homo_723,AD.vs.MCI; bulk RNA-seq,Homo_723,MCI.vs.control; bulk RNA-seq,Homo_714,AD.vs.MCI; bulk RNA-seq,Homo_714,MCI.vs.control; bulk RNA-seq,Homo_633,AD.vs.control	6
CC	GO:0098857	membrane microdomain	bulk RNA-seq,Homo_723,AD.vs.control; bulk RNA-seq,Homo_723,AD.vs.MCI; bulk RNA-seq,Homo_723,MCI.vs.control; bulk RNA-seq,Homo_714,AD.vs.MCI; bulk RNA-seq,Homo_633,AD.vs.control; bulk RNA-seq,Homo_633,AD.vs.MCI	6
BP	GO:0030199	collagen fibril organization	bulk RNA-seq,Homo_723,AD.vs.control; bulk RNA-seq,Homo_723,AD.vs.MCI; bulk RNA-seq,Homo_723,MCI.vs.control; bulk RNA-seq,Homo_714,AD.vs.MCI; bulk RNA-seq,Homo_714,MCI.vs.control; bulk RNA-seq,SRP223445,AD.vs.control	6
MF	GO:0022851	GABA-gated chloride ion channel activity	bulk RNA-seq,Homo_723,AD.vs.control; bulk RNA-seq,Homo_723,AD.vs.MCI; bulk RNA-seq,Homo_714,AD.vs.control; bulk RNA-seq,Homo_714,AD.vs.MCI	4
BP	GO:0050795	regulation of behavior	bulk RNA-seq,Homo_723,AD.vs.control; bulk RNA-seq,Homo_723,AD.vs.MCI; bulk RNA-seq,Homo_723,MCI.vs.control; bulk RNA-seq,Homo_714,AD.vs.control; bulk RNA-seq,Homo_714,AD.vs.MCI	5
BP	GO:0007274	neuromuscular synaptic transmission	bulk RNA-seq,Homo_723,AD.vs.control; bulk RNA-seq,Homo_723,AD.vs.MCI; bulk RNA-seq,Homo_723,MCI.vs.control; bulk RNA-seq,Homo_714,AD.vs.MCI	4
MF	GO:0043023	ribosomal large subunit binding	bulk RNA-seq,Homo_723,AD.vs.control; bulk RNA-seq,Homo_723,AD.vs.MCI; bulk RNA-seq,Homo_723,MCI.vs.control; bulk RNA-seq,Homo_714,AD.vs.control; bulk RNA-seq,Homo_714,MCI.vs.control; bulk RNA-seq,Homo_633,AD.vs.control; bulk RNA-seq,Homo_633,AD.vs.MCI; bulk	8
MF	GO:0016758	hexosyltransferase activity	bulk RNA-seq,Homo_723,AD.vs.control; bulk RNA-seq,Homo_723,AD.vs.MCI; bulk RNA-seq,Homo_723,MCI.vs.control; bulk RNA-seq,Homo_714,AD.vs.control; bulk RNA-seq,Homo_714,AD.vs.MCI; bulk RNA-seq,Homo_633,AD.vs.control; bulk RNA-seq,Homo_633,AD.vs.MCI	7
CC	GO:0016592	mediator complex	bulk RNA-seq,Homo_723,AD.vs.control; bulk RNA-seq,Homo_723,MCI.vs.control; bulk RNA-seq,Homo_714,AD.vs.control; bulk RNA-seq,Homo_714,MCI.vs.control; bulk RNA-seq,Homo_633,AD.vs.control; bulk RNA-seq,Homo_633,AD.vs.MCI	6
CC	GO:0030687	preribosome, large subunit precursor	bulk RNA-seq,Homo_723,AD.vs.control; bulk RNA-seq,Homo_723,MCI.vs.control; bulk RNA-seq,Homo_714,AD.vs.control; bulk RNA-seq,Homo_714,MCI.vs.control; bulk RNA-seq,Homo_633,AD.vs.control; bulk RNA-seq,Homo_633,AD.vs.MCI; bulk RNA-seq,Homo_633,MCI.vs.control	7
BP	GO:1905039	carboxylic acid transmembrane transport	bulk RNA-seq,Homo_723,AD.vs.control; bulk RNA-seq,Homo_723,AD.vs.MCI; bulk RNA-seq,Homo_723,MCI.vs.control; bulk RNA-seq,Homo_714,AD.vs.MCI; bulk RNA-seq,Homo_633,AD.vs.control; bulk RNA-seq,Homo_633,AD.vs.MCI	6
CC	GO:0002080	acrosomal membrane	bulk RNA-seq,Homo_723,AD.vs.control; bulk RNA-seq,Homo_723,AD.vs.MCI; bulk RNA-seq,Homo_714,AD.vs.control; bulk RNA-seq,Homo_714,AD.vs.MCI	4
BP	GO:0014013	regulation of gliogenesis	bulk RNA-seq,Homo_723,AD.vs.control; bulk RNA-seq,Homo_723,AD.vs.MCI; bulk RNA-seq,Homo_723,MCI.vs.control; bulk RNA-seq,Homo_714,AD.vs.MCI; bulk RNA-seq,Homo_714,MCI.vs.control; bulk RNA-seq,Homo_633,AD.vs.control	6
BP	GO:0021892	cerebral cortex GABAergic interneuron differentiation	bulk RNA-seq,Homo_723,AD.vs.control; bulk RNA-seq,Homo_723,AD.vs.MCI; bulk RNA-seq,Homo_723,MCI.vs.control; bulk RNA-seq,Homo_714,AD.vs.control; bulk RNA-seq,Homo_714,AD.vs.MCI	5
BP	GO:0090190	positive regulation of branching involved in ureteric bud	bulk RNA-seq,Homo_723,AD.vs.control; bulk RNA-seq,Homo_723,AD.vs.MCI; bulk RNA-seq,Homo_723,MCI.vs.control; bulk RNA-seq,Homo_714,AD.vs.MCI	4
BP	GO:0003281	ventricular septum development	bulk RNA-seq,Homo_723,AD.vs.control; bulk RNA-seq,Homo_723,AD.vs.MCI; bulk RNA-seq,Homo_723,MCI.vs.control; bulk RNA-seq,Homo_714,AD.vs.MCI; bulk RNA-seq,Homo_714,MCI.vs.control; bulk RNA-seq,Homo_633,AD.vs.control	6
CC	GO:0005697	telomerase holoenzyme complex	bulk RNA-seq,Homo_723,AD.vs.control; bulk RNA-seq,Homo_723,AD.vs.MCI; bulk RNA-seq,Homo_723,MCI.vs.control; bulk RNA-seq,Homo_714,AD.vs.control; bulk RNA-seq,Homo_714,MCI.vs.control; bulk RNA-seq,Homo_633,AD.vs.control; bulk RNA-seq,Homo_633,AD.vs.MCI; bulk	8
BP	GO:0048672	positive regulation of collateral sprouting	bulk RNA-seq,Homo_723,AD.vs.control; bulk RNA-seq,Homo_723,AD.vs.MCI; bulk RNA-seq,Homo_723,MCI.vs.control	3
MF	GO:0008013	beta-catenin binding	bulk RNA-seq,Homo_723,AD.vs.control; bulk RNA-seq,Homo_723,AD.vs.MCI; bulk RNA-seq,Homo_723,MCI.vs.control; bulk RNA-seq,Homo_714,AD.vs.MCI; bulk RNA-seq,Homo_714,MCI.vs.control; bulk RNA-seq,Homo_633,AD.vs.control	6
BP	GO:0000184	nuclear-transcribed mRNA catabolic process, nonsense-mediated decay	bulk RNA-seq,Homo_723,AD.vs.control; bulk RNA-seq,Homo_723,AD.vs.MCI; bulk RNA-seq,Homo_714,AD.vs.control; bulk RNA-seq,Homo_633,AD.vs.control; bulk RNA-seq,Homo_633,AD.vs.MCI	5
BP	GO:0019081	viral translation	bulk RNA-seq,Homo_723,AD.vs.control; bulk RNA-seq,Homo_723,AD.vs.MCI; bulk RNA-seq,Homo_714,AD.vs.control; bulk RNA-seq,Homo_714,AD.vs.MCI; bulk RNA-seq,Homo_633,AD.vs.control; bulk RNA-seq,Homo_633,AD.vs.MCI	6
BP	GO:1904889	regulation of excitatory synapse assembly	bulk RNA-seq,Homo_723,AD.vs.control; bulk RNA-seq,Homo_723,AD.vs.MCI; bulk RNA-seq,Homo_723,MCI.vs.control; bulk RNA-seq,Homo_714,AD.vs.MCI	4
BP	GO:0035850	epithelial cell differentiation involved in kidney development	bulk RNA-seq,Homo_723,AD.vs.control; bulk RNA-seq,Homo_723,AD.vs.MCI; bulk RNA-seq,Homo_723,MCI.vs.control; bulk RNA-seq,Homo_714,AD.vs.control; bulk RNA-seq,Homo_714,AD.vs.MCI; bulk RNA-seq,Homo_714,MCI.vs.control; bulk RNA-seq,Homo_633,MCI.vs.control	7
BP	GO:0002495	antigen processing and presentation of peptide antigen via MHC class II	bulk RNA-seq,Homo_723,AD.vs.control; bulk RNA-seq,Homo_723,MCI.vs.control; bulk RNA-seq,Homo_714,AD.vs.control; bulk RNA-seq,Homo_714,MCI.vs.control; bulk RNA-seq,Homo_633,AD.vs.control; bulk RNA-seq,Homo_633,AD.vs.MCI; bulk RNA-seq,Homo_633,MCI.vs.control; scRNA-seq,SRP330776,Naive CD8+ T cell_2-AD.vs.control; scRNA-seq,SRP215507,CD8+ T cell_3-AD.vs.control; scRNA-seq,SRP215507,CD8+ T cell_3-	10
BP	GO:0009152	purine ribonucleotide biosynthetic process	bulk RNA-seq,Homo_723,AD.vs.control; bulk RNA-seq,Homo_723,MCI.vs.control; bulk RNA-seq,Homo_714,AD.vs.control; bulk RNA-seq,Homo_633,AD.vs.control; bulk RNA-seq,Homo_633,AD.vs.MCI	5
MF	GO:0042923	neuropeptide binding	bulk RNA-seq,Homo_723,AD.vs.control; bulk RNA-seq,Homo_723,AD.vs.MCI; bulk RNA-seq,Homo_714,AD.vs.control; bulk RNA-seq,Homo_714,AD.vs.MCI; bulk RNA-seq,Homo_633,MCI.vs.control	5

BP	GO:0097154	GABAergic neuron differentiation	bulk RNA-seq,Homo_723,AD.vs.control; bulk RNA-seq,Homo_723,AD.vs.MCI; bulk RNA-seq,Homo_723,MCI.vs.control; bulk RNA-seq,Homo_714,AD.vs.control; bulk RNA-seq,Homo_714,AD.vs.MCI	5
BP	GO:0000470	maturation of LSU-rRNA	bulk RNA-seq,Homo_723,AD.vs.control; bulk RNA-seq,Homo_723,MCI.vs.control; bulk RNA-seq,Homo_714,AD.vs.control; bulk RNA-seq,Homo_714,MCI.vs.control; bulk RNA-seq,Homo_633,AD.vs.control; bulk RNA-seq,Homo_633,AD.vs.MCI; bulk RNA-seq,Homo_633,MCI.vs.control	7
BP	GO:0021936	regulation of cerebellar granule cell precursor proliferation	bulk RNA-seq,Homo_723,AD.vs.control; bulk RNA-seq,Homo_723,AD.vs.MCI; bulk RNA-seq,Homo_723,MCI.vs.control; bulk RNA-seq,Homo_714,AD.vs.MCI	4
BP	GO:1900271	regulation of long-term synaptic potentiation	bulk RNA-seq,Homo_723,AD.vs.control; bulk RNA-seq,Homo_723,AD.vs.MCI; bulk RNA-seq,Homo_723,MCI.vs.control; bulk RNA-seq,Homo_714,AD.vs.control; bulk RNA-seq,Homo_714,AD.vs.MCI; bulk RNA-seq,Homo_714,MCI.vs.control; bulk RNA-seq,Homo_633,AD.vs.control; bulk	8
CC	GO:0031045	dense core granule	bulk RNA-seq,Homo_723,AD.vs.control; bulk RNA-seq,Homo_723,AD.vs.MCI; bulk RNA-seq,Homo_723,MCI.vs.control; bulk RNA-seq,Homo_714,AD.vs.control; bulk RNA-seq,Homo_714,AD.vs.MCI	5
BP	GO:1903039	positive regulation of leukocyte cell-cell adhesion	bulk RNA-seq,Homo_723,AD.vs.control; bulk RNA-seq,Homo_723,MCI.vs.control; bulk RNA-seq,Homo_714,AD.vs.control; bulk RNA-seq,Homo_714,AD.vs.MCI; bulk RNA-seq,Homo_633,AD.vs.control; bulk RNA-seq,Homo_633,AD.vs.MCI; scRNA-seq,SRP215507,CD8+ T cell_3-	8
BP	GO:0003016	respiratory system process	bulk RNA-seq,Homo_723,AD.vs.control; bulk RNA-seq,Homo_723,AD.vs.MCI; bulk RNA-seq,Homo_723,MCI.vs.control; bulk RNA-seq,Homo_714,AD.vs.MCI	4
BP	GO:0019884	antigen processing and presentation of exogenous antigen	bulk RNA-seq,Homo_723,AD.vs.control; bulk RNA-seq,Homo_723,MCI.vs.control; bulk RNA-seq,Homo_714,AD.vs.control; bulk RNA-seq,Homo_714,MCI.vs.control; bulk RNA-seq,Homo_633,AD.vs.control; bulk RNA-seq,Homo_633,AD.vs.MCI; scRNA-seq,SRP330776,Naive CD8+ T cell_2-AD.vs.control; scRNA-seq,SRP215507,CD8+ T cell_3-AD.vs.control; scRNA-seq,SRP215507,CD8+ T cell_3-MCI.vs.control	9
BP	GO:0055008	cardiac muscle tissue morphogenesis	bulk RNA-seq,Homo_723,AD.vs.control; bulk RNA-seq,Homo_723,AD.vs.MCI; bulk RNA-seq,Homo_723,MCI.vs.control; bulk RNA-seq,Homo_714,AD.vs.control; bulk RNA-seq,Homo_714,AD.vs.MCI; bulk RNA-seq,Homo_714,MCI.vs.control	6
CC	GO:0030669	clathrin-coated endocytic vesicle membrane	bulk RNA-seq,Homo_723,AD.vs.control; bulk RNA-seq,Homo_723,AD.vs.MCI; bulk RNA-seq,Homo_723,MCI.vs.control; bulk RNA-seq,Homo_714,AD.vs.MCI; bulk RNA-seq,Homo_714,MCI.vs.control; scRNA-seq,SRP309935,Dendritic cell-AD.vs.control; scRNA-seq,SRP215507,CD8+ T cell_3-AD.vs.control	7
MF	GO:0042813	Wnt receptor activity	bulk RNA-seq,Homo_723,AD.vs.control; bulk RNA-seq,Homo_723,AD.vs.MCI; bulk RNA-seq,Homo_714,AD.vs.MCI; bulk RNA-seq,SRP223445,AD.vs.control	4
BP	GO:0014902	myotube differentiation	bulk RNA-seq,Homo_723,AD.vs.control; bulk RNA-seq,Homo_723,AD.vs.MCI; bulk RNA-seq,Homo_723,MCI.vs.control; bulk RNA-seq,Homo_714,AD.vs.MCI; bulk RNA-seq,Homo_633,AD.vs.control; bulk RNA-seq,Homo_633,AD.vs.MCI	6
CC	GO:0008023	transcription elongation factor complex	bulk RNA-seq,Homo_723,AD.vs.control; bulk RNA-seq,Homo_723,MCI.vs.control; bulk RNA-seq,Homo_714,AD.vs.control; bulk RNA-seq,Homo_714,MCI.vs.control; bulk RNA-seq,Homo_633,AD.vs.control; bulk RNA-seq,Homo_633,AD.vs.MCI	6
BP	GO:0001976	nervous system process involved in regulation of systemic arterial blood pressure	bulk RNA-seq,Homo_723,AD.vs.control; bulk RNA-seq,Homo_723,AD.vs.MCI; bulk RNA-seq,Homo_714,AD.vs.MCI	3
BP	GO:0021694	cerebellar Purkinje cell layer formation	bulk RNA-seq,Homo_723,AD.vs.control; bulk RNA-seq,Homo_723,AD.vs.MCI; bulk RNA-seq,Homo_723,MCI.vs.control; bulk RNA-seq,Homo_633,AD.vs.control; bulk RNA-seq,Homo_633,AD.vs.MCI	5
BP	GO:0045214	sarcomere organization	bulk RNA-seq,Homo_723,AD.vs.control; bulk RNA-seq,Homo_723,AD.vs.MCI; bulk RNA-seq,Homo_723,MCI.vs.control; bulk RNA-seq,Homo_714,AD.vs.MCI; bulk RNA-seq,Homo_714,MCI.vs.control	5
MF	GO:0098632	cell-cell adhesion mediator activity	bulk RNA-seq,Homo_723,AD.vs.control; bulk RNA-seq,Homo_723,AD.vs.MCI; bulk RNA-seq,Homo_723,MCI.vs.control; bulk RNA-seq,Homo_714,AD.vs.MCI; bulk RNA-seq,Homo_714,MCI.vs.control	5
BP	GO:0007032	endosome organization	bulk RNA-seq,Homo_723,AD.vs.control; bulk RNA-seq,Homo_723,MCI.vs.control; bulk RNA-seq,Homo_714,MCI.vs.control; bulk RNA-seq,Homo_633,AD.vs.control; bulk RNA-seq,Homo_633,AD.vs.MCI	5
BP	GO:0051236	establishment of RNA localization	bulk RNA-seq,Homo_723,AD.vs.control; bulk RNA-seq,Homo_723,MCI.vs.control; bulk RNA-seq,Homo_714,AD.vs.control; bulk RNA-seq,Homo_633,AD.vs.control; bulk RNA-seq,Homo_633,AD.vs.MCI	5
BP	GO:0042773	ATP synthesis coupled electron transport	bulk RNA-seq,Homo_723,AD.vs.control; bulk RNA-seq,Homo_723,MCI.vs.control; bulk RNA-seq,Homo_714,AD.vs.control; bulk RNA-seq,Homo_714,MCI.vs.control; bulk RNA-seq,Homo_633,AD.vs.control; bulk RNA-seq,Homo_633,AD.vs.MCI	6
BP	GO:0042775	mitochondrial ATP synthesis coupled electron transport	bulk RNA-seq,Homo_723,AD.vs.control; bulk RNA-seq,Homo_723,MCI.vs.control; bulk RNA-seq,Homo_714,AD.vs.control; bulk RNA-seq,Homo_714,MCI.vs.control; bulk RNA-seq,Homo_633,AD.vs.control; bulk RNA-seq,Homo_633,AD.vs.MCI	6
BP	GO:0006720	isoprenoid metabolic process	bulk RNA-seq,Homo_723,AD.vs.control; bulk RNA-seq,Homo_723,AD.vs.MCI; bulk RNA-seq,Homo_723,MCI.vs.control; bulk RNA-seq,Homo_714,AD.vs.control; bulk RNA-seq,Homo_714,AD.vs.MCI; bulk RNA-seq,Homo_714,MCI.vs.control; bulk RNA-seq,Homo_633,AD.vs.control; bulk	8
BP	GO:0048488	synaptic vesicle endocytosis	bulk RNA-seq,Homo_723,AD.vs.control; bulk RNA-seq,Homo_723,AD.vs.MCI; bulk RNA-seq,Homo_723,MCI.vs.control; bulk RNA-seq,Homo_714,AD.vs.MCI; bulk RNA-seq,Homo_714,MCI.vs.control; bulk RNA-seq,Homo_633,AD.vs.control; bulk RNA-seq,Homo_633,AD.vs.MCI	7
BP	GO:0140238	presynaptic endocytosis	bulk RNA-seq,Homo_723,AD.vs.control; bulk RNA-seq,Homo_723,AD.vs.MCI; bulk RNA-seq,Homo_723,MCI.vs.control; bulk RNA-seq,Homo_714,AD.vs.MCI; bulk RNA-seq,Homo_714,MCI.vs.control; bulk RNA-seq,Homo_633,AD.vs.control; bulk RNA-seq,Homo_633,AD.vs.MCI	7
BP	GO:0002707	negative regulation of lymphocyte mediated immunity	bulk RNA-seq,Homo_723,AD.vs.control; bulk RNA-seq,Homo_723,MCI.vs.control; bulk RNA-seq,Homo_714,AD.vs.control; bulk RNA-seq,Homo_714,MCI.vs.control; bulk RNA-seq,Homo_633,AD.vs.control; bulk RNA-seq,Homo_633,AD.vs.MCI	6
MF	GO:0035254	glutamate receptor binding	bulk RNA-seq,Homo_723,AD.vs.control; bulk RNA-seq,Homo_723,AD.vs.MCI; bulk RNA-seq,Homo_723,MCI.vs.control; bulk RNA-seq,Homo_714,AD.vs.MCI; bulk RNA-seq,Homo_714,MCI.vs.control	5
CC	GO:0060205	cytoplasmic vesicle lumen	bulk RNA-seq,Homo_723,AD.vs.control; bulk RNA-seq,Homo_723,AD.vs.MCI; bulk RNA-seq,Homo_723,MCI.vs.control; bulk RNA-seq,Homo_714,AD.vs.control; bulk RNA-seq,Homo_714,AD.vs.MCI; bulk RNA-seq,Homo_633,AD.vs.control; bulk RNA-seq,Homo_633,AD.vs.MCI; bulk RNA-seq,MCSA,MCI.vs.control; bulk RNA-seq,SRP223445,AD.vs.control	9
BP	GO:0046620	regulation of organ growth	bulk RNA-seq,Homo_723,AD.vs.control; bulk RNA-seq,Homo_723,AD.vs.MCI; bulk RNA-seq,Homo_723,MCI.vs.control; bulk RNA-seq,Homo_714,AD.vs.MCI; bulk RNA-seq,Homo_714,MCI.vs.control	5
BP	GO:0034332	adherens junction organization	bulk RNA-seq,Homo_723,AD.vs.control; bulk RNA-seq,Homo_723,AD.vs.MCI; bulk RNA-seq,Homo_723,MCI.vs.control; bulk RNA-seq,Homo_714,AD.vs.MCI; bulk RNA-seq,Homo_714,MCI.vs.control	5
BP	GO:0021559	trigeminal nerve development	bulk RNA-seq,Homo_723,AD.vs.control; bulk RNA-seq,Homo_723,AD.vs.MCI; bulk RNA-seq,Homo_723,MCI.vs.control; bulk RNA-seq,Homo_714,AD.vs.control; bulk RNA-seq,Homo_714,AD.vs.MCI	5
CC	GO:0000151	ubiquitin ligase complex	bulk RNA-seq,Homo_723,AD.vs.control; bulk RNA-seq,Homo_723,MCI.vs.control; bulk RNA-seq,Homo_714,AD.vs.control; bulk RNA-seq,Homo_633,AD.vs.control; bulk RNA-seq,Homo_633,AD.vs.MCI	5
BP	GO:0003170	heart valve development	bulk RNA-seq,Homo_723,AD.vs.control; bulk RNA-seq,Homo_723,AD.vs.MCI; bulk RNA-seq,Homo_723,MCI.vs.control; bulk RNA-seq,Homo_714,AD.vs.MCI; bulk RNA-seq,Homo_714,MCI.vs.control; bulk RNA-seq,SRP223445,AD.vs.control	6
BP	GO:0030239	myofibril assembly	bulk RNA-seq,Homo_723,AD.vs.control; bulk RNA-seq,Homo_723,AD.vs.MCI; bulk RNA-seq,Homo_723,MCI.vs.control; bulk RNA-seq,Homo_714,AD.vs.MCI; bulk RNA-seq,Homo_714,MCI.vs.control; bulk RNA-seq,Homo_633,AD.vs.control	6

BP	GO:0000028	ribosomal small subunit assembly	bulk RNA-seq,Homo_723,AD.vs.control; bulk RNA-seq,Homo_723,AD.vs.MCI; bulk RNA-seq,Homo_723,MCI.vs.control; bulk RNA-seq,Homo_714,AD.vs.control; bulk RNA-seq,Homo_714,MCI.vs.control; bulk RNA-seq,Homo_633,AD.vs.control; bulk RNA-seq,Homo_633,AD.vs.MCI; bulk	8
BP	GO:0019882	antigen processing and presentation	bulk RNA-seq,Homo_723,AD.vs.control; bulk RNA-seq,Homo_723,MCI.vs.control; bulk RNA-seq,Homo_714,AD.vs.control; bulk RNA-seq,Homo_714,MCI.vs.control; bulk RNA-seq,Homo_633,AD.vs.control; bulk RNA-seq,Homo_633,AD.vs.MCI; scRNA-seq,SRP215507,CD8+ T cell_3-	7
BP	GO:0061035	regulation of cartilage development	bulk RNA-seq,Homo_723,AD.vs.control; bulk RNA-seq,Homo_723,AD.vs.MCI; bulk RNA-seq,Homo_723,MCI.vs.control; bulk RNA-seq,Homo_714,AD.vs.MCI; bulk RNA-seq,Homo_714,MCI.vs.control; bulk RNA-seq,Homo_633,AD.vs.control; bulk RNA-seq,Homo_633,AD.vs.MCI	7
BP	GO:2000463	positive regulation of excitatory postsynaptic potential	bulk RNA-seq,Homo_723,AD.vs.control; bulk RNA-seq,Homo_723,AD.vs.MCI; bulk RNA-seq,Homo_723,MCI.vs.control; bulk RNA-seq,Homo_714,AD.vs.control; bulk RNA-seq,Homo_714,AD.vs.MCI; bulk RNA-seq,Homo_714,MCI.vs.control; bulk RNA-seq,ROSMAP,AD.vs.control; bulk	8
BP	GO:1901379	regulation of potassium ion transmembrane transport	bulk RNA-seq,Homo_723,AD.vs.control; bulk RNA-seq,Homo_723,AD.vs.MCI; bulk RNA-seq,Homo_714,AD.vs.MCI	3
BP	GO:0050684	regulation of mRNA processing	bulk RNA-seq,Homo_723,AD.vs.control; bulk RNA-seq,Homo_723,MCI.vs.control; bulk RNA-seq,Homo_714,AD.vs.control; bulk RNA-seq,Homo_633,AD.vs.control; bulk RNA-seq,Homo_633,AD.vs.MCI	5
BP	GO:0021587	cerebellum morphogenesis	bulk RNA-seq,Homo_723,AD.vs.control; bulk RNA-seq,Homo_723,AD.vs.MCI; bulk RNA-seq,Homo_723,MCI.vs.control; bulk RNA-seq,Homo_714,AD.vs.MCI; bulk RNA-seq,Homo_714,MCI.vs.control; bulk RNA-seq,Homo_633,AD.vs.control; bulk RNA-seq,Homo_633,AD.vs.MCI	7
BP	GO:0000288	nuclear-transcribed mRNA catabolic process, deadenylation-dependent decay	bulk RNA-seq,Homo_723,AD.vs.control; bulk RNA-seq,Homo_714,AD.vs.control; bulk RNA-seq,Homo_714,MCI.vs.control; bulk RNA-seq,Homo_633,AD.vs.control; bulk RNA-seq,Homo_633,AD.vs.MCI	5
BP	GO:0051155	positive regulation of striated muscle cell differentiation	bulk RNA-seq,Homo_723,AD.vs.control; bulk RNA-seq,Homo_723,AD.vs.MCI; bulk RNA-seq,Homo_723,MCI.vs.control; bulk RNA-seq,Homo_714,AD.vs.control; bulk RNA-seq,Homo_714,AD.vs.MCI	5
BP	GO:0006282	regulation of DNA repair	bulk RNA-seq,Homo_723,AD.vs.control; bulk RNA-seq,Homo_723,MCI.vs.control; bulk RNA-seq,Homo_714,AD.vs.control; bulk RNA-seq,Homo_633,AD.vs.control; bulk RNA-seq,Homo_633,AD.vs.MCI	5
BP	GO:0002714	positive regulation of B cell mediated immunity	bulk RNA-seq,Homo_723,AD.vs.control; bulk RNA-seq,Homo_633,AD.vs.control; bulk RNA-seq,Homo_633,AD.vs.MCI	3
BP	GO:0002891	positive regulation of immunoglobulin mediated immune	bulk RNA-seq,Homo_723,AD.vs.control; bulk RNA-seq,Homo_633,AD.vs.control; bulk RNA-seq,Homo_633,AD.vs.MCI	3
BP	GO:0061037	negative regulation of cartilage development	bulk RNA-seq,Homo_723,AD.vs.control; bulk RNA-seq,Homo_723,AD.vs.MCI; bulk RNA-seq,Homo_723,MCI.vs.control; bulk RNA-seq,Homo_714,AD.vs.MCI; bulk RNA-seq,Homo_714,MCI.vs.control	5
BP	GO:0002230	positive regulation of defense response to virus by host	bulk RNA-seq,Homo_723,AD.vs.control; bulk RNA-seq,Homo_723,AD.vs.MCI; bulk RNA-seq,Homo_723,MCI.vs.control; bulk RNA-seq,Homo_714,AD.vs.control; bulk RNA-seq,Homo_714,MCI.vs.control; bulk RNA-seq,Homo_633,AD.vs.control; bulk RNA-seq,Homo_633,MCI.vs.control	7
BP	GO:0021846	cell proliferation in forebrain	bulk RNA-seq,Homo_723,AD.vs.control; bulk RNA-seq,Homo_723,AD.vs.MCI; bulk RNA-seq,Homo_723,MCI.vs.control; bulk RNA-seq,Homo_714,AD.vs.MCI	4
CC	GO:0009295	nucleoid	bulk RNA-seq,Homo_723,AD.vs.control; bulk RNA-seq,Homo_723,MCI.vs.control; bulk RNA-seq,Homo_714,AD.vs.control; bulk RNA-seq,Homo_714,MCI.vs.control; bulk RNA-seq,Homo_633,AD.vs.control; bulk RNA-seq,Homo_633,AD.vs.MCI	6
CC	GO:0042645	mitochondrial nucleoid	bulk RNA-seq,Homo_723,AD.vs.control; bulk RNA-seq,Homo_723,MCI.vs.control; bulk RNA-seq,Homo_714,AD.vs.control; bulk RNA-seq,Homo_714,MCI.vs.control; bulk RNA-seq,Homo_633,AD.vs.control; bulk RNA-seq,Homo_633,AD.vs.MCI	6
BP	GO:0002053	positive regulation of mesenchymal cell proliferation	bulk RNA-seq,Homo_723,AD.vs.control; bulk RNA-seq,Homo_723,AD.vs.MCI; bulk RNA-seq,Homo_723,MCI.vs.control; bulk RNA-seq,Homo_714,AD.vs.MCI; bulk RNA-seq,Homo_714,MCI.vs.control	5
BP	GO:0010821	regulation of mitochondrion organization	bulk RNA-seq,Homo_723,AD.vs.control; bulk RNA-seq,Homo_723,MCI.vs.control; bulk RNA-seq,Homo_714,AD.vs.control; bulk RNA-seq,Homo_633,AD.vs.control; bulk RNA-seq,Homo_633,AD.vs.MCI	5
BP	GO:0010226	response to lithium ion	bulk RNA-seq,Homo_723,AD.vs.control; bulk RNA-seq,Homo_723,AD.vs.MCI; bulk RNA-seq,Homo_723,MCI.vs.control; bulk RNA-seq,Homo_714,AD.vs.control; bulk RNA-seq,Homo_714,AD.vs.MCI; bulk RNA-seq,Homo_714,MCI.vs.control	6
BP	GO:0098760	response to interleukin-7	bulk RNA-seq,Homo_723,AD.vs.control; bulk RNA-seq,Homo_723,MCI.vs.control; bulk RNA-seq,Homo_714,AD.vs.control; bulk RNA-seq,Homo_714,MCI.vs.control; bulk RNA-seq,Homo_633,AD.vs.control; bulk RNA-seq,Homo_633,AD.vs.MCI; bulk RNA-seq,Homo_633,MCI.vs.control	7
BP	GO:0098761	cellular response to interleukin-7	bulk RNA-seq,Homo_723,AD.vs.control; bulk RNA-seq,Homo_723,MCI.vs.control; bulk RNA-seq,Homo_714,AD.vs.control; bulk RNA-seq,Homo_714,MCI.vs.control; bulk RNA-seq,Homo_633,AD.vs.control; bulk RNA-seq,Homo_633,AD.vs.MCI; bulk RNA-seq,Homo_633,MCI.vs.control	7
MF	GO:0005272	sodium channel activity	bulk RNA-seq,Homo_723,AD.vs.control; bulk RNA-seq,Homo_723,AD.vs.MCI; bulk RNA-seq,Homo_723,MCI.vs.control; bulk RNA-seq,Homo_714,AD.vs.control; bulk RNA-seq,Homo_714,AD.vs.MCI; bulk RNA-seq,Homo_714,MCI.vs.control; bulk RNA-seq,ROSMAP,AD.vs.control; bulk	8
CC	GO:0033268	node of Ranvier	bulk RNA-seq,Homo_723,AD.vs.control; bulk RNA-seq,Homo_723,AD.vs.MCI; bulk RNA-seq,Homo_723,MCI.vs.control; bulk RNA-seq,Homo_714,AD.vs.MCI	4
BP	GO:0048002	antigen processing and presentation of peptide antigen	bulk RNA-seq,Homo_723,AD.vs.control; bulk RNA-seq,Homo_723,MCI.vs.control; bulk RNA-seq,Homo_714,AD.vs.control; bulk RNA-seq,Homo_714,MCI.vs.control; bulk RNA-seq,Homo_633,AD.vs.control; bulk RNA-seq,Homo_633,AD.vs.MCI; scRNA-seq,SRP215507,CD8+ T cell_4-	7
MF	GO:0016918	retinal binding	bulk RNA-seq,Homo_723,AD.vs.control; bulk RNA-seq,Homo_723,AD.vs.MCI; bulk RNA-seq,Homo_723,MCI.vs.control; bulk RNA-seq,Homo_714,AD.vs.control; bulk RNA-seq,Homo_714,AD.vs.MCI; bulk RNA-seq,SRP223445,AD.vs.control	6
BP	GO:0021516	dorsal spinal cord development	bulk RNA-seq,Homo_723,AD.vs.control; bulk RNA-seq,Homo_723,AD.vs.MCI; bulk RNA-seq,Homo_723,MCI.vs.control; bulk RNA-seq,Homo_714,AD.vs.control; bulk RNA-seq,Homo_714,AD.vs.MCI; bulk RNA-seq,SRP223445,AD.vs.control	6
BP	GO:0030325	adrenal gland development	bulk RNA-seq,Homo_723,AD.vs.control; bulk RNA-seq,Homo_723,AD.vs.MCI; bulk RNA-seq,Homo_723,MCI.vs.control; bulk RNA-seq,Homo_714,AD.vs.MCI; bulk RNA-seq,Homo_714,MCI.vs.control; bulk RNA-seq,Homo_633,AD.vs.control; bulk RNA-seq,Homo_633,AD.vs.MCI	7
BP	GO:0006490	oligosaccharide-lipid intermediate biosynthetic process	bulk RNA-seq,Homo_723,AD.vs.control; bulk RNA-seq,Homo_723,AD.vs.MCI; bulk RNA-seq,Homo_714,AD.vs.control; bulk RNA-seq,Homo_714,AD.vs.MCI; bulk RNA-seq,Homo_633,AD.vs.control; bulk RNA-seq,Homo_633,AD.vs.MCI	6
BP	GO:0021783	preganglionic parasympathetic fiber development	bulk RNA-seq,Homo_723,AD.vs.control; bulk RNA-seq,Homo_723,AD.vs.MCI; bulk RNA-seq,Homo_723,MCI.vs.control; bulk RNA-seq,Homo_714,AD.vs.MCI	4
BP	GO:0031223	auditory behavior	bulk RNA-seq,Homo_723,AD.vs.control; bulk RNA-seq,Homo_723,AD.vs.MCI; bulk RNA-seq,Homo_723,MCI.vs.control; bulk RNA-seq,Homo_714,AD.vs.control; bulk RNA-seq,Homo_714,AD.vs.MCI; bulk RNA-seq,ROSMAP,MCI.vs.control	6
BP	GO:0030166	proteoglycan biosynthetic process	bulk RNA-seq,Homo_723,AD.vs.control; bulk RNA-seq,Homo_723,AD.vs.MCI; bulk RNA-seq,Homo_723,MCI.vs.control; bulk RNA-seq,Homo_714,AD.vs.MCI; bulk RNA-seq,Homo_714,MCI.vs.control; bulk RNA-seq,Homo_633,AD.vs.control; bulk RNA-seq,Homo_633,AD.vs.MCI	7
BP	GO:0006488	dolichol-linked oligosaccharide biosynthetic process	bulk RNA-seq,Homo_723,AD.vs.control; bulk RNA-seq,Homo_723,AD.vs.MCI; bulk RNA-seq,Homo_714,AD.vs.control; bulk RNA-seq,Homo_714,AD.vs.MCI; bulk RNA-seq,Homo_633,AD.vs.control; bulk RNA-seq,Homo_633,AD.vs.MCI	6
BP	GO:0055006	cardiac cell development	bulk RNA-seq,Homo_723,AD.vs.control; bulk RNA-seq,Homo_723,AD.vs.MCI; bulk RNA-seq,Homo_723,MCI.vs.control; bulk RNA-seq,Homo_714,AD.vs.control; bulk RNA-seq,Homo_714,AD.vs.MCI; bulk RNA-seq,Homo_714,MCI.vs.control	6
BP	GO:0001504	neurotransmitter uptake	bulk RNA-seq,Homo_723,AD.vs.control; bulk RNA-seq,Homo_723,AD.vs.MCI; bulk RNA-seq,Homo_723,MCI.vs.control; bulk RNA-seq,Homo_714,AD.vs.control; bulk RNA-seq,Homo_714,AD.vs.MCI; bulk RNA-seq,ROSMAP,AD.vs.control; bulk RNA-seq,ROSMAP,MCI.vs.control	7

MF	GO:0000993	RNA polymerase II complex binding	bulk RNA-seq,Homo_723,AD.vs.control; bulk RNA-seq,Homo_723,MCI.vs.control; bulk RNA-seq,Homo_714,AD.vs.control; bulk RNA-seq,Homo_714,MCI.vs.control; bulk RNA-seq,Homo_633,AD.vs.control; bulk RNA-seq,Homo_633,AD.vs.MCI	6
CC	GO:0101031	chaperone complex	bulk RNA-seq,Homo_723,AD.vs.control; bulk RNA-seq,Homo_723,MCI.vs.control; bulk RNA-seq,Homo_714,AD.vs.control; bulk RNA-seq,Homo_714,MCI.vs.control; bulk RNA-seq,Homo_633,AD.vs.control; bulk RNA-seq,Homo_633,AD.vs.MCI	6
CC	GO:0034774	secretory granule lumen	bulk RNA-seq,Homo_723,AD.vs.control; bulk RNA-seq,Homo_723,AD.vs.MCI; bulk RNA-seq,Homo_723,MCI.vs.control; bulk RNA-seq,Homo_714,AD.vs.control; bulk RNA-seq,Homo_714,AD.vs.MCI; bulk RNA-seq,Homo_633,AD.vs.control; bulk RNA-seq,Homo_633,AD.vs.MCI; bulk RNA-seq,MCSA,MCI.vs.control; bulk RNA-seq,SRP223445,AD.vs.control	9
MF	GO:0046943	carboxylic acid transmembrane transporter activity	bulk RNA-seq,Homo_723,AD.vs.control; bulk RNA-seq,Homo_723,AD.vs.MCI; bulk RNA-seq,Homo_723,MCI.vs.control; bulk RNA-seq,Homo_714,AD.vs.control; bulk RNA-seq,Homo_714,AD.vs.MCI; bulk RNA-seq,Homo_633,AD.vs.control; bulk RNA-seq,Homo_633,AD.vs.MCI	7
BP	GO:0072202	cell differentiation involved in metanephros development	bulk RNA-seq,Homo_723,AD.vs.control; bulk RNA-seq,Homo_723,AD.vs.MCI; bulk RNA-seq,Homo_723,MCI.vs.control; bulk RNA-seq,Homo_714,AD.vs.control; bulk RNA-seq,Homo_714,AD.vs.MCI; bulk RNA-seq,Homo_714,MCI.vs.control; bulk RNA-seq,Homo_633,MCI.vs.control	7
BP	GO:0015807	L-amino acid transport	bulk RNA-seq,Homo_723,AD.vs.control; bulk RNA-seq,Homo_723,AD.vs.MCI; bulk RNA-seq,Homo_723,MCI.vs.control; bulk RNA-seq,Homo_714,AD.vs.MCI; bulk RNA-seq,Homo_633,AD.vs.control; bulk RNA-seq,Homo_633,AD.vs.MCI	6
MF	GO:0005104	fibroblast growth factor receptor binding	bulk RNA-seq,Homo_723,AD.vs.control; bulk RNA-seq,Homo_723,AD.vs.MCI; bulk RNA-seq,Homo_723,MCI.vs.control; bulk RNA-seq,Homo_714,AD.vs.control; bulk RNA-seq,Homo_714,AD.vs.MCI	5
BP	GO:0031102	neuron projection regeneration	bulk RNA-seq,Homo_723,AD.vs.control; bulk RNA-seq,Homo_723,AD.vs.MCI; bulk RNA-seq,Homo_723,MCI.vs.control; bulk RNA-seq,Homo_714,AD.vs.MCI; bulk RNA-seq,Homo_714,MCI.vs.control; bulk RNA-seq,Homo_633,AD.vs.control	6
BP	GO:0072010	glomerular epithelium development	bulk RNA-seq,Homo_723,AD.vs.control; bulk RNA-seq,Homo_723,AD.vs.MCI; bulk RNA-seq,Homo_723,MCI.vs.control; bulk RNA-seq,Homo_714,AD.vs.MCI	4
BP	GO:0043407	negative regulation of MAP kinase activity	bulk RNA-seq,Homo_723,AD.vs.control; bulk RNA-seq,Homo_723,AD.vs.MCI; bulk RNA-seq,Homo_723,MCI.vs.control; bulk RNA-seq,Homo_714,AD.vs.MCI; bulk RNA-seq,Homo_714,MCI.vs.control; bulk RNA-seq,Homo_633,AD.vs.control	6
MF	GO:0004993	G protein-coupled serotonin receptor activity	bulk RNA-seq,Homo_723,AD.vs.control; bulk RNA-seq,Homo_723,AD.vs.MCI; bulk RNA-seq,Homo_714,AD.vs.control; bulk RNA-seq,Homo_714,AD.vs.MCI; bulk RNA-seq,Homo_633,MCI.vs.control	5
MF	GO:0099589	serotonin receptor activity	bulk RNA-seq,Homo_723,AD.vs.control; bulk RNA-seq,Homo_723,AD.vs.MCI; bulk RNA-seq,Homo_714,AD.vs.control; bulk RNA-seq,Homo_714,AD.vs.MCI; bulk RNA-seq,Homo_633,MCI.vs.control	5
MF	GO:0005184	neuropeptide hormone activity	bulk RNA-seq,Homo_723,AD.vs.control; bulk RNA-seq,Homo_723,AD.vs.MCI; bulk RNA-seq,Homo_723,MCI.vs.control; bulk RNA-seq,Homo_714,AD.vs.control; bulk RNA-seq,Homo_714,AD.vs.MCI	5
CC	GO:0070603	SWI/SNF superfamily-type complex	bulk RNA-seq,Homo_723,AD.vs.control; bulk RNA-seq,Homo_723,MCI.vs.control; bulk RNA-seq,Homo_714,AD.vs.control; bulk RNA-seq,Homo_714,MCI.vs.control; bulk RNA-seq,Homo_633,AD.vs.control; bulk RNA-seq,Homo_633,AD.vs.MCI	6
BP	GO:0048730	epidermis morphogenesis	bulk RNA-seq,Homo_723,AD.vs.control; bulk RNA-seq,Homo_723,AD.vs.MCI; bulk RNA-seq,Homo_723,MCI.vs.control; bulk RNA-seq,Homo_714,AD.vs.control; bulk RNA-seq,Homo_714,AD.vs.MCI	5
BP	GO:0055010	ventricular cardiac muscle tissue morphogenesis	bulk RNA-seq,Homo_723,AD.vs.control; bulk RNA-seq,Homo_723,AD.vs.MCI; bulk RNA-seq,Homo_723,MCI.vs.control; bulk RNA-seq,Homo_714,AD.vs.control; bulk RNA-seq,Homo_714,AD.vs.MCI; bulk RNA-seq,Homo_714,MCI.vs.control; bulk RNA-seq,Homo_633,AD.vs.control; scRNA-	8
MF	GO:0033612	receptor serine/threonine kinase binding	bulk RNA-seq,Homo_723,AD.vs.control; bulk RNA-seq,Homo_723,AD.vs.MCI; bulk RNA-seq,Homo_723,MCI.vs.control; bulk RNA-seq,Homo_714,AD.vs.control; bulk RNA-seq,Homo_714,AD.vs.MCI; bulk RNA-seq,Homo_714,MCI.vs.control	6
BP	GO:0060996	dendritic spine development	bulk RNA-seq,Homo_723,AD.vs.control; bulk RNA-seq,Homo_723,AD.vs.MCI; bulk RNA-seq,Homo_723,MCI.vs.control; bulk RNA-seq,Homo_714,AD.vs.MCI; bulk RNA-seq,Homo_714,MCI.vs.control; bulk RNA-seq,Homo_633,AD.vs.control; bulk RNA-seq,Homo_633,AD.vs.MCI; scRNA-seq,SRP330776,Naive CD8+ T	8
BP	GO:0060271	cilium assembly	bulk RNA-seq,Homo_723,AD.vs.control; bulk RNA-seq,Homo_723,AD.vs.MCI; bulk RNA-seq,Homo_714,AD.vs.MCI; bulk RNA-seq,Homo_633,AD.vs.control; bulk RNA-seq,Homo_633,AD.vs.MCI; bulk RNA-seq,ROSMAP,AD.vs.control; bulk RNA-seq,ROSMAP,MCI.vs.control	7
CC	GO:0043083	synaptic cleft	bulk RNA-seq,Homo_723,AD.vs.control; bulk RNA-seq,Homo_723,AD.vs.MCI; bulk RNA-seq,Homo_723,MCI.vs.control; bulk RNA-seq,Homo_714,AD.vs.control; bulk RNA-seq,Homo_714,AD.vs.MCI	5
MF	GO:0003713	transcription coactivator activity	bulk RNA-seq,Homo_723,AD.vs.control; bulk RNA-seq,Homo_723,MCI.vs.control; bulk RNA-seq,Homo_714,AD.vs.control; bulk RNA-seq,Homo_633,AD.vs.control; bulk RNA-seq,Homo_633,AD.vs.MCI	5
MF	GO:0008170	N-methyltransferase activity	bulk RNA-seq,Homo_723,AD.vs.control; bulk RNA-seq,Homo_723,MCI.vs.control; bulk RNA-seq,Homo_714,AD.vs.control; bulk RNA-seq,Homo_714,MCI.vs.control; bulk RNA-seq,Homo_633,AD.vs.control; bulk RNA-seq,Homo_633,AD.vs.MCI	6
BP	GO:0006744	ubiquinone biosynthetic process	bulk RNA-seq,Homo_723,AD.vs.control; bulk RNA-seq,Homo_723,AD.vs.MCI; bulk RNA-seq,Homo_714,AD.vs.control; bulk RNA-seq,Homo_714,AD.vs.MCI; bulk RNA-seq,Homo_633,AD.vs.control; bulk RNA-seq,Homo_633,AD.vs.MCI	6
BP	GO:1901663	quinone biosynthetic process	bulk RNA-seq,Homo_723,AD.vs.control; bulk RNA-seq,Homo_723,AD.vs.MCI; bulk RNA-seq,Homo_714,AD.vs.control; bulk RNA-seq,Homo_714,AD.vs.MCI; bulk RNA-seq,Homo_633,AD.vs.control; bulk RNA-seq,Homo_633,AD.vs.MCI	6
BP	GO:0050926	regulation of positive chemotaxis	bulk RNA-seq,Homo_723,AD.vs.control; bulk RNA-seq,Homo_723,AD.vs.MCI; bulk RNA-seq,Homo_723,MCI.vs.control; bulk RNA-seq,Homo_714,AD.vs.control; bulk RNA-seq,Homo_714,AD.vs.MCI; bulk RNA-seq,Homo_714,MCI.vs.control	6
BP	GO:0007204	positive regulation of cytosolic calcium ion concentration	bulk RNA-seq,Homo_723,AD.vs.control; bulk RNA-seq,Homo_723,AD.vs.MCI; bulk RNA-seq,Homo_723,MCI.vs.control; bulk RNA-seq,Homo_714,AD.vs.control; bulk RNA-seq,Homo_714,AD.vs.MCI; bulk RNA-seq,Homo_633,AD.vs.control; bulk RNA-seq,Homo_633,AD.vs.MCI	7
BP	GO:0035767	endothelial cell chemotaxis	bulk RNA-seq,Homo_723,AD.vs.control; bulk RNA-seq,Homo_723,AD.vs.MCI; bulk RNA-seq,Homo_723,MCI.vs.control; bulk RNA-seq,Homo_714,AD.vs.control; bulk RNA-seq,Homo_714,AD.vs.MCI; bulk RNA-seq,Homo_714,MCI.vs.control	6
CC	GO:0031527	filopodium membrane	bulk RNA-seq,Homo_723,AD.vs.control; bulk RNA-seq,Homo_723,AD.vs.MCI; bulk RNA-seq,Homo_723,MCI.vs.control; bulk RNA-seq,Homo_714,AD.vs.control; bulk RNA-seq,Homo_714,AD.vs.MCI; bulk RNA-seq,Homo_714,MCI.vs.control	6
BP	GO:0002228	natural killer cell mediated immunity	bulk RNA-seq,Homo_723,AD.vs.control; bulk RNA-seq,Homo_714,AD.vs.control; bulk RNA-seq,Homo_714,MCI.vs.control; bulk RNA-seq,Homo_633,AD.vs.control; bulk RNA-seq,Homo_633,AD.vs.MCI; scRNA-seq,SRP309935,B cell_1-AD.vs.control	6
BP	GO:0071027	nuclear RNA surveillance	bulk RNA-seq,Homo_723,AD.vs.control; bulk RNA-seq,Homo_723,AD.vs.MCI; bulk RNA-seq,Homo_714,AD.vs.control; bulk RNA-seq,Homo_714,AD.vs.MCI; bulk RNA-seq,Homo_633,AD.vs.control; bulk RNA-seq,Homo_633,AD.vs.MCI	6
BP	GO:0000394	RNA splicing, via endonucleolytic cleavage and ligation	bulk RNA-seq,Homo_723,AD.vs.control; bulk RNA-seq,Homo_723,AD.vs.MCI; bulk RNA-seq,Homo_723,MCI.vs.control; bulk RNA-seq,Homo_714,AD.vs.control; bulk RNA-seq,Homo_714,MCI.vs.control; bulk RNA-seq,Homo_633,AD.vs.control; bulk RNA-seq,Homo_633,AD.vs.MCI; bulk	8
BP	GO:0000291	nuclear-transcribed mRNA catabolic process, exonucleolytic	bulk RNA-seq,Homo_723,AD.vs.control; bulk RNA-seq,Homo_723,AD.vs.MCI; bulk RNA-seq,Homo_714,AD.vs.control; bulk RNA-seq,Homo_714,AD.vs.MCI; bulk RNA-seq,Homo_633,AD.vs.control; bulk RNA-seq,Homo_633,AD.vs.MCI	6
MF	GO:0061578	Lys63-specific deubiquitinase activity	bulk RNA-seq,Homo_723,AD.vs.control; bulk RNA-seq,Homo_723,AD.vs.MCI; bulk RNA-seq,Homo_723,MCI.vs.control; bulk RNA-seq,Homo_714,AD.vs.control; bulk RNA-seq,Homo_714,AD.vs.MCI; bulk RNA-seq,Homo_714,MCI.vs.control; bulk RNA-seq,Homo_633,AD.vs.control; bulk	8

BP	GO:0031018	endocrine pancreas development	bulk RNA-seq,Homo_723,AD.vs.control; bulk RNA-seq,Homo_723,AD.vs.MCI; bulk RNA-seq,Homo_723,MCI.vs.control; bulk RNA-seq,Homo_714,AD.vs.MCI; bulk RNA-seq,Homo_633,MCI.vs.control	5
BP	GO:1990090	cellular response to nerve growth factor stimulus	bulk RNA-seq,Homo_723,AD.vs.control; bulk RNA-seq,Homo_723,AD.vs.MCI; bulk RNA-seq,Homo_723,MCI.vs.control; bulk RNA-seq,Homo_714,AD.vs.MCI	4
BP	GO:0002088	lens development in camera-type eye	bulk RNA-seq,Homo_723,AD.vs.control; bulk RNA-seq,Homo_723,AD.vs.MCI; bulk RNA-seq,Homo_723,MCI.vs.control; bulk RNA-seq,Homo_714,AD.vs.MCI; bulk RNA-seq,Homo_714,MCI.vs.control; bulk RNA-seq,Homo_633,AD.vs.control	6
BP	GO:0071867	response to monoamine	bulk RNA-seq,Homo_723,AD.vs.control; bulk RNA-seq,Homo_723,AD.vs.MCI; bulk RNA-seq,Homo_723,MCI.vs.control; bulk RNA-seq,Homo_714,AD.vs.control; bulk RNA-seq,Homo_714,AD.vs.MCI; bulk RNA-seq,Homo_714,MCI.vs.control; bulk RNA-seq,Homo_633,AD.vs.control; bulk	8
BP	GO:0071869	response to catecholamine	bulk RNA-seq,Homo_723,AD.vs.control; bulk RNA-seq,Homo_723,AD.vs.MCI; bulk RNA-seq,Homo_723,MCI.vs.control; bulk RNA-seq,Homo_714,AD.vs.control; bulk RNA-seq,Homo_714,AD.vs.MCI; bulk RNA-seq,Homo_714,MCI.vs.control; bulk RNA-seq,Homo_633,AD.vs.control; bulk	8
BP	GO:0007219	Notch signaling pathway	bulk RNA-seq,Homo_723,AD.vs.control; bulk RNA-seq,Homo_723,AD.vs.MCI; bulk RNA-seq,Homo_723,MCI.vs.control; bulk RNA-seq,Homo_714,AD.vs.control; bulk RNA-seq,Homo_714,AD.vs.MCI; bulk RNA-seq,Homo_633,AD.vs.control; bulk RNA-seq,Homo_633,AD.vs.MCI; bulk RNA-	8
BP	GO:0010996	response to auditory stimulus	bulk RNA-seq,Homo_723,AD.vs.control; bulk RNA-seq,Homo_723,AD.vs.MCI; bulk RNA-seq,Homo_723,MCI.vs.control; bulk RNA-seq,Homo_714,AD.vs.MCI	4
BP	GO:0016078	tRNA catabolic process	bulk RNA-seq,Homo_723,AD.vs.control; bulk RNA-seq,Homo_723,AD.vs.MCI; bulk RNA-seq,Homo_714,AD.vs.control; bulk RNA-seq,Homo_714,AD.vs.MCI; bulk RNA-seq,Homo_633,AD.vs.control; bulk RNA-seq,Homo_633,AD.vs.MCI	6
BP	GO:0018209	peptidyl-serine modification	bulk RNA-seq,Homo_723,AD.vs.control; bulk RNA-seq,Homo_723,AD.vs.MCI; bulk RNA-seq,Homo_723,MCI.vs.control; bulk RNA-seq,Homo_714,AD.vs.control; bulk RNA-seq,Homo_714,AD.vs.MCI; bulk RNA-seq,Homo_633,AD.vs.control; bulk RNA-seq,Homo_633,AD.vs.MCI	7
BP	GO:0060055	angiogenesis involved in wound healing	bulk RNA-seq,Homo_723,AD.vs.control; bulk RNA-seq,Homo_723,AD.vs.MCI; bulk RNA-seq,Homo_723,MCI.vs.control; bulk RNA-seq,Homo_714,AD.vs.control; bulk RNA-seq,Homo_714,AD.vs.MCI; bulk RNA-seq,Homo_714,MCI.vs.control; bulk RNA-seq,Homo_633,AD.vs.control; bulk	8
BP	GO:0043542	endothelial cell migration	bulk RNA-seq,Homo_723,AD.vs.control; bulk RNA-seq,Homo_723,AD.vs.MCI; bulk RNA-seq,Homo_723,MCI.vs.control; bulk RNA-seq,Homo_714,AD.vs.control; bulk RNA-seq,Homo_714,AD.vs.MCI; bulk RNA-seq,Homo_633,AD.vs.control; bulk RNA-seq,Homo_633,AD.vs.MCI	7
BP	GO:0003228	atrial cardiac muscle tissue development	bulk RNA-seq,Homo_723,AD.vs.control; bulk RNA-seq,Homo_723,AD.vs.MCI; bulk RNA-seq,Homo_723,MCI.vs.control; bulk RNA-seq,Homo_714,AD.vs.control; bulk RNA-seq,Homo_714,AD.vs.MCI; bulk RNA-seq,Homo_714,MCI.vs.control; bulk RNA-seq,Homo_633,MCI.vs.control	7
BP	GO:0050657	nucleic acid transport	bulk RNA-seq,Homo_723,AD.vs.control; bulk RNA-seq,Homo_723,MCI.vs.control; bulk RNA-seq,Homo_714,AD.vs.control; bulk RNA-seq,Homo_633,AD.vs.control; bulk RNA-seq,Homo_633,AD.vs.MCI	5
BP	GO:0050658	RNA transport	bulk RNA-seq,Homo_723,AD.vs.control; bulk RNA-seq,Homo_723,MCI.vs.control; bulk RNA-seq,Homo_714,AD.vs.control; bulk RNA-seq,Homo_633,AD.vs.control; bulk RNA-seq,Homo_633,AD.vs.MCI	5
BP	GO:0061572	actin filament bundle organization	bulk RNA-seq,Homo_723,AD.vs.control; bulk RNA-seq,Homo_723,AD.vs.MCI; bulk RNA-seq,Homo_723,MCI.vs.control; bulk RNA-seq,Homo_714,AD.vs.MCI; bulk RNA-seq,Homo_633,AD.vs.control; bulk RNA-seq,Homo_633,AD.vs.MCI	6
BP	GO:0060601	lateral sprouting from an epithelium	bulk RNA-seq,Homo_723,AD.vs.control; bulk RNA-seq,Homo_723,AD.vs.MCI; bulk RNA-seq,Homo_723,MCI.vs.control; bulk RNA-seq,Homo_714,AD.vs.MCI	4
BP	GO:0031341	regulation of cell killing	bulk RNA-seq,Homo_723,AD.vs.control; bulk RNA-seq,Homo_723,MCI.vs.control; bulk RNA-seq,Homo_714,AD.vs.control; bulk RNA-seq,Homo_714,MCI.vs.control; bulk RNA-seq,Homo_633,AD.vs.control; bulk RNA-seq,Homo_633,AD.vs.MCI; scRNA-seq,SRP309935,B cell_1-AD.vs.control;	8
BP	GO:0045191	regulation of isotype switching	bulk RNA-seq,Homo_723,AD.vs.control; bulk RNA-seq,Homo_723,MCI.vs.control; bulk RNA-seq,Homo_714,AD.vs.control; bulk RNA-seq,Homo_714,MCI.vs.control; bulk RNA-seq,Homo_633,AD.vs.control; bulk RNA-seq,Homo_633,AD.vs.MCI	6
BP	GO:0043405	regulation of MAP kinase activity	bulk RNA-seq,Homo_723,AD.vs.control; bulk RNA-seq,Homo_723,AD.vs.MCI; bulk RNA-seq,Homo_723,MCI.vs.control; bulk RNA-seq,Homo_714,AD.vs.MCI; bulk RNA-seq,Homo_633,AD.vs.control; bulk RNA-seq,Homo_633,AD.vs.MCI	6
CC	GO:0005583	fibrillar collagen trimer	bulk RNA-seq,Homo_723,AD.vs.control; bulk RNA-seq,Homo_723,AD.vs.MCI; bulk RNA-seq,Homo_723,MCI.vs.control; bulk RNA-seq,Homo_714,AD.vs.MCI; bulk RNA-seq,ROSMAP,AD.vs.control	5
CC	GO:0098643	banded collagen fibril	bulk RNA-seq,Homo_723,AD.vs.control; bulk RNA-seq,Homo_723,AD.vs.MCI; bulk RNA-seq,Homo_723,MCI.vs.control; bulk RNA-seq,Homo_714,AD.vs.MCI; bulk RNA-seq,ROSMAP,AD.vs.control	5
MF	GO:0003743	translation initiation factor activity	bulk RNA-seq,Homo_723,AD.vs.control; bulk RNA-seq,Homo_723,AD.vs.MCI; bulk RNA-seq,Homo_714,AD.vs.control; bulk RNA-seq,Homo_714,AD.vs.MCI; bulk RNA-seq,Homo_714,MCI.vs.control; bulk RNA-seq,Homo_633,AD.vs.control; bulk RNA-seq,Homo_633,AD.vs.MCI	7
BP	GO:0035743	CD4-positive, alpha-beta T cell cytokine production	bulk RNA-seq,Homo_723,AD.vs.control; bulk RNA-seq,Homo_723,AD.vs.MCI; bulk RNA-seq,Homo_723,MCI.vs.control; bulk RNA-seq,Homo_714,AD.vs.control; bulk RNA-seq,Homo_714,MCI.vs.control; bulk RNA-seq,Homo_633,AD.vs.control; bulk RNA-seq,Homo_633,AD.vs.MCI; bulk	8
BP	GO:0044743	protein transmembrane import into intracellular organelle	bulk RNA-seq,Homo_723,AD.vs.control; bulk RNA-seq,Homo_723,MCI.vs.control; bulk RNA-seq,Homo_714,AD.vs.control; bulk RNA-seq,Homo_714,MCI.vs.control; bulk RNA-seq,Homo_633,AD.vs.control; bulk RNA-seq,Homo_633,AD.vs.MCI	6
BP	GO:2000648	positive regulation of stem cell proliferation	bulk RNA-seq,Homo_723,AD.vs.control; bulk RNA-seq,Homo_723,AD.vs.MCI; bulk RNA-seq,Homo_723,MCI.vs.control; bulk RNA-seq,Homo_714,AD.vs.control; bulk RNA-seq,Homo_714,AD.vs.MCI; bulk RNA-seq,Homo_714,MCI.vs.control	6
BP	GO:0050686	negative regulation of mRNA processing	bulk RNA-seq,Homo_723,AD.vs.control; bulk RNA-seq,Homo_723,AD.vs.MCI; bulk RNA-seq,Homo_723,MCI.vs.control; bulk RNA-seq,Homo_714,AD.vs.control; bulk RNA-seq,Homo_714,AD.vs.MCI; bulk RNA-seq,Homo_714,MCI.vs.control; bulk RNA-seq,Homo_633,AD.vs.control	7
BP	GO:0015931	nucleobase-containing compound transport	bulk RNA-seq,Homo_723,AD.vs.control; bulk RNA-seq,Homo_723,MCI.vs.control; bulk RNA-seq,Homo_714,AD.vs.control; bulk RNA-seq,Homo_633,AD.vs.control; bulk RNA-seq,Homo_633,AD.vs.MCI	5
BP	GO:0033617	mitochondrial cytochrome c oxidase assembly	bulk RNA-seq,Homo_723,AD.vs.control; bulk RNA-seq,Homo_723,MCI.vs.control; bulk RNA-seq,Homo_714,AD.vs.control; bulk RNA-seq,Homo_714,MCI.vs.control; bulk RNA-seq,Homo_633,AD.vs.control; bulk RNA-seq,Homo_633,AD.vs.MCI; bulk RNA-seq,Homo_633,MCI.vs.control	7
CC	GO:1904724	tertiary granule lumen	bulk RNA-seq,Homo_723,AD.vs.control; bulk RNA-seq,Homo_723,AD.vs.MCI; bulk RNA-seq,Homo_633,AD.vs.control; bulk RNA-seq,Homo_633,AD.vs.MCI; bulk RNA-seq,SRP223445,AD.vs.control	5
BP	GO:0043487	regulation of RNA stability	bulk RNA-seq,Homo_723,AD.vs.control; bulk RNA-seq,Homo_723,MCI.vs.control; bulk RNA-seq,Homo_714,AD.vs.control; bulk RNA-seq,Homo_633,AD.vs.control; bulk RNA-seq,Homo_633,AD.vs.MCI	5
BP	GO:0040013	negative regulation of locomotion	bulk RNA-seq,Homo_723,AD.vs.control; bulk RNA-seq,Homo_723,AD.vs.MCI; bulk RNA-seq,Homo_714,AD.vs.control; bulk RNA-seq,Homo_714,AD.vs.MCI; bulk RNA-seq,Homo_633,AD.vs.control; bulk RNA-seq,Homo_633,AD.vs.MCI; scRNA-seq,SRP330776,Naive CD8+ T cell_2-AD.vs.control	7
MF	GO:0043022	ribosome binding	bulk RNA-seq,Homo_723,AD.vs.control; bulk RNA-seq,Homo_723,MCI.vs.control; bulk RNA-seq,Homo_714,AD.vs.control; bulk RNA-seq,Homo_714,MCI.vs.control; bulk RNA-seq,Homo_633,AD.vs.control; bulk RNA-seq,Homo_633,AD.vs.MCI	6
BP	GO:0051017	actin filament bundle assembly	bulk RNA-seq,Homo_723,AD.vs.control; bulk RNA-seq,Homo_723,AD.vs.MCI; bulk RNA-seq,Homo_723,MCI.vs.control; bulk RNA-seq,Homo_714,AD.vs.MCI; bulk RNA-seq,Homo_633,AD.vs.control; bulk RNA-seq,Homo_633,AD.vs.MCI	6
BP	GO:2001054	negative regulation of mesenchymal cell apoptotic process	bulk RNA-seq,Homo_723,AD.vs.control; bulk RNA-seq,Homo_723,AD.vs.MCI; bulk RNA-seq,Homo_723,MCI.vs.control; bulk RNA-seq,Homo_714,AD.vs.control; bulk RNA-seq,Homo_714,AD.vs.MCI; bulk RNA-seq,Homo_633,MCI.vs.control	6

BP	GO:0045911	positive regulation of DNA recombination	bulk RNA-seq,Homo_723,AD.vs.control; bulk RNA-seq,Homo_723,MCI.vs.control; bulk RNA-seq,Homo_714,AD.vs.control; bulk RNA-seq,Homo_714,MCI.vs.control; bulk RNA-seq,Homo_633,AD.vs.control; bulk RNA-seq,Homo_633,AD.vs.MCI	6
BP	GO:0006287	base-excision repair, gap-filling	bulk RNA-seq,Homo_723,AD.vs.control; bulk RNA-seq,Homo_723,AD.vs.MCI; bulk RNA-seq,Homo_723,MCI.vs.control; bulk RNA-seq,Homo_714,AD.vs.control; bulk RNA-seq,Homo_714,MCI.vs.control; bulk RNA-seq,Homo_633,AD.vs.control; bulk RNA-seq,Homo_633,MCI.vs.control	7
BP	GO:0045604	regulation of epidermal cell differentiation	bulk RNA-seq,Homo_723,AD.vs.control; bulk RNA-seq,Homo_723,AD.vs.MCI; bulk RNA-seq,Homo_723,MCI.vs.control; bulk RNA-seq,Homo_714,AD.vs.control; bulk RNA-seq,Homo_714,AD.vs.MCI; bulk RNA-seq,Homo_714,MCI.vs.control; bulk RNA-seq,Homo_633,AD.vs.control	7
BP	GO:0110154	RNA decapping	bulk RNA-seq,Homo_723,AD.vs.control; bulk RNA-seq,Homo_723,AD.vs.MCI; bulk RNA-seq,Homo_714,AD.vs.control; bulk RNA-seq,Homo_714,AD.vs.MCI; bulk RNA-seq,Homo_633,AD.vs.control	5
BP	GO:1904321	response to forskolin	bulk RNA-seq,Homo_723,AD.vs.control; bulk RNA-seq,Homo_723,AD.vs.MCI; bulk RNA-seq,Homo_633,MCI.vs.control	3
BP	GO:1904322	cellular response to forskolin	bulk RNA-seq,Homo_723,AD.vs.control; bulk RNA-seq,Homo_723,AD.vs.MCI; bulk RNA-seq,Homo_633,MCI.vs.control	3
BP	GO:0099116	tRNA 5'-end processing	bulk RNA-seq,Homo_723,AD.vs.control; bulk RNA-seq,Homo_723,AD.vs.MCI; bulk RNA-seq,Homo_714,AD.vs.control; bulk RNA-seq,Homo_633,AD.vs.control; bulk RNA-seq,Homo_633,AD.vs.MCI	5
BP	GO:0050730	regulation of peptidyl-tyrosine phosphorylation	bulk RNA-seq,Homo_723,AD.vs.control; bulk RNA-seq,Homo_723,AD.vs.MCI; bulk RNA-seq,Homo_723,MCI.vs.control; bulk RNA-seq,Homo_714,AD.vs.control; bulk RNA-seq,Homo_714,AD.vs.MCI; bulk RNA-seq,Homo_633,AD.vs.control; bulk RNA-seq,Homo_633,AD.vs.MCI	7
BP	GO:0060976	coronary vasculature development	bulk RNA-seq,Homo_723,AD.vs.control; bulk RNA-seq,Homo_723,AD.vs.MCI; bulk RNA-seq,Homo_723,MCI.vs.control; bulk RNA-seq,Homo_714,AD.vs.MCI; bulk RNA-seq,Homo_714,MCI.vs.control; bulk RNA-seq,Homo_633,AD.vs.control	6
BP	GO:0032210	regulation of telomere maintenance via telomerase	bulk RNA-seq,Homo_723,AD.vs.control; bulk RNA-seq,Homo_723,MCI.vs.control; bulk RNA-seq,Homo_714,AD.vs.control; bulk RNA-seq,Homo_714,MCI.vs.control; bulk RNA-seq,Homo_633,AD.vs.control; bulk RNA-seq,Homo_633,AD.vs.MCI	6
BP	GO:0006900	vesicle budding from membrane	bulk RNA-seq,Homo_723,AD.vs.control; bulk RNA-seq,Homo_723,MCI.vs.control; bulk RNA-seq,Homo_714,AD.vs.control; bulk RNA-seq,Homo_714,MCI.vs.control; bulk RNA-seq,Homo_633,AD.vs.control; bulk RNA-seq,Homo_633,AD.vs.MCI	6
BP	GO:0045926	negative regulation of growth	bulk RNA-seq,Homo_723,AD.vs.control; bulk RNA-seq,Homo_723,AD.vs.MCI; bulk RNA-seq,Homo_723,MCI.vs.control; bulk RNA-seq,Homo_714,AD.vs.MCI; bulk RNA-seq,Homo_633,AD.vs.control; bulk RNA-seq,Homo_633,AD.vs.MCI	6
BP	GO:1903350	response to dopamine	bulk RNA-seq,Homo_723,AD.vs.control; bulk RNA-seq,Homo_723,AD.vs.MCI; bulk RNA-seq,Homo_723,MCI.vs.control; bulk RNA-seq,Homo_714,AD.vs.control; bulk RNA-seq,Homo_714,AD.vs.MCI; bulk RNA-seq,Homo_633,AD.vs.control	6
MF	GO:0008195	phosphatidate phosphatase activity	bulk RNA-seq,Homo_723,AD.vs.control; bulk RNA-seq,Homo_723,AD.vs.MCI; bulk RNA-seq,Homo_723,MCI.vs.control; bulk RNA-seq,Homo_714,AD.vs.MCI	4
BP	GO:0021979	hypothalamus cell differentiation	bulk RNA-seq,Homo_723,AD.vs.control; bulk RNA-seq,Homo_723,AD.vs.MCI; bulk RNA-seq,Homo_714,AD.vs.MCI	3
BP	GO:0097164	ammonium ion metabolic process	bulk RNA-seq,Homo_723,AD.vs.control; bulk RNA-seq,Homo_723,AD.vs.MCI; bulk RNA-seq,Homo_723,MCI.vs.control; bulk RNA-seq,Homo_714,AD.vs.control; bulk RNA-seq,Homo_714,AD.vs.MCI	5
CC	GO:0005777	peroxisome	bulk RNA-seq,Homo_723,AD.vs.control; bulk RNA-seq,Homo_723,MCI.vs.control; bulk RNA-seq,Homo_714,AD.vs.control; bulk RNA-seq,Homo_633,AD.vs.control; bulk RNA-seq,Homo_633,AD.vs.MCI	5
CC	GO:0042579	microbody	bulk RNA-seq,Homo_723,AD.vs.control; bulk RNA-seq,Homo_723,MCI.vs.control; bulk RNA-seq,Homo_714,AD.vs.control; bulk RNA-seq,Homo_633,AD.vs.control; bulk RNA-seq,Homo_633,AD.vs.MCI	5
BP	GO:0002040	sprouting angiogenesis	bulk RNA-seq,Homo_723,AD.vs.control; bulk RNA-seq,Homo_723,AD.vs.MCI; bulk RNA-seq,Homo_723,MCI.vs.control; bulk RNA-seq,Homo_714,AD.vs.control; bulk RNA-seq,Homo_714,AD.vs.MCI	5
BP	GO:0032479	regulation of type I interferon production	bulk RNA-seq,Homo_723,AD.vs.control; bulk RNA-seq,Homo_723,MCI.vs.control; bulk RNA-seq,Homo_714,AD.vs.control; bulk RNA-seq,Homo_714,MCI.vs.control; bulk RNA-seq,Homo_633,AD.vs.control; bulk RNA-seq,Homo_633,AD.vs.MCI	6
BP	GO:0032606	type I interferon production	bulk RNA-seq,Homo_723,AD.vs.control; bulk RNA-seq,Homo_723,MCI.vs.control; bulk RNA-seq,Homo_714,AD.vs.control; bulk RNA-seq,Homo_714,MCI.vs.control; bulk RNA-seq,Homo_633,AD.vs.control; bulk RNA-seq,Homo_633,AD.vs.MCI	6
CC	GO:1904949	ATPase complex	bulk RNA-seq,Homo_723,AD.vs.control; bulk RNA-seq,Homo_723,MCI.vs.control; bulk RNA-seq,Homo_714,AD.vs.control; bulk RNA-seq,Homo_714,MCI.vs.control; bulk RNA-seq,Homo_633,AD.vs.control; bulk RNA-seq,Homo_633,AD.vs.MCI	6
BP	GO:0071230	cellular response to amino acid stimulus	bulk RNA-seq,Homo_723,AD.vs.control; bulk RNA-seq,Homo_723,AD.vs.MCI; bulk RNA-seq,Homo_723,MCI.vs.control; bulk RNA-seq,Homo_714,AD.vs.MCI; bulk RNA-seq,Homo_714,MCI.vs.control; bulk RNA-seq,Homo_633,AD.vs.control; bulk RNA-seq,Homo_633,AD.vs.MCI; scRNA-seq,SRP330776,CD8+ T cell_1-AD.vs.control; scRNA-seq,SRP330776,Naive CD8+ T cell_1-AD.vs.control	9
BP	GO:1904874	positive regulation of telomerase RNA localization to Cajal body	bulk RNA-seq,Homo_723,AD.vs.control; bulk RNA-seq,Homo_723,AD.vs.MCI; bulk RNA-seq,Homo_723,MCI.vs.control; bulk RNA-seq,Homo_714,AD.vs.control; bulk RNA-seq,Homo_714,AD.vs.MCI; bulk RNA-seq,Homo_714,MCI.vs.control; bulk RNA-seq,Homo_633,AD.vs.control; bulk RNA-seq,Homo_633,AD.vs.MCI; bulk RNA-seq,Homo_633,MCI.vs.control	9
BP	GO:0048640	negative regulation of developmental growth	bulk RNA-seq,Homo_723,AD.vs.control; bulk RNA-seq,Homo_723,AD.vs.MCI; bulk RNA-seq,Homo_723,MCI.vs.control; bulk RNA-seq,Homo_714,AD.vs.MCI; bulk RNA-seq,Homo_714,MCI.vs.control; bulk RNA-seq,Homo_633,AD.vs.control; bulk RNA-seq,Homo_633,AD.vs.MCI	7
BP	GO:0010822	positive regulation of mitochondrion organization	bulk RNA-seq,Homo_723,AD.vs.control; bulk RNA-seq,Homo_723,MCI.vs.control; bulk RNA-seq,Homo_714,AD.vs.control; bulk RNA-seq,Homo_714,MCI.vs.control; bulk RNA-seq,Homo_633,AD.vs.control; bulk RNA-seq,Homo_633,AD.vs.MCI	6
BP	GO:0038063	collagen-activated tyrosine kinase receptor signaling pathway	bulk RNA-seq,Homo_723,AD.vs.control; bulk RNA-seq,Homo_723,AD.vs.MCI; bulk RNA-seq,Homo_723,MCI.vs.control; bulk RNA-seq,Homo_714,AD.vs.control; bulk RNA-seq,Homo_714,AD.vs.MCI	5
BP	GO:0014888	striated muscle adaptation	bulk RNA-seq,Homo_723,AD.vs.control; bulk RNA-seq,Homo_723,AD.vs.MCI; bulk RNA-seq,Homo_723,MCI.vs.control; bulk RNA-seq,Homo_714,AD.vs.control; bulk RNA-seq,Homo_714,AD.vs.MCI; bulk RNA-seq,Homo_714,MCI.vs.control	6
BP	GO:0021522	spinal cord motor neuron differentiation	bulk RNA-seq,Homo_723,AD.vs.control; bulk RNA-seq,Homo_723,AD.vs.MCI; bulk RNA-seq,Homo_723,MCI.vs.control; bulk RNA-seq,Homo_714,AD.vs.MCI	4
BP	GO:1901654	response to ketone	bulk RNA-seq,Homo_723,AD.vs.control; bulk RNA-seq,Homo_723,AD.vs.MCI; bulk RNA-seq,Homo_723,MCI.vs.control; bulk RNA-seq,Homo_714,AD.vs.control; bulk RNA-seq,Homo_714,AD.vs.MCI; bulk RNA-seq,Homo_633,AD.vs.control; bulk RNA-seq,Homo_633,AD.vs.MCI	7
BP	GO:0086091	regulation of heart rate by cardiac conduction	bulk RNA-seq,Homo_723,AD.vs.control; bulk RNA-seq,Homo_723,AD.vs.MCI; bulk RNA-seq,Homo_723,MCI.vs.control; bulk RNA-seq,Homo_714,AD.vs.MCI; bulk RNA-seq,Homo_714,MCI.vs.control	5
BP	GO:0006898	receptor-mediated endocytosis	bulk RNA-seq,Homo_723,AD.vs.control; bulk RNA-seq,Homo_723,AD.vs.MCI; bulk RNA-seq,Homo_723,MCI.vs.control; bulk RNA-seq,Homo_714,AD.vs.control; bulk RNA-seq,Homo_714,AD.vs.MCI; bulk RNA-seq,Homo_633,AD.vs.control; bulk RNA-seq,Homo_633,AD.vs.MCI; scRNA-	8
BP	GO:0042093	T-helper cell differentiation	bulk RNA-seq,Homo_723,AD.vs.control; bulk RNA-seq,Homo_723,MCI.vs.control; bulk RNA-seq,Homo_714,AD.vs.control; bulk RNA-seq,Homo_714,MCI.vs.control; bulk RNA-seq,Homo_633,AD.vs.control; bulk RNA-seq,Homo_633,AD.vs.MCI	6
BP	GO:0098900	regulation of action potential	bulk RNA-seq,Homo_723,AD.vs.control; bulk RNA-seq,Homo_723,AD.vs.MCI; bulk RNA-seq,Homo_723,MCI.vs.control; bulk RNA-seq,Homo_714,AD.vs.MCI; bulk RNA-seq,Homo_714,MCI.vs.control	5

BP	GO:0007163	establishment or maintenance of cell polarity	bulk RNA-seq,Homo_723,AD.vs.control; bulk RNA-seq,Homo_723,AD.vs.MCI; bulk RNA-seq,Homo_723,MCI.vs.control; bulk RNA-seq,Homo_714,AD.vs.MCI; bulk RNA-seq,Homo_633,AD.vs.control; bulk RNA-seq,Homo_633,AD.vs.MCI	6
BP	GO:0071229	cellular response to acid chemical	bulk RNA-seq,Homo_723,AD.vs.control; bulk RNA-seq,Homo_723,AD.vs.MCI; bulk RNA-seq,Homo_723,MCI.vs.control; bulk RNA-seq,Homo_714,AD.vs.MCI; bulk RNA-seq,Homo_714,MCI.vs.control; bulk RNA-seq,Homo_633,AD.vs.control; bulk RNA-seq,Homo_633,AD.vs.MCI	7
BP	GO:0045069	regulation of viral genome replication	bulk RNA-seq,Homo_723,AD.vs.control; bulk RNA-seq,Homo_723,MCI.vs.control; bulk RNA-seq,Homo_714,AD.vs.control; bulk RNA-seq,Homo_714,MCI.vs.control; bulk RNA-seq,Homo_633,AD.vs.control	5
BP	GO:0090101	negative regulation of transmembrane receptor protein serine/threonine kinase signaling pathway	bulk RNA-seq,Homo_723,AD.vs.control; bulk RNA-seq,Homo_723,AD.vs.MCI; bulk RNA-seq,Homo_723,MCI.vs.control; bulk RNA-seq,Homo_714,AD.vs.MCI; bulk RNA-seq,Homo_633,AD.vs.control; bulk RNA-seq,Homo_633,AD.vs.MCI	6
BP	GO:0042790	nucleolar large rRNA transcription by RNA polymerase I	bulk RNA-seq,Homo_723,AD.vs.control; bulk RNA-seq,Homo_723,AD.vs.MCI; bulk RNA-seq,Homo_723,MCI.vs.control; bulk RNA-seq,Homo_714,AD.vs.control; bulk RNA-seq,Homo_714,MCI.vs.control; bulk RNA-seq,Homo_633,AD.vs.control; bulk RNA-seq,Homo_633,AD.vs.MCI; bulk	8
BP	GO:0006942	regulation of striated muscle contraction	bulk RNA-seq,Homo_723,AD.vs.control; bulk RNA-seq,Homo_723,AD.vs.MCI; bulk RNA-seq,Homo_723,MCI.vs.control; bulk RNA-seq,Homo_714,AD.vs.control; bulk RNA-seq,Homo_714,AD.vs.MCI; bulk RNA-seq,Homo_714,MCI.vs.control	6
BP	GO:0010632	regulation of epithelial cell migration	bulk RNA-seq,Homo_723,AD.vs.control; bulk RNA-seq,Homo_723,AD.vs.MCI; bulk RNA-seq,Homo_723,MCI.vs.control; bulk RNA-seq,Homo_714,AD.vs.control; bulk RNA-seq,Homo_714,AD.vs.MCI; bulk RNA-seq,Homo_633,AD.vs.control; bulk RNA-seq,Homo_633,AD.vs.MCI; scRNA-	8
BP	GO:0030038	contractile actin filament bundle assembly	bulk RNA-seq,Homo_723,AD.vs.control; bulk RNA-seq,Homo_723,AD.vs.MCI; bulk RNA-seq,Homo_723,MCI.vs.control; bulk RNA-seq,Homo_714,AD.vs.MCI; bulk RNA-seq,Homo_714,MCI.vs.control; bulk RNA-seq,Homo_633,AD.vs.control; bulk RNA-seq,Homo_633,AD.vs.MCI	7
BP	GO:0043149	stress fiber assembly	bulk RNA-seq,Homo_723,AD.vs.control; bulk RNA-seq,Homo_723,AD.vs.MCI; bulk RNA-seq,Homo_723,MCI.vs.control; bulk RNA-seq,Homo_714,AD.vs.MCI; bulk RNA-seq,Homo_714,MCI.vs.control; bulk RNA-seq,Homo_633,AD.vs.control; bulk RNA-seq,Homo_633,AD.vs.MCI	7
BP	GO:0040036	regulation of fibroblast growth factor receptor signaling pathway	bulk RNA-seq,Homo_723,AD.vs.control; bulk RNA-seq,Homo_723,AD.vs.MCI; bulk RNA-seq,Homo_723,MCI.vs.control; bulk RNA-seq,Homo_714,AD.vs.MCI; bulk RNA-seq,Homo_714,MCI.vs.control	5
BP	GO:0060324	face development	bulk RNA-seq,Homo_723,AD.vs.control; bulk RNA-seq,Homo_723,AD.vs.MCI; bulk RNA-seq,Homo_723,MCI.vs.control; bulk RNA-seq,Homo_714,AD.vs.MCI; bulk RNA-seq,Homo_714,MCI.vs.control; bulk RNA-seq,Homo_633,AD.vs.control; bulk RNA-seq,Homo_633,AD.vs.MCI	7
MF	GO:0030276	clathrin binding	bulk RNA-seq,Homo_723,AD.vs.control; bulk RNA-seq,Homo_723,AD.vs.MCI; bulk RNA-seq,Homo_723,MCI.vs.control; bulk RNA-seq,Homo_714,AD.vs.MCI; bulk RNA-seq,Homo_714,MCI.vs.control; bulk RNA-seq,Homo_633,AD.vs.control; bulk RNA-seq,Homo_633,AD.vs.MCI	7
CC	GO:0000176	nuclear exosome (RNase complex)	bulk RNA-seq,Homo_723,AD.vs.control; bulk RNA-seq,Homo_723,AD.vs.MCI; bulk RNA-seq,Homo_714,AD.vs.control; bulk RNA-seq,Homo_714,AD.vs.MCI; bulk RNA-seq,Homo_633,AD.vs.control; bulk RNA-seq,Homo_633,AD.vs.MCI	6
BP	GO:0098810	neurotransmitter reuptake	bulk RNA-seq,Homo_723,AD.vs.control; bulk RNA-seq,Homo_723,AD.vs.MCI; bulk RNA-seq,Homo_723,MCI.vs.control; bulk RNA-seq,Homo_714,AD.vs.MCI	4
BP	GO:0060603	mammary gland duct morphogenesis	bulk RNA-seq,Homo_723,AD.vs.control; bulk RNA-seq,Homo_723,AD.vs.MCI; bulk RNA-seq,Homo_723,MCI.vs.control; bulk RNA-seq,Homo_714,AD.vs.MCI; bulk RNA-seq,Homo_633,AD.vs.control; bulk RNA-seq,Homo_633,AD.vs.MCI	6
BP	GO:0001732	formation of cytoplasmic translation initiation complex	bulk RNA-seq,Homo_723,AD.vs.control; bulk RNA-seq,Homo_723,AD.vs.MCI; bulk RNA-seq,Homo_714,AD.vs.control; bulk RNA-	4
CC	GO:0031968	organelle outer membrane	bulk RNA-seq,Homo_723,AD.vs.control; bulk RNA-seq,Homo_723,MCI.vs.control; bulk RNA-seq,Homo_714,AD.vs.control; bulk RNA-seq,Homo_633,AD.vs.control; bulk RNA-seq,Homo_633,AD.vs.MCI	5
BP	GO:0010594	regulation of endothelial cell migration	bulk RNA-seq,Homo_723,AD.vs.control; bulk RNA-seq,Homo_723,AD.vs.MCI; bulk RNA-seq,Homo_723,MCI.vs.control; bulk RNA-seq,Homo_714,AD.vs.control; bulk RNA-seq,Homo_714,AD.vs.MCI; bulk RNA-seq,Homo_633,AD.vs.control; bulk RNA-seq,Homo_633,AD.vs.MCI	7
BP	GO:1904816	positive regulation of protein localization to chromosome, telomeric region	bulk RNA-seq,Homo_723,AD.vs.control; bulk RNA-seq,Homo_723,AD.vs.MCI; bulk RNA-seq,Homo_723,MCI.vs.control; bulk RNA-seq,Homo_714,AD.vs.control; bulk RNA-seq,Homo_714,MCI.vs.control; bulk RNA-seq,Homo_633,AD.vs.control; bulk RNA-seq,Homo_633,AD.vs.MCI; bulk	8
CC	GO:0140534	endoplasmic reticulum protein-containing complex	bulk RNA-seq,Homo_723,AD.vs.control; bulk RNA-seq,Homo_723,MCI.vs.control; bulk RNA-seq,Homo_714,AD.vs.control; bulk RNA-seq,Homo_714,MCI.vs.control; bulk RNA-seq,Homo_633,AD.vs.control; bulk RNA-seq,Homo_633,AD.vs.MCI	6
MF	GO:0000149	SNARE binding	bulk RNA-seq,Homo_723,AD.vs.control; bulk RNA-seq,Homo_723,AD.vs.MCI; bulk RNA-seq,Homo_723,MCI.vs.control; bulk RNA-seq,Homo_714,AD.vs.MCI; bulk RNA-seq,Homo_714,MCI.vs.control; bulk RNA-seq,Homo_633,AD.vs.control; bulk RNA-seq,Homo_633,AD.vs.MCI	7
BP	GO:0019886	antigen processing and presentation of exogenous peptide antigen via MHC class II	bulk RNA-seq,Homo_723,AD.vs.control; bulk RNA-seq,Homo_723,MCI.vs.control; bulk RNA-seq,Homo_714,AD.vs.control; bulk RNA-seq,Homo_714,MCI.vs.control; bulk RNA-seq,Homo_633,AD.vs.control; bulk RNA-seq,Homo_633,AD.vs.MCI; bulk RNA-seq,Homo_633,MCI.vs.control; scRNA-seq,SRP330776,Naive CD8+ T cell_2-AD.vs.control; scRNA-seq,SRP215507,CD8+ T cell_3-AD.vs.control; scRNA-seq,SRP215507,CD8+ T cell_3-	10
BP	GO:0002704	negative regulation of leukocyte mediated immunity	bulk RNA-seq,Homo_723,AD.vs.control; bulk RNA-seq,Homo_723,MCI.vs.control; bulk RNA-seq,Homo_714,AD.vs.control; bulk RNA-seq,Homo_714,MCI.vs.control; bulk RNA-seq,Homo_633,AD.vs.control; bulk RNA-seq,Homo_633,AD.vs.MCI	6
CC	GO:0005884	actin filament	bulk RNA-seq,Homo_723,AD.vs.control; bulk RNA-seq,Homo_723,AD.vs.MCI; bulk RNA-seq,Homo_723,MCI.vs.control; bulk RNA-seq,Homo_714,AD.vs.MCI; bulk RNA-seq,Homo_714,MCI.vs.control; bulk RNA-seq,Homo_633,AD.vs.control; bulk RNA-seq,Homo_633,AD.vs.MCI	7
CC	GO:0005839	proteasome core complex	bulk RNA-seq,Homo_723,AD.vs.control; bulk RNA-seq,Homo_723,AD.vs.MCI; bulk RNA-seq,Homo_714,AD.vs.control; bulk RNA-seq,Homo_633,AD.vs.control; bulk RNA-seq,Homo_633,AD.vs.MCI	5
BP	GO:0006388	tRNA splicing, via endonucleolytic cleavage and ligation	bulk RNA-seq,Homo_723,AD.vs.control; bulk RNA-seq,Homo_723,AD.vs.MCI; bulk RNA-seq,Homo_723,MCI.vs.control; bulk RNA-seq,Homo_714,AD.vs.control; bulk RNA-seq,Homo_714,MCI.vs.control; bulk RNA-seq,Homo_633,AD.vs.control; bulk RNA-seq,Homo_633,AD.vs.MCI	7
BP	GO:0030202	heparin metabolic process	bulk RNA-seq,Homo_723,AD.vs.control; bulk RNA-seq,Homo_723,AD.vs.MCI; bulk RNA-seq,Homo_723,MCI.vs.control; bulk RNA-seq,Homo_714,AD.vs.control; bulk RNA-seq,Homo_714,AD.vs.MCI; bulk RNA-seq,Homo_714,MCI.vs.control; bulk RNA-seq,Homo_633,AD.vs.control	7
BP	GO:0048532	anatomical structure arrangement	bulk RNA-seq,Homo_723,AD.vs.control; bulk RNA-seq,Homo_723,AD.vs.MCI; bulk RNA-seq,Homo_723,MCI.vs.control; bulk RNA-seq,Homo_714,AD.vs.MCI	4
BP	GO:0048278	vesicle docking	bulk RNA-seq,Homo_723,AD.vs.control; bulk RNA-seq,Homo_723,AD.vs.MCI; bulk RNA-seq,Homo_723,MCI.vs.control; bulk RNA-seq,Homo_714,AD.vs.MCI; bulk RNA-seq,Homo_714,MCI.vs.control; bulk RNA-seq,Homo_633,AD.vs.control; bulk RNA-seq,Homo_633,AD.vs.MCI	7
MF	GO:0031593	polyubiquitin modification-dependent protein binding	bulk RNA-seq,Homo_723,AD.vs.control; bulk RNA-seq,Homo_723,MCI.vs.control; bulk RNA-seq,Homo_714,AD.vs.control; bulk RNA-seq,Homo_714,MCI.vs.control; bulk RNA-seq,Homo_633,AD.vs.control; bulk RNA-seq,Homo_633,AD.vs.MCI	6
BP	GO:0042267	natural killer cell mediated cytotoxicity	bulk RNA-seq,Homo_723,AD.vs.control; bulk RNA-seq,Homo_714,AD.vs.control; bulk RNA-seq,Homo_714,MCI.vs.control; bulk RNA-seq,Homo_633,AD.vs.control; bulk RNA-seq,Homo_633,AD.vs.MCI; scRNA-seq,SRP309935,B cell_1-AD.vs.control	6
BP	GO:0045727	positive regulation of translation	bulk RNA-seq,Homo_723,AD.vs.control; bulk RNA-seq,Homo_723,MCI.vs.control; bulk RNA-seq,Homo_714,AD.vs.control; bulk RNA-seq,Homo_633,AD.vs.control; bulk RNA-seq,Homo_633,AD.vs.MCI	5
BP	GO:0110149	regulation of biomineralization	bulk RNA-seq,Homo_723,AD.vs.control; bulk RNA-seq,Homo_723,AD.vs.MCI; bulk RNA-seq,Homo_723,MCI.vs.control; bulk RNA-seq,Homo_714,AD.vs.control; bulk RNA-seq,Homo_714,AD.vs.MCI; bulk RNA-seq,Homo_714,MCI.vs.control; bulk RNA-seq,Homo_633,AD.vs.control; bulk	8

BP	GO:0008277	regulation of G protein-coupled receptor signaling pathway	bulk RNA-seq,Homo_723,AD.vs.control; bulk RNA-seq,Homo_723,AD.vs.MCI; bulk RNA-seq,Homo_723,MCI.vs.control; bulk RNA-seq,Homo_714,AD.vs.control; bulk RNA-seq,Homo_714,AD.vs.MCI; bulk RNA-seq,Homo_714,MCI.vs.control; bulk RNA-seq,Homo_633,AD.vs.control	7
BP	GO:0002294	CD4-positive, alpha-beta T cell differentiation involved in immune response	bulk RNA-seq,Homo_723,AD.vs.control; bulk RNA-seq,Homo_723,MCI.vs.control; bulk RNA-seq,Homo_714,AD.vs.control; bulk RNA-seq,Homo_714,MCI.vs.control; bulk RNA-seq,Homo_633,AD.vs.control; bulk RNA-seq,Homo_633,AD.vs.MCI	6
BP	GO:0045939	negative regulation of steroid metabolic process	bulk RNA-seq,Homo_723,AD.vs.control; bulk RNA-seq,Homo_723,AD.vs.MCI; bulk RNA-seq,Homo_723,MCI.vs.control; bulk RNA-seq,Homo_714,AD.vs.control; bulk RNA-seq,Homo_714,AD.vs.MCI; bulk RNA-seq,Homo_714,MCI.vs.control	6
CC	GO:0098831	presynaptic active zone cytoplasmic component	bulk RNA-seq,Homo_723,AD.vs.control; bulk RNA-seq,Homo_723,AD.vs.MCI; bulk RNA-seq,Homo_723,MCI.vs.control; bulk RNA-seq,Homo_714,AD.vs.MCI; bulk RNA-seq,Homo_714,MCI.vs.control; bulk RNA-seq,Homo_633,AD.vs.control	6
BP	GO:0003416	endochondral bone growth	bulk RNA-seq,Homo_723,AD.vs.control; bulk RNA-seq,Homo_723,AD.vs.MCI; bulk RNA-seq,Homo_723,MCI.vs.control; bulk RNA-seq,Homo_714,AD.vs.MCI; bulk RNA-seq,Homo_714,MCI.vs.control; bulk RNA-seq,Homo_633,AD.vs.control	6
BP	GO:0021859	pyramidal neuron differentiation	bulk RNA-seq,Homo_723,AD.vs.control; bulk RNA-seq,Homo_723,AD.vs.MCI; bulk RNA-seq,Homo_723,MCI.vs.control; bulk RNA-	4
BP	GO:0009396	folic acid-containing compound biosynthetic process	bulk RNA-seq,Homo_723,AD.vs.control; bulk RNA-seq,Homo_723,AD.vs.MCI; bulk RNA-seq,Homo_723,MCI.vs.control; bulk RNA-seq,Homo_714,AD.vs.control; bulk RNA-seq,Homo_714,MCI.vs.control; bulk RNA-seq,Homo_633,AD.vs.control; bulk RNA-seq,Homo_633,AD.vs.MCI; bulk	8
BP	GO:0042698	ovulation cycle	bulk RNA-seq,Homo_723,AD.vs.control; bulk RNA-seq,Homo_723,AD.vs.MCI; bulk RNA-seq,Homo_723,MCI.vs.control; bulk RNA-seq,Homo_714,AD.vs.control; bulk RNA-seq,Homo_714,AD.vs.MCI; bulk RNA-seq,Homo_714,MCI.vs.control; bulk RNA-seq,Homo_633,AD.vs.control	7
CC	GO:0005643	nuclear pore	bulk RNA-seq,Homo_723,AD.vs.control; bulk RNA-seq,Homo_723,MCI.vs.control; bulk RNA-seq,Homo_714,AD.vs.control; bulk RNA-seq,Homo_714,MCI.vs.control; bulk RNA-seq,Homo_633,AD.vs.control; bulk RNA-seq,Homo_633,AD.vs.MCI	6
BP	GO:0010719	negative regulation of epithelial to mesenchymal transition	bulk RNA-seq,Homo_723,AD.vs.control; bulk RNA-seq,Homo_723,AD.vs.MCI; bulk RNA-seq,Homo_723,MCI.vs.control; bulk RNA-seq,Homo_714,AD.vs.MCI; bulk RNA-seq,Homo_714,MCI.vs.control	5
BP	GO:0090114	COPII-coated vesicle budding	bulk RNA-seq,Homo_723,AD.vs.control; bulk RNA-seq,Homo_723,MCI.vs.control; bulk RNA-seq,Homo_714,AD.vs.control; bulk RNA-seq,Homo_714,MCI.vs.control; bulk RNA-seq,Homo_633,AD.vs.control; bulk RNA-seq,Homo_633,AD.vs.MCI	6
BP	GO:0072311	glomerular epithelial cell differentiation	bulk RNA-seq,Homo_723,AD.vs.control; bulk RNA-seq,Homo_723,AD.vs.MCI; bulk RNA-seq,Homo_723,MCI.vs.control; bulk RNA-seq,Homo_714,AD.vs.MCI	4
MF	GO:0070696	transmembrane receptor protein serine/threonine kinase binding	bulk RNA-seq,Homo_723,AD.vs.control; bulk RNA-seq,Homo_723,AD.vs.MCI; bulk RNA-seq,Homo_723,MCI.vs.control; bulk RNA-seq,Homo_714,AD.vs.control; bulk RNA-seq,Homo_714,AD.vs.MCI; bulk RNA-seq,Homo_714,MCI.vs.control	6
BP	GO:0019083	viral transcription	bulk RNA-seq,Homo_723,AD.vs.control; bulk RNA-seq,Homo_723,MCI.vs.control; bulk RNA-seq,Homo_714,AD.vs.control; bulk RNA-seq,Homo_714,MCI.vs.control; bulk RNA-seq,Homo_633,AD.vs.control; bulk RNA-seq,Homo_633,AD.vs.MCI	6
MF	GO:0005251	delayed rectifier potassium channel activity	bulk RNA-seq,Homo_723,AD.vs.control; bulk RNA-seq,Homo_723,AD.vs.MCI; bulk RNA-seq,Homo_714,AD.vs.control; bulk RNA-seq,Homo_714,AD.vs.MCI	4
BP	GO:0048708	astrocyte differentiation	bulk RNA-seq,Homo_723,AD.vs.control; bulk RNA-seq,Homo_723,AD.vs.MCI; bulk RNA-seq,Homo_723,MCI.vs.control; bulk RNA-seq,Homo_714,AD.vs.MCI; bulk RNA-seq,Homo_714,MCI.vs.control; bulk RNA-seq,Homo_633,AD.vs.control	6
BP	GO:1904356	regulation of telomere maintenance via telomere lengthening	bulk RNA-seq,Homo_723,AD.vs.control; bulk RNA-seq,Homo_723,MCI.vs.control; bulk RNA-seq,Homo_714,AD.vs.control; bulk RNA-seq,Homo_714,MCI.vs.control; bulk RNA-seq,Homo_633,AD.vs.control; bulk RNA-seq,Homo_633,AD.vs.MCI	6
CC	GO:0019867	outer membrane	bulk RNA-seq,Homo_723,AD.vs.control; bulk RNA-seq,Homo_723,MCI.vs.control; bulk RNA-seq,Homo_714,AD.vs.control; bulk RNA-seq,Homo_633,AD.vs.control; bulk RNA-seq,Homo_633,AD.vs.MCI	5
MF	GO:0003954	NADH dehydrogenase activity	bulk RNA-seq,Homo_723,AD.vs.control; bulk RNA-seq,Homo_723,MCI.vs.control; bulk RNA-seq,Homo_714,AD.vs.control; bulk RNA-seq,Homo_714,MCI.vs.control; bulk RNA-seq,Homo_633,AD.vs.control; bulk RNA-seq,Homo_633,AD.vs.MCI	6
BP	GO:0010464	regulation of mesenchymal cell proliferation	bulk RNA-seq,Homo_723,AD.vs.control; bulk RNA-seq,Homo_723,AD.vs.MCI; bulk RNA-seq,Homo_723,MCI.vs.control; bulk RNA-seq,Homo_714,AD.vs.MCI; bulk RNA-seq,Homo_714,MCI.vs.control	5
MF	GO:0004659	prenyltransferase activity	bulk RNA-seq,Homo_723,AD.vs.control; bulk RNA-seq,Homo_723,AD.vs.MCI; bulk RNA-seq,Homo_714,AD.vs.control; bulk RNA-seq,Homo_714,AD.vs.MCI; bulk RNA-seq,Homo_633,AD.vs.control; bulk RNA-seq,Homo_633,AD.vs.MCI	6
MF	GO:0005544	calcium-dependent phospholipid binding	bulk RNA-seq,Homo_723,AD.vs.control; bulk RNA-seq,Homo_723,AD.vs.MCI; bulk RNA-seq,Homo_723,MCI.vs.control; bulk RNA-seq,Homo_714,AD.vs.MCI; bulk RNA-seq,Homo_633,AD.vs.control; bulk RNA-seq,Homo_633,AD.vs.MCI	6
BP	GO:0072538	T-helper 17 type immune response	bulk RNA-seq,Homo_723,AD.vs.control; bulk RNA-seq,Homo_723,MCI.vs.control; bulk RNA-seq,Homo_714,AD.vs.control; bulk RNA-seq,Homo_714,MCI.vs.control; bulk RNA-seq,Homo_633,AD.vs.control	5
BP	GO:0006390	mitochondrial transcription	bulk RNA-seq,Homo_723,AD.vs.control; bulk RNA-seq,Homo_723,MCI.vs.control; bulk RNA-seq,Homo_714,AD.vs.control; bulk RNA-seq,Homo_714,MCI.vs.control; bulk RNA-seq,Homo_633,AD.vs.control; bulk RNA-seq,Homo_633,MCI.vs.control	6
BP	GO:0043488	regulation of mRNA stability	bulk RNA-seq,Homo_723,AD.vs.control; bulk RNA-seq,Homo_723,MCI.vs.control; bulk RNA-seq,Homo_714,AD.vs.control; bulk RNA-seq,Homo_633,AD.vs.control; bulk RNA-seq,Homo_633,AD.vs.MCI	5
BP	GO:0061299	retina vasculature morphogenesis in camera-type eye	bulk RNA-seq,Homo_723,AD.vs.control; bulk RNA-seq,Homo_723,AD.vs.MCI; bulk RNA-seq,Homo_723,MCI.vs.control; bulk RNA-seq,Homo_714,AD.vs.control; bulk RNA-seq,Homo_714,AD.vs.MCI; bulk RNA-seq,Homo_714,MCI.vs.control	6
BP	GO:0006743	ubiquinone metabolic process	bulk RNA-seq,Homo_723,AD.vs.control; bulk RNA-seq,Homo_723,AD.vs.MCI; bulk RNA-seq,Homo_714,AD.vs.control; bulk RNA-seq,Homo_714,AD.vs.MCI; bulk RNA-seq,Homo_633,AD.vs.control; bulk RNA-seq,Homo_633,AD.vs.MCI	6
BP	GO:0072283	metanephric renal vesicle morphogenesis	bulk RNA-seq,Homo_723,AD.vs.control; bulk RNA-seq,Homo_723,AD.vs.MCI; bulk RNA-seq,Homo_723,MCI.vs.control; bulk RNA-seq,Homo_714,AD.vs.MCI; bulk RNA-seq,Homo_633,MCI.vs.control	5
BP	GO:0070131	positive regulation of mitochondrial translation	bulk RNA-seq,Homo_723,AD.vs.control; bulk RNA-seq,Homo_723,AD.vs.MCI; bulk RNA-seq,Homo_714,AD.vs.control; bulk RNA-seq,Homo_633,AD.vs.control; bulk RNA-seq,Homo_633,AD.vs.MCI	5
BP	GO:0001910	regulation of leukocyte mediated cytotoxicity	bulk RNA-seq,Homo_723,AD.vs.control; bulk RNA-seq,Homo_714,AD.vs.control; bulk RNA-seq,Homo_714,MCI.vs.control; bulk RNA-seq,Homo_633,AD.vs.control; bulk RNA-seq,Homo_633,AD.vs.MCI; scRNA-seq,SRP309935,B cell_1-AD.vs.control; scRNA-seq,SRP215507,CD8+ T cell_3-	7
BP	GO:0045830	positive regulation of isotype switching	bulk RNA-seq,Homo_723,AD.vs.control; bulk RNA-seq,Homo_723,MCI.vs.control; bulk RNA-seq,Homo_714,AD.vs.control; bulk RNA-seq,Homo_714,MCI.vs.control; bulk RNA-seq,Homo_633,AD.vs.control; bulk RNA-seq,Homo_633,AD.vs.MCI; bulk RNA-seq,Homo_633,MCI.vs.control	7
BP	GO:1901096	regulation of autophagosome maturation	bulk RNA-seq,Homo_723,AD.vs.control; bulk RNA-seq,Homo_723,AD.vs.MCI; bulk RNA-seq,Homo_714,AD.vs.control; bulk RNA-seq,Homo_714,AD.vs.MCI; bulk RNA-seq,Homo_633,AD.vs.control; bulk RNA-seq,Homo_633,AD.vs.MCI	6
MF	GO:0042043	neurexin family protein binding	bulk RNA-seq,Homo_723,AD.vs.control; bulk RNA-seq,Homo_723,AD.vs.MCI; bulk RNA-seq,Homo_723,MCI.vs.control; bulk RNA-seq,Homo_714,AD.vs.MCI; bulk RNA-seq,Homo_714,MCI.vs.control	5
BP	GO:0060575	intestinal epithelial cell differentiation	bulk RNA-seq,Homo_723,AD.vs.control; bulk RNA-seq,Homo_723,AD.vs.MCI; bulk RNA-seq,Homo_723,MCI.vs.control; bulk RNA-seq,Homo_714,AD.vs.MCI; bulk RNA-seq,Homo_714,MCI.vs.control	5

BP	GO:0045619	regulation of lymphocyte differentiation	bulk RNA-seq,Homo_723,AD.vs.control; bulk RNA-seq,Homo_723,MCI.vs.control; bulk RNA-seq,Homo_714,AD.vs.control; bulk RNA-seq,Homo_633,AD.vs.control; bulk RNA-seq,Homo_633,AD.vs.MCI	5
CC	GO:0014704	intercalated disc	bulk RNA-seq,Homo_723,AD.vs.control; bulk RNA-seq,Homo_723,AD.vs.MCI; bulk RNA-seq,Homo_723,MCI.vs.control; bulk RNA-seq,Homo_714,AD.vs.MCI; bulk RNA-seq,Homo_714,MCI.vs.control	5
BP	GO:0030539	male genitalia development	bulk RNA-seq,Homo_723,AD.vs.control; bulk RNA-seq,Homo_723,AD.vs.MCI; bulk RNA-seq,Homo_723,MCI.vs.control; bulk RNA-seq,Homo_714,AD.vs.control; bulk RNA-seq,Homo_714,AD.vs.MCI	5
MF	GO:0008146	sulfotransferase activity	bulk RNA-seq,Homo_723,AD.vs.control; bulk RNA-seq,Homo_723,AD.vs.MCI; bulk RNA-seq,Homo_723,MCI.vs.control; bulk RNA-seq,Homo_714,AD.vs.control; bulk RNA-seq,Homo_714,AD.vs.MCI	5
BP	GO:0097120	receptor localization to synapse	bulk RNA-seq,Homo_723,AD.vs.control; bulk RNA-seq,Homo_723,AD.vs.MCI; bulk RNA-seq,Homo_723,MCI.vs.control; bulk RNA-seq,Homo_714,AD.vs.MCI; bulk RNA-seq,Homo_714,MCI.vs.control; bulk RNA-seq,Homo_633,AD.vs.control; bulk RNA-seq,Homo_633,AD.vs.MCI	7
MF	GO:0009055	electron transfer activity	bulk RNA-seq,Homo_723,AD.vs.control; bulk RNA-seq,Homo_723,MCI.vs.control; bulk RNA-seq,Homo_714,AD.vs.control; bulk RNA-seq,Homo_714,MCI.vs.control; bulk RNA-seq,Homo_633,AD.vs.control; bulk RNA-seq,Homo_633,AD.vs.MCI	6
BP	GO:0052548	regulation of endopeptidase activity	bulk RNA-seq,Homo_723,AD.vs.control; bulk RNA-seq,Homo_723,AD.vs.MCI; bulk RNA-seq,Homo_714,AD.vs.control; bulk RNA-seq,Homo_714,AD.vs.MCI; bulk RNA-seq,Homo_633,AD.vs.control; bulk RNA-seq,Homo_633,AD.vs.MCI; bulk RNA-seq,SRP223445,AD.vs.control	7
BP	GO:0051899	membrane depolarization	bulk RNA-seq,Homo_723,AD.vs.control; bulk RNA-seq,Homo_723,AD.vs.MCI; bulk RNA-seq,Homo_723,MCI.vs.control; bulk RNA-seq,Homo_714,AD.vs.MCI; bulk RNA-seq,Homo_714,MCI.vs.control; bulk RNA-seq,Homo_633,AD.vs.control; bulk RNA-seq,Homo_633,AD.vs.MCI	7
BP	GO:0021533	cell differentiation in hindbrain	bulk RNA-seq,Homo_723,AD.vs.control; bulk RNA-seq,Homo_723,AD.vs.MCI; bulk RNA-seq,Homo_723,MCI.vs.control; bulk RNA-seq,Homo_714,AD.vs.MCI; bulk RNA-seq,Homo_633,AD.vs.control; bulk RNA-seq,Homo_633,AD.vs.MCI	6
CC	GO:0016327	apicolateral plasma membrane	bulk RNA-seq,Homo_723,AD.vs.control; bulk RNA-seq,Homo_723,AD.vs.MCI; bulk RNA-seq,Homo_723,MCI.vs.control; bulk RNA-seq,Homo_714,AD.vs.control; bulk RNA-seq,Homo_714,AD.vs.MCI	5
BP	GO:0089718	amino acid import across plasma membrane	bulk RNA-seq,Homo_723,AD.vs.control; bulk RNA-seq,Homo_723,AD.vs.MCI; bulk RNA-seq,Homo_723,MCI.vs.control; bulk RNA-seq,Homo_714,AD.vs.MCI	4
BP	GO:0070202	regulation of establishment of protein localization to chromosome	bulk RNA-seq,Homo_723,AD.vs.control; bulk RNA-seq,Homo_723,AD.vs.MCI; bulk RNA-seq,Homo_723,MCI.vs.control; bulk RNA-seq,Homo_714,AD.vs.control; bulk RNA-seq,Homo_714,AD.vs.MCI; bulk RNA-seq,Homo_714,MCI.vs.control; bulk RNA-seq,Homo_633,AD.vs.control; bulk RNA-seq,Homo_633,AD.vs.MCI; bulk RNA-seq,Homo_633,MCI.vs.control	9
BP	GO:0060444	branching involved in mammary gland duct morphogenesis	bulk RNA-seq,Homo_723,AD.vs.control; bulk RNA-seq,Homo_723,AD.vs.MCI; bulk RNA-seq,Homo_714,AD.vs.control; bulk RNA-seq,Homo_714,AD.vs.MCI	4
MF	GO:0015459	potassium channel regulator activity	bulk RNA-seq,Homo_723,AD.vs.control; bulk RNA-seq,Homo_723,AD.vs.MCI; bulk RNA-seq,Homo_723,MCI.vs.control; bulk RNA-seq,Homo_714,AD.vs.MCI; bulk RNA-seq,Homo_714,MCI.vs.control	5
BP	GO:0060384	innervation	bulk RNA-seq,Homo_723,AD.vs.control; bulk RNA-seq,Homo_723,AD.vs.MCI; bulk RNA-seq,Homo_723,MCI.vs.control; bulk RNA-seq,Homo_714,AD.vs.MCI; bulk RNA-seq,Homo_714,MCI.vs.control	5
BP	GO:0072087	renal vesicle development	bulk RNA-seq,Homo_723,AD.vs.control; bulk RNA-seq,Homo_723,AD.vs.MCI; bulk RNA-seq,Homo_723,MCI.vs.control; bulk RNA-seq,Homo_714,AD.vs.MCI; bulk RNA-seq,Homo_714,MCI.vs.control; bulk RNA-seq,Homo_633,MCI.vs.control	6
BP	GO:0044331	cell-cell adhesion mediated by cadherin	bulk RNA-seq,Homo_723,AD.vs.control; bulk RNA-seq,Homo_723,AD.vs.MCI; bulk RNA-seq,Homo_723,MCI.vs.control; bulk RNA-seq,Homo_714,AD.vs.MCI; bulk RNA-seq,Homo_714,MCI.vs.control; bulk RNA-seq,Homo_633,AD.vs.control	6
CC	GO:0045334	clathrin-coated endocytic vesicle	bulk RNA-seq,Homo_723,AD.vs.control; bulk RNA-seq,Homo_723,AD.vs.MCI; bulk RNA-seq,Homo_723,MCI.vs.control; bulk RNA-seq,Homo_714,AD.vs.MCI; bulk RNA-seq,Homo_714,MCI.vs.control; bulk RNA-seq,Homo_633,AD.vs.control; scRNA-seq,SRP215507,CD8+ T cell_3-AD.vs.control	7
BP	GO:0001541	ovarian follicle development	bulk RNA-seq,Homo_723,AD.vs.control; bulk RNA-seq,Homo_723,AD.vs.MCI; bulk RNA-seq,Homo_723,MCI.vs.control; bulk RNA-seq,Homo_714,AD.vs.control; bulk RNA-seq,Homo_714,AD.vs.MCI; bulk RNA-seq,Homo_714,MCI.vs.control	6
BP	GO:0021695	cerebellar cortex development	bulk RNA-seq,Homo_723,AD.vs.control; bulk RNA-seq,Homo_723,AD.vs.MCI; bulk RNA-seq,Homo_723,MCI.vs.control; bulk RNA-seq,Homo_714,AD.vs.MCI; bulk RNA-seq,Homo_714,MCI.vs.control; bulk RNA-seq,Homo_633,AD.vs.control; bulk RNA-seq,Homo_633,AD.vs.MCI	7
BP	GO:0046632	alpha-beta T cell differentiation	bulk RNA-seq,Homo_723,AD.vs.control; bulk RNA-seq,Homo_723,MCI.vs.control; bulk RNA-seq,Homo_714,AD.vs.control; bulk RNA-seq,Homo_714,MCI.vs.control; bulk RNA-seq,Homo_633,AD.vs.control; bulk RNA-seq,Homo_633,AD.vs.MCI	6
MF	GO:0004713	protein tyrosine kinase activity	bulk RNA-seq,Homo_723,AD.vs.control; bulk RNA-seq,Homo_723,AD.vs.MCI; bulk RNA-seq,Homo_723,MCI.vs.control; bulk RNA-seq,Homo_714,AD.vs.MCI; bulk RNA-seq,Homo_714,MCI.vs.control; bulk RNA-seq,Homo_633,AD.vs.control; bulk RNA-seq,Homo_633,AD.vs.MCI; bulk RNA-	8
BP	GO:0006367	transcription initiation at RNA polymerase II promoter	bulk RNA-seq,Homo_723,AD.vs.control; bulk RNA-seq,Homo_723,MCI.vs.control; bulk RNA-seq,Homo_714,AD.vs.control; bulk RNA-seq,Homo_714,MCI.vs.control; bulk RNA-seq,Homo_633,AD.vs.control; bulk RNA-seq,Homo_633,AD.vs.MCI	6
BP	GO:0033622	integrin activation	bulk RNA-seq,Homo_723,AD.vs.control; bulk RNA-seq,Homo_723,AD.vs.MCI; bulk RNA-seq,Homo_723,MCI.vs.control; bulk RNA-seq,Homo_714,AD.vs.control; bulk RNA-seq,Homo_714,AD.vs.MCI; bulk RNA-seq,Homo_714,MCI.vs.control; bulk RNA-seq,Homo_633,AD.vs.control	7
BP	GO:0060561	apoptotic process involved in morphogenesis	bulk RNA-seq,Homo_723,AD.vs.control; bulk RNA-seq,Homo_723,AD.vs.MCI; bulk RNA-seq,Homo_714,AD.vs.control; bulk RNA-seq,Homo_714,AD.vs.MCI; bulk RNA-seq,Homo_633,MCI.vs.control	5
MF	GO:0003777	microtubule motor activity	bulk RNA-seq,Homo_723,AD.vs.control; bulk RNA-seq,Homo_723,AD.vs.MCI; bulk RNA-seq,Homo_723,MCI.vs.control; bulk RNA-seq,Homo_714,AD.vs.MCI; bulk RNA-seq,Homo_714,MCI.vs.control; bulk RNA-seq,Homo_633,AD.vs.control; bulk RNA-seq,Homo_633,AD.vs.MCI; bulk RNA-	8
BP	GO:0034331	cell junction maintenance	bulk RNA-seq,Homo_723,AD.vs.control; bulk RNA-seq,Homo_723,AD.vs.MCI; bulk RNA-seq,Homo_723,MCI.vs.control; bulk RNA-seq,Homo_714,AD.vs.MCI; bulk RNA-seq,Homo_714,MCI.vs.control; bulk RNA-seq,ROSMAP,AD.vs.control	6
CC	GO:0005852	eukaryotic translation initiation factor 3 complex	bulk RNA-seq,Homo_723,AD.vs.control; bulk RNA-seq,Homo_723,AD.vs.MCI; bulk RNA-seq,Homo_714,AD.vs.control; bulk RNA-seq,Homo_714,AD.vs.MCI; bulk RNA-seq,Homo_633,AD.vs.control	5
BP	GO:0030509	BMP signaling pathway	bulk RNA-seq,Homo_723,AD.vs.control; bulk RNA-seq,Homo_723,AD.vs.MCI; bulk RNA-seq,Homo_723,MCI.vs.control; bulk RNA-seq,Homo_714,AD.vs.MCI; bulk RNA-seq,Homo_633,AD.vs.control; bulk RNA-seq,SRP223445,AD.vs.control	6
BP	GO:0001838	embryonic epithelial tube formation	bulk RNA-seq,Homo_723,AD.vs.control; bulk RNA-seq,Homo_723,AD.vs.MCI; bulk RNA-seq,Homo_723,MCI.vs.control; bulk RNA-seq,Homo_714,AD.vs.MCI; bulk RNA-seq,Homo_714,MCI.vs.control; bulk RNA-seq,Homo_633,AD.vs.control; bulk RNA-seq,Homo_633,AD.vs.MCI	7
BP	GO:0051412	response to corticosterone	bulk RNA-seq,Homo_723,AD.vs.control; bulk RNA-seq,Homo_723,AD.vs.MCI; bulk RNA-seq,Homo_723,MCI.vs.control; bulk RNA-seq,Homo_714,AD.vs.control; bulk RNA-seq,Homo_714,AD.vs.MCI	5
BP	GO:0001909	leukocyte mediated cytotoxicity	bulk RNA-seq,Homo_723,AD.vs.control; bulk RNA-seq,Homo_723,MCI.vs.control; bulk RNA-seq,Homo_714,AD.vs.control; bulk RNA-seq,Homo_633,AD.vs.control; bulk RNA-seq,Homo_633,AD.vs.MCI; scRNA-seq,SRP309935,B cell_1-AD.vs.control; scRNA-seq,SRP215507,CD8+ T cell_3-	7

BP	GO:0002823	negative regulation of adaptive immune response based on somatic recombination of immune receptors built from immunoglobulin superfamily domains	bulk RNA-seq,Homo_723,AD.vs.control; bulk RNA-seq,Homo_723,MCI.vs.control; bulk RNA-seq,Homo_714,AD.vs.control; bulk RNA-seq,Homo_714,MCI.vs.control; bulk RNA-seq,Homo_633,AD.vs.control; bulk RNA-seq,Homo_633,AD.vs.MCI	6
CC	GO:0098992	neuronal dense core vesicle	bulk RNA-seq,Homo_723,AD.vs.control; bulk RNA-seq,Homo_723,AD.vs.MCI; bulk RNA-seq,Homo_723,MCI.vs.control; bulk RNA-seq,Homo_714,AD.vs.MCI	4
BP	GO:0051957	positive regulation of amino acid transport	bulk RNA-seq,Homo_723,AD.vs.control; bulk RNA-seq,Homo_723,AD.vs.MCI; bulk RNA-seq,Homo_723,MCI.vs.control; bulk RNA-seq,Homo_714,AD.vs.control; bulk RNA-seq,Homo_714,AD.vs.MCI	5
BP	GO:0099072	regulation of postsynaptic membrane neurotransmitter receptor levels	bulk RNA-seq,Homo_723,AD.vs.control; bulk RNA-seq,Homo_723,AD.vs.MCI; bulk RNA-seq,Homo_723,MCI.vs.control; bulk RNA-seq,Homo_714,AD.vs.MCI; bulk RNA-seq,Homo_714,MCI.vs.control; bulk RNA-seq,Homo_633,AD.vs.control	6
BP	GO:0071868	cellular response to monoamine stimulus	bulk RNA-seq,Homo_723,AD.vs.control; bulk RNA-seq,Homo_723,AD.vs.MCI; bulk RNA-seq,Homo_723,MCI.vs.control; bulk RNA-seq,Homo_714,AD.vs.control; bulk RNA-seq,Homo_714,AD.vs.MCI; bulk RNA-seq,Homo_714,MCI.vs.control; bulk RNA-seq,Homo_633,AD.vs.control; bulk	8
BP	GO:0071870	cellular response to catecholamine stimulus	bulk RNA-seq,Homo_723,AD.vs.control; bulk RNA-seq,Homo_723,AD.vs.MCI; bulk RNA-seq,Homo_723,MCI.vs.control; bulk RNA-seq,Homo_714,AD.vs.control; bulk RNA-seq,Homo_714,AD.vs.MCI; bulk RNA-seq,Homo_714,MCI.vs.control; bulk RNA-seq,Homo_633,AD.vs.control; bulk	8
BP	GO:0050792	regulation of viral process	bulk RNA-seq,Homo_723,AD.vs.control; bulk RNA-seq,Homo_723,MCI.vs.control; bulk RNA-seq,Homo_714,AD.vs.control; bulk RNA-seq,Homo_633,AD.vs.control; bulk RNA-seq,Homo_633,AD.vs.MCI	5
BP	GO:0030432	peristalsis	bulk RNA-seq,Homo_723,AD.vs.control; bulk RNA-seq,Homo_723,AD.vs.MCI; bulk RNA-seq,Homo_723,MCI.vs.control; bulk RNA-seq,Homo_714,AD.vs.control; bulk RNA-seq,Homo_714,AD.vs.MCI; bulk RNA-seq,Homo_633,MCI.vs.control	6
BP	GO:0071466	cellular response to xenobiotic stimulus	bulk RNA-seq,Homo_723,AD.vs.control; bulk RNA-seq,Homo_723,AD.vs.MCI; bulk RNA-seq,Homo_723,MCI.vs.control; bulk RNA-seq,Homo_714,AD.vs.control; bulk RNA-seq,Homo_714,AD.vs.MCI; bulk RNA-seq,Homo_633,AD.vs.control; bulk RNA-seq,Homo_633,AD.vs.MCI	7
BP	GO:0061013	regulation of mRNA catabolic process	bulk RNA-seq,Homo_723,AD.vs.control; bulk RNA-seq,Homo_723,MCI.vs.control; bulk RNA-seq,Homo_714,AD.vs.control; bulk RNA-seq,Homo_633,AD.vs.control; bulk RNA-seq,Homo_633,AD.vs.MCI	5
MF	GO:0001637	G protein-coupled chemoattractant receptor activity	bulk RNA-seq,Homo_723,AD.vs.control; bulk RNA-seq,Homo_723,AD.vs.MCI; bulk RNA-seq,Homo_633,AD.vs.control	3
MF	GO:0004950	chemokine receptor activity	bulk RNA-seq,Homo_723,AD.vs.control; bulk RNA-seq,Homo_723,AD.vs.MCI; bulk RNA-seq,Homo_633,AD.vs.control	3
BP	GO:0036257	multivesicular body organization	bulk RNA-seq,Homo_723,AD.vs.control; bulk RNA-seq,Homo_723,MCI.vs.control; bulk RNA-seq,Homo_714,AD.vs.control; bulk RNA-seq,Homo_714,MCI.vs.control; bulk RNA-seq,Homo_633,AD.vs.control; bulk RNA-seq,Homo_633,AD.vs.MCI; bulk RNA-seq,Homo_633,MCI.vs.control	7
BP	GO:0001787	natural killer cell proliferation	bulk RNA-seq,Homo_723,AD.vs.control; bulk RNA-seq,Homo_723,AD.vs.MCI; bulk RNA-seq,Homo_714,MCI.vs.control; bulk RNA-	4
BP	GO:0048041	focal adhesion assembly	bulk RNA-seq,Homo_723,AD.vs.control; bulk RNA-seq,Homo_723,AD.vs.MCI; bulk RNA-seq,Homo_723,MCI.vs.control; bulk RNA-seq,Homo_714,AD.vs.MCI; bulk RNA-seq,Homo_714,MCI.vs.control; bulk RNA-seq,Homo_633,AD.vs.control; bulk RNA-seq,Homo_633,AD.vs.MCI	7
BP	GO:0048148	behavioral response to cocaine	bulk RNA-seq,Homo_723,AD.vs.control; bulk RNA-seq,Homo_723,AD.vs.MCI; bulk RNA-seq,Homo_714,AD.vs.control; bulk RNA-seq,Homo_714,AD.vs.MCI	4
CC	GO:0070847	core mediator complex	bulk RNA-seq,Homo_723,AD.vs.control; bulk RNA-seq,Homo_723,MCI.vs.control; bulk RNA-seq,Homo_714,AD.vs.control; bulk RNA-seq,Homo_714,MCI.vs.control; bulk RNA-seq,Homo_633,AD.vs.control; bulk RNA-seq,Homo_633,AD.vs.MCI; bulk RNA-seq,Homo_633,MCI.vs.control	7
BP	GO:0097090	presynaptic membrane organization	bulk RNA-seq,Homo_723,AD.vs.control; bulk RNA-seq,Homo_723,AD.vs.MCI; bulk RNA-seq,Homo_723,MCI.vs.control; bulk RNA-seq,Homo_714,AD.vs.MCI; bulk RNA-seq,Homo_714,MCI.vs.control; bulk RNA-seq,SRP223445,AD.vs.control	6
CC	GO:0030027	lamellipodium	bulk RNA-seq,Homo_723,AD.vs.control; bulk RNA-seq,Homo_723,AD.vs.MCI; bulk RNA-seq,Homo_723,MCI.vs.control; bulk RNA-seq,Homo_714,AD.vs.MCI; bulk RNA-seq,Homo_633,AD.vs.control; bulk RNA-seq,Homo_633,AD.vs.MCI	6
BP	GO:0006901	vesicle coating	bulk RNA-seq,Homo_723,AD.vs.control; bulk RNA-seq,Homo_714,AD.vs.control; bulk RNA-seq,Homo_714,MCI.vs.control; bulk RNA-seq,Homo_633,AD.vs.control; bulk RNA-seq,Homo_633,AD.vs.MCI	5
CC	GO:0005849	mRNA cleavage factor complex	bulk RNA-seq,Homo_723,AD.vs.control; bulk RNA-seq,Homo_723,AD.vs.MCI; bulk RNA-seq,Homo_714,AD.vs.control; bulk RNA-seq,Homo_714,AD.vs.MCI; bulk RNA-seq,Homo_633,AD.vs.control; bulk RNA-seq,Homo_633,AD.vs.MCI	6
MF	GO:0048407	platelet-derived growth factor binding	bulk RNA-seq,Homo_723,AD.vs.control; bulk RNA-seq,Homo_723,AD.vs.MCI; bulk RNA-seq,Homo_723,MCI.vs.control; bulk RNA-seq,Homo_714,AD.vs.MCI; bulk RNA-seq,Homo_714,MCI.vs.control	5
BP	GO:0070167	regulation of biomineral tissue development	bulk RNA-seq,Homo_723,AD.vs.control; bulk RNA-seq,Homo_723,AD.vs.MCI; bulk RNA-seq,Homo_723,MCI.vs.control; bulk RNA-seq,Homo_714,AD.vs.control; bulk RNA-seq,Homo_714,AD.vs.MCI; bulk RNA-seq,Homo_714,MCI.vs.control; bulk RNA-seq,Homo_633,AD.vs.control; bulk	8
BP	GO:0032411	positive regulation of transporter activity	bulk RNA-seq,Homo_723,AD.vs.control; bulk RNA-seq,Homo_723,AD.vs.MCI; bulk RNA-seq,Homo_723,MCI.vs.control; bulk RNA-seq,Homo_714,AD.vs.MCI; bulk RNA-seq,Homo_714,MCI.vs.control; bulk RNA-seq,Homo_633,AD.vs.control	6
MF	GO:0032182	ubiquitin-like protein binding	bulk RNA-seq,Homo_723,AD.vs.control; bulk RNA-seq,Homo_723,MCI.vs.control; bulk RNA-seq,Homo_714,AD.vs.control; bulk RNA-seq,Homo_714,MCI.vs.control; bulk RNA-seq,Homo_633,AD.vs.control; bulk RNA-seq,Homo_633,AD.vs.MCI	6
BP	GO:0045446	endothelial cell differentiation	bulk RNA-seq,Homo_723,AD.vs.control; bulk RNA-seq,Homo_723,AD.vs.MCI; bulk RNA-seq,Homo_723,MCI.vs.control; bulk RNA-seq,Homo_714,AD.vs.control; bulk RNA-seq,Homo_714,AD.vs.MCI; bulk RNA-seq,Homo_714,MCI.vs.control; bulk RNA-seq,Homo_633,AD.vs.control; bulk	8
BP	GO:0061318	renal filtration cell differentiation	bulk RNA-seq,Homo_723,AD.vs.control; bulk RNA-seq,Homo_723,AD.vs.MCI; bulk RNA-seq,Homo_723,MCI.vs.control; bulk RNA-seq,Homo_714,AD.vs.MCI	4
BP	GO:0072112	podocyte differentiation	bulk RNA-seq,Homo_723,AD.vs.control; bulk RNA-seq,Homo_723,AD.vs.MCI; bulk RNA-seq,Homo_723,MCI.vs.control; bulk RNA-seq,Homo_714,AD.vs.MCI	4
BP	GO:2001259	positive regulation of cation channel activity	bulk RNA-seq,Homo_723,AD.vs.control; bulk RNA-seq,Homo_723,AD.vs.MCI; bulk RNA-seq,Homo_723,MCI.vs.control; bulk RNA-seq,Homo_714,AD.vs.MCI; bulk RNA-seq,Homo_714,MCI.vs.control	5
BP	GO:0045880	positive regulation of smoothened signaling pathway	bulk RNA-seq,Homo_723,AD.vs.control; bulk RNA-seq,Homo_723,AD.vs.MCI; bulk RNA-seq,Homo_723,MCI.vs.control; bulk RNA-seq,Homo_714,AD.vs.MCI; bulk RNA-seq,Homo_714,MCI.vs.control; bulk RNA-seq,Homo_633,AD.vs.control; bulk RNA-seq,SRP223445,AD.vs.control	7
BP	GO:0006903	vesicle targeting	bulk RNA-seq,Homo_723,AD.vs.control; bulk RNA-seq,Homo_723,MCI.vs.control; bulk RNA-seq,Homo_714,AD.vs.control; bulk RNA-seq,Homo_714,MCI.vs.control; bulk RNA-seq,Homo_633,AD.vs.control; bulk RNA-seq,Homo_633,AD.vs.MCI	6
CC	GO:0030863	cortical cytoskeleton	bulk RNA-seq,Homo_723,AD.vs.control; bulk RNA-seq,Homo_723,AD.vs.MCI; bulk RNA-seq,Homo_723,MCI.vs.control; bulk RNA-seq,Homo_714,AD.vs.MCI; bulk RNA-seq,Homo_714,MCI.vs.control; bulk RNA-seq,Homo_633,AD.vs.control; bulk RNA-seq,Homo_633,AD.vs.MCI; bulk RNA-	8
BP	GO:0000819	sister chromatid segregation	bulk RNA-seq,Homo_723,AD.vs.control; bulk RNA-seq,Homo_723,MCI.vs.control; bulk RNA-seq,Homo_714,AD.vs.control; bulk RNA-seq,Homo_633,AD.vs.control; bulk RNA-seq,Homo_633,AD.vs.MCI	5
BP	GO:0048569	post-embryonic animal organ development	bulk RNA-seq,Homo_723,AD.vs.control; bulk RNA-seq,Homo_723,AD.vs.MCI; bulk RNA-seq,Homo_723,MCI.vs.control; bulk RNA-seq,Homo_714,AD.vs.MCI; bulk RNA-seq,Homo_714,MCI.vs.control	5
BP	GO:1905207	regulation of cardiocyte differentiation	bulk RNA-seq,Homo_723,AD.vs.control; bulk RNA-seq,Homo_723,AD.vs.MCI; bulk RNA-seq,Homo_723,MCI.vs.control; bulk RNA-seq,Homo_714,AD.vs.control; bulk RNA-seq,Homo_714,AD.vs.MCI; bulk RNA-seq,Homo_714,MCI.vs.control	6

BP	GO:0035459	vesicle cargo loading	bulk RNA-seq,Homo_723,AD.vs.control; bulk RNA-seq,Homo_723,MCI.vs.control; bulk RNA-seq,Homo_714,AD.vs.control; bulk RNA-seq,Homo_714,MCI.vs.control; bulk RNA-seq,Homo_633,AD.vs.control; bulk RNA-seq,Homo_633,AD.vs.MCI; bulk RNA-seq,Homo_633,MCI.vs.control	7
BP	GO:0050909	sensory perception of taste	bulk RNA-seq,Homo_723,AD.vs.control; bulk RNA-seq,Homo_723,AD.vs.MCI; bulk RNA-seq,Homo_714,AD.vs.control; bulk RNA-seq,Homo_714,AD.vs.MCI; bulk RNA-seq,ROSMAP,AD.vs.control; bulk RNA-seq,SRP223445,AD.vs.control	6
BP	GO:0045933	positive regulation of muscle contraction	bulk RNA-seq,Homo_723,AD.vs.control; bulk RNA-seq,Homo_723,AD.vs.MCI; bulk RNA-seq,Homo_714,AD.vs.control; bulk RNA-seq,Homo_714,AD.vs.MCI; bulk RNA-seq,SRP223445,AD.vs.control	5
BP	GO:0021781	glial cell fate commitment	bulk RNA-seq,Homo_723,AD.vs.control; bulk RNA-seq,Homo_723,AD.vs.MCI; bulk RNA-seq,Homo_723,MCI.vs.control; bulk RNA-	4
MF	GO:0043236	laminin binding	bulk RNA-seq,Homo_723,AD.vs.control; bulk RNA-seq,Homo_723,AD.vs.MCI; bulk RNA-seq,Homo_723,MCI.vs.control; bulk RNA-seq,Homo_714,AD.vs.MCI; bulk RNA-seq,Homo_714,MCI.vs.control; bulk RNA-seq,Homo_633,AD.vs.control	6
BP	GO:0030278	regulation of ossification	bulk RNA-seq,Homo_723,AD.vs.control; bulk RNA-seq,Homo_723,AD.vs.MCI; bulk RNA-seq,Homo_723,MCI.vs.control; bulk RNA-seq,Homo_714,AD.vs.MCI; bulk RNA-seq,Homo_714,MCI.vs.control; bulk RNA-seq,Homo_633,AD.vs.control; bulk RNA-seq,Homo_633,AD.vs.MCI	7
MF	GO:0008135	translation factor activity, RNA binding	bulk RNA-seq,Homo_723,AD.vs.control; bulk RNA-seq,Homo_723,MCI.vs.control; bulk RNA-seq,Homo_714,AD.vs.control; bulk RNA-seq,Homo_714,MCI.vs.control; bulk RNA-seq,Homo_633,AD.vs.control; bulk RNA-seq,Homo_633,AD.vs.MCI	6
MF	GO:0043394	proteoglycan binding	bulk RNA-seq,Homo_723,AD.vs.control; bulk RNA-seq,Homo_723,AD.vs.MCI; bulk RNA-seq,Homo_723,MCI.vs.control; bulk RNA-seq,Homo_714,AD.vs.control; bulk RNA-seq,Homo_714,AD.vs.MCI; bulk RNA-seq,Homo_714,MCI.vs.control	6
BP	GO:0002820	negative regulation of adaptive immune response	bulk RNA-seq,Homo_723,AD.vs.control; bulk RNA-seq,Homo_723,MCI.vs.control; bulk RNA-seq,Homo_714,AD.vs.control; bulk RNA-seq,Homo_714,MCI.vs.control; bulk RNA-seq,Homo_633,AD.vs.control; bulk RNA-seq,Homo_633,AD.vs.MCI	6
BP	GO:2000779	regulation of double-strand break repair	bulk RNA-seq,Homo_723,AD.vs.control; bulk RNA-seq,Homo_723,MCI.vs.control; bulk RNA-seq,Homo_714,AD.vs.control; bulk RNA-seq,Homo_633,AD.vs.control; bulk RNA-seq,Homo_633,AD.vs.MCI	5
BP	GO:0044703	multi-organism reproductive process	bulk RNA-seq,Homo_723,AD.vs.control; bulk RNA-seq,Homo_723,AD.vs.MCI; bulk RNA-seq,Homo_723,MCI.vs.control; bulk RNA-seq,Homo_714,AD.vs.control; bulk RNA-seq,Homo_714,AD.vs.MCI; bulk RNA-seq,Homo_633,AD.vs.control; bulk RNA-seq,Homo_633,AD.vs.MCI	7
BP	GO:0035458	cellular response to interferon-beta	bulk RNA-seq,Homo_723,AD.vs.control; bulk RNA-seq,Homo_714,AD.vs.control; bulk RNA-seq,Homo_633,AD.vs.control; bulk RNA-seq,Homo_633,AD.vs.MCI	4
CC	GO:0097346	INO80-type complex	bulk RNA-seq,Homo_723,AD.vs.control; bulk RNA-seq,Homo_723,MCI.vs.control; bulk RNA-seq,Homo_714,AD.vs.control; bulk RNA-seq,Homo_714,MCI.vs.control; bulk RNA-seq,Homo_633,AD.vs.control; bulk RNA-seq,Homo_633,AD.vs.MCI; bulk RNA-seq,Homo_633,MCI.vs.control	7
BP	GO:0006284	base-excision repair	bulk RNA-seq,Homo_723,AD.vs.control; bulk RNA-seq,Homo_723,MCI.vs.control; bulk RNA-seq,Homo_714,AD.vs.control; bulk RNA-seq,Homo_714,MCI.vs.control; bulk RNA-seq,Homo_633,AD.vs.control; bulk RNA-seq,Homo_633,AD.vs.MCI	6
BP	GO:1903405	protein localization to nuclear body	bulk RNA-seq,Homo_723,AD.vs.control; bulk RNA-seq,Homo_723,MCI.vs.control; bulk RNA-seq,Homo_714,AD.vs.control; bulk RNA-seq,Homo_714,MCI.vs.control; bulk RNA-seq,Homo_633,AD.vs.control; bulk RNA-seq,Homo_633,AD.vs.MCI; bulk RNA-seq,Homo_633,MCI.vs.control	7
BP	GO:1904867	protein localization to Cajal body	bulk RNA-seq,Homo_723,AD.vs.control; bulk RNA-seq,Homo_723,MCI.vs.control; bulk RNA-seq,Homo_714,AD.vs.control; bulk RNA-seq,Homo_714,MCI.vs.control; bulk RNA-seq,Homo_633,AD.vs.control; bulk RNA-seq,Homo_633,AD.vs.MCI; bulk RNA-seq,Homo_633,MCI.vs.control	7
BP	GO:0070527	platelet aggregation	bulk RNA-seq,Homo_723,AD.vs.control; bulk RNA-seq,Homo_723,AD.vs.MCI; bulk RNA-seq,Homo_723,MCI.vs.control; bulk RNA-seq,Homo_714,AD.vs.control; bulk RNA-seq,Homo_714,AD.vs.MCI; bulk RNA-seq,Homo_714,MCI.vs.control; bulk RNA-seq,Homo_633,AD.vs.control; bulk	8
BP	GO:1903351	cellular response to dopamine	bulk RNA-seq,Homo_723,AD.vs.control; bulk RNA-seq,Homo_723,AD.vs.MCI; bulk RNA-seq,Homo_723,MCI.vs.control; bulk RNA-seq,Homo_714,AD.vs.control; bulk RNA-seq,Homo_714,AD.vs.MCI; bulk RNA-seq,Homo_633,AD.vs.control	6
BP	GO:0034250	positive regulation of cellular amide metabolic process	bulk RNA-seq,Homo_723,AD.vs.control; bulk RNA-seq,Homo_723,MCI.vs.control; bulk RNA-seq,Homo_714,AD.vs.control; bulk RNA-seq,Homo_633,AD.vs.control; bulk RNA-seq,Homo_633,AD.vs.MCI	5
BP	GO:0042440	pigment metabolic process	bulk RNA-seq,Homo_723,AD.vs.control; bulk RNA-seq,Homo_723,AD.vs.MCI; bulk RNA-seq,Homo_723,MCI.vs.control; bulk RNA-seq,Homo_714,AD.vs.MCI; bulk RNA-seq,Homo_714,MCI.vs.control; bulk RNA-seq,Homo_633,AD.vs.control; bulk RNA-seq,Homo_633,AD.vs.MCI	7
BP	GO:0032608	interferon-beta production	bulk RNA-seq,Homo_723,AD.vs.control; bulk RNA-seq,Homo_723,MCI.vs.control; bulk RNA-seq,Homo_714,AD.vs.control; bulk RNA-seq,Homo_714,MCI.vs.control; bulk RNA-seq,Homo_633,AD.vs.control; bulk RNA-seq,Homo_633,AD.vs.MCI	6
BP	GO:0032648	regulation of interferon-beta production	bulk RNA-seq,Homo_723,AD.vs.control; bulk RNA-seq,Homo_723,MCI.vs.control; bulk RNA-seq,Homo_714,AD.vs.control; bulk RNA-seq,Homo_714,MCI.vs.control; bulk RNA-seq,Homo_633,AD.vs.control; bulk RNA-seq,Homo_633,AD.vs.MCI	6
BP	GO:0001710	mesodermal cell fate commitment	bulk RNA-seq,Homo_723,AD.vs.control; bulk RNA-seq,Homo_723,AD.vs.MCI; bulk RNA-seq,Homo_714,AD.vs.MCI	3
BP	GO:0051147	regulation of muscle cell differentiation	bulk RNA-seq,Homo_723,AD.vs.control; bulk RNA-seq,Homo_723,AD.vs.MCI; bulk RNA-seq,Homo_723,MCI.vs.control; bulk RNA-seq,Homo_714,AD.vs.MCI	4
BP	GO:0072498	embryonic skeletal joint development	bulk RNA-seq,Homo_723,AD.vs.control; bulk RNA-seq,Homo_723,AD.vs.MCI; bulk RNA-seq,Homo_723,MCI.vs.control; bulk RNA-seq,Homo_714,AD.vs.MCI; bulk RNA-seq,SRP223445,AD.vs.control	5
BP	GO:0007565	female pregnancy	bulk RNA-seq,Homo_723,AD.vs.control; bulk RNA-seq,Homo_723,AD.vs.MCI; bulk RNA-seq,Homo_723,MCI.vs.control; bulk RNA-seq,Homo_714,AD.vs.control; bulk RNA-seq,Homo_714,AD.vs.MCI; bulk RNA-seq,Homo_633,AD.vs.control; bulk RNA-seq,Homo_633,AD.vs.MCI	7
BP	GO:0035455	response to interferon-alpha	bulk RNA-seq,Homo_723,AD.vs.control; bulk RNA-seq,Homo_723,AD.vs.MCI; bulk RNA-seq,Homo_714,AD.vs.control; bulk RNA-seq,Homo_714,AD.vs.MCI; bulk RNA-seq,Homo_633,AD.vs.control; bulk RNA-seq,Homo_633,AD.vs.MCI	6
BP	GO:0002712	regulation of B cell mediated immunity	bulk RNA-seq,Homo_723,AD.vs.control; bulk RNA-seq,Homo_723,MCI.vs.control; bulk RNA-seq,Homo_714,AD.vs.control; bulk RNA-seq,Homo_714,MCI.vs.control; bulk RNA-seq,Homo_633,AD.vs.control; bulk RNA-seq,Homo_633,AD.vs.MCI	6
BP	GO:0002889	regulation of immunoglobulin mediated immune response	bulk RNA-seq,Homo_723,AD.vs.control; bulk RNA-seq,Homo_723,MCI.vs.control; bulk RNA-seq,Homo_714,AD.vs.control; bulk RNA-seq,Homo_714,MCI.vs.control; bulk RNA-seq,Homo_633,AD.vs.control; bulk RNA-seq,Homo_633,AD.vs.MCI	6
CC	GO:0031233	intrinsic component of external side of plasma membrane	bulk RNA-seq,Homo_723,AD.vs.control; bulk RNA-seq,Homo_723,AD.vs.MCI; bulk RNA-seq,Homo_723,MCI.vs.control; bulk RNA-seq,Homo_714,AD.vs.control; bulk RNA-seq,Homo_714,AD.vs.MCI	5
BP	GO:0060571	morphogenesis of an epithelial fold	bulk RNA-seq,Homo_723,AD.vs.control; bulk RNA-seq,Homo_723,AD.vs.MCI; bulk RNA-seq,Homo_723,MCI.vs.control; bulk RNA-seq,Homo_714,AD.vs.control; bulk RNA-seq,Homo_714,AD.vs.MCI; bulk RNA-seq,Homo_714,MCI.vs.control; bulk RNA-seq,Homo_633,MCI.vs.control	7
CC	GO:0070160	tight junction	bulk RNA-seq,Homo_723,AD.vs.control; bulk RNA-seq,Homo_723,AD.vs.MCI; bulk RNA-seq,Homo_723,MCI.vs.control; bulk RNA-seq,Homo_714,AD.vs.control; bulk RNA-seq,Homo_714,AD.vs.MCI; bulk RNA-seq,Homo_633,AD.vs.control; bulk RNA-seq,Homo_633,AD.vs.MCI	7
BP	GO:0015812	gamma-aminobutyric acid transport	bulk RNA-seq,Homo_723,AD.vs.control; bulk RNA-seq,Homo_723,AD.vs.MCI; bulk RNA-seq,Homo_723,MCI.vs.control; bulk RNA-seq,Homo_714,AD.vs.MCI	4
BP	GO:0044804	autophagy of nucleus	bulk RNA-seq,Homo_723,AD.vs.control; bulk RNA-seq,Homo_723,AD.vs.MCI; bulk RNA-seq,Homo_714,AD.vs.control; bulk RNA-seq,Homo_633,AD.vs.control; bulk RNA-seq,Homo_633,AD.vs.MCI	5
BP	GO:0050731	positive regulation of peptidyl-tyrosine phosphorylation	bulk RNA-seq,Homo_723,AD.vs.control; bulk RNA-seq,Homo_723,AD.vs.MCI; bulk RNA-seq,Homo_723,MCI.vs.control; bulk RNA-seq,Homo_714,AD.vs.control; bulk RNA-seq,Homo_714,AD.vs.MCI; bulk RNA-seq,Homo_633,AD.vs.control; bulk RNA-seq,Homo_633,AD.vs.MCI	7

BP	GO:0035710	CD4-positive, alpha-beta T cell activation	bulk RNA-seq,Homo_723,AD.vs.control; bulk RNA-seq,Homo_723,MCI.vs.control; bulk RNA-seq,Homo_714,AD.vs.control; bulk RNA-seq,Homo_714,MCI.vs.control; bulk RNA-seq,Homo_633,AD.vs.control; bulk RNA-seq,Homo_633,AD.vs.MCI	6
CC	GO:0000974	Prp19 complex	bulk RNA-seq,Homo_723,AD.vs.control; bulk RNA-seq,Homo_723,AD.vs.MCI; bulk RNA-seq,Homo_714,AD.vs.control; bulk RNA-seq,Homo_714,AD.vs.MCI; bulk RNA-seq,Homo_633,AD.vs.control; bulk RNA-seq,Homo_633,AD.vs.MCI	6
BP	GO:0003203	endocardial cushion morphogenesis	bulk RNA-seq,Homo_723,AD.vs.control; bulk RNA-seq,Homo_723,AD.vs.MCI; bulk RNA-seq,Homo_723,MCI.vs.control; bulk RNA-seq,Homo_714,AD.vs.control; bulk RNA-seq,Homo_714,AD.vs.MCI; bulk RNA-seq,Homo_714,MCI.vs.control	6
BP	GO:0021603	cranial nerve formation	bulk RNA-seq,Homo_723,AD.vs.control; bulk RNA-seq,Homo_723,AD.vs.MCI; bulk RNA-seq,Homo_714,AD.vs.control; bulk RNA-seq,Homo_714,AD.vs.MCI	4
BP	GO:0002218	activation of innate immune response	bulk RNA-seq,Homo_723,AD.vs.control; bulk RNA-seq,Homo_723,MCI.vs.control; bulk RNA-seq,Homo_714,AD.vs.control; bulk RNA-seq,Homo_714,MCI.vs.control; bulk RNA-seq,Homo_633,AD.vs.control; bulk RNA-seq,Homo_633,AD.vs.MCI; scRNA-seq,SRP330776,Naive CD8+ T cell_2-AD.vs.control; scRNA-seq,SRP330776,Natural killer cell_1-AD.vs.control	8
BP	GO:0006356	regulation of transcription by RNA polymerase I	bulk RNA-seq,Homo_723,AD.vs.control; bulk RNA-seq,Homo_723,MCI.vs.control; bulk RNA-seq,Homo_714,AD.vs.control; bulk RNA-seq,Homo_714,MCI.vs.control; bulk RNA-seq,Homo_633,AD.vs.control; bulk RNA-seq,Homo_633,AD.vs.MCI	6
BP	GO:0031532	actin cytoskeleton reorganization	bulk RNA-seq,Homo_723,AD.vs.control; bulk RNA-seq,Homo_723,AD.vs.MCI; bulk RNA-seq,Homo_723,MCI.vs.control; bulk RNA-seq,Homo_714,AD.vs.control; bulk RNA-seq,Homo_714,AD.vs.MCI; bulk RNA-seq,Homo_714,MCI.vs.control; bulk RNA-seq,Homo_633,AD.vs.control; bulk	8
BP	GO:0090277	positive regulation of peptide hormone secretion	bulk RNA-seq,Homo_723,AD.vs.control; bulk RNA-seq,Homo_723,AD.vs.MCI; bulk RNA-seq,Homo_723,MCI.vs.control; bulk RNA-seq,Homo_714,AD.vs.control; bulk RNA-seq,Homo_714,AD.vs.MCI; bulk RNA-seq,Homo_714,MCI.vs.control; bulk RNA-seq,Homo_633,AD.vs.control	7
BP	GO:1904851	positive regulation of establishment of protein localization to telomere	bulk RNA-seq,Homo_723,AD.vs.control; bulk RNA-seq,Homo_723,AD.vs.MCI; bulk RNA-seq,Homo_723,MCI.vs.control; bulk RNA-seq,Homo_714,AD.vs.control; bulk RNA-seq,Homo_714,MCI.vs.control; bulk RNA-seq,Homo_633,AD.vs.control; bulk RNA-seq,Homo_633,AD.vs.MCI; bulk	8
MF	GO:0015179	L-amino acid transmembrane transporter activity	bulk RNA-seq,Homo_723,AD.vs.control; bulk RNA-seq,Homo_723,AD.vs.MCI; bulk RNA-seq,Homo_723,MCI.vs.control; bulk RNA-seq,Homo_714,AD.vs.MCI; bulk RNA-seq,Homo_714,MCI.vs.control; bulk RNA-seq,Homo_633,AD.vs.control; bulk RNA-seq,Homo_633,AD.vs.MCI	7
BP	GO:1904814	regulation of protein localization to chromosome, telomeric region	bulk RNA-seq,Homo_723,AD.vs.control; bulk RNA-seq,Homo_723,MCI.vs.control; bulk RNA-seq,Homo_714,AD.vs.control; bulk RNA-seq,Homo_714,MCI.vs.control; bulk RNA-seq,Homo_633,AD.vs.control; bulk RNA-seq,Homo_633,AD.vs.MCI; bulk RNA-seq,Homo_633,MCI.vs.control	7
MF	GO:0015291	secondary active transmembrane transporter activity	bulk RNA-seq,Homo_723,AD.vs.control; bulk RNA-seq,Homo_723,AD.vs.MCI; bulk RNA-seq,Homo_723,MCI.vs.control; bulk RNA-seq,Homo_714,AD.vs.control; bulk RNA-seq,Homo_714,AD.vs.MCI; bulk RNA-seq,Homo_633,AD.vs.control; bulk RNA-seq,Homo_633,AD.vs.MCI; bulk RNA-seq,ROSMAP,AD.vs.control; bulk RNA-seq,ROSMAP,MCI.vs.control	9
BP	GO:0016241	regulation of macroautophagy	bulk RNA-seq,Homo_723,AD.vs.control; bulk RNA-seq,Homo_723,MCI.vs.control; bulk RNA-seq,Homo_714,AD.vs.control; bulk RNA-seq,Homo_633,AD.vs.control; bulk RNA-seq,Homo_633,AD.vs.MCI	5
CC	GO:0005770	late endosome	bulk RNA-seq,Homo_723,AD.vs.control; bulk RNA-seq,Homo_723,MCI.vs.control; bulk RNA-seq,Homo_714,AD.vs.control; bulk RNA-seq,Homo_633,AD.vs.control; bulk RNA-seq,Homo_633,AD.vs.MCI; scRNA-seq,SRP330776,Naive CD8+ T cell_2-AD.vs.control	6
BP	GO:1901019	regulation of calcium ion transmembrane transporter activity	bulk RNA-seq,Homo_723,AD.vs.control; bulk RNA-seq,Homo_723,AD.vs.MCI; bulk RNA-seq,Homo_723,MCI.vs.control; bulk RNA-seq,Homo_714,AD.vs.control; bulk RNA-seq,Homo_714,AD.vs.MCI; bulk RNA-seq,Homo_633,AD.vs.control; bulk RNA-seq,Homo_633,AD.vs.MCI	7
MF	GO:0005247	voltage-gated chloride channel activity	bulk RNA-seq,Homo_723,AD.vs.control; bulk RNA-seq,Homo_723,AD.vs.MCI	2
BP	GO:0008654	phospholipid biosynthetic process	bulk RNA-seq,Homo_723,AD.vs.control; bulk RNA-seq,Homo_723,MCI.vs.control; bulk RNA-seq,Homo_714,AD.vs.control; bulk RNA-seq,Homo_633,AD.vs.control; bulk RNA-seq,Homo_633,AD.vs.MCI; scRNA-seq,SRP330776,Naive CD8+ T cell_2-AD.vs.control	6
BP	GO:0035881	amacrine cell differentiation	bulk RNA-seq,Homo_723,AD.vs.control; bulk RNA-seq,Homo_723,AD.vs.MCI; bulk RNA-seq,Homo_723,MCI.vs.control; bulk RNA-seq,Homo_714,AD.vs.MCI	4
BP	GO:1901136	carbohydrate derivative catabolic process	bulk RNA-seq,Homo_723,AD.vs.control; bulk RNA-seq,Homo_723,MCI.vs.control; bulk RNA-seq,Homo_714,AD.vs.control; bulk RNA-seq,Homo_633,AD.vs.control; bulk RNA-seq,Homo_633,AD.vs.MCI	5
BP	GO:0030901	midbrain development	bulk RNA-seq,Homo_723,AD.vs.control; bulk RNA-seq,Homo_723,AD.vs.MCI; bulk RNA-seq,Homo_723,MCI.vs.control; bulk RNA-seq,Homo_714,AD.vs.MCI; bulk RNA-seq,Homo_633,AD.vs.control; bulk RNA-seq,Homo_633,AD.vs.MCI	6
BP	GO:2001234	negative regulation of apoptotic signaling pathway	bulk RNA-seq,Homo_723,AD.vs.control; bulk RNA-seq,Homo_723,AD.vs.MCI; bulk RNA-seq,Homo_723,MCI.vs.control; bulk RNA-seq,Homo_714,AD.vs.MCI; bulk RNA-seq,Homo_633,AD.vs.control; bulk RNA-seq,Homo_633,AD.vs.MCI	6
BP	GO:0003009	skeletal muscle contraction	bulk RNA-seq,Homo_723,AD.vs.control; bulk RNA-seq,Homo_723,AD.vs.MCI; bulk RNA-seq,Homo_714,AD.vs.control; bulk RNA-seq,Homo_714,AD.vs.MCI	4
BP	GO:2001022	positive regulation of response to DNA damage stimulus	bulk RNA-seq,Homo_723,AD.vs.control; bulk RNA-seq,Homo_723,MCI.vs.control; bulk RNA-seq,Homo_714,AD.vs.control; bulk RNA-seq,Homo_633,AD.vs.control; bulk RNA-seq,Homo_633,AD.vs.MCI	5
MF	GO:0016493	C-C chemokine receptor activity	bulk RNA-seq,Homo_723,AD.vs.control; bulk RNA-seq,Homo_633,AD.vs.control; bulk RNA-seq,Homo_633,AD.vs.MCI	3
BP	GO:0098976	excitatory chemical synaptic transmission	bulk RNA-seq,Homo_723,AD.vs.control; bulk RNA-seq,Homo_723,AD.vs.MCI; bulk RNA-seq,Homo_723,MCI.vs.control; bulk RNA-seq,Homo_714,AD.vs.MCI	4
BP	GO:0005513	detection of calcium ion	bulk RNA-seq,Homo_723,AD.vs.control; bulk RNA-seq,Homo_723,AD.vs.MCI; bulk RNA-seq,Homo_714,AD.vs.control; bulk RNA-seq,Homo_714,AD.vs.MCI; bulk RNA-seq,ROSMAP,MCI.vs.control	5
BP	GO:0003272	endocardial cushion formation	bulk RNA-seq,Homo_723,AD.vs.control; bulk RNA-seq,Homo_723,AD.vs.MCI; bulk RNA-seq,Homo_723,MCI.vs.control; bulk RNA-seq,Homo_714,AD.vs.control; bulk RNA-seq,Homo_714,AD.vs.MCI; bulk RNA-seq,Homo_714,MCI.vs.control; bulk RNA-seq,Homo_633,AD.vs.control; bulk	8
BP	GO:0106027	neuron projection organization	bulk RNA-seq,Homo_723,AD.vs.control; bulk RNA-seq,Homo_723,AD.vs.MCI; bulk RNA-seq,Homo_723,MCI.vs.control; bulk RNA-seq,Homo_714,AD.vs.MCI; bulk RNA-seq,Homo_714,MCI.vs.control; bulk RNA-seq,Homo_633,AD.vs.control; bulk RNA-seq,Homo_633,AD.vs.MCI; bulk RNA-seq,ROSMAP,AD.vs.control; scRNA-seq,SRP330776,Naive CD8+ T cell_2-AD.vs.control	9
BP	GO:0060046	regulation of acrosome reaction	bulk RNA-seq,Homo_723,AD.vs.control; bulk RNA-seq,Homo_723,AD.vs.MCI	2
BP	GO:0050930	induction of positive chemotaxis	bulk RNA-seq,Homo_723,AD.vs.control; bulk RNA-seq,Homo_723,AD.vs.MCI; bulk RNA-seq,Homo_723,MCI.vs.control; bulk RNA-seq,Homo_714,AD.vs.control; bulk RNA-seq,Homo_714,AD.vs.MCI; bulk RNA-seq,Homo_714,MCI.vs.control	6
MF	GO:0016799	hydrolase activity, hydrolyzing N-glycosyl compounds	bulk RNA-seq,Homo_723,AD.vs.control; bulk RNA-seq,Homo_723,MCI.vs.control; bulk RNA-seq,Homo_714,AD.vs.control; bulk RNA-seq,Homo_714,MCI.vs.control; bulk RNA-seq,Homo_633,AD.vs.control; bulk RNA-seq,Homo_633,AD.vs.MCI	6
BP	GO:0002286	T cell activation involved in immune response	bulk RNA-seq,Homo_723,AD.vs.control; bulk RNA-seq,Homo_723,MCI.vs.control; bulk RNA-seq,Homo_714,MCI.vs.control; bulk RNA-seq,Homo_633,AD.vs.control; bulk RNA-seq,Homo_633,AD.vs.MCI; bulk RNA-seq,ROSMAP,AD.vs.control	6
BP	GO:0014855	striated muscle cell proliferation	bulk RNA-seq,Homo_723,AD.vs.control; bulk RNA-seq,Homo_723,AD.vs.MCI; bulk RNA-seq,Homo_723,MCI.vs.control; bulk RNA-seq,Homo_714,AD.vs.MCI; bulk RNA-seq,Homo_714,MCI.vs.control; bulk RNA-seq,Homo_633,AD.vs.control; bulk RNA-seq,Homo_633,AD.vs.MCI	7
BP	GO:1902285	semaphorin-plexin signaling pathway involved in neuron projection guidance	bulk RNA-seq,Homo_723,AD.vs.control; bulk RNA-seq,Homo_723,AD.vs.MCI; bulk RNA-seq,Homo_723,MCI.vs.control; bulk RNA-seq,Homo_714,AD.vs.MCI; bulk RNA-seq,Homo_714,MCI.vs.control	5

BP	GO:0007588	excretion	bulk RNA-seq,Homo_723,AD.vs.control; bulk RNA-seq,Homo_723,AD.vs.MCI; bulk RNA-seq,Homo_714,AD.vs.control; bulk RNA-seq,Homo_714,AD.vs.MCI; bulk RNA-seq,Homo_633,MCI.vs.control	5
CC	GO:0035861	site of double-strand break	bulk RNA-seq,Homo_723,AD.vs.control; bulk RNA-seq,Homo_723,MCI.vs.control; bulk RNA-seq,Homo_714,AD.vs.control; bulk RNA-seq,Homo_714,MCI.vs.control; bulk RNA-seq,Homo_633,AD.vs.control; bulk RNA-seq,Homo_633,AD.vs.MCI	6
BP	GO:0048538	thymus development	bulk RNA-seq,Homo_723,AD.vs.control; bulk RNA-seq,Homo_723,AD.vs.MCI; bulk RNA-seq,Homo_723,MCI.vs.control; bulk RNA-seq,Homo_714,AD.vs.MCI; bulk RNA-seq,Homo_633,AD.vs.control	5
BP	GO:2000234	positive regulation of rRNA processing	bulk RNA-seq,Homo_723,AD.vs.control; bulk RNA-seq,Homo_723,AD.vs.MCI; bulk RNA-seq,Homo_714,AD.vs.control; bulk RNA-seq,Homo_714,AD.vs.MCI; bulk RNA-seq,Homo_633,AD.vs.control; bulk RNA-seq,Homo_633,AD.vs.MCI	6
BP	GO:0034109	homotypic cell-cell adhesion	bulk RNA-seq,Homo_723,AD.vs.control; bulk RNA-seq,Homo_723,AD.vs.MCI; bulk RNA-seq,Homo_723,MCI.vs.control; bulk RNA-seq,Homo_714,AD.vs.control; bulk RNA-seq,Homo_714,AD.vs.MCI; bulk RNA-seq,Homo_714,MCI.vs.control; bulk RNA-seq,Homo_633,AD.vs.control; bulk	8
BP	GO:0021702	cerebellar Purkinje cell differentiation	bulk RNA-seq,Homo_723,AD.vs.control; bulk RNA-seq,Homo_723,AD.vs.MCI; bulk RNA-seq,Homo_723,MCI.vs.control; bulk RNA-seq,Homo_633,AD.vs.control; bulk RNA-seq,Homo_633,AD.vs.MCI	5
CC	GO:0060198	clathrin-sculpted vesicle	bulk RNA-seq,Homo_723,AD.vs.control; bulk RNA-seq,Homo_723,AD.vs.MCI; bulk RNA-seq,Homo_723,MCI.vs.control; bulk RNA-seq,Homo_714,AD.vs.MCI	4
BP	GO:2001171	positive regulation of ATP biosynthetic process	bulk RNA-seq,Homo_723,AD.vs.control; bulk RNA-seq,Homo_723,MCI.vs.control; bulk RNA-seq,Homo_714,AD.vs.control; bulk RNA-seq,Homo_714,MCI.vs.control; bulk RNA-seq,Homo_633,AD.vs.control; bulk RNA-seq,Homo_633,AD.vs.MCI	6
MF	GO:1901618	organic hydroxy compound transmembrane transporter activity	bulk RNA-seq,Homo_723,AD.vs.control; bulk RNA-seq,Homo_723,AD.vs.MCI; bulk RNA-seq,Homo_723,MCI.vs.control; bulk RNA-seq,Homo_714,AD.vs.control; bulk RNA-seq,Homo_714,AD.vs.MCI; bulk RNA-seq,Homo_714,MCI.vs.control	6
BP	GO:0009187	cyclic nucleotide metabolic process	bulk RNA-seq,Homo_723,AD.vs.control; bulk RNA-seq,Homo_723,AD.vs.MCI; bulk RNA-seq,Homo_723,MCI.vs.control; bulk RNA-seq,Homo_714,AD.vs.MCI; bulk RNA-seq,Homo_714,MCI.vs.control	5
BP	GO:1904948	midbrain dopaminergic neuron differentiation	bulk RNA-seq,Homo_723,AD.vs.control; bulk RNA-seq,Homo_723,AD.vs.MCI; bulk RNA-seq,Homo_723,MCI.vs.control; bulk RNA-seq,Homo_714,AD.vs.control; bulk RNA-seq,Homo_714,AD.vs.MCI	5
MF	GO:0048038	quinone binding	bulk RNA-seq,Homo_723,AD.vs.control; bulk RNA-seq,Homo_723,AD.vs.MCI; bulk RNA-seq,Homo_714,AD.vs.control; bulk RNA-seq,Homo_633,AD.vs.control	4
CC	GO:0005741	mitochondrial outer membrane	bulk RNA-seq,Homo_723,AD.vs.control; bulk RNA-seq,Homo_723,MCI.vs.control; bulk RNA-seq,Homo_714,AD.vs.control; bulk RNA-seq,Homo_714,AD.vs.MCI; bulk RNA-seq,Homo_633,AD.vs.control; bulk RNA-seq,Homo_633,AD.vs.MCI	6
BP	GO:0051580	regulation of neurotransmitter uptake	bulk RNA-seq,Homo_723,AD.vs.control; bulk RNA-seq,Homo_723,AD.vs.MCI; bulk RNA-seq,Homo_723,MCI.vs.control; bulk RNA-	4
MF	GO:0043130	ubiquitin binding	bulk RNA-seq,Homo_723,AD.vs.control; bulk RNA-seq,Homo_723,MCI.vs.control; bulk RNA-seq,Homo_714,AD.vs.control; bulk RNA-seq,Homo_714,MCI.vs.control; bulk RNA-seq,Homo_633,AD.vs.control; bulk RNA-seq,Homo_633,AD.vs.MCI	6
BP	GO:0051149	positive regulation of muscle cell differentiation	bulk RNA-seq,Homo_723,AD.vs.control; bulk RNA-seq,Homo_723,AD.vs.MCI; bulk RNA-seq,Homo_723,MCI.vs.control; bulk RNA-seq,Homo_714,AD.vs.control; bulk RNA-seq,Homo_714,AD.vs.MCI	5
BP	GO:0045667	regulation of osteoblast differentiation	bulk RNA-seq,Homo_723,AD.vs.control; bulk RNA-seq,Homo_723,AD.vs.MCI; bulk RNA-seq,Homo_723,MCI.vs.control; bulk RNA-seq,Homo_714,AD.vs.MCI; bulk RNA-seq,Homo_633,AD.vs.control; bulk RNA-seq,Homo_633,AD.vs.MCI; scRNA-seq,SRP330776,CD8+ T cell_2-AD.vs.control	7
BP	GO:0032729	positive regulation of interferon-gamma production	bulk RNA-seq,Homo_723,AD.vs.control; bulk RNA-seq,Homo_723,MCI.vs.control; bulk RNA-seq,Homo_714,AD.vs.control; bulk RNA-seq,Homo_714,MCI.vs.control; bulk RNA-seq,Homo_633,AD.vs.control; bulk RNA-seq,Homo_633,AD.vs.MCI	6
CC	GO:0016328	lateral plasma membrane	bulk RNA-seq,Homo_723,AD.vs.control; bulk RNA-seq,Homo_723,AD.vs.MCI; bulk RNA-seq,Homo_723,MCI.vs.control; bulk RNA-seq,Homo_714,AD.vs.MCI; bulk RNA-seq,Homo_714,MCI.vs.control; bulk RNA-seq,Homo_633,AD.vs.control	6
BP	GO:0016197	endosomal transport	bulk RNA-seq,Homo_723,AD.vs.control; bulk RNA-seq,Homo_723,MCI.vs.control; bulk RNA-seq,Homo_714,AD.vs.control; bulk RNA-seq,Homo_633,AD.vs.control; bulk RNA-seq,Homo_633,AD.vs.MCI; scRNA-seq,SRP330776,Naive CD8+ T cell_2-AD.vs.control	6
BP	GO:0021692	cerebellar Purkinje cell layer morphogenesis	bulk RNA-seq,Homo_723,AD.vs.control; bulk RNA-seq,Homo_723,AD.vs.MCI; bulk RNA-seq,Homo_723,MCI.vs.control; bulk RNA-seq,Homo_633,AD.vs.control; bulk RNA-seq,Homo_633,AD.vs.MCI	5
CC	GO:0033290	eukaryotic 48S preinitiation complex	bulk RNA-seq,Homo_723,AD.vs.control; bulk RNA-seq,Homo_723,AD.vs.MCI; bulk RNA-seq,Homo_714,AD.vs.control; bulk RNA-seq,Homo_714,AD.vs.MCI; bulk RNA-seq,Homo_633,AD.vs.control	5
BP	GO:0002068	glandular epithelial cell development	bulk RNA-seq,Homo_723,AD.vs.control; bulk RNA-seq,Homo_723,AD.vs.MCI; bulk RNA-seq,Homo_723,MCI.vs.control; bulk RNA-seq,Homo_714,AD.vs.MCI	4
BP	GO:1902547	regulation of cellular response to vascular endothelial growth factor stimulus	bulk RNA-seq,Homo_723,AD.vs.control; bulk RNA-seq,Homo_723,AD.vs.MCI; bulk RNA-seq,Homo_723,MCI.vs.control; bulk RNA-seq,Homo_714,AD.vs.MCI; bulk RNA-seq,Homo_714,MCI.vs.control	5
CC	GO:0005923	bicellular tight junction	bulk RNA-seq,Homo_723,AD.vs.control; bulk RNA-seq,Homo_723,AD.vs.MCI; bulk RNA-seq,Homo_723,MCI.vs.control; bulk RNA-seq,Homo_714,AD.vs.control; bulk RNA-seq,Homo_714,AD.vs.MCI; bulk RNA-seq,Homo_714,MCI.vs.control; bulk RNA-seq,Homo_633,AD.vs.control; bulk	8
BP	GO:0007431	salivary gland development	bulk RNA-seq,Homo_723,AD.vs.control; bulk RNA-seq,Homo_723,AD.vs.MCI; bulk RNA-seq,Homo_723,MCI.vs.control; bulk RNA-seq,Homo_714,AD.vs.MCI; bulk RNA-seq,Homo_714,MCI.vs.control; bulk RNA-seq,Homo_633,AD.vs.control; bulk RNA-seq,Homo_633,AD.vs.MCI	7
MF	GO:0019905	syntaxin binding	bulk RNA-seq,Homo_723,AD.vs.control; bulk RNA-seq,Homo_723,AD.vs.MCI; bulk RNA-seq,Homo_723,MCI.vs.control; bulk RNA-seq,Homo_714,AD.vs.MCI; bulk RNA-seq,Homo_714,MCI.vs.control; bulk RNA-seq,Homo_633,AD.vs.control; bulk RNA-seq,Homo_633,AD.vs.MCI	7
BP	GO:0050951	sensory perception of temperature stimulus	bulk RNA-seq,Homo_723,AD.vs.control; bulk RNA-seq,Homo_723,AD.vs.MCI; bulk RNA-seq,Homo_723,MCI.vs.control; bulk RNA-seq,Homo_714,AD.vs.control; bulk RNA-seq,Homo_714,AD.vs.MCI	5
CC	GO:0030904	retromer complex	bulk RNA-seq,Homo_723,AD.vs.control; bulk RNA-seq,Homo_714,AD.vs.control; bulk RNA-seq,Homo_633,AD.vs.control; bulk RNA-seq,Homo_633,AD.vs.MCI	4
CC	GO:0019897	extrinsic component of plasma membrane	bulk RNA-seq,Homo_723,AD.vs.control; bulk RNA-seq,Homo_723,AD.vs.MCI; bulk RNA-seq,Homo_723,MCI.vs.control; bulk RNA-seq,Homo_714,AD.vs.MCI; bulk RNA-seq,Homo_633,AD.vs.control; bulk RNA-seq,Homo_633,AD.vs.MCI; bulk RNA-seq,ROSMAP,AD.vs.control	7
MF	GO:0004549	tRNA-specific ribonuclease activity	bulk RNA-seq,Homo_723,AD.vs.control; bulk RNA-seq,Homo_723,AD.vs.MCI; bulk RNA-seq,Homo_714,AD.vs.control; bulk RNA-seq,Homo_633,AD.vs.control	4
BP	GO:2000826	regulation of heart morphogenesis	bulk RNA-seq,Homo_723,AD.vs.control; bulk RNA-seq,Homo_723,AD.vs.MCI; bulk RNA-seq,Homo_723,MCI.vs.control; bulk RNA-seq,Homo_714,AD.vs.control; bulk RNA-seq,Homo_714,AD.vs.MCI; bulk RNA-seq,Homo_714,MCI.vs.control	6
MF	GO:0008171	O-methyltransferase activity	bulk RNA-seq,Homo_723,AD.vs.control; bulk RNA-seq,Homo_723,MCI.vs.control; bulk RNA-seq,Homo_714,AD.vs.control; bulk RNA-seq,Homo_714,MCI.vs.control; bulk RNA-seq,Homo_633,AD.vs.control; bulk RNA-seq,Homo_633,AD.vs.MCI; bulk RNA-seq,Homo_633,MCI.vs.control	7
BP	GO:0048664	neuron fate determination	bulk RNA-seq,Homo_723,AD.vs.control; bulk RNA-seq,Homo_723,AD.vs.MCI; bulk RNA-seq,Homo_723,MCI.vs.control; bulk RNA-seq,Homo_714,AD.vs.control; bulk RNA-seq,Homo_714,AD.vs.MCI	5
BP	GO:0110053	regulation of actin filament organization	bulk RNA-seq,Homo_723,AD.vs.control; bulk RNA-seq,Homo_723,AD.vs.MCI; bulk RNA-seq,Homo_723,MCI.vs.control; bulk RNA-seq,Homo_714,AD.vs.MCI; bulk RNA-seq,Homo_633,AD.vs.control; bulk RNA-seq,Homo_633,AD.vs.MCI	6

BP	GO:0007202	activation of phospholipase C activity	bulk RNA-seq,Homo_723,AD.vs.control; bulk RNA-seq,Homo_723,AD.vs.MCI; bulk RNA-seq,Homo_723,MCI.vs.control; bulk RNA-seq,Homo_714,AD.vs.MCI	4
BP	GO:0001964	startle response	bulk RNA-seq,Homo_723,AD.vs.control; bulk RNA-seq,Homo_723,AD.vs.MCI; bulk RNA-seq,Homo_723,MCI.vs.control; bulk RNA-seq,Homo_714,AD.vs.control; bulk RNA-seq,Homo_714,AD.vs.MCI; bulk RNA-seq,SRP223445,AD.vs.control	6
BP	GO:0055117	regulation of cardiac muscle contraction	bulk RNA-seq,Homo_723,AD.vs.control; bulk RNA-seq,Homo_723,AD.vs.MCI; bulk RNA-seq,Homo_723,MCI.vs.control; bulk RNA-seq,Homo_714,AD.vs.control; bulk RNA-seq,Homo_714,AD.vs.MCI; bulk RNA-seq,Homo_714,MCI.vs.control	6
BP	GO:0051204	protein insertion into mitochondrial membrane	bulk RNA-seq,Homo_723,AD.vs.control; bulk RNA-seq,Homo_723,MCI.vs.control; bulk RNA-seq,Homo_714,AD.vs.control; bulk RNA-seq,Homo_714,MCI.vs.control; bulk RNA-seq,Homo_633,AD.vs.control; bulk RNA-seq,Homo_633,AD.vs.MCI; bulk RNA-seq,Homo_633,MCI.vs.control	7
BP	GO:0006779	porphyrin-containing compound biosynthetic process	bulk RNA-seq,Homo_723,AD.vs.control; bulk RNA-seq,Homo_723,MCI.vs.control; bulk RNA-seq,Homo_714,AD.vs.control; bulk RNA-seq,Homo_714,MCI.vs.control; bulk RNA-seq,Homo_633,AD.vs.control; bulk RNA-seq,Homo_633,AD.vs.MCI; bulk RNA-seq,Homo_633,MCI.vs.control	7
BP	GO:0033014	tetrapyrrole biosynthetic process	bulk RNA-seq,Homo_723,AD.vs.control; bulk RNA-seq,Homo_723,MCI.vs.control; bulk RNA-seq,Homo_714,AD.vs.control; bulk RNA-seq,Homo_714,MCI.vs.control; bulk RNA-seq,Homo_633,AD.vs.control; bulk RNA-seq,Homo_633,AD.vs.MCI; bulk RNA-seq,Homo_633,MCI.vs.control	7
BP	GO:0046034	ATP metabolic process	bulk RNA-seq,Homo_723,AD.vs.control; bulk RNA-seq,Homo_723,MCI.vs.control; bulk RNA-seq,Homo_714,AD.vs.control; bulk RNA-seq,Homo_633,AD.vs.control; bulk RNA-seq,Homo_633,AD.vs.MCI	5
BP	GO:0010837	regulation of keratinocyte proliferation	bulk RNA-seq,Homo_723,AD.vs.control; bulk RNA-seq,Homo_723,AD.vs.MCI; bulk RNA-seq,Homo_723,MCI.vs.control; bulk RNA-seq,Homo_714,AD.vs.control; bulk RNA-seq,Homo_714,AD.vs.MCI; bulk RNA-seq,Homo_714,MCI.vs.control; bulk RNA-seq,Homo_633,AD.vs.control	7
BP	GO:0043628	small regulatory ncRNA 3'-end processing	bulk RNA-seq,Homo_723,AD.vs.control; bulk RNA-seq,Homo_723,AD.vs.MCI; bulk RNA-seq,Homo_714,AD.vs.control; bulk RNA-seq,Homo_633,AD.vs.control; bulk RNA-seq,Homo_633,AD.vs.MCI	5
BP	GO:0006119	oxidative phosphorylation	bulk RNA-seq,Homo_723,AD.vs.control; bulk RNA-seq,Homo_723,MCI.vs.control; bulk RNA-seq,Homo_714,AD.vs.control; bulk RNA-seq,Homo_633,AD.vs.control; bulk RNA-seq,Homo_633,AD.vs.MCI	5
BP	GO:0033138	positive regulation of peptidyl-serine phosphorylation	bulk RNA-seq,Homo_723,AD.vs.control; bulk RNA-seq,Homo_723,AD.vs.MCI; bulk RNA-seq,Homo_723,MCI.vs.control; bulk RNA-seq,Homo_714,AD.vs.control; bulk RNA-seq,Homo_714,AD.vs.MCI; bulk RNA-seq,Homo_714,MCI.vs.control; bulk RNA-seq,Homo_633,AD.vs.control	7
BP	GO:0000478	endonucleolytic cleavage involved in rRNA processing	bulk RNA-seq,Homo_723,AD.vs.control; bulk RNA-seq,Homo_723,MCI.vs.control; bulk RNA-seq,Homo_714,AD.vs.control; bulk RNA-seq,Homo_714,MCI.vs.control; bulk RNA-seq,Homo_633,AD.vs.control; bulk RNA-seq,Homo_633,MCI.vs.control	6
BP	GO:0000479	endonucleolytic cleavage of tricistronic rRNA transcript (SSU-rRNA, 5.8S rRNA, LSU-rRNA)	bulk RNA-seq,Homo_723,AD.vs.control; bulk RNA-seq,Homo_723,MCI.vs.control; bulk RNA-seq,Homo_714,AD.vs.control; bulk RNA-seq,Homo_714,MCI.vs.control; bulk RNA-seq,Homo_633,AD.vs.control; bulk RNA-seq,Homo_633,MCI.vs.control	6
BP	GO:0051480	regulation of cytosolic calcium ion concentration	bulk RNA-seq,Homo_723,AD.vs.control; bulk RNA-seq,Homo_723,AD.vs.MCI; bulk RNA-seq,Homo_723,MCI.vs.control; bulk RNA-seq,Homo_714,AD.vs.MCI; bulk RNA-seq,Homo_714,MCI.vs.control	5
BP	GO:2000671	regulation of motor neuron apoptotic process	bulk RNA-seq,Homo_723,AD.vs.control; bulk RNA-seq,Homo_723,AD.vs.MCI; bulk RNA-seq,Homo_723,MCI.vs.control	3
BP	GO:0060338	regulation of type I interferon-mediated signaling pathway	bulk RNA-seq,Homo_723,AD.vs.control; bulk RNA-seq,Homo_723,MCI.vs.control; bulk RNA-seq,Homo_714,AD.vs.control; bulk RNA-seq,Homo_714,MCI.vs.control; bulk RNA-seq,Homo_633,AD.vs.control	5
CC	GO:0016591	RNA polymerase II, holoenzyme	bulk RNA-seq,Homo_723,AD.vs.control; bulk RNA-seq,Homo_723,MCI.vs.control; bulk RNA-seq,Homo_714,AD.vs.control; bulk RNA-seq,Homo_714,MCI.vs.control; bulk RNA-seq,Homo_633,AD.vs.control; bulk RNA-seq,Homo_633,AD.vs.MCI	6
BP	GO:0003073	regulation of systemic arterial blood pressure	bulk RNA-seq,Homo_723,AD.vs.control; bulk RNA-seq,Homo_723,AD.vs.MCI; bulk RNA-seq,Homo_723,MCI.vs.control; bulk RNA-seq,Homo_714,AD.vs.control; bulk RNA-seq,Homo_714,AD.vs.MCI; bulk RNA-seq,Homo_714,MCI.vs.control	6
MF	GO:0016853	isomerase activity	bulk RNA-seq,Homo_723,AD.vs.control; bulk RNA-seq,Homo_723,MCI.vs.control; bulk RNA-seq,Homo_714,AD.vs.control; bulk RNA-seq,Homo_633,AD.vs.control; bulk RNA-seq,Homo_633,AD.vs.MCI	5
BP	GO:0042438	melanin biosynthetic process	bulk RNA-seq,Homo_723,AD.vs.control; bulk RNA-seq,Homo_723,AD.vs.MCI; bulk RNA-seq,Homo_723,MCI.vs.control; bulk RNA-seq,Homo_633,AD.vs.control; bulk RNA-seq,Homo_633,AD.vs.MCI	5
BP	GO:2000316	regulation of T-helper 17 type immune response	bulk RNA-seq,Homo_723,AD.vs.control; bulk RNA-seq,Homo_723,MCI.vs.control; bulk RNA-seq,Homo_714,AD.vs.control; bulk RNA-seq,Homo_714,MCI.vs.control; bulk RNA-seq,Homo_633,MCI.vs.control	5
BP	GO:0044065	regulation of respiratory system process	bulk RNA-seq,Homo_723,AD.vs.control; bulk RNA-seq,Homo_723,AD.vs.MCI; bulk RNA-seq,Homo_723,MCI.vs.control; bulk RNA-seq,Homo_714,AD.vs.MCI	4
MF	GO:0043175	RNA polymerase core enzyme binding	bulk RNA-seq,Homo_723,AD.vs.control; bulk RNA-seq,Homo_723,MCI.vs.control; bulk RNA-seq,Homo_714,AD.vs.control; bulk RNA-seq,Homo_714,MCI.vs.control; bulk RNA-seq,Homo_633,AD.vs.control; bulk RNA-seq,Homo_633,AD.vs.MCI	6
BP	GO:0003179	heart valve morphogenesis	bulk RNA-seq,Homo_723,AD.vs.control; bulk RNA-seq,Homo_723,AD.vs.MCI; bulk RNA-seq,Homo_723,MCI.vs.control; bulk RNA-seq,Homo_714,AD.vs.MCI; bulk RNA-seq,Homo_714,MCI.vs.control; bulk RNA-seq,SRP223445,AD.vs.control	6
BP	GO:0009312	oligosaccharide biosynthetic process	bulk RNA-seq,Homo_723,AD.vs.control; bulk RNA-seq,Homo_723,AD.vs.MCI; bulk RNA-seq,Homo_633,AD.vs.control; bulk RNA-seq,Homo_633,AD.vs.MCI	4
BP	GO:0090646	mitochondrial tRNA processing	bulk RNA-seq,Homo_723,AD.vs.control; bulk RNA-seq,Homo_723,AD.vs.MCI; bulk RNA-seq,Homo_714,AD.vs.control; bulk RNA-seq,Homo_633,AD.vs.control; bulk RNA-seq,Homo_633,AD.vs.MCI	5
BP	GO:0043116	negative regulation of vascular permeability	bulk RNA-seq,Homo_723,AD.vs.control; bulk RNA-seq,Homo_723,AD.vs.MCI; bulk RNA-seq,Homo_723,MCI.vs.control; bulk RNA-seq,Homo_714,AD.vs.control; bulk RNA-seq,Homo_714,AD.vs.MCI; bulk RNA-seq,Homo_714,MCI.vs.control; bulk RNA-seq,Homo_633,AD.vs.control	7
BP	GO:0038084	vascular endothelial growth factor signaling pathway	bulk RNA-seq,Homo_723,AD.vs.control; bulk RNA-seq,Homo_723,AD.vs.MCI; bulk RNA-seq,Homo_723,MCI.vs.control; bulk RNA-seq,Homo_714,AD.vs.MCI; bulk RNA-seq,Homo_714,MCI.vs.control	5
BP	GO:0051145	smooth muscle cell differentiation	bulk RNA-seq,Homo_723,AD.vs.control; bulk RNA-seq,Homo_723,AD.vs.MCI; bulk RNA-seq,Homo_723,MCI.vs.control; bulk RNA-seq,Homo_714,AD.vs.control; bulk RNA-seq,Homo_714,AD.vs.MCI; bulk RNA-seq,Homo_714,MCI.vs.control	6
BP	GO:0001958	endochondral ossification	bulk RNA-seq,Homo_723,AD.vs.control; bulk RNA-seq,Homo_723,AD.vs.MCI; bulk RNA-seq,Homo_723,MCI.vs.control; bulk RNA-seq,Homo_714,AD.vs.MCI; bulk RNA-seq,Homo_633,AD.vs.control; bulk RNA-seq,Homo_633,AD.vs.MCI	6
BP	GO:0036075	replacement ossification	bulk RNA-seq,Homo_723,AD.vs.control; bulk RNA-seq,Homo_723,AD.vs.MCI; bulk RNA-seq,Homo_723,MCI.vs.control; bulk RNA-seq,Homo_714,AD.vs.MCI; bulk RNA-seq,Homo_633,AD.vs.control; bulk RNA-seq,Homo_633,AD.vs.MCI	6
BP	GO:0035272	exocrine system development	bulk RNA-seq,Homo_723,AD.vs.control; bulk RNA-seq,Homo_723,AD.vs.MCI; bulk RNA-seq,Homo_723,MCI.vs.control; bulk RNA-seq,Homo_714,AD.vs.MCI; bulk RNA-seq,Homo_714,MCI.vs.control; bulk RNA-seq,Homo_633,AD.vs.control; bulk RNA-seq,Homo_633,AD.vs.MCI; bulk RNA-	8
MF	GO:0016757	glycosyltransferase activity	bulk RNA-seq,Homo_723,AD.vs.control; bulk RNA-seq,Homo_723,AD.vs.MCI; bulk RNA-seq,Homo_723,MCI.vs.control; bulk RNA-seq,Homo_714,AD.vs.control; bulk RNA-seq,Homo_714,AD.vs.MCI; bulk RNA-seq,Homo_633,AD.vs.control; bulk RNA-seq,Homo_633,AD.vs.MCI	7
BP	GO:1901976	regulation of cell cycle checkpoint	bulk RNA-seq,Homo_723,AD.vs.control; bulk RNA-seq,Homo_723,MCI.vs.control; bulk RNA-seq,Homo_714,AD.vs.control; bulk RNA-seq,Homo_714,MCI.vs.control; bulk RNA-seq,Homo_633,AD.vs.control; bulk RNA-seq,Homo_633,AD.vs.MCI	6
BP	GO:0001913	T cell mediated cytotoxicity	bulk RNA-seq,Homo_723,AD.vs.control; bulk RNA-seq,Homo_633,AD.vs.control; bulk RNA-seq,Homo_633,AD.vs.MCI	3

BP	GO:0060997	dendritic spine morphogenesis	bulk RNA-seq,Homo_723,AD.vs.control; bulk RNA-seq,Homo_723,AD.vs.MCI; bulk RNA-seq,Homo_723,MCI.vs.control; bulk RNA-seq,Homo_714,AD.vs.MCI; bulk RNA-seq,Homo_714,MCI.vs.control; bulk RNA-seq,Homo_633,AD.vs.control; bulk RNA-seq,ROSMAP,AD.vs.control; scRNA-seq,SRP330776,Naive CD8+	8
BP	GO:0097152	mesenchymal cell apoptotic process	bulk RNA-seq,Homo_723,AD.vs.control; bulk RNA-seq,Homo_723,AD.vs.MCI; bulk RNA-seq,Homo_723,MCI.vs.control; bulk RNA-seq,Homo_714,AD.vs.MCI; bulk RNA-seq,Homo_633,MCI.vs.control	5
BP	GO:0097061	dendritic spine organization	bulk RNA-seq,Homo_723,AD.vs.control; bulk RNA-seq,Homo_723,AD.vs.MCI; bulk RNA-seq,Homo_723,MCI.vs.control; bulk RNA-seq,Homo_714,AD.vs.MCI; bulk RNA-seq,Homo_714,MCI.vs.control; bulk RNA-seq,Homo_633,AD.vs.control; bulk RNA-seq,Homo_633,AD.vs.MCI; bulk RNA-	8
BP	GO:0006379	mRNA cleavage	bulk RNA-seq,Homo_723,AD.vs.control; bulk RNA-seq,Homo_723,AD.vs.MCI; bulk RNA-seq,Homo_723,MCI.vs.control; bulk RNA-seq,Homo_714,AD.vs.control; bulk RNA-seq,Homo_714,AD.vs.MCI; bulk RNA-seq,Homo_633,AD.vs.control; bulk RNA-seq,Homo_633,AD.vs.MCI; bulk RNA-	8
BP	GO:0035510	DNA dealkylation	bulk RNA-seq,Homo_723,AD.vs.control; bulk RNA-seq,Homo_723,MCI.vs.control; bulk RNA-seq,Homo_714,AD.vs.control; bulk RNA-seq,Homo_714,MCI.vs.control; bulk RNA-seq,Homo_633,AD.vs.control	5
BP	GO:0007530	sex determination	bulk RNA-seq,Homo_723,AD.vs.control; bulk RNA-seq,Homo_723,AD.vs.MCI; bulk RNA-seq,Homo_723,MCI.vs.control; bulk RNA-seq,Homo_714,AD.vs.control; bulk RNA-seq,Homo_714,AD.vs.MCI; bulk RNA-seq,Homo_633,MCI.vs.control; bulk RNA-seq,SRP223445,AD.vs.control	7
BP	GO:1904036	negative regulation of epithelial cell apoptotic process	bulk RNA-seq,Homo_723,AD.vs.control; bulk RNA-seq,Homo_723,AD.vs.MCI; bulk RNA-seq,Homo_723,MCI.vs.control; bulk RNA-seq,Homo_714,AD.vs.control; bulk RNA-seq,Homo_714,AD.vs.MCI; bulk RNA-seq,Homo_714,MCI.vs.control	6
BP	GO:0048469	cell maturation	bulk RNA-seq,Homo_723,AD.vs.control; bulk RNA-seq,Homo_723,AD.vs.MCI; bulk RNA-seq,Homo_723,MCI.vs.control; bulk RNA-seq,Homo_714,AD.vs.MCI; bulk RNA-seq,Homo_633,AD.vs.control; bulk RNA-seq,Homo_633,AD.vs.MCI	6
BP	GO:0035914	skeletal muscle cell differentiation	bulk RNA-seq,Homo_723,AD.vs.control; bulk RNA-seq,Homo_723,AD.vs.MCI; bulk RNA-seq,Homo_723,MCI.vs.control; bulk RNA-seq,Homo_714,AD.vs.MCI; bulk RNA-seq,Homo_714,MCI.vs.control	5
BP	GO:0018958	phenol-containing compound metabolic process	bulk RNA-seq,Homo_723,AD.vs.control; bulk RNA-seq,Homo_723,AD.vs.MCI; bulk RNA-seq,Homo_723,MCI.vs.control; bulk RNA-seq,Homo_714,AD.vs.control; bulk RNA-seq,Homo_714,AD.vs.MCI; bulk RNA-seq,Homo_714,MCI.vs.control; bulk RNA-seq,Homo_633,AD.vs.control; bulk	8
BP	GO:0060419	heart growth	bulk RNA-seq,Homo_723,AD.vs.control; bulk RNA-seq,Homo_723,AD.vs.MCI; bulk RNA-seq,Homo_723,MCI.vs.control; bulk RNA-seq,Homo_714,AD.vs.MCI; bulk RNA-seq,Homo_714,MCI.vs.control	5
BP	GO:0008334	histone mRNA metabolic process	bulk RNA-seq,Homo_723,AD.vs.control; bulk RNA-seq,Homo_723,AD.vs.MCI; bulk RNA-seq,Homo_714,AD.vs.control; bulk RNA-seq,Homo_633,AD.vs.control; bulk RNA-seq,Homo_633,AD.vs.MCI	5
CC	GO:0031672	A band	bulk RNA-seq,Homo_723,AD.vs.control; bulk RNA-seq,Homo_723,AD.vs.MCI; bulk RNA-seq,Homo_714,AD.vs.control; bulk RNA-seq,Homo_714,AD.vs.MCI	4
BP	GO:0061043	regulation of vascular wound healing	bulk RNA-seq,Homo_723,AD.vs.control; bulk RNA-seq,Homo_723,AD.vs.MCI; bulk RNA-seq,Homo_714,AD.vs.control; bulk RNA-seq,Homo_714,AD.vs.MCI	4
MF	GO:0017070	U6 snRNA binding	bulk RNA-seq,Homo_723,AD.vs.control; bulk RNA-seq,Homo_723,MCI.vs.control; bulk RNA-seq,Homo_714,AD.vs.control; bulk RNA-seq,Homo_714,MCI.vs.control; bulk RNA-seq,Homo_633,AD.vs.control; bulk RNA-seq,Homo_633,AD.vs.MCI; bulk RNA-seq,Homo_633,MCI.vs.control	7
BP	GO:0009190	cyclic nucleotide biosynthetic process	bulk RNA-seq,Homo_723,AD.vs.control; bulk RNA-seq,Homo_723,AD.vs.MCI; bulk RNA-seq,Homo_723,MCI.vs.control; bulk RNA-seq,Homo_714,AD.vs.MCI	4
BP	GO:0052652	cyclic purine nucleotide metabolic process	bulk RNA-seq,Homo_723,AD.vs.control; bulk RNA-seq,Homo_723,AD.vs.MCI; bulk RNA-seq,Homo_723,MCI.vs.control; bulk RNA-seq,Homo_714,AD.vs.MCI	4
BP	GO:0050671	positive regulation of lymphocyte proliferation	bulk RNA-seq,Homo_723,AD.vs.control; bulk RNA-seq,Homo_723,MCI.vs.control; bulk RNA-seq,Homo_633,AD.vs.control; bulk RNA-	4
BP	GO:0023035	CD40 signaling pathway	bulk RNA-seq,Homo_723,AD.vs.control; bulk RNA-seq,Homo_723,MCI.vs.control; bulk RNA-seq,Homo_714,MCI.vs.control; bulk RNA-seq,Homo_633,AD.vs.control; bulk RNA-seq,Homo_633,MCI.vs.control	5
BP	GO:0014074	response to purine-containing compound	bulk RNA-seq,Homo_723,AD.vs.control; bulk RNA-seq,Homo_723,AD.vs.MCI; bulk RNA-seq,Homo_723,MCI.vs.control; bulk RNA-seq,Homo_714,AD.vs.MCI; bulk RNA-seq,Homo_633,AD.vs.control; bulk RNA-seq,Homo_633,AD.vs.MCI	6
BP	GO:1905939	regulation of gonad development	bulk RNA-seq,Homo_723,AD.vs.control; bulk RNA-seq,Homo_723,AD.vs.MCI; bulk RNA-seq,Homo_723,MCI.vs.control; bulk RNA-seq,Homo_714,AD.vs.control; bulk RNA-seq,Homo_714,AD.vs.MCI; bulk RNA-seq,Homo_714,MCI.vs.control	6
BP	GO:0043271	negative regulation of ion transport	bulk RNA-seq,Homo_723,AD.vs.control; bulk RNA-seq,Homo_723,AD.vs.MCI; bulk RNA-seq,Homo_723,MCI.vs.control; bulk RNA-seq,Homo_714,AD.vs.control; bulk RNA-seq,Homo_714,AD.vs.MCI; bulk RNA-seq,Homo_633,AD.vs.control; bulk RNA-seq,Homo_633,AD.vs.MCI	7
BP	GO:0006622	protein targeting to lysosome	bulk RNA-seq,Homo_723,AD.vs.control; bulk RNA-seq,Homo_714,AD.vs.control; bulk RNA-seq,Homo_633,AD.vs.control; bulk RNA-seq,Homo_633,AD.vs.MCI	4
BP	GO:1905314	semi-lunar valve development	bulk RNA-seq,Homo_723,AD.vs.control; bulk RNA-seq,Homo_723,AD.vs.MCI; bulk RNA-seq,Homo_723,MCI.vs.control; bulk RNA-seq,Homo_714,AD.vs.MCI; bulk RNA-seq,Homo_714,MCI.vs.control; bulk RNA-seq,SRP223445,AD.vs.control	6
BP	GO:0043928	exonucleolytic catabolism of deadenylated mRNA	bulk RNA-seq,Homo_723,AD.vs.control; bulk RNA-seq,Homo_723,AD.vs.MCI; bulk RNA-seq,Homo_714,AD.vs.control; bulk RNA-seq,Homo_714,AD.vs.MCI	4
BP	GO:0090502	RNA phosphodiester bond hydrolysis, endonucleolytic	bulk RNA-seq,Homo_723,AD.vs.control; bulk RNA-seq,Homo_714,AD.vs.control; bulk RNA-seq,Homo_714,MCI.vs.control; bulk RNA-seq,Homo_633,AD.vs.control; bulk RNA-seq,Homo_633,AD.vs.MCI	5
BP	GO:0071880	adenylate cyclase-activating adrenergic receptor signaling pathway	bulk RNA-seq,Homo_723,AD.vs.control; bulk RNA-seq,Homo_723,AD.vs.MCI; bulk RNA-seq,Homo_714,AD.vs.control; bulk RNA-seq,Homo_714,AD.vs.MCI; bulk RNA-seq,Homo_633,MCI.vs.control	5
BP	GO:0070327	thyroid hormone transport	bulk RNA-seq,Homo_723,AD.vs.control; bulk RNA-seq,Homo_723,AD.vs.MCI; bulk RNA-seq,Homo_723,MCI.vs.control; bulk RNA-seq,Homo_714,AD.vs.MCI	4
BP	GO:0000054	ribosomal subunit export from nucleus	bulk RNA-seq,Homo_723,AD.vs.control; bulk RNA-seq,Homo_714,AD.vs.control; bulk RNA-seq,Homo_633,AD.vs.control; bulk RNA-seq,Homo_633,AD.vs.MCI	4
BP	GO:0033750	ribosome localization	bulk RNA-seq,Homo_723,AD.vs.control; bulk RNA-seq,Homo_714,AD.vs.control; bulk RNA-seq,Homo_633,AD.vs.control; bulk RNA-seq,Homo_633,AD.vs.MCI	4
BP	GO:0000070	mitotic sister chromatid segregation	bulk RNA-seq,Homo_723,AD.vs.control; bulk RNA-seq,Homo_723,MCI.vs.control; bulk RNA-seq,Homo_714,AD.vs.control; bulk RNA-seq,Homo_633,AD.vs.control; bulk RNA-seq,Homo_633,AD.vs.MCI	5
BP	GO:0072527	pyrimidine-containing compound metabolic process	bulk RNA-seq,Homo_723,AD.vs.control; bulk RNA-seq,Homo_723,MCI.vs.control; bulk RNA-seq,Homo_714,AD.vs.control; bulk RNA-seq,Homo_714,MCI.vs.control; bulk RNA-seq,Homo_633,AD.vs.control; bulk RNA-seq,Homo_633,AD.vs.MCI	6
MF	GO:0140662	ATP-dependent protein folding chaperone	bulk RNA-seq,Homo_723,AD.vs.control; bulk RNA-seq,Homo_723,MCI.vs.control; bulk RNA-seq,Homo_714,AD.vs.control; bulk RNA-seq,Homo_714,MCI.vs.control; bulk RNA-seq,Homo_633,AD.vs.control; bulk RNA-seq,Homo_633,AD.vs.MCI	6
BP	GO:0007171	activation of transmembrane receptor protein tyrosine kinase activity	bulk RNA-seq,Homo_723,AD.vs.control; bulk RNA-seq,Homo_723,AD.vs.MCI; bulk RNA-seq,Homo_723,MCI.vs.control; bulk RNA-seq,Homo_714,AD.vs.MCI; bulk RNA-seq,Homo_714,MCI.vs.control; bulk RNA-seq,Homo_633,AD.vs.control	6
BP	GO:0072079	nephron tubule formation	bulk RNA-seq,Homo_723,AD.vs.control; bulk RNA-seq,Homo_723,AD.vs.MCI; bulk RNA-seq,Homo_723,MCI.vs.control; bulk RNA-seq,Homo_714,AD.vs.MCI; bulk RNA-seq,Homo_714,MCI.vs.control; bulk RNA-seq,Homo_633,MCI.vs.control	6
BP	GO:0030308	negative regulation of cell growth	bulk RNA-seq,Homo_723,AD.vs.control; bulk RNA-seq,Homo_723,AD.vs.MCI; bulk RNA-seq,Homo_723,MCI.vs.control; bulk RNA-seq,Homo_714,AD.vs.MCI; bulk RNA-seq,Homo_633,AD.vs.control; bulk RNA-seq,Homo_633,AD.vs.MCI; scRNA-seq,SRP330776,Naive CD8+ T cell_2-AD.vs.control	7
BP	GO:0043500	muscle adaptation	bulk RNA-seq,Homo_723,AD.vs.control; bulk RNA-seq,Homo_723,AD.vs.MCI; bulk RNA-seq,Homo_723,MCI.vs.control; bulk RNA-seq,Homo_714,AD.vs.control; bulk RNA-seq,Homo_714,AD.vs.MCI; bulk RNA-seq,Homo_714,MCI.vs.control; bulk RNA-seq,Homo_633,AD.vs.control; bulk	8

BP	GO:0014051	gamma-aminobutyric acid secretion	bulk RNA-seq,Homo_723,AD.vs.control; bulk RNA-seq,Homo_723,AD.vs.MCI; bulk RNA-seq,Homo_723,MCI.vs.control; bulk RNA-seq,Homo_714,AD.vs.MCI	4
BP	GO:0086015	SA node cell action potential	bulk RNA-seq,Homo_723,AD.vs.control; bulk RNA-seq,Homo_723,AD.vs.MCI; bulk RNA-seq,Homo_723,MCI.vs.control; bulk RNA-seq,Homo_714,AD.vs.MCI; bulk RNA-seq,Homo_714,MCI.vs.control	5
BP	GO:0086018	SA node cell to atrial cardiac muscle cell signaling	bulk RNA-seq,Homo_723,AD.vs.control; bulk RNA-seq,Homo_723,AD.vs.MCI; bulk RNA-seq,Homo_723,MCI.vs.control; bulk RNA-seq,Homo_714,AD.vs.MCI; bulk RNA-seq,Homo_714,MCI.vs.control	5
MF	GO:0070063	RNA polymerase binding	bulk RNA-seq,Homo_723,AD.vs.control; bulk RNA-seq,Homo_723,MCI.vs.control; bulk RNA-seq,Homo_714,AD.vs.control; bulk RNA-seq,Homo_714,MCI.vs.control; bulk RNA-seq,Homo_633,AD.vs.control; bulk RNA-seq,Homo_633,AD.vs.MCI	6
BP	GO:0045778	positive regulation of ossification	bulk RNA-seq,Homo_723,AD.vs.control; bulk RNA-seq,Homo_723,AD.vs.MCI; bulk RNA-seq,Homo_723,MCI.vs.control; bulk RNA-seq,Homo_714,AD.vs.MCI; bulk RNA-seq,Homo_633,AD.vs.control; bulk RNA-seq,Homo_633,AD.vs.MCI	6
BP	GO:0048025	negative regulation of mRNA splicing, via spliceosome	bulk RNA-seq,Homo_723,AD.vs.control; bulk RNA-seq,Homo_723,AD.vs.MCI; bulk RNA-seq,Homo_714,AD.vs.control; bulk RNA-seq,Homo_714,AD.vs.MCI; bulk RNA-seq,Homo_633,AD.vs.control; bulk RNA-seq,Homo_633,AD.vs.MCI	6
BP	GO:0050773	regulation of dendrite development	bulk RNA-seq,Homo_723,AD.vs.control; bulk RNA-seq,Homo_723,AD.vs.MCI; bulk RNA-seq,Homo_723,MCI.vs.control; bulk RNA-seq,Homo_714,AD.vs.MCI; bulk RNA-seq,Homo_714,MCI.vs.control; bulk RNA-seq,Homo_633,AD.vs.control; bulk RNA-seq,Homo_633,AD.vs.MCI	7
BP	GO:0031099	regeneration	bulk RNA-seq,Homo_723,AD.vs.control; bulk RNA-seq,Homo_723,AD.vs.MCI; bulk RNA-seq,Homo_723,MCI.vs.control; bulk RNA-seq,Homo_714,AD.vs.MCI; bulk RNA-seq,Homo_633,AD.vs.control; bulk RNA-seq,Homo_633,AD.vs.MCI	6
BP	GO:0048670	regulation of collateral sprouting	bulk RNA-seq,Homo_723,AD.vs.control; bulk RNA-seq,Homo_723,AD.vs.MCI; bulk RNA-seq,Homo_723,MCI.vs.control	3
BP	GO:0003148	outflow tract septum morphogenesis	bulk RNA-seq,Homo_723,AD.vs.control; bulk RNA-seq,Homo_723,AD.vs.MCI; bulk RNA-seq,Homo_723,MCI.vs.control; bulk RNA-seq,Homo_714,AD.vs.MCI; bulk RNA-seq,Homo_714,MCI.vs.control	5
BP	GO:0110151	positive regulation of biomineralization	bulk RNA-seq,Homo_723,AD.vs.control; bulk RNA-seq,Homo_723,AD.vs.MCI; bulk RNA-seq,Homo_723,MCI.vs.control; bulk RNA-seq,Homo_714,AD.vs.MCI; bulk RNA-seq,Homo_633,AD.vs.control; bulk RNA-seq,Homo_633,AD.vs.MCI	6
BP	GO:0006505	GPI anchor metabolic process	bulk RNA-seq,Homo_723,AD.vs.control; bulk RNA-seq,Homo_723,MCI.vs.control; bulk RNA-seq,Homo_714,AD.vs.control; bulk RNA-seq,Homo_714,MCI.vs.control; bulk RNA-seq,Homo_633,AD.vs.control; bulk RNA-seq,Homo_633,AD.vs.MCI; bulk RNA-seq,Homo_633,MCI.vs.control	7
BP	GO:0007034	vacuolar transport	bulk RNA-seq,Homo_723,AD.vs.control; bulk RNA-seq,Homo_723,MCI.vs.control; bulk RNA-seq,Homo_714,AD.vs.control; bulk RNA-seq,Homo_633,AD.vs.control; bulk RNA-seq,Homo_633,AD.vs.MCI	5
BP	GO:0006913	nucleocytoplasmic transport	bulk RNA-seq,Homo_723,AD.vs.control; bulk RNA-seq,Homo_723,MCI.vs.control; bulk RNA-seq,Homo_714,AD.vs.control; bulk RNA-seq,Homo_633,AD.vs.control; bulk RNA-seq,Homo_633,AD.vs.MCI	5
BP	GO:0051169	nuclear transport	bulk RNA-seq,Homo_723,AD.vs.control; bulk RNA-seq,Homo_723,MCI.vs.control; bulk RNA-seq,Homo_714,AD.vs.control; bulk RNA-seq,Homo_633,AD.vs.control; bulk RNA-seq,Homo_633,AD.vs.MCI	5
BP	GO:0070169	positive regulation of biomineral tissue development	bulk RNA-seq,Homo_723,AD.vs.control; bulk RNA-seq,Homo_723,AD.vs.MCI; bulk RNA-seq,Homo_723,MCI.vs.control; bulk RNA-seq,Homo_714,AD.vs.MCI; bulk RNA-seq,Homo_633,AD.vs.control; bulk RNA-seq,Homo_633,AD.vs.MCI	6
MF	GO:0071855	neuropeptide receptor binding	bulk RNA-seq,Homo_723,AD.vs.control; bulk RNA-seq,Homo_723,AD.vs.MCI; bulk RNA-seq,Homo_714,AD.vs.control; bulk RNA-seq,Homo_714,AD.vs.MCI	4
BP	GO:0045622	regulation of T-helper cell differentiation	bulk RNA-seq,Homo_723,AD.vs.control; bulk RNA-seq,Homo_723,MCI.vs.control; bulk RNA-seq,Homo_714,MCI.vs.control; bulk RNA-	4
BP	GO:0043249	erythrocyte maturation	bulk RNA-seq,Homo_723,AD.vs.control; bulk RNA-seq,Homo_723,MCI.vs.control; bulk RNA-seq,Homo_714,AD.vs.control; bulk RNA-seq,Homo_714,MCI.vs.control; bulk RNA-seq,Homo_633,AD.vs.control	5
BP	GO:0007620	copulation	bulk RNA-seq,Homo_723,AD.vs.control; bulk RNA-seq,Homo_723,AD.vs.MCI; bulk RNA-seq,Homo_714,AD.vs.control; bulk RNA-seq,Homo_714,AD.vs.MCI; bulk RNA-seq,Homo_633,AD.vs.control	5
BP	GO:0031069	hair follicle morphogenesis	bulk RNA-seq,Homo_723,AD.vs.control; bulk RNA-seq,Homo_723,AD.vs.MCI; bulk RNA-seq,Homo_723,MCI.vs.control; bulk RNA-seq,Homo_714,AD.vs.MCI	4
BP	GO:0110156	methylguanosine-cap decapping	bulk RNA-seq,Homo_723,AD.vs.control; bulk RNA-seq,Homo_723,AD.vs.MCI; bulk RNA-seq,Homo_714,AD.vs.control; bulk RNA-seq,Homo_714,AD.vs.MCI; bulk RNA-seq,Homo_633,AD.vs.control	5
BP	GO:0003161	cardiac conduction system development	bulk RNA-seq,Homo_723,AD.vs.control; bulk RNA-seq,Homo_723,AD.vs.MCI; bulk RNA-seq,Homo_723,MCI.vs.control; bulk RNA-seq,Homo_714,AD.vs.MCI; bulk RNA-seq,Homo_714,MCI.vs.control	5
CC	GO:0042383	sarcolemma	bulk RNA-seq,Homo_723,AD.vs.control; bulk RNA-seq,Homo_723,AD.vs.MCI; bulk RNA-seq,Homo_723,MCI.vs.control; bulk RNA-seq,Homo_714,AD.vs.control; bulk RNA-seq,Homo_714,AD.vs.MCI; bulk RNA-seq,Homo_633,AD.vs.control; bulk RNA-seq,Homo_633,AD.vs.MCI	7
BP	GO:0002833	positive regulation of response to biotic stimulus	bulk RNA-seq,Homo_723,AD.vs.control; bulk RNA-seq,Homo_723,MCI.vs.control; bulk RNA-seq,Homo_714,AD.vs.control; bulk RNA-seq,Homo_633,AD.vs.control; bulk RNA-seq,Homo_633,AD.vs.MCI	5
BP	GO:0086005	ventricular cardiac muscle cell action potential	bulk RNA-seq,Homo_723,AD.vs.control; bulk RNA-seq,Homo_723,AD.vs.MCI; bulk RNA-seq,Homo_723,MCI.vs.control; bulk RNA-seq,Homo_714,AD.vs.MCI; bulk RNA-seq,Homo_714,MCI.vs.control	5
BP	GO:0043114	regulation of vascular permeability	bulk RNA-seq,Homo_723,AD.vs.control; bulk RNA-seq,Homo_723,AD.vs.MCI; bulk RNA-seq,Homo_723,MCI.vs.control; bulk RNA-seq,Homo_714,AD.vs.control; bulk RNA-seq,Homo_714,AD.vs.MCI; bulk RNA-seq,Homo_714,MCI.vs.control; bulk RNA-seq,Homo_633,AD.vs.control; bulk	8
CC	GO:0043256	laminin complex	bulk RNA-seq,Homo_723,AD.vs.control; bulk RNA-seq,Homo_723,AD.vs.MCI; bulk RNA-seq,Homo_723,MCI.vs.control; bulk RNA-seq,Homo_714,AD.vs.MCI	4
BP	GO:0045071	negative regulation of viral genome replication	bulk RNA-seq,Homo_723,AD.vs.control; bulk RNA-seq,Homo_723,MCI.vs.control; bulk RNA-seq,Homo_714,AD.vs.control; bulk RNA-seq,Homo_714,MCI.vs.control; bulk RNA-seq,Homo_633,AD.vs.control	5
MF	GO:0019841	retinol binding	bulk RNA-seq,Homo_723,AD.vs.control; bulk RNA-seq,Homo_723,AD.vs.MCI; bulk RNA-seq,Homo_723,MCI.vs.control; bulk RNA-seq,Homo_714,AD.vs.MCI	4
BP	GO:0001569	branching involved in blood vessel morphogenesis	bulk RNA-seq,Homo_723,AD.vs.control; bulk RNA-seq,Homo_723,AD.vs.MCI; bulk RNA-seq,Homo_723,MCI.vs.control; bulk RNA-seq,Homo_714,AD.vs.control; bulk RNA-seq,Homo_714,AD.vs.MCI; bulk RNA-seq,Homo_714,MCI.vs.control	6
BP	GO:0042551	neuron maturation	bulk RNA-seq,Homo_723,AD.vs.control; bulk RNA-seq,Homo_723,AD.vs.MCI; bulk RNA-seq,Homo_723,MCI.vs.control; bulk RNA-seq,Homo_714,AD.vs.MCI; bulk RNA-seq,Homo_714,MCI.vs.control; bulk RNA-seq,Homo_633,AD.vs.control	6
BP	GO:0070203	regulation of establishment of protein localization to telomere	bulk RNA-seq,Homo_723,AD.vs.control; bulk RNA-seq,Homo_723,MCI.vs.control; bulk RNA-seq,Homo_714,AD.vs.control; bulk RNA-seq,Homo_714,MCI.vs.control; bulk RNA-seq,Homo_633,AD.vs.control; bulk RNA-seq,Homo_633,AD.vs.MCI; bulk RNA-seq,Homo_633,MCI.vs.control	7
BP	GO:1902903	regulation of supramolecular fiber organization	bulk RNA-seq,Homo_723,AD.vs.control; bulk RNA-seq,Homo_723,AD.vs.MCI; bulk RNA-seq,Homo_714,AD.vs.MCI; bulk RNA-seq,Homo_633,AD.vs.control; bulk RNA-seq,Homo_633,AD.vs.MCI	5
BP	GO:0002715	regulation of natural killer cell mediated immunity	bulk RNA-seq,Homo_723,AD.vs.control; bulk RNA-seq,Homo_714,AD.vs.control; bulk RNA-seq,Homo_714,MCI.vs.control; bulk RNA-seq,Homo_633,AD.vs.control; bulk RNA-seq,Homo_633,AD.vs.MCI; scRNA-seq,SRP309935,B cell_1-AD.vs.control	6

MF	GO:0034511	U3 snoRNA binding	bulk RNA-seq,Homo_723,AD.vs.control; bulk RNA-seq,Homo_723,AD.vs.MCI; bulk RNA-seq,Homo_714,AD.vs.control; bulk RNA-seq,Homo_714,AD.vs.MCI; bulk RNA-seq,Homo_714,MCI.vs.control; bulk RNA-seq,Homo_633,AD.vs.control; bulk RNA-seq,Homo_633,AD.vs.MCI	7
CC	GO:0045263	proton-transporting ATP synthase complex, coupling factor F(o)	bulk RNA-seq,Homo_723,AD.vs.control; bulk RNA-seq,Homo_723,AD.vs.MCI; bulk RNA-seq,Homo_714,AD.vs.control; bulk RNA-seq,Homo_714,MCI.vs.control; bulk RNA-seq,Homo_633,MCI.vs.control	5
BP	GO:0002087	regulation of respiratory gaseous exchange by nervous system	bulk RNA-seq,Homo_723,AD.vs.control; bulk RNA-seq,Homo_723,AD.vs.MCI; bulk RNA-seq,Homo_723,MCI.vs.control; bulk RNA-seq,Homo_714,AD.vs.MCI	4
BP	GO:2001239	regulation of extrinsic apoptotic signaling pathway in absence of ligand	bulk RNA-seq,Homo_723,AD.vs.control; bulk RNA-seq,Homo_723,AD.vs.MCI; bulk RNA-seq,Homo_723,MCI.vs.control; bulk RNA-seq,Homo_714,AD.vs.MCI; bulk RNA-seq,Homo_714,MCI.vs.control	5
BP	GO:1903169	regulation of calcium ion transmembrane transport	bulk RNA-seq,Homo_723,AD.vs.control; bulk RNA-seq,Homo_723,AD.vs.MCI; bulk RNA-seq,Homo_723,MCI.vs.control; bulk RNA-seq,Homo_714,AD.vs.MCI; bulk RNA-seq,Homo_633,AD.vs.control; bulk RNA-seq,Homo_633,AD.vs.MCI	6
BP	GO:0032230	positive regulation of synaptic transmission, GABAergic	bulk RNA-seq,Homo_723,AD.vs.control; bulk RNA-seq,Homo_723,AD.vs.MCI; bulk RNA-seq,Homo_714,AD.vs.MCI	3
BP	GO:0042088	T-helper 1 type immune response	bulk RNA-seq,Homo_723,AD.vs.control; bulk RNA-seq,Homo_723,MCI.vs.control; bulk RNA-seq,Homo_714,MCI.vs.control; bulk RNA-	4
MF	GO:0072341	modified amino acid binding	bulk RNA-seq,Homo_723,AD.vs.control; bulk RNA-seq,Homo_723,AD.vs.MCI; bulk RNA-seq,Homo_723,MCI.vs.control; bulk RNA-seq,Homo_714,AD.vs.MCI; bulk RNA-seq,Homo_633,AD.vs.control; bulk RNA-seq,Homo_633,AD.vs.MCI	6
BP	GO:0045444	fat cell differentiation	bulk RNA-seq,Homo_723,AD.vs.control; bulk RNA-seq,Homo_723,AD.vs.MCI; bulk RNA-seq,Homo_723,MCI.vs.control; bulk RNA-seq,Homo_714,AD.vs.MCI; bulk RNA-seq,Homo_633,AD.vs.control; bulk RNA-seq,Homo_633,AD.vs.MCI	6
BP	GO:0045686	negative regulation of glial cell differentiation	bulk RNA-seq,Homo_723,AD.vs.control; bulk RNA-seq,Homo_723,AD.vs.MCI; bulk RNA-seq,Homo_723,MCI.vs.control; bulk RNA-seq,Homo_714,AD.vs.MCI	4
BP	GO:1904019	epithelial cell apoptotic process	bulk RNA-seq,Homo_723,AD.vs.control; bulk RNA-seq,Homo_723,AD.vs.MCI; bulk RNA-seq,Homo_723,MCI.vs.control; bulk RNA-seq,Homo_714,AD.vs.control; bulk RNA-seq,Homo_714,AD.vs.MCI; bulk RNA-seq,Homo_633,AD.vs.control	6
MF	GO:0030551	cyclic nucleotide binding	bulk RNA-seq,Homo_723,AD.vs.control; bulk RNA-seq,Homo_723,AD.vs.MCI; bulk RNA-seq,Homo_723,MCI.vs.control; bulk RNA-seq,Homo_714,AD.vs.MCI; bulk RNA-seq,Homo_714,MCI.vs.control	5
BP	GO:0007413	axonal fasciculation	bulk RNA-seq,Homo_723,AD.vs.control; bulk RNA-seq,Homo_723,AD.vs.MCI; bulk RNA-seq,Homo_723,MCI.vs.control; bulk RNA-seq,Homo_714,AD.vs.MCI; bulk RNA-seq,Homo_714,MCI.vs.control; bulk RNA-seq,Homo_633,AD.vs.control	6
BP	GO:0106030	neuron projection fasciculation	bulk RNA-seq,Homo_723,AD.vs.control; bulk RNA-seq,Homo_723,AD.vs.MCI; bulk RNA-seq,Homo_723,MCI.vs.control; bulk RNA-seq,Homo_714,AD.vs.MCI; bulk RNA-seq,Homo_714,MCI.vs.control; bulk RNA-seq,Homo_633,AD.vs.control	6
BP	GO:0016578	histone deubiquitination	bulk RNA-seq,Homo_723,AD.vs.control; bulk RNA-seq,Homo_723,MCI.vs.control; bulk RNA-seq,Homo_714,MCI.vs.control; bulk RNA-seq,Homo_633,AD.vs.control; bulk RNA-seq,Homo_633,AD.vs.MCI	5
BP	GO:2001251	negative regulation of chromosome organization	bulk RNA-seq,Homo_723,AD.vs.control; bulk RNA-seq,Homo_723,MCI.vs.control; bulk RNA-seq,Homo_714,AD.vs.control; bulk RNA-seq,Homo_714,MCI.vs.control; bulk RNA-seq,Homo_633,AD.vs.control; bulk RNA-seq,Homo_633,AD.vs.MCI	6
BP	GO:2000181	negative regulation of blood vessel morphogenesis	bulk RNA-seq,Homo_723,AD.vs.control; bulk RNA-seq,Homo_723,AD.vs.MCI; bulk RNA-seq,Homo_723,MCI.vs.control; bulk RNA-seq,Homo_714,AD.vs.control; bulk RNA-seq,Homo_714,AD.vs.MCI; scRNA-seq,SRP330776,B cell_1-AD.vs.control	6
BP	GO:0009583	detection of light stimulus	bulk RNA-seq,Homo_723,AD.vs.control; bulk RNA-seq,Homo_723,AD.vs.MCI; bulk RNA-seq,Homo_714,AD.vs.control; bulk RNA-seq,Homo_714,AD.vs.MCI	4
BP	GO:0046622	positive regulation of organ growth	bulk RNA-seq,Homo_723,AD.vs.control; bulk RNA-seq,Homo_723,AD.vs.MCI; bulk RNA-seq,Homo_723,MCI.vs.control; bulk RNA-seq,Homo_714,AD.vs.control; bulk RNA-seq,Homo_714,AD.vs.MCI; bulk RNA-seq,Homo_714,MCI.vs.control	6
BP	GO:0032695	negative regulation of interleukin-12 production	bulk RNA-seq,Homo_723,AD.vs.control; bulk RNA-seq,Homo_723,MCI.vs.control; bulk RNA-seq,Homo_714,MCI.vs.control; bulk RNA-seq,Homo_633,AD.vs.control; bulk RNA-seq,Homo_633,AD.vs.MCI; bulk RNA-seq,Homo_633,MCI.vs.control	6
BP	GO:0045860	positive regulation of protein kinase activity	bulk RNA-seq,Homo_723,AD.vs.control; bulk RNA-seq,Homo_723,AD.vs.MCI; bulk RNA-seq,Homo_714,AD.vs.MCI; bulk RNA-seq,Homo_633,AD.vs.control; bulk RNA-seq,Homo_633,AD.vs.MCI	5
BP	GO:1901099	negative regulation of signal transduction in absence of ligand	bulk RNA-seq,Homo_723,AD.vs.control; bulk RNA-seq,Homo_723,AD.vs.MCI; bulk RNA-seq,Homo_723,MCI.vs.control; bulk RNA-seq,Homo_714,AD.vs.MCI; bulk RNA-seq,Homo_714,MCI.vs.control	5
BP	GO:2001240	negative regulation of extrinsic apoptotic signaling pathway in absence of ligand	bulk RNA-seq,Homo_723,AD.vs.control; bulk RNA-seq,Homo_723,AD.vs.MCI; bulk RNA-seq,Homo_723,MCI.vs.control; bulk RNA-seq,Homo_714,AD.vs.MCI; bulk RNA-seq,Homo_714,MCI.vs.control	5
BP	GO:0035633	maintenance of blood-brain barrier	bulk RNA-seq,Homo_723,AD.vs.control; bulk RNA-seq,Homo_723,AD.vs.MCI; bulk RNA-seq,Homo_723,MCI.vs.control; bulk RNA-seq,Homo_714,AD.vs.MCI; bulk RNA-seq,Homo_714,MCI.vs.control; bulk RNA-seq,Homo_633,AD.vs.control	6
CC	GO:0030864	cortical actin cytoskeleton	bulk RNA-seq,Homo_723,AD.vs.control; bulk RNA-seq,Homo_723,AD.vs.MCI; bulk RNA-seq,Homo_723,MCI.vs.control; bulk RNA-seq,Homo_714,AD.vs.MCI; bulk RNA-seq,Homo_714,MCI.vs.control; bulk RNA-seq,Homo_633,AD.vs.control; bulk RNA-seq,Homo_633,AD.vs.MCI	7
MF	GO:0042277	peptide binding	bulk RNA-seq,Homo_723,AD.vs.control; bulk RNA-seq,Homo_723,AD.vs.MCI; bulk RNA-seq,Homo_723,MCI.vs.control; bulk RNA-seq,Homo_714,AD.vs.MCI; bulk RNA-seq,Homo_633,AD.vs.control; bulk RNA-seq,Homo_633,AD.vs.MCI; scRNA-seq,SRP330776,Natural killer cell_1-AD.vs.control	7
MF	GO:0008514	organic anion transmembrane transporter activity	bulk RNA-seq,Homo_723,AD.vs.control; bulk RNA-seq,Homo_723,AD.vs.MCI; bulk RNA-seq,Homo_723,MCI.vs.control; bulk RNA-seq,Homo_714,AD.vs.MCI; bulk RNA-seq,Homo_633,AD.vs.control; bulk RNA-seq,Homo_633,AD.vs.MCI	6
BP	GO:0006582	melanin metabolic process	bulk RNA-seq,Homo_723,AD.vs.control; bulk RNA-seq,Homo_723,AD.vs.MCI; bulk RNA-seq,Homo_723,MCI.vs.control; bulk RNA-seq,Homo_633,AD.vs.control; bulk RNA-seq,Homo_633,AD.vs.MCI	5
BP	GO:2000095	regulation of Wnt signaling pathway, planar cell polarity pathway	bulk RNA-seq,Homo_723,AD.vs.control; bulk RNA-seq,Homo_723,AD.vs.MCI; bulk RNA-seq,Homo_723,MCI.vs.control; bulk RNA-seq,Homo_714,AD.vs.MCI; bulk RNA-seq,Homo_714,MCI.vs.control	5
BP	GO:0060037	pharyngeal system development	bulk RNA-seq,Homo_723,AD.vs.control; bulk RNA-seq,Homo_723,AD.vs.MCI; bulk RNA-seq,Homo_723,MCI.vs.control; bulk RNA-seq,Homo_714,AD.vs.MCI; bulk RNA-seq,Homo_714,MCI.vs.control	5
BP	GO:0048265	response to pain	bulk RNA-seq,Homo_723,AD.vs.control; bulk RNA-seq,Homo_723,AD.vs.MCI; bulk RNA-seq,Homo_723,MCI.vs.control; bulk RNA-seq,Homo_714,AD.vs.MCI; bulk RNA-seq,Homo_633,AD.vs.control	5
BP	GO:0002291	T cell activation via T cell receptor contact with antigen bound to MHC molecule on antigen presenting cell	bulk RNA-seq,Homo_723,AD.vs.control; bulk RNA-seq,Homo_723,AD.vs.MCI; bulk RNA-seq,Homo_714,AD.vs.control; bulk RNA-seq,Homo_714,AD.vs.MCI; bulk RNA-seq,Homo_633,AD.vs.control; bulk RNA-seq,Homo_633,AD.vs.MCI	6
BP	GO:0006471	protein ADP-ribosylation	bulk RNA-seq,Homo_723,AD.vs.control; bulk RNA-seq,Homo_723,MCI.vs.control; bulk RNA-seq,Homo_714,AD.vs.control; bulk RNA-seq,Homo_714,MCI.vs.control; bulk RNA-seq,Homo_633,AD.vs.control	5
MF	GO:0030297	transmembrane receptor protein tyrosine kinase activator	bulk RNA-seq,Homo_723,AD.vs.control; bulk RNA-seq,Homo_723,AD.vs.MCI; bulk RNA-seq,Homo_723,MCI.vs.control	3
BP	GO:0010894	negative regulation of steroid biosynthetic process	bulk RNA-seq,Homo_723,AD.vs.control; bulk RNA-seq,Homo_723,AD.vs.MCI; bulk RNA-seq,Homo_723,MCI.vs.control; bulk RNA-seq,Homo_714,AD.vs.MCI; bulk RNA-seq,Homo_714,MCI.vs.control	5

BP	GO:0035036	sperm-egg recognition	bulk RNA-seq,Homo_723,AD.vs.control; bulk RNA-seq,Homo_714,AD.vs.control; bulk RNA-seq,Homo_714,AD.vs.MCI	3
MF	GO:0050840	extracellular matrix binding	bulk RNA-seq,Homo_723,AD.vs.control; bulk RNA-seq,Homo_723,AD.vs.MCI; bulk RNA-seq,Homo_723,MCI.vs.control; bulk RNA-seq,Homo_714,AD.vs.control; bulk RNA-seq,Homo_714,AD.vs.MCI; bulk RNA-seq,Homo_714,MCI.vs.control; bulk RNA-seq,Homo_633,AD.vs.control	7
CC	GO:0000242	pericentriolar material	bulk RNA-seq,Homo_723,AD.vs.control; bulk RNA-seq,Homo_714,AD.vs.control; bulk RNA-seq,Homo_633,AD.vs.control; bulk RNA-seq,Homo_633,AD.vs.MCI	4
CC	GO:0005742	mitochondrial outer membrane translocase complex	bulk RNA-seq,Homo_723,AD.vs.control; bulk RNA-seq,Homo_714,AD.vs.control; bulk RNA-seq,Homo_633,AD.vs.control; bulk RNA-seq,Homo_633,AD.vs.MCI	4
BP	GO:0033275	actin-myosin filament sliding	bulk RNA-seq,Homo_723,AD.vs.control; bulk RNA-seq,Homo_723,AD.vs.MCI; bulk RNA-seq,Homo_714,AD.vs.control; bulk RNA-seq,Homo_714,AD.vs.MCI	4
BP	GO:0002097	tRNA wobble base modification	bulk RNA-seq,Homo_723,AD.vs.control; bulk RNA-seq,Homo_723,MCI.vs.control; bulk RNA-seq,Homo_714,AD.vs.control; bulk RNA-seq,Homo_714,MCI.vs.control; bulk RNA-seq,Homo_633,AD.vs.control; bulk RNA-seq,Homo_633,MCI.vs.control	6
BP	GO:0090009	primitive streak formation	bulk RNA-seq,Homo_723,AD.vs.control; bulk RNA-seq,Homo_723,AD.vs.MCI; bulk RNA-seq,Homo_723,MCI.vs.control	3
CC	GO:0034704	calcium channel complex	bulk RNA-seq,Homo_723,AD.vs.control; bulk RNA-seq,Homo_723,AD.vs.MCI; bulk RNA-seq,Homo_723,MCI.vs.control; bulk RNA-seq,Homo_714,AD.vs.MCI; bulk RNA-seq,Homo_633,AD.vs.control; bulk RNA-seq,ROSMAP,AD.vs.control	6
CC	GO:0008180	COP9 signalosome	bulk RNA-seq,Homo_723,AD.vs.control; bulk RNA-seq,Homo_723,AD.vs.MCI; bulk RNA-seq,Homo_714,AD.vs.control; bulk RNA-seq,Homo_633,AD.vs.control; bulk RNA-seq,Homo_633,AD.vs.MCI	5
BP	GO:0007589	body fluid secretion	bulk RNA-seq,Homo_723,AD.vs.control; bulk RNA-seq,Homo_723,AD.vs.MCI; bulk RNA-seq,Homo_723,MCI.vs.control; bulk RNA-seq,Homo_714,AD.vs.MCI; bulk RNA-seq,Homo_714,MCI.vs.control; bulk RNA-seq,Homo_633,AD.vs.control	6
MF	GO:0030552	cAMP binding	bulk RNA-seq,Homo_723,AD.vs.control; bulk RNA-seq,Homo_723,AD.vs.MCI; bulk RNA-seq,Homo_723,MCI.vs.control; bulk RNA-seq,Homo_714,AD.vs.control; bulk RNA-seq,Homo_714,AD.vs.MCI; bulk RNA-seq,Homo_714,MCI.vs.control	6
BP	GO:0048524	positive regulation of viral process	bulk RNA-seq,Homo_723,AD.vs.control; bulk RNA-seq,Homo_723,MCI.vs.control; bulk RNA-seq,Homo_714,AD.vs.control; bulk RNA-seq,Homo_714,MCI.vs.control; bulk RNA-seq,Homo_633,AD.vs.control; bulk RNA-seq,Homo_633,AD.vs.MCI	6
BP	GO:0097352	autophagosome maturation	bulk RNA-seq,Homo_723,AD.vs.control; bulk RNA-seq,Homo_714,AD.vs.control; bulk RNA-seq,Homo_714,MCI.vs.control; bulk RNA-seq,Homo_633,AD.vs.control; bulk RNA-seq,Homo_633,AD.vs.MCI	5
BP	GO:0051225	spindle assembly	bulk RNA-seq,Homo_723,AD.vs.control; bulk RNA-seq,Homo_723,MCI.vs.control; bulk RNA-seq,Homo_714,AD.vs.control; bulk RNA-seq,Homo_714,MCI.vs.control; bulk RNA-seq,Homo_633,AD.vs.control; bulk RNA-seq,Homo_633,AD.vs.MCI	6
BP	GO:0003180	aortic valve morphogenesis	bulk RNA-seq,Homo_723,AD.vs.control; bulk RNA-seq,Homo_723,AD.vs.MCI; bulk RNA-seq,Homo_723,MCI.vs.control; bulk RNA-seq,Homo_714,AD.vs.MCI; bulk RNA-seq,Homo_714,MCI.vs.control; bulk RNA-seq,SRP223445,AD.vs.control	6
BP	GO:0014904	myotube cell development	bulk RNA-seq,Homo_723,AD.vs.control; bulk RNA-seq,Homo_723,AD.vs.MCI; bulk RNA-seq,Homo_723,MCI.vs.control; bulk RNA-seq,Homo_714,AD.vs.MCI; bulk RNA-seq,Homo_714,MCI.vs.control; bulk RNA-seq,Homo_633,AD.vs.control; bulk RNA-seq,Homo_633,AD.vs.MCI	7
BP	GO:1990778	protein localization to cell periphery	bulk RNA-seq,Homo_723,AD.vs.control; bulk RNA-seq,Homo_723,AD.vs.MCI; bulk RNA-seq,Homo_714,AD.vs.MCI; bulk RNA-seq,Homo_633,AD.vs.control; bulk RNA-seq,Homo_633,AD.vs.MCI	5
BP	GO:0051851	modulation by host of symbiont process	bulk RNA-seq,Homo_723,AD.vs.control; bulk RNA-seq,Homo_723,MCI.vs.control; bulk RNA-seq,Homo_714,AD.vs.control; bulk RNA-seq,Homo_714,MCI.vs.control; bulk RNA-seq,Homo_633,AD.vs.control; bulk RNA-seq,Homo_633,AD.vs.MCI	6
BP	GO:0021604	cranial nerve structural organization	bulk RNA-seq,Homo_723,AD.vs.control; bulk RNA-seq,Homo_723,AD.vs.MCI; bulk RNA-seq,Homo_723,MCI.vs.control; bulk RNA-seq,Homo_714,AD.vs.MCI	4
MF	GO:0008179	adenylate cyclase binding	bulk RNA-seq,Homo_723,AD.vs.control; bulk RNA-seq,Homo_723,AD.vs.MCI; bulk RNA-seq,Homo_723,MCI.vs.control	3
CC	GO:0071006	U2-type catalytic step 1 spliceosome	bulk RNA-seq,Homo_723,AD.vs.control; bulk RNA-seq,Homo_723,AD.vs.MCI; bulk RNA-seq,Homo_723,MCI.vs.control; bulk RNA-seq,Homo_714,AD.vs.control; bulk RNA-seq,Homo_714,MCI.vs.control; bulk RNA-seq,Homo_633,MCI.vs.control	6
CC	GO:0071012	catalytic step 1 spliceosome	bulk RNA-seq,Homo_723,AD.vs.control; bulk RNA-seq,Homo_723,AD.vs.MCI; bulk RNA-seq,Homo_723,MCI.vs.control; bulk RNA-seq,Homo_714,AD.vs.control; bulk RNA-seq,Homo_714,MCI.vs.control; bulk RNA-seq,Homo_633,MCI.vs.control	6
CC	GO:0005750	mitochondrial respiratory chain complex III	bulk RNA-seq,Homo_723,AD.vs.control; bulk RNA-seq,Homo_723,MCI.vs.control; bulk RNA-seq,Homo_714,AD.vs.control; bulk RNA-seq,Homo_714,MCI.vs.control; bulk RNA-seq,Homo_633,AD.vs.control; bulk RNA-seq,Homo_633,AD.vs.MCI; bulk RNA-seq,Homo_633,MCI.vs.control	7
CC	GO:0045275	respiratory chain complex III	bulk RNA-seq,Homo_723,AD.vs.control; bulk RNA-seq,Homo_723,MCI.vs.control; bulk RNA-seq,Homo_714,AD.vs.control; bulk RNA-seq,Homo_714,MCI.vs.control; bulk RNA-seq,Homo_633,AD.vs.control; bulk RNA-seq,Homo_633,AD.vs.MCI; bulk RNA-seq,Homo_633,MCI.vs.control	7
BP	GO:0060259	regulation of feeding behavior	bulk RNA-seq,Homo_723,AD.vs.control; bulk RNA-seq,Homo_723,AD.vs.MCI; bulk RNA-seq,Homo_714,AD.vs.control	3
BP	GO:0071559	response to transforming growth factor beta	bulk RNA-seq,Homo_723,AD.vs.control; bulk RNA-seq,Homo_723,AD.vs.MCI; bulk RNA-seq,Homo_723,MCI.vs.control; bulk RNA-seq,Homo_714,AD.vs.MCI; bulk RNA-seq,Homo_633,AD.vs.control; bulk RNA-seq,Homo_633,AD.vs.MCI; scRNA-seq,SRP330776,CD8+ T cell_2-AD.vs.control; scRNA-seq,SRP330776,Naive CD8+ T cell_1-AD.vs.control; scRNA-seq,SRP330776,Naive CD8+ T cell_2-AD.vs.control; scRNA-seq,SRP330776,Natural killer cell_1-	10
BP	GO:0006904	vesicle docking involved in exocytosis	bulk RNA-seq,Homo_723,AD.vs.control; bulk RNA-seq,Homo_723,AD.vs.MCI; bulk RNA-seq,Homo_723,MCI.vs.control; bulk RNA-seq,Homo_714,AD.vs.MCI; bulk RNA-seq,Homo_714,MCI.vs.control; bulk RNA-seq,Homo_633,AD.vs.control; bulk RNA-seq,Homo_633,AD.vs.MCI	7
BP	GO:0046641	positive regulation of alpha-beta T cell proliferation	bulk RNA-seq,Homo_723,AD.vs.control; bulk RNA-seq,Homo_714,AD.vs.control; bulk RNA-seq,Homo_714,MCI.vs.control; bulk RNA-seq,Homo_633,AD.vs.control; bulk RNA-seq,Homo_633,MCI.vs.control	5
BP	GO:0032728	positive regulation of interferon-beta production	bulk RNA-seq,Homo_723,AD.vs.control; bulk RNA-seq,Homo_723,MCI.vs.control; bulk RNA-seq,Homo_714,MCI.vs.control; bulk RNA-seq,Homo_633,AD.vs.control; bulk RNA-seq,Homo_633,AD.vs.MCI	5
BP	GO:0032438	melanosome organization	bulk RNA-seq,Homo_723,AD.vs.control; bulk RNA-seq,Homo_723,MCI.vs.control; bulk RNA-seq,Homo_714,AD.vs.control; bulk RNA-seq,Homo_714,MCI.vs.control; bulk RNA-seq,Homo_633,AD.vs.control; bulk RNA-seq,Homo_633,AD.vs.MCI	6
MF	GO:0016505	peptidase activator activity involved in apoptotic process	bulk RNA-seq,Homo_723,AD.vs.control; bulk RNA-seq,Homo_723,MCI.vs.control; bulk RNA-seq,Homo_714,MCI.vs.control; bulk RNA-seq,Homo_633,AD.vs.control; bulk RNA-seq,Homo_633,AD.vs.MCI; bulk RNA-seq,Homo_633,MCI.vs.control	6
BP	GO:0060339	negative regulation of type I interferon-mediated signaling pathway	bulk RNA-seq,Homo_723,AD.vs.control; bulk RNA-seq,Homo_723,MCI.vs.control; bulk RNA-seq,Homo_714,AD.vs.control; bulk RNA-seq,Homo_714,MCI.vs.control; bulk RNA-seq,Homo_633,AD.vs.control; bulk RNA-seq,Homo_633,MCI.vs.control	6
BP	GO:0010752	regulation of cGMP-mediated signaling	bulk RNA-seq,Homo_723,AD.vs.control; bulk RNA-seq,Homo_723,AD.vs.MCI; bulk RNA-seq,Homo_723,MCI.vs.control; bulk RNA-seq,Homo_714,AD.vs.control; bulk RNA-seq,Homo_714,AD.vs.MCI; bulk RNA-seq,Homo_714,MCI.vs.control	6
BP	GO:0048799	animal organ maturation	bulk RNA-seq,Homo_723,AD.vs.control; bulk RNA-seq,Homo_723,AD.vs.MCI; bulk RNA-seq,Homo_723,MCI.vs.control	3
BP	GO:0050680	negative regulation of epithelial cell proliferation	bulk RNA-seq,Homo_723,AD.vs.control; bulk RNA-seq,Homo_723,AD.vs.MCI; bulk RNA-seq,Homo_723,MCI.vs.control; bulk RNA-seq,Homo_714,AD.vs.control; bulk RNA-seq,Homo_714,AD.vs.MCI; bulk RNA-seq,Homo_633,AD.vs.control; bulk RNA-seq,Homo_633,AD.vs.MCI	7
BP	GO:0061077	chaperone-mediated protein folding	bulk RNA-seq,Homo_723,AD.vs.control; bulk RNA-seq,Homo_723,MCI.vs.control; bulk RNA-seq,Homo_714,AD.vs.control; bulk RNA-seq,Homo_714,MCI.vs.control; bulk RNA-seq,Homo_633,AD.vs.control; bulk RNA-seq,Homo_633,AD.vs.MCI	6

CC	GO:0001533	cornified envelope	bulk RNA-seq,Homo_723,AD.vs.control; bulk RNA-seq,Homo_723,AD.vs.MCI; bulk RNA-seq,Homo_714,AD.vs.control; bulk RNA-seq,Homo_714,AD.vs.MCI; bulk RNA-seq,Homo_714,MCI.vs.control	5
MF	GO:0042056	chemoattractant activity	bulk RNA-seq,Homo_723,AD.vs.control; bulk RNA-seq,Homo_723,AD.vs.MCI; bulk RNA-seq,Homo_714,AD.vs.control; bulk RNA-seq,Homo_714,AD.vs.MCI	4
BP	GO:0090307	mitotic spindle assembly	bulk RNA-seq,Homo_723,AD.vs.control; bulk RNA-seq,Homo_723,MCI.vs.control; bulk RNA-seq,Homo_714,MCI.vs.control; bulk RNA-seq,Homo_633,AD.vs.control; bulk RNA-seq,Homo_633,AD.vs.MCI	5
MF	GO:0008276	protein methyltransferase activity	bulk RNA-seq,Homo_723,AD.vs.control; bulk RNA-seq,Homo_723,MCI.vs.control; bulk RNA-seq,Homo_714,AD.vs.control; bulk RNA-seq,Homo_714,MCI.vs.control; bulk RNA-seq,Homo_633,AD.vs.control; bulk RNA-seq,Homo_633,AD.vs.MCI	6
BP	GO:0051153	regulation of striated muscle cell differentiation	bulk RNA-seq,Homo_723,AD.vs.control; bulk RNA-seq,Homo_723,AD.vs.MCI; bulk RNA-seq,Homo_723,MCI.vs.control; bulk RNA-seq,Homo_714,AD.vs.MCI; bulk RNA-seq,Homo_714,MCI.vs.control	5
BP	GO:0036258	multivesicular body assembly	bulk RNA-seq,Homo_723,AD.vs.control; bulk RNA-seq,Homo_723,MCI.vs.control; bulk RNA-seq,Homo_714,AD.vs.control; bulk RNA-seq,Homo_714,MCI.vs.control; bulk RNA-seq,Homo_633,AD.vs.control; bulk RNA-seq,Homo_633,AD.vs.MCI; bulk RNA-seq,Homo_633,MCI.vs.control	7
BP	GO:0048714	positive regulation of oligodendrocyte differentiation	bulk RNA-seq,Homo_723,AD.vs.control; bulk RNA-seq,Homo_723,AD.vs.MCI; bulk RNA-seq,Homo_723,MCI.vs.control; bulk RNA-seq,Homo_714,AD.vs.MCI; bulk RNA-seq,Homo_714,MCI.vs.control	5
CC	GO:0030127	COPII vesicle coat	bulk RNA-seq,Homo_723,AD.vs.control; bulk RNA-seq,Homo_723,MCI.vs.control; bulk RNA-seq,Homo_714,AD.vs.control; bulk RNA-seq,Homo_714,MCI.vs.control; bulk RNA-seq,Homo_633,AD.vs.control; bulk RNA-seq,Homo_633,MCI.vs.control	6
BP	GO:0002637	regulation of immunoglobulin production	bulk RNA-seq,Homo_723,AD.vs.control; bulk RNA-seq,Homo_723,MCI.vs.control; bulk RNA-seq,Homo_714,MCI.vs.control; bulk RNA-seq,Homo_633,AD.vs.control; bulk RNA-seq,Homo_633,AD.vs.MCI	5
CC	GO:0000118	histone deacetylase complex	bulk RNA-seq,Homo_723,AD.vs.control; bulk RNA-seq,Homo_723,MCI.vs.control; bulk RNA-seq,Homo_714,AD.vs.control; bulk RNA-seq,Homo_714,MCI.vs.control; bulk RNA-seq,Homo_633,AD.vs.control; bulk RNA-seq,Homo_633,AD.vs.MCI	6
BP	GO:0051054	positive regulation of DNA metabolic process	bulk RNA-seq,Homo_723,AD.vs.control; bulk RNA-seq,Homo_723,MCI.vs.control; bulk RNA-seq,Homo_714,AD.vs.control; bulk RNA-seq,Homo_633,AD.vs.control; bulk RNA-seq,Homo_633,AD.vs.MCI	5
BP	GO:0060973	cell migration involved in heart development	bulk RNA-seq,Homo_723,AD.vs.control; bulk RNA-seq,Homo_723,AD.vs.MCI; bulk RNA-seq,Homo_723,MCI.vs.control; bulk RNA-seq,Homo_714,AD.vs.control; bulk RNA-seq,Homo_714,AD.vs.MCI; bulk RNA-seq,Homo_714,MCI.vs.control; bulk RNA-seq,SRP223445,AD.vs.control	7
BP	GO:0045598	regulation of fat cell differentiation	bulk RNA-seq,Homo_723,AD.vs.control; bulk RNA-seq,Homo_723,AD.vs.MCI; bulk RNA-seq,Homo_723,MCI.vs.control; bulk RNA-seq,Homo_714,AD.vs.MCI	4
BP	GO:0060038	cardiac muscle cell proliferation	bulk RNA-seq,Homo_723,AD.vs.control; bulk RNA-seq,Homo_723,AD.vs.MCI; bulk RNA-seq,Homo_723,MCI.vs.control; bulk RNA-seq,Homo_714,AD.vs.MCI; bulk RNA-seq,Homo_714,MCI.vs.control; bulk RNA-seq,Homo_633,AD.vs.control; bulk RNA-seq,Homo_633,AD.vs.MCI	7
BP	GO:0001682	tRNA 5'-leader removal	bulk RNA-seq,Homo_723,AD.vs.control; bulk RNA-seq,Homo_723,AD.vs.MCI; bulk RNA-seq,Homo_714,AD.vs.control; bulk RNA-seq,Homo_633,AD.vs.control; bulk RNA-seq,Homo_633,AD.vs.MCI	5
MF	GO:0097602	cullin family protein binding	bulk RNA-seq,Homo_723,AD.vs.control; bulk RNA-seq,Homo_723,MCI.vs.control; bulk RNA-seq,Homo_714,AD.vs.control; bulk RNA-seq,Homo_714,MCI.vs.control; bulk RNA-seq,Homo_633,AD.vs.control; bulk RNA-seq,Homo_633,AD.vs.MCI; bulk RNA-seq,Homo_633,MCI.vs.control	7
CC	GO:0005732	sno(s)RNA-containing ribonucleoprotein complex	bulk RNA-seq,Homo_723,AD.vs.control; bulk RNA-seq,Homo_714,AD.vs.control; bulk RNA-seq,Homo_633,AD.vs.control; bulk RNA-seq,Homo_633,AD.vs.MCI	4
BP	GO:0009165	nucleotide biosynthetic process	bulk RNA-seq,Homo_723,AD.vs.control; bulk RNA-seq,Homo_723,MCI.vs.control; bulk RNA-seq,Homo_714,AD.vs.control; bulk RNA-seq,Homo_633,AD.vs.control; bulk RNA-seq,Homo_633,AD.vs.MCI	5
MF	GO:0008191	metalloendopeptidase inhibitor activity	bulk RNA-seq,Homo_723,AD.vs.control; bulk RNA-seq,Homo_723,AD.vs.MCI	2
BP	GO:0043367	CD4-positive, alpha-beta T cell differentiation	bulk RNA-seq,Homo_723,AD.vs.control; bulk RNA-seq,Homo_723,MCI.vs.control; bulk RNA-seq,Homo_714,AD.vs.control; bulk RNA-seq,Homo_714,MCI.vs.control; bulk RNA-seq,Homo_633,AD.vs.control; bulk RNA-seq,Homo_633,AD.vs.MCI	6
BP	GO:0098801	regulation of renal system process	bulk RNA-seq,Homo_723,AD.vs.control; bulk RNA-seq,Homo_723,AD.vs.MCI; bulk RNA-seq,Homo_723,MCI.vs.control; bulk RNA-seq,Homo_714,AD.vs.control; bulk RNA-seq,Homo_714,AD.vs.MCI	5
BP	GO:1900006	positive regulation of dendrite development	bulk RNA-seq,Homo_723,AD.vs.control; bulk RNA-seq,Homo_723,AD.vs.MCI; bulk RNA-seq,Homo_723,MCI.vs.control; bulk RNA-seq,Homo_714,AD.vs.MCI; bulk RNA-seq,Homo_714,MCI.vs.control	5
BP	GO:0070593	dendrite self-avoidance	bulk RNA-seq,Homo_723,AD.vs.control; bulk RNA-seq,Homo_723,AD.vs.MCI; bulk RNA-seq,Homo_723,MCI.vs.control; bulk RNA-seq,Homo_714,AD.vs.MCI	4
BP	GO:0006783	heme biosynthetic process	bulk RNA-seq,Homo_723,AD.vs.control; bulk RNA-seq,Homo_723,MCI.vs.control; bulk RNA-seq,Homo_714,AD.vs.control; bulk RNA-seq,Homo_714,MCI.vs.control; bulk RNA-seq,Homo_633,AD.vs.control; bulk RNA-seq,Homo_633,AD.vs.MCI; bulk RNA-seq,Homo_633,MCI.vs.control	7
BP	GO:0045823	positive regulation of heart contraction	bulk RNA-seq,Homo_723,AD.vs.control; bulk RNA-seq,Homo_723,AD.vs.MCI; bulk RNA-seq,Homo_723,MCI.vs.control; bulk RNA-seq,Homo_714,AD.vs.MCI	4
BP	GO:0060272	embryonic skeletal joint morphogenesis	bulk RNA-seq,Homo_723,AD.vs.control; bulk RNA-seq,Homo_723,AD.vs.MCI; bulk RNA-seq,Homo_723,MCI.vs.control; bulk RNA-seq,Homo_714,AD.vs.MCI	4
MF	GO:0035064	methylated histone binding	bulk RNA-seq,Homo_723,AD.vs.control; bulk RNA-seq,Homo_723,MCI.vs.control; bulk RNA-seq,Homo_714,AD.vs.control; bulk RNA-seq,Homo_714,MCI.vs.control; bulk RNA-seq,Homo_633,AD.vs.control; bulk RNA-seq,Homo_633,AD.vs.MCI	6
MF	GO:0140034	methylation-dependent protein binding	bulk RNA-seq,Homo_723,AD.vs.control; bulk RNA-seq,Homo_723,MCI.vs.control; bulk RNA-seq,Homo_714,AD.vs.control; bulk RNA-seq,Homo_714,MCI.vs.control; bulk RNA-seq,Homo_633,AD.vs.control; bulk RNA-seq,Homo_633,AD.vs.MCI	6
BP	GO:0033135	regulation of peptidyl-serine phosphorylation	bulk RNA-seq,Homo_723,AD.vs.control; bulk RNA-seq,Homo_723,AD.vs.MCI; bulk RNA-seq,Homo_723,MCI.vs.control; bulk RNA-seq,Homo_714,AD.vs.control; bulk RNA-seq,Homo_714,AD.vs.MCI; bulk RNA-seq,Homo_633,AD.vs.control; bulk RNA-seq,Homo_633,AD.vs.MCI	7
BP	GO:0098868	bone growth	bulk RNA-seq,Homo_723,AD.vs.control; bulk RNA-seq,Homo_723,AD.vs.MCI; bulk RNA-seq,Homo_723,MCI.vs.control; bulk RNA-seq,Homo_714,AD.vs.MCI; bulk RNA-seq,Homo_714,MCI.vs.control; bulk RNA-seq,Homo_633,AD.vs.control	6
MF	GO:0008237	metallopeptidase activity	bulk RNA-seq,Homo_723,AD.vs.control; bulk RNA-seq,Homo_723,AD.vs.MCI; bulk RNA-seq,Homo_723,MCI.vs.control; bulk RNA-seq,Homo_714,AD.vs.control; bulk RNA-seq,Homo_714,AD.vs.MCI; bulk RNA-seq,Homo_633,AD.vs.control; bulk RNA-seq,Homo_633,AD.vs.MCI	7
BP	GO:0000075	cell cycle checkpoint signaling	bulk RNA-seq,Homo_723,AD.vs.control; bulk RNA-seq,Homo_723,MCI.vs.control; bulk RNA-seq,Homo_714,AD.vs.control; bulk RNA-seq,Homo_633,AD.vs.control; bulk RNA-seq,Homo_633,AD.vs.MCI	5
CC	GO:0030894	replisome	bulk RNA-seq,Homo_723,AD.vs.control; bulk RNA-seq,Homo_723,MCI.vs.control; bulk RNA-seq,Homo_714,AD.vs.control; bulk RNA-seq,Homo_714,MCI.vs.control; bulk RNA-seq,Homo_633,AD.vs.control; bulk RNA-seq,Homo_633,MCI.vs.control	6
BP	GO:1901343	negative regulation of vasculature development	bulk RNA-seq,Homo_723,AD.vs.control; bulk RNA-seq,Homo_723,AD.vs.MCI; bulk RNA-seq,Homo_723,MCI.vs.control; bulk RNA-seq,Homo_714,AD.vs.control; bulk RNA-seq,Homo_714,AD.vs.MCI; scRNA-seq,SRP330776,B cell_1-AD.vs.control	6
CC	GO:0005912	adherens junction	bulk RNA-seq,Homo_723,AD.vs.control; bulk RNA-seq,Homo_723,AD.vs.MCI; bulk RNA-seq,Homo_723,MCI.vs.control; bulk RNA-seq,Homo_714,AD.vs.MCI; bulk RNA-seq,Homo_633,AD.vs.control; bulk RNA-seq,Homo_633,AD.vs.MCI; bulk RNA-seq,ROSMAP,AD.vs.control; bulk RNA-seq,SRP223445,AD.vs.control	8

BP	GO:0042269	regulation of natural killer cell mediated cytotoxicity	bulk RNA-seq,Homo_723,AD.vs.control; bulk RNA-seq,Homo_714,AD.vs.control; bulk RNA-seq,Homo_714,MCI.vs.control; bulk RNA-seq,Homo_633,AD.vs.control; bulk RNA-seq,Homo_633,AD.vs.MCI; scRNA-seq,SRP309935,B cell_1-AD.vs.control	6
BP	GO:0021955	central nervous system neuron axonogenesis	bulk RNA-seq,Homo_723,AD.vs.control; bulk RNA-seq,Homo_723,AD.vs.MCI; bulk RNA-seq,Homo_723,MCI.vs.control; bulk RNA-seq,Homo_714,AD.vs.MCI; bulk RNA-seq,Homo_714,MCI.vs.control; bulk RNA-seq,Homo_633,AD.vs.control; bulk RNA-seq,Homo_633,AD.vs.MCI	7
CC	GO:0070993	translation preinitiation complex	bulk RNA-seq,Homo_723,AD.vs.control; bulk RNA-seq,Homo_714,AD.vs.control; bulk RNA-seq,Homo_714,AD.vs.MCI; bulk RNA-seq,Homo_633,AD.vs.control	4
BP	GO:0060351	cartilage development involved in endochondral bone	bulk RNA-seq,Homo_723,AD.vs.control; bulk RNA-seq,Homo_723,AD.vs.MCI; bulk RNA-seq,Homo_723,MCI.vs.control; bulk RNA-seq,Homo_714,AD.vs.MCI	4
BP	GO:0042755	eating behavior	bulk RNA-seq,Homo_723,AD.vs.control; bulk RNA-seq,Homo_723,AD.vs.MCI; bulk RNA-seq,Homo_714,AD.vs.control	3
BP	GO:2001252	positive regulation of chromosome organization	bulk RNA-seq,Homo_723,AD.vs.control; bulk RNA-seq,Homo_723,MCI.vs.control; bulk RNA-seq,Homo_714,AD.vs.control; bulk RNA-seq,Homo_714,MCI.vs.control; bulk RNA-seq,Homo_633,AD.vs.control; bulk RNA-seq,Homo_633,AD.vs.MCI	6
BP	GO:2000319	regulation of T-helper 17 cell differentiation	bulk RNA-seq,Homo_723,AD.vs.control; bulk RNA-seq,Homo_723,MCI.vs.control; bulk RNA-seq,Homo_714,MCI.vs.control; bulk RNA-	4
BP	GO:0040037	negative regulation of fibroblast growth factor receptor signaling pathway	bulk RNA-seq,Homo_723,AD.vs.control; bulk RNA-seq,Homo_723,AD.vs.MCI; bulk RNA-seq,Homo_723,MCI.vs.control; bulk RNA-seq,Homo_714,AD.vs.MCI; bulk RNA-seq,Homo_714,MCI.vs.control	5
MF	GO:0035255	ionotropic glutamate receptor binding	bulk RNA-seq,Homo_723,AD.vs.control; bulk RNA-seq,Homo_723,AD.vs.MCI; bulk RNA-seq,Homo_723,MCI.vs.control; bulk RNA-seq,Homo_714,AD.vs.MCI; bulk RNA-seq,Homo_714,MCI.vs.control	5
BP	GO:0003351	epithelial cilium movement involved in extracellular fluid	bulk RNA-seq,Homo_723,AD.vs.control; bulk RNA-seq,Homo_723,AD.vs.MCI; bulk RNA-seq,Homo_723,MCI.vs.control	3
CC	GO:0101019	nucleolar exosome (RNase complex)	bulk RNA-seq,Homo_723,AD.vs.control; bulk RNA-seq,Homo_723,AD.vs.MCI; bulk RNA-seq,Homo_714,AD.vs.control; bulk RNA-seq,Homo_714,AD.vs.MCI; bulk RNA-seq,Homo_633,AD.vs.control; bulk RNA-seq,Homo_633,AD.vs.MCI	6
BP	GO:0033119	negative regulation of RNA splicing	bulk RNA-seq,Homo_723,AD.vs.control; bulk RNA-seq,Homo_723,AD.vs.MCI; bulk RNA-seq,Homo_714,AD.vs.control; bulk RNA-seq,Homo_714,AD.vs.MCI; bulk RNA-seq,Homo_633,AD.vs.control; bulk RNA-seq,SRP310421,AD.vs.control	6
BP	GO:0090075	relaxation of muscle	bulk RNA-seq,Homo_723,AD.vs.control; bulk RNA-seq,Homo_723,AD.vs.MCI	2
BP	GO:0060402	calcium ion transport into cytosol	bulk RNA-seq,Homo_723,AD.vs.control; bulk RNA-seq,Homo_723,AD.vs.MCI; bulk RNA-seq,Homo_723,MCI.vs.control; bulk RNA-seq,Homo_714,AD.vs.MCI	4
BP	GO:0007339	binding of sperm to zona pellucida	bulk RNA-seq,Homo_723,AD.vs.control; bulk RNA-seq,Homo_714,AD.vs.control; bulk RNA-seq,Homo_714,AD.vs.MCI; bulk RNA-seq,Homo_633,AD.vs.control	4
BP	GO:0032007	negative regulation of TOR signaling	bulk RNA-seq,Homo_723,AD.vs.control; bulk RNA-seq,Homo_723,MCI.vs.control; bulk RNA-seq,Homo_714,AD.vs.control; bulk RNA-seq,Homo_714,MCI.vs.control; bulk RNA-seq,Homo_633,AD.vs.control; bulk RNA-seq,Homo_633,AD.vs.MCI	6
BP	GO:0048484	enteric nervous system development	bulk RNA-seq,Homo_723,AD.vs.control; bulk RNA-seq,Homo_723,AD.vs.MCI; bulk RNA-seq,Homo_723,MCI.vs.control; bulk RNA-seq,Homo_714,AD.vs.control; bulk RNA-seq,Homo_714,AD.vs.MCI	5
BP	GO:0032006	regulation of TOR signaling	bulk RNA-seq,Homo_723,AD.vs.control; bulk RNA-seq,Homo_723,MCI.vs.control; bulk RNA-seq,Homo_714,AD.vs.control; bulk RNA-seq,Homo_714,MCI.vs.control; bulk RNA-seq,Homo_633,AD.vs.control; bulk RNA-seq,Homo_633,AD.vs.MCI	6
CC	GO:0030665	clathrin-coated vesicle membrane	bulk RNA-seq,Homo_723,AD.vs.control; bulk RNA-seq,Homo_723,AD.vs.MCI; bulk RNA-seq,Homo_723,MCI.vs.control; bulk RNA-seq,Homo_714,AD.vs.MCI; bulk RNA-seq,Homo_633,AD.vs.control; bulk RNA-seq,Homo_633,AD.vs.MCI; scRNA-seq,SRP215507,CD8+ T cell_3-AD.vs.control	7
BP	GO:0007585	respiratory gaseous exchange by respiratory system	bulk RNA-seq,Homo_723,AD.vs.control; bulk RNA-seq,Homo_723,AD.vs.MCI; bulk RNA-seq,Homo_723,MCI.vs.control; bulk RNA-seq,Homo_714,AD.vs.MCI; bulk RNA-seq,Homo_714,MCI.vs.control	5
MF	GO:0015370	solute:sodium symporter activity	bulk RNA-seq,Homo_723,AD.vs.control; bulk RNA-seq,Homo_723,AD.vs.MCI; bulk RNA-seq,Homo_723,MCI.vs.control; bulk RNA-seq,Homo_714,AD.vs.control; bulk RNA-seq,Homo_714,AD.vs.MCI; bulk RNA-seq,Homo_714,MCI.vs.control; bulk RNA-seq,ROSMAP,MCI.vs.control	7
BP	GO:0072207	metanephric epithelium development	bulk RNA-seq,Homo_723,AD.vs.control; bulk RNA-seq,Homo_723,AD.vs.MCI; bulk RNA-seq,Homo_723,MCI.vs.control; bulk RNA-seq,Homo_714,AD.vs.MCI; bulk RNA-seq,Homo_714,MCI.vs.control; bulk RNA-seq,Homo_633,MCI.vs.control	6
BP	GO:2000647	negative regulation of stem cell proliferation	bulk RNA-seq,Homo_723,AD.vs.control; bulk RNA-seq,Homo_723,AD.vs.MCI; bulk RNA-seq,Homo_723,MCI.vs.control; bulk RNA-seq,Homo_714,AD.vs.control; bulk RNA-seq,Homo_714,AD.vs.MCI; bulk RNA-seq,Homo_714,MCI.vs.control	6
BP	GO:0045600	positive regulation of fat cell differentiation	bulk RNA-seq,Homo_723,AD.vs.control; bulk RNA-seq,Homo_723,AD.vs.MCI; bulk RNA-seq,Homo_723,MCI.vs.control; bulk RNA-seq,Homo_714,AD.vs.MCI; bulk RNA-seq,Homo_714,MCI.vs.control	5
BP	GO:0018105	peptidyl-serine phosphorylation	bulk RNA-seq,Homo_723,AD.vs.control; bulk RNA-seq,Homo_723,AD.vs.MCI; bulk RNA-seq,Homo_723,MCI.vs.control; bulk RNA-seq,Homo_714,AD.vs.control; bulk RNA-seq,Homo_714,AD.vs.MCI; bulk RNA-seq,Homo_633,AD.vs.control; bulk RNA-seq,Homo_633,AD.vs.MCI	7
MF	GO:0009975	cyclase activity	bulk RNA-seq,Homo_723,AD.vs.control; bulk RNA-seq,Homo_723,AD.vs.MCI; bulk RNA-seq,Homo_723,MCI.vs.control; bulk RNA-seq,Homo_714,AD.vs.MCI; bulk RNA-seq,Homo_714,MCI.vs.control; bulk RNA-seq,Homo_633,MCI.vs.control	6
BP	GO:0002710	negative regulation of T cell mediated immunity	bulk RNA-seq,Homo_723,AD.vs.control; bulk RNA-seq,Homo_714,MCI.vs.control; bulk RNA-seq,Homo_633,MCI.vs.control	3
BP	GO:1901077	regulation of relaxation of muscle	bulk RNA-seq,Homo_723,AD.vs.control; bulk RNA-seq,Homo_723,AD.vs.MCI	2
BP	GO:0033046	negative regulation of sister chromatid segregation	bulk RNA-seq,Homo_723,AD.vs.control; bulk RNA-seq,Homo_723,MCI.vs.control; bulk RNA-seq,Homo_714,AD.vs.control; bulk RNA-seq,Homo_714,MCI.vs.control; bulk RNA-seq,Homo_633,AD.vs.control; bulk RNA-seq,Homo_633,AD.vs.MCI	6
BP	GO:0033048	negative regulation of mitotic sister chromatid segregation	bulk RNA-seq,Homo_723,AD.vs.control; bulk RNA-seq,Homo_723,MCI.vs.control; bulk RNA-seq,Homo_714,AD.vs.control; bulk RNA-seq,Homo_714,MCI.vs.control; bulk RNA-seq,Homo_633,AD.vs.control; bulk RNA-seq,Homo_633,AD.vs.MCI	6
BP	GO:2000816	negative regulation of mitotic sister chromatid separation	bulk RNA-seq,Homo_723,AD.vs.control; bulk RNA-seq,Homo_723,MCI.vs.control; bulk RNA-seq,Homo_714,AD.vs.control; bulk RNA-seq,Homo_714,MCI.vs.control; bulk RNA-seq,Homo_633,AD.vs.control; bulk RNA-seq,Homo_633,AD.vs.MCI	6
BP	GO:0010811	positive regulation of cell-substrate adhesion	bulk RNA-seq,Homo_723,AD.vs.control; bulk RNA-seq,Homo_723,AD.vs.MCI; bulk RNA-seq,Homo_723,MCI.vs.control; bulk RNA-seq,Homo_714,AD.vs.MCI; bulk RNA-seq,Homo_714,MCI.vs.control; bulk RNA-seq,Homo_633,AD.vs.control; bulk RNA-seq,Homo_633,AD.vs.MCI	7
BP	GO:0043248	proteasome assembly	bulk RNA-seq,Homo_723,AD.vs.control; bulk RNA-seq,Homo_723,AD.vs.MCI; bulk RNA-seq,Homo_723,MCI.vs.control; bulk RNA-seq,Homo_714,AD.vs.control; bulk RNA-seq,Homo_714,MCI.vs.control; bulk RNA-seq,Homo_633,AD.vs.control; bulk RNA-seq,Homo_633,AD.vs.MCI; bulk	8
MF	GO:0019957	C-C chemokine binding	bulk RNA-seq,Homo_723,AD.vs.control; bulk RNA-seq,Homo_633,AD.vs.control; bulk RNA-seq,Homo_633,AD.vs.MCI	3
BP	GO:0045943	positive regulation of transcription by RNA polymerase I	bulk RNA-seq,Homo_723,AD.vs.control; bulk RNA-seq,Homo_723,MCI.vs.control; bulk RNA-seq,Homo_714,AD.vs.control; bulk RNA-seq,Homo_714,MCI.vs.control; bulk RNA-seq,Homo_633,AD.vs.control; bulk RNA-seq,Homo_633,AD.vs.MCI; bulk RNA-seq,Homo_633,MCI.vs.control	7
CC	GO:0032591	dendritic spine membrane	bulk RNA-seq,Homo_723,AD.vs.control; bulk RNA-seq,Homo_723,AD.vs.MCI	2
MF	GO:0030215	semaphorin receptor binding	bulk RNA-seq,Homo_723,AD.vs.control; bulk RNA-seq,Homo_723,AD.vs.MCI; bulk RNA-seq,Homo_723,MCI.vs.control; bulk RNA-seq,Homo_714,AD.vs.MCI; bulk RNA-seq,Homo_714,MCI.vs.control; bulk RNA-seq,Homo_633,AD.vs.control	6

MF	GO:0015293	symporter activity	bulk RNA-seq,Homo_723,AD.vs.control; bulk RNA-seq,Homo_723,AD.vs.MCI; bulk RNA-seq,Homo_723,MCI.vs.control; bulk RNA-seq,Homo_714,AD.vs.control; bulk RNA-seq,Homo_714,AD.vs.MCI; bulk RNA-seq,Homo_633,AD.vs.control	6
BP	GO:0006506	GPI anchor biosynthetic process	bulk RNA-seq,Homo_723,AD.vs.control; bulk RNA-seq,Homo_723,MCI.vs.control; bulk RNA-seq,Homo_714,AD.vs.control; bulk RNA-seq,Homo_714,MCI.vs.control; bulk RNA-seq,Homo_633,AD.vs.control; bulk RNA-seq,Homo_633,AD.vs.MCI; bulk RNA-seq,Homo_633,MCI.vs.control	7
BP	GO:0090660	cerebrospinal fluid circulation	bulk RNA-seq,Homo_723,AD.vs.control; bulk RNA-seq,Homo_723,AD.vs.MCI	2
BP	GO:0051591	response to cAMP	bulk RNA-seq,Homo_723,AD.vs.control; bulk RNA-seq,Homo_723,AD.vs.MCI; bulk RNA-seq,Homo_723,MCI.vs.control; bulk RNA-seq,Homo_714,AD.vs.MCI; bulk RNA-seq,Homo_714,MCI.vs.control; bulk RNA-seq,Homo_633,AD.vs.control; bulk RNA-seq,Homo_633,AD.vs.MCI	7
BP	GO:0006858	extracellular transport	bulk RNA-seq,Homo_723,AD.vs.control; bulk RNA-seq,Homo_723,AD.vs.MCI; bulk RNA-seq,Homo_723,MCI.vs.control	3
BP	GO:0006164	purine nucleotide biosynthetic process	bulk RNA-seq,Homo_723,AD.vs.control; bulk RNA-seq,Homo_723,MCI.vs.control; bulk RNA-seq,Homo_714,AD.vs.control; bulk RNA-seq,Homo_633,AD.vs.control; bulk RNA-seq,Homo_633,AD.vs.MCI	5
BP	GO:0032042	mitochondrial DNA metabolic process	bulk RNA-seq,Homo_723,AD.vs.control; bulk RNA-seq,Homo_723,MCI.vs.control; bulk RNA-seq,Homo_714,AD.vs.control; bulk RNA-seq,Homo_714,MCI.vs.control; bulk RNA-seq,Homo_633,AD.vs.control; bulk RNA-seq,Homo_633,AD.vs.MCI; bulk RNA-seq,Homo_633,MCI.vs.control	7
BP	GO:0016482	cytosolic transport	bulk RNA-seq,Homo_723,AD.vs.control; bulk RNA-seq,Homo_723,MCI.vs.control; bulk RNA-seq,Homo_714,AD.vs.control; bulk RNA-seq,Homo_633,AD.vs.control; bulk RNA-seq,Homo_633,AD.vs.MCI; scRNA-seq,SRP330776,Naive CD8+ T cell_2-AD.vs.control	6
MF	GO:0070325	lipoprotein particle receptor binding	bulk RNA-seq,Homo_723,AD.vs.control; bulk RNA-seq,Homo_723,AD.vs.MCI; bulk RNA-seq,Homo_723,MCI.vs.control; bulk RNA-seq,Homo_714,AD.vs.MCI; bulk RNA-seq,Homo_714,MCI.vs.control; bulk RNA-seq,Homo_633,AD.vs.control; bulk RNA-seq,Homo_633,AD.vs.MCI	7
BP	GO:0072539	T-helper 17 cell differentiation	bulk RNA-seq,Homo_723,AD.vs.control; bulk RNA-seq,Homo_723,MCI.vs.control; bulk RNA-seq,Homo_714,AD.vs.control; bulk RNA-seq,Homo_714,MCI.vs.control; bulk RNA-seq,Homo_633,AD.vs.control; bulk RNA-seq,Homo_633,MCI.vs.control	6
BP	GO:0019933	cAMP-mediated signaling	bulk RNA-seq,Homo_723,AD.vs.control; bulk RNA-seq,Homo_723,AD.vs.MCI; bulk RNA-seq,Homo_723,MCI.vs.control; bulk RNA-seq,Homo_714,AD.vs.MCI; bulk RNA-seq,Homo_714,MCI.vs.control	5
BP	GO:0090184	positive regulation of kidney development	bulk RNA-seq,Homo_723,AD.vs.control; bulk RNA-seq,Homo_723,AD.vs.MCI; bulk RNA-seq,Homo_714,AD.vs.MCI	3
MF	GO:0042162	telomeric DNA binding	bulk RNA-seq,Homo_723,AD.vs.control; bulk RNA-seq,Homo_723,MCI.vs.control; bulk RNA-seq,Homo_714,AD.vs.control; bulk RNA-seq,Homo_714,MCI.vs.control; bulk RNA-seq,Homo_633,AD.vs.control; bulk RNA-seq,Homo_633,AD.vs.MCI	6
BP	GO:0033047	regulation of mitotic sister chromatid segregation	bulk RNA-seq,Homo_723,AD.vs.control; bulk RNA-seq,Homo_723,MCI.vs.control; bulk RNA-seq,Homo_714,AD.vs.control; bulk RNA-seq,Homo_714,MCI.vs.control; bulk RNA-seq,Homo_633,AD.vs.control; bulk RNA-seq,Homo_633,AD.vs.MCI	6
BP	GO:0032205	negative regulation of telomere maintenance	bulk RNA-seq,Homo_723,AD.vs.control; bulk RNA-seq,Homo_714,AD.vs.control; bulk RNA-seq,Homo_633,AD.vs.control; bulk RNA-seq,Homo_633,AD.vs.MCI	4
MF	GO:0005200	structural constituent of cytoskeleton	bulk RNA-seq,Homo_723,AD.vs.control; bulk RNA-seq,Homo_723,AD.vs.MCI; bulk RNA-seq,Homo_723,MCI.vs.control; bulk RNA-seq,Homo_714,AD.vs.control; bulk RNA-seq,Homo_714,AD.vs.MCI; bulk RNA-seq,Homo_714,MCI.vs.control; bulk RNA-seq,Homo_633,AD.vs.control	7
CC	GO:0044291	cell-cell contact zone	bulk RNA-seq,Homo_723,AD.vs.control; bulk RNA-seq,Homo_723,AD.vs.MCI; bulk RNA-seq,Homo_723,MCI.vs.control; bulk RNA-seq,Homo_714,AD.vs.MCI; bulk RNA-seq,Homo_714,MCI.vs.control; bulk RNA-seq,Homo_633,AD.vs.control	6
BP	GO:0010965	regulation of mitotic sister chromatid separation	bulk RNA-seq,Homo_723,AD.vs.control; bulk RNA-seq,Homo_723,MCI.vs.control; bulk RNA-seq,Homo_714,AD.vs.control; bulk RNA-seq,Homo_714,MCI.vs.control; bulk RNA-seq,Homo_633,AD.vs.control; bulk RNA-seq,Homo_633,AD.vs.MCI	6
BP	GO:0006415	translational termination	bulk RNA-seq,Homo_723,AD.vs.control; bulk RNA-seq,Homo_723,MCI.vs.control; bulk RNA-seq,Homo_633,AD.vs.control	3
BP	GO:2001053	regulation of mesenchymal cell apoptotic process	bulk RNA-seq,Homo_723,AD.vs.control; bulk RNA-seq,Homo_723,AD.vs.MCI; bulk RNA-seq,Homo_723,MCI.vs.control; bulk RNA-seq,Homo_714,AD.vs.MCI	4
BP	GO:0033605	positive regulation of catecholamine secretion	bulk RNA-seq,Homo_723,AD.vs.control; bulk RNA-seq,Homo_723,AD.vs.MCI; bulk RNA-seq,Homo_723,MCI.vs.control	3
BP	GO:0099550	trans-synaptic signaling, modulating synaptic transmission	bulk RNA-seq,Homo_723,AD.vs.control; bulk RNA-seq,Homo_723,AD.vs.MCI; bulk RNA-seq,Homo_723,MCI.vs.control; bulk RNA-seq,Homo_714,AD.vs.MCI	4
BP	GO:0010830	regulation of myotube differentiation	bulk RNA-seq,Homo_723,AD.vs.control; bulk RNA-seq,Homo_723,AD.vs.MCI; bulk RNA-seq,Homo_714,AD.vs.control; bulk RNA-seq,Homo_714,AD.vs.MCI	4
BP	GO:0048259	regulation of receptor-mediated endocytosis	bulk RNA-seq,Homo_723,AD.vs.control; bulk RNA-seq,Homo_723,AD.vs.MCI; bulk RNA-seq,Homo_723,MCI.vs.control; bulk RNA-seq,Homo_714,AD.vs.control; bulk RNA-seq,Homo_714,AD.vs.MCI; bulk RNA-seq,Homo_714,MCI.vs.control; bulk RNA-seq,Homo_633,AD.vs.control; bulk RNA-seq,Homo_633,AD.vs.MCI; scRNA-seq,SRP330776,Naive CD8+ T cell_2-AD.vs.control	9
MF	GO:0004869	cysteine-type endopeptidase inhibitor activity	bulk RNA-seq,Homo_723,AD.vs.control; bulk RNA-seq,Homo_723,AD.vs.MCI; bulk RNA-seq,Homo_714,AD.vs.control; bulk RNA-seq,Homo_714,AD.vs.MCI	4
BP	GO:0006584	catecholamine metabolic process	bulk RNA-seq,Homo_723,AD.vs.control; bulk RNA-seq,Homo_723,AD.vs.MCI; bulk RNA-seq,Homo_723,MCI.vs.control; bulk RNA-seq,Homo_714,AD.vs.control; bulk RNA-seq,Homo_714,AD.vs.MCI; bulk RNA-seq,Homo_714,MCI.vs.control; bulk RNA-seq,Homo_633,MCI.vs.control	7
BP	GO:0009712	catechol-containing compound metabolic process	bulk RNA-seq,Homo_723,AD.vs.control; bulk RNA-seq,Homo_723,AD.vs.MCI; bulk RNA-seq,Homo_723,MCI.vs.control; bulk RNA-seq,Homo_714,AD.vs.control; bulk RNA-seq,Homo_714,AD.vs.MCI; bulk RNA-seq,Homo_714,MCI.vs.control; bulk RNA-seq,Homo_633,MCI.vs.control	7
BP	GO:0098787	mRNA cleavage involved in mRNA processing	bulk RNA-seq,Homo_723,AD.vs.control; bulk RNA-seq,Homo_723,AD.vs.MCI; bulk RNA-seq,Homo_714,AD.vs.control; bulk RNA-seq,Homo_633,AD.vs.control; bulk RNA-seq,Homo_633,AD.vs.MCI; bulk RNA-seq,Homo_633,MCI.vs.control	6
BP	GO:0051048	negative regulation of secretion	bulk RNA-seq,Homo_723,AD.vs.control; bulk RNA-seq,Homo_723,AD.vs.MCI; bulk RNA-seq,Homo_723,MCI.vs.control; bulk RNA-seq,Homo_714,AD.vs.control; bulk RNA-seq,Homo_714,AD.vs.MCI; bulk RNA-seq,Homo_633,AD.vs.control; bulk RNA-seq,Homo_633,AD.vs.MCI	7
CC	GO:0043596	nuclear replication fork	bulk RNA-seq,Homo_723,AD.vs.control; bulk RNA-seq,Homo_723,MCI.vs.control; bulk RNA-seq,Homo_714,AD.vs.control; bulk RNA-seq,Homo_714,MCI.vs.control; bulk RNA-seq,Homo_633,AD.vs.control; bulk RNA-seq,Homo_633,AD.vs.MCI; bulk RNA-seq,Homo_633,MCI.vs.control	7
BP	GO:1903793	positive regulation of anion transport	bulk RNA-seq,Homo_723,AD.vs.control; bulk RNA-seq,Homo_723,AD.vs.MCI; bulk RNA-seq,Homo_723,MCI.vs.control; bulk RNA-seq,Homo_714,AD.vs.control; bulk RNA-seq,Homo_714,AD.vs.MCI; bulk RNA-seq,Homo_633,AD.vs.control; bulk RNA-seq,Homo_633,AD.vs.MCI; bulk RNA-	8
BP	GO:0090231	regulation of spindle checkpoint	bulk RNA-seq,Homo_723,AD.vs.control; bulk RNA-seq,Homo_723,MCI.vs.control; bulk RNA-seq,Homo_714,AD.vs.control; bulk RNA-seq,Homo_714,MCI.vs.control; bulk RNA-seq,Homo_633,AD.vs.control; bulk RNA-seq,Homo_633,AD.vs.MCI; bulk RNA-seq,Homo_633,MCI.vs.control	7
BP	GO:0090266	regulation of mitotic cell cycle spindle assembly checkpoint	bulk RNA-seq,Homo_723,AD.vs.control; bulk RNA-seq,Homo_723,MCI.vs.control; bulk RNA-seq,Homo_714,AD.vs.control; bulk RNA-seq,Homo_714,MCI.vs.control; bulk RNA-seq,Homo_633,AD.vs.control; bulk RNA-seq,Homo_633,AD.vs.MCI; bulk RNA-seq,Homo_633,MCI.vs.control	7
BP	GO:1903504	regulation of mitotic spindle checkpoint	bulk RNA-seq,Homo_723,AD.vs.control; bulk RNA-seq,Homo_723,MCI.vs.control; bulk RNA-seq,Homo_714,AD.vs.control; bulk RNA-seq,Homo_714,MCI.vs.control; bulk RNA-seq,Homo_633,AD.vs.control; bulk RNA-seq,Homo_633,AD.vs.MCI; bulk RNA-seq,Homo_633,MCI.vs.control	7
BP	GO:0010749	regulation of nitric oxide mediated signal transduction	bulk RNA-seq,Homo_723,AD.vs.control; bulk RNA-seq,Homo_723,AD.vs.MCI; bulk RNA-seq,Homo_714,AD.vs.MCI	3
BP	GO:1900118	negative regulation of execution phase of apoptosis	bulk RNA-seq,Homo_723,AD.vs.control; bulk RNA-seq,Homo_723,MCI.vs.control; bulk RNA-seq,Homo_714,AD.vs.control; bulk RNA-seq,Homo_714,MCI.vs.control; bulk RNA-seq,Homo_633,MCI.vs.control	5
MF	GO:0008174	mRNA methyltransferase activity	bulk RNA-seq,Homo_723,AD.vs.control; bulk RNA-seq,Homo_723,MCI.vs.control; bulk RNA-seq,Homo_714,AD.vs.control; bulk RNA-seq,Homo_714,MCI.vs.control; bulk RNA-seq,Homo_633,AD.vs.control; bulk RNA-seq,Homo_633,AD.vs.MCI; bulk RNA-seq,Homo_633,MCI.vs.control	7

BP	GO:0086011	membrane repolarization during action potential	bulk RNA-seq,Homo_723,AD.vs.control; bulk RNA-seq,Homo_723,AD.vs.MCI; bulk RNA-seq,Homo_723,MCI.vs.control; bulk RNA-seq,Homo_714,AD.vs.control; bulk RNA-seq,Homo_714,AD.vs.MCI; bulk RNA-seq,Homo_714,MCI.vs.control	6
BP	GO:0097254	renal tubular secretion	bulk RNA-seq,Homo_723,AD.vs.control; bulk RNA-seq,Homo_723,AD.vs.MCI; bulk RNA-seq,Homo_714,AD.vs.control; bulk RNA-seq,Homo_714,AD.vs.MCI; bulk RNA-seq,Homo_633,MCI.vs.control	5
BP	GO:0006260	DNA replication	bulk RNA-seq,Homo_723,AD.vs.control; bulk RNA-seq,Homo_723,MCI.vs.control; bulk RNA-seq,Homo_714,AD.vs.control; bulk RNA-seq,Homo_633,AD.vs.control; bulk RNA-seq,Homo_633,AD.vs.MCI	5
BP	GO:0010517	regulation of phospholipase activity	bulk RNA-seq,Homo_723,AD.vs.control; bulk RNA-seq,Homo_723,AD.vs.MCI; bulk RNA-seq,Homo_723,MCI.vs.control; bulk RNA-seq,Homo_714,AD.vs.MCI; bulk RNA-seq,Homo_714,MCI.vs.control; bulk RNA-seq,Homo_633,AD.vs.control; bulk RNA-seq,Homo_633,AD.vs.MCI	7
BP	GO:0120254	olefinic compound metabolic process	bulk RNA-seq,Homo_723,AD.vs.control; bulk RNA-seq,Homo_723,AD.vs.MCI; bulk RNA-seq,Homo_723,MCI.vs.control; bulk RNA-seq,Homo_714,AD.vs.control; bulk RNA-seq,Homo_714,AD.vs.MCI; bulk RNA-seq,Homo_633,AD.vs.control; scRNA-seq,SRP330776,Naive CD8+ T cell_3-	7
MF	GO:0047485	protein N-terminus binding	bulk RNA-seq,Homo_723,AD.vs.control; bulk RNA-seq,Homo_723,MCI.vs.control; bulk RNA-seq,Homo_714,AD.vs.control; bulk RNA-seq,Homo_714,MCI.vs.control; bulk RNA-seq,Homo_633,AD.vs.control; bulk RNA-seq,Homo_633,AD.vs.MCI	6
BP	GO:0048024	regulation of mRNA splicing, via spliceosome	bulk RNA-seq,Homo_723,AD.vs.control; bulk RNA-seq,Homo_723,MCI.vs.control; bulk RNA-seq,Homo_714,AD.vs.control; bulk RNA-seq,Homo_714,MCI.vs.control; bulk RNA-seq,Homo_633,AD.vs.control; bulk RNA-seq,Homo_633,AD.vs.MCI	6
BP	GO:0080009	mRNA methylation	bulk RNA-seq,Homo_723,AD.vs.control; bulk RNA-seq,Homo_723,MCI.vs.control; bulk RNA-seq,Homo_714,AD.vs.control; bulk RNA-seq,Homo_714,MCI.vs.control; bulk RNA-seq,Homo_633,AD.vs.control; bulk RNA-seq,Homo_633,AD.vs.MCI; bulk RNA-seq,Homo_633,MCI.vs.control	7
BP	GO:0001963	synaptic transmission, dopaminergic	bulk RNA-seq,Homo_723,AD.vs.control; bulk RNA-seq,Homo_723,AD.vs.MCI; bulk RNA-seq,Homo_714,AD.vs.control; bulk RNA-seq,Homo_714,AD.vs.MCI; bulk RNA-seq,Homo_633,MCI.vs.control	5
BP	GO:0006893	Golgi to plasma membrane transport	bulk RNA-seq,Homo_723,AD.vs.control; bulk RNA-seq,Homo_723,MCI.vs.control; bulk RNA-seq,Homo_714,AD.vs.control; bulk RNA-seq,Homo_714,MCI.vs.control; bulk RNA-seq,Homo_633,AD.vs.control; bulk RNA-seq,Homo_633,AD.vs.MCI	6
BP	GO:1902570	protein localization to nucleolus	bulk RNA-seq,Homo_723,AD.vs.control; bulk RNA-seq,Homo_723,MCI.vs.control; bulk RNA-seq,Homo_714,AD.vs.control; bulk RNA-seq,Homo_714,MCI.vs.control; bulk RNA-seq,Homo_633,AD.vs.control; bulk RNA-seq,Homo_633,AD.vs.MCI; bulk RNA-seq,Homo_633,MCI.vs.control	7
BP	GO:0002827	positive regulation of T-helper 1 type immune response	bulk RNA-seq,Homo_723,AD.vs.control; bulk RNA-seq,Homo_723,MCI.vs.control; bulk RNA-seq,Homo_714,MCI.vs.control	3
BP	GO:0075522	IRES-dependent viral translational initiation	bulk RNA-seq,Homo_723,AD.vs.control; bulk RNA-seq,Homo_723,AD.vs.MCI; bulk RNA-seq,Homo_714,AD.vs.control; bulk RNA-seq,Homo_714,AD.vs.MCI; bulk RNA-seq,Homo_633,AD.vs.control; bulk RNA-seq,Homo_633,AD.vs.MCI	6
MF	GO:0008175	tRNA methyltransferase activity	bulk RNA-seq,Homo_723,AD.vs.control; bulk RNA-seq,Homo_723,MCI.vs.control; bulk RNA-seq,Homo_714,AD.vs.control; bulk RNA-seq,Homo_714,MCI.vs.control; bulk RNA-seq,Homo_633,AD.vs.control; bulk RNA-seq,Homo_633,AD.vs.MCI; bulk RNA-seq,Homo_633,MCI.vs.control	7
BP	GO:0051084	'de novo' post-translational protein folding	bulk RNA-seq,Homo_723,AD.vs.control; bulk RNA-seq,Homo_723,MCI.vs.control; bulk RNA-seq,Homo_714,AD.vs.control; bulk RNA-seq,Homo_714,MCI.vs.control; bulk RNA-seq,Homo_633,AD.vs.control; bulk RNA-seq,Homo_633,AD.vs.MCI	6
BP	GO:1905508	protein localization to microtubule organizing center	bulk RNA-seq,Homo_723,AD.vs.control; bulk RNA-seq,Homo_723,MCI.vs.control; bulk RNA-seq,Homo_714,AD.vs.control; bulk RNA-seq,Homo_714,MCI.vs.control; bulk RNA-seq,Homo_633,AD.vs.control; bulk RNA-seq,Homo_633,AD.vs.MCI	6
BP	GO:0086009	membrane repolarization	bulk RNA-seq,Homo_723,AD.vs.control; bulk RNA-seq,Homo_723,AD.vs.MCI; bulk RNA-seq,Homo_723,MCI.vs.control; bulk RNA-seq,Homo_714,AD.vs.control; bulk RNA-seq,Homo_714,AD.vs.MCI; bulk RNA-seq,Homo_714,MCI.vs.control	6
BP	GO:0098840	protein transport along microtubule	bulk RNA-seq,Homo_723,AD.vs.control; bulk RNA-seq,Homo_723,AD.vs.MCI; bulk RNA-seq,Homo_723,MCI.vs.control; bulk RNA-	4
BP	GO:0099118	microtubule-based protein transport	bulk RNA-seq,Homo_723,AD.vs.control; bulk RNA-seq,Homo_723,AD.vs.MCI; bulk RNA-seq,Homo_723,MCI.vs.control; bulk RNA-	4
BP	GO:0098927	vesicle-mediated transport between endosomal compartments	bulk RNA-seq,Homo_723,AD.vs.control; bulk RNA-seq,Homo_723,MCI.vs.control; bulk RNA-seq,Homo_714,AD.vs.control; bulk RNA-seq,Homo_714,MCI.vs.control; bulk RNA-seq,Homo_633,AD.vs.control; bulk RNA-seq,Homo_633,AD.vs.MCI	6
BP	GO:0002639	positive regulation of immunoglobulin production	bulk RNA-seq,Homo_723,AD.vs.control; bulk RNA-seq,Homo_723,MCI.vs.control; bulk RNA-seq,Homo_714,MCI.vs.control; bulk RNA-seq,Homo_633,AD.vs.control; bulk RNA-seq,Homo_633,AD.vs.MCI	5
BP	GO:2000514	regulation of CD4-positive, alpha-beta T cell activation	bulk RNA-seq,Homo_723,AD.vs.control; bulk RNA-seq,Homo_723,MCI.vs.control; bulk RNA-seq,Homo_714,AD.vs.control; bulk RNA-seq,Homo_714,MCI.vs.control; bulk RNA-seq,Homo_633,AD.vs.control; bulk RNA-seq,Homo_633,AD.vs.MCI	6
BP	GO:0061053	somite development	bulk RNA-seq,Homo_723,AD.vs.control; bulk RNA-seq,Homo_723,AD.vs.MCI; bulk RNA-seq,Homo_723,MCI.vs.control; bulk RNA-seq,Homo_714,AD.vs.MCI; bulk RNA-seq,Homo_714,MCI.vs.control; bulk RNA-seq,Homo_633,AD.vs.control; bulk RNA-seq,Homo_633,AD.vs.MCI; bulk RNA-	8
BP	GO:0007435	salivary gland morphogenesis	bulk RNA-seq,Homo_723,AD.vs.control; bulk RNA-seq,Homo_723,AD.vs.MCI; bulk RNA-seq,Homo_723,MCI.vs.control; bulk RNA-seq,Homo_714,AD.vs.MCI; bulk RNA-seq,Homo_714,MCI.vs.control; bulk RNA-seq,Homo_633,AD.vs.MCI	6
BP	GO:0021527	spinal cord association neuron differentiation	bulk RNA-seq,Homo_723,AD.vs.control; bulk RNA-seq,Homo_723,AD.vs.MCI; bulk RNA-seq,Homo_723,MCI.vs.control; bulk RNA-seq,Homo_714,AD.vs.control; bulk RNA-seq,Homo_714,AD.vs.MCI; bulk RNA-seq,SRP223445,AD.vs.control	6
MF	GO:0090079	translation regulator activity, nucleic acid binding	bulk RNA-seq,Homo_723,AD.vs.control; bulk RNA-seq,Homo_723,MCI.vs.control; bulk RNA-seq,Homo_714,AD.vs.control; bulk RNA-seq,Homo_714,MCI.vs.control; bulk RNA-seq,Homo_633,AD.vs.control; bulk RNA-seq,Homo_633,AD.vs.MCI	6
BP	GO:0021984	adenohypophysis development	bulk RNA-seq,Homo_723,AD.vs.control; bulk RNA-seq,Homo_723,AD.vs.MCI; bulk RNA-seq,Homo_714,AD.vs.MCI; bulk RNA-seq,SRP223445,AD.vs.control	4
BP	GO:0060412	ventricular septum morphogenesis	bulk RNA-seq,Homo_723,AD.vs.control; bulk RNA-seq,Homo_723,AD.vs.MCI; bulk RNA-seq,Homo_723,MCI.vs.control; bulk RNA-seq,Homo_714,AD.vs.MCI; bulk RNA-seq,Homo_714,MCI.vs.control; bulk RNA-seq,Homo_633,AD.vs.control	6
BP	GO:0006352	DNA-templated transcription initiation	bulk RNA-seq,Homo_723,AD.vs.control; bulk RNA-seq,Homo_723,MCI.vs.control; bulk RNA-seq,Homo_714,AD.vs.control; bulk RNA-seq,Homo_633,AD.vs.control; bulk RNA-seq,Homo_633,AD.vs.MCI	5
CC	GO:0098799	outer mitochondrial membrane protein complex	bulk RNA-seq,Homo_723,AD.vs.control; bulk RNA-seq,Homo_714,AD.vs.control; bulk RNA-seq,Homo_714,MCI.vs.control; bulk RNA-seq,Homo_633,AD.vs.control; bulk RNA-seq,Homo_633,AD.vs.MCI; bulk RNA-seq,Homo_633,MCI.vs.control	6
BP	GO:0086014	atrial cardiac muscle cell action potential	bulk RNA-seq,Homo_723,AD.vs.control; bulk RNA-seq,Homo_723,AD.vs.MCI; bulk RNA-seq,Homo_723,MCI.vs.control; bulk RNA-seq,Homo_714,AD.vs.MCI; bulk RNA-seq,Homo_714,MCI.vs.control	5
BP	GO:0086026	atrial cardiac muscle cell to AV node cell signaling	bulk RNA-seq,Homo_723,AD.vs.control; bulk RNA-seq,Homo_723,AD.vs.MCI; bulk RNA-seq,Homo_723,MCI.vs.control; bulk RNA-seq,Homo_714,AD.vs.MCI; bulk RNA-seq,Homo_714,MCI.vs.control	5
BP	GO:0086066	atrial cardiac muscle cell to AV node cell communication	bulk RNA-seq,Homo_723,AD.vs.control; bulk RNA-seq,Homo_723,AD.vs.MCI; bulk RNA-seq,Homo_723,MCI.vs.control; bulk RNA-seq,Homo_714,AD.vs.MCI; bulk RNA-seq,Homo_714,MCI.vs.control	5
BP	GO:0007625	grooming behavior	bulk RNA-seq,Homo_723,AD.vs.control; bulk RNA-seq,Homo_723,AD.vs.MCI; bulk RNA-seq,Homo_714,AD.vs.control	3
BP	GO:0042310	vasoconstriction	bulk RNA-seq,Homo_723,AD.vs.control; bulk RNA-seq,Homo_723,AD.vs.MCI; bulk RNA-seq,Homo_723,MCI.vs.control; bulk RNA-seq,Homo_714,AD.vs.MCI; bulk RNA-seq,Homo_714,MCI.vs.control	5

BP	GO:0016525	negative regulation of angiogenesis	bulk RNA-seq,Homo_723,AD.vs.control; bulk RNA-seq,Homo_723,AD.vs.MCI; bulk RNA-seq,Homo_723,MCI.vs.control; bulk RNA-seq,Homo_714,AD.vs.control; bulk RNA-seq,Homo_714,AD.vs.MCI; scRNA-seq,SRP330776,B cell_1-AD.vs.control	6
BP	GO:0003176	aortic valve development	bulk RNA-seq,Homo_723,AD.vs.control; bulk RNA-seq,Homo_723,AD.vs.MCI; bulk RNA-seq,Homo_723,MCI.vs.control; bulk RNA-seq,Homo_714,AD.vs.MCI; bulk RNA-seq,Homo_714,MCI.vs.control; bulk RNA-seq,SRP223445,AD.vs.control	6
BP	GO:0014812	muscle cell migration	bulk RNA-seq,Homo_723,AD.vs.control; bulk RNA-seq,Homo_723,AD.vs.MCI; bulk RNA-seq,Homo_723,MCI.vs.control; bulk RNA-seq,Homo_714,AD.vs.control; bulk RNA-seq,Homo_714,AD.vs.MCI; bulk RNA-seq,Homo_714,MCI.vs.control; bulk RNA-seq,Homo_633,AD.vs.control; bulk	8
BP	GO:0035315	hair cell differentiation	bulk RNA-seq,Homo_723,AD.vs.control; bulk RNA-seq,Homo_723,AD.vs.MCI; bulk RNA-seq,Homo_723,MCI.vs.control; bulk RNA-seq,Homo_714,AD.vs.MCI; bulk RNA-seq,Homo_714,MCI.vs.control; bulk RNA-seq,SRP223445,AD.vs.control	6
BP	GO:0003128	heart field specification	bulk RNA-seq,Homo_723,AD.vs.control; bulk RNA-seq,Homo_723,AD.vs.MCI; bulk RNA-seq,Homo_723,MCI.vs.control; bulk RNA-seq,Homo_714,AD.vs.MCI	4
BP	GO:0015813	L-glutamate transmembrane transport	bulk RNA-seq,Homo_723,AD.vs.control; bulk RNA-seq,Homo_723,AD.vs.MCI; bulk RNA-seq,Homo_723,MCI.vs.control	3
BP	GO:0051938	L-glutamate import	bulk RNA-seq,Homo_723,AD.vs.control; bulk RNA-seq,Homo_723,AD.vs.MCI; bulk RNA-seq,Homo_723,MCI.vs.control	3
BP	GO:0001975	response to amphetamine	bulk RNA-seq,Homo_723,AD.vs.control; bulk RNA-seq,Homo_723,AD.vs.MCI; bulk RNA-seq,Homo_714,AD.vs.control; bulk RNA-seq,Homo_714,AD.vs.MCI; bulk RNA-seq,Homo_633,MCI.vs.control	5
BP	GO:2001236	regulation of extrinsic apoptotic signaling pathway	bulk RNA-seq,Homo_723,AD.vs.control; bulk RNA-seq,Homo_723,AD.vs.MCI; bulk RNA-seq,Homo_723,MCI.vs.control; bulk RNA-seq,Homo_714,AD.vs.MCI; bulk RNA-seq,Homo_633,AD.vs.control; bulk RNA-seq,Homo_633,AD.vs.MCI	6
CC	GO:0071735	IgG immunoglobulin complex	bulk RNA-seq,Homo_723,AD.vs.control; bulk RNA-seq,Homo_723,AD.vs.MCI; bulk RNA-seq,Homo_714,AD.vs.control; bulk RNA-seq,Homo_714,AD.vs.MCI; bulk RNA-seq,Homo_633,AD.vs.control	5
BP	GO:0050961	detection of temperature stimulus involved in sensory perception	bulk RNA-seq,Homo_723,AD.vs.control; bulk RNA-seq,Homo_723,AD.vs.MCI; bulk RNA-seq,Homo_723,MCI.vs.control; bulk RNA-seq,Homo_714,AD.vs.control; bulk RNA-seq,Homo_714,AD.vs.MCI	5
BP	GO:0038180	nerve growth factor signaling pathway	bulk RNA-seq,Homo_723,AD.vs.control; bulk RNA-seq,Homo_723,AD.vs.MCI; bulk RNA-seq,Homo_723,MCI.vs.control; bulk RNA-	4
BP	GO:0050854	regulation of antigen receptor-mediated signaling pathway	bulk RNA-seq,Homo_723,AD.vs.control; bulk RNA-seq,Homo_723,MCI.vs.control; bulk RNA-seq,Homo_714,MCI.vs.control; bulk RNA-seq,Homo_633,AD.vs.control; bulk RNA-seq,Homo_633,AD.vs.MCI; scRNA-seq,SRP330776,Naive CD8+ T cell_2-AD.vs.control	6
BP	GO:0006446	regulation of translational initiation	bulk RNA-seq,Homo_723,AD.vs.control; bulk RNA-seq,Homo_714,AD.vs.control; bulk RNA-seq,Homo_714,MCI.vs.control; bulk RNA-seq,Homo_633,AD.vs.control; bulk RNA-seq,Homo_633,AD.vs.MCI	5
BP	GO:0002793	positive regulation of peptide secretion	bulk RNA-seq,Homo_723,AD.vs.control; bulk RNA-seq,Homo_723,AD.vs.MCI; bulk RNA-seq,Homo_723,MCI.vs.control; bulk RNA-seq,Homo_714,AD.vs.control; bulk RNA-seq,Homo_714,AD.vs.MCI; bulk RNA-seq,Homo_714,MCI.vs.control	6
BP	GO:0032252	secretory granule localization	bulk RNA-seq,Homo_723,AD.vs.control; bulk RNA-seq,Homo_723,AD.vs.MCI; bulk RNA-seq,Homo_723,MCI.vs.control	3
BP	GO:0045762	positive regulation of adenylate cyclase activity	bulk RNA-seq,Homo_723,AD.vs.control; bulk RNA-seq,Homo_723,AD.vs.MCI; bulk RNA-seq,Homo_723,MCI.vs.control; bulk RNA-seq,Homo_714,AD.vs.MCI	4
BP	GO:0046683	response to organophosphorus	bulk RNA-seq,Homo_723,AD.vs.control; bulk RNA-seq,Homo_723,AD.vs.MCI; bulk RNA-seq,Homo_723,MCI.vs.control; bulk RNA-seq,Homo_714,AD.vs.MCI; bulk RNA-seq,Homo_714,MCI.vs.control; bulk RNA-seq,Homo_633,AD.vs.control; bulk RNA-seq,Homo_633,AD.vs.MCI	7
CC	GO:0099569	presynaptic cytoskeleton	bulk RNA-seq,Homo_723,AD.vs.control; bulk RNA-seq,Homo_723,AD.vs.MCI; bulk RNA-seq,Homo_723,MCI.vs.control; bulk RNA-seq,Homo_714,AD.vs.MCI; bulk RNA-seq,ROSMAP,AD.vs.control	5
MF	GO:0070700	BMP receptor binding	bulk RNA-seq,Homo_723,AD.vs.control; bulk RNA-seq,Homo_723,AD.vs.MCI; bulk RNA-seq,Homo_723,MCI.vs.control; bulk RNA-seq,Homo_714,AD.vs.MCI; bulk RNA-seq,Homo_714,MCI.vs.control	5
BP	GO:0060998	regulation of dendritic spine development	bulk RNA-seq,Homo_723,AD.vs.control; bulk RNA-seq,Homo_723,AD.vs.MCI; bulk RNA-seq,Homo_723,MCI.vs.control; bulk RNA-seq,Homo_714,AD.vs.MCI; bulk RNA-seq,Homo_714,MCI.vs.control; bulk RNA-seq,Homo_633,AD.vs.control; bulk RNA-seq,Homo_633,AD.vs.MCI	7
BP	GO:0006405	RNA export from nucleus	bulk RNA-seq,Homo_723,AD.vs.control; bulk RNA-seq,Homo_723,MCI.vs.control; bulk RNA-seq,Homo_714,AD.vs.control; bulk RNA-seq,Homo_714,MCI.vs.control; bulk RNA-seq,Homo_633,AD.vs.control; bulk RNA-seq,Homo_633,AD.vs.MCI	6
MF	GO:0005262	calcium channel activity	bulk RNA-seq,Homo_723,AD.vs.control; bulk RNA-seq,Homo_723,AD.vs.MCI; bulk RNA-seq,Homo_723,MCI.vs.control; bulk RNA-seq,Homo_714,AD.vs.MCI; bulk RNA-seq,Homo_714,MCI.vs.control; bulk RNA-seq,Homo_633,AD.vs.control; bulk RNA-seq,Homo_633,AD.vs.MCI; bulk RNA-	8
BP	GO:1901292	nucleoside phosphate catabolic process	bulk RNA-seq,Homo_723,AD.vs.control; bulk RNA-seq,Homo_723,MCI.vs.control; bulk RNA-seq,Homo_714,AD.vs.control; bulk RNA-seq,Homo_714,MCI.vs.control; bulk RNA-seq,Homo_633,AD.vs.control; bulk RNA-seq,Homo_633,AD.vs.MCI	6
BP	GO:0035810	positive regulation of urine volume	bulk RNA-seq,Homo_723,AD.vs.control; bulk RNA-seq,Homo_723,AD.vs.MCI; bulk RNA-seq,Homo_723,MCI.vs.control; bulk RNA-seq,Homo_714,AD.vs.control; bulk RNA-seq,Homo_714,AD.vs.MCI	5
MF	GO:0043121	neurotrophin binding	bulk RNA-seq,Homo_723,AD.vs.control; bulk RNA-seq,Homo_723,AD.vs.MCI	2
BP	GO:0055078	sodium ion homeostasis	bulk RNA-seq,Homo_723,AD.vs.control; bulk RNA-seq,Homo_723,AD.vs.MCI; bulk RNA-seq,Homo_723,MCI.vs.control; bulk RNA-seq,Homo_714,AD.vs.control; bulk RNA-seq,Homo_714,AD.vs.MCI	5
CC	GO:0016282	eukaryotic 43S preinitiation complex	bulk RNA-seq,Homo_723,AD.vs.control; bulk RNA-seq,Homo_723,AD.vs.MCI; bulk RNA-seq,Homo_714,AD.vs.control; bulk RNA-seq,Homo_714,AD.vs.MCI; bulk RNA-seq,Homo_633,AD.vs.control	5
BP	GO:0051350	negative regulation of lyase activity	bulk RNA-seq,Homo_723,AD.vs.control; bulk RNA-seq,Homo_723,AD.vs.MCI; bulk RNA-seq,Homo_723,MCI.vs.control	3
BP	GO:0021952	central nervous system projection neuron axonogenesis	bulk RNA-seq,Homo_723,AD.vs.control; bulk RNA-seq,Homo_723,AD.vs.MCI; bulk RNA-seq,Homo_723,MCI.vs.control; bulk RNA-seq,Homo_714,AD.vs.MCI; bulk RNA-seq,Homo_714,MCI.vs.control; bulk RNA-seq,Homo_633,AD.vs.control; bulk RNA-seq,Homo_633,AD.vs.MCI	7
BP	GO:0016574	histone ubiquitination	bulk RNA-seq,Homo_723,AD.vs.control; bulk RNA-seq,Homo_723,MCI.vs.control; bulk RNA-seq,Homo_714,MCI.vs.control; bulk RNA-seq,Homo_633,AD.vs.control; bulk RNA-seq,Homo_633,AD.vs.MCI	5
BP	GO:0060043	regulation of cardiac muscle cell proliferation	bulk RNA-seq,Homo_723,AD.vs.control; bulk RNA-seq,Homo_723,AD.vs.MCI; bulk RNA-seq,Homo_723,MCI.vs.control; bulk RNA-seq,Homo_714,AD.vs.MCI; bulk RNA-seq,Homo_714,MCI.vs.control	5
BP	GO:0033045	regulation of sister chromatid segregation	bulk RNA-seq,Homo_723,AD.vs.control; bulk RNA-seq,Homo_723,MCI.vs.control; bulk RNA-seq,Homo_714,AD.vs.control; bulk RNA-seq,Homo_714,MCI.vs.control; bulk RNA-seq,Homo_633,AD.vs.control; bulk RNA-seq,Homo_633,AD.vs.MCI	6
BP	GO:0002098	tRNA wobble uridine modification	bulk RNA-seq,Homo_723,AD.vs.control; bulk RNA-seq,Homo_723,MCI.vs.control; bulk RNA-seq,Homo_714,AD.vs.control; bulk RNA-seq,Homo_714,MCI.vs.control; bulk RNA-seq,Homo_633,MCI.vs.control	5
MF	GO:0008028	monocarboxylic acid transmembrane transporter activity	bulk RNA-seq,Homo_723,AD.vs.control; bulk RNA-seq,Homo_723,AD.vs.MCI; bulk RNA-seq,Homo_723,MCI.vs.control; bulk RNA-seq,Homo_714,AD.vs.control; bulk RNA-seq,Homo_714,AD.vs.MCI; bulk RNA-seq,Homo_714,MCI.vs.control	6
BP	GO:0035904	aorta development	bulk RNA-seq,Homo_723,AD.vs.control; bulk RNA-seq,Homo_723,AD.vs.MCI; bulk RNA-seq,Homo_723,MCI.vs.control; bulk RNA-seq,Homo_714,AD.vs.control; bulk RNA-seq,Homo_714,AD.vs.MCI; bulk RNA-seq,Homo_714,MCI.vs.control	6

BP	GO:0090085	regulation of protein deubiquitination	bulk RNA-seq,Homo_723,AD.vs.control; bulk RNA-seq,Homo_723,AD.vs.MCI; bulk RNA-seq,Homo_714,AD.vs.control; bulk RNA-seq,Homo_714,MCI.vs.control; bulk RNA-seq,Homo_633,AD.vs.control; bulk RNA-seq,Homo_633,AD.vs.MCI	6
CC	GO:0030426	growth cone	bulk RNA-seq,Homo_723,AD.vs.control; bulk RNA-seq,Homo_723,AD.vs.MCI; bulk RNA-seq,Homo_723,MCI.vs.control; bulk RNA-seq,Homo_714,AD.vs.MCI; bulk RNA-seq,Homo_633,AD.vs.control; bulk RNA-seq,Homo_633,AD.vs.MCI	6
MF	GO:0098918	structural constituent of synapse	bulk RNA-seq,Homo_723,AD.vs.control; bulk RNA-seq,Homo_723,AD.vs.MCI; bulk RNA-seq,Homo_723,MCI.vs.control; bulk RNA-seq,Homo_714,AD.vs.control; bulk RNA-seq,Homo_714,AD.vs.MCI	5
BP	GO:0042832	defense response to protozoan	bulk RNA-seq,Homo_723,AD.vs.control; bulk RNA-seq,Homo_723,MCI.vs.control; bulk RNA-seq,Homo_714,AD.vs.control; bulk RNA-seq,Homo_714,MCI.vs.control; bulk RNA-seq,Homo_633,AD.vs.control; bulk RNA-seq,Homo_633,MCI.vs.control	6
BP	GO:1990573	potassium ion import across plasma membrane	bulk RNA-seq,Homo_723,AD.vs.control; bulk RNA-seq,Homo_723,AD.vs.MCI; bulk RNA-seq,Homo_723,MCI.vs.control; bulk RNA-seq,Homo_714,AD.vs.MCI; bulk RNA-seq,SRP223445,AD.vs.control	5
MF	GO:0017154	semaphorin receptor activity	bulk RNA-seq,Homo_723,AD.vs.control; bulk RNA-seq,Homo_723,AD.vs.MCI; bulk RNA-seq,Homo_723,MCI.vs.control; bulk RNA-seq,Homo_714,AD.vs.MCI; bulk RNA-seq,Homo_714,MCI.vs.control; bulk RNA-seq,Homo_633,AD.vs.control	6
BP	GO:0008053	mitochondrial fusion	bulk RNA-seq,Homo_723,AD.vs.control; bulk RNA-seq,Homo_714,AD.vs.control; bulk RNA-seq,Homo_633,AD.vs.control; bulk RNA-seq,Homo_633,AD.vs.MCI	4
BP	GO:0051095	regulation of helicase activity	bulk RNA-seq,Homo_723,AD.vs.control; bulk RNA-seq,Homo_723,AD.vs.MCI; bulk RNA-seq,Homo_723,MCI.vs.control; bulk RNA-seq,Homo_714,AD.vs.control; bulk RNA-seq,Homo_714,AD.vs.MCI; bulk RNA-seq,Homo_714,MCI.vs.control; bulk RNA-seq,Homo_633,AD.vs.control; bulk RNA-seq,Homo_633,AD.vs.MCI; bulk RNA-seq,Homo_633,MCI.vs.control	9
BP	GO:0015780	nucleotide-sugar transmembrane transport	bulk RNA-seq,Homo_723,AD.vs.control; bulk RNA-seq,Homo_714,AD.vs.control; bulk RNA-seq,Homo_633,AD.vs.control; bulk RNA-seq,Homo_633,AD.vs.MCI	4
BP	GO:0031126	sno(s)RNA 3'-end processing	bulk RNA-seq,Homo_723,AD.vs.control; bulk RNA-seq,Homo_633,AD.vs.control; bulk RNA-seq,Homo_633,AD.vs.MCI	3
BP	GO:0050821	protein stabilization	bulk RNA-seq,Homo_723,AD.vs.control; bulk RNA-seq,Homo_723,MCI.vs.control; bulk RNA-seq,Homo_714,AD.vs.control; bulk RNA-seq,Homo_633,AD.vs.control; bulk RNA-seq,Homo_633,AD.vs.MCI	5
BP	GO:1904869	regulation of protein localization to Cajal body	bulk RNA-seq,Homo_723,AD.vs.control; bulk RNA-seq,Homo_723,MCI.vs.control; bulk RNA-seq,Homo_714,AD.vs.control; bulk RNA-seq,Homo_714,MCI.vs.control; bulk RNA-seq,Homo_633,AD.vs.control; bulk RNA-seq,Homo_633,AD.vs.MCI; bulk RNA-seq,Homo_633,MCI.vs.control	7
BP	GO:1904871	positive regulation of protein localization to Cajal body	bulk RNA-seq,Homo_723,AD.vs.control; bulk RNA-seq,Homo_723,MCI.vs.control; bulk RNA-seq,Homo_714,AD.vs.control; bulk RNA-seq,Homo_714,MCI.vs.control; bulk RNA-seq,Homo_633,AD.vs.control; bulk RNA-seq,Homo_633,AD.vs.MCI; bulk RNA-seq,Homo_633,MCI.vs.control	7
MF	GO:0071813	lipoprotein particle binding	bulk RNA-seq,Homo_723,AD.vs.control; bulk RNA-seq,Homo_723,AD.vs.MCI; bulk RNA-seq,Homo_723,MCI.vs.control; bulk RNA-seq,Homo_714,AD.vs.MCI; bulk RNA-seq,Homo_714,MCI.vs.control; bulk RNA-seq,Homo_633,AD.vs.control; bulk RNA-seq,Homo_633,AD.vs.MCI	7
MF	GO:0071814	protein-lipid complex binding	bulk RNA-seq,Homo_723,AD.vs.control; bulk RNA-seq,Homo_723,AD.vs.MCI; bulk RNA-seq,Homo_723,MCI.vs.control; bulk RNA-seq,Homo_714,AD.vs.MCI; bulk RNA-seq,Homo_714,MCI.vs.control; bulk RNA-seq,Homo_633,AD.vs.control; bulk RNA-seq,Homo_633,AD.vs.MCI	7
BP	GO:0007194	negative regulation of adenylate cyclase activity	bulk RNA-seq,Homo_723,AD.vs.control; bulk RNA-seq,Homo_723,AD.vs.MCI; bulk RNA-seq,Homo_723,MCI.vs.control	3
BP	GO:0048753	pigment granule organization	bulk RNA-seq,Homo_723,AD.vs.control; bulk RNA-seq,Homo_723,MCI.vs.control; bulk RNA-seq,Homo_714,AD.vs.control; bulk RNA-seq,Homo_714,MCI.vs.control; bulk RNA-seq,Homo_633,AD.vs.control; bulk RNA-seq,Homo_633,AD.vs.MCI	6
CC	GO:0045335	phagocytic vesicle	bulk RNA-seq,Homo_723,AD.vs.control; bulk RNA-seq,Homo_723,MCI.vs.control; bulk RNA-seq,Homo_633,AD.vs.control; bulk RNA-seq,Homo_633,AD.vs.MCI; bulk RNA-seq,SRP223445,AD.vs.control	5
BP	GO:0006891	intra-Golgi vesicle-mediated transport	bulk RNA-seq,Homo_723,AD.vs.control; bulk RNA-seq,Homo_723,MCI.vs.control; bulk RNA-seq,Homo_714,AD.vs.control; bulk RNA-seq,Homo_714,MCI.vs.control; bulk RNA-seq,Homo_633,AD.vs.control; bulk RNA-seq,Homo_633,AD.vs.MCI; bulk RNA-seq,Homo_633,MCI.vs.control	7
CC	GO:1990071	TRAPPII protein complex	bulk RNA-seq,Homo_723,AD.vs.control; bulk RNA-seq,Homo_723,AD.vs.MCI; bulk RNA-seq,Homo_714,AD.vs.control; bulk RNA-seq,Homo_714,AD.vs.MCI; bulk RNA-seq,Homo_633,AD.vs.control; bulk RNA-seq,Homo_633,AD.vs.MCI	6
BP	GO:0060033	anatomical structure regression	bulk RNA-seq,Homo_723,AD.vs.control; bulk RNA-seq,Homo_723,AD.vs.MCI; bulk RNA-seq,Homo_723,MCI.vs.control; bulk RNA-seq,Homo_714,AD.vs.control; bulk RNA-seq,Homo_714,AD.vs.MCI	5
MF	GO:0005338	nucleotide-sugar transmembrane transporter activity	bulk RNA-seq,Homo_723,AD.vs.control; bulk RNA-seq,Homo_714,AD.vs.control; bulk RNA-seq,Homo_633,AD.vs.control; bulk RNA-seq,Homo_633,AD.vs.MCI	4
CC	GO:0030427	site of polarized growth	bulk RNA-seq,Homo_723,AD.vs.control; bulk RNA-seq,Homo_723,AD.vs.MCI; bulk RNA-seq,Homo_723,MCI.vs.control; bulk RNA-seq,Homo_714,AD.vs.MCI; bulk RNA-seq,Homo_633,AD.vs.control; bulk RNA-seq,Homo_633,AD.vs.MCI	6
BP	GO:0003129	heart induction	bulk RNA-seq,Homo_723,AD.vs.control; bulk RNA-seq,Homo_723,AD.vs.MCI; bulk RNA-seq,Homo_723,MCI.vs.control; bulk RNA-seq,Homo_714,AD.vs.MCI	4
MF	GO:0005248	voltage-gated sodium channel activity	bulk RNA-seq,Homo_723,AD.vs.control; bulk RNA-seq,Homo_723,AD.vs.MCI; bulk RNA-seq,Homo_723,MCI.vs.control; bulk RNA-seq,Homo_714,AD.vs.MCI; bulk RNA-seq,Homo_633,MCI.vs.control; bulk RNA-seq,ROSMAP,AD.vs.control	6
BP	GO:0000027	ribosomal large subunit assembly	bulk RNA-seq,Homo_723,AD.vs.control; bulk RNA-seq,Homo_723,MCI.vs.control; bulk RNA-seq,Homo_714,AD.vs.control; bulk RNA-seq,Homo_714,MCI.vs.control; bulk RNA-seq,Homo_633,AD.vs.control; bulk RNA-seq,Homo_633,AD.vs.MCI; bulk RNA-seq,Homo_633,MCI.vs.control	7
BP	GO:0035924	cellular response to vascular endothelial growth factor stimulus	bulk RNA-seq,Homo_723,AD.vs.control; bulk RNA-seq,Homo_723,AD.vs.MCI; bulk RNA-seq,Homo_723,MCI.vs.control; bulk RNA-seq,Homo_714,AD.vs.control; bulk RNA-seq,Homo_714,AD.vs.MCI; bulk RNA-seq,Homo_714,MCI.vs.control	6
BP	GO:0030049	muscle filament sliding	bulk RNA-seq,Homo_723,AD.vs.control; bulk RNA-seq,Homo_723,AD.vs.MCI; bulk RNA-seq,Homo_714,AD.vs.control; bulk RNA-seq,Homo_714,AD.vs.MCI	4
BP	GO:0007041	lysosomal transport	bulk RNA-seq,Homo_723,AD.vs.control; bulk RNA-seq,Homo_723,MCI.vs.control; bulk RNA-seq,Homo_714,AD.vs.control; bulk RNA-seq,Homo_633,AD.vs.control; bulk RNA-seq,Homo_633,AD.vs.MCI	5
CC	GO:1990498	mitotic spindle microtubule	bulk RNA-seq,Homo_723,AD.vs.control; bulk RNA-seq,Homo_723,AD.vs.MCI; bulk RNA-seq,Homo_723,MCI.vs.control; bulk RNA-seq,Homo_714,AD.vs.control; bulk RNA-seq,Homo_714,MCI.vs.control; bulk RNA-seq,Homo_633,AD.vs.control; bulk RNA-seq,Homo_633,AD.vs.MCI; bulk	8
BP	GO:0048715	negative regulation of oligodendrocyte differentiation	bulk RNA-seq,Homo_723,AD.vs.control; bulk RNA-seq,Homo_723,AD.vs.MCI; bulk RNA-seq,Homo_723,MCI.vs.control; bulk RNA-seq,Homo_714,AD.vs.MCI; bulk RNA-seq,Homo_714,MCI.vs.control	5
BP	GO:0008045	motor neuron axon guidance	bulk RNA-seq,Homo_723,AD.vs.control; bulk RNA-seq,Homo_723,AD.vs.MCI; bulk RNA-seq,Homo_723,MCI.vs.control; bulk RNA-seq,Homo_714,AD.vs.MCI; bulk RNA-seq,Homo_714,MCI.vs.control; bulk RNA-seq,Homo_633,AD.vs.control	6
BP	GO:0072224	metanephric glomerulus development	bulk RNA-seq,Homo_723,AD.vs.control; bulk RNA-seq,Homo_723,AD.vs.MCI; bulk RNA-seq,Homo_723,MCI.vs.control; bulk RNA-seq,Homo_714,AD.vs.MCI; bulk RNA-seq,Homo_714,MCI.vs.control	5
BP	GO:0072522	purine-containing compound biosynthetic process	bulk RNA-seq,Homo_723,AD.vs.control; bulk RNA-seq,Homo_723,MCI.vs.control; bulk RNA-seq,Homo_714,AD.vs.control; bulk RNA-seq,Homo_633,AD.vs.control; bulk RNA-seq,Homo_633,AD.vs.MCI	5
BP	GO:0032069	regulation of nuclease activity	bulk RNA-seq,Homo_723,AD.vs.control; bulk RNA-seq,Homo_723,MCI.vs.control; bulk RNA-seq,Homo_714,AD.vs.control; bulk RNA-seq,Homo_714,MCI.vs.control; bulk RNA-seq,Homo_633,MCI.vs.control	5

BP	GO:0009166	nucleotide catabolic process	bulk RNA-seq,Homo_723,AD.vs.control; bulk RNA-seq,Homo_723,MCI.vs.control; bulk RNA-seq,Homo_714,AD.vs.control; bulk RNA-seq,Homo_714,MCI.vs.control; bulk RNA-seq,Homo_633,AD.vs.control; bulk RNA-seq,Homo_633,AD.vs.MCI	6
BP	GO:0036344	platelet morphogenesis	bulk RNA-seq,Homo_723,AD.vs.control; bulk RNA-seq,Homo_723,MCI.vs.control; bulk RNA-seq,Homo_714,AD.vs.control; bulk RNA-seq,Homo_714,MCI.vs.control; bulk RNA-seq,Homo_633,AD.vs.control; bulk RNA-seq,Homo_633,AD.vs.MCI; bulk RNA-seq,Homo_633,MCI.vs.control	7
BP	GO:0000578	embryonic axis specification	bulk RNA-seq,Homo_723,AD.vs.control; bulk RNA-seq,Homo_723,AD.vs.MCI; bulk RNA-seq,Homo_723,MCI.vs.control; bulk RNA-seq,Homo_714,AD.vs.control; bulk RNA-seq,Homo_714,AD.vs.MCI; bulk RNA-seq,Homo_714,MCI.vs.control	6
BP	GO:0051055	negative regulation of lipid biosynthetic process	bulk RNA-seq,Homo_723,AD.vs.control; bulk RNA-seq,Homo_723,AD.vs.MCI; bulk RNA-seq,Homo_723,MCI.vs.control; bulk RNA-seq,Homo_714,AD.vs.control; bulk RNA-seq,Homo_714,AD.vs.MCI; bulk RNA-seq,Homo_714,MCI.vs.control; bulk RNA-seq,Homo_633,AD.vs.control	7
BP	GO:0051953	negative regulation of amine transport	bulk RNA-seq,Homo_723,AD.vs.control; bulk RNA-seq,Homo_723,AD.vs.MCI; bulk RNA-seq,Homo_723,MCI.vs.control; bulk RNA-seq,Homo_714,AD.vs.control; bulk RNA-seq,Homo_714,AD.vs.MCI	5
BP	GO:0060382	regulation of DNA strand elongation	bulk RNA-seq,Homo_723,AD.vs.control; bulk RNA-seq,Homo_714,AD.vs.control; bulk RNA-seq,Homo_633,AD.vs.control; bulk RNA-seq,Homo_633,AD.vs.MCI	4
BP	GO:0036342	post-anal tail morphogenesis	bulk RNA-seq,Homo_723,AD.vs.control; bulk RNA-seq,Homo_723,AD.vs.MCI; bulk RNA-seq,Homo_723,MCI.vs.control; bulk RNA-seq,Homo_714,AD.vs.MCI	4
MF	GO:0005109	frizzled binding	bulk RNA-seq,Homo_723,AD.vs.control; bulk RNA-seq,Homo_723,AD.vs.MCI; bulk RNA-seq,Homo_723,MCI.vs.control; bulk RNA-seq,Homo_714,AD.vs.MCI	4
BP	GO:1900746	regulation of vascular endothelial growth factor signaling pathway	bulk RNA-seq,Homo_723,AD.vs.control; bulk RNA-seq,Homo_723,AD.vs.MCI; bulk RNA-seq,Homo_723,MCI.vs.control; bulk RNA-seq,Homo_714,AD.vs.MCI; bulk RNA-seq,Homo_714,MCI.vs.control	5
BP	GO:0022602	ovulation cycle process	bulk RNA-seq,Homo_723,AD.vs.control; bulk RNA-seq,Homo_723,AD.vs.MCI; bulk RNA-seq,Homo_723,MCI.vs.control; bulk RNA-seq,Homo_714,AD.vs.control; bulk RNA-seq,Homo_714,AD.vs.MCI; bulk RNA-seq,Homo_714,MCI.vs.control; bulk RNA-seq,Homo_633,AD.vs.control	7
BP	GO:0002825	regulation of T-helper 1 type immune response	bulk RNA-seq,Homo_723,AD.vs.control; bulk RNA-seq,Homo_723,MCI.vs.control; bulk RNA-seq,Homo_714,MCI.vs.control; bulk RNA-seq,Homo_633,AD.vs.control; bulk RNA-seq,Homo_633,MCI.vs.control	5
BP	GO:0032946	positive regulation of mononuclear cell proliferation	bulk RNA-seq,Homo_723,AD.vs.control; bulk RNA-seq,Homo_723,MCI.vs.control; bulk RNA-seq,Homo_633,AD.vs.control; bulk RNA-	4
BP	GO:0043001	Golgi to plasma membrane protein transport	bulk RNA-seq,Homo_723,AD.vs.control; bulk RNA-seq,Homo_723,MCI.vs.control; bulk RNA-seq,Homo_714,AD.vs.control; bulk RNA-seq,Homo_714,MCI.vs.control; bulk RNA-seq,Homo_633,AD.vs.control; bulk RNA-seq,Homo_633,AD.vs.MCI	6
BP	GO:0032890	regulation of organic acid transport	bulk RNA-seq,Homo_723,AD.vs.control; bulk RNA-seq,Homo_723,AD.vs.MCI; bulk RNA-seq,Homo_723,MCI.vs.control; bulk RNA-seq,Homo_714,AD.vs.MCI; bulk RNA-seq,Homo_633,AD.vs.control	5
BP	GO:0045841	negative regulation of mitotic metaphase/anaphase transition	bulk RNA-seq,Homo_723,AD.vs.control; bulk RNA-seq,Homo_723,MCI.vs.control; bulk RNA-seq,Homo_714,AD.vs.control; bulk RNA-seq,Homo_714,MCI.vs.control; bulk RNA-seq,Homo_633,AD.vs.control; bulk RNA-seq,Homo_633,AD.vs.MCI	6
BP	GO:0060008	Sertoli cell differentiation	bulk RNA-seq,Homo_723,AD.vs.control; bulk RNA-seq,Homo_723,AD.vs.MCI; bulk RNA-seq,Homo_723,MCI.vs.control; bulk RNA-seq,Homo_714,AD.vs.control; bulk RNA-seq,Homo_714,AD.vs.MCI; bulk RNA-seq,Homo_714,MCI.vs.control	6
BP	GO:0045197	establishment or maintenance of epithelial cell apical/basal polarity	bulk RNA-seq,Homo_723,AD.vs.control; bulk RNA-seq,Homo_723,AD.vs.MCI; bulk RNA-seq,Homo_723,MCI.vs.control; bulk RNA-seq,Homo_714,AD.vs.MCI; bulk RNA-seq,Homo_714,MCI.vs.control; bulk RNA-seq,Homo_633,AD.vs.control; bulk RNA-seq,Homo_633,AD.vs.MCI	7
BP	GO:0072243	metanephric nephron epithelium development	bulk RNA-seq,Homo_723,AD.vs.control; bulk RNA-seq,Homo_723,AD.vs.MCI; bulk RNA-seq,Homo_723,MCI.vs.control; bulk RNA-seq,Homo_714,AD.vs.MCI; bulk RNA-seq,Homo_714,MCI.vs.control	5
MF	GO:0005343	organic acid:sodium symporter activity	bulk RNA-seq,Homo_723,AD.vs.control; bulk RNA-seq,Homo_723,AD.vs.MCI; bulk RNA-seq,Homo_723,MCI.vs.control; bulk RNA-seq,Homo_714,AD.vs.control; bulk RNA-seq,Homo_714,AD.vs.MCI; bulk RNA-seq,Homo_633,MCI.vs.control	6
BP	GO:0030510	regulation of BMP signaling pathway	bulk RNA-seq,Homo_723,AD.vs.control; bulk RNA-seq,Homo_723,AD.vs.MCI; bulk RNA-seq,Homo_723,MCI.vs.control; bulk RNA-seq,Homo_714,AD.vs.MCI; bulk RNA-seq,Homo_714,MCI.vs.control; bulk RNA-seq,SRP223445,AD.vs.control	6
MF	GO:0044325	transmembrane transporter binding	bulk RNA-seq,Homo_723,AD.vs.control; bulk RNA-seq,Homo_723,AD.vs.MCI; bulk RNA-seq,Homo_723,MCI.vs.control; bulk RNA-seq,Homo_714,AD.vs.MCI; bulk RNA-seq,Homo_714,MCI.vs.control; bulk RNA-seq,Homo_633,AD.vs.control; bulk RNA-seq,Homo_633,AD.vs.MCI	7
BP	GO:0002263	cell activation involved in immune response	bulk RNA-seq,Homo_723,AD.vs.control; bulk RNA-seq,Homo_723,MCI.vs.control; bulk RNA-seq,Homo_714,AD.vs.MCI; bulk RNA-seq,Homo_633,AD.vs.control; bulk RNA-seq,Homo_633,AD.vs.MCI; scRNA-seq,SRP330776,Naive CD8+ T cell_2-AD.vs.control; scRNA-seq,SRP215507,CD8+	7
BP	GO:1901293	nucleoside phosphate biosynthetic process	bulk RNA-seq,Homo_723,AD.vs.control; bulk RNA-seq,Homo_723,MCI.vs.control; bulk RNA-seq,Homo_714,AD.vs.control; bulk RNA-seq,Homo_633,AD.vs.control; bulk RNA-seq,Homo_633,AD.vs.MCI	5
BP	GO:0090128	regulation of synapse maturation	bulk RNA-seq,Homo_723,AD.vs.control; bulk RNA-seq,Homo_723,AD.vs.MCI; bulk RNA-seq,Homo_723,MCI.vs.control; bulk RNA-seq,Homo_714,AD.vs.MCI; bulk RNA-seq,Homo_714,MCI.vs.control	5
BP	GO:0070206	protein trimerization	bulk RNA-seq,Homo_723,AD.vs.control; bulk RNA-seq,Homo_723,AD.vs.MCI; bulk RNA-seq,Homo_723,MCI.vs.control; bulk RNA-	4
BP	GO:0045833	negative regulation of lipid metabolic process	bulk RNA-seq,Homo_723,AD.vs.control; bulk RNA-seq,Homo_723,AD.vs.MCI; bulk RNA-seq,Homo_723,MCI.vs.control; bulk RNA-seq,Homo_714,AD.vs.control; bulk RNA-seq,Homo_714,AD.vs.MCI; bulk RNA-seq,Homo_714,MCI.vs.control; bulk RNA-seq,Homo_633,AD.vs.control	7
BP	GO:0003230	cardiac atrium development	bulk RNA-seq,Homo_723,AD.vs.control; bulk RNA-seq,Homo_723,AD.vs.MCI; bulk RNA-seq,Homo_723,MCI.vs.control; bulk RNA-seq,Homo_714,AD.vs.control; bulk RNA-seq,Homo_714,AD.vs.MCI; bulk RNA-seq,Homo_714,MCI.vs.control	6
BP	GO:1901016	regulation of potassium ion transmembrane transporter	bulk RNA-seq,Homo_723,AD.vs.control; bulk RNA-seq,Homo_723,AD.vs.MCI; bulk RNA-seq,Homo_714,AD.vs.MCI	3
BP	GO:0000725	recombinational repair	bulk RNA-seq,Homo_723,AD.vs.control; bulk RNA-seq,Homo_723,MCI.vs.control; bulk RNA-seq,Homo_714,AD.vs.control; bulk RNA-seq,Homo_633,AD.vs.control; bulk RNA-seq,Homo_633,AD.vs.MCI	5
MF	GO:0015085	calcium ion transmembrane transporter activity	bulk RNA-seq,Homo_723,AD.vs.control; bulk RNA-seq,Homo_723,AD.vs.MCI; bulk RNA-seq,Homo_723,MCI.vs.control; bulk RNA-seq,Homo_714,AD.vs.MCI; bulk RNA-seq,Homo_633,AD.vs.control; bulk RNA-seq,Homo_633,AD.vs.MCI; bulk RNA-seq,ROSMAP,AD.vs.control	7
BP	GO:0014909	smooth muscle cell migration	bulk RNA-seq,Homo_723,AD.vs.control; bulk RNA-seq,Homo_723,AD.vs.MCI; bulk RNA-seq,Homo_723,MCI.vs.control; bulk RNA-seq,Homo_714,AD.vs.control; bulk RNA-seq,Homo_714,AD.vs.MCI; bulk RNA-seq,Homo_714,MCI.vs.control; bulk RNA-seq,Homo_633,AD.vs.control; bulk	8
BP	GO:0045064	T-helper 2 cell differentiation	bulk RNA-seq,Homo_723,AD.vs.control; bulk RNA-seq,Homo_633,AD.vs.control; bulk RNA-seq,Homo_633,AD.vs.MCI; bulk RNA-	4
BP	GO:0120193	tight junction organization	bulk RNA-seq,Homo_723,AD.vs.control; bulk RNA-seq,Homo_723,AD.vs.MCI; bulk RNA-seq,Homo_723,MCI.vs.control; bulk RNA-seq,Homo_714,AD.vs.control; bulk RNA-seq,Homo_714,AD.vs.MCI; bulk RNA-seq,Homo_714,MCI.vs.control; bulk RNA-seq,Homo_633,AD.vs.control; bulk	8
MF	GO:0008376	acetylgalactosaminyltransferase activity	bulk RNA-seq,Homo_723,AD.vs.control; bulk RNA-seq,Homo_723,AD.vs.MCI; bulk RNA-seq,Homo_723,MCI.vs.control; bulk RNA-seq,Homo_714,AD.vs.MCI; bulk RNA-seq,Homo_633,AD.vs.control	5
BP	GO:0003163	sinoatrial node development	bulk RNA-seq,Homo_723,AD.vs.control; bulk RNA-seq,Homo_723,AD.vs.MCI; bulk RNA-seq,Homo_723,MCI.vs.control; bulk RNA-seq,Homo_714,AD.vs.MCI	4
CC	GO:0034709	methylosome	bulk RNA-seq,Homo_723,AD.vs.control; bulk RNA-seq,Homo_723,MCI.vs.control; bulk RNA-seq,Homo_714,AD.vs.control; bulk RNA-seq,Homo_714,MCI.vs.control; bulk RNA-seq,Homo_633,AD.vs.control; bulk RNA-seq,Homo_633,AD.vs.MCI; bulk RNA-seq,Homo_633,MCI.vs.control	7

BP	GO:0006465	signal peptide processing	bulk RNA-seq,Homo_723,AD.vs.control; bulk RNA-seq,Homo_723,MCI.vs.control; bulk RNA-seq,Homo_714,AD.vs.control; bulk RNA-seq,Homo_714,MCI.vs.control; bulk RNA-seq,Homo_633,AD.vs.control; bulk RNA-seq,Homo_633,AD.vs.MCI	6
CC	GO:0097381	photoreceptor disc membrane	bulk RNA-seq,Homo_723,AD.vs.control; bulk RNA-seq,Homo_723,AD.vs.MCI; bulk RNA-seq,Homo_714,AD.vs.control; bulk RNA-seq,Homo_714,AD.vs.MCI	4
BP	GO:0051928	positive regulation of calcium ion transport	bulk RNA-seq,Homo_723,AD.vs.control; bulk RNA-seq,Homo_723,AD.vs.MCI; bulk RNA-seq,Homo_723,MCI.vs.control; bulk RNA-seq,Homo_714,AD.vs.MCI; bulk RNA-seq,Homo_633,AD.vs.control; bulk RNA-seq,Homo_633,AD.vs.MCI	6
BP	GO:0050856	regulation of T cell receptor signaling pathway	bulk RNA-seq,Homo_723,AD.vs.control; bulk RNA-seq,Homo_723,MCI.vs.control; bulk RNA-seq,Homo_714,MCI.vs.control; bulk RNA-seq,Homo_633,AD.vs.control; bulk RNA-seq,Homo_633,AD.vs.MCI	5
CC	GO:0030134	COPII-coated ER to Golgi transport vesicle	bulk RNA-seq,Homo_723,AD.vs.control; bulk RNA-seq,Homo_723,MCI.vs.control; bulk RNA-seq,Homo_714,AD.vs.control; bulk RNA-seq,Homo_714,MCI.vs.control; bulk RNA-seq,Homo_633,AD.vs.control; bulk RNA-seq,Homo_633,AD.vs.MCI	6
MF	GO:0005326	neurotransmitter transmembrane transporter activity	bulk RNA-seq,Homo_723,AD.vs.control; bulk RNA-seq,Homo_723,AD.vs.MCI; bulk RNA-seq,Homo_723,MCI.vs.control; bulk RNA-seq,Homo_714,AD.vs.MCI	4
BP	GO:0035094	response to nicotine	bulk RNA-seq,Homo_723,AD.vs.control; bulk RNA-seq,Homo_723,AD.vs.MCI; bulk RNA-seq,Homo_723,MCI.vs.control; bulk RNA-seq,Homo_714,AD.vs.MCI; bulk RNA-seq,Homo_714,MCI.vs.control	5
BP	GO:0002366	leukocyte activation involved in immune response	bulk RNA-seq,Homo_723,AD.vs.control; bulk RNA-seq,Homo_723,MCI.vs.control; bulk RNA-seq,Homo_633,AD.vs.control; bulk RNA-seq,Homo_633,AD.vs.MCI; scRNA-seq,SRP215507,CD8+ T cell_3-AD.vs.control	5
BP	GO:0021860	pyramidal neuron development	bulk RNA-seq,Homo_723,AD.vs.control; bulk RNA-seq,Homo_723,MCI.vs.control; bulk RNA-seq,Homo_633,AD.vs.control	3
BP	GO:0030501	positive regulation of bone mineralization	bulk RNA-seq,Homo_723,AD.vs.control; bulk RNA-seq,Homo_723,AD.vs.MCI; bulk RNA-seq,Homo_723,MCI.vs.control; bulk RNA-seq,Homo_714,AD.vs.MCI; bulk RNA-seq,Homo_714,MCI.vs.control; bulk RNA-seq,Homo_633,AD.vs.control; bulk RNA-seq,Homo_633,AD.vs.MCI	7
MF	GO:0005246	calcium channel regulator activity	bulk RNA-seq,Homo_723,AD.vs.control; bulk RNA-seq,Homo_723,AD.vs.MCI; bulk RNA-seq,Homo_723,MCI.vs.control; bulk RNA-seq,Homo_714,AD.vs.MCI; bulk RNA-seq,Homo_714,MCI.vs.control; bulk RNA-seq,Homo_633,AD.vs.control	6
BP	GO:0007091	metaphase/anaphase transition of mitotic cell cycle	bulk RNA-seq,Homo_723,AD.vs.control; bulk RNA-seq,Homo_723,MCI.vs.control; bulk RNA-seq,Homo_714,AD.vs.control; bulk RNA-seq,Homo_714,MCI.vs.control; bulk RNA-seq,Homo_633,AD.vs.control; bulk RNA-seq,Homo_633,AD.vs.MCI	6
BP	GO:0061136	regulation of proteasomal protein catabolic process	bulk RNA-seq,Homo_723,AD.vs.control; bulk RNA-seq,Homo_723,MCI.vs.control; bulk RNA-seq,Homo_714,AD.vs.control; bulk RNA-seq,Homo_633,AD.vs.control; bulk RNA-seq,Homo_633,AD.vs.MCI	5
BP	GO:0033141	positive regulation of peptidyl-serine phosphorylation of	bulk RNA-seq,Homo_723,AD.vs.control; bulk RNA-seq,Homo_714,AD.vs.control; bulk RNA-seq,Homo_714,AD.vs.MCI	3
BP	GO:1901863	positive regulation of muscle tissue development	bulk RNA-seq,Homo_723,AD.vs.control; bulk RNA-seq,Homo_723,AD.vs.MCI; bulk RNA-seq,Homo_723,MCI.vs.control; bulk RNA-seq,Homo_714,AD.vs.MCI	4
BP	GO:0050908	detection of light stimulus involved in visual perception	bulk RNA-seq,Homo_723,AD.vs.control	1
BP	GO:0050962	detection of light stimulus involved in sensory perception	bulk RNA-seq,Homo_723,AD.vs.control	1
BP	GO:0035019	somatic stem cell population maintenance	bulk RNA-seq,Homo_723,AD.vs.control; bulk RNA-seq,Homo_723,AD.vs.MCI; bulk RNA-seq,Homo_723,MCI.vs.control; bulk RNA-seq,Homo_714,AD.vs.MCI; bulk RNA-seq,Homo_714,MCI.vs.control; bulk RNA-seq,Homo_633,AD.vs.control; bulk RNA-seq,Homo_633,AD.vs.MCI; scRNA-seq,SRP330776,Naive CD8+ T	8
CC	GO:0043601	nuclear replisome	bulk RNA-seq,Homo_723,AD.vs.control; bulk RNA-seq,Homo_723,MCI.vs.control; bulk RNA-seq,Homo_714,MCI.vs.control; bulk RNA-	4
BP	GO:0010927	cellular component assembly involved in morphogenesis	bulk RNA-seq,Homo_723,AD.vs.control; bulk RNA-seq,Homo_723,AD.vs.MCI; bulk RNA-seq,Homo_723,MCI.vs.control; bulk RNA-seq,Homo_714,AD.vs.MCI; bulk RNA-seq,Homo_714,MCI.vs.control; bulk RNA-seq,Homo_633,AD.vs.control; bulk RNA-seq,Homo_633,AD.vs.MCI	7
BP	GO:0043406	positive regulation of MAP kinase activity	bulk RNA-seq,Homo_723,AD.vs.control; bulk RNA-seq,Homo_723,AD.vs.MCI; bulk RNA-seq,Homo_723,MCI.vs.control; bulk RNA-seq,Homo_714,AD.vs.MCI; bulk RNA-seq,Homo_714,MCI.vs.control; bulk RNA-seq,Homo_633,AD.vs.control; bulk RNA-seq,Homo_633,AD.vs.MCI; bulk RNA-seq,ROSMAP,AD.vs.control; bulk RNA-seq,ROSMAP,MCI.vs.control	9
CC	GO:0034045	phagophore assembly site membrane	bulk RNA-seq,Homo_723,AD.vs.control; bulk RNA-seq,Homo_723,AD.vs.MCI; bulk RNA-seq,Homo_714,AD.vs.control; bulk RNA-seq,Homo_633,AD.vs.control; bulk RNA-seq,Homo_633,AD.vs.MCI	5
BP	GO:0035883	enteroendocrine cell differentiation	bulk RNA-seq,Homo_723,AD.vs.control; bulk RNA-seq,Homo_723,AD.vs.MCI; bulk RNA-seq,Homo_723,MCI.vs.control; bulk RNA-seq,Homo_714,AD.vs.MCI	4
BP	GO:0000463	maturation of LSU-rRNA from tricistronic rRNA transcript (SSU-rRNA, 5.8S rRNA, LSU-rRNA)	bulk RNA-seq,Homo_723,AD.vs.control; bulk RNA-seq,Homo_723,MCI.vs.control; bulk RNA-seq,Homo_714,AD.vs.control; bulk RNA-seq,Homo_714,MCI.vs.control; bulk RNA-seq,Homo_633,AD.vs.control; bulk RNA-seq,Homo_633,AD.vs.MCI; bulk RNA-seq,Homo_633,MCI.vs.control	7
BP	GO:0002504	antigen processing and presentation of peptide or polysaccharide antigen via MHC class II	bulk RNA-seq,Homo_723,AD.vs.control; bulk RNA-seq,Homo_723,MCI.vs.control; bulk RNA-seq,Homo_714,AD.vs.control; bulk RNA-seq,Homo_714,MCI.vs.control; bulk RNA-seq,Homo_633,AD.vs.control; bulk RNA-seq,Homo_633,AD.vs.MCI; scRNA-seq,SRP330776,Naive CD8+ T cell_2-AD.vs.control; scRNA-seq,SRP215507,CD8+ T cell_3-AD.vs.control; scRNA-seq,SRP215507,CD8+ T cell_3-MCI.vs.control	9
BP	GO:0045669	positive regulation of osteoblast differentiation	bulk RNA-seq,Homo_723,AD.vs.control; bulk RNA-seq,Homo_723,AD.vs.MCI; bulk RNA-seq,Homo_723,MCI.vs.control; bulk RNA-seq,Homo_714,AD.vs.MCI; bulk RNA-seq,Homo_714,MCI.vs.control; bulk RNA-seq,Homo_633,AD.vs.control; bulk RNA-seq,Homo_633,AD.vs.MCI; scRNA-seq,SRP330776,CD8+ T	8
CC	GO:0030126	COPI vesicle coat	bulk RNA-seq,Homo_723,AD.vs.control; bulk RNA-seq,Homo_714,AD.vs.control; bulk RNA-seq,Homo_714,MCI.vs.control; bulk RNA-seq,Homo_633,AD.vs.control; bulk RNA-seq,Homo_633,AD.vs.MCI; bulk RNA-seq,Homo_633,MCI.vs.control	6
BP	GO:1903818	positive regulation of voltage-gated potassium channel	bulk RNA-seq,Homo_723,AD.vs.control; bulk RNA-seq,Homo_723,AD.vs.MCI	2
BP	GO:0046887	positive regulation of hormone secretion	bulk RNA-seq,Homo_723,AD.vs.control; bulk RNA-seq,Homo_723,AD.vs.MCI; bulk RNA-seq,Homo_723,MCI.vs.control; bulk RNA-seq,Homo_714,AD.vs.control; bulk RNA-seq,Homo_714,AD.vs.MCI; bulk RNA-seq,Homo_714,MCI.vs.control; bulk RNA-seq,Homo_633,AD.vs.control; bulk	8
BP	GO:0019218	regulation of steroid metabolic process	bulk RNA-seq,Homo_723,AD.vs.control; bulk RNA-seq,Homo_723,AD.vs.MCI; bulk RNA-seq,Homo_723,MCI.vs.control; bulk RNA-seq,Homo_714,AD.vs.control; bulk RNA-seq,Homo_714,AD.vs.MCI; bulk RNA-seq,Homo_714,MCI.vs.control; bulk RNA-seq,Homo_633,AD.vs.control; bulk	8
BP	GO:0042552	myelination	bulk RNA-seq,Homo_723,AD.vs.control; bulk RNA-seq,Homo_723,AD.vs.MCI; bulk RNA-seq,Homo_723,MCI.vs.control; bulk RNA-seq,Homo_714,AD.vs.MCI; bulk RNA-seq,Homo_633,AD.vs.control; bulk RNA-seq,Homo_633,AD.vs.MCI	6
BP	GO:1902904	negative regulation of supramolecular fiber organization	bulk RNA-seq,Homo_723,AD.vs.control; bulk RNA-seq,Homo_723,AD.vs.MCI; bulk RNA-seq,Homo_723,MCI.vs.control; bulk RNA-seq,Homo_714,AD.vs.MCI; bulk RNA-seq,Homo_633,AD.vs.control; bulk RNA-seq,Homo_633,AD.vs.MCI	6
CC	GO:0034719	SMN-Sm protein complex	bulk RNA-seq,Homo_723,AD.vs.control; bulk RNA-seq,Homo_723,MCI.vs.control; bulk RNA-seq,Homo_714,AD.vs.control; bulk RNA-seq,Homo_714,MCI.vs.control; bulk RNA-seq,Homo_633,AD.vs.control; bulk RNA-seq,Homo_633,AD.vs.MCI; bulk RNA-seq,Homo_633,MCI.vs.control	7
BP	GO:0050879	multicellular organismal movement	bulk RNA-seq,Homo_723,AD.vs.control; bulk RNA-seq,Homo_723,AD.vs.MCI; bulk RNA-seq,Homo_723,MCI.vs.control; bulk RNA-seq,Homo_714,AD.vs.control; bulk RNA-seq,Homo_714,AD.vs.MCI; bulk RNA-seq,Homo_714,MCI.vs.control	6
BP	GO:0050881	musculoskeletal movement	bulk RNA-seq,Homo_723,AD.vs.control; bulk RNA-seq,Homo_723,AD.vs.MCI; bulk RNA-seq,Homo_723,MCI.vs.control; bulk RNA-seq,Homo_714,AD.vs.control; bulk RNA-seq,Homo_714,AD.vs.MCI; bulk RNA-seq,Homo_714,MCI.vs.control	6
CC	GO:0012507	ER to Golgi transport vesicle membrane	bulk RNA-seq,Homo_723,AD.vs.control; bulk RNA-seq,Homo_723,MCI.vs.control; bulk RNA-seq,Homo_714,AD.vs.control; bulk RNA-seq,Homo_714,MCI.vs.control; bulk RNA-seq,Homo_633,AD.vs.control; bulk RNA-seq,Homo_633,AD.vs.MCI; scRNA-seq,SRP215507,CD8+ T cell_3-	7

CC	GO:0030136	clathrin-coated vesicle	bulk RNA-seq,Homo_723,AD.vs.control; bulk RNA-seq,Homo_723,AD.vs.MCI; bulk RNA-seq,Homo_723,MCI.vs.control; bulk RNA-seq,Homo_714,AD.vs.MCI; bulk RNA-seq,Homo_633,AD.vs.control; bulk RNA-seq,Homo_633,AD.vs.MCI	6
BP	GO:0042756	drinking behavior	bulk RNA-seq,Homo_723,AD.vs.control; bulk RNA-seq,Homo_714,AD.vs.control	2
BP	GO:0002478	antigen processing and presentation of exogenous peptide antigen	bulk RNA-seq,Homo_723,AD.vs.control; bulk RNA-seq,Homo_723,MCI.vs.control; bulk RNA-seq,Homo_714,MCI.vs.control; bulk RNA-seq,Homo_633,AD.vs.control; bulk RNA-seq,Homo_633,AD.vs.MCI; scRNA-seq,SRP330776,Naive CD8+ T cell_2-AD.vs.control; scRNA-seq,SRP215507,CD8+ T cell_3-AD.vs.control; scRNA-seq,SRP215507,CD8+ T cell_3-MCI.vs.control	8
BP	GO:0060445	branching involved in salivary gland morphogenesis	bulk RNA-seq,Homo_723,AD.vs.control; bulk RNA-seq,Homo_723,AD.vs.MCI; bulk RNA-seq,Homo_723,MCI.vs.control	3
BP	GO:0060260	regulation of transcription initiation by RNA polymerase II	bulk RNA-seq,Homo_723,AD.vs.control; bulk RNA-seq,Homo_723,MCI.vs.control; bulk RNA-seq,Homo_714,AD.vs.control; bulk RNA-seq,Homo_714,MCI.vs.control; bulk RNA-seq,Homo_633,AD.vs.control; bulk RNA-seq,Homo_633,AD.vs.MCI	6
BP	GO:0045006	DNA deamination	bulk RNA-seq,Homo_723,AD.vs.control; bulk RNA-seq,Homo_723,AD.vs.MCI; bulk RNA-seq,Homo_714,AD.vs.control	3
BP	GO:2000672	negative regulation of motor neuron apoptotic process	bulk RNA-seq,Homo_723,AD.vs.control; bulk RNA-seq,Homo_723,AD.vs.MCI; bulk RNA-seq,Homo_723,MCI.vs.control	3
BP	GO:0043966	histone H3 acetylation	bulk RNA-seq,Homo_723,AD.vs.control; bulk RNA-seq,Homo_723,MCI.vs.control; bulk RNA-seq,Homo_714,AD.vs.control; bulk RNA-seq,Homo_714,MCI.vs.control; bulk RNA-seq,Homo_633,AD.vs.control; bulk RNA-seq,Homo_633,AD.vs.MCI	6
MF	GO:0022853	active ion transmembrane transporter activity	bulk RNA-seq,Homo_723,AD.vs.control; bulk RNA-seq,Homo_723,AD.vs.MCI; bulk RNA-seq,Homo_723,MCI.vs.control; bulk RNA-seq,Homo_714,AD.vs.control; bulk RNA-seq,Homo_714,AD.vs.MCI; bulk RNA-seq,Homo_633,AD.vs.control; bulk RNA-seq,Homo_633,AD.vs.MCI	7
CC	GO:0035578	azurophil granule lumen	bulk RNA-seq,Homo_723,AD.vs.control; bulk RNA-seq,Homo_723,MCI.vs.control; bulk RNA-seq,Homo_714,AD.vs.control; bulk RNA-seq,Homo_714,MCI.vs.control; bulk RNA-seq,Homo_633,AD.vs.control; bulk RNA-seq,Homo_633,AD.vs.MCI	6
BP	GO:0031343	positive regulation of cell killing	bulk RNA-seq,Homo_723,AD.vs.control; bulk RNA-seq,Homo_714,AD.vs.control; bulk RNA-seq,Homo_714,MCI.vs.control; bulk RNA-seq,Homo_633,AD.vs.control; bulk RNA-seq,Homo_633,AD.vs.MCI; scRNA-seq,SRP330776,Naive CD8+ T cell_2-AD.vs.control	6
BP	GO:0060536	cartilage morphogenesis	bulk RNA-seq,Homo_723,AD.vs.control; bulk RNA-seq,Homo_723,AD.vs.MCI; bulk RNA-seq,Homo_723,MCI.vs.control; bulk RNA-seq,Homo_714,AD.vs.MCI	4
BP	GO:0043574	peroxisomal transport	bulk RNA-seq,Homo_723,AD.vs.control; bulk RNA-seq,Homo_723,MCI.vs.control; bulk RNA-seq,Homo_714,AD.vs.control; bulk RNA-seq,Homo_714,MCI.vs.control; bulk RNA-seq,Homo_633,AD.vs.control; bulk RNA-seq,Homo_633,AD.vs.MCI; bulk RNA-seq,Homo_633,MCI.vs.control	7
BP	GO:2000516	positive regulation of CD4-positive, alpha-beta T cell activation	bulk RNA-seq,Homo_723,AD.vs.control; bulk RNA-seq,Homo_714,AD.vs.control; bulk RNA-seq,Homo_714,MCI.vs.control; bulk RNA-seq,Homo_633,AD.vs.control; bulk RNA-seq,Homo_633,AD.vs.MCI	5
BP	GO:0045682	regulation of epidermis development	bulk RNA-seq,Homo_723,AD.vs.control; bulk RNA-seq,Homo_723,AD.vs.MCI; bulk RNA-seq,Homo_723,MCI.vs.control; bulk RNA-seq,Homo_714,AD.vs.MCI; bulk RNA-seq,Homo_714,MCI.vs.control; bulk RNA-seq,Homo_633,AD.vs.control; bulk RNA-seq,Homo_633,AD.vs.MCI	7
BP	GO:0045582	positive regulation of T cell differentiation	bulk RNA-seq,Homo_723,AD.vs.control; bulk RNA-seq,Homo_723,MCI.vs.control; bulk RNA-seq,Homo_714,AD.vs.control; bulk RNA-seq,Homo_714,MCI.vs.control; bulk RNA-seq,Homo_633,AD.vs.control; bulk RNA-seq,Homo_633,AD.vs.MCI	6
BP	GO:0021782	glial cell development	bulk RNA-seq,Homo_723,AD.vs.control; bulk RNA-seq,Homo_723,AD.vs.MCI; bulk RNA-seq,Homo_723,MCI.vs.control; bulk RNA-seq,Homo_714,AD.vs.MCI; bulk RNA-seq,Homo_714,MCI.vs.control; bulk RNA-seq,Homo_633,AD.vs.control; bulk RNA-seq,Homo_633,AD.vs.MCI	7
BP	GO:0007501	mesodermal cell fate specification	bulk RNA-seq,Homo_723,AD.vs.control; bulk RNA-seq,Homo_723,AD.vs.MCI; bulk RNA-seq,Homo_714,AD.vs.MCI	3
BP	GO:0098935	dendritic transport	bulk RNA-seq,Homo_723,AD.vs.control; bulk RNA-seq,Homo_723,AD.vs.MCI; bulk RNA-seq,Homo_723,MCI.vs.control; bulk RNA-seq,Homo_714,AD.vs.MCI; bulk RNA-seq,Homo_714,MCI.vs.control	5
BP	GO:0086004	regulation of cardiac muscle cell contraction	bulk RNA-seq,Homo_723,AD.vs.control; bulk RNA-seq,Homo_723,AD.vs.MCI; bulk RNA-seq,Homo_723,MCI.vs.control; bulk RNA-seq,Homo_714,AD.vs.control; bulk RNA-seq,Homo_714,AD.vs.MCI; bulk RNA-seq,Homo_714,MCI.vs.control	6
CC	GO:0031011	Ino80 complex	bulk RNA-seq,Homo_723,AD.vs.control; bulk RNA-seq,Homo_714,AD.vs.control; bulk RNA-seq,Homo_633,AD.vs.control; bulk RNA-seq,Homo_633,AD.vs.MCI	4
BP	GO:0060420	regulation of heart growth	bulk RNA-seq,Homo_723,AD.vs.control; bulk RNA-seq,Homo_723,AD.vs.MCI; bulk RNA-seq,Homo_723,MCI.vs.control; bulk RNA-seq,Homo_714,AD.vs.MCI; bulk RNA-seq,Homo_714,MCI.vs.control	5
BP	GO:0010508	positive regulation of autophagy	bulk RNA-seq,Homo_723,AD.vs.control; bulk RNA-seq,Homo_723,MCI.vs.control; bulk RNA-seq,Homo_714,AD.vs.control; bulk RNA-seq,Homo_633,AD.vs.control; bulk RNA-seq,Homo_633,AD.vs.MCI	5
BP	GO:0070365	hepatocyte differentiation	bulk RNA-seq,Homo_723,AD.vs.control; bulk RNA-seq,Homo_723,AD.vs.MCI; bulk RNA-seq,Homo_723,MCI.vs.control; bulk RNA-seq,Homo_714,AD.vs.MCI	4
BP	GO:0043117	positive regulation of vascular permeability	bulk RNA-seq,Homo_723,AD.vs.control; bulk RNA-seq,Homo_723,AD.vs.MCI; bulk RNA-seq,Homo_723,MCI.vs.control; bulk RNA-seq,Homo_714,AD.vs.control; bulk RNA-seq,Homo_714,AD.vs.MCI; bulk RNA-seq,Homo_714,MCI.vs.control; bulk RNA-seq,Homo_633,AD.vs.control; bulk	8
BP	GO:1902287	semaphorin-plexin signaling pathway involved in axon guidance	bulk RNA-seq,Homo_723,AD.vs.control; bulk RNA-seq,Homo_723,AD.vs.MCI; bulk RNA-seq,Homo_723,MCI.vs.control; bulk RNA-seq,Homo_714,AD.vs.MCI; bulk RNA-seq,Homo_714,MCI.vs.control	5
BP	GO:2000146	negative regulation of cell motility	bulk RNA-seq,Homo_723,AD.vs.control; bulk RNA-seq,Homo_723,AD.vs.MCI; bulk RNA-seq,Homo_714,AD.vs.control; bulk RNA-seq,Homo_714,AD.vs.MCI; bulk RNA-seq,Homo_633,AD.vs.control; bulk RNA-seq,Homo_633,AD.vs.MCI; scRNA-seq,SRP330776,Naive CD8+ T cell_2-AD.vs.control	7
BP	GO:0098789	pre-mRNA cleavage required for polyadenylation	bulk RNA-seq,Homo_723,AD.vs.control; bulk RNA-seq,Homo_723,AD.vs.MCI; bulk RNA-seq,Homo_714,AD.vs.control; bulk RNA-seq,Homo_633,AD.vs.control; bulk RNA-seq,Homo_633,AD.vs.MCI	5
MF	GO:0004535	poly(A)-specific ribonuclease activity	bulk RNA-seq,Homo_723,AD.vs.control; bulk RNA-seq,Homo_714,AD.vs.control; bulk RNA-seq,Homo_714,MCI.vs.control; bulk RNA-	4
CC	GO:0030677	ribonuclease P complex	bulk RNA-seq,Homo_723,AD.vs.control; bulk RNA-seq,Homo_723,AD.vs.MCI; bulk RNA-seq,Homo_714,AD.vs.control; bulk RNA-seq,Homo_633,AD.vs.control; bulk RNA-seq,Homo_633,AD.vs.MCI	5
BP	GO:0014821	phasic smooth muscle contraction	bulk RNA-seq,Homo_723,AD.vs.control; bulk RNA-seq,Homo_723,AD.vs.MCI; bulk RNA-seq,Homo_723,MCI.vs.control; bulk RNA-seq,Homo_714,AD.vs.control; bulk RNA-seq,Homo_714,AD.vs.MCI; bulk RNA-seq,Homo_633,MCI.vs.control	6
MF	GO:0008097	5S rRNA binding	bulk RNA-seq,Homo_723,AD.vs.control; bulk RNA-seq,Homo_723,AD.vs.MCI; bulk RNA-seq,Homo_723,MCI.vs.control; bulk RNA-seq,Homo_714,AD.vs.control; bulk RNA-seq,Homo_714,MCI.vs.control; bulk RNA-seq,Homo_633,AD.vs.control; bulk RNA-seq,Homo_633,AD.vs.MCI; bulk	8
BP	GO:0007602	phototransduction	bulk RNA-seq,Homo_723,AD.vs.control; bulk RNA-seq,Homo_723,AD.vs.MCI; bulk RNA-seq,Homo_714,AD.vs.control; bulk RNA-seq,Homo_714,AD.vs.MCI	4
BP	GO:0042481	regulation of odontogenesis	bulk RNA-seq,Homo_723,AD.vs.control; bulk RNA-seq,Homo_723,AD.vs.MCI; bulk RNA-seq,Homo_723,MCI.vs.control; bulk RNA-seq,Homo_714,AD.vs.MCI	4
BP	GO:1903524	positive regulation of blood circulation	bulk RNA-seq,Homo_723,AD.vs.control; bulk RNA-seq,Homo_723,AD.vs.MCI; bulk RNA-seq,Homo_723,MCI.vs.control; bulk RNA-seq,Homo_714,AD.vs.MCI	4
BP	GO:0048199	vesicle targeting, to, from or within Golgi	bulk RNA-seq,Homo_723,AD.vs.control; bulk RNA-seq,Homo_714,AD.vs.control; bulk RNA-seq,Homo_714,MCI.vs.control; bulk RNA-seq,Homo_633,AD.vs.control; bulk RNA-seq,Homo_633,AD.vs.MCI; bulk RNA-seq,Homo_633,MCI.vs.control	6
BP	GO:0070977	bone maturation	bulk RNA-seq,Homo_723,AD.vs.control; bulk RNA-seq,Homo_723,AD.vs.MCI; bulk RNA-seq,Homo_723,MCI.vs.control	3

BP	GO:0008210	estrogen metabolic process	bulk RNA-seq,Homo_723,AD.vs.control; bulk RNA-seq,Homo_723,AD.vs.MCI; bulk RNA-seq,Homo_723,MCI.vs.control; bulk RNA-seq,Homo_714,AD.vs.control; bulk RNA-seq,Homo_714,AD.vs.MCI; bulk RNA-seq,Homo_714,MCI.vs.control	6
BP	GO:0051893	regulation of focal adhesion assembly	bulk RNA-seq,Homo_723,AD.vs.control; bulk RNA-seq,Homo_723,AD.vs.MCI; bulk RNA-seq,Homo_723,MCI.vs.control; bulk RNA-seq,Homo_714,AD.vs.MCI; bulk RNA-seq,Homo_714,MCI.vs.control; bulk RNA-seq,Homo_633,AD.vs.control; bulk RNA-seq,Homo_633,AD.vs.MCI	7
BP	GO:0090109	regulation of cell-substrate junction assembly	bulk RNA-seq,Homo_723,AD.vs.control; bulk RNA-seq,Homo_723,AD.vs.MCI; bulk RNA-seq,Homo_723,MCI.vs.control; bulk RNA-seq,Homo_714,AD.vs.MCI; bulk RNA-seq,Homo_714,MCI.vs.control; bulk RNA-seq,Homo_633,AD.vs.control; bulk RNA-seq,Homo_633,AD.vs.MCI	7
BP	GO:0050687	negative regulation of defense response to virus	bulk RNA-seq,Homo_723,AD.vs.control; bulk RNA-seq,Homo_714,AD.vs.control; bulk RNA-seq,Homo_633,AD.vs.control; bulk RNA-seq,Homo_633,AD.vs.MCI	4
BP	GO:0046634	regulation of alpha-beta T cell activation	bulk RNA-seq,Homo_723,AD.vs.control; bulk RNA-seq,Homo_723,MCI.vs.control; bulk RNA-seq,Homo_714,AD.vs.control; bulk RNA-seq,Homo_714,MCI.vs.control; bulk RNA-seq,Homo_633,AD.vs.control; bulk RNA-seq,Homo_633,AD.vs.MCI	6
BP	GO:0080111	DNA demethylation	bulk RNA-seq,Homo_723,AD.vs.control; bulk RNA-seq,Homo_723,MCI.vs.control; bulk RNA-seq,Homo_714,AD.vs.control; bulk RNA-	4
BP	GO:0030514	negative regulation of BMP signaling pathway	bulk RNA-seq,Homo_723,AD.vs.control; bulk RNA-seq,Homo_723,AD.vs.MCI; bulk RNA-seq,Homo_723,MCI.vs.control; bulk RNA-seq,Homo_714,AD.vs.MCI; bulk RNA-seq,Homo_714,MCI.vs.control	5
BP	GO:0072529	pyrimidine-containing compound catabolic process	bulk RNA-seq,Homo_723,AD.vs.control; bulk RNA-seq,Homo_714,AD.vs.control; bulk RNA-seq,Homo_633,AD.vs.control; bulk RNA-seq,Homo_633,AD.vs.MCI	4
BP	GO:0060732	positive regulation of inositol phosphate biosynthetic process	bulk RNA-seq,Homo_723,AD.vs.control; bulk RNA-seq,Homo_723,AD.vs.MCI	2
BP	GO:0090200	positive regulation of release of cytochrome c from	bulk RNA-seq,Homo_723,AD.vs.control; bulk RNA-seq,Homo_633,AD.vs.control; bulk RNA-seq,Homo_633,AD.vs.MCI	3
BP	GO:0048741	skeletal muscle fiber development	bulk RNA-seq,Homo_723,AD.vs.control; bulk RNA-seq,Homo_723,AD.vs.MCI; bulk RNA-seq,Homo_723,MCI.vs.control; bulk RNA-seq,Homo_714,AD.vs.MCI; bulk RNA-seq,Homo_714,MCI.vs.control; bulk RNA-seq,Homo_633,AD.vs.control	6
MF	GO:0031369	translation initiation factor binding	bulk RNA-seq,Homo_723,AD.vs.control; bulk RNA-seq,Homo_723,MCI.vs.control; bulk RNA-seq,Homo_714,AD.vs.control; bulk RNA-seq,Homo_714,MCI.vs.control; bulk RNA-seq,Homo_633,AD.vs.control; bulk RNA-seq,Homo_633,AD.vs.MCI	6
BP	GO:0006414	translational elongation	bulk RNA-seq,Homo_723,AD.vs.control; bulk RNA-seq,Homo_723,MCI.vs.control; bulk RNA-seq,Homo_714,AD.vs.control; bulk RNA-seq,Homo_714,MCI.vs.control; bulk RNA-seq,Homo_633,AD.vs.control; bulk RNA-seq,Homo_633,AD.vs.MCI	6
BP	GO:0006775	fat-soluble vitamin metabolic process	bulk RNA-seq,Homo_723,AD.vs.control; bulk RNA-seq,Homo_723,AD.vs.MCI; bulk RNA-seq,Homo_723,MCI.vs.control; bulk RNA-seq,Homo_714,AD.vs.MCI; bulk RNA-seq,Homo_714,MCI.vs.control	5
CC	GO:0005891	voltage-gated calcium channel complex	bulk RNA-seq,Homo_723,AD.vs.control; bulk RNA-seq,Homo_723,AD.vs.MCI; bulk RNA-seq,Homo_723,MCI.vs.control; bulk RNA-seq,Homo_714,AD.vs.MCI; bulk RNA-seq,ROSMAP,AD.vs.control	5
MF	GO:0140375	immune receptor activity	bulk RNA-seq,Homo_723,AD.vs.control; bulk RNA-seq,Homo_723,MCI.vs.control; bulk RNA-seq,Homo_633,AD.vs.control; scRNA-seq,SRP215507,CD8+ T	4
CC	GO:1990391	DNA repair complex	bulk RNA-seq,Homo_723,AD.vs.control; bulk RNA-seq,Homo_723,MCI.vs.control; bulk RNA-seq,Homo_714,AD.vs.control; bulk RNA-seq,Homo_633,AD.vs.control; bulk RNA-seq,Homo_633,AD.vs.MCI	5
BP	GO:0061101	neuroendocrine cell differentiation	bulk RNA-seq,Homo_723,AD.vs.control; bulk RNA-seq,Homo_723,AD.vs.MCI; bulk RNA-seq,Homo_714,AD.vs.MCI	3
MF	GO:0045182	translation regulator activity	bulk RNA-seq,Homo_723,AD.vs.control; bulk RNA-seq,Homo_723,MCI.vs.control; bulk RNA-seq,Homo_714,AD.vs.control; bulk RNA-	4
BP	GO:0032817	regulation of natural killer cell proliferation	bulk RNA-seq,Homo_723,AD.vs.control; bulk RNA-seq,Homo_723,AD.vs.MCI	2
BP	GO:0006929	substrate-dependent cell migration	bulk RNA-seq,Homo_723,AD.vs.control; bulk RNA-seq,Homo_723,AD.vs.MCI; bulk RNA-seq,Homo_723,MCI.vs.control; bulk RNA-seq,Homo_714,AD.vs.MCI; bulk RNA-seq,Homo_714,MCI.vs.control; bulk RNA-seq,Homo_633,AD.vs.control	6
BP	GO:0002026	regulation of the force of heart contraction	bulk RNA-seq,Homo_723,AD.vs.control; bulk RNA-seq,Homo_723,AD.vs.MCI; bulk RNA-seq,Homo_723,MCI.vs.control; bulk RNA-seq,Homo_714,AD.vs.control; bulk RNA-seq,Homo_714,AD.vs.MCI	5
BP	GO:0009584	detection of visible light	bulk RNA-seq,Homo_723,AD.vs.control; bulk RNA-seq,Homo_723,AD.vs.MCI; bulk RNA-seq,Homo_714,AD.vs.control; bulk RNA-seq,Homo_714,AD.vs.MCI	4
BP	GO:2001135	regulation of endocytic recycling	bulk RNA-seq,Homo_723,AD.vs.control; bulk RNA-seq,Homo_723,AD.vs.MCI; bulk RNA-seq,Homo_723,MCI.vs.control; bulk RNA-seq,Homo_714,AD.vs.MCI; bulk RNA-seq,Homo_714,MCI.vs.control	5
MF	GO:0005001	transmembrane receptor protein tyrosine phosphatase activity	bulk RNA-seq,Homo_723,AD.vs.control; bulk RNA-seq,Homo_723,AD.vs.MCI; bulk RNA-seq,Homo_723,MCI.vs.control; bulk RNA-seq,Homo_714,AD.vs.MCI; bulk RNA-seq,Homo_714,MCI.vs.control; bulk RNA-seq,ROSMAP,MCI.vs.control; bulk RNA-seq,SRP223445,AD.vs.control	7
MF	GO:0019198	transmembrane receptor protein phosphatase activity	bulk RNA-seq,Homo_723,AD.vs.control; bulk RNA-seq,Homo_723,AD.vs.MCI; bulk RNA-seq,Homo_723,MCI.vs.control; bulk RNA-seq,Homo_714,AD.vs.MCI; bulk RNA-seq,Homo_714,MCI.vs.control; bulk RNA-seq,ROSMAP,MCI.vs.control; bulk RNA-seq,SRP223445,AD.vs.control	7
BP	GO:0032206	positive regulation of telomere maintenance	bulk RNA-seq,Homo_723,AD.vs.control; bulk RNA-seq,Homo_723,MCI.vs.control; bulk RNA-seq,Homo_714,AD.vs.control; bulk RNA-seq,Homo_714,MCI.vs.control; bulk RNA-seq,Homo_633,AD.vs.control; bulk RNA-seq,Homo_633,AD.vs.MCI	6
BP	GO:0016056	rhodopsin mediated signaling pathway	bulk RNA-seq,Homo_723,AD.vs.control; bulk RNA-seq,Homo_714,AD.vs.control	2
BP	GO:0140059	dendrite arborization	bulk RNA-seq,Homo_723,AD.vs.control; bulk RNA-seq,Homo_723,AD.vs.MCI; bulk RNA-seq,Homo_723,MCI.vs.control; bulk RNA-seq,Homo_714,AD.vs.control; bulk RNA-seq,Homo_714,AD.vs.MCI	5
MF	GO:0030280	structural constituent of skin epidermis	bulk RNA-seq,Homo_723,AD.vs.control; bulk RNA-seq,Homo_723,AD.vs.MCI; bulk RNA-seq,Homo_714,AD.vs.control; bulk RNA-seq,Homo_714,AD.vs.MCI; bulk RNA-seq,SRP223445,AD.vs.control	5
BP	GO:0034982	mitochondrial protein processing	bulk RNA-seq,Homo_723,AD.vs.control; bulk RNA-seq,Homo_714,AD.vs.control; bulk RNA-seq,Homo_633,AD.vs.control; bulk RNA-seq,Homo_633,AD.vs.MCI	4
BP	GO:0038203	TORC2 signaling	bulk RNA-seq,Homo_723,AD.vs.control; bulk RNA-seq,Homo_723,MCI.vs.control; bulk RNA-seq,Homo_714,AD.vs.control; bulk RNA-seq,Homo_714,MCI.vs.control; bulk RNA-seq,Homo_633,MCI.vs.control	5
MF	GO:0019104	DNA N-glycosylase activity	bulk RNA-seq,Homo_723,AD.vs.control; bulk RNA-seq,Homo_723,AD.vs.MCI; bulk RNA-seq,Homo_714,AD.vs.control; bulk RNA-seq,Homo_633,AD.vs.control; bulk RNA-seq,Homo_633,AD.vs.MCI	5
MF	GO:0004653	polypeptide N-acetylgalactosaminyltransferase activity	bulk RNA-seq,Homo_723,AD.vs.control; bulk RNA-seq,Homo_723,AD.vs.MCI; bulk RNA-seq,Homo_723,MCI.vs.control; bulk RNA-seq,Homo_714,MCI.vs.control; bulk RNA-seq,Homo_633,AD.vs.control	5
BP	GO:0099558	maintenance of synapse structure	bulk RNA-seq,Homo_723,AD.vs.control; bulk RNA-seq,Homo_723,AD.vs.MCI; bulk RNA-seq,Homo_723,MCI.vs.control; bulk RNA-seq,Homo_714,AD.vs.MCI	4
MF	GO:0005132	type I interferon receptor binding	bulk RNA-seq,Homo_723,AD.vs.control; bulk RNA-seq,Homo_714,AD.vs.control; bulk RNA-seq,Homo_714,AD.vs.MCI; bulk RNA-seq,Homo_633,AD.vs.control; bulk RNA-seq,Homo_633,AD.vs.MCI; bulk RNA-seq,ROSMAP,AD.vs.control	6
BP	GO:0031281	positive regulation of cyclase activity	bulk RNA-seq,Homo_723,AD.vs.control; bulk RNA-seq,Homo_723,AD.vs.MCI; bulk RNA-seq,Homo_723,MCI.vs.control; bulk RNA-seq,Homo_714,AD.vs.control; bulk RNA-seq,Homo_714,AD.vs.MCI; bulk RNA-seq,SRP223445,AD.vs.control	6
BP	GO:0030071	regulation of mitotic metaphase/anaphase transition	bulk RNA-seq,Homo_723,AD.vs.control; bulk RNA-seq,Homo_723,MCI.vs.control; bulk RNA-seq,Homo_714,AD.vs.control; bulk RNA-seq,Homo_714,MCI.vs.control; bulk RNA-seq,Homo_633,AD.vs.control; bulk RNA-seq,Homo_633,AD.vs.MCI	6

MF	GO:0015347	sodium-independent organic anion transmembrane	bulk RNA-seq,Homo_723,AD.vs.control; bulk RNA-seq,Homo_723,AD.vs.MCI; bulk RNA-seq,Homo_714,AD.vs.control; bulk RNA-seq,Homo_714,AD.vs.MCI	4
BP	GO:0030033	microvillus assembly	bulk RNA-seq,Homo_723,AD.vs.control; bulk RNA-seq,Homo_723,MCI.vs.control; bulk RNA-seq,Homo_714,AD.vs.control; bulk RNA-seq,Homo_714,MCI.vs.control; bulk RNA-seq,Homo_633,AD.vs.control; bulk RNA-seq,Homo_633,AD.vs.MCI; bulk RNA-seq,Homo_633,MCI.vs.control	7
BP	GO:0010518	positive regulation of phospholipase activity	bulk RNA-seq,Homo_723,AD.vs.control; bulk RNA-seq,Homo_723,AD.vs.MCI; bulk RNA-seq,Homo_723,MCI.vs.control; bulk RNA-seq,Homo_714,AD.vs.MCI; bulk RNA-seq,Homo_714,MCI.vs.control; bulk RNA-seq,Homo_633,AD.vs.control; bulk RNA-seq,Homo_633,AD.vs.MCI	7
MF	GO:0005504	fatty acid binding	bulk RNA-seq,Homo_723,AD.vs.control; bulk RNA-seq,Homo_723,AD.vs.MCI; bulk RNA-seq,Homo_723,MCI.vs.control; bulk RNA-seq,Homo_714,AD.vs.MCI; bulk RNA-seq,Homo_714,MCI.vs.control	5
BP	GO:0038034	signal transduction in absence of ligand	bulk RNA-seq,Homo_723,AD.vs.control; bulk RNA-seq,Homo_723,AD.vs.MCI; bulk RNA-seq,Homo_723,MCI.vs.control; bulk RNA-seq,Homo_714,AD.vs.MCI; bulk RNA-seq,Homo_714,MCI.vs.control	5
BP	GO:0097192	extrinsic apoptotic signaling pathway in absence of ligand	bulk RNA-seq,Homo_723,AD.vs.control; bulk RNA-seq,Homo_723,AD.vs.MCI; bulk RNA-seq,Homo_723,MCI.vs.control; bulk RNA-seq,Homo_714,AD.vs.MCI; bulk RNA-seq,Homo_714,MCI.vs.control	5
BP	GO:0043370	regulation of CD4-positive, alpha-beta T cell differentiation	bulk RNA-seq,Homo_723,AD.vs.control; bulk RNA-seq,Homo_723,MCI.vs.control; bulk RNA-seq,Homo_714,AD.vs.control; bulk RNA-seq,Homo_714,MCI.vs.control; bulk RNA-seq,Homo_633,AD.vs.control; bulk RNA-seq,Homo_633,AD.vs.MCI	6
MF	GO:0051059	NF-kappaB binding	bulk RNA-seq,Homo_723,AD.vs.control; bulk RNA-seq,Homo_723,MCI.vs.control; bulk RNA-seq,Homo_714,MCI.vs.control; bulk RNA-seq,Homo_633,AD.vs.control; bulk RNA-seq,Homo_633,AD.vs.MCI; bulk RNA-seq,Homo_633,MCI.vs.control	6
BP	GO:0042147	retrograde transport, endosome to Golgi	bulk RNA-seq,Homo_723,AD.vs.control; bulk RNA-seq,Homo_723,MCI.vs.control; bulk RNA-seq,Homo_714,AD.vs.control; bulk RNA-seq,Homo_714,MCI.vs.control; bulk RNA-seq,Homo_633,AD.vs.control; bulk RNA-seq,Homo_633,AD.vs.MCI	6
BP	GO:0030500	regulation of bone mineralization	bulk RNA-seq,Homo_723,AD.vs.control; bulk RNA-seq,Homo_723,AD.vs.MCI; bulk RNA-seq,Homo_723,MCI.vs.control; bulk RNA-seq,Homo_714,AD.vs.MCI; bulk RNA-seq,Homo_714,MCI.vs.control; bulk RNA-seq,Homo_633,AD.vs.control; bulk RNA-seq,Homo_633,AD.vs.MCI	7
BP	GO:0045579	positive regulation of B cell differentiation	bulk RNA-seq,Homo_723,AD.vs.control; bulk RNA-seq,Homo_633,AD.vs.control; bulk RNA-seq,Homo_633,AD.vs.MCI	3
CC	GO:0048787	presynaptic active zone membrane	bulk RNA-seq,Homo_723,AD.vs.control; bulk RNA-seq,Homo_723,AD.vs.MCI; bulk RNA-seq,Homo_723,MCI.vs.control; bulk RNA-	4
BP	GO:0030168	platelet activation	bulk RNA-seq,Homo_723,AD.vs.control; bulk RNA-seq,Homo_723,AD.vs.MCI; bulk RNA-seq,Homo_723,MCI.vs.control; bulk RNA-seq,Homo_714,AD.vs.control; bulk RNA-seq,Homo_714,AD.vs.MCI; bulk RNA-seq,Homo_633,AD.vs.control; bulk RNA-seq,Homo_633,AD.vs.MCI	7
BP	GO:0002089	lens morphogenesis in camera-type eye	bulk RNA-seq,Homo_723,AD.vs.control; bulk RNA-seq,Homo_723,AD.vs.MCI; bulk RNA-seq,Homo_723,MCI.vs.control; bulk RNA-seq,Homo_714,AD.vs.MCI	4
BP	GO:0007190	activation of adenylate cyclase activity	bulk RNA-seq,Homo_723,AD.vs.control; bulk RNA-seq,Homo_723,AD.vs.MCI; bulk RNA-seq,Homo_714,AD.vs.control; bulk RNA-seq,Homo_714,AD.vs.MCI	4
BP	GO:0045987	positive regulation of smooth muscle contraction	bulk RNA-seq,Homo_723,AD.vs.control; bulk RNA-seq,Homo_723,AD.vs.MCI; bulk RNA-seq,Homo_723,MCI.vs.control; bulk RNA-seq,Homo_714,AD.vs.control; bulk RNA-seq,Homo_714,AD.vs.MCI	5
BP	GO:0048339	paraxial mesoderm development	bulk RNA-seq,Homo_723,AD.vs.control; bulk RNA-seq,Homo_723,AD.vs.MCI; bulk RNA-seq,Homo_723,MCI.vs.control; bulk RNA-seq,Homo_714,AD.vs.MCI	4
BP	GO:0030858	positive regulation of epithelial cell differentiation	bulk RNA-seq,Homo_723,AD.vs.control; bulk RNA-seq,Homo_723,AD.vs.MCI; bulk RNA-seq,Homo_723,MCI.vs.control; bulk RNA-seq,Homo_714,AD.vs.MCI	4
BP	GO:0035088	establishment or maintenance of apical/basal cell polarity	bulk RNA-seq,Homo_723,AD.vs.control; bulk RNA-seq,Homo_723,AD.vs.MCI; bulk RNA-seq,Homo_723,MCI.vs.control; bulk RNA-seq,Homo_714,AD.vs.MCI; bulk RNA-seq,Homo_714,MCI.vs.control; bulk RNA-seq,Homo_633,AD.vs.control; bulk RNA-seq,Homo_633,AD.vs.MCI	7
BP	GO:0061245	establishment or maintenance of bipolar cell polarity	bulk RNA-seq,Homo_723,AD.vs.control; bulk RNA-seq,Homo_723,AD.vs.MCI; bulk RNA-seq,Homo_723,MCI.vs.control; bulk RNA-seq,Homo_714,AD.vs.MCI; bulk RNA-seq,Homo_714,MCI.vs.control; bulk RNA-seq,Homo_633,AD.vs.control; bulk RNA-seq,Homo_633,AD.vs.MCI	7
BP	GO:0031577	spindle checkpoint signaling	bulk RNA-seq,Homo_723,AD.vs.control; bulk RNA-seq,Homo_723,MCI.vs.control; bulk RNA-seq,Homo_714,AD.vs.control; bulk RNA-seq,Homo_714,MCI.vs.control; bulk RNA-seq,Homo_633,AD.vs.control; bulk RNA-seq,Homo_633,AD.vs.MCI	6
BP	GO:0014897	striated muscle hypertrophy	bulk RNA-seq,Homo_723,AD.vs.control; bulk RNA-seq,Homo_723,AD.vs.MCI; bulk RNA-seq,Homo_723,MCI.vs.control; bulk RNA-seq,Homo_714,AD.vs.control; bulk RNA-seq,Homo_714,AD.vs.MCI; bulk RNA-seq,Homo_714,MCI.vs.control; bulk RNA-seq,Homo_633,AD.vs.control	7
BP	GO:0032963	collagen metabolic process	bulk RNA-seq,Homo_723,AD.vs.control; bulk RNA-seq,Homo_723,AD.vs.MCI; bulk RNA-seq,Homo_723,MCI.vs.control; bulk RNA-seq,Homo_714,AD.vs.control; bulk RNA-seq,Homo_714,AD.vs.MCI; bulk RNA-seq,Homo_714,MCI.vs.control; bulk RNA-seq,Homo_633,AD.vs.control; bulk	8
BP	GO:0099171	presynaptic modulation of chemical synaptic transmission	bulk RNA-seq,Homo_723,AD.vs.control; bulk RNA-seq,Homo_723,AD.vs.MCI; bulk RNA-seq,Homo_723,MCI.vs.control	3
CC	GO:0097730	non-motile cilium	bulk RNA-seq,Homo_723,AD.vs.control; bulk RNA-seq,Homo_723,AD.vs.MCI; bulk RNA-seq,Homo_723,MCI.vs.control; bulk RNA-seq,Homo_714,AD.vs.control; bulk RNA-seq,Homo_714,AD.vs.MCI; bulk RNA-seq,Homo_633,AD.vs.control; bulk RNA-seq,Homo_633,AD.vs.MCI	7
MF	GO:0034062	5'-3' RNA polymerase activity	bulk RNA-seq,Homo_723,AD.vs.control; bulk RNA-seq,Homo_714,AD.vs.control; bulk RNA-seq,Homo_633,AD.vs.control; bulk RNA-seq,Homo_633,AD.vs.MCI; bulk RNA-seq,Homo_633,MCI.vs.control	5
MF	GO:0097747	RNA polymerase activity	bulk RNA-seq,Homo_723,AD.vs.control; bulk RNA-seq,Homo_714,AD.vs.control; bulk RNA-seq,Homo_633,AD.vs.control; bulk RNA-seq,Homo_633,AD.vs.MCI; bulk RNA-seq,Homo_633,MCI.vs.control	5
BP	GO:0002828	regulation of type 2 immune response	bulk RNA-seq,Homo_723,AD.vs.control; bulk RNA-seq,Homo_723,MCI.vs.control; bulk RNA-seq,Homo_714,MCI.vs.control; bulk RNA-seq,Homo_633,AD.vs.control; bulk RNA-seq,Homo_633,AD.vs.MCI; bulk RNA-seq,Homo_633,MCI.vs.control	6
BP	GO:0007094	mitotic spindle assembly checkpoint signaling	bulk RNA-seq,Homo_723,AD.vs.control; bulk RNA-seq,Homo_723,MCI.vs.control; bulk RNA-seq,Homo_714,AD.vs.control; bulk RNA-seq,Homo_714,MCI.vs.control; bulk RNA-seq,Homo_633,AD.vs.control; bulk RNA-seq,Homo_633,AD.vs.MCI	6
BP	GO:0071173	spindle assembly checkpoint signaling	bulk RNA-seq,Homo_723,AD.vs.control; bulk RNA-seq,Homo_723,MCI.vs.control; bulk RNA-seq,Homo_714,AD.vs.control; bulk RNA-seq,Homo_714,MCI.vs.control; bulk RNA-seq,Homo_633,AD.vs.control; bulk RNA-seq,Homo_633,AD.vs.MCI	6
BP	GO:0071174	mitotic spindle checkpoint signaling	bulk RNA-seq,Homo_723,AD.vs.control; bulk RNA-seq,Homo_723,MCI.vs.control; bulk RNA-seq,Homo_714,AD.vs.control; bulk RNA-seq,Homo_714,MCI.vs.control; bulk RNA-seq,Homo_633,AD.vs.control; bulk RNA-seq,Homo_633,AD.vs.MCI	6
BP	GO:0051385	response to mineralocorticoid	bulk RNA-seq,Homo_723,AD.vs.control; bulk RNA-seq,Homo_723,AD.vs.MCI; bulk RNA-seq,Homo_723,MCI.vs.control; bulk RNA-seq,Homo_714,AD.vs.control; bulk RNA-seq,Homo_714,AD.vs.MCI; bulk RNA-seq,Homo_714,MCI.vs.control	6
BP	GO:0002830	positive regulation of type 2 immune response	bulk RNA-seq,Homo_723,AD.vs.control; bulk RNA-seq,Homo_723,MCI.vs.control; bulk RNA-seq,Homo_714,MCI.vs.control; bulk RNA-seq,Homo_633,AD.vs.control; bulk RNA-seq,Homo_633,MCI.vs.control	5
BP	GO:0048291	isotype switching to IgG isotypes	bulk RNA-seq,Homo_723,AD.vs.control; bulk RNA-seq,Homo_723,MCI.vs.control; bulk RNA-seq,Homo_714,MCI.vs.control; bulk RNA-seq,Homo_633,AD.vs.control; bulk RNA-seq,Homo_633,AD.vs.MCI; bulk RNA-seq,Homo_633,MCI.vs.control	6
CC	GO:0044305	calyx of Held	bulk RNA-seq,Homo_723,AD.vs.control; bulk RNA-seq,Homo_723,AD.vs.MCI; bulk RNA-seq,Homo_723,MCI.vs.control; bulk RNA-seq,Homo_714,AD.vs.MCI	4
BP	GO:0006304	DNA modification	bulk RNA-seq,Homo_723,AD.vs.control; bulk RNA-seq,Homo_723,MCI.vs.control; bulk RNA-seq,Homo_714,AD.vs.control; bulk RNA-seq,Homo_714,MCI.vs.control; bulk RNA-seq,Homo_633,AD.vs.control; bulk RNA-seq,Homo_633,AD.vs.MCI	6

BP	GO:0140289	protein mono-ADP-ribosylation	bulk RNA-seq,Homo_723,AD.vs.control; bulk RNA-seq,Homo_723,MCI.vs.control; bulk RNA-seq,Homo_714,AD.vs.control; bulk RNA-seq,Homo_714,MCI.vs.control; bulk RNA-seq,Homo_633,AD.vs.control; bulk RNA-seq,Homo_633,MCI.vs.control	6
BP	GO:0006285	base-excision repair, AP site formation	bulk RNA-seq,Homo_723,AD.vs.control; bulk RNA-seq,Homo_723,AD.vs.MCI; bulk RNA-seq,Homo_714,AD.vs.control; bulk RNA-seq,Homo_633,AD.vs.control; bulk RNA-seq,Homo_633,AD.vs.MCI	5
BP	GO:0044784	metaphase/anaphase transition of cell cycle	bulk RNA-seq,Homo_723,AD.vs.control; bulk RNA-seq,Homo_723,MCI.vs.control; bulk RNA-seq,Homo_714,AD.vs.control; bulk RNA-seq,Homo_714,MCI.vs.control; bulk RNA-seq,Homo_633,AD.vs.control; bulk RNA-seq,Homo_633,AD.vs.MCI	6
BP	GO:0009235	cobalamin metabolic process	bulk RNA-seq,Homo_723,AD.vs.control; bulk RNA-seq,Homo_723,AD.vs.MCI; bulk RNA-seq,Homo_714,AD.vs.control; bulk RNA-seq,Homo_633,AD.vs.control	4
BP	GO:0061709	reticulophagy	bulk RNA-seq,Homo_723,AD.vs.control; bulk RNA-seq,Homo_714,AD.vs.control; bulk RNA-seq,Homo_633,AD.vs.control; bulk RNA-seq,Homo_633,AD.vs.MCI	4
BP	GO:0006479	protein methylation	bulk RNA-seq,Homo_723,AD.vs.control; bulk RNA-seq,Homo_723,MCI.vs.control; bulk RNA-seq,Homo_714,AD.vs.control; bulk RNA-seq,Homo_633,AD.vs.control; bulk RNA-seq,Homo_633,AD.vs.MCI	5
BP	GO:0008213	protein alkylation	bulk RNA-seq,Homo_723,AD.vs.control; bulk RNA-seq,Homo_723,MCI.vs.control; bulk RNA-seq,Homo_714,AD.vs.control; bulk RNA-seq,Homo_633,AD.vs.control; bulk RNA-seq,Homo_633,AD.vs.MCI	5
BP	GO:0030010	establishment of cell polarity	bulk RNA-seq,Homo_723,AD.vs.control; bulk RNA-seq,Homo_723,AD.vs.MCI; bulk RNA-seq,Homo_723,MCI.vs.control; bulk RNA-seq,Homo_714,AD.vs.MCI; bulk RNA-seq,Homo_633,AD.vs.control; bulk RNA-seq,Homo_633,AD.vs.MCI	6
BP	GO:0097094	craniofacial suture morphogenesis	bulk RNA-seq,Homo_723,AD.vs.control; bulk RNA-seq,Homo_723,AD.vs.MCI; bulk RNA-seq,Homo_723,MCI.vs.control; bulk RNA-seq,Homo_714,AD.vs.MCI	4
BP	GO:1902774	late endosome to lysosome transport	bulk RNA-seq,Homo_723,AD.vs.control; bulk RNA-seq,Homo_723,MCI.vs.control; bulk RNA-seq,Homo_714,AD.vs.control; bulk RNA-seq,Homo_714,MCI.vs.control; bulk RNA-seq,Homo_633,AD.vs.control; bulk RNA-seq,Homo_633,AD.vs.MCI; bulk RNA-seq,Homo_633,MCI.vs.control	7
BP	GO:0071560	cellular response to transforming growth factor beta stimulus	bulk RNA-seq,Homo_723,AD.vs.control; bulk RNA-seq,Homo_723,AD.vs.MCI; bulk RNA-seq,Homo_723,MCI.vs.control; bulk RNA-seq,Homo_714,AD.vs.MCI; bulk RNA-seq,Homo_633,AD.vs.control; bulk RNA-seq,Homo_633,AD.vs.MCI; scRNA-seq,SRP330776,CD8+ T cell_2-AD.vs.control; scRNA-seq,SRP330776,Naive CD8+ T cell_1-AD.vs.control; scRNA-seq,SRP330776,Naive CD8+ T cell_2-AD.vs.control; scRNA-seq,SRP330776,Natural killer cell_1-	10
MF	GO:0035197	siRNA binding	bulk RNA-seq,Homo_723,AD.vs.control; bulk RNA-seq,Homo_723,AD.vs.MCI; bulk RNA-seq,Homo_714,AD.vs.control; bulk RNA-seq,Homo_633,AD.vs.control; bulk RNA-seq,Homo_633,AD.vs.MCI	5
BP	GO:0043252	sodium-independent organic anion transport	bulk RNA-seq,Homo_723,AD.vs.control; bulk RNA-seq,Homo_723,AD.vs.MCI; bulk RNA-seq,Homo_714,AD.vs.control; bulk RNA-seq,Homo_714,AD.vs.MCI	4
BP	GO:0055081	anion homeostasis	bulk RNA-seq,Homo_723,AD.vs.control; bulk RNA-seq,Homo_723,AD.vs.MCI; bulk RNA-seq,Homo_723,MCI.vs.control; bulk RNA-seq,Homo_714,AD.vs.MCI	4
BP	GO:1904035	regulation of epithelial cell apoptotic process	bulk RNA-seq,Homo_723,AD.vs.control; bulk RNA-seq,Homo_723,AD.vs.MCI; bulk RNA-seq,Homo_723,MCI.vs.control; bulk RNA-seq,Homo_714,AD.vs.control; bulk RNA-seq,Homo_714,AD.vs.MCI; bulk RNA-seq,Homo_714,MCI.vs.control; bulk RNA-seq,Homo_633,AD.vs.control	7
BP	GO:0098876	vesicle-mediated transport to the plasma membrane	bulk RNA-seq,Homo_723,AD.vs.control; bulk RNA-seq,Homo_723,MCI.vs.control; bulk RNA-seq,Homo_714,AD.vs.control; bulk RNA-seq,Homo_633,AD.vs.control; bulk RNA-seq,Homo_633,AD.vs.MCI; scRNA-seq,SRP330776,Naive CD8+ T cell_2-AD.vs.control	6
MF	GO:0003899	DNA-directed 5'-3' RNA polymerase activity	bulk RNA-seq,Homo_723,AD.vs.control; bulk RNA-seq,Homo_714,AD.vs.control; bulk RNA-seq,Homo_633,AD.vs.control; bulk RNA-seq,Homo_633,AD.vs.MCI	4
BP	GO:0034101	erythrocyte homeostasis	bulk RNA-seq,Homo_723,AD.vs.control; bulk RNA-seq,Homo_723,MCI.vs.control; bulk RNA-seq,Homo_714,AD.vs.control; bulk RNA-seq,Homo_633,AD.vs.control; bulk RNA-seq,Homo_633,AD.vs.MCI	5
BP	GO:0002323	natural killer cell activation involved in immune response	bulk RNA-seq,Homo_723,AD.vs.control; bulk RNA-seq,Homo_714,AD.vs.control; bulk RNA-seq,ROSMAP,AD.vs.control; bulk RNA-	4
BP	GO:0048384	retinoic acid receptor signaling pathway	bulk RNA-seq,Homo_723,AD.vs.control; bulk RNA-seq,Homo_723,AD.vs.MCI; bulk RNA-seq,Homo_723,MCI.vs.control; bulk RNA-seq,Homo_714,AD.vs.MCI; bulk RNA-seq,Homo_633,AD.vs.control	5
BP	GO:0140058	neuron projection arborization	bulk RNA-seq,Homo_723,AD.vs.control; bulk RNA-seq,Homo_723,AD.vs.MCI; bulk RNA-seq,Homo_723,MCI.vs.control; bulk RNA-seq,Homo_633,AD.vs.control; bulk RNA-seq,Homo_633,AD.vs.MCI	5
CC	GO:0000781	chromosome, telomeric region	bulk RNA-seq,Homo_723,AD.vs.control; bulk RNA-seq,Homo_723,MCI.vs.control; bulk RNA-seq,Homo_714,AD.vs.control; bulk RNA-seq,Homo_633,AD.vs.control; bulk RNA-seq,Homo_633,AD.vs.MCI	5
BP	GO:0001782	B cell homeostasis	bulk RNA-seq,Homo_723,AD.vs.control; bulk RNA-seq,Homo_723,MCI.vs.control; bulk RNA-seq,Homo_714,AD.vs.control; bulk RNA-seq,Homo_714,MCI.vs.control; bulk RNA-seq,Homo_633,AD.vs.control; bulk RNA-seq,Homo_633,AD.vs.MCI; bulk RNA-seq,Homo_633,MCI.vs.control	7
BP	GO:0045777	positive regulation of blood pressure	bulk RNA-seq,Homo_723,AD.vs.control; bulk RNA-seq,Homo_723,AD.vs.MCI; bulk RNA-seq,Homo_723,MCI.vs.control; bulk RNA-seq,Homo_714,AD.vs.control; bulk RNA-seq,Homo_714,AD.vs.MCI	5
BP	GO:2001212	regulation of vasculogenesis	bulk RNA-seq,Homo_723,AD.vs.control; bulk RNA-seq,Homo_723,AD.vs.MCI; bulk RNA-seq,Homo_723,MCI.vs.control; bulk RNA-seq,Homo_714,AD.vs.control; bulk RNA-seq,Homo_714,AD.vs.MCI; bulk RNA-seq,Homo_714,MCI.vs.control	6
CC	GO:0002116	semaphorin receptor complex	bulk RNA-seq,Homo_723,AD.vs.control; bulk RNA-seq,Homo_723,AD.vs.MCI; bulk RNA-seq,Homo_714,AD.vs.MCI; bulk RNA-seq,Homo_633,AD.vs.control; bulk RNA-seq,Homo_633,AD.vs.MCI	5
BP	GO:0003309	type B pancreatic cell differentiation	bulk RNA-seq,Homo_723,AD.vs.control; bulk RNA-seq,Homo_723,AD.vs.MCI; bulk RNA-seq,Homo_723,MCI.vs.control; bulk RNA-seq,Homo_714,AD.vs.MCI	4
MF	GO:0005313	L-glutamate transmembrane transporter activity	bulk RNA-seq,Homo_723,AD.vs.control; bulk RNA-seq,Homo_723,AD.vs.MCI; bulk RNA-seq,Homo_723,MCI.vs.control; bulk RNA-seq,Homo_714,AD.vs.MCI	4
BP	GO:0097479	synaptic vesicle localization	bulk RNA-seq,Homo_723,AD.vs.control; bulk RNA-seq,Homo_723,AD.vs.MCI; bulk RNA-seq,Homo_723,MCI.vs.control; bulk RNA-seq,Homo_714,MCI.vs.control; bulk RNA-seq,Homo_633,AD.vs.control; bulk RNA-seq,Homo_633,AD.vs.MCI	6
BP	GO:0060572	morphogenesis of an epithelial bud	bulk RNA-seq,Homo_723,AD.vs.control; bulk RNA-seq,Homo_723,AD.vs.MCI; bulk RNA-seq,Homo_723,MCI.vs.control; bulk RNA-seq,Homo_714,AD.vs.MCI; bulk RNA-seq,Homo_714,MCI.vs.control	5
BP	GO:0098962	regulation of postsynaptic neurotransmitter receptor activity	bulk RNA-seq,Homo_723,AD.vs.control; bulk RNA-seq,Homo_723,AD.vs.MCI; bulk RNA-seq,Homo_723,MCI.vs.control	3
BP	GO:0048302	regulation of isotype switching to IgG isotypes	bulk RNA-seq,Homo_723,AD.vs.control; bulk RNA-seq,Homo_723,MCI.vs.control; bulk RNA-seq,Homo_714,MCI.vs.control; bulk RNA-seq,Homo_633,AD.vs.control; bulk RNA-seq,Homo_633,AD.vs.MCI; bulk RNA-seq,Homo_633,MCI.vs.control	6
BP	GO:0043589	skin morphogenesis	bulk RNA-seq,Homo_723,AD.vs.control; bulk RNA-seq,Homo_723,AD.vs.MCI; bulk RNA-seq,Homo_723,MCI.vs.control; bulk RNA-seq,Homo_714,AD.vs.control; bulk RNA-seq,Homo_714,AD.vs.MCI; bulk RNA-seq,Homo_714,MCI.vs.control	6
BP	GO:0031440	regulation of mRNA 3'-end processing	bulk RNA-seq,Homo_723,AD.vs.control; bulk RNA-seq,Homo_723,MCI.vs.control; bulk RNA-seq,Homo_714,AD.vs.control; bulk RNA-seq,Homo_714,MCI.vs.control; bulk RNA-seq,Homo_633,AD.vs.control; bulk RNA-seq,Homo_633,AD.vs.MCI; bulk RNA-seq,Homo_633,MCI.vs.control	7
BP	GO:0043650	dicarboxylic acid biosynthetic process	bulk RNA-seq,Homo_723,AD.vs.control; bulk RNA-seq,Homo_633,AD.vs.control	2
MF	GO:0031435	mitogen-activated protein kinase kinase kinase binding	bulk RNA-seq,Homo_723,AD.vs.control; bulk RNA-seq,Homo_723,AD.vs.MCI; bulk RNA-seq,Homo_723,MCI.vs.control; bulk RNA-seq,Homo_714,AD.vs.MCI; bulk RNA-seq,Homo_714,MCI.vs.control	5
BP	GO:0021544	subpallium development	bulk RNA-seq,Homo_723,AD.vs.control; bulk RNA-seq,Homo_723,AD.vs.MCI; bulk RNA-seq,Homo_723,MCI.vs.control; bulk RNA-seq,Homo_714,AD.vs.MCI; bulk RNA-seq,Homo_714,MCI.vs.control	5

CC	GO:0005776	autophagosome	bulk RNA-seq,Homo_723,AD.vs.control; bulk RNA-seq,Homo_723,MCI.vs.control; bulk RNA-seq,Homo_714,AD.vs.control; bulk RNA-seq,Homo_714,MCI.vs.control; bulk RNA-seq,Homo_633,AD.vs.control; bulk RNA-seq,Homo_633,AD.vs.MCI	6
CC	GO:0120114	Sm-like protein family complex	bulk RNA-seq,Homo_723,AD.vs.control; bulk RNA-seq,Homo_714,AD.vs.control; bulk RNA-seq,Homo_714,MCI.vs.control; bulk RNA-seq,Homo_633,AD.vs.control; bulk RNA-seq,Homo_633,AD.vs.MCI	5
CC	GO:0005744	TIM23 mitochondrial import inner membrane translocase complex	bulk RNA-seq,Homo_723,AD.vs.control; bulk RNA-seq,Homo_723,MCI.vs.control; bulk RNA-seq,Homo_714,AD.vs.control; bulk RNA-seq,Homo_714,MCI.vs.control; bulk RNA-seq,Homo_633,AD.vs.control; bulk RNA-seq,Homo_633,AD.vs.MCI; bulk RNA-seq,Homo_633,MCI.vs.control	7
BP	GO:0048857	neural nucleus development	bulk RNA-seq,Homo_723,AD.vs.control; bulk RNA-seq,Homo_723,AD.vs.MCI; bulk RNA-seq,Homo_723,MCI.vs.control; bulk RNA-seq,Homo_714,AD.vs.control; bulk RNA-seq,Homo_714,AD.vs.MCI; bulk RNA-seq,Homo_714,MCI.vs.control; bulk RNA-seq,Homo_633,AD.vs.control; bulk	8
BP	GO:0032211	negative regulation of telomere maintenance via telomerase	bulk RNA-seq,Homo_723,AD.vs.control; bulk RNA-seq,Homo_723,MCI.vs.control; bulk RNA-seq,Homo_714,AD.vs.control; bulk RNA-seq,Homo_633,AD.vs.control; bulk RNA-seq,Homo_633,AD.vs.MCI	5
BP	GO:0021562	vestibulocochlear nerve development	bulk RNA-seq,Homo_723,AD.vs.control; bulk RNA-seq,Homo_723,AD.vs.MCI; bulk RNA-seq,Homo_723,MCI.vs.control; bulk RNA-seq,Homo_714,AD.vs.control; bulk RNA-seq,Homo_714,AD.vs.MCI	5
BP	GO:1902667	regulation of axon guidance	bulk RNA-seq,Homo_723,AD.vs.control; bulk RNA-seq,Homo_723,AD.vs.MCI; bulk RNA-seq,Homo_723,MCI.vs.control; bulk RNA-seq,Homo_714,AD.vs.MCI	4
BP	GO:0001938	positive regulation of endothelial cell proliferation	bulk RNA-seq,Homo_723,AD.vs.control; bulk RNA-seq,Homo_723,AD.vs.MCI; bulk RNA-seq,Homo_723,MCI.vs.control; bulk RNA-seq,Homo_714,AD.vs.MCI; bulk RNA-seq,Homo_714,MCI.vs.control; bulk RNA-seq,Homo_633,AD.vs.control	6
MF	GO:0016854	racemase and epimerase activity	bulk RNA-seq,Homo_723,AD.vs.control; bulk RNA-seq,Homo_714,AD.vs.control; bulk RNA-seq,Homo_633,AD.vs.control; bulk RNA-seq,Homo_633,AD.vs.MCI	4
BP	GO:0014910	regulation of smooth muscle cell migration	bulk RNA-seq,Homo_723,AD.vs.control; bulk RNA-seq,Homo_723,AD.vs.MCI; bulk RNA-seq,Homo_723,MCI.vs.control; bulk RNA-seq,Homo_714,AD.vs.control; bulk RNA-seq,Homo_714,AD.vs.MCI; bulk RNA-seq,Homo_714,MCI.vs.control	6
CC	GO:0099738	cell cortex region	bulk RNA-seq,Homo_723,AD.vs.control; bulk RNA-seq,Homo_723,AD.vs.MCI; bulk RNA-seq,Homo_723,MCI.vs.control; bulk RNA-seq,Homo_714,AD.vs.MCI; bulk RNA-seq,Homo_714,MCI.vs.control; bulk RNA-seq,Homo_633,AD.vs.control; bulk RNA-seq,Homo_633,AD.vs.MCI	7
BP	GO:0030220	platelet formation	bulk RNA-seq,Homo_723,AD.vs.control; bulk RNA-seq,Homo_723,MCI.vs.control; bulk RNA-seq,Homo_714,AD.vs.control; bulk RNA-seq,Homo_714,MCI.vs.control; bulk RNA-seq,Homo_633,AD.vs.control; bulk RNA-seq,Homo_633,AD.vs.MCI; bulk RNA-seq,Homo_633,MCI.vs.control	7
BP	GO:0086010	membrane depolarization during action potential	bulk RNA-seq,Homo_723,AD.vs.control; bulk RNA-seq,Homo_723,AD.vs.MCI; bulk RNA-seq,Homo_723,MCI.vs.control; bulk RNA-seq,Homo_714,AD.vs.MCI; bulk RNA-seq,ROSMAP,AD.vs.control; bulk RNA-seq,SRP223445,AD.vs.control	6
BP	GO:0006890	retrograde vesicle-mediated transport, Golgi to endoplasmic reticulum	bulk RNA-seq,Homo_723,AD.vs.control; bulk RNA-seq,Homo_723,MCI.vs.control; bulk RNA-seq,Homo_714,AD.vs.control; bulk RNA-seq,Homo_714,MCI.vs.control; bulk RNA-seq,Homo_633,AD.vs.control; bulk RNA-seq,Homo_633,AD.vs.MCI	6
BP	GO:0045616	regulation of keratinocyte differentiation	bulk RNA-seq,Homo_723,AD.vs.control; bulk RNA-seq,Homo_723,AD.vs.MCI; bulk RNA-seq,Homo_723,MCI.vs.control; bulk RNA-seq,Homo_714,AD.vs.control; bulk RNA-seq,Homo_714,AD.vs.MCI; bulk RNA-seq,Homo_714,MCI.vs.control	6
MF	GO:0045295	gamma-catenin binding	bulk RNA-seq,Homo_723,AD.vs.control; bulk RNA-seq,Homo_723,AD.vs.MCI; bulk RNA-seq,Homo_723,MCI.vs.control	3
BP	GO:0032147	activation of protein kinase activity	bulk RNA-seq,Homo_723,AD.vs.control; bulk RNA-seq,Homo_723,AD.vs.MCI; bulk RNA-seq,Homo_723,MCI.vs.control; bulk RNA-seq,Homo_714,AD.vs.MCI; bulk RNA-seq,Homo_633,AD.vs.control; bulk RNA-seq,Homo_633,AD.vs.MCI	6
BP	GO:0098901	regulation of cardiac muscle cell action potential	bulk RNA-seq,Homo_723,AD.vs.control; bulk RNA-seq,Homo_723,AD.vs.MCI; bulk RNA-seq,Homo_723,MCI.vs.control; bulk RNA-seq,Homo_714,AD.vs.MCI; bulk RNA-seq,Homo_714,MCI.vs.control	5
BP	GO:0010919	regulation of inositol phosphate biosynthetic process	bulk RNA-seq,Homo_723,AD.vs.control	1
MF	GO:0070016	armadillo repeat domain binding	bulk RNA-seq,Homo_723,AD.vs.control; bulk RNA-seq,Homo_723,AD.vs.MCI; bulk RNA-seq,Homo_723,MCI.vs.control; bulk RNA-seq,Homo_714,MCI.vs.control; bulk RNA-seq,Homo_633,MCI.vs.control	5
BP	GO:0032231	regulation of actin filament bundle assembly	bulk RNA-seq,Homo_723,AD.vs.control; bulk RNA-seq,Homo_723,AD.vs.MCI; bulk RNA-seq,Homo_723,MCI.vs.control; bulk RNA-seq,Homo_714,AD.vs.MCI; bulk RNA-seq,Homo_714,MCI.vs.control; bulk RNA-seq,Homo_633,AD.vs.control; bulk RNA-seq,Homo_633,AD.vs.MCI	7
BP	GO:0099633	protein localization to postsynaptic specialization membrane	bulk RNA-seq,Homo_723,AD.vs.control; bulk RNA-seq,Homo_723,AD.vs.MCI; bulk RNA-seq,Homo_723,MCI.vs.control; bulk RNA-seq,Homo_714,AD.vs.MCI	4
BP	GO:0099645	neurotransmitter receptor localization to postsynaptic specialization membrane	bulk RNA-seq,Homo_723,AD.vs.control; bulk RNA-seq,Homo_723,AD.vs.MCI; bulk RNA-seq,Homo_723,MCI.vs.control; bulk RNA-seq,Homo_714,AD.vs.MCI	4
MF	GO:0051287	NAD binding	bulk RNA-seq,Homo_723,AD.vs.control; bulk RNA-seq,Homo_723,MCI.vs.control; bulk RNA-seq,Homo_714,AD.vs.control; bulk RNA-seq,Homo_714,MCI.vs.control; bulk RNA-seq,Homo_633,AD.vs.control; bulk RNA-seq,Homo_633,AD.vs.MCI	6
MF	GO:0016530	metallochaperone activity	bulk RNA-seq,Homo_723,AD.vs.control; bulk RNA-seq,Homo_714,AD.vs.control; bulk RNA-seq,Homo_633,AD.vs.control; bulk RNA-seq,Homo_633,AD.vs.MCI	4
BP	GO:0031641	regulation of myelination	bulk RNA-seq,Homo_723,AD.vs.control; bulk RNA-seq,Homo_723,AD.vs.MCI; bulk RNA-seq,Homo_723,MCI.vs.control; bulk RNA-seq,Homo_714,AD.vs.MCI; bulk RNA-seq,Homo_714,MCI.vs.control; bulk RNA-seq,Homo_633,AD.vs.control	6
BP	GO:0016081	synaptic vesicle docking	bulk RNA-seq,Homo_723,AD.vs.control; bulk RNA-seq,Homo_723,AD.vs.MCI; bulk RNA-seq,Homo_723,MCI.vs.control; bulk RNA-seq,Homo_714,AD.vs.MCI	4
MF	GO:0051427	hormone receptor binding	bulk RNA-seq,Homo_723,AD.vs.control; bulk RNA-seq,Homo_723,AD.vs.MCI; bulk RNA-seq,Homo_723,MCI.vs.control; bulk RNA-seq,Homo_714,AD.vs.control; bulk RNA-seq,Homo_714,AD.vs.MCI	5
BP	GO:0043368	positive T cell selection	bulk RNA-seq,Homo_723,AD.vs.control; bulk RNA-seq,Homo_714,AD.vs.control; bulk RNA-seq,Homo_714,MCI.vs.control; bulk RNA-	4
BP	GO:0016073	snRNA metabolic process	bulk RNA-seq,Homo_723,AD.vs.control; bulk RNA-seq,Homo_723,AD.vs.MCI; bulk RNA-seq,Homo_633,AD.vs.control; bulk RNA-seq,Homo_633,AD.vs.MCI	4
BP	GO:0006544	glycine metabolic process	bulk RNA-seq,Homo_723,AD.vs.control; bulk RNA-seq,Homo_723,AD.vs.MCI; bulk RNA-seq,Homo_723,MCI.vs.control; bulk RNA-seq,Homo_714,AD.vs.control; bulk RNA-seq,Homo_714,AD.vs.MCI; bulk RNA-seq,Homo_714,MCI.vs.control	6
BP	GO:0098712	L-glutamate import across plasma membrane	bulk RNA-seq,Homo_723,AD.vs.control; bulk RNA-seq,Homo_723,AD.vs.MCI; bulk RNA-seq,Homo_714,AD.vs.MCI	3
BP	GO:0007033	vacuole organization	bulk RNA-seq,Homo_723,AD.vs.control; bulk RNA-seq,Homo_723,MCI.vs.control; bulk RNA-seq,Homo_714,AD.vs.control; bulk RNA-seq,Homo_633,AD.vs.control; bulk RNA-seq,Homo_633,AD.vs.MCI	5
BP	GO:0042491	inner ear auditory receptor cell differentiation	bulk RNA-seq,Homo_723,AD.vs.control; bulk RNA-seq,Homo_723,AD.vs.MCI; bulk RNA-seq,Homo_723,MCI.vs.control; bulk RNA-seq,Homo_714,AD.vs.MCI; bulk RNA-seq,Homo_714,MCI.vs.control	5
BP	GO:0030540	female genitalia development	bulk RNA-seq,Homo_723,AD.vs.control; bulk RNA-seq,Homo_723,AD.vs.MCI; bulk RNA-seq,Homo_723,MCI.vs.control; bulk RNA-seq,Homo_714,AD.vs.MCI; bulk RNA-seq,Homo_714,MCI.vs.control	5
BP	GO:0019082	viral protein processing	bulk RNA-seq,Homo_723,AD.vs.control; bulk RNA-seq,Homo_723,MCI.vs.control; bulk RNA-seq,Homo_714,AD.vs.control; bulk RNA-seq,Homo_714,MCI.vs.control; bulk RNA-seq,Homo_633,AD.vs.control; bulk RNA-seq,Homo_633,AD.vs.MCI; bulk RNA-seq,Homo_633,MCI.vs.control	7
BP	GO:0044070	regulation of anion transport	bulk RNA-seq,Homo_723,AD.vs.control; bulk RNA-seq,Homo_723,AD.vs.MCI; bulk RNA-seq,Homo_723,MCI.vs.control; bulk RNA-seq,Homo_714,AD.vs.MCI; bulk RNA-seq,Homo_633,AD.vs.control	5

BP	GO:0043616	keratinocyte proliferation	bulk RNA-seq,Homo_723,AD.vs.control; bulk RNA-seq,Homo_723,AD.vs.MCI; bulk RNA-seq,Homo_723,MCI.vs.control; bulk RNA-seq,Homo_714,AD.vs.control; bulk RNA-seq,Homo_714,AD.vs.MCI; bulk RNA-seq,Homo_714,MCI.vs.control; bulk RNA-seq,Homo_633,AD.vs.control; bulk	8
CC	GO:0044665	MLL1/2 complex	bulk RNA-seq,Homo_723,AD.vs.control; bulk RNA-seq,Homo_723,MCI.vs.control; bulk RNA-seq,Homo_714,AD.vs.control; bulk RNA-seq,Homo_633,AD.vs.control; bulk RNA-seq,Homo_633,AD.vs.MCI	5
MF	GO:0008656	cysteine-type endopeptidase activator activity involved in apoptotic process	bulk RNA-seq,Homo_723,AD.vs.control; bulk RNA-seq,Homo_723,MCI.vs.control; bulk RNA-seq,Homo_714,MCI.vs.control; bulk RNA-seq,Homo_633,AD.vs.control; bulk RNA-seq,Homo_633,AD.vs.MCI; bulk RNA-seq,Homo_633,MCI.vs.control	6
BP	GO:0055017	cardiac muscle tissue growth	bulk RNA-seq,Homo_723,AD.vs.control; bulk RNA-seq,Homo_723,AD.vs.MCI; bulk RNA-seq,Homo_723,MCI.vs.control; bulk RNA-seq,Homo_714,AD.vs.MCI; bulk RNA-seq,Homo_714,MCI.vs.control	5
BP	GO:0050810	regulation of steroid biosynthetic process	bulk RNA-seq,Homo_723,AD.vs.control; bulk RNA-seq,Homo_723,AD.vs.MCI; bulk RNA-seq,Homo_723,MCI.vs.control; bulk RNA-seq,Homo_714,AD.vs.control; bulk RNA-seq,Homo_714,AD.vs.MCI; bulk RNA-seq,Homo_714,MCI.vs.control; bulk RNA-seq,Homo_633,AD.vs.control; bulk	8
BP	GO:0050862	positive regulation of T cell receptor signaling pathway	bulk RNA-seq,Homo_723,AD.vs.control; bulk RNA-seq,Homo_723,MCI.vs.control; bulk RNA-seq,Homo_714,MCI.vs.control; bulk RNA-seq,Homo_633,AD.vs.control; bulk RNA-seq,Homo_633,MCI.vs.control	5
BP	GO:0060911	cardiac cell fate commitment	bulk RNA-seq,Homo_723,AD.vs.control; bulk RNA-seq,Homo_723,AD.vs.MCI; bulk RNA-seq,Homo_723,MCI.vs.control; bulk RNA-seq,Homo_714,AD.vs.MCI; bulk RNA-seq,Homo_714,MCI.vs.control	5
BP	GO:0090049	regulation of cell migration involved in sprouting angiogenesis	bulk RNA-seq,Homo_723,AD.vs.control; bulk RNA-seq,Homo_723,AD.vs.MCI; bulk RNA-seq,Homo_723,MCI.vs.control; bulk RNA-seq,Homo_714,AD.vs.control; bulk RNA-seq,Homo_714,AD.vs.MCI; bulk RNA-seq,Homo_714,MCI.vs.control	6
BP	GO:0030218	erythrocyte differentiation	bulk RNA-seq,Homo_723,AD.vs.control; bulk RNA-seq,Homo_723,MCI.vs.control; bulk RNA-seq,Homo_714,AD.vs.control; bulk RNA-seq,Homo_714,MCI.vs.control; bulk RNA-seq,Homo_633,AD.vs.control; bulk RNA-seq,Homo_633,AD.vs.MCI	6
BP	GO:0072173	metanephric tubule morphogenesis	bulk RNA-seq,Homo_723,AD.vs.control; bulk RNA-seq,Homo_723,AD.vs.MCI; bulk RNA-seq,Homo_723,MCI.vs.control; bulk RNA-seq,Homo_714,AD.vs.MCI; bulk RNA-seq,Homo_714,MCI.vs.control	5
BP	GO:0015718	monocarboxylic acid transport	bulk RNA-seq,Homo_723,AD.vs.control; bulk RNA-seq,Homo_723,AD.vs.MCI; bulk RNA-seq,Homo_723,MCI.vs.control; bulk RNA-seq,Homo_714,AD.vs.control; bulk RNA-seq,Homo_714,AD.vs.MCI; bulk RNA-seq,Homo_714,MCI.vs.control; bulk RNA-seq,Homo_633,AD.vs.control	7
CC	GO:0005758	mitochondrial intermembrane space	bulk RNA-seq,Homo_723,AD.vs.control; bulk RNA-seq,Homo_723,MCI.vs.control; bulk RNA-seq,Homo_714,AD.vs.control; bulk RNA-seq,Homo_714,MCI.vs.control; bulk RNA-seq,Homo_633,AD.vs.control; bulk RNA-seq,Homo_633,AD.vs.MCI; scRNA-seq,SRP215507,CD8+ T cell_8-	7
BP	GO:0014848	urinary tract smooth muscle contraction	bulk RNA-seq,Homo_723,AD.vs.control; bulk RNA-seq,Homo_723,AD.vs.MCI; bulk RNA-seq,Homo_723,MCI.vs.control; bulk RNA-seq,Homo_714,AD.vs.MCI	4
BP	GO:0061307	cardiac neural crest cell differentiation involved in heart	bulk RNA-seq,Homo_723,AD.vs.control; bulk RNA-seq,Homo_723,AD.vs.MCI; bulk RNA-seq,Homo_723,MCI.vs.control; bulk RNA-seq,Homo_714,AD.vs.MCI	4
BP	GO:0061308	cardiac neural crest cell development involved in heart	bulk RNA-seq,Homo_723,AD.vs.control; bulk RNA-seq,Homo_723,AD.vs.MCI; bulk RNA-seq,Homo_723,MCI.vs.control; bulk RNA-seq,Homo_714,AD.vs.MCI	4
BP	GO:0006605	protein targeting	bulk RNA-seq,Homo_723,AD.vs.control; bulk RNA-seq,Homo_723,MCI.vs.control; bulk RNA-seq,Homo_714,AD.vs.control; bulk RNA-seq,Homo_633,AD.vs.control; bulk RNA-seq,Homo_633,AD.vs.MCI	5
BP	GO:0001756	somitogenesis	bulk RNA-seq,Homo_723,AD.vs.control; bulk RNA-seq,Homo_723,AD.vs.MCI; bulk RNA-seq,Homo_723,MCI.vs.control; bulk RNA-seq,Homo_714,AD.vs.MCI; bulk RNA-seq,Homo_714,MCI.vs.control; bulk RNA-seq,Homo_633,AD.vs.control; bulk RNA-seq,Homo_633,AD.vs.MCI; bulk RNA-	8
BP	GO:1905383	protein localization to presynapse	bulk RNA-seq,Homo_723,AD.vs.control; bulk RNA-seq,Homo_723,AD.vs.MCI; bulk RNA-seq,Homo_723,MCI.vs.control; bulk RNA-	4
BP	GO:0035745	T-helper 2 cell cytokine production	bulk RNA-seq,Homo_723,AD.vs.control; bulk RNA-seq,Homo_723,MCI.vs.control; bulk RNA-seq,Homo_714,MCI.vs.control	3
BP	GO:2000551	regulation of T-helper 2 cell cytokine production	bulk RNA-seq,Homo_723,AD.vs.control; bulk RNA-seq,Homo_723,MCI.vs.control; bulk RNA-seq,Homo_714,MCI.vs.control	3
BP	GO:0044550	secondary metabolite biosynthetic process	bulk RNA-seq,Homo_723,AD.vs.control; bulk RNA-seq,Homo_723,AD.vs.MCI; bulk RNA-seq,Homo_723,MCI.vs.control; bulk RNA-seq,Homo_633,AD.vs.control; bulk RNA-seq,Homo_633,AD.vs.MCI; scRNA-seq,SRP309935,Monocyte_3-AD.vs.control	6
BP	GO:0042487	regulation of odontogenesis of dentin-containing tooth	bulk RNA-seq,Homo_723,AD.vs.control; bulk RNA-seq,Homo_723,AD.vs.MCI; bulk RNA-seq,Homo_723,MCI.vs.control	3
BP	GO:0003337	mesenchymal to epithelial transition involved in metanephros morphogenesis	bulk RNA-seq,Homo_723,AD.vs.control; bulk RNA-seq,Homo_723,AD.vs.MCI; bulk RNA-seq,Homo_723,MCI.vs.control; bulk RNA-seq,Homo_714,AD.vs.MCI; bulk RNA-seq,Homo_633,MCI.vs.control	5
BP	GO:0042102	positive regulation of T cell proliferation	bulk RNA-seq,Homo_723,AD.vs.control; bulk RNA-seq,Homo_723,MCI.vs.control; bulk RNA-seq,Homo_714,MCI.vs.control; bulk RNA-seq,Homo_633,AD.vs.control; bulk RNA-seq,Homo_633,AD.vs.MCI	5
MF	GO:0045159	myosin II binding	bulk RNA-seq,Homo_723,AD.vs.control; bulk RNA-seq,Homo_723,AD.vs.MCI; bulk RNA-seq,Homo_723,MCI.vs.control; bulk RNA-seq,Homo_714,AD.vs.control; bulk RNA-seq,Homo_714,AD.vs.MCI; bulk RNA-seq,Homo_714,MCI.vs.control	6
BP	GO:0010835	regulation of protein ADP-ribosylation	bulk RNA-seq,Homo_723,AD.vs.control; bulk RNA-seq,Homo_723,MCI.vs.control; bulk RNA-seq,Homo_714,AD.vs.control; bulk RNA-seq,Homo_714,MCI.vs.control; bulk RNA-seq,Homo_633,MCI.vs.control	5
BP	GO:0060900	embryonic camera-type eye formation	bulk RNA-seq,Homo_723,AD.vs.control; bulk RNA-seq,Homo_723,AD.vs.MCI; bulk RNA-seq,Homo_723,MCI.vs.control; bulk RNA-seq,Homo_714,AD.vs.MCI	4
MF	GO:0016248	channel inhibitor activity	bulk RNA-seq,Homo_723,AD.vs.control; bulk RNA-seq,Homo_723,AD.vs.MCI; bulk RNA-seq,Homo_723,MCI.vs.control; bulk RNA-seq,Homo_714,AD.vs.MCI; bulk RNA-seq,Homo_714,MCI.vs.control; bulk RNA-seq,Homo_633,AD.vs.control	6
BP	GO:1902905	positive regulation of supramolecular fiber organization	bulk RNA-seq,Homo_723,AD.vs.control; bulk RNA-seq,Homo_723,AD.vs.MCI; bulk RNA-seq,Homo_723,MCI.vs.control; bulk RNA-seq,Homo_714,AD.vs.MCI; bulk RNA-seq,Homo_633,AD.vs.control; bulk RNA-seq,Homo_633,AD.vs.MCI	6
BP	GO:0043201	response to leucine	bulk RNA-seq,Homo_723,AD.vs.control; bulk RNA-seq,Homo_633,AD.vs.control; bulk RNA-seq,Homo_633,AD.vs.MCI	3
BP	GO:0006625	protein targeting to peroxisome	bulk RNA-seq,Homo_723,AD.vs.control; bulk RNA-seq,Homo_723,MCI.vs.control; bulk RNA-seq,Homo_714,AD.vs.control; bulk RNA-seq,Homo_714,MCI.vs.control; bulk RNA-seq,Homo_633,AD.vs.control; bulk RNA-seq,Homo_633,AD.vs.MCI; bulk RNA-seq,Homo_633,MCI.vs.control	7
BP	GO:0072662	protein localization to peroxisome	bulk RNA-seq,Homo_723,AD.vs.control; bulk RNA-seq,Homo_723,MCI.vs.control; bulk RNA-seq,Homo_714,AD.vs.control; bulk RNA-seq,Homo_714,MCI.vs.control; bulk RNA-seq,Homo_633,AD.vs.control; bulk RNA-seq,Homo_633,AD.vs.MCI; bulk RNA-seq,Homo_633,MCI.vs.control	7
BP	GO:0072663	establishment of protein localization to peroxisome	bulk RNA-seq,Homo_723,AD.vs.control; bulk RNA-seq,Homo_723,MCI.vs.control; bulk RNA-seq,Homo_714,AD.vs.control; bulk RNA-seq,Homo_714,MCI.vs.control; bulk RNA-seq,Homo_633,AD.vs.control; bulk RNA-seq,Homo_633,AD.vs.MCI; bulk RNA-seq,Homo_633,MCI.vs.control	7
BP	GO:0051028	mRNA transport	bulk RNA-seq,Homo_723,AD.vs.control; bulk RNA-seq,Homo_723,MCI.vs.control; bulk RNA-seq,Homo_714,AD.vs.control; bulk RNA-seq,Homo_714,MCI.vs.control; bulk RNA-seq,Homo_633,AD.vs.control; bulk RNA-seq,Homo_633,AD.vs.MCI	6
BP	GO:1903510	mucopolysaccharide metabolic process	bulk RNA-seq,Homo_723,AD.vs.control; bulk RNA-seq,Homo_723,AD.vs.MCI; bulk RNA-seq,Homo_723,MCI.vs.control; bulk RNA-seq,Homo_714,AD.vs.control; bulk RNA-seq,Homo_714,AD.vs.MCI; bulk RNA-seq,Homo_714,MCI.vs.control; bulk RNA-seq,Homo_633,AD.vs.control; bulk	8
MF	GO:0004715	non-membrane spanning protein tyrosine kinase activity	bulk RNA-seq,Homo_723,AD.vs.control; bulk RNA-seq,Homo_723,MCI.vs.control; bulk RNA-seq,Homo_714,MCI.vs.control; bulk RNA-	4
BP	GO:0000209	protein polyubiquitination	bulk RNA-seq,Homo_723,AD.vs.control; bulk RNA-seq,Homo_723,MCI.vs.control; bulk RNA-seq,Homo_633,AD.vs.control; bulk RNA-	4

MF	GO:0022804	active transmembrane transporter activity	bulk RNA-seq,Homo_723,AD.vs.control; bulk RNA-seq,Homo_723,AD.vs.MCI; bulk RNA-seq,Homo_714,AD.vs.control; bulk RNA-seq,Homo_714,AD.vs.MCI; bulk RNA-seq,Homo_633,AD.vs.control; bulk RNA-seq,Homo_633,AD.vs.MCI	6
MF	GO:0004721	phosphoprotein phosphatase activity	bulk RNA-seq,Homo_723,AD.vs.control; bulk RNA-seq,Homo_723,AD.vs.MCI; bulk RNA-seq,Homo_723,MCI.vs.control; bulk RNA-seq,Homo_714,AD.vs.MCI; bulk RNA-seq,Homo_633,AD.vs.control; bulk RNA-seq,Homo_633,AD.vs.MCI; scRNA-seq,SRP330776,CD8+ T cell_1-AD.vs.control; scRNA-seq,SRP330776,Naive CD8+ T cell_1-AD.vs.control	8
CC	GO:1990229	iron-sulfur cluster assembly complex	bulk RNA-seq,Homo_723,AD.vs.control; bulk RNA-seq,Homo_723,MCI.vs.control; bulk RNA-seq,Homo_714,AD.vs.control; bulk RNA-seq,Homo_714,MCI.vs.control; bulk RNA-seq,Homo_633,AD.vs.control; bulk RNA-seq,Homo_633,MCI.vs.control	6
BP	GO:0035112	genitalia morphogenesis	bulk RNA-seq,Homo_723,AD.vs.control; bulk RNA-seq,Homo_723,AD.vs.MCI; bulk RNA-seq,Homo_723,MCI.vs.control; bulk RNA-seq,Homo_714,AD.vs.control; bulk RNA-seq,Homo_714,AD.vs.MCI	5
BP	GO:0002029	desensitization of G protein-coupled receptor signaling	bulk RNA-seq,Homo_723,AD.vs.control; bulk RNA-seq,Homo_723,AD.vs.MCI; bulk RNA-seq,Homo_723,MCI.vs.control	3
BP	GO:0022401	negative adaptation of signaling pathway	bulk RNA-seq,Homo_723,AD.vs.control; bulk RNA-seq,Homo_723,AD.vs.MCI; bulk RNA-seq,Homo_723,MCI.vs.control	3
BP	GO:0001952	regulation of cell-matrix adhesion	bulk RNA-seq,Homo_723,AD.vs.control; bulk RNA-seq,Homo_723,AD.vs.MCI; bulk RNA-seq,Homo_723,MCI.vs.control; bulk RNA-seq,Homo_714,AD.vs.MCI; bulk RNA-seq,Homo_714,MCI.vs.control; bulk RNA-seq,Homo_633,AD.vs.control; bulk RNA-seq,Homo_633,AD.vs.MCI	7
BP	GO:0061014	positive regulation of mRNA catabolic process	bulk RNA-seq,Homo_723,AD.vs.control; bulk RNA-seq,Homo_723,MCI.vs.control; bulk RNA-seq,Homo_714,AD.vs.control; bulk RNA-seq,Homo_714,MCI.vs.control; bulk RNA-seq,Homo_633,AD.vs.control; bulk RNA-seq,Homo_633,AD.vs.MCI	6
CC	GO:0030532	small nuclear ribonucleoprotein complex	bulk RNA-seq,Homo_723,AD.vs.control; bulk RNA-seq,Homo_714,MCI.vs.control; bulk RNA-seq,Homo_633,AD.vs.control; bulk RNA-	4
BP	GO:0010043	response to zinc ion	bulk RNA-seq,Homo_723,AD.vs.control; bulk RNA-seq,Homo_723,AD.vs.MCI; bulk RNA-seq,Homo_714,AD.vs.MCI; bulk RNA-seq,Homo_633,AD.vs.control	4
BP	GO:0042574	retinal metabolic process	bulk RNA-seq,Homo_723,AD.vs.control; bulk RNA-seq,Homo_723,AD.vs.MCI; bulk RNA-seq,Homo_723,MCI.vs.control	3
CC	GO:0098945	intrinsic component of presynaptic active zone membrane	bulk RNA-seq,Homo_723,AD.vs.control; bulk RNA-seq,Homo_723,AD.vs.MCI; bulk RNA-seq,Homo_723,MCI.vs.control; bulk RNA-	4
BP	GO:0051495	positive regulation of cytoskeleton organization	bulk RNA-seq,Homo_723,AD.vs.control; bulk RNA-seq,Homo_723,AD.vs.MCI; bulk RNA-seq,Homo_723,MCI.vs.control; bulk RNA-seq,Homo_714,AD.vs.MCI; bulk RNA-seq,Homo_633,AD.vs.control; bulk RNA-seq,Homo_633,AD.vs.MCI	6
CC	GO:0038201	TOR complex	bulk RNA-seq,Homo_723,AD.vs.control; bulk RNA-seq,Homo_714,MCI.vs.control; bulk RNA-seq,Homo_633,AD.vs.control; bulk RNA-seq,Homo_633,AD.vs.MCI; bulk RNA-seq,Homo_633,MCI.vs.control	5
BP	GO:0071679	commissural neuron axon guidance	bulk RNA-seq,Homo_723,AD.vs.control; bulk RNA-seq,Homo_723,AD.vs.MCI; bulk RNA-seq,Homo_723,MCI.vs.control; bulk RNA-seq,Homo_714,AD.vs.MCI; bulk RNA-seq,Homo_714,MCI.vs.control	5
BP	GO:1904338	regulation of dopaminergic neuron differentiation	bulk RNA-seq,Homo_723,AD.vs.control; bulk RNA-seq,Homo_723,AD.vs.MCI; bulk RNA-seq,Homo_714,AD.vs.MCI; bulk RNA-seq,SRP223445,AD.vs.control	4
MF	GO:0030296	protein tyrosine kinase activator activity	bulk RNA-seq,Homo_723,AD.vs.control; bulk RNA-seq,Homo_723,AD.vs.MCI; bulk RNA-seq,Homo_723,MCI.vs.control; bulk RNA-seq,Homo_714,AD.vs.MCI; bulk RNA-seq,Homo_714,MCI.vs.control	5
CC	GO:0005689	U12-type spliceosomal complex	bulk RNA-seq,Homo_723,AD.vs.control; bulk RNA-seq,Homo_714,AD.vs.control; bulk RNA-seq,Homo_633,AD.vs.control; bulk RNA-seq,Homo_633,AD.vs.MCI	4
BP	GO:0045605	negative regulation of epidermal cell differentiation	bulk RNA-seq,Homo_723,AD.vs.control; bulk RNA-seq,Homo_723,AD.vs.MCI; bulk RNA-seq,Homo_723,MCI.vs.control; bulk RNA-seq,Homo_714,AD.vs.MCI	4
BP	GO:0045683	negative regulation of epidermis development	bulk RNA-seq,Homo_723,AD.vs.control; bulk RNA-seq,Homo_723,AD.vs.MCI; bulk RNA-seq,Homo_723,MCI.vs.control; bulk RNA-seq,Homo_714,AD.vs.MCI	4
MF	GO:0032395	MHC class II receptor activity	bulk RNA-seq,Homo_723,AD.vs.control; bulk RNA-seq,Homo_723,AD.vs.MCI; bulk RNA-seq,Homo_723,MCI.vs.control; bulk RNA-seq,Homo_714,AD.vs.control; bulk RNA-seq,Homo_714,MCI.vs.control; bulk RNA-seq,Homo_633,MCI.vs.control	6
BP	GO:0034497	protein localization to phagophore assembly site	bulk RNA-seq,Homo_723,AD.vs.control; bulk RNA-seq,Homo_723,AD.vs.MCI; bulk RNA-seq,Homo_714,AD.vs.control; bulk RNA-seq,Homo_633,AD.vs.control; bulk RNA-seq,Homo_633,AD.vs.MCI	5
BP	GO:1902099	regulation of metaphase/anaphase transition of cell cycle	bulk RNA-seq,Homo_723,AD.vs.control; bulk RNA-seq,Homo_723,MCI.vs.control; bulk RNA-seq,Homo_714,AD.vs.control; bulk RNA-seq,Homo_714,MCI.vs.control; bulk RNA-seq,Homo_633,AD.vs.control; bulk RNA-seq,Homo_633,AD.vs.MCI	6
BP	GO:0022617	extracellular matrix disassembly	bulk RNA-seq,Homo_723,AD.vs.control; bulk RNA-seq,Homo_723,AD.vs.MCI; bulk RNA-seq,Homo_723,MCI.vs.control; bulk RNA-seq,Homo_714,AD.vs.MCI	4
BP	GO:0051983	regulation of chromosome segregation	bulk RNA-seq,Homo_723,AD.vs.control; bulk RNA-seq,Homo_723,MCI.vs.control; bulk RNA-seq,Homo_714,AD.vs.control; bulk RNA-seq,Homo_633,AD.vs.control; bulk RNA-seq,Homo_633,AD.vs.MCI	5
BP	GO:0051967	negative regulation of synaptic transmission, glutamatergic	bulk RNA-seq,Homo_723,AD.vs.control; bulk RNA-seq,Homo_723,AD.vs.MCI; bulk RNA-seq,Homo_723,MCI.vs.control	3
BP	GO:0032271	regulation of protein polymerization	bulk RNA-seq,Homo_723,AD.vs.control; bulk RNA-seq,Homo_723,AD.vs.MCI; bulk RNA-seq,Homo_723,MCI.vs.control; bulk RNA-seq,Homo_714,AD.vs.MCI; bulk RNA-seq,Homo_633,AD.vs.control; bulk RNA-seq,Homo_633,AD.vs.MCI	6
BP	GO:0009791	post-embryonic development	bulk RNA-seq,Homo_723,AD.vs.control; bulk RNA-seq,Homo_723,AD.vs.MCI; bulk RNA-seq,Homo_723,MCI.vs.control; bulk RNA-seq,Homo_714,AD.vs.MCI; bulk RNA-seq,Homo_714,MCI.vs.control; bulk RNA-seq,Homo_633,AD.vs.control; bulk RNA-seq,Homo_633,AD.vs.MCI	7
BP	GO:0015695	organic cation transport	bulk RNA-seq,Homo_723,AD.vs.control; bulk RNA-seq,Homo_723,AD.vs.MCI; bulk RNA-seq,Homo_714,AD.vs.control; bulk RNA-seq,Homo_714,AD.vs.MCI	4
MF	GO:0004519	endonuclease activity	bulk RNA-seq,Homo_723,AD.vs.control; bulk RNA-seq,Homo_723,MCI.vs.control; bulk RNA-seq,Homo_714,MCI.vs.control; bulk RNA-seq,Homo_633,AD.vs.control; bulk RNA-seq,Homo_633,AD.vs.MCI	5
BP	GO:0014014	negative regulation of gliogenesis	bulk RNA-seq,Homo_723,AD.vs.control; bulk RNA-seq,Homo_723,AD.vs.MCI; bulk RNA-seq,Homo_723,MCI.vs.control; bulk RNA-seq,Homo_714,AD.vs.MCI; bulk RNA-seq,SRP223445,AD.vs.control	5
BP	GO:1901836	regulation of transcription of nucleolar large rRNA by RNA polymerase I	bulk RNA-seq,Homo_723,AD.vs.control; bulk RNA-seq,Homo_723,MCI.vs.control; bulk RNA-seq,Homo_714,AD.vs.control; bulk RNA-seq,Homo_714,MCI.vs.control; bulk RNA-seq,Homo_633,AD.vs.control; bulk RNA-seq,Homo_633,AD.vs.MCI; bulk RNA-seq,Homo_633,MCI.vs.control	7
MF	GO:0030374	nuclear receptor coactivator activity	bulk RNA-seq,Homo_723,AD.vs.control; bulk RNA-seq,Homo_723,MCI.vs.control; bulk RNA-seq,Homo_714,AD.vs.control; bulk RNA-seq,Homo_714,MCI.vs.control; bulk RNA-seq,Homo_633,AD.vs.control; bulk RNA-seq,Homo_633,AD.vs.MCI	6
BP	GO:0042407	cristae formation	bulk RNA-seq,Homo_723,AD.vs.control; bulk RNA-seq,Homo_714,AD.vs.control; bulk RNA-seq,Homo_633,AD.vs.control; bulk RNA-seq,Homo_633,AD.vs.MCI	4
MF	GO:0035198	miRNA binding	bulk RNA-seq,Homo_723,AD.vs.control; bulk RNA-seq,Homo_714,AD.vs.control; bulk RNA-seq,Homo_714,MCI.vs.control; bulk RNA-seq,Homo_633,AD.vs.control; bulk RNA-seq,Homo_633,AD.vs.MCI	5
BP	GO:1903313	positive regulation of mRNA metabolic process	bulk RNA-seq,Homo_723,AD.vs.control; bulk RNA-seq,Homo_723,MCI.vs.control; bulk RNA-seq,Homo_714,AD.vs.control; bulk RNA-seq,Homo_633,AD.vs.control; bulk RNA-seq,Homo_633,AD.vs.MCI	5
CC	GO:0071564	npBAF complex	bulk RNA-seq,Homo_723,AD.vs.control; bulk RNA-seq,Homo_723,MCI.vs.control; bulk RNA-seq,Homo_714,AD.vs.control; bulk RNA-seq,Homo_633,AD.vs.control; bulk RNA-seq,Homo_633,AD.vs.MCI	5
BP	GO:0033363	secretory granule organization	bulk RNA-seq,Homo_723,AD.vs.control; bulk RNA-seq,Homo_723,MCI.vs.control; bulk RNA-seq,Homo_714,AD.vs.control; bulk RNA-seq,Homo_714,MCI.vs.control; bulk RNA-seq,Homo_633,AD.vs.control; bulk RNA-seq,Homo_633,AD.vs.MCI	6

CC	GO:0005847	mRNA cleavage and polyadenylation specificity factor complex	bulk RNA-seq,Homo_723,AD.vs.control; bulk RNA-seq,Homo_723,AD.vs.MCI; bulk RNA-seq,Homo_714,AD.vs.control; bulk RNA-seq,Homo_633,AD.vs.control; bulk RNA-seq,Homo_633,AD.vs.MCI	5
BP	GO:0006271	DNA strand elongation involved in DNA replication	bulk RNA-seq,Homo_723,AD.vs.control; bulk RNA-seq,Homo_723,MCI.vs.control; bulk RNA-seq,Homo_714,MCI.vs.control; bulk RNA-seq,Homo_633,AD.vs.control; bulk RNA-seq,Homo_633,MCI.vs.control	5
BP	GO:0032790	ribosome disassembly	bulk RNA-seq,Homo_723,AD.vs.control; bulk RNA-seq,Homo_723,AD.vs.MCI; bulk RNA-seq,Homo_714,AD.vs.control; bulk RNA-seq,Homo_714,MCI.vs.control; bulk RNA-seq,Homo_633,AD.vs.control; bulk RNA-seq,Homo_633,AD.vs.MCI; bulk RNA-seq,Homo_633,MCI.vs.control	7
BP	GO:0015801	aromatic amino acid transport	bulk RNA-seq,Homo_723,AD.vs.control; bulk RNA-seq,Homo_723,AD.vs.MCI; bulk RNA-seq,Homo_633,AD.vs.control	3
BP	GO:0060479	lung cell differentiation	bulk RNA-seq,Homo_723,AD.vs.control; bulk RNA-seq,Homo_723,AD.vs.MCI; bulk RNA-seq,Homo_723,MCI.vs.control; bulk RNA-seq,Homo_714,AD.vs.MCI; bulk RNA-seq,Homo_714,MCI.vs.control	5
BP	GO:0060487	lung epithelial cell differentiation	bulk RNA-seq,Homo_723,AD.vs.control; bulk RNA-seq,Homo_723,AD.vs.MCI; bulk RNA-seq,Homo_723,MCI.vs.control; bulk RNA-seq,Homo_714,AD.vs.MCI; bulk RNA-seq,Homo_714,MCI.vs.control	5
MF	GO:0019842	vitamin binding	bulk RNA-seq,Homo_723,AD.vs.control; bulk RNA-seq,Homo_723,AD.vs.MCI; bulk RNA-seq,Homo_723,MCI.vs.control; bulk RNA-seq,Homo_714,AD.vs.MCI; bulk RNA-seq,Homo_633,AD.vs.control; bulk RNA-seq,Homo_633,AD.vs.MCI	6
CC	GO:0097386	glial cell projection	bulk RNA-seq,Homo_723,AD.vs.control; bulk RNA-seq,Homo_723,AD.vs.MCI; bulk RNA-seq,Homo_723,MCI.vs.control; bulk RNA-seq,Homo_714,AD.vs.MCI; bulk RNA-seq,Homo_714,MCI.vs.control	5
BP	GO:0010800	positive regulation of peptidyl-threonine phosphorylation	bulk RNA-seq,Homo_723,AD.vs.control; bulk RNA-seq,Homo_723,AD.vs.MCI; bulk RNA-seq,Homo_723,MCI.vs.control; bulk RNA-seq,Homo_714,AD.vs.MCI; bulk RNA-seq,Homo_714,MCI.vs.control	5
BP	GO:1900273	positive regulation of long-term synaptic potentiation	bulk RNA-seq,Homo_723,AD.vs.control; bulk RNA-seq,Homo_723,AD.vs.MCI; bulk RNA-seq,Homo_723,MCI.vs.control; bulk RNA-seq,Homo_714,AD.vs.control; bulk RNA-seq,Homo_714,AD.vs.MCI; bulk RNA-seq,Homo_714,MCI.vs.control	6
MF	GO:0015125	bile acid transmembrane transporter activity	bulk RNA-seq,Homo_723,AD.vs.control; bulk RNA-seq,Homo_723,AD.vs.MCI; bulk RNA-seq,Homo_723,MCI.vs.control; bulk RNA-seq,Homo_714,AD.vs.control; bulk RNA-seq,Homo_714,AD.vs.MCI; bulk RNA-seq,Homo_714,MCI.vs.control	6
BP	GO:0090036	regulation of protein kinase C signaling	bulk RNA-seq,Homo_723,AD.vs.control; bulk RNA-seq,Homo_723,AD.vs.MCI; bulk RNA-seq,Homo_633,AD.vs.control	3
BP	GO:0110020	regulation of actomyosin structure organization	bulk RNA-seq,Homo_723,AD.vs.control; bulk RNA-seq,Homo_723,AD.vs.MCI; bulk RNA-seq,Homo_723,MCI.vs.control; bulk RNA-seq,Homo_714,AD.vs.MCI; bulk RNA-seq,Homo_714,MCI.vs.control; bulk RNA-seq,Homo_633,AD.vs.control; bulk RNA-seq,Homo_633,AD.vs.MCI	7
MF	GO:0016810	hydrolase activity, acting on carbon-nitrogen (but not peptide) bonds	bulk RNA-seq,Homo_723,AD.vs.control; bulk RNA-seq,Homo_723,MCI.vs.control; bulk RNA-seq,Homo_714,MCI.vs.control; bulk RNA-seq,Homo_633,AD.vs.control; bulk RNA-seq,Homo_633,AD.vs.MCI	5
BP	GO:0010958	regulation of amino acid import across plasma membrane	bulk RNA-seq,Homo_723,AD.vs.control	1
BP	GO:1903789	regulation of amino acid transmembrane transport	bulk RNA-seq,Homo_723,AD.vs.control	1
CC	GO:0001750	photoreceptor outer segment	bulk RNA-seq,Homo_723,AD.vs.control; bulk RNA-seq,Homo_723,AD.vs.MCI; bulk RNA-seq,Homo_723,MCI.vs.control; bulk RNA-seq,Homo_714,AD.vs.control; bulk RNA-seq,Homo_714,AD.vs.MCI; bulk RNA-seq,Homo_714,MCI.vs.control; bulk RNA-seq,Homo_633,AD.vs.control	7
MF	GO:0050699	WW domain binding	bulk RNA-seq,Homo_723,AD.vs.control; bulk RNA-seq,Homo_723,AD.vs.MCI; bulk RNA-seq,Homo_723,MCI.vs.control; bulk RNA-seq,Homo_714,AD.vs.control; bulk RNA-seq,Homo_714,AD.vs.MCI; bulk RNA-seq,Homo_714,MCI.vs.control; bulk RNA-seq,Homo_633,AD.vs.control; bulk	8
BP	GO:0045132	meiotic chromosome segregation	bulk RNA-seq,Homo_723,AD.vs.control; bulk RNA-seq,Homo_723,AD.vs.MCI; bulk RNA-seq,Homo_723,MCI.vs.control; bulk RNA-seq,Homo_714,AD.vs.MCI; bulk RNA-seq,Homo_714,MCI.vs.control; bulk RNA-seq,Homo_633,AD.vs.control; bulk RNA-seq,Homo_633,AD.vs.MCI	7
BP	GO:1902337	regulation of apoptotic process involved in morphogenesis	bulk RNA-seq,Homo_723,AD.vs.control; bulk RNA-seq,Homo_633,MCI.vs.control	2
BP	GO:0090481	pyrimidine nucleotide-sugar transmembrane transport	bulk RNA-seq,Homo_723,AD.vs.control; bulk RNA-seq,Homo_714,AD.vs.control; bulk RNA-seq,Homo_714,MCI.vs.control; bulk RNA-seq,Homo_633,AD.vs.control; bulk RNA-seq,Homo_633,MCI.vs.control	5
BP	GO:0051306	mitotic sister chromatid separation	bulk RNA-seq,Homo_723,AD.vs.control; bulk RNA-seq,Homo_723,MCI.vs.control; bulk RNA-seq,Homo_714,AD.vs.control; bulk RNA-seq,Homo_714,MCI.vs.control; bulk RNA-seq,Homo_633,AD.vs.control; bulk RNA-seq,Homo_633,AD.vs.MCI	6
BP	GO:0003254	regulation of membrane depolarization	bulk RNA-seq,Homo_723,AD.vs.control; bulk RNA-seq,Homo_723,AD.vs.MCI; bulk RNA-seq,Homo_723,MCI.vs.control; bulk RNA-seq,Homo_714,AD.vs.MCI; bulk RNA-seq,Homo_714,MCI.vs.control; bulk RNA-seq,Homo_633,AD.vs.control; bulk RNA-seq,Homo_633,AD.vs.MCI	7
BP	GO:0060421	positive regulation of heart growth	bulk RNA-seq,Homo_723,AD.vs.control; bulk RNA-seq,Homo_723,AD.vs.MCI; bulk RNA-seq,Homo_723,MCI.vs.control; bulk RNA-seq,Homo_714,AD.vs.control; bulk RNA-seq,Homo_714,AD.vs.MCI; bulk RNA-seq,Homo_714,MCI.vs.control	6
BP	GO:0019730	antimicrobial humoral response	bulk RNA-seq,Homo_723,AD.vs.control; bulk RNA-seq,Homo_723,AD.vs.MCI; bulk RNA-seq,Homo_723,MCI.vs.control; bulk RNA-seq,Homo_714,AD.vs.control; bulk RNA-seq,Homo_714,AD.vs.MCI; bulk RNA-seq,Homo_714,MCI.vs.control; bulk RNA-seq,SRP325058,AD.vs.control	7
BP	GO:0051349	positive regulation of lyase activity	bulk RNA-seq,Homo_723,AD.vs.control; bulk RNA-seq,Homo_723,AD.vs.MCI; bulk RNA-seq,Homo_723,MCI.vs.control; bulk RNA-seq,Homo_714,AD.vs.control; bulk RNA-seq,Homo_714,AD.vs.MCI; bulk RNA-seq,SRP223445,AD.vs.control	6
BP	GO:0010769	regulation of cell morphogenesis involved in differentiation	bulk RNA-seq,Homo_723,AD.vs.control; bulk RNA-seq,Homo_723,AD.vs.MCI; bulk RNA-seq,Homo_723,MCI.vs.control; bulk RNA-seq,Homo_714,AD.vs.MCI; bulk RNA-seq,Homo_714,MCI.vs.control; bulk RNA-seq,Homo_633,AD.vs.control; bulk RNA-seq,Homo_633,AD.vs.MCI	7
BP	GO:0021978	telencephalon regionalization	bulk RNA-seq,Homo_723,AD.vs.control; bulk RNA-seq,Homo_723,AD.vs.MCI; bulk RNA-seq,Homo_723,MCI.vs.control	3
BP	GO:0045058	T cell selection	bulk RNA-seq,Homo_723,AD.vs.control; bulk RNA-seq,Homo_723,MCI.vs.control; bulk RNA-seq,Homo_714,AD.vs.control; bulk RNA-seq,Homo_714,MCI.vs.control; bulk RNA-seq,Homo_633,AD.vs.control	5
BP	GO:0015919	peroxisomal membrane transport	bulk RNA-seq,Homo_723,AD.vs.control; bulk RNA-seq,Homo_723,MCI.vs.control; bulk RNA-seq,Homo_714,AD.vs.control; bulk RNA-seq,Homo_714,MCI.vs.control; bulk RNA-seq,Homo_633,AD.vs.control; bulk RNA-seq,Homo_633,AD.vs.MCI; bulk RNA-seq,Homo_633,MCI.vs.control	7
BP	GO:0048821	erythrocyte development	bulk RNA-seq,Homo_723,AD.vs.control; bulk RNA-seq,Homo_723,MCI.vs.control; bulk RNA-seq,Homo_714,MCI.vs.control; bulk RNA-seq,Homo_633,AD.vs.control; bulk RNA-seq,SRP325058,AD.vs.control	5
MF	GO:0070402	NADPH binding	bulk RNA-seq,Homo_723,AD.vs.control; bulk RNA-seq,Homo_714,AD.vs.control; bulk RNA-seq,Homo_633,AD.vs.control; bulk RNA-seq,Homo_633,AD.vs.MCI; bulk RNA-seq,Homo_633,MCI.vs.control	5
BP	GO:0030204	chondroitin sulfate metabolic process	bulk RNA-seq,Homo_723,AD.vs.control; bulk RNA-seq,Homo_723,AD.vs.MCI; bulk RNA-seq,Homo_723,MCI.vs.control; bulk RNA-seq,Homo_714,AD.vs.MCI; bulk RNA-seq,Homo_714,MCI.vs.control	5
BP	GO:0060261	positive regulation of transcription initiation by RNA polymerase II	bulk RNA-seq,Homo_723,AD.vs.control; bulk RNA-seq,Homo_723,MCI.vs.control; bulk RNA-seq,Homo_714,AD.vs.control; bulk RNA-seq,Homo_714,MCI.vs.control; bulk RNA-seq,Homo_633,AD.vs.control; bulk RNA-seq,Homo_633,AD.vs.MCI	6
MF	GO:0008200	ion channel inhibitor activity	bulk RNA-seq,Homo_723,AD.vs.control; bulk RNA-seq,Homo_723,AD.vs.MCI; bulk RNA-seq,Homo_723,MCI.vs.control; bulk RNA-seq,Homo_714,AD.vs.MCI; bulk RNA-seq,Homo_714,MCI.vs.control; bulk RNA-seq,Homo_633,AD.vs.control	6

BP	GO:0150116	regulation of cell-substrate junction organization	bulk RNA-seq,Homo_723,AD.vs.control; bulk RNA-seq,Homo_723,AD.vs.MCI; bulk RNA-seq,Homo_723,MCI.vs.control; bulk RNA-seq,Homo_714,AD.vs.MCI; bulk RNA-seq,Homo_714,MCI.vs.control; bulk RNA-seq,Homo_633,AD.vs.control; bulk RNA-seq,Homo_633,AD.vs.MCI	7
BP	GO:0036303	lymph vessel morphogenesis	bulk RNA-seq,Homo_723,AD.vs.control; bulk RNA-seq,Homo_723,AD.vs.MCI; bulk RNA-seq,Homo_723,MCI.vs.control; bulk RNA-seq,Homo_714,AD.vs.MCI; bulk RNA-seq,Homo_714,MCI.vs.control	5
BP	GO:0043392	negative regulation of DNA binding	bulk RNA-seq,Homo_723,AD.vs.control; bulk RNA-seq,Homo_723,AD.vs.MCI; bulk RNA-seq,Homo_723,MCI.vs.control; bulk RNA-seq,Homo_714,AD.vs.MCI; bulk RNA-seq,Homo_714,MCI.vs.control; bulk RNA-seq,Homo_633,AD.vs.control; bulk RNA-seq,Homo_633,AD.vs.MCI	7
BP	GO:2000144	positive regulation of DNA-templated transcription initiation	bulk RNA-seq,Homo_723,AD.vs.control; bulk RNA-seq,Homo_723,MCI.vs.control; bulk RNA-seq,Homo_714,AD.vs.control; bulk RNA-seq,Homo_714,MCI.vs.control; bulk RNA-seq,Homo_633,AD.vs.control; bulk RNA-seq,Homo_633,AD.vs.MCI	6
BP	GO:0019395	fatty acid oxidation	bulk RNA-seq,Homo_723,AD.vs.control; bulk RNA-seq,Homo_723,MCI.vs.control; bulk RNA-seq,Homo_714,AD.vs.control; bulk RNA-seq,Homo_714,MCI.vs.control; bulk RNA-seq,Homo_633,AD.vs.control; bulk RNA-seq,Homo_633,AD.vs.MCI	6
MF	GO:0016423	tRNA (guanine) methyltransferase activity	bulk RNA-seq,Homo_723,AD.vs.control; bulk RNA-seq,Homo_714,AD.vs.control; bulk RNA-seq,Homo_714,MCI.vs.control; bulk RNA-seq,Homo_633,AD.vs.control; bulk RNA-seq,Homo_633,AD.vs.MCI	5
BP	GO:0021532	neural tube patterning	bulk RNA-seq,Homo_723,AD.vs.control; bulk RNA-seq,Homo_723,AD.vs.MCI; bulk RNA-seq,Homo_723,MCI.vs.control; bulk RNA-seq,Homo_714,AD.vs.MCI; bulk RNA-seq,Homo_714,MCI.vs.control; bulk RNA-seq,Homo_633,AD.vs.control; bulk RNA-seq,Homo_633,AD.vs.MCI	7
BP	GO:0043267	negative regulation of potassium ion transport	bulk RNA-seq,Homo_723,AD.vs.control; bulk RNA-seq,Homo_723,AD.vs.MCI; bulk RNA-seq,Homo_714,AD.vs.control; bulk RNA-seq,Homo_714,AD.vs.MCI	4
BP	GO:1902932	positive regulation of alcohol biosynthetic process	bulk RNA-seq,Homo_723,AD.vs.control; bulk RNA-seq,Homo_723,AD.vs.MCI; bulk RNA-seq,Homo_714,AD.vs.MCI	3
BP	GO:0032148	activation of protein kinase B activity	bulk RNA-seq,Homo_723,AD.vs.control; bulk RNA-seq,Homo_723,AD.vs.MCI; bulk RNA-seq,Homo_714,AD.vs.MCI; bulk RNA-seq,Homo_633,AD.vs.control; bulk RNA-seq,Homo_633,AD.vs.MCI	5
CC	GO:0031082	BLOC complex	bulk RNA-seq,Homo_723,AD.vs.control; bulk RNA-seq,Homo_723,MCI.vs.control; bulk RNA-seq,Homo_714,AD.vs.control; bulk RNA-seq,Homo_714,MCI.vs.control; bulk RNA-seq,Homo_633,AD.vs.control; bulk RNA-seq,Homo_633,AD.vs.MCI; bulk RNA-seq,Homo_633,MCI.vs.control	7
BP	GO:0045022	early endosome to late endosome transport	bulk RNA-seq,Homo_723,AD.vs.control; bulk RNA-seq,Homo_723,MCI.vs.control; bulk RNA-seq,Homo_714,AD.vs.control; bulk RNA-seq,Homo_714,MCI.vs.control; bulk RNA-seq,Homo_633,AD.vs.control; bulk RNA-seq,Homo_633,AD.vs.MCI	6
MF	GO:0017046	peptide hormone binding	bulk RNA-seq,Homo_723,AD.vs.control; bulk RNA-seq,Homo_723,AD.vs.MCI; bulk RNA-seq,Homo_723,MCI.vs.control; bulk RNA-seq,Homo_714,AD.vs.control; bulk RNA-seq,Homo_714,AD.vs.MCI; bulk RNA-seq,Homo_714,MCI.vs.control	6
BP	GO:0001731	formation of translation preinitiation complex	bulk RNA-seq,Homo_723,AD.vs.control; bulk RNA-seq,Homo_714,AD.vs.control; bulk RNA-seq,Homo_714,MCI.vs.control; bulk RNA-seq,Homo_633,AD.vs.control; bulk RNA-seq,Homo_633,AD.vs.MCI	5
BP	GO:1905475	regulation of protein localization to membrane	bulk RNA-seq,Homo_723,AD.vs.control; bulk RNA-seq,Homo_723,AD.vs.MCI; bulk RNA-seq,Homo_723,MCI.vs.control; bulk RNA-seq,Homo_714,AD.vs.MCI; bulk RNA-seq,Homo_633,AD.vs.control; bulk RNA-seq,Homo_633,AD.vs.MCI	6
BP	GO:0000422	autophagy of mitochondrion	bulk RNA-seq,Homo_723,AD.vs.control; bulk RNA-seq,Homo_723,MCI.vs.control; bulk RNA-seq,Homo_714,AD.vs.control; bulk RNA-seq,Homo_714,MCI.vs.control; bulk RNA-seq,Homo_633,AD.vs.control; bulk RNA-seq,Homo_633,AD.vs.MCI	6
BP	GO:0061726	mitochondrion disassembly	bulk RNA-seq,Homo_723,AD.vs.control; bulk RNA-seq,Homo_723,MCI.vs.control; bulk RNA-seq,Homo_714,AD.vs.control; bulk RNA-seq,Homo_714,MCI.vs.control; bulk RNA-seq,Homo_633,AD.vs.control; bulk RNA-seq,Homo_633,AD.vs.MCI	6
BP	GO:0046189	phenol-containing compound biosynthetic process	bulk RNA-seq,Homo_723,AD.vs.control; bulk RNA-seq,Homo_723,AD.vs.MCI; bulk RNA-seq,Homo_723,MCI.vs.control; bulk RNA-seq,Homo_714,AD.vs.MCI; bulk RNA-seq,Homo_633,AD.vs.control; bulk RNA-seq,Homo_633,AD.vs.MCI	6
BP	GO:0051590	positive regulation of neurotransmitter transport	bulk RNA-seq,Homo_723,AD.vs.control; bulk RNA-seq,Homo_723,AD.vs.MCI; bulk RNA-seq,Homo_723,MCI.vs.control	3
MF	GO:0033218	amide binding	bulk RNA-seq,Homo_723,AD.vs.control; bulk RNA-seq,Homo_723,AD.vs.MCI; bulk RNA-seq,Homo_714,AD.vs.MCI; bulk RNA-seq,Homo_633,AD.vs.control; bulk RNA-seq,Homo_633,AD.vs.MCI	5
BP	GO:0043247	telomere maintenance in response to DNA damage	bulk RNA-seq,Homo_723,AD.vs.control; bulk RNA-seq,Homo_714,AD.vs.control; bulk RNA-seq,Homo_633,AD.vs.control; bulk RNA-seq,Homo_633,AD.vs.MCI	4
BP	GO:0008593	regulation of Notch signaling pathway	bulk RNA-seq,Homo_723,AD.vs.control; bulk RNA-seq,Homo_723,AD.vs.MCI; bulk RNA-seq,Homo_723,MCI.vs.control; bulk RNA-seq,Homo_714,AD.vs.MCI; bulk RNA-seq,Homo_714,MCI.vs.control; bulk RNA-seq,Homo_633,AD.vs.control	6
BP	GO:0048563	post-embryonic animal organ morphogenesis	bulk RNA-seq,Homo_723,AD.vs.control; bulk RNA-seq,Homo_723,AD.vs.MCI; bulk RNA-seq,Homo_723,MCI.vs.control; bulk RNA-seq,Homo_714,AD.vs.MCI; bulk RNA-seq,Homo_714,MCI.vs.control	5
BP	GO:0060134	prepulse inhibition	bulk RNA-seq,Homo_723,AD.vs.control; bulk RNA-seq,Homo_723,AD.vs.MCI; bulk RNA-seq,Homo_723,MCI.vs.control	3
BP	GO:0070373	negative regulation of ERK1 and ERK2 cascade	bulk RNA-seq,Homo_723,AD.vs.control; bulk RNA-seq,Homo_723,AD.vs.MCI; bulk RNA-seq,Homo_723,MCI.vs.control; bulk RNA-seq,Homo_714,AD.vs.MCI; bulk RNA-seq,Homo_714,MCI.vs.control; bulk RNA-seq,Homo_633,AD.vs.control; bulk RNA-seq,Homo_633,AD.vs.MCI	7
BP	GO:0031115	negative regulation of microtubule polymerization	bulk RNA-seq,Homo_723,AD.vs.control; bulk RNA-seq,Homo_723,AD.vs.MCI; bulk RNA-seq,Homo_723,MCI.vs.control; bulk RNA-seq,Homo_714,AD.vs.MCI; bulk RNA-seq,Homo_714,MCI.vs.control; bulk RNA-seq,Homo_633,AD.vs.control	6
BP	GO:0033273	response to vitamin	bulk RNA-seq,Homo_723,AD.vs.control; bulk RNA-seq,Homo_723,AD.vs.MCI; bulk RNA-seq,Homo_723,MCI.vs.control; bulk RNA-seq,Homo_714,AD.vs.MCI; bulk RNA-seq,Homo_714,MCI.vs.control; bulk RNA-seq,Homo_633,AD.vs.control; bulk RNA-seq,Homo_633,AD.vs.MCI	7
BP	GO:1904357	negative regulation of telomere maintenance via telomere	bulk RNA-seq,Homo_723,AD.vs.control; bulk RNA-seq,Homo_714,AD.vs.control; bulk RNA-seq,Homo_633,AD.vs.control; bulk RNA-seq,Homo_633,AD.vs.MCI	4
BP	GO:0007162	negative regulation of cell adhesion	bulk RNA-seq,Homo_723,AD.vs.control; bulk RNA-seq,Homo_723,AD.vs.MCI; bulk RNA-seq,Homo_723,MCI.vs.control; bulk RNA-seq,Homo_714,AD.vs.MCI; bulk RNA-seq,Homo_633,AD.vs.control; bulk RNA-seq,Homo_633,AD.vs.MCI	6
BP	GO:1902224	ketone body metabolic process	bulk RNA-seq,Homo_723,AD.vs.control; bulk RNA-seq,Homo_723,AD.vs.MCI; bulk RNA-seq,Homo_723,MCI.vs.control; bulk RNA-seq,Homo_714,AD.vs.MCI	4
BP	GO:0051492	regulation of stress fiber assembly	bulk RNA-seq,Homo_723,AD.vs.control; bulk RNA-seq,Homo_723,AD.vs.MCI; bulk RNA-seq,Homo_723,MCI.vs.control; bulk RNA-seq,Homo_714,AD.vs.MCI; bulk RNA-seq,Homo_714,MCI.vs.control; bulk RNA-seq,Homo_633,AD.vs.control; bulk RNA-seq,Homo_633,AD.vs.MCI	7
CC	GO:0071004	U2-type prespliceosome	bulk RNA-seq,Homo_723,AD.vs.control; bulk RNA-seq,Homo_714,AD.vs.control	2
CC	GO:0071010	prespliceosome	bulk RNA-seq,Homo_723,AD.vs.control; bulk RNA-seq,Homo_714,AD.vs.control	2
MF	GO:0042288	MHC class I protein binding	bulk RNA-seq,Homo_723,AD.vs.control; bulk RNA-seq,Homo_723,MCI.vs.control; bulk RNA-seq,Homo_714,AD.vs.control; bulk RNA-seq,Homo_714,MCI.vs.control; bulk RNA-seq,Homo_633,AD.vs.control; bulk RNA-seq,Homo_633,MCI.vs.control	6
MF	GO:0015280	ligand-gated sodium channel activity	bulk RNA-seq,Homo_723,AD.vs.control; bulk RNA-seq,Homo_723,AD.vs.MCI; bulk RNA-seq,Homo_723,MCI.vs.control; bulk RNA-seq,Homo_714,AD.vs.MCI	4
CC	GO:0044853	plasma membrane raft	bulk RNA-seq,Homo_723,AD.vs.control; bulk RNA-seq,Homo_723,AD.vs.MCI; bulk RNA-seq,Homo_723,MCI.vs.control; bulk RNA-seq,Homo_714,AD.vs.MCI; bulk RNA-seq,Homo_714,MCI.vs.control; bulk RNA-seq,Homo_633,AD.vs.control; bulk RNA-seq,Homo_633,AD.vs.MCI	7
MF	GO:0004529	exodeoxyribonuclease activity	bulk RNA-seq,Homo_723,AD.vs.control; bulk RNA-seq,Homo_723,MCI.vs.control; bulk RNA-seq,Homo_714,AD.vs.control; bulk RNA-seq,Homo_633,AD.vs.control; bulk RNA-seq,Homo_633,AD.vs.MCI	5

MF	GO:0016895	exodeoxyribonuclease activity, producing 5'-phosphomonoesters	bulk RNA-seq,Homo_723,AD.vs.control; bulk RNA-seq,Homo_723,MCI.vs.control; bulk RNA-seq,Homo_714,AD.vs.control; bulk RNA-seq,Homo_633,AD.vs.control; bulk RNA-seq,Homo_633,AD.vs.MCI	5
BP	GO:0046777	protein autophosphorylation	bulk RNA-seq,Homo_723,AD.vs.control; bulk RNA-seq,Homo_723,AD.vs.MCI; bulk RNA-seq,Homo_723,MCI.vs.control; bulk RNA-seq,Homo_714,AD.vs.MCI; bulk RNA-seq,Homo_633,AD.vs.control; bulk RNA-seq,Homo_633,AD.vs.MCI	6
BP	GO:0042074	cell migration involved in gastrulation	bulk RNA-seq,Homo_723,AD.vs.control; bulk RNA-seq,Homo_723,AD.vs.MCI; bulk RNA-seq,Homo_723,MCI.vs.control; bulk RNA-seq,Homo_714,AD.vs.MCI	4
BP	GO:0048643	positive regulation of skeletal muscle tissue development	bulk RNA-seq,Homo_723,AD.vs.control; bulk RNA-seq,Homo_723,AD.vs.MCI; bulk RNA-seq,Homo_723,MCI.vs.control; bulk RNA-seq,Homo_714,AD.vs.MCI	4
BP	GO:0085029	extracellular matrix assembly	bulk RNA-seq,Homo_723,AD.vs.control; bulk RNA-seq,Homo_723,AD.vs.MCI; bulk RNA-seq,Homo_723,MCI.vs.control; bulk RNA-seq,Homo_714,AD.vs.MCI; bulk RNA-seq,Homo_714,MCI.vs.control; bulk RNA-seq,Homo_633,AD.vs.control; bulk RNA-seq,Homo_633,AD.vs.MCI	7
BP	GO:0031290	retinal ganglion cell axon guidance	bulk RNA-seq,Homo_723,AD.vs.control; bulk RNA-seq,Homo_723,AD.vs.MCI; bulk RNA-seq,Homo_723,MCI.vs.control; bulk RNA-seq,Homo_633,AD.vs.control; bulk RNA-seq,Homo_633,AD.vs.MCI	5
BP	GO:0030150	protein import into mitochondrial matrix	bulk RNA-seq,Homo_723,AD.vs.control; bulk RNA-seq,Homo_723,MCI.vs.control; bulk RNA-seq,Homo_714,MCI.vs.control; bulk RNA-seq,Homo_633,AD.vs.control; bulk RNA-seq,Homo_633,AD.vs.MCI; bulk RNA-seq,Homo_633,MCI.vs.control	6
BP	GO:0010644	cell communication by electrical coupling	bulk RNA-seq,Homo_723,AD.vs.control; bulk RNA-seq,Homo_723,AD.vs.MCI; bulk RNA-seq,Homo_723,MCI.vs.control; bulk RNA-seq,Homo_714,AD.vs.MCI; bulk RNA-seq,Homo_714,MCI.vs.control	5
BP	GO:0034058	endosomal vesicle fusion	bulk RNA-seq,Homo_723,AD.vs.control; bulk RNA-seq,Homo_723,MCI.vs.control; bulk RNA-seq,Homo_714,MCI.vs.control; bulk RNA-seq,Homo_633,AD.vs.control; bulk RNA-seq,Homo_633,MCI.vs.control	5
CC	GO:0097449	astrocyte projection	bulk RNA-seq,Homo_723,AD.vs.control; bulk RNA-seq,Homo_723,AD.vs.MCI; bulk RNA-seq,Homo_723,MCI.vs.control; bulk RNA-seq,Homo_714,AD.vs.MCI; bulk RNA-seq,Homo_714,MCI.vs.control	5
BP	GO:1901894	regulation of ATPase-coupled calcium transmembrane transporter activity	bulk RNA-seq,Homo_723,AD.vs.control; bulk RNA-seq,Homo_723,AD.vs.MCI	2
BP	GO:0031952	regulation of protein autophosphorylation	bulk RNA-seq,Homo_723,AD.vs.control; bulk RNA-seq,Homo_723,AD.vs.MCI; bulk RNA-seq,Homo_723,MCI.vs.control; bulk RNA-seq,Homo_714,AD.vs.control; bulk RNA-seq,Homo_714,AD.vs.MCI; bulk RNA-seq,Homo_714,MCI.vs.control; bulk RNA-seq,Homo_633,AD.vs.control	7
BP	GO:0031954	positive regulation of protein autophosphorylation	bulk RNA-seq,Homo_723,AD.vs.control; bulk RNA-seq,Homo_723,AD.vs.MCI; bulk RNA-seq,Homo_723,MCI.vs.control; bulk RNA-seq,Homo_714,AD.vs.control; bulk RNA-seq,Homo_714,AD.vs.MCI; bulk RNA-seq,Homo_714,MCI.vs.control; bulk RNA-seq,Homo_633,AD.vs.control	7
BP	GO:0021535	cell migration in hindbrain	bulk RNA-seq,Homo_723,AD.vs.control; bulk RNA-seq,Homo_723,AD.vs.MCI; bulk RNA-seq,Homo_723,MCI.vs.control	3
BP	GO:2000317	negative regulation of T-helper 17 type immune response	bulk RNA-seq,Homo_723,AD.vs.control; bulk RNA-seq,Homo_723,MCI.vs.control; bulk RNA-seq,Homo_714,MCI.vs.control; bulk RNA-	4
MF	GO:0005436	sodium:phosphate symporter activity	bulk RNA-seq,Homo_723,AD.vs.control; bulk RNA-seq,Homo_723,AD.vs.MCI; bulk RNA-seq,Homo_723,MCI.vs.control; bulk RNA-seq,Homo_714,AD.vs.MCI	4
BP	GO:1902305	regulation of sodium ion transmembrane transport	bulk RNA-seq,Homo_723,AD.vs.control; bulk RNA-seq,Homo_723,AD.vs.MCI; bulk RNA-seq,Homo_723,MCI.vs.control; bulk RNA-seq,Homo_714,AD.vs.MCI; bulk RNA-seq,Homo_714,MCI.vs.control; bulk RNA-seq,Homo_633,AD.vs.control	6
BP	GO:0021903	rostrocaudal neural tube patterning	bulk RNA-seq,Homo_723,AD.vs.control; bulk RNA-seq,Homo_723,AD.vs.MCI; bulk RNA-seq,Homo_723,MCI.vs.control; bulk RNA-seq,Homo_714,AD.vs.MCI	4
BP	GO:0043371	negative regulation of CD4-positive, alpha-beta T cell differentiation	bulk RNA-seq,Homo_723,AD.vs.control; bulk RNA-seq,Homo_723,MCI.vs.control; bulk RNA-seq,Homo_714,AD.vs.control; bulk RNA-seq,Homo_714,MCI.vs.control; bulk RNA-seq,Homo_633,AD.vs.control; bulk RNA-seq,Homo_633,AD.vs.MCI; bulk RNA-seq,Homo_633,MCI.vs.control	7
BP	GO:0001912	positive regulation of leukocyte mediated cytotoxicity	bulk RNA-seq,Homo_723,AD.vs.control; bulk RNA-seq,Homo_714,AD.vs.control; bulk RNA-seq,Homo_633,AD.vs.control; bulk RNA-seq,Homo_633,AD.vs.MCI	4
BP	GO:1904262	negative regulation of TORC1 signaling	bulk RNA-seq,Homo_723,AD.vs.control; bulk RNA-seq,Homo_723,MCI.vs.control; bulk RNA-seq,Homo_714,MCI.vs.control; bulk RNA-seq,Homo_633,AD.vs.control; bulk RNA-seq,Homo_633,AD.vs.MCI; bulk RNA-seq,Homo_633,MCI.vs.control	6
BP	GO:0002566	somatic diversification of immune receptors via somatic mutation	bulk RNA-seq,Homo_723,AD.vs.control; bulk RNA-seq,Homo_714,AD.vs.control; bulk RNA-seq,Homo_714,MCI.vs.control; bulk RNA-seq,Homo_633,AD.vs.control; bulk RNA-seq,Homo_633,AD.vs.MCI; bulk RNA-seq,Homo_633,MCI.vs.control	6
BP	GO:0060452	positive regulation of cardiac muscle contraction	bulk RNA-seq,Homo_723,AD.vs.control	1
BP	GO:0071294	cellular response to zinc ion	bulk RNA-seq,Homo_723,AD.vs.control; bulk RNA-seq,Homo_633,AD.vs.MCI	2
BP	GO:1903050	regulation of proteolysis involved in protein catabolic process	bulk RNA-seq,Homo_723,AD.vs.control; bulk RNA-seq,Homo_723,MCI.vs.control; bulk RNA-seq,Homo_714,AD.vs.control; bulk RNA-seq,Homo_714,AD.vs.MCI; bulk RNA-seq,Homo_633,AD.vs.control; bulk RNA-seq,Homo_633,AD.vs.MCI	6
MF	GO:0042577	lipid phosphatase activity	bulk RNA-seq,Homo_723,AD.vs.control; bulk RNA-seq,Homo_723,AD.vs.MCI; bulk RNA-seq,Homo_723,MCI.vs.control	3
BP	GO:1902100	negative regulation of metaphase/anaphase transition of cell cycle	bulk RNA-seq,Homo_723,AD.vs.control; bulk RNA-seq,Homo_723,MCI.vs.control; bulk RNA-seq,Homo_714,AD.vs.control; bulk RNA-seq,Homo_714,MCI.vs.control; bulk RNA-seq,Homo_633,AD.vs.control; bulk RNA-seq,Homo_633,AD.vs.MCI	6
BP	GO:0090037	positive regulation of protein kinase C signaling	bulk RNA-seq,Homo_723,AD.vs.control; bulk RNA-seq,Homo_723,AD.vs.MCI	2
BP	GO:0019722	calcium-mediated signaling	bulk RNA-seq,Homo_723,AD.vs.control; bulk RNA-seq,Homo_723,AD.vs.MCI; bulk RNA-seq,Homo_723,MCI.vs.control; bulk RNA-seq,Homo_714,AD.vs.MCI; bulk RNA-seq,Homo_633,AD.vs.control; bulk RNA-seq,Homo_633,AD.vs.MCI	6
CC	GO:0005875	microtubule associated complex	bulk RNA-seq,Homo_723,AD.vs.control; bulk RNA-seq,Homo_723,AD.vs.MCI; bulk RNA-seq,Homo_723,MCI.vs.control; bulk RNA-seq,Homo_714,AD.vs.MCI; bulk RNA-seq,Homo_633,AD.vs.control; bulk RNA-seq,Homo_633,AD.vs.MCI	6
BP	GO:0061001	regulation of dendritic spine morphogenesis	bulk RNA-seq,Homo_723,AD.vs.control; bulk RNA-seq,Homo_723,AD.vs.MCI; bulk RNA-seq,Homo_723,MCI.vs.control; bulk RNA-seq,Homo_714,AD.vs.MCI; bulk RNA-seq,Homo_714,MCI.vs.control; bulk RNA-seq,Homo_633,AD.vs.control; bulk RNA-seq,Homo_633,AD.vs.MCI	7
BP	GO:0003310	pancreatic A cell differentiation	bulk RNA-seq,Homo_723,AD.vs.control; bulk RNA-seq,Homo_723,AD.vs.MCI; bulk RNA-seq,Homo_723,MCI.vs.control; bulk RNA-seq,Homo_714,AD.vs.MCI	4
BP	GO:0099149	regulation of postsynaptic neurotransmitter receptor	bulk RNA-seq,Homo_723,AD.vs.control; bulk RNA-seq,Homo_723,AD.vs.MCI; bulk RNA-seq,Homo_723,MCI.vs.control	3
BP	GO:0016446	somatic hypermutation of immunoglobulin genes	bulk RNA-seq,Homo_723,AD.vs.control; bulk RNA-seq,Homo_723,MCI.vs.control; bulk RNA-seq,Homo_714,AD.vs.control; bulk RNA-seq,Homo_714,MCI.vs.control; bulk RNA-seq,Homo_633,AD.vs.control; bulk RNA-seq,Homo_633,AD.vs.MCI; bulk RNA-seq,Homo_633,MCI.vs.control	7
BP	GO:0035520	monoubiquitinated protein deubiquitination	bulk RNA-seq,Homo_723,AD.vs.control; bulk RNA-seq,Homo_723,MCI.vs.control; bulk RNA-seq,Homo_714,AD.vs.control; bulk RNA-seq,Homo_714,MCI.vs.control; bulk RNA-seq,Homo_633,AD.vs.control; bulk RNA-seq,Homo_633,AD.vs.MCI; bulk RNA-seq,Homo_633,MCI.vs.control	7
BP	GO:1903312	negative regulation of mRNA metabolic process	bulk RNA-seq,Homo_723,AD.vs.control; bulk RNA-seq,Homo_723,MCI.vs.control; bulk RNA-seq,Homo_714,AD.vs.control; bulk RNA-seq,Homo_714,MCI.vs.control; bulk RNA-seq,Homo_633,AD.vs.control; bulk RNA-seq,Homo_633,AD.vs.MCI	6
BP	GO:0000290	deadenylation-dependent decapping of nuclear-transcribed	bulk RNA-seq,Homo_723,AD.vs.control; bulk RNA-seq,Homo_723,AD.vs.MCI; bulk RNA-seq,Homo_714,AD.vs.control	3
MF	GO:0070888	E-box binding	bulk RNA-seq,Homo_723,AD.vs.control; bulk RNA-seq,Homo_723,AD.vs.MCI; bulk RNA-seq,Homo_723,MCI.vs.control; bulk RNA-seq,Homo_714,AD.vs.MCI; bulk RNA-seq,Homo_714,MCI.vs.control	5

CC	GO:0005669	transcription factor TFIID complex	bulk RNA-seq,Homo_723,AD.vs.control; bulk RNA-seq,Homo_723,MCI.vs.control; bulk RNA-seq,Homo_714,AD.vs.control; bulk RNA-seq,Homo_714,MCI.vs.control; bulk RNA-seq,Homo_633,AD.vs.control; bulk RNA-seq,Homo_633,AD.vs.MCI; bulk RNA-seq,Homo_633,MCI.vs.control	7
CC	GO:0071745	IgA immunoglobulin complex	bulk RNA-seq,Homo_723,AD.vs.control; bulk RNA-seq,Homo_723,AD.vs.MCI; bulk RNA-seq,Homo_633,AD.vs.control	3
BP	GO:0017062	respiratory chain complex III assembly	bulk RNA-seq,Homo_723,AD.vs.control; bulk RNA-seq,Homo_723,AD.vs.MCI; bulk RNA-seq,Homo_723,MCI.vs.control; bulk RNA-seq,Homo_714,AD.vs.control; bulk RNA-seq,Homo_633,MCI.vs.control	5
BP	GO:0034551	mitochondrial respiratory chain complex III assembly	bulk RNA-seq,Homo_723,AD.vs.control; bulk RNA-seq,Homo_723,AD.vs.MCI; bulk RNA-seq,Homo_723,MCI.vs.control; bulk RNA-seq,Homo_714,AD.vs.control; bulk RNA-seq,Homo_633,MCI.vs.control	5
BP	GO:1904748	regulation of apoptotic process involved in development	bulk RNA-seq,Homo_723,AD.vs.control; bulk RNA-seq,Homo_723,AD.vs.MCI	2
BP	GO:0003352	regulation of cilium movement	bulk RNA-seq,Homo_723,AD.vs.control; bulk RNA-seq,Homo_723,AD.vs.MCI	2
BP	GO:0071600	otic vesicle morphogenesis	bulk RNA-seq,Homo_723,AD.vs.control; bulk RNA-seq,Homo_723,AD.vs.MCI; bulk RNA-seq,Homo_723,MCI.vs.control; bulk RNA-seq,Homo_714,AD.vs.MCI; bulk RNA-seq,Homo_714,MCI.vs.control	5
BP	GO:0051985	negative regulation of chromosome segregation	bulk RNA-seq,Homo_723,AD.vs.control; bulk RNA-seq,Homo_723,MCI.vs.control; bulk RNA-seq,Homo_714,MCI.vs.control; bulk RNA-seq,Homo_633,AD.vs.control; bulk RNA-seq,Homo_633,AD.vs.MCI	5
BP	GO:1905819	negative regulation of chromosome separation	bulk RNA-seq,Homo_723,AD.vs.control; bulk RNA-seq,Homo_723,MCI.vs.control; bulk RNA-seq,Homo_714,MCI.vs.control; bulk RNA-seq,Homo_633,AD.vs.control; bulk RNA-seq,Homo_633,AD.vs.MCI	5
MF	GO:0005227	calcium activated cation channel activity	bulk RNA-seq,Homo_723,AD.vs.control; bulk RNA-seq,Homo_723,AD.vs.MCI; bulk RNA-seq,Homo_723,MCI.vs.control; bulk RNA-seq,Homo_714,AD.vs.MCI; bulk RNA-seq,Homo_714,MCI.vs.control	5
MF	GO:0005217	intracellular ligand-gated ion channel activity	bulk RNA-seq,Homo_723,AD.vs.control; bulk RNA-seq,Homo_723,AD.vs.MCI; bulk RNA-seq,Homo_723,MCI.vs.control; bulk RNA-seq,Homo_714,AD.vs.MCI; bulk RNA-seq,Homo_714,MCI.vs.control; bulk RNA-seq,Homo_633,AD.vs.control	6
CC	GO:0000276	mitochondrial proton-transporting ATP synthase complex, coupling factor F(o)	bulk RNA-seq,Homo_723,AD.vs.control; bulk RNA-seq,Homo_714,AD.vs.control; bulk RNA-seq,Homo_714,MCI.vs.control; bulk RNA-seq,Homo_633,MCI.vs.control	4
BP	GO:0016485	protein processing	bulk RNA-seq,Homo_723,AD.vs.control; bulk RNA-seq,Homo_723,AD.vs.MCI; bulk RNA-seq,Homo_723,MCI.vs.control; bulk RNA-seq,Homo_714,AD.vs.MCI; bulk RNA-seq,Homo_633,AD.vs.control; bulk RNA-seq,Homo_633,AD.vs.MCI	6
BP	GO:0030336	negative regulation of cell migration	bulk RNA-seq,Homo_723,AD.vs.control; bulk RNA-seq,Homo_723,AD.vs.MCI; bulk RNA-seq,Homo_714,AD.vs.control; bulk RNA-seq,Homo_714,AD.vs.MCI; bulk RNA-seq,Homo_633,AD.vs.control; bulk RNA-seq,Homo_633,AD.vs.MCI; scRNA-seq,SRP330776,Naive CD8+ T cell_2-AD.vs.control	7
CC	GO:0034706	sodium channel complex	bulk RNA-seq,Homo_723,AD.vs.control; bulk RNA-seq,Homo_723,AD.vs.MCI; bulk RNA-seq,Homo_723,MCI.vs.control; bulk RNA-seq,Homo_714,AD.vs.MCI; bulk RNA-seq,Homo_714,MCI.vs.control; bulk RNA-seq,ROSMAP,AD.vs.control	6
BP	GO:0003253	cardiac neural crest cell migration involved in outflow tract morphogenesis	bulk RNA-seq,Homo_723,AD.vs.control; bulk RNA-seq,Homo_723,AD.vs.MCI; bulk RNA-seq,Homo_723,MCI.vs.control; bulk RNA-seq,Homo_714,AD.vs.MCI	4
BP	GO:1903531	negative regulation of secretion by cell	bulk RNA-seq,Homo_723,AD.vs.control; bulk RNA-seq,Homo_723,AD.vs.MCI; bulk RNA-seq,Homo_723,MCI.vs.control; bulk RNA-seq,Homo_714,AD.vs.MCI; bulk RNA-seq,Homo_633,AD.vs.control; bulk RNA-seq,Homo_633,AD.vs.MCI	6
BP	GO:1905209	positive regulation of cardiocyte differentiation	bulk RNA-seq,Homo_723,AD.vs.control; bulk RNA-seq,Homo_723,AD.vs.MCI; bulk RNA-seq,Homo_714,AD.vs.MCI	3
CC	GO:0042405	nuclear inclusion body	bulk RNA-seq,Homo_723,AD.vs.control; bulk RNA-seq,Homo_714,AD.vs.control; bulk RNA-seq,Homo_633,AD.vs.control; bulk RNA-seq,Homo_633,AD.vs.MCI	4
BP	GO:0051209	release of sequestered calcium ion into cytosol	bulk RNA-seq,Homo_723,AD.vs.control; bulk RNA-seq,Homo_723,AD.vs.MCI; bulk RNA-seq,Homo_723,MCI.vs.control; bulk RNA-seq,Homo_714,AD.vs.MCI; bulk RNA-seq,Homo_714,MCI.vs.control; bulk RNA-seq,Homo_633,AD.vs.control; bulk RNA-seq,Homo_633,AD.vs.MCI	7
BP	GO:0038128	ERBB2 signaling pathway	bulk RNA-seq,Homo_723,AD.vs.control; bulk RNA-seq,Homo_723,AD.vs.MCI; bulk RNA-seq,Homo_723,MCI.vs.control; bulk RNA-seq,Homo_714,AD.vs.MCI; bulk RNA-seq,Homo_714,MCI.vs.control	5
BP	GO:0045445	myoblast differentiation	bulk RNA-seq,Homo_723,AD.vs.control; bulk RNA-seq,Homo_723,AD.vs.MCI; bulk RNA-seq,Homo_723,MCI.vs.control; bulk RNA-seq,Homo_714,AD.vs.MCI; bulk RNA-seq,Homo_714,MCI.vs.control; bulk RNA-seq,Homo_633,AD.vs.control; bulk RNA-seq,Homo_633,AD.vs.MCI	7
BP	GO:0051261	protein depolymerization	bulk RNA-seq,Homo_723,AD.vs.control; bulk RNA-seq,Homo_723,AD.vs.MCI; bulk RNA-seq,Homo_723,MCI.vs.control; bulk RNA-seq,Homo_714,AD.vs.MCI; bulk RNA-seq,Homo_714,MCI.vs.control; bulk RNA-seq,Homo_633,AD.vs.control; bulk RNA-seq,Homo_633,AD.vs.MCI	7
BP	GO:0032615	interleukin-12 production	bulk RNA-seq,Homo_723,AD.vs.control; bulk RNA-seq,Homo_723,MCI.vs.control; bulk RNA-seq,Homo_714,MCI.vs.control; bulk RNA-seq,Homo_633,AD.vs.control; bulk RNA-seq,Homo_633,AD.vs.MCI	5
BP	GO:0032655	regulation of interleukin-12 production	bulk RNA-seq,Homo_723,AD.vs.control; bulk RNA-seq,Homo_723,MCI.vs.control; bulk RNA-seq,Homo_714,MCI.vs.control; bulk RNA-seq,Homo_633,AD.vs.control; bulk RNA-seq,Homo_633,AD.vs.MCI	5
MF	GO:0016651	oxidoreductase activity, acting on NAD(P)H	bulk RNA-seq,Homo_723,AD.vs.control; bulk RNA-seq,Homo_723,MCI.vs.control; bulk RNA-seq,Homo_714,AD.vs.control; bulk RNA-seq,Homo_714,MCI.vs.control; bulk RNA-seq,Homo_633,AD.vs.control; bulk RNA-seq,Homo_633,AD.vs.MCI	6
BP	GO:1905818	regulation of chromosome separation	bulk RNA-seq,Homo_723,AD.vs.control; bulk RNA-seq,Homo_723,MCI.vs.control; bulk RNA-seq,Homo_714,AD.vs.control; bulk RNA-seq,Homo_714,MCI.vs.control; bulk RNA-seq,Homo_633,AD.vs.control; bulk RNA-seq,Homo_633,AD.vs.MCI	6
BP	GO:0050922	negative regulation of chemotaxis	bulk RNA-seq,Homo_723,AD.vs.control; bulk RNA-seq,Homo_723,AD.vs.MCI; bulk RNA-seq,Homo_723,MCI.vs.control; bulk RNA-seq,Homo_714,AD.vs.MCI; bulk RNA-seq,Homo_714,MCI.vs.control; bulk RNA-seq,Homo_633,AD.vs.control; bulk RNA-seq,ROSMAP,AD.vs.control; scRNA-seq,SRP330776,Naive CD8+	8
BP	GO:0009988	cell-cell recognition	bulk RNA-seq,Homo_723,AD.vs.control; bulk RNA-seq,Homo_714,AD.vs.control; bulk RNA-seq,Homo_714,AD.vs.MCI; bulk RNA-seq,Homo_714,MCI.vs.control; bulk RNA-seq,Homo_633,AD.vs.control; bulk RNA-seq,Homo_633,AD.vs.MCI	6
BP	GO:0060795	cell fate commitment involved in formation of primary germ	bulk RNA-seq,Homo_723,AD.vs.control; bulk RNA-seq,Homo_723,AD.vs.MCI; bulk RNA-seq,Homo_723,MCI.vs.control; bulk RNA-seq,Homo_714,AD.vs.MCI	4
BP	GO:0003209	cardiac atrium morphogenesis	bulk RNA-seq,Homo_723,AD.vs.control; bulk RNA-seq,Homo_723,AD.vs.MCI; bulk RNA-seq,Homo_723,MCI.vs.control; bulk RNA-seq,Homo_714,AD.vs.control; bulk RNA-seq,Homo_714,AD.vs.MCI; bulk RNA-seq,Homo_714,MCI.vs.control	6
BP	GO:0046068	cGMP metabolic process	bulk RNA-seq,Homo_723,AD.vs.control; bulk RNA-seq,Homo_723,AD.vs.MCI; bulk RNA-seq,Homo_723,MCI.vs.control; bulk RNA-seq,Homo_714,AD.vs.MCI; bulk RNA-seq,Homo_714,MCI.vs.control	5
BP	GO:2000142	regulation of DNA-templated transcription initiation	bulk RNA-seq,Homo_723,AD.vs.control; bulk RNA-seq,Homo_723,MCI.vs.control; bulk RNA-seq,Homo_714,MCI.vs.control; bulk RNA-seq,Homo_633,AD.vs.control; bulk RNA-seq,Homo_633,AD.vs.MCI	5
BP	GO:0032008	positive regulation of TOR signaling	bulk RNA-seq,Homo_723,AD.vs.control; bulk RNA-seq,Homo_723,MCI.vs.control; bulk RNA-seq,Homo_714,AD.vs.control; bulk RNA-seq,Homo_714,MCI.vs.control; bulk RNA-seq,Homo_633,AD.vs.control; bulk RNA-seq,Homo_633,AD.vs.MCI	6
BP	GO:0051933	amino acid neurotransmitter reuptake	bulk RNA-seq,Homo_723,AD.vs.control; bulk RNA-seq,Homo_723,AD.vs.MCI; bulk RNA-seq,Homo_723,MCI.vs.control; bulk RNA-	4

BP	GO:2001224	positive regulation of neuron migration	bulk RNA-seq,Homo_723,AD.vs.control; bulk RNA-seq,Homo_723,AD.vs.MCI; bulk RNA-seq,Homo_723,MCI.vs.control; bulk RNA-seq,Homo_714,AD.vs.MCI; bulk RNA-seq,Homo_714,MCI.vs.control; bulk RNA-seq,Homo_633,AD.vs.control	6
BP	GO:0016264	gap junction assembly	bulk RNA-seq,Homo_723,AD.vs.control; bulk RNA-seq,Homo_723,AD.vs.MCI; bulk RNA-seq,Homo_723,MCI.vs.control; bulk RNA-seq,Homo_714,AD.vs.MCI; bulk RNA-seq,Homo_714,MCI.vs.control	5
BP	GO:0002028	regulation of sodium ion transport	bulk RNA-seq,Homo_723,AD.vs.control; bulk RNA-seq,Homo_723,AD.vs.MCI; bulk RNA-seq,Homo_723,MCI.vs.control; bulk RNA-seq,Homo_714,AD.vs.MCI; bulk RNA-seq,Homo_714,MCI.vs.control; bulk RNA-seq,Homo_633,AD.vs.control	6
BP	GO:0014896	muscle hypertrophy	bulk RNA-seq,Homo_723,AD.vs.control; bulk RNA-seq,Homo_723,AD.vs.MCI; bulk RNA-seq,Homo_723,MCI.vs.control; bulk RNA-seq,Homo_714,AD.vs.control; bulk RNA-seq,Homo_714,AD.vs.MCI; bulk RNA-seq,Homo_714,MCI.vs.control; bulk RNA-seq,Homo_633,AD.vs.control	7
CC	GO:0031932	TORC2 complex	bulk RNA-seq,Homo_723,AD.vs.control; bulk RNA-seq,Homo_723,MCI.vs.control; bulk RNA-seq,Homo_633,AD.vs.control; bulk RNA-seq,Homo_633,AD.vs.MCI; bulk RNA-seq,Homo_633,MCI.vs.control	5
BP	GO:0060442	branching involved in prostate gland morphogenesis	bulk RNA-seq,Homo_723,AD.vs.control; bulk RNA-seq,Homo_723,AD.vs.MCI; bulk RNA-seq,Homo_633,MCI.vs.control	3
BP	GO:0090331	negative regulation of platelet aggregation	bulk RNA-seq,Homo_723,AD.vs.control; bulk RNA-seq,Homo_723,AD.vs.MCI; bulk RNA-seq,Homo_723,MCI.vs.control; bulk RNA-seq,Homo_714,AD.vs.control; bulk RNA-seq,Homo_714,AD.vs.MCI; bulk RNA-seq,Homo_714,MCI.vs.control	6
BP	GO:0021795	cerebral cortex cell migration	bulk RNA-seq,Homo_723,AD.vs.control; bulk RNA-seq,Homo_723,AD.vs.MCI; bulk RNA-seq,Homo_723,MCI.vs.control; bulk RNA-seq,Homo_714,AD.vs.MCI; bulk RNA-seq,Homo_714,MCI.vs.control; bulk RNA-seq,Homo_633,AD.vs.control; bulk RNA-seq,Homo_633,AD.vs.MCI	7
MF	GO:0004708	MAP kinase kinase activity	bulk RNA-seq,Homo_723,AD.vs.control; bulk RNA-seq,Homo_723,AD.vs.MCI; bulk RNA-seq,Homo_723,MCI.vs.control; bulk RNA-	4
BP	GO:0032892	positive regulation of organic acid transport	bulk RNA-seq,Homo_723,AD.vs.control; bulk RNA-seq,Homo_723,AD.vs.MCI; bulk RNA-seq,Homo_723,MCI.vs.control; bulk RNA-seq,Homo_714,AD.vs.MCI	4
BP	GO:0045732	positive regulation of protein catabolic process	bulk RNA-seq,Homo_723,AD.vs.control; bulk RNA-seq,Homo_723,AD.vs.MCI; bulk RNA-seq,Homo_723,MCI.vs.control; bulk RNA-seq,Homo_714,AD.vs.control; bulk RNA-seq,Homo_714,AD.vs.MCI; bulk RNA-seq,Homo_633,AD.vs.control; bulk RNA-seq,Homo_633,AD.vs.MCI	7
CC	GO:0031463	Cul3-RING ubiquitin ligase complex	bulk RNA-seq,Homo_723,AD.vs.control; bulk RNA-seq,Homo_714,AD.vs.control; bulk RNA-seq,Homo_633,AD.vs.control; bulk RNA-seq,Homo_633,AD.vs.MCI	4
MF	GO:0004526	ribonuclease P activity	bulk RNA-seq,Homo_723,AD.vs.control; bulk RNA-seq,Homo_633,AD.vs.control; bulk RNA-seq,Homo_633,AD.vs.MCI	3
BP	GO:0097049	motor neuron apoptotic process	bulk RNA-seq,Homo_723,AD.vs.control; bulk RNA-seq,Homo_723,AD.vs.MCI; bulk RNA-seq,Homo_723,MCI.vs.control; bulk RNA-	4
BP	GO:0043373	CD4-positive, alpha-beta T cell lineage commitment	bulk RNA-seq,Homo_723,AD.vs.control; bulk RNA-seq,Homo_714,AD.vs.control; bulk RNA-seq,Homo_714,MCI.vs.control; bulk RNA-	4
BP	GO:1902459	positive regulation of stem cell population maintenance	bulk RNA-seq,Homo_723,AD.vs.control; bulk RNA-seq,Homo_723,MCI.vs.control; bulk RNA-seq,Homo_714,AD.vs.control; bulk RNA-seq,Homo_714,MCI.vs.control; bulk RNA-seq,Homo_633,AD.vs.control; bulk RNA-seq,Homo_633,AD.vs.MCI	6
BP	GO:0090713	immunological memory process	bulk RNA-seq,Homo_723,AD.vs.control; bulk RNA-seq,Homo_723,MCI.vs.control; bulk RNA-seq,Homo_714,MCI.vs.control	3
BP	GO:0002698	negative regulation of immune effector process	bulk RNA-seq,Homo_723,AD.vs.control; bulk RNA-seq,Homo_723,MCI.vs.control; bulk RNA-seq,Homo_714,AD.vs.MCI; bulk RNA-seq,Homo_714,MCI.vs.control; bulk RNA-seq,Homo_633,AD.vs.control; bulk RNA-seq,Homo_633,AD.vs.MCI	6
BP	GO:0072234	metanephric nephron tubule development	bulk RNA-seq,Homo_723,AD.vs.control; bulk RNA-seq,Homo_723,AD.vs.MCI; bulk RNA-seq,Homo_723,MCI.vs.control; bulk RNA-seq,Homo_714,AD.vs.MCI; bulk RNA-seq,Homo_714,MCI.vs.control	5
BP	GO:0046058	cAMP metabolic process	bulk RNA-seq,Homo_723,AD.vs.control; bulk RNA-seq,Homo_723,AD.vs.MCI; bulk RNA-seq,Homo_723,MCI.vs.control; bulk RNA-seq,Homo_714,AD.vs.MCI; bulk RNA-seq,Homo_714,MCI.vs.control	5
BP	GO:0033572	transferrin transport	bulk RNA-seq,Homo_723,AD.vs.control; bulk RNA-seq,Homo_714,AD.vs.control; bulk RNA-seq,Homo_633,AD.vs.control	3
BP	GO:0051132	NK T cell activation	bulk RNA-seq,Homo_723,AD.vs.control	1
BP	GO:0009886	post-embryonic animal morphogenesis	bulk RNA-seq,Homo_723,AD.vs.control; bulk RNA-seq,Homo_723,AD.vs.MCI; bulk RNA-seq,Homo_723,MCI.vs.control; bulk RNA-seq,Homo_714,AD.vs.MCI; bulk RNA-seq,Homo_714,MCI.vs.control	5
BP	GO:0010595	positive regulation of endothelial cell migration	bulk RNA-seq,Homo_723,AD.vs.control; bulk RNA-seq,Homo_723,AD.vs.MCI; bulk RNA-seq,Homo_723,MCI.vs.control; bulk RNA-seq,Homo_714,AD.vs.control; bulk RNA-seq,Homo_714,AD.vs.MCI; bulk RNA-seq,Homo_633,AD.vs.control; bulk RNA-seq,Homo_633,AD.vs.MCI	7
BP	GO:0018023	peptidyl-lysine trimethylation	bulk RNA-seq,Homo_723,AD.vs.control; bulk RNA-seq,Homo_723,MCI.vs.control; bulk RNA-seq,Homo_714,AD.vs.control; bulk RNA-seq,Homo_714,MCI.vs.control; bulk RNA-seq,Homo_633,AD.vs.control; bulk RNA-seq,Homo_633,AD.vs.MCI	6
BP	GO:1904888	cranial skeletal system development	bulk RNA-seq,Homo_723,AD.vs.control; bulk RNA-seq,Homo_723,AD.vs.MCI; bulk RNA-seq,Homo_723,MCI.vs.control; bulk RNA-seq,Homo_714,AD.vs.MCI; bulk RNA-seq,Homo_714,MCI.vs.control; bulk RNA-seq,Homo_633,AD.vs.control	6
CC	GO:0031362	anchored component of external side of plasma membrane	bulk RNA-seq,Homo_723,AD.vs.control; bulk RNA-seq,Homo_723,AD.vs.MCI; bulk RNA-seq,Homo_714,AD.vs.MCI	3
BP	GO:0007512	adult heart development	bulk RNA-seq,Homo_723,AD.vs.control; bulk RNA-seq,Homo_723,AD.vs.MCI	2
BP	GO:0021794	thalamus development	bulk RNA-seq,Homo_723,AD.vs.control; bulk RNA-seq,Homo_723,AD.vs.MCI; bulk RNA-seq,Homo_723,MCI.vs.control; bulk RNA-	4
BP	GO:1903587	regulation of blood vessel endothelial cell proliferation involved in sprouting angiogenesis	bulk RNA-seq,Homo_723,AD.vs.control; bulk RNA-seq,Homo_723,AD.vs.MCI; bulk RNA-seq,Homo_714,AD.vs.control; bulk RNA-seq,Homo_714,AD.vs.MCI	4
BP	GO:0006458	'de novo' protein folding	bulk RNA-seq,Homo_723,AD.vs.control; bulk RNA-seq,Homo_723,MCI.vs.control; bulk RNA-seq,Homo_714,AD.vs.control; bulk RNA-seq,Homo_714,MCI.vs.control; bulk RNA-seq,Homo_633,AD.vs.control; bulk RNA-seq,Homo_633,AD.vs.MCI	6
BP	GO:0014047	glutamate secretion	bulk RNA-seq,Homo_723,AD.vs.control; bulk RNA-seq,Homo_723,AD.vs.MCI; bulk RNA-seq,Homo_723,MCI.vs.control; bulk RNA-seq,Homo_714,AD.vs.MCI	4
BP	GO:0140014	mitotic nuclear division	bulk RNA-seq,Homo_723,AD.vs.control; bulk RNA-seq,Homo_723,AD.vs.MCI; bulk RNA-seq,Homo_723,MCI.vs.control; bulk RNA-seq,Homo_714,AD.vs.control; bulk RNA-seq,Homo_714,AD.vs.MCI; bulk RNA-seq,Homo_633,AD.vs.control; bulk RNA-seq,Homo_633,AD.vs.MCI	7
CC	GO:0099023	vesicle tethering complex	bulk RNA-seq,Homo_723,AD.vs.control; bulk RNA-seq,Homo_723,MCI.vs.control; bulk RNA-seq,Homo_714,AD.vs.control; bulk RNA-seq,Homo_714,MCI.vs.control; bulk RNA-seq,Homo_633,AD.vs.control; bulk RNA-seq,Homo_633,AD.vs.MCI	6
BP	GO:0045623	negative regulation of T-helper cell differentiation	bulk RNA-seq,Homo_723,AD.vs.control; bulk RNA-seq,Homo_723,MCI.vs.control; bulk RNA-seq,Homo_714,MCI.vs.control; bulk RNA-seq,Homo_633,AD.vs.control; bulk RNA-seq,Homo_633,AD.vs.MCI; bulk RNA-seq,Homo_633,MCI.vs.control	6
CC	GO:0034399	nuclear periphery	bulk RNA-seq,Homo_723,AD.vs.control; bulk RNA-seq,Homo_723,MCI.vs.control; bulk RNA-seq,Homo_714,AD.vs.control; bulk RNA-seq,Homo_633,AD.vs.control; bulk RNA-seq,Homo_633,AD.vs.MCI	5
BP	GO:2000649	regulation of sodium ion transmembrane transporter activity	bulk RNA-seq,Homo_723,AD.vs.control; bulk RNA-seq,Homo_723,AD.vs.MCI; bulk RNA-seq,Homo_723,MCI.vs.control; bulk RNA-seq,Homo_714,AD.vs.MCI; bulk RNA-seq,Homo_633,AD.vs.control	5
BP	GO:0120192	tight junction assembly	bulk RNA-seq,Homo_723,AD.vs.control; bulk RNA-seq,Homo_723,AD.vs.MCI; bulk RNA-seq,Homo_723,MCI.vs.control; bulk RNA-seq,Homo_714,AD.vs.control; bulk RNA-seq,Homo_714,AD.vs.MCI; bulk RNA-seq,Homo_714,MCI.vs.control	6

MF	GO:0015294	solute:cation symporter activity	bulk RNA-seq,Homo_723,AD.vs.control; bulk RNA-seq,Homo_723,AD.vs.MCI; bulk RNA-seq,Homo_723,MCI.vs.control; bulk RNA-seq,Homo_714,AD.vs.control; bulk RNA-seq,Homo_714,AD.vs.MCI; bulk RNA-seq,Homo_714,MCI.vs.control	6
CC	GO:0000152	nuclear ubiquitin ligase complex	bulk RNA-seq,Homo_723,AD.vs.control; bulk RNA-seq,Homo_723,MCI.vs.control; bulk RNA-seq,Homo_714,AD.vs.control; bulk RNA-seq,Homo_714,MCI.vs.control; bulk RNA-seq,Homo_633,AD.vs.control; bulk RNA-seq,Homo_633,AD.vs.MCI	6
BP	GO:0045907	positive regulation of vasoconstriction	bulk RNA-seq,Homo_723,AD.vs.control; bulk RNA-seq,Homo_723,AD.vs.MCI; bulk RNA-seq,Homo_723,MCI.vs.control; bulk RNA-seq,Homo_714,AD.vs.MCI; bulk RNA-seq,Homo_714,MCI.vs.control	5
MF	GO:0005245	voltage-gated calcium channel activity	bulk RNA-seq,Homo_723,AD.vs.control; bulk RNA-seq,Homo_723,AD.vs.MCI; bulk RNA-seq,Homo_723,MCI.vs.control; bulk RNA-seq,Homo_714,AD.vs.MCI	4
BP	GO:0061178	regulation of insulin secretion involved in cellular response to glucose stimulus	bulk RNA-seq,Homo_723,AD.vs.control; bulk RNA-seq,Homo_723,AD.vs.MCI; bulk RNA-seq,Homo_723,MCI.vs.control; bulk RNA-seq,Homo_714,AD.vs.MCI; bulk RNA-seq,Homo_714,MCI.vs.control; bulk RNA-seq,Homo_633,AD.vs.control; bulk RNA-seq,Homo_633,AD.vs.MCI	7
BP	GO:0045143	homologous chromosome segregation	bulk RNA-seq,Homo_723,AD.vs.control; bulk RNA-seq,Homo_723,AD.vs.MCI; bulk RNA-seq,Homo_723,MCI.vs.control; bulk RNA-seq,Homo_714,AD.vs.MCI; bulk RNA-seq,Homo_714,MCI.vs.control; bulk RNA-seq,Homo_633,AD.vs.control; bulk RNA-seq,Homo_633,AD.vs.MCI	7
BP	GO:0022400	regulation of rhodopsin mediated signaling pathway	bulk RNA-seq,Homo_723,AD.vs.control; bulk RNA-seq,Homo_714,AD.vs.control	2
BP	GO:0048172	regulation of short-term neuronal synaptic plasticity	bulk RNA-seq,Homo_723,AD.vs.control; bulk RNA-seq,Homo_723,AD.vs.MCI; bulk RNA-seq,Homo_723,MCI.vs.control	3
MF	GO:0008417	fucosyltransferase activity	bulk RNA-seq,Homo_723,AD.vs.control; bulk RNA-seq,Homo_723,AD.vs.MCI; bulk RNA-seq,Homo_723,MCI.vs.control	3
MF	GO:0016893	endonuclease activity, active with either ribo- or deoxyribonucleic acids and producing 5'-phosphomonoesters	bulk RNA-seq,Homo_723,AD.vs.control; bulk RNA-seq,Homo_723,MCI.vs.control; bulk RNA-seq,Homo_714,AD.vs.control; bulk RNA-seq,Homo_714,MCI.vs.control; bulk RNA-seq,Homo_633,AD.vs.control; bulk RNA-seq,Homo_633,AD.vs.MCI	6
BP	GO:0034440	lipid oxidation	bulk RNA-seq,Homo_723,AD.vs.control; bulk RNA-seq,Homo_723,MCI.vs.control; bulk RNA-seq,Homo_714,AD.vs.control; bulk RNA-seq,Homo_714,MCI.vs.control; bulk RNA-seq,Homo_633,AD.vs.control; bulk RNA-seq,Homo_633,AD.vs.MCI	6
MF	GO:0044183	protein folding chaperone	bulk RNA-seq,Homo_723,AD.vs.control; bulk RNA-seq,Homo_723,MCI.vs.control; bulk RNA-seq,Homo_714,AD.vs.control; bulk RNA-seq,Homo_714,MCI.vs.control; bulk RNA-seq,Homo_633,AD.vs.control; bulk RNA-seq,Homo_633,AD.vs.MCI	6
BP	GO:0032703	negative regulation of interleukin-2 production	bulk RNA-seq,Homo_723,AD.vs.control; bulk RNA-seq,Homo_723,MCI.vs.control; bulk RNA-seq,Homo_714,MCI.vs.control	3
MF	GO:0030983	mismatched DNA binding	bulk RNA-seq,Homo_723,AD.vs.control; bulk RNA-seq,Homo_723,MCI.vs.control; bulk RNA-seq,Homo_714,AD.vs.control; bulk RNA-seq,Homo_714,MCI.vs.control; bulk RNA-seq,Homo_633,AD.vs.control; bulk RNA-seq,Homo_633,AD.vs.MCI; bulk RNA-seq,Homo_633,MCI.vs.control	7
BP	GO:0097191	extrinsic apoptotic signaling pathway	bulk RNA-seq,Homo_723,AD.vs.control; bulk RNA-seq,Homo_723,AD.vs.MCI; bulk RNA-seq,Homo_723,MCI.vs.control; bulk RNA-seq,Homo_714,AD.vs.MCI; bulk RNA-seq,Homo_633,AD.vs.control; bulk RNA-seq,Homo_633,AD.vs.MCI	6
BP	GO:0060576	intestinal epithelial cell development	bulk RNA-seq,Homo_723,AD.vs.control; bulk RNA-seq,Homo_714,AD.vs.MCI	2
CC	GO:0097225	sperm midpiece	bulk RNA-seq,Homo_723,AD.vs.control; bulk RNA-seq,Homo_723,AD.vs.MCI; bulk RNA-seq,Homo_714,AD.vs.MCI; bulk RNA-seq,Homo_633,AD.vs.control; bulk RNA-seq,Homo_633,AD.vs.MCI	5
BP	GO:0010887	negative regulation of cholesterol storage	bulk RNA-seq,Homo_723,AD.vs.control; bulk RNA-seq,Homo_723,MCI.vs.control; bulk RNA-seq,Homo_714,MCI.vs.control; bulk RNA-seq,Homo_633,AD.vs.control; bulk RNA-seq,Homo_633,AD.vs.MCI	5
BP	GO:0060174	limb bud formation	bulk RNA-seq,Homo_723,AD.vs.control; bulk RNA-seq,Homo_723,AD.vs.MCI	2
BP	GO:0007614	short-term memory	bulk RNA-seq,Homo_723,AD.vs.control; bulk RNA-seq,Homo_723,MCI.vs.control	2
BP	GO:0044788	modulation by host of viral process	bulk RNA-seq,Homo_723,AD.vs.control; bulk RNA-seq,Homo_714,AD.vs.control; bulk RNA-seq,Homo_633,AD.vs.control; bulk RNA-seq,Homo_633,AD.vs.MCI	4
BP	GO:0048641	regulation of skeletal muscle tissue development	bulk RNA-seq,Homo_723,AD.vs.control; bulk RNA-seq,Homo_723,AD.vs.MCI; bulk RNA-seq,Homo_723,MCI.vs.control	3
BP	GO:0034134	toll-like receptor 2 signaling pathway	bulk RNA-seq,Homo_723,AD.vs.control; bulk RNA-seq,Homo_723,MCI.vs.control; bulk RNA-seq,Homo_714,MCI.vs.control; bulk RNA-seq,Homo_633,AD.vs.control; bulk RNA-seq,Homo_633,MCI.vs.control	5
BP	GO:0140029	exocytic process	bulk RNA-seq,Homo_723,AD.vs.control; bulk RNA-seq,Homo_723,AD.vs.MCI; bulk RNA-seq,Homo_723,MCI.vs.control; bulk RNA-seq,Homo_714,AD.vs.MCI; bulk RNA-seq,Homo_714,MCI.vs.control; bulk RNA-seq,Homo_633,AD.vs.control; bulk RNA-seq,Homo_633,AD.vs.MCI	7
BP	GO:0006266	DNA ligation	bulk RNA-seq,Homo_723,AD.vs.control; bulk RNA-seq,Homo_723,MCI.vs.control; bulk RNA-seq,Homo_714,MCI.vs.control; bulk RNA-seq,Homo_633,AD.vs.control; bulk RNA-seq,Homo_633,MCI.vs.control	5
BP	GO:1901800	positive regulation of proteasomal protein catabolic process	bulk RNA-seq,Homo_723,AD.vs.control; bulk RNA-seq,Homo_723,MCI.vs.control; bulk RNA-seq,Homo_714,MCI.vs.control; bulk RNA-seq,Homo_633,AD.vs.control; bulk RNA-seq,Homo_633,AD.vs.MCI	5
BP	GO:0071353	cellular response to interleukin-4	bulk RNA-seq,Homo_723,AD.vs.control; bulk RNA-seq,Homo_723,MCI.vs.control; bulk RNA-seq,Homo_714,MCI.vs.control; bulk RNA-	4
BP	GO:0006497	protein lipidation	bulk RNA-seq,Homo_723,AD.vs.control; bulk RNA-seq,Homo_723,MCI.vs.control; bulk RNA-seq,Homo_714,AD.vs.control; bulk RNA-seq,Homo_714,MCI.vs.control; bulk RNA-seq,Homo_633,AD.vs.control; bulk RNA-seq,Homo_633,AD.vs.MCI	6
BP	GO:0045910	negative regulation of DNA recombination	bulk RNA-seq,Homo_723,AD.vs.control; bulk RNA-seq,Homo_723,MCI.vs.control; bulk RNA-seq,Homo_714,AD.vs.control; bulk RNA-seq,Homo_714,MCI.vs.control; bulk RNA-seq,Homo_633,AD.vs.control; bulk RNA-seq,Homo_633,AD.vs.MCI	6
BP	GO:0031645	negative regulation of nervous system process	bulk RNA-seq,Homo_723,AD.vs.control	1
BP	GO:0033238	regulation of cellular amine metabolic process	bulk RNA-seq,Homo_723,AD.vs.control; bulk RNA-seq,Homo_723,AD.vs.MCI; bulk RNA-seq,Homo_723,MCI.vs.control; bulk RNA-seq,Homo_714,AD.vs.control; bulk RNA-seq,Homo_714,AD.vs.MCI	5
BP	GO:0061309	cardiac neural crest cell development involved in outflow tract morphogenesis	bulk RNA-seq,Homo_723,AD.vs.control; bulk RNA-seq,Homo_723,AD.vs.MCI; bulk RNA-seq,Homo_723,MCI.vs.control; bulk RNA-seq,Homo_714,AD.vs.MCI	4
BP	GO:0033523	histone H2B ubiquitination	bulk RNA-seq,Homo_723,AD.vs.control; bulk RNA-seq,Homo_633,AD.vs.control; bulk RNA-seq,Homo_633,AD.vs.MCI	3
MF	GO:0005229	intracellular calcium activated chloride channel activity	bulk RNA-seq,Homo_723,AD.vs.control; bulk RNA-seq,Homo_723,AD.vs.MCI; bulk RNA-seq,Homo_723,MCI.vs.control; bulk RNA-seq,Homo_714,AD.vs.control; bulk RNA-seq,Homo_714,AD.vs.MCI	5
MF	GO:0061778	intracellular chloride channel activity	bulk RNA-seq,Homo_723,AD.vs.control; bulk RNA-seq,Homo_723,AD.vs.MCI; bulk RNA-seq,Homo_723,MCI.vs.control; bulk RNA-seq,Homo_714,AD.vs.control; bulk RNA-seq,Homo_714,AD.vs.MCI	5
BP	GO:0051650	establishment of vesicle localization	bulk RNA-seq,Homo_723,AD.vs.control; bulk RNA-seq,Homo_723,AD.vs.MCI; bulk RNA-seq,Homo_723,MCI.vs.control; bulk RNA-seq,Homo_714,AD.vs.control; bulk RNA-seq,Homo_633,AD.vs.control; bulk RNA-seq,Homo_633,AD.vs.MCI	6
BP	GO:0030890	positive regulation of B cell proliferation	bulk RNA-seq,Homo_723,AD.vs.control; bulk RNA-seq,Homo_723,MCI.vs.control; bulk RNA-seq,Homo_714,MCI.vs.control; bulk RNA-seq,Homo_633,AD.vs.control; bulk RNA-seq,Homo_633,AD.vs.MCI	5
BP	GO:0032233	positive regulation of actin filament bundle assembly	bulk RNA-seq,Homo_723,AD.vs.control; bulk RNA-seq,Homo_723,AD.vs.MCI; bulk RNA-seq,Homo_723,MCI.vs.control; bulk RNA-seq,Homo_714,AD.vs.MCI; bulk RNA-seq,Homo_714,MCI.vs.control; bulk RNA-seq,Homo_633,AD.vs.control; bulk RNA-seq,Homo_633,AD.vs.MCI	7

BP	GO:1900024	regulation of substrate adhesion-dependent cell spreading	bulk RNA-seq,Homo_723,AD.vs.control; bulk RNA-seq,Homo_723,AD.vs.MCI; bulk RNA-seq,Homo_723,MCI.vs.control; bulk RNA-seq,Homo_714,AD.vs.MCI; bulk RNA-seq,Homo_714,MCI.vs.control; bulk RNA-seq,Homo_633,AD.vs.control; bulk RNA-seq,Homo_633,AD.vs.MCI	7
BP	GO:0061157	mRNA destabilization	bulk RNA-seq,Homo_723,AD.vs.control; bulk RNA-seq,Homo_723,MCI.vs.control; bulk RNA-seq,Homo_714,AD.vs.control; bulk RNA-seq,Homo_714,MCI.vs.control; bulk RNA-seq,Homo_633,AD.vs.control; bulk RNA-seq,Homo_633,AD.vs.MCI	6
CC	GO:0005667	transcription regulator complex	bulk RNA-seq,Homo_723,AD.vs.control; bulk RNA-seq,Homo_723,AD.vs.MCI; bulk RNA-seq,Homo_714,AD.vs.MCI; bulk RNA-seq,Homo_633,AD.vs.control; bulk RNA-seq,Homo_633,AD.vs.MCI; bulk RNA-seq,SRP223445,AD.vs.control; scRNA-seq,SRP330776,Naive CD8+ T cell_2-AD.vs.control	7
BP	GO:0010838	positive regulation of keratinocyte proliferation	bulk RNA-seq,Homo_723,AD.vs.control; bulk RNA-seq,Homo_723,AD.vs.MCI; bulk RNA-seq,Homo_714,AD.vs.control; bulk RNA-seq,Homo_714,AD.vs.MCI	4
BP	GO:0060972	left/right pattern formation	bulk RNA-seq,Homo_723,AD.vs.control; bulk RNA-seq,Homo_723,AD.vs.MCI; bulk RNA-seq,Homo_723,MCI.vs.control; bulk RNA-seq,Homo_714,AD.vs.MCI; bulk RNA-seq,Homo_714,MCI.vs.control	5
MF	GO:0030548	acetylcholine receptor regulator activity	bulk RNA-seq,Homo_723,AD.vs.control; bulk RNA-seq,Homo_723,AD.vs.MCI; bulk RNA-seq,Homo_723,MCI.vs.control; bulk RNA-	4
MF	GO:0099602	neurotransmitter receptor regulator activity	bulk RNA-seq,Homo_723,AD.vs.control; bulk RNA-seq,Homo_723,AD.vs.MCI; bulk RNA-seq,Homo_723,MCI.vs.control; bulk RNA-	4
MF	GO:0016627	oxidoreductase activity, acting on the CH-CH group of donors	bulk RNA-seq,Homo_723,AD.vs.control; bulk RNA-seq,Homo_714,AD.vs.control; bulk RNA-seq,Homo_714,MCI.vs.control; bulk RNA-seq,Homo_633,AD.vs.control; bulk RNA-seq,Homo_633,AD.vs.MCI	5
BP	GO:0000387	spliceosomal snRNP assembly	bulk RNA-seq,Homo_723,AD.vs.control; bulk RNA-seq,Homo_723,MCI.vs.control; bulk RNA-seq,Homo_714,MCI.vs.control; bulk RNA-	4
CC	GO:0097731	9+0 non-motile cilium	bulk RNA-seq,Homo_723,AD.vs.control; bulk RNA-seq,Homo_723,AD.vs.MCI; bulk RNA-seq,Homo_723,MCI.vs.control; bulk RNA-seq,Homo_714,AD.vs.control; bulk RNA-seq,Homo_714,AD.vs.MCI; bulk RNA-seq,Homo_633,AD.vs.control; bulk RNA-seq,Homo_633,AD.vs.MCI	7
BP	GO:0051969	regulation of transmission of nerve impulse	bulk RNA-seq,Homo_723,AD.vs.control; bulk RNA-seq,Homo_723,AD.vs.MCI; bulk RNA-seq,Homo_723,MCI.vs.control	3
BP	GO:0033627	cell adhesion mediated by integrin	bulk RNA-seq,Homo_723,AD.vs.control; bulk RNA-seq,Homo_723,AD.vs.MCI; bulk RNA-seq,Homo_723,MCI.vs.control; bulk RNA-seq,Homo_714,AD.vs.MCI; bulk RNA-seq,Homo_714,MCI.vs.control; bulk RNA-seq,Homo_633,AD.vs.control; bulk RNA-seq,Homo_633,AD.vs.MCI	7
BP	GO:0018149	peptide cross-linking	bulk RNA-seq,Homo_723,AD.vs.control; bulk RNA-seq,Homo_723,AD.vs.MCI; bulk RNA-seq,Homo_723,MCI.vs.control; bulk RNA-seq,Homo_714,AD.vs.control; bulk RNA-seq,Homo_714,AD.vs.MCI; bulk RNA-seq,Homo_714,MCI.vs.control; bulk RNA-seq,Homo_633,AD.vs.control	7
BP	GO:0048771	tissue remodeling	bulk RNA-seq,Homo_723,AD.vs.control; bulk RNA-seq,Homo_723,AD.vs.MCI; bulk RNA-seq,Homo_723,MCI.vs.control; bulk RNA-seq,Homo_714,AD.vs.control; bulk RNA-seq,Homo_714,AD.vs.MCI; bulk RNA-seq,Homo_633,AD.vs.control; bulk RNA-seq,Homo_633,AD.vs.MCI	7
BP	GO:0035794	positive regulation of mitochondrial membrane permeability	bulk RNA-seq,Homo_723,AD.vs.control; bulk RNA-seq,Homo_714,AD.vs.control; bulk RNA-seq,Homo_714,MCI.vs.control; bulk RNA-seq,Homo_633,AD.vs.control; bulk RNA-seq,Homo_633,AD.vs.MCI	5
BP	GO:0045773	positive regulation of axon extension	bulk RNA-seq,Homo_723,AD.vs.control; bulk RNA-seq,Homo_723,AD.vs.MCI; bulk RNA-seq,Homo_723,MCI.vs.control; bulk RNA-seq,Homo_714,AD.vs.MCI; bulk RNA-seq,Homo_714,MCI.vs.control	5
MF	GO:0016504	peptidase activator activity	bulk RNA-seq,Homo_723,AD.vs.control; bulk RNA-seq,Homo_723,MCI.vs.control; bulk RNA-seq,Homo_714,MCI.vs.control; bulk RNA-seq,Homo_633,AD.vs.control; bulk RNA-seq,Homo_633,AD.vs.MCI	5
MF	GO:0016874	ligase activity	bulk RNA-seq,Homo_723,AD.vs.control; bulk RNA-seq,Homo_723,MCI.vs.control; bulk RNA-seq,Homo_714,AD.vs.control; bulk RNA-seq,Homo_633,AD.vs.control; bulk RNA-seq,Homo_633,AD.vs.MCI	5
MF	GO:0003727	single-stranded RNA binding	bulk RNA-seq,Homo_723,AD.vs.control; bulk RNA-seq,Homo_723,MCI.vs.control; bulk RNA-seq,Homo_714,AD.vs.control; bulk RNA-seq,Homo_714,MCI.vs.control; bulk RNA-seq,Homo_633,AD.vs.control; bulk RNA-seq,Homo_633,AD.vs.MCI	6
BP	GO:0007635	chemosensory behavior	bulk RNA-seq,Homo_723,AD.vs.control; bulk RNA-seq,Homo_723,AD.vs.MCI; bulk RNA-seq,Homo_723,MCI.vs.control	3
BP	GO:0050779	RNA destabilization	bulk RNA-seq,Homo_723,AD.vs.control; bulk RNA-seq,Homo_723,MCI.vs.control; bulk RNA-seq,Homo_714,AD.vs.control; bulk RNA-seq,Homo_714,MCI.vs.control; bulk RNA-seq,Homo_633,AD.vs.control; bulk RNA-seq,Homo_633,AD.vs.MCI	6
BP	GO:0016338	calcium-independent cell-cell adhesion via plasma membrane cell-adhesion molecules	bulk RNA-seq,Homo_723,AD.vs.control; bulk RNA-seq,Homo_723,AD.vs.MCI; bulk RNA-seq,Homo_714,AD.vs.MCI	3
BP	GO:0016556	mRNA modification	bulk RNA-seq,Homo_723,AD.vs.control; bulk RNA-seq,Homo_723,MCI.vs.control; bulk RNA-seq,Homo_714,MCI.vs.control; bulk RNA-seq,Homo_633,AD.vs.control; bulk RNA-seq,Homo_633,AD.vs.MCI; bulk RNA-seq,Homo_633,MCI.vs.control	6
BP	GO:0015908	fatty acid transport	bulk RNA-seq,Homo_723,AD.vs.control; bulk RNA-seq,Homo_723,AD.vs.MCI; bulk RNA-seq,Homo_723,MCI.vs.control; bulk RNA-seq,Homo_714,AD.vs.control; bulk RNA-seq,Homo_714,AD.vs.MCI; bulk RNA-seq,Homo_714,MCI.vs.control; bulk RNA-seq,Homo_633,AD.vs.control; bulk	8
BP	GO:0086103	G protein-coupled receptor signaling pathway involved in	bulk RNA-seq,Homo_723,AD.vs.control; bulk RNA-seq,Homo_723,AD.vs.MCI; bulk RNA-seq,Homo_714,AD.vs.control; bulk RNA-seq,Homo_714,AD.vs.MCI	4
BP	GO:0046717	acid secretion	bulk RNA-seq,Homo_723,AD.vs.control; bulk RNA-seq,Homo_723,AD.vs.MCI	2
BP	GO:1903959	regulation of anion transmembrane transport	bulk RNA-seq,Homo_723,AD.vs.control; bulk RNA-seq,Homo_723,AD.vs.MCI; bulk RNA-seq,Homo_723,MCI.vs.control; bulk RNA-seq,Homo_714,AD.vs.MCI; bulk RNA-seq,Homo_633,AD.vs.control	5
BP	GO:0070198	protein localization to chromosome, telomeric region	bulk RNA-seq,Homo_723,AD.vs.control; bulk RNA-seq,Homo_714,AD.vs.control; bulk RNA-seq,Homo_714,MCI.vs.control; bulk RNA-seq,Homo_633,AD.vs.control; bulk RNA-seq,Homo_633,AD.vs.MCI; bulk RNA-seq,Homo_633,MCI.vs.control	6
CC	GO:0060170	ciliary membrane	bulk RNA-seq,Homo_723,AD.vs.control; bulk RNA-seq,Homo_723,AD.vs.MCI; bulk RNA-seq,Homo_723,MCI.vs.control; bulk RNA-seq,Homo_714,AD.vs.MCI; bulk RNA-seq,Homo_714,MCI.vs.control; bulk RNA-seq,Homo_633,AD.vs.control; bulk RNA-seq,Homo_633,AD.vs.MCI	7
BP	GO:0032819	positive regulation of natural killer cell proliferation	bulk RNA-seq,Homo_723,AD.vs.control	1
BP	GO:0010888	negative regulation of lipid storage	bulk RNA-seq,Homo_723,AD.vs.control; bulk RNA-seq,Homo_723,MCI.vs.control; bulk RNA-seq,Homo_714,MCI.vs.control; bulk RNA-seq,Homo_633,AD.vs.control; bulk RNA-seq,Homo_633,AD.vs.MCI; bulk RNA-seq,Homo_633,MCI.vs.control	6
BP	GO:0007031	peroxisome organization	bulk RNA-seq,Homo_723,AD.vs.control; bulk RNA-seq,Homo_723,MCI.vs.control; bulk RNA-seq,Homo_714,AD.vs.control; bulk RNA-seq,Homo_714,MCI.vs.control; bulk RNA-seq,Homo_633,AD.vs.control; bulk RNA-seq,Homo_633,AD.vs.MCI; bulk RNA-seq,Homo_633,MCI.vs.control	7
BP	GO:0070534	protein K63-linked ubiquitination	bulk RNA-seq,Homo_723,AD.vs.control; bulk RNA-seq,Homo_723,MCI.vs.control; bulk RNA-seq,Homo_714,MCI.vs.control; bulk RNA-seq,Homo_633,AD.vs.control; bulk RNA-seq,Homo_633,AD.vs.MCI	5
CC	GO:0042470	melanosome	bulk RNA-seq,Homo_723,AD.vs.control; bulk RNA-seq,Homo_723,MCI.vs.control; bulk RNA-seq,Homo_714,AD.vs.control; bulk RNA-seq,Homo_714,MCI.vs.control; bulk RNA-seq,Homo_633,AD.vs.control; bulk RNA-seq,Homo_633,AD.vs.MCI	6
CC	GO:0048770	pigment granule	bulk RNA-seq,Homo_723,AD.vs.control; bulk RNA-seq,Homo_723,MCI.vs.control; bulk RNA-seq,Homo_714,AD.vs.control; bulk RNA-seq,Homo_714,MCI.vs.control; bulk RNA-seq,Homo_633,AD.vs.control; bulk RNA-seq,Homo_633,AD.vs.MCI	6
BP	GO:0006661	phosphatidylinositol biosynthetic process	bulk RNA-seq,Homo_723,AD.vs.control; bulk RNA-seq,Homo_723,MCI.vs.control; bulk RNA-seq,Homo_714,AD.vs.control; bulk RNA-seq,Homo_633,AD.vs.control; bulk RNA-seq,Homo_633,AD.vs.MCI; scRNA-seq,SRP330776,CD8+ T cell_2-AD.vs.control	6

BP	GO:2000050	regulation of non-canonical Wnt signaling pathway	bulk RNA-seq,Homo_723,AD.vs.control; bulk RNA-seq,Homo_723,AD.vs.MCI; bulk RNA-seq,Homo_723,MCI.vs.control; bulk RNA-seq,Homo_714,AD.vs.MCI; bulk RNA-seq,Homo_714,MCI.vs.control; bulk RNA-seq,Homo_633,AD.vs.control; bulk RNA-seq,Homo_633,AD.vs.MCI	7
CC	GO:0005778	peroxisomal membrane	bulk RNA-seq,Homo_723,AD.vs.control; bulk RNA-seq,Homo_723,MCI.vs.control; bulk RNA-seq,Homo_714,AD.vs.control; bulk RNA-seq,Homo_714,MCI.vs.control; bulk RNA-seq,Homo_633,AD.vs.control; bulk RNA-seq,Homo_633,AD.vs.MCI	6
CC	GO:0031903	microbody membrane	bulk RNA-seq,Homo_723,AD.vs.control; bulk RNA-seq,Homo_723,MCI.vs.control; bulk RNA-seq,Homo_714,AD.vs.control; bulk RNA-seq,Homo_714,MCI.vs.control; bulk RNA-seq,Homo_633,AD.vs.control; bulk RNA-seq,Homo_633,AD.vs.MCI	6
BP	GO:0070830	bicellular tight junction assembly	bulk RNA-seq,Homo_723,AD.vs.control; bulk RNA-seq,Homo_723,AD.vs.MCI; bulk RNA-seq,Homo_723,MCI.vs.control; bulk RNA-seq,Homo_714,AD.vs.control; bulk RNA-seq,Homo_714,AD.vs.MCI; bulk RNA-seq,Homo_714,MCI.vs.control	6
MF	GO:0016782	transferase activity, transferring sulphur-containing groups	bulk RNA-seq,Homo_723,AD.vs.control; bulk RNA-seq,Homo_723,AD.vs.MCI; bulk RNA-seq,Homo_723,MCI.vs.control	3
MF	GO:0005221	intracellular cyclic nucleotide activated cation channel	bulk RNA-seq,Homo_723,AD.vs.control; bulk RNA-seq,Homo_723,AD.vs.MCI; bulk RNA-seq,Homo_723,MCI.vs.control; bulk RNA-seq,Homo_714,AD.vs.MCI	4
MF	GO:0043855	cyclic nucleotide-gated ion channel activity	bulk RNA-seq,Homo_723,AD.vs.control; bulk RNA-seq,Homo_723,AD.vs.MCI; bulk RNA-seq,Homo_723,MCI.vs.control; bulk RNA-seq,Homo_714,AD.vs.MCI	4
BP	GO:0046006	regulation of activated T cell proliferation	bulk RNA-seq,Homo_723,AD.vs.control; bulk RNA-seq,Homo_714,MCI.vs.control; bulk RNA-seq,Homo_633,AD.vs.control; bulk RNA-	4
BP	GO:0071103	DNA conformation change	bulk RNA-seq,Homo_723,AD.vs.control; bulk RNA-seq,Homo_723,MCI.vs.control; bulk RNA-seq,Homo_714,AD.vs.control; bulk RNA-seq,Homo_714,MCI.vs.control; bulk RNA-seq,Homo_633,AD.vs.control; bulk RNA-seq,Homo_633,AD.vs.MCI	6
BP	GO:0008037	cell recognition	bulk RNA-seq,Homo_723,AD.vs.control; bulk RNA-seq,Homo_723,AD.vs.MCI; bulk RNA-seq,Homo_723,MCI.vs.control; bulk RNA-seq,Homo_714,AD.vs.control; bulk RNA-seq,Homo_714,AD.vs.MCI; bulk RNA-seq,Homo_633,AD.vs.control; bulk RNA-seq,Homo_633,AD.vs.MCI; bulk RNA-seq,SRP223445,AD.vs.control; scRNA-seq,SRP309935,Monocyte_2-AD.vs.control	9
BP	GO:0071599	otic vesicle development	bulk RNA-seq,Homo_723,AD.vs.control; bulk RNA-seq,Homo_723,AD.vs.MCI; bulk RNA-seq,Homo_723,MCI.vs.control; bulk RNA-seq,Homo_714,AD.vs.MCI; bulk RNA-seq,Homo_714,MCI.vs.control	5
BP	GO:0015803	branched-chain amino acid transport	bulk RNA-seq,Homo_723,AD.vs.control; bulk RNA-seq,Homo_723,AD.vs.MCI	2
CC	GO:0055037	recycling endosome	bulk RNA-seq,Homo_723,AD.vs.control; bulk RNA-seq,Homo_723,MCI.vs.control; bulk RNA-seq,Homo_714,AD.vs.control; bulk RNA-seq,Homo_714,AD.vs.MCI; bulk RNA-seq,Homo_633,AD.vs.control; bulk RNA-seq,Homo_633,AD.vs.MCI	6
MF	GO:0015165	pyrimidine nucleotide-sugar transmembrane transporter activity	bulk RNA-seq,Homo_723,AD.vs.control; bulk RNA-seq,Homo_723,MCI.vs.control; bulk RNA-seq,Homo_714,AD.vs.control; bulk RNA-seq,Homo_714,MCI.vs.control; bulk RNA-seq,Homo_633,AD.vs.control; bulk RNA-seq,Homo_633,MCI.vs.control	6
BP	GO:0001562	response to protozoan	bulk RNA-seq,Homo_723,AD.vs.control; bulk RNA-seq,Homo_723,MCI.vs.control; bulk RNA-seq,Homo_714,MCI.vs.control; bulk RNA-	4
BP	GO:0006171	cAMP biosynthetic process	bulk RNA-seq,Homo_723,AD.vs.control; bulk RNA-seq,Homo_723,AD.vs.MCI; bulk RNA-seq,Homo_723,MCI.vs.control	3
BP	GO:0014015	positive regulation of gliogenesis	bulk RNA-seq,Homo_723,AD.vs.control; bulk RNA-seq,Homo_723,AD.vs.MCI; bulk RNA-seq,Homo_723,MCI.vs.control; bulk RNA-seq,Homo_714,AD.vs.MCI; bulk RNA-seq,Homo_714,MCI.vs.control; bulk RNA-seq,Homo_633,AD.vs.control	6
CC	GO:0005779	integral component of peroxisomal membrane	bulk RNA-seq,Homo_723,AD.vs.control; bulk RNA-seq,Homo_714,AD.vs.control; bulk RNA-seq,Homo_633,AD.vs.control; bulk RNA-seq,Homo_633,AD.vs.MCI	4
CC	GO:0031231	intrinsic component of peroxisomal membrane	bulk RNA-seq,Homo_723,AD.vs.control; bulk RNA-seq,Homo_714,AD.vs.control; bulk RNA-seq,Homo_633,AD.vs.control; bulk RNA-seq,Homo_633,AD.vs.MCI	4
BP	GO:0042133	neurotransmitter metabolic process	bulk RNA-seq,Homo_723,AD.vs.control; bulk RNA-seq,Homo_723,AD.vs.MCI; bulk RNA-seq,Homo_723,MCI.vs.control; bulk RNA-seq,Homo_714,AD.vs.control; bulk RNA-seq,Homo_714,AD.vs.MCI	5
BP	GO:0006283	transcription-coupled nucleotide-excision repair	bulk RNA-seq,Homo_723,AD.vs.control; bulk RNA-seq,Homo_723,MCI.vs.control; bulk RNA-seq,Homo_714,AD.vs.control; bulk RNA-seq,Homo_714,MCI.vs.control; bulk RNA-seq,Homo_633,AD.vs.control; bulk RNA-seq,Homo_633,MCI.vs.control	6
MF	GO:0004300	enoyl-CoA hydratase activity	bulk RNA-seq,Homo_723,AD.vs.control; bulk RNA-seq,Homo_723,AD.vs.MCI; bulk RNA-seq,Homo_723,MCI.vs.control; bulk RNA-seq,Homo_714,AD.vs.control; bulk RNA-seq,Homo_714,MCI.vs.control; bulk RNA-seq,Homo_633,MCI.vs.control	6
BP	GO:0090303	positive regulation of wound healing	bulk RNA-seq,Homo_723,AD.vs.control; bulk RNA-seq,Homo_723,AD.vs.MCI; bulk RNA-seq,Homo_723,MCI.vs.control; bulk RNA-seq,Homo_714,AD.vs.control; bulk RNA-seq,Homo_714,AD.vs.MCI; bulk RNA-seq,Homo_714,MCI.vs.control; bulk RNA-seq,Homo_633,AD.vs.control; bulk	8
MF	GO:0031386	protein tag	bulk RNA-seq,Homo_723,AD.vs.control; bulk RNA-seq,Homo_633,AD.vs.control	2
BP	GO:0006953	acute-phase response	bulk RNA-seq,Homo_723,AD.vs.control; bulk RNA-seq,Homo_723,AD.vs.MCI; bulk RNA-seq,Homo_723,MCI.vs.control; bulk RNA-seq,Homo_714,AD.vs.control; bulk RNA-seq,Homo_714,AD.vs.MCI; bulk RNA-seq,Homo_714,MCI.vs.control	6
BP	GO:0046653	tetrahydrofolate metabolic process	bulk RNA-seq,Homo_723,AD.vs.control; bulk RNA-seq,Homo_723,MCI.vs.control; bulk RNA-seq,Homo_714,MCI.vs.control; bulk RNA-seq,Homo_633,AD.vs.control; bulk RNA-seq,Homo_633,AD.vs.MCI; bulk RNA-seq,Homo_633,MCI.vs.control	6
MF	GO:0005520	insulin-like growth factor binding	bulk RNA-seq,Homo_723,AD.vs.control; bulk RNA-seq,Homo_723,AD.vs.MCI; bulk RNA-seq,Homo_723,MCI.vs.control; bulk RNA-seq,Homo_714,AD.vs.MCI; bulk RNA-seq,Homo_633,AD.vs.control	5
BP	GO:0002092	positive regulation of receptor internalization	bulk RNA-seq,Homo_723,AD.vs.control; bulk RNA-seq,Homo_723,AD.vs.MCI; bulk RNA-seq,Homo_723,MCI.vs.control; bulk RNA-seq,Homo_714,AD.vs.MCI; bulk RNA-seq,Homo_714,MCI.vs.control; bulk RNA-seq,Homo_633,AD.vs.control; bulk RNA-seq,Homo_633,AD.vs.MCI	7
BP	GO:0048520	positive regulation of behavior	bulk RNA-seq,Homo_723,AD.vs.control; bulk RNA-seq,Homo_723,AD.vs.MCI; bulk RNA-seq,Homo_714,AD.vs.control; bulk RNA-seq,Homo_714,AD.vs.MCI; bulk RNA-seq,SRP223445,AD.vs.control	5
BP	GO:0086064	cell communication by electrical coupling involved in cardiac conduction	bulk RNA-seq,Homo_723,AD.vs.control; bulk RNA-seq,Homo_723,AD.vs.MCI; bulk RNA-seq,Homo_723,MCI.vs.control; bulk RNA-seq,Homo_714,AD.vs.MCI; bulk RNA-seq,Homo_714,MCI.vs.control	5
BP	GO:0050654	chondroitin sulfate proteoglycan metabolic process	bulk RNA-seq,Homo_723,AD.vs.control; bulk RNA-seq,Homo_723,AD.vs.MCI; bulk RNA-seq,Homo_723,MCI.vs.control; bulk RNA-seq,Homo_714,AD.vs.MCI; bulk RNA-seq,Homo_714,MCI.vs.control	5
BP	GO:0055075	potassium ion homeostasis	bulk RNA-seq,Homo_723,AD.vs.control; bulk RNA-seq,Homo_723,AD.vs.MCI; bulk RNA-seq,Homo_714,AD.vs.MCI	3
MF	GO:0099186	structural constituent of postsynapse	bulk RNA-seq,Homo_723,AD.vs.control; bulk RNA-seq,Homo_723,AD.vs.MCI; bulk RNA-seq,Homo_723,MCI.vs.control; bulk RNA-seq,Homo_714,AD.vs.control; bulk RNA-seq,Homo_714,AD.vs.MCI	5
BP	GO:0051304	chromosome separation	bulk RNA-seq,Homo_723,AD.vs.control; bulk RNA-seq,Homo_723,MCI.vs.control; bulk RNA-seq,Homo_714,AD.vs.control; bulk RNA-seq,Homo_633,AD.vs.control; bulk RNA-seq,Homo_633,AD.vs.MCI	5
MF	GO:0031996	thioesterase binding	bulk RNA-seq,Homo_723,AD.vs.control; bulk RNA-seq,Homo_723,AD.vs.MCI; bulk RNA-seq,Homo_714,MCI.vs.control; bulk RNA-seq,Homo_633,AD.vs.control; bulk RNA-seq,Homo_633,MCI.vs.control	5
BP	GO:0060155	platelet dense granule organization	bulk RNA-seq,Homo_723,AD.vs.control; bulk RNA-seq,Homo_723,MCI.vs.control; bulk RNA-seq,Homo_714,AD.vs.control; bulk RNA-seq,Homo_714,MCI.vs.control; bulk RNA-seq,Homo_633,AD.vs.control; bulk RNA-seq,Homo_633,AD.vs.MCI; bulk RNA-seq,Homo_633,MCI.vs.control	7
BP	GO:0010718	positive regulation of epithelial to mesenchymal transition	bulk RNA-seq,Homo_723,AD.vs.control; bulk RNA-seq,Homo_723,AD.vs.MCI; bulk RNA-seq,Homo_723,MCI.vs.control; bulk RNA-seq,Homo_714,AD.vs.MCI; bulk RNA-seq,Homo_714,MCI.vs.control; bulk RNA-seq,Homo_633,AD.vs.control; bulk RNA-seq,Homo_633,AD.vs.MCI	7

BP	GO:0006261	DNA-templated DNA replication	bulk RNA-seq,Homo_723,AD.vs.control; bulk RNA-seq,Homo_723,MCI.vs.control; bulk RNA-seq,Homo_714,AD.vs.control; bulk RNA-seq,Homo_633,AD.vs.control; bulk RNA-seq,Homo_633,AD.vs.MCI	5
CC	GO:0071339	MLL1 complex	bulk RNA-seq,Homo_723,AD.vs.control; bulk RNA-seq,Homo_714,AD.vs.control; bulk RNA-seq,Homo_633,AD.vs.control; bulk RNA-seq,Homo_633,AD.vs.MCI	4
BP	GO:0007272	ensheathment of neurons	bulk RNA-seq,Homo_723,AD.vs.control; bulk RNA-seq,Homo_723,AD.vs.MCI; bulk RNA-seq,Homo_723,MCI.vs.control; bulk RNA-seq,Homo_714,AD.vs.MCI; bulk RNA-seq,Homo_633,AD.vs.control; bulk RNA-seq,Homo_633,AD.vs.MCI	6
BP	GO:0008366	axon ensheathment	bulk RNA-seq,Homo_723,AD.vs.control; bulk RNA-seq,Homo_723,AD.vs.MCI; bulk RNA-seq,Homo_723,MCI.vs.control; bulk RNA-seq,Homo_714,AD.vs.MCI; bulk RNA-seq,Homo_633,AD.vs.control; bulk RNA-seq,Homo_633,AD.vs.MCI	6
BP	GO:0071257	cellular response to electrical stimulus	bulk RNA-seq,Homo_723,AD.vs.control; bulk RNA-seq,Homo_723,AD.vs.MCI; bulk RNA-seq,Homo_723,MCI.vs.control	3
BP	GO:0032212	positive regulation of telomere maintenance via telomerase	bulk RNA-seq,Homo_723,AD.vs.control; bulk RNA-seq,Homo_723,MCI.vs.control; bulk RNA-seq,Homo_714,AD.vs.control; bulk RNA-seq,Homo_714,MCI.vs.control; bulk RNA-seq,Homo_633,AD.vs.control; bulk RNA-seq,Homo_633,AD.vs.MCI; bulk RNA-seq,Homo_633,MCI.vs.control	7
BP	GO:0055003	cardiac myofibril assembly	bulk RNA-seq,Homo_723,AD.vs.control; bulk RNA-seq,Homo_723,AD.vs.MCI; bulk RNA-seq,Homo_723,MCI.vs.control; bulk RNA-seq,Homo_714,AD.vs.control; bulk RNA-seq,Homo_714,AD.vs.MCI	5
BP	GO:1901381	positive regulation of potassium ion transmembrane transport	bulk RNA-seq,Homo_723,AD.vs.control; bulk RNA-seq,Homo_723,AD.vs.MCI	2
CC	GO:0097525	spliceosomal snRNP complex	bulk RNA-seq,Homo_723,AD.vs.control; bulk RNA-seq,Homo_714,MCI.vs.control; bulk RNA-seq,Homo_633,AD.vs.control; bulk RNA-	4
BP	GO:0000724	double-strand break repair via homologous recombination	bulk RNA-seq,Homo_723,AD.vs.control; bulk RNA-seq,Homo_723,MCI.vs.control; bulk RNA-seq,Homo_714,AD.vs.control; bulk RNA-seq,Homo_633,AD.vs.control; bulk RNA-seq,Homo_633,AD.vs.MCI	5
BP	GO:0003171	atrioventricular valve development	bulk RNA-seq,Homo_723,AD.vs.control; bulk RNA-seq,Homo_723,AD.vs.MCI; bulk RNA-seq,Homo_723,MCI.vs.control; bulk RNA-seq,Homo_714,AD.vs.MCI; bulk RNA-seq,Homo_714,MCI.vs.control; bulk RNA-seq,SRP223445,AD.vs.control	6
BP	GO:0002363	alpha-beta T cell lineage commitment	bulk RNA-seq,Homo_723,AD.vs.control; bulk RNA-seq,Homo_714,AD.vs.control; bulk RNA-seq,Homo_714,MCI.vs.control; bulk RNA-	4
BP	GO:0097428	protein maturation by iron-sulfur cluster transfer	bulk RNA-seq,Homo_723,AD.vs.control; bulk RNA-seq,Homo_723,MCI.vs.control; bulk RNA-seq,Homo_714,AD.vs.control; bulk RNA-seq,Homo_714,MCI.vs.control; bulk RNA-seq,Homo_633,AD.vs.control; bulk RNA-seq,Homo_633,AD.vs.MCI; bulk RNA-seq,Homo_633,MCI.vs.control	7
BP	GO:2000819	regulation of nucleotide-excision repair	bulk RNA-seq,Homo_723,AD.vs.control; bulk RNA-seq,Homo_723,MCI.vs.control; bulk RNA-seq,Homo_714,AD.vs.control; bulk RNA-seq,Homo_714,MCI.vs.control; bulk RNA-seq,Homo_633,AD.vs.control; bulk RNA-seq,Homo_633,AD.vs.MCI; bulk RNA-seq,Homo_633,MCI.vs.control	7
BP	GO:0033625	positive regulation of integrin activation	bulk RNA-seq,Homo_723,AD.vs.control; bulk RNA-seq,Homo_723,AD.vs.MCI; bulk RNA-seq,Homo_723,MCI.vs.control; bulk RNA-seq,Homo_714,AD.vs.control; bulk RNA-seq,Homo_714,AD.vs.MCI; bulk RNA-seq,Homo_714,MCI.vs.control	6
BP	GO:0006220	pyrimidine nucleotide metabolic process	bulk RNA-seq,Homo_723,AD.vs.control; bulk RNA-seq,Homo_723,MCI.vs.control; bulk RNA-seq,Homo_714,MCI.vs.control; bulk RNA-seq,Homo_633,AD.vs.control; bulk RNA-seq,Homo_633,AD.vs.MCI	5
BP	GO:0097113	AMPA glutamate receptor clustering	bulk RNA-seq,Homo_723,AD.vs.control; bulk RNA-seq,Homo_723,AD.vs.MCI; bulk RNA-seq,Homo_723,MCI.vs.control; bulk RNA-seq,Homo_714,AD.vs.MCI; bulk RNA-seq,Homo_714,MCI.vs.control	5
BP	GO:0097688	glutamate receptor clustering	bulk RNA-seq,Homo_723,AD.vs.control; bulk RNA-seq,Homo_723,AD.vs.MCI; bulk RNA-seq,Homo_723,MCI.vs.control; bulk RNA-seq,Homo_714,AD.vs.MCI; bulk RNA-seq,Homo_714,MCI.vs.control	5
BP	GO:0048340	paraxial mesoderm morphogenesis	bulk RNA-seq,Homo_723,AD.vs.control; bulk RNA-seq,Homo_714,AD.vs.control	2
BP	GO:0090494	dopamine uptake	bulk RNA-seq,Homo_723,AD.vs.control; bulk RNA-seq,Homo_723,AD.vs.MCI; bulk RNA-seq,Homo_714,AD.vs.control	3
CC	GO:0005782	peroxisomal matrix	bulk RNA-seq,Homo_723,AD.vs.control; bulk RNA-seq,Homo_723,MCI.vs.control; bulk RNA-seq,Homo_714,AD.vs.control; bulk RNA-seq,Homo_714,MCI.vs.control; bulk RNA-seq,Homo_633,AD.vs.control; bulk RNA-seq,Homo_633,AD.vs.MCI	6
CC	GO:0031907	microbody lumen	bulk RNA-seq,Homo_723,AD.vs.control; bulk RNA-seq,Homo_723,MCI.vs.control; bulk RNA-seq,Homo_714,AD.vs.control; bulk RNA-seq,Homo_714,MCI.vs.control; bulk RNA-seq,Homo_633,AD.vs.control; bulk RNA-seq,Homo_633,AD.vs.MCI	6
BP	GO:0051053	negative regulation of DNA metabolic process	bulk RNA-seq,Homo_723,AD.vs.control; bulk RNA-seq,Homo_723,MCI.vs.control; bulk RNA-seq,Homo_714,AD.vs.control; bulk RNA-seq,Homo_633,AD.vs.control; bulk RNA-seq,Homo_633,AD.vs.MCI	5
BP	GO:0003283	atrial septum development	bulk RNA-seq,Homo_723,AD.vs.control; bulk RNA-seq,Homo_723,AD.vs.MCI; bulk RNA-seq,Homo_723,MCI.vs.control; bulk RNA-seq,Homo_714,AD.vs.MCI; bulk RNA-seq,Homo_714,MCI.vs.control	5
BP	GO:1901017	negative regulation of potassium ion transmembrane	bulk RNA-seq,Homo_723,AD.vs.control; bulk RNA-seq,Homo_723,AD.vs.MCI; bulk RNA-seq,Homo_714,AD.vs.control; bulk RNA-seq,Homo_714,AD.vs.MCI	4
BP	GO:0061323	cell proliferation involved in heart morphogenesis	bulk RNA-seq,Homo_723,AD.vs.control; bulk RNA-seq,Homo_723,AD.vs.MCI; bulk RNA-seq,Homo_723,MCI.vs.control; bulk RNA-seq,Homo_714,AD.vs.MCI; bulk RNA-seq,Homo_714,MCI.vs.control	5
BP	GO:0072170	metanephric tubule development	bulk RNA-seq,Homo_723,AD.vs.control; bulk RNA-seq,Homo_723,AD.vs.MCI; bulk RNA-seq,Homo_723,MCI.vs.control; bulk RNA-seq,Homo_714,AD.vs.MCI; bulk RNA-seq,Homo_714,MCI.vs.control	5
BP	GO:0002718	regulation of cytokine production involved in immune response	bulk RNA-seq,Homo_723,AD.vs.control; bulk RNA-seq,Homo_723,MCI.vs.control; bulk RNA-seq,Homo_714,MCI.vs.control; bulk RNA-seq,Homo_633,AD.vs.control; bulk RNA-seq,Homo_633,AD.vs.MCI; scRNA-seq,SRP330776,CD8+ T cell_2-AD.vs.control	6
BP	GO:1903115	regulation of actin filament-based movement	bulk RNA-seq,Homo_723,AD.vs.control; bulk RNA-seq,Homo_723,AD.vs.MCI; bulk RNA-seq,Homo_723,MCI.vs.control; bulk RNA-seq,Homo_714,AD.vs.control; bulk RNA-seq,Homo_714,AD.vs.MCI; bulk RNA-seq,Homo_714,MCI.vs.control	6
BP	GO:0140747	regulation of ncRNA transcription	bulk RNA-seq,Homo_723,AD.vs.control; bulk RNA-seq,Homo_723,MCI.vs.control; bulk RNA-seq,Homo_714,AD.vs.control; bulk RNA-seq,Homo_714,MCI.vs.control; bulk RNA-seq,Homo_633,AD.vs.control; bulk RNA-seq,Homo_633,AD.vs.MCI	6
MF	GO:0008569	minus-end-directed microtubule motor activity	bulk RNA-seq,Homo_723,AD.vs.control; bulk RNA-seq,Homo_723,AD.vs.MCI; bulk RNA-seq,Homo_723,MCI.vs.control; bulk RNA-seq,Homo_714,AD.vs.MCI	4
BP	GO:0010458	exit from mitosis	bulk RNA-seq,Homo_723,AD.vs.control; bulk RNA-seq,Homo_723,MCI.vs.control; bulk RNA-seq,Homo_714,AD.vs.control; bulk RNA-seq,Homo_714,MCI.vs.control; bulk RNA-seq,Homo_633,AD.vs.control; bulk RNA-seq,Homo_633,AD.vs.MCI; bulk RNA-seq,Homo_633,MCI.vs.control	7
BP	GO:0055021	regulation of cardiac muscle tissue growth	bulk RNA-seq,Homo_723,AD.vs.control; bulk RNA-seq,Homo_723,AD.vs.MCI; bulk RNA-seq,Homo_723,MCI.vs.control; bulk RNA-seq,Homo_714,AD.vs.MCI; bulk RNA-seq,Homo_714,MCI.vs.control	5
BP	GO:1900274	regulation of phospholipase C activity	bulk RNA-seq,Homo_723,AD.vs.control; bulk RNA-seq,Homo_723,AD.vs.MCI; bulk RNA-seq,Homo_723,MCI.vs.control; bulk RNA-seq,Homo_714,AD.vs.MCI; bulk RNA-seq,Homo_633,AD.vs.control; bulk RNA-seq,Homo_633,AD.vs.MCI	6
MF	GO:0140296	general transcription initiation factor binding	bulk RNA-seq,Homo_723,AD.vs.control; bulk RNA-seq,Homo_723,MCI.vs.control; bulk RNA-seq,Homo_714,MCI.vs.control; bulk RNA-seq,Homo_633,AD.vs.control; bulk RNA-seq,Homo_633,AD.vs.MCI	5
MF	GO:0061133	endopeptidase activator activity	bulk RNA-seq,Homo_723,AD.vs.control; bulk RNA-seq,Homo_714,AD.vs.control; bulk RNA-seq,Homo_633,AD.vs.control; bulk RNA-seq,Homo_633,AD.vs.MCI	4
BP	GO:0060292	long-term synaptic depression	bulk RNA-seq,Homo_723,AD.vs.control; bulk RNA-seq,Homo_723,AD.vs.MCI; bulk RNA-seq,Homo_723,MCI.vs.control; bulk RNA-seq,Homo_714,AD.vs.MCI; bulk RNA-seq,Homo_714,MCI.vs.control	5

MF	GO:0008022	protein C-terminus binding	bulk RNA-seq,Homo_723,AD.vs.control; bulk RNA-seq,Homo_723,AD.vs.MCI; bulk RNA-seq,Homo_723,MCI.vs.control; bulk RNA-seq,Homo_714,AD.vs.MCI; bulk RNA-seq,Homo_633,AD.vs.control; bulk RNA-seq,Homo_633,AD.vs.MCI	6
BP	GO:0051291	protein heterooligomerization	bulk RNA-seq,Homo_723,AD.vs.control; bulk RNA-seq,Homo_633,AD.vs.control; bulk RNA-seq,Homo_633,AD.vs.MCI	3
MF	GO:0008392	arachidonic acid epoxygenase activity	bulk RNA-seq,Homo_723,AD.vs.control; bulk RNA-seq,Homo_723,AD.vs.MCI; bulk RNA-seq,Homo_714,AD.vs.control; bulk RNA-seq,Homo_714,AD.vs.MCI	4
MF	GO:0045504	dynein heavy chain binding	bulk RNA-seq,Homo_723,AD.vs.control; bulk RNA-seq,Homo_723,AD.vs.MCI; bulk RNA-seq,Homo_723,MCI.vs.control	3
BP	GO:0045687	positive regulation of glial cell differentiation	bulk RNA-seq,Homo_723,AD.vs.control; bulk RNA-seq,Homo_723,AD.vs.MCI; bulk RNA-seq,Homo_723,MCI.vs.control; bulk RNA-seq,Homo_714,AD.vs.MCI; bulk RNA-seq,Homo_714,MCI.vs.control	5
BP	GO:0010863	positive regulation of phospholipase C activity	bulk RNA-seq,Homo_723,AD.vs.control; bulk RNA-seq,Homo_723,AD.vs.MCI; bulk RNA-seq,Homo_723,MCI.vs.control; bulk RNA-seq,Homo_714,AD.vs.MCI; bulk RNA-seq,Homo_714,MCI.vs.control; bulk RNA-seq,Homo_633,AD.vs.control	6
CC	GO:0005902	microvillus	bulk RNA-seq,Homo_723,AD.vs.control; bulk RNA-seq,Homo_723,AD.vs.MCI; bulk RNA-seq,Homo_723,MCI.vs.control; bulk RNA-seq,Homo_714,AD.vs.control; bulk RNA-seq,Homo_714,AD.vs.MCI; bulk RNA-seq,Homo_714,MCI.vs.control; bulk RNA-seq,Homo_633,AD.vs.control; bulk	8
BP	GO:0043374	CD8-positive, alpha-beta T cell differentiation	bulk RNA-seq,Homo_723,AD.vs.control	1
BP	GO:0090162	establishment of epithelial cell polarity	bulk RNA-seq,Homo_723,AD.vs.control; bulk RNA-seq,Homo_723,AD.vs.MCI; bulk RNA-seq,Homo_723,MCI.vs.control; bulk RNA-	4
BP	GO:0006406	mRNA export from nucleus	bulk RNA-seq,Homo_723,AD.vs.control; bulk RNA-seq,Homo_723,MCI.vs.control; bulk RNA-seq,Homo_714,AD.vs.control; bulk RNA-seq,Homo_714,MCI.vs.control; bulk RNA-seq,Homo_633,AD.vs.control; bulk RNA-seq,Homo_633,AD.vs.MCI	6
BP	GO:0001933	negative regulation of protein phosphorylation	bulk RNA-seq,Homo_723,AD.vs.control; bulk RNA-seq,Homo_723,AD.vs.MCI; bulk RNA-seq,Homo_714,AD.vs.MCI; bulk RNA-seq,Homo_633,AD.vs.control; bulk RNA-seq,Homo_633,AD.vs.MCI	5
BP	GO:0030203	glycosaminoglycan metabolic process	bulk RNA-seq,Homo_723,AD.vs.control; bulk RNA-seq,Homo_723,AD.vs.MCI; bulk RNA-seq,Homo_723,MCI.vs.control; bulk RNA-seq,Homo_714,AD.vs.MCI; bulk RNA-seq,Homo_714,MCI.vs.control; bulk RNA-seq,Homo_633,AD.vs.control; bulk RNA-seq,Homo_633,AD.vs.MCI	7
CC	GO:0000172	ribonuclease MRP complex	bulk RNA-seq,Homo_723,AD.vs.control; bulk RNA-seq,Homo_723,AD.vs.MCI	2
MF	GO:0016702	oxidoreductase activity, acting on single donors with incorporation of molecular oxygen, incorporation of two	bulk RNA-seq,Homo_723,AD.vs.control; bulk RNA-seq,Homo_723,AD.vs.MCI; bulk RNA-seq,Homo_723,MCI.vs.control; bulk RNA-seq,Homo_714,AD.vs.control; bulk RNA-seq,Homo_714,AD.vs.MCI	5
BP	GO:1904358	positive regulation of telomere maintenance via telomere lengthening	bulk RNA-seq,Homo_723,AD.vs.control; bulk RNA-seq,Homo_723,MCI.vs.control; bulk RNA-seq,Homo_714,AD.vs.control; bulk RNA-seq,Homo_714,MCI.vs.control; bulk RNA-seq,Homo_633,AD.vs.control; bulk RNA-seq,Homo_633,AD.vs.MCI	6
BP	GO:0014048	regulation of glutamate secretion	bulk RNA-seq,Homo_723,AD.vs.control; bulk RNA-seq,Homo_723,AD.vs.MCI; bulk RNA-seq,Homo_723,MCI.vs.control	3
CC	GO:0032280	symmetric synapse	bulk RNA-seq,Homo_723,AD.vs.control; bulk RNA-seq,Homo_723,AD.vs.MCI; bulk RNA-seq,Homo_723,MCI.vs.control; bulk RNA-	4
BP	GO:0007051	spindle organization	bulk RNA-seq,Homo_723,AD.vs.control; bulk RNA-seq,Homo_723,MCI.vs.control; bulk RNA-seq,Homo_633,AD.vs.control; bulk RNA-	4
BP	GO:0003356	regulation of cilium beat frequency	bulk RNA-seq,Homo_723,AD.vs.control; bulk RNA-seq,Homo_723,AD.vs.MCI; bulk RNA-seq,Homo_723,MCI.vs.control	3
BP	GO:0042092	type 2 immune response	bulk RNA-seq,Homo_723,AD.vs.control; bulk RNA-seq,Homo_723,MCI.vs.control; bulk RNA-seq,Homo_714,MCI.vs.control; bulk RNA-seq,Homo_633,AD.vs.control; bulk RNA-seq,Homo_633,AD.vs.MCI	5
CC	GO:0090575	RNA polymerase II transcription regulator complex	bulk RNA-seq,Homo_723,AD.vs.control; bulk RNA-seq,Homo_723,AD.vs.MCI; bulk RNA-seq,Homo_723,MCI.vs.control; bulk RNA-seq,Homo_714,AD.vs.MCI; bulk RNA-seq,Homo_633,AD.vs.control; bulk RNA-seq,Homo_633,AD.vs.MCI; scRNA-seq,SRP330776,Naive CD8+ T cell_2-AD.vs.control	7
BP	GO:0055023	positive regulation of cardiac muscle tissue growth	bulk RNA-seq,Homo_723,AD.vs.control; bulk RNA-seq,Homo_723,AD.vs.MCI; bulk RNA-seq,Homo_723,MCI.vs.control; bulk RNA-seq,Homo_714,AD.vs.MCI; bulk RNA-seq,Homo_714,MCI.vs.control	5
MF	GO:0001848	complement binding	bulk RNA-seq,Homo_723,AD.vs.control	1
BP	GO:0060020	Bergmann glial cell differentiation	bulk RNA-seq,Homo_723,AD.vs.control	1
BP	GO:0045580	regulation of T cell differentiation	bulk RNA-seq,Homo_723,AD.vs.control; bulk RNA-seq,Homo_723,MCI.vs.control; bulk RNA-seq,Homo_714,AD.vs.control; bulk RNA-seq,Homo_633,AD.vs.control; bulk RNA-seq,Homo_633,AD.vs.MCI	5
BP	GO:2000725	regulation of cardiac muscle cell differentiation	bulk RNA-seq,Homo_723,AD.vs.control; bulk RNA-seq,Homo_723,AD.vs.MCI; bulk RNA-seq,Homo_714,AD.vs.MCI	3
MF	GO:0034483	heparan sulfate sulfotransferase activity	bulk RNA-seq,Homo_723,AD.vs.control; bulk RNA-seq,Homo_723,AD.vs.MCI; bulk RNA-seq,Homo_723,MCI.vs.control; bulk RNA-seq,Homo_714,AD.vs.MCI	4
BP	GO:0043144	sno(s)RNA processing	bulk RNA-seq,Homo_723,AD.vs.control; bulk RNA-seq,Homo_633,AD.vs.control; bulk RNA-seq,Homo_633,AD.vs.MCI	3
BP	GO:0071285	cellular response to lithium ion	bulk RNA-seq,Homo_723,AD.vs.control; bulk RNA-seq,Homo_723,AD.vs.MCI; bulk RNA-seq,Homo_723,MCI.vs.control; bulk RNA-	4
BP	GO:0036065	fucosylation	bulk RNA-seq,Homo_723,AD.vs.control; bulk RNA-seq,Homo_723,AD.vs.MCI; bulk RNA-seq,Homo_723,MCI.vs.control	3
BP	GO:0007009	plasma membrane organization	bulk RNA-seq,Homo_723,AD.vs.control; bulk RNA-seq,Homo_723,AD.vs.MCI; bulk RNA-seq,Homo_723,MCI.vs.control; bulk RNA-seq,Homo_714,AD.vs.MCI; bulk RNA-seq,Homo_633,AD.vs.control; bulk RNA-seq,Homo_633,AD.vs.MCI	6
BP	GO:0001776	leukocyte homeostasis	bulk RNA-seq,Homo_723,AD.vs.control; bulk RNA-seq,Homo_723,MCI.vs.control; bulk RNA-seq,Homo_714,MCI.vs.control; bulk RNA-seq,Homo_633,AD.vs.control; bulk RNA-seq,Homo_633,AD.vs.MCI	5
BP	GO:0051547	regulation of keratinocyte migration	bulk RNA-seq,Homo_723,AD.vs.control; bulk RNA-seq,Homo_723,AD.vs.MCI; bulk RNA-seq,Homo_723,MCI.vs.control; bulk RNA-seq,Homo_714,AD.vs.control; bulk RNA-seq,Homo_714,AD.vs.MCI; bulk RNA-seq,Homo_633,AD.vs.MCI	6
CC	GO:0070971	endoplasmic reticulum exit site	bulk RNA-seq,Homo_723,AD.vs.control; bulk RNA-seq,Homo_723,MCI.vs.control; bulk RNA-seq,Homo_714,MCI.vs.control; bulk RNA-seq,Homo_633,AD.vs.control; bulk RNA-seq,Homo_633,MCI.vs.control	5
BP	GO:2000434	regulation of protein neddylation	bulk RNA-seq,Homo_723,AD.vs.control; bulk RNA-seq,Homo_723,MCI.vs.control; bulk RNA-seq,Homo_714,AD.vs.control; bulk RNA-seq,Homo_633,AD.vs.control; bulk RNA-seq,Homo_633,AD.vs.MCI	5
BP	GO:0070141	response to UV-A	bulk RNA-seq,Homo_723,AD.vs.control; bulk RNA-seq,Homo_723,AD.vs.MCI; bulk RNA-seq,Homo_723,MCI.vs.control; bulk RNA-seq,Homo_714,AD.vs.MCI; bulk RNA-seq,Homo_714,MCI.vs.control; bulk RNA-seq,Homo_633,MCI.vs.control	6
CC	GO:0070820	tertiary granule	bulk RNA-seq,Homo_723,AD.vs.control; bulk RNA-seq,Homo_723,MCI.vs.control; bulk RNA-seq,Homo_633,AD.vs.control; bulk RNA-seq,Homo_633,AD.vs.MCI; bulk RNA-seq,SRP223445,AD.vs.control	5
BP	GO:0071108	protein K48-linked deubiquitination	bulk RNA-seq,Homo_723,AD.vs.control; bulk RNA-seq,Homo_723,MCI.vs.control; bulk RNA-seq,Homo_714,AD.vs.control; bulk RNA-seq,Homo_714,MCI.vs.control; bulk RNA-seq,Homo_633,AD.vs.control; bulk RNA-seq,Homo_633,AD.vs.MCI	6
BP	GO:0045954	positive regulation of natural killer cell mediated cytotoxicity	bulk RNA-seq,Homo_723,AD.vs.control; bulk RNA-seq,Homo_714,AD.vs.control; bulk RNA-seq,Homo_633,AD.vs.control; bulk RNA-seq,Homo_633,AD.vs.MCI; bulk RNA-seq,Homo_633,MCI.vs.control; scRNA-seq,SRP309935,B cell_1-AD.vs.control	6
BP	GO:1904505	regulation of telomere maintenance in response to DNA	bulk RNA-seq,Homo_723,AD.vs.control; bulk RNA-seq,Homo_714,AD.vs.control; bulk RNA-seq,Homo_633,AD.vs.control; bulk RNA-seq,Homo_633,AD.vs.MCI	4

BP	GO:0035909	aorta morphogenesis	bulk RNA-seq,Homo_723,AD.vs.control; bulk RNA-seq,Homo_723,AD.vs.MCI; bulk RNA-seq,Homo_723,MCI.vs.control; bulk RNA-seq,Homo_714,AD.vs.MCI; bulk RNA-seq,Homo_714,MCI.vs.control	5
BP	GO:0001937	negative regulation of endothelial cell proliferation	bulk RNA-seq,Homo_723,AD.vs.control; bulk RNA-seq,Homo_723,AD.vs.MCI; bulk RNA-seq,Homo_714,AD.vs.control; bulk RNA-seq,Homo_714,AD.vs.MCI	4
BP	GO:0072310	glomerular epithelial cell development	bulk RNA-seq,Homo_723,AD.vs.control; bulk RNA-seq,Homo_723,AD.vs.MCI; bulk RNA-seq,Homo_714,AD.vs.MCI	3
CC	GO:0005736	RNA polymerase I complex	bulk RNA-seq,Homo_723,AD.vs.control; bulk RNA-seq,Homo_723,MCI.vs.control; bulk RNA-seq,Homo_714,MCI.vs.control; bulk RNA-seq,Homo_633,AD.vs.control; bulk RNA-seq,Homo_633,MCI.vs.control	5
BP	GO:0060920	cardiac pacemaker cell differentiation	bulk RNA-seq,Homo_723,AD.vs.control; bulk RNA-seq,Homo_723,AD.vs.MCI; bulk RNA-seq,Homo_723,MCI.vs.control; bulk RNA-seq,Homo_714,AD.vs.MCI	4
BP	GO:0071028	nuclear mRNA surveillance	bulk RNA-seq,Homo_723,AD.vs.control; bulk RNA-seq,Homo_723,AD.vs.MCI; bulk RNA-seq,Homo_714,AD.vs.control; bulk RNA-seq,Homo_633,AD.vs.control; bulk RNA-seq,Homo_633,AD.vs.MCI	5
MF	GO:0140142	nucleocytoplasmic carrier activity	bulk RNA-seq,Homo_723,AD.vs.control; bulk RNA-seq,Homo_723,MCI.vs.control; bulk RNA-seq,Homo_714,AD.vs.control; bulk RNA-seq,Homo_714,MCI.vs.control; bulk RNA-seq,Homo_633,AD.vs.control; bulk RNA-seq,Homo_633,AD.vs.MCI	6
BP	GO:0010633	negative regulation of epithelial cell migration	bulk RNA-seq,Homo_723,AD.vs.control; bulk RNA-seq,Homo_723,AD.vs.MCI; bulk RNA-seq,Homo_723,MCI.vs.control; bulk RNA-seq,Homo_714,AD.vs.control; bulk RNA-seq,Homo_714,AD.vs.MCI; bulk RNA-seq,Homo_714,MCI.vs.control	6
CC	GO:0001527	microfibril	bulk RNA-seq,Homo_723,AD.vs.control; bulk RNA-seq,Homo_723,AD.vs.MCI; bulk RNA-seq,Homo_723,MCI.vs.control; bulk RNA-seq,Homo_714,AD.vs.MCI; bulk RNA-seq,Homo_714,MCI.vs.control	5
BP	GO:0015804	neutral amino acid transport	bulk RNA-seq,Homo_723,AD.vs.control; bulk RNA-seq,Homo_723,AD.vs.MCI; bulk RNA-seq,Homo_723,MCI.vs.control; bulk RNA-seq,Homo_714,AD.vs.MCI; bulk RNA-seq,Homo_633,AD.vs.control; bulk RNA-seq,Homo_633,AD.vs.MCI	6
BP	GO:0030041	actin filament polymerization	bulk RNA-seq,Homo_723,AD.vs.control; bulk RNA-seq,Homo_723,AD.vs.MCI; bulk RNA-seq,Homo_723,MCI.vs.control; bulk RNA-seq,Homo_714,AD.vs.MCI; bulk RNA-seq,Homo_633,AD.vs.control; bulk RNA-seq,Homo_633,AD.vs.MCI	6
BP	GO:2000781	positive regulation of double-strand break repair	bulk RNA-seq,Homo_723,AD.vs.control; bulk RNA-seq,Homo_723,MCI.vs.control; bulk RNA-seq,Homo_714,AD.vs.control; bulk RNA-seq,Homo_714,MCI.vs.control; bulk RNA-seq,Homo_633,AD.vs.control; bulk RNA-seq,Homo_633,AD.vs.MCI	6
BP	GO:0007052	mitotic spindle organization	bulk RNA-seq,Homo_723,AD.vs.control; bulk RNA-seq,Homo_723,MCI.vs.control; bulk RNA-seq,Homo_714,AD.vs.MCI; bulk RNA-seq,Homo_633,AD.vs.control; bulk RNA-seq,Homo_633,AD.vs.MCI	5
CC	GO:0030062	mitochondrial tricarboxylic acid cycle enzyme complex	bulk RNA-seq,Homo_723,AD.vs.control; bulk RNA-seq,Homo_723,MCI.vs.control; bulk RNA-seq,Homo_714,AD.vs.control; bulk RNA-seq,Homo_714,MCI.vs.control; bulk RNA-seq,Homo_633,AD.vs.control; bulk RNA-seq,Homo_633,AD.vs.MCI; bulk RNA-seq,Homo_633,MCI.vs.control	7
BP	GO:0060231	mesenchymal to epithelial transition	bulk RNA-seq,Homo_723,AD.vs.control; bulk RNA-seq,Homo_723,AD.vs.MCI; bulk RNA-seq,Homo_723,MCI.vs.control; bulk RNA-seq,Homo_714,AD.vs.MCI; bulk RNA-seq,Homo_714,MCI.vs.control; bulk RNA-seq,Homo_633,MCI.vs.control	6
BP	GO:0016242	negative regulation of macroautophagy	bulk RNA-seq,Homo_723,AD.vs.control; bulk RNA-seq,Homo_723,MCI.vs.control; bulk RNA-seq,Homo_714,AD.vs.control; bulk RNA-seq,Homo_714,MCI.vs.control; bulk RNA-seq,Homo_633,AD.vs.control; bulk RNA-seq,Homo_633,AD.vs.MCI	6
BP	GO:1904738	vascular associated smooth muscle cell migration	bulk RNA-seq,Homo_723,AD.vs.control; bulk RNA-seq,Homo_723,AD.vs.MCI; bulk RNA-seq,Homo_714,AD.vs.control; bulk RNA-seq,Homo_714,AD.vs.MCI	4
MF	GO:0004016	adenylate cyclase activity	bulk RNA-seq,Homo_723,AD.vs.control; bulk RNA-seq,Homo_723,AD.vs.MCI; bulk RNA-seq,Homo_723,MCI.vs.control	3
MF	GO:0016891	endoribonuclease activity, producing 5'-phosphomonoesters	bulk RNA-seq,Homo_723,AD.vs.control; bulk RNA-seq,Homo_714,AD.vs.control; bulk RNA-seq,Homo_633,AD.vs.control; bulk RNA-seq,Homo_633,AD.vs.MCI	4
BP	GO:0045879	negative regulation of smoothened signaling pathway	bulk RNA-seq,Homo_723,AD.vs.control; bulk RNA-seq,Homo_723,AD.vs.MCI; bulk RNA-seq,Homo_723,MCI.vs.control; bulk RNA-seq,Homo_633,AD.vs.control; bulk RNA-seq,Homo_633,AD.vs.MCI	5
BP	GO:1903900	regulation of viral life cycle	bulk RNA-seq,Homo_723,AD.vs.control; bulk RNA-seq,Homo_723,MCI.vs.control; bulk RNA-seq,Homo_714,AD.vs.control; bulk RNA-seq,Homo_633,AD.vs.control; bulk RNA-seq,Homo_633,AD.vs.MCI	5
BP	GO:0071539	protein localization to centrosome	bulk RNA-seq,Homo_723,AD.vs.control; bulk RNA-seq,Homo_714,AD.vs.control; bulk RNA-seq,Homo_633,AD.vs.control; bulk RNA-seq,Homo_633,AD.vs.MCI	4
MF	GO:0015355	secondary active monocarboxylate transmembrane	bulk RNA-seq,Homo_723,AD.vs.control; bulk RNA-seq,Homo_723,AD.vs.MCI; bulk RNA-seq,Homo_714,AD.vs.MCI	3
BP	GO:0070665	positive regulation of leukocyte proliferation	bulk RNA-seq,Homo_723,AD.vs.control; bulk RNA-seq,Homo_723,MCI.vs.control; bulk RNA-seq,Homo_633,AD.vs.control; bulk RNA-	4
BP	GO:0060219	camera-type eye photoreceptor cell differentiation	bulk RNA-seq,Homo_723,AD.vs.control; bulk RNA-seq,Homo_723,AD.vs.MCI; bulk RNA-seq,Homo_723,MCI.vs.control	3
BP	GO:0002262	myeloid cell homeostasis	bulk RNA-seq,Homo_723,AD.vs.control; bulk RNA-seq,Homo_723,MCI.vs.control; bulk RNA-seq,Homo_714,AD.vs.MCI; bulk RNA-seq,Homo_633,AD.vs.control; bulk RNA-seq,Homo_633,AD.vs.MCI	5
MF	GO:0030169	low-density lipoprotein particle binding	bulk RNA-seq,Homo_723,AD.vs.control; bulk RNA-seq,Homo_723,AD.vs.MCI; bulk RNA-seq,Homo_723,MCI.vs.control; bulk RNA-seq,Homo_714,AD.vs.MCI; bulk RNA-seq,Homo_633,AD.vs.control	5
BP	GO:0060333	interferon-gamma-mediated signaling pathway	bulk RNA-seq,Homo_723,AD.vs.control; bulk RNA-seq,Homo_633,AD.vs.control	2
BP	GO:0032456	endocytic recycling	bulk RNA-seq,Homo_723,AD.vs.control; bulk RNA-seq,Homo_723,MCI.vs.control; bulk RNA-seq,Homo_714,MCI.vs.control; bulk RNA-seq,Homo_633,AD.vs.control; bulk RNA-seq,Homo_633,AD.vs.MCI; scRNA-seq,SRP330776,Naive CD8+ T cell_2-AD.vs.control	6
BP	GO:1901018	positive regulation of potassium ion transmembrane	bulk RNA-seq,Homo_723,AD.vs.control; bulk RNA-seq,Homo_723,AD.vs.MCI	2
BP	GO:0090493	catecholamine uptake	bulk RNA-seq,Homo_723,AD.vs.control; bulk RNA-seq,Homo_723,AD.vs.MCI; bulk RNA-seq,Homo_714,AD.vs.control	3
BP	GO:0043931	ossification involved in bone maturation	bulk RNA-seq,Homo_723,AD.vs.control	1
BP	GO:0050858	negative regulation of antigen receptor-mediated signaling	bulk RNA-seq,Homo_723,AD.vs.control; bulk RNA-seq,Homo_633,AD.vs.control; bulk RNA-seq,Homo_633,AD.vs.MCI	3
BP	GO:2001233	regulation of apoptotic signaling pathway	bulk RNA-seq,Homo_723,AD.vs.control; bulk RNA-seq,Homo_723,AD.vs.MCI; bulk RNA-seq,Homo_714,AD.vs.MCI; bulk RNA-seq,Homo_633,AD.vs.control; bulk RNA-seq,Homo_633,AD.vs.MCI	5
CC	GO:0043034	costamere	bulk RNA-seq,Homo_723,AD.vs.control; bulk RNA-seq,Homo_723,AD.vs.MCI; bulk RNA-seq,Homo_723,MCI.vs.control; bulk RNA-seq,Homo_714,AD.vs.MCI; bulk RNA-seq,Homo_714,MCI.vs.control	5
BP	GO:0019748	secondary metabolic process	bulk RNA-seq,Homo_723,AD.vs.control; bulk RNA-seq,Homo_723,AD.vs.MCI; bulk RNA-seq,Homo_723,MCI.vs.control; bulk RNA-seq,Homo_714,AD.vs.MCI; bulk RNA-seq,Homo_633,AD.vs.control; bulk RNA-seq,Homo_633,AD.vs.MCI	6
BP	GO:0014911	positive regulation of smooth muscle cell migration	bulk RNA-seq,Homo_723,AD.vs.control; bulk RNA-seq,Homo_723,AD.vs.MCI; bulk RNA-seq,Homo_723,MCI.vs.control; bulk RNA-seq,Homo_714,AD.vs.control; bulk RNA-seq,Homo_714,AD.vs.MCI; bulk RNA-seq,Homo_714,MCI.vs.control; bulk RNA-seq,Homo_633,AD.vs.control	7
MF	GO:0017134	fibroblast growth factor binding	bulk RNA-seq,Homo_723,AD.vs.control; bulk RNA-seq,Homo_723,AD.vs.MCI; bulk RNA-seq,Homo_723,MCI.vs.control; bulk RNA-seq,Homo_714,AD.vs.MCI; bulk RNA-seq,Homo_714,MCI.vs.control; bulk RNA-seq,Homo_633,AD.vs.control	6
BP	GO:0031294	lymphocyte costimulation	bulk RNA-seq,Homo_723,AD.vs.control; bulk RNA-seq,Homo_723,MCI.vs.control; bulk RNA-seq,Homo_714,AD.vs.control; bulk RNA-seq,Homo_714,MCI.vs.control; bulk RNA-seq,Homo_633,AD.vs.control	5

BP	GO:0003214	cardiac left ventricle morphogenesis	bulk RNA-seq,Homo_723,AD.vs.control; bulk RNA-seq,Homo_723,AD.vs.MCI; bulk RNA-seq,Homo_723,MCI.vs.control; bulk RNA-seq,Homo_714,AD.vs.MCI	4
BP	GO:0060295	regulation of cilium movement involved in cell motility	bulk RNA-seq,Homo_723,AD.vs.control; bulk RNA-seq,Homo_723,AD.vs.MCI	2
BP	GO:1902019	regulation of cilium-dependent cell motility	bulk RNA-seq,Homo_723,AD.vs.control; bulk RNA-seq,Homo_723,AD.vs.MCI	2
BP	GO:0007271	synaptic transmission, cholinergic	bulk RNA-seq,Homo_723,AD.vs.control; bulk RNA-seq,Homo_723,AD.vs.MCI; bulk RNA-seq,Homo_723,MCI.vs.control; bulk RNA-seq,Homo_714,AD.vs.MCI	4
BP	GO:0038007	netrin-activated signaling pathway	bulk RNA-seq,Homo_723,AD.vs.control; bulk RNA-seq,Homo_723,AD.vs.MCI	2
BP	GO:0055062	phosphate ion homeostasis	bulk RNA-seq,Homo_723,AD.vs.control; bulk RNA-seq,Homo_723,AD.vs.MCI; bulk RNA-seq,Homo_723,MCI.vs.control; bulk RNA-seq,Homo_714,AD.vs.MCI	4
BP	GO:0072506	trivalent inorganic anion homeostasis	bulk RNA-seq,Homo_723,AD.vs.control; bulk RNA-seq,Homo_723,AD.vs.MCI; bulk RNA-seq,Homo_723,MCI.vs.control; bulk RNA-seq,Homo_714,AD.vs.MCI	4
BP	GO:0038127	ERBB signaling pathway	bulk RNA-seq,Homo_723,AD.vs.control; bulk RNA-seq,Homo_723,AD.vs.MCI; bulk RNA-seq,Homo_723,MCI.vs.control; bulk RNA-seq,Homo_714,AD.vs.MCI; bulk RNA-seq,Homo_714,MCI.vs.control; bulk RNA-seq,Homo_633,AD.vs.control; bulk RNA-seq,Homo_633,AD.vs.MCI	7
BP	GO:0006892	post-Golgi vesicle-mediated transport	bulk RNA-seq,Homo_723,AD.vs.control; bulk RNA-seq,Homo_723,MCI.vs.control; bulk RNA-seq,Homo_714,AD.vs.control; bulk RNA-seq,Homo_714,MCI.vs.control; bulk RNA-seq,Homo_633,AD.vs.control; bulk RNA-seq,Homo_633,AD.vs.MCI	6
BP	GO:0009950	dorsal/ventral axis specification	bulk RNA-seq,Homo_723,AD.vs.control; bulk RNA-seq,Homo_723,AD.vs.MCI; bulk RNA-seq,Homo_723,MCI.vs.control; bulk RNA-seq,Homo_714,AD.vs.MCI	4
BP	GO:0086067	AV node cell to bundle of His cell communication	bulk RNA-seq,Homo_723,AD.vs.control; bulk RNA-seq,Homo_723,AD.vs.MCI; bulk RNA-seq,Homo_723,MCI.vs.control; bulk RNA-seq,Homo_714,AD.vs.MCI; bulk RNA-seq,Homo_714,MCI.vs.control	5
BP	GO:0010634	positive regulation of epithelial cell migration	bulk RNA-seq,Homo_723,AD.vs.control; bulk RNA-seq,Homo_723,AD.vs.MCI; bulk RNA-seq,Homo_723,MCI.vs.control; bulk RNA-seq,Homo_714,AD.vs.control; bulk RNA-seq,Homo_714,AD.vs.MCI; bulk RNA-seq,Homo_633,AD.vs.control; bulk RNA-seq,Homo_633,AD.vs.MCI; scRNA-	8
BP	GO:0097502	mannosylation	bulk RNA-seq,Homo_723,AD.vs.control; bulk RNA-seq,Homo_723,MCI.vs.control; bulk RNA-seq,Homo_714,AD.vs.control; bulk RNA-seq,Homo_714,MCI.vs.control; bulk RNA-seq,Homo_633,AD.vs.control; bulk RNA-seq,Homo_633,MCI.vs.control	6
BP	GO:0097531	mast cell migration	bulk RNA-seq,Homo_723,AD.vs.control; bulk RNA-seq,Homo_723,MCI.vs.control	2
BP	GO:0021801	cerebral cortex radial glia-guided migration	bulk RNA-seq,Homo_723,AD.vs.control; bulk RNA-seq,Homo_723,AD.vs.MCI; bulk RNA-seq,Homo_723,MCI.vs.control; bulk RNA-seq,Homo_714,MCI.vs.control; bulk RNA-seq,Homo_633,AD.vs.control; bulk RNA-seq,Homo_633,AD.vs.MCI	6
BP	GO:0022030	telencephalon glial cell migration	bulk RNA-seq,Homo_723,AD.vs.control; bulk RNA-seq,Homo_723,AD.vs.MCI; bulk RNA-seq,Homo_723,MCI.vs.control; bulk RNA-seq,Homo_714,MCI.vs.control; bulk RNA-seq,Homo_633,AD.vs.control; bulk RNA-seq,Homo_633,AD.vs.MCI	6
BP	GO:0048668	collateral sprouting	bulk RNA-seq,Homo_723,AD.vs.control; bulk RNA-seq,Homo_723,AD.vs.MCI; bulk RNA-seq,Homo_723,MCI.vs.control; bulk RNA-seq,Homo_714,AD.vs.MCI	4
MF	GO:0031419	cobalamin binding	bulk RNA-seq,Homo_723,AD.vs.control; bulk RNA-seq,Homo_714,AD.vs.control; bulk RNA-seq,Homo_633,AD.vs.control	3
BP	GO:0014020	primary neural tube formation	bulk RNA-seq,Homo_723,AD.vs.control; bulk RNA-seq,Homo_723,AD.vs.MCI; bulk RNA-seq,Homo_723,MCI.vs.control; bulk RNA-seq,Homo_714,AD.vs.MCI; bulk RNA-seq,Homo_714,MCI.vs.control; bulk RNA-seq,Homo_633,AD.vs.control; bulk RNA-seq,Homo_633,AD.vs.MCI	7
BP	GO:1905710	positive regulation of membrane permeability	bulk RNA-seq,Homo_723,AD.vs.control; bulk RNA-seq,Homo_714,AD.vs.control; bulk RNA-seq,Homo_714,MCI.vs.control; bulk RNA-seq,Homo_633,AD.vs.control; bulk RNA-seq,Homo_633,AD.vs.MCI	5
BP	GO:0032908	regulation of transforming growth factor beta1 production	bulk RNA-seq,Homo_723,AD.vs.control; bulk RNA-seq,Homo_723,AD.vs.MCI; bulk RNA-seq,Homo_723,MCI.vs.control; bulk RNA-seq,Homo_714,AD.vs.MCI; bulk RNA-seq,Homo_714,MCI.vs.control	5
BP	GO:0071044	histone mRNA catabolic process	bulk RNA-seq,Homo_723,AD.vs.control; bulk RNA-seq,Homo_714,AD.vs.control; bulk RNA-seq,Homo_633,AD.vs.control; bulk RNA-seq,Homo_633,AD.vs.MCI	4
BP	GO:0002507	tolerance induction	bulk RNA-seq,Homo_723,AD.vs.control; bulk RNA-seq,Homo_714,AD.vs.control; bulk RNA-seq,Homo_714,MCI.vs.control; bulk RNA-seq,Homo_633,AD.vs.control; bulk RNA-seq,Homo_633,MCI.vs.control; scRNA-seq,SRP215507,CD8+ T cell_3-MCI.vs.control	6
CC	GO:0005686	U2 snRNP	bulk RNA-seq,Homo_723,AD.vs.control; bulk RNA-seq,Homo_714,AD.vs.control; bulk RNA-seq,Homo_633,AD.vs.control; bulk RNA-seq,Homo_633,AD.vs.MCI	4
BP	GO:0016074	sno(s)RNA metabolic process	bulk RNA-seq,Homo_723,AD.vs.control; bulk RNA-seq,Homo_633,AD.vs.control; bulk RNA-seq,Homo_633,AD.vs.MCI	3
BP	GO:0045807	positive regulation of endocytosis	bulk RNA-seq,Homo_723,AD.vs.control; bulk RNA-seq,Homo_723,AD.vs.MCI; bulk RNA-seq,Homo_723,MCI.vs.control; bulk RNA-seq,Homo_714,AD.vs.control; bulk RNA-seq,Homo_714,AD.vs.MCI; bulk RNA-seq,Homo_714,MCI.vs.control; bulk RNA-seq,Homo_633,AD.vs.control; bulk	8
BP	GO:0002367	cytokine production involved in immune response	bulk RNA-seq,Homo_723,AD.vs.control; bulk RNA-seq,Homo_723,MCI.vs.control; bulk RNA-seq,Homo_714,MCI.vs.control; bulk RNA-seq,Homo_633,AD.vs.control; bulk RNA-seq,Homo_633,AD.vs.MCI; scRNA-seq,SRP330776,CD8+ T cell_2-AD.vs.control	6
BP	GO:0031125	rRNA 3'-end processing	bulk RNA-seq,Homo_723,AD.vs.control; bulk RNA-seq,Homo_723,AD.vs.MCI; bulk RNA-seq,Homo_714,AD.vs.control; bulk RNA-seq,Homo_714,AD.vs.MCI	4
BP	GO:0042117	monocyte activation	bulk RNA-seq,Homo_723,AD.vs.control; bulk RNA-seq,Homo_723,AD.vs.MCI; bulk RNA-seq,Homo_714,AD.vs.MCI; bulk RNA-seq,Homo_714,MCI.vs.control	4
MF	GO:0005126	cytokine receptor binding	bulk RNA-seq,Homo_723,AD.vs.control; bulk RNA-seq,Homo_723,AD.vs.MCI; bulk RNA-seq,Homo_723,MCI.vs.control; bulk RNA-seq,Homo_714,AD.vs.control; bulk RNA-seq,Homo_714,AD.vs.MCI; bulk RNA-seq,Homo_633,AD.vs.control	6
BP	GO:0001771	immunological synapse formation	bulk RNA-seq,Homo_723,AD.vs.control; bulk RNA-seq,Homo_723,MCI.vs.control; bulk RNA-seq,Homo_714,MCI.vs.control; bulk RNA-seq,Homo_633,AD.vs.control; bulk RNA-seq,Homo_633,AD.vs.MCI	5
BP	GO:0060742	epithelial cell differentiation involved in prostate gland	bulk RNA-seq,Homo_723,AD.vs.control; bulk RNA-seq,Homo_723,AD.vs.MCI; bulk RNA-seq,Homo_714,AD.vs.control; bulk RNA-	4
BP	GO:0072148	epithelial cell fate commitment	bulk RNA-seq,Homo_723,AD.vs.control; bulk RNA-seq,Homo_723,AD.vs.MCI; bulk RNA-seq,Homo_723,MCI.vs.control; bulk RNA-seq,Homo_714,AD.vs.MCI	4
BP	GO:0090030	regulation of steroid hormone biosynthetic process	bulk RNA-seq,Homo_723,AD.vs.control; bulk RNA-seq,Homo_723,AD.vs.MCI; bulk RNA-seq,Homo_723,MCI.vs.control	3
BP	GO:0071392	cellular response to estradiol stimulus	bulk RNA-seq,Homo_723,AD.vs.control; bulk RNA-seq,Homo_723,AD.vs.MCI; bulk RNA-seq,Homo_723,MCI.vs.control; bulk RNA-seq,Homo_714,AD.vs.MCI; bulk RNA-seq,Homo_714,MCI.vs.control	5
BP	GO:0098732	macromolecule deacylation	bulk RNA-seq,Homo_723,AD.vs.control; bulk RNA-seq,Homo_723,MCI.vs.control; bulk RNA-seq,Homo_714,AD.vs.control; bulk RNA-seq,Homo_633,AD.vs.control; bulk RNA-seq,Homo_633,AD.vs.MCI	5
BP	GO:0032102	negative regulation of response to external stimulus	bulk RNA-seq,Homo_723,AD.vs.control; bulk RNA-seq,Homo_723,AD.vs.MCI; bulk RNA-seq,Homo_714,AD.vs.control; bulk RNA-seq,Homo_714,AD.vs.MCI; bulk RNA-seq,Homo_633,AD.vs.control; bulk RNA-seq,Homo_633,AD.vs.MCI	6
BP	GO:0007030	Golgi organization	bulk RNA-seq,Homo_723,AD.vs.control; bulk RNA-seq,Homo_723,MCI.vs.control; bulk RNA-seq,Homo_633,AD.vs.control; bulk RNA-	4
BP	GO:1901617	organic hydroxy compound biosynthetic process	bulk RNA-seq,Homo_723,AD.vs.control; bulk RNA-seq,Homo_723,AD.vs.MCI; bulk RNA-seq,Homo_723,MCI.vs.control; bulk RNA-seq,Homo_714,AD.vs.MCI; bulk RNA-seq,Homo_633,AD.vs.control; bulk RNA-seq,Homo_633,AD.vs.MCI	6
BP	GO:0002643	regulation of tolerance induction	bulk RNA-seq,Homo_723,AD.vs.control; bulk RNA-seq,Homo_714,AD.vs.control; bulk RNA-seq,Homo_714,MCI.vs.control; bulk RNA-	4
BP	GO:0099625	ventricular cardiac muscle cell membrane repolarization	bulk RNA-seq,Homo_723,AD.vs.control; bulk RNA-seq,Homo_723,AD.vs.MCI; bulk RNA-seq,Homo_723,MCI.vs.control; bulk RNA-seq,Homo_714,AD.vs.control; bulk RNA-seq,Homo_714,AD.vs.MCI; bulk RNA-seq,Homo_714,MCI.vs.control	6

BP	GO:0046348	amino sugar catabolic process	bulk RNA-seq,Homo_723,AD.vs.control; bulk RNA-seq,Homo_633,AD.vs.control; bulk RNA-seq,Homo_633,AD.vs.MCI	3
BP	GO:0050798	activated T cell proliferation	bulk RNA-seq,Homo_723,AD.vs.control; bulk RNA-seq,Homo_714,MCI.vs.control; bulk RNA-seq,Homo_633,AD.vs.control; bulk RNA-	4
CC	GO:0036019	endolysosome	bulk RNA-seq,Homo_723,AD.vs.control; bulk RNA-seq,Homo_723,MCI.vs.control; bulk RNA-seq,Homo_714,MCI.vs.control; bulk RNA-seq,Homo_633,AD.vs.control; bulk RNA-seq,Homo_633,AD.vs.MCI	5
BP	GO:0009100	glycoprotein metabolic process	bulk RNA-seq,Homo_723,AD.vs.control; bulk RNA-seq,Homo_723,AD.vs.MCI; bulk RNA-seq,Homo_714,AD.vs.MCI; bulk RNA-seq,Homo_633,AD.vs.control; bulk RNA-seq,Homo_633,AD.vs.MCI; scRNA-seq,SRP330776,CD8+ T cell_1-AD.vs.control	6
BP	GO:0048008	platelet-derived growth factor receptor signaling pathway	bulk RNA-seq,Homo_723,AD.vs.control; bulk RNA-seq,Homo_723,AD.vs.MCI; bulk RNA-seq,Homo_723,MCI.vs.control; bulk RNA-seq,Homo_714,AD.vs.MCI; bulk RNA-seq,Homo_714,MCI.vs.control; bulk RNA-seq,Homo_633,AD.vs.control; bulk RNA-seq,Homo_633,AD.vs.MCI	7
BP	GO:0072659	protein localization to plasma membrane	bulk RNA-seq,Homo_723,AD.vs.control; bulk RNA-seq,Homo_723,AD.vs.MCI; bulk RNA-seq,Homo_723,MCI.vs.control; bulk RNA-seq,Homo_714,AD.vs.MCI; bulk RNA-seq,Homo_633,AD.vs.control; bulk RNA-seq,Homo_633,AD.vs.MCI	6
MF	GO:0032404	mismatch repair complex binding	bulk RNA-seq,Homo_723,AD.vs.control; bulk RNA-seq,Homo_633,AD.vs.control; bulk RNA-seq,Homo_633,AD.vs.MCI	3
BP	GO:0014850	response to muscle activity	bulk RNA-seq,Homo_723,AD.vs.control; bulk RNA-seq,Homo_723,AD.vs.MCI; bulk RNA-seq,Homo_723,MCI.vs.control; bulk RNA-seq,Homo_714,AD.vs.MCI	4
BP	GO:0060065	uterus development	bulk RNA-seq,Homo_723,AD.vs.control; bulk RNA-seq,Homo_723,AD.vs.MCI; bulk RNA-seq,Homo_723,MCI.vs.control	3
BP	GO:0007342	fusion of sperm to egg plasma membrane involved in single	bulk RNA-seq,Homo_723,AD.vs.control; bulk RNA-seq,Homo_714,AD.vs.control; bulk RNA-seq,Homo_714,AD.vs.MCI	3
MF	GO:0050811	GABA receptor binding	bulk RNA-seq,Homo_723,AD.vs.control; bulk RNA-seq,Homo_723,AD.vs.MCI	2
BP	GO:0002517	T cell tolerance induction	bulk RNA-seq,Homo_723,AD.vs.control; bulk RNA-seq,Homo_714,MCI.vs.control; bulk RNA-seq,Homo_633,AD.vs.control	3
BP	GO:1904427	positive regulation of calcium ion transmembrane transport	bulk RNA-seq,Homo_723,AD.vs.control; bulk RNA-seq,Homo_723,AD.vs.MCI; bulk RNA-seq,Homo_723,MCI.vs.control; bulk RNA-seq,Homo_714,AD.vs.MCI; bulk RNA-seq,Homo_714,MCI.vs.control; bulk RNA-seq,Homo_633,AD.vs.control; bulk RNA-seq,Homo_633,AD.vs.MCI	7
BP	GO:0045026	plasma membrane fusion	bulk RNA-seq,Homo_723,AD.vs.control; bulk RNA-seq,Homo_714,AD.vs.control; bulk RNA-seq,Homo_714,AD.vs.MCI	3
BP	GO:0048853	forebrain morphogenesis	bulk RNA-seq,Homo_723,AD.vs.control; bulk RNA-seq,Homo_723,AD.vs.MCI; bulk RNA-seq,Homo_723,MCI.vs.control	3
BP	GO:0090336	positive regulation of brown fat cell differentiation	bulk RNA-seq,Homo_723,AD.vs.control; bulk RNA-seq,Homo_723,AD.vs.MCI	2
BP	GO:0051279	regulation of release of sequestered calcium ion into cytosol	bulk RNA-seq,Homo_723,AD.vs.control; bulk RNA-seq,Homo_723,AD.vs.MCI; bulk RNA-seq,Homo_723,MCI.vs.control; bulk RNA-seq,Homo_714,AD.vs.MCI; bulk RNA-seq,Homo_714,MCI.vs.control; bulk RNA-seq,Homo_633,AD.vs.control; bulk RNA-seq,Homo_633,AD.vs.MCI	7
BP	GO:0050974	detection of mechanical stimulus involved in sensory	bulk RNA-seq,Homo_723,AD.vs.control; bulk RNA-seq,Homo_723,AD.vs.MCI; bulk RNA-seq,Homo_723,MCI.vs.control; bulk RNA-seq,Homo_714,AD.vs.MCI	4
BP	GO:0009261	ribonucleotide catabolic process	bulk RNA-seq,Homo_723,AD.vs.control; bulk RNA-seq,Homo_723,MCI.vs.control; bulk RNA-seq,Homo_714,AD.vs.control; bulk RNA-seq,Homo_714,MCI.vs.control; bulk RNA-seq,Homo_633,AD.vs.control; bulk RNA-seq,Homo_633,AD.vs.MCI; scRNA-seq,SRP330776,Naive CD8+ T cell_1-	7
BP	GO:0050913	sensory perception of bitter taste	bulk RNA-seq,Homo_723,AD.vs.control; bulk RNA-seq,Homo_714,AD.vs.control; bulk RNA-seq,Homo_714,AD.vs.MCI; bulk RNA-seq,Homo_633,AD.vs.control; bulk RNA-seq,Homo_633,AD.vs.MCI; bulk RNA-seq,ROSMAP,AD.vs.control; bulk RNA-seq,SRP223445,AD.vs.control	7
CC	GO:0033391	chromatoid body	bulk RNA-seq,Homo_723,AD.vs.control; bulk RNA-seq,Homo_723,AD.vs.MCI; bulk RNA-seq,Homo_714,AD.vs.MCI	3
BP	GO:0030259	lipid glycosylation	bulk RNA-seq,Homo_723,AD.vs.control	1
CC	GO:0045259	proton-transporting ATP synthase complex	bulk RNA-seq,Homo_723,AD.vs.control; bulk RNA-seq,Homo_723,MCI.vs.control; bulk RNA-seq,Homo_714,AD.vs.control; bulk RNA-seq,Homo_714,MCI.vs.control; bulk RNA-seq,Homo_633,AD.vs.control; bulk RNA-seq,Homo_633,MCI.vs.control	6
BP	GO:2001169	regulation of ATP biosynthetic process	bulk RNA-seq,Homo_723,AD.vs.control; bulk RNA-seq,Homo_723,MCI.vs.control; bulk RNA-seq,Homo_714,MCI.vs.control; bulk RNA-seq,Homo_633,AD.vs.control; bulk RNA-seq,Homo_633,AD.vs.MCI; bulk RNA-seq,Homo_633,MCI.vs.control	6
BP	GO:0031348	negative regulation of defense response	bulk RNA-seq,Homo_723,AD.vs.control; bulk RNA-seq,Homo_723,MCI.vs.control; bulk RNA-seq,Homo_714,AD.vs.MCI; bulk RNA-seq,Homo_633,AD.vs.control; bulk RNA-seq,Homo_633,AD.vs.MCI	5
BP	GO:0048011	neurotrophin TRK receptor signaling pathway	bulk RNA-seq,Homo_723,AD.vs.control; bulk RNA-seq,Homo_723,AD.vs.MCI; bulk RNA-seq,Homo_723,MCI.vs.control; bulk RNA-seq,Homo_714,AD.vs.MCI; bulk RNA-seq,Homo_714,MCI.vs.control; bulk RNA-seq,Homo_633,AD.vs.control	6
CC	GO:0001650	fibrillar center	bulk RNA-seq,Homo_723,AD.vs.control; bulk RNA-seq,Homo_723,MCI.vs.control; bulk RNA-seq,Homo_714,AD.vs.MCI; bulk RNA-seq,Homo_633,AD.vs.control; bulk RNA-seq,Homo_633,AD.vs.MCI	5
BP	GO:0051127	positive regulation of actin nucleation	bulk RNA-seq,Homo_723,AD.vs.control; bulk RNA-seq,Homo_723,AD.vs.MCI; bulk RNA-seq,Homo_723,MCI.vs.control; bulk RNA-seq,Homo_714,AD.vs.control; bulk RNA-seq,Homo_633,AD.vs.control	5
BP	GO:0048712	negative regulation of astrocyte differentiation	bulk RNA-seq,Homo_723,AD.vs.control; bulk RNA-seq,Homo_723,AD.vs.MCI; bulk RNA-seq,Homo_723,MCI.vs.control; bulk RNA-seq,Homo_714,AD.vs.MCI; bulk RNA-seq,Homo_714,MCI.vs.control	5
BP	GO:0045638	negative regulation of myeloid cell differentiation	bulk RNA-seq,Homo_723,AD.vs.control; bulk RNA-seq,Homo_723,AD.vs.MCI; bulk RNA-seq,Homo_723,MCI.vs.control; bulk RNA-seq,Homo_714,AD.vs.control; bulk RNA-seq,Homo_714,AD.vs.MCI; bulk RNA-seq,Homo_714,MCI.vs.control; bulk RNA-seq,Homo_633,AD.vs.control; bulk	8
BP	GO:0014003	oligodendrocyte development	bulk RNA-seq,Homo_723,AD.vs.control; bulk RNA-seq,Homo_723,AD.vs.MCI; bulk RNA-seq,Homo_723,MCI.vs.control; bulk RNA-seq,Homo_714,MCI.vs.control; bulk RNA-seq,Homo_633,AD.vs.control	5
BP	GO:0001945	lymph vessel development	bulk RNA-seq,Homo_723,AD.vs.control; bulk RNA-seq,Homo_723,AD.vs.MCI; bulk RNA-seq,Homo_723,MCI.vs.control; bulk RNA-seq,Homo_714,AD.vs.MCI; bulk RNA-seq,Homo_714,MCI.vs.control	5
BP	GO:1904263	positive regulation of TORC1 signaling	bulk RNA-seq,Homo_723,AD.vs.control; bulk RNA-seq,Homo_723,MCI.vs.control; bulk RNA-seq,Homo_633,AD.vs.control; bulk RNA-	4
BP	GO:0044794	positive regulation by host of viral process	bulk RNA-seq,Homo_723,AD.vs.control; bulk RNA-seq,Homo_714,AD.vs.control; bulk RNA-seq,Homo_714,AD.vs.MCI; bulk RNA-seq,Homo_633,AD.vs.control; bulk RNA-seq,Homo_633,AD.vs.MCI	5
MF	GO:0004521	endoribonuclease activity	bulk RNA-seq,Homo_723,AD.vs.control; bulk RNA-seq,Homo_633,AD.vs.control; bulk RNA-seq,Homo_633,AD.vs.MCI	3
BP	GO:0043923	positive regulation by host of viral transcription	bulk RNA-seq,Homo_723,AD.vs.control; bulk RNA-seq,Homo_723,MCI.vs.control; bulk RNA-seq,Homo_714,MCI.vs.control; bulk RNA-seq,Homo_633,AD.vs.control; bulk RNA-seq,Homo_633,AD.vs.MCI; bulk RNA-seq,Homo_633,MCI.vs.control	6
MF	GO:0016783	sulfurtransferase activity	bulk RNA-seq,Homo_723,AD.vs.control	1
BP	GO:1903232	melanosome assembly	bulk RNA-seq,Homo_723,AD.vs.control; bulk RNA-seq,Homo_723,MCI.vs.control; bulk RNA-seq,Homo_714,MCI.vs.control; bulk RNA-seq,Homo_633,AD.vs.control; bulk RNA-seq,Homo_633,AD.vs.MCI; bulk RNA-seq,Homo_633,MCI.vs.control	6
BP	GO:0003096	renal sodium ion transport	bulk RNA-seq,Homo_723,AD.vs.control; bulk RNA-seq,Homo_723,AD.vs.MCI; bulk RNA-seq,Homo_714,AD.vs.control; bulk RNA-seq,Homo_714,AD.vs.MCI	4
BP	GO:0002295	T-helper cell lineage commitment	bulk RNA-seq,Homo_723,AD.vs.control; bulk RNA-seq,Homo_714,MCI.vs.control; bulk RNA-seq,Homo_633,MCI.vs.control	3

BP	GO:1901028	regulation of mitochondrial outer membrane permeabilization involved in apoptotic signaling pathway	bulk RNA-seq,Homo_723,AD.vs.control; bulk RNA-seq,Homo_723,MCI.vs.control; bulk RNA-seq,Homo_714,AD.vs.control; bulk RNA-seq,Homo_714,MCI.vs.control; bulk RNA-seq,Homo_633,AD.vs.control; bulk RNA-seq,Homo_633,AD.vs.MCI; bulk RNA-seq,Homo_633,MCI.vs.control	7
BP	GO:0030838	positive regulation of actin filament polymerization	bulk RNA-seq,Homo_723,AD.vs.control; bulk RNA-seq,Homo_723,AD.vs.MCI; bulk RNA-seq,Homo_723,MCI.vs.control; bulk RNA-seq,Homo_714,AD.vs.MCI; bulk RNA-seq,Homo_714,MCI.vs.control; bulk RNA-seq,Homo_633,AD.vs.control; bulk RNA-seq,Homo_633,AD.vs.MCI	7
CC	GO:0030681	multimeric ribonuclease P complex	bulk RNA-seq,Homo_723,AD.vs.control; bulk RNA-seq,Homo_723,AD.vs.MCI	2
MF	GO:0033204	ribonuclease P RNA binding	bulk RNA-seq,Homo_723,AD.vs.control; bulk RNA-seq,Homo_723,AD.vs.MCI	2
BP	GO:0001914	regulation of T cell mediated cytotoxicity	bulk RNA-seq,Homo_723,AD.vs.control; bulk RNA-seq,Homo_633,AD.vs.control	2
BP	GO:0090330	regulation of platelet aggregation	bulk RNA-seq,Homo_723,AD.vs.control; bulk RNA-seq,Homo_723,AD.vs.MCI; bulk RNA-seq,Homo_723,MCI.vs.control; bulk RNA-seq,Homo_714,AD.vs.control; bulk RNA-seq,Homo_714,AD.vs.MCI; bulk RNA-seq,Homo_714,MCI.vs.control	6
CC	GO:0032806	carboxy-terminal domain protein kinase complex	bulk RNA-seq,Homo_723,AD.vs.control; bulk RNA-seq,Homo_723,MCI.vs.control; bulk RNA-seq,Homo_714,AD.vs.control; bulk RNA-seq,Homo_714,MCI.vs.control; bulk RNA-seq,Homo_633,AD.vs.control; bulk RNA-seq,Homo_633,AD.vs.MCI; bulk RNA-seq,Homo_633,MCI.vs.control	7
BP	GO:0008300	isoprenoid catabolic process	bulk RNA-seq,Homo_723,AD.vs.control; bulk RNA-seq,Homo_723,AD.vs.MCI; bulk RNA-seq,Homo_723,MCI.vs.control	3
BP	GO:0090100	positive regulation of transmembrane receptor protein serine/threonine kinase signaling pathway	bulk RNA-seq,Homo_723,AD.vs.control; bulk RNA-seq,Homo_723,AD.vs.MCI; bulk RNA-seq,Homo_723,MCI.vs.control; bulk RNA-seq,Homo_714,AD.vs.MCI; bulk RNA-seq,Homo_714,MCI.vs.control; bulk RNA-seq,Homo_633,AD.vs.control; bulk RNA-seq,Homo_633,AD.vs.MCI	7
MF	GO:0005159	insulin-like growth factor receptor binding	bulk RNA-seq,Homo_723,AD.vs.control	1
BP	GO:0048385	regulation of retinoic acid receptor signaling pathway	bulk RNA-seq,Homo_723,AD.vs.control; bulk RNA-seq,Homo_723,AD.vs.MCI; bulk RNA-seq,Homo_723,MCI.vs.control	3
BP	GO:0090110	COPII-coated vesicle cargo loading	bulk RNA-seq,Homo_723,AD.vs.control; bulk RNA-seq,Homo_723,MCI.vs.control; bulk RNA-seq,Homo_714,MCI.vs.control; bulk RNA-	4
BP	GO:0010831	positive regulation of myotube differentiation	bulk RNA-seq,Homo_723,AD.vs.control; bulk RNA-seq,Homo_714,AD.vs.control; bulk RNA-seq,Homo_714,AD.vs.MCI	3
BP	GO:1905037	autophagosome organization	bulk RNA-seq,Homo_723,AD.vs.control; bulk RNA-seq,Homo_714,AD.vs.control; bulk RNA-seq,Homo_714,MCI.vs.control; bulk RNA-seq,Homo_633,AD.vs.control; bulk RNA-seq,Homo_633,AD.vs.MCI	5
BP	GO:0043297	apical junction assembly	bulk RNA-seq,Homo_723,AD.vs.control; bulk RNA-seq,Homo_723,AD.vs.MCI; bulk RNA-seq,Homo_723,MCI.vs.control; bulk RNA-seq,Homo_714,AD.vs.control; bulk RNA-seq,Homo_714,AD.vs.MCI; bulk RNA-seq,Homo_714,MCI.vs.control	6
BP	GO:0045577	regulation of B cell differentiation	bulk RNA-seq,Homo_723,AD.vs.control; bulk RNA-seq,Homo_723,MCI.vs.control; bulk RNA-seq,Homo_714,MCI.vs.control; bulk RNA-	4
BP	GO:2001034	positive regulation of double-strand break repair via nonhomologous end joining	bulk RNA-seq,Homo_723,AD.vs.control; bulk RNA-seq,Homo_714,MCI.vs.control; bulk RNA-seq,Homo_633,MCI.vs.control	3
BP	GO:0003071	renal system process involved in regulation of systemic arterial blood pressure	bulk RNA-seq,Homo_723,AD.vs.control; bulk RNA-seq,Homo_723,AD.vs.MCI; bulk RNA-seq,Homo_723,MCI.vs.control; bulk RNA-seq,Homo_714,AD.vs.control; bulk RNA-seq,Homo_714,AD.vs.MCI; bulk RNA-seq,Homo_714,MCI.vs.control	6
BP	GO:0072584	caveolin-mediated endocytosis	bulk RNA-seq,Homo_723,AD.vs.control; bulk RNA-seq,Homo_723,AD.vs.MCI; bulk RNA-seq,Homo_714,AD.vs.MCI; bulk RNA-seq,Homo_714,MCI.vs.control	4
MF	GO:0015026	coreceptor activity	bulk RNA-seq,Homo_723,AD.vs.control; bulk RNA-seq,Homo_723,AD.vs.MCI; bulk RNA-seq,Homo_723,MCI.vs.control; bulk RNA-seq,Homo_714,AD.vs.MCI; bulk RNA-seq,Homo_714,MCI.vs.control; bulk RNA-seq,Homo_633,AD.vs.control; bulk RNA-seq,Homo_633,AD.vs.MCI	7
BP	GO:0072044	collecting duct development	bulk RNA-seq,Homo_723,AD.vs.control; bulk RNA-seq,Homo_723,AD.vs.MCI; bulk RNA-seq,Homo_633,MCI.vs.control	3
BP	GO:0034308	primary alcohol metabolic process	bulk RNA-seq,Homo_723,AD.vs.control; bulk RNA-seq,Homo_723,AD.vs.MCI; bulk RNA-seq,Homo_723,MCI.vs.control; bulk RNA-seq,Homo_714,AD.vs.MCI; bulk RNA-seq,Homo_714,MCI.vs.control; bulk RNA-seq,Homo_633,AD.vs.control; bulk RNA-seq,Homo_633,AD.vs.MCI	7
BP	GO:0002043	blood vessel endothelial cell proliferation involved in sprouting angiogenesis	bulk RNA-seq,Homo_723,AD.vs.control; bulk RNA-seq,Homo_723,AD.vs.MCI; bulk RNA-seq,Homo_714,AD.vs.control; bulk RNA-seq,Homo_714,AD.vs.MCI	4
MF	GO:0004536	deoxyribonuclease activity	bulk RNA-seq,Homo_723,AD.vs.control; bulk RNA-seq,Homo_723,MCI.vs.control; bulk RNA-seq,Homo_714,MCI.vs.control; bulk RNA-seq,Homo_633,AD.vs.control; bulk RNA-seq,Homo_633,AD.vs.MCI	5
BP	GO:1903321	negative regulation of protein modification by small protein conjugation or removal	bulk RNA-seq,Homo_723,AD.vs.control; bulk RNA-seq,Homo_723,MCI.vs.control; bulk RNA-seq,Homo_714,MCI.vs.control; bulk RNA-seq,Homo_633,AD.vs.control; bulk RNA-seq,Homo_633,AD.vs.MCI	5
BP	GO:0060395	SMAD protein signal transduction	bulk RNA-seq,Homo_723,AD.vs.control; bulk RNA-seq,Homo_723,AD.vs.MCI; bulk RNA-seq,Homo_723,MCI.vs.control; bulk RNA-seq,Homo_714,AD.vs.MCI; bulk RNA-seq,Homo_714,MCI.vs.control; bulk RNA-seq,Homo_633,AD.vs.control; bulk RNA-seq,Homo_633,AD.vs.MCI	7
BP	GO:1901185	negative regulation of ERBB signaling pathway	bulk RNA-seq,Homo_723,AD.vs.control; bulk RNA-seq,Homo_723,AD.vs.MCI; bulk RNA-seq,Homo_723,MCI.vs.control; bulk RNA-seq,Homo_714,AD.vs.MCI; bulk RNA-seq,Homo_714,MCI.vs.control	5
MF	GO:1990841	promoter-specific chromatin binding	bulk RNA-seq,Homo_723,AD.vs.control; bulk RNA-seq,Homo_723,MCI.vs.control; bulk RNA-seq,Homo_714,MCI.vs.control; bulk RNA-seq,Homo_633,AD.vs.control; bulk RNA-seq,Homo_633,AD.vs.MCI	5
CC	GO:0019774	proteasome core complex, beta-subunit complex	bulk RNA-seq,Homo_723,AD.vs.control; bulk RNA-seq,Homo_633,AD.vs.control; bulk RNA-seq,Homo_633,AD.vs.MCI	3
BP	GO:0010596	negative regulation of endothelial cell migration	bulk RNA-seq,Homo_723,AD.vs.control; bulk RNA-seq,Homo_723,AD.vs.MCI; bulk RNA-seq,Homo_723,MCI.vs.control; bulk RNA-seq,Homo_714,AD.vs.control; bulk RNA-seq,Homo_714,AD.vs.MCI; bulk RNA-seq,Homo_714,MCI.vs.control	6
MF	GO:0019902	phosphatase binding	bulk RNA-seq,Homo_723,AD.vs.control; bulk RNA-seq,Homo_723,MCI.vs.control; bulk RNA-seq,Homo_714,AD.vs.MCI; bulk RNA-seq,Homo_633,AD.vs.control; bulk RNA-seq,Homo_633,AD.vs.MCI	5
BP	GO:0031998	regulation of fatty acid beta-oxidation	bulk RNA-seq,Homo_723,AD.vs.control; bulk RNA-seq,Homo_723,MCI.vs.control; bulk RNA-seq,Homo_714,MCI.vs.control; bulk RNA-seq,Homo_633,AD.vs.control; bulk RNA-seq,Homo_633,AD.vs.MCI	5
BP	GO:1990928	response to amino acid starvation	bulk RNA-seq,Homo_723,AD.vs.control; bulk RNA-seq,Homo_723,MCI.vs.control; bulk RNA-seq,Homo_714,MCI.vs.control; bulk RNA-seq,Homo_633,AD.vs.control; bulk RNA-seq,Homo_633,AD.vs.MCI	5
BP	GO:0072074	kidney mesenchyme development	bulk RNA-seq,Homo_723,AD.vs.control; bulk RNA-seq,Homo_723,AD.vs.MCI; bulk RNA-seq,Homo_723,MCI.vs.control; bulk RNA-seq,Homo_714,AD.vs.MCI; bulk RNA-seq,Homo_633,MCI.vs.control	5
BP	GO:0070863	positive regulation of protein exit from endoplasmic	bulk RNA-seq,Homo_723,AD.vs.control; bulk RNA-seq,Homo_633,AD.vs.control	2
BP	GO:0006469	negative regulation of protein kinase activity	bulk RNA-seq,Homo_723,AD.vs.control; bulk RNA-seq,Homo_723,AD.vs.MCI; bulk RNA-seq,Homo_723,MCI.vs.control; bulk RNA-seq,Homo_714,AD.vs.MCI; bulk RNA-seq,Homo_633,AD.vs.control; bulk RNA-seq,Homo_633,AD.vs.MCI	6
BP	GO:0015936	coenzyme A metabolic process	bulk RNA-seq,Homo_723,AD.vs.control; bulk RNA-seq,Homo_723,MCI.vs.control; bulk RNA-seq,Homo_714,MCI.vs.control; bulk RNA-seq,Homo_633,AD.vs.control; bulk RNA-seq,Homo_633,AD.vs.MCI; bulk RNA-seq,Homo_633,MCI.vs.control	6
BP	GO:0060736	prostate gland growth	bulk RNA-seq,Homo_723,AD.vs.control; bulk RNA-seq,Homo_723,AD.vs.MCI; bulk RNA-seq,Homo_723,MCI.vs.control; bulk RNA-seq,Homo_714,AD.vs.MCI	4

BP	GO:0021680	cerebellar Purkinje cell layer development	bulk RNA-seq,Homo_723,AD.vs.control; bulk RNA-seq,Homo_723,AD.vs.MCI; bulk RNA-seq,Homo_723,MCI.vs.control; bulk RNA-seq,Homo_633,AD.vs.control; bulk RNA-seq,Homo_633,AD.vs.MCI	5
BP	GO:0035601	protein deacylation	bulk RNA-seq,Homo_723,AD.vs.control; bulk RNA-seq,Homo_723,MCI.vs.control; bulk RNA-seq,Homo_714,AD.vs.control; bulk RNA-seq,Homo_633,AD.vs.control; bulk RNA-seq,Homo_633,AD.vs.MCI	5
BP	GO:0006835	dicarboxylic acid transport	bulk RNA-seq,Homo_723,AD.vs.control; bulk RNA-seq,Homo_723,AD.vs.MCI; bulk RNA-seq,Homo_723,MCI.vs.control; bulk RNA-seq,Homo_714,AD.vs.MCI; bulk RNA-seq,Homo_633,AD.vs.control; bulk RNA-seq,Homo_633,AD.vs.MCI; bulk RNA-seq,ROSMAP,AD.vs.control	7
BP	GO:0071634	regulation of transforming growth factor beta production	bulk RNA-seq,Homo_723,AD.vs.control; bulk RNA-seq,Homo_723,AD.vs.MCI; bulk RNA-seq,Homo_723,MCI.vs.control; bulk RNA-seq,Homo_714,AD.vs.MCI; bulk RNA-seq,Homo_714,MCI.vs.control; bulk RNA-seq,Homo_633,AD.vs.control; bulk RNA-seq,Homo_633,AD.vs.MCI	7
MF	GO:0050998	nitric-oxide synthase binding	bulk RNA-seq,Homo_723,AD.vs.control; bulk RNA-seq,Homo_723,AD.vs.MCI; bulk RNA-seq,Homo_723,MCI.vs.control; bulk RNA-seq,Homo_714,AD.vs.control; bulk RNA-seq,Homo_714,AD.vs.MCI; bulk RNA-seq,Homo_714,MCI.vs.control	6
MF	GO:0016798	hydrolase activity, acting on glycosyl bonds	bulk RNA-seq,Homo_723,AD.vs.control; bulk RNA-seq,Homo_723,MCI.vs.control; bulk RNA-seq,Homo_633,AD.vs.control; bulk RNA-	4
BP	GO:1903670	regulation of sprouting angiogenesis	bulk RNA-seq,Homo_723,AD.vs.control; bulk RNA-seq,Homo_723,AD.vs.MCI; bulk RNA-seq,Homo_723,MCI.vs.control; bulk RNA-seq,Homo_714,AD.vs.control; bulk RNA-seq,Homo_714,AD.vs.MCI; bulk RNA-seq,Homo_714,MCI.vs.control	6
BP	GO:0060039	pericardium development	bulk RNA-seq,Homo_723,AD.vs.control; bulk RNA-seq,Homo_723,AD.vs.MCI; bulk RNA-seq,Homo_723,MCI.vs.control; bulk RNA-seq,Homo_714,AD.vs.MCI	4
BP	GO:1901844	regulation of cell communication by electrical coupling involved in cardiac conduction	bulk RNA-seq,Homo_723,AD.vs.control; bulk RNA-seq,Homo_723,AD.vs.MCI; bulk RNA-seq,Homo_723,MCI.vs.control; bulk RNA-seq,Homo_714,AD.vs.control; bulk RNA-seq,Homo_714,AD.vs.MCI; bulk RNA-seq,Homo_714,MCI.vs.control	6
CC	GO:0001401	SAM complex	bulk RNA-seq,Homo_723,AD.vs.control; bulk RNA-seq,Homo_714,AD.vs.control; bulk RNA-seq,Homo_633,AD.vs.control; bulk RNA-seq,Homo_633,AD.vs.MCI	4
CC	GO:0140275	MIB complex	bulk RNA-seq,Homo_723,AD.vs.control; bulk RNA-seq,Homo_714,AD.vs.control; bulk RNA-seq,Homo_633,AD.vs.control; bulk RNA-seq,Homo_633,AD.vs.MCI	4
MF	GO:0015464	acetylcholine receptor activity	bulk RNA-seq,Homo_723,AD.vs.control; bulk RNA-seq,Homo_723,AD.vs.MCI; bulk RNA-seq,Homo_723,MCI.vs.control; bulk RNA-seq,Homo_714,AD.vs.MCI; bulk RNA-seq,Homo_633,MCI.vs.control	5
BP	GO:0002755	MyD88-dependent toll-like receptor signaling pathway	bulk RNA-seq,Homo_723,AD.vs.control; bulk RNA-seq,Homo_633,AD.vs.control; bulk RNA-seq,Homo_633,AD.vs.MCI	3
BP	GO:0042593	glucose homeostasis	bulk RNA-seq,Homo_723,AD.vs.control; bulk RNA-seq,Homo_723,AD.vs.MCI; bulk RNA-seq,Homo_723,MCI.vs.control; bulk RNA-seq,Homo_714,AD.vs.MCI; bulk RNA-seq,Homo_633,AD.vs.control; bulk RNA-seq,Homo_633,AD.vs.MCI	6
CC	GO:0030673	axolemma	bulk RNA-seq,Homo_723,AD.vs.control; bulk RNA-seq,Homo_723,AD.vs.MCI; bulk RNA-seq,Homo_723,MCI.vs.control; bulk RNA-	4
BP	GO:0030318	melanocyte differentiation	bulk RNA-seq,Homo_723,AD.vs.control; bulk RNA-seq,Homo_723,AD.vs.MCI; bulk RNA-seq,Homo_723,MCI.vs.control; bulk RNA-seq,Homo_714,AD.vs.MCI; bulk RNA-seq,Homo_714,MCI.vs.control	5
BP	GO:0045839	negative regulation of mitotic nuclear division	bulk RNA-seq,Homo_723,AD.vs.control; bulk RNA-seq,Homo_723,MCI.vs.control; bulk RNA-seq,Homo_714,MCI.vs.control; bulk RNA-seq,Homo_633,AD.vs.control; bulk RNA-seq,Homo_633,AD.vs.MCI	5
CC	GO:0031970	organelle envelope lumen	bulk RNA-seq,Homo_723,AD.vs.control; bulk RNA-seq,Homo_723,MCI.vs.control; bulk RNA-seq,Homo_714,AD.vs.control; bulk RNA-seq,Homo_714,MCI.vs.control; bulk RNA-seq,Homo_633,AD.vs.control; bulk RNA-seq,Homo_633,AD.vs.MCI; scRNA-seq,SRP215507,CD8+ T cell_8-	7
BP	GO:0006623	protein targeting to vacuole	bulk RNA-seq,Homo_723,AD.vs.control; bulk RNA-seq,Homo_723,MCI.vs.control; bulk RNA-seq,Homo_714,AD.vs.control; bulk RNA-seq,Homo_714,MCI.vs.control; bulk RNA-seq,Homo_633,AD.vs.control; bulk RNA-seq,Homo_633,AD.vs.MCI	6
CC	GO:0090533	cation-transporting ATPase complex	bulk RNA-seq,Homo_723,AD.vs.control; bulk RNA-seq,Homo_723,AD.vs.MCI	2
BP	GO:0033500	carbohydrate homeostasis	bulk RNA-seq,Homo_723,AD.vs.control; bulk RNA-seq,Homo_723,AD.vs.MCI; bulk RNA-seq,Homo_723,MCI.vs.control; bulk RNA-seq,Homo_714,AD.vs.MCI; bulk RNA-seq,Homo_633,AD.vs.control; bulk RNA-seq,Homo_633,AD.vs.MCI	6
BP	GO:0051283	negative regulation of sequestering of calcium ion	bulk RNA-seq,Homo_723,AD.vs.control; bulk RNA-seq,Homo_723,AD.vs.MCI; bulk RNA-seq,Homo_723,MCI.vs.control; bulk RNA-seq,Homo_714,AD.vs.MCI; bulk RNA-seq,Homo_714,MCI.vs.control; bulk RNA-seq,Homo_633,AD.vs.control; bulk RNA-seq,Homo_633,AD.vs.MCI	7
BP	GO:0015012	heparan sulfate proteoglycan biosynthetic process	bulk RNA-seq,Homo_723,AD.vs.control; bulk RNA-seq,Homo_723,AD.vs.MCI; bulk RNA-seq,Homo_723,MCI.vs.control; bulk RNA-seq,Homo_714,AD.vs.MCI; bulk RNA-seq,Homo_633,AD.vs.control; bulk RNA-seq,Homo_633,AD.vs.MCI	6
CC	GO:0030315	T-tubule	bulk RNA-seq,Homo_723,AD.vs.control; bulk RNA-seq,Homo_723,AD.vs.MCI; bulk RNA-seq,Homo_723,MCI.vs.control; bulk RNA-seq,Homo_714,AD.vs.MCI; bulk RNA-seq,Homo_714,MCI.vs.control	5
BP	GO:0035766	cell chemotaxis to fibroblast growth factor	bulk RNA-seq,Homo_723,AD.vs.control; bulk RNA-seq,Homo_714,AD.vs.control; bulk RNA-seq,Homo_714,AD.vs.MCI	3
BP	GO:1904847	regulation of cell chemotaxis to fibroblast growth factor	bulk RNA-seq,Homo_723,AD.vs.control; bulk RNA-seq,Homo_714,AD.vs.control; bulk RNA-seq,Homo_714,AD.vs.MCI	3
BP	GO:0071492	cellular response to UV-A	bulk RNA-seq,Homo_723,AD.vs.control	1
CC	GO:1990454	L-type voltage-gated calcium channel complex	bulk RNA-seq,Homo_723,AD.vs.control; bulk RNA-seq,Homo_723,AD.vs.MCI; bulk RNA-seq,Homo_723,MCI.vs.control; bulk RNA-seq,Homo_714,AD.vs.MCI	4
BP	GO:0030210	heparin biosynthetic process	bulk RNA-seq,Homo_723,AD.vs.control; bulk RNA-seq,Homo_723,AD.vs.MCI; bulk RNA-seq,Homo_723,MCI.vs.control; bulk RNA-seq,Homo_714,AD.vs.control; bulk RNA-seq,Homo_714,MCI.vs.control; bulk RNA-seq,Homo_633,AD.vs.control	6
BP	GO:0061311	cell surface receptor signaling pathway involved in heart development	bulk RNA-seq,Homo_723,AD.vs.control; bulk RNA-seq,Homo_723,AD.vs.MCI; bulk RNA-seq,Homo_723,MCI.vs.control; bulk RNA-seq,Homo_714,AD.vs.MCI; bulk RNA-seq,Homo_714,MCI.vs.control	5
BP	GO:0031118	rRNA pseudouridine synthesis	bulk RNA-seq,Homo_723,AD.vs.control; bulk RNA-seq,Homo_723,MCI.vs.control; bulk RNA-seq,Homo_714,AD.vs.control; bulk RNA-seq,Homo_714,MCI.vs.control; bulk RNA-seq,Homo_633,AD.vs.control; bulk RNA-seq,Homo_633,AD.vs.MCI; bulk RNA-seq,Homo_633,MCI.vs.control	7
BP	GO:0043268	positive regulation of potassium ion transport	bulk RNA-seq,Homo_723,AD.vs.control; bulk RNA-seq,Homo_723,AD.vs.MCI; bulk RNA-seq,Homo_723,MCI.vs.control; bulk RNA-seq,Homo_714,AD.vs.MCI	4
BP	GO:0006383	transcription by RNA polymerase III	bulk RNA-seq,Homo_723,AD.vs.control; bulk RNA-seq,Homo_723,MCI.vs.control; bulk RNA-seq,Homo_714,MCI.vs.control; bulk RNA-seq,Homo_633,AD.vs.control; bulk RNA-seq,Homo_633,AD.vs.MCI	5
BP	GO:1903523	negative regulation of blood circulation	bulk RNA-seq,Homo_723,AD.vs.control	1
BP	GO:0072524	pyridine-containing compound metabolic process	bulk RNA-seq,Homo_723,AD.vs.control; bulk RNA-seq,Homo_723,MCI.vs.control; bulk RNA-seq,Homo_714,AD.vs.control; bulk RNA-seq,Homo_714,MCI.vs.control; bulk RNA-seq,Homo_633,AD.vs.control; bulk RNA-seq,Homo_633,AD.vs.MCI	6
BP	GO:0046637	regulation of alpha-beta T cell differentiation	bulk RNA-seq,Homo_723,AD.vs.control; bulk RNA-seq,Homo_723,MCI.vs.control; bulk RNA-seq,Homo_714,AD.vs.control; bulk RNA-seq,Homo_714,MCI.vs.control; bulk RNA-seq,Homo_633,AD.vs.control; bulk RNA-seq,Homo_633,AD.vs.MCI	6
BP	GO:0050857	positive regulation of antigen receptor-mediated signaling pathway	bulk RNA-seq,Homo_723,AD.vs.control; bulk RNA-seq,Homo_723,MCI.vs.control; bulk RNA-seq,Homo_714,MCI.vs.control; bulk RNA-seq,Homo_633,AD.vs.control; bulk RNA-seq,Homo_633,MCI.vs.control	5
CC	GO:0005828	kinetochore microtubule	bulk RNA-seq,Homo_723,AD.vs.control; bulk RNA-seq,Homo_723,MCI.vs.control; bulk RNA-seq,Homo_714,MCI.vs.control; bulk RNA-seq,Homo_633,AD.vs.control; bulk RNA-seq,Homo_633,MCI.vs.control	5

BP	GO:0050966	detection of mechanical stimulus involved in sensory perception of pain	bulk RNA-seq,Homo_723,AD.vs.control; bulk RNA-seq,Homo_723,AD.vs.MCI; bulk RNA-seq,Homo_723,MCI.vs.control; bulk RNA-seq,Homo_714,AD.vs.MCI; bulk RNA-seq,Homo_714,MCI.vs.control	5
MF	GO:0005172	vascular endothelial growth factor receptor binding	bulk RNA-seq,Homo_723,AD.vs.control; bulk RNA-seq,Homo_723,AD.vs.MCI; bulk RNA-seq,Homo_723,MCI.vs.control; bulk RNA-seq,Homo_714,AD.vs.MCI; bulk RNA-seq,Homo_714,MCI.vs.control	5
BP	GO:0009812	flavonoid metabolic process	bulk RNA-seq,Homo_723,AD.vs.control; bulk RNA-seq,Homo_723,AD.vs.MCI; bulk RNA-seq,Homo_714,AD.vs.control; bulk RNA-seq,Homo_714,AD.vs.MCI	4
BP	GO:0065002	intracellular protein transmembrane transport	bulk RNA-seq,Homo_723,AD.vs.control; bulk RNA-seq,Homo_723,MCI.vs.control; bulk RNA-seq,Homo_714,MCI.vs.control; bulk RNA-seq,Homo_633,AD.vs.control; bulk RNA-seq,Homo_633,AD.vs.MCI	5
BP	GO:0030574	collagen catabolic process	bulk RNA-seq,Homo_723,AD.vs.control; bulk RNA-seq,Homo_723,AD.vs.MCI; bulk RNA-seq,Homo_714,AD.vs.control; bulk RNA-seq,Homo_714,AD.vs.MCI	4
CC	GO:0030286	dynein complex	bulk RNA-seq,Homo_723,AD.vs.control; bulk RNA-seq,Homo_723,AD.vs.MCI; bulk RNA-seq,Homo_723,MCI.vs.control; bulk RNA-seq,Homo_714,AD.vs.MCI; bulk RNA-seq,Homo_714,MCI.vs.control; bulk RNA-seq,Homo_633,AD.vs.control	6
BP	GO:1903826	L-arginine transmembrane transport	bulk RNA-seq,Homo_723,AD.vs.control; bulk RNA-seq,Homo_723,AD.vs.MCI; bulk RNA-seq,Homo_723,MCI.vs.control	3
BP	GO:0070200	establishment of protein localization to telomere	bulk RNA-seq,Homo_723,AD.vs.control; bulk RNA-seq,Homo_723,MCI.vs.control; bulk RNA-seq,Homo_714,AD.vs.control; bulk RNA-seq,Homo_714,MCI.vs.control; bulk RNA-seq,Homo_633,AD.vs.control; bulk RNA-seq,Homo_633,AD.vs.MCI; bulk RNA-seq,Homo_633,MCI.vs.control	7
BP	GO:0046549	retinal cone cell development	bulk RNA-seq,Homo_723,AD.vs.control; bulk RNA-seq,Homo_723,AD.vs.MCI	2
BP	GO:0031365	N-terminal protein amino acid modification	bulk RNA-seq,Homo_723,AD.vs.control; bulk RNA-seq,Homo_723,MCI.vs.control; bulk RNA-seq,Homo_714,AD.vs.control; bulk RNA-seq,Homo_714,MCI.vs.control; bulk RNA-seq,Homo_633,AD.vs.control; bulk RNA-seq,Homo_633,AD.vs.MCI; bulk RNA-seq,Homo_633,MCI.vs.control	7
BP	GO:0070914	UV-damage excision repair	bulk RNA-seq,Homo_723,AD.vs.control; bulk RNA-seq,Homo_714,MCI.vs.control; bulk RNA-seq,Homo_633,AD.vs.control; bulk RNA-	4
CC	GO:0005766	primary lysosome	bulk RNA-seq,Homo_723,AD.vs.control; bulk RNA-seq,Homo_723,MCI.vs.control; bulk RNA-seq,Homo_633,AD.vs.control; bulk RNA-seq,Homo_633,AD.vs.MCI; bulk RNA-seq,MCSA,MCI.vs.control; bulk RNA-seq,SRP223445,AD.vs.control	6
CC	GO:0042582	azurophil granule	bulk RNA-seq,Homo_723,AD.vs.control; bulk RNA-seq,Homo_723,MCI.vs.control; bulk RNA-seq,Homo_633,AD.vs.control; bulk RNA-seq,Homo_633,AD.vs.MCI; bulk RNA-seq,MCSA,MCI.vs.control; bulk RNA-seq,SRP223445,AD.vs.control	6
BP	GO:0002260	lymphocyte homeostasis	bulk RNA-seq,Homo_723,AD.vs.control; bulk RNA-seq,Homo_723,MCI.vs.control; bulk RNA-seq,Homo_714,MCI.vs.control; bulk RNA-seq,Homo_633,AD.vs.control; bulk RNA-seq,Homo_633,AD.vs.MCI	5
MF	GO:0008308	voltage-gated anion channel activity	bulk RNA-seq,Homo_723,AD.vs.control; bulk RNA-seq,Homo_723,AD.vs.MCI; bulk RNA-seq,Homo_723,MCI.vs.control	3
MF	GO:0050815	phosphoserine residue binding	bulk RNA-seq,Homo_723,AD.vs.control; bulk RNA-seq,Homo_723,MCI.vs.control; bulk RNA-seq,Homo_714,AD.vs.control; bulk RNA-seq,Homo_714,MCI.vs.control; bulk RNA-seq,Homo_633,AD.vs.control; bulk RNA-seq,Homo_633,AD.vs.MCI; bulk RNA-seq,Homo_633,MCI.vs.control	7
MF	GO:0005165	neurotrophin receptor binding	bulk RNA-seq,Homo_723,AD.vs.control; bulk RNA-seq,Homo_723,AD.vs.MCI	2
BP	GO:0042053	regulation of dopamine metabolic process	bulk RNA-seq,Homo_723,AD.vs.control; bulk RNA-seq,Homo_723,AD.vs.MCI; bulk RNA-seq,Homo_714,AD.vs.control; bulk RNA-seq,Homo_714,AD.vs.MCI	4
BP	GO:0042069	regulation of catecholamine metabolic process	bulk RNA-seq,Homo_723,AD.vs.control; bulk RNA-seq,Homo_723,AD.vs.MCI; bulk RNA-seq,Homo_714,AD.vs.control; bulk RNA-seq,Homo_714,AD.vs.MCI	4
BP	GO:0007603	phototransduction, visible light	bulk RNA-seq,Homo_723,AD.vs.control	1
BP	GO:0006476	protein deacetylation	bulk RNA-seq,Homo_723,AD.vs.control; bulk RNA-seq,Homo_723,MCI.vs.control; bulk RNA-seq,Homo_714,AD.vs.control; bulk RNA-seq,Homo_714,MCI.vs.control; bulk RNA-seq,Homo_633,AD.vs.control; bulk RNA-seq,Homo_633,AD.vs.MCI	6
BP	GO:0010826	negative regulation of centrosome duplication	bulk RNA-seq,Homo_723,AD.vs.control; bulk RNA-seq,Homo_723,MCI.vs.control; bulk RNA-seq,Homo_714,MCI.vs.control	3
BP	GO:0046606	negative regulation of centrosome cycle	bulk RNA-seq,Homo_723,AD.vs.control; bulk RNA-seq,Homo_723,MCI.vs.control; bulk RNA-seq,Homo_714,MCI.vs.control	3
BP	GO:2001023	regulation of response to drug	bulk RNA-seq,Homo_723,AD.vs.control; bulk RNA-seq,Homo_714,AD.vs.control; bulk RNA-seq,Homo_633,AD.vs.control; bulk RNA-seq,Homo_633,AD.vs.MCI	4
CC	GO:0005753	mitochondrial proton-transporting ATP synthase complex	bulk RNA-seq,Homo_723,AD.vs.control; bulk RNA-seq,Homo_714,AD.vs.control; bulk RNA-seq,Homo_714,MCI.vs.control; bulk RNA-seq,Homo_633,AD.vs.control; bulk RNA-seq,Homo_633,MCI.vs.control	5
BP	GO:0006766	vitamin metabolic process	bulk RNA-seq,Homo_723,AD.vs.control; bulk RNA-seq,Homo_723,AD.vs.MCI; bulk RNA-seq,Homo_723,MCI.vs.control; bulk RNA-seq,Homo_714,AD.vs.MCI; bulk RNA-seq,Homo_714,MCI.vs.control; bulk RNA-seq,Homo_633,AD.vs.control; bulk RNA-seq,Homo_633,AD.vs.MCI	7
BP	GO:0002042	cell migration involved in sprouting angiogenesis	bulk RNA-seq,Homo_723,AD.vs.control; bulk RNA-seq,Homo_723,AD.vs.MCI; bulk RNA-seq,Homo_723,MCI.vs.control; bulk RNA-seq,Homo_714,AD.vs.control; bulk RNA-seq,Homo_714,AD.vs.MCI; bulk RNA-seq,Homo_714,MCI.vs.control	6
BP	GO:0021591	ventricular system development	bulk RNA-seq,Homo_723,AD.vs.control; bulk RNA-seq,Homo_723,AD.vs.MCI; bulk RNA-seq,Homo_723,MCI.vs.control; bulk RNA-seq,Homo_714,AD.vs.MCI; bulk RNA-seq,Homo_633,AD.vs.control; bulk RNA-seq,Homo_633,AD.vs.MCI	6
MF	GO:0005416	amino acid:cation symporter activity	bulk RNA-seq,Homo_723,AD.vs.control; bulk RNA-seq,Homo_723,AD.vs.MCI; bulk RNA-seq,Homo_714,AD.vs.MCI; bulk RNA-seq,Homo_633,MCI.vs.control	4
BP	GO:0033623	regulation of integrin activation	bulk RNA-seq,Homo_723,AD.vs.control; bulk RNA-seq,Homo_723,AD.vs.MCI; bulk RNA-seq,Homo_723,MCI.vs.control; bulk RNA-seq,Homo_714,AD.vs.control; bulk RNA-seq,Homo_714,AD.vs.MCI; bulk RNA-seq,Homo_714,MCI.vs.control; bulk RNA-seq,Homo_633,AD.vs.control	7
BP	GO:0051496	positive regulation of stress fiber assembly	bulk RNA-seq,Homo_723,AD.vs.control; bulk RNA-seq,Homo_723,AD.vs.MCI; bulk RNA-seq,Homo_723,MCI.vs.control; bulk RNA-seq,Homo_714,AD.vs.MCI; bulk RNA-seq,Homo_714,MCI.vs.control; bulk RNA-seq,Homo_633,AD.vs.control; bulk RNA-seq,Homo_633,AD.vs.MCI	7
BP	GO:0046635	positive regulation of alpha-beta T cell activation	bulk RNA-seq,Homo_723,AD.vs.control; bulk RNA-seq,Homo_714,AD.vs.control; bulk RNA-seq,Homo_714,MCI.vs.control; bulk RNA-seq,Homo_633,AD.vs.control; bulk RNA-seq,Homo_633,AD.vs.MCI	5
BP	GO:0050891	multicellular organismal water homeostasis	bulk RNA-seq,Homo_723,AD.vs.control; bulk RNA-seq,Homo_723,AD.vs.MCI; bulk RNA-seq,Homo_723,MCI.vs.control; bulk RNA-seq,Homo_714,AD.vs.control; bulk RNA-seq,Homo_714,AD.vs.MCI; bulk RNA-seq,Homo_714,MCI.vs.control; bulk RNA-seq,Homo_633,AD.vs.MCI; bulk RNA-	8
BP	GO:1901216	positive regulation of neuron death	bulk RNA-seq,Homo_723,AD.vs.control; bulk RNA-seq,Homo_723,AD.vs.MCI; bulk RNA-seq,Homo_723,MCI.vs.control; bulk RNA-seq,Homo_714,MCI.vs.control; bulk RNA-seq,Homo_633,AD.vs.control; bulk RNA-seq,Homo_633,AD.vs.MCI	6
BP	GO:0002227	innate immune response in mucosa	bulk RNA-seq,Homo_723,AD.vs.control; bulk RNA-seq,SRP325058,AD.vs.control	2
BP	GO:0042670	retinal cone cell differentiation	bulk RNA-seq,Homo_723,AD.vs.control; bulk RNA-seq,Homo_723,AD.vs.MCI	2
BP	GO:0030201	heparan sulfate proteoglycan metabolic process	bulk RNA-seq,Homo_723,AD.vs.control; bulk RNA-seq,Homo_723,AD.vs.MCI; bulk RNA-seq,Homo_723,MCI.vs.control; bulk RNA-seq,Homo_714,MCI.vs.control; bulk RNA-seq,Homo_633,AD.vs.control; bulk RNA-seq,Homo_633,AD.vs.MCI	6
BP	GO:0043501	skeletal muscle adaptation	bulk RNA-seq,Homo_723,AD.vs.control; bulk RNA-seq,Homo_723,AD.vs.MCI; bulk RNA-seq,Homo_723,MCI.vs.control; bulk RNA-seq,Homo_714,AD.vs.MCI	4
BP	GO:0003184	pulmonary valve morphogenesis	bulk RNA-seq,Homo_723,AD.vs.control; bulk RNA-seq,Homo_723,AD.vs.MCI; bulk RNA-seq,Homo_723,MCI.vs.control; bulk RNA-seq,Homo_714,AD.vs.MCI; bulk RNA-seq,Homo_714,MCI.vs.control	5
BP	GO:1904752	regulation of vascular associated smooth muscle cell	bulk RNA-seq,Homo_723,AD.vs.control; bulk RNA-seq,Homo_723,AD.vs.MCI; bulk RNA-seq,Homo_714,AD.vs.control; bulk RNA-seq,Homo_714,AD.vs.MCI	4

BP	GO:0032609	interferon-gamma production	bulk RNA-seq,Homo_723,AD.vs.control; bulk RNA-seq,Homo_723,MCI.vs.control; bulk RNA-seq,Homo_714,MCI.vs.control; bulk RNA-seq,Homo_633,AD.vs.control; bulk RNA-seq,Homo_633,AD.vs.MCI	5
BP	GO:0032649	regulation of interferon-gamma production	bulk RNA-seq,Homo_723,AD.vs.control; bulk RNA-seq,Homo_723,MCI.vs.control; bulk RNA-seq,Homo_714,MCI.vs.control; bulk RNA-seq,Homo_633,AD.vs.control; bulk RNA-seq,Homo_633,AD.vs.MCI	5
BP	GO:0032481	positive regulation of type I interferon production	bulk RNA-seq,Homo_723,AD.vs.control; bulk RNA-seq,Homo_723,MCI.vs.control; bulk RNA-seq,Homo_714,MCI.vs.control; bulk RNA-seq,Homo_633,AD.vs.control; bulk RNA-seq,Homo_633,AD.vs.MCI	5
BP	GO:0035768	endothelial cell chemotaxis to fibroblast growth factor	bulk RNA-seq,Homo_723,AD.vs.control; bulk RNA-seq,Homo_723,AD.vs.MCI; bulk RNA-seq,Homo_714,AD.vs.control; bulk RNA-seq,Homo_714,AD.vs.MCI	4
BP	GO:2000544	regulation of endothelial cell chemotaxis to fibroblast growth	bulk RNA-seq,Homo_723,AD.vs.control; bulk RNA-seq,Homo_723,AD.vs.MCI; bulk RNA-seq,Homo_714,AD.vs.control; bulk RNA-seq,Homo_714,AD.vs.MCI	4
CC	GO:0005790	smooth endoplasmic reticulum	bulk RNA-seq,Homo_723,AD.vs.control; bulk RNA-seq,Homo_723,AD.vs.MCI; bulk RNA-seq,Homo_723,MCI.vs.control; bulk RNA-seq,Homo_714,AD.vs.MCI; bulk RNA-seq,Homo_714,MCI.vs.control; bulk RNA-seq,Homo_633,AD.vs.control	6
BP	GO:0070213	protein auto-ADP-ribosylation	bulk RNA-seq,Homo_723,AD.vs.control; bulk RNA-seq,Homo_723,MCI.vs.control; bulk RNA-seq,Homo_714,MCI.vs.control; bulk RNA-seq,Homo_633,AD.vs.control; bulk RNA-seq,Homo_633,MCI.vs.control	5
BP	GO:0010984	regulation of lipoprotein particle clearance	bulk RNA-seq,Homo_723,AD.vs.control; bulk RNA-seq,Homo_723,AD.vs.MCI; bulk RNA-seq,Homo_723,MCI.vs.control; bulk RNA-seq,Homo_714,AD.vs.control; bulk RNA-seq,Homo_714,AD.vs.MCI	5
BP	GO:0044827	modulation by host of viral genome replication	bulk RNA-seq,Homo_723,AD.vs.control; bulk RNA-seq,Homo_714,AD.vs.control; bulk RNA-seq,Homo_714,AD.vs.MCI; bulk RNA-seq,Homo_633,AD.vs.control; bulk RNA-seq,Homo_633,AD.vs.MCI	5
BP	GO:2000178	negative regulation of neural precursor cell proliferation	bulk RNA-seq,Homo_723,AD.vs.control; bulk RNA-seq,Homo_723,AD.vs.MCI; bulk RNA-seq,Homo_723,MCI.vs.control; bulk RNA-seq,Homo_714,AD.vs.MCI	4
BP	GO:2000052	positive regulation of non-canonical Wnt signaling pathway	bulk RNA-seq,Homo_723,AD.vs.control; bulk RNA-seq,Homo_723,AD.vs.MCI; bulk RNA-seq,Homo_723,MCI.vs.control; bulk RNA-seq,Homo_714,AD.vs.MCI; bulk RNA-seq,Homo_714,MCI.vs.control	5
MF	GO:0005243	gap junction channel activity	bulk RNA-seq,Homo_723,AD.vs.control; bulk RNA-seq,Homo_723,AD.vs.MCI; bulk RNA-seq,Homo_723,MCI.vs.control; bulk RNA-seq,Homo_714,AD.vs.control; bulk RNA-seq,Homo_714,AD.vs.MCI	5
CC	GO:0005922	connexin complex	bulk RNA-seq,Homo_723,AD.vs.control; bulk RNA-seq,Homo_723,AD.vs.MCI; bulk RNA-seq,Homo_723,MCI.vs.control; bulk RNA-seq,Homo_714,AD.vs.control; bulk RNA-seq,Homo_714,AD.vs.MCI	5
BP	GO:0033604	negative regulation of catecholamine secretion	bulk RNA-seq,Homo_723,AD.vs.control; bulk RNA-seq,Homo_723,AD.vs.MCI; bulk RNA-seq,Homo_723,MCI.vs.control	3
CC	GO:0005879	axonemal microtubule	bulk RNA-seq,Homo_723,AD.vs.control; bulk RNA-seq,Homo_723,AD.vs.MCI; bulk RNA-seq,Homo_723,MCI.vs.control; bulk RNA-seq,Homo_714,AD.vs.MCI; bulk RNA-seq,Homo_714,MCI.vs.control	5
BP	GO:0048512	circadian behavior	bulk RNA-seq,Homo_723,AD.vs.control; bulk RNA-seq,Homo_723,AD.vs.MCI	2
CC	GO:0019898	extrinsic component of membrane	bulk RNA-seq,Homo_723,AD.vs.control; bulk RNA-seq,Homo_723,AD.vs.MCI; bulk RNA-seq,Homo_723,MCI.vs.control; bulk RNA-seq,Homo_714,AD.vs.MCI; bulk RNA-seq,Homo_633,AD.vs.control; bulk RNA-seq,Homo_633,AD.vs.MCI	6
MF	GO:0045236	CXCR chemokine receptor binding	bulk RNA-seq,Homo_723,AD.vs.control; bulk RNA-seq,Homo_723,AD.vs.MCI; bulk RNA-seq,Homo_723,MCI.vs.control; bulk RNA-seq,Homo_714,AD.vs.control; bulk RNA-seq,Homo_714,AD.vs.MCI; bulk RNA-seq,Homo_714,MCI.vs.control	6
MF	GO:0016712	oxidoreductase activity, acting on paired donors, with incorporation or reduction of molecular oxygen, reduced flavin or flavoprotein as one donor, and incorporation of one	bulk RNA-seq,Homo_723,AD.vs.control; bulk RNA-seq,Homo_723,AD.vs.MCI; bulk RNA-seq,Homo_714,AD.vs.control; bulk RNA-seq,Homo_714,AD.vs.MCI; bulk RNA-seq,Homo_714,MCI.vs.control	5
BP	GO:0001886	endothelial cell morphogenesis	bulk RNA-seq,Homo_723,AD.vs.control; bulk RNA-seq,Homo_723,AD.vs.MCI; bulk RNA-seq,Homo_723,MCI.vs.control; bulk RNA-seq,Homo_714,AD.vs.MCI; bulk RNA-seq,Homo_714,MCI.vs.control	5
MF	GO:0051139	metal cation:proton antiporter activity	bulk RNA-seq,Homo_723,AD.vs.control; bulk RNA-seq,Homo_723,AD.vs.MCI; bulk RNA-seq,Homo_723,MCI.vs.control; bulk RNA-seq,Homo_714,AD.vs.MCI	4
BP	GO:0030104	water homeostasis	bulk RNA-seq,Homo_723,AD.vs.control; bulk RNA-seq,Homo_723,AD.vs.MCI; bulk RNA-seq,Homo_723,MCI.vs.control; bulk RNA-seq,Homo_714,AD.vs.control; bulk RNA-seq,Homo_714,AD.vs.MCI; bulk RNA-seq,Homo_714,MCI.vs.control; bulk RNA-seq,SRP223445,AD.vs.control	7
BP	GO:0034198	cellular response to amino acid starvation	bulk RNA-seq,Homo_723,AD.vs.control; bulk RNA-seq,Homo_723,MCI.vs.control; bulk RNA-seq,Homo_714,MCI.vs.control; bulk RNA-seq,Homo_633,AD.vs.control; bulk RNA-seq,Homo_633,AD.vs.MCI	5
BP	GO:0048865	stem cell fate commitment	bulk RNA-seq,Homo_723,AD.vs.control; bulk RNA-seq,Homo_723,AD.vs.MCI; bulk RNA-seq,Homo_723,MCI.vs.control; bulk RNA-seq,Homo_714,AD.vs.MCI	4
BP	GO:1903707	negative regulation of hemopoiesis	bulk RNA-seq,Homo_723,AD.vs.control; bulk RNA-seq,Homo_723,AD.vs.MCI; bulk RNA-seq,Homo_723,MCI.vs.control; bulk RNA-seq,Homo_714,AD.vs.MCI; bulk RNA-seq,Homo_714,MCI.vs.control; bulk RNA-seq,Homo_633,AD.vs.control; bulk RNA-seq,Homo_633,AD.vs.MCI; bulk RNA-	8
BP	GO:0043029	T cell homeostasis	bulk RNA-seq,Homo_723,AD.vs.control; bulk RNA-seq,Homo_633,AD.vs.control; bulk RNA-seq,Homo_633,AD.vs.MCI	3
CC	GO:0042575	DNA polymerase complex	bulk RNA-seq,Homo_723,AD.vs.control; bulk RNA-seq,Homo_723,MCI.vs.control; bulk RNA-seq,Homo_714,MCI.vs.control; bulk RNA-seq,Homo_633,AD.vs.control; bulk RNA-seq,Homo_633,AD.vs.MCI; bulk RNA-seq,Homo_633,MCI.vs.control	6
BP	GO:0010543	regulation of platelet activation	bulk RNA-seq,Homo_723,AD.vs.control; bulk RNA-seq,Homo_723,AD.vs.MCI; bulk RNA-seq,Homo_723,MCI.vs.control; bulk RNA-seq,Homo_714,AD.vs.control; bulk RNA-seq,Homo_714,AD.vs.MCI; bulk RNA-seq,Homo_714,MCI.vs.control; bulk RNA-seq,Homo_633,AD.vs.control; bulk	8
BP	GO:0010544	negative regulation of platelet activation	bulk RNA-seq,Homo_723,AD.vs.control; bulk RNA-seq,Homo_723,AD.vs.MCI; bulk RNA-seq,Homo_723,MCI.vs.control; bulk RNA-seq,Homo_714,AD.vs.control; bulk RNA-seq,Homo_714,AD.vs.MCI; bulk RNA-seq,Homo_714,MCI.vs.control	6
BP	GO:0006837	serotonin transport	bulk RNA-seq,Homo_723,AD.vs.control; bulk RNA-seq,Homo_723,AD.vs.MCI	2
BP	GO:0034135	regulation of toll-like receptor 2 signaling pathway	bulk RNA-seq,Homo_723,AD.vs.control; bulk RNA-seq,Homo_723,MCI.vs.control; bulk RNA-seq,Homo_714,MCI.vs.control; bulk RNA-	4
BP	GO:1904507	positive regulation of telomere maintenance in response to DNA damage	bulk RNA-seq,Homo_723,AD.vs.control; bulk RNA-seq,Homo_633,AD.vs.control; bulk RNA-seq,Homo_633,AD.vs.MCI	3
BP	GO:0033540	fatty acid beta-oxidation using acyl-CoA oxidase	bulk RNA-seq,Homo_723,AD.vs.control; bulk RNA-seq,Homo_714,AD.vs.control	2
BP	GO:0055057	neuroblast division	bulk RNA-seq,Homo_723,AD.vs.control; bulk RNA-seq,Homo_723,AD.vs.MCI; bulk RNA-seq,Homo_723,MCI.vs.control	3
BP	GO:0055012	ventricular cardiac muscle cell differentiation	bulk RNA-seq,Homo_723,AD.vs.control; bulk RNA-seq,Homo_723,AD.vs.MCI; bulk RNA-seq,Homo_723,MCI.vs.control; bulk RNA-seq,Homo_714,AD.vs.MCI	4
BP	GO:2000347	positive regulation of hepatocyte proliferation	bulk RNA-seq,Homo_723,AD.vs.control; bulk RNA-seq,Homo_723,AD.vs.MCI; bulk RNA-seq,Homo_723,MCI.vs.control; bulk RNA-seq,Homo_714,AD.vs.control; bulk RNA-seq,Homo_714,AD.vs.MCI; bulk RNA-seq,Homo_714,MCI.vs.control	6
BP	GO:1903747	regulation of establishment of protein localization to mitochondrion	bulk RNA-seq,Homo_723,AD.vs.control; bulk RNA-seq,Homo_723,MCI.vs.control; bulk RNA-seq,Homo_714,AD.vs.control; bulk RNA-seq,Homo_714,MCI.vs.control; bulk RNA-seq,Homo_633,AD.vs.control; bulk RNA-seq,Homo_633,AD.vs.MCI	6

BP	GO:0007080	mitotic metaphase plate congression	bulk RNA-seq,Homo_723,AD.vs.control; bulk RNA-seq,Homo_723,MCI.vs.control; bulk RNA-seq,Homo_714,MCI.vs.control; bulk RNA-seq,Homo_633,AD.vs.control; bulk RNA-seq,Homo_633,AD.vs.MCI	5
CC	GO:0016528	sarcoplasm	bulk RNA-seq,Homo_723,AD.vs.control; bulk RNA-seq,Homo_723,AD.vs.MCI; bulk RNA-seq,Homo_723,MCI.vs.control; bulk RNA-seq,Homo_714,AD.vs.MCI; bulk RNA-seq,Homo_714,MCI.vs.control; bulk RNA-seq,Homo_633,AD.vs.control; bulk RNA-seq,Homo_633,AD.vs.MCI	7
MF	GO:0030957	Tat protein binding	bulk RNA-seq,Homo_723,AD.vs.control; bulk RNA-seq,Homo_723,MCI.vs.control; bulk RNA-seq,Homo_714,MCI.vs.control; bulk RNA-	4
CC	GO:0032300	mismatch repair complex	bulk RNA-seq,Homo_723,AD.vs.control; bulk RNA-seq,Homo_723,MCI.vs.control; bulk RNA-seq,Homo_714,MCI.vs.control; bulk RNA-seq,Homo_633,AD.vs.control; bulk RNA-seq,Homo_633,AD.vs.MCI; bulk RNA-seq,Homo_633,MCI.vs.control	6
BP	GO:0010656	negative regulation of muscle cell apoptotic process	bulk RNA-seq,Homo_723,AD.vs.control; bulk RNA-seq,Homo_723,AD.vs.MCI; bulk RNA-seq,Homo_723,MCI.vs.control; bulk RNA-seq,Homo_714,AD.vs.MCI	4
BP	GO:0016575	histone deacetylation	bulk RNA-seq,Homo_723,AD.vs.control; bulk RNA-seq,Homo_723,MCI.vs.control; bulk RNA-seq,Homo_714,AD.vs.control; bulk RNA-seq,Homo_714,MCI.vs.control; bulk RNA-seq,Homo_633,AD.vs.control; bulk RNA-seq,Homo_633,AD.vs.MCI	6
CC	GO:0099059	integral component of presynaptic active zone membrane	bulk RNA-seq,Homo_723,AD.vs.control; bulk RNA-seq,Homo_723,AD.vs.MCI; bulk RNA-seq,Homo_723,MCI.vs.control	3
CC	GO:0098637	protein complex involved in cell-matrix adhesion	bulk RNA-seq,Homo_723,AD.vs.control; bulk RNA-seq,Homo_723,AD.vs.MCI; bulk RNA-seq,Homo_723,MCI.vs.control; bulk RNA-seq,Homo_714,AD.vs.MCI	4
BP	GO:0043687	post-translational protein modification	bulk RNA-seq,Homo_723,AD.vs.control; bulk RNA-seq,Homo_714,AD.vs.control; bulk RNA-seq,Homo_714,MCI.vs.control; bulk RNA-seq,Homo_633,AD.vs.control; bulk RNA-seq,Homo_633,AD.vs.MCI	5
CC	GO:0044194	cytolytic granule	bulk RNA-seq,Homo_723,AD.vs.control; bulk RNA-seq,Homo_714,MCI.vs.control; bulk RNA-seq,Homo_633,AD.vs.control; bulk RNA-seq,Homo_633,AD.vs.MCI; bulk RNA-seq,Homo_633,MCI.vs.control	5
BP	GO:0099622	cardiac muscle cell membrane repolarization	bulk RNA-seq,Homo_723,AD.vs.control; bulk RNA-seq,Homo_723,AD.vs.MCI; bulk RNA-seq,Homo_723,MCI.vs.control; bulk RNA-seq,Homo_714,AD.vs.MCI; bulk RNA-seq,Homo_714,MCI.vs.control	5
BP	GO:0010171	body morphogenesis	bulk RNA-seq,Homo_723,AD.vs.control; bulk RNA-seq,Homo_723,AD.vs.MCI; bulk RNA-seq,Homo_723,MCI.vs.control; bulk RNA-seq,Homo_714,AD.vs.MCI; bulk RNA-seq,Homo_714,MCI.vs.control; bulk RNA-seq,Homo_633,AD.vs.control; bulk RNA-seq,Homo_633,AD.vs.MCI	7
BP	GO:0002221	pattern recognition receptor signaling pathway	bulk RNA-seq,Homo_723,AD.vs.control; bulk RNA-seq,Homo_723,MCI.vs.control; bulk RNA-seq,Homo_714,AD.vs.MCI; bulk RNA-seq,Homo_633,AD.vs.control; bulk RNA-seq,Homo_633,AD.vs.MCI	5
CC	GO:0035631	CD40 receptor complex	bulk RNA-seq,Homo_723,AD.vs.control; bulk RNA-seq,Homo_723,MCI.vs.control; bulk RNA-seq,Homo_714,MCI.vs.control; bulk RNA-seq,Homo_633,AD.vs.control; bulk RNA-seq,Homo_633,AD.vs.MCI; bulk RNA-seq,Homo_633,MCI.vs.control	6
BP	GO:0070570	regulation of neuron projection regeneration	bulk RNA-seq,Homo_723,AD.vs.control; bulk RNA-seq,Homo_723,AD.vs.MCI; bulk RNA-seq,Homo_723,MCI.vs.control; bulk RNA-seq,Homo_714,AD.vs.MCI; bulk RNA-seq,Homo_714,MCI.vs.control	5
BP	GO:0003299	muscle hypertrophy in response to stress	bulk RNA-seq,Homo_723,AD.vs.control; bulk RNA-seq,Homo_723,AD.vs.MCI; bulk RNA-seq,Homo_714,AD.vs.MCI	3
BP	GO:0014887	cardiac muscle adaptation	bulk RNA-seq,Homo_723,AD.vs.control; bulk RNA-seq,Homo_723,AD.vs.MCI; bulk RNA-seq,Homo_714,AD.vs.MCI	3
BP	GO:0014898	cardiac muscle hypertrophy in response to stress	bulk RNA-seq,Homo_723,AD.vs.control; bulk RNA-seq,Homo_723,AD.vs.MCI; bulk RNA-seq,Homo_714,AD.vs.MCI	3
BP	GO:0018202	peptidyl-histidine modification	bulk RNA-seq,Homo_723,AD.vs.control; bulk RNA-seq,Homo_723,MCI.vs.control; bulk RNA-seq,Homo_714,MCI.vs.control; bulk RNA-seq,Homo_633,AD.vs.control; bulk RNA-seq,Homo_633,AD.vs.MCI; bulk RNA-seq,Homo_633,MCI.vs.control	6
MF	GO:0004622	lysophospholipase activity	bulk RNA-seq,Homo_723,AD.vs.control; bulk RNA-seq,Homo_723,AD.vs.MCI; bulk RNA-seq,Homo_633,AD.vs.control; bulk RNA-seq,Homo_633,AD.vs.MCI	4
BP	GO:0010922	positive regulation of phosphatase activity	bulk RNA-seq,Homo_723,AD.vs.control; bulk RNA-seq,Homo_723,AD.vs.MCI; bulk RNA-seq,Homo_714,AD.vs.control; bulk RNA-seq,Homo_714,AD.vs.MCI; bulk RNA-seq,Homo_633,AD.vs.control	5
BP	GO:0060122	inner ear receptor cell stereocilium organization	bulk RNA-seq,Homo_723,AD.vs.control; bulk RNA-seq,Homo_723,AD.vs.MCI; bulk RNA-seq,Homo_723,MCI.vs.control; bulk RNA-seq,Homo_714,AD.vs.MCI	4
CC	GO:0046930	pore complex	bulk RNA-seq,Homo_723,AD.vs.control; bulk RNA-seq,Homo_723,MCI.vs.control; bulk RNA-seq,Homo_714,AD.vs.MCI; bulk RNA-seq,Homo_714,MCI.vs.control; bulk RNA-seq,Homo_633,AD.vs.control	5
MF	GO:0003730	mRNA 3'-UTR binding	bulk RNA-seq,Homo_723,AD.vs.control; bulk RNA-seq,Homo_714,AD.vs.control; bulk RNA-seq,Homo_714,AD.vs.MCI	3
MF	GO:0016595	glutamate binding	bulk RNA-seq,Homo_723,AD.vs.control; bulk RNA-seq,Homo_723,AD.vs.MCI; bulk RNA-seq,Homo_723,MCI.vs.control	3
MF	GO:0015271	outward rectifier potassium channel activity	bulk RNA-seq,Homo_723,AD.vs.control; bulk RNA-seq,Homo_723,AD.vs.MCI; bulk RNA-seq,Homo_723,MCI.vs.control; bulk RNA-seq,Homo_714,AD.vs.MCI	4
BP	GO:1902116	negative regulation of organelle assembly	bulk RNA-seq,Homo_723,AD.vs.control; bulk RNA-seq,Homo_723,MCI.vs.control; bulk RNA-seq,Homo_714,AD.vs.control; bulk RNA-seq,Homo_714,MCI.vs.control; bulk RNA-seq,Homo_633,AD.vs.control; bulk RNA-seq,Homo_633,AD.vs.MCI	6
BP	GO:0071985	multivesicular body sorting pathway	bulk RNA-seq,Homo_723,AD.vs.control; bulk RNA-seq,Homo_723,MCI.vs.control; bulk RNA-seq,Homo_714,MCI.vs.control; bulk RNA-seq,Homo_633,AD.vs.control; bulk RNA-seq,Homo_633,AD.vs.MCI	5
MF	GO:0042809	nuclear vitamin D receptor binding	bulk RNA-seq,Homo_723,AD.vs.control; bulk RNA-seq,Homo_723,MCI.vs.control; bulk RNA-seq,Homo_714,MCI.vs.control; bulk RNA-seq,Homo_633,AD.vs.control; bulk RNA-seq,Homo_633,AD.vs.MCI; bulk RNA-seq,Homo_633,MCI.vs.control	6
CC	GO:0005874	microtubule	bulk RNA-seq,Homo_723,AD.vs.control; bulk RNA-seq,Homo_723,AD.vs.MCI; bulk RNA-seq,Homo_714,AD.vs.MCI; bulk RNA-seq,Homo_633,AD.vs.control; bulk RNA-seq,Homo_633,AD.vs.MCI; bulk RNA-seq,ROSMAP,AD.vs.control	6
BP	GO:0032886	regulation of microtubule-based process	bulk RNA-seq,Homo_723,AD.vs.control; bulk RNA-seq,Homo_723,AD.vs.MCI; bulk RNA-seq,Homo_723,MCI.vs.control; bulk RNA-seq,Homo_714,AD.vs.MCI; bulk RNA-seq,Homo_633,AD.vs.control; bulk RNA-seq,Homo_633,AD.vs.MCI	6
BP	GO:0007020	microtubule nucleation	bulk RNA-seq,Homo_723,AD.vs.control; bulk RNA-seq,Homo_723,MCI.vs.control; bulk RNA-seq,Homo_633,AD.vs.control; bulk RNA-	4
BP	GO:0060502	epithelial cell proliferation involved in lung morphogenesis	bulk RNA-seq,Homo_723,AD.vs.control; bulk RNA-seq,Homo_723,AD.vs.MCI; bulk RNA-seq,Homo_723,MCI.vs.control; bulk RNA-seq,Homo_714,AD.vs.MCI; bulk RNA-seq,Homo_714,MCI.vs.control	5
BP	GO:0023058	adaptation of signaling pathway	bulk RNA-seq,Homo_723,AD.vs.control; bulk RNA-seq,Homo_723,AD.vs.MCI; bulk RNA-seq,Homo_723,MCI.vs.control	3
BP	GO:0050931	pigment cell differentiation	bulk RNA-seq,Homo_723,AD.vs.control; bulk RNA-seq,Homo_723,AD.vs.MCI; bulk RNA-seq,Homo_723,MCI.vs.control; bulk RNA-seq,Homo_714,AD.vs.MCI; bulk RNA-seq,Homo_714,MCI.vs.control	5
BP	GO:0030832	regulation of actin filament length	bulk RNA-seq,Homo_723,AD.vs.control; bulk RNA-seq,Homo_723,AD.vs.MCI; bulk RNA-seq,Homo_723,MCI.vs.control; bulk RNA-seq,Homo_714,AD.vs.MCI; bulk RNA-seq,Homo_633,AD.vs.control; bulk RNA-seq,Homo_633,AD.vs.MCI	6
MF	GO:0008009	chemokine activity	bulk RNA-seq,Homo_723,AD.vs.control; bulk RNA-seq,Homo_723,AD.vs.MCI; bulk RNA-seq,Homo_723,MCI.vs.control; bulk RNA-seq,Homo_714,AD.vs.control; bulk RNA-seq,Homo_714,AD.vs.MCI; bulk RNA-seq,Homo_714,MCI.vs.control; scRNA-seq,SRP309935,Naive CD8+ T cell_1-	7
BP	GO:0097345	mitochondrial outer membrane permeabilization	bulk RNA-seq,Homo_723,AD.vs.control; bulk RNA-seq,Homo_714,AD.vs.control; bulk RNA-seq,Homo_714,MCI.vs.control; bulk RNA-seq,Homo_633,AD.vs.control; bulk RNA-seq,Homo_633,AD.vs.MCI	5
MF	GO:0004407	histone deacetylase activity	bulk RNA-seq,Homo_723,AD.vs.control; bulk RNA-seq,Homo_633,AD.vs.control; bulk RNA-seq,Homo_633,AD.vs.MCI	3

CC	GO:0005881	cytoplasmic microtubule	bulk RNA-seq,Homo_723,AD.vs.control; bulk RNA-seq,Homo_723,AD.vs.MCI; bulk RNA-seq,Homo_723,MCI.vs.control; bulk RNA-seq,Homo_714,AD.vs.MCI; bulk RNA-seq,Homo_714,MCI.vs.control; bulk RNA-seq,Homo_633,AD.vs.control; bulk RNA-seq,Homo_633,AD.vs.MCI	7
BP	GO:0001890	placenta development	bulk RNA-seq,Homo_723,AD.vs.control; bulk RNA-seq,Homo_723,AD.vs.MCI; bulk RNA-seq,Homo_723,MCI.vs.control; bulk RNA-seq,Homo_714,AD.vs.MCI; bulk RNA-seq,Homo_633,AD.vs.control; bulk RNA-seq,Homo_633,AD.vs.MCI	6
MF	GO:0001846	opsonin binding	bulk RNA-seq,Homo_723,AD.vs.control	1
BP	GO:0006213	pyrimidine nucleoside metabolic process	bulk RNA-seq,Homo_723,AD.vs.control; bulk RNA-seq,Homo_723,AD.vs.MCI; bulk RNA-seq,Homo_633,AD.vs.control	3
CC	GO:0000782	telomere cap complex	bulk RNA-seq,Homo_723,AD.vs.control; bulk RNA-seq,Homo_723,AD.vs.MCI; bulk RNA-seq,Homo_714,AD.vs.MCI	3
CC	GO:0000783	nuclear telomere cap complex	bulk RNA-seq,Homo_723,AD.vs.control; bulk RNA-seq,Homo_723,AD.vs.MCI; bulk RNA-seq,Homo_714,AD.vs.MCI	3
BP	GO:0061844	antimicrobial humoral immune response mediated by antimicrobial peptide	bulk RNA-seq,Homo_723,AD.vs.control; bulk RNA-seq,Homo_723,AD.vs.MCI; bulk RNA-seq,Homo_714,AD.vs.control; bulk RNA-seq,Homo_714,AD.vs.MCI; bulk RNA-seq,SRP325058,AD.vs.control	5
BP	GO:0002762	negative regulation of myeloid leukocyte differentiation	bulk RNA-seq,Homo_723,AD.vs.control; bulk RNA-seq,Homo_723,AD.vs.MCI; bulk RNA-seq,Homo_723,MCI.vs.control; bulk RNA-seq,Homo_714,AD.vs.control; bulk RNA-seq,Homo_714,AD.vs.MCI; bulk RNA-seq,Homo_714,MCI.vs.control; bulk RNA-seq,Homo_633,AD.vs.control; bulk	8
BP	GO:0003417	growth plate cartilage development	bulk RNA-seq,Homo_723,AD.vs.control; bulk RNA-seq,Homo_723,AD.vs.MCI; bulk RNA-seq,Homo_723,MCI.vs.control; bulk RNA-seq,Homo_633,AD.vs.control; bulk RNA-seq,Homo_633,AD.vs.MCI	5
BP	GO:0035630	bone mineralization involved in bone maturation	bulk RNA-seq,Homo_723,AD.vs.control	1
BP	GO:0006099	tricarboxylic acid cycle	bulk RNA-seq,Homo_723,AD.vs.control; bulk RNA-seq,Homo_723,MCI.vs.control; bulk RNA-seq,Homo_714,AD.vs.control; bulk RNA-seq,Homo_714,MCI.vs.control; bulk RNA-seq,Homo_633,AD.vs.control; bulk RNA-seq,Homo_633,AD.vs.MCI	6
MF	GO:0003697	single-stranded DNA binding	bulk RNA-seq,Homo_723,AD.vs.control; bulk RNA-seq,Homo_723,MCI.vs.control; bulk RNA-seq,Homo_714,AD.vs.control; bulk RNA-seq,Homo_714,MCI.vs.control; bulk RNA-seq,Homo_633,AD.vs.control; bulk RNA-seq,Homo_633,AD.vs.MCI	6
BP	GO:0048634	regulation of muscle organ development	bulk RNA-seq,Homo_723,AD.vs.control; bulk RNA-seq,Homo_723,MCI.vs.control	2
BP	GO:0002775	antimicrobial peptide production	bulk RNA-seq,Homo_723,AD.vs.control; bulk RNA-seq,Homo_714,AD.vs.control; bulk RNA-seq,Homo_714,AD.vs.MCI	3
BP	GO:0044091	membrane biogenesis	bulk RNA-seq,Homo_723,AD.vs.control; bulk RNA-seq,Homo_723,AD.vs.MCI; bulk RNA-seq,Homo_723,MCI.vs.control; bulk RNA-seq,Homo_714,AD.vs.MCI; bulk RNA-seq,Homo_714,MCI.vs.control; bulk RNA-seq,Homo_633,AD.vs.control; bulk RNA-seq,Homo_633,AD.vs.MCI	7
BP	GO:0051494	negative regulation of cytoskeleton organization	bulk RNA-seq,Homo_723,AD.vs.control; bulk RNA-seq,Homo_723,AD.vs.MCI; bulk RNA-seq,Homo_723,MCI.vs.control; bulk RNA-seq,Homo_714,AD.vs.MCI; bulk RNA-seq,Homo_633,AD.vs.control; bulk RNA-seq,Homo_633,AD.vs.MCI	6
MF	GO:0017069	snRNA binding	bulk RNA-seq,Homo_723,AD.vs.control; bulk RNA-seq,Homo_723,MCI.vs.control; bulk RNA-seq,Homo_714,MCI.vs.control; bulk RNA-seq,Homo_633,AD.vs.control; bulk RNA-seq,Homo_633,AD.vs.MCI	5
BP	GO:0010459	negative regulation of heart rate	bulk RNA-seq,Homo_723,AD.vs.control	1
BP	GO:1901380	negative regulation of potassium ion transmembrane	bulk RNA-seq,Homo_723,AD.vs.control; bulk RNA-seq,Homo_723,AD.vs.MCI; bulk RNA-seq,Homo_714,AD.vs.control; bulk RNA-seq,Homo_714,AD.vs.MCI	4
BP	GO:0060326	cell chemotaxis	bulk RNA-seq,Homo_723,AD.vs.control; bulk RNA-seq,Homo_723,AD.vs.MCI; bulk RNA-seq,Homo_723,MCI.vs.control; bulk RNA-seq,Homo_714,AD.vs.control; bulk RNA-seq,Homo_714,AD.vs.MCI; bulk RNA-seq,Homo_633,AD.vs.control; bulk RNA-seq,Homo_633,AD.vs.MCI; scRNA-	8
CC	GO:1990072	TRAPPIII protein complex	bulk RNA-seq,Homo_723,AD.vs.control; bulk RNA-seq,Homo_714,MCI.vs.control; bulk RNA-seq,Homo_633,AD.vs.control; bulk RNA-seq,Homo_633,AD.vs.MCI; bulk RNA-seq,Homo_633,MCI.vs.control	5
BP	GO:1901659	glycosyl compound biosynthetic process	bulk RNA-seq,Homo_723,AD.vs.control; bulk RNA-seq,Homo_633,AD.vs.control; bulk RNA-seq,Homo_633,AD.vs.MCI	3
BP	GO:0009755	hormone-mediated signaling pathway	bulk RNA-seq,Homo_723,AD.vs.control; bulk RNA-seq,Homo_723,AD.vs.MCI; bulk RNA-seq,Homo_723,MCI.vs.control; bulk RNA-seq,Homo_714,AD.vs.MCI; bulk RNA-seq,Homo_633,AD.vs.control; bulk RNA-seq,Homo_633,AD.vs.MCI	6
BP	GO:0030224	monocyte differentiation	bulk RNA-seq,Homo_723,AD.vs.control; bulk RNA-seq,Homo_723,AD.vs.MCI; bulk RNA-seq,Homo_723,MCI.vs.control; bulk RNA-seq,Homo_714,AD.vs.MCI; bulk RNA-seq,Homo_714,MCI.vs.control	5
BP	GO:0002418	immune response to tumor cell	bulk RNA-seq,Homo_723,AD.vs.control; bulk RNA-seq,Homo_723,MCI.vs.control; bulk RNA-seq,Homo_714,AD.vs.control; bulk RNA-seq,Homo_714,MCI.vs.control; bulk RNA-seq,Homo_633,AD.vs.control; bulk RNA-seq,Homo_633,AD.vs.MCI; bulk RNA-seq,Homo_633,MCI.vs.control	7
BP	GO:0007019	microtubule depolymerization	bulk RNA-seq,Homo_723,AD.vs.control; bulk RNA-seq,Homo_723,AD.vs.MCI; bulk RNA-seq,Homo_723,MCI.vs.control; bulk RNA-seq,Homo_714,AD.vs.MCI; bulk RNA-seq,Homo_714,MCI.vs.control; bulk RNA-seq,Homo_633,AD.vs.control	6
BP	GO:1901838	positive regulation of transcription of nucleolar large rRNA by RNA polymerase I	bulk RNA-seq,Homo_723,AD.vs.control; bulk RNA-seq,Homo_723,MCI.vs.control; bulk RNA-seq,Homo_714,MCI.vs.control; bulk RNA-seq,Homo_633,AD.vs.control; bulk RNA-seq,Homo_633,AD.vs.MCI; bulk RNA-seq,Homo_633,MCI.vs.control	6
MF	GO:0061608	nuclear import signal receptor activity	bulk RNA-seq,Homo_723,AD.vs.control; bulk RNA-seq,Homo_714,AD.vs.control; bulk RNA-seq,Homo_633,AD.vs.control; bulk RNA-seq,Homo_633,AD.vs.MCI	4
BP	GO:0045947	negative regulation of translational initiation	bulk RNA-seq,Homo_723,AD.vs.control; bulk RNA-seq,Homo_714,MCI.vs.control; bulk RNA-seq,Homo_633,AD.vs.control	3
BP	GO:0070296	sarcoplasmic reticulum calcium ion transport	bulk RNA-seq,Homo_723,AD.vs.control	1
MF	GO:0030553	cGMP binding	bulk RNA-seq,Homo_723,AD.vs.control; bulk RNA-seq,Homo_723,AD.vs.MCI; bulk RNA-seq,Homo_723,MCI.vs.control; bulk RNA-seq,Homo_714,AD.vs.MCI; bulk RNA-seq,Homo_714,MCI.vs.control	5
BP	GO:0035641	locomotory exploration behavior	bulk RNA-seq,Homo_723,AD.vs.control; bulk RNA-seq,Homo_723,AD.vs.MCI; bulk RNA-seq,Homo_723,MCI.vs.control	3
BP	GO:0032623	interleukin-2 production	bulk RNA-seq,Homo_723,AD.vs.control; bulk RNA-seq,Homo_723,MCI.vs.control; bulk RNA-seq,Homo_714,MCI.vs.control; bulk RNA-seq,Homo_633,AD.vs.control; bulk RNA-seq,Homo_633,AD.vs.MCI	5
BP	GO:0032663	regulation of interleukin-2 production	bulk RNA-seq,Homo_723,AD.vs.control; bulk RNA-seq,Homo_723,MCI.vs.control; bulk RNA-seq,Homo_714,MCI.vs.control; bulk RNA-seq,Homo_633,AD.vs.control; bulk RNA-seq,Homo_633,AD.vs.MCI	5
BP	GO:0030888	regulation of B cell proliferation	bulk RNA-seq,Homo_723,AD.vs.control; bulk RNA-seq,Homo_723,MCI.vs.control; bulk RNA-seq,Homo_714,MCI.vs.control; bulk RNA-seq,Homo_633,AD.vs.control; bulk RNA-seq,Homo_633,AD.vs.MCI	5
BP	GO:0035812	renal sodium excretion	bulk RNA-seq,Homo_723,AD.vs.control; bulk RNA-seq,Homo_723,AD.vs.MCI; bulk RNA-seq,Homo_714,AD.vs.control; bulk RNA-seq,Homo_714,AD.vs.MCI	4
MF	GO:0045503	dynein light chain binding	bulk RNA-seq,Homo_723,AD.vs.control; bulk RNA-seq,Homo_723,AD.vs.MCI; bulk RNA-seq,Homo_723,MCI.vs.control	3
BP	GO:0048241	epinephrine transport	bulk RNA-seq,Homo_723,AD.vs.control; bulk RNA-seq,Homo_723,AD.vs.MCI; bulk RNA-seq,Homo_723,MCI.vs.control	3
MF	GO:0070330	aromatase activity	bulk RNA-seq,Homo_723,AD.vs.control; bulk RNA-seq,Homo_723,AD.vs.MCI; bulk RNA-seq,Homo_723,MCI.vs.control; bulk RNA-seq,Homo_714,AD.vs.control; bulk RNA-seq,Homo_714,AD.vs.MCI; bulk RNA-seq,Homo_714,MCI.vs.control	6

CC	GO:0016529	sarcoplasmic reticulum	bulk RNA-seq,Homo_723,AD.vs.control; bulk RNA-seq,Homo_723,AD.vs.MCI; bulk RNA-seq,Homo_723,MCI.vs.control; bulk RNA-seq,Homo_714,AD.vs.MCI; bulk RNA-seq,Homo_714,MCI.vs.control; bulk RNA-seq,Homo_633,AD.vs.control; bulk RNA-seq,Homo_633,AD.vs.MCI	7
BP	GO:0010332	response to gamma radiation	bulk RNA-seq,Homo_723,AD.vs.control; bulk RNA-seq,Homo_723,MCI.vs.control; bulk RNA-seq,Homo_714,MCI.vs.control; bulk RNA-seq,Homo_633,AD.vs.control; bulk RNA-seq,Homo_633,AD.vs.MCI	5
BP	GO:0051549	positive regulation of keratinocyte migration	bulk RNA-seq,Homo_723,AD.vs.control; bulk RNA-seq,Homo_723,AD.vs.MCI; bulk RNA-seq,Homo_723,MCI.vs.control; bulk RNA-seq,Homo_714,AD.vs.MCI	4
BP	GO:0051639	actin filament network formation	bulk RNA-seq,Homo_723,AD.vs.control; bulk RNA-seq,Homo_723,AD.vs.MCI; bulk RNA-seq,Homo_723,MCI.vs.control; bulk RNA-seq,Homo_714,AD.vs.MCI; bulk RNA-seq,Homo_714,MCI.vs.control	5
BP	GO:0060606	tube closure	bulk RNA-seq,Homo_723,AD.vs.control; bulk RNA-seq,Homo_723,AD.vs.MCI; bulk RNA-seq,Homo_723,MCI.vs.control; bulk RNA-seq,Homo_714,AD.vs.MCI; bulk RNA-seq,Homo_714,MCI.vs.control; bulk RNA-seq,Homo_633,AD.vs.control; bulk RNA-seq,Homo_633,AD.vs.MCI	7
BP	GO:1901317	regulation of flagellated sperm motility	bulk RNA-seq,Homo_723,AD.vs.control; bulk RNA-seq,Homo_723,AD.vs.MCI	2
BP	GO:0046541	saliva secretion	bulk RNA-seq,Homo_723,AD.vs.control; bulk RNA-seq,Homo_723,AD.vs.MCI	2
BP	GO:1902930	regulation of alcohol biosynthetic process	bulk RNA-seq,Homo_723,AD.vs.control; bulk RNA-seq,Homo_723,AD.vs.MCI; bulk RNA-seq,Homo_723,MCI.vs.control; bulk RNA-seq,Homo_714,AD.vs.MCI; bulk RNA-seq,Homo_714,MCI.vs.control	5
CC	GO:0098636	protein complex involved in cell adhesion	bulk RNA-seq,Homo_723,AD.vs.control; bulk RNA-seq,Homo_723,AD.vs.MCI; bulk RNA-seq,Homo_723,MCI.vs.control; bulk RNA-seq,Homo_714,AD.vs.MCI; bulk RNA-seq,Homo_714,MCI.vs.control; bulk RNA-seq,Homo_633,AD.vs.control; bulk RNA-seq,Homo_633,AD.vs.MCI	7
MF	GO:0005080	protein kinase C binding	bulk RNA-seq,Homo_723,AD.vs.control; bulk RNA-seq,Homo_723,AD.vs.MCI; bulk RNA-seq,Homo_723,MCI.vs.control; bulk RNA-seq,Homo_633,AD.vs.control; bulk RNA-seq,Homo_633,AD.vs.MCI	5
BP	GO:0051085	chaperone cofactor-dependent protein refolding	bulk RNA-seq,Homo_723,AD.vs.control; bulk RNA-seq,Homo_723,MCI.vs.control; bulk RNA-seq,Homo_714,AD.vs.control; bulk RNA-seq,Homo_714,MCI.vs.control; bulk RNA-seq,Homo_633,AD.vs.control; bulk RNA-seq,Homo_633,AD.vs.MCI; bulk RNA-seq,Homo_633,MCI.vs.control	7
BP	GO:0140718	facultative heterochromatin formation	bulk RNA-seq,Homo_723,AD.vs.control; bulk RNA-seq,Homo_723,MCI.vs.control; bulk RNA-seq,Homo_714,MCI.vs.control; bulk RNA-seq,Homo_633,AD.vs.control; bulk RNA-seq,Homo_633,MCI.vs.control	5
BP	GO:0002834	regulation of response to tumor cell	bulk RNA-seq,Homo_723,AD.vs.control; bulk RNA-seq,Homo_723,MCI.vs.control; bulk RNA-seq,Homo_714,AD.vs.control; bulk RNA-seq,Homo_714,MCI.vs.control; bulk RNA-seq,Homo_633,AD.vs.control	5
BP	GO:0001836	release of cytochrome c from mitochondria	bulk RNA-seq,Homo_723,AD.vs.control; bulk RNA-seq,Homo_714,MCI.vs.control; bulk RNA-seq,Homo_633,AD.vs.control; bulk RNA-	4
BP	GO:0098792	xenophagy	bulk RNA-seq,Homo_723,AD.vs.control; bulk RNA-seq,Homo_633,AD.vs.control; bulk RNA-seq,Homo_633,AD.vs.MCI	3
CC	GO:0030131	clathrin adaptor complex	bulk RNA-seq,Homo_723,AD.vs.control; bulk RNA-seq,Homo_723,MCI.vs.control; bulk RNA-seq,Homo_714,MCI.vs.control; bulk RNA-	4
BP	GO:0034063	stress granule assembly	bulk RNA-seq,Homo_723,AD.vs.control; bulk RNA-seq,Homo_723,MCI.vs.control; bulk RNA-seq,Homo_714,AD.vs.control; bulk RNA-seq,Homo_714,MCI.vs.control; bulk RNA-seq,Homo_633,AD.vs.control; bulk RNA-seq,Homo_633,AD.vs.MCI; bulk RNA-seq,Homo_633,MCI.vs.control	7
MF	GO:0016922	nuclear receptor binding	bulk RNA-seq,Homo_723,AD.vs.control; bulk RNA-seq,Homo_723,MCI.vs.control; bulk RNA-seq,Homo_714,AD.vs.MCI; bulk RNA-seq,Homo_633,AD.vs.control; bulk RNA-seq,Homo_633,AD.vs.MCI	5
MF	GO:0016835	carbon-oxygen lyase activity	bulk RNA-seq,Homo_723,AD.vs.control; bulk RNA-seq,Homo_723,MCI.vs.control; bulk RNA-seq,Homo_714,AD.vs.control; bulk RNA-seq,Homo_714,MCI.vs.control; bulk RNA-seq,Homo_633,AD.vs.control; bulk RNA-seq,Homo_633,AD.vs.MCI	6
CC	GO:0016514	SWI/SNF complex	bulk RNA-seq,Homo_723,AD.vs.control; bulk RNA-seq,Homo_723,MCI.vs.control; bulk RNA-seq,Homo_714,AD.vs.control; bulk RNA-seq,Homo_714,MCI.vs.control; bulk RNA-seq,Homo_633,AD.vs.control; bulk RNA-seq,Homo_633,AD.vs.MCI; bulk RNA-seq,Homo_633,MCI.vs.control	7
BP	GO:0007040	lysosome organization	bulk RNA-seq,Homo_723,AD.vs.control; bulk RNA-seq,Homo_723,MCI.vs.control; bulk RNA-seq,Homo_714,AD.vs.control; bulk RNA-seq,Homo_714,MCI.vs.control; bulk RNA-seq,Homo_633,AD.vs.control; bulk RNA-seq,Homo_633,AD.vs.MCI	6
BP	GO:0080171	lytic vacuole organization	bulk RNA-seq,Homo_723,AD.vs.control; bulk RNA-seq,Homo_723,MCI.vs.control; bulk RNA-seq,Homo_714,AD.vs.control; bulk RNA-seq,Homo_714,MCI.vs.control; bulk RNA-seq,Homo_633,AD.vs.control; bulk RNA-seq,Homo_633,AD.vs.MCI	6
BP	GO:1902622	regulation of neutrophil migration	bulk RNA-seq,Homo_723,AD.vs.control; bulk RNA-seq,Homo_723,MCI.vs.control; bulk RNA-seq,Homo_714,MCI.vs.control; bulk RNA-	4
BP	GO:0033327	Leydig cell differentiation	bulk RNA-seq,Homo_723,AD.vs.control; bulk RNA-seq,Homo_723,AD.vs.MCI; bulk RNA-seq,Homo_723,MCI.vs.control; bulk RNA-seq,Homo_714,AD.vs.control; bulk RNA-seq,Homo_714,AD.vs.MCI; bulk RNA-seq,Homo_714,MCI.vs.control	6
BP	GO:0003353	positive regulation of cilium movement	bulk RNA-seq,Homo_723,AD.vs.control; bulk RNA-seq,Homo_723,AD.vs.MCI	2
MF	GO:0033130	acetylcholine receptor binding	bulk RNA-seq,Homo_723,AD.vs.control; bulk RNA-seq,Homo_723,AD.vs.MCI; bulk RNA-seq,Homo_723,MCI.vs.control	3
BP	GO:0033673	negative regulation of kinase activity	bulk RNA-seq,Homo_723,AD.vs.control; bulk RNA-seq,Homo_723,AD.vs.MCI; bulk RNA-seq,Homo_723,MCI.vs.control; bulk RNA-seq,Homo_714,AD.vs.MCI; bulk RNA-seq,Homo_633,AD.vs.control; bulk RNA-seq,Homo_633,AD.vs.MCI	6
BP	GO:0035640	exploration behavior	bulk RNA-seq,Homo_723,AD.vs.control; bulk RNA-seq,Homo_723,AD.vs.MCI; bulk RNA-seq,Homo_723,MCI.vs.control	3
BP	GO:0032958	inositol phosphate biosynthetic process	bulk RNA-seq,Homo_723,AD.vs.control; bulk RNA-seq,Homo_723,AD.vs.MCI	2
BP	GO:0031342	negative regulation of cell killing	bulk RNA-seq,Homo_723,AD.vs.control; bulk RNA-seq,Homo_633,AD.vs.control; bulk RNA-seq,Homo_633,AD.vs.MCI	3
BP	GO:0043534	blood vessel endothelial cell migration	bulk RNA-seq,Homo_723,AD.vs.control; bulk RNA-seq,Homo_723,AD.vs.MCI; bulk RNA-seq,Homo_723,MCI.vs.control; bulk RNA-seq,Homo_714,AD.vs.control; bulk RNA-seq,Homo_714,AD.vs.MCI; bulk RNA-seq,Homo_633,AD.vs.control	6
MF	GO:0032050	clathrin heavy chain binding	bulk RNA-seq,Homo_723,AD.vs.control; bulk RNA-seq,Homo_633,AD.vs.control; bulk RNA-seq,Homo_633,AD.vs.MCI	3
BP	GO:0003266	regulation of secondary heart field cardioblast proliferation	bulk RNA-seq,Homo_723,AD.vs.control; bulk RNA-seq,Homo_723,AD.vs.MCI	2
BP	GO:0021561	facial nerve development	bulk RNA-seq,Homo_723,AD.vs.control; bulk RNA-seq,Homo_723,AD.vs.MCI; bulk RNA-seq,Homo_723,MCI.vs.control; bulk RNA-seq,Homo_714,AD.vs.MCI	4
BP	GO:0021610	facial nerve morphogenesis	bulk RNA-seq,Homo_723,AD.vs.control; bulk RNA-seq,Homo_723,AD.vs.MCI; bulk RNA-seq,Homo_723,MCI.vs.control; bulk RNA-seq,Homo_714,AD.vs.MCI	4
BP	GO:0032508	DNA duplex unwinding	bulk RNA-seq,Homo_723,AD.vs.control; bulk RNA-seq,Homo_723,MCI.vs.control; bulk RNA-seq,Homo_714,AD.vs.control; bulk RNA-seq,Homo_714,MCI.vs.control; bulk RNA-seq,Homo_633,AD.vs.control; bulk RNA-seq,Homo_633,AD.vs.MCI	6
BP	GO:0051036	regulation of endosome size	bulk RNA-seq,Homo_723,AD.vs.control; bulk RNA-seq,Homo_723,MCI.vs.control; bulk RNA-seq,Homo_714,MCI.vs.control; bulk RNA-seq,Homo_633,AD.vs.control; bulk RNA-seq,Homo_633,AD.vs.MCI; bulk RNA-seq,Homo_633,MCI.vs.control	6
CC	GO:0140445	chromosome, telomeric repeat region	bulk RNA-seq,Homo_723,AD.vs.control; bulk RNA-seq,Homo_723,AD.vs.MCI; bulk RNA-seq,Homo_714,AD.vs.MCI	3
BP	GO:0007008	outer mitochondrial membrane organization	bulk RNA-seq,Homo_723,AD.vs.control; bulk RNA-seq,Homo_633,AD.vs.control; bulk RNA-seq,Homo_633,AD.vs.MCI; bulk RNA-	4
BP	GO:0045040	protein insertion into mitochondrial outer membrane	bulk RNA-seq,Homo_723,AD.vs.control; bulk RNA-seq,Homo_633,AD.vs.control; bulk RNA-seq,Homo_633,AD.vs.MCI; bulk RNA-	4

MF	GO:0032977	membrane insertase activity	bulk RNA-seq,Homo_723,AD.vs.control; bulk RNA-seq,Homo_723,MCI.vs.control; bulk RNA-seq,Homo_714,MCI.vs.control; bulk RNA-seq,Homo_633,AD.vs.control; bulk RNA-seq,Homo_633,AD.vs.MCI	5
BP	GO:0006703	estrogen biosynthetic process	bulk RNA-seq,Homo_723,AD.vs.control; bulk RNA-seq,Homo_723,AD.vs.MCI; bulk RNA-seq,Homo_714,AD.vs.control	3
BP	GO:0032341	aldosterone metabolic process	bulk RNA-seq,Homo_723,AD.vs.control; bulk RNA-seq,Homo_723,AD.vs.MCI; bulk RNA-seq,Homo_723,MCI.vs.control; bulk RNA-seq,Homo_714,AD.vs.MCI; bulk RNA-seq,Homo_714,MCI.vs.control	5
BP	GO:0060393	regulation of pathway-restricted SMAD protein phosphorylation	bulk RNA-seq,Homo_723,AD.vs.control; bulk RNA-seq,Homo_723,AD.vs.MCI; bulk RNA-seq,Homo_723,MCI.vs.control; bulk RNA-seq,Homo_714,AD.vs.control; bulk RNA-seq,Homo_714,AD.vs.MCI; bulk RNA-seq,Homo_714,MCI.vs.control; bulk RNA-seq,Homo_633,AD.vs.control	7
CC	GO:0030119	AP-type membrane coat adaptor complex	bulk RNA-seq,Homo_723,AD.vs.control; bulk RNA-seq,Homo_723,MCI.vs.control; bulk RNA-seq,Homo_714,AD.vs.control; bulk RNA-seq,Homo_714,MCI.vs.control; bulk RNA-seq,Homo_633,AD.vs.control; bulk RNA-seq,Homo_633,AD.vs.MCI; bulk RNA-seq,Homo_633,MCI.vs.control	7
BP	GO:1990253	cellular response to leucine starvation	bulk RNA-seq,Homo_723,AD.vs.control; bulk RNA-seq,Homo_723,MCI.vs.control; bulk RNA-seq,Homo_714,MCI.vs.control; bulk RNA-	4
BP	GO:0072350	tricarboxylic acid metabolic process	bulk RNA-seq,Homo_723,AD.vs.control; bulk RNA-seq,Homo_723,MCI.vs.control; bulk RNA-seq,Homo_714,AD.vs.control; bulk RNA-seq,Homo_714,MCI.vs.control; bulk RNA-seq,Homo_633,AD.vs.control; bulk RNA-seq,Homo_633,AD.vs.MCI	6
MF	GO:0016866	intramolecular transferase activity	bulk RNA-seq,Homo_723,AD.vs.control; bulk RNA-seq,Homo_714,AD.vs.control; bulk RNA-seq,Homo_633,AD.vs.control; bulk RNA-seq,Homo_633,AD.vs.MCI	4
CC	GO:0005871	kinesin complex	bulk RNA-seq,Homo_723,AD.vs.control; bulk RNA-seq,Homo_723,AD.vs.MCI; bulk RNA-seq,Homo_723,MCI.vs.control; bulk RNA-seq,Homo_714,AD.vs.MCI; bulk RNA-seq,Homo_714,MCI.vs.control; bulk RNA-seq,Homo_633,AD.vs.control	6
MF	GO:0008641	ubiquitin-like modifier activating enzyme activity	bulk RNA-seq,Homo_723,AD.vs.control; bulk RNA-seq,Homo_633,AD.vs.control; bulk RNA-seq,Homo_633,MCI.vs.control	3
BP	GO:0098884	postsynaptic neurotransmitter receptor internalization	bulk RNA-seq,Homo_723,AD.vs.control; bulk RNA-seq,Homo_723,AD.vs.MCI; bulk RNA-seq,Homo_723,MCI.vs.control; bulk RNA-	4
BP	GO:0140239	postsynaptic endocytosis	bulk RNA-seq,Homo_723,AD.vs.control; bulk RNA-seq,Homo_723,AD.vs.MCI; bulk RNA-seq,Homo_723,MCI.vs.control; bulk RNA-	4
MF	GO:0008391	arachidonic acid monooxygenase activity	bulk RNA-seq,Homo_723,AD.vs.control; bulk RNA-seq,Homo_723,AD.vs.MCI; bulk RNA-seq,Homo_723,MCI.vs.control; bulk RNA-seq,Homo_714,AD.vs.control; bulk RNA-seq,Homo_714,AD.vs.MCI	5
MF	GO:0036312	phosphatidylinositol 3-kinase regulatory subunit binding	bulk RNA-seq,Homo_723,AD.vs.control; bulk RNA-seq,Homo_723,AD.vs.MCI; bulk RNA-seq,Homo_714,MCI.vs.control	3
CC	GO:0005832	chaperonin-containing T-complex	bulk RNA-seq,Homo_723,AD.vs.control; bulk RNA-seq,Homo_723,MCI.vs.control; bulk RNA-seq,Homo_633,AD.vs.control; bulk RNA-	4
BP	GO:0071696	ectodermal placode development	bulk RNA-seq,Homo_723,AD.vs.control; bulk RNA-seq,Homo_723,AD.vs.MCI; bulk RNA-seq,Homo_723,MCI.vs.control; bulk RNA-seq,Homo_714,AD.vs.MCI; bulk RNA-seq,Homo_714,MCI.vs.control; bulk RNA-seq,Homo_633,MCI.vs.control	6
BP	GO:0090199	regulation of release of cytochrome c from mitochondria	bulk RNA-seq,Homo_723,AD.vs.control; bulk RNA-seq,Homo_714,MCI.vs.control; bulk RNA-seq,Homo_633,AD.vs.control; bulk RNA-	4
CC	GO:0044615	nuclear pore nuclear basket	bulk RNA-seq,Homo_723,AD.vs.control; bulk RNA-seq,Homo_723,MCI.vs.control; bulk RNA-seq,Homo_714,MCI.vs.control; bulk RNA-seq,Homo_633,AD.vs.control; bulk RNA-seq,Homo_633,AD.vs.MCI; bulk RNA-seq,Homo_633,MCI.vs.control	6
CC	GO:0005675	transcription factor TFIIH holo complex	bulk RNA-seq,Homo_723,AD.vs.control; bulk RNA-seq,Homo_723,MCI.vs.control; bulk RNA-seq,Homo_714,AD.vs.control; bulk RNA-seq,Homo_714,MCI.vs.control; bulk RNA-seq,Homo_633,AD.vs.control; bulk RNA-seq,Homo_633,AD.vs.MCI; bulk RNA-seq,Homo_633,MCI.vs.control	7
MF	GO:0005161	platelet-derived growth factor receptor binding	bulk RNA-seq,Homo_723,AD.vs.control; bulk RNA-seq,Homo_723,AD.vs.MCI; bulk RNA-seq,Homo_723,MCI.vs.control; bulk RNA-seq,Homo_714,MCI.vs.control; bulk RNA-seq,Homo_633,AD.vs.control; bulk RNA-seq,Homo_633,AD.vs.MCI	6
BP	GO:0045648	positive regulation of erythrocyte differentiation	bulk RNA-seq,Homo_723,AD.vs.control; bulk RNA-seq,Homo_723,MCI.vs.control; bulk RNA-seq,Homo_714,MCI.vs.control; bulk RNA-seq,Homo_633,AD.vs.control; bulk RNA-seq,Homo_633,AD.vs.MCI; bulk RNA-seq,Homo_633,MCI.vs.control	6
BP	GO:0060389	pathway-restricted SMAD protein phosphorylation	bulk RNA-seq,Homo_723,AD.vs.control; bulk RNA-seq,Homo_723,AD.vs.MCI; bulk RNA-seq,Homo_723,MCI.vs.control; bulk RNA-seq,Homo_714,AD.vs.control; bulk RNA-seq,Homo_714,AD.vs.MCI; bulk RNA-seq,Homo_714,MCI.vs.control; bulk RNA-seq,Homo_633,AD.vs.control	7
BP	GO:0035065	regulation of histone acetylation	bulk RNA-seq,Homo_723,AD.vs.control; bulk RNA-seq,Homo_723,MCI.vs.control; bulk RNA-seq,Homo_714,MCI.vs.control; bulk RNA-seq,Homo_633,AD.vs.control; bulk RNA-seq,Homo_633,AD.vs.MCI	5
BP	GO:0010569	regulation of double-strand break repair via homologous recombination	bulk RNA-seq,Homo_723,AD.vs.control; bulk RNA-seq,Homo_723,MCI.vs.control; bulk RNA-seq,Homo_714,AD.vs.control; bulk RNA-seq,Homo_714,MCI.vs.control; bulk RNA-seq,Homo_633,AD.vs.control; bulk RNA-seq,Homo_633,AD.vs.MCI	6
BP	GO:0086016	AV node cell action potential	bulk RNA-seq,Homo_723,AD.vs.control; bulk RNA-seq,Homo_723,AD.vs.MCI; bulk RNA-seq,Homo_714,AD.vs.MCI; bulk RNA-seq,Homo_714,MCI.vs.control	4
BP	GO:0086027	AV node cell to bundle of His cell signaling	bulk RNA-seq,Homo_723,AD.vs.control; bulk RNA-seq,Homo_723,AD.vs.MCI; bulk RNA-seq,Homo_714,AD.vs.MCI; bulk RNA-seq,Homo_714,MCI.vs.control	4
CC	GO:0005614	interstitial matrix	bulk RNA-seq,Homo_723,AD.vs.control; bulk RNA-seq,Homo_723,AD.vs.MCI; bulk RNA-seq,Homo_723,MCI.vs.control; bulk RNA-seq,Homo_714,AD.vs.MCI; bulk RNA-seq,Homo_714,MCI.vs.control	5
BP	GO:0035279	miRNA-mediated gene silencing by mRNA destabilization	bulk RNA-seq,Homo_723,AD.vs.control	1
BP	GO:0040019	positive regulation of embryonic development	bulk RNA-seq,Homo_723,AD.vs.control; bulk RNA-seq,Homo_723,AD.vs.MCI; bulk RNA-seq,Homo_723,MCI.vs.control; bulk RNA-seq,Homo_714,AD.vs.MCI; bulk RNA-seq,SRP223445,AD.vs.control	5
CC	GO:0015030	Cajal body	bulk RNA-seq,Homo_723,AD.vs.control; bulk RNA-seq,Homo_723,MCI.vs.control; bulk RNA-seq,Homo_714,MCI.vs.control; bulk RNA-seq,Homo_633,AD.vs.control; bulk RNA-seq,Homo_633,AD.vs.MCI	5
BP	GO:2000320	negative regulation of T-helper 17 cell differentiation	bulk RNA-seq,Homo_723,AD.vs.control; bulk RNA-seq,Homo_723,MCI.vs.control; bulk RNA-seq,Homo_714,MCI.vs.control; bulk RNA-	4
BP	GO:0070475	rRNA base methylation	bulk RNA-seq,Homo_723,AD.vs.control; bulk RNA-seq,Homo_633,AD.vs.control; bulk RNA-seq,Homo_633,AD.vs.MCI	3
BP	GO:0002725	negative regulation of T cell cytokine production	bulk RNA-seq,Homo_723,AD.vs.control; bulk RNA-seq,Homo_714,MCI.vs.control	2
BP	GO:0001967	suckling behavior	bulk RNA-seq,Homo_723,AD.vs.control; bulk RNA-seq,Homo_723,AD.vs.MCI; bulk RNA-seq,Homo_723,MCI.vs.control; bulk RNA-seq,Homo_633,AD.vs.control; bulk RNA-seq,Homo_633,AD.vs.MCI	5
BP	GO:0044341	sodium-dependent phosphate transport	bulk RNA-seq,Homo_723,AD.vs.control; bulk RNA-seq,Homo_723,AD.vs.MCI; bulk RNA-seq,Homo_723,MCI.vs.control; bulk RNA-seq,Homo_714,AD.vs.control; bulk RNA-seq,Homo_714,AD.vs.MCI	5
BP	GO:0070199	establishment of protein localization to chromosome	bulk RNA-seq,Homo_723,AD.vs.control; bulk RNA-seq,Homo_714,MCI.vs.control; bulk RNA-seq,Homo_633,AD.vs.control; bulk RNA-seq,Homo_633,AD.vs.MCI; bulk RNA-seq,Homo_633,MCI.vs.control	5
BP	GO:0014733	regulation of skeletal muscle adaptation	bulk RNA-seq,Homo_723,AD.vs.control; bulk RNA-seq,Homo_723,MCI.vs.control	2
BP	GO:0042136	neurotransmitter biosynthetic process	bulk RNA-seq,Homo_723,AD.vs.control; bulk RNA-seq,Homo_723,AD.vs.MCI; bulk RNA-seq,Homo_714,AD.vs.control; bulk RNA-seq,Homo_714,AD.vs.MCI	4
BP	GO:0046488	phosphatidylinositol metabolic process	bulk RNA-seq,Homo_723,AD.vs.control; bulk RNA-seq,Homo_723,MCI.vs.control; bulk RNA-seq,Homo_714,AD.vs.control; bulk RNA-seq,Homo_633,AD.vs.control; bulk RNA-seq,Homo_633,AD.vs.MCI; scRNA-seq,SRP330776,CD8+ T cell_2-AD.vs.control	6

BP	GO:0007093	mitotic cell cycle checkpoint signaling	bulk RNA-seq,Homo_723,AD.vs.control; bulk RNA-seq,Homo_723,MCI.vs.control; bulk RNA-seq,Homo_714,AD.vs.control; bulk RNA-seq,Homo_633,AD.vs.control; bulk RNA-seq,Homo_633,AD.vs.MCI	5
BP	GO:0045820	negative regulation of glycolytic process	bulk RNA-seq,Homo_723,AD.vs.control; bulk RNA-seq,Homo_633,AD.vs.control	2
BP	GO:0048755	branching morphogenesis of a nerve	bulk RNA-seq,Homo_723,AD.vs.control; bulk RNA-seq,Homo_723,AD.vs.MCI; bulk RNA-seq,Homo_723,MCI.vs.control	3
BP	GO:2000727	positive regulation of cardiac muscle cell differentiation	bulk RNA-seq,Homo_723,AD.vs.control; bulk RNA-seq,Homo_714,AD.vs.MCI	2
BP	GO:2000042	negative regulation of double-strand break repair via homologous recombination	bulk RNA-seq,Homo_723,AD.vs.control; bulk RNA-seq,Homo_723,MCI.vs.control; bulk RNA-seq,Homo_714,MCI.vs.control; bulk RNA-seq,Homo_633,AD.vs.control; bulk RNA-seq,Homo_633,MCI.vs.control	5
BP	GO:0060746	parental behavior	bulk RNA-seq,Homo_723,AD.vs.control	1
BP	GO:0071357	cellular response to type I interferon	bulk RNA-seq,Homo_723,AD.vs.control; bulk RNA-seq,Homo_723,MCI.vs.control; bulk RNA-seq,Homo_714,MCI.vs.control; bulk RNA-	4
BP	GO:0006650	glycerophospholipid metabolic process	bulk RNA-seq,Homo_723,AD.vs.control; bulk RNA-seq,Homo_723,MCI.vs.control; bulk RNA-seq,Homo_714,AD.vs.control; bulk RNA-seq,Homo_714,AD.vs.MCI; bulk RNA-seq,Homo_633,AD.vs.control; bulk RNA-seq,Homo_633,AD.vs.MCI; scRNA-seq,SRP330776,CD8+ T cell_1-	8
BP	GO:0045822	negative regulation of heart contraction	bulk RNA-seq,Homo_723,AD.vs.control; bulk RNA-seq,Homo_714,AD.vs.MCI	2
BP	GO:0009411	response to UV	bulk RNA-seq,Homo_723,AD.vs.control; bulk RNA-seq,Homo_723,MCI.vs.control; bulk RNA-seq,Homo_633,AD.vs.control; bulk RNA-	4
MF	GO:0000030	mannosyltransferase activity	bulk RNA-seq,Homo_723,AD.vs.control; bulk RNA-seq,Homo_714,AD.vs.control; bulk RNA-seq,Homo_714,MCI.vs.control; bulk RNA-seq,Homo_633,AD.vs.control; bulk RNA-seq,Homo_633,MCI.vs.control	5
MF	GO:0015172	acidic amino acid transmembrane transporter activity	bulk RNA-seq,Homo_723,AD.vs.control; bulk RNA-seq,Homo_723,AD.vs.MCI; bulk RNA-seq,Homo_723,MCI.vs.control; bulk RNA-	4
BP	GO:0007622	rhythmic behavior	bulk RNA-seq,Homo_723,AD.vs.control; bulk RNA-seq,Homo_723,AD.vs.MCI	2
BP	GO:0007292	female gamete generation	bulk RNA-seq,Homo_723,AD.vs.control; bulk RNA-seq,Homo_723,AD.vs.MCI; bulk RNA-seq,Homo_723,MCI.vs.control; bulk RNA-seq,Homo_714,AD.vs.MCI; bulk RNA-seq,Homo_633,AD.vs.control; bulk RNA-seq,Homo_633,AD.vs.MCI	6
BP	GO:0006047	UDP-N-acetylglucosamine metabolic process	bulk RNA-seq,Homo_723,AD.vs.control; bulk RNA-seq,Homo_714,AD.vs.control; bulk RNA-seq,Homo_633,AD.vs.control; bulk RNA-seq,Homo_633,AD.vs.MCI	4
CC	GO:0030008	TRAPP complex	bulk RNA-seq,Homo_723,AD.vs.control; bulk RNA-seq,Homo_723,MCI.vs.control; bulk RNA-seq,Homo_714,MCI.vs.control; bulk RNA-seq,Homo_633,AD.vs.control; bulk RNA-seq,Homo_633,AD.vs.MCI; bulk RNA-seq,Homo_633,MCI.vs.control	6
MF	GO:0017075	syntaxin-1 binding	bulk RNA-seq,Homo_723,AD.vs.control; bulk RNA-seq,Homo_723,AD.vs.MCI; bulk RNA-seq,Homo_723,MCI.vs.control; bulk RNA-seq,Homo_714,MCI.vs.control; bulk RNA-seq,Homo_633,AD.vs.control	5
BP	GO:0015909	long-chain fatty acid transport	bulk RNA-seq,Homo_723,AD.vs.control; bulk RNA-seq,Homo_723,AD.vs.MCI; bulk RNA-seq,Homo_723,MCI.vs.control; bulk RNA-seq,Homo_714,AD.vs.MCI; bulk RNA-seq,Homo_714,MCI.vs.control; bulk RNA-seq,Homo_633,AD.vs.control; bulk RNA-seq,Homo_633,AD.vs.MCI	7
BP	GO:0007266	Rho protein signal transduction	bulk RNA-seq,Homo_723,AD.vs.control; bulk RNA-seq,Homo_723,AD.vs.MCI; bulk RNA-seq,Homo_723,MCI.vs.control; bulk RNA-seq,Homo_714,AD.vs.MCI; bulk RNA-seq,Homo_633,AD.vs.control; bulk RNA-seq,Homo_633,AD.vs.MCI	6
MF	GO:0004181	metallocarboxypeptidase activity	bulk RNA-seq,Homo_723,AD.vs.control; bulk RNA-seq,Homo_723,AD.vs.MCI	2
BP	GO:1901657	glycosyl compound metabolic process	bulk RNA-seq,Homo_723,AD.vs.control; bulk RNA-seq,Homo_723,MCI.vs.control; bulk RNA-seq,Homo_714,MCI.vs.control; bulk RNA-seq,Homo_633,AD.vs.control; bulk RNA-seq,Homo_633,AD.vs.MCI	5
BP	GO:0001678	cellular glucose homeostasis	bulk RNA-seq,Homo_723,AD.vs.control; bulk RNA-seq,Homo_723,AD.vs.MCI; bulk RNA-seq,Homo_723,MCI.vs.control; bulk RNA-seq,Homo_714,AD.vs.MCI; bulk RNA-seq,Homo_633,AD.vs.control; bulk RNA-seq,Homo_633,AD.vs.MCI	6
BP	GO:0046148	pigment biosynthetic process	bulk RNA-seq,Homo_723,AD.vs.control; bulk RNA-seq,Homo_723,AD.vs.MCI; bulk RNA-seq,Homo_723,MCI.vs.control; bulk RNA-seq,Homo_633,AD.vs.control; bulk RNA-seq,Homo_633,AD.vs.MCI	5
BP	GO:0070979	protein K11-linked ubiquitination	bulk RNA-seq,Homo_723,AD.vs.control; bulk RNA-seq,Homo_723,MCI.vs.control; bulk RNA-seq,Homo_714,MCI.vs.control; bulk RNA-seq,Homo_633,AD.vs.control; bulk RNA-seq,Homo_633,AD.vs.MCI; bulk RNA-seq,Homo_633,MCI.vs.control	6
MF	GO:0050750	low-density lipoprotein particle receptor binding	bulk RNA-seq,Homo_723,AD.vs.control; bulk RNA-seq,Homo_723,AD.vs.MCI; bulk RNA-seq,Homo_723,MCI.vs.control; bulk RNA-seq,Homo_714,AD.vs.MCI; bulk RNA-seq,Homo_714,MCI.vs.control; bulk RNA-seq,Homo_633,AD.vs.control; bulk RNA-seq,Homo_633,AD.vs.MCI	7
BP	GO:0035860	glial cell-derived neurotrophic factor receptor signaling	bulk RNA-seq,Homo_723,AD.vs.control; bulk RNA-seq,Homo_723,AD.vs.MCI; bulk RNA-seq,Homo_714,AD.vs.MCI	3
BP	GO:0060525	prostate glandular acinus development	bulk RNA-seq,Homo_723,AD.vs.control; bulk RNA-seq,Homo_633,MCI.vs.control	2
BP	GO:1903596	regulation of gap junction assembly	bulk RNA-seq,Homo_723,AD.vs.control; bulk RNA-seq,Homo_723,AD.vs.MCI; bulk RNA-seq,Homo_723,MCI.vs.control; bulk RNA-seq,Homo_714,AD.vs.MCI; bulk RNA-seq,Homo_714,MCI.vs.control	5
BP	GO:0000002	mitochondrial genome maintenance	bulk RNA-seq,Homo_723,AD.vs.control; bulk RNA-seq,Homo_723,MCI.vs.control; bulk RNA-seq,Homo_714,AD.vs.control; bulk RNA-seq,Homo_714,MCI.vs.control; bulk RNA-seq,Homo_633,AD.vs.control; bulk RNA-seq,Homo_633,AD.vs.MCI; bulk RNA-seq,Homo_633,MCI.vs.control	7
BP	GO:1904667	negative regulation of ubiquitin protein ligase activity	bulk RNA-seq,Homo_723,AD.vs.control; bulk RNA-seq,Homo_723,MCI.vs.control; bulk RNA-seq,Homo_714,MCI.vs.control; bulk RNA-seq,Homo_633,AD.vs.control; bulk RNA-seq,Homo_633,AD.vs.MCI; bulk RNA-seq,Homo_633,MCI.vs.control	6
MF	GO:0097110	scaffold protein binding	bulk RNA-seq,Homo_723,AD.vs.control; bulk RNA-seq,Homo_723,AD.vs.MCI; bulk RNA-seq,Homo_723,MCI.vs.control; bulk RNA-seq,Homo_714,AD.vs.MCI; bulk RNA-seq,Homo_714,MCI.vs.control; bulk RNA-seq,Homo_633,AD.vs.control; bulk RNA-seq,Homo_633,AD.vs.MCI	7
CC	GO:0030663	COPI-coated vesicle membrane	bulk RNA-seq,Homo_723,AD.vs.control; bulk RNA-seq,Homo_633,AD.vs.control; bulk RNA-seq,Homo_633,AD.vs.MCI; bulk RNA-	4
BP	GO:0008347	glial cell migration	bulk RNA-seq,Homo_723,AD.vs.control; bulk RNA-seq,Homo_723,AD.vs.MCI; bulk RNA-seq,Homo_723,MCI.vs.control; bulk RNA-seq,Homo_714,AD.vs.MCI; bulk RNA-seq,Homo_714,MCI.vs.control; bulk RNA-seq,Homo_633,AD.vs.control; bulk RNA-seq,Homo_633,AD.vs.MCI	7
BP	GO:0009116	nucleoside metabolic process	bulk RNA-seq,Homo_723,AD.vs.control; bulk RNA-seq,Homo_633,AD.vs.control; bulk RNA-seq,Homo_633,AD.vs.MCI	3
BP	GO:0071359	cellular response to dsRNA	bulk RNA-seq,Homo_723,AD.vs.control; bulk RNA-seq,Homo_723,MCI.vs.control; bulk RNA-seq,Homo_714,MCI.vs.control; bulk RNA-seq,Homo_633,AD.vs.control; bulk RNA-seq,Homo_633,MCI.vs.control	5
BP	GO:0032070	regulation of deoxyribonuclease activity	bulk RNA-seq,Homo_723,AD.vs.control; bulk RNA-seq,Homo_714,AD.vs.control	2
BP	GO:0030878	thyroid gland development	bulk RNA-seq,Homo_723,AD.vs.control; bulk RNA-seq,Homo_723,AD.vs.MCI; bulk RNA-seq,Homo_714,AD.vs.MCI	3
BP	GO:1900026	positive regulation of substrate adhesion-dependent cell spreading	bulk RNA-seq,Homo_723,AD.vs.control; bulk RNA-seq,Homo_723,AD.vs.MCI; bulk RNA-seq,Homo_723,MCI.vs.control; bulk RNA-seq,Homo_714,AD.vs.MCI; bulk RNA-seq,Homo_714,MCI.vs.control; bulk RNA-seq,Homo_633,AD.vs.control; bulk RNA-seq,Homo_633,AD.vs.MCI	7
CC	GO:0000812	Swr1 complex	bulk RNA-seq,Homo_723,AD.vs.control; bulk RNA-seq,Homo_723,MCI.vs.control; bulk RNA-seq,Homo_714,AD.vs.control; bulk RNA-	4
BP	GO:0003357	noradrenergic neuron differentiation	bulk RNA-seq,Homo_723,AD.vs.control; bulk RNA-seq,Homo_723,AD.vs.MCI; bulk RNA-seq,Homo_714,AD.vs.MCI	3
BP	GO:0045063	T-helper 1 cell differentiation	bulk RNA-seq,Homo_723,AD.vs.control; bulk RNA-seq,Homo_723,MCI.vs.control; bulk RNA-seq,Homo_633,AD.vs.control; bulk RNA-	4

BP	GO:0070507	regulation of microtubule cytoskeleton organization	bulk RNA-seq,Homo_723,AD.vs.control; bulk RNA-seq,Homo_723,AD.vs.MCI; bulk RNA-seq,Homo_723,MCI.vs.control; bulk RNA-seq,Homo_714,AD.vs.MCI; bulk RNA-seq,Homo_633,AD.vs.control; bulk RNA-seq,Homo_633,AD.vs.MCI	6
BP	GO:0032392	DNA geometric change	bulk RNA-seq,Homo_723,AD.vs.control; bulk RNA-seq,Homo_723,MCI.vs.control; bulk RNA-seq,Homo_714,AD.vs.control; bulk RNA-seq,Homo_714,MCI.vs.control; bulk RNA-seq,Homo_633,AD.vs.control; bulk RNA-seq,Homo_633,AD.vs.MCI	6
BP	GO:0072329	monocarboxylic acid catabolic process	bulk RNA-seq,Homo_723,AD.vs.control; bulk RNA-seq,Homo_723,MCI.vs.control; bulk RNA-seq,Homo_714,AD.vs.control; bulk RNA-seq,Homo_714,MCI.vs.control; bulk RNA-seq,Homo_633,AD.vs.control; bulk RNA-seq,Homo_633,AD.vs.MCI	6
BP	GO:0002031	G protein-coupled receptor internalization	bulk RNA-seq,Homo_723,AD.vs.control	1
CC	GO:0030055	cell-substrate junction	bulk RNA-seq,Homo_723,AD.vs.control; bulk RNA-seq,Homo_723,AD.vs.MCI; bulk RNA-seq,Homo_714,AD.vs.MCI; bulk RNA-seq,Homo_633,AD.vs.control; bulk RNA-seq,Homo_633,AD.vs.MCI	5
BP	GO:0097494	regulation of vesicle size	bulk RNA-seq,Homo_723,AD.vs.control; bulk RNA-seq,Homo_723,MCI.vs.control; bulk RNA-seq,Homo_714,AD.vs.control; bulk RNA-seq,Homo_633,AD.vs.control; bulk RNA-seq,Homo_633,AD.vs.MCI	5
BP	GO:0060713	labyrinthine layer morphogenesis	bulk RNA-seq,Homo_723,AD.vs.control; bulk RNA-seq,Homo_723,AD.vs.MCI; bulk RNA-seq,Homo_723,MCI.vs.control	3
MF	GO:0038187	pattern recognition receptor activity	bulk RNA-seq,Homo_723,AD.vs.control; bulk RNA-seq,Homo_633,AD.vs.control; bulk RNA-seq,Homo_633,AD.vs.MCI	3
BP	GO:0046755	viral budding	bulk RNA-seq,Homo_723,AD.vs.control; bulk RNA-seq,Homo_723,MCI.vs.control; bulk RNA-seq,Homo_714,AD.vs.control; bulk RNA-seq,Homo_633,AD.vs.control; bulk RNA-seq,Homo_633,AD.vs.MCI	5
BP	GO:0045646	regulation of erythrocyte differentiation	bulk RNA-seq,Homo_723,AD.vs.control; bulk RNA-seq,Homo_723,MCI.vs.control; bulk RNA-seq,Homo_714,MCI.vs.control; bulk RNA-seq,Homo_633,AD.vs.control; bulk RNA-seq,Homo_633,AD.vs.MCI	5
CC	GO:0097228	sperm principal piece	bulk RNA-seq,Homo_723,AD.vs.control; bulk RNA-seq,Homo_723,AD.vs.MCI; bulk RNA-seq,Homo_714,AD.vs.MCI	3
BP	GO:1902275	regulation of chromatin organization	bulk RNA-seq,Homo_723,AD.vs.control; bulk RNA-seq,Homo_723,MCI.vs.control; bulk RNA-seq,Homo_714,MCI.vs.control; bulk RNA-seq,Homo_633,AD.vs.control; bulk RNA-seq,Homo_633,AD.vs.MCI	5
BP	GO:1905208	negative regulation of cardiocyte differentiation	bulk RNA-seq,Homo_723,AD.vs.control; bulk RNA-seq,Homo_723,AD.vs.MCI; bulk RNA-seq,Homo_723,MCI.vs.control; bulk RNA-seq,Homo_714,AD.vs.MCI; bulk RNA-seq,Homo_714,MCI.vs.control	5
BP	GO:0035329	hippo signaling	bulk RNA-seq,Homo_723,AD.vs.control; bulk RNA-seq,Homo_723,AD.vs.MCI; bulk RNA-seq,Homo_723,MCI.vs.control; bulk RNA-seq,Homo_714,AD.vs.MCI; bulk RNA-seq,Homo_714,MCI.vs.control; bulk RNA-seq,Homo_633,AD.vs.control; bulk RNA-seq,Homo_633,AD.vs.MCI	7
BP	GO:0048066	developmental pigmentation	bulk RNA-seq,Homo_723,AD.vs.control; bulk RNA-seq,Homo_723,AD.vs.MCI; bulk RNA-seq,Homo_723,MCI.vs.control; bulk RNA-seq,Homo_714,AD.vs.MCI; bulk RNA-seq,Homo_714,MCI.vs.control; bulk RNA-seq,Homo_633,AD.vs.control	6
BP	GO:1902107	positive regulation of leukocyte differentiation	bulk RNA-seq,Homo_723,AD.vs.control; bulk RNA-seq,Homo_723,MCI.vs.control; bulk RNA-seq,Homo_633,AD.vs.control; bulk RNA-seq,Homo_633,AD.vs.MCI; scRNA-seq,SRP215507,CD8+ T cell_3-AD.vs.control	5
BP	GO:1903708	positive regulation of hemopoiesis	bulk RNA-seq,Homo_723,AD.vs.control; bulk RNA-seq,Homo_723,MCI.vs.control; bulk RNA-seq,Homo_633,AD.vs.control; bulk RNA-seq,Homo_633,AD.vs.MCI; scRNA-seq,SRP215507,CD8+ T cell_3-AD.vs.control	5
BP	GO:2001014	regulation of skeletal muscle cell differentiation	bulk RNA-seq,Homo_723,AD.vs.control; bulk RNA-seq,Homo_723,AD.vs.MCI; bulk RNA-seq,Homo_723,MCI.vs.control; bulk RNA-seq,Homo_714,AD.vs.MCI	4
MF	GO:0035374	chondroitin sulfate binding	bulk RNA-seq,Homo_723,AD.vs.control; bulk RNA-seq,Homo_723,AD.vs.MCI; bulk RNA-seq,Homo_723,MCI.vs.control; bulk RNA-seq,Homo_714,AD.vs.MCI; bulk RNA-seq,Homo_714,MCI.vs.control	5
BP	GO:0006022	aminoglycan metabolic process	bulk RNA-seq,Homo_723,AD.vs.control; bulk RNA-seq,Homo_723,AD.vs.MCI; bulk RNA-seq,Homo_723,MCI.vs.control; bulk RNA-seq,Homo_714,AD.vs.MCI; bulk RNA-seq,Homo_714,MCI.vs.control; bulk RNA-seq,Homo_633,AD.vs.control; bulk RNA-seq,Homo_633,AD.vs.MCI; scRNA-seq,SRP330776,Memory T	8
BP	GO:0000447	endonucleolytic cleavage in ITS1 to separate SSU-rRNA from 5.8S rRNA and LSU-rRNA from tricistronic rRNA transcript (SSU-rRNA, 5.8S rRNA, LSU-rRNA)	bulk RNA-seq,Homo_723,AD.vs.control	1
BP	GO:0043084	penile erection	bulk RNA-seq,Homo_723,AD.vs.control; bulk RNA-seq,Homo_714,AD.vs.control	2
BP	GO:0032413	negative regulation of ion transmembrane transporter activity	bulk RNA-seq,Homo_723,AD.vs.control; bulk RNA-seq,Homo_723,AD.vs.MCI; bulk RNA-seq,Homo_714,AD.vs.MCI; bulk RNA-seq,Homo_633,AD.vs.control	4
BP	GO:0019068	virion assembly	bulk RNA-seq,Homo_723,AD.vs.control; bulk RNA-seq,Homo_723,MCI.vs.control; bulk RNA-seq,Homo_714,MCI.vs.control; bulk RNA-seq,Homo_633,AD.vs.control; bulk RNA-seq,Homo_633,AD.vs.MCI	5
BP	GO:0009313	oligosaccharide catabolic process	bulk RNA-seq,Homo_723,AD.vs.control; bulk RNA-seq,Homo_633,AD.vs.MCI	2
BP	GO:0071472	cellular response to salt stress	bulk RNA-seq,Homo_723,AD.vs.control; bulk RNA-seq,Homo_633,AD.vs.control; bulk RNA-seq,Homo_633,AD.vs.MCI	3
MF	GO:0017166	vinculin binding	bulk RNA-seq,Homo_723,AD.vs.control; bulk RNA-seq,Homo_723,AD.vs.MCI; bulk RNA-seq,Homo_723,MCI.vs.control; bulk RNA-seq,Homo_714,AD.vs.MCI	4
BP	GO:0072376	protein activation cascade	bulk RNA-seq,Homo_723,AD.vs.control; bulk RNA-seq,Homo_723,AD.vs.MCI; bulk RNA-seq,Homo_723,MCI.vs.control; bulk RNA-seq,Homo_714,AD.vs.control; bulk RNA-seq,Homo_714,AD.vs.MCI; bulk RNA-seq,Homo_714,MCI.vs.control	6
BP	GO:0051781	positive regulation of cell division	bulk RNA-seq,Homo_723,AD.vs.control; bulk RNA-seq,Homo_723,AD.vs.MCI; bulk RNA-seq,Homo_723,MCI.vs.control; bulk RNA-seq,Homo_714,AD.vs.MCI; bulk RNA-seq,Homo_714,MCI.vs.control; bulk RNA-seq,Homo_633,AD.vs.control; bulk RNA-seq,Homo_633,AD.vs.MCI	7
MF	GO:0042578	phosphoric ester hydrolase activity	bulk RNA-seq,Homo_723,AD.vs.control; bulk RNA-seq,Homo_723,AD.vs.MCI; bulk RNA-seq,Homo_714,AD.vs.MCI; bulk RNA-seq,Homo_633,AD.vs.control; bulk RNA-seq,Homo_633,AD.vs.MCI; scRNA-seq,SRP330776,B cell_1-AD.vs.control; scRNA-seq,SRP330776,CD8+ T cell_1-AD.vs.control; scRNA-seq,SRP330776,Naive CD8+ T cell_1-AD.vs.control; scRNA-seq,SRP330776,Natural killer cell_1-AD.vs.control	9
BP	GO:0003044	regulation of systemic arterial blood pressure mediated by a chemical signal	bulk RNA-seq,Homo_723,AD.vs.control; bulk RNA-seq,Homo_723,AD.vs.MCI; bulk RNA-seq,Homo_714,AD.vs.MCI	3
BP	GO:0008154	actin polymerization or depolymerization	bulk RNA-seq,Homo_723,AD.vs.control; bulk RNA-seq,Homo_723,AD.vs.MCI; bulk RNA-seq,Homo_723,MCI.vs.control; bulk RNA-seq,Homo_714,AD.vs.MCI; bulk RNA-seq,Homo_633,AD.vs.control; bulk RNA-seq,Homo_633,AD.vs.MCI	6
BP	GO:0061097	regulation of protein tyrosine kinase activity	bulk RNA-seq,Homo_723,AD.vs.control; bulk RNA-seq,Homo_723,AD.vs.MCI; bulk RNA-seq,Homo_723,MCI.vs.control; bulk RNA-seq,Homo_714,AD.vs.MCI; bulk RNA-seq,Homo_714,MCI.vs.control; bulk RNA-seq,Homo_633,AD.vs.control; bulk RNA-seq,Homo_633,AD.vs.MCI	7
BP	GO:0006576	cellular biogenic amine metabolic process	bulk RNA-seq,Homo_723,AD.vs.control; bulk RNA-seq,Homo_723,AD.vs.MCI; bulk RNA-seq,Homo_723,MCI.vs.control; bulk RNA-seq,Homo_714,AD.vs.control; bulk RNA-seq,Homo_714,AD.vs.MCI; bulk RNA-seq,Homo_714,MCI.vs.control; bulk RNA-seq,Homo_633,AD.vs.control; bulk	8
BP	GO:0002717	positive regulation of natural killer cell mediated immunity	bulk RNA-seq,Homo_723,AD.vs.control; bulk RNA-seq,Homo_633,AD.vs.control; bulk RNA-seq,Homo_633,AD.vs.MCI	3
BP	GO:0046849	bone remodeling	bulk RNA-seq,Homo_723,AD.vs.control; bulk RNA-seq,Homo_723,AD.vs.MCI; bulk RNA-seq,Homo_723,MCI.vs.control; bulk RNA-seq,Homo_714,AD.vs.MCI; bulk RNA-seq,Homo_714,MCI.vs.control; bulk RNA-seq,Homo_633,AD.vs.control; bulk RNA-seq,Homo_633,AD.vs.MCI	7

BP	GO:1902106	negative regulation of leukocyte differentiation	bulk RNA-seq,Homo_723,AD.vs.control; bulk RNA-seq,Homo_723,AD.vs.MCI; bulk RNA-seq,Homo_723,MCI.vs.control; bulk RNA-seq,Homo_714,AD.vs.MCI; bulk RNA-seq,Homo_714,MCI.vs.control; bulk RNA-seq,Homo_633,AD.vs.control; bulk RNA-seq,Homo_633,AD.vs.MCI	7
BP	GO:0048207	vesicle targeting, rough ER to cis-Golgi	bulk RNA-seq,Homo_723,AD.vs.control; bulk RNA-seq,Homo_633,AD.vs.control; bulk RNA-seq,Homo_633,AD.vs.MCI; bulk RNA-	4
BP	GO:0048208	COPII vesicle coating	bulk RNA-seq,Homo_723,AD.vs.control; bulk RNA-seq,Homo_633,AD.vs.control; bulk RNA-seq,Homo_633,AD.vs.MCI; bulk RNA-	4
MF	GO:0033558	protein lysine deacetylase activity	bulk RNA-seq,Homo_723,AD.vs.control; bulk RNA-seq,Homo_633,AD.vs.control; bulk RNA-seq,Homo_633,AD.vs.MCI	3
BP	GO:0033687	osteoblast proliferation	bulk RNA-seq,Homo_723,AD.vs.control; bulk RNA-seq,Homo_723,AD.vs.MCI; bulk RNA-seq,Homo_633,AD.vs.control	3
BP	GO:0007320	insemination	bulk RNA-seq,Homo_723,AD.vs.control; bulk RNA-seq,Homo_633,AD.vs.control	2
MF	GO:0004126	cytidine deaminase activity	bulk RNA-seq,Homo_723,AD.vs.control; bulk RNA-seq,Homo_723,AD.vs.MCI	2
BP	GO:0006216	cytidine catabolic process	bulk RNA-seq,Homo_723,AD.vs.control; bulk RNA-seq,Homo_723,AD.vs.MCI	2
BP	GO:0009972	cytidine deamination	bulk RNA-seq,Homo_723,AD.vs.control; bulk RNA-seq,Homo_723,AD.vs.MCI	2
BP	GO:0046087	cytidine metabolic process	bulk RNA-seq,Homo_723,AD.vs.control; bulk RNA-seq,Homo_723,AD.vs.MCI	2
BP	GO:1990535	neuron projection maintenance	bulk RNA-seq,Homo_723,AD.vs.control; bulk RNA-seq,Homo_723,AD.vs.MCI; bulk RNA-seq,Homo_633,AD.vs.control; bulk RNA-seq,Homo_633,AD.vs.MCI	4
BP	GO:0006182	cGMP biosynthetic process	bulk RNA-seq,Homo_723,AD.vs.control; bulk RNA-seq,Homo_723,AD.vs.MCI	2
BP	GO:0045739	positive regulation of DNA repair	bulk RNA-seq,Homo_723,AD.vs.control; bulk RNA-seq,Homo_723,MCI.vs.control; bulk RNA-seq,Homo_714,AD.vs.control; bulk RNA-seq,Homo_714,MCI.vs.control; bulk RNA-seq,Homo_633,AD.vs.control; bulk RNA-seq,Homo_633,AD.vs.MCI	6
BP	GO:0019373	epoxygenase P450 pathway	bulk RNA-seq,Homo_723,AD.vs.control; bulk RNA-seq,Homo_723,AD.vs.MCI; bulk RNA-seq,Homo_714,AD.vs.control; bulk RNA-seq,Homo_714,AD.vs.MCI	4
BP	GO:0032941	secretion by tissue	bulk RNA-seq,Homo_723,AD.vs.control; bulk RNA-seq,Homo_723,AD.vs.MCI; bulk RNA-seq,Homo_723,MCI.vs.control; bulk RNA-seq,Homo_714,AD.vs.MCI; bulk RNA-seq,Homo_714,MCI.vs.control	5
BP	GO:0009069	serine family amino acid metabolic process	bulk RNA-seq,Homo_723,AD.vs.control; bulk RNA-seq,Homo_723,AD.vs.MCI; bulk RNA-seq,Homo_723,MCI.vs.control; bulk RNA-seq,Homo_633,AD.vs.control; bulk RNA-seq,Homo_633,AD.vs.MCI	5
BP	GO:0031295	T cell costimulation	bulk RNA-seq,Homo_723,AD.vs.control; bulk RNA-seq,Homo_723,MCI.vs.control; bulk RNA-seq,Homo_714,AD.vs.control; bulk RNA-seq,Homo_714,MCI.vs.control; bulk RNA-seq,Homo_633,AD.vs.control	5
BP	GO:0035815	positive regulation of renal sodium excretion	bulk RNA-seq,Homo_723,AD.vs.control	1
MF	GO:0016594	glycine binding	bulk RNA-seq,Homo_723,AD.vs.control; bulk RNA-seq,Homo_723,AD.vs.MCI; bulk RNA-seq,Homo_723,MCI.vs.control; bulk RNA-seq,Homo_714,AD.vs.MCI	4
BP	GO:0032434	regulation of proteasomal ubiquitin-dependent protein	bulk RNA-seq,Homo_723,AD.vs.control; bulk RNA-seq,Homo_723,MCI.vs.control; bulk RNA-seq,Homo_633,AD.vs.control; bulk RNA-	4
CC	GO:0000922	spindle pole	bulk RNA-seq,Homo_723,AD.vs.control; bulk RNA-seq,Homo_723,MCI.vs.control; bulk RNA-seq,Homo_714,AD.vs.MCI; bulk RNA-seq,Homo_633,AD.vs.control; bulk RNA-seq,Homo_633,AD.vs.MCI	5
BP	GO:1903365	regulation of fear response	bulk RNA-seq,Homo_723,AD.vs.control; bulk RNA-seq,Homo_723,AD.vs.MCI; bulk RNA-seq,Homo_723,MCI.vs.control; bulk RNA-seq,Homo_714,AD.vs.MCI	4
BP	GO:0060075	regulation of resting membrane potential	bulk RNA-seq,Homo_723,AD.vs.control	1
BP	GO:0042059	negative regulation of epidermal growth factor receptor signaling pathway	bulk RNA-seq,Homo_723,AD.vs.control; bulk RNA-seq,Homo_723,AD.vs.MCI; bulk RNA-seq,Homo_723,MCI.vs.control; bulk RNA-seq,Homo_714,AD.vs.MCI; bulk RNA-seq,Homo_714,MCI.vs.control	5
BP	GO:1902850	microtubule cytoskeleton organization involved in mitosis	bulk RNA-seq,Homo_723,AD.vs.control; bulk RNA-seq,Homo_723,AD.vs.MCI; bulk RNA-seq,Homo_723,MCI.vs.control; bulk RNA-seq,Homo_714,AD.vs.MCI; bulk RNA-seq,Homo_633,AD.vs.control; bulk RNA-seq,Homo_633,AD.vs.MCI	6
CC	GO:0032432	actin filament bundle	bulk RNA-seq,Homo_723,AD.vs.control; bulk RNA-seq,Homo_723,AD.vs.MCI; bulk RNA-seq,Homo_723,MCI.vs.control; bulk RNA-seq,Homo_714,AD.vs.control; bulk RNA-seq,Homo_714,AD.vs.MCI; bulk RNA-seq,Homo_714,MCI.vs.control; bulk RNA-seq,Homo_633,AD.vs.control	7
BP	GO:0045649	regulation of macrophage differentiation	bulk RNA-seq,Homo_723,AD.vs.control; bulk RNA-seq,Homo_723,AD.vs.MCI; bulk RNA-seq,Homo_714,AD.vs.MCI	3
BP	GO:1905144	response to acetylcholine	bulk RNA-seq,Homo_723,AD.vs.control; bulk RNA-seq,Homo_723,AD.vs.MCI; bulk RNA-seq,Homo_723,MCI.vs.control; bulk RNA-seq,Homo_714,AD.vs.MCI; bulk RNA-seq,Homo_714,MCI.vs.control	5
BP	GO:0045005	DNA-templated DNA replication maintenance of fidelity	bulk RNA-seq,Homo_723,AD.vs.control; bulk RNA-seq,Homo_723,MCI.vs.control; bulk RNA-seq,Homo_714,MCI.vs.control; bulk RNA-seq,Homo_633,AD.vs.control; bulk RNA-seq,Homo_633,AD.vs.MCI	5
CC	GO:0001518	voltage-gated sodium channel complex	bulk RNA-seq,Homo_723,AD.vs.control; bulk RNA-seq,Homo_723,AD.vs.MCI; bulk RNA-seq,Homo_723,MCI.vs.control; bulk RNA-seq,Homo_714,AD.vs.MCI; bulk RNA-seq,Homo_714,MCI.vs.control; bulk RNA-seq,ROSMAP,AD.vs.control	6
MF	GO:0031005	filamin binding	bulk RNA-seq,Homo_723,AD.vs.control; bulk RNA-seq,Homo_723,AD.vs.MCI; bulk RNA-seq,Homo_723,MCI.vs.control; bulk RNA-seq,Homo_714,AD.vs.MCI; bulk RNA-seq,Homo_714,MCI.vs.control	5
BP	GO:0035988	chondrocyte proliferation	bulk RNA-seq,Homo_723,AD.vs.control; bulk RNA-seq,Homo_723,AD.vs.MCI; bulk RNA-seq,Homo_723,MCI.vs.control; bulk RNA-seq,Homo_714,AD.vs.MCI; bulk RNA-seq,Homo_714,MCI.vs.control	5
BP	GO:0046854	phosphatidylinositol phosphate biosynthetic process	bulk RNA-seq,Homo_723,AD.vs.control; bulk RNA-seq,Homo_723,MCI.vs.control; bulk RNA-seq,Homo_714,AD.vs.control; bulk RNA-seq,Homo_714,MCI.vs.control; bulk RNA-seq,Homo_633,AD.vs.control; bulk RNA-seq,Homo_633,AD.vs.MCI	6
BP	GO:0045730	respiratory burst	bulk RNA-seq,Homo_723,AD.vs.control; bulk RNA-seq,Homo_633,AD.vs.control; bulk RNA-seq,Homo_633,AD.vs.MCI	3
BP	GO:0050965	detection of temperature stimulus involved in sensory perception of pain	bulk RNA-seq,Homo_723,AD.vs.control; bulk RNA-seq,Homo_723,AD.vs.MCI; bulk RNA-seq,Homo_723,MCI.vs.control; bulk RNA-seq,Homo_714,AD.vs.MCI; bulk RNA-seq,Homo_714,MCI.vs.control	5
BP	GO:0060632	regulation of microtubule-based movement	bulk RNA-seq,Homo_723,AD.vs.control; bulk RNA-seq,Homo_723,AD.vs.MCI; bulk RNA-seq,Homo_723,MCI.vs.control; bulk RNA-	4
BP	GO:0010918	positive regulation of mitochondrial membrane potential	bulk RNA-seq,Homo_723,AD.vs.control; bulk RNA-seq,Homo_714,AD.vs.control; bulk RNA-seq,Homo_633,AD.vs.control	3
BP	GO:0010566	regulation of ketone biosynthetic process	bulk RNA-seq,Homo_723,AD.vs.control; bulk RNA-seq,Homo_723,AD.vs.MCI; bulk RNA-seq,Homo_723,MCI.vs.control; bulk RNA-	4
BP	GO:0045747	positive regulation of Notch signaling pathway	bulk RNA-seq,Homo_723,AD.vs.control; bulk RNA-seq,Homo_723,AD.vs.MCI; bulk RNA-seq,Homo_723,MCI.vs.control; bulk RNA-seq,Homo_714,AD.vs.MCI; bulk RNA-seq,Homo_714,MCI.vs.control; bulk RNA-seq,Homo_633,AD.vs.control	6
BP	GO:0051788	response to misfolded protein	bulk RNA-seq,Homo_723,AD.vs.control; bulk RNA-seq,Homo_723,MCI.vs.control; bulk RNA-seq,Homo_714,MCI.vs.control; bulk RNA-seq,Homo_633,AD.vs.control; bulk RNA-seq,Homo_633,AD.vs.MCI; bulk RNA-seq,Homo_633,MCI.vs.control	6
BP	GO:0070571	negative regulation of neuron projection regeneration	bulk RNA-seq,Homo_723,AD.vs.control; bulk RNA-seq,Homo_723,AD.vs.MCI; bulk RNA-seq,Homo_723,MCI.vs.control	3
CC	GO:0097733	photoreceptor cell cilium	bulk RNA-seq,Homo_723,AD.vs.control; bulk RNA-seq,Homo_723,AD.vs.MCI; bulk RNA-seq,Homo_723,MCI.vs.control; bulk RNA-seq,Homo_714,AD.vs.control; bulk RNA-seq,Homo_714,AD.vs.MCI; bulk RNA-seq,Homo_714,MCI.vs.control; bulk RNA-seq,Homo_633,AD.vs.control; bulk	8

BP	GO:0001843	neural tube closure	bulk RNA-seq,Homo_723,AD.vs.control; bulk RNA-seq,Homo_723,AD.vs.MCI; bulk RNA-seq,Homo_723,MCI.vs.control; bulk RNA-seq,Homo_714,AD.vs.MCI; bulk RNA-seq,Homo_714,MCI.vs.control; bulk RNA-seq,Homo_633,AD.vs.control; bulk RNA-seq,Homo_633,AD.vs.MCI	7
BP	GO:0002076	osteoblast development	bulk RNA-seq,Homo_723,AD.vs.control; bulk RNA-seq,Homo_723,AD.vs.MCI; bulk RNA-seq,Homo_633,AD.vs.control	3
BP	GO:0031643	positive regulation of myelination	bulk RNA-seq,Homo_723,AD.vs.control; bulk RNA-seq,Homo_723,AD.vs.MCI; bulk RNA-seq,Homo_723,MCI.vs.control; bulk RNA-seq,Homo_714,AD.vs.MCI; bulk RNA-seq,Homo_714,MCI.vs.control; bulk RNA-seq,Homo_633,AD.vs.control	6
CC	GO:0031941	filamentous actin	bulk RNA-seq,Homo_723,AD.vs.control; bulk RNA-seq,Homo_723,AD.vs.MCI; bulk RNA-seq,Homo_723,MCI.vs.control; bulk RNA-seq,Homo_714,AD.vs.MCI; bulk RNA-seq,Homo_714,MCI.vs.control; bulk RNA-seq,Homo_633,AD.vs.control	6
BP	GO:0030238	male sex determination	bulk RNA-seq,Homo_723,AD.vs.control; bulk RNA-seq,Homo_723,AD.vs.MCI; bulk RNA-seq,Homo_723,MCI.vs.control; bulk RNA-seq,Homo_714,AD.vs.MCI	4
MF	GO:0016801	hydrolase activity, acting on ether bonds	bulk RNA-seq,Homo_723,AD.vs.control; bulk RNA-seq,Homo_723,AD.vs.MCI; bulk RNA-seq,Homo_633,AD.vs.control; bulk RNA-seq,Homo_633,AD.vs.MCI	4
BP	GO:0006167	AMP biosynthetic process	bulk RNA-seq,Homo_723,AD.vs.control; bulk RNA-seq,Homo_723,MCI.vs.control; bulk RNA-seq,Homo_714,MCI.vs.control; bulk RNA-seq,Homo_633,AD.vs.control; bulk RNA-seq,Homo_633,MCI.vs.control	5
BP	GO:0010225	response to UV-C	bulk RNA-seq,Homo_723,AD.vs.control; bulk RNA-seq,Homo_723,MCI.vs.control; bulk RNA-seq,Homo_714,MCI.vs.control; bulk RNA-seq,Homo_633,AD.vs.control; bulk RNA-seq,Homo_633,AD.vs.MCI; bulk RNA-seq,Homo_633,MCI.vs.control	6
BP	GO:0042326	negative regulation of phosphorylation	bulk RNA-seq,Homo_723,AD.vs.control; bulk RNA-seq,Homo_723,AD.vs.MCI; bulk RNA-seq,Homo_714,AD.vs.MCI; bulk RNA-seq,Homo_633,AD.vs.control; bulk RNA-seq,Homo_633,AD.vs.MCI	5
BP	GO:0071900	regulation of protein serine/threonine kinase activity	bulk RNA-seq,Homo_723,AD.vs.control; bulk RNA-seq,Homo_723,AD.vs.MCI; bulk RNA-seq,Homo_714,AD.vs.MCI; bulk RNA-seq,Homo_633,AD.vs.control; bulk RNA-seq,Homo_633,AD.vs.MCI	5
BP	GO:0015802	basic amino acid transport	bulk RNA-seq,Homo_723,AD.vs.control; bulk RNA-seq,Homo_723,AD.vs.MCI; bulk RNA-seq,Homo_723,MCI.vs.control	3
BP	GO:0060009	Sertoli cell development	bulk RNA-seq,Homo_723,AD.vs.control; bulk RNA-seq,Homo_723,AD.vs.MCI; bulk RNA-seq,Homo_723,MCI.vs.control; bulk RNA-seq,Homo_714,AD.vs.MCI; bulk RNA-seq,Homo_714,MCI.vs.control	5
BP	GO:0050714	positive regulation of protein secretion	bulk RNA-seq,Homo_723,AD.vs.control; bulk RNA-seq,Homo_723,AD.vs.MCI; bulk RNA-seq,Homo_723,MCI.vs.control; bulk RNA-seq,Homo_714,AD.vs.MCI; bulk RNA-seq,Homo_633,AD.vs.control; bulk RNA-seq,Homo_633,AD.vs.MCI	6
BP	GO:0051208	sequestering of calcium ion	bulk RNA-seq,Homo_723,AD.vs.control; bulk RNA-seq,Homo_723,AD.vs.MCI; bulk RNA-seq,Homo_723,MCI.vs.control; bulk RNA-seq,Homo_714,AD.vs.MCI; bulk RNA-seq,Homo_633,AD.vs.control; bulk RNA-seq,Homo_633,AD.vs.MCI	6
CC	GO:0035097	histone methyltransferase complex	bulk RNA-seq,Homo_723,AD.vs.control; bulk RNA-seq,Homo_723,MCI.vs.control; bulk RNA-seq,Homo_714,MCI.vs.control; bulk RNA-seq,Homo_633,AD.vs.control; bulk RNA-seq,Homo_633,AD.vs.MCI	5
BP	GO:2000846	regulation of corticosteroid hormone secretion	bulk RNA-seq,Homo_723,AD.vs.control; bulk RNA-seq,Homo_723,AD.vs.MCI; bulk RNA-seq,Homo_714,AD.vs.control; bulk RNA-seq,Homo_714,AD.vs.MCI	4
BP	GO:2000696	regulation of epithelial cell differentiation involved in kidney development	bulk RNA-seq,Homo_723,AD.vs.control; bulk RNA-seq,Homo_723,AD.vs.MCI; bulk RNA-seq,Homo_723,MCI.vs.control; bulk RNA-seq,Homo_714,AD.vs.MCI; bulk RNA-seq,Homo_714,MCI.vs.control	5
BP	GO:0000077	DNA damage checkpoint signaling	bulk RNA-seq,Homo_723,AD.vs.control; bulk RNA-seq,Homo_723,MCI.vs.control; bulk RNA-seq,Homo_714,AD.vs.control; bulk RNA-seq,Homo_714,MCI.vs.control; bulk RNA-seq,Homo_633,AD.vs.control; bulk RNA-seq,Homo_633,AD.vs.MCI	6
MF	GO:0042805	actinin binding	bulk RNA-seq,Homo_723,AD.vs.control; bulk RNA-seq,Homo_723,AD.vs.MCI; bulk RNA-seq,Homo_723,MCI.vs.control; bulk RNA-seq,Homo_714,AD.vs.MCI; bulk RNA-seq,Homo_714,MCI.vs.control; bulk RNA-seq,SRP325058,AD.vs.control	6
BP	GO:0007007	inner mitochondrial membrane organization	bulk RNA-seq,Homo_723,AD.vs.control; bulk RNA-seq,Homo_723,MCI.vs.control; bulk RNA-seq,Homo_714,AD.vs.control; bulk RNA-seq,Homo_714,MCI.vs.control; bulk RNA-seq,Homo_633,AD.vs.control; bulk RNA-seq,Homo_633,AD.vs.MCI	6
MF	GO:0008409	5'-3' exonuclease activity	bulk RNA-seq,Homo_723,AD.vs.control; bulk RNA-seq,Homo_723,MCI.vs.control; bulk RNA-seq,Homo_714,MCI.vs.control; bulk RNA-seq,Homo_633,AD.vs.control; bulk RNA-seq,Homo_633,AD.vs.MCI	5
BP	GO:0032342	aldosterone biosynthetic process	bulk RNA-seq,Homo_723,AD.vs.control; bulk RNA-seq,Homo_723,AD.vs.MCI; bulk RNA-seq,Homo_714,AD.vs.MCI	3
BP	GO:0032272	negative regulation of protein polymerization	bulk RNA-seq,Homo_723,AD.vs.control; bulk RNA-seq,Homo_723,AD.vs.MCI; bulk RNA-seq,Homo_723,MCI.vs.control; bulk RNA-seq,Homo_714,AD.vs.MCI; bulk RNA-seq,Homo_714,MCI.vs.control; bulk RNA-seq,Homo_633,AD.vs.control	6
BP	GO:0045785	positive regulation of cell adhesion	bulk RNA-seq,Homo_723,AD.vs.control; bulk RNA-seq,Homo_723,AD.vs.MCI; bulk RNA-seq,Homo_714,AD.vs.MCI; bulk RNA-seq,Homo_633,AD.vs.control; bulk RNA-seq,Homo_633,AD.vs.MCI; scRNA-seq,SRP215507,CD8+ T cell_3-AD.vs.control	6
BP	GO:0086069	bundle of His cell to Purkinje myocyte communication	bulk RNA-seq,Homo_723,AD.vs.control; bulk RNA-seq,Homo_723,AD.vs.MCI; bulk RNA-seq,Homo_723,MCI.vs.control; bulk RNA-seq,Homo_714,AD.vs.MCI; bulk RNA-seq,Homo_714,MCI.vs.control	5
BP	GO:0022010	central nervous system myelination	bulk RNA-seq,Homo_723,AD.vs.control; bulk RNA-seq,Homo_723,AD.vs.MCI; bulk RNA-seq,Homo_723,MCI.vs.control; bulk RNA-seq,Homo_714,AD.vs.MCI; bulk RNA-seq,Homo_714,MCI.vs.control	5
BP	GO:0032291	axon ensheathment in central nervous system	bulk RNA-seq,Homo_723,AD.vs.control; bulk RNA-seq,Homo_723,AD.vs.MCI; bulk RNA-seq,Homo_723,MCI.vs.control; bulk RNA-seq,Homo_714,AD.vs.MCI; bulk RNA-seq,Homo_714,MCI.vs.control	5
BP	GO:0015721	bile acid and bile salt transport	bulk RNA-seq,Homo_723,AD.vs.control; bulk RNA-seq,Homo_723,AD.vs.MCI; bulk RNA-seq,Homo_723,MCI.vs.control; bulk RNA-seq,Homo_714,AD.vs.control; bulk RNA-seq,Homo_714,AD.vs.MCI; bulk RNA-seq,Homo_714,MCI.vs.control	6
BP	GO:0017015	regulation of transforming growth factor beta receptor signaling pathway	bulk RNA-seq,Homo_723,AD.vs.control; bulk RNA-seq,Homo_723,AD.vs.MCI; bulk RNA-seq,Homo_723,MCI.vs.control; bulk RNA-seq,Homo_714,AD.vs.MCI; bulk RNA-seq,Homo_633,AD.vs.control; bulk RNA-seq,Homo_633,AD.vs.MCI	6
BP	GO:0043369	CD4-positive or CD8-positive, alpha-beta T cell lineage	bulk RNA-seq,Homo_723,AD.vs.control; bulk RNA-seq,Homo_714,AD.vs.control; bulk RNA-seq,Homo_714,MCI.vs.control; bulk RNA-	4
BP	GO:2001044	regulation of integrin-mediated signaling pathway	bulk RNA-seq,Homo_723,AD.vs.control; bulk RNA-seq,Homo_723,AD.vs.MCI; bulk RNA-seq,Homo_723,MCI.vs.control	3
BP	GO:0072182	regulation of nephron tubule epithelial cell differentiation	bulk RNA-seq,Homo_723,AD.vs.control; bulk RNA-seq,Homo_723,AD.vs.MCI; bulk RNA-seq,Homo_723,MCI.vs.control; bulk RNA-seq,Homo_714,AD.vs.MCI; bulk RNA-seq,Homo_714,MCI.vs.control	5
CC	GO:0031074	nucleocytoplasmic transport complex	bulk RNA-seq,Homo_723,AD.vs.control; bulk RNA-seq,Homo_714,AD.vs.control	2
CC	GO:0031314	extrinsic component of mitochondrial inner membrane	bulk RNA-seq,Homo_723,AD.vs.control; bulk RNA-seq,Homo_723,MCI.vs.control; bulk RNA-seq,Homo_714,MCI.vs.control; bulk RNA-seq,Homo_633,AD.vs.control; bulk RNA-seq,Homo_633,MCI.vs.control	5
BP	GO:0072540	T-helper 17 cell lineage commitment	bulk RNA-seq,Homo_723,AD.vs.control; bulk RNA-seq,Homo_714,MCI.vs.control; bulk RNA-seq,Homo_633,MCI.vs.control	3
BP	GO:0010460	positive regulation of heart rate	bulk RNA-seq,Homo_723,AD.vs.control; bulk RNA-seq,Homo_723,AD.vs.MCI; bulk RNA-seq,Homo_714,AD.vs.MCI	3
BP	GO:0042249	establishment of planar polarity of embryonic epithelium	bulk RNA-seq,Homo_723,AD.vs.control; bulk RNA-seq,Homo_723,AD.vs.MCI; bulk RNA-seq,Homo_723,MCI.vs.control; bulk RNA-seq,Homo_714,AD.vs.MCI	4
MF	GO:0050780	dopamine receptor binding	bulk RNA-seq,Homo_723,AD.vs.control; bulk RNA-seq,Homo_723,AD.vs.MCI; bulk RNA-seq,Homo_633,AD.vs.control; bulk RNA-seq,Homo_633,AD.vs.MCI	4

BP	GO:0006450	regulation of translational fidelity	bulk RNA-seq,Homo_723,AD.vs.control; bulk RNA-seq,Homo_723,MCI.vs.control; bulk RNA-seq,Homo_714,AD.vs.control; bulk RNA-seq,Homo_714,MCI.vs.control; bulk RNA-seq,Homo_633,AD.vs.control; bulk RNA-seq,Homo_633,AD.vs.MCI; bulk RNA-seq,Homo_633,MCI.vs.control	7
BP	GO:0070294	renal sodium ion absorption	bulk RNA-seq,Homo_723,AD.vs.control; bulk RNA-seq,Homo_723,AD.vs.MCI; bulk RNA-seq,Homo_714,AD.vs.MCI	3
BP	GO:1903844	regulation of cellular response to transforming growth factor beta stimulus	bulk RNA-seq,Homo_723,AD.vs.control; bulk RNA-seq,Homo_723,AD.vs.MCI; bulk RNA-seq,Homo_723,MCI.vs.control; bulk RNA-seq,Homo_714,AD.vs.MCI; bulk RNA-seq,Homo_633,AD.vs.control; bulk RNA-seq,Homo_633,AD.vs.MCI; scRNA-seq,SRP330776,Natural killer cell_1-AD.vs.control	7
BP	GO:1904645	response to amyloid-beta	bulk RNA-seq,Homo_723,AD.vs.control; bulk RNA-seq,Homo_723,AD.vs.MCI; bulk RNA-seq,Homo_723,MCI.vs.control; bulk RNA-seq,Homo_714,AD.vs.MCI; bulk RNA-seq,Homo_714,MCI.vs.control	5
BP	GO:1903036	positive regulation of response to wounding	bulk RNA-seq,Homo_723,AD.vs.control; bulk RNA-seq,Homo_723,AD.vs.MCI; bulk RNA-seq,Homo_723,MCI.vs.control; bulk RNA-seq,Homo_714,AD.vs.control; bulk RNA-seq,Homo_714,AD.vs.MCI; bulk RNA-seq,Homo_714,MCI.vs.control; bulk RNA-seq,Homo_633,AD.vs.control; bulk	8
BP	GO:0051282	regulation of sequestering of calcium ion	bulk RNA-seq,Homo_723,AD.vs.control; bulk RNA-seq,Homo_723,AD.vs.MCI; bulk RNA-seq,Homo_723,MCI.vs.control; bulk RNA-seq,Homo_714,AD.vs.MCI; bulk RNA-seq,Homo_714,MCI.vs.control; bulk RNA-seq,Homo_633,AD.vs.control; bulk RNA-seq,Homo_633,AD.vs.MCI	7
MF	GO:1990380	Lys48-specific deubiquitinase activity	bulk RNA-seq,Homo_723,AD.vs.control; bulk RNA-seq,Homo_714,MCI.vs.control; bulk RNA-seq,Homo_633,AD.vs.control; bulk RNA-	4
BP	GO:0005984	disaccharide metabolic process	bulk RNA-seq,Homo_723,AD.vs.control; bulk RNA-seq,Homo_714,AD.vs.control; bulk RNA-seq,Homo_633,AD.vs.control; bulk RNA-seq,Homo_633,AD.vs.MCI	4
BP	GO:0140056	organelle localization by membrane tethering	bulk RNA-seq,Homo_723,AD.vs.control; bulk RNA-seq,Homo_723,AD.vs.MCI; bulk RNA-seq,Homo_723,MCI.vs.control; bulk RNA-seq,Homo_714,MCI.vs.control; bulk RNA-seq,Homo_633,AD.vs.control; bulk RNA-seq,Homo_633,AD.vs.MCI	6
BP	GO:0042048	olfactory behavior	bulk RNA-seq,Homo_723,AD.vs.control; bulk RNA-seq,Homo_723,AD.vs.MCI; bulk RNA-seq,Homo_723,MCI.vs.control	3
BP	GO:0043627	response to estrogen	bulk RNA-seq,Homo_723,AD.vs.control; bulk RNA-seq,Homo_723,AD.vs.MCI; bulk RNA-seq,Homo_723,MCI.vs.control; bulk RNA-seq,Homo_714,AD.vs.MCI; bulk RNA-seq,Homo_714,MCI.vs.control; bulk RNA-seq,Homo_633,AD.vs.control	6
CC	GO:0000109	nucleotide-excision repair complex	bulk RNA-seq,Homo_723,AD.vs.control; bulk RNA-seq,Homo_723,MCI.vs.control; bulk RNA-seq,Homo_714,MCI.vs.control; bulk RNA-seq,Homo_633,AD.vs.control; bulk RNA-seq,Homo_633,AD.vs.MCI; bulk RNA-seq,Homo_633,MCI.vs.control	6
BP	GO:0002759	regulation of antimicrobial humoral response	bulk RNA-seq,Homo_723,AD.vs.control; bulk RNA-seq,Homo_714,AD.vs.control; bulk RNA-seq,Homo_714,AD.vs.MCI	3
BP	GO:1901224	positive regulation of NIK/NF-kappaB signaling	bulk RNA-seq,Homo_723,AD.vs.control; bulk RNA-seq,Homo_723,MCI.vs.control; bulk RNA-seq,Homo_714,MCI.vs.control; bulk RNA-seq,Homo_633,AD.vs.control; bulk RNA-seq,Homo_633,AD.vs.MCI	5
BP	GO:0050829	defense response to Gram-negative bacterium	bulk RNA-seq,Homo_723,AD.vs.control; bulk RNA-seq,Homo_714,AD.vs.control; bulk RNA-seq,Homo_714,AD.vs.MCI; bulk RNA-	4
BP	GO:0048368	lateral mesoderm development	bulk RNA-seq,Homo_723,AD.vs.control; bulk RNA-seq,Homo_723,AD.vs.MCI; bulk RNA-seq,Homo_714,AD.vs.MCI	3
BP	GO:0001841	neural tube formation	bulk RNA-seq,Homo_723,AD.vs.control; bulk RNA-seq,Homo_723,AD.vs.MCI; bulk RNA-seq,Homo_723,MCI.vs.control; bulk RNA-seq,Homo_714,AD.vs.MCI; bulk RNA-seq,Homo_714,MCI.vs.control; bulk RNA-seq,Homo_633,AD.vs.control; bulk RNA-seq,Homo_633,AD.vs.MCI	7
BP	GO:0016558	protein import into peroxisome matrix	bulk RNA-seq,Homo_723,AD.vs.control; bulk RNA-seq,Homo_723,MCI.vs.control; bulk RNA-seq,Homo_714,AD.vs.control; bulk RNA-seq,Homo_714,MCI.vs.control; bulk RNA-seq,Homo_633,AD.vs.control; bulk RNA-seq,Homo_633,AD.vs.MCI; bulk RNA-seq,Homo_633,MCI.vs.control	7
BP	GO:0060045	positive regulation of cardiac muscle cell proliferation	bulk RNA-seq,Homo_723,AD.vs.control; bulk RNA-seq,Homo_723,AD.vs.MCI; bulk RNA-seq,Homo_723,MCI.vs.control; bulk RNA-seq,Homo_714,AD.vs.MCI; bulk RNA-seq,Homo_714,MCI.vs.control	5
CC	GO:0030688	preribosome, small subunit precursor	bulk RNA-seq,Homo_723,AD.vs.control; bulk RNA-seq,Homo_633,AD.vs.control; bulk RNA-seq,Homo_633,AD.vs.MCI	3
CC	GO:0097504	Gemini of coiled bodies	bulk RNA-seq,Homo_723,AD.vs.control; bulk RNA-seq,Homo_723,MCI.vs.control; bulk RNA-seq,Homo_714,MCI.vs.control; bulk RNA-seq,Homo_633,AD.vs.control; bulk RNA-seq,Homo_633,MCI.vs.control	5
BP	GO:0071901	negative regulation of protein serine/threonine kinase activity	bulk RNA-seq,Homo_723,AD.vs.control; bulk RNA-seq,Homo_723,AD.vs.MCI; bulk RNA-seq,Homo_723,MCI.vs.control; bulk RNA-seq,Homo_714,AD.vs.MCI; bulk RNA-seq,Homo_714,MCI.vs.control; bulk RNA-seq,Homo_633,AD.vs.control; bulk RNA-seq,Homo_633,AD.vs.MCI	7
BP	GO:0071875	adrenergic receptor signaling pathway	bulk RNA-seq,Homo_723,AD.vs.control; bulk RNA-seq,Homo_723,AD.vs.MCI	2
BP	GO:2000780	negative regulation of double-strand break repair	bulk RNA-seq,Homo_723,AD.vs.control; bulk RNA-seq,Homo_723,MCI.vs.control; bulk RNA-seq,Homo_714,MCI.vs.control; bulk RNA-seq,Homo_633,AD.vs.control; bulk RNA-seq,Homo_633,AD.vs.MCI; bulk RNA-seq,Homo_633,MCI.vs.control	6
BP	GO:0002070	epithelial cell maturation	bulk RNA-seq,Homo_723,AD.vs.control; bulk RNA-seq,Homo_723,MCI.vs.control; bulk RNA-seq,Homo_714,AD.vs.MCI; bulk RNA-seq,Homo_714,MCI.vs.control; bulk RNA-seq,Homo_633,AD.vs.control; bulk RNA-seq,Homo_633,AD.vs.MCI	6
BP	GO:0046135	pyrimidine nucleoside catabolic process	bulk RNA-seq,Homo_723,AD.vs.control	1
BP	GO:0060337	type I interferon signaling pathway	bulk RNA-seq,Homo_723,AD.vs.control; bulk RNA-seq,Homo_723,MCI.vs.control; bulk RNA-seq,Homo_714,MCI.vs.control; bulk RNA-	4
BP	GO:0006515	protein quality control for misfolded or incompletely synthesized proteins	bulk RNA-seq,Homo_723,AD.vs.control; bulk RNA-seq,Homo_633,AD.vs.control; bulk RNA-seq,Homo_633,AD.vs.MCI	3
MF	GO:0016701	oxidoreductase activity, acting on single donors with incorporation of molecular oxygen	bulk RNA-seq,Homo_723,AD.vs.control; bulk RNA-seq,Homo_723,AD.vs.MCI; bulk RNA-seq,Homo_723,MCI.vs.control; bulk RNA-seq,Homo_714,AD.vs.MCI	4
BP	GO:0008207	C21-steroid hormone metabolic process	bulk RNA-seq,Homo_723,AD.vs.control; bulk RNA-seq,Homo_723,AD.vs.MCI; bulk RNA-seq,Homo_723,MCI.vs.control; bulk RNA-seq,Homo_714,AD.vs.MCI; bulk RNA-seq,Homo_714,MCI.vs.control; bulk RNA-seq,Homo_633,AD.vs.control	6
BP	GO:0006949	syncytium formation	bulk RNA-seq,Homo_723,AD.vs.control; bulk RNA-seq,Homo_723,AD.vs.MCI; bulk RNA-seq,Homo_723,MCI.vs.control; bulk RNA-seq,Homo_714,AD.vs.control; bulk RNA-seq,Homo_714,AD.vs.MCI; bulk RNA-seq,Homo_714,MCI.vs.control; bulk RNA-seq,Homo_633,AD.vs.control	7
CC	GO:0044214	spanning component of plasma membrane	bulk RNA-seq,Homo_723,AD.vs.control; bulk RNA-seq,Homo_723,AD.vs.MCI; bulk RNA-seq,Homo_723,MCI.vs.control; bulk RNA-seq,Homo_714,AD.vs.MCI	4
BP	GO:0001580	detection of chemical stimulus involved in sensory perception of bitter taste	bulk RNA-seq,Homo_723,AD.vs.control; bulk RNA-seq,Homo_714,AD.vs.control; bulk RNA-seq,Homo_714,AD.vs.MCI; bulk RNA-seq,Homo_633,AD.vs.control; bulk RNA-seq,Homo_633,AD.vs.MCI; bulk RNA-seq,ROSMAP,AD.vs.control	6
BP	GO:1904377	positive regulation of protein localization to cell periphery	bulk RNA-seq,Homo_723,AD.vs.control; bulk RNA-seq,Homo_723,AD.vs.MCI; bulk RNA-seq,Homo_723,MCI.vs.control; bulk RNA-seq,Homo_714,AD.vs.MCI; bulk RNA-seq,Homo_714,MCI.vs.control; bulk RNA-seq,Homo_633,AD.vs.control	6
BP	GO:0009083	branched-chain amino acid catabolic process	bulk RNA-seq,Homo_723,AD.vs.control; bulk RNA-seq,Homo_723,MCI.vs.control; bulk RNA-seq,Homo_714,MCI.vs.control; bulk RNA-seq,Homo_633,AD.vs.control; bulk RNA-seq,Homo_633,AD.vs.MCI	5
BP	GO:0042558	pteridine-containing compound metabolic process	bulk RNA-seq,Homo_723,AD.vs.control; bulk RNA-seq,Homo_723,MCI.vs.control; bulk RNA-seq,Homo_714,MCI.vs.control; bulk RNA-seq,Homo_633,AD.vs.control; bulk RNA-seq,Homo_633,AD.vs.MCI; bulk RNA-seq,Homo_633,MCI.vs.control	6
BP	GO:0032480	negative regulation of type I interferon production	bulk RNA-seq,Homo_723,AD.vs.control; bulk RNA-seq,Homo_723,MCI.vs.control; bulk RNA-seq,Homo_714,MCI.vs.control; bulk RNA-seq,Homo_633,AD.vs.control; bulk RNA-seq,Homo_633,MCI.vs.control	5

BP	GO:0000768	syncytium formation by plasma membrane fusion	bulk RNA-seq,Homo_723,AD.vs.control; bulk RNA-seq,Homo_723,AD.vs.MCI; bulk RNA-seq,Homo_723,MCI.vs.control; bulk RNA-seq,Homo_714,AD.vs.control; bulk RNA-seq,Homo_714,AD.vs.MCI; bulk RNA-seq,Homo_714,MCI.vs.control	6
BP	GO:0140253	cell-cell fusion	bulk RNA-seq,Homo_723,AD.vs.control; bulk RNA-seq,Homo_723,AD.vs.MCI; bulk RNA-seq,Homo_723,MCI.vs.control; bulk RNA-seq,Homo_714,AD.vs.control; bulk RNA-seq,Homo_714,AD.vs.MCI; bulk RNA-seq,Homo_714,MCI.vs.control	6
CC	GO:0000439	transcription factor TFIIH core complex	bulk RNA-seq,Homo_723,AD.vs.control; bulk RNA-seq,Homo_723,MCI.vs.control; bulk RNA-seq,Homo_714,AD.vs.control; bulk RNA-seq,Homo_714,MCI.vs.control; bulk RNA-seq,Homo_633,AD.vs.control; bulk RNA-seq,Homo_633,AD.vs.MCI; bulk RNA-seq,Homo_633,MCI.vs.control	7
BP	GO:0072075	metanephric mesenchyme development	bulk RNA-seq,Homo_723,AD.vs.control; bulk RNA-seq,Homo_723,AD.vs.MCI; bulk RNA-seq,Homo_723,MCI.vs.control; bulk RNA-seq,Homo_714,AD.vs.MCI; bulk RNA-seq,Homo_633,MCI.vs.control	5
BP	GO:0061003	positive regulation of dendritic spine morphogenesis	bulk RNA-seq,Homo_723,AD.vs.control; bulk RNA-seq,Homo_723,AD.vs.MCI; bulk RNA-seq,Homo_723,MCI.vs.control; bulk RNA-seq,Homo_714,AD.vs.MCI; bulk RNA-seq,Homo_633,AD.vs.control	5
MF	GO:0043395	heparan sulfate proteoglycan binding	bulk RNA-seq,Homo_723,AD.vs.control; bulk RNA-seq,Homo_723,AD.vs.MCI; bulk RNA-seq,Homo_723,MCI.vs.control; bulk RNA-seq,Homo_714,AD.vs.MCI; bulk RNA-seq,Homo_714,MCI.vs.control	5
BP	GO:0002699	positive regulation of immune effector process	bulk RNA-seq,Homo_723,AD.vs.control; bulk RNA-seq,Homo_723,MCI.vs.control; bulk RNA-seq,Homo_714,AD.vs.MCI; bulk RNA-seq,Homo_633,AD.vs.control; bulk RNA-seq,Homo_633,AD.vs.MCI; scRNA-seq,SRP215507,CD8+ T cell_3-AD.vs.control	6
BP	GO:0090051	negative regulation of cell migration involved in sprouting	bulk RNA-seq,Homo_723,AD.vs.control; bulk RNA-seq,Homo_723,AD.vs.MCI; bulk RNA-seq,Homo_714,AD.vs.control; bulk RNA-seq,Homo_714,AD.vs.MCI	4
BP	GO:1903955	positive regulation of protein targeting to mitochondrion	bulk RNA-seq,Homo_723,AD.vs.control; bulk RNA-seq,Homo_723,MCI.vs.control; bulk RNA-seq,Homo_714,MCI.vs.control; bulk RNA-seq,Homo_633,AD.vs.control; bulk RNA-seq,Homo_633,MCI.vs.control	5
BP	GO:0061912	selective autophagy	bulk RNA-seq,Homo_723,AD.vs.control; bulk RNA-seq,Homo_723,MCI.vs.control; bulk RNA-seq,Homo_714,AD.vs.control; bulk RNA-seq,Homo_714,MCI.vs.control; bulk RNA-seq,Homo_633,AD.vs.control; bulk RNA-seq,Homo_633,AD.vs.MCI	6
BP	GO:1902686	mitochondrial outer membrane permeabilization involved in programmed cell death	bulk RNA-seq,Homo_723,AD.vs.control; bulk RNA-seq,Homo_714,AD.vs.control; bulk RNA-seq,Homo_714,MCI.vs.control; bulk RNA-seq,Homo_633,AD.vs.control; bulk RNA-seq,Homo_633,AD.vs.MCI	5
MF	GO:0008301	DNA binding, bending	bulk RNA-seq,Homo_723,AD.vs.control; bulk RNA-seq,Homo_633,AD.vs.control; bulk RNA-seq,Homo_633,AD.vs.MCI	3
BP	GO:0034113	heterotypic cell-cell adhesion	bulk RNA-seq,Homo_723,AD.vs.control; bulk RNA-seq,Homo_723,AD.vs.MCI; bulk RNA-seq,Homo_723,MCI.vs.control; bulk RNA-seq,Homo_714,AD.vs.MCI; bulk RNA-seq,Homo_714,MCI.vs.control; bulk RNA-seq,Homo_633,AD.vs.control; bulk RNA-seq,Homo_633,AD.vs.MCI	7
BP	GO:0010390	histone monoubiquitination	bulk RNA-seq,Homo_723,AD.vs.control; bulk RNA-seq,Homo_723,MCI.vs.control; bulk RNA-seq,Homo_714,MCI.vs.control; bulk RNA-seq,Homo_633,AD.vs.control; bulk RNA-seq,Homo_633,AD.vs.MCI; bulk RNA-seq,Homo_633,MCI.vs.control	6
BP	GO:1903749	positive regulation of establishment of protein localization to mitochondrion	bulk RNA-seq,Homo_723,AD.vs.control; bulk RNA-seq,Homo_723,MCI.vs.control; bulk RNA-seq,Homo_714,AD.vs.control; bulk RNA-seq,Homo_714,MCI.vs.control; bulk RNA-seq,Homo_633,AD.vs.control	5
CC	GO:0001931	uropod	bulk RNA-seq,Homo_723,AD.vs.control	1
CC	GO:0031254	cell trailing edge	bulk RNA-seq,Homo_723,AD.vs.control	1
MF	GO:0015386	potassium:proton antiporter activity	bulk RNA-seq,Homo_723,AD.vs.control; bulk RNA-seq,Homo_723,AD.vs.MCI; bulk RNA-seq,Homo_723,MCI.vs.control	3
BP	GO:0045668	negative regulation of osteoblast differentiation	bulk RNA-seq,Homo_723,AD.vs.control; bulk RNA-seq,Homo_723,AD.vs.MCI; bulk RNA-seq,Homo_723,MCI.vs.control; bulk RNA-seq,Homo_714,AD.vs.MCI; bulk RNA-seq,Homo_714,MCI.vs.control	5
MF	GO:0001091	RNA polymerase II general transcription initiation factor binding	bulk RNA-seq,Homo_723,AD.vs.control; bulk RNA-seq,Homo_723,MCI.vs.control; bulk RNA-seq,Homo_714,MCI.vs.control; bulk RNA-seq,Homo_633,AD.vs.control; bulk RNA-seq,Homo_633,AD.vs.MCI; bulk RNA-seq,Homo_633,MCI.vs.control	6
MF	GO:0036122	BMP binding	bulk RNA-seq,Homo_723,AD.vs.control; bulk RNA-seq,Homo_723,AD.vs.MCI; bulk RNA-seq,Homo_723,MCI.vs.control; bulk RNA-seq,Homo_714,AD.vs.control; bulk RNA-seq,Homo_714,AD.vs.MCI	5
BP	GO:0006739	NADP metabolic process	bulk RNA-seq,Homo_723,AD.vs.control; bulk RNA-seq,Homo_723,MCI.vs.control; bulk RNA-seq,Homo_714,AD.vs.control; bulk RNA-seq,Homo_714,MCI.vs.control; bulk RNA-seq,Homo_633,AD.vs.control; bulk RNA-seq,Homo_633,AD.vs.MCI	6
BP	GO:0032925	regulation of activin receptor signaling pathway	bulk RNA-seq,Homo_723,AD.vs.control; bulk RNA-seq,Homo_723,AD.vs.MCI; bulk RNA-seq,Homo_723,MCI.vs.control; bulk RNA-seq,Homo_714,AD.vs.MCI	4
BP	GO:1900117	regulation of execution phase of apoptosis	bulk RNA-seq,Homo_723,AD.vs.control	1
BP	GO:0045738	negative regulation of DNA repair	bulk RNA-seq,Homo_723,AD.vs.control; bulk RNA-seq,Homo_723,MCI.vs.control; bulk RNA-seq,Homo_714,MCI.vs.control; bulk RNA-seq,Homo_633,AD.vs.control; bulk RNA-seq,Homo_633,AD.vs.MCI	5
BP	GO:0051205	protein insertion into membrane	bulk RNA-seq,Homo_723,AD.vs.control; bulk RNA-seq,Homo_723,MCI.vs.control; bulk RNA-seq,Homo_714,MCI.vs.control; bulk RNA-seq,Homo_633,AD.vs.control; bulk RNA-seq,Homo_633,AD.vs.MCI	5
BP	GO:0042711	maternal behavior	bulk RNA-seq,Homo_723,AD.vs.control	1
BP	GO:1903214	regulation of protein targeting to mitochondrion	bulk RNA-seq,Homo_723,AD.vs.control; bulk RNA-seq,Homo_723,MCI.vs.control; bulk RNA-seq,Homo_714,AD.vs.control; bulk RNA-seq,Homo_714,MCI.vs.control; bulk RNA-seq,Homo_633,AD.vs.control	5
BP	GO:0099566	regulation of postsynaptic cytosolic calcium ion	bulk RNA-seq,Homo_723,AD.vs.control; bulk RNA-seq,Homo_723,AD.vs.MCI; bulk RNA-seq,Homo_723,MCI.vs.control	3
BP	GO:0046633	alpha-beta T cell proliferation	bulk RNA-seq,Homo_723,AD.vs.control; bulk RNA-seq,Homo_723,MCI.vs.control; bulk RNA-seq,Homo_714,MCI.vs.control; bulk RNA-	4
MF	GO:0034061	DNA polymerase activity	bulk RNA-seq,Homo_723,AD.vs.control; bulk RNA-seq,Homo_723,MCI.vs.control; bulk RNA-seq,Homo_714,MCI.vs.control; bulk RNA-seq,Homo_633,AD.vs.control; bulk RNA-seq,Homo_633,AD.vs.MCI; bulk RNA-seq,Homo_633,MCI.vs.control	6
MF	GO:0003953	NAD+ nucleosidase activity	bulk RNA-seq,Homo_723,AD.vs.control; bulk RNA-seq,Homo_723,MCI.vs.control; bulk RNA-seq,Homo_633,AD.vs.control; bulk RNA-	4
MF	GO:0050135	NAD(P)+ nucleosidase activity	bulk RNA-seq,Homo_723,AD.vs.control; bulk RNA-seq,Homo_723,MCI.vs.control; bulk RNA-seq,Homo_633,AD.vs.control; bulk RNA-	4
MF	GO:0061809	NAD+ nucleotidase, cyclic ADP-ribose generating	bulk RNA-seq,Homo_723,AD.vs.control; bulk RNA-seq,Homo_723,MCI.vs.control; bulk RNA-seq,Homo_633,AD.vs.control; bulk RNA-	4
BP	GO:0033866	nucleoside bisphosphate biosynthetic process	bulk RNA-seq,Homo_723,AD.vs.control; bulk RNA-seq,Homo_723,MCI.vs.control; bulk RNA-seq,Homo_714,MCI.vs.control; bulk RNA-seq,Homo_633,AD.vs.control; bulk RNA-seq,Homo_633,AD.vs.MCI	5
BP	GO:0034030	ribonucleoside bisphosphate biosynthetic process	bulk RNA-seq,Homo_723,AD.vs.control; bulk RNA-seq,Homo_723,MCI.vs.control; bulk RNA-seq,Homo_714,MCI.vs.control; bulk RNA-seq,Homo_633,AD.vs.control; bulk RNA-seq,Homo_633,AD.vs.MCI	5
BP	GO:0034033	purine nucleoside bisphosphate biosynthetic process	bulk RNA-seq,Homo_723,AD.vs.control; bulk RNA-seq,Homo_723,MCI.vs.control; bulk RNA-seq,Homo_714,MCI.vs.control; bulk RNA-seq,Homo_633,AD.vs.control; bulk RNA-seq,Homo_633,AD.vs.MCI	5
BP	GO:0048304	positive regulation of isotype switching to IgG isotypes	bulk RNA-seq,Homo_723,AD.vs.control; bulk RNA-seq,Homo_723,MCI.vs.control; bulk RNA-seq,Homo_633,AD.vs.control	3
BP	GO:0003323	type B pancreatic cell development	bulk RNA-seq,Homo_723,AD.vs.control; bulk RNA-seq,Homo_723,AD.vs.MCI; bulk RNA-seq,Homo_723,MCI.vs.control	3

BP	GO:0009048	dosage compensation by inactivation of X chromosome	bulk RNA-seq,Homo_723,AD.vs.control; bulk RNA-seq,Homo_714,MCI.vs.control; bulk RNA-seq,Homo_633,AD.vs.control; bulk RNA-seq,Homo_633,AD.vs.MCI; bulk RNA-seq,SRP325058,AD.vs.control	5
MF	GO:0070064	proline-rich region binding	bulk RNA-seq,Homo_723,AD.vs.control; bulk RNA-seq,Homo_723,AD.vs.MCI; bulk RNA-seq,Homo_723,MCI.vs.control; bulk RNA-seq,Homo_714,AD.vs.MCI; bulk RNA-seq,Homo_714,MCI.vs.control	5
BP	GO:0097484	dendrite extension	bulk RNA-seq,Homo_723,AD.vs.control; bulk RNA-seq,Homo_723,AD.vs.MCI; bulk RNA-seq,Homo_723,MCI.vs.control; bulk RNA-seq,Homo_714,AD.vs.MCI; bulk RNA-seq,Homo_633,AD.vs.control	5
BP	GO:0008212	mineralocorticoid metabolic process	bulk RNA-seq,Homo_723,AD.vs.control; bulk RNA-seq,Homo_723,AD.vs.MCI; bulk RNA-seq,Homo_723,MCI.vs.control; bulk RNA-seq,Homo_714,AD.vs.MCI; bulk RNA-seq,Homo_714,MCI.vs.control	5
BP	GO:0032332	positive regulation of chondrocyte differentiation	bulk RNA-seq,Homo_723,AD.vs.control; bulk RNA-seq,Homo_723,AD.vs.MCI; bulk RNA-seq,Homo_723,MCI.vs.control; bulk RNA-seq,Homo_714,AD.vs.MCI; bulk RNA-seq,Homo_714,MCI.vs.control	5
BP	GO:0045725	positive regulation of glycogen biosynthetic process	bulk RNA-seq,Homo_723,AD.vs.control	1
BP	GO:0070831	basement membrane assembly	bulk RNA-seq,Homo_723,AD.vs.control; bulk RNA-seq,Homo_723,AD.vs.MCI; bulk RNA-seq,Homo_723,MCI.vs.control; bulk RNA-seq,Homo_714,AD.vs.MCI; bulk RNA-seq,Homo_714,MCI.vs.control; bulk RNA-seq,Homo_633,AD.vs.control	6
BP	GO:0033522	histone H2A ubiquitination	bulk RNA-seq,Homo_723,AD.vs.control; bulk RNA-seq,Homo_723,MCI.vs.control; bulk RNA-seq,Homo_714,MCI.vs.control; bulk RNA-seq,Homo_633,AD.vs.control; bulk RNA-seq,Homo_633,MCI.vs.control	5
BP	GO:0006607	NLS-bearing protein import into nucleus	bulk RNA-seq,Homo_723,AD.vs.control; bulk RNA-seq,Homo_633,AD.vs.control; bulk RNA-seq,Homo_633,AD.vs.MCI	3
BP	GO:0061036	positive regulation of cartilage development	bulk RNA-seq,Homo_723,AD.vs.control; bulk RNA-seq,Homo_723,AD.vs.MCI; bulk RNA-seq,Homo_723,MCI.vs.control; bulk RNA-seq,Homo_714,AD.vs.MCI; bulk RNA-seq,Homo_714,MCI.vs.control; bulk RNA-seq,Homo_633,AD.vs.control	6
BP	GO:0002720	positive regulation of cytokine production involved in immune response	bulk RNA-seq,Homo_723,AD.vs.control; bulk RNA-seq,Homo_723,MCI.vs.control; bulk RNA-seq,Homo_714,MCI.vs.control; bulk RNA-seq,Homo_633,AD.vs.control; bulk RNA-seq,Homo_633,AD.vs.MCI	5
BP	GO:0015874	norepinephrine transport	bulk RNA-seq,Homo_723,AD.vs.control	1
MF	GO:0140767	enzyme-substrate adaptor activity	bulk RNA-seq,Homo_723,AD.vs.control; bulk RNA-seq,Homo_723,AD.vs.MCI; bulk RNA-seq,Homo_723,MCI.vs.control; bulk RNA-seq,Homo_714,AD.vs.MCI; bulk RNA-seq,Homo_714,MCI.vs.control; bulk RNA-seq,Homo_633,AD.vs.control	6
BP	GO:0001573	ganglioside metabolic process	bulk RNA-seq,Homo_723,AD.vs.control; bulk RNA-seq,Homo_723,AD.vs.MCI; bulk RNA-seq,Homo_723,MCI.vs.control; bulk RNA-	4
MF	GO:0004177	aminopeptidase activity	bulk RNA-seq,Homo_723,AD.vs.control; bulk RNA-seq,Homo_723,MCI.vs.control; bulk RNA-seq,Homo_714,MCI.vs.control; bulk RNA-seq,Homo_633,AD.vs.control; bulk RNA-seq,Homo_633,AD.vs.MCI	5
MF	GO:0043295	glutathione binding	bulk RNA-seq,Homo_723,AD.vs.control; bulk RNA-seq,Homo_633,AD.vs.control; bulk RNA-seq,Homo_633,AD.vs.MCI	3
CC	GO:0098576	lumenal side of membrane	bulk RNA-seq,Homo_723,AD.vs.control; bulk RNA-seq,Homo_723,MCI.vs.control; bulk RNA-seq,Homo_714,MCI.vs.control; bulk RNA-seq,Homo_633,AD.vs.control; scRNA-seq,SRP215507,CD8+ T cell_3-AD.vs.control; scRNA-seq,SRP215507,CD8+ T cell_3-MCI.vs.control	6
BP	GO:0003181	atrioventricular valve morphogenesis	bulk RNA-seq,Homo_723,AD.vs.control; bulk RNA-seq,Homo_723,AD.vs.MCI; bulk RNA-seq,Homo_723,MCI.vs.control; bulk RNA-seq,Homo_714,AD.vs.MCI; bulk RNA-seq,Homo_714,MCI.vs.control	5
MF	GO:0031267	small GTPase binding	bulk RNA-seq,Homo_723,AD.vs.control; bulk RNA-seq,Homo_723,AD.vs.MCI; bulk RNA-seq,Homo_723,MCI.vs.control; bulk RNA-seq,Homo_714,AD.vs.MCI; bulk RNA-seq,Homo_633,AD.vs.control; bulk RNA-seq,Homo_633,AD.vs.MCI	6
BP	GO:2000344	positive regulation of acrosome reaction	bulk RNA-seq,Homo_723,AD.vs.control	1
BP	GO:0060788	ectodermal placode formation	bulk RNA-seq,Homo_723,AD.vs.control; bulk RNA-seq,Homo_723,AD.vs.MCI; bulk RNA-seq,Homo_723,MCI.vs.control; bulk RNA-seq,Homo_714,AD.vs.MCI; bulk RNA-seq,Homo_714,MCI.vs.control; bulk RNA-seq,Homo_633,MCI.vs.control	6
BP	GO:0071697	ectodermal placode morphogenesis	bulk RNA-seq,Homo_723,AD.vs.control; bulk RNA-seq,Homo_723,AD.vs.MCI; bulk RNA-seq,Homo_723,MCI.vs.control; bulk RNA-seq,Homo_714,AD.vs.MCI; bulk RNA-seq,Homo_714,MCI.vs.control; bulk RNA-seq,Homo_633,MCI.vs.control	6
BP	GO:0010739	positive regulation of protein kinase A signaling	bulk RNA-seq,Homo_723,AD.vs.control; bulk RNA-seq,Homo_723,AD.vs.MCI	2
BP	GO:0007379	segment specification	bulk RNA-seq,Homo_723,AD.vs.control; bulk RNA-seq,Homo_723,AD.vs.MCI; bulk RNA-seq,Homo_723,MCI.vs.control; bulk RNA-seq,Homo_714,AD.vs.MCI; bulk RNA-seq,Homo_714,MCI.vs.control	5
BP	GO:0045198	establishment of epithelial cell apical/basal polarity	bulk RNA-seq,Homo_723,AD.vs.control; bulk RNA-seq,Homo_723,AD.vs.MCI; bulk RNA-seq,Homo_723,MCI.vs.control	3
MF	GO:0051879	Hsp90 protein binding	bulk RNA-seq,Homo_723,AD.vs.control; bulk RNA-seq,Homo_723,MCI.vs.control; bulk RNA-seq,Homo_714,AD.vs.control; bulk RNA-seq,Homo_714,MCI.vs.control; bulk RNA-seq,Homo_633,AD.vs.control; bulk RNA-seq,Homo_633,AD.vs.MCI	6
BP	GO:0048814	regulation of dendrite morphogenesis	bulk RNA-seq,Homo_723,AD.vs.control; bulk RNA-seq,Homo_723,AD.vs.MCI; bulk RNA-seq,Homo_723,MCI.vs.control; bulk RNA-seq,Homo_714,AD.vs.MCI; bulk RNA-seq,Homo_714,MCI.vs.control; bulk RNA-seq,Homo_633,AD.vs.control; bulk RNA-seq,Homo_633,AD.vs.MCI	7
CC	GO:0106068	SUMO ligase complex	bulk RNA-seq,Homo_723,AD.vs.control	1
BP	GO:0001522	pseudouridine synthesis	bulk RNA-seq,Homo_723,AD.vs.control; bulk RNA-seq,Homo_723,MCI.vs.control; bulk RNA-seq,Homo_714,MCI.vs.control; bulk RNA-seq,Homo_633,AD.vs.control; bulk RNA-seq,Homo_633,AD.vs.MCI; bulk RNA-seq,Homo_633,MCI.vs.control	6
MF	GO:0016860	intramolecular oxidoreductase activity	bulk RNA-seq,Homo_723,AD.vs.control; bulk RNA-seq,Homo_723,MCI.vs.control; bulk RNA-seq,Homo_714,MCI.vs.control; bulk RNA-seq,Homo_633,AD.vs.control; bulk RNA-seq,Homo_633,AD.vs.MCI; scRNA-seq,SRP330776,Natural killer cell_1-AD.vs.control	6
BP	GO:0008360	regulation of cell shape	bulk RNA-seq,Homo_723,AD.vs.control; bulk RNA-seq,Homo_723,AD.vs.MCI; bulk RNA-seq,Homo_723,MCI.vs.control; bulk RNA-seq,Homo_714,AD.vs.MCI; bulk RNA-seq,Homo_633,AD.vs.control; bulk RNA-seq,Homo_633,AD.vs.MCI	6
BP	GO:0006098	pentose-phosphate shunt	bulk RNA-seq,Homo_723,AD.vs.control	1
CC	GO:0005921	gap junction	bulk RNA-seq,Homo_723,AD.vs.control; bulk RNA-seq,Homo_723,AD.vs.MCI; bulk RNA-seq,Homo_723,MCI.vs.control; bulk RNA-seq,Homo_714,AD.vs.MCI; bulk RNA-seq,Homo_714,MCI.vs.control	5
BP	GO:0002224	toll-like receptor signaling pathway	bulk RNA-seq,Homo_723,AD.vs.control; bulk RNA-seq,Homo_723,MCI.vs.control; bulk RNA-seq,Homo_714,MCI.vs.control; bulk RNA-seq,Homo_633,AD.vs.control; bulk RNA-seq,Homo_633,AD.vs.MCI	5
CC	GO:1902911	protein kinase complex	bulk RNA-seq,Homo_723,AD.vs.control; bulk RNA-seq,Homo_723,MCI.vs.control; bulk RNA-seq,Homo_633,AD.vs.control; bulk RNA-	4
BP	GO:0055091	phospholipid homeostasis	bulk RNA-seq,Homo_723,AD.vs.control; bulk RNA-seq,Homo_723,AD.vs.MCI; bulk RNA-seq,Homo_723,MCI.vs.control; bulk RNA-seq,Homo_714,AD.vs.MCI	4
MF	GO:0004861	cyclin-dependent protein serine/threonine kinase inhibitor	bulk RNA-seq,Homo_723,AD.vs.control; bulk RNA-seq,Homo_633,AD.vs.control; bulk RNA-seq,Homo_633,AD.vs.MCI	3
BP	GO:1905699	regulation of xenobiotic detoxification by transmembrane export across the plasma membrane	bulk RNA-seq,Homo_723,AD.vs.control; bulk RNA-seq,Homo_714,AD.vs.control; bulk RNA-seq,Homo_633,AD.vs.control; bulk RNA-seq,Homo_633,AD.vs.MCI	4

BP	GO:1905700	negative regulation of xenobiotic detoxification by transmembrane export across the plasma membrane	bulk RNA-seq,Homo_723,AD.vs.control; bulk RNA-seq,Homo_714,AD.vs.control; bulk RNA-seq,Homo_633,AD.vs.control; bulk RNA-seq,Homo_633,AD.vs.MCI	4
BP	GO:2001024	negative regulation of response to drug	bulk RNA-seq,Homo_723,AD.vs.control; bulk RNA-seq,Homo_714,AD.vs.control; bulk RNA-seq,Homo_633,AD.vs.control; bulk RNA-seq,Homo_633,AD.vs.MCI	4
BP	GO:0060749	mammary gland alveolus development	bulk RNA-seq,Homo_723,AD.vs.control; bulk RNA-seq,Homo_723,AD.vs.MCI; bulk RNA-seq,Homo_723,MCI.vs.control; bulk RNA-seq,Homo_714,AD.vs.MCI; bulk RNA-seq,Homo_714,MCI.vs.control; bulk RNA-seq,Homo_633,AD.vs.control	6
BP	GO:0061377	mammary gland lobule development	bulk RNA-seq,Homo_723,AD.vs.control; bulk RNA-seq,Homo_723,AD.vs.MCI; bulk RNA-seq,Homo_723,MCI.vs.control; bulk RNA-seq,Homo_714,AD.vs.MCI; bulk RNA-seq,Homo_714,MCI.vs.control; bulk RNA-seq,Homo_633,AD.vs.control	6
BP	GO:0071233	cellular response to leucine	bulk RNA-seq,Homo_723,AD.vs.control; bulk RNA-seq,Homo_723,MCI.vs.control; bulk RNA-seq,Homo_633,AD.vs.control; bulk RNA-	4
MF	GO:0019955	cytokine binding	bulk RNA-seq,Homo_723,AD.vs.control; bulk RNA-seq,Homo_723,AD.vs.MCI; bulk RNA-seq,Homo_723,MCI.vs.control; bulk RNA-seq,Homo_714,AD.vs.control; bulk RNA-seq,Homo_714,AD.vs.MCI; bulk RNA-seq,Homo_633,AD.vs.control; bulk RNA-seq,Homo_633,AD.vs.MCI	7
BP	GO:1903441	protein localization to ciliary membrane	bulk RNA-seq,Homo_723,AD.vs.control; bulk RNA-seq,Homo_723,MCI.vs.control; bulk RNA-seq,Homo_714,AD.vs.control; bulk RNA-seq,Homo_714,MCI.vs.control; bulk RNA-seq,Homo_633,AD.vs.control; bulk RNA-seq,Homo_633,AD.vs.MCI; bulk RNA-seq,Homo_633,MCI.vs.control	7
BP	GO:0046890	regulation of lipid biosynthetic process	bulk RNA-seq,Homo_723,AD.vs.control; bulk RNA-seq,Homo_723,AD.vs.MCI; bulk RNA-seq,Homo_723,MCI.vs.control; bulk RNA-seq,Homo_714,AD.vs.control; bulk RNA-seq,Homo_714,AD.vs.MCI; bulk RNA-seq,Homo_633,AD.vs.control; bulk RNA-seq,Homo_633,AD.vs.MCI	7
BP	GO:0032984	protein-containing complex disassembly	bulk RNA-seq,Homo_723,AD.vs.control; bulk RNA-seq,Homo_723,AD.vs.MCI; bulk RNA-seq,Homo_723,MCI.vs.control; bulk RNA-seq,Homo_714,AD.vs.control; bulk RNA-seq,Homo_633,AD.vs.control; bulk RNA-seq,Homo_633,AD.vs.MCI	6
BP	GO:0001946	lymphangiogenesis	bulk RNA-seq,Homo_723,AD.vs.control; bulk RNA-seq,Homo_723,AD.vs.MCI; bulk RNA-seq,Homo_723,MCI.vs.control; bulk RNA-seq,Homo_714,AD.vs.MCI; bulk RNA-seq,Homo_714,MCI.vs.control	5
BP	GO:0061162	establishment of monopolar cell polarity	bulk RNA-seq,Homo_723,AD.vs.control; bulk RNA-seq,Homo_723,AD.vs.MCI; bulk RNA-seq,Homo_723,MCI.vs.control	3
BP	GO:0010770	positive regulation of cell morphogenesis involved in differentiation	bulk RNA-seq,Homo_723,AD.vs.control; bulk RNA-seq,Homo_723,AD.vs.MCI; bulk RNA-seq,Homo_723,MCI.vs.control; bulk RNA-seq,Homo_714,AD.vs.MCI; bulk RNA-seq,Homo_714,MCI.vs.control; bulk RNA-seq,Homo_633,AD.vs.control; bulk RNA-seq,Homo_633,AD.vs.MCI	7
BP	GO:0060245	detection of cell density	bulk RNA-seq,Homo_723,AD.vs.control; bulk RNA-seq,Homo_723,AD.vs.MCI; bulk RNA-seq,Homo_723,MCI.vs.control; bulk RNA-seq,Homo_714,AD.vs.MCI; bulk RNA-seq,Homo_714,MCI.vs.control	5
BP	GO:0042149	cellular response to glucose starvation	bulk RNA-seq,Homo_723,AD.vs.control; bulk RNA-seq,Homo_723,MCI.vs.control; bulk RNA-seq,Homo_714,AD.vs.control; bulk RNA-seq,Homo_714,MCI.vs.control; bulk RNA-seq,Homo_633,AD.vs.control; bulk RNA-seq,Homo_633,AD.vs.MCI	6
BP	GO:0060977	coronary vasculature morphogenesis	bulk RNA-seq,Homo_723,AD.vs.control; bulk RNA-seq,Homo_723,AD.vs.MCI; bulk RNA-seq,Homo_723,MCI.vs.control; bulk RNA-seq,Homo_714,AD.vs.MCI; bulk RNA-seq,Homo_714,MCI.vs.control; bulk RNA-seq,Homo_633,AD.vs.control; bulk RNA-seq,Homo_633,AD.vs.MCI	7
BP	GO:0008340	determination of adult lifespan	bulk RNA-seq,Homo_723,AD.vs.control; bulk RNA-seq,Homo_723,MCI.vs.control; bulk RNA-seq,Homo_714,MCI.vs.control; bulk RNA-seq,Homo_633,AD.vs.control; bulk RNA-seq,Homo_633,MCI.vs.control	5
MF	GO:0008574	plus-end-directed microtubule motor activity	bulk RNA-seq,Homo_723,AD.vs.control; bulk RNA-seq,Homo_723,AD.vs.MCI; bulk RNA-seq,Homo_723,MCI.vs.control; bulk RNA-	4
BP	GO:0046033	AMP metabolic process	bulk RNA-seq,Homo_723,AD.vs.control; bulk RNA-seq,Homo_723,MCI.vs.control; bulk RNA-seq,Homo_714,MCI.vs.control; bulk RNA-seq,Homo_633,AD.vs.control; bulk RNA-seq,Homo_633,AD.vs.MCI; bulk RNA-seq,Homo_633,MCI.vs.control	6
BP	GO:1903588	negative regulation of blood vessel endothelial cell proliferation involved in sprouting angiogenesis	bulk RNA-seq,Homo_723,AD.vs.control; bulk RNA-seq,Homo_714,AD.vs.control; bulk RNA-seq,Homo_714,AD.vs.MCI	3
CC	GO:0046540	U4/U6 x U5 tri-snRNP complex	bulk RNA-seq,Homo_723,AD.vs.control; bulk RNA-seq,Homo_714,MCI.vs.control; bulk RNA-seq,Homo_633,AD.vs.control; bulk RNA-	4
CC	GO:0097526	spliceosomal tri-snRNP complex	bulk RNA-seq,Homo_723,AD.vs.control; bulk RNA-seq,Homo_714,MCI.vs.control; bulk RNA-seq,Homo_633,AD.vs.control; bulk RNA-	4
BP	GO:0061098	positive regulation of protein tyrosine kinase activity	bulk RNA-seq,Homo_723,AD.vs.control; bulk RNA-seq,Homo_723,AD.vs.MCI; bulk RNA-seq,Homo_723,MCI.vs.control; bulk RNA-seq,Homo_714,AD.vs.MCI; bulk RNA-seq,Homo_714,MCI.vs.control; bulk RNA-seq,Homo_633,AD.vs.control; bulk RNA-seq,Homo_633,AD.vs.MCI	7
BP	GO:1903817	negative regulation of voltage-gated potassium channel	bulk RNA-seq,Homo_723,AD.vs.control; bulk RNA-seq,Homo_714,AD.vs.control; bulk RNA-seq,Homo_714,AD.vs.MCI	3
BP	GO:0043484	regulation of RNA splicing	bulk RNA-seq,Homo_723,AD.vs.control; bulk RNA-seq,Homo_723,MCI.vs.control; bulk RNA-seq,Homo_714,AD.vs.control; bulk RNA-seq,Homo_633,AD.vs.control; bulk RNA-seq,Homo_633,AD.vs.MCI	5
BP	GO:0070207	protein homotrimerization	bulk RNA-seq,Homo_723,AD.vs.control; bulk RNA-seq,Homo_723,AD.vs.MCI; bulk RNA-seq,Homo_723,MCI.vs.control; bulk RNA-	4
BP	GO:0002716	negative regulation of natural killer cell mediated immunity	bulk RNA-seq,Homo_723,AD.vs.control; bulk RNA-seq,Homo_633,AD.vs.control	2
BP	GO:0010524	positive regulation of calcium ion transport into cytosol	bulk RNA-seq,Homo_723,AD.vs.control; bulk RNA-seq,Homo_723,AD.vs.MCI; bulk RNA-seq,Homo_723,MCI.vs.control	3
BP	GO:0051386	regulation of neurotrophin TRK receptor signaling pathway	bulk RNA-seq,Homo_723,AD.vs.control	1
BP	GO:1902093	positive regulation of flagellated sperm motility	bulk RNA-seq,Homo_723,AD.vs.control; bulk RNA-seq,Homo_723,AD.vs.MCI	2
BP	GO:2000155	positive regulation of cilium-dependent cell motility	bulk RNA-seq,Homo_723,AD.vs.control; bulk RNA-seq,Homo_723,AD.vs.MCI	2
BP	GO:0062149	detection of stimulus involved in sensory perception of pain	bulk RNA-seq,Homo_723,AD.vs.control; bulk RNA-seq,Homo_723,AD.vs.MCI; bulk RNA-seq,Homo_723,MCI.vs.control; bulk RNA-seq,Homo_714,AD.vs.MCI; bulk RNA-seq,Homo_714,MCI.vs.control	5
BP	GO:0060218	hematopoietic stem cell differentiation	bulk RNA-seq,Homo_723,AD.vs.control; bulk RNA-seq,Homo_723,MCI.vs.control; bulk RNA-seq,Homo_714,MCI.vs.control; bulk RNA-seq,Homo_633,AD.vs.control; bulk RNA-seq,Homo_633,AD.vs.MCI	5
BP	GO:0034340	response to type I interferon	bulk RNA-seq,Homo_723,AD.vs.control; bulk RNA-seq,Homo_723,MCI.vs.control; bulk RNA-seq,Homo_714,MCI.vs.control; bulk RNA-	4
BP	GO:0051133	regulation of NK T cell activation	bulk RNA-seq,Homo_723,AD.vs.control	1
MF	GO:0016653	oxidoreductase activity, acting on NAD(P)H, heme protein as	bulk RNA-seq,Homo_723,AD.vs.control; bulk RNA-seq,Homo_723,MCI.vs.control; bulk RNA-seq,Homo_714,MCI.vs.control; bulk RNA-	4
BP	GO:0051583	dopamine uptake involved in synaptic transmission	bulk RNA-seq,Homo_723,AD.vs.control; bulk RNA-seq,Homo_633,MCI.vs.control	2
BP	GO:0051934	catecholamine uptake involved in synaptic transmission	bulk RNA-seq,Homo_723,AD.vs.control; bulk RNA-seq,Homo_633,MCI.vs.control	2
BP	GO:0072070	loop of Henle development	bulk RNA-seq,Homo_723,AD.vs.control; bulk RNA-seq,Homo_723,AD.vs.MCI; bulk RNA-seq,Homo_723,MCI.vs.control; bulk RNA-seq,Homo_714,AD.vs.MCI	4
BP	GO:0001990	regulation of systemic arterial blood pressure by hormone	bulk RNA-seq,Homo_723,AD.vs.control; bulk RNA-seq,Homo_723,AD.vs.MCI; bulk RNA-seq,Homo_714,AD.vs.MCI	3
BP	GO:0008637	apoptotic mitochondrial changes	bulk RNA-seq,Homo_723,AD.vs.control; bulk RNA-seq,Homo_723,MCI.vs.control; bulk RNA-seq,Homo_714,AD.vs.control; bulk RNA-seq,Homo_714,MCI.vs.control; bulk RNA-seq,Homo_633,AD.vs.control; bulk RNA-seq,Homo_633,AD.vs.MCI	6
BP	GO:0060999	positive regulation of dendritic spine development	bulk RNA-seq,Homo_723,AD.vs.control; bulk RNA-seq,Homo_723,AD.vs.MCI; bulk RNA-seq,Homo_723,MCI.vs.control; bulk RNA-seq,Homo_714,AD.vs.MCI; bulk RNA-seq,Homo_714,MCI.vs.control; bulk RNA-seq,Homo_633,AD.vs.control; bulk RNA-seq,Homo_633,AD.vs.MCI	7

CC	GO:0099144	anchored component of synaptic membrane	bulk RNA-seq,Homo_723,AD.vs.control; bulk RNA-seq,Homo_723,AD.vs.MCI	2
BP	GO:0002474	antigen processing and presentation of peptide antigen via MHC class I	bulk RNA-seq,Homo_723,AD.vs.control; bulk RNA-seq,Homo_723,MCI.vs.control; bulk RNA-seq,Homo_714,MCI.vs.control; bulk RNA-seq,Homo_633,AD.vs.control; bulk RNA-seq,Homo_633,AD.vs.MCI; bulk RNA-seq,Homo_633,MCI.vs.control	6
MF	GO:0005540	hyaluronic acid binding	bulk RNA-seq,Homo_723,AD.vs.control; bulk RNA-seq,Homo_723,AD.vs.MCI; bulk RNA-seq,Homo_723,MCI.vs.control; bulk RNA-seq,Homo_714,AD.vs.MCI	4
BP	GO:0030859	polarized epithelial cell differentiation	bulk RNA-seq,Homo_723,AD.vs.control; bulk RNA-seq,Homo_723,AD.vs.MCI; bulk RNA-seq,Homo_723,MCI.vs.control; bulk RNA-	4
BP	GO:2000563	positive regulation of CD4-positive, alpha-beta T cell	bulk RNA-seq,Homo_723,AD.vs.control; bulk RNA-seq,Homo_714,MCI.vs.control; bulk RNA-seq,Homo_633,AD.vs.control; bulk RNA-	4
BP	GO:1904749	regulation of protein localization to nucleolus	bulk RNA-seq,Homo_723,AD.vs.control; bulk RNA-seq,Homo_633,AD.vs.control	2
CC	GO:0000228	nuclear chromosome	bulk RNA-seq,Homo_723,AD.vs.control; bulk RNA-seq,Homo_723,MCI.vs.control; bulk RNA-seq,Homo_714,AD.vs.control; bulk RNA-seq,Homo_633,AD.vs.control; bulk RNA-seq,Homo_633,AD.vs.MCI	5
BP	GO:0061951	establishment of protein localization to plasma membrane	bulk RNA-seq,Homo_723,AD.vs.control; bulk RNA-seq,Homo_723,MCI.vs.control; bulk RNA-seq,Homo_714,AD.vs.control; bulk RNA-seq,Homo_714,MCI.vs.control; bulk RNA-seq,Homo_633,AD.vs.control; bulk RNA-seq,Homo_633,AD.vs.MCI	6
MF	GO:0008307	structural constituent of muscle	bulk RNA-seq,Homo_723,AD.vs.control; bulk RNA-seq,Homo_723,AD.vs.MCI; bulk RNA-seq,Homo_723,MCI.vs.control; bulk RNA-seq,Homo_714,AD.vs.MCI; bulk RNA-seq,Homo_714,MCI.vs.control; bulk RNA-seq,Homo_633,AD.vs.control; bulk RNA-seq,Homo_633,AD.vs.MCI	7
MF	GO:0051393	alpha-actinin binding	bulk RNA-seq,Homo_723,AD.vs.control; bulk RNA-seq,Homo_723,AD.vs.MCI; bulk RNA-seq,Homo_723,MCI.vs.control; bulk RNA-seq,Homo_714,AD.vs.MCI; bulk RNA-seq,Homo_714,MCI.vs.control	5
BP	GO:1903428	positive regulation of reactive oxygen species biosynthetic process	bulk RNA-seq,Homo_723,AD.vs.control; bulk RNA-seq,Homo_723,AD.vs.MCI; bulk RNA-seq,Homo_723,MCI.vs.control; bulk RNA-seq,Homo_714,AD.vs.MCI; bulk RNA-seq,Homo_714,MCI.vs.control; bulk RNA-seq,Homo_633,AD.vs.control; bulk RNA-seq,Homo_633,AD.vs.MCI	7
BP	GO:0061339	establishment or maintenance of monopolar cell polarity	bulk RNA-seq,Homo_723,AD.vs.control; bulk RNA-seq,Homo_723,AD.vs.MCI; bulk RNA-seq,Homo_723,MCI.vs.control	3
BP	GO:0034114	regulation of heterotypic cell-cell adhesion	bulk RNA-seq,Homo_723,AD.vs.control; bulk RNA-seq,Homo_723,AD.vs.MCI; bulk RNA-seq,Homo_723,MCI.vs.control; bulk RNA-seq,Homo_714,AD.vs.MCI; bulk RNA-seq,Homo_714,MCI.vs.control; bulk RNA-seq,Homo_633,AD.vs.MCI	6
BP	GO:1905563	negative regulation of vascular endothelial cell proliferation	bulk RNA-seq,Homo_723,AD.vs.control; bulk RNA-seq,Homo_714,AD.vs.control	2
BP	GO:1903320	regulation of protein modification by small protein conjugation or removal	bulk RNA-seq,Homo_723,AD.vs.control; bulk RNA-seq,Homo_723,MCI.vs.control; bulk RNA-seq,Homo_633,AD.vs.control; bulk RNA-seq,Homo_633,AD.vs.MCI	4
CC	GO:0005925	focal adhesion	bulk RNA-seq,Homo_723,AD.vs.MCI; bulk RNA-seq,Homo_714,AD.vs.MCI; bulk RNA-seq,Homo_633,AD.vs.control; bulk RNA-seq,Homo_633,AD.vs.MCI	4
BP	GO:0051056	regulation of small GTPase mediated signal transduction	bulk RNA-seq,Homo_723,AD.vs.MCI; bulk RNA-seq,Homo_723,MCI.vs.control; bulk RNA-seq,Homo_714,AD.vs.MCI; bulk RNA-seq,Homo_633,AD.vs.control; bulk RNA-seq,Homo_633,AD.vs.MCI; bulk RNA-seq,SRP223445,AD.vs.control	6
BP	GO:0007265	Ras protein signal transduction	bulk RNA-seq,Homo_723,AD.vs.MCI; bulk RNA-seq,Homo_714,AD.vs.MCI; bulk RNA-seq,Homo_633,AD.vs.control; bulk RNA-seq,Homo_633,AD.vs.MCI	4
MF	GO:0005543	phospholipid binding	bulk RNA-seq,Homo_723,AD.vs.MCI; bulk RNA-seq,Homo_714,AD.vs.MCI; bulk RNA-seq,Homo_633,AD.vs.control; bulk RNA-seq,Homo_633,AD.vs.MCI	4
BP	GO:0043087	regulation of GTPase activity	bulk RNA-seq,Homo_723,AD.vs.MCI; bulk RNA-seq,Homo_714,AD.vs.MCI; bulk RNA-seq,Homo_633,AD.vs.control; bulk RNA-seq,Homo_633,AD.vs.MCI	4
MF	GO:0015631	tubulin binding	bulk RNA-seq,Homo_723,AD.vs.MCI; bulk RNA-seq,Homo_714,AD.vs.MCI; bulk RNA-seq,Homo_633,AD.vs.control; bulk RNA-seq,Homo_633,AD.vs.MCI	4
BP	GO:0051258	protein polymerization	bulk RNA-seq,Homo_723,AD.vs.MCI; bulk RNA-seq,Homo_723,MCI.vs.control; bulk RNA-seq,Homo_714,AD.vs.MCI; bulk RNA-seq,Homo_633,AD.vs.control; bulk RNA-seq,Homo_633,AD.vs.MCI	5
BP	GO:0070482	response to oxygen levels	bulk RNA-seq,Homo_723,AD.vs.MCI; bulk RNA-seq,Homo_714,AD.vs.MCI; bulk RNA-seq,Homo_633,AD.vs.control; bulk RNA-seq,Homo_633,AD.vs.MCI	4
BP	GO:1901653	cellular response to peptide	bulk RNA-seq,Homo_723,AD.vs.MCI; bulk RNA-seq,Homo_714,AD.vs.MCI; bulk RNA-seq,Homo_633,AD.vs.control; bulk RNA-seq,Homo_633,AD.vs.MCI	4
BP	GO:0001701	in utero embryonic development	bulk RNA-seq,Homo_723,AD.vs.MCI; bulk RNA-seq,Homo_714,AD.vs.MCI; bulk RNA-seq,Homo_633,AD.vs.control; bulk RNA-seq,Homo_633,AD.vs.MCI	4
BP	GO:0022407	regulation of cell-cell adhesion	bulk RNA-seq,Homo_723,AD.vs.MCI; bulk RNA-seq,Homo_714,AD.vs.MCI; bulk RNA-seq,Homo_633,AD.vs.control; bulk RNA-seq,Homo_633,AD.vs.MCI; scRNA-seq,SRP330776,CD8+ T cell_1-AD.vs.control; scRNA-seq,SRP215507,CD8+ T cell_3-AD.vs.control	6
BP	GO:0045936	negative regulation of phosphate metabolic process	bulk RNA-seq,Homo_723,AD.vs.MCI; bulk RNA-seq,Homo_714,AD.vs.MCI; bulk RNA-seq,Homo_633,AD.vs.control; bulk RNA-seq,Homo_633,AD.vs.MCI	4
BP	GO:0022613	ribonucleoprotein complex biogenesis	bulk RNA-seq,Homo_723,AD.vs.MCI; bulk RNA-seq,Homo_714,AD.vs.control; bulk RNA-seq,Homo_714,AD.vs.MCI; bulk RNA-seq,Homo_633,AD.vs.control; bulk RNA-seq,Homo_633,AD.vs.MCI; bulk RNA-seq,SRP223445,AD.vs.control; scRNA-seq,SRP309935,Megakaryocyte_2-AD.vs.control	7
MF	GO:0030695	GTPase regulator activity	bulk RNA-seq,Homo_723,AD.vs.MCI; bulk RNA-seq,Homo_714,AD.vs.MCI; bulk RNA-seq,Homo_633,AD.vs.control; bulk RNA-seq,Homo_633,AD.vs.MCI	4
MF	GO:0060589	nucleoside-triphosphatase regulator activity	bulk RNA-seq,Homo_723,AD.vs.MCI; bulk RNA-seq,Homo_714,AD.vs.MCI; bulk RNA-seq,Homo_633,AD.vs.control; bulk RNA-seq,Homo_633,AD.vs.MCI	4
BP	GO:0010563	negative regulation of phosphorus metabolic process	bulk RNA-seq,Homo_723,AD.vs.MCI; bulk RNA-seq,Homo_714,AD.vs.MCI; bulk RNA-seq,Homo_633,AD.vs.control; bulk RNA-seq,Homo_633,AD.vs.MCI	4
BP	GO:1901605	alpha-amino acid metabolic process	bulk RNA-seq,Homo_723,AD.vs.MCI; bulk RNA-seq,Homo_723,MCI.vs.control; bulk RNA-seq,Homo_714,AD.vs.MCI; bulk RNA-seq,Homo_633,AD.vs.control; bulk RNA-seq,Homo_633,AD.vs.MCI	5
BP	GO:0051223	regulation of protein transport	bulk RNA-seq,Homo_723,AD.vs.MCI; bulk RNA-seq,Homo_714,AD.vs.MCI; bulk RNA-seq,Homo_633,AD.vs.control; bulk RNA-seq,Homo_633,AD.vs.MCI	4
BP	GO:0043254	regulation of protein-containing complex assembly	bulk RNA-seq,Homo_723,AD.vs.MCI; bulk RNA-seq,Homo_714,AD.vs.MCI; bulk RNA-seq,Homo_633,AD.vs.control; bulk RNA-seq,Homo_633,AD.vs.MCI	4
BP	GO:0034470	ncRNA processing	bulk RNA-seq,Homo_723,AD.vs.MCI; bulk RNA-seq,Homo_714,AD.vs.control; bulk RNA-seq,Homo_714,AD.vs.MCI; bulk RNA-seq,Homo_633,AD.vs.control; bulk RNA-seq,Homo_633,AD.vs.MCI; bulk RNA-seq,SRP223445,AD.vs.control; scRNA-seq,SRP309935,Megakaryocyte_2-AD.vs.control	7
BP	GO:1903829	positive regulation of protein localization	bulk RNA-seq,Homo_723,AD.vs.MCI; bulk RNA-seq,Homo_714,AD.vs.MCI; bulk RNA-seq,Homo_633,AD.vs.control; bulk RNA-seq,Homo_633,AD.vs.MCI	4
BP	GO:0002460	adaptive immune response based on somatic recombination of immune receptors built from immunoglobulin superfamily	bulk RNA-seq,Homo_723,AD.vs.MCI; bulk RNA-seq,Homo_714,AD.vs.control; bulk RNA-seq,Homo_633,AD.vs.control; bulk RNA-seq,Homo_633,AD.vs.MCI; scRNA-seq,SRP309935,Monocyte_2-AD.vs.control; scRNA-seq,SRP215507,CD8+ T cell_3-AD.vs.control; scRNA-seq,SRP215507,CD8+ T cell_3-MCI.vs.control	7
BP	GO:0009314	response to radiation	bulk RNA-seq,Homo_723,AD.vs.MCI; bulk RNA-seq,Homo_714,AD.vs.MCI; bulk RNA-seq,Homo_633,AD.vs.control; bulk RNA-seq,Homo_633,AD.vs.MCI	4
MF	GO:0008017	microtubule binding	bulk RNA-seq,Homo_723,AD.vs.MCI; bulk RNA-seq,Homo_723,MCI.vs.control; bulk RNA-seq,Homo_714,AD.vs.MCI; bulk RNA-seq,Homo_633,AD.vs.control; bulk RNA-seq,Homo_633,AD.vs.MCI	5
MF	GO:0004674	protein serine/threonine kinase activity	bulk RNA-seq,Homo_723,AD.vs.MCI; bulk RNA-seq,Homo_714,AD.vs.MCI; bulk RNA-seq,Homo_633,AD.vs.control; bulk RNA-seq,Homo_633,AD.vs.MCI; scRNA-seq,SRP330776,B cell_1-AD.vs.control; scRNA-seq,SRP330776,Naive CD8+ T cell_1-AD.vs.control; scRNA-seq,SRP330776,Natural killer cell_1-	7
MF	GO:0045296	cadherin binding	bulk RNA-seq,Homo_723,AD.vs.MCI; bulk RNA-seq,Homo_723,MCI.vs.control; bulk RNA-seq,Homo_714,AD.vs.MCI; bulk RNA-seq,Homo_633,AD.vs.control; bulk RNA-seq,Homo_633,AD.vs.MCI	5
BP	GO:0045787	positive regulation of cell cycle	bulk RNA-seq,Homo_723,AD.vs.MCI; bulk RNA-seq,Homo_714,AD.vs.MCI; bulk RNA-seq,Homo_633,AD.vs.control; bulk RNA-seq,Homo_633,AD.vs.MCI	4

CC	GO:0005769	early endosome	bulk RNA-seq,Homo_723,AD.vs.MCI; bulk RNA-seq,Homo_714,AD.vs.control; bulk RNA-seq,Homo_714,AD.vs.MCI; bulk RNA-seq,Homo_633,AD.vs.control; bulk RNA-seq,Homo_633,AD.vs.MCI; scRNA-seq,SRP330776,Naive CD8+ T cell_2-AD.vs.control	6
CC	GO:0030139	endocytic vesicle	bulk RNA-seq,Homo_723,AD.vs.MCI; bulk RNA-seq,Homo_714,AD.vs.MCI; bulk RNA-seq,Homo_633,AD.vs.control; bulk RNA-seq,Homo_633,AD.vs.MCI; scRNA-seq,SRP330776,Naive CD8+ T cell_2-AD.vs.control	5
BP	GO:0051098	regulation of binding	bulk RNA-seq,Homo_723,AD.vs.MCI; bulk RNA-seq,Homo_714,AD.vs.MCI; bulk RNA-seq,Homo_633,AD.vs.control; bulk RNA-seq,Homo_633,AD.vs.MCI	4
MF	GO:0016705	oxidoreductase activity, acting on paired donors, with incorporation or reduction of molecular oxygen	bulk RNA-seq,Homo_723,AD.vs.MCI; bulk RNA-seq,Homo_723,MCI.vs.control; bulk RNA-seq,Homo_714,AD.vs.MCI; bulk RNA-seq,Homo_633,AD.vs.control; bulk RNA-seq,Homo_633,AD.vs.MCI; bulk RNA-seq,SRP223445,AD.vs.control	6
BP	GO:0006694	steroid biosynthetic process	bulk RNA-seq,Homo_723,AD.vs.MCI; bulk RNA-seq,Homo_723,MCI.vs.control; bulk RNA-seq,Homo_714,AD.vs.control; bulk RNA-seq,Homo_714,AD.vs.MCI; bulk RNA-seq,Homo_633,AD.vs.control; bulk RNA-seq,Homo_633,AD.vs.MCI	6
BP	GO:0071214	cellular response to abiotic stimulus	bulk RNA-seq,Homo_723,AD.vs.MCI; bulk RNA-seq,Homo_723,MCI.vs.control; bulk RNA-seq,Homo_714,AD.vs.MCI; bulk RNA-seq,Homo_633,AD.vs.control; bulk RNA-seq,Homo_633,AD.vs.MCI	5
BP	GO:0104004	cellular response to environmental stimulus	bulk RNA-seq,Homo_723,AD.vs.MCI; bulk RNA-seq,Homo_723,MCI.vs.control; bulk RNA-seq,Homo_714,AD.vs.MCI; bulk RNA-seq,Homo_633,AD.vs.control; bulk RNA-seq,Homo_633,AD.vs.MCI	5
CC	GO:0001726	ruffle	bulk RNA-seq,Homo_723,AD.vs.MCI; bulk RNA-seq,Homo_723,MCI.vs.control; bulk RNA-seq,Homo_714,AD.vs.MCI; bulk RNA-seq,Homo_633,AD.vs.control; bulk RNA-seq,Homo_633,AD.vs.MCI	5
BP	GO:0098727	maintenance of cell number	bulk RNA-seq,Homo_723,AD.vs.MCI; bulk RNA-seq,Homo_723,MCI.vs.control; bulk RNA-seq,Homo_714,AD.vs.MCI; bulk RNA-seq,Homo_633,AD.vs.control; bulk RNA-seq,Homo_633,AD.vs.MCI	5
BP	GO:0042113	B cell activation	bulk RNA-seq,Homo_723,AD.vs.MCI; bulk RNA-seq,Homo_714,AD.vs.control; bulk RNA-seq,Homo_633,AD.vs.control; bulk RNA-seq,Homo_633,AD.vs.MCI; scRNA-seq,SRP330776,Naive CD8+ T cell_2-AD.vs.control; scRNA-seq,SRP309935,Megakaryocyte_2-AD.vs.control; scRNA-seq,SRP309935,Monocyte_2-	7
MF	GO:0060090	molecular adaptor activity	bulk RNA-seq,Homo_723,AD.vs.MCI; bulk RNA-seq,Homo_714,AD.vs.control; bulk RNA-seq,Homo_714,AD.vs.MCI; bulk RNA-seq,Homo_633,AD.vs.control; bulk RNA-seq,Homo_633,AD.vs.MCI	5
BP	GO:0071383	cellular response to steroid hormone stimulus	bulk RNA-seq,Homo_723,AD.vs.MCI; bulk RNA-seq,Homo_723,MCI.vs.control; bulk RNA-seq,Homo_714,AD.vs.MCI; bulk RNA-seq,Homo_633,AD.vs.control; bulk RNA-seq,Homo_633,AD.vs.MCI	5
MF	GO:0003924	GTPase activity	bulk RNA-seq,Homo_723,AD.vs.MCI; bulk RNA-seq,Homo_714,AD.vs.MCI; bulk RNA-seq,Homo_633,AD.vs.control; bulk RNA-seq,Homo_633,AD.vs.MCI	4
CC	GO:0030135	coated vesicle	bulk RNA-seq,Homo_723,AD.vs.MCI; bulk RNA-seq,Homo_723,MCI.vs.control; bulk RNA-seq,Homo_714,AD.vs.MCI; bulk RNA-seq,Homo_633,AD.vs.control; bulk RNA-seq,Homo_633,AD.vs.MCI; bulk RNA-seq,SRP310421,AD.vs.control	6
MF	GO:0016791	phosphatase activity	bulk RNA-seq,Homo_723,AD.vs.MCI; bulk RNA-seq,Homo_723,MCI.vs.control; bulk RNA-seq,Homo_714,AD.vs.MCI; bulk RNA-seq,Homo_633,AD.vs.control; bulk RNA-seq,Homo_633,AD.vs.MCI; scRNA-seq,SRP330776,B cell_1-AD.vs.control; scRNA-seq,SRP330776,Naive CD8+ T cell_1-AD.vs.control	7
BP	GO:0071902	positive regulation of protein serine/threonine kinase activity	bulk RNA-seq,Homo_723,AD.vs.MCI; bulk RNA-seq,Homo_723,MCI.vs.control; bulk RNA-seq,Homo_714,AD.vs.MCI; bulk RNA-seq,Homo_633,AD.vs.control; bulk RNA-seq,Homo_633,AD.vs.MCI	5
CC	GO:0032587	ruffle membrane	bulk RNA-seq,Homo_723,AD.vs.MCI; bulk RNA-seq,Homo_723,MCI.vs.control; bulk RNA-seq,Homo_714,AD.vs.MCI; bulk RNA-seq,Homo_714,MCI.vs.control; bulk RNA-seq,Homo_633,AD.vs.control; bulk RNA-seq,Homo_633,AD.vs.MCI	6
BP	GO:0007179	transforming growth factor beta receptor signaling pathway	bulk RNA-seq,Homo_723,AD.vs.MCI; bulk RNA-seq,Homo_723,MCI.vs.control; bulk RNA-seq,Homo_714,AD.vs.MCI; bulk RNA-seq,Homo_633,AD.vs.control; bulk RNA-seq,Homo_633,AD.vs.MCI; scRNA-seq,SRP330776,Naive CD8+ T cell_1-AD.vs.control; scRNA-seq,SRP330776,Natural killer cell_1-AD.vs.control	7
BP	GO:0045995	regulation of embryonic development	bulk RNA-seq,Homo_723,AD.vs.MCI; bulk RNA-seq,Homo_723,MCI.vs.control; bulk RNA-seq,Homo_714,AD.vs.MCI; bulk RNA-seq,Homo_714,MCI.vs.control; bulk RNA-seq,Homo_633,AD.vs.control; bulk RNA-seq,Homo_633,AD.vs.MCI; bulk RNA-seq,SRP223445,AD.vs.control	7
BP	GO:0046578	regulation of Ras protein signal transduction	bulk RNA-seq,Homo_723,AD.vs.MCI; bulk RNA-seq,Homo_723,MCI.vs.control; bulk RNA-seq,Homo_714,AD.vs.MCI; bulk RNA-seq,Homo_633,AD.vs.control; bulk RNA-seq,Homo_633,AD.vs.MCI	5
BP	GO:0042180	cellular ketone metabolic process	bulk RNA-seq,Homo_723,AD.vs.MCI; bulk RNA-seq,Homo_723,MCI.vs.control; bulk RNA-seq,Homo_714,AD.vs.MCI; bulk RNA-seq,Homo_633,AD.vs.control; bulk RNA-seq,Homo_633,AD.vs.MCI	5
BP	GO:0051348	negative regulation of transferase activity	bulk RNA-seq,Homo_723,AD.vs.MCI; bulk RNA-seq,Homo_723,MCI.vs.control; bulk RNA-seq,Homo_714,AD.vs.MCI; bulk RNA-seq,Homo_633,AD.vs.control; bulk RNA-seq,Homo_633,AD.vs.MCI	5
BP	GO:0022409	positive regulation of cell-cell adhesion	bulk RNA-seq,Homo_723,AD.vs.MCI; bulk RNA-seq,Homo_723,MCI.vs.control; bulk RNA-seq,Homo_714,AD.vs.MCI; bulk RNA-seq,Homo_633,AD.vs.control; bulk RNA-seq,Homo_633,AD.vs.MCI; scRNA-seq,SRP215507,CD8+ T cell_3-AD.vs.control; scRNA-seq,SRP215507,CD8+ T cell_3-MCI.vs.control	7
BP	GO:0051259	protein complex oligomerization	bulk RNA-seq,Homo_723,AD.vs.MCI; bulk RNA-seq,Homo_723,MCI.vs.control; bulk RNA-seq,Homo_714,AD.vs.MCI; bulk RNA-seq,Homo_633,AD.vs.control; bulk RNA-seq,Homo_633,AD.vs.MCI	5
BP	GO:0002573	myeloid leukocyte differentiation	bulk RNA-seq,Homo_723,AD.vs.MCI; bulk RNA-seq,Homo_723,MCI.vs.control; bulk RNA-seq,Homo_714,AD.vs.MCI; bulk RNA-seq,Homo_633,AD.vs.control; bulk RNA-seq,Homo_633,AD.vs.MCI; scRNA-seq,SRP215507,CD8+ T cell_3-AD.vs.control	6
BP	GO:0000280	nuclear division	bulk RNA-seq,Homo_723,AD.vs.MCI; bulk RNA-seq,Homo_714,AD.vs.MCI; bulk RNA-seq,Homo_633,AD.vs.control; bulk RNA-seq,Homo_633,AD.vs.MCI	4
MF	GO:0106310	protein serine kinase activity	bulk RNA-seq,Homo_723,AD.vs.MCI; bulk RNA-seq,Homo_714,AD.vs.MCI; bulk RNA-seq,Homo_633,AD.vs.control; bulk RNA-seq,Homo_633,AD.vs.MCI; scRNA-seq,SRP330776,Naive CD8+ T cell_1-AD.vs.control; scRNA-seq,SRP330776,Natural killer cell_1-AD.vs.control	6
BP	GO:0043547	positive regulation of GTPase activity	bulk RNA-seq,Homo_723,AD.vs.MCI; bulk RNA-seq,Homo_723,MCI.vs.control; bulk RNA-seq,Homo_714,AD.vs.MCI; bulk RNA-seq,Homo_633,AD.vs.control; bulk RNA-seq,Homo_633,AD.vs.MCI	5
BP	GO:0071496	cellular response to external stimulus	bulk RNA-seq,Homo_723,AD.vs.MCI; bulk RNA-seq,Homo_723,MCI.vs.control; bulk RNA-seq,Homo_714,AD.vs.MCI; bulk RNA-seq,Homo_633,AD.vs.control; bulk RNA-seq,Homo_633,AD.vs.MCI	5
MF	GO:0005096	GTPase activator activity	bulk RNA-seq,Homo_723,AD.vs.MCI; bulk RNA-seq,Homo_723,MCI.vs.control; bulk RNA-seq,Homo_714,AD.vs.MCI; bulk RNA-seq,Homo_633,AD.vs.control; bulk RNA-seq,Homo_633,AD.vs.MCI	5
BP	GO:0051302	regulation of cell division	bulk RNA-seq,Homo_723,AD.vs.MCI; bulk RNA-seq,Homo_723,MCI.vs.control; bulk RNA-seq,Homo_714,AD.vs.MCI; bulk RNA-seq,Homo_633,AD.vs.control; bulk RNA-seq,Homo_633,AD.vs.MCI	5
MF	GO:0140297	DNA-binding transcription factor binding	bulk RNA-seq,Homo_723,AD.vs.MCI; bulk RNA-seq,Homo_714,AD.vs.MCI; bulk RNA-seq,Homo_633,AD.vs.control; bulk RNA-seq,Homo_633,AD.vs.MCI	4
BP	GO:0006520	cellular amino acid metabolic process	bulk RNA-seq,Homo_723,AD.vs.MCI; bulk RNA-seq,Homo_723,MCI.vs.control; bulk RNA-seq,Homo_714,AD.vs.MCI; bulk RNA-seq,Homo_633,AD.vs.control; bulk RNA-seq,Homo_633,AD.vs.MCI	5
MF	GO:0017124	SH3 domain binding	bulk RNA-seq,Homo_723,AD.vs.MCI; bulk RNA-seq,Homo_723,MCI.vs.control; bulk RNA-seq,Homo_714,AD.vs.MCI; bulk RNA-seq,Homo_714,MCI.vs.control; bulk RNA-seq,Homo_633,AD.vs.control; bulk RNA-seq,Homo_633,AD.vs.MCI	6

BP	GO:0034612	response to tumor necrosis factor	bulk RNA-seq,Homo_723,AD.vs.MCI; bulk RNA-seq,Homo_723,MCI.vs.control; bulk RNA-seq,Homo_714,AD.vs.MCI; bulk RNA-seq,Homo_633,AD.vs.control; bulk RNA-seq,Homo_633,AD.vs.MCI; scRNA-seq,SRP330776,Naive CD8+ T cell_2-AD.vs.control	6
BP	GO:0022411	cellular component disassembly	bulk RNA-seq,Homo_723,AD.vs.MCI; bulk RNA-seq,Homo_714,AD.vs.MCI; bulk RNA-seq,Homo_633,AD.vs.control; bulk RNA-seq,Homo_633,AD.vs.MCI	4
BP	GO:0009416	response to light stimulus	bulk RNA-seq,Homo_723,AD.vs.MCI; bulk RNA-seq,Homo_723,MCI.vs.control; bulk RNA-seq,Homo_714,AD.vs.MCI; bulk RNA-seq,Homo_633,AD.vs.control; bulk RNA-seq,Homo_633,AD.vs.MCI	5
BP	GO:0062014	negative regulation of small molecule metabolic process	bulk RNA-seq,Homo_723,AD.vs.MCI; bulk RNA-seq,Homo_723,MCI.vs.control; bulk RNA-seq,Homo_714,AD.vs.MCI; bulk RNA-seq,Homo_714,MCI.vs.control; bulk RNA-seq,Homo_633,AD.vs.control; bulk RNA-seq,Homo_633,AD.vs.MCI	6
BP	GO:0002443	leukocyte mediated immunity	bulk RNA-seq,Homo_723,AD.vs.MCI; bulk RNA-seq,Homo_714,AD.vs.control; bulk RNA-seq,Homo_633,AD.vs.control; bulk RNA-seq,Homo_633,AD.vs.MCI; scRNA-seq,SRP330776,Naive CD8+ T cell_2-AD.vs.control; scRNA-seq,SRP309935,Monocyte_2-AD.vs.control; scRNA-seq,SRP215507,CD8+ T cell_3-	7
MF	GO:0042626	ATPase-coupled transmembrane transporter activity	bulk RNA-seq,Homo_723,AD.vs.MCI; bulk RNA-seq,Homo_723,MCI.vs.control; bulk RNA-seq,Homo_714,AD.vs.MCI; bulk RNA-seq,Homo_714,MCI.vs.control; bulk RNA-seq,Homo_633,AD.vs.control; bulk RNA-seq,Homo_633,AD.vs.MCI; scRNA-seq,SRP330776,Naive CD8+ T cell_2-	7
BP	GO:0002253	activation of immune response	bulk RNA-seq,Homo_723,AD.vs.MCI; bulk RNA-seq,Homo_714,AD.vs.control; bulk RNA-seq,Homo_633,AD.vs.control; bulk RNA-seq,Homo_633,AD.vs.MCI; scRNA-seq,SRP330776,Naive CD8+ T cell_2-AD.vs.control; scRNA-seq,SRP309935,Monocyte_2-AD.vs.control	6
BP	GO:0051090	regulation of DNA-binding transcription factor activity	bulk RNA-seq,Homo_723,AD.vs.MCI; bulk RNA-seq,Homo_714,AD.vs.MCI; bulk RNA-seq,Homo_633,AD.vs.control; bulk RNA-seq,Homo_633,AD.vs.MCI	4
BP	GO:0070555	response to interleukin-1	bulk RNA-seq,Homo_723,AD.vs.MCI; bulk RNA-seq,Homo_723,MCI.vs.control; bulk RNA-seq,Homo_714,AD.vs.MCI; bulk RNA-seq,Homo_633,AD.vs.control; bulk RNA-seq,Homo_633,AD.vs.MCI	5
BP	GO:0099111	microtubule-based transport	bulk RNA-seq,Homo_723,AD.vs.MCI; bulk RNA-seq,Homo_723,MCI.vs.control; bulk RNA-seq,Homo_714,AD.vs.MCI; bulk RNA-seq,Homo_633,AD.vs.control; bulk RNA-seq,Homo_633,AD.vs.MCI; bulk RNA-seq,ROSMAP,AD.vs.control	6
BP	GO:0055067	monovalent inorganic cation homeostasis	bulk RNA-seq,Homo_723,AD.vs.MCI; bulk RNA-seq,Homo_723,MCI.vs.control; bulk RNA-seq,Homo_714,AD.vs.control; bulk RNA-seq,Homo_714,AD.vs.MCI; bulk RNA-seq,Homo_633,AD.vs.control; bulk RNA-seq,Homo_633,AD.vs.MCI	6
MF	GO:0019001	guanyl nucleotide binding	bulk RNA-seq,Homo_723,AD.vs.MCI; bulk RNA-seq,Homo_714,AD.vs.MCI; bulk RNA-seq,Homo_633,AD.vs.control; bulk RNA-seq,Homo_633,AD.vs.MCI	4
MF	GO:0032561	guanyl ribonucleotide binding	bulk RNA-seq,Homo_723,AD.vs.MCI; bulk RNA-seq,Homo_714,AD.vs.MCI; bulk RNA-seq,Homo_633,AD.vs.control; bulk RNA-seq,Homo_633,AD.vs.MCI	4
BP	GO:0051235	maintenance of location	bulk RNA-seq,Homo_723,AD.vs.MCI; bulk RNA-seq,Homo_714,AD.vs.MCI; bulk RNA-seq,Homo_633,AD.vs.control; bulk RNA-seq,Homo_633,AD.vs.MCI	4
BP	GO:0010639	negative regulation of organelle organization	bulk RNA-seq,Homo_723,AD.vs.MCI; bulk RNA-seq,Homo_714,AD.vs.control; bulk RNA-seq,Homo_714,AD.vs.MCI; bulk RNA-seq,Homo_633,AD.vs.control; bulk RNA-seq,Homo_633,AD.vs.MCI	5
BP	GO:0009101	glycoprotein biosynthetic process	bulk RNA-seq,Homo_723,AD.vs.MCI; bulk RNA-seq,Homo_723,MCI.vs.control; bulk RNA-seq,Homo_714,AD.vs.MCI; bulk RNA-seq,Homo_633,AD.vs.control; bulk RNA-seq,Homo_633,AD.vs.MCI	5
MF	GO:0030246	carbohydrate binding	bulk RNA-seq,Homo_723,AD.vs.MCI; bulk RNA-seq,Homo_723,MCI.vs.control; bulk RNA-seq,Homo_714,AD.vs.MCI; bulk RNA-seq,Homo_633,AD.vs.control; bulk RNA-seq,Homo_633,AD.vs.MCI	5
BP	GO:0071356	cellular response to tumor necrosis factor	bulk RNA-seq,Homo_723,AD.vs.MCI; bulk RNA-seq,Homo_723,MCI.vs.control; bulk RNA-seq,Homo_714,AD.vs.MCI; bulk RNA-seq,Homo_633,AD.vs.control; bulk RNA-seq,Homo_633,AD.vs.MCI; scRNA-seq,SRP330776,Naive CD8+ T cell_2-AD.vs.control	6
BP	GO:0006470	protein dephosphorylation	bulk RNA-seq,Homo_723,AD.vs.MCI; bulk RNA-seq,Homo_723,MCI.vs.control; bulk RNA-seq,Homo_714,AD.vs.MCI; bulk RNA-seq,Homo_633,AD.vs.control; bulk RNA-seq,Homo_633,AD.vs.MCI; scRNA-seq,SRP330776,CD8+ T cell_1-AD.vs.control; scRNA-seq,SRP330776,Naive CD8+ T cell_1-AD.vs.control; scRNA-seq,SRP330776,Natural killer cell_1-AD.vs.control	8
BP	GO:0051260	protein homooligomerization	bulk RNA-seq,Homo_723,AD.vs.MCI; bulk RNA-seq,Homo_723,MCI.vs.control; bulk RNA-seq,Homo_714,AD.vs.MCI; bulk RNA-seq,Homo_633,AD.vs.control; bulk RNA-seq,Homo_633,AD.vs.MCI; bulk RNA-seq,SRP223445,AD.vs.control	6
BP	GO:0010812	negative regulation of cell-substrate adhesion	bulk RNA-seq,Homo_723,AD.vs.MCI; bulk RNA-seq,Homo_723,MCI.vs.control; bulk RNA-seq,Homo_714,AD.vs.MCI; bulk RNA-seq,Homo_714,MCI.vs.control; bulk RNA-seq,Homo_633,AD.vs.control; bulk RNA-seq,Homo_633,AD.vs.MCI	6
BP	GO:0051251	positive regulation of lymphocyte activation	bulk RNA-seq,Homo_723,AD.vs.MCI; bulk RNA-seq,Homo_714,AD.vs.control; bulk RNA-seq,Homo_633,AD.vs.control; bulk RNA-seq,Homo_633,AD.vs.MCI; scRNA-seq,SRP330776,Naive CD8+ T cell_2-AD.vs.control; scRNA-seq,SRP309935,Monocyte_2-AD.vs.control; scRNA-seq,SRP215507,CD8+ T cell_3-AD.vs.control; scRNA-seq,SRP215507,CD8+ T cell_3-MCI.vs.control	8
BP	GO:0010565	regulation of cellular ketone metabolic process	bulk RNA-seq,Homo_723,AD.vs.MCI; bulk RNA-seq,Homo_723,MCI.vs.control; bulk RNA-seq,Homo_714,AD.vs.MCI; bulk RNA-seq,Homo_633,AD.vs.control; bulk RNA-seq,Homo_633,AD.vs.MCI	5
BP	GO:0009266	response to temperature stimulus	bulk RNA-seq,Homo_723,AD.vs.MCI; bulk RNA-seq,Homo_723,MCI.vs.control; bulk RNA-seq,Homo_714,AD.vs.MCI; bulk RNA-seq,Homo_633,AD.vs.control; bulk RNA-seq,Homo_633,AD.vs.MCI	5
BP	GO:0019827	stem cell population maintenance	bulk RNA-seq,Homo_723,AD.vs.MCI; bulk RNA-seq,Homo_723,MCI.vs.control; bulk RNA-seq,Homo_714,AD.vs.MCI; bulk RNA-seq,Homo_633,AD.vs.control; bulk RNA-seq,Homo_633,AD.vs.MCI; bulk RNA-seq,SRP223445,AD.vs.control	6
BP	GO:0032496	response to lipopolysaccharide	bulk RNA-seq,Homo_723,AD.vs.MCI; bulk RNA-seq,Homo_714,AD.vs.MCI; bulk RNA-seq,Homo_633,AD.vs.control; bulk RNA-seq,Homo_633,AD.vs.MCI	4
BP	GO:0031110	regulation of microtubule polymerization or depolymerization	bulk RNA-seq,Homo_723,AD.vs.MCI; bulk RNA-seq,Homo_723,MCI.vs.control; bulk RNA-seq,Homo_714,AD.vs.MCI; bulk RNA-seq,Homo_714,MCI.vs.control; bulk RNA-seq,Homo_633,AD.vs.control; bulk RNA-seq,Homo_633,AD.vs.MCI	6
BP	GO:0045786	negative regulation of cell cycle	bulk RNA-seq,Homo_723,AD.vs.MCI; bulk RNA-seq,Homo_714,AD.vs.MCI; bulk RNA-seq,Homo_633,AD.vs.control; bulk RNA-seq,Homo_633,AD.vs.MCI	4
BP	GO:0002237	response to molecule of bacterial origin	bulk RNA-seq,Homo_723,AD.vs.MCI; bulk RNA-seq,Homo_714,AD.vs.MCI; bulk RNA-seq,Homo_633,AD.vs.control; bulk RNA-seq,Homo_633,AD.vs.MCI	4
CC	GO:0030662	coated vesicle membrane	bulk RNA-seq,Homo_723,AD.vs.MCI; bulk RNA-seq,Homo_723,MCI.vs.control; bulk RNA-seq,Homo_714,AD.vs.MCI; bulk RNA-seq,Homo_633,AD.vs.control; bulk RNA-seq,Homo_633,AD.vs.MCI	5
BP	GO:0048144	fibroblast proliferation	bulk RNA-seq,Homo_723,AD.vs.MCI; bulk RNA-seq,Homo_723,MCI.vs.control; bulk RNA-seq,Homo_714,AD.vs.MCI; bulk RNA-seq,Homo_714,MCI.vs.control; bulk RNA-seq,Homo_633,AD.vs.control; bulk RNA-seq,Homo_633,AD.vs.MCI	6
BP	GO:0090068	positive regulation of cell cycle process	bulk RNA-seq,Homo_723,AD.vs.MCI; bulk RNA-seq,Homo_723,MCI.vs.control; bulk RNA-seq,Homo_714,AD.vs.MCI; bulk RNA-seq,Homo_633,AD.vs.control; bulk RNA-seq,Homo_633,AD.vs.MCI	5
BP	GO:0016053	organic acid biosynthetic process	bulk RNA-seq,Homo_723,AD.vs.MCI; bulk RNA-seq,Homo_723,MCI.vs.control; bulk RNA-seq,Homo_714,AD.vs.MCI; bulk RNA-seq,Homo_633,AD.vs.control; bulk RNA-seq,Homo_633,AD.vs.MCI	5
BP	GO:0030705	cytoskeleton-dependent intracellular transport	bulk RNA-seq,Homo_723,AD.vs.MCI; bulk RNA-seq,Homo_723,MCI.vs.control; bulk RNA-seq,Homo_714,AD.vs.MCI; bulk RNA-seq,Homo_633,AD.vs.control; bulk RNA-seq,Homo_633,AD.vs.MCI; bulk RNA-seq,ROSMAP,AD.vs.control	6
BP	GO:0001892	embryonic placenta development	bulk RNA-seq,Homo_723,AD.vs.MCI; bulk RNA-seq,Homo_723,MCI.vs.control; bulk RNA-seq,Homo_714,AD.vs.MCI; bulk RNA-seq,Homo_714,MCI.vs.control; bulk RNA-seq,Homo_633,AD.vs.control; bulk RNA-seq,Homo_633,AD.vs.MCI; scRNA-seq,SRP215507,CD8+ T cell_3-	7

BP	GO:0046889	positive regulation of lipid biosynthetic process	bulk RNA-seq,Homo_723,AD.vs.MCI; bulk RNA-seq,Homo_723,MCI.vs.control; bulk RNA-seq,Homo_714,AD.vs.MCI; bulk RNA-seq,Homo_714,MCI.vs.control; bulk RNA-seq,Homo_633,AD.vs.control	5
BP	GO:0120032	regulation of plasma membrane bounded cell projection assembly	bulk RNA-seq,Homo_723,AD.vs.MCI; bulk RNA-seq,Homo_723,MCI.vs.control; bulk RNA-seq,Homo_714,AD.vs.MCI; bulk RNA-seq,Homo_633,AD.vs.control; bulk RNA-seq,Homo_633,AD.vs.MCI	5
BP	GO:0051222	positive regulation of protein transport	bulk RNA-seq,Homo_723,AD.vs.MCI; bulk RNA-seq,Homo_723,MCI.vs.control; bulk RNA-seq,Homo_714,AD.vs.MCI; bulk RNA-seq,Homo_633,AD.vs.control; bulk RNA-seq,Homo_633,AD.vs.MCI	5
MF	GO:0140098	catalytic activity, acting on RNA	bulk RNA-seq,Homo_723,AD.vs.MCI; bulk RNA-seq,Homo_714,AD.vs.control; bulk RNA-seq,Homo_714,AD.vs.MCI; bulk RNA-seq,Homo_633,AD.vs.control; bulk RNA-seq,Homo_633,AD.vs.MCI	5
CC	GO:0042641	actomyosin	bulk RNA-seq,Homo_723,AD.vs.MCI; bulk RNA-seq,Homo_723,MCI.vs.control; bulk RNA-seq,Homo_714,AD.vs.control; bulk RNA-seq,Homo_714,AD.vs.MCI; bulk RNA-seq,Homo_714,MCI.vs.control; bulk RNA-seq,Homo_633,AD.vs.control	6
BP	GO:0008064	regulation of actin polymerization or depolymerization	bulk RNA-seq,Homo_723,AD.vs.MCI; bulk RNA-seq,Homo_723,MCI.vs.control; bulk RNA-seq,Homo_714,AD.vs.MCI; bulk RNA-seq,Homo_633,AD.vs.control; bulk RNA-seq,Homo_633,AD.vs.MCI	5
BP	GO:0062013	positive regulation of small molecule metabolic process	bulk RNA-seq,Homo_723,AD.vs.MCI; bulk RNA-seq,Homo_723,MCI.vs.control; bulk RNA-seq,Homo_714,AD.vs.MCI; bulk RNA-seq,Homo_633,AD.vs.control; bulk RNA-seq,Homo_633,AD.vs.MCI	5
BP	GO:0032874	positive regulation of stress-activated MAPK cascade	bulk RNA-seq,Homo_723,AD.vs.MCI; bulk RNA-seq,Homo_723,MCI.vs.control; bulk RNA-seq,Homo_714,AD.vs.MCI; bulk RNA-seq,Homo_633,AD.vs.control; bulk RNA-seq,Homo_633,AD.vs.MCI	5
BP	GO:0034116	positive regulation of heterotypic cell-cell adhesion	bulk RNA-seq,Homo_723,AD.vs.MCI; bulk RNA-seq,Homo_723,MCI.vs.control; bulk RNA-seq,Homo_714,AD.vs.MCI; bulk RNA-seq,Homo_714,MCI.vs.control	4
BP	GO:0060491	regulation of cell projection assembly	bulk RNA-seq,Homo_723,AD.vs.MCI; bulk RNA-seq,Homo_723,MCI.vs.control; bulk RNA-seq,Homo_714,AD.vs.MCI; bulk RNA-seq,Homo_633,AD.vs.control; bulk RNA-seq,Homo_633,AD.vs.MCI	5
BP	GO:0009743	response to carbohydrate	bulk RNA-seq,Homo_723,AD.vs.MCI; bulk RNA-seq,Homo_723,MCI.vs.control; bulk RNA-seq,Homo_714,AD.vs.MCI; bulk RNA-seq,Homo_633,AD.vs.control; bulk RNA-seq,Homo_633,AD.vs.MCI	5
BP	GO:0097529	myeloid leukocyte migration	bulk RNA-seq,Homo_723,AD.vs.MCI; bulk RNA-seq,Homo_723,MCI.vs.control; bulk RNA-seq,Homo_714,AD.vs.MCI; bulk RNA-seq,Homo_633,AD.vs.control; bulk RNA-seq,Homo_633,AD.vs.MCI; bulk RNA-seq,SRP223445,AD.vs.control; scRNA-seq,SRP330776,Naive CD8+ T cell_2-AD.vs.control	7
BP	GO:1903046	meiotic cell cycle process	bulk RNA-seq,Homo_723,AD.vs.MCI; bulk RNA-seq,Homo_723,MCI.vs.control; bulk RNA-seq,Homo_714,AD.vs.MCI; bulk RNA-seq,Homo_633,AD.vs.control; bulk RNA-seq,Homo_633,AD.vs.MCI	5
BP	GO:0031098	stress-activated protein kinase signaling cascade	bulk RNA-seq,Homo_723,AD.vs.MCI; bulk RNA-seq,Homo_723,MCI.vs.control; bulk RNA-seq,Homo_714,AD.vs.MCI; bulk RNA-seq,Homo_633,AD.vs.control; bulk RNA-seq,Homo_633,AD.vs.MCI	5
MF	GO:0030674	protein-macromolecule adaptor activity	bulk RNA-seq,Homo_723,AD.vs.MCI; bulk RNA-seq,Homo_714,AD.vs.control; bulk RNA-seq,Homo_714,AD.vs.MCI; bulk RNA-seq,Homo_633,AD.vs.control; bulk RNA-seq,Homo_633,AD.vs.MCI	5
BP	GO:0051100	negative regulation of binding	bulk RNA-seq,Homo_723,AD.vs.MCI; bulk RNA-seq,Homo_723,MCI.vs.control; bulk RNA-seq,Homo_714,AD.vs.MCI; bulk RNA-seq,Homo_633,AD.vs.control; bulk RNA-seq,Homo_633,AD.vs.MCI	5
BP	GO:0051321	meiotic cell cycle	bulk RNA-seq,Homo_723,AD.vs.MCI; bulk RNA-seq,Homo_723,MCI.vs.control; bulk RNA-seq,Homo_714,AD.vs.MCI; bulk RNA-seq,Homo_633,AD.vs.control; bulk RNA-seq,Homo_633,AD.vs.MCI	5
BP	GO:0050886	endocrine process	bulk RNA-seq,Homo_723,AD.vs.MCI; bulk RNA-seq,Homo_723,MCI.vs.control; bulk RNA-seq,Homo_714,AD.vs.MCI; bulk RNA-seq,Homo_714,MCI.vs.control; bulk RNA-seq,Homo_633,AD.vs.control	5
BP	GO:0048660	regulation of smooth muscle cell proliferation	bulk RNA-seq,Homo_723,AD.vs.MCI; bulk RNA-seq,Homo_723,MCI.vs.control; bulk RNA-seq,Homo_714,AD.vs.control; bulk RNA-seq,Homo_714,AD.vs.MCI; bulk RNA-seq,Homo_633,AD.vs.control	5
BP	GO:0046394	carboxylic acid biosynthetic process	bulk RNA-seq,Homo_723,AD.vs.MCI; bulk RNA-seq,Homo_723,MCI.vs.control; bulk RNA-seq,Homo_714,AD.vs.MCI; bulk RNA-seq,Homo_633,AD.vs.control; bulk RNA-seq,Homo_633,AD.vs.MCI	5
BP	GO:0051651	maintenance of location in cell	bulk RNA-seq,Homo_723,AD.vs.MCI; bulk RNA-seq,Homo_723,MCI.vs.control; bulk RNA-seq,Homo_714,AD.vs.MCI; bulk RNA-seq,Homo_633,AD.vs.control; bulk RNA-seq,Homo_633,AD.vs.MCI	5
BP	GO:0140013	meiotic nuclear division	bulk RNA-seq,Homo_723,AD.vs.MCI; bulk RNA-seq,Homo_723,MCI.vs.control; bulk RNA-seq,Homo_714,AD.vs.MCI; bulk RNA-seq,Homo_633,AD.vs.control; bulk RNA-seq,Homo_633,AD.vs.MCI	5
BP	GO:0031668	cellular response to extracellular stimulus	bulk RNA-seq,Homo_723,AD.vs.MCI; bulk RNA-seq,Homo_723,MCI.vs.control; bulk RNA-seq,Homo_714,AD.vs.MCI; bulk RNA-seq,Homo_633,AD.vs.control; bulk RNA-seq,Homo_633,AD.vs.MCI	5
BP	GO:0006790	sulfur compound metabolic process	bulk RNA-seq,Homo_723,AD.vs.MCI; bulk RNA-seq,Homo_714,AD.vs.MCI; bulk RNA-seq,Homo_633,AD.vs.control; bulk RNA-seq,Homo_633,AD.vs.MCI	4
BP	GO:0006575	cellular modified amino acid metabolic process	bulk RNA-seq,Homo_723,AD.vs.MCI; bulk RNA-seq,Homo_723,MCI.vs.control; bulk RNA-seq,Homo_714,AD.vs.MCI; bulk RNA-seq,Homo_633,AD.vs.control; bulk RNA-seq,Homo_633,AD.vs.MCI	5
BP	GO:0031333	negative regulation of protein-containing complex assembly	bulk RNA-seq,Homo_723,AD.vs.MCI; bulk RNA-seq,Homo_723,MCI.vs.control; bulk RNA-seq,Homo_714,AD.vs.MCI; bulk RNA-seq,Homo_633,AD.vs.control; bulk RNA-seq,Homo_633,AD.vs.MCI	5
CC	GO:0001725	stress fiber	bulk RNA-seq,Homo_723,AD.vs.MCI; bulk RNA-seq,Homo_723,MCI.vs.control; bulk RNA-seq,Homo_714,AD.vs.MCI; bulk RNA-seq,Homo_714,MCI.vs.control	4
CC	GO:0097517	contractile actin filament bundle	bulk RNA-seq,Homo_723,AD.vs.MCI; bulk RNA-seq,Homo_723,MCI.vs.control; bulk RNA-seq,Homo_714,AD.vs.MCI; bulk RNA-seq,Homo_714,MCI.vs.control	4
BP	GO:0048145	regulation of fibroblast proliferation	bulk RNA-seq,Homo_723,AD.vs.MCI; bulk RNA-seq,Homo_723,MCI.vs.control; bulk RNA-seq,Homo_714,AD.vs.MCI; bulk RNA-seq,Homo_714,MCI.vs.control; bulk RNA-seq,Homo_633,AD.vs.control; bulk RNA-seq,Homo_633,AD.vs.MCI	6
BP	GO:0071384	cellular response to corticosteroid stimulus	bulk RNA-seq,Homo_723,AD.vs.MCI; bulk RNA-seq,Homo_723,MCI.vs.control; bulk RNA-seq,Homo_714,AD.vs.MCI; bulk RNA-seq,Homo_714,MCI.vs.control; bulk RNA-seq,Homo_633,AD.vs.control; bulk RNA-seq,Homo_633,AD.vs.MCI	6
BP	GO:0070304	positive regulation of stress-activated protein kinase signaling cascade	bulk RNA-seq,Homo_723,AD.vs.MCI; bulk RNA-seq,Homo_723,MCI.vs.control; bulk RNA-seq,Homo_714,AD.vs.MCI; bulk RNA-seq,Homo_633,AD.vs.control; bulk RNA-seq,Homo_633,AD.vs.MCI	5
MF	GO:0140272	exogenous protein binding	bulk RNA-seq,Homo_723,AD.vs.MCI; bulk RNA-seq,Homo_723,MCI.vs.control; bulk RNA-seq,Homo_714,AD.vs.MCI; bulk RNA-seq,Homo_714,MCI.vs.control; bulk RNA-seq,Homo_633,AD.vs.control; bulk RNA-seq,Homo_633,AD.vs.MCI	6
BP	GO:1904951	positive regulation of establishment of protein localization	bulk RNA-seq,Homo_723,AD.vs.MCI; bulk RNA-seq,Homo_723,MCI.vs.control; bulk RNA-seq,Homo_714,AD.vs.MCI; bulk RNA-seq,Homo_633,AD.vs.control; bulk RNA-seq,Homo_633,AD.vs.MCI	5
MF	GO:0051020	GTPase binding	bulk RNA-seq,Homo_723,AD.vs.MCI; bulk RNA-seq,Homo_723,MCI.vs.control; bulk RNA-seq,Homo_714,AD.vs.MCI; bulk RNA-seq,Homo_633,AD.vs.control; bulk RNA-seq,Homo_633,AD.vs.MCI; scRNA-seq,SRP330776,Naive CD8+ T cell_2-AD.vs.control	6

BP	GO:0030595	leukocyte chemotaxis	bulk RNA-seq,Homo_723,AD.vs.MCI; bulk RNA-seq,Homo_723,MCI.vs.control; bulk RNA-seq,Homo_714,AD.vs.MCI; bulk RNA-seq,Homo_633,AD.vs.control; bulk RNA-seq,Homo_633,AD.vs.MCI; bulk RNA-seq,SRP223445,AD.vs.control; scRNA-seq,SRP330776,Naive CD8+ T cell_2-AD.vs.control	7
MF	GO:1902936	phosphatidylinositol bisphosphate binding	bulk RNA-seq,Homo_723,AD.vs.MCI; bulk RNA-seq,Homo_723,MCI.vs.control; bulk RNA-seq,Homo_714,AD.vs.MCI; bulk RNA-seq,Homo_714,MCI.vs.control; bulk RNA-seq,Homo_633,AD.vs.control	5
BP	GO:0002244	hematopoietic progenitor cell differentiation	bulk RNA-seq,Homo_723,AD.vs.MCI; bulk RNA-seq,Homo_723,MCI.vs.control; bulk RNA-seq,Homo_714,AD.vs.MCI; bulk RNA-seq,Homo_633,AD.vs.control; bulk RNA-seq,Homo_633,AD.vs.MCI; scRNA-seq,SRP330776,Naive CD8+ T cell_2-AD.vs.control	6
BP	GO:0006631	fatty acid metabolic process	bulk RNA-seq,Homo_723,AD.vs.MCI; bulk RNA-seq,Homo_714,AD.vs.MCI; bulk RNA-seq,Homo_633,AD.vs.control; bulk RNA-seq,Homo_633,AD.vs.MCI; bulk RNA-seq,SRP325058,AD.vs.control	5
BP	GO:2000736	regulation of stem cell differentiation	bulk RNA-seq,Homo_723,AD.vs.MCI; bulk RNA-seq,Homo_723,MCI.vs.control; bulk RNA-seq,Homo_714,AD.vs.MCI; bulk RNA-seq,Homo_714,MCI.vs.control; bulk RNA-seq,Homo_633,AD.vs.control; bulk RNA-seq,Homo_633,AD.vs.MCI	6
MF	GO:0042379	chemokine receptor binding	bulk RNA-seq,Homo_723,AD.vs.MCI; bulk RNA-seq,Homo_723,MCI.vs.control; bulk RNA-seq,Homo_714,AD.vs.control; bulk RNA-seq,Homo_714,AD.vs.MCI; bulk RNA-seq,Homo_714,MCI.vs.control; scRNA-seq,SRP309935,Naive CD8+ T cell_1-AD.vs.control	6
BP	GO:0002764	immune response-regulating signaling pathway	bulk RNA-seq,Homo_723,AD.vs.MCI; bulk RNA-seq,Homo_714,AD.vs.control; bulk RNA-seq,Homo_633,AD.vs.control; bulk RNA-seq,Homo_633,AD.vs.MCI; scRNA-seq,SRP330776,Naive CD8+ T cell_2-AD.vs.control	5
BP	GO:0072330	monocarboxylic acid biosynthetic process	bulk RNA-seq,Homo_723,AD.vs.MCI; bulk RNA-seq,Homo_723,MCI.vs.control; bulk RNA-seq,Homo_714,AD.vs.MCI; bulk RNA-seq,Homo_633,AD.vs.control; bulk RNA-seq,Homo_633,AD.vs.MCI	5
BP	GO:0030833	regulation of actin filament polymerization	bulk RNA-seq,Homo_723,AD.vs.MCI; bulk RNA-seq,Homo_723,MCI.vs.control; bulk RNA-seq,Homo_714,AD.vs.MCI; bulk RNA-seq,Homo_633,AD.vs.control; bulk RNA-seq,Homo_633,AD.vs.MCI	5
BP	GO:0061383	trabecula morphogenesis	bulk RNA-seq,Homo_723,AD.vs.MCI; bulk RNA-seq,Homo_723,MCI.vs.control; bulk RNA-seq,Homo_714,AD.vs.MCI; bulk RNA-seq,Homo_714,MCI.vs.control	4
BP	GO:0016311	dephosphorylation	bulk RNA-seq,Homo_723,AD.vs.MCI; bulk RNA-seq,Homo_714,AD.vs.MCI; bulk RNA-seq,Homo_633,AD.vs.control; bulk RNA-seq,Homo_633,AD.vs.MCI; scRNA-seq,SRP330776,B cell_1-AD.vs.control; scRNA-seq,SRP330776,CD8+ T cell_1-AD.vs.control; scRNA-seq,SRP330776,Naive CD8+ T cell_1-AD.vs.control; scRNA-seq,SRP330776,Natural killer cell_1-AD.vs.control	8
BP	GO:0030522	intracellular receptor signaling pathway	bulk RNA-seq,Homo_723,AD.vs.MCI; bulk RNA-seq,Homo_723,MCI.vs.control; bulk RNA-seq,Homo_714,AD.vs.MCI; bulk RNA-seq,Homo_633,AD.vs.control; bulk RNA-seq,Homo_633,AD.vs.MCI	5
MF	GO:0061629	RNA polymerase II-specific DNA-binding transcription	bulk RNA-seq,Homo_723,AD.vs.MCI; bulk RNA-seq,Homo_714,AD.vs.MCI; bulk RNA-seq,Homo_633,AD.vs.control; bulk RNA-seq,Homo_633,AD.vs.MCI	4
CC	GO:0098862	cluster of actin-based cell projections	bulk RNA-seq,Homo_723,AD.vs.MCI; bulk RNA-seq,Homo_723,MCI.vs.control; bulk RNA-seq,Homo_714,AD.vs.MCI; bulk RNA-seq,Homo_633,AD.vs.control; bulk RNA-seq,Homo_633,AD.vs.MCI	5
MF	GO:0050997	quaternary ammonium group binding	bulk RNA-seq,Homo_723,AD.vs.MCI; bulk RNA-seq,Homo_723,MCI.vs.control; bulk RNA-seq,Homo_714,AD.vs.MCI; bulk RNA-seq,Homo_714,MCI.vs.control; bulk RNA-seq,Homo_633,AD.vs.control; bulk RNA-seq,Homo_633,AD.vs.MCI	6
BP	GO:0097581	lamellipodium organization	bulk RNA-seq,Homo_723,AD.vs.MCI; bulk RNA-seq,Homo_723,MCI.vs.control; bulk RNA-seq,Homo_714,AD.vs.MCI; bulk RNA-seq,Homo_714,MCI.vs.control; bulk RNA-seq,Homo_633,AD.vs.control; bulk RNA-seq,Homo_633,AD.vs.MCI	6
BP	GO:0006024	glycosaminoglycan biosynthetic process	bulk RNA-seq,Homo_723,AD.vs.MCI; bulk RNA-seq,Homo_723,MCI.vs.control; bulk RNA-seq,Homo_714,AD.vs.MCI; bulk RNA-seq,Homo_714,MCI.vs.control; bulk RNA-seq,Homo_633,AD.vs.control; bulk RNA-seq,Homo_633,AD.vs.MCI	6
CC	GO:0005819	spindle	bulk RNA-seq,Homo_723,AD.vs.MCI; bulk RNA-seq,Homo_714,AD.vs.MCI; bulk RNA-seq,Homo_633,AD.vs.control; bulk RNA-seq,Homo_633,AD.vs.MCI	4
BP	GO:0007173	epidermal growth factor receptor signaling pathway	bulk RNA-seq,Homo_723,AD.vs.MCI; bulk RNA-seq,Homo_723,MCI.vs.control; bulk RNA-seq,Homo_714,AD.vs.MCI; bulk RNA-seq,Homo_714,MCI.vs.control; bulk RNA-seq,Homo_633,AD.vs.control; bulk RNA-seq,Homo_633,AD.vs.MCI	6
BP	GO:0051091	positive regulation of DNA-binding transcription factor activity	bulk RNA-seq,Homo_723,AD.vs.MCI; bulk RNA-seq,Homo_723,MCI.vs.control; bulk RNA-seq,Homo_714,AD.vs.MCI; bulk RNA-seq,Homo_633,AD.vs.control; bulk RNA-seq,Homo_633,AD.vs.MCI	5
BP	GO:0031503	protein-containing complex localization	bulk RNA-seq,Homo_723,AD.vs.MCI; bulk RNA-seq,Homo_723,MCI.vs.control; bulk RNA-seq,Homo_714,AD.vs.MCI; bulk RNA-seq,Homo_633,AD.vs.control; bulk RNA-seq,Homo_633,AD.vs.MCI	5
BP	GO:0010862	positive regulation of pathway-restricted SMAD protein phosphorylation	bulk RNA-seq,Homo_723,AD.vs.MCI; bulk RNA-seq,Homo_723,MCI.vs.control; bulk RNA-seq,Homo_714,AD.vs.MCI; bulk RNA-seq,Homo_714,MCI.vs.control	4
BP	GO:0031109	microtubule polymerization or depolymerization	bulk RNA-seq,Homo_723,AD.vs.MCI; bulk RNA-seq,Homo_723,MCI.vs.control; bulk RNA-seq,Homo_714,AD.vs.MCI; bulk RNA-seq,Homo_714,MCI.vs.control; bulk RNA-seq,Homo_633,AD.vs.control; bulk RNA-seq,Homo_633,AD.vs.MCI	6
BP	GO:0002761	regulation of myeloid leukocyte differentiation	bulk RNA-seq,Homo_723,AD.vs.MCI; bulk RNA-seq,Homo_723,MCI.vs.control; bulk RNA-seq,Homo_714,AD.vs.control; bulk RNA-seq,Homo_714,AD.vs.MCI; bulk RNA-seq,Homo_714,MCI.vs.control; bulk RNA-seq,Homo_633,AD.vs.control; scRNA-seq,SRP215507,CD8+ T cell_3-AD.vs.control	7
MF	GO:0005085	guanyl-nucleotide exchange factor activity	bulk RNA-seq,Homo_723,AD.vs.MCI; bulk RNA-seq,Homo_723,MCI.vs.control; bulk RNA-seq,Homo_714,AD.vs.MCI; bulk RNA-seq,Homo_633,AD.vs.control; bulk RNA-seq,Homo_633,AD.vs.MCI	5
BP	GO:0071347	cellular response to interleukin-1	bulk RNA-seq,Homo_723,AD.vs.MCI; bulk RNA-seq,Homo_723,MCI.vs.control; bulk RNA-seq,Homo_714,AD.vs.MCI; bulk RNA-seq,Homo_714,MCI.vs.control; bulk RNA-seq,Homo_633,AD.vs.control; bulk RNA-seq,Homo_633,AD.vs.MCI	6
BP	GO:0048659	smooth muscle cell proliferation	bulk RNA-seq,Homo_723,AD.vs.MCI; bulk RNA-seq,Homo_723,MCI.vs.control; bulk RNA-seq,Homo_714,AD.vs.MCI; bulk RNA-seq,Homo_633,AD.vs.control	4
BP	GO:0002683	negative regulation of immune system process	bulk RNA-seq,Homo_723,AD.vs.MCI; bulk RNA-seq,Homo_714,AD.vs.MCI; bulk RNA-seq,Homo_633,AD.vs.control; bulk RNA-seq,Homo_633,AD.vs.MCI	4
BP	GO:0051656	establishment of organelle localization	bulk RNA-seq,Homo_723,AD.vs.MCI; bulk RNA-seq,Homo_714,AD.vs.control; bulk RNA-seq,Homo_714,AD.vs.MCI; bulk RNA-seq,Homo_633,AD.vs.control; bulk RNA-seq,Homo_633,AD.vs.MCI	5
BP	GO:0051783	regulation of nuclear division	bulk RNA-seq,Homo_723,AD.vs.MCI; bulk RNA-seq,Homo_723,MCI.vs.control; bulk RNA-seq,Homo_714,AD.vs.MCI; bulk RNA-seq,Homo_633,AD.vs.control; bulk RNA-seq,Homo_633,AD.vs.MCI	5
BP	GO:0072577	endothelial cell apoptotic process	bulk RNA-seq,Homo_723,AD.vs.MCI; bulk RNA-seq,Homo_723,MCI.vs.control; bulk RNA-seq,Homo_714,AD.vs.MCI; bulk RNA-seq,Homo_714,MCI.vs.control	4
CC	GO:0098791	Golgi apparatus subcompartment	bulk RNA-seq,Homo_723,AD.vs.MCI; bulk RNA-seq,Homo_714,AD.vs.control; bulk RNA-seq,Homo_714,AD.vs.MCI; bulk RNA-seq,Homo_633,AD.vs.control; bulk RNA-seq,Homo_633,AD.vs.MCI; scRNA-seq,SRP330776,Naive CD8+ T cell_2-AD.vs.control	6
MF	GO:0019829	ATPase-coupled cation transmembrane transporter activity	bulk RNA-seq,Homo_723,AD.vs.MCI; bulk RNA-seq,Homo_723,MCI.vs.control; bulk RNA-seq,Homo_714,AD.vs.MCI; bulk RNA-seq,Homo_633,AD.vs.control; bulk RNA-seq,Homo_633,AD.vs.MCI; scRNA-seq,SRP330776,Naive CD8+ T cell_2-AD.vs.control	6
MF	GO:0015297	antiporter activity	bulk RNA-seq,Homo_723,AD.vs.MCI; bulk RNA-seq,Homo_723,MCI.vs.control; bulk RNA-seq,Homo_714,AD.vs.MCI; bulk RNA-seq,Homo_633,AD.vs.control; bulk RNA-seq,Homo_633,AD.vs.MCI	5
BP	GO:0097306	cellular response to alcohol	bulk RNA-seq,Homo_723,AD.vs.MCI; bulk RNA-seq,Homo_723,MCI.vs.control; bulk RNA-seq,Homo_714,AD.vs.MCI; bulk RNA-seq,Homo_714,MCI.vs.control; bulk RNA-seq,Homo_633,AD.vs.control; bulk RNA-seq,Homo_633,AD.vs.MCI	6

BP	GO:0051403	stress-activated MAPK cascade	bulk RNA-seq,Homo_723,AD.vs.MCI; bulk RNA-seq,Homo_723,MCI.vs.control; bulk RNA-seq,Homo_714,AD.vs.MCI; bulk RNA-seq,Homo_633,AD.vs.control; bulk RNA-seq,Homo_633,AD.vs.MCI	5
BP	GO:0043473	pigmentation	bulk RNA-seq,Homo_723,AD.vs.MCI; bulk RNA-seq,Homo_723,MCI.vs.control; bulk RNA-seq,Homo_714,AD.vs.MCI; bulk RNA-seq,Homo_714,MCI.vs.control; bulk RNA-seq,Homo_633,AD.vs.control; bulk RNA-seq,Homo_633,AD.vs.MCI	6
BP	GO:0044282	small molecule catabolic process	bulk RNA-seq,Homo_723,AD.vs.MCI; bulk RNA-seq,Homo_714,AD.vs.control; bulk RNA-seq,Homo_714,AD.vs.MCI; bulk RNA-seq,Homo_633,AD.vs.control; bulk RNA-seq,Homo_633,AD.vs.MCI; bulk RNA-seq,SRP223445,AD.vs.control	6
BP	GO:0044409	entry into host	bulk RNA-seq,Homo_723,AD.vs.MCI; bulk RNA-seq,Homo_723,MCI.vs.control; bulk RNA-seq,Homo_714,AD.vs.MCI; bulk RNA-seq,Homo_633,AD.vs.control; bulk RNA-seq,Homo_633,AD.vs.MCI	5
BP	GO:0006081	cellular aldehyde metabolic process	bulk RNA-seq,Homo_723,AD.vs.MCI; bulk RNA-seq,Homo_723,MCI.vs.control; bulk RNA-seq,Homo_714,AD.vs.MCI; bulk RNA-seq,Homo_714,MCI.vs.control; bulk RNA-seq,Homo_633,AD.vs.control; bulk RNA-seq,Homo_633,AD.vs.MCI	6
BP	GO:0042572	retinol metabolic process	bulk RNA-seq,Homo_723,AD.vs.MCI; bulk RNA-seq,Homo_723,MCI.vs.control; bulk RNA-seq,Homo_714,AD.vs.MCI; bulk RNA-seq,Homo_714,MCI.vs.control; bulk RNA-seq,Homo_633,AD.vs.control; bulk RNA-seq,Homo_633,AD.vs.MCI	6
BP	GO:0007566	embryo implantation	bulk RNA-seq,Homo_723,AD.vs.MCI; bulk RNA-seq,Homo_723,MCI.vs.control; bulk RNA-seq,Homo_714,AD.vs.MCI; bulk RNA-seq,Homo_714,MCI.vs.control; bulk RNA-seq,Homo_633,AD.vs.control; bulk RNA-seq,Homo_633,AD.vs.MCI	6
CC	GO:0005759	mitochondrial matrix	bulk RNA-seq,Homo_723,AD.vs.MCI; bulk RNA-seq,Homo_714,AD.vs.control; bulk RNA-seq,Homo_714,AD.vs.MCI; bulk RNA-seq,Homo_633,AD.vs.control; bulk RNA-seq,Homo_633,AD.vs.MCI; bulk RNA-seq,SRP223445,AD.vs.control	6
BP	GO:0006163	purine nucleotide metabolic process	bulk RNA-seq,Homo_723,AD.vs.MCI; bulk RNA-seq,Homo_714,AD.vs.control; bulk RNA-seq,Homo_714,AD.vs.MCI; bulk RNA-seq,Homo_633,AD.vs.control; bulk RNA-seq,Homo_633,AD.vs.MCI	5
BP	GO:1901879	regulation of protein depolymerization	bulk RNA-seq,Homo_723,AD.vs.MCI; bulk RNA-seq,Homo_723,MCI.vs.control; bulk RNA-seq,Homo_714,AD.vs.MCI; bulk RNA-seq,Homo_714,MCI.vs.control; bulk RNA-seq,Homo_633,AD.vs.control	5
BP	GO:0032872	regulation of stress-activated MAPK cascade	bulk RNA-seq,Homo_723,AD.vs.MCI; bulk RNA-seq,Homo_723,MCI.vs.control; bulk RNA-seq,Homo_714,AD.vs.MCI; bulk RNA-seq,Homo_633,AD.vs.control; bulk RNA-seq,Homo_633,AD.vs.MCI	5
BP	GO:1901606	alpha-amino acid catabolic process	bulk RNA-seq,Homo_723,AD.vs.MCI; bulk RNA-seq,Homo_723,MCI.vs.control; bulk RNA-seq,Homo_714,AD.vs.MCI; bulk RNA-seq,Homo_714,MCI.vs.control; bulk RNA-seq,Homo_633,AD.vs.control; bulk RNA-seq,Homo_633,AD.vs.MCI; bulk RNA-seq,SRP223445,AD.vs.control	7
MF	GO:0005525	GTP binding	bulk RNA-seq,Homo_723,AD.vs.MCI; bulk RNA-seq,Homo_714,AD.vs.MCI; bulk RNA-seq,Homo_633,AD.vs.control; bulk RNA-seq,Homo_633,AD.vs.MCI	4
BP	GO:0048010	vascular endothelial growth factor receptor signaling pathway	bulk RNA-seq,Homo_723,AD.vs.MCI; bulk RNA-seq,Homo_723,MCI.vs.control; bulk RNA-seq,Homo_714,AD.vs.MCI; bulk RNA-seq,Homo_714,MCI.vs.control; bulk RNA-seq,Homo_633,AD.vs.control; bulk RNA-seq,Homo_633,AD.vs.MCI	6
BP	GO:1901889	negative regulation of cell junction assembly	bulk RNA-seq,Homo_723,AD.vs.MCI; bulk RNA-seq,Homo_723,MCI.vs.control; bulk RNA-seq,Homo_714,AD.vs.MCI; bulk RNA-seq,Homo_714,MCI.vs.control; bulk RNA-seq,Homo_633,AD.vs.control; bulk RNA-seq,Homo_633,AD.vs.MCI	6
BP	GO:0009063	cellular amino acid catabolic process	bulk RNA-seq,Homo_723,AD.vs.MCI; bulk RNA-seq,Homo_723,MCI.vs.control; bulk RNA-seq,Homo_714,AD.vs.MCI; bulk RNA-seq,Homo_714,MCI.vs.control; bulk RNA-seq,Homo_633,AD.vs.control; bulk RNA-seq,Homo_633,AD.vs.MCI; bulk RNA-seq,SRP223445,AD.vs.control	7
MF	GO:1990782	protein tyrosine kinase binding	bulk RNA-seq,Homo_723,AD.vs.MCI; bulk RNA-seq,Homo_723,MCI.vs.control; bulk RNA-seq,Homo_714,AD.vs.MCI; bulk RNA-seq,Homo_714,MCI.vs.control; bulk RNA-seq,Homo_633,AD.vs.control; bulk RNA-seq,Homo_633,AD.vs.MCI; scRNA-seq,SRP215507,CD8+ T cell_3-	7
MF	GO:0001618	virus receptor activity	bulk RNA-seq,Homo_723,AD.vs.MCI; bulk RNA-seq,Homo_723,MCI.vs.control; bulk RNA-seq,Homo_714,AD.vs.MCI; bulk RNA-seq,Homo_714,MCI.vs.control; bulk RNA-seq,Homo_633,AD.vs.control; bulk RNA-seq,Homo_633,AD.vs.MCI	6
BP	GO:0032924	activin receptor signaling pathway	bulk RNA-seq,Homo_723,AD.vs.MCI; bulk RNA-seq,Homo_723,MCI.vs.control; bulk RNA-seq,Homo_714,AD.vs.MCI; bulk RNA-seq,Homo_714,MCI.vs.control	4
BP	GO:0031638	zymogen activation	bulk RNA-seq,Homo_723,AD.vs.MCI; bulk RNA-seq,Homo_723,MCI.vs.control; bulk RNA-seq,Homo_714,AD.vs.MCI; bulk RNA-seq,Homo_714,MCI.vs.control; bulk RNA-seq,Homo_633,AD.vs.control	5
BP	GO:0045940	positive regulation of steroid metabolic process	bulk RNA-seq,Homo_723,AD.vs.MCI; bulk RNA-seq,Homo_723,MCI.vs.control; bulk RNA-seq,Homo_714,AD.vs.MCI; bulk RNA-seq,Homo_714,MCI.vs.control	4
CC	GO:0005791	rough endoplasmic reticulum	bulk RNA-seq,Homo_723,AD.vs.MCI; bulk RNA-seq,Homo_723,MCI.vs.control; bulk RNA-seq,Homo_714,AD.vs.MCI; bulk RNA-seq,Homo_714,MCI.vs.control; bulk RNA-seq,Homo_633,AD.vs.control; bulk RNA-seq,Homo_633,AD.vs.MCI	6
BP	GO:0035023	regulation of Rho protein signal transduction	bulk RNA-seq,Homo_723,AD.vs.MCI; bulk RNA-seq,Homo_723,MCI.vs.control; bulk RNA-seq,Homo_714,AD.vs.MCI; bulk RNA-seq,Homo_714,MCI.vs.control; bulk RNA-seq,Homo_633,AD.vs.control; bulk RNA-seq,Homo_633,AD.vs.MCI	6
BP	GO:0002687	positive regulation of leukocyte migration	bulk RNA-seq,Homo_723,AD.vs.MCI; bulk RNA-seq,Homo_723,MCI.vs.control; bulk RNA-seq,Homo_714,AD.vs.MCI; bulk RNA-seq,Homo_633,AD.vs.control; bulk RNA-seq,Homo_633,AD.vs.MCI	5
MF	GO:0005518	collagen binding	bulk RNA-seq,Homo_723,AD.vs.MCI; bulk RNA-seq,Homo_723,MCI.vs.control; bulk RNA-seq,Homo_714,AD.vs.MCI; bulk RNA-seq,Homo_714,MCI.vs.control; bulk RNA-seq,Homo_633,AD.vs.control; bulk RNA-seq,Homo_633,AD.vs.MCI	6
MF	GO:0005546	phosphatidylinositol-4,5-bisphosphate binding	bulk RNA-seq,Homo_723,AD.vs.MCI; bulk RNA-seq,Homo_723,MCI.vs.control; bulk RNA-seq,Homo_714,AD.vs.MCI; bulk RNA-seq,Homo_714,MCI.vs.control; bulk RNA-seq,Homo_633,AD.vs.control	5
BP	GO:0007088	regulation of mitotic nuclear division	bulk RNA-seq,Homo_723,AD.vs.MCI; bulk RNA-seq,Homo_723,MCI.vs.control; bulk RNA-seq,Homo_714,AD.vs.MCI; bulk RNA-seq,Homo_714,MCI.vs.control; bulk RNA-seq,Homo_633,AD.vs.control; bulk RNA-seq,Homo_633,AD.vs.MCI	6
CC	GO:0032153	cell division site	bulk RNA-seq,Homo_723,AD.vs.MCI; bulk RNA-seq,Homo_723,MCI.vs.control; bulk RNA-seq,Homo_714,AD.vs.MCI; bulk RNA-seq,Homo_714,MCI.vs.control; bulk RNA-seq,Homo_633,AD.vs.control; bulk RNA-seq,Homo_633,AD.vs.MCI	6
CC	GO:0000793	condensed chromosome	bulk RNA-seq,Homo_723,AD.vs.MCI; bulk RNA-seq,Homo_723,MCI.vs.control; bulk RNA-seq,Homo_714,AD.vs.MCI; bulk RNA-seq,Homo_633,AD.vs.control; bulk RNA-seq,Homo_633,AD.vs.MCI	5
BP	GO:0030099	myeloid cell differentiation	bulk RNA-seq,Homo_723,AD.vs.MCI; bulk RNA-seq,Homo_714,AD.vs.MCI; bulk RNA-seq,Homo_633,AD.vs.control; bulk RNA-seq,Homo_633,AD.vs.MCI; scRNA-seq,SRP330776,Naive CD8+ T cell_2-AD.vs.control	5
BP	GO:0032232	negative regulation of actin filament bundle assembly	bulk RNA-seq,Homo_723,AD.vs.MCI; bulk RNA-seq,Homo_723,MCI.vs.control; bulk RNA-seq,Homo_714,AD.vs.MCI; bulk RNA-seq,Homo_714,MCI.vs.control	4
MF	GO:0002020	protease binding	bulk RNA-seq,Homo_723,AD.vs.MCI; bulk RNA-seq,Homo_723,MCI.vs.control; bulk RNA-seq,Homo_714,AD.vs.MCI; bulk RNA-seq,Homo_714,MCI.vs.control; bulk RNA-seq,Homo_633,AD.vs.control; bulk RNA-seq,Homo_633,AD.vs.MCI	6
BP	GO:0060317	cardiac epithelial to mesenchymal transition	bulk RNA-seq,Homo_723,AD.vs.MCI; bulk RNA-seq,Homo_723,MCI.vs.control; bulk RNA-seq,Homo_714,AD.vs.MCI; bulk RNA-seq,Homo_714,MCI.vs.control; bulk RNA-seq,Homo_633,AD.vs.control	5
BP	GO:0001954	positive regulation of cell-matrix adhesion	bulk RNA-seq,Homo_723,AD.vs.MCI; bulk RNA-seq,Homo_723,MCI.vs.control; bulk RNA-seq,Homo_714,AD.vs.MCI; bulk RNA-seq,Homo_714,MCI.vs.control; bulk RNA-seq,Homo_633,AD.vs.control; bulk RNA-seq,Homo_633,AD.vs.MCI	6

BP	GO:0045862	positive regulation of proteolysis	bulk RNA-seq,Homo_723,AD.vs.MCI; bulk RNA-seq,Homo_714,AD.vs.MCI; bulk RNA-seq,Homo_633,AD.vs.control; bulk RNA-seq,Homo_633,AD.vs.MCI; scRNA-seq,SRP330776,Naive CD8+ T cell_2-AD.vs.control	5
MF	GO:0005044	scavenger receptor activity	bulk RNA-seq,Homo_723,AD.vs.MCI; bulk RNA-seq,Homo_723,MCI.vs.control; bulk RNA-seq,Homo_714,AD.vs.MCI; bulk RNA-seq,Homo_714,MCI.vs.control; bulk RNA-seq,Homo_633,AD.vs.control	5
MF	GO:0046332	SMAD binding	bulk RNA-seq,Homo_723,AD.vs.MCI; bulk RNA-seq,Homo_723,MCI.vs.control; bulk RNA-seq,Homo_714,AD.vs.MCI; bulk RNA-seq,Homo_714,MCI.vs.control; bulk RNA-seq,Homo_633,AD.vs.control; bulk RNA-seq,Homo_633,AD.vs.MCI	6
BP	GO:0070302	regulation of stress-activated protein kinase signaling cascade	bulk RNA-seq,Homo_723,AD.vs.MCI; bulk RNA-seq,Homo_723,MCI.vs.control; bulk RNA-seq,Homo_714,AD.vs.MCI; bulk RNA-seq,Homo_633,AD.vs.control; bulk RNA-seq,Homo_633,AD.vs.MCI	5
BP	GO:2000273	positive regulation of signaling receptor activity	bulk RNA-seq,Homo_723,AD.vs.MCI; bulk RNA-seq,Homo_723,MCI.vs.control; bulk RNA-seq,Homo_714,AD.vs.MCI; bulk RNA-seq,Homo_714,MCI.vs.control; bulk RNA-seq,Homo_633,AD.vs.control; bulk RNA-seq,Homo_633,AD.vs.MCI	6
BP	GO:0150117	positive regulation of cell-substrate junction organization	bulk RNA-seq,Homo_723,AD.vs.MCI; bulk RNA-seq,Homo_723,MCI.vs.control; bulk RNA-seq,Homo_714,AD.vs.MCI; bulk RNA-seq,Homo_714,MCI.vs.control; bulk RNA-seq,Homo_633,AD.vs.control; bulk RNA-seq,Homo_633,AD.vs.MCI	6
BP	GO:0007127	meiosis I	bulk RNA-seq,Homo_723,AD.vs.MCI; bulk RNA-seq,Homo_723,MCI.vs.control; bulk RNA-seq,Homo_714,AD.vs.MCI; bulk RNA-seq,Homo_633,AD.vs.control; bulk RNA-seq,Homo_633,AD.vs.MCI	5
BP	GO:0050727	regulation of inflammatory response	bulk RNA-seq,Homo_723,AD.vs.MCI; bulk RNA-seq,Homo_714,AD.vs.MCI; bulk RNA-seq,Homo_633,AD.vs.control; bulk RNA-seq,Homo_633,AD.vs.MCI	4
BP	GO:0050729	positive regulation of inflammatory response	bulk RNA-seq,Homo_723,AD.vs.MCI; bulk RNA-seq,Homo_723,MCI.vs.control; bulk RNA-seq,Homo_714,AD.vs.MCI; bulk RNA-seq,Homo_633,AD.vs.control; bulk RNA-seq,Homo_633,AD.vs.MCI	5
BP	GO:0071385	cellular response to glucocorticoid stimulus	bulk RNA-seq,Homo_723,AD.vs.MCI; bulk RNA-seq,Homo_723,MCI.vs.control; bulk RNA-seq,Homo_714,AD.vs.MCI; bulk RNA-seq,Homo_714,MCI.vs.control; bulk RNA-seq,Homo_633,AD.vs.control; bulk RNA-seq,Homo_633,AD.vs.MCI	6
BP	GO:0061640	cytoskeleton-dependent cytokinesis	bulk RNA-seq,Homo_723,AD.vs.MCI; bulk RNA-seq,Homo_723,MCI.vs.control; bulk RNA-seq,Homo_714,AD.vs.MCI; bulk RNA-seq,Homo_714,MCI.vs.control; bulk RNA-seq,Homo_633,AD.vs.control; bulk RNA-seq,Homo_633,AD.vs.MCI	6
BP	GO:1900180	regulation of protein localization to nucleus	bulk RNA-seq,Homo_723,AD.vs.MCI; bulk RNA-seq,Homo_723,MCI.vs.control; bulk RNA-seq,Homo_714,AD.vs.MCI; bulk RNA-seq,Homo_633,AD.vs.control; bulk RNA-seq,Homo_633,AD.vs.MCI	5
BP	GO:0033143	regulation of intracellular steroid hormone receptor signaling pathway	bulk RNA-seq,Homo_723,AD.vs.MCI; bulk RNA-seq,Homo_723,MCI.vs.control; bulk RNA-seq,Homo_714,AD.vs.MCI; bulk RNA-seq,Homo_714,MCI.vs.control; bulk RNA-seq,Homo_633,AD.vs.control; bulk RNA-seq,Homo_633,AD.vs.MCI	6
BP	GO:0010970	transport along microtubule	bulk RNA-seq,Homo_723,AD.vs.MCI; bulk RNA-seq,Homo_723,MCI.vs.control; bulk RNA-seq,Homo_714,AD.vs.MCI; bulk RNA-seq,Homo_633,AD.vs.control; bulk RNA-seq,Homo_633,AD.vs.MCI	5
MF	GO:0016709	oxidoreductase activity, acting on paired donors, with incorporation or reduction of molecular oxygen, NAD(P)H as one donor, and incorporation of one atom of oxygen	bulk RNA-seq,Homo_723,AD.vs.MCI; bulk RNA-seq,Homo_723,MCI.vs.control; bulk RNA-seq,Homo_714,AD.vs.MCI; bulk RNA-seq,Homo_714,MCI.vs.control; bulk RNA-seq,Homo_633,AD.vs.control; bulk RNA-seq,Homo_633,AD.vs.MCI; bulk RNA-seq,SRP223445,AD.vs.control	7
MF	GO:0008081	phosphoric diester hydrolase activity	bulk RNA-seq,Homo_723,AD.vs.MCI; bulk RNA-seq,Homo_723,MCI.vs.control; bulk RNA-seq,Homo_714,AD.vs.MCI; bulk RNA-seq,Homo_714,MCI.vs.control; bulk RNA-seq,Homo_633,AD.vs.control; bulk RNA-seq,Homo_633,AD.vs.MCI; bulk RNA-seq,ROSMAP,AD.vs.control	7
BP	GO:0071604	transforming growth factor beta production	bulk RNA-seq,Homo_723,AD.vs.MCI; bulk RNA-seq,Homo_723,MCI.vs.control; bulk RNA-seq,Homo_714,AD.vs.MCI; bulk RNA-seq,Homo_714,MCI.vs.control; bulk RNA-seq,Homo_633,AD.vs.control; bulk RNA-seq,Homo_633,AD.vs.MCI	6
BP	GO:0023019	signal transduction involved in regulation of gene expression	bulk RNA-seq,Homo_723,AD.vs.MCI; bulk RNA-seq,Homo_723,MCI.vs.control; bulk RNA-seq,Homo_714,AD.vs.MCI; bulk RNA-seq,Homo_633,MCI.vs.control	4
BP	GO:1990868	response to chemokine	bulk RNA-seq,Homo_723,AD.vs.MCI; bulk RNA-seq,Homo_723,MCI.vs.control; bulk RNA-seq,Homo_714,AD.vs.control; bulk RNA-seq,Homo_714,AD.vs.MCI; bulk RNA-seq,Homo_714,MCI.vs.control	5
BP	GO:1990869	cellular response to chemokine	bulk RNA-seq,Homo_723,AD.vs.MCI; bulk RNA-seq,Homo_723,MCI.vs.control; bulk RNA-seq,Homo_714,AD.vs.control; bulk RNA-seq,Homo_714,AD.vs.MCI; bulk RNA-seq,Homo_714,MCI.vs.control	5
BP	GO:0010799	regulation of peptidyl-threonine phosphorylation	bulk RNA-seq,Homo_723,AD.vs.MCI; bulk RNA-seq,Homo_723,MCI.vs.control; bulk RNA-seq,Homo_714,AD.vs.MCI; bulk RNA-seq,Homo_714,MCI.vs.control; bulk RNA-seq,Homo_633,AD.vs.control	5
BP	GO:0051894	positive regulation of focal adhesion assembly	bulk RNA-seq,Homo_723,AD.vs.MCI; bulk RNA-seq,Homo_723,MCI.vs.control; bulk RNA-seq,Homo_714,AD.vs.MCI; bulk RNA-seq,Homo_714,MCI.vs.control; bulk RNA-seq,Homo_633,AD.vs.control; bulk RNA-seq,Homo_633,AD.vs.MCI	6
BP	GO:0031111	negative regulation of microtubule polymerization or depolymerization	bulk RNA-seq,Homo_723,AD.vs.MCI; bulk RNA-seq,Homo_723,MCI.vs.control; bulk RNA-seq,Homo_714,AD.vs.MCI; bulk RNA-seq,Homo_714,MCI.vs.control; bulk RNA-seq,Homo_633,AD.vs.control	5
BP	GO:0006023	aminoglycan biosynthetic process	bulk RNA-seq,Homo_723,AD.vs.MCI; bulk RNA-seq,Homo_723,MCI.vs.control; bulk RNA-seq,Homo_714,AD.vs.MCI; bulk RNA-seq,Homo_714,MCI.vs.control; bulk RNA-seq,Homo_633,AD.vs.control; bulk RNA-seq,Homo_633,AD.vs.MCI	6
BP	GO:1904375	regulation of protein localization to cell periphery	bulk RNA-seq,Homo_723,AD.vs.MCI; bulk RNA-seq,Homo_723,MCI.vs.control; bulk RNA-seq,Homo_714,AD.vs.MCI; bulk RNA-seq,Homo_714,MCI.vs.control; bulk RNA-seq,Homo_633,AD.vs.control; bulk RNA-seq,Homo_633,AD.vs.MCI	6
BP	GO:0045806	negative regulation of endocytosis	bulk RNA-seq,Homo_723,AD.vs.MCI; bulk RNA-seq,Homo_723,MCI.vs.control; bulk RNA-seq,Homo_714,AD.vs.control; bulk RNA-seq,Homo_714,AD.vs.MCI; bulk RNA-seq,Homo_633,AD.vs.control; bulk RNA-seq,Homo_633,AD.vs.MCI	6
BP	GO:0046718	viral entry into host cell	bulk RNA-seq,Homo_723,AD.vs.MCI; bulk RNA-seq,Homo_723,MCI.vs.control; bulk RNA-seq,Homo_714,AD.vs.MCI; bulk RNA-seq,Homo_633,AD.vs.control; bulk RNA-seq,Homo_633,AD.vs.MCI	5
MF	GO:0046982	protein heterodimerization activity	bulk RNA-seq,Homo_723,AD.vs.MCI; bulk RNA-seq,Homo_723,MCI.vs.control; bulk RNA-seq,Homo_714,AD.vs.MCI; bulk RNA-seq,Homo_633,AD.vs.control; bulk RNA-seq,Homo_633,AD.vs.MCI	5
BP	GO:1901655	cellular response to ketone	bulk RNA-seq,Homo_723,AD.vs.MCI; bulk RNA-seq,Homo_723,MCI.vs.control; bulk RNA-seq,Homo_714,AD.vs.MCI; bulk RNA-seq,Homo_714,MCI.vs.control; bulk RNA-seq,Homo_633,AD.vs.control; bulk RNA-seq,Homo_633,AD.vs.MCI	6
BP	GO:0045637	regulation of myeloid cell differentiation	bulk RNA-seq,Homo_723,AD.vs.MCI; bulk RNA-seq,Homo_723,MCI.vs.control; bulk RNA-seq,Homo_714,AD.vs.MCI; bulk RNA-seq,Homo_633,AD.vs.control; bulk RNA-seq,Homo_633,AD.vs.MCI	5
BP	GO:0051057	positive regulation of small GTPase mediated signal transduction	bulk RNA-seq,Homo_723,AD.vs.MCI; bulk RNA-seq,Homo_723,MCI.vs.control; bulk RNA-seq,Homo_714,AD.vs.MCI; bulk RNA-seq,Homo_714,MCI.vs.control; bulk RNA-seq,Homo_633,AD.vs.control	5
BP	GO:0071621	granulocyte chemotaxis	bulk RNA-seq,Homo_723,AD.vs.MCI; bulk RNA-seq,Homo_723,MCI.vs.control; bulk RNA-seq,Homo_714,AD.vs.MCI; bulk RNA-seq,Homo_633,AD.vs.control; bulk RNA-seq,SRP223445,AD.vs.control	5
BP	GO:0030512	negative regulation of transforming growth factor beta receptor signaling pathway	bulk RNA-seq,Homo_723,AD.vs.MCI; bulk RNA-seq,Homo_723,MCI.vs.control; bulk RNA-seq,Homo_714,AD.vs.MCI; bulk RNA-seq,Homo_714,MCI.vs.control; bulk RNA-seq,Homo_633,AD.vs.control; bulk RNA-seq,Homo_633,AD.vs.MCI	6

MF	GO:0046906	tetrapyrrole binding	bulk RNA-seq,Homo_723,AD.vs.MCI; bulk RNA-seq,Homo_723,MCI.vs.control; bulk RNA-seq,Homo_714,AD.vs.MCI; bulk RNA-seq,Homo_633,AD.vs.control; bulk RNA-seq,Homo_633,AD.vs.MCI; bulk RNA-seq,SRP223445,AD.vs.control	6
MF	GO:0035091	phosphatidylinositol binding	bulk RNA-seq,Homo_723,AD.vs.MCI; bulk RNA-seq,Homo_723,MCI.vs.control; bulk RNA-seq,Homo_714,AD.vs.control; bulk RNA-seq,Homo_714,AD.vs.MCI; bulk RNA-seq,Homo_633,AD.vs.control; bulk RNA-seq,Homo_633,AD.vs.MCI	6
MF	GO:0031490	chromatin DNA binding	bulk RNA-seq,Homo_723,AD.vs.MCI; bulk RNA-seq,Homo_723,MCI.vs.control; bulk RNA-seq,Homo_714,AD.vs.MCI; bulk RNA-seq,Homo_714,MCI.vs.control; bulk RNA-seq,Homo_633,AD.vs.control; bulk RNA-seq,Homo_633,AD.vs.MCI	6
BP	GO:0071453	cellular response to oxygen levels	bulk RNA-seq,Homo_723,AD.vs.MCI; bulk RNA-seq,Homo_723,MCI.vs.control; bulk RNA-seq,Homo_714,AD.vs.MCI; bulk RNA-seq,Homo_633,AD.vs.control; bulk RNA-seq,Homo_633,AD.vs.MCI	5
BP	GO:0034384	high-density lipoprotein particle clearance	bulk RNA-seq,Homo_723,AD.vs.MCI; bulk RNA-seq,Homo_723,MCI.vs.control; bulk RNA-seq,Homo_714,AD.vs.control; bulk RNA-seq,Homo_714,AD.vs.MCI; bulk RNA-seq,Homo_714,MCI.vs.control	5
BP	GO:2000352	negative regulation of endothelial cell apoptotic process	bulk RNA-seq,Homo_723,AD.vs.MCI; bulk RNA-seq,Homo_723,MCI.vs.control; bulk RNA-seq,Homo_714,AD.vs.MCI; bulk RNA-seq,Homo_714,MCI.vs.control	4
MF	GO:0038024	cargo receptor activity	bulk RNA-seq,Homo_723,AD.vs.MCI; bulk RNA-seq,Homo_723,MCI.vs.control; bulk RNA-seq,Homo_714,AD.vs.MCI; bulk RNA-seq,Homo_714,MCI.vs.control; bulk RNA-seq,Homo_633,AD.vs.control; bulk RNA-seq,Homo_633,AD.vs.MCI	6
BP	GO:2001214	positive regulation of vasculogenesis	bulk RNA-seq,Homo_723,AD.vs.MCI; bulk RNA-seq,Homo_723,MCI.vs.control; bulk RNA-seq,Homo_714,AD.vs.control; bulk RNA-seq,Homo_714,AD.vs.MCI; bulk RNA-seq,Homo_714,MCI.vs.control	5
BP	GO:0034284	response to monosaccharide	bulk RNA-seq,Homo_723,AD.vs.MCI; bulk RNA-seq,Homo_723,MCI.vs.control; bulk RNA-seq,Homo_714,AD.vs.MCI; bulk RNA-seq,Homo_633,AD.vs.control; bulk RNA-seq,Homo_633,AD.vs.MCI	5
BP	GO:1901881	positive regulation of protein depolymerization	bulk RNA-seq,Homo_723,AD.vs.MCI; bulk RNA-seq,Homo_723,MCI.vs.control; bulk RNA-seq,Homo_714,AD.vs.MCI; bulk RNA-seq,Homo_714,MCI.vs.control	4
MF	GO:0048306	calcium-dependent protein binding	bulk RNA-seq,Homo_723,AD.vs.MCI; bulk RNA-seq,Homo_723,MCI.vs.control; bulk RNA-seq,Homo_714,AD.vs.MCI; bulk RNA-seq,Homo_714,MCI.vs.control; bulk RNA-seq,Homo_633,AD.vs.control	5
BP	GO:0032273	positive regulation of protein polymerization	bulk RNA-seq,Homo_723,AD.vs.MCI; bulk RNA-seq,Homo_723,MCI.vs.control; bulk RNA-seq,Homo_714,AD.vs.MCI; bulk RNA-seq,Homo_714,MCI.vs.control; bulk RNA-seq,Homo_633,AD.vs.control; bulk RNA-seq,Homo_633,AD.vs.MCI	6
CC	GO:0000775	chromosome, centromeric region	bulk RNA-seq,Homo_723,AD.vs.MCI; bulk RNA-seq,Homo_723,MCI.vs.control; bulk RNA-seq,Homo_714,AD.vs.MCI; bulk RNA-seq,Homo_633,AD.vs.control; bulk RNA-seq,Homo_633,AD.vs.MCI	5
CC	GO:0005905	clathrin-coated pit	bulk RNA-seq,Homo_723,AD.vs.MCI; bulk RNA-seq,Homo_723,MCI.vs.control; bulk RNA-seq,Homo_714,AD.vs.MCI; bulk RNA-seq,Homo_714,MCI.vs.control; bulk RNA-seq,Homo_633,AD.vs.control	5
BP	GO:0009150	purine ribonucleotide metabolic process	bulk RNA-seq,Homo_723,AD.vs.MCI; bulk RNA-seq,Homo_714,AD.vs.control; bulk RNA-seq,Homo_714,AD.vs.MCI; bulk RNA-seq,Homo_633,AD.vs.control; bulk RNA-seq,Homo_633,AD.vs.MCI	5
BP	GO:0071456	cellular response to hypoxia	bulk RNA-seq,Homo_723,AD.vs.MCI; bulk RNA-seq,Homo_723,MCI.vs.control; bulk RNA-seq,Homo_714,AD.vs.MCI; bulk RNA-seq,Homo_633,AD.vs.control; bulk RNA-seq,Homo_633,AD.vs.MCI	5
BP	GO:0070192	chromosome organization involved in meiotic cell cycle	bulk RNA-seq,Homo_723,AD.vs.MCI; bulk RNA-seq,Homo_723,MCI.vs.control; bulk RNA-seq,Homo_714,AD.vs.MCI; bulk RNA-seq,Homo_714,MCI.vs.control; bulk RNA-seq,Homo_633,AD.vs.control; bulk RNA-seq,Homo_633,AD.vs.MCI	6
CC	GO:0098562	cytoplasmic side of membrane	bulk RNA-seq,Homo_723,AD.vs.MCI; bulk RNA-seq,Homo_723,MCI.vs.control; bulk RNA-seq,Homo_714,AD.vs.MCI; bulk RNA-seq,Homo_633,AD.vs.control; bulk RNA-seq,Homo_633,AD.vs.MCI	5
BP	GO:0062197	cellular response to chemical stress	bulk RNA-seq,Homo_723,AD.vs.MCI; bulk RNA-seq,Homo_714,AD.vs.MCI; bulk RNA-seq,Homo_633,AD.vs.control; bulk RNA-seq,Homo_633,AD.vs.MCI	4
BP	GO:2000345	regulation of hepatocyte proliferation	bulk RNA-seq,Homo_723,AD.vs.MCI; bulk RNA-seq,Homo_723,MCI.vs.control; bulk RNA-seq,Homo_714,AD.vs.control; bulk RNA-seq,Homo_714,AD.vs.MCI; bulk RNA-seq,Homo_714,MCI.vs.control	5
BP	GO:0050775	positive regulation of dendrite morphogenesis	bulk RNA-seq,Homo_723,AD.vs.MCI; bulk RNA-seq,Homo_723,MCI.vs.control; bulk RNA-seq,Homo_714,AD.vs.MCI; bulk RNA-seq,Homo_714,MCI.vs.control; bulk RNA-seq,Homo_633,AD.vs.control; bulk RNA-seq,Homo_633,AD.vs.MCI	6
MF	GO:0015174	basic amino acid transmembrane transporter activity	bulk RNA-seq,Homo_723,AD.vs.MCI; bulk RNA-seq,Homo_723,MCI.vs.control	2
MF	GO:0003714	transcription corepressor activity	bulk RNA-seq,Homo_723,AD.vs.MCI; bulk RNA-seq,Homo_723,MCI.vs.control; bulk RNA-seq,Homo_714,AD.vs.MCI; bulk RNA-seq,Homo_633,AD.vs.control; bulk RNA-seq,Homo_633,AD.vs.MCI; bulk RNA-seq,SRP310421,AD.vs.control	6
MF	GO:0035591	signaling adaptor activity	bulk RNA-seq,Homo_723,AD.vs.MCI; bulk RNA-seq,Homo_723,MCI.vs.control; bulk RNA-seq,Homo_714,AD.vs.MCI; bulk RNA-seq,Homo_714,MCI.vs.control; bulk RNA-seq,Homo_633,AD.vs.control; bulk RNA-seq,Homo_633,AD.vs.MCI	6
MF	GO:0005506	iron ion binding	bulk RNA-seq,Homo_723,AD.vs.MCI; bulk RNA-seq,Homo_723,MCI.vs.control; bulk RNA-seq,Homo_714,AD.vs.MCI; bulk RNA-seq,Homo_633,AD.vs.control; bulk RNA-seq,Homo_633,AD.vs.MCI; bulk RNA-seq,SRP223445,AD.vs.control	6
BP	GO:0031334	positive regulation of protein-containing complex assembly	bulk RNA-seq,Homo_723,AD.vs.MCI; bulk RNA-seq,Homo_723,MCI.vs.control; bulk RNA-seq,Homo_714,AD.vs.MCI; bulk RNA-seq,Homo_633,AD.vs.control; bulk RNA-seq,Homo_633,AD.vs.MCI	5
MF	GO:1901981	phosphatidylinositol phosphate binding	bulk RNA-seq,Homo_723,AD.vs.MCI; bulk RNA-seq,Homo_723,MCI.vs.control; bulk RNA-seq,Homo_714,AD.vs.MCI; bulk RNA-seq,Homo_633,AD.vs.control; bulk RNA-seq,Homo_633,AD.vs.MCI	5
CC	GO:0030667	secretory granule membrane	bulk RNA-seq,Homo_723,AD.vs.MCI; bulk RNA-seq,Homo_723,MCI.vs.control; bulk RNA-seq,Homo_714,AD.vs.MCI; bulk RNA-seq,Homo_633,AD.vs.control; bulk RNA-seq,Homo_633,AD.vs.MCI; bulk RNA-seq,SRP223445,AD.vs.control	6
BP	GO:1901616	organic hydroxy compound catabolic process	bulk RNA-seq,Homo_723,AD.vs.MCI; bulk RNA-seq,Homo_723,MCI.vs.control; bulk RNA-seq,Homo_714,AD.vs.MCI; bulk RNA-seq,Homo_714,MCI.vs.control; bulk RNA-seq,Homo_633,AD.vs.control; bulk RNA-seq,Homo_633,AD.vs.MCI	6
BP	GO:0098926	postsynaptic signal transduction	bulk RNA-seq,Homo_723,AD.vs.MCI; bulk RNA-seq,Homo_723,MCI.vs.control; bulk RNA-seq,Homo_714,AD.vs.MCI	3
BP	GO:0014009	glial cell proliferation	bulk RNA-seq,Homo_723,AD.vs.MCI; bulk RNA-seq,Homo_723,MCI.vs.control; bulk RNA-seq,Homo_714,AD.vs.MCI; bulk RNA-seq,Homo_714,MCI.vs.control; bulk RNA-seq,Homo_633,AD.vs.control	5
BP	GO:1990822	basic amino acid transmembrane transport	bulk RNA-seq,Homo_723,AD.vs.MCI; bulk RNA-seq,Homo_723,MCI.vs.control	2
BP	GO:0032964	collagen biosynthetic process	bulk RNA-seq,Homo_723,AD.vs.MCI; bulk RNA-seq,Homo_723,MCI.vs.control; bulk RNA-seq,Homo_714,AD.vs.MCI; bulk RNA-seq,Homo_714,MCI.vs.control	4
BP	GO:2000351	regulation of endothelial cell apoptotic process	bulk RNA-seq,Homo_723,AD.vs.MCI; bulk RNA-seq,Homo_723,MCI.vs.control; bulk RNA-seq,Homo_714,AD.vs.MCI; bulk RNA-seq,Homo_714,MCI.vs.control	4
BP	GO:0009112	nucleobase metabolic process	bulk RNA-seq,Homo_723,AD.vs.MCI; bulk RNA-seq,Homo_723,MCI.vs.control; bulk RNA-seq,Homo_714,AD.vs.MCI; bulk RNA-seq,Homo_714,MCI.vs.control	4
BP	GO:0016054	organic acid catabolic process	bulk RNA-seq,Homo_723,AD.vs.MCI; bulk RNA-seq,Homo_723,MCI.vs.control; bulk RNA-seq,Homo_714,AD.vs.control; bulk RNA-seq,Homo_714,AD.vs.MCI; bulk RNA-seq,Homo_633,AD.vs.control; bulk RNA-seq,Homo_633,AD.vs.MCI; bulk RNA-seq,SRP223445,AD.vs.control	7
BP	GO:0048661	positive regulation of smooth muscle cell proliferation	bulk RNA-seq,Homo_723,AD.vs.MCI; bulk RNA-seq,Homo_723,MCI.vs.control; bulk RNA-seq,Homo_714,AD.vs.MCI; bulk RNA-seq,Homo_714,MCI.vs.control	4

BP	GO:2001046	positive regulation of integrin-mediated signaling pathway	bulk RNA-seq,Homo_723,AD.vs.MCI; bulk RNA-seq,Homo_723,MCI.vs.control; bulk RNA-seq,Homo_714,AD.vs.MCI	3
BP	GO:0018210	peptidyl-threonine modification	bulk RNA-seq,Homo_723,AD.vs.MCI; bulk RNA-seq,Homo_723,MCI.vs.control; bulk RNA-seq,Homo_714,AD.vs.MCI; bulk RNA-seq,Homo_714,MCI.vs.control; bulk RNA-seq,Homo_633,AD.vs.control; bulk RNA-seq,Homo_633,AD.vs.MCI	6
BP	GO:0044772	mitotic cell cycle phase transition	bulk RNA-seq,Homo_723,AD.vs.MCI; bulk RNA-seq,Homo_714,AD.vs.control; bulk RNA-seq,Homo_714,AD.vs.MCI; bulk RNA-seq,Homo_633,AD.vs.control; bulk RNA-seq,Homo_633,AD.vs.MCI	5
BP	GO:0050867	positive regulation of cell activation	bulk RNA-seq,Homo_723,AD.vs.MCI; bulk RNA-seq,Homo_714,AD.vs.control; bulk RNA-seq,Homo_633,AD.vs.control; bulk RNA-seq,Homo_633,AD.vs.MCI; scRNA-seq,SRP330776,Naive CD8+ T cell_2-AD.vs.control; scRNA-seq,SRP309935,Monocyte_2-AD.vs.control; scRNA-seq,SRP215507,CD8+ T cell_3-	7
BP	GO:0002696	positive regulation of leukocyte activation	bulk RNA-seq,Homo_723,AD.vs.MCI; bulk RNA-seq,Homo_714,AD.vs.control; bulk RNA-seq,Homo_633,AD.vs.control; bulk RNA-seq,Homo_633,AD.vs.MCI; scRNA-seq,SRP330776,Naive CD8+ T cell_2-AD.vs.control; scRNA-seq,SRP309935,Monocyte_2-AD.vs.control; scRNA-seq,SRP215507,CD8+ T cell_3-	7
BP	GO:0000910	cytokinesis	bulk RNA-seq,Homo_723,AD.vs.MCI; bulk RNA-seq,Homo_723,MCI.vs.control; bulk RNA-seq,Homo_714,AD.vs.MCI; bulk RNA-seq,Homo_633,AD.vs.control; bulk RNA-seq,Homo_633,AD.vs.MCI	5
BP	GO:0006509	membrane protein ectodomain proteolysis	bulk RNA-seq,Homo_723,AD.vs.MCI; bulk RNA-seq,Homo_723,MCI.vs.control; bulk RNA-seq,Homo_714,AD.vs.MCI; bulk RNA-seq,Homo_714,MCI.vs.control; bulk RNA-seq,Homo_633,AD.vs.control; bulk RNA-seq,Homo_633,AD.vs.MCI	6
BP	GO:0030004	cellular monovalent inorganic cation homeostasis	bulk RNA-seq,Homo_723,AD.vs.MCI; bulk RNA-seq,Homo_723,MCI.vs.control; bulk RNA-seq,Homo_714,AD.vs.MCI; bulk RNA-seq,Homo_714,MCI.vs.control; bulk RNA-seq,Homo_633,AD.vs.control; bulk RNA-seq,Homo_633,AD.vs.MCI	6
BP	GO:0061982	meiosis I cell cycle process	bulk RNA-seq,Homo_723,AD.vs.MCI; bulk RNA-seq,Homo_723,MCI.vs.control; bulk RNA-seq,Homo_714,AD.vs.MCI; bulk RNA-seq,Homo_633,AD.vs.control; bulk RNA-seq,Homo_633,AD.vs.MCI	5
CC	GO:0043198	dendritic shaft	bulk RNA-seq,Homo_723,AD.vs.MCI; bulk RNA-seq,Homo_723,MCI.vs.control; bulk RNA-seq,Homo_714,AD.vs.MCI; bulk RNA-seq,Homo_714,MCI.vs.control; bulk RNA-seq,Homo_633,AD.vs.control	5
BP	GO:0048477	oogenesis	bulk RNA-seq,Homo_723,AD.vs.MCI; bulk RNA-seq,Homo_723,MCI.vs.control; bulk RNA-seq,Homo_714,AD.vs.MCI; bulk RNA-seq,Homo_714,MCI.vs.control; bulk RNA-seq,Homo_633,AD.vs.control; bulk RNA-seq,Homo_633,AD.vs.MCI; bulk RNA-seq,SRP325058,AD.vs.control	7
BP	GO:0045124	regulation of bone resorption	bulk RNA-seq,Homo_723,AD.vs.MCI; bulk RNA-seq,Homo_723,MCI.vs.control; bulk RNA-seq,Homo_714,AD.vs.MCI; bulk RNA-seq,Homo_714,MCI.vs.control; bulk RNA-seq,Homo_633,AD.vs.control	5
CC	GO:0044295	axonal growth cone	bulk RNA-seq,Homo_723,AD.vs.MCI; bulk RNA-seq,Homo_723,MCI.vs.control; bulk RNA-seq,Homo_714,MCI.vs.control; bulk RNA-seq,Homo_633,AD.vs.control; bulk RNA-seq,Homo_633,AD.vs.MCI	5
MF	GO:0004879	nuclear receptor activity	bulk RNA-seq,Homo_723,AD.vs.MCI; bulk RNA-seq,Homo_723,MCI.vs.control; bulk RNA-seq,Homo_714,AD.vs.MCI; bulk RNA-seq,Homo_714,MCI.vs.control; bulk RNA-seq,Homo_633,AD.vs.control	5
MF	GO:0098531	ligand-activated transcription factor activity	bulk RNA-seq,Homo_723,AD.vs.MCI; bulk RNA-seq,Homo_723,MCI.vs.control; bulk RNA-seq,Homo_714,AD.vs.MCI; bulk RNA-seq,Homo_714,MCI.vs.control; bulk RNA-seq,Homo_633,AD.vs.control	5
BP	GO:0010470	regulation of gastrulation	bulk RNA-seq,Homo_723,AD.vs.MCI; bulk RNA-seq,Homo_723,MCI.vs.control	2
BP	GO:0048070	regulation of developmental pigmentation	bulk RNA-seq,Homo_723,AD.vs.MCI; bulk RNA-seq,Homo_723,MCI.vs.control; bulk RNA-seq,Homo_714,AD.vs.MCI; bulk RNA-seq,Homo_714,MCI.vs.control	4
BP	GO:0120305	regulation of pigmentation	bulk RNA-seq,Homo_723,AD.vs.MCI; bulk RNA-seq,Homo_723,MCI.vs.control; bulk RNA-seq,Homo_714,AD.vs.MCI; bulk RNA-seq,Homo_714,MCI.vs.control	4
BP	GO:0060343	trabecula formation	bulk RNA-seq,Homo_723,AD.vs.MCI; bulk RNA-seq,Homo_723,MCI.vs.control; bulk RNA-seq,Homo_714,AD.vs.MCI; bulk RNA-seq,Homo_714,MCI.vs.control	4
BP	GO:1905954	positive regulation of lipid localization	bulk RNA-seq,Homo_723,AD.vs.MCI; bulk RNA-seq,Homo_723,MCI.vs.control; bulk RNA-seq,Homo_714,AD.vs.MCI; bulk RNA-seq,Homo_714,MCI.vs.control; bulk RNA-seq,Homo_633,AD.vs.control; bulk RNA-seq,Homo_633,AD.vs.MCI	6
BP	GO:0009749	response to glucose	bulk RNA-seq,Homo_723,AD.vs.MCI; bulk RNA-seq,Homo_723,MCI.vs.control; bulk RNA-seq,Homo_714,AD.vs.MCI; bulk RNA-seq,Homo_633,AD.vs.control; bulk RNA-seq,Homo_633,AD.vs.MCI	5
BP	GO:0051099	positive regulation of binding	bulk RNA-seq,Homo_723,AD.vs.MCI; bulk RNA-seq,Homo_723,MCI.vs.control; bulk RNA-seq,Homo_714,AD.vs.MCI; bulk RNA-seq,Homo_633,AD.vs.control; bulk RNA-seq,Homo_633,AD.vs.MCI	5
BP	GO:0045453	bone resorption	bulk RNA-seq,Homo_723,AD.vs.MCI; bulk RNA-seq,Homo_723,MCI.vs.control; bulk RNA-seq,Homo_714,AD.vs.MCI; bulk RNA-seq,Homo_714,MCI.vs.control; bulk RNA-seq,Homo_633,AD.vs.control; bulk RNA-seq,Homo_633,AD.vs.MCI	6
BP	GO:0009746	response to hexose	bulk RNA-seq,Homo_723,AD.vs.MCI; bulk RNA-seq,Homo_723,MCI.vs.control; bulk RNA-seq,Homo_714,AD.vs.MCI; bulk RNA-seq,Homo_633,AD.vs.control; bulk RNA-seq,Homo_633,AD.vs.MCI	5
BP	GO:1905477	positive regulation of protein localization to membrane	bulk RNA-seq,Homo_723,AD.vs.MCI; bulk RNA-seq,Homo_723,MCI.vs.control; bulk RNA-seq,Homo_714,AD.vs.MCI; bulk RNA-seq,Homo_714,MCI.vs.control; bulk RNA-seq,Homo_633,AD.vs.control; bulk RNA-seq,Homo_633,AD.vs.MCI	6
BP	GO:0090630	activation of GTPase activity	bulk RNA-seq,Homo_723,AD.vs.MCI; bulk RNA-seq,Homo_723,MCI.vs.control; bulk RNA-seq,Homo_714,AD.vs.MCI; bulk RNA-seq,Homo_714,MCI.vs.control; bulk RNA-seq,Homo_633,AD.vs.control; bulk RNA-seq,Homo_633,AD.vs.MCI	6
BP	GO:0044000	movement in host	bulk RNA-seq,Homo_723,AD.vs.MCI; bulk RNA-seq,Homo_723,MCI.vs.control; bulk RNA-seq,Homo_714,AD.vs.MCI; bulk RNA-seq,Homo_633,AD.vs.control; bulk RNA-seq,Homo_633,AD.vs.MCI	5
BP	GO:0046850	regulation of bone remodeling	bulk RNA-seq,Homo_723,AD.vs.MCI; bulk RNA-seq,Homo_723,MCI.vs.control; bulk RNA-seq,Homo_714,AD.vs.MCI; bulk RNA-seq,Homo_714,MCI.vs.control; bulk RNA-seq,Homo_633,AD.vs.control; bulk RNA-seq,SRP325058,AD.vs.control	6
BP	GO:0003382	epithelial cell morphogenesis	bulk RNA-seq,Homo_723,AD.vs.MCI; bulk RNA-seq,Homo_723,MCI.vs.control; bulk RNA-seq,Homo_714,AD.vs.MCI; bulk RNA-seq,Homo_714,MCI.vs.control; bulk RNA-seq,Homo_633,AD.vs.control	5
BP	GO:0051043	regulation of membrane protein ectodomain proteolysis	bulk RNA-seq,Homo_723,AD.vs.MCI; bulk RNA-seq,Homo_723,MCI.vs.control; bulk RNA-seq,Homo_714,AD.vs.MCI; bulk RNA-seq,Homo_714,MCI.vs.control; bulk RNA-seq,Homo_633,AD.vs.control	5
BP	GO:2000738	positive regulation of stem cell differentiation	bulk RNA-seq,Homo_723,AD.vs.MCI; bulk RNA-seq,Homo_723,MCI.vs.control; bulk RNA-seq,Homo_714,AD.vs.MCI; bulk RNA-seq,Homo_714,MCI.vs.control	4
BP	GO:0036294	cellular response to decreased oxygen levels	bulk RNA-seq,Homo_723,AD.vs.MCI; bulk RNA-seq,Homo_723,MCI.vs.control; bulk RNA-seq,Homo_714,AD.vs.MCI; bulk RNA-seq,Homo_633,AD.vs.control; bulk RNA-seq,Homo_633,AD.vs.MCI	5
BP	GO:0006979	response to oxidative stress	bulk RNA-seq,Homo_723,AD.vs.MCI; bulk RNA-seq,Homo_714,AD.vs.control; bulk RNA-seq,Homo_714,AD.vs.MCI; bulk RNA-seq,Homo_633,AD.vs.control; bulk RNA-seq,Homo_633,AD.vs.MCI	5
BP	GO:0061028	establishment of endothelial barrier	bulk RNA-seq,Homo_723,AD.vs.MCI; bulk RNA-seq,Homo_723,MCI.vs.control; bulk RNA-seq,Homo_714,AD.vs.MCI; bulk RNA-seq,Homo_714,MCI.vs.control; bulk RNA-seq,Homo_633,AD.vs.control; bulk RNA-seq,Homo_633,AD.vs.MCI	6
BP	GO:0048710	regulation of astrocyte differentiation	bulk RNA-seq,Homo_723,AD.vs.MCI; bulk RNA-seq,Homo_723,MCI.vs.control; bulk RNA-seq,Homo_714,AD.vs.MCI; bulk RNA-seq,Homo_714,MCI.vs.control	4

BP	GO:0001953	negative regulation of cell-matrix adhesion	bulk RNA-seq,Homo_723,AD.vs.MCI; bulk RNA-seq,Homo_723,MCI.vs.control; bulk RNA-seq,Homo_714,AD.vs.MCI; bulk RNA-seq,Homo_714,MCI.vs.control; bulk RNA-seq,Homo_633,AD.vs.control; bulk RNA-seq,Homo_633,AD.vs.MCI	6
BP	GO:0045671	negative regulation of osteoclast differentiation	bulk RNA-seq,Homo_723,AD.vs.MCI; bulk RNA-seq,Homo_723,MCI.vs.control; bulk RNA-seq,Homo_714,AD.vs.MCI; bulk RNA-seq,Homo_714,MCI.vs.control; bulk RNA-seq,Homo_633,AD.vs.control; bulk RNA-seq,SRP325058,AD.vs.control	6
BP	GO:1905145	cellular response to acetylcholine	bulk RNA-seq,Homo_723,AD.vs.MCI; bulk RNA-seq,Homo_723,MCI.vs.control	2
BP	GO:0000302	response to reactive oxygen species	bulk RNA-seq,Homo_723,AD.vs.MCI; bulk RNA-seq,Homo_723,MCI.vs.control; bulk RNA-seq,Homo_714,AD.vs.MCI; bulk RNA-seq,Homo_633,AD.vs.control; bulk RNA-seq,Homo_633,AD.vs.MCI	5
CC	GO:0009898	cytoplasmic side of plasma membrane	bulk RNA-seq,Homo_723,AD.vs.MCI; bulk RNA-seq,Homo_723,MCI.vs.control; bulk RNA-seq,Homo_714,AD.vs.MCI; bulk RNA-seq,Homo_633,AD.vs.control; bulk RNA-seq,Homo_633,AD.vs.MCI	5
MF	GO:0019207	kinase regulator activity	bulk RNA-seq,Homo_723,AD.vs.MCI; bulk RNA-seq,Homo_723,MCI.vs.control; bulk RNA-seq,Homo_714,AD.vs.MCI; bulk RNA-seq,Homo_633,AD.vs.control; bulk RNA-seq,Homo_633,AD.vs.MCI	5
BP	GO:0150146	cell junction disassembly	bulk RNA-seq,Homo_723,AD.vs.MCI; bulk RNA-seq,Homo_723,MCI.vs.control; bulk RNA-seq,Homo_714,AD.vs.MCI; bulk RNA-seq,Homo_714,MCI.vs.control; bulk RNA-seq,Homo_633,AD.vs.control; bulk RNA-seq,Homo_633,AD.vs.MCI	6
BP	GO:1901880	negative regulation of protein depolymerization	bulk RNA-seq,Homo_723,AD.vs.MCI; bulk RNA-seq,Homo_723,MCI.vs.control; bulk RNA-seq,Homo_714,AD.vs.MCI; bulk RNA-seq,Homo_714,MCI.vs.control; bulk RNA-seq,Homo_633,AD.vs.control	5
BP	GO:0070633	transepithelial transport	bulk RNA-seq,Homo_723,AD.vs.MCI; bulk RNA-seq,Homo_723,MCI.vs.control; bulk RNA-seq,Homo_714,AD.vs.MCI; bulk RNA-seq,Homo_714,MCI.vs.control; bulk RNA-seq,Homo_633,AD.vs.control	5
BP	GO:0031669	cellular response to nutrient levels	bulk RNA-seq,Homo_723,AD.vs.MCI; bulk RNA-seq,Homo_723,MCI.vs.control; bulk RNA-seq,Homo_714,AD.vs.MCI; bulk RNA-seq,Homo_633,AD.vs.control; bulk RNA-seq,Homo_633,AD.vs.MCI	5
BP	GO:0046395	carboxylic acid catabolic process	bulk RNA-seq,Homo_723,AD.vs.MCI; bulk RNA-seq,Homo_723,MCI.vs.control; bulk RNA-seq,Homo_714,AD.vs.control; bulk RNA-seq,Homo_714,AD.vs.MCI; bulk RNA-seq,Homo_633,AD.vs.control; bulk RNA-seq,Homo_633,AD.vs.MCI; bulk RNA-seq,SRP223445,AD.vs.control	7
MF	GO:0031210	phosphatidylcholine binding	bulk RNA-seq,Homo_723,AD.vs.MCI; bulk RNA-seq,Homo_723,MCI.vs.control; bulk RNA-seq,Homo_714,AD.vs.MCI; bulk RNA-seq,Homo_714,MCI.vs.control; bulk RNA-seq,Homo_633,AD.vs.control; bulk RNA-seq,Homo_633,AD.vs.MCI	6
BP	GO:0120255	olefinic compound biosynthetic process	bulk RNA-seq,Homo_723,AD.vs.MCI; bulk RNA-seq,Homo_723,MCI.vs.control; bulk RNA-seq,Homo_714,AD.vs.MCI	3
BP	GO:0061384	heart trabecula morphogenesis	bulk RNA-seq,Homo_723,AD.vs.MCI; bulk RNA-seq,Homo_723,MCI.vs.control; bulk RNA-seq,Homo_714,AD.vs.MCI; bulk RNA-seq,Homo_714,MCI.vs.control	4
MF	GO:0043548	phosphatidylinositol 3-kinase binding	bulk RNA-seq,Homo_723,AD.vs.MCI; bulk RNA-seq,Homo_723,MCI.vs.control; bulk RNA-seq,Homo_714,MCI.vs.control	3
BP	GO:0032386	regulation of intracellular transport	bulk RNA-seq,Homo_723,AD.vs.MCI; bulk RNA-seq,Homo_714,AD.vs.MCI; bulk RNA-seq,Homo_633,AD.vs.control; bulk RNA-seq,Homo_633,AD.vs.MCI	4
BP	GO:0051489	regulation of filopodium assembly	bulk RNA-seq,Homo_723,AD.vs.MCI; bulk RNA-seq,Homo_723,MCI.vs.control; bulk RNA-seq,Homo_714,AD.vs.MCI; bulk RNA-seq,Homo_714,MCI.vs.control; bulk RNA-seq,Homo_633,AD.vs.control; bulk RNA-seq,Homo_633,AD.vs.MCI	6
BP	GO:0048701	embryonic cranial skeleton morphogenesis	bulk RNA-seq,Homo_723,AD.vs.MCI; bulk RNA-seq,Homo_723,MCI.vs.control; bulk RNA-seq,Homo_714,AD.vs.MCI; bulk RNA-seq,Homo_714,MCI.vs.control; bulk RNA-seq,Homo_633,AD.vs.control	5
BP	GO:0030279	negative regulation of ossification	bulk RNA-seq,Homo_723,AD.vs.MCI; bulk RNA-seq,Homo_723,MCI.vs.control; bulk RNA-seq,Homo_714,AD.vs.MCI; bulk RNA-seq,Homo_714,MCI.vs.control	4
BP	GO:0007520	myoblast fusion	bulk RNA-seq,Homo_723,AD.vs.MCI; bulk RNA-seq,Homo_723,MCI.vs.control; bulk RNA-seq,Homo_714,AD.vs.MCI; bulk RNA-seq,Homo_714,MCI.vs.control	4
CC	GO:0090665	glycoprotein complex	bulk RNA-seq,Homo_723,AD.vs.MCI; bulk RNA-seq,Homo_714,AD.vs.control; bulk RNA-seq,Homo_714,AD.vs.MCI; bulk RNA-seq,Homo_633,AD.vs.control	4
BP	GO:1901032	negative regulation of response to reactive oxygen species	bulk RNA-seq,Homo_723,AD.vs.MCI; bulk RNA-seq,Homo_723,MCI.vs.control; bulk RNA-seq,Homo_714,AD.vs.MCI; bulk RNA-seq,Homo_714,MCI.vs.control; bulk RNA-seq,Homo_633,AD.vs.control	5
MF	GO:0046875	ephrin receptor binding	bulk RNA-seq,Homo_723,AD.vs.MCI; bulk RNA-seq,Homo_723,MCI.vs.control; bulk RNA-seq,Homo_714,MCI.vs.control; bulk RNA-seq,Homo_633,AD.vs.control; bulk RNA-seq,Homo_633,AD.vs.MCI	5
MF	GO:0001968	fibronectin binding	bulk RNA-seq,Homo_723,AD.vs.MCI; bulk RNA-seq,Homo_723,MCI.vs.control; bulk RNA-seq,Homo_714,AD.vs.MCI; bulk RNA-seq,Homo_714,MCI.vs.control; bulk RNA-seq,Homo_633,AD.vs.control	5
BP	GO:0061437	renal system vasculature development	bulk RNA-seq,Homo_723,AD.vs.MCI; bulk RNA-seq,Homo_723,MCI.vs.control; bulk RNA-seq,Homo_714,AD.vs.MCI; bulk RNA-seq,Homo_714,MCI.vs.control	4
BP	GO:0061440	kidney vasculature development	bulk RNA-seq,Homo_723,AD.vs.MCI; bulk RNA-seq,Homo_723,MCI.vs.control; bulk RNA-seq,Homo_714,AD.vs.MCI; bulk RNA-seq,Homo_714,MCI.vs.control	4
BP	GO:0050994	regulation of lipid catabolic process	bulk RNA-seq,Homo_723,AD.vs.MCI; bulk RNA-seq,Homo_723,MCI.vs.control; bulk RNA-seq,Homo_714,AD.vs.MCI; bulk RNA-seq,Homo_714,MCI.vs.control; bulk RNA-seq,Homo_633,AD.vs.control; bulk RNA-seq,Homo_633,AD.vs.MCI	6
CC	GO:0045171	intercellular bridge	bulk RNA-seq,Homo_723,AD.vs.MCI; bulk RNA-seq,Homo_723,MCI.vs.control; bulk RNA-seq,Homo_714,AD.vs.MCI; bulk RNA-seq,Homo_714,MCI.vs.control; bulk RNA-seq,Homo_633,AD.vs.control; bulk RNA-seq,Homo_633,AD.vs.MCI	6
BP	GO:0016051	carbohydrate biosynthetic process	bulk RNA-seq,Homo_723,AD.vs.MCI; bulk RNA-seq,Homo_723,MCI.vs.control; bulk RNA-seq,Homo_714,AD.vs.MCI; bulk RNA-seq,Homo_633,AD.vs.control; bulk RNA-seq,Homo_633,AD.vs.MCI	5
BP	GO:0042446	hormone biosynthetic process	bulk RNA-seq,Homo_723,AD.vs.MCI; bulk RNA-seq,Homo_723,MCI.vs.control; bulk RNA-seq,Homo_714,AD.vs.MCI; bulk RNA-seq,Homo_714,MCI.vs.control; bulk RNA-seq,Homo_633,AD.vs.control	5
BP	GO:0097530	granulocyte migration	bulk RNA-seq,Homo_723,AD.vs.MCI; bulk RNA-seq,Homo_723,MCI.vs.control; bulk RNA-seq,Homo_714,AD.vs.MCI; bulk RNA-seq,Homo_633,AD.vs.control; bulk RNA-seq,SRP223445,AD.vs.control	5
BP	GO:0034103	regulation of tissue remodeling	bulk RNA-seq,Homo_723,AD.vs.MCI; bulk RNA-seq,Homo_723,MCI.vs.control; bulk RNA-seq,Homo_714,AD.vs.MCI; bulk RNA-seq,Homo_714,MCI.vs.control; bulk RNA-seq,Homo_633,AD.vs.control	5
MF	GO:0016410	N-acyltransferase activity	bulk RNA-seq,Homo_723,AD.vs.MCI; bulk RNA-seq,Homo_723,MCI.vs.control; bulk RNA-seq,Homo_714,AD.vs.MCI; bulk RNA-seq,Homo_714,MCI.vs.control; bulk RNA-seq,Homo_633,AD.vs.control; bulk RNA-seq,Homo_633,AD.vs.MCI	6
BP	GO:0001659	temperature homeostasis	bulk RNA-seq,Homo_723,AD.vs.MCI; bulk RNA-seq,Homo_723,MCI.vs.control; bulk RNA-seq,Homo_714,AD.vs.MCI; bulk RNA-seq,Homo_633,AD.vs.control; bulk RNA-seq,Homo_633,AD.vs.MCI	5
BP	GO:1904705	regulation of vascular associated smooth muscle cell	bulk RNA-seq,Homo_723,AD.vs.MCI; bulk RNA-seq,Homo_714,AD.vs.control; bulk RNA-seq,Homo_714,AD.vs.MCI; bulk RNA-seq,Homo_714,MCI.vs.control	4
BP	GO:0046579	positive regulation of Ras protein signal transduction	bulk RNA-seq,Homo_723,AD.vs.MCI; bulk RNA-seq,Homo_723,MCI.vs.control; bulk RNA-seq,Homo_714,AD.vs.MCI; bulk RNA-seq,Homo_714,MCI.vs.control; bulk RNA-seq,Homo_633,AD.vs.control; bulk RNA-seq,Homo_633,AD.vs.MCI	6
BP	GO:0009259	ribonucleotide metabolic process	bulk RNA-seq,Homo_723,AD.vs.MCI; bulk RNA-seq,Homo_714,AD.vs.control; bulk RNA-seq,Homo_714,AD.vs.MCI; bulk RNA-seq,Homo_633,AD.vs.control; bulk RNA-seq,Homo_633,AD.vs.MCI	5

BP	GO:0048260	positive regulation of receptor-mediated endocytosis	bulk RNA-seq,Homo_723,AD.vs.MCI; bulk RNA-seq,Homo_723,MCI.vs.control; bulk RNA-seq,Homo_714,AD.vs.control; bulk RNA-seq,Homo_714,AD.vs.MCI; bulk RNA-seq,Homo_714,MCI.vs.control; bulk RNA-seq,Homo_633,AD.vs.control; bulk RNA-seq,Homo_633,AD.vs.MCI	7
BP	GO:1903076	regulation of protein localization to plasma membrane	bulk RNA-seq,Homo_723,AD.vs.MCI; bulk RNA-seq,Homo_723,MCI.vs.control; bulk RNA-seq,Homo_714,AD.vs.MCI; bulk RNA-seq,Homo_714,MCI.vs.control; bulk RNA-seq,Homo_633,AD.vs.control; bulk RNA-seq,Homo_633,AD.vs.MCI	6
CC	GO:0005775	vacuolar lumen	bulk RNA-seq,Homo_723,AD.vs.MCI; bulk RNA-seq,Homo_723,MCI.vs.control; bulk RNA-seq,Homo_633,AD.vs.control; bulk RNA-seq,Homo_633,AD.vs.MCI	4
BP	GO:0051701	biological process involved in interaction with host	bulk RNA-seq,Homo_723,AD.vs.MCI; bulk RNA-seq,Homo_723,MCI.vs.control; bulk RNA-seq,Homo_714,AD.vs.MCI; bulk RNA-seq,Homo_633,AD.vs.control; bulk RNA-seq,Homo_633,AD.vs.MCI	5
BP	GO:0021681	cerebellar granular layer development	bulk RNA-seq,Homo_723,AD.vs.MCI; bulk RNA-seq,Homo_723,MCI.vs.control; bulk RNA-seq,Homo_714,AD.vs.MCI; bulk RNA-seq,Homo_714,MCI.vs.control	4
BP	GO:0010259	multicellular organism aging	bulk RNA-seq,Homo_723,AD.vs.MCI; bulk RNA-seq,Homo_723,MCI.vs.control; bulk RNA-seq,Homo_714,AD.vs.MCI	3
BP	GO:1903409	reactive oxygen species biosynthetic process	bulk RNA-seq,Homo_723,AD.vs.MCI; bulk RNA-seq,Homo_714,AD.vs.MCI; bulk RNA-seq,Homo_714,MCI.vs.control; bulk RNA-seq,Homo_633,AD.vs.control; bulk RNA-seq,Homo_633,AD.vs.MCI	5
BP	GO:0008088	axo-dendritic transport	bulk RNA-seq,Homo_723,AD.vs.MCI; bulk RNA-seq,Homo_723,MCI.vs.control; bulk RNA-seq,Homo_714,MCI.vs.control; bulk RNA-seq,Homo_633,AD.vs.control; bulk RNA-seq,Homo_633,AD.vs.MCI	5
BP	GO:0043535	regulation of blood vessel endothelial cell migration	bulk RNA-seq,Homo_723,AD.vs.MCI; bulk RNA-seq,Homo_723,MCI.vs.control; bulk RNA-seq,Homo_714,AD.vs.control; bulk RNA-seq,Homo_714,AD.vs.MCI; bulk RNA-seq,Homo_633,AD.vs.control	5
BP	GO:0006665	sphingolipid metabolic process	bulk RNA-seq,Homo_723,AD.vs.MCI; bulk RNA-seq,Homo_723,MCI.vs.control; bulk RNA-seq,Homo_633,AD.vs.control; bulk RNA-seq,Homo_633,AD.vs.MCI	4
BP	GO:0070098	chemokine-mediated signaling pathway	bulk RNA-seq,Homo_723,AD.vs.MCI; bulk RNA-seq,Homo_723,MCI.vs.control; bulk RNA-seq,Homo_714,AD.vs.control; bulk RNA-seq,Homo_714,AD.vs.MCI; bulk RNA-seq,Homo_714,MCI.vs.control	5
BP	GO:0048146	positive regulation of fibroblast proliferation	bulk RNA-seq,Homo_723,AD.vs.MCI; bulk RNA-seq,Homo_723,MCI.vs.control; bulk RNA-seq,Homo_714,AD.vs.MCI; bulk RNA-seq,Homo_714,MCI.vs.control; bulk RNA-seq,Homo_633,AD.vs.control; bulk RNA-seq,Homo_633,AD.vs.MCI	6
BP	GO:0032869	cellular response to insulin stimulus	bulk RNA-seq,Homo_723,AD.vs.MCI; bulk RNA-seq,Homo_723,MCI.vs.control; bulk RNA-seq,Homo_714,AD.vs.MCI; bulk RNA-seq,Homo_633,AD.vs.control; bulk RNA-seq,Homo_633,AD.vs.MCI	5
CC	GO:0036064	ciliary basal body	bulk RNA-seq,Homo_723,AD.vs.MCI; bulk RNA-seq,Homo_723,MCI.vs.control; bulk RNA-seq,Homo_714,AD.vs.MCI; bulk RNA-seq,Homo_633,AD.vs.control; bulk RNA-seq,Homo_633,AD.vs.MCI	5
BP	GO:0097696	receptor signaling pathway via STAT	bulk RNA-seq,Homo_723,AD.vs.MCI; bulk RNA-seq,Homo_723,MCI.vs.control; bulk RNA-seq,Homo_714,AD.vs.control; bulk RNA-seq,Homo_714,AD.vs.MCI; bulk RNA-seq,Homo_633,AD.vs.control; bulk RNA-seq,Homo_633,AD.vs.MCI	6
BP	GO:0060711	labyrinthine layer development	bulk RNA-seq,Homo_723,AD.vs.MCI; bulk RNA-seq,Homo_723,MCI.vs.control; bulk RNA-seq,Homo_714,AD.vs.MCI; bulk RNA-seq,Homo_714,MCI.vs.control; bulk RNA-seq,Homo_633,AD.vs.control	5
BP	GO:1903861	positive regulation of dendrite extension	bulk RNA-seq,Homo_723,AD.vs.MCI; bulk RNA-seq,Homo_723,MCI.vs.control; bulk RNA-seq,Homo_714,AD.vs.MCI	3
BP	GO:0045930	negative regulation of mitotic cell cycle	bulk RNA-seq,Homo_723,AD.vs.MCI; bulk RNA-seq,Homo_723,MCI.vs.control; bulk RNA-seq,Homo_714,AD.vs.MCI; bulk RNA-seq,Homo_633,AD.vs.control; bulk RNA-seq,Homo_633,AD.vs.MCI	5
BP	GO:1904953	Wnt signaling pathway involved in midbrain dopaminergic neuron differentiation	bulk RNA-seq,Homo_723,AD.vs.MCI; bulk RNA-seq,Homo_723,MCI.vs.control; bulk RNA-seq,Homo_714,AD.vs.MCI	3
CC	GO:0043202	lysosomal lumen	bulk RNA-seq,Homo_723,AD.vs.MCI; bulk RNA-seq,Homo_723,MCI.vs.control; bulk RNA-seq,Homo_714,AD.vs.MCI; bulk RNA-seq,Homo_714,MCI.vs.control; bulk RNA-seq,Homo_633,AD.vs.control; bulk RNA-seq,Homo_633,AD.vs.MCI	6
BP	GO:0007254	JNK cascade	bulk RNA-seq,Homo_723,AD.vs.MCI; bulk RNA-seq,Homo_723,MCI.vs.control; bulk RNA-seq,Homo_714,AD.vs.MCI; bulk RNA-seq,Homo_633,AD.vs.control; bulk RNA-seq,Homo_633,AD.vs.MCI	5
BP	GO:0042594	response to starvation	bulk RNA-seq,Homo_723,AD.vs.MCI; bulk RNA-seq,Homo_723,MCI.vs.control; bulk RNA-seq,Homo_714,AD.vs.MCI; bulk RNA-seq,Homo_633,AD.vs.control; bulk RNA-seq,Homo_633,AD.vs.MCI	5
BP	GO:0051497	negative regulation of stress fiber assembly	bulk RNA-seq,Homo_723,AD.vs.MCI; bulk RNA-seq,Homo_723,MCI.vs.control; bulk RNA-seq,Homo_714,AD.vs.MCI; bulk RNA-seq,Homo_714,MCI.vs.control	4
BP	GO:1902893	regulation of miRNA transcription	bulk RNA-seq,Homo_723,AD.vs.MCI; bulk RNA-seq,Homo_723,MCI.vs.control; bulk RNA-seq,Homo_714,AD.vs.MCI; bulk RNA-seq,Homo_714,MCI.vs.control; bulk RNA-seq,Homo_633,AD.vs.control; bulk RNA-seq,Homo_633,AD.vs.MCI	6
BP	GO:0072576	liver morphogenesis	bulk RNA-seq,Homo_723,AD.vs.MCI; bulk RNA-seq,Homo_723,MCI.vs.control; bulk RNA-seq,Homo_714,AD.vs.control; bulk RNA-seq,Homo_714,AD.vs.MCI; bulk RNA-seq,Homo_714,MCI.vs.control	5
BP	GO:0031529	ruffle organization	bulk RNA-seq,Homo_723,AD.vs.MCI; bulk RNA-seq,Homo_723,MCI.vs.control; bulk RNA-seq,Homo_714,AD.vs.MCI; bulk RNA-seq,Homo_714,MCI.vs.control; bulk RNA-seq,Homo_633,AD.vs.control	5
BP	GO:0072012	glomerulus vasculature development	bulk RNA-seq,Homo_723,AD.vs.MCI; bulk RNA-seq,Homo_723,MCI.vs.control; bulk RNA-seq,Homo_714,AD.vs.MCI; bulk RNA-seq,Homo_714,MCI.vs.control	4
BP	GO:0007259	receptor signaling pathway via JAK-STAT	bulk RNA-seq,Homo_723,AD.vs.MCI; bulk RNA-seq,Homo_723,MCI.vs.control; bulk RNA-seq,Homo_714,AD.vs.control; bulk RNA-seq,Homo_714,AD.vs.MCI; bulk RNA-seq,Homo_633,AD.vs.control; bulk RNA-seq,Homo_633,AD.vs.MCI	6
BP	GO:0042306	regulation of protein import into nucleus	bulk RNA-seq,Homo_723,AD.vs.MCI; bulk RNA-seq,Homo_723,MCI.vs.control; bulk RNA-seq,Homo_714,AD.vs.MCI; bulk RNA-seq,Homo_714,MCI.vs.control; bulk RNA-seq,Homo_633,AD.vs.control; bulk RNA-seq,Homo_633,AD.vs.MCI	6
MF	GO:0048156	tau protein binding	bulk RNA-seq,Homo_723,AD.vs.MCI; bulk RNA-seq,Homo_723,MCI.vs.control; bulk RNA-seq,Homo_714,MCI.vs.control; bulk RNA-seq,Homo_633,AD.vs.control; bulk RNA-seq,Homo_633,AD.vs.MCI	5
BP	GO:0046330	positive regulation of JNK cascade	bulk RNA-seq,Homo_723,AD.vs.MCI; bulk RNA-seq,Homo_723,MCI.vs.control; bulk RNA-seq,Homo_714,AD.vs.MCI; bulk RNA-seq,Homo_714,MCI.vs.control; bulk RNA-seq,Homo_633,AD.vs.control; bulk RNA-seq,Homo_633,AD.vs.MCI	6
CC	GO:0097542	ciliary tip	bulk RNA-seq,Homo_723,AD.vs.MCI; bulk RNA-seq,Homo_723,MCI.vs.control; bulk RNA-seq,Homo_714,AD.vs.MCI; bulk RNA-seq,Homo_714,MCI.vs.control; bulk RNA-seq,Homo_633,AD.vs.control; bulk RNA-seq,Homo_633,AD.vs.MCI	6
MF	GO:0019894	kinesin binding	bulk RNA-seq,Homo_723,AD.vs.MCI; bulk RNA-seq,Homo_723,MCI.vs.control; bulk RNA-seq,Homo_714,MCI.vs.control; bulk RNA-seq,Homo_633,AD.vs.control; bulk RNA-seq,Homo_633,AD.vs.MCI	5
CC	GO:0042827	platelet dense granule	bulk RNA-seq,Homo_723,AD.vs.MCI; bulk RNA-seq,Homo_723,MCI.vs.control; bulk RNA-seq,Homo_714,AD.vs.MCI; bulk RNA-seq,Homo_714,MCI.vs.control	4
BP	GO:0033619	membrane protein proteolysis	bulk RNA-seq,Homo_723,AD.vs.MCI; bulk RNA-seq,Homo_723,MCI.vs.control; bulk RNA-seq,Homo_714,AD.vs.MCI; bulk RNA-seq,Homo_714,MCI.vs.control; bulk RNA-seq,Homo_633,AD.vs.control; bulk RNA-seq,Homo_633,AD.vs.MCI	6
BP	GO:0031113	regulation of microtubule polymerization	bulk RNA-seq,Homo_723,AD.vs.MCI; bulk RNA-seq,Homo_723,MCI.vs.control; bulk RNA-seq,Homo_714,AD.vs.MCI; bulk RNA-seq,Homo_714,MCI.vs.control; bulk RNA-seq,Homo_633,AD.vs.control; bulk RNA-seq,Homo_633,AD.vs.MCI	6

BP	GO:0007129	homologous chromosome pairing at meiosis	bulk RNA-seq,Homo_723,AD.vs.MCI; bulk RNA-seq,Homo_723,MCI.vs.control; bulk RNA-seq,Homo_714,AD.vs.MCI; bulk RNA-seq,Homo_714,MCI.vs.control; bulk RNA-seq,Homo_633,AD.vs.control	5
BP	GO:0046888	negative regulation of hormone secretion	bulk RNA-seq,Homo_723,AD.vs.MCI; bulk RNA-seq,Homo_723,MCI.vs.control; bulk RNA-seq,Homo_633,AD.vs.control; bulk RNA-seq,Homo_633,AD.vs.MCI	4
BP	GO:0061614	miRNA transcription	bulk RNA-seq,Homo_723,AD.vs.MCI; bulk RNA-seq,Homo_723,MCI.vs.control; bulk RNA-seq,Homo_714,AD.vs.MCI; bulk RNA-seq,Homo_714,MCI.vs.control; bulk RNA-seq,Homo_633,AD.vs.control; bulk RNA-seq,Homo_633,AD.vs.MCI	6
BP	GO:0045599	negative regulation of fat cell differentiation	bulk RNA-seq,Homo_723,AD.vs.MCI; bulk RNA-seq,Homo_723,MCI.vs.control; bulk RNA-seq,Homo_714,AD.vs.MCI; bulk RNA-seq,Homo_714,MCI.vs.control	4
BP	GO:0009066	aspartate family amino acid metabolic process	bulk RNA-seq,Homo_723,AD.vs.MCI; bulk RNA-seq,Homo_723,MCI.vs.control; bulk RNA-seq,Homo_714,AD.vs.MCI; bulk RNA-seq,Homo_714,MCI.vs.control; bulk RNA-seq,Homo_633,AD.vs.control; bulk RNA-seq,Homo_633,AD.vs.MCI	6
CC	GO:0005903	brush border	bulk RNA-seq,Homo_723,AD.vs.MCI; bulk RNA-seq,Homo_723,MCI.vs.control; bulk RNA-seq,Homo_714,AD.vs.MCI; bulk RNA-seq,Homo_714,MCI.vs.control; bulk RNA-seq,Homo_633,AD.vs.control; bulk RNA-seq,Homo_633,AD.vs.MCI	6
BP	GO:0003177	pulmonary valve development	bulk RNA-seq,Homo_723,AD.vs.MCI; bulk RNA-seq,Homo_723,MCI.vs.control; bulk RNA-seq,Homo_714,AD.vs.MCI; bulk RNA-seq,Homo_714,MCI.vs.control	4
BP	GO:0032868	response to insulin	bulk RNA-seq,Homo_723,AD.vs.MCI; bulk RNA-seq,Homo_723,MCI.vs.control; bulk RNA-seq,Homo_714,AD.vs.MCI; bulk RNA-seq,Homo_633,AD.vs.control; bulk RNA-seq,Homo_633,AD.vs.MCI	5
BP	GO:1904672	regulation of somatic stem cell population maintenance	bulk RNA-seq,Homo_723,AD.vs.MCI; bulk RNA-seq,Homo_714,AD.vs.MCI	2
BP	GO:0000096	sulfur amino acid metabolic process	bulk RNA-seq,Homo_723,AD.vs.MCI; bulk RNA-seq,Homo_723,MCI.vs.control; bulk RNA-seq,Homo_714,AD.vs.MCI; bulk RNA-seq,Homo_714,MCI.vs.control; bulk RNA-seq,Homo_633,AD.vs.control; bulk RNA-seq,Homo_633,AD.vs.MCI	6
BP	GO:0071331	cellular response to hexose stimulus	bulk RNA-seq,Homo_723,AD.vs.MCI; bulk RNA-seq,Homo_723,MCI.vs.control; bulk RNA-seq,Homo_714,AD.vs.MCI; bulk RNA-seq,Homo_714,MCI.vs.control; bulk RNA-seq,Homo_633,AD.vs.control; bulk RNA-seq,Homo_633,AD.vs.MCI	6
BP	GO:0030513	positive regulation of BMP signaling pathway	bulk RNA-seq,Homo_723,AD.vs.MCI; bulk RNA-seq,Homo_723,MCI.vs.control; bulk RNA-seq,Homo_714,AD.vs.MCI; bulk RNA-seq,Homo_714,MCI.vs.control	4
BP	GO:0034504	protein localization to nucleus	bulk RNA-seq,Homo_723,AD.vs.MCI; bulk RNA-seq,Homo_723,MCI.vs.control; bulk RNA-seq,Homo_714,AD.vs.MCI; bulk RNA-seq,Homo_633,AD.vs.control; bulk RNA-seq,Homo_633,AD.vs.MCI	5
BP	GO:0034599	cellular response to oxidative stress	bulk RNA-seq,Homo_723,AD.vs.MCI; bulk RNA-seq,Homo_723,MCI.vs.control; bulk RNA-seq,Homo_714,AD.vs.MCI; bulk RNA-seq,Homo_633,AD.vs.control; bulk RNA-seq,Homo_633,AD.vs.MCI	5
BP	GO:0050764	regulation of phagocytosis	bulk RNA-seq,Homo_723,AD.vs.MCI; bulk RNA-seq,Homo_723,MCI.vs.control; bulk RNA-seq,Homo_714,AD.vs.MCI; bulk RNA-seq,Homo_714,MCI.vs.control; bulk RNA-seq,Homo_633,AD.vs.control; bulk RNA-seq,Homo_633,AD.vs.MCI	6
BP	GO:0070509	calcium ion import	bulk RNA-seq,Homo_723,AD.vs.MCI; bulk RNA-seq,Homo_714,AD.vs.MCI	2
BP	GO:0010744	positive regulation of macrophage derived foam cell	bulk RNA-seq,Homo_723,AD.vs.MCI; bulk RNA-seq,Homo_723,MCI.vs.control; bulk RNA-seq,Homo_714,AD.vs.MCI; bulk RNA-seq,Homo_714,MCI.vs.control	4
BP	GO:0002931	response to ischemia	bulk RNA-seq,Homo_723,AD.vs.MCI; bulk RNA-seq,Homo_723,MCI.vs.control; bulk RNA-seq,Homo_714,MCI.vs.control; bulk RNA-seq,Homo_633,AD.vs.control; bulk RNA-seq,Homo_633,AD.vs.MCI	5
BP	GO:0022408	negative regulation of cell-cell adhesion	bulk RNA-seq,Homo_723,AD.vs.MCI; bulk RNA-seq,Homo_723,MCI.vs.control; bulk RNA-seq,Homo_714,AD.vs.MCI; bulk RNA-seq,Homo_633,AD.vs.control; bulk RNA-seq,Homo_633,AD.vs.MCI	5
BP	GO:0019693	ribose phosphate metabolic process	bulk RNA-seq,Homo_723,AD.vs.MCI; bulk RNA-seq,Homo_714,AD.vs.control; bulk RNA-seq,Homo_714,AD.vs.MCI; bulk RNA-seq,Homo_633,AD.vs.control; bulk RNA-seq,Homo_633,AD.vs.MCI	5
BP	GO:0007059	chromosome segregation	bulk RNA-seq,Homo_723,AD.vs.MCI; bulk RNA-seq,Homo_714,AD.vs.control; bulk RNA-seq,Homo_714,AD.vs.MCI; bulk RNA-seq,Homo_633,AD.vs.control; bulk RNA-seq,Homo_633,AD.vs.MCI	5
BP	GO:0030316	osteoclast differentiation	bulk RNA-seq,Homo_723,AD.vs.MCI; bulk RNA-seq,Homo_723,MCI.vs.control; bulk RNA-seq,Homo_714,AD.vs.MCI; bulk RNA-seq,Homo_714,MCI.vs.control; bulk RNA-seq,Homo_633,AD.vs.control; bulk RNA-seq,Homo_633,AD.vs.MCI; bulk RNA-seq,SRP325058,AD.vs.control	7
BP	GO:0010893	positive regulation of steroid biosynthetic process	bulk RNA-seq,Homo_723,AD.vs.MCI; bulk RNA-seq,Homo_714,AD.vs.MCI	2
BP	GO:1903859	regulation of dendrite extension	bulk RNA-seq,Homo_723,AD.vs.MCI; bulk RNA-seq,Homo_723,MCI.vs.control	2
BP	GO:0095500	acetylcholine receptor signaling pathway	bulk RNA-seq,Homo_723,AD.vs.MCI; bulk RNA-seq,Homo_723,MCI.vs.control	2
MF	GO:0019887	protein kinase regulator activity	bulk RNA-seq,Homo_723,AD.vs.MCI; bulk RNA-seq,Homo_723,MCI.vs.control; bulk RNA-seq,Homo_714,AD.vs.MCI; bulk RNA-seq,Homo_633,AD.vs.control; bulk RNA-seq,Homo_633,AD.vs.MCI	5
BP	GO:0032370	positive regulation of lipid transport	bulk RNA-seq,Homo_723,AD.vs.MCI; bulk RNA-seq,Homo_723,MCI.vs.control; bulk RNA-seq,Homo_714,AD.vs.MCI; bulk RNA-seq,Homo_714,MCI.vs.control; bulk RNA-seq,Homo_633,AD.vs.control; bulk RNA-seq,Homo_633,AD.vs.MCI	6
BP	GO:0050766	positive regulation of phagocytosis	bulk RNA-seq,Homo_723,AD.vs.MCI; bulk RNA-seq,Homo_723,MCI.vs.control; bulk RNA-seq,Homo_714,AD.vs.MCI; bulk RNA-seq,Homo_714,MCI.vs.control; bulk RNA-seq,Homo_633,AD.vs.control; bulk RNA-seq,Homo_633,AD.vs.MCI	6
BP	GO:0000381	regulation of alternative mRNA splicing, via spliceosome	bulk RNA-seq,Homo_723,AD.vs.MCI; bulk RNA-seq,Homo_723,MCI.vs.control; bulk RNA-seq,Homo_714,MCI.vs.control; bulk RNA-seq,Homo_633,AD.vs.control; bulk RNA-seq,Homo_633,AD.vs.MCI	5
BP	GO:0019221	cytokine-mediated signaling pathway	bulk RNA-seq,Homo_723,AD.vs.MCI; bulk RNA-seq,Homo_714,AD.vs.control; bulk RNA-seq,Homo_714,AD.vs.MCI; bulk RNA-seq,Homo_633,AD.vs.control; bulk RNA-seq,Homo_633,AD.vs.MCI; scRNA-seq,SRP215507,CD8+ T cell_3-AD.vs.control	6
BP	GO:0010948	negative regulation of cell cycle process	bulk RNA-seq,Homo_723,AD.vs.MCI; bulk RNA-seq,Homo_723,MCI.vs.control; bulk RNA-seq,Homo_714,AD.vs.MCI; bulk RNA-seq,Homo_633,AD.vs.control; bulk RNA-seq,Homo_633,AD.vs.MCI	5
MF	GO:0016849	phosphorus-oxygen lyase activity	bulk RNA-seq,Homo_723,AD.vs.MCI; bulk RNA-seq,Homo_723,MCI.vs.control; bulk RNA-seq,Homo_714,MCI.vs.control	3
BP	GO:0007632	visual behavior	bulk RNA-seq,Homo_723,AD.vs.MCI; bulk RNA-seq,Homo_723,MCI.vs.control; bulk RNA-seq,Homo_714,AD.vs.MCI; bulk RNA-seq,Homo_633,AD.vs.control; bulk RNA-seq,Homo_633,AD.vs.MCI	5
BP	GO:0009408	response to heat	bulk RNA-seq,Homo_723,AD.vs.MCI; bulk RNA-seq,Homo_723,MCI.vs.control; bulk RNA-seq,Homo_714,AD.vs.MCI; bulk RNA-seq,Homo_714,MCI.vs.control; bulk RNA-seq,Homo_633,AD.vs.control; bulk RNA-seq,Homo_633,AD.vs.MCI	6
BP	GO:0110011	regulation of basement membrane organization	bulk RNA-seq,Homo_723,AD.vs.MCI; bulk RNA-seq,Homo_723,MCI.vs.control; bulk RNA-seq,Homo_633,AD.vs.control; bulk RNA-seq,Homo_633,AD.vs.MCI	4
BP	GO:0097421	liver regeneration	bulk RNA-seq,Homo_723,AD.vs.MCI; bulk RNA-seq,Homo_723,MCI.vs.control; bulk RNA-seq,Homo_714,AD.vs.MCI; bulk RNA-seq,Homo_714,MCI.vs.control; bulk RNA-seq,Homo_633,AD.vs.control	5
BP	GO:0010560	positive regulation of glycoprotein biosynthetic process	bulk RNA-seq,Homo_723,AD.vs.MCI; bulk RNA-seq,Homo_723,MCI.vs.control; bulk RNA-seq,Homo_714,AD.vs.MCI; bulk RNA-seq,Homo_714,MCI.vs.control; bulk RNA-seq,Homo_633,AD.vs.control	5

BP	GO:0042176	regulation of protein catabolic process	bulk RNA-seq,Homo_723,AD.vs.MCI; bulk RNA-seq,Homo_714,AD.vs.control; bulk RNA-seq,Homo_714,AD.vs.MCI; bulk RNA-seq,Homo_633,AD.vs.control; bulk RNA-seq,Homo_633,AD.vs.MCI	5
MF	GO:0140678	molecular function inhibitor activity	bulk RNA-seq,Homo_723,AD.vs.MCI; bulk RNA-seq,Homo_723,MCI.vs.control; bulk RNA-seq,Homo_714,AD.vs.MCI; bulk RNA-seq,Homo_714,MCI.vs.control; bulk RNA-seq,Homo_633,AD.vs.control; bulk RNA-seq,Homo_633,AD.vs.MCI	6
BP	GO:0098813	nuclear chromosome segregation	bulk RNA-seq,Homo_723,AD.vs.MCI; bulk RNA-seq,Homo_723,MCI.vs.control; bulk RNA-seq,Homo_714,AD.vs.MCI; bulk RNA-seq,Homo_633,AD.vs.control; bulk RNA-seq,Homo_633,AD.vs.MCI	5
MF	GO:0003785	actin monomer binding	bulk RNA-seq,Homo_723,AD.vs.MCI; bulk RNA-seq,Homo_723,MCI.vs.control; bulk RNA-seq,Homo_714,AD.vs.MCI; bulk RNA-seq,Homo_714,MCI.vs.control	4
MF	GO:0070279	vitamin B6 binding	bulk RNA-seq,Homo_723,AD.vs.MCI; bulk RNA-seq,Homo_723,MCI.vs.control; bulk RNA-seq,Homo_714,AD.vs.MCI; bulk RNA-seq,Homo_714,MCI.vs.control	4
BP	GO:2000116	regulation of cysteine-type endopeptidase activity	bulk RNA-seq,Homo_723,AD.vs.MCI; bulk RNA-seq,Homo_723,MCI.vs.control; bulk RNA-seq,Homo_714,AD.vs.MCI; bulk RNA-seq,Homo_633,AD.vs.control; bulk RNA-seq,Homo_633,AD.vs.MCI	5
BP	GO:1902041	regulation of extrinsic apoptotic signaling pathway via death domain receptors	bulk RNA-seq,Homo_723,AD.vs.MCI; bulk RNA-seq,Homo_723,MCI.vs.control; bulk RNA-seq,Homo_714,AD.vs.MCI; bulk RNA-seq,Homo_714,MCI.vs.control; bulk RNA-seq,Homo_633,AD.vs.control; bulk RNA-seq,Homo_633,AD.vs.MCI	6
BP	GO:0032967	positive regulation of collagen biosynthetic process	bulk RNA-seq,Homo_723,AD.vs.MCI; bulk RNA-seq,Homo_714,AD.vs.MCI	2
BP	GO:0002011	morphogenesis of an epithelial sheet	bulk RNA-seq,Homo_723,AD.vs.MCI; bulk RNA-seq,Homo_723,MCI.vs.control; bulk RNA-seq,Homo_714,AD.vs.MCI; bulk RNA-seq,Homo_714,MCI.vs.control; bulk RNA-seq,Homo_633,AD.vs.control	5
MF	GO:0030170	pyridoxal phosphate binding	bulk RNA-seq,Homo_723,AD.vs.MCI; bulk RNA-seq,Homo_723,MCI.vs.control; bulk RNA-seq,Homo_714,AD.vs.MCI; bulk RNA-seq,Homo_714,MCI.vs.control	4
MF	GO:0030971	receptor tyrosine kinase binding	bulk RNA-seq,Homo_723,AD.vs.MCI; bulk RNA-seq,Homo_723,MCI.vs.control; bulk RNA-seq,Homo_714,AD.vs.MCI; bulk RNA-seq,Homo_714,MCI.vs.control; bulk RNA-seq,Homo_633,AD.vs.control; bulk RNA-seq,Homo_633,AD.vs.MCI	6
MF	GO:0005451	monovalent cation:proton antiporter activity	bulk RNA-seq,Homo_723,AD.vs.MCI; bulk RNA-seq,Homo_723,MCI.vs.control	2
MF	GO:0015299	solute:proton antiporter activity	bulk RNA-seq,Homo_723,AD.vs.MCI; bulk RNA-seq,Homo_723,MCI.vs.control	2
BP	GO:0010447	response to acidic pH	bulk RNA-seq,Homo_723,AD.vs.MCI; bulk RNA-seq,Homo_723,MCI.vs.control; bulk RNA-seq,Homo_714,AD.vs.MCI; bulk RNA-seq,Homo_714,MCI.vs.control	4
BP	GO:0097150	neuronal stem cell population maintenance	bulk RNA-seq,Homo_723,AD.vs.MCI; bulk RNA-seq,Homo_723,MCI.vs.control; bulk RNA-seq,Homo_714,AD.vs.MCI; bulk RNA-seq,Homo_714,MCI.vs.control; bulk RNA-seq,Homo_633,AD.vs.control	5
BP	GO:0002690	positive regulation of leukocyte chemotaxis	bulk RNA-seq,Homo_723,AD.vs.MCI; bulk RNA-seq,Homo_723,MCI.vs.control; bulk RNA-seq,Homo_714,AD.vs.MCI; bulk RNA-seq,Homo_714,MCI.vs.control; bulk RNA-seq,Homo_633,AD.vs.control; scRNA-seq,SRP330776,Naive CD8+ T cell_2-AD.vs.control	6
CC	GO:0016010	dystrophin-associated glycoprotein complex	bulk RNA-seq,Homo_723,AD.vs.MCI; bulk RNA-seq,Homo_714,AD.vs.MCI	2
BP	GO:0071326	cellular response to monosaccharide stimulus	bulk RNA-seq,Homo_723,AD.vs.MCI; bulk RNA-seq,Homo_723,MCI.vs.control; bulk RNA-seq,Homo_714,AD.vs.MCI; bulk RNA-seq,Homo_633,AD.vs.control; bulk RNA-seq,Homo_633,AD.vs.MCI	5
MF	GO:0015298	solute:cation antiporter activity	bulk RNA-seq,Homo_723,AD.vs.MCI; bulk RNA-seq,Homo_723,MCI.vs.control	2
CC	GO:1990023	mitotic spindle midzone	bulk RNA-seq,Homo_723,AD.vs.MCI; bulk RNA-seq,Homo_723,MCI.vs.control; bulk RNA-seq,Homo_714,AD.vs.MCI; bulk RNA-seq,Homo_714,MCI.vs.control; bulk RNA-seq,Homo_633,AD.vs.control	5
BP	GO:0010712	regulation of collagen metabolic process	bulk RNA-seq,Homo_723,AD.vs.MCI; bulk RNA-seq,Homo_723,MCI.vs.control; bulk RNA-seq,Homo_714,AD.vs.MCI	3
BP	GO:0070168	negative regulation of biomineral tissue development	bulk RNA-seq,Homo_723,AD.vs.MCI; bulk RNA-seq,Homo_723,MCI.vs.control; bulk RNA-seq,Homo_714,AD.vs.MCI; bulk RNA-seq,Homo_714,MCI.vs.control	4
MF	GO:0140828	metal cation:monoatomic cation antiporter activity	bulk RNA-seq,Homo_723,AD.vs.MCI; bulk RNA-seq,Homo_723,MCI.vs.control; bulk RNA-seq,Homo_714,AD.vs.MCI	3
BP	GO:0097178	ruffle assembly	bulk RNA-seq,Homo_723,AD.vs.MCI; bulk RNA-seq,Homo_723,MCI.vs.control; bulk RNA-seq,Homo_714,AD.vs.MCI; bulk RNA-seq,Homo_714,MCI.vs.control; bulk RNA-seq,Homo_633,AD.vs.control	5
BP	GO:0010714	positive regulation of collagen metabolic process	bulk RNA-seq,Homo_723,AD.vs.MCI; bulk RNA-seq,Homo_723,MCI.vs.control; bulk RNA-seq,Homo_714,AD.vs.MCI	3
BP	GO:0046165	alcohol biosynthetic process	bulk RNA-seq,Homo_723,AD.vs.MCI; bulk RNA-seq,Homo_723,MCI.vs.control; bulk RNA-seq,Homo_714,AD.vs.MCI; bulk RNA-seq,Homo_633,AD.vs.control; bulk RNA-seq,Homo_633,AD.vs.MCI	5
BP	GO:0071322	cellular response to carbohydrate stimulus	bulk RNA-seq,Homo_723,AD.vs.MCI; bulk RNA-seq,Homo_723,MCI.vs.control; bulk RNA-seq,Homo_714,AD.vs.MCI; bulk RNA-seq,Homo_633,AD.vs.control; bulk RNA-seq,Homo_633,AD.vs.MCI	5
BP	GO:0035886	vascular associated smooth muscle cell differentiation	bulk RNA-seq,Homo_723,AD.vs.MCI; bulk RNA-seq,Homo_723,MCI.vs.control; bulk RNA-seq,Homo_714,AD.vs.MCI; bulk RNA-seq,Homo_714,MCI.vs.control	4
MF	GO:0042169	SH2 domain binding	bulk RNA-seq,Homo_723,AD.vs.MCI; bulk RNA-seq,Homo_723,MCI.vs.control; bulk RNA-seq,Homo_714,AD.vs.MCI; bulk RNA-seq,Homo_714,MCI.vs.control; bulk RNA-seq,Homo_633,AD.vs.control; bulk RNA-seq,Homo_633,AD.vs.MCI	6
BP	GO:0120178	steroid hormone biosynthetic process	bulk RNA-seq,Homo_723,AD.vs.MCI; bulk RNA-seq,Homo_723,MCI.vs.control; bulk RNA-seq,Homo_714,AD.vs.MCI	3
MF	GO:0030295	protein kinase activator activity	bulk RNA-seq,Homo_723,AD.vs.MCI; bulk RNA-seq,Homo_723,MCI.vs.control; bulk RNA-seq,Homo_714,AD.vs.MCI; bulk RNA-seq,Homo_714,MCI.vs.control; bulk RNA-seq,Homo_633,AD.vs.control; bulk RNA-seq,Homo_633,AD.vs.MCI	6
BP	GO:0086012	membrane depolarization during cardiac muscle cell action	bulk RNA-seq,Homo_723,AD.vs.MCI; bulk RNA-seq,Homo_723,MCI.vs.control; bulk RNA-seq,Homo_714,AD.vs.MCI	3
CC	GO:0000779	condensed chromosome, centromeric region	bulk RNA-seq,Homo_723,AD.vs.MCI; bulk RNA-seq,Homo_723,MCI.vs.control; bulk RNA-seq,Homo_714,AD.vs.MCI; bulk RNA-seq,Homo_633,AD.vs.control; bulk RNA-seq,Homo_633,AD.vs.MCI	5
MF	GO:0008235	metalloexopeptidase activity	bulk RNA-seq,Homo_723,AD.vs.MCI; bulk RNA-seq,Homo_723,MCI.vs.control; bulk RNA-seq,Homo_714,AD.vs.MCI; bulk RNA-seq,Homo_714,MCI.vs.control; bulk RNA-seq,Homo_633,AD.vs.control	5
BP	GO:0006486	protein glycosylation	bulk RNA-seq,Homo_723,AD.vs.MCI; bulk RNA-seq,Homo_723,MCI.vs.control; bulk RNA-seq,Homo_633,AD.vs.control; bulk RNA-seq,Homo_633,AD.vs.MCI	4
BP	GO:0043413	macromolecule glycosylation	bulk RNA-seq,Homo_723,AD.vs.MCI; bulk RNA-seq,Homo_723,MCI.vs.control; bulk RNA-seq,Homo_633,AD.vs.control; bulk RNA-seq,Homo_633,AD.vs.MCI	4
BP	GO:0006705	mineralocorticoid biosynthetic process	bulk RNA-seq,Homo_723,AD.vs.MCI; bulk RNA-seq,Homo_714,AD.vs.MCI	2
CC	GO:0120111	neuron projection cytoplasm	bulk RNA-seq,Homo_723,AD.vs.MCI; bulk RNA-seq,Homo_723,MCI.vs.control; bulk RNA-seq,Homo_714,MCI.vs.control; bulk RNA-seq,Homo_633,AD.vs.control; bulk RNA-seq,Homo_633,AD.vs.MCI	5
BP	GO:0010586	miRNA metabolic process	bulk RNA-seq,Homo_723,AD.vs.MCI; bulk RNA-seq,Homo_723,MCI.vs.control; bulk RNA-seq,Homo_714,AD.vs.MCI; bulk RNA-seq,Homo_714,MCI.vs.control; bulk RNA-seq,Homo_633,AD.vs.control; bulk RNA-seq,Homo_633,AD.vs.MCI	6
MF	GO:0008395	steroid hydroxylase activity	bulk RNA-seq,Homo_723,AD.vs.MCI; bulk RNA-seq,Homo_714,AD.vs.MCI	2
CC	GO:0032839	dendrite cytoplasm	bulk RNA-seq,Homo_723,AD.vs.MCI; bulk RNA-seq,Homo_723,MCI.vs.control; bulk RNA-seq,Homo_714,AD.vs.MCI; bulk RNA-seq,Homo_714,MCI.vs.control; bulk RNA-seq,Homo_633,AD.vs.control	5

BP	GO:0071260	cellular response to mechanical stimulus	bulk RNA-seq,Homo_723,AD.vs.MCI; bulk RNA-seq,Homo_723,MCI.vs.control; bulk RNA-seq,Homo_714,AD.vs.MCI; bulk RNA-seq,Homo_714,MCI.vs.control; bulk RNA-seq,Homo_633,AD.vs.control; bulk RNA-seq,Homo_633,AD.vs.MCI	6
MF	GO:0140358	P-type transmembrane transporter activity	bulk RNA-seq,Homo_723,AD.vs.MCI; bulk RNA-seq,Homo_723,MCI.vs.control; bulk RNA-seq,Homo_633,AD.vs.control; bulk RNA-seq,Homo_633,AD.vs.MCI	4
BP	GO:0072160	nephron tubule epithelial cell differentiation	bulk RNA-seq,Homo_723,AD.vs.MCI; bulk RNA-seq,Homo_723,MCI.vs.control; bulk RNA-seq,Homo_714,AD.vs.MCI; bulk RNA-seq,Homo_714,MCI.vs.control	4
BP	GO:0008206	bile acid metabolic process	bulk RNA-seq,Homo_723,AD.vs.MCI; bulk RNA-seq,Homo_723,MCI.vs.control; bulk RNA-seq,Homo_714,AD.vs.MCI; bulk RNA-seq,Homo_714,MCI.vs.control; bulk RNA-seq,Homo_633,AD.vs.control; bulk RNA-seq,Homo_633,AD.vs.MCI	6
BP	GO:0060390	regulation of SMAD protein signal transduction	bulk RNA-seq,Homo_723,AD.vs.MCI; bulk RNA-seq,Homo_723,MCI.vs.control; bulk RNA-seq,Homo_714,AD.vs.MCI; bulk RNA-seq,Homo_714,MCI.vs.control; bulk RNA-seq,Homo_633,AD.vs.control; bulk RNA-seq,Homo_633,AD.vs.MCI	6
BP	GO:0001974	blood vessel remodeling	bulk RNA-seq,Homo_723,AD.vs.MCI; bulk RNA-seq,Homo_723,MCI.vs.control; bulk RNA-seq,Homo_714,AD.vs.MCI; bulk RNA-seq,Homo_714,MCI.vs.control; bulk RNA-seq,Homo_633,AD.vs.control; bulk RNA-seq,Homo_633,AD.vs.MCI	6
BP	GO:0071548	response to dexamethasone	bulk RNA-seq,Homo_723,AD.vs.MCI; bulk RNA-seq,Homo_723,MCI.vs.control; bulk RNA-seq,Homo_714,AD.vs.MCI; bulk RNA-seq,Homo_714,MCI.vs.control; bulk RNA-seq,Homo_633,AD.vs.control; bulk RNA-seq,Homo_633,AD.vs.MCI	6
BP	GO:1903020	positive regulation of glycoprotein metabolic process	bulk RNA-seq,Homo_723,AD.vs.MCI; bulk RNA-seq,Homo_723,MCI.vs.control; bulk RNA-seq,Homo_714,AD.vs.MCI; bulk RNA-seq,Homo_714,MCI.vs.control; bulk RNA-seq,Homo_633,AD.vs.control	5
CC	GO:0032421	stereocilium bundle	bulk RNA-seq,Homo_723,AD.vs.MCI; bulk RNA-seq,Homo_723,MCI.vs.control; bulk RNA-seq,Homo_714,AD.vs.MCI; bulk RNA-seq,Homo_714,MCI.vs.control	4
CC	GO:0005802	trans-Golgi network	bulk RNA-seq,Homo_723,AD.vs.MCI; bulk RNA-seq,Homo_723,MCI.vs.control; bulk RNA-seq,Homo_714,AD.vs.control; bulk RNA-seq,Homo_633,AD.vs.control; bulk RNA-seq,Homo_633,AD.vs.MCI; scRNA-seq,SRP330776,Naive CD8+ T cell_2-AD.vs.control	6
BP	GO:0030865	cortical cytoskeleton organization	bulk RNA-seq,Homo_723,AD.vs.MCI; bulk RNA-seq,Homo_723,MCI.vs.control; bulk RNA-seq,Homo_714,AD.vs.MCI; bulk RNA-seq,Homo_714,MCI.vs.control; bulk RNA-seq,Homo_633,AD.vs.control; bulk RNA-seq,Homo_633,AD.vs.MCI	6
CC	GO:0005795	Golgi stack	bulk RNA-seq,Homo_723,AD.vs.MCI; bulk RNA-seq,Homo_723,MCI.vs.control; bulk RNA-seq,Homo_714,AD.vs.MCI; bulk RNA-seq,Homo_633,AD.vs.control; bulk RNA-seq,Homo_633,AD.vs.MCI	5
BP	GO:0034614	cellular response to reactive oxygen species	bulk RNA-seq,Homo_723,AD.vs.MCI; bulk RNA-seq,Homo_723,MCI.vs.control; bulk RNA-seq,Homo_714,AD.vs.MCI; bulk RNA-seq,Homo_633,AD.vs.control; bulk RNA-seq,Homo_633,AD.vs.MCI	5
BP	GO:0002274	myeloid leukocyte activation	bulk RNA-seq,Homo_723,AD.vs.MCI; bulk RNA-seq,Homo_723,MCI.vs.control; bulk RNA-seq,Homo_714,AD.vs.MCI; bulk RNA-seq,Homo_633,AD.vs.control; bulk RNA-seq,Homo_633,AD.vs.MCI	5
BP	GO:1901987	regulation of cell cycle phase transition	bulk RNA-seq,Homo_723,AD.vs.MCI; bulk RNA-seq,Homo_714,AD.vs.MCI; bulk RNA-seq,Homo_633,AD.vs.control; bulk RNA-seq,Homo_633,AD.vs.MCI	4
BP	GO:0030032	lamellipodium assembly	bulk RNA-seq,Homo_723,AD.vs.MCI; bulk RNA-seq,Homo_723,MCI.vs.control; bulk RNA-seq,Homo_714,AD.vs.MCI; bulk RNA-seq,Homo_714,MCI.vs.control; bulk RNA-seq,Homo_633,AD.vs.control; bulk RNA-seq,Homo_633,AD.vs.MCI	6
BP	GO:0022406	membrane docking	bulk RNA-seq,Homo_723,AD.vs.MCI; bulk RNA-seq,Homo_723,MCI.vs.control; bulk RNA-seq,Homo_714,MCI.vs.control; bulk RNA-seq,Homo_633,AD.vs.control; bulk RNA-seq,Homo_633,AD.vs.MCI	5
BP	GO:0071222	cellular response to lipopolysaccharide	bulk RNA-seq,Homo_723,AD.vs.MCI; bulk RNA-seq,Homo_723,MCI.vs.control; bulk RNA-seq,Homo_714,AD.vs.MCI; bulk RNA-seq,Homo_633,AD.vs.control; bulk RNA-seq,Homo_633,AD.vs.MCI	5
BP	GO:0018107	peptidyl-threonine phosphorylation	bulk RNA-seq,Homo_723,AD.vs.MCI; bulk RNA-seq,Homo_723,MCI.vs.control; bulk RNA-seq,Homo_714,AD.vs.MCI; bulk RNA-seq,Homo_714,MCI.vs.control; bulk RNA-seq,Homo_633,AD.vs.control; bulk RNA-seq,Homo_633,AD.vs.MCI	6
BP	GO:0032026	response to magnesium ion	bulk RNA-seq,Homo_723,AD.vs.MCI; bulk RNA-seq,Homo_723,MCI.vs.control; bulk RNA-seq,Homo_714,AD.vs.MCI; bulk RNA-seq,Homo_714,MCI.vs.control; bulk RNA-seq,Homo_633,AD.vs.control; bulk RNA-seq,Homo_633,AD.vs.MCI	6
CC	GO:0000776	kinetochore	bulk RNA-seq,Homo_723,AD.vs.MCI; bulk RNA-seq,Homo_723,MCI.vs.control; bulk RNA-seq,Homo_714,AD.vs.MCI; bulk RNA-seq,Homo_633,AD.vs.control; bulk RNA-seq,Homo_633,AD.vs.MCI	5
BP	GO:0043243	positive regulation of protein-containing complex disassembly	bulk RNA-seq,Homo_723,AD.vs.MCI; bulk RNA-seq,Homo_723,MCI.vs.control; bulk RNA-seq,Homo_714,AD.vs.MCI; bulk RNA-seq,Homo_714,MCI.vs.control; bulk RNA-seq,Homo_633,AD.vs.control	5
BP	GO:0072574	hepatocyte proliferation	bulk RNA-seq,Homo_723,AD.vs.MCI; bulk RNA-seq,Homo_723,MCI.vs.control; bulk RNA-seq,Homo_714,AD.vs.control; bulk RNA-seq,Homo_714,AD.vs.MCI; bulk RNA-seq,Homo_714,MCI.vs.control	5
BP	GO:0072575	epithelial cell proliferation involved in liver morphogenesis	bulk RNA-seq,Homo_723,AD.vs.MCI; bulk RNA-seq,Homo_723,MCI.vs.control; bulk RNA-seq,Homo_714,AD.vs.control; bulk RNA-seq,Homo_714,AD.vs.MCI; bulk RNA-seq,Homo_714,MCI.vs.control	5
BP	GO:0021756	striatum development	bulk RNA-seq,Homo_723,AD.vs.MCI; bulk RNA-seq,Homo_723,MCI.vs.control; bulk RNA-seq,Homo_714,AD.vs.MCI; bulk RNA-seq,Homo_714,MCI.vs.control	4
BP	GO:0007131	reciprocal meiotic recombination	bulk RNA-seq,Homo_723,AD.vs.MCI; bulk RNA-seq,Homo_723,MCI.vs.control; bulk RNA-seq,Homo_714,AD.vs.MCI; bulk RNA-seq,Homo_714,MCI.vs.control; bulk RNA-seq,Homo_633,AD.vs.control	5
BP	GO:0140527	reciprocal homologous recombination	bulk RNA-seq,Homo_723,AD.vs.MCI; bulk RNA-seq,Homo_723,MCI.vs.control; bulk RNA-seq,Homo_714,AD.vs.MCI; bulk RNA-seq,Homo_714,MCI.vs.control; bulk RNA-seq,Homo_633,AD.vs.control	5
BP	GO:0030728	ovulation	bulk RNA-seq,Homo_723,AD.vs.MCI; bulk RNA-seq,Homo_723,MCI.vs.control; bulk RNA-seq,Homo_714,AD.vs.MCI	3
CC	GO:0032420	stereocilium	bulk RNA-seq,Homo_723,AD.vs.MCI; bulk RNA-seq,Homo_714,AD.vs.MCI; bulk RNA-seq,Homo_714,MCI.vs.control	3
BP	GO:1903078	positive regulation of protein localization to plasma membrane	bulk RNA-seq,Homo_723,AD.vs.MCI; bulk RNA-seq,Homo_723,MCI.vs.control; bulk RNA-seq,Homo_714,AD.vs.MCI; bulk RNA-seq,Homo_714,MCI.vs.control; bulk RNA-seq,Homo_633,AD.vs.control	5
BP	GO:0045661	regulation of myoblast differentiation	bulk RNA-seq,Homo_723,AD.vs.MCI; bulk RNA-seq,Homo_723,MCI.vs.control; bulk RNA-seq,Homo_714,AD.vs.MCI; bulk RNA-seq,Homo_714,MCI.vs.control; bulk RNA-seq,Homo_633,AD.vs.control; bulk RNA-seq,Homo_633,AD.vs.MCI	6
BP	GO:0014037	Schwann cell differentiation	bulk RNA-seq,Homo_723,AD.vs.MCI; bulk RNA-seq,Homo_723,MCI.vs.control; bulk RNA-seq,Homo_714,AD.vs.MCI; bulk RNA-seq,Homo_714,MCI.vs.control; bulk RNA-seq,Homo_633,AD.vs.control	5
MF	GO:0090482	vitamin transmembrane transporter activity	bulk RNA-seq,Homo_723,AD.vs.MCI; bulk RNA-seq,Homo_723,MCI.vs.control; bulk RNA-seq,Homo_714,AD.vs.MCI; bulk RNA-seq,Homo_714,MCI.vs.control	4
BP	GO:0060707	trophoblast giant cell differentiation	bulk RNA-seq,Homo_723,AD.vs.MCI; bulk RNA-seq,Homo_723,MCI.vs.control; bulk RNA-seq,Homo_714,AD.vs.MCI	3
BP	GO:0051648	vesicle localization	bulk RNA-seq,Homo_723,AD.vs.MCI; bulk RNA-seq,Homo_723,MCI.vs.control; bulk RNA-seq,Homo_714,AD.vs.control; bulk RNA-seq,Homo_633,AD.vs.control; bulk RNA-seq,Homo_633,AD.vs.MCI	5
MF	GO:0022821	solute:potassium antiporter activity	bulk RNA-seq,Homo_723,AD.vs.MCI; bulk RNA-seq,Homo_723,MCI.vs.control	2
BP	GO:1903236	regulation of leukocyte tethering or rolling	bulk RNA-seq,Homo_723,AD.vs.MCI; bulk RNA-seq,Homo_723,MCI.vs.control; bulk RNA-seq,Homo_714,AD.vs.MCI; bulk RNA-seq,Homo_714,MCI.vs.control; bulk RNA-seq,Homo_633,AD.vs.control	5

BP	GO:0032024	positive regulation of insulin secretion	bulk RNA-seq,Homo_723,AD.vs.MCI; bulk RNA-seq,Homo_723,MCI.vs.control; bulk RNA-seq,Homo_714,AD.vs.MCI; bulk RNA-seq,Homo_714,MCI.vs.control; bulk RNA-seq,Homo_633,AD.vs.control; bulk RNA-seq,Homo_633,AD.vs.MCI	6
BP	GO:0071333	cellular response to glucose stimulus	bulk RNA-seq,Homo_723,AD.vs.MCI; bulk RNA-seq,Homo_723,MCI.vs.control; bulk RNA-seq,Homo_714,AD.vs.MCI; bulk RNA-seq,Homo_633,AD.vs.control; bulk RNA-seq,Homo_633,AD.vs.MCI	5
MF	GO:0016746	acyltransferase activity	bulk RNA-seq,Homo_723,AD.vs.MCI; bulk RNA-seq,Homo_723,MCI.vs.control; bulk RNA-seq,Homo_714,AD.vs.MCI; bulk RNA-seq,Homo_633,AD.vs.control; bulk RNA-seq,Homo_633,AD.vs.MCI	5
MF	GO:0031690	adrenergic receptor binding	bulk RNA-seq,Homo_723,AD.vs.MCI; bulk RNA-seq,Homo_723,MCI.vs.control; bulk RNA-seq,Homo_714,AD.vs.MCI; bulk RNA-seq,Homo_714,MCI.vs.control; bulk RNA-seq,Homo_633,AD.vs.control	5
CC	GO:0008305	integrin complex	bulk RNA-seq,Homo_723,AD.vs.MCI; bulk RNA-seq,Homo_723,MCI.vs.control; bulk RNA-seq,Homo_714,AD.vs.MCI; bulk RNA-seq,Homo_714,MCI.vs.control; bulk RNA-seq,Homo_633,AD.vs.control; bulk RNA-seq,Homo_633,AD.vs.MCI	6
BP	GO:0008542	visual learning	bulk RNA-seq,Homo_723,AD.vs.MCI; bulk RNA-seq,Homo_723,MCI.vs.control; bulk RNA-seq,Homo_714,AD.vs.MCI; bulk RNA-seq,Homo_633,AD.vs.control; bulk RNA-seq,Homo_633,AD.vs.MCI	5
BP	GO:0061469	regulation of type B pancreatic cell proliferation	bulk RNA-seq,Homo_723,AD.vs.MCI; bulk RNA-seq,Homo_723,MCI.vs.control; bulk RNA-seq,Homo_714,AD.vs.MCI; bulk RNA-seq,Homo_714,MCI.vs.control	4
BP	GO:0010952	positive regulation of peptidase activity	bulk RNA-seq,Homo_723,AD.vs.MCI; bulk RNA-seq,Homo_723,MCI.vs.control; bulk RNA-seq,Homo_714,AD.vs.MCI; bulk RNA-seq,Homo_633,AD.vs.control; bulk RNA-seq,Homo_633,AD.vs.MCI	5
BP	GO:1990874	vascular associated smooth muscle cell proliferation	bulk RNA-seq,Homo_723,AD.vs.MCI; bulk RNA-seq,Homo_714,AD.vs.control; bulk RNA-seq,Homo_714,AD.vs.MCI; bulk RNA-seq,Homo_714,MCI.vs.control	4
MF	GO:0055056	D-glucose transmembrane transporter activity	bulk RNA-seq,Homo_723,AD.vs.MCI; bulk RNA-seq,Homo_723,MCI.vs.control	2
MF	GO:0097493	structural molecule activity conferring elasticity	bulk RNA-seq,Homo_723,AD.vs.MCI; bulk RNA-seq,Homo_723,MCI.vs.control; bulk RNA-seq,Homo_714,AD.vs.MCI; bulk RNA-seq,Homo_714,MCI.vs.control; bulk RNA-seq,Homo_633,AD.vs.control	5
MF	GO:0019209	kinase activator activity	bulk RNA-seq,Homo_723,AD.vs.MCI; bulk RNA-seq,Homo_723,MCI.vs.control; bulk RNA-seq,Homo_714,AD.vs.MCI; bulk RNA-seq,Homo_714,MCI.vs.control; bulk RNA-seq,Homo_633,AD.vs.control; bulk RNA-seq,Homo_633,AD.vs.MCI	6
CC	GO:1905286	serine-type peptidase complex	bulk RNA-seq,Homo_723,AD.vs.MCI; bulk RNA-seq,Homo_723,MCI.vs.control; bulk RNA-seq,Homo_714,AD.vs.MCI; bulk RNA-seq,Homo_714,MCI.vs.control	4
BP	GO:1900181	negative regulation of protein localization to nucleus	bulk RNA-seq,Homo_723,AD.vs.MCI; bulk RNA-seq,Homo_723,MCI.vs.control; bulk RNA-seq,Homo_714,AD.vs.MCI; bulk RNA-seq,Homo_714,MCI.vs.control; bulk RNA-seq,Homo_633,AD.vs.control; bulk RNA-seq,Homo_633,AD.vs.MCI	6
CC	GO:0031300	intrinsic component of organelle membrane	bulk RNA-seq,Homo_723,AD.vs.MCI; bulk RNA-seq,Homo_714,AD.vs.control; bulk RNA-seq,Homo_633,AD.vs.control; bulk RNA-seq,Homo_633,AD.vs.MCI	4
MF	GO:0015399	primary active transmembrane transporter activity	bulk RNA-seq,Homo_723,AD.vs.MCI; bulk RNA-seq,Homo_723,MCI.vs.control; bulk RNA-seq,Homo_714,AD.vs.MCI; bulk RNA-seq,Homo_633,AD.vs.control; bulk RNA-seq,Homo_633,AD.vs.MCI	5
BP	GO:0044403	biological process involved in symbiotic interaction	bulk RNA-seq,Homo_723,AD.vs.MCI; bulk RNA-seq,Homo_723,MCI.vs.control; bulk RNA-seq,Homo_714,AD.vs.MCI; bulk RNA-seq,Homo_633,AD.vs.control; bulk RNA-seq,Homo_633,AD.vs.MCI	5
BP	GO:0003174	mitral valve development	bulk RNA-seq,Homo_723,AD.vs.MCI; bulk RNA-seq,Homo_723,MCI.vs.control; bulk RNA-seq,Homo_714,AD.vs.MCI; bulk RNA-seq,Homo_714,MCI.vs.control	4
CC	GO:0032156	septin cytoskeleton	bulk RNA-seq,Homo_723,AD.vs.MCI; bulk RNA-seq,Homo_723,MCI.vs.control; bulk RNA-seq,Homo_714,AD.vs.MCI; bulk RNA-seq,Homo_714,MCI.vs.control; bulk RNA-seq,Homo_633,AD.vs.control; bulk RNA-seq,Homo_633,AD.vs.MCI	6
BP	GO:0046328	regulation of JNK cascade	bulk RNA-seq,Homo_723,AD.vs.MCI; bulk RNA-seq,Homo_723,MCI.vs.control; bulk RNA-seq,Homo_714,AD.vs.MCI; bulk RNA-seq,Homo_633,AD.vs.control; bulk RNA-seq,Homo_633,AD.vs.MCI	5
BP	GO:0031023	microtubule organizing center organization	bulk RNA-seq,Homo_723,AD.vs.MCI; bulk RNA-seq,Homo_723,MCI.vs.control; bulk RNA-seq,Homo_714,AD.vs.MCI; bulk RNA-seq,Homo_633,AD.vs.control; bulk RNA-seq,Homo_633,AD.vs.MCI	5
BP	GO:0033688	regulation of osteoblast proliferation	bulk RNA-seq,Homo_723,AD.vs.MCI; bulk RNA-seq,Homo_633,AD.vs.control	2
BP	GO:0032091	negative regulation of protein binding	bulk RNA-seq,Homo_723,AD.vs.MCI; bulk RNA-seq,Homo_723,MCI.vs.control; bulk RNA-seq,Homo_714,AD.vs.MCI; bulk RNA-seq,Homo_714,MCI.vs.control; bulk RNA-seq,Homo_633,AD.vs.control; bulk RNA-seq,Homo_633,AD.vs.MCI	6
BP	GO:0006970	response to osmotic stress	bulk RNA-seq,Homo_723,AD.vs.MCI; bulk RNA-seq,Homo_723,MCI.vs.control; bulk RNA-seq,Homo_714,AD.vs.MCI; bulk RNA-seq,Homo_714,MCI.vs.control; bulk RNA-seq,Homo_633,AD.vs.control; bulk RNA-seq,Homo_633,AD.vs.MCI	6
BP	GO:1901568	fatty acid derivative metabolic process	bulk RNA-seq,Homo_723,AD.vs.MCI; bulk RNA-seq,Homo_723,MCI.vs.control; bulk RNA-seq,Homo_714,AD.vs.MCI; bulk RNA-seq,Homo_714,MCI.vs.control; bulk RNA-seq,Homo_633,AD.vs.control; bulk RNA-seq,Homo_633,AD.vs.MCI	6
CC	GO:0031526	brush border membrane	bulk RNA-seq,Homo_723,AD.vs.MCI; bulk RNA-seq,Homo_723,MCI.vs.control; bulk RNA-seq,Homo_714,AD.vs.MCI; bulk RNA-seq,Homo_633,AD.vs.control	4
BP	GO:0043525	positive regulation of neuron apoptotic process	bulk RNA-seq,Homo_723,AD.vs.MCI; bulk RNA-seq,Homo_723,MCI.vs.control; bulk RNA-seq,Homo_714,MCI.vs.control; bulk RNA-seq,Homo_633,AD.vs.control; bulk RNA-seq,Homo_633,AD.vs.MCI	5
BP	GO:0050866	negative regulation of cell activation	bulk RNA-seq,Homo_723,AD.vs.MCI; bulk RNA-seq,Homo_723,MCI.vs.control; bulk RNA-seq,Homo_714,AD.vs.MCI; bulk RNA-seq,Homo_633,AD.vs.control; bulk RNA-seq,Homo_633,AD.vs.MCI	5
BP	GO:0090177	establishment of planar polarity involved in neural tube	bulk RNA-seq,Homo_723,AD.vs.MCI; bulk RNA-seq,Homo_723,MCI.vs.control	2
BP	GO:0031338	regulation of vesicle fusion	bulk RNA-seq,Homo_723,AD.vs.MCI; bulk RNA-seq,Homo_723,MCI.vs.control	2
BP	GO:1903706	regulation of hemopoiesis	bulk RNA-seq,Homo_723,AD.vs.MCI; bulk RNA-seq,Homo_714,AD.vs.MCI; bulk RNA-seq,Homo_633,AD.vs.control; bulk RNA-seq,Homo_633,AD.vs.MCI; scRNA-seq,SRP330776,Naive CD8+ T cell_2-AD.vs.control; scRNA-seq,SRP215507,CD8+ T cell_3-AD.vs.control	6
MF	GO:0070697	activin receptor binding	bulk RNA-seq,Homo_723,AD.vs.MCI; bulk RNA-seq,Homo_714,AD.vs.MCI	2
BP	GO:0050995	negative regulation of lipid catabolic process	bulk RNA-seq,Homo_723,AD.vs.MCI; bulk RNA-seq,Homo_723,MCI.vs.control; bulk RNA-seq,Homo_714,AD.vs.MCI; bulk RNA-seq,Homo_714,MCI.vs.control	4
BP	GO:0034309	primary alcohol biosynthetic process	bulk RNA-seq,Homo_723,AD.vs.MCI; bulk RNA-seq,Homo_714,AD.vs.MCI	2
BP	GO:0030593	neutrophil chemotaxis	bulk RNA-seq,Homo_723,AD.vs.MCI; bulk RNA-seq,Homo_723,MCI.vs.control; bulk RNA-seq,Homo_714,AD.vs.MCI; bulk RNA-seq,Homo_714,MCI.vs.control; bulk RNA-seq,SRP223445,AD.vs.control	5
BP	GO:0030258	lipid modification	bulk RNA-seq,Homo_723,AD.vs.MCI; bulk RNA-seq,Homo_723,MCI.vs.control; bulk RNA-seq,Homo_714,AD.vs.MCI; bulk RNA-seq,Homo_633,AD.vs.control; bulk RNA-seq,Homo_633,AD.vs.MCI	5
CC	GO:0005743	mitochondrial inner membrane	bulk RNA-seq,Homo_723,AD.vs.MCI; bulk RNA-seq,Homo_714,AD.vs.control; bulk RNA-seq,Homo_633,AD.vs.control; bulk RNA-seq,Homo_633,AD.vs.MCI; scRNA-seq,SRP330776,Natural killer cell_1-AD.vs.control	5
MF	GO:0045505	dynein intermediate chain binding	bulk RNA-seq,Homo_723,AD.vs.MCI; bulk RNA-seq,Homo_723,MCI.vs.control	2
BP	GO:0090279	regulation of calcium ion import	bulk RNA-seq,Homo_723,AD.vs.MCI; bulk RNA-seq,Homo_714,AD.vs.MCI	2

BP	GO:0051281	positive regulation of release of sequestered calcium ion into	bulk RNA-seq,Homo_723,AD.vs.MCI; bulk RNA-seq,Homo_723,MCI.vs.control; bulk RNA-seq,Homo_714,AD.vs.MCI; bulk RNA-seq,Homo_714,MCI.vs.control	4
BP	GO:0042219	cellular modified amino acid catabolic process	bulk RNA-seq,Homo_723,AD.vs.MCI; bulk RNA-seq,Homo_723,MCI.vs.control; bulk RNA-seq,Homo_714,MCI.vs.control; bulk RNA-	4
BP	GO:0038066	p38MAPK cascade	bulk RNA-seq,Homo_723,AD.vs.MCI; bulk RNA-seq,Homo_723,MCI.vs.control; bulk RNA-seq,Homo_714,MCI.vs.control; bulk RNA-	4
BP	GO:0043507	positive regulation of JUN kinase activity	bulk RNA-seq,Homo_723,AD.vs.MCI; bulk RNA-seq,Homo_723,MCI.vs.control; bulk RNA-seq,Homo_714,AD.vs.MCI; bulk RNA-seq,Homo_714,MCI.vs.control; bulk RNA-seq,Homo_633,AD.vs.control	5
BP	GO:0035825	homologous recombination	bulk RNA-seq,Homo_723,AD.vs.MCI; bulk RNA-seq,Homo_723,MCI.vs.control; bulk RNA-seq,Homo_714,AD.vs.MCI; bulk RNA-seq,Homo_714,MCI.vs.control; bulk RNA-seq,Homo_633,AD.vs.control	5
BP	GO:0032350	regulation of hormone metabolic process	bulk RNA-seq,Homo_723,AD.vs.MCI; bulk RNA-seq,Homo_723,MCI.vs.control; bulk RNA-seq,Homo_714,MCI.vs.control; bulk RNA-seq,Homo_633,AD.vs.control; bulk RNA-seq,Homo_633,AD.vs.MCI	5
CC	GO:0005858	axonemal dynein complex	bulk RNA-seq,Homo_723,AD.vs.MCI; bulk RNA-seq,Homo_723,MCI.vs.control; bulk RNA-seq,Homo_633,AD.vs.control; bulk RNA-seq,Homo_633,AD.vs.MCI	4
MF	GO:0015078	proton transmembrane transporter activity	bulk RNA-seq,Homo_723,AD.vs.MCI; bulk RNA-seq,Homo_723,MCI.vs.control; bulk RNA-seq,Homo_714,AD.vs.MCI; bulk RNA-seq,Homo_633,AD.vs.control; bulk RNA-seq,Homo_633,AD.vs.MCI	5
BP	GO:0006521	regulation of cellular amino acid metabolic process	bulk RNA-seq,Homo_723,AD.vs.MCI; bulk RNA-seq,Homo_714,AD.vs.MCI	2
BP	GO:0042246	tissue regeneration	bulk RNA-seq,Homo_723,AD.vs.MCI; bulk RNA-seq,Homo_723,MCI.vs.control; bulk RNA-seq,Homo_714,AD.vs.MCI; bulk RNA-seq,Homo_714,MCI.vs.control; bulk RNA-seq,Homo_633,AD.vs.control; bulk RNA-seq,Homo_633,AD.vs.MCI	6
BP	GO:0097048	dendritic cell apoptotic process	bulk RNA-seq,Homo_723,AD.vs.MCI; bulk RNA-seq,Homo_723,MCI.vs.control; bulk RNA-seq,Homo_714,AD.vs.MCI; bulk RNA-seq,Homo_714,MCI.vs.control	4
BP	GO:2000668	regulation of dendritic cell apoptotic process	bulk RNA-seq,Homo_723,AD.vs.MCI; bulk RNA-seq,Homo_723,MCI.vs.control; bulk RNA-seq,Homo_714,AD.vs.MCI; bulk RNA-seq,Homo_714,MCI.vs.control	4
BP	GO:2000036	regulation of stem cell population maintenance	bulk RNA-seq,Homo_723,AD.vs.MCI; bulk RNA-seq,Homo_723,MCI.vs.control; bulk RNA-seq,Homo_714,MCI.vs.control; bulk RNA-seq,Homo_633,AD.vs.control; bulk RNA-seq,Homo_633,AD.vs.MCI	5
BP	GO:0098930	axonal transport	bulk RNA-seq,Homo_723,AD.vs.MCI; bulk RNA-seq,Homo_723,MCI.vs.control; bulk RNA-seq,Homo_714,MCI.vs.control; bulk RNA-seq,Homo_633,AD.vs.control; bulk RNA-seq,Homo_633,AD.vs.MCI	5
BP	GO:0009267	cellular response to starvation	bulk RNA-seq,Homo_723,AD.vs.MCI; bulk RNA-seq,Homo_723,MCI.vs.control; bulk RNA-seq,Homo_633,AD.vs.control; bulk RNA-seq,Homo_633,AD.vs.MCI	4
BP	GO:0060923	cardiac muscle cell fate commitment	bulk RNA-seq,Homo_723,AD.vs.MCI; bulk RNA-seq,Homo_723,MCI.vs.control; bulk RNA-seq,Homo_714,AD.vs.MCI; bulk RNA-seq,Homo_714,MCI.vs.control; bulk RNA-seq,Homo_633,AD.vs.MCI	5
MF	GO:0004745	NAD-retinol dehydrogenase activity	bulk RNA-seq,Homo_723,AD.vs.MCI; bulk RNA-seq,Homo_723,MCI.vs.control; bulk RNA-seq,Homo_714,AD.vs.MCI; bulk RNA-seq,Homo_714,MCI.vs.control	4
BP	GO:0071320	cellular response to cAMP	bulk RNA-seq,Homo_723,AD.vs.MCI; bulk RNA-seq,Homo_723,MCI.vs.control; bulk RNA-seq,Homo_714,AD.vs.MCI; bulk RNA-seq,Homo_714,MCI.vs.control; bulk RNA-seq,Homo_633,AD.vs.control; bulk RNA-seq,Homo_633,AD.vs.MCI	6
BP	GO:0070542	response to fatty acid	bulk RNA-seq,Homo_723,AD.vs.MCI; bulk RNA-seq,Homo_723,MCI.vs.control; bulk RNA-seq,Homo_714,AD.vs.MCI; bulk RNA-seq,Homo_714,MCI.vs.control; bulk RNA-seq,Homo_633,AD.vs.control; bulk RNA-seq,Homo_633,AD.vs.MCI	6
BP	GO:0061626	pharyngeal arch artery morphogenesis	bulk RNA-seq,Homo_723,AD.vs.MCI; bulk RNA-seq,Homo_723,MCI.vs.control; bulk RNA-seq,Homo_714,AD.vs.MCI; bulk RNA-seq,Homo_714,MCI.vs.control	4
BP	GO:0110150	negative regulation of biomineralization	bulk RNA-seq,Homo_723,AD.vs.MCI; bulk RNA-seq,Homo_723,MCI.vs.control; bulk RNA-seq,Homo_714,AD.vs.MCI; bulk RNA-seq,Homo_714,MCI.vs.control	4
BP	GO:0062237	protein localization to postsynapse	bulk RNA-seq,Homo_723,AD.vs.MCI; bulk RNA-seq,Homo_723,MCI.vs.control; bulk RNA-seq,Homo_714,AD.vs.MCI; bulk RNA-seq,Homo_633,AD.vs.control	4
BP	GO:0008625	extrinsic apoptotic signaling pathway via death domain receptors	bulk RNA-seq,Homo_723,AD.vs.MCI; bulk RNA-seq,Homo_723,MCI.vs.control; bulk RNA-seq,Homo_714,AD.vs.MCI; bulk RNA-seq,Homo_714,MCI.vs.control; bulk RNA-seq,Homo_633,AD.vs.control; bulk RNA-seq,Homo_633,AD.vs.MCI	6
BP	GO:0045670	regulation of osteoclast differentiation	bulk RNA-seq,Homo_723,AD.vs.MCI; bulk RNA-seq,Homo_723,MCI.vs.control; bulk RNA-seq,Homo_714,AD.vs.MCI; bulk RNA-seq,Homo_714,MCI.vs.control; bulk RNA-seq,Homo_633,AD.vs.control; bulk RNA-seq,SRP325058,AD.vs.control	6
BP	GO:0031670	cellular response to nutrient	bulk RNA-seq,Homo_723,AD.vs.MCI; bulk RNA-seq,Homo_723,MCI.vs.control; bulk RNA-seq,Homo_714,AD.vs.MCI; bulk RNA-seq,Homo_714,MCI.vs.control; bulk RNA-seq,Homo_633,AD.vs.control	5
BP	GO:0120034	positive regulation of plasma membrane bounded cell projection assembly	bulk RNA-seq,Homo_723,AD.vs.MCI; bulk RNA-seq,Homo_723,MCI.vs.control; bulk RNA-seq,Homo_714,AD.vs.MCI; bulk RNA-seq,Homo_714,MCI.vs.control; bulk RNA-seq,Homo_633,AD.vs.control; bulk RNA-seq,Homo_633,AD.vs.MCI	6
BP	GO:0033280	response to vitamin D	bulk RNA-seq,Homo_723,AD.vs.MCI; bulk RNA-seq,Homo_723,MCI.vs.control; bulk RNA-seq,Homo_714,MCI.vs.control; bulk RNA-	4
BP	GO:0048742	regulation of skeletal muscle fiber development	bulk RNA-seq,Homo_723,AD.vs.MCI; bulk RNA-seq,Homo_714,AD.vs.MCI	2
BP	GO:0070528	protein kinase C signaling	bulk RNA-seq,Homo_723,AD.vs.MCI; bulk RNA-seq,Homo_723,MCI.vs.control	2
BP	GO:0044342	type B pancreatic cell proliferation	bulk RNA-seq,Homo_723,AD.vs.MCI; bulk RNA-seq,Homo_723,MCI.vs.control; bulk RNA-seq,Homo_714,AD.vs.MCI; bulk RNA-seq,Homo_714,MCI.vs.control	4
BP	GO:0033574	response to testosterone	bulk RNA-seq,Homo_723,AD.vs.MCI; bulk RNA-seq,Homo_714,AD.vs.control; bulk RNA-seq,Homo_714,AD.vs.MCI; bulk RNA-seq,Homo_633,AD.vs.control; bulk RNA-seq,Homo_633,AD.vs.MCI	5
BP	GO:0030007	cellular potassium ion homeostasis	bulk RNA-seq,Homo_723,AD.vs.MCI; bulk RNA-seq,Homo_723,MCI.vs.control; bulk RNA-seq,Homo_714,AD.vs.MCI	3
BP	GO:0006687	glycosphingolipid metabolic process	bulk RNA-seq,Homo_723,AD.vs.MCI; bulk RNA-seq,Homo_723,MCI.vs.control; bulk RNA-seq,Homo_714,MCI.vs.control; bulk RNA-seq,Homo_633,AD.vs.control; bulk RNA-seq,Homo_633,AD.vs.MCI	5
BP	GO:0001893	maternal placenta development	bulk RNA-seq,Homo_723,AD.vs.MCI; bulk RNA-seq,Homo_723,MCI.vs.control; bulk RNA-seq,Homo_714,AD.vs.MCI	3
CC	GO:0032982	myosin filament	bulk RNA-seq,Homo_723,AD.vs.MCI; bulk RNA-seq,Homo_723,MCI.vs.control; bulk RNA-seq,Homo_714,AD.vs.MCI; bulk RNA-seq,Homo_633,MCI.vs.control	4
BP	GO:0051770	positive regulation of nitric-oxide synthase biosynthetic process	bulk RNA-seq,Homo_723,AD.vs.MCI; bulk RNA-seq,Homo_723,MCI.vs.control; bulk RNA-seq,Homo_714,AD.vs.MCI; bulk RNA-seq,Homo_714,MCI.vs.control; bulk RNA-seq,Homo_633,AD.vs.control	5
BP	GO:0033628	regulation of cell adhesion mediated by integrin	bulk RNA-seq,Homo_723,AD.vs.MCI; bulk RNA-seq,Homo_723,MCI.vs.control; bulk RNA-seq,Homo_714,AD.vs.MCI; bulk RNA-seq,Homo_714,MCI.vs.control; bulk RNA-seq,Homo_633,AD.vs.control; bulk RNA-seq,Homo_633,AD.vs.MCI	6
BP	GO:0034698	response to gonadotropin	bulk RNA-seq,Homo_723,AD.vs.MCI; bulk RNA-seq,Homo_714,AD.vs.MCI	2
BP	GO:0097720	calcineurin-mediated signaling	bulk RNA-seq,Homo_723,AD.vs.MCI; bulk RNA-seq,Homo_723,MCI.vs.control; bulk RNA-seq,Homo_714,AD.vs.MCI; bulk RNA-seq,Homo_714,MCI.vs.control; bulk RNA-seq,Homo_633,AD.vs.control	5
BP	GO:0042135	neurotransmitter catabolic process	bulk RNA-seq,Homo_723,AD.vs.MCI; bulk RNA-seq,Homo_723,MCI.vs.control	2
CC	GO:0035869	ciliary transition zone	bulk RNA-seq,Homo_723,AD.vs.MCI; bulk RNA-seq,Homo_723,MCI.vs.control; bulk RNA-seq,Homo_714,AD.vs.MCI; bulk RNA-seq,Homo_714,MCI.vs.control; bulk RNA-seq,Homo_633,AD.vs.control; bulk RNA-seq,Homo_633,AD.vs.MCI	6

BP	GO:0000380	alternative mRNA splicing, via spliceosome	bulk RNA-seq,Homo_723,AD.vs.MCI; bulk RNA-seq,Homo_723,MCI.vs.control; bulk RNA-seq,Homo_714,MCI.vs.control; bulk RNA-seq,Homo_633,AD.vs.control; bulk RNA-seq,Homo_633,AD.vs.MCI	5
BP	GO:0016540	protein autoprocessing	bulk RNA-seq,Homo_723,AD.vs.MCI; bulk RNA-seq,Homo_723,MCI.vs.control; bulk RNA-seq,Homo_714,AD.vs.MCI; bulk RNA-seq,Homo_633,AD.vs.control; bulk RNA-seq,Homo_633,AD.vs.MCI	5
CC	GO:0032426	stereocilium tip	bulk RNA-seq,Homo_723,AD.vs.MCI	1
BP	GO:1902548	negative regulation of cellular response to vascular endothelial growth factor stimulus	bulk RNA-seq,Homo_723,AD.vs.MCI; bulk RNA-seq,Homo_723,MCI.vs.control; bulk RNA-seq,Homo_714,AD.vs.MCI; bulk RNA-seq,Homo_714,MCI.vs.control	4
BP	GO:0051785	positive regulation of nuclear division	bulk RNA-seq,Homo_723,AD.vs.MCI; bulk RNA-seq,Homo_723,MCI.vs.control; bulk RNA-seq,Homo_714,AD.vs.MCI; bulk RNA-seq,Homo_714,MCI.vs.control; bulk RNA-seq,Homo_633,AD.vs.control; bulk RNA-seq,Homo_633,AD.vs.MCI	6
BP	GO:0060135	maternal process involved in female pregnancy	bulk RNA-seq,Homo_723,AD.vs.MCI; bulk RNA-seq,Homo_723,MCI.vs.control; bulk RNA-seq,Homo_714,AD.vs.MCI; bulk RNA-seq,Homo_714,MCI.vs.control	4
BP	GO:0019217	regulation of fatty acid metabolic process	bulk RNA-seq,Homo_723,AD.vs.MCI; bulk RNA-seq,Homo_723,MCI.vs.control; bulk RNA-seq,Homo_714,AD.vs.MCI; bulk RNA-seq,Homo_714,MCI.vs.control; bulk RNA-seq,Homo_633,AD.vs.control; bulk RNA-seq,Homo_633,AD.vs.MCI	6
MF	GO:0003712	transcription coregulator activity	bulk RNA-seq,Homo_723,AD.vs.MCI; bulk RNA-seq,Homo_714,AD.vs.control; bulk RNA-seq,Homo_714,AD.vs.MCI; bulk RNA-seq,Homo_633,AD.vs.control; bulk RNA-seq,Homo_633,AD.vs.MCI	5
BP	GO:0014044	Schwann cell development	bulk RNA-seq,Homo_723,AD.vs.MCI; bulk RNA-seq,Homo_723,MCI.vs.control; bulk RNA-seq,Homo_714,AD.vs.MCI; bulk RNA-seq,Homo_714,MCI.vs.control; bulk RNA-seq,Homo_633,AD.vs.control	5
BP	GO:0002792	negative regulation of peptide secretion	bulk RNA-seq,Homo_723,AD.vs.MCI; bulk RNA-seq,Homo_723,MCI.vs.control; bulk RNA-seq,Homo_633,AD.vs.control; bulk RNA-seq,Homo_633,AD.vs.MCI	4
MF	GO:0015662	P-type ion transporter activity	bulk RNA-seq,Homo_723,AD.vs.MCI; bulk RNA-seq,Homo_723,MCI.vs.control	2
BP	GO:0008211	glucocorticoid metabolic process	bulk RNA-seq,Homo_723,AD.vs.MCI; bulk RNA-seq,Homo_723,MCI.vs.control	2
BP	GO:0032104	regulation of response to extracellular stimulus	bulk RNA-seq,Homo_723,AD.vs.MCI; bulk RNA-seq,Homo_723,MCI.vs.control; bulk RNA-seq,Homo_714,MCI.vs.control; bulk RNA-seq,Homo_633,AD.vs.control; bulk RNA-seq,Homo_633,AD.vs.MCI	5
BP	GO:0032107	regulation of response to nutrient levels	bulk RNA-seq,Homo_723,AD.vs.MCI; bulk RNA-seq,Homo_723,MCI.vs.control; bulk RNA-seq,Homo_714,MCI.vs.control; bulk RNA-seq,Homo_633,AD.vs.control; bulk RNA-seq,Homo_633,AD.vs.MCI	5
BP	GO:0042730	fibrinolysis	bulk RNA-seq,Homo_723,AD.vs.MCI; bulk RNA-seq,Homo_723,MCI.vs.control; bulk RNA-seq,Homo_714,AD.vs.MCI; bulk RNA-seq,Homo_714,MCI.vs.control	4
BP	GO:0006633	fatty acid biosynthetic process	bulk RNA-seq,Homo_723,AD.vs.MCI; bulk RNA-seq,Homo_723,MCI.vs.control; bulk RNA-seq,Homo_714,AD.vs.MCI; bulk RNA-seq,Homo_633,AD.vs.control; bulk RNA-seq,Homo_633,AD.vs.MCI	5
CC	GO:0005940	septin ring	bulk RNA-seq,Homo_723,AD.vs.MCI; bulk RNA-seq,Homo_723,MCI.vs.control; bulk RNA-seq,Homo_714,AD.vs.MCI; bulk RNA-seq,Homo_633,AD.vs.control; bulk RNA-seq,Homo_633,AD.vs.MCI	5
CC	GO:0031105	septin complex	bulk RNA-seq,Homo_723,AD.vs.MCI; bulk RNA-seq,Homo_723,MCI.vs.control; bulk RNA-seq,Homo_714,AD.vs.MCI; bulk RNA-seq,Homo_633,AD.vs.control; bulk RNA-seq,Homo_633,AD.vs.MCI	5
BP	GO:0097194	execution phase of apoptosis	bulk RNA-seq,Homo_723,AD.vs.MCI; bulk RNA-seq,Homo_714,AD.vs.MCI; bulk RNA-seq,Homo_633,AD.vs.control; bulk RNA-seq,Homo_633,AD.vs.MCI	4
BP	GO:0034405	response to fluid shear stress	bulk RNA-seq,Homo_723,AD.vs.MCI; bulk RNA-seq,Homo_723,MCI.vs.control; bulk RNA-seq,Homo_714,AD.vs.MCI; bulk RNA-seq,Homo_714,MCI.vs.control; bulk RNA-seq,Homo_633,AD.vs.control; bulk RNA-seq,Homo_633,AD.vs.MCI	6
BP	GO:1990776	response to angiotensin	bulk RNA-seq,Homo_723,AD.vs.MCI; bulk RNA-seq,Homo_723,MCI.vs.control; bulk RNA-seq,Homo_714,AD.vs.MCI; bulk RNA-seq,Homo_714,MCI.vs.control; scRNA-seq,SRP215507,CD8+ T cell_3-AD.vs.control	5
BP	GO:0031639	plasminogen activation	bulk RNA-seq,Homo_723,AD.vs.MCI; bulk RNA-seq,Homo_723,MCI.vs.control; bulk RNA-seq,Homo_714,AD.vs.MCI; bulk RNA-seq,Homo_714,MCI.vs.control	4
CC	GO:0031089	platelet dense granule lumen	bulk RNA-seq,Homo_723,AD.vs.MCI; bulk RNA-seq,Homo_723,MCI.vs.control; bulk RNA-seq,Homo_714,AD.vs.MCI; bulk RNA-seq,Homo_714,MCI.vs.control	4
MF	GO:0004114	3',5'-cyclic-nucleotide phosphodiesterase activity	bulk RNA-seq,Homo_723,AD.vs.MCI; bulk RNA-seq,Homo_723,MCI.vs.control; bulk RNA-seq,Homo_714,AD.vs.MCI; bulk RNA-seq,Homo_714,MCI.vs.control; bulk RNA-seq,Homo_633,AD.vs.control	5
BP	GO:0071371	cellular response to gonadotropin stimulus	bulk RNA-seq,Homo_723,AD.vs.MCI; bulk RNA-seq,Homo_723,MCI.vs.control; bulk RNA-seq,Homo_714,AD.vs.MCI	3
BP	GO:0038083	peptidyl-tyrosine autophosphorylation	bulk RNA-seq,Homo_723,AD.vs.MCI; bulk RNA-seq,Homo_723,MCI.vs.control; bulk RNA-seq,Homo_714,AD.vs.MCI; bulk RNA-seq,Homo_714,MCI.vs.control; bulk RNA-seq,Homo_633,AD.vs.control	5
BP	GO:0048662	negative regulation of smooth muscle cell proliferation	bulk RNA-seq,Homo_723,AD.vs.MCI; bulk RNA-seq,Homo_723,MCI.vs.control; bulk RNA-seq,Homo_714,AD.vs.control; bulk RNA-seq,Homo_714,AD.vs.MCI; bulk RNA-seq,Homo_714,MCI.vs.control; bulk RNA-seq,Homo_633,AD.vs.control; bulk RNA-seq,Homo_633,AD.vs.MCI	7
BP	GO:0032965	regulation of collagen biosynthetic process	bulk RNA-seq,Homo_723,AD.vs.MCI; bulk RNA-seq,Homo_714,AD.vs.MCI	2
CC	GO:0032154	cleavage furrow	bulk RNA-seq,Homo_723,AD.vs.MCI; bulk RNA-seq,Homo_723,MCI.vs.control; bulk RNA-seq,Homo_714,AD.vs.MCI; bulk RNA-seq,Homo_714,MCI.vs.control; bulk RNA-seq,Homo_633,AD.vs.control	5
MF	GO:0050431	transforming growth factor beta binding	bulk RNA-seq,Homo_723,AD.vs.MCI; bulk RNA-seq,Homo_723,MCI.vs.control; bulk RNA-seq,Homo_714,AD.vs.MCI; bulk RNA-seq,Homo_714,MCI.vs.control; bulk RNA-seq,Homo_633,AD.vs.control	5
BP	GO:0062009	secondary palate development	bulk RNA-seq,Homo_723,AD.vs.MCI; bulk RNA-seq,Homo_723,MCI.vs.control; bulk RNA-seq,Homo_714,AD.vs.MCI; bulk RNA-seq,Homo_633,AD.vs.control; bulk RNA-seq,Homo_633,AD.vs.MCI	5
BP	GO:0000271	polysaccharide biosynthetic process	bulk RNA-seq,Homo_723,AD.vs.MCI; bulk RNA-seq,Homo_723,MCI.vs.control; bulk RNA-seq,Homo_714,AD.vs.MCI; bulk RNA-seq,Homo_714,MCI.vs.control; bulk RNA-seq,Homo_633,AD.vs.control	5
BP	GO:0000086	G2/M transition of mitotic cell cycle	bulk RNA-seq,Homo_723,AD.vs.MCI; bulk RNA-seq,Homo_723,MCI.vs.control; bulk RNA-seq,Homo_714,AD.vs.MCI; bulk RNA-seq,Homo_633,AD.vs.control; bulk RNA-seq,Homo_633,AD.vs.MCI	5
BP	GO:0071216	cellular response to biotic stimulus	bulk RNA-seq,Homo_723,AD.vs.MCI; bulk RNA-seq,Homo_723,MCI.vs.control; bulk RNA-seq,Homo_714,AD.vs.MCI; bulk RNA-seq,Homo_633,AD.vs.control; bulk RNA-seq,Homo_633,AD.vs.MCI; scRNA-seq,SRP330776,Naive CD8+ T cell_2-AD.vs.control	6
BP	GO:0009895	negative regulation of catabolic process	bulk RNA-seq,Homo_723,AD.vs.MCI; bulk RNA-seq,Homo_723,MCI.vs.control; bulk RNA-seq,Homo_714,AD.vs.MCI; bulk RNA-seq,Homo_633,AD.vs.control; bulk RNA-seq,Homo_633,AD.vs.MCI	5
BP	GO:0043393	regulation of protein binding	bulk RNA-seq,Homo_723,AD.vs.MCI; bulk RNA-seq,Homo_723,MCI.vs.control; bulk RNA-seq,Homo_714,AD.vs.MCI; bulk RNA-seq,Homo_633,AD.vs.control; bulk RNA-seq,Homo_633,AD.vs.MCI	5
BP	GO:0009068	aspartate family amino acid catabolic process	bulk RNA-seq,Homo_723,AD.vs.MCI; bulk RNA-seq,Homo_723,MCI.vs.control; bulk RNA-seq,Homo_714,MCI.vs.control; bulk RNA-	4

BP	GO:1903306	negative regulation of regulated secretory pathway	bulk RNA-seq,Homo_723,AD.vs.MCI; bulk RNA-seq,Homo_723,MCI.vs.control; bulk RNA-seq,Homo_714,MCI.vs.control; bulk RNA-seq,Homo_633,AD.vs.control; bulk RNA-seq,Homo_633,AD.vs.MCI	5
BP	GO:0030837	negative regulation of actin filament polymerization	bulk RNA-seq,Homo_723,AD.vs.MCI; bulk RNA-seq,Homo_723,MCI.vs.control; bulk RNA-seq,Homo_714,MCI.vs.control; bulk RNA-	4
BP	GO:2000628	regulation of miRNA metabolic process	bulk RNA-seq,Homo_723,AD.vs.MCI; bulk RNA-seq,Homo_723,MCI.vs.control; bulk RNA-seq,Homo_714,AD.vs.MCI; bulk RNA-seq,Homo_714,MCI.vs.control; bulk RNA-seq,Homo_633,AD.vs.control; bulk RNA-seq,Homo_633,AD.vs.MCI	6
BP	GO:0051918	negative regulation of fibrinolysis	bulk RNA-seq,Homo_723,AD.vs.MCI; bulk RNA-seq,Homo_723,MCI.vs.control; bulk RNA-seq,Homo_714,AD.vs.MCI; bulk RNA-seq,Homo_714,MCI.vs.control	4
BP	GO:0061029	eyelid development in camera-type eye	bulk RNA-seq,Homo_723,AD.vs.MCI; bulk RNA-seq,Homo_723,MCI.vs.control; bulk RNA-seq,Homo_714,AD.vs.MCI	3
BP	GO:0050667	homocysteine metabolic process	bulk RNA-seq,Homo_723,AD.vs.MCI; bulk RNA-seq,Homo_723,MCI.vs.control; bulk RNA-seq,Homo_714,AD.vs.MCI; bulk RNA-seq,Homo_714,MCI.vs.control	4
BP	GO:0048261	negative regulation of receptor-mediated endocytosis	bulk RNA-seq,Homo_723,AD.vs.MCI; bulk RNA-seq,Homo_714,AD.vs.MCI; bulk RNA-seq,Homo_633,AD.vs.control; bulk RNA-seq,Homo_633,AD.vs.MCI; scRNA-seq,SRP330776,Naive CD8+ T cell_2-AD.vs.control	5
BP	GO:1990266	neutrophil migration	bulk RNA-seq,Homo_723,AD.vs.MCI; bulk RNA-seq,Homo_723,MCI.vs.control; bulk RNA-seq,Homo_714,AD.vs.MCI; bulk RNA-seq,SRP223445,AD.vs.control	4
BP	GO:1902230	negative regulation of intrinsic apoptotic signaling pathway in response to DNA damage	bulk RNA-seq,Homo_723,AD.vs.MCI; bulk RNA-seq,Homo_723,MCI.vs.control; bulk RNA-seq,Homo_714,AD.vs.MCI; bulk RNA-seq,Homo_714,MCI.vs.control; bulk RNA-seq,Homo_633,AD.vs.control; bulk RNA-seq,Homo_633,AD.vs.MCI	6
MF	GO:0016747	acyltransferase activity, transferring groups other than amino-acyl groups	bulk RNA-seq,Homo_723,AD.vs.MCI; bulk RNA-seq,Homo_723,MCI.vs.control; bulk RNA-seq,Homo_714,AD.vs.MCI; bulk RNA-seq,Homo_633,AD.vs.control; bulk RNA-seq,Homo_633,AD.vs.MCI	5
BP	GO:0051764	actin crosslink formation	bulk RNA-seq,Homo_723,AD.vs.MCI; bulk RNA-seq,Homo_723,MCI.vs.control; bulk RNA-seq,Homo_714,AD.vs.MCI; bulk RNA-seq,Homo_714,MCI.vs.control	4
BP	GO:0043584	nose development	bulk RNA-seq,Homo_723,AD.vs.MCI; bulk RNA-seq,Homo_723,MCI.vs.control; bulk RNA-seq,Homo_714,AD.vs.MCI	3
BP	GO:0070085	glycosylation	bulk RNA-seq,Homo_723,AD.vs.MCI; bulk RNA-seq,Homo_723,MCI.vs.control; bulk RNA-seq,Homo_633,AD.vs.control; bulk RNA-seq,Homo_633,AD.vs.MCI	4
BP	GO:0006570	tyrosine metabolic process	bulk RNA-seq,Homo_723,AD.vs.MCI; bulk RNA-seq,Homo_723,MCI.vs.control; bulk RNA-seq,Homo_714,AD.vs.MCI	3
BP	GO:0045744	negative regulation of G protein-coupled receptor signaling	bulk RNA-seq,Homo_723,AD.vs.MCI; bulk RNA-seq,Homo_723,MCI.vs.control; bulk RNA-seq,Homo_714,AD.vs.MCI; bulk RNA-seq,Homo_714,MCI.vs.control	4
BP	GO:0006817	phosphate ion transport	bulk RNA-seq,Homo_723,AD.vs.MCI; bulk RNA-seq,Homo_723,MCI.vs.control; bulk RNA-seq,Homo_633,AD.vs.control; bulk RNA-seq,Homo_633,AD.vs.MCI	4
BP	GO:0030836	positive regulation of actin filament depolymerization	bulk RNA-seq,Homo_723,AD.vs.MCI; bulk RNA-seq,Homo_723,MCI.vs.control; bulk RNA-seq,Homo_714,AD.vs.MCI; bulk RNA-seq,Homo_714,MCI.vs.control	4
MF	GO:0004693	cyclin-dependent protein serine/threonine kinase activity	bulk RNA-seq,Homo_723,AD.vs.MCI; bulk RNA-seq,Homo_714,AD.vs.MCI; bulk RNA-seq,Homo_633,AD.vs.control; bulk RNA-seq,Homo_633,AD.vs.MCI	4
MF	GO:0097472	cyclin-dependent protein kinase activity	bulk RNA-seq,Homo_723,AD.vs.MCI; bulk RNA-seq,Homo_714,AD.vs.MCI; bulk RNA-seq,Homo_633,AD.vs.control; bulk RNA-seq,Homo_633,AD.vs.MCI	4
MF	GO:0099604	ligand-gated calcium channel activity	bulk RNA-seq,Homo_723,AD.vs.MCI; bulk RNA-seq,Homo_723,MCI.vs.control; bulk RNA-seq,Homo_714,AD.vs.MCI; bulk RNA-seq,Homo_633,AD.vs.control; bulk RNA-seq,Homo_633,AD.vs.MCI	5
MF	GO:0004112	cyclic-nucleotide phosphodiesterase activity	bulk RNA-seq,Homo_723,AD.vs.MCI; bulk RNA-seq,Homo_723,MCI.vs.control; bulk RNA-seq,Homo_714,AD.vs.MCI; bulk RNA-seq,Homo_714,MCI.vs.control; bulk RNA-seq,Homo_633,AD.vs.control	5
BP	GO:0043401	steroid hormone mediated signaling pathway	bulk RNA-seq,Homo_723,AD.vs.MCI; bulk RNA-seq,Homo_723,MCI.vs.control; bulk RNA-seq,Homo_714,AD.vs.MCI; bulk RNA-seq,Homo_633,AD.vs.control; bulk RNA-seq,Homo_633,AD.vs.MCI	5
BP	GO:0051895	negative regulation of focal adhesion assembly	bulk RNA-seq,Homo_723,AD.vs.MCI; bulk RNA-seq,Homo_723,MCI.vs.control; bulk RNA-seq,Homo_714,AD.vs.MCI; bulk RNA-seq,Homo_714,MCI.vs.control; bulk RNA-seq,Homo_633,AD.vs.control	5
BP	GO:0150118	negative regulation of cell-substrate junction organization	bulk RNA-seq,Homo_723,AD.vs.MCI; bulk RNA-seq,Homo_723,MCI.vs.control; bulk RNA-seq,Homo_714,AD.vs.MCI; bulk RNA-seq,Homo_714,MCI.vs.control; bulk RNA-seq,Homo_633,AD.vs.control	5
BP	GO:0048679	regulation of axon regeneration	bulk RNA-seq,Homo_723,AD.vs.MCI; bulk RNA-seq,Homo_723,MCI.vs.control; bulk RNA-seq,Homo_714,AD.vs.MCI; bulk RNA-seq,Homo_714,MCI.vs.control	4
MF	GO:0030021	extracellular matrix structural constituent conferring compression resistance	bulk RNA-seq,Homo_723,AD.vs.MCI; bulk RNA-seq,Homo_723,MCI.vs.control; bulk RNA-seq,Homo_714,AD.vs.control; bulk RNA-seq,Homo_714,AD.vs.MCI	4
BP	GO:0042363	fat-soluble vitamin catabolic process	bulk RNA-seq,Homo_723,AD.vs.MCI; bulk RNA-seq,Homo_723,MCI.vs.control; bulk RNA-seq,SRP223445,AD.vs.control	3
BP	GO:0042542	response to hydrogen peroxide	bulk RNA-seq,Homo_723,AD.vs.MCI; bulk RNA-seq,Homo_723,MCI.vs.control; bulk RNA-seq,Homo_714,AD.vs.MCI; bulk RNA-seq,Homo_633,AD.vs.control; bulk RNA-seq,Homo_633,AD.vs.MCI	5
BP	GO:0032885	regulation of polysaccharide biosynthetic process	bulk RNA-seq,Homo_723,AD.vs.MCI; bulk RNA-seq,Homo_723,MCI.vs.control; bulk RNA-seq,Homo_714,AD.vs.MCI; bulk RNA-seq,Homo_714,MCI.vs.control	4
BP	GO:1904646	cellular response to amyloid-beta	bulk RNA-seq,Homo_723,AD.vs.MCI; bulk RNA-seq,Homo_723,MCI.vs.control; bulk RNA-seq,Homo_714,AD.vs.MCI; bulk RNA-seq,Homo_714,MCI.vs.control	4
MF	GO:0140323	solute:anion antiporter activity	bulk RNA-seq,Homo_723,AD.vs.MCI; bulk RNA-seq,Homo_723,MCI.vs.control; bulk RNA-seq,Homo_633,AD.vs.control; bulk RNA-seq,Homo_633,AD.vs.MCI	4
BP	GO:0090527	actin filament reorganization	bulk RNA-seq,Homo_723,AD.vs.MCI; bulk RNA-seq,Homo_723,MCI.vs.control; bulk RNA-seq,Homo_714,MCI.vs.control; bulk RNA-seq,Homo_633,AD.vs.control; bulk RNA-seq,Homo_633,AD.vs.MCI	5
BP	GO:0035813	regulation of renal sodium excretion	bulk RNA-seq,Homo_723,AD.vs.MCI; bulk RNA-seq,Homo_714,AD.vs.control; bulk RNA-seq,Homo_714,AD.vs.MCI	3
BP	GO:1903828	negative regulation of protein localization	bulk RNA-seq,Homo_723,AD.vs.MCI; bulk RNA-seq,Homo_723,MCI.vs.control; bulk RNA-seq,Homo_633,AD.vs.control; bulk RNA-seq,Homo_633,AD.vs.MCI	4
BP	GO:0045920	negative regulation of exocytosis	bulk RNA-seq,Homo_723,AD.vs.MCI; bulk RNA-seq,Homo_723,MCI.vs.control; bulk RNA-seq,Homo_714,MCI.vs.control; bulk RNA-seq,Homo_633,AD.vs.control; bulk RNA-seq,Homo_633,AD.vs.MCI	5
BP	GO:1904707	positive regulation of vascular associated smooth muscle cell proliferation	bulk RNA-seq,Homo_723,AD.vs.MCI; bulk RNA-seq,Homo_723,MCI.vs.control; bulk RNA-seq,Homo_714,AD.vs.MCI; bulk RNA-seq,Homo_714,MCI.vs.control	4
BP	GO:0071715	icosanoid transport	bulk RNA-seq,Homo_723,AD.vs.MCI; bulk RNA-seq,Homo_714,AD.vs.control; bulk RNA-seq,Homo_714,AD.vs.MCI; bulk RNA-seq,Homo_633,AD.vs.control; bulk RNA-seq,Homo_633,AD.vs.MCI	5
BP	GO:0009111	vitamin catabolic process	bulk RNA-seq,Homo_723,AD.vs.MCI; bulk RNA-seq,Homo_723,MCI.vs.control; bulk RNA-seq,Homo_714,AD.vs.MCI	3
BP	GO:0043242	negative regulation of protein-containing complex	bulk RNA-seq,Homo_723,AD.vs.MCI; bulk RNA-seq,Homo_723,MCI.vs.control; bulk RNA-seq,Homo_714,MCI.vs.control; bulk RNA-	4
BP	GO:0032309	icosanoid secretion	bulk RNA-seq,Homo_723,AD.vs.MCI; bulk RNA-seq,Homo_714,AD.vs.MCI	2
BP	GO:0019058	viral life cycle	bulk RNA-seq,Homo_723,AD.vs.MCI; bulk RNA-seq,Homo_723,MCI.vs.control; bulk RNA-seq,Homo_714,AD.vs.control; bulk RNA-seq,Homo_714,AD.vs.MCI; bulk RNA-seq,Homo_633,AD.vs.control; bulk RNA-seq,Homo_633,AD.vs.MCI	6
BP	GO:0060347	heart trabecula formation	bulk RNA-seq,Homo_723,AD.vs.MCI; bulk RNA-seq,Homo_723,MCI.vs.control; bulk RNA-seq,Homo_714,AD.vs.MCI; bulk RNA-seq,Homo_714,MCI.vs.control	4
BP	GO:0006643	membrane lipid metabolic process	bulk RNA-seq,Homo_723,AD.vs.MCI; bulk RNA-seq,Homo_723,MCI.vs.control; bulk RNA-seq,Homo_714,AD.vs.control; bulk RNA-seq,Homo_633,AD.vs.control; bulk RNA-seq,Homo_633,AD.vs.MCI	5

BP	GO:0009268	response to pH	bulk RNA-seq,Homo_723,AD.vs.MCI; bulk RNA-seq,Homo_723,MCI.vs.control; bulk RNA-seq,Homo_714,AD.vs.MCI; bulk RNA-seq,Homo_714,MCI.vs.control	4
BP	GO:0033561	regulation of water loss via skin	bulk RNA-seq,Homo_723,AD.vs.MCI; bulk RNA-seq,Homo_723,MCI.vs.control; bulk RNA-seq,Homo_714,AD.vs.MCI; bulk RNA-seq,Homo_714,MCI.vs.control; bulk RNA-seq,SRP223445,AD.vs.control	5
BP	GO:0090278	negative regulation of peptide hormone secretion	bulk RNA-seq,Homo_723,AD.vs.MCI; bulk RNA-seq,Homo_723,MCI.vs.control; bulk RNA-seq,Homo_633,AD.vs.control; bulk RNA-seq,Homo_633,AD.vs.MCI	4
MF	GO:0051117	ATPase binding	bulk RNA-seq,Homo_723,AD.vs.MCI; bulk RNA-seq,Homo_723,MCI.vs.control; bulk RNA-seq,Homo_714,AD.vs.MCI; bulk RNA-seq,Homo_714,MCI.vs.control; bulk RNA-seq,Homo_633,AD.vs.control; bulk RNA-seq,Homo_633,AD.vs.MCI	6
MF	GO:0009678	pyrophosphate hydrolysis-driven proton transmembrane transporter activity	bulk RNA-seq,Homo_723,AD.vs.MCI; bulk RNA-seq,Homo_633,AD.vs.control; bulk RNA-seq,Homo_633,AD.vs.MCI	3
BP	GO:1902883	negative regulation of response to oxidative stress	bulk RNA-seq,Homo_723,AD.vs.MCI; bulk RNA-seq,Homo_723,MCI.vs.control; bulk RNA-seq,Homo_714,AD.vs.MCI; bulk RNA-seq,Homo_714,MCI.vs.control; bulk RNA-seq,Homo_633,AD.vs.control; bulk RNA-seq,Homo_633,AD.vs.MCI	6
BP	GO:0022011	myelination in peripheral nervous system	bulk RNA-seq,Homo_723,AD.vs.MCI; bulk RNA-seq,Homo_723,MCI.vs.control; bulk RNA-seq,Homo_714,AD.vs.MCI; bulk RNA-seq,Homo_714,MCI.vs.control; bulk RNA-seq,Homo_633,AD.vs.control	5
BP	GO:0032292	peripheral nervous system axon ensheathment	bulk RNA-seq,Homo_723,AD.vs.MCI; bulk RNA-seq,Homo_723,MCI.vs.control; bulk RNA-seq,Homo_714,AD.vs.MCI; bulk RNA-seq,Homo_714,MCI.vs.control; bulk RNA-seq,Homo_633,AD.vs.control	5
BP	GO:0033146	regulation of intracellular estrogen receptor signaling pathway	bulk RNA-seq,Homo_723,AD.vs.MCI; bulk RNA-seq,Homo_723,MCI.vs.control; bulk RNA-seq,Homo_714,AD.vs.MCI; bulk RNA-seq,Homo_714,MCI.vs.control; bulk RNA-seq,Homo_633,AD.vs.control	5
BP	GO:0044062	regulation of excretion	bulk RNA-seq,Homo_723,AD.vs.MCI; bulk RNA-seq,Homo_723,MCI.vs.control; bulk RNA-seq,Homo_714,AD.vs.control; bulk RNA-seq,Homo_714,AD.vs.MCI	4
BP	GO:1902105	regulation of leukocyte differentiation	bulk RNA-seq,Homo_723,AD.vs.MCI; bulk RNA-seq,Homo_714,AD.vs.MCI; bulk RNA-seq,Homo_633,AD.vs.control; bulk RNA-seq,Homo_633,AD.vs.MCI; bulk RNA-seq,SRP325058,AD.vs.control; scRNA-seq,SRP215507,CD8+ T cell_3-AD.vs.control	6
BP	GO:1902743	regulation of lamellipodium organization	bulk RNA-seq,Homo_723,AD.vs.MCI; bulk RNA-seq,Homo_723,MCI.vs.control; bulk RNA-seq,Homo_714,AD.vs.MCI; bulk RNA-seq,Homo_714,MCI.vs.control; bulk RNA-seq,Homo_633,AD.vs.control; bulk RNA-seq,Homo_633,AD.vs.MCI	6
BP	GO:0051567	histone H3-K9 methylation	bulk RNA-seq,Homo_723,AD.vs.MCI; bulk RNA-seq,Homo_723,MCI.vs.control; bulk RNA-seq,Homo_714,AD.vs.MCI; bulk RNA-seq,Homo_714,MCI.vs.control; bulk RNA-seq,Homo_633,AD.vs.control; bulk RNA-seq,Homo_633,AD.vs.MCI	6
BP	GO:0007595	lactation	bulk RNA-seq,Homo_723,AD.vs.MCI; bulk RNA-seq,Homo_723,MCI.vs.control; bulk RNA-seq,Homo_714,AD.vs.MCI; bulk RNA-seq,Homo_714,MCI.vs.control; bulk RNA-seq,Homo_633,AD.vs.control	5
BP	GO:0006144	purine nucleobase metabolic process	bulk RNA-seq,Homo_723,AD.vs.MCI; bulk RNA-seq,Homo_723,MCI.vs.control; bulk RNA-seq,Homo_714,MCI.vs.control	3
BP	GO:0051782	negative regulation of cell division	bulk RNA-seq,Homo_723,AD.vs.MCI; bulk RNA-seq,Homo_723,MCI.vs.control; bulk RNA-seq,Homo_714,AD.vs.MCI; bulk RNA-seq,Homo_714,MCI.vs.control	4
BP	GO:2001243	negative regulation of intrinsic apoptotic signaling pathway	bulk RNA-seq,Homo_723,AD.vs.MCI; bulk RNA-seq,Homo_723,MCI.vs.control; bulk RNA-seq,Homo_714,AD.vs.MCI; bulk RNA-seq,Homo_714,MCI.vs.control; bulk RNA-seq,Homo_633,AD.vs.control; bulk RNA-seq,Homo_633,AD.vs.MCI	6
BP	GO:0005976	polysaccharide metabolic process	bulk RNA-seq,Homo_723,AD.vs.MCI; bulk RNA-seq,Homo_723,MCI.vs.control; bulk RNA-seq,Homo_714,AD.vs.MCI; bulk RNA-seq,Homo_714,MCI.vs.control; bulk RNA-seq,Homo_633,AD.vs.control; bulk RNA-seq,Homo_633,AD.vs.MCI	6
BP	GO:1903539	protein localization to postsynaptic membrane	bulk RNA-seq,Homo_723,AD.vs.MCI; bulk RNA-seq,Homo_723,MCI.vs.control; bulk RNA-seq,Homo_714,AD.vs.MCI; bulk RNA-seq,Homo_633,AD.vs.control	4
MF	GO:0019208	phosphatase regulator activity	bulk RNA-seq,Homo_723,AD.vs.MCI; bulk RNA-seq,Homo_723,MCI.vs.control; bulk RNA-seq,Homo_714,AD.vs.MCI; bulk RNA-seq,Homo_714,MCI.vs.control; bulk RNA-seq,Homo_633,AD.vs.control; bulk RNA-seq,Homo_633,AD.vs.MCI	6
CC	GO:0005635	nuclear envelope	bulk RNA-seq,Homo_723,AD.vs.MCI; bulk RNA-seq,Homo_714,AD.vs.control; bulk RNA-seq,Homo_714,AD.vs.MCI; bulk RNA-seq,Homo_633,AD.vs.control; bulk RNA-seq,Homo_633,AD.vs.MCI; scRNA-seq,SRP330776,Naive CD8+ T cell_2-AD.vs.control	6
BP	GO:0034651	cortisol biosynthetic process	bulk RNA-seq,Homo_723,AD.vs.MCI; bulk RNA-seq,Homo_723,MCI.vs.control	2
BP	GO:1902645	tertiary alcohol biosynthetic process	bulk RNA-seq,Homo_723,AD.vs.MCI; bulk RNA-seq,Homo_723,MCI.vs.control	2
BP	GO:0001542	ovulation from ovarian follicle	bulk RNA-seq,Homo_723,AD.vs.MCI; bulk RNA-seq,Homo_723,MCI.vs.control; bulk RNA-seq,Homo_714,AD.vs.MCI	3
BP	GO:0006699	bile acid biosynthetic process	bulk RNA-seq,Homo_723,AD.vs.MCI; bulk RNA-seq,Homo_723,MCI.vs.control; bulk RNA-seq,Homo_714,AD.vs.MCI; bulk RNA-seq,Homo_714,MCI.vs.control; bulk RNA-seq,Homo_633,AD.vs.control; bulk RNA-seq,Homo_633,AD.vs.MCI	6
BP	GO:0071709	membrane assembly	bulk RNA-seq,Homo_723,AD.vs.MCI; bulk RNA-seq,Homo_723,MCI.vs.control; bulk RNA-seq,Homo_714,AD.vs.MCI; bulk RNA-seq,Homo_714,MCI.vs.control; bulk RNA-seq,Homo_633,AD.vs.control; bulk RNA-seq,Homo_633,AD.vs.MCI	6
MF	GO:0051018	protein kinase A binding	bulk RNA-seq,Homo_723,AD.vs.MCI; bulk RNA-seq,Homo_723,MCI.vs.control; bulk RNA-seq,Homo_714,AD.vs.MCI; bulk RNA-seq,Homo_714,MCI.vs.control; bulk RNA-seq,Homo_633,AD.vs.control; bulk RNA-seq,Homo_633,AD.vs.MCI	6
BP	GO:0031114	regulation of microtubule depolymerization	bulk RNA-seq,Homo_723,AD.vs.MCI; bulk RNA-seq,Homo_723,MCI.vs.control; bulk RNA-seq,Homo_714,MCI.vs.control; bulk RNA-	4
BP	GO:1900272	negative regulation of long-term synaptic potentiation	bulk RNA-seq,Homo_723,AD.vs.MCI; bulk RNA-seq,Homo_723,MCI.vs.control; bulk RNA-seq,Homo_714,AD.vs.MCI; bulk RNA-seq,Homo_714,MCI.vs.control; bulk RNA-seq,Homo_633,AD.vs.control	5
BP	GO:0033692	cellular polysaccharide biosynthetic process	bulk RNA-seq,Homo_723,AD.vs.MCI; bulk RNA-seq,Homo_723,MCI.vs.control; bulk RNA-seq,Homo_714,AD.vs.MCI; bulk RNA-seq,Homo_714,MCI.vs.control; bulk RNA-seq,Homo_633,AD.vs.control	5
BP	GO:0043552	positive regulation of phosphatidylinositol 3-kinase activity	bulk RNA-seq,Homo_723,AD.vs.MCI; bulk RNA-seq,Homo_723,MCI.vs.control; bulk RNA-seq,Homo_714,AD.vs.MCI; bulk RNA-seq,Homo_714,MCI.vs.control; bulk RNA-seq,Homo_633,AD.vs.control	5
BP	GO:0071219	cellular response to molecule of bacterial origin	bulk RNA-seq,Homo_723,AD.vs.MCI; bulk RNA-seq,Homo_723,MCI.vs.control; bulk RNA-seq,Homo_714,AD.vs.MCI; bulk RNA-seq,Homo_633,AD.vs.control; bulk RNA-seq,Homo_633,AD.vs.MCI	5
BP	GO:0021799	cerebral cortex radially oriented cell migration	bulk RNA-seq,Homo_723,AD.vs.MCI; bulk RNA-seq,Homo_723,MCI.vs.control; bulk RNA-seq,Homo_714,AD.vs.MCI; bulk RNA-seq,Homo_714,MCI.vs.control; bulk RNA-seq,Homo_633,AD.vs.control; bulk RNA-seq,Homo_633,AD.vs.MCI; bulk RNA-seq,ROSMAP,AD.vs.control	7
BP	GO:0070091	glucagon secretion	bulk RNA-seq,Homo_723,AD.vs.MCI; bulk RNA-seq,Homo_723,MCI.vs.control	2
BP	GO:0070092	regulation of glucagon secretion	bulk RNA-seq,Homo_723,AD.vs.MCI; bulk RNA-seq,Homo_723,MCI.vs.control	2
BP	GO:2000404	regulation of T cell migration	bulk RNA-seq,Homo_723,AD.vs.MCI; bulk RNA-seq,Homo_723,MCI.vs.control; bulk RNA-seq,Homo_714,AD.vs.MCI; bulk RNA-seq,Homo_714,MCI.vs.control; bulk RNA-seq,Homo_633,AD.vs.control	5
BP	GO:0035865	cellular response to potassium ion	bulk RNA-seq,Homo_723,AD.vs.MCI; bulk RNA-seq,Homo_714,AD.vs.MCI	2
CC	GO:0072686	mitotic spindle	bulk RNA-seq,Homo_723,AD.vs.MCI; bulk RNA-seq,Homo_723,MCI.vs.control; bulk RNA-seq,Homo_714,AD.vs.MCI; bulk RNA-seq,Homo_633,AD.vs.control; bulk RNA-seq,Homo_633,AD.vs.MCI	5

BP	GO:0010657	muscle cell apoptotic process	bulk RNA-seq,Homo_723,AD.vs.MCI; bulk RNA-seq,Homo_714,AD.vs.MCI; bulk RNA-seq,Homo_633,AD.vs.control; bulk RNA-seq,Homo_633,AD.vs.MCI	4
BP	GO:0097084	vascular associated smooth muscle cell development	bulk RNA-seq,Homo_723,AD.vs.MCI; bulk RNA-seq,Homo_723,MCI.vs.control; bulk RNA-seq,Homo_714,AD.vs.MCI; bulk RNA-seq,Homo_714,MCI.vs.control	4
BP	GO:1904892	regulation of receptor signaling pathway via STAT	bulk RNA-seq,Homo_723,AD.vs.MCI; bulk RNA-seq,Homo_714,AD.vs.control; bulk RNA-seq,Homo_714,AD.vs.MCI	3
BP	GO:0043244	regulation of protein-containing complex disassembly	bulk RNA-seq,Homo_723,AD.vs.MCI; bulk RNA-seq,Homo_723,MCI.vs.control; bulk RNA-seq,Homo_714,MCI.vs.control; bulk RNA-seq,Homo_633,AD.vs.control; bulk RNA-seq,Homo_633,AD.vs.MCI	5
BP	GO:0046697	decidualization	bulk RNA-seq,Homo_723,AD.vs.MCI; bulk RNA-seq,Homo_723,MCI.vs.control; bulk RNA-seq,Homo_714,AD.vs.MCI	3
CC	GO:0031901	early endosome membrane	bulk RNA-seq,Homo_723,AD.vs.MCI; bulk RNA-seq,Homo_723,MCI.vs.control; bulk RNA-seq,Homo_714,AD.vs.control; bulk RNA-seq,Homo_633,AD.vs.control; bulk RNA-seq,Homo_633,AD.vs.MCI	5
MF	GO:0047555	3',5'-cyclic-GMP phosphodiesterase activity	bulk RNA-seq,Homo_723,AD.vs.MCI; bulk RNA-seq,Homo_723,MCI.vs.control; bulk RNA-seq,Homo_714,AD.vs.MCI; bulk RNA-seq,Homo_714,MCI.vs.control	4
BP	GO:2000249	regulation of actin cytoskeleton reorganization	bulk RNA-seq,Homo_723,AD.vs.MCI; bulk RNA-seq,Homo_723,MCI.vs.control; bulk RNA-seq,Homo_714,AD.vs.MCI; bulk RNA-seq,Homo_714,MCI.vs.control; bulk RNA-seq,Homo_633,AD.vs.control	5
BP	GO:0014002	astrocyte development	bulk RNA-seq,Homo_723,AD.vs.MCI; bulk RNA-seq,Homo_723,MCI.vs.control; bulk RNA-seq,Homo_714,AD.vs.MCI; bulk RNA-seq,Homo_633,AD.vs.control	4
BP	GO:0043455	regulation of secondary metabolic process	bulk RNA-seq,Homo_723,AD.vs.MCI; bulk RNA-seq,Homo_723,MCI.vs.control; bulk RNA-seq,Homo_633,AD.vs.control; bulk RNA-seq,Homo_633,AD.vs.MCI	4
BP	GO:0048021	regulation of melanin biosynthetic process	bulk RNA-seq,Homo_723,AD.vs.MCI; bulk RNA-seq,Homo_723,MCI.vs.control; bulk RNA-seq,Homo_633,AD.vs.control; bulk RNA-seq,Homo_633,AD.vs.MCI	4
BP	GO:1900376	regulation of secondary metabolite biosynthetic process	bulk RNA-seq,Homo_723,AD.vs.MCI; bulk RNA-seq,Homo_723,MCI.vs.control; bulk RNA-seq,Homo_633,AD.vs.control; bulk RNA-seq,Homo_633,AD.vs.MCI	4
BP	GO:0032905	transforming growth factor beta1 production	bulk RNA-seq,Homo_723,AD.vs.MCI; bulk RNA-seq,Homo_723,MCI.vs.control; bulk RNA-seq,Homo_714,AD.vs.MCI; bulk RNA-seq,Homo_714,MCI.vs.control	4
MF	GO:0051213	dioxygenase activity	bulk RNA-seq,Homo_723,AD.vs.MCI; bulk RNA-seq,Homo_723,MCI.vs.control; bulk RNA-seq,Homo_714,AD.vs.MCI; bulk RNA-seq,Homo_714,MCI.vs.control; bulk RNA-seq,Homo_633,AD.vs.control; bulk RNA-seq,Homo_633,AD.vs.MCI	6
BP	GO:1904385	cellular response to angiotensin	bulk RNA-seq,Homo_723,AD.vs.MCI; bulk RNA-seq,Homo_723,MCI.vs.control; bulk RNA-seq,Homo_714,AD.vs.MCI; bulk RNA-seq,Homo_714,MCI.vs.control; scRNA-seq,SRP215507,CD8+ T cell_3-AD.vs.control	5
BP	GO:0002638	negative regulation of immunoglobulin production	bulk RNA-seq,Homo_723,AD.vs.MCI; bulk RNA-seq,Homo_723,MCI.vs.control; bulk RNA-seq,Homo_714,AD.vs.MCI; bulk RNA-seq,Homo_714,MCI.vs.control	4
BP	GO:0045779	negative regulation of bone resorption	bulk RNA-seq,Homo_723,AD.vs.MCI; bulk RNA-seq,Homo_723,MCI.vs.control; bulk RNA-seq,Homo_714,AD.vs.MCI; bulk RNA-seq,Homo_714,MCI.vs.control; bulk RNA-seq,Homo_633,MCI.vs.control	5
BP	GO:0006338	chromatin remodeling	bulk RNA-seq,Homo_723,AD.vs.MCI; bulk RNA-seq,Homo_714,AD.vs.MCI; bulk RNA-seq,Homo_633,AD.vs.control; bulk RNA-seq,Homo_633,AD.vs.MCI	4
BP	GO:0044060	regulation of endocrine process	bulk RNA-seq,Homo_723,AD.vs.MCI; bulk RNA-seq,Homo_714,AD.vs.MCI	2
BP	GO:0006833	water transport	bulk RNA-seq,Homo_723,AD.vs.MCI; bulk RNA-seq,Homo_723,MCI.vs.control; bulk RNA-seq,Homo_714,AD.vs.MCI	3
MF	GO:0016887	ATP hydrolysis activity	bulk RNA-seq,Homo_723,AD.vs.MCI; bulk RNA-seq,Homo_714,AD.vs.control; bulk RNA-seq,Homo_714,AD.vs.MCI; bulk RNA-seq,Homo_633,AD.vs.control; bulk RNA-seq,Homo_633,AD.vs.MCI	5
BP	GO:2000010	positive regulation of protein localization to cell surface	bulk RNA-seq,Homo_723,AD.vs.MCI; bulk RNA-seq,Homo_723,MCI.vs.control	2
BP	GO:0032881	regulation of polysaccharide metabolic process	bulk RNA-seq,Homo_723,AD.vs.MCI; bulk RNA-seq,Homo_723,MCI.vs.control; bulk RNA-seq,Homo_714,AD.vs.MCI; bulk RNA-seq,Homo_714,MCI.vs.control	4
MF	GO:0008080	N-acetyltransferase activity	bulk RNA-seq,Homo_723,AD.vs.MCI; bulk RNA-seq,Homo_723,MCI.vs.control; bulk RNA-seq,Homo_714,AD.vs.MCI; bulk RNA-seq,Homo_714,MCI.vs.control; bulk RNA-seq,Homo_633,AD.vs.control; bulk RNA-seq,Homo_633,AD.vs.MCI	6
BP	GO:0072331	signal transduction by p53 class mediator	bulk RNA-seq,Homo_723,AD.vs.MCI; bulk RNA-seq,Homo_723,MCI.vs.control; bulk RNA-seq,Homo_714,AD.vs.MCI; bulk RNA-seq,Homo_633,AD.vs.control; bulk RNA-seq,Homo_633,AD.vs.MCI	5
BP	GO:0006672	ceramide metabolic process	bulk RNA-seq,Homo_723,AD.vs.MCI; bulk RNA-seq,Homo_723,MCI.vs.control; bulk RNA-seq,Homo_714,MCI.vs.control; bulk RNA-seq,Homo_633,AD.vs.control; bulk RNA-seq,Homo_633,AD.vs.MCI	5
MF	GO:0017080	sodium channel regulator activity	bulk RNA-seq,Homo_723,AD.vs.MCI; bulk RNA-seq,Homo_723,MCI.vs.control; bulk RNA-seq,Homo_714,AD.vs.MCI; bulk RNA-seq,Homo_714,MCI.vs.control; bulk RNA-seq,Homo_633,AD.vs.control	5
BP	GO:0090181	regulation of cholesterol metabolic process	bulk RNA-seq,Homo_723,AD.vs.MCI; bulk RNA-seq,Homo_723,MCI.vs.control; bulk RNA-seq,Homo_714,AD.vs.MCI; bulk RNA-seq,Homo_714,MCI.vs.control; bulk RNA-seq,Homo_633,AD.vs.control; bulk RNA-seq,Homo_633,AD.vs.MCI	6
BP	GO:0006308	DNA catabolic process	bulk RNA-seq,Homo_723,AD.vs.MCI; bulk RNA-seq,Homo_723,MCI.vs.control; bulk RNA-seq,Homo_714,AD.vs.MCI; bulk RNA-seq,Homo_633,AD.vs.control	4
BP	GO:0043543	protein acylation	bulk RNA-seq,Homo_723,AD.vs.MCI; bulk RNA-seq,Homo_723,MCI.vs.control; bulk RNA-seq,Homo_714,AD.vs.MCI; bulk RNA-seq,Homo_633,AD.vs.control; bulk RNA-seq,Homo_633,AD.vs.MCI	5
BP	GO:0032373	positive regulation of sterol transport	bulk RNA-seq,Homo_723,AD.vs.MCI; bulk RNA-seq,Homo_723,MCI.vs.control; bulk RNA-seq,Homo_714,AD.vs.MCI; bulk RNA-seq,Homo_714,MCI.vs.control; bulk RNA-seq,Homo_633,AD.vs.control; bulk RNA-seq,Homo_633,AD.vs.MCI	6
BP	GO:0032376	positive regulation of cholesterol transport	bulk RNA-seq,Homo_723,AD.vs.MCI; bulk RNA-seq,Homo_723,MCI.vs.control; bulk RNA-seq,Homo_714,AD.vs.MCI; bulk RNA-seq,Homo_714,MCI.vs.control; bulk RNA-seq,Homo_633,AD.vs.control; bulk RNA-seq,Homo_633,AD.vs.MCI	6
BP	GO:0090077	foam cell differentiation	bulk RNA-seq,Homo_723,AD.vs.MCI; bulk RNA-seq,Homo_723,MCI.vs.control; bulk RNA-seq,Homo_714,AD.vs.MCI; bulk RNA-seq,Homo_714,MCI.vs.control; bulk RNA-seq,Homo_633,AD.vs.control; bulk RNA-seq,Homo_633,AD.vs.MCI	6
BP	GO:0044264	cellular polysaccharide metabolic process	bulk RNA-seq,Homo_723,AD.vs.MCI; bulk RNA-seq,Homo_723,MCI.vs.control; bulk RNA-seq,Homo_714,AD.vs.MCI; bulk RNA-seq,Homo_714,MCI.vs.control; bulk RNA-seq,Homo_633,AD.vs.control; bulk RNA-seq,Homo_633,AD.vs.MCI	6
BP	GO:0060306	regulation of membrane repolarization	bulk RNA-seq,Homo_723,AD.vs.MCI; bulk RNA-seq,Homo_714,AD.vs.MCI	2
BP	GO:0034637	cellular carbohydrate biosynthetic process	bulk RNA-seq,Homo_723,AD.vs.MCI; bulk RNA-seq,Homo_723,MCI.vs.control; bulk RNA-seq,Homo_714,AD.vs.MCI; bulk RNA-seq,Homo_714,MCI.vs.control; bulk RNA-seq,Homo_633,AD.vs.control	5
BP	GO:1901990	regulation of mitotic cell cycle phase transition	bulk RNA-seq,Homo_723,AD.vs.MCI; bulk RNA-seq,Homo_714,AD.vs.MCI; bulk RNA-seq,Homo_633,AD.vs.control; bulk RNA-seq,Homo_633,AD.vs.MCI	4
BP	GO:0043154	negative regulation of cysteine-type endopeptidase activity involved in apoptotic process	bulk RNA-seq,Homo_723,AD.vs.MCI; bulk RNA-seq,Homo_723,MCI.vs.control; bulk RNA-seq,Homo_714,AD.vs.MCI; bulk RNA-seq,Homo_714,MCI.vs.control; bulk RNA-seq,Homo_633,AD.vs.control; bulk RNA-seq,Homo_633,AD.vs.MCI	6
BP	GO:0090557	establishment of endothelial intestinal barrier	bulk RNA-seq,Homo_723,AD.vs.MCI; bulk RNA-seq,Homo_723,MCI.vs.control; bulk RNA-seq,Homo_714,AD.vs.MCI; bulk RNA-seq,Homo_714,MCI.vs.control; bulk RNA-seq,Homo_633,AD.vs.MCI	5
BP	GO:0044262	cellular carbohydrate metabolic process	bulk RNA-seq,Homo_723,AD.vs.MCI; bulk RNA-seq,Homo_723,MCI.vs.control; bulk RNA-seq,Homo_714,AD.vs.MCI; bulk RNA-seq,Homo_633,AD.vs.control; bulk RNA-seq,Homo_633,AD.vs.MCI	5
MF	GO:0005154	epidermal growth factor receptor binding	bulk RNA-seq,Homo_723,AD.vs.MCI; bulk RNA-seq,Homo_723,MCI.vs.control; bulk RNA-seq,Homo_714,AD.vs.MCI; bulk RNA-seq,Homo_714,MCI.vs.control	4

BP	GO:0006885	regulation of pH	bulk RNA-seq,Homo_723,AD.vs.MCI; bulk RNA-seq,Homo_714,AD.vs.MCI; bulk RNA-seq,Homo_633,AD.vs.control; bulk RNA-seq,Homo_633,AD.vs.MCI	4
MF	GO:0005112	Notch binding	bulk RNA-seq,Homo_723,AD.vs.MCI; bulk RNA-seq,Homo_723,MCI.vs.control; bulk RNA-seq,Homo_714,MCI.vs.control	3
BP	GO:0060837	blood vessel endothelial cell differentiation	bulk RNA-seq,Homo_723,AD.vs.MCI; bulk RNA-seq,Homo_723,MCI.vs.control; bulk RNA-seq,Homo_714,AD.vs.MCI; bulk RNA-seq,Homo_714,MCI.vs.control	4
BP	GO:0032922	circadian regulation of gene expression	bulk RNA-seq,Homo_723,AD.vs.MCI; bulk RNA-seq,Homo_723,MCI.vs.control; bulk RNA-seq,Homo_633,AD.vs.control; bulk RNA-seq,Homo_633,AD.vs.MCI; bulk RNA-seq,SRP325058,AD.vs.control	5
MF	GO:0050660	flavin adenine dinucleotide binding	bulk RNA-seq,Homo_723,AD.vs.MCI; bulk RNA-seq,Homo_723,MCI.vs.control; bulk RNA-seq,Homo_714,AD.vs.MCI; bulk RNA-seq,Homo_714,MCI.vs.control; bulk RNA-seq,Homo_633,AD.vs.control; bulk RNA-seq,Homo_633,AD.vs.MCI	6
BP	GO:0071674	mononuclear cell migration	bulk RNA-seq,Homo_723,AD.vs.MCI; bulk RNA-seq,Homo_723,MCI.vs.control; bulk RNA-seq,Homo_714,AD.vs.MCI; bulk RNA-seq,Homo_633,AD.vs.control; scRNA-seq,SRP309935,B cell_1-AD.vs.control	5
BP	GO:0099637	neurotransmitter receptor transport	bulk RNA-seq,Homo_723,AD.vs.MCI; bulk RNA-seq,Homo_723,MCI.vs.control; bulk RNA-seq,Homo_714,AD.vs.MCI; bulk RNA-seq,Homo_633,AD.vs.control	4
BP	GO:0060669	embryonic placenta morphogenesis	bulk RNA-seq,Homo_723,AD.vs.MCI; bulk RNA-seq,Homo_723,MCI.vs.control; bulk RNA-seq,Homo_714,MCI.vs.control	3
BP	GO:1904063	negative regulation of cation transmembrane transport	bulk RNA-seq,Homo_723,AD.vs.MCI; bulk RNA-seq,Homo_714,AD.vs.control; bulk RNA-seq,Homo_714,AD.vs.MCI; bulk RNA-seq,Homo_633,AD.vs.control; bulk RNA-seq,Homo_633,AD.vs.MCI	5
BP	GO:0046885	regulation of hormone biosynthetic process	bulk RNA-seq,Homo_723,AD.vs.MCI; bulk RNA-seq,Homo_723,MCI.vs.control; bulk RNA-seq,Homo_714,MCI.vs.control; bulk RNA-seq,Homo_633,AD.vs.control; bulk RNA-seq,Homo_633,AD.vs.MCI; bulk RNA-seq,Homo_633,MCI.vs.control	6
BP	GO:0050901	leukocyte tethering or rolling	bulk RNA-seq,Homo_723,AD.vs.MCI; bulk RNA-seq,Homo_723,MCI.vs.control; bulk RNA-seq,Homo_714,AD.vs.MCI; bulk RNA-seq,Homo_714,MCI.vs.control; bulk RNA-seq,Homo_633,AD.vs.control	5
BP	GO:0032570	response to progesterone	bulk RNA-seq,Homo_723,AD.vs.MCI; bulk RNA-seq,Homo_723,MCI.vs.control; bulk RNA-seq,Homo_714,AD.vs.MCI; bulk RNA-seq,Homo_714,MCI.vs.control; bulk RNA-seq,Homo_633,AD.vs.control; bulk RNA-seq,Homo_633,AD.vs.MCI	6
BP	GO:0048875	chemical homeostasis within a tissue	bulk RNA-seq,Homo_723,AD.vs.MCI; bulk RNA-seq,Homo_723,MCI.vs.control; bulk RNA-seq,Homo_714,AD.vs.MCI; bulk RNA-seq,Homo_714,MCI.vs.control	4
MF	GO:0015269	calcium-activated potassium channel activity	bulk RNA-seq,Homo_723,AD.vs.MCI; bulk RNA-seq,Homo_723,MCI.vs.control; bulk RNA-seq,Homo_714,AD.vs.MCI	3
BP	GO:0032926	negative regulation of activin receptor signaling pathway	bulk RNA-seq,Homo_723,AD.vs.MCI; bulk RNA-seq,Homo_723,MCI.vs.control	2
BP	GO:0061140	lung secretory cell differentiation	bulk RNA-seq,Homo_723,AD.vs.MCI; bulk RNA-seq,Homo_723,MCI.vs.control; bulk RNA-seq,Homo_714,AD.vs.MCI	3
CC	GO:0031234	extrinsic component of cytoplasmic side of plasma membrane	bulk RNA-seq,Homo_723,AD.vs.MCI; bulk RNA-seq,Homo_723,MCI.vs.control; bulk RNA-seq,Homo_714,AD.vs.MCI; bulk RNA-seq,Homo_714,MCI.vs.control; bulk RNA-seq,Homo_633,AD.vs.control; bulk RNA-seq,Homo_633,AD.vs.MCI	6
BP	GO:0060986	endocrine hormone secretion	bulk RNA-seq,Homo_723,AD.vs.MCI; bulk RNA-seq,Homo_723,MCI.vs.control; bulk RNA-seq,Homo_714,AD.vs.MCI; bulk RNA-seq,Homo_714,MCI.vs.control; bulk RNA-seq,Homo_633,AD.vs.control; bulk RNA-seq,Homo_633,AD.vs.MCI	6
BP	GO:0070050	neuron cellular homeostasis	bulk RNA-seq,Homo_723,AD.vs.MCI; bulk RNA-seq,Homo_723,MCI.vs.control; bulk RNA-seq,Homo_714,AD.vs.MCI; bulk RNA-seq,Homo_714,MCI.vs.control; bulk RNA-seq,Homo_633,AD.vs.control; bulk RNA-seq,Homo_633,AD.vs.MCI	6
BP	GO:0033144	negative regulation of intracellular steroid hormone receptor signaling pathway	bulk RNA-seq,Homo_723,AD.vs.MCI; bulk RNA-seq,Homo_723,MCI.vs.control; bulk RNA-seq,Homo_714,AD.vs.MCI; bulk RNA-seq,Homo_714,MCI.vs.control; bulk RNA-seq,Homo_633,AD.vs.control	5
CC	GO:0098993	anchored component of synaptic vesicle membrane	bulk RNA-seq,Homo_723,AD.vs.MCI	1
BP	GO:0010896	regulation of triglyceride catabolic process	bulk RNA-seq,Homo_723,AD.vs.MCI; bulk RNA-seq,Homo_723,MCI.vs.control; bulk RNA-seq,Homo_714,AD.vs.MCI; bulk RNA-seq,Homo_714,MCI.vs.control	4
BP	GO:0010269	response to selenium ion	bulk RNA-seq,Homo_723,AD.vs.MCI; bulk RNA-seq,Homo_723,MCI.vs.control; bulk RNA-seq,Homo_714,AD.vs.MCI; bulk RNA-seq,Homo_714,MCI.vs.control	4
MF	GO:0031994	insulin-like growth factor I binding	bulk RNA-seq,Homo_723,AD.vs.MCI; bulk RNA-seq,Homo_723,MCI.vs.control; bulk RNA-seq,Homo_714,AD.vs.MCI; bulk RNA-seq,Homo_714,MCI.vs.control; bulk RNA-seq,Homo_633,AD.vs.control	5
BP	GO:0007260	tyrosine phosphorylation of STAT protein	bulk RNA-seq,Homo_723,AD.vs.MCI; bulk RNA-seq,Homo_723,MCI.vs.control; bulk RNA-seq,Homo_714,AD.vs.MCI	3
BP	GO:0030299	intestinal cholesterol absorption	bulk RNA-seq,Homo_723,AD.vs.MCI; bulk RNA-seq,Homo_723,MCI.vs.control; bulk RNA-seq,Homo_714,AD.vs.MCI; bulk RNA-seq,Homo_714,MCI.vs.control	4
BP	GO:0030949	positive regulation of vascular endothelial growth factor receptor signaling pathway	bulk RNA-seq,Homo_723,AD.vs.MCI; bulk RNA-seq,Homo_723,MCI.vs.control; bulk RNA-seq,Homo_714,AD.vs.MCI; bulk RNA-seq,Homo_714,MCI.vs.control; bulk RNA-seq,Homo_633,AD.vs.control; bulk RNA-seq,Homo_633,AD.vs.MCI	6
MF	GO:0016830	carbon-carbon lyase activity	bulk RNA-seq,Homo_723,AD.vs.MCI; bulk RNA-seq,Homo_723,MCI.vs.control; bulk RNA-seq,Homo_714,AD.vs.MCI; bulk RNA-seq,Homo_714,MCI.vs.control; bulk RNA-seq,Homo_633,AD.vs.control; bulk RNA-seq,Homo_633,AD.vs.MCI	6
BP	GO:0003198	epithelial to mesenchymal transition involved in endocardial cushion formation	bulk RNA-seq,Homo_723,AD.vs.MCI; bulk RNA-seq,Homo_723,MCI.vs.control; bulk RNA-seq,Homo_714,AD.vs.MCI; bulk RNA-seq,Homo_714,MCI.vs.control; bulk RNA-seq,Homo_633,AD.vs.control; bulk RNA-seq,Homo_633,AD.vs.MCI	6
BP	GO:0040034	regulation of development, heterochronic	bulk RNA-seq,Homo_723,AD.vs.MCI; bulk RNA-seq,Homo_723,MCI.vs.control	2
BP	GO:0106106	cold-induced thermogenesis	bulk RNA-seq,Homo_723,AD.vs.MCI; bulk RNA-seq,Homo_723,MCI.vs.control; bulk RNA-seq,Homo_714,AD.vs.MCI; bulk RNA-seq,Homo_633,AD.vs.control; bulk RNA-seq,Homo_633,AD.vs.MCI	5
BP	GO:0120161	regulation of cold-induced thermogenesis	bulk RNA-seq,Homo_723,AD.vs.MCI; bulk RNA-seq,Homo_723,MCI.vs.control; bulk RNA-seq,Homo_714,AD.vs.MCI; bulk RNA-seq,Homo_633,AD.vs.control; bulk RNA-seq,Homo_633,AD.vs.MCI	5
BP	GO:0071478	cellular response to radiation	bulk RNA-seq,Homo_723,AD.vs.MCI; bulk RNA-seq,Homo_723,MCI.vs.control; bulk RNA-seq,Homo_714,AD.vs.MCI; bulk RNA-seq,Homo_633,AD.vs.control; bulk RNA-seq,Homo_633,AD.vs.MCI	5
BP	GO:0010985	negative regulation of lipoprotein particle clearance	bulk RNA-seq,Homo_723,AD.vs.MCI; bulk RNA-seq,Homo_723,MCI.vs.control; bulk RNA-seq,Homo_714,AD.vs.MCI	3
CC	GO:0005892	acetylcholine-gated channel complex	bulk RNA-seq,Homo_723,AD.vs.MCI; bulk RNA-seq,Homo_723,MCI.vs.control; bulk RNA-seq,Homo_714,AD.vs.MCI	3
BP	GO:0006706	steroid catabolic process	bulk RNA-seq,Homo_723,AD.vs.MCI; bulk RNA-seq,Homo_723,MCI.vs.control	2
BP	GO:0044839	cell cycle G2/M phase transition	bulk RNA-seq,Homo_723,AD.vs.MCI; bulk RNA-seq,Homo_723,MCI.vs.control; bulk RNA-seq,Homo_714,AD.vs.MCI; bulk RNA-seq,Homo_633,AD.vs.control; bulk RNA-seq,Homo_633,AD.vs.MCI	5
BP	GO:0006067	ethanol metabolic process	bulk RNA-seq,Homo_723,AD.vs.MCI; bulk RNA-seq,Homo_723,MCI.vs.control; bulk RNA-seq,Homo_714,AD.vs.MCI; bulk RNA-seq,Homo_714,MCI.vs.control; bulk RNA-seq,Homo_633,AD.vs.control; bulk RNA-seq,Homo_633,AD.vs.MCI	6
CC	GO:0051233	spindle midzone	bulk RNA-seq,Homo_723,AD.vs.MCI; bulk RNA-seq,Homo_723,MCI.vs.control; bulk RNA-seq,Homo_714,AD.vs.MCI; bulk RNA-seq,Homo_714,MCI.vs.control; bulk RNA-seq,Homo_633,AD.vs.control; bulk RNA-seq,Homo_633,AD.vs.MCI	6
BP	GO:1903672	positive regulation of sprouting angiogenesis	bulk RNA-seq,Homo_723,AD.vs.MCI; bulk RNA-seq,Homo_723,MCI.vs.control; bulk RNA-seq,Homo_714,AD.vs.MCI; bulk RNA-seq,Homo_714,MCI.vs.control	4

BP	GO:0018205	peptidyl-lysine modification	bulk RNA-seq,Homo_723,AD.vs.MCI; bulk RNA-seq,Homo_714,AD.vs.control; bulk RNA-seq,Homo_714,AD.vs.MCI; bulk RNA-seq,Homo_633,AD.vs.control; bulk RNA-seq,Homo_633,AD.vs.MCI	5
MF	GO:0019869	chloride channel inhibitor activity	bulk RNA-seq,Homo_723,AD.vs.MCI; bulk RNA-seq,Homo_723,MCI.vs.control	2
BP	GO:1903018	regulation of glycoprotein metabolic process	bulk RNA-seq,Homo_723,AD.vs.MCI; bulk RNA-seq,Homo_723,MCI.vs.control; bulk RNA-seq,Homo_714,AD.vs.MCI; bulk RNA-seq,Homo_714,MCI.vs.control; bulk RNA-seq,Homo_633,AD.vs.control; bulk RNA-seq,Homo_633,AD.vs.MCI	6
BP	GO:0009994	oocyte differentiation	bulk RNA-seq,Homo_723,AD.vs.MCI; bulk RNA-seq,Homo_723,MCI.vs.control; bulk RNA-seq,Homo_714,AD.vs.MCI; bulk RNA-seq,Homo_714,MCI.vs.control; bulk RNA-seq,Homo_633,AD.vs.control	5
BP	GO:0097193	intrinsic apoptotic signaling pathway	bulk RNA-seq,Homo_723,AD.vs.MCI; bulk RNA-seq,Homo_723,MCI.vs.control; bulk RNA-seq,Homo_714,AD.vs.MCI; bulk RNA-seq,Homo_633,AD.vs.control; bulk RNA-seq,Homo_633,AD.vs.MCI	5
MF	GO:0022829	wide pore channel activity	bulk RNA-seq,Homo_723,AD.vs.MCI; bulk RNA-seq,Homo_723,MCI.vs.control; bulk RNA-seq,Homo_714,AD.vs.MCI; bulk RNA-seq,Homo_714,MCI.vs.control	4
BP	GO:0032754	positive regulation of interleukin-5 production	bulk RNA-seq,Homo_723,AD.vs.MCI; bulk RNA-seq,Homo_723,MCI.vs.control; bulk RNA-seq,Homo_714,AD.vs.MCI; bulk RNA-seq,Homo_714,MCI.vs.control	4
MF	GO:0015175	neutral amino acid transmembrane transporter activity	bulk RNA-seq,Homo_723,AD.vs.MCI; bulk RNA-seq,Homo_723,MCI.vs.control; bulk RNA-seq,Homo_633,AD.vs.control; bulk RNA-seq,Homo_633,AD.vs.MCI	4
BP	GO:0001553	luteinization	bulk RNA-seq,Homo_723,AD.vs.MCI; bulk RNA-seq,Homo_723,MCI.vs.control; bulk RNA-seq,Homo_714,AD.vs.MCI; bulk RNA-seq,Homo_714,MCI.vs.control	4
BP	GO:0006493	protein O-linked glycosylation	bulk RNA-seq,Homo_723,AD.vs.MCI; bulk RNA-seq,Homo_723,MCI.vs.control; bulk RNA-seq,Homo_714,AD.vs.MCI; bulk RNA-seq,Homo_714,MCI.vs.control; bulk RNA-seq,Homo_633,AD.vs.control; bulk RNA-seq,Homo_633,AD.vs.MCI	6
BP	GO:0046184	aldehyde biosynthetic process	bulk RNA-seq,Homo_723,AD.vs.MCI; bulk RNA-seq,Homo_723,MCI.vs.control; bulk RNA-seq,Homo_714,MCI.vs.control	3
BP	GO:0046851	negative regulation of bone remodeling	bulk RNA-seq,Homo_723,AD.vs.MCI; bulk RNA-seq,Homo_723,MCI.vs.control; bulk RNA-seq,Homo_714,AD.vs.MCI	3
BP	GO:0043281	regulation of cysteine-type endopeptidase activity involved in apoptotic process	bulk RNA-seq,Homo_723,AD.vs.MCI; bulk RNA-seq,Homo_723,MCI.vs.control; bulk RNA-seq,Homo_714,AD.vs.MCI; bulk RNA-seq,Homo_633,AD.vs.control; bulk RNA-seq,Homo_633,AD.vs.MCI	5
CC	GO:0033162	melanosome membrane	bulk RNA-seq,Homo_723,AD.vs.MCI; bulk RNA-seq,Homo_723,MCI.vs.control; bulk RNA-seq,Homo_633,AD.vs.control; bulk RNA-seq,Homo_633,AD.vs.MCI	4
CC	GO:0045009	chitosome	bulk RNA-seq,Homo_723,AD.vs.MCI; bulk RNA-seq,Homo_723,MCI.vs.control; bulk RNA-seq,Homo_633,AD.vs.control; bulk RNA-seq,Homo_633,AD.vs.MCI	4
CC	GO:0090741	pigment granule membrane	bulk RNA-seq,Homo_723,AD.vs.MCI; bulk RNA-seq,Homo_723,MCI.vs.control; bulk RNA-seq,Homo_633,AD.vs.control; bulk RNA-seq,Homo_633,AD.vs.MCI	4
BP	GO:0140115	export across plasma membrane	bulk RNA-seq,Homo_723,AD.vs.MCI; bulk RNA-seq,Homo_714,AD.vs.MCI; bulk RNA-seq,Homo_633,AD.vs.control	3
BP	GO:0050848	regulation of calcium-mediated signaling	bulk RNA-seq,Homo_723,AD.vs.MCI; bulk RNA-seq,Homo_723,MCI.vs.control; bulk RNA-seq,Homo_714,MCI.vs.control; bulk RNA-seq,Homo_633,AD.vs.control; bulk RNA-seq,Homo_633,AD.vs.MCI	5
BP	GO:0098814	spontaneous synaptic transmission	bulk RNA-seq,Homo_723,AD.vs.MCI; bulk RNA-seq,Homo_723,MCI.vs.control; bulk RNA-seq,Homo_714,AD.vs.MCI; bulk RNA-seq,Homo_633,AD.vs.control	4
MF	GO:0031404	chloride ion binding	bulk RNA-seq,Homo_723,AD.vs.MCI; bulk RNA-seq,Homo_723,MCI.vs.control; bulk RNA-seq,Homo_714,AD.vs.MCI; bulk RNA-seq,ROSMAP,AD.vs.control	4
BP	GO:0033762	response to glucagon	bulk RNA-seq,Homo_723,AD.vs.MCI; bulk RNA-seq,Homo_723,MCI.vs.control; bulk RNA-seq,Homo_714,AD.vs.MCI; bulk RNA-seq,Homo_714,MCI.vs.control	4
MF	GO:0016500	protein-hormone receptor activity	bulk RNA-seq,Homo_723,AD.vs.MCI; bulk RNA-seq,Homo_723,MCI.vs.control	2
MF	GO:0051087	chaperone binding	bulk RNA-seq,Homo_723,AD.vs.MCI; bulk RNA-seq,Homo_723,MCI.vs.control; bulk RNA-seq,Homo_714,AD.vs.MCI; bulk RNA-seq,Homo_714,MCI.vs.control; bulk RNA-seq,Homo_633,AD.vs.control; bulk RNA-seq,Homo_633,AD.vs.MCI; scRNA-seq,SRP215507,CD8+ T cell_8-	7
BP	GO:0071636	positive regulation of transforming growth factor beta	bulk RNA-seq,Homo_723,AD.vs.MCI; bulk RNA-seq,Homo_723,MCI.vs.control; bulk RNA-seq,Homo_714,AD.vs.MCI; bulk RNA-seq,Homo_714,MCI.vs.control	4
BP	GO:0072203	cell proliferation involved in metanephros development	bulk RNA-seq,Homo_723,AD.vs.MCI; bulk RNA-seq,Homo_723,MCI.vs.control	2
BP	GO:0045123	cellular extravasation	bulk RNA-seq,Homo_723,AD.vs.MCI; bulk RNA-seq,Homo_723,MCI.vs.control; bulk RNA-seq,Homo_714,AD.vs.MCI; bulk RNA-seq,Homo_714,MCI.vs.control; bulk RNA-seq,Homo_633,AD.vs.control; bulk RNA-seq,SRP223445,AD.vs.control	6
MF	GO:0004675	transmembrane receptor protein serine/threonine kinase activity	bulk RNA-seq,Homo_723,AD.vs.MCI; bulk RNA-seq,Homo_723,MCI.vs.control; bulk RNA-seq,Homo_714,AD.vs.MCI; bulk RNA-seq,Homo_714,MCI.vs.control; bulk RNA-seq,Homo_633,AD.vs.control; bulk RNA-seq,Homo_633,AD.vs.MCI	6
BP	GO:0045913	positive regulation of carbohydrate metabolic process	bulk RNA-seq,Homo_723,AD.vs.MCI; bulk RNA-seq,Homo_723,MCI.vs.control; bulk RNA-seq,Homo_714,AD.vs.MCI; bulk RNA-seq,Homo_714,MCI.vs.control; bulk RNA-seq,Homo_633,AD.vs.control; bulk RNA-seq,Homo_633,AD.vs.MCI	6
BP	GO:1904181	positive regulation of membrane depolarization	bulk RNA-seq,Homo_723,AD.vs.MCI; bulk RNA-seq,Homo_723,MCI.vs.control; bulk RNA-seq,Homo_714,AD.vs.MCI; bulk RNA-seq,Homo_714,MCI.vs.control; bulk RNA-seq,Homo_633,AD.vs.control; bulk RNA-seq,Homo_633,AD.vs.MCI	6
BP	GO:0032507	maintenance of protein location in cell	bulk RNA-seq,Homo_723,AD.vs.MCI; bulk RNA-seq,Homo_723,MCI.vs.control; bulk RNA-seq,Homo_714,AD.vs.MCI; bulk RNA-seq,Homo_714,MCI.vs.control; bulk RNA-seq,Homo_633,AD.vs.control; bulk RNA-seq,Homo_633,AD.vs.MCI	6
BP	GO:1903037	regulation of leukocyte cell-cell adhesion	bulk RNA-seq,Homo_723,AD.vs.MCI; bulk RNA-seq,Homo_714,AD.vs.control; bulk RNA-seq,Homo_714,AD.vs.MCI; bulk RNA-seq,Homo_633,AD.vs.control; bulk RNA-seq,Homo_633,AD.vs.MCI; scRNA-seq,SRP215507,CD8+ T cell_3-AD.vs.control; scRNA-seq,SRP215507,CD8+ T cell_3-MCI.vs.control	7
MF	GO:1901338	catecholamine binding	bulk RNA-seq,Homo_723,AD.vs.MCI; bulk RNA-seq,Homo_633,MCI.vs.control	2
BP	GO:1901731	positive regulation of platelet aggregation	bulk RNA-seq,Homo_723,AD.vs.MCI; bulk RNA-seq,Homo_723,MCI.vs.control; bulk RNA-seq,Homo_714,AD.vs.control; bulk RNA-seq,Homo_714,AD.vs.MCI; bulk RNA-seq,Homo_714,MCI.vs.control	5
BP	GO:0032467	positive regulation of cytokinesis	bulk RNA-seq,Homo_723,AD.vs.MCI; bulk RNA-seq,Homo_714,AD.vs.MCI; bulk RNA-seq,Homo_633,AD.vs.control; bulk RNA-seq,Homo_633,AD.vs.MCI	4
BP	GO:1901184	regulation of ERBB signaling pathway	bulk RNA-seq,Homo_723,AD.vs.MCI; bulk RNA-seq,Homo_723,MCI.vs.control; bulk RNA-seq,Homo_714,AD.vs.MCI; bulk RNA-seq,Homo_714,MCI.vs.control; bulk RNA-seq,Homo_633,AD.vs.control; bulk RNA-seq,Homo_633,AD.vs.MCI	6
BP	GO:0008298	intracellular mRNA localization	bulk RNA-seq,Homo_723,AD.vs.MCI; bulk RNA-seq,Homo_723,MCI.vs.control; bulk RNA-seq,Homo_714,AD.vs.MCI; bulk RNA-seq,Homo_714,MCI.vs.control; bulk RNA-seq,Homo_633,AD.vs.control; bulk RNA-seq,Homo_633,AD.vs.MCI	6
BP	GO:0048505	regulation of timing of cell differentiation	bulk RNA-seq,Homo_723,AD.vs.MCI; bulk RNA-seq,Homo_723,MCI.vs.control	2
BP	GO:0046164	alcohol catabolic process	bulk RNA-seq,Homo_723,AD.vs.MCI; bulk RNA-seq,Homo_723,MCI.vs.control; bulk RNA-seq,Homo_714,AD.vs.MCI; bulk RNA-seq,Homo_714,MCI.vs.control; bulk RNA-seq,Homo_633,AD.vs.control; bulk RNA-seq,Homo_633,AD.vs.MCI	6
BP	GO:1903426	regulation of reactive oxygen species biosynthetic process	bulk RNA-seq,Homo_723,AD.vs.MCI; bulk RNA-seq,Homo_714,AD.vs.MCI; bulk RNA-seq,Homo_633,AD.vs.control; bulk RNA-seq,Homo_633,AD.vs.MCI	4
BP	GO:0060706	cell differentiation involved in embryonic placenta	bulk RNA-seq,Homo_723,AD.vs.MCI; bulk RNA-seq,Homo_723,MCI.vs.control; bulk RNA-seq,Homo_714,AD.vs.MCI; bulk RNA-seq,Homo_714,MCI.vs.control	4
BP	GO:0033145	positive regulation of intracellular steroid hormone receptor signaling pathway	bulk RNA-seq,Homo_723,AD.vs.MCI; bulk RNA-seq,Homo_723,MCI.vs.control; bulk RNA-seq,Homo_714,AD.vs.MCI; bulk RNA-seq,Homo_714,MCI.vs.control	4
BP	GO:0046425	regulation of receptor signaling pathway via JAK-STAT	bulk RNA-seq,Homo_723,AD.vs.MCI; bulk RNA-seq,Homo_714,AD.vs.MCI	2

BP	GO:0007026	negative regulation of microtubule depolymerization	bulk RNA-seq,Homo_723,AD.vs.MCI; bulk RNA-seq,Homo_723,MCI.vs.control; bulk RNA-seq,Homo_714,MCI.vs.control	3
BP	GO:0033630	positive regulation of cell adhesion mediated by integrin	bulk RNA-seq,Homo_723,AD.vs.MCI; bulk RNA-seq,Homo_723,MCI.vs.control; bulk RNA-seq,Homo_714,AD.vs.MCI; bulk RNA-seq,Homo_714,MCI.vs.control	4
BP	GO:0070486	leukocyte aggregation	bulk RNA-seq,Homo_723,AD.vs.MCI; bulk RNA-seq,Homo_723,MCI.vs.control; bulk RNA-seq,Homo_714,MCI.vs.control; bulk RNA-	4
BP	GO:0008652	cellular amino acid biosynthetic process	bulk RNA-seq,Homo_723,AD.vs.MCI; bulk RNA-seq,Homo_723,MCI.vs.control; bulk RNA-seq,Homo_714,AD.vs.MCI; bulk RNA-seq,Homo_714,MCI.vs.control; bulk RNA-seq,Homo_633,AD.vs.control; bulk RNA-seq,Homo_633,AD.vs.MCI	6
BP	GO:0010766	negative regulation of sodium ion transport	bulk RNA-seq,Homo_723,AD.vs.MCI; bulk RNA-seq,Homo_723,MCI.vs.control	2
BP	GO:0003094	glomerular filtration	bulk RNA-seq,Homo_723,AD.vs.MCI; bulk RNA-seq,Homo_723,MCI.vs.control; bulk RNA-seq,Homo_714,AD.vs.MCI; bulk RNA-seq,Homo_714,MCI.vs.control; bulk RNA-seq,Homo_633,AD.vs.control; bulk RNA-seq,Homo_633,AD.vs.MCI	6
MF	GO:0022848	acetylcholine-gated cation-selective channel activity	bulk RNA-seq,Homo_723,AD.vs.MCI; bulk RNA-seq,Homo_723,MCI.vs.control; bulk RNA-seq,Homo_714,AD.vs.MCI	3
MF	GO:0042826	histone deacetylase binding	bulk RNA-seq,Homo_723,AD.vs.MCI; bulk RNA-seq,Homo_723,MCI.vs.control; bulk RNA-seq,Homo_714,AD.vs.MCI; bulk RNA-seq,Homo_714,MCI.vs.control; bulk RNA-seq,Homo_633,AD.vs.control; bulk RNA-seq,Homo_633,AD.vs.MCI	6
BP	GO:0001676	long-chain fatty acid metabolic process	bulk RNA-seq,Homo_723,AD.vs.MCI; bulk RNA-seq,Homo_723,MCI.vs.control; bulk RNA-seq,Homo_714,AD.vs.MCI; bulk RNA-seq,Homo_714,MCI.vs.control; bulk RNA-seq,Homo_633,AD.vs.control	5
BP	GO:0045922	negative regulation of fatty acid metabolic process	bulk RNA-seq,Homo_723,AD.vs.MCI; bulk RNA-seq,Homo_723,MCI.vs.control; bulk RNA-seq,Homo_714,AD.vs.MCI; bulk RNA-seq,Homo_714,MCI.vs.control; bulk RNA-seq,Homo_633,AD.vs.control	5
MF	GO:0005507	copper ion binding	bulk RNA-seq,Homo_723,AD.vs.MCI; bulk RNA-seq,Homo_723,MCI.vs.control; bulk RNA-seq,Homo_714,MCI.vs.control; bulk RNA-seq,Homo_633,AD.vs.control; bulk RNA-seq,Homo_633,AD.vs.MCI	5
BP	GO:0010743	regulation of macrophage derived foam cell differentiation	bulk RNA-seq,Homo_723,AD.vs.MCI; bulk RNA-seq,Homo_723,MCI.vs.control; bulk RNA-seq,Homo_714,AD.vs.MCI; bulk RNA-seq,Homo_714,MCI.vs.control; bulk RNA-seq,Homo_633,AD.vs.control; bulk RNA-seq,Homo_633,AD.vs.MCI	6
MF	GO:0030159	signaling receptor complex adaptor activity	bulk RNA-seq,Homo_723,AD.vs.MCI; bulk RNA-seq,Homo_723,MCI.vs.control; bulk RNA-seq,Homo_714,AD.vs.MCI; bulk RNA-seq,Homo_714,MCI.vs.control; bulk RNA-seq,Homo_633,AD.vs.control; bulk RNA-seq,Homo_633,AD.vs.MCI	6
CC	GO:0098687	chromosomal region	bulk RNA-seq,Homo_723,AD.vs.MCI; bulk RNA-seq,Homo_714,AD.vs.MCI; bulk RNA-seq,Homo_633,AD.vs.control; bulk RNA-seq,Homo_633,AD.vs.MCI	4
BP	GO:0016322	neuron remodeling	bulk RNA-seq,Homo_723,AD.vs.MCI; bulk RNA-seq,Homo_723,MCI.vs.control; bulk RNA-seq,Homo_714,AD.vs.MCI; bulk RNA-seq,Homo_714,MCI.vs.control	4
BP	GO:0007398	ectoderm development	bulk RNA-seq,Homo_723,AD.vs.MCI	1
CC	GO:0031528	microvillus membrane	bulk RNA-seq,Homo_723,AD.vs.MCI; bulk RNA-seq,Homo_723,MCI.vs.control; bulk RNA-seq,Homo_633,AD.vs.control	3
BP	GO:0042415	norepinephrine metabolic process	bulk RNA-seq,Homo_723,AD.vs.MCI; bulk RNA-seq,Homo_723,MCI.vs.control; bulk RNA-seq,Homo_714,AD.vs.MCI	3
BP	GO:0043502	regulation of muscle adaptation	bulk RNA-seq,Homo_723,AD.vs.MCI; bulk RNA-seq,Homo_723,MCI.vs.control; bulk RNA-seq,Homo_714,AD.vs.MCI; bulk RNA-seq,Homo_714,MCI.vs.control; bulk RNA-seq,Homo_633,AD.vs.control	5
BP	GO:0031349	positive regulation of defense response	bulk RNA-seq,Homo_723,AD.vs.MCI; bulk RNA-seq,Homo_723,MCI.vs.control; bulk RNA-seq,Homo_714,AD.vs.MCI; bulk RNA-seq,Homo_633,AD.vs.control; bulk RNA-seq,Homo_633,AD.vs.MCI	5
BP	GO:0045746	negative regulation of Notch signaling pathway	bulk RNA-seq,Homo_723,AD.vs.MCI; bulk RNA-seq,Homo_723,MCI.vs.control; bulk RNA-seq,Homo_714,AD.vs.MCI; bulk RNA-seq,Homo_714,MCI.vs.control; scRNA-seq,SRP309935,Natural killer cell_1-AD.vs.control	5
BP	GO:0010664	negative regulation of striated muscle cell apoptotic process	bulk RNA-seq,Homo_723,AD.vs.MCI; bulk RNA-seq,Homo_714,AD.vs.MCI	2
BP	GO:1902894	negative regulation of miRNA transcription	bulk RNA-seq,Homo_723,AD.vs.MCI; bulk RNA-seq,Homo_723,MCI.vs.control; bulk RNA-seq,Homo_714,MCI.vs.control; bulk RNA-	4
MF	GO:0043176	amine binding	bulk RNA-seq,Homo_723,AD.vs.MCI; bulk RNA-seq,Homo_714,AD.vs.MCI	2
BP	GO:0051445	regulation of meiotic cell cycle	bulk RNA-seq,Homo_723,AD.vs.MCI; bulk RNA-seq,Homo_723,MCI.vs.control; bulk RNA-seq,Homo_714,AD.vs.MCI; bulk RNA-seq,Homo_714,MCI.vs.control; bulk RNA-seq,Homo_633,AD.vs.control	5
BP	GO:0014820	tonic smooth muscle contraction	bulk RNA-seq,Homo_723,AD.vs.MCI; bulk RNA-seq,Homo_714,AD.vs.MCI	2
BP	GO:0031282	regulation of guanylate cyclase activity	bulk RNA-seq,Homo_723,AD.vs.MCI; bulk RNA-seq,Homo_714,AD.vs.MCI	2
BP	GO:0045185	maintenance of protein location	bulk RNA-seq,Homo_723,AD.vs.MCI; bulk RNA-seq,Homo_723,MCI.vs.control; bulk RNA-seq,Homo_714,AD.vs.MCI; bulk RNA-seq,Homo_714,MCI.vs.control; bulk RNA-seq,Homo_633,AD.vs.control; bulk RNA-seq,Homo_633,AD.vs.MCI	6
BP	GO:0033559	unsaturated fatty acid metabolic process	bulk RNA-seq,Homo_723,AD.vs.MCI; bulk RNA-seq,Homo_723,MCI.vs.control; bulk RNA-seq,Homo_714,AD.vs.MCI; bulk RNA-seq,Homo_714,MCI.vs.control; bulk RNA-seq,Homo_633,AD.vs.control; bulk RNA-seq,Homo_633,AD.vs.MCI	6
CC	GO:0043073	germ cell nucleus	bulk RNA-seq,Homo_723,AD.vs.MCI; bulk RNA-seq,Homo_723,MCI.vs.control; bulk RNA-seq,Homo_714,AD.vs.MCI; bulk RNA-seq,Homo_714,MCI.vs.control; bulk RNA-seq,Homo_633,AD.vs.control; bulk RNA-seq,Homo_633,AD.vs.MCI	6
BP	GO:0010649	regulation of cell communication by electrical coupling	bulk RNA-seq,Homo_723,AD.vs.MCI; bulk RNA-seq,Homo_723,MCI.vs.control; bulk RNA-seq,Homo_714,AD.vs.MCI; bulk RNA-seq,Homo_714,MCI.vs.control	4
BP	GO:0003298	physiological muscle hypertrophy	bulk RNA-seq,Homo_723,AD.vs.MCI; bulk RNA-seq,Homo_714,AD.vs.MCI; bulk RNA-seq,Homo_714,MCI.vs.control	3
BP	GO:0003301	physiological cardiac muscle hypertrophy	bulk RNA-seq,Homo_723,AD.vs.MCI; bulk RNA-seq,Homo_714,AD.vs.MCI; bulk RNA-seq,Homo_714,MCI.vs.control	3
BP	GO:0061049	cell growth involved in cardiac muscle cell development	bulk RNA-seq,Homo_723,AD.vs.MCI; bulk RNA-seq,Homo_714,AD.vs.MCI; bulk RNA-seq,Homo_714,MCI.vs.control	3
BP	GO:0034260	negative regulation of GTPase activity	bulk RNA-seq,Homo_723,AD.vs.MCI; bulk RNA-seq,Homo_723,MCI.vs.control; bulk RNA-seq,Homo_714,AD.vs.MCI; bulk RNA-seq,Homo_714,MCI.vs.control; bulk RNA-seq,Homo_633,AD.vs.control; bulk RNA-seq,Homo_633,AD.vs.MCI	6
BP	GO:0018198	peptidyl-cysteine modification	bulk RNA-seq,Homo_723,AD.vs.MCI; bulk RNA-seq,Homo_633,AD.vs.control; bulk RNA-seq,Homo_633,AD.vs.MCI	3
BP	GO:0043301	negative regulation of leukocyte degranulation	bulk RNA-seq,Homo_723,AD.vs.MCI; bulk RNA-seq,Homo_723,MCI.vs.control; bulk RNA-seq,Homo_714,AD.vs.MCI; bulk RNA-seq,Homo_714,MCI.vs.control; bulk RNA-seq,Homo_633,AD.vs.MCI	5
BP	GO:0071295	cellular response to vitamin	bulk RNA-seq,Homo_723,AD.vs.MCI; bulk RNA-seq,Homo_723,MCI.vs.control; bulk RNA-seq,Homo_714,AD.vs.MCI; bulk RNA-seq,Homo_714,MCI.vs.control; bulk RNA-seq,Homo_633,AD.vs.control	5
BP	GO:1990845	adaptive thermogenesis	bulk RNA-seq,Homo_723,AD.vs.MCI; bulk RNA-seq,Homo_723,MCI.vs.control; bulk RNA-seq,Homo_714,AD.vs.MCI; bulk RNA-seq,Homo_633,AD.vs.control; bulk RNA-seq,Homo_633,AD.vs.MCI	5
BP	GO:0098911	regulation of ventricular cardiac muscle cell action potential	bulk RNA-seq,Homo_723,AD.vs.MCI; bulk RNA-seq,Homo_723,MCI.vs.control; bulk RNA-seq,Homo_714,AD.vs.MCI; bulk RNA-seq,Homo_714,MCI.vs.control	4
BP	GO:1901607	alpha-amino acid biosynthetic process	bulk RNA-seq,Homo_723,AD.vs.MCI; bulk RNA-seq,Homo_723,MCI.vs.control; bulk RNA-seq,Homo_714,MCI.vs.control; bulk RNA-seq,Homo_633,AD.vs.control; bulk RNA-seq,Homo_633,AD.vs.MCI	5

BP	GO:0150076	neuroinflammatory response	bulk RNA-seq,Homo_723,AD.vs.MCI; bulk RNA-seq,Homo_723,MCI.vs.control; bulk RNA-seq,Homo_714,AD.vs.MCI; bulk RNA-seq,Homo_714,MCI.vs.control; bulk RNA-seq,Homo_633,AD.vs.control; bulk RNA-seq,Homo_633,AD.vs.MCI	6
BP	GO:0035930	corticosteroid hormone secretion	bulk RNA-seq,Homo_723,AD.vs.MCI; bulk RNA-seq,Homo_714,AD.vs.control; bulk RNA-seq,Homo_714,AD.vs.MCI; bulk RNA-seq,Homo_633,AD.vs.MCI	4
BP	GO:0009404	toxin metabolic process	bulk RNA-seq,Homo_723,AD.vs.MCI; bulk RNA-seq,Homo_723,MCI.vs.control	2
BP	GO:0003263	cardioblast proliferation	bulk RNA-seq,Homo_723,AD.vs.MCI	1
BP	GO:0003264	regulation of cardioblast proliferation	bulk RNA-seq,Homo_723,AD.vs.MCI	1
BP	GO:0034104	negative regulation of tissue remodeling	bulk RNA-seq,Homo_723,AD.vs.MCI; bulk RNA-seq,Homo_723,MCI.vs.control; bulk RNA-seq,Homo_714,AD.vs.MCI	3
BP	GO:0090026	positive regulation of monocyte chemotaxis	bulk RNA-seq,Homo_723,AD.vs.MCI; bulk RNA-seq,Homo_714,AD.vs.MCI	2
BP	GO:0044843	cell cycle G1/S phase transition	bulk RNA-seq,Homo_723,AD.vs.MCI; bulk RNA-seq,Homo_723,MCI.vs.control; bulk RNA-seq,Homo_714,AD.vs.MCI; bulk RNA-seq,Homo_633,AD.vs.control; bulk RNA-seq,Homo_633,AD.vs.MCI; scRNA-seq,SRP330776,Naive CD8+ T cell_2-AD.vs.control	6
CC	GO:1904115	axon cytoplasm	bulk RNA-seq,Homo_723,AD.vs.MCI; bulk RNA-seq,Homo_723,MCI.vs.control; bulk RNA-seq,Homo_714,MCI.vs.control; bulk RNA-seq,Homo_633,AD.vs.control; bulk RNA-seq,Homo_633,AD.vs.MCI	5
BP	GO:0008630	intrinsic apoptotic signaling pathway in response to DNA damage	bulk RNA-seq,Homo_723,AD.vs.MCI; bulk RNA-seq,Homo_723,MCI.vs.control; bulk RNA-seq,Homo_714,AD.vs.MCI; bulk RNA-seq,Homo_714,MCI.vs.control; bulk RNA-seq,Homo_633,AD.vs.control; bulk RNA-seq,Homo_633,AD.vs.MCI	6
BP	GO:0014743	regulation of muscle hypertrophy	bulk RNA-seq,Homo_723,AD.vs.MCI; bulk RNA-seq,Homo_723,MCI.vs.control; bulk RNA-seq,Homo_714,AD.vs.MCI; bulk RNA-seq,Homo_714,MCI.vs.control; bulk RNA-seq,Homo_633,AD.vs.control	5
BP	GO:0097205	renal filtration	bulk RNA-seq,Homo_723,AD.vs.MCI; bulk RNA-seq,Homo_723,MCI.vs.control; bulk RNA-seq,Homo_714,AD.vs.MCI; bulk RNA-seq,Homo_714,MCI.vs.control; bulk RNA-seq,Homo_633,AD.vs.control; bulk RNA-seq,Homo_633,AD.vs.MCI	6
BP	GO:2000737	negative regulation of stem cell differentiation	bulk RNA-seq,Homo_723,AD.vs.MCI; bulk RNA-seq,Homo_723,MCI.vs.control; bulk RNA-seq,Homo_714,AD.vs.MCI; bulk RNA-seq,Homo_714,MCI.vs.control; bulk RNA-seq,Homo_633,AD.vs.control; bulk RNA-seq,Homo_633,AD.vs.MCI	6
BP	GO:0090205	positive regulation of cholesterol metabolic process	bulk RNA-seq,Homo_723,AD.vs.MCI; bulk RNA-seq,Homo_723,MCI.vs.control; bulk RNA-seq,Homo_714,AD.vs.MCI; bulk RNA-seq,Homo_714,MCI.vs.control	4
BP	GO:2000117	negative regulation of cysteine-type endopeptidase activity	bulk RNA-seq,Homo_723,AD.vs.MCI; bulk RNA-seq,Homo_714,AD.vs.MCI; bulk RNA-seq,Homo_714,MCI.vs.control; bulk RNA-seq,Homo_633,AD.vs.control; bulk RNA-seq,Homo_633,AD.vs.MCI	5
BP	GO:0090050	positive regulation of cell migration involved in sprouting angiogenesis	bulk RNA-seq,Homo_723,AD.vs.MCI; bulk RNA-seq,Homo_723,MCI.vs.control; bulk RNA-seq,Homo_714,AD.vs.control; bulk RNA-seq,Homo_714,AD.vs.MCI; bulk RNA-seq,Homo_714,MCI.vs.control	5
BP	GO:0071398	cellular response to fatty acid	bulk RNA-seq,Homo_723,AD.vs.MCI; bulk RNA-seq,Homo_723,MCI.vs.control; bulk RNA-seq,Homo_714,AD.vs.MCI; bulk RNA-seq,Homo_714,MCI.vs.control; bulk RNA-seq,Homo_633,AD.vs.control; bulk RNA-seq,Homo_633,AD.vs.MCI	6
BP	GO:0002544	chronic inflammatory response	bulk RNA-seq,Homo_723,AD.vs.MCI; bulk RNA-seq,Homo_723,MCI.vs.control; bulk RNA-seq,Homo_714,AD.vs.MCI; bulk RNA-seq,Homo_714,MCI.vs.control	4
BP	GO:0033504	floor plate development	bulk RNA-seq,Homo_723,AD.vs.MCI	1
MF	GO:0019855	calcium channel inhibitor activity	bulk RNA-seq,Homo_723,AD.vs.MCI; bulk RNA-seq,Homo_723,MCI.vs.control; bulk RNA-seq,Homo_714,MCI.vs.control; bulk RNA-	4
BP	GO:0030148	sphingolipid biosynthetic process	bulk RNA-seq,Homo_723,AD.vs.MCI; bulk RNA-seq,Homo_723,MCI.vs.control; bulk RNA-seq,Homo_714,MCI.vs.control; bulk RNA-seq,Homo_633,AD.vs.control; bulk RNA-seq,Homo_633,AD.vs.MCI	5
BP	GO:0006704	glucocorticoid biosynthetic process	bulk RNA-seq,Homo_723,AD.vs.MCI; bulk RNA-seq,Homo_723,MCI.vs.control	2
BP	GO:0071361	cellular response to ethanol	bulk RNA-seq,Homo_723,AD.vs.MCI	1
CC	GO:0031258	lamellipodium membrane	bulk RNA-seq,Homo_723,AD.vs.MCI; bulk RNA-seq,Homo_723,MCI.vs.control; bulk RNA-seq,Homo_633,AD.vs.control; bulk RNA-seq,Homo_633,AD.vs.MCI	4
BP	GO:0010559	regulation of glycoprotein biosynthetic process	bulk RNA-seq,Homo_723,AD.vs.MCI; bulk RNA-seq,Homo_723,MCI.vs.control; bulk RNA-seq,Homo_714,AD.vs.MCI; bulk RNA-seq,Homo_714,MCI.vs.control; bulk RNA-seq,Homo_633,AD.vs.control	5
BP	GO:0098719	sodium ion import across plasma membrane	bulk RNA-seq,Homo_723,AD.vs.MCI; bulk RNA-seq,Homo_723,MCI.vs.control; bulk RNA-seq,Homo_714,AD.vs.MCI	3
CC	GO:0043209	myelin sheath	bulk RNA-seq,Homo_723,AD.vs.MCI; bulk RNA-seq,Homo_723,MCI.vs.control; bulk RNA-seq,Homo_714,AD.vs.MCI; bulk RNA-seq,Homo_714,MCI.vs.control; bulk RNA-seq,Homo_633,AD.vs.control; bulk RNA-seq,Homo_633,AD.vs.MCI	6
BP	GO:1903727	positive regulation of phospholipid metabolic process	bulk RNA-seq,Homo_723,AD.vs.MCI; bulk RNA-seq,Homo_723,MCI.vs.control; bulk RNA-seq,Homo_714,AD.vs.MCI; bulk RNA-seq,Homo_714,MCI.vs.control; bulk RNA-seq,Homo_633,AD.vs.control; bulk RNA-seq,Homo_633,AD.vs.MCI	6
BP	GO:0010658	striated muscle cell apoptotic process	bulk RNA-seq,Homo_723,AD.vs.MCI; bulk RNA-seq,Homo_714,AD.vs.MCI; bulk RNA-seq,Homo_633,AD.vs.control; bulk RNA-seq,Homo_633,AD.vs.MCI	4
BP	GO:0010742	macrophage derived foam cell differentiation	bulk RNA-seq,Homo_723,AD.vs.MCI; bulk RNA-seq,Homo_723,MCI.vs.control; bulk RNA-seq,Homo_714,AD.vs.MCI; bulk RNA-seq,Homo_714,MCI.vs.control; bulk RNA-seq,Homo_633,AD.vs.control; bulk RNA-seq,Homo_633,AD.vs.MCI	6
BP	GO:0015816	glycine transport	bulk RNA-seq,Homo_723,AD.vs.MCI; bulk RNA-seq,Homo_714,AD.vs.MCI	2
MF	GO:0005452	solute:inorganic anion antiporter activity	bulk RNA-seq,Homo_723,AD.vs.MCI; bulk RNA-seq,Homo_723,MCI.vs.control	2
BP	GO:0045931	positive regulation of mitotic cell cycle	bulk RNA-seq,Homo_723,AD.vs.MCI; bulk RNA-seq,Homo_723,MCI.vs.control; bulk RNA-seq,Homo_714,AD.vs.MCI; bulk RNA-seq,Homo_633,AD.vs.control; bulk RNA-seq,Homo_633,AD.vs.MCI	5
BP	GO:0010660	regulation of muscle cell apoptotic process	bulk RNA-seq,Homo_723,AD.vs.MCI; bulk RNA-seq,Homo_714,AD.vs.MCI; bulk RNA-seq,Homo_633,AD.vs.control	3
MF	GO:0039706	co-receptor binding	bulk RNA-seq,Homo_723,AD.vs.MCI; bulk RNA-seq,Homo_723,MCI.vs.control; bulk RNA-seq,Homo_714,MCI.vs.control	3
MF	GO:0019888	protein phosphatase regulator activity	bulk RNA-seq,Homo_723,AD.vs.MCI; bulk RNA-seq,Homo_723,MCI.vs.control; bulk RNA-seq,Homo_714,AD.vs.MCI; bulk RNA-seq,Homo_714,MCI.vs.control; bulk RNA-seq,Homo_633,AD.vs.control; bulk RNA-seq,Homo_633,AD.vs.MCI	6
BP	GO:0060026	convergent extension	bulk RNA-seq,Homo_723,AD.vs.MCI; bulk RNA-seq,Homo_723,MCI.vs.control; bulk RNA-seq,Homo_633,AD.vs.control	3
CC	GO:0097546	ciliary base	bulk RNA-seq,Homo_723,AD.vs.MCI; bulk RNA-seq,Homo_723,MCI.vs.control; bulk RNA-seq,Homo_714,AD.vs.MCI; bulk RNA-seq,Homo_714,MCI.vs.control; bulk RNA-seq,Homo_633,AD.vs.control; bulk RNA-seq,Homo_633,AD.vs.MCI	6
BP	GO:0016114	terpenoid biosynthetic process	bulk RNA-seq,Homo_723,AD.vs.MCI; bulk RNA-seq,Homo_723,MCI.vs.control; bulk RNA-seq,Homo_714,MCI.vs.control; bulk RNA-seq,Homo_633,AD.vs.control; bulk RNA-seq,Homo_633,AD.vs.MCI	5
BP	GO:0008090	retrograde axonal transport	bulk RNA-seq,Homo_723,AD.vs.MCI; bulk RNA-seq,Homo_723,MCI.vs.control; bulk RNA-seq,Homo_714,MCI.vs.control; bulk RNA-	4
BP	GO:0002548	monocyte chemotaxis	bulk RNA-seq,Homo_723,AD.vs.MCI; bulk RNA-seq,Homo_723,MCI.vs.control; bulk RNA-seq,Homo_714,AD.vs.MCI; bulk RNA-seq,Homo_714,MCI.vs.control	4

BP	GO:0045663	positive regulation of myoblast differentiation	bulk RNA-seq,Homo_723,AD.vs.MCI; bulk RNA-seq,Homo_723,MCI.vs.control; bulk RNA-seq,Homo_714,MCI.vs.control; bulk RNA-seq,Homo_633,AD.vs.control; bulk RNA-seq,Homo_633,AD.vs.MCI; scRNA-seq,SRP330776,Naive CD8+ T cell_2-AD.vs.control	6
BP	GO:0042509	regulation of tyrosine phosphorylation of STAT protein	bulk RNA-seq,Homo_723,AD.vs.MCI; bulk RNA-seq,Homo_723,MCI.vs.control; bulk RNA-seq,Homo_714,AD.vs.MCI	3
CC	GO:0033017	sarcoplasmic reticulum membrane	bulk RNA-seq,Homo_723,AD.vs.MCI; bulk RNA-seq,Homo_714,AD.vs.MCI	2
BP	GO:0051491	positive regulation of filopodium assembly	bulk RNA-seq,Homo_723,AD.vs.MCI; bulk RNA-seq,Homo_723,MCI.vs.control; bulk RNA-seq,Homo_714,AD.vs.MCI; bulk RNA-seq,Homo_714,MCI.vs.control; bulk RNA-seq,Homo_633,AD.vs.control	5
BP	GO:0061436	establishment of skin barrier	bulk RNA-seq,Homo_723,AD.vs.MCI; bulk RNA-seq,Homo_723,MCI.vs.control; bulk RNA-seq,Homo_714,AD.vs.MCI; bulk RNA-seq,Homo_714,MCI.vs.control; bulk RNA-seq,SRP223445,AD.vs.control	5
BP	GO:0003306	Wnt signaling pathway involved in heart development	bulk RNA-seq,Homo_723,AD.vs.MCI; bulk RNA-seq,Homo_723,MCI.vs.control; bulk RNA-seq,Homo_714,AD.vs.MCI	3
BP	GO:0097009	energy homeostasis	bulk RNA-seq,Homo_723,AD.vs.MCI; bulk RNA-seq,Homo_723,MCI.vs.control; bulk RNA-seq,Homo_714,MCI.vs.control; bulk RNA-	4
BP	GO:0008286	insulin receptor signaling pathway	bulk RNA-seq,Homo_723,AD.vs.MCI; bulk RNA-seq,Homo_723,MCI.vs.control; bulk RNA-seq,Homo_714,AD.vs.MCI; bulk RNA-seq,Homo_714,MCI.vs.control; bulk RNA-seq,Homo_633,AD.vs.control; bulk RNA-seq,Homo_633,AD.vs.MCI	6
BP	GO:0006690	icosanoid metabolic process	bulk RNA-seq,Homo_723,AD.vs.MCI; bulk RNA-seq,Homo_714,AD.vs.MCI; bulk RNA-seq,Homo_633,AD.vs.control; bulk RNA-seq,Homo_633,AD.vs.MCI	4
BP	GO:0061430	bone trabecula morphogenesis	bulk RNA-seq,Homo_723,AD.vs.MCI; bulk RNA-seq,Homo_723,MCI.vs.control	2
MF	GO:0016831	carboxy-lyase activity	bulk RNA-seq,Homo_723,AD.vs.MCI; bulk RNA-seq,Homo_723,MCI.vs.control; bulk RNA-seq,Homo_714,AD.vs.MCI; bulk RNA-seq,Homo_714,MCI.vs.control; bulk RNA-seq,Homo_633,AD.vs.control; bulk RNA-seq,Homo_633,AD.vs.MCI	6
BP	GO:0032634	interleukin-5 production	bulk RNA-seq,Homo_723,AD.vs.MCI; bulk RNA-seq,Homo_723,MCI.vs.control; bulk RNA-seq,Homo_714,AD.vs.MCI; bulk RNA-seq,Homo_714,MCI.vs.control	4
BP	GO:0032674	regulation of interleukin-5 production	bulk RNA-seq,Homo_723,AD.vs.MCI; bulk RNA-seq,Homo_723,MCI.vs.control; bulk RNA-seq,Homo_714,AD.vs.MCI; bulk RNA-seq,Homo_714,MCI.vs.control	4
BP	GO:0043536	positive regulation of blood vessel endothelial cell migration	bulk RNA-seq,Homo_723,AD.vs.MCI; bulk RNA-seq,Homo_723,MCI.vs.control; bulk RNA-seq,Homo_714,AD.vs.control; bulk RNA-seq,Homo_714,AD.vs.MCI; bulk RNA-seq,Homo_714,MCI.vs.control; bulk RNA-seq,Homo_633,AD.vs.control; bulk RNA-seq,Homo_633,AD.vs.MCI	7
BP	GO:0010623	programmed cell death involved in cell development	bulk RNA-seq,Homo_723,AD.vs.MCI; bulk RNA-seq,Homo_723,MCI.vs.control; bulk RNA-seq,Homo_714,AD.vs.MCI; bulk RNA-seq,Homo_714,MCI.vs.control	4
BP	GO:0051262	protein tetramerization	bulk RNA-seq,Homo_723,AD.vs.MCI; bulk RNA-seq,Homo_723,MCI.vs.control; bulk RNA-seq,Homo_714,AD.vs.MCI; bulk RNA-seq,Homo_714,MCI.vs.control; bulk RNA-seq,Homo_633,AD.vs.control; bulk RNA-seq,Homo_633,AD.vs.MCI	6
BP	GO:2000406	positive regulation of T cell migration	bulk RNA-seq,Homo_723,AD.vs.MCI; bulk RNA-seq,Homo_723,MCI.vs.control; bulk RNA-seq,Homo_714,AD.vs.MCI; bulk RNA-seq,Homo_714,MCI.vs.control; bulk RNA-seq,Homo_633,AD.vs.control	5
CC	GO:1990752	microtubule end	bulk RNA-seq,Homo_723,AD.vs.MCI; bulk RNA-seq,Homo_723,MCI.vs.control; bulk RNA-seq,Homo_714,AD.vs.MCI; bulk RNA-seq,Homo_714,MCI.vs.control; bulk RNA-seq,Homo_633,AD.vs.control	5
MF	GO:0055102	lipase inhibitor activity	bulk RNA-seq,Homo_723,AD.vs.MCI; bulk RNA-seq,Homo_723,MCI.vs.control; bulk RNA-seq,Homo_714,AD.vs.control; bulk RNA-seq,Homo_714,AD.vs.MCI; bulk RNA-seq,Homo_714,MCI.vs.control; bulk RNA-seq,Homo_633,AD.vs.control; bulk RNA-seq,Homo_633,AD.vs.MCI	7
BP	GO:0044272	sulfur compound biosynthetic process	bulk RNA-seq,Homo_723,AD.vs.MCI; bulk RNA-seq,Homo_723,MCI.vs.control; bulk RNA-seq,Homo_714,AD.vs.MCI; bulk RNA-seq,Homo_633,AD.vs.control; bulk RNA-seq,Homo_633,AD.vs.MCI	5
BP	GO:1901031	regulation of response to reactive oxygen species	bulk RNA-seq,Homo_723,AD.vs.MCI; bulk RNA-seq,Homo_723,MCI.vs.control; bulk RNA-seq,Homo_714,AD.vs.MCI; bulk RNA-seq,Homo_714,MCI.vs.control; bulk RNA-seq,Homo_633,AD.vs.control	5
BP	GO:0098703	calcium ion import across plasma membrane	bulk RNA-seq,Homo_723,AD.vs.MCI; bulk RNA-seq,Homo_723,MCI.vs.control; bulk RNA-seq,Homo_714,AD.vs.MCI	3
BP	GO:0007176	regulation of epidermal growth factor-activated receptor activity	bulk RNA-seq,Homo_723,AD.vs.MCI; bulk RNA-seq,Homo_723,MCI.vs.control; bulk RNA-seq,Homo_714,AD.vs.MCI; bulk RNA-seq,Homo_714,MCI.vs.control; bulk RNA-seq,Homo_633,AD.vs.control	5
BP	GO:1900407	regulation of cellular response to oxidative stress	bulk RNA-seq,Homo_723,AD.vs.MCI; bulk RNA-seq,Homo_723,MCI.vs.control; bulk RNA-seq,Homo_714,AD.vs.MCI; bulk RNA-seq,Homo_714,MCI.vs.control; bulk RNA-seq,Homo_633,AD.vs.control; bulk RNA-seq,Homo_633,AD.vs.MCI	6
BP	GO:0006473	protein acetylation	bulk RNA-seq,Homo_723,AD.vs.MCI; bulk RNA-seq,Homo_723,MCI.vs.control; bulk RNA-seq,Homo_714,AD.vs.MCI; bulk RNA-seq,Homo_633,AD.vs.control; bulk RNA-seq,Homo_633,AD.vs.MCI	5
BP	GO:0000082	G1/S transition of mitotic cell cycle	bulk RNA-seq,Homo_723,AD.vs.MCI; bulk RNA-seq,Homo_723,MCI.vs.control; bulk RNA-seq,Homo_714,AD.vs.MCI; bulk RNA-seq,Homo_633,AD.vs.control; bulk RNA-seq,Homo_633,AD.vs.MCI	5
BP	GO:0051451	myoblast migration	bulk RNA-seq,Homo_723,AD.vs.MCI; bulk RNA-seq,Homo_723,MCI.vs.control; bulk RNA-seq,Homo_633,AD.vs.control	3
BP	GO:0007159	leukocyte cell-cell adhesion	bulk RNA-seq,Homo_723,AD.vs.MCI; bulk RNA-seq,Homo_714,AD.vs.control; bulk RNA-seq,Homo_714,AD.vs.MCI; bulk RNA-seq,Homo_633,AD.vs.control; bulk RNA-seq,Homo_633,AD.vs.MCI; scRNA-seq,SRP215507,CD8+ T cell_3-AD.vs.control	6
BP	GO:0070613	regulation of protein processing	bulk RNA-seq,Homo_723,AD.vs.MCI; bulk RNA-seq,Homo_723,MCI.vs.control; bulk RNA-seq,Homo_714,AD.vs.MCI; bulk RNA-seq,Homo_714,MCI.vs.control; bulk RNA-seq,Homo_633,AD.vs.control; bulk RNA-seq,Homo_633,AD.vs.MCI	6
BP	GO:0014912	negative regulation of smooth muscle cell migration	bulk RNA-seq,Homo_723,AD.vs.MCI; bulk RNA-seq,Homo_714,AD.vs.control; bulk RNA-seq,Homo_714,AD.vs.MCI	3
BP	GO:0002691	regulation of cellular extravasation	bulk RNA-seq,Homo_723,AD.vs.MCI; bulk RNA-seq,Homo_723,MCI.vs.control; bulk RNA-seq,Homo_714,AD.vs.MCI; bulk RNA-seq,Homo_714,MCI.vs.control; bulk RNA-seq,Homo_633,AD.vs.control	5
BP	GO:0034394	protein localization to cell surface	bulk RNA-seq,Homo_723,AD.vs.MCI; bulk RNA-seq,Homo_723,MCI.vs.control; bulk RNA-seq,Homo_714,MCI.vs.control; bulk RNA-seq,Homo_633,AD.vs.control; bulk RNA-seq,Homo_633,AD.vs.MCI	5
BP	GO:0048599	oocyte development	bulk RNA-seq,Homo_723,AD.vs.MCI; bulk RNA-seq,Homo_723,MCI.vs.control; bulk RNA-seq,Homo_714,AD.vs.MCI; bulk RNA-seq,Homo_714,MCI.vs.control; bulk RNA-seq,Homo_633,AD.vs.control	5
BP	GO:0042742	defense response to bacterium	bulk RNA-seq,Homo_723,AD.vs.MCI; bulk RNA-seq,Homo_714,AD.vs.control; bulk RNA-seq,Homo_714,AD.vs.MCI; bulk RNA-seq,Homo_633,AD.vs.control; bulk RNA-seq,Homo_633,AD.vs.MCI; bulk RNA-seq,ROSMAP,AD.vs.MCI; bulk RNA-seq,SRP325058,AD.vs.control; scRNA-seq,SRP309935,Monocyte_2-	8
BP	GO:0043277	apoptotic cell clearance	bulk RNA-seq,Homo_723,AD.vs.MCI; bulk RNA-seq,Homo_723,MCI.vs.control; bulk RNA-seq,Homo_714,AD.vs.MCI; bulk RNA-seq,Homo_714,MCI.vs.control; bulk RNA-seq,Homo_633,AD.vs.control; bulk RNA-seq,Homo_633,AD.vs.MCI	6
BP	GO:0045176	apical protein localization	bulk RNA-seq,Homo_723,AD.vs.MCI; bulk RNA-seq,Homo_723,MCI.vs.control; bulk RNA-seq,Homo_714,AD.vs.MCI; bulk RNA-seq,Homo_714,MCI.vs.control; bulk RNA-seq,Homo_633,AD.vs.control	5
BP	GO:0001829	trophectodermal cell differentiation	bulk RNA-seq,Homo_723,AD.vs.MCI; bulk RNA-seq,Homo_723,MCI.vs.control; bulk RNA-seq,Homo_714,AD.vs.MCI; bulk RNA-seq,Homo_714,MCI.vs.control	4
BP	GO:0000281	mitotic cytokinesis	bulk RNA-seq,Homo_723,AD.vs.MCI; bulk RNA-seq,Homo_723,MCI.vs.control; bulk RNA-seq,Homo_714,MCI.vs.control; bulk RNA-seq,Homo_633,AD.vs.control; bulk RNA-seq,Homo_633,AD.vs.MCI	5

BP	GO:0045840	positive regulation of mitotic nuclear division	bulk RNA-seq,Homo_723,AD.vs.MCI; bulk RNA-seq,Homo_723,MCI.vs.control; bulk RNA-seq,Homo_714,AD.vs.MCI; bulk RNA-seq,Homo_714,MCI.vs.control; bulk RNA-seq,Homo_633,AD.vs.control; bulk RNA-seq,Homo_633,AD.vs.MCI	6
BP	GO:1900747	negative regulation of vascular endothelial growth factor signaling pathway	bulk RNA-seq,Homo_723,AD.vs.MCI; bulk RNA-seq,Homo_723,MCI.vs.control; bulk RNA-seq,Homo_714,AD.vs.MCI; bulk RNA-seq,Homo_714,MCI.vs.control	4
BP	GO:0034766	negative regulation of ion transmembrane transport	bulk RNA-seq,Homo_723,AD.vs.MCI; bulk RNA-seq,Homo_714,AD.vs.MCI; bulk RNA-seq,Homo_633,AD.vs.control; bulk RNA-seq,Homo_633,AD.vs.MCI	4
BP	GO:0006112	energy reserve metabolic process	bulk RNA-seq,Homo_723,AD.vs.MCI; bulk RNA-seq,Homo_723,MCI.vs.control; bulk RNA-seq,Homo_714,AD.vs.MCI; bulk RNA-seq,Homo_714,MCI.vs.control; bulk RNA-seq,Homo_633,AD.vs.control	5
BP	GO:0045217	cell-cell junction maintenance	bulk RNA-seq,Homo_723,AD.vs.MCI; bulk RNA-seq,Homo_723,MCI.vs.control; bulk RNA-seq,Homo_714,MCI.vs.control	3
BP	GO:1904994	regulation of leukocyte adhesion to vascular endothelial cell	bulk RNA-seq,Homo_723,AD.vs.MCI; bulk RNA-seq,Homo_723,MCI.vs.control; bulk RNA-seq,Homo_714,AD.vs.MCI; bulk RNA-seq,Homo_714,MCI.vs.control; bulk RNA-seq,Homo_633,AD.vs.control; bulk RNA-seq,Homo_633,AD.vs.MCI	6
BP	GO:0033629	negative regulation of cell adhesion mediated by integrin	bulk RNA-seq,Homo_723,AD.vs.MCI; bulk RNA-seq,Homo_723,MCI.vs.control; bulk RNA-seq,Homo_714,AD.vs.MCI; bulk RNA-seq,Homo_714,MCI.vs.control	4
BP	GO:0002688	regulation of leukocyte chemotaxis	bulk RNA-seq,Homo_723,AD.vs.MCI; bulk RNA-seq,Homo_723,MCI.vs.control; bulk RNA-seq,Homo_714,AD.vs.MCI; bulk RNA-seq,Homo_714,MCI.vs.control; bulk RNA-seq,Homo_633,AD.vs.control; bulk RNA-seq,Homo_633,AD.vs.MCI; scRNA-seq,SRP330776,Naive CD8+ T cell_2-	7
BP	GO:0035306	positive regulation of dephosphorylation	bulk RNA-seq,Homo_723,AD.vs.MCI; bulk RNA-seq,Homo_723,MCI.vs.control; bulk RNA-seq,Homo_714,AD.vs.MCI; bulk RNA-seq,Homo_714,MCI.vs.control; bulk RNA-seq,Homo_633,AD.vs.control; bulk RNA-seq,Homo_633,AD.vs.MCI	6
BP	GO:0051450	myoblast proliferation	bulk RNA-seq,Homo_723,AD.vs.MCI; bulk RNA-seq,Homo_714,AD.vs.MCI	2
BP	GO:1903792	negative regulation of anion transport	bulk RNA-seq,Homo_723,AD.vs.MCI; bulk RNA-seq,Homo_723,MCI.vs.control; bulk RNA-seq,Homo_714,AD.vs.control; bulk RNA-seq,Homo_714,AD.vs.MCI	4
BP	GO:0045745	positive regulation of G protein-coupled receptor signaling	bulk RNA-seq,Homo_723,AD.vs.MCI	1
MF	GO:0046790	virion binding	bulk RNA-seq,Homo_723,AD.vs.MCI; bulk RNA-seq,Homo_723,MCI.vs.control; bulk RNA-seq,Homo_714,AD.vs.MCI; bulk RNA-seq,Homo_714,MCI.vs.control; bulk RNA-seq,Homo_633,AD.vs.control; bulk RNA-seq,Homo_633,AD.vs.MCI	6
BP	GO:0010522	regulation of calcium ion transport into cytosol	bulk RNA-seq,Homo_723,AD.vs.MCI; bulk RNA-seq,Homo_723,MCI.vs.control; bulk RNA-seq,Homo_633,AD.vs.control	3
BP	GO:0061025	membrane fusion	bulk RNA-seq,Homo_723,AD.vs.MCI; bulk RNA-seq,Homo_723,MCI.vs.control; bulk RNA-seq,Homo_714,AD.vs.MCI; bulk RNA-seq,Homo_633,AD.vs.control; bulk RNA-seq,Homo_633,AD.vs.MCI	5
BP	GO:0120162	positive regulation of cold-induced thermogenesis	bulk RNA-seq,Homo_723,AD.vs.MCI; bulk RNA-seq,Homo_723,MCI.vs.control; bulk RNA-seq,Homo_714,AD.vs.MCI; bulk RNA-seq,Homo_714,MCI.vs.control; bulk RNA-seq,Homo_633,AD.vs.control; bulk RNA-seq,Homo_633,AD.vs.MCI	6
BP	GO:0090178	regulation of establishment of planar polarity involved in neural tube closure	bulk RNA-seq,Homo_723,AD.vs.MCI; bulk RNA-seq,Homo_723,MCI.vs.control	2
BP	GO:0042177	negative regulation of protein catabolic process	bulk RNA-seq,Homo_723,AD.vs.MCI; bulk RNA-seq,Homo_723,MCI.vs.control; bulk RNA-seq,Homo_714,AD.vs.MCI; bulk RNA-seq,Homo_714,MCI.vs.control; bulk RNA-seq,Homo_633,AD.vs.control; bulk RNA-seq,Homo_633,AD.vs.MCI	6
BP	GO:0000076	DNA replication checkpoint signaling	bulk RNA-seq,Homo_723,AD.vs.MCI; bulk RNA-seq,Homo_723,MCI.vs.control; bulk RNA-seq,Homo_714,MCI.vs.control; bulk RNA-seq,Homo_633,AD.vs.control; bulk RNA-seq,Homo_633,AD.vs.MCI; bulk RNA-seq,Homo_633,MCI.vs.control	6
BP	GO:0055089	fatty acid homeostasis	bulk RNA-seq,Homo_723,AD.vs.MCI; bulk RNA-seq,Homo_723,MCI.vs.control; bulk RNA-seq,Homo_714,MCI.vs.control	3
MF	GO:0004435	phosphatidylinositol phospholipase C activity	bulk RNA-seq,Homo_723,AD.vs.MCI; bulk RNA-seq,Homo_723,MCI.vs.control; bulk RNA-seq,Homo_714,AD.vs.MCI; bulk RNA-seq,Homo_714,MCI.vs.control	4
MF	GO:0035256	G protein-coupled glutamate receptor binding	bulk RNA-seq,Homo_723,AD.vs.MCI; bulk RNA-seq,Homo_723,MCI.vs.control; bulk RNA-seq,Homo_714,MCI.vs.control	3
BP	GO:0005979	regulation of glycogen biosynthetic process	bulk RNA-seq,Homo_723,AD.vs.MCI; bulk RNA-seq,Homo_723,MCI.vs.control; bulk RNA-seq,Homo_714,AD.vs.MCI	3
BP	GO:0010962	regulation of glucan biosynthetic process	bulk RNA-seq,Homo_723,AD.vs.MCI; bulk RNA-seq,Homo_723,MCI.vs.control; bulk RNA-seq,Homo_714,AD.vs.MCI	3
BP	GO:0010971	positive regulation of G2/M transition of mitotic cell cycle	bulk RNA-seq,Homo_723,AD.vs.MCI; bulk RNA-seq,Homo_723,MCI.vs.control; bulk RNA-seq,Homo_714,AD.vs.MCI; bulk RNA-seq,Homo_714,MCI.vs.control; bulk RNA-seq,Homo_633,AD.vs.control; bulk RNA-seq,Homo_633,AD.vs.MCI; bulk RNA-seq,Homo_633,MCI.vs.control	7
BP	GO:0046580	negative regulation of Ras protein signal transduction	bulk RNA-seq,Homo_723,AD.vs.MCI; bulk RNA-seq,Homo_723,MCI.vs.control; bulk RNA-seq,Homo_714,MCI.vs.control; bulk RNA-seq,Homo_633,AD.vs.control; bulk RNA-seq,Homo_633,AD.vs.MCI	5
MF	GO:0030955	potassium ion binding	bulk RNA-seq,Homo_723,AD.vs.MCI; bulk RNA-seq,Homo_723,MCI.vs.control; bulk RNA-seq,Homo_714,MCI.vs.control	3
BP	GO:0006688	glycosphingolipid biosynthetic process	bulk RNA-seq,Homo_723,AD.vs.MCI; bulk RNA-seq,Homo_723,MCI.vs.control; bulk RNA-seq,Homo_633,AD.vs.control	3
BP	GO:0070286	axonemal dynein complex assembly	bulk RNA-seq,Homo_723,AD.vs.MCI; bulk RNA-seq,Homo_723,MCI.vs.control; bulk RNA-seq,Homo_633,AD.vs.control	3
BP	GO:0030225	macrophage differentiation	bulk RNA-seq,Homo_723,AD.vs.MCI; bulk RNA-seq,Homo_723,MCI.vs.control; bulk RNA-seq,Homo_714,AD.vs.MCI; bulk RNA-seq,Homo_714,MCI.vs.control; bulk RNA-seq,Homo_633,AD.vs.control; bulk RNA-seq,Homo_633,AD.vs.MCI; scRNA-seq,SRP215507,CD8+ T cell_3-	7
MF	GO:0004712	protein serine/threonine/tyrosine kinase activity	bulk RNA-seq,Homo_723,AD.vs.MCI; bulk RNA-seq,Homo_723,MCI.vs.control; bulk RNA-seq,Homo_714,MCI.vs.control; bulk RNA-seq,Homo_633,AD.vs.control; scRNA-seq,SRP330776,Naive CD8+ T cell_1-AD.vs.control; scRNA-seq,SRP330776,Natural killer cell_1-AD.vs.control	6
BP	GO:0034392	negative regulation of smooth muscle cell apoptotic process	bulk RNA-seq,Homo_723,AD.vs.MCI; bulk RNA-seq,Homo_714,AD.vs.MCI	2
BP	GO:0045019	negative regulation of nitric oxide biosynthetic process	bulk RNA-seq,Homo_723,AD.vs.MCI; bulk RNA-seq,Homo_723,MCI.vs.control; bulk RNA-seq,Homo_714,AD.vs.MCI; bulk RNA-seq,Homo_714,MCI.vs.control; bulk RNA-seq,Homo_633,AD.vs.control	5
BP	GO:1904406	negative regulation of nitric oxide metabolic process	bulk RNA-seq,Homo_723,AD.vs.MCI; bulk RNA-seq,Homo_723,MCI.vs.control; bulk RNA-seq,Homo_714,AD.vs.MCI; bulk RNA-seq,Homo_714,MCI.vs.control; bulk RNA-seq,Homo_633,AD.vs.control	5
BP	GO:0051646	mitochondrion localization	bulk RNA-seq,Homo_723,AD.vs.MCI; bulk RNA-seq,Homo_723,MCI.vs.control; bulk RNA-seq,Homo_714,MCI.vs.control; bulk RNA-seq,Homo_633,AD.vs.control; bulk RNA-seq,Homo_633,AD.vs.MCI	5
BP	GO:0033173	calcineurin-NFAT signaling cascade	bulk RNA-seq,Homo_723,AD.vs.MCI; bulk RNA-seq,Homo_723,MCI.vs.control; bulk RNA-seq,Homo_714,MCI.vs.control; bulk RNA-	4
BP	GO:0035994	response to muscle stretch	bulk RNA-seq,Homo_723,AD.vs.MCI; bulk RNA-seq,Homo_723,MCI.vs.control; bulk RNA-seq,Homo_714,MCI.vs.control; bulk RNA-	4
BP	GO:0043129	surfactant homeostasis	bulk RNA-seq,Homo_723,AD.vs.MCI; bulk RNA-seq,Homo_723,MCI.vs.control; bulk RNA-seq,Homo_714,MCI.vs.control	3
BP	GO:0010457	centriole-centriole cohesion	bulk RNA-seq,Homo_723,AD.vs.MCI; bulk RNA-seq,Homo_723,MCI.vs.control; bulk RNA-seq,Homo_714,AD.vs.MCI; bulk RNA-seq,Homo_714,MCI.vs.control; bulk RNA-seq,Homo_633,AD.vs.control	5
BP	GO:0045776	negative regulation of blood pressure	bulk RNA-seq,Homo_723,AD.vs.MCI; bulk RNA-seq,Homo_723,MCI.vs.control; bulk RNA-seq,Homo_714,AD.vs.MCI; bulk RNA-seq,Homo_714,MCI.vs.control	4
BP	GO:1990823	response to leukemia inhibitory factor	bulk RNA-seq,Homo_723,AD.vs.MCI; bulk RNA-seq,Homo_723,MCI.vs.control; bulk RNA-seq,Homo_714,AD.vs.MCI; bulk RNA-seq,Homo_714,MCI.vs.control; bulk RNA-seq,Homo_633,AD.vs.control; bulk RNA-seq,Homo_633,AD.vs.MCI	6

BP	GO:1900182	positive regulation of protein localization to nucleus	bulk RNA-seq,Homo_723,AD.vs.MCI; bulk RNA-seq,Homo_723,MCI.vs.control; bulk RNA-seq,Homo_714,MCI.vs.control; bulk RNA-seq,Homo_633,AD.vs.control; bulk RNA-seq,Homo_633,AD.vs.MCI	5
CC	GO:1905370	serine-type endopeptidase complex	bulk RNA-seq,Homo_723,AD.vs.MCI; bulk RNA-seq,Homo_723,MCI.vs.control; bulk RNA-seq,Homo_714,AD.vs.MCI; bulk RNA-seq,Homo_714,MCI.vs.control	4
BP	GO:0099518	vesicle cytoskeletal trafficking	bulk RNA-seq,Homo_723,AD.vs.MCI; bulk RNA-seq,Homo_723,MCI.vs.control; bulk RNA-seq,Homo_714,AD.vs.control; bulk RNA-seq,Homo_714,MCI.vs.control; bulk RNA-seq,Homo_633,AD.vs.control; bulk RNA-seq,Homo_633,AD.vs.MCI; bulk RNA-seq,ROSMAP,AD.vs.control	7
BP	GO:0042759	long-chain fatty acid biosynthetic process	bulk RNA-seq,Homo_723,AD.vs.MCI; bulk RNA-seq,Homo_723,MCI.vs.control; bulk RNA-seq,Homo_714,AD.vs.MCI; bulk RNA-seq,Homo_714,MCI.vs.control	4
BP	GO:0033598	mammary gland epithelial cell proliferation	bulk RNA-seq,Homo_723,AD.vs.MCI; bulk RNA-seq,Homo_723,MCI.vs.control; bulk RNA-seq,Homo_714,AD.vs.MCI; bulk RNA-seq,Homo_714,MCI.vs.control; bulk RNA-seq,Homo_633,MCI.vs.control	5
BP	GO:0006883	cellular sodium ion homeostasis	bulk RNA-seq,Homo_723,AD.vs.MCI; bulk RNA-seq,Homo_723,MCI.vs.control; bulk RNA-seq,Homo_714,AD.vs.MCI	3
BP	GO:0002887	negative regulation of myeloid leukocyte mediated immunity	bulk RNA-seq,Homo_723,AD.vs.MCI; bulk RNA-seq,Homo_723,MCI.vs.control; bulk RNA-seq,Homo_714,AD.vs.MCI; bulk RNA-seq,Homo_714,MCI.vs.control; bulk RNA-seq,Homo_633,AD.vs.MCI	5
BP	GO:0051610	serotonin uptake	bulk RNA-seq,Homo_723,AD.vs.MCI	1
BP	GO:0060433	bronchus development	bulk RNA-seq,Homo_723,AD.vs.MCI; bulk RNA-seq,Homo_723,MCI.vs.control; bulk RNA-seq,Homo_714,AD.vs.MCI	3
BP	GO:2000058	regulation of ubiquitin-dependent protein catabolic process	bulk RNA-seq,Homo_723,AD.vs.MCI; bulk RNA-seq,Homo_723,MCI.vs.control; bulk RNA-seq,Homo_714,AD.vs.MCI; bulk RNA-seq,Homo_633,AD.vs.control; bulk RNA-seq,Homo_633,AD.vs.MCI	5
BP	GO:0070986	left/right axis specification	bulk RNA-seq,Homo_723,AD.vs.MCI; bulk RNA-seq,Homo_723,MCI.vs.control; bulk RNA-seq,Homo_714,AD.vs.MCI; bulk RNA-seq,Homo_714,MCI.vs.control	4
BP	GO:1900542	regulation of purine nucleotide metabolic process	bulk RNA-seq,Homo_723,AD.vs.MCI; bulk RNA-seq,Homo_723,MCI.vs.control; bulk RNA-seq,Homo_714,AD.vs.MCI; bulk RNA-seq,Homo_714,MCI.vs.control; bulk RNA-seq,Homo_633,AD.vs.control; bulk RNA-seq,Homo_633,AD.vs.MCI	6
BP	GO:0072593	reactive oxygen species metabolic process	bulk RNA-seq,Homo_723,AD.vs.MCI; bulk RNA-seq,Homo_723,MCI.vs.control; bulk RNA-seq,Homo_714,AD.vs.MCI; bulk RNA-seq,Homo_633,AD.vs.control; bulk RNA-seq,Homo_633,AD.vs.MCI	5
BP	GO:0007212	dopamine receptor signaling pathway	bulk RNA-seq,Homo_723,AD.vs.MCI; bulk RNA-seq,Homo_723,MCI.vs.control; bulk RNA-seq,Homo_714,MCI.vs.control; bulk RNA-seq,Homo_633,AD.vs.control; bulk RNA-seq,Homo_633,AD.vs.MCI	5
BP	GO:0060413	atrial septum morphogenesis	bulk RNA-seq,Homo_723,AD.vs.MCI; bulk RNA-seq,Homo_723,MCI.vs.control; bulk RNA-seq,Homo_714,AD.vs.MCI	3
BP	GO:0031642	negative regulation of myelination	bulk RNA-seq,Homo_723,AD.vs.MCI	1
CC	GO:0097470	ribbon synapse	bulk RNA-seq,Homo_723,AD.vs.MCI; bulk RNA-seq,Homo_723,MCI.vs.control	2
BP	GO:0061517	macrophage proliferation	bulk RNA-seq,Homo_723,AD.vs.MCI; bulk RNA-seq,Homo_723,MCI.vs.control; bulk RNA-seq,Homo_714,AD.vs.MCI; bulk RNA-seq,Homo_714,MCI.vs.control	4
BP	GO:0035728	response to hepatocyte growth factor	bulk RNA-seq,Homo_723,AD.vs.MCI; bulk RNA-seq,Homo_723,MCI.vs.control; bulk RNA-seq,Homo_714,AD.vs.MCI; bulk RNA-seq,Homo_714,MCI.vs.control; bulk RNA-seq,Homo_633,AD.vs.control; bulk RNA-seq,Homo_633,AD.vs.MCI	6
MF	GO:0001221	transcription coregulator binding	bulk RNA-seq,Homo_723,AD.vs.MCI; bulk RNA-seq,Homo_723,MCI.vs.control; bulk RNA-seq,Homo_714,AD.vs.MCI; bulk RNA-seq,Homo_714,MCI.vs.control; bulk RNA-seq,Homo_633,AD.vs.control; bulk RNA-seq,Homo_633,AD.vs.MCI	6
BP	GO:0006986	response to unfolded protein	bulk RNA-seq,Homo_723,AD.vs.MCI; bulk RNA-seq,Homo_723,MCI.vs.control; bulk RNA-seq,Homo_714,AD.vs.MCI; bulk RNA-seq,Homo_633,AD.vs.control; bulk RNA-seq,Homo_633,AD.vs.MCI	5
BP	GO:0035773	insulin secretion involved in cellular response to glucose stimulus	bulk RNA-seq,Homo_723,AD.vs.MCI; bulk RNA-seq,Homo_723,MCI.vs.control; bulk RNA-seq,Homo_714,MCI.vs.control; bulk RNA-seq,Homo_633,AD.vs.control; bulk RNA-seq,Homo_633,AD.vs.MCI	5
BP	GO:0030262	apoptotic nuclear changes	bulk RNA-seq,Homo_723,AD.vs.MCI; bulk RNA-seq,Homo_714,AD.vs.MCI	2
BP	GO:0090179	planar cell polarity pathway involved in neural tube closure	bulk RNA-seq,Homo_723,AD.vs.MCI; bulk RNA-seq,Homo_723,MCI.vs.control	2
BP	GO:0032410	negative regulation of transporter activity	bulk RNA-seq,Homo_723,AD.vs.MCI; bulk RNA-seq,Homo_714,AD.vs.control; bulk RNA-seq,Homo_714,AD.vs.MCI	3
BP	GO:0006695	cholesterol biosynthetic process	bulk RNA-seq,Homo_723,AD.vs.MCI; bulk RNA-seq,Homo_723,MCI.vs.control; bulk RNA-seq,Homo_714,AD.vs.MCI; bulk RNA-seq,Homo_714,MCI.vs.control; bulk RNA-seq,Homo_633,AD.vs.control; bulk RNA-seq,Homo_633,AD.vs.MCI	6
BP	GO:1902653	secondary alcohol biosynthetic process	bulk RNA-seq,Homo_723,AD.vs.MCI; bulk RNA-seq,Homo_723,MCI.vs.control; bulk RNA-seq,Homo_714,AD.vs.MCI; bulk RNA-seq,Homo_714,MCI.vs.control; bulk RNA-seq,Homo_633,AD.vs.control; bulk RNA-seq,Homo_633,AD.vs.MCI	6
BP	GO:0010453	regulation of cell fate commitment	bulk RNA-seq,Homo_723,AD.vs.MCI; bulk RNA-seq,Homo_723,MCI.vs.control; bulk RNA-seq,Homo_714,AD.vs.MCI; bulk RNA-seq,Homo_714,MCI.vs.control	4
CC	GO:0098691	dopaminergic synapse	bulk RNA-seq,Homo_723,AD.vs.MCI; bulk RNA-seq,Homo_723,MCI.vs.control	2
BP	GO:0090219	negative regulation of lipid kinase activity	bulk RNA-seq,Homo_723,AD.vs.MCI; bulk RNA-seq,Homo_723,MCI.vs.control; bulk RNA-seq,Homo_714,AD.vs.MCI; bulk RNA-seq,Homo_714,MCI.vs.control; bulk RNA-seq,Homo_633,AD.vs.control; bulk RNA-seq,Homo_633,AD.vs.MCI	6
CC	GO:0031301	integral component of organelle membrane	bulk RNA-seq,Homo_723,AD.vs.MCI; bulk RNA-seq,Homo_714,AD.vs.control; bulk RNA-seq,Homo_633,AD.vs.control; bulk RNA-seq,Homo_633,AD.vs.MCI	4
BP	GO:0072583	clathrin-dependent endocytosis	bulk RNA-seq,Homo_723,AD.vs.MCI; bulk RNA-seq,Homo_723,MCI.vs.control; bulk RNA-seq,Homo_714,MCI.vs.control; bulk RNA-seq,Homo_633,AD.vs.control; bulk RNA-seq,Homo_633,AD.vs.MCI	5
BP	GO:1904251	regulation of bile acid metabolic process	bulk RNA-seq,Homo_723,AD.vs.MCI; bulk RNA-seq,Homo_714,AD.vs.MCI	2
CC	GO:0005798	Golgi-associated vesicle	bulk RNA-seq,Homo_723,AD.vs.MCI; bulk RNA-seq,Homo_723,MCI.vs.control; bulk RNA-seq,Homo_714,AD.vs.MCI; bulk RNA-seq,Homo_714,MCI.vs.control; bulk RNA-seq,Homo_633,AD.vs.control; bulk RNA-seq,Homo_633,AD.vs.MCI	6
BP	GO:0044321	response to leptin	bulk RNA-seq,Homo_723,AD.vs.MCI; bulk RNA-seq,Homo_723,MCI.vs.control; bulk RNA-seq,Homo_714,MCI.vs.control	3
BP	GO:0003084	positive regulation of systemic arterial blood pressure	bulk RNA-seq,Homo_723,AD.vs.MCI; bulk RNA-seq,Homo_714,AD.vs.MCI	2
BP	GO:0061314	Notch signaling involved in heart development	bulk RNA-seq,Homo_723,AD.vs.MCI; bulk RNA-seq,Homo_723,MCI.vs.control; bulk RNA-seq,Homo_714,AD.vs.MCI; bulk RNA-seq,Homo_714,MCI.vs.control	4
BP	GO:0042531	positive regulation of tyrosine phosphorylation of STAT	bulk RNA-seq,Homo_723,AD.vs.MCI; bulk RNA-seq,Homo_723,MCI.vs.control; bulk RNA-seq,Homo_714,AD.vs.MCI	3
BP	GO:0035929	steroid hormone secretion	bulk RNA-seq,Homo_723,AD.vs.MCI; bulk RNA-seq,Homo_714,AD.vs.MCI; bulk RNA-seq,Homo_633,AD.vs.control; bulk RNA-seq,Homo_633,AD.vs.MCI	4
BP	GO:0009070	serine family amino acid biosynthetic process	bulk RNA-seq,Homo_723,AD.vs.MCI; bulk RNA-seq,Homo_633,AD.vs.control; bulk RNA-seq,Homo_633,AD.vs.MCI	3
MF	GO:0016614	oxidoreductase activity, acting on CH-OH group of donors	bulk RNA-seq,Homo_723,AD.vs.MCI; bulk RNA-seq,Homo_723,MCI.vs.control; bulk RNA-seq,Homo_714,AD.vs.MCI; bulk RNA-seq,Homo_633,AD.vs.control; bulk RNA-seq,Homo_633,AD.vs.MCI	5
BP	GO:0042474	middle ear morphogenesis	bulk RNA-seq,Homo_723,AD.vs.MCI; bulk RNA-seq,Homo_723,MCI.vs.control; bulk RNA-seq,Homo_714,AD.vs.MCI	3

BP	GO:0006921	cellular component disassembly involved in execution phase of apoptosis	bulk RNA-seq,Homo_723,AD.vs.MCI; bulk RNA-seq,Homo_714,AD.vs.MCI	2
BP	GO:2000379	positive regulation of reactive oxygen species metabolic process	bulk RNA-seq,Homo_723,AD.vs.MCI; bulk RNA-seq,Homo_723,MCI.vs.control; bulk RNA-seq,Homo_714,AD.vs.MCI; bulk RNA-seq,Homo_714,MCI.vs.control; bulk RNA-seq,Homo_633,AD.vs.control; bulk RNA-seq,Homo_633,AD.vs.MCI	6
BP	GO:0030947	regulation of vascular endothelial growth factor receptor signaling pathway	bulk RNA-seq,Homo_723,AD.vs.MCI; bulk RNA-seq,Homo_723,MCI.vs.control; bulk RNA-seq,Homo_714,AD.vs.MCI; bulk RNA-seq,Homo_714,MCI.vs.control; bulk RNA-seq,Homo_633,AD.vs.control; bulk RNA-seq,Homo_633,AD.vs.MCI; bulk RNA-seq,Homo_633,MCI.vs.control	7
MF	GO:0051219	phosphoprotein binding	bulk RNA-seq,Homo_723,AD.vs.MCI; bulk RNA-seq,Homo_723,MCI.vs.control; bulk RNA-seq,Homo_714,MCI.vs.control; bulk RNA-seq,Homo_633,AD.vs.control; bulk RNA-seq,Homo_633,AD.vs.MCI	5
BP	GO:0051446	positive regulation of meiotic cell cycle	bulk RNA-seq,Homo_723,AD.vs.MCI; bulk RNA-seq,Homo_714,AD.vs.MCI	2
BP	GO:0034383	low-density lipoprotein particle clearance	bulk RNA-seq,Homo_723,AD.vs.MCI; bulk RNA-seq,Homo_714,AD.vs.MCI; bulk RNA-seq,Homo_633,AD.vs.MCI	3
BP	GO:0000737	DNA catabolic process, endonucleolytic	bulk RNA-seq,Homo_723,AD.vs.MCI; bulk RNA-seq,Homo_714,AD.vs.MCI	2
BP	GO:0032096	negative regulation of response to food	bulk RNA-seq,Homo_723,AD.vs.MCI; bulk RNA-seq,Homo_723,MCI.vs.control; bulk RNA-seq,Homo_633,AD.vs.control; bulk RNA-seq,Homo_633,AD.vs.MCI	4
BP	GO:0032099	negative regulation of appetite	bulk RNA-seq,Homo_723,AD.vs.MCI; bulk RNA-seq,Homo_723,MCI.vs.control; bulk RNA-seq,Homo_633,AD.vs.control; bulk RNA-seq,Homo_633,AD.vs.MCI	4
BP	GO:0032105	negative regulation of response to extracellular stimulus	bulk RNA-seq,Homo_723,AD.vs.MCI; bulk RNA-seq,Homo_723,MCI.vs.control; bulk RNA-seq,Homo_633,AD.vs.control; bulk RNA-seq,Homo_633,AD.vs.MCI	4
BP	GO:0032108	negative regulation of response to nutrient levels	bulk RNA-seq,Homo_723,AD.vs.MCI; bulk RNA-seq,Homo_723,MCI.vs.control; bulk RNA-seq,Homo_633,AD.vs.control; bulk RNA-seq,Homo_633,AD.vs.MCI	4
BP	GO:0010765	positive regulation of sodium ion transport	bulk RNA-seq,Homo_723,AD.vs.MCI; bulk RNA-seq,Homo_723,MCI.vs.control; bulk RNA-seq,Homo_714,MCI.vs.control	3
BP	GO:0032372	negative regulation of sterol transport	bulk RNA-seq,Homo_723,AD.vs.MCI; bulk RNA-seq,Homo_723,MCI.vs.control; bulk RNA-seq,Homo_714,AD.vs.MCI; bulk RNA-seq,Homo_714,MCI.vs.control	4
BP	GO:0032375	negative regulation of cholesterol transport	bulk RNA-seq,Homo_723,AD.vs.MCI; bulk RNA-seq,Homo_723,MCI.vs.control; bulk RNA-seq,Homo_714,AD.vs.MCI; bulk RNA-seq,Homo_714,MCI.vs.control	4
BP	GO:1901385	regulation of voltage-gated calcium channel activity	bulk RNA-seq,Homo_723,AD.vs.MCI	1
BP	GO:0140353	lipid export from cell	bulk RNA-seq,Homo_723,AD.vs.MCI; bulk RNA-seq,Homo_714,AD.vs.MCI; bulk RNA-seq,Homo_633,AD.vs.control; bulk RNA-seq,Homo_633,AD.vs.MCI	4
MF	GO:0034185	apolipoprotein binding	bulk RNA-seq,Homo_723,AD.vs.MCI; bulk RNA-seq,Homo_723,MCI.vs.control; bulk RNA-seq,Homo_714,MCI.vs.control	3
BP	GO:0071389	cellular response to mineralocorticoid stimulus	bulk RNA-seq,Homo_723,AD.vs.MCI; bulk RNA-seq,Homo_723,MCI.vs.control; bulk RNA-seq,Homo_714,AD.vs.MCI; bulk RNA-seq,Homo_714,MCI.vs.control	4
BP	GO:0014824	artery smooth muscle contraction	bulk RNA-seq,Homo_723,AD.vs.MCI; bulk RNA-seq,Homo_723,MCI.vs.control	2
BP	GO:0007440	foregut morphogenesis	bulk RNA-seq,Homo_723,AD.vs.MCI; bulk RNA-seq,Homo_723,MCI.vs.control	2
BP	GO:0030502	negative regulation of bone mineralization	bulk RNA-seq,Homo_723,AD.vs.MCI; bulk RNA-seq,Homo_723,MCI.vs.control	2
BP	GO:0019627	urea metabolic process	bulk RNA-seq,Homo_723,AD.vs.MCI; bulk RNA-seq,Homo_723,MCI.vs.control; bulk RNA-seq,Homo_714,AD.vs.MCI; bulk RNA-seq,Homo_714,MCI.vs.control	4
BP	GO:0071941	nitrogen cycle metabolic process	bulk RNA-seq,Homo_723,AD.vs.MCI; bulk RNA-seq,Homo_723,MCI.vs.control; bulk RNA-seq,Homo_714,AD.vs.MCI; bulk RNA-seq,Homo_714,MCI.vs.control	4
BP	GO:0002269	leukocyte activation involved in inflammatory response	bulk RNA-seq,Homo_723,AD.vs.MCI; bulk RNA-seq,Homo_723,MCI.vs.control; bulk RNA-seq,Homo_714,AD.vs.MCI; bulk RNA-seq,Homo_714,MCI.vs.control; bulk RNA-seq,Homo_633,AD.vs.control; bulk RNA-seq,Homo_633,AD.vs.MCI	6
BP	GO:0030011	maintenance of cell polarity	bulk RNA-seq,Homo_723,AD.vs.MCI; bulk RNA-seq,Homo_723,MCI.vs.control; bulk RNA-seq,Homo_633,AD.vs.control; bulk RNA-seq,Homo_633,AD.vs.MCI	4
BP	GO:0038092	nodal signaling pathway	bulk RNA-seq,Homo_723,AD.vs.MCI	1
BP	GO:0030261	chromosome condensation	bulk RNA-seq,Homo_723,AD.vs.MCI; bulk RNA-seq,Homo_633,AD.vs.control; bulk RNA-seq,Homo_633,AD.vs.MCI	3
BP	GO:0071459	protein localization to chromosome, centromeric region	bulk RNA-seq,Homo_723,AD.vs.MCI; bulk RNA-seq,Homo_723,MCI.vs.control; bulk RNA-seq,Homo_714,AD.vs.MCI; bulk RNA-seq,Homo_714,MCI.vs.control; bulk RNA-seq,Homo_633,AD.vs.control; bulk RNA-seq,Homo_633,AD.vs.MCI	6
BP	GO:0060251	regulation of glial cell proliferation	bulk RNA-seq,Homo_723,AD.vs.MCI; bulk RNA-seq,Homo_714,AD.vs.MCI; bulk RNA-seq,Homo_633,AD.vs.control	3
BP	GO:0070857	regulation of bile acid biosynthetic process	bulk RNA-seq,Homo_723,AD.vs.MCI; bulk RNA-seq,Homo_723,MCI.vs.control; bulk RNA-seq,Homo_714,AD.vs.MCI	3
BP	GO:0044351	macropinocytosis	bulk RNA-seq,Homo_723,AD.vs.MCI; bulk RNA-seq,Homo_714,AD.vs.MCI; bulk RNA-seq,Homo_633,AD.vs.control	3
BP	GO:0033157	regulation of intracellular protein transport	bulk RNA-seq,Homo_723,AD.vs.MCI; bulk RNA-seq,Homo_723,MCI.vs.control; bulk RNA-seq,Homo_714,AD.vs.MCI; bulk RNA-seq,Homo_633,AD.vs.control; bulk RNA-seq,Homo_633,AD.vs.MCI	5
MF	GO:0019003	GDP binding	bulk RNA-seq,Homo_723,AD.vs.MCI; bulk RNA-seq,Homo_723,MCI.vs.control; bulk RNA-seq,Homo_714,MCI.vs.control; bulk RNA-seq,Homo_633,AD.vs.control; bulk RNA-seq,Homo_633,AD.vs.MCI	5
BP	GO:0002082	regulation of oxidative phosphorylation	bulk RNA-seq,Homo_723,AD.vs.MCI; bulk RNA-seq,Homo_723,MCI.vs.control; bulk RNA-seq,Homo_714,MCI.vs.control; bulk RNA-seq,Homo_633,AD.vs.control; bulk RNA-seq,Homo_633,AD.vs.MCI	5
MF	GO:1990825	sequence-specific mRNA binding	bulk RNA-seq,Homo_723,AD.vs.MCI; bulk RNA-seq,Homo_723,MCI.vs.control; bulk RNA-seq,Homo_714,MCI.vs.control; bulk RNA-	4
BP	GO:0048012	hepatocyte growth factor receptor signaling pathway	bulk RNA-seq,Homo_723,AD.vs.MCI; bulk RNA-seq,Homo_723,MCI.vs.control; bulk RNA-seq,Homo_714,AD.vs.MCI; bulk RNA-seq,Homo_714,MCI.vs.control	4
BP	GO:1900452	regulation of long-term synaptic depression	bulk RNA-seq,Homo_723,AD.vs.MCI; bulk RNA-seq,Homo_723,MCI.vs.control; bulk RNA-seq,Homo_714,MCI.vs.control	3
BP	GO:1902042	negative regulation of extrinsic apoptotic signaling pathway via death domain receptors	bulk RNA-seq,Homo_723,AD.vs.MCI; bulk RNA-seq,Homo_723,MCI.vs.control; bulk RNA-seq,Homo_714,AD.vs.MCI; bulk RNA-seq,Homo_714,MCI.vs.control	4
BP	GO:0070661	leukocyte proliferation	bulk RNA-seq,Homo_723,AD.vs.MCI; bulk RNA-seq,Homo_714,AD.vs.MCI; bulk RNA-seq,Homo_633,AD.vs.control; bulk RNA-seq,Homo_633,AD.vs.MCI	4
BP	GO:1901992	positive regulation of mitotic cell cycle phase transition	bulk RNA-seq,Homo_723,AD.vs.MCI; bulk RNA-seq,Homo_723,MCI.vs.control; bulk RNA-seq,Homo_714,AD.vs.MCI; bulk RNA-seq,Homo_714,MCI.vs.control; bulk RNA-seq,Homo_633,AD.vs.control; bulk RNA-seq,Homo_633,AD.vs.MCI	6
BP	GO:0003183	mitral valve morphogenesis	bulk RNA-seq,Homo_723,AD.vs.MCI; bulk RNA-seq,Homo_723,MCI.vs.control; bulk RNA-seq,Homo_714,MCI.vs.control	3
BP	GO:0048016	inositol phosphate-mediated signaling	bulk RNA-seq,Homo_723,AD.vs.MCI; bulk RNA-seq,Homo_723,MCI.vs.control; bulk RNA-seq,Homo_714,AD.vs.MCI; bulk RNA-seq,Homo_714,MCI.vs.control; bulk RNA-seq,Homo_633,AD.vs.control	5
BP	GO:0097499	protein localization to non-motile cilium	bulk RNA-seq,Homo_723,AD.vs.MCI; bulk RNA-seq,Homo_723,MCI.vs.control	2
CC	GO:1904090	peptidase inhibitor complex	bulk RNA-seq,Homo_723,AD.vs.MCI; bulk RNA-seq,Homo_714,AD.vs.MCI	2
BP	GO:0033033	negative regulation of myeloid cell apoptotic process	bulk RNA-seq,Homo_723,AD.vs.MCI; bulk RNA-seq,Homo_723,MCI.vs.control; bulk RNA-seq,Homo_714,AD.vs.MCI; bulk RNA-seq,Homo_714,MCI.vs.control; bulk RNA-seq,Homo_633,AD.vs.control; bulk RNA-seq,Homo_633,AD.vs.MCI	6
BP	GO:0035372	protein localization to microtubule	bulk RNA-seq,Homo_723,AD.vs.MCI; bulk RNA-seq,Homo_723,MCI.vs.control; bulk RNA-seq,Homo_714,AD.vs.MCI; bulk RNA-seq,Homo_714,MCI.vs.control; bulk RNA-seq,Homo_633,AD.vs.control; bulk RNA-seq,Homo_633,AD.vs.MCI	6

BP	GO:0070301	cellular response to hydrogen peroxide	bulk RNA-seq,Homo_723,AD.vs.MCI; bulk RNA-seq,Homo_723,MCI.vs.control; bulk RNA-seq,Homo_714,AD.vs.MCI; bulk RNA-seq,Homo_714,MCI.vs.control; bulk RNA-seq,Homo_633,AD.vs.control; bulk RNA-seq,Homo_633,AD.vs.MCI	6
BP	GO:0010828	positive regulation of glucose transmembrane transport	bulk RNA-seq,Homo_723,AD.vs.MCI; bulk RNA-seq,Homo_723,MCI.vs.control; bulk RNA-seq,Homo_714,AD.vs.MCI; bulk RNA-seq,Homo_633,AD.vs.control	4
MF	GO:0004629	phospholipase C activity	bulk RNA-seq,Homo_723,AD.vs.MCI; bulk RNA-seq,Homo_723,MCI.vs.control; bulk RNA-seq,Homo_714,AD.vs.MCI; bulk RNA-seq,Homo_714,MCI.vs.control	4
BP	GO:0045428	regulation of nitric oxide biosynthetic process	bulk RNA-seq,Homo_723,AD.vs.MCI; bulk RNA-seq,Homo_723,MCI.vs.control; bulk RNA-seq,Homo_714,AD.vs.MCI; bulk RNA-seq,Homo_714,MCI.vs.control; bulk RNA-seq,Homo_633,AD.vs.control; bulk RNA-seq,Homo_633,AD.vs.MCI	6
BP	GO:2000629	negative regulation of miRNA metabolic process	bulk RNA-seq,Homo_723,AD.vs.MCI; bulk RNA-seq,Homo_723,MCI.vs.control; bulk RNA-seq,Homo_714,MCI.vs.control; bulk RNA-	4
BP	GO:1900017	positive regulation of cytokine production involved in inflammatory response	bulk RNA-seq,Homo_723,AD.vs.MCI; bulk RNA-seq,Homo_714,AD.vs.MCI	2
MF	GO:0004620	phospholipase activity	bulk RNA-seq,Homo_723,AD.vs.MCI; bulk RNA-seq,Homo_723,MCI.vs.control; bulk RNA-seq,Homo_714,AD.vs.MCI; bulk RNA-seq,Homo_714,MCI.vs.control; bulk RNA-seq,Homo_633,AD.vs.control; bulk RNA-seq,Homo_633,AD.vs.MCI	6
BP	GO:0034650	cortisol metabolic process	bulk RNA-seq,Homo_723,AD.vs.MCI; bulk RNA-seq,Homo_723,MCI.vs.control	2
BP	GO:1902115	regulation of organelle assembly	bulk RNA-seq,Homo_723,AD.vs.MCI; bulk RNA-seq,Homo_723,MCI.vs.control; bulk RNA-seq,Homo_633,AD.vs.control; bulk RNA-seq,Homo_633,AD.vs.MCI	4
BP	GO:0006305	DNA alkylation	bulk RNA-seq,Homo_723,AD.vs.MCI; bulk RNA-seq,Homo_723,MCI.vs.control; bulk RNA-seq,Homo_714,AD.vs.MCI; bulk RNA-seq,Homo_714,MCI.vs.control; bulk RNA-seq,Homo_633,AD.vs.control; bulk RNA-seq,Homo_633,AD.vs.MCI	6
BP	GO:0006306	DNA methylation	bulk RNA-seq,Homo_723,AD.vs.MCI; bulk RNA-seq,Homo_723,MCI.vs.control; bulk RNA-seq,Homo_714,AD.vs.MCI; bulk RNA-seq,Homo_714,MCI.vs.control; bulk RNA-seq,Homo_633,AD.vs.control; bulk RNA-seq,Homo_633,AD.vs.MCI	6
BP	GO:0043304	regulation of mast cell degranulation	bulk RNA-seq,Homo_723,AD.vs.MCI; bulk RNA-seq,Homo_723,MCI.vs.control; bulk RNA-seq,Homo_714,AD.vs.MCI; bulk RNA-seq,Homo_714,MCI.vs.control; bulk RNA-seq,Homo_633,AD.vs.control; bulk RNA-seq,Homo_633,AD.vs.MCI	6
BP	GO:0002275	myeloid cell activation involved in immune response	bulk RNA-seq,Homo_723,AD.vs.MCI; bulk RNA-seq,Homo_723,MCI.vs.control; bulk RNA-seq,Homo_714,AD.vs.MCI; bulk RNA-seq,Homo_714,MCI.vs.control; bulk RNA-seq,Homo_633,AD.vs.control; bulk RNA-seq,Homo_633,AD.vs.MCI; scRNA-seq,SRP215507,CD8+ T cell_1-	7
BP	GO:0042770	signal transduction in response to DNA damage	bulk RNA-seq,Homo_723,AD.vs.MCI; bulk RNA-seq,Homo_723,MCI.vs.control; bulk RNA-seq,Homo_714,AD.vs.control; bulk RNA-seq,Homo_714,AD.vs.MCI; bulk RNA-seq,Homo_633,AD.vs.control; bulk RNA-seq,Homo_633,AD.vs.MCI	6
BP	GO:0044771	meiotic cell cycle phase transition	bulk RNA-seq,Homo_723,AD.vs.MCI; bulk RNA-seq,Homo_714,AD.vs.MCI	2
BP	GO:2001241	positive regulation of extrinsic apoptotic signaling pathway in absence of ligand	bulk RNA-seq,Homo_723,AD.vs.MCI; bulk RNA-seq,Homo_714,AD.vs.MCI; bulk RNA-seq,Homo_714,MCI.vs.control	3
BP	GO:0042634	regulation of hair cycle	bulk RNA-seq,Homo_723,AD.vs.MCI	1
BP	GO:0034620	cellular response to unfolded protein	bulk RNA-seq,Homo_723,AD.vs.MCI; bulk RNA-seq,Homo_723,MCI.vs.control; bulk RNA-seq,Homo_714,AD.vs.MCI; bulk RNA-seq,Homo_714,MCI.vs.control; bulk RNA-seq,Homo_633,AD.vs.control; bulk RNA-seq,Homo_633,AD.vs.MCI	6
BP	GO:0003159	morphogenesis of an endothelium	bulk RNA-seq,Homo_723,AD.vs.MCI; bulk RNA-seq,Homo_723,MCI.vs.control; bulk RNA-seq,Homo_714,AD.vs.MCI; bulk RNA-seq,Homo_714,MCI.vs.control	4
BP	GO:0061154	endothelial tube morphogenesis	bulk RNA-seq,Homo_723,AD.vs.MCI; bulk RNA-seq,Homo_723,MCI.vs.control; bulk RNA-seq,Homo_714,AD.vs.MCI; bulk RNA-seq,Homo_714,MCI.vs.control	4
BP	GO:0034110	regulation of homotypic cell-cell adhesion	bulk RNA-seq,Homo_723,AD.vs.MCI; bulk RNA-seq,Homo_723,MCI.vs.control; bulk RNA-seq,Homo_714,AD.vs.control; bulk RNA-seq,Homo_714,AD.vs.MCI; bulk RNA-seq,Homo_714,MCI.vs.control; bulk RNA-seq,Homo_633,AD.vs.control	6
BP	GO:0034508	centromere complex assembly	bulk RNA-seq,Homo_723,AD.vs.MCI; bulk RNA-seq,Homo_723,MCI.vs.control; bulk RNA-seq,Homo_714,MCI.vs.control; bulk RNA-seq,Homo_633,AD.vs.control; bulk RNA-seq,Homo_633,AD.vs.MCI; bulk RNA-seq,Homo_633,MCI.vs.control	6
BP	GO:0090398	cellular senescence	bulk RNA-seq,Homo_723,AD.vs.MCI; bulk RNA-seq,Homo_723,MCI.vs.control; bulk RNA-seq,Homo_714,AD.vs.MCI; bulk RNA-seq,Homo_714,MCI.vs.control; bulk RNA-seq,Homo_633,AD.vs.control; bulk RNA-seq,Homo_633,AD.vs.MCI; scRNA-seq,SRP330776,Memory T cell_2-AD.vs.control; scRNA-seq,SRP330776,Naive CD8+ T cell_2-AD.vs.control; scRNA-seq,SRP330776,Naive CD8+ T cell_3-AD.vs.control	9
BP	GO:1902895	positive regulation of miRNA transcription	bulk RNA-seq,Homo_723,AD.vs.MCI; bulk RNA-seq,Homo_723,MCI.vs.control; bulk RNA-seq,Homo_714,AD.vs.MCI; bulk RNA-seq,Homo_714,MCI.vs.control; bulk RNA-seq,Homo_633,AD.vs.control	5
CC	GO:1990909	Wnt signalosome	bulk RNA-seq,Homo_723,AD.vs.MCI; bulk RNA-seq,Homo_723,MCI.vs.control; bulk RNA-seq,Homo_714,MCI.vs.control	3
BP	GO:0036315	cellular response to sterol	bulk RNA-seq,Homo_723,AD.vs.MCI; bulk RNA-seq,Homo_714,AD.vs.MCI; bulk RNA-seq,Homo_633,AD.vs.control	3
CC	GO:0000794	condensed nuclear chromosome	bulk RNA-seq,Homo_723,AD.vs.MCI; bulk RNA-seq,Homo_723,MCI.vs.control; bulk RNA-seq,Homo_714,AD.vs.MCI; bulk RNA-seq,Homo_714,MCI.vs.control; bulk RNA-seq,Homo_633,AD.vs.control; bulk RNA-seq,Homo_633,AD.vs.MCI	6
BP	GO:0060841	venous blood vessel development	bulk RNA-seq,Homo_723,AD.vs.MCI; bulk RNA-seq,Homo_723,MCI.vs.control; bulk RNA-seq,Homo_714,AD.vs.MCI; bulk RNA-seq,Homo_714,MCI.vs.control; bulk RNA-seq,Homo_633,AD.vs.control	5
MF	GO:0004303	estradiol 17-beta-dehydrogenase activity	bulk RNA-seq,Homo_723,AD.vs.MCI; bulk RNA-seq,Homo_723,MCI.vs.control; bulk RNA-seq,Homo_714,AD.vs.MCI	3
BP	GO:0098869	cellular oxidant detoxification	bulk RNA-seq,Homo_723,AD.vs.MCI; bulk RNA-seq,Homo_723,MCI.vs.control; bulk RNA-seq,Homo_714,AD.vs.MCI; bulk RNA-seq,Homo_714,MCI.vs.control; bulk RNA-seq,Homo_633,AD.vs.control; bulk RNA-seq,Homo_633,AD.vs.MCI; bulk RNA-seq,ROSMAP,MCI.vs.control	7
MF	GO:0140326	ATPase-coupled intramembrane lipid transporter activity	bulk RNA-seq,Homo_723,AD.vs.MCI; bulk RNA-seq,Homo_723,MCI.vs.control; bulk RNA-seq,Homo_714,AD.vs.MCI; bulk RNA-seq,Homo_714,MCI.vs.control; bulk RNA-seq,Homo_633,AD.vs.control; bulk RNA-seq,Homo_633,AD.vs.MCI	6
BP	GO:0072676	lymphocyte migration	bulk RNA-seq,Homo_723,AD.vs.MCI; bulk RNA-seq,Homo_723,MCI.vs.control; bulk RNA-seq,Homo_714,AD.vs.MCI; bulk RNA-seq,Homo_714,MCI.vs.control; bulk RNA-seq,Homo_633,AD.vs.control	5
BP	GO:0003081	regulation of systemic arterial blood pressure by renin-	bulk RNA-seq,Homo_723,AD.vs.MCI; bulk RNA-seq,Homo_723,MCI.vs.control; bulk RNA-seq,Homo_714,AD.vs.MCI; bulk RNA-seq,Homo_714,MCI.vs.control	4
BP	GO:0045540	regulation of cholesterol biosynthetic process	bulk RNA-seq,Homo_723,AD.vs.MCI; bulk RNA-seq,Homo_723,MCI.vs.control; bulk RNA-seq,Homo_714,AD.vs.MCI	3
BP	GO:0106118	regulation of sterol biosynthetic process	bulk RNA-seq,Homo_723,AD.vs.MCI; bulk RNA-seq,Homo_723,MCI.vs.control; bulk RNA-seq,Homo_714,AD.vs.MCI	3
MF	GO:0008093	cytoskeletal anchor activity	bulk RNA-seq,Homo_723,AD.vs.MCI; bulk RNA-seq,Homo_723,MCI.vs.control; bulk RNA-seq,Homo_714,MCI.vs.control	3
BP	GO:1901988	negative regulation of cell cycle phase transition	bulk RNA-seq,Homo_723,AD.vs.MCI; bulk RNA-seq,Homo_723,MCI.vs.control; bulk RNA-seq,Homo_714,AD.vs.MCI; bulk RNA-seq,Homo_633,AD.vs.control; bulk RNA-seq,Homo_633,AD.vs.MCI	5
BP	GO:0031054	pre-miRNA processing	bulk RNA-seq,Homo_723,AD.vs.MCI; bulk RNA-seq,Homo_633,AD.vs.control; bulk RNA-seq,Homo_633,AD.vs.MCI	3
CC	GO:0000792	heterochromatin	bulk RNA-seq,Homo_723,AD.vs.MCI; bulk RNA-seq,Homo_723,MCI.vs.control; bulk RNA-seq,Homo_714,MCI.vs.control; bulk RNA-seq,Homo_633,AD.vs.control; bulk RNA-seq,Homo_633,AD.vs.MCI	5

BP	GO:0008299	isoprenoid biosynthetic process	bulk RNA-seq,Homo_723,AD.vs.MCI; bulk RNA-seq,Homo_723,MCI.vs.control; bulk RNA-seq,Homo_714,MCI.vs.control	3
BP	GO:0086013	membrane repolarization during cardiac muscle cell action	bulk RNA-seq,Homo_723,AD.vs.MCI; bulk RNA-seq,Homo_714,AD.vs.MCI	2
BP	GO:0035331	negative regulation of hippo signaling	bulk RNA-seq,Homo_723,AD.vs.MCI; bulk RNA-seq,Homo_633,AD.vs.control	2
BP	GO:0051546	keratinocyte migration	bulk RNA-seq,Homo_723,AD.vs.MCI; bulk RNA-seq,Homo_723,MCI.vs.control; bulk RNA-seq,Homo_714,AD.vs.MCI; bulk RNA-seq,Homo_714,MCI.vs.control	4
BP	GO:0006707	cholesterol catabolic process	bulk RNA-seq,Homo_723,AD.vs.MCI; bulk RNA-seq,Homo_723,MCI.vs.control	2
BP	GO:0016127	sterol catabolic process	bulk RNA-seq,Homo_723,AD.vs.MCI; bulk RNA-seq,Homo_723,MCI.vs.control	2
BP	GO:0051917	regulation of fibrinolysis	bulk RNA-seq,Homo_723,AD.vs.MCI; bulk RNA-seq,Homo_723,MCI.vs.control; bulk RNA-seq,Homo_714,AD.vs.MCI; bulk RNA-seq,Homo_714,MCI.vs.control	4
BP	GO:0051044	positive regulation of membrane protein ectodomain proteolysis	bulk RNA-seq,Homo_723,AD.vs.MCI; bulk RNA-seq,Homo_723,MCI.vs.control; bulk RNA-seq,Homo_714,AD.vs.MCI; bulk RNA-seq,Homo_714,MCI.vs.control; bulk RNA-seq,Homo_633,AD.vs.control; bulk RNA-seq,Homo_633,AD.vs.MCI	6
MF	GO:0004089	carbonate dehydratase activity	bulk RNA-seq,Homo_723,AD.vs.MCI; bulk RNA-seq,Homo_723,MCI.vs.control; bulk RNA-seq,Homo_714,MCI.vs.control	3
BP	GO:1990830	cellular response to leukemia inhibitory factor	bulk RNA-seq,Homo_723,AD.vs.MCI; bulk RNA-seq,Homo_723,MCI.vs.control; bulk RNA-seq,Homo_714,AD.vs.MCI; bulk RNA-seq,Homo_714,MCI.vs.control; bulk RNA-seq,Homo_633,AD.vs.control; bulk RNA-seq,Homo_633,AD.vs.MCI	6
BP	GO:0099509	regulation of presynaptic cytosolic calcium ion concentration	bulk RNA-seq,Homo_723,AD.vs.MCI	1
CC	GO:0098533	ATPase dependent transmembrane transport complex	bulk RNA-seq,Homo_723,AD.vs.MCI	1
BP	GO:0048147	negative regulation of fibroblast proliferation	bulk RNA-seq,Homo_723,AD.vs.MCI; bulk RNA-seq,Homo_723,MCI.vs.control; bulk RNA-seq,Homo_714,MCI.vs.control; bulk RNA-seq,Homo_633,AD.vs.control; bulk RNA-seq,Homo_633,AD.vs.MCI	5
BP	GO:0032353	negative regulation of hormone biosynthetic process	bulk RNA-seq,Homo_723,AD.vs.MCI; bulk RNA-seq,Homo_723,MCI.vs.control; bulk RNA-seq,Homo_714,MCI.vs.control	3
BP	GO:2000369	regulation of clathrin-dependent endocytosis	bulk RNA-seq,Homo_723,AD.vs.MCI; bulk RNA-seq,Homo_723,MCI.vs.control; bulk RNA-seq,Homo_633,AD.vs.control; bulk RNA-seq,Homo_633,AD.vs.MCI	4
BP	GO:0006637	acyl-CoA metabolic process	bulk RNA-seq,Homo_723,AD.vs.MCI; bulk RNA-seq,Homo_723,MCI.vs.control; bulk RNA-seq,Homo_714,AD.vs.MCI; bulk RNA-seq,Homo_714,MCI.vs.control; bulk RNA-seq,Homo_633,AD.vs.control; bulk RNA-seq,Homo_633,AD.vs.MCI	6
BP	GO:0035383	thioester metabolic process	bulk RNA-seq,Homo_723,AD.vs.MCI; bulk RNA-seq,Homo_723,MCI.vs.control; bulk RNA-seq,Homo_714,AD.vs.MCI; bulk RNA-seq,Homo_714,MCI.vs.control; bulk RNA-seq,Homo_633,AD.vs.control; bulk RNA-seq,Homo_633,AD.vs.MCI	6
BP	GO:0006140	regulation of nucleotide metabolic process	bulk RNA-seq,Homo_723,AD.vs.MCI; bulk RNA-seq,Homo_723,MCI.vs.control; bulk RNA-seq,Homo_714,AD.vs.MCI; bulk RNA-seq,Homo_714,MCI.vs.control; bulk RNA-seq,Homo_633,AD.vs.control; bulk RNA-seq,Homo_633,AD.vs.MCI	6
CC	GO:0031092	platelet alpha granule membrane	bulk RNA-seq,Homo_723,AD.vs.MCI; bulk RNA-seq,Homo_714,AD.vs.control; bulk RNA-seq,Homo_714,AD.vs.MCI; bulk RNA-seq,Homo_714,MCI.vs.control; bulk RNA-seq,Homo_633,AD.vs.control; bulk RNA-seq,Homo_633,AD.vs.MCI; bulk RNA-seq,SRP223445,AD.vs.control	7
BP	GO:1900037	regulation of cellular response to hypoxia	bulk RNA-seq,Homo_723,AD.vs.MCI; bulk RNA-seq,Homo_723,MCI.vs.control; bulk RNA-seq,Homo_714,AD.vs.MCI; bulk RNA-seq,Homo_714,MCI.vs.control; bulk RNA-seq,Homo_633,AD.vs.control	5
MF	GO:0140161	monocarboxylate:sodium symporter activity	bulk RNA-seq,Homo_723,AD.vs.MCI; bulk RNA-seq,Homo_714,AD.vs.MCI	2
BP	GO:0071635	negative regulation of transforming growth factor beta	bulk RNA-seq,Homo_723,AD.vs.MCI; bulk RNA-seq,Homo_723,MCI.vs.control; bulk RNA-seq,Homo_714,AD.vs.MCI; bulk RNA-seq,Homo_714,MCI.vs.control	4
BP	GO:0006787	porphyrin-containing compound catabolic process	bulk RNA-seq,Homo_723,AD.vs.MCI; bulk RNA-seq,Homo_723,MCI.vs.control	2
BP	GO:0033015	tetrapyrrole catabolic process	bulk RNA-seq,Homo_723,AD.vs.MCI; bulk RNA-seq,Homo_723,MCI.vs.control	2
BP	GO:0048872	homeostasis of number of cells	bulk RNA-seq,Homo_723,AD.vs.MCI; bulk RNA-seq,Homo_723,MCI.vs.control; bulk RNA-seq,Homo_714,AD.vs.MCI; bulk RNA-seq,Homo_633,AD.vs.control; bulk RNA-seq,Homo_633,AD.vs.MCI; scRNA-seq,SRP330776,Naive CD8+ T cell_2-AD.vs.control	6
BP	GO:0010389	regulation of G2/M transition of mitotic cell cycle	bulk RNA-seq,Homo_723,AD.vs.MCI; bulk RNA-seq,Homo_723,MCI.vs.control; bulk RNA-seq,Homo_714,AD.vs.MCI; bulk RNA-seq,Homo_714,MCI.vs.control; bulk RNA-seq,Homo_633,AD.vs.control; bulk RNA-seq,Homo_633,AD.vs.MCI	6
BP	GO:0051602	response to electrical stimulus	bulk RNA-seq,Homo_723,AD.vs.MCI; bulk RNA-seq,Homo_723,MCI.vs.control	2
BP	GO:0045981	positive regulation of nucleotide metabolic process	bulk RNA-seq,Homo_723,AD.vs.MCI; bulk RNA-seq,Homo_723,MCI.vs.control; bulk RNA-seq,Homo_714,AD.vs.MCI; bulk RNA-seq,Homo_714,MCI.vs.control; bulk RNA-seq,Homo_633,AD.vs.control	5
BP	GO:1900544	positive regulation of purine nucleotide metabolic process	bulk RNA-seq,Homo_723,AD.vs.MCI; bulk RNA-seq,Homo_723,MCI.vs.control; bulk RNA-seq,Homo_714,AD.vs.MCI; bulk RNA-seq,Homo_714,MCI.vs.control; bulk RNA-seq,Homo_633,AD.vs.control	5
BP	GO:0090209	negative regulation of triglyceride metabolic process	bulk RNA-seq,Homo_723,AD.vs.MCI; bulk RNA-seq,Homo_723,MCI.vs.control; bulk RNA-seq,Homo_714,AD.vs.control; bulk RNA-seq,Homo_714,AD.vs.MCI	4
BP	GO:2001223	negative regulation of neuron migration	bulk RNA-seq,Homo_723,AD.vs.MCI; bulk RNA-seq,Homo_723,MCI.vs.control	2
MF	GO:0005548	phospholipid transporter activity	bulk RNA-seq,Homo_723,AD.vs.MCI; bulk RNA-seq,Homo_723,MCI.vs.control; bulk RNA-seq,Homo_714,AD.vs.MCI; bulk RNA-seq,Homo_714,MCI.vs.control; bulk RNA-seq,Homo_633,AD.vs.control; bulk RNA-seq,Homo_633,AD.vs.MCI	6
BP	GO:0072678	T cell migration	bulk RNA-seq,Homo_723,AD.vs.MCI; bulk RNA-seq,Homo_723,MCI.vs.control; bulk RNA-seq,Homo_714,AD.vs.MCI; bulk RNA-seq,Homo_714,MCI.vs.control; bulk RNA-seq,Homo_633,AD.vs.control	5
BP	GO:0048169	regulation of long-term neuronal synaptic plasticity	bulk RNA-seq,Homo_723,AD.vs.MCI; bulk RNA-seq,Homo_723,MCI.vs.control; bulk RNA-seq,Homo_714,AD.vs.MCI; bulk RNA-seq,Homo_714,MCI.vs.control	4
BP	GO:1900120	regulation of receptor binding	bulk RNA-seq,Homo_723,AD.vs.MCI; bulk RNA-seq,Homo_714,AD.vs.MCI	2
BP	GO:0032352	positive regulation of hormone metabolic process	bulk RNA-seq,Homo_723,AD.vs.MCI; bulk RNA-seq,Homo_723,MCI.vs.control; bulk RNA-seq,Homo_714,MCI.vs.control; bulk RNA-seq,Homo_633,AD.vs.control; bulk RNA-seq,Homo_633,MCI.vs.control	5
BP	GO:0042308	negative regulation of protein import into nucleus	bulk RNA-seq,Homo_723,AD.vs.MCI; bulk RNA-seq,Homo_723,MCI.vs.control; bulk RNA-seq,Homo_714,AD.vs.MCI; bulk RNA-seq,Homo_714,MCI.vs.control; bulk RNA-seq,Homo_633,AD.vs.control	5
BP	GO:0042058	regulation of epidermal growth factor receptor signaling pathway	bulk RNA-seq,Homo_723,AD.vs.MCI; bulk RNA-seq,Homo_723,MCI.vs.control; bulk RNA-seq,Homo_714,AD.vs.MCI; bulk RNA-seq,Homo_714,MCI.vs.control; bulk RNA-seq,Homo_633,AD.vs.control	5
BP	GO:0033081	regulation of T cell differentiation in thymus	bulk RNA-seq,Homo_723,AD.vs.MCI; bulk RNA-seq,Homo_723,MCI.vs.control; bulk RNA-seq,Homo_714,MCI.vs.control	3
MF	GO:1990756	ubiquitin ligase-substrate adaptor activity	bulk RNA-seq,Homo_723,AD.vs.MCI; bulk RNA-seq,Homo_723,MCI.vs.control; bulk RNA-seq,Homo_714,AD.vs.MCI; bulk RNA-seq,Homo_714,MCI.vs.control; bulk RNA-seq,Homo_633,AD.vs.control; bulk RNA-seq,Homo_633,MCI.vs.control	6
BP	GO:0007205	protein kinase C-activating G protein-coupled receptor signaling pathway	bulk RNA-seq,Homo_723,AD.vs.MCI; bulk RNA-seq,Homo_633,AD.vs.control; bulk RNA-seq,Homo_633,AD.vs.MCI	3

BP	GO:0033077	T cell differentiation in thymus	bulk RNA-seq,Homo_723,AD.vs.MCI; bulk RNA-seq,Homo_723,MCI.vs.control; bulk RNA-seq,Homo_714,MCI.vs.control; bulk RNA-seq,Homo_633,AD.vs.control; bulk RNA-seq,Homo_633,AD.vs.MCI	5
MF	GO:0017081	chloride channel regulator activity	bulk RNA-seq,Homo_723,AD.vs.MCI; bulk RNA-seq,Homo_723,MCI.vs.control	2
BP	GO:2000291	regulation of myoblast proliferation	bulk RNA-seq,Homo_723,AD.vs.MCI; bulk RNA-seq,Homo_714,AD.vs.MCI	2
BP	GO:0036473	cell death in response to oxidative stress	bulk RNA-seq,Homo_723,AD.vs.MCI; bulk RNA-seq,Homo_723,MCI.vs.control; bulk RNA-seq,Homo_714,AD.vs.MCI; bulk RNA-seq,Homo_714,MCI.vs.control; bulk RNA-seq,Homo_633,AD.vs.control; bulk RNA-seq,Homo_633,AD.vs.MCI	6
BP	GO:0006612	protein targeting to membrane	bulk RNA-seq,Homo_723,AD.vs.MCI; bulk RNA-seq,Homo_723,MCI.vs.control; bulk RNA-seq,Homo_714,MCI.vs.control; bulk RNA-seq,Homo_633,AD.vs.control; bulk RNA-seq,Homo_633,AD.vs.MCI	5
BP	GO:0050910	detection of mechanical stimulus involved in sensory perception of sound	bulk RNA-seq,Homo_723,AD.vs.MCI	1
BP	GO:0014856	skeletal muscle cell proliferation	bulk RNA-seq,Homo_723,AD.vs.MCI; bulk RNA-seq,Homo_723,MCI.vs.control; bulk RNA-seq,Homo_714,AD.vs.MCI; bulk RNA-seq,Homo_714,MCI.vs.control; bulk RNA-seq,Homo_633,AD.vs.control; bulk RNA-seq,Homo_633,AD.vs.MCI	6
BP	GO:0014841	skeletal muscle satellite cell proliferation	bulk RNA-seq,Homo_723,AD.vs.MCI; bulk RNA-seq,Homo_723,MCI.vs.control; bulk RNA-seq,Homo_714,AD.vs.MCI; bulk RNA-seq,Homo_714,MCI.vs.control	4
BP	GO:0048009	insulin-like growth factor receptor signaling pathway	bulk RNA-seq,Homo_723,AD.vs.MCI; bulk RNA-seq,Homo_723,MCI.vs.control; bulk RNA-seq,Homo_714,AD.vs.MCI; bulk RNA-seq,Homo_714,MCI.vs.control	4
MF	GO:0048020	CCR chemokine receptor binding	bulk RNA-seq,Homo_723,AD.vs.MCI; bulk RNA-seq,Homo_723,MCI.vs.control; bulk RNA-seq,Homo_714,AD.vs.MCI; bulk RNA-seq,Homo_714,MCI.vs.control; scRNA-seq,SRP309935,Naive CD8+ T cell_1-AD.vs.control	5
BP	GO:0051926	negative regulation of calcium ion transport	bulk RNA-seq,Homo_723,AD.vs.MCI; bulk RNA-seq,Homo_714,AD.vs.MCI	2
BP	GO:0090174	organelle membrane fusion	bulk RNA-seq,Homo_723,AD.vs.MCI; bulk RNA-seq,Homo_723,MCI.vs.control; bulk RNA-seq,Homo_714,MCI.vs.control; bulk RNA-seq,Homo_633,AD.vs.control; bulk RNA-seq,Homo_633,AD.vs.MCI	5
BP	GO:0010755	regulation of plasminogen activation	bulk RNA-seq,Homo_723,AD.vs.MCI; bulk RNA-seq,Homo_723,MCI.vs.control; bulk RNA-seq,Homo_714,AD.vs.MCI; bulk RNA-seq,Homo_714,MCI.vs.control; bulk RNA-seq,Homo_633,AD.vs.control	5
BP	GO:0003188	heart valve formation	bulk RNA-seq,Homo_723,AD.vs.MCI; bulk RNA-seq,Homo_723,MCI.vs.control; bulk RNA-seq,Homo_714,MCI.vs.control	3
BP	GO:0070741	response to interleukin-6	bulk RNA-seq,Homo_723,AD.vs.MCI; bulk RNA-seq,Homo_723,MCI.vs.control; bulk RNA-seq,Homo_714,AD.vs.MCI; bulk RNA-seq,Homo_714,MCI.vs.control; bulk RNA-seq,Homo_633,AD.vs.control; bulk RNA-seq,Homo_633,AD.vs.MCI	6
BP	GO:0006165	nucleoside diphosphate phosphorylation	bulk RNA-seq,Homo_723,AD.vs.MCI; bulk RNA-seq,Homo_723,MCI.vs.control; bulk RNA-seq,Homo_714,MCI.vs.control; bulk RNA-seq,Homo_633,AD.vs.control; bulk RNA-seq,Homo_633,AD.vs.MCI	5
BP	GO:0060044	negative regulation of cardiac muscle cell proliferation	bulk RNA-seq,Homo_723,AD.vs.MCI; bulk RNA-seq,Homo_714,AD.vs.MCI	2
BP	GO:0106056	regulation of calcineurin-mediated signaling	bulk RNA-seq,Homo_723,AD.vs.MCI; bulk RNA-seq,Homo_723,MCI.vs.control; bulk RNA-seq,Homo_714,MCI.vs.control; bulk RNA-	4
MF	GO:0004864	protein phosphatase inhibitor activity	bulk RNA-seq,Homo_723,AD.vs.MCI; bulk RNA-seq,Homo_723,MCI.vs.control; bulk RNA-seq,Homo_714,MCI.vs.control	3
MF	GO:0031420	alkali metal ion binding	bulk RNA-seq,Homo_723,AD.vs.MCI; bulk RNA-seq,Homo_723,MCI.vs.control; bulk RNA-seq,Homo_714,MCI.vs.control	3
BP	GO:0060314	regulation of ryanodine-sensitive calcium-release channel	bulk RNA-seq,Homo_723,AD.vs.MCI; bulk RNA-seq,Homo_714,AD.vs.MCI	2
BP	GO:1900744	regulation of p38MAPK cascade	bulk RNA-seq,Homo_723,AD.vs.MCI; bulk RNA-seq,Homo_714,MCI.vs.control	2
BP	GO:0008356	asymmetric cell division	bulk RNA-seq,Homo_723,AD.vs.MCI; bulk RNA-seq,Homo_723,MCI.vs.control; bulk RNA-seq,Homo_633,AD.vs.control; bulk RNA-seq,Homo_633,AD.vs.MCI	4
MF	GO:0016829	lyase activity	bulk RNA-seq,Homo_723,AD.vs.MCI; bulk RNA-seq,Homo_723,MCI.vs.control; bulk RNA-seq,Homo_714,AD.vs.control; bulk RNA-seq,Homo_714,AD.vs.MCI; bulk RNA-seq,Homo_633,AD.vs.control; bulk RNA-seq,Homo_633,AD.vs.MCI	6
BP	GO:0061756	leukocyte adhesion to vascular endothelial cell	bulk RNA-seq,Homo_723,AD.vs.MCI; bulk RNA-seq,Homo_723,MCI.vs.control; bulk RNA-seq,Homo_714,AD.vs.MCI; bulk RNA-seq,Homo_714,MCI.vs.control; bulk RNA-seq,Homo_633,AD.vs.control	5
BP	GO:2001238	positive regulation of extrinsic apoptotic signaling pathway	bulk RNA-seq,Homo_723,AD.vs.MCI; bulk RNA-seq,Homo_723,MCI.vs.control; bulk RNA-seq,Homo_714,AD.vs.MCI; bulk RNA-seq,Homo_714,MCI.vs.control; bulk RNA-seq,Homo_633,AD.vs.control; bulk RNA-seq,Homo_633,AD.vs.MCI	6
BP	GO:0042448	progesterone metabolic process	bulk RNA-seq,Homo_723,AD.vs.MCI; bulk RNA-seq,Homo_723,MCI.vs.control	2
BP	GO:1902931	negative regulation of alcohol biosynthetic process	bulk RNA-seq,Homo_723,AD.vs.MCI; bulk RNA-seq,Homo_723,MCI.vs.control; bulk RNA-seq,Homo_714,MCI.vs.control	3
BP	GO:0006730	one-carbon metabolic process	bulk RNA-seq,Homo_723,AD.vs.MCI; bulk RNA-seq,Homo_723,MCI.vs.control; bulk RNA-seq,Homo_714,MCI.vs.control; bulk RNA-seq,Homo_633,AD.vs.control; bulk RNA-seq,Homo_633,AD.vs.MCI	5
CC	GO:0032391	photoreceptor connecting cilium	bulk RNA-seq,Homo_723,AD.vs.MCI; bulk RNA-seq,Homo_723,MCI.vs.control; bulk RNA-seq,Homo_714,MCI.vs.control; bulk RNA-seq,Homo_633,AD.vs.control; bulk RNA-seq,Homo_633,AD.vs.MCI	5
BP	GO:0010839	negative regulation of keratinocyte proliferation	bulk RNA-seq,Homo_723,AD.vs.MCI; bulk RNA-seq,Homo_723,MCI.vs.control; bulk RNA-seq,Homo_714,MCI.vs.control; bulk RNA-seq,Homo_633,AD.vs.control; bulk RNA-seq,Homo_633,AD.vs.MCI	5
CC	GO:0032580	Golgi cisterna membrane	bulk RNA-seq,Homo_723,AD.vs.MCI; bulk RNA-seq,Homo_723,MCI.vs.control; bulk RNA-seq,Homo_714,MCI.vs.control; bulk RNA-seq,Homo_633,AD.vs.control; bulk RNA-seq,Homo_633,AD.vs.MCI	5
MF	GO:0004364	glutathione transferase activity	bulk RNA-seq,Homo_723,AD.vs.MCI	1
BP	GO:0001825	blastocyst formation	bulk RNA-seq,Homo_723,AD.vs.MCI; bulk RNA-seq,Homo_723,MCI.vs.control; bulk RNA-seq,Homo_714,AD.vs.MCI; bulk RNA-seq,Homo_714,MCI.vs.control; bulk RNA-seq,Homo_633,AD.vs.control; bulk RNA-seq,Homo_633,AD.vs.MCI	6
MF	GO:0034212	peptide N-acetyltransferase activity	bulk RNA-seq,Homo_723,AD.vs.MCI; bulk RNA-seq,Homo_714,AD.vs.MCI; bulk RNA-seq,Homo_633,AD.vs.control; bulk RNA-seq,Homo_633,AD.vs.MCI	4
BP	GO:1903206	negative regulation of hydrogen peroxide-induced cell death	bulk RNA-seq,Homo_723,AD.vs.MCI; bulk RNA-seq,Homo_633,AD.vs.control	2
BP	GO:0036119	response to platelet-derived growth factor	bulk RNA-seq,Homo_723,AD.vs.MCI; bulk RNA-seq,Homo_723,MCI.vs.control; bulk RNA-seq,Homo_714,MCI.vs.control; bulk RNA-seq,Homo_633,AD.vs.control; bulk RNA-seq,Homo_633,AD.vs.MCI	5
MF	GO:0004180	carboxypeptidase activity	bulk RNA-seq,Homo_723,AD.vs.MCI; bulk RNA-seq,Homo_723,MCI.vs.control; bulk RNA-seq,Homo_714,AD.vs.MCI; bulk RNA-seq,Homo_633,AD.vs.control	4
BP	GO:0018394	peptidyl-lysine acetylation	bulk RNA-seq,Homo_723,AD.vs.MCI; bulk RNA-seq,Homo_723,MCI.vs.control; bulk RNA-seq,Homo_714,AD.vs.MCI; bulk RNA-seq,Homo_633,AD.vs.control; bulk RNA-seq,Homo_633,AD.vs.MCI	5
BP	GO:0044774	mitotic DNA integrity checkpoint signaling	bulk RNA-seq,Homo_723,AD.vs.MCI; bulk RNA-seq,Homo_723,MCI.vs.control; bulk RNA-seq,Homo_714,MCI.vs.control; bulk RNA-seq,Homo_633,AD.vs.control; bulk RNA-seq,Homo_633,AD.vs.MCI	5

BP	GO:0032722	positive regulation of chemokine production	bulk RNA-seq,Homo_723,AD.vs.MCI; bulk RNA-seq,Homo_723,MCI.vs.control; bulk RNA-seq,Homo_714,AD.vs.MCI; bulk RNA-seq,Homo_714,MCI.vs.control; bulk RNA-seq,Homo_633,AD.vs.control	5
MF	GO:0022840	leak channel activity	bulk RNA-seq,Homo_723,AD.vs.MCI; bulk RNA-seq,SRP223445,AD.vs.control	2
MF	GO:0022842	narrow pore channel activity	bulk RNA-seq,Homo_723,AD.vs.MCI; bulk RNA-seq,SRP223445,AD.vs.control	2
BP	GO:0002468	dendritic cell antigen processing and presentation	bulk RNA-seq,Homo_723,AD.vs.MCI; bulk RNA-seq,Homo_723,MCI.vs.control; bulk RNA-seq,Homo_714,AD.vs.MCI; bulk RNA-seq,Homo_714,MCI.vs.control	4
CC	GO:0030057	desmosome	bulk RNA-seq,Homo_723,AD.vs.MCI; bulk RNA-seq,Homo_723,MCI.vs.control; bulk RNA-seq,Homo_714,AD.vs.MCI; bulk RNA-seq,Homo_714,MCI.vs.control	4
BP	GO:0034643	establishment of mitochondrion localization, microtubule-	bulk RNA-seq,Homo_723,AD.vs.MCI; bulk RNA-seq,Homo_723,MCI.vs.control; bulk RNA-seq,Homo_633,AD.vs.control; bulk RNA-seq,Homo_633,AD.vs.MCI	4
BP	GO:0047497	mitochondrion transport along microtubule	bulk RNA-seq,Homo_723,AD.vs.MCI; bulk RNA-seq,Homo_723,MCI.vs.control; bulk RNA-seq,Homo_633,AD.vs.control; bulk RNA-seq,Homo_633,AD.vs.MCI	4
MF	GO:0002162	dystroglycan binding	bulk RNA-seq,Homo_723,AD.vs.MCI; bulk RNA-seq,Homo_723,MCI.vs.control; bulk RNA-seq,Homo_714,MCI.vs.control	3
MF	GO:0005078	MAP-kinase scaffold activity	bulk RNA-seq,Homo_723,AD.vs.MCI; bulk RNA-seq,Homo_723,MCI.vs.control; bulk RNA-seq,Homo_714,MCI.vs.control; bulk RNA-	4
BP	GO:0015824	proline transport	bulk RNA-seq,Homo_723,AD.vs.MCI	1
BP	GO:0035025	positive regulation of Rho protein signal transduction	bulk RNA-seq,Homo_723,AD.vs.MCI; bulk RNA-seq,Homo_723,MCI.vs.control; bulk RNA-seq,Homo_714,AD.vs.MCI; bulk RNA-seq,Homo_714,MCI.vs.control	4
BP	GO:0071377	cellular response to glucagon stimulus	bulk RNA-seq,Homo_723,AD.vs.MCI; bulk RNA-seq,Homo_723,MCI.vs.control; bulk RNA-seq,Homo_714,AD.vs.MCI; bulk RNA-seq,ROSMAP,AD.vs.control	4
BP	GO:0070242	thymocyte apoptotic process	bulk RNA-seq,Homo_723,AD.vs.MCI; bulk RNA-seq,Homo_723,MCI.vs.control	2
BP	GO:0034111	negative regulation of homotypic cell-cell adhesion	bulk RNA-seq,Homo_723,AD.vs.MCI; bulk RNA-seq,Homo_723,MCI.vs.control; bulk RNA-seq,Homo_714,AD.vs.MCI; bulk RNA-seq,Homo_714,MCI.vs.control	4
CC	GO:0017053	transcription repressor complex	bulk RNA-seq,Homo_723,AD.vs.MCI; bulk RNA-seq,Homo_723,MCI.vs.control; bulk RNA-seq,Homo_714,AD.vs.MCI; bulk RNA-seq,Homo_714,MCI.vs.control; bulk RNA-seq,Homo_633,AD.vs.control; bulk RNA-seq,Homo_633,AD.vs.MCI	6
BP	GO:0034976	response to endoplasmic reticulum stress	bulk RNA-seq,Homo_723,AD.vs.MCI; bulk RNA-seq,Homo_723,MCI.vs.control; bulk RNA-seq,Homo_714,AD.vs.MCI; bulk RNA-seq,Homo_633,AD.vs.control; bulk RNA-seq,Homo_633,AD.vs.MCI	5
BP	GO:0060352	cell adhesion molecule production	bulk RNA-seq,Homo_723,AD.vs.MCI; bulk RNA-seq,Homo_723,MCI.vs.control; bulk RNA-seq,Homo_714,AD.vs.MCI	3
BP	GO:0090218	positive regulation of lipid kinase activity	bulk RNA-seq,Homo_723,AD.vs.MCI; bulk RNA-seq,Homo_723,MCI.vs.control; bulk RNA-seq,Homo_714,AD.vs.MCI; bulk RNA-seq,Homo_714,MCI.vs.control; bulk RNA-seq,Homo_633,AD.vs.control	5
BP	GO:0032328	alanine transport	bulk RNA-seq,Homo_723,AD.vs.MCI	1
BP	GO:0030575	nuclear body organization	bulk RNA-seq,Homo_723,AD.vs.MCI; bulk RNA-seq,Homo_723,MCI.vs.control; bulk RNA-seq,Homo_714,MCI.vs.control; bulk RNA-seq,Homo_633,AD.vs.control; bulk RNA-seq,Homo_633,AD.vs.MCI	5
MF	GO:0032452	histone demethylase activity	bulk RNA-seq,Homo_723,AD.vs.MCI; bulk RNA-seq,Homo_723,MCI.vs.control; bulk RNA-seq,Homo_714,AD.vs.MCI; bulk RNA-seq,Homo_714,MCI.vs.control; bulk RNA-seq,Homo_633,AD.vs.control; bulk RNA-seq,Homo_633,AD.vs.MCI; bulk RNA-seq,Homo_633,MCI.vs.control	7
MF	GO:0140457	protein demethylase activity	bulk RNA-seq,Homo_723,AD.vs.MCI; bulk RNA-seq,Homo_723,MCI.vs.control; bulk RNA-seq,Homo_714,AD.vs.MCI; bulk RNA-seq,Homo_714,MCI.vs.control; bulk RNA-seq,Homo_633,AD.vs.control; bulk RNA-seq,Homo_633,AD.vs.MCI; bulk RNA-seq,Homo_633,MCI.vs.control	7
BP	GO:2000650	negative regulation of sodium ion transmembrane transporter	bulk RNA-seq,Homo_723,AD.vs.MCI	1
BP	GO:0035196	miRNA processing	bulk RNA-seq,Homo_723,AD.vs.MCI; bulk RNA-seq,Homo_723,MCI.vs.control; bulk RNA-seq,Homo_714,MCI.vs.control; bulk RNA-seq,Homo_633,AD.vs.control; bulk RNA-seq,Homo_633,AD.vs.MCI	5
BP	GO:0002604	regulation of dendritic cell antigen processing and presentation	bulk RNA-seq,Homo_723,AD.vs.MCI; bulk RNA-seq,Homo_723,MCI.vs.control; bulk RNA-seq,Homo_714,AD.vs.MCI; bulk RNA-seq,Homo_714,MCI.vs.control; bulk RNA-seq,Homo_633,AD.vs.MCI	5
BP	GO:0048245	eosinophil chemotaxis	bulk RNA-seq,Homo_723,AD.vs.MCI; bulk RNA-seq,Homo_723,MCI.vs.control; bulk RNA-seq,Homo_714,AD.vs.MCI; bulk RNA-seq,Homo_714,MCI.vs.control	4
BP	GO:0097623	potassium ion export across plasma membrane	bulk RNA-seq,Homo_723,AD.vs.MCI; bulk RNA-seq,Homo_714,AD.vs.MCI; bulk RNA-seq,Homo_633,AD.vs.control	3
BP	GO:1902170	cellular response to reactive nitrogen species	bulk RNA-seq,Homo_723,AD.vs.MCI; bulk RNA-seq,Homo_723,MCI.vs.control; bulk RNA-seq,Homo_633,AD.vs.control	3
BP	GO:2000008	regulation of protein localization to cell surface	bulk RNA-seq,Homo_723,AD.vs.MCI; bulk RNA-seq,Homo_723,MCI.vs.control; bulk RNA-seq,Homo_633,AD.vs.control	3
BP	GO:0003157	endocardium development	bulk RNA-seq,Homo_723,AD.vs.MCI; bulk RNA-seq,Homo_723,MCI.vs.control; bulk RNA-seq,Homo_714,AD.vs.MCI; bulk RNA-seq,Homo_714,MCI.vs.control; bulk RNA-seq,Homo_633,AD.vs.control; bulk RNA-seq,Homo_633,AD.vs.MCI	6
CC	GO:0005641	nuclear envelope lumen	bulk RNA-seq,Homo_723,AD.vs.MCI; bulk RNA-seq,Homo_723,MCI.vs.control; bulk RNA-seq,Homo_633,AD.vs.control	3
BP	GO:0043506	regulation of JUN kinase activity	bulk RNA-seq,Homo_723,AD.vs.MCI; bulk RNA-seq,Homo_723,MCI.vs.control; bulk RNA-seq,Homo_714,MCI.vs.control; bulk RNA-	4
BP	GO:0001991	regulation of systemic arterial blood pressure by circulatory renin-angiotensin	bulk RNA-seq,Homo_723,AD.vs.MCI; bulk RNA-seq,Homo_723,MCI.vs.control; bulk RNA-seq,Homo_714,AD.vs.MCI; bulk RNA-seq,Homo_714,MCI.vs.control	4
BP	GO:0010950	positive regulation of endopeptidase activity	bulk RNA-seq,Homo_723,AD.vs.MCI; bulk RNA-seq,Homo_723,MCI.vs.control; bulk RNA-seq,Homo_633,AD.vs.control; bulk RNA-seq,Homo_633,AD.vs.MCI	4
BP	GO:0071675	regulation of mononuclear cell migration	bulk RNA-seq,Homo_723,AD.vs.MCI; bulk RNA-seq,Homo_723,MCI.vs.control; bulk RNA-seq,Homo_714,AD.vs.MCI; bulk RNA-seq,Homo_714,MCI.vs.control; bulk RNA-seq,Homo_633,AD.vs.control; bulk RNA-seq,Homo_633,AD.vs.MCI; scRNA-seq,SRP330776,Naive CD8+ T cell_2-	7
BP	GO:0032768	regulation of monooxygenase activity	bulk RNA-seq,Homo_723,AD.vs.MCI; bulk RNA-seq,Homo_723,MCI.vs.control; bulk RNA-seq,Homo_714,AD.vs.MCI; bulk RNA-seq,Homo_714,MCI.vs.control; bulk RNA-seq,Homo_633,AD.vs.control; bulk RNA-seq,Homo_633,AD.vs.MCI	6
BP	GO:0043303	mast cell degranulation	bulk RNA-seq,Homo_723,AD.vs.MCI; bulk RNA-seq,Homo_723,MCI.vs.control; bulk RNA-seq,Homo_714,AD.vs.MCI; bulk RNA-seq,Homo_714,MCI.vs.control; bulk RNA-seq,Homo_633,AD.vs.control; bulk RNA-seq,Homo_633,AD.vs.MCI; scRNA-seq,SRP215507,CD8+ T cell_1-	7
BP	GO:1901985	positive regulation of protein acetylation	bulk RNA-seq,Homo_723,AD.vs.MCI; bulk RNA-seq,Homo_723,MCI.vs.control; bulk RNA-seq,Homo_714,AD.vs.MCI; bulk RNA-seq,Homo_714,MCI.vs.control	4
MF	GO:0051378	serotonin binding	bulk RNA-seq,Homo_723,AD.vs.MCI; bulk RNA-seq,Homo_714,AD.vs.MCI; bulk RNA-seq,Homo_633,MCI.vs.control	3
BP	GO:0009713	catechol-containing compound biosynthetic process	bulk RNA-seq,Homo_723,AD.vs.MCI; bulk RNA-seq,Homo_714,AD.vs.MCI	2
BP	GO:0042423	catecholamine biosynthetic process	bulk RNA-seq,Homo_723,AD.vs.MCI; bulk RNA-seq,Homo_714,AD.vs.MCI	2
BP	GO:0000050	urea cycle	bulk RNA-seq,Homo_723,AD.vs.MCI; bulk RNA-seq,Homo_723,MCI.vs.control; bulk RNA-seq,Homo_714,AD.vs.MCI; bulk RNA-seq,Homo_714,MCI.vs.control	4
BP	GO:0072172	mesonephric tubule formation	bulk RNA-seq,Homo_723,AD.vs.MCI	1
BP	GO:0032801	receptor catabolic process	bulk RNA-seq,Homo_723,AD.vs.MCI; bulk RNA-seq,Homo_723,MCI.vs.control; bulk RNA-seq,Homo_633,AD.vs.control; bulk RNA-seq,Homo_633,AD.vs.MCI	4
BP	GO:0006906	vesicle fusion	bulk RNA-seq,Homo_723,AD.vs.MCI; bulk RNA-seq,Homo_723,MCI.vs.control; bulk RNA-seq,Homo_714,MCI.vs.control; bulk RNA-seq,Homo_633,AD.vs.control; bulk RNA-seq,Homo_633,AD.vs.MCI	5

BP	GO:0140694	non-membrane-bounded organelle assembly	bulk RNA-seq,Homo_723,AD.vs.MCI; bulk RNA-seq,Homo_714,AD.vs.control; bulk RNA-seq,Homo_714,AD.vs.MCI; bulk RNA-seq,Homo_633,AD.vs.control; bulk RNA-seq,Homo_633,AD.vs.MCI	5
BP	GO:0061365	positive regulation of triglyceride lipase activity	bulk RNA-seq,Homo_723,AD.vs.MCI; bulk RNA-seq,Homo_723,MCI.vs.control; bulk RNA-seq,Homo_714,AD.vs.MCI; bulk RNA-seq,Homo_714,MCI.vs.control	4
MF	GO:0008331	high voltage-gated calcium channel activity	bulk RNA-seq,Homo_723,AD.vs.MCI	1
BP	GO:0042359	vitamin D metabolic process	bulk RNA-seq,Homo_723,AD.vs.MCI; bulk RNA-seq,Homo_723,MCI.vs.control; bulk RNA-seq,Homo_714,AD.vs.MCI; bulk RNA-seq,Homo_714,MCI.vs.control	4
BP	GO:2001056	positive regulation of cysteine-type endopeptidase activity	bulk RNA-seq,Homo_723,AD.vs.MCI; bulk RNA-seq,Homo_723,MCI.vs.control; bulk RNA-seq,Homo_633,AD.vs.control; bulk RNA-seq,Homo_633,AD.vs.MCI	4
BP	GO:0050872	white fat cell differentiation	bulk RNA-seq,Homo_723,AD.vs.MCI; bulk RNA-seq,Homo_723,MCI.vs.control; bulk RNA-seq,Homo_714,AD.vs.MCI; bulk RNA-seq,Homo_714,MCI.vs.control; bulk RNA-seq,Homo_633,AD.vs.MCI	5
CC	GO:0070938	contractile ring	bulk RNA-seq,Homo_723,AD.vs.MCI; bulk RNA-seq,Homo_714,AD.vs.MCI; bulk RNA-seq,Homo_633,AD.vs.control	3
BP	GO:0099623	regulation of cardiac muscle cell membrane repolarization	bulk RNA-seq,Homo_723,AD.vs.MCI; bulk RNA-seq,Homo_714,AD.vs.MCI	2
BP	GO:0043586	tongue development	bulk RNA-seq,Homo_723,AD.vs.MCI; bulk RNA-seq,Homo_723,MCI.vs.control	2
BP	GO:0046621	negative regulation of organ growth	bulk RNA-seq,Homo_723,AD.vs.MCI; bulk RNA-seq,Homo_723,MCI.vs.control; bulk RNA-seq,Homo_714,AD.vs.MCI; bulk RNA-seq,Homo_714,MCI.vs.control; scRNA-seq,SRP330776,CD8+ T cell_1-AD.vs.control; scRNA-seq,SRP330776,Naive CD8+ T cell_1-AD.vs.control	6
CC	GO:0000137	Golgi cis cisterna	bulk RNA-seq,Homo_723,AD.vs.MCI; bulk RNA-seq,Homo_723,MCI.vs.control; bulk RNA-seq,Homo_633,AD.vs.control	3
BP	GO:0033032	regulation of myeloid cell apoptotic process	bulk RNA-seq,Homo_723,AD.vs.MCI; bulk RNA-seq,Homo_723,MCI.vs.control; bulk RNA-seq,Homo_714,AD.vs.MCI; bulk RNA-seq,Homo_714,MCI.vs.control; bulk RNA-seq,Homo_633,AD.vs.control; bulk RNA-seq,Homo_633,AD.vs.MCI; bulk RNA-seq,Homo_633,MCI.vs.control	7
BP	GO:0010874	regulation of cholesterol efflux	bulk RNA-seq,Homo_723,AD.vs.MCI; bulk RNA-seq,Homo_714,AD.vs.MCI	2
BP	GO:0002758	innate immune response-activating signal transduction	bulk RNA-seq,Homo_723,AD.vs.MCI; bulk RNA-seq,Homo_723,MCI.vs.control; bulk RNA-seq,Homo_714,AD.vs.MCI; bulk RNA-seq,Homo_714,MCI.vs.control; bulk RNA-seq,Homo_633,AD.vs.control; scRNA-seq,SRP330776,Natural killer cell_1-AD.vs.control	6
MF	GO:0042166	acetylcholine binding	bulk RNA-seq,Homo_723,AD.vs.MCI	1
MF	GO:0015173	aromatic amino acid transmembrane transporter activity	bulk RNA-seq,Homo_723,AD.vs.MCI; bulk RNA-seq,Homo_633,AD.vs.control; bulk RNA-seq,Homo_633,AD.vs.MCI	3
BP	GO:0003139	secondary heart field specification	bulk RNA-seq,Homo_723,AD.vs.MCI	1
BP	GO:0035437	maintenance of protein localization in endoplasmic reticulum	bulk RNA-seq,Homo_723,AD.vs.MCI	1
BP	GO:1900121	negative regulation of receptor binding	bulk RNA-seq,Homo_723,AD.vs.MCI; bulk RNA-seq,Homo_714,AD.vs.MCI	2
BP	GO:0061900	glial cell activation	bulk RNA-seq,Homo_723,AD.vs.MCI; bulk RNA-seq,Homo_723,MCI.vs.control; bulk RNA-seq,Homo_714,AD.vs.MCI; bulk RNA-seq,Homo_714,MCI.vs.control; bulk RNA-seq,Homo_633,AD.vs.control; bulk RNA-seq,Homo_633,AD.vs.MCI	6
BP	GO:0042304	regulation of fatty acid biosynthetic process	bulk RNA-seq,Homo_723,AD.vs.MCI; bulk RNA-seq,Homo_723,MCI.vs.control; bulk RNA-seq,Homo_714,AD.vs.MCI; bulk RNA-seq,Homo_714,MCI.vs.control; bulk RNA-seq,Homo_633,AD.vs.control; bulk RNA-seq,Homo_633,AD.vs.MCI	6
BP	GO:0032098	regulation of appetite	bulk RNA-seq,Homo_723,AD.vs.MCI; bulk RNA-seq,Homo_723,MCI.vs.control	2
CC	GO:0032588	trans-Golgi network membrane	bulk RNA-seq,Homo_723,AD.vs.MCI; bulk RNA-seq,Homo_723,MCI.vs.control; bulk RNA-seq,Homo_714,MCI.vs.control; bulk RNA-seq,Homo_633,AD.vs.control; bulk RNA-seq,Homo_633,AD.vs.MCI; scRNA-seq,SRP330776,Naive CD8+ T cell_2-AD.vs.control	6
BP	GO:1903725	regulation of phospholipid metabolic process	bulk RNA-seq,Homo_723,AD.vs.MCI; bulk RNA-seq,Homo_723,MCI.vs.control; bulk RNA-seq,Homo_714,AD.vs.MCI; bulk RNA-seq,Homo_714,MCI.vs.control; bulk RNA-seq,Homo_633,AD.vs.control; bulk RNA-seq,Homo_633,AD.vs.MCI	6
BP	GO:0031330	negative regulation of cellular catabolic process	bulk RNA-seq,Homo_723,AD.vs.MCI; bulk RNA-seq,Homo_723,MCI.vs.control; bulk RNA-seq,Homo_714,AD.vs.MCI; bulk RNA-seq,Homo_633,AD.vs.control; bulk RNA-seq,Homo_633,AD.vs.MCI	5
BP	GO:0008089	anterograde axonal transport	bulk RNA-seq,Homo_723,AD.vs.MCI; bulk RNA-seq,Homo_723,MCI.vs.control; bulk RNA-seq,Homo_714,MCI.vs.control; bulk RNA-seq,Homo_633,AD.vs.control; bulk RNA-seq,Homo_633,AD.vs.MCI	5
BP	GO:0003413	chondrocyte differentiation involved in endochondral bone morphogenesis	bulk RNA-seq,Homo_723,AD.vs.MCI; bulk RNA-seq,Homo_723,MCI.vs.control; bulk RNA-seq,Homo_714,MCI.vs.control; bulk RNA-seq,Homo_633,AD.vs.control	4
BP	GO:2000630	positive regulation of miRNA metabolic process	bulk RNA-seq,Homo_723,AD.vs.MCI; bulk RNA-seq,Homo_723,MCI.vs.control; bulk RNA-seq,Homo_714,MCI.vs.control; bulk RNA-seq,Homo_633,AD.vs.control; bulk RNA-seq,Homo_633,AD.vs.MCI	5
BP	GO:0070873	regulation of glycogen metabolic process	bulk RNA-seq,Homo_723,AD.vs.MCI	1
BP	GO:0043032	positive regulation of macrophage activation	bulk RNA-seq,Homo_723,AD.vs.MCI; bulk RNA-seq,Homo_723,MCI.vs.control; bulk RNA-seq,Homo_714,MCI.vs.control; bulk RNA-	4
MF	GO:0140318	protein transporter activity	bulk RNA-seq,Homo_723,AD.vs.MCI; bulk RNA-seq,Homo_723,MCI.vs.control; bulk RNA-seq,Homo_714,MCI.vs.control; bulk RNA-seq,Homo_633,AD.vs.control; bulk RNA-seq,Homo_633,AD.vs.MCI; bulk RNA-seq,Homo_633,MCI.vs.control	6
BP	GO:0035729	cellular response to hepatocyte growth factor stimulus	bulk RNA-seq,Homo_723,AD.vs.MCI; bulk RNA-seq,Homo_723,MCI.vs.control; bulk RNA-seq,Homo_714,AD.vs.MCI; bulk RNA-seq,Homo_714,MCI.vs.control; bulk RNA-seq,Homo_633,AD.vs.control; bulk RNA-seq,Homo_633,AD.vs.MCI	6
BP	GO:0051341	regulation of oxidoreductase activity	bulk RNA-seq,Homo_723,AD.vs.MCI; bulk RNA-seq,Homo_723,MCI.vs.control; bulk RNA-seq,Homo_714,AD.vs.MCI; bulk RNA-seq,Homo_714,MCI.vs.control; bulk RNA-seq,Homo_633,AD.vs.control; bulk RNA-seq,Homo_633,AD.vs.MCI	6
BP	GO:0071346	cellular response to interferon-gamma	bulk RNA-seq,Homo_723,AD.vs.MCI; bulk RNA-seq,Homo_723,MCI.vs.control; bulk RNA-seq,Homo_714,AD.vs.MCI; bulk RNA-seq,Homo_714,MCI.vs.control; bulk RNA-seq,Homo_633,AD.vs.control; bulk RNA-seq,Homo_633,AD.vs.MCI	6
BP	GO:0019369	arachidonic acid metabolic process	bulk RNA-seq,Homo_723,AD.vs.MCI; bulk RNA-seq,Homo_714,AD.vs.MCI; bulk RNA-seq,Homo_714,MCI.vs.control	3
BP	GO:0034655	nucleobase-containing compound catabolic process	bulk RNA-seq,Homo_723,AD.vs.MCI; bulk RNA-seq,Homo_714,AD.vs.control; bulk RNA-seq,Homo_633,AD.vs.control; bulk RNA-seq,Homo_633,AD.vs.MCI	4
BP	GO:1905907	negative regulation of amyloid fibril formation	bulk RNA-seq,Homo_723,AD.vs.MCI	1
BP	GO:0006309	apoptotic DNA fragmentation	bulk RNA-seq,Homo_723,AD.vs.MCI; bulk RNA-seq,Homo_714,AD.vs.MCI	2
BP	GO:0034112	positive regulation of homotypic cell-cell adhesion	bulk RNA-seq,Homo_723,AD.vs.MCI; bulk RNA-seq,Homo_723,MCI.vs.control; bulk RNA-seq,Homo_714,AD.vs.MCI; bulk RNA-seq,Homo_714,MCI.vs.control	4
CC	GO:0001673	male germ cell nucleus	bulk RNA-seq,Homo_723,AD.vs.MCI; bulk RNA-seq,Homo_723,MCI.vs.control; bulk RNA-seq,Homo_714,AD.vs.MCI; bulk RNA-seq,Homo_714,MCI.vs.control; bulk RNA-seq,Homo_633,AD.vs.control; bulk RNA-seq,Homo_633,AD.vs.MCI	6
MF	GO:0071837	HMG box domain binding	bulk RNA-seq,Homo_723,AD.vs.MCI; bulk RNA-seq,Homo_723,MCI.vs.control	2
BP	GO:0016126	sterol biosynthetic process	bulk RNA-seq,Homo_723,AD.vs.MCI; bulk RNA-seq,Homo_723,MCI.vs.control; bulk RNA-seq,Homo_714,AD.vs.MCI; bulk RNA-seq,Homo_714,MCI.vs.control; bulk RNA-seq,Homo_633,AD.vs.control; bulk RNA-seq,Homo_633,AD.vs.MCI	6

BP	GO:0001977	renal system process involved in regulation of blood volume	bulk RNA-seq,Homo_723,AD.vs.MCI; bulk RNA-seq,Homo_723,MCI.vs.control; bulk RNA-seq,Homo_714,AD.vs.MCI; bulk RNA-seq,Homo_714,MCI.vs.control	4
BP	GO:2000251	positive regulation of actin cytoskeleton reorganization	bulk RNA-seq,Homo_723,AD.vs.MCI; bulk RNA-seq,Homo_723,MCI.vs.control; bulk RNA-seq,Homo_714,AD.vs.MCI	3
MF	GO:0003746	translation elongation factor activity	bulk RNA-seq,Homo_723,AD.vs.MCI; bulk RNA-seq,Homo_723,MCI.vs.control; bulk RNA-seq,Homo_714,MCI.vs.control	3
BP	GO:0010002	cardioblast differentiation	bulk RNA-seq,Homo_723,AD.vs.MCI; bulk RNA-seq,Homo_714,AD.vs.MCI; bulk RNA-seq,Homo_633,MCI.vs.control	3
BP	GO:0033085	negative regulation of T cell differentiation in thymus	bulk RNA-seq,Homo_723,AD.vs.MCI	1
BP	GO:0046322	negative regulation of fatty acid oxidation	bulk RNA-seq,Homo_723,AD.vs.MCI; bulk RNA-seq,Homo_723,MCI.vs.control; bulk RNA-seq,Homo_714,MCI.vs.control	3
BP	GO:0014857	regulation of skeletal muscle cell proliferation	bulk RNA-seq,Homo_723,AD.vs.MCI; bulk RNA-seq,Homo_723,MCI.vs.control; bulk RNA-seq,Homo_714,AD.vs.MCI; bulk RNA-seq,Homo_714,MCI.vs.control; bulk RNA-seq,Homo_633,AD.vs.control	5
BP	GO:0051150	regulation of smooth muscle cell differentiation	bulk RNA-seq,Homo_723,AD.vs.MCI	1
BP	GO:0045010	actin nucleation	bulk RNA-seq,Homo_723,AD.vs.MCI; bulk RNA-seq,Homo_723,MCI.vs.control; bulk RNA-seq,Homo_714,MCI.vs.control; bulk RNA-seq,Homo_633,AD.vs.control; bulk RNA-seq,Homo_633,AD.vs.MCI	5
BP	GO:0098903	regulation of membrane repolarization during action potential	bulk RNA-seq,Homo_723,AD.vs.MCI; bulk RNA-seq,Homo_714,AD.vs.MCI	2
MF	GO:0070097	delta-catenin binding	bulk RNA-seq,Homo_723,AD.vs.MCI; bulk RNA-seq,Homo_723,MCI.vs.control	2
BP	GO:0006749	glutathione metabolic process	bulk RNA-seq,Homo_723,AD.vs.MCI; bulk RNA-seq,Homo_633,AD.vs.control; bulk RNA-seq,Homo_633,AD.vs.MCI	3
BP	GO:1900745	positive regulation of p38MAPK cascade	bulk RNA-seq,Homo_723,AD.vs.MCI	1
BP	GO:0070918	small regulatory ncRNA processing	bulk RNA-seq,Homo_723,AD.vs.MCI; bulk RNA-seq,Homo_723,MCI.vs.control; bulk RNA-seq,Homo_714,MCI.vs.control; bulk RNA-seq,Homo_633,AD.vs.control; bulk RNA-seq,Homo_633,AD.vs.MCI	5
BP	GO:0046470	phosphatidylcholine metabolic process	bulk RNA-seq,Homo_723,AD.vs.MCI; bulk RNA-seq,Homo_723,MCI.vs.control; bulk RNA-seq,Homo_714,AD.vs.MCI; bulk RNA-seq,Homo_714,MCI.vs.control; bulk RNA-seq,Homo_633,AD.vs.control; bulk RNA-seq,Homo_633,AD.vs.MCI	6
CC	GO:0043505	CENP-A containing nucleosome	bulk RNA-seq,Homo_723,AD.vs.MCI; bulk RNA-seq,Homo_714,AD.vs.MCI	2
CC	GO:0061638	CENP-A containing chromatin	bulk RNA-seq,Homo_723,AD.vs.MCI; bulk RNA-seq,Homo_714,AD.vs.MCI	2
BP	GO:0061644	protein localization to CENP-A containing chromatin	bulk RNA-seq,Homo_723,AD.vs.MCI; bulk RNA-seq,Homo_714,AD.vs.MCI	2
BP	GO:0050999	regulation of nitric-oxide synthase activity	bulk RNA-seq,Homo_723,AD.vs.MCI; bulk RNA-seq,Homo_723,MCI.vs.control; bulk RNA-seq,Homo_714,AD.vs.MCI; bulk RNA-seq,Homo_714,MCI.vs.control; bulk RNA-seq,Homo_633,AD.vs.control; scRNA-seq,SRP309935,Megakaryocyte_2-AD.vs.control	6
BP	GO:0046785	microtubule polymerization	bulk RNA-seq,Homo_723,AD.vs.MCI; bulk RNA-seq,Homo_723,MCI.vs.control; bulk RNA-seq,Homo_714,MCI.vs.control; bulk RNA-seq,Homo_633,AD.vs.control; bulk RNA-seq,Homo_633,AD.vs.MCI	5
BP	GO:0046823	negative regulation of nucleocytoplasmic transport	bulk RNA-seq,Homo_723,AD.vs.MCI; bulk RNA-seq,Homo_723,MCI.vs.control; bulk RNA-seq,Homo_714,AD.vs.MCI; bulk RNA-seq,Homo_714,MCI.vs.control; bulk RNA-seq,Homo_633,AD.vs.control; bulk RNA-seq,Homo_633,AD.vs.MCI	6
BP	GO:2000644	regulation of receptor catabolic process	bulk RNA-seq,Homo_723,AD.vs.MCI; bulk RNA-seq,Homo_723,MCI.vs.control; bulk RNA-seq,Homo_714,AD.vs.MCI; bulk RNA-seq,Homo_714,MCI.vs.control; bulk RNA-seq,Homo_633,AD.vs.control	5
BP	GO:1901989	positive regulation of cell cycle phase transition	bulk RNA-seq,Homo_723,AD.vs.MCI; bulk RNA-seq,Homo_723,MCI.vs.control; bulk RNA-seq,Homo_714,AD.vs.MCI; bulk RNA-seq,Homo_714,MCI.vs.control; bulk RNA-seq,Homo_633,AD.vs.control; bulk RNA-seq,Homo_633,AD.vs.MCI	6
BP	GO:0006809	nitric oxide biosynthetic process	bulk RNA-seq,Homo_723,AD.vs.MCI; bulk RNA-seq,Homo_723,MCI.vs.control; bulk RNA-seq,Homo_714,AD.vs.MCI; bulk RNA-seq,Homo_714,MCI.vs.control; bulk RNA-seq,Homo_633,AD.vs.control; bulk RNA-seq,Homo_633,AD.vs.MCI	6
BP	GO:0007063	regulation of sister chromatid cohesion	bulk RNA-seq,Homo_723,AD.vs.MCI; bulk RNA-seq,SRP310421,AD.vs.control	2
BP	GO:0051170	import into nucleus	bulk RNA-seq,Homo_723,AD.vs.MCI; bulk RNA-seq,Homo_723,MCI.vs.control; bulk RNA-seq,Homo_714,AD.vs.MCI; bulk RNA-seq,Homo_633,AD.vs.control; bulk RNA-seq,Homo_633,AD.vs.MCI	5
BP	GO:0035089	establishment of apical/basal cell polarity	bulk RNA-seq,Homo_723,AD.vs.MCI; bulk RNA-seq,Homo_723,MCI.vs.control	2
BP	GO:0051006	positive regulation of lipoprotein lipase activity	bulk RNA-seq,Homo_723,AD.vs.MCI; bulk RNA-seq,Homo_723,MCI.vs.control; bulk RNA-seq,Homo_714,AD.vs.MCI; bulk RNA-seq,Homo_714,MCI.vs.control	4
BP	GO:0060147	regulation of post-transcriptional gene silencing	bulk RNA-seq,Homo_723,AD.vs.MCI; bulk RNA-seq,Homo_723,MCI.vs.control; bulk RNA-seq,Homo_714,MCI.vs.control; bulk RNA-	4
BP	GO:0060391	positive regulation of SMAD protein signal transduction	bulk RNA-seq,Homo_723,AD.vs.MCI; bulk RNA-seq,Homo_714,AD.vs.MCI; bulk RNA-seq,Homo_633,AD.vs.control	3
BP	GO:0045542	positive regulation of cholesterol biosynthetic process	bulk RNA-seq,Homo_723,AD.vs.MCI; bulk RNA-seq,Homo_723,MCI.vs.control; bulk RNA-seq,Homo_633,AD.vs.control	3
BP	GO:0106120	positive regulation of sterol biosynthetic process	bulk RNA-seq,Homo_723,AD.vs.MCI; bulk RNA-seq,Homo_723,MCI.vs.control; bulk RNA-seq,Homo_633,AD.vs.control	3
BP	GO:0015740	C4-dicarboxylate transport	bulk RNA-seq,Homo_723,AD.vs.MCI; bulk RNA-seq,Homo_723,MCI.vs.control; bulk RNA-seq,Homo_633,AD.vs.control	3
CC	GO:0016471	vacuolar proton-transporting V-type ATPase complex	bulk RNA-seq,Homo_723,AD.vs.MCI; bulk RNA-seq,Homo_633,AD.vs.control; bulk RNA-seq,Homo_633,AD.vs.MCI	3
BP	GO:1904478	regulation of intestinal absorption	bulk RNA-seq,Homo_723,AD.vs.MCI; bulk RNA-seq,Homo_723,MCI.vs.control; bulk RNA-seq,Homo_714,AD.vs.MCI; bulk RNA-seq,Homo_714,MCI.vs.control	4
BP	GO:0060966	regulation of gene silencing by RNA	bulk RNA-seq,Homo_723,AD.vs.MCI; bulk RNA-seq,Homo_723,MCI.vs.control; bulk RNA-seq,Homo_714,MCI.vs.control; bulk RNA-seq,Homo_633,AD.vs.control; bulk RNA-seq,Homo_633,AD.vs.MCI	5
BP	GO:0031284	positive regulation of guanylate cyclase activity	bulk RNA-seq,Homo_723,AD.vs.MCI	1
BP	GO:0071897	DNA biosynthetic process	bulk RNA-seq,Homo_723,AD.vs.MCI; bulk RNA-seq,Homo_723,MCI.vs.control; bulk RNA-seq,Homo_714,AD.vs.MCI; bulk RNA-seq,Homo_633,AD.vs.control; bulk RNA-seq,Homo_633,AD.vs.MCI	5
BP	GO:0032095	regulation of response to food	bulk RNA-seq,Homo_723,AD.vs.MCI; bulk RNA-seq,Homo_723,MCI.vs.control; bulk RNA-seq,Homo_633,AD.vs.control	3
MF	GO:0043425	bHLH transcription factor binding	bulk RNA-seq,Homo_723,AD.vs.MCI; bulk RNA-seq,Homo_723,MCI.vs.control; bulk RNA-seq,Homo_714,AD.vs.MCI; bulk RNA-seq,Homo_714,MCI.vs.control; bulk RNA-seq,Homo_633,AD.vs.control; bulk RNA-seq,Homo_633,AD.vs.MCI	6
BP	GO:0045723	positive regulation of fatty acid biosynthetic process	bulk RNA-seq,Homo_723,AD.vs.MCI; bulk RNA-seq,Homo_723,MCI.vs.control; bulk RNA-seq,Homo_714,AD.vs.MCI; bulk RNA-seq,Homo_714,MCI.vs.control; bulk RNA-seq,Homo_633,AD.vs.control; bulk RNA-seq,Homo_633,AD.vs.MCI	6
CC	GO:0097224	sperm connecting piece	bulk RNA-seq,Homo_723,AD.vs.MCI	1
BP	GO:0016102	diterpenoid biosynthetic process	bulk RNA-seq,Homo_723,AD.vs.MCI; bulk RNA-seq,Homo_723,MCI.vs.control; bulk RNA-seq,Homo_714,MCI.vs.control	3
BP	GO:0046209	nitric oxide metabolic process	bulk RNA-seq,Homo_723,AD.vs.MCI; bulk RNA-seq,Homo_723,MCI.vs.control; bulk RNA-seq,Homo_714,AD.vs.MCI; bulk RNA-seq,Homo_714,MCI.vs.control; bulk RNA-seq,Homo_633,AD.vs.control; bulk RNA-seq,Homo_633,AD.vs.MCI	6

BP	GO:0061577	calcium ion transmembrane transport via high voltage-gated calcium channel	bulk RNA-seq,Homo_723,AD.vs.MCI; bulk RNA-seq,Homo_714,AD.vs.MCI	2
BP	GO:0051180	vitamin transport	bulk RNA-seq,Homo_723,AD.vs.MCI; bulk RNA-seq,Homo_723,MCI.vs.control; bulk RNA-seq,Homo_714,AD.vs.MCI	3
BP	GO:0042401	cellular biogenic amine biosynthetic process	bulk RNA-seq,Homo_723,AD.vs.MCI; bulk RNA-seq,Homo_723,MCI.vs.control; bulk RNA-seq,Homo_714,AD.vs.MCI; bulk RNA-seq,Homo_714,MCI.vs.control; bulk RNA-seq,Homo_633,AD.vs.control	5
BP	GO:0033028	myeloid cell apoptotic process	bulk RNA-seq,Homo_723,AD.vs.MCI; bulk RNA-seq,Homo_723,MCI.vs.control; bulk RNA-seq,Homo_714,AD.vs.MCI; bulk RNA-seq,Homo_714,MCI.vs.control; bulk RNA-seq,Homo_633,AD.vs.control; bulk RNA-seq,Homo_633,AD.vs.MCI	6
BP	GO:0032351	negative regulation of hormone metabolic process	bulk RNA-seq,Homo_723,AD.vs.MCI; bulk RNA-seq,Homo_723,MCI.vs.control; bulk RNA-seq,Homo_714,MCI.vs.control; bulk RNA-	4
BP	GO:0006109	regulation of carbohydrate metabolic process	bulk RNA-seq,Homo_723,AD.vs.MCI; bulk RNA-seq,Homo_723,MCI.vs.control; bulk RNA-seq,Homo_714,AD.vs.MCI; bulk RNA-seq,Homo_633,AD.vs.control; bulk RNA-seq,Homo_633,AD.vs.MCI	5
BP	GO:0014829	vascular associated smooth muscle contraction	bulk RNA-seq,Homo_723,AD.vs.MCI; bulk RNA-seq,Homo_723,MCI.vs.control; bulk RNA-seq,Homo_714,AD.vs.MCI; bulk RNA-seq,Homo_714,MCI.vs.control; bulk RNA-seq,Homo_633,AD.vs.control	5
BP	GO:0001574	ganglioside biosynthetic process	bulk RNA-seq,Homo_723,AD.vs.MCI; bulk RNA-seq,Homo_723,MCI.vs.control; bulk RNA-seq,Homo_633,MCI.vs.control	3
BP	GO:1903317	regulation of protein maturation	bulk RNA-seq,Homo_723,AD.vs.MCI; bulk RNA-seq,Homo_723,MCI.vs.control; bulk RNA-seq,Homo_714,AD.vs.MCI; bulk RNA-seq,Homo_714,MCI.vs.control; bulk RNA-seq,Homo_633,AD.vs.control; bulk RNA-seq,Homo_633,AD.vs.MCI	6
BP	GO:0022038	corpus callosum development	bulk RNA-seq,Homo_723,AD.vs.MCI; bulk RNA-seq,Homo_633,AD.vs.control; bulk RNA-seq,Homo_633,AD.vs.MCI	3
BP	GO:0033007	negative regulation of mast cell activation involved in	bulk RNA-seq,Homo_723,AD.vs.MCI; bulk RNA-seq,Homo_723,MCI.vs.control; bulk RNA-seq,Homo_714,AD.vs.MCI; bulk RNA-seq,Homo_714,MCI.vs.control	4
BP	GO:0002448	mast cell mediated immunity	bulk RNA-seq,Homo_723,AD.vs.MCI; bulk RNA-seq,Homo_723,MCI.vs.control; bulk RNA-seq,Homo_714,AD.vs.MCI; bulk RNA-seq,Homo_714,MCI.vs.control; bulk RNA-seq,Homo_633,AD.vs.control; bulk RNA-seq,Homo_633,AD.vs.MCI; scRNA-seq,SRP215507,CD8+ T cell_1-	7
BP	GO:0071732	cellular response to nitric oxide	bulk RNA-seq,Homo_723,AD.vs.MCI; bulk RNA-seq,Homo_723,MCI.vs.control; bulk RNA-seq,Homo_714,MCI.vs.control; bulk RNA-	4
BP	GO:0033189	response to vitamin A	bulk RNA-seq,Homo_723,AD.vs.MCI; bulk RNA-seq,Homo_723,MCI.vs.control; bulk RNA-seq,Homo_714,AD.vs.MCI; bulk RNA-seq,Homo_714,MCI.vs.control	4
BP	GO:0034341	response to interferon-gamma	bulk RNA-seq,Homo_723,AD.vs.MCI; bulk RNA-seq,Homo_723,MCI.vs.control; bulk RNA-seq,Homo_714,AD.vs.MCI; bulk RNA-seq,Homo_633,AD.vs.control; bulk RNA-seq,Homo_633,AD.vs.MCI	5
BP	GO:0036296	response to increased oxygen levels	bulk RNA-seq,Homo_723,AD.vs.MCI; bulk RNA-seq,Homo_723,MCI.vs.control; bulk RNA-seq,Homo_714,AD.vs.MCI; bulk RNA-seq,Homo_714,MCI.vs.control; bulk RNA-seq,Homo_633,AD.vs.control; bulk RNA-seq,Homo_633,AD.vs.MCI	6
BP	GO:0071372	cellular response to follicle-stimulating hormone stimulus	bulk RNA-seq,Homo_723,AD.vs.MCI	1
BP	GO:1902882	regulation of response to oxidative stress	bulk RNA-seq,Homo_723,AD.vs.MCI; bulk RNA-seq,Homo_723,MCI.vs.control; bulk RNA-seq,Homo_714,AD.vs.MCI; bulk RNA-seq,Homo_714,MCI.vs.control; bulk RNA-seq,Homo_633,AD.vs.control; bulk RNA-seq,Homo_633,AD.vs.MCI	6
CC	GO:0031965	nuclear membrane	bulk RNA-seq,Homo_723,AD.vs.MCI; bulk RNA-seq,Homo_723,MCI.vs.control; bulk RNA-seq,Homo_714,AD.vs.control; bulk RNA-seq,Homo_633,AD.vs.control; bulk RNA-seq,Homo_633,AD.vs.MCI; scRNA-seq,SRP330776,Naive CD8+ T cell_2-AD.vs.control	6
BP	GO:0043537	negative regulation of blood vessel endothelial cell migration	bulk RNA-seq,Homo_723,AD.vs.MCI; bulk RNA-seq,Homo_714,AD.vs.control; bulk RNA-seq,Homo_714,AD.vs.MCI	3
BP	GO:0033137	negative regulation of peptidyl-serine phosphorylation	bulk RNA-seq,Homo_723,AD.vs.MCI; bulk RNA-seq,Homo_723,MCI.vs.control; bulk RNA-seq,Homo_714,AD.vs.MCI; bulk RNA-seq,Homo_714,MCI.vs.control; bulk RNA-seq,Homo_633,AD.vs.control; bulk RNA-seq,Homo_633,AD.vs.MCI	6
CC	GO:0030496	midbody	bulk RNA-seq,Homo_723,AD.vs.MCI; bulk RNA-seq,Homo_723,MCI.vs.control; bulk RNA-seq,Homo_714,AD.vs.control; bulk RNA-seq,Homo_633,AD.vs.control; bulk RNA-seq,Homo_633,AD.vs.MCI	5
BP	GO:0010611	regulation of cardiac muscle hypertrophy	bulk RNA-seq,Homo_723,AD.vs.MCI; bulk RNA-seq,Homo_714,AD.vs.MCI; bulk RNA-seq,Homo_714,MCI.vs.control; bulk RNA-seq,Homo_633,AD.vs.control	4
BP	GO:0010955	negative regulation of protein processing	bulk RNA-seq,Homo_723,AD.vs.MCI	1
BP	GO:1903318	negative regulation of protein maturation	bulk RNA-seq,Homo_723,AD.vs.MCI	1
BP	GO:0043949	regulation of cAMP-mediated signaling	bulk RNA-seq,Homo_723,AD.vs.MCI	1
BP	GO:2000392	regulation of lamellipodium morphogenesis	bulk RNA-seq,Homo_723,AD.vs.MCI; bulk RNA-seq,Homo_723,MCI.vs.control; bulk RNA-seq,Homo_714,AD.vs.MCI	3
MF	GO:0019903	protein phosphatase binding	bulk RNA-seq,Homo_723,AD.vs.MCI; bulk RNA-seq,Homo_723,MCI.vs.control; bulk RNA-seq,Homo_714,AD.vs.MCI; bulk RNA-seq,Homo_633,AD.vs.control; bulk RNA-seq,Homo_633,AD.vs.MCI	5
BP	GO:0042307	positive regulation of protein import into nucleus	bulk RNA-seq,Homo_723,AD.vs.MCI; bulk RNA-seq,Homo_723,MCI.vs.control; bulk RNA-seq,Homo_714,MCI.vs.control; bulk RNA-seq,Homo_633,AD.vs.control; bulk RNA-seq,Homo_633,AD.vs.MCI	5
MF	GO:0005041	low-density lipoprotein particle receptor activity	bulk RNA-seq,Homo_723,AD.vs.MCI; bulk RNA-seq,Homo_633,AD.vs.control; bulk RNA-seq,ROSMAP,AD.vs.control; bulk RNA-seq,ROSMAP,MCI.vs.control	4
BP	GO:0033006	regulation of mast cell activation involved in immune response	bulk RNA-seq,Homo_723,AD.vs.MCI; bulk RNA-seq,Homo_723,MCI.vs.control; bulk RNA-seq,Homo_714,AD.vs.MCI; bulk RNA-seq,Homo_714,MCI.vs.control; bulk RNA-seq,Homo_633,AD.vs.control; bulk RNA-seq,Homo_633,AD.vs.MCI	6
BP	GO:0016050	vesicle organization	bulk RNA-seq,Homo_723,AD.vs.MCI; bulk RNA-seq,Homo_714,AD.vs.control; bulk RNA-seq,Homo_633,AD.vs.control; bulk RNA-seq,Homo_633,AD.vs.MCI	4
MF	GO:0019215	intermediate filament binding	bulk RNA-seq,Homo_723,AD.vs.MCI; bulk RNA-seq,Homo_723,MCI.vs.control; bulk RNA-seq,Homo_714,AD.vs.MCI; bulk RNA-seq,Homo_714,MCI.vs.control	4
BP	GO:0046467	membrane lipid biosynthetic process	bulk RNA-seq,Homo_723,AD.vs.MCI; bulk RNA-seq,Homo_723,MCI.vs.control; bulk RNA-seq,Homo_714,AD.vs.control; bulk RNA-seq,Homo_633,AD.vs.control; bulk RNA-seq,Homo_633,AD.vs.MCI	5
BP	GO:0031570	DNA integrity checkpoint signaling	bulk RNA-seq,Homo_723,MCI.vs.control; bulk RNA-seq,Homo_714,AD.vs.control; bulk RNA-seq,Homo_633,AD.vs.control; bulk RNA-	4
BP	GO:1902749	regulation of cell cycle G2/M phase transition	bulk RNA-seq,Homo_723,MCI.vs.control; bulk RNA-seq,Homo_714,AD.vs.MCI; bulk RNA-seq,Homo_714,MCI.vs.control; bulk RNA-seq,Homo_633,AD.vs.control; bulk RNA-seq,Homo_633,AD.vs.MCI	5
BP	GO:0018393	internal peptidyl-lysine acetylation	bulk RNA-seq,Homo_723,MCI.vs.control; bulk RNA-seq,Homo_714,AD.vs.MCI; bulk RNA-seq,Homo_633,AD.vs.control; bulk RNA-seq,Homo_633,AD.vs.MCI	4
BP	GO:0044773	mitotic DNA damage checkpoint signaling	bulk RNA-seq,Homo_723,MCI.vs.control; bulk RNA-seq,Homo_714,MCI.vs.control; bulk RNA-seq,Homo_633,AD.vs.control; bulk RNA-	4
BP	GO:0016573	histone acetylation	bulk RNA-seq,Homo_723,MCI.vs.control; bulk RNA-seq,Homo_714,AD.vs.MCI; bulk RNA-seq,Homo_633,AD.vs.control; bulk RNA-seq,Homo_633,AD.vs.MCI	4
BP	GO:0006475	internal protein amino acid acetylation	bulk RNA-seq,Homo_723,MCI.vs.control; bulk RNA-seq,Homo_714,AD.vs.MCI; bulk RNA-seq,Homo_633,AD.vs.control; bulk RNA-seq,Homo_633,AD.vs.MCI	4
MF	GO:0008094	ATP-dependent activity, acting on DNA	bulk RNA-seq,Homo_723,MCI.vs.control; bulk RNA-seq,Homo_714,AD.vs.MCI; bulk RNA-seq,Homo_714,MCI.vs.control; bulk RNA-seq,Homo_633,AD.vs.control; bulk RNA-seq,Homo_633,AD.vs.MCI	5
BP	GO:0040029	epigenetic regulation of gene expression	bulk RNA-seq,Homo_723,MCI.vs.control; bulk RNA-seq,Homo_714,AD.vs.MCI; bulk RNA-seq,Homo_633,AD.vs.control; bulk RNA-seq,Homo_633,AD.vs.MCI	4

BP	GO:0032388	positive regulation of intracellular transport	bulk RNA-seq,Homo_723,MCI.vs.control; bulk RNA-seq,Homo_633,AD.vs.control; bulk RNA-seq,Homo_633,AD.vs.MCI	3
BP	GO:0031056	regulation of histone modification	bulk RNA-seq,Homo_723,MCI.vs.control; bulk RNA-seq,Homo_633,AD.vs.control; bulk RNA-seq,Homo_633,AD.vs.MCI	3
BP	GO:0046474	glycerophospholipid biosynthetic process	bulk RNA-seq,Homo_723,MCI.vs.control; bulk RNA-seq,Homo_714,AD.vs.control; bulk RNA-seq,Homo_633,AD.vs.control; bulk RNA-seq,Homo_633,AD.vs.MCI; scRNA-seq,SRP330776,B cell_1-AD.vs.control; scRNA-seq,SRP330776,CD8+ T cell_2-AD.vs.control	6
BP	GO:0090150	establishment of protein localization to membrane	bulk RNA-seq,Homo_723,MCI.vs.control; bulk RNA-seq,Homo_714,AD.vs.control; bulk RNA-seq,Homo_633,AD.vs.control; bulk RNA-	4
BP	GO:0045017	glycerolipid biosynthetic process	bulk RNA-seq,Homo_723,MCI.vs.control; bulk RNA-seq,Homo_714,AD.vs.control; bulk RNA-seq,Homo_633,AD.vs.control; bulk RNA-seq,Homo_633,AD.vs.MCI; scRNA-seq,SRP330776,B cell_1-AD.vs.control; scRNA-seq,SRP330776,CD8+ T cell_2-AD.vs.control; scRNA-seq,SRP330776,Naive	7
MF	GO:0044389	ubiquitin-like protein ligase binding	bulk RNA-seq,Homo_723,MCI.vs.control; bulk RNA-seq,Homo_633,AD.vs.control; bulk RNA-seq,Homo_633,AD.vs.MCI	3
BP	GO:0090316	positive regulation of intracellular protein transport	bulk RNA-seq,Homo_723,MCI.vs.control; bulk RNA-seq,Homo_633,AD.vs.control; bulk RNA-seq,Homo_633,AD.vs.MCI	3
BP	GO:0010212	response to ionizing radiation	bulk RNA-seq,Homo_723,MCI.vs.control; bulk RNA-seq,Homo_714,AD.vs.MCI; bulk RNA-seq,Homo_633,AD.vs.control; bulk RNA-seq,Homo_633,AD.vs.MCI	4
MF	GO:0000287	magnesium ion binding	bulk RNA-seq,Homo_723,MCI.vs.control; bulk RNA-seq,Homo_714,AD.vs.MCI; bulk RNA-seq,Homo_633,AD.vs.control; bulk RNA-seq,Homo_633,AD.vs.MCI	4
BP	GO:0006275	regulation of DNA replication	bulk RNA-seq,Homo_723,MCI.vs.control; bulk RNA-seq,Homo_633,AD.vs.control; bulk RNA-seq,Homo_633,AD.vs.MCI	3
BP	GO:0007249	I-kappaB kinase/NF-kappaB signaling	bulk RNA-seq,Homo_723,MCI.vs.control; bulk RNA-seq,Homo_714,AD.vs.MCI; bulk RNA-seq,Homo_633,AD.vs.control; bulk RNA-seq,Homo_633,AD.vs.MCI	4
BP	GO:2000401	regulation of lymphocyte migration	bulk RNA-seq,Homo_723,MCI.vs.control; bulk RNA-seq,Homo_714,AD.vs.MCI; bulk RNA-seq,Homo_714,MCI.vs.control; bulk RNA-	4
MF	GO:0031625	ubiquitin protein ligase binding	bulk RNA-seq,Homo_723,MCI.vs.control; bulk RNA-seq,Homo_633,AD.vs.control; bulk RNA-seq,Homo_633,AD.vs.MCI	3
BP	GO:1901991	negative regulation of mitotic cell cycle phase transition	bulk RNA-seq,Homo_723,MCI.vs.control; bulk RNA-seq,Homo_714,AD.vs.MCI; bulk RNA-seq,Homo_633,AD.vs.control; bulk RNA-seq,Homo_633,AD.vs.MCI	4
CC	GO:0005814	centriole	bulk RNA-seq,Homo_723,MCI.vs.control; bulk RNA-seq,Homo_633,AD.vs.control; bulk RNA-seq,Homo_633,AD.vs.MCI	3
CC	GO:0055038	recycling endosome membrane	bulk RNA-seq,Homo_723,MCI.vs.control; bulk RNA-seq,Homo_714,MCI.vs.control; bulk RNA-seq,Homo_633,AD.vs.control; bulk RNA-	4
BP	GO:0062207	regulation of pattern recognition receptor signaling pathway	bulk RNA-seq,Homo_723,MCI.vs.control; bulk RNA-seq,Homo_714,MCI.vs.control; bulk RNA-seq,Homo_633,AD.vs.control; bulk RNA-	4
BP	GO:0030866	cortical actin cytoskeleton organization	bulk RNA-seq,Homo_723,MCI.vs.control; bulk RNA-seq,Homo_714,MCI.vs.control; bulk RNA-seq,Homo_633,AD.vs.control; bulk RNA-	4
MF	GO:0031072	heat shock protein binding	bulk RNA-seq,Homo_723,MCI.vs.control; bulk RNA-seq,Homo_714,MCI.vs.control; bulk RNA-seq,Homo_633,AD.vs.control; bulk RNA-	4
BP	GO:0071806	protein transmembrane transport	bulk RNA-seq,Homo_723,MCI.vs.control; bulk RNA-seq,Homo_714,MCI.vs.control; bulk RNA-seq,Homo_633,AD.vs.control; bulk RNA-	4
BP	GO:0002700	regulation of production of molecular mediator of immune	bulk RNA-seq,Homo_723,MCI.vs.control; bulk RNA-seq,Homo_714,AD.vs.MCI; bulk RNA-seq,Homo_633,AD.vs.control; bulk RNA-seq,Homo_633,AD.vs.MCI	4
BP	GO:0031058	positive regulation of histone modification	bulk RNA-seq,Homo_723,MCI.vs.control; bulk RNA-seq,Homo_714,AD.vs.MCI; bulk RNA-seq,Homo_714,MCI.vs.control; bulk RNA-seq,Homo_633,AD.vs.control; bulk RNA-seq,Homo_633,AD.vs.MCI	5
BP	GO:0030518	intracellular steroid hormone receptor signaling pathway	bulk RNA-seq,Homo_723,MCI.vs.control; bulk RNA-seq,Homo_714,AD.vs.MCI; bulk RNA-seq,Homo_714,MCI.vs.control; bulk RNA-seq,Homo_633,AD.vs.control; bulk RNA-seq,Homo_633,AD.vs.MCI	5
BP	GO:0043123	positive regulation of I-kappaB kinase/NF-kappaB signaling	bulk RNA-seq,Homo_723,MCI.vs.control; bulk RNA-seq,Homo_633,AD.vs.control; bulk RNA-seq,Homo_633,AD.vs.MCI	3
BP	GO:1903052	positive regulation of proteolysis involved in protein	bulk RNA-seq,Homo_723,MCI.vs.control; bulk RNA-seq,Homo_633,AD.vs.control; bulk RNA-seq,Homo_633,AD.vs.MCI	3
BP	GO:1903578	regulation of ATP metabolic process	bulk RNA-seq,Homo_723,MCI.vs.control; bulk RNA-seq,Homo_714,AD.vs.MCI; bulk RNA-seq,Homo_714,MCI.vs.control; bulk RNA-seq,Homo_633,AD.vs.control; bulk RNA-seq,Homo_633,AD.vs.MCI	5
BP	GO:0031396	regulation of protein ubiquitination	bulk RNA-seq,Homo_723,MCI.vs.control; bulk RNA-seq,Homo_714,AD.vs.MCI; bulk RNA-seq,Homo_633,AD.vs.control; bulk RNA-seq,Homo_633,AD.vs.MCI	4
BP	GO:0016571	histone methylation	bulk RNA-seq,Homo_723,MCI.vs.control; bulk RNA-seq,Homo_633,AD.vs.control; bulk RNA-seq,Homo_633,AD.vs.MCI	3
BP	GO:0070663	regulation of leukocyte proliferation	bulk RNA-seq,Homo_723,MCI.vs.control; bulk RNA-seq,Homo_714,AD.vs.MCI; bulk RNA-seq,Homo_633,AD.vs.control; bulk RNA-seq,Homo_633,AD.vs.MCI	4
MF	GO:0016616	oxidoreductase activity, acting on the CH-OH group of donors, NAD or NADP as acceptor	bulk RNA-seq,Homo_723,MCI.vs.control; bulk RNA-seq,Homo_714,AD.vs.MCI; bulk RNA-seq,Homo_714,MCI.vs.control; bulk RNA-seq,Homo_633,AD.vs.control; bulk RNA-seq,Homo_633,AD.vs.MCI	5
BP	GO:0045814	negative regulation of gene expression, epigenetic	bulk RNA-seq,Homo_723,MCI.vs.control; bulk RNA-seq,Homo_714,AD.vs.MCI; bulk RNA-seq,Homo_714,MCI.vs.control; bulk RNA-seq,Homo_633,AD.vs.control; bulk RNA-seq,Homo_633,AD.vs.MCI	5
BP	GO:0042098	T cell proliferation	bulk RNA-seq,Homo_723,MCI.vs.control; bulk RNA-seq,Homo_714,AD.vs.MCI; bulk RNA-seq,Homo_633,AD.vs.control; bulk RNA-seq,Homo_633,AD.vs.MCI	4
BP	GO:0007098	centrosome cycle	bulk RNA-seq,Homo_723,MCI.vs.control; bulk RNA-seq,Homo_714,AD.vs.MCI; bulk RNA-seq,Homo_633,AD.vs.control; bulk RNA-seq,Homo_633,AD.vs.MCI	4
BP	GO:0043967	histone H4 acetylation	bulk RNA-seq,Homo_723,MCI.vs.control; bulk RNA-seq,Homo_714,MCI.vs.control; bulk RNA-seq,Homo_633,AD.vs.control; bulk RNA-	4
BP	GO:0046822	regulation of nucleocytoplasmic transport	bulk RNA-seq,Homo_723,MCI.vs.control; bulk RNA-seq,Homo_714,AD.vs.MCI; bulk RNA-seq,Homo_714,MCI.vs.control; bulk RNA-seq,Homo_633,AD.vs.control; bulk RNA-seq,Homo_633,AD.vs.MCI	5
BP	GO:0044728	DNA methylation or demethylation	bulk RNA-seq,Homo_723,MCI.vs.control; bulk RNA-seq,Homo_714,MCI.vs.control; bulk RNA-seq,Homo_633,AD.vs.control; bulk RNA-	4
BP	GO:0048284	organelle fusion	bulk RNA-seq,Homo_723,MCI.vs.control; bulk RNA-seq,Homo_633,AD.vs.control; bulk RNA-seq,Homo_633,AD.vs.MCI	3
BP	GO:0070828	heterochromatin organization	bulk RNA-seq,Homo_723,MCI.vs.control; bulk RNA-seq,Homo_714,AD.vs.MCI; bulk RNA-seq,Homo_714,MCI.vs.control; bulk RNA-seq,Homo_633,AD.vs.control; bulk RNA-seq,Homo_633,AD.vs.MCI	5
BP	GO:0048525	negative regulation of viral process	bulk RNA-seq,Homo_723,MCI.vs.control; bulk RNA-seq,Homo_714,AD.vs.MCI; bulk RNA-seq,Homo_714,MCI.vs.control; bulk RNA-	4
BP	GO:0043122	regulation of I-kappaB kinase/NF-kappaB signaling	bulk RNA-seq,Homo_723,MCI.vs.control; bulk RNA-seq,Homo_633,AD.vs.control; bulk RNA-seq,Homo_633,AD.vs.MCI	3
CC	GO:0010494	cytoplasmic stress granule	bulk RNA-seq,Homo_723,MCI.vs.control; bulk RNA-seq,Homo_714,AD.vs.control; bulk RNA-seq,Homo_714,MCI.vs.control; bulk RNA-seq,Homo_633,AD.vs.control; bulk RNA-seq,Homo_633,AD.vs.MCI	5
BP	GO:2000278	regulation of DNA biosynthetic process	bulk RNA-seq,Homo_723,MCI.vs.control; bulk RNA-seq,Homo_714,AD.vs.control; bulk RNA-seq,Homo_714,MCI.vs.control; bulk RNA-seq,Homo_633,AD.vs.control; bulk RNA-seq,Homo_633,AD.vs.MCI; bulk RNA-seq,SRP223445,AD.vs.control	6
CC	GO:1902554	serine/threonine protein kinase complex	bulk RNA-seq,Homo_723,MCI.vs.control; bulk RNA-seq,Homo_714,MCI.vs.control; bulk RNA-seq,Homo_633,AD.vs.control; bulk RNA-	4
BP	GO:0006606	protein import into nucleus	bulk RNA-seq,Homo_723,MCI.vs.control; bulk RNA-seq,Homo_714,AD.vs.MCI; bulk RNA-seq,Homo_633,AD.vs.control; bulk RNA-seq,Homo_633,AD.vs.MCI	4
BP	GO:0051784	negative regulation of nuclear division	bulk RNA-seq,Homo_723,MCI.vs.control; bulk RNA-seq,Homo_714,MCI.vs.control; bulk RNA-seq,Homo_633,AD.vs.control; bulk RNA-	4
BP	GO:0018022	peptidyl-lysine methylation	bulk RNA-seq,Homo_723,MCI.vs.control; bulk RNA-seq,Homo_633,AD.vs.control; bulk RNA-seq,Homo_633,AD.vs.MCI	3
BP	GO:0032943	mononuclear cell proliferation	bulk RNA-seq,Homo_723,MCI.vs.control; bulk RNA-seq,Homo_714,AD.vs.MCI; bulk RNA-seq,Homo_633,AD.vs.control; bulk RNA-seq,Homo_633,AD.vs.MCI	4

BP	GO:1902751	positive regulation of cell cycle G2/M phase transition	bulk RNA-seq,Homo_723,MCI.vs.control; bulk RNA-seq,Homo_714,AD.vs.MCI; bulk RNA-seq,Homo_714,MCI.vs.control; bulk RNA-seq,Homo_633,AD.vs.control; bulk RNA-seq,Homo_633,AD.vs.MCI; bulk RNA-seq,Homo_633,MCI.vs.control	6
BP	GO:0016239	positive regulation of macroautophagy	bulk RNA-seq,Homo_723,MCI.vs.control; bulk RNA-seq,Homo_714,MCI.vs.control; bulk RNA-seq,Homo_633,AD.vs.control; bulk RNA-	4
CC	GO:0034451	centriolar satellite	bulk RNA-seq,Homo_723,MCI.vs.control; bulk RNA-seq,Homo_714,AD.vs.MCI; bulk RNA-seq,Homo_714,MCI.vs.control; bulk RNA-seq,Homo_633,AD.vs.control; bulk RNA-seq,Homo_633,AD.vs.MCI	5
MF	GO:0003678	DNA helicase activity	bulk RNA-seq,Homo_723,MCI.vs.control; bulk RNA-seq,Homo_714,MCI.vs.control; bulk RNA-seq,Homo_633,AD.vs.control; bulk RNA-	4
BP	GO:0034968	histone lysine methylation	bulk RNA-seq,Homo_723,MCI.vs.control; bulk RNA-seq,Homo_714,AD.vs.MCI; bulk RNA-seq,Homo_633,AD.vs.control; bulk RNA-seq,Homo_633,AD.vs.MCI	4
CC	GO:0016363	nuclear matrix	bulk RNA-seq,Homo_723,MCI.vs.control; bulk RNA-seq,Homo_714,MCI.vs.control; bulk RNA-seq,Homo_633,AD.vs.control; bulk RNA-	4
BP	GO:0046939	nucleotide phosphorylation	bulk RNA-seq,Homo_723,MCI.vs.control; bulk RNA-seq,Homo_714,MCI.vs.control; bulk RNA-seq,Homo_633,AD.vs.control; bulk RNA-	4
BP	GO:0010972	negative regulation of G2/M transition of mitotic cell cycle	bulk RNA-seq,Homo_723,MCI.vs.control; bulk RNA-seq,Homo_714,MCI.vs.control; bulk RNA-seq,Homo_633,AD.vs.control; bulk RNA-	4
BP	GO:0042129	regulation of T cell proliferation	bulk RNA-seq,Homo_723,MCI.vs.control; bulk RNA-seq,Homo_714,AD.vs.MCI; bulk RNA-seq,Homo_633,AD.vs.control; bulk RNA-seq,Homo_633,AD.vs.MCI	4
BP	GO:0046651	lymphocyte proliferation	bulk RNA-seq,Homo_723,MCI.vs.control; bulk RNA-seq,Homo_714,AD.vs.MCI; bulk RNA-seq,Homo_633,AD.vs.control; bulk RNA-seq,Homo_633,AD.vs.MCI	4
BP	GO:0043648	dicarboxylic acid metabolic process	bulk RNA-seq,Homo_723,MCI.vs.control; bulk RNA-seq,Homo_714,MCI.vs.control; bulk RNA-seq,Homo_633,AD.vs.control; bulk RNA-	4
BP	GO:0009132	nucleoside diphosphate metabolic process	bulk RNA-seq,Homo_723,MCI.vs.control; bulk RNA-seq,Homo_633,AD.vs.control; bulk RNA-seq,Homo_633,AD.vs.MCI	3
BP	GO:0032944	regulation of mononuclear cell proliferation	bulk RNA-seq,Homo_723,MCI.vs.control; bulk RNA-seq,Homo_714,AD.vs.MCI; bulk RNA-seq,Homo_633,AD.vs.control; bulk RNA-seq,Homo_633,AD.vs.MCI; scRNA-seq,SRP215507,CD8+ T cell_1-MCI.vs.control	5
BP	GO:0009135	purine nucleoside diphosphate metabolic process	bulk RNA-seq,Homo_723,MCI.vs.control; bulk RNA-seq,Homo_714,MCI.vs.control; bulk RNA-seq,Homo_633,AD.vs.control; bulk RNA-	4
BP	GO:0009179	purine ribonucleoside diphosphate metabolic process	bulk RNA-seq,Homo_723,MCI.vs.control; bulk RNA-seq,Homo_714,MCI.vs.control; bulk RNA-seq,Homo_633,AD.vs.control; bulk RNA-	4
BP	GO:0032760	positive regulation of tumor necrosis factor production	bulk RNA-seq,Homo_723,MCI.vs.control; bulk RNA-seq,Homo_714,AD.vs.MCI; bulk RNA-seq,Homo_714,MCI.vs.control; bulk RNA-seq,Homo_633,AD.vs.control; bulk RNA-seq,Homo_633,AD.vs.MCI	5
BP	GO:0034502	protein localization to chromosome	bulk RNA-seq,Homo_723,MCI.vs.control; bulk RNA-seq,Homo_714,AD.vs.MCI; bulk RNA-seq,Homo_714,MCI.vs.control; bulk RNA-seq,Homo_633,AD.vs.control; bulk RNA-seq,Homo_633,AD.vs.MCI	5
BP	GO:0009185	ribonucleoside diphosphate metabolic process	bulk RNA-seq,Homo_723,MCI.vs.control; bulk RNA-seq,Homo_714,MCI.vs.control; bulk RNA-seq,Homo_633,AD.vs.control; bulk RNA-	4
BP	GO:0044818	mitotic G2/M transition checkpoint	bulk RNA-seq,Homo_723,MCI.vs.control; bulk RNA-seq,Homo_714,MCI.vs.control; bulk RNA-seq,Homo_633,AD.vs.control; bulk RNA-	4
BP	GO:0032418	lysosome localization	bulk RNA-seq,Homo_723,MCI.vs.control; bulk RNA-seq,Homo_714,MCI.vs.control; bulk RNA-seq,Homo_633,AD.vs.control; bulk RNA-	4
BP	GO:1990849	vacuolar localization	bulk RNA-seq,Homo_723,MCI.vs.control; bulk RNA-seq,Homo_714,MCI.vs.control; bulk RNA-seq,Homo_633,AD.vs.control; bulk RNA-	4
BP	GO:0072384	organelle transport along microtubule	bulk RNA-seq,Homo_723,MCI.vs.control; bulk RNA-seq,Homo_714,MCI.vs.control; bulk RNA-seq,Homo_633,AD.vs.control; bulk RNA-	4
MF	GO:0043621	protein self-association	bulk RNA-seq,Homo_723,MCI.vs.control; bulk RNA-seq,Homo_714,AD.vs.MCI; bulk RNA-seq,Homo_714,MCI.vs.control; bulk RNA-seq,Homo_633,AD.vs.control; bulk RNA-seq,Homo_633,AD.vs.MCI	5
BP	GO:0031507	heterochromatin formation	bulk RNA-seq,Homo_723,MCI.vs.control; bulk RNA-seq,Homo_714,AD.vs.MCI; bulk RNA-seq,Homo_714,MCI.vs.control; bulk RNA-seq,Homo_633,AD.vs.control; bulk RNA-seq,Homo_633,AD.vs.MCI	5
CC	GO:0005876	spindle microtubule	bulk RNA-seq,Homo_723,MCI.vs.control; bulk RNA-seq,Homo_714,MCI.vs.control; bulk RNA-seq,Homo_633,AD.vs.control; bulk RNA-	4
BP	GO:0061484	hematopoietic stem cell homeostasis	bulk RNA-seq,Homo_723,MCI.vs.control; bulk RNA-seq,Homo_714,MCI.vs.control; bulk RNA-seq,Homo_633,AD.vs.control	3
BP	GO:0090329	regulation of DNA-templated DNA replication	bulk RNA-seq,Homo_723,MCI.vs.control; bulk RNA-seq,Homo_714,MCI.vs.control; bulk RNA-seq,Homo_633,AD.vs.control; bulk RNA-	4
BP	GO:0007095	mitotic G2 DNA damage checkpoint signaling	bulk RNA-seq,Homo_723,MCI.vs.control; bulk RNA-seq,Homo_714,MCI.vs.control; bulk RNA-seq,Homo_633,AD.vs.control; bulk RNA-seq,Homo_633,AD.vs.MCI; bulk RNA-seq,Homo_633,MCI.vs.control	5
BP	GO:0006757	ATP generation from ADP	bulk RNA-seq,Homo_723,MCI.vs.control; bulk RNA-seq,Homo_714,AD.vs.MCI; bulk RNA-seq,Homo_714,MCI.vs.control; bulk RNA-seq,Homo_633,AD.vs.control; bulk RNA-seq,Homo_633,AD.vs.MCI	5
BP	GO:0006513	protein monoubiquitination	bulk RNA-seq,Homo_723,MCI.vs.control; bulk RNA-seq,Homo_714,MCI.vs.control; bulk RNA-seq,Homo_633,AD.vs.control; bulk RNA-	4
BP	GO:0035966	response to topologically incorrect protein	bulk RNA-seq,Homo_723,MCI.vs.control; bulk RNA-seq,Homo_714,AD.vs.MCI; bulk RNA-seq,Homo_633,AD.vs.control; bulk RNA-seq,Homo_633,AD.vs.MCI	4
CC	GO:0005811	lipid droplet	bulk RNA-seq,Homo_723,MCI.vs.control; bulk RNA-seq,Homo_714,AD.vs.MCI; bulk RNA-seq,Homo_714,MCI.vs.control; bulk RNA-seq,Homo_633,AD.vs.control; bulk RNA-seq,Homo_633,AD.vs.MCI	5
BP	GO:1903580	positive regulation of ATP metabolic process	bulk RNA-seq,Homo_723,MCI.vs.control; bulk RNA-seq,Homo_714,MCI.vs.control; bulk RNA-seq,Homo_633,AD.vs.control; bulk RNA-seq,Homo_633,AD.vs.MCI; bulk RNA-seq,Homo_633,MCI.vs.control	5
BP	GO:0006090	pyruvate metabolic process	bulk RNA-seq,Homo_723,MCI.vs.control; bulk RNA-seq,Homo_714,AD.vs.MCI; bulk RNA-seq,Homo_714,MCI.vs.control; bulk RNA-seq,Homo_633,AD.vs.control; bulk RNA-seq,Homo_633,AD.vs.MCI	5
CC	GO:0000123	histone acetyltransferase complex	bulk RNA-seq,Homo_723,MCI.vs.control; bulk RNA-seq,Homo_714,MCI.vs.control; bulk RNA-seq,Homo_633,AD.vs.control; bulk RNA-	4
BP	GO:0036336	dendritic cell migration	bulk RNA-seq,Homo_723,MCI.vs.control; bulk RNA-seq,Homo_714,AD.vs.MCI; bulk RNA-seq,Homo_714,MCI.vs.control; bulk RNA-seq,Homo_633,AD.vs.control; bulk RNA-seq,Homo_633,AD.vs.MCI; bulk RNA-seq,Homo_633,MCI.vs.control	6
MF	GO:0061659	ubiquitin-like protein ligase activity	bulk RNA-seq,Homo_723,MCI.vs.control; bulk RNA-seq,Homo_633,AD.vs.control; bulk RNA-seq,Homo_633,AD.vs.MCI; scRNA-seq,SRP330776,B cell_1-AD.vs.control; scRNA-seq,SRP330776,B cell_2-AD.vs.control; scRNA-seq,SRP330776,CD8+ T cell_1-AD.vs.control; scRNA-seq,SRP330776,Memory T cell_2-	7
CC	GO:0031461	cullin-RING ubiquitin ligase complex	bulk RNA-seq,Homo_723,MCI.vs.control; bulk RNA-seq,Homo_714,AD.vs.control; bulk RNA-seq,Homo_633,AD.vs.control; bulk RNA-	4
MF	GO:0097718	disordered domain specific binding	bulk RNA-seq,Homo_723,MCI.vs.control; bulk RNA-seq,Homo_714,MCI.vs.control; bulk RNA-seq,Homo_633,AD.vs.control; bulk RNA-	4
MF	GO:0061630	ubiquitin protein ligase activity	bulk RNA-seq,Homo_723,MCI.vs.control; bulk RNA-seq,Homo_633,AD.vs.control; bulk RNA-seq,Homo_633,AD.vs.MCI; scRNA-seq,SRP330776,B cell_1-AD.vs.control; scRNA-seq,SRP330776,B cell_2-AD.vs.control; scRNA-seq,SRP330776,CD8+ T cell_1-AD.vs.control; scRNA-seq,SRP330776,Memory T cell_2-	7
BP	GO:0002701	negative regulation of production of molecular mediator of immune response	bulk RNA-seq,Homo_723,MCI.vs.control; bulk RNA-seq,Homo_714,AD.vs.MCI; bulk RNA-seq,Homo_714,MCI.vs.control	3
BP	GO:0007143	female meiotic nuclear division	bulk RNA-seq,Homo_723,MCI.vs.control; bulk RNA-seq,Homo_714,MCI.vs.control; bulk RNA-seq,Homo_633,AD.vs.control; bulk RNA-	4
BP	GO:1903322	positive regulation of protein modification by small protein conjugation or removal	bulk RNA-seq,Homo_723,MCI.vs.control; bulk RNA-seq,Homo_714,AD.vs.MCI; bulk RNA-seq,Homo_633,AD.vs.control; bulk RNA-seq,Homo_633,AD.vs.MCI	4

BP	GO:1990748	cellular detoxification	bulk RNA-seq,Homo_723,MCI.vs.control; bulk RNA-seq,Homo_714,AD.vs.MCI; bulk RNA-seq,Homo_714,MCI.vs.control; bulk RNA-seq,Homo_633,AD.vs.control; bulk RNA-seq,Homo_633,AD.vs.MCI; bulk RNA-seq,ROSMAP,MCI.vs.control	6
BP	GO:1902750	negative regulation of cell cycle G2/M phase transition	bulk RNA-seq,Homo_723,MCI.vs.control; bulk RNA-seq,Homo_714,MCI.vs.control; bulk RNA-seq,Homo_633,AD.vs.control; bulk RNA-	4
BP	GO:0034644	cellular response to UV	bulk RNA-seq,Homo_723,MCI.vs.control; bulk RNA-seq,Homo_714,MCI.vs.control; bulk RNA-seq,Homo_633,AD.vs.control; bulk RNA-	4
BP	GO:0050670	regulation of lymphocyte proliferation	bulk RNA-seq,Homo_723,MCI.vs.control; bulk RNA-seq,Homo_714,AD.vs.MCI; bulk RNA-seq,Homo_633,AD.vs.control; bulk RNA-seq,Homo_633,AD.vs.MCI; scRNA-seq,SRP215507,CD8+ T cell_1-MCI.vs.control	5
MF	GO:0004722	protein serine/threonine phosphatase activity	bulk RNA-seq,Homo_723,MCI.vs.control; bulk RNA-seq,Homo_714,MCI.vs.control; bulk RNA-seq,Homo_633,AD.vs.control; bulk RNA-	4
BP	GO:0001767	establishment of lymphocyte polarity	bulk RNA-seq,Homo_723,MCI.vs.control; bulk RNA-seq,Homo_714,MCI.vs.control; bulk RNA-seq,Homo_633,MCI.vs.control	3
BP	GO:2000756	regulation of peptidyl-lysine acetylation	bulk RNA-seq,Homo_723,MCI.vs.control; bulk RNA-seq,Homo_714,MCI.vs.control; bulk RNA-seq,Homo_633,AD.vs.control; bulk RNA-	4
BP	GO:1903557	positive regulation of tumor necrosis factor superfamily cytokine production	bulk RNA-seq,Homo_723,MCI.vs.control; bulk RNA-seq,Homo_714,AD.vs.MCI; bulk RNA-seq,Homo_714,MCI.vs.control; bulk RNA-seq,Homo_633,AD.vs.control; bulk RNA-seq,Homo_633,AD.vs.MCI	5
BP	GO:2000060	positive regulation of ubiquitin-dependent protein catabolic	bulk RNA-seq,Homo_723,MCI.vs.control; bulk RNA-seq,Homo_714,MCI.vs.control; bulk RNA-seq,Homo_633,AD.vs.control; bulk RNA-	4
BP	GO:0034501	protein localization to kinetochore	bulk RNA-seq,Homo_723,MCI.vs.control; bulk RNA-seq,Homo_714,MCI.vs.control; bulk RNA-seq,Homo_633,AD.vs.control; bulk RNA-seq,Homo_633,AD.vs.MCI; bulk RNA-seq,Homo_633,MCI.vs.control	5
BP	GO:1903083	protein localization to condensed chromosome	bulk RNA-seq,Homo_723,MCI.vs.control; bulk RNA-seq,Homo_714,MCI.vs.control; bulk RNA-seq,Homo_633,AD.vs.control; bulk RNA-seq,Homo_633,AD.vs.MCI; bulk RNA-seq,Homo_633,MCI.vs.control	5
BP	GO:0002753	cytoplasmic pattern recognition receptor signaling pathway	bulk RNA-seq,Homo_723,MCI.vs.control; bulk RNA-seq,Homo_714,MCI.vs.control; bulk RNA-seq,Homo_633,AD.vs.control; bulk RNA-	4
BP	GO:0006096	glycolytic process	bulk RNA-seq,Homo_723,MCI.vs.control; bulk RNA-seq,Homo_714,AD.vs.MCI; bulk RNA-seq,Homo_714,MCI.vs.control; bulk RNA-seq,Homo_633,AD.vs.control; bulk RNA-seq,Homo_633,AD.vs.MCI	5
BP	GO:0030520	intracellular estrogen receptor signaling pathway	bulk RNA-seq,Homo_723,MCI.vs.control; bulk RNA-seq,Homo_714,AD.vs.MCI; bulk RNA-seq,Homo_714,MCI.vs.control; bulk RNA-seq,Homo_633,AD.vs.control; bulk RNA-seq,Homo_633,AD.vs.MCI; bulk RNA-seq,SRP310421,AD.vs.control	6
BP	GO:0032436	positive regulation of proteasomal ubiquitin-dependent protein catabolic process	bulk RNA-seq,Homo_723,MCI.vs.control; bulk RNA-seq,Homo_714,MCI.vs.control; bulk RNA-seq,Homo_633,AD.vs.control; bulk RNA-seq,Homo_633,AD.vs.MCI	4
BP	GO:0009110	vitamin biosynthetic process	bulk RNA-seq,Homo_723,MCI.vs.control; bulk RNA-seq,Homo_714,MCI.vs.control; bulk RNA-seq,Homo_633,AD.vs.control	3
BP	GO:0050000	chromosome localization	bulk RNA-seq,Homo_723,MCI.vs.control; bulk RNA-seq,Homo_714,AD.vs.MCI; bulk RNA-seq,Homo_714,MCI.vs.control; bulk RNA-seq,Homo_633,AD.vs.control; bulk RNA-seq,Homo_633,AD.vs.MCI	5
MF	GO:0048027	mRNA 5'-UTR binding	bulk RNA-seq,Homo_723,MCI.vs.control; bulk RNA-seq,Homo_714,MCI.vs.control; bulk RNA-seq,Homo_633,AD.vs.control; bulk RNA-seq,Homo_633,AD.vs.MCI; bulk RNA-seq,Homo_633,MCI.vs.control	5
BP	GO:0002686	negative regulation of leukocyte migration	bulk RNA-seq,Homo_723,MCI.vs.control; bulk RNA-seq,Homo_714,AD.vs.MCI; bulk RNA-seq,Homo_714,MCI.vs.control; bulk RNA-	4
BP	GO:0046031	ADP metabolic process	bulk RNA-seq,Homo_723,MCI.vs.control; bulk RNA-seq,Homo_714,MCI.vs.control; bulk RNA-seq,Homo_633,AD.vs.control; bulk RNA-	4
MF	GO:0048029	monosaccharide binding	bulk RNA-seq,Homo_723,MCI.vs.control; bulk RNA-seq,Homo_714,AD.vs.MCI; bulk RNA-seq,Homo_714,MCI.vs.control; bulk RNA-seq,Homo_633,AD.vs.control; bulk RNA-seq,Homo_633,AD.vs.MCI	5
BP	GO:0032092	positive regulation of protein binding	bulk RNA-seq,Homo_723,MCI.vs.control; bulk RNA-seq,Homo_714,AD.vs.MCI; bulk RNA-seq,Homo_714,MCI.vs.control; bulk RNA-seq,Homo_633,AD.vs.control; bulk RNA-seq,Homo_633,AD.vs.MCI	5
BP	GO:0031060	regulation of histone methylation	bulk RNA-seq,Homo_723,MCI.vs.control; bulk RNA-seq,Homo_714,MCI.vs.control; bulk RNA-seq,Homo_633,AD.vs.control; bulk RNA-	4
BP	GO:0071824	protein-DNA complex subunit organization	bulk RNA-seq,Homo_723,MCI.vs.control; bulk RNA-seq,Homo_714,AD.vs.MCI; bulk RNA-seq,Homo_633,AD.vs.control; bulk RNA-seq,Homo_633,AD.vs.MCI	4
BP	GO:0035066	positive regulation of histone acetylation	bulk RNA-seq,Homo_723,MCI.vs.control; bulk RNA-seq,Homo_714,AD.vs.MCI; bulk RNA-seq,Homo_714,MCI.vs.control; bulk RNA-	4
MF	GO:0004896	cytokine receptor activity	bulk RNA-seq,Homo_723,MCI.vs.control; bulk RNA-seq,Homo_714,AD.vs.MCI; bulk RNA-seq,Homo_714,MCI.vs.control; bulk RNA-	4
BP	GO:0071887	leukocyte apoptotic process	bulk RNA-seq,Homo_723,MCI.vs.control; bulk RNA-seq,Homo_714,AD.vs.MCI; bulk RNA-seq,Homo_714,MCI.vs.control; bulk RNA-seq,Homo_633,AD.vs.control; bulk RNA-seq,Homo_633,AD.vs.MCI	5
CC	GO:0031248	protein acetyltransferase complex	bulk RNA-seq,Homo_723,MCI.vs.control; bulk RNA-seq,Homo_714,MCI.vs.control; bulk RNA-seq,Homo_633,AD.vs.control; bulk RNA-	4
CC	GO:1902493	acetyltransferase complex	bulk RNA-seq,Homo_723,MCI.vs.control; bulk RNA-seq,Homo_714,MCI.vs.control; bulk RNA-seq,Homo_633,AD.vs.control; bulk RNA-	4
BP	GO:2000106	regulation of leukocyte apoptotic process	bulk RNA-seq,Homo_723,MCI.vs.control; bulk RNA-seq,Homo_714,AD.vs.MCI; bulk RNA-seq,Homo_714,MCI.vs.control; bulk RNA-seq,Homo_633,AD.vs.control; bulk RNA-seq,Homo_633,AD.vs.MCI	5
BP	GO:0080182	histone H3-K4 trimethylation	bulk RNA-seq,Homo_723,MCI.vs.control; bulk RNA-seq,Homo_714,MCI.vs.control; bulk RNA-seq,Homo_633,AD.vs.control	3
BP	GO:1905168	positive regulation of double-strand break repair via homologous recombination	bulk RNA-seq,Homo_723,MCI.vs.control; bulk RNA-seq,Homo_714,MCI.vs.control; bulk RNA-seq,Homo_633,AD.vs.control; bulk RNA-seq,Homo_633,AD.vs.MCI	4
BP	GO:0030212	hyaluronan metabolic process	bulk RNA-seq,Homo_723,MCI.vs.control; bulk RNA-seq,Homo_714,AD.vs.MCI; bulk RNA-seq,Homo_714,MCI.vs.control; bulk RNA-seq,Homo_633,AD.vs.control; bulk RNA-seq,Homo_633,AD.vs.MCI	5
BP	GO:0034121	regulation of toll-like receptor signaling pathway	bulk RNA-seq,Homo_723,MCI.vs.control; bulk RNA-seq,Homo_714,MCI.vs.control; bulk RNA-seq,Homo_633,AD.vs.control; bulk RNA-	4
MF	GO:0017018	myosin phosphatase activity	bulk RNA-seq,Homo_723,MCI.vs.control; bulk RNA-seq,Homo_714,AD.vs.MCI; bulk RNA-seq,Homo_714,MCI.vs.control; bulk RNA-seq,Homo_633,AD.vs.control; bulk RNA-seq,Homo_633,AD.vs.MCI	5
MF	GO:0070717	poly-purine tract binding	bulk RNA-seq,Homo_723,MCI.vs.control; bulk RNA-seq,Homo_714,MCI.vs.control; bulk RNA-seq,Homo_633,AD.vs.control; bulk RNA-seq,Homo_633,AD.vs.MCI; bulk RNA-seq,Homo_633,MCI.vs.control	5
MF	GO:0005527	macrolide binding	bulk RNA-seq,Homo_723,MCI.vs.control; bulk RNA-seq,Homo_714,MCI.vs.control; bulk RNA-seq,Homo_633,MCI.vs.control	3
BP	GO:1905515	non-motile cilium assembly	bulk RNA-seq,Homo_723,MCI.vs.control; bulk RNA-seq,Homo_714,MCI.vs.control; bulk RNA-seq,Homo_633,AD.vs.control; bulk RNA-	4
BP	GO:0034315	regulation of Arp2/3 complex-mediated actin nucleation	bulk RNA-seq,Homo_723,MCI.vs.control; bulk RNA-seq,Homo_714,MCI.vs.control; bulk RNA-seq,Homo_633,AD.vs.control; bulk RNA-seq,Homo_633,AD.vs.MCI; bulk RNA-seq,Homo_633,MCI.vs.control	5
BP	GO:0033003	regulation of mast cell activation	bulk RNA-seq,Homo_723,MCI.vs.control; bulk RNA-seq,Homo_714,AD.vs.MCI; bulk RNA-seq,Homo_714,MCI.vs.control; bulk RNA-seq,Homo_633,AD.vs.control; bulk RNA-seq,Homo_633,AD.vs.MCI	5
BP	GO:1900027	regulation of ruffle assembly	bulk RNA-seq,Homo_723,MCI.vs.control; bulk RNA-seq,Homo_714,MCI.vs.control; bulk RNA-seq,Homo_633,AD.vs.control	3

CC	GO:0031519	PcG protein complex	bulk RNA-seq,Homo_723,MCI.vs.control; bulk RNA-seq,Homo_714,MCI.vs.control; bulk RNA-seq,Homo_633,AD.vs.control; bulk RNA-seq,Homo_633,AD.vs.MCI; bulk RNA-seq,Homo_633,MCI.vs.control	5
BP	GO:1901796	regulation of signal transduction by p53 class mediator	bulk RNA-seq,Homo_723,MCI.vs.control; bulk RNA-seq,Homo_714,MCI.vs.control; bulk RNA-seq,Homo_633,AD.vs.control; bulk RNA-	4
BP	GO:0065004	protein-DNA complex assembly	bulk RNA-seq,Homo_723,MCI.vs.control; bulk RNA-seq,Homo_714,AD.vs.MCI; bulk RNA-seq,Homo_633,AD.vs.control; bulk RNA-seq,Homo_633,AD.vs.MCI	4
BP	GO:1901532	regulation of hematopoietic progenitor cell differentiation	bulk RNA-seq,Homo_723,MCI.vs.control; bulk RNA-seq,Homo_714,AD.vs.MCI; bulk RNA-seq,Homo_714,MCI.vs.control; bulk RNA-seq,Homo_633,AD.vs.control; bulk RNA-seq,Homo_633,AD.vs.MCI	5
BP	GO:0045740	positive regulation of DNA replication	bulk RNA-seq,Homo_723,MCI.vs.control; bulk RNA-seq,Homo_714,MCI.vs.control; bulk RNA-seq,Homo_633,AD.vs.control; bulk RNA-	4
BP	GO:2000107	negative regulation of leukocyte apoptotic process	bulk RNA-seq,Homo_723,MCI.vs.control; bulk RNA-seq,Homo_714,AD.vs.MCI; bulk RNA-seq,Homo_714,MCI.vs.control; bulk RNA-seq,Homo_633,AD.vs.control; bulk RNA-seq,Homo_633,AD.vs.MCI; scRNA-seq,SRP330776,Megakaryocyte_2-AD.vs.control	6
BP	GO:0016052	carbohydrate catabolic process	bulk RNA-seq,Homo_723,MCI.vs.control; bulk RNA-seq,Homo_633,AD.vs.control; bulk RNA-seq,Homo_633,AD.vs.MCI	3
BP	GO:0001824	blastocyst development	bulk RNA-seq,Homo_723,MCI.vs.control; bulk RNA-seq,Homo_714,MCI.vs.control; bulk RNA-seq,Homo_633,AD.vs.control; bulk RNA-	4
MF	GO:0070566	adenylyltransferase activity	bulk RNA-seq,Homo_723,MCI.vs.control; bulk RNA-seq,Homo_714,MCI.vs.control; bulk RNA-seq,Homo_633,AD.vs.control; bulk RNA-seq,Homo_633,AD.vs.MCI; bulk RNA-seq,Homo_633,MCI.vs.control	5
CC	GO:0005771	multivesicular body	bulk RNA-seq,Homo_723,MCI.vs.control; bulk RNA-seq,Homo_714,MCI.vs.control; bulk RNA-seq,Homo_633,AD.vs.control; bulk RNA-	4
MF	GO:0035925	mRNA 3'-UTR AU-rich region binding	bulk RNA-seq,Homo_723,MCI.vs.control; bulk RNA-seq,Homo_714,MCI.vs.control; bulk RNA-seq,Homo_633,AD.vs.control; bulk RNA-seq,Homo_633,AD.vs.MCI; bulk RNA-seq,Homo_633,MCI.vs.control; bulk RNA-seq,SRP310421,AD.vs.control	6
BP	GO:0035303	regulation of dephosphorylation	bulk RNA-seq,Homo_723,MCI.vs.control; bulk RNA-seq,Homo_714,AD.vs.MCI; bulk RNA-seq,Homo_633,AD.vs.control; bulk RNA-seq,Homo_633,AD.vs.MCI	4
BP	GO:0051310	metaphase plate congression	bulk RNA-seq,Homo_723,MCI.vs.control; bulk RNA-seq,Homo_714,MCI.vs.control; bulk RNA-seq,Homo_633,AD.vs.control; bulk RNA-	4
MF	GO:0008143	poly(A) binding	bulk RNA-seq,Homo_723,MCI.vs.control; bulk RNA-seq,Homo_714,MCI.vs.control; bulk RNA-seq,Homo_633,AD.vs.control	3
MF	GO:0070530	K63-linked polyubiquitin modification-dependent protein	bulk RNA-seq,Homo_723,MCI.vs.control; bulk RNA-seq,Homo_714,MCI.vs.control; bulk RNA-seq,Homo_633,AD.vs.control; bulk RNA-	4
MF	GO:0035615	clathrin adaptor activity	bulk RNA-seq,Homo_723,MCI.vs.control; bulk RNA-seq,Homo_714,MCI.vs.control; bulk RNA-seq,Homo_633,AD.vs.control; bulk RNA-	4
BP	GO:0070988	demethylation	bulk RNA-seq,Homo_723,MCI.vs.control; bulk RNA-seq,Homo_714,MCI.vs.control; bulk RNA-seq,Homo_633,AD.vs.control; bulk RNA-	4
BP	GO:1903533	regulation of protein targeting	bulk RNA-seq,Homo_723,MCI.vs.control; bulk RNA-seq,Homo_714,MCI.vs.control; bulk RNA-seq,Homo_633,AD.vs.control; bulk RNA-	4
BP	GO:0071479	cellular response to ionizing radiation	bulk RNA-seq,Homo_723,MCI.vs.control; bulk RNA-seq,Homo_714,AD.vs.MCI; bulk RNA-seq,Homo_714,MCI.vs.control; bulk RNA-seq,Homo_633,AD.vs.control; bulk RNA-seq,Homo_633,AD.vs.MCI	5
MF	GO:0051082	unfolded protein binding	bulk RNA-seq,Homo_723,MCI.vs.control; bulk RNA-seq,Homo_714,AD.vs.control; bulk RNA-seq,Homo_714,MCI.vs.control; bulk RNA-seq,Homo_633,AD.vs.control; bulk RNA-seq,Homo_633,AD.vs.MCI; bulk RNA-seq,ROSMAP,MCI.vs.control	6
BP	GO:0042073	intraciliary transport	bulk RNA-seq,Homo_723,MCI.vs.control; bulk RNA-seq,Homo_714,MCI.vs.control; bulk RNA-seq,Homo_633,AD.vs.control; bulk RNA-	4
BP	GO:0031398	positive regulation of protein ubiquitination	bulk RNA-seq,Homo_723,MCI.vs.control; bulk RNA-seq,Homo_714,AD.vs.MCI; bulk RNA-seq,Homo_714,MCI.vs.control; bulk RNA-seq,Homo_633,AD.vs.control; bulk RNA-seq,Homo_633,AD.vs.MCI	5
BP	GO:0002279	mast cell activation involved in immune response	bulk RNA-seq,Homo_723,MCI.vs.control; bulk RNA-seq,Homo_714,AD.vs.MCI; bulk RNA-seq,Homo_714,MCI.vs.control; bulk RNA-seq,Homo_633,AD.vs.control; bulk RNA-seq,Homo_633,AD.vs.MCI; scRNA-seq,SRP215507,CD8+ T cell_1-AD.vs.control	6
MF	GO:0051019	mitogen-activated protein kinase binding	bulk RNA-seq,Homo_723,MCI.vs.control; bulk RNA-seq,Homo_714,MCI.vs.control; bulk RNA-seq,Homo_633,AD.vs.control; bulk RNA-	4
CC	GO:0031227	intrinsic component of endoplasmic reticulum membrane	bulk RNA-seq,Homo_723,MCI.vs.control; bulk RNA-seq,Homo_633,AD.vs.control; bulk RNA-seq,Homo_633,AD.vs.MCI	3
BP	GO:0033599	regulation of mammary gland epithelial cell proliferation	bulk RNA-seq,Homo_723,MCI.vs.control; bulk RNA-seq,Homo_714,MCI.vs.control; bulk RNA-seq,Homo_633,MCI.vs.control	3
BP	GO:0032640	tumor necrosis factor production	bulk RNA-seq,Homo_723,MCI.vs.control; bulk RNA-seq,Homo_714,AD.vs.MCI; bulk RNA-seq,Homo_633,AD.vs.control; bulk RNA-seq,Homo_633,AD.vs.MCI	4
BP	GO:0032680	regulation of tumor necrosis factor production	bulk RNA-seq,Homo_723,MCI.vs.control; bulk RNA-seq,Homo_714,AD.vs.MCI; bulk RNA-seq,Homo_633,AD.vs.control; bulk RNA-seq,Homo_633,AD.vs.MCI	4
BP	GO:0043467	regulation of generation of precursor metabolites and energy	bulk RNA-seq,Homo_723,MCI.vs.control; bulk RNA-seq,Homo_633,AD.vs.control; bulk RNA-seq,Homo_633,AD.vs.MCI	3
BP	GO:0032736	positive regulation of interleukin-13 production	bulk RNA-seq,Homo_723,MCI.vs.control; bulk RNA-seq,Homo_714,MCI.vs.control; bulk RNA-seq,Homo_633,MCI.vs.control	3
CC	GO:0002102	podosome	bulk RNA-seq,Homo_723,MCI.vs.control; bulk RNA-seq,Homo_714,AD.vs.MCI; bulk RNA-seq,Homo_714,MCI.vs.control; bulk RNA-	4
BP	GO:0031269	pseudopodium assembly	bulk RNA-seq,Homo_723,MCI.vs.control; bulk RNA-seq,Homo_714,MCI.vs.control; bulk RNA-seq,Homo_633,AD.vs.control; bulk RNA-	4
BP	GO:0061647	histone H3-K9 modification	bulk RNA-seq,Homo_723,MCI.vs.control; bulk RNA-seq,Homo_714,MCI.vs.control; bulk RNA-seq,Homo_633,AD.vs.control; bulk RNA-	4
BP	GO:0038095	Fc-epsilon receptor signaling pathway	bulk RNA-seq,Homo_723,MCI.vs.control; bulk RNA-seq,Homo_714,MCI.vs.control; bulk RNA-seq,Homo_633,AD.vs.control; bulk RNA-seq,Homo_633,AD.vs.MCI; bulk RNA-seq,Homo_633,MCI.vs.control	5
BP	GO:0035162	embryonic hemopoiesis	bulk RNA-seq,Homo_723,MCI.vs.control; bulk RNA-seq,Homo_714,AD.vs.MCI; bulk RNA-seq,Homo_714,MCI.vs.control; bulk RNA-seq,Homo_633,AD.vs.control; bulk RNA-seq,Homo_633,AD.vs.MCI	5
BP	GO:0071677	positive regulation of mononuclear cell migration	bulk RNA-seq,Homo_723,MCI.vs.control; bulk RNA-seq,Homo_714,AD.vs.MCI; bulk RNA-seq,Homo_714,MCI.vs.control; bulk RNA-seq,Homo_633,AD.vs.control; bulk RNA-seq,Homo_633,AD.vs.MCI	5
CC	GO:0005793	endoplasmic reticulum-Golgi intermediate compartment	bulk RNA-seq,Homo_723,MCI.vs.control; bulk RNA-seq,Homo_633,AD.vs.control; bulk RNA-seq,Homo_633,AD.vs.MCI	3
BP	GO:0043620	regulation of DNA-templated transcription in response to	bulk RNA-seq,Homo_723,MCI.vs.control; bulk RNA-seq,Homo_714,MCI.vs.control; bulk RNA-seq,Homo_633,AD.vs.control; bulk RNA-	4
BP	GO:0019915	lipid storage	bulk RNA-seq,Homo_723,MCI.vs.control; bulk RNA-seq,Homo_714,AD.vs.MCI; bulk RNA-seq,Homo_714,MCI.vs.control; bulk RNA-seq,Homo_633,AD.vs.control; bulk RNA-seq,Homo_633,AD.vs.MCI	5
BP	GO:0039528	cytoplasmic pattern recognition receptor signaling pathway in response to virus	bulk RNA-seq,Homo_723,MCI.vs.control; bulk RNA-seq,Homo_714,MCI.vs.control; bulk RNA-seq,Homo_633,AD.vs.control; bulk RNA-seq,Homo_633,MCI.vs.control	4
BP	GO:0051125	regulation of actin nucleation	bulk RNA-seq,Homo_723,MCI.vs.control; bulk RNA-seq,Homo_714,MCI.vs.control; bulk RNA-seq,Homo_633,AD.vs.control; bulk RNA-seq,Homo_633,AD.vs.MCI; scRNA-seq,SRP309935,Monocyte_2-AD.vs.control	5
BP	GO:0071425	hematopoietic stem cell proliferation	bulk RNA-seq,Homo_723,MCI.vs.control; bulk RNA-seq,Homo_714,MCI.vs.control	2
BP	GO:0002702	positive regulation of production of molecular mediator of immune response	bulk RNA-seq,Homo_723,MCI.vs.control; bulk RNA-seq,Homo_633,AD.vs.control; bulk RNA-seq,Homo_633,AD.vs.MCI	3
MF	GO:0140612	DNA damage sensor activity	bulk RNA-seq,Homo_723,MCI.vs.control; bulk RNA-seq,Homo_714,MCI.vs.control; bulk RNA-seq,Homo_633,AD.vs.control; bulk RNA-seq,Homo_633,AD.vs.MCI; bulk RNA-seq,Homo_633,MCI.vs.control	5

BP	GO:0071622	regulation of granulocyte chemotaxis	bulk RNA-seq,Homo_723,MCI.vs.control; bulk RNA-seq,Homo_714,AD.vs.MCI; bulk RNA-seq,Homo_714,MCI.vs.control; bulk RNA-	4
BP	GO:0048247	lymphocyte chemotaxis	bulk RNA-seq,Homo_723,MCI.vs.control; bulk RNA-seq,Homo_714,AD.vs.MCI; bulk RNA-seq,Homo_714,MCI.vs.control	3
BP	GO:0006884	cell volume homeostasis	bulk RNA-seq,Homo_723,MCI.vs.control; bulk RNA-seq,Homo_714,MCI.vs.control; bulk RNA-seq,Homo_633,AD.vs.control; bulk RNA-	4
BP	GO:2000045	regulation of G1/S transition of mitotic cell cycle	bulk RNA-seq,Homo_723,MCI.vs.control; bulk RNA-seq,Homo_714,AD.vs.MCI; bulk RNA-seq,Homo_633,AD.vs.control; bulk RNA-seq,Homo_633,AD.vs.MCI	4
CC	GO:0005844	polysome	bulk RNA-seq,Homo_723,MCI.vs.control; bulk RNA-seq,Homo_714,MCI.vs.control; bulk RNA-seq,Homo_633,AD.vs.control; bulk RNA-	4
MF	GO:0000217	DNA secondary structure binding	bulk RNA-seq,Homo_723,MCI.vs.control; bulk RNA-seq,Homo_714,MCI.vs.control; bulk RNA-seq,Homo_633,AD.vs.control; bulk RNA-	4
BP	GO:0051298	centrosome duplication	bulk RNA-seq,Homo_723,MCI.vs.control; bulk RNA-seq,Homo_714,MCI.vs.control; bulk RNA-seq,Homo_633,AD.vs.control; bulk RNA-	4
BP	GO:0030330	DNA damage response, signal transduction by p53 class	bulk RNA-seq,Homo_723,MCI.vs.control; bulk RNA-seq,Homo_714,MCI.vs.control; bulk RNA-seq,Homo_633,AD.vs.control; bulk RNA-	4
BP	GO:0071706	tumor necrosis factor superfamily cytokine production	bulk RNA-seq,Homo_723,MCI.vs.control; bulk RNA-seq,Homo_714,AD.vs.MCI; bulk RNA-seq,Homo_633,AD.vs.control; bulk RNA-seq,Homo_633,AD.vs.MCI	4
BP	GO:1903555	regulation of tumor necrosis factor superfamily cytokine	bulk RNA-seq,Homo_723,MCI.vs.control; bulk RNA-seq,Homo_714,AD.vs.MCI; bulk RNA-seq,Homo_633,AD.vs.control; bulk RNA-seq,Homo_633,AD.vs.MCI	4
BP	GO:0002695	negative regulation of leukocyte activation	bulk RNA-seq,Homo_723,MCI.vs.control; bulk RNA-seq,Homo_714,AD.vs.MCI; bulk RNA-seq,Homo_633,AD.vs.control; bulk RNA-seq,Homo_633,AD.vs.MCI	4
BP	GO:0043618	regulation of transcription from RNA polymerase II promoter in response to stress	bulk RNA-seq,Homo_723,MCI.vs.control; bulk RNA-seq,Homo_714,MCI.vs.control; bulk RNA-seq,Homo_633,AD.vs.control; bulk RNA-seq,Homo_633,AD.vs.MCI	4
BP	GO:0097284	hepatocyte apoptotic process	bulk RNA-seq,Homo_723,MCI.vs.control; bulk RNA-seq,Homo_714,AD.vs.MCI; bulk RNA-seq,Homo_714,MCI.vs.control	3
BP	GO:0061515	myeloid cell development	bulk RNA-seq,Homo_723,MCI.vs.control; bulk RNA-seq,Homo_714,MCI.vs.control; bulk RNA-seq,Homo_633,AD.vs.control; bulk RNA-seq,Homo_633,AD.vs.MCI; bulk RNA-seq,SRP325058,AD.vs.control	5
BP	GO:0033865	nucleoside bisphosphate metabolic process	bulk RNA-seq,Homo_723,MCI.vs.control; bulk RNA-seq,Homo_714,AD.vs.MCI; bulk RNA-seq,Homo_714,MCI.vs.control; bulk RNA-seq,Homo_633,AD.vs.control; bulk RNA-seq,Homo_633,AD.vs.MCI	5
BP	GO:0033875	ribonucleoside bisphosphate metabolic process	bulk RNA-seq,Homo_723,MCI.vs.control; bulk RNA-seq,Homo_714,AD.vs.MCI; bulk RNA-seq,Homo_714,MCI.vs.control; bulk RNA-seq,Homo_633,AD.vs.control; bulk RNA-seq,Homo_633,AD.vs.MCI	5
BP	GO:0034032	purine nucleoside bisphosphate metabolic process	bulk RNA-seq,Homo_723,MCI.vs.control; bulk RNA-seq,Homo_714,AD.vs.MCI; bulk RNA-seq,Homo_714,MCI.vs.control; bulk RNA-seq,Homo_633,AD.vs.control; bulk RNA-seq,Homo_633,AD.vs.MCI	5
BP	GO:2000403	positive regulation of lymphocyte migration	bulk RNA-seq,Homo_723,MCI.vs.control; bulk RNA-seq,Homo_714,MCI.vs.control; bulk RNA-seq,Homo_633,AD.vs.control; bulk RNA-	4
BP	GO:0042368	vitamin D biosynthetic process	bulk RNA-seq,Homo_723,MCI.vs.control; bulk RNA-seq,Homo_714,MCI.vs.control	2
BP	GO:0002577	regulation of antigen processing and presentation	bulk RNA-seq,Homo_723,MCI.vs.control; bulk RNA-seq,Homo_714,MCI.vs.control	2
BP	GO:0051882	mitochondrial depolarization	bulk RNA-seq,Homo_723,MCI.vs.control; bulk RNA-seq,Homo_714,AD.vs.MCI; bulk RNA-seq,Homo_714,MCI.vs.control; bulk RNA-seq,Homo_633,AD.vs.control; bulk RNA-seq,Homo_633,AD.vs.MCI	5
BP	GO:1902806	regulation of cell cycle G1/S phase transition	bulk RNA-seq,Homo_723,MCI.vs.control; bulk RNA-seq,Homo_714,AD.vs.MCI; bulk RNA-seq,Homo_633,AD.vs.control; bulk RNA-seq,Homo_633,AD.vs.MCI; scRNA-seq,SRP330776,Naive CD8+ T cell_2-AD.vs.control	5
BP	GO:0045576	mast cell activation	bulk RNA-seq,Homo_723,MCI.vs.control; bulk RNA-seq,Homo_714,AD.vs.MCI; bulk RNA-seq,Homo_714,MCI.vs.control; bulk RNA-seq,Homo_633,AD.vs.control; bulk RNA-seq,Homo_633,AD.vs.MCI; scRNA-seq,SRP215507,CD8+ T cell_1-AD.vs.control	6
BP	GO:0044819	mitotic G1/S transition checkpoint signaling	bulk RNA-seq,Homo_723,MCI.vs.control; bulk RNA-seq,Homo_714,MCI.vs.control; bulk RNA-seq,Homo_633,AD.vs.control; bulk RNA-seq,Homo_633,AD.vs.MCI; bulk RNA-seq,Homo_633,MCI.vs.control	5
CC	GO:0030990	intraciliary transport particle	bulk RNA-seq,Homo_723,MCI.vs.control; bulk RNA-seq,Homo_714,MCI.vs.control; bulk RNA-seq,Homo_633,AD.vs.control; bulk RNA-seq,Homo_633,AD.vs.MCI; bulk RNA-seq,Homo_633,MCI.vs.control	5
MF	GO:0140658	ATP-dependent chromatin remodeler activity	bulk RNA-seq,Homo_723,MCI.vs.control; bulk RNA-seq,Homo_714,AD.vs.MCI; bulk RNA-seq,Homo_714,MCI.vs.control; bulk RNA-seq,Homo_633,AD.vs.control; bulk RNA-seq,Homo_633,AD.vs.MCI; bulk RNA-seq,Homo_633,MCI.vs.control	6
BP	GO:1904950	negative regulation of establishment of protein localization	bulk RNA-seq,Homo_723,MCI.vs.control; bulk RNA-seq,Homo_633,AD.vs.control; bulk RNA-seq,Homo_633,AD.vs.MCI	3
BP	GO:0006482	protein demethylation	bulk RNA-seq,Homo_723,MCI.vs.control; bulk RNA-seq,Homo_714,AD.vs.MCI; bulk RNA-seq,Homo_714,MCI.vs.control; bulk RNA-seq,Homo_633,AD.vs.control; bulk RNA-seq,Homo_633,AD.vs.MCI; bulk RNA-seq,Homo_633,MCI.vs.control	6
BP	GO:0008214	protein dealkylation	bulk RNA-seq,Homo_723,MCI.vs.control; bulk RNA-seq,Homo_714,AD.vs.MCI; bulk RNA-seq,Homo_714,MCI.vs.control; bulk RNA-seq,Homo_633,AD.vs.control; bulk RNA-seq,Homo_633,AD.vs.MCI; bulk RNA-seq,Homo_633,MCI.vs.control	6
BP	GO:0051900	regulation of mitochondrial depolarization	bulk RNA-seq,Homo_723,MCI.vs.control; bulk RNA-seq,Homo_714,AD.vs.MCI; bulk RNA-seq,Homo_714,MCI.vs.control; bulk RNA-seq,Homo_633,AD.vs.control; bulk RNA-seq,Homo_633,AD.vs.MCI	5
MF	GO:0016706	2-oxoglutarate-dependent dioxygenase activity	bulk RNA-seq,Homo_723,MCI.vs.control; bulk RNA-seq,Homo_714,MCI.vs.control; bulk RNA-seq,Homo_633,AD.vs.control; bulk RNA-	4
BP	GO:1901983	regulation of protein acetylation	bulk RNA-seq,Homo_723,MCI.vs.control; bulk RNA-seq,Homo_714,MCI.vs.control; bulk RNA-seq,Homo_633,AD.vs.control; bulk RNA-	4
BP	GO:2001021	negative regulation of response to DNA damage stimulus	bulk RNA-seq,Homo_723,MCI.vs.control; bulk RNA-seq,Homo_714,MCI.vs.control; bulk RNA-seq,Homo_633,AD.vs.control; bulk RNA-	4
BP	GO:0010761	fibroblast migration	bulk RNA-seq,Homo_723,MCI.vs.control; bulk RNA-seq,Homo_714,MCI.vs.control; bulk RNA-seq,Homo_633,AD.vs.control; bulk RNA-	4
BP	GO:0051092	positive regulation of NF-kappaB transcription factor activity	bulk RNA-seq,Homo_723,MCI.vs.control; bulk RNA-seq,Homo_714,AD.vs.MCI; bulk RNA-seq,Homo_633,AD.vs.control; bulk RNA-seq,Homo_633,AD.vs.MCI	4
BP	GO:2001242	regulation of intrinsic apoptotic signaling pathway	bulk RNA-seq,Homo_723,MCI.vs.control; bulk RNA-seq,Homo_714,AD.vs.MCI; bulk RNA-seq,Homo_633,AD.vs.control; bulk RNA-seq,Homo_633,AD.vs.MCI	4
BP	GO:0043276	anoikis	bulk RNA-seq,Homo_723,MCI.vs.control; bulk RNA-seq,Homo_714,AD.vs.MCI; bulk RNA-seq,Homo_714,MCI.vs.control; bulk RNA-seq,Homo_633,AD.vs.control; bulk RNA-seq,Homo_633,AD.vs.MCI	5
BP	GO:1902624	positive regulation of neutrophil migration	bulk RNA-seq,Homo_723,MCI.vs.control; bulk RNA-seq,Homo_714,AD.vs.MCI; bulk RNA-seq,Homo_714,MCI.vs.control; bulk RNA-seq,Homo_633,MCI.vs.control; bulk RNA-seq,SRP325058,AD.vs.control	5
BP	GO:0044786	cell cycle DNA replication	bulk RNA-seq,Homo_723,MCI.vs.control; bulk RNA-seq,Homo_714,MCI.vs.control; bulk RNA-seq,Homo_633,AD.vs.control; bulk RNA-	4
BP	GO:0006959	humoral immune response	bulk RNA-seq,Homo_723,MCI.vs.control; bulk RNA-seq,Homo_714,AD.vs.control; bulk RNA-seq,Homo_714,AD.vs.MCI; bulk RNA-seq,Homo_633,AD.vs.control; bulk RNA-seq,Homo_633,AD.vs.MCI; bulk RNA-seq,SRP325058,AD.vs.control; scRNA-seq,SRP330776,Naive CD8+ T cell_2-AD.vs.control; scRNA-seq,SRP309935,Monocyte_2-AD.vs.control	8
MF	GO:0001223	transcription coactivator binding	bulk RNA-seq,Homo_723,MCI.vs.control; bulk RNA-seq,Homo_714,MCI.vs.control; bulk RNA-seq,Homo_633,AD.vs.control; bulk RNA-	4
BP	GO:0019751	polyol metabolic process	bulk RNA-seq,Homo_723,MCI.vs.control; bulk RNA-seq,Homo_714,MCI.vs.control; bulk RNA-seq,Homo_633,AD.vs.control; bulk RNA-	4
MF	GO:0002039	p53 binding	bulk RNA-seq,Homo_723,MCI.vs.control; bulk RNA-seq,Homo_714,MCI.vs.control; bulk RNA-seq,Homo_633,AD.vs.control; bulk RNA-	4

BP	GO:0016601	Rac protein signal transduction	bulk RNA-seq,Homo_723,MCI.vs.control; bulk RNA-seq,Homo_714,MCI.vs.control; bulk RNA-seq,Homo_633,AD.vs.control; bulk RNA-	4
BP	GO:0031297	replication fork processing	bulk RNA-seq,Homo_723,MCI.vs.control; bulk RNA-seq,Homo_714,MCI.vs.control; bulk RNA-seq,Homo_633,AD.vs.control; bulk RNA-	4
BP	GO:1903509	liposaccharide metabolic process	bulk RNA-seq,Homo_723,MCI.vs.control; bulk RNA-seq,Homo_714,MCI.vs.control; bulk RNA-seq,Homo_633,AD.vs.control; bulk RNA-	4
BP	GO:0090311	regulation of protein deacetylation	bulk RNA-seq,Homo_723,MCI.vs.control; bulk RNA-seq,Homo_714,AD.vs.MCI; bulk RNA-seq,Homo_714,MCI.vs.control; bulk RNA-seq,Homo_633,AD.vs.control; bulk RNA-seq,Homo_633,AD.vs.MCI	5
BP	GO:0006611	protein export from nucleus	bulk RNA-seq,Homo_723,MCI.vs.control; bulk RNA-seq,Homo_714,MCI.vs.control; bulk RNA-seq,Homo_633,AD.vs.control; bulk RNA-	4
BP	GO:0002886	regulation of myeloid leukocyte mediated immunity	bulk RNA-seq,Homo_723,MCI.vs.control; bulk RNA-seq,Homo_714,AD.vs.MCI; bulk RNA-seq,Homo_714,MCI.vs.control; bulk RNA-seq,Homo_633,AD.vs.control; bulk RNA-seq,Homo_633,AD.vs.MCI; scRNA-seq,SRP215507,CD8+ T cell_1-AD.vs.control	6
BP	GO:0006997	nucleus organization	bulk RNA-seq,Homo_723,MCI.vs.control; bulk RNA-seq,Homo_633,AD.vs.control; bulk RNA-seq,Homo_633,AD.vs.MCI	3
CC	GO:0030176	integral component of endoplasmic reticulum membrane	bulk RNA-seq,Homo_723,MCI.vs.control; bulk RNA-seq,Homo_633,AD.vs.control; bulk RNA-seq,Homo_633,AD.vs.MCI	3
BP	GO:2000508	regulation of dendritic cell chemotaxis	bulk RNA-seq,Homo_723,MCI.vs.control; bulk RNA-seq,Homo_714,MCI.vs.control; bulk RNA-seq,Homo_633,MCI.vs.control	3
BP	GO:0035518	histone H2A monoubiquitination	bulk RNA-seq,Homo_723,MCI.vs.control; bulk RNA-seq,Homo_714,AD.vs.MCI; bulk RNA-seq,Homo_714,MCI.vs.control; bulk RNA-seq,Homo_633,AD.vs.control; bulk RNA-seq,Homo_633,MCI.vs.control	5
BP	GO:0018206	peptidyl-methionine modification	bulk RNA-seq,Homo_723,MCI.vs.control; bulk RNA-seq,Homo_714,MCI.vs.control; bulk RNA-seq,Homo_633,AD.vs.control; bulk RNA-	4
BP	GO:0002444	myeloid leukocyte mediated immunity	bulk RNA-seq,Homo_723,MCI.vs.control; bulk RNA-seq,Homo_714,AD.vs.MCI; bulk RNA-seq,Homo_714,MCI.vs.control; bulk RNA-seq,Homo_633,AD.vs.control; bulk RNA-seq,Homo_633,AD.vs.MCI	5
BP	GO:0001768	establishment of T cell polarity	bulk RNA-seq,Homo_723,MCI.vs.control; bulk RNA-seq,Homo_714,MCI.vs.control; bulk RNA-seq,Homo_633,MCI.vs.control	3
BP	GO:0008608	attachment of spindle microtubules to kinetochore	bulk RNA-seq,Homo_723,MCI.vs.control; bulk RNA-seq,Homo_714,MCI.vs.control; bulk RNA-seq,Homo_633,AD.vs.control; bulk RNA-	4
BP	GO:0043030	regulation of macrophage activation	bulk RNA-seq,Homo_723,MCI.vs.control; bulk RNA-seq,Homo_714,MCI.vs.control; bulk RNA-seq,Homo_633,AD.vs.control; bulk RNA-	4
MF	GO:0003707	nuclear steroid receptor activity	bulk RNA-seq,Homo_723,MCI.vs.control; bulk RNA-seq,Homo_714,MCI.vs.control; bulk RNA-seq,Homo_633,AD.vs.control; bulk RNA-	4
BP	GO:0047496	vesicle transport along microtubule	bulk RNA-seq,Homo_723,MCI.vs.control; bulk RNA-seq,Homo_714,AD.vs.control; bulk RNA-seq,Homo_714,MCI.vs.control; bulk RNA-seq,Homo_633,AD.vs.control; bulk RNA-seq,Homo_633,AD.vs.MCI	5
BP	GO:0032633	interleukin-4 production	bulk RNA-seq,Homo_723,MCI.vs.control; bulk RNA-seq,Homo_714,MCI.vs.control; bulk RNA-seq,Homo_633,AD.vs.control; bulk RNA-	4
BP	GO:0032673	regulation of interleukin-4 production	bulk RNA-seq,Homo_723,MCI.vs.control; bulk RNA-seq,Homo_714,MCI.vs.control; bulk RNA-seq,Homo_633,AD.vs.control; bulk RNA-	4
BP	GO:0048268	clathrin coat assembly	bulk RNA-seq,Homo_723,MCI.vs.control; bulk RNA-seq,Homo_714,MCI.vs.control; bulk RNA-seq,Homo_633,AD.vs.control; bulk RNA-seq,Homo_633,AD.vs.MCI; bulk RNA-seq,Homo_633,MCI.vs.control	5
MF	GO:0035173	histone kinase activity	bulk RNA-seq,Homo_723,MCI.vs.control; bulk RNA-seq,Homo_714,MCI.vs.control; bulk RNA-seq,Homo_633,AD.vs.control	3
MF	GO:0016903	oxidoreductase activity, acting on the aldehyde or oxo group	bulk RNA-seq,Homo_723,MCI.vs.control; bulk RNA-seq,Homo_714,MCI.vs.control; bulk RNA-seq,Homo_633,AD.vs.control; bulk RNA-	4
CC	GO:0030173	integral component of Golgi membrane	bulk RNA-seq,Homo_723,MCI.vs.control; bulk RNA-seq,Homo_714,MCI.vs.control; bulk RNA-seq,Homo_633,AD.vs.control; bulk RNA-	4
BP	GO:0019362	pyridine nucleotide metabolic process	bulk RNA-seq,Homo_723,MCI.vs.control; bulk RNA-seq,Homo_714,AD.vs.control; bulk RNA-seq,Homo_714,MCI.vs.control; bulk RNA-seq,Homo_633,AD.vs.control; bulk RNA-seq,Homo_633,AD.vs.MCI	5
BP	GO:0046496	nicotinamide nucleotide metabolic process	bulk RNA-seq,Homo_723,MCI.vs.control; bulk RNA-seq,Homo_714,AD.vs.control; bulk RNA-seq,Homo_714,MCI.vs.control; bulk RNA-seq,Homo_633,AD.vs.control; bulk RNA-seq,Homo_633,AD.vs.MCI	5
BP	GO:0008209	androgen metabolic process	bulk RNA-seq,Homo_723,MCI.vs.control; bulk RNA-seq,Homo_714,AD.vs.MCI; bulk RNA-seq,Homo_714,MCI.vs.control	3
BP	GO:0009299	mRNA transcription	bulk RNA-seq,Homo_723,MCI.vs.control; bulk RNA-seq,Homo_714,MCI.vs.control; bulk RNA-seq,Homo_633,AD.vs.control; bulk RNA-	4
BP	GO:0002407	dendritic cell chemotaxis	bulk RNA-seq,Homo_723,MCI.vs.control; bulk RNA-seq,Homo_714,MCI.vs.control; bulk RNA-seq,Homo_633,AD.vs.control; bulk RNA-seq,Homo_633,AD.vs.MCI; bulk RNA-seq,Homo_633,MCI.vs.control	5
BP	GO:0072665	protein localization to vacuole	bulk RNA-seq,Homo_723,MCI.vs.control; bulk RNA-seq,Homo_714,MCI.vs.control; bulk RNA-seq,Homo_633,AD.vs.control; bulk RNA-	4
BP	GO:0051568	histone H3-K4 methylation	bulk RNA-seq,Homo_723,MCI.vs.control; bulk RNA-seq,Homo_714,MCI.vs.control; bulk RNA-seq,Homo_633,AD.vs.control; bulk RNA-	4
BP	GO:0061512	protein localization to cilium	bulk RNA-seq,Homo_723,MCI.vs.control; bulk RNA-seq,Homo_714,MCI.vs.control; bulk RNA-seq,Homo_633,AD.vs.control; bulk RNA-	4
BP	GO:0062208	positive regulation of pattern recognition receptor signaling	bulk RNA-seq,Homo_723,MCI.vs.control; bulk RNA-seq,Homo_714,MCI.vs.control; bulk RNA-seq,Homo_633,AD.vs.control; bulk RNA-	4
MF	GO:0000979	RNA polymerase II core promoter sequence-specific DNA	bulk RNA-seq,Homo_723,MCI.vs.control; bulk RNA-seq,Homo_633,AD.vs.control; bulk RNA-seq,Homo_633,AD.vs.MCI	3
MF	GO:0019210	kinase inhibitor activity	bulk RNA-seq,Homo_723,MCI.vs.control; bulk RNA-seq,Homo_714,AD.vs.MCI; bulk RNA-seq,Homo_714,MCI.vs.control; bulk RNA-seq,Homo_633,AD.vs.control; bulk RNA-seq,Homo_633,AD.vs.MCI	5
BP	GO:0044380	protein localization to cytoskeleton	bulk RNA-seq,Homo_723,MCI.vs.control; bulk RNA-seq,Homo_714,MCI.vs.control; bulk RNA-seq,Homo_633,AD.vs.control; bulk RNA-	4
MF	GO:0140664	ATP-dependent DNA damage sensor activity	bulk RNA-seq,Homo_723,MCI.vs.control; bulk RNA-seq,Homo_714,MCI.vs.control; bulk RNA-seq,Homo_633,AD.vs.control; bulk RNA-seq,Homo_633,AD.vs.MCI; bulk RNA-seq,Homo_633,MCI.vs.control	5
MF	GO:0030332	cyclin binding	bulk RNA-seq,Homo_723,MCI.vs.control; bulk RNA-seq,Homo_714,MCI.vs.control; bulk RNA-seq,Homo_633,AD.vs.control; bulk RNA-	4
BP	GO:0035020	regulation of Rac protein signal transduction	bulk RNA-seq,Homo_723,MCI.vs.control; bulk RNA-seq,Homo_714,AD.vs.control; bulk RNA-seq,Homo_714,MCI.vs.control; bulk RNA-seq,Homo_633,AD.vs.control; bulk RNA-seq,Homo_633,AD.vs.MCI; bulk RNA-seq,Homo_633,MCI.vs.control	6
BP	GO:0006312	mitotic recombination	bulk RNA-seq,Homo_723,MCI.vs.control; bulk RNA-seq,Homo_714,MCI.vs.control; bulk RNA-seq,Homo_633,AD.vs.control; bulk RNA-seq,Homo_633,AD.vs.MCI; bulk RNA-seq,Homo_633,MCI.vs.control	5
MF	GO:0015643	toxic substance binding	bulk RNA-seq,Homo_723,MCI.vs.control; bulk RNA-seq,Homo_714,AD.vs.MCI; bulk RNA-seq,Homo_714,MCI.vs.control	3
BP	GO:0046459	short-chain fatty acid metabolic process	bulk RNA-seq,Homo_723,MCI.vs.control; bulk RNA-seq,Homo_633,AD.vs.control; bulk RNA-seq,Homo_633,AD.vs.MCI	3
BP	GO:0006978	DNA damage response, signal transduction by p53 class mediator resulting in transcription of p21 class mediator	bulk RNA-seq,Homo_723,MCI.vs.control; bulk RNA-seq,Homo_714,AD.vs.control; bulk RNA-seq,Homo_714,MCI.vs.control; bulk RNA-seq,Homo_633,AD.vs.control; bulk RNA-seq,Homo_633,AD.vs.MCI; bulk RNA-seq,Homo_633,MCI.vs.control	6
MF	GO:0050661	NADP binding	bulk RNA-seq,Homo_723,MCI.vs.control; bulk RNA-seq,Homo_714,MCI.vs.control; bulk RNA-seq,Homo_633,AD.vs.control; bulk RNA-	4
BP	GO:0042045	epithelial fluid transport	bulk RNA-seq,Homo_723,MCI.vs.control; bulk RNA-seq,Homo_714,MCI.vs.control; bulk RNA-seq,Homo_633,AD.vs.control; bulk RNA-	4
MF	GO:0016645	oxidoreductase activity, acting on the CH-NH group of	bulk RNA-seq,Homo_723,MCI.vs.control; bulk RNA-seq,Homo_714,MCI.vs.control; bulk RNA-seq,Homo_633,AD.vs.control; bulk RNA-	4
BP	GO:0001959	regulation of cytokine-mediated signaling pathway	bulk RNA-seq,Homo_723,MCI.vs.control; bulk RNA-seq,Homo_633,AD.vs.control; bulk RNA-seq,Homo_633,AD.vs.MCI	3

BP	GO:0017145	stem cell division	bulk RNA-seq,Homo_723,MCI.vs.control; bulk RNA-seq,Homo_714,MCI.vs.control; bulk RNA-seq,Homo_633,AD.vs.control; bulk RNA-	4
MF	GO:0140312	cargo adaptor activity	bulk RNA-seq,Homo_723,MCI.vs.control; bulk RNA-seq,Homo_714,MCI.vs.control; bulk RNA-seq,Homo_633,AD.vs.control; bulk RNA-	4
BP	GO:0072523	purine-containing compound catabolic process	bulk RNA-seq,Homo_723,MCI.vs.control; bulk RNA-seq,Homo_714,MCI.vs.control; bulk RNA-seq,Homo_633,AD.vs.control; bulk RNA-	4
BP	GO:2000001	regulation of DNA damage checkpoint	bulk RNA-seq,Homo_723,MCI.vs.control; bulk RNA-seq,Homo_714,MCI.vs.control; bulk RNA-seq,Homo_633,AD.vs.control; bulk RNA-seq,Homo_633,AD.vs.MCI; bulk RNA-seq,Homo_633,MCI.vs.control	5
BP	GO:0032365	intracellular lipid transport	bulk RNA-seq,Homo_723,MCI.vs.control; bulk RNA-seq,Homo_714,MCI.vs.control; bulk RNA-seq,Homo_633,AD.vs.control; bulk RNA-	4
BP	GO:0051058	negative regulation of small GTPase mediated signal transduction	bulk RNA-seq,Homo_723,MCI.vs.control; bulk RNA-seq,Homo_714,AD.vs.control; bulk RNA-seq,Homo_714,MCI.vs.control; bulk RNA-seq,Homo_633,AD.vs.control; bulk RNA-seq,Homo_633,AD.vs.MCI	5
BP	GO:2000510	positive regulation of dendritic cell chemotaxis	bulk RNA-seq,Homo_723,MCI.vs.control; bulk RNA-seq,Homo_714,AD.vs.MCI; bulk RNA-seq,Homo_714,MCI.vs.control	3
MF	GO:0031491	nucleosome binding	bulk RNA-seq,Homo_723,MCI.vs.control; bulk RNA-seq,Homo_714,MCI.vs.control; bulk RNA-seq,Homo_633,AD.vs.control; bulk RNA-	4
BP	GO:0046321	positive regulation of fatty acid oxidation	bulk RNA-seq,Homo_723,MCI.vs.control; bulk RNA-seq,Homo_714,MCI.vs.control; bulk RNA-seq,Homo_633,AD.vs.control; bulk RNA-	4
BP	GO:0007097	nuclear migration	bulk RNA-seq,Homo_723,MCI.vs.control; bulk RNA-seq,Homo_714,MCI.vs.control; bulk RNA-seq,Homo_633,AD.vs.control	3
BP	GO:0072698	protein localization to microtubule cytoskeleton	bulk RNA-seq,Homo_723,MCI.vs.control; bulk RNA-seq,Homo_714,MCI.vs.control; bulk RNA-seq,Homo_633,AD.vs.control; bulk RNA-	4
BP	GO:1905523	positive regulation of macrophage migration	bulk RNA-seq,Homo_723,MCI.vs.control; bulk RNA-seq,Homo_714,AD.vs.MCI; bulk RNA-seq,Homo_714,MCI.vs.control	3
BP	GO:2000758	positive regulation of peptidyl-lysine acetylation	bulk RNA-seq,Homo_723,MCI.vs.control; bulk RNA-seq,Homo_714,AD.vs.MCI; bulk RNA-seq,Homo_714,MCI.vs.control; bulk RNA-	4
BP	GO:0031122	cytoplasmic microtubule organization	bulk RNA-seq,Homo_723,MCI.vs.control; bulk RNA-seq,Homo_714,MCI.vs.control; bulk RNA-seq,Homo_633,AD.vs.control; bulk RNA-	4
BP	GO:0035304	regulation of protein dephosphorylation	bulk RNA-seq,Homo_723,MCI.vs.control; bulk RNA-seq,Homo_714,MCI.vs.control; bulk RNA-seq,Homo_633,AD.vs.control; bulk RNA-	4
BP	GO:0051589	negative regulation of neurotransmitter transport	bulk RNA-seq,Homo_723,MCI.vs.control; bulk RNA-seq,Homo_633,AD.vs.control	2
MF	GO:0016742	hydroxymethyl-, formyl- and related transferase activity	bulk RNA-seq,Homo_723,MCI.vs.control; bulk RNA-seq,Homo_714,MCI.vs.control; bulk RNA-seq,Homo_633,MCI.vs.control	3
BP	GO:2000811	negative regulation of anoikis	bulk RNA-seq,Homo_723,MCI.vs.control; bulk RNA-seq,Homo_714,AD.vs.MCI; bulk RNA-seq,Homo_714,MCI.vs.control	3
BP	GO:0046824	positive regulation of nucleocytoplasmic transport	bulk RNA-seq,Homo_723,MCI.vs.control; bulk RNA-seq,Homo_714,MCI.vs.control; bulk RNA-seq,Homo_633,AD.vs.control; bulk RNA-	4
BP	GO:0019430	removal of superoxide radicals	bulk RNA-seq,Homo_723,MCI.vs.control; bulk RNA-seq,Homo_714,MCI.vs.control; bulk RNA-seq,Homo_633,AD.vs.control; bulk RNA-	4
MF	GO:0032451	demethylase activity	bulk RNA-seq,Homo_723,MCI.vs.control; bulk RNA-seq,Homo_714,MCI.vs.control; bulk RNA-seq,Homo_633,AD.vs.control; bulk RNA-seq,Homo_633,AD.vs.MCI; scRNA-seq,SRP330776,Naive CD8+ T cell_1-AD.vs.control	5
CC	GO:1902562	H4 histone acetyltransferase complex	bulk RNA-seq,Homo_723,MCI.vs.control; bulk RNA-seq,Homo_714,MCI.vs.control; bulk RNA-seq,Homo_633,AD.vs.control; bulk RNA-	4
BP	GO:0072666	establishment of protein localization to vacuole	bulk RNA-seq,Homo_723,MCI.vs.control; bulk RNA-seq,Homo_714,MCI.vs.control; bulk RNA-seq,Homo_633,AD.vs.control; bulk RNA-	4
BP	GO:0006103	2-oxoglutarate metabolic process	bulk RNA-seq,Homo_723,MCI.vs.control; bulk RNA-seq,Homo_714,MCI.vs.control	2
BP	GO:0034138	toll-like receptor 3 signaling pathway	bulk RNA-seq,Homo_723,MCI.vs.control; bulk RNA-seq,Homo_714,MCI.vs.control; bulk RNA-seq,Homo_633,MCI.vs.control	3
BP	GO:0031648	protein destabilization	bulk RNA-seq,Homo_723,MCI.vs.control; bulk RNA-seq,Homo_714,MCI.vs.control; bulk RNA-seq,Homo_633,AD.vs.control; bulk RNA-	4
BP	GO:0051224	negative regulation of protein transport	bulk RNA-seq,Homo_723,MCI.vs.control; bulk RNA-seq,Homo_633,AD.vs.control; bulk RNA-seq,Homo_633,AD.vs.MCI	3
BP	GO:0035024	negative regulation of Rho protein signal transduction	bulk RNA-seq,Homo_723,MCI.vs.control; bulk RNA-seq,Homo_714,MCI.vs.control; bulk RNA-seq,Homo_633,AD.vs.control	3
BP	GO:0001556	oocyte maturation	bulk RNA-seq,Homo_723,MCI.vs.control; bulk RNA-seq,Homo_714,MCI.vs.control; bulk RNA-seq,Homo_633,AD.vs.control	3
BP	GO:0000303	response to superoxide	bulk RNA-seq,Homo_723,MCI.vs.control; bulk RNA-seq,Homo_714,MCI.vs.control; bulk RNA-seq,Homo_633,AD.vs.control; bulk RNA-seq,Homo_633,AD.vs.MCI; bulk RNA-seq,SRP310421,AD.vs.control	5
BP	GO:0045342	MHC class II biosynthetic process	bulk RNA-seq,Homo_723,MCI.vs.control; bulk RNA-seq,Homo_714,MCI.vs.control	2
BP	GO:0051303	establishment of chromosome localization	bulk RNA-seq,Homo_723,MCI.vs.control; bulk RNA-seq,Homo_714,MCI.vs.control; bulk RNA-seq,Homo_633,AD.vs.control; bulk RNA-	4
BP	GO:0033260	nuclear DNA replication	bulk RNA-seq,Homo_723,MCI.vs.control; bulk RNA-seq,Homo_714,MCI.vs.control; bulk RNA-seq,Homo_633,AD.vs.control; bulk RNA-	4
BP	GO:0006555	methionine metabolic process	bulk RNA-seq,Homo_723,MCI.vs.control; bulk RNA-seq,Homo_714,AD.vs.MCI; bulk RNA-seq,Homo_714,MCI.vs.control	3
BP	GO:0060644	mammary gland epithelial cell differentiation	bulk RNA-seq,Homo_723,MCI.vs.control; bulk RNA-seq,Homo_714,MCI.vs.control	2
BP	GO:0042789	mRNA transcription by RNA polymerase II	bulk RNA-seq,Homo_723,MCI.vs.control; bulk RNA-seq,Homo_714,MCI.vs.control; bulk RNA-seq,Homo_633,AD.vs.control; bulk RNA-	4
BP	GO:0002523	leukocyte migration involved in inflammatory response	bulk RNA-seq,Homo_723,MCI.vs.control; bulk RNA-seq,Homo_714,AD.vs.MCI; bulk RNA-seq,Homo_714,MCI.vs.control; bulk RNA-	4
MF	GO:0005310	dicarboxylic acid transmembrane transporter activity	bulk RNA-seq,Homo_723,MCI.vs.control; bulk RNA-seq,Homo_714,MCI.vs.control; bulk RNA-seq,Homo_633,AD.vs.control; bulk RNA-	4
CC	GO:0000803	sex chromosome	bulk RNA-seq,Homo_723,MCI.vs.control; bulk RNA-seq,Homo_714,AD.vs.MCI; bulk RNA-seq,Homo_714,MCI.vs.control; bulk RNA-seq,Homo_633,AD.vs.control; bulk RNA-seq,Homo_633,MCI.vs.control; bulk RNA-seq,SRP325058,AD.vs.control; scRNA-seq,SRP330776,Naive CD8+ T cell_2-	7
BP	GO:0090314	positive regulation of protein targeting to membrane	bulk RNA-seq,Homo_723,MCI.vs.control; bulk RNA-seq,Homo_714,MCI.vs.control; bulk RNA-seq,Homo_633,AD.vs.control; bulk RNA-	4
BP	GO:1903038	negative regulation of leukocyte cell-cell adhesion	bulk RNA-seq,Homo_723,MCI.vs.control; bulk RNA-seq,Homo_714,AD.vs.MCI; bulk RNA-seq,Homo_633,AD.vs.control; bulk RNA-seq,Homo_633,AD.vs.MCI	4
BP	GO:0034333	adherens junction assembly	bulk RNA-seq,Homo_723,MCI.vs.control; bulk RNA-seq,Homo_714,MCI.vs.control; bulk RNA-seq,Homo_633,MCI.vs.control	3
BP	GO:0010762	regulation of fibroblast migration	bulk RNA-seq,Homo_723,MCI.vs.control; bulk RNA-seq,Homo_714,MCI.vs.control; bulk RNA-seq,Homo_633,AD.vs.control	3
BP	GO:1903421	regulation of synaptic vesicle recycling	bulk RNA-seq,Homo_723,MCI.vs.control; bulk RNA-seq,Homo_714,MCI.vs.control; bulk RNA-seq,Homo_633,AD.vs.control; bulk RNA-	4
MF	GO:0018024	histone-lysine N-methyltransferase activity	bulk RNA-seq,Homo_723,MCI.vs.control; bulk RNA-seq,Homo_714,MCI.vs.control; bulk RNA-seq,Homo_633,AD.vs.control; bulk RNA-	4
MF	GO:0016646	oxidoreductase activity, acting on the CH-NH group of donors, NAD or NADP as acceptor	bulk RNA-seq,Homo_723,MCI.vs.control; bulk RNA-seq,Homo_714,MCI.vs.control	2
BP	GO:0036507	protein demannosylation	bulk RNA-seq,Homo_723,MCI.vs.control; bulk RNA-seq,Homo_714,MCI.vs.control; bulk RNA-seq,Homo_633,AD.vs.control; bulk RNA-seq,Homo_633,AD.vs.MCI; bulk RNA-seq,Homo_633,MCI.vs.control	5
BP	GO:0036508	protein alpha-1,2-demannosylation	bulk RNA-seq,Homo_723,MCI.vs.control; bulk RNA-seq,Homo_714,MCI.vs.control; bulk RNA-seq,Homo_633,AD.vs.control; bulk RNA-seq,Homo_633,AD.vs.MCI; bulk RNA-seq,Homo_633,MCI.vs.control	5
BP	GO:0032753	positive regulation of interleukin-4 production	bulk RNA-seq,Homo_723,MCI.vs.control; bulk RNA-seq,Homo_714,MCI.vs.control; bulk RNA-seq,Homo_633,AD.vs.control; bulk RNA-	4
BP	GO:1901741	positive regulation of myoblast fusion	bulk RNA-seq,Homo_723,MCI.vs.control; bulk RNA-seq,Homo_714,MCI.vs.control	2

MF	GO:0030247	polysaccharide binding	bulk RNA-seq,Homo_723,MCI.vs.control; bulk RNA-seq,Homo_714,MCI.vs.control; bulk RNA-seq,Homo_633,AD.vs.control	3
CC	GO:0016234	inclusion body	bulk RNA-seq,Homo_723,MCI.vs.control; bulk RNA-seq,Homo_714,MCI.vs.control; bulk RNA-seq,Homo_633,AD.vs.control; bulk RNA-	4
BP	GO:0036503	ERAD pathway	bulk RNA-seq,Homo_723,MCI.vs.control; bulk RNA-seq,Homo_714,MCI.vs.control; bulk RNA-seq,Homo_633,AD.vs.control; bulk RNA-	4
BP	GO:0009156	ribonucleoside monophosphate biosynthetic process	bulk RNA-seq,Homo_723,MCI.vs.control; bulk RNA-seq,Homo_714,MCI.vs.control; bulk RNA-seq,Homo_633,AD.vs.control; bulk RNA-seq,Homo_633,AD.vs.MCI; bulk RNA-seq,Homo_633,MCI.vs.control	5
MF	GO:0008157	protein phosphatase 1 binding	bulk RNA-seq,Homo_723,MCI.vs.control; bulk RNA-seq,Homo_714,AD.vs.MCI; bulk RNA-seq,Homo_714,MCI.vs.control; bulk RNA-seq,Homo_633,AD.vs.control; bulk RNA-seq,Homo_633,AD.vs.MCI	5
BP	GO:0051642	centrosome localization	bulk RNA-seq,Homo_723,MCI.vs.control; bulk RNA-seq,Homo_714,MCI.vs.control; bulk RNA-seq,Homo_633,AD.vs.control; bulk RNA-	4
BP	GO:1903203	regulation of oxidative stress-induced neuron death	bulk RNA-seq,Homo_723,MCI.vs.control; bulk RNA-seq,Homo_714,MCI.vs.control; bulk RNA-seq,Homo_633,AD.vs.control; bulk RNA-	4
BP	GO:0060759	regulation of response to cytokine stimulus	bulk RNA-seq,Homo_723,MCI.vs.control; bulk RNA-seq,Homo_633,AD.vs.control; bulk RNA-seq,Homo_633,AD.vs.MCI	3
BP	GO:0006664	glycolipid metabolic process	bulk RNA-seq,Homo_723,MCI.vs.control; bulk RNA-seq,Homo_714,MCI.vs.control; bulk RNA-seq,Homo_633,AD.vs.control; bulk RNA-	4
BP	GO:0031445	regulation of heterochromatin formation	bulk RNA-seq,Homo_723,MCI.vs.control; bulk RNA-seq,Homo_714,MCI.vs.control; bulk RNA-seq,Homo_633,AD.vs.control; bulk RNA-	4
BP	GO:0120261	regulation of heterochromatin organization	bulk RNA-seq,Homo_723,MCI.vs.control; bulk RNA-seq,Homo_714,MCI.vs.control; bulk RNA-seq,Homo_633,AD.vs.control; bulk RNA-	4
CC	GO:0031228	intrinsic component of Golgi membrane	bulk RNA-seq,Homo_723,MCI.vs.control; bulk RNA-seq,Homo_714,MCI.vs.control; bulk RNA-seq,Homo_633,AD.vs.control; bulk RNA-	4
CC	GO:0031307	integral component of mitochondrial outer membrane	bulk RNA-seq,Homo_723,MCI.vs.control; bulk RNA-seq,Homo_714,MCI.vs.control; bulk RNA-seq,Homo_633,AD.vs.control; bulk RNA-seq,Homo_633,AD.vs.MCI; bulk RNA-seq,Homo_633,MCI.vs.control	5
BP	GO:0006760	folic acid-containing compound metabolic process	bulk RNA-seq,Homo_723,MCI.vs.control; bulk RNA-seq,Homo_714,MCI.vs.control; bulk RNA-seq,Homo_633,AD.vs.control; bulk RNA-seq,Homo_633,AD.vs.MCI; bulk RNA-seq,Homo_633,MCI.vs.control	5
MF	GO:0045309	protein phosphorylated amino acid binding	bulk RNA-seq,Homo_723,MCI.vs.control; bulk RNA-seq,Homo_714,MCI.vs.control; bulk RNA-seq,Homo_633,AD.vs.control; bulk RNA-	4
MF	GO:0070006	metalloaminopeptidase activity	bulk RNA-seq,Homo_723,MCI.vs.control; bulk RNA-seq,Homo_714,MCI.vs.control; bulk RNA-seq,Homo_633,AD.vs.control	3
BP	GO:0035967	cellular response to topologically incorrect protein	bulk RNA-seq,Homo_723,MCI.vs.control; bulk RNA-seq,Homo_714,AD.vs.MCI; bulk RNA-seq,Homo_714,MCI.vs.control; bulk RNA-seq,Homo_633,AD.vs.control; bulk RNA-seq,Homo_633,AD.vs.MCI	5
MF	GO:0016279	protein-lysine N-methyltransferase activity	bulk RNA-seq,Homo_723,MCI.vs.control; bulk RNA-seq,Homo_714,MCI.vs.control; bulk RNA-seq,Homo_633,AD.vs.control; bulk RNA-	4
BP	GO:0061462	protein localization to lysosome	bulk RNA-seq,Homo_723,MCI.vs.control; bulk RNA-seq,Homo_714,MCI.vs.control; bulk RNA-seq,Homo_633,AD.vs.control; bulk RNA-	4
BP	GO:0043280	positive regulation of cysteine-type endopeptidase activity involved in apoptotic process	bulk RNA-seq,Homo_723,MCI.vs.control; bulk RNA-seq,Homo_714,MCI.vs.control; bulk RNA-seq,Homo_633,AD.vs.control; bulk RNA-seq,Homo_633,AD.vs.MCI	4
MF	GO:0004438	phosphatidylinositol-3-phosphatase activity	bulk RNA-seq,Homo_723,MCI.vs.control; bulk RNA-seq,Homo_714,MCI.vs.control; bulk RNA-seq,Homo_633,AD.vs.control; bulk RNA-seq,Homo_633,AD.vs.MCI; bulk RNA-seq,Homo_633,MCI.vs.control	5
CC	GO:0005721	pericentric heterochromatin	bulk RNA-seq,Homo_723,MCI.vs.control; bulk RNA-seq,Homo_714,MCI.vs.control; bulk RNA-seq,Homo_633,AD.vs.control; bulk RNA-	4
BP	GO:0071549	cellular response to dexamethasone stimulus	bulk RNA-seq,Homo_723,MCI.vs.control; bulk RNA-seq,Homo_714,MCI.vs.control; bulk RNA-seq,Homo_633,AD.vs.control; bulk RNA-seq,Homo_633,AD.vs.MCI; bulk RNA-seq,Homo_633,MCI.vs.control	5
BP	GO:0034122	negative regulation of toll-like receptor signaling pathway	bulk RNA-seq,Homo_723,MCI.vs.control; bulk RNA-seq,Homo_714,MCI.vs.control; bulk RNA-seq,Homo_633,AD.vs.control	3
MF	GO:0016209	antioxidant activity	bulk RNA-seq,Homo_723,MCI.vs.control; bulk RNA-seq,Homo_714,AD.vs.MCI; bulk RNA-seq,Homo_714,MCI.vs.control; bulk RNA-seq,Homo_633,AD.vs.control; bulk RNA-seq,Homo_633,AD.vs.MCI; bulk RNA-seq,ROSMAP,MCI.vs.control	6
MF	GO:0008172	S-methyltransferase activity	bulk RNA-seq,Homo_723,MCI.vs.control; bulk RNA-seq,Homo_714,MCI.vs.control	2
CC	GO:0008287	protein serine/threonine phosphatase complex	bulk RNA-seq,Homo_723,MCI.vs.control; bulk RNA-seq,Homo_714,MCI.vs.control; bulk RNA-seq,Homo_633,AD.vs.control; bulk RNA-	4
CC	GO:0031985	Golgi cisterna	bulk RNA-seq,Homo_723,MCI.vs.control; bulk RNA-seq,Homo_714,MCI.vs.control; bulk RNA-seq,Homo_633,AD.vs.control; bulk RNA-	4
BP	GO:0046605	regulation of centrosome cycle	bulk RNA-seq,Homo_723,MCI.vs.control; bulk RNA-seq,Homo_714,MCI.vs.control; bulk RNA-seq,Homo_633,AD.vs.control; bulk RNA-	4
MF	GO:0140223	general transcription initiation factor activity	bulk RNA-seq,Homo_723,MCI.vs.control; bulk RNA-seq,Homo_714,MCI.vs.control; bulk RNA-seq,Homo_633,AD.vs.control; bulk RNA-	4
BP	GO:0060964	regulation of miRNA-mediated gene silencing	bulk RNA-seq,Homo_723,MCI.vs.control; bulk RNA-seq,Homo_714,MCI.vs.control; bulk RNA-seq,Homo_633,AD.vs.control	3
BP	GO:0072497	mesenchymal stem cell differentiation	bulk RNA-seq,Homo_723,MCI.vs.control; bulk RNA-seq,Homo_714,MCI.vs.control	2
BP	GO:0010640	regulation of platelet-derived growth factor receptor signaling	bulk RNA-seq,Homo_723,MCI.vs.control; bulk RNA-seq,Homo_714,MCI.vs.control; bulk RNA-seq,Homo_633,AD.vs.control; bulk RNA-	4
CC	GO:1903293	phosphatase complex	bulk RNA-seq,Homo_723,MCI.vs.control; bulk RNA-seq,Homo_714,MCI.vs.control; bulk RNA-seq,Homo_633,AD.vs.control; bulk RNA-	4
BP	GO:0071450	cellular response to oxygen radical	bulk RNA-seq,Homo_723,MCI.vs.control; bulk RNA-seq,Homo_714,MCI.vs.control; bulk RNA-seq,Homo_633,AD.vs.control; bulk RNA-	4
BP	GO:0071451	cellular response to superoxide	bulk RNA-seq,Homo_723,MCI.vs.control; bulk RNA-seq,Homo_714,MCI.vs.control; bulk RNA-seq,Homo_633,AD.vs.control; bulk RNA-	4
BP	GO:0006491	N-glycan processing	bulk RNA-seq,Homo_723,MCI.vs.control; bulk RNA-seq,Homo_714,MCI.vs.control; bulk RNA-seq,Homo_633,AD.vs.control; bulk RNA-	4
BP	GO:2000105	positive regulation of DNA-templated DNA replication	bulk RNA-seq,Homo_723,MCI.vs.control; bulk RNA-seq,Homo_714,MCI.vs.control; bulk RNA-seq,Homo_633,AD.vs.control; bulk RNA-	4
MF	GO:0016620	oxidoreductase activity, acting on the aldehyde or oxo group of donors, NAD or NADP as acceptor	bulk RNA-seq,Homo_723,MCI.vs.control; bulk RNA-seq,Homo_714,MCI.vs.control; bulk RNA-seq,Homo_633,AD.vs.control; bulk RNA-seq,Homo_633,AD.vs.MCI	4
BP	GO:0006298	mismatch repair	bulk RNA-seq,Homo_723,MCI.vs.control; bulk RNA-seq,Homo_714,MCI.vs.control; bulk RNA-seq,Homo_633,AD.vs.control; bulk RNA-	4
BP	GO:0090022	regulation of neutrophil chemotaxis	bulk RNA-seq,Homo_723,MCI.vs.control; bulk RNA-seq,Homo_714,MCI.vs.control; bulk RNA-seq,Homo_633,MCI.vs.control; bulk RNA-	4
MF	GO:0043495	protein-membrane adaptor activity	bulk RNA-seq,Homo_723,MCI.vs.control; bulk RNA-seq,Homo_714,AD.vs.control; bulk RNA-seq,Homo_714,MCI.vs.control; bulk RNA-	4
MF	GO:0140299	small molecule sensor activity	bulk RNA-seq,Homo_723,MCI.vs.control; bulk RNA-seq,Homo_714,MCI.vs.control; bulk RNA-seq,Homo_633,AD.vs.control; bulk RNA-	4
MF	GO:0042054	histone methyltransferase activity	bulk RNA-seq,Homo_723,MCI.vs.control; bulk RNA-seq,Homo_714,MCI.vs.control; bulk RNA-seq,Homo_633,AD.vs.control; bulk RNA-	4
BP	GO:0033194	response to hydroperoxide	bulk RNA-seq,Homo_723,MCI.vs.control; bulk RNA-seq,Homo_714,MCI.vs.control; bulk RNA-seq,Homo_633,AD.vs.control; bulk RNA-seq,Homo_633,AD.vs.MCI; bulk RNA-seq,Homo_633,MCI.vs.control	5
BP	GO:0008343	adult feeding behavior	bulk RNA-seq,Homo_723,MCI.vs.control	1
BP	GO:2001057	reactive nitrogen species metabolic process	bulk RNA-seq,Homo_723,MCI.vs.control; bulk RNA-seq,Homo_714,AD.vs.MCI; bulk RNA-seq,Homo_714,MCI.vs.control; bulk RNA-seq,Homo_633,AD.vs.control; bulk RNA-seq,Homo_633,AD.vs.MCI	5

BP	GO:1902017	regulation of cilium assembly	bulk RNA-seq,Homo_723,MCI.vs.control; bulk RNA-seq,Homo_714,MCI.vs.control; bulk RNA-seq,Homo_633,AD.vs.control; bulk RNA-	4
BP	GO:0046839	phospholipid dephosphorylation	bulk RNA-seq,Homo_723,MCI.vs.control; bulk RNA-seq,Homo_714,MCI.vs.control; bulk RNA-seq,Homo_633,AD.vs.control; bulk RNA-	4
BP	GO:0033004	negative regulation of mast cell activation	bulk RNA-seq,Homo_723,MCI.vs.control; bulk RNA-seq,Homo_714,MCI.vs.control	2
BP	GO:0035234	ectopic germ cell programmed cell death	bulk RNA-seq,Homo_723,MCI.vs.control; bulk RNA-seq,Homo_714,AD.vs.MCI; bulk RNA-seq,Homo_714,MCI.vs.control; bulk RNA-	4
MF	GO:0016278	lysine N-methyltransferase activity	bulk RNA-seq,Homo_723,MCI.vs.control; bulk RNA-seq,Homo_714,MCI.vs.control; bulk RNA-seq,Homo_633,AD.vs.control; bulk RNA-	4
BP	GO:0043649	dicarboxylic acid catabolic process	bulk RNA-seq,Homo_723,MCI.vs.control; bulk RNA-seq,Homo_714,MCI.vs.control; bulk RNA-seq,Homo_633,AD.vs.control	3
BP	GO:0030433	ubiquitin-dependent ERAD pathway	bulk RNA-seq,Homo_723,MCI.vs.control; bulk RNA-seq,Homo_714,MCI.vs.control; bulk RNA-seq,Homo_633,AD.vs.control; bulk RNA-	4
MF	GO:0008320	protein transmembrane transporter activity	bulk RNA-seq,Homo_723,MCI.vs.control; bulk RNA-seq,Homo_714,MCI.vs.control; bulk RNA-seq,Homo_633,AD.vs.control; bulk RNA-seq,Homo_633,AD.vs.MCI; bulk RNA-seq,Homo_633,MCI.vs.control	5
CC	GO:0030660	Golgi-associated vesicle membrane	bulk RNA-seq,Homo_723,MCI.vs.control; bulk RNA-seq,Homo_714,MCI.vs.control; bulk RNA-seq,Homo_633,AD.vs.control; bulk RNA-	4
BP	GO:0009072	aromatic amino acid family metabolic process	bulk RNA-seq,Homo_723,MCI.vs.control; bulk RNA-seq,Homo_714,AD.vs.MCI; bulk RNA-seq,Homo_714,MCI.vs.control; bulk RNA-	4
BP	GO:1902117	positive regulation of organelle assembly	bulk RNA-seq,Homo_723,MCI.vs.control; bulk RNA-seq,Homo_714,MCI.vs.control; bulk RNA-seq,Homo_633,AD.vs.control; bulk RNA-	4
BP	GO:0043299	leukocyte degranulation	bulk RNA-seq,Homo_723,MCI.vs.control; bulk RNA-seq,Homo_714,AD.vs.MCI; bulk RNA-seq,Homo_714,MCI.vs.control; bulk RNA-seq,Homo_633,AD.vs.control; bulk RNA-seq,Homo_633,AD.vs.MCI; bulk RNA-seq,SRP223445,AD.vs.control; scRNA-seq,SRP330776,Naive CD8+ T cell_2-	7
BP	GO:0035278	miRNA-mediated gene silencing by inhibition of translation	bulk RNA-seq,Homo_723,MCI.vs.control; bulk RNA-seq,Homo_714,AD.vs.control; bulk RNA-seq,Homo_714,MCI.vs.control	3
BP	GO:0051569	regulation of histone H3-K4 methylation	bulk RNA-seq,Homo_723,MCI.vs.control; bulk RNA-seq,Homo_714,MCI.vs.control; bulk RNA-seq,Homo_633,AD.vs.control; bulk RNA-	4
BP	GO:0030149	sphingolipid catabolic process	bulk RNA-seq,Homo_723,MCI.vs.control; bulk RNA-seq,Homo_714,MCI.vs.control; bulk RNA-seq,Homo_633,AD.vs.control	3
BP	GO:0009161	ribonucleoside monophosphate metabolic process	bulk RNA-seq,Homo_723,MCI.vs.control; bulk RNA-seq,Homo_714,MCI.vs.control; bulk RNA-seq,Homo_633,AD.vs.control; bulk RNA-	4
BP	GO:0001780	neutrophil homeostasis	bulk RNA-seq,Homo_723,MCI.vs.control; bulk RNA-seq,Homo_714,MCI.vs.control; bulk RNA-seq,Homo_633,AD.vs.control; bulk RNA-seq,Homo_633,AD.vs.MCI; bulk RNA-seq,Homo_633,MCI.vs.control	5
BP	GO:0042116	macrophage activation	bulk RNA-seq,Homo_723,MCI.vs.control; bulk RNA-seq,Homo_714,AD.vs.MCI; bulk RNA-seq,Homo_714,MCI.vs.control; bulk RNA-seq,Homo_633,AD.vs.control; bulk RNA-seq,Homo_633,AD.vs.MCI	5
BP	GO:0031571	mitotic G1 DNA damage checkpoint signaling	bulk RNA-seq,Homo_723,MCI.vs.control; bulk RNA-seq,Homo_714,MCI.vs.control; bulk RNA-seq,Homo_633,AD.vs.control; bulk RNA-seq,Homo_633,AD.vs.MCI; bulk RNA-seq,Homo_633,MCI.vs.control	5
BP	GO:0006270	DNA replication initiation	bulk RNA-seq,Homo_723,MCI.vs.control; bulk RNA-seq,Homo_714,MCI.vs.control; bulk RNA-seq,Homo_633,AD.vs.control; bulk RNA-	4
BP	GO:0055093	response to hyperoxia	bulk RNA-seq,Homo_723,MCI.vs.control; bulk RNA-seq,Homo_714,AD.vs.MCI; bulk RNA-seq,Homo_714,MCI.vs.control; bulk RNA-seq,Homo_633,AD.vs.MCI	4
MF	GO:0019212	phosphatase inhibitor activity	bulk RNA-seq,Homo_723,MCI.vs.control; bulk RNA-seq,Homo_714,MCI.vs.control	2
BP	GO:0043300	regulation of leukocyte degranulation	bulk RNA-seq,Homo_723,MCI.vs.control; bulk RNA-seq,Homo_714,AD.vs.MCI; bulk RNA-seq,Homo_714,MCI.vs.control; bulk RNA-seq,Homo_633,AD.vs.control; bulk RNA-seq,Homo_633,AD.vs.MCI; bulk RNA-seq,SRP223445,AD.vs.control	6
MF	GO:0005092	GDP-dissociation inhibitor activity	bulk RNA-seq,Homo_723,MCI.vs.control; bulk RNA-seq,Homo_633,AD.vs.control	2
BP	GO:0039531	regulation of viral-induced cytoplasmic pattern recognition receptor signaling pathway	bulk RNA-seq,Homo_723,MCI.vs.control; bulk RNA-seq,Homo_714,MCI.vs.control; bulk RNA-seq,Homo_633,AD.vs.control; bulk RNA-seq,Homo_633,MCI.vs.control	4
BP	GO:0046685	response to arsenic-containing substance	bulk RNA-seq,Homo_723,MCI.vs.control; bulk RNA-seq,Homo_714,MCI.vs.control; bulk RNA-seq,Homo_633,AD.vs.control; bulk RNA-seq,Homo_633,AD.vs.MCI; bulk RNA-seq,Homo_633,MCI.vs.control	5
BP	GO:1904292	regulation of ERAD pathway	bulk RNA-seq,Homo_723,MCI.vs.control; bulk RNA-seq,Homo_714,MCI.vs.control; bulk RNA-seq,Homo_633,AD.vs.control; bulk RNA-	4
MF	GO:0003887	DNA-directed DNA polymerase activity	bulk RNA-seq,Homo_723,MCI.vs.control; bulk RNA-seq,Homo_714,MCI.vs.control; bulk RNA-seq,Homo_633,AD.vs.control; bulk RNA-seq,Homo_633,AD.vs.MCI; bulk RNA-seq,Homo_633,MCI.vs.control	5
BP	GO:0002347	response to tumor cell	bulk RNA-seq,Homo_723,MCI.vs.control; bulk RNA-seq,Homo_714,AD.vs.control; bulk RNA-seq,Homo_714,MCI.vs.control; bulk RNA-seq,Homo_633,AD.vs.control; bulk RNA-seq,Homo_633,AD.vs.MCI; scRNA-seq,SRP309935,B cell_1-AD.vs.control	6
BP	GO:0045923	positive regulation of fatty acid metabolic process	bulk RNA-seq,Homo_723,MCI.vs.control; bulk RNA-seq,Homo_714,MCI.vs.control; bulk RNA-seq,Homo_633,AD.vs.control; bulk RNA-	4
BP	GO:1903051	negative regulation of proteolysis involved in protein	bulk RNA-seq,Homo_723,MCI.vs.control; bulk RNA-seq,Homo_714,MCI.vs.control; bulk RNA-seq,Homo_633,AD.vs.control; bulk RNA-	4
BP	GO:0002719	negative regulation of cytokine production involved in	bulk RNA-seq,Homo_723,MCI.vs.control; bulk RNA-seq,Homo_714,MCI.vs.control	2
BP	GO:2000310	regulation of NMDA receptor activity	bulk RNA-seq,Homo_723,MCI.vs.control; bulk RNA-seq,Homo_714,AD.vs.MCI; bulk RNA-seq,Homo_714,MCI.vs.control	3
BP	GO:0002934	desmosome organization	bulk RNA-seq,Homo_723,MCI.vs.control; bulk RNA-seq,Homo_714,AD.vs.MCI; bulk RNA-seq,Homo_714,MCI.vs.control	3
BP	GO:0000305	response to oxygen radical	bulk RNA-seq,Homo_723,MCI.vs.control; bulk RNA-seq,Homo_714,MCI.vs.control; bulk RNA-seq,Homo_633,AD.vs.control; bulk RNA-seq,Homo_633,AD.vs.MCI; bulk RNA-seq,SRP310421,AD.vs.control	5
BP	GO:0090312	positive regulation of protein deacetylation	bulk RNA-seq,Homo_723,MCI.vs.control; bulk RNA-seq,Homo_714,MCI.vs.control; bulk RNA-seq,Homo_633,AD.vs.control; bulk RNA-	4
BP	GO:0070584	mitochondrion morphogenesis	bulk RNA-seq,Homo_723,MCI.vs.control; bulk RNA-seq,Homo_714,MCI.vs.control; bulk RNA-seq,Homo_633,AD.vs.control; bulk RNA-	4
MF	GO:0030331	nuclear estrogen receptor binding	bulk RNA-seq,Homo_723,MCI.vs.control; bulk RNA-seq,Homo_714,MCI.vs.control	2
MF	GO:0004030	aldehyde dehydrogenase [NAD(P)+] activity	bulk RNA-seq,Homo_723,MCI.vs.control; bulk RNA-seq,Homo_714,MCI.vs.control	2
CC	GO:0044322	endoplasmic reticulum quality control compartment	bulk RNA-seq,Homo_723,MCI.vs.control; bulk RNA-seq,Homo_714,MCI.vs.control; bulk RNA-seq,Homo_633,AD.vs.control; bulk RNA-seq,Homo_633,AD.vs.MCI; bulk RNA-seq,Homo_633,MCI.vs.control	5
BP	GO:1900368	regulation of post-transcriptional gene silencing by RNA	bulk RNA-seq,Homo_723,MCI.vs.control; bulk RNA-seq,Homo_714,MCI.vs.control; bulk RNA-seq,Homo_633,AD.vs.control	3
BP	GO:0098883	synapse pruning	bulk RNA-seq,Homo_723,MCI.vs.control; bulk RNA-seq,Homo_714,AD.vs.MCI; bulk RNA-seq,Homo_714,MCI.vs.control	3
BP	GO:0042119	neutrophil activation	bulk RNA-seq,Homo_723,MCI.vs.control; bulk RNA-seq,Homo_714,MCI.vs.control; bulk RNA-seq,Homo_633,AD.vs.control; bulk RNA-seq,Homo_633,AD.vs.MCI; bulk RNA-seq,SRP223445,AD.vs.control	5
BP	GO:0071676	negative regulation of mononuclear cell migration	bulk RNA-seq,Homo_723,MCI.vs.control; bulk RNA-seq,Homo_714,MCI.vs.control; bulk RNA-seq,Homo_633,MCI.vs.control	3
CC	GO:0120293	dynein axonemal particle	bulk RNA-seq,Homo_723,MCI.vs.control; bulk RNA-seq,Homo_633,AD.vs.control	2
BP	GO:1902369	negative regulation of RNA catabolic process	bulk RNA-seq,Homo_723,MCI.vs.control; bulk RNA-seq,Homo_714,MCI.vs.control; bulk RNA-seq,Homo_633,AD.vs.control; bulk RNA-	4

CC	GO:0000307	cyclin-dependent protein kinase holoenzyme complex	bulk RNA-seq,Homo_723,MCI.vs.control; bulk RNA-seq,Homo_714,MCI.vs.control; bulk RNA-seq,Homo_633,AD.vs.control; bulk RNA-	4
BP	GO:0046931	pore complex assembly	bulk RNA-seq,Homo_723,MCI.vs.control; bulk RNA-seq,Homo_714,MCI.vs.control; bulk RNA-seq,Homo_633,AD.vs.control; bulk RNA-seq,Homo_633,AD.vs.MCI; bulk RNA-seq,Homo_633,MCI.vs.control	5
BP	GO:0036230	granulocyte activation	bulk RNA-seq,Homo_723,MCI.vs.control; bulk RNA-seq,Homo_714,MCI.vs.control; bulk RNA-seq,Homo_633,AD.vs.control; bulk RNA-seq,Homo_633,AD.vs.MCI; bulk RNA-seq,SRP223445,AD.vs.control	5
BP	GO:0031397	negative regulation of protein ubiquitination	bulk RNA-seq,Homo_723,MCI.vs.control; bulk RNA-seq,Homo_714,MCI.vs.control; bulk RNA-seq,Homo_633,AD.vs.control; bulk RNA-	4
CC	GO:0140092	bBAF complex	bulk RNA-seq,Homo_723,MCI.vs.control; bulk RNA-seq,Homo_633,AD.vs.control; bulk RNA-seq,Homo_633,AD.vs.MCI	3
MF	GO:0033691	sialic acid binding	bulk RNA-seq,Homo_723,MCI.vs.control; bulk RNA-seq,Homo_714,MCI.vs.control	2
BP	GO:0042362	fat-soluble vitamin biosynthetic process	bulk RNA-seq,Homo_723,MCI.vs.control; bulk RNA-seq,Homo_714,MCI.vs.control	2
CC	GO:0035267	NuA4 histone acetyltransferase complex	bulk RNA-seq,Homo_723,MCI.vs.control; bulk RNA-seq,Homo_714,MCI.vs.control; bulk RNA-seq,Homo_633,AD.vs.control; bulk RNA-seq,Homo_633,AD.vs.MCI; bulk RNA-seq,Homo_633,MCI.vs.control	5
CC	GO:0043189	H4/H2A histone acetyltransferase complex	bulk RNA-seq,Homo_723,MCI.vs.control; bulk RNA-seq,Homo_714,MCI.vs.control; bulk RNA-seq,Homo_633,AD.vs.control; bulk RNA-seq,Homo_633,AD.vs.MCI; bulk RNA-seq,Homo_633,MCI.vs.control	5
BP	GO:0043112	receptor metabolic process	bulk RNA-seq,Homo_723,MCI.vs.control; bulk RNA-seq,Homo_714,MCI.vs.control; bulk RNA-seq,Homo_633,AD.vs.control; bulk RNA-	4
BP	GO:0060236	regulation of mitotic spindle organization	bulk RNA-seq,Homo_723,MCI.vs.control; bulk RNA-seq,Homo_714,MCI.vs.control; bulk RNA-seq,Homo_633,AD.vs.control; bulk RNA-	4
BP	GO:0034311	diol metabolic process	bulk RNA-seq,Homo_723,MCI.vs.control; bulk RNA-seq,Homo_714,MCI.vs.control; bulk RNA-seq,Homo_633,AD.vs.control; bulk RNA-seq,Homo_633,AD.vs.MCI; bulk RNA-seq,Homo_633,MCI.vs.control	5
MF	GO:0005528	FK506 binding	bulk RNA-seq,Homo_723,MCI.vs.control; bulk RNA-seq,Homo_714,MCI.vs.control	2
MF	GO:0009931	calcium-dependent protein serine/threonine kinase activity	bulk RNA-seq,Homo_723,MCI.vs.control; bulk RNA-seq,Homo_714,MCI.vs.control; bulk RNA-seq,Homo_633,AD.vs.control	3
CC	GO:0090543	Flemming body	bulk RNA-seq,Homo_723,MCI.vs.control; bulk RNA-seq,Homo_714,MCI.vs.control; bulk RNA-seq,Homo_633,AD.vs.control; bulk RNA-seq,Homo_633,AD.vs.MCI; bulk RNA-seq,Homo_633,MCI.vs.control	5
BP	GO:0043470	regulation of carbohydrate catabolic process	bulk RNA-seq,Homo_723,MCI.vs.control; bulk RNA-seq,Homo_714,MCI.vs.control; bulk RNA-seq,Homo_633,AD.vs.control; bulk RNA-	4
BP	GO:1904424	regulation of GTP binding	bulk RNA-seq,Homo_723,MCI.vs.control; bulk RNA-seq,Homo_714,MCI.vs.control; bulk RNA-seq,Homo_633,AD.vs.control; bulk RNA-	4
MF	GO:0042800	histone methyltransferase activity (H3-K4 specific)	bulk RNA-seq,Homo_723,MCI.vs.control; bulk RNA-seq,Homo_714,MCI.vs.control; bulk RNA-seq,Homo_633,AD.vs.control; bulk RNA-	4
BP	GO:0030183	B cell differentiation	bulk RNA-seq,Homo_723,MCI.vs.control; bulk RNA-seq,Homo_633,AD.vs.control; bulk RNA-seq,Homo_633,AD.vs.MCI; scRNA-seq,SRP330776,Naive CD8+ T cell_2-AD.vs.control	4
BP	GO:0006646	phosphatidylethanolamine biosynthetic process	bulk RNA-seq,Homo_723,MCI.vs.control; bulk RNA-seq,Homo_714,MCI.vs.control	2
BP	GO:1900029	positive regulation of ruffle assembly	bulk RNA-seq,Homo_723,MCI.vs.control; bulk RNA-seq,Homo_714,MCI.vs.control	2
BP	GO:0032239	regulation of nucleobase-containing compound transport	bulk RNA-seq,Homo_723,MCI.vs.control; bulk RNA-seq,Homo_714,MCI.vs.control; bulk RNA-seq,Homo_633,AD.vs.control; bulk RNA-seq,Homo_633,AD.vs.MCI; bulk RNA-seq,Homo_633,MCI.vs.control	5
BP	GO:0006195	purine nucleotide catabolic process	bulk RNA-seq,Homo_723,MCI.vs.control; bulk RNA-seq,Homo_714,MCI.vs.control; bulk RNA-seq,Homo_633,AD.vs.control; bulk RNA-seq,Homo_633,AD.vs.MCI; scRNA-seq,SRP330776,Naive CD8+ T cell_1-AD.vs.control	5
BP	GO:1900371	regulation of purine nucleotide biosynthetic process	bulk RNA-seq,Homo_723,MCI.vs.control; bulk RNA-seq,Homo_714,MCI.vs.control; bulk RNA-seq,Homo_633,AD.vs.control	3
CC	GO:0045239	tricarboxylic acid cycle enzyme complex	bulk RNA-seq,Homo_723,MCI.vs.control; bulk RNA-seq,Homo_714,MCI.vs.control; bulk RNA-seq,Homo_633,AD.vs.control; bulk RNA-seq,Homo_633,AD.vs.MCI; bulk RNA-seq,Homo_633,MCI.vs.control	5
BP	GO:0090042	tubulin deacetylation	bulk RNA-seq,Homo_723,MCI.vs.control; bulk RNA-seq,Homo_714,MCI.vs.control; bulk RNA-seq,Homo_633,AD.vs.control; bulk RNA-	4
BP	GO:0120033	negative regulation of plasma membrane bounded cell projection assembly	bulk RNA-seq,Homo_723,MCI.vs.control; bulk RNA-seq,Homo_714,MCI.vs.control; bulk RNA-seq,Homo_633,AD.vs.control; bulk RNA-seq,Homo_633,AD.vs.MCI	4
BP	GO:0043433	negative regulation of DNA-binding transcription factor	bulk RNA-seq,Homo_723,MCI.vs.control; bulk RNA-seq,Homo_714,AD.vs.MCI; bulk RNA-seq,Homo_633,AD.vs.control; bulk RNA-seq,Homo_633,AD.vs.MCI	4
BP	GO:0009123	nucleoside monophosphate metabolic process	bulk RNA-seq,Homo_723,MCI.vs.control; bulk RNA-seq,Homo_714,MCI.vs.control; bulk RNA-seq,Homo_633,AD.vs.control; bulk RNA-	4
MF	GO:1901682	sulfur compound transmembrane transporter activity	bulk RNA-seq,Homo_723,MCI.vs.control; bulk RNA-seq,Homo_714,MCI.vs.control; bulk RNA-seq,Homo_633,AD.vs.control; bulk RNA-	4
BP	GO:0030213	hyaluronan biosynthetic process	bulk RNA-seq,Homo_723,MCI.vs.control; bulk RNA-seq,Homo_714,MCI.vs.control; bulk RNA-seq,Homo_633,AD.vs.control; bulk RNA-	4
MF	GO:0004520	endodeoxyribonuclease activity	bulk RNA-seq,Homo_723,MCI.vs.control; bulk RNA-seq,Homo_714,MCI.vs.control; bulk RNA-seq,Homo_633,AD.vs.control; bulk RNA-	4
BP	GO:0002182	cytoplasmic translational elongation	bulk RNA-seq,Homo_723,MCI.vs.control; bulk RNA-seq,Homo_714,MCI.vs.control; bulk RNA-seq,Homo_633,AD.vs.control; bulk RNA-seq,Homo_633,AD.vs.MCI; bulk RNA-seq,Homo_633,MCI.vs.control	5
BP	GO:0008333	endosome to lysosome transport	bulk RNA-seq,Homo_723,MCI.vs.control; bulk RNA-seq,Homo_714,MCI.vs.control; bulk RNA-seq,Homo_633,AD.vs.control; bulk RNA-	4
BP	GO:0045639	positive regulation of myeloid cell differentiation	bulk RNA-seq,Homo_723,MCI.vs.control; bulk RNA-seq,Homo_714,MCI.vs.control; bulk RNA-seq,Homo_633,AD.vs.control; bulk RNA-	4
MF	GO:0016836	hydro-lyase activity	bulk RNA-seq,Homo_723,MCI.vs.control; bulk RNA-seq,Homo_714,MCI.vs.control; bulk RNA-seq,Homo_633,AD.vs.control; bulk RNA-	4
MF	GO:0051010	microtubule plus-end binding	bulk RNA-seq,Homo_723,MCI.vs.control; bulk RNA-seq,Homo_714,MCI.vs.control; bulk RNA-seq,Homo_633,AD.vs.control; bulk RNA-	4
BP	GO:0039692	single stranded viral RNA replication via double stranded DNA intermediate	bulk RNA-seq,Homo_723,MCI.vs.control; bulk RNA-seq,Homo_714,MCI.vs.control	2
MF	GO:0022884	macromolecule transmembrane transporter activity	bulk RNA-seq,Homo_723,MCI.vs.control; bulk RNA-seq,Homo_714,MCI.vs.control; bulk RNA-seq,Homo_633,AD.vs.control; bulk RNA-seq,Homo_633,AD.vs.MCI; bulk RNA-seq,Homo_633,MCI.vs.control	5
BP	GO:0035493	SNARE complex assembly	bulk RNA-seq,Homo_723,MCI.vs.control; bulk RNA-seq,Homo_714,MCI.vs.control; bulk RNA-seq,Homo_633,AD.vs.control; bulk RNA-seq,Homo_633,AD.vs.MCI; bulk RNA-seq,Homo_633,MCI.vs.control	5
BP	GO:0035338	long-chain fatty-acyl-CoA biosynthetic process	bulk RNA-seq,Homo_723,MCI.vs.control; bulk RNA-seq,Homo_714,MCI.vs.control; bulk RNA-seq,Homo_633,AD.vs.control; bulk RNA-	4
BP	GO:0006801	superoxide metabolic process	bulk RNA-seq,Homo_723,MCI.vs.control; bulk RNA-seq,Homo_714,AD.vs.MCI; bulk RNA-seq,Homo_714,MCI.vs.control; bulk RNA-seq,Homo_633,AD.vs.control; bulk RNA-seq,Homo_633,AD.vs.MCI	5
MF	GO:0008526	phosphatidylinositol transfer activity	bulk RNA-seq,Homo_723,MCI.vs.control; bulk RNA-seq,Homo_714,MCI.vs.control	2
BP	GO:1905898	positive regulation of response to endoplasmic reticulum	bulk RNA-seq,Homo_723,MCI.vs.control; bulk RNA-seq,Homo_714,AD.vs.MCI; bulk RNA-seq,Homo_714,MCI.vs.control	3

BP	GO:1901799	negative regulation of proteasomal protein catabolic process	bulk RNA-seq,Homo_723,MCI.vs.control; bulk RNA-seq,Homo_714,MCI.vs.control; bulk RNA-seq,Homo_633,AD.vs.control; bulk RNA-	4
BP	GO:0043462	regulation of ATP-dependent activity	bulk RNA-seq,Homo_723,MCI.vs.control; bulk RNA-seq,Homo_714,MCI.vs.control; bulk RNA-seq,Homo_633,AD.vs.control; bulk RNA-	4
MF	GO:0008187	poly-pyrimidine tract binding	bulk RNA-seq,Homo_723,MCI.vs.control; bulk RNA-seq,Homo_633,AD.vs.control; bulk RNA-seq,Homo_633,AD.vs.MCI	3
BP	GO:0032781	positive regulation of ATP-dependent activity	bulk RNA-seq,Homo_723,MCI.vs.control; bulk RNA-seq,Homo_714,MCI.vs.control; bulk RNA-seq,Homo_633,AD.vs.control; bulk RNA-	4
BP	GO:0006999	nuclear pore organization	bulk RNA-seq,Homo_723,MCI.vs.control; bulk RNA-seq,Homo_714,MCI.vs.control; bulk RNA-seq,Homo_633,AD.vs.control; bulk RNA-seq,Homo_633,AD.vs.MCI; bulk RNA-seq,Homo_633,MCI.vs.control	5
MF	GO:0050321	tau-protein kinase activity	bulk RNA-seq,Homo_723,MCI.vs.control; bulk RNA-seq,Homo_714,MCI.vs.control; bulk RNA-seq,Homo_633,AD.vs.control	3
MF	GO:0070182	DNA polymerase binding	bulk RNA-seq,Homo_723,MCI.vs.control; bulk RNA-seq,Homo_714,AD.vs.control; bulk RNA-seq,Homo_714,MCI.vs.control; bulk RNA-seq,Homo_633,AD.vs.control; bulk RNA-seq,Homo_633,AD.vs.MCI; bulk RNA-seq,Homo_633,MCI.vs.control	6
CC	GO:0016605	PML body	bulk RNA-seq,Homo_723,MCI.vs.control; bulk RNA-seq,Homo_714,MCI.vs.control; bulk RNA-seq,Homo_633,AD.vs.control; bulk RNA-	4
BP	GO:0016486	peptide hormone processing	bulk RNA-seq,Homo_723,MCI.vs.control; bulk RNA-seq,Homo_633,AD.vs.control	2
BP	GO:0006907	pinocytosis	bulk RNA-seq,Homo_723,MCI.vs.control; bulk RNA-seq,Homo_714,MCI.vs.control; bulk RNA-seq,Homo_633,AD.vs.control	3
BP	GO:1905517	macrophage migration	bulk RNA-seq,Homo_723,MCI.vs.control; bulk RNA-seq,Homo_714,AD.vs.MCI; bulk RNA-seq,Homo_714,MCI.vs.control; bulk RNA-seq,Homo_633,AD.vs.control; bulk RNA-seq,Homo_633,AD.vs.MCI	5
BP	GO:0031272	regulation of pseudopodium assembly	bulk RNA-seq,Homo_723,MCI.vs.control; bulk RNA-seq,Homo_714,MCI.vs.control	2
BP	GO:0031274	positive regulation of pseudopodium assembly	bulk RNA-seq,Homo_723,MCI.vs.control; bulk RNA-seq,Homo_714,MCI.vs.control	2
BP	GO:0048489	synaptic vesicle transport	bulk RNA-seq,Homo_723,MCI.vs.control; bulk RNA-seq,Homo_714,AD.vs.control; bulk RNA-seq,Homo_714,MCI.vs.control; bulk RNA-seq,Homo_633,AD.vs.control; bulk RNA-seq,Homo_633,AD.vs.MCI	5
BP	GO:0051702	biological process involved in interaction with symbiont	bulk RNA-seq,Homo_723,MCI.vs.control; bulk RNA-seq,Homo_714,MCI.vs.control; bulk RNA-seq,Homo_633,AD.vs.control; bulk RNA-	4
BP	GO:0046949	fatty-acyl-CoA biosynthetic process	bulk RNA-seq,Homo_723,MCI.vs.control; bulk RNA-seq,Homo_714,MCI.vs.control; bulk RNA-seq,Homo_633,AD.vs.control; bulk RNA-	4
BP	GO:0042158	lipoprotein biosynthetic process	bulk RNA-seq,Homo_723,MCI.vs.control; bulk RNA-seq,Homo_714,AD.vs.control; bulk RNA-seq,Homo_714,MCI.vs.control; bulk RNA-seq,Homo_633,AD.vs.control; bulk RNA-seq,Homo_633,AD.vs.MCI	5
BP	GO:0010507	negative regulation of autophagy	bulk RNA-seq,Homo_723,MCI.vs.control; bulk RNA-seq,Homo_714,MCI.vs.control; bulk RNA-seq,Homo_633,AD.vs.control; bulk RNA-	4
BP	GO:0031650	regulation of heat generation	bulk RNA-seq,Homo_723,MCI.vs.control; bulk RNA-seq,Homo_714,MCI.vs.control	2
MF	GO:0018455	alcohol dehydrogenase [NAD(P)+] activity	bulk RNA-seq,Homo_723,MCI.vs.control; bulk RNA-seq,Homo_714,AD.vs.MCI; bulk RNA-seq,Homo_714,MCI.vs.control	3
BP	GO:1902373	negative regulation of mRNA catabolic process	bulk RNA-seq,Homo_723,MCI.vs.control; bulk RNA-seq,Homo_714,MCI.vs.control; bulk RNA-seq,Homo_633,AD.vs.control; bulk RNA-	4
BP	GO:1902018	negative regulation of cilium assembly	bulk RNA-seq,Homo_723,MCI.vs.control; bulk RNA-seq,Homo_714,MCI.vs.control; bulk RNA-seq,Homo_633,AD.vs.control; bulk RNA-	4
MF	GO:0140333	glycerophospholipid flippase activity	bulk RNA-seq,Homo_723,MCI.vs.control; bulk RNA-seq,Homo_714,AD.vs.MCI; bulk RNA-seq,Homo_714,MCI.vs.control; bulk RNA-	4
CC	GO:0032993	protein-DNA complex	bulk RNA-seq,Homo_723,MCI.vs.control; bulk RNA-seq,Homo_714,AD.vs.MCI; bulk RNA-seq,Homo_633,AD.vs.control; bulk RNA-seq,Homo_633,AD.vs.MCI	4
BP	GO:0002138	retinoic acid biosynthetic process	bulk RNA-seq,Homo_723,MCI.vs.control; bulk RNA-seq,Homo_714,MCI.vs.control; bulk RNA-seq,Homo_633,AD.vs.control	3
CC	GO:0019005	SCF ubiquitin ligase complex	bulk RNA-seq,Homo_723,MCI.vs.control; bulk RNA-seq,Homo_714,MCI.vs.control; bulk RNA-seq,Homo_633,AD.vs.control; bulk RNA-	4
BP	GO:0042159	lipoprotein catabolic process	bulk RNA-seq,Homo_723,MCI.vs.control; bulk RNA-seq,Homo_633,AD.vs.control; bulk RNA-seq,Homo_633,AD.vs.MCI	3
BP	GO:0032495	response to muramyl dipeptide	bulk RNA-seq,Homo_723,MCI.vs.control; bulk RNA-seq,Homo_714,MCI.vs.control; bulk RNA-seq,Homo_633,MCI.vs.control	3
BP	GO:0035791	platelet-derived growth factor receptor-beta signaling	bulk RNA-seq,Homo_723,MCI.vs.control; bulk RNA-seq,Homo_714,MCI.vs.control; bulk RNA-seq,Homo_633,AD.vs.control	3
MF	GO:0017002	activin receptor activity	bulk RNA-seq,Homo_723,MCI.vs.control; bulk RNA-seq,Homo_714,MCI.vs.control; bulk RNA-seq,Homo_633,AD.vs.control; bulk RNA-	4
BP	GO:0033314	mitotic DNA replication checkpoint signaling	bulk RNA-seq,Homo_723,MCI.vs.control; bulk RNA-seq,Homo_714,MCI.vs.control; bulk RNA-seq,Homo_633,AD.vs.control	3
CC	GO:0031209	SCAR complex	bulk RNA-seq,Homo_723,MCI.vs.control; bulk RNA-seq,Homo_714,MCI.vs.control	2
BP	GO:0045348	positive regulation of MHC class II biosynthetic process	bulk RNA-seq,Homo_723,MCI.vs.control; bulk RNA-seq,Homo_714,MCI.vs.control; bulk RNA-seq,Homo_633,MCI.vs.control	3
BP	GO:0042772	DNA damage response, signal transduction resulting in transcription	bulk RNA-seq,Homo_723,MCI.vs.control; bulk RNA-seq,Homo_714,AD.vs.control; bulk RNA-seq,Homo_714,MCI.vs.control; bulk RNA-seq,Homo_633,AD.vs.control; bulk RNA-seq,Homo_633,AD.vs.MCI; bulk RNA-seq,Homo_633,MCI.vs.control	6
BP	GO:0060143	positive regulation of syncytium formation by plasma	bulk RNA-seq,Homo_723,MCI.vs.control; bulk RNA-seq,Homo_714,MCI.vs.control	2
BP	GO:0071482	cellular response to light stimulus	bulk RNA-seq,Homo_723,MCI.vs.control; bulk RNA-seq,Homo_714,MCI.vs.control; bulk RNA-seq,Homo_633,AD.vs.control; bulk RNA-	4
BP	GO:0006517	protein deglycosylation	bulk RNA-seq,Homo_723,MCI.vs.control; bulk RNA-seq,Homo_714,MCI.vs.control; bulk RNA-seq,Homo_633,AD.vs.control; bulk RNA-seq,Homo_633,AD.vs.MCI; bulk RNA-seq,Homo_633,MCI.vs.control	5
BP	GO:0031065	positive regulation of histone deacetylation	bulk RNA-seq,Homo_723,MCI.vs.control; bulk RNA-seq,Homo_714,MCI.vs.control; bulk RNA-seq,Homo_633,AD.vs.control; bulk RNA-	4
BP	GO:0007029	endoplasmic reticulum organization	bulk RNA-seq,Homo_723,MCI.vs.control; bulk RNA-seq,Homo_714,MCI.vs.control; bulk RNA-seq,Homo_633,AD.vs.control; bulk RNA-	4
BP	GO:0000289	nuclear-transcribed mRNA poly(A) tail shortening	bulk RNA-seq,Homo_723,MCI.vs.control; bulk RNA-seq,Homo_714,MCI.vs.control; bulk RNA-seq,Homo_633,AD.vs.control; bulk RNA-seq,Homo_633,AD.vs.MCI; bulk RNA-seq,Homo_633,MCI.vs.control	5
BP	GO:0051571	positive regulation of histone H3-K4 methylation	bulk RNA-seq,Homo_723,MCI.vs.control; bulk RNA-seq,Homo_714,MCI.vs.control; bulk RNA-seq,Homo_633,AD.vs.control; bulk RNA-	4
BP	GO:0051570	regulation of histone H3-K9 methylation	bulk RNA-seq,Homo_723,MCI.vs.control; bulk RNA-seq,Homo_714,MCI.vs.control; bulk RNA-seq,Homo_633,AD.vs.control; bulk RNA-	4
BP	GO:0031112	positive regulation of microtubule polymerization or	bulk RNA-seq,Homo_723,MCI.vs.control; bulk RNA-seq,Homo_714,MCI.vs.control; bulk RNA-seq,Homo_633,AD.vs.control; bulk RNA-	4
CC	GO:0005687	U4 snRNP	bulk RNA-seq,Homo_723,MCI.vs.control; bulk RNA-seq,Homo_714,MCI.vs.control; bulk RNA-seq,Homo_633,AD.vs.control; bulk RNA-	4
BP	GO:1903867	extraembryonic membrane development	bulk RNA-seq,Homo_723,MCI.vs.control; bulk RNA-seq,Homo_714,MCI.vs.control	2
BP	GO:0009168	purine ribonucleoside monophosphate biosynthetic process	bulk RNA-seq,Homo_723,MCI.vs.control; bulk RNA-seq,Homo_714,MCI.vs.control; bulk RNA-seq,Homo_633,AD.vs.control; bulk RNA-	4
BP	GO:0034312	diol biosynthetic process	bulk RNA-seq,Homo_723,MCI.vs.control; bulk RNA-seq,Homo_714,MCI.vs.control; bulk RNA-seq,Homo_633,AD.vs.control; bulk RNA-	4
CC	GO:0035102	PRC1 complex	bulk RNA-seq,Homo_723,MCI.vs.control; bulk RNA-seq,Homo_714,MCI.vs.control; bulk RNA-seq,Homo_633,AD.vs.control; bulk RNA-	4
BP	GO:0051340	regulation of ligase activity	bulk RNA-seq,Homo_723,MCI.vs.control; bulk RNA-seq,Homo_714,MCI.vs.control; bulk RNA-seq,Homo_633,AD.vs.control; bulk RNA-	4

BP	GO:0009154	purine ribonucleotide catabolic process	bulk RNA-seq,Homo_723,MCI.vs.control; bulk RNA-seq,Homo_714,MCI.vs.control; bulk RNA-seq,Homo_633,AD.vs.control; bulk RNA-seq,Homo_633,AD.vs.MCI; scRNA-seq,SRP330776,Naive CD8+ T cell_1-AD.vs.control	5
BP	GO:0043011	myeloid dendritic cell differentiation	bulk RNA-seq,Homo_723,MCI.vs.control; bulk RNA-seq,Homo_714,MCI.vs.control	2
BP	GO:0034314	Arp2/3 complex-mediated actin nucleation	bulk RNA-seq,Homo_723,MCI.vs.control; bulk RNA-seq,Homo_714,MCI.vs.control; bulk RNA-seq,Homo_633,AD.vs.control; bulk RNA-	4
BP	GO:0070816	phosphorylation of RNA polymerase II C-terminal domain	bulk RNA-seq,Homo_723,MCI.vs.control; bulk RNA-seq,Homo_714,MCI.vs.control; bulk RNA-seq,Homo_633,AD.vs.control; bulk RNA-seq,Homo_633,AD.vs.MCI; bulk RNA-seq,Homo_633,MCI.vs.control	5
BP	GO:0006073	cellular glucan metabolic process	bulk RNA-seq,Homo_723,MCI.vs.control; bulk RNA-seq,Homo_714,MCI.vs.control; bulk RNA-seq,Homo_633,AD.vs.control; bulk RNA-	4
BP	GO:0044042	glucan metabolic process	bulk RNA-seq,Homo_723,MCI.vs.control; bulk RNA-seq,Homo_714,MCI.vs.control; bulk RNA-seq,Homo_633,AD.vs.control; bulk RNA-	4
BP	GO:0006778	porphyrin-containing compound metabolic process	bulk RNA-seq,Homo_723,MCI.vs.control; bulk RNA-seq,Homo_714,MCI.vs.control; bulk RNA-seq,Homo_633,AD.vs.control; bulk RNA-	4
BP	GO:0032366	intracellular sterol transport	bulk RNA-seq,Homo_723,MCI.vs.control; bulk RNA-seq,Homo_714,MCI.vs.control; bulk RNA-seq,Homo_633,AD.vs.control; bulk RNA-	4
MF	GO:0106018	phosphatidylinositol-3,5-bisphosphate phosphatase activity	bulk RNA-seq,Homo_723,MCI.vs.control; bulk RNA-seq,Homo_714,MCI.vs.control; bulk RNA-seq,Homo_633,AD.vs.control; bulk RNA-seq,Homo_633,AD.vs.MCI; bulk RNA-seq,Homo_633,MCI.vs.control	5
MF	GO:0004860	protein kinase inhibitor activity	bulk RNA-seq,Homo_723,MCI.vs.control; bulk RNA-seq,Homo_714,MCI.vs.control; bulk RNA-seq,Homo_633,AD.vs.control; bulk RNA-	4
BP	GO:0072567	chemokine (C-X-C motif) ligand 2 production	bulk RNA-seq,Homo_723,MCI.vs.control; bulk RNA-seq,Homo_714,MCI.vs.control; bulk RNA-seq,Homo_633,AD.vs.control; bulk RNA-	4
BP	GO:2000341	regulation of chemokine (C-X-C motif) ligand 2 production	bulk RNA-seq,Homo_723,MCI.vs.control; bulk RNA-seq,Homo_714,MCI.vs.control; bulk RNA-seq,Homo_633,AD.vs.control; bulk RNA-	4
BP	GO:0006110	regulation of glycolytic process	bulk RNA-seq,Homo_723,MCI.vs.control; bulk RNA-seq,Homo_714,MCI.vs.control; bulk RNA-seq,Homo_633,AD.vs.control; bulk RNA-	4
MF	GO:0005355	glucose transmembrane transporter activity	bulk RNA-seq,Homo_723,MCI.vs.control; bulk RNA-seq,Homo_633,AD.vs.control	2
MF	GO:0015149	hexose transmembrane transporter activity	bulk RNA-seq,Homo_723,MCI.vs.control; bulk RNA-seq,Homo_633,AD.vs.control	2
BP	GO:0032527	protein exit from endoplasmic reticulum	bulk RNA-seq,Homo_723,MCI.vs.control; bulk RNA-seq,Homo_714,MCI.vs.control; bulk RNA-seq,Homo_633,AD.vs.control; bulk RNA-	4
BP	GO:0031268	pseudopodium organization	bulk RNA-seq,Homo_723,MCI.vs.control; bulk RNA-seq,Homo_714,MCI.vs.control; bulk RNA-seq,Homo_633,AD.vs.control	3
BP	GO:0003093	regulation of glomerular filtration	bulk RNA-seq,Homo_723,MCI.vs.control; bulk RNA-seq,Homo_714,MCI.vs.control	2
BP	GO:0006268	DNA unwinding involved in DNA replication	bulk RNA-seq,Homo_723,MCI.vs.control; bulk RNA-seq,Homo_714,MCI.vs.control; bulk RNA-seq,Homo_633,AD.vs.control; bulk RNA-	4
MF	GO:0010857	calcium-dependent protein kinase activity	bulk RNA-seq,Homo_723,MCI.vs.control; bulk RNA-seq,Homo_714,MCI.vs.control; bulk RNA-seq,Homo_633,AD.vs.control	3
BP	GO:0090224	regulation of spindle organization	bulk RNA-seq,Homo_723,MCI.vs.control; bulk RNA-seq,Homo_714,MCI.vs.control; bulk RNA-seq,Homo_633,AD.vs.control; bulk RNA-	4
BP	GO:0001919	regulation of receptor recycling	bulk RNA-seq,Homo_723,MCI.vs.control; bulk RNA-seq,Homo_714,MCI.vs.control; bulk RNA-seq,Homo_633,AD.vs.control; bulk RNA-	4
BP	GO:0048246	macrophage chemotaxis	bulk RNA-seq,Homo_723,MCI.vs.control; bulk RNA-seq,Homo_714,AD.vs.MCI; bulk RNA-seq,Homo_714,MCI.vs.control	3
BP	GO:0032733	positive regulation of interleukin-10 production	bulk RNA-seq,Homo_723,MCI.vs.control; bulk RNA-seq,Homo_714,MCI.vs.control; bulk RNA-seq,Homo_633,AD.vs.control	3
BP	GO:0090313	regulation of protein targeting to membrane	bulk RNA-seq,Homo_723,MCI.vs.control; bulk RNA-seq,Homo_714,MCI.vs.control; bulk RNA-seq,Homo_633,AD.vs.control; bulk RNA-	4
BP	GO:0051220	cytoplasmic sequestering of protein	bulk RNA-seq,Homo_723,MCI.vs.control; bulk RNA-seq,Homo_714,MCI.vs.control; bulk RNA-seq,Homo_633,AD.vs.control; bulk RNA-	4
CC	GO:0070821	tertiary granule membrane	bulk RNA-seq,Homo_723,MCI.vs.control; bulk RNA-seq,Homo_714,MCI.vs.control; bulk RNA-seq,Homo_633,AD.vs.control; bulk RNA-seq,Homo_633,AD.vs.MCI; bulk RNA-seq,SRP223445,AD.vs.control	5
BP	GO:0034694	response to prostaglandin	bulk RNA-seq,Homo_723,MCI.vs.control; bulk RNA-seq,Homo_714,MCI.vs.control	2
BP	GO:1901998	toxin transport	bulk RNA-seq,Homo_723,MCI.vs.control; bulk RNA-seq,Homo_714,AD.vs.control; bulk RNA-seq,Homo_714,MCI.vs.control; bulk RNA-seq,Homo_633,AD.vs.control; bulk RNA-seq,Homo_633,AD.vs.MCI	5
BP	GO:0051593	response to folic acid	bulk RNA-seq,Homo_723,MCI.vs.control	1
BP	GO:0030834	regulation of actin filament depolymerization	bulk RNA-seq,Homo_723,MCI.vs.control; bulk RNA-seq,Homo_714,MCI.vs.control; bulk RNA-seq,Homo_633,AD.vs.control	3
BP	GO:0051767	nitric-oxide synthase biosynthetic process	bulk RNA-seq,Homo_723,MCI.vs.control; bulk RNA-seq,Homo_714,MCI.vs.control	2
BP	GO:0051769	regulation of nitric-oxide synthase biosynthetic process	bulk RNA-seq,Homo_723,MCI.vs.control; bulk RNA-seq,Homo_714,MCI.vs.control	2
BP	GO:0055022	negative regulation of cardiac muscle tissue growth	bulk RNA-seq,Homo_723,MCI.vs.control; bulk RNA-seq,Homo_714,AD.vs.MCI; bulk RNA-seq,Homo_714,MCI.vs.control; scRNA-seq,SRP330776,CD8+ T cell_1-AD.vs.control; scRNA-seq,SRP330776,Naive CD8+ T cell_1-AD.vs.control	5
BP	GO:0061117	negative regulation of heart growth	bulk RNA-seq,Homo_723,MCI.vs.control; bulk RNA-seq,Homo_714,AD.vs.MCI; bulk RNA-seq,Homo_714,MCI.vs.control; scRNA-seq,SRP330776,CD8+ T cell_1-AD.vs.control; scRNA-seq,SRP330776,Naive CD8+ T cell_1-AD.vs.control	5
MF	GO:0072349	modified amino acid transmembrane transporter activity	bulk RNA-seq,Homo_723,MCI.vs.control; bulk RNA-seq,Homo_714,MCI.vs.control; bulk RNA-seq,Homo_633,MCI.vs.control	3
BP	GO:1900242	regulation of synaptic vesicle endocytosis	bulk RNA-seq,Homo_723,MCI.vs.control; bulk RNA-seq,Homo_714,MCI.vs.control; bulk RNA-seq,Homo_633,AD.vs.control	3
BP	GO:0030300	regulation of intestinal cholesterol absorption	bulk RNA-seq,Homo_723,MCI.vs.control; bulk RNA-seq,Homo_714,AD.vs.MCI; bulk RNA-seq,Homo_714,MCI.vs.control	3
CC	GO:0098845	postsynaptic endosome	bulk RNA-seq,Homo_723,MCI.vs.control; bulk RNA-seq,Homo_714,MCI.vs.control; bulk RNA-seq,Homo_633,AD.vs.control; bulk RNA-	4
MF	GO:0042887	amide transmembrane transporter activity	bulk RNA-seq,Homo_723,MCI.vs.control; bulk RNA-seq,Homo_714,MCI.vs.control; bulk RNA-seq,Homo_633,AD.vs.control; bulk RNA-	4
BP	GO:0030656	regulation of vitamin metabolic process	bulk RNA-seq,Homo_723,MCI.vs.control; bulk RNA-seq,Homo_714,MCI.vs.control; bulk RNA-seq,Homo_633,AD.vs.control	3
MF	GO:0008266	poly(U) RNA binding	bulk RNA-seq,Homo_723,MCI.vs.control; bulk RNA-seq,Homo_633,AD.vs.control; bulk RNA-seq,Homo_633,AD.vs.MCI	3
BP	GO:0150078	positive regulation of neuroinflammatory response	bulk RNA-seq,Homo_723,MCI.vs.control; bulk RNA-seq,Homo_714,MCI.vs.control	2
BP	GO:0030948	negative regulation of vascular endothelial growth factor receptor signaling pathway	bulk RNA-seq,Homo_723,MCI.vs.control; bulk RNA-seq,Homo_714,MCI.vs.control; bulk RNA-seq,Homo_633,AD.vs.control; bulk RNA-seq,Homo_633,MCI.vs.control	4
BP	GO:0007144	female meiosis I	bulk RNA-seq,Homo_723,MCI.vs.control; bulk RNA-seq,Homo_714,MCI.vs.control; bulk RNA-seq,Homo_633,AD.vs.control; bulk RNA-	4
BP	GO:0018126	protein hydroxylation	bulk RNA-seq,Homo_723,MCI.vs.control; bulk RNA-seq,Homo_714,AD.vs.control; bulk RNA-seq,Homo_714,MCI.vs.control; bulk RNA-seq,Homo_633,AD.vs.control; bulk RNA-seq,Homo_633,MCI.vs.control	5
BP	GO:0090169	regulation of spindle assembly	bulk RNA-seq,Homo_723,MCI.vs.control; bulk RNA-seq,Homo_714,MCI.vs.control; bulk RNA-seq,Homo_633,AD.vs.control; bulk RNA-seq,Homo_633,AD.vs.MCI; bulk RNA-seq,Homo_633,MCI.vs.control	5
BP	GO:0035872	nucleotide-binding domain, leucine rich repeat containing receptor signaling pathway	bulk RNA-seq,Homo_723,MCI.vs.control; bulk RNA-seq,Homo_714,MCI.vs.control; bulk RNA-seq,Homo_633,AD.vs.control; bulk RNA-seq,Homo_633,AD.vs.MCI; bulk RNA-seq,Homo_633,MCI.vs.control	5

BP	GO:0030953	astral microtubule organization	bulk RNA-seq,Homo_723,MCI.vs.control; bulk RNA-seq,Homo_633,AD.vs.control	2
BP	GO:1900247	regulation of cytoplasmic translational elongation	bulk RNA-seq,Homo_723,MCI.vs.control; bulk RNA-seq,Homo_714,MCI.vs.control; bulk RNA-seq,Homo_633,AD.vs.control; bulk RNA-seq,Homo_633,AD.vs.MCI; bulk RNA-seq,Homo_633,MCI.vs.control	5
CC	GO:0071682	endocytic vesicle lumen	bulk RNA-seq,Homo_723,MCI.vs.control; bulk RNA-seq,Homo_714,MCI.vs.control; bulk RNA-seq,Homo_633,AD.vs.control; bulk RNA-	4
BP	GO:0045724	positive regulation of cilium assembly	bulk RNA-seq,Homo_723,MCI.vs.control; bulk RNA-seq,Homo_714,MCI.vs.control; bulk RNA-seq,Homo_633,AD.vs.control; bulk RNA-	4
BP	GO:0002693	positive regulation of cellular extravasation	bulk RNA-seq,Homo_723,MCI.vs.control; bulk RNA-seq,Homo_714,MCI.vs.control	2
BP	GO:0003418	growth plate cartilage chondrocyte differentiation	bulk RNA-seq,Homo_723,MCI.vs.control; bulk RNA-seq,Homo_633,AD.vs.control; bulk RNA-seq,Homo_633,AD.vs.MCI	3
MF	GO:0046624	sphingolipid transporter activity	bulk RNA-seq,Homo_723,MCI.vs.control; bulk RNA-seq,Homo_633,AD.vs.control	2
BP	GO:2000641	regulation of early endosome to late endosome transport	bulk RNA-seq,Homo_723,MCI.vs.control; bulk RNA-seq,Homo_714,MCI.vs.control; bulk RNA-seq,Homo_633,AD.vs.control; bulk RNA-	4
BP	GO:0016577	histone demethylation	bulk RNA-seq,Homo_723,MCI.vs.control; bulk RNA-seq,Homo_714,AD.vs.MCI; bulk RNA-seq,Homo_714,MCI.vs.control; bulk RNA-seq,Homo_633,AD.vs.control; bulk RNA-seq,Homo_633,AD.vs.MCI; bulk RNA-seq,Homo_633,MCI.vs.control	6
BP	GO:2000121	regulation of removal of superoxide radicals	bulk RNA-seq,Homo_723,MCI.vs.control; bulk RNA-seq,Homo_714,MCI.vs.control; bulk RNA-seq,Homo_633,AD.vs.control; bulk RNA-	4
BP	GO:0039529	RIG-I signaling pathway	bulk RNA-seq,Homo_723,MCI.vs.control; bulk RNA-seq,Homo_714,MCI.vs.control; bulk RNA-seq,Homo_633,AD.vs.control; bulk RNA-	4
BP	GO:0009074	aromatic amino acid family catabolic process	bulk RNA-seq,Homo_723,MCI.vs.control; bulk RNA-seq,Homo_714,MCI.vs.control	2
MF	GO:0017025	TBP-class protein binding	bulk RNA-seq,Homo_723,MCI.vs.control; bulk RNA-seq,Homo_714,AD.vs.control; bulk RNA-seq,Homo_714,MCI.vs.control; bulk RNA-seq,Homo_633,AD.vs.control; bulk RNA-seq,Homo_633,AD.vs.MCI; bulk RNA-seq,Homo_633,MCI.vs.control	6
BP	GO:0035337	fatty-acyl-CoA metabolic process	bulk RNA-seq,Homo_723,MCI.vs.control; bulk RNA-seq,Homo_714,MCI.vs.control; bulk RNA-seq,Homo_633,AD.vs.control; bulk RNA-	4
BP	GO:0051865	protein autoubiquitination	bulk RNA-seq,Homo_723,MCI.vs.control; bulk RNA-seq,Homo_714,MCI.vs.control; bulk RNA-seq,Homo_633,AD.vs.control; bulk RNA-	4
BP	GO:1901387	positive regulation of voltage-gated calcium channel activity	bulk RNA-seq,Homo_723,MCI.vs.control	1
BP	GO:0039535	regulation of RIG-I signaling pathway	bulk RNA-seq,Homo_723,MCI.vs.control; bulk RNA-seq,Homo_714,MCI.vs.control; bulk RNA-seq,Homo_633,AD.vs.control; bulk RNA-	4
BP	GO:0046794	transport of virus	bulk RNA-seq,Homo_723,MCI.vs.control; bulk RNA-seq,Homo_714,MCI.vs.control; bulk RNA-seq,Homo_633,AD.vs.control; bulk RNA-seq,Homo_633,AD.vs.MCI; bulk RNA-seq,Homo_633,MCI.vs.control	5
BP	GO:0090322	regulation of superoxide metabolic process	bulk RNA-seq,Homo_723,MCI.vs.control; bulk RNA-seq,Homo_714,MCI.vs.control; bulk RNA-seq,Homo_633,AD.vs.control; bulk RNA-	4
BP	GO:0006596	polyamine biosynthetic process	bulk RNA-seq,Homo_723,MCI.vs.control; bulk RNA-seq,Homo_714,MCI.vs.control; bulk RNA-seq,Homo_633,AD.vs.control; bulk RNA-	4
BP	GO:2000402	negative regulation of lymphocyte migration	bulk RNA-seq,Homo_723,MCI.vs.control; bulk RNA-seq,Homo_714,MCI.vs.control; bulk RNA-seq,Homo_633,MCI.vs.control	3
CC	GO:0044224	juxtaparanode region of axon	bulk RNA-seq,Homo_723,MCI.vs.control	1
BP	GO:0006919	activation of cysteine-type endopeptidase activity involved in apoptotic process	bulk RNA-seq,Homo_723,MCI.vs.control; bulk RNA-seq,Homo_714,MCI.vs.control; bulk RNA-seq,Homo_633,AD.vs.control; bulk RNA-seq,Homo_633,AD.vs.MCI	4
CC	GO:0071565	nBAF complex	bulk RNA-seq,Homo_723,MCI.vs.control; bulk RNA-seq,Homo_633,AD.vs.control; bulk RNA-seq,Homo_633,AD.vs.MCI	3
CC	GO:0031306	intrinsic component of mitochondrial outer membrane	bulk RNA-seq,Homo_723,MCI.vs.control; bulk RNA-seq,Homo_714,MCI.vs.control; bulk RNA-seq,Homo_633,AD.vs.control; bulk RNA-seq,Homo_633,AD.vs.MCI; bulk RNA-seq,Homo_633,MCI.vs.control	5
BP	GO:0006536	glutamate metabolic process	bulk RNA-seq,Homo_723,MCI.vs.control; bulk RNA-seq,Homo_633,AD.vs.control; bulk RNA-seq,Homo_633,AD.vs.MCI	3
BP	GO:0015886	heme transport	bulk RNA-seq,Homo_723,MCI.vs.control; bulk RNA-seq,Homo_714,MCI.vs.control	2
BP	GO:0035542	regulation of SNARE complex assembly	bulk RNA-seq,Homo_723,MCI.vs.control; bulk RNA-seq,Homo_714,MCI.vs.control; bulk RNA-seq,Homo_633,AD.vs.control; bulk RNA-	4
BP	GO:1904294	positive regulation of ERAD pathway	bulk RNA-seq,Homo_723,MCI.vs.control; bulk RNA-seq,Homo_714,MCI.vs.control; bulk RNA-seq,Homo_633,MCI.vs.control	3
BP	GO:0050709	negative regulation of protein secretion	bulk RNA-seq,Homo_723,MCI.vs.control; bulk RNA-seq,Homo_633,AD.vs.control; bulk RNA-seq,Homo_633,AD.vs.MCI	3
MF	GO:0140328	floppase activity	bulk RNA-seq,Homo_723,MCI.vs.control; bulk RNA-seq,Homo_714,AD.vs.MCI; bulk RNA-seq,Homo_714,MCI.vs.control; bulk RNA-seq,Homo_633,AD.vs.control; bulk RNA-seq,Homo_633,AD.vs.MCI; bulk RNA-seq,Homo_633,MCI.vs.control	6
BP	GO:0031629	synaptic vesicle fusion to presynaptic active zone membrane	bulk RNA-seq,Homo_723,MCI.vs.control; bulk RNA-seq,Homo_714,MCI.vs.control	2
BP	GO:0044154	histone H3-K14 acetylation	bulk RNA-seq,Homo_723,MCI.vs.control; bulk RNA-seq,Homo_714,MCI.vs.control; bulk RNA-seq,Homo_633,AD.vs.control; bulk RNA-	4
BP	GO:2000810	regulation of bicellular tight junction assembly	bulk RNA-seq,Homo_723,MCI.vs.control; bulk RNA-seq,Homo_714,AD.vs.MCI; bulk RNA-seq,Homo_714,MCI.vs.control	3
BP	GO:0045346	regulation of MHC class II biosynthetic process	bulk RNA-seq,Homo_723,MCI.vs.control; bulk RNA-seq,Homo_714,MCI.vs.control	2
BP	GO:1901533	negative regulation of hematopoietic progenitor cell	bulk RNA-seq,Homo_723,MCI.vs.control; bulk RNA-seq,Homo_714,AD.vs.MCI; bulk RNA-seq,Homo_714,MCI.vs.control	3
BP	GO:0045161	neuronal ion channel clustering	bulk RNA-seq,Homo_723,MCI.vs.control; bulk RNA-seq,Homo_714,MCI.vs.control	2
MF	GO:0031418	L-ascorbic acid binding	bulk RNA-seq,Homo_723,MCI.vs.control; bulk RNA-seq,Homo_714,AD.vs.control; bulk RNA-seq,Homo_714,MCI.vs.control; bulk RNA-	4
BP	GO:0006670	sphingosine metabolic process	bulk RNA-seq,Homo_723,MCI.vs.control; bulk RNA-seq,Homo_714,MCI.vs.control; bulk RNA-seq,Homo_633,AD.vs.control; bulk RNA-	4
BP	GO:0005977	glycogen metabolic process	bulk RNA-seq,Homo_723,MCI.vs.control; bulk RNA-seq,Homo_714,MCI.vs.control; bulk RNA-seq,Homo_633,AD.vs.control	3
BP	GO:0035307	positive regulation of protein dephosphorylation	bulk RNA-seq,Homo_723,MCI.vs.control; bulk RNA-seq,Homo_714,AD.vs.MCI; bulk RNA-seq,Homo_714,MCI.vs.control; bulk RNA-seq,Homo_633,AD.vs.control; bulk RNA-seq,Homo_633,AD.vs.MCI	5
BP	GO:0045821	positive regulation of glycolytic process	bulk RNA-seq,Homo_723,MCI.vs.control; bulk RNA-seq,Homo_714,AD.vs.MCI; bulk RNA-seq,Homo_714,MCI.vs.control; bulk RNA-	4
BP	GO:0042537	benzene-containing compound metabolic process	bulk RNA-seq,Homo_723,MCI.vs.control; bulk RNA-seq,Homo_714,MCI.vs.control; bulk RNA-seq,Homo_633,AD.vs.control; bulk RNA-	4
BP	GO:1905897	regulation of response to endoplasmic reticulum stress	bulk RNA-seq,Homo_723,MCI.vs.control; bulk RNA-seq,Homo_714,MCI.vs.control; bulk RNA-seq,Homo_633,AD.vs.control; bulk RNA-	4
BP	GO:0014842	regulation of skeletal muscle satellite cell proliferation	bulk RNA-seq,Homo_723,MCI.vs.control; bulk RNA-seq,Homo_714,AD.vs.MCI; bulk RNA-seq,Homo_714,MCI.vs.control	3
BP	GO:0060716	labyrinthine layer blood vessel development	bulk RNA-seq,Homo_723,MCI.vs.control; bulk RNA-seq,Homo_714,MCI.vs.control	2
BP	GO:0031958	corticosteroid receptor signaling pathway	bulk RNA-seq,Homo_723,MCI.vs.control; bulk RNA-seq,Homo_714,AD.vs.control; bulk RNA-seq,Homo_714,MCI.vs.control; bulk RNA-	4
BP	GO:0032607	interferon-alpha production	bulk RNA-seq,Homo_723,MCI.vs.control; bulk RNA-seq,Homo_714,MCI.vs.control; bulk RNA-seq,Homo_633,AD.vs.control; bulk RNA-seq,Homo_633,AD.vs.MCI; bulk RNA-seq,Homo_633,MCI.vs.control	5

BP	GO:0032647	regulation of interferon-alpha production	bulk RNA-seq,Homo_723,MCI.vs.control; bulk RNA-seq,Homo_714,MCI.vs.control; bulk RNA-seq,Homo_633,AD.vs.control; bulk RNA-seq,Homo_633,AD.vs.MCI; bulk RNA-seq,Homo_633,MCI.vs.control	5
BP	GO:0021762	substantia nigra development	bulk RNA-seq,Homo_723,MCI.vs.control; bulk RNA-seq,Homo_714,MCI.vs.control; bulk RNA-seq,Homo_633,AD.vs.control; bulk RNA-	4
BP	GO:0090023	positive regulation of neutrophil chemotaxis	bulk RNA-seq,Homo_723,MCI.vs.control; bulk RNA-seq,Homo_714,MCI.vs.control; bulk RNA-seq,Homo_633,MCI.vs.control; bulk RNA-	4
BP	GO:0034605	cellular response to heat	bulk RNA-seq,Homo_723,MCI.vs.control; bulk RNA-seq,Homo_714,AD.vs.MCI; bulk RNA-seq,Homo_714,MCI.vs.control; bulk RNA-seq,Homo_633,AD.vs.control; bulk RNA-seq,Homo_633,AD.vs.MCI	5
BP	GO:0035584	calcium-mediated signaling using intracellular calcium	bulk RNA-seq,Homo_723,MCI.vs.control; bulk RNA-seq,Homo_714,MCI.vs.control	2
BP	GO:0010820	positive regulation of T cell chemotaxis	bulk RNA-seq,Homo_723,MCI.vs.control; bulk RNA-seq,Homo_714,MCI.vs.control	2
BP	GO:2000209	regulation of anoikis	bulk RNA-seq,Homo_723,MCI.vs.control; bulk RNA-seq,Homo_714,AD.vs.MCI; bulk RNA-seq,Homo_714,MCI.vs.control; bulk RNA-	4
BP	GO:0000245	spliceosomal complex assembly	bulk RNA-seq,Homo_723,MCI.vs.control; bulk RNA-seq,Homo_714,AD.vs.control; bulk RNA-seq,Homo_714,MCI.vs.control; bulk RNA-seq,Homo_633,AD.vs.control; bulk RNA-seq,Homo_633,AD.vs.MCI	5
CC	GO:0000791	euchromatin	bulk RNA-seq,Homo_723,MCI.vs.control; bulk RNA-seq,Homo_714,MCI.vs.control; bulk RNA-seq,Homo_633,AD.vs.control; bulk RNA-seq,Homo_633,AD.vs.MCI; bulk RNA-seq,ROSMAP,MCI.vs.control	5
MF	GO:1990247	N6-methyladenosine-containing RNA binding	bulk RNA-seq,Homo_723,MCI.vs.control; bulk RNA-seq,Homo_633,AD.vs.control	2
BP	GO:0072673	lamellipodium morphogenesis	bulk RNA-seq,Homo_723,MCI.vs.control; bulk RNA-seq,Homo_714,AD.vs.MCI; bulk RNA-seq,Homo_714,MCI.vs.control	3
BP	GO:0043981	histone H4-K5 acetylation	bulk RNA-seq,Homo_723,MCI.vs.control; bulk RNA-seq,Homo_714,MCI.vs.control; bulk RNA-seq,Homo_633,AD.vs.control; bulk RNA-	4
BP	GO:0043982	histone H4-K8 acetylation	bulk RNA-seq,Homo_723,MCI.vs.control; bulk RNA-seq,Homo_714,MCI.vs.control; bulk RNA-seq,Homo_633,AD.vs.control; bulk RNA-	4
BP	GO:0030388	fructose 1,6-bisphosphate metabolic process	bulk RNA-seq,Homo_723,MCI.vs.control; bulk RNA-seq,Homo_714,MCI.vs.control	2
MF	GO:0070410	co-SMAD binding	bulk RNA-seq,Homo_723,MCI.vs.control; bulk RNA-seq,Homo_714,MCI.vs.control	2
BP	GO:0045655	regulation of monocyte differentiation	bulk RNA-seq,Homo_723,MCI.vs.control; bulk RNA-seq,Homo_714,AD.vs.MCI; bulk RNA-seq,Homo_714,MCI.vs.control	3
BP	GO:0090309	positive regulation of DNA methylation-dependent heterochromatin formation	bulk RNA-seq,Homo_723,MCI.vs.control; bulk RNA-seq,Homo_714,AD.vs.MCI; bulk RNA-seq,Homo_714,MCI.vs.control; bulk RNA-seq,Homo_633,MCI.vs.control	4
BP	GO:0009081	branched-chain amino acid metabolic process	bulk RNA-seq,Homo_723,MCI.vs.control; bulk RNA-seq,Homo_714,MCI.vs.control; bulk RNA-seq,Homo_633,AD.vs.control; bulk RNA-seq,Homo_633,AD.vs.MCI; bulk RNA-seq,Homo_633,MCI.vs.control	5
BP	GO:0030968	endoplasmic reticulum unfolded protein response	bulk RNA-seq,Homo_723,MCI.vs.control; bulk RNA-seq,Homo_714,MCI.vs.control; bulk RNA-seq,Homo_633,AD.vs.control; bulk RNA-	4
BP	GO:0036297	interstrand cross-link repair	bulk RNA-seq,Homo_723,MCI.vs.control; bulk RNA-seq,Homo_714,MCI.vs.control; bulk RNA-seq,Homo_633,AD.vs.control; bulk RNA-	4
BP	GO:1901673	regulation of mitotic spindle assembly	bulk RNA-seq,Homo_723,MCI.vs.control; bulk RNA-seq,Homo_714,MCI.vs.control; bulk RNA-seq,Homo_633,AD.vs.control; bulk RNA-seq,Homo_633,AD.vs.MCI; bulk RNA-seq,Homo_633,MCI.vs.control	5
BP	GO:1902001	fatty acid transmembrane transport	bulk RNA-seq,Homo_723,MCI.vs.control; bulk RNA-seq,Homo_714,MCI.vs.control; bulk RNA-seq,Homo_633,AD.vs.control; bulk RNA-	4
BP	GO:0042402	cellular biogenic amine catabolic process	bulk RNA-seq,Homo_723,MCI.vs.control; bulk RNA-seq,Homo_714,MCI.vs.control; bulk RNA-seq,Homo_633,AD.vs.control	3
CC	GO:0032585	multivesicular body membrane	bulk RNA-seq,Homo_723,MCI.vs.control; bulk RNA-seq,Homo_714,MCI.vs.control; bulk RNA-seq,Homo_633,AD.vs.control; bulk RNA-seq,Homo_633,AD.vs.MCI; bulk RNA-seq,Homo_633,MCI.vs.control	5
BP	GO:1902946	protein localization to early endosome	bulk RNA-seq,Homo_723,MCI.vs.control; bulk RNA-seq,Homo_714,MCI.vs.control; bulk RNA-seq,Homo_633,AD.vs.control; bulk RNA-	4
BP	GO:0044320	cellular response to leptin stimulus	bulk RNA-seq,Homo_723,MCI.vs.control; bulk RNA-seq,Homo_714,MCI.vs.control	2
MF	GO:0043274	phospholipase binding	bulk RNA-seq,Homo_723,MCI.vs.control; bulk RNA-seq,Homo_714,AD.vs.MCI; bulk RNA-seq,Homo_714,MCI.vs.control; bulk RNA-seq,Homo_633,AD.vs.control; bulk RNA-seq,Homo_633,AD.vs.MCI	5
BP	GO:0001881	receptor recycling	bulk RNA-seq,Homo_723,MCI.vs.control; bulk RNA-seq,Homo_714,MCI.vs.control; bulk RNA-seq,Homo_633,AD.vs.control; bulk RNA-seq,Homo_633,AD.vs.MCI; bulk RNA-seq,Homo_633,MCI.vs.control	5
BP	GO:0071624	positive regulation of granulocyte chemotaxis	bulk RNA-seq,Homo_723,MCI.vs.control; bulk RNA-seq,Homo_714,MCI.vs.control; bulk RNA-seq,Homo_633,MCI.vs.control; bulk RNA-	4
BP	GO:0021904	dorsal/ventral neural tube patterning	bulk RNA-seq,Homo_723,MCI.vs.control; bulk RNA-seq,Homo_714,MCI.vs.control; bulk RNA-seq,Homo_633,AD.vs.control	3
MF	GO:0140416	transcription regulator inhibitor activity	bulk RNA-seq,Homo_723,MCI.vs.control; bulk RNA-seq,Homo_714,MCI.vs.control; bulk RNA-seq,Homo_633,AD.vs.control; bulk RNA-	4
MF	GO:0005522	profilin binding	bulk RNA-seq,Homo_723,MCI.vs.control; bulk RNA-seq,Homo_714,MCI.vs.control; bulk RNA-seq,Homo_633,AD.vs.control	3
BP	GO:1901978	positive regulation of cell cycle checkpoint	bulk RNA-seq,Homo_723,MCI.vs.control; bulk RNA-seq,Homo_714,MCI.vs.control; bulk RNA-seq,Homo_633,AD.vs.control; bulk RNA-seq,Homo_633,AD.vs.MCI; bulk RNA-seq,Homo_633,MCI.vs.control	5
MF	GO:0052744	phosphatidylinositol monophosphate phosphatase activity	bulk RNA-seq,Homo_723,MCI.vs.control; bulk RNA-seq,Homo_714,MCI.vs.control; bulk RNA-seq,Homo_633,AD.vs.control; bulk RNA-seq,Homo_633,AD.vs.MCI; bulk RNA-seq,Homo_633,MCI.vs.control	5
BP	GO:2000035	regulation of stem cell division	bulk RNA-seq,Homo_723,MCI.vs.control; bulk RNA-seq,Homo_714,MCI.vs.control; bulk RNA-seq,Homo_633,AD.vs.control; bulk RNA-	4
BP	GO:0070884	regulation of calcineurin-NFAT signaling cascade	bulk RNA-seq,Homo_723,MCI.vs.control; bulk RNA-seq,Homo_714,MCI.vs.control; bulk RNA-seq,Homo_633,AD.vs.control	3
CC	GO:0140672	ATAC complex	bulk RNA-seq,Homo_723,MCI.vs.control; bulk RNA-seq,Homo_714,AD.vs.MCI; bulk RNA-seq,Homo_714,MCI.vs.control; bulk RNA-seq,Homo_633,AD.vs.control; bulk RNA-seq,Homo_633,AD.vs.MCI; bulk RNA-seq,Homo_633,MCI.vs.control	6
BP	GO:1902033	regulation of hematopoietic stem cell proliferation	bulk RNA-seq,Homo_723,MCI.vs.control; bulk RNA-seq,Homo_714,MCI.vs.control	2
BP	GO:0034349	glial cell apoptotic process	bulk RNA-seq,Homo_723,MCI.vs.control; bulk RNA-seq,Homo_714,MCI.vs.control; bulk RNA-seq,Homo_633,MCI.vs.control	3
CC	GO:0030867	rough endoplasmic reticulum membrane	bulk RNA-seq,Homo_723,MCI.vs.control; bulk RNA-seq,Homo_714,MCI.vs.control; bulk RNA-seq,Homo_633,AD.vs.control; bulk RNA-seq,Homo_633,AD.vs.MCI; bulk RNA-seq,Homo_633,MCI.vs.control	5
BP	GO:0001867	complement activation, lectin pathway	bulk RNA-seq,Homo_723,MCI.vs.control; bulk RNA-seq,Homo_714,AD.vs.MCI; bulk RNA-seq,Homo_714,MCI.vs.control	3
BP	GO:0033083	regulation of immature T cell proliferation	bulk RNA-seq,Homo_723,MCI.vs.control; bulk RNA-seq,Homo_714,MCI.vs.control; bulk RNA-seq,Homo_633,AD.vs.control	3
BP	GO:0033084	regulation of immature T cell proliferation in thymus	bulk RNA-seq,Homo_723,MCI.vs.control; bulk RNA-seq,Homo_714,MCI.vs.control; bulk RNA-seq,Homo_633,AD.vs.control	3
BP	GO:0045048	protein insertion into ER membrane	bulk RNA-seq,Homo_723,MCI.vs.control; bulk RNA-seq,Homo_714,MCI.vs.control; bulk RNA-seq,Homo_633,AD.vs.control; bulk RNA-seq,Homo_633,AD.vs.MCI; bulk RNA-seq,Homo_633,MCI.vs.control	5
BP	GO:0046513	ceramide biosynthetic process	bulk RNA-seq,Homo_723,MCI.vs.control; bulk RNA-seq,Homo_714,MCI.vs.control; bulk RNA-seq,Homo_633,AD.vs.control	3

MF	GO:0016298	lipase activity	bulk RNA-seq,Homo_723,MCI.vs.control; bulk RNA-seq,Homo_714,AD.vs.MCI; bulk RNA-seq,Homo_633,AD.vs.control; bulk RNA-seq,Homo_633,AD.vs.MCI	4
BP	GO:0019400	alditol metabolic process	bulk RNA-seq,Homo_723,MCI.vs.control; bulk RNA-seq,Homo_714,MCI.vs.control	2
BP	GO:2000573	positive regulation of DNA biosynthetic process	bulk RNA-seq,Homo_723,MCI.vs.control; bulk RNA-seq,Homo_714,AD.vs.control; bulk RNA-seq,Homo_714,MCI.vs.control; bulk RNA-seq,Homo_633,AD.vs.control; bulk RNA-seq,Homo_633,AD.vs.MCI	5
MF	GO:0120014	phospholipid transfer activity	bulk RNA-seq,Homo_723,MCI.vs.control; bulk RNA-seq,Homo_714,MCI.vs.control	2
BP	GO:0070664	negative regulation of leukocyte proliferation	bulk RNA-seq,Homo_723,MCI.vs.control; bulk RNA-seq,Homo_714,AD.vs.MCI; bulk RNA-seq,Homo_714,MCI.vs.control; bulk RNA-seq,Homo_633,AD.vs.control; bulk RNA-seq,Homo_633,AD.vs.MCI	5
BP	GO:0032616	interleukin-13 production	bulk RNA-seq,Homo_723,MCI.vs.control; bulk RNA-seq,Homo_714,AD.vs.MCI; bulk RNA-seq,Homo_714,MCI.vs.control	3
BP	GO:0032656	regulation of interleukin-13 production	bulk RNA-seq,Homo_723,MCI.vs.control; bulk RNA-seq,Homo_714,AD.vs.MCI; bulk RNA-seq,Homo_714,MCI.vs.control	3
BP	GO:0032465	regulation of cytokinesis	bulk RNA-seq,Homo_723,MCI.vs.control; bulk RNA-seq,Homo_714,AD.vs.MCI; bulk RNA-seq,Homo_714,MCI.vs.control; bulk RNA-seq,Homo_633,AD.vs.control; bulk RNA-seq,Homo_633,AD.vs.MCI	5
BP	GO:0140448	signaling receptor ligand precursor processing	bulk RNA-seq,Homo_723,MCI.vs.control; bulk RNA-seq,Homo_633,AD.vs.control	2
MF	GO:0005048	signal sequence binding	bulk RNA-seq,Homo_723,MCI.vs.control; bulk RNA-seq,Homo_714,MCI.vs.control; bulk RNA-seq,Homo_633,AD.vs.control; bulk RNA-	4
BP	GO:0046466	membrane lipid catabolic process	bulk RNA-seq,Homo_723,MCI.vs.control; bulk RNA-seq,Homo_714,MCI.vs.control; bulk RNA-seq,Homo_633,AD.vs.control; bulk RNA-	4
BP	GO:0006301	postreplication repair	bulk RNA-seq,Homo_723,MCI.vs.control; bulk RNA-seq,Homo_714,MCI.vs.control; bulk RNA-seq,Homo_633,AD.vs.control; bulk RNA-seq,Homo_633,AD.vs.MCI; bulk RNA-seq,Homo_633,MCI.vs.control	5
BP	GO:0006968	cellular defense response	bulk RNA-seq,Homo_723,MCI.vs.control; bulk RNA-seq,Homo_714,AD.vs.MCI; bulk RNA-seq,Homo_714,MCI.vs.control; bulk RNA-seq,Homo_633,AD.vs.control; bulk RNA-seq,Homo_633,AD.vs.MCI	5
BP	GO:2000637	positive regulation of miRNA-mediated gene silencing	bulk RNA-seq,Homo_723,MCI.vs.control; bulk RNA-seq,Homo_714,MCI.vs.control; bulk RNA-seq,Homo_633,AD.vs.control	3
BP	GO:0043486	histone exchange	bulk RNA-seq,Homo_723,MCI.vs.control; bulk RNA-seq,Homo_714,MCI.vs.control; bulk RNA-seq,Homo_633,AD.vs.control; bulk RNA-	4
CC	GO:0034506	chromosome, centromeric core domain	bulk RNA-seq,Homo_723,MCI.vs.control; bulk RNA-seq,Homo_714,AD.vs.MCI; bulk RNA-seq,Homo_714,MCI.vs.control	3
BP	GO:0072337	modified amino acid transport	bulk RNA-seq,Homo_723,MCI.vs.control; bulk RNA-seq,Homo_714,MCI.vs.control; bulk RNA-seq,Homo_633,AD.vs.control	3
BP	GO:1903579	negative regulation of ATP metabolic process	bulk RNA-seq,Homo_723,MCI.vs.control; bulk RNA-seq,Homo_714,MCI.vs.control; bulk RNA-seq,Homo_633,AD.vs.control; bulk RNA-	4
BP	GO:1903975	regulation of glial cell migration	bulk RNA-seq,Homo_723,MCI.vs.control; bulk RNA-seq,Homo_714,MCI.vs.control	2
CC	GO:0005834	heterotrimeric G-protein complex	bulk RNA-seq,Homo_723,MCI.vs.control; bulk RNA-seq,Homo_714,AD.vs.MCI; bulk RNA-seq,Homo_714,MCI.vs.control; bulk RNA-seq,Homo_633,AD.vs.control; bulk RNA-seq,Homo_633,AD.vs.MCI	5
BP	GO:0021819	layer formation in cerebral cortex	bulk RNA-seq,Homo_723,MCI.vs.control	1
BP	GO:1900543	negative regulation of purine nucleotide metabolic process	bulk RNA-seq,Homo_723,MCI.vs.control; bulk RNA-seq,Homo_714,MCI.vs.control; bulk RNA-seq,Homo_633,AD.vs.control; bulk RNA-	4
MF	GO:0034593	phosphatidylinositol bisphosphate phosphatase activity	bulk RNA-seq,Homo_723,MCI.vs.control; bulk RNA-seq,Homo_714,MCI.vs.control; bulk RNA-seq,Homo_633,AD.vs.control; bulk RNA-seq,Homo_633,AD.vs.MCI; bulk RNA-seq,Homo_633,MCI.vs.control	5
BP	GO:0048255	mRNA stabilization	bulk RNA-seq,Homo_723,MCI.vs.control; bulk RNA-seq,Homo_633,AD.vs.control; bulk RNA-seq,Homo_633,AD.vs.MCI	3
BP	GO:0031146	SCF-dependent proteasomal ubiquitin-dependent protein catabolic process	bulk RNA-seq,Homo_723,MCI.vs.control; bulk RNA-seq,Homo_714,MCI.vs.control; bulk RNA-seq,Homo_633,AD.vs.control; bulk RNA-seq,Homo_633,AD.vs.MCI	4
MF	GO:0070567	cytidylyltransferase activity	bulk RNA-seq,Homo_723,MCI.vs.control; bulk RNA-seq,Homo_714,MCI.vs.control; bulk RNA-seq,Homo_633,MCI.vs.control	3
BP	GO:0009247	glycolipid biosynthetic process	bulk RNA-seq,Homo_723,MCI.vs.control; bulk RNA-seq,Homo_714,MCI.vs.control; bulk RNA-seq,Homo_633,AD.vs.control; bulk RNA-	4
MF	GO:0032356	oxidized DNA binding	bulk RNA-seq,Homo_723,MCI.vs.control; bulk RNA-seq,Homo_714,MCI.vs.control; bulk RNA-seq,Homo_633,AD.vs.control; bulk RNA-seq,Homo_633,AD.vs.MCI; bulk RNA-seq,Homo_633,MCI.vs.control	5
BP	GO:0030042	actin filament depolymerization	bulk RNA-seq,Homo_723,MCI.vs.control; bulk RNA-seq,Homo_714,MCI.vs.control; bulk RNA-seq,Homo_633,AD.vs.control	3
BP	GO:0035561	regulation of chromatin binding	bulk RNA-seq,Homo_723,MCI.vs.control; bulk RNA-seq,Homo_714,MCI.vs.control; bulk RNA-seq,Homo_633,AD.vs.control; bulk RNA-	4
BP	GO:1902473	regulation of protein localization to synapse	bulk RNA-seq,Homo_723,MCI.vs.control; bulk RNA-seq,Homo_633,AD.vs.control	2
BP	GO:0006221	pyrimidine nucleotide biosynthetic process	bulk RNA-seq,Homo_723,MCI.vs.control; bulk RNA-seq,Homo_714,MCI.vs.control; bulk RNA-seq,Homo_633,AD.vs.control; bulk RNA-seq,Homo_633,AD.vs.MCI; bulk RNA-seq,Homo_633,MCI.vs.control	5
MF	GO:0005402	carbohydrate:cation symporter activity	bulk RNA-seq,Homo_723,MCI.vs.control; bulk RNA-seq,Homo_714,MCI.vs.control	2
BP	GO:0070306	lens fiber cell differentiation	bulk RNA-seq,Homo_723,MCI.vs.control; bulk RNA-seq,Homo_714,MCI.vs.control	2
BP	GO:0031063	regulation of histone deacetylation	bulk RNA-seq,Homo_723,MCI.vs.control; bulk RNA-seq,Homo_714,AD.vs.MCI; bulk RNA-seq,Homo_714,MCI.vs.control; bulk RNA-seq,Homo_633,AD.vs.control; bulk RNA-seq,Homo_633,AD.vs.MCI	5
CC	GO:0044292	dendrite terminus	bulk RNA-seq,Homo_723,MCI.vs.control; bulk RNA-seq,Homo_714,AD.vs.MCI; bulk RNA-seq,Homo_714,MCI.vs.control; bulk RNA-	4
MF	GO:0030507	spectrin binding	bulk RNA-seq,Homo_723,MCI.vs.control; bulk RNA-seq,Homo_714,MCI.vs.control	2
BP	GO:0006896	Golgi to vacuole transport	bulk RNA-seq,Homo_723,MCI.vs.control; bulk RNA-seq,Homo_714,MCI.vs.control; bulk RNA-seq,Homo_633,AD.vs.control; bulk RNA-seq,Homo_633,AD.vs.MCI; bulk RNA-seq,Homo_633,MCI.vs.control	5
BP	GO:1905269	positive regulation of chromatin organization	bulk RNA-seq,Homo_723,MCI.vs.control; bulk RNA-seq,Homo_714,AD.vs.MCI; bulk RNA-seq,Homo_714,MCI.vs.control; bulk RNA-seq,Homo_633,AD.vs.control; bulk RNA-seq,Homo_633,MCI.vs.control	5
CC	GO:0071014	post-mRNA release spliceosomal complex	bulk RNA-seq,Homo_723,MCI.vs.control; bulk RNA-seq,Homo_714,MCI.vs.control; bulk RNA-seq,Homo_633,AD.vs.control; bulk RNA-seq,Homo_633,AD.vs.MCI; bulk RNA-seq,Homo_633,MCI.vs.control	5
BP	GO:0098586	cellular response to virus	bulk RNA-seq,Homo_723,MCI.vs.control; bulk RNA-seq,Homo_714,MCI.vs.control; bulk RNA-seq,Homo_633,AD.vs.control	3
BP	GO:0042430	indole-containing compound metabolic process	bulk RNA-seq,Homo_723,MCI.vs.control	1
MF	GO:0070412	R-SMAD binding	bulk RNA-seq,Homo_723,MCI.vs.control; bulk RNA-seq,Homo_633,AD.vs.control	2
BP	GO:2000279	negative regulation of DNA biosynthetic process	bulk RNA-seq,Homo_723,MCI.vs.control; bulk RNA-seq,Homo_714,MCI.vs.control; bulk RNA-seq,Homo_633,AD.vs.control; bulk RNA-	4
BP	GO:0032288	myelin assembly	bulk RNA-seq,Homo_723,MCI.vs.control; bulk RNA-seq,Homo_714,MCI.vs.control	2

BP	GO:0043968	histone H2A acetylation	bulk RNA-seq,Homo_723,MCI.vs.control; bulk RNA-seq,Homo_714,MCI.vs.control; bulk RNA-seq,Homo_633,AD.vs.control; bulk RNA-seq,Homo_633,AD.vs.MCI; bulk RNA-seq,Homo_633,MCI.vs.control	5
BP	GO:1905521	regulation of macrophage migration	bulk RNA-seq,Homo_723,MCI.vs.control; bulk RNA-seq,Homo_714,MCI.vs.control; bulk RNA-seq,Homo_633,AD.vs.control	3
BP	GO:0051383	kinetochore organization	bulk RNA-seq,Homo_723,MCI.vs.control; bulk RNA-seq,Homo_714,MCI.vs.control; bulk RNA-seq,Homo_633,AD.vs.control; bulk RNA-	4
CC	GO:0035371	microtubule plus-end	bulk RNA-seq,Homo_723,MCI.vs.control; bulk RNA-seq,Homo_714,MCI.vs.control	2
BP	GO:0033013	tetrapyrrole metabolic process	bulk RNA-seq,Homo_723,MCI.vs.control; bulk RNA-seq,Homo_714,MCI.vs.control; bulk RNA-seq,Homo_633,AD.vs.control; bulk RNA-	4
CC	GO:0030125	clathrin vesicle coat	bulk RNA-seq,Homo_723,MCI.vs.control; bulk RNA-seq,Homo_714,MCI.vs.control; bulk RNA-seq,Homo_633,MCI.vs.control	3
BP	GO:0006346	DNA methylation-dependent heterochromatin formation	bulk RNA-seq,Homo_723,MCI.vs.control; bulk RNA-seq,Homo_714,MCI.vs.control; bulk RNA-seq,Homo_633,MCI.vs.control	3
BP	GO:0090232	positive regulation of spindle checkpoint	bulk RNA-seq,Homo_723,MCI.vs.control; bulk RNA-seq,Homo_714,MCI.vs.control; bulk RNA-seq,Homo_633,AD.vs.control; bulk RNA-seq,Homo_633,AD.vs.MCI; bulk RNA-seq,Homo_633,MCI.vs.control	5
BP	GO:0090267	positive regulation of mitotic cell cycle spindle assembly checkpoint	bulk RNA-seq,Homo_723,MCI.vs.control; bulk RNA-seq,Homo_714,MCI.vs.control; bulk RNA-seq,Homo_633,AD.vs.control; bulk RNA-seq,Homo_633,AD.vs.MCI; bulk RNA-seq,Homo_633,MCI.vs.control	5
BP	GO:1904029	regulation of cyclin-dependent protein kinase activity	bulk RNA-seq,Homo_723,MCI.vs.control; bulk RNA-seq,Homo_714,MCI.vs.control; bulk RNA-seq,Homo_633,AD.vs.control; bulk RNA-	4
MF	GO:0070411	I-SMAD binding	bulk RNA-seq,Homo_723,MCI.vs.control; bulk RNA-seq,Homo_714,MCI.vs.control; bulk RNA-seq,Homo_633,AD.vs.control	3
MF	GO:0019200	carbohydrate kinase activity	bulk RNA-seq,Homo_723,MCI.vs.control; bulk RNA-seq,Homo_714,MCI.vs.control; bulk RNA-seq,Homo_633,AD.vs.control; bulk RNA-seq,Homo_633,AD.vs.MCI; bulk RNA-seq,Homo_633,MCI.vs.control	5
BP	GO:0140374	antiviral innate immune response	bulk RNA-seq,Homo_723,MCI.vs.control; bulk RNA-seq,Homo_714,MCI.vs.control	2
BP	GO:0051881	regulation of mitochondrial membrane potential	bulk RNA-seq,Homo_723,MCI.vs.control; bulk RNA-seq,Homo_714,AD.vs.control; bulk RNA-seq,Homo_714,MCI.vs.control; bulk RNA-seq,Homo_633,AD.vs.control; bulk RNA-seq,Homo_633,AD.vs.MCI	5
BP	GO:0046112	nucleobase biosynthetic process	bulk RNA-seq,Homo_723,MCI.vs.control; bulk RNA-seq,Homo_714,MCI.vs.control; bulk RNA-seq,Homo_633,AD.vs.control; bulk RNA-	4
BP	GO:0006071	glycerol metabolic process	bulk RNA-seq,Homo_723,MCI.vs.control; bulk RNA-seq,Homo_714,MCI.vs.control	2
BP	GO:0070071	proton-transporting two-sector ATPase complex assembly	bulk RNA-seq,Homo_723,MCI.vs.control	1
BP	GO:0035970	peptidyl-threonine dephosphorylation	bulk RNA-seq,Homo_723,MCI.vs.control; bulk RNA-seq,Homo_714,MCI.vs.control; bulk RNA-seq,Homo_633,AD.vs.control; bulk RNA-seq,Homo_633,AD.vs.MCI; bulk RNA-seq,Homo_633,MCI.vs.control	5
BP	GO:0097202	activation of cysteine-type endopeptidase activity	bulk RNA-seq,Homo_723,MCI.vs.control; bulk RNA-seq,Homo_714,MCI.vs.control; bulk RNA-seq,Homo_633,AD.vs.control; bulk RNA-seq,Homo_633,AD.vs.MCI; bulk RNA-seq,Homo_633,MCI.vs.control	5
BP	GO:0035268	protein mannosylation	bulk RNA-seq,Homo_723,MCI.vs.control; bulk RNA-seq,Homo_714,MCI.vs.control; bulk RNA-seq,Homo_633,MCI.vs.control	3
BP	GO:0032506	cytokinetic process	bulk RNA-seq,Homo_723,MCI.vs.control; bulk RNA-seq,Homo_714,MCI.vs.control; bulk RNA-seq,Homo_633,AD.vs.control; bulk RNA-	4
MF	GO:0005547	phosphatidylinositol-3,4,5-trisphosphate binding	bulk RNA-seq,Homo_723,MCI.vs.control; bulk RNA-seq,Homo_714,MCI.vs.control; bulk RNA-seq,Homo_633,AD.vs.control	3
BP	GO:0034163	regulation of toll-like receptor 9 signaling pathway	bulk RNA-seq,Homo_723,MCI.vs.control; bulk RNA-seq,Homo_714,MCI.vs.control; bulk RNA-seq,Homo_633,AD.vs.control; bulk RNA-seq,Homo_633,AD.vs.MCI; bulk RNA-seq,Homo_633,MCI.vs.control	5
BP	GO:0045332	phospholipid translocation	bulk RNA-seq,Homo_723,MCI.vs.control; bulk RNA-seq,Homo_714,MCI.vs.control; bulk RNA-seq,Homo_633,AD.vs.control; bulk RNA-	4
MF	GO:0030544	Hsp70 protein binding	bulk RNA-seq,Homo_723,MCI.vs.control; bulk RNA-seq,Homo_714,MCI.vs.control; bulk RNA-seq,Homo_633,AD.vs.control; bulk RNA-	4
BP	GO:0031062	positive regulation of histone methylation	bulk RNA-seq,Homo_723,MCI.vs.control; bulk RNA-seq,Homo_714,MCI.vs.control; bulk RNA-seq,Homo_633,AD.vs.control; bulk RNA-	4
BP	GO:0080164	regulation of nitric oxide metabolic process	bulk RNA-seq,Homo_723,MCI.vs.control; bulk RNA-seq,Homo_714,AD.vs.MCI; bulk RNA-seq,Homo_714,MCI.vs.control; bulk RNA-seq,Homo_633,AD.vs.control; bulk RNA-seq,Homo_633,AD.vs.MCI	5
BP	GO:0043489	RNA stabilization	bulk RNA-seq,Homo_723,MCI.vs.control; bulk RNA-seq,Homo_714,MCI.vs.control; bulk RNA-seq,Homo_633,AD.vs.control; bulk RNA-	4
BP	GO:0070972	protein localization to endoplasmic reticulum	bulk RNA-seq,Homo_723,MCI.vs.control; bulk RNA-seq,Homo_714,MCI.vs.control; bulk RNA-seq,Homo_633,AD.vs.control; bulk RNA-	4
BP	GO:0030835	negative regulation of actin filament depolymerization	bulk RNA-seq,Homo_723,MCI.vs.control; bulk RNA-seq,Homo_714,MCI.vs.control	2
BP	GO:0046514	ceramide catabolic process	bulk RNA-seq,Homo_723,MCI.vs.control; bulk RNA-seq,Homo_714,MCI.vs.control; bulk RNA-seq,Homo_633,AD.vs.control	3
BP	GO:0014049	positive regulation of glutamate secretion	bulk RNA-seq,Homo_723,MCI.vs.control	1
BP	GO:0030808	regulation of nucleotide biosynthetic process	bulk RNA-seq,Homo_723,MCI.vs.control; bulk RNA-seq,Homo_714,MCI.vs.control; bulk RNA-seq,Homo_633,AD.vs.control	3
CC	GO:0035579	specific granule membrane	bulk RNA-seq,Homo_723,MCI.vs.control; bulk RNA-seq,Homo_714,MCI.vs.control; bulk RNA-seq,Homo_633,AD.vs.control; bulk RNA-seq,Homo_633,AD.vs.MCI; bulk RNA-seq,SRP223445,AD.vs.control	5
CC	GO:0016235	aggresome	bulk RNA-seq,Homo_723,MCI.vs.control; bulk RNA-seq,Homo_714,MCI.vs.control; bulk RNA-seq,Homo_633,AD.vs.control	3
MF	GO:0035497	cAMP response element binding	bulk RNA-seq,Homo_723,MCI.vs.control; bulk RNA-seq,Homo_714,MCI.vs.control; bulk RNA-seq,Homo_633,AD.vs.control	3
CC	GO:0097197	tetraspanin-enriched microdomain	bulk RNA-seq,Homo_723,MCI.vs.control	1
MF	GO:0043539	protein serine/threonine kinase activator activity	bulk RNA-seq,Homo_723,MCI.vs.control; bulk RNA-seq,Homo_714,MCI.vs.control; bulk RNA-seq,Homo_633,AD.vs.control; bulk RNA-	4
MF	GO:0070840	dynein complex binding	bulk RNA-seq,Homo_723,MCI.vs.control; bulk RNA-seq,Homo_714,MCI.vs.control; bulk RNA-seq,Homo_633,AD.vs.control	3
BP	GO:0031053	primary miRNA processing	bulk RNA-seq,Homo_723,MCI.vs.control; bulk RNA-seq,Homo_633,AD.vs.control	2
BP	GO:0031453	positive regulation of heterochromatin formation	bulk RNA-seq,Homo_723,MCI.vs.control; bulk RNA-seq,Homo_714,AD.vs.MCI; bulk RNA-seq,Homo_714,MCI.vs.control; bulk RNA-seq,Homo_633,AD.vs.control; bulk RNA-seq,Homo_633,MCI.vs.control	5
BP	GO:0120263	positive regulation of heterochromatin organization	bulk RNA-seq,Homo_723,MCI.vs.control; bulk RNA-seq,Homo_714,AD.vs.MCI; bulk RNA-seq,Homo_714,MCI.vs.control; bulk RNA-seq,Homo_633,AD.vs.control; bulk RNA-seq,Homo_633,MCI.vs.control	5
BP	GO:0048873	homeostasis of number of cells within a tissue	bulk RNA-seq,Homo_723,MCI.vs.control; bulk RNA-seq,Homo_714,MCI.vs.control; bulk RNA-seq,Homo_633,AD.vs.control	3
BP	GO:0002220	innate immune response activating cell surface receptor signaling pathway	bulk RNA-seq,Homo_723,MCI.vs.control; bulk RNA-seq,Homo_714,AD.vs.MCI; bulk RNA-seq,Homo_714,MCI.vs.control; bulk RNA-seq,Homo_633,AD.vs.control; scRNA-seq,SRP330776,Natural killer cell_1-AD.vs.control	5
BP	GO:0090140	regulation of mitochondrial fission	bulk RNA-seq,Homo_723,MCI.vs.control; bulk RNA-seq,Homo_714,MCI.vs.control; bulk RNA-seq,Homo_633,AD.vs.control; bulk RNA-seq,Homo_633,AD.vs.MCI; bulk RNA-seq,SRP325058,AD.vs.control	5
BP	GO:1901678	iron coordination entity transport	bulk RNA-seq,Homo_723,MCI.vs.control; bulk RNA-seq,Homo_714,MCI.vs.control; bulk RNA-seq,Homo_633,AD.vs.control	3

BP	GO:1902969	mitotic DNA replication	bulk RNA-seq,Homo_723,MCI.vs.control; bulk RNA-seq,Homo_714,MCI.vs.control; bulk RNA-seq,Homo_633,MCI.vs.control	3
MF	GO:0017116	single-stranded DNA helicase activity	bulk RNA-seq,Homo_723,MCI.vs.control; bulk RNA-seq,Homo_714,MCI.vs.control; bulk RNA-seq,Homo_633,AD.vs.control; bulk RNA-	4
BP	GO:1903649	regulation of cytoplasmic transport	bulk RNA-seq,Homo_723,MCI.vs.control; bulk RNA-seq,Homo_714,MCI.vs.control; bulk RNA-seq,Homo_633,AD.vs.control; bulk RNA-	4
BP	GO:0033483	gas homeostasis	bulk RNA-seq,Homo_723,MCI.vs.control	1
BP	GO:0010824	regulation of centrosome duplication	bulk RNA-seq,Homo_723,MCI.vs.control; bulk RNA-seq,Homo_714,MCI.vs.control; bulk RNA-seq,Homo_633,AD.vs.control; bulk RNA-	4
BP	GO:0036010	protein localization to endosome	bulk RNA-seq,Homo_723,MCI.vs.control; bulk RNA-seq,Homo_714,MCI.vs.control; bulk RNA-seq,Homo_633,AD.vs.control; bulk RNA-	4
MF	GO:0070273	phosphatidylinositol-4-phosphate binding	bulk RNA-seq,Homo_723,MCI.vs.control; bulk RNA-seq,Homo_714,MCI.vs.control; bulk RNA-seq,Homo_633,AD.vs.control; bulk RNA-	4
MF	GO:0032266	phosphatidylinositol-3-phosphate binding	bulk RNA-seq,Homo_723,MCI.vs.control; bulk RNA-seq,Homo_714,MCI.vs.control; bulk RNA-seq,Homo_633,AD.vs.control; bulk RNA-	4
MF	GO:0004709	MAP kinase kinase kinase activity	bulk RNA-seq,Homo_723,MCI.vs.control; bulk RNA-seq,Homo_714,MCI.vs.control; bulk RNA-seq,Homo_633,AD.vs.control; bulk RNA-	4
BP	GO:0033151	V(D)J recombination	bulk RNA-seq,Homo_723,MCI.vs.control; bulk RNA-seq,Homo_714,MCI.vs.control; bulk RNA-seq,Homo_633,AD.vs.control; bulk RNA-seq,Homo_633,AD.vs.MCI; bulk RNA-seq,Homo_633,MCI.vs.control	5
BP	GO:0090308	regulation of DNA methylation-dependent heterochromatin	bulk RNA-seq,Homo_723,MCI.vs.control; bulk RNA-seq,Homo_714,AD.vs.MCI; bulk RNA-seq,Homo_714,MCI.vs.control; bulk RNA-	4
BP	GO:0051647	nucleus localization	bulk RNA-seq,Homo_723,MCI.vs.control; bulk RNA-seq,Homo_714,MCI.vs.control; bulk RNA-seq,Homo_633,AD.vs.control; bulk RNA-	4
BP	GO:0060142	regulation of syncytium formation by plasma membrane	bulk RNA-seq,Homo_723,MCI.vs.control; bulk RNA-seq,Homo_714,MCI.vs.control	2
BP	GO:0042989	sequestering of actin monomers	bulk RNA-seq,Homo_723,MCI.vs.control; bulk RNA-seq,Homo_714,MCI.vs.control	2
BP	GO:0018026	peptidyl-lysine monomethylation	bulk RNA-seq,Homo_723,MCI.vs.control; bulk RNA-seq,Homo_714,MCI.vs.control; bulk RNA-seq,Homo_633,AD.vs.control; bulk RNA-seq,Homo_633,AD.vs.MCI; bulk RNA-seq,Homo_633,MCI.vs.control	5
BP	GO:0051573	negative regulation of histone H3-K9 methylation	bulk RNA-seq,Homo_723,MCI.vs.control; bulk RNA-seq,Homo_714,MCI.vs.control; bulk RNA-seq,Homo_633,AD.vs.control; bulk RNA-	4
BP	GO:0046479	glycosphingolipid catabolic process	bulk RNA-seq,Homo_723,MCI.vs.control; bulk RNA-seq,Homo_714,MCI.vs.control; bulk RNA-seq,Homo_633,AD.vs.control; bulk RNA-	4
BP	GO:0061952	midbody abscission	bulk RNA-seq,Homo_723,MCI.vs.control; bulk RNA-seq,Homo_714,MCI.vs.control; bulk RNA-seq,Homo_633,AD.vs.control; bulk RNA-	4
MF	GO:0051537	2 iron, 2 sulfur cluster binding	bulk RNA-seq,Homo_723,MCI.vs.control; bulk RNA-seq,Homo_714,MCI.vs.control; bulk RNA-seq,Homo_633,AD.vs.control; bulk RNA-	4
BP	GO:0051353	positive regulation of oxidoreductase activity	bulk RNA-seq,Homo_723,MCI.vs.control; bulk RNA-seq,Homo_714,MCI.vs.control; bulk RNA-seq,Homo_633,AD.vs.control; bulk RNA-	4
CC	GO:0070461	SAGA-type complex	bulk RNA-seq,Homo_723,MCI.vs.control; bulk RNA-seq,Homo_714,MCI.vs.control; bulk RNA-seq,Homo_633,AD.vs.control; bulk RNA-	4
BP	GO:0033198	response to ATP	bulk RNA-seq,Homo_723,MCI.vs.control; bulk RNA-seq,Homo_633,AD.vs.control; bulk RNA-seq,Homo_633,AD.vs.MCI	3
CC	GO:0030992	intraciliary transport particle B	bulk RNA-seq,Homo_723,MCI.vs.control; bulk RNA-seq,Homo_714,MCI.vs.control; bulk RNA-seq,Homo_633,AD.vs.control; bulk RNA-seq,Homo_633,AD.vs.MCI; bulk RNA-seq,Homo_633,MCI.vs.control	5
BP	GO:0010818	T cell chemotaxis	bulk RNA-seq,Homo_723,MCI.vs.control; bulk RNA-seq,Homo_714,AD.vs.MCI; bulk RNA-seq,Homo_714,MCI.vs.control	3
CC	GO:0012510	trans-Golgi network transport vesicle membrane	bulk RNA-seq,Homo_723,MCI.vs.control; bulk RNA-seq,Homo_714,MCI.vs.control	2
BP	GO:1902410	mitotic cytokinetic process	bulk RNA-seq,Homo_723,MCI.vs.control; bulk RNA-seq,Homo_714,MCI.vs.control; bulk RNA-seq,Homo_633,AD.vs.control; bulk RNA-seq,Homo_633,AD.vs.MCI; bulk RNA-seq,Homo_633,MCI.vs.control	5
MF	GO:0097157	pre-mRNA intronic binding	bulk RNA-seq,Homo_723,MCI.vs.control; bulk RNA-seq,Homo_714,MCI.vs.control; bulk RNA-seq,Homo_633,AD.vs.control	3
MF	GO:0045294	alpha-catenin binding	bulk RNA-seq,Homo_723,MCI.vs.control	1
CC	GO:0005801	cis-Golgi network	bulk RNA-seq,Homo_723,MCI.vs.control; bulk RNA-seq,Homo_714,MCI.vs.control; bulk RNA-seq,Homo_633,AD.vs.control; bulk RNA-	4
CC	GO:0000145	exocyst	bulk RNA-seq,Homo_723,MCI.vs.control; bulk RNA-seq,Homo_714,MCI.vs.control; bulk RNA-seq,Homo_633,AD.vs.control	3
CC	GO:0099522	cytosolic region	bulk RNA-seq,Homo_723,MCI.vs.control; bulk RNA-seq,Homo_714,MCI.vs.control	2
BP	GO:0045039	protein insertion into mitochondrial inner membrane	bulk RNA-seq,Homo_723,MCI.vs.control; bulk RNA-seq,Homo_714,MCI.vs.control; bulk RNA-seq,Homo_633,AD.vs.control; bulk RNA-seq,Homo_633,AD.vs.MCI; bulk RNA-seq,Homo_633,MCI.vs.control	5
BP	GO:0010758	regulation of macrophage chemotaxis	bulk RNA-seq,Homo_723,MCI.vs.control; bulk RNA-seq,Homo_714,MCI.vs.control; bulk RNA-seq,Homo_633,AD.vs.control	3
MF	GO:0003756	protein disulfide isomerase activity	bulk RNA-seq,Homo_723,MCI.vs.control; bulk RNA-seq,Homo_714,MCI.vs.control; bulk RNA-seq,Homo_633,AD.vs.control; bulk RNA-	4
MF	GO:0016864	intramolecular oxidoreductase activity, transposing S-S	bulk RNA-seq,Homo_723,MCI.vs.control; bulk RNA-seq,Homo_714,MCI.vs.control; bulk RNA-seq,Homo_633,AD.vs.control; bulk RNA-	4
BP	GO:0035720	intraciliary anterograde transport	bulk RNA-seq,Homo_723,MCI.vs.control; bulk RNA-seq,Homo_714,MCI.vs.control; bulk RNA-seq,Homo_633,AD.vs.control; bulk RNA-seq,Homo_633,AD.vs.MCI; bulk RNA-seq,Homo_633,MCI.vs.control	5
BP	GO:0031573	mitotic intra-S DNA damage checkpoint signaling	bulk RNA-seq,Homo_723,MCI.vs.control; bulk RNA-seq,Homo_714,MCI.vs.control; bulk RNA-seq,Homo_633,AD.vs.control; bulk RNA-	4
BP	GO:0070431	nucleotide-binding oligomerization domain containing 2 signaling pathway	bulk RNA-seq,Homo_723,MCI.vs.control; bulk RNA-seq,Homo_714,MCI.vs.control; bulk RNA-seq,Homo_633,AD.vs.control; bulk RNA-seq,Homo_633,AD.vs.MCI; bulk RNA-seq,Homo_633,MCI.vs.control	5
BP	GO:0000079	regulation of cyclin-dependent protein serine/threonine	bulk RNA-seq,Homo_723,MCI.vs.control; bulk RNA-seq,Homo_714,MCI.vs.control; bulk RNA-seq,Homo_633,AD.vs.control; bulk RNA-	4
BP	GO:0060761	negative regulation of response to cytokine stimulus	bulk RNA-seq,Homo_723,MCI.vs.control; bulk RNA-seq,Homo_714,MCI.vs.control; bulk RNA-seq,Homo_633,AD.vs.control	3
BP	GO:0035384	thioester biosynthetic process	bulk RNA-seq,Homo_723,MCI.vs.control; bulk RNA-seq,Homo_714,MCI.vs.control; bulk RNA-seq,Homo_633,AD.vs.control; bulk RNA-	4
BP	GO:0071616	acyl-CoA biosynthetic process	bulk RNA-seq,Homo_723,MCI.vs.control; bulk RNA-seq,Homo_714,MCI.vs.control; bulk RNA-seq,Homo_633,AD.vs.control; bulk RNA-	4
BP	GO:0036376	sodium ion export across plasma membrane	bulk RNA-seq,Homo_723,MCI.vs.control	1
BP	GO:0001921	positive regulation of receptor recycling	bulk RNA-seq,Homo_723,MCI.vs.control; bulk RNA-seq,Homo_714,MCI.vs.control; bulk RNA-seq,Homo_633,AD.vs.control; bulk RNA-seq,Homo_633,AD.vs.MCI; bulk RNA-seq,Homo_633,MCI.vs.control	5
BP	GO:0090141	positive regulation of mitochondrial fission	bulk RNA-seq,Homo_723,MCI.vs.control; bulk RNA-seq,Homo_714,MCI.vs.control; bulk RNA-seq,Homo_633,AD.vs.control; bulk RNA-	4
BP	GO:0010771	negative regulation of cell morphogenesis involved in	bulk RNA-seq,Homo_723,MCI.vs.control; bulk RNA-seq,Homo_714,MCI.vs.control; bulk RNA-seq,Homo_633,AD.vs.control	3
BP	GO:1900025	negative regulation of substrate adhesion-dependent cell	bulk RNA-seq,Homo_723,MCI.vs.control; bulk RNA-seq,Homo_714,MCI.vs.control; bulk RNA-seq,Homo_633,AD.vs.control	3
BP	GO:0045091	regulation of single stranded viral RNA replication via double stranded DNA intermediate	bulk RNA-seq,Homo_723,MCI.vs.control; bulk RNA-seq,Homo_714,MCI.vs.control	2
BP	GO:0018242	protein O-linked glycosylation via serine	bulk RNA-seq,Homo_723,MCI.vs.control	1

BP	GO:0150105	protein localization to cell-cell junction	bulk RNA-seq,Homo_723,MCI.vs.control; bulk RNA-seq,Homo_714,AD.vs.MCI; bulk RNA-seq,Homo_714,MCI.vs.control	3
BP	GO:0046173	polyol biosynthetic process	bulk RNA-seq,Homo_723,MCI.vs.control; bulk RNA-seq,Homo_714,MCI.vs.control; bulk RNA-seq,Homo_633,AD.vs.control; bulk RNA-	4
BP	GO:0046825	regulation of protein export from nucleus	bulk RNA-seq,Homo_723,MCI.vs.control; bulk RNA-seq,Homo_714,MCI.vs.control; bulk RNA-seq,Homo_633,AD.vs.control; bulk RNA-seq,Homo_633,AD.vs.MCI; bulk RNA-seq,Homo_633,MCI.vs.control	5
CC	GO:1990907	beta-catenin-TCF complex	bulk RNA-seq,Homo_723,MCI.vs.control; bulk RNA-seq,Homo_714,MCI.vs.control; bulk RNA-seq,Homo_633,MCI.vs.control	3
MF	GO:0016780	phosphotransferase activity, for other substituted phosphate groups	bulk RNA-seq,Homo_723,MCI.vs.control; bulk RNA-seq,Homo_714,AD.vs.control; bulk RNA-seq,Homo_714,MCI.vs.control; bulk RNA-seq,Homo_633,AD.vs.control; bulk RNA-seq,Homo_633,AD.vs.MCI; bulk RNA-seq,Homo_633,MCI.vs.control	6
MF	GO:0052866	phosphatidylinositol phosphate phosphatase activity	bulk RNA-seq,Homo_723,MCI.vs.control; bulk RNA-seq,Homo_714,MCI.vs.control; bulk RNA-seq,Homo_633,AD.vs.control; bulk RNA-seq,Homo_633,AD.vs.MCI; bulk RNA-seq,Homo_633,MCI.vs.control	5
MF	GO:0016812	hydrolase activity, acting on carbon-nitrogen (but not peptide) bonds, in cyclic amides	bulk RNA-seq,Homo_723,MCI.vs.control; bulk RNA-seq,Homo_714,MCI.vs.control; bulk RNA-seq,Homo_633,MCI.vs.control	3
BP	GO:0009310	amine catabolic process	bulk RNA-seq,Homo_723,MCI.vs.control; bulk RNA-seq,Homo_714,MCI.vs.control; bulk RNA-seq,Homo_633,AD.vs.control	3
MF	GO:0048185	activin binding	bulk RNA-seq,Homo_723,MCI.vs.control; bulk RNA-seq,Homo_714,MCI.vs.control; bulk RNA-seq,Homo_633,AD.vs.control	3
BP	GO:1901030	positive regulation of mitochondrial outer membrane permeabilization involved in apoptotic signaling pathway	bulk RNA-seq,Homo_723,MCI.vs.control; bulk RNA-seq,Homo_714,MCI.vs.control; bulk RNA-seq,Homo_633,AD.vs.control; bulk RNA-seq,Homo_633,MCI.vs.control	4
MF	GO:0052689	carboxylic ester hydrolase activity	bulk RNA-seq,Homo_723,MCI.vs.control; bulk RNA-seq,Homo_714,AD.vs.MCI; bulk RNA-seq,Homo_633,AD.vs.control; bulk RNA-seq,Homo_633,AD.vs.MCI	4
BP	GO:0032727	positive regulation of interferon-alpha production	bulk RNA-seq,Homo_723,MCI.vs.control; bulk RNA-seq,Homo_714,MCI.vs.control; bulk RNA-seq,Homo_633,AD.vs.control; bulk RNA-	4
BP	GO:0038093	Fc receptor signaling pathway	bulk RNA-seq,Homo_723,MCI.vs.control; bulk RNA-seq,Homo_714,MCI.vs.control; bulk RNA-seq,Homo_633,AD.vs.control; bulk RNA-	4
BP	GO:0010288	response to lead ion	bulk RNA-seq,Homo_723,MCI.vs.control; bulk RNA-seq,Homo_714,MCI.vs.control; bulk RNA-seq,Homo_633,AD.vs.control; bulk RNA-	4
BP	GO:0032785	negative regulation of DNA-templated transcription, elongation	bulk RNA-seq,Homo_723,MCI.vs.control; bulk RNA-seq,Homo_714,MCI.vs.control; bulk RNA-seq,Homo_633,AD.vs.control; bulk RNA-seq,Homo_633,AD.vs.MCI; bulk RNA-seq,Homo_633,MCI.vs.control	5
BP	GO:0061842	microtubule organizing center localization	bulk RNA-seq,Homo_723,MCI.vs.control; bulk RNA-seq,Homo_714,MCI.vs.control; bulk RNA-seq,Homo_633,AD.vs.control; bulk RNA-	4
BP	GO:0006525	arginine metabolic process	bulk RNA-seq,Homo_723,MCI.vs.control; bulk RNA-seq,Homo_714,AD.vs.MCI; bulk RNA-seq,Homo_714,MCI.vs.control	3
BP	GO:0046040	IMP metabolic process	bulk RNA-seq,Homo_723,MCI.vs.control; bulk RNA-seq,Homo_714,MCI.vs.control	2
CC	GO:0031235	intrinsic component of the cytoplasmic side of the plasma	bulk RNA-seq,Homo_723,MCI.vs.control; bulk RNA-seq,Homo_714,AD.vs.MCI; bulk RNA-seq,Homo_714,MCI.vs.control	3
MF	GO:0032036	myosin heavy chain binding	bulk RNA-seq,Homo_723,MCI.vs.control	1
CC	GO:0005666	RNA polymerase III complex	bulk RNA-seq,Homo_723,MCI.vs.control; bulk RNA-seq,Homo_714,MCI.vs.control; bulk RNA-seq,Homo_633,AD.vs.control; bulk RNA-	4
BP	GO:0019883	antigen processing and presentation of endogenous antigen	bulk RNA-seq,Homo_723,MCI.vs.control; bulk RNA-seq,Homo_714,MCI.vs.control; bulk RNA-seq,Homo_633,AD.vs.control; bulk RNA-seq,Homo_633,MCI.vs.control; scRNA-seq,SRP215507,CD8+ T cell_8-MCI.vs.control	5
MF	GO:0015166	polyol transmembrane transporter activity	bulk RNA-seq,Homo_723,MCI.vs.control; bulk RNA-seq,Homo_714,MCI.vs.control	2
MF	GO:0050145	nucleoside monophosphate kinase activity	bulk RNA-seq,Homo_723,MCI.vs.control; bulk RNA-seq,Homo_714,MCI.vs.control; bulk RNA-seq,Homo_633,AD.vs.control	3
BP	GO:0019377	glycolipid catabolic process	bulk RNA-seq,Homo_723,MCI.vs.control; bulk RNA-seq,Homo_633,AD.vs.control; bulk RNA-seq,Homo_633,AD.vs.MCI	3
BP	GO:0070841	inclusion body assembly	bulk RNA-seq,Homo_723,MCI.vs.control; bulk RNA-seq,Homo_714,MCI.vs.control	2
BP	GO:1904666	regulation of ubiquitin protein ligase activity	bulk RNA-seq,Homo_723,MCI.vs.control; bulk RNA-seq,Homo_633,AD.vs.control; bulk RNA-seq,Homo_633,AD.vs.MCI	3
BP	GO:0006448	regulation of translational elongation	bulk RNA-seq,Homo_723,MCI.vs.control; bulk RNA-seq,Homo_714,AD.vs.control; bulk RNA-seq,Homo_714,MCI.vs.control; bulk RNA-seq,Homo_633,AD.vs.control; bulk RNA-seq,Homo_633,AD.vs.MCI	5
BP	GO:0051231	spindle elongation	bulk RNA-seq,Homo_723,MCI.vs.control; bulk RNA-seq,Homo_714,MCI.vs.control; bulk RNA-seq,Homo_633,MCI.vs.control	3
BP	GO:2000134	negative regulation of G1/S transition of mitotic cell cycle	bulk RNA-seq,Homo_723,MCI.vs.control; bulk RNA-seq,Homo_714,AD.vs.MCI; bulk RNA-seq,Homo_714,MCI.vs.control; bulk RNA-seq,Homo_633,AD.vs.control; bulk RNA-seq,Homo_633,AD.vs.MCI	5
BP	GO:0046337	phosphatidylethanolamine metabolic process	bulk RNA-seq,Homo_723,MCI.vs.control; bulk RNA-seq,Homo_714,MCI.vs.control; bulk RNA-seq,Homo_633,AD.vs.control; bulk RNA-	4
BP	GO:0090161	Golgi ribbon formation	bulk RNA-seq,Homo_723,MCI.vs.control; bulk RNA-seq,Homo_714,MCI.vs.control; bulk RNA-seq,Homo_633,MCI.vs.control	3
MF	GO:0030291	protein serine/threonine kinase inhibitor activity	bulk RNA-seq,Homo_723,MCI.vs.control; bulk RNA-seq,Homo_714,MCI.vs.control; bulk RNA-seq,Homo_633,AD.vs.control; bulk RNA-	4
MF	GO:0140597	protein carrier chaperone	bulk RNA-seq,Homo_723,MCI.vs.control; bulk RNA-seq,Homo_714,MCI.vs.control; bulk RNA-seq,Homo_633,AD.vs.control; bulk RNA-seq,Homo_633,AD.vs.MCI; bulk RNA-seq,Homo_633,MCI.vs.control	5
BP	GO:0070423	nucleotide-binding oligomerization domain containing signaling pathway	bulk RNA-seq,Homo_723,MCI.vs.control; bulk RNA-seq,Homo_714,MCI.vs.control; bulk RNA-seq,Homo_633,AD.vs.control; bulk RNA-seq,Homo_633,AD.vs.MCI; bulk RNA-seq,Homo_633,MCI.vs.control	5
BP	GO:0042168	heme metabolic process	bulk RNA-seq,Homo_723,MCI.vs.control; bulk RNA-seq,Homo_714,MCI.vs.control; bulk RNA-seq,Homo_633,AD.vs.control; bulk RNA-	4
BP	GO:0006826	iron ion transport	bulk RNA-seq,Homo_723,MCI.vs.control; bulk RNA-seq,Homo_714,MCI.vs.control; bulk RNA-seq,Homo_633,AD.vs.control; bulk RNA-	4
BP	GO:0001960	negative regulation of cytokine-mediated signaling pathway	bulk RNA-seq,Homo_723,MCI.vs.control; bulk RNA-seq,Homo_714,AD.vs.MCI; bulk RNA-seq,Homo_714,MCI.vs.control; bulk RNA-	4
BP	GO:0001774	microglial cell activation	bulk RNA-seq,Homo_723,MCI.vs.control; bulk RNA-seq,Homo_714,AD.vs.MCI; bulk RNA-seq,Homo_714,MCI.vs.control; bulk RNA-seq,Homo_633,AD.vs.control; bulk RNA-seq,Homo_633,AD.vs.MCI	5
BP	GO:0051988	regulation of attachment of spindle microtubules to	bulk RNA-seq,Homo_723,MCI.vs.control; bulk RNA-seq,Homo_633,AD.vs.control	2
BP	GO:0039694	viral RNA genome replication	bulk RNA-seq,Homo_723,MCI.vs.control; bulk RNA-seq,Homo_714,MCI.vs.control; bulk RNA-seq,Homo_633,MCI.vs.control	3
BP	GO:0071467	cellular response to pH	bulk RNA-seq,Homo_723,MCI.vs.control; bulk RNA-seq,Homo_714,MCI.vs.control	2
MF	GO:0140677	molecular function activator activity	bulk RNA-seq,Homo_723,MCI.vs.control; bulk RNA-seq,Homo_714,MCI.vs.control; bulk RNA-seq,Homo_633,AD.vs.control; bulk RNA-	4
BP	GO:0014741	negative regulation of muscle hypertrophy	bulk RNA-seq,Homo_723,MCI.vs.control; bulk RNA-seq,Homo_714,AD.vs.MCI; bulk RNA-seq,Homo_714,MCI.vs.control; bulk RNA-	4
BP	GO:0048266	behavioral response to pain	bulk RNA-seq,Homo_723,MCI.vs.control; bulk RNA-seq,Homo_633,AD.vs.control	2
MF	GO:0010181	FMN binding	bulk RNA-seq,Homo_723,MCI.vs.control; bulk RNA-seq,Homo_714,AD.vs.MCI; bulk RNA-seq,Homo_714,MCI.vs.control	3
BP	GO:0031649	heat generation	bulk RNA-seq,Homo_723,MCI.vs.control; bulk RNA-seq,Homo_714,MCI.vs.control	2

BP	GO:0070099	regulation of chemokine-mediated signaling pathway	bulk RNA-seq,Homo_723,MCI.vs.control	1
BP	GO:1901739	regulation of myoblast fusion	bulk RNA-seq,Homo_723,MCI.vs.control; bulk RNA-seq,Homo_714,MCI.vs.control	2
MF	GO:0030742	GTP-dependent protein binding	bulk RNA-seq,Homo_723,MCI.vs.control; bulk RNA-seq,Homo_714,AD.vs.MCI; bulk RNA-seq,Homo_714,MCI.vs.control; bulk RNA-seq,Homo_633,AD.vs.control; bulk RNA-seq,Homo_633,AD.vs.MCI	5
CC	GO:0032433	filopodium tip	bulk RNA-seq,Homo_723,MCI.vs.control; bulk RNA-seq,Homo_714,MCI.vs.control; bulk RNA-seq,Homo_633,AD.vs.control	3
BP	GO:0070935	3'-UTR-mediated mRNA stabilization	bulk RNA-seq,Homo_723,MCI.vs.control; bulk RNA-seq,Homo_633,AD.vs.control; bulk RNA-seq,Homo_633,AD.vs.MCI; bulk RNA-seq,ROSMAP,AD.vs.control; bulk RNA-seq,ROSMAP,MCI.vs.control	5
CC	GO:0034362	low-density lipoprotein particle	bulk RNA-seq,Homo_723,MCI.vs.control; bulk RNA-seq,Homo_714,MCI.vs.control; bulk RNA-seq,Homo_633,AD.vs.control	3
BP	GO:0051693	actin filament capping	bulk RNA-seq,Homo_723,MCI.vs.control; bulk RNA-seq,Homo_714,MCI.vs.control	2
BP	GO:0097242	amyloid-beta clearance	bulk RNA-seq,Homo_723,MCI.vs.control; bulk RNA-seq,Homo_714,MCI.vs.control; bulk RNA-seq,Homo_633,AD.vs.control; bulk RNA-	4
MF	GO:0016251	RNA polymerase II general transcription initiation factor	bulk RNA-seq,Homo_723,MCI.vs.control; bulk RNA-seq,Homo_714,MCI.vs.control; bulk RNA-seq,Homo_633,AD.vs.control; bulk RNA-	4
BP	GO:0006972	hyperosmotic response	bulk RNA-seq,Homo_723,MCI.vs.control; bulk RNA-seq,Homo_633,AD.vs.control; bulk RNA-seq,Homo_633,AD.vs.MCI	3
BP	GO:0016137	glycoside metabolic process	bulk RNA-seq,Homo_723,MCI.vs.control; bulk RNA-seq,Homo_633,AD.vs.control; bulk RNA-seq,Homo_633,AD.vs.MCI	3
BP	GO:0043517	positive regulation of DNA damage response, signal transduction by p53 class mediator	bulk RNA-seq,Homo_723,MCI.vs.control; bulk RNA-seq,Homo_714,MCI.vs.control; bulk RNA-seq,Homo_633,AD.vs.control; bulk RNA-seq,Homo_633,AD.vs.MCI	4
CC	GO:0030118	clathrin coat	bulk RNA-seq,Homo_723,MCI.vs.control; bulk RNA-seq,Homo_714,MCI.vs.control; bulk RNA-seq,Homo_633,AD.vs.control; bulk RNA-	4
BP	GO:0060394	negative regulation of pathway-restricted SMAD protein phosphorylation	bulk RNA-seq,Homo_723,MCI.vs.control; bulk RNA-seq,Homo_714,MCI.vs.control	2
BP	GO:0070936	protein K48-linked ubiquitination	bulk RNA-seq,Homo_723,MCI.vs.control; bulk RNA-seq,Homo_714,MCI.vs.control; bulk RNA-seq,Homo_633,AD.vs.control; bulk RNA-	4
BP	GO:0009127	purine nucleoside monophosphate biosynthetic process	bulk RNA-seq,Homo_723,MCI.vs.control; bulk RNA-seq,Homo_714,MCI.vs.control; bulk RNA-seq,Homo_633,AD.vs.control; bulk RNA-	4
BP	GO:0033147	negative regulation of intracellular estrogen receptor	bulk RNA-seq,Homo_723,MCI.vs.control; bulk RNA-seq,Homo_714,MCI.vs.control	2
MF	GO:0001784	phosphotyrosine residue binding	bulk RNA-seq,Homo_723,MCI.vs.control; bulk RNA-seq,Homo_633,AD.vs.control; bulk RNA-seq,Homo_633,AD.vs.MCI	3
BP	GO:0002320	lymphoid progenitor cell differentiation	bulk RNA-seq,Homo_723,MCI.vs.control; bulk RNA-seq,Homo_714,MCI.vs.control; bulk RNA-seq,Homo_633,AD.vs.control; bulk RNA-	4
BP	GO:2000059	negative regulation of ubiquitin-dependent protein catabolic	bulk RNA-seq,Homo_723,MCI.vs.control; bulk RNA-seq,Homo_714,MCI.vs.control; bulk RNA-seq,Homo_633,AD.vs.control; bulk RNA-	4
BP	GO:0006734	NADH metabolic process	bulk RNA-seq,Homo_723,MCI.vs.control; bulk RNA-seq,Homo_714,MCI.vs.control; bulk RNA-seq,Homo_633,AD.vs.control	3
BP	GO:0060766	negative regulation of androgen receptor signaling pathway	bulk RNA-seq,Homo_723,MCI.vs.control; bulk RNA-seq,Homo_714,MCI.vs.control	2
BP	GO:2001140	positive regulation of phospholipid transport	bulk RNA-seq,Homo_723,MCI.vs.control; bulk RNA-seq,Homo_714,AD.vs.MCI; bulk RNA-seq,Homo_714,MCI.vs.control	3
BP	GO:0006264	mitochondrial DNA replication	bulk RNA-seq,Homo_723,MCI.vs.control; bulk RNA-seq,Homo_714,MCI.vs.control; bulk RNA-seq,Homo_633,AD.vs.control; bulk RNA-seq,Homo_633,AD.vs.MCI; bulk RNA-seq,Homo_633,MCI.vs.control	5
BP	GO:0045980	negative regulation of nucleotide metabolic process	bulk RNA-seq,Homo_723,MCI.vs.control; bulk RNA-seq,Homo_714,MCI.vs.control; bulk RNA-seq,Homo_633,AD.vs.control; bulk RNA-	4
BP	GO:0048251	elastic fiber assembly	bulk RNA-seq,Homo_723,MCI.vs.control; bulk RNA-seq,Homo_714,MCI.vs.control	2
MF	GO:0008603	cAMP-dependent protein kinase regulator activity	bulk RNA-seq,Homo_723,MCI.vs.control; bulk RNA-seq,Homo_714,MCI.vs.control; bulk RNA-seq,Homo_633,MCI.vs.control	3
BP	GO:1902307	positive regulation of sodium ion transmembrane transport	bulk RNA-seq,Homo_723,MCI.vs.control; bulk RNA-seq,Homo_714,MCI.vs.control	2
BP	GO:0043046	DNA methylation involved in gamete generation	bulk RNA-seq,Homo_723,MCI.vs.control; bulk RNA-seq,Homo_714,MCI.vs.control	2
BP	GO:0071379	cellular response to prostaglandin stimulus	bulk RNA-seq,Homo_723,MCI.vs.control; bulk RNA-seq,Homo_714,MCI.vs.control	2
MF	GO:0004698	calcium-dependent protein kinase C activity	bulk RNA-seq,Homo_723,MCI.vs.control; bulk RNA-seq,Homo_714,MCI.vs.control; bulk RNA-seq,Homo_633,AD.vs.control	3
MF	GO:0052813	phosphatidylinositol bisphosphate kinase activity	bulk RNA-seq,Homo_723,MCI.vs.control; bulk RNA-seq,Homo_714,MCI.vs.control; bulk RNA-seq,Homo_633,AD.vs.control; bulk RNA-seq,Homo_633,AD.vs.MCI; bulk RNA-seq,Homo_633,MCI.vs.control	5
MF	GO:0043208	glycosphingolipid binding	bulk RNA-seq,Homo_723,MCI.vs.control	1
BP	GO:0070316	regulation of G0 to G1 transition	bulk RNA-seq,Homo_723,MCI.vs.control; bulk RNA-seq,Homo_633,AD.vs.control; bulk RNA-seq,Homo_633,AD.vs.MCI	3
BP	GO:0010452	histone H3-K36 methylation	bulk RNA-seq,Homo_723,MCI.vs.control; bulk RNA-seq,Homo_714,MCI.vs.control	2
MF	GO:0005523	tropomyosin binding	bulk RNA-seq,Homo_723,MCI.vs.control; bulk RNA-seq,Homo_714,MCI.vs.control	2
MF	GO:0140327	flippase activity	bulk RNA-seq,Homo_723,MCI.vs.control; bulk RNA-seq,Homo_714,MCI.vs.control; bulk RNA-seq,Homo_633,AD.vs.control	3
BP	GO:1904729	regulation of intestinal lipid absorption	bulk RNA-seq,Homo_723,MCI.vs.control; bulk RNA-seq,Homo_714,AD.vs.MCI; bulk RNA-seq,Homo_714,MCI.vs.control	3
BP	GO:1903715	regulation of aerobic respiration	bulk RNA-seq,Homo_723,MCI.vs.control; bulk RNA-seq,Homo_714,MCI.vs.control; bulk RNA-seq,Homo_633,AD.vs.control; bulk RNA-	4
MF	GO:0003925	G protein activity	bulk RNA-seq,Homo_723,MCI.vs.control; bulk RNA-seq,Homo_714,MCI.vs.control; bulk RNA-seq,Homo_633,AD.vs.control; bulk RNA-	4
BP	GO:0042398	cellular modified amino acid biosynthetic process	bulk RNA-seq,Homo_723,MCI.vs.control; bulk RNA-seq,Homo_714,MCI.vs.control; bulk RNA-seq,Homo_633,AD.vs.control; bulk RNA-	4
BP	GO:0006595	polyamine metabolic process	bulk RNA-seq,Homo_723,MCI.vs.control; bulk RNA-seq,Homo_714,MCI.vs.control; bulk RNA-seq,Homo_633,AD.vs.control	3
BP	GO:0034616	response to laminar fluid shear stress	bulk RNA-seq,Homo_723,MCI.vs.control; bulk RNA-seq,Homo_714,AD.vs.MCI; bulk RNA-seq,Homo_714,MCI.vs.control	3
BP	GO:1903798	regulation of miRNA maturation	bulk RNA-seq,Homo_723,MCI.vs.control	1
MF	GO:0051861	glycolipid binding	bulk RNA-seq,Homo_723,MCI.vs.control	1
BP	GO:0051382	kinetochore assembly	bulk RNA-seq,Homo_723,MCI.vs.control; bulk RNA-seq,Homo_714,MCI.vs.control; bulk RNA-seq,Homo_633,AD.vs.control	3
MF	GO:0001046	core promoter sequence-specific DNA binding	bulk RNA-seq,Homo_723,MCI.vs.control; bulk RNA-seq,Homo_714,MCI.vs.control; bulk RNA-seq,Homo_633,AD.vs.control; bulk RNA-	4
BP	GO:0051972	regulation of telomerase activity	bulk RNA-seq,Homo_723,MCI.vs.control; bulk RNA-seq,Homo_714,MCI.vs.control; bulk RNA-seq,Homo_633,AD.vs.control; bulk RNA-	4
BP	GO:2000377	regulation of reactive oxygen species metabolic process	bulk RNA-seq,Homo_723,MCI.vs.control; bulk RNA-seq,Homo_633,AD.vs.control; bulk RNA-seq,Homo_633,AD.vs.MCI	3
BP	GO:0000038	very long-chain fatty acid metabolic process	bulk RNA-seq,Homo_723,MCI.vs.control; bulk RNA-seq,Homo_714,MCI.vs.control; bulk RNA-seq,Homo_633,AD.vs.control; bulk RNA-	4
BP	GO:0150077	regulation of neuroinflammatory response	bulk RNA-seq,Homo_723,MCI.vs.control; bulk RNA-seq,Homo_714,MCI.vs.control	2

BP	GO:1904044	response to aldosterone	bulk RNA-seq,Homo_723,MCI.vs.control; bulk RNA-seq,Homo_714,MCI.vs.control	2
BP	GO:0044849	estrous cycle	bulk RNA-seq,Homo_723,MCI.vs.control	1
MF	GO:0044594	17-beta-hydroxysteroid dehydrogenase (NAD+) activity	bulk RNA-seq,Homo_723,MCI.vs.control; bulk RNA-seq,Homo_714,MCI.vs.control	2
MF	GO:0052740	1-acyl-2-lysophosphatidylserine acylhydrolase activity	bulk RNA-seq,Homo_723,MCI.vs.control; bulk RNA-seq,Homo_633,AD.vs.control	2
BP	GO:1901021	positive regulation of calcium ion transmembrane transporter	bulk RNA-seq,Homo_723,MCI.vs.control; bulk RNA-seq,Homo_714,MCI.vs.control; bulk RNA-seq,Homo_633,AD.vs.control	3
BP	GO:0036475	neuron death in response to oxidative stress	bulk RNA-seq,Homo_723,MCI.vs.control; bulk RNA-seq,Homo_714,MCI.vs.control; bulk RNA-seq,Homo_633,AD.vs.control; bulk RNA-	4
CC	GO:0016586	RSC-type complex	bulk RNA-seq,Homo_723,MCI.vs.control; bulk RNA-seq,Homo_633,AD.vs.control; bulk RNA-seq,Homo_633,AD.vs.MCI	3
MF	GO:0016884	carbon-nitrogen ligase activity, with glutamine as amido-N-donor	bulk RNA-seq,Homo_723,MCI.vs.control; bulk RNA-seq,Homo_714,MCI.vs.control; bulk RNA-seq,Homo_633,AD.vs.control; bulk RNA-seq,Homo_633,AD.vs.MCI; bulk RNA-seq,Homo_633,MCI.vs.control	5
BP	GO:0045662	negative regulation of myoblast differentiation	bulk RNA-seq,Homo_723,MCI.vs.control; bulk RNA-seq,Homo_714,MCI.vs.control	2
BP	GO:0045869	negative regulation of single stranded viral RNA replication via double stranded DNA intermediate	bulk RNA-seq,Homo_723,MCI.vs.control; bulk RNA-seq,Homo_714,MCI.vs.control	2
BP	GO:0010039	response to iron ion	bulk RNA-seq,Homo_723,MCI.vs.control; bulk RNA-seq,Homo_714,AD.vs.MCI; bulk RNA-seq,Homo_633,AD.vs.control; bulk RNA-seq,Homo_633,AD.vs.MCI	4
BP	GO:0070424	regulation of nucleotide-binding oligomerization domain containing signaling pathway	bulk RNA-seq,Homo_723,MCI.vs.control; bulk RNA-seq,Homo_714,MCI.vs.control; bulk RNA-seq,Homo_633,AD.vs.control; bulk RNA-seq,Homo_633,MCI.vs.control	4
BP	GO:0051973	positive regulation of telomerase activity	bulk RNA-seq,Homo_723,MCI.vs.control; bulk RNA-seq,Homo_714,MCI.vs.control; bulk RNA-seq,Homo_633,AD.vs.control; bulk RNA-	4
BP	GO:0090043	regulation of tubulin deacetylation	bulk RNA-seq,Homo_723,MCI.vs.control; bulk RNA-seq,Homo_714,MCI.vs.control; bulk RNA-seq,Homo_633,AD.vs.control; bulk RNA-	4
MF	GO:0097001	ceramide binding	bulk RNA-seq,Homo_723,MCI.vs.control	1
BP	GO:0045741	positive regulation of epidermal growth factor-activated receptor activity	bulk RNA-seq,Homo_723,MCI.vs.control; bulk RNA-seq,Homo_714,MCI.vs.control; bulk RNA-seq,Homo_633,AD.vs.control	3
BP	GO:0062099	negative regulation of programmed necrotic cell death	bulk RNA-seq,Homo_723,MCI.vs.control; bulk RNA-seq,Homo_714,MCI.vs.control; bulk RNA-seq,Homo_633,AD.vs.control	3
BP	GO:0097028	dendritic cell differentiation	bulk RNA-seq,Homo_723,MCI.vs.control; bulk RNA-seq,Homo_714,MCI.vs.control; bulk RNA-seq,Homo_633,AD.vs.control; scRNA-seq,SRP330776,Naive CD8+ T cell_2-AD.vs.control	4
BP	GO:0051351	positive regulation of ligase activity	bulk RNA-seq,Homo_723,MCI.vs.control; bulk RNA-seq,Homo_714,MCI.vs.control; bulk RNA-seq,Homo_633,MCI.vs.control	3
MF	GO:0004683	calmodulin-dependent protein kinase activity	bulk RNA-seq,Homo_723,MCI.vs.control; bulk RNA-seq,Homo_633,AD.vs.control; bulk RNA-seq,Homo_633,AD.vs.MCI	3
BP	GO:0032367	intracellular cholesterol transport	bulk RNA-seq,Homo_723,MCI.vs.control; bulk RNA-seq,Homo_714,MCI.vs.control; bulk RNA-seq,Homo_633,AD.vs.control; bulk RNA-	4
BP	GO:0000041	transition metal ion transport	bulk RNA-seq,Homo_723,MCI.vs.control; bulk RNA-seq,Homo_714,MCI.vs.control; bulk RNA-seq,Homo_633,AD.vs.control; bulk RNA-	4
BP	GO:0032743	positive regulation of interleukin-2 production	bulk RNA-seq,Homo_723,MCI.vs.control; bulk RNA-seq,Homo_714,MCI.vs.control; bulk RNA-seq,Homo_633,AD.vs.control; bulk RNA-	4
BP	GO:0000083	regulation of transcription involved in G1/S transition of mitotic cell cycle	bulk RNA-seq,Homo_723,MCI.vs.control; bulk RNA-seq,Homo_714,MCI.vs.control; bulk RNA-seq,Homo_633,AD.vs.control; bulk RNA-seq,Homo_633,AD.vs.MCI	4
BP	GO:0009148	pyrimidine nucleoside triphosphate biosynthetic process	bulk RNA-seq,Homo_723,MCI.vs.control; bulk RNA-seq,Homo_714,MCI.vs.control; bulk RNA-seq,Homo_633,AD.vs.control; bulk RNA-	4
BP	GO:0034162	toll-like receptor 9 signaling pathway	bulk RNA-seq,Homo_723,MCI.vs.control; bulk RNA-seq,Homo_714,MCI.vs.control; bulk RNA-seq,Homo_633,AD.vs.control; bulk RNA-seq,Homo_633,AD.vs.MCI; bulk RNA-seq,Homo_633,MCI.vs.control	5
MF	GO:0004029	aldehyde dehydrogenase (NAD+) activity	bulk RNA-seq,Homo_723,MCI.vs.control; bulk RNA-seq,Homo_714,MCI.vs.control; bulk RNA-seq,Homo_633,AD.vs.control	3
BP	GO:2000601	positive regulation of Arp2/3 complex-mediated actin	bulk RNA-seq,Homo_723,MCI.vs.control; bulk RNA-seq,Homo_714,MCI.vs.control	2
MF	GO:0034596	phosphatidylinositol phosphate 4-phosphatase activity	bulk RNA-seq,Homo_723,MCI.vs.control; bulk RNA-seq,Homo_714,MCI.vs.control; bulk RNA-seq,Homo_633,AD.vs.control; bulk RNA-seq,Homo_633,AD.vs.MCI; bulk RNA-seq,Homo_633,MCI.vs.control	5
MF	GO:1990404	NAD+-protein ADP-ribosyltransferase activity	bulk RNA-seq,Homo_723,MCI.vs.control; bulk RNA-seq,Homo_714,MCI.vs.control; bulk RNA-seq,Homo_633,AD.vs.control; bulk RNA-	4
BP	GO:0070886	positive regulation of calcineurin-NFAT signaling cascade	bulk RNA-seq,Homo_723,MCI.vs.control; bulk RNA-seq,Homo_714,MCI.vs.control	2
BP	GO:0106058	positive regulation of calcineurin-mediated signaling	bulk RNA-seq,Homo_723,MCI.vs.control; bulk RNA-seq,Homo_714,MCI.vs.control	2
BP	GO:1902455	negative regulation of stem cell population maintenance	bulk RNA-seq,Homo_723,MCI.vs.control; bulk RNA-seq,Homo_633,AD.vs.control; bulk RNA-seq,Homo_633,AD.vs.MCI	3
BP	GO:1903540	establishment of protein localization to postsynaptic	bulk RNA-seq,Homo_723,MCI.vs.control; bulk RNA-seq,Homo_714,MCI.vs.control; bulk RNA-seq,Homo_633,AD.vs.control	3
BP	GO:0043162	ubiquitin-dependent protein catabolic process via the multivesicular body sorting pathway	bulk RNA-seq,Homo_723,MCI.vs.control; bulk RNA-seq,Homo_714,MCI.vs.control; bulk RNA-seq,Homo_633,AD.vs.control; bulk RNA-seq,Homo_633,AD.vs.MCI	4
BP	GO:0043255	regulation of carbohydrate biosynthetic process	bulk RNA-seq,Homo_723,MCI.vs.control; bulk RNA-seq,Homo_714,MCI.vs.control; bulk RNA-seq,Homo_633,AD.vs.control	3
MF	GO:0008373	sialyltransferase activity	bulk RNA-seq,Homo_723,MCI.vs.control; bulk RNA-seq,Homo_714,AD.vs.MCI; bulk RNA-seq,Homo_714,MCI.vs.control; bulk RNA-seq,Homo_633,AD.vs.control; bulk RNA-seq,Homo_633,AD.vs.MCI	5
BP	GO:0097503	sialylation	bulk RNA-seq,Homo_723,MCI.vs.control; bulk RNA-seq,Homo_714,AD.vs.MCI; bulk RNA-seq,Homo_714,MCI.vs.control; bulk RNA-seq,Homo_633,AD.vs.control; bulk RNA-seq,Homo_633,AD.vs.MCI	5
BP	GO:0007140	male meiotic nuclear division	bulk RNA-seq,Homo_723,MCI.vs.control; bulk RNA-seq,Homo_714,MCI.vs.control; bulk RNA-seq,Homo_633,AD.vs.control	3
BP	GO:0042492	gamma-delta T cell differentiation	bulk RNA-seq,Homo_723,MCI.vs.control; bulk RNA-seq,Homo_714,MCI.vs.control	2
BP	GO:0071305	cellular response to vitamin D	bulk RNA-seq,Homo_723,MCI.vs.control; bulk RNA-seq,Homo_714,MCI.vs.control; bulk RNA-seq,Homo_633,AD.vs.control	3
BP	GO:0001835	blastocyst hatching	bulk RNA-seq,Homo_723,MCI.vs.control; bulk RNA-seq,Homo_633,AD.vs.control	2
BP	GO:0035188	hatching	bulk RNA-seq,Homo_723,MCI.vs.control; bulk RNA-seq,Homo_633,AD.vs.control	2
BP	GO:0071684	organism emergence from protective structure	bulk RNA-seq,Homo_723,MCI.vs.control; bulk RNA-seq,Homo_633,AD.vs.control	2
CC	GO:0042788	polysomal ribosome	bulk RNA-seq,Homo_723,MCI.vs.control; bulk RNA-seq,Homo_714,MCI.vs.control; bulk RNA-seq,Homo_633,AD.vs.control; bulk RNA-seq,Homo_633,AD.vs.MCI; bulk RNA-seq,Homo_633,MCI.vs.control	5
BP	GO:0010921	regulation of phosphatase activity	bulk RNA-seq,Homo_723,MCI.vs.control; bulk RNA-seq,Homo_714,AD.vs.MCI; bulk RNA-seq,Homo_714,MCI.vs.control; bulk RNA-seq,Homo_633,AD.vs.control; bulk RNA-seq,Homo_633,AD.vs.MCI	5

CC	GO:0000835	ER ubiquitin ligase complex	bulk RNA-seq,Homo_723,MCI.vs.control; bulk RNA-seq,Homo_714,MCI.vs.control; bulk RNA-seq,Homo_633,AD.vs.control; bulk RNA-	4
BP	GO:0007213	G protein-coupled acetylcholine receptor signaling pathway	bulk RNA-seq,Homo_723,MCI.vs.control; bulk RNA-seq,Homo_714,MCI.vs.control	2
CC	GO:0031312	extrinsic component of organelle membrane	bulk RNA-seq,Homo_723,MCI.vs.control; bulk RNA-seq,Homo_633,AD.vs.control; bulk RNA-seq,Homo_633,AD.vs.MCI	3
BP	GO:0075509	endocytosis involved in viral entry into host cell	bulk RNA-seq,Homo_723,MCI.vs.control; bulk RNA-seq,Homo_714,MCI.vs.control; bulk RNA-seq,Homo_633,AD.vs.control; bulk RNA-	4
MF	GO:0003995	acyl-CoA dehydrogenase activity	bulk RNA-seq,Homo_723,MCI.vs.control	1
MF	GO:0052890	oxidoreductase activity, acting on the CH-CH group of donors, with a flavin as acceptor	bulk RNA-seq,Homo_723,MCI.vs.control	1
BP	GO:0071340	skeletal muscle acetylcholine-gated channel clustering	bulk RNA-seq,Homo_723,MCI.vs.control	1
MF	GO:0098641	cadherin binding involved in cell-cell adhesion	bulk RNA-seq,Homo_723,MCI.vs.control; bulk RNA-seq,Homo_714,MCI.vs.control	2
BP	GO:2001235	positive regulation of apoptotic signaling pathway	bulk RNA-seq,Homo_723,MCI.vs.control; bulk RNA-seq,Homo_714,AD.vs.MCI; bulk RNA-seq,Homo_633,AD.vs.control; bulk RNA-seq,Homo_633,AD.vs.MCI	4
BP	GO:0070561	vitamin D receptor signaling pathway	bulk RNA-seq,Homo_723,MCI.vs.control; bulk RNA-seq,Homo_714,MCI.vs.control	2
BP	GO:0000212	meiotic spindle organization	bulk RNA-seq,Homo_723,MCI.vs.control; bulk RNA-seq,Homo_714,MCI.vs.control	2
BP	GO:0046831	regulation of RNA export from nucleus	bulk RNA-seq,Homo_723,MCI.vs.control; bulk RNA-seq,Homo_714,MCI.vs.control; bulk RNA-seq,Homo_633,AD.vs.control; bulk RNA-seq,Homo_633,AD.vs.MCI; bulk RNA-seq,Homo_633,MCI.vs.control	5
BP	GO:0052372	modulation by symbiont of entry into host	bulk RNA-seq,Homo_723,MCI.vs.control; bulk RNA-seq,Homo_714,MCI.vs.control; bulk RNA-seq,Homo_633,AD.vs.control; bulk RNA-	4
MF	GO:0004033	aldo-keto reductase (NADP) activity	bulk RNA-seq,Homo_723,MCI.vs.control; bulk RNA-seq,Homo_714,MCI.vs.control; bulk RNA-seq,Homo_633,AD.vs.control; bulk RNA-	4
BP	GO:0006177	GMP biosynthetic process	bulk RNA-seq,Homo_723,MCI.vs.control; bulk RNA-seq,Homo_714,MCI.vs.control; bulk RNA-seq,Homo_633,MCI.vs.control	3
CC	GO:0035060	brahma complex	bulk RNA-seq,Homo_723,MCI.vs.control; bulk RNA-seq,Homo_633,AD.vs.control; bulk RNA-seq,Homo_633,AD.vs.MCI	3
BP	GO:0036093	germ cell proliferation	bulk RNA-seq,Homo_723,MCI.vs.control	1
BP	GO:0070307	lens fiber cell development	bulk RNA-seq,Homo_723,MCI.vs.control	1
BP	GO:0033631	cell-cell adhesion mediated by integrin	bulk RNA-seq,Homo_723,MCI.vs.control; bulk RNA-seq,Homo_714,MCI.vs.control	2
BP	GO:0032528	microvillus organization	bulk RNA-seq,Homo_723,MCI.vs.control; bulk RNA-seq,Homo_714,MCI.vs.control; bulk RNA-seq,Homo_633,AD.vs.control; bulk RNA-	4
BP	GO:0006925	inflammatory cell apoptotic process	bulk RNA-seq,Homo_723,MCI.vs.control; bulk RNA-seq,Homo_714,AD.vs.control; bulk RNA-seq,Homo_714,MCI.vs.control; bulk RNA-seq,Homo_633,AD.vs.control; bulk RNA-seq,Homo_633,AD.vs.MCI; bulk RNA-seq,Homo_633,MCI.vs.control	6
MF	GO:0031624	ubiquitin conjugating enzyme binding	bulk RNA-seq,Homo_723,MCI.vs.control; bulk RNA-seq,Homo_714,MCI.vs.control; bulk RNA-seq,Homo_633,AD.vs.control; bulk RNA-	4
BP	GO:0086100	endothelin receptor signaling pathway	bulk RNA-seq,Homo_723,MCI.vs.control; bulk RNA-seq,Homo_714,MCI.vs.control	2
BP	GO:0042996	regulation of Golgi to plasma membrane protein transport	bulk RNA-seq,Homo_723,MCI.vs.control; bulk RNA-seq,Homo_714,MCI.vs.control; bulk RNA-seq,Homo_633,AD.vs.control	3
MF	GO:0005381	iron ion transmembrane transporter activity	bulk RNA-seq,Homo_723,MCI.vs.control	1
BP	GO:0042178	xenobiotic catabolic process	bulk RNA-seq,Homo_723,MCI.vs.control; bulk RNA-seq,Homo_714,MCI.vs.control	2
BP	GO:0006577	amino-acid betaine metabolic process	bulk RNA-seq,Homo_723,MCI.vs.control; bulk RNA-seq,Homo_714,MCI.vs.control	2
CC	GO:0140288	GBAF complex	bulk RNA-seq,Homo_723,MCI.vs.control; bulk RNA-seq,Homo_633,AD.vs.control; bulk RNA-seq,Homo_633,AD.vs.MCI	3
BP	GO:0048490	anterograde synaptic vesicle transport	bulk RNA-seq,Homo_723,MCI.vs.control; bulk RNA-seq,Homo_714,AD.vs.control; bulk RNA-seq,Homo_714,MCI.vs.control; bulk RNA-seq,Homo_633,AD.vs.control; bulk RNA-seq,Homo_633,AD.vs.MCI	5
BP	GO:0099514	synaptic vesicle cytoskeletal transport	bulk RNA-seq,Homo_723,MCI.vs.control; bulk RNA-seq,Homo_714,AD.vs.control; bulk RNA-seq,Homo_714,MCI.vs.control; bulk RNA-seq,Homo_633,AD.vs.control; bulk RNA-seq,Homo_633,AD.vs.MCI	5
BP	GO:0099517	synaptic vesicle transport along microtubule	bulk RNA-seq,Homo_723,MCI.vs.control; bulk RNA-seq,Homo_714,AD.vs.control; bulk RNA-seq,Homo_714,MCI.vs.control; bulk RNA-seq,Homo_633,AD.vs.control; bulk RNA-seq,Homo_633,AD.vs.MCI	5
CC	GO:0097431	mitotic spindle pole	bulk RNA-seq,Homo_723,MCI.vs.control; bulk RNA-seq,Homo_714,MCI.vs.control; bulk RNA-seq,Homo_633,AD.vs.control; bulk RNA-	4
MF	GO:0000900	mRNA regulatory element binding translation repressor	bulk RNA-seq,Homo_723,MCI.vs.control; bulk RNA-seq,Homo_714,MCI.vs.control; bulk RNA-seq,Homo_633,MCI.vs.control	3
BP	GO:0048499	synaptic vesicle membrane organization	bulk RNA-seq,Homo_723,MCI.vs.control; bulk RNA-seq,Homo_714,MCI.vs.control; bulk RNA-seq,Homo_633,AD.vs.control	3
BP	GO:0044088	regulation of vacuole organization	bulk RNA-seq,Homo_723,MCI.vs.control; bulk RNA-seq,Homo_714,MCI.vs.control; bulk RNA-seq,Homo_633,AD.vs.control; bulk RNA-	4
BP	GO:0050728	negative regulation of inflammatory response	bulk RNA-seq,Homo_723,MCI.vs.control; bulk RNA-seq,Homo_714,AD.vs.MCI; bulk RNA-seq,Homo_633,AD.vs.control	3
BP	GO:0070142	synaptic vesicle budding	bulk RNA-seq,Homo_723,MCI.vs.control; bulk RNA-seq,Homo_633,AD.vs.control; bulk RNA-seq,Homo_633,AD.vs.MCI	3
BP	GO:0002676	regulation of chronic inflammatory response	bulk RNA-seq,Homo_723,MCI.vs.control; bulk RNA-seq,Homo_714,MCI.vs.control; bulk RNA-seq,Homo_633,MCI.vs.control	3
MF	GO:0052742	phosphatidylinositol kinase activity	bulk RNA-seq,Homo_723,MCI.vs.control; bulk RNA-seq,Homo_714,MCI.vs.control; bulk RNA-seq,Homo_633,AD.vs.control; bulk RNA-	4
BP	GO:0030050	vesicle transport along actin filament	bulk RNA-seq,Homo_723,MCI.vs.control; bulk RNA-seq,Homo_714,MCI.vs.control; bulk RNA-seq,Homo_633,AD.vs.control	3
BP	GO:0030157	pancreatic juice secretion	bulk RNA-seq,Homo_723,MCI.vs.control; bulk RNA-seq,Homo_714,MCI.vs.control	2
BP	GO:1905666	regulation of protein localization to endosome	bulk RNA-seq,Homo_723,MCI.vs.control; bulk RNA-seq,Homo_714,MCI.vs.control; bulk RNA-seq,Homo_633,AD.vs.control; bulk RNA-	4
BP	GO:0048548	regulation of pinocytosis	bulk RNA-seq,Homo_723,MCI.vs.control; bulk RNA-seq,Homo_714,MCI.vs.control	2
BP	GO:0140354	lipid import into cell	bulk RNA-seq,Homo_723,MCI.vs.control; bulk RNA-seq,Homo_714,MCI.vs.control	2
CC	GO:1905360	GTPase complex	bulk RNA-seq,Homo_723,MCI.vs.control; bulk RNA-seq,Homo_714,MCI.vs.control; bulk RNA-seq,Homo_633,AD.vs.control; bulk RNA-	4
MF	GO:0016765	transferase activity, transferring alkyl or aryl (other than	bulk RNA-seq,Homo_723,MCI.vs.control; bulk RNA-seq,Homo_714,MCI.vs.control; bulk RNA-seq,Homo_633,AD.vs.control; bulk RNA-	4
MF	GO:0015924	mannosyl-oligosaccharide mannosidase activity	bulk RNA-seq,Homo_723,MCI.vs.control; bulk RNA-seq,Homo_714,MCI.vs.control; bulk RNA-seq,Homo_633,AD.vs.control; bulk RNA-seq,Homo_633,AD.vs.MCI; bulk RNA-seq,Homo_633,MCI.vs.control	5
BP	GO:0001778	plasma membrane repair	bulk RNA-seq,Homo_723,MCI.vs.control; bulk RNA-seq,Homo_714,MCI.vs.control; bulk RNA-seq,Homo_633,AD.vs.control; bulk RNA-	4
BP	GO:0071468	cellular response to acidic pH	bulk RNA-seq,Homo_723,MCI.vs.control; bulk RNA-seq,Homo_714,MCI.vs.control	2
BP	GO:0046929	negative regulation of neurotransmitter secretion	bulk RNA-seq,Homo_723,MCI.vs.control; bulk RNA-seq,Homo_633,AD.vs.control	2

BP	GO:0043951	negative regulation of cAMP-mediated signaling	bulk RNA-seq,Homo_723,MCI.vs.control; bulk RNA-seq,Homo_714,MCI.vs.control	2
BP	GO:0002467	germinal center formation	bulk RNA-seq,Homo_723,MCI.vs.control; bulk RNA-seq,Homo_714,MCI.vs.control; bulk RNA-seq,Homo_633,AD.vs.control; bulk RNA-seq,Homo_633,AD.vs.MCI; bulk RNA-seq,Homo_633,MCI.vs.control	5
BP	GO:0009167	purine ribonucleoside monophosphate metabolic process	bulk RNA-seq,Homo_723,MCI.vs.control; bulk RNA-seq,Homo_714,MCI.vs.control; bulk RNA-seq,Homo_633,AD.vs.control; bulk RNA-	4
BP	GO:0090136	epithelial cell-cell adhesion	bulk RNA-seq,Homo_723,MCI.vs.control; bulk RNA-seq,Homo_714,MCI.vs.control	2
MF	GO:0070403	NAD+ binding	bulk RNA-seq,Homo_723,MCI.vs.control; bulk RNA-seq,Homo_714,MCI.vs.control; bulk RNA-seq,Homo_633,MCI.vs.control	3
BP	GO:0000097	sulfur amino acid biosynthetic process	bulk RNA-seq,Homo_723,MCI.vs.control; bulk RNA-seq,Homo_633,AD.vs.control; bulk RNA-seq,Homo_633,AD.vs.MCI	3
BP	GO:2000288	positive regulation of myoblast proliferation	bulk RNA-seq,Homo_723,MCI.vs.control; bulk RNA-seq,Homo_714,MCI.vs.control; bulk RNA-seq,Homo_633,AD.vs.control	3
BP	GO:0007062	sister chromatid cohesion	bulk RNA-seq,Homo_723,MCI.vs.control; bulk RNA-seq,Homo_714,MCI.vs.control; bulk RNA-seq,Homo_633,AD.vs.control; bulk RNA-	4
MF	GO:0005537	mannose binding	bulk RNA-seq,Homo_723,MCI.vs.control; bulk RNA-seq,Homo_714,AD.vs.MCI; bulk RNA-seq,Homo_714,MCI.vs.control	3
BP	GO:0090239	regulation of histone H4 acetylation	bulk RNA-seq,Homo_723,MCI.vs.control; bulk RNA-seq,Homo_714,MCI.vs.control; bulk RNA-seq,Homo_633,AD.vs.control; bulk RNA-seq,Homo_633,AD.vs.MCI; bulk RNA-seq,Homo_633,MCI.vs.control	5
BP	GO:0032755	positive regulation of interleukin-6 production	bulk RNA-seq,Homo_723,MCI.vs.control; bulk RNA-seq,Homo_714,MCI.vs.control; bulk RNA-seq,Homo_633,AD.vs.control; bulk RNA-	4
BP	GO:1901623	regulation of lymphocyte chemotaxis	bulk RNA-seq,Homo_723,MCI.vs.control; bulk RNA-seq,Homo_714,MCI.vs.control; bulk RNA-seq,Homo_633,AD.vs.control	3
BP	GO:0014029	neural crest formation	bulk RNA-seq,Homo_723,MCI.vs.control; bulk RNA-seq,Homo_714,AD.vs.MCI; bulk RNA-seq,Homo_714,MCI.vs.control	3
MF	GO:0016805	dipeptidase activity	bulk RNA-seq,Homo_723,MCI.vs.control	1
CC	GO:0000159	protein phosphatase type 2A complex	bulk RNA-seq,Homo_723,MCI.vs.control; bulk RNA-seq,Homo_714,MCI.vs.control; bulk RNA-seq,Homo_633,AD.vs.control	3
BP	GO:0002483	antigen processing and presentation of endogenous peptide	bulk RNA-seq,Homo_723,MCI.vs.control; bulk RNA-seq,Homo_714,MCI.vs.control; scRNA-seq,SRP215507,CD8+ T cell_8-MCI.vs.control	3
BP	GO:0006991	response to sterol depletion	bulk RNA-seq,Homo_723,MCI.vs.control; bulk RNA-seq,Homo_714,MCI.vs.control; bulk RNA-seq,Homo_633,AD.vs.control; bulk RNA-	4
BP	GO:0001660	fever generation	bulk RNA-seq,Homo_723,MCI.vs.control; bulk RNA-seq,Homo_714,MCI.vs.control	2
MF	GO:0008106	alcohol dehydrogenase (NADP+) activity	bulk RNA-seq,Homo_723,MCI.vs.control; bulk RNA-seq,Homo_714,MCI.vs.control; bulk RNA-seq,Homo_633,AD.vs.control; bulk RNA-seq,Homo_633,AD.vs.MCI; scRNA-seq,SRP330776,Naive CD8+ T cell_3-AD.vs.control	5
BP	GO:1902600	proton transmembrane transport	bulk RNA-seq,Homo_723,MCI.vs.control; bulk RNA-seq,Homo_633,AD.vs.control; bulk RNA-seq,Homo_633,AD.vs.MCI	3
BP	GO:1904526	regulation of microtubule binding	bulk RNA-seq,Homo_723,MCI.vs.control; bulk RNA-seq,Homo_714,MCI.vs.control	2
BP	GO:0098534	centriole assembly	bulk RNA-seq,Homo_723,MCI.vs.control; bulk RNA-seq,Homo_714,MCI.vs.control; bulk RNA-seq,Homo_633,AD.vs.control; bulk RNA-	4
BP	GO:2000269	regulation of fibroblast apoptotic process	bulk RNA-seq,Homo_723,MCI.vs.control; bulk RNA-seq,Homo_714,MCI.vs.control	2
BP	GO:1903358	regulation of Golgi organization	bulk RNA-seq,Homo_723,MCI.vs.control; bulk RNA-seq,Homo_714,MCI.vs.control; bulk RNA-seq,Homo_633,AD.vs.control; bulk RNA-	4
BP	GO:0045070	positive regulation of viral genome replication	bulk RNA-seq,Homo_723,MCI.vs.control; bulk RNA-seq,Homo_714,MCI.vs.control; bulk RNA-seq,Homo_633,AD.vs.control	3
BP	GO:0015732	prostaglandin transport	bulk RNA-seq,Homo_723,MCI.vs.control; bulk RNA-seq,Homo_633,AD.vs.control; bulk RNA-seq,Homo_633,AD.vs.MCI	3
MF	GO:0004550	nucleoside diphosphate kinase activity	bulk RNA-seq,Homo_723,MCI.vs.control; bulk RNA-seq,Homo_714,MCI.vs.control; bulk RNA-seq,Homo_633,AD.vs.control	3
BP	GO:0051984	positive regulation of chromosome segregation	bulk RNA-seq,Homo_723,MCI.vs.control; bulk RNA-seq,Homo_714,MCI.vs.control; bulk RNA-seq,Homo_633,AD.vs.control; bulk RNA-seq,Homo_633,AD.vs.MCI; bulk RNA-seq,Homo_633,MCI.vs.control	5
BP	GO:0006004	fucose metabolic process	bulk RNA-seq,Homo_723,MCI.vs.control	1
BP	GO:0001773	myeloid dendritic cell activation	bulk RNA-seq,Homo_723,MCI.vs.control; bulk RNA-seq,Homo_714,MCI.vs.control; bulk RNA-seq,Homo_633,AD.vs.control; bulk RNA-	4
BP	GO:0072528	pyrimidine-containing compound biosynthetic process	bulk RNA-seq,Homo_723,MCI.vs.control; bulk RNA-seq,Homo_714,MCI.vs.control; bulk RNA-seq,Homo_633,AD.vs.control; bulk RNA-	4
BP	GO:0035907	dorsal aorta development	bulk RNA-seq,Homo_723,MCI.vs.control	1
BP	GO:1902043	positive regulation of extrinsic apoptotic signaling pathway via death domain receptors	bulk RNA-seq,Homo_723,MCI.vs.control; bulk RNA-seq,Homo_633,AD.vs.control	2
MF	GO:0000995	RNA polymerase III general transcription initiation factor activity	bulk RNA-seq,Homo_723,MCI.vs.control; bulk RNA-seq,Homo_714,MCI.vs.control; bulk RNA-seq,Homo_633,AD.vs.control; bulk RNA-seq,Homo_633,AD.vs.MCI; bulk RNA-seq,Homo_633,MCI.vs.control	5
BP	GO:0014745	negative regulation of muscle adaptation	bulk RNA-seq,Homo_723,MCI.vs.control; bulk RNA-seq,Homo_714,AD.vs.MCI; bulk RNA-seq,Homo_714,MCI.vs.control	3
BP	GO:0035336	long-chain fatty-acyl-CoA metabolic process	bulk RNA-seq,Homo_723,MCI.vs.control; bulk RNA-seq,Homo_714,MCI.vs.control; bulk RNA-seq,Homo_633,AD.vs.control; bulk RNA-	4
MF	GO:0031957	very long-chain fatty acid-CoA ligase activity	bulk RNA-seq,Homo_723,MCI.vs.control	1
CC	GO:0030140	trans-Golgi network transport vesicle	bulk RNA-seq,Homo_723,MCI.vs.control; bulk RNA-seq,Homo_714,MCI.vs.control; bulk RNA-seq,Homo_633,AD.vs.control	3
MF	GO:0016879	ligase activity, forming carbon-nitrogen bonds	bulk RNA-seq,Homo_723,MCI.vs.control; bulk RNA-seq,Homo_714,MCI.vs.control; bulk RNA-seq,Homo_633,AD.vs.control; bulk RNA-	4
BP	GO:0031116	positive regulation of microtubule polymerization	bulk RNA-seq,Homo_723,MCI.vs.control; bulk RNA-seq,Homo_714,MCI.vs.control; bulk RNA-seq,Homo_633,AD.vs.control; bulk RNA-	4
BP	GO:0046629	gamma-delta T cell activation	bulk RNA-seq,Homo_723,MCI.vs.control; bulk RNA-seq,Homo_714,MCI.vs.control; bulk RNA-seq,Homo_633,AD.vs.control; bulk RNA-seq,Homo_633,AD.vs.MCI; bulk RNA-seq,Homo_633,MCI.vs.control	5
BP	GO:1901658	glycosyl compound catabolic process	bulk RNA-seq,Homo_723,MCI.vs.control; bulk RNA-seq,Homo_714,MCI.vs.control; bulk RNA-seq,Homo_633,AD.vs.control; bulk RNA-	4
BP	GO:0010819	regulation of T cell chemotaxis	bulk RNA-seq,Homo_723,MCI.vs.control; bulk RNA-seq,Homo_714,MCI.vs.control	2
MF	GO:0015556	C4-dicarboxylate transmembrane transporter activity	bulk RNA-seq,Homo_723,MCI.vs.control; bulk RNA-seq,Homo_633,AD.vs.control	2
BP	GO:0010675	regulation of cellular carbohydrate metabolic process	bulk RNA-seq,Homo_723,MCI.vs.control; bulk RNA-seq,Homo_633,AD.vs.control; bulk RNA-seq,Homo_633,AD.vs.MCI	3
BP	GO:0032088	negative regulation of NF-kappaB transcription factor activity	bulk RNA-seq,Homo_723,MCI.vs.control; bulk RNA-seq,Homo_714,MCI.vs.control; bulk RNA-seq,Homo_633,AD.vs.control; bulk RNA-	4
BP	GO:0071470	cellular response to osmotic stress	bulk RNA-seq,Homo_723,MCI.vs.control; bulk RNA-seq,Homo_714,MCI.vs.control; bulk RNA-seq,Homo_633,AD.vs.control; bulk RNA-	4
BP	GO:0044346	fibroblast apoptotic process	bulk RNA-seq,Homo_723,MCI.vs.control; bulk RNA-seq,Homo_714,MCI.vs.control; bulk RNA-seq,Homo_633,AD.vs.control	3
MF	GO:0031543	peptidyl-proline dioxygenase activity	bulk RNA-seq,Homo_723,MCI.vs.control; bulk RNA-seq,Homo_714,AD.vs.control; bulk RNA-seq,Homo_714,MCI.vs.control; bulk RNA-	4
BP	GO:0046415	urate metabolic process	bulk RNA-seq,Homo_723,MCI.vs.control; bulk RNA-seq,Homo_714,MCI.vs.control	2

BP	GO:1902855	regulation of non-motile cilium assembly	bulk RNA-seq,Homo_723,MCI.vs.control; bulk RNA-seq,Homo_714,MCI.vs.control; bulk RNA-seq,Homo_633,AD.vs.control; bulk RNA-	4
BP	GO:0032482	Rab protein signal transduction	bulk RNA-seq,Homo_723,MCI.vs.control; bulk RNA-seq,Homo_714,MCI.vs.control; bulk RNA-seq,Homo_633,AD.vs.control; bulk RNA-seq,Homo_633,AD.vs.MCI; bulk RNA-seq,Homo_633,MCI.vs.control	5
BP	GO:0033079	immature T cell proliferation	bulk RNA-seq,Homo_723,MCI.vs.control; bulk RNA-seq,Homo_714,MCI.vs.control; bulk RNA-seq,Homo_633,AD.vs.control	3
BP	GO:0033080	immature T cell proliferation in thymus	bulk RNA-seq,Homo_723,MCI.vs.control; bulk RNA-seq,Homo_714,MCI.vs.control; bulk RNA-seq,Homo_633,AD.vs.control	3
CC	GO:0005677	chromatin silencing complex	bulk RNA-seq,Homo_723,MCI.vs.control; bulk RNA-seq,Homo_714,MCI.vs.control; bulk RNA-seq,Homo_633,AD.vs.control; bulk RNA-seq,Homo_633,AD.vs.MCI; bulk RNA-seq,Homo_633,MCI.vs.control	5
MF	GO:0004707	MAP kinase activity	bulk RNA-seq,Homo_723,MCI.vs.control	1
MF	GO:0071889	14-3-3 protein binding	bulk RNA-seq,Homo_723,MCI.vs.control; bulk RNA-seq,Homo_714,AD.vs.MCI; bulk RNA-seq,Homo_714,MCI.vs.control; bulk RNA-	4
BP	GO:0046856	phosphatidylinositol dephosphorylation	bulk RNA-seq,Homo_723,MCI.vs.control; bulk RNA-seq,Homo_714,MCI.vs.control; bulk RNA-seq,Homo_633,AD.vs.control; bulk RNA-seq,Homo_633,AD.vs.MCI; bulk RNA-seq,Homo_633,MCI.vs.control	5
BP	GO:0010759	positive regulation of macrophage chemotaxis	bulk RNA-seq,Homo_723,MCI.vs.control; bulk RNA-seq,Homo_714,AD.vs.MCI; bulk RNA-seq,Homo_714,MCI.vs.control	3
BP	GO:0036092	phosphatidylinositol-3-phosphate biosynthetic process	bulk RNA-seq,Homo_723,MCI.vs.control; bulk RNA-seq,Homo_714,AD.vs.control; bulk RNA-seq,Homo_714,MCI.vs.control; bulk RNA-seq,Homo_633,AD.vs.control; bulk RNA-seq,Homo_633,AD.vs.MCI	5
BP	GO:1900424	regulation of defense response to bacterium	bulk RNA-seq,Homo_723,MCI.vs.control; bulk RNA-seq,Homo_714,MCI.vs.control; bulk RNA-seq,Homo_633,AD.vs.control	3
CC	GO:0031010	ISWI-type complex	bulk RNA-seq,Homo_723,MCI.vs.control; bulk RNA-seq,Homo_714,MCI.vs.control; bulk RNA-seq,Homo_633,AD.vs.control; bulk RNA-	4
BP	GO:2000615	regulation of histone H3-K9 acetylation	bulk RNA-seq,Homo_723,MCI.vs.control; bulk RNA-seq,Homo_714,MCI.vs.control; bulk RNA-seq,Homo_633,AD.vs.control; bulk RNA-	4
BP	GO:0000022	mitotic spindle elongation	bulk RNA-seq,Homo_723,MCI.vs.control; bulk RNA-seq,Homo_714,MCI.vs.control	2
BP	GO:0000727	double-strand break repair via break-induced replication	bulk RNA-seq,Homo_723,MCI.vs.control; bulk RNA-seq,Homo_714,MCI.vs.control; bulk RNA-seq,Homo_633,MCI.vs.control	3
BP	GO:0070670	response to interleukin-4	bulk RNA-seq,Homo_723,MCI.vs.control	1
BP	GO:0010165	response to X-ray	bulk RNA-seq,Homo_723,MCI.vs.control; bulk RNA-seq,Homo_633,AD.vs.control; bulk RNA-seq,Homo_633,AD.vs.MCI	3
CC	GO:0033116	endoplasmic reticulum-Golgi intermediate compartment membrane	bulk RNA-seq,Homo_723,MCI.vs.control; bulk RNA-seq,Homo_714,AD.vs.MCI; bulk RNA-seq,Homo_714,MCI.vs.control; bulk RNA-seq,Homo_633,AD.vs.control; bulk RNA-seq,Homo_633,AD.vs.MCI	5
BP	GO:0099500	vesicle fusion to plasma membrane	bulk RNA-seq,Homo_723,MCI.vs.control; bulk RNA-seq,Homo_714,MCI.vs.control	2
BP	GO:0099515	actin filament-based transport	bulk RNA-seq,Homo_723,MCI.vs.control; bulk RNA-seq,Homo_714,MCI.vs.control; bulk RNA-seq,Homo_633,AD.vs.control	3
BP	GO:0072595	maintenance of protein localization in organelle	bulk RNA-seq,Homo_723,MCI.vs.control; bulk RNA-seq,Homo_714,MCI.vs.control; bulk RNA-seq,Homo_633,AD.vs.control; bulk RNA-	4
BP	GO:0046886	positive regulation of hormone biosynthetic process	bulk RNA-seq,Homo_723,MCI.vs.control; bulk RNA-seq,Homo_714,MCI.vs.control; bulk RNA-seq,Homo_633,AD.vs.control	3
CC	GO:0001939	female pronucleus	bulk RNA-seq,Homo_723,MCI.vs.control; bulk RNA-seq,Homo_714,MCI.vs.control	2
BP	GO:0098877	neurotransmitter receptor transport to plasma membrane	bulk RNA-seq,Homo_723,MCI.vs.control; bulk RNA-seq,Homo_714,MCI.vs.control; bulk RNA-seq,Homo_633,AD.vs.control	3
BP	GO:0060148	positive regulation of post-transcriptional gene silencing	bulk RNA-seq,Homo_723,MCI.vs.control; bulk RNA-seq,Homo_714,MCI.vs.control; bulk RNA-seq,Homo_633,AD.vs.control	3
BP	GO:1900370	positive regulation of post-transcriptional gene silencing by	bulk RNA-seq,Homo_723,MCI.vs.control; bulk RNA-seq,Homo_714,MCI.vs.control; bulk RNA-seq,Homo_633,AD.vs.control	3
MF	GO:0034236	protein kinase A catalytic subunit binding	bulk RNA-seq,Homo_723,MCI.vs.control; bulk RNA-seq,Homo_714,MCI.vs.control; bulk RNA-seq,Homo_633,AD.vs.control	3
BP	GO:0072525	pyridine-containing compound biosynthetic process	bulk RNA-seq,Homo_723,MCI.vs.control; bulk RNA-seq,Homo_714,MCI.vs.control; bulk RNA-seq,Homo_633,AD.vs.control	3
BP	GO:0032613	interleukin-10 production	bulk RNA-seq,Homo_723,MCI.vs.control; bulk RNA-seq,Homo_714,MCI.vs.control; bulk RNA-seq,Homo_633,AD.vs.control	3
BP	GO:0032653	regulation of interleukin-10 production	bulk RNA-seq,Homo_723,MCI.vs.control; bulk RNA-seq,Homo_714,MCI.vs.control; bulk RNA-seq,Homo_633,AD.vs.control	3
BP	GO:0032435	negative regulation of proteasomal ubiquitin-dependent protein catabolic process	bulk RNA-seq,Homo_723,MCI.vs.control; bulk RNA-seq,Homo_714,MCI.vs.control; bulk RNA-seq,Homo_633,AD.vs.control; bulk RNA-seq,Homo_633,AD.vs.MCI	4
CC	GO:0000940	outer kinetochore	bulk RNA-seq,Homo_723,MCI.vs.control; bulk RNA-seq,Homo_714,MCI.vs.control	2
BP	GO:0070778	L-aspartate transmembrane transport	bulk RNA-seq,Homo_723,MCI.vs.control	1
BP	GO:0051307	meiotic chromosome separation	bulk RNA-seq,Homo_723,MCI.vs.control; bulk RNA-seq,Homo_714,MCI.vs.control; bulk RNA-seq,Homo_633,AD.vs.control	3
MF	GO:0044548	S100 protein binding	bulk RNA-seq,Homo_723,MCI.vs.control; bulk RNA-seq,Homo_633,AD.vs.control; bulk RNA-seq,Homo_633,AD.vs.MCI	3
MF	GO:0070577	lysine-acetylated histone binding	bulk RNA-seq,Homo_723,MCI.vs.control; bulk RNA-seq,Homo_714,MCI.vs.control; bulk RNA-seq,Homo_633,AD.vs.control; bulk RNA-	4
MF	GO:0140033	acetylation-dependent protein binding	bulk RNA-seq,Homo_723,MCI.vs.control; bulk RNA-seq,Homo_714,MCI.vs.control; bulk RNA-seq,Homo_633,AD.vs.control; bulk RNA-	4
BP	GO:1903204	negative regulation of oxidative stress-induced neuron death	bulk RNA-seq,Homo_723,MCI.vs.control; bulk RNA-seq,Homo_714,MCI.vs.control; bulk RNA-seq,Homo_633,AD.vs.control; bulk RNA-	4
BP	GO:0071073	positive regulation of phospholipid biosynthetic process	bulk RNA-seq,Homo_723,MCI.vs.control	1
BP	GO:0046834	lipid phosphorylation	bulk RNA-seq,Homo_723,MCI.vs.control; bulk RNA-seq,Homo_714,MCI.vs.control; bulk RNA-seq,Homo_633,AD.vs.control	3
BP	GO:1902229	regulation of intrinsic apoptotic signaling pathway in response to DNA damage	bulk RNA-seq,Homo_723,MCI.vs.control; bulk RNA-seq,Homo_714,MCI.vs.control; bulk RNA-seq,Homo_633,AD.vs.control; bulk RNA-seq,Homo_633,AD.vs.MCI	4
MF	GO:0003906	DNA-(apurinic or apyrimidinic site) endonuclease activity	bulk RNA-seq,Homo_723,MCI.vs.control; bulk RNA-seq,Homo_714,MCI.vs.control; bulk RNA-seq,Homo_633,AD.vs.control; bulk RNA-	4
MF	GO:0032794	GTPase activating protein binding	bulk RNA-seq,Homo_723,MCI.vs.control; bulk RNA-seq,Homo_714,MCI.vs.control; bulk RNA-seq,Homo_633,AD.vs.control; bulk RNA-seq,Homo_633,AD.vs.MCI; bulk RNA-seq,Homo_633,MCI.vs.control	5
BP	GO:1902807	negative regulation of cell cycle G1/S phase transition	bulk RNA-seq,Homo_723,MCI.vs.control; bulk RNA-seq,Homo_714,AD.vs.MCI; bulk RNA-seq,Homo_714,MCI.vs.control; bulk RNA-seq,Homo_633,AD.vs.control; bulk RNA-seq,Homo_633,AD.vs.MCI	5
BP	GO:0090715	immunological memory formation process	bulk RNA-seq,Homo_723,MCI.vs.control; bulk RNA-seq,Homo_714,MCI.vs.control	2
MF	GO:0046975	histone methyltransferase activity (H3-K36 specific)	bulk RNA-seq,Homo_723,MCI.vs.control; bulk RNA-seq,Homo_714,MCI.vs.control	2
BP	GO:1905953	negative regulation of lipid localization	bulk RNA-seq,Homo_723,MCI.vs.control; bulk RNA-seq,Homo_714,AD.vs.MCI; bulk RNA-seq,Homo_714,MCI.vs.control; bulk RNA-seq,Homo_633,AD.vs.control; bulk RNA-seq,Homo_633,AD.vs.MCI	5
CC	GO:1990531	phospholipid-translocating ATPase complex	bulk RNA-seq,Homo_723,MCI.vs.control; bulk RNA-seq,Homo_714,MCI.vs.control	2

BP	GO:0033169	histone H3-K9 demethylation	bulk RNA-seq,Homo_723,MCI.vs.control; bulk RNA-seq,Homo_714,MCI.vs.control; bulk RNA-seq,Homo_633,AD.vs.control; bulk RNA-	4
BP	GO:0031061	negative regulation of histone methylation	bulk RNA-seq,Homo_723,MCI.vs.control; bulk RNA-seq,Homo_714,MCI.vs.control; bulk RNA-seq,Homo_633,AD.vs.control; bulk RNA-	4
BP	GO:0002921	negative regulation of humoral immune response	bulk RNA-seq,Homo_723,MCI.vs.control; bulk RNA-seq,Homo_714,MCI.vs.control; bulk RNA-seq,Homo_633,AD.vs.control; bulk RNA-	4
BP	GO:0050868	negative regulation of T cell activation	bulk RNA-seq,Homo_723,MCI.vs.control; bulk RNA-seq,Homo_714,MCI.vs.control; bulk RNA-seq,Homo_633,AD.vs.control; bulk RNA-	4
BP	GO:0072677	eosinophil migration	bulk RNA-seq,Homo_723,MCI.vs.control; bulk RNA-seq,Homo_714,AD.vs.MCI; bulk RNA-seq,Homo_714,MCI.vs.control	3
BP	GO:0048143	astrocyte activation	bulk RNA-seq,Homo_723,MCI.vs.control; bulk RNA-seq,Homo_633,AD.vs.control	2
BP	GO:0007076	mitotic chromosome condensation	bulk RNA-seq,Homo_723,MCI.vs.control; bulk RNA-seq,Homo_714,MCI.vs.control; bulk RNA-seq,Homo_633,AD.vs.control; bulk RNA-	4
BP	GO:0021670	lateral ventricle development	bulk RNA-seq,Homo_723,MCI.vs.control; bulk RNA-seq,Homo_714,MCI.vs.control; bulk RNA-seq,Homo_633,AD.vs.control; bulk RNA-	4
BP	GO:1904037	positive regulation of epithelial cell apoptotic process	bulk RNA-seq,Homo_723,MCI.vs.control; bulk RNA-seq,Homo_714,AD.vs.MCI; bulk RNA-seq,Homo_714,MCI.vs.control; bulk RNA-seq,Homo_633,AD.vs.control; bulk RNA-seq,Homo_633,AD.vs.MCI	5
BP	GO:0071218	cellular response to misfolded protein	bulk RNA-seq,Homo_723,MCI.vs.control; bulk RNA-seq,Homo_714,MCI.vs.control; bulk RNA-seq,Homo_633,AD.vs.control; bulk RNA-seq,Homo_633,AD.vs.MCI; bulk RNA-seq,Homo_633,MCI.vs.control	5
MF	GO:0016407	acetyltransferase activity	bulk RNA-seq,Homo_723,MCI.vs.control; bulk RNA-seq,Homo_714,MCI.vs.control; bulk RNA-seq,Homo_633,AD.vs.control; bulk RNA-	4
BP	GO:0010906	regulation of glucose metabolic process	bulk RNA-seq,Homo_723,MCI.vs.control; bulk RNA-seq,Homo_714,MCI.vs.control; bulk RNA-seq,Homo_633,AD.vs.control; bulk RNA-	4
BP	GO:0051131	chaperone-mediated protein complex assembly	bulk RNA-seq,Homo_723,MCI.vs.control; bulk RNA-seq,Homo_633,AD.vs.control	2
BP	GO:0006977	DNA damage response, signal transduction by p53 class mediator resulting in cell cycle arrest	bulk RNA-seq,Homo_723,MCI.vs.control; bulk RNA-seq,Homo_714,MCI.vs.control; bulk RNA-seq,Homo_633,AD.vs.control; bulk RNA-seq,Homo_633,AD.vs.MCI	4
BP	GO:0046519	sphingoid metabolic process	bulk RNA-seq,Homo_723,MCI.vs.control; bulk RNA-seq,Homo_633,AD.vs.control; bulk RNA-seq,Homo_633,MCI.vs.control	3
MF	GO:0036002	pre-mRNA binding	bulk RNA-seq,Homo_723,MCI.vs.control; bulk RNA-seq,Homo_633,AD.vs.control; bulk RNA-seq,Homo_633,AD.vs.MCI	3
CC	GO:0030670	phagocytic vesicle membrane	bulk RNA-seq,Homo_723,MCI.vs.control; bulk RNA-seq,Homo_714,MCI.vs.control; bulk RNA-seq,Homo_633,AD.vs.control; bulk RNA-seq,Homo_633,AD.vs.MCI; bulk RNA-seq,SRP223445,AD.vs.control	5
BP	GO:0009086	methionine biosynthetic process	bulk RNA-seq,Homo_723,MCI.vs.control; bulk RNA-seq,Homo_633,AD.vs.control; bulk RNA-seq,Homo_633,AD.vs.MCI	3
MF	GO:0008483	transaminase activity	bulk RNA-seq,Homo_723,MCI.vs.control	1
BP	GO:0030214	hyaluronan catabolic process	bulk RNA-seq,Homo_723,MCI.vs.control; bulk RNA-seq,Homo_714,MCI.vs.control; bulk RNA-seq,Homo_633,AD.vs.control	3
BP	GO:0045023	G0 to G1 transition	bulk RNA-seq,Homo_723,MCI.vs.control; bulk RNA-seq,Homo_633,AD.vs.control; bulk RNA-seq,Homo_633,AD.vs.MCI	3
BP	GO:1904380	endoplasmic reticulum mannose trimming	bulk RNA-seq,Homo_723,MCI.vs.control; bulk RNA-seq,Homo_714,MCI.vs.control; bulk RNA-seq,Homo_633,AD.vs.control; bulk RNA-seq,Homo_633,AD.vs.MCI; bulk RNA-seq,Homo_633,MCI.vs.control	5
BP	GO:0048711	positive regulation of astrocyte differentiation	bulk RNA-seq,Homo_723,MCI.vs.control	1
BP	GO:2000785	regulation of autophagosome assembly	bulk RNA-seq,Homo_723,MCI.vs.control; bulk RNA-seq,Homo_714,MCI.vs.control; bulk RNA-seq,Homo_633,AD.vs.control; bulk RNA-	4
BP	GO:0097320	plasma membrane tubulation	bulk RNA-seq,Homo_723,MCI.vs.control; bulk RNA-seq,Homo_714,MCI.vs.control; bulk RNA-seq,Homo_633,AD.vs.control; bulk RNA-	4
BP	GO:0019885	antigen processing and presentation of endogenous peptide antigen via MHC class I	bulk RNA-seq,Homo_723,MCI.vs.control; bulk RNA-seq,Homo_714,MCI.vs.control; bulk RNA-seq,Homo_633,MCI.vs.control; scRNA-seq,SRP215507,CD8+ T cell_8-MCI.vs.control	4
BP	GO:0051444	negative regulation of ubiquitin-protein transferase activity	bulk RNA-seq,Homo_723,MCI.vs.control; bulk RNA-seq,Homo_714,MCI.vs.control; bulk RNA-seq,Homo_633,AD.vs.control; bulk RNA-	4
MF	GO:0000099	sulfur amino acid transmembrane transporter activity	bulk RNA-seq,Homo_723,MCI.vs.control; bulk RNA-seq,Homo_714,MCI.vs.control	2
BP	GO:2000773	negative regulation of cellular senescence	bulk RNA-seq,Homo_723,MCI.vs.control	1
BP	GO:0009262	deoxyribonucleotide metabolic process	bulk RNA-seq,Homo_723,MCI.vs.control; bulk RNA-seq,Homo_714,MCI.vs.control; bulk RNA-seq,Homo_633,AD.vs.control; bulk RNA-	4
BP	GO:0019692	deoxyribose phosphate metabolic process	bulk RNA-seq,Homo_723,MCI.vs.control; bulk RNA-seq,Homo_714,MCI.vs.control; bulk RNA-seq,Homo_633,AD.vs.control; bulk RNA-	4
BP	GO:1902074	response to salt	bulk RNA-seq,Homo_723,MCI.vs.control	1
BP	GO:0030810	positive regulation of nucleotide biosynthetic process	bulk RNA-seq,Homo_723,MCI.vs.control; bulk RNA-seq,Homo_714,MCI.vs.control	2
BP	GO:1900373	positive regulation of purine nucleotide biosynthetic process	bulk RNA-seq,Homo_723,MCI.vs.control; bulk RNA-seq,Homo_714,MCI.vs.control	2
BP	GO:0036003	positive regulation of transcription from RNA polymerase II promoter in response to stress	bulk RNA-seq,Homo_723,MCI.vs.control; bulk RNA-seq,Homo_633,AD.vs.control; bulk RNA-seq,Homo_633,AD.vs.MCI	3
BP	GO:0032770	positive regulation of monooxygenase activity	bulk RNA-seq,Homo_723,MCI.vs.control; bulk RNA-seq,Homo_714,MCI.vs.control; bulk RNA-seq,Homo_633,AD.vs.control; bulk RNA-	4
BP	GO:0061620	glycolytic process through glucose-6-phosphate	bulk RNA-seq,Homo_723,MCI.vs.control; bulk RNA-seq,Homo_714,MCI.vs.control; bulk RNA-seq,Homo_633,AD.vs.control; bulk RNA-	4
BP	GO:0000712	resolution of meiotic recombination intermediates	bulk RNA-seq,Homo_723,MCI.vs.control; bulk RNA-seq,Homo_714,MCI.vs.control; bulk RNA-seq,Homo_633,AD.vs.control	3
MF	GO:0000182	rDNA binding	bulk RNA-seq,Homo_723,MCI.vs.control	1
BP	GO:0051250	negative regulation of lymphocyte activation	bulk RNA-seq,Homo_723,MCI.vs.control; bulk RNA-seq,Homo_633,AD.vs.control; bulk RNA-seq,Homo_633,AD.vs.MCI	3
BP	GO:1900221	regulation of amyloid-beta clearance	bulk RNA-seq,Homo_723,MCI.vs.control	1
BP	GO:0043970	histone H3-K9 acetylation	bulk RNA-seq,Homo_723,MCI.vs.control; bulk RNA-seq,Homo_714,MCI.vs.control; bulk RNA-seq,Homo_633,AD.vs.control; bulk RNA-	4
MF	GO:1990459	transferrin receptor binding	bulk RNA-seq,Homo_723,MCI.vs.control; bulk RNA-seq,Homo_714,MCI.vs.control; bulk RNA-seq,Homo_633,AD.vs.control; bulk RNA-	4
MF	GO:0043560	insulin receptor substrate binding	bulk RNA-seq,Homo_723,MCI.vs.control; bulk RNA-seq,Homo_714,MCI.vs.control; bulk RNA-seq,Homo_633,AD.vs.control; bulk RNA-	4
BP	GO:0035721	intraciliary retrograde transport	bulk RNA-seq,Homo_723,MCI.vs.control; bulk RNA-seq,Homo_714,MCI.vs.control; bulk RNA-seq,Homo_633,AD.vs.control; bulk RNA-seq,Homo_633,AD.vs.MCI; bulk RNA-seq,Homo_633,MCI.vs.control	5
BP	GO:0072332	intrinsic apoptotic signaling pathway by p53 class mediator	bulk RNA-seq,Homo_723,MCI.vs.control; bulk RNA-seq,Homo_714,MCI.vs.control; bulk RNA-seq,Homo_633,AD.vs.control; bulk RNA-	4
BP	GO:0009126	purine nucleoside monophosphate metabolic process	bulk RNA-seq,Homo_723,MCI.vs.control; bulk RNA-seq,Homo_714,MCI.vs.control; bulk RNA-seq,Homo_633,AD.vs.control; bulk RNA-	4
BP	GO:0090394	negative regulation of excitatory postsynaptic potential	bulk RNA-seq,Homo_723,MCI.vs.control	1
BP	GO:1901798	positive regulation of signal transduction by p53 class	bulk RNA-seq,Homo_723,MCI.vs.control; bulk RNA-seq,Homo_714,MCI.vs.control; bulk RNA-seq,Homo_633,AD.vs.control; bulk RNA-	4
BP	GO:0051016	barbed-end actin filament capping	bulk RNA-seq,Homo_723,MCI.vs.control; bulk RNA-seq,Homo_714,MCI.vs.control	2

BP	GO:0010575	positive regulation of vascular endothelial growth factor	bulk RNA-seq,Homo_723,MCI.vs.control; bulk RNA-seq,Homo_714,MCI.vs.control; bulk RNA-seq,Homo_633,AD.vs.control; bulk RNA-	4
BP	GO:0060612	adipose tissue development	bulk RNA-seq,Homo_723,MCI.vs.control; bulk RNA-seq,Homo_633,AD.vs.control; bulk RNA-seq,Homo_633,AD.vs.MCI	3
MF	GO:0031432	titin binding	bulk RNA-seq,Homo_723,MCI.vs.control; bulk RNA-seq,Homo_714,MCI.vs.control; bulk RNA-seq,Homo_633,AD.vs.control	3
BP	GO:0010528	regulation of transposition	bulk RNA-seq,Homo_723,MCI.vs.control; bulk RNA-seq,Homo_714,MCI.vs.control; bulk RNA-seq,Homo_633,AD.vs.control	3
BP	GO:0010529	negative regulation of transposition	bulk RNA-seq,Homo_723,MCI.vs.control; bulk RNA-seq,Homo_714,MCI.vs.control; bulk RNA-seq,Homo_633,AD.vs.control	3
MF	GO:0019213	deacetylase activity	bulk RNA-seq,Homo_723,MCI.vs.control; bulk RNA-seq,Homo_714,MCI.vs.control; bulk RNA-seq,Homo_633,AD.vs.control; bulk RNA-	4
BP	GO:0010763	positive regulation of fibroblast migration	bulk RNA-seq,Homo_723,MCI.vs.control; bulk RNA-seq,Homo_714,MCI.vs.control	2
BP	GO:0002713	negative regulation of B cell mediated immunity	bulk RNA-seq,Homo_723,MCI.vs.control; bulk RNA-seq,Homo_714,MCI.vs.control; bulk RNA-seq,Homo_633,AD.vs.control; bulk RNA-	4
BP	GO:0002890	negative regulation of immunoglobulin mediated immune	bulk RNA-seq,Homo_723,MCI.vs.control; bulk RNA-seq,Homo_714,MCI.vs.control; bulk RNA-seq,Homo_633,AD.vs.control; bulk RNA-	4
BP	GO:0045657	positive regulation of monocyte differentiation	bulk RNA-seq,Homo_723,MCI.vs.control; bulk RNA-seq,Homo_714,MCI.vs.control	2
BP	GO:1990700	nucleolar chromatin organization	bulk RNA-seq,Homo_723,MCI.vs.control; bulk RNA-seq,Homo_714,MCI.vs.control; bulk RNA-seq,Homo_633,AD.vs.control; bulk RNA-seq,Homo_633,AD.vs.MCI; bulk RNA-seq,Homo_633,MCI.vs.control	5
BP	GO:0036474	cell death in response to hydrogen peroxide	bulk RNA-seq,Homo_723,MCI.vs.control; bulk RNA-seq,Homo_714,MCI.vs.control; bulk RNA-seq,Homo_633,AD.vs.control	3
BP	GO:0002328	pro-B cell differentiation	bulk RNA-seq,Homo_723,MCI.vs.control; bulk RNA-seq,Homo_714,MCI.vs.control; bulk RNA-seq,Homo_633,AD.vs.control	3
BP	GO:0010454	negative regulation of cell fate commitment	bulk RNA-seq,Homo_723,MCI.vs.control; bulk RNA-seq,SRP223445,AD.vs.control	2
BP	GO:0006084	acetyl-CoA metabolic process	bulk RNA-seq,Homo_723,MCI.vs.control; bulk RNA-seq,Homo_714,MCI.vs.control; bulk RNA-seq,Homo_633,AD.vs.control; bulk RNA-	4
BP	GO:0060216	definitive hemopoiesis	bulk RNA-seq,Homo_723,MCI.vs.control; bulk RNA-seq,Homo_714,MCI.vs.control; bulk RNA-seq,Homo_633,AD.vs.control	3
CC	GO:0032045	guanyl-nucleotide exchange factor complex	bulk RNA-seq,Homo_723,MCI.vs.control; bulk RNA-seq,Homo_714,MCI.vs.control; bulk RNA-seq,Homo_633,AD.vs.control; bulk RNA-	4
BP	GO:0090083	regulation of inclusion body assembly	bulk RNA-seq,Homo_723,MCI.vs.control; bulk RNA-seq,Homo_714,MCI.vs.control	2
BP	GO:0070572	positive regulation of neuron projection regeneration	bulk RNA-seq,Homo_723,MCI.vs.control	1
CC	GO:0005639	integral component of nuclear inner membrane	bulk RNA-seq,Homo_723,MCI.vs.control; bulk RNA-seq,Homo_633,AD.vs.control; bulk RNA-seq,Homo_633,AD.vs.MCI	3
CC	GO:0031229	intrinsic component of nuclear inner membrane	bulk RNA-seq,Homo_723,MCI.vs.control; bulk RNA-seq,Homo_633,AD.vs.control; bulk RNA-seq,Homo_633,AD.vs.MCI	3
BP	GO:0030219	megakaryocyte differentiation	bulk RNA-seq,Homo_723,MCI.vs.control; bulk RNA-seq,Homo_714,MCI.vs.control; bulk RNA-seq,Homo_633,AD.vs.control; bulk RNA-	4
BP	GO:0070189	kynurenine metabolic process	bulk RNA-seq,Homo_723,MCI.vs.control; bulk RNA-seq,Homo_714,MCI.vs.control; bulk RNA-seq,Homo_633,AD.vs.control	3
BP	GO:0048681	negative regulation of axon regeneration	bulk RNA-seq,Homo_723,MCI.vs.control	1
BP	GO:0150172	regulation of phosphatidylcholine metabolic process	bulk RNA-seq,Homo_723,MCI.vs.control; bulk RNA-seq,Homo_633,AD.vs.control	2
BP	GO:0090559	regulation of membrane permeability	bulk RNA-seq,Homo_723,MCI.vs.control; bulk RNA-seq,Homo_714,MCI.vs.control; bulk RNA-seq,Homo_633,AD.vs.control; bulk RNA-	4
BP	GO:0071071	regulation of phospholipid biosynthetic process	bulk RNA-seq,Homo_723,MCI.vs.control; bulk RNA-seq,Homo_714,MCI.vs.control; bulk RNA-seq,Homo_633,AD.vs.control	3
CC	GO:0045120	pronucleus	bulk RNA-seq,Homo_723,MCI.vs.control; bulk RNA-seq,Homo_714,MCI.vs.control	2
MF	GO:0015923	mannosidase activity	bulk RNA-seq,Homo_723,MCI.vs.control; bulk RNA-seq,Homo_714,MCI.vs.control; bulk RNA-seq,Homo_633,AD.vs.control; bulk RNA-seq,Homo_633,AD.vs.MCI; bulk RNA-seq,Homo_633,MCI.vs.control	5
BP	GO:1902004	positive regulation of amyloid-beta formation	bulk RNA-seq,Homo_723,MCI.vs.control; bulk RNA-seq,Homo_714,MCI.vs.control; bulk RNA-seq,Homo_633,AD.vs.control; bulk RNA-	4
MF	GO:0046625	sphingolipid binding	bulk RNA-seq,Homo_723,MCI.vs.control; bulk RNA-seq,Homo_633,AD.vs.control	2
BP	GO:0009263	deoxyribonucleotide biosynthetic process	bulk RNA-seq,Homo_723,MCI.vs.control; bulk RNA-seq,Homo_714,MCI.vs.control; bulk RNA-seq,Homo_633,AD.vs.control	3
BP	GO:0009265	2'-deoxyribonucleotide biosynthetic process	bulk RNA-seq,Homo_723,MCI.vs.control; bulk RNA-seq,Homo_714,MCI.vs.control; bulk RNA-seq,Homo_633,AD.vs.control	3
BP	GO:0046385	deoxyribose phosphate biosynthetic process	bulk RNA-seq,Homo_723,MCI.vs.control; bulk RNA-seq,Homo_714,MCI.vs.control; bulk RNA-seq,Homo_633,AD.vs.control	3
MF	GO:0019211	phosphatase activator activity	bulk RNA-seq,Homo_723,MCI.vs.control; bulk RNA-seq,Homo_714,MCI.vs.control; bulk RNA-seq,Homo_633,AD.vs.control; bulk RNA-	4
MF	GO:0003950	NAD+ ADP-ribosyltransferase activity	bulk RNA-seq,Homo_723,MCI.vs.control; bulk RNA-seq,Homo_714,MCI.vs.control; bulk RNA-seq,Homo_633,AD.vs.control; bulk RNA-	4
BP	GO:0051657	maintenance of organelle location	bulk RNA-seq,Homo_723,MCI.vs.control; bulk RNA-seq,Homo_714,MCI.vs.control; bulk RNA-seq,Homo_633,AD.vs.control; bulk RNA-	4
BP	GO:0042921	glucocorticoid receptor signaling pathway	bulk RNA-seq,Homo_723,MCI.vs.control; bulk RNA-seq,Homo_714,AD.vs.control; bulk RNA-seq,Homo_714,MCI.vs.control; bulk RNA-	4
BP	GO:0051660	establishment of centrosome localization	bulk RNA-seq,Homo_723,MCI.vs.control; bulk RNA-seq,Homo_633,AD.vs.control	2
CC	GO:0043220	Schmidt-Lanterman incisure	bulk RNA-seq,Homo_723,MCI.vs.control; bulk RNA-seq,Homo_714,MCI.vs.control; bulk RNA-seq,Homo_633,AD.vs.control	3
BP	GO:0019320	hexose catabolic process	bulk RNA-seq,Homo_723,MCI.vs.control; bulk RNA-seq,Homo_714,MCI.vs.control; bulk RNA-seq,Homo_633,AD.vs.control	3
BP	GO:0007252	I-kappaB phosphorylation	bulk RNA-seq,Homo_723,MCI.vs.control; bulk RNA-seq,Homo_714,MCI.vs.control; bulk RNA-seq,Homo_633,AD.vs.control	3
BP	GO:0032486	Rap protein signal transduction	bulk RNA-seq,Homo_723,MCI.vs.control; bulk RNA-seq,Homo_714,MCI.vs.control; bulk RNA-seq,Homo_633,AD.vs.control	3
BP	GO:0060547	negative regulation of necrotic cell death	bulk RNA-seq,Homo_723,MCI.vs.control; bulk RNA-seq,Homo_714,AD.vs.MCI; bulk RNA-seq,Homo_714,MCI.vs.control; bulk RNA-	4
BP	GO:0032878	regulation of establishment or maintenance of cell polarity	bulk RNA-seq,Homo_723,MCI.vs.control; bulk RNA-seq,Homo_714,MCI.vs.control; bulk RNA-seq,Homo_633,AD.vs.control; bulk RNA-	4
BP	GO:0070920	regulation of production of small RNA involved in gene silencing by RNA	bulk RNA-seq,Homo_723,MCI.vs.control	1
BP	GO:0072111	cell proliferation involved in kidney development	bulk RNA-seq,Homo_723,MCI.vs.control	1
BP	GO:0009219	pyrimidine deoxyribonucleotide metabolic process	bulk RNA-seq,Homo_723,MCI.vs.control; bulk RNA-seq,Homo_714,MCI.vs.control; bulk RNA-seq,Homo_633,AD.vs.control; bulk RNA-seq,Homo_633,AD.vs.MCI; bulk RNA-seq,Homo_633,MCI.vs.control	5
MF	GO:0034237	protein kinase A regulatory subunit binding	bulk RNA-seq,Homo_723,MCI.vs.control; bulk RNA-seq,Homo_633,AD.vs.control; bulk RNA-seq,Homo_633,AD.vs.MCI	3
MF	GO:0140776	protein-containing complex destabilizing activity	bulk RNA-seq,Homo_723,MCI.vs.control; bulk RNA-seq,Homo_633,AD.vs.control	2
MF	GO:0016638	oxidoreductase activity, acting on the CH-NH2 group of	bulk RNA-seq,Homo_723,MCI.vs.control	1
MF	GO:0005158	insulin receptor binding	bulk RNA-seq,Homo_723,MCI.vs.control	1

CC	GO:0031904	endosome lumen	bulk RNA-seq,Homo_723,MCI.vs.control; bulk RNA-seq,Homo_714,MCI.vs.control	2
BP	GO:0046039	GTP metabolic process	bulk RNA-seq,Homo_723,MCI.vs.control; bulk RNA-seq,Homo_633,AD.vs.control; bulk RNA-seq,Homo_633,AD.vs.MCI	3
BP	GO:0006677	glycosylceramide metabolic process	bulk RNA-seq,Homo_723,MCI.vs.control; bulk RNA-seq,Homo_714,MCI.vs.control; bulk RNA-seq,Homo_633,AD.vs.control; bulk RNA-seq,Homo_633,AD.vs.MCI; bulk RNA-seq,Homo_633,MCI.vs.control	5
MF	GO:0005372	water transmembrane transporter activity	bulk RNA-seq,Homo_723,MCI.vs.control	1
BP	GO:0051014	actin filament severing	bulk RNA-seq,Homo_723,MCI.vs.control; bulk RNA-seq,Homo_714,MCI.vs.control; bulk RNA-seq,Homo_633,MCI.vs.control	3
BP	GO:0051000	positive regulation of nitric-oxide synthase activity	bulk RNA-seq,Homo_723,MCI.vs.control	1
BP	GO:0015701	bicarbonate transport	bulk RNA-seq,Homo_723,MCI.vs.control; bulk RNA-seq,Homo_633,AD.vs.control; bulk RNA-seq,Homo_633,AD.vs.MCI	3
BP	GO:0003215	cardiac right ventricle morphogenesis	bulk RNA-seq,Homo_723,MCI.vs.control	1
MF	GO:0015035	protein-disulfide reductase activity	bulk RNA-seq,Homo_723,MCI.vs.control; bulk RNA-seq,Homo_714,MCI.vs.control; bulk RNA-seq,Homo_633,AD.vs.control; bulk RNA-	4
BP	GO:0034067	protein localization to Golgi apparatus	bulk RNA-seq,Homo_723,MCI.vs.control; bulk RNA-seq,Homo_714,MCI.vs.control; bulk RNA-seq,Homo_633,AD.vs.control	3
BP	GO:0002283	neutrophil activation involved in immune response	bulk RNA-seq,Homo_723,MCI.vs.control; bulk RNA-seq,Homo_714,MCI.vs.control; bulk RNA-seq,Homo_633,AD.vs.control; bulk RNA-	4
BP	GO:0043045	DNA methylation involved in embryo development	bulk RNA-seq,Homo_723,MCI.vs.control; bulk RNA-seq,Homo_714,MCI.vs.control	2
BP	GO:1901538	changes to DNA methylation involved in embryo	bulk RNA-seq,Homo_723,MCI.vs.control; bulk RNA-seq,Homo_714,MCI.vs.control	2
BP	GO:1901862	negative regulation of muscle tissue development	bulk RNA-seq,Homo_723,MCI.vs.control; bulk RNA-seq,Homo_714,MCI.vs.control; bulk RNA-seq,Homo_633,AD.vs.control; bulk RNA-	4
BP	GO:0061052	negative regulation of cell growth involved in cardiac muscle cell development	bulk RNA-seq,Homo_723,MCI.vs.control; bulk RNA-seq,Homo_714,MCI.vs.control	2
MF	GO:0004312	fatty acid synthase activity	bulk RNA-seq,Homo_723,MCI.vs.control; bulk RNA-seq,Homo_633,AD.vs.control; bulk RNA-seq,Homo_633,AD.vs.MCI	3
BP	GO:0071501	cellular response to sterol depletion	bulk RNA-seq,Homo_723,MCI.vs.control; bulk RNA-seq,Homo_714,MCI.vs.control; bulk RNA-seq,Homo_633,AD.vs.control; bulk RNA-seq,Homo_633,AD.vs.MCI; bulk RNA-seq,Homo_633,MCI.vs.control	5
BP	GO:1903027	regulation of opsonization	bulk RNA-seq,Homo_723,MCI.vs.control; bulk RNA-seq,Homo_714,MCI.vs.control	2
BP	GO:0120163	negative regulation of cold-induced thermogenesis	bulk RNA-seq,Homo_723,MCI.vs.control; bulk RNA-seq,Homo_714,MCI.vs.control; bulk RNA-seq,Homo_633,AD.vs.control; bulk RNA-	4
MF	GO:0035004	phosphatidylinositol 3-kinase activity	bulk RNA-seq,Homo_723,MCI.vs.control; bulk RNA-seq,Homo_714,MCI.vs.control; bulk RNA-seq,Homo_633,AD.vs.control; bulk RNA-	4
BP	GO:0016188	synaptic vesicle maturation	bulk RNA-seq,Homo_723,MCI.vs.control	1
BP	GO:0009394	2'-deoxyribonucleotide metabolic process	bulk RNA-seq,Homo_723,MCI.vs.control; bulk RNA-seq,Homo_714,MCI.vs.control; bulk RNA-seq,Homo_633,AD.vs.control; bulk RNA-	4
CC	GO:0098981	cholinergic synapse	bulk RNA-seq,Homo_723,MCI.vs.control	1
BP	GO:1900246	positive regulation of RIG-I signaling pathway	bulk RNA-seq,Homo_723,MCI.vs.control	1
BP	GO:0000266	mitochondrial fission	bulk RNA-seq,Homo_723,MCI.vs.control; bulk RNA-seq,Homo_714,MCI.vs.control; bulk RNA-seq,Homo_633,AD.vs.control; bulk RNA-seq,Homo_633,AD.vs.MCI; bulk RNA-seq,SRP325058,AD.vs.control	5
BP	GO:1990000	amyloid fibril formation	bulk RNA-seq,Homo_723,MCI.vs.control; bulk RNA-seq,Homo_714,AD.vs.MCI; bulk RNA-seq,Homo_714,MCI.vs.control; bulk RNA-seq,Homo_633,AD.vs.control; bulk RNA-seq,Homo_633,AD.vs.MCI	5
BP	GO:1903977	positive regulation of glial cell migration	bulk RNA-seq,Homo_723,MCI.vs.control; bulk RNA-seq,Homo_714,MCI.vs.control	2
MF	GO:0055106	ubiquitin-protein transferase regulator activity	bulk RNA-seq,Homo_723,MCI.vs.control; bulk RNA-seq,Homo_633,AD.vs.control; bulk RNA-seq,Homo_633,AD.vs.MCI	3
MF	GO:0030283	testosterone dehydrogenase [NAD(P)] activity	bulk RNA-seq,Homo_723,MCI.vs.control; bulk RNA-seq,Homo_714,MCI.vs.control	2
BP	GO:0070314	G1 to G0 transition	bulk RNA-seq,Homo_723,MCI.vs.control; bulk RNA-seq,Homo_633,AD.vs.control; bulk RNA-seq,Homo_633,AD.vs.MCI	3
BP	GO:0035090	maintenance of apical/basal cell polarity	bulk RNA-seq,Homo_723,MCI.vs.control; bulk RNA-seq,Homo_633,AD.vs.control; bulk RNA-seq,Homo_633,AD.vs.MCI	3
BP	GO:0045199	maintenance of epithelial cell apical/basal polarity	bulk RNA-seq,Homo_723,MCI.vs.control; bulk RNA-seq,Homo_633,AD.vs.control; bulk RNA-seq,Homo_633,AD.vs.MCI	3
BP	GO:0034380	high-density lipoprotein particle assembly	bulk RNA-seq,Homo_723,MCI.vs.control; bulk RNA-seq,Homo_714,AD.vs.MCI; bulk RNA-seq,Homo_714,MCI.vs.control	3
MF	GO:0016888	endodeoxyribonuclease activity, producing 5'-	bulk RNA-seq,Homo_723,MCI.vs.control; bulk RNA-seq,Homo_714,MCI.vs.control; bulk RNA-seq,Homo_633,AD.vs.control; bulk RNA-	4
MF	GO:0051787	misfolded protein binding	bulk RNA-seq,Homo_723,MCI.vs.control; bulk RNA-seq,Homo_714,MCI.vs.control; bulk RNA-seq,Homo_633,AD.vs.control; bulk RNA-seq,Homo_633,AD.vs.MCI; bulk RNA-seq,Homo_633,MCI.vs.control	5
BP	GO:1905820	positive regulation of chromosome separation	bulk RNA-seq,Homo_723,MCI.vs.control; bulk RNA-seq,Homo_714,MCI.vs.control; bulk RNA-seq,Homo_633,AD.vs.control; bulk RNA-seq,Homo_633,AD.vs.MCI; bulk RNA-seq,Homo_633,MCI.vs.control	5
MF	GO:0046527	glucosyltransferase activity	bulk RNA-seq,Homo_723,MCI.vs.control; bulk RNA-seq,Homo_714,MCI.vs.control; bulk RNA-seq,Homo_633,AD.vs.control; bulk RNA-seq,Homo_633,AD.vs.MCI; bulk RNA-seq,Homo_633,MCI.vs.control	5
MF	GO:0016229	steroid dehydrogenase activity	bulk RNA-seq,Homo_723,MCI.vs.control; bulk RNA-seq,Homo_714,AD.vs.MCI; bulk RNA-seq,Homo_714,MCI.vs.control	3
CC	GO:0036020	endolysosome membrane	bulk RNA-seq,Homo_723,MCI.vs.control; bulk RNA-seq,Homo_714,MCI.vs.control; bulk RNA-seq,Homo_633,AD.vs.control; bulk RNA-	4
BP	GO:1904046	negative regulation of vascular endothelial growth factor	bulk RNA-seq,Homo_723,MCI.vs.control; bulk RNA-seq,Homo_714,MCI.vs.control; bulk RNA-seq,Homo_633,AD.vs.control; bulk RNA-	4
MF	GO:0030506	ankyrin binding	bulk RNA-seq,Homo_723,MCI.vs.control	1
MF	GO:0046974	histone methyltransferase activity (H3-K9 specific)	bulk RNA-seq,Homo_723,MCI.vs.control	1
BP	GO:0036158	outer dynein arm assembly	bulk RNA-seq,Homo_723,MCI.vs.control	1
BP	GO:0032891	negative regulation of organic acid transport	bulk RNA-seq,Homo_723,MCI.vs.control	1
BP	GO:2001032	regulation of double-strand break repair via nonhomologous	bulk RNA-seq,Homo_723,MCI.vs.control; bulk RNA-seq,Homo_714,MCI.vs.control; bulk RNA-seq,Homo_633,AD.vs.control	3
BP	GO:0003334	keratinocyte development	bulk RNA-seq,Homo_723,MCI.vs.control; bulk RNA-seq,Homo_714,MCI.vs.control	2
BP	GO:0051148	negative regulation of muscle cell differentiation	bulk RNA-seq,Homo_723,MCI.vs.control; bulk RNA-seq,Homo_714,AD.vs.MCI; bulk RNA-seq,Homo_714,MCI.vs.control	3
BP	GO:1901550	regulation of endothelial cell development	bulk RNA-seq,Homo_723,MCI.vs.control; bulk RNA-seq,Homo_714,MCI.vs.control; bulk RNA-seq,Homo_633,AD.vs.control; bulk RNA-	4
BP	GO:1903140	regulation of establishment of endothelial barrier	bulk RNA-seq,Homo_723,MCI.vs.control; bulk RNA-seq,Homo_714,MCI.vs.control; bulk RNA-seq,Homo_633,AD.vs.control; bulk RNA-	4

MF	GO:0043015	gamma-tubulin binding	bulk RNA-seq,Homo_723,MCI.vs.control; bulk RNA-seq,Homo_714,AD.vs.control; bulk RNA-seq,Homo_714,MCI.vs.control; bulk RNA-seq,Homo_633,AD.vs.control; bulk RNA-seq,Homo_633,AD.vs.MCI; bulk RNA-seq,Homo_633,MCI.vs.control	6
BP	GO:0097250	mitochondrial respirasome assembly	bulk RNA-seq,Homo_723,MCI.vs.control	1
BP	GO:0006662	glycerol ether metabolic process	bulk RNA-seq,Homo_723,MCI.vs.control; bulk RNA-seq,Homo_714,MCI.vs.control; bulk RNA-seq,Homo_633,AD.vs.control; bulk RNA-seq,Homo_633,AD.vs.MCI; bulk RNA-seq,Homo_633,MCI.vs.control	5
BP	GO:0046485	ether lipid metabolic process	bulk RNA-seq,Homo_723,MCI.vs.control; bulk RNA-seq,Homo_714,MCI.vs.control; bulk RNA-seq,Homo_633,AD.vs.control; bulk RNA-seq,Homo_633,AD.vs.MCI; bulk RNA-seq,Homo_633,MCI.vs.control	5
BP	GO:0009650	UV protection	bulk RNA-seq,Homo_723,MCI.vs.control; bulk RNA-seq,Homo_714,MCI.vs.control; bulk RNA-seq,Homo_633,AD.vs.control	3
BP	GO:0061000	negative regulation of dendritic spine development	bulk RNA-seq,Homo_723,MCI.vs.control; bulk RNA-seq,Homo_633,AD.vs.control	2
MF	GO:0005024	transforming growth factor beta receptor activity	bulk RNA-seq,Homo_723,MCI.vs.control; bulk RNA-seq,Homo_714,MCI.vs.control; bulk RNA-seq,Homo_633,AD.vs.control; bulk RNA-	4
CC	GO:0043240	Fanconi anaemia nuclear complex	bulk RNA-seq,Homo_723,MCI.vs.control; bulk RNA-seq,Homo_714,MCI.vs.control; bulk RNA-seq,Homo_633,AD.vs.control; bulk RNA-	4
BP	GO:0009218	pyrimidine ribonucleotide metabolic process	bulk RNA-seq,Homo_723,MCI.vs.control; bulk RNA-seq,Homo_633,AD.vs.control; bulk RNA-seq,Homo_633,AD.vs.MCI	3
MF	GO:0042910	xenobiotic transmembrane transporter activity	bulk RNA-seq,Homo_723,MCI.vs.control; bulk RNA-seq,Homo_714,MCI.vs.control	2
BP	GO:0030970	retrograde protein transport, ER to cytosol	bulk RNA-seq,Homo_723,MCI.vs.control; bulk RNA-seq,Homo_714,MCI.vs.control; bulk RNA-seq,Homo_633,AD.vs.control; bulk RNA-seq,Homo_633,AD.vs.MCI; bulk RNA-seq,Homo_633,MCI.vs.control	5
BP	GO:1903513	endoplasmic reticulum to cytosol transport	bulk RNA-seq,Homo_723,MCI.vs.control; bulk RNA-seq,Homo_714,MCI.vs.control; bulk RNA-seq,Homo_633,AD.vs.control; bulk RNA-seq,Homo_633,AD.vs.MCI; bulk RNA-seq,Homo_633,MCI.vs.control	5
BP	GO:0036120	cellular response to platelet-derived growth factor stimulus	bulk RNA-seq,Homo_723,MCI.vs.control; bulk RNA-seq,Homo_633,AD.vs.control; bulk RNA-seq,Homo_633,AD.vs.MCI	3
MF	GO:0016763	pentosyltransferase activity	bulk RNA-seq,Homo_723,MCI.vs.control; bulk RNA-seq,Homo_714,MCI.vs.control; bulk RNA-seq,Homo_633,AD.vs.control; bulk RNA-	4
BP	GO:0055119	relaxation of cardiac muscle	bulk RNA-seq,Homo_723,MCI.vs.control	1
MF	GO:0070628	proteasome binding	bulk RNA-seq,Homo_723,MCI.vs.control; bulk RNA-seq,Homo_714,MCI.vs.control; bulk RNA-seq,Homo_633,AD.vs.control; bulk RNA-	4
MF	GO:0004697	protein kinase C activity	bulk RNA-seq,Homo_723,MCI.vs.control; bulk RNA-seq,Homo_714,MCI.vs.control; bulk RNA-seq,Homo_633,AD.vs.control	3
BP	GO:0008228	opsonization	bulk RNA-seq,Homo_723,MCI.vs.control; bulk RNA-seq,Homo_714,AD.vs.MCI; bulk RNA-seq,Homo_714,MCI.vs.control	3
MF	GO:0016417	S-acyltransferase activity	bulk RNA-seq,Homo_723,MCI.vs.control; bulk RNA-seq,Homo_714,MCI.vs.control; bulk RNA-seq,Homo_633,AD.vs.control; bulk RNA-	4
BP	GO:1901570	fatty acid derivative biosynthetic process	bulk RNA-seq,Homo_723,MCI.vs.control; bulk RNA-seq,Homo_714,MCI.vs.control; bulk RNA-seq,Homo_633,AD.vs.control; bulk RNA-	4
BP	GO:0009133	nucleoside diphosphate biosynthetic process	bulk RNA-seq,Homo_723,MCI.vs.control; bulk RNA-seq,Homo_714,AD.vs.control; bulk RNA-seq,Homo_633,AD.vs.control	3
BP	GO:0030497	fatty acid elongation	bulk RNA-seq,Homo_723,MCI.vs.control; bulk RNA-seq,Homo_714,MCI.vs.control; bulk RNA-seq,Homo_633,AD.vs.control; bulk RNA-	4
BP	GO:0016082	synaptic vesicle priming	bulk RNA-seq,Homo_723,MCI.vs.control; scRNA-seq,SRP309935,B cell_1-AD.vs.control	2
BP	GO:0070254	mucus secretion	bulk RNA-seq,Homo_723,MCI.vs.control; bulk RNA-seq,Homo_714,MCI.vs.control	2
BP	GO:0018208	peptidyl-proline modification	bulk RNA-seq,Homo_723,MCI.vs.control; bulk RNA-seq,Homo_714,AD.vs.control; bulk RNA-seq,Homo_714,MCI.vs.control; bulk RNA-seq,Homo_633,AD.vs.control; bulk RNA-seq,Homo_633,AD.vs.MCI	5
MF	GO:0052739	phosphatidylserine 1-acylhydrolase activity	bulk RNA-seq,Homo_723,MCI.vs.control; bulk RNA-seq,Homo_714,MCI.vs.control; bulk RNA-seq,Homo_633,AD.vs.control	3
BP	GO:0002837	regulation of immune response to tumor cell	bulk RNA-seq,Homo_723,MCI.vs.control; bulk RNA-seq,Homo_714,MCI.vs.control; bulk RNA-seq,Homo_633,AD.vs.control	3
BP	GO:1902563	regulation of neutrophil activation	bulk RNA-seq,Homo_723,MCI.vs.control; bulk RNA-seq,Homo_714,MCI.vs.control	2
BP	GO:0006376	mRNA splice site selection	bulk RNA-seq,Homo_723,MCI.vs.control; bulk RNA-seq,Homo_633,AD.vs.control; bulk RNA-seq,Homo_633,AD.vs.MCI	3
BP	GO:0034123	positive regulation of toll-like receptor signaling pathway	bulk RNA-seq,Homo_723,MCI.vs.control; bulk RNA-seq,Homo_714,MCI.vs.control; bulk RNA-seq,Homo_633,AD.vs.control; bulk RNA-seq,Homo_633,AD.vs.MCI; bulk RNA-seq,Homo_633,MCI.vs.control	5
MF	GO:0016878	acid-thiol ligase activity	bulk RNA-seq,Homo_723,MCI.vs.control; bulk RNA-seq,Homo_714,MCI.vs.control; bulk RNA-seq,Homo_633,AD.vs.control; bulk RNA-	4
BP	GO:0009309	amine biosynthetic process	bulk RNA-seq,Homo_723,MCI.vs.control; bulk RNA-seq,Homo_714,AD.vs.MCI; bulk RNA-seq,Homo_714,MCI.vs.control; bulk RNA-	4
BP	GO:0033005	positive regulation of mast cell activation	bulk RNA-seq,Homo_723,MCI.vs.control; bulk RNA-seq,Homo_714,AD.vs.MCI	2
BP	GO:2001225	regulation of chloride transport	bulk RNA-seq,Homo_723,MCI.vs.control	1
CC	GO:0097440	apical dendrite	bulk RNA-seq,Homo_723,MCI.vs.control; bulk RNA-seq,Homo_714,MCI.vs.control; bulk RNA-seq,Homo_633,AD.vs.control	3
BP	GO:1902036	regulation of hematopoietic stem cell differentiation	bulk RNA-seq,Homo_723,MCI.vs.control; bulk RNA-seq,Homo_714,MCI.vs.control; bulk RNA-seq,Homo_633,AD.vs.control; bulk RNA-	4
MF	GO:0008330	protein tyrosine/threonine phosphatase activity	bulk RNA-seq,Homo_723,MCI.vs.control	1
CC	GO:0099243	extrinsic component of synaptic membrane	bulk RNA-seq,Homo_723,MCI.vs.control; bulk RNA-seq,Homo_633,AD.vs.control; bulk RNA-seq,Homo_633,AD.vs.MCI	3
BP	GO:1903205	regulation of hydrogen peroxide-induced cell death	bulk RNA-seq,Homo_723,MCI.vs.control; bulk RNA-seq,Homo_714,AD.vs.MCI; bulk RNA-seq,Homo_714,MCI.vs.control	3
BP	GO:0035461	vitamin transmembrane transport	bulk RNA-seq,Homo_723,MCI.vs.control	1
BP	GO:2000651	positive regulation of sodium ion transmembrane transporter	bulk RNA-seq,Homo_723,MCI.vs.control	1
BP	GO:0036302	atrioventricular canal development	bulk RNA-seq,Homo_723,MCI.vs.control; bulk RNA-seq,Homo_714,MCI.vs.control; bulk RNA-seq,Homo_633,AD.vs.MCI	3
BP	GO:0046512	sphingosine biosynthetic process	bulk RNA-seq,Homo_723,MCI.vs.control; bulk RNA-seq,Homo_714,MCI.vs.control; bulk RNA-seq,Homo_633,AD.vs.control; bulk RNA-	4
BP	GO:0046520	sphingoid biosynthetic process	bulk RNA-seq,Homo_723,MCI.vs.control; bulk RNA-seq,Homo_714,MCI.vs.control; bulk RNA-seq,Homo_633,AD.vs.control; bulk RNA-	4
BP	GO:2000515	negative regulation of CD4-positive, alpha-beta T cell activation	bulk RNA-seq,Homo_723,MCI.vs.control; bulk RNA-seq,Homo_714,MCI.vs.control; bulk RNA-seq,Homo_633,AD.vs.control; bulk RNA-seq,Homo_633,AD.vs.MCI; bulk RNA-seq,Homo_633,MCI.vs.control	5
BP	GO:0051354	negative regulation of oxidoreductase activity	bulk RNA-seq,Homo_723,MCI.vs.control; bulk RNA-seq,Homo_714,MCI.vs.control	2
BP	GO:0043587	tongue morphogenesis	bulk RNA-seq,Homo_723,MCI.vs.control	1
BP	GO:0071605	monocyte chemotactic protein-1 production	bulk RNA-seq,Homo_723,MCI.vs.control; bulk RNA-seq,Homo_714,MCI.vs.control	2
BP	GO:0071637	regulation of monocyte chemotactic protein-1 production	bulk RNA-seq,Homo_723,MCI.vs.control; bulk RNA-seq,Homo_714,MCI.vs.control	2
BP	GO:0140131	positive regulation of lymphocyte chemotaxis	bulk RNA-seq,Homo_723,MCI.vs.control; bulk RNA-seq,Homo_714,MCI.vs.control; bulk RNA-seq,Homo_633,AD.vs.control; bulk RNA-	4

CC	GO:0017101	aminoacyl-tRNA synthetase multienzyme complex	bulk RNA-seq,Homo_723,MCI.vs.control; bulk RNA-seq,Homo_633,AD.vs.control; bulk RNA-seq,Homo_633,AD.vs.MCI	3
BP	GO:0010248	establishment or maintenance of transmembrane electrochemical gradient	bulk RNA-seq,Homo_723,MCI.vs.control	1
BP	GO:0032310	prostaglandin secretion	bulk RNA-seq,Homo_723,MCI.vs.control; bulk RNA-seq,Homo_633,AD.vs.control	2
MF	GO:0009982	pseudouridine synthase activity	bulk RNA-seq,Homo_723,MCI.vs.control; bulk RNA-seq,Homo_714,MCI.vs.control; bulk RNA-seq,Homo_633,AD.vs.control; bulk RNA-seq,Homo_633,AD.vs.MCI; bulk RNA-seq,Homo_633,MCI.vs.control	5
MF	GO:0106019	phosphatidylinositol-4,5-bisphosphate phosphatase activity	bulk RNA-seq,Homo_723,MCI.vs.control; bulk RNA-seq,Homo_633,AD.vs.control; bulk RNA-seq,Homo_633,AD.vs.MCI; bulk RNA-	4
MF	GO:0008190	eukaryotic initiation factor 4E binding	bulk RNA-seq,Homo_723,MCI.vs.control	1
MF	GO:0005542	folic acid binding	bulk RNA-seq,Homo_723,MCI.vs.control; bulk RNA-seq,Homo_714,MCI.vs.control; bulk RNA-seq,Homo_633,AD.vs.control; bulk RNA-	4
BP	GO:0043403	skeletal muscle tissue regeneration	bulk RNA-seq,Homo_723,MCI.vs.control; bulk RNA-seq,Homo_714,MCI.vs.control; bulk RNA-seq,Homo_633,AD.vs.control; bulk RNA-	4
BP	GO:0051289	protein homotetramerization	bulk RNA-seq,Homo_723,MCI.vs.control; bulk RNA-seq,Homo_714,AD.vs.MCI; bulk RNA-seq,Homo_714,MCI.vs.control; bulk RNA-	4
BP	GO:0010224	response to UV-B	bulk RNA-seq,Homo_723,MCI.vs.control; bulk RNA-seq,Homo_714,MCI.vs.control; bulk RNA-seq,Homo_633,AD.vs.control; bulk RNA-	4
BP	GO:0006735	NADH regeneration	bulk RNA-seq,Homo_723,MCI.vs.control; bulk RNA-seq,Homo_714,MCI.vs.control; bulk RNA-seq,Homo_633,MCI.vs.control	3
BP	GO:0061621	canonical glycolysis	bulk RNA-seq,Homo_723,MCI.vs.control; bulk RNA-seq,Homo_714,MCI.vs.control; bulk RNA-seq,Homo_633,MCI.vs.control	3
BP	GO:0061718	glucose catabolic process to pyruvate	bulk RNA-seq,Homo_723,MCI.vs.control; bulk RNA-seq,Homo_714,MCI.vs.control; bulk RNA-seq,Homo_633,MCI.vs.control	3
BP	GO:0032635	interleukin-6 production	bulk RNA-seq,Homo_723,MCI.vs.control; bulk RNA-seq,Homo_714,AD.vs.MCI; bulk RNA-seq,Homo_633,AD.vs.control; bulk RNA-seq,Homo_633,AD.vs.MCI	4
BP	GO:0032675	regulation of interleukin-6 production	bulk RNA-seq,Homo_723,MCI.vs.control; bulk RNA-seq,Homo_714,AD.vs.MCI; bulk RNA-seq,Homo_633,AD.vs.control; bulk RNA-seq,Homo_633,AD.vs.MCI	4
BP	GO:0005978	glycogen biosynthetic process	bulk RNA-seq,Homo_723,MCI.vs.control; bulk RNA-seq,Homo_714,MCI.vs.control	2
BP	GO:0009250	glucan biosynthetic process	bulk RNA-seq,Homo_723,MCI.vs.control; bulk RNA-seq,Homo_714,MCI.vs.control	2
BP	GO:0007000	nucleolus organization	bulk RNA-seq,Homo_723,MCI.vs.control; bulk RNA-seq,Homo_714,MCI.vs.control; bulk RNA-seq,Homo_633,AD.vs.control; bulk RNA-seq,Homo_633,AD.vs.MCI; bulk RNA-seq,Homo_633,MCI.vs.control	5
CC	GO:0005637	nuclear inner membrane	bulk RNA-seq,Homo_723,MCI.vs.control; bulk RNA-seq,Homo_714,MCI.vs.control; bulk RNA-seq,Homo_633,AD.vs.control; bulk RNA-	4
CC	GO:0000153	cytoplasmic ubiquitin ligase complex	bulk RNA-seq,Homo_723,MCI.vs.control; bulk RNA-seq,Homo_714,MCI.vs.control; bulk RNA-seq,Homo_633,AD.vs.control; bulk RNA-seq,Homo_633,AD.vs.MCI; bulk RNA-seq,Homo_633,MCI.vs.control	5
BP	GO:0051482	positive regulation of cytosolic calcium ion concentration involved in phospholipase C-activating G protein-coupled	bulk RNA-seq,Homo_723,MCI.vs.control	1
BP	GO:0044539	long-chain fatty acid import into cell	bulk RNA-seq,Homo_723,MCI.vs.control; bulk RNA-seq,Homo_714,MCI.vs.control; bulk RNA-seq,Homo_633,AD.vs.control; bulk RNA-	4
MF	GO:0051721	protein phosphatase 2A binding	bulk RNA-seq,Homo_723,MCI.vs.control; bulk RNA-seq,Homo_714,MCI.vs.control	2
BP	GO:0051457	maintenance of protein location in nucleus	bulk RNA-seq,Homo_723,MCI.vs.control; bulk RNA-seq,Homo_714,MCI.vs.control; bulk RNA-seq,Homo_633,AD.vs.control; bulk RNA-	4
MF	GO:0017017	MAP kinase tyrosine/serine/threonine phosphatase activity	bulk RNA-seq,Homo_723,MCI.vs.control	1
CC	GO:0035253	ciliary rootlet	bulk RNA-seq,Homo_723,MCI.vs.control; bulk RNA-seq,Homo_633,AD.vs.control	2
BP	GO:0043457	regulation of cellular respiration	bulk RNA-seq,Homo_723,MCI.vs.control; bulk RNA-seq,Homo_714,MCI.vs.control; bulk RNA-seq,Homo_633,AD.vs.control; bulk RNA-	4
BP	GO:0071354	cellular response to interleukin-6	bulk RNA-seq,Homo_723,MCI.vs.control; bulk RNA-seq,Homo_714,AD.vs.MCI; bulk RNA-seq,Homo_633,AD.vs.control; bulk RNA-seq,Homo_633,AD.vs.MCI	4
CC	GO:0005942	phosphatidylinositol 3-kinase complex	bulk RNA-seq,Homo_723,MCI.vs.control; bulk RNA-seq,Homo_714,AD.vs.control; bulk RNA-seq,Homo_714,MCI.vs.control; bulk RNA-seq,Homo_633,AD.vs.control; bulk RNA-seq,Homo_633,AD.vs.MCI	5
BP	GO:0010878	cholesterol storage	bulk RNA-seq,Homo_723,MCI.vs.control; bulk RNA-seq,Homo_714,MCI.vs.control; bulk RNA-seq,Homo_633,AD.vs.control; bulk RNA-	4
BP	GO:0002924	negative regulation of humoral immune response mediated by circulating immunoglobulin	bulk RNA-seq,Homo_723,MCI.vs.control	1
CC	GO:0031261	DNA replication preinitiation complex	bulk RNA-seq,Homo_723,MCI.vs.control; bulk RNA-seq,Homo_714,MCI.vs.control; bulk RNA-seq,Homo_633,MCI.vs.control	3
MF	GO:0060590	ATPase regulator activity	bulk RNA-seq,Homo_723,MCI.vs.control; bulk RNA-seq,Homo_714,MCI.vs.control; bulk RNA-seq,Homo_633,AD.vs.control	3
BP	GO:1903909	regulation of receptor clustering	bulk RNA-seq,Homo_723,MCI.vs.control	1
CC	GO:0031616	spindle pole centrosome	bulk RNA-seq,Homo_723,MCI.vs.control; bulk RNA-seq,Homo_714,MCI.vs.control	2
MF	GO:0043138	3'-5' DNA helicase activity	bulk RNA-seq,Homo_723,MCI.vs.control; bulk RNA-seq,Homo_633,AD.vs.control; bulk RNA-seq,Homo_633,AD.vs.MCI	3
BP	GO:0034695	response to prostaglandin E	bulk RNA-seq,Homo_723,MCI.vs.control; bulk RNA-seq,Homo_714,MCI.vs.control	2
BP	GO:0072109	glomerular mesangium development	bulk RNA-seq,Homo_723,MCI.vs.control	1
BP	GO:0034453	microtubule anchoring	bulk RNA-seq,Homo_723,MCI.vs.control; bulk RNA-seq,Homo_633,AD.vs.control; bulk RNA-seq,Homo_633,AD.vs.MCI	3
BP	GO:1903830	magnesium ion transmembrane transport	bulk RNA-seq,Homo_723,MCI.vs.control; bulk RNA-seq,Homo_714,MCI.vs.control	2
CC	GO:0000164	protein phosphatase type 1 complex	bulk RNA-seq,Homo_723,MCI.vs.control	1
MF	GO:0035014	phosphatidylinositol 3-kinase regulator activity	bulk RNA-seq,Homo_723,MCI.vs.control; bulk RNA-seq,Homo_714,MCI.vs.control	2
BP	GO:0032536	regulation of cell projection size	bulk RNA-seq,Homo_723,MCI.vs.control	1
BP	GO:1904995	negative regulation of leukocyte adhesion to vascular	bulk RNA-seq,Homo_723,MCI.vs.control; bulk RNA-seq,Homo_714,AD.vs.MCI; bulk RNA-seq,Homo_714,MCI.vs.control	3
BP	GO:0033262	regulation of nuclear cell cycle DNA replication	bulk RNA-seq,Homo_723,MCI.vs.control; bulk RNA-seq,Homo_714,MCI.vs.control; bulk RNA-seq,Homo_633,MCI.vs.control	3
CC	GO:0099524	postsynaptic cytosol	bulk RNA-seq,Homo_723,MCI.vs.control	1
BP	GO:0009147	pyrimidine nucleoside triphosphate metabolic process	bulk RNA-seq,Homo_723,MCI.vs.control; bulk RNA-seq,Homo_714,MCI.vs.control; bulk RNA-seq,Homo_633,AD.vs.control; bulk RNA-	4
BP	GO:0045061	thymic T cell selection	bulk RNA-seq,Homo_723,MCI.vs.control; bulk RNA-seq,Homo_714,MCI.vs.control	2
BP	GO:0030521	androgen receptor signaling pathway	bulk RNA-seq,Homo_723,MCI.vs.control; bulk RNA-seq,Homo_714,MCI.vs.control; bulk RNA-seq,Homo_633,AD.vs.control; bulk RNA-	4
BP	GO:0050746	regulation of lipoprotein metabolic process	bulk RNA-seq,Homo_723,MCI.vs.control; bulk RNA-seq,Homo_633,AD.vs.control; bulk RNA-seq,Homo_633,AD.vs.MCI	3

CC	GO:0031201	SNARE complex	bulk RNA-seq,Homo_723,MCI.vs.control; bulk RNA-seq,Homo_633,AD.vs.control; bulk RNA-seq,Homo_633,AD.vs.MCI	3
MF	GO:0001965	G-protein alpha-subunit binding	bulk RNA-seq,Homo_723,MCI.vs.control	1
BP	GO:0071243	cellular response to arsenic-containing substance	bulk RNA-seq,Homo_723,MCI.vs.control; bulk RNA-seq,Homo_714,MCI.vs.control; bulk RNA-seq,Homo_633,AD.vs.control	3
BP	GO:0035269	protein O-linked mannosylation	bulk RNA-seq,Homo_723,MCI.vs.control; bulk RNA-seq,Homo_714,MCI.vs.control; bulk RNA-seq,Homo_633,MCI.vs.control	3
BP	GO:0019985	translesion synthesis	bulk RNA-seq,Homo_723,MCI.vs.control; bulk RNA-seq,Homo_714,MCI.vs.control; bulk RNA-seq,Homo_633,AD.vs.control; bulk RNA-seq,Homo_633,AD.vs.MCI; bulk RNA-seq,Homo_633,MCI.vs.control	5
BP	GO:1904587	response to glycoprotein	bulk RNA-seq,Homo_723,MCI.vs.control; bulk RNA-seq,Homo_633,AD.vs.control; bulk RNA-seq,Homo_633,MCI.vs.control	3
MF	GO:0070878	primary miRNA binding	bulk RNA-seq,Homo_723,MCI.vs.control; bulk RNA-seq,Homo_714,MCI.vs.control; bulk RNA-seq,Homo_633,AD.vs.control; bulk RNA-	4
BP	GO:0032469	endoplasmic reticulum calcium ion homeostasis	bulk RNA-seq,Homo_723,MCI.vs.control; bulk RNA-seq,Homo_714,MCI.vs.control; bulk RNA-seq,Homo_633,AD.vs.control; bulk RNA-seq,Homo_633,AD.vs.MCI; bulk RNA-seq,Homo_633,MCI.vs.control	5
MF	GO:0004706	JUN kinase kinase kinase activity	bulk RNA-seq,Homo_723,MCI.vs.control	1
BP	GO:0010992	ubiquitin recycling	bulk RNA-seq,Homo_723,MCI.vs.control; bulk RNA-seq,Homo_714,MCI.vs.control; bulk RNA-seq,Homo_633,AD.vs.control; bulk RNA-	4
BP	GO:0042181	ketone biosynthetic process	bulk RNA-seq,Homo_723,MCI.vs.control; bulk RNA-seq,Homo_714,MCI.vs.control; bulk RNA-seq,Homo_633,AD.vs.control	3
BP	GO:0043555	regulation of translation in response to stress	bulk RNA-seq,Homo_723,MCI.vs.control; bulk RNA-seq,Homo_714,MCI.vs.control; bulk RNA-seq,Homo_633,AD.vs.control; bulk RNA-	4
CC	GO:0005665	RNA polymerase II, core complex	bulk RNA-seq,Homo_723,MCI.vs.control; bulk RNA-seq,Homo_714,MCI.vs.control; bulk RNA-seq,Homo_633,AD.vs.control; bulk RNA-seq,Homo_633,AD.vs.MCI; bulk RNA-seq,Homo_633,MCI.vs.control	5
BP	GO:1904353	regulation of telomere capping	bulk RNA-seq,Homo_723,MCI.vs.control; bulk RNA-seq,Homo_714,MCI.vs.control; bulk RNA-seq,Homo_633,AD.vs.control; bulk RNA-	4
BP	GO:0002357	defense response to tumor cell	bulk RNA-seq,Homo_723,MCI.vs.control; bulk RNA-seq,Homo_633,AD.vs.control	2
BP	GO:0044030	regulation of DNA methylation	bulk RNA-seq,Homo_723,MCI.vs.control; bulk RNA-seq,Homo_714,MCI.vs.control; bulk RNA-seq,Homo_633,AD.vs.control; bulk RNA-	4
BP	GO:1902644	tertiary alcohol metabolic process	bulk RNA-seq,Homo_723,MCI.vs.control	1
BP	GO:0035809	regulation of urine volume	bulk RNA-seq,Homo_723,MCI.vs.control; bulk RNA-seq,Homo_633,MCI.vs.control	2
CC	GO:0033270	paranode region of axon	bulk RNA-seq,Homo_723,MCI.vs.control; bulk RNA-seq,Homo_714,MCI.vs.control	2
BP	GO:1905668	positive regulation of protein localization to endosome	bulk RNA-seq,Homo_723,MCI.vs.control; bulk RNA-seq,Homo_714,MCI.vs.control; bulk RNA-seq,Homo_633,AD.vs.control; bulk RNA-	4
MF	GO:0022889	serine transmembrane transporter activity	bulk RNA-seq,Homo_723,MCI.vs.control; bulk RNA-seq,Homo_714,MCI.vs.control; bulk RNA-seq,Homo_633,AD.vs.control; bulk RNA-seq,Homo_633,AD.vs.MCI; bulk RNA-seq,Homo_633,MCI.vs.control	5
BP	GO:0032329	serine transport	bulk RNA-seq,Homo_723,MCI.vs.control; bulk RNA-seq,Homo_714,MCI.vs.control; bulk RNA-seq,Homo_633,AD.vs.control; bulk RNA-seq,Homo_633,AD.vs.MCI; bulk RNA-seq,Homo_633,MCI.vs.control	5
BP	GO:0019065	receptor-mediated endocytosis of virus by host cell	bulk RNA-seq,Homo_723,MCI.vs.control; bulk RNA-seq,Homo_714,MCI.vs.control; bulk RNA-seq,Homo_633,AD.vs.control	3
CC	GO:0005868	cytoplasmic dynein complex	bulk RNA-seq,Homo_723,MCI.vs.control; bulk RNA-seq,Homo_714,MCI.vs.control	2
MF	GO:0000400	four-way junction DNA binding	bulk RNA-seq,Homo_723,MCI.vs.control; bulk RNA-seq,Homo_714,MCI.vs.control; bulk RNA-seq,Homo_633,AD.vs.control; bulk RNA-seq,Homo_633,AD.vs.MCI; bulk RNA-seq,Homo_633,MCI.vs.control	5
BP	GO:0033008	positive regulation of mast cell activation involved in	bulk RNA-seq,Homo_723,MCI.vs.control; bulk RNA-seq,Homo_714,MCI.vs.control	2
BP	GO:0043306	positive regulation of mast cell degranulation	bulk RNA-seq,Homo_723,MCI.vs.control; bulk RNA-seq,Homo_714,MCI.vs.control	2
BP	GO:0034144	negative regulation of toll-like receptor 4 signaling pathway	bulk RNA-seq,Homo_723,MCI.vs.control; bulk RNA-seq,Homo_714,MCI.vs.control	2
MF	GO:0051011	microtubule minus-end binding	bulk RNA-seq,Homo_723,MCI.vs.control; bulk RNA-seq,Homo_714,MCI.vs.control; bulk RNA-seq,Homo_633,AD.vs.control; bulk RNA-	4
MF	GO:0005035	death receptor activity	bulk RNA-seq,Homo_723,MCI.vs.control	1
MF	GO:0016409	palmitoyltransferase activity	bulk RNA-seq,Homo_723,MCI.vs.control; bulk RNA-seq,Homo_714,MCI.vs.control; bulk RNA-seq,Homo_633,AD.vs.control; bulk RNA-	4
BP	GO:0035563	positive regulation of chromatin binding	bulk RNA-seq,Homo_723,MCI.vs.control	1
CC	GO:0044753	amphisome	bulk RNA-seq,Homo_723,MCI.vs.control; bulk RNA-seq,Homo_714,MCI.vs.control; bulk RNA-seq,Homo_633,AD.vs.control; bulk RNA-seq,Homo_633,AD.vs.MCI; bulk RNA-seq,Homo_633,MCI.vs.control	5
MF	GO:0052629	phosphatidylinositol-3,5-bisphosphate 3-phosphatase activity	bulk RNA-seq,Homo_723,MCI.vs.control; bulk RNA-seq,Homo_714,MCI.vs.control; bulk RNA-seq,Homo_633,AD.vs.control; bulk RNA-seq,Homo_633,AD.vs.MCI; bulk RNA-seq,Homo_633,MCI.vs.control	5
MF	GO:0035251	UDP-glucosyltransferase activity	bulk RNA-seq,Homo_723,MCI.vs.control; bulk RNA-seq,Homo_714,MCI.vs.control; bulk RNA-seq,Homo_633,AD.vs.control; bulk RNA-seq,Homo_633,AD.vs.MCI; bulk RNA-seq,Homo_633,MCI.vs.control	5
BP	GO:1904238	pericyte cell differentiation	bulk RNA-seq,Homo_723,MCI.vs.control; bulk RNA-seq,Homo_714,MCI.vs.control	2
BP	GO:0046643	regulation of gamma-delta T cell activation	bulk RNA-seq,Homo_723,MCI.vs.control; bulk RNA-seq,Homo_714,MCI.vs.control	2
BP	GO:0043031	negative regulation of macrophage activation	bulk RNA-seq,Homo_723,MCI.vs.control; bulk RNA-seq,Homo_714,MCI.vs.control	2
MF	GO:0016776	phosphotransferase activity, phosphate group as acceptor	bulk RNA-seq,Homo_723,MCI.vs.control; bulk RNA-seq,Homo_633,AD.vs.control; bulk RNA-seq,Homo_633,AD.vs.MCI	3
MF	GO:0048487	beta-tubulin binding	bulk RNA-seq,Homo_723,MCI.vs.control; bulk RNA-seq,Homo_714,MCI.vs.control; bulk RNA-seq,Homo_633,AD.vs.control; bulk RNA-	4
CC	GO:0031143	pseudopodium	bulk RNA-seq,Homo_723,MCI.vs.control; bulk RNA-seq,Homo_633,AD.vs.control; bulk RNA-seq,Homo_633,AD.vs.MCI	3
BP	GO:1903429	regulation of cell maturation	bulk RNA-seq,Homo_723,MCI.vs.control; bulk RNA-seq,Homo_714,AD.vs.MCI; bulk RNA-seq,Homo_714,MCI.vs.control	3
MF	GO:0015106	bicarbonate transmembrane transporter activity	bulk RNA-seq,Homo_723,MCI.vs.control; bulk RNA-seq,Homo_633,AD.vs.control; bulk RNA-seq,Homo_633,AD.vs.MCI	3
BP	GO:0043379	memory T cell differentiation	bulk RNA-seq,Homo_723,MCI.vs.control; bulk RNA-seq,Homo_714,MCI.vs.control	2
BP	GO:0043922	negative regulation by host of viral transcription	bulk RNA-seq,Homo_723,MCI.vs.control; bulk RNA-seq,Homo_633,AD.vs.control; bulk RNA-seq,Homo_633,AD.vs.MCI	3
BP	GO:0043516	regulation of DNA damage response, signal transduction by p53 class mediator	bulk RNA-seq,Homo_723,MCI.vs.control; bulk RNA-seq,Homo_714,MCI.vs.control; bulk RNA-seq,Homo_633,AD.vs.control; bulk RNA-seq,Homo_633,AD.vs.MCI	4
BP	GO:0033539	fatty acid beta-oxidation using acyl-CoA dehydrogenase	bulk RNA-seq,Homo_723,MCI.vs.control; bulk RNA-seq,Homo_633,AD.vs.control	2
BP	GO:0061099	negative regulation of protein tyrosine kinase activity	bulk RNA-seq,Homo_723,MCI.vs.control; bulk RNA-seq,Homo_714,MCI.vs.control	2

MF	GO:0015645	fatty acid ligase activity	bulk RNA-seq,Homo_723,MCI.vs.control	1
MF	GO:0004865	protein serine/threonine phosphatase inhibitor activity	bulk RNA-seq,Homo_723,MCI.vs.control	1
BP	GO:0061158	3'-UTR-mediated mRNA destabilization	bulk RNA-seq,Homo_723,MCI.vs.control; bulk RNA-seq,Homo_714,MCI.vs.control; bulk RNA-seq,Homo_633,AD.vs.control	3
BP	GO:0009435	NAD biosynthetic process	bulk RNA-seq,Homo_723,MCI.vs.control	1
BP	GO:0018027	peptidyl-lysine dimethylation	bulk RNA-seq,Homo_723,MCI.vs.control; bulk RNA-seq,Homo_714,MCI.vs.control; bulk RNA-seq,Homo_633,AD.vs.control; bulk RNA-	4
BP	GO:0000491	small nucleolar ribonucleoprotein complex assembly	bulk RNA-seq,Homo_723,MCI.vs.control; bulk RNA-seq,Homo_714,MCI.vs.control; bulk RNA-seq,Homo_633,AD.vs.control; bulk RNA-seq,Homo_633,AD.vs.MCI; bulk RNA-seq,Homo_633,MCI.vs.control	5
BP	GO:1903201	regulation of oxidative stress-induced cell death	bulk RNA-seq,Homo_723,MCI.vs.control; bulk RNA-seq,Homo_714,AD.vs.MCI; bulk RNA-seq,Homo_714,MCI.vs.control; bulk RNA-seq,Homo_633,AD.vs.control; bulk RNA-seq,Homo_633,AD.vs.MCI	5
MF	GO:0016307	phosphatidylinositol phosphate kinase activity	bulk RNA-seq,Homo_723,MCI.vs.control; bulk RNA-seq,Homo_714,MCI.vs.control; bulk RNA-seq,Homo_633,AD.vs.control	3
BP	GO:0030511	positive regulation of transforming growth factor beta receptor signaling pathway	bulk RNA-seq,Homo_723,MCI.vs.control; bulk RNA-seq,Homo_714,MCI.vs.control; bulk RNA-seq,Homo_633,AD.vs.control; bulk RNA-seq,Homo_633,AD.vs.MCI	4
BP	GO:1903846	positive regulation of cellular response to transforming growth factor beta stimulus	bulk RNA-seq,Homo_723,MCI.vs.control; bulk RNA-seq,Homo_714,MCI.vs.control; bulk RNA-seq,Homo_633,AD.vs.control; bulk RNA-seq,Homo_633,AD.vs.MCI	4
MF	GO:0032052	bile acid binding	bulk RNA-seq,Homo_723,MCI.vs.control; bulk RNA-seq,Homo_714,AD.vs.MCI; bulk RNA-seq,Homo_714,MCI.vs.control	3
BP	GO:0035627	ceramide transport	bulk RNA-seq,Homo_723,MCI.vs.control; bulk RNA-seq,Homo_633,AD.vs.control; bulk RNA-seq,Homo_633,AD.vs.MCI	3
BP	GO:0071318	cellular response to ATP	bulk RNA-seq,Homo_723,MCI.vs.control; bulk RNA-seq,Homo_633,AD.vs.control	2
BP	GO:2001138	regulation of phospholipid transport	bulk RNA-seq,Homo_723,MCI.vs.control; bulk RNA-seq,Homo_714,AD.vs.MCI; bulk RNA-seq,Homo_714,MCI.vs.control	3
BP	GO:0010883	regulation of lipid storage	bulk RNA-seq,Homo_723,MCI.vs.control; bulk RNA-seq,Homo_714,MCI.vs.control; bulk RNA-seq,Homo_633,AD.vs.control; bulk RNA-	4
BP	GO:0036363	transforming growth factor beta activation	bulk RNA-seq,Homo_723,MCI.vs.control; bulk RNA-seq,Homo_714,MCI.vs.control	2
BP	GO:0009084	glutamine family amino acid biosynthetic process	bulk RNA-seq,Homo_723,MCI.vs.control; bulk RNA-seq,Homo_714,MCI.vs.control	2
BP	GO:2000271	positive regulation of fibroblast apoptotic process	bulk RNA-seq,Homo_723,MCI.vs.control	1
BP	GO:0040015	negative regulation of multicellular organism growth	bulk RNA-seq,Homo_723,MCI.vs.control; bulk RNA-seq,Homo_633,AD.vs.control; bulk RNA-seq,Homo_633,AD.vs.MCI	3
BP	GO:0046640	regulation of alpha-beta T cell proliferation	bulk RNA-seq,Homo_723,MCI.vs.control; bulk RNA-seq,Homo_714,MCI.vs.control; bulk RNA-seq,Homo_633,AD.vs.control	3
BP	GO:0035521	monoubiquitinated histone deubiquitination	bulk RNA-seq,Homo_723,MCI.vs.control; bulk RNA-seq,Homo_714,MCI.vs.control; bulk RNA-seq,Homo_633,AD.vs.control; bulk RNA-seq,Homo_633,AD.vs.MCI; bulk RNA-seq,Homo_633,MCI.vs.control	5
BP	GO:0035522	monoubiquitinated histone H2A deubiquitination	bulk RNA-seq,Homo_723,MCI.vs.control; bulk RNA-seq,Homo_714,MCI.vs.control; bulk RNA-seq,Homo_633,AD.vs.control; bulk RNA-seq,Homo_633,AD.vs.MCI; bulk RNA-seq,Homo_633,MCI.vs.control	5
BP	GO:2000757	negative regulation of peptidyl-lysine acetylation	bulk RNA-seq,Homo_723,MCI.vs.control; bulk RNA-seq,Homo_633,AD.vs.control; bulk RNA-seq,Homo_633,AD.vs.MCI	3
BP	GO:0090148	membrane fission	bulk RNA-seq,Homo_723,MCI.vs.control; bulk RNA-seq,Homo_714,MCI.vs.control; bulk RNA-seq,Homo_633,AD.vs.control; bulk RNA-	4
BP	GO:0032196	transposition	bulk RNA-seq,Homo_723,MCI.vs.control; bulk RNA-seq,Homo_714,MCI.vs.control; bulk RNA-seq,Homo_633,AD.vs.control	3
MF	GO:0015278	calcium-release channel activity	bulk RNA-seq,Homo_723,MCI.vs.control; bulk RNA-seq,Homo_714,MCI.vs.control; bulk RNA-seq,Homo_633,AD.vs.control; bulk RNA-seq,Homo_633,AD.vs.MCI; bulk RNA-seq,Homo_633,MCI.vs.control	5
MF	GO:0140693	molecular condensate scaffold activity	bulk RNA-seq,Homo_723,MCI.vs.control; bulk RNA-seq,Homo_633,AD.vs.control; bulk RNA-seq,Homo_633,AD.vs.MCI	3
BP	GO:0032689	negative regulation of interferon-gamma production	bulk RNA-seq,Homo_723,MCI.vs.control; bulk RNA-seq,Homo_714,MCI.vs.control	2
BP	GO:0007549	dosage compensation	bulk RNA-seq,Homo_723,MCI.vs.control; bulk RNA-seq,Homo_714,MCI.vs.control; bulk RNA-seq,Homo_633,AD.vs.control; bulk RNA-seq,Homo_633,AD.vs.MCI; bulk RNA-seq,SRP325058,AD.vs.control	5
BP	GO:1900262	regulation of DNA-directed DNA polymerase activity	bulk RNA-seq,Homo_723,MCI.vs.control; bulk RNA-seq,Homo_714,MCI.vs.control; bulk RNA-seq,Homo_633,AD.vs.control	3
BP	GO:1900264	positive regulation of DNA-directed DNA polymerase	bulk RNA-seq,Homo_723,MCI.vs.control; bulk RNA-seq,Homo_714,MCI.vs.control; bulk RNA-seq,Homo_633,AD.vs.control	3
MF	GO:0016004	phospholipase activator activity	bulk RNA-seq,Homo_723,MCI.vs.control; bulk RNA-seq,Homo_633,AD.vs.control; bulk RNA-seq,Homo_633,AD.vs.MCI	3
BP	GO:0034975	protein folding in endoplasmic reticulum	bulk RNA-seq,Homo_723,MCI.vs.control; bulk RNA-seq,Homo_714,MCI.vs.control; bulk RNA-seq,Homo_633,AD.vs.control; bulk RNA-	4
CC	GO:0031094	platelet dense tubular network	bulk RNA-seq,Homo_723,MCI.vs.control; bulk RNA-seq,Homo_714,MCI.vs.control; bulk RNA-seq,Homo_633,AD.vs.control; bulk RNA-	4
BP	GO:0032836	glomerular basement membrane development	bulk RNA-seq,Homo_723,MCI.vs.control; bulk RNA-seq,Homo_714,MCI.vs.control; bulk RNA-seq,Homo_633,AD.vs.control	3
BP	GO:0046037	GMP metabolic process	bulk RNA-seq,Homo_723,MCI.vs.control; bulk RNA-seq,Homo_714,MCI.vs.control; bulk RNA-seq,Homo_633,AD.vs.control	3
BP	GO:0034154	toll-like receptor 7 signaling pathway	bulk RNA-seq,Homo_723,MCI.vs.control; bulk RNA-seq,Homo_714,MCI.vs.control; bulk RNA-seq,Homo_633,AD.vs.control; bulk RNA-seq,Homo_633,AD.vs.MCI; bulk RNA-seq,Homo_633,MCI.vs.control	5
BP	GO:0036159	inner dynein arm assembly	bulk RNA-seq,Homo_723,MCI.vs.control	1
BP	GO:0033089	positive regulation of T cell differentiation in thymus	bulk RNA-seq,Homo_723,MCI.vs.control; bulk RNA-seq,Homo_714,MCI.vs.control	2
MF	GO:0033764	steroid dehydrogenase activity, acting on the CH-OH group of donors, NAD or NADP as acceptor	bulk RNA-seq,Homo_723,MCI.vs.control; bulk RNA-seq,Homo_714,AD.vs.MCI	2
BP	GO:0046473	phosphatidic acid metabolic process	bulk RNA-seq,Homo_723,MCI.vs.control; bulk RNA-seq,Homo_633,AD.vs.control; bulk RNA-seq,Homo_633,AD.vs.MCI	3
BP	GO:0061615	glycolytic process through fructose-6-phosphate	bulk RNA-seq,Homo_723,MCI.vs.control; bulk RNA-seq,Homo_714,MCI.vs.control; bulk RNA-seq,Homo_633,AD.vs.control; bulk RNA-	4
BP	GO:0075733	intracellular transport of virus	bulk RNA-seq,Homo_723,MCI.vs.control; bulk RNA-seq,Homo_714,MCI.vs.control; bulk RNA-seq,Homo_633,AD.vs.control; bulk RNA-	4
BP	GO:1903978	regulation of microglial cell activation	bulk RNA-seq,Homo_723,MCI.vs.control	1
BP	GO:0046475	glycerophospholipid catabolic process	bulk RNA-seq,Homo_723,MCI.vs.control; bulk RNA-seq,Homo_714,MCI.vs.control; bulk RNA-seq,Homo_633,AD.vs.control; bulk RNA-	4
CC	GO:0044232	organelle membrane contact site	bulk RNA-seq,Homo_723,MCI.vs.control; bulk RNA-seq,Homo_714,MCI.vs.control; bulk RNA-seq,Homo_633,AD.vs.control; bulk RNA-	4
BP	GO:0001956	positive regulation of neurotransmitter secretion	bulk RNA-seq,Homo_723,MCI.vs.control	1
BP	GO:0050850	positive regulation of calcium-mediated signaling	bulk RNA-seq,Homo_723,MCI.vs.control; bulk RNA-seq,Homo_714,MCI.vs.control; bulk RNA-seq,Homo_633,AD.vs.control	3

BP	GO:0015937	coenzyme A biosynthetic process	bulk RNA-seq,Homo_723,MCI.vs.control; bulk RNA-seq,Homo_714,MCI.vs.control; bulk RNA-seq,Homo_633,AD.vs.control; bulk RNA-seq,Homo_633,AD.vs.MCI; bulk RNA-seq,Homo_633,MCI.vs.control	5
BP	GO:0010885	regulation of cholesterol storage	bulk RNA-seq,Homo_723,MCI.vs.control; bulk RNA-seq,Homo_714,MCI.vs.control; bulk RNA-seq,Homo_633,AD.vs.control; bulk RNA-	4
MF	GO:0043325	phosphatidylinositol-3,4-bisphosphate binding	bulk RNA-seq,Homo_723,MCI.vs.control; bulk RNA-seq,Homo_714,MCI.vs.control	2
MF	GO:0003688	DNA replication origin binding	bulk RNA-seq,Homo_723,MCI.vs.control; bulk RNA-seq,Homo_633,AD.vs.control; bulk RNA-seq,Homo_633,AD.vs.MCI; bulk RNA-	4
BP	GO:0070243	regulation of thymocyte apoptotic process	bulk RNA-seq,Homo_723,MCI.vs.control	1
BP	GO:1903077	negative regulation of protein localization to plasma	bulk RNA-seq,Homo_723,MCI.vs.control; bulk RNA-seq,Homo_633,AD.vs.control; bulk RNA-seq,Homo_633,AD.vs.MCI	3
BP	GO:0070942	neutrophil mediated cytotoxicity	bulk RNA-seq,Homo_723,MCI.vs.control; bulk RNA-seq,Homo_714,AD.vs.MCI; bulk RNA-seq,Homo_714,MCI.vs.control; bulk RNA-	4
BP	GO:0071107	response to parathyroid hormone	bulk RNA-seq,Homo_723,MCI.vs.control	1
CC	GO:0017119	Golgi transport complex	bulk RNA-seq,Homo_723,MCI.vs.control; bulk RNA-seq,Homo_714,MCI.vs.control; bulk RNA-seq,Homo_633,AD.vs.control; bulk RNA-	4
BP	GO:0015791	polyol transmembrane transport	bulk RNA-seq,Homo_723,MCI.vs.control; bulk RNA-seq,Homo_714,MCI.vs.control; bulk RNA-seq,Homo_633,AD.vs.control	3
BP	GO:1900151	regulation of nuclear-transcribed mRNA catabolic process, deadenylation-dependent decay	bulk RNA-seq,Homo_723,MCI.vs.control; bulk RNA-seq,Homo_633,AD.vs.control; bulk RNA-seq,Homo_633,AD.vs.MCI	3
MF	GO:0015295	solute:proton symporter activity	bulk RNA-seq,Homo_723,MCI.vs.control; bulk RNA-seq,Homo_633,AD.vs.control	2
CC	GO:0031464	Cul4A-RING E3 ubiquitin ligase complex	bulk RNA-seq,Homo_723,MCI.vs.control; bulk RNA-seq,Homo_714,MCI.vs.control; bulk RNA-seq,Homo_633,AD.vs.control; bulk RNA-	4
CC	GO:0001741	XY body	bulk RNA-seq,Homo_723,MCI.vs.control; bulk RNA-seq,Homo_714,MCI.vs.control; bulk RNA-seq,Homo_633,MCI.vs.control	3
BP	GO:0007250	activation of NF-kappaB-inducing kinase activity	bulk RNA-seq,Homo_723,MCI.vs.control; bulk RNA-seq,Homo_714,MCI.vs.control; bulk RNA-seq,Homo_633,AD.vs.control	3
BP	GO:0046174	polyol catabolic process	bulk RNA-seq,Homo_723,MCI.vs.control; bulk RNA-seq,Homo_714,MCI.vs.control; bulk RNA-seq,Homo_633,AD.vs.control; bulk RNA-seq,Homo_633,AD.vs.MCI; bulk RNA-seq,Homo_633,MCI.vs.control	5
BP	GO:0070341	fat cell proliferation	bulk RNA-seq,Homo_723,MCI.vs.control; bulk RNA-seq,Homo_633,AD.vs.control; bulk RNA-seq,Homo_633,AD.vs.MCI	3
BP	GO:0060159	regulation of dopamine receptor signaling pathway	bulk RNA-seq,Homo_723,MCI.vs.control; bulk RNA-seq,Homo_633,AD.vs.control	2
BP	GO:0140112	extracellular vesicle biogenesis	bulk RNA-seq,Homo_723,MCI.vs.control; bulk RNA-seq,Homo_633,AD.vs.control; bulk RNA-seq,Homo_633,AD.vs.MCI	3
CC	GO:0031617	NMS complex	bulk RNA-seq,Homo_723,MCI.vs.control; bulk RNA-seq,Homo_633,AD.vs.control	2
MF	GO:0032454	histone H3-methyl-lysine-9 demethylase activity	bulk RNA-seq,Homo_723,MCI.vs.control; bulk RNA-seq,Homo_714,MCI.vs.control; bulk RNA-seq,Homo_633,AD.vs.control; bulk RNA-	4
BP	GO:2000765	regulation of cytoplasmic translation	bulk RNA-seq,Homo_723,MCI.vs.control; bulk RNA-seq,Homo_633,AD.vs.control; bulk RNA-seq,Homo_633,AD.vs.MCI	3
BP	GO:0071480	cellular response to gamma radiation	bulk RNA-seq,Homo_723,MCI.vs.control; bulk RNA-seq,Homo_633,AD.vs.control; bulk RNA-seq,Homo_633,AD.vs.MCI	3
MF	GO:0106222	lncRNA binding	bulk RNA-seq,Homo_723,MCI.vs.control; bulk RNA-seq,Homo_714,MCI.vs.control; bulk RNA-seq,Homo_633,AD.vs.control; bulk RNA-	4
BP	GO:0046597	negative regulation of viral entry into host cell	bulk RNA-seq,Homo_723,MCI.vs.control	1
BP	GO:0035313	wound healing, spreading of epidermal cells	bulk RNA-seq,Homo_723,MCI.vs.control; bulk RNA-seq,Homo_633,AD.vs.control	2
BP	GO:0010954	positive regulation of protein processing	bulk RNA-seq,Homo_723,MCI.vs.control; bulk RNA-seq,Homo_633,AD.vs.control	2
CC	GO:0044233	mitochondria-associated endoplasmic reticulum membrane	bulk RNA-seq,Homo_723,MCI.vs.control; bulk RNA-seq,Homo_714,MCI.vs.control; bulk RNA-seq,Homo_633,AD.vs.control; bulk RNA-seq,Homo_633,AD.vs.MCI; bulk RNA-seq,Homo_633,MCI.vs.control	5
MF	GO:0016769	transferase activity, transferring nitrogenous groups	bulk RNA-seq,Homo_723,MCI.vs.control	1
BP	GO:0010827	regulation of glucose transmembrane transport	bulk RNA-seq,Homo_723,MCI.vs.control; bulk RNA-seq,Homo_633,AD.vs.control; bulk RNA-seq,Homo_633,AD.vs.MCI	3
BP	GO:0048671	negative regulation of collateral sprouting	bulk RNA-seq,Homo_723,MCI.vs.control	1
BP	GO:0030449	regulation of complement activation	bulk RNA-seq,Homo_723,MCI.vs.control; bulk RNA-seq,Homo_633,AD.vs.control; bulk RNA-seq,Homo_633,AD.vs.MCI	3
BP	GO:0051791	medium-chain fatty acid metabolic process	bulk RNA-seq,Homo_723,MCI.vs.control; bulk RNA-seq,Homo_714,MCI.vs.control	2
BP	GO:0048680	positive regulation of axon regeneration	bulk RNA-seq,Homo_723,MCI.vs.control	1
MF	GO:0002161	aminoacyl-tRNA editing activity	bulk RNA-seq,Homo_723,MCI.vs.control; bulk RNA-seq,Homo_714,MCI.vs.control; bulk RNA-seq,Homo_633,AD.vs.control; bulk RNA-seq,Homo_633,AD.vs.MCI; bulk RNA-seq,Homo_633,MCI.vs.control	5
BP	GO:0106074	aminoacyl-tRNA metabolism involved in translational fidelity	bulk RNA-seq,Homo_723,MCI.vs.control; bulk RNA-seq,Homo_714,MCI.vs.control; bulk RNA-seq,Homo_633,AD.vs.control; bulk RNA-seq,Homo_633,AD.vs.MCI; bulk RNA-seq,Homo_633,MCI.vs.control	5
BP	GO:1903651	positive regulation of cytoplasmic transport	bulk RNA-seq,Homo_723,MCI.vs.control; bulk RNA-seq,Homo_633,AD.vs.control; bulk RNA-seq,Homo_633,AD.vs.MCI	3
BP	GO:0031000	response to caffeine	bulk RNA-seq,Homo_723,MCI.vs.control	1
BP	GO:0010614	negative regulation of cardiac muscle hypertrophy	bulk RNA-seq,Homo_723,MCI.vs.control; bulk RNA-seq,Homo_714,MCI.vs.control; bulk RNA-seq,Homo_633,AD.vs.control	3
BP	GO:0019835	cytolysis	bulk RNA-seq,Homo_723,MCI.vs.control; bulk RNA-seq,Homo_714,AD.vs.MCI; bulk RNA-seq,Homo_714,MCI.vs.control; bulk RNA-seq,Homo_633,AD.vs.control; bulk RNA-seq,Homo_633,AD.vs.MCI	5
BP	GO:0002920	regulation of humoral immune response	bulk RNA-seq,Homo_723,MCI.vs.control; bulk RNA-seq,Homo_714,AD.vs.MCI; bulk RNA-seq,Homo_714,MCI.vs.control; bulk RNA-seq,Homo_633,AD.vs.control; bulk RNA-seq,Homo_633,AD.vs.MCI	5
BP	GO:1903589	positive regulation of blood vessel endothelial cell proliferation involved in sprouting angiogenesis	bulk RNA-seq,Homo_723,MCI.vs.control; bulk RNA-seq,Homo_714,AD.vs.control; bulk RNA-seq,Homo_714,AD.vs.MCI	3
MF	GO:0015250	water channel activity	bulk RNA-seq,Homo_723,MCI.vs.control	1
BP	GO:0071236	cellular response to antibiotic	bulk RNA-seq,Homo_723,MCI.vs.control; bulk RNA-seq,Homo_633,AD.vs.control; bulk RNA-seq,Homo_633,AD.vs.MCI	3
BP	GO:0007175	negative regulation of epidermal growth factor-activated receptor activity	bulk RNA-seq,Homo_723,MCI.vs.control	1
BP	GO:0061744	motor behavior	bulk RNA-seq,Homo_723,MCI.vs.control	1
MF	GO:0008401	retinoic acid 4-hydroxylase activity	bulk RNA-seq,Homo_723,MCI.vs.control; bulk RNA-seq,SRP223445,AD.vs.control	2
BP	GO:0009651	response to salt stress	bulk RNA-seq,Homo_723,MCI.vs.control; bulk RNA-seq,Homo_633,AD.vs.control; bulk RNA-seq,Homo_633,AD.vs.MCI	3

MF	GO:0019205	nucleobase-containing compound kinase activity	bulk RNA-seq,Homo_723,MCI.vs.control; bulk RNA-seq,Homo_714,MCI.vs.control; bulk RNA-seq,Homo_633,AD.vs.control; bulk RNA-	4
MF	GO:0016408	C-acyltransferase activity	bulk RNA-seq,Homo_723,MCI.vs.control	1
BP	GO:0032000	positive regulation of fatty acid beta-oxidation	bulk RNA-seq,Homo_723,MCI.vs.control; bulk RNA-seq,Homo_714,MCI.vs.control; bulk RNA-seq,Homo_633,AD.vs.control; bulk RNA-	4
BP	GO:0034350	regulation of glial cell apoptotic process	bulk RNA-seq,Homo_723,MCI.vs.control; bulk RNA-seq,Homo_714,MCI.vs.control	2
CC	GO:0097550	transcription preinitiation complex	bulk RNA-seq,Homo_723,MCI.vs.control; bulk RNA-seq,Homo_714,MCI.vs.control; bulk RNA-seq,Homo_633,AD.vs.control; bulk RNA-	4
CC	GO:0005688	U6 snRNP	bulk RNA-seq,Homo_723,MCI.vs.control; bulk RNA-seq,Homo_714,MCI.vs.control; bulk RNA-seq,Homo_633,AD.vs.control; bulk RNA-	4
BP	GO:0010172	embryonic body morphogenesis	bulk RNA-seq,Homo_723,MCI.vs.control; bulk RNA-seq,Homo_633,AD.vs.control	2
MF	GO:0034595	phosphatidylinositol phosphate 5-phosphatase activity	bulk RNA-seq,Homo_723,MCI.vs.control; bulk RNA-seq,Homo_714,MCI.vs.control; bulk RNA-seq,Homo_633,AD.vs.control; bulk RNA-seq,Homo_633,AD.vs.MCI; bulk RNA-seq,Homo_633,MCI.vs.control	5
MF	GO:0034450	ubiquitin-ubiquitin ligase activity	bulk RNA-seq,Homo_723,MCI.vs.control; bulk RNA-seq,Homo_633,AD.vs.control; bulk RNA-seq,Homo_633,AD.vs.MCI; bulk RNA-	4
MF	GO:0005123	death receptor binding	bulk RNA-seq,Homo_723,MCI.vs.control; bulk RNA-seq,Homo_633,AD.vs.control	2
BP	GO:0009067	aspartate family amino acid biosynthetic process	bulk RNA-seq,Homo_723,MCI.vs.control; bulk RNA-seq,Homo_633,AD.vs.control; bulk RNA-seq,Homo_633,AD.vs.MCI	3
CC	GO:0097038	perinuclear endoplasmic reticulum	bulk RNA-seq,Homo_723,MCI.vs.control; bulk RNA-seq,Homo_714,MCI.vs.control; bulk RNA-seq,Homo_633,AD.vs.control; bulk RNA-	4
BP	GO:2000772	regulation of cellular senescence	bulk RNA-seq,Homo_723,MCI.vs.control; bulk RNA-seq,Homo_633,AD.vs.control	2
BP	GO:0009065	glutamine family amino acid catabolic process	bulk RNA-seq,Homo_723,MCI.vs.control; bulk RNA-seq,Homo_714,AD.vs.MCI	2
BP	GO:0002763	positive regulation of myeloid leukocyte differentiation	bulk RNA-seq,Homo_723,MCI.vs.control; scRNA-seq,SRP215507,CD8+ T cell_3-AD.vs.control	2
BP	GO:0032303	regulation of icosanoid secretion	bulk RNA-seq,Homo_723,MCI.vs.control	1
BP	GO:0032369	negative regulation of lipid transport	bulk RNA-seq,Homo_723,MCI.vs.control; bulk RNA-seq,Homo_714,AD.vs.MCI; bulk RNA-seq,Homo_714,MCI.vs.control	3
BP	GO:0007099	centriole replication	bulk RNA-seq,Homo_723,MCI.vs.control; bulk RNA-seq,Homo_714,MCI.vs.control; bulk RNA-seq,Homo_633,AD.vs.control; bulk RNA-	4
MF	GO:0008138	protein tyrosine/serine/threonine phosphatase activity	bulk RNA-seq,Homo_723,MCI.vs.control; bulk RNA-seq,Homo_714,MCI.vs.control; bulk RNA-seq,Homo_633,AD.vs.control; bulk RNA-	4
MF	GO:0015377	cation:chloride symporter activity	bulk RNA-seq,Homo_723,MCI.vs.control; bulk RNA-seq,ROSMAP,AD.vs.control	2
BP	GO:0032873	negative regulation of stress-activated MAPK cascade	bulk RNA-seq,Homo_723,MCI.vs.control; bulk RNA-seq,Homo_633,AD.vs.control; bulk RNA-seq,Homo_633,AD.vs.MCI	3
BP	GO:0070303	negative regulation of stress-activated protein kinase	bulk RNA-seq,Homo_723,MCI.vs.control; bulk RNA-seq,Homo_633,AD.vs.control; bulk RNA-seq,Homo_633,AD.vs.MCI	3
BP	GO:0050884	neuromuscular process controlling posture	bulk RNA-seq,Homo_723,MCI.vs.control	1
BP	GO:0032060	bleb assembly	bulk RNA-seq,Homo_723,MCI.vs.control; bulk RNA-seq,Homo_714,MCI.vs.control; bulk RNA-seq,Homo_633,AD.vs.control	3
BP	GO:0000423	mitophagy	bulk RNA-seq,Homo_723,MCI.vs.control; bulk RNA-seq,Homo_714,AD.vs.control; bulk RNA-seq,Homo_714,MCI.vs.control; bulk RNA-seq,Homo_633,AD.vs.control; bulk RNA-seq,Homo_633,AD.vs.MCI	5
BP	GO:0002281	macrophage activation involved in immune response	bulk RNA-seq,Homo_723,MCI.vs.control; bulk RNA-seq,Homo_714,AD.vs.MCI; bulk RNA-seq,Homo_633,AD.vs.control	3
BP	GO:0010801	negative regulation of peptidyl-threonine phosphorylation	bulk RNA-seq,Homo_723,MCI.vs.control	1
BP	GO:0046639	negative regulation of alpha-beta T cell differentiation	bulk RNA-seq,Homo_723,MCI.vs.control; bulk RNA-seq,Homo_714,MCI.vs.control; bulk RNA-seq,Homo_633,AD.vs.control; bulk RNA-	4
MF	GO:0140666	annealing activity	bulk RNA-seq,Homo_723,MCI.vs.control; bulk RNA-seq,Homo_633,AD.vs.control	2
MF	GO:0035259	nuclear glucocorticoid receptor binding	bulk RNA-seq,Homo_723,MCI.vs.control; bulk RNA-seq,Homo_714,MCI.vs.control	2
BP	GO:0090435	protein localization to nuclear envelope	bulk RNA-seq,Homo_723,MCI.vs.control; bulk RNA-seq,Homo_633,AD.vs.control; bulk RNA-seq,Homo_633,AD.vs.MCI	3
CC	GO:0010369	chromocenter	bulk RNA-seq,Homo_723,MCI.vs.control	1
MF	GO:0015562	efflux transmembrane transporter activity	bulk RNA-seq,Homo_723,MCI.vs.control	1
BP	GO:0006000	fructose metabolic process	bulk RNA-seq,Homo_723,MCI.vs.control; bulk RNA-seq,Homo_633,AD.vs.control; bulk RNA-seq,Homo_633,AD.vs.MCI	3
MF	GO:0004553	hydrolase activity, hydrolyzing O-glycosyl compounds	bulk RNA-seq,Homo_723,MCI.vs.control; bulk RNA-seq,Homo_714,MCI.vs.control; bulk RNA-seq,Homo_633,AD.vs.control; bulk RNA-	4
BP	GO:0097734	extracellular exosome biogenesis	bulk RNA-seq,Homo_723,MCI.vs.control; bulk RNA-seq,Homo_633,AD.vs.control; bulk RNA-seq,Homo_633,AD.vs.MCI	3
MF	GO:0016814	hydrolase activity, acting on carbon-nitrogen (but not peptide) bonds, in cyclic amidines	bulk RNA-seq,Homo_723,MCI.vs.control; bulk RNA-seq,Homo_714,MCI.vs.control; bulk RNA-seq,Homo_633,AD.vs.control	3
CC	GO:0043218	compact myelin	bulk RNA-seq,Homo_723,MCI.vs.control; bulk RNA-seq,Homo_714,MCI.vs.control; bulk RNA-seq,Homo_633,AD.vs.control	3
BP	GO:1905214	regulation of RNA binding	bulk RNA-seq,Homo_723,MCI.vs.control; bulk RNA-seq,Homo_714,AD.vs.control; bulk RNA-seq,Homo_714,MCI.vs.control	3
BP	GO:0046185	aldehyde catabolic process	bulk RNA-seq,Homo_723,MCI.vs.control; bulk RNA-seq,Homo_633,AD.vs.control; bulk RNA-seq,Homo_633,AD.vs.MCI	3
BP	GO:1901841	regulation of high voltage-gated calcium channel activity	bulk RNA-seq,Homo_723,MCI.vs.control	1
BP	GO:0071415	cellular response to purine-containing compound	bulk RNA-seq,Homo_723,MCI.vs.control	1
MF	GO:0004065	arylsulfatase activity	bulk RNA-seq,Homo_723,MCI.vs.control	1
BP	GO:0046323	glucose import	bulk RNA-seq,Homo_723,MCI.vs.control; bulk RNA-seq,Homo_633,AD.vs.control; bulk RNA-seq,Homo_633,AD.vs.MCI	3
MF	GO:0016725	oxidoreductase activity, acting on CH or CH2 groups	bulk RNA-seq,Homo_723,MCI.vs.control; bulk RNA-seq,Homo_714,AD.vs.MCI; bulk RNA-seq,Homo_714,MCI.vs.control	3
MF	GO:0030275	LRR domain binding	bulk RNA-seq,Homo_723,MCI.vs.control; bulk RNA-seq,Homo_633,AD.vs.control; bulk RNA-seq,Homo_633,AD.vs.MCI	3
BP	GO:0034755	iron ion transmembrane transport	bulk RNA-seq,Homo_723,MCI.vs.control	1
BP	GO:0060831	smoothened signaling pathway involved in dorsal/ventral neural tube patterning	bulk RNA-seq,Homo_723,MCI.vs.control; bulk RNA-seq,Homo_633,AD.vs.control; bulk RNA-seq,Homo_633,AD.vs.MCI	3
BP	GO:0006188	IMP biosynthetic process	bulk RNA-seq,Homo_723,MCI.vs.control; bulk RNA-seq,Homo_714,MCI.vs.control; bulk RNA-seq,Homo_633,AD.vs.control; bulk RNA-	4
BP	GO:0006656	phosphatidylcholine biosynthetic process	bulk RNA-seq,Homo_723,MCI.vs.control; bulk RNA-seq,Homo_714,MCI.vs.control; bulk RNA-seq,Homo_633,AD.vs.control; bulk RNA-	4
BP	GO:0032682	negative regulation of chemokine production	bulk RNA-seq,Homo_723,MCI.vs.control; bulk RNA-seq,Homo_714,MCI.vs.control; scRNA-seq,SRP309935,B cell_1-AD.vs.control	3
BP	GO:0046838	phosphorylated carbohydrate dephosphorylation	bulk RNA-seq,Homo_723,MCI.vs.control; bulk RNA-seq,Homo_714,MCI.vs.control; bulk RNA-seq,Homo_633,AD.vs.control; bulk RNA-seq,Homo_633,AD.vs.MCI; bulk RNA-seq,Homo_633,MCI.vs.control	5

BP	GO:0046855	inositol phosphate dephosphorylation	bulk RNA-seq,Homo_723,MCI.vs.control; bulk RNA-seq,Homo_714,MCI.vs.control; bulk RNA-seq,Homo_633,AD.vs.control; bulk RNA-seq,Homo_633,AD.vs.MCI; bulk RNA-seq,Homo_633,MCI.vs.control	5
MF	GO:0016859	cis-trans isomerase activity	bulk RNA-seq,Homo_723,MCI.vs.control; bulk RNA-seq,Homo_633,AD.vs.control; bulk RNA-seq,Homo_633,AD.vs.MCI	3
BP	GO:0044319	wound healing, spreading of cells	bulk RNA-seq,Homo_723,MCI.vs.control; bulk RNA-seq,Homo_633,AD.vs.control	2
BP	GO:0090505	epiboly involved in wound healing	bulk RNA-seq,Homo_723,MCI.vs.control; bulk RNA-seq,Homo_633,AD.vs.control	2
BP	GO:2000114	regulation of establishment of cell polarity	bulk RNA-seq,Homo_723,MCI.vs.control; bulk RNA-seq,Homo_714,MCI.vs.control; bulk RNA-seq,Homo_633,AD.vs.control; bulk RNA-	4
BP	GO:0045620	negative regulation of lymphocyte differentiation	bulk RNA-seq,Homo_723,MCI.vs.control; bulk RNA-seq,Homo_714,MCI.vs.control	2
BP	GO:2000303	regulation of ceramide biosynthetic process	bulk RNA-seq,Homo_723,MCI.vs.control; bulk RNA-seq,Homo_714,MCI.vs.control	2
BP	GO:0014831	gastro-intestinal system smooth muscle contraction	bulk RNA-seq,Homo_723,MCI.vs.control; bulk RNA-seq,Homo_714,MCI.vs.control	2
BP	GO:0044650	adhesion of symbiont to host cell	bulk RNA-seq,Homo_723,MCI.vs.control; bulk RNA-seq,Homo_714,MCI.vs.control; bulk RNA-seq,Homo_633,AD.vs.control	3
BP	GO:0018158	protein oxidation	bulk RNA-seq,Homo_723,MCI.vs.control	1
CC	GO:0019908	nuclear cyclin-dependent protein kinase holoenzyme complex	bulk RNA-seq,Homo_723,MCI.vs.control; bulk RNA-seq,Homo_633,AD.vs.control; bulk RNA-seq,Homo_633,AD.vs.MCI; bulk RNA-	4
MF	GO:0008525	phosphatidylcholine transporter activity	bulk RNA-seq,Homo_723,MCI.vs.control; bulk RNA-seq,Homo_633,AD.vs.control	2
CC	GO:0071556	integral component of lumenal side of endoplasmic reticulum membrane	bulk RNA-seq,Homo_723,MCI.vs.control; bulk RNA-seq,Homo_714,MCI.vs.control; bulk RNA-seq,Homo_633,AD.vs.control; bulk RNA-seq,Homo_633,MCI.vs.control; scRNA-seq,SRP215507,CD8+ T cell_3-AD.vs.control; scRNA-seq,SRP215507,CD8+ T cell_3-MCI.vs.control	6
CC	GO:0098553	lumenal side of endoplasmic reticulum membrane	bulk RNA-seq,Homo_723,MCI.vs.control; bulk RNA-seq,Homo_714,MCI.vs.control; bulk RNA-seq,Homo_633,AD.vs.control; bulk RNA-seq,Homo_633,MCI.vs.control; scRNA-seq,SRP215507,CD8+ T cell_3-AD.vs.control; scRNA-seq,SRP215507,CD8+ T cell_3-MCI.vs.control	6
CC	GO:0080008	Cul4-RING E3 ubiquitin ligase complex	bulk RNA-seq,Homo_723,MCI.vs.control; bulk RNA-seq,Homo_714,MCI.vs.control; bulk RNA-seq,Homo_633,AD.vs.control; bulk RNA-	4
BP	GO:0090160	Golgi to lysosome transport	bulk RNA-seq,Homo_723,MCI.vs.control; bulk RNA-seq,Homo_714,MCI.vs.control; bulk RNA-seq,Homo_633,AD.vs.control; bulk RNA-	4
BP	GO:0039530	MDA-5 signaling pathway	bulk RNA-seq,Homo_723,MCI.vs.control; bulk RNA-seq,Homo_714,MCI.vs.control; bulk RNA-seq,Homo_633,MCI.vs.control	3
BP	GO:0000731	DNA synthesis involved in DNA repair	bulk RNA-seq,Homo_723,MCI.vs.control; bulk RNA-seq,Homo_714,MCI.vs.control; bulk RNA-seq,Homo_633,AD.vs.control; bulk RNA-	4
CC	GO:0036452	ESCRT complex	bulk RNA-seq,Homo_723,MCI.vs.control; bulk RNA-seq,Homo_714,MCI.vs.control; bulk RNA-seq,Homo_633,AD.vs.control; bulk RNA-seq,Homo_633,AD.vs.MCI; bulk RNA-seq,Homo_633,MCI.vs.control	5
MF	GO:0031681	G-protein beta-subunit binding	bulk RNA-seq,Homo_723,MCI.vs.control; bulk RNA-seq,Homo_714,AD.vs.MCI; bulk RNA-seq,Homo_633,AD.vs.control; bulk RNA-seq,Homo_633,AD.vs.MCI	4
BP	GO:0006040	amino sugar metabolic process	bulk RNA-seq,Homo_723,MCI.vs.control; bulk RNA-seq,Homo_714,MCI.vs.control; bulk RNA-seq,Homo_633,AD.vs.control; bulk RNA-	4
BP	GO:0051875	pigment granule localization	bulk RNA-seq,Homo_723,MCI.vs.control; bulk RNA-seq,Homo_714,MCI.vs.control; bulk RNA-seq,Homo_633,AD.vs.control; bulk RNA-	4
BP	GO:1904355	positive regulation of telomere capping	bulk RNA-seq,Homo_723,MCI.vs.control	1
BP	GO:0016202	regulation of striated muscle tissue development	bulk RNA-seq,Homo_723,MCI.vs.control	1
BP	GO:0071360	cellular response to exogenous dsRNA	bulk RNA-seq,Homo_723,MCI.vs.control; bulk RNA-seq,Homo_714,MCI.vs.control	2
BP	GO:1903800	positive regulation of miRNA maturation	bulk RNA-seq,Homo_723,MCI.vs.control	1
BP	GO:0032511	late endosome to vacuole transport via multivesicular body sorting pathway	bulk RNA-seq,Homo_723,MCI.vs.control; bulk RNA-seq,Homo_714,MCI.vs.control; bulk RNA-seq,Homo_633,AD.vs.control; bulk RNA-seq,Homo_633,AD.vs.MCI; bulk RNA-seq,Homo_633,MCI.vs.control	5
BP	GO:0060346	bone trabecula formation	bulk RNA-seq,Homo_723,MCI.vs.control	1
MF	GO:0019956	chemokine binding	bulk RNA-seq,Homo_723,MCI.vs.control; bulk RNA-seq,Homo_633,AD.vs.control; bulk RNA-seq,Homo_633,AD.vs.MCI	3
BP	GO:0039702	viral budding via host ESCRT complex	bulk RNA-seq,Homo_723,MCI.vs.control; bulk RNA-seq,Homo_633,AD.vs.control; bulk RNA-seq,Homo_633,AD.vs.MCI	3
BP	GO:0072672	neutrophil extravasation	bulk RNA-seq,Homo_723,MCI.vs.control; bulk RNA-seq,Homo_714,MCI.vs.control; bulk RNA-seq,SRP223445,AD.vs.control	3
BP	GO:1902993	positive regulation of amyloid precursor protein catabolic	bulk RNA-seq,Homo_723,MCI.vs.control; bulk RNA-seq,Homo_714,MCI.vs.control; bulk RNA-seq,Homo_633,AD.vs.control; bulk RNA-	4
MF	GO:0051959	dynein light intermediate chain binding	bulk RNA-seq,Homo_723,MCI.vs.control; bulk RNA-seq,ROSMAP,AD.vs.control	2
BP	GO:0045581	negative regulation of T cell differentiation	bulk RNA-seq,Homo_723,MCI.vs.control; bulk RNA-seq,Homo_714,MCI.vs.control; bulk RNA-seq,Homo_633,AD.vs.control	3
MF	GO:0034713	type I transforming growth factor beta receptor binding	bulk RNA-seq,Homo_723,MCI.vs.control; bulk RNA-seq,Homo_714,MCI.vs.control	2
MF	GO:1902282	voltage-gated potassium channel activity involved in ventricular cardiac muscle cell action potential repolarization	bulk RNA-seq,Homo_723,MCI.vs.control; bulk RNA-seq,Homo_714,AD.vs.MCI	2
BP	GO:0061635	regulation of protein complex stability	bulk RNA-seq,Homo_723,MCI.vs.control; bulk RNA-seq,Homo_633,AD.vs.control; bulk RNA-seq,Homo_633,AD.vs.MCI	3
BP	GO:1905456	regulation of lymphoid progenitor cell differentiation	bulk RNA-seq,Homo_723,MCI.vs.control	1
BP	GO:0043302	positive regulation of leukocyte degranulation	bulk RNA-seq,Homo_723,MCI.vs.control; bulk RNA-seq,Homo_633,AD.vs.control; bulk RNA-seq,Homo_633,AD.vs.MCI; bulk RNA-	4
BP	GO:0006658	phosphatidylserine metabolic process	bulk RNA-seq,Homo_723,MCI.vs.control; bulk RNA-seq,Homo_714,MCI.vs.control; bulk RNA-seq,Homo_633,AD.vs.control; bulk RNA-	4
BP	GO:0006359	regulation of transcription by RNA polymerase III	bulk RNA-seq,Homo_723,MCI.vs.control; bulk RNA-seq,Homo_714,MCI.vs.control; bulk RNA-seq,Homo_633,AD.vs.control; bulk RNA-	4
BP	GO:0032769	negative regulation of monooxygenase activity	bulk RNA-seq,Homo_723,MCI.vs.control	1
BP	GO:0046676	negative regulation of insulin secretion	bulk RNA-seq,Homo_723,MCI.vs.control; bulk RNA-seq,Homo_633,AD.vs.control; bulk RNA-seq,Homo_633,AD.vs.MCI	3
BP	GO:1903238	positive regulation of leukocyte tethering or rolling	bulk RNA-seq,Homo_723,MCI.vs.control; bulk RNA-seq,Homo_714,MCI.vs.control; bulk RNA-seq,Homo_633,AD.vs.control	3
MF	GO:0004652	polynucleotide adenylyltransferase activity	bulk RNA-seq,Homo_723,MCI.vs.control; bulk RNA-seq,Homo_633,AD.vs.control; bulk RNA-seq,Homo_633,AD.vs.MCI	3
CC	GO:0101003	ficolin-1-rich granule membrane	bulk RNA-seq,Homo_723,MCI.vs.control; bulk RNA-seq,Homo_714,MCI.vs.control; bulk RNA-seq,Homo_633,AD.vs.control; bulk RNA-seq,Homo_633,AD.vs.MCI; bulk RNA-seq,SRP223445,AD.vs.control	5
BP	GO:0046638	positive regulation of alpha-beta T cell differentiation	bulk RNA-seq,Homo_723,MCI.vs.control; bulk RNA-seq,Homo_714,MCI.vs.control; bulk RNA-seq,Homo_633,AD.vs.control; bulk RNA-	4
BP	GO:0040001	establishment of mitotic spindle localization	bulk RNA-seq,Homo_723,MCI.vs.control; bulk RNA-seq,Homo_633,AD.vs.control; bulk RNA-seq,Homo_633,AD.vs.MCI	3
BP	GO:0060149	negative regulation of post-transcriptional gene silencing	bulk RNA-seq,Homo_723,MCI.vs.control	1
BP	GO:0060967	negative regulation of gene silencing by RNA	bulk RNA-seq,Homo_723,MCI.vs.control	1

BP	GO:1900369	negative regulation of post-transcriptional gene silencing by	bulk RNA-seq,Homo_723,MCI.vs.control	1
MF	GO:0004439	phosphatidylinositol-4,5-bisphosphate 5-phosphatase activity	bulk RNA-seq,Homo_723,MCI.vs.control; bulk RNA-seq,Homo_633,AD.vs.control; bulk RNA-seq,Homo_633,AD.vs.MCI	3
MF	GO:0035613	RNA stem-loop binding	bulk RNA-seq,Homo_723,MCI.vs.control; bulk RNA-seq,Homo_714,MCI.vs.control; bulk RNA-seq,Homo_633,AD.vs.control	3
BP	GO:0060287	epithelial cilium movement involved in determination of left/right asymmetry	bulk RNA-seq,Homo_723,MCI.vs.control	1
MF	GO:0019706	protein-cysteine S-palmitoyltransferase activity	bulk RNA-seq,Homo_723,MCI.vs.control; bulk RNA-seq,Homo_633,AD.vs.control; bulk RNA-seq,Homo_633,AD.vs.MCI	3
MF	GO:0019707	protein-cysteine S-acyltransferase activity	bulk RNA-seq,Homo_723,MCI.vs.control; bulk RNA-seq,Homo_633,AD.vs.control; bulk RNA-seq,Homo_633,AD.vs.MCI	3
BP	GO:0045454	cell redox homeostasis	bulk RNA-seq,Homo_723,MCI.vs.control; bulk RNA-seq,Homo_633,AD.vs.control; bulk RNA-seq,Homo_633,AD.vs.MCI	3
MF	GO:0016877	ligase activity, forming carbon-sulfur bonds	bulk RNA-seq,Homo_723,MCI.vs.control; bulk RNA-seq,Homo_714,MCI.vs.control; bulk RNA-seq,Homo_633,AD.vs.control; bulk RNA-	4
BP	GO:0071168	protein localization to chromatin	bulk RNA-seq,Homo_723,MCI.vs.control; bulk RNA-seq,Homo_714,AD.vs.MCI	2
BP	GO:0006534	cysteine metabolic process	bulk RNA-seq,Homo_723,MCI.vs.control; bulk RNA-seq,Homo_633,AD.vs.control; bulk RNA-seq,Homo_633,AD.vs.MCI	3
BP	GO:0006767	water-soluble vitamin metabolic process	bulk RNA-seq,Homo_723,MCI.vs.control; bulk RNA-seq,Homo_714,AD.vs.control; bulk RNA-seq,Homo_714,MCI.vs.control; bulk RNA-seq,Homo_633,AD.vs.control; bulk RNA-seq,Homo_633,AD.vs.MCI	5
BP	GO:0046835	carbohydrate phosphorylation	bulk RNA-seq,Homo_723,MCI.vs.control; bulk RNA-seq,Homo_714,MCI.vs.control; bulk RNA-seq,Homo_633,AD.vs.control; bulk RNA-seq,Homo_633,AD.vs.MCI; bulk RNA-seq,Homo_633,MCI.vs.control	5
CC	GO:0031430	M band	bulk RNA-seq,Homo_723,MCI.vs.control; bulk RNA-seq,Homo_714,MCI.vs.control	2
BP	GO:0009124	nucleoside monophosphate biosynthetic process	bulk RNA-seq,Homo_723,MCI.vs.control; bulk RNA-seq,Homo_714,MCI.vs.control; bulk RNA-seq,Homo_633,AD.vs.control; bulk RNA-	4
MF	GO:0016538	cyclin-dependent protein serine/threonine kinase regulator	bulk RNA-seq,Homo_723,MCI.vs.control; bulk RNA-seq,Homo_714,MCI.vs.control; bulk RNA-seq,Homo_633,AD.vs.control; bulk RNA-	4
BP	GO:0038061	NIK/NF-kappaB signaling	bulk RNA-seq,Homo_723,MCI.vs.control; bulk RNA-seq,Homo_633,AD.vs.control; bulk RNA-seq,Homo_633,AD.vs.MCI; bulk RNA-	4
BP	GO:0060397	growth hormone receptor signaling pathway via JAK-STAT	bulk RNA-seq,Homo_723,MCI.vs.control; bulk RNA-seq,Homo_714,MCI.vs.control; bulk RNA-seq,Homo_633,AD.vs.MCI; bulk RNA-	4
MF	GO:0004129	cytochrome-c oxidase activity	bulk RNA-seq,Homo_723,MCI.vs.control; bulk RNA-seq,Homo_714,MCI.vs.control; bulk RNA-seq,Homo_633,AD.vs.control	3
BP	GO:0046940	nucleoside monophosphate phosphorylation	bulk RNA-seq,Homo_723,MCI.vs.control; bulk RNA-seq,Homo_714,MCI.vs.control; bulk RNA-seq,Homo_633,AD.vs.control	3
BP	GO:2000480	negative regulation of cAMP-dependent protein kinase	bulk RNA-seq,Homo_723,MCI.vs.control; bulk RNA-seq,Homo_714,AD.vs.MCI; bulk RNA-seq,Homo_714,MCI.vs.control	3
CC	GO:0030877	beta-catenin destruction complex	bulk RNA-seq,Homo_723,MCI.vs.control	1
BP	GO:1904031	positive regulation of cyclin-dependent protein kinase activity	bulk RNA-seq,Homo_723,MCI.vs.control; bulk RNA-seq,Homo_714,MCI.vs.control; bulk RNA-seq,Homo_633,AD.vs.control; bulk RNA-	4
MF	GO:0008234	cysteine-type peptidase activity	bulk RNA-seq,Homo_723,MCI.vs.control; bulk RNA-seq,Homo_633,AD.vs.control; bulk RNA-seq,Homo_633,AD.vs.MCI	3
BP	GO:1902808	positive regulation of cell cycle G1/S phase transition	bulk RNA-seq,Homo_723,MCI.vs.control; bulk RNA-seq,Homo_714,AD.vs.MCI; bulk RNA-seq,Homo_714,MCI.vs.control; bulk RNA-seq,Homo_633,AD.vs.control; bulk RNA-seq,Homo_633,AD.vs.MCI	5
BP	GO:0050435	amyloid-beta metabolic process	bulk RNA-seq,Homo_723,MCI.vs.control; bulk RNA-seq,Homo_714,MCI.vs.control; bulk RNA-seq,Homo_633,AD.vs.control; bulk RNA-	4
BP	GO:0034244	negative regulation of transcription elongation by RNA	bulk RNA-seq,Homo_723,MCI.vs.control; bulk RNA-seq,Homo_633,AD.vs.control; bulk RNA-seq,Homo_633,AD.vs.MCI	3
BP	GO:0071545	inositol phosphate catabolic process	bulk RNA-seq,Homo_723,MCI.vs.control; bulk RNA-seq,Homo_714,MCI.vs.control; bulk RNA-seq,Homo_633,AD.vs.control; bulk RNA-	4
MF	GO:0071949	FAD binding	bulk RNA-seq,Homo_723,MCI.vs.control; bulk RNA-seq,Homo_633,AD.vs.control; bulk RNA-seq,Homo_633,AD.vs.MCI	3
BP	GO:0031579	membrane raft organization	bulk RNA-seq,Homo_723,MCI.vs.control; bulk RNA-seq,Homo_714,MCI.vs.control; bulk RNA-seq,Homo_633,AD.vs.control	3
BP	GO:0090504	epiboly	bulk RNA-seq,Homo_723,MCI.vs.control; bulk RNA-seq,Homo_633,AD.vs.control	2
BP	GO:0015693	magnesium ion transport	bulk RNA-seq,Homo_723,MCI.vs.control	1
BP	GO:0055070	copper ion homeostasis	bulk RNA-seq,Homo_723,MCI.vs.control; bulk RNA-seq,Homo_714,AD.vs.control; bulk RNA-seq,Homo_714,MCI.vs.control; bulk RNA-seq,Homo_633,AD.vs.control; bulk RNA-seq,Homo_633,AD.vs.MCI	5
CC	GO:0000346	transcription export complex	bulk RNA-seq,Homo_723,MCI.vs.control; bulk RNA-seq,Homo_714,MCI.vs.control; bulk RNA-seq,Homo_633,AD.vs.control; bulk RNA-	4
BP	GO:0110096	cellular response to aldehyde	bulk RNA-seq,Homo_723,MCI.vs.control; bulk RNA-seq,Homo_633,AD.vs.control; bulk RNA-seq,Homo_633,AD.vs.MCI	3
BP	GO:0071816	tail-anchored membrane protein insertion into ER membrane	bulk RNA-seq,Homo_723,MCI.vs.control; bulk RNA-seq,Homo_714,MCI.vs.control; bulk RNA-seq,Homo_633,AD.vs.control; bulk RNA-	4
BP	GO:0006700	C21-steroid hormone biosynthetic process	bulk RNA-seq,Homo_723,MCI.vs.control	1
BP	GO:0035067	negative regulation of histone acetylation	bulk RNA-seq,Homo_723,MCI.vs.control; bulk RNA-seq,Homo_633,AD.vs.control; bulk RNA-seq,Homo_633,AD.vs.MCI	3
BP	GO:0045945	positive regulation of transcription by RNA polymerase III	bulk RNA-seq,Homo_723,MCI.vs.control; bulk RNA-seq,Homo_633,AD.vs.control	2
BP	GO:0036035	osteoclast development	bulk RNA-seq,Homo_723,MCI.vs.control; bulk RNA-seq,Homo_714,MCI.vs.control; bulk RNA-seq,Homo_633,AD.vs.control	3
BP	GO:0035435	phosphate ion transmembrane transport	bulk RNA-seq,Homo_723,MCI.vs.control; bulk RNA-seq,Homo_633,AD.vs.control; bulk RNA-seq,Homo_633,AD.vs.MCI	3
MF	GO:0051371	muscle alpha-actinin binding	bulk RNA-seq,Homo_723,MCI.vs.control; bulk RNA-seq,Homo_714,MCI.vs.control	2
BP	GO:0098969	neurotransmitter receptor transport to postsynaptic membrane	bulk RNA-seq,Homo_723,MCI.vs.control; bulk RNA-seq,Homo_714,MCI.vs.control; bulk RNA-seq,Homo_633,AD.vs.control	3
MF	GO:0071933	Arp2/3 complex binding	bulk RNA-seq,Homo_723,MCI.vs.control; bulk RNA-seq,Homo_714,MCI.vs.control; bulk RNA-seq,Homo_633,AD.vs.control; bulk RNA-	4
BP	GO:0002475	antigen processing and presentation via MHC class Ib	bulk RNA-seq,Homo_723,MCI.vs.control	1
BP	GO:0051654	establishment of mitochondrion localization	bulk RNA-seq,Homo_723,MCI.vs.control; bulk RNA-seq,Homo_633,AD.vs.control; bulk RNA-seq,Homo_633,AD.vs.MCI	3
BP	GO:0015911	long-chain fatty acid import across plasma membrane	bulk RNA-seq,Homo_723,MCI.vs.control	1
BP	GO:0008380	RNA splicing	bulk RNA-seq,Homo_714,AD.vs.control; bulk RNA-seq,Homo_633,AD.vs.control; bulk RNA-seq,Homo_633,AD.vs.MCI; scRNA-	4
BP	GO:0044270	cellular nitrogen compound catabolic process	bulk RNA-seq,Homo_714,AD.vs.control; bulk RNA-seq,Homo_633,AD.vs.control; bulk RNA-seq,Homo_633,AD.vs.MCI	3
BP	GO:0046700	heterocycle catabolic process	bulk RNA-seq,Homo_714,AD.vs.control; bulk RNA-seq,Homo_633,AD.vs.control; bulk RNA-seq,Homo_633,AD.vs.MCI	3
BP	GO:0019439	aromatic compound catabolic process	bulk RNA-seq,Homo_714,AD.vs.control; bulk RNA-seq,Homo_633,AD.vs.control; bulk RNA-seq,Homo_633,AD.vs.MCI	3
BP	GO:0043161	proteasome-mediated ubiquitin-dependent protein catabolic	bulk RNA-seq,Homo_714,AD.vs.control; bulk RNA-seq,Homo_633,AD.vs.control; bulk RNA-seq,Homo_633,AD.vs.MCI	3
BP	GO:0016032	viral process	bulk RNA-seq,Homo_714,AD.vs.control; bulk RNA-seq,Homo_714,AD.vs.MCI; bulk RNA-seq,Homo_633,AD.vs.control; bulk RNA-seq,Homo_633,AD.vs.MCI	4

CC	GO:0016607	nuclear speck	bulk RNA-seq,Homo_714,AD.vs.control; bulk RNA-seq,Homo_633,AD.vs.control; bulk RNA-seq,Homo_633,AD.vs.MCI; scRNA-	4
BP	GO:0002831	regulation of response to biotic stimulus	bulk RNA-seq,Homo_714,AD.vs.control; bulk RNA-seq,Homo_633,AD.vs.control; bulk RNA-seq,Homo_633,AD.vs.MCI; scRNA-	4
BP	GO:0072594	establishment of protein localization to organelle	bulk RNA-seq,Homo_714,AD.vs.control; bulk RNA-seq,Homo_633,AD.vs.control; bulk RNA-seq,Homo_633,AD.vs.MCI	3
BP	GO:0030098	lymphocyte differentiation	bulk RNA-seq,Homo_714,AD.vs.control; bulk RNA-seq,Homo_633,AD.vs.control; bulk RNA-seq,Homo_633,AD.vs.MCI; scRNA-seq,SRP330776,Naive CD8+ T cell_2-AD.vs.control; scRNA-seq,SRP309935,Megakaryocyte_2-AD.vs.control	5
CC	GO:0005774	vacuolar membrane	bulk RNA-seq,Homo_714,AD.vs.control; bulk RNA-seq,Homo_633,AD.vs.control; bulk RNA-seq,Homo_633,AD.vs.MCI; scRNA-seq,SRP330776,Naive CD8+ T	4
CC	GO:0005765	lysosomal membrane	bulk RNA-seq,Homo_714,AD.vs.control; bulk RNA-seq,Homo_633,AD.vs.control; bulk RNA-seq,Homo_633,AD.vs.MCI; scRNA-seq,SRP330776,Naive CD8+ T	4
CC	GO:0098852	lytic vacuole membrane	bulk RNA-seq,Homo_714,AD.vs.control; bulk RNA-seq,Homo_633,AD.vs.control; bulk RNA-seq,Homo_633,AD.vs.MCI; scRNA-seq,SRP330776,Naive CD8+ T	4
BP	GO:0043414	macromolecule methylation	bulk RNA-seq,Homo_714,AD.vs.control; bulk RNA-seq,Homo_633,AD.vs.control; bulk RNA-seq,Homo_633,AD.vs.MCI	3
BP	GO:1903131	mononuclear cell differentiation	bulk RNA-seq,Homo_714,AD.vs.control; bulk RNA-seq,Homo_633,AD.vs.control; bulk RNA-seq,Homo_633,AD.vs.MCI; scRNA-seq,SRP330776,Naive CD8+ T cell_2-AD.vs.control; scRNA-seq,SRP215507,CD8+ T cell_3-AD.vs.control	5
BP	GO:0010506	regulation of autophagy	bulk RNA-seq,Homo_714,AD.vs.control; bulk RNA-seq,Homo_633,AD.vs.control; bulk RNA-seq,Homo_633,AD.vs.MCI	3
BP	GO:0032259	methylation	bulk RNA-seq,Homo_714,AD.vs.control; bulk RNA-seq,Homo_633,AD.vs.control; bulk RNA-seq,Homo_633,AD.vs.MCI	3
BP	GO:0009615	response to virus	bulk RNA-seq,Homo_714,AD.vs.control; bulk RNA-seq,Homo_714,AD.vs.MCI; bulk RNA-seq,Homo_633,AD.vs.control; bulk RNA-seq,Homo_633,AD.vs.MCI; scRNA-seq,SRP215507,CD8+ T cell_3-AD.vs.control	5
BP	GO:0031640	killing of cells of another organism	bulk RNA-seq,Homo_714,AD.vs.control	1
BP	GO:0050830	defense response to Gram-positive bacterium	bulk RNA-seq,Homo_714,AD.vs.control; bulk RNA-seq,Homo_714,AD.vs.MCI; bulk RNA-seq,Homo_633,AD.vs.control; bulk RNA-seq,Homo_633,AD.vs.MCI; bulk RNA-seq,SRP325058,AD.vs.control	5
BP	GO:0016570	histone modification	bulk RNA-seq,Homo_714,AD.vs.control; bulk RNA-seq,Homo_714,AD.vs.MCI; bulk RNA-seq,Homo_633,AD.vs.control; bulk RNA-seq,Homo_633,AD.vs.MCI	4
BP	GO:0050863	regulation of T cell activation	bulk RNA-seq,Homo_714,AD.vs.control; bulk RNA-seq,Homo_714,AD.vs.MCI; bulk RNA-seq,Homo_633,AD.vs.control; bulk RNA-seq,Homo_633,AD.vs.MCI; scRNA-seq,SRP215507,CD8+ T cell_3-AD.vs.control; scRNA-seq,SRP215507,CD8+ T cell_3-MCI.vs.control	6
BP	GO:1905651	regulation of artery morphogenesis	bulk RNA-seq,Homo_714,AD.vs.control; bulk RNA-seq,Homo_633,AD.vs.control	2
BP	GO:0001833	inner cell mass cell proliferation	bulk RNA-seq,Homo_714,AD.vs.control	1
BP	GO:0042501	serine phosphorylation of STAT protein	bulk RNA-seq,Homo_714,AD.vs.control	1
BP	GO:0050912	detection of chemical stimulus involved in sensory perception	bulk RNA-seq,Homo_714,AD.vs.control; bulk RNA-seq,Homo_714,AD.vs.MCI; bulk RNA-seq,ROSMAP,AD.vs.control; bulk RNA-seq,SRP223445,AD.vs.control	4
BP	GO:0019511	peptidyl-proline hydroxylation	bulk RNA-seq,Homo_714,AD.vs.control; bulk RNA-seq,Homo_714,MCI.vs.control; bulk RNA-seq,Homo_633,MCI.vs.control	3
BP	GO:0033139	regulation of peptidyl-serine phosphorylation of STAT	bulk RNA-seq,Homo_714,AD.vs.control; bulk RNA-seq,ROSMAP,AD.vs.control	2
BP	GO:1904753	negative regulation of vascular associated smooth muscle cell	bulk RNA-seq,Homo_714,AD.vs.control; bulk RNA-seq,Homo_714,AD.vs.MCI	2
BP	GO:0051873	killing by host of symbiont cells	bulk RNA-seq,Homo_714,AD.vs.control; bulk RNA-seq,Homo_714,AD.vs.MCI	2
MF	GO:0042301	phosphate ion binding	bulk RNA-seq,Homo_714,AD.vs.control; bulk RNA-seq,Homo_714,AD.vs.MCI	2
BP	GO:0002335	mature B cell differentiation	bulk RNA-seq,Homo_714,AD.vs.control; bulk RNA-seq,Homo_714,MCI.vs.control; bulk RNA-seq,Homo_633,AD.vs.control	3
MF	GO:0061980	regulatory RNA binding	bulk RNA-seq,Homo_714,AD.vs.control; bulk RNA-seq,Homo_714,MCI.vs.control; bulk RNA-seq,Homo_633,AD.vs.control; bulk RNA-	4
BP	GO:2001258	negative regulation of cation channel activity	bulk RNA-seq,Homo_714,AD.vs.control	1
BP	GO:0009620	response to fungus	bulk RNA-seq,Homo_714,AD.vs.control; bulk RNA-seq,Homo_633,AD.vs.control; bulk RNA-seq,Homo_633,AD.vs.MCI; bulk RNA-seq,SRP325058,AD.vs.control; scRNA-seq,SRP330776,Naive CD8+ T cell_2-AD.vs.control	5
BP	GO:0042745	circadian sleep/wake cycle	bulk RNA-seq,Homo_714,AD.vs.control	1
MF	GO:0008527	taste receptor activity	bulk RNA-seq,Homo_714,AD.vs.control; bulk RNA-seq,Homo_714,AD.vs.MCI; bulk RNA-seq,Homo_633,AD.vs.MCI; bulk RNA-seq,ROSMAP,AD.vs.control; bulk RNA-seq,ROSMAP,AD.vs.MCI	5
BP	GO:1904706	negative regulation of vascular associated smooth muscle cell proliferation	bulk RNA-seq,Homo_714,AD.vs.control; bulk RNA-seq,Homo_714,AD.vs.MCI	2
BP	GO:1902110	positive regulation of mitochondrial membrane permeability involved in apoptotic process	bulk RNA-seq,Homo_714,AD.vs.control; bulk RNA-seq,Homo_633,AD.vs.control; bulk RNA-seq,Homo_633,AD.vs.MCI	3
BP	GO:1902260	negative regulation of delayed rectifier potassium channel	bulk RNA-seq,Homo_714,AD.vs.control	1
BP	GO:0050832	defense response to fungus	bulk RNA-seq,Homo_714,AD.vs.control; bulk RNA-seq,SRP325058,AD.vs.control	2
MF	GO:0036435	K48-linked polyubiquitin modification-dependent protein	bulk RNA-seq,Homo_714,AD.vs.control; bulk RNA-seq,Homo_633,AD.vs.control; bulk RNA-seq,Homo_633,AD.vs.MCI	3
MF	GO:0005131	growth hormone receptor binding	bulk RNA-seq,Homo_714,AD.vs.control	1
BP	GO:0016233	telomere capping	bulk RNA-seq,Homo_714,AD.vs.control; bulk RNA-seq,Homo_714,MCI.vs.control; bulk RNA-seq,Homo_633,AD.vs.control; bulk RNA-	4
BP	GO:1902415	regulation of mRNA binding	bulk RNA-seq,Homo_714,AD.vs.control; bulk RNA-seq,Homo_714,MCI.vs.control	2
MF	GO:0019203	carbohydrate phosphatase activity	bulk RNA-seq,Homo_714,AD.vs.control	1
MF	GO:0050308	sugar-phosphatase activity	bulk RNA-seq,Homo_714,AD.vs.control	1
CC	GO:0061827	sperm head	bulk RNA-seq,Homo_714,AD.vs.control; bulk RNA-seq,Homo_714,AD.vs.MCI	2
BP	GO:0032025	response to cobalt ion	bulk RNA-seq,Homo_714,AD.vs.control; bulk RNA-seq,Homo_633,AD.vs.control; bulk RNA-seq,Homo_633,AD.vs.MCI	3
BP	GO:0001818	negative regulation of cytokine production	bulk RNA-seq,Homo_714,AD.vs.MCI; bulk RNA-seq,Homo_633,AD.vs.control; bulk RNA-seq,Homo_633,AD.vs.MCI	3
CC	GO:0000786	nucleosome	bulk RNA-seq,Homo_714,AD.vs.MCI; bulk RNA-seq,ROSMAP,AD.vs.control; bulk RNA-seq,ROSMAP,MCI.vs.control	3
BP	GO:0006334	nucleosome assembly	bulk RNA-seq,Homo_714,AD.vs.MCI	1
BP	GO:2000479	regulation of cAMP-dependent protein kinase activity	bulk RNA-seq,Homo_714,AD.vs.MCI	1
BP	GO:0034728	nucleosome organization	bulk RNA-seq,Homo_714,AD.vs.MCI; bulk RNA-seq,Homo_633,AD.vs.control; bulk RNA-seq,Homo_633,AD.vs.MCI	3
BP	GO:0010988	regulation of low-density lipoprotein particle clearance	bulk RNA-seq,Homo_714,AD.vs.MCI	1

BP	GO:0060353	regulation of cell adhesion molecule production	bulk RNA-seq,Homo_714,AD.vs.MCI	1
BP	GO:0045601	regulation of endothelial cell differentiation	bulk RNA-seq,Homo_714,AD.vs.MCI; bulk RNA-seq,Homo_714,MCI.vs.control; bulk RNA-seq,Homo_633,AD.vs.control	3
BP	GO:0014742	positive regulation of muscle hypertrophy	bulk RNA-seq,Homo_714,AD.vs.MCI	1
BP	GO:1904754	positive regulation of vascular associated smooth muscle cell	bulk RNA-seq,Homo_714,AD.vs.MCI	1
BP	GO:0036314	response to sterol	bulk RNA-seq,Homo_714,AD.vs.MCI; bulk RNA-seq,Homo_633,AD.vs.control; bulk RNA-seq,Homo_633,AD.vs.MCI	3
BP	GO:0034763	negative regulation of transmembrane transport	bulk RNA-seq,Homo_714,AD.vs.MCI; bulk RNA-seq,Homo_633,AD.vs.control; bulk RNA-seq,Homo_633,AD.vs.MCI	3
BP	GO:1902915	negative regulation of protein polyubiquitination	bulk RNA-seq,Homo_714,AD.vs.MCI; bulk RNA-seq,Homo_633,AD.vs.control; bulk RNA-seq,Homo_633,AD.vs.MCI	3
BP	GO:0032387	negative regulation of intracellular transport	bulk RNA-seq,Homo_714,AD.vs.MCI; bulk RNA-seq,Homo_714,MCI.vs.control; bulk RNA-seq,Homo_633,AD.vs.control; bulk RNA-seq,Homo_633,AD.vs.MCI	4
BP	GO:0032611	interleukin-1 beta production	bulk RNA-seq,Homo_714,AD.vs.MCI; bulk RNA-seq,Homo_714,MCI.vs.control; bulk RNA-seq,Homo_633,AD.vs.control	3
BP	GO:0032651	regulation of interleukin-1 beta production	bulk RNA-seq,Homo_714,AD.vs.MCI; bulk RNA-seq,Homo_714,MCI.vs.control; bulk RNA-seq,Homo_633,AD.vs.control	3
BP	GO:0042026	protein refolding	bulk RNA-seq,Homo_714,AD.vs.MCI; bulk RNA-seq,Homo_633,AD.vs.control	2
BP	GO:0000353	formation of quadruple SL/U4/U5/U6 snRNP	bulk RNA-seq,Homo_714,AD.vs.MCI	1
BP	GO:0000365	mRNA trans splicing, via spliceosome	bulk RNA-seq,Homo_714,AD.vs.MCI	1
BP	GO:0045291	mRNA trans splicing, SL addition	bulk RNA-seq,Homo_714,AD.vs.MCI	1
BP	GO:0032612	interleukin-1 production	bulk RNA-seq,Homo_714,AD.vs.MCI; bulk RNA-seq,Homo_633,AD.vs.control; bulk RNA-seq,Homo_633,AD.vs.MCI; scRNA-seq,SRP215507,CD8+ T cell_3-	4
BP	GO:0032652	regulation of interleukin-1 production	bulk RNA-seq,Homo_714,AD.vs.MCI; bulk RNA-seq,Homo_633,AD.vs.control; bulk RNA-seq,Homo_633,AD.vs.MCI; scRNA-seq,SRP215507,CD8+ T cell_3-	4
BP	GO:0043330	response to exogenous dsRNA	bulk RNA-seq,Homo_714,AD.vs.MCI	1
BP	GO:0060354	negative regulation of cell adhesion molecule production	bulk RNA-seq,Homo_714,AD.vs.MCI	1
BP	GO:0009595	detection of biotic stimulus	bulk RNA-seq,Homo_714,AD.vs.MCI; bulk RNA-seq,Homo_633,AD.vs.control; bulk RNA-seq,Homo_633,AD.vs.MCI	3
BP	GO:0010659	cardiac muscle cell apoptotic process	bulk RNA-seq,Homo_714,AD.vs.MCI; bulk RNA-seq,Homo_633,AD.vs.control	2
BP	GO:0046426	negative regulation of receptor signaling pathway via JAK-	bulk RNA-seq,Homo_714,AD.vs.MCI; bulk RNA-seq,Homo_633,AD.vs.control; bulk RNA-seq,Homo_633,AD.vs.MCI	3
BP	GO:0099624	atrial cardiac muscle cell membrane repolarization	bulk RNA-seq,Homo_714,AD.vs.MCI	1
MF	GO:0019865	immunoglobulin binding	bulk RNA-seq,Homo_714,AD.vs.MCI	1
MF	GO:0086008	voltage-gated potassium channel activity involved in cardiac muscle cell action potential repolarization	bulk RNA-seq,Homo_714,AD.vs.MCI	1
BP	GO:0006617	SRP-dependent cotranslational protein targeting to membrane, signal sequence recognition	bulk RNA-seq,Homo_714,AD.vs.MCI	1
BP	GO:1903055	positive regulation of extracellular matrix organization	bulk RNA-seq,Homo_714,AD.vs.MCI; bulk RNA-seq,Homo_633,AD.vs.control; bulk RNA-seq,Homo_633,AD.vs.MCI	3
BP	GO:0010591	regulation of lamellipodium assembly	bulk RNA-seq,Homo_714,AD.vs.MCI; bulk RNA-seq,Homo_714,MCI.vs.control; bulk RNA-seq,Homo_633,AD.vs.control; bulk RNA-seq,Homo_633,AD.vs.MCI	4
BP	GO:0014823	response to activity	bulk RNA-seq,Homo_714,AD.vs.MCI; bulk RNA-seq,Homo_633,AD.vs.control; bulk RNA-seq,Homo_633,AD.vs.MCI	3
BP	GO:0010667	negative regulation of cardiac muscle cell apoptotic process	bulk RNA-seq,Homo_714,AD.vs.MCI	1
MF	GO:0070492	oligosaccharide binding	bulk RNA-seq,Homo_714,AD.vs.MCI	1
BP	GO:0090084	negative regulation of inclusion body assembly	bulk RNA-seq,Homo_714,AD.vs.MCI; bulk RNA-seq,Homo_714,MCI.vs.control	2
MF	GO:0015926	glucosidase activity	bulk RNA-seq,Homo_714,AD.vs.MCI	1
BP	GO:0010613	positive regulation of cardiac muscle hypertrophy	bulk RNA-seq,Homo_714,AD.vs.MCI	1
BP	GO:0050665	hydrogen peroxide biosynthetic process	bulk RNA-seq,Homo_714,AD.vs.MCI	1
BP	GO:0072015	podocyte development	bulk RNA-seq,Homo_714,AD.vs.MCI	1
BP	GO:0014819	regulation of skeletal muscle contraction	bulk RNA-seq,Homo_714,AD.vs.MCI	1
MF	GO:0051428	peptide hormone receptor binding	bulk RNA-seq,Homo_714,AD.vs.MCI	1
BP	GO:0010662	regulation of striated muscle cell apoptotic process	bulk RNA-seq,Homo_714,AD.vs.MCI	1
BP	GO:0055015	ventricular cardiac muscle cell development	bulk RNA-seq,Homo_714,AD.vs.MCI	1
MF	GO:0030527	structural constituent of chromatin	bulk RNA-seq,Homo_714,AD.vs.MCI; bulk RNA-seq,ROSMAP,AD.vs.control; bulk RNA-seq,ROSMAP,MCI.vs.control	3
MF	GO:0004402	histone acetyltransferase activity	bulk RNA-seq,Homo_714,AD.vs.MCI; bulk RNA-seq,Homo_633,AD.vs.control; bulk RNA-seq,Homo_633,AD.vs.MCI	3
CC	GO:0001674	female germ cell nucleus	bulk RNA-seq,Homo_714,AD.vs.MCI	1
BP	GO:1904996	positive regulation of leukocyte adhesion to vascular endothelial cell	bulk RNA-seq,Homo_714,AD.vs.MCI; bulk RNA-seq,Homo_714,MCI.vs.control; bulk RNA-seq,Homo_633,AD.vs.control; bulk RNA-seq,Homo_633,AD.vs.MCI; bulk RNA-seq,Homo_633,MCI.vs.control	5
BP	GO:0071397	cellular response to cholesterol	bulk RNA-seq,Homo_714,AD.vs.MCI	1
BP	GO:0045780	positive regulation of bone resorption	bulk RNA-seq,Homo_714,AD.vs.MCI	1
MF	GO:0061733	peptide-lysine-N-acetyltransferase activity	bulk RNA-seq,Homo_714,AD.vs.MCI; bulk RNA-seq,Homo_633,AD.vs.control; bulk RNA-seq,Homo_633,AD.vs.MCI	3
BP	GO:0097186	amelogenesis	bulk RNA-seq,Homo_714,AD.vs.MCI	1
BP	GO:0090317	negative regulation of intracellular protein transport	bulk RNA-seq,Homo_714,AD.vs.MCI; bulk RNA-seq,Homo_714,MCI.vs.control; bulk RNA-seq,Homo_633,AD.vs.control; bulk RNA-seq,Homo_633,AD.vs.MCI	4
BP	GO:0045653	negative regulation of megakaryocyte differentiation	bulk RNA-seq,Homo_714,AD.vs.MCI	1
BP	GO:2000831	regulation of steroid hormone secretion	bulk RNA-seq,Homo_714,AD.vs.MCI; bulk RNA-seq,Homo_633,AD.vs.control; bulk RNA-seq,Homo_633,AD.vs.MCI	3
MF	GO:0005160	transforming growth factor beta receptor binding	bulk RNA-seq,Homo_714,AD.vs.MCI; bulk RNA-seq,Homo_633,AD.vs.control	2
BP	GO:0010574	regulation of vascular endothelial growth factor production	bulk RNA-seq,Homo_714,AD.vs.MCI; bulk RNA-seq,Homo_633,AD.vs.MCI	2
BP	GO:0043331	response to dsRNA	bulk RNA-seq,Homo_714,AD.vs.MCI; bulk RNA-seq,Homo_714,MCI.vs.control	2

BP	GO:0010989	negative regulation of low-density lipoprotein particle	bulk RNA-seq,Homo_714,AD.vs.MCI	1
BP	GO:0032945	negative regulation of mononuclear cell proliferation	bulk RNA-seq,Homo_714,AD.vs.MCI; bulk RNA-seq,Homo_714,MCI.vs.control; bulk RNA-seq,Homo_633,AD.vs.control; bulk RNA-seq,Homo_633,AD.vs.MCI	4
BP	GO:0002534	cytokine production involved in inflammatory response	bulk RNA-seq,Homo_714,AD.vs.MCI	1
BP	GO:1900015	regulation of cytokine production involved in inflammatory	bulk RNA-seq,Homo_714,AD.vs.MCI	1
BP	GO:1905906	regulation of amyloid fibril formation	bulk RNA-seq,Homo_714,AD.vs.MCI	1
BP	GO:0097267	omega-hydroxylase P450 pathway	bulk RNA-seq,Homo_714,AD.vs.MCI	1
BP	GO:1903961	positive regulation of anion transmembrane transport	bulk RNA-seq,Homo_714,AD.vs.MCI	1
BP	GO:1904893	negative regulation of receptor signaling pathway via STAT	bulk RNA-seq,Homo_714,AD.vs.MCI	1
BP	GO:0090280	positive regulation of calcium ion import	bulk RNA-seq,Homo_714,AD.vs.MCI; bulk RNA-seq,Homo_714,MCI.vs.control	2
MF	GO:0010314	phosphatidylinositol-5-phosphate binding	bulk RNA-seq,Homo_714,AD.vs.MCI; bulk RNA-seq,Homo_633,AD.vs.control; bulk RNA-seq,Homo_633,AD.vs.MCI	3
BP	GO:0034505	tooth mineralization	bulk RNA-seq,Homo_714,AD.vs.MCI	1
BP	GO:0070723	response to cholesterol	bulk RNA-seq,Homo_714,AD.vs.MCI; bulk RNA-seq,Homo_633,AD.vs.control; bulk RNA-seq,Homo_633,AD.vs.MCI	3
MF	GO:0043027	cysteine-type endopeptidase inhibitor activity involved in apoptotic process	bulk RNA-seq,Homo_714,AD.vs.MCI	1
MF	GO:0001530	lipopolysaccharide binding	bulk RNA-seq,Homo_714,AD.vs.MCI; bulk RNA-seq,Homo_633,AD.vs.control; bulk RNA-seq,Homo_633,AD.vs.MCI	3
MF	GO:0015385	sodium:proton antiporter activity	bulk RNA-seq,Homo_714,AD.vs.MCI	1
BP	GO:0042403	thyroid hormone metabolic process	bulk RNA-seq,Homo_714,AD.vs.MCI	1
BP	GO:0010573	vascular endothelial growth factor production	bulk RNA-seq,Homo_714,AD.vs.MCI	1
BP	GO:0010665	regulation of cardiac muscle cell apoptotic process	bulk RNA-seq,Homo_714,AD.vs.MCI	1
BP	GO:1903671	negative regulation of sprouting angiogenesis	bulk RNA-seq,Homo_714,AD.vs.MCI	1
BP	GO:0006971	hypotonic response	bulk RNA-seq,Homo_714,AD.vs.MCI	1
BP	GO:0002922	positive regulation of humoral immune response	bulk RNA-seq,Homo_714,AD.vs.MCI	1
BP	GO:1901523	icosanoid catabolic process	bulk RNA-seq,Homo_714,AD.vs.MCI	1
MF	GO:0019787	ubiquitin-like protein transferase activity	bulk RNA-seq,Homo_714,AD.vs.MCI; bulk RNA-seq,Homo_633,AD.vs.control; bulk RNA-seq,Homo_633,AD.vs.MCI; scRNA-seq,SRP330776,Memory T cell_2-AD.vs.control; scRNA-seq,SRP330776,Naive CD8+ T cell_3-AD.vs.control; scRNA-seq,SRP330776,Natural killer cell_1-AD.vs.control	6
BP	GO:0002675	positive regulation of acute inflammatory response	bulk RNA-seq,Homo_714,AD.vs.MCI; bulk RNA-seq,Homo_633,AD.vs.control; bulk RNA-seq,SRP223445,AD.vs.control	3
BP	GO:0051154	negative regulation of striated muscle cell differentiation	bulk RNA-seq,Homo_714,AD.vs.MCI; bulk RNA-seq,Homo_714,MCI.vs.control	2
CC	GO:0090571	RNA polymerase II transcription repressor complex	bulk RNA-seq,Homo_714,AD.vs.MCI; bulk RNA-seq,Homo_714,MCI.vs.control; bulk RNA-seq,Homo_633,AD.vs.control; bulk RNA-seq,Homo_633,AD.vs.MCI	4
BP	GO:0060054	positive regulation of epithelial cell proliferation involved in wound healing	bulk RNA-seq,Homo_714,AD.vs.MCI	1
BP	GO:0019731	antibacterial humoral response	bulk RNA-seq,Homo_714,AD.vs.MCI; bulk RNA-seq,Homo_633,AD.vs.control; bulk RNA-seq,SRP325058,AD.vs.control	3
BP	GO:1901223	negative regulation of NIK/NF-kappaB signaling	bulk RNA-seq,Homo_714,AD.vs.MCI	1
BP	GO:0002645	positive regulation of tolerance induction	bulk RNA-seq,Homo_714,MCI.vs.control; bulk RNA-seq,Homo_633,MCI.vs.control	2
BP	GO:0006998	nuclear envelope organization	bulk RNA-seq,Homo_714,MCI.vs.control; bulk RNA-seq,Homo_633,AD.vs.control; bulk RNA-seq,Homo_633,AD.vs.MCI	3
BP	GO:0042130	negative regulation of T cell proliferation	bulk RNA-seq,Homo_714,MCI.vs.control; bulk RNA-seq,Homo_633,AD.vs.control; bulk RNA-seq,Homo_633,AD.vs.MCI	3
BP	GO:0000045	autophagosome assembly	bulk RNA-seq,Homo_714,MCI.vs.control; bulk RNA-seq,Homo_633,AD.vs.control; bulk RNA-seq,Homo_633,AD.vs.MCI	3
BP	GO:0042100	B cell proliferation	bulk RNA-seq,Homo_714,MCI.vs.control; bulk RNA-seq,Homo_633,AD.vs.control; bulk RNA-seq,Homo_633,AD.vs.MCI	3
BP	GO:0035739	CD4-positive, alpha-beta T cell proliferation	bulk RNA-seq,Homo_714,MCI.vs.control; bulk RNA-seq,Homo_633,AD.vs.control; bulk RNA-seq,Homo_633,AD.vs.MCI	3
BP	GO:2000561	regulation of CD4-positive, alpha-beta T cell proliferation	bulk RNA-seq,Homo_714,MCI.vs.control; bulk RNA-seq,Homo_633,AD.vs.control; bulk RNA-seq,Homo_633,AD.vs.MCI	3
MF	GO:0016667	oxidoreductase activity, acting on a sulfur group of donors	bulk RNA-seq,Homo_714,MCI.vs.control; bulk RNA-seq,Homo_633,AD.vs.control; bulk RNA-seq,Homo_633,AD.vs.MCI	3
BP	GO:0033632	regulation of cell-cell adhesion mediated by integrin	bulk RNA-seq,Homo_714,MCI.vs.control; bulk RNA-seq,Homo_633,AD.vs.control; bulk RNA-seq,Homo_633,AD.vs.MCI; bulk RNA-	4
BP	GO:0002360	T cell lineage commitment	bulk RNA-seq,Homo_714,MCI.vs.control	1
BP	GO:0042771	intrinsic apoptotic signaling pathway in response to DNA damage by p53 class mediator	bulk RNA-seq,Homo_714,MCI.vs.control; bulk RNA-seq,Homo_633,AD.vs.control; bulk RNA-seq,Homo_633,AD.vs.MCI	3
BP	GO:0060211	regulation of nuclear-transcribed mRNA poly(A) tail	bulk RNA-seq,Homo_714,MCI.vs.control; bulk RNA-seq,Homo_633,AD.vs.control; bulk RNA-seq,Homo_633,AD.vs.MCI; bulk RNA-	4
CC	GO:0005797	Golgi medial cisterna	bulk RNA-seq,Homo_714,MCI.vs.control; bulk RNA-seq,Homo_633,AD.vs.control; bulk RNA-seq,Homo_633,AD.vs.MCI; bulk RNA-	4
BP	GO:0002664	regulation of T cell tolerance induction	bulk RNA-seq,Homo_714,MCI.vs.control	1
MF	GO:0036041	long-chain fatty acid binding	bulk RNA-seq,Homo_714,MCI.vs.control	1
BP	GO:0070265	necrotic cell death	bulk RNA-seq,Homo_714,MCI.vs.control; bulk RNA-seq,Homo_633,AD.vs.control; bulk RNA-seq,Homo_633,AD.vs.MCI	3
BP	GO:0010939	regulation of necrotic cell death	bulk RNA-seq,Homo_714,MCI.vs.control; bulk RNA-seq,Homo_633,AD.vs.control; bulk RNA-seq,Homo_633,AD.vs.MCI	3
BP	GO:0050672	negative regulation of lymphocyte proliferation	bulk RNA-seq,Homo_714,MCI.vs.control; bulk RNA-seq,Homo_633,AD.vs.control; bulk RNA-seq,Homo_633,AD.vs.MCI	3
CC	GO:0070069	cytochrome complex	bulk RNA-seq,Homo_714,MCI.vs.control; bulk RNA-seq,Homo_633,AD.vs.control; bulk RNA-seq,Homo_633,AD.vs.MCI	3
BP	GO:0071763	nuclear membrane organization	bulk RNA-seq,Homo_714,MCI.vs.control; bulk RNA-seq,Homo_633,AD.vs.control; bulk RNA-seq,Homo_633,AD.vs.MCI	3
BP	GO:0070227	lymphocyte apoptotic process	bulk RNA-seq,Homo_714,MCI.vs.control; bulk RNA-seq,Homo_633,AD.vs.control	2
BP	GO:0006094	gluconeogenesis	bulk RNA-seq,Homo_714,MCI.vs.control; bulk RNA-seq,Homo_633,AD.vs.control	2
BP	GO:0060760	positive regulation of response to cytokine stimulus	bulk RNA-seq,Homo_714,MCI.vs.control; bulk RNA-seq,Homo_633,AD.vs.control; bulk RNA-seq,Homo_633,AD.vs.MCI	3

BP	GO:0042987	amyloid precursor protein catabolic process	bulk RNA-seq,Homo_714,MCI.vs.control; bulk RNA-seq,Homo_633,AD.vs.control; bulk RNA-seq,Homo_633,AD.vs.MCI	3
BP	GO:0032740	positive regulation of interleukin-17 production	bulk RNA-seq,Homo_714,MCI.vs.control; bulk RNA-seq,Homo_633,MCI.vs.control	2
BP	GO:0043903	regulation of biological process involved in symbiotic	bulk RNA-seq,Homo_714,MCI.vs.control; bulk RNA-seq,Homo_633,AD.vs.control; bulk RNA-seq,Homo_633,AD.vs.MCI	3
BP	GO:0001961	positive regulation of cytokine-mediated signaling pathway	bulk RNA-seq,Homo_714,MCI.vs.control; bulk RNA-seq,Homo_633,AD.vs.control; bulk RNA-seq,Homo_633,AD.vs.MCI	3
BP	GO:0034205	amyloid-beta formation	bulk RNA-seq,Homo_714,MCI.vs.control; bulk RNA-seq,Homo_633,AD.vs.control; bulk RNA-seq,Homo_633,AD.vs.MCI	3
MF	GO:0000774	adenyl-nucleotide exchange factor activity	bulk RNA-seq,Homo_714,MCI.vs.control; bulk RNA-seq,Homo_633,AD.vs.control; bulk RNA-seq,Homo_633,AD.vs.MCI; bulk RNA-	4
MF	GO:0004559	alpha-mannosidase activity	bulk RNA-seq,Homo_714,MCI.vs.control; bulk RNA-seq,Homo_633,AD.vs.control; bulk RNA-seq,Homo_633,AD.vs.MCI; bulk RNA-	4
BP	GO:0051560	mitochondrial calcium ion homeostasis	bulk RNA-seq,Homo_714,MCI.vs.control; bulk RNA-seq,Homo_633,AD.vs.control; bulk RNA-seq,Homo_633,AD.vs.MCI; scRNA-seq,SRP330776,Naive CD8+ T cell_3-AD.vs.control	4
BP	GO:0072683	T cell extravasation	bulk RNA-seq,Homo_714,MCI.vs.control	1
BP	GO:0098751	bone cell development	bulk RNA-seq,Homo_714,MCI.vs.control; bulk RNA-seq,Homo_633,AD.vs.control; bulk RNA-seq,Homo_633,AD.vs.MCI	3
BP	GO:1901186	positive regulation of ERBB signaling pathway	bulk RNA-seq,Homo_714,MCI.vs.control; bulk RNA-seq,Homo_633,AD.vs.control	2
BP	GO:0044275	cellular carbohydrate catabolic process	bulk RNA-seq,Homo_714,MCI.vs.control; bulk RNA-seq,Homo_633,AD.vs.control; bulk RNA-seq,Homo_633,AD.vs.MCI	3
BP	GO:0046007	negative regulation of activated T cell proliferation	bulk RNA-seq,Homo_714,MCI.vs.control; bulk RNA-seq,Homo_633,AD.vs.control	2
MF	GO:0044390	ubiquitin-like protein conjugating enzyme binding	bulk RNA-seq,Homo_714,MCI.vs.control; bulk RNA-seq,Homo_633,AD.vs.control; bulk RNA-seq,Homo_633,AD.vs.MCI	3
MF	GO:0042608	T cell receptor binding	bulk RNA-seq,Homo_714,MCI.vs.control; bulk RNA-seq,Homo_633,AD.vs.control; bulk RNA-seq,Homo_633,MCI.vs.control; scRNA-seq,SRP215507,CD8+ T	4
MF	GO:0005049	nuclear export signal receptor activity	bulk RNA-seq,Homo_714,MCI.vs.control; bulk RNA-seq,Homo_633,AD.vs.control; bulk RNA-seq,Homo_633,AD.vs.MCI	3
BP	GO:0006206	pyrimidine nucleobase metabolic process	bulk RNA-seq,Homo_714,MCI.vs.control	1
BP	GO:0050869	negative regulation of B cell activation	bulk RNA-seq,Homo_714,MCI.vs.control; bulk RNA-seq,Homo_633,AD.vs.control; bulk RNA-seq,Homo_633,AD.vs.MCI	3
BP	GO:0006984	ER-nucleus signaling pathway	bulk RNA-seq,Homo_714,MCI.vs.control; bulk RNA-seq,Homo_633,AD.vs.control; bulk RNA-seq,Homo_633,AD.vs.MCI	3
BP	GO:0031663	lipopolysaccharide-mediated signaling pathway	bulk RNA-seq,Homo_714,MCI.vs.control; bulk RNA-seq,Homo_633,AD.vs.control; bulk RNA-seq,Homo_633,AD.vs.MCI	3
BP	GO:0019319	hexose biosynthetic process	bulk RNA-seq,Homo_714,MCI.vs.control; bulk RNA-seq,Homo_633,AD.vs.control	2
BP	GO:0043124	negative regulation of I-kappaB kinase/NF-kappaB signaling	bulk RNA-seq,Homo_714,MCI.vs.control; bulk RNA-seq,Homo_633,AD.vs.control; bulk RNA-seq,Homo_633,AD.vs.MCI	3
BP	GO:0046636	negative regulation of alpha-beta T cell activation	bulk RNA-seq,Homo_714,MCI.vs.control; bulk RNA-seq,Homo_633,AD.vs.control; bulk RNA-seq,Homo_633,AD.vs.MCI	3
MF	GO:0015036	disulfide oxidoreductase activity	bulk RNA-seq,Homo_714,MCI.vs.control; bulk RNA-seq,Homo_633,AD.vs.control; bulk RNA-seq,Homo_633,AD.vs.MCI	3
BP	GO:0045060	negative thymic T cell selection	bulk RNA-seq,Homo_714,MCI.vs.control	1
BP	GO:0046364	monosaccharide biosynthetic process	bulk RNA-seq,Homo_714,MCI.vs.control; bulk RNA-seq,Homo_633,AD.vs.control	2
MF	GO:0016670	oxidoreductase activity, acting on a sulfur group of donors, oxygen as acceptor	bulk RNA-seq,Homo_714,MCI.vs.control; bulk RNA-seq,Homo_633,AD.vs.control; bulk RNA-seq,Homo_633,AD.vs.MCI; bulk RNA-seq,Homo_633,MCI.vs.control	4
BP	GO:0060213	positive regulation of nuclear-transcribed mRNA poly(A) tail	bulk RNA-seq,Homo_714,MCI.vs.control; bulk RNA-seq,Homo_633,AD.vs.control	2
BP	GO:0090153	regulation of sphingolipid biosynthetic process	bulk RNA-seq,Homo_714,MCI.vs.control	1
BP	GO:1905038	regulation of membrane lipid metabolic process	bulk RNA-seq,Homo_714,MCI.vs.control	1
BP	GO:1903573	negative regulation of response to endoplasmic reticulum	bulk RNA-seq,Homo_714,MCI.vs.control; bulk RNA-seq,Homo_633,AD.vs.control; bulk RNA-seq,Homo_633,AD.vs.MCI	3
BP	GO:0016266	O-glycan processing	bulk RNA-seq,Homo_714,MCI.vs.control; bulk RNA-seq,Homo_633,AD.vs.control; bulk RNA-seq,Homo_633,AD.vs.MCI	3
MF	GO:0016684	oxidoreductase activity, acting on peroxide as acceptor	bulk RNA-seq,Homo_714,MCI.vs.control; bulk RNA-seq,Homo_633,AD.vs.control; bulk RNA-seq,Homo_633,AD.vs.MCI; bulk RNA-seq,ROSMAP,AD.vs.MCI; bulk RNA-seq,ROSMAP,MCI.vs.control	5
BP	GO:0070059	intrinsic apoptotic signaling pathway in response to endoplasmic reticulum stress	bulk RNA-seq,Homo_714,MCI.vs.control; bulk RNA-seq,Homo_633,AD.vs.control; bulk RNA-seq,Homo_633,AD.vs.MCI; scRNA-seq,SRP330776,Naive CD8+ T cell_2-AD.vs.control	4
BP	GO:0071712	ER-associated misfolded protein catabolic process	bulk RNA-seq,Homo_714,MCI.vs.control; bulk RNA-seq,Homo_633,AD.vs.control; bulk RNA-seq,Homo_633,AD.vs.MCI; bulk RNA-	4
BP	GO:0043383	negative T cell selection	bulk RNA-seq,Homo_714,MCI.vs.control	1
BP	GO:2000104	negative regulation of DNA-templated DNA replication	bulk RNA-seq,Homo_714,MCI.vs.control; bulk RNA-seq,Homo_633,AD.vs.control; bulk RNA-seq,Homo_633,AD.vs.MCI; bulk RNA-	4
BP	GO:0033209	tumor necrosis factor-mediated signaling pathway	bulk RNA-seq,Homo_714,MCI.vs.control; bulk RNA-seq,Homo_633,AD.vs.control; bulk RNA-seq,Homo_633,AD.vs.MCI	3
CC	GO:0030014	CCR4-NOT complex	bulk RNA-seq,Homo_714,MCI.vs.control; bulk RNA-seq,Homo_633,AD.vs.control	2
BP	GO:0002313	mature B cell differentiation involved in immune response	bulk RNA-seq,Homo_714,MCI.vs.control; bulk RNA-seq,Homo_633,AD.vs.control; scRNA-seq,SRP330776,Naive CD8+ T cell_2-AD.vs.control	3
BP	GO:0046365	monosaccharide catabolic process	bulk RNA-seq,Homo_714,MCI.vs.control; bulk RNA-seq,Homo_633,AD.vs.control	2
BP	GO:0001783	B cell apoptotic process	bulk RNA-seq,Homo_714,MCI.vs.control; bulk RNA-seq,Homo_633,AD.vs.control; bulk RNA-seq,Homo_633,MCI.vs.control	3
CC	GO:0061702	inflammasome complex	bulk RNA-seq,Homo_714,MCI.vs.control; bulk RNA-seq,Homo_633,AD.vs.control; bulk RNA-seq,Homo_633,AD.vs.MCI	3
BP	GO:0032720	negative regulation of tumor necrosis factor production	bulk RNA-seq,Homo_714,MCI.vs.control	1
BP	GO:0032509	endosome transport via multivesicular body sorting pathway	bulk RNA-seq,Homo_714,MCI.vs.control; bulk RNA-seq,Homo_633,AD.vs.control; bulk RNA-seq,Homo_633,AD.vs.MCI	3
BP	GO:0018904	ether metabolic process	bulk RNA-seq,Homo_714,MCI.vs.control; bulk RNA-seq,Homo_633,AD.vs.control; bulk RNA-seq,Homo_633,AD.vs.MCI; bulk RNA-	4
BP	GO:0006620	post-translational protein targeting to endoplasmic reticulum	bulk RNA-seq,Homo_714,MCI.vs.control; bulk RNA-seq,Homo_633,AD.vs.control; bulk RNA-seq,Homo_633,AD.vs.MCI; bulk RNA-	4
BP	GO:0071474	cellular hyperosmotic response	bulk RNA-seq,Homo_714,MCI.vs.control; bulk RNA-seq,Homo_633,AD.vs.control; bulk RNA-seq,Homo_633,AD.vs.MCI; bulk RNA-	4
BP	GO:0006054	N-acetylneuraminate metabolic process	bulk RNA-seq,Homo_714,MCI.vs.control; bulk RNA-seq,Homo_633,AD.vs.control; bulk RNA-seq,Homo_633,AD.vs.MCI; bulk RNA-	4
BP	GO:1905216	positive regulation of RNA binding	bulk RNA-seq,Homo_714,MCI.vs.control	1
BP	GO:0050860	negative regulation of T cell receptor signaling pathway	bulk RNA-seq,Homo_714,MCI.vs.control; bulk RNA-seq,Homo_633,AD.vs.control; bulk RNA-seq,Homo_633,AD.vs.MCI	3
BP	GO:1900034	regulation of cellular response to heat	bulk RNA-seq,Homo_714,MCI.vs.control; bulk RNA-seq,Homo_633,AD.vs.control; bulk RNA-seq,Homo_633,AD.vs.MCI	3

BP	GO:0035774	positive regulation of insulin secretion involved in cellular response to glucose stimulus	bulk RNA-seq,Homo_714,MCI.vs.control; bulk RNA-seq,Homo_633,AD.vs.control; bulk RNA-seq,Homo_633,AD.vs.MCI	3
BP	GO:0045742	positive regulation of epidermal growth factor receptor signaling pathway	bulk RNA-seq,Homo_714,MCI.vs.control; bulk RNA-seq,Homo_633,AD.vs.control	2
MF	GO:0004726	non-membrane spanning protein tyrosine phosphatase	bulk RNA-seq,Homo_714,MCI.vs.control; bulk RNA-seq,Homo_633,AD.vs.control; bulk RNA-seq,Homo_633,AD.vs.MCI	3
BP	GO:0039532	negative regulation of viral-induced cytoplasmic pattern recognition receptor signaling pathway	bulk RNA-seq,Homo_714,MCI.vs.control	1
BP	GO:0062098	regulation of programmed necrotic cell death	bulk RNA-seq,Homo_714,MCI.vs.control; bulk RNA-seq,Homo_633,AD.vs.control; bulk RNA-seq,Homo_633,AD.vs.MCI	3
BP	GO:1903556	negative regulation of tumor necrosis factor superfamily cytokine production	bulk RNA-seq,Homo_714,MCI.vs.control	1
CC	GO:0000815	ESCRT III complex	bulk RNA-seq,Homo_714,MCI.vs.control; bulk RNA-seq,Homo_633,AD.vs.control; bulk RNA-seq,Homo_633,AD.vs.MCI	3
BP	GO:0042762	regulation of sulfur metabolic process	bulk RNA-seq,Homo_714,MCI.vs.control	1
BP	GO:0045737	positive regulation of cyclin-dependent protein serine/threonine kinase activity	bulk RNA-seq,Homo_714,MCI.vs.control; bulk RNA-seq,Homo_633,AD.vs.control; bulk RNA-seq,Homo_633,AD.vs.MCI	3
BP	GO:1901222	regulation of NIK/NF-kappaB signaling	bulk RNA-seq,Homo_714,MCI.vs.control; bulk RNA-seq,Homo_633,AD.vs.control; bulk RNA-seq,Homo_633,AD.vs.MCI	3
BP	GO:1902991	regulation of amyloid precursor protein catabolic process	bulk RNA-seq,Homo_714,MCI.vs.control; bulk RNA-seq,Homo_633,AD.vs.control; bulk RNA-seq,Homo_633,AD.vs.MCI	3
BP	GO:0002836	positive regulation of response to tumor cell	bulk RNA-seq,Homo_714,MCI.vs.control	1
BP	GO:0002839	positive regulation of immune response to tumor cell	bulk RNA-seq,Homo_714,MCI.vs.control	1
MF	GO:0019107	myristoyltransferase activity	bulk RNA-seq,Homo_714,MCI.vs.control; bulk RNA-seq,Homo_633,MCI.vs.control	2
BP	GO:0051256	mitotic spindle midzone assembly	bulk RNA-seq,Homo_714,MCI.vs.control	1
BP	GO:0019062	virion attachment to host cell	bulk RNA-seq,Homo_714,MCI.vs.control; bulk RNA-seq,Homo_633,AD.vs.control; bulk RNA-seq,Homo_633,AD.vs.MCI	3
BP	GO:0070228	regulation of lymphocyte apoptotic process	bulk RNA-seq,Homo_714,MCI.vs.control; bulk RNA-seq,Homo_633,AD.vs.control	2
MF	GO:0001222	transcription corepressor binding	bulk RNA-seq,Homo_714,MCI.vs.control; bulk RNA-seq,Homo_633,AD.vs.control; bulk RNA-seq,Homo_633,AD.vs.MCI	3
CC	GO:0071162	CMG complex	bulk RNA-seq,Homo_714,MCI.vs.control; bulk RNA-seq,Homo_633,MCI.vs.control	2
BP	GO:2001185	regulation of CD8-positive, alpha-beta T cell activation	bulk RNA-seq,Homo_714,MCI.vs.control	1
BP	GO:0046599	regulation of centriole replication	bulk RNA-seq,Homo_714,MCI.vs.control	1
BP	GO:0045793	positive regulation of cell size	bulk RNA-seq,Homo_714,MCI.vs.control	1
BP	GO:1903202	negative regulation of oxidative stress-induced cell death	bulk RNA-seq,Homo_714,MCI.vs.control; bulk RNA-seq,Homo_633,AD.vs.control; bulk RNA-seq,Homo_633,AD.vs.MCI	3
BP	GO:0090201	negative regulation of release of cytochrome c from	bulk RNA-seq,Homo_714,MCI.vs.control; bulk RNA-seq,Homo_633,AD.vs.control; bulk RNA-seq,Homo_633,AD.vs.MCI	3
BP	GO:1902003	regulation of amyloid-beta formation	bulk RNA-seq,Homo_714,MCI.vs.control; bulk RNA-seq,Homo_633,AD.vs.control; bulk RNA-seq,Homo_633,AD.vs.MCI	3
BP	GO:0032933	SREBP signaling pathway	bulk RNA-seq,Homo_714,MCI.vs.control; bulk RNA-seq,Homo_633,AD.vs.control; bulk RNA-seq,Homo_633,AD.vs.MCI; bulk RNA-	4
BP	GO:0031293	membrane protein intracellular domain proteolysis	bulk RNA-seq,Homo_714,MCI.vs.control; bulk RNA-seq,Homo_633,AD.vs.control; bulk RNA-seq,Homo_633,AD.vs.MCI; bulk RNA-	4
BP	GO:1901797	negative regulation of signal transduction by p53 class	bulk RNA-seq,Homo_714,MCI.vs.control; bulk RNA-seq,Homo_633,AD.vs.control; bulk RNA-seq,Homo_633,AD.vs.MCI	3
BP	GO:0046677	response to antibiotic	bulk RNA-seq,Homo_714,MCI.vs.control; bulk RNA-seq,Homo_633,AD.vs.control; bulk RNA-seq,Homo_633,AD.vs.MCI	3
BP	GO:0071402	cellular response to lipoprotein particle stimulus	bulk RNA-seq,Homo_714,MCI.vs.control; bulk RNA-seq,Homo_633,AD.vs.control	2
BP	GO:0060623	regulation of chromosome condensation	bulk RNA-seq,Homo_714,MCI.vs.control; bulk RNA-seq,Homo_633,AD.vs.control; bulk RNA-seq,Homo_633,AD.vs.MCI	3
BP	GO:0006895	Golgi to endosome transport	bulk RNA-seq,Homo_714,MCI.vs.control; bulk RNA-seq,Homo_633,AD.vs.control; bulk RNA-seq,Homo_633,AD.vs.MCI	3
BP	GO:0048820	hair follicle maturation	bulk RNA-seq,Homo_714,MCI.vs.control; bulk RNA-seq,Homo_633,AD.vs.control	2
MF	GO:0043422	protein kinase B binding	bulk RNA-seq,Homo_714,MCI.vs.control; bulk RNA-seq,Homo_633,AD.vs.control	2
BP	GO:0060767	epithelial cell proliferation involved in prostate gland	bulk RNA-seq,Homo_714,MCI.vs.control; bulk RNA-seq,Homo_633,AD.vs.control; bulk RNA-seq,Homo_633,AD.vs.MCI	3
BP	GO:0045116	protein neddylation	bulk RNA-seq,Homo_714,MCI.vs.control; bulk RNA-seq,Homo_633,AD.vs.control; bulk RNA-seq,Homo_633,AD.vs.MCI	3
CC	GO:0035145	exon-exon junction complex	bulk RNA-seq,Homo_714,MCI.vs.control	1
MF	GO:0042974	nuclear retinoic acid receptor binding	bulk RNA-seq,Homo_714,MCI.vs.control; bulk RNA-seq,Homo_633,AD.vs.control	2
BP	GO:0046902	regulation of mitochondrial membrane permeability	bulk RNA-seq,Homo_714,MCI.vs.control; bulk RNA-seq,Homo_633,AD.vs.control; bulk RNA-seq,Homo_633,AD.vs.MCI	3
BP	GO:0097212	lysosomal membrane organization	bulk RNA-seq,Homo_714,MCI.vs.control; bulk RNA-seq,Homo_633,AD.vs.control	2
MF	GO:0010485	H4 histone acetyltransferase activity	bulk RNA-seq,Homo_714,MCI.vs.control; bulk RNA-seq,Homo_633,AD.vs.control; bulk RNA-seq,Homo_633,AD.vs.MCI	3
BP	GO:0070229	negative regulation of lymphocyte apoptotic process	bulk RNA-seq,Homo_714,MCI.vs.control; bulk RNA-seq,Homo_633,AD.vs.control; bulk RNA-seq,Homo_633,AD.vs.MCI	3
BP	GO:0043666	regulation of phosphoprotein phosphatase activity	bulk RNA-seq,Homo_714,MCI.vs.control; bulk RNA-seq,Homo_633,AD.vs.control; bulk RNA-seq,Homo_633,AD.vs.MCI	3
BP	GO:0000244	spliceosomal tri-snRNP complex assembly	bulk RNA-seq,Homo_714,MCI.vs.control; bulk RNA-seq,Homo_633,MCI.vs.control	2
BP	GO:1904407	positive regulation of nitric oxide metabolic process	bulk RNA-seq,Homo_714,MCI.vs.control; bulk RNA-seq,Homo_633,AD.vs.control; bulk RNA-seq,Homo_633,AD.vs.MCI	3
BP	GO:2000109	regulation of macrophage apoptotic process	bulk RNA-seq,Homo_714,MCI.vs.control; bulk RNA-seq,Homo_633,AD.vs.control; bulk RNA-seq,Homo_633,MCI.vs.control	3
BP	GO:0071514	genomic imprinting	bulk RNA-seq,Homo_714,MCI.vs.control; bulk RNA-seq,SRP325058,AD.vs.control	2
BP	GO:0097278	complement-dependent cytotoxicity	bulk RNA-seq,Homo_714,MCI.vs.control; bulk RNA-seq,Homo_633,AD.vs.control; bulk RNA-seq,Homo_633,AD.vs.MCI	3
CC	GO:1990124	messenger ribonucleoprotein complex	bulk RNA-seq,Homo_714,MCI.vs.control	1
BP	GO:0070673	response to interleukin-18	bulk RNA-seq,Homo_714,MCI.vs.control	1
BP	GO:0071888	macrophage apoptotic process	bulk RNA-seq,Homo_714,MCI.vs.control; bulk RNA-seq,Homo_633,AD.vs.control; bulk RNA-seq,Homo_633,MCI.vs.control	3
BP	GO:0006878	cellular copper ion homeostasis	bulk RNA-seq,Homo_714,MCI.vs.control; bulk RNA-seq,Homo_633,AD.vs.control; bulk RNA-seq,Homo_633,AD.vs.MCI	3

BP	GO:0046753	non-lytic viral release	bulk RNA-seq,Homo_714,MCI.vs.control; bulk RNA-seq,Homo_633,AD.vs.control; bulk RNA-seq,Homo_633,AD.vs.MCI	3
BP	GO:0051469	vesicle fusion with vacuole	bulk RNA-seq,Homo_714,MCI.vs.control; bulk RNA-seq,Homo_633,AD.vs.control; bulk RNA-seq,Homo_633,AD.vs.MCI	3
BP	GO:0061763	multivesicular body-lysosome fusion	bulk RNA-seq,Homo_714,MCI.vs.control; bulk RNA-seq,Homo_633,AD.vs.control; bulk RNA-seq,Homo_633,AD.vs.MCI	3
BP	GO:0072599	establishment of protein localization to endoplasmic	bulk RNA-seq,Homo_714,MCI.vs.control; bulk RNA-seq,Homo_633,AD.vs.control; bulk RNA-seq,Homo_633,AD.vs.MCI	3
BP	GO:0042982	amyloid precursor protein metabolic process	bulk RNA-seq,Homo_714,MCI.vs.control; bulk RNA-seq,Homo_633,AD.vs.control; bulk RNA-seq,Homo_633,AD.vs.MCI	3
CC	GO:1904930	amphisome membrane	bulk RNA-seq,Homo_714,MCI.vs.control; bulk RNA-seq,Homo_633,AD.vs.control; bulk RNA-seq,Homo_633,AD.vs.MCI	3
BP	GO:0000722	telomere maintenance via recombination	bulk RNA-seq,Homo_714,MCI.vs.control; bulk RNA-seq,Homo_633,AD.vs.control; bulk RNA-seq,Homo_633,AD.vs.MCI	3
BP	GO:1901661	quinone metabolic process	bulk RNA-seq,Homo_714,MCI.vs.control; bulk RNA-seq,Homo_633,AD.vs.control; bulk RNA-seq,Homo_633,AD.vs.MCI; scRNA-seq,SRP330776,Natural killer	4
BP	GO:0031468	nuclear membrane reassembly	bulk RNA-seq,Homo_714,MCI.vs.control; bulk RNA-seq,Homo_633,AD.vs.control; bulk RNA-seq,Homo_633,AD.vs.MCI; bulk RNA-	4
BP	GO:0006007	glucose catabolic process	bulk RNA-seq,Homo_714,MCI.vs.control; bulk RNA-seq,Homo_633,AD.vs.control; bulk RNA-seq,Homo_633,MCI.vs.control	3
CC	GO:0000421	autophagosome membrane	bulk RNA-seq,Homo_633,AD.vs.control; bulk RNA-seq,Homo_633,AD.vs.MCI	2
BP	GO:2001244	positive regulation of intrinsic apoptotic signaling pathway	bulk RNA-seq,Homo_633,AD.vs.control; bulk RNA-seq,Homo_633,AD.vs.MCI	2
BP	GO:0006487	protein N-linked glycosylation	bulk RNA-seq,Homo_633,AD.vs.control; bulk RNA-seq,Homo_633,AD.vs.MCI	2
BP	GO:0070646	protein modification by small protein removal	bulk RNA-seq,Homo_633,AD.vs.control; bulk RNA-seq,Homo_633,AD.vs.MCI	2
BP	GO:0016579	protein deubiquitination	bulk RNA-seq,Homo_633,AD.vs.control; bulk RNA-seq,Homo_633,AD.vs.MCI	2
MF	GO:0004842	ubiquitin-protein transferase activity	bulk RNA-seq,Homo_633,AD.vs.control; bulk RNA-seq,Homo_633,AD.vs.MCI; scRNA-seq,SRP330776,CD8+ T cell_1-AD.vs.control; scRNA-seq,SRP330776,Memory T cell_2-AD.vs.control; scRNA-seq,SRP330776,Naive CD8+ T cell_2-AD.vs.control; scRNA-seq,SRP330776,Naive CD8+ T cell_3-AD.vs.control; scRNA-seq,SRP330776,Natural killer cell_1-AD.vs.control	7
BP	GO:0055076	transition metal ion homeostasis	bulk RNA-seq,Homo_633,AD.vs.control; bulk RNA-seq,Homo_633,AD.vs.MCI	2
BP	GO:0006417	regulation of translation	bulk RNA-seq,Homo_633,AD.vs.control; bulk RNA-seq,Homo_633,AD.vs.MCI	2
MF	GO:0019783	ubiquitin-like protein peptidase activity	bulk RNA-seq,Homo_633,AD.vs.control; bulk RNA-seq,Homo_633,AD.vs.MCI; bulk RNA-seq,SRP223445,AD.vs.control	3
BP	GO:0001906	cell killing	bulk RNA-seq,Homo_633,AD.vs.control; bulk RNA-seq,Homo_633,AD.vs.MCI; scRNA-seq,SRP309935,B cell_1-AD.vs.control; scRNA-seq,SRP215507,CD8+ T	4
BP	GO:0034249	negative regulation of cellular amide metabolic process	bulk RNA-seq,Homo_633,AD.vs.control; bulk RNA-seq,Homo_633,AD.vs.MCI	2
BP	GO:0017148	negative regulation of translation	bulk RNA-seq,Homo_633,AD.vs.control; bulk RNA-seq,Homo_633,AD.vs.MCI	2
BP	GO:2000113	negative regulation of cellular macromolecule biosynthetic	bulk RNA-seq,Homo_633,AD.vs.control; bulk RNA-seq,Homo_633,AD.vs.MCI	2
BP	GO:0016925	protein sumoylation	bulk RNA-seq,Homo_633,AD.vs.control; bulk RNA-seq,Homo_633,AD.vs.MCI	2
BP	GO:0035751	regulation of lysosomal lumen pH	bulk RNA-seq,Homo_633,AD.vs.control; bulk RNA-seq,Homo_633,AD.vs.MCI	2
MF	GO:0005484	SNAP receptor activity	bulk RNA-seq,Homo_633,AD.vs.control; bulk RNA-seq,Homo_633,AD.vs.MCI	2
BP	GO:0055072	iron ion homeostasis	bulk RNA-seq,Homo_633,AD.vs.control; bulk RNA-seq,Homo_633,AD.vs.MCI; scRNA-seq,SRP215507,CD8+ T cell_8-MCI.vs.control	3
BP	GO:0051438	regulation of ubiquitin-protein transferase activity	bulk RNA-seq,Homo_633,AD.vs.control; bulk RNA-seq,Homo_633,AD.vs.MCI	2
CC	GO:0016469	proton-transporting two-sector ATPase complex	bulk RNA-seq,Homo_633,AD.vs.control; bulk RNA-seq,Homo_633,AD.vs.MCI	2
BP	GO:0045324	late endosome to vacuole transport	bulk RNA-seq,Homo_633,AD.vs.control; bulk RNA-seq,Homo_633,AD.vs.MCI	2
BP	GO:0007035	vacuolar acidification	bulk RNA-seq,Homo_633,AD.vs.control; bulk RNA-seq,Homo_633,AD.vs.MCI	2
MF	GO:0017056	structural constituent of nuclear pore	bulk RNA-seq,Homo_633,AD.vs.control; bulk RNA-seq,Homo_633,AD.vs.MCI	2
MF	GO:0101005	deubiquitinase activity	bulk RNA-seq,Homo_633,AD.vs.control; bulk RNA-seq,Homo_633,AD.vs.MCI; bulk RNA-seq,SRP223445,AD.vs.control	3
BP	GO:0007042	lysosomal lumen acidification	bulk RNA-seq,Homo_633,AD.vs.control; bulk RNA-seq,Homo_633,AD.vs.MCI	2
CC	GO:0035577	azurophil granule membrane	bulk RNA-seq,Homo_633,AD.vs.control; bulk RNA-seq,Homo_633,AD.vs.MCI; bulk RNA-seq,SRP223445,AD.vs.control	3
BP	GO:2000378	negative regulation of reactive oxygen species metabolic	bulk RNA-seq,Homo_633,AD.vs.control; bulk RNA-seq,Homo_633,AD.vs.MCI	2
BP	GO:0006122	mitochondrial electron transport, ubiquinol to cytochrome c	bulk RNA-seq,Homo_633,AD.vs.control; bulk RNA-seq,Homo_633,AD.vs.MCI	2
BP	GO:0000413	protein peptidyl-prolyl isomerization	bulk RNA-seq,Homo_633,AD.vs.control; bulk RNA-seq,Homo_633,AD.vs.MCI	2
BP	GO:1901264	carbohydrate derivative transport	bulk RNA-seq,Homo_633,AD.vs.control; bulk RNA-seq,Homo_633,AD.vs.MCI	2
MF	GO:0030145	manganese ion binding	bulk RNA-seq,Homo_633,AD.vs.control; bulk RNA-seq,Homo_633,AD.vs.MCI	2
BP	GO:1903146	regulation of autophagy of mitochondrion	bulk RNA-seq,Homo_633,AD.vs.control; bulk RNA-seq,Homo_633,AD.vs.MCI	2
MF	GO:0042171	lysophosphatidic acid acyltransferase activity	bulk RNA-seq,Homo_633,AD.vs.control; bulk RNA-seq,Homo_633,AD.vs.MCI	2
MF	GO:0071617	lysophospholipid acyltransferase activity	bulk RNA-seq,Homo_633,AD.vs.control; bulk RNA-seq,Homo_633,AD.vs.MCI	2
BP	GO:0046916	cellular transition metal ion homeostasis	bulk RNA-seq,Homo_633,AD.vs.control; bulk RNA-seq,Homo_633,AD.vs.MCI	2
MF	GO:0003841	1-acylglycerol-3-phosphate O-acyltransferase activity	bulk RNA-seq,Homo_633,AD.vs.control; bulk RNA-seq,Homo_633,AD.vs.MCI	2
MF	GO:0061650	ubiquitin-like protein conjugating enzyme activity	bulk RNA-seq,Homo_633,AD.vs.control; bulk RNA-seq,Homo_633,AD.vs.MCI	2
BP	GO:0051453	regulation of intracellular pH	bulk RNA-seq,Homo_633,AD.vs.control; bulk RNA-seq,Homo_633,AD.vs.MCI	2
BP	GO:0030641	regulation of cellular pH	bulk RNA-seq,Homo_633,AD.vs.control; bulk RNA-seq,Homo_633,AD.vs.MCI	2
BP	GO:0070498	interleukin-1-mediated signaling pathway	bulk RNA-seq,Homo_633,AD.vs.control; bulk RNA-seq,Homo_633,AD.vs.MCI	2
CC	GO:0033276	transcription factor TFTC complex	bulk RNA-seq,Homo_633,AD.vs.control; bulk RNA-seq,Homo_633,AD.vs.MCI	2
MF	GO:0016811	hydrolase activity, acting on carbon-nitrogen (but not peptide) bonds, in linear amides	bulk RNA-seq,Homo_633,AD.vs.control; bulk RNA-seq,Homo_633,AD.vs.MCI	2
BP	GO:0051452	intracellular pH reduction	bulk RNA-seq,Homo_633,AD.vs.control; bulk RNA-seq,Homo_633,AD.vs.MCI	2
BP	GO:0010823	negative regulation of mitochondrion organization	bulk RNA-seq,Homo_633,AD.vs.control; bulk RNA-seq,Homo_633,AD.vs.MCI	2

CC	GO:0044754	autolysosome	bulk RNA-seq,Homo_633,AD.vs.control; bulk RNA-seq,Homo_633,AD.vs.MCI	2
BP	GO:0002888	positive regulation of myeloid leukocyte mediated immunity	bulk RNA-seq,Homo_633,AD.vs.control; bulk RNA-seq,Homo_633,AD.vs.MCI; bulk RNA-seq,SRP223445,AD.vs.control	3
CC	GO:0033176	proton-transporting V-type ATPase complex	bulk RNA-seq,Homo_633,AD.vs.control; bulk RNA-seq,Homo_633,AD.vs.MCI	2
CC	GO:0000124	SAGA complex	bulk RNA-seq,Homo_633,AD.vs.control; bulk RNA-seq,Homo_633,AD.vs.MCI	2
BP	GO:0006654	phosphatidic acid biosynthetic process	bulk RNA-seq,Homo_633,AD.vs.control; bulk RNA-seq,Homo_633,AD.vs.MCI	2
BP	GO:1902108	regulation of mitochondrial membrane permeability involved in apoptotic process	bulk RNA-seq,Homo_633,AD.vs.control; bulk RNA-seq,Homo_633,AD.vs.MCI	2
MF	GO:0061631	ubiquitin conjugating enzyme activity	bulk RNA-seq,Homo_633,AD.vs.control; bulk RNA-seq,Homo_633,AD.vs.MCI	2
BP	GO:0044090	positive regulation of vacuole organization	bulk RNA-seq,Homo_633,AD.vs.control; bulk RNA-seq,Homo_633,AD.vs.MCI	2
BP	GO:0061082	myeloid leukocyte cytokine production	bulk RNA-seq,Homo_633,AD.vs.control; bulk RNA-seq,Homo_633,AD.vs.MCI	2
MF	GO:0004197	cysteine-type endopeptidase activity	bulk RNA-seq,Homo_633,AD.vs.control; bulk RNA-seq,Homo_633,AD.vs.MCI; bulk RNA-seq,SRP223445,AD.vs.control	3
BP	GO:0032897	negative regulation of viral transcription	bulk RNA-seq,Homo_633,AD.vs.control; bulk RNA-seq,Homo_633,AD.vs.MCI	2
MF	GO:0016018	cyclosporin A binding	bulk RNA-seq,Homo_633,AD.vs.control; bulk RNA-seq,Homo_633,AD.vs.MCI	2
BP	GO:0044790	suppression of viral release by host	bulk RNA-seq,Homo_633,AD.vs.control; bulk RNA-seq,Homo_633,AD.vs.MCI	2
BP	GO:0002437	inflammatory response to antigenic stimulus	bulk RNA-seq,Homo_633,AD.vs.control; bulk RNA-seq,Homo_633,AD.vs.MCI	2
BP	GO:1901522	positive regulation of transcription from RNA polymerase II promoter involved in cellular response to chemical stimulus	bulk RNA-seq,Homo_633,AD.vs.control; bulk RNA-seq,Homo_633,AD.vs.MCI	2
BP	GO:0018195	peptidyl-arginine modification	bulk RNA-seq,Homo_633,AD.vs.control; bulk RNA-seq,Homo_633,AD.vs.MCI	2
BP	GO:1902745	positive regulation of lamellipodium organization	bulk RNA-seq,Homo_633,AD.vs.control; bulk RNA-seq,Homo_633,AD.vs.MCI	2
BP	GO:0032637	interleukin-8 production	bulk RNA-seq,Homo_633,AD.vs.control; bulk RNA-seq,Homo_633,AD.vs.MCI; bulk RNA-seq,SRP325058,AD.vs.control	3
BP	GO:2000786	positive regulation of autophagosome assembly	bulk RNA-seq,Homo_633,AD.vs.control; bulk RNA-seq,Homo_633,AD.vs.MCI	2
BP	GO:0046782	regulation of viral transcription	bulk RNA-seq,Homo_633,AD.vs.control; bulk RNA-seq,Homo_633,AD.vs.MCI	2
MF	GO:0008374	O-acyltransferase activity	bulk RNA-seq,Homo_633,AD.vs.control; bulk RNA-seq,Homo_633,AD.vs.MCI	2
BP	GO:0032732	positive regulation of interleukin-1 production	bulk RNA-seq,Homo_633,AD.vs.control; bulk RNA-seq,Homo_633,AD.vs.MCI	2
BP	GO:0045047	protein targeting to ER	bulk RNA-seq,Homo_633,AD.vs.control; bulk RNA-seq,Homo_633,AD.vs.MCI	2
BP	GO:0098780	response to mitochondrial depolarisation	bulk RNA-seq,Homo_633,AD.vs.control; bulk RNA-seq,Homo_633,AD.vs.MCI	2
BP	GO:0010042	response to manganese ion	bulk RNA-seq,Homo_633,AD.vs.control; bulk RNA-seq,Homo_633,AD.vs.MCI	2
BP	GO:0031057	negative regulation of histone modification	bulk RNA-seq,Homo_633,AD.vs.control; bulk RNA-seq,Homo_633,AD.vs.MCI	2
BP	GO:0032677	regulation of interleukin-8 production	bulk RNA-seq,Homo_633,AD.vs.control; bulk RNA-seq,Homo_633,AD.vs.MCI; bulk RNA-seq,SRP325058,AD.vs.control	3
BP	GO:0016973	poly(A)+ mRNA export from nucleus	bulk RNA-seq,Homo_633,AD.vs.control; bulk RNA-seq,Homo_633,AD.vs.MCI	2
MF	GO:0003755	peptidyl-prolyl cis-trans isomerase activity	bulk RNA-seq,Homo_633,AD.vs.control; bulk RNA-seq,Homo_633,AD.vs.MCI	2
BP	GO:0032731	positive regulation of interleukin-1 beta production	bulk RNA-seq,Homo_633,AD.vs.control; bulk RNA-seq,Homo_633,AD.vs.MCI	2
MF	GO:0004843	cysteine-type deubiquitinase activity	bulk RNA-seq,Homo_633,AD.vs.control; bulk RNA-seq,Homo_633,AD.vs.MCI; bulk RNA-seq,Homo_633,MCI.vs.control; bulk RNA-	4
BP	GO:0008631	intrinsic apoptotic signaling pathway in response to oxidative	bulk RNA-seq,Homo_633,AD.vs.control; bulk RNA-seq,Homo_633,AD.vs.MCI	2
MF	GO:0016411	acylglycerol O-acyltransferase activity	bulk RNA-seq,Homo_633,AD.vs.control; bulk RNA-seq,Homo_633,AD.vs.MCI	2
BP	GO:0070266	necroptotic process	bulk RNA-seq,Homo_633,AD.vs.control; bulk RNA-seq,Homo_633,AD.vs.MCI	2
CC	GO:0000930	gamma-tubulin complex	bulk RNA-seq,Homo_633,AD.vs.control; bulk RNA-seq,Homo_633,AD.vs.MCI; bulk RNA-seq,Homo_633,MCI.vs.control	3
BP	GO:0033233	regulation of protein sumoylation	bulk RNA-seq,Homo_633,AD.vs.control; bulk RNA-seq,Homo_633,AD.vs.MCI	2
BP	GO:0008156	negative regulation of DNA replication	bulk RNA-seq,Homo_633,AD.vs.control; bulk RNA-seq,Homo_633,AD.vs.MCI	2
BP	GO:0061081	positive regulation of myeloid leukocyte cytokine production involved in immune response	bulk RNA-seq,Homo_633,AD.vs.control; bulk RNA-seq,Homo_633,AD.vs.MCI	2
CC	GO:0000220	vacuolar proton-transporting V-type ATPase, V0 domain	bulk RNA-seq,Homo_633,AD.vs.control; bulk RNA-seq,Homo_633,AD.vs.MCI	2
BP	GO:0010592	positive regulation of lamellipodium assembly	bulk RNA-seq,Homo_633,AD.vs.control; bulk RNA-seq,Homo_633,AD.vs.MCI	2
BP	GO:0006244	pyrimidine nucleotide catabolic process	bulk RNA-seq,Homo_633,AD.vs.control; bulk RNA-seq,Homo_633,AD.vs.MCI	2
BP	GO:0097300	programmed necrotic cell death	bulk RNA-seq,Homo_633,AD.vs.control; bulk RNA-seq,Homo_633,AD.vs.MCI	2
BP	GO:0032757	positive regulation of interleukin-8 production	bulk RNA-seq,Homo_633,AD.vs.control; bulk RNA-seq,Homo_633,AD.vs.MCI; bulk RNA-seq,SRP223445,AD.vs.control; bulk RNA-	4
BP	GO:0061795	Golgi lumen acidification	bulk RNA-seq,Homo_633,AD.vs.control; bulk RNA-seq,Homo_633,AD.vs.MCI	2
BP	GO:1903902	positive regulation of viral life cycle	bulk RNA-seq,Homo_633,AD.vs.control; bulk RNA-seq,Homo_633,AD.vs.MCI	2
CC	GO:0033177	proton-transporting two-sector ATPase complex, proton-transporting domain	bulk RNA-seq,Homo_633,AD.vs.control; bulk RNA-seq,Homo_633,AD.vs.MCI	2
MF	GO:0008327	methyl-CpG binding	bulk RNA-seq,Homo_633,AD.vs.control; bulk RNA-seq,Homo_633,AD.vs.MCI	2
BP	GO:0050685	positive regulation of mRNA processing	bulk RNA-seq,Homo_633,AD.vs.control; bulk RNA-seq,Homo_633,AD.vs.MCI	2
BP	GO:0051443	positive regulation of ubiquitin-protein transferase activity	bulk RNA-seq,Homo_633,AD.vs.control; bulk RNA-seq,Homo_633,AD.vs.MCI	2
MF	GO:0019789	SUMO transferase activity	bulk RNA-seq,Homo_633,AD.vs.control; bulk RNA-seq,Homo_633,AD.vs.MCI	2
BP	GO:0070262	peptidyl-serine dephosphorylation	bulk RNA-seq,Homo_633,AD.vs.control; bulk RNA-seq,Homo_633,AD.vs.MCI	2
BP	GO:1900152	negative regulation of nuclear-transcribed mRNA catabolic process, deadenylation-dependent decay	bulk RNA-seq,Homo_633,AD.vs.control; bulk RNA-seq,Homo_633,AD.vs.MCI	2

BP	GO:0030851	granulocyte differentiation	bulk RNA-seq,Homo_633,AD.vs.control; bulk RNA-seq,Homo_633,AD.vs.MCI	2
BP	GO:0045736	negative regulation of cyclin-dependent protein serine/threonine kinase activity	bulk RNA-seq,Homo_633,AD.vs.control; bulk RNA-seq,Homo_633,AD.vs.MCI	2
BP	GO:1904030	negative regulation of cyclin-dependent protein kinase	bulk RNA-seq,Homo_633,AD.vs.control; bulk RNA-seq,Homo_633,AD.vs.MCI	2
BP	GO:0043647	inositol phosphate metabolic process	bulk RNA-seq,Homo_633,AD.vs.control; bulk RNA-seq,Homo_633,AD.vs.MCI	2
BP	GO:0008611	ether lipid biosynthetic process	bulk RNA-seq,Homo_633,AD.vs.control; bulk RNA-seq,Homo_633,AD.vs.MCI	2
BP	GO:0046504	glycerol ether biosynthetic process	bulk RNA-seq,Homo_633,AD.vs.control; bulk RNA-seq,Homo_633,AD.vs.MCI	2
BP	GO:0097384	cellular lipid biosynthetic process	bulk RNA-seq,Homo_633,AD.vs.control; bulk RNA-seq,Homo_633,AD.vs.MCI	2
BP	GO:0070862	negative regulation of protein exit from endoplasmic	bulk RNA-seq,Homo_633,AD.vs.control; bulk RNA-seq,Homo_633,AD.vs.MCI	2
BP	GO:0048388	endosomal lumen acidification	bulk RNA-seq,Homo_633,AD.vs.control; bulk RNA-seq,Homo_633,AD.vs.MCI	2
BP	GO:0046596	regulation of viral entry into host cell	bulk RNA-seq,Homo_633,AD.vs.control; bulk RNA-seq,Homo_633,AD.vs.MCI	2
BP	GO:0035305	negative regulation of dephosphorylation	bulk RNA-seq,Homo_633,AD.vs.control; bulk RNA-seq,Homo_633,AD.vs.MCI	2
MF	GO:0015605	organophosphate ester transmembrane transporter activity	bulk RNA-seq,Homo_633,AD.vs.control; bulk RNA-seq,Homo_633,AD.vs.MCI	2
BP	GO:0070269	pyroptosis	bulk RNA-seq,Homo_633,AD.vs.control; bulk RNA-seq,Homo_633,AD.vs.MCI	2
BP	GO:1905146	lysosomal protein catabolic process	bulk RNA-seq,Homo_633,AD.vs.control; bulk RNA-seq,Homo_633,AD.vs.MCI	2
MF	GO:0008139	nuclear localization sequence binding	bulk RNA-seq,Homo_633,AD.vs.control; bulk RNA-seq,Homo_633,AD.vs.MCI	2
BP	GO:1901503	ether biosynthetic process	bulk RNA-seq,Homo_633,AD.vs.control; bulk RNA-seq,Homo_633,AD.vs.MCI	2
CC	GO:0005767	secondary lysosome	bulk RNA-seq,Homo_633,AD.vs.control; bulk RNA-seq,Homo_633,AD.vs.MCI	2
BP	GO:1905476	negative regulation of protein localization to membrane	bulk RNA-seq,Homo_633,AD.vs.control; bulk RNA-seq,Homo_633,AD.vs.MCI	2
MF	GO:0016790	thiolester hydrolase activity	bulk RNA-seq,Homo_633,AD.vs.control; bulk RNA-seq,Homo_633,AD.vs.MCI	2
MF	GO:0046933	proton-transporting ATP synthase activity, rotational	bulk RNA-seq,Homo_633,AD.vs.control; bulk RNA-seq,Homo_633,AD.vs.MCI; bulk RNA-seq,Homo_633,MCI.vs.control	3
BP	GO:0010155	regulation of proton transport	bulk RNA-seq,Homo_633,AD.vs.control; bulk RNA-seq,Homo_633,AD.vs.MCI	2
MF	GO:0043014	alpha-tubulin binding	bulk RNA-seq,Homo_633,AD.vs.control; bulk RNA-seq,Homo_633,AD.vs.MCI	2
BP	GO:1902902	negative regulation of autophagosome assembly	bulk RNA-seq,Homo_633,AD.vs.control; bulk RNA-seq,Homo_633,AD.vs.MCI	2
BP	GO:0018216	peptidyl-arginine methylation	bulk RNA-seq,Homo_633,AD.vs.control; bulk RNA-seq,Homo_633,AD.vs.MCI	2
BP	GO:0070934	CRD-mediated mRNA stabilization	bulk RNA-seq,Homo_633,AD.vs.control; bulk RNA-seq,Homo_633,AD.vs.MCI	2
BP	GO:0051315	attachment of mitotic spindle microtubules to kinetochore	bulk RNA-seq,Homo_633,AD.vs.control; bulk RNA-seq,Homo_633,AD.vs.MCI	2
CC	GO:0033179	proton-transporting V-type ATPase, V0 domain	bulk RNA-seq,Homo_633,AD.vs.control; bulk RNA-seq,Homo_633,AD.vs.MCI	2
BP	GO:0045948	positive regulation of translational initiation	bulk RNA-seq,Homo_633,AD.vs.control; bulk RNA-seq,Homo_633,AD.vs.MCI; bulk RNA-seq,ROSMAP,AD.vs.control; bulk RNA-seq,ROSMAP,MCI.vs.control	4
BP	GO:0007096	regulation of exit from mitosis	bulk RNA-seq,Homo_633,AD.vs.control; bulk RNA-seq,Homo_633,AD.vs.MCI	2
BP	GO:0046598	positive regulation of viral entry into host cell	bulk RNA-seq,Homo_633,AD.vs.control; bulk RNA-seq,Homo_633,AD.vs.MCI	2
BP	GO:0075294	positive regulation by symbiont of entry into host	bulk RNA-seq,Homo_633,AD.vs.control; bulk RNA-seq,Homo_633,AD.vs.MCI	2
BP	GO:0002431	Fc receptor mediated stimulatory signaling pathway	bulk RNA-seq,Homo_633,AD.vs.control; bulk RNA-seq,Homo_633,AD.vs.MCI	2
BP	GO:0033120	positive regulation of RNA splicing	bulk RNA-seq,Homo_633,AD.vs.control; bulk RNA-seq,Homo_633,AD.vs.MCI	2
BP	GO:0140467	integrated stress response signaling	bulk RNA-seq,Homo_633,AD.vs.control; bulk RNA-seq,Homo_633,AD.vs.MCI	2
BP	GO:1903599	positive regulation of autophagy of mitochondrion	bulk RNA-seq,Homo_633,AD.vs.control; bulk RNA-seq,Homo_633,AD.vs.MCI	2
BP	GO:0007039	protein catabolic process in the vacuole	bulk RNA-seq,Homo_633,AD.vs.control; bulk RNA-seq,Homo_633,AD.vs.MCI	2
BP	GO:0070861	regulation of protein exit from endoplasmic reticulum	bulk RNA-seq,Homo_633,AD.vs.control; bulk RNA-seq,Homo_633,AD.vs.MCI	2
BP	GO:0018196	peptidyl-asparagine modification	bulk RNA-seq,Homo_633,AD.vs.control; bulk RNA-seq,Homo_633,AD.vs.MCI	2
MF	GO:1901505	carbohydrate derivative transmembrane transporter activity	bulk RNA-seq,Homo_633,AD.vs.control; bulk RNA-seq,Homo_633,AD.vs.MCI	2
BP	GO:0009226	nucleotide-sugar biosynthetic process	bulk RNA-seq,Homo_633,AD.vs.control; bulk RNA-seq,Homo_633,AD.vs.MCI	2
MF	GO:0008375	acetylglucosaminyltransferase activity	bulk RNA-seq,Homo_633,AD.vs.control; bulk RNA-seq,Homo_633,AD.vs.MCI	2
CC	GO:0000138	Golgi trans cisterna	bulk RNA-seq,Homo_633,AD.vs.control; bulk RNA-seq,Homo_633,AD.vs.MCI	2
BP	GO:0009264	deoxyribonucleotide catabolic process	bulk RNA-seq,Homo_633,AD.vs.control; bulk RNA-seq,Homo_633,AD.vs.MCI	2
BP	GO:0046386	deoxyribose phosphate catabolic process	bulk RNA-seq,Homo_633,AD.vs.control; bulk RNA-seq,Homo_633,AD.vs.MCI	2
BP	GO:0043653	mitochondrial fragmentation involved in apoptotic process	bulk RNA-seq,Homo_633,AD.vs.control; bulk RNA-seq,Homo_633,AD.vs.MCI	2
MF	GO:0140035	ubiquitination-like modification-dependent protein binding	bulk RNA-seq,Homo_633,AD.vs.control; bulk RNA-seq,Homo_633,AD.vs.MCI	2
BP	GO:0006879	cellular iron ion homeostasis	bulk RNA-seq,Homo_633,AD.vs.control; bulk RNA-seq,Homo_633,AD.vs.MCI; scRNA-seq,SRP215507,CD8+ T cell_8-MCI.vs.control	3
BP	GO:0046341	CDP-diacylglycerol metabolic process	bulk RNA-seq,Homo_633,AD.vs.control; bulk RNA-seq,Homo_633,AD.vs.MCI	2
BP	GO:0044273	sulfur compound catabolic process	bulk RNA-seq,Homo_633,AD.vs.control; bulk RNA-seq,Homo_633,AD.vs.MCI	2
CC	GO:0034518	RNA cap binding complex	bulk RNA-seq,Homo_633,AD.vs.control; bulk RNA-seq,Homo_633,AD.vs.MCI	2
BP	GO:0035308	negative regulation of protein dephosphorylation	bulk RNA-seq,Homo_633,AD.vs.control; bulk RNA-seq,Homo_633,AD.vs.MCI	2
BP	GO:0032986	protein-DNA complex disassembly	bulk RNA-seq,Homo_633,AD.vs.control; bulk RNA-seq,Homo_633,AD.vs.MCI	2
MF	GO:0001614	purinergic nucleotide receptor activity	bulk RNA-seq,Homo_633,AD.vs.control; bulk RNA-seq,Homo_633,AD.vs.MCI	2
MF	GO:0016502	nucleotide receptor activity	bulk RNA-seq,Homo_633,AD.vs.control; bulk RNA-seq,Homo_633,AD.vs.MCI	2

BP	GO:1904376	negative regulation of protein localization to cell periphery	bulk RNA-seq,Homo_633,AD.vs.control; bulk RNA-seq,Homo_633,AD.vs.MCI	2
BP	GO:0043312	neutrophil degranulation	bulk RNA-seq,Homo_633,AD.vs.control; bulk RNA-seq,Homo_633,AD.vs.MCI	2
BP	GO:0042994	cytoplasmic sequestering of transcription factor	bulk RNA-seq,Homo_633,AD.vs.control; bulk RNA-seq,Homo_633,AD.vs.MCI	2
BP	GO:0006862	nucleotide transport	bulk RNA-seq,Homo_633,AD.vs.control; bulk RNA-seq,Homo_633,AD.vs.MCI	2
BP	GO:0001325	formation of extrachromosomal circular DNA	bulk RNA-seq,Homo_633,AD.vs.control; bulk RNA-seq,Homo_633,AD.vs.MCI	2
BP	GO:0090656	t-circle formation	bulk RNA-seq,Homo_633,AD.vs.control; bulk RNA-seq,Homo_633,AD.vs.MCI	2
BP	GO:0090737	telomere maintenance via telomere trimming	bulk RNA-seq,Homo_633,AD.vs.control; bulk RNA-seq,Homo_633,AD.vs.MCI	2
CC	GO:0005845	mRNA cap binding complex	bulk RNA-seq,Homo_633,AD.vs.control; bulk RNA-seq,Homo_633,AD.vs.MCI	2
BP	GO:0032048	cardiolipin metabolic process	bulk RNA-seq,Homo_633,AD.vs.control; bulk RNA-seq,Homo_633,AD.vs.MCI	2
CC	GO:0031414	N-terminal protein acetyltransferase complex	bulk RNA-seq,Homo_633,AD.vs.control; bulk RNA-seq,Homo_633,AD.vs.MCI	2
CC	GO:0005680	anaphase-promoting complex	bulk RNA-seq,Homo_633,AD.vs.control; bulk RNA-seq,Homo_633,AD.vs.MCI	2
BP	GO:0090657	telomeric loop disassembly	bulk RNA-seq,Homo_633,AD.vs.control; bulk RNA-seq,Homo_633,AD.vs.MCI	2
BP	GO:0140632	inflammasome complex assembly	bulk RNA-seq,Homo_633,AD.vs.control; bulk RNA-seq,Homo_633,AD.vs.MCI	2
BP	GO:0009251	glucan catabolic process	bulk RNA-seq,Homo_633,AD.vs.control; bulk RNA-seq,Homo_633,AD.vs.MCI	2
MF	GO:0042625	ATPase-coupled ion transmembrane transporter activity	bulk RNA-seq,Homo_633,AD.vs.control; bulk RNA-seq,Homo_633,AD.vs.MCI	2
MF	GO:0044769	ATPase activity, coupled to transmembrane movement of ions, rotational mechanism	bulk RNA-seq,Homo_633,AD.vs.control; bulk RNA-seq,Homo_633,AD.vs.MCI	2
MF	GO:0046961	proton-transporting ATPase activity, rotational mechanism	bulk RNA-seq,Homo_633,AD.vs.control; bulk RNA-seq,Homo_633,AD.vs.MCI	2
BP	GO:0009208	pyrimidine ribonucleoside triphosphate metabolic process	bulk RNA-seq,Homo_633,AD.vs.control; bulk RNA-seq,Homo_633,AD.vs.MCI	2
BP	GO:0010803	regulation of tumor necrosis factor-mediated signaling	bulk RNA-seq,Homo_633,AD.vs.control; bulk RNA-seq,Homo_633,AD.vs.MCI	2
BP	GO:0051290	protein heterotetramerization	bulk RNA-seq,Homo_633,AD.vs.control; bulk RNA-seq,Homo_633,AD.vs.MCI	2
BP	GO:0010587	miRNA catabolic process	bulk RNA-seq,Homo_633,AD.vs.control; bulk RNA-seq,Homo_633,AD.vs.MCI	2
BP	GO:0018279	protein N-linked glycosylation via asparagine	bulk RNA-seq,Homo_633,AD.vs.control; bulk RNA-seq,Homo_633,AD.vs.MCI	2
BP	GO:0000338	protein deneddylation	bulk RNA-seq,Homo_633,AD.vs.control; bulk RNA-seq,Homo_633,AD.vs.MCI	2
BP	GO:0006655	phosphatidylglycerol biosynthetic process	bulk RNA-seq,Homo_633,AD.vs.control; bulk RNA-seq,Homo_633,AD.vs.MCI	2
CC	GO:0016281	eukaryotic translation initiation factor 4F complex	bulk RNA-seq,Homo_633,AD.vs.control; bulk RNA-seq,Homo_633,AD.vs.MCI	2
MF	GO:0004596	peptide alpha-N-acetyltransferase activity	bulk RNA-seq,Homo_633,AD.vs.control; bulk RNA-seq,Homo_633,AD.vs.MCI	2
BP	GO:0090399	replicative senescence	bulk RNA-seq,Homo_633,AD.vs.control; bulk RNA-seq,Homo_633,AD.vs.MCI	2
MF	GO:0001671	ATPase activator activity	bulk RNA-seq,Homo_633,AD.vs.control; bulk RNA-seq,Homo_633,AD.vs.MCI	2
BP	GO:1903059	regulation of protein lipidation	bulk RNA-seq,Homo_633,AD.vs.control; bulk RNA-seq,Homo_633,AD.vs.MCI	2
BP	GO:0019076	viral release from host cell	bulk RNA-seq,Homo_633,AD.vs.control; bulk RNA-seq,Homo_633,AD.vs.MCI	2
BP	GO:0035891	exit from host cell	bulk RNA-seq,Homo_633,AD.vs.control; bulk RNA-seq,Homo_633,AD.vs.MCI	2
MF	GO:0015215	nucleotide transmembrane transporter activity	bulk RNA-seq,Homo_633,AD.vs.control; bulk RNA-seq,Homo_633,AD.vs.MCI	2
CC	GO:0001891	phagocytic cup	bulk RNA-seq,Homo_633,AD.vs.control; bulk RNA-seq,Homo_633,AD.vs.MCI	2
MF	GO:0016628	oxidoreductase activity, acting on the CH-CH group of donors, NAD or NADP as acceptor	bulk RNA-seq,Homo_633,AD.vs.control; bulk RNA-seq,Homo_633,AD.vs.MCI	2
CC	GO:0045277	respiratory chain complex IV	bulk RNA-seq,Homo_633,AD.vs.control; bulk RNA-seq,Homo_633,AD.vs.MCI	2
BP	GO:0009220	pyrimidine ribonucleotide biosynthetic process	bulk RNA-seq,Homo_633,AD.vs.control; bulk RNA-seq,Homo_633,AD.vs.MCI	2
BP	GO:0007064	mitotic sister chromatid cohesion	bulk RNA-seq,Homo_633,AD.vs.control; bulk RNA-seq,Homo_633,AD.vs.MCI	2
BP	GO:0045429	positive regulation of nitric oxide biosynthetic process	bulk RNA-seq,Homo_633,AD.vs.control; bulk RNA-seq,Homo_633,AD.vs.MCI	2
BP	GO:0001911	negative regulation of leukocyte mediated cytotoxicity	bulk RNA-seq,Homo_633,AD.vs.control; bulk RNA-seq,Homo_633,AD.vs.MCI	2
MF	GO:0016671	oxidoreductase activity, acting on a sulfur group of donors, disulfide as acceptor	bulk RNA-seq,Homo_633,AD.vs.control; bulk RNA-seq,Homo_633,AD.vs.MCI	2
BP	GO:1901679	nucleotide transmembrane transport	bulk RNA-seq,Homo_633,AD.vs.control; bulk RNA-seq,Homo_633,AD.vs.MCI	2
BP	GO:0006613	cotranslational protein targeting to membrane	bulk RNA-seq,Homo_633,AD.vs.control; bulk RNA-seq,Homo_633,AD.vs.MCI	2
BP	GO:0043984	histone H4-K16 acetylation	bulk RNA-seq,Homo_633,AD.vs.control; bulk RNA-seq,Homo_633,AD.vs.MCI	2
BP	GO:0043372	positive regulation of CD4-positive, alpha-beta T cell	bulk RNA-seq,Homo_633,AD.vs.control; bulk RNA-seq,Homo_633,AD.vs.MCI	2
BP	GO:0010934	macrophage cytokine production	bulk RNA-seq,Homo_633,AD.vs.control; bulk RNA-seq,Homo_633,AD.vs.MCI	2
BP	GO:0010935	regulation of macrophage cytokine production	bulk RNA-seq,Homo_633,AD.vs.control; bulk RNA-seq,Homo_633,AD.vs.MCI	2
BP	GO:0045292	mRNA cis splicing, via spliceosome	bulk RNA-seq,Homo_633,AD.vs.control; bulk RNA-seq,Homo_633,AD.vs.MCI	2
BP	GO:1901984	negative regulation of protein acetylation	bulk RNA-seq,Homo_633,AD.vs.control; bulk RNA-seq,Homo_633,AD.vs.MCI	2
BP	GO:0006516	glycoprotein catabolic process	bulk RNA-seq,Homo_633,AD.vs.control; bulk RNA-seq,Homo_633,AD.vs.MCI	2
BP	GO:0018342	protein prenylation	bulk RNA-seq,Homo_633,AD.vs.control; bulk RNA-seq,Homo_633,AD.vs.MCI	2
BP	GO:0097354	prenylation	bulk RNA-seq,Homo_633,AD.vs.control; bulk RNA-seq,Homo_633,AD.vs.MCI	2
BP	GO:1903427	negative regulation of reactive oxygen species biosynthetic	bulk RNA-seq,Homo_633,AD.vs.control; bulk RNA-seq,Homo_633,AD.vs.MCI	2
BP	GO:0000272	polysaccharide catabolic process	bulk RNA-seq,Homo_633,AD.vs.control; bulk RNA-seq,Homo_633,AD.vs.MCI	2

BP	GO:0046460	neutral lipid biosynthetic process	bulk RNA-seq,Homo_633,AD.vs.control; bulk RNA-seq,Homo_633,AD.vs.MCI	2
BP	GO:0046463	acylglycerol biosynthetic process	bulk RNA-seq,Homo_633,AD.vs.control; bulk RNA-seq,Homo_633,AD.vs.MCI	2
BP	GO:0006337	nucleosome disassembly	bulk RNA-seq,Homo_633,AD.vs.control; bulk RNA-seq,Homo_633,AD.vs.MCI	2
BP	GO:1904350	regulation of protein catabolic process in the vacuole	bulk RNA-seq,Homo_633,AD.vs.control; bulk RNA-seq,Homo_633,AD.vs.MCI	2
BP	GO:1990182	exosomal secretion	bulk RNA-seq,Homo_633,AD.vs.control; bulk RNA-seq,Homo_633,AD.vs.MCI	2
BP	GO:0002861	regulation of inflammatory response to antigenic stimulus	bulk RNA-seq,Homo_633,AD.vs.control; bulk RNA-seq,Homo_633,AD.vs.MCI	2
MF	GO:0034452	dynactin binding	bulk RNA-seq,Homo_633,AD.vs.control; bulk RNA-seq,Homo_633,AD.vs.MCI	2
BP	GO:0097576	vacuole fusion	bulk RNA-seq,Homo_633,AD.vs.control; bulk RNA-seq,Homo_633,AD.vs.MCI	2
BP	GO:1902914	regulation of protein polyubiquitination	bulk RNA-seq,Homo_633,AD.vs.control; bulk RNA-seq,Homo_633,AD.vs.MCI	2
BP	GO:0060368	regulation of Fc receptor mediated stimulatory signaling	bulk RNA-seq,Homo_633,AD.vs.control; bulk RNA-seq,Homo_633,AD.vs.MCI	2
BP	GO:0006026	aminoglycan catabolic process	bulk RNA-seq,Homo_633,AD.vs.control; bulk RNA-seq,Homo_633,AD.vs.MCI	2
CC	GO:0033178	proton-transporting two-sector ATPase complex, catalytic	bulk RNA-seq,Homo_633,AD.vs.control; bulk RNA-seq,Homo_633,AD.vs.MCI	2
BP	GO:0006851	mitochondrial calcium ion transmembrane transport	bulk RNA-seq,Homo_633,AD.vs.control; bulk RNA-seq,Homo_633,AD.vs.MCI	2
BP	GO:1904152	regulation of retrograde protein transport, ER to cytosol	bulk RNA-seq,Homo_633,AD.vs.control; bulk RNA-seq,Homo_633,AD.vs.MCI	2
CC	GO:0098574	cytoplasmic side of lysosomal membrane	bulk RNA-seq,Homo_633,AD.vs.control; bulk RNA-seq,Homo_633,AD.vs.MCI	2
BP	GO:0016180	snRNA processing	bulk RNA-seq,Homo_633,AD.vs.control; bulk RNA-seq,Homo_633,AD.vs.MCI	2
BP	GO:0070932	histone H3 deacetylation	bulk RNA-seq,Homo_633,AD.vs.control; bulk RNA-seq,Homo_633,AD.vs.MCI	2
MF	GO:0004601	peroxidase activity	bulk RNA-seq,Homo_633,AD.vs.control; bulk RNA-seq,Homo_633,AD.vs.MCI; bulk RNA-seq,ROSMAP,AD.vs.MCI; bulk RNA-seq,ROSMAP,MCI.vs.control	4
BP	GO:2000767	positive regulation of cytoplasmic translation	bulk RNA-seq,Homo_633,AD.vs.control; bulk RNA-seq,Homo_633,AD.vs.MCI	2
BP	GO:0009223	pyrimidine deoxyribonucleotide catabolic process	bulk RNA-seq,Homo_633,AD.vs.control; bulk RNA-seq,Homo_633,AD.vs.MCI	2
MF	GO:0140036	ubiquitin-dependent protein binding	bulk RNA-seq,Homo_633,AD.vs.control; bulk RNA-seq,Homo_633,AD.vs.MCI	2
BP	GO:0051409	response to nitrosative stress	bulk RNA-seq,Homo_633,AD.vs.control; bulk RNA-seq,Homo_633,AD.vs.MCI	2
BP	GO:1900095	regulation of dosage compensation by inactivation of X	bulk RNA-seq,Homo_633,AD.vs.control; bulk RNA-seq,Homo_633,AD.vs.MCI	2
BP	GO:0031664	regulation of lipopolysaccharide-mediated signaling pathway	bulk RNA-seq,Homo_633,AD.vs.control; bulk RNA-seq,Homo_633,AD.vs.MCI	2
BP	GO:0044247	cellular polysaccharide catabolic process	bulk RNA-seq,Homo_633,AD.vs.control; bulk RNA-seq,Homo_633,AD.vs.MCI	2
BP	GO:0010968	regulation of microtubule nucleation	bulk RNA-seq,Homo_633,AD.vs.control; bulk RNA-seq,Homo_633,AD.vs.MCI	2
BP	GO:0031442	positive regulation of mRNA 3'-end processing	bulk RNA-seq,Homo_633,AD.vs.control; bulk RNA-seq,Homo_633,AD.vs.MCI	2
BP	GO:0006636	unsaturated fatty acid biosynthetic process	bulk RNA-seq,Homo_633,AD.vs.control; bulk RNA-seq,Homo_633,AD.vs.MCI	2
MF	GO:0046915	transition metal ion transmembrane transporter activity	bulk RNA-seq,Homo_633,AD.vs.control; bulk RNA-seq,Homo_633,AD.vs.MCI	2
BP	GO:0046036	CTP metabolic process	bulk RNA-seq,Homo_633,AD.vs.control; bulk RNA-seq,Homo_633,AD.vs.MCI	2
BP	GO:0097066	response to thyroid hormone	bulk RNA-seq,Homo_633,AD.vs.control; bulk RNA-seq,Homo_633,AD.vs.MCI	2
BP	GO:0046655	folic acid metabolic process	bulk RNA-seq,Homo_633,AD.vs.control; bulk RNA-seq,Homo_633,AD.vs.MCI	2
MF	GO:0070300	phosphatidic acid binding	bulk RNA-seq,Homo_633,AD.vs.control; bulk RNA-seq,Homo_633,AD.vs.MCI	2
BP	GO:0090382	phagosome maturation	bulk RNA-seq,Homo_633,AD.vs.control; bulk RNA-seq,Homo_633,AD.vs.MCI	2
BP	GO:1902253	regulation of intrinsic apoptotic signaling pathway by p53	bulk RNA-seq,Homo_633,AD.vs.control; bulk RNA-seq,Homo_633,AD.vs.MCI	2
BP	GO:0030101	natural killer cell activation	bulk RNA-seq,Homo_633,AD.vs.control; bulk RNA-seq,Homo_633,AD.vs.MCI	2
BP	GO:0016024	CDP-diacylglycerol biosynthetic process	bulk RNA-seq,Homo_633,AD.vs.control; bulk RNA-seq,Homo_633,AD.vs.MCI	2
BP	GO:0051503	adenine nucleotide transport	bulk RNA-seq,Homo_633,AD.vs.control; bulk RNA-seq,Homo_633,AD.vs.MCI	2
BP	GO:0031145	anaphase-promoting complex-dependent catabolic process	bulk RNA-seq,Homo_633,AD.vs.control; bulk RNA-seq,Homo_633,AD.vs.MCI	2
MF	GO:0051864	histone H3-methyl-lysine-36 demethylase activity	bulk RNA-seq,Homo_633,AD.vs.control; bulk RNA-seq,Homo_633,AD.vs.MCI	2
BP	GO:0070544	histone H3-K36 demethylation	bulk RNA-seq,Homo_633,AD.vs.control; bulk RNA-seq,Homo_633,AD.vs.MCI	2
BP	GO:0043558	regulation of translational initiation in response to stress	bulk RNA-seq,Homo_633,AD.vs.control; bulk RNA-seq,Homo_633,AD.vs.MCI	2
BP	GO:0015868	purine ribonucleotide transport	bulk RNA-seq,Homo_633,AD.vs.control; bulk RNA-seq,Homo_633,AD.vs.MCI	2
MF	GO:0000340	RNA 7-methylguanosine cap binding	bulk RNA-seq,Homo_633,AD.vs.control; bulk RNA-seq,Homo_633,AD.vs.MCI	2
BP	GO:0009130	pyrimidine nucleoside monophosphate biosynthetic process	bulk RNA-seq,Homo_633,AD.vs.control; bulk RNA-seq,Homo_633,AD.vs.MCI	2
BP	GO:0044406	adhesion of symbiont to host	bulk RNA-seq,Homo_633,AD.vs.control; bulk RNA-seq,Homo_633,AD.vs.MCI	2
BP	GO:1901524	regulation of mitophagy	bulk RNA-seq,Homo_633,AD.vs.control; bulk RNA-seq,Homo_633,AD.vs.MCI	2
BP	GO:0007289	spermatid nucleus differentiation	bulk RNA-seq,Homo_633,AD.vs.control; bulk RNA-seq,Homo_633,AD.vs.MCI	2
MF	GO:0005545	1-phosphatidylinositol binding	bulk RNA-seq,Homo_633,AD.vs.control; bulk RNA-seq,Homo_633,AD.vs.MCI	2
BP	GO:1900076	regulation of cellular response to insulin stimulus	bulk RNA-seq,Homo_633,AD.vs.control; bulk RNA-seq,Homo_633,AD.vs.MCI	2
BP	GO:0050849	negative regulation of calcium-mediated signaling	bulk RNA-seq,Homo_633,AD.vs.control; bulk RNA-seq,Homo_633,AD.vs.MCI	2
BP	GO:0032814	regulation of natural killer cell activation	bulk RNA-seq,Homo_633,AD.vs.control; bulk RNA-seq,Homo_633,AD.vs.MCI	2
BP	GO:0050765	negative regulation of phagocytosis	bulk RNA-seq,Homo_633,AD.vs.control; bulk RNA-seq,Homo_633,AD.vs.MCI	2
MF	GO:0016868	intramolecular transferase activity, phosphotransferases	bulk RNA-seq,Homo_633,AD.vs.control; bulk RNA-seq,Homo_633,AD.vs.MCI	2
BP	GO:0043094	cellular metabolic compound salvage	bulk RNA-seq,Homo_633,AD.vs.control; bulk RNA-seq,Homo_633,AD.vs.MCI	2

BP	GO:0045628	regulation of T-helper 2 cell differentiation	bulk RNA-seq,Homo_633,AD.vs.control; bulk RNA-seq,Homo_633,AD.vs.MCI	2
CC	GO:0016581	NuRD complex	bulk RNA-seq,Homo_633,AD.vs.control; bulk RNA-seq,Homo_633,AD.vs.MCI	2
CC	GO:0090545	CHD-type complex	bulk RNA-seq,Homo_633,AD.vs.control; bulk RNA-seq,Homo_633,AD.vs.MCI	2
BP	GO:0001865	NK T cell differentiation	bulk RNA-seq,Homo_633,AD.vs.control; bulk RNA-seq,Homo_633,AD.vs.MCI	2
BP	GO:0005980	glycogen catabolic process	bulk RNA-seq,Homo_633,AD.vs.control; bulk RNA-seq,Homo_633,AD.vs.MCI	2
BP	GO:0018410	C-terminal protein amino acid modification	bulk RNA-seq,Homo_633,AD.vs.control; bulk RNA-seq,Homo_633,AD.vs.MCI	2
MF	GO:0035325	Toll-like receptor binding	bulk RNA-seq,Homo_633,AD.vs.control; bulk RNA-seq,Homo_633,AD.vs.MCI	2
BP	GO:0002446	neutrophil mediated immunity	bulk RNA-seq,Homo_633,AD.vs.control; bulk RNA-seq,Homo_633,AD.vs.MCI	2
BP	GO:0007183	SMAD protein complex assembly	bulk RNA-seq,Homo_633,AD.vs.control; bulk RNA-seq,Homo_633,AD.vs.MCI	2
BP	GO:0042118	endothelial cell activation	bulk RNA-seq,Homo_633,AD.vs.control; bulk RNA-seq,Homo_633,AD.vs.MCI	2
MF	GO:0016273	arginine N-methyltransferase activity	bulk RNA-seq,Homo_633,AD.vs.control; bulk RNA-seq,Homo_633,AD.vs.MCI	2
MF	GO:0016274	protein-arginine N-methyltransferase activity	bulk RNA-seq,Homo_633,AD.vs.control; bulk RNA-seq,Homo_633,AD.vs.MCI	2
BP	GO:0009409	response to cold	bulk RNA-seq,Homo_633,AD.vs.control; bulk RNA-seq,Homo_633,AD.vs.MCI	2
BP	GO:0038094	Fc-gamma receptor signaling pathway	bulk RNA-seq,Homo_633,AD.vs.control; bulk RNA-seq,Homo_633,AD.vs.MCI	2
MF	GO:0032183	SUMO binding	bulk RNA-seq,Homo_633,AD.vs.control	1
BP	GO:0048308	organelle inheritance	bulk RNA-seq,Homo_633,AD.vs.control; bulk RNA-seq,Homo_633,AD.vs.MCI	2
BP	GO:0048313	Golgi inheritance	bulk RNA-seq,Homo_633,AD.vs.control; bulk RNA-seq,Homo_633,AD.vs.MCI	2
BP	GO:0046827	positive regulation of protein export from nucleus	bulk RNA-seq,Homo_633,AD.vs.control; bulk RNA-seq,Homo_633,AD.vs.MCI	2
BP	GO:0015865	purine nucleotide transport	bulk RNA-seq,Homo_633,AD.vs.control; bulk RNA-seq,Homo_633,AD.vs.MCI	2
MF	GO:0001094	TFIID-class transcription factor complex binding	bulk RNA-seq,Homo_633,AD.vs.control; bulk RNA-seq,Homo_633,AD.vs.MCI; bulk RNA-seq,Homo_633,MCI.vs.control	3
BP	GO:0031666	positive regulation of lipopolysaccharide-mediated signaling	bulk RNA-seq,Homo_633,AD.vs.control; bulk RNA-seq,Homo_633,AD.vs.MCI	2
MF	GO:0080025	phosphatidylinositol-3,5-bisphosphate binding	bulk RNA-seq,Homo_633,AD.vs.control; bulk RNA-seq,Homo_633,AD.vs.MCI	2
BP	GO:0036462	TRAIL-activated apoptotic signaling pathway	bulk RNA-seq,Homo_633,AD.vs.control; bulk RNA-seq,Homo_633,AD.vs.MCI	2
BP	GO:0002327	immature B cell differentiation	bulk RNA-seq,Homo_633,AD.vs.control; bulk RNA-seq,Homo_633,AD.vs.MCI	2
BP	GO:0019432	triglyceride biosynthetic process	bulk RNA-seq,Homo_633,AD.vs.control; bulk RNA-seq,Homo_633,AD.vs.MCI	2
MF	GO:0008353	RNA polymerase II CTD heptapeptide repeat kinase activity	bulk RNA-seq,Homo_633,AD.vs.control; bulk RNA-seq,Homo_633,AD.vs.MCI	2
BP	GO:1905709	negative regulation of membrane permeability	bulk RNA-seq,Homo_633,AD.vs.control; bulk RNA-seq,Homo_633,AD.vs.MCI	2
CC	GO:0031313	extrinsic component of endosome membrane	bulk RNA-seq,Homo_633,AD.vs.control; bulk RNA-seq,Homo_633,AD.vs.MCI	2
BP	GO:0006027	glycosaminoglycan catabolic process	bulk RNA-seq,Homo_633,AD.vs.control; bulk RNA-seq,Homo_633,AD.vs.MCI	2
CC	GO:0005652	nuclear lamina	bulk RNA-seq,Homo_633,AD.vs.control; bulk RNA-seq,Homo_633,AD.vs.MCI	2
BP	GO:0010866	regulation of triglyceride biosynthetic process	bulk RNA-seq,Homo_633,AD.vs.control; bulk RNA-seq,Homo_633,AD.vs.MCI	2
CC	GO:0000813	ESCRT I complex	bulk RNA-seq,Homo_633,AD.vs.control; bulk RNA-seq,Homo_633,AD.vs.MCI	2
BP	GO:0035330	regulation of hippo signaling	bulk RNA-seq,Homo_633,AD.vs.control	1
BP	GO:0009129	pyrimidine nucleoside monophosphate metabolic process	bulk RNA-seq,Homo_633,AD.vs.control; bulk RNA-seq,Homo_633,AD.vs.MCI	2
BP	GO:1905634	regulation of protein localization to chromatin	bulk RNA-seq,Homo_633,AD.vs.control; bulk RNA-seq,Homo_633,AD.vs.MCI	2
BP	GO:0006995	cellular response to nitrogen starvation	bulk RNA-seq,Homo_633,AD.vs.control; bulk RNA-seq,Homo_633,AD.vs.MCI	2
BP	GO:0043562	cellular response to nitrogen levels	bulk RNA-seq,Homo_633,AD.vs.control; bulk RNA-seq,Homo_633,AD.vs.MCI	2
MF	GO:0015932	nucleobase-containing compound transmembrane transporter	bulk RNA-seq,Homo_633,AD.vs.control; bulk RNA-seq,Homo_633,AD.vs.MCI	2
BP	GO:0045842	positive regulation of mitotic metaphase/anaphase transition	bulk RNA-seq,Homo_633,AD.vs.control; bulk RNA-seq,Homo_633,AD.vs.MCI	2
BP	GO:0003056	regulation of vascular associated smooth muscle contraction	bulk RNA-seq,Homo_633,AD.vs.control; bulk RNA-seq,Homo_633,AD.vs.MCI	2
MF	GO:0005068	transmembrane receptor protein tyrosine kinase adaptor	bulk RNA-seq,Homo_633,AD.vs.control; bulk RNA-seq,Homo_633,AD.vs.MCI	2
BP	GO:0048026	positive regulation of mRNA splicing, via spliceosome	bulk RNA-seq,Homo_633,AD.vs.control; bulk RNA-seq,Homo_633,AD.vs.MCI	2
BP	GO:0009173	pyrimidine ribonucleoside monophosphate metabolic process	bulk RNA-seq,Homo_633,AD.vs.control; bulk RNA-seq,Homo_633,AD.vs.MCI	2
BP	GO:0046049	UMP metabolic process	bulk RNA-seq,Homo_633,AD.vs.control; bulk RNA-seq,Homo_633,AD.vs.MCI	2
MF	GO:0035250	UDP-galactosyltransferase activity	bulk RNA-seq,Homo_633,AD.vs.control; bulk RNA-seq,Homo_633,AD.vs.MCI	2
BP	GO:0000729	DNA double-strand break processing	bulk RNA-seq,Homo_633,AD.vs.control; bulk RNA-seq,Homo_633,AD.vs.MCI	2
BP	GO:0032816	positive regulation of natural killer cell activation	bulk RNA-seq,Homo_633,AD.vs.control; bulk RNA-seq,Homo_633,AD.vs.MCI	2
BP	GO:0046456	icosanoid biosynthetic process	bulk RNA-seq,Homo_633,AD.vs.control; bulk RNA-seq,Homo_633,AD.vs.MCI; scRNA-seq,SRP330776,Monocyte_1-AD.vs.control	3
CC	GO:0005751	mitochondrial respiratory chain complex IV	bulk RNA-seq,Homo_633,AD.vs.control; bulk RNA-seq,Homo_633,AD.vs.MCI	2
MF	GO:0004859	phospholipase inhibitor activity	bulk RNA-seq,Homo_633,AD.vs.control; bulk RNA-seq,Homo_633,AD.vs.MCI	2
BP	GO:0034969	histone arginine methylation	bulk RNA-seq,Homo_633,AD.vs.control; bulk RNA-seq,Homo_633,AD.vs.MCI	2
MF	GO:0008296	3'-5'-exodeoxyribonuclease activity	bulk RNA-seq,Homo_633,AD.vs.control; bulk RNA-seq,Homo_633,AD.vs.MCI	2
BP	GO:0072710	response to hydroxyurea	bulk RNA-seq,Homo_633,AD.vs.control; bulk RNA-seq,Homo_633,AD.vs.MCI	2
BP	GO:0002438	acute inflammatory response to antigenic stimulus	bulk RNA-seq,Homo_633,AD.vs.control; bulk RNA-seq,Homo_633,AD.vs.MCI	2
BP	GO:0051645	Golgi localization	bulk RNA-seq,Homo_633,AD.vs.control; bulk RNA-seq,Homo_633,AD.vs.MCI	2

BP	GO:0060768	regulation of epithelial cell proliferation involved in prostate gland development	bulk RNA-seq,Homo_633,AD.vs.control; bulk RNA-seq,Homo_633,AD.vs.MCI	2
BP	GO:0044546	NLRP3 inflammasome complex assembly	bulk RNA-seq,Homo_633,AD.vs.control; bulk RNA-seq,Homo_633,AD.vs.MCI	2
BP	GO:0032780	negative regulation of ATP-dependent activity	bulk RNA-seq,Homo_633,AD.vs.control; bulk RNA-seq,Homo_633,AD.vs.MCI	2
BP	GO:0043320	natural killer cell degranulation	bulk RNA-seq,Homo_633,AD.vs.control; bulk RNA-seq,Homo_633,AD.vs.MCI	2
BP	GO:0048311	mitochondrion distribution	bulk RNA-seq,Homo_633,AD.vs.control; bulk RNA-seq,Homo_633,AD.vs.MCI	2
BP	GO:0090385	phagosome-lysosome fusion	bulk RNA-seq,Homo_633,AD.vs.control; bulk RNA-seq,Homo_633,AD.vs.MCI	2
BP	GO:0042339	keratan sulfate metabolic process	bulk RNA-seq,Homo_633,AD.vs.control; bulk RNA-seq,Homo_633,AD.vs.MCI	2
BP	GO:1902992	negative regulation of amyloid precursor protein catabolic	bulk RNA-seq,Homo_633,AD.vs.control; bulk RNA-seq,Homo_633,AD.vs.MCI	2
MF	GO:0016289	CoA hydrolase activity	bulk RNA-seq,Homo_633,AD.vs.control; bulk RNA-seq,Homo_633,AD.vs.MCI	2
CC	GO:0030137	COPI-coated vesicle	bulk RNA-seq,Homo_633,AD.vs.control; bulk RNA-seq,Homo_633,AD.vs.MCI	2
BP	GO:0046761	viral budding from plasma membrane	bulk RNA-seq,Homo_633,AD.vs.control; bulk RNA-seq,Homo_633,AD.vs.MCI	2
BP	GO:0001845	phagolysosome assembly	bulk RNA-seq,Homo_633,AD.vs.control; bulk RNA-seq,Homo_633,AD.vs.MCI	2
BP	GO:0043328	protein transport to vacuole involved in ubiquitin-dependent protein catabolic process via the multivesicular body sorting	bulk RNA-seq,Homo_633,AD.vs.control; bulk RNA-seq,Homo_633,AD.vs.MCI	2
BP	GO:1901856	negative regulation of cellular respiration	bulk RNA-seq,Homo_633,AD.vs.control; bulk RNA-seq,Homo_633,AD.vs.MCI	2
BP	GO:0046501	protoporphyrinogen IX metabolic process	bulk RNA-seq,Homo_633,AD.vs.control; bulk RNA-seq,Homo_633,AD.vs.MCI	2
BP	GO:0060099	regulation of phagocytosis, engulfment	bulk RNA-seq,Homo_633,AD.vs.control; bulk RNA-seq,Homo_633,AD.vs.MCI	2
BP	GO:1905153	regulation of membrane invagination	bulk RNA-seq,Homo_633,AD.vs.control; bulk RNA-seq,Homo_633,AD.vs.MCI	2
BP	GO:0006085	acetyl-CoA biosynthetic process	bulk RNA-seq,Homo_633,AD.vs.control; bulk RNA-seq,Homo_633,AD.vs.MCI	2
MF	GO:0052745	inositol phosphate phosphatase activity	bulk RNA-seq,Homo_633,AD.vs.control; bulk RNA-seq,Homo_633,AD.vs.MCI	2
BP	GO:0072711	cellular response to hydroxyurea	bulk RNA-seq,Homo_633,AD.vs.control; bulk RNA-seq,Homo_633,AD.vs.MCI	2
BP	GO:0009174	pyrimidine ribonucleoside monophosphate biosynthetic	bulk RNA-seq,Homo_633,AD.vs.control; bulk RNA-seq,Homo_633,AD.vs.MCI	2
CC	GO:0031462	Cul2-RING ubiquitin ligase complex	bulk RNA-seq,Homo_633,AD.vs.control; bulk RNA-seq,Homo_633,AD.vs.MCI	2
BP	GO:0046324	regulation of glucose import	bulk RNA-seq,Homo_633,AD.vs.control; bulk RNA-seq,Homo_633,AD.vs.MCI	2
BP	GO:0035590	purinergic nucleotide receptor signaling pathway	bulk RNA-seq,Homo_633,AD.vs.control; bulk RNA-seq,Homo_633,AD.vs.MCI	2
BP	GO:0046688	response to copper ion	bulk RNA-seq,Homo_633,AD.vs.control; bulk RNA-seq,Homo_633,AD.vs.MCI	2
BP	GO:0045624	positive regulation of T-helper cell differentiation	bulk RNA-seq,Homo_633,AD.vs.control; bulk RNA-seq,Homo_633,AD.vs.MCI	2
BP	GO:0000012	single strand break repair	bulk RNA-seq,Homo_633,AD.vs.control; bulk RNA-seq,Homo_633,AD.vs.MCI	2
BP	GO:0010884	positive regulation of lipid storage	bulk RNA-seq,Homo_633,AD.vs.control; bulk RNA-seq,Homo_633,AD.vs.MCI	2
MF	GO:0008239	dipeptidyl-peptidase activity	bulk RNA-seq,Homo_633,AD.vs.control; bulk RNA-seq,Homo_633,AD.vs.MCI; bulk RNA-seq,ROSMAP,AD.vs.control	3
BP	GO:0035795	negative regulation of mitochondrial membrane permeability	bulk RNA-seq,Homo_633,AD.vs.control; bulk RNA-seq,Homo_633,AD.vs.MCI	2
BP	GO:0006614	SRP-dependent cotranslational protein targeting to membrane	bulk RNA-seq,Homo_633,AD.vs.control; bulk RNA-seq,Homo_633,AD.vs.MCI	2
BP	GO:0002433	immune response-regulating cell surface receptor signaling pathway involved in phagocytosis	bulk RNA-seq,Homo_633,AD.vs.control; bulk RNA-seq,Homo_633,AD.vs.MCI	2
BP	GO:0038096	Fc-gamma receptor signaling pathway involved in	bulk RNA-seq,Homo_633,AD.vs.control; bulk RNA-seq,Homo_633,AD.vs.MCI	2
MF	GO:0097153	cysteine-type endopeptidase activity involved in apoptotic	bulk RNA-seq,Homo_633,AD.vs.control; bulk RNA-seq,Homo_633,AD.vs.MCI	2
BP	GO:0033034	positive regulation of myeloid cell apoptotic process	bulk RNA-seq,Homo_633,AD.vs.control; bulk RNA-seq,Homo_633,AD.vs.MCI	2
BP	GO:0006241	CTP biosynthetic process	bulk RNA-seq,Homo_633,AD.vs.control; bulk RNA-seq,Homo_633,AD.vs.MCI	2
MF	GO:0008378	galactosyltransferase activity	bulk RNA-seq,Homo_633,AD.vs.control; bulk RNA-seq,Homo_633,AD.vs.MCI	2
BP	GO:1901970	positive regulation of mitotic sister chromatid separation	bulk RNA-seq,Homo_633,AD.vs.control	1
BP	GO:0010998	regulation of translational initiation by eIF2 alpha	bulk RNA-seq,Homo_633,AD.vs.control; bulk RNA-seq,Homo_633,AD.vs.MCI	2
MF	GO:0043531	ADP binding	bulk RNA-seq,Homo_633,AD.vs.control	1
BP	GO:0032400	melanosome localization	bulk RNA-seq,Homo_633,AD.vs.control; bulk RNA-seq,Homo_633,AD.vs.MCI	2
MF	GO:0000014	single-stranded DNA endodeoxyribonuclease activity	bulk RNA-seq,Homo_633,AD.vs.control; bulk RNA-seq,Homo_633,AD.vs.MCI	2
CC	GO:1990062	RPAP3/R2TP/prefoldin-like complex	bulk RNA-seq,Homo_633,AD.vs.control	1
MF	GO:0004190	aspartic-type endopeptidase activity	bulk RNA-seq,Homo_633,AD.vs.control; bulk RNA-seq,Homo_633,AD.vs.MCI	2
BP	GO:0030730	sequestering of triglyceride	bulk RNA-seq,Homo_633,AD.vs.control; bulk RNA-seq,Homo_633,AD.vs.MCI	2
BP	GO:0097067	cellular response to thyroid hormone stimulus	bulk RNA-seq,Homo_633,AD.vs.control; bulk RNA-seq,Homo_633,AD.vs.MCI	2
BP	GO:2000659	regulation of interleukin-1-mediated signaling pathway	bulk RNA-seq,Homo_633,AD.vs.control	1
BP	GO:0006222	UMP biosynthetic process	bulk RNA-seq,Homo_633,AD.vs.control; bulk RNA-seq,Homo_633,AD.vs.MCI	2
MF	GO:1900750	oligopeptide binding	bulk RNA-seq,Homo_633,AD.vs.control; bulk RNA-seq,Homo_633,AD.vs.MCI	2
BP	GO:0150065	regulation of deacetylase activity	bulk RNA-seq,Homo_633,AD.vs.control; bulk RNA-seq,Homo_633,AD.vs.MCI	2
MF	GO:0097199	cysteine-type endopeptidase activity involved in apoptotic signaling pathway	bulk RNA-seq,Homo_633,AD.vs.control; bulk RNA-seq,Homo_633,AD.vs.MCI	2
CC	GO:0120103	centriolar subdistal appendage	bulk RNA-seq,Homo_633,AD.vs.control; bulk RNA-seq,Homo_633,AD.vs.MCI	2

BP	GO:0033235	positive regulation of protein sumoylation	bulk RNA-seq,Homo_633,AD.vs.control; bulk RNA-seq,Homo_633,AD.vs.MCI	2
MF	GO:0046030	inositol trisphosphate phosphatase activity	bulk RNA-seq,Homo_633,AD.vs.control; bulk RNA-seq,Homo_633,AD.vs.MCI	2
BP	GO:0071287	cellular response to manganese ion	bulk RNA-seq,Homo_633,AD.vs.control; bulk RNA-seq,Homo_633,AD.vs.MCI	2
BP	GO:0002524	hypersensitivity	bulk RNA-seq,Homo_633,AD.vs.control	1
BP	GO:0002756	MyD88-independent toll-like receptor signaling pathway	bulk RNA-seq,Homo_633,AD.vs.control	1
CC	GO:0071782	endoplasmic reticulum tubular network	bulk RNA-seq,Homo_633,AD.vs.control; bulk RNA-seq,Homo_633,AD.vs.MCI	2
MF	GO:0000295	adenine nucleotide transmembrane transporter activity	bulk RNA-seq,Homo_633,AD.vs.control; bulk RNA-seq,Homo_633,AD.vs.MCI	2
MF	GO:0005346	purine ribonucleotide transmembrane transporter activity	bulk RNA-seq,Homo_633,AD.vs.control; bulk RNA-seq,Homo_633,AD.vs.MCI	2
BP	GO:0009209	pyrimidine ribonucleoside triphosphate biosynthetic process	bulk RNA-seq,Homo_633,AD.vs.control; bulk RNA-seq,Homo_633,AD.vs.MCI	2
BP	GO:0000132	establishment of mitotic spindle orientation	bulk RNA-seq,Homo_633,AD.vs.control; bulk RNA-seq,Homo_633,AD.vs.MCI	2
BP	GO:0070601	centromeric sister chromatid cohesion	bulk RNA-seq,Homo_633,AD.vs.control; bulk RNA-seq,Homo_633,AD.vs.MCI	2
BP	GO:0030889	negative regulation of B cell proliferation	bulk RNA-seq,Homo_633,AD.vs.control; bulk RNA-seq,Homo_633,AD.vs.MCI	2
BP	GO:0006474	N-terminal protein amino acid acetylation	bulk RNA-seq,Homo_633,AD.vs.control	1
BP	GO:0030174	regulation of DNA-templated DNA replication initiation	bulk RNA-seq,Homo_633,AD.vs.control; bulk RNA-seq,Homo_633,AD.vs.MCI	2
BP	GO:0010867	positive regulation of triglyceride biosynthetic process	bulk RNA-seq,Homo_633,AD.vs.control; bulk RNA-seq,Homo_633,AD.vs.MCI	2
BP	GO:0002730	regulation of dendritic cell cytokine production	bulk RNA-seq,Homo_633,AD.vs.control; bulk RNA-seq,Homo_633,AD.vs.MCI	2
BP	GO:0070233	negative regulation of T cell apoptotic process	bulk RNA-seq,Homo_633,AD.vs.control; bulk RNA-seq,Homo_633,AD.vs.MCI	2
BP	GO:0036444	calcium import into the mitochondrion	bulk RNA-seq,Homo_633,AD.vs.control; bulk RNA-seq,Homo_633,AD.vs.MCI	2
BP	GO:0072393	microtubule anchoring at microtubule organizing center	bulk RNA-seq,Homo_633,AD.vs.control; bulk RNA-seq,Homo_633,AD.vs.MCI	2
BP	GO:0030223	neutrophil differentiation	bulk RNA-seq,Homo_633,AD.vs.control; bulk RNA-seq,Homo_633,AD.vs.MCI	2
MF	GO:0019992	diacylglycerol binding	bulk RNA-seq,Homo_633,AD.vs.control	1
MF	GO:0016881	acid-amino acid ligase activity	bulk RNA-seq,Homo_633,AD.vs.control	1
BP	GO:0003222	ventricular trabecula myocardium morphogenesis	bulk RNA-seq,Homo_633,AD.vs.control; bulk RNA-seq,Homo_633,AD.vs.MCI	2
CC	GO:0098554	cytoplasmic side of endoplasmic reticulum membrane	bulk RNA-seq,Homo_633,AD.vs.control; bulk RNA-seq,Homo_633,AD.vs.MCI	2
BP	GO:0047484	regulation of response to osmotic stress	bulk RNA-seq,Homo_633,AD.vs.control; bulk RNA-seq,Homo_633,AD.vs.MCI	2
CC	GO:0036038	MKS complex	bulk RNA-seq,Homo_633,AD.vs.control; bulk RNA-seq,Homo_633,AD.vs.MCI	2
BP	GO:0048535	lymph node development	bulk RNA-seq,Homo_633,AD.vs.control; bulk RNA-seq,Homo_633,AD.vs.MCI	2
BP	GO:1904923	regulation of autophagy of mitochondrion in response to mitochondrial depolarization	bulk RNA-seq,Homo_633,AD.vs.control; bulk RNA-seq,Homo_633,AD.vs.MCI	2
BP	GO:0035845	photoreceptor cell outer segment organization	bulk RNA-seq,Homo_633,AD.vs.control; bulk RNA-seq,Homo_633,AD.vs.MCI	2
BP	GO:0035855	megakaryocyte development	bulk RNA-seq,Homo_633,AD.vs.control; bulk RNA-seq,Homo_633,AD.vs.MCI	2
BP	GO:0002371	dendritic cell cytokine production	bulk RNA-seq,Homo_633,AD.vs.control; bulk RNA-seq,Homo_633,AD.vs.MCI	2
MF	GO:0050700	CARD domain binding	bulk RNA-seq,Homo_633,AD.vs.control; bulk RNA-seq,Homo_633,AD.vs.MCI	2
BP	GO:0002732	positive regulation of dendritic cell cytokine production	bulk RNA-seq,Homo_633,AD.vs.control; bulk RNA-seq,Homo_633,AD.vs.MCI	2
BP	GO:1903319	positive regulation of protein maturation	bulk RNA-seq,Homo_633,AD.vs.control; bulk RNA-seq,Homo_633,AD.vs.MCI	2
BP	GO:0033869	nucleoside bisphosphate catabolic process	bulk RNA-seq,Homo_633,AD.vs.control	1
BP	GO:0034031	ribonucleoside bisphosphate catabolic process	bulk RNA-seq,Homo_633,AD.vs.control	1
BP	GO:0034034	purine nucleoside bisphosphate catabolic process	bulk RNA-seq,Homo_633,AD.vs.control	1
BP	GO:0060100	positive regulation of phagocytosis, engulfment	bulk RNA-seq,Homo_633,AD.vs.control; bulk RNA-seq,Homo_633,AD.vs.MCI	2
BP	GO:1905155	positive regulation of membrane invagination	bulk RNA-seq,Homo_633,AD.vs.control; bulk RNA-seq,Homo_633,AD.vs.MCI	2
BP	GO:1903624	regulation of DNA catabolic process	bulk RNA-seq,Homo_633,AD.vs.control; bulk RNA-seq,Homo_633,AD.vs.MCI	2
MF	GO:0015216	purine nucleotide transmembrane transporter activity	bulk RNA-seq,Homo_633,AD.vs.control; bulk RNA-seq,Homo_633,AD.vs.MCI	2
BP	GO:0036124	histone H3-K9 trimethylation	bulk RNA-seq,Homo_633,AD.vs.control; bulk RNA-seq,Homo_633,AD.vs.MCI	2
BP	GO:0006002	fructose 6-phosphate metabolic process	bulk RNA-seq,Homo_633,AD.vs.control; bulk RNA-seq,Homo_633,AD.vs.MCI	2
BP	GO:0046686	response to cadmium ion	bulk RNA-seq,Homo_633,AD.vs.control; bulk RNA-seq,Homo_633,AD.vs.MCI	2
MF	GO:0017110	nucleoside diphosphate phosphatase activity	bulk RNA-seq,Homo_633,AD.vs.control; bulk RNA-seq,Homo_633,AD.vs.MCI	2
BP	GO:0006361	transcription initiation at RNA polymerase I promoter	bulk RNA-seq,Homo_633,AD.vs.control; bulk RNA-seq,Homo_633,AD.vs.MCI	2
BP	GO:0002084	protein depalmitoylation	bulk RNA-seq,Homo_633,AD.vs.control; bulk RNA-seq,Homo_633,AD.vs.MCI	2
BP	GO:0097201	negative regulation of transcription from RNA polymerase II promoter in response to stress	bulk RNA-seq,Homo_633,AD.vs.control; bulk RNA-seq,Homo_633,AD.vs.MCI	2
BP	GO:0002223	stimulatory C-type lectin receptor signaling pathway	bulk RNA-seq,Homo_633,AD.vs.control; bulk RNA-seq,Homo_633,AD.vs.MCI; scRNA-seq,SRP330776,Natural killer cell_1-AD.vs.control	3
BP	GO:1990840	response to lectin	bulk RNA-seq,Homo_633,AD.vs.control; bulk RNA-seq,Homo_633,AD.vs.MCI; scRNA-seq,SRP330776,Natural killer cell_1-AD.vs.control	3
BP	GO:1990858	cellular response to lectin	bulk RNA-seq,Homo_633,AD.vs.control; bulk RNA-seq,Homo_633,AD.vs.MCI; scRNA-seq,SRP330776,Natural killer cell_1-AD.vs.control	3
MF	GO:0051880	G-quadruplex DNA binding	bulk RNA-seq,Homo_633,AD.vs.control; bulk RNA-seq,Homo_633,AD.vs.MCI	2
MF	GO:0047617	acyl-CoA hydrolase activity	bulk RNA-seq,Homo_633,AD.vs.control; bulk RNA-seq,Homo_633,AD.vs.MCI	2
BP	GO:0001779	natural killer cell differentiation	bulk RNA-seq,Homo_633,AD.vs.control; bulk RNA-seq,Homo_633,AD.vs.MCI	2

BP	GO:0006068	ethanol catabolic process	bulk RNA-seq,Homo_633,AD.vs.control; bulk RNA-seq,Homo_633,AD.vs.MCI	2
BP	GO:0032268	regulation of cellular protein metabolic process	bulk RNA-seq,Homo_633,AD.vs.control; bulk RNA-seq,Homo_633,AD.vs.MCI	2
BP	GO:0006089	lactate metabolic process	bulk RNA-seq,Homo_633,AD.vs.control; bulk RNA-seq,Homo_633,AD.vs.MCI	2
MF	GO:0005521	lamin binding	bulk RNA-seq,Homo_633,AD.vs.control; bulk RNA-seq,Homo_633,AD.vs.MCI	2
MF	GO:0061665	SUMO ligase activity	bulk RNA-seq,Homo_633,AD.vs.control; bulk RNA-seq,Homo_633,AD.vs.MCI	2
BP	GO:0070231	T cell apoptotic process	bulk RNA-seq,Homo_633,AD.vs.control	1
BP	GO:0036151	phosphatidylcholine acyl-chain remodeling	bulk RNA-seq,Homo_633,AD.vs.control; bulk RNA-seq,Homo_633,AD.vs.MCI	2
MF	GO:1990226	histone methyltransferase binding	bulk RNA-seq,Homo_633,AD.vs.control; bulk RNA-seq,Homo_633,AD.vs.MCI	2
MF	GO:0017128	phospholipid scramblase activity	bulk RNA-seq,Homo_633,AD.vs.control; bulk RNA-seq,Homo_633,AD.vs.MCI	2
BP	GO:1905665	positive regulation of calcium ion import across plasma	bulk RNA-seq,Homo_633,AD.vs.control	1
BP	GO:0060296	regulation of cilium beat frequency involved in ciliary	bulk RNA-seq,Homo_633,AD.vs.control	1
BP	GO:0018345	protein palmitoylation	bulk RNA-seq,Homo_633,AD.vs.control; bulk RNA-seq,Homo_633,AD.vs.MCI	2
MF	GO:0016846	carbon-sulfur lyase activity	bulk RNA-seq,Homo_633,AD.vs.control; bulk RNA-seq,Homo_633,AD.vs.MCI	2
BP	GO:1900225	regulation of NLRP3 inflammasome complex assembly	bulk RNA-seq,Homo_633,AD.vs.control	1
MF	GO:0005149	interleukin-1 receptor binding	bulk RNA-seq,Homo_633,AD.vs.control; bulk RNA-seq,Homo_633,AD.vs.MCI	2
BP	GO:0097264	self proteolysis	bulk RNA-seq,Homo_633,AD.vs.control	1
MF	GO:0016857	racemase and epimerase activity, acting on carbohydrates and derivatives	bulk RNA-seq,Homo_633,AD.vs.control; bulk RNA-seq,Homo_633,AD.vs.MCI	2
BP	GO:0002863	positive regulation of inflammatory response to antigenic	bulk RNA-seq,Homo_633,AD.vs.control; bulk RNA-seq,Homo_633,AD.vs.MCI	2
BP	GO:1902101	positive regulation of metaphase/anaphase transition of cell	bulk RNA-seq,Homo_633,AD.vs.control; bulk RNA-seq,Homo_633,AD.vs.MCI	2
BP	GO:1900112	regulation of histone H3-K9 trimethylation	bulk RNA-seq,Homo_633,AD.vs.control; bulk RNA-seq,Homo_633,AD.vs.MCI	2
CC	GO:0000221	vacuolar proton-transporting V-type ATPase, V1 domain	bulk RNA-seq,Homo_633,AD.vs.control; bulk RNA-seq,Homo_633,AD.vs.MCI	2
CC	GO:0033180	proton-transporting V-type ATPase, V1 domain	bulk RNA-seq,Homo_633,AD.vs.control; bulk RNA-seq,Homo_633,AD.vs.MCI	2
BP	GO:0060253	negative regulation of glial cell proliferation	bulk RNA-seq,Homo_633,AD.vs.control	1
BP	GO:0060907	positive regulation of macrophage cytokine production	bulk RNA-seq,Homo_633,AD.vs.control; bulk RNA-seq,Homo_633,AD.vs.MCI	2
BP	GO:0051775	response to redox state	bulk RNA-seq,Homo_633,AD.vs.control; bulk RNA-seq,Homo_633,AD.vs.MCI	2
BP	GO:0140719	constitutive heterochromatin formation	bulk RNA-seq,Homo_633,AD.vs.control; bulk RNA-seq,Homo_633,AD.vs.MCI	2
BP	GO:0033234	negative regulation of protein sumoylation	bulk RNA-seq,Homo_633,AD.vs.control; bulk RNA-seq,Homo_633,AD.vs.MCI	2
MF	GO:0031683	G-protein beta/gamma-subunit complex binding	bulk RNA-seq,Homo_633,AD.vs.control; bulk RNA-seq,Homo_633,AD.vs.MCI	2
BP	GO:0034472	snRNA 3'-end processing	bulk RNA-seq,Homo_633,AD.vs.control; bulk RNA-seq,Homo_633,AD.vs.MCI	2
MF	GO:0070001	aspartic-type peptidase activity	bulk RNA-seq,Homo_633,AD.vs.control; bulk RNA-seq,Homo_633,AD.vs.MCI	2
BP	GO:0018146	keratan sulfate biosynthetic process	bulk RNA-seq,Homo_633,AD.vs.control	1
CC	GO:0099091	postsynaptic specialization, intracellular component	bulk RNA-seq,Homo_633,AD.vs.control	1
CC	GO:0140268	endoplasmic reticulum-plasma membrane contact site	bulk RNA-seq,Homo_633,AD.vs.control	1
BP	GO:0019184	nonribosomal peptide biosynthetic process	bulk RNA-seq,Homo_633,AD.vs.control	1
BP	GO:0010923	negative regulation of phosphatase activity	bulk RNA-seq,Homo_633,AD.vs.control; bulk RNA-seq,Homo_633,AD.vs.MCI	2
BP	GO:0010635	regulation of mitochondrial fusion	bulk RNA-seq,Homo_633,AD.vs.control; bulk RNA-seq,Homo_633,AD.vs.MCI	2
BP	GO:0034454	microtubule anchoring at centrosome	bulk RNA-seq,Homo_633,AD.vs.control; bulk RNA-seq,Homo_633,AD.vs.MCI	2
MF	GO:0015252	proton channel activity	bulk RNA-seq,Homo_633,AD.vs.control; bulk RNA-seq,Homo_633,AD.vs.MCI	2
BP	GO:0042278	purine nucleoside metabolic process	bulk RNA-seq,Homo_633,AD.vs.control; bulk RNA-seq,Homo_633,AD.vs.MCI	2
BP	GO:0006684	sphingomyelin metabolic process	bulk RNA-seq,Homo_633,AD.vs.control; bulk RNA-seq,Homo_633,AD.vs.MCI	2
BP	GO:0046329	negative regulation of JNK cascade	bulk RNA-seq,Homo_633,AD.vs.control; bulk RNA-seq,Homo_633,AD.vs.MCI	2
BP	GO:0035646	endosome to melanosome transport	bulk RNA-seq,Homo_633,AD.vs.control; bulk RNA-seq,Homo_633,AD.vs.MCI	2
BP	GO:0043485	endosome to pigment granule transport	bulk RNA-seq,Homo_633,AD.vs.control; bulk RNA-seq,Homo_633,AD.vs.MCI	2
BP	GO:0048757	pigment granule maturation	bulk RNA-seq,Homo_633,AD.vs.control; bulk RNA-seq,Homo_633,AD.vs.MCI	2
BP	GO:0001915	negative regulation of T cell mediated cytotoxicity	bulk RNA-seq,Homo_633,AD.vs.control	1
BP	GO:1900044	regulation of protein K63-linked ubiquitination	bulk RNA-seq,Homo_633,AD.vs.control; bulk RNA-seq,Homo_633,AD.vs.MCI	2
BP	GO:0006123	mitochondrial electron transport, cytochrome c to oxygen	bulk RNA-seq,Homo_633,AD.vs.control; bulk RNA-seq,Homo_633,AD.vs.MCI	2
MF	GO:0102545	phosphatidyl phospholipase B activity	bulk RNA-seq,Homo_633,AD.vs.control; bulk RNA-seq,Homo_633,AD.vs.MCI	2
MF	GO:0031492	nucleosomal DNA binding	bulk RNA-seq,Homo_633,AD.vs.control	1
MF	GO:0140313	molecular sequestering activity	bulk RNA-seq,Homo_633,AD.vs.control	1
BP	GO:1903543	positive regulation of exosomal secretion	bulk RNA-seq,Homo_633,AD.vs.control	1
BP	GO:0099638	endosome to plasma membrane protein transport	bulk RNA-seq,Homo_633,AD.vs.control	1
BP	GO:0043471	regulation of cellular carbohydrate catabolic process	bulk RNA-seq,Homo_633,AD.vs.control	1
MF	GO:0050786	RAGE receptor binding	bulk RNA-seq,Homo_633,AD.vs.control; bulk RNA-seq,Homo_633,AD.vs.MCI	2

BP	GO:0050732	negative regulation of peptidyl-tyrosine phosphorylation	bulk RNA-seq,Homo_633,AD.vs.control	1
BP	GO:0010889	regulation of sequestering of triglyceride	bulk RNA-seq,Homo_633,AD.vs.control; bulk RNA-seq,Homo_633,AD.vs.MCI	2
BP	GO:0048840	otolith development	bulk RNA-seq,Homo_633,AD.vs.control; bulk RNA-seq,Homo_633,AD.vs.MCI	2
MF	GO:0097027	ubiquitin-protein transferase activator activity	bulk RNA-seq,Homo_633,AD.vs.control; bulk RNA-seq,Homo_633,AD.vs.MCI	2
BP	GO:0002424	T cell mediated immune response to tumor cell	bulk RNA-seq,Homo_633,AD.vs.control	1
BP	GO:0097062	dendritic spine maintenance	bulk RNA-seq,Homo_633,AD.vs.control	1
MF	GO:0005384	manganese ion transmembrane transporter activity	bulk RNA-seq,Homo_633,AD.vs.control	1
BP	GO:0071421	manganese ion transmembrane transport	bulk RNA-seq,Homo_633,AD.vs.control	1
MF	GO:0043028	cysteine-type endopeptidase regulator activity involved in apoptotic process	bulk RNA-seq,Homo_633,AD.vs.control	1
BP	GO:0060544	regulation of necroptotic process	bulk RNA-seq,Homo_633,AD.vs.control; bulk RNA-seq,Homo_633,AD.vs.MCI	2
BP	GO:0030397	membrane disassembly	bulk RNA-seq,Homo_633,AD.vs.control	1
BP	GO:2001279	regulation of unsaturated fatty acid biosynthetic process	bulk RNA-seq,Homo_633,AD.vs.control; bulk RNA-seq,Homo_633,AD.vs.MCI	2
BP	GO:0090235	regulation of metaphase plate congression	bulk RNA-seq,Homo_633,AD.vs.control	1
BP	GO:0045916	negative regulation of complement activation	bulk RNA-seq,Homo_633,AD.vs.control; bulk RNA-seq,Homo_633,AD.vs.MCI	2
BP	GO:0043476	pigment accumulation	bulk RNA-seq,Homo_633,AD.vs.control	1
BP	GO:0043482	cellular pigment accumulation	bulk RNA-seq,Homo_633,AD.vs.control	1
BP	GO:1904896	ESCRT complex disassembly	bulk RNA-seq,Homo_633,AD.vs.control; bulk RNA-seq,Homo_633,AD.vs.MCI	2
BP	GO:1904903	ESCRT III complex disassembly	bulk RNA-seq,Homo_633,AD.vs.control; bulk RNA-seq,Homo_633,AD.vs.MCI	2
BP	GO:1902235	regulation of endoplasmic reticulum stress-induced intrinsic apoptotic signaling pathway	bulk RNA-seq,Homo_633,AD.vs.control	1
CC	GO:0044815	DNA packaging complex	bulk RNA-seq,Homo_633,AD.vs.control; bulk RNA-seq,ROSMAP,AD.vs.control	2
BP	GO:1902430	negative regulation of amyloid-beta formation	bulk RNA-seq,Homo_633,AD.vs.control	1
BP	GO:1900078	positive regulation of cellular response to insulin stimulus	bulk RNA-seq,Homo_633,AD.vs.control; bulk RNA-seq,Homo_633,AD.vs.MCI	2
BP	GO:1903025	regulation of RNA polymerase II regulatory region sequence-specific DNA binding	bulk RNA-seq,Homo_633,AD.vs.control; bulk RNA-seq,Homo_633,AD.vs.MCI	2
BP	GO:1902683	regulation of receptor localization to synapse	bulk RNA-seq,Homo_633,AD.vs.control; bulk RNA-seq,Homo_633,AD.vs.MCI	2
BP	GO:0016559	peroxisome fission	bulk RNA-seq,Homo_633,AD.vs.control; bulk RNA-seq,Homo_633,MCI.vs.control	2
CC	GO:0032797	SMN complex	bulk RNA-seq,Homo_633,AD.vs.control	1
BP	GO:1904925	positive regulation of autophagy of mitochondrion in response to mitochondrial depolarization	bulk RNA-seq,Homo_633,AD.vs.control	1
BP	GO:0046716	muscle cell cellular homeostasis	bulk RNA-seq,Homo_633,AD.vs.control; bulk RNA-seq,Homo_633,AD.vs.MCI	2
MF	GO:0005347	ATP transmembrane transporter activity	bulk RNA-seq,Homo_633,AD.vs.control; bulk RNA-seq,Homo_633,AD.vs.MCI	2
BP	GO:0010940	positive regulation of necrotic cell death	bulk RNA-seq,Homo_633,AD.vs.control; bulk RNA-seq,Homo_633,AD.vs.MCI	2
BP	GO:0009162	deoxyribonucleoside monophosphate metabolic process	bulk RNA-seq,Homo_633,AD.vs.control; bulk RNA-seq,Homo_633,AD.vs.MCI	2
BP	GO:1903541	regulation of exosomal secretion	bulk RNA-seq,Homo_633,AD.vs.control; bulk RNA-seq,Homo_633,AD.vs.MCI	2
BP	GO:0071786	endoplasmic reticulum tubular network organization	bulk RNA-seq,Homo_633,AD.vs.control	1
MF	GO:0050664	oxidoreductase activity, acting on NAD(P)H, oxygen as	bulk RNA-seq,Homo_633,AD.vs.control; bulk RNA-seq,Homo_633,AD.vs.MCI	2
CC	GO:0005640	nuclear outer membrane	bulk RNA-seq,Homo_633,AD.vs.control; bulk RNA-seq,Homo_633,AD.vs.MCI	2
BP	GO:0017121	plasma membrane phospholipid scrambling	bulk RNA-seq,Homo_633,AD.vs.control; bulk RNA-seq,Homo_633,AD.vs.MCI	2
BP	GO:0051561	positive regulation of mitochondrial calcium ion	bulk RNA-seq,Homo_633,AD.vs.control; bulk RNA-seq,Homo_633,AD.vs.MCI	2
MF	GO:0046935	1-phosphatidylinositol-3-kinase regulator activity	bulk RNA-seq,Homo_633,AD.vs.control; bulk RNA-seq,Homo_633,AD.vs.MCI	2
MF	GO:0004953	icosanoid receptor activity	bulk RNA-seq,Homo_633,AD.vs.control; bulk RNA-seq,Homo_633,AD.vs.MCI	2
BP	GO:0098887	neurotransmitter receptor transport, endosome to postsynaptic membrane	bulk RNA-seq,Homo_633,AD.vs.control	1
BP	GO:0099639	neurotransmitter receptor transport, endosome to plasma	bulk RNA-seq,Homo_633,AD.vs.control	1
BP	GO:0031848	protection from non-homologous end joining at telomere	bulk RNA-seq,Homo_633,AD.vs.control; bulk RNA-seq,Homo_633,AD.vs.MCI	2
BP	GO:0071394	cellular response to testosterone stimulus	bulk RNA-seq,Homo_633,AD.vs.control	1
BP	GO:0034397	telomere localization	bulk RNA-seq,Homo_633,AD.vs.control; bulk RNA-seq,Homo_633,AD.vs.MCI	2
BP	GO:0090220	chromosome localization to nuclear envelope involved in homologous chromosome segregation	bulk RNA-seq,Homo_633,AD.vs.control; bulk RNA-seq,Homo_633,AD.vs.MCI	2
BP	GO:0034310	primary alcohol catabolic process	bulk RNA-seq,Homo_633,AD.vs.control; bulk RNA-seq,Homo_633,AD.vs.MCI	2
BP	GO:0009120	deoxyribonucleoside metabolic process	bulk RNA-seq,Homo_633,AD.vs.control; bulk RNA-seq,Homo_633,AD.vs.MCI	2
BP	GO:1903265	positive regulation of tumor necrosis factor-mediated	bulk RNA-seq,Homo_633,AD.vs.control	1
BP	GO:0018200	peptidyl-glutamic acid modification	bulk RNA-seq,Homo_633,AD.vs.control	1
MF	GO:0008198	ferrous iron binding	bulk RNA-seq,Homo_633,AD.vs.control; bulk RNA-seq,Homo_633,AD.vs.MCI	2
BP	GO:0070344	regulation of fat cell proliferation	bulk RNA-seq,Homo_633,AD.vs.control; bulk RNA-seq,Homo_633,AD.vs.MCI	2

BP	GO:1902175	regulation of oxidative stress-induced intrinsic apoptotic signaling pathway	bulk RNA-seq,Homo_633,AD.vs.control; bulk RNA-seq,Homo_633,AD.vs.MCI	2
BP	GO:1900087	positive regulation of G1/S transition of mitotic cell cycle	bulk RNA-seq,Homo_633,AD.vs.control	1
BP	GO:0048262	determination of dorsal/ventral asymmetry	bulk RNA-seq,Homo_633,AD.vs.control	1
BP	GO:0051974	negative regulation of telomerase activity	bulk RNA-seq,Homo_633,AD.vs.control	1
BP	GO:1902254	negative regulation of intrinsic apoptotic signaling pathway by p53 class mediator	bulk RNA-seq,Homo_633,AD.vs.control	1
BP	GO:0071280	cellular response to copper ion	bulk RNA-seq,Homo_633,AD.vs.control; bulk RNA-seq,Homo_633,AD.vs.MCI	2
MF	GO:0033038	bitter taste receptor activity	bulk RNA-seq,Homo_633,AD.vs.control; bulk RNA-seq,Homo_633,AD.vs.MCI; bulk RNA-seq,ROSMAP,AD.vs.control; bulk RNA-seq,ROSMAP,AD.vs.MCI	4
BP	GO:0015867	ATP transport	bulk RNA-seq,Homo_633,AD.vs.control; bulk RNA-seq,Homo_633,AD.vs.MCI	2
BP	GO:0043173	nucleotide salvage	bulk RNA-seq,Homo_633,AD.vs.control; bulk RNA-seq,Homo_633,AD.vs.MCI	2
MF	GO:0015929	hexosaminidase activity	bulk RNA-seq,Homo_633,AD.vs.control	1
BP	GO:1905664	regulation of calcium ion import across plasma membrane	bulk RNA-seq,Homo_633,AD.vs.control	1
BP	GO:0009301	snRNA transcription	bulk RNA-seq,Homo_633,AD.vs.control; bulk RNA-seq,Homo_633,AD.vs.MCI	2
CC	GO:0032009	early phagosome	bulk RNA-seq,Homo_633,AD.vs.control	1
BP	GO:0006692	prostanoid metabolic process	bulk RNA-seq,Homo_633,AD.vs.control; bulk RNA-seq,Homo_633,AD.vs.MCI; scRNA-seq,SRP330776,Monocyte_1-AD.vs.control	3
BP	GO:0006693	prostaglandin metabolic process	bulk RNA-seq,Homo_633,AD.vs.control; bulk RNA-seq,Homo_633,AD.vs.MCI; scRNA-seq,SRP330776,Monocyte_1-AD.vs.control	3
CC	GO:0032039	integrator complex	bulk RNA-seq,Homo_633,AD.vs.control; bulk RNA-seq,Homo_633,AD.vs.MCI	2
BP	GO:0051238	sequestering of metal ion	bulk RNA-seq,Homo_633,AD.vs.control; bulk RNA-seq,Homo_633,AD.vs.MCI	2
MF	GO:0031489	myosin V binding	bulk RNA-seq,Homo_633,AD.vs.control	1
BP	GO:0001832	blastocyst growth	bulk RNA-seq,Homo_633,AD.vs.control	1
MF	GO:0102991	myristoyl-CoA hydrolase activity	bulk RNA-seq,Homo_633,AD.vs.control; bulk RNA-seq,Homo_633,AD.vs.MCI	2
BP	GO:0036109	alpha-linolenic acid metabolic process	bulk RNA-seq,Homo_633,AD.vs.control; bulk RNA-seq,Homo_633,AD.vs.MCI	2
BP	GO:0046349	amino sugar biosynthetic process	bulk RNA-seq,Homo_633,AD.vs.control	1
BP	GO:0036152	phosphatidylethanolamine acyl-chain remodeling	bulk RNA-seq,Homo_633,AD.vs.control; bulk RNA-seq,Homo_633,AD.vs.MCI	2
BP	GO:1903170	negative regulation of calcium ion transmembrane transport	bulk RNA-seq,Homo_633,AD.vs.control; bulk RNA-seq,Homo_633,AD.vs.MCI	2
BP	GO:0106049	regulation of cellular response to osmotic stress	bulk RNA-seq,Homo_633,AD.vs.control; bulk RNA-seq,Homo_633,AD.vs.MCI	2
BP	GO:0042276	error-prone translesion synthesis	bulk RNA-seq,Homo_633,AD.vs.control; bulk RNA-seq,Homo_633,AD.vs.MCI	2
CC	GO:0048188	Set1C/COMPASS complex	bulk RNA-seq,Homo_633,AD.vs.control; bulk RNA-seq,Homo_633,AD.vs.MCI	2
MF	GO:0047429	nucleoside triphosphate diphosphatase activity	bulk RNA-seq,Homo_633,AD.vs.control	1
MF	GO:0004602	glutathione peroxidase activity	bulk RNA-seq,Homo_633,AD.vs.control; bulk RNA-seq,Homo_633,AD.vs.MCI	2
MF	GO:0032813	tumor necrosis factor receptor superfamily binding	bulk RNA-seq,Homo_633,AD.vs.control	1
MF	GO:0008484	sulfuric ester hydrolase activity	bulk RNA-seq,Homo_633,AD.vs.control; bulk RNA-seq,Homo_633,AD.vs.MCI	2
BP	GO:0060628	regulation of ER to Golgi vesicle-mediated transport	bulk RNA-seq,Homo_633,AD.vs.control	1
BP	GO:0051255	spindle midzone assembly	bulk RNA-seq,Homo_633,AD.vs.control	1
MF	GO:0052650	NADP-retinol dehydrogenase activity	bulk RNA-seq,Homo_633,AD.vs.control; bulk RNA-seq,Homo_633,AD.vs.MCI	2
BP	GO:0046326	positive regulation of glucose import	bulk RNA-seq,Homo_633,AD.vs.control	1
BP	GO:0002673	regulation of acute inflammatory response	bulk RNA-seq,Homo_633,AD.vs.control	1
BP	GO:0032510	endosome to lysosome transport via multivesicular body sorting pathway	bulk RNA-seq,Homo_633,AD.vs.control; bulk RNA-seq,Homo_633,AD.vs.MCI	2
BP	GO:0046339	diacylglycerol metabolic process	bulk RNA-seq,Homo_633,AD.vs.control; bulk RNA-seq,Homo_633,AD.vs.MCI	2
BP	GO:0034389	lipid droplet organization	bulk RNA-seq,Homo_633,AD.vs.control	1
BP	GO:0006541	glutamine metabolic process	bulk RNA-seq,Homo_633,AD.vs.control; bulk RNA-seq,Homo_633,AD.vs.MCI	2
MF	GO:0008970	phospholipase A1 activity	bulk RNA-seq,Homo_633,AD.vs.control; bulk RNA-seq,Homo_633,AD.vs.MCI	2
BP	GO:2001267	regulation of cysteine-type endopeptidase activity involved in apoptotic signaling pathway	bulk RNA-seq,Homo_633,AD.vs.control; bulk RNA-seq,Homo_633,AD.vs.MCI	2
BP	GO:0010764	negative regulation of fibroblast migration	bulk RNA-seq,Homo_633,AD.vs.control	1
MF	GO:0061575	cyclin-dependent protein serine/threonine kinase activator	bulk RNA-seq,Homo_633,AD.vs.control; bulk RNA-seq,Homo_633,AD.vs.MCI	2
BP	GO:0044872	lipoprotein localization	bulk RNA-seq,Homo_633,AD.vs.control	1
BP	GO:0009113	purine nucleobase biosynthetic process	bulk RNA-seq,Homo_633,AD.vs.control	1
BP	GO:1902065	response to L-glutamate	bulk RNA-seq,Homo_633,AD.vs.control; bulk RNA-seq,Homo_633,AD.vs.MCI	2
BP	GO:0046051	UTP metabolic process	bulk RNA-seq,Homo_633,AD.vs.control; bulk RNA-seq,Homo_633,AD.vs.MCI	2
MF	GO:0035252	UDP-xylosyltransferase activity	bulk RNA-seq,Homo_633,AD.vs.control	1
MF	GO:0042285	xylosyltransferase activity	bulk RNA-seq,Homo_633,AD.vs.control	1
MF	GO:1990381	ubiquitin-specific protease binding	bulk RNA-seq,Homo_633,AD.vs.control	1
BP	GO:0034085	establishment of sister chromatid cohesion	bulk RNA-seq,Homo_633,AD.vs.control; bulk RNA-seq,Homo_633,MCI.vs.control	2

BP	GO:0045838	positive regulation of membrane potential	bulk RNA-seq,Homo_633,AD.vs.control	1
BP	GO:0033690	positive regulation of osteoblast proliferation	bulk RNA-seq,Homo_633,AD.vs.control; bulk RNA-seq,Homo_633,AD.vs.MCI	2
BP	GO:0009134	nucleoside diphosphate catabolic process	bulk RNA-seq,Homo_633,AD.vs.control; bulk RNA-seq,Homo_633,AD.vs.MCI	2
MF	GO:0016290	palmitoyl-CoA hydrolase activity	bulk RNA-seq,Homo_633,AD.vs.control; bulk RNA-seq,Homo_633,AD.vs.MCI	2
BP	GO:0020027	hemoglobin metabolic process	bulk RNA-seq,Homo_633,AD.vs.control; bulk RNA-seq,Homo_633,AD.vs.MCI	2
BP	GO:1901163	regulation of trophoblast cell migration	bulk RNA-seq,Homo_633,AD.vs.control; bulk RNA-seq,Homo_633,AD.vs.MCI	2
CC	GO:0030130	clathrin coat of trans-Golgi network vesicle	bulk RNA-seq,Homo_633,AD.vs.control	1
BP	GO:2000833	positive regulation of steroid hormone secretion	bulk RNA-seq,Homo_633,AD.vs.control; bulk RNA-seq,Homo_633,AD.vs.MCI	2
CC	GO:0034663	endoplasmic reticulum chaperone complex	bulk RNA-seq,Homo_633,AD.vs.control; bulk RNA-seq,Homo_633,AD.vs.MCI	2
CC	GO:1905103	integral component of lysosomal membrane	bulk RNA-seq,Homo_633,AD.vs.control	1
BP	GO:0042104	positive regulation of activated T cell proliferation	bulk RNA-seq,Homo_633,AD.vs.control	1
BP	GO:0046471	phosphatidylglycerol metabolic process	bulk RNA-seq,Homo_633,AD.vs.control; bulk RNA-seq,Homo_633,AD.vs.MCI	2
MF	GO:0000339	RNA cap binding	bulk RNA-seq,Homo_633,AD.vs.control	1
BP	GO:0045059	positive thymic T cell selection	bulk RNA-seq,Homo_633,AD.vs.control; bulk RNA-seq,Homo_633,AD.vs.MCI	2
CC	GO:0005682	U5 snRNP	bulk RNA-seq,Homo_633,AD.vs.control; bulk RNA-seq,Homo_633,MCI.vs.control	2
BP	GO:0030252	growth hormone secretion	bulk RNA-seq,Homo_633,AD.vs.control; bulk RNA-seq,Homo_633,AD.vs.MCI	2
BP	GO:1901029	negative regulation of mitochondrial outer membrane permeabilization involved in apoptotic signaling pathway	bulk RNA-seq,Homo_633,AD.vs.control; bulk RNA-seq,Homo_633,AD.vs.MCI	2
BP	GO:0051987	positive regulation of attachment of spindle microtubules to	bulk RNA-seq,Homo_633,AD.vs.control	1
BP	GO:0050855	regulation of B cell receptor signaling pathway	bulk RNA-seq,Homo_633,AD.vs.control	1
CC	GO:0031166	integral component of vacuolar membrane	bulk RNA-seq,Homo_633,AD.vs.control	1
CC	GO:0031310	intrinsic component of vacuolar membrane	bulk RNA-seq,Homo_633,AD.vs.control	1
BP	GO:1902510	regulation of apoptotic DNA fragmentation	bulk RNA-seq,Homo_633,AD.vs.control; bulk RNA-seq,Homo_633,AD.vs.MCI	2
MF	GO:0005114	type II transforming growth factor beta receptor binding	bulk RNA-seq,Homo_633,AD.vs.control	1
BP	GO:0001516	prostaglandin biosynthetic process	bulk RNA-seq,Homo_633,AD.vs.control; scRNA-seq,SRP330776,Monocyte_1-AD.vs.control	2
BP	GO:0046457	prostanoid biosynthetic process	bulk RNA-seq,Homo_633,AD.vs.control; scRNA-seq,SRP330776,Monocyte_1-AD.vs.control	2
BP	GO:0002923	regulation of humoral immune response mediated by circulating immunoglobulin	bulk RNA-seq,Homo_633,AD.vs.control	1
BP	GO:2001204	regulation of osteoclast development	bulk RNA-seq,Homo_633,AD.vs.control	1
BP	GO:0072697	protein localization to cell cortex	bulk RNA-seq,Homo_633,AD.vs.control; bulk RNA-seq,Homo_633,AD.vs.MCI	2
MF	GO:0015116	sulfate transmembrane transporter activity	bulk RNA-seq,Homo_633,AD.vs.control; bulk RNA-seq,Homo_633,AD.vs.MCI	2
BP	GO:1902358	sulfate transmembrane transport	bulk RNA-seq,Homo_633,AD.vs.control; bulk RNA-seq,Homo_633,AD.vs.MCI	2
BP	GO:0048733	sebaceous gland development	bulk RNA-seq,Homo_633,AD.vs.control	1
BP	GO:0006750	glutathione biosynthetic process	bulk RNA-seq,Homo_633,AD.vs.control	1
BP	GO:0051081	nuclear membrane disassembly	bulk RNA-seq,Homo_633,AD.vs.control	1
BP	GO:0052646	alditol phosphate metabolic process	bulk RNA-seq,Homo_633,AD.vs.control	1
BP	GO:1901620	regulation of smoothened signaling pathway involved in dorsal/ventral neural tube patterning	bulk RNA-seq,Homo_633,AD.vs.control	1
BP	GO:0032306	regulation of prostaglandin secretion	bulk RNA-seq,Homo_633,AD.vs.control	1
BP	GO:0032308	positive regulation of prostaglandin secretion	bulk RNA-seq,Homo_633,AD.vs.control	1
MF	GO:0016208	AMP binding	bulk RNA-seq,Homo_633,AD.vs.control; bulk RNA-seq,Homo_633,MCI.vs.control	2
BP	GO:0002864	regulation of acute inflammatory response to antigenic	bulk RNA-seq,Homo_633,AD.vs.control	1
BP	GO:0010737	protein kinase A signaling	bulk RNA-seq,Homo_633,AD.vs.control; bulk RNA-seq,Homo_633,AD.vs.MCI	2
BP	GO:0002423	natural killer cell mediated immune response to tumor cell	bulk RNA-seq,Homo_633,AD.vs.control	1
BP	GO:0010661	positive regulation of muscle cell apoptotic process	bulk RNA-seq,Homo_633,AD.vs.control	1
BP	GO:0036295	cellular response to increased oxygen levels	bulk RNA-seq,Homo_633,AD.vs.control; bulk RNA-seq,Homo_633,AD.vs.MCI	2
BP	GO:0010917	negative regulation of mitochondrial membrane potential	bulk RNA-seq,Homo_633,AD.vs.control	1
BP	GO:0045837	negative regulation of membrane potential	bulk RNA-seq,Homo_633,AD.vs.control	1
BP	GO:0042182	ketone catabolic process	bulk RNA-seq,Homo_633,AD.vs.control; bulk RNA-seq,Homo_633,AD.vs.MCI	2
BP	GO:0032401	establishment of melanosome localization	bulk RNA-seq,Homo_633,AD.vs.control	1
BP	GO:0032402	melanosome transport	bulk RNA-seq,Homo_633,AD.vs.control	1
BP	GO:0032515	negative regulation of phosphoprotein phosphatase activity	bulk RNA-seq,Homo_633,AD.vs.control; bulk RNA-seq,Homo_633,AD.vs.MCI	2
MF	GO:0016894	endonuclease activity, active with either ribo- or deoxyribonucleic acids and producing 3'-phosphomonoesters	bulk RNA-seq,Homo_633,AD.vs.control	1
BP	GO:0030638	polyketide metabolic process	bulk RNA-seq,Homo_633,AD.vs.control	1
BP	GO:0044598	doxorubicin metabolic process	bulk RNA-seq,Homo_633,AD.vs.control	1

BP	GO:0006686	sphingomyelin biosynthetic process	bulk RNA-seq,Homo_633,AD.vs.control	1
BP	GO:0051898	negative regulation of protein kinase B signaling	bulk RNA-seq,Homo_633,AD.vs.control	1
BP	GO:0060546	negative regulation of necroptotic process	bulk RNA-seq,Homo_633,AD.vs.control	1
BP	GO:0032011	ARF protein signal transduction	bulk RNA-seq,Homo_633,AD.vs.control	1
BP	GO:0032012	regulation of ARF protein signal transduction	bulk RNA-seq,Homo_633,AD.vs.control	1
BP	GO:0006825	copper ion transport	bulk RNA-seq,Homo_633,AD.vs.control	1
MF	GO:0043522	leucine zipper domain binding	bulk RNA-seq,Homo_633,AD.vs.control	1
MF	GO:0120227	acyl-CoA binding	bulk RNA-seq,Homo_633,AD.vs.control	1
BP	GO:2000108	positive regulation of leukocyte apoptotic process	bulk RNA-seq,Homo_633,AD.vs.control; scRNA-seq,SRP215507,CD8+ T cell_3-MCI.vs.control	2
BP	GO:0046626	regulation of insulin receptor signaling pathway	bulk RNA-seq,Homo_633,AD.vs.control	1
BP	GO:0034638	phosphatidylcholine catabolic process	bulk RNA-seq,Homo_633,AD.vs.control; bulk RNA-seq,Homo_633,AD.vs.MCI	2
BP	GO:0046642	negative regulation of alpha-beta T cell proliferation	bulk RNA-seq,Homo_633,AD.vs.control	1
MF	GO:0005385	zinc ion transmembrane transporter activity	bulk RNA-seq,Homo_633,AD.vs.control	1
BP	GO:0070232	regulation of T cell apoptotic process	bulk RNA-seq,Homo_633,AD.vs.control	1
BP	GO:0061684	chaperone-mediated autophagy	bulk RNA-seq,Homo_633,AD.vs.control	1
BP	GO:0006957	complement activation, alternative pathway	bulk RNA-seq,Homo_633,AD.vs.control; bulk RNA-seq,Homo_633,AD.vs.MCI	2
BP	GO:1990034	calcium ion export across plasma membrane	bulk RNA-seq,Homo_633,AD.vs.control	1
BP	GO:0048703	embryonic viscerocranium morphogenesis	bulk RNA-seq,Homo_633,AD.vs.control	1
BP	GO:0019359	nicotinamide nucleotide biosynthetic process	bulk RNA-seq,Homo_633,AD.vs.control	1
BP	GO:0019363	pyridine nucleotide biosynthetic process	bulk RNA-seq,Homo_633,AD.vs.control	1
BP	GO:0070278	extracellular matrix constituent secretion	bulk RNA-seq,Homo_633,AD.vs.control	1
BP	GO:0042416	dopamine biosynthetic process	bulk RNA-seq,Homo_633,AD.vs.control; bulk RNA-seq,Homo_633,AD.vs.MCI	2
BP	GO:0032735	positive regulation of interleukin-12 production	bulk RNA-seq,Homo_633,AD.vs.control	1
MF	GO:0016675	oxidoreductase activity, acting on a heme group of donors	bulk RNA-seq,Homo_633,AD.vs.control	1
BP	GO:0042761	very long-chain fatty acid biosynthetic process	bulk RNA-seq,Homo_633,AD.vs.control; bulk RNA-seq,Homo_633,AD.vs.MCI	2
CC	GO:0002177	manchette	bulk RNA-seq,Homo_633,AD.vs.control	1
MF	GO:0004198	calcium-dependent cysteine-type endopeptidase activity	bulk RNA-seq,Homo_633,AD.vs.control	1
BP	GO:0010663	positive regulation of striated muscle cell apoptotic process	bulk RNA-seq,Homo_633,AD.vs.control; bulk RNA-seq,Homo_633,AD.vs.MCI	2
BP	GO:0010666	positive regulation of cardiac muscle cell apoptotic process	bulk RNA-seq,Homo_633,AD.vs.control; bulk RNA-seq,Homo_633,AD.vs.MCI	2
BP	GO:1902412	regulation of mitotic cytokinesis	bulk RNA-seq,Homo_633,AD.vs.control	1
BP	GO:0150011	regulation of neuron projection arborization	bulk RNA-seq,Homo_633,AD.vs.control	1
BP	GO:0045815	epigenetic maintenance of chromatin in transcription-competent conformation	bulk RNA-seq,Homo_633,AD.vs.control	1
BP	GO:0010745	negative regulation of macrophage derived foam cell	bulk RNA-seq,Homo_633,AD.vs.control; bulk RNA-seq,Homo_633,AD.vs.MCI; scRNA-seq,SRP309935,B cell_1-AD.vs.control	3
BP	GO:1900227	positive regulation of NLRP3 inflammasome complex	bulk RNA-seq,Homo_633,AD.vs.control	1
BP	GO:0046689	response to mercury ion	bulk RNA-seq,Homo_633,AD.vs.control	1
BP	GO:1901725	regulation of histone deacetylase activity	bulk RNA-seq,Homo_633,AD.vs.control	1
BP	GO:0010572	positive regulation of platelet activation	bulk RNA-seq,Homo_633,AD.vs.control	1
BP	GO:0046710	GDP metabolic process	bulk RNA-seq,Homo_633,AD.vs.control	1
BP	GO:0045056	transcytosis	bulk RNA-seq,Homo_633,AD.vs.control	1
BP	GO:0071476	cellular hypotonic response	bulk RNA-seq,Homo_633,AD.vs.control	1
BP	GO:0001821	histamine secretion	bulk RNA-seq,Homo_633,AD.vs.control	1
MF	GO:0008474	palmitoyl-(protein) hydrolase activity	bulk RNA-seq,Homo_633,AD.vs.control	1
MF	GO:0098599	palmitoyl hydrolase activity	bulk RNA-seq,Homo_633,AD.vs.control	1
BP	GO:0098734	macromolecule depalmitoylation	bulk RNA-seq,Homo_633,AD.vs.control	1
BP	GO:0009125	nucleoside monophosphate catabolic process	bulk RNA-seq,Homo_633,AD.vs.control	1
BP	GO:2001198	regulation of dendritic cell differentiation	bulk RNA-seq,Homo_633,AD.vs.control	1
BP	GO:0061450	trophoblast cell migration	bulk RNA-seq,Homo_633,AD.vs.control	1
BP	GO:0001916	positive regulation of T cell mediated cytotoxicity	bulk RNA-seq,Homo_633,AD.vs.control	1
BP	GO:2000009	negative regulation of protein localization to cell surface	bulk RNA-seq,Homo_633,AD.vs.control	1
BP	GO:1902513	regulation of organelle transport along microtubule	bulk RNA-seq,Homo_633,AD.vs.control	1
BP	GO:1902959	regulation of aspartic-type endopeptidase activity involved in amyloid precursor protein catabolic process	bulk RNA-seq,Homo_633,AD.vs.control; bulk RNA-seq,Homo_633,AD.vs.MCI	2
BP	GO:0043217	myelin maintenance	bulk RNA-seq,Homo_633,AD.vs.control	1
BP	GO:0010829	negative regulation of glucose transmembrane transport	bulk RNA-seq,Homo_633,AD.vs.control	1
BP	GO:0015810	aspartate transmembrane transport	bulk RNA-seq,Homo_633,AD.vs.control	1

BP	GO:0009176	pyrimidine deoxyribonucleoside monophosphate metabolic	bulk RNA-seq,Homo_633,AD.vs.control; bulk RNA-seq,Homo_633,AD.vs.MCI	2
BP	GO:0042795	snRNA transcription by RNA polymerase II	bulk RNA-seq,Homo_633,AD.vs.control	1
MF	GO:0008519	ammonium transmembrane transporter activity	bulk RNA-seq,Homo_633,AD.vs.control	1
BP	GO:0072488	ammonium transmembrane transport	bulk RNA-seq,Homo_633,AD.vs.control	1
BP	GO:0009158	ribonucleoside monophosphate catabolic process	bulk RNA-seq,Homo_633,AD.vs.control	1
BP	GO:0034086	maintenance of sister chromatid cohesion	bulk RNA-seq,Homo_633,AD.vs.control; bulk RNA-seq,Homo_633,AD.vs.MCI	2
BP	GO:0034088	maintenance of mitotic sister chromatid cohesion	bulk RNA-seq,Homo_633,AD.vs.control; bulk RNA-seq,Homo_633,AD.vs.MCI	2
MF	GO:0004143	diacylglycerol kinase activity	bulk RNA-seq,Homo_633,AD.vs.control	1
BP	GO:0018230	peptidyl-L-cysteine S-palmitoylation	bulk RNA-seq,Homo_633,AD.vs.control	1
BP	GO:0018231	peptidyl-S-diacylglycerol-L-cysteine biosynthetic process from peptidyl-cysteine	bulk RNA-seq,Homo_633,AD.vs.control	1
BP	GO:0071404	cellular response to low-density lipoprotein particle stimulus	bulk RNA-seq,Homo_633,AD.vs.control	1
BP	GO:0051904	pigment granule transport	bulk RNA-seq,Homo_633,AD.vs.control	1
BP	GO:1905522	negative regulation of macrophage migration	bulk RNA-seq,Homo_633,AD.vs.control	1
BP	GO:0010738	regulation of protein kinase A signaling	bulk RNA-seq,Homo_633,AD.vs.control; bulk RNA-seq,Homo_633,AD.vs.MCI	2
BP	GO:1902916	positive regulation of protein polyubiquitination	bulk RNA-seq,Homo_633,AD.vs.control	1
BP	GO:0044793	negative regulation by host of viral process	bulk RNA-seq,Homo_633,AD.vs.control	1
BP	GO:0030647	aminoglycoside antibiotic metabolic process	bulk RNA-seq,Homo_633,AD.vs.control	1
MF	GO:1901567	fatty acid derivative binding	bulk RNA-seq,Homo_633,AD.vs.control	1
MF	GO:0070008	serine-type exopeptidase activity	bulk RNA-seq,Homo_633,AD.vs.control	1
BP	GO:0060263	regulation of respiratory burst	bulk RNA-seq,Homo_633,AD.vs.control	1
MF	GO:0030228	lipoprotein particle receptor activity	bulk RNA-seq,Homo_633,AD.vs.control; bulk RNA-seq,ROSMAP,AD.vs.control; bulk RNA-seq,ROSMAP,MCI.vs.control	3
BP	GO:0009143	nucleoside triphosphate catabolic process	bulk RNA-seq,Homo_633,AD.vs.control	1
CC	GO:0036379	myofilament	bulk RNA-seq,Homo_633,AD.vs.control	1
CC	GO:0000795	synaptonemal complex	bulk RNA-seq,Homo_633,AD.vs.control	1
CC	GO:0099086	synaptonemal structure	bulk RNA-seq,Homo_633,AD.vs.control	1
BP	GO:0036490	regulation of translation in response to endoplasmic	bulk RNA-seq,Homo_633,AD.vs.control; bulk RNA-seq,Homo_633,MCI.vs.control	2
BP	GO:0070586	cell-cell adhesion involved in gastrulation	bulk RNA-seq,Homo_633,AD.vs.control; bulk RNA-seq,Homo_633,AD.vs.MCI	2
BP	GO:0051905	establishment of pigment granule localization	bulk RNA-seq,Homo_633,AD.vs.control	1
BP	GO:0009163	nucleoside biosynthetic process	bulk RNA-seq,Homo_633,AD.vs.control	1
BP	GO:0034404	nucleobase-containing small molecule biosynthetic process	bulk RNA-seq,Homo_633,AD.vs.control	1
BP	GO:0045953	negative regulation of natural killer cell mediated cytotoxicity	bulk RNA-seq,Homo_633,AD.vs.control	1
BP	GO:0042541	hemoglobin biosynthetic process	bulk RNA-seq,Homo_633,AD.vs.control; bulk RNA-seq,Homo_633,AD.vs.MCI	2
BP	GO:1990403	embryonic brain development	bulk RNA-seq,Homo_633,AD.vs.control	1
BP	GO:0002689	negative regulation of leukocyte chemotaxis	bulk RNA-seq,Homo_633,AD.vs.control	1
MF	GO:0000062	fatty-acyl-CoA binding	bulk RNA-seq,Homo_633,AD.vs.control	1
BP	GO:0006857	oligopeptide transport	bulk RNA-seq,Homo_633,AD.vs.control	1
BP	GO:2000353	positive regulation of endothelial cell apoptotic process	bulk RNA-seq,Homo_633,AD.vs.control	1
BP	GO:0014067	negative regulation of phosphatidylinositol 3-kinase signaling	bulk RNA-seq,Homo_633,AD.vs.control	1
CC	GO:0005685	U1 snRNP	bulk RNA-seq,Homo_633,AD.vs.control	1
BP	GO:1902514	regulation of calcium ion transmembrane transport via high voltage-gated calcium channel	bulk RNA-seq,Homo_633,AD.vs.control	1
BP	GO:0046628	positive regulation of insulin receptor signaling pathway	bulk RNA-seq,Homo_633,AD.vs.control	1
BP	GO:0006111	regulation of gluconeogenesis	bulk RNA-seq,Homo_633,AD.vs.control	1
MF	GO:0001161	intronic transcription regulatory region sequence-specific	bulk RNA-seq,Homo_633,AD.vs.control; bulk RNA-seq,Homo_633,AD.vs.MCI	2
BP	GO:1900426	positive regulation of defense response to bacterium	bulk RNA-seq,Homo_633,AD.vs.control	1
BP	GO:1901020	negative regulation of calcium ion transmembrane transporter	bulk RNA-seq,Homo_633,AD.vs.control	1
BP	GO:2000678	negative regulation of transcription regulatory region DNA	bulk RNA-seq,Homo_633,AD.vs.control; bulk RNA-seq,Homo_633,AD.vs.MCI	2
BP	GO:0002862	negative regulation of inflammatory response to antigenic	bulk RNA-seq,Homo_633,AD.vs.control	1
BP	GO:1900101	regulation of endoplasmic reticulum unfolded protein	bulk RNA-seq,Homo_633,AD.vs.control	1
BP	GO:0045066	regulatory T cell differentiation	bulk RNA-seq,Homo_633,AD.vs.control; scRNA-seq,SRP330776,Naive CD8+ T cell_2-AD.vs.control	2
BP	GO:0070885	negative regulation of calcineurin-NFAT signaling cascade	bulk RNA-seq,Homo_633,AD.vs.control	1
BP	GO:0106057	negative regulation of calcineurin-mediated signaling	bulk RNA-seq,Homo_633,AD.vs.control	1
BP	GO:0010907	positive regulation of glucose metabolic process	bulk RNA-seq,Homo_633,AD.vs.control	1
BP	GO:0035933	glucocorticoid secretion	bulk RNA-seq,Homo_633,AD.vs.control	1
BP	GO:1905245	regulation of aspartic-type peptidase activity	bulk RNA-seq,Homo_633,AD.vs.control	1

MF	GO:0140103	catalytic activity, acting on a glycoprotein	bulk RNA-seq,Homo_633,AD.vs.control	1
BP	GO:0042538	hyperosmotic salinity response	bulk RNA-seq,Homo_633,AD.vs.control; bulk RNA-seq,Homo_633,AD.vs.MCI	2
BP	GO:0003091	renal water homeostasis	bulk RNA-seq,Homo_633,AD.vs.MCI	1
BP	GO:0034115	negative regulation of heterotypic cell-cell adhesion	bulk RNA-seq,Homo_633,AD.vs.MCI	1
BP	GO:0070587	regulation of cell-cell adhesion involved in gastrulation	bulk RNA-seq,Homo_633,AD.vs.MCI	1
BP	GO:0021554	optic nerve development	bulk RNA-seq,Homo_633,AD.vs.MCI	1
BP	GO:0060123	regulation of growth hormone secretion	bulk RNA-seq,Homo_633,AD.vs.MCI	1
BP	GO:0007191	adenylate cyclase-activating dopamine receptor signaling	bulk RNA-seq,Homo_633,AD.vs.MCI	1
BP	GO:0006563	L-serine metabolic process	bulk RNA-seq,Homo_633,AD.vs.MCI	1
BP	GO:1901203	positive regulation of extracellular matrix assembly	bulk RNA-seq,Homo_633,AD.vs.MCI	1
BP	GO:0035878	nail development	bulk RNA-seq,Homo_633,AD.vs.MCI	1
BP	GO:0031392	regulation of prostaglandin biosynthetic process	bulk RNA-seq,Homo_633,AD.vs.MCI	1
MF	GO:0004623	phospholipase A2 activity	bulk RNA-seq,Homo_633,AD.vs.MCI	1
BP	GO:1901201	regulation of extracellular matrix assembly	bulk RNA-seq,Homo_633,AD.vs.MCI	1
MF	GO:0008494	translation activator activity	bulk RNA-seq,Homo_633,AD.vs.MCI; bulk RNA-seq,ROSMAP,AD.vs.control; bulk RNA-seq,ROSMAP,MCI.vs.control	3
MF	GO:1904047	S-adenosyl-L-methionine binding	bulk RNA-seq,Homo_633,AD.vs.MCI	1
BP	GO:0051673	membrane disruption in another organism	bulk RNA-seq,Homo_633,AD.vs.MCI	1
MF	GO:1901611	phosphatidylglycerol binding	bulk RNA-seq,Homo_633,AD.vs.MCI	1
BP	GO:0035821	modulation of process of another organism	bulk RNA-seq,Homo_633,AD.vs.MCI	1
BP	GO:0048194	Golgi vesicle budding	bulk RNA-seq,Homo_633,MCI.vs.control	1
BP	GO:0009415	response to water	bulk RNA-seq,Homo_633,MCI.vs.control	1
BP	GO:0002902	regulation of B cell apoptotic process	bulk RNA-seq,Homo_633,MCI.vs.control	1
BP	GO:0019321	pentose metabolic process	bulk RNA-seq,Homo_633,MCI.vs.control	1
BP	GO:0072017	distal tubule development	bulk RNA-seq,Homo_633,MCI.vs.control	1
BP	GO:0060379	cardiac muscle cell myoblast differentiation	bulk RNA-seq,Homo_633,MCI.vs.control	1
CC	GO:0002178	palmitoyltransferase complex	bulk RNA-seq,Homo_633,MCI.vs.control	1
BP	GO:0034765	regulation of ion transmembrane transport	bulk RNA-seq,ROSMAP,AD.vs.control; bulk RNA-seq,ROSMAP,MCI.vs.control	2
MF	GO:0015296	anion:cation symporter activity	bulk RNA-seq,ROSMAP,AD.vs.control	1
BP	GO:0006820	anion transport	bulk RNA-seq,ROSMAP,AD.vs.control	1
MF	GO:0015373	anion:sodium symporter activity	bulk RNA-seq,ROSMAP,AD.vs.control	1
MF	GO:0015378	sodium:chloride symporter activity	bulk RNA-seq,ROSMAP,AD.vs.control	1
BP	GO:0050906	detection of stimulus involved in sensory perception	bulk RNA-seq,ROSMAP,AD.vs.MCI	1
BP	GO:0007606	sensory perception of chemical stimulus	bulk RNA-seq,ROSMAP,AD.vs.MCI	1
BP	GO:0019755	one-carbon compound transport	bulk RNA-seq,ROSMAP,AD.vs.MCI	1
BP	GO:0015670	carbon dioxide transport	bulk RNA-seq,ROSMAP,AD.vs.MCI; bulk RNA-seq,ROSMAP,MCI.vs.control	2
BP	GO:0015669	gas transport	bulk RNA-seq,ROSMAP,AD.vs.MCI; scRNA-seq,SRP309935,Erythroid cell-AD.vs.control	2
MF	GO:0005344	oxygen carrier activity	bulk RNA-seq,ROSMAP,AD.vs.MCI; scRNA-seq,SRP309935,Erythroid cell-AD.vs.control	2
BP	GO:0015671	oxygen transport	bulk RNA-seq,ROSMAP,AD.vs.MCI; scRNA-seq,SRP309935,Erythroid cell-AD.vs.control	2
BP	GO:0010880	regulation of release of sequestered calcium ion into cytosol by sarcoplasmic reticulum	bulk RNA-seq,ROSMAP,MCI.vs.control	1
BP	GO:0007343	egg activation	bulk RNA-seq,SRP223445,AD.vs.control	1
BP	GO:0007028	cytoplasm organization	bulk RNA-seq,SRP223445,AD.vs.control	1
MF	GO:0042165	neurotransmitter binding	bulk RNA-seq,SRP223445,AD.vs.control	1
BP	GO:0030322	stabilization of membrane potential	bulk RNA-seq,SRP223445,AD.vs.control	1
MF	GO:0022841	potassium ion leak channel activity	bulk RNA-seq,SRP223445,AD.vs.control	1
MF	GO:0004875	complement receptor activity	bulk RNA-seq,SRP223445,AD.vs.control	1
BP	GO:0002430	complement receptor mediated signaling pathway	bulk RNA-seq,SRP223445,AD.vs.control	1
BP	GO:0070943	neutrophil-mediated killing of symbiont cell	bulk RNA-seq,SRP223445,AD.vs.control	1
MF	GO:0032396	inhibitory MHC class I receptor activity	bulk RNA-seq,SRP223445,AD.vs.control	1
BP	GO:0002385	mucosal immune response	bulk RNA-seq,SRP325058,AD.vs.control	1
KEGG	hsa04979	Cholesterol metabolism	bulk RNA-seq,ACOM,AD.vs.control; bulk RNA-seq,Homo_723,AD.vs.MCI; bulk RNA-seq,Homo_723,MCI.vs.control; bulk RNA-seq,Homo_714,AD.vs.MCI; bulk RNA-seq,Homo_714,MCI.vs.control; bulk RNA-seq,Homo_633,AD.vs.control; bulk RNA-seq,Homo_633,AD.vs.MCI	7
KEGG	hsa04080	Neuroactive ligand-receptor interaction	bulk RNA-seq,ACOM,AD.vs.control; bulk RNA-seq,Emory,MCI.vs.control; bulk RNA-seq,Homo_723,AD.vs.control; bulk RNA-seq,Homo_723,AD.vs.MCI; bulk RNA-seq,Homo_714,AD.vs.control; bulk RNA-seq,Homo_714,AD.vs.MCI; bulk RNA-seq,Homo_633,MCI.vs.control; bulk RNA-seq,ROSMAP,AD.vs.MCI; bulk RNA-seq,SRP223445,AD.vs.control	9

KEGG	hsa04975	Fat digestion and absorption	bulk RNA-seq,ACOM,AD.vs.control; bulk RNA-seq,Homo_723,AD.vs.MCI; bulk RNA-seq,Homo_723,MCI.vs.control; bulk RNA-seq,Homo_714,AD.vs.MCI; bulk RNA-seq,Homo_714,MCI.vs.control	5
KEGG	hsa04610	Complement and coagulation cascades	bulk RNA-seq,ACOM,AD.vs.control; bulk RNA-seq,Homo_723,AD.vs.MCI; bulk RNA-seq,Homo_723,MCI.vs.control; bulk RNA-seq,Homo_714,AD.vs.control; bulk RNA-seq,Homo_714,AD.vs.MCI; bulk RNA-seq,Homo_714,MCI.vs.control; bulk RNA-seq,Homo_633,AD.vs.control; bulk RNA-seq,Homo_633,AD.vs.MCI	8
KEGG	hsa00970	Aminoacyl-tRNA biosynthesis	bulk RNA-seq,Emory,MCI.vs.control; bulk RNA-seq,Homo_723,AD.vs.control; bulk RNA-seq,Homo_723,MCI.vs.control; bulk RNA-seq,Homo_714,AD.vs.control; bulk RNA-seq,Homo_714,AD.vs.MCI; bulk RNA-seq,Homo_714,MCI.vs.control; bulk RNA-seq,Homo_633,AD.vs.control; bulk	8
KEGG	hsa04024	cAMP signaling pathway	bulk RNA-seq,Emory,MCI.vs.control; bulk RNA-seq,Homo_723,AD.vs.control; bulk RNA-seq,Homo_723,AD.vs.MCI; bulk RNA-seq,Homo_723,MCI.vs.control; bulk RNA-seq,Homo_714,AD.vs.control; bulk RNA-seq,Homo_714,AD.vs.MCI; bulk RNA-seq,Homo_633,AD.vs.control; bulk RNA-seq,Homo_633,AD.vs.MCI; bulk RNA-seq,ROSMAP,AD.vs.MCI	9
KEGG	hsa04713	Circadian entrainment	bulk RNA-seq,Emory,MCI.vs.control; bulk RNA-seq,Homo_723,AD.vs.control; bulk RNA-seq,Homo_723,AD.vs.MCI; bulk RNA-seq,Homo_723,MCI.vs.control; bulk RNA-seq,Homo_714,AD.vs.MCI; bulk RNA-seq,Homo_714,MCI.vs.control; bulk RNA-seq,Homo_633,AD.vs.control; bulk RNA-seq,Homo_633,AD.vs.MCI; bulk RNA-seq,ROSMAP,AD.vs.control	9
KEGG	hsa04912	GnRH signaling pathway	bulk RNA-seq,Emory,MCI.vs.control; bulk RNA-seq,Homo_723,AD.vs.MCI; bulk RNA-seq,Homo_723,MCI.vs.control; bulk RNA-seq,Homo_714,AD.vs.MCI; bulk RNA-seq,Homo_714,MCI.vs.control; bulk RNA-seq,Homo_633,AD.vs.control; bulk RNA-seq,Homo_633,AD.vs.MCI	7
KEGG	hsa04020	Calcium signaling pathway	bulk RNA-seq,Emory,MCI.vs.control; bulk RNA-seq,Homo_723,AD.vs.control; bulk RNA-seq,Homo_723,AD.vs.MCI; bulk RNA-seq,Homo_723,MCI.vs.control; bulk RNA-seq,Homo_714,AD.vs.control; bulk RNA-seq,Homo_714,AD.vs.MCI; bulk RNA-seq,Homo_633,AD.vs.control; bulk RNA-seq,Homo_633,AD.vs.MCI; bulk RNA-seq,ROSMAP,AD.vs.control; bulk RNA-seq,SRP223445,AD.vs.control	10
KEGG	hsa05032	Morphine addiction	bulk RNA-seq,Emory,MCI.vs.control; bulk RNA-seq,Homo_723,AD.vs.control; bulk RNA-seq,Homo_723,AD.vs.MCI; bulk RNA-seq,Homo_723,MCI.vs.control; bulk RNA-seq,Homo_714,AD.vs.control; bulk RNA-seq,Homo_714,AD.vs.MCI; bulk RNA-seq,Homo_714,MCI.vs.control; bulk RNA-seq,Homo_633,AD.vs.control; bulk RNA-seq,Homo_633,AD.vs.MCI; bulk RNA-seq,ROSMAP,AD.vs.control	10
KEGG	hsa04727	GABAergic synapse	bulk RNA-seq,Emory,MCI.vs.control; bulk RNA-seq,Homo_723,AD.vs.control; bulk RNA-seq,Homo_723,AD.vs.MCI; bulk RNA-seq,Homo_723,MCI.vs.control; bulk RNA-seq,Homo_714,AD.vs.control; bulk RNA-seq,Homo_714,AD.vs.MCI; bulk RNA-seq,Homo_714,MCI.vs.control; bulk RNA-seq,Homo_633,AD.vs.control; bulk RNA-seq,Homo_633,AD.vs.MCI; bulk RNA-seq,Homo_633,MCI.vs.control; bulk RNA-seq,ROSMAP,AD.vs.control; bulk	12
KEGG	hsa03010	Ribosome	bulk RNA-seq,Emory,MCI.vs.control; bulk RNA-seq,Homo_723,AD.vs.control; bulk RNA-seq,Homo_723,MCI.vs.control; bulk RNA-seq,Homo_714,AD.vs.control; bulk RNA-seq,Homo_633,AD.vs.control; bulk RNA-seq,Homo_633,AD.vs.MCI; bulk RNA-seq,ROSMAP,MCI.vs.control; scRNA-seq,SRP330776,B cell_4-AD.vs.control; scRNA-seq,SRP330776,Megakaryocyte_2-AD.vs.control; scRNA-seq,SRP215507,CD8+ T cell_4-AD.vs.control	10
KEGG	hsa04934	Cushing syndrome	bulk RNA-seq,Emory,MCI.vs.control; bulk RNA-seq,Homo_723,AD.vs.control; bulk RNA-seq,Homo_723,AD.vs.MCI; bulk RNA-seq,Homo_723,MCI.vs.control; bulk RNA-seq,Homo_714,AD.vs.MCI; bulk RNA-seq,Homo_633,AD.vs.control; bulk RNA-seq,Homo_633,AD.vs.MCI; bulk RNA-seq,SRP223445,AD.vs.control	8
KEGG	hsa04723	Retrograde endocannabinoid signaling	bulk RNA-seq,Emory,MCI.vs.control; bulk RNA-seq,Homo_723,AD.vs.control; bulk RNA-seq,Homo_723,AD.vs.MCI; bulk RNA-seq,Homo_723,MCI.vs.control; bulk RNA-seq,Homo_714,AD.vs.MCI; bulk RNA-seq,Homo_633,AD.vs.control; bulk RNA-seq,Homo_633,AD.vs.MCI	7
KEGG	hsa04921	Oxytocin signaling pathway	bulk RNA-seq,Emory,MCI.vs.control; bulk RNA-seq,Homo_723,AD.vs.control; bulk RNA-seq,Homo_723,AD.vs.MCI; bulk RNA-seq,Homo_723,MCI.vs.control; bulk RNA-seq,Homo_714,AD.vs.MCI; bulk RNA-seq,Homo_633,AD.vs.control; bulk RNA-seq,Homo_633,AD.vs.MCI; bulk RNA-seq,ROSMAP,AD.vs.control; bulk RNA-seq,ROSMAP,AD.vs.MCI	9
KEGG	hsa02010	ABC transporters	bulk RNA-seq,Emory,MCI.vs.control; bulk RNA-seq,Homo_723,AD.vs.MCI; bulk RNA-seq,Homo_723,MCI.vs.control; bulk RNA-seq,Homo_714,AD.vs.MCI; bulk RNA-seq,Homo_714,MCI.vs.control; bulk RNA-seq,Homo_633,AD.vs.control; bulk RNA-seq,Homo_633,AD.vs.MCI	7
KEGG	hsa04911	Insulin secretion	bulk RNA-seq,Emory,MCI.vs.control; bulk RNA-seq,Homo_723,AD.vs.control; bulk RNA-seq,Homo_723,AD.vs.MCI; bulk RNA-seq,Homo_723,MCI.vs.control; bulk RNA-seq,Homo_714,AD.vs.control; bulk RNA-seq,Homo_714,AD.vs.MCI; bulk RNA-seq,Homo_714,MCI.vs.control	7
KEGG	hsa04925	Aldosterone synthesis and secretion	bulk RNA-seq,Emory,MCI.vs.control; bulk RNA-seq,Homo_723,AD.vs.control; bulk RNA-seq,Homo_723,AD.vs.MCI; bulk RNA-seq,Homo_723,MCI.vs.control; bulk RNA-seq,Homo_714,AD.vs.MCI; bulk RNA-seq,Homo_714,MCI.vs.control; bulk RNA-seq,Homo_633,AD.vs.control; bulk RNA-seq,Homo_633,AD.vs.MCI; bulk RNA-seq,ROSMAP,AD.vs.MCI	9
KEGG	hsa04970	Salivary secretion	bulk RNA-seq,Emory,MCI.vs.control; bulk RNA-seq,Homo_723,AD.vs.control; bulk RNA-seq,Homo_723,AD.vs.MCI; bulk RNA-seq,Homo_723,MCI.vs.control; bulk RNA-seq,Homo_714,AD.vs.MCI; bulk RNA-seq,Homo_714,MCI.vs.control; bulk RNA-seq,Homo_633,AD.vs.control; bulk RNA-seq,ROSMAP,AD.vs.control; bulk RNA-seq,SRP223445,AD.vs.control	9
KEGG	hsa04927	Cortisol synthesis and secretion	bulk RNA-seq,Emory,MCI.vs.control; bulk RNA-seq,Homo_723,AD.vs.MCI; bulk RNA-seq,Homo_723,MCI.vs.control; bulk RNA-seq,Homo_714,AD.vs.MCI; bulk RNA-seq,Homo_714,MCI.vs.control; bulk RNA-seq,Homo_633,AD.vs.control; bulk RNA-seq,Homo_633,AD.vs.MCI	7
KEGG	hsa04261	Adrenergic signaling in cardiomyocytes	bulk RNA-seq,Emory,MCI.vs.control; bulk RNA-seq,Homo_723,AD.vs.control; bulk RNA-seq,Homo_723,AD.vs.MCI; bulk RNA-seq,Homo_723,MCI.vs.control; bulk RNA-seq,Homo_714,AD.vs.control; bulk RNA-seq,Homo_714,AD.vs.MCI; bulk RNA-seq,Homo_633,AD.vs.control; bulk RNA-seq,ROSMAP,AD.vs.MCI	8
KEGG	hsa04926	Relaxin signaling pathway	bulk RNA-seq,Emory,MCI.vs.control; bulk RNA-seq,Homo_723,AD.vs.control; bulk RNA-seq,Homo_723,AD.vs.MCI; bulk RNA-seq,Homo_723,MCI.vs.control; bulk RNA-seq,Homo_714,AD.vs.MCI; bulk RNA-seq,Homo_714,MCI.vs.control; bulk RNA-seq,Homo_633,AD.vs.control; bulk RNA-seq,Homo_633,AD.vs.MCI; bulk RNA-seq,ROSMAP,AD.vs.MCI	9
KEGG	hsa04728	Dopaminergic synapse	bulk RNA-seq,Emory,MCI.vs.control; bulk RNA-seq,Homo_723,AD.vs.control; bulk RNA-seq,Homo_723,AD.vs.MCI; bulk RNA-seq,Homo_723,MCI.vs.control; bulk RNA-seq,Homo_714,AD.vs.MCI; bulk RNA-seq,Homo_714,MCI.vs.control; bulk RNA-seq,Homo_633,AD.vs.control; bulk RNA-seq,Homo_633,AD.vs.MCI; bulk RNA-seq,ROSMAP,AD.vs.control	9
KEGG	hsa00430	Taurine and hypotaurine metabolism	bulk RNA-seq,Emory,MCI.vs.control; bulk RNA-seq,Homo_723,AD.vs.control; bulk RNA-seq,Homo_723,AD.vs.MCI; bulk RNA-seq,Homo_723,MCI.vs.control; bulk RNA-seq,Homo_714,AD.vs.MCI; bulk RNA-seq,Homo_714,MCI.vs.control	6
KEGG	hsa04720	Long-term potentiation	bulk RNA-seq,Emory,MCI.vs.control; bulk RNA-seq,Homo_723,AD.vs.control; bulk RNA-seq,Homo_723,AD.vs.MCI; bulk RNA-seq,Homo_723,MCI.vs.control; bulk RNA-seq,Homo_714,AD.vs.MCI; bulk RNA-seq,Homo_714,MCI.vs.control; bulk RNA-seq,Homo_633,AD.vs.control; bulk RNA-seq,Homo_633,AD.vs.MCI	8
KEGG	hsa04512	ECM-receptor interaction	bulk RNA-seq,Emory,MCI.vs.control; bulk RNA-seq,Homo_723,AD.vs.control; bulk RNA-seq,Homo_723,AD.vs.MCI; bulk RNA-seq,Homo_723,MCI.vs.control; bulk RNA-seq,Homo_714,AD.vs.control; bulk RNA-seq,Homo_714,AD.vs.MCI; bulk RNA-seq,Homo_714,MCI.vs.control; bulk RNA-seq,Homo_633,AD.vs.control; bulk RNA-seq,Homo_633,AD.vs.MCI; bulk RNA-seq,ROSMAP,AD.vs.control; scRNA-seq,SRP309935,Monocyte_1-AD.vs.control;	12
KEGG	hsa04725	Cholinergic synapse	bulk RNA-seq,Emory,MCI.vs.control; bulk RNA-seq,Homo_723,AD.vs.control; bulk RNA-seq,Homo_723,AD.vs.MCI; bulk RNA-seq,Homo_723,MCI.vs.control; bulk RNA-seq,Homo_714,AD.vs.MCI; bulk RNA-seq,Homo_714,MCI.vs.control; bulk RNA-seq,Homo_633,AD.vs.control; bulk RNA-seq,Homo_633,AD.vs.MCI	8
KEGG	hsa04820	Cytoskeleton in muscle cells	bulk RNA-seq,Emory,MCI.vs.control; bulk RNA-seq,Homo_723,AD.vs.control; bulk RNA-seq,Homo_723,AD.vs.MCI; bulk RNA-seq,Homo_723,MCI.vs.control; bulk RNA-seq,Homo_714,AD.vs.control; bulk RNA-seq,Homo_714,AD.vs.MCI; bulk RNA-seq,Homo_633,AD.vs.control; bulk RNA-seq,Homo_633,AD.vs.MCI; bulk RNA-seq,ROSMAP,AD.vs.control; bulk RNA-seq,SRP223445,AD.vs.control	10

KEGG	hsa04924	Renin secretion	bulk RNA-seq,Emory,MCI.vs.control; bulk RNA-seq,Homo_723,AD.vs.control; bulk RNA-seq,Homo_723,AD.vs.MCI; bulk RNA-seq,Homo_723,MCI.vs.control; bulk RNA-seq,Homo_714,AD.vs.control; bulk RNA-seq,Homo_714,AD.vs.MCI; bulk RNA-seq,Homo_714,MCI.vs.control; bulk RNA-seq,Homo_633,AD.vs.control; bulk RNA-seq,Homo_633,AD.vs.MCI; scRNA-seq,SRP215507,CD8+ T cell_8-MCI.vs.control	10
KEGG	hsa04724	Glutamatergic synapse	bulk RNA-seq,Emory,MCI.vs.control; bulk RNA-seq,Homo_723,AD.vs.control; bulk RNA-seq,Homo_723,AD.vs.MCI; bulk RNA-seq,Homo_723,MCI.vs.control; bulk RNA-seq,Homo_714,AD.vs.control; bulk RNA-seq,Homo_714,AD.vs.MCI; bulk RNA-seq,Homo_714,MCI.vs.control; bulk RNA-seq,Homo_633,AD.vs.control; bulk RNA-seq,Homo_633,AD.vs.MCI; bulk RNA-seq,ROSMAP,AD.vs.control	10
KEGG	hsa04915	Estrogen signaling pathway	bulk RNA-seq,Emory,MCI.vs.control; bulk RNA-seq,Homo_723,AD.vs.control; bulk RNA-seq,Homo_723,AD.vs.MCI; bulk RNA-seq,Homo_723,MCI.vs.control; bulk RNA-seq,Homo_714,AD.vs.control; bulk RNA-seq,Homo_714,AD.vs.MCI; bulk RNA-seq,Homo_633,AD.vs.control; bulk RNA-seq,Homo_633,AD.vs.MCI; bulk RNA-seq,SRP223445,AD.vs.control	9
KEGG	hsa03050	Proteasome	bulk RNA-seq,Homo_723,AD.vs.control; bulk RNA-seq,Homo_723,AD.vs.MCI; bulk RNA-seq,Homo_714,AD.vs.control; bulk RNA-seq,Homo_714,AD.vs.MCI; bulk RNA-seq,Homo_633,AD.vs.control; bulk RNA-seq,Homo_633,AD.vs.MCI; scRNA-seq,SRP330776,B cell_4-AD.vs.control; scRNA-seq,SRP215507,CD8+ T	8
KEGG	hsa04740	Olfactory transduction	bulk RNA-seq,Homo_723,AD.vs.control; bulk RNA-seq,Homo_723,AD.vs.MCI; bulk RNA-seq,Homo_723,MCI.vs.control; bulk RNA-seq,Homo_714,AD.vs.control; bulk RNA-seq,Homo_714,AD.vs.MCI; bulk RNA-seq,Homo_714,MCI.vs.control; bulk RNA-seq,Homo_633,MCI.vs.control; bulk RNA-seq,ROSMAP,AD.vs.MCI; bulk RNA-seq,SRP223445,AD.vs.control	9
KEGG	hsa05033	Nicotine addiction	bulk RNA-seq,Homo_723,AD.vs.control; bulk RNA-seq,Homo_723,AD.vs.MCI; bulk RNA-seq,Homo_723,MCI.vs.control; bulk RNA-seq,Homo_714,AD.vs.control; bulk RNA-seq,Homo_714,AD.vs.MCI; bulk RNA-seq,ROSMAP,MCI.vs.control	6
KEGG	hsa04360	Axon guidance	bulk RNA-seq,Homo_723,AD.vs.control; bulk RNA-seq,Homo_723,AD.vs.MCI; bulk RNA-seq,Homo_723,MCI.vs.control; bulk RNA-seq,Homo_714,AD.vs.control; bulk RNA-seq,Homo_714,AD.vs.MCI; bulk RNA-seq,Homo_633,AD.vs.control; bulk RNA-seq,Homo_633,AD.vs.MCI; bulk RNA-seq,ROSMAP,AD.vs.control; scRNA-seq,SRP309935,Dendritic cell-AD.vs.control	9
KEGG	hsa04015	Rap1 signaling pathway	bulk RNA-seq,Homo_723,AD.vs.control; bulk RNA-seq,Homo_723,AD.vs.MCI; bulk RNA-seq,Homo_723,MCI.vs.control; bulk RNA-seq,Homo_714,AD.vs.control; bulk RNA-seq,Homo_714,AD.vs.MCI; bulk RNA-seq,Homo_633,AD.vs.control; bulk RNA-seq,Homo_633,AD.vs.MCI; bulk RNA-seq,ROSMAP,AD.vs.control; bulk RNA-seq,ROSMAP,AD.vs.MCI	9
KEGG	hsa04510	Focal adhesion	bulk RNA-seq,Homo_723,AD.vs.control; bulk RNA-seq,Homo_723,AD.vs.MCI; bulk RNA-seq,Homo_723,MCI.vs.control; bulk RNA-seq,Homo_714,AD.vs.control; bulk RNA-seq,Homo_714,AD.vs.MCI; bulk RNA-seq,Homo_633,AD.vs.control; bulk RNA-seq,Homo_633,AD.vs.MCI; bulk RNA-	8
KEGG	hsa04151	PI3K-Akt signaling pathway	bulk RNA-seq,Homo_723,AD.vs.control; bulk RNA-seq,Homo_723,AD.vs.MCI; bulk RNA-seq,Homo_714,AD.vs.control; bulk RNA-seq,Homo_714,AD.vs.MCI; bulk RNA-seq,Homo_633,AD.vs.control; bulk RNA-seq,Homo_633,AD.vs.MCI	6
KEGG	hsa04976	Bile secretion	bulk RNA-seq,Homo_723,AD.vs.control; bulk RNA-seq,Homo_723,AD.vs.MCI; bulk RNA-seq,Homo_723,MCI.vs.control; bulk RNA-seq,Homo_714,AD.vs.control; bulk RNA-seq,Homo_714,AD.vs.MCI; bulk RNA-seq,Homo_714,MCI.vs.control	6
KEGG	hsa04810	Regulation of actin cytoskeleton	bulk RNA-seq,Homo_723,AD.vs.control; bulk RNA-seq,Homo_723,AD.vs.MCI; bulk RNA-seq,Homo_723,MCI.vs.control; bulk RNA-seq,Homo_714,AD.vs.control; bulk RNA-seq,Homo_714,AD.vs.MCI; bulk RNA-seq,Homo_633,AD.vs.control; bulk RNA-seq,Homo_633,AD.vs.MCI	7
KEGG	hsa03040	Spliceosome	bulk RNA-seq,Homo_723,AD.vs.control; bulk RNA-seq,Homo_723,AD.vs.MCI; bulk RNA-seq,Homo_723,MCI.vs.control; bulk RNA-seq,Homo_714,AD.vs.control; bulk RNA-seq,Homo_633,AD.vs.control; bulk RNA-seq,Homo_633,AD.vs.MCI; scRNA-seq,SRP309935,Megakaryocyte_2-	7
KEGG	hsa04659	Th17 cell differentiation	bulk RNA-seq,Homo_723,AD.vs.control; bulk RNA-seq,Homo_723,MCI.vs.control; bulk RNA-seq,Homo_714,AD.vs.control; bulk RNA-seq,Homo_714,MCI.vs.control; bulk RNA-seq,Homo_633,AD.vs.MCI; scRNA-seq,SRP330776,CD8+ T cell_1-AD.vs.control; scRNA-seq,SRP215507,CD8+ T cell_3-AD.vs.control; scRNA-seq,SRP215507,CD8+ T cell_3-MCI.vs.control; scRNA-seq,SRP215507,CD8+ T	11
KEGG	hsa03250	Viral life cycle - HIV-1	bulk RNA-seq,Homo_723,AD.vs.control; bulk RNA-seq,Homo_723,AD.vs.MCI; bulk RNA-seq,Homo_723,MCI.vs.control; bulk RNA-seq,Homo_714,AD.vs.control; bulk RNA-seq,Homo_714,AD.vs.MCI; bulk RNA-seq,Homo_714,MCI.vs.control; bulk RNA-seq,Homo_633,AD.vs.control; bulk	8
KEGG	hsa04260	Cardiac muscle contraction	bulk RNA-seq,Homo_723,AD.vs.control; bulk RNA-seq,Homo_723,AD.vs.MCI; bulk RNA-seq,Homo_723,MCI.vs.control; bulk RNA-seq,Homo_714,AD.vs.control; bulk RNA-seq,Homo_714,AD.vs.MCI; bulk RNA-seq,Homo_714,MCI.vs.control; scRNA-seq,SRP215507,CD8+ T cell_8-	7
KEGG	hsa04514	Cell adhesion molecules	bulk RNA-seq,Homo_723,AD.vs.control; bulk RNA-seq,Homo_723,AD.vs.MCI; bulk RNA-seq,Homo_723,MCI.vs.control; bulk RNA-seq,Homo_714,AD.vs.MCI; bulk RNA-seq,Homo_633,AD.vs.control; bulk RNA-seq,Homo_633,AD.vs.MCI; bulk RNA-seq,ROSMAP,AD.vs.control; scRNA-seq,SRP215507,CD8+ T cell_3-AD.vs.control; scRNA-seq,SRP215507,CD8+ T cell_3-MCI.vs.control	9
KEGG	hsa03410	Base excision repair	bulk RNA-seq,Homo_723,AD.vs.control; bulk RNA-seq,Homo_723,AD.vs.MCI; bulk RNA-seq,Homo_723,MCI.vs.control; bulk RNA-seq,Homo_714,AD.vs.control; bulk RNA-seq,Homo_714,MCI.vs.control; bulk RNA-seq,Homo_633,AD.vs.control; bulk RNA-seq,Homo_633,AD.vs.MCI	7
KEGG	hsa04662	B cell receptor signaling pathway	bulk RNA-seq,Homo_723,AD.vs.control; bulk RNA-seq,Homo_723,AD.vs.MCI; bulk RNA-seq,Homo_723,MCI.vs.control; bulk RNA-seq,Homo_714,AD.vs.control; bulk RNA-seq,Homo_714,MCI.vs.control; bulk RNA-seq,Homo_633,AD.vs.control; bulk RNA-seq,Homo_633,AD.vs.MCI; scRNA-	8
KEGG	hsa05207	Chemical carcinogenesis - receptor activation	bulk RNA-seq,Homo_723,AD.vs.control; bulk RNA-seq,Homo_723,AD.vs.MCI; bulk RNA-seq,Homo_723,MCI.vs.control; bulk RNA-seq,Homo_714,AD.vs.control; bulk RNA-seq,Homo_714,AD.vs.MCI; bulk RNA-seq,Homo_633,AD.vs.control	6
KEGG	hsa04014	Ras signaling pathway	bulk RNA-seq,Homo_723,AD.vs.control; bulk RNA-seq,Homo_723,AD.vs.MCI; bulk RNA-seq,Homo_723,MCI.vs.control; bulk RNA-seq,Homo_714,AD.vs.control; bulk RNA-seq,Homo_714,AD.vs.MCI; bulk RNA-seq,Homo_633,AD.vs.control; bulk RNA-seq,Homo_633,AD.vs.MCI; bulk RNA-seq,ROSMAP,AD.vs.control; scRNA-seq,SRP215507,CD8+ T cell_8-MCI.vs.control	9
KEGG	hsa04721	Synaptic vesicle cycle	bulk RNA-seq,Homo_723,AD.vs.control; bulk RNA-seq,Homo_723,AD.vs.MCI; bulk RNA-seq,Homo_723,MCI.vs.control; bulk RNA-seq,Homo_714,AD.vs.MCI; bulk RNA-seq,Homo_714,MCI.vs.control; bulk RNA-seq,Homo_633,AD.vs.control; bulk RNA-seq,Homo_633,AD.vs.MCI; bulk RNA-	8
KEGG	hsa05226	Gastric cancer	bulk RNA-seq,Homo_723,AD.vs.control; bulk RNA-seq,Homo_723,AD.vs.MCI; bulk RNA-seq,Homo_723,MCI.vs.control; bulk RNA-seq,Homo_714,AD.vs.MCI; bulk RNA-seq,Homo_633,AD.vs.control; bulk RNA-seq,Homo_633,AD.vs.MCI; bulk RNA-seq,SRP223445,AD.vs.control	7
KEGG	hsa04390	Hippo signaling pathway	bulk RNA-seq,Homo_723,AD.vs.control; bulk RNA-seq,Homo_723,AD.vs.MCI; bulk RNA-seq,Homo_723,MCI.vs.control; bulk RNA-seq,Homo_714,AD.vs.MCI; bulk RNA-seq,Homo_633,AD.vs.control; bulk RNA-seq,Homo_633,AD.vs.MCI; bulk RNA-seq,SRP223445,AD.vs.control	7
KEGG	hsa05414	Dilated cardiomyopathy	bulk RNA-seq,Homo_723,AD.vs.control; bulk RNA-seq,Homo_723,AD.vs.MCI; bulk RNA-seq,Homo_723,MCI.vs.control; bulk RNA-seq,Homo_714,AD.vs.control; bulk RNA-seq,Homo_714,AD.vs.MCI; bulk RNA-seq,Homo_714,MCI.vs.control; bulk RNA-seq,Homo_633,AD.vs.control; bulk RNA-seq,Homo_633,AD.vs.MCI; bulk RNA-seq,ROSMAP,AD.vs.control; bulk RNA-seq,ROSMAP,AD.vs.MCI	10
KEGG	hsa05224	Breast cancer	bulk RNA-seq,Homo_723,AD.vs.control; bulk RNA-seq,Homo_723,AD.vs.MCI; bulk RNA-seq,Homo_723,MCI.vs.control; bulk RNA-seq,Homo_714,AD.vs.MCI; bulk RNA-seq,Homo_633,AD.vs.control; bulk RNA-seq,Homo_633,AD.vs.MCI	6
KEGG	hsa03420	Nucleotide excision repair	bulk RNA-seq,Homo_723,AD.vs.control; bulk RNA-seq,Homo_723,MCI.vs.control; bulk RNA-seq,Homo_714,AD.vs.control; bulk RNA-seq,Homo_714,MCI.vs.control; bulk RNA-seq,Homo_633,AD.vs.control; bulk RNA-seq,Homo_633,AD.vs.MCI	6

KEGG	hsa04612	Antigen processing and presentation	bulk RNA-seq,Homo_723,AD.vs.control; bulk RNA-seq,Homo_723,AD.vs.MCI; bulk RNA-seq,Homo_714,AD.vs.control; bulk RNA-seq,Homo_633,AD.vs.control; bulk RNA-seq,Homo_633,AD.vs.MCI; scRNA-seq,SRP330776,Natural killer cell_1-AD.vs.control; scRNA-seq,SRP309935,B cell_1-AD.vs.control; scRNA-seq,SRP309935,CD8+ T cell_1-AD.vs.control; scRNA-seq,SRP309935,Monocyte_2-AD.vs.control; scRNA-seq,SRP215507,CD8+ T	11
KEGG	hsa00980	Metabolism of xenobiotics by cytochrome P450	bulk RNA-seq,Homo_723,AD.vs.control; bulk RNA-seq,Homo_723,AD.vs.MCI; bulk RNA-seq,Homo_723,MCI.vs.control; bulk RNA-seq,Homo_714,AD.vs.control; bulk RNA-seq,Homo_714,AD.vs.MCI; bulk RNA-seq,Homo_714,MCI.vs.control; bulk RNA-seq,ROSMAP,AD.vs.MCI	7
KEGG	hsa04550	Signaling pathways regulating pluripotency of stem cells	bulk RNA-seq,Homo_723,AD.vs.control; bulk RNA-seq,Homo_723,AD.vs.MCI; bulk RNA-seq,Homo_723,MCI.vs.control; bulk RNA-seq,Homo_714,AD.vs.MCI; bulk RNA-seq,Homo_633,AD.vs.control; bulk RNA-seq,Homo_633,AD.vs.MCI; bulk RNA-seq,SRP223445,AD.vs.control	7
KEGG	hsa04640	Hematopoietic cell lineage	bulk RNA-seq,Homo_723,AD.vs.control; bulk RNA-seq,Homo_723,MCI.vs.control; bulk RNA-seq,Homo_714,AD.vs.control; bulk RNA-seq,Homo_714,MCI.vs.control; bulk RNA-seq,Homo_633,AD.vs.control; bulk RNA-seq,Homo_633,AD.vs.MCI; scRNA-seq,SRP330776,Monocyte_2-AD.vs.control; scRNA-seq,SRP330776,Naive CD8+ T cell_2-AD.vs.control; scRNA-seq,SRP309935,Monocyte_2-AD.vs.control; scRNA-seq,SRP215507,CD8+ T	11
KEGG	hsa00982	Drug metabolism - cytochrome P450	bulk RNA-seq,Homo_723,AD.vs.control; bulk RNA-seq,Homo_723,AD.vs.MCI; bulk RNA-seq,Homo_723,MCI.vs.control; bulk RNA-seq,Homo_714,AD.vs.control; bulk RNA-seq,Homo_714,AD.vs.MCI; bulk RNA-seq,Homo_714,MCI.vs.control; bulk RNA-seq,ROSMAP,AD.vs.MCI	7
KEGG	hsa04010	MAPK signaling pathway	bulk RNA-seq,Homo_723,AD.vs.control; bulk RNA-seq,Homo_723,AD.vs.MCI; bulk RNA-seq,Homo_714,AD.vs.MCI; bulk RNA-seq,Homo_633,AD.vs.control; bulk RNA-seq,Homo_633,AD.vs.MCI; scRNA-seq,SRP330776,CD8+ T cell_3-AD.vs.control; scRNA-seq,SRP330776,Natural killer cell_1-AD.vs.control; scRNA-seq,SRP215507,CD8+ T cell_8-MCI.vs.control	8
KEGG	hsa05321	Inflammatory bowel disease	bulk RNA-seq,Homo_723,AD.vs.control; bulk RNA-seq,Homo_723,AD.vs.MCI; bulk RNA-seq,Homo_723,MCI.vs.control; bulk RNA-seq,Homo_714,AD.vs.control; bulk RNA-seq,Homo_714,MCI.vs.control; bulk RNA-seq,Homo_633,AD.vs.control; scRNA-seq,SRP215507,CD8+ T cell_3-AD.vs.control; scRNA-seq,SRP215507,CD8+ T cell_3-MCI.vs.control; scRNA-seq,SRP215507,CD8+ T cell_4-MCI.vs.control	9
KEGG	hsa04310	Wnt signaling pathway	bulk RNA-seq,Homo_723,AD.vs.control; bulk RNA-seq,Homo_723,AD.vs.MCI; bulk RNA-seq,Homo_723,MCI.vs.control; bulk RNA-seq,Homo_714,AD.vs.MCI; bulk RNA-seq,Homo_633,AD.vs.control; bulk RNA-seq,Homo_633,AD.vs.MCI; bulk RNA-seq,SRP223445,AD.vs.control	7
KEGG	hsa04971	Gastric acid secretion	bulk RNA-seq,Homo_723,AD.vs.control; bulk RNA-seq,Homo_723,AD.vs.MCI; bulk RNA-seq,Homo_723,MCI.vs.control; bulk RNA-seq,Homo_714,AD.vs.MCI; bulk RNA-seq,Homo_714,MCI.vs.control; bulk RNA-seq,Homo_633,AD.vs.control; bulk RNA-seq,Homo_633,AD.vs.MCI	7
KEGG	hsa05330	Allograft rejection	bulk RNA-seq,Homo_723,AD.vs.control; bulk RNA-seq,Homo_723,AD.vs.MCI; bulk RNA-seq,Homo_723,MCI.vs.control; bulk RNA-seq,Homo_714,AD.vs.control; bulk RNA-seq,Homo_714,MCI.vs.control; bulk RNA-seq,Homo_633,AD.vs.control; bulk RNA-seq,Homo_633,AD.vs.MCI; scRNA-seq,SRP330776,Megakaryocyte_1-AD.vs.control; scRNA-seq,SRP215507,CD8+ T cell_3-AD.vs.control; scRNA-seq,SRP215507,CD8+ T cell_3-MCI.vs.control; scRNA-seq,SRP215507,CD8+ T cell_4-AD.vs.control; scRNA-seq,SRP215507,CD8+ T cell_8-MCI.vs.control	12
KEGG	hsa05169	Epstein-Barr virus infection	bulk RNA-seq,Homo_723,AD.vs.control; bulk RNA-seq,Homo_723,MCI.vs.control; bulk RNA-seq,Homo_714,AD.vs.control; bulk RNA-seq,Homo_633,AD.vs.control; bulk RNA-seq,Homo_633,AD.vs.MCI; scRNA-seq,SRP215507,CD8+ T cell_3-AD.vs.control; scRNA-seq,SRP215507,CD8+ T cell_3-MCI.vs.control; scRNA-seq,SRP215507,CD8+ T cell_8-MCI.vs.control	8
KEGG	hsa04974	Protein digestion and absorption	bulk RNA-seq,Homo_723,AD.vs.control; bulk RNA-seq,Homo_723,AD.vs.MCI; bulk RNA-seq,Homo_723,MCI.vs.control; bulk RNA-seq,Homo_714,AD.vs.MCI; bulk RNA-seq,Homo_714,MCI.vs.control; bulk RNA-seq,Homo_633,AD.vs.control; bulk RNA-seq,ROSMAP,AD.vs.control	7
KEGG	hsa04742	Taste transduction	bulk RNA-seq,Homo_723,AD.vs.control; bulk RNA-seq,Homo_723,AD.vs.MCI; bulk RNA-seq,Homo_723,MCI.vs.control; bulk RNA-seq,Homo_714,AD.vs.control; bulk RNA-seq,Homo_714,AD.vs.MCI; bulk RNA-seq,SRP223445,AD.vs.control	6
KEGG	hsa04714	Thermogenesis	bulk RNA-seq,Homo_723,AD.vs.control; bulk RNA-seq,Homo_723,MCI.vs.control; bulk RNA-seq,Homo_714,AD.vs.control; bulk RNA-seq,Homo_633,AD.vs.control; bulk RNA-seq,Homo_633,AD.vs.MCI; scRNA-seq,SRP215507,CD8+ T cell_8-MCI.vs.control	6
KEGG	hsa05100	Bacterial invasion of epithelial cells	bulk RNA-seq,Homo_723,AD.vs.control; bulk RNA-seq,Homo_723,AD.vs.MCI; bulk RNA-seq,Homo_723,MCI.vs.control; bulk RNA-seq,Homo_714,AD.vs.control; bulk RNA-seq,Homo_714,AD.vs.MCI; bulk RNA-seq,Homo_714,MCI.vs.control; bulk RNA-seq,Homo_633,AD.vs.control; bulk	8
KEGG	hsa05031	Amphetamine addiction	bulk RNA-seq,Homo_723,AD.vs.control; bulk RNA-seq,Homo_723,AD.vs.MCI; bulk RNA-seq,Homo_723,MCI.vs.control; bulk RNA-seq,Homo_714,AD.vs.control; bulk RNA-seq,Homo_714,AD.vs.MCI; bulk RNA-seq,Homo_633,AD.vs.control; bulk RNA-seq,Homo_633,AD.vs.MCI	7
KEGG	hsa05030	Cocaine addiction	bulk RNA-seq,Homo_723,AD.vs.control; bulk RNA-seq,Homo_723,AD.vs.MCI; bulk RNA-seq,Homo_723,MCI.vs.control; bulk RNA-seq,Homo_714,AD.vs.control; bulk RNA-seq,Homo_714,AD.vs.MCI; bulk RNA-seq,Homo_714,MCI.vs.control; bulk RNA-seq,Homo_633,AD.vs.control; bulk RNA-seq,Homo_633,AD.vs.MCI; bulk RNA-seq,ROSMAP,AD.vs.control	9
KEGG	hsa04950	Maturity onset diabetes of the young	bulk RNA-seq,Homo_723,AD.vs.control; bulk RNA-seq,Homo_723,AD.vs.MCI; bulk RNA-seq,Homo_714,AD.vs.control; bulk RNA-seq,Homo_714,AD.vs.MCI; bulk RNA-seq,Homo_633,MCI.vs.control	5
KEGG	hsa04672	Intestinal immune network for IgA production	bulk RNA-seq,Homo_723,AD.vs.control; bulk RNA-seq,Homo_723,AD.vs.MCI; bulk RNA-seq,Homo_723,MCI.vs.control; bulk RNA-seq,Homo_714,AD.vs.control; bulk RNA-seq,Homo_714,MCI.vs.control; bulk RNA-seq,Homo_633,AD.vs.control; bulk RNA-seq,Homo_633,AD.vs.MCI; scRNA-seq,SRP215507,CD8+ T cell_3-AD.vs.control; scRNA-seq,SRP215507,CD8+ T cell_3-MCI.vs.control	9
KEGG	hsa04814	Motor proteins	bulk RNA-seq,Homo_723,AD.vs.control; bulk RNA-seq,Homo_723,AD.vs.MCI; bulk RNA-seq,Homo_723,MCI.vs.control; bulk RNA-seq,Homo_714,AD.vs.control; bulk RNA-seq,Homo_714,AD.vs.MCI; bulk RNA-seq,Homo_633,AD.vs.control; bulk RNA-seq,ROSMAP,AD.vs.control; bulk	8
KEGG	hsa03018	RNA degradation	bulk RNA-seq,Homo_723,AD.vs.control; bulk RNA-seq,Homo_723,MCI.vs.control; bulk RNA-seq,Homo_714,AD.vs.control; bulk RNA-seq,Homo_714,MCI.vs.control; bulk RNA-seq,Homo_633,AD.vs.control; bulk RNA-seq,Homo_633,AD.vs.MCI	6
KEGG	hsa05340	Primary immunodeficiency	bulk RNA-seq,Homo_723,AD.vs.control; bulk RNA-seq,Homo_723,AD.vs.MCI; bulk RNA-seq,Homo_723,MCI.vs.control; bulk RNA-seq,Homo_714,AD.vs.control; bulk RNA-seq,Homo_714,MCI.vs.control; bulk RNA-seq,Homo_633,AD.vs.control; bulk RNA-seq,Homo_633,AD.vs.MCI; scRNA-	8
KEGG	hsa05410	Hypertrophic cardiomyopathy	bulk RNA-seq,Homo_723,AD.vs.control; bulk RNA-seq,Homo_723,AD.vs.MCI; bulk RNA-seq,Homo_723,MCI.vs.control; bulk RNA-seq,Homo_714,AD.vs.control; bulk RNA-seq,Homo_714,AD.vs.MCI; bulk RNA-seq,Homo_714,MCI.vs.control; bulk RNA-seq,Homo_633,AD.vs.control; bulk RNA-seq,ROSMAP,AD.vs.control; bulk RNA-seq,ROSMAP,AD.vs.MCI	9
KEGG	hsa04658	Th1 and Th2 cell differentiation	bulk RNA-seq,Homo_723,AD.vs.control; bulk RNA-seq,Homo_723,MCI.vs.control; bulk RNA-seq,Homo_714,AD.vs.control; bulk RNA-seq,Homo_714,MCI.vs.control; bulk RNA-seq,Homo_633,AD.vs.control; bulk RNA-seq,Homo_633,AD.vs.MCI; scRNA-seq,SRP215507,CD8+ T cell_3-	8
KEGG	hsa00830	Retinol metabolism	bulk RNA-seq,Homo_723,AD.vs.control; bulk RNA-seq,Homo_723,AD.vs.MCI; bulk RNA-seq,Homo_723,MCI.vs.control; bulk RNA-seq,Homo_714,AD.vs.control; bulk RNA-seq,Homo_714,AD.vs.MCI; bulk RNA-seq,Homo_714,MCI.vs.control	6
KEGG	hsa05412	Arrhythmogenic right ventricular cardiomyopathy	bulk RNA-seq,Homo_723,AD.vs.control; bulk RNA-seq,Homo_723,AD.vs.MCI; bulk RNA-seq,Homo_723,MCI.vs.control; bulk RNA-seq,Homo_714,AD.vs.control; bulk RNA-seq,Homo_714,AD.vs.MCI; bulk RNA-seq,Homo_714,MCI.vs.control; bulk RNA-seq,Homo_633,AD.vs.control; bulk	8
KEGG	hsa04520	Adherens junction	bulk RNA-seq,Homo_723,AD.vs.control; bulk RNA-seq,Homo_723,AD.vs.MCI; bulk RNA-seq,Homo_723,MCI.vs.control; bulk RNA-seq,Homo_714,AD.vs.control; bulk RNA-seq,Homo_714,AD.vs.MCI; bulk RNA-seq,Homo_714,MCI.vs.control; bulk RNA-seq,Homo_633,AD.vs.control; bulk	8
KEGG	hsa03430	Mismatch repair	bulk RNA-seq,Homo_723,AD.vs.control; bulk RNA-seq,Homo_723,MCI.vs.control; bulk RNA-seq,Homo_714,AD.vs.control; bulk RNA-seq,Homo_714,MCI.vs.control; bulk RNA-seq,Homo_633,AD.vs.control; bulk RNA-seq,Homo_633,AD.vs.MCI; bulk RNA-seq,Homo_633,MCI.vs.control	7

KEGG	hsa04916	Melanogenesis	bulk RNA-seq,Homo_723,AD.vs.control; bulk RNA-seq,Homo_723,AD.vs.MCI; bulk RNA-seq,Homo_723,MCI.vs.control; bulk RNA-seq,Homo_714,AD.vs.MCI; bulk RNA-seq,Homo_714,MCI.vs.control; bulk RNA-seq,Homo_633,AD.vs.control; bulk RNA-seq,Homo_633,AD.vs.MCI; bulk RNA-	8
KEGG	hsa04022	cGMP-PKG signaling pathway	bulk RNA-seq,Homo_723,AD.vs.control; bulk RNA-seq,Homo_723,AD.vs.MCI; bulk RNA-seq,Homo_723,MCI.vs.control; bulk RNA-seq,Homo_714,AD.vs.MCI; bulk RNA-seq,Homo_633,AD.vs.control; bulk RNA-seq,Homo_633,AD.vs.MCI; bulk RNA-seq,ROSMAP,AD.vs.MCI	7
KEGG	hsa05204	Chemical carcinogenesis - DNA adducts	bulk RNA-seq,Homo_723,AD.vs.control; bulk RNA-seq,Homo_723,AD.vs.MCI; bulk RNA-seq,Homo_723,MCI.vs.control; bulk RNA-seq,Homo_714,AD.vs.control; bulk RNA-seq,Homo_714,AD.vs.MCI; bulk RNA-seq,Homo_714,MCI.vs.control; bulk RNA-seq,ROSMAP,AD.vs.MCI	7
KEGG	hsa00053	Ascorbate and aldarate metabolism	bulk RNA-seq,Homo_723,AD.vs.control; bulk RNA-seq,Homo_723,AD.vs.MCI; bulk RNA-seq,Homo_714,AD.vs.control; bulk RNA-seq,Homo_714,AD.vs.MCI; bulk RNA-seq,ROSMAP,AD.vs.MCI	5
KEGG	hsa03060	Protein export	bulk RNA-seq,Homo_723,AD.vs.control; bulk RNA-seq,Homo_723,MCI.vs.control; bulk RNA-seq,Homo_714,AD.vs.control; bulk RNA-seq,Homo_714,MCI.vs.control; bulk RNA-seq,Homo_633,AD.vs.control; bulk RNA-seq,Homo_633,AD.vs.MCI; bulk RNA-seq,Homo_633,MCI.vs.control	7
KEGG	hsa05217	Basal cell carcinoma	bulk RNA-seq,Homo_723,AD.vs.control; bulk RNA-seq,Homo_723,AD.vs.MCI; bulk RNA-seq,Homo_723,MCI.vs.control; bulk RNA-seq,Homo_714,AD.vs.MCI; bulk RNA-seq,Homo_714,MCI.vs.control; bulk RNA-seq,Homo_633,AD.vs.control; bulk RNA-seq,Homo_633,AD.vs.MCI; bulk RNA-	8
KEGG	hsa04146	Peroxisome	bulk RNA-seq,Homo_723,AD.vs.control; bulk RNA-seq,Homo_723,MCI.vs.control; bulk RNA-seq,Homo_714,AD.vs.control; bulk RNA-seq,Homo_714,MCI.vs.control; bulk RNA-seq,Homo_633,AD.vs.control; bulk RNA-seq,Homo_633,AD.vs.MCI	6
KEGG	hsa05205	Proteoglycans in cancer	bulk RNA-seq,Homo_723,AD.vs.control; bulk RNA-seq,Homo_723,AD.vs.MCI; bulk RNA-seq,Homo_723,MCI.vs.control; bulk RNA-seq,Homo_714,AD.vs.MCI; bulk RNA-seq,Homo_633,AD.vs.control; bulk RNA-seq,Homo_633,AD.vs.MCI	6
KEGG	hsa04140	Autophagy - animal	bulk RNA-seq,Homo_723,AD.vs.control; bulk RNA-seq,Homo_723,MCI.vs.control; bulk RNA-seq,Homo_714,AD.vs.control; bulk RNA-seq,Homo_633,AD.vs.control; bulk RNA-seq,Homo_633,AD.vs.MCI; scRNA-seq,SRP215507,CD8+ T cell_8-MCI.vs.control	6
KEGG	hsa04540	Gap junction	bulk RNA-seq,Homo_723,AD.vs.control; bulk RNA-seq,Homo_723,AD.vs.MCI; bulk RNA-seq,Homo_723,MCI.vs.control; bulk RNA-seq,Homo_714,AD.vs.MCI; bulk RNA-seq,Homo_714,MCI.vs.control; bulk RNA-seq,Homo_633,AD.vs.control; bulk RNA-seq,Homo_633,AD.vs.MCI	7
KEGG	hsa04142	Lysosome	bulk RNA-seq,Homo_723,AD.vs.control; bulk RNA-seq,Homo_723,MCI.vs.control; bulk RNA-seq,Homo_714,AD.vs.control; bulk RNA-seq,Homo_714,MCI.vs.control; bulk RNA-seq,Homo_633,AD.vs.control; bulk RNA-seq,Homo_633,AD.vs.MCI; bulk RNA-seq,SRP223445,AD.vs.control	7
KEGG	hsa04660	T cell receptor signaling pathway	bulk RNA-seq,Homo_723,AD.vs.control; bulk RNA-seq,Homo_723,MCI.vs.control; bulk RNA-seq,Homo_714,AD.vs.control; bulk RNA-seq,Homo_714,MCI.vs.control; bulk RNA-seq,Homo_633,AD.vs.control; bulk RNA-seq,Homo_633,AD.vs.MCI; scRNA-seq,SRP215507,CD8+ T cell_9-	7
KEGG	hsa04726	Serotonergic synapse	bulk RNA-seq,Homo_723,AD.vs.control; bulk RNA-seq,Homo_723,AD.vs.MCI; bulk RNA-seq,Homo_723,MCI.vs.control; bulk RNA-seq,Homo_714,AD.vs.control; bulk RNA-seq,Homo_714,AD.vs.MCI; bulk RNA-seq,Homo_714,MCI.vs.control; bulk RNA-seq,Homo_633,AD.vs.control; bulk RNA-seq,Homo_633,AD.vs.MCI; bulk RNA-seq,ROSMAP,AD.vs.control	9
KEGG	hsa04928	Parathyroid hormone synthesis, secretion and action	bulk RNA-seq,Homo_723,AD.vs.control; bulk RNA-seq,Homo_723,AD.vs.MCI; bulk RNA-seq,Homo_723,MCI.vs.control; bulk RNA-seq,Homo_714,AD.vs.MCI; bulk RNA-seq,Homo_714,MCI.vs.control; bulk RNA-seq,Homo_633,AD.vs.control; bulk RNA-seq,Homo_633,AD.vs.MCI	7
KEGG	hsa05218	Melanoma	bulk RNA-seq,Homo_723,AD.vs.control; bulk RNA-seq,Homo_723,AD.vs.MCI; bulk RNA-seq,Homo_723,MCI.vs.control; bulk RNA-seq,Homo_714,AD.vs.control; bulk RNA-seq,Homo_714,AD.vs.MCI; bulk RNA-seq,Homo_714,MCI.vs.control; bulk RNA-seq,Homo_633,AD.vs.control; bulk	8
KEGG	hsa03030	DNA replication	bulk RNA-seq,Homo_723,AD.vs.control; bulk RNA-seq,Homo_723,MCI.vs.control; bulk RNA-seq,Homo_714,AD.vs.control; bulk RNA-seq,Homo_714,MCI.vs.control; bulk RNA-seq,Homo_633,AD.vs.control; bulk RNA-seq,Homo_633,AD.vs.MCI	6
KEGG	hsa04072	Phospholipase D signaling pathway	bulk RNA-seq,Homo_723,AD.vs.control; bulk RNA-seq,Homo_723,AD.vs.MCI; bulk RNA-seq,Homo_723,MCI.vs.control; bulk RNA-seq,Homo_714,AD.vs.MCI; bulk RNA-seq,Homo_633,AD.vs.control; bulk RNA-seq,Homo_633,AD.vs.MCI; scRNA-seq,SRP330776,B cell_2-AD.vs.control; scRNA-seq,SRP215507,CD8+ T	8
KEGG	hsa00040	Pentose and glucuronate interconversions	bulk RNA-seq,Homo_723,AD.vs.control; bulk RNA-seq,Homo_723,AD.vs.MCI; bulk RNA-seq,Homo_714,AD.vs.control; bulk RNA-seq,Homo_714,AD.vs.MCI	4
KEGG	hsa05235	PD-L1 expression and PD-1 checkpoint pathway in cancer	bulk RNA-seq,Homo_723,AD.vs.control; bulk RNA-seq,Homo_723,MCI.vs.control; bulk RNA-seq,Homo_714,AD.vs.control; bulk RNA-seq,Homo_714,MCI.vs.control; bulk RNA-seq,Homo_633,AD.vs.control; bulk RNA-seq,Homo_633,AD.vs.MCI	6
KEGG	hsa05140	Leishmaniasis	bulk RNA-seq,Homo_723,AD.vs.control; bulk RNA-seq,Homo_723,MCI.vs.control; bulk RNA-seq,Homo_714,AD.vs.control; bulk RNA-seq,Homo_714,MCI.vs.control; bulk RNA-seq,Homo_633,AD.vs.control; bulk RNA-seq,Homo_633,AD.vs.MCI; scRNA-seq,SRP330776,Megakaryocyte_1-AD.vs.control; scRNA-seq,SRP215507,CD8+ T cell_3-AD.vs.control; scRNA-seq,SRP215507,CD8+ T cell_3-MCI.vs.control; scRNA-seq,SRP215507,CD8+ T	11
KEGG	hsa05332	Graft-versus-host disease	bulk RNA-seq,Homo_723,AD.vs.control; bulk RNA-seq,Homo_723,AD.vs.MCI; bulk RNA-seq,Homo_633,AD.vs.control; bulk RNA-seq,Homo_633,AD.vs.MCI; scRNA-seq,SRP330776,Megakaryocyte_1-AD.vs.control; scRNA-seq,SRP330776,Monocyte_2-AD.vs.control; scRNA-seq,SRP309935,B cell_1-AD.vs.control; scRNA-seq,SRP215507,CD8+ T cell_3-AD.vs.control; scRNA-seq,SRP215507,CD8+ T cell_3-MCI.vs.control; scRNA-seq,SRP215507,CD8+ T cell_4-	10
KEGG	hsa03450	Non-homologous end-joining	bulk RNA-seq,Homo_723,AD.vs.control; bulk RNA-seq,Homo_723,AD.vs.MCI; bulk RNA-seq,Homo_714,AD.vs.control; bulk RNA-seq,Homo_714,AD.vs.MCI; bulk RNA-seq,Homo_633,AD.vs.control; bulk RNA-seq,Homo_633,AD.vs.MCI	6
KEGG	hsa04932	Non-alcoholic fatty liver disease	bulk RNA-seq,Homo_723,AD.vs.control; bulk RNA-seq,Homo_723,MCI.vs.control; bulk RNA-seq,Homo_714,AD.vs.control; bulk RNA-seq,Homo_633,AD.vs.control; bulk RNA-seq,Homo_633,AD.vs.MCI	5
KEGG	hsa04972	Pancreatic secretion	bulk RNA-seq,Homo_723,AD.vs.control; bulk RNA-seq,Homo_723,AD.vs.MCI; bulk RNA-seq,Homo_723,MCI.vs.control; bulk RNA-seq,Homo_714,AD.vs.control; bulk RNA-seq,Homo_714,AD.vs.MCI; bulk RNA-seq,Homo_714,MCI.vs.control; bulk RNA-seq,Homo_633,AD.vs.control; bulk	8
KEGG	hsa05164	Influenza A	bulk RNA-seq,Homo_723,AD.vs.control; bulk RNA-seq,Homo_723,MCI.vs.control; bulk RNA-seq,Homo_714,AD.vs.control; bulk RNA-seq,Homo_633,AD.vs.control; bulk RNA-seq,Homo_633,AD.vs.MCI; scRNA-seq,SRP215507,CD8+ T cell_3-AD.vs.control; scRNA-seq,SRP215507,CD8+ T cell_3-MCI.vs.control; scRNA-seq,SRP215507,CD8+ T cell_4-AD.vs.control; scRNA-seq,SRP215507,CD8+ T cell_8-MCI.vs.control	9
KEGG	hsa05034	Alcoholism	bulk RNA-seq,Homo_723,AD.vs.control; bulk RNA-seq,Homo_723,AD.vs.MCI; bulk RNA-seq,Homo_723,MCI.vs.control; bulk RNA-seq,Homo_714,AD.vs.control; bulk RNA-seq,Homo_714,AD.vs.MCI; bulk RNA-seq,Homo_633,AD.vs.control	6
KEGG	hsa04120	Ubiquitin mediated proteolysis	bulk RNA-seq,Homo_723,AD.vs.control; bulk RNA-seq,Homo_723,MCI.vs.control; bulk RNA-seq,Homo_714,AD.vs.control; bulk RNA-seq,Homo_633,AD.vs.control; bulk RNA-seq,Homo_633,AD.vs.MCI	5
KEGG	hsa04670	Leukocyte transendothelial migration	bulk RNA-seq,Homo_723,AD.vs.control; bulk RNA-seq,Homo_723,AD.vs.MCI; bulk RNA-seq,Homo_723,MCI.vs.control; bulk RNA-seq,Homo_714,AD.vs.MCI; bulk RNA-seq,Homo_714,MCI.vs.control; bulk RNA-seq,Homo_633,AD.vs.control; bulk RNA-seq,Homo_633,AD.vs.MCI	7
KEGG	hsa03020	RNA polymerase	bulk RNA-seq,Homo_723,AD.vs.control; bulk RNA-seq,Homo_723,MCI.vs.control; bulk RNA-seq,Homo_714,AD.vs.control; bulk RNA-seq,Homo_714,MCI.vs.control; bulk RNA-seq,Homo_633,AD.vs.control; bulk RNA-seq,Homo_633,AD.vs.MCI	6
KEGG	hsa03082	ATP-dependent chromatin remodeling	bulk RNA-seq,Homo_723,AD.vs.control; bulk RNA-seq,Homo_723,MCI.vs.control; bulk RNA-seq,Homo_714,AD.vs.control; bulk RNA-seq,Homo_714,MCI.vs.control; bulk RNA-seq,Homo_633,AD.vs.control; bulk RNA-seq,Homo_633,AD.vs.MCI	6
KEGG	hsa04980	Cobalamin transport and metabolism	bulk RNA-seq,Homo_723,AD.vs.control; bulk RNA-seq,Homo_723,AD.vs.MCI; bulk RNA-seq,Homo_714,AD.vs.control; bulk RNA-seq,Homo_714,AD.vs.MCI; bulk RNA-seq,Homo_633,AD.vs.control; bulk RNA-seq,Homo_633,AD.vs.MCI	6

KEGG	hsa01521	EGFR tyrosine kinase inhibitor resistance	bulk RNA-seq,Homo_723,AD.vs.control; bulk RNA-seq,Homo_723,AD.vs.MCI; bulk RNA-seq,Homo_723,MCI.vs.control; bulk RNA-seq,Homo_714,AD.vs.MCI; bulk RNA-seq,Homo_714,MCI.vs.control; bulk RNA-seq,Homo_633,AD.vs.control; bulk RNA-seq,Homo_633,AD.vs.MCI	7
KEGG	hsa05166	Human T-cell leukemia virus 1 infection	bulk RNA-seq,Homo_723,AD.vs.control; bulk RNA-seq,Homo_723,MCI.vs.control; bulk RNA-seq,Homo_714,AD.vs.control; bulk RNA-seq,Homo_633,AD.vs.control; bulk RNA-seq,Homo_633,AD.vs.MCI; scRNA-seq,SRP215507,CD8+ T cell_3-AD.vs.control; scRNA-seq,SRP215507,CD8+ T	7
KEGG	hsa04935	Growth hormone synthesis, secretion and action	bulk RNA-seq,Homo_723,AD.vs.control; bulk RNA-seq,Homo_723,AD.vs.MCI; bulk RNA-seq,Homo_723,MCI.vs.control; bulk RNA-seq,Homo_714,AD.vs.MCI; bulk RNA-seq,Homo_714,MCI.vs.control; bulk RNA-seq,Homo_633,AD.vs.control; bulk RNA-seq,Homo_633,AD.vs.MCI; bulk RNA-seq,ROSMAP,AD.vs.MCI; scRNA-seq,SRP330776,CD8+ T cell_1-AD.vs.control; scRNA-seq,SRP330776,CD8+ T cell_3-AD.vs.control	10
KEGG	hsa00563	Glycosylphosphatidylinositol (GPI)-anchor biosynthesis	bulk RNA-seq,Homo_723,AD.vs.control; bulk RNA-seq,Homo_723,MCI.vs.control; bulk RNA-seq,Homo_714,AD.vs.control; bulk RNA-seq,Homo_714,AD.vs.MCI; bulk RNA-seq,Homo_714,MCI.vs.control; bulk RNA-seq,Homo_633,AD.vs.control; bulk RNA-seq,Homo_633,AD.vs.MCI; bulk RNA-	8
KEGG	hsa04064	NF-kappa B signaling pathway	bulk RNA-seq,Homo_723,AD.vs.control; bulk RNA-seq,Homo_723,MCI.vs.control; bulk RNA-seq,Homo_714,AD.vs.MCI; bulk RNA-seq,Homo_714,MCI.vs.control; bulk RNA-seq,Homo_633,AD.vs.control; bulk RNA-seq,Homo_633,AD.vs.MCI	6
KEGG	hsa04270	Vascular smooth muscle contraction	bulk RNA-seq,Homo_723,AD.vs.control; bulk RNA-seq,Homo_723,AD.vs.MCI; bulk RNA-seq,Homo_723,MCI.vs.control; bulk RNA-seq,Homo_714,AD.vs.control; bulk RNA-seq,Homo_714,AD.vs.MCI; bulk RNA-seq,Homo_633,AD.vs.control; bulk RNA-seq,Homo_633,AD.vs.MCI; bulk RNA-seq,ROSMAP,AD.vs.control; bulk RNA-seq,ROSMAP,AD.vs.MCI	9
KEGG	hsa04136	Autophagy - other	bulk RNA-seq,Homo_723,AD.vs.control; bulk RNA-seq,Homo_714,AD.vs.control; bulk RNA-seq,Homo_633,AD.vs.control; bulk RNA-seq,Homo_633,AD.vs.MCI	4
KEGG	hsa05146	Amoebiasis	bulk RNA-seq,Homo_723,AD.vs.control; bulk RNA-seq,Homo_723,AD.vs.MCI; bulk RNA-seq,Homo_723,MCI.vs.control; bulk RNA-seq,Homo_714,AD.vs.MCI; bulk RNA-seq,Homo_714,MCI.vs.control; bulk RNA-seq,Homo_633,AD.vs.control; bulk RNA-seq,Homo_633,AD.vs.MCI	7
KEGG	hsa03015	mRNA surveillance pathway	bulk RNA-seq,Homo_723,AD.vs.control; bulk RNA-seq,Homo_723,MCI.vs.control; bulk RNA-seq,Homo_714,AD.vs.control; bulk RNA-seq,Homo_714,MCI.vs.control; bulk RNA-seq,Homo_633,AD.vs.control; bulk RNA-seq,Homo_633,AD.vs.MCI	6
KEGG	hsa00983	Drug metabolism - other enzymes	bulk RNA-seq,Homo_723,AD.vs.control; bulk RNA-seq,Homo_723,AD.vs.MCI; bulk RNA-seq,Homo_723,MCI.vs.control; bulk RNA-seq,Homo_714,AD.vs.control; bulk RNA-seq,Homo_714,AD.vs.MCI; bulk RNA-seq,Homo_714,MCI.vs.control; bulk RNA-seq,Homo_633,AD.vs.control; bulk	8
KEGG	hsa03440	Homologous recombination	bulk RNA-seq,Homo_723,AD.vs.control; bulk RNA-seq,Homo_723,MCI.vs.control; bulk RNA-seq,Homo_714,AD.vs.control; bulk RNA-seq,Homo_714,MCI.vs.control; bulk RNA-seq,Homo_633,AD.vs.control; bulk RNA-seq,Homo_633,AD.vs.MCI	6
KEGG	hsa00860	Porphyrin metabolism	bulk RNA-seq,Homo_723,AD.vs.control; bulk RNA-seq,Homo_723,AD.vs.MCI; bulk RNA-seq,Homo_714,AD.vs.control; bulk RNA-seq,Homo_714,AD.vs.MCI; bulk RNA-seq,Homo_714,MCI.vs.control	5
KEGG	hsa03460	Fanconi anemia pathway	bulk RNA-seq,Homo_723,AD.vs.control; bulk RNA-seq,Homo_723,MCI.vs.control; bulk RNA-seq,Homo_714,AD.vs.control; bulk RNA-seq,Homo_714,MCI.vs.control; bulk RNA-seq,Homo_633,AD.vs.control; bulk RNA-seq,Homo_633,AD.vs.MCI	6
KEGG	hsa05206	MicroRNAs in cancer	bulk RNA-seq,Homo_723,AD.vs.control; bulk RNA-seq,Homo_723,AD.vs.MCI; bulk RNA-seq,Homo_714,AD.vs.control; bulk RNA-seq,Homo_714,AD.vs.MCI	4
KEGG	hsa04918	Thyroid hormone synthesis	bulk RNA-seq,Homo_723,AD.vs.control; bulk RNA-seq,Homo_723,AD.vs.MCI; bulk RNA-seq,Homo_723,MCI.vs.control; bulk RNA-seq,Homo_714,AD.vs.MCI; bulk RNA-seq,Homo_714,MCI.vs.control; bulk RNA-seq,Homo_633,AD.vs.control	6
KEGG	hsa05132	Salmonella infection	bulk RNA-seq,Homo_723,AD.vs.control; bulk RNA-seq,Homo_723,MCI.vs.control; bulk RNA-seq,Homo_714,AD.vs.control; bulk RNA-seq,Homo_633,AD.vs.control; bulk RNA-seq,Homo_633,AD.vs.MCI	5
KEGG	hsa05310	Asthma	bulk RNA-seq,Homo_723,AD.vs.control; bulk RNA-seq,Homo_633,AD.vs.control; scRNA-seq,SRP330776,Megakaryocyte_1-AD.vs.control; scRNA-seq,SRP215507,CD8+ T cell_1-MCI.vs.control; scRNA-seq,SRP215507,CD8+ T cell_3-AD.vs.control; scRNA-seq,SRP215507,CD8+ T cell_3-MCI.vs.control	6
KEGG	hsa04144	Endocytosis	bulk RNA-seq,Homo_723,AD.vs.control; bulk RNA-seq,Homo_723,MCI.vs.control; bulk RNA-seq,Homo_714,AD.vs.control; bulk RNA-seq,Homo_633,AD.vs.control; bulk RNA-seq,Homo_633,AD.vs.MCI	5
KEGG	hsa04621	NOD-like receptor signaling pathway	bulk RNA-seq,Homo_723,AD.vs.control; bulk RNA-seq,Homo_723,MCI.vs.control; bulk RNA-seq,Homo_633,AD.vs.control; bulk RNA-	4
KEGG	hsa00190	Oxidative phosphorylation	bulk RNA-seq,Homo_723,AD.vs.control; bulk RNA-seq,Homo_723,MCI.vs.control; bulk RNA-seq,Homo_714,AD.vs.control; bulk RNA-seq,Homo_633,AD.vs.control; bulk RNA-seq,Homo_633,AD.vs.MCI; scRNA-seq,SRP215507,CD8+ T cell_8-MCI.vs.control	6
KEGG	hsa04060	Cytokine-cytokine receptor interaction	bulk RNA-seq,Homo_723,AD.vs.control; bulk RNA-seq,Homo_723,AD.vs.MCI; bulk RNA-seq,Homo_714,AD.vs.control; bulk RNA-seq,Homo_714,AD.vs.MCI; scRNA-seq,SRP330776,Monocyte_2-AD.vs.control; scRNA-seq,SRP215507,CD8+ T cell_3-AD.vs.control	6
KEGG	hsa05135	Yersinia infection	bulk RNA-seq,Homo_723,AD.vs.control; bulk RNA-seq,Homo_723,MCI.vs.control; bulk RNA-seq,Homo_714,AD.vs.control; bulk RNA-seq,Homo_633,AD.vs.control; bulk RNA-seq,Homo_633,AD.vs.MCI	5
KEGG	hsa04380	Osteoclast differentiation	bulk RNA-seq,Homo_723,AD.vs.control; bulk RNA-seq,Homo_723,MCI.vs.control; bulk RNA-seq,Homo_633,AD.vs.control; bulk RNA-seq,Homo_633,AD.vs.MCI; bulk RNA-seq,SRP223445,AD.vs.control; scRNA-seq,SRP330776,CD8+ T cell_1-AD.vs.control; scRNA-seq,SRP330776,Naive CD8+ T cell_2-AD.vs.control; scRNA-seq,SRP215507,CD8+ T cell_8-MCI.vs.control	8
KEGG	hsa04929	GnRH secretion	bulk RNA-seq,Homo_723,AD.vs.control; bulk RNA-seq,Homo_723,AD.vs.MCI; bulk RNA-seq,Homo_723,MCI.vs.control; bulk RNA-seq,Homo_714,AD.vs.MCI; bulk RNA-seq,Homo_714,MCI.vs.control; bulk RNA-seq,Homo_633,AD.vs.control; bulk RNA-seq,ROSMAP,AD.vs.MCI	7
KEGG	hsa00900	Terpenoid backbone biosynthesis	bulk RNA-seq,Homo_723,AD.vs.control; bulk RNA-seq,Homo_723,MCI.vs.control; bulk RNA-seq,Homo_714,AD.vs.control; bulk RNA-seq,Homo_714,MCI.vs.control; bulk RNA-seq,Homo_633,AD.vs.control; bulk RNA-seq,Homo_633,AD.vs.MCI	6
KEGG	hsa05165	Human papillomavirus infection	bulk RNA-seq,Homo_723,AD.vs.control; bulk RNA-seq,Homo_723,AD.vs.MCI; bulk RNA-seq,Homo_714,AD.vs.MCI; bulk RNA-seq,Homo_633,AD.vs.control; bulk RNA-seq,Homo_633,AD.vs.MCI	5
KEGG	hsa03022	Basal transcription factors	bulk RNA-seq,Homo_723,AD.vs.control; bulk RNA-seq,Homo_723,MCI.vs.control; bulk RNA-seq,Homo_714,AD.vs.control; bulk RNA-seq,Homo_714,MCI.vs.control; bulk RNA-seq,Homo_633,AD.vs.control; bulk RNA-seq,Homo_633,AD.vs.MCI	6
KEGG	hsa04611	Platelet activation	bulk RNA-seq,Homo_723,AD.vs.control; bulk RNA-seq,Homo_723,AD.vs.MCI; bulk RNA-seq,Homo_723,MCI.vs.control; bulk RNA-seq,Homo_714,AD.vs.MCI; bulk RNA-seq,Homo_714,MCI.vs.control; bulk RNA-seq,Homo_633,AD.vs.control; bulk RNA-seq,Homo_633,AD.vs.MCI; scRNA-seq,SRP309935,Monocyte_2-	8
KEGG	hsa00670	One carbon pool by folate	bulk RNA-seq,Homo_723,AD.vs.control; bulk RNA-seq,Homo_723,MCI.vs.control; bulk RNA-seq,Homo_714,AD.vs.control; bulk RNA-seq,Homo_714,MCI.vs.control; bulk RNA-seq,Homo_633,AD.vs.control; bulk RNA-seq,Homo_633,AD.vs.MCI; bulk RNA-seq,Homo_633,MCI.vs.control	7
KEGG	hsa00520	Amino sugar and nucleotide sugar metabolism	bulk RNA-seq,Homo_723,AD.vs.control; bulk RNA-seq,Homo_714,AD.vs.control; bulk RNA-seq,Homo_633,AD.vs.control; bulk RNA-seq,Homo_633,AD.vs.MCI	4
KEGG	hsa00140	Steroid hormone biosynthesis	bulk RNA-seq,Homo_723,AD.vs.control; bulk RNA-seq,Homo_723,AD.vs.MCI; bulk RNA-seq,Homo_723,MCI.vs.control; bulk RNA-seq,Homo_714,AD.vs.control; bulk RNA-seq,Homo_714,AD.vs.MCI; bulk RNA-seq,Homo_714,MCI.vs.control	6
KEGG	hsa04012	ErbB signaling pathway	bulk RNA-seq,Homo_723,AD.vs.control; bulk RNA-seq,Homo_723,AD.vs.MCI; bulk RNA-seq,Homo_723,MCI.vs.control; bulk RNA-seq,Homo_714,AD.vs.MCI; bulk RNA-seq,Homo_714,MCI.vs.control; bulk RNA-seq,Homo_633,AD.vs.control; bulk RNA-seq,Homo_633,AD.vs.MCI	7
KEGG	hsa03008	Ribosome biogenesis in eukaryotes	bulk RNA-seq,Homo_723,AD.vs.control; bulk RNA-seq,Homo_723,AD.vs.MCI; bulk RNA-seq,Homo_714,AD.vs.control; bulk RNA-seq,Homo_714,AD.vs.MCI; bulk RNA-seq,Homo_633,AD.vs.control; bulk RNA-seq,Homo_633,AD.vs.MCI	6

KEGG	hsa04913	Ovarian steroidogenesis	bulk RNA-seq,Homo_723,AD.vs.control; bulk RNA-seq,Homo_723,AD.vs.MCI; bulk RNA-seq,Homo_714,AD.vs.MCI	3
KEGG	hsa04210	Apoptosis	bulk RNA-seq,Homo_723,AD.vs.control; bulk RNA-seq,Homo_723,MCI.vs.control; bulk RNA-seq,Homo_714,AD.vs.control; bulk RNA-seq,Homo_633,AD.vs.control; bulk RNA-seq,Homo_633,AD.vs.MCI	5
KEGG	hsa01232	Nucleotide metabolism	bulk RNA-seq,Homo_723,AD.vs.control; bulk RNA-seq,Homo_723,MCI.vs.control; bulk RNA-seq,Homo_714,AD.vs.control; bulk RNA-seq,Homo_714,MCI.vs.control; bulk RNA-seq,Homo_633,AD.vs.control; bulk RNA-seq,Homo_633,AD.vs.MCI	6
KEGG	hsa04392	Hippo signaling pathway - multiple species	bulk RNA-seq,Homo_723,AD.vs.control; bulk RNA-seq,Homo_723,AD.vs.MCI; bulk RNA-seq,Homo_723,MCI.vs.control; bulk RNA-seq,Homo_714,AD.vs.MCI; bulk RNA-seq,Homo_714,MCI.vs.control; bulk RNA-seq,Homo_633,AD.vs.control; bulk RNA-seq,Homo_633,AD.vs.MCI	7
KEGG	hsa00534	Glycosaminoglycan biosynthesis - heparan sulfate / heparin	bulk RNA-seq,Homo_723,AD.vs.control; bulk RNA-seq,Homo_723,AD.vs.MCI; bulk RNA-seq,Homo_723,MCI.vs.control; bulk RNA-	4
KEGG	hsa04933	AGE-RAGE signaling pathway in diabetic complications	bulk RNA-seq,Homo_723,AD.vs.control; bulk RNA-seq,Homo_723,AD.vs.MCI; bulk RNA-seq,Homo_723,MCI.vs.control; bulk RNA-seq,Homo_714,AD.vs.MCI; bulk RNA-seq,Homo_714,MCI.vs.control; bulk RNA-seq,Homo_633,AD.vs.control; scRNA-seq,SRP330776,CD8+ T cell_1-AD.vs.control	7
KEGG	hsa05208	Chemical carcinogenesis - reactive oxygen species	bulk RNA-seq,Homo_723,AD.vs.control; bulk RNA-seq,Homo_723,MCI.vs.control; bulk RNA-seq,Homo_714,AD.vs.control; bulk RNA-seq,Homo_714,AD.vs.MCI; bulk RNA-seq,Homo_633,AD.vs.control; bulk RNA-seq,Homo_633,AD.vs.MCI; scRNA-seq,SRP215507,CD8+ T cell_8-	7
KEGG	hsa00640	Propanoate metabolism	bulk RNA-seq,Homo_723,AD.vs.control; bulk RNA-seq,Homo_714,AD.vs.control; bulk RNA-seq,Homo_714,MCI.vs.control; bulk RNA-seq,Homo_633,AD.vs.control; bulk RNA-seq,Homo_633,AD.vs.MCI	5
KEGG	hsa04940	Type I diabetes mellitus	bulk RNA-seq,Homo_723,AD.vs.control; bulk RNA-seq,Homo_723,MCI.vs.control; bulk RNA-seq,Homo_714,MCI.vs.control; bulk RNA-seq,Homo_633,AD.vs.control; bulk RNA-seq,Homo_633,AD.vs.MCI; scRNA-seq,SRP330776,Megakaryocyte_1-AD.vs.control; scRNA-seq,SRP215507,CD8+ T cell_3-AD.vs.control; scRNA-seq,SRP215507,CD8+ T cell_3-MCI.vs.control; scRNA-seq,SRP215507,CD8+ T cell_4-AD.vs.control; scRNA-	10
KEGG	hsa00514	Other types of O-glycan biosynthesis	bulk RNA-seq,Homo_723,AD.vs.control; bulk RNA-seq,Homo_723,AD.vs.MCI; bulk RNA-seq,Homo_723,MCI.vs.control; bulk RNA-seq,Homo_714,AD.vs.MCI; bulk RNA-seq,Homo_714,MCI.vs.control; bulk RNA-seq,Homo_633,AD.vs.control; bulk RNA-seq,Homo_633,AD.vs.MCI	7
KEGG	hsa04930	Type II diabetes mellitus	bulk RNA-seq,Homo_723,AD.vs.control; bulk RNA-seq,Homo_723,AD.vs.MCI; bulk RNA-seq,Homo_723,MCI.vs.control; bulk RNA-seq,Homo_714,AD.vs.MCI; bulk RNA-seq,Homo_633,AD.vs.control; bulk RNA-seq,Homo_633,AD.vs.MCI	6
KEGG	hsa04130	SNARE interactions in vesicular transport	bulk RNA-seq,Homo_723,AD.vs.control; bulk RNA-seq,Homo_714,MCI.vs.control; bulk RNA-seq,Homo_633,AD.vs.control; bulk RNA-	4
KEGG	hsa04110	Cell cycle	bulk RNA-seq,Homo_723,AD.vs.control; bulk RNA-seq,Homo_723,MCI.vs.control; bulk RNA-seq,Homo_714,AD.vs.control; bulk RNA-seq,Homo_633,AD.vs.control; bulk RNA-seq,Homo_633,AD.vs.MCI	5
KEGG	hsa00650	Butanoate metabolism	bulk RNA-seq,Homo_723,AD.vs.control; bulk RNA-seq,Homo_723,AD.vs.MCI; bulk RNA-seq,Homo_723,MCI.vs.control; bulk RNA-seq,Homo_714,AD.vs.MCI; bulk RNA-seq,Homo_714,MCI.vs.control; bulk RNA-seq,Homo_633,AD.vs.MCI	6
KEGG	hsa04530	Tight junction	bulk RNA-seq,Homo_723,AD.vs.control; bulk RNA-seq,Homo_723,AD.vs.MCI; bulk RNA-seq,Homo_723,MCI.vs.control; bulk RNA-seq,Homo_714,AD.vs.MCI; bulk RNA-seq,Homo_633,AD.vs.control; bulk RNA-seq,Homo_633,AD.vs.MCI	6
KEGG	hsa04141	Protein processing in endoplasmic reticulum	bulk RNA-seq,Homo_723,AD.vs.control; bulk RNA-seq,Homo_723,MCI.vs.control; bulk RNA-seq,Homo_714,AD.vs.control; bulk RNA-seq,Homo_633,AD.vs.control; bulk RNA-seq,Homo_633,AD.vs.MCI; scRNA-seq,SRP215507,CD8+ T cell_7-MCI.vs.control; scRNA-seq,SRP215507,CD8+ T	7
KEGG	hsa00020	Citrate cycle (TCA cycle)	bulk RNA-seq,Homo_723,AD.vs.control; bulk RNA-seq,Homo_723,MCI.vs.control; bulk RNA-seq,Homo_714,AD.vs.control; bulk RNA-seq,Homo_714,MCI.vs.control; bulk RNA-seq,Homo_633,AD.vs.control; bulk RNA-seq,Homo_633,AD.vs.MCI	6
KEGG	hsa05222	Small cell lung cancer	bulk RNA-seq,Homo_723,AD.vs.control; bulk RNA-seq,Homo_723,AD.vs.MCI; bulk RNA-seq,Homo_723,MCI.vs.control; bulk RNA-seq,Homo_714,AD.vs.MCI; bulk RNA-seq,Homo_714,MCI.vs.control; bulk RNA-seq,Homo_633,AD.vs.control; bulk RNA-seq,Homo_633,AD.vs.MCI	7
KEGG	hsa04978	Mineral absorption	bulk RNA-seq,Homo_723,AD.vs.control; bulk RNA-seq,Homo_723,AD.vs.MCI	2
KEGG	hsa00562	Inositol phosphate metabolism	bulk RNA-seq,Homo_723,AD.vs.control; bulk RNA-seq,Homo_723,MCI.vs.control; bulk RNA-seq,Homo_714,AD.vs.control; bulk RNA-seq,Homo_714,MCI.vs.control; bulk RNA-seq,Homo_633,AD.vs.control; bulk RNA-seq,Homo_633,AD.vs.MCI; scRNA-seq,SRP309935,Monocyte_2-	7
KEGG	hsa01523	Antifolate resistance	bulk RNA-seq,Homo_723,AD.vs.control; bulk RNA-seq,Homo_723,MCI.vs.control; bulk RNA-seq,Homo_714,MCI.vs.control; bulk RNA-seq,Homo_633,AD.vs.control; bulk RNA-seq,Homo_633,AD.vs.MCI; bulk RNA-seq,Homo_633,MCI.vs.control	6
KEGG	hsa04730	Long-term depression	bulk RNA-seq,Homo_723,AD.vs.control; bulk RNA-seq,Homo_723,AD.vs.MCI; bulk RNA-seq,Homo_723,MCI.vs.control; bulk RNA-seq,Homo_714,AD.vs.MCI; bulk RNA-seq,Homo_714,MCI.vs.control; bulk RNA-seq,Homo_633,AD.vs.control; bulk RNA-seq,Homo_633,AD.vs.MCI	7
KEGG	hsa04625	C-type lectin receptor signaling pathway	bulk RNA-seq,Homo_723,AD.vs.control; bulk RNA-seq,Homo_723,MCI.vs.control; bulk RNA-seq,Homo_714,AD.vs.MCI; bulk RNA-seq,Homo_714,MCI.vs.control; bulk RNA-seq,Homo_633,AD.vs.control; bulk RNA-seq,Homo_633,AD.vs.MCI; scRNA-seq,SRP215507,CD8+ T cell_9-	7
KEGG	hsa04340	Hedgehog signaling pathway	bulk RNA-seq,Homo_723,AD.vs.control; bulk RNA-seq,Homo_723,AD.vs.MCI; bulk RNA-seq,Homo_723,MCI.vs.control; bulk RNA-seq,Homo_714,AD.vs.MCI; bulk RNA-seq,Homo_714,MCI.vs.control; bulk RNA-seq,Homo_633,AD.vs.control; bulk RNA-seq,Homo_633,AD.vs.MCI	7
KEGG	hsa01212	Fatty acid metabolism	bulk RNA-seq,Homo_723,AD.vs.control; bulk RNA-seq,Homo_723,MCI.vs.control; bulk RNA-seq,Homo_714,AD.vs.control; bulk RNA-seq,Homo_714,MCI.vs.control; bulk RNA-seq,Homo_633,AD.vs.control; bulk RNA-seq,Homo_633,AD.vs.MCI	6
KEGG	hsa00601	Glycosphingolipid biosynthesis - lacto and neolacto series	bulk RNA-seq,Homo_723,AD.vs.control; bulk RNA-seq,Homo_723,AD.vs.MCI; bulk RNA-seq,Homo_633,AD.vs.control; bulk RNA-seq,Homo_633,AD.vs.MCI	4
KEGG	hsa04350	TGF-beta signaling pathway	bulk RNA-seq,Homo_723,AD.vs.MCI; bulk RNA-seq,Homo_723,MCI.vs.control; bulk RNA-seq,Homo_714,AD.vs.MCI; bulk RNA-seq,Homo_714,MCI.vs.control; bulk RNA-seq,Homo_633,AD.vs.control; bulk RNA-seq,Homo_633,AD.vs.MCI	6
KEGG	hsa05418	Fluid shear stress and atherosclerosis	bulk RNA-seq,Homo_723,AD.vs.MCI; bulk RNA-seq,Homo_723,MCI.vs.control; bulk RNA-seq,Homo_714,AD.vs.MCI; bulk RNA-seq,Homo_633,AD.vs.control; bulk RNA-seq,Homo_633,AD.vs.MCI	5
KEGG	hsa04062	Chemokine signaling pathway	bulk RNA-seq,Homo_723,AD.vs.MCI; bulk RNA-seq,Homo_723,MCI.vs.control; bulk RNA-seq,Homo_714,AD.vs.MCI; bulk RNA-seq,Homo_633,AD.vs.control; bulk RNA-seq,Homo_633,AD.vs.MCI	5
KEGG	hsa05022	Pathways of neurodegeneration - multiple diseases	bulk RNA-seq,Homo_723,AD.vs.MCI; bulk RNA-seq,Homo_714,AD.vs.control; bulk RNA-seq,Homo_714,AD.vs.MCI; bulk RNA-seq,Homo_633,AD.vs.control; bulk RNA-seq,Homo_633,AD.vs.MCI; scRNA-seq,SRP215507,CD8+ T cell_7-MCI.vs.control; scRNA-seq,SRP215507,CD8+ T cell_8-MCI.vs.control	7
KEGG	hsa00350	Tyrosine metabolism	bulk RNA-seq,Homo_723,AD.vs.MCI; bulk RNA-seq,Homo_723,MCI.vs.control; bulk RNA-seq,Homo_714,AD.vs.MCI; bulk RNA-seq,Homo_714,MCI.vs.control	4
KEGG	hsa05215	Prostate cancer	bulk RNA-seq,Homo_723,AD.vs.MCI; bulk RNA-seq,Homo_723,MCI.vs.control; bulk RNA-seq,Homo_714,AD.vs.MCI; bulk RNA-seq,Homo_714,MCI.vs.control; bulk RNA-seq,Homo_633,AD.vs.control; bulk RNA-seq,Homo_633,AD.vs.MCI	6
KEGG	hsa05225	Hepatocellular carcinoma	bulk RNA-seq,Homo_723,AD.vs.MCI; bulk RNA-seq,Homo_723,MCI.vs.control; bulk RNA-seq,Homo_714,AD.vs.MCI; bulk RNA-seq,Homo_633,AD.vs.control; bulk RNA-seq,Homo_633,AD.vs.MCI; scRNA-seq,SRP215507,CD8+ T cell_8-MCI.vs.control	6
KEGG	hsa05202	Transcriptional misregulation in cancer	bulk RNA-seq,Homo_723,AD.vs.MCI; bulk RNA-seq,Homo_723,MCI.vs.control; bulk RNA-seq,Homo_714,AD.vs.MCI; bulk RNA-seq,Homo_633,AD.vs.control; bulk RNA-seq,Homo_633,AD.vs.MCI	5
KEGG	hsa04750	Inflammatory mediator regulation of TRP channels	bulk RNA-seq,Homo_723,AD.vs.MCI; bulk RNA-seq,Homo_723,MCI.vs.control; bulk RNA-seq,Homo_714,AD.vs.MCI; bulk RNA-seq,Homo_714,MCI.vs.control; bulk RNA-seq,Homo_633,AD.vs.control; bulk RNA-seq,Homo_633,AD.vs.MCI; bulk RNA-seq,ROSMAP,AD.vs.MCI	7

KEGG	hsa03320	PPAR signaling pathway	bulk RNA-seq,Homo_723,AD.vs.MCI; bulk RNA-seq,Homo_723,MCI.vs.control; bulk RNA-seq,Homo_714,AD.vs.MCI; bulk RNA-seq,Homo_714,MCI.vs.control; bulk RNA-seq,Homo_633,AD.vs.control; bulk RNA-seq,Homo_633,AD.vs.MCI	6
KEGG	hsa01522	Endocrine resistance	bulk RNA-seq,Homo_723,AD.vs.MCI; bulk RNA-seq,Homo_723,MCI.vs.control; bulk RNA-seq,Homo_714,AD.vs.MCI; bulk RNA-seq,Homo_714,MCI.vs.control; bulk RNA-seq,Homo_633,AD.vs.control; bulk RNA-seq,Homo_633,AD.vs.MCI; bulk RNA-seq,ROSMAP,AD.vs.MCI	7
KEGG	hsa04371	Apelin signaling pathway	bulk RNA-seq,Homo_723,AD.vs.MCI; bulk RNA-seq,Homo_723,MCI.vs.control; bulk RNA-seq,Homo_714,AD.vs.MCI; bulk RNA-seq,Homo_633,AD.vs.control; bulk RNA-seq,Homo_633,AD.vs.MCI; bulk RNA-seq,ROSMAP,AD.vs.control; bulk RNA-seq,SRP223445,AD.vs.control	7
KEGG	hsa05223	Non-small cell lung cancer	bulk RNA-seq,Homo_723,AD.vs.MCI; bulk RNA-seq,Homo_723,MCI.vs.control; bulk RNA-seq,Homo_714,AD.vs.MCI; bulk RNA-seq,Homo_714,MCI.vs.control; bulk RNA-seq,Homo_633,AD.vs.control; bulk RNA-seq,Homo_633,AD.vs.MCI	6
KEGG	hsa05120	Epithelial cell signaling in Helicobacter pylori infection	bulk RNA-seq,Homo_723,AD.vs.MCI; bulk RNA-seq,Homo_723,MCI.vs.control; bulk RNA-seq,Homo_714,AD.vs.MCI; bulk RNA-seq,Homo_714,MCI.vs.control; bulk RNA-seq,Homo_633,AD.vs.control; bulk RNA-seq,Homo_633,AD.vs.MCI	6
KEGG	hsa05130	Pathogenic Escherichia coli infection	bulk RNA-seq,Homo_723,AD.vs.MCI; bulk RNA-seq,Homo_723,MCI.vs.control; bulk RNA-seq,Homo_714,AD.vs.MCI; bulk RNA-seq,Homo_633,AD.vs.control; bulk RNA-seq,Homo_633,AD.vs.MCI	5
KEGG	hsa05163	Human cytomegalovirus infection	bulk RNA-seq,Homo_723,AD.vs.MCI; bulk RNA-seq,Homo_723,MCI.vs.control; bulk RNA-seq,Homo_714,AD.vs.MCI; bulk RNA-seq,Homo_633,AD.vs.control; bulk RNA-seq,Homo_633,AD.vs.MCI; scRNA-seq,SRP215507,CD8+ T cell_8-MCI.vs.control	6
KEGG	hsa05214	Glioma	bulk RNA-seq,Homo_723,AD.vs.MCI; bulk RNA-seq,Homo_723,MCI.vs.control; bulk RNA-seq,Homo_714,AD.vs.MCI; bulk RNA-seq,Homo_714,MCI.vs.control; bulk RNA-seq,Homo_633,AD.vs.control; bulk RNA-seq,Homo_633,AD.vs.MCI	6
KEGG	hsa04923	Regulation of lipolysis in adipocytes	bulk RNA-seq,Homo_723,AD.vs.MCI; bulk RNA-seq,Homo_723,MCI.vs.control; bulk RNA-seq,Homo_714,AD.vs.MCI; bulk RNA-seq,Homo_714,MCI.vs.control	4
KEGG	hsa04148	Efferocytosis	bulk RNA-seq,Homo_723,AD.vs.MCI; bulk RNA-seq,Homo_723,MCI.vs.control; bulk RNA-seq,Homo_714,AD.vs.MCI; bulk RNA-seq,Homo_633,AD.vs.control; bulk RNA-seq,Homo_633,AD.vs.MCI	5
KEGG	hsa05417	Lipid and atherosclerosis	bulk RNA-seq,Homo_723,AD.vs.MCI; bulk RNA-seq,Homo_723,MCI.vs.control; bulk RNA-seq,Homo_714,AD.vs.MCI; bulk RNA-seq,Homo_633,AD.vs.control; bulk RNA-seq,Homo_633,AD.vs.MCI	5
KEGG	hsa04722	Neurotrophin signaling pathway	bulk RNA-seq,Homo_723,AD.vs.MCI; bulk RNA-seq,Homo_723,MCI.vs.control; bulk RNA-seq,Homo_714,AD.vs.MCI; bulk RNA-seq,Homo_714,MCI.vs.control; bulk RNA-seq,Homo_633,AD.vs.control; bulk RNA-seq,Homo_633,AD.vs.MCI; bulk RNA-seq,ROSMAP,AD.vs.MCI	7
KEGG	hsa04917	Prolactin signaling pathway	bulk RNA-seq,Homo_723,AD.vs.MCI; bulk RNA-seq,Homo_723,MCI.vs.control; bulk RNA-seq,Homo_714,AD.vs.MCI; bulk RNA-seq,Homo_714,MCI.vs.control; bulk RNA-seq,Homo_633,AD.vs.control; bulk RNA-seq,Homo_633,AD.vs.MCI	6
KEGG	hsa04919	Thyroid hormone signaling pathway	bulk RNA-seq,Homo_723,AD.vs.MCI; bulk RNA-seq,Homo_723,MCI.vs.control; bulk RNA-seq,Homo_714,AD.vs.MCI; bulk RNA-seq,Homo_714,MCI.vs.control; bulk RNA-seq,Homo_633,AD.vs.control; bulk RNA-seq,Homo_633,AD.vs.MCI	6
KEGG	hsa04973	Carbohydrate digestion and absorption	bulk RNA-seq,Homo_723,AD.vs.MCI; bulk RNA-seq,Homo_723,MCI.vs.control; bulk RNA-seq,Homo_714,AD.vs.MCI; bulk RNA-seq,Homo_714,MCI.vs.control; bulk RNA-seq,Homo_633,AD.vs.control	5
KEGG	hsa05142	Chagas disease	bulk RNA-seq,Homo_723,AD.vs.MCI; bulk RNA-seq,Homo_723,MCI.vs.control; bulk RNA-seq,Homo_714,AD.vs.MCI; bulk RNA-seq,Homo_714,MCI.vs.control; bulk RNA-seq,Homo_633,AD.vs.control; bulk RNA-seq,Homo_633,AD.vs.MCI; scRNA-seq,SRP215507,CD8+ T cell_3-	7
KEGG	hsa04960	Aldosterone-regulated sodium reabsorption	bulk RNA-seq,Homo_723,AD.vs.MCI; bulk RNA-seq,Homo_723,MCI.vs.control; bulk RNA-seq,Homo_714,AD.vs.MCI; bulk RNA-seq,Homo_714,MCI.vs.control; bulk RNA-seq,ROSMAP,AD.vs.MCI	5
KEGG	hsa05213	Endometrial cancer	bulk RNA-seq,Homo_723,AD.vs.MCI; bulk RNA-seq,Homo_723,MCI.vs.control; bulk RNA-seq,Homo_714,AD.vs.MCI; bulk RNA-seq,Homo_714,MCI.vs.control; bulk RNA-seq,Homo_633,AD.vs.control; bulk RNA-seq,Homo_633,AD.vs.MCI	6
KEGG	hsa00330	Arginine and proline metabolism	bulk RNA-seq,Homo_723,AD.vs.MCI; bulk RNA-seq,Homo_723,MCI.vs.control; bulk RNA-seq,Homo_633,AD.vs.control	3
KEGG	hsa05144	Malaria	bulk RNA-seq,Homo_723,AD.vs.MCI; bulk RNA-seq,Homo_723,MCI.vs.control; bulk RNA-seq,Homo_714,AD.vs.MCI; bulk RNA-seq,Homo_714,MCI.vs.control; bulk RNA-seq,Homo_633,AD.vs.control; bulk RNA-seq,Homo_633,AD.vs.MCI	6
KEGG	hsa05231	Choline metabolism in cancer	bulk RNA-seq,Homo_723,AD.vs.MCI; bulk RNA-seq,Homo_723,MCI.vs.control; bulk RNA-seq,Homo_714,AD.vs.MCI; bulk RNA-seq,Homo_714,MCI.vs.control; bulk RNA-seq,Homo_633,AD.vs.control; bulk RNA-seq,Homo_633,AD.vs.MCI	6
KEGG	hsa05171	Coronavirus disease - COVID-19	bulk RNA-seq,Homo_723,AD.vs.MCI; bulk RNA-seq,Homo_723,MCI.vs.control; bulk RNA-seq,Homo_714,AD.vs.MCI; bulk RNA-seq,Homo_633,AD.vs.control; bulk RNA-seq,Homo_633,AD.vs.MCI; scRNA-seq,SRP330776,Megakaryocyte_2-AD.vs.control; scRNA-seq,SRP215507,CD8+ T cell_4-AD.vs.control	7
KEGG	hsa00260	Glycine, serine and threonine metabolism	bulk RNA-seq,Homo_723,AD.vs.MCI; bulk RNA-seq,Homo_723,MCI.vs.control; bulk RNA-seq,Homo_714,MCI.vs.control; bulk RNA-seq,Homo_633,AD.vs.control; bulk RNA-seq,Homo_633,AD.vs.MCI	5
KEGG	hsa04961	Endocrine and other factor-regulated calcium reabsorption	bulk RNA-seq,Homo_723,AD.vs.MCI; bulk RNA-seq,Homo_723,MCI.vs.control; bulk RNA-seq,Homo_714,AD.vs.MCI; bulk RNA-seq,Homo_714,MCI.vs.control	4
KEGG	hsa04966	Collecting duct acid secretion	bulk RNA-seq,Homo_723,AD.vs.MCI; bulk RNA-seq,Homo_633,AD.vs.control; bulk RNA-seq,Homo_633,AD.vs.MCI	3
KEGG	hsa05219	Bladder cancer	bulk RNA-seq,Homo_723,AD.vs.MCI; bulk RNA-seq,Homo_723,MCI.vs.control; bulk RNA-seq,Homo_714,AD.vs.MCI; bulk RNA-seq,Homo_714,MCI.vs.control; bulk RNA-seq,Homo_633,AD.vs.control; bulk RNA-seq,Homo_633,AD.vs.MCI	6
KEGG	hsa04061	Viral protein interaction with cytokine and cytokine receptor	bulk RNA-seq,Homo_723,AD.vs.MCI; bulk RNA-seq,Homo_723,MCI.vs.control; bulk RNA-seq,Homo_714,AD.vs.MCI; bulk RNA-seq,Homo_714,MCI.vs.control; scRNA-seq,SRP330776,Monocyte_2-AD.vs.control; scRNA-seq,SRP215507,CD8+ T cell_8-MCI.vs.control	6
KEGG	hsa04936	Alcoholic liver disease	bulk RNA-seq,Homo_723,AD.vs.MCI; bulk RNA-seq,Homo_723,MCI.vs.control; bulk RNA-seq,Homo_714,AD.vs.MCI; bulk RNA-seq,Homo_633,AD.vs.control; bulk RNA-seq,Homo_633,AD.vs.MCI	5
KEGG	hsa00010	Glycolysis / Gluconeogenesis	bulk RNA-seq,Homo_723,AD.vs.MCI; bulk RNA-seq,Homo_723,MCI.vs.control; bulk RNA-seq,Homo_714,AD.vs.MCI; bulk RNA-seq,Homo_714,MCI.vs.control; bulk RNA-seq,Homo_633,AD.vs.control; bulk RNA-seq,Homo_633,AD.vs.MCI	6
KEGG	hsa04931	Insulin resistance	bulk RNA-seq,Homo_723,AD.vs.MCI; bulk RNA-seq,Homo_723,MCI.vs.control; bulk RNA-seq,Homo_714,AD.vs.MCI; bulk RNA-seq,Homo_714,MCI.vs.control; bulk RNA-seq,Homo_633,AD.vs.control; bulk RNA-seq,Homo_633,AD.vs.MCI	6
KEGG	hsa05110	Vibrio cholerae infection	bulk RNA-seq,Homo_723,AD.vs.MCI; bulk RNA-seq,Homo_723,MCI.vs.control; bulk RNA-seq,Homo_714,MCI.vs.control; bulk RNA-seq,Homo_633,AD.vs.control; bulk RNA-seq,Homo_633,AD.vs.MCI; scRNA-seq,SRP215507,CD8+ T cell_8-MCI.vs.control	6
KEGG	hsa00380	Tryptophan metabolism	bulk RNA-seq,Homo_723,AD.vs.MCI; bulk RNA-seq,Homo_723,MCI.vs.control; bulk RNA-seq,Homo_714,AD.vs.MCI; bulk RNA-seq,Homo_714,MCI.vs.control; bulk RNA-seq,Homo_633,AD.vs.control; scRNA-seq,SRP215507,CD8+ T cell_8-AD.vs.control	6
KEGG	hsa04066	HIF-1 signaling pathway	bulk RNA-seq,Homo_723,AD.vs.MCI; bulk RNA-seq,Homo_723,MCI.vs.control; bulk RNA-seq,Homo_714,AD.vs.MCI; bulk RNA-seq,Homo_714,MCI.vs.control; bulk RNA-seq,Homo_633,AD.vs.control; bulk RNA-seq,Homo_633,AD.vs.MCI	6
KEGG	hsa04630	JAK-STAT signaling pathway	bulk RNA-seq,Homo_723,AD.vs.MCI; bulk RNA-seq,Homo_723,MCI.vs.control; bulk RNA-seq,Homo_714,AD.vs.control; bulk RNA-seq,Homo_714,AD.vs.MCI	4
KEGG	hsa04068	FoxO signaling pathway	bulk RNA-seq,Homo_723,AD.vs.MCI; bulk RNA-seq,Homo_723,MCI.vs.control; bulk RNA-seq,Homo_714,MCI.vs.control; bulk RNA-seq,Homo_633,AD.vs.control; bulk RNA-seq,Homo_633,AD.vs.MCI	5

KEGG	hsa04910	Insulin signaling pathway	bulk RNA-seq,Homo_723,AD.vs.MCI; bulk RNA-seq,Homo_723,MCI.vs.control; bulk RNA-seq,Homo_714,AD.vs.MCI; bulk RNA-seq,Homo_633,AD.vs.control; bulk RNA-seq,Homo_633,AD.vs.MCI	5
KEGG	hsa04668	TNF signaling pathway	bulk RNA-seq,Homo_723,AD.vs.MCI; bulk RNA-seq,Homo_723,MCI.vs.control; bulk RNA-seq,Homo_714,AD.vs.MCI; bulk RNA-seq,Homo_714,MCI.vs.control; bulk RNA-seq,Homo_633,AD.vs.control; bulk RNA-seq,Homo_633,AD.vs.MCI; scRNA-seq,SRP330776,CD8+ T cell_1-	8
KEGG	hsa04914	Progesterone-mediated oocyte maturation	bulk RNA-seq,Homo_723,AD.vs.MCI; bulk RNA-seq,Homo_723,MCI.vs.control; bulk RNA-seq,Homo_714,MCI.vs.control; bulk RNA-seq,Homo_633,AD.vs.control; bulk RNA-seq,Homo_633,AD.vs.MCI	5
KEGG	hsa04657	IL-17 signaling pathway	bulk RNA-seq,Homo_723,AD.vs.MCI; bulk RNA-seq,Homo_723,MCI.vs.control; bulk RNA-seq,Homo_714,AD.vs.control; bulk RNA-seq,Homo_714,AD.vs.MCI; bulk RNA-seq,Homo_714,MCI.vs.control; bulk RNA-seq,Homo_633,AD.vs.control; bulk RNA-seq,Homo_633,AD.vs.MCI; bulk RNA-	8
KEGG	hsa05010	Alzheimer disease	bulk RNA-seq,Homo_723,AD.vs.MCI; bulk RNA-seq,Homo_714,AD.vs.control; bulk RNA-seq,Homo_633,AD.vs.control; bulk RNA-seq,Homo_633,AD.vs.MCI; scRNA-seq,SRP215507,CD8+ T cell_7-MCI.vs.control; scRNA-seq,SRP215507,CD8+ T cell_8-MCI.vs.control	6
KEGG	hsa05143	African trypanosomiasis	bulk RNA-seq,Homo_723,AD.vs.MCI; bulk RNA-seq,Homo_723,MCI.vs.control; bulk RNA-seq,Homo_714,AD.vs.MCI; bulk RNA-seq,Homo_714,MCI.vs.control	4
KEGG	hsa00340	Histidine metabolism	bulk RNA-seq,Homo_723,AD.vs.MCI; bulk RNA-seq,Homo_723,MCI.vs.control; bulk RNA-seq,Homo_714,AD.vs.MCI; bulk RNA-seq,Homo_714,MCI.vs.control; bulk RNA-seq,Homo_633,AD.vs.control	5
KEGG	hsa00230	Purine metabolism	bulk RNA-seq,Homo_723,AD.vs.MCI; bulk RNA-seq,Homo_723,MCI.vs.control; bulk RNA-seq,Homo_714,AD.vs.MCI; bulk RNA-seq,Homo_714,MCI.vs.control; bulk RNA-seq,Homo_633,AD.vs.control; bulk RNA-seq,Homo_633,AD.vs.MCI	6
KEGG	hsa05145	Toxoplasmosis	bulk RNA-seq,Homo_723,AD.vs.MCI; bulk RNA-seq,Homo_723,MCI.vs.control; bulk RNA-seq,Homo_714,MCI.vs.control; bulk RNA-seq,Homo_633,AD.vs.control; bulk RNA-seq,Homo_633,AD.vs.MCI; scRNA-seq,SRP330776,Megakaryocyte_1-AD.vs.control; scRNA-seq,SRP215507,CD8+ T cell_3-AD.vs.control; scRNA-seq,SRP215507,CD8+ T cell_3-MCI.vs.control	8
KEGG	hsa04071	Sphingolipid signaling pathway	bulk RNA-seq,Homo_723,AD.vs.MCI; bulk RNA-seq,Homo_723,MCI.vs.control; bulk RNA-seq,Homo_714,MCI.vs.control; bulk RNA-seq,Homo_633,AD.vs.control; bulk RNA-seq,Homo_633,AD.vs.MCI; scRNA-seq,SRP330776,CD8+ T cell_1-AD.vs.control; scRNA-seq,SRP330776,Natural killer	7
KEGG	hsa00561	Glycerolipid metabolism	bulk RNA-seq,Homo_723,AD.vs.MCI; bulk RNA-seq,Homo_714,AD.vs.MCI; bulk RNA-seq,Homo_633,AD.vs.control; bulk RNA-seq,Homo_633,AD.vs.MCI	4
KEGG	hsa00250	Alanine, aspartate and glutamate metabolism	bulk RNA-seq,Homo_723,AD.vs.MCI; bulk RNA-seq,Homo_723,MCI.vs.control; bulk RNA-seq,Homo_714,MCI.vs.control; bulk RNA-seq,Homo_633,AD.vs.control; bulk RNA-seq,Homo_633,AD.vs.MCI	5
KEGG	hsa04922	Glucagon signaling pathway	bulk RNA-seq,Homo_723,AD.vs.MCI; bulk RNA-seq,Homo_723,MCI.vs.control; bulk RNA-seq,Homo_714,AD.vs.MCI; bulk RNA-seq,Homo_714,MCI.vs.control; bulk RNA-seq,Homo_633,AD.vs.control; bulk RNA-seq,Homo_633,AD.vs.MCI	6
KEGG	hsa05230	Central carbon metabolism in cancer	bulk RNA-seq,Homo_723,AD.vs.MCI; bulk RNA-seq,Homo_723,MCI.vs.control; bulk RNA-seq,Homo_714,MCI.vs.control; bulk RNA-seq,Homo_633,AD.vs.control; bulk RNA-seq,Homo_633,AD.vs.MCI	5
KEGG	hsa05212	Pancreatic cancer	bulk RNA-seq,Homo_723,AD.vs.MCI; bulk RNA-seq,Homo_723,MCI.vs.control; bulk RNA-seq,Homo_714,AD.vs.MCI; bulk RNA-seq,Homo_714,MCI.vs.control; bulk RNA-seq,Homo_633,AD.vs.control; bulk RNA-seq,Homo_633,AD.vs.MCI	6
KEGG	hsa05416	Viral myocarditis	bulk RNA-seq,Homo_723,AD.vs.MCI; bulk RNA-seq,Homo_723,MCI.vs.control; bulk RNA-seq,Homo_714,AD.vs.MCI; bulk RNA-seq,Homo_714,MCI.vs.control; bulk RNA-seq,Homo_633,AD.vs.control; bulk RNA-seq,Homo_633,AD.vs.MCI; scRNA-seq,SRP330776,Megakaryocyte_1-AD.vs.control; scRNA-seq,SRP215507,CD8+ T cell_3-AD.vs.control; scRNA-seq,SRP215507,CD8+ T cell_3-MCI.vs.control	9
KEGG	hsa05161	Hepatitis B	bulk RNA-seq,Homo_723,AD.vs.MCI; bulk RNA-seq,Homo_723,MCI.vs.control; bulk RNA-seq,Homo_714,AD.vs.MCI; bulk RNA-seq,Homo_633,AD.vs.control; bulk RNA-seq,Homo_633,AD.vs.MCI	5
KEGG	hsa04150	mTOR signaling pathway	bulk RNA-seq,Homo_723,AD.vs.MCI; bulk RNA-seq,Homo_723,MCI.vs.control; bulk RNA-seq,Homo_714,AD.vs.control; bulk RNA-seq,Homo_633,AD.vs.control; bulk RNA-seq,Homo_633,AD.vs.MCI	5
KEGG	hsa00310	Lysine degradation	bulk RNA-seq,Homo_723,AD.vs.MCI; bulk RNA-seq,Homo_723,MCI.vs.control; bulk RNA-seq,Homo_714,AD.vs.MCI; bulk RNA-seq,Homo_714,MCI.vs.control; bulk RNA-seq,Homo_633,AD.vs.control; bulk RNA-seq,Homo_633,AD.vs.MCI	6
KEGG	hsa04152	AMPK signaling pathway	bulk RNA-seq,Homo_723,AD.vs.MCI; bulk RNA-seq,Homo_723,MCI.vs.control; bulk RNA-seq,Homo_714,AD.vs.control; bulk RNA-seq,Homo_714,MCI.vs.control; bulk RNA-seq,Homo_633,AD.vs.control; bulk RNA-seq,Homo_633,AD.vs.MCI	6
KEGG	hsa04114	Oocyte meiosis	bulk RNA-seq,Homo_723,AD.vs.MCI; bulk RNA-seq,Homo_723,MCI.vs.control; bulk RNA-seq,Homo_633,AD.vs.control; bulk RNA-seq,Homo_633,AD.vs.MCI	4
KEGG	hsa05131	Shigellosis	bulk RNA-seq,Homo_723,MCI.vs.control; bulk RNA-seq,Homo_714,AD.vs.control; bulk RNA-seq,Homo_714,AD.vs.MCI; bulk RNA-seq,Homo_633,AD.vs.control; bulk RNA-seq,Homo_633,AD.vs.MCI	5
KEGG	hsa04218	Cellular senescence	bulk RNA-seq,Homo_723,MCI.vs.control; bulk RNA-seq,Homo_633,AD.vs.control; bulk RNA-seq,Homo_633,AD.vs.MCI; scRNA-seq,SRP215507,CD8+ T	4
KEGG	hsa05170	Human immunodeficiency virus 1 infection	bulk RNA-seq,Homo_723,MCI.vs.control; bulk RNA-seq,Homo_633,AD.vs.control; bulk RNA-seq,Homo_633,AD.vs.MCI	3
KEGG	hsa03083	Polycomb repressive complex	bulk RNA-seq,Homo_723,MCI.vs.control; bulk RNA-seq,Homo_714,AD.vs.MCI; bulk RNA-seq,Homo_714,MCI.vs.control; bulk RNA-seq,Homo_633,AD.vs.control; bulk RNA-seq,Homo_633,AD.vs.MCI	5
KEGG	hsa05162	Measles	bulk RNA-seq,Homo_723,MCI.vs.control; bulk RNA-seq,Homo_714,AD.vs.MCI; bulk RNA-seq,Homo_633,AD.vs.control; bulk RNA-seq,Homo_633,AD.vs.MCI; scRNA-seq,SRP215507,CD8+ T cell_8-MCI.vs.control	5
KEGG	hsa05020	Prion disease	bulk RNA-seq,Homo_723,MCI.vs.control; bulk RNA-seq,Homo_714,AD.vs.control; bulk RNA-seq,Homo_633,AD.vs.control; bulk RNA-seq,Homo_633,AD.vs.MCI; scRNA-seq,SRP215507,CD8+ T cell_8-MCI.vs.control	5
KEGG	hsa05167	Kaposi sarcoma-associated herpesvirus infection	bulk RNA-seq,Homo_723,MCI.vs.control; bulk RNA-seq,Homo_714,AD.vs.MCI; bulk RNA-seq,Homo_633,AD.vs.control; bulk RNA-seq,Homo_633,AD.vs.MCI; scRNA-seq,SRP215507,CD8+ T cell_8-MCI.vs.control	5
KEGG	hsa04145	Phagosome	bulk RNA-seq,Homo_723,MCI.vs.control; bulk RNA-seq,Homo_714,AD.vs.MCI; bulk RNA-seq,Homo_633,AD.vs.control; bulk RNA-seq,Homo_633,AD.vs.MCI; bulk RNA-seq,SRP223445,AD.vs.control; scRNA-seq,SRP215507,CD8+ T cell_8-MCI.vs.control	6
KEGG	hsa01240	Biosynthesis of cofactors	bulk RNA-seq,Homo_723,MCI.vs.control; bulk RNA-seq,Homo_714,AD.vs.MCI; bulk RNA-seq,Homo_633,AD.vs.control; bulk RNA-seq,Homo_633,AD.vs.MCI; scRNA-seq,SRP330776,Megakaryocyte_1-AD.vs.control	5
KEGG	hsa00760	Nicotinate and nicotinamide metabolism	bulk RNA-seq,Homo_723,MCI.vs.control; bulk RNA-seq,Homo_714,MCI.vs.control; bulk RNA-seq,Homo_633,AD.vs.control; bulk RNA-	4
KEGG	hsa05203	Viral carcinogenesis	bulk RNA-seq,Homo_723,MCI.vs.control; bulk RNA-seq,Homo_714,AD.vs.MCI; bulk RNA-seq,Homo_633,AD.vs.control; bulk RNA-seq,Homo_633,AD.vs.MCI	4
KEGG	hsa03013	Nucleocytoplasmic transport	bulk RNA-seq,Homo_723,MCI.vs.control; bulk RNA-seq,Homo_714,MCI.vs.control; bulk RNA-seq,Homo_633,AD.vs.control; bulk RNA-	4
KEGG	hsa05216	Thyroid cancer	bulk RNA-seq,Homo_723,MCI.vs.control; bulk RNA-seq,Homo_714,MCI.vs.control; bulk RNA-seq,Homo_633,AD.vs.control; bulk RNA-seq,Homo_633,AD.vs.MCI; scRNA-seq,SRP215507,CD8+ T cell_3-AD.vs.control	5
KEGG	hsa04370	VEGF signaling pathway	bulk RNA-seq,Homo_723,MCI.vs.control; bulk RNA-seq,Homo_714,MCI.vs.control; bulk RNA-seq,Homo_633,AD.vs.control; bulk RNA-	4

KEGG	hsa04920	Adipocytokine signaling pathway	bulk RNA-seq,Homo_723,MCI.vs.control; bulk RNA-seq,Homo_714,MCI.vs.control; bulk RNA-seq,Homo_633,AD.vs.control; bulk RNA-seq,Homo_633,AD.vs.MCI; scRNA-seq,SRP215507,CD8+ T cell_8-AD.vs.MCI	5
KEGG	hsa05210	Colorectal cancer	bulk RNA-seq,Homo_723,MCI.vs.control; bulk RNA-seq,Homo_714,MCI.vs.control; bulk RNA-seq,Homo_633,AD.vs.control; bulk RNA-seq,Homo_633,AD.vs.MCI; scRNA-seq,SRP215507,CD8+ T cell_8-MCI.vs.control	5
KEGG	hsa05160	Hepatitis C	bulk RNA-seq,Homo_723,MCI.vs.control; bulk RNA-seq,Homo_714,AD.vs.MCI; bulk RNA-seq,Homo_633,AD.vs.control; bulk RNA-seq,Homo_633,AD.vs.MCI	4
KEGG	hsa04330	Notch signaling pathway	bulk RNA-seq,Homo_723,MCI.vs.control; bulk RNA-seq,Homo_714,MCI.vs.control; bulk RNA-seq,Homo_633,AD.vs.control; bulk RNA-	4
KEGG	hsa00620	Pyruvate metabolism	bulk RNA-seq,Homo_723,MCI.vs.control; bulk RNA-seq,Homo_714,AD.vs.MCI; bulk RNA-seq,Homo_714,MCI.vs.control; bulk RNA-seq,Homo_633,AD.vs.control; bulk RNA-seq,Homo_633,AD.vs.MCI	5
KEGG	hsa05012	Parkinson disease	bulk RNA-seq,Homo_723,MCI.vs.control; bulk RNA-seq,Homo_714,AD.vs.control; bulk RNA-seq,Homo_633,AD.vs.control; bulk RNA-seq,Homo_633,AD.vs.MCI; scRNA-seq,SRP215507,CD8+ T cell_7-MCI.vs.control; scRNA-seq,SRP215507,CD8+ T cell_8-MCI.vs.control	6
KEGG	hsa05134	Legionellosis	bulk RNA-seq,Homo_723,MCI.vs.control; bulk RNA-seq,Homo_714,MCI.vs.control; bulk RNA-seq,Homo_633,AD.vs.control; bulk RNA-seq,Homo_633,AD.vs.MCI; bulk RNA-seq,ROSMAP,MCI.vs.control	5
KEGG	hsa04070	Phosphatidylinositol signaling system	bulk RNA-seq,Homo_723,MCI.vs.control; bulk RNA-seq,Homo_714,MCI.vs.control; bulk RNA-seq,Homo_633,AD.vs.control; bulk RNA-seq,Homo_633,AD.vs.MCI; scRNA-seq,SRP309935,Monocyte_2-AD.vs.control	5
KEGG	hsa04613	Neutrophil extracellular trap formation	bulk RNA-seq,Homo_723,MCI.vs.control; bulk RNA-seq,Homo_714,AD.vs.MCI; bulk RNA-seq,Homo_633,AD.vs.control; bulk RNA-seq,Homo_633,AD.vs.MCI; bulk RNA-seq,SRP223445,AD.vs.control	5
KEGG	hsa05415	Diabetic cardiomyopathy	bulk RNA-seq,Homo_723,MCI.vs.control; bulk RNA-seq,Homo_633,AD.vs.control; bulk RNA-seq,Homo_633,AD.vs.MCI; scRNA-seq,SRP215507,CD8+ T	4
KEGG	hsa04211	Longevity regulating pathway	bulk RNA-seq,Homo_723,MCI.vs.control; bulk RNA-seq,Homo_714,AD.vs.control; bulk RNA-seq,Homo_714,MCI.vs.control; bulk RNA-seq,Homo_633,AD.vs.control; bulk RNA-seq,Homo_633,AD.vs.MCI	5
KEGG	hsa05133	Pertussis	bulk RNA-seq,Homo_723,MCI.vs.control; bulk RNA-seq,Homo_714,MCI.vs.control; bulk RNA-seq,Homo_633,AD.vs.control; bulk RNA-	4
KEGG	hsa04666	Fc gamma R-mediated phagocytosis	bulk RNA-seq,Homo_723,MCI.vs.control; bulk RNA-seq,Homo_714,MCI.vs.control; bulk RNA-seq,Homo_633,AD.vs.control; bulk RNA-seq,Homo_633,AD.vs.MCI; scRNA-seq,SRP330776,B cell_2-AD.vs.control	5
KEGG	hsa05221	Acute myeloid leukemia	bulk RNA-seq,Homo_723,MCI.vs.control; bulk RNA-seq,Homo_714,MCI.vs.control; bulk RNA-seq,Homo_633,AD.vs.control; bulk RNA-	4
KEGG	hsa00280	Valine, leucine and isoleucine degradation	bulk RNA-seq,Homo_723,MCI.vs.control; bulk RNA-seq,Homo_714,MCI.vs.control; bulk RNA-seq,Homo_633,AD.vs.control; bulk RNA-	4
KEGG	hsa00071	Fatty acid degradation	bulk RNA-seq,Homo_723,MCI.vs.control; bulk RNA-seq,Homo_714,MCI.vs.control; bulk RNA-seq,Homo_633,AD.vs.control; bulk RNA-	4
KEGG	hsa04115	p53 signaling pathway	bulk RNA-seq,Homo_723,MCI.vs.control; bulk RNA-seq,Homo_714,AD.vs.MCI; bulk RNA-seq,Homo_714,MCI.vs.control; bulk RNA-seq,Homo_633,AD.vs.control; bulk RNA-seq,Homo_633,AD.vs.MCI	5
KEGG	hsa01230	Biosynthesis of amino acids	bulk RNA-seq,Homo_723,MCI.vs.control; bulk RNA-seq,Homo_714,MCI.vs.control; bulk RNA-seq,Homo_633,AD.vs.control; bulk RNA-	4
KEGG	hsa01200	Carbon metabolism	bulk RNA-seq,Homo_723,MCI.vs.control; bulk RNA-seq,Homo_714,AD.vs.control; bulk RNA-seq,Homo_714,MCI.vs.control; bulk RNA-seq,Homo_633,AD.vs.control; bulk RNA-seq,Homo_633,AD.vs.MCI	5
KEGG	hsa05323	Rheumatoid arthritis	bulk RNA-seq,Homo_723,MCI.vs.control; bulk RNA-seq,Homo_714,AD.vs.MCI; bulk RNA-seq,Homo_714,MCI.vs.control; bulk RNA-seq,Homo_633,AD.vs.control; bulk RNA-seq,Homo_633,AD.vs.MCI; bulk RNA-seq,SRP223445,AD.vs.control; scRNA-seq,SRP330776,Monocyte_2-AD.vs.control; scRNA-seq,SRP309935,Naive CD8+ T cell_1-AD.vs.control; scRNA-seq,SRP215507,CD8+ T cell_3-AD.vs.control; scRNA-seq,SRP215507,CD8+	11
KEGG	hsa00910	Nitrogen metabolism	bulk RNA-seq,Homo_723,MCI.vs.control; bulk RNA-seq,Homo_714,MCI.vs.control	2
KEGG	hsa05220	Chronic myeloid leukemia	bulk RNA-seq,Homo_723,MCI.vs.control; bulk RNA-seq,Homo_714,MCI.vs.control; bulk RNA-seq,Homo_633,AD.vs.control; bulk RNA-	4
KEGG	hsa00510	N-Glycan biosynthesis	bulk RNA-seq,Homo_723,MCI.vs.control; bulk RNA-seq,Homo_714,MCI.vs.control; bulk RNA-seq,Homo_633,AD.vs.control; bulk RNA-	4
KEGG	hsa04710	Circadian rhythm	bulk RNA-seq,Homo_723,MCI.vs.control; bulk RNA-seq,Homo_714,MCI.vs.control; bulk RNA-seq,Homo_633,AD.vs.control; bulk RNA-	4
KEGG	hsa05017	Spinocerebellar ataxia	bulk RNA-seq,Homo_723,MCI.vs.control; bulk RNA-seq,Homo_714,AD.vs.control; bulk RNA-seq,Homo_633,AD.vs.control; bulk RNA-	4
KEGG	hsa00270	Cysteine and methionine metabolism	bulk RNA-seq,Homo_723,MCI.vs.control; bulk RNA-seq,Homo_714,MCI.vs.control; bulk RNA-seq,Homo_633,AD.vs.control; bulk RNA-	4
KEGG	hsa05211	Renal cell carcinoma	bulk RNA-seq,Homo_723,MCI.vs.control; bulk RNA-seq,Homo_714,MCI.vs.control; bulk RNA-seq,Homo_633,AD.vs.control; bulk RNA-	4
KEGG	hsa00240	Pyrimidine metabolism	bulk RNA-seq,Homo_723,MCI.vs.control; bulk RNA-seq,Homo_714,MCI.vs.control; bulk RNA-seq,Homo_633,AD.vs.control; bulk RNA-	4
KEGG	hsa00600	Sphingolipid metabolism	bulk RNA-seq,Homo_723,MCI.vs.control; bulk RNA-seq,Homo_714,MCI.vs.control; bulk RNA-seq,Homo_633,AD.vs.control; bulk RNA-	4
KEGG	hsa00450	Selenocompound metabolism	bulk RNA-seq,Homo_723,MCI.vs.control; bulk RNA-seq,Homo_714,MCI.vs.control; bulk RNA-seq,Homo_633,AD.vs.control; bulk RNA-seq,Homo_633,AD.vs.MCI; bulk RNA-seq,Homo_633,MCI.vs.control	5
KEGG	hsa00120	Primary bile acid biosynthesis	bulk RNA-seq,Homo_723,MCI.vs.control; bulk RNA-seq,Homo_714,MCI.vs.control	2
KEGG	hsa04137	Mitophagy - animal	bulk RNA-seq,Homo_723,MCI.vs.control; bulk RNA-seq,Homo_714,MCI.vs.control; bulk RNA-seq,Homo_633,AD.vs.control; bulk RNA-	4
KEGG	hsa00564	Glycerophospholipid metabolism	bulk RNA-seq,Homo_723,MCI.vs.control; bulk RNA-seq,Homo_714,MCI.vs.control; bulk RNA-seq,Homo_633,AD.vs.control; bulk RNA-	4
KEGG	hsa00515	Mannose type O-glycan biosynthesis	bulk RNA-seq,Homo_723,MCI.vs.control; bulk RNA-seq,Homo_714,MCI.vs.control; bulk RNA-seq,Homo_633,AD.vs.control; bulk RNA-	4
KEGG	hsa00220	Arginine biosynthesis	bulk RNA-seq,Homo_723,MCI.vs.control; bulk RNA-seq,Homo_714,AD.vs.MCI; bulk RNA-seq,Homo_714,MCI.vs.control; bulk RNA-seq,Homo_633,AD.vs.control; bulk RNA-seq,Homo_633,AD.vs.MCI	5
KEGG	hsa04964	Proximal tubule bicarbonate reclamation	bulk RNA-seq,Homo_723,MCI.vs.control; bulk RNA-seq,Homo_633,AD.vs.control; bulk RNA-seq,Homo_633,AD.vs.MCI	3
KEGG	hsa01210	2-Oxocarboxylic acid metabolism	bulk RNA-seq,Homo_723,MCI.vs.control; bulk RNA-seq,Homo_714,MCI.vs.control; bulk RNA-seq,Homo_633,AD.vs.control; bulk RNA-seq,Homo_633,AD.vs.MCI; bulk RNA-seq,SRP223445,AD.vs.control	5
KEGG	hsa04962	Vasopressin-regulated water reabsorption	bulk RNA-seq,Homo_723,MCI.vs.control; bulk RNA-seq,Homo_714,AD.vs.MCI; bulk RNA-seq,Homo_714,MCI.vs.control; bulk RNA-seq,Homo_633,AD.vs.control; bulk RNA-seq,Homo_633,AD.vs.MCI	5
KEGG	hsa04620	Toll-like receptor signaling pathway	bulk RNA-seq,Homo_723,MCI.vs.control; bulk RNA-seq,Homo_714,MCI.vs.control; bulk RNA-seq,Homo_633,AD.vs.control; bulk RNA-	4
KEGG	hsa00062	Fatty acid elongation	bulk RNA-seq,Homo_723,MCI.vs.control; bulk RNA-seq,Homo_714,MCI.vs.control; bulk RNA-seq,Homo_633,AD.vs.control; bulk RNA-	4
KEGG	hsa00565	Ether lipid metabolism	bulk RNA-seq,Homo_723,MCI.vs.control; bulk RNA-seq,Homo_714,MCI.vs.control; bulk RNA-seq,Homo_633,AD.vs.control; bulk RNA-	4
KEGG	hsa05152	Tuberculosis	bulk RNA-seq,Homo_723,MCI.vs.control; bulk RNA-seq,Homo_633,AD.vs.control; bulk RNA-seq,Homo_633,AD.vs.MCI; scRNA-seq,SRP330776,CD8+ T cell_1-AD.vs.control; scRNA-seq,SRP215507,CD8+ T cell_3-AD.vs.control	5
KEGG	hsa03265	Virion - Ebolavirus, Lyssavirus and Morbillivirus	bulk RNA-seq,Homo_723,MCI.vs.control; bulk RNA-seq,Homo_714,MCI.vs.control	2

KEGG	hsa00030	Pentose phosphate pathway	bulk RNA-seq,Homo_723,MCI.vs.control; bulk RNA-seq,Homo_714,MCI.vs.control; bulk RNA-seq,Homo_633,AD.vs.control; bulk RNA-	4
KEGG	hsa04664	Fc epsilon RI signaling pathway	bulk RNA-seq,Homo_723,MCI.vs.control; bulk RNA-seq,Homo_714,MCI.vs.control; bulk RNA-seq,Homo_633,AD.vs.control; bulk RNA-	4
KEGG	hsa00051	Fructose and mannose metabolism	bulk RNA-seq,Homo_723,MCI.vs.control; bulk RNA-seq,Homo_714,MCI.vs.control; bulk RNA-seq,Homo_633,AD.vs.control; bulk RNA-	4
KEGG	hsa00511	Other glycan degradation	bulk RNA-seq,Homo_723,MCI.vs.control; bulk RNA-seq,Homo_714,MCI.vs.control; bulk RNA-seq,Homo_633,AD.vs.control; bulk RNA-	4
KEGG	hsa04623	Cytosolic DNA-sensing pathway	bulk RNA-seq,Homo_723,MCI.vs.control; bulk RNA-seq,Homo_714,MCI.vs.control; bulk RNA-seq,Homo_633,AD.vs.control; bulk RNA-	4
KEGG	hsa04213	Longevity regulating pathway - multiple species	bulk RNA-seq,Homo_723,MCI.vs.control; bulk RNA-seq,Homo_714,MCI.vs.control; bulk RNA-seq,Homo_633,AD.vs.control; bulk RNA-	4
KEGG	hsa00630	Glyoxylate and dicarboxylate metabolism	bulk RNA-seq,Homo_723,MCI.vs.control; bulk RNA-seq,Homo_714,MCI.vs.control; bulk RNA-seq,Homo_633,AD.vs.control; bulk RNA-	4
KEGG	hsa01040	Biosynthesis of unsaturated fatty acids	bulk RNA-seq,Homo_723,MCI.vs.control; bulk RNA-seq,Homo_714,MCI.vs.control; bulk RNA-seq,Homo_633,AD.vs.control; bulk RNA-	4
KEGG	hsa04622	RIG-I-like receptor signaling pathway	bulk RNA-seq,Homo_723,MCI.vs.control; bulk RNA-seq,Homo_714,AD.vs.MCI; bulk RNA-seq,Homo_714,MCI.vs.control; bulk RNA-seq,Homo_633,AD.vs.control; bulk RNA-seq,Homo_633,AD.vs.MCI	5
KEGG	hsa00591	Linoleic acid metabolism	bulk RNA-seq,Homo_723,MCI.vs.control; bulk RNA-seq,Homo_714,AD.vs.MCI; bulk RNA-seq,Homo_714,MCI.vs.control; scRNA-seq,SRP215507,CD8+ T	4
KEGG	hsa04217	Necroptosis	bulk RNA-seq,Homo_723,MCI.vs.control; bulk RNA-seq,Homo_633,AD.vs.control; bulk RNA-seq,Homo_633,AD.vs.MCI; scRNA-seq,SRP215507,CD8+ T	4
KEGG	hsa00100	Steroid biosynthesis	bulk RNA-seq,Homo_723,MCI.vs.control; bulk RNA-seq,Homo_714,AD.vs.control; bulk RNA-seq,Homo_714,MCI.vs.control; bulk RNA-	4
KEGG	hsa00130	Ubiquinone and other terpenoid-quinone biosynthesis	bulk RNA-seq,Homo_723,MCI.vs.control; bulk RNA-seq,Homo_714,MCI.vs.control	2
KEGG	hsa00480	Glutathione metabolism	bulk RNA-seq,Homo_723,MCI.vs.control; bulk RNA-seq,Homo_714,MCI.vs.control; bulk RNA-seq,Homo_633,AD.vs.control; bulk RNA-	4
KEGG	hsa00590	Arachidonic acid metabolism	bulk RNA-seq,Homo_723,MCI.vs.control; bulk RNA-seq,Homo_714,MCI.vs.control	2
KEGG	hsa00360	Phenylalanine metabolism	bulk RNA-seq,Homo_723,MCI.vs.control	1
KEGG	hsa00785	Lipoic acid metabolism	bulk RNA-seq,Homo_723,MCI.vs.control; bulk RNA-seq,Homo_633,AD.vs.control; bulk RNA-seq,Homo_633,AD.vs.MCI	3
KEGG	hsa01524	Platinum drug resistance	bulk RNA-seq,Homo_723,MCI.vs.control; bulk RNA-seq,Homo_714,MCI.vs.control; bulk RNA-seq,Homo_633,AD.vs.control; bulk RNA-	4
KEGG	hsa00061	Fatty acid biosynthesis	bulk RNA-seq,Homo_723,MCI.vs.control; bulk RNA-seq,Homo_714,MCI.vs.control; bulk RNA-seq,Homo_633,AD.vs.control	3
KEGG	hsa00513	Various types of N-glycan biosynthesis	bulk RNA-seq,Homo_723,MCI.vs.control; bulk RNA-seq,Homo_633,AD.vs.control; bulk RNA-seq,Homo_633,AD.vs.MCI	3
KEGG	hsa05016	Huntington disease	bulk RNA-seq,Homo_714,AD.vs.control; bulk RNA-seq,Homo_633,AD.vs.control; bulk RNA-seq,Homo_633,AD.vs.MCI; scRNA-seq,SRP215507,CD8+ T cell_8-	4
KEGG	hsa05014	Amyotrophic lateral sclerosis	bulk RNA-seq,Homo_714,AD.vs.control; bulk RNA-seq,Homo_633,AD.vs.control; bulk RNA-seq,Homo_633,AD.vs.MCI; scRNA-seq,SRP215507,CD8+ T cell_8-	4
KEGG	hsa05150	Staphylococcus aureus infection	bulk RNA-seq,Homo_714,AD.vs.control; bulk RNA-seq,Homo_714,AD.vs.MCI; scRNA-seq,SRP215507,CD8+ T cell_3-MCI.vs.control	3
KEGG	hsa01250	Biosynthesis of nucleotide sugars	bulk RNA-seq,Homo_714,AD.vs.control; bulk RNA-seq,Homo_633,AD.vs.control; bulk RNA-seq,Homo_633,AD.vs.MCI	3
KEGG	hsa04614	Renin-angiotensin system	bulk RNA-seq,Homo_714,AD.vs.MCI	1
KEGG	hsa05322	Systemic lupus erythematosus	bulk RNA-seq,Homo_714,MCI.vs.control; bulk RNA-seq,ROSMAP,MCI.vs.control; scRNA-seq,SRP215507,CD8+ T cell_3-AD.vs.control; scRNA-seq,SRP215507,CD8+ T cell_3-MCI.vs.control	4
KEGG	hsa00532	Glycosaminoglycan biosynthesis - chondroitin sulfate /	bulk RNA-seq,Homo_714,MCI.vs.control	1
KEGG	hsa00052	Galactose metabolism	bulk RNA-seq,Homo_633,AD.vs.control; bulk RNA-seq,Homo_633,AD.vs.MCI	2
KEGG	hsa04650	Natural killer cell mediated cytotoxicity	bulk RNA-seq,Homo_633,AD.vs.control; bulk RNA-seq,Homo_633,AD.vs.MCI; scRNA-seq,SRP215507,CD8+ T cell_3-AD.vs.control; scRNA-seq,SRP215507,CD8+ T cell_4-AD.vs.MCI	4
KEGG	hsa04215	Apoptosis - multiple species	bulk RNA-seq,Homo_633,AD.vs.control; bulk RNA-seq,Homo_633,AD.vs.MCI	2
KEGG	hsa04216	Ferroptosis	bulk RNA-seq,Homo_633,AD.vs.control; bulk RNA-seq,Homo_633,AD.vs.MCI	2
KEGG	hsa00920	Sulfur metabolism	bulk RNA-seq,Homo_633,AD.vs.control; bulk RNA-seq,Homo_633,AD.vs.MCI; scRNA-seq,SRP215507,CD8+ T cell_3-MCI.vs.control	3
KEGG	hsa00531	Glycosaminoglycan degradation	bulk RNA-seq,Homo_633,AD.vs.control; bulk RNA-seq,Homo_633,AD.vs.MCI	2
KEGG	hsa00500	Starch and sucrose metabolism	bulk RNA-seq,Homo_633,AD.vs.control; bulk RNA-seq,Homo_633,AD.vs.MCI	2
KEGG	hsa00512	Mucin type O-glycan biosynthesis	bulk RNA-seq,Homo_633,AD.vs.control	1
KEGG	hsa00770	Pantothenate and CoA biosynthesis	bulk RNA-seq,Homo_633,AD.vs.control; bulk RNA-seq,Homo_633,AD.vs.MCI	2
KEGG	hsa00410	beta-Alanine metabolism	bulk RNA-seq,Homo_633,AD.vs.control; bulk RNA-seq,Homo_633,AD.vs.MCI	2
KEGG	hsa00533	Glycosaminoglycan biosynthesis - keratan sulfate	bulk RNA-seq,Homo_633,AD.vs.control; bulk RNA-seq,Homo_633,AD.vs.MCI	2
KEGG	hsa00790	Folate biosynthesis	bulk RNA-seq,Homo_633,AD.vs.control; bulk RNA-seq,Homo_633,AD.vs.MCI	2
KEGG	hsa05320	Autoimmune thyroid disease	bulk RNA-seq,Homo_633,AD.vs.control; bulk RNA-seq,ROSMAP,MCI.vs.control; scRNA-seq,SRP215507,CD8+ T cell_3-AD.vs.control; scRNA-seq,SRP215507,CD8+ T cell_3-MCI.vs.control	4
KEGG	hsa00604	Glycosphingolipid biosynthesis - ganglio series	bulk RNA-seq,Homo_633,AD.vs.control	1
KEGG	mmu03010	Ribosome	bulk RNA-seq,SRP325058,AD.vs.control	1
KEGG	mmu05168	Herpes simplex virus 1 infection	bulk RNA-seq,SRP325058,AD.vs.control	1
BP	GO:0051093	negative regulation of developmental process	scRNA-seq,SRP330776,B cell_1-AD.vs.control; scRNA-seq,SRP330776,CD8+ T cell_1-AD.vs.control; scRNA-seq,SRP330776,CD8+ T cell_2-AD.vs.control; scRNA-seq,SRP330776,Memory T cell_2-AD.vs.control; scRNA-seq,SRP330776,Naive CD8+ T cell_1-AD.vs.control; scRNA-seq,SRP330776,Naive CD8+ T cell_2-AD.vs.control; scRNA-seq,SRP330776,Natural killer cell_1-AD.vs.control; scRNA-seq,SRP330776,Natural killer T cell-AD.vs.control	8
BP	GO:0071363	cellular response to growth factor stimulus	scRNA-seq,SRP330776,B cell_1-AD.vs.control; scRNA-seq,SRP330776,CD8+ T cell_1-AD.vs.control; scRNA-seq,SRP330776,CD8+ T cell_2-AD.vs.control; scRNA-seq,SRP330776,Naive CD8+ T cell_1-AD.vs.control; scRNA-seq,SRP330776,Naive CD8+ T cell_2-AD.vs.control; scRNA-seq,SRP330776,Natural killer	6
BP	GO:0008610	lipid biosynthetic process	scRNA-seq,SRP330776,B cell_1-AD.vs.control	1
BP	GO:0007169	transmembrane receptor protein tyrosine kinase signaling	scRNA-seq,SRP330776,B cell_1-AD.vs.control; scRNA-seq,SRP330776,Naive CD8+ T cell_2-AD.vs.control	2
BP	GO:0045597	positive regulation of cell differentiation	scRNA-seq,SRP330776,CD8+ T cell_1-AD.vs.control; scRNA-seq,SRP330776,CD8+ T cell_2-AD.vs.control; scRNA-seq,SRP330776,Memory T cell_2-AD.vs.control; scRNA-seq,SRP330776,Naive CD8+ T cell_1-AD.vs.control	4
BP	GO:0070848	response to growth factor	scRNA-seq,SRP330776,CD8+ T cell_1-AD.vs.control; scRNA-seq,SRP330776,CD8+ T cell_2-AD.vs.control; scRNA-seq,SRP330776,Naive CD8+ T cell_1-AD.vs.control; scRNA-seq,SRP330776,Naive CD8+ T cell_2-AD.vs.control	4

BP	GO:0040008	regulation of growth	scRNA-seq,SRP330776,CD8+ T cell_1-AD.vs.control; scRNA-seq,SRP330776,Naive CD8+ T cell_1-AD.vs.control; scRNA-seq,SRP330776,Naive CD8+ T cell_2-	3
BP	GO:0030155	regulation of cell adhesion	scRNA-seq,SRP330776,CD8+ T cell_1-AD.vs.control; scRNA-seq,SRP330776,Naive CD8+ T cell_2-AD.vs.control; scRNA-seq,SRP215507,CD8+ T cell_3-	3
BP	GO:0061061	muscle structure development	scRNA-seq,SRP330776,CD8+ T cell_1-AD.vs.control	1
BP	GO:0071417	cellular response to organonitrogen compound	scRNA-seq,SRP330776,CD8+ T cell_1-AD.vs.control	1
BP	GO:0045596	negative regulation of cell differentiation	scRNA-seq,SRP330776,CD8+ T cell_1-AD.vs.control; scRNA-seq,SRP330776,Naive CD8+ T cell_2-AD.vs.control; scRNA-seq,SRP330776,Natural killer T cell-	3
BP	GO:0090407	organophosphate biosynthetic process	scRNA-seq,SRP330776,CD8+ T cell_2-AD.vs.control; scRNA-seq,SRP330776,Naive CD8+ T cell_3-AD.vs.control	2
CC	GO:1990904	ribonucleoprotein complex	scRNA-seq,SRP330776,Megakaryocyte_2-AD.vs.control; scRNA-seq,SRP309935,Megakaryocyte_2-AD.vs.control	2
BP	GO:0006412	translation	scRNA-seq,SRP330776,Megakaryocyte_2-AD.vs.control	1
BP	GO:0006518	peptide metabolic process	scRNA-seq,SRP330776,Megakaryocyte_2-AD.vs.control	1
BP	GO:0043043	peptide biosynthetic process	scRNA-seq,SRP330776,Megakaryocyte_2-AD.vs.control	1
BP	GO:0009887	animal organ morphogenesis	scRNA-seq,SRP330776,Memory T cell_2-AD.vs.control; scRNA-seq,SRP330776,Naive CD8+ T cell_1-AD.vs.control; scRNA-seq,SRP330776,Natural killer	3
MF	GO:0004672	protein kinase activity	scRNA-seq,SRP330776,Naive CD8+ T cell_1-AD.vs.control; scRNA-seq,SRP330776,Naive CD8+ T cell_2-AD.vs.control; scRNA-seq,SRP330776,Natural killer	3
MF	GO:0016773	phosphotransferase activity, alcohol group as acceptor	scRNA-seq,SRP330776,Naive CD8+ T cell_1-AD.vs.control; scRNA-seq,SRP330776,Naive CD8+ T cell_2-AD.vs.control; scRNA-seq,SRP330776,Natural killer	3
BP	GO:0003013	circulatory system process	scRNA-seq,SRP330776,Naive CD8+ T cell_1-AD.vs.control	1
BP	GO:0046649	lymphocyte activation	scRNA-seq,SRP330776,Naive CD8+ T cell_2-AD.vs.control; scRNA-seq,SRP309935,Megakaryocyte_2-AD.vs.control	2
BP	GO:0050778	positive regulation of immune response	scRNA-seq,SRP330776,Naive CD8+ T cell_2-AD.vs.control; scRNA-seq,SRP215507,CD8+ T cell_3-AD.vs.control	2
CC	GO:0010008	endosome membrane	scRNA-seq,SRP330776,Naive CD8+ T cell_2-AD.vs.control	1
MF	GO:0019900	kinase binding	scRNA-seq,SRP330776,Naive CD8+ T cell_2-AD.vs.control	1
BP	GO:0051270	regulation of cellular component movement	scRNA-seq,SRP330776,Naive CD8+ T cell_2-AD.vs.control	1
BP	GO:0032101	regulation of response to external stimulus	scRNA-seq,SRP330776,Naive CD8+ T cell_2-AD.vs.control	1
BP	GO:0051272	positive regulation of cellular component movement	scRNA-seq,SRP330776,Naive CD8+ T cell_2-AD.vs.control	1
CC	GO:0098552	side of membrane	scRNA-seq,SRP330776,Naive CD8+ T cell_2-AD.vs.control; scRNA-seq,SRP309935,Monocyte_2-AD.vs.control; scRNA-seq,SRP215507,CD8+ T cell_3-	3
CC	GO:0000139	Golgi membrane	scRNA-seq,SRP330776,Naive CD8+ T cell_2-AD.vs.control	1
BP	GO:0042330	taxis	scRNA-seq,SRP330776,Naive CD8+ T cell_2-AD.vs.control	1
BP	GO:0030335	positive regulation of cell migration	scRNA-seq,SRP330776,Naive CD8+ T cell_2-AD.vs.control	1
BP	GO:2000145	regulation of cell motility	scRNA-seq,SRP330776,Naive CD8+ T cell_2-AD.vs.control	1
MF	GO:0019901	protein kinase binding	scRNA-seq,SRP330776,Naive CD8+ T cell_2-AD.vs.control	1
BP	GO:2000147	positive regulation of cell motility	scRNA-seq,SRP330776,Naive CD8+ T cell_2-AD.vs.control	1
BP	GO:0006935	chemotaxis	scRNA-seq,SRP330776,Naive CD8+ T cell_2-AD.vs.control	1
BP	GO:0040017	positive regulation of locomotion	scRNA-seq,SRP330776,Naive CD8+ T cell_2-AD.vs.control	1
BP	GO:0002252	immune effector process	scRNA-seq,SRP330776,Naive CD8+ T cell_2-AD.vs.control; scRNA-seq,SRP215507,CD8+ T cell_3-AD.vs.control	2
BP	GO:0030334	regulation of cell migration	scRNA-seq,SRP330776,Naive CD8+ T cell_2-AD.vs.control	1
BP	GO:0051249	regulation of lymphocyte activation	scRNA-seq,SRP330776,Naive CD8+ T cell_2-AD.vs.control; scRNA-seq,SRP309935,Monocyte_2-AD.vs.control; scRNA-seq,SRP215507,CD8+ T cell_1-MCI.vs.control; scRNA-seq,SRP215507,CD8+ T cell_3-AD.vs.control	4
BP	GO:0040012	regulation of locomotion	scRNA-seq,SRP330776,Naive CD8+ T cell_2-AD.vs.control	1
BP	GO:0060627	regulation of vesicle-mediated transport	scRNA-seq,SRP330776,Naive CD8+ T cell_2-AD.vs.control	1
BP	GO:0002694	regulation of leukocyte activation	scRNA-seq,SRP330776,Naive CD8+ T cell_2-AD.vs.control; scRNA-seq,SRP215507,CD8+ T cell_3-AD.vs.control	2
BP	GO:0001816	cytokine production	scRNA-seq,SRP330776,Naive CD8+ T cell_2-AD.vs.control	1
BP	GO:0019725	cellular homeostasis	scRNA-seq,SRP330776,Naive CD8+ T cell_2-AD.vs.control	1
BP	GO:0001817	regulation of cytokine production	scRNA-seq,SRP330776,Naive CD8+ T cell_2-AD.vs.control	1
BP	GO:0002521	leukocyte differentiation	scRNA-seq,SRP330776,Naive CD8+ T cell_2-AD.vs.control; scRNA-seq,SRP215507,CD8+ T cell_3-AD.vs.control	2
BP	GO:0007167	enzyme linked receptor protein signaling pathway	scRNA-seq,SRP330776,Naive CD8+ T cell_2-AD.vs.control	1
BP	GO:0006897	endocytosis	scRNA-seq,SRP330776,Naive CD8+ T cell_2-AD.vs.control	1
BP	GO:0055082	cellular chemical homeostasis	scRNA-seq,SRP330776,Naive CD8+ T cell_2-AD.vs.control	1
BP	GO:0031175	neuron projection development	scRNA-seq,SRP330776,Naive CD8+ T cell_2-AD.vs.control	1
BP	GO:0051050	positive regulation of transport	scRNA-seq,SRP330776,Naive CD8+ T cell_2-AD.vs.control	1
BP	GO:0006954	inflammatory response	scRNA-seq,SRP330776,Naive CD8+ T cell_2-AD.vs.control; scRNA-seq,SRP215507,CD8+ T cell_3-AD.vs.control	2
MF	GO:0038023	signaling receptor activity	scRNA-seq,SRP330776,Naive CD8+ T cell_2-AD.vs.control; scRNA-seq,SRP309935,B cell_2-AD.vs.control; scRNA-seq,SRP215507,CD8+ T cell_3-AD.vs.control; scRNA-seq,SRP215507,CD8+ T cell_4-AD.vs.MCI	4
MF	GO:0060089	molecular transducer activity	scRNA-seq,SRP330776,Naive CD8+ T cell_2-AD.vs.control; scRNA-seq,SRP309935,B cell_2-AD.vs.control; scRNA-seq,SRP215507,CD8+ T cell_3-AD.vs.control; scRNA-seq,SRP215507,CD8+ T cell_4-AD.vs.MCI	4
BP	GO:0010942	positive regulation of cell death	scRNA-seq,SRP330776,Naive CD8+ T cell_2-AD.vs.control	1
MF	GO:0016301	kinase activity	scRNA-seq,SRP330776,Naive CD8+ T cell_2-AD.vs.control; scRNA-seq,SRP330776,Natural killer cell_1-AD.vs.control	2
BP	GO:0061024	membrane organization	scRNA-seq,SRP330776,Naive CD8+ T cell_2-AD.vs.control	1
CC	GO:0000323	lytic vacuole	scRNA-seq,SRP330776,Naive CD8+ T cell_2-AD.vs.control	1
CC	GO:0005764	lysosome	scRNA-seq,SRP330776,Naive CD8+ T cell_2-AD.vs.control	1

BP	GO:0048666	neuron development	scRNA-seq,SRP330776,Naive CD8+ T cell_2-AD.vs.control	1
BP	GO:0140352	export from cell	scRNA-seq,SRP330776,Naive CD8+ T cell_2-AD.vs.control	1
BP	GO:0040007	growth	scRNA-seq,SRP330776,Naive CD8+ T cell_2-AD.vs.control	1
BP	GO:0098771	inorganic ion homeostasis	scRNA-seq,SRP330776,Naive CD8+ T cell_2-AD.vs.control	1
BP	GO:0006873	cellular ion homeostasis	scRNA-seq,SRP330776,Naive CD8+ T cell_2-AD.vs.control	1
BP	GO:0098655	cation transmembrane transport	scRNA-seq,SRP330776,Naive CD8+ T cell_2-AD.vs.control	1
BP	GO:0050865	regulation of cell activation	scRNA-seq,SRP330776,Naive CD8+ T cell_2-AD.vs.control; scRNA-seq,SRP215507,CD8+ T cell_1-MCI.vs.control; scRNA-seq,SRP215507,CD8+ T cell_3-	3
BP	GO:0022603	regulation of anatomical structure morphogenesis	scRNA-seq,SRP330776,Naive CD8+ T cell_2-AD.vs.control	1
BP	GO:0050801	ion homeostasis	scRNA-seq,SRP330776,Naive CD8+ T cell_2-AD.vs.control	1
BP	GO:0034097	response to cytokine	scRNA-seq,SRP330776,Naive CD8+ T cell_2-AD.vs.control; scRNA-seq,SRP215507,CD8+ T cell_8-MCI.vs.control	2
BP	GO:0055080	cation homeostasis	scRNA-seq,SRP330776,Naive CD8+ T cell_2-AD.vs.control	1
BP	GO:0051241	negative regulation of multicellular organismal process	scRNA-seq,SRP330776,Naive CD8+ T cell_2-AD.vs.control	1
BP	GO:0120035	regulation of plasma membrane bounded cell projection	scRNA-seq,SRP330776,Naive CD8+ T cell_2-AD.vs.control	1
BP	GO:0031344	regulation of cell projection organization	scRNA-seq,SRP330776,Naive CD8+ T cell_2-AD.vs.control	1
BP	GO:0032940	secretion by cell	scRNA-seq,SRP330776,Naive CD8+ T cell_2-AD.vs.control	1
BP	GO:0072359	circulatory system development	scRNA-seq,SRP330776,Naive CD8+ T cell_2-AD.vs.control	1
BP	GO:0008284	positive regulation of cell population proliferation	scRNA-seq,SRP330776,Naive CD8+ T cell_2-AD.vs.control	1
BP	GO:0045591	positive regulation of regulatory T cell differentiation	scRNA-seq,SRP330776,Naive CD8+ T cell_2-AD.vs.control	1
MF	GO:0019904	protein domain specific binding	scRNA-seq,SRP330776,Naive CD8+ T cell_2-AD.vs.control	1
BP	GO:0034220	ion transmembrane transport	scRNA-seq,SRP330776,Naive CD8+ T cell_2-AD.vs.control	1
BP	GO:0043068	positive regulation of programmed cell death	scRNA-seq,SRP330776,Naive CD8+ T cell_2-AD.vs.control	1
MF	GO:0004888	transmembrane signaling receptor activity	scRNA-seq,SRP330776,Naive CD8+ T cell_2-AD.vs.control; scRNA-seq,SRP215507,CD8+ T cell_3-AD.vs.control	2
BP	GO:2000191	regulation of fatty acid transport	scRNA-seq,SRP330776,Naive CD8+ T cell_2-AD.vs.control	1
MF	GO:0008324	cation transmembrane transporter activity	scRNA-seq,SRP330776,Naive CD8+ T cell_2-AD.vs.control	1
BP	GO:0051271	negative regulation of cellular component movement	scRNA-seq,SRP330776,Naive CD8+ T cell_2-AD.vs.control	1
BP	GO:0010035	response to inorganic substance	scRNA-seq,SRP330776,Naive CD8+ T cell_2-AD.vs.control	1
BP	GO:0046903	secretion	scRNA-seq,SRP330776,Naive CD8+ T cell_2-AD.vs.control	1
MF	GO:0015075	ion transmembrane transporter activity	scRNA-seq,SRP330776,Naive CD8+ T cell_2-AD.vs.control	1
BP	GO:0051640	organelle localization	scRNA-seq,SRP330776,Naive CD8+ T cell_2-AD.vs.control	1
BP	GO:0048598	embryonic morphogenesis	scRNA-seq,SRP330776,Naive CD8+ T cell_3-AD.vs.control	1
BP	GO:0043009	chordate embryonic development	scRNA-seq,SRP330776,Naive CD8+ T cell_3-AD.vs.control	1
BP	GO:0048646	anatomical structure formation involved in morphogenesis	scRNA-seq,SRP330776,Naive CD8+ T cell_3-AD.vs.control	1
BP	GO:0099536	synaptic signaling	scRNA-seq,SRP330776,Naive CD8+ T cell_3-AD.vs.control	1
BP	GO:0009792	embryo development ending in birth or egg hatching	scRNA-seq,SRP330776,Naive CD8+ T cell_3-AD.vs.control	1
BP	GO:0007268	chemical synaptic transmission	scRNA-seq,SRP330776,Naive CD8+ T cell_3-AD.vs.control	1
BP	GO:0098916	anterograde trans-synaptic signaling	scRNA-seq,SRP330776,Naive CD8+ T cell_3-AD.vs.control	1
BP	GO:0002250	adaptive immune response	scRNA-seq,SRP330776,Natural killer cell_1-AD.vs.control; scRNA-seq,SRP309935,Monocyte_2-AD.vs.control; scRNA-seq,SRP215507,CD8+ T cell_1-AD.vs.control; scRNA-seq,SRP215507,CD8+ T cell_1-AD.vs.MCI; scRNA-seq,SRP215507,CD8+ T cell_1-MCI.vs.control; scRNA-seq,SRP215507,CD8+ T cell_3-AD.vs.control; scRNA-seq,SRP215507,CD8+ T cell_4-AD.vs.MCI; scRNA-seq,SRP215507,CD8+ T cell_4-MCI.vs.control	8
CC	GO:0098797	plasma membrane protein complex	scRNA-seq,SRP330776,Natural killer cell_1-AD.vs.control; scRNA-seq,SRP309935,Natural killer cell_1-AD.vs.control; scRNA-seq,SRP215507,CD8+ T cell_1-AD.vs.control; scRNA-seq,SRP215507,CD8+ T cell_1-AD.vs.MCI; scRNA-seq,SRP215507,CD8+ T cell_1-MCI.vs.control; scRNA-seq,SRP215507,CD8+ T cell_3-AD.vs.control; scRNA-seq,SRP215507,CD8+ T cell_4-MCI.vs.control	7
CC	GO:0019866	organelle inner membrane	scRNA-seq,SRP330776,Natural killer cell_1-AD.vs.control	1
BP	GO:0007005	mitochondrion organization	scRNA-seq,SRP330776,Natural killer cell_1-AD.vs.control	1
BP	GO:0005975	carbohydrate metabolic process	scRNA-seq,SRP330776,Natural killer cell_1-AD.vs.control	1
MF	GO:0016491	oxidoreductase activity	scRNA-seq,SRP330776,Natural killer cell_1-AD.vs.control	1
MF	GO:0003700	DNA-binding transcription factor activity	scRNA-seq,SRP309935,B cell_3-AD.vs.control; scRNA-seq,SRP215507,CD8+ T cell_7-MCI.vs.control; scRNA-seq,SRP215507,CD8+ T cell_8-MCI.vs.control	3
CC	GO:0005833	hemoglobin complex	scRNA-seq,SRP309935,Erythroid cell-AD.vs.control	1
CC	GO:0005730	nucleolus	scRNA-seq,SRP309935,Megakaryocyte_2-AD.vs.control	1
BP	GO:0006397	mRNA processing	scRNA-seq,SRP309935,Megakaryocyte_2-AD.vs.control	1
BP	GO:0034660	ncRNA metabolic process	scRNA-seq,SRP309935,Megakaryocyte_2-AD.vs.control	1
BP	GO:0045087	innate immune response	scRNA-seq,SRP309935,Megakaryocyte_2-AD.vs.control	1
CC	GO:0016604	nuclear body	scRNA-seq,SRP309935,Megakaryocyte_2-AD.vs.control	1
BP	GO:0016071	mRNA metabolic process	scRNA-seq,SRP309935,Megakaryocyte_2-AD.vs.control	1
MF	GO:0000981	DNA-binding transcription factor activity, RNA polymerase	scRNA-seq,SRP309935,Plasmacytoid dendritic cell_2-AD.vs.control; scRNA-seq,SRP215507,CD8+ T cell_7-MCI.vs.control; scRNA-seq,SRP215507,CD8+ T	3

MF	GO:0000978	RNA polymerase II cis-regulatory region sequence-specific DNA binding	scRNA-seq,SRP309935,Plasmacytoid dendritic cell_2-AD.vs.control	1
MF	GO:0000987	cis-regulatory region sequence-specific DNA binding	scRNA-seq,SRP309935,Plasmacytoid dendritic cell_2-AD.vs.control	1
KEGG	hsa05168	Herpes simplex virus 1 infection	scRNA-seq,SRP309935,B cell_1-AD.vs.control; scRNA-seq,SRP309935,CD4+ T cell-AD.vs.control; scRNA-seq,SRP309935,Dendritic cell-AD.vs.control; scRNA-seq,SRP309935,Megakaryocyte_1-AD.vs.control; scRNA-seq,SRP309935,Monocyte_1-AD.vs.control; scRNA-seq,SRP309935,Plasmacytoid dendritic cell_2-AD.vs.control; scRNA-seq,SRP215507,CD8+ T cell_1-AD.vs.control; scRNA-seq,SRP215507,CD8+ T cell_5-AD.vs.control; scRNA-seq,SRP215507,CD8+ T cell_7-MCI.vs.control; scRNA-seq,SRP215507,CD8+ T cell_8-MCI.vs.control	10
BP	GO:0098609	cell-cell adhesion	scRNA-seq,SRP215507,CD8+ T cell_3-AD.vs.control	1
CC	GO:0009986	cell surface	scRNA-seq,SRP215507,CD8+ T cell_3-AD.vs.control	1
BP	GO:0042110	T cell activation	scRNA-seq,SRP215507,CD8+ T cell_3-AD.vs.control	1
BP	GO:0030031	cell projection assembly	scRNA-seq,SRP215507,CD8+ T cell_3-AD.vs.control	1
BP	GO:0070230	positive regulation of lymphocyte apoptotic process	scRNA-seq,SRP215507,CD8+ T cell_3-AD.vs.control	1
MF	GO:0004930	G protein-coupled receptor activity	scRNA-seq,SRP215507,CD8+ T cell_3-AD.vs.MCI	1
MF	GO:0003682	chromatin binding	scRNA-seq,SRP215507,CD8+ T cell_6-AD.vs.control	1
MF	GO:0000977	RNA polymerase II transcription regulatory region sequence-specific DNA binding	scRNA-seq,SRP215507,CD8+ T cell_7-MCI.vs.control; scRNA-seq,SRP215507,CD8+ T cell_8-MCI.vs.control	2
MF	GO:0000976	transcription cis-regulatory region binding	scRNA-seq,SRP215507,CD8+ T cell_8-MCI.vs.control	1
MF	GO:0001067	transcription regulatory region nucleic acid binding	scRNA-seq,SRP215507,CD8+ T cell_8-MCI.vs.control	1
KEGG	hsa03264	Virion - Flavivirus	scRNA-seq,SRP215507,CD8+ T cell_5-AD.vs.control	1
KEGG	hsa05200	Pathways in cancer	scRNA-seq,SRP215507,CD8+ T cell_8-MCI.vs.control	1
KEGG	hsa00440	Phosphonate and phosphinate metabolism	scRNA-seq,SRP215507,CD8+ T cell_9-AD.vs.control	1
GO	organelle	organelle	miRNA-seq;SRP022043;mirPath;AD.vs.control;up regulated miRNAs; miRNA-seq;SRP022043;mirPath;AD.vs.control;down regulated miRNAs	10
GO	ion binding	ion binding	miRNA-seq;SRP022043;mirPath;AD.vs.control;up regulated miRNAs; miRNA-seq;SRP022043;mirPath;AD.vs.control;down regulated miRNAs	10
GO	cellular nitrogen compound metabolic process	cellular nitrogen compound metabolic process	miRNA-seq;SRP022043;mirPath;AD.vs.control;up regulated miRNAs; miRNA-seq;SRP022043;mirPath;AD.vs.control;down regulated miRNAs	10
GO	biosynthetic	biosynthetic process	miRNA-seq;SRP022043;mirPath;AD.vs.control;up regulated miRNAs; miRNA-seq;SRP022043;mirPath;AD.vs.control;down regulated miRNAs	10
GO	cellular protein modification	cellular protein modification process	miRNA-seq;SRP022043;mirPath;AD.vs.control;up regulated miRNAs; miRNA-seq;SRP022043;mirPath;AD.vs.control;down regulated miRNAs	10
GO	neurotrophin TRK receptor signaling pathway	neurotrophin TRK receptor signaling pathway	miRNA-seq;SRP022043;mirPath;AD.vs.control;up regulated miRNAs; miRNA-seq;SRP022043;mirPath;AD.vs.control;down regulated miRNAs	10
GO	small molecule metabolic process	small molecule metabolic process	miRNA-seq;SRP022043;mirPath;AD.vs.control;up regulated miRNAs; miRNA-seq;SRP022043;mirPath;AD.vs.control;down regulated miRNAs	10
GO	Fc-epsilon receptor signaling	Fc-epsilon receptor signaling pathway	miRNA-seq;SRP022043;mirPath;AD.vs.control;up regulated miRNAs; miRNA-seq;SRP022043;mirPath;AD.vs.control;down regulated miRNAs	10
GO	gene expression	gene expression	miRNA-seq;SRP022043;mirPath;AD.vs.control;up regulated miRNAs; miRNA-seq;SRP022043;mirPath;AD.vs.control;down regulated miRNAs	10
GO	nucleic acid binding transcription	nucleic acid binding transcription factor activity	miRNA-seq;SRP022043;mirPath;AD.vs.control;up regulated miRNAs; miRNA-seq;SRP022043;mirPath;AD.vs.control;down regulated miRNAs	10
GO	molecular_functi	molecular_function	miRNA-seq;SRP022043;mirPath;AD.vs.control;up regulated miRNAs; miRNA-seq;SRP022043;mirPath;AD.vs.control;down regulated miRNAs	10
GO	viral process	viral process	miRNA-seq;SRP022043;mirPath;AD.vs.control;up regulated miRNAs; miRNA-seq;SRP022043;mirPath;AD.vs.control;down regulated miRNAs	10
GO	symbiosis, encompassing mutualism through	symbiosis, encompassing mutualism through parasitism	miRNA-seq;SRP022043;mirPath;AD.vs.control;up regulated miRNAs; miRNA-seq;SRP022043;mirPath;AD.vs.control;down regulated miRNAs	10
GO	catabolic process	catabolic process	miRNA-seq;SRP022043;mirPath;AD.vs.control;up regulated miRNAs; miRNA-seq;SRP022043;mirPath;AD.vs.control;down regulated miRNAs	10
GO	cellular_compone	cellular_component	miRNA-seq;SRP022043;mirPath;AD.vs.control;up regulated miRNAs; miRNA-seq;SRP022043;mirPath;AD.vs.control;down regulated miRNAs	10
GO	blood coagulation	blood coagulation	miRNA-seq;SRP022043;mirPath;AD.vs.control;up regulated miRNAs; miRNA-seq;SRP022043;mirPath;AD.vs.control;down regulated miRNAs	10
GO	enzyme binding	enzyme binding	miRNA-seq;SRP022043;mirPath;AD.vs.control;up regulated miRNAs; miRNA-seq;SRP022043;mirPath;AD.vs.control;down regulated miRNAs	10
GO	mitotic cell cycle	mitotic cell cycle	miRNA-seq;SRP022043;mirPath;AD.vs.control;up regulated miRNAs; miRNA-seq;SRP022043;mirPath;AD.vs.control;down regulated miRNAs	10
GO	cellular component	cellular component assembly	miRNA-seq;SRP022043;mirPath;AD.vs.control;up regulated miRNAs; miRNA-seq;SRP022043;mirPath;AD.vs.control;down regulated miRNAs	10
GO	protein complex	protein complex	miRNA-seq;SRP022043;mirPath;AD.vs.control;up regulated miRNAs; miRNA-seq;SRP022043;mirPath;AD.vs.control;down regulated miRNAs	10
GO	protein binding transcription factor activity	protein binding transcription factor activity	miRNA-seq;SRP022043;mirPath;AD.vs.control;up regulated miRNAs; miRNA-seq;SRP022043;mirPath;AD.vs.control;down regulated miRNAs	10
GO	post-translational protein	post-translational protein modification	miRNA-seq;SRP022043;mirPath;AD.vs.control;up regulated miRNAs; miRNA-seq;SRP022043;mirPath;AD.vs.control;down regulated miRNAs	10

GO	nucleobase-containing compound catabolic process	nucleobase-containing compound catabolic process	miRNA-seq;SRP022043;mirPath;AD.vs.control;up regulated miRNAs; miRNA-seq;SRP022043;mirPath;AD.vs.control;down regulated miRNAs	10
GO	nucleoplasm	nucleoplasm	miRNA-seq;SRP022043;mirPath;AD.vs.control;up regulated miRNAs; miRNA-seq;SRP022043;mirPath;AD.vs.control;down regulated miRNAs	10
GO	cytosol	cytosol	miRNA-seq;SRP022043;mirPath;AD.vs.control;up regulated miRNAs; miRNA-seq;SRP022043;mirPath;AD.vs.control;down regulated miRNAs	10
GO	cytoskeletal protein binding	cytoskeletal protein binding	miRNA-seq;SRP022043;mirPath;AD.vs.control;up regulated miRNAs; miRNA-seq;SRP022043;mirPath;AD.vs.control;down regulated miRNAs	10
GO	cellular protein metabolic process	cellular protein metabolic process	miRNA-seq;SRP022043;mirPath;AD.vs.control;up regulated miRNAs; miRNA-seq;SRP022043;mirPath;AD.vs.control;down regulated miRNAs	10
GO	epidermal growth factor receptor signaling pathway	epidermal growth factor receptor signaling pathway	miRNA-seq;SRP022043;mirPath;AD.vs.control;up regulated miRNAs; miRNA-seq;SRP022043;mirPath;AD.vs.control;down regulated miRNAs	10
GO	macromolecular complex	macromolecular complex assembly	miRNA-seq;SRP022043;mirPath;AD.vs.control;up regulated miRNAs; miRNA-seq;SRP022043;mirPath;AD.vs.control;down regulated miRNAs	10
GO	membrane organization	membrane organization	miRNA-seq;SRP022043;mirPath;AD.vs.control;up regulated miRNAs; miRNA-seq;SRP022043;mirPath;AD.vs.control;down regulated miRNAs	10
GO	cell death	cell death	miRNA-seq;SRP022043;mirPath;AD.vs.control;up regulated miRNAs; miRNA-seq;SRP022043;mirPath;AD.vs.control;down regulated miRNAs	10
GO	response to stress	response to stress	miRNA-seq;SRP022043;mirPath;AD.vs.control;up regulated miRNAs; miRNA-seq;SRP022043;mirPath;AD.vs.control;down regulated miRNAs	10
GO	toll-like receptor 10 signaling	toll-like receptor 10 signaling pathway	miRNA-seq;SRP022043;mirPath;AD.vs.control;up regulated miRNAs; miRNA-seq;SRP022043;mirPath;AD.vs.control;down regulated miRNAs	10
GO	platelet activation	platelet activation	miRNA-seq;SRP022043;mirPath;AD.vs.control;up regulated miRNAs; miRNA-seq;SRP022043;mirPath;AD.vs.control;down regulated miRNAs	10
GO	toll-like receptor TLR1:TLR2 signaling pathway	toll-like receptor TLR1:TLR2 signaling pathway	miRNA-seq;SRP022043;mirPath;AD.vs.control;up regulated miRNAs; miRNA-seq;SRP022043;mirPath;AD.vs.control;down regulated miRNAs	10
GO	toll-like receptor TLR6:TLR2 signaling pathway	toll-like receptor TLR6:TLR2 signaling pathway	miRNA-seq;SRP022043;mirPath;AD.vs.control;up regulated miRNAs; miRNA-seq;SRP022043;mirPath;AD.vs.control;down regulated miRNAs	10
GO	protein complex assembly	protein complex assembly	miRNA-seq;SRP022043;mirPath;AD.vs.control;up regulated miRNAs; miRNA-seq;SRP022043;mirPath;AD.vs.control;down regulated miRNAs	10
GO	cellular lipid metabolic process	cellular lipid metabolic process	miRNA-seq;SRP022043;mirPath;AD.vs.control;up regulated miRNAs; miRNA-seq;SRP022043;mirPath;AD.vs.control;down regulated miRNAs	10
GO	fibroblast growth factor receptor signaling pathway	fibroblast growth factor receptor signaling pathway	miRNA-seq;SRP022043;mirPath;AD.vs.control;up regulated miRNAs; miRNA-seq;SRP022043;mirPath;AD.vs.control;down regulated miRNAs	10
GO	synaptic	synaptic transmission	miRNA-seq;SRP022043;mirPath;AD.vs.control;up regulated miRNAs; miRNA-seq;SRP022043;mirPath;AD.vs.control;down regulated miRNAs	10
GO	phosphatidylinositol-mediated	phosphatidylinositol-mediated signaling	miRNA-seq;SRP022043;mirPath;AD.vs.control;up regulated miRNAs; miRNA-seq;SRP022043;mirPath;AD.vs.control;down regulated miRNAs	10
GO	toll-like receptor 5 signaling	toll-like receptor 5 signaling pathway	miRNA-seq;SRP022043;mirPath;AD.vs.control;up regulated miRNAs; miRNA-seq;SRP022043;mirPath;AD.vs.control;down regulated miRNAs	10
GO	toll-like receptor 9 signaling	toll-like receptor 9 signaling pathway	miRNA-seq;SRP022043;mirPath;AD.vs.control;up regulated miRNAs; miRNA-seq;SRP022043;mirPath;AD.vs.control;down regulated miRNAs	10
GO	enzyme regulator activity	enzyme regulator activity	miRNA-seq;SRP022043;mirPath;AD.vs.control;up regulated miRNAs; miRNA-seq;SRP022043;mirPath;AD.vs.control;down regulated miRNAs	10
GO	TRIF-dependent toll-like receptor signaling pathway	TRIF-dependent toll-like receptor signaling pathway	miRNA-seq;SRP022043;mirPath;AD.vs.control;up regulated miRNAs; miRNA-seq;SRP022043;mirPath;AD.vs.control;down regulated miRNAs	10
GO	protein N-linked glycosylation via asparagine	protein N-linked glycosylation via asparagine	miRNA-seq;SRP022043;mirPath;AD.vs.control;up regulated miRNAs; miRNA-seq;SRP022043;mirPath;AD.vs.control;down regulated miRNAs	10
GO	DNA metabolic process	DNA metabolic process	miRNA-seq;SRP022043;mirPath;AD.vs.control;up regulated miRNAs; miRNA-seq;SRP022043;mirPath;AD.vs.control;down regulated miRNAs	10
GO	Fc-gamma receptor signaling pathway involved in phagocytosis	Fc-gamma receptor signaling pathway involved in phagocytosis	miRNA-seq;SRP022043;mirPath;AD.vs.control;up regulated miRNAs; miRNA-seq;SRP022043;mirPath;AD.vs.control;down regulated miRNAs	10
GO	biological_proces	biological_process	miRNA-seq;SRP022043;mirPath;AD.vs.control;up regulated miRNAs; miRNA-seq;SRP022043;mirPath;AD.vs.control;down regulated miRNAs	10
GO	platelet	platelet degranulation	miRNA-seq;SRP022043;mirPath;AD.vs.control;up regulated miRNAs; miRNA-seq;SRP022043;mirPath;AD.vs.control;down regulated miRNAs	10
GO	immune system process	immune system process	miRNA-seq;SRP022043;mirPath;AD.vs.control;up regulated miRNAs; miRNA-seq;SRP022043;mirPath;AD.vs.control;down regulated miRNAs	10
GO	cell motility	cell motility	miRNA-seq;SRP022043;mirPath;AD.vs.control;up regulated miRNAs; miRNA-seq;SRP022043;mirPath;AD.vs.control;down regulated miRNAs	10
GO	MyD88-independent toll-like receptor	MyD88-independent toll-like receptor signaling pathway	miRNA-seq;SRP022043;mirPath;AD.vs.control;up regulated miRNAs; miRNA-seq;SRP022043;mirPath;AD.vs.control;down regulated miRNAs	10

GO	axon guidance	axon guidance	miRNA-seq;SRP022043;mirPath;AD.vs.control;up regulated miRNAs; miRNA-seq;SRP022043;mirPath;AD.vs.control;down regulated miRNAs	10
GO	toll-like receptor 2 signaling	toll-like receptor 2 signaling pathway	miRNA-seq;SRP022043;mirPath;AD.vs.control;up regulated miRNAs; miRNA-seq;SRP022043;mirPath;AD.vs.control;down regulated miRNAs	10
GO	leukocyte	leukocyte migration	miRNA-seq;SRP022043;mirPath;AD.vs.control;up regulated miRNAs; miRNA-seq;SRP022043;mirPath;AD.vs.control;down regulated miRNAs	10
GO	glycosaminoglycan metabolic	glycosaminoglycan metabolic process	miRNA-seq;SRP022043;mirPath;AD.vs.control;up regulated miRNAs; miRNA-seq;SRP022043;mirPath;AD.vs.control;down regulated miRNAs	10
GO	cell junction organization	cell junction organization	miRNA-seq;SRP022043;mirPath;AD.vs.control;up regulated miRNAs; miRNA-seq;SRP022043;mirPath;AD.vs.control;down regulated miRNAs	10
GO	cell-cell signaling	cell-cell signaling	miRNA-seq;SRP022043;mirPath;AD.vs.control;up regulated miRNAs; miRNA-seq;SRP022043;mirPath;AD.vs.control;down regulated miRNAs	10
GO	microtubule organizing center	microtubule organizing center	miRNA-seq;SRP022043;mirPath;AD.vs.control;up regulated miRNAs; miRNA-seq;SRP022043;mirPath;AD.vs.control;down regulated miRNAs	10
GO	cell junction assembly	cell junction assembly	miRNA-seq;SRP022043;mirPath;AD.vs.control;up regulated miRNAs; miRNA-seq;SRP022043;mirPath;AD.vs.control;down regulated miRNAs	10
GO	toll-like receptor 4 signaling	toll-like receptor 4 signaling pathway	miRNA-seq;SRP022043;mirPath;AD.vs.control;up regulated miRNAs; miRNA-seq;SRP022043;mirPath;AD.vs.control;down regulated miRNAs	10
GO	G2/M transition of mitotic cell	G2/M transition of mitotic cell cycle	miRNA-seq;SRP022043;mirPath;AD.vs.control;up regulated miRNAs; miRNA-seq;SRP022043;mirPath;AD.vs.control;down regulated miRNAs	10
GO	energy reserve metabolic process	energy reserve metabolic process	miRNA-seq;SRP022043;mirPath;AD.vs.control;up regulated miRNAs; miRNA-seq;SRP022043;mirPath;AD.vs.control;down regulated miRNAs	10
GO	toll-like receptor 3 signaling	toll-like receptor 3 signaling pathway	miRNA-seq;SRP022043;mirPath;AD.vs.control;up regulated miRNAs; miRNA-seq;SRP022043;mirPath;AD.vs.control;down regulated miRNAs	10
GO	post-Golgi vesicle-mediated	post-Golgi vesicle-mediated transport	miRNA-seq;SRP022043;mirPath;AD.vs.control;up regulated miRNAs; miRNA-seq;SRP022043;mirPath;AD.vs.control;down regulated miRNAs	10
GO	transcription, DNA-templated	transcription, DNA-templated	miRNA-seq;SRP022043;mirPath;AD.vs.control;up regulated miRNAs; miRNA-seq;SRP022043;mirPath;AD.vs.control;down regulated miRNAs	10
GO	platelet alpha granule lumen	platelet alpha granule lumen	miRNA-seq;SRP022043;mirPath;AD.vs.control;up regulated miRNAs; miRNA-seq;SRP022043;mirPath;AD.vs.control;down regulated miRNAs	10
GO	RNA binding	RNA binding	miRNA-seq;SRP022043;mirPath;AD.vs.control;up regulated miRNAs; miRNA-seq;SRP022043;mirPath;AD.vs.control;down regulated miRNAs	10
GO	toll-like receptor signaling pathway	toll-like receptor signaling pathway	miRNA-seq;SRP022043;mirPath;AD.vs.control;up regulated miRNAs; miRNA-seq;SRP022043;mirPath;AD.vs.control;down regulated miRNAs	10
GO	anatomical structure	anatomical structure morphogenesis	miRNA-seq;SRP022043;mirPath;AD.vs.control;up regulated miRNAs; miRNA-seq;SRP022043;mirPath;AD.vs.control;down regulated miRNAs	10
GO	mRNA metabolic process	mRNA metabolic process	miRNA-seq;SRP022043;mirPath;AD.vs.control;up regulated miRNAs; miRNA-seq;SRP022043;mirPath;AD.vs.control;down regulated miRNAs	10
GO	transcription initiation from RNA polymerase II promoter	transcription initiation from RNA polymerase II promoter	miRNA-seq;SRP022043;mirPath;AD.vs.control;up regulated miRNAs; miRNA-seq;SRP022043;mirPath;AD.vs.control;down regulated miRNAs	10
GO	cellular component disassembly involved in	cellular component disassembly involved in execution phase of apoptosis	miRNA-seq;SRP022043;mirPath;AD.vs.control;up regulated miRNAs; miRNA-seq;SRP022043;mirPath;AD.vs.control;down regulated miRNAs	10
GO	extracellular matrix	extracellular matrix disassembly	miRNA-seq;SRP022043;mirPath;AD.vs.control;up regulated miRNAs; miRNA-seq;SRP022043;mirPath;AD.vs.control;down regulated miRNAs	10
GO	stress-activated MAPK cascade	stress-activated MAPK cascade	miRNA-seq;SRP022043;mirPath;AD.vs.control;up regulated miRNAs; miRNA-seq;SRP022043;mirPath;AD.vs.control;down regulated miRNAs	10
GO	extracellular matrix	extracellular matrix organization	miRNA-seq;SRP022043;mirPath;AD.vs.control;up regulated miRNAs; miRNA-seq;SRP022043;mirPath;AD.vs.control;down regulated miRNAs	10
GO	regulation of defense response to virus by virus	regulation of defense response to virus by virus	miRNA-seq;SRP022043;mirPath;AD.vs.control;up regulated miRNAs; miRNA-seq;SRP022043;mirPath;AD.vs.control;down regulated miRNAs	10
GO	intrinsic apoptotic signaling pathway	intrinsic apoptotic signaling pathway	miRNA-seq;SRP022043;mirPath;AD.vs.control;up regulated miRNAs; miRNA-seq;SRP022043;mirPath;AD.vs.control;down regulated miRNAs	10
GO	nucleobase-containing small molecule metabolic process	nucleobase-containing small molecule metabolic process	miRNA-seq;SRP022043;mirPath;AD.vs.control;up regulated miRNAs; miRNA-seq;SRP022043;mirPath;AD.vs.control;down regulated miRNAs	10
GO	MyD88-dependent toll-like receptor	MyD88-dependent toll-like receptor signaling pathway	miRNA-seq;SRP022043;mirPath;AD.vs.control;up regulated miRNAs; miRNA-seq;SRP022043;mirPath;AD.vs.control;down regulated miRNAs	10
GO	hexose transport	hexose transport	miRNA-seq;SRP022043;mirPath;AD.vs.control;up regulated miRNAs; miRNA-seq;SRP022043;mirPath;AD.vs.control;down regulated miRNAs	10
GO	nucleotide-binding oligomerization domain	nucleotide-binding oligomerization domain containing signaling pathway	miRNA-seq;SRP022043;mirPath;AD.vs.control;up regulated miRNAs; miRNA-seq;SRP022043;mirPath;AD.vs.control;down regulated miRNAs	10

GO	water-soluble vitamin metabolic process	water-soluble vitamin metabolic process	miRNA-seq;SRP022043;mirPath;AD.vs.control;up regulated miRNAs; miRNA-seq;SRP022043;mirPath;AD.vs.control;down regulated miRNAs	10
GO	vitamin metabolic process	vitamin metabolic process	miRNA-seq;SRP022043;mirPath;AD.vs.control;up regulated miRNAs; miRNA-seq;SRP022043;mirPath;AD.vs.control;down regulated miRNAs	10
GO	positive regulation of protein insertion into mitochondrial membrane	positive regulation of protein insertion into mitochondrial membrane involved in apoptotic signaling pathway	miRNA-seq;SRP022043;mirPath;AD.vs.control;up regulated miRNAs; miRNA-seq;SRP022043;mirPath;AD.vs.control;down regulated miRNAs	10
GO	insulin receptor signaling pathway	insulin receptor signaling pathway	miRNA-seq;SRP022043;mirPath;AD.vs.control;up regulated miRNAs; miRNA-seq;SRP022043;mirPath;AD.vs.control;down regulated miRNAs	10
GO	innate immune response	innate immune response	miRNA-seq;SRP022043;mirPath;AD.vs.control;up regulated miRNAs; miRNA-seq;SRP022043;mirPath;AD.vs.control;down regulated miRNAs	10
GO	chromatin organization	chromatin organization	miRNA-seq;SRP022043;mirPath;AD.vs.control;up regulated miRNAs; miRNA-seq;SRP022043;mirPath;AD.vs.control;down regulated miRNAs	10
GO	nucleotide-binding domain, leucine rich repeat containing receptor signaling	nucleotide-binding domain, leucine rich repeat containing receptor signaling pathway	miRNA-seq;SRP022043;mirPath;AD.vs.control;up regulated miRNAs; miRNA-seq;SRP022043;mirPath;AD.vs.control;down regulated miRNAs	10
GO	phospholipid metabolic process	phospholipid metabolic process	miRNA-seq;SRP022043;mirPath;AD.vs.control;up regulated miRNAs; miRNA-seq;SRP022043;mirPath;AD.vs.control;down regulated miRNAs	10
GO	protein binding, bridging	protein binding, bridging	miRNA-seq;SRP022043;mirPath;AD.vs.control;up regulated miRNAs; miRNA-seq;SRP022043;mirPath;AD.vs.control;down regulated miRNAs	10
GO	nucleocytoplasmi c transport	nucleocytoplasmic transport	miRNA-seq;SRP022043;mirPath;AD.vs.control;up regulated miRNAs; miRNA-seq;SRP022043;mirPath;AD.vs.control;down regulated miRNAs	10
GO	O-glycan	O-glycan processing	miRNA-seq;SRP022043;mirPath;AD.vs.control;up regulated miRNAs	5
GO	sulfur compound metabolic process	sulfur compound metabolic process	miRNA-seq;SRP022043;mirPath;AD.vs.control;up regulated miRNAs; miRNA-seq;SRP022043;mirPath;AD.vs.control;down regulated miRNAs	10
GO	glycerophospholi pid biosynthetic	glycerophospholipid biosynthetic process	miRNA-seq;SRP022043;mirPath;AD.vs.control;up regulated miRNAs; miRNA-seq;SRP022043;mirPath;AD.vs.control;down regulated miRNAs	10
GO	xenobiotic metabolic process	xenobiotic metabolic process	miRNA-seq;SRP022043;mirPath;AD.vs.control;up regulated miRNAs; miRNA-seq;SRP022043;mirPath;AD.vs.control;down regulated miRNAs	10
GO	regulation of transcription from RNA polymerase II promoter in response to	regulation of transcription from RNA polymerase II promoter in response to hypoxia	miRNA-seq;SRP022043;mirPath;AD.vs.control;up regulated miRNAs; miRNA-seq;SRP022043;mirPath;AD.vs.control;down regulated miRNAs	10
GO	phosphatidylinosi tol biosynthetic	phosphatidylinositol biosynthetic process	miRNA-seq;SRP022043;mirPath;AD.vs.control;up regulated miRNAs; miRNA-seq;SRP022043;mirPath;AD.vs.control;down regulated miRNAs	10
GO	RNA metabolic process	RNA metabolic process	miRNA-seq;SRP022043;mirPath;AD.vs.control;up regulated miRNAs; miRNA-seq;SRP022043;mirPath;AD.vs.control;down regulated miRNAs	10
GO	sulfur amino acid metabolic process	sulfur amino acid metabolic process	miRNA-seq;SRP022043;mirPath;AD.vs.control;up regulated miRNAs	5
GO	activation of phospholipase C activity	activation of phospholipase C activity	miRNA-seq;SRP022043;mirPath;AD.vs.control;up regulated miRNAs	5
GO	regulation of ubiquitin-protein ligase activity involved in mitotic cell cycle	regulation of ubiquitin-protein ligase activity involved in mitotic cell cycle	miRNA-seq;SRP022043;mirPath;AD.vs.control;up regulated miRNAs; miRNA-seq;SRP022043;mirPath;AD.vs.control;down regulated miRNAs	10
GO	cytoskeleton-dependent intracellular	cytoskeleton-dependent intracellular transport	miRNA-seq;SRP022043;mirPath;AD.vs.control;up regulated miRNAs; miRNA-seq;SRP022043;mirPath;AD.vs.control;down regulated miRNAs	10
GO	regulation of glucose transport	regulation of glucose transport	miRNA-seq;SRP022043;mirPath;AD.vs.control;up regulated miRNAs	5
GO	small conjugating protein binding	small conjugating protein binding	miRNA-seq;SRP022043;mirPath;AD.vs.control;up regulated miRNAs; miRNA-seq;SRP022043;mirPath;AD.vs.control;down regulated miRNAs	10
GO	mitotic nuclear envelope disassembly	mitotic nuclear envelope disassembly	miRNA-seq;SRP022043;mirPath;AD.vs.control;up regulated miRNAs; miRNA-seq;SRP022043;mirPath;AD.vs.control;down regulated miRNAs	10
GO	vesicle-mediated transport	vesicle-mediated transport	miRNA-seq;SRP022043;mirPath;AD.vs.control;up regulated miRNAs; miRNA-seq;SRP022043;mirPath;AD.vs.control;down regulated miRNAs	10

GO	branched-chain amino acid catabolic process	branched-chain amino acid catabolic process	miRNA-seq;SRP022043;mirPath;AD.vs.control;up regulated miRNAs	5
GO	JAK-STAT cascade involved in growth hormone	JAK-STAT cascade involved in growth hormone signaling pathway	miRNA-seq;SRP022043;mirPath;AD.vs.control;up regulated miRNAs; miRNA-seq;SRP022043;mirPath;AD.vs.control;down regulated miRNAs	10
GO	regulation of interferon-gamma-mediated signaling pathway	regulation of interferon-gamma-mediated signaling pathway	miRNA-seq;SRP022043;mirPath;AD.vs.control;up regulated miRNAs	5
GO	cytoskeleton organization	cytoskeleton organization	miRNA-seq;SRP022043;mirPath;AD.vs.control;up regulated miRNAs; miRNA-seq;SRP022043;mirPath;AD.vs.control;down regulated miRNAs	10
GO	inositol phosphate	inositol phosphate metabolic process	miRNA-seq;SRP022043;mirPath;AD.vs.control;up regulated miRNAs; miRNA-seq;SRP022043;mirPath;AD.vs.control;down regulated miRNAs	10
GO	cell-cell junction organization	cell-cell junction organization	miRNA-seq;SRP022043;mirPath;AD.vs.control;up regulated miRNAs; miRNA-seq;SRP022043;mirPath;AD.vs.control;down regulated miRNAs	10
GO	positive regulation of ubiquitin-protein ligase activity involved in	positive regulation of ubiquitin-protein ligase activity involved in mitotic cell cycle	miRNA-seq;SRP022043;mirPath;AD.vs.control;up regulated miRNAs; miRNA-seq;SRP022043;mirPath;AD.vs.control;down regulated miRNAs	10
GO	regulation of insulin secretion	regulation of insulin secretion	miRNA-seq;SRP022043;mirPath;AD.vs.control;up regulated miRNAs	5
GO	keratan sulfate biosynthetic	keratan sulfate biosynthetic process	miRNA-seq;SRP022043;mirPath;AD.vs.control;up regulated miRNAs	5
GO	chondroitin sulfate metabolic	chondroitin sulfate metabolic process	miRNA-seq;SRP022043;mirPath;AD.vs.control;up regulated miRNAs; miRNA-seq;SRP022043;mirPath;AD.vs.control;down regulated miRNAs	10
GO	nuclear-transcribed mRNA catabolic process, deadenylation-	nuclear-transcribed mRNA catabolic process, deadenylation-dependent decay	miRNA-seq;SRP022043;mirPath;AD.vs.control;up regulated miRNAs; miRNA-seq;SRP022043;mirPath;AD.vs.control;down regulated miRNAs	10
GO	generation of precursor metabolites and energy	generation of precursor metabolites and energy	miRNA-seq;SRP022043;mirPath;AD.vs.control;up regulated miRNAs	5
GO	keratan sulfate metabolic process	keratan sulfate metabolic process	miRNA-seq;SRP022043;mirPath;AD.vs.control;up regulated miRNAs	5
GO	termination of RNA polymerase II transcription	termination of RNA polymerase II transcription	miRNA-seq;SRP022043;mirPath;AD.vs.control;up regulated miRNAs; miRNA-seq;SRP022043;mirPath;AD.vs.control;down regulated miRNAs	10
GO	protein polyubiquitinatio	protein polyubiquitination	miRNA-seq;SRP022043;mirPath;AD.vs.control;up regulated miRNAs	5
GO	cellular response to glucagon	cellular response to glucagon stimulus	miRNA-seq;SRP022043;mirPath;AD.vs.control;up regulated miRNAs	5
GO	homeostatic	homeostatic process	miRNA-seq;SRP022043;mirPath;AD.vs.control;up regulated miRNAs; miRNA-seq;SRP022043;mirPath;AD.vs.control;down regulated miRNAs	10
GO	nitric oxide metabolic process	nitric oxide metabolic process	miRNA-seq;SRP022043;mirPath;AD.vs.control;up regulated miRNAs	5
GO	regulation of cardiac muscle contraction by regulation of the release of sequestered	regulation of cardiac muscle contraction by regulation of the release of sequestered calcium ion	miRNA-seq;SRP022043;mirPath;AD.vs.control;up regulated miRNAs	5
GO	transforming growth factor beta receptor	transforming growth factor beta receptor signaling pathway	miRNA-seq;SRP022043;mirPath;AD.vs.control;up regulated miRNAs; miRNA-seq;SRP022043;mirPath;AD.vs.control;down regulated miRNAs	10
GO	mRNA 3'-end processing	mRNA 3'-end processing	miRNA-seq;SRP022043;mirPath;AD.vs.control;up regulated miRNAs; miRNA-seq;SRP022043;mirPath;AD.vs.control;down regulated miRNAs	10
GO	nervous system development	nervous system development	miRNA-seq;SRP022043;mirPath;AD.vs.control;up regulated miRNAs; miRNA-seq;SRP022043;mirPath;AD.vs.control;down regulated miRNAs	10
GO	regulation of nitric-oxide synthase activity	regulation of nitric-oxide synthase activity	miRNA-seq;SRP022043;mirPath;AD.vs.control;up regulated miRNAs	5

GO	transcription elongation from RNA polymerase I promoter	transcription elongation from RNA polymerase I promoter	miRNA-seq;SRP022043;mirPath;AD.vs.control;up regulated miRNAs; miRNA-seq;SRP022043;mirPath;AD.vs.control;down regulated miRNAs	10
GO	regulation of small GTPase mediated signal	regulation of small GTPase mediated signal transduction	miRNA-seq;SRP022043;mirPath;AD.vs.control;up regulated miRNAs; miRNA-seq;SRP022043;mirPath;AD.vs.control;down regulated miRNAs	10
GO	muscle cell differentiation	muscle cell differentiation	miRNA-seq;SRP022043;mirPath;AD.vs.control;up regulated miRNAs	5
GO	negative regulation of ubiquitin-protein ligase activity involved in	negative regulation of ubiquitin-protein ligase activity involved in mitotic cell cycle	miRNA-seq;SRP022043;mirPath;AD.vs.control;up regulated miRNAs	5
GO	unsaturated fatty acid metabolic process	unsaturated fatty acid metabolic process	miRNA-seq;SRP022043;mirPath;AD.vs.control;up regulated miRNAs	5
GO	alpha-linolenic acid metabolic	alpha-linolenic acid metabolic process	miRNA-seq;SRP022043;mirPath;AD.vs.control;up regulated miRNAs	5
GO	phosphatidylinositol 3-kinase	phosphatidylinositol 3-kinase activity	miRNA-seq;SRP022043;mirPath;AD.vs.control;up regulated miRNAs	5
GO	negative regulation of	negative regulation of axonogenesis	miRNA-seq;SRP022043;mirPath;AD.vs.control;up regulated miRNAs	5
GO	ribonucleoprotein complex	ribonucleoprotein complex assembly	miRNA-seq;SRP022043;mirPath;AD.vs.control;up regulated miRNAs	5
GO	G1/S transition of mitotic cell cycle	G1/S transition of mitotic cell cycle	miRNA-seq;SRP022043;mirPath;AD.vs.control;up regulated miRNAs; miRNA-seq;SRP022043;mirPath;AD.vs.control;down regulated miRNAs	10
GO	transmembrane transporter	transmembrane transporter activity	miRNA-seq;SRP022043;mirPath;AD.vs.control;down regulated miRNAs	5
GO	cellular component	cellular component movement	miRNA-seq;SRP022043;mirPath;AD.vs.control;down regulated miRNAs	5
GO	endoplasmic reticulum lumen	endoplasmic reticulum lumen	miRNA-seq;SRP022043;mirPath;AD.vs.control;down regulated miRNAs	5
GO	lipid binding	lipid binding	miRNA-seq;SRP022043;mirPath;AD.vs.control;down regulated miRNAs	5
GO	apoptotic signaling pathway	apoptotic signaling pathway	miRNA-seq;SRP022043;mirPath;AD.vs.control;down regulated miRNAs	5
GO	regulation of Ras GTPase activity	regulation of Ras GTPase activity	miRNA-seq;SRP022043;mirPath;AD.vs.control;down regulated miRNAs	5
GO	positive regulation of	positive regulation of signal transduction	miRNA-seq;SRP022043;mirPath;AD.vs.control;down regulated miRNAs	5
GO	activation of signaling protein activity involved in unfolded	activation of signaling protein activity involved in unfolded protein response	miRNA-seq;SRP022043;mirPath;AD.vs.control;down regulated miRNAs	5
GO	SH3/SH2 adaptor activity	SH3/SH2 adaptor activity	miRNA-seq;SRP022043;mirPath;AD.vs.control;down regulated miRNAs	5
GO	regulation of rhodopsin mediated	regulation of rhodopsin mediated signaling pathway	miRNA-seq;SRP022043;mirPath;AD.vs.control;down regulated miRNAs	5
GO	protein	protein maturation	miRNA-seq;SRP022043;mirPath;AD.vs.control;down regulated miRNAs	5
GO	C-terminal protein lipidation	C-terminal protein lipidation	miRNA-seq;SRP022043;mirPath;AD.vs.control;down regulated miRNAs	5
GO	histone binding	histone binding	miRNA-seq;SRP022043;mirPath;AD.vs.control;down regulated miRNAs	5
GO	Ras protein signal transduction	Ras protein signal transduction	miRNA-seq;SRP022043;mirPath;AD.vs.control;down regulated miRNAs	5
GO	collagen catabolic process	collagen catabolic process	miRNA-seq;SRP022043;mirPath;AD.vs.control;down regulated miRNAs	5
GO	lysosomal lumen	lysosomal lumen	miRNA-seq;SRP022043;mirPath;AD.vs.control;down regulated miRNAs	5
GO	telomere maintenance via semi-conservative replication	telomere maintenance via semi-conservative replication	miRNA-seq;SRP022043;mirPath;AD.vs.control;down regulated miRNAs	5
GO	transcription-coupled nucleotide-	transcription-coupled nucleotide-excision repair	miRNA-seq;SRP022043;mirPath;AD.vs.control;down regulated miRNAs	5

GO	dermatan sulfate biosynthetic	dermatan sulfate biosynthetic process	miRNA-seq;SRP022043;mirPath;AD.vs.control;down regulated miRNAs	5
GO	DNA damage response, signal transduction by p53 class mediator resulting in cell	DNA damage response, signal transduction by p53 class mediator resulting in cell cycle arrest	miRNA-seq;SRP022043;mirPath;AD.vs.control;down regulated miRNAs	5
GO	in utero embryonic	in utero embryonic development	miRNA-seq;SRP022043;mirPath;AD.vs.control;down regulated miRNAs	5
GO	regulation of cellular amino acid metabolic	regulation of cellular amino acid metabolic process	miRNA-seq;SRP022043;mirPath;AD.vs.control;down regulated miRNAs	5
GO	cell proliferation	cell proliferation	miRNA-seq;SRP022043;mirPath;AD.vs.control;down regulated miRNAs	5
GO	sphingolipid biosynthetic	sphingolipid biosynthetic process	miRNA-seq;SRP022043;mirPath;AD.vs.control;down regulated miRNAs	5
GO	transmembrane receptor protein tyrosine phosphatase	transmembrane receptor protein tyrosine phosphatase activity	miRNA-seq;SRP022043;mirPath;AD.vs.control;down regulated miRNAs	5
GO	glutamate	glutamate secretion	miRNA-seq;SRP022043;mirPath;AD.vs.control;down regulated miRNAs	5
GO	endosome	endosome	miRNA-seq;SRP022043;mirPath;AD.vs.control;down regulated miRNAs	5
GO	neuron cell-cell adhesion	neuron cell-cell adhesion	miRNA-seq;SRP022043;mirPath;AD.vs.control;down regulated miRNAs	5
GO	androgen receptor signaling pathway	androgen receptor signaling pathway	miRNA-seq;SRP022043;mirPath;AD.vs.control;down regulated miRNAs	5
KEGG	Proteoglycans in cancer	Proteoglycans in cancer	miRNA-seq;SRP022043;mirPath;AD.vs.control;up regulated miRNAs; miRNA-seq;SRP022043;mirPath;AD.vs.control;down regulated miRNAs; miRNA-seq;SRP022043;miEAA;AD.vs.control;down regulated miRNAs	15
KEGG	Mucin type O-Glycan	Mucin type O-Glycan biosynthesis	miRNA-seq;SRP022043;mirPath;AD.vs.control;up regulated miRNAs	5
KEGG	Axon guidance	Axon guidance	miRNA-seq;SRP022043;mirPath;AD.vs.control;up regulated miRNAs; miRNA-seq;SRP022043;mirPath;AD.vs.control;down regulated miRNAs; miRNA-seq;SRP022043;miEAA;AD.vs.control;down regulated miRNAs	15
KEGG	Neurotrophin signaling pathway	Neurotrophin signaling pathway	miRNA-seq;SRP022043;mirPath;AD.vs.control;up regulated miRNAs; miRNA-seq;SRP022043;mirPath;AD.vs.control;down regulated miRNAs; miRNA-seq;SRP022043;miEAA;AD.vs.control;down regulated miRNAs	15
KEGG	Ubiquitin mediated	Ubiquitin mediated proteolysis	miRNA-seq;SRP022043;mirPath;AD.vs.control;up regulated miRNAs; miRNA-seq;SRP022043;mirPath;AD.vs.control;down regulated miRNAs; miRNA-seq;SRP022043;miEAA;AD.vs.control;down regulated miRNAs	15
KEGG	Glioma	Glioma	miRNA-seq;SRP022043;mirPath;AD.vs.control;up regulated miRNAs; miRNA-seq;SRP022043;mirPath;AD.vs.control;down regulated miRNAs; miRNA-seq;SRP022043;miEAA;AD.vs.control;down regulated miRNAs; miRNA-seq;SRP022043;miEAA;AD.vs.control;down regulated miRNAs	20
KEGG	TGF-beta signaling pathway	TGF-beta signaling pathway	miRNA-seq;SRP022043;mirPath;AD.vs.control;up regulated miRNAs; miRNA-seq;SRP022043;mirPath;AD.vs.control;down regulated miRNAs; miRNA-seq;SRP022043;miEAA;AD.vs.control;down regulated miRNAs	15
KEGG	Rap1 signaling pathway	Rap1 signaling pathway	miRNA-seq;SRP022043;mirPath;AD.vs.control;up regulated miRNAs; miRNA-seq;SRP022043;mirPath;AD.vs.control;down regulated miRNAs; miRNA-seq;SRP022043;miEAA;AD.vs.control;up regulated miRNAs; miRNA-seq;SRP022043;miEAA;AD.vs.control;down regulated miRNAs	20
KEGG	Focal adhesion	Focal adhesion	miRNA-seq;SRP022043;mirPath;AD.vs.control;up regulated miRNAs; miRNA-seq;SRP022043;mirPath;AD.vs.control;down regulated miRNAs; miRNA-seq;SRP022043;miEAA;AD.vs.control;down regulated miRNAs	15
KEGG	PI3K-Akt signaling pathway	PI3K-Akt signaling pathway	miRNA-seq;SRP022043;mirPath;AD.vs.control;up regulated miRNAs; miRNA-seq;SRP022043;mirPath;AD.vs.control;down regulated miRNAs; miRNA-seq;SRP022043;miEAA;AD.vs.control;down regulated miRNAs	15
KEGG	FoxO signaling pathway	FoxO signaling pathway	miRNA-seq;SRP022043;mirPath;AD.vs.control;up regulated miRNAs; miRNA-seq;SRP022043;mirPath;AD.vs.control;down regulated miRNAs; miRNA-seq;SRP022043;miEAA;AD.vs.control;up regulated miRNAs; miRNA-seq;SRP022043;miEAA;AD.vs.control;down regulated miRNAs	20
KEGG	ErbB signaling pathway	ErbB signaling pathway	miRNA-seq;SRP022043;mirPath;AD.vs.control;up regulated miRNAs; miRNA-seq;SRP022043;mirPath;AD.vs.control;down regulated miRNAs; miRNA-seq;SRP022043;miEAA;AD.vs.control;up regulated miRNAs; miRNA-seq;SRP022043;miEAA;AD.vs.control;down regulated miRNAs	20
KEGG	Prion diseases	Prion diseases	miRNA-seq;SRP022043;mirPath;AD.vs.control;up regulated miRNAs; miRNA-seq;SRP022043;mirPath;AD.vs.control;down regulated miRNAs	10
KEGG	Morphine	Morphine addiction	miRNA-seq;SRP022043;mirPath;AD.vs.control;up regulated miRNAs; miRNA-seq;SRP022043;miEAA;AD.vs.control;down regulated miRNAs	10
KEGG	Adherens junction	Adherens junction	miRNA-seq;SRP022043;mirPath;AD.vs.control;up regulated miRNAs; miRNA-seq;SRP022043;mirPath;AD.vs.control;down regulated miRNAs; miRNA-seq;SRP022043;miEAA;AD.vs.control;down regulated miRNAs	15
KEGG	GABAergic	GABAergic synapse	miRNA-seq;SRP022043;mirPath;AD.vs.control;up regulated miRNAs; miRNA-seq;SRP022043;miEAA;AD.vs.control;down regulated miRNAs	10
KEGG	Protein processing in endoplasmic	Protein processing in endoplasmic reticulum	miRNA-seq;SRP022043;mirPath;AD.vs.control;up regulated miRNAs; miRNA-seq;SRP022043;mirPath;AD.vs.control;down regulated miRNAs; miRNA-seq;SRP022043;miEAA;AD.vs.control;down regulated miRNAs	15
KEGG	Ras signaling pathway	Ras signaling pathway	miRNA-seq;SRP022043;mirPath;AD.vs.control;up regulated miRNAs; miRNA-seq;SRP022043;mirPath;AD.vs.control;down regulated miRNAs; miRNA-seq;SRP022043;miEAA;AD.vs.control;down regulated miRNAs	15
KEGG	Renal cell carcinoma	Renal cell carcinoma	miRNA-seq;SRP022043;mirPath;AD.vs.control;up regulated miRNAs; miRNA-seq;SRP022043;mirPath;AD.vs.control;down regulated miRNAs; miRNA-seq;SRP022043;miEAA;AD.vs.control;up regulated miRNAs; miRNA-seq;SRP022043;miEAA;AD.vs.control;down regulated miRNAs; miRNA-seq;SRP022043;miEAA;AD.vs.control;down regulated miRNAs	25
KEGG	Hippo signaling pathway	Hippo signaling pathway	miRNA-seq;SRP022043;mirPath;AD.vs.control;up regulated miRNAs; miRNA-seq;SRP022043;mirPath;AD.vs.control;down regulated miRNAs; miRNA-seq;SRP022043;miEAA;AD.vs.control;down regulated miRNAs	15

KEGG	Prostate cancer	Prostate cancer	miRNA-seq;SRP022043;mirPath;AD.vs.control;up regulated miRNAs; miRNA-seq;SRP022043;mirPath;AD.vs.control;down regulated miRNAs; miRNA-seq;SRP022043;miEAA;AD.vs.control;up regulated miRNAs; miRNA-seq;SRP022043;miEAA;AD.vs.control;down regulated miRNAs; miRNA-seq;SRP022043;miEAA;AD.vs.control;down regulated miRNAs	25
KEGG	Circadian entrainment	Circadian entrainment	miRNA-seq;SRP022043;mirPath;AD.vs.control;up regulated miRNAs; miRNA-seq;SRP022043;miEAA;AD.vs.control;down regulated miRNAs	10
KEGG	N-Glycan biosynthesis	N-Glycan biosynthesis	miRNA-seq;SRP022043;mirPath;AD.vs.control;up regulated miRNAs; miRNA-seq;SRP022043;miEAA;AD.vs.control;down regulated miRNAs	10
KEGG	Arrhythmogenic right ventricular cardiomyopathy (ARVC)	Arrhythmogenic right ventricular cardiomyopathy (ARVC)	miRNA-seq;SRP022043;mirPath;AD.vs.control;up regulated miRNAs	5
KEGG	AMPK signaling pathway	AMPK signaling pathway	miRNA-seq;SRP022043;mirPath;AD.vs.control;up regulated miRNAs; miRNA-seq;SRP022043;mirPath;AD.vs.control;down regulated miRNAs; miRNA-seq;SRP022043;miEAA;AD.vs.control;up regulated miRNAs; miRNA-seq;SRP022043;miEAA;AD.vs.control;down regulated miRNAs	20
KEGG	Lysine degradation	Lysine degradation	miRNA-seq;SRP022043;mirPath;AD.vs.control;up regulated miRNAs; miRNA-seq;SRP022043;mirPath;AD.vs.control;down regulated miRNAs; miRNA-seq;SRP022043;miEAA;AD.vs.control;down regulated miRNAs	15
KEGG	T cell receptor signaling pathway	T cell receptor signaling pathway	miRNA-seq;SRP022043;mirPath;AD.vs.control;up regulated miRNAs; miRNA-seq;SRP022043;miEAA;AD.vs.control;down regulated miRNAs	10
KEGG	Pathways in	Pathways in cancer	miRNA-seq;SRP022043;mirPath;AD.vs.control;up regulated miRNAs; miRNA-seq;SRP022043;mirPath;AD.vs.control;down regulated miRNAs	10
KEGG	ECM-receptor interaction	ECM-receptor interaction	miRNA-seq;SRP022043;mirPath;AD.vs.control;up regulated miRNAs; miRNA-seq;SRP022043;mirPath;AD.vs.control;down regulated miRNAs; miRNA-seq;SRP022043;miEAA;AD.vs.control;down regulated miRNAs	15
KEGG	Biotin	Biotin metabolism	miRNA-seq;SRP022043;mirPath;AD.vs.control;up regulated miRNAs	5
KEGG	Glutamatergic synapse	Glutamatergic synapse	miRNA-seq;SRP022043;mirPath;AD.vs.control;up regulated miRNAs; miRNA-seq;SRP022043;miEAA;AD.vs.control;down regulated miRNAs	10
KEGG	Adrenergic signaling in	Adrenergic signaling in cardiomyocytes	miRNA-seq;SRP022043;mirPath;AD.vs.control;up regulated miRNAs; miRNA-seq;SRP022043;miEAA;AD.vs.control;up regulated miRNAs; miRNA-seq;SRP022043;miEAA;AD.vs.control;down regulated miRNAs	15
KEGG	Melanoma	Melanoma	miRNA-seq;SRP022043;mirPath;AD.vs.control;up regulated miRNAs; miRNA-seq;SRP022043;mirPath;AD.vs.control;down regulated miRNAs; miRNA-seq;SRP022043;miEAA;AD.vs.control;up regulated miRNAs; miRNA-seq;SRP022043;miEAA;AD.vs.control;down regulated miRNAs; miRNA-seq;SRP022043;miEAA;AD.vs.control;down regulated miRNAs	25
KEGG	Amphetamine addiction	Amphetamine addiction	miRNA-seq;SRP022043;mirPath;AD.vs.control;up regulated miRNAs	5
KEGG	Regulation of actin cytoskeleton	Regulation of actin cytoskeleton	miRNA-seq;SRP022043;mirPath;AD.vs.control;up regulated miRNAs; miRNA-seq;SRP022043;mirPath;AD.vs.control;down regulated miRNAs; miRNA-seq;SRP022043;miEAA;AD.vs.control;down regulated miRNAs	15
KEGG	Retrograde endocannabinoid signaling	Retrograde endocannabinoid signaling	miRNA-seq;SRP022043;mirPath;AD.vs.control;up regulated miRNAs; miRNA-seq;SRP022043;miEAA;AD.vs.control;down regulated miRNAs	10
KEGG	Bacterial invasion of epithelial cells	Bacterial invasion of epithelial cells	miRNA-seq;SRP022043;mirPath;AD.vs.control;up regulated miRNAs; miRNA-seq;SRP022043;miEAA;AD.vs.control;down regulated miRNAs	10
KEGG	Chronic myeloid leukemia	Chronic myeloid leukemia	miRNA-seq;SRP022043;mirPath;AD.vs.control;up regulated miRNAs; miRNA-seq;SRP022043;mirPath;AD.vs.control;down regulated miRNAs; miRNA-seq;SRP022043;miEAA;AD.vs.control;down regulated miRNAs; miRNA-seq;SRP022043;miEAA;AD.vs.control;down regulated miRNAs	20
KEGG	Endocytosis	Endocytosis	miRNA-seq;SRP022043;mirPath;AD.vs.control;up regulated miRNAs; miRNA-seq;SRP022043;mirPath;AD.vs.control;down regulated miRNAs	10
KEGG	Long-term potentiation	Long-term potentiation	miRNA-seq;SRP022043;mirPath;AD.vs.control;up regulated miRNAs	5
KEGG	Sphingolipid signaling pathway	Sphingolipid signaling pathway	miRNA-seq;SRP022043;mirPath;AD.vs.control;up regulated miRNAs; miRNA-seq;SRP022043;mirPath;AD.vs.control;down regulated miRNAs; miRNA-seq;SRP022043;miEAA;AD.vs.control;up regulated miRNAs; miRNA-seq;SRP022043;miEAA;AD.vs.control;down regulated miRNAs	20
KEGG	Insulin signaling pathway	Insulin signaling pathway	miRNA-seq;SRP022043;mirPath;AD.vs.control;up regulated miRNAs; miRNA-seq;SRP022043;mirPath;AD.vs.control;down regulated miRNAs; miRNA-seq;SRP022043;miEAA;AD.vs.control;up regulated miRNAs; miRNA-seq;SRP022043;miEAA;AD.vs.control;down regulated miRNAs	20
KEGG	Thyroid hormone signaling pathway	Thyroid hormone signaling pathway	miRNA-seq;SRP022043;mirPath;AD.vs.control;up regulated miRNAs; miRNA-seq;SRP022043;mirPath;AD.vs.control;down regulated miRNAs; miRNA-seq;SRP022043;miEAA;AD.vs.control;down regulated miRNAs	15
KEGG	mTOR signaling pathway	mTOR signaling pathway	miRNA-seq;SRP022043;mirPath;AD.vs.control;up regulated miRNAs; miRNA-seq;SRP022043;mirPath;AD.vs.control;down regulated miRNAs; miRNA-seq;SRP022043;miEAA;AD.vs.control;down regulated miRNAs	15
KEGG	Ubiquinone and other terpenoid-quinone	Ubiquinone and other terpenoid-quinone biosynthesis	miRNA-seq;SRP022043;mirPath;AD.vs.control;up regulated miRNAs	5
KEGG	Taurine and hypotaurine metabolism	Taurine and hypotaurine metabolism	miRNA-seq;SRP022043;mirPath;AD.vs.control;up regulated miRNAs; miRNA-seq;SRP022043;miEAA;AD.vs.control;down regulated miRNAs	10
KEGG	Pancreatic cancer	Pancreatic cancer	miRNA-seq;SRP022043;mirPath;AD.vs.control;up regulated miRNAs; miRNA-seq;SRP022043;mirPath;AD.vs.control;down regulated miRNAs; miRNA-seq;SRP022043;miEAA;AD.vs.control;down regulated miRNAs; miRNA-seq;SRP022043;miEAA;AD.vs.control;down regulated miRNAs	20
KEGG	B cell receptor signaling pathway	B cell receptor signaling pathway	miRNA-seq;SRP022043;mirPath;AD.vs.control;up regulated miRNAs; miRNA-seq;SRP022043;miEAA;AD.vs.control;down regulated miRNAs	10
KEGG	Tight junction	Tight junction	miRNA-seq;SRP022043;mirPath;AD.vs.control;up regulated miRNAs; miRNA-seq;SRP022043;miEAA;AD.vs.control;down regulated miRNAs	10
KEGG	Long-term depression	Long-term depression	miRNA-seq;SRP022043;mirPath;AD.vs.control;up regulated miRNAs; miRNA-seq;SRP022043;mirPath;AD.vs.control;down regulated miRNAs; miRNA-seq;SRP022043;miEAA;AD.vs.control;up regulated miRNAs; miRNA-seq;SRP022043;miEAA;AD.vs.control;down regulated miRNAs	20

KEGG	Signaling pathways regulating pluripotency of	Signaling pathways regulating pluripotency of stem cells	miRNA-seq;SRP022043;mirPath;AD.vs.control;up regulated miRNAs; miRNA-seq;SRP022043;mirPath;AD.vs.control;down regulated miRNAs; miRNA-seq;SRP022043;miEAA;AD.vs.control;down regulated miRNAs	15
KEGG	Dopaminergic synapse	Dopaminergic synapse	miRNA-seq;SRP022043;mirPath;AD.vs.control;up regulated miRNAs; miRNA-seq;SRP022043;miEAA;AD.vs.control;up regulated miRNAs; miRNA-seq;SRP022043;miEAA;AD.vs.control;down regulated miRNAs	15
KEGG	Non-small cell lung cancer	Non-small cell lung cancer	miRNA-seq;SRP022043;mirPath;AD.vs.control;up regulated miRNAs; miRNA-seq;SRP022043;mirPath;AD.vs.control;down regulated miRNAs; miRNA-seq;SRP022043;miEAA;AD.vs.control;down regulated miRNAs; miRNA-seq;SRP022043;miEAA;AD.vs.control;down regulated miRNAs	20
KEGG	Wnt signaling pathway	Wnt signaling pathway	miRNA-seq;SRP022043;mirPath;AD.vs.control;up regulated miRNAs; miRNA-seq;SRP022043;mirPath;AD.vs.control;down regulated miRNAs; miRNA-seq;SRP022043;miEAA;AD.vs.control;down regulated miRNAs	15
KEGG	Gap junction	Gap junction	miRNA-seq;SRP022043;mirPath;AD.vs.control;up regulated miRNAs; miRNA-seq;SRP022043;miEAA;AD.vs.control;down regulated miRNAs	10
KEGG	MAPK signaling pathway	MAPK signaling pathway	miRNA-seq;SRP022043;mirPath;AD.vs.control;up regulated miRNAs; miRNA-seq;SRP022043;mirPath;AD.vs.control;down regulated miRNAs; miRNA-seq;SRP022043;miEAA;AD.vs.control;down regulated miRNAs	15
KEGG	cGMP-PKG signaling pathway	cGMP-PKG signaling pathway	miRNA-seq;SRP022043;mirPath;AD.vs.control;up regulated miRNAs; miRNA-seq;SRP022043;miEAA;AD.vs.control;down regulated miRNAs	10
KEGG	Oxytocin signaling pathway	Oxytocin signaling pathway	miRNA-seq;SRP022043;mirPath;AD.vs.control;up regulated miRNAs; miRNA-seq;SRP022043;miEAA;AD.vs.control;down regulated miRNAs	10
KEGG	cAMP signaling pathway	cAMP signaling pathway	miRNA-seq;SRP022043;mirPath;AD.vs.control;up regulated miRNAs; miRNA-seq;SRP022043;miEAA;AD.vs.control;down regulated miRNAs	10
KEGG	Shigellosis	Shigellosis	miRNA-seq;SRP022043;mirPath;AD.vs.control;up regulated miRNAs	5
KEGG	Fatty acid biosynthesis	Fatty acid biosynthesis	miRNA-seq;SRP022043;mirPath;AD.vs.control;down regulated miRNAs; miRNA-seq;SRP022043;miEAA;AD.vs.control;down regulated miRNAs	10
KEGG	Small cell lung cancer	Small cell lung cancer	miRNA-seq;SRP022043;mirPath;AD.vs.control;down regulated miRNAs; miRNA-seq;SRP022043;miEAA;AD.vs.control;up regulated miRNAs; miRNA-seq;SRP022043;miEAA;AD.vs.control;down regulated miRNAs; miRNA-seq;SRP022043;miEAA;AD.vs.control;down regulated miRNAs	20
KEGG	Glycosaminoglycan biosynthesis - heparan sulfate / heparin	Glycosaminoglycan biosynthesis - heparan sulfate / heparin	miRNA-seq;SRP022043;mirPath;AD.vs.control;down regulated miRNAs	5
KEGG	Colorectal cancer	Colorectal cancer	miRNA-seq;SRP022043;mirPath;AD.vs.control;down regulated miRNAs; miRNA-seq;SRP022043;miEAA;AD.vs.control;up regulated miRNAs; miRNA-seq;SRP022043;miEAA;AD.vs.control;down regulated miRNAs; miRNA-seq;SRP022043;miEAA;AD.vs.control;down regulated miRNAs	20
KEGG	Acute myeloid leukemia	Acute myeloid leukemia	miRNA-seq;SRP022043;mirPath;AD.vs.control;down regulated miRNAs; miRNA-seq;SRP022043;miEAA;AD.vs.control;down regulated miRNAs; miRNA-seq;SRP022043;miEAA;AD.vs.control;down regulated miRNAs	15
KEGG	Glycosaminoglycan biosynthesis - chondroitin sulfate / dermatan	Glycosaminoglycan biosynthesis - chondroitin sulfate / dermatan sulfate	miRNA-seq;SRP022043;mirPath;AD.vs.control;down regulated miRNAs	5
KEGG	Choline metabolism in	Choline metabolism in cancer	miRNA-seq;SRP022043;mirPath;AD.vs.control;down regulated miRNAs; miRNA-seq;SRP022043;miEAA;AD.vs.control;down regulated miRNAs	10
KEGG	Endometrial cancer	Endometrial cancer	miRNA-seq;SRP022043;mirPath;AD.vs.control;down regulated miRNAs; miRNA-seq;SRP022043;miEAA;AD.vs.control;up regulated miRNAs; miRNA-seq;SRP022043;miEAA;AD.vs.control;down regulated miRNAs; miRNA-seq;SRP022043;miEAA;AD.vs.control;down regulated miRNAs	20
KEGG	Prolactin signaling pathway	Prolactin signaling pathway	miRNA-seq;SRP022043;mirPath;AD.vs.control;down regulated miRNAs; miRNA-seq;SRP022043;miEAA;AD.vs.control;up regulated miRNAs; miRNA-seq;SRP022043;miEAA;AD.vs.control;down regulated miRNAs	15
KEGG	Transcriptional misregulation in cancer	Transcriptional misregulation in cancer	miRNA-seq;SRP022043;mirPath;AD.vs.control;down regulated miRNAs	5
KEGG	Amoebiasis	Amoebiasis	miRNA-seq;SRP022043;mirPath;AD.vs.control;down regulated miRNAs; miRNA-seq;SRP022043;miEAA;AD.vs.control;down regulated miRNAs	10
KEGG	Thyroid cancer	Thyroid cancer	miRNA-seq;SRP022043;mirPath;AD.vs.control;down regulated miRNAs; miRNA-seq;SRP022043;miEAA;AD.vs.control;up regulated miRNAs; miRNA-seq;SRP022043;miEAA;AD.vs.control;down regulated miRNAs; miRNA-seq;SRP022043;miEAA;AD.vs.control;down regulated miRNAs	20
KEGG	Hepatitis B	Hepatitis B	miRNA-seq;SRP022043;mirPath;AD.vs.control;down regulated miRNAs; miRNA-seq;SRP022043;miEAA;AD.vs.control;down regulated miRNAs; miRNA-seq;SRP022043;miEAA;AD.vs.control;down regulated miRNAs	15
KEGG	Dorso-ventral axis formation	Dorso-ventral axis formation	miRNA-seq;SRP022043;mirPath;AD.vs.control;down regulated miRNAs	5
KEGG	p53 signaling pathway	p53 signaling pathway	miRNA-seq;SRP022043;mirPath;AD.vs.control;down regulated miRNAs	5
KEGG	Basal cell carcinoma	Basal cell carcinoma	miRNA-seq;SRP022043;mirPath;AD.vs.control;down regulated miRNAs; miRNA-seq;SRP022043;miEAA;AD.vs.control;down regulated miRNAs; miRNA-seq;SRP022043;miEAA;AD.vs.control;down regulated miRNAs	15
KEGG	Phosphatidylinositol signaling	Phosphatidylinositol signaling system	miRNA-seq;SRP022043;mirPath;AD.vs.control;down regulated miRNAs	5
KEGG	Adipocytokine signaling pathway	Adipocytokine signaling pathway	miRNA-seq;SRP022043;mirPath;AD.vs.control;down regulated miRNAs; miRNA-seq;SRP022043;miEAA;AD.vs.control;down regulated miRNAs	10
KEGG	Viral	Viral carcinogenesis	miRNA-seq;SRP022043;mirPath;AD.vs.control;down regulated miRNAs; miRNA-seq;SRP022043;miEAA;AD.vs.control;down regulated miRNAs	10
KEGG	Circadian rhythm	Circadian rhythm	miRNA-seq;SRP022043;mirPath;AD.vs.control;down regulated miRNAs	5
KEGG	Alanine, aspartate and glutamate metabolism	Alanine, aspartate and glutamate metabolism	miRNA-seq;SRP022043;mirPath;AD.vs.control;down regulated miRNAs; miRNA-seq;SRP022043;miEAA;AD.vs.control;down regulated miRNAs	10

Diseases (MNDR)	Cardiovascular disease	Cardiovascular disease	miRNA-seq;SRP022043;miEAA;AD.vs.control;up regulated miRNAs; miRNA-seq;SRP022043;miEAA;AD.vs.control;down regulated miRNAs	10
Diseases (MNDR)	Breast invasive carcinoma	Breast invasive carcinoma	miRNA-seq;SRP022043;miEAA;AD.vs.control;up regulated miRNAs; miRNA-seq;SRP022043;miEAA;AD.vs.control;down regulated miRNAs	10
Diseases (MNDR)	Carcinoma ductal breast	Carcinoma ductal breast	miRNA-seq;SRP022043;miEAA;AD.vs.control;up regulated miRNAs; miRNA-seq;SRP022043;miEAA;AD.vs.control;down regulated miRNAs	10
Diseases (MNDR)	Rheumatoid arthritis	Rheumatoid arthritis	miRNA-seq;SRP022043;miEAA;AD.vs.control;up regulated miRNAs; miRNA-seq;SRP022043;miEAA;AD.vs.control;down regulated miRNAs; miRNA-seq;SRP022043;miEAA;AD.vs.control;down regulated miRNAs	15
Diseases (MNDR)	Alzheimer	Alzheimer disease	miRNA-seq;SRP022043;miEAA;AD.vs.control;up regulated miRNAs; miRNA-seq;SRP022043;miEAA;AD.vs.control;down regulated miRNAs	10
Diseases (MNDR)	Carcinoma renal	Carcinoma renal cell	miRNA-seq;SRP022043;miEAA;AD.vs.control;up regulated miRNAs; miRNA-seq;SRP022043;miEAA;AD.vs.control;down regulated miRNAs	10
Diseases (MNDR)	Kidney chromophobe cancer	Kidney chromophobe cancer	miRNA-seq;SRP022043;miEAA;AD.vs.control;up regulated miRNAs; miRNA-seq;SRP022043;miEAA;AD.vs.control;down regulated miRNAs	10
Diseases (MNDR)	Renal clear cell carcinoma	Renal clear cell carcinoma	miRNA-seq;SRP022043;miEAA;AD.vs.control;up regulated miRNAs; miRNA-seq;SRP022043;miEAA;AD.vs.control;down regulated miRNAs	10
Diseases (MNDR)	Bladder urothelial carcinoma	Bladder urothelial carcinoma	miRNA-seq;SRP022043;miEAA;AD.vs.control;up regulated miRNAs; miRNA-seq;SRP022043;miEAA;AD.vs.control;down regulated miRNAs	10
Diseases (MNDR)	Pancreatic adenocarcinoma	Pancreatic adenocarcinoma	miRNA-seq;SRP022043;miEAA;AD.vs.control;up regulated miRNAs; miRNA-seq;SRP022043;miEAA;AD.vs.control;down regulated miRNAs	10
Diseases (MNDR)	Colon adenocarcinoma	Colon adenocarcinoma	miRNA-seq;SRP022043;miEAA;AD.vs.control;up regulated miRNAs; miRNA-seq;SRP022043;miEAA;AD.vs.control;down regulated miRNAs	10
Diseases (MNDR)	Lung squamous cell carcinoma	Lung squamous cell carcinoma	miRNA-seq;SRP022043;miEAA;AD.vs.control;up regulated miRNAs; miRNA-seq;SRP022043;miEAA;AD.vs.control;down regulated miRNAs	10
Diseases (MNDR)	Lymphoma	Lymphoma	miRNA-seq;SRP022043;miEAA;AD.vs.control;up regulated miRNAs; miRNA-seq;SRP022043;miEAA;AD.vs.control;down regulated miRNAs; miRNA-seq;SRP022043;miEAA;AD.vs.control;down regulated miRNAs	15
Diseases (MNDR)	Prostate adenocarcinoma	Prostate adenocarcinoma	miRNA-seq;SRP022043;miEAA;AD.vs.control;up regulated miRNAs; miRNA-seq;SRP022043;miEAA;AD.vs.control;down regulated miRNAs	10
Diseases (MNDR)	Thyroid	Thyroid carcinoma	miRNA-seq;SRP022043;miEAA;AD.vs.control;up regulated miRNAs; miRNA-seq;SRP022043;miEAA;AD.vs.control;down regulated miRNAs	10
Diseases (MNDR)	Head and neck squamous cell carcinoma	Head and neck squamous cell carcinoma	miRNA-seq;SRP022043;miEAA;AD.vs.control;up regulated miRNAs; miRNA-seq;SRP022043;miEAA;AD.vs.control;down regulated miRNAs	10
Diseases (MNDR)	Multiple sclerosis relapsing-	Multiple sclerosis relapsing-remitting	miRNA-seq;SRP022043;miEAA;AD.vs.control;up regulated miRNAs; miRNA-seq;SRP022043;miEAA;AD.vs.control;down regulated miRNAs	10
Diseases (MNDR)	Seizures	Seizures	miRNA-seq;SRP022043;miEAA;AD.vs.control;up regulated miRNAs	5
Diseases (MNDR)	Vascular diseases	Vascular diseases	miRNA-seq;SRP022043;miEAA;AD.vs.control;up regulated miRNAs; miRNA-seq;SRP022043;miEAA;AD.vs.control;down regulated miRNAs	10
Diseases (MNDR)	Neurodegenerative diseases	Neurodegenerative diseases	miRNA-seq;SRP022043;miEAA;AD.vs.control;up regulated miRNAs; miRNA-seq;SRP022043;miEAA;AD.vs.control;down regulated miRNAs	10
Diseases (MNDR)	Gastric adenocarcinoma	Gastric adenocarcinoma	miRNA-seq;SRP022043;miEAA;AD.vs.control;up regulated miRNAs; miRNA-seq;SRP022043;miEAA;AD.vs.control;down regulated miRNAs	10
Diseases (MNDR)	Rectum adenocarcinoma	Rectum adenocarcinoma	miRNA-seq;SRP022043;miEAA;AD.vs.control;up regulated miRNAs; miRNA-seq;SRP022043;miEAA;AD.vs.control;down regulated miRNAs	10
Diseases (MNDR)	Osteosarcoma	Osteosarcoma	miRNA-seq;SRP022043;miEAA;AD.vs.control;up regulated miRNAs; miRNA-seq;SRP022043;miEAA;AD.vs.control;down regulated miRNAs	10
Diseases (MNDR)	Stomach adenocarcinoma	Stomach adenocarcinoma	miRNA-seq;SRP022043;miEAA;AD.vs.control;up regulated miRNAs; miRNA-seq;SRP022043;miEAA;AD.vs.control;down regulated miRNAs	10
Diseases (MNDR)	Uterine corpus endometrial carcinoma	Uterine corpus endometrial carcinoma	miRNA-seq;SRP022043;miEAA;AD.vs.control;up regulated miRNAs; miRNA-seq;SRP022043;miEAA;AD.vs.control;down regulated miRNAs	10
Diseases (MNDR)	Stroke lacunar	Stroke lacunar	miRNA-seq;SRP022043;miEAA;AD.vs.control;up regulated miRNAs; miRNA-seq;SRP022043;miEAA;AD.vs.control;down regulated miRNAs	10
Diseases (MNDR)	Breast cancer	Breast cancer	miRNA-seq;SRP022043;miEAA;AD.vs.control;up regulated miRNAs; miRNA-seq;SRP022043;miEAA;AD.vs.control;down regulated miRNAs; miRNA-seq;SRP022043;miEAA;AD.vs.control;down regulated miRNAs	15
Diseases (MNDR)	Papillary renal cell carcinoma	Papillary renal cell carcinoma	miRNA-seq;SRP022043;miEAA;AD.vs.control;up regulated miRNAs; miRNA-seq;SRP022043;miEAA;AD.vs.control;down regulated miRNAs	10
Diseases (MNDR)	B-cell lymphoma	B-cell lymphoma	miRNA-seq;SRP022043;miEAA;AD.vs.control;up regulated miRNAs; miRNA-seq;SRP022043;miEAA;AD.vs.control;down regulated miRNAs	10
Diseases (MNDR)	Bladder cancer	Bladder cancer	miRNA-seq;SRP022043;miEAA;AD.vs.control;up regulated miRNAs; miRNA-seq;SRP022043;miEAA;AD.vs.control;down regulated miRNAs	10
Diseases (MNDR)	Cervical squamous cell	Cervical squamous cell carcinoma	miRNA-seq;SRP022043;miEAA;AD.vs.control;up regulated miRNAs; miRNA-seq;SRP022043;miEAA;AD.vs.control;down regulated miRNAs	10
Diseases (MNDR)	Hepatocellular carcinoma	Hepatocellular carcinoma	miRNA-seq;SRP022043;miEAA;AD.vs.control;up regulated miRNAs; miRNA-seq;SRP022043;miEAA;AD.vs.control;down regulated miRNAs; miRNA-seq;SRP022043;miEAA;AD.vs.control;down regulated miRNAs	15
Diseases (MNDR)	Hodgkin	Hodgkin lymphoma	miRNA-seq;SRP022043;miEAA;AD.vs.control;up regulated miRNAs; miRNA-seq;SRP022043;miEAA;AD.vs.control;down regulated miRNAs	10
Diseases (MNDR)	Brain disease	Brain disease	miRNA-seq;SRP022043;miEAA;AD.vs.control;up regulated miRNAs; miRNA-seq;SRP022043;miEAA;AD.vs.control;down regulated miRNAs	10
Diseases (MNDR)	Kidney renal papillary cell carcinoma	Kidney renal papillary cell carcinoma	miRNA-seq;SRP022043;miEAA;AD.vs.control;up regulated miRNAs; miRNA-seq;SRP022043;miEAA;AD.vs.control;down regulated miRNAs	10

Diseases (MNDR)	Progesterone-receptor negative breast cancer	Progesterone-receptor negative breast cancer	miRNA-seq;SRP022043;miEAA;AD.vs.control;up regulated miRNAs; miRNA-seq;SRP022043;miEAA;AD.vs.control;down regulated miRNAs	10
Diseases (MNDR)	Pancreatitis	Pancreatitis chronic	miRNA-seq;SRP022043;miEAA;AD.vs.control;up regulated miRNAs	5
Diseases (MNDR)	Glioblastoma	Glioblastoma	miRNA-seq;SRP022043;miEAA;AD.vs.control;up regulated miRNAs; miRNA-seq;SRP022043;miEAA;AD.vs.control;down regulated miRNAs	10
Diseases (MNDR)	Amyotrophic lateral sclerosis	Amyotrophic lateral sclerosis	miRNA-seq;SRP022043;miEAA;AD.vs.control;up regulated miRNAs; miRNA-seq;SRP022043;miEAA;AD.vs.control;down regulated miRNAs	10
Diseases (MNDR)	Cholangiocarcino	Cholangiocarcinoma	miRNA-seq;SRP022043;miEAA;AD.vs.control;up regulated miRNAs; miRNA-seq;SRP022043;miEAA;AD.vs.control;down regulated miRNAs	10
Diseases (MNDR)	Ataxia	Ataxia telangiectasia	miRNA-seq;SRP022043;miEAA;AD.vs.control;up regulated miRNAs; miRNA-seq;SRP022043;miEAA;AD.vs.control;down regulated miRNAs	10
Diseases (MNDR)	Lung cancer	Lung cancer	miRNA-seq;SRP022043;miEAA;AD.vs.control;up regulated miRNAs; miRNA-seq;SRP022043;miEAA;AD.vs.control;down regulated miRNAs	10
Diseases (MNDR)	Chronic obstructive	Chronic obstructive pulmonary disease	miRNA-seq;SRP022043;miEAA;AD.vs.control;up regulated miRNAs; miRNA-seq;SRP022043;miEAA;AD.vs.control;down regulated miRNAs	10
Diseases (MNDR)	Burkitt	Burkitt lymphoma	miRNA-seq;SRP022043;miEAA;AD.vs.control;up regulated miRNAs; miRNA-seq;SRP022043;miEAA;AD.vs.control;down regulated miRNAs	10
Tissue specific (isomiRdb)	cerebrospinal fluid specific	cerebrospinal fluid specific	miRNA-seq;SRP022043;miEAA;AD.vs.control;up regulated miRNAs; miRNA-seq;SRP022043;miEAA;AD.vs.control;down regulated miRNAs	10
Tissue specific (isomiRdb)	sperm specific	sperm specific	miRNA-seq;SRP022043;miEAA;AD.vs.control;up regulated miRNAs	5
Diseases (MNDR)	Esophageal carcinoma	Esophageal carcinoma	miRNA-seq;SRP022043;miEAA;AD.vs.control;up regulated miRNAs; miRNA-seq;SRP022043;miEAA;AD.vs.control;down regulated miRNAs	10
Diseases (MNDR)	Dysautonomia familial	Dysautonomia familial	miRNA-seq;SRP022043;miEAA;AD.vs.control;up regulated miRNAs; miRNA-seq;SRP022043;miEAA;AD.vs.control;down regulated miRNAs	10
Pathways (KEGG)	Progesterone-mediated oocyte maturation	Progesterone-mediated oocyte maturation	miRNA-seq;SRP022043;miEAA;AD.vs.control;up regulated miRNAs; miRNA-seq;SRP022043;miEAA;AD.vs.control;down regulated miRNAs	10
Diseases (MNDR)	Intellectual	Intellectual disability	miRNA-seq;SRP022043;miEAA;AD.vs.control;up regulated miRNAs; miRNA-seq;SRP022043;miEAA;AD.vs.control;down regulated miRNAs	10
Seed family (miRBase)	ACCCGUA	ACCCGUA	miRNA-seq;SRP022043;miEAA;AD.vs.control;up regulated miRNAs	5
Seed family (miRBase)	UUGGCAC	UUGGCAC	miRNA-seq;SRP022043;miEAA;AD.vs.control;up regulated miRNAs	5
Published Diseases	Alzheimers Disease	Alzheimers Disease upregulated	miRNA-seq;SRP022043;miEAA;AD.vs.control;up regulated miRNAs	5
Published Diseases	melanoma upregulated	melanoma upregulated	miRNA-seq;SRP022043;miEAA;AD.vs.control;up regulated miRNAs	5
Diseases (MNDR)	Colonic	Colonic adenomas	miRNA-seq;SRP022043;miEAA;AD.vs.control;up regulated miRNAs	5
Diseases (MNDR)	Bone-invasive pituitary adenoma	Bone-invasive pituitary adenoma	miRNA-seq;SRP022043;miEAA;AD.vs.control;up regulated miRNAs	5
Diseases (MNDR)	Familiar ovarian carcinoma	Familiar ovarian carcinoma	miRNA-seq;SRP022043;miEAA;AD.vs.control;up regulated miRNAs; miRNA-seq;SRP022043;miEAA;AD.vs.control;down regulated miRNAs	10
Pathways (KEGG)	Neomycin, kanamycin and gentamicin biosynthesis	Neomycin, kanamycin and gentamicin biosynthesis	miRNA-seq;SRP022043;miEAA;AD.vs.control;up regulated miRNAs; miRNA-seq;SRP022043;miEAA;AD.vs.control;down regulated miRNAs	10
Diseases (MNDR)	Solid-pseudopapillary neoplasm of pancreas	Solid-pseudopapillary neoplasm of pancreas	miRNA-seq;SRP022043;miEAA;AD.vs.control;up regulated miRNAs; miRNA-seq;SRP022043;miEAA;AD.vs.control;down regulated miRNAs	10
Diseases (MNDR)	Lung adenocarcinoma	Lung adenocarcinoma	miRNA-seq;SRP022043;miEAA;AD.vs.control;up regulated miRNAs; miRNA-seq;SRP022043;miEAA;AD.vs.control;down regulated miRNAs	10
Diseases (MNDR)	Synovial sarcoma	Synovial sarcoma	miRNA-seq;SRP022043;miEAA;AD.vs.control;up regulated miRNAs; miRNA-seq;SRP022043;miEAA;AD.vs.control;down regulated miRNAs	10
Diseases (MNDR)	Sleep deprivation	Sleep deprivation	miRNA-seq;SRP022043;miEAA;AD.vs.control;up regulated miRNAs; miRNA-seq;SRP022043;miEAA;AD.vs.control;down regulated miRNAs	10
Diseases (MNDR)	Carcinoma	Carcinoma	miRNA-seq;SRP022043;miEAA;AD.vs.control;up regulated miRNAs; miRNA-seq;SRP022043;miEAA;AD.vs.control;down regulated miRNAs	10
Diseases (MNDR)	Estrogen-receptor negative breast cancer	Estrogen-receptor negative breast cancer	miRNA-seq;SRP022043;miEAA;AD.vs.control;up regulated miRNAs; miRNA-seq;SRP022043;miEAA;AD.vs.control;down regulated miRNAs	10
Diseases (MNDR)	Meningioma	Meningioma	miRNA-seq;SRP022043;miEAA;AD.vs.control;up regulated miRNAs; miRNA-seq;SRP022043;miEAA;AD.vs.control;down regulated miRNAs	10
Diseases (MNDR)	Gastric	Gastric lymphoma	miRNA-seq;SRP022043;miEAA;AD.vs.control;up regulated miRNAs; miRNA-seq;SRP022043;miEAA;AD.vs.control;down regulated miRNAs	10
Diseases (MNDR)	Low grade dysplastic nodule	Low grade dysplastic nodule	miRNA-seq;SRP022043;miEAA;AD.vs.control;up regulated miRNAs; miRNA-seq;SRP022043;miEAA;AD.vs.control;down regulated miRNAs	10
Diseases (MNDR)	T-cell leukemia	T-cell leukemia	miRNA-seq;SRP022043;miEAA;AD.vs.control;up regulated miRNAs; miRNA-seq;SRP022043;miEAA;AD.vs.control;down regulated miRNAs	10
Localization (RNALocate)	microvesicle	microvesicle	miRNA-seq;SRP022043;miEAA;AD.vs.control;up regulated miRNAs; miRNA-seq;SRP022043;miEAA;AD.vs.control;down regulated miRNAs	10
Diseases (MNDR)	Breast neoplasms	Breast neoplasms	miRNA-seq;SRP022043;miEAA;AD.vs.control;up regulated miRNAs; miRNA-seq;SRP022043;miEAA;AD.vs.control;down regulated miRNAs	10

Diseases (MNDR)	Clear cell renal cell carcinoma	Clear cell renal cell carcinoma	miRNA-seq;SRP022043;miEAA;AD.vs.control;up regulated miRNAs; miRNA-seq;SRP022043;miEAA;AD.vs.control;down regulated miRNAs	10
Diseases (MNDR)	Malignant pleural mesothelioma	Malignant pleural mesothelioma	miRNA-seq;SRP022043;miEAA;AD.vs.control;up regulated miRNAs; miRNA-seq;SRP022043;miEAA;AD.vs.control;down regulated miRNAs	10
Localization (RNALocate)	Circulating	Circulating	miRNA-seq;SRP022043;miEAA;AD.vs.control;up regulated miRNAs; miRNA-seq;SRP022043;miEAA;AD.vs.control;down regulated miRNAs	10
Diseases (MNDR)	Infection	Infection	miRNA-seq;SRP022043;miEAA;AD.vs.control;up regulated miRNAs; miRNA-seq;SRP022043;miEAA;AD.vs.control;down regulated miRNAs; miRNA-seq;SRP022043;miEAA;AD.vs.control;down regulated miRNAs	15
Diseases (MNDR)	Familial ovarian cancer	Familial ovarian cancer	miRNA-seq;SRP022043;miEAA;AD.vs.control;up regulated miRNAs; miRNA-seq;SRP022043;miEAA;AD.vs.control;down regulated miRNAs	10
Diseases (MNDR)	Cervical adenocarcinoma	Cervical adenocarcinoma	miRNA-seq;SRP022043;miEAA;AD.vs.control;up regulated miRNAs; miRNA-seq;SRP022043;miEAA;AD.vs.control;down regulated miRNAs	10
Diseases (MNDR)	Leukemia	Leukemia	miRNA-seq;SRP022043;miEAA;AD.vs.control;up regulated miRNAs; miRNA-seq;SRP022043;miEAA;AD.vs.control;down regulated miRNAs	10
Diseases (MNDR)	Psoriasis	Psoriasis	miRNA-seq;SRP022043;miEAA;AD.vs.control;up regulated miRNAs; miRNA-seq;SRP022043;miEAA;AD.vs.control;down regulated miRNAs	10
Cell-type specific (Cellular	Fibroblast eye specific	Fibroblast eye specific	miRNA-seq;SRP022043;miEAA;AD.vs.control;up regulated miRNAs	5
Cell-type specific (Cellular	Megakaryocyte derived specific	Megakaryocyte derived specific	miRNA-seq;SRP022043;miEAA;AD.vs.control;up regulated miRNAs	5
Diseases (MNDR)	Fibrosis	Fibrosis	miRNA-seq;SRP022043;miEAA;AD.vs.control;up regulated miRNAs; miRNA-seq;SRP022043;miEAA;AD.vs.control;down regulated miRNAs; miRNA-seq;SRP022043;miEAA;AD.vs.control;down regulated miRNAs	15
Diseases (MNDR)	Neoplasms glandular and	Neoplasms glandular and epithelial	miRNA-seq;SRP022043;miEAA;AD.vs.control;up regulated miRNAs; miRNA-seq;SRP022043;miEAA;AD.vs.control;down regulated miRNAs	10
Diseases (MNDR)	Duke C	Duke C	miRNA-seq;SRP022043;miEAA;AD.vs.control;up regulated miRNAs; miRNA-seq;SRP022043;miEAA;AD.vs.control;down regulated miRNAs	10
Diseases (MNDR)	Estrogen-receptor positive breast cancer	Estrogen-receptor positive breast cancer	miRNA-seq;SRP022043;miEAA;AD.vs.control;up regulated miRNAs; miRNA-seq;SRP022043;miEAA;AD.vs.control;down regulated miRNAs	10
Diseases (MNDR)	Urinary bladder cancer	Urinary bladder cancer	miRNA-seq;SRP022043;miEAA;AD.vs.control;up regulated miRNAs; miRNA-seq;SRP022043;miEAA;AD.vs.control;down regulated miRNAs	10
Diseases (MNDR)	Lung small cell carcinoma	Lung small cell carcinoma	miRNA-seq;SRP022043;miEAA;AD.vs.control;up regulated miRNAs; miRNA-seq;SRP022043;miEAA;AD.vs.control;down regulated miRNAs	10
Diseases (MNDR)	Fibrosarcoma	Fibrosarcoma	miRNA-seq;SRP022043;miEAA;AD.vs.control;up regulated miRNAs; miRNA-seq;SRP022043;miEAA;AD.vs.control;down regulated miRNAs	10
Diseases (MNDR)	Uterine cancer	Uterine cancer	miRNA-seq;SRP022043;miEAA;AD.vs.control;up regulated miRNAs; miRNA-seq;SRP022043;miEAA;AD.vs.control;down regulated miRNAs	10
Diseases (MNDR)	Lipid metabolism disorder	Lipid metabolism disorder	miRNA-seq;SRP022043;miEAA;AD.vs.control;up regulated miRNAs; miRNA-seq;SRP022043;miEAA;AD.vs.control;down regulated miRNAs	10
Tissue specific (isomiRdb)	umbilical cord specific	umbilical cord specific	miRNA-seq;SRP022043;miEAA;AD.vs.control;up regulated miRNAs	5
Diseases (MNDR)	Skin cancer	Skin cancer	miRNA-seq;SRP022043;miEAA;AD.vs.control;up regulated miRNAs; miRNA-seq;SRP022043;miEAA;AD.vs.control;down regulated miRNAs	10
Diseases (MNDR)	Gout	Gout	miRNA-seq;SRP022043;miEAA;AD.vs.control;up regulated miRNAs; miRNA-seq;SRP022043;miEAA;AD.vs.control;down regulated miRNAs	10
Diseases (MNDR)	Neuroendocrine neoplasia	Neuroendocrine neoplasia	miRNA-seq;SRP022043;miEAA;AD.vs.control;up regulated miRNAs	5
Diseases (MNDR)	Chordoma	Chordoma	miRNA-seq;SRP022043;miEAA;AD.vs.control;up regulated miRNAs; miRNA-seq;SRP022043;miEAA;AD.vs.control;down regulated miRNAs	10
Cell-type specific (Cellular	Mast cell specific	Mast cell specific	miRNA-seq;SRP022043;miEAA;AD.vs.control;up regulated miRNAs	5
Pathways (KEGG)	Carbohydrate digestion and absorption	Carbohydrate digestion and absorption	miRNA-seq;SRP022043;miEAA;AD.vs.control;up regulated miRNAs; miRNA-seq;SRP022043;miEAA;AD.vs.control;down regulated miRNAs	10
Pathways (KEGG)	Cholinergic	Cholinergic synapse	miRNA-seq;SRP022043;miEAA;AD.vs.control;up regulated miRNAs; miRNA-seq;SRP022043;miEAA;AD.vs.control;down regulated miRNAs	10
Pathways (KEGG)	Glucagon signaling pathway	Glucagon signaling pathway	miRNA-seq;SRP022043;miEAA;AD.vs.control;up regulated miRNAs; miRNA-seq;SRP022043;miEAA;AD.vs.control;down regulated miRNAs	10
Pathways (KEGG)	Longevity regulating pathway -	Longevity regulating pathway - multiple species	miRNA-seq;SRP022043;miEAA;AD.vs.control;up regulated miRNAs; miRNA-seq;SRP022043;miEAA;AD.vs.control;down regulated miRNAs	10
Pathways (KEGG)	Platelet activation	Platelet activation	miRNA-seq;SRP022043;miEAA;AD.vs.control;up regulated miRNAs; miRNA-seq;SRP022043;miEAA;AD.vs.control;down regulated miRNAs	10
Pathways (KEGG)	Renin secretion	Renin secretion	miRNA-seq;SRP022043;miEAA;AD.vs.control;up regulated miRNAs; miRNA-seq;SRP022043;miEAA;AD.vs.control;down regulated miRNAs	10
Diseases (MNDR)	Neuroendocrine tumors	Neuroendocrine tumors	miRNA-seq;SRP022043;miEAA;AD.vs.control;up regulated miRNAs	5
Annotations derived over miRTarBase (Gene Ontology)	cellular response to osmotic stress GO0071470	cellular response to osmotic stress GO0071470	miRNA-seq;SRP022043;miEAA;AD.vs.control;up regulated miRNAs	5

Annotations derived over miRTarBase (Gene Ontology)	membrane lipid catabolic process GO0046466	membrane lipid catabolic process GO0046466	miRNA-seq;SRP022043;miEAA;AD.vs.control;up regulated miRNAs	5
Annotations derived over miRTarBase (Gene Ontology)	positive regulation of cell migration involved in sprouting	positive regulation of cell migration involved in sprouting angiogenesis GO0090050	miRNA-seq;SRP022043;miEAA;AD.vs.control;up regulated miRNAs; miRNA-seq;SRP022043;miEAA;AD.vs.control;down regulated miRNAs	10
Annotations derived over miRTarBase (Gene Ontology)	positive regulation of intracellular protein transport	positive regulation of intracellular protein transport GO0090316	miRNA-seq;SRP022043;miEAA;AD.vs.control;up regulated miRNAs	5
Annotations derived over miRTarBase (Gene Ontology)	protein serine kinase activity GO0106310	protein serine kinase activity GO0106310	miRNA-seq;SRP022043;miEAA;AD.vs.control;up regulated miRNAs	5
Pathways (KEGG)	Endocrine and other factor-regulated calcium	Endocrine and other factor-regulated calcium reabsorption	miRNA-seq;SRP022043;miEAA;AD.vs.control;up regulated miRNAs	5
Localization (RNALocate)	exosome	exosome	miRNA-seq;SRP022043;miEAA;AD.vs.control;up regulated miRNAs; miRNA-seq;SRP022043;miEAA;AD.vs.control;down regulated miRNAs; miRNA-seq;SRP022043;miEAA;AD.vs.control;down regulated miRNAs	15
Diseases (MNDR)	Oligodendroglio	Oligodendroglioma	miRNA-seq;SRP022043;miEAA;AD.vs.control;up regulated miRNAs; miRNA-seq;SRP022043;miEAA;AD.vs.control;down regulated miRNAs	10
Diseases (MNDR)	Ectopic	Ectopic pregnancy	miRNA-seq;SRP022043;miEAA;AD.vs.control;up regulated miRNAs; miRNA-seq;SRP022043;miEAA;AD.vs.control;down regulated miRNAs	10
Diseases (MNDR)	Breast cancer luminal	Breast cancer luminal	miRNA-seq;SRP022043;miEAA;AD.vs.control;up regulated miRNAs; miRNA-seq;SRP022043;miEAA;AD.vs.control;down regulated miRNAs	10
Diseases (MNDR)	Multiple	Multiple myeloma	miRNA-seq;SRP022043;miEAA;AD.vs.control;up regulated miRNAs; miRNA-seq;SRP022043;miEAA;AD.vs.control;down regulated miRNAs	10
Diseases (MNDR)	Barretts adenocarcinoma	Barretts adenocarcinoma	miRNA-seq;SRP022043;miEAA;AD.vs.control;up regulated miRNAs	5
Diseases (MNDR)	Neuromyelitis	Neuromyelitis optica	miRNA-seq;SRP022043;miEAA;AD.vs.control;up regulated miRNAs; miRNA-seq;SRP022043;miEAA;AD.vs.control;down regulated miRNAs	10
Pathways (KEGG)	Hedgehog signaling pathway	Hedgehog signaling pathway	miRNA-seq;SRP022043;miEAA;AD.vs.control;up regulated miRNAs; miRNA-seq;SRP022043;miEAA;AD.vs.control;down regulated miRNAs	10
Diseases (MNDR)	Barretts carcinogenesis	Barretts carcinogenesis	miRNA-seq;SRP022043;miEAA;AD.vs.control;up regulated miRNAs; miRNA-seq;SRP022043;miEAA;AD.vs.control;down regulated miRNAs	10
Annotations derived over miRTarBase (Gene Ontology)	blood vessel morphogenesis GO0048514	blood vessel morphogenesis GO0048514	miRNA-seq;SRP022043;miEAA;AD.vs.control;up regulated miRNAs; miRNA-seq;SRP022043;miEAA;AD.vs.control;down regulated miRNAs	10
Annotations derived over miRTarBase (Gene Ontology)	fructokinase activity GO0008865	fructokinase activity GO0008865	miRNA-seq;SRP022043;miEAA;AD.vs.control;up regulated miRNAs	5
Annotations derived over miRTarBase (Gene Ontology)	glucokinase activity GO0004340	glucokinase activity GO0004340	miRNA-seq;SRP022043;miEAA;AD.vs.control;up regulated miRNAs	5
Annotations derived over miRTarBase (Gene Ontology)	maintenance of protein location in mitochondrion GO0072656	maintenance of protein location in mitochondrion GO0072656	miRNA-seq;SRP022043;miEAA;AD.vs.control;up regulated miRNAs; miRNA-seq;SRP022043;miEAA;AD.vs.control;down regulated miRNAs	10
Annotations derived over miRTarBase (Gene Ontology)	nitric oxide biosynthetic process GO0006809	nitric oxide biosynthetic process GO0006809	miRNA-seq;SRP022043;miEAA;AD.vs.control;up regulated miRNAs; miRNA-seq;SRP022043;miEAA;AD.vs.control;down regulated miRNAs	10
Annotations derived over miRTarBase (Gene Ontology)	hexokinase activity GO0004396	hexokinase activity GO0004396	miRNA-seq;SRP022043;miEAA;AD.vs.control;up regulated miRNAs; miRNA-seq;SRP022043;miEAA;AD.vs.control;down regulated miRNAs	10
Diseases (MNDR)	Bone disease	Bone disease	miRNA-seq;SRP022043;miEAA;AD.vs.control;up regulated miRNAs; miRNA-seq;SRP022043;miEAA;AD.vs.control;down regulated miRNAs	10
[truncated: 1,740,055 more chars]
