# Supplementary material for: RBAD: The first database dedicated alterations of blood RNA in individuals with Alzheimer’s disease and their clinical relevance
Source: Neural Regen Res. 2025 Mar 25;21(6):2553–62. doi: 10.4103/NRR.NRR-D-24-01165 (PMC13211806; doi:10.4103/NRR.NRR-D-24-01165)
Supplement: Supplementary file 2 [file NRR-21-2553_Suppl1.pdf]

Additional Table 4. Disease-associated DEGs detected in RBAD

Symbol	Group (data type, dataset, comparison)	Strength of evidence
A1BG	bulk RNA-seq,Homo_723,AD.vs.control; bulk RNA-seq,Homo_633,MCI.vs.control; bulk RNA-seq,Homo_714,AD.vs.control; bulk RNA-seq,Homo_714,AD.vs.MCI; bulk RNA-seq,Homo_714,MCI.vs.control; scRNA-seq,SRP215507,CD8+ T cell_5-AD.vs.MCI; scRNA-seq,SRP215507,CD8+ T cell_1-MCI.vs.control; scRNA-seq,SRP330776,γδ T cell-AD.vs.control; scRNA-seq,SRP330776,B cell_1-AD.vs.control; scRNA-seq,SRP330776,Memory T cell_1-AD.vs.control; scRNA-seq,SRP330776,Naive CD8+ T cell_3-AD.vs.control; scRNA-seq,SRP330776,Monocyte_2-	12
A1BG-AS1	scRNA-seq, SRP215507, CD8+ T cell_5-AD. vs.MCI; scRNA-seq, SRP215507, CD8+ T cell_8-MCI. vs.control; scRNA-seq,SRP330776,CD8+ T cell_1-AD.vs.control; scRNA-seq,SRP330776,γδ T cell-AD.vs.control; scRNA-seq,SRP330776,Memory T cell_2-AD.vs.control; scRNA-seq,SRP330776,Monocyte_2-AD.vs.control; scRNA-seq,SRP330776,Natural killer cell_2-AD.vs.control	7
A1CF	bulk RNA-seq,ACOM,AD.vs.control; bulk RNA-seq,Homo_723,AD.vs.control; bulk RNA-seq,Homo_723,AD.vs.MCI; bulk RNA-seq,Homo_633,AD.vs.control; bulk RNA-seq,Homo_633,AD.vs.MCI; bulk RNA-seq,Homo_714,MCI.vs.control	6
A2M	bulk RNA-seq,ACOM,AD.vs.control; bulk RNA-seq,Homo_723,AD.vs.MCI; bulk RNA-seq,Homo_633,AD.vs.MCI; bulk RNA-seq,ROSMAP,MCI.vs.control; bulk RNA-seq,SRP223445,AD.vs.control; scRNA-seq,SRP215507,CD8+ T cell_1-AD.vs.control; scRNA-seq,SRP215507,CD8+ T cell_1-AD.vs.MCI; scRNA-seq,SRP330776,γδ T cell-AD.vs.control; scRNA-seq,SRP330776,Memory T cell_2-AD.vs.control;	10
A2M-AS1	scRNA-seq,SRP215507,CD8+ T cell_2-AD.vs.control; scRNA-seq,SRP215507,CD8+ T cell_3-AD.vs.control; scRNA-seq,SRP215507,CD8+ T cell_1-AD.vs.control; scRNA-seq,SRP215507,CD8+ T cell_6-AD.vs.control; scRNA-seq,SRP215507,CD8+ T cell_2-AD.vs.MCI; scRNA-seq,SRP215507,CD8+ T cell_3-AD.vs.MCI; scRNA-seq,SRP309935,Monocyte_1-AD.vs.control; scRNA-seq,SRP330776,CD8+ T cell_1-AD.vs.control; scRNA-seq,SRP330776, γδ T cell-AD.vs.control; scRNA-seq,SRP330776,CD8+ T cell_2-AD.vs.control; scRNA-seq,SRP330776,Natural killer cell_1-AD.vs.control; scRNA-seq,SRP330776,Memory T cell_1-AD.vs.control; scRNA-seq,SRP330776,Natural killer T cell-AD.vs.control; scRNA-seq,SRP330776,CD8+ T cell_3-	14
A2ML1	bulk RNA-seq,Homo_723,AD.vs.control; bulk RNA-seq,Homo_723,AD.vs.MCI; bulk RNA-seq,Homo_723,MCI.vs.control; bulk RNA-seq,Homo_633,AD.vs.control; bulk RNA-seq,Homo_633,AD.vs.MCI; bulk RNA-seq,Homo_633,MCI.vs.control; bulk RNA-seq,Homo_714,MCI.vs.control; bulk RNA-seq,ROSMAP,AD.vs.control; bulk RNA-seq,ROSMAP,MCI.vs.control	9
A4GALT	bulk RNA-seq,Homo_723,AD.vs.control; bulk RNA-seq,Homo_723,AD.vs.MCI; bulk RNA-seq,Homo_633,AD.vs.control; bulk RNA-seq,Homo_633,AD.vs.MCI; bulk RNA-seq,Homo_714,AD.vs.MCI; bulk RNA-seq,Homo_714,MCI.vs.control	6
A4GNT	bulk RNA-seq,Homo_723,AD.vs.control; bulk RNA-seq,Homo_633,AD.vs.control	2
AAAS	bulk RNA-seq,Homo_723,AD.vs.control; bulk RNA-seq,Homo_723,AD.vs.MCI; bulk RNA-seq,Homo_633,AD.vs.control; bulk RNA-seq,Homo_633,AD.vs.MCI; bulk RNA-seq,Homo_714,AD.vs.control; bulk RNA-seq,Homo_714,AD.vs.MCI; bulk RNA-seq,Homo_714,MCI.vs.control; scRNA-seq,SRP215507,CD8+ T cell_2-AD.vs.control; scRNA-seq,SRP330776,CD8+ T cell_1-AD.vs.control; scRNA-seq,SRP330776,Natural killer cell_1-AD.vs.control; scRNA-seq,SRP330776,Natural killer T cell-AD.vs.control; scRNA-seq,SRP330776,Naive CD8+ T cell_3-AD.vs.control; scRNA-seq,SRP330776,B cell_2-AD.vs.control; scRNA-seq,SRP330776,CD8+ T cell_3-AD.vs.control; scRNA-seq,SRP330776,Monocyte_2-AD.vs.control	15
AACS	bulk RNA-seq,Homo_633,AD.vs.control; bulk RNA-seq,Homo_714,AD.vs.control; bulk RNA-seq,Homo_714,AD.vs.MCI; scRNA-seq,SRP215507,CD8+ T cell_2-AD.vs.MCI; scRNA-seq,SRP215507,CD8+ T cell_4-MCI.vs.control; scRNA-seq,SRP215507,CD8+ T cell_2-MCI.vs.control; scRNA-seq,SRP330776,CD8+ T cell_1-AD.vs.control; scRNA-seq,SRP330776,CD8+ T cell_2-AD.vs.control; scRNA-seq,SRP330776,Naive CD8+ T cell_1-AD.vs.control; scRNA-seq,SRP330776,Memory T cell_1-AD.vs.control; scRNA-seq,SRP330776,Naive CD8+ T cell_3-AD.vs.control; scRNA-seq,SRP330776,B cell_2-AD.vs.control; scRNA-seq,SRP330776,T cell-AD.vs.control	13
AADACL3	bulk RNA-seq,Homo_714,MCI.vs.control	1
AADACL4	bulk RNA-seq,Homo_723,AD.vs.control; bulk RNA-seq,Homo_723,MCI.vs.control; bulk RNA-seq,Homo_633,AD.vs.control; bulk RNA-seq,Homo_633,MCI.vs.control; bulk RNA-seq,Homo_714,MCI.vs.control	5
AADAT	bulk RNA-seq,Homo_723,AD.vs.control; bulk RNA-seq,Homo_723,AD.vs.MCI	2
AAGAB	scRNA-seq,SRP215507,CD8+ T cell_3-AD.vs.control; scRNA-seq,SRP215507,CD8+ T cell_7-AD.vs.control; scRNA-seq,SRP330776,B cell_2-AD.vs.control; scRNA-seq,SRP330776,CD8+ T cell_3-AD.vs.control; scRNA-seq,SRP330776,B cell_3-AD.vs.control; scRNA-seq,SRP330776,Natural	6

AAK1	scRNA-seq,SRP215507,CD8+ T cell_3-MCI.vs.control; scRNA-seq,SRP309935,Natural killer cell_1-AD.vs.control; scRNA-seq,SRP330776,CD8+ T cell_1-AD.vs.control; scRNA-seq,SRP330776,γδ T cell-AD.vs.control; scRNA-seq,SRP330776,Memory T cell_2-AD.vs.control; scRNA-seq,SRP330776,CD8+ T cell_2-AD.vs.control; scRNA-seq,SRP330776,B cell_1-AD.vs.control; scRNA-seq,SRP330776,Naive CD8+ T cell_1-AD.vs.control; scRNA-seq,SRP330776,Natural killer cell_1-AD.vs.control; scRNA-seq,SRP330776,Memory T cell_1-AD.vs.control; scRNA-seq,SRP330776,Natural killer T cell-AD.vs.control; scRNA-seq,SRP330776,Naive CD8+ T cell_3-AD.vs.control; scRNA-seq,SRP330776,B cell_2-AD.vs.control; scRNA-seq,SRP330776,T cell-AD.vs.control; scRNA-seq,SRP330776,Naive CD8+ T cell_2-AD.vs.control; scRNA-seq,SRP330776,CD8+ T cell_3-AD.vs.control; scRNA-seq,SRP330776,B cell_3-AD.vs.control; scRNA-seq,SRP330776,Regulatory T cell-AD.vs.control; scRNA- [illegible]	19
AAMDC	bulk RNA-seq,Homo_633,AD.vs.control; bulk RNA-seq,Homo_714,AD.vs.control; bulk RNA-seq,Homo_714,AD.vs.MCI; scRNA-seq,SRP215507,CD8+ T cell_2-AD.vs.MCI; scRNA-seq,SRP330776,γδ T cell-AD.vs.control; scRNA-seq,SRP330776,CD8+ T cell_2-AD.vs.control; scRNA-seq,SRP330776,B cell_1-AD.vs.control; scRNA-seq,SRP330776,Naive CD8+ T cell_1-AD.vs.control; scRNA-seq,SRP330776,Natural killer cell_1-AD.vs.control; scRNA-seq,SRP330776,Memory T cell_1-AD.vs.control; scRNA-seq,SRP330776,Natural killer T cell-AD.vs.control; scRNA-seq,SRP330776,Naive CD8+ T cell_3-AD.vs.control; scRNA-seq,SRP330776,T cell-AD.vs.control; scRNA-seq,SRP330776,CD8+ T cell_3-AD.vs.control; scRNA-	15
AAMP	bulk RNA-seq,Homo_723,AD.vs.control; bulk RNA-seq,Homo_723,AD.vs.MCI; bulk RNA-seq,Homo_723,MCI.vs.control; bulk RNA-seq,Homo_633,AD.vs.control; bulk RNA-seq,Homo_633,AD.vs.MCI; bulk RNA-seq,Homo_633,MCI.vs.control; scRNA-seq,SRP330776,CD8+ T cell_1-AD.vs.control; scRNA-seq,SRP330776,γδ T cell-AD.vs.control; scRNA-seq,SRP330776,Memory T cell_2-AD.vs.control; scRNA-seq,SRP330776,CD8+ T cell_2-AD.vs.control; scRNA-seq,SRP330776,B cell_1-AD.vs.control; scRNA-seq,SRP330776,Naive CD8+ T cell_1-AD.vs.control; scRNA-seq,SRP330776,Natural killer cell_1-AD.vs.control; scRNA-seq,SRP330776,Memory T cell_1-AD.vs.control; scRNA-seq,SRP330776,Natural killer T cell-AD.vs.control; scRNA-seq,SRP330776,B cell_2-AD.vs.control; scRNA-seq,SRP330776,T cell-AD.vs.control; scRNA-seq,SRP330776,CD8+ T cell_3- [illegible]	19
AANAT	bulk RNA-seq,Homo_723,AD.vs.MCI; bulk RNA-seq,Homo_723,MCI.vs.control; bulk RNA-seq,Homo_633,AD.vs.MCI; bulk RNA-seq,Homo_633,MCI.vs.control; bulk RNA-seq,Homo_714,AD.vs.control; bulk RNA-seq,Homo_714,AD.vs.MCI; bulk RNA-seq,Homo_714,MCI.vs.control; scRNA-seq,SRP215507,CD8+ T cell_1-MCI.vs.control	8
AAR2	bulk RNA-seq,Homo_723,AD.vs.control; bulk RNA-seq,Homo_723,AD.vs.MCI; bulk RNA-seq,Homo_723,MCI.vs.control; bulk RNA-seq,Homo_633,AD.vs.control; bulk RNA-seq,Homo_633,MCI.vs.control; scRNA-seq,SRP215507,CD8+ T cell_7-AD.vs.control; scRNA-seq,SRP215507,CD8+ T cell_4-AD.vs.MCI; scRNA-seq,SRP215507,CD8+ T cell_4-MCI.vs.control; scRNA-seq,SRP309935,Naive CD8+ T cell_1-	10
AARD	bulk RNA-seq,Homo_723,AD.vs.control; bulk RNA-seq,Homo_723,AD.vs.MCI; bulk RNA-seq,Homo_633,AD.vs.MCI; scRNA-seq,SRP215507,CD8+ T cell_1-AD.vs.MCI; scRNA-seq,SRP330776,Memory T cell_1-AD.vs.control	5
AARS	scRNA-seq,SRP215507,CD8+ T cell_2-AD.vs.control; scRNA-seq,SRP215507,CD8+ T cell_2-MCI.vs.control; scRNA-seq,SRP309935,Monocyte_2-AD.vs.control; scRNA-seq,SRP309935,CD8+ T cell_1-AD.vs.control; scRNA-seq,SRP330776,Memory T cell_2-AD.vs.control; scRNA-seq,SRP330776,Naive CD8+ T cell_1-AD.vs.control; scRNA-seq,SRP330776,B cell_2-AD.vs.control; scRNA-seq,SRP330776,T cell-AD.vs.control; scRNA-seq,SRP330776,CD8+ T cell_3-AD.vs.control; scRNA-seq,SRP330776,B cell_3-AD.vs.control	10
AARS1	bulk RNA-seq,Homo_723,AD.vs.control; bulk RNA-seq,Homo_723,MCI.vs.control; bulk RNA-seq,Homo_633,AD.vs.control; bulk RNA-seq,Homo_633,MCI.vs.control; bulk RNA-seq,Homo_714,AD.vs.control; bulk RNA-seq,Homo_714,AD.vs.MCI; bulk RNA-	8
AARS2	scRNA-seq,SRP215507,CD8+ T cell_1-AD.vs.control; scRNA-seq,SRP309935,Naive CD8+ T cell_1-AD.vs.control; scRNA-seq,SRP330776,CD8+ T cell_1-AD.vs.control; scRNA-seq,SRP330776,CD8+ T cell_2-AD.vs.control; scRNA-seq,SRP330776,B cell_1-AD.vs.control; scRNA-seq,SRP330776,Naive CD8+ T cell_3-AD.vs.control; scRNA-seq,SRP330776,B cell_2-AD.vs.control	7
AARSD1	bulk RNA-seq,Homo_723,AD.vs.control; bulk RNA-seq,Homo_723,AD.vs.MCI; bulk RNA-seq,Homo_633,AD.vs.control; bulk RNA-seq,Homo_633,AD.vs.MCI; bulk RNA-seq,Homo_714,AD.vs.control; bulk RNA-seq,Homo_714,AD.vs.MCI; scRNA-seq,SRP215507,CD8+ T cell_5-	7
AASDH	bulk RNA-seq,Homo_714,AD.vs.control; bulk RNA-seq,Homo_714,AD.vs.MCI; scRNA-seq,SRP215507,CD8+ T cell_3-AD.vs.MCI; scRNA-seq,SRP215507,CD8+ T cell_5-AD.vs.MCI; scRNA-seq,SRP215507,CD8+ T cell_5-MCI.vs.control; scRNA-seq,SRP215507,CD8+ T cell_6-MCI.vs.control; scRNA-seq,SRP330776,CD8+ T cell_1-AD.vs.control; scRNA-seq,SRP330776,γδ T cell-AD.vs.control; scRNA-seq,SRP330776,Memory T cell_2-AD.vs.control; scRNA-seq,SRP330776,Natural killer T cell-AD.vs.control; scRNA-seq,SRP330776,Naive CD8+ T cell_3-AD.vs.control; scRNA-seq,SRP330776,B cell_2-AD.vs.control; scRNA-seq,SRP330776,CD8+ T cell_3-AD.vs.control; scRNA-seq,SRP330776,Monocyte_2-AD.vs.control;	16

AASDHPPT	bulk RNA-seq,Homo_714,AD.vs.control; bulk RNA-seq,Homo_714,AD.vs.MCI; scRNA-seq,SRP215507,CD8+ T cell_7-AD.vs.control; scRNA-seq,SRP309935,Erythroid cell-AD.vs.control; scRNA-seq,SRP309935,CD8+ T cell_2-AD.vs.control; scRNA-seq,SRP330776,Memory T cell_2-AD.vs.control; scRNA-seq,SRP330776,CD8+ T cell_3-AD.vs.control; scRNA-seq,SRP330776,Monocyte_2-AD.vs.control; scRNA-seq,SRP330776,Regulatory T cell-AD.vs.control; scRNA-seq,SRP330776,Megakaryocyte_1-AD.vs.control	10
AASS	bulk RNA-seq,Homo_723,AD.vs.control; bulk RNA-seq,Homo_723,AD.vs.MCI; bulk RNA-seq,Homo_723,MCI.vs.control; bulk RNA-seq,Homo_633,AD.vs.control; bulk RNA-seq,Homo_633,AD.vs.MCI; bulk RNA-seq,Homo_633,MCI.vs.control; bulk RNA-seq,Homo_714,AD.vs.control; bulk RNA-seq,Homo_714,AD.vs.MCI; scRNA-seq,SRP215507,CD8+ T cell_2-AD.vs.control; scRNA-seq,SRP215507,CD8+ T cell_1-AD.vs.control; scRNA-seq,SRP215507,CD8+ T cell_8-MCI.vs.control; scRNA-seq,SRP309935,CD8+ T cell_1-AD.vs.control; scRNA-seq,SRP330776,CD8+ T cell_1-	14
AATBC	scRNA-seq,SRP330776,Monocyte_2-AD.vs.control	1
AATF	bulk RNA-seq,Homo_723,AD.vs.control; bulk RNA-seq,Homo_723,AD.vs.MCI; bulk RNA-seq,Homo_633,AD.vs.control; bulk RNA-seq,Homo_633,AD.vs.MCI; bulk RNA-seq,Homo_714,AD.vs.control; bulk RNA-seq,Homo_714,AD.vs.MCI; scRNA-seq,SRP215507,CD8+ T cell_7-AD.vs.control; scRNA-seq,SRP215507,CD8+ T cell_4-AD.vs.MCI	8
AATK	scRNA-seq,SRP309935,Monocyte_2-AD.vs.control	1
ABALON	scRNA-seq,SRP215507,CD8+ T cell_3-AD.vs.MCI; scRNA-seq,SRP215507,CD8+ T cell_5-AD.vs.MCI; scRNA-seq,SRP215507,CD8+ T cell_3-MCI.vs.control; scRNA-seq,SRP330776,γδ T cell-AD.vs.control; scRNA-seq,SRP330776,B cell_1-AD.vs.control; scRNA-seq,SRP330776,Naive CD8+ T cell_1-AD.vs.control; scRNA-seq,SRP330776,Natural killer cell_1-AD.vs.control	7
ABAT	scRNA-seq,SRP215507,CD8+ T cell_5-MCI.vs.control; scRNA-seq,SRP215507,CD8+ T cell_2-MCI.vs.control; scRNA-seq,SRP215507,CD8+ T cell_3-MCI.vs.control; scRNA-seq,SRP330776,γδ T cell-AD.vs.control; scRNA-seq,SRP330776,Naive CD8+ T cell_1-AD.vs.control; scRNA-seq,SRP330776,Natural killer cell_1-AD.vs.control; scRNA-seq,SRP330776,Memory T cell_1-AD.vs.control; scRNA-seq,SRP330776,CD8+ T cell_3-	8
ABCA1	bulk RNA-seq,Homo_723,AD.vs.MCI; bulk RNA-seq,Homo_633,AD.vs.MCI; bulk RNA-seq,Homo_633,MCI.vs.control; bulk RNA-seq,Homo_714,AD.vs.control; bulk RNA-seq,Homo_714,AD.vs.MCI; scRNA-seq,SRP215507,CD8+ T cell_3-AD.vs.control; scRNA-seq,SRP215507,CD8+ T cell_5-AD.vs.MCI; scRNA-seq,SRP215507,CD8+ T cell_5-MCI.vs.control; scRNA-seq,SRP215507,CD8+ T cell_3-MCI.vs.control; scRNA-seq,SRP330776,Memory T cell_2-AD.vs.control; scRNA-seq,SRP330776,B cell_2-AD.vs.control; scRNA-seq,SRP330776,B	12
ABCA10	bulk RNA-seq,Homo_723,AD.vs.MCI; bulk RNA-seq,Homo_723,MCI.vs.control; bulk RNA-seq,Homo_633,AD.vs.MCI; bulk RNA-seq,Homo_633,MCI.vs.control; bulk RNA-seq,Homo_714,AD.vs.control; bulk RNA-seq,Homo_714,AD.vs.MCI; bulk RNA-seq,Homo_714,MCI.vs.control; scRNA-seq,SRP330776,Naive CD8+ T cell_1-AD.vs.control	8
ABCA12	bulk RNA-seq,ROSMAP,AD.vs.control; bulk RNA-seq,ROSMAP,MCI.vs.control	2
ABCA13	bulk RNA-seq,Homo_723,AD.vs.control; bulk RNA-seq,Homo_723,AD.vs.MCI; bulk RNA-seq,Homo_633,AD.vs.control; bulk RNA-seq,Homo_633,AD.vs.MCI; bulk RNA-seq,Homo_714,AD.vs.control; bulk RNA-seq,Homo_714,AD.vs.MCI; bulk RNA-seq,ROSMAP,AD.vs.control;	8
Abca13	bulk RNA-seq,SRP325058,AD.vs.control	1
ABCA2	scRNA-seq,SRP309935,Natural killer cell_2-AD.vs.control; scRNA-seq,SRP330776,CD8+ T cell_1-AD.vs.control; scRNA-seq,SRP330776,B cell_1-AD.vs.control; scRNA-seq,SRP330776,B cell_2-AD.vs.control; scRNA-seq,SRP330776,T cell-AD.vs.control; scRNA-seq,SRP330776,CD8+ T cell_3-	6
ABCA3	bulk RNA-seq,Homo_723,MCI.vs.control; bulk RNA-seq,Homo_633,MCI.vs.control; bulk RNA-seq,Homo_714,AD.vs.control; bulk RNA-seq,Homo_714,MCI.vs.control; bulk RNA-seq,ROSMAP,MCI.vs.control; scRNA-seq,SRP215507,CD8+ T cell_1-AD.vs.control; scRNA-seq,SRP215507,CD8+ T cell_1-AD.vs.MCI; scRNA-seq,SRP309935,Naive CD8+ T cell_1-AD.vs.control; scRNA-seq,SRP309935,Natural killer cell_1-AD.vs.control; scRNA-seq,SRP330776,CD8+ T cell_1-AD.vs.control; scRNA-seq,SRP330776,γδ T cell-AD.vs.control; scRNA-seq,SRP330776,Memory T cell_2-AD.vs.control; scRNA-seq,SRP330776,CD8+ T cell_2-AD.vs.control; scRNA-seq,SRP330776,Memory T cell_1-AD.vs.control	14
ABCA4	bulk RNA-seq,Homo_723,AD.vs.control; bulk RNA-seq,Homo_723,AD.vs.MCI; bulk RNA-seq,Homo_714,MCI.vs.control; bulk RNA-seq,ROSMAP,AD.vs.control; bulk RNA-seq,ROSMAP,MCI.vs.control	5

ABCA5	bulk RNA-seq,Homo_633,AD.vs.control; bulk RNA-seq,Homo_633,AD.vs.MCI; bulk RNA-seq,Homo_714,AD.vs.control; bulk RNA-seq,Homo_714,AD.vs.MCI; scRNA-seq,SRP215507,CD8+ T cell_3-AD.vs.control; scRNA-seq,SRP215507,CD8+ T cell_5-AD.vs.control; scRNA-seq,SRP215507,CD8+ T cell_4-AD.vs.MCI; scRNA-seq,SRP215507,CD8+ T cell_2-MCI.vs.control; scRNA-seq,SRP215507,CD8+ T cell_3-MCI.vs.control; scRNA-seq,SRP215507,CD8+ T cell_6-MCI.vs.control; scRNA-seq,SRP330776,CD8+ T cell_1-AD.vs.control; scRNA-seq,SRP330776,B cell_1-AD.vs.control; scRNA-seq,SRP330776,Memory T cell_1-AD.vs.control; scRNA-seq,SRP330776,T cell-AD.vs.control; scRNA-seq,SRP330776,B	15
ABCA6	bulk RNA-seq,Homo_723,AD.vs.control; bulk RNA-seq,Homo_723,AD.vs.MCI; bulk RNA-seq,Homo_633,AD.vs.control; bulk RNA-seq,Homo_633,AD.vs.MCI; scRNA-seq,SRP330776,B cell_1-AD.vs.control	5
ABCA7	scRNA-seq,SRP215507,CD8+ T cell_5-AD.vs.MCI; scRNA-seq,SRP215507,CD8+ T cell_5-MCI.vs.control; scRNA-seq,SRP309935,Naive CD8+ T cell_2-AD.vs.control; scRNA-seq,SRP330776,CD8+ T cell_1-AD.vs.control; scRNA-seq,SRP330776,Memory T cell_2-AD.vs.control; scRNA-seq,SRP330776,Natural killer T cell-AD.vs.control; scRNA-seq,SRP330776,T cell-AD.vs.control; scRNA-seq,SRP330776,CD8+ T cell_3-AD.vs.control; scRNA-seq,SRP330776,Monocyte_1-AD.vs.control; scRNA-seq,SRP330776,Monocyte_2-AD.vs.control; scRNA-seq,SRP330776,Regulatory T cell-	11
ABCA8	bulk RNA-seq,Homo_723,AD.vs.control; bulk RNA-seq,Homo_714,MCI.vs.control; bulk RNA-seq,ROSMAP,AD.vs.control; bulk RNA-	4
ABCA9	bulk RNA-seq,Homo_723,AD.vs.control; bulk RNA-seq,Homo_723,AD.vs.MCI; bulk RNA-seq,Homo_723,MCI.vs.control; bulk RNA-seq,Homo_633,AD.vs.control; bulk RNA-seq,Homo_633,AD.vs.MCI; bulk RNA-seq,Homo_633,MCI.vs.control; bulk RNA-seq,Homo_714,AD.vs.control;	8
ABCB1	bulk RNA-seq,Homo_723,AD.vs.control; bulk RNA-seq,Homo_723,AD.vs.MCI; bulk RNA-seq,Homo_633,AD.vs.control; bulk RNA-seq,Homo_633,AD.vs.MCI; bulk RNA-seq,Homo_714,AD.vs.control; bulk RNA-seq,Homo_714,AD.vs.MCI; bulk RNA-seq,Homo_714,MCI.vs.control; scRNA-seq,SRP215507,CD8+ T cell_1-AD.vs.MCI; scRNA-seq,SRP215507,CD8+ T cell_1-MCI.vs.control; scRNA-seq,SRP215507,CD8+ T cell_2-MCI.vs.control; scRNA-seq,SRP330776,CD8+ T cell_1-AD.vs.control; scRNA-seq,SRP330776,γδ T cell-AD.vs.control; scRNA-seq,SRP330776,B cell_1-AD.vs.control; scRNA-seq,SRP330776,Natural killer cell_1-AD.vs.control; scRNA-seq,SRP330776,B cell_4-AD.vs.control	15
ABCB10	bulk RNA-seq,Homo_723,AD.vs.MCI; bulk RNA-seq,Homo_633,AD.vs.control; bulk RNA-seq,Homo_633,AD.vs.MCI; bulk RNA-seq,Homo_714,AD.vs.control; bulk RNA-seq,Homo_714,AD.vs.MCI; scRNA-seq,SRP309935,CD8+ T cell_1-AD.vs.control; scRNA-seq,SRP330776,CD8+ T cell_1-AD.vs.control; scRNA-seq,SRP330776,γδ T cell-AD.vs.control; scRNA-seq,SRP330776,Memory T cell_2-AD.vs.control; scRNA-seq,SRP330776,B cell_1-AD.vs.control; scRNA-seq,SRP330776,Naive CD8+ T cell_1-AD.vs.control; scRNA-seq,SRP330776,Natural killer cell_1-AD.vs.control; scRNA-seq,SRP330776,T cell-AD.vs.control; scRNA-seq,SRP330776,CD8+ T cell_3-AD.vs.control	14
ABCB11	bulk RNA-seq,ROSMAP,AD.vs.control; bulk RNA-seq,ROSMAP,MCI.vs.control	2
ABCB4	bulk RNA-seq,Homo_723,MCI.vs.control; bulk RNA-seq,Homo_633,MCI.vs.control; bulk RNA-seq,Homo_714,AD.vs.control; bulk RNA-seq,Homo_714,AD.vs.MCI; bulk RNA-seq,Homo_714,MCI.vs.control; scRNA-seq,SRP330776,B cell_1-AD.vs.control; scRNA-seq,SRP330776,B cell_2-	7
ABCB5	bulk RNA-seq,Homo_723,AD.vs.control; bulk RNA-seq,Homo_723,AD.vs.MCI; bulk RNA-seq,Homo_633,AD.vs.control; bulk RNA-	4
ABCB6	scRNA-seq,SRP330776,CD8+ T cell_1-AD.vs.control; scRNA-seq,SRP330776,Memory T cell_2-AD.vs.control; scRNA-seq,SRP330776,Naive CD8+ T cell_1-AD.vs.control; scRNA-seq,SRP330776,Memory T cell_1-AD.vs.control	4
Abcb7	bulk RNA-seq,SRP325058,AD.vs.control	1
ABCB7	scRNA-seq,SRP309935,Monocyte_1-AD.vs.control; scRNA-seq,SRP330776,Naive CD8+ T cell_3-AD.vs.control; scRNA-seq,SRP330776,B cell_3-	3
ABCB8	bulk RNA-seq,Homo_723,AD.vs.control; bulk RNA-seq,Homo_723,AD.vs.MCI; bulk RNA-seq,Homo_723,MCI.vs.control; bulk RNA-seq,Homo_633,AD.vs.control; bulk RNA-seq,Homo_633,AD.vs.MCI; bulk RNA-seq,Homo_633,MCI.vs.control; bulk RNA-seq,Homo_714,AD.vs.control; bulk RNA-seq,Homo_714,AD.vs.MCI; bulk RNA-seq,Homo_714,MCI.vs.control; scRNA-seq,SRP215507,CD8+ T cell_7-AD.vs.MCI; scRNA-seq,SRP309935,Natural killer cell_2-AD.vs.control; scRNA-seq,SRP309935,Monocyte_1-AD.vs.control; scRNA-seq,SRP330776,CD8+ T cell_1-AD.vs.control; scRNA-seq,SRP330776,Memory T cell_2-AD.vs.control; scRNA-seq,SRP330776,Naive CD8+ T cell_1-AD.vs.control; scRNA-seq,SRP330776,Natural killer cell_1-AD.vs.control; scRNA-seq,SRP330776,Naive CD8+ T cell_3-AD.vs.control; scRNA-seq,SRP330776,Natural killer	18
ABCB9	bulk RNA-seq,Homo_723,AD.vs.control; bulk RNA-seq,Homo_723,MCI.vs.control; bulk RNA-seq,Homo_633,AD.vs.control; bulk RNA-seq,Homo_633,AD.vs.MCI; bulk RNA-seq,Homo_633,MCI.vs.control; bulk RNA-seq,Homo_714,AD.vs.control; bulk RNA-seq,Homo_714,AD.vs.MCI; bulk RNA-seq,Homo_714,MCI.vs.control; scRNA-seq,SRP215507,CD8+ T cell_5-AD.vs.control; scRNA-seq,SRP215507,CD8+ T cell_5-MCI.vs.control; scRNA-seq,SRP330776,CD8+ T cell_2-AD.vs.control; scRNA-seq,SRP330776,Memory T cell_1-AD.vs.control	12

ABCC1	scRNA-seq,SRP215507,CD8+ T cell_5-AD.vs.MCI; scRNA-seq,SRP215507,CD8+ T cell_5-MCI.vs.control; scRNA-seq,SRP309935,Naive CD8+ T cell_1-AD.vs.control; scRNA-seq,SRP330776,γδ T cell-AD.vs.control; scRNA-seq,SRP330776,Memory T cell_2-AD.vs.control; scRNA-seq,SRP330776,B cell_1-AD.vs.control; scRNA-seq,SRP330776,Naive CD8+ T cell_1-AD.vs.control; scRNA-seq,SRP330776,Memory T cell_1-AD.vs.control; scRNA-seq,SRP330776,Natural killer T cell-AD.vs.control; scRNA-seq,SRP330776,Naive CD8+ T cell_3-AD.vs.control; scRNA-seq,SRP330776,B cell_2-AD.vs.control; scRNA-seq,SRP330776,T cell-AD.vs.control; scRNA-seq,SRP330776,CD8+ T cell_3-AD.vs.control; scRNA-seq,SRP330776,Monocyte_2-	14
ABCC10	scRNA-seq,SRP215507,CD8+ T cell_3-AD.vs.control; scRNA-seq,SRP215507,CD8+ T cell_3-AD.vs.MCI; scRNA-seq,SRP215507,CD8+ T cell_1-AD.vs.MCI; scRNA-seq,SRP215507,CD8+ T cell_6-MCI.vs.control; scRNA-seq,SRP330776,γδ T cell-AD.vs.control; scRNA-seq,SRP330776,Natural killer T cell-AD.vs.control; scRNA-seq,SRP330776,Naive CD8+ T cell_3-AD.vs.control; scRNA-seq,SRP330776,CD8+ T cell_3-AD.vs.control; scRNA-	9
ABCC11	bulk RNA-seq,Homo_723,AD.vs.MCI; bulk RNA-seq,Homo_723,MCI.vs.control; bulk RNA-seq,Homo_633,AD.vs.MCI; bulk RNA-seq,Homo_633,MCI.vs.control; bulk RNA-seq,Homo_714,AD.vs.MCI; bulk RNA-seq,Homo_714,MCI.vs.control; scRNA-seq,SRP330776,CD8+ T cell_2-AD.vs.control; scRNA-seq,SRP330776,B cell_1-AD.vs.control; scRNA-seq,SRP330776,B cell_2-AD.vs.control	9
ABCC2	bulk RNA-seq,Homo_723,AD.vs.control; bulk RNA-seq,Homo_723,AD.vs.MCI; bulk RNA-seq,Homo_723,MCI.vs.control; bulk RNA-seq,Homo_633,AD.vs.control; bulk RNA-seq,Homo_633,AD.vs.MCI; bulk RNA-seq,Homo_633,MCI.vs.control; bulk RNA-seq,Homo_714,AD.vs.control; bulk RNA-seq,Homo_714,AD.vs.MCI; bulk RNA-seq,Homo_714,MCI.vs.control; scRNA-seq,SRP215507,CD8+ T cell_4-AD.vs.control; scRNA-seq,SRP215507,CD8+ T cell_2-AD.vs.MCI; scRNA-seq,SRP215507,CD8+ T cell_2-MCI.vs.control; scRNA-seq,SRP330776,Naive CD8+ T cell_1-	13
ABCC3	bulk RNA-seq,Homo_723,AD.vs.control; bulk RNA-seq,Homo_723,AD.vs.MCI; bulk RNA-seq,Homo_723,MCI.vs.control; bulk RNA-seq,Homo_633,AD.vs.control; bulk RNA-seq,Homo_633,AD.vs.MCI; bulk RNA-seq,Homo_633,MCI.vs.control; bulk RNA-seq,Homo_714,AD.vs.control; bulk RNA-seq,Homo_714,AD.vs.MCI; scRNA-seq,SRP309935,Megakaryocyte_1-AD.vs.control; scRNA-seq,SRP330776,Monocyte_2-AD.vs.control;	11
ABCC4	bulk RNA-seq,Homo_723,AD.vs.control; bulk RNA-seq,Homo_723,AD.vs.MCI; bulk RNA-seq,Homo_723,MCI.vs.control; bulk RNA-seq,Homo_633,AD.vs.control; bulk RNA-seq,Homo_633,AD.vs.MCI; bulk RNA-seq,Homo_633,MCI.vs.control; bulk RNA-seq,Homo_714,AD.vs.control; bulk RNA-seq,Homo_714,AD.vs.MCI; scRNA-seq,SRP215507,CD8+ T cell_1-AD.vs.control; scRNA-seq,SRP215507,CD8+ T cell_5-AD.vs.MCI; scRNA-seq,SRP215507,CD8+ T cell_1-MCI.vs.control; scRNA-seq,SRP215507,CD8+ T cell_5-MCI.vs.control; scRNA-seq,SRP309935,Megakaryocyte_2-AD.vs.control; scRNA-seq,SRP330776,γδ T cell-AD.vs.control; scRNA-seq,SRP330776,B cell_2-AD.vs.control;	16
Abcc4	bulk RNA-seq,SRP325058,AD.vs.control	1
ABCC5	scRNA-seq,SRP215507,CD8+ T cell_5-AD.vs.control; scRNA-seq,SRP330776,Naive CD8+ T cell_3-AD.vs.control	2
ABCC6	bulk RNA-seq,Homo_723,AD.vs.control; bulk RNA-seq,Homo_723,AD.vs.MCI; bulk RNA-seq,Homo_633,AD.vs.control; bulk RNA-seq,Homo_633,AD.vs.MCI; bulk RNA-seq,Homo_714,AD.vs.control; bulk RNA-seq,Homo_714,AD.vs.MCI	6
ABCC8	bulk RNA-seq,Homo_723,AD.vs.control; bulk RNA-seq,Homo_723,AD.vs.MCI; bulk RNA-seq,Homo_714,MCI.vs.control; bulk RNA-	4
ABCC9	bulk RNA-seq,Homo_723,AD.vs.control; bulk RNA-seq,Homo_723,AD.vs.MCI; bulk RNA-seq,Homo_633,AD.vs.control; bulk RNA-seq,Homo_633,AD.vs.MCI; bulk RNA-seq,Homo_714,MCI.vs.control	5
ABCD1	scRNA-seq,SRP215507,CD8+ T cell_3-AD.vs.MCI; scRNA-seq,SRP215507,CD8+ T cell_5-AD.vs.MCI; scRNA-seq,SRP215507,CD8+ T cell_5-MCI.vs.control; scRNA-seq,SRP215507,CD8+ T cell_3-MCI.vs.control; scRNA-seq,SRP330776,CD8+ T cell_2-AD.vs.control; scRNA-seq,SRP330776,B cell_1-AD.vs.control; scRNA-seq,SRP330776,Naive CD8+ T cell_1-AD.vs.control; scRNA-seq,SRP330776,B cell_2-AD.vs.control; scRNA-seq,SRP330776,CD8+ T cell_3-AD.vs.control; scRNA-seq,SRP330776,Megakaryocyte_1-AD.vs.control; scRNA-seq,SRP330776,Natural killer cell_2-	12
ABCD2	bulk RNA-seq,Homo_714,AD.vs.control; bulk RNA-seq,Homo_714,AD.vs.MCI; bulk RNA-seq,Homo_714,MCI.vs.control; scRNA-seq,SRP215507,CD8+ T cell_3-AD.vs.MCI; scRNA-seq,SRP330776,B cell_4-AD.vs.control	5
ABCD3	bulk RNA-seq,Homo_714,AD.vs.control; bulk RNA-seq,Homo_714,AD.vs.MCI; scRNA-seq,SRP215507,CD8+ T cell_5-MCI.vs.control; scRNA-seq,SRP309935,Naive CD8+ T cell_1-AD.vs.control; scRNA-seq,SRP330776,CD8+ T cell_1-AD.vs.control; scRNA-seq,SRP330776,Memory T cell_2-AD.vs.control; scRNA-seq,SRP330776,Naive CD8+ T cell_1-AD.vs.control; scRNA-seq,SRP330776,Natural killer T cell-AD.vs.control; scRNA-seq,SRP330776,B cell_3-AD.vs.control; scRNA-seq,SRP330776,Natural killer cell_2-AD.vs.control	10
ABCD4	scRNA-seq,SRP215507,CD8+ T cell_7-AD.vs.control; scRNA-seq,SRP215507,CD8+ T cell_3-AD.vs.MCI; scRNA-seq,SRP215507,CD8+ T cell_4-AD.vs.MCI; scRNA-seq,SRP215507,CD8+ T cell_5-AD.vs.MCI; scRNA-seq,SRP215507,CD8+ T cell_2-MCI.vs.control; scRNA-seq,SRP215507,CD8+ T cell_7-MCI.vs.control; scRNA-seq,SRP330776,Memory T cell_1-AD.vs.control	7

ABCE1	scRNA-seq,SRP215507,CD8+ T cell_3-MCI.vs.control; scRNA-seq,SRP309935,Monocyte_2-AD.vs.control; scRNA-seq,SRP330776,B cell_3-	3
ABCF1	bulk RNA-seq,Homo_723,AD.vs.MCI; bulk RNA-seq,Homo_723,MCI.vs.control; bulk RNA-seq,Homo_633,AD.vs.MCI; bulk RNA-seq,Homo_633,MCI.vs.control; bulk RNA-seq,Homo_714,AD.vs.control; bulk RNA-seq,Homo_714,AD.vs.MCI; bulk RNA-seq,Homo_714,MCI.vs.control; scRNA-seq,SRP330776,γδ T cell-AD.vs.control; scRNA-seq,SRP330776,Memory T cell_1-AD.vs.control; scRNA-seq,SRP330776,Naive CD8+ T cell_3-AD.vs.control; scRNA-seq,SRP330776,B cell_2-AD.vs.control; scRNA-seq,SRP330776,Megakaryocyte_1-	12
ABCF2	bulk RNA-seq,Homo_723,AD.vs.MCI; bulk RNA-seq,Homo_723,MCI.vs.control; bulk RNA-seq,Homo_633,AD.vs.MCI; bulk RNA-seq,Homo_633,MCI.vs.control; bulk RNA-seq,Homo_714,AD.vs.control; bulk RNA-seq,Homo_714,AD.vs.MCI; bulk RNA-seq,Homo_714,MCI.vs.control; scRNA-seq,SRP330776,Naive CD8+ T cell_3-AD.vs.control; scRNA-seq,SRP330776,CD8+ T cell_3-AD.vs.control;	11
ABCF2-H2BE1	bulk RNA-seq,Homo_723,AD.vs.control; bulk RNA-seq,Homo_723,AD.vs.MCI; bulk RNA-seq,Homo_633,AD.vs.control; bulk RNA-seq,Homo_633,AD.vs.MCI; bulk RNA-seq,Homo_714,AD.vs.control; bulk RNA-seq,Homo_714,AD.vs.MCI	6
ABCF3	scRNA-seq,SRP309935,CD8+ T cell_2-AD.vs.control; scRNA-seq,SRP330776,γδ T cell-AD.vs.control; scRNA-seq,SRP330776,CD8+ T cell_3-AD.vs.control; scRNA-seq,SRP330776,Megakaryocyte_1-AD.vs.control	4
ABCG1	scRNA-seq,SRP215507,CD8+ T cell_3-AD.vs.MCI; scRNA-seq,SRP215507,CD8+ T cell_1-AD.vs.MCI; scRNA-seq,SRP215507,CD8+ T cell_1-MCI.vs.control; scRNA-seq,SRP309935,Naive CD8+ T cell_1-AD.vs.control; scRNA-seq,SRP309935,CD8+ T cell_2-AD.vs.control; scRNA-seq,SRP330776,CD8+ T cell_1-AD.vs.control; scRNA-seq,SRP330776,γδ T cell-AD.vs.control; scRNA-seq,SRP330776,Memory T cell_2-AD.vs.control; scRNA-seq,SRP330776,CD8+ T cell_2-AD.vs.control; scRNA-seq,SRP330776,B cell_1-AD.vs.control; scRNA-seq,SRP330776,Naive CD8+ T cell_1-AD.vs.control; scRNA-seq,SRP330776,Memory T cell_1-AD.vs.control; scRNA-seq,SRP330776,Natural killer T cell-AD.vs.control; scRNA-seq,SRP330776,Naive CD8+ T cell_3-AD.vs.control; scRNA-seq,SRP330776,B cell_2-AD.vs.control; scRNA-seq,SRP330776,T cell-AD.vs.control; scRNA-seq,SRP330776,Naive CD8+ T cell_2-AD.vs.control; scRNA-seq,SRP330776,CD8+ T cell_3-AD.vs.control; scRNA-seq,SRP330776,B cell_3-	19
ABCG2	bulk RNA-seq,Homo_723,AD.vs.control; bulk RNA-seq,Homo_723,AD.vs.MCI; bulk RNA-seq,Homo_633,AD.vs.control; bulk RNA-seq,Homo_633,AD.vs.MCI; bulk RNA-seq,Homo_714,AD.vs.control; bulk RNA-seq,Homo_714,AD.vs.MCI; bulk RNA-seq,ROSMAP,AD.vs.MCI; bulk RNA-seq,ROSMAP,MCI.vs.control; scRNA-seq,SRP215507,CD8+ T cell_1-AD.vs.control; scRNA-seq,SRP215507,CD8+ T cell_5-AD.vs.control; scRNA-seq,SRP215507,CD8+ T cell_2-AD.vs.MCI; scRNA-seq,SRP215507,CD8+ T cell_5-AD.vs.MCI; scRNA-seq,SRP215507,CD8+ T cell_1-MCI.vs.control; scRNA-seq,SRP215507,CD8+ T cell_2-MCI.vs.control; scRNA-seq,SRP330776,CD8+ T cell_1-AD.vs.control; scRNA-	17
ABCG4	bulk RNA-seq,Homo_723,AD.vs.control; bulk RNA-seq,Homo_723,AD.vs.MCI; bulk RNA-seq,Homo_714,MCI.vs.control	3
ABCG5	bulk RNA-seq,Homo_714,MCI.vs.control	1
ABHD1	scRNA-seq,SRP215507,CD8+ T cell_2-AD.vs.control; scRNA-seq,SRP215507,CD8+ T cell_2-AD.vs.MCI; scRNA-seq,SRP330776,CD8+ T cell_1-AD.vs.control; scRNA-seq,SRP330776,CD8+ T cell_2-AD.vs.control	4
ABHD10	scRNA-seq,SRP215507,CD8+ T cell_4-AD.vs.control; scRNA-seq,SRP215507,CD8+ T cell_3-AD.vs.MCI; scRNA-seq,SRP215507,CD8+ T cell_4-AD.vs.MCI; scRNA-seq,SRP215507,CD8+ T cell_3-MCI.vs.control; scRNA-seq,SRP215507,CD8+ T cell_6-MCI.vs.control; scRNA-seq,SRP309935,Monocyte_1-AD.vs.control; scRNA-seq,SRP330776,B cell_2-AD.vs.control; scRNA-seq,SRP330776,Regulatory T cell-AD.vs.control	8
ABHD11	scRNA-seq,SRP215507,CD8+ T cell_1-AD.vs.MCI; scRNA-seq,SRP215507,CD8+ T cell_5-AD.vs.MCI; scRNA-seq,SRP215507,CD8+ T cell_6-AD.vs.MCI; scRNA-seq,SRP215507,CD8+ T cell_5-MCI.vs.control; scRNA-seq,SRP215507,CD8+ T cell_4-MCI.vs.control; scRNA-seq,SRP330776,CD8+ T cell_2-AD.vs.control; scRNA-seq,SRP330776,Memory T cell_1-AD.vs.control; scRNA-seq,SRP330776,B cell_2-AD.vs.control;	9
ABHD12	scRNA-seq,SRP215507,CD8+ T cell_4-AD.vs.control; scRNA-seq,SRP215507,CD8+ T cell_10-AD.vs.control; scRNA-seq,SRP309935,Natural killer cell_2-AD.vs.control; scRNA-seq,SRP309935,Naive CD8+ T cell_2-AD.vs.control; scRNA-seq,SRP309935,CD8+ T cell_1-AD.vs.control; scRNA-seq,SRP330776,CD8+ T cell_1-AD.vs.control; scRNA-seq,SRP330776,γδ T cell-AD.vs.control; scRNA-seq,SRP330776,Memory T cell_2-AD.vs.control; scRNA-seq,SRP330776,CD8+ T cell_2-AD.vs.control; scRNA-seq,SRP330776,B cell_1-AD.vs.control; scRNA-seq,SRP330776,Naive CD8+ T cell_1-AD.vs.control; scRNA-seq,SRP330776,Natural killer cell_1-AD.vs.control; scRNA-seq,SRP330776,Memory T cell_1-AD.vs.control; scRNA-seq,SRP330776,Natural killer T cell-AD.vs.control; scRNA-seq,SRP330776,Naive CD8+ T cell_3-AD.vs.control; scRNA-seq,SRP330776,B cell_2-AD.vs.control; scRNA-seq,SRP330776,T cell-AD.vs.control; scRNA-seq,SRP330776,CD8+ T cell_3-AD.vs.control; scRNA-seq,SRP330776,Monocyte_1-AD.vs.control; scRNA-seq,SRP330776,Monocyte_2-AD.vs.control; scRNA-seq,SRP330776,B cell_3-AD.vs.control; scRNA-seq,SRP330776,B cell_4-	22

ABHD12B	bulk RNA-seq,Homo_723,AD.vs.control; bulk RNA-seq,Homo_723,AD.vs.MCI; bulk RNA-seq,Homo_723,MCI.vs.control; bulk RNA-seq,Homo_633,AD.vs.control; bulk RNA-seq,Homo_633,AD.vs.MCI; bulk RNA-seq,Homo_633,MCI.vs.control; bulk RNA-seq,Homo_714,AD.vs.control; bulk RNA-seq,Homo_714,AD.vs.MCI; bulk RNA-seq,Homo_714,MCI.vs.control	9
ABHD13	scRNA-seq,SRP215507,CD8+ T cell_10-AD.vs.control; scRNA-seq,SRP215507,CD8+ T cell_1-AD.vs.MCI; scRNA-seq,SRP215507,CD8+ T cell_4-AD.vs.MCI; scRNA-seq,SRP215507,CD8+ T cell_7-AD.vs.MCI; scRNA-seq,SRP215507,CD8+ T cell_1-MCI.vs.control; scRNA-seq,SRP215507,CD8+ T cell_4-MCI.vs.control; scRNA-seq,SRP215507,CD8+ T cell_2-MCI.vs.control; scRNA-seq,SRP330776,Naive CD8+ T cell_3-AD.vs.control; scRNA-	9
ABHD14A	scRNA-seq,SRP215507,CD8+ T cell_4-AD.vs.control; scRNA-seq,SRP215507,CD8+ T cell_7-AD.vs.control; scRNA-seq,SRP330776,Natural killer T cell-AD.vs.control; scRNA-seq,SRP330776,CD8+ T cell_3-AD.vs.control; scRNA-seq,SRP330776,Natural killer cell_2-AD.vs.control	5
ABHD14B	scRNA-seq,SRP215507,CD8+ T cell_9-AD.vs.control; scRNA-seq,SRP215507,CD8+ T cell_6-MCI.vs.control; scRNA-seq,SRP330776,Monocyte_1-	3
ABHD15	bulk RNA-seq,SRP223445,AD.vs.control; scRNA-seq,SRP215507,CD8+ T cell_3-AD.vs.control; scRNA-seq,SRP215507,CD8+ T cell_2-AD.vs.MCI; scRNA-seq,SRP215507,CD8+ T cell_5-AD.vs.MCI; scRNA-seq,SRP215507,CD8+ T cell_5-MCI.vs.control; scRNA-seq,SRP309935,Natural killer cell_1-AD.vs.control; scRNA-seq,SRP309935,CD8+ T cell_2-AD.vs.control; scRNA-seq,SRP309935,Plasmacytoid dendritic cell_1-AD.vs.control; scRNA-seq,SRP330776,CD8+ T cell_1-AD.vs.control; scRNA-seq,SRP330776,γδ T cell-AD.vs.control; scRNA-seq,SRP330776,Memory T cell_2-AD.vs.control; scRNA-seq,SRP330776,CD8+ T cell_2-AD.vs.control; scRNA-seq,SRP330776,Naive CD8+ T cell_1-AD.vs.control; scRNA-seq,SRP330776,Natural killer cell_1-AD.vs.control; scRNA-seq,SRP330776,Memory T cell_1-AD.vs.control; scRNA-seq,SRP330776,CD8+ T cell_3-AD.vs.control; scRNA- [illegible]	17
ABHD16A	bulk RNA-seq,Homo_723,AD.vs.control; bulk RNA-seq,Homo_723,AD.vs.MCI; bulk RNA-seq,Homo_633,AD.vs.control; bulk RNA-seq,Homo_633,AD.vs.MCI; scRNA-seq,SRP215507,CD8+ T cell_3-MCI.vs.control; scRNA-seq,SRP215507,CD8+ T cell_6-MCI.vs.control; scRNA-seq,SRP309935,Monocyte_2-AD.vs.control; scRNA-seq,SRP330776,Naive CD8+ T cell_1-AD.vs.control; scRNA-seq,SRP330776,B cell_2-AD.vs.control; scRNA-seq,SRP330776,T cell-AD.vs.control; scRNA-seq,SRP330776,B cell_3-AD.vs.control; scRNA-seq,SRP330776,Natural killer cell_2-AD.vs.control	12
ABHD16B	bulk RNA-seq,Homo_723,AD.vs.MCI; bulk RNA-seq,Homo_723,MCI.vs.control; bulk RNA-seq,Homo_633,AD.vs.MCI; bulk RNA-	4
ABHD17A	scRNA-seq,SRP215507,CD8+ T cell_7-AD.vs.control; scRNA-seq,SRP215507,CD8+ T cell_4-AD.vs.MCI; scRNA-seq,SRP215507,CD8+ T cell_7-AD.vs.MCI; scRNA-seq,SRP215507,CD8+ T cell_5-AD.vs.MCI; scRNA-seq,SRP215507,CD8+ T cell_5-MCI.vs.control; scRNA-seq,SRP309935,Erythroid cell-AD.vs.control; scRNA-seq,SRP330776,CD8+ T cell_1-AD.vs.control; scRNA-seq,SRP330776,γδ T cell-AD.vs.control; scRNA-seq,SRP330776,Memory T cell_2-AD.vs.control; scRNA-seq,SRP330776,CD8+ T cell_2-AD.vs.control; scRNA-seq,SRP330776,B cell_1-AD.vs.control; scRNA-seq,SRP330776,Naive CD8+ T cell_1-AD.vs.control; scRNA-seq,SRP330776,Natural killer cell_1-AD.vs.control; scRNA-seq,SRP330776,Memory T cell_1-AD.vs.control; scRNA-seq,SRP330776,Natural killer T cell-AD.vs.control; scRNA-seq,SRP330776,Naive CD8+ T cell_3-AD.vs.control; scRNA-seq,SRP330776,B cell_2-AD.vs.control; scRNA-seq,SRP330776,T cell-AD.vs.control; scRNA-seq,SRP330776,Naive CD8+ T cell_2-AD.vs.control; scRNA-seq,SRP330776,CD8+ T cell_3-AD.vs.control; scRNA-seq,SRP330776,Monocyte_2-AD.vs.control; scRNA- [illegible]	24
ABHD17B	bulk RNA-seq,Homo_723,AD.vs.MCI; bulk RNA-seq,Homo_633,AD.vs.MCI; bulk RNA-seq,Homo_714,AD.vs.control; bulk RNA-seq,Homo_714,AD.vs.MCI; scRNA-seq,SRP215507,CD8+ T cell_7-AD.vs.control; scRNA-seq,SRP215507,CD8+ T cell_2-AD.vs.MCI; scRNA-seq,SRP215507,CD8+ T cell_1-AD.vs.MCI; scRNA-seq,SRP215507,CD8+ T cell_1-MCI.vs.control; scRNA-seq,SRP215507,CD8+ T cell_2-MCI.vs.control; scRNA-seq,SRP309935,B cell_2-AD.vs.control; scRNA-seq,SRP330776,CD8+ T cell_1-AD.vs.control; scRNA-seq,SRP330776,Memory T cell_2-AD.vs.control; scRNA-seq,SRP330776,Naive CD8+ T cell_1-AD.vs.control; scRNA-seq,SRP330776,Natural killer cell_1-AD.vs.control; scRNA-seq,SRP330776,Memory T cell_1-AD.vs.control; scRNA-seq,SRP330776,T cell-AD.vs.control; scRNA-seq,SRP330776,CD8+ T cell_3-AD.vs.control	17
ABHD17C	bulk RNA-seq,Homo_633,AD.vs.MCI; bulk RNA-seq,Homo_714,AD.vs.control; bulk RNA-seq,Homo_714,AD.vs.MCI; scRNA-seq,SRP215507,CD8+ T cell_1-MCI.vs.control; scRNA-seq,SRP330776,Memory T cell_1-AD.vs.control	5
ABHD18	scRNA-seq,SRP215507,CD8+ T cell_7-AD.vs.MCI; scRNA-seq,SRP215507,CD8+ T cell_7-MCI.vs.control; scRNA-seq,SRP309935,Megakaryocyte_1-AD.vs.control; scRNA-seq,SRP309935,Dendritic cell-AD.vs.control; scRNA-seq,SRP330776,γδ T cell-AD.vs.control; scRNA-seq,SRP330776,Memory T cell_1-AD.vs.control; scRNA-seq,SRP330776,Regulatory T cell-AD.vs.control	7
ABHD2	bulk RNA-seq,Homo_723,AD.vs.control; bulk RNA-seq,Homo_723,AD.vs.MCI; bulk RNA-seq,Homo_633,AD.vs.control; bulk RNA-seq,Homo_633,AD.vs.MCI; bulk RNA-seq,SRP223445,AD.vs.control; scRNA-seq,SRP215507,CD8+ T cell_3-AD.vs.control; scRNA-seq,SRP215507,CD8+ T cell_5-AD.vs.MCI; scRNA-seq,SRP215507,CD8+ T cell_5-MCI.vs.control; scRNA-seq,SRP309935,Natural killer cell_2-AD.vs.control; scRNA-seq,SRP309935,Natural killer cell_1-AD.vs.control; scRNA-seq,SRP309935,Monocyte_2-AD.vs.control; scRNA-seq,SRP330776,γδ	13

ABHD3	bulk RNA-seq,Homo_723,AD.vs.control; bulk RNA-seq,Homo_723,AD.vs.MCI; bulk RNA-seq,Homo_633,AD.vs.control; bulk RNA-seq,Homo_633,AD.vs.MCI; bulk RNA-seq,Homo_714,AD.vs.control; bulk RNA-seq,Homo_714,AD.vs.MCI; scRNA-seq,SRP215507,CD8+ T cell_10-AD.vs.control; scRNA-seq,SRP215507,CD8+ T cell_3-AD.vs.MCI; scRNA-seq,SRP215507,CD8+ T cell_4-AD.vs.MCI; scRNA-seq,SRP215507,CD8+ T cell_5-AD.vs.MCI; scRNA-seq,SRP215507,CD8+ T cell_5-MCI.vs.control; scRNA-seq,SRP309935,Naive CD8+ T cell_2-AD.vs.control; scRNA-seq,SRP309935,B cell_1-AD.vs.control; scRNA-seq,SRP330776,CD8+ T cell_1-AD.vs.control; scRNA-seq,SRP330776,γδ T cell-AD.vs.control; scRNA-seq,SRP330776,Memory T cell_2-AD.vs.control; scRNA-seq,SRP330776,CD8+ T cell_2-AD.vs.control; scRNA-seq,SRP330776,B cell_1-AD.vs.control; scRNA-seq,SRP330776,Naive CD8+ T cell_1-AD.vs.control; scRNA-seq,SRP330776,Natural killer cell_1-AD.vs.control; scRNA-seq,SRP330776,Memory T cell_1-AD.vs.control; scRNA-seq,SRP330776,Natural killer T cell-AD.vs.control; scRNA-seq,SRP330776,Naive CD8+ T cell_3-AD.vs.control; scRNA-seq,SRP330776,B cell_2-AD.vs.control; scRNA-seq,SRP330776,CD8+ T cell_3-AD.vs.control; scRNA-seq,SRP330776,Natural killer cell_2-AD.vs.control	26
ABHD4	bulk RNA-seq,Homo_723,AD.vs.control; bulk RNA-seq,Homo_723,AD.vs.MCI; bulk RNA-seq,Homo_714,AD.vs.control; bulk RNA-seq,Homo_714,AD.vs.MCI; bulk RNA-seq,SRP310421,AD.vs.control; scRNA-seq,SRP215507,CD8+ T cell_3-AD.vs.control; scRNA-seq,SRP215507,CD8+ T cell_2-AD.vs.MCI; scRNA-seq,SRP215507,CD8+ T cell_5-AD.vs.MCI; scRNA-seq,SRP215507,CD8+ T cell_5-MCI.vs.control; scRNA-seq,SRP215507,CD8+ T cell_6-MCI.vs.control; scRNA-seq,SRP330776,Naive CD8+ T cell_3-AD.vs.control	11
ABHD5	bulk RNA-seq,Homo_723,AD.vs.control; bulk RNA-seq,Homo_723,AD.vs.MCI; bulk RNA-seq,Homo_633,AD.vs.control; bulk RNA-seq,Homo_633,AD.vs.MCI; bulk RNA-seq,Homo_714,AD.vs.control; bulk RNA-seq,Homo_714,AD.vs.MCI; bulk RNA-seq,Homo_714,MCI.vs.control; scRNA-seq,SRP215507,CD8+ T cell_1-AD.vs.MCI; scRNA-seq,SRP215507,CD8+ T cell_5-AD.vs.MCI; scRNA-seq,SRP215507,CD8+ T cell_5-MCI.vs.control; scRNA-seq,SRP215507,CD8+ T cell_2-MCI.vs.control; scRNA-seq,SRP330776,Naive CD8+ T cell_1-AD.vs.control; scRNA-seq,SRP330776,Naive CD8+ T cell_3-AD.vs.control; scRNA-seq,SRP330776,B cell_2-AD.vs.control; scRNA-seq,SRP330776,CD8+ T cell_3-	16
Abhd5	bulk RNA-seq,SRP325058,AD.vs.control	1
ABHD6	bulk RNA-seq,Homo_633,AD.vs.control; bulk RNA-seq,Homo_633,AD.vs.MCI; scRNA-seq,SRP215507,CD8+ T cell_2-AD.vs.control; scRNA-seq,SRP215507,CD8+ T cell_2-AD.vs.MCI; scRNA-seq,SRP215507,CD8+ T cell_3-AD.vs.MCI; scRNA-seq,SRP215507,CD8+ T cell_5-AD.vs.MCI; scRNA-seq,SRP215507,CD8+ T cell_2-MCI.vs.control; scRNA-seq,SRP215507,CD8+ T cell_3-MCI.vs.control; scRNA-seq,SRP330776,CD8+ T cell_1-AD.vs.control; scRNA-seq,SRP330776,γδ T cell-AD.vs.control; scRNA-seq,SRP330776,Memory T cell_2-AD.vs.control; scRNA-seq,SRP330776,CD8+ T cell_2-AD.vs.control; scRNA-seq,SRP330776,B cell_1-AD.vs.control; scRNA-seq,SRP330776,Naive CD8+ T cell_1-AD.vs.control; scRNA-seq,SRP330776,Natural killer cell_1-AD.vs.control; scRNA-seq,SRP330776,Memory T cell_1-AD.vs.control; scRNA-seq,SRP330776,Natural killer T cell-AD.vs.control; scRNA-seq,SRP330776,Naive CD8+ T cell_3-AD.vs.control; scRNA-seq,SRP330776,B cell_2-AD.vs.control; scRNA-seq,SRP330776,T cell-AD.vs.control; scRNA-seq,SRP330776,CD8+ T cell_3-AD.vs.control; scRNA-seq,SRP330776,Monocyte_1-AD.vs.control	22
ABHD8	bulk RNA-seq,Homo_723,MCI.vs.control; bulk RNA-seq,Homo_633,AD.vs.MCI; bulk RNA-seq,Homo_633,MCI.vs.control; bulk RNA-seq,Homo_714,AD.vs.control; bulk RNA-seq,Homo_714,AD.vs.MCI; bulk RNA-seq,Homo_714,MCI.vs.control; scRNA-seq,SRP215507,CD8+ T cell_1-AD.vs.control; scRNA-seq,SRP215507,CD8+ T cell_3-AD.vs.MCI; scRNA-seq,SRP215507,CD8+ T cell_1-AD.vs.MCI; scRNA-seq,SRP215507,CD8+ T cell_3-MCI.vs.control; scRNA-seq,SRP330776,CD8+ T cell_1-AD.vs.control; scRNA-seq,SRP330776,γδ T cell-AD.vs.control; scRNA-seq,SRP330776,Memory T cell_2-AD.vs.control; scRNA-seq,SRP330776,B cell_1-AD.vs.control; scRNA-seq,SRP330776,Naive CD8+ T cell_1-AD.vs.control; scRNA-seq,SRP330776,Memory T cell_1-AD.vs.control; scRNA-seq,SRP330776,Natural killer T cell-AD.vs.control; scRNA-seq,SRP330776,Naive CD8+ T cell_3-AD.vs.control; scRNA-seq,SRP330776,B cell_2-AD.vs.control; scRNA-seq,SRP330776,T cell-AD.vs.control; [illegible]	22
ABI1	scRNA-seq,SRP215507,CD8+ T cell_5-AD.vs.control; scRNA-seq,SRP215507,CD8+ T cell_9-AD.vs.control; scRNA-seq,SRP215507,CD8+ T cell_5-MCI.vs.control; scRNA-seq,SRP309935,Megakaryocyte_2-AD.vs.control	4
ABI2	bulk RNA-seq,Homo_723,AD.vs.control; bulk RNA-seq,Homo_723,AD.vs.MCI; bulk RNA-seq,Homo_633,AD.vs.control; bulk RNA-seq,Homo_633,AD.vs.MCI; scRNA-seq,SRP215507,CD8+ T cell_2-AD.vs.MCI; scRNA-seq,SRP215507,CD8+ T cell_4-MCI.vs.control; scRNA-seq,SRP309935,Monocyte_2-AD.vs.control; scRNA-seq,SRP330776,γδ T cell-AD.vs.control; scRNA-seq,SRP330776,Naive CD8+ T cell_3-AD.vs.control; scRNA-seq,SRP330776,T cell-AD.vs.control; scRNA-seq,SRP330776,CD8+ T cell_3-AD.vs.control; scRNA-seq,SRP330776,Monocyte_2-AD.vs.control	12

ABI3	bulk RNA-seq,Homo_723,AD.vs.control; bulk RNA-seq,Homo_633,AD.vs.control; bulk RNA-seq,Homo_633,MCI.vs.control; scRNA-seq,SRP215507,CD8+ T cell_3-AD.vs.control; scRNA-seq,SRP215507,CD8+ T cell_6-AD.vs.control; scRNA-seq,SRP215507,CD8+ T cell_4-AD.vs.MCI; scRNA-seq,SRP215507,CD8+ T cell_3-MCI.vs.control; scRNA-seq,SRP215507,CD8+ T cell_6-MCI.vs.control; scRNA-seq,SRP309935,Erythroid cell-AD.vs.control; scRNA-seq,SRP330776,CD8+ T cell_1-AD.vs.control; scRNA-seq,SRP330776,γδ T cell-AD.vs.control; scRNA-seq,SRP330776,CD8+ T cell_2-AD.vs.control; scRNA-seq,SRP330776,B cell_1-AD.vs.control; scRNA-seq,SRP330776,Natural killer cell_1-AD.vs.control; scRNA-seq,SRP330776,Memory T cell_1-AD.vs.control; scRNA-seq,SRP330776,Naive CD8+ T cell_3-AD.vs.control; scRNA-seq,SRP330776,Naive CD8+ T [illegible]	17
Abi3	bulk RNA-seq,SRP325058,AD.vs.control	1
ABI3BP	bulk RNA-seq,Homo_723,AD.vs.control; bulk RNA-seq,Homo_723,AD.vs.MCI; bulk RNA-seq,Homo_723,MCI.vs.control; bulk RNA-seq,Homo_633,AD.vs.control; bulk RNA-seq,Homo_633,AD.vs.MCI; bulk RNA-seq,Homo_633,MCI.vs.control; bulk RNA-seq,ROSMAP,AD.vs.control;	8
ABITRAM	bulk RNA-seq,Homo_714,AD.vs.control; bulk RNA-seq,Homo_714,AD.vs.MCI; scRNA-seq,SRP215507,CD8+ T cell_3-AD.vs.MCI; scRNA-seq,SRP215507,CD8+ T cell_3-MCI.vs.control; scRNA-seq,SRP330776,Natural killer T cell-AD.vs.control; scRNA-seq,SRP330776,Regulatory T cell-AD.vs.control; scRNA-seq,SRP330776,Natural killer cell_2-AD.vs.control	7
ABL1	bulk RNA-seq,Homo_723,AD.vs.control; bulk RNA-seq,Homo_723,AD.vs.MCI; bulk RNA-seq,Homo_633,AD.vs.control; bulk RNA-seq,Homo_633,AD.vs.MCI; bulk RNA-seq,Homo_714,AD.vs.control; bulk RNA-seq,Homo_714,AD.vs.MCI; bulk RNA-seq,Homo_714,MCI.vs.control; scRNA-seq,SRP215507,CD8+ T cell_2-AD.vs.control; scRNA-seq,SRP215507,CD8+ T cell_8-AD.vs.control; scRNA-seq,SRP215507,CD8+ T cell_3-AD.vs.MCI; scRNA-seq,SRP215507,CD8+ T cell_5-AD.vs.MCI; scRNA-seq,SRP215507,CD8+ T cell_5-MCI.vs.control; scRNA-seq,SRP215507,CD8+ T cell_3-MCI.vs.control; scRNA-seq,SRP330776,Natural killer cell_1-AD.vs.control; scRNA-seq,SRP330776,Memory T cell_1-AD.vs.control; scRNA-seq,SRP330776,Naive CD8+ T cell_3-AD.vs.control; scRNA-seq,SRP330776,Monocyte_2-AD.vs.control; scRNA-seq,SRP330776,Natural killer cell_2-[illegible]	19
ABL2	bulk RNA-seq,Homo_723,AD.vs.control; bulk RNA-seq,Homo_723,AD.vs.MCI; bulk RNA-seq,Homo_723,MCI.vs.control; bulk RNA-seq,Homo_633,AD.vs.control; bulk RNA-seq,Homo_633,AD.vs.MCI; bulk RNA-seq,Homo_714,AD.vs.control; scRNA-seq,SRP215507,CD8+ T cell_8-AD.vs.control; scRNA-seq,SRP215507,CD8+ T cell_4-AD.vs.MCI; scRNA-seq,SRP215507,CD8+ T cell_5-MCI.vs.control; scRNA-seq,SRP215507,CD8+ T cell_2-MCI.vs.control; scRNA-seq,SRP330776,CD8+ T cell_1-AD.vs.control; scRNA-seq,SRP330776,γδ T cell-AD.vs.control; scRNA-seq,SRP330776,Memory T cell_2-AD.vs.control; scRNA-seq,SRP330776,CD8+ T cell_2-AD.vs.control; scRNA-seq,SRP330776,B cell_1-AD.vs.control; scRNA-seq,SRP330776,Naive CD8+ T cell_1-AD.vs.control; scRNA-seq,SRP330776,Natural killer cell_1-AD.vs.control; scRNA-seq,SRP330776,Memory T cell_1-AD.vs.control; scRNA-seq,SRP330776,Naive CD8+ T cell_3-AD.vs.control; scRNA-seq,SRP330776,B cell_2-AD.vs.control; scRNA-[illegible]	21
ABLIM1	bulk RNA-seq,Homo_723,AD.vs.control; bulk RNA-seq,Homo_633,AD.vs.control; bulk RNA-seq,Homo_633,MCI.vs.control; scRNA-seq,SRP215507,CD8+ T cell_4-AD.vs.MCI; scRNA-seq,SRP309935,Natural killer cell_1-AD.vs.control; scRNA-seq,SRP330776,γδ T cell-AD.vs.control; scRNA-seq,SRP330776,Natural killer cell_1-AD.vs.control	7
ABLIM2	bulk RNA-seq,Homo_723,AD.vs.control; bulk RNA-seq,Homo_723,AD.vs.MCI; bulk RNA-seq,Homo_633,AD.vs.control; bulk RNA-seq,Homo_633,AD.vs.MCI; bulk RNA-seq,Homo_714,AD.vs.control; bulk RNA-seq,Homo_714,AD.vs.MCI; bulk RNA-seq,ROSMAP,AD.vs.MCI; bulk RNA-seq,ROSMAP,MCI.vs.control; scRNA-seq,SRP215507,CD8+ T cell_1-AD.vs.control; scRNA-seq,SRP215507,CD8+ T cell_6-AD.vs.control; scRNA-seq,SRP215507,CD8+ T cell_5-AD.vs.MCI; scRNA-seq,SRP330776,CD8+ T cell_1-AD.vs.control; scRNA-seq,SRP330776,γδ T cell-AD.vs.control; scRNA-seq,SRP330776,Memory T cell_2-AD.vs.control; scRNA-seq,SRP330776,CD8+ T cell_2-AD.vs.control; scRNA-seq,SRP330776,B cell_1-AD.vs.control; scRNA-seq,SRP330776,Naive CD8+ T cell_1-AD.vs.control; scRNA-seq,SRP330776,Natural killer cell_1-AD.vs.control; scRNA-[illegible]	20
ABLIM3	bulk RNA-seq,Homo_723,AD.vs.control; bulk RNA-seq,Homo_723,AD.vs.MCI; bulk RNA-seq,Homo_723,MCI.vs.control; bulk RNA-seq,Homo_633,AD.vs.MCI; bulk RNA-seq,Homo_633,MCI.vs.control; scRNA-seq,SRP309935,Monocyte_1-AD.vs.control	6
ABO	bulk RNA-seq,Homo_723,AD.vs.control; bulk RNA-seq,Homo_723,MCI.vs.control; bulk RNA-seq,Homo_633,AD.vs.control; bulk RNA-seq,Homo_633,MCI.vs.control; bulk RNA-seq,Homo_714,AD.vs.control; bulk RNA-seq,Homo_714,AD.vs.MCI; bulk RNA-seq,Homo_714,MCI.vs.control; scRNA-seq,SRP330776,CD8+ T cell_1-AD.vs.control; scRNA-seq,SRP330776,Memory T cell_2-AD.vs.control; scRNA-seq,SRP330776,B cell_1-AD.vs.control; scRNA-seq,SRP330776,Naive CD8+ T cell_1-AD.vs.control; scRNA-seq,SRP330776,T cell-AD.vs.control	12
ABR	scRNA-seq,SRP215507,CD8+ T cell_4-AD.vs.MCI; scRNA-seq,SRP215507,CD8+ T cell_3-MCI.vs.control; scRNA-seq,SRP309935,Natural killer cell_2-AD.vs.control; scRNA-seq,SRP309935,Monocyte_2-AD.vs.control; scRNA-seq,SRP309935,CD8+ T cell_2-AD.vs.control; scRNA-seq,SRP330776,CD8+ T cell_1-AD.vs.control; scRNA-seq,SRP330776,γδ T cell-AD.vs.control; scRNA-seq,SRP330776,Naive CD8+ T cell_3-AD.vs.control; scRNA-seq,SRP330776,Monocyte_1-AD.vs.control; scRNA-seq,SRP330776,Monocyte_2-AD.vs.control	10

ABRA	bulk RNA-seq,Homo_723,MCI.vs.control; bulk RNA-seq,Homo_714,MCI.vs.control	2
ABRACL	bulk RNA-seq,Homo_633,AD.vs.MCI; bulk RNA-seq,Homo_714,AD.vs.control; bulk RNA-seq,Homo_714,AD.vs.MCI; scRNA-seq,SRP215507,CD8+ T cell_4-AD.vs.control; scRNA-seq,SRP215507,CD8+ T cell_7-AD.vs.control; scRNA-seq,SRP215507,CD8+ T cell_4-MCI.vs.control; scRNA-seq,SRP309935,Erythroid cell-AD.vs.control; scRNA-seq,SRP330776,CD8+ T cell_1-AD.vs.control; scRNA-seq,SRP330776,γδ T cell-AD.vs.control; scRNA-seq,SRP330776,Memory T cell_2-AD.vs.control; scRNA-seq,SRP330776,CD8+ T cell_2-AD.vs.control; scRNA-seq,SRP330776,Naive CD8+ T cell_1-AD.vs.control; scRNA-seq,SRP330776,Natural killer cell_1-AD.vs.control; scRNA-seq,SRP330776,Memory T cell_1-AD.vs.control; scRNA-seq,SRP330776,Natural killer T cell-AD.vs.control; scRNA-seq,SRP330776,Naive CD8+ T cell_3-AD.vs.control; scRNA-seq,SRP330776,B cell_2-AD.vs.control; scRNA-seq,SRP330776,T cell-AD.vs.control; scRNA-seq,SRP330776,CD8+ T cell_3-AD.vs.control; scRNA-seq,SRP330776,B cell_3- [illegible]	21
ABRAXAS1	bulk RNA-seq,Homo_723,AD.vs.MCI; bulk RNA-seq,Homo_723,MCI.vs.control; bulk RNA-seq,Homo_633,AD.vs.MCI; bulk RNA-seq,Homo_633,MCI.vs.control; bulk RNA-seq,Homo_714,AD.vs.control; bulk RNA-seq,Homo_714,AD.vs.MCI; bulk RNA-seq,Homo_714,MCI.vs.control; scRNA-seq,SRP215507,CD8+ T cell_7-MCI.vs.control; scRNA-seq,SRP309935,Natural killer cell_2-AD.vs.control; scRNA-seq,SRP309935,Monocyte_1-AD.vs.control; scRNA-seq,SRP330776,γδ T cell-AD.vs.control; scRNA-seq,SRP330776,B cell_1-AD.vs.control; scRNA-seq,SRP330776,Naive CD8+ T cell_3-AD.vs.control; scRNA-seq,SRP330776,Monocyte_1-AD.vs.control; scRNA-seq,SRP330776,B cell_3-	16
ABRAXAS2	bulk RNA-seq,Homo_723,AD.vs.control; bulk RNA-seq,Homo_723,MCI.vs.control; bulk RNA-seq,Homo_633,AD.vs.control; bulk RNA-seq,Homo_633,MCI.vs.control; bulk RNA-seq,Homo_714,AD.vs.control; bulk RNA-seq,Homo_714,AD.vs.MCI; bulk RNA-seq,Homo_714,MCI.vs.control; scRNA-seq,SRP330776,Memory T cell_2-AD.vs.control; scRNA-seq,SRP330776,Naive CD8+ T cell_1-AD.vs.control; scRNA-seq,SRP330776,Naive CD8+ T cell_3-AD.vs.control; scRNA-seq,SRP330776,B cell_3-AD.vs.control	11
ABT1	bulk RNA-seq,Homo_723,AD.vs.control; bulk RNA-seq,Homo_723,AD.vs.MCI; bulk RNA-seq,Homo_633,AD.vs.control; bulk RNA-seq,Homo_633,AD.vs.MCI; bulk RNA-seq,Homo_714,AD.vs.control; bulk RNA-seq,Homo_714,AD.vs.MCI; scRNA-seq,SRP330776,B cell_2-AD.vs.control; scRNA-seq,SRP330776,Monocyte_2-AD.vs.control; scRNA-seq,SRP330776,B cell_3-AD.vs.control	9
ABTB1	bulk RNA-seq,SRP223445,AD.vs.control; scRNA-seq,SRP215507,CD8+ T cell_7-AD.vs.control; scRNA-seq,SRP215507,CD8+ T cell_9-AD.vs.control; scRNA-seq,SRP330776,Natural killer T cell-AD.vs.control; scRNA-seq,SRP330776,B cell_3-AD.vs.control; scRNA-seq,SRP330776,Regulatory T cell-	6
Abtb1	bulk RNA-seq,SRP325058,AD.vs.control	1
ABTB2	scRNA-seq,SRP215507,CD8+ T cell_2-AD.vs.control; scRNA-seq,SRP215507,CD8+ T cell_3-AD.vs.control; scRNA-seq,SRP215507,CD8+ T cell_1-AD.vs.MCI; scRNA-seq,SRP215507,CD8+ T cell_5-AD.vs.MCI; scRNA-seq,SRP215507,CD8+ T cell_1-MCI.vs.control; scRNA-seq,SRP215507,CD8+ T cell_5-MCI.vs.control; scRNA-seq,SRP215507,CD8+ T cell_2-MCI.vs.control; scRNA-seq,SRP215507,CD8+ T cell_3-MCI.vs.control; scRNA-	9
AC000032.1	scRNA-seq,SRP330776,CD8+ T cell_1-AD.vs.control; scRNA-seq,SRP330776,Memory T cell_2-AD.vs.control; scRNA-seq,SRP330776,Naive CD8+ T cell_1-AD.vs.control; scRNA-seq,SRP330776,Natural killer T cell-AD.vs.control; scRNA-seq,SRP330776,Naive CD8+ T cell_3-AD.vs.control	5
AC000065.2	scRNA-seq,SRP215507,CD8+ T cell_1-MCI.vs.control	1
AC000068.1	scRNA-seq,SRP215507,CD8+ T cell_3-AD.vs.MCI; scRNA-seq,SRP330776,CD8+ T cell_1-AD.vs.control; scRNA-seq,SRP330776,B cell_1-AD.vs.control; scRNA-seq,SRP330776,Naive CD8+ T cell_1-AD.vs.control; scRNA-seq,SRP330776,CD8+ T cell_3-AD.vs.control	5
AC000085.1	scRNA-seq,SRP330776,Naive CD8+ T cell_1-AD.vs.control	1
AC002091.2	scRNA-seq,SRP215507,CD8+ T cell_1-AD.vs.control; scRNA-seq,SRP215507,CD8+ T cell_1-MCI.vs.control; scRNA-seq,SRP330776,Memory T cell_2-AD.vs.control; scRNA-seq,SRP330776,B cell_2-AD.vs.control	4
AC002094.2	scRNA-seq,SRP330776,Natural killer cell_1-AD.vs.control; scRNA-seq,SRP330776,T cell-AD.vs.control	2
AC002094.4	scRNA-seq,SRP215507,CD8+ T cell_2-MCI.vs.control; scRNA-seq,SRP215507,CD8+ T cell_3-MCI.vs.control; scRNA-seq,SRP330776,CD8+ T cell_1-AD.vs.control; scRNA-seq,SRP330776,Memory T cell_1-AD.vs.control	4
AC002116.2	scRNA-seq,SRP215507,CD8+ T cell_1-AD.vs.MCI; scRNA-seq,SRP330776,Memory T cell_2-AD.vs.control; scRNA-seq,SRP330776,CD8+ T cell_2-AD.vs.control; scRNA-seq,SRP330776,B cell_1-AD.vs.control; scRNA-seq,SRP330776,Naive CD8+ T cell_1-AD.vs.control; scRNA-seq,SRP330776,B	6
AC002310.1	scRNA-seq,SRP215507,CD8+ T cell_2-AD.vs.control; scRNA-seq,SRP215507,CD8+ T cell_3-AD.vs.MCI; scRNA-seq,SRP309935,Naive CD8+ T cell_2-AD.vs.control; scRNA-seq,SRP330776,Memory T cell_2-AD.vs.control; scRNA-seq,SRP330776,CD8+ T cell_2-AD.vs.control; scRNA-seq,SRP330776,B cell_1-AD.vs.control; scRNA-seq,SRP330776,Memory T cell_1-AD.vs.control; scRNA-seq,SRP330776,Natural killer cell_2-AD.vs.control	8
AC002310.2	scRNA-seq,SRP330776,Naive CD8+ T cell_1-AD.vs.control	1

AC002463.1	scRNA-seq,SRP330776,Naive CD8+ T cell_2-AD.vs.control	1
AC002467.1	scRNA-seq,SRP330776,CD8+ T cell_1-AD.vs.control; scRNA-seq,SRP330776,Memory T cell_2-AD.vs.control; scRNA-seq,SRP330776,B cell_1-AD.vs.control; scRNA-seq,SRP330776,Naive CD8+ T cell_1-AD.vs.control; scRNA-seq,SRP330776,Natural killer cell_1-AD.vs.control; scRNA-	6
AC002553.1	scRNA-seq,SRP330776,Naive CD8+ T cell_1-AD.vs.control	1
AC002553.2	scRNA-seq,SRP215507,CD8+ T cell_1-MCI.vs.control	1
AC002558.3	scRNA-seq,SRP215507,CD8+ T cell_3-AD.vs.MCI; scRNA-seq,SRP215507,CD8+ T cell_5-AD.vs.MCI; scRNA-seq,SRP215507,CD8+ T cell_1-MCI.vs.control; scRNA-seq,SRP215507,CD8+ T cell_5-MCI.vs.control; scRNA-seq,SRP309935,Natural killer cell_2-AD.vs.control; scRNA-seq,SRP309935,Naive CD8+ T cell_1-AD.vs.control; scRNA-seq,SRP330776,CD8+ T cell_1-AD.vs.control; scRNA-seq,SRP330776,γδ T cell-AD.vs.control; scRNA-seq,SRP330776,Memory T cell_2-AD.vs.control; scRNA-seq,SRP330776,Naive CD8+ T cell_1-AD.vs.control; scRNA-seq,SRP330776,Natural killer cell_1-AD.vs.control; scRNA-seq,SRP330776,Memory T cell_1-AD.vs.control; scRNA-seq,SRP330776,Naive CD8+ T	15
AC003086.1	scRNA-seq,SRP215507,CD8+ T cell_1-AD.vs.control; scRNA-seq,SRP215507,CD8+ T cell_3-AD.vs.MCI; scRNA-seq,SRP215507,CD8+ T cell_3-	3
AC003093.1	scRNA-seq,SRP215507,CD8+ T cell_4-AD.vs.control	1
AC003101.2	scRNA-seq,SRP309935,Monocyte_1-AD.vs.control	1
AC003102.1	scRNA-seq,SRP215507,CD8+ T cell_1-AD.vs.MCI; scRNA-seq,SRP215507,CD8+ T cell_1-MCI.vs.control; scRNA-seq,SRP215507,CD8+ T cell_3-MCI.vs.control; scRNA-seq,SRP330776,CD8+ T cell_1-AD.vs.control; scRNA-seq,SRP330776,γδ T cell-AD.vs.control; scRNA-seq,SRP330776,Memory T cell_2-AD.vs.control; scRNA-seq,SRP330776,Naive CD8+ T cell_1-AD.vs.control; scRNA-seq,SRP330776,Memory T cell_1-AD.vs.control; scRNA-seq,SRP330776,T cell-AD.vs.control; scRNA-seq,SRP330776,CD8+ T cell_3-AD.vs.control; scRNA-seq,SRP330776,Natural killer cell_2-AD.vs.control	11
AC003681.1	scRNA-seq,SRP215507,CD8+ T cell_1-AD.vs.control; scRNA-seq,SRP215507,CD8+ T cell_1-AD.vs.MCI; scRNA-seq,SRP309935,Natural killer cell_1-AD.vs.control; scRNA-seq,SRP330776,Naive CD8+ T cell_2-AD.vs.control	4
AC004000.1	scRNA-seq,SRP215507,CD8+ T cell_6-MCI.vs.control	1
AC004039.1	scRNA-seq,SRP215507,CD8+ T cell_1-MCI.vs.control	1
AC004067.1	scRNA-seq,SRP215507,CD8+ T cell_2-AD.vs.MCI; scRNA-seq,SRP215507,CD8+ T cell_2-MCI.vs.control	2
AC004130.2	scRNA-seq,SRP330776,B cell_4-AD.vs.control	1
AC004223.4	scRNA-seq,SRP215507,CD8+ T cell_4-MCI.vs.control	1
AC004241.1	scRNA-seq,SRP215507,CD8+ T cell_3-AD.vs.control; scRNA-seq,SRP215507,CD8+ T cell_1-AD.vs.control; scRNA-seq,SRP215507,CD8+ T cell_1-MCI.vs.control; scRNA-seq,SRP330776,Natural killer T cell-AD.vs.control; scRNA-seq,SRP330776,T cell-AD.vs.control	5
AC004253.1	scRNA-seq,SRP215507,CD8+ T cell_1-MCI.vs.control; scRNA-seq,SRP330776,CD8+ T cell_1-AD.vs.control; scRNA-seq,SRP330776,B cell_1-AD.vs.control; scRNA-seq,SRP330776,Memory T cell_1-AD.vs.control	4
AC004263.2	scRNA-seq,SRP330776,γδ T cell-AD.vs.control	1
AC004381.1	scRNA-seq,SRP215507,CD8+ T cell_2-AD.vs.MCI; scRNA-seq,SRP215507,CD8+ T cell_1-MCI.vs.control; scRNA-seq,SRP215507,CD8+ T cell_2-	3
AC004466.1	scRNA-seq,SRP330776,CD8+ T cell_1-AD.vs.control; scRNA-seq,SRP330776,Memory T cell_2-AD.vs.control; scRNA-seq,SRP330776,Naive CD8+ T	3
AC004477.1	scRNA-seq,SRP215507,CD8+ T cell_3-AD.vs.control; scRNA-seq,SRP215507,CD8+ T cell_2-AD.vs.MCI; scRNA-seq,SRP215507,CD8+ T cell_1-MCI.vs.control; scRNA-seq,SRP215507,CD8+ T cell_3-MCI.vs.control; scRNA-seq,SRP330776,CD8+ T cell_1-AD.vs.control; scRNA-	6
AC004492.1	scRNA-seq,SRP330776,Memory T cell_2-AD.vs.control; scRNA-seq,SRP330776,B cell_1-AD.vs.control; scRNA-seq,SRP330776,Naive CD8+ T cell_1-	3
AC004494.1	scRNA-seq,SRP330776,Naive CD8+ T cell_1-AD.vs.control	1
AC004540.2	scRNA-seq,SRP330776,CD8+ T cell_1-AD.vs.control; scRNA-seq,SRP330776,CD8+ T cell_2-AD.vs.control	2

AC004556.3	scRNA-seq,SRP215507,CD8+ T cell_3-AD.vs.control; scRNA-seq,SRP215507,CD8+ T cell_1-AD.vs.control; scRNA-seq,SRP215507,CD8+ T cell_5-AD.vs.control; scRNA-seq,SRP215507,CD8+ T cell_2-AD.vs.MCI; scRNA-seq,SRP215507,CD8+ T cell_3-AD.vs.MCI; scRNA-seq,SRP215507,CD8+ T cell_1-AD.vs.MCI; scRNA-seq,SRP215507,CD8+ T cell_2-MCI.vs.control; scRNA-seq,SRP309935,Natural killer cell_2-AD.vs.control; scRNA-seq,SRP309935,Monocyte_1-AD.vs.control; scRNA-seq,SRP309935,Megakaryocyte_1-AD.vs.control; scRNA-seq,SRP309935,Naive CD8+ T cell_1-AD.vs.control; scRNA-seq,SRP309935,Natural killer cell_1-AD.vs.control; scRNA-seq,SRP309935,Monocyte_2-AD.vs.control; scRNA-seq,SRP309935,Naive CD8+ T cell_2-AD.vs.control; scRNA-seq,SRP309935,CD8+ T cell_1-AD.vs.control; scRNA-seq,SRP309935,B cell_1-AD.vs.control; scRNA-seq,SRP309935,CD8+ T cell_2-AD.vs.control; scRNA-seq,SRP309935,Plasmacytoid dendritic cell_1-AD.vs.control; scRNA-[illegible]	20
AC004585.1	scRNA-seq,SRP215507,CD8+ T cell_1-AD.vs.MCI; scRNA-seq,SRP215507,CD8+ T cell_6-AD.vs.MCI; scRNA-seq,SRP215507,CD8+ T cell_1-MCI.vs.control; scRNA-seq,SRP215507,CD8+ T cell_2-MCI.vs.control; scRNA-seq,SRP330776,CD8+ T cell_1-AD.vs.control; scRNA-seq,SRP330776,Memory T cell_2-AD.vs.control; scRNA-seq,SRP330776,CD8+ T cell_2-AD.vs.control; scRNA-seq,SRP330776,B cell_1-AD.vs.control; scRNA-seq,SRP330776,Natural killer cell_1-AD.vs.control; scRNA-seq,SRP330776,Natural killer T cell-AD.vs.control; scRNA-seq,SRP330776,CD8+ T	11
AC004687.1	scRNA-seq,SRP215507,CD8+ T cell_2-AD.vs.MCI; scRNA-seq,SRP215507,CD8+ T cell_3-AD.vs.MCI; scRNA-seq,SRP215507,CD8+ T cell_4-AD.vs.MCI; scRNA-seq,SRP215507,CD8+ T cell_1-MCI.vs.control; scRNA-seq,SRP215507,CD8+ T cell_5-MCI.vs.control; scRNA-seq,SRP215507,CD8+ T cell_4-MCI.vs.control; scRNA-seq,SRP215507,CD8+ T cell_2-MCI.vs.control; scRNA-seq,SRP215507,CD8+ T cell_3-MCI.vs.control; scRNA-seq,SRP215507,CD8+ T cell_6-MCI.vs.control; scRNA-seq,SRP215507,CD8+ T cell_8-MCI.vs.control; scRNA-seq,SRP330776,Naive CD8+ T cell_1-AD.vs.control; scRNA-seq,SRP330776,Naive CD8+ T cell_3-AD.vs.control; scRNA-seq,SRP330776,Natural killer	13
AC004771.3	scRNA-seq,SRP215507,CD8+ T cell_3-AD.vs.MCI; scRNA-seq,SRP215507,CD8+ T cell_3-MCI.vs.control; scRNA-seq,SRP330776,B cell_1-AD.vs.control; scRNA-seq,SRP330776,T cell-AD.vs.control	4
AC004771.4	scRNA-seq,SRP330776,CD8+ T cell_1-AD.vs.control	1
AC004771.5	scRNA-seq,SRP215507,CD8+ T cell_4-MCI.vs.control	1
AC004797.1	scRNA-seq,SRP330776,CD8+ T cell_1-AD.vs.control; scRNA-seq,SRP330776,γδ T cell-AD.vs.control; scRNA-seq,SRP330776,Memory T cell_2-AD.vs.control; scRNA-seq,SRP330776,CD8+ T cell_2-AD.vs.control; scRNA-seq,SRP330776,B cell_1-AD.vs.control; scRNA-seq,SRP330776,Natural killer cell_1-AD.vs.control; scRNA-seq,SRP330776,Memory T cell_1-AD.vs.control; scRNA-seq,SRP330776,Natural killer T cell-AD.vs.control; scRNA-seq,SRP330776,Naive CD8+ T cell_3-AD.vs.control; scRNA-seq,SRP330776,B cell_2-AD.vs.control; scRNA-seq,SRP330776,T cell-AD.vs.control; scRNA-seq,SRP330776,CD8+ T cell_3-AD.vs.control; scRNA-seq,SRP330776,B cell_3-AD.vs.control	13
AC004801.6	scRNA-seq,SRP215507,CD8+ T cell_1-MCI.vs.control	1
AC004803.1	scRNA-seq,SRP215507,CD8+ T cell_1-MCI.vs.control; scRNA-seq,SRP330776,Memory T cell_1-AD.vs.control	2
AC004812.2	scRNA-seq,SRP215507,CD8+ T cell_2-AD.vs.MCI; scRNA-seq,SRP215507,CD8+ T cell_2-MCI.vs.control; scRNA-seq,SRP330776,CD8+ T cell_1-AD.vs.control; scRNA-seq,SRP330776,γδ T cell-AD.vs.control; scRNA-seq,SRP330776,Memory T cell_2-AD.vs.control; scRNA-seq,SRP330776,CD8+ T cell_2-AD.vs.control; scRNA-seq,SRP330776,B cell_1-AD.vs.control; scRNA-seq,SRP330776,Naive CD8+ T cell_1-AD.vs.control; scRNA-seq,SRP330776,Natural killer cell_1-AD.vs.control; scRNA-seq,SRP330776,Memory T cell_1-AD.vs.control; scRNA-seq,SRP330776,Natural killer T cell-AD.vs.control; scRNA-seq,SRP330776,B cell_2-AD.vs.control; scRNA-seq,SRP330776,T cell-AD.vs.control; scRNA-seq,SRP330776,CD8+ T cell_3-AD.vs.control; scRNA-seq,SRP330776,Natural killer cell_2-AD.vs.control; scRNA-seq,SRP330776,B cell_4-AD.vs.control	16
AC004816.1	scRNA-seq,SRP215507,CD8+ T cell_2-AD.vs.MCI; scRNA-seq,SRP215507,CD8+ T cell_2-MCI.vs.control; scRNA-seq,SRP330776,B cell_1-	3
AC004817.3	scRNA-seq,SRP215507,CD8+ T cell_2-AD.vs.control; scRNA-seq,SRP215507,CD8+ T cell_3-AD.vs.control; scRNA-seq,SRP215507,CD8+ T cell_4-AD.vs.control; scRNA-seq,SRP215507,CD8+ T cell_2-AD.vs.MCI; scRNA-seq,SRP215507,CD8+ T cell_4-AD.vs.MCI; scRNA-seq,SRP330776,T cell-	6
AC004817.5	scRNA-seq,SRP215507,CD8+ T cell_2-AD.vs.control; scRNA-seq,SRP215507,CD8+ T cell_3-AD.vs.control; scRNA-seq,SRP215507,CD8+ T cell_1-MCI.vs.control; scRNA-seq,SRP309935,Natural killer cell_2-AD.vs.control	4
AC004825.2	scRNA-seq,SRP215507,CD8+ T cell_5-MCI.vs.control; scRNA-seq,SRP215507,CD8+ T cell_2-MCI.vs.control	2
AC004825.3	scRNA-seq,SRP215507,CD8+ T cell_2-MCI.vs.control	1
AC004832.4	scRNA-seq,SRP215507,CD8+ T cell_1-MCI.vs.control	1

AC004832.6	scRNA-seq,SRP215507,CD8+ T cell_1-AD.vs.control; scRNA-seq,SRP215507,CD8+ T cell_1-AD.vs.MCI; scRNA-seq,SRP215507,CD8+ T cell_2-MCI.vs.control; scRNA-seq,SRP215507,CD8+ T cell_3-MCI.vs.control; scRNA-seq,SRP309935,CD8+ T cell_2-AD.vs.control; scRNA-seq,SRP330776,CD8+ T cell_2-AD.vs.control; scRNA-seq,SRP330776,B cell_1-AD.vs.control; scRNA-seq,SRP330776,Naive CD8+ T cell_1-AD.vs.control; scRNA-seq,SRP330776,Naive CD8+ T cell_3-AD.vs.control; scRNA-seq,SRP330776,CD8+ T cell_3-AD.vs.control	10
AC004834.1	scRNA-seq,SRP215507,CD8+ T cell_3-AD.vs.control; scRNA-seq,SRP215507,CD8+ T cell_2-MCI.vs.control	2
AC004839.2	scRNA-seq,SRP215507,CD8+ T cell_1-MCI.vs.control; scRNA-seq,SRP215507,CD8+ T cell_5-MCI.vs.control; scRNA-seq,SRP330776,Memory T cell_1-	3
AC004846.2	scRNA-seq,SRP309935,B cell_1-AD.vs.control	1
AC004847.1	scRNA-seq,SRP215507,CD8+ T cell_4-AD.vs.control; scRNA-seq,SRP215507,CD8+ T cell_5-AD.vs.control; scRNA-seq,SRP215507,CD8+ T cell_3-AD.vs.MCI; scRNA-seq,SRP215507,CD8+ T cell_5-MCI.vs.control; scRNA-seq,SRP215507,CD8+ T cell_3-MCI.vs.control; scRNA-seq,SRP309935,CD8+ T cell_2-AD.vs.control; scRNA-seq,SRP330776,CD8+ T cell_1-AD.vs.control; scRNA-seq,SRP330776,Memory T cell_2-AD.vs.control; scRNA-seq,SRP330776,CD8+ T cell_2-AD.vs.control; scRNA-seq,SRP330776,B cell_1-AD.vs.control; scRNA-seq,SRP330776,Naive CD8+ T cell_1-AD.vs.control; scRNA-seq,SRP330776,Natural killer cell_1-AD.vs.control; scRNA-seq,SRP330776,Memory T cell_1-AD.vs.control;	14
AC004854.2	scRNA-seq,SRP215507,CD8+ T cell_3-AD.vs.control; scRNA-seq,SRP215507,CD8+ T cell_1-AD.vs.MCI; scRNA-seq,SRP215507,CD8+ T cell_1-MCI.vs.control; scRNA-seq,SRP330776,CD8+ T cell_2-AD.vs.control; scRNA-seq,SRP330776,B cell_1-AD.vs.control; scRNA-seq,SRP330776,Naive CD8+ T cell_1-AD.vs.control; scRNA-seq,SRP330776,Memory T cell_1-AD.vs.control; scRNA-seq,SRP330776,Naive CD8+ T cell_3-AD.vs.control;	9
AC004865.2	scRNA-seq,SRP330776,CD8+ T cell_2-AD.vs.control; scRNA-seq,SRP330776,Naive CD8+ T cell_1-AD.vs.control; scRNA-seq,SRP330776,Memory T cell_1-AD.vs.control; scRNA-seq,SRP330776,B cell_2-AD.vs.control	4
AC004877.1	scRNA-seq,SRP215507,CD8+ T cell_3-AD.vs.control	1
AC004889.1	scRNA-seq,SRP215507,CD8+ T cell_2-AD.vs.control; scRNA-seq,SRP215507,CD8+ T cell_1-AD.vs.control; scRNA-seq,SRP215507,CD8+ T cell_4-AD.vs.control; scRNA-seq,SRP215507,CD8+ T cell_2-AD.vs.MCI; scRNA-seq,SRP215507,CD8+ T cell_1-AD.vs.MCI; scRNA-seq,SRP215507,CD8+ T cell_4-AD.vs.MCI; scRNA-seq,SRP309935,Naive CD8+ T cell_2-AD.vs.control; scRNA-seq,SRP309935,CD8+ T cell_1-AD.vs.control; scRNA-seq,SRP309935,CD8+ T cell_2-AD.vs.control; scRNA-seq,SRP330776,CD8+ T cell_1-AD.vs.control; scRNA-seq,SRP330776,Memory T cell_2-AD.vs.control; scRNA-seq,SRP330776,CD8+ T cell_2-AD.vs.control; scRNA-seq,SRP330776,B cell_1-AD.vs.control; scRNA-seq,SRP330776,Naive CD8+ T cell_1-AD.vs.control; scRNA-seq,SRP330776,Natural killer cell_1-AD.vs.control; scRNA-seq,SRP330776,Memory T cell_1-AD.vs.control; scRNA-seq,SRP330776,Natural killer T cell-AD.vs.control; scRNA-seq,SRP330776,Naive CD8+ T cell_3-AD.vs.control; scRNA-seq,SRP330776,B cell_2- [illegible]	21
AC004893.3	scRNA-seq,SRP330776,Naive CD8+ T cell_1-AD.vs.control	1
AC004906.1	scRNA-seq,SRP330776,CD8+ T cell_1-AD.vs.control; scRNA-seq,SRP330776,Memory T cell_2-AD.vs.control; scRNA-seq,SRP330776,Naive CD8+ T	3
AC004908.2	scRNA-seq,SRP330776,Naive CD8+ T cell_1-AD.vs.control	1
AC004918.1	scRNA-seq,SRP215507,CD8+ T cell_3-AD.vs.MCI; scRNA-seq,SRP215507,CD8+ T cell_3-MCI.vs.control; scRNA-seq,SRP309935,Natural killer cell_1-AD.vs.control; scRNA-seq,SRP309935,CD8+ T cell_1-AD.vs.control; scRNA-seq,SRP330776,CD8+ T cell_1-AD.vs.control; scRNA-seq,SRP330776,γδ T cell-AD.vs.control; scRNA-seq,SRP330776,Naive CD8+ T cell_1-AD.vs.control; scRNA-seq,SRP330776,Memory T cell_1-AD.vs.control	8
AC004918.4	scRNA-seq,SRP215507,CD8+ T cell_1-AD.vs.MCI; scRNA-seq,SRP215507,CD8+ T cell_5-AD.vs.MCI; scRNA-seq,SRP215507,CD8+ T cell_5-MCI.vs.control; scRNA-seq,SRP330776,CD8+ T cell_1-AD.vs.control; scRNA-seq,SRP330776,CD8+ T cell_2-AD.vs.control	5
AC004938.2	scRNA-seq,SRP215507,CD8+ T cell_1-AD.vs.control; scRNA-seq,SRP215507,CD8+ T cell_1-MCI.vs.control; scRNA-seq,SRP330776,Natural killer	3
AC004943.2	scRNA-seq,SRP215507,CD8+ T cell_3-AD.vs.MCI; scRNA-seq,SRP215507,CD8+ T cell_3-MCI.vs.control; scRNA-seq,SRP330776,CD8+ T cell_1-AD.vs.control; scRNA-seq,SRP330776,Memory T cell_2-AD.vs.control; scRNA-seq,SRP330776,B cell_1-AD.vs.control; scRNA-seq,SRP330776,Naive CD8+ T cell_1-AD.vs.control; scRNA-seq,SRP330776,B cell_2-AD.vs.control; scRNA-seq,SRP330776,Monocyte_2-AD.vs.control	8
AC004951.1	scRNA-seq,SRP215507,CD8+ T cell_1-AD.vs.MCI; scRNA-seq,SRP215507,CD8+ T cell_1-MCI.vs.control; scRNA-seq,SRP215507,CD8+ T cell_2-MCI.vs.control; scRNA-seq,SRP215507,CD8+ T cell_3-MCI.vs.control; scRNA-seq,SRP330776,CD8+ T cell_1-AD.vs.control; scRNA-seq,SRP330776,γδ T cell-AD.vs.control; scRNA-seq,SRP330776,Naive CD8+ T cell_1-AD.vs.control; scRNA-seq,SRP330776,Natural killer T cell-AD.vs.control; scRNA-	9

AC004974.1	scRNA-seq,SRP215507,CD8+ T cell_3-AD.vs.MCI; scRNA-seq,SRP215507,CD8+ T cell_1-MCI.vs.control; scRNA-seq,SRP215507,CD8+ T cell_3-MCI.vs.control; scRNA-seq,SRP330776,γδ T cell-AD.vs.control; scRNA-seq,SRP330776,Naive CD8+ T cell_1-AD.vs.control; scRNA-seq,SRP330776,Naive CD8+ T cell_3-AD.vs.control; scRNA-seq,SRP330776,B cell_3-AD.vs.control; scRNA-seq,SRP330776,Natural killer cell_2-	8
AC004982.2	scRNA-seq,SRP215507,CD8+ T cell_2-AD.vs.control; scRNA-seq,SRP215507,CD8+ T cell_5-AD.vs.control; scRNA-seq,SRP215507,CD8+ T cell_1-AD.vs.MCI; scRNA-seq,SRP215507,CD8+ T cell_1-MCI.vs.control; scRNA-seq,SRP215507,CD8+ T cell_5-MCI.vs.control; scRNA-seq,SRP215507,CD8+ T cell_3-MCI.vs.control; scRNA-seq,SRP330776,CD8+ T cell_1-AD.vs.control; scRNA-seq,SRP330776,γδ T cell-AD.vs.control; scRNA-seq,SRP330776,CD8+ T cell_2-AD.vs.control; scRNA-seq,SRP330776,Naive CD8+ T cell_1-AD.vs.control; scRNA-seq,SRP330776,Memory T	12
AC005021.1	scRNA-seq,SRP330776,CD8+ T cell_1-AD.vs.control	1
AC005034.6	scRNA-seq,SRP215507,CD8+ T cell_3-AD.vs.MCI; scRNA-seq,SRP215507,CD8+ T cell_1-MCI.vs.control; scRNA-seq,SRP330776,Naive CD8+ T	3
AC005037.1	scRNA-seq,SRP215507,CD8+ T cell_5-MCI.vs.control; scRNA-seq,SRP215507,CD8+ T cell_4-MCI.vs.control; scRNA-seq,SRP215507,CD8+ T cell_3-	3
AC005041.5	scRNA-seq,SRP215507,CD8+ T cell_2-AD.vs.control; scRNA-seq,SRP215507,CD8+ T cell_3-AD.vs.control; scRNA-seq,SRP215507,CD8+ T cell_3-MCI.vs.control; scRNA-seq,SRP330776,Naive CD8+ T cell_1-AD.vs.control; scRNA-seq,SRP330776,Memory T cell_1-AD.vs.control	5
AC005052.2	scRNA-seq,SRP330776,Naive CD8+ T cell_1-AD.vs.control	1
AC005062.1	scRNA-seq,SRP215507,CD8+ T cell_1-AD.vs.control	1
AC005064.1	scRNA-seq,SRP215507,CD8+ T cell_2-AD.vs.control; scRNA-seq,SRP215507,CD8+ T cell_1-AD.vs.control; scRNA-seq,SRP215507,CD8+ T cell_5-MCI.vs.control; scRNA-seq,SRP215507,CD8+ T cell_2-MCI.vs.control; scRNA-seq,SRP215507,CD8+ T cell_3-MCI.vs.control; scRNA-	6
AC005070.3	scRNA-seq,SRP215507,CD8+ T cell_2-AD.vs.MCI; scRNA-seq,SRP215507,CD8+ T cell_4-MCI.vs.control; scRNA-seq,SRP215507,CD8+ T cell_2-MCI.vs.control; scRNA-seq,SRP330776,B cell_1-AD.vs.control; scRNA-seq,SRP330776,Naive CD8+ T cell_1-AD.vs.control	5
AC005072.1	scRNA-seq,SRP215507,CD8+ T cell_1-MCI.vs.control; scRNA-seq,SRP215507,CD8+ T cell_4-MCI.vs.control; scRNA-seq,SRP309935,Monocyte_1-AD.vs.control; scRNA-seq,SRP309935,Natural killer cell_1-AD.vs.control	4
AC005076.1	scRNA-seq,SRP215507,CD8+ T cell_5-AD.vs.MCI; scRNA-seq,SRP215507,CD8+ T cell_1-MCI.vs.control; scRNA-seq,SRP215507,CD8+ T cell_5-	3
AC005082.1	scRNA-seq,SRP215507,CD8+ T cell_3-AD.vs.MCI; scRNA-seq,SRP215507,CD8+ T cell_2-MCI.vs.control; scRNA-seq,SRP215507,CD8+ T cell_3-MCI.vs.control; scRNA-seq,SRP330776,Naive CD8+ T cell_1-AD.vs.control	4
AC005083.1	scRNA-seq,SRP215507,CD8+ T cell_3-AD.vs.control; scRNA-seq,SRP330776,Natural killer cell_2-AD.vs.control	2
AC005165.1	scRNA-seq,SRP215507,CD8+ T cell_3-MCI.vs.control	1
AC005224.2	scRNA-seq,SRP215507,CD8+ T cell_3-AD.vs.control; scRNA-seq,SRP215507,CD8+ T cell_5-AD.vs.MCI; scRNA-seq,SRP215507,CD8+ T cell_5-MCI.vs.control; scRNA-seq,SRP215507,CD8+ T cell_3-MCI.vs.control; scRNA-seq,SRP309935,Naive CD8+ T cell_1-AD.vs.control; scRNA-seq,SRP309935,Natural killer cell_1-AD.vs.control; scRNA-seq,SRP309935,CD8+ T cell_1-AD.vs.control; scRNA-seq,SRP309935,CD8+ T cell_2-AD.vs.control; scRNA-seq,SRP330776,Memory T cell_2-AD.vs.control; scRNA-seq,SRP330776,CD8+ T cell_2-AD.vs.control; scRNA-seq,SRP330776,Naive CD8+ T cell_1-AD.vs.control; scRNA-seq,SRP330776,Natural killer cell_1-AD.vs.control; scRNA-seq,SRP330776,Memory T	13
AC005224.3	scRNA-seq,SRP330776,CD8+ T cell_1-AD.vs.control	1
AC005229.4	scRNA-seq,SRP215507,CD8+ T cell_5-AD.vs.MCI; scRNA-seq,SRP215507,CD8+ T cell_5-MCI.vs.control; scRNA-seq,SRP309935,Monocyte_1-AD.vs.control; scRNA-seq,SRP330776,CD8+ T cell_1-AD.vs.control; scRNA-seq,SRP330776,γδ T cell-AD.vs.control; scRNA-seq,SRP330776,Memory T cell_2-AD.vs.control; scRNA-seq,SRP330776,Naive CD8+ T cell_1-AD.vs.control; scRNA-seq,SRP330776,Memory T cell_1-AD.vs.control; scRNA-	9
AC005253.1	scRNA-seq,SRP215507,CD8+ T cell_5-MCI.vs.control	1
AC005261.1	scRNA-seq,SRP215507,CD8+ T cell_3-AD.vs.control; scRNA-seq,SRP215507,CD8+ T cell_1-AD.vs.MCI; scRNA-seq,SRP215507,CD8+ T cell_5-AD.vs.MCI; scRNA-seq,SRP215507,CD8+ T cell_1-MCI.vs.control; scRNA-seq,SRP215507,CD8+ T cell_5-MCI.vs.control; scRNA-seq,SRP309935,Natural killer cell_2-AD.vs.control; scRNA-seq,SRP309935,Naive CD8+ T cell_3-AD.vs.control; scRNA-seq,SRP330776,γδ T cell-AD.vs.control; scRNA-seq,SRP330776,CD8+ T cell_2-AD.vs.control; scRNA-seq,SRP330776,B cell_1-AD.vs.control; scRNA-seq,SRP330776,Naive CD8+ T cell_1-AD.vs.control; scRNA-seq,SRP330776,Memory T cell_1-AD.vs.control; scRNA-seq,SRP330776,Natural killer T cell-AD.vs.control	13
AC005280.2	scRNA-seq,SRP330776,Monocyte_2-AD.vs.control	1

AC005288.1	scRNA-seq,SRP215507,CD8+ T cell_1-AD.vs.control; scRNA-seq,SRP215507,CD8+ T cell_1-MCI.vs.control; scRNA-seq,SRP330776,CD8+ T cell_1-AD.vs.control; scRNA-seq,SRP330776,Naive CD8+ T cell_1-AD.vs.control	4
AC005306.1	scRNA-seq,SRP215507,CD8+ T cell_2-AD.vs.MCI; scRNA-seq,SRP215507,CD8+ T cell_4-AD.vs.MCI; scRNA-seq,SRP215507,CD8+ T cell_4-MCI.vs.control; scRNA-seq,SRP215507,CD8+ T cell_2-MCI.vs.control; scRNA-seq,SRP330776,CD8+ T cell_1-AD.vs.control; scRNA-seq,SRP330776,Memory T cell_2-AD.vs.control; scRNA-seq,SRP330776,CD8+ T cell_2-AD.vs.control; scRNA-seq,SRP330776,B cell_1-AD.vs.control	8
AC005329.1	scRNA-seq,SRP215507,CD8+ T cell_1-AD.vs.control; scRNA-seq,SRP330776,Memory T cell_1-AD.vs.control	2
AC005332.1	scRNA-seq,SRP215507,CD8+ T cell_1-AD.vs.MCI; scRNA-seq,SRP215507,CD8+ T cell_5-AD.vs.MCI; scRNA-seq,SRP215507,CD8+ T cell_1-MCI.vs.control; scRNA-seq,SRP215507,CD8+ T cell_5-MCI.vs.control; scRNA-seq,SRP309935,CD8+ T cell_2-AD.vs.control; scRNA-seq,SRP330776,Natural killer cell_1-AD.vs.control; scRNA-seq,SRP330776,Naive CD8+ T cell_3-AD.vs.control; scRNA-seq,SRP330776,B cell_2-	9
AC005332.2	scRNA-seq,SRP215507,CD8+ T cell_1-AD.vs.MCI; scRNA-seq,SRP215507,CD8+ T cell_1-MCI.vs.control; scRNA-seq,SRP330776,CD8+ T cell_1-	3
AC005332.3	scRNA-seq,SRP215507,CD8+ T cell_1-AD.vs.control; scRNA-seq,SRP215507,CD8+ T cell_1-MCI.vs.control	2
AC005332.4	scRNA-seq,SRP215507,CD8+ T cell_5-AD.vs.MCI; scRNA-seq,SRP330776,CD8+ T cell_1-AD.vs.control; scRNA-seq,SRP330776,γδ T cell-AD.vs.control; scRNA-seq,SRP330776,Memory T cell_2-AD.vs.control; scRNA-seq,SRP330776,CD8+ T cell_2-AD.vs.control; scRNA-seq,SRP330776,B cell_1-AD.vs.control; scRNA-seq,SRP330776,Naive CD8+ T cell_1-AD.vs.control; scRNA-seq,SRP330776,Natural killer cell_1-AD.vs.control; scRNA-seq,SRP330776,Memory T cell_1-AD.vs.control; scRNA-seq,SRP330776,Natural killer T cell-AD.vs.control; scRNA-seq,SRP330776,Naive CD8+ T cell_3-AD.vs.control; scRNA-seq,SRP330776,B cell_2-AD.vs.control; scRNA-seq,SRP330776,T cell-AD.vs.control; scRNA-seq,SRP330776,CD8+ T cell_3-AD.vs.control; scRNA-seq,SRP330776,B cell_3-AD.vs.control; scRNA-seq,SRP330776,Natural killer cell_2-AD.vs.control	16
AC005332.5	scRNA-seq,SRP215507,CD8+ T cell_3-AD.vs.MCI; scRNA-seq,SRP215507,CD8+ T cell_5-AD.vs.MCI; scRNA-seq,SRP330776,CD8+ T cell_1-AD.vs.control; scRNA-seq,SRP330776,CD8+ T cell_2-AD.vs.control; scRNA-seq,SRP330776,Natural killer T cell-AD.vs.control; scRNA-	6
AC005332.6	scRNA-seq,SRP215507,CD8+ T cell_2-AD.vs.control; scRNA-seq,SRP215507,CD8+ T cell_3-AD.vs.control; scRNA-seq,SRP215507,CD8+ T cell_3-AD.vs.MCI; scRNA-seq,SRP215507,CD8+ T cell_4-AD.vs.MCI; scRNA-seq,SRP215507,CD8+ T cell_4-MCI.vs.control; scRNA-seq,SRP215507,CD8+ T cell_6-MCI.vs.control; scRNA-seq,SRP309935,Naive CD8+ T cell_1-AD.vs.control; scRNA-seq,SRP330776,γδ T cell-AD.vs.control; scRNA-seq,SRP330776,Memory T cell_2-AD.vs.control; scRNA-seq,SRP330776,B cell_1-AD.vs.control	10
AC005332.7	scRNA-seq,SRP215507,CD8+ T cell_4-MCI.vs.control	1
AC005355.1	scRNA-seq,SRP330776,Memory T cell_1-AD.vs.control	1
AC005363.2	scRNA-seq,SRP215507,CD8+ T cell_3-AD.vs.control; scRNA-seq,SRP215507,CD8+ T cell_1-AD.vs.control; scRNA-seq,SRP215507,CD8+ T cell_4-AD.vs.control; scRNA-seq,SRP215507,CD8+ T cell_5-AD.vs.control; scRNA-seq,SRP215507,CD8+ T cell_4-MCI.vs.control; scRNA-seq,SRP215507,CD8+ T cell_3-MCI.vs.control; scRNA-seq,SRP330776,Naive CD8+ T cell_1-AD.vs.control; scRNA-seq,SRP330776,B cell_2-	8
AC005392.2	scRNA-seq,SRP330776,Monocyte_2-AD.vs.control	1
AC005480.1	scRNA-seq,SRP215507,CD8+ T cell_2-MCI.vs.control; scRNA-seq,SRP330776,B cell_1-AD.vs.control	2
AC005498.2	scRNA-seq,SRP330776,CD8+ T cell_1-AD.vs.control	1
AC005498.3	scRNA-seq,SRP215507,CD8+ T cell_2-AD.vs.MCI; scRNA-seq,SRP215507,CD8+ T cell_5-AD.vs.MCI; scRNA-seq,SRP215507,CD8+ T cell_2-MCI.vs.control; scRNA-seq,SRP330776,CD8+ T cell_1-AD.vs.control; scRNA-seq,SRP330776,Naive CD8+ T cell_2-AD.vs.control	5
AC005519.1	scRNA-seq,SRP215507,CD8+ T cell_3-AD.vs.control; scRNA-seq,SRP215507,CD8+ T cell_3-MCI.vs.control	2
AC005520.2	scRNA-seq,SRP215507,CD8+ T cell_3-AD.vs.MCI; scRNA-seq,SRP215507,CD8+ T cell_5-AD.vs.MCI; scRNA-seq,SRP215507,CD8+ T cell_1-MCI.vs.control; scRNA-seq,SRP215507,CD8+ T cell_5-MCI.vs.control; scRNA-seq,SRP309935,Naive CD8+ T cell_1-AD.vs.control; scRNA-seq,SRP330776,CD8+ T cell_1-AD.vs.control; scRNA-seq,SRP330776,Memory T cell_2-AD.vs.control; scRNA-seq,SRP330776,Naive CD8+ T cell_1-	9
AC005520.5	scRNA-seq,SRP215507,CD8+ T cell_4-AD.vs.control; scRNA-seq,SRP215507,CD8+ T cell_5-MCI.vs.control; scRNA-seq,SRP215507,CD8+ T cell_3-	3
AC005523.1	scRNA-seq,SRP215507,CD8+ T cell_1-MCI.vs.control; scRNA-seq,SRP215507,CD8+ T cell_5-MCI.vs.control	2
AC005523.2	scRNA-seq,SRP309935,Dendritic cell-AD.vs.control; scRNA-seq,SRP330776,CD8+ T cell_1-AD.vs.control; scRNA-seq,SRP330776,Naive CD8+ T cell_1-AD.vs.control; scRNA-seq,SRP330776,Memory T cell_1-AD.vs.control	4

AC005532.1	scRNA-seq,SRP215507,CD8+ T cell_2-MCI.vs.control	1
AC005534.1	scRNA-seq,SRP330776,Naive CD8+ T cell_1-AD.vs.control	1
AC005562.1	scRNA-seq,SRP309935,Natural killer cell_2-AD.vs.control; scRNA-seq,SRP309935,CD8+ T cell_1-AD.vs.control; scRNA-seq,SRP330776,CD8+ T cell_1-AD.vs.control; scRNA-seq,SRP330776,γδ T cell-AD.vs.control; scRNA-seq,SRP330776,Memory T cell_2-AD.vs.control; scRNA-seq,SRP330776,CD8+ T cell_2-AD.vs.control; scRNA-seq,SRP330776,B cell_1-AD.vs.control; scRNA-seq,SRP330776,Naive CD8+ T cell_1-AD.vs.control; scRNA-seq,SRP330776,Memory T cell_1-AD.vs.control; scRNA-seq,SRP330776,B cell_2-AD.vs.control	10
AC005586.2	scRNA-seq,SRP215507,CD8+ T cell_3-MCI.vs.control	1
AC005614.1	scRNA-seq,SRP215507,CD8+ T cell_3-AD.vs.control; scRNA-seq,SRP330776,CD8+ T cell_1-AD.vs.control	2
AC005618.1	scRNA-seq,SRP215507,CD8+ T cell_3-MCI.vs.control	1
AC005632.3	scRNA-seq,SRP215507,CD8+ T cell_3-AD.vs.control; scRNA-seq,SRP215507,CD8+ T cell_3-AD.vs.MCI; scRNA-seq,SRP330776,CD8+ T cell_1-AD.vs.control; scRNA-seq,SRP330776,CD8+ T cell_2-AD.vs.control	4
AC005670.3	scRNA-seq,SRP215507,CD8+ T cell_1-AD.vs.MCI; scRNA-seq,SRP309935,Naive CD8+ T cell_1-AD.vs.control; scRNA-seq,SRP309935,Natural killer cell_1-AD.vs.control; scRNA-seq,SRP309935,Naive CD8+ T cell_2-AD.vs.control; scRNA-seq,SRP330776,γδ T cell-AD.vs.control; scRNA-seq,SRP330776,Memory T cell_2-AD.vs.control; scRNA-seq,SRP330776,B cell_1-AD.vs.control; scRNA-seq,SRP330776,Naive CD8+ T cell_1-AD.vs.control; scRNA-seq,SRP330776,Natural killer cell_1-AD.vs.control; scRNA-seq,SRP330776,Naive CD8+ T cell_3-AD.vs.control; scRNA-	12
AC005674.2	scRNA-seq,SRP215507,CD8+ T cell_2-AD.vs.control; scRNA-seq,SRP215507,CD8+ T cell_3-AD.vs.control; scRNA-seq,SRP215507,CD8+ T cell_1-AD.vs.control; scRNA-seq,SRP215507,CD8+ T cell_5-MCI.vs.control; scRNA-seq,SRP215507,CD8+ T cell_4-MCI.vs.control; scRNA-seq,SRP330776,CD8+ T cell_1-AD.vs.control; scRNA-seq,SRP330776,Memory T cell_2-AD.vs.control; scRNA-seq,SRP330776,Naive CD8+ T cell_1-AD.vs.control; scRNA-seq,SRP330776,Memory T cell_1-AD.vs.control	9
AC005682.2	scRNA-seq,SRP330776,CD8+ T cell_1-AD.vs.control	1
AC005726.1	scRNA-seq,SRP215507,CD8+ T cell_1-MCI.vs.control; scRNA-seq,SRP330776,CD8+ T cell_1-AD.vs.control; scRNA-seq,SRP330776,B cell_1-AD.vs.control; scRNA-seq,SRP330776,Naive CD8+ T cell_1-AD.vs.control; scRNA-seq,SRP330776,Memory T cell_1-AD.vs.control	5
AC005726.2	scRNA-seq,SRP330776,Memory T cell_1-AD.vs.control	1
AC005775.1	scRNA-seq,SRP215507,CD8+ T cell_2-AD.vs.MCI; scRNA-seq,SRP215507,CD8+ T cell_3-AD.vs.MCI; scRNA-seq,SRP215507,CD8+ T cell_2-MCI.vs.control; scRNA-seq,SRP215507,CD8+ T cell_3-MCI.vs.control; scRNA-seq,SRP330776,Memory T cell_2-AD.vs.control	5
AC005803.1	scRNA-seq,SRP215507,CD8+ T cell_2-MCI.vs.control; scRNA-seq,SRP330776,γδ T cell-AD.vs.control; scRNA-seq,SRP330776,T cell-AD.vs.control	3
AC005837.1	scRNA-seq,SRP215507,CD8+ T cell_2-AD.vs.control; scRNA-seq,SRP215507,CD8+ T cell_1-AD.vs.control; scRNA-seq,SRP215507,CD8+ T cell_5-AD.vs.control; scRNA-seq,SRP215507,CD8+ T cell_1-MCI.vs.control; scRNA-seq,SRP330776,Memory T cell_1-AD.vs.control	5
AC005838.2	scRNA-seq,SRP215507,CD8+ T cell_3-AD.vs.control; scRNA-seq,SRP309935,Naive CD8+ T cell_1-AD.vs.control	2
AC005842.1	scRNA-seq,SRP215507,CD8+ T cell_2-AD.vs.control; scRNA-seq,SRP215507,CD8+ T cell_3-AD.vs.MCI; scRNA-seq,SRP215507,CD8+ T cell_2-MCI.vs.control; scRNA-seq,SRP215507,CD8+ T cell_3-MCI.vs.control; scRNA-seq,SRP309935,Naive CD8+ T cell_1-AD.vs.control; scRNA-seq,SRP309935,CD8+ T cell_2-AD.vs.control; scRNA-seq,SRP330776,γδ T cell-AD.vs.control; scRNA-seq,SRP330776,Naive CD8+ T cell_1-	9
AC005856.1	scRNA-seq,SRP215507,CD8+ T cell_2-MCI.vs.control	1
AC005899.5	scRNA-seq,SRP309935,Natural killer cell_1-AD.vs.control	1
AC005899.6	scRNA-seq,SRP330776,Memory T cell_1-AD.vs.control	1
AC005899.7	scRNA-seq,SRP309935,Natural killer cell_1-AD.vs.control	1
AC005911.1	scRNA-seq,SRP309935,Natural killer cell_1-AD.vs.control	1
AC005920.1	scRNA-seq,SRP330776,CD8+ T cell_1-AD.vs.control; scRNA-seq,SRP330776,Memory T cell_2-AD.vs.control; scRNA-seq,SRP330776,Naive CD8+ T cell_1-AD.vs.control; scRNA-seq,SRP330776,Naive CD8+ T cell_3-AD.vs.control	4
AC005921.2	scRNA-seq,SRP309935,Natural killer cell_2-AD.vs.control	1

AC005944.1	scRNA-seq,SRP215507,CD8+ T cell_2-AD.vs.MCI; scRNA-seq,SRP215507,CD8+ T cell_3-AD.vs.MCI; scRNA-seq,SRP215507,CD8+ T cell_2-MCI.vs.control; scRNA-seq,SRP215507,CD8+ T cell_3-MCI.vs.control; scRNA-seq,SRP330776,Natural killer cell_1-AD.vs.control; scRNA-seq,SRP330776,Natural killer T cell-AD.vs.control; scRNA-seq,SRP330776,Regulatory T cell-AD.vs.control	7
AC005962.2	scRNA-seq,SRP215507,CD8+ T cell_2-AD.vs.control; scRNA-seq,SRP215507,CD8+ T cell_5-MCI.vs.control	2
AC006001.2	scRNA-seq,SRP215507,CD8+ T cell_2-AD.vs.control; scRNA-seq,SRP215507,CD8+ T cell_1-AD.vs.control; scRNA-seq,SRP215507,CD8+ T cell_2-AD.vs.MCI; scRNA-seq,SRP215507,CD8+ T cell_1-MCI.vs.control; scRNA-seq,SRP330776,γδ T cell-AD.vs.control; scRNA-seq,SRP330776,Memory T cell_2-AD.vs.control; scRNA-seq,SRP330776,CD8+ T cell_2-AD.vs.control	7
AC006008.1	scRNA-seq,SRP330776,CD8+ T cell_1-AD.vs.control; scRNA-seq,SRP330776,γδ T cell-AD.vs.control; scRNA-seq,SRP330776,B cell_1-AD.vs.control	3
AC006033.2	scRNA-seq,SRP215507,CD8+ T cell_1-AD.vs.control; scRNA-seq,SRP215507,CD8+ T cell_2-AD.vs.MCI; scRNA-seq,SRP215507,CD8+ T cell_2-MCI.vs.control; scRNA-seq,SRP330776,γδ T cell-AD.vs.control; scRNA-seq,SRP330776,CD8+ T cell_2-AD.vs.control; scRNA-seq,SRP330776,Memory	6
AC006042.2	scRNA-seq,SRP330776,Memory T cell_1-AD.vs.control	1
AC006042.4	scRNA-seq,SRP215507,CD8+ T cell_3-AD.vs.control; scRNA-seq,SRP330776,Natural killer cell_1-AD.vs.control	2
AC006059.1	scRNA-seq,SRP215507,CD8+ T cell_2-AD.vs.MCI; scRNA-seq,SRP215507,CD8+ T cell_1-MCI.vs.control; scRNA-seq,SRP215507,CD8+ T cell_2-MCI.vs.control; scRNA-seq,SRP330776,Naive CD8+ T cell_1-AD.vs.control	4
AC006064.2	scRNA-seq,SRP215507,CD8+ T cell_2-AD.vs.control; scRNA-seq,SRP215507,CD8+ T cell_2-MCI.vs.control	2
AC006064.4	scRNA-seq,SRP330776,B cell_1-AD.vs.control; scRNA-seq,SRP330776,Natural killer T cell-AD.vs.control; scRNA-seq,SRP330776,Naive CD8+ T cell_3-AD.vs.control; scRNA-seq,SRP330776,Regulatory T cell-AD.vs.control	4
AC006064.5	scRNA-seq,SRP215507,CD8+ T cell_3-MCI.vs.control; scRNA-seq,SRP215507,CD8+ T cell_6-MCI.vs.control; scRNA-seq,SRP330776,CD8+ T cell_1-	3
AC006111.2	scRNA-seq,SRP215507,CD8+ T cell_2-MCI.vs.control	1
AC006115.2	scRNA-seq,SRP215507,CD8+ T cell_3-AD.vs.MCI; scRNA-seq,SRP215507,CD8+ T cell_3-MCI.vs.control; scRNA-seq,SRP309935,Naive CD8+ T cell_1-AD.vs.control; scRNA-seq,SRP309935,B cell_1-AD.vs.control; scRNA-seq,SRP330776,CD8+ T cell_1-AD.vs.control; scRNA-seq,SRP330776,Naive CD8+ T cell_1-AD.vs.control; scRNA-seq,SRP330776,B cell_2-AD.vs.control	7
AC006116.10	scRNA-seq,SRP330776,B cell_2-AD.vs.control	1
AC006116.8	scRNA-seq,SRP215507,CD8+ T cell_2-AD.vs.control; scRNA-seq,SRP215507,CD8+ T cell_3-AD.vs.MCI	2
AC006141.1	scRNA-seq,SRP215507,CD8+ T cell_1-AD.vs.control; scRNA-seq,SRP309935,Natural killer cell_1-AD.vs.control; scRNA-seq,SRP309935,B cell_1-AD.vs.control; scRNA-seq,SRP330776,CD8+ T cell_1-AD.vs.control; scRNA-seq,SRP330776,γδ T cell-AD.vs.control; scRNA-seq,SRP330776,Memory T cell_2-AD.vs.control; scRNA-seq,SRP330776,CD8+ T cell_2-AD.vs.control; scRNA-seq,SRP330776,B cell_1-AD.vs.control; scRNA-seq,SRP330776,Naive CD8+ T cell_1-AD.vs.control; scRNA-seq,SRP330776,Natural killer cell_1-AD.vs.control; scRNA-seq,SRP330776,Memory T cell_1-AD.vs.control; scRNA-seq,SRP330776,T cell-AD.vs.control; scRNA-seq,SRP330776,CD8+ T cell_3-AD.vs.control	13
AC006207.1	scRNA-seq,SRP215507,CD8+ T cell_1-AD.vs.MCI; scRNA-seq,SRP215507,CD8+ T cell_1-MCI.vs.control; scRNA-seq,SRP215507,CD8+ T cell_6-	3
AC006213.2	scRNA-seq,SRP215507,CD8+ T cell_5-AD.vs.control; scRNA-seq,SRP215507,CD8+ T cell_5-MCI.vs.control; scRNA-seq,SRP215507,CD8+ T cell_8-MCI.vs.control; scRNA-seq,SRP309935,B cell_1-AD.vs.control; scRNA-seq,SRP330776,Memory T cell_2-AD.vs.control; scRNA-seq,SRP330776,Naive CD8+ T cell_1-AD.vs.control; scRNA-seq,SRP330776,Natural killer cell_1-AD.vs.control; scRNA-seq,SRP330776,Natural killer T cell-AD.vs.control; scRNA-seq,SRP330776,T cell-AD.vs.control; scRNA-seq,SRP330776,Naive CD8+ T cell_2-AD.vs.control; scRNA-seq,SRP330776,Natural killer cell_2-	11
AC006213.3	scRNA-seq,SRP215507,CD8+ T cell_1-AD.vs.control	1
AC006230.1	scRNA-seq,SRP330776,B cell_2-AD.vs.control	1
AC006237.1	scRNA-seq,SRP215507,CD8+ T cell_1-AD.vs.control	1
AC006252.1	scRNA-seq,SRP215507,CD8+ T cell_1-AD.vs.control; scRNA-seq,SRP215507,CD8+ T cell_5-AD.vs.control; scRNA-seq,SRP215507,CD8+ T cell_1-AD.vs.MCI; scRNA-seq,SRP215507,CD8+ T cell_5-MCI.vs.control; scRNA-seq,SRP330776,B cell_1-AD.vs.control; scRNA-seq,SRP330776,Naive CD8+ T cell_1-AD.vs.control; scRNA-seq,SRP330776,Memory T cell_1-AD.vs.control; scRNA-seq,SRP330776,Naive CD8+ T cell_3-AD.vs.control	8

AC006299.1	scRNA-seq,SRP215507,CD8+ T cell_4-MCI.vs.control; scRNA-seq,SRP330776,CD8+ T cell_1-AD.vs.control; scRNA-seq,SRP330776,γδ T cell-AD.vs.control; scRNA-seq,SRP330776,Memory T cell_2-AD.vs.control; scRNA-seq,SRP330776,CD8+ T cell_2-AD.vs.control; scRNA-seq,SRP330776,Naive CD8+ T cell_1-AD.vs.control; scRNA-seq,SRP330776,Memory T cell_1-AD.vs.control; scRNA-seq,SRP330776,Naive CD8+ T	8
AC006329.1	scRNA-seq,SRP215507,CD8+ T cell_2-AD.vs.control; scRNA-seq,SRP215507,CD8+ T cell_3-AD.vs.MCI; scRNA-seq,SRP215507,CD8+ T cell_2-MCI.vs.control; scRNA-seq,SRP215507,CD8+ T cell_3-MCI.vs.control; scRNA-seq,SRP330776,CD8+ T cell_1-AD.vs.control	5
AC006333.2	scRNA-seq,SRP215507,CD8+ T cell_2-AD.vs.control; scRNA-seq,SRP215507,CD8+ T cell_1-AD.vs.control; scRNA-seq,SRP215507,CD8+ T cell_4-AD.vs.MCI; scRNA-seq,SRP215507,CD8+ T cell_1-MCI.vs.control; scRNA-seq,SRP215507,CD8+ T cell_5-MCI.vs.control; scRNA-seq,SRP330776,CD8+ T cell_1-AD.vs.control; scRNA-seq,SRP330776,γδ T cell-AD.vs.control; scRNA-seq,SRP330776,CD8+ T cell_2-AD.vs.control; scRNA-seq,SRP330776,Naive CD8+ T cell_1-AD.vs.control; scRNA-seq,SRP330776,Naive CD8+ T cell_3-AD.vs.control; scRNA-seq,SRP330776,CD8+	11
AC006364.1	scRNA-seq,SRP309935,B cell_1-AD.vs.control	1
AC006369.1	scRNA-seq,SRP309935,Natural killer cell_1-AD.vs.control; scRNA-seq,SRP309935,CD8+ T cell_1-AD.vs.control; scRNA-seq,SRP309935,CD8+ T cell_2-AD.vs.control; scRNA-seq,SRP330776,CD8+ T cell_1-AD.vs.control; scRNA-seq,SRP330776,Memory T cell_2-AD.vs.control; scRNA-seq,SRP330776,CD8+ T cell_2-AD.vs.control; scRNA-seq,SRP330776,B cell_1-AD.vs.control; scRNA-seq,SRP330776,Natural killer cell_1-AD.vs.control; scRNA-seq,SRP330776,Memory T cell_1-AD.vs.control; scRNA-seq,SRP330776,Natural killer T cell-AD.vs.control; scRNA-seq,SRP330776,Naive CD8+ T cell_3-AD.vs.control; scRNA-seq,SRP330776,T cell-AD.vs.control; scRNA-seq,SRP330776,CD8+ T cell_3-AD.vs.control	13
AC006449.6	scRNA-seq,SRP215507,CD8+ T cell_5-AD.vs.control; scRNA-seq,SRP215507,CD8+ T cell_4-AD.vs.MCI; scRNA-seq,SRP215507,CD8+ T cell_4-MCI.vs.control; scRNA-seq,SRP309935,Natural killer cell_2-AD.vs.control; scRNA-seq,SRP330776,CD8+ T cell_2-AD.vs.control; scRNA-seq,SRP330776,Naive CD8+ T cell_1-AD.vs.control; scRNA-seq,SRP330776,Natural killer T cell-AD.vs.control; scRNA-seq,SRP330776,CD8+ T cell_3-	9
AC006460.2	scRNA-seq,SRP215507,CD8+ T cell_3-AD.vs.control; scRNA-seq,SRP215507,CD8+ T cell_1-AD.vs.control; scRNA-seq,SRP215507,CD8+ T cell_5-AD.vs.control; scRNA-seq,SRP215507,CD8+ T cell_1-MCI.vs.control; scRNA-seq,SRP215507,CD8+ T cell_5-MCI.vs.control; scRNA-seq,SRP215507,CD8+ T cell_6-MCI.vs.control; scRNA-seq,SRP330776,Memory T cell_2-AD.vs.control; scRNA-seq,SRP330776,Naive CD8+ T cell_1-AD.vs.control; scRNA-seq,SRP330776,Natural killer cell_1-AD.vs.control	9
AC006480.2	scRNA-seq,SRP215507,CD8+ T cell_1-MCI.vs.control; scRNA-seq,SRP309935,Naive CD8+ T cell_1-AD.vs.control; scRNA-seq,SRP309935,Natural killer cell_1-AD.vs.control; scRNA-seq,SRP330776,CD8+ T cell_1-AD.vs.control	4
AC006504.5	scRNA-seq,SRP215507,CD8+ T cell_2-AD.vs.MCI; scRNA-seq,SRP215507,CD8+ T cell_1-MCI.vs.control; scRNA-seq,SRP215507,CD8+ T cell_2-MCI.vs.control; scRNA-seq,SRP309935,CD8+ T cell_1-AD.vs.control; scRNA-seq,SRP330776,γδ T cell-AD.vs.control; scRNA-seq,SRP330776,Memory T cell_2-AD.vs.control; scRNA-seq,SRP330776,CD8+ T cell_2-AD.vs.control; scRNA-seq,SRP330776,B cell_1-AD.vs.control; scRNA-seq,SRP330776,Naive CD8+ T cell_1-AD.vs.control; scRNA-seq,SRP330776,Memory T cell_1-AD.vs.control; scRNA-seq,SRP330776,Natural killer T	11
AC006538.3	scRNA-seq,SRP330776,B cell_1-AD.vs.control	1
AC006942.1	scRNA-seq,SRP330776,Memory T cell_2-AD.vs.control; scRNA-seq,SRP330776,B cell_1-AD.vs.control	2
AC006994.2	scRNA-seq,SRP215507,CD8+ T cell_2-AD.vs.control	1
AC007029.1	scRNA-seq,SRP215507,CD8+ T cell_1-MCI.vs.control; scRNA-seq,SRP330776,Naive CD8+ T cell_1-AD.vs.control	2
AC007032.1	scRNA-seq,SRP215507,CD8+ T cell_2-AD.vs.control; scRNA-seq,SRP215507,CD8+ T cell_1-AD.vs.MCI; scRNA-seq,SRP215507,CD8+ T cell_1-MCI.vs.control; scRNA-seq,SRP330776,Memory T cell_2-AD.vs.control; scRNA-seq,SRP330776,Memory T cell_1-AD.vs.control	5
AC007038.1	scRNA-seq,SRP215507,CD8+ T cell_3-AD.vs.MCI; scRNA-seq,SRP215507,CD8+ T cell_1-MCI.vs.control; scRNA-seq,SRP330776,Naive CD8+ T cell_1-AD.vs.control; scRNA-seq,SRP330776,Memory T cell_1-AD.vs.control	4
AC007038.2	scRNA-seq,SRP215507,CD8+ T cell_3-AD.vs.control; scRNA-seq,SRP309935,Natural killer cell_2-AD.vs.control; scRNA-seq,SRP330776,CD8+ T cell_1-AD.vs.control; scRNA-seq,SRP330776,Naive CD8+ T cell_1-AD.vs.control; scRNA-seq,SRP330776,B cell_2-AD.vs.control	5
AC007066.2	scRNA-seq,SRP215507,CD8+ T cell_3-AD.vs.MCI; scRNA-seq,SRP215507,CD8+ T cell_4-MCI.vs.control; scRNA-seq,SRP215507,CD8+ T cell_2-MCI.vs.control; scRNA-seq,SRP215507,CD8+ T cell_3-MCI.vs.control	4
AC007098.1	scRNA-seq,SRP309935,Naive CD8+ T cell_1-AD.vs.control; scRNA-seq,SRP330776,CD8+ T cell_1-AD.vs.control	2

AC007114.1	scRNA-seq,SRP215507,CD8+ T cell_3-AD.vs.MCI; scRNA-seq,SRP215507,CD8+ T cell_3-MCI.vs.control; scRNA-seq,SRP215507,CD8+ T cell_6-MCI.vs.control; scRNA-seq,SRP330776,Memory T cell_2-AD.vs.control; scRNA-seq,SRP330776,B cell_1-AD.vs.control; scRNA-seq,SRP330776,Naive CD8+ T cell_1-AD.vs.control; scRNA-seq,SRP330776,Natural killer T cell-AD.vs.control; scRNA-seq,SRP330776,T cell-AD.vs.control; scRNA-	9
AC007163.1	scRNA-seq,SRP215507,CD8+ T cell_2-AD.vs.MCI; scRNA-seq,SRP215507,CD8+ T cell_1-AD.vs.MCI; scRNA-seq,SRP215507,CD8+ T cell_1-MCI.vs.control; scRNA-seq,SRP215507,CD8+ T cell_2-MCI.vs.control	4
AC007192.2	scRNA-seq,SRP330776,Naive CD8+ T cell_1-AD.vs.control	1
AC007216.3	scRNA-seq,SRP215507,CD8+ T cell_3-AD.vs.control; scRNA-seq,SRP309935,Natural killer cell_2-AD.vs.control	2
AC007216.4	scRNA-seq,SRP215507,CD8+ T cell_5-AD.vs.MCI; scRNA-seq,SRP215507,CD8+ T cell_5-MCI.vs.control; scRNA-seq,SRP215507,CD8+ T cell_3-MCI.vs.control; scRNA-seq,SRP330776,γδ T cell-AD.vs.control; scRNA-seq,SRP330776,Memory T cell_2-AD.vs.control; scRNA-seq,SRP330776,CD8+ T cell_2-AD.vs.control; scRNA-seq,SRP330776,B cell_1-AD.vs.control; scRNA-seq,SRP330776,Natural killer T cell-AD.vs.control	8
AC007262.2	scRNA-seq,SRP330776,Memory T cell_2-AD.vs.control	1
AC007278.1	scRNA-seq,SRP215507,CD8+ T cell_5-AD.vs.control; scRNA-seq,SRP309935,Natural killer cell_2-AD.vs.control; scRNA-seq,SRP309935,Natural killer	3
AC007278.2	scRNA-seq,SRP215507,CD8+ T cell_5-AD.vs.control; scRNA-seq,SRP330776,Memory T cell_1-AD.vs.control	2
AC007285.1	scRNA-seq,SRP215507,CD8+ T cell_5-MCI.vs.control; scRNA-seq,SRP330776,B cell_1-AD.vs.control	2
AC007292.2	scRNA-seq,SRP309935,CD8+ T cell_1-AD.vs.control	1
AC007314.1	scRNA-seq,SRP215507,CD8+ T cell_1-MCI.vs.control	1
AC007325.4	scRNA-seq,SRP309935,Monocyte_1-AD.vs.control; scRNA-seq,SRP309935,CD8+ T cell_1-AD.vs.control	2
AC007342.1	scRNA-seq,SRP215507,CD8+ T cell_2-AD.vs.control; scRNA-seq,SRP215507,CD8+ T cell_1-AD.vs.MCI; scRNA-seq,SRP215507,CD8+ T cell_4-AD.vs.MCI; scRNA-seq,SRP309935,Naive CD8+ T cell_1-AD.vs.control; scRNA-seq,SRP330776,CD8+ T cell_1-AD.vs.control	5
AC007342.4	scRNA-seq,SRP215507,CD8+ T cell_2-AD.vs.control; scRNA-seq,SRP215507,CD8+ T cell_3-AD.vs.MCI; scRNA-seq,SRP215507,CD8+ T cell_4-MCI.vs.control; scRNA-seq,SRP215507,CD8+ T cell_3-MCI.vs.control; scRNA-seq,SRP330776,Memory T cell_2-AD.vs.control	5
AC007364.1	scRNA-seq,SRP215507,CD8+ T cell_1-AD.vs.control; scRNA-seq,SRP330776,CD8+ T cell_1-AD.vs.control; scRNA-seq,SRP330776,γδ T cell-	3
AC007365.1	scRNA-seq,SRP215507,CD8+ T cell_4-MCI.vs.control; scRNA-seq,SRP330776,Memory T cell_1-AD.vs.control	2
AC007368.1	scRNA-seq,SRP330776,B cell_1-AD.vs.control	1
AC007381.1	scRNA-seq,SRP330776,B cell_2-AD.vs.control	1
AC007383.2	scRNA-seq,SRP215507,CD8+ T cell_4-AD.vs.control; scRNA-seq,SRP215507,CD8+ T cell_3-AD.vs.MCI; scRNA-seq,SRP309935,Naive CD8+ T cell_1-AD.vs.control; scRNA-seq,SRP330776,CD8+ T cell_1-AD.vs.control; scRNA-seq,SRP330776,γδ T cell-AD.vs.control; scRNA-seq,SRP330776,CD8+ T cell_2-AD.vs.control; scRNA-seq,SRP330776,Natural killer cell_1-AD.vs.control; scRNA-seq,SRP330776,Memory T cell_1-AD.vs.control; scRNA-seq,SRP330776,Natural killer T cell-AD.vs.control; scRNA-seq,SRP330776,Natural killer cell_2-AD.vs.control	10
AC007384.1	scRNA-seq,SRP215507,CD8+ T cell_4-AD.vs.control; scRNA-seq,SRP215507,CD8+ T cell_4-MCI.vs.control; scRNA-seq,SRP330776,Memory T cell_2-AD.vs.control; scRNA-seq,SRP330776,Naive CD8+ T cell_1-AD.vs.control; scRNA-seq,SRP330776,Memory T cell_1-AD.vs.control; scRNA-seq,SRP330776,Natural killer T cell-AD.vs.control; scRNA-seq,SRP330776,Naive CD8+ T cell_2-AD.vs.control	7
AC007390.2	scRNA-seq,SRP215507,CD8+ T cell_4-AD.vs.control; scRNA-seq,SRP215507,CD8+ T cell_1-AD.vs.MCI; scRNA-seq,SRP215507,CD8+ T cell_1-MCI.vs.control; scRNA-seq,SRP215507,CD8+ T cell_4-MCI.vs.control; scRNA-seq,SRP330776,CD8+ T cell_1-AD.vs.control; scRNA-	6
AC007405.3	scRNA-seq,SRP215507,CD8+ T cell_3-AD.vs.control; scRNA-seq,SRP215507,CD8+ T cell_1-AD.vs.control; scRNA-seq,SRP215507,CD8+ T cell_2-AD.vs.MCI; scRNA-seq,SRP215507,CD8+ T cell_1-MCI.vs.control; scRNA-seq,SRP215507,CD8+ T cell_2-MCI.vs.control; scRNA-seq,SRP330776,γδ T cell-AD.vs.control; scRNA-seq,SRP330776,CD8+ T cell_2-AD.vs.control; scRNA-seq,SRP330776,Memory T cell_1-AD.vs.control	8
AC007406.5	scRNA-seq,SRP215507,CD8+ T cell_3-AD.vs.control; scRNA-seq,SRP215507,CD8+ T cell_3-MCI.vs.control; scRNA-seq,SRP330776,CD8+ T cell_1-AD.vs.control; scRNA-seq,SRP330776,γδ T cell-AD.vs.control; scRNA-seq,SRP330776,CD8+ T cell_2-AD.vs.control; scRNA-seq,SRP330776,Naive CD8+ T cell_1-AD.vs.control; scRNA-seq,SRP330776,Memory T cell_1-AD.vs.control; scRNA-seq,SRP330776,B cell_2-AD.vs.control; scRNA-	9

AC007448.4	scRNA-seq,SRP215507,CD8+ T cell_1-AD.vs.MCI; scRNA-seq,SRP215507,CD8+ T cell_5-AD.vs.MCI; scRNA-seq,SRP215507,CD8+ T cell_1-MCI.vs.control; scRNA-seq,SRP215507,CD8+ T cell_5-MCI.vs.control; scRNA-seq,SRP215507,CD8+ T cell_4-MCI.vs.control; scRNA-seq,SRP215507,CD8+ T cell_6-MCI.vs.control; scRNA-seq,SRP330776,γδ T cell-AD.vs.control; scRNA-seq,SRP330776,CD8+ T cell_2-AD.vs.control;	9
AC007497.2	scRNA-seq,SRP215507,CD8+ T cell_1-AD.vs.control; scRNA-seq,SRP330776,T cell-AD.vs.control; scRNA-seq,SRP330776,Naive CD8+ T cell_2-	3
AC007541.1	scRNA-seq,SRP215507,CD8+ T cell_3-AD.vs.MCI; scRNA-seq,SRP215507,CD8+ T cell_1-AD.vs.MCI; scRNA-seq,SRP215507,CD8+ T cell_4-AD.vs.MCI; scRNA-seq,SRP215507,CD8+ T cell_1-MCI.vs.control; scRNA-seq,SRP309935,Natural killer cell_1-AD.vs.control; scRNA-seq,SRP330776,CD8+ T cell_1-AD.vs.control; scRNA-seq,SRP330776,Memory T cell_2-AD.vs.control; scRNA-seq,SRP330776,CD8+ T cell_2-AD.vs.control; scRNA-seq,SRP330776,Naive CD8+ T cell_1-AD.vs.control; scRNA-seq,SRP330776,CD8+ T cell_3-AD.vs.control	10
AC007566.1	scRNA-seq,SRP215507,CD8+ T cell_5-AD.vs.MCI; scRNA-seq,SRP215507,CD8+ T cell_5-MCI.vs.control; scRNA-seq,SRP330776,CD8+ T cell_2-AD.vs.control; scRNA-seq,SRP330776,Naive CD8+ T cell_1-AD.vs.control; scRNA-seq,SRP330776,Memory T cell_1-AD.vs.control	5
AC007569.1	scRNA-seq,SRP215507,CD8+ T cell_5-MCI.vs.control	1
AC007608.3	scRNA-seq,SRP215507,CD8+ T cell_2-AD.vs.MCI; scRNA-seq,SRP330776,Natural killer cell_1-AD.vs.control; scRNA-seq,SRP330776,CD8+ T cell_3-	3
AC007611.1	scRNA-seq,SRP215507,CD8+ T cell_3-AD.vs.MCI; scRNA-seq,SRP215507,CD8+ T cell_3-MCI.vs.control	2
AC007613.1	scRNA-seq,SRP215507,CD8+ T cell_1-AD.vs.control; scRNA-seq,SRP215507,CD8+ T cell_1-AD.vs.MCI; scRNA-seq,SRP215507,CD8+ T cell_4-MCI.vs.control; scRNA-seq,SRP330776,CD8+ T cell_1-AD.vs.control; scRNA-seq,SRP330776,Memory T cell_2-AD.vs.control; scRNA-	6
AC007620.2	scRNA-seq,SRP215507,CD8+ T cell_4-MCI.vs.control; scRNA-seq,SRP330776,B cell_2-AD.vs.control	2
AC007637.1	scRNA-seq,SRP215507,CD8+ T cell_3-AD.vs.control; scRNA-seq,SRP215507,CD8+ T cell_5-AD.vs.control; scRNA-seq,SRP215507,CD8+ T cell_1-AD.vs.MCI; scRNA-seq,SRP330776,Memory T cell_2-AD.vs.control; scRNA-seq,SRP330776,B cell_1-AD.vs.control; scRNA-seq,SRP330776,Naive CD8+ T cell_1-AD.vs.control; scRNA-seq,SRP330776,Natural killer cell_1-AD.vs.control	7
AC007686.3	scRNA-seq,SRP309935,Monocyte_1-AD.vs.control; scRNA-seq,SRP309935,Naive CD8+ T cell_1-AD.vs.control; scRNA-seq,SRP330776,γδ T cell-AD.vs.control; scRNA-seq,SRP330776,Memory T cell_2-AD.vs.control; scRNA-seq,SRP330776,CD8+ T cell_2-AD.vs.control; scRNA-seq,SRP330776,Naive CD8+ T cell_1-AD.vs.control; scRNA-seq,SRP330776,Memory T cell_1-AD.vs.control	7
AC007695.1	scRNA-seq,SRP215507,CD8+ T cell_3-AD.vs.control; scRNA-seq,SRP330776,CD8+ T cell_1-AD.vs.control; scRNA-seq,SRP330776,γδ T cell-AD.vs.control; scRNA-seq,SRP330776,Memory T cell_2-AD.vs.control; scRNA-seq,SRP330776,CD8+ T cell_2-AD.vs.control; scRNA-seq,SRP330776,B cell_1-AD.vs.control; scRNA-seq,SRP330776,Naive CD8+ T cell_1-AD.vs.control; scRNA-seq,SRP330776,Naive CD8+ T cell_3-AD.vs.control	8
AC007728.1	scRNA-seq,SRP330776,CD8+ T cell_1-AD.vs.control; scRNA-seq,SRP330776,γδ T cell-AD.vs.control; scRNA-seq,SRP330776,Memory T cell_2-AD.vs.control; scRNA-seq,SRP330776,CD8+ T cell_2-AD.vs.control; scRNA-seq,SRP330776,Naive CD8+ T cell_1-AD.vs.control	5
AC007728.2	scRNA-seq,SRP215507,CD8+ T cell_1-AD.vs.control; scRNA-seq,SRP215507,CD8+ T cell_1-AD.vs.MCI; scRNA-seq,SRP330776,Memory T cell_1-	3
AC007773.1	scRNA-seq,SRP215507,CD8+ T cell_3-AD.vs.control; scRNA-seq,SRP215507,CD8+ T cell_3-MCI.vs.control; scRNA-seq,SRP330776,CD8+ T cell_1-AD.vs.control; scRNA-seq,SRP330776,γδ T cell-AD.vs.control; scRNA-seq,SRP330776,CD8+ T cell_2-AD.vs.control; scRNA-seq,SRP330776,Memory T	6
AC007780.1	scRNA-seq,SRP215507,CD8+ T cell_4-MCI.vs.control; scRNA-seq,SRP330776,CD8+ T cell_1-AD.vs.control; scRNA-seq,SRP330776,CD8+ T cell_2-AD.vs.control; scRNA-seq,SRP330776,Naive CD8+ T cell_1-AD.vs.control; scRNA-seq,SRP330776,Natural killer cell_1-AD.vs.control; scRNA-seq,SRP330776,CD8+ T cell_3-AD.vs.control	6
AC007878.1	scRNA-seq,SRP215507,CD8+ T cell_3-AD.vs.MCI; scRNA-seq,SRP215507,CD8+ T cell_3-MCI.vs.control; scRNA-seq,SRP330776,CD8+ T cell_1-	3
AC007881.3	scRNA-seq,SRP215507,CD8+ T cell_1-AD.vs.control; scRNA-seq,SRP215507,CD8+ T cell_1-MCI.vs.control; scRNA-seq,SRP330776,Naive CD8+ T cell_1-AD.vs.control; scRNA-seq,SRP330776,Memory T cell_1-AD.vs.control	4
AC007950.2	scRNA-seq,SRP215507,CD8+ T cell_2-AD.vs.MCI	1

AC007952.4	scRNA-seq,SRP215507,CD8+ T cell_4-AD.vs.control; scRNA-seq,SRP215507,CD8+ T cell_10-AD.vs.control; scRNA-seq,SRP215507,CD8+ T cell_2-AD.vs.MCI; scRNA-seq,SRP215507,CD8+ T cell_3-AD.vs.MCI; scRNA-seq,SRP215507,CD8+ T cell_1-AD.vs.MCI; scRNA-seq,SRP215507,CD8+ T cell_4-AD.vs.MCI; scRNA-seq,SRP215507,CD8+ T cell_7-AD.vs.MCI; scRNA-seq,SRP215507,CD8+ T cell_5-AD.vs.MCI; scRNA-seq,SRP215507,CD8+ T cell_1-MCI.vs.control; scRNA-seq,SRP215507,CD8+ T cell_5-MCI.vs.control; scRNA-seq,SRP215507,CD8+ T cell_2-MCI.vs.control; scRNA-seq,SRP215507,CD8+ T cell_3-MCI.vs.control; scRNA-seq,SRP309935,Natural killer cell_2-AD.vs.control; scRNA-seq,SRP309935,Erythroid cell-AD.vs.control; scRNA-seq,SRP309935,Natural killer cell_1-AD.vs.control; scRNA-seq,SRP309935,CD8+ T cell_1-AD.vs.control; scRNA-seq,SRP330776,Memory T cell_2-AD.vs.control; scRNA-seq,SRP330776,B cell_1-AD.vs.control; scRNA-seq,SRP330776,Naive [illegible]	20
AC007993.2	scRNA-seq,SRP215507,CD8+ T cell_3-MCI.vs.control	1
AC007993.3	scRNA-seq,SRP215507,CD8+ T cell_2-AD.vs.control; scRNA-seq,SRP215507,CD8+ T cell_3-AD.vs.control; scRNA-seq,SRP215507,CD8+ T cell_6-AD.vs.control; scRNA-seq,SRP330776,CD8+ T cell_1-AD.vs.control; scRNA-seq,SRP330776,Naive CD8+ T cell_1-AD.vs.control	5
AC008014.1	scRNA-seq,SRP215507,CD8+ T cell_5-AD.vs.control; scRNA-seq,SRP215507,CD8+ T cell_5-MCI.vs.control; scRNA-seq,SRP215507,CD8+ T cell_4-MCI.vs.control; scRNA-seq,SRP330776,γδ T cell-AD.vs.control; scRNA-seq,SRP330776,B cell_1-AD.vs.control; scRNA-seq,SRP330776,Memory T	6
AC008026.3	scRNA-seq,SRP215507,CD8+ T cell_1-MCI.vs.control	1
AC008033.3	scRNA-seq,SRP215507,CD8+ T cell_1-AD.vs.MCI; scRNA-seq,SRP215507,CD8+ T cell_1-MCI.vs.control; scRNA-seq,SRP309935,Natural killer cell_1-	3
AC008035.1	scRNA-seq,SRP215507,CD8+ T cell_4-AD.vs.control; scRNA-seq,SRP215507,CD8+ T cell_1-AD.vs.MCI; scRNA-seq,SRP330776,γδ T cell-AD.vs.control; scRNA-seq,SRP330776,CD8+ T cell_2-AD.vs.control; scRNA-seq,SRP330776,Memory T cell_1-AD.vs.control	5
AC008040.1	scRNA-seq,SRP215507,CD8+ T cell_2-AD.vs.control; scRNA-seq,SRP215507,CD8+ T cell_1-AD.vs.MCI; scRNA-seq,SRP215507,CD8+ T cell_1-MCI.vs.control; scRNA-seq,SRP330776,B cell_1-AD.vs.control; scRNA-seq,SRP330776,Naive CD8+ T cell_1-AD.vs.control	5
AC008063.1	scRNA-seq,SRP215507,CD8+ T cell_5-AD.vs.control; scRNA-seq,SRP215507,CD8+ T cell_5-AD.vs.MCI; scRNA-seq,SRP330776,CD8+ T cell_1-AD.vs.control; scRNA-seq,SRP330776,Memory T cell_2-AD.vs.control; scRNA-seq,SRP330776,Naive CD8+ T cell_1-AD.vs.control	5
AC008074.2	scRNA-seq,SRP215507,CD8+ T cell_2-AD.vs.MCI; scRNA-seq,SRP215507,CD8+ T cell_2-MCI.vs.control; scRNA-seq,SRP330776,CD8+ T cell_2-AD.vs.control; scRNA-seq,SRP330776,B cell_1-AD.vs.control; scRNA-seq,SRP330776,Memory T cell_1-AD.vs.control	5
AC008083.3	scRNA-seq,SRP215507,CD8+ T cell_1-AD.vs.control; scRNA-seq,SRP215507,CD8+ T cell_1-AD.vs.MCI; scRNA-seq,SRP215507,CD8+ T cell_5-AD.vs.MCI; scRNA-seq,SRP215507,CD8+ T cell_5-MCI.vs.control	4
AC008105.1	scRNA-seq,SRP330776,CD8+ T cell_1-AD.vs.control; scRNA-seq,SRP330776,γδ T cell-AD.vs.control; scRNA-seq,SRP330776,Memory T cell_2-AD.vs.control; scRNA-seq,SRP330776,CD8+ T cell_2-AD.vs.control; scRNA-seq,SRP330776,B cell_1-AD.vs.control; scRNA-seq,SRP330776,Naive CD8+ T cell_1-AD.vs.control; scRNA-seq,SRP330776,Natural killer cell_1-AD.vs.control; scRNA-seq,SRP330776,Memory T cell_1-AD.vs.control;	9
AC008105.3	scRNA-seq,SRP215507,CD8+ T cell_5-AD.vs.control; scRNA-seq,SRP215507,CD8+ T cell_1-AD.vs.MCI; scRNA-seq,SRP309935,Naive CD8+ T cell_1-AD.vs.control; scRNA-seq,SRP309935,Monocyte_2-AD.vs.control; scRNA-seq,SRP309935,Naive CD8+ T cell_2-AD.vs.control; scRNA-seq,SRP309935,CD8+ T cell_1-AD.vs.control; scRNA-seq,SRP309935,CD8+ T cell_2-AD.vs.control; scRNA-seq,SRP330776,CD8+ T cell_1-AD.vs.control; scRNA-seq,SRP330776,γδ T cell-AD.vs.control; scRNA-seq,SRP330776,Memory T cell_2-AD.vs.control; scRNA-seq,SRP330776,CD8+ T cell_2-AD.vs.control; scRNA-seq,SRP330776,B cell_1-AD.vs.control; scRNA-seq,SRP330776,Naive CD8+ T cell_1-AD.vs.control; scRNA-seq,SRP330776,Natural killer cell_1-AD.vs.control; scRNA-seq,SRP330776,Memory T cell_1-AD.vs.control; scRNA-seq,SRP330776,Natural killer T cell-AD.vs.control; scRNA-seq,SRP330776,Naive CD8+ T cell_3-AD.vs.control; scRNA-seq,SRP330776,T cell-AD.vs.control; scRNA-seq,SRP330776,Naive CD8+ T cell_2-AD.vs.control; scRNA-seq,SRP330776,CD8+ T cell_3-AD.vs.control; scRNA-seq,SRP330776,Monocyte_1-AD.vs.control; scRNA- [illegible]	24
AC008115.3	scRNA-seq,SRP215507,CD8+ T cell_3-AD.vs.control; scRNA-seq,SRP215507,CD8+ T cell_1-AD.vs.control; scRNA-seq,SRP309935,Naive CD8+ T	3
AC008115.4	scRNA-seq,SRP215507,CD8+ T cell_2-MCI.vs.control	1
AC008119.1	scRNA-seq,SRP330776,CD8+ T cell_2-AD.vs.control	1
AC008124.1	scRNA-seq,SRP215507,CD8+ T cell_3-AD.vs.MCI; scRNA-seq,SRP215507,CD8+ T cell_3-MCI.vs.control; scRNA-seq,SRP330776,Natural killer cell_1-AD.vs.control; scRNA-seq,SRP330776,Natural killer T cell-AD.vs.control; scRNA-seq,SRP330776,Naive CD8+ T cell_3-AD.vs.control; scRNA-seq,SRP330776,CD8+ T cell_3-AD.vs.control; scRNA-seq,SRP330776,Monocyte_2-AD.vs.control; scRNA-seq,SRP330776,B cell_4-AD.vs.control	8
AC008149.2	scRNA-seq,SRP309935,Monocyte_1-AD.vs.control	1

AC008264.2	scRNA-seq,SRP330776,Naive CD8+ T cell_1-AD.vs.control	1
AC008267.5	scRNA-seq,SRP215507,CD8+ T cell_8-AD.vs.control; scRNA-seq,SRP330776,γδ T cell-AD.vs.control; scRNA-seq,SRP330776,Memory T cell_2-AD.vs.control; scRNA-seq,SRP330776,CD8+ T cell_2-AD.vs.control; scRNA-seq,SRP330776,B cell_1-AD.vs.control; scRNA-seq,SRP330776,Natural killer cell_1-AD.vs.control; scRNA-seq,SRP330776,Naive CD8+ T cell_3-AD.vs.control; scRNA-seq,SRP330776,B cell_2-AD.vs.control	8
AC008269.1	scRNA-seq,SRP215507,CD8+ T cell_4-AD.vs.control; scRNA-seq,SRP215507,CD8+ T cell_2-AD.vs.MCI; scRNA-seq,SRP215507,CD8+ T cell_2-	3
AC008378.1	scRNA-seq,SRP215507,CD8+ T cell_1-MCI.vs.control	1
AC008393.1	scRNA-seq,SRP215507,CD8+ T cell_1-AD.vs.control; scRNA-seq,SRP215507,CD8+ T cell_4-AD.vs.control; scRNA-seq,SRP215507,CD8+ T cell_1-AD.vs.MCI; scRNA-seq,SRP215507,CD8+ T cell_4-AD.vs.MCI; scRNA-seq,SRP215507,CD8+ T cell_1-MCI.vs.control; scRNA-seq,SRP330776,γδ T cell-AD.vs.control; scRNA-seq,SRP330776,Naive CD8+ T cell_1-AD.vs.control; scRNA-seq,SRP330776,B cell_2-AD.vs.control; scRNA-	9
AC008397.1	scRNA-seq,SRP215507,CD8+ T cell_2-AD.vs.control	1
AC008403.3	scRNA-seq,SRP215507,CD8+ T cell_1-AD.vs.control	1
AC008438.1	scRNA-seq,SRP215507,CD8+ T cell_1-MCI.vs.control; scRNA-seq,SRP330776,CD8+ T cell_1-AD.vs.control; scRNA-seq,SRP330776,Memory T cell_2-AD.vs.control; scRNA-seq,SRP330776,B cell_1-AD.vs.control; scRNA-seq,SRP330776,Naive CD8+ T cell_1-AD.vs.control; scRNA-seq,SRP330776,Natural killer cell_1-AD.vs.control; scRNA-seq,SRP330776,Natural killer T cell-AD.vs.control; scRNA-seq,SRP330776,B cell_2-AD.vs.control; scRNA-seq,SRP330776,CD8+ T cell_3-AD.vs.control; scRNA-seq,SRP330776,B cell_4-AD.vs.control	10
AC008440.1	scRNA-seq,SRP215507,CD8+ T cell_1-AD.vs.MCI; scRNA-seq,SRP215507,CD8+ T cell_5-AD.vs.MCI; scRNA-seq,SRP215507,CD8+ T cell_1-MCI.vs.control; scRNA-seq,SRP215507,CD8+ T cell_5-MCI.vs.control; scRNA-seq,SRP330776,Memory T cell_1-AD.vs.control	5
AC008440.2	scRNA-seq,SRP330776,CD8+ T cell_1-AD.vs.control; scRNA-seq,SRP330776,Natural killer cell_1-AD.vs.control	2
AC008443.1	scRNA-seq,SRP215507,CD8+ T cell_6-AD.vs.control; scRNA-seq,SRP215507,CD8+ T cell_5-AD.vs.MCI; scRNA-seq,SRP215507,CD8+ T cell_6-AD.vs.MCI; scRNA-seq,SRP215507,CD8+ T cell_5-MCI.vs.control; scRNA-seq,SRP215507,CD8+ T cell_2-MCI.vs.control; scRNA-seq,SRP330776,CD8+ T cell_1-AD.vs.control; scRNA-seq,SRP330776,Memory T cell_2-AD.vs.control; scRNA-seq,SRP330776,CD8+ T cell_2-AD.vs.control; scRNA-seq,SRP330776,B cell_1-AD.vs.control; scRNA-seq,SRP330776,Naive CD8+ T cell_1-AD.vs.control; scRNA-seq,SRP330776,Memory T cell_1-AD.vs.control; scRNA-seq,SRP330776,B cell_2-AD.vs.control; scRNA-seq,SRP330776,T cell-AD.vs.control; scRNA-seq,SRP330776,CD8+ T cell_3-AD.vs.control; scRNA-seq,SRP330776,B cell_3-AD.vs.control; scRNA-seq,SRP330776,Natural killer cell_2-AD.vs.control	16
AC008443.5	scRNA-seq,SRP215507,CD8+ T cell_3-AD.vs.MCI; scRNA-seq,SRP215507,CD8+ T cell_1-MCI.vs.control; scRNA-seq,SRP330776,CD8+ T cell_1-AD.vs.control; scRNA-seq,SRP330776,Memory T cell_2-AD.vs.control; scRNA-seq,SRP330776,B cell_1-AD.vs.control; scRNA-seq,SRP330776,Memory T cell_1-AD.vs.control; scRNA-seq,SRP330776,B cell_2-AD.vs.control	7
AC008443.9	scRNA-seq,SRP330776,CD8+ T cell_1-AD.vs.control; scRNA-seq,SRP330776,B cell_1-AD.vs.control; scRNA-seq,SRP330776,CD8+ T cell_3-	3
AC008462.1	scRNA-seq,SRP215507,CD8+ T cell_1-MCI.vs.control	1
AC008467.1	scRNA-seq,SRP215507,CD8+ T cell_2-AD.vs.control; scRNA-seq,SRP215507,CD8+ T cell_3-AD.vs.control; scRNA-seq,SRP215507,CD8+ T cell_4-MCI.vs.control; scRNA-seq,SRP330776,B cell_1-AD.vs.control; scRNA-seq,SRP330776,Naive CD8+ T cell_1-AD.vs.control	5
AC008522.1	scRNA-seq,SRP330776,CD8+ T cell_1-AD.vs.control; scRNA-seq,SRP330776,Memory T cell_2-AD.vs.control; scRNA-seq,SRP330776,B cell_1-AD.vs.control; scRNA-seq,SRP330776,Naive CD8+ T cell_1-AD.vs.control; scRNA-seq,SRP330776,T cell-AD.vs.control	5
AC008537.4	scRNA-seq,SRP330776,CD8+ T cell_2-AD.vs.control; scRNA-seq,SRP330776,Natural killer T cell-AD.vs.control; scRNA-seq,SRP330776,Naive CD8+ T cell_3-AD.vs.control	3
AC008549.2	scRNA-seq,SRP215507,CD8+ T cell_5-MCI.vs.control; scRNA-seq,SRP309935,Naive CD8+ T cell_2-AD.vs.control; scRNA-seq,SRP309935,CD8+ T	3
AC008555.1	scRNA-seq,SRP215507,CD8+ T cell_1-AD.vs.MCI	1
AC008555.4	scRNA-seq,SRP215507,CD8+ T cell_7-AD.vs.control; scRNA-seq,SRP215507,CD8+ T cell_4-AD.vs.MCI; scRNA-seq,SRP215507,CD8+ T cell_4-	3
AC008569.2	scRNA-seq,SRP215507,CD8+ T cell_5-MCI.vs.control; scRNA-seq,SRP309935,Monocyte_2-AD.vs.control; scRNA-seq,SRP330776,CD8+ T cell_2-	3
AC008608.2	scRNA-seq,SRP215507,CD8+ T cell_2-AD.vs.control; scRNA-seq,SRP215507,CD8+ T cell_3-AD.vs.control; scRNA-seq,SRP330776,Memory T cell_2-AD.vs.control; scRNA-seq,SRP330776,CD8+ T cell_2-AD.vs.control; scRNA-seq,SRP330776,Naive CD8+ T cell_1-AD.vs.control; scRNA-	6

AC008610.1	scRNA-seq,SRP330776,Naive CD8+ T cell_1-AD.vs.control	1
AC008622.2	scRNA-seq,SRP215507,CD8+ T cell_5-MCI.vs.control; scRNA-seq,SRP330776,CD8+ T cell_2-AD.vs.control	2
AC008629.1	scRNA-seq,SRP215507,CD8+ T cell_3-AD.vs.control; scRNA-seq,SRP215507,CD8+ T cell_1-AD.vs.control	2
AC008655.2	scRNA-seq,SRP215507,CD8+ T cell_2-AD.vs.MCI; scRNA-seq,SRP330776,CD8+ T cell_1-AD.vs.control; scRNA-seq,SRP330776,Naive CD8+ T cell_1-AD.vs.control; scRNA-seq,SRP330776,Memory T cell_1-AD.vs.control	4
AC008686.1	scRNA-seq,SRP330776,CD8+ T cell_1-AD.vs.control	1
AC008687.4	scRNA-seq,SRP215507,CD8+ T cell_2-AD.vs.control; scRNA-seq,SRP215507,CD8+ T cell_1-AD.vs.control	2
AC008691.1	scRNA-seq,SRP215507,CD8+ T cell_5-MCI.vs.control; scRNA-seq,SRP330776,CD8+ T cell_1-AD.vs.control; scRNA-seq,SRP330776,B cell_1-	3
AC008735.1	scRNA-seq,SRP215507,CD8+ T cell_4-MCI.vs.control; scRNA-seq,SRP330776,Memory T cell_1-AD.vs.control	2
AC008735.2	scRNA-seq,SRP215507,CD8+ T cell_4-AD.vs.MCI; scRNA-seq,SRP215507,CD8+ T cell_4-MCI.vs.control; scRNA-seq,SRP330776,Naive CD8+ T	3
AC008736.1	scRNA-seq,SRP309935,Naive CD8+ T cell_1-AD.vs.control	1
AC008750.1	scRNA-seq,SRP330776,B cell_1-AD.vs.control; scRNA-seq,SRP330776,Memory T cell_1-AD.vs.control	2
AC008750.2	scRNA-seq,SRP215507,CD8+ T cell_1-AD.vs.MCI; scRNA-seq,SRP215507,CD8+ T cell_4-MCI.vs.control; scRNA-seq,SRP215507,CD8+ T cell_2-MCI.vs.control; scRNA-seq,SRP215507,CD8+ T cell_6-MCI.vs.control; scRNA-seq,SRP330776,B cell_1-AD.vs.control; scRNA-seq,SRP330776,Memory	6
AC008750.7	scRNA-seq,SRP215507,CD8+ T cell_1-MCI.vs.control	1
AC008752.2	scRNA-seq,SRP215507,CD8+ T cell_3-MCI.vs.control; scRNA-seq,SRP330776,CD8+ T cell_2-AD.vs.control	2
AC008760.1	scRNA-seq,SRP215507,CD8+ T cell_3-MCI.vs.control	1
AC008763.1	scRNA-seq,SRP215507,CD8+ T cell_4-AD.vs.MCI; scRNA-seq,SRP330776,CD8+ T cell_1-AD.vs.control; scRNA-seq,SRP330776,CD8+ T cell_3-	3
AC008764.6	scRNA-seq,SRP215507,CD8+ T cell_2-AD.vs.MCI; scRNA-seq,SRP215507,CD8+ T cell_4-MCI.vs.control; scRNA-seq,SRP215507,CD8+ T cell_2-MCI.vs.control; scRNA-seq,SRP215507,CD8+ T cell_3-MCI.vs.control; scRNA-seq,SRP330776,Natural killer cell_1-AD.vs.control; scRNA-	6
AC008764.7	scRNA-seq,SRP215507,CD8+ T cell_6-AD.vs.control; scRNA-seq,SRP215507,CD8+ T cell_2-AD.vs.MCI; scRNA-seq,SRP215507,CD8+ T cell_2-MCI.vs.control; scRNA-seq,SRP215507,CD8+ T cell_6-MCI.vs.control; scRNA-seq,SRP330776,γδ T cell-AD.vs.control; scRNA-seq,SRP330776,CD8+ T cell_2-AD.vs.control; scRNA-seq,SRP330776,T cell-AD.vs.control	7
AC008764.8	scRNA-seq,SRP215507,CD8+ T cell_4-AD.vs.control; scRNA-seq,SRP215507,CD8+ T cell_4-AD.vs.MCI; scRNA-seq,SRP215507,CD8+ T cell_5-MCI.vs.control; scRNA-seq,SRP215507,CD8+ T cell_4-MCI.vs.control; scRNA-seq,SRP215507,CD8+ T cell_3-MCI.vs.control; scRNA-seq,SRP330776,Natural killer cell_1-AD.vs.control; scRNA-seq,SRP330776,Natural killer T cell-AD.vs.control; scRNA-seq,SRP330776,B cell_2-	8
AC008771.1	scRNA-seq,SRP330776,CD8+ T cell_1-AD.vs.control; scRNA-seq,SRP330776,Naive CD8+ T cell_1-AD.vs.control; scRNA-seq,SRP330776,Natural killer cell_1-AD.vs.control	3
AC008840.1	scRNA-seq,SRP330776,Memory T cell_1-AD.vs.control	1
AC008875.1	scRNA-seq,SRP215507,CD8+ T cell_1-MCI.vs.control	1
AC008875.2	scRNA-seq,SRP215507,CD8+ T cell_2-AD.vs.control; scRNA-seq,SRP215507,CD8+ T cell_2-AD.vs.MCI; scRNA-seq,SRP215507,CD8+ T cell_6-MCI.vs.control; scRNA-seq,SRP309935,CD8+ T cell_2-AD.vs.control; scRNA-seq,SRP330776,CD8+ T cell_1-AD.vs.control; scRNA-seq,SRP330776,CD8+ T cell_2-AD.vs.control; scRNA-seq,SRP330776,Naive CD8+ T cell_1-AD.vs.control; scRNA-seq,SRP330776,Memory T cell_1-	9
AC008875.3	scRNA-seq,SRP215507,CD8+ T cell_5-AD.vs.MCI; scRNA-seq,SRP215507,CD8+ T cell_5-MCI.vs.control; scRNA-seq,SRP215507,CD8+ T cell_6-MCI.vs.control; scRNA-seq,SRP330776,CD8+ T cell_2-AD.vs.control; scRNA-seq,SRP330776,B cell_1-AD.vs.control; scRNA-seq,SRP330776,Natural killer cell_1-AD.vs.control; scRNA-seq,SRP330776,T cell-AD.vs.control; scRNA-seq,SRP330776,CD8+ T cell_3-AD.vs.control; scRNA-	9
AC008892.1	scRNA-seq,SRP330776,Naive CD8+ T cell_1-AD.vs.control; scRNA-seq,SRP330776,T cell-AD.vs.control	2
AC008894.2	scRNA-seq,SRP215507,CD8+ T cell_1-AD.vs.control; scRNA-seq,SRP215507,CD8+ T cell_5-AD.vs.control; scRNA-seq,SRP215507,CD8+ T cell_1-	3
AC008906.1	scRNA-seq,SRP309935,CD8+ T cell_1-AD.vs.control; scRNA-seq,SRP330776,Naive CD8+ T cell_2-AD.vs.control	2
AC008906.2	scRNA-seq,SRP215507,CD8+ T cell_1-AD.vs.control; scRNA-seq,SRP330776,Memory T cell_2-AD.vs.control	2

AC008914.1	scRNA-seq,SRP215507,CD8+ T cell_2-AD.vs.control; scRNA-seq,SRP330776,Naive CD8+ T cell_1-AD.vs.control	2
AC008915.1	scRNA-seq,SRP215507,CD8+ T cell_1-AD.vs.control	1
AC008915.2	scRNA-seq,SRP330776,CD8+ T cell_1-AD.vs.control; scRNA-seq,SRP330776,γδ T cell-AD.vs.control	2
AC008937.2	scRNA-seq,SRP330776,CD8+ T cell_1-AD.vs.control; scRNA-seq,SRP330776,B cell_1-AD.vs.control; scRNA-seq,SRP330776,B cell_2-AD.vs.control	3
AC008957.1	scRNA-seq,SRP215507,CD8+ T cell_3-AD.vs.control; scRNA-seq,SRP215507,CD8+ T cell_1-AD.vs.control; scRNA-seq,SRP215507,CD8+ T cell_2-AD.vs.MCI; scRNA-seq,SRP215507,CD8+ T cell_1-AD.vs.MCI; scRNA-seq,SRP215507,CD8+ T cell_4-MCI.vs.control; scRNA-seq,SRP215507,CD8+ T cell_2-MCI.vs.control; scRNA-seq,SRP215507,CD8+ T cell_3-MCI.vs.control; scRNA-seq,SRP330776,B cell_1-AD.vs.control; scRNA-	9
AC008964.1	scRNA-seq,SRP215507,CD8+ T cell_3-AD.vs.control	1
AC008966.1	scRNA-seq,SRP215507,CD8+ T cell_1-AD.vs.MCI; scRNA-seq,SRP330776,Memory T cell_2-AD.vs.control	2
AC008969.1	scRNA-seq,SRP215507,CD8+ T cell_2-AD.vs.control; scRNA-seq,SRP215507,CD8+ T cell_3-AD.vs.control; scRNA-seq,SRP215507,CD8+ T cell_1-AD.vs.control; scRNA-seq,SRP215507,CD8+ T cell_1-MCI.vs.control; scRNA-seq,SRP330776,B cell_1-AD.vs.control	5
AC008982.2	scRNA-seq,SRP215507,CD8+ T cell_2-AD.vs.MCI; scRNA-seq,SRP215507,CD8+ T cell_2-MCI.vs.control; scRNA-seq,SRP309935,Megakaryocyte_1-AD.vs.control; scRNA-seq,SRP309935,CD8+ T cell_2-AD.vs.control; scRNA-seq,SRP330776,CD8+ T cell_1-AD.vs.control; scRNA-seq,SRP330776,Memory T cell_2-AD.vs.control; scRNA-seq,SRP330776,B cell_1-AD.vs.control; scRNA-seq,SRP330776,Naive CD8+ T cell_1-AD.vs.control; scRNA-seq,SRP330776,B cell_2-AD.vs.control; scRNA-seq,SRP330776,Naive CD8+ T cell_2-AD.vs.control	10
AC009005.1	scRNA-seq,SRP215507,CD8+ T cell_1-AD.vs.control; scRNA-seq,SRP215507,CD8+ T cell_5-AD.vs.control; scRNA-seq,SRP215507,CD8+ T cell_5-AD.vs.MCI; scRNA-seq,SRP330776,CD8+ T cell_1-AD.vs.control; scRNA-seq,SRP330776,γδ T cell-AD.vs.control; scRNA-seq,SRP330776,Naive CD8+	6
AC009021.1	scRNA-seq,SRP215507,CD8+ T cell_2-MCI.vs.control	1
AC009032.1	scRNA-seq,SRP330776,Memory T cell_1-AD.vs.control	1
AC009041.1	scRNA-seq,SRP215507,CD8+ T cell_1-AD.vs.MCI; scRNA-seq,SRP215507,CD8+ T cell_3-MCI.vs.control; scRNA-seq,SRP330776,CD8+ T cell_1-AD.vs.control; scRNA-seq,SRP330776,Memory T cell_2-AD.vs.control; scRNA-seq,SRP330776,CD8+ T cell_2-AD.vs.control; scRNA-seq,SRP330776,Natural killer T cell-AD.vs.control; scRNA-seq,SRP330776,Naive CD8+ T cell_3-AD.vs.control; scRNA-seq,SRP330776,T cell-	9
AC009041.2	scRNA-seq,SRP215507,CD8+ T cell_2-AD.vs.control; scRNA-seq,SRP215507,CD8+ T cell_4-AD.vs.control; scRNA-seq,SRP215507,CD8+ T cell_3-AD.vs.MCI; scRNA-seq,SRP215507,CD8+ T cell_4-AD.vs.MCI; scRNA-seq,SRP215507,CD8+ T cell_3-MCI.vs.control	5
AC009053.2	scRNA-seq,SRP215507,CD8+ T cell_8-AD.vs.control; scRNA-seq,SRP215507,CD8+ T cell_3-AD.vs.MCI; scRNA-seq,SRP215507,CD8+ T cell_1-AD.vs.MCI; scRNA-seq,SRP215507,CD8+ T cell_5-AD.vs.MCI; scRNA-seq,SRP215507,CD8+ T cell_1-MCI.vs.control; scRNA-seq,SRP215507,CD8+ T cell_5-MCI.vs.control; scRNA-seq,SRP215507,CD8+ T cell_3-MCI.vs.control; scRNA-seq,SRP215507,CD8+ T cell_6-MCI.vs.control; scRNA-seq,SRP309935,Naive CD8+ T cell_1-AD.vs.control; scRNA-seq,SRP330776,Memory T cell_2-AD.vs.control; scRNA-seq,SRP330776,CD8+ T cell_2-AD.vs.control; scRNA-seq,SRP330776,Naive CD8+ T cell_1-AD.vs.control; scRNA-seq,SRP330776,Naive CD8+ T cell_3-AD.vs.control; scRNA-seq,SRP330776,B cell_2-AD.vs.control; scRNA-seq,SRP330776,T cell-AD.vs.control; scRNA-seq,SRP330776,CD8+ T cell_3-AD.vs.control	16
AC009060.1	scRNA-seq,SRP309935,Monocyte_1-AD.vs.control; scRNA-seq,SRP309935,Natural killer cell_1-AD.vs.control; scRNA-seq,SRP309935,Monocyte_2-AD.vs.control; scRNA-seq,SRP309935,Naive CD8+ T cell_2-AD.vs.control; scRNA-seq,SRP309935,CD8+ T cell_1-AD.vs.control; scRNA-seq,SRP309935,CD8+ T cell_2-AD.vs.control; scRNA-seq,SRP330776,Memory T cell_1-AD.vs.control	7
AC009061.2	scRNA-seq,SRP215507,CD8+ T cell_5-AD.vs.MCI; scRNA-seq,SRP215507,CD8+ T cell_4-MCI.vs.control; scRNA-seq,SRP309935,Naive CD8+ T cell_1-AD.vs.control; scRNA-seq,SRP330776,CD8+ T cell_1-AD.vs.control; scRNA-seq,SRP330776,γδ T cell-AD.vs.control; scRNA-seq,SRP330776,B cell_1-AD.vs.control; scRNA-seq,SRP330776,Natural killer cell_1-AD.vs.control; scRNA-seq,SRP330776,Memory T cell_1-AD.vs.control; scRNA-seq,SRP330776,Natural killer T cell-AD.vs.control; scRNA-seq,SRP330776,Naive CD8+ T cell_3-AD.vs.control; scRNA-seq,SRP330776,B cell_2-AD.vs.control; scRNA-seq,SRP330776,B cell_3-AD.vs.control; scRNA-seq,SRP330776,B cell_4-AD.vs.control	13
AC009063.2	scRNA-seq,SRP309935,Monocyte_1-AD.vs.control	1
AC009065.4	scRNA-seq,SRP215507,CD8+ T cell_3-AD.vs.control; scRNA-seq,SRP215507,CD8+ T cell_1-AD.vs.control; scRNA-seq,SRP215507,CD8+ T cell_3-AD.vs.MCI; scRNA-seq,SRP215507,CD8+ T cell_4-AD.vs.MCI; scRNA-seq,SRP215507,CD8+ T cell_4-MCI.vs.control; scRNA-seq,SRP330776,CD8+ T cell_1-AD.vs.control; scRNA-seq,SRP330776,CD8+ T cell_2-AD.vs.control; scRNA-seq,SRP330776,Naive CD8+ T cell_1-AD.vs.control; scRNA-	9

AC009088.1	scRNA-seq,SRP309935,Natural killer cell_2-AD.vs.control	1
AC009090.1	scRNA-seq,SRP330776,γδ T cell-AD.vs.control; scRNA-seq,SRP330776,Naive CD8+ T cell_1-AD.vs.control; scRNA-seq,SRP330776,Memory T cell_1-	3
AC009090.6	scRNA-seq,SRP330776,γδ T cell-AD.vs.control; scRNA-seq,SRP330776,Memory T cell_2-AD.vs.control; scRNA-seq,SRP330776,Memory T cell_1-	3
AC009093.1	scRNA-seq,SRP330776,CD8+ T cell_1-AD.vs.control; scRNA-seq,SRP330776,B cell_1-AD.vs.control; scRNA-seq,SRP330776,Naive CD8+ T cell_1-	3
AC009093.2	scRNA-seq,SRP215507,CD8+ T cell_4-AD.vs.control; scRNA-seq,SRP215507,CD8+ T cell_2-AD.vs.MCI; scRNA-seq,SRP215507,CD8+ T cell_1-AD.vs.MCI; scRNA-seq,SRP215507,CD8+ T cell_5-MCI.vs.control; scRNA-seq,SRP215507,CD8+ T cell_2-MCI.vs.control; scRNA-seq,SRP309935,Natural killer cell_2-AD.vs.control; scRNA-seq,SRP309935,CD8+ T cell_1-AD.vs.control; scRNA-seq,SRP330776,Naive CD8+ T cell_1-	9
AC009107.2	scRNA-seq,SRP215507,CD8+ T cell_1-AD.vs.MCI; scRNA-seq,SRP215507,CD8+ T cell_1-MCI.vs.control; scRNA-seq,SRP215507,CD8+ T cell_6-MCI.vs.control; scRNA-seq,SRP330776,CD8+ T cell_1-AD.vs.control; scRNA-seq,SRP330776,γδ T cell-AD.vs.control; scRNA-seq,SRP330776,Memory T cell_2-AD.vs.control; scRNA-seq,SRP330776,CD8+ T cell_2-AD.vs.control; scRNA-seq,SRP330776,B cell_1-AD.vs.control; scRNA-seq,SRP330776,Naive CD8+ T cell_1-AD.vs.control; scRNA-seq,SRP330776,Memory T cell_1-AD.vs.control; scRNA-seq,SRP330776,Natural killer T cell-AD.vs.control; scRNA-seq,SRP330776,B cell_2-AD.vs.control; scRNA-seq,SRP330776,CD8+ T cell_3-AD.vs.control	13
AC009113.1	scRNA-seq,SRP215507,CD8+ T cell_2-AD.vs.MCI; scRNA-seq,SRP215507,CD8+ T cell_1-MCI.vs.control; scRNA-seq,SRP215507,CD8+ T cell_2-MCI.vs.control; scRNA-seq,SRP330776,CD8+ T cell_2-AD.vs.control; scRNA-seq,SRP330776,Natural killer cell_1-AD.vs.control	5
AC009118.2	scRNA-seq,SRP215507,CD8+ T cell_1-AD.vs.MCI; scRNA-seq,SRP215507,CD8+ T cell_4-AD.vs.MCI; scRNA-seq,SRP215507,CD8+ T cell_6-MCI.vs.control; scRNA-seq,SRP330776,Memory T cell_2-AD.vs.control; scRNA-seq,SRP330776,B cell_2-AD.vs.control	5
AC009118.3	scRNA-seq,SRP215507,CD8+ T cell_2-AD.vs.control; scRNA-seq,SRP215507,CD8+ T cell_3-AD.vs.MCI; scRNA-seq,SRP215507,CD8+ T cell_3-MCI.vs.control; scRNA-seq,SRP330776,CD8+ T cell_1-AD.vs.control; scRNA-seq,SRP330776,CD8+ T cell_2-AD.vs.control; scRNA-seq,SRP330776,B cell_1-AD.vs.control; scRNA-seq,SRP330776,Naive CD8+ T cell_1-AD.vs.control; scRNA-seq,SRP330776,Memory T cell_1-AD.vs.control	8
AC009119.1	scRNA-seq,SRP330776,B cell_1-AD.vs.control	1
AC009120.2	scRNA-seq,SRP215507,CD8+ T cell_2-AD.vs.MCI; scRNA-seq,SRP330776,CD8+ T cell_2-AD.vs.control; scRNA-seq,SRP330776,Naive CD8+ T cell_3-	3
AC009123.1	scRNA-seq,SRP215507,CD8+ T cell_3-AD.vs.control; scRNA-seq,SRP215507,CD8+ T cell_3-AD.vs.MCI; scRNA-seq,SRP215507,CD8+ T cell_3-	3
AC009126.1	scRNA-seq,SRP215507,CD8+ T cell_5-AD.vs.control; scRNA-seq,SRP215507,CD8+ T cell_2-AD.vs.MCI; scRNA-seq,SRP215507,CD8+ T cell_5-AD.vs.MCI; scRNA-seq,SRP215507,CD8+ T cell_2-MCI.vs.control; scRNA-seq,SRP330776,B cell_1-AD.vs.control; scRNA-seq,SRP330776,Natural killer cell_1-AD.vs.control; scRNA-seq,SRP330776,Natural killer T cell-AD.vs.control	7
AC009133.1	scRNA-seq,SRP215507,CD8+ T cell_3-MCI.vs.control; scRNA-seq,SRP330776,CD8+ T cell_2-AD.vs.control; scRNA-seq,SRP330776,Natural killer cell_1-AD.vs.control; scRNA-seq,SRP330776,Memory T cell_1-AD.vs.control	4
AC009133.3	scRNA-seq,SRP215507,CD8+ T cell_5-AD.vs.MCI; scRNA-seq,SRP215507,CD8+ T cell_4-MCI.vs.control; scRNA-seq,SRP330776,Memory T cell_2-AD.vs.control; scRNA-seq,SRP330776,B cell_1-AD.vs.control; scRNA-seq,SRP330776,CD8+ T cell_3-AD.vs.control	5
AC009133.4	scRNA-seq,SRP330776,Naive CD8+ T cell_1-AD.vs.control; scRNA-seq,SRP330776,Memory T cell_1-AD.vs.control; scRNA-seq,SRP330776,CD8+ T	3
AC009137.1	scRNA-seq,SRP330776,CD8+ T cell_2-AD.vs.control	1
AC009137.2	scRNA-seq,SRP215507,CD8+ T cell_1-MCI.vs.control	1
AC009152.3	scRNA-seq,SRP330776,Memory T cell_2-AD.vs.control	1
AC009159.3	scRNA-seq,SRP215507,CD8+ T cell_1-MCI.vs.control	1
AC009163.7	scRNA-seq,SRP215507,CD8+ T cell_5-MCI.vs.control	1
AC009185.1	scRNA-seq,SRP215507,CD8+ T cell_3-AD.vs.MCI; scRNA-seq,SRP215507,CD8+ T cell_3-MCI.vs.control	2
AC009226.1	scRNA-seq,SRP215507,CD8+ T cell_3-AD.vs.MCI; scRNA-seq,SRP215507,CD8+ T cell_3-MCI.vs.control	2
AC009228.1	scRNA-seq,SRP215507,CD8+ T cell_1-AD.vs.control; scRNA-seq,SRP215507,CD8+ T cell_1-AD.vs.MCI; scRNA-seq,SRP330776,Memory T cell_1-	3
AC009237.14	scRNA-seq,SRP215507,CD8+ T cell_1-AD.vs.MCI; scRNA-seq,SRP215507,CD8+ T cell_1-MCI.vs.control; scRNA-seq,SRP330776,Naive CD8+ T	3
AC009275.1	scRNA-seq,SRP215507,CD8+ T cell_5-AD.vs.MCI	1

AC009283.1	scRNA-seq,SRP215507,CD8+ T cell_3-AD.vs.control; scRNA-seq,SRP215507,CD8+ T cell_5-AD.vs.MCI; scRNA-seq,SRP215507,CD8+ T cell_5-MCI.vs.control; scRNA-seq,SRP330776,CD8+ T cell_1-AD.vs.control	4
AC009299.3	scRNA-seq,SRP215507,CD8+ T cell_3-AD.vs.control	1
AC009303.2	scRNA-seq,SRP330776,CD8+ T cell_1-AD.vs.control	1
AC009309.1	scRNA-seq,SRP215507,CD8+ T cell_3-AD.vs.control; scRNA-seq,SRP215507,CD8+ T cell_1-AD.vs.control; scRNA-seq,SRP215507,CD8+ T cell_1-AD.vs.MCI; scRNA-seq,SRP215507,CD8+ T cell_2-MCI.vs.control; scRNA-seq,SRP215507,CD8+ T cell_3-MCI.vs.control; scRNA-seq,SRP215507,CD8+ T cell_6-MCI.vs.control; scRNA-seq,SRP309935,CD8+ T cell_1-AD.vs.control; scRNA-seq,SRP330776,CD8+ T cell_1-AD.vs.control; scRNA-seq,SRP330776,Memory T cell_2-AD.vs.control; scRNA-seq,SRP330776,Naive CD8+ T cell_1-AD.vs.control; scRNA-	12
AC009312.1	scRNA-seq,SRP215507,CD8+ T cell_2-AD.vs.control	1
AC009315.1	scRNA-seq,SRP215507,CD8+ T cell_3-AD.vs.MCI; scRNA-seq,SRP215507,CD8+ T cell_3-MCI.vs.control; scRNA-seq,SRP330776,B cell_1-AD.vs.control; scRNA-seq,SRP330776,Naive CD8+ T cell_1-AD.vs.control	4
AC009318.1	scRNA-seq,SRP215507,CD8+ T cell_3-AD.vs.MCI; scRNA-seq,SRP215507,CD8+ T cell_3-MCI.vs.control; scRNA-seq,SRP330776,CD8+ T cell_1-AD.vs.control; scRNA-seq,SRP330776,Memory T cell_2-AD.vs.control; scRNA-seq,SRP330776,Naive CD8+ T cell_1-AD.vs.control	5
AC009318.2	scRNA-seq,SRP215507,CD8+ T cell_3-MCI.vs.control	1
AC009403.1	scRNA-seq,SRP330776,γδ T cell-AD.vs.control; scRNA-seq,SRP330776,Memory T cell_2-AD.vs.control; scRNA-seq,SRP330776,CD8+ T cell_2-AD.vs.control; scRNA-seq,SRP330776,Naive CD8+ T cell_1-AD.vs.control	4
AC009403.2	scRNA-seq,SRP215507,CD8+ T cell_2-AD.vs.control; scRNA-seq,SRP215507,CD8+ T cell_5-AD.vs.control; scRNA-seq,SRP330776,B cell_1-AD.vs.control; scRNA-seq,SRP330776,B cell_4-AD.vs.control	4
AC009404.1	scRNA-seq,SRP215507,CD8+ T cell_4-MCI.vs.control; scRNA-seq,SRP215507,CD8+ T cell_3-MCI.vs.control; scRNA-seq,SRP330776,Memory T cell_2-AD.vs.control; scRNA-seq,SRP330776,Naive CD8+ T cell_1-AD.vs.control; scRNA-seq,SRP330776,Memory T cell_1-AD.vs.control; scRNA-	6
AC009486.2	scRNA-seq,SRP215507,CD8+ T cell_5-MCI.vs.control	1
AC009495.3	scRNA-seq,SRP215507,CD8+ T cell_1-AD.vs.control; scRNA-seq,SRP215507,CD8+ T cell_1-AD.vs.MCI; scRNA-seq,SRP215507,CD8+ T cell_5-AD.vs.MCI; scRNA-seq,SRP330776,B cell_2-AD.vs.control	4
AC009509.1	scRNA-seq,SRP309935,CD8+ T cell_1-AD.vs.control; scRNA-seq,SRP330776,CD8+ T cell_1-AD.vs.control; scRNA-seq,SRP330776,Memory T cell_2-AD.vs.control; scRNA-seq,SRP330776,CD8+ T cell_2-AD.vs.control; scRNA-seq,SRP330776,B cell_1-AD.vs.control; scRNA-seq,SRP330776,Naive CD8+ T cell_1-AD.vs.control; scRNA-seq,SRP330776,Natural killer T cell-AD.vs.control; scRNA-seq,SRP330776,B cell_2-AD.vs.control; scRNA-	9
AC009542.1	scRNA-seq,SRP330776,Naive CD8+ T cell_1-AD.vs.control	1
AC009549.1	scRNA-seq,SRP215507,CD8+ T cell_2-MCI.vs.control	1
AC009630.1	scRNA-seq,SRP215507,CD8+ T cell_2-AD.vs.control; scRNA-seq,SRP215507,CD8+ T cell_3-AD.vs.MCI; scRNA-seq,SRP215507,CD8+ T cell_3-MCI.vs.control; scRNA-seq,SRP330776,Naive CD8+ T cell_3-AD.vs.control	4
AC009630.3	scRNA-seq,SRP215507,CD8+ T cell_1-AD.vs.MCI; scRNA-seq,SRP330776,Memory T cell_2-AD.vs.control	2
AC009630.4	scRNA-seq,SRP330776,CD8+ T cell_1-AD.vs.control	1
AC009690.2	scRNA-seq,SRP215507,CD8+ T cell_1-AD.vs.MCI; scRNA-seq,SRP215507,CD8+ T cell_1-MCI.vs.control; scRNA-seq,SRP215507,CD8+ T cell_3-MCI.vs.control; scRNA-seq,SRP330776,CD8+ T cell_1-AD.vs.control; scRNA-seq,SRP330776,γδ T cell-AD.vs.control	5
AC009779.2	scRNA-seq,SRP215507,CD8+ T cell_3-AD.vs.control; scRNA-seq,SRP215507,CD8+ T cell_2-AD.vs.MCI; scRNA-seq,SRP215507,CD8+ T cell_5-AD.vs.MCI; scRNA-seq,SRP215507,CD8+ T cell_5-MCI.vs.control; scRNA-seq,SRP215507,CD8+ T cell_2-MCI.vs.control; scRNA-seq,SRP215507,CD8+ T cell_3-MCI.vs.control; scRNA-seq,SRP309935,CD4+ T cell-AD.vs.control; scRNA-seq,SRP330776,CD8+ T cell_1-AD.vs.control; scRNA-seq,SRP330776,Naive CD8+ T cell_1-AD.vs.control; scRNA-seq,SRP330776,Natural killer cell_1-AD.vs.control; scRNA-seq,SRP330776,B cell_2-AD.vs.control; scRNA-seq,SRP330776,T cell-AD.vs.control; scRNA-seq,SRP330776,Natural killer cell_2-AD.vs.control	13

AC009812.1	scRNA-seq,SRP215507,CD8+ T cell_3-AD.vs.MCI; scRNA-seq,SRP215507,CD8+ T cell_1-AD.vs.MCI; scRNA-seq,SRP215507,CD8+ T cell_5-AD.vs.MCI; scRNA-seq,SRP215507,CD8+ T cell_1-MCI.vs.control; scRNA-seq,SRP215507,CD8+ T cell_5-MCI.vs.control; scRNA-seq,SRP215507,CD8+ T cell_3-MCI.vs.control; scRNA-seq,SRP330776,CD8+ T cell_1-AD.vs.control; scRNA-seq,SRP330776,Memory T cell_2-AD.vs.control; scRNA-seq,SRP330776,CD8+ T cell_2-AD.vs.control; scRNA-seq,SRP330776,B cell_1-AD.vs.control; scRNA-seq,SRP330776,Memory T	12
AC009812.4	scRNA-seq,SRP215507,CD8+ T cell_3-AD.vs.control; scRNA-seq,SRP215507,CD8+ T cell_3-MCI.vs.control	2
AC009831.1	scRNA-seq,SRP215507,CD8+ T cell_4-AD.vs.control; scRNA-seq,SRP215507,CD8+ T cell_1-AD.vs.MCI; scRNA-seq,SRP215507,CD8+ T cell_5-AD.vs.MCI; scRNA-seq,SRP215507,CD8+ T cell_5-MCI.vs.control; scRNA-seq,SRP330776,Memory T cell_2-AD.vs.control; scRNA-seq,SRP330776,CD8+ T cell_2-AD.vs.control; scRNA-seq,SRP330776,Naive CD8+ T cell_3-AD.vs.control; scRNA-seq,SRP330776,CD8+ T cell_3-	8
AC009831.3	scRNA-seq,SRP330776,CD8+ T cell_1-AD.vs.control; scRNA-seq,SRP330776,Naive CD8+ T cell_1-AD.vs.control; scRNA-seq,SRP330776,Natural killer cell_1-AD.vs.control	3
AC009879.4	scRNA-seq,SRP215507,CD8+ T cell_2-AD.vs.MCI; scRNA-seq,SRP215507,CD8+ T cell_1-MCI.vs.control	2
AC009950.1	scRNA-seq,SRP215507,CD8+ T cell_4-AD.vs.control; scRNA-seq,SRP215507,CD8+ T cell_1-AD.vs.MCI; scRNA-seq,SRP215507,CD8+ T cell_1-MCI.vs.control; scRNA-seq,SRP215507,CD8+ T cell_2-MCI.vs.control; scRNA-seq,SRP330776,CD8+ T cell_1-AD.vs.control; scRNA-seq,SRP330776,γδ T cell-AD.vs.control; scRNA-seq,SRP330776,CD8+ T cell_2-AD.vs.control; scRNA-seq,SRP330776,B cell_1-AD.vs.control; scRNA-seq,SRP330776,Naive CD8+ T cell_1-AD.vs.control; scRNA-seq,SRP330776,Natural killer cell_1-AD.vs.control; scRNA-seq,SRP330776,Memory T	12
AC009951.6	scRNA-seq,SRP330776,Memory T cell_1-AD.vs.control; scRNA-seq,SRP330776,Monocyte_1-AD.vs.control	2
AC009961.1	scRNA-seq,SRP215507,CD8+ T cell_3-AD.vs.MCI; scRNA-seq,SRP215507,CD8+ T cell_1-AD.vs.MCI; scRNA-seq,SRP215507,CD8+ T cell_1-MCI.vs.control; scRNA-seq,SRP215507,CD8+ T cell_2-MCI.vs.control; scRNA-seq,SRP330776,Memory T cell_2-AD.vs.control; scRNA-seq,SRP330776,CD8+ T cell_2-AD.vs.control; scRNA-seq,SRP330776,B cell_1-AD.vs.control; scRNA-seq,SRP330776,Natural killer cell_1-	9
AC009996.1	scRNA-seq,SRP215507,CD8+ T cell_1-AD.vs.MCI; scRNA-seq,SRP330776,Naive CD8+ T cell_1-AD.vs.control	2
AC010148.1	scRNA-seq,SRP215507,CD8+ T cell_3-AD.vs.control; scRNA-seq,SRP215507,CD8+ T cell_2-MCI.vs.control	2
AC010168.2	scRNA-seq,SRP215507,CD8+ T cell_4-AD.vs.control; scRNA-seq,SRP215507,CD8+ T cell_2-AD.vs.MCI; scRNA-seq,SRP215507,CD8+ T cell_5-MCI.vs.control; scRNA-seq,SRP215507,CD8+ T cell_2-MCI.vs.control; scRNA-seq,SRP330776,B cell_1-AD.vs.control; scRNA-seq,SRP330776,Naive	6
AC010175.1	scRNA-seq,SRP215507,CD8+ T cell_1-AD.vs.control; scRNA-seq,SRP330776,γδ T cell-AD.vs.control; scRNA-seq,SRP330776,CD8+ T cell_2-AD.vs.control; scRNA-seq,SRP330776,Natural killer cell_1-AD.vs.control	4
AC010186.3	scRNA-seq,SRP215507,CD8+ T cell_6-MCI.vs.control; scRNA-seq,SRP330776,CD8+ T cell_1-AD.vs.control; scRNA-seq,SRP330776,γδ T cell-AD.vs.control; scRNA-seq,SRP330776,CD8+ T cell_2-AD.vs.control; scRNA-seq,SRP330776,Naive CD8+ T cell_1-AD.vs.control; scRNA-seq,SRP330776,Memory T cell_1-AD.vs.control; scRNA-seq,SRP330776,B cell_2-AD.vs.control	7
AC010198.1	scRNA-seq,SRP215507,CD8+ T cell_2-MCI.vs.control; scRNA-seq,SRP330776,CD8+ T cell_1-AD.vs.control; scRNA-seq,SRP330776,Naive CD8+ T	3
AC010198.2	scRNA-seq,SRP215507,CD8+ T cell_1-AD.vs.MCI	1
AC010210.1	scRNA-seq,SRP215507,CD8+ T cell_1-AD.vs.control; scRNA-seq,SRP330776,Memory T cell_1-AD.vs.control	2
AC010226.1	scRNA-seq,SRP215507,CD8+ T cell_1-AD.vs.MCI; scRNA-seq,SRP215507,CD8+ T cell_1-MCI.vs.control; scRNA-seq,SRP330776,CD8+ T cell_1-AD.vs.control; scRNA-seq,SRP330776,γδ T cell-AD.vs.control; scRNA-seq,SRP330776,Memory T cell_2-AD.vs.control; scRNA-seq,SRP330776,CD8+ T cell_2-AD.vs.control; scRNA-seq,SRP330776,B cell_1-AD.vs.control; scRNA-seq,SRP330776,Naive CD8+ T cell_1-AD.vs.control; scRNA-seq,SRP330776,Natural killer cell_1-AD.vs.control; scRNA-seq,SRP330776,Memory T cell_1-AD.vs.control; scRNA-seq,SRP330776,Naive CD8+ T	12
AC010240.3	scRNA-seq,SRP215507,CD8+ T cell_2-AD.vs.control; scRNA-seq,SRP215507,CD8+ T cell_2-MCI.vs.control; scRNA-seq,SRP330776,CD8+ T cell_2-AD.vs.control; scRNA-seq,SRP330776,B cell_1-AD.vs.control	4
AC010247.1	scRNA-seq,SRP215507,CD8+ T cell_8-MCI.vs.control; scRNA-seq,SRP330776,Memory T cell_2-AD.vs.control; scRNA-seq,SRP330776,CD8+ T cell_2-AD.vs.control; scRNA-seq,SRP330776,B cell_1-AD.vs.control; scRNA-seq,SRP330776,Natural killer cell_1-AD.vs.control; scRNA-	6
AC010247.2	scRNA-seq,SRP215507,CD8+ T cell_1-AD.vs.control; scRNA-seq,SRP215507,CD8+ T cell_2-AD.vs.MCI; scRNA-seq,SRP215507,CD8+ T cell_1-AD.vs.MCI; scRNA-seq,SRP215507,CD8+ T cell_4-MCI.vs.control; scRNA-seq,SRP330776,γδ T cell-AD.vs.control; scRNA-seq,SRP330776,B cell_1-	6
AC010260.1	scRNA-seq,SRP215507,CD8+ T cell_3-AD.vs.control	1

AC010271.1	scRNA-seq,SRP215507,CD8+ T cell_1-MCI.vs.control	1
AC010320.2	scRNA-seq,SRP330776,Naive CD8+ T cell_1-AD.vs.control	1
AC010326.3	scRNA-seq,SRP215507,CD8+ T cell_5-MCI.vs.control; scRNA-seq,SRP330776,Memory T cell_1-AD.vs.control	2
AC010327.8	scRNA-seq,SRP215507,CD8+ T cell_2-AD.vs.control; scRNA-seq,SRP215507,CD8+ T cell_2-MCI.vs.control	2
AC010331.1	scRNA-seq,SRP215507,CD8+ T cell_3-AD.vs.control; scRNA-seq,SRP330776,Naive CD8+ T cell_1-AD.vs.control	2
AC010332.3	scRNA-seq,SRP215507,CD8+ T cell_1-AD.vs.control; scRNA-seq,SRP309935,Natural killer cell_2-AD.vs.control; scRNA-seq,SRP309935,Naive CD8+ T cell_1-AD.vs.control; scRNA-seq,SRP309935,Natural killer cell_1-AD.vs.control; scRNA-seq,SRP309935,B cell_1-AD.vs.control; scRNA-seq,SRP309935,CD4+ T cell-AD.vs.control; scRNA-seq,SRP330776,CD8+ T cell_1-AD.vs.control; scRNA-seq,SRP330776,Memory T cell_1-AD.vs.control; scRNA-seq,SRP330776,B cell_2-AD.vs.control	9
AC010336.1	scRNA-seq,SRP330776,Naive CD8+ T cell_1-AD.vs.control	1
AC010336.5	scRNA-seq,SRP330776,CD8+ T cell_1-AD.vs.control; scRNA-seq,SRP330776,Naive CD8+ T cell_1-AD.vs.control	2
AC010336.8	scRNA-seq,SRP215507,CD8+ T cell_3-AD.vs.control; scRNA-seq,SRP215507,CD8+ T cell_1-AD.vs.MCI; scRNA-seq,SRP215507,CD8+ T cell_5-AD.vs.MCI; scRNA-seq,SRP215507,CD8+ T cell_1-MCI.vs.control	4
AC010359.3	scRNA-seq,SRP215507,CD8+ T cell_1-AD.vs.control; scRNA-seq,SRP215507,CD8+ T cell_5-AD.vs.MCI; scRNA-seq,SRP215507,CD8+ T cell_1-MCI.vs.control; scRNA-seq,SRP215507,CD8+ T cell_5-MCI.vs.control; scRNA-seq,SRP215507,CD8+ T cell_4-MCI.vs.control; scRNA-seq,SRP309935,Monocyte_1-AD.vs.control; scRNA-seq,SRP330776,CD8+ T cell_2-AD.vs.control; scRNA-seq,SRP330776,Naive CD8+ T cell_1-	9
AC010401.1	scRNA-seq,SRP330776,Memory T cell_2-AD.vs.control	1
AC010422.4	scRNA-seq,SRP215507,CD8+ T cell_3-MCI.vs.control	1
AC010442.1	scRNA-seq,SRP215507,CD8+ T cell_3-AD.vs.control; scRNA-seq,SRP215507,CD8+ T cell_1-MCI.vs.control; scRNA-seq,SRP330776,CD8+ T cell_2-AD.vs.control; scRNA-seq,SRP330776,B cell_1-AD.vs.control; scRNA-seq,SRP330776,T cell-AD.vs.control	5
AC010491.1	scRNA-seq,SRP215507,CD8+ T cell_3-MCI.vs.control; scRNA-seq,SRP330776,γδ T cell-AD.vs.control; scRNA-seq,SRP330776,Naive CD8+ T cell_1-	3
AC010501.2	scRNA-seq,SRP215507,CD8+ T cell_3-AD.vs.control	1
AC010503.1	scRNA-seq,SRP330776,Memory T cell_1-AD.vs.control	1
AC010531.6	scRNA-seq,SRP215507,CD8+ T cell_2-AD.vs.control; scRNA-seq,SRP215507,CD8+ T cell_3-AD.vs.control; scRNA-seq,SRP330776,CD8+ T cell_1-AD.vs.control; scRNA-seq,SRP330776,Memory T cell_2-AD.vs.control; scRNA-seq,SRP330776,CD8+ T cell_2-AD.vs.control; scRNA-seq,SRP330776,B cell_1-AD.vs.control; scRNA-seq,SRP330776,Memory T cell_1-AD.vs.control	7
AC010536.1	scRNA-seq,SRP215507,CD8+ T cell_4-MCI.vs.control	1
AC010536.3	scRNA-seq,SRP215507,CD8+ T cell_2-MCI.vs.control	1
AC010547.1	scRNA-seq,SRP215507,CD8+ T cell_2-MCI.vs.control; scRNA-seq,SRP215507,CD8+ T cell_3-MCI.vs.control	2
AC010618.3	scRNA-seq,SRP215507,CD8+ T cell_2-AD.vs.control; scRNA-seq,SRP215507,CD8+ T cell_3-AD.vs.control; scRNA-seq,SRP330776,CD8+ T cell_1-AD.vs.control; scRNA-seq,SRP330776,γδ T cell-AD.vs.control; scRNA-seq,SRP330776,Memory T cell_2-AD.vs.control; scRNA-seq,SRP330776,Memory T cell_1-AD.vs.control; scRNA-seq,SRP330776,Natural killer T cell-AD.vs.control	7
AC010623.1	scRNA-seq,SRP309935,Naive CD8+ T cell_1-AD.vs.control	1
AC010624.1	scRNA-seq,SRP215507,CD8+ T cell_1-AD.vs.MCI; scRNA-seq,SRP215507,CD8+ T cell_1-MCI.vs.control	2
AC010624.3	scRNA-seq,SRP215507,CD8+ T cell_3-AD.vs.control; scRNA-seq,SRP215507,CD8+ T cell_1-AD.vs.control; scRNA-seq,SRP215507,CD8+ T cell_4-MCI.vs.control; scRNA-seq,SRP330776,CD8+ T cell_1-AD.vs.control; scRNA-seq,SRP330776,Memory T cell_2-AD.vs.control; scRNA-seq,SRP330776,Memory T cell_1-AD.vs.control; scRNA-seq,SRP330776,T cell-AD.vs.control	7
AC010624.5	scRNA-seq,SRP215507,CD8+ T cell_5-MCI.vs.control	1
AC010632.1	scRNA-seq,SRP330776,Naive CD8+ T cell_1-AD.vs.control	1

AC010642.2	scRNA-seq,SRP215507,CD8+ T cell_4-AD.vs.control; scRNA-seq,SRP215507,CD8+ T cell_5-AD.vs.control; scRNA-seq,SRP215507,CD8+ T cell_4-AD.vs.MCI; scRNA-seq,SRP215507,CD8+ T cell_5-AD.vs.MCI; scRNA-seq,SRP330776,CD8+ T cell_1-AD.vs.control; scRNA-seq,SRP330776,γδ T cell-AD.vs.control; scRNA-seq,SRP330776,Memory T cell_2-AD.vs.control; scRNA-seq,SRP330776,CD8+ T cell_2-AD.vs.control; scRNA-seq,SRP330776,B cell_1-AD.vs.control; scRNA-seq,SRP330776,Naive CD8+ T cell_1-AD.vs.control; scRNA-seq,SRP330776,Natural killer cell_1-AD.vs.control; scRNA-seq,SRP330776,Memory T cell_1-AD.vs.control; scRNA-seq,SRP330776,Natural killer T cell-AD.vs.control; scRNA-seq,SRP330776,Naive CD8+ T cell_3-AD.vs.control; scRNA-seq,SRP330776,T cell-AD.vs.control; scRNA-seq,SRP330776,CD8+ T cell_3-AD.vs.control; scRNA-	17
AC010654.1	scRNA-seq,SRP330776,Natural killer cell_1-AD.vs.control; scRNA-seq,SRP330776,Natural killer T cell-AD.vs.control; scRNA-seq,SRP330776,Naive CD8+ T cell_3-AD.vs.control; scRNA-seq,SRP330776,CD8+ T cell_3-AD.vs.control	4
AC010680.2	scRNA-seq,SRP215507,CD8+ T cell_1-MCI.vs.control; scRNA-seq,SRP330776,B cell_1-AD.vs.control; scRNA-seq,SRP330776,Naive CD8+ T cell_1-	3
AC010680.3	scRNA-seq,SRP215507,CD8+ T cell_2-AD.vs.control	1
AC010746.2	scRNA-seq,SRP215507,CD8+ T cell_3-AD.vs.control	1
AC010754.1	scRNA-seq,SRP330776,CD8+ T cell_1-AD.vs.control; scRNA-seq,SRP330776,Memory T cell_2-AD.vs.control	2
AC010809.2	scRNA-seq,SRP215507,CD8+ T cell_1-MCI.vs.control	1
AC010864.1	scRNA-seq,SRP330776,CD8+ T cell_1-AD.vs.control; scRNA-seq,SRP330776,CD8+ T cell_2-AD.vs.control; scRNA-seq,SRP330776,Memory T cell_1-	3
AC010883.1	scRNA-seq,SRP215507,CD8+ T cell_5-MCI.vs.control; scRNA-seq,SRP330776,B cell_1-AD.vs.control; scRNA-seq,SRP330776,Naive CD8+ T cell_1-	3
AC010883.2	scRNA-seq,SRP330776,Naive CD8+ T cell_3-AD.vs.control	1
AC010883.3	scRNA-seq,SRP215507,CD8+ T cell_2-AD.vs.MCI; scRNA-seq,SRP215507,CD8+ T cell_5-MCI.vs.control; scRNA-seq,SRP215507,CD8+ T cell_2-MCI.vs.control; scRNA-seq,SRP330776,CD8+ T cell_1-AD.vs.control; scRNA-seq,SRP330776,Memory T cell_2-AD.vs.control; scRNA-seq,SRP330776,CD8+ T cell_2-AD.vs.control; scRNA-seq,SRP330776,B cell_1-AD.vs.control; scRNA-seq,SRP330776,Naive CD8+ T cell_1-AD.vs.control; scRNA-seq,SRP330776,Natural killer cell_1-AD.vs.control; scRNA-seq,SRP330776,Memory T cell_1-AD.vs.control; scRNA-	12
AC010889.2	scRNA-seq,SRP215507,CD8+ T cell_4-MCI.vs.control; scRNA-seq,SRP330776,Naive CD8+ T cell_1-AD.vs.control	2
AC010894.2	scRNA-seq,SRP215507,CD8+ T cell_3-AD.vs.control; scRNA-seq,SRP215507,CD8+ T cell_3-MCI.vs.control; scRNA-seq,SRP309935,Natural killer	3
AC010894.4	scRNA-seq,SRP215507,CD8+ T cell_3-MCI.vs.control	1
AC010896.1	scRNA-seq,SRP330776,CD8+ T cell_1-AD.vs.control; scRNA-seq,SRP330776,Naive CD8+ T cell_1-AD.vs.control	2
AC010913.1	scRNA-seq,SRP215507,CD8+ T cell_1-AD.vs.MCI; scRNA-seq,SRP215507,CD8+ T cell_1-MCI.vs.control	2
AC010967.1	scRNA-seq,SRP215507,CD8+ T cell_5-AD.vs.control; scRNA-seq,SRP215507,CD8+ T cell_5-AD.vs.MCI; scRNA-seq,SRP215507,CD8+ T cell_5-	3
AC010969.2	scRNA-seq,SRP330776,B cell_1-AD.vs.control; scRNA-seq,SRP330776,Naive CD8+ T cell_1-AD.vs.control; scRNA-seq,SRP330776,Memory T cell_1-	3
AC010978.1	scRNA-seq,SRP330776,CD8+ T cell_1-AD.vs.control; scRNA-seq,SRP330776,Memory T cell_2-AD.vs.control; scRNA-seq,SRP330776,CD8+ T cell_3-	3
AC010996.1	scRNA-seq,SRP215507,CD8+ T cell_2-MCI.vs.control	1
AC011043.1	scRNA-seq,SRP215507,CD8+ T cell_1-AD.vs.control; scRNA-seq,SRP215507,CD8+ T cell_1-MCI.vs.control; scRNA-seq,SRP309935,Monocyte_1-	3
AC011313.1	scRNA-seq,SRP215507,CD8+ T cell_1-AD.vs.MCI; scRNA-seq,SRP215507,CD8+ T cell_1-MCI.vs.control	2
AC011330.2	scRNA-seq,SRP215507,CD8+ T cell_1-AD.vs.MCI	1
AC011337.1	scRNA-seq,SRP215507,CD8+ T cell_1-AD.vs.MCI; scRNA-seq,SRP215507,CD8+ T cell_1-MCI.vs.control	2
AC011346.1	scRNA-seq,SRP309935,Naive CD8+ T cell_1-AD.vs.control; scRNA-seq,SRP330776,CD8+ T cell_1-AD.vs.control; scRNA-seq,SRP330776,Naive CD8+ T cell_1-AD.vs.control	3
AC011374.2	scRNA-seq,SRP215507,CD8+ T cell_5-MCI.vs.control	1
AC011379.2	scRNA-seq,SRP215507,CD8+ T cell_1-AD.vs.control; scRNA-seq,SRP215507,CD8+ T cell_1-MCI.vs.control; scRNA-seq,SRP309935,CD8+ T cell_1-AD.vs.control; scRNA-seq,SRP330776,B cell_1-AD.vs.control; scRNA-seq,SRP330776,Naive CD8+ T cell_1-AD.vs.control	5
AC011416.3	scRNA-seq,SRP215507,CD8+ T cell_2-AD.vs.control; scRNA-seq,SRP215507,CD8+ T cell_3-AD.vs.MCI; scRNA-seq,SRP215507,CD8+ T cell_5-MCI.vs.control; scRNA-seq,SRP215507,CD8+ T cell_3-MCI.vs.control; scRNA-seq,SRP330776,Memory T cell_2-AD.vs.control	5

AC011444.1	scRNA-seq,SRP215507,CD8+ T cell_1-AD.vs.control	1
AC011445.1	scRNA-seq,SRP330776,CD8+ T cell_2-AD.vs.control	1
AC011445.2	scRNA-seq,SRP215507,CD8+ T cell_1-AD.vs.control; scRNA-seq,SRP330776,Natural killer cell_1-AD.vs.control; scRNA-seq,SRP330776,Naive CD8+ T cell_2-AD.vs.control	3
AC011446.2	scRNA-seq,SRP215507,CD8+ T cell_2-AD.vs.MCI; scRNA-seq,SRP215507,CD8+ T cell_1-AD.vs.MCI; scRNA-seq,SRP215507,CD8+ T cell_1-MCI.vs.control; scRNA-seq,SRP215507,CD8+ T cell_5-MCI.vs.control; scRNA-seq,SRP330776,CD8+ T cell_1-AD.vs.control; scRNA-seq,SRP330776,γδ T cell-AD.vs.control; scRNA-seq,SRP330776,Memory T cell_2-AD.vs.control; scRNA-seq,SRP330776,CD8+ T cell_2-AD.vs.control; scRNA-seq,SRP330776,B cell_1-AD.vs.control; scRNA-seq,SRP330776,Natural killer T cell-AD.vs.control; scRNA-seq,SRP330776,Naive CD8+ T cell_3-AD.vs.control; scRNA-seq,SRP330776,B cell_2-AD.vs.control; scRNA-seq,SRP330776,CD8+ T cell_3-AD.vs.control; scRNA-	14
AC011447.3	scRNA-seq,SRP215507,CD8+ T cell_2-MCI.vs.control; scRNA-seq,SRP330776,Memory T cell_2-AD.vs.control; scRNA-seq,SRP330776,B cell_2-	3
AC011450.1	scRNA-seq,SRP215507,CD8+ T cell_2-AD.vs.control; scRNA-seq,SRP215507,CD8+ T cell_8-AD.vs.control; scRNA-seq,SRP215507,CD8+ T cell_1-AD.vs.control; scRNA-seq,SRP215507,CD8+ T cell_2-AD.vs.MCI; scRNA-seq,SRP215507,CD8+ T cell_1-AD.vs.MCI; scRNA-seq,SRP215507,CD8+ T cell_5-AD.vs.MCI; scRNA-seq,SRP215507,CD8+ T cell_2-MCI.vs.control	7
AC011462.5	scRNA-seq,SRP215507,CD8+ T cell_2-AD.vs.MCI; scRNA-seq,SRP215507,CD8+ T cell_1-AD.vs.MCI; scRNA-seq,SRP215507,CD8+ T cell_1-MCI.vs.control; scRNA-seq,SRP215507,CD8+ T cell_2-MCI.vs.control; scRNA-seq,SRP330776,B cell_1-AD.vs.control	5
AC011466.1	scRNA-seq,SRP215507,CD8+ T cell_2-MCI.vs.control; scRNA-seq,SRP330776,CD8+ T cell_1-AD.vs.control; scRNA-seq,SRP330776,Naive CD8+ T	3
AC011468.5	scRNA-seq,SRP215507,CD8+ T cell_1-AD.vs.control; scRNA-seq,SRP215507,CD8+ T cell_2-AD.vs.MCI; scRNA-seq,SRP215507,CD8+ T cell_1-MCI.vs.control; scRNA-seq,SRP215507,CD8+ T cell_2-MCI.vs.control; scRNA-seq,SRP330776,Memory T cell_1-AD.vs.control	5
AC011471.2	scRNA-seq,SRP309935,Natural killer cell_1-AD.vs.control; scRNA-seq,SRP309935,Monocyte_2-AD.vs.control	2
AC011474.2	scRNA-seq,SRP215507,CD8+ T cell_1-MCI.vs.control	1
AC011476.3	scRNA-seq,SRP215507,CD8+ T cell_3-AD.vs.MCI; scRNA-seq,SRP215507,CD8+ T cell_1-AD.vs.MCI; scRNA-seq,SRP215507,CD8+ T cell_4-AD.vs.MCI; scRNA-seq,SRP215507,CD8+ T cell_4-MCI.vs.control; scRNA-seq,SRP215507,CD8+ T cell_3-MCI.vs.control; scRNA-seq,SRP330776,CD8+ T cell_1-AD.vs.control; scRNA-seq,SRP330776,CD8+ T cell_2-AD.vs.control; scRNA-seq,SRP330776,Naive CD8+ T cell_1-	8
AC011477.2	scRNA-seq,SRP215507,CD8+ T cell_2-AD.vs.MCI; scRNA-seq,SRP330776,CD8+ T cell_1-AD.vs.control; scRNA-seq,SRP330776,CD8+ T cell_2-AD.vs.control; scRNA-seq,SRP330776,Naive CD8+ T cell_1-AD.vs.control	4
AC011481.2	scRNA-seq,SRP215507,CD8+ T cell_3-AD.vs.control; scRNA-seq,SRP330776,B cell_1-AD.vs.control	2
AC011484.1	scRNA-seq,SRP309935,Naive CD8+ T cell_1-AD.vs.control	1
AC011498.1	scRNA-seq,SRP215507,CD8+ T cell_3-AD.vs.control; scRNA-seq,SRP215507,CD8+ T cell_2-MCI.vs.control; scRNA-seq,SRP330776,Naive CD8+ T	3
AC011511.2	scRNA-seq,SRP215507,CD8+ T cell_5-AD.vs.MCI	1
AC011603.2	scRNA-seq,SRP215507,CD8+ T cell_3-AD.vs.control; scRNA-seq,SRP330776,B cell_1-AD.vs.control; scRNA-seq,SRP330776,Memory T cell_1-AD.vs.control; scRNA-seq,SRP330776,Natural killer T cell-AD.vs.control; scRNA-seq,SRP330776,CD8+ T cell_3-AD.vs.control; scRNA-seq,SRP330776,Monocyte_2-AD.vs.control; scRNA-seq,SRP330776,Natural killer cell_2-AD.vs.control	7
AC011611.3	scRNA-seq,SRP215507,CD8+ T cell_5-AD.vs.MCI; scRNA-seq,SRP215507,CD8+ T cell_5-MCI.vs.control; scRNA-seq,SRP330776,Memory T cell_1-	3
AC011611.4	scRNA-seq,SRP215507,CD8+ T cell_1-AD.vs.MCI; scRNA-seq,SRP215507,CD8+ T cell_1-MCI.vs.control	2
AC011726.3	scRNA-seq,SRP215507,CD8+ T cell_3-AD.vs.control	1
AC011773.4	scRNA-seq,SRP330776,B cell_1-AD.vs.control	1
AC011815.1	scRNA-seq,SRP215507,CD8+ T cell_1-AD.vs.control; scRNA-seq,SRP215507,CD8+ T cell_5-AD.vs.control; scRNA-seq,SRP215507,CD8+ T cell_1-MCI.vs.control; scRNA-seq,SRP330776,Naive CD8+ T cell_1-AD.vs.control	4
AC011825.4	scRNA-seq,SRP330776,Naive CD8+ T cell_1-AD.vs.control	1
AC011899.3	scRNA-seq,SRP309935,Monocyte_2-AD.vs.control	1

AC011912.1	scRNA-seq,SRP215507,CD8+ T cell_3-MCI.vs.control; scRNA-seq,SRP330776,CD8+ T cell_1-AD.vs.control; scRNA-seq,SRP330776,Naive CD8+ T	3
AC011921.1	scRNA-seq,SRP215507,CD8+ T cell_2-AD.vs.control; scRNA-seq,SRP215507,CD8+ T cell_1-AD.vs.control; scRNA-seq,SRP215507,CD8+ T cell_2-AD.vs.MCI; scRNA-seq,SRP215507,CD8+ T cell_1-MCI.vs.control	4
AC011933.2	scRNA-seq,SRP330776,CD8+ T cell_1-AD.vs.control	1
AC011939.3	scRNA-seq,SRP215507,CD8+ T cell_1-AD.vs.control; scRNA-seq,SRP215507,CD8+ T cell_1-AD.vs.MCI; scRNA-seq,SRP215507,CD8+ T cell_5-MCI.vs.control; scRNA-seq,SRP330776,Naive CD8+ T cell_1-AD.vs.control	4
AC012020.1	scRNA-seq,SRP215507,CD8+ T cell_1-AD.vs.control; scRNA-seq,SRP215507,CD8+ T cell_4-AD.vs.MCI; scRNA-seq,SRP215507,CD8+ T cell_1-MCI.vs.control; scRNA-seq,SRP215507,CD8+ T cell_4-MCI.vs.control; scRNA-seq,SRP309935,Natural killer cell_2-AD.vs.control; scRNA-seq,SRP309935,CD4+ T cell-AD.vs.control; scRNA-seq,SRP330776,CD8+ T cell_1-AD.vs.control; scRNA-seq,SRP330776,γδ T cell-AD.vs.control; scRNA-seq,SRP330776,Memory T cell_2-AD.vs.control; scRNA-seq,SRP330776,B cell_1-AD.vs.control; scRNA-seq,SRP330776,Naive CD8+ T cell_1-AD.vs.control; scRNA-seq,SRP330776,Natural killer cell_1-AD.vs.control; scRNA-seq,SRP330776,B cell_2-AD.vs.control; scRNA-seq,SRP330776,T cell-AD.vs.control; scRNA-seq,SRP330776,CD8+ T cell_3-AD.vs.control; scRNA-seq,SRP330776,B cell_3-AD.vs.control	16
AC012063.1	scRNA-seq,SRP215507,CD8+ T cell_3-AD.vs.control; scRNA-seq,SRP330776,Naive CD8+ T cell_1-AD.vs.control	2
AC012074.1	scRNA-seq,SRP215507,CD8+ T cell_6-AD.vs.control; scRNA-seq,SRP215507,CD8+ T cell_3-MCI.vs.control	2
AC012146.1	scRNA-seq,SRP215507,CD8+ T cell_7-AD.vs.control; scRNA-seq,SRP330776,CD8+ T cell_1-AD.vs.control; scRNA-seq,SRP330776,γδ T cell-AD.vs.control; scRNA-seq,SRP330776,Memory T cell_1-AD.vs.control; scRNA-seq,SRP330776,Natural killer T cell-AD.vs.control; scRNA-seq,SRP330776,Naive CD8+ T cell_3-AD.vs.control; scRNA-seq,SRP330776,B cell_2-AD.vs.control; scRNA-seq,SRP330776,T cell-AD.vs.control;	9
AC012173.1	scRNA-seq,SRP215507,CD8+ T cell_2-AD.vs.control; scRNA-seq,SRP215507,CD8+ T cell_1-MCI.vs.control	2
AC012181.1	scRNA-seq,SRP215507,CD8+ T cell_4-MCI.vs.control	1
AC012184.3	scRNA-seq,SRP215507,CD8+ T cell_6-AD.vs.control; scRNA-seq,SRP330776,CD8+ T cell_1-AD.vs.control; scRNA-seq,SRP330776,B cell_1-AD.vs.control; scRNA-seq,SRP330776,Memory T cell_1-AD.vs.control	4
AC012184.4	scRNA-seq,SRP330776,CD8+ T cell_3-AD.vs.control	1
AC012236.1	scRNA-seq,SRP330776,B cell_4-AD.vs.control	1
AC012291.3	scRNA-seq,SRP215507,CD8+ T cell_1-AD.vs.control; scRNA-seq,SRP215507,CD8+ T cell_5-MCI.vs.control; scRNA-seq,SRP309935,Naive CD8+ T cell_1-AD.vs.control; scRNA-seq,SRP330776,Naive CD8+ T cell_1-AD.vs.control	4
AC012306.2	scRNA-seq,SRP215507,CD8+ T cell_1-AD.vs.control; scRNA-seq,SRP215507,CD8+ T cell_1-AD.vs.MCI; scRNA-seq,SRP330776,CD8+ T cell_1-AD.vs.control; scRNA-seq,SRP330776,Memory T cell_2-AD.vs.control; scRNA-seq,SRP330776,CD8+ T cell_2-AD.vs.control	5
AC012306.3	scRNA-seq,SRP215507,CD8+ T cell_1-AD.vs.MCI; scRNA-seq,SRP215507,CD8+ T cell_1-MCI.vs.control	2
AC012313.1	scRNA-seq,SRP330776,B cell_1-AD.vs.control; scRNA-seq,SRP330776,CD8+ T cell_3-AD.vs.control	2
AC012313.2	scRNA-seq,SRP215507,CD8+ T cell_3-AD.vs.control; scRNA-seq,SRP215507,CD8+ T cell_3-MCI.vs.control	2
AC012313.5	scRNA-seq,SRP330776,Naive CD8+ T cell_1-AD.vs.control	1
AC012313.6	scRNA-seq,SRP215507,CD8+ T cell_3-MCI.vs.control; scRNA-seq,SRP330776,B cell_1-AD.vs.control	2
AC012313.7	scRNA-seq,SRP215507,CD8+ T cell_1-MCI.vs.control	1
AC012358.3	scRNA-seq,SRP330776,CD8+ T cell_1-AD.vs.control; scRNA-seq,SRP330776,B cell_1-AD.vs.control; scRNA-seq,SRP330776,Naive CD8+ T cell_1-	3
AC012360.1	scRNA-seq,SRP215507,CD8+ T cell_3-MCI.vs.control	1
AC012360.3	scRNA-seq,SRP215507,CD8+ T cell_4-AD.vs.MCI; scRNA-seq,SRP330776,CD8+ T cell_1-AD.vs.control; scRNA-seq,SRP330776,Memory T cell_2-AD.vs.control; scRNA-seq,SRP330776,CD8+ T cell_2-AD.vs.control; scRNA-seq,SRP330776,Naive CD8+ T cell_1-AD.vs.control; scRNA-seq,SRP330776,Natural killer cell_1-AD.vs.control; scRNA-seq,SRP330776,Memory T cell_1-AD.vs.control; scRNA-seq,SRP330776,Naive CD8+ T	8
AC012368.1	scRNA-seq,SRP330776,Memory T cell_2-AD.vs.control; scRNA-seq,SRP330776,B cell_1-AD.vs.control	2
AC012447.1	scRNA-seq,SRP215507,CD8+ T cell_3-MCI.vs.control	1

AC012467.2	scRNA-seq,SRP215507,CD8+ T cell_4-AD.vs.control; scRNA-seq,SRP215507,CD8+ T cell_4-MCI.vs.control; scRNA-seq,SRP309935,CD8+ T cell_2-	3
AC012485.3	scRNA-seq,SRP215507,CD8+ T cell_2-AD.vs.MCI; scRNA-seq,SRP215507,CD8+ T cell_3-MCI.vs.control; scRNA-seq,SRP330776,Memory T cell_1-	3
AC012510.1	scRNA-seq,SRP215507,CD8+ T cell_4-MCI.vs.control	1
AC012557.2	scRNA-seq,SRP330776,CD8+ T cell_2-AD.vs.control	1
AC012615.1	scRNA-seq,SRP215507,CD8+ T cell_3-AD.vs.control; scRNA-seq,SRP215507,CD8+ T cell_4-AD.vs.MCI; scRNA-seq,SRP215507,CD8+ T cell_5-AD.vs.MCI; scRNA-seq,SRP215507,CD8+ T cell_5-MCI.vs.control; scRNA-seq,SRP215507,CD8+ T cell_4-MCI.vs.control; scRNA-seq,SRP330776,B cell_1-AD.vs.control; scRNA-seq,SRP330776,Monocyte_2-AD.vs.control; scRNA-seq,SRP330776,Natural killer cell_2-AD.vs.control	8
AC012615.5	scRNA-seq,SRP215507,CD8+ T cell_3-AD.vs.control; scRNA-seq,SRP215507,CD8+ T cell_1-MCI.vs.control	2
AC012615.6	scRNA-seq,SRP330776,CD8+ T cell_1-AD.vs.control; scRNA-seq,SRP330776,Naive CD8+ T cell_1-AD.vs.control	2
AC012636.1	scRNA-seq,SRP215507,CD8+ T cell_3-AD.vs.MCI; scRNA-seq,SRP215507,CD8+ T cell_3-MCI.vs.control; scRNA-seq,SRP330776,Naive CD8+ T	3
AC012640.2	scRNA-seq,SRP215507,CD8+ T cell_1-MCI.vs.control; scRNA-seq,SRP330776,CD8+ T cell_1-AD.vs.control; scRNA-seq,SRP330776,Memory T cell_2-AD.vs.control; scRNA-seq,SRP330776,CD8+ T cell_2-AD.vs.control; scRNA-seq,SRP330776,B cell_1-AD.vs.control; scRNA-	6
AC012645.1	scRNA-seq,SRP215507,CD8+ T cell_1-MCI.vs.control; scRNA-seq,SRP330776,CD8+ T cell_1-AD.vs.control; scRNA-seq,SRP330776,Naive CD8+ T	3
AC012645.3	scRNA-seq,SRP215507,CD8+ T cell_4-AD.vs.control; scRNA-seq,SRP215507,CD8+ T cell_4-AD.vs.MCI; scRNA-seq,SRP215507,CD8+ T cell_5-AD.vs.MCI; scRNA-seq,SRP309935,Monocyte_1-AD.vs.control; scRNA-seq,SRP309935,Megakaryocyte_1-AD.vs.control; scRNA-seq,SRP309935,Naive CD8+ T cell_1-AD.vs.control; scRNA-seq,SRP309935,Natural killer cell_1-AD.vs.control; scRNA-seq,SRP309935,CD8+ T cell_2-AD.vs.control; scRNA-seq,SRP309935,Naive CD8+ T cell_3-AD.vs.control; scRNA-seq,SRP330776,CD8+ T cell_1-AD.vs.control; scRNA-seq,SRP330776,Memory T cell_2-AD.vs.control; scRNA-seq,SRP330776,CD8+ T cell_2-AD.vs.control; scRNA-seq,SRP330776,B cell_1-AD.vs.control; scRNA-seq,SRP330776,Naive CD8+ T cell_1-AD.vs.control; scRNA-seq,SRP330776,Memory T cell_1-AD.vs.control; scRNA-seq,SRP330776,Natural killer T cell-AD.vs.control; scRNA-seq,SRP330776,Naive CD8+ T cell_3-AD.vs.control; scRNA-seq,SRP330776,B cell_2-AD.vs.control; scRNA-seq,SRP330776,T cell-AD.vs.control; scRNA-seq,SRP330776,Naive CD8+ T cell_2-AD.vs.control; scRNA-seq,SRP330776,CD8+ T cell_3-AD.vs.control; scRNA- [illegible]	23
AC012673.1	scRNA-seq,SRP215507,CD8+ T cell_2-AD.vs.control	1
AC013264.1	scRNA-seq,SRP215507,CD8+ T cell_2-AD.vs.MCI; scRNA-seq,SRP215507,CD8+ T cell_3-AD.vs.MCI; scRNA-seq,SRP215507,CD8+ T cell_1-AD.vs.MCI; scRNA-seq,SRP215507,CD8+ T cell_1-MCI.vs.control; scRNA-seq,SRP215507,CD8+ T cell_2-MCI.vs.control; scRNA-seq,SRP215507,CD8+ T cell_3-MCI.vs.control; scRNA-seq,SRP330776,CD8+ T cell_1-AD.vs.control; scRNA-seq,SRP330776,γδ T cell-AD.vs.control; scRNA-seq,SRP330776,Memory T cell_2-AD.vs.control; scRNA-seq,SRP330776,CD8+ T cell_2-AD.vs.control; scRNA-seq,SRP330776,Naive CD8+ T cell_1-AD.vs.control; scRNA-seq,SRP330776,Memory T cell_1-AD.vs.control; scRNA-seq,SRP330776,Natural killer T cell-AD.vs.control; scRNA-seq,SRP330776,T cell-AD.vs.control; scRNA-seq,SRP330776,CD8+ T cell_3-AD.vs.control; scRNA-seq,SRP330776,Natural killer cell_2-AD.vs.control	16
AC013400.1	scRNA-seq,SRP215507,CD8+ T cell_2-AD.vs.control; scRNA-seq,SRP215507,CD8+ T cell_3-AD.vs.control; scRNA-seq,SRP215507,CD8+ T cell_4-AD.vs.MCI; scRNA-seq,SRP215507,CD8+ T cell_4-MCI.vs.control; scRNA-seq,SRP215507,CD8+ T cell_2-MCI.vs.control; scRNA-seq,SRP330776,B cell_2-AD.vs.control; scRNA-seq,SRP330776,CD8+ T cell_3-AD.vs.control	7
AC013403.2	scRNA-seq,SRP215507,CD8+ T cell_4-AD.vs.control; scRNA-seq,SRP215507,CD8+ T cell_4-MCI.vs.control; scRNA-seq,SRP215507,CD8+ T cell_2-MCI.vs.control; scRNA-seq,SRP330776,CD8+ T cell_1-AD.vs.control	4
AC013460.1	scRNA-seq,SRP215507,CD8+ T cell_1-AD.vs.MCI; scRNA-seq,SRP215507,CD8+ T cell_1-MCI.vs.control	2
AC013486.1	scRNA-seq,SRP330776,CD8+ T cell_1-AD.vs.control; scRNA-seq,SRP330776,CD8+ T cell_2-AD.vs.control; scRNA-seq,SRP330776,B cell_1-AD.vs.control; scRNA-seq,SRP330776,Memory T cell_1-AD.vs.control	4
AC013565.3	scRNA-seq,SRP215507,CD8+ T cell_2-MCI.vs.control	1
AC013731.1	scRNA-seq,SRP309935,CD8+ T cell_2-AD.vs.control	1
AC015712.1	scRNA-seq,SRP330776,CD8+ T cell_1-AD.vs.control; scRNA-seq,SRP330776,B cell_1-AD.vs.control	2
AC015726.1	scRNA-seq,SRP215507,CD8+ T cell_1-AD.vs.control	1
AC015802.4	scRNA-seq,SRP215507,CD8+ T cell_2-AD.vs.control; scRNA-seq,SRP215507,CD8+ T cell_5-AD.vs.MCI	2

AC015802.6	scRNA-seq,SRP215507,CD8+ T cell_3-AD.vs.MCI; scRNA-seq,SRP330776,CD8+ T cell_1-AD.vs.control; scRNA-seq,SRP330776,Naive CD8+ T cell_1-	3
AC015813.1	scRNA-seq,SRP215507,CD8+ T cell_4-AD.vs.control; scRNA-seq,SRP215507,CD8+ T cell_6-MCI.vs.control; scRNA-seq,SRP330776,Memory T cell_1-AD.vs.control; scRNA-seq,SRP330776,Naive CD8+ T cell_3-AD.vs.control	4
AC015819.1	scRNA-seq,SRP215507,CD8+ T cell_3-AD.vs.control; scRNA-seq,SRP215507,CD8+ T cell_1-AD.vs.MCI; scRNA-seq,SRP215507,CD8+ T cell_3-	3
AC015819.2	scRNA-seq,SRP215507,CD8+ T cell_3-MCI.vs.control	1
AC015849.1	scRNA-seq,SRP215507,CD8+ T cell_1-AD.vs.control; scRNA-seq,SRP215507,CD8+ T cell_1-AD.vs.MCI; scRNA-seq,SRP215507,CD8+ T cell_5-	3
AC015871.1	scRNA-seq,SRP215507,CD8+ T cell_1-MCI.vs.control; scRNA-seq,SRP215507,CD8+ T cell_5-MCI.vs.control; scRNA-seq,SRP330776,CD8+ T cell_1-AD.vs.control; scRNA-seq,SRP330776,γδ T cell-AD.vs.control; scRNA-seq,SRP330776,Memory T cell_2-AD.vs.control; scRNA-seq,SRP330776,CD8+ T cell_2-AD.vs.control; scRNA-seq,SRP330776,Naive CD8+ T cell_1-AD.vs.control; scRNA-seq,SRP330776,Natural killer cell_1-AD.vs.control; scRNA-seq,SRP330776,Natural killer T cell-AD.vs.control; scRNA-seq,SRP330776,Natural killer cell_2-AD.vs.control	10
AC015871.4	scRNA-seq,SRP215507,CD8+ T cell_1-AD.vs.control; scRNA-seq,SRP330776,CD8+ T cell_1-AD.vs.control; scRNA-seq,SRP330776,γδ T cell-AD.vs.control; scRNA-seq,SRP330776,CD8+ T cell_2-AD.vs.control	4
AC015871.7	scRNA-seq,SRP215507,CD8+ T cell_4-AD.vs.control; scRNA-seq,SRP215507,CD8+ T cell_4-AD.vs.MCI; scRNA-seq,SRP309935,Monocyte_2-AD.vs.control; scRNA-seq,SRP330776,CD8+ T cell_1-AD.vs.control; scRNA-seq,SRP330776,Memory T cell_2-AD.vs.control; scRNA-seq,SRP330776,CD8+ T cell_2-AD.vs.control; scRNA-seq,SRP330776,Naive CD8+ T cell_1-AD.vs.control; scRNA-seq,SRP330776,Memory T cell_1-	8
AC015911.3	scRNA-seq,SRP215507,CD8+ T cell_1-AD.vs.MCI; scRNA-seq,SRP215507,CD8+ T cell_5-AD.vs.MCI; scRNA-seq,SRP215507,CD8+ T cell_1-MCI.vs.control; scRNA-seq,SRP215507,CD8+ T cell_5-MCI.vs.control; scRNA-seq,SRP330776,CD8+ T cell_1-AD.vs.control; scRNA-seq,SRP330776,Naive CD8+ T cell_1-AD.vs.control; scRNA-seq,SRP330776,Memory T cell_1-AD.vs.control	7
AC015911.5	scRNA-seq,SRP330776,Memory T cell_1-AD.vs.control	1
AC015911.6	scRNA-seq,SRP215507,CD8+ T cell_4-AD.vs.control; scRNA-seq,SRP215507,CD8+ T cell_3-AD.vs.MCI; scRNA-seq,SRP215507,CD8+ T cell_4-MCI.vs.control; scRNA-seq,SRP215507,CD8+ T cell_3-MCI.vs.control	4
AC015912.1	scRNA-seq,SRP330776,CD8+ T cell_3-AD.vs.control	1
AC015912.3	scRNA-seq,SRP215507,CD8+ T cell_3-AD.vs.control; scRNA-seq,SRP215507,CD8+ T cell_1-AD.vs.MCI; scRNA-seq,SRP215507,CD8+ T cell_5-AD.vs.MCI; scRNA-seq,SRP215507,CD8+ T cell_1-MCI.vs.control; scRNA-seq,SRP215507,CD8+ T cell_5-MCI.vs.control; scRNA-seq,SRP215507,CD8+ T cell_3-MCI.vs.control; scRNA-seq,SRP309935,Monocyte_1-AD.vs.control	7
AC015921.1	scRNA-seq,SRP330776,CD8+ T cell_2-AD.vs.control; scRNA-seq,SRP330776,Memory T cell_1-AD.vs.control	2
AC015936.1	scRNA-seq,SRP215507,CD8+ T cell_1-AD.vs.control; scRNA-seq,SRP330776,B cell_1-AD.vs.control	2
AC015982.2	scRNA-seq,SRP215507,CD8+ T cell_2-AD.vs.MCI; scRNA-seq,SRP215507,CD8+ T cell_2-MCI.vs.control; scRNA-seq,SRP215507,CD8+ T cell_3-MCI.vs.control; scRNA-seq,SRP330776,Memory T cell_2-AD.vs.control; scRNA-seq,SRP330776,CD8+ T cell_2-AD.vs.control; scRNA-seq,SRP330776,B cell_1-AD.vs.control; scRNA-seq,SRP330776,B cell_2-AD.vs.control; scRNA-seq,SRP330776,CD8+ T cell_3-AD.vs.control	8
AC015987.1	scRNA-seq,SRP309935,Natural killer cell_1-AD.vs.control	1
AC016027.1	scRNA-seq,SRP215507,CD8+ T cell_2-AD.vs.control; scRNA-seq,SRP215507,CD8+ T cell_3-AD.vs.control; scRNA-seq,SRP215507,CD8+ T cell_1-AD.vs.control; scRNA-seq,SRP215507,CD8+ T cell_2-AD.vs.MCI; scRNA-seq,SRP330776,CD8+ T cell_2-AD.vs.control; scRNA-seq,SRP330776,B cell_1-AD.vs.control; scRNA-seq,SRP330776,Naive CD8+ T cell_1-AD.vs.control; scRNA-seq,SRP330776,Memory T cell_1-AD.vs.control	8
AC016065.1	scRNA-seq,SRP215507,CD8+ T cell_3-AD.vs.MCI; scRNA-seq,SRP215507,CD8+ T cell_2-MCI.vs.control; scRNA-seq,SRP330776,CD8+ T cell_1-AD.vs.control; scRNA-seq,SRP330776,Memory T cell_2-AD.vs.control; scRNA-seq,SRP330776,CD8+ T cell_2-AD.vs.control; scRNA-seq,SRP330776,Naive CD8+ T cell_1-AD.vs.control; scRNA-seq,SRP330776,Natural killer T cell-AD.vs.control; scRNA-seq,SRP330776,Naive CD8+ T cell_3-AD.vs.control; scRNA-seq,SRP330776,T cell-AD.vs.control; scRNA-seq,SRP330776,Monocyte_1-AD.vs.control; scRNA-seq,SRP330776,B cell_3-	12
AC016074.2	scRNA-seq,SRP215507,CD8+ T cell_1-AD.vs.control; scRNA-seq,SRP215507,CD8+ T cell_2-AD.vs.MCI; scRNA-seq,SRP215507,CD8+ T cell_5-MCI.vs.control; scRNA-seq,SRP215507,CD8+ T cell_2-MCI.vs.control; scRNA-seq,SRP330776,CD8+ T cell_1-AD.vs.control; scRNA-seq,SRP330776,γδ T cell-AD.vs.control; scRNA-seq,SRP330776,Naive CD8+ T cell_1-AD.vs.control	7
AC016168.2	scRNA-seq,SRP330776,Memory T cell_2-AD.vs.control	1

AC016245.1	scRNA-seq,SRP330776,Natural killer cell_1-AD.vs.control	1
AC016355.1	scRNA-seq,SRP215507,CD8+ T cell_1-AD.vs.control; scRNA-seq,SRP215507,CD8+ T cell_5-AD.vs.control; scRNA-seq,SRP215507,CD8+ T cell_1-AD.vs.MCI; scRNA-seq,SRP215507,CD8+ T cell_3-MCI.vs.control; scRNA-seq,SRP309935,Monocyte_2-AD.vs.control; scRNA-seq,SRP309935,CD8+ T cell_2-AD.vs.control; scRNA-seq,SRP330776,CD8+ T cell_1-AD.vs.control; scRNA-seq,SRP330776,Memory T cell_2-AD.vs.control	8
AC016394.1	scRNA-seq,SRP215507,CD8+ T cell_5-AD.vs.control; scRNA-seq,SRP309935,Natural killer cell_1-AD.vs.control; scRNA-seq,SRP330776,CD8+ T cell_1-AD.vs.control; scRNA-seq,SRP330776,γδ T cell-AD.vs.control; scRNA-seq,SRP330776,Memory T cell_2-AD.vs.control; scRNA-seq,SRP330776,B cell_1-AD.vs.control; scRNA-seq,SRP330776,Naive CD8+ T cell_1-AD.vs.control; scRNA-seq,SRP330776,Memory T cell_1-AD.vs.control; scRNA-seq,SRP330776,Natural killer T cell-AD.vs.control; scRNA-seq,SRP330776,Naive CD8+ T cell_3-AD.vs.control; scRNA-seq,SRP330776,T cell-	11
AC016405.3	scRNA-seq,SRP215507,CD8+ T cell_6-AD.vs.control; scRNA-seq,SRP215507,CD8+ T cell_1-AD.vs.MCI; scRNA-seq,SRP215507,CD8+ T cell_4-AD.vs.MCI; scRNA-seq,SRP215507,CD8+ T cell_1-MCI.vs.control; scRNA-seq,SRP215507,CD8+ T cell_4-MCI.vs.control; scRNA-seq,SRP330776,Memory T cell_2-AD.vs.control; scRNA-seq,SRP330776,CD8+ T cell_2-AD.vs.control; scRNA-seq,SRP330776,Naive CD8+ T cell_3-	9
AC016588.1	scRNA-seq,SRP330776,CD8+ T cell_1-AD.vs.control	1
AC016590.1	scRNA-seq,SRP330776,CD8+ T cell_1-AD.vs.control	1
AC016596.1	scRNA-seq,SRP215507,CD8+ T cell_1-AD.vs.MCI; scRNA-seq,SRP215507,CD8+ T cell_5-AD.vs.MCI; scRNA-seq,SRP215507,CD8+ T cell_1-MCI.vs.control; scRNA-seq,SRP215507,CD8+ T cell_5-MCI.vs.control; scRNA-seq,SRP215507,CD8+ T cell_4-MCI.vs.control; scRNA-seq,SRP330776,CD8+ T cell_2-AD.vs.control; scRNA-seq,SRP330776,Memory T cell_1-AD.vs.control; scRNA-seq,SRP330776,Naive CD8+ T cell_3-	8
AC016683.1	scRNA-seq,SRP330776,B cell_1-AD.vs.control	1
AC016722.2	scRNA-seq,SRP215507,CD8+ T cell_2-MCI.vs.control; scRNA-seq,SRP330776,Natural killer cell_1-AD.vs.control	2
AC016727.1	scRNA-seq,SRP215507,CD8+ T cell_4-AD.vs.control; scRNA-seq,SRP215507,CD8+ T cell_1-AD.vs.MCI; scRNA-seq,SRP215507,CD8+ T cell_4-AD.vs.MCI; scRNA-seq,SRP330776,γδ T cell-AD.vs.control; scRNA-seq,SRP330776,Memory T cell_2-AD.vs.control; scRNA-seq,SRP330776,Natural killer cell_1-AD.vs.control; scRNA-seq,SRP330776,Natural killer T cell-AD.vs.control; scRNA-seq,SRP330776,CD8+ T cell_3-AD.vs.control; scRNA-	9
AC016745.1	scRNA-seq,SRP215507,CD8+ T cell_1-AD.vs.control; scRNA-seq,SRP215507,CD8+ T cell_4-AD.vs.control; scRNA-seq,SRP215507,CD8+ T cell_2-AD.vs.MCI; scRNA-seq,SRP215507,CD8+ T cell_1-AD.vs.MCI; scRNA-seq,SRP215507,CD8+ T cell_2-MCI.vs.control	5
AC016745.2	scRNA-seq,SRP330776,B cell_1-AD.vs.control	1
AC016747.1	scRNA-seq,SRP215507,CD8+ T cell_1-AD.vs.control; scRNA-seq,SRP215507,CD8+ T cell_3-MCI.vs.control; scRNA-seq,SRP330776,CD8+ T cell_2-AD.vs.control; scRNA-seq,SRP330776,B cell_1-AD.vs.control; scRNA-seq,SRP330776,T cell-AD.vs.control	5
AC016773.1	scRNA-seq,SRP215507,CD8+ T cell_5-AD.vs.control; scRNA-seq,SRP215507,CD8+ T cell_4-AD.vs.MCI; scRNA-seq,SRP330776,γδ T cell-AD.vs.control; scRNA-seq,SRP330776,Memory T cell_1-AD.vs.control	4
AC016831.1	scRNA-seq,SRP215507,CD8+ T cell_4-AD.vs.MCI; scRNA-seq,SRP215507,CD8+ T cell_5-AD.vs.MCI; scRNA-seq,SRP215507,CD8+ T cell_5-MCI.vs.control; scRNA-seq,SRP330776,B cell_1-AD.vs.control	4
AC016831.5	scRNA-seq,SRP215507,CD8+ T cell_1-AD.vs.MCI; scRNA-seq,SRP215507,CD8+ T cell_4-AD.vs.MCI; scRNA-seq,SRP215507,CD8+ T cell_1-MCI.vs.control; scRNA-seq,SRP330776,CD8+ T cell_1-AD.vs.control; scRNA-seq,SRP330776,CD8+ T cell_2-AD.vs.control; scRNA-seq,SRP330776,Naive CD8+ T cell_1-AD.vs.control; scRNA-seq,SRP330776,B cell_2-AD.vs.control	7
AC016831.7	scRNA-seq,SRP215507,CD8+ T cell_1-AD.vs.control; scRNA-seq,SRP215507,CD8+ T cell_2-AD.vs.MCI; scRNA-seq,SRP215507,CD8+ T cell_1-MCI.vs.control; scRNA-seq,SRP215507,CD8+ T cell_2-MCI.vs.control; scRNA-seq,SRP330776,Naive CD8+ T cell_1-AD.vs.control; scRNA-seq,SRP330776,Memory T cell_1-AD.vs.control; scRNA-seq,SRP330776,CD8+ T cell_3-AD.vs.control	7
AC016876.1	scRNA-seq,SRP215507,CD8+ T cell_5-AD.vs.MCI; scRNA-seq,SRP215507,CD8+ T cell_5-MCI.vs.control; scRNA-seq,SRP330776,CD8+ T cell_1-AD.vs.control; scRNA-seq,SRP330776,γδ T cell-AD.vs.control; scRNA-seq,SRP330776,Memory T cell_2-AD.vs.control; scRNA-seq,SRP330776,CD8+ T cell_2-AD.vs.control; scRNA-seq,SRP330776,B cell_1-AD.vs.control; scRNA-seq,SRP330776,Naive CD8+ T cell_1-AD.vs.control; scRNA-seq,SRP330776,Memory T cell_1-AD.vs.control; scRNA-seq,SRP330776,Naive CD8+ T cell_3-AD.vs.control; scRNA-seq,SRP330776,B cell_2-	12
AC016888.1	scRNA-seq,SRP215507,CD8+ T cell_1-MCI.vs.control	1
AC016949.1	scRNA-seq,SRP215507,CD8+ T cell_3-AD.vs.MCI; scRNA-seq,SRP215507,CD8+ T cell_3-MCI.vs.control; scRNA-seq,SRP330776,Naive CD8+ T	3

AC016957.2	scRNA-seq,SRP215507,CD8+ T cell_3-AD.vs.control; scRNA-seq,SRP215507,CD8+ T cell_3-AD.vs.MCI; scRNA-seq,SRP215507,CD8+ T cell_5-MCI.vs.control; scRNA-seq,SRP215507,CD8+ T cell_3-MCI.vs.control; scRNA-seq,SRP309935,CD8+ T cell_1-AD.vs.control; scRNA-seq,SRP330776,CD8+ T cell_1-AD.vs.control; scRNA-seq,SRP330776,B cell_1-AD.vs.control; scRNA-seq,SRP330776,Naive CD8+ T cell_1-	8
AC017002.3	scRNA-seq,SRP330776,Natural killer cell_1-AD.vs.control; scRNA-seq,SRP330776,Natural killer T cell-AD.vs.control	2
AC017028.1	scRNA-seq,SRP330776,CD8+ T cell_1-AD.vs.control; scRNA-seq,SRP330776,Memory T cell_2-AD.vs.control	2
AC017083.1	scRNA-seq,SRP215507,CD8+ T cell_2-AD.vs.MCI; scRNA-seq,SRP215507,CD8+ T cell_3-AD.vs.MCI; scRNA-seq,SRP215507,CD8+ T cell_1-AD.vs.MCI; scRNA-seq,SRP215507,CD8+ T cell_1-MCI.vs.control; scRNA-seq,SRP215507,CD8+ T cell_3-MCI.vs.control; scRNA-seq,SRP330776,Naive CD8+ T cell_3-AD.vs.control; scRNA-seq,SRP330776,B cell_2-AD.vs.control; scRNA-seq,SRP330776,Natural killer cell_2-	8
AC017083.2	scRNA-seq,SRP215507,CD8+ T cell_1-AD.vs.MCI; scRNA-seq,SRP330776,CD8+ T cell_1-AD.vs.control; scRNA-seq,SRP330776,Memory T cell_2-AD.vs.control; scRNA-seq,SRP330776,CD8+ T cell_2-AD.vs.control; scRNA-seq,SRP330776,Naive CD8+ T cell_1-AD.vs.control; scRNA-seq,SRP330776,Natural killer cell_1-AD.vs.control; scRNA-seq,SRP330776,Memory T cell_1-AD.vs.control; scRNA-seq,SRP330776,Natural killer T cell-	8
AC017104.1	scRNA-seq,SRP215507,CD8+ T cell_1-AD.vs.MCI; scRNA-seq,SRP215507,CD8+ T cell_1-MCI.vs.control; scRNA-seq,SRP330776,γδ T cell-AD.vs.control; scRNA-seq,SRP330776,B cell_1-AD.vs.control; scRNA-seq,SRP330776,B cell_2-AD.vs.control; scRNA-seq,SRP330776,B cell_4-	6
AC017104.6	scRNA-seq,SRP215507,CD8+ T cell_1-AD.vs.MCI; scRNA-seq,SRP309935,Monocyte_1-AD.vs.control; scRNA-seq,SRP330776,CD8+ T cell_2-AD.vs.control; scRNA-seq,SRP330776,Memory T cell_1-AD.vs.control	4
AC018362.1	scRNA-seq,SRP215507,CD8+ T cell_1-AD.vs.control; scRNA-seq,SRP215507,CD8+ T cell_1-MCI.vs.control; scRNA-seq,SRP330776,Memory T cell_2-	3
AC018413.1	scRNA-seq,SRP215507,CD8+ T cell_2-AD.vs.control	1
AC018521.5	scRNA-seq,SRP215507,CD8+ T cell_2-AD.vs.control; scRNA-seq,SRP215507,CD8+ T cell_2-AD.vs.MCI; scRNA-seq,SRP215507,CD8+ T cell_3-AD.vs.MCI; scRNA-seq,SRP215507,CD8+ T cell_3-MCI.vs.control; scRNA-seq,SRP309935,Natural killer cell_2-AD.vs.control; scRNA-seq,SRP330776,Naive CD8+ T cell_1-AD.vs.control; scRNA-seq,SRP330776,Natural killer cell_1-AD.vs.control; scRNA-seq,SRP330776,B cell_2-AD.vs.control; scRNA-seq,SRP330776,CD8+ T cell_3-AD.vs.control; scRNA-seq,SRP330776,Natural killer cell_2-AD.vs.control; scRNA-	11
AC018557.3	scRNA-seq,SRP215507,CD8+ T cell_2-AD.vs.control; scRNA-seq,SRP215507,CD8+ T cell_2-MCI.vs.control; scRNA-seq,SRP330776,Natural killer	3
AC018638.7	scRNA-seq,SRP215507,CD8+ T cell_1-AD.vs.control; scRNA-seq,SRP215507,CD8+ T cell_2-AD.vs.MCI; scRNA-seq,SRP215507,CD8+ T cell_2-	3
AC018647.2	scRNA-seq,SRP215507,CD8+ T cell_5-MCI.vs.control; scRNA-seq,SRP309935,Naive CD8+ T cell_1-AD.vs.control; scRNA-seq,SRP309935,CD8+ T cell_1-AD.vs.control; scRNA-seq,SRP309935,CD8+ T cell_2-AD.vs.control; scRNA-seq,SRP330776,CD8+ T cell_1-AD.vs.control; scRNA-seq,SRP330776,B cell_1-AD.vs.control; scRNA-seq,SRP330776,Natural killer cell_1-AD.vs.control; scRNA-seq,SRP330776,Memory T cell_1-AD.vs.control; scRNA-seq,SRP330776,Naive CD8+ T cell_3-AD.vs.control; scRNA-seq,SRP330776,B cell_2-AD.vs.control	10
AC018742.1	scRNA-seq,SRP215507,CD8+ T cell_2-MCI.vs.control	1
AC018754.1	scRNA-seq,SRP330776,Memory T cell_2-AD.vs.control; scRNA-seq,SRP330776,Memory T cell_1-AD.vs.control; scRNA-seq,SRP330776,CD8+ T	3
AC018755.4	scRNA-seq,SRP309935,Monocyte_1-AD.vs.control; scRNA-seq,SRP309935,Monocyte_2-AD.vs.control	2
AC018761.2	scRNA-seq,SRP330776,CD8+ T cell_1-AD.vs.control; scRNA-seq,SRP330776,γδ T cell-AD.vs.control; scRNA-seq,SRP330776,CD8+ T cell_2-	3
AC018809.1	scRNA-seq,SRP309935,Naive CD8+ T cell_1-AD.vs.control	1
AC018816.1	scRNA-seq,SRP215507,CD8+ T cell_2-AD.vs.control; scRNA-seq,SRP215507,CD8+ T cell_5-AD.vs.control; scRNA-seq,SRP215507,CD8+ T cell_5-AD.vs.MCI; scRNA-seq,SRP215507,CD8+ T cell_2-MCI.vs.control; scRNA-seq,SRP215507,CD8+ T cell_3-MCI.vs.control; scRNA-seq,SRP330776,CD8+ T cell_1-AD.vs.control; scRNA-seq,SRP330776,γδ T cell-AD.vs.control; scRNA-seq,SRP330776,Naive CD8+ T cell_1-	8
AC018946.1	scRNA-seq,SRP330776,Memory T cell_1-AD.vs.control	1
AC019080.6	scRNA-seq,SRP215507,CD8+ T cell_2-MCI.vs.control	1
AC019129.2	scRNA-seq,SRP215507,CD8+ T cell_2-AD.vs.MCI; scRNA-seq,SRP215507,CD8+ T cell_3-AD.vs.MCI; scRNA-seq,SRP215507,CD8+ T cell_1-AD.vs.MCI; scRNA-seq,SRP215507,CD8+ T cell_1-MCI.vs.control; scRNA-seq,SRP215507,CD8+ T cell_2-MCI.vs.control; scRNA-seq,SRP215507,CD8+ T cell_3-MCI.vs.control; scRNA-seq,SRP330776,CD8+ T cell_1-AD.vs.control; scRNA-seq,SRP330776,Memory T cell_2-AD.vs.control; scRNA-seq,SRP330776,CD8+ T cell_2-AD.vs.control; scRNA-seq,SRP330776,Memory T cell_1-AD.vs.control	10

AC019131.2	scRNA-seq,SRP215507,CD8+ T cell_2-AD.vs.control; scRNA-seq,SRP215507,CD8+ T cell_3-AD.vs.MCI; scRNA-seq,SRP215507,CD8+ T cell_3-	3
AC019163.1	scRNA-seq,SRP215507,CD8+ T cell_2-AD.vs.control; scRNA-seq,SRP215507,CD8+ T cell_4-AD.vs.control; scRNA-seq,SRP215507,CD8+ T cell_2-AD.vs.MCI; scRNA-seq,SRP215507,CD8+ T cell_4-AD.vs.MCI; scRNA-seq,SRP215507,CD8+ T cell_5-AD.vs.MCI; scRNA-seq,SRP215507,CD8+ T cell_5-MCI.vs.control; scRNA-seq,SRP330776,Memory T cell_1-AD.vs.control; scRNA-seq,SRP330776,B cell_2-AD.vs.control	8
AC019197.1	scRNA-seq,SRP330776,B cell_1-AD.vs.control	1
AC019205.1	scRNA-seq,SRP330776,B cell_1-AD.vs.control; scRNA-seq,SRP330776,Naive CD8+ T cell_1-AD.vs.control; scRNA-seq,SRP330776,Natural killer T cell-	3
AC019257.1	scRNA-seq,SRP309935,Naive CD8+ T cell_2-AD.vs.control	1
AC019257.9	scRNA-seq,SRP330776,CD8+ T cell_1-AD.vs.control	1
AC020550.2	scRNA-seq,SRP215507,CD8+ T cell_5-MCI.vs.control	1
AC020558.1	scRNA-seq,SRP330776,CD8+ T cell_1-AD.vs.control; scRNA-seq,SRP330776,Naive CD8+ T cell_1-AD.vs.control	2
AC020656.1	scRNA-seq,SRP309935,Monocyte_1-AD.vs.control; scRNA-seq,SRP309935,Monocyte_2-AD.vs.control; scRNA-seq,SRP309935,Monocyte_3-AD.vs.control; scRNA-seq,SRP330776,Monocyte_2-AD.vs.control	4
AC020656.2	scRNA-seq,SRP330776,CD8+ T cell_1-AD.vs.control; scRNA-seq,SRP330776,Naive CD8+ T cell_1-AD.vs.control	2
AC020659.1	scRNA-seq,SRP215507,CD8+ T cell_3-AD.vs.control; scRNA-seq,SRP215507,CD8+ T cell_1-AD.vs.control; scRNA-seq,SRP215507,CD8+ T cell_4-AD.vs.control; scRNA-seq,SRP215507,CD8+ T cell_3-AD.vs.MCI; scRNA-seq,SRP215507,CD8+ T cell_1-AD.vs.MCI; scRNA-seq,SRP215507,CD8+ T cell_4-MCI.vs.control; scRNA-seq,SRP215507,CD8+ T cell_2-MCI.vs.control; scRNA-seq,SRP215507,CD8+ T cell_6-MCI.vs.control; scRNA-seq,SRP309935,Natural killer cell_2-AD.vs.control; scRNA-seq,SRP309935,Natural killer cell_1-AD.vs.control; scRNA-seq,SRP309935,CD8+ T cell_1-AD.vs.control; scRNA-seq,SRP330776,Memory T cell_1-AD.vs.control; scRNA-seq,SRP330776,B cell_2-AD.vs.control; scRNA-seq,SRP330776,T cell-	14
AC020663.3	scRNA-seq,SRP215507,CD8+ T cell_2-AD.vs.control; scRNA-seq,SRP215507,CD8+ T cell_4-AD.vs.control; scRNA-seq,SRP215507,CD8+ T cell_4-AD.vs.MCI; scRNA-seq,SRP215507,CD8+ T cell_1-MCI.vs.control; scRNA-seq,SRP330776,B cell_1-AD.vs.control; scRNA-seq,SRP330776,Memory T	6
AC020741.1	scRNA-seq,SRP309935,Natural killer cell_1-AD.vs.control	1
AC020765.2	scRNA-seq,SRP215507,CD8+ T cell_2-AD.vs.control; scRNA-seq,SRP215507,CD8+ T cell_2-AD.vs.MCI; scRNA-seq,SRP215507,CD8+ T cell_1-AD.vs.MCI; scRNA-seq,SRP215507,CD8+ T cell_5-AD.vs.MCI; scRNA-seq,SRP215507,CD8+ T cell_5-MCI.vs.control; scRNA-seq,SRP215507,CD8+ T cell_6-MCI.vs.control; scRNA-seq,SRP330776,CD8+ T cell_1-AD.vs.control; scRNA-seq,SRP330776,γδ T cell-AD.vs.control; scRNA-seq,SRP330776,Naive CD8+ T cell_1-AD.vs.control; scRNA-seq,SRP330776,CD8+ T cell_3-AD.vs.control	10
AC020910.5	scRNA-seq,SRP215507,CD8+ T cell_3-AD.vs.control; scRNA-seq,SRP215507,CD8+ T cell_1-AD.vs.MCI; scRNA-seq,SRP309935,Naive CD8+ T cell_3-AD.vs.control; scRNA-seq,SRP330776,CD8+ T cell_1-AD.vs.control; scRNA-seq,SRP330776,γδ T cell-AD.vs.control; scRNA-seq,SRP330776,Memory T cell_2-AD.vs.control; scRNA-seq,SRP330776,Naive CD8+ T cell_1-AD.vs.control; scRNA-seq,SRP330776,Memory T cell_1-AD.vs.control	8
AC020911.2	scRNA-seq,SRP215507,CD8+ T cell_7-AD.vs.control; scRNA-seq,SRP215507,CD8+ T cell_3-AD.vs.MCI; scRNA-seq,SRP215507,CD8+ T cell_5-AD.vs.MCI; scRNA-seq,SRP215507,CD8+ T cell_5-MCI.vs.control; scRNA-seq,SRP215507,CD8+ T cell_4-MCI.vs.control; scRNA-seq,SRP309935,CD8+ T cell_2-AD.vs.control; scRNA-seq,SRP330776,Memory T cell_2-AD.vs.control; scRNA-seq,SRP330776,CD8+ T cell_2-AD.vs.control; scRNA-seq,SRP330776,B cell_1-AD.vs.control; scRNA-seq,SRP330776,Naive CD8+ T cell_1-AD.vs.control; scRNA-seq,SRP330776,Memory T cell_1-AD.vs.control; scRNA-seq,SRP330776,B cell_2-AD.vs.control; scRNA-seq,SRP330776,T cell-AD.vs.control; scRNA-	15
AC020914.1	scRNA-seq,SRP330776,B cell_1-AD.vs.control	1
AC020915.2	scRNA-seq,SRP215507,CD8+ T cell_4-AD.vs.control; scRNA-seq,SRP215507,CD8+ T cell_6-AD.vs.control; scRNA-seq,SRP215507,CD8+ T cell_3-AD.vs.MCI; scRNA-seq,SRP215507,CD8+ T cell_3-MCI.vs.control; scRNA-seq,SRP330776,CD8+ T cell_1-AD.vs.control; scRNA-seq,SRP330776,γδ T cell-AD.vs.control; scRNA-seq,SRP330776,Memory T cell_2-AD.vs.control; scRNA-seq,SRP330776,CD8+ T cell_2-AD.vs.control; scRNA-seq,SRP330776,B cell_1-AD.vs.control; scRNA-seq,SRP330776,Naive CD8+ T cell_1-AD.vs.control; scRNA-seq,SRP330776,Natural killer cell_1-AD.vs.control; scRNA-seq,SRP330776,Memory T cell_1-AD.vs.control; scRNA-seq,SRP330776,B cell_2-AD.vs.control; scRNA-seq,SRP330776,T cell-AD.vs.control; scRNA-seq,SRP330776,Naive CD8+ T cell_2-AD.vs.control; scRNA-seq,SRP330776,CD8+ T cell_3-AD.vs.control; scRNA-	17

AC020916.1	scRNA-seq,SRP215507,CD8+ T cell_8-AD.vs.control; scRNA-seq,SRP215507,CD8+ T cell_2-AD.vs.MCI; scRNA-seq,SRP215507,CD8+ T cell_1-AD.vs.MCI; scRNA-seq,SRP215507,CD8+ T cell_5-AD.vs.MCI; scRNA-seq,SRP215507,CD8+ T cell_1-MCI.vs.control; scRNA-seq,SRP215507,CD8+ T cell_5-MCI.vs.control; scRNA-seq,SRP215507,CD8+ T cell_2-MCI.vs.control; scRNA-seq,SRP309935,Erythroid cell-AD.vs.control; scRNA-seq,SRP309935,CD4+ T cell-AD.vs.control; scRNA-seq,SRP330776,B cell_1-AD.vs.control; scRNA-seq,SRP330776,Naive CD8+ T cell_1-AD.vs.control; scRNA-seq,SRP330776,B cell_2-AD.vs.control; scRNA-seq,SRP330776,Monocyte_2-AD.vs.control; scRNA-seq,SRP330776,B cell_3-AD.vs.control	14
AC020917.3	scRNA-seq,SRP309935,Naive CD8+ T cell_2-AD.vs.control	1
AC020922.4	scRNA-seq,SRP330776,Naive CD8+ T cell_1-AD.vs.control; scRNA-seq,SRP330776,Memory T cell_1-AD.vs.control	2
AC020928.1	scRNA-seq,SRP215507,CD8+ T cell_3-AD.vs.control; scRNA-seq,SRP215507,CD8+ T cell_1-AD.vs.MCI; scRNA-seq,SRP215507,CD8+ T cell_1-MCI.vs.control; scRNA-seq,SRP330776,CD8+ T cell_1-AD.vs.control	4
AC020978.5	scRNA-seq,SRP330776,Memory T cell_2-AD.vs.control; scRNA-seq,SRP330776,CD8+ T cell_2-AD.vs.control; scRNA-seq,SRP330776,Naive CD8+ T cell_1-AD.vs.control; scRNA-seq,SRP330776,Memory T cell_1-AD.vs.control	4
AC020978.7	scRNA-seq,SRP215507,CD8+ T cell_2-AD.vs.control; scRNA-seq,SRP215507,CD8+ T cell_1-AD.vs.control; scRNA-seq,SRP215507,CD8+ T cell_3-AD.vs.MCI; scRNA-seq,SRP215507,CD8+ T cell_3-MCI.vs.control; scRNA-seq,SRP330776,CD8+ T cell_1-AD.vs.control; scRNA-seq,SRP330776,Memory T cell_2-AD.vs.control; scRNA-seq,SRP330776,CD8+ T cell_2-AD.vs.control; scRNA-seq,SRP330776,B cell_1-AD.vs.control; scRNA-seq,SRP330776,Naive CD8+ T cell_1-AD.vs.control; scRNA-seq,SRP330776,Memory T cell_1-AD.vs.control; scRNA-seq,SRP330776,CD8+ T	11
AC021016.2	scRNA-seq,SRP215507,CD8+ T cell_4-AD.vs.MCI; scRNA-seq,SRP330776,CD8+ T cell_1-AD.vs.control; scRNA-seq,SRP330776,Memory T cell_2-AD.vs.control; scRNA-seq,SRP330776,Naive CD8+ T cell_1-AD.vs.control	4
AC021016.3	scRNA-seq,SRP330776,Memory T cell_1-AD.vs.control	1
AC021028.1	scRNA-seq,SRP215507,CD8+ T cell_1-AD.vs.control; scRNA-seq,SRP215507,CD8+ T cell_4-AD.vs.control; scRNA-seq,SRP215507,CD8+ T cell_1-AD.vs.MCI; scRNA-seq,SRP330776,CD8+ T cell_1-AD.vs.control; scRNA-seq,SRP330776,Memory T cell_2-AD.vs.control; scRNA-seq,SRP330776,CD8+ T cell_2-AD.vs.control; scRNA-seq,SRP330776,B cell_1-AD.vs.control; scRNA-seq,SRP330776,Naive CD8+ T cell_1-AD.vs.control; scRNA-seq,SRP330776,Naive CD8+ T cell_3-AD.vs.control; scRNA-seq,SRP330776,CD8+ T cell_3-AD.vs.control	10
AC021054.1	scRNA-seq,SRP215507,CD8+ T cell_2-AD.vs.control; scRNA-seq,SRP215507,CD8+ T cell_4-AD.vs.control; scRNA-seq,SRP215507,CD8+ T cell_3-AD.vs.MCI; scRNA-seq,SRP215507,CD8+ T cell_4-MCI.vs.control; scRNA-seq,SRP330776,Memory T cell_2-AD.vs.control; scRNA-seq,SRP330776,B cell_1-AD.vs.control; scRNA-seq,SRP330776,Natural killer T cell-AD.vs.control; scRNA-seq,SRP330776,B cell_3-AD.vs.control	8
AC021086.1	scRNA-seq,SRP215507,CD8+ T cell_3-AD.vs.MCI; scRNA-seq,SRP215507,CD8+ T cell_3-MCI.vs.control	2
AC021092.1	scRNA-seq,SRP215507,CD8+ T cell_3-AD.vs.MCI; scRNA-seq,SRP215507,CD8+ T cell_3-MCI.vs.control; scRNA-seq,SRP330776,Natural killer cell_1-AD.vs.control; scRNA-seq,SRP330776,B cell_3-AD.vs.control; scRNA-seq,SRP330776,Regulatory T cell-AD.vs.control	5
AC021151.1	scRNA-seq,SRP215507,CD8+ T cell_5-MCI.vs.control	1
AC021188.1	scRNA-seq,SRP215507,CD8+ T cell_1-MCI.vs.control; scRNA-seq,SRP330776,γδ T cell-AD.vs.control; scRNA-seq,SRP330776,B cell_1-AD.vs.control; scRNA-seq,SRP330776,T cell-AD.vs.control	4
AC021236.1	scRNA-seq,SRP330776,CD8+ T cell_1-AD.vs.control; scRNA-seq,SRP330776,CD8+ T cell_2-AD.vs.control; scRNA-seq,SRP330776,Naive CD8+ T	3
AC021242.3	scRNA-seq,SRP215507,CD8+ T cell_2-AD.vs.MCI; scRNA-seq,SRP215507,CD8+ T cell_1-AD.vs.MCI; scRNA-seq,SRP215507,CD8+ T cell_1-MCI.vs.control; scRNA-seq,SRP330776,Memory T cell_1-AD.vs.control	4
AC021269.3	scRNA-seq,SRP215507,CD8+ T cell_1-MCI.vs.control	1
AC021321.1	scRNA-seq,SRP215507,CD8+ T cell_3-AD.vs.MCI; scRNA-seq,SRP215507,CD8+ T cell_3-MCI.vs.control	2
AC021678.2	scRNA-seq,SRP330776,B cell_1-AD.vs.control	1
AC021683.5	scRNA-seq,SRP215507,CD8+ T cell_2-AD.vs.control; scRNA-seq,SRP215507,CD8+ T cell_1-AD.vs.control; scRNA-seq,SRP215507,CD8+ T cell_1-MCI.vs.control; scRNA-seq,SRP215507,CD8+ T cell_2-MCI.vs.control; scRNA-seq,SRP330776,CD8+ T cell_1-AD.vs.control; scRNA-seq,SRP330776,γδ T cell-AD.vs.control; scRNA-seq,SRP330776,Memory T cell_2-AD.vs.control; scRNA-seq,SRP330776,CD8+ T cell_2-AD.vs.control; scRNA-seq,SRP330776,Naive CD8+ T cell_1-AD.vs.control; scRNA-seq,SRP330776,T cell-AD.vs.control	10
AC021701.1	scRNA-seq,SRP330776,Naive CD8+ T cell_1-AD.vs.control	1

AC021739.4	scRNA-seq,SRP330776,γδ T cell-AD.vs.control; scRNA-seq,SRP330776,T cell-AD.vs.control; scRNA-seq,SRP330776,CD8+ T cell_3-AD.vs.control; scRNA-seq,SRP330776,Natural killer cell_2-AD.vs.control	4
AC021752.1	scRNA-seq,SRP215507,CD8+ T cell_1-AD.vs.MCI; scRNA-seq,SRP215507,CD8+ T cell_4-MCI.vs.control; scRNA-seq,SRP330776,Naive CD8+ T	3
AC022031.2	scRNA-seq,SRP215507,CD8+ T cell_3-MCI.vs.control	1
AC022075.1	scRNA-seq,SRP215507,CD8+ T cell_3-AD.vs.control; scRNA-seq,SRP215507,CD8+ T cell_5-AD.vs.MCI; scRNA-seq,SRP215507,CD8+ T cell_5-MCI.vs.control; scRNA-seq,SRP330776,B cell_1-AD.vs.control; scRNA-seq,SRP330776,Natural killer cell_1-AD.vs.control; scRNA-seq,SRP330776,Natural killer T cell-AD.vs.control; scRNA-seq,SRP330776,B cell_2-AD.vs.control; scRNA-seq,SRP330776,B cell_3-AD.vs.control	8
AC022098.1	scRNA-seq,SRP215507,CD8+ T cell_2-AD.vs.MCI; scRNA-seq,SRP215507,CD8+ T cell_3-AD.vs.MCI; scRNA-seq,SRP215507,CD8+ T cell_7-AD.vs.MCI; scRNA-seq,SRP215507,CD8+ T cell_3-MCI.vs.control; scRNA-seq,SRP330776,Natural killer T cell-AD.vs.control; scRNA-seq,SRP330776,CD8+ T cell_3-AD.vs.control; scRNA-seq,SRP330776,Monocyte_2-AD.vs.control; scRNA-seq,SRP330776,B cell_4-AD.vs.control	8
AC022098.2	scRNA-seq,SRP330776,Natural killer cell_1-AD.vs.control	1
AC022098.4	scRNA-seq,SRP215507,CD8+ T cell_1-AD.vs.control; scRNA-seq,SRP215507,CD8+ T cell_1-AD.vs.MCI; scRNA-seq,SRP330776,Memory T cell_2-AD.vs.control; scRNA-seq,SRP330776,CD8+ T cell_2-AD.vs.control; scRNA-seq,SRP330776,B cell_1-AD.vs.control; scRNA-seq,SRP330776,Naive	6
AC022126.1	scRNA-seq,SRP309935,Naive CD8+ T cell_1-AD.vs.control; scRNA-seq,SRP309935,Natural killer cell_1-AD.vs.control; scRNA-seq,SRP309935,CD8+ T cell_2-AD.vs.control	3
AC022137.4	scRNA-seq,SRP309935,Natural killer cell_2-AD.vs.control	1
AC022144.1	scRNA-seq,SRP215507,CD8+ T cell_2-AD.vs.MCI; scRNA-seq,SRP215507,CD8+ T cell_1-MCI.vs.control	2
AC022150.4	scRNA-seq,SRP215507,CD8+ T cell_1-AD.vs.control; scRNA-seq,SRP215507,CD8+ T cell_4-MCI.vs.control; scRNA-seq,SRP215507,CD8+ T cell_6-MCI.vs.control; scRNA-seq,SRP309935,Natural killer cell_1-AD.vs.control; scRNA-seq,SRP330776,CD8+ T cell_1-AD.vs.control; scRNA-seq,SRP330776,γδ T cell-AD.vs.control; scRNA-seq,SRP330776,B cell_1-AD.vs.control	7
AC022154.1	scRNA-seq,SRP330776,Memory T cell_1-AD.vs.control	1
AC022167.2	scRNA-seq,SRP215507,CD8+ T cell_2-AD.vs.MCI	1
AC022167.3	scRNA-seq,SRP215507,CD8+ T cell_2-AD.vs.control; scRNA-seq,SRP215507,CD8+ T cell_1-MCI.vs.control; scRNA-seq,SRP330776,Memory T cell_2-AD.vs.control; scRNA-seq,SRP330776,B cell_2-AD.vs.control; scRNA-seq,SRP330776,B cell_4-AD.vs.control	5
AC022182.1	scRNA-seq,SRP215507,CD8+ T cell_1-AD.vs.control; scRNA-seq,SRP215507,CD8+ T cell_5-AD.vs.control; scRNA-seq,SRP330776,CD8+ T cell_2-AD.vs.control; scRNA-seq,SRP330776,B cell_1-AD.vs.control; scRNA-seq,SRP330776,B cell_3-AD.vs.control	5
AC022182.2	scRNA-seq,SRP215507,CD8+ T cell_1-AD.vs.control; scRNA-seq,SRP215507,CD8+ T cell_5-AD.vs.control; scRNA-seq,SRP215507,CD8+ T cell_3-AD.vs.MCI; scRNA-seq,SRP330776,γδ T cell-AD.vs.control; scRNA-seq,SRP330776,Memory T cell_2-AD.vs.control; scRNA-seq,SRP330776,Naive CD8+ T cell_1-AD.vs.control; scRNA-seq,SRP330776,Naive CD8+ T cell_3-AD.vs.control; scRNA-seq,SRP330776,B cell_2-AD.vs.control; scRNA-	9
AC022201.2	scRNA-seq,SRP215507,CD8+ T cell_2-AD.vs.control; scRNA-seq,SRP215507,CD8+ T cell_1-AD.vs.control; scRNA-seq,SRP215507,CD8+ T cell_2-	3
AC022210.2	scRNA-seq,SRP215507,CD8+ T cell_2-AD.vs.control; scRNA-seq,SRP215507,CD8+ T cell_1-AD.vs.control; scRNA-seq,SRP215507,CD8+ T cell_1-AD.vs.MCI; scRNA-seq,SRP215507,CD8+ T cell_5-AD.vs.MCI; scRNA-seq,SRP215507,CD8+ T cell_1-MCI.vs.control; scRNA-seq,SRP330776,γδ T cell-AD.vs.control; scRNA-seq,SRP330776,B cell_2-AD.vs.control; scRNA-seq,SRP330776,CD8+ T cell_3-AD.vs.control	8
AC022211.2	scRNA-seq,SRP215507,CD8+ T cell_5-AD.vs.MCI; scRNA-seq,SRP215507,CD8+ T cell_5-MCI.vs.control; scRNA-seq,SRP330776,Naive CD8+ T	3
AC022217.3	scRNA-seq,SRP215507,CD8+ T cell_2-MCI.vs.control; scRNA-seq,SRP330776,CD8+ T cell_1-AD.vs.control; scRNA-seq,SRP330776,Memory T cell_1-	3
AC022239.1	scRNA-seq,SRP330776,Naive CD8+ T cell_2-AD.vs.control	1
AC022306.2	scRNA-seq,SRP215507,CD8+ T cell_2-AD.vs.control; scRNA-seq,SRP215507,CD8+ T cell_4-MCI.vs.control; scRNA-seq,SRP215507,CD8+ T cell_2-	3
AC022364.1	scRNA-seq,SRP215507,CD8+ T cell_2-AD.vs.control; scRNA-seq,SRP215507,CD8+ T cell_1-AD.vs.control; scRNA-seq,SRP215507,CD8+ T cell_3-AD.vs.MCI; scRNA-seq,SRP215507,CD8+ T cell_2-MCI.vs.control; scRNA-seq,SRP215507,CD8+ T cell_3-MCI.vs.control; scRNA-seq,SRP330776,CD8+ T cell_2-AD.vs.control; scRNA-seq,SRP330776,Memory T cell_1-AD.vs.control; scRNA-seq,SRP330776,Naive CD8+ T cell_3-	9
AC022392.1	scRNA-seq,SRP215507,CD8+ T cell_5-AD.vs.MCI; scRNA-seq,SRP215507,CD8+ T cell_5-MCI.vs.control	2

AC022400.4	scRNA-seq,SRP215507,CD8+ T cell_6-AD.vs.control; scRNA-seq,SRP215507,CD8+ T cell_2-MCI.vs.control; scRNA-seq,SRP330776,γδ T cell-	3
AC022506.2	scRNA-seq,SRP215507,CD8+ T cell_2-AD.vs.MCI; scRNA-seq,SRP215507,CD8+ T cell_1-AD.vs.MCI; scRNA-seq,SRP215507,CD8+ T cell_1-MCI.vs.control; scRNA-seq,SRP330776,CD8+ T cell_2-AD.vs.control; scRNA-seq,SRP330776,B cell_1-AD.vs.control	5
AC022509.3	scRNA-seq,SRP330776,B cell_1-AD.vs.control	1
AC022706.1	scRNA-seq,SRP215507,CD8+ T cell_2-AD.vs.control; scRNA-seq,SRP215507,CD8+ T cell_3-AD.vs.control; scRNA-seq,SRP215507,CD8+ T cell_4-AD.vs.control; scRNA-seq,SRP215507,CD8+ T cell_5-AD.vs.control; scRNA-seq,SRP215507,CD8+ T cell_6-AD.vs.control; scRNA-seq,SRP215507,CD8+ T cell_3-AD.vs.MCI; scRNA-seq,SRP215507,CD8+ T cell_5-AD.vs.MCI; scRNA-seq,SRP330776,Natural killer cell_1-	9
AC022726.2	scRNA-seq,SRP215507,CD8+ T cell_3-MCI.vs.control	1
AC022784.1	scRNA-seq,SRP330776,B cell_1-AD.vs.control; scRNA-seq,SRP330776,Naive CD8+ T cell_1-AD.vs.control	2
AC022868.2	scRNA-seq,SRP215507,CD8+ T cell_1-AD.vs.control; scRNA-seq,SRP215507,CD8+ T cell_1-AD.vs.MCI; scRNA-seq,SRP215507,CD8+ T cell_2-	3
AC022893.1	scRNA-seq,SRP215507,CD8+ T cell_2-MCI.vs.control	1
AC022916.1	scRNA-seq,SRP215507,CD8+ T cell_2-AD.vs.control; scRNA-seq,SRP215507,CD8+ T cell_6-MCI.vs.control; scRNA-seq,SRP330776,CD8+ T cell_1-AD.vs.control; scRNA-seq,SRP330776,T cell-AD.vs.control; scRNA-seq,SRP330776,CD8+ T cell_3-AD.vs.control; scRNA-seq,SRP330776,B cell_3-	6
AC022973.3	scRNA-seq,SRP330776,Memory T cell_1-AD.vs.control	1
AC022973.4	scRNA-seq,SRP215507,CD8+ T cell_1-AD.vs.control; scRNA-seq,SRP330776,CD8+ T cell_1-AD.vs.control; scRNA-seq,SRP330776,Memory T cell_2-AD.vs.control; scRNA-seq,SRP330776,Naive CD8+ T cell_1-AD.vs.control; scRNA-seq,SRP330776,CD8+ T cell_3-AD.vs.control	5
AC023024.1	scRNA-seq,SRP330776,CD8+ T cell_2-AD.vs.control; scRNA-seq,SRP330776,Naive CD8+ T cell_3-AD.vs.control	2
AC023157.3	scRNA-seq,SRP215507,CD8+ T cell_2-MCI.vs.control; scRNA-seq,SRP330776,γδ T cell-AD.vs.control; scRNA-seq,SRP330776,Naive CD8+ T cell_1-AD.vs.control; scRNA-seq,SRP330776,Natural killer T cell-AD.vs.control; scRNA-seq,SRP330776,T cell-AD.vs.control; scRNA-seq,SRP330776,CD8+ T cell_3-AD.vs.control; scRNA-seq,SRP330776,Monocyte_1-AD.vs.control	7
AC023355.1	scRNA-seq,SRP215507,CD8+ T cell_3-AD.vs.control; scRNA-seq,SRP330776,γδ T cell-AD.vs.control; scRNA-seq,SRP330776,CD8+ T cell_2-AD.vs.control; scRNA-seq,SRP330776,B cell_1-AD.vs.control; scRNA-seq,SRP330776,Naive CD8+ T cell_1-AD.vs.control	5
AC023389.1	scRNA-seq,SRP330776,Naive CD8+ T cell_1-AD.vs.control	1
AC023424.3	scRNA-seq,SRP330776,CD8+ T cell_2-AD.vs.control	1
AC023449.2	scRNA-seq,SRP215507,CD8+ T cell_2-AD.vs.control; scRNA-seq,SRP215507,CD8+ T cell_3-AD.vs.control; scRNA-seq,SRP215507,CD8+ T cell_3-AD.vs.MCI; scRNA-seq,SRP215507,CD8+ T cell_5-AD.vs.MCI; scRNA-seq,SRP215507,CD8+ T cell_5-MCI.vs.control; scRNA-seq,SRP330776,CD8+ T cell_1-AD.vs.control; scRNA-seq,SRP330776,CD8+ T cell_2-AD.vs.control; scRNA-seq,SRP330776,Naive CD8+ T cell_1-AD.vs.control; scRNA-	9
AC023509.3	scRNA-seq,SRP215507,CD8+ T cell_1-AD.vs.MCI; scRNA-seq,SRP215507,CD8+ T cell_1-MCI.vs.control	2
AC023510.2	scRNA-seq,SRP330776,B cell_1-AD.vs.control	1
AC023632.2	scRNA-seq,SRP215507,CD8+ T cell_1-AD.vs.control; scRNA-seq,SRP330776,CD8+ T cell_1-AD.vs.control; scRNA-seq,SRP330776,Memory T cell_2-	3
AC023794.3	scRNA-seq,SRP330776,CD8+ T cell_1-AD.vs.control	1
AC023813.3	scRNA-seq,SRP215507,CD8+ T cell_3-AD.vs.control	1
AC023830.3	scRNA-seq,SRP215507,CD8+ T cell_2-MCI.vs.control	1
AC023906.3	scRNA-seq,SRP330776,B cell_3-AD.vs.control	1
AC023908.3	scRNA-seq,SRP330776,Memory T cell_1-AD.vs.control	1
AC024028.1	scRNA-seq,SRP215507,CD8+ T cell_1-AD.vs.control; scRNA-seq,SRP330776,Memory T cell_1-AD.vs.control	2
AC024060.2	scRNA-seq,SRP215507,CD8+ T cell_1-AD.vs.control; scRNA-seq,SRP215507,CD8+ T cell_1-MCI.vs.control	2
AC024075.1	scRNA-seq,SRP215507,CD8+ T cell_1-AD.vs.MCI; scRNA-seq,SRP330776,CD8+ T cell_1-AD.vs.control; scRNA-seq,SRP330776,B cell_1-AD.vs.control; scRNA-seq,SRP330776,Naive CD8+ T cell_1-AD.vs.control	4

AC024075.2	scRNA-seq,SRP215507,CD8+ T cell_3-AD.vs.MCI; scRNA-seq,SRP215507,CD8+ T cell_3-MCI.vs.control; scRNA-seq,SRP330776,CD8+ T cell_1-AD.vs.control; scRNA-seq,SRP330776,B cell_1-AD.vs.control; scRNA-seq,SRP330776,Naive CD8+ T cell_1-AD.vs.control; scRNA-	6
AC024084.1	scRNA-seq,SRP215507,CD8+ T cell_2-AD.vs.control; scRNA-seq,SRP215507,CD8+ T cell_3-AD.vs.control; scRNA-seq,SRP215507,CD8+ T cell_1-AD.vs.MCI; scRNA-seq,SRP215507,CD8+ T cell_1-MCI.vs.control; scRNA-seq,SRP330776,Memory T cell_1-AD.vs.control	5
AC024257.3	scRNA-seq,SRP215507,CD8+ T cell_2-AD.vs.control; scRNA-seq,SRP215507,CD8+ T cell_1-AD.vs.control; scRNA-seq,SRP215507,CD8+ T cell_4-AD.vs.control; scRNA-seq,SRP215507,CD8+ T cell_1-AD.vs.MCI; scRNA-seq,SRP215507,CD8+ T cell_1-MCI.vs.control; scRNA-seq,SRP309935,CD8+ T cell_2-AD.vs.control; scRNA-seq,SRP330776,B cell_1-AD.vs.control; scRNA-seq,SRP330776,Naive CD8+ T cell_1-AD.vs.control; scRNA-	9
AC024267.6	scRNA-seq,SRP330776,CD8+ T cell_1-AD.vs.control; scRNA-seq,SRP330776,γδ T cell-AD.vs.control; scRNA-seq,SRP330776,Naive CD8+ T cell_1-AD.vs.control; scRNA-seq,SRP330776,Memory T cell_1-AD.vs.control	4
AC024451.4	scRNA-seq,SRP215507,CD8+ T cell_2-AD.vs.control; scRNA-seq,SRP215507,CD8+ T cell_4-MCI.vs.control	2
AC024560.5	scRNA-seq,SRP330776,CD8+ T cell_2-AD.vs.control; scRNA-seq,SRP330776,B cell_1-AD.vs.control; scRNA-seq,SRP330776,Naive CD8+ T cell_1-AD.vs.control; scRNA-seq,SRP330776,Memory T cell_1-AD.vs.control; scRNA-seq,SRP330776,CD8+ T cell_3-AD.vs.control	5
AC024575.1	scRNA-seq,SRP215507,CD8+ T cell_2-MCI.vs.control; scRNA-seq,SRP215507,CD8+ T cell_3-MCI.vs.control; scRNA-seq,SRP330776,CD8+ T cell_1-AD.vs.control; scRNA-seq,SRP330776,γδ T cell-AD.vs.control; scRNA-seq,SRP330776,Naive CD8+ T cell_1-AD.vs.control	5
AC024619.3	scRNA-seq,SRP330776,Natural killer cell_1-AD.vs.control	1
AC024909.1	scRNA-seq,SRP215507,CD8+ T cell_1-AD.vs.control; scRNA-seq,SRP215507,CD8+ T cell_1-AD.vs.MCI; scRNA-seq,SRP330776,γδ T cell-AD.vs.control; scRNA-seq,SRP330776,B cell_1-AD.vs.control; scRNA-seq,SRP330776,Monocyte_1-AD.vs.control	5
AC024940.1	scRNA-seq,SRP215507,CD8+ T cell_3-AD.vs.control; scRNA-seq,SRP215507,CD8+ T cell_5-AD.vs.MCI; scRNA-seq,SRP330776,CD8+ T cell_1-AD.vs.control; scRNA-seq,SRP330776,Naive CD8+ T cell_1-AD.vs.control; scRNA-seq,SRP330776,CD8+ T cell_3-AD.vs.control	5
AC025031.3	scRNA-seq,SRP330776,Naive CD8+ T cell_1-AD.vs.control; scRNA-seq,SRP330776,Natural killer cell_1-AD.vs.control	2
AC025048.2	scRNA-seq,SRP215507,CD8+ T cell_1-MCI.vs.control; scRNA-seq,SRP215507,CD8+ T cell_4-MCI.vs.control; scRNA-seq,SRP330776,Memory T cell_2-	3
AC025159.1	scRNA-seq,SRP215507,CD8+ T cell_4-AD.vs.MCI; scRNA-seq,SRP215507,CD8+ T cell_5-AD.vs.MCI; scRNA-seq,SRP215507,CD8+ T cell_4-MCI.vs.control; scRNA-seq,SRP330776,CD8+ T cell_3-AD.vs.control	4
AC025164.1	scRNA-seq,SRP215507,CD8+ T cell_7-AD.vs.control; scRNA-seq,SRP215507,CD8+ T cell_9-AD.vs.control; scRNA-seq,SRP215507,CD8+ T cell_4-AD.vs.MCI; scRNA-seq,SRP215507,CD8+ T cell_4-MCI.vs.control; scRNA-seq,SRP330776,T cell-AD.vs.control; scRNA-seq,SRP330776,Regulatory T cell-AD.vs.control; scRNA-seq,SRP330776,Megakaryocyte_1-AD.vs.control; scRNA-seq,SRP330776,Natural killer cell_2-AD.vs.control	8
AC025165.2	scRNA-seq,SRP330776,B cell_1-AD.vs.control	1
AC025171.1	scRNA-seq,SRP215507,CD8+ T cell_1-AD.vs.MCI; scRNA-seq,SRP215507,CD8+ T cell_5-AD.vs.MCI; scRNA-seq,SRP215507,CD8+ T cell_5-MCI.vs.control; scRNA-seq,SRP309935,Natural killer cell_2-AD.vs.control; scRNA-seq,SRP330776,Memory T cell_2-AD.vs.control; scRNA-seq,SRP330776,CD8+ T cell_3-AD.vs.control; scRNA-seq,SRP330776,Regulatory T cell-AD.vs.control	7
AC025171.2	scRNA-seq,SRP215507,CD8+ T cell_1-AD.vs.MCI; scRNA-seq,SRP215507,CD8+ T cell_5-AD.vs.MCI; scRNA-seq,SRP215507,CD8+ T cell_1-MCI.vs.control; scRNA-seq,SRP330776,Monocyte_1-AD.vs.control; scRNA-seq,SRP330776,B cell_3-AD.vs.control	5
AC025171.3	scRNA-seq,SRP215507,CD8+ T cell_3-AD.vs.control; scRNA-seq,SRP215507,CD8+ T cell_1-AD.vs.MCI; scRNA-seq,SRP215507,CD8+ T cell_4-AD.vs.MCI; scRNA-seq,SRP215507,CD8+ T cell_5-AD.vs.MCI; scRNA-seq,SRP215507,CD8+ T cell_5-MCI.vs.control; scRNA-seq,SRP215507,CD8+ T cell_4-MCI.vs.control; scRNA-seq,SRP309935,Naive CD8+ T cell_1-AD.vs.control; scRNA-seq,SRP330776,CD8+ T cell_1-AD.vs.control; scRNA-seq,SRP330776,CD8+ T cell_2-AD.vs.control; scRNA-seq,SRP330776,Naive CD8+ T cell_1-AD.vs.control; scRNA-seq,SRP330776,Natural killer cell_1-AD.vs.control; scRNA-seq,SRP330776,Memory T cell_1-AD.vs.control; scRNA-seq,SRP330776,Natural killer T cell-AD.vs.control	13
AC025171.4	scRNA-seq,SRP215507,CD8+ T cell_4-AD.vs.control; scRNA-seq,SRP215507,CD8+ T cell_4-MCI.vs.control; scRNA-seq,SRP309935,B cell_1-AD.vs.control; scRNA-seq,SRP330776,CD8+ T cell_1-AD.vs.control	4
AC025171.5	scRNA-seq,SRP215507,CD8+ T cell_1-AD.vs.control; scRNA-seq,SRP215507,CD8+ T cell_1-AD.vs.MCI; scRNA-seq,SRP330776,Naive CD8+ T cell_1-	3
AC025175.2	scRNA-seq,SRP330776,Memory T cell_2-AD.vs.control; scRNA-seq,SRP330776,Memory T cell_1-AD.vs.control	2

AC025181.2	scRNA-seq,SRP215507,CD8+ T cell_3-AD.vs.control; scRNA-seq,SRP309935,Monocyte_1-AD.vs.control; scRNA-seq,SRP330776,CD8+ T cell_2-AD.vs.control; scRNA-seq,SRP330776,T cell-AD.vs.control	4
AC025253.2	scRNA-seq,SRP215507,CD8+ T cell_2-MCI.vs.control; scRNA-seq,SRP215507,CD8+ T cell_3-MCI.vs.control	2
AC025257.1	scRNA-seq,SRP215507,CD8+ T cell_2-AD.vs.control; scRNA-seq,SRP215507,CD8+ T cell_6-AD.vs.control; scRNA-seq,SRP215507,CD8+ T cell_1-AD.vs.MCI; scRNA-seq,SRP215507,CD8+ T cell_1-MCI.vs.control; scRNA-seq,SRP215507,CD8+ T cell_3-MCI.vs.control; scRNA-seq,SRP330776,Memory T cell_1-AD.vs.control; scRNA-seq,SRP330776,Naive CD8+ T cell_3-AD.vs.control; scRNA-seq,SRP330776,Monocyte_1-	8
AC025259.3	scRNA-seq,SRP330776,Monocyte_1-AD.vs.control	1
AC025263.1	scRNA-seq,SRP215507,CD8+ T cell_5-MCI.vs.control	1
AC025283.2	scRNA-seq,SRP330776,CD8+ T cell_1-AD.vs.control	1
AC025423.1	scRNA-seq,SRP309935,Natural killer cell_2-AD.vs.control; scRNA-seq,SRP309935,Natural killer cell_1-AD.vs.control	2
AC025423.4	scRNA-seq,SRP330776,CD8+ T cell_1-AD.vs.control; scRNA-seq,SRP330776,Memory T cell_2-AD.vs.control; scRNA-seq,SRP330776,CD8+ T cell_2-AD.vs.control; scRNA-seq,SRP330776,B cell_1-AD.vs.control; scRNA-seq,SRP330776,Naive CD8+ T cell_1-AD.vs.control; scRNA-seq,SRP330776,Memory T cell_1-AD.vs.control; scRNA-seq,SRP330776,T cell-AD.vs.control; scRNA-seq,SRP330776,B cell_3-AD.vs.control	8
AC025442.1	scRNA-seq,SRP330776,CD8+ T cell_1-AD.vs.control; scRNA-seq,SRP330776,CD8+ T cell_3-AD.vs.control	2
AC025449.2	scRNA-seq,SRP215507,CD8+ T cell_5-AD.vs.MCI; scRNA-seq,SRP215507,CD8+ T cell_1-MCI.vs.control; scRNA-seq,SRP330776,B cell_1-	3
AC025682.1	scRNA-seq,SRP215507,CD8+ T cell_4-AD.vs.control; scRNA-seq,SRP330776,CD8+ T cell_1-AD.vs.control; scRNA-seq,SRP330776,Memory T cell_2-AD.vs.control; scRNA-seq,SRP330776,Naive CD8+ T cell_1-AD.vs.control; scRNA-seq,SRP330776,Natural killer T cell-AD.vs.control; scRNA-seq,SRP330776,Naive CD8+ T cell_2-AD.vs.control	6
AC025917.1	scRNA-seq,SRP330776,CD8+ T cell_3-AD.vs.control	1
AC026124.2	scRNA-seq,SRP215507,CD8+ T cell_2-AD.vs.control; scRNA-seq,SRP215507,CD8+ T cell_3-AD.vs.control; scRNA-seq,SRP215507,CD8+ T cell_1-AD.vs.control; scRNA-seq,SRP215507,CD8+ T cell_3-MCI.vs.control; scRNA-seq,SRP309935,Natural killer cell_1-AD.vs.control; scRNA-seq,SRP309935,Naive CD8+ T cell_2-AD.vs.control	6
AC026191.1	scRNA-seq,SRP215507,CD8+ T cell_2-AD.vs.control; scRNA-seq,SRP215507,CD8+ T cell_1-AD.vs.MCI; scRNA-seq,SRP215507,CD8+ T cell_1-MCI.vs.control; scRNA-seq,SRP330776,CD8+ T cell_1-AD.vs.control; scRNA-seq,SRP330776,Naive CD8+ T cell_1-AD.vs.control; scRNA-	6
AC026202.3	scRNA-seq,SRP215507,CD8+ T cell_1-MCI.vs.control; scRNA-seq,SRP215507,CD8+ T cell_2-MCI.vs.control; scRNA-seq,SRP330776,CD8+ T cell_1-	3
AC026304.1	scRNA-seq,SRP215507,CD8+ T cell_3-AD.vs.control; scRNA-seq,SRP215507,CD8+ T cell_3-AD.vs.MCI; scRNA-seq,SRP215507,CD8+ T cell_4-MCI.vs.control; scRNA-seq,SRP330776,CD8+ T cell_1-AD.vs.control; scRNA-seq,SRP330776,T cell-AD.vs.control	5
AC026341.1	scRNA-seq,SRP215507,CD8+ T cell_2-AD.vs.control; scRNA-seq,SRP330776,γδ T cell-AD.vs.control; scRNA-seq,SRP330776,Memory T cell_2-AD.vs.control; scRNA-seq,SRP330776,B cell_1-AD.vs.control; scRNA-seq,SRP330776,Naive CD8+ T cell_3-AD.vs.control	5
AC026347.1	scRNA-seq,SRP330776,B cell_1-AD.vs.control	1
AC026356.2	scRNA-seq,SRP215507,CD8+ T cell_3-MCI.vs.control; scRNA-seq,SRP330776,CD8+ T cell_2-AD.vs.control; scRNA-seq,SRP330776,B cell_1-AD.vs.control; scRNA-seq,SRP330776,Naive CD8+ T cell_1-AD.vs.control	4
AC026369.3	scRNA-seq,SRP330776,Monocyte_1-AD.vs.control	1
AC026401.1	scRNA-seq,SRP215507,CD8+ T cell_3-AD.vs.MCI; scRNA-seq,SRP215507,CD8+ T cell_3-MCI.vs.control	2
AC026401.3	scRNA-seq,SRP215507,CD8+ T cell_3-AD.vs.control; scRNA-seq,SRP215507,CD8+ T cell_3-AD.vs.MCI; scRNA-seq,SRP215507,CD8+ T cell_1-MCI.vs.control; scRNA-seq,SRP309935,Natural killer cell_2-AD.vs.control; scRNA-seq,SRP309935,Monocyte_1-AD.vs.control; scRNA-seq,SRP330776,B cell_1-AD.vs.control; scRNA-seq,SRP330776,Naive CD8+ T cell_1-AD.vs.control	7
AC026412.3	scRNA-seq,SRP330776,B cell_1-AD.vs.control	1

AC026471.1	scRNA-seq,SRP215507,CD8+ T cell_4-AD.vs.control; scRNA-seq,SRP215507,CD8+ T cell_5-AD.vs.control; scRNA-seq,SRP215507,CD8+ T cell_6-AD.vs.control; scRNA-seq,SRP215507,CD8+ T cell_6-MCI.vs.control; scRNA-seq,SRP330776,γδ T cell-AD.vs.control; scRNA-seq,SRP330776,Memory T cell_2-AD.vs.control; scRNA-seq,SRP330776,B cell_1-AD.vs.control; scRNA-seq,SRP330776,Natural killer cell_1-AD.vs.control; scRNA-seq,SRP330776,Natural killer T cell-AD.vs.control; scRNA-seq,SRP330776,Naive CD8+ T cell_3-AD.vs.control; scRNA-seq,SRP330776,B cell_2-	12
AC026471.2	scRNA-seq,SRP215507,CD8+ T cell_2-AD.vs.control; scRNA-seq,SRP215507,CD8+ T cell_3-AD.vs.control; scRNA-seq,SRP215507,CD8+ T cell_1-AD.vs.control; scRNA-seq,SRP215507,CD8+ T cell_2-AD.vs.MCI; scRNA-seq,SRP215507,CD8+ T cell_3-AD.vs.MCI; scRNA-seq,SRP330776,CD8+ T cell_1-AD.vs.control; scRNA-seq,SRP330776,γδ T cell-AD.vs.control; scRNA-seq,SRP330776,CD8+ T cell_2-AD.vs.control; scRNA-seq,SRP330776,Natural killer T cell-AD.vs.control; scRNA-seq,SRP330776,CD8+ T cell_3-AD.vs.control	10
AC026471.3	scRNA-seq,SRP215507,CD8+ T cell_2-AD.vs.control; scRNA-seq,SRP215507,CD8+ T cell_3-AD.vs.control; scRNA-seq,SRP215507,CD8+ T cell_2-AD.vs.MCI; scRNA-seq,SRP215507,CD8+ T cell_5-AD.vs.MCI; scRNA-seq,SRP330776,CD8+ T cell_1-AD.vs.control; scRNA-seq,SRP330776,Memory T cell_2-AD.vs.control; scRNA-seq,SRP330776,Naive CD8+ T cell_3-AD.vs.control; scRNA-seq,SRP330776,Naive CD8+ T cell_2-AD.vs.control;	9
AC026748.3	scRNA-seq,SRP215507,CD8+ T cell_3-AD.vs.control; scRNA-seq,SRP215507,CD8+ T cell_3-MCI.vs.control; scRNA-seq,SRP330776,CD8+ T cell_1-AD.vs.control; scRNA-seq,SRP330776,γδ T cell-AD.vs.control; scRNA-seq,SRP330776,B cell_1-AD.vs.control; scRNA-seq,SRP330776,Naive CD8+ T cell_1-AD.vs.control; scRNA-seq,SRP330776,Memory T cell_1-AD.vs.control	7
AC026771.1	scRNA-seq,SRP330776,Naive CD8+ T cell_1-AD.vs.control	1
AC026801.2	scRNA-seq,SRP215507,CD8+ T cell_5-AD.vs.control; scRNA-seq,SRP215507,CD8+ T cell_1-AD.vs.MCI; scRNA-seq,SRP215507,CD8+ T cell_1-MCI.vs.control; scRNA-seq,SRP215507,CD8+ T cell_2-MCI.vs.control; scRNA-seq,SRP330776,CD8+ T cell_1-AD.vs.control; scRNA-seq,SRP330776,Memory T cell_2-AD.vs.control; scRNA-seq,SRP330776,B cell_1-AD.vs.control; scRNA-seq,SRP330776,Naive CD8+ T cell_1-	9
AC026904.1	scRNA-seq,SRP215507,CD8+ T cell_1-AD.vs.MCI; scRNA-seq,SRP215507,CD8+ T cell_1-MCI.vs.control; scRNA-seq,SRP215507,CD8+ T cell_2-	3
AC026979.2	scRNA-seq,SRP215507,CD8+ T cell_4-AD.vs.control; scRNA-seq,SRP215507,CD8+ T cell_6-AD.vs.control; scRNA-seq,SRP215507,CD8+ T cell_5-AD.vs.MCI; scRNA-seq,SRP309935,Natural killer cell_2-AD.vs.control; scRNA-seq,SRP330776,CD8+ T cell_1-AD.vs.control; scRNA-seq,SRP330776,Memory T cell_2-AD.vs.control; scRNA-seq,SRP330776,CD8+ T cell_2-AD.vs.control; scRNA-seq,SRP330776,B cell_1-AD.vs.control; scRNA-seq,SRP330776,Naive CD8+ T cell_1-AD.vs.control; scRNA-seq,SRP330776,Natural killer cell_1-AD.vs.control; scRNA-seq,SRP330776,Memory T cell_1-AD.vs.control; scRNA-seq,SRP330776,Natural killer T cell-AD.vs.control; scRNA-seq,SRP330776,Naive CD8+ T cell_3-AD.vs.control; scRNA-seq,SRP330776,B cell_2-AD.vs.control; scRNA-seq,SRP330776,T cell-AD.vs.control; scRNA-seq,SRP330776,CD8+ T cell_3-AD.vs.control; scRNA- [illegible]	17
AC026979.4	scRNA-seq,SRP215507,CD8+ T cell_3-AD.vs.control; scRNA-seq,SRP215507,CD8+ T cell_1-MCI.vs.control; scRNA-seq,SRP215507,CD8+ T cell_3-MCI.vs.control; scRNA-seq,SRP330776,CD8+ T cell_1-AD.vs.control	4
AC027020.2	scRNA-seq,SRP215507,CD8+ T cell_4-AD.vs.control; scRNA-seq,SRP215507,CD8+ T cell_5-AD.vs.control; scRNA-seq,SRP215507,CD8+ T cell_4-AD.vs.MCI; scRNA-seq,SRP215507,CD8+ T cell_3-MCI.vs.control; scRNA-seq,SRP330776,CD8+ T cell_1-AD.vs.control; scRNA-seq,SRP330776,Naive	6
AC027031.2	scRNA-seq,SRP215507,CD8+ T cell_3-AD.vs.control; scRNA-seq,SRP215507,CD8+ T cell_1-AD.vs.MCI; scRNA-seq,SRP215507,CD8+ T cell_1-MCI.vs.control; scRNA-seq,SRP309935,Naive CD8+ T cell_2-AD.vs.control; scRNA-seq,SRP330776,CD8+ T cell_1-AD.vs.control; scRNA-seq,SRP330776,Memory T cell_2-AD.vs.control; scRNA-seq,SRP330776,Naive CD8+ T cell_1-AD.vs.control	7
AC027097.1	scRNA-seq,SRP215507,CD8+ T cell_4-AD.vs.MCI; scRNA-seq,SRP309935,Naive CD8+ T cell_2-AD.vs.control; scRNA-seq,SRP330776,Memory T cell_1-AD.vs.control; scRNA-seq,SRP330776,CD8+ T cell_3-AD.vs.control	4
AC027097.2	scRNA-seq,SRP215507,CD8+ T cell_1-AD.vs.control; scRNA-seq,SRP215507,CD8+ T cell_5-AD.vs.control; scRNA-seq,SRP215507,CD8+ T cell_1-AD.vs.MCI; scRNA-seq,SRP215507,CD8+ T cell_4-AD.vs.MCI; scRNA-seq,SRP215507,CD8+ T cell_5-MCI.vs.control; scRNA-seq,SRP309935,CD8+ T cell_2-AD.vs.control; scRNA-seq,SRP330776,Naive CD8+ T cell_1-AD.vs.control; scRNA-seq,SRP330776,B cell_2-AD.vs.control	8
AC027279.1	scRNA-seq,SRP215507,CD8+ T cell_2-AD.vs.control; scRNA-seq,SRP215507,CD8+ T cell_5-AD.vs.control; scRNA-seq,SRP215507,CD8+ T cell_5-AD.vs.MCI; scRNA-seq,SRP215507,CD8+ T cell_3-MCI.vs.control; scRNA-seq,SRP330776,CD8+ T cell_1-AD.vs.control; scRNA-seq,SRP330776,CD8+ T cell_2-AD.vs.control; scRNA-seq,SRP330776,B cell_1-AD.vs.control; scRNA-seq,SRP330776,Naive CD8+ T cell_1-AD.vs.control; scRNA-seq,SRP330776,Memory T cell_1-AD.vs.control; scRNA-seq,SRP330776,Natural killer T cell-AD.vs.control; scRNA-seq,SRP330776,CD8+ T cell_3-	11
AC027279.2	scRNA-seq,SRP215507,CD8+ T cell_2-MCI.vs.control	1

AC027290.1	scRNA-seq,SRP215507,CD8+ T cell_5-AD.vs.MCI; scRNA-seq,SRP330776,CD8+ T cell_1-AD.vs.control; scRNA-seq,SRP330776,Memory T cell_2-AD.vs.control; scRNA-seq,SRP330776,CD8+ T cell_2-AD.vs.control; scRNA-seq,SRP330776,B cell_1-AD.vs.control; scRNA-seq,SRP330776,Natural killer T cell-AD.vs.control; scRNA-seq,SRP330776,B cell_3-AD.vs.control; scRNA-seq,SRP330776,Regulatory T cell-AD.vs.control; scRNA-	9
AC027307.1	scRNA-seq,SRP215507,CD8+ T cell_3-AD.vs.control; scRNA-seq,SRP215507,CD8+ T cell_3-MCI.vs.control	2
AC027307.2	scRNA-seq,SRP215507,CD8+ T cell_1-AD.vs.MCI	1
AC027307.3	scRNA-seq,SRP215507,CD8+ T cell_3-AD.vs.MCI; scRNA-seq,SRP309935,Naive CD8+ T cell_1-AD.vs.control	2
AC027449.1	scRNA-seq,SRP309935,Natural killer cell_2-AD.vs.control; scRNA-seq,SRP309935,Naive CD8+ T cell_1-AD.vs.control	2
AC027514.1	scRNA-seq,SRP215507,CD8+ T cell_1-AD.vs.MCI; scRNA-seq,SRP215507,CD8+ T cell_4-MCI.vs.control	2
AC027601.4	scRNA-seq,SRP215507,CD8+ T cell_1-MCI.vs.control	1
AC027644.3	scRNA-seq,SRP215507,CD8+ T cell_7-AD.vs.MCI; scRNA-seq,SRP330776,γδ T cell-AD.vs.control; scRNA-seq,SRP330776,CD8+ T cell_2-AD.vs.control; scRNA-seq,SRP330776,Naive CD8+ T cell_1-AD.vs.control; scRNA-seq,SRP330776,Memory T cell_1-AD.vs.control; scRNA-	6
AC027682.1	scRNA-seq,SRP215507,CD8+ T cell_1-AD.vs.control; scRNA-seq,SRP215507,CD8+ T cell_5-AD.vs.control; scRNA-seq,SRP215507,CD8+ T cell_1-MCI.vs.control; scRNA-seq,SRP215507,CD8+ T cell_5-MCI.vs.control; scRNA-seq,SRP330776,Natural killer cell_1-AD.vs.control; scRNA-	6
AC027682.2	scRNA-seq,SRP215507,CD8+ T cell_1-AD.vs.MCI; scRNA-seq,SRP215507,CD8+ T cell_1-MCI.vs.control	2
AC027682.4	scRNA-seq,SRP215507,CD8+ T cell_2-AD.vs.control; scRNA-seq,SRP215507,CD8+ T cell_3-AD.vs.control; scRNA-seq,SRP215507,CD8+ T cell_5-AD.vs.control; scRNA-seq,SRP215507,CD8+ T cell_5-MCI.vs.control; scRNA-seq,SRP309935,Naive CD8+ T cell_1-AD.vs.control; scRNA-seq,SRP330776,γδ T cell-AD.vs.control; scRNA-seq,SRP330776,Memory T cell_2-AD.vs.control; scRNA-seq,SRP330776,Naive CD8+ T cell_1-AD.vs.control; scRNA-seq,SRP330776,Memory T cell_1-AD.vs.control; scRNA-seq,SRP330776,T cell-AD.vs.control	10
AC027682.6	scRNA-seq,SRP215507,CD8+ T cell_2-AD.vs.MCI; scRNA-seq,SRP215507,CD8+ T cell_2-MCI.vs.control	2
AC027702.1	scRNA-seq,SRP215507,CD8+ T cell_3-AD.vs.MCI; scRNA-seq,SRP215507,CD8+ T cell_1-AD.vs.MCI; scRNA-seq,SRP215507,CD8+ T cell_5-AD.vs.MCI; scRNA-seq,SRP215507,CD8+ T cell_5-MCI.vs.control; scRNA-seq,SRP215507,CD8+ T cell_2-MCI.vs.control; scRNA-seq,SRP215507,CD8+ T cell_3-MCI.vs.control; scRNA-seq,SRP330776,B cell_1-AD.vs.control; scRNA-seq,SRP330776,B cell_3-AD.vs.control	8
AC034102.3	scRNA-seq,SRP215507,CD8+ T cell_3-AD.vs.MCI; scRNA-seq,SRP215507,CD8+ T cell_3-MCI.vs.control	2
AC034102.5	scRNA-seq,SRP330776,Naive CD8+ T cell_1-AD.vs.control	1
AC034102.6	scRNA-seq,SRP215507,CD8+ T cell_2-AD.vs.control; scRNA-seq,SRP215507,CD8+ T cell_1-AD.vs.MCI; scRNA-seq,SRP330776,B cell_1-AD.vs.control; scRNA-seq,SRP330776,Natural killer cell_1-AD.vs.control; scRNA-seq,SRP330776,Naive CD8+ T cell_3-AD.vs.control	5
AC034111.1	scRNA-seq,SRP215507,CD8+ T cell_2-MCI.vs.control	1
AC034193.1	scRNA-seq,SRP215507,CD8+ T cell_5-AD.vs.MCI; scRNA-seq,SRP215507,CD8+ T cell_1-MCI.vs.control; scRNA-seq,SRP215507,CD8+ T cell_5-	3
AC034231.1	scRNA-seq,SRP215507,CD8+ T cell_3-AD.vs.control; scRNA-seq,SRP215507,CD8+ T cell_3-AD.vs.MCI	2
AC034236.2	scRNA-seq,SRP215507,CD8+ T cell_1-AD.vs.control; scRNA-seq,SRP215507,CD8+ T cell_4-AD.vs.MCI; scRNA-seq,SRP215507,CD8+ T cell_1-MCI.vs.control; scRNA-seq,SRP215507,CD8+ T cell_4-MCI.vs.control; scRNA-seq,SRP330776,γδ T cell-AD.vs.control; scRNA-seq,SRP330776,Memory T cell_2-AD.vs.control; scRNA-seq,SRP330776,Memory T cell_1-AD.vs.control; scRNA-seq,SRP330776,Naive CD8+ T cell_3-AD.vs.control; scRNA-	9
AC034238.1	scRNA-seq,SRP215507,CD8+ T cell_6-AD.vs.control; scRNA-seq,SRP215507,CD8+ T cell_2-AD.vs.MCI; scRNA-seq,SRP215507,CD8+ T cell_3-AD.vs.MCI; scRNA-seq,SRP215507,CD8+ T cell_5-MCI.vs.control; scRNA-seq,SRP215507,CD8+ T cell_3-MCI.vs.control; scRNA-seq,SRP215507,CD8+ T cell_6-MCI.vs.control; scRNA-seq,SRP330776,Memory T cell_2-AD.vs.control; scRNA-seq,SRP330776,CD8+ T cell_3-	8
AC035140.1	scRNA-seq,SRP215507,CD8+ T cell_1-MCI.vs.control	1
AC036108.2	scRNA-seq,SRP330776,Natural killer cell_1-AD.vs.control; scRNA-seq,SRP330776,Memory T cell_1-AD.vs.control	2
AC036108.3	scRNA-seq,SRP215507,CD8+ T cell_1-MCI.vs.control	1
AC036176.1	scRNA-seq,SRP215507,CD8+ T cell_3-AD.vs.MCI; scRNA-seq,SRP215507,CD8+ T cell_5-AD.vs.MCI; scRNA-seq,SRP215507,CD8+ T cell_5-MCI.vs.control; scRNA-seq,SRP330776,γδ T cell-AD.vs.control; scRNA-seq,SRP330776,T cell-AD.vs.control	5

AC036214.2	scRNA-seq,SRP215507,CD8+ T cell_4-AD.vs.control	1
AC037198.1	scRNA-seq,SRP309935,Monocyte_1-AD.vs.control; scRNA-seq,SRP309935,Monocyte_2-AD.vs.control	2
AC037198.2	scRNA-seq,SRP330776,Monocyte_2-AD.vs.control	1
AC037459.2	scRNA-seq,SRP215507,CD8+ T cell_5-AD.vs.MCI; scRNA-seq,SRP330776,CD8+ T cell_1-AD.vs.control; scRNA-seq,SRP330776,Memory T cell_2-AD.vs.control; scRNA-seq,SRP330776,Naive CD8+ T cell_1-AD.vs.control; scRNA-seq,SRP330776,Natural killer cell_1-AD.vs.control; scRNA-seq,SRP330776,Memory T cell_1-AD.vs.control; scRNA-seq,SRP330776,Naive CD8+ T cell_3-AD.vs.control	7
AC037459.4	scRNA-seq,SRP215507,CD8+ T cell_2-AD.vs.control	1
AC040168.1	scRNA-seq,SRP215507,CD8+ T cell_1-AD.vs.MCI; scRNA-seq,SRP215507,CD8+ T cell_1-MCI.vs.control	2
AC040169.1	scRNA-seq,SRP215507,CD8+ T cell_3-AD.vs.control; scRNA-seq,SRP215507,CD8+ T cell_2-AD.vs.MCI; scRNA-seq,SRP215507,CD8+ T cell_2-MCI.vs.control; scRNA-seq,SRP330776,CD8+ T cell_1-AD.vs.control; scRNA-seq,SRP330776,B cell_1-AD.vs.control; scRNA-seq,SRP330776,Naive CD8+ T cell_1-AD.vs.control; scRNA-seq,SRP330776,Memory T cell_1-AD.vs.control; scRNA-seq,SRP330776,CD8+ T cell_3-AD.vs.control	8
AC040169.3	scRNA-seq,SRP215507,CD8+ T cell_2-AD.vs.MCI; scRNA-seq,SRP215507,CD8+ T cell_1-AD.vs.MCI; scRNA-seq,SRP215507,CD8+ T cell_1-MCI.vs.control; scRNA-seq,SRP215507,CD8+ T cell_2-MCI.vs.control	4
AC040970.1	scRNA-seq,SRP215507,CD8+ T cell_1-AD.vs.MCI; scRNA-seq,SRP215507,CD8+ T cell_5-AD.vs.MCI; scRNA-seq,SRP215507,CD8+ T cell_1-MCI.vs.control; scRNA-seq,SRP215507,CD8+ T cell_5-MCI.vs.control; scRNA-seq,SRP330776,CD8+ T cell_1-AD.vs.control; scRNA-seq,SRP330776,B cell_1-AD.vs.control; scRNA-seq,SRP330776,Naive CD8+ T cell_1-AD.vs.control; scRNA-seq,SRP330776,Memory T cell_1-AD.vs.control; scRNA-seq,SRP330776,Natural killer T cell-AD.vs.control; scRNA-seq,SRP330776,B cell_2-AD.vs.control	10
AC040977.1	scRNA-seq,SRP215507,CD8+ T cell_2-AD.vs.control; scRNA-seq,SRP309935,Natural killer cell_1-AD.vs.control; scRNA-seq,SRP309935,Naive CD8+ T cell_2-AD.vs.control; scRNA-seq,SRP309935,CD8+ T cell_1-AD.vs.control	4
AC044849.1	scRNA-seq,SRP215507,CD8+ T cell_1-AD.vs.control; scRNA-seq,SRP215507,CD8+ T cell_4-AD.vs.control; scRNA-seq,SRP215507,CD8+ T cell_1-AD.vs.MCI; scRNA-seq,SRP215507,CD8+ T cell_5-AD.vs.MCI; scRNA-seq,SRP215507,CD8+ T cell_6-AD.vs.MCI; scRNA-seq,SRP215507,CD8+ T cell_5-MCI.vs.control; scRNA-seq,SRP215507,CD8+ T cell_4-MCI.vs.control; scRNA-seq,SRP215507,CD8+ T cell_6-MCI.vs.control; scRNA-seq,SRP309935,Monocyte_1-AD.vs.control; scRNA-seq,SRP309935,Megakaryocyte_1-AD.vs.control; scRNA-seq,SRP309935,CD8+ T cell_1-AD.vs.control; scRNA-seq,SRP309935,Naive CD8+ T cell_3-AD.vs.control; scRNA-seq,SRP330776,CD8+ T cell_1-AD.vs.control; scRNA-seq,SRP330776,γδ T cell-AD.vs.control; scRNA-seq,SRP330776,Memory T cell_2-AD.vs.control; scRNA-seq,SRP330776,CD8+ T cell_2-AD.vs.control; scRNA-seq,SRP330776,B cell_1-AD.vs.control; scRNA-seq,SRP330776,Naive CD8+ T cell_1-AD.vs.control; scRNA-seq,SRP330776,Memory T cell_1-AD.vs.control; scRNA-seq,SRP330776,Natural killer T cell-AD.vs.control; scRNA-seq,SRP330776,Naive CD8+ T cell_3-AD.vs.control; scRNA-[illegible]	23
AC046134.2	scRNA-seq,SRP215507,CD8+ T cell_5-MCI.vs.control; scRNA-seq,SRP330776,CD8+ T cell_1-AD.vs.control; scRNA-seq,SRP330776,Memory T cell_2-AD.vs.control; scRNA-seq,SRP330776,Naive CD8+ T cell_1-AD.vs.control; scRNA-seq,SRP330776,T cell-AD.vs.control; scRNA-seq,SRP330776,CD8+	6
AC048338.2	scRNA-seq,SRP215507,CD8+ T cell_1-MCI.vs.control; scRNA-seq,SRP330776,CD8+ T cell_2-AD.vs.control	2
AC048341.1	scRNA-seq,SRP330776,CD8+ T cell_1-AD.vs.control; scRNA-seq,SRP330776,CD8+ T cell_2-AD.vs.control; scRNA-seq,SRP330776,Naive CD8+ T cell_1-AD.vs.control; scRNA-seq,SRP330776,B cell_2-AD.vs.control	4
AC048341.2	scRNA-seq,SRP215507,CD8+ T cell_2-AD.vs.control; scRNA-seq,SRP215507,CD8+ T cell_5-AD.vs.control; scRNA-seq,SRP330776,B cell_2-	3
AC048382.5	scRNA-seq,SRP215507,CD8+ T cell_5-AD.vs.control; scRNA-seq,SRP215507,CD8+ T cell_5-AD.vs.MCI; scRNA-seq,SRP330776,CD8+ T cell_1-AD.vs.control; scRNA-seq,SRP330776,γδ T cell-AD.vs.control; scRNA-seq,SRP330776,CD8+ T cell_2-AD.vs.control; scRNA-seq,SRP330776,B cell_1-AD.vs.control; scRNA-seq,SRP330776,Naive CD8+ T cell_1-AD.vs.control; scRNA-seq,SRP330776,Natural killer cell_1-AD.vs.control; scRNA-seq,SRP330776,Memory T cell_1-AD.vs.control; scRNA-seq,SRP330776,CD8+ T cell_3-AD.vs.control	10
AC048382.6	scRNA-seq,SRP215507,CD8+ T cell_3-MCI.vs.control; scRNA-seq,SRP330776,γδ T cell-AD.vs.control; scRNA-seq,SRP330776,CD8+ T cell_2-	3
AC051619.5	scRNA-seq,SRP215507,CD8+ T cell_2-AD.vs.control; scRNA-seq,SRP215507,CD8+ T cell_3-AD.vs.control; scRNA-seq,SRP215507,CD8+ T cell_1-AD.vs.control; scRNA-seq,SRP215507,CD8+ T cell_3-AD.vs.MCI; scRNA-seq,SRP215507,CD8+ T cell_1-AD.vs.MCI; scRNA-seq,SRP330776,CD8+ T	6
AC051619.6	scRNA-seq,SRP215507,CD8+ T cell_4-MCI.vs.control; scRNA-seq,SRP330776,γδ T cell-AD.vs.control; scRNA-seq,SRP330776,Memory T cell_2-AD.vs.control; scRNA-seq,SRP330776,T cell-AD.vs.control	4

AC051619.8	scRNA-seq,SRP215507,CD8+ T cell_2-AD.vs.control; scRNA-seq,SRP330776,Memory T cell_1-AD.vs.control	2
AC053513.1	scRNA-seq,SRP330776,CD8+ T cell_3-AD.vs.control	1
AC053527.2	scRNA-seq,SRP215507,CD8+ T cell_3-AD.vs.control; scRNA-seq,SRP215507,CD8+ T cell_4-MCI.vs.control; scRNA-seq,SRP330776,Natural killer	3
AC055839.2	scRNA-seq,SRP215507,CD8+ T cell_1-AD.vs.control; scRNA-seq,SRP215507,CD8+ T cell_3-AD.vs.MCI; scRNA-seq,SRP215507,CD8+ T cell_5-AD.vs.MCI; scRNA-seq,SRP215507,CD8+ T cell_5-MCI.vs.control; scRNA-seq,SRP330776,CD8+ T cell_1-AD.vs.control; scRNA-seq,SRP330776,Memory T cell_2-AD.vs.control; scRNA-seq,SRP330776,Naive CD8+ T cell_1-AD.vs.control; scRNA-seq,SRP330776,Memory T cell_1-	8
AC058791.1	scRNA-seq,SRP215507,CD8+ T cell_4-AD.vs.control; scRNA-seq,SRP215507,CD8+ T cell_6-AD.vs.control; scRNA-seq,SRP215507,CD8+ T cell_4-MCI.vs.control; scRNA-seq,SRP309935,Monocyte_1-AD.vs.control; scRNA-seq,SRP309935,Erythroid cell-AD.vs.control; scRNA-seq,SRP330776,CD8+ T cell_1-AD.vs.control; scRNA-seq,SRP330776,Memory T cell_2-AD.vs.control; scRNA-seq,SRP330776,Naive CD8+ T cell_1-AD.vs.control; scRNA-	9
AC060780.1	scRNA-seq,SRP309935,Naive CD8+ T cell_2-AD.vs.control; scRNA-seq,SRP330776,CD8+ T cell_1-AD.vs.control; scRNA-seq,SRP330776,Memory T cell_2-AD.vs.control; scRNA-seq,SRP330776,Naive CD8+ T cell_1-AD.vs.control; scRNA-seq,SRP330776,T cell-AD.vs.control; scRNA-seq,SRP330776,CD8+ T cell_3-AD.vs.control; scRNA-seq,SRP330776,B cell_3-AD.vs.control	7
AC062028.1	scRNA-seq,SRP330776,CD8+ T cell_1-AD.vs.control; scRNA-seq,SRP330776,Naive CD8+ T cell_1-AD.vs.control	2
AC063977.6	scRNA-seq,SRP330776,Monocyte_2-AD.vs.control; scRNA-seq,SRP330776,Natural killer cell_2-AD.vs.control	2
AC064801.1	scRNA-seq,SRP215507,CD8+ T cell_2-AD.vs.control	1
AC064805.1	scRNA-seq,SRP330776,Monocyte_2-AD.vs.control	1
AC064807.1	scRNA-seq,SRP215507,CD8+ T cell_4-AD.vs.control; scRNA-seq,SRP215507,CD8+ T cell_2-AD.vs.MCI; scRNA-seq,SRP215507,CD8+ T cell_4-MCI.vs.control; scRNA-seq,SRP215507,CD8+ T cell_2-MCI.vs.control; scRNA-seq,SRP215507,CD8+ T cell_3-MCI.vs.control; scRNA-seq,SRP309935,Naive CD8+ T cell_1-AD.vs.control; scRNA-seq,SRP309935,Natural killer cell_1-AD.vs.control; scRNA-seq,SRP330776,Natural killer	8
AC064836.3	scRNA-seq,SRP215507,CD8+ T cell_3-AD.vs.MCI; scRNA-seq,SRP215507,CD8+ T cell_2-MCI.vs.control; scRNA-seq,SRP215507,CD8+ T cell_3-MCI.vs.control; scRNA-seq,SRP330776,Naive CD8+ T cell_1-AD.vs.control	4
AC064836.4	scRNA-seq,SRP215507,CD8+ T cell_2-MCI.vs.control; scRNA-seq,SRP215507,CD8+ T cell_3-MCI.vs.control; scRNA-seq,SRP330776,CD8+ T cell_1-AD.vs.control; scRNA-seq,SRP330776,γδ T cell-AD.vs.control; scRNA-seq,SRP330776,Memory T cell_2-AD.vs.control; scRNA-seq,SRP330776,CD8+ T cell_2-AD.vs.control; scRNA-seq,SRP330776,Naive CD8+ T cell_1-AD.vs.control; scRNA-seq,SRP330776,Natural killer cell_1-AD.vs.control; scRNA-seq,SRP330776,Memory T cell_1-AD.vs.control; scRNA-seq,SRP330776,Naive CD8+ T cell_3-AD.vs.control; scRNA-seq,SRP330776,B cell_2-AD.vs.control; scRNA-seq,SRP330776,T cell-AD.vs.control; scRNA-seq,SRP330776,CD8+ T cell_3-AD.vs.control; scRNA-seq,SRP330776,Natural killer	14
AC067852.2	scRNA-seq,SRP215507,CD8+ T cell_2-AD.vs.control; scRNA-seq,SRP309935,Naive CD8+ T cell_1-AD.vs.control; scRNA-seq,SRP309935,Naive CD8+ T cell_2-AD.vs.control; scRNA-seq,SRP330776,CD8+ T cell_1-AD.vs.control; scRNA-seq,SRP330776,γδ T cell-AD.vs.control; scRNA-seq,SRP330776,Memory T cell_2-AD.vs.control; scRNA-seq,SRP330776,Memory T cell_1-AD.vs.control; scRNA-seq,SRP330776,Natural killer T cell-	8
AC067852.5	scRNA-seq,SRP215507,CD8+ T cell_5-AD.vs.control; scRNA-seq,SRP215507,CD8+ T cell_1-AD.vs.MCI; scRNA-seq,SRP215507,CD8+ T cell_1-MCI.vs.control; scRNA-seq,SRP330776,CD8+ T cell_2-AD.vs.control	4
AC067930.3	scRNA-seq,SRP330776,CD8+ T cell_1-AD.vs.control	1
AC067930.8	scRNA-seq,SRP215507,CD8+ T cell_3-AD.vs.MCI; scRNA-seq,SRP215507,CD8+ T cell_1-AD.vs.MCI; scRNA-seq,SRP215507,CD8+ T cell_1-MCI.vs.control; scRNA-seq,SRP215507,CD8+ T cell_4-MCI.vs.control; scRNA-seq,SRP309935,Natural killer cell_2-AD.vs.control; scRNA-seq,SRP309935,Monocyte_1-AD.vs.control; scRNA-seq,SRP309935,Naive CD8+ T cell_3-AD.vs.control	7
AC067945.4	scRNA-seq,SRP330776,CD8+ T cell_1-AD.vs.control; scRNA-seq,SRP330776,CD8+ T cell_2-AD.vs.control; scRNA-seq,SRP330776,Natural killer cell_1-AD.vs.control; scRNA-seq,SRP330776,Memory T cell_1-AD.vs.control	4
AC068051.1	scRNA-seq,SRP215507,CD8+ T cell_3-MCI.vs.control; scRNA-seq,SRP330776,CD8+ T cell_1-AD.vs.control; scRNA-seq,SRP330776,γδ T cell-AD.vs.control; scRNA-seq,SRP330776,B cell_1-AD.vs.control; scRNA-seq,SRP330776,Naive CD8+ T cell_1-AD.vs.control	5
AC068193.1	scRNA-seq,SRP215507,CD8+ T cell_3-AD.vs.control; scRNA-seq,SRP215507,CD8+ T cell_5-MCI.vs.control; scRNA-seq,SRP330776,CD8+ T cell_1-AD.vs.control; scRNA-seq,SRP330776,CD8+ T cell_2-AD.vs.control; scRNA-seq,SRP330776,B cell_1-AD.vs.control; scRNA-seq,SRP330776,Naive CD8+ T cell_1-AD.vs.control; scRNA-seq,SRP330776,Memory T cell_1-AD.vs.control	7

AC068205.2	scRNA-seq,SRP215507,CD8+ T cell_2-MCI.vs.control; scRNA-seq,SRP309935,Naive CD8+ T cell_1-AD.vs.control; scRNA-seq,SRP330776,Memory T cell_1-AD.vs.control	3
AC068279.2	scRNA-seq,SRP330776,T cell-AD.vs.control	1
AC068282.1	scRNA-seq,SRP215507,CD8+ T cell_2-AD.vs.control; scRNA-seq,SRP215507,CD8+ T cell_2-MCI.vs.control	2
AC068338.2	scRNA-seq,SRP215507,CD8+ T cell_4-AD.vs.control; scRNA-seq,SRP215507,CD8+ T cell_5-AD.vs.MCI; scRNA-seq,SRP215507,CD8+ T cell_5-MCI.vs.control; scRNA-seq,SRP330776,Memory T cell_2-AD.vs.control; scRNA-seq,SRP330776,B cell_1-AD.vs.control; scRNA-seq,SRP330776,Memory T cell_1-AD.vs.control; scRNA-seq,SRP330776,Natural killer T cell-AD.vs.control; scRNA-seq,SRP330776,CD8+ T cell_3-AD.vs.control; scRNA-seq,SRP330776,Natural killer cell_2-AD.vs.control; scRNA-seq,SRP330776,B cell_4-AD.vs.control	10
AC068338.3	scRNA-seq,SRP330776,CD8+ T cell_1-AD.vs.control; scRNA-seq,SRP330776,B cell_1-AD.vs.control; scRNA-seq,SRP330776,B cell_2-AD.vs.control	3
AC068389.3	scRNA-seq,SRP215507,CD8+ T cell_6-AD.vs.control; scRNA-seq,SRP330776,CD8+ T cell_1-AD.vs.control; scRNA-seq,SRP330776,γδ T cell-AD.vs.control; scRNA-seq,SRP330776,Memory T cell_2-AD.vs.control; scRNA-seq,SRP330776,CD8+ T cell_2-AD.vs.control; scRNA-seq,SRP330776,B cell_1-AD.vs.control; scRNA-seq,SRP330776,Naive CD8+ T cell_1-AD.vs.control; scRNA-seq,SRP330776,Natural killer cell_1-AD.vs.control; scRNA-seq,SRP330776,Memory T cell_1-AD.vs.control; scRNA-seq,SRP330776,Natural killer T cell-AD.vs.control; scRNA-seq,SRP330776,Naive CD8+ T	11
AC068418.1	scRNA-seq,SRP215507,CD8+ T cell_1-MCI.vs.control	1
AC068446.2	scRNA-seq,SRP330776,Memory T cell_1-AD.vs.control	1
AC068473.5	scRNA-seq,SRP215507,CD8+ T cell_5-AD.vs.control; scRNA-seq,SRP215507,CD8+ T cell_2-AD.vs.MCI; scRNA-seq,SRP330776,CD8+ T cell_1-AD.vs.control; scRNA-seq,SRP330776,γδ T cell-AD.vs.control; scRNA-seq,SRP330776,CD8+ T cell_2-AD.vs.control; scRNA-seq,SRP330776,B cell_2-AD.vs.control; scRNA-seq,SRP330776,Monocyte_2-AD.vs.control	7
AC068580.1	scRNA-seq,SRP215507,CD8+ T cell_1-MCI.vs.control; scRNA-seq,SRP215507,CD8+ T cell_2-MCI.vs.control	2
AC068587.4	scRNA-seq,SRP215507,CD8+ T cell_1-AD.vs.MCI; scRNA-seq,SRP215507,CD8+ T cell_1-MCI.vs.control; scRNA-seq,SRP309935,Monocyte_1-AD.vs.control; scRNA-seq,SRP309935,CD4+ T cell-AD.vs.control; scRNA-seq,SRP330776,Memory T cell_2-AD.vs.control; scRNA-seq,SRP330776,CD8+ T cell_2-AD.vs.control; scRNA-seq,SRP330776,T cell-AD.vs.control	7
AC068620.1	scRNA-seq,SRP215507,CD8+ T cell_3-AD.vs.MCI; scRNA-seq,SRP215507,CD8+ T cell_1-AD.vs.MCI; scRNA-seq,SRP215507,CD8+ T cell_3-MCI.vs.control; scRNA-seq,SRP330776,Naive CD8+ T cell_1-AD.vs.control	4
AC068631.1	scRNA-seq,SRP215507,CD8+ T cell_2-AD.vs.control; scRNA-seq,SRP330776,γδ T cell-AD.vs.control; scRNA-seq,SRP330776,Naive CD8+ T cell_1-AD.vs.control; scRNA-seq,SRP330776,Natural killer cell_1-AD.vs.control	4
AC068633.1	scRNA-seq,SRP330776,CD8+ T cell_1-AD.vs.control	1
AC068643.1	scRNA-seq,SRP215507,CD8+ T cell_3-MCI.vs.control	1
AC068724.1	scRNA-seq,SRP215507,CD8+ T cell_4-AD.vs.control	1
AC068790.7	scRNA-seq,SRP215507,CD8+ T cell_4-MCI.vs.control	1
AC068831.4	scRNA-seq,SRP215507,CD8+ T cell_1-AD.vs.control; scRNA-seq,SRP215507,CD8+ T cell_1-MCI.vs.control; scRNA-seq,SRP330776,B cell_2-	3
AC068888.1	scRNA-seq,SRP215507,CD8+ T cell_1-AD.vs.MCI; scRNA-seq,SRP215507,CD8+ T cell_1-MCI.vs.control; scRNA-seq,SRP330776,Natural killer cell_1-AD.vs.control; scRNA-seq,SRP330776,CD8+ T cell_3-AD.vs.control	4
AC068888.2	scRNA-seq,SRP309935,Natural killer cell_1-AD.vs.control; scRNA-seq,SRP330776,Naive CD8+ T cell_1-AD.vs.control; scRNA-seq,SRP330776,Memory T cell_1-AD.vs.control	3
AC068989.1	scRNA-seq,SRP215507,CD8+ T cell_3-AD.vs.control; scRNA-seq,SRP215507,CD8+ T cell_4-AD.vs.control	2
AC069185.1	scRNA-seq,SRP215507,CD8+ T cell_3-AD.vs.MCI; scRNA-seq,SRP215507,CD8+ T cell_1-AD.vs.MCI; scRNA-seq,SRP215507,CD8+ T cell_1-MCI.vs.control; scRNA-seq,SRP215507,CD8+ T cell_3-MCI.vs.control; scRNA-seq,SRP309935,Natural killer cell_1-AD.vs.control; scRNA-seq,SRP330776,CD8+ T cell_1-AD.vs.control; scRNA-seq,SRP330776,Natural killer T cell-AD.vs.control; scRNA-seq,SRP330776,T cell-AD.vs.control	8
AC069213.1	scRNA-seq,SRP215507,CD8+ T cell_1-AD.vs.MCI; scRNA-seq,SRP215507,CD8+ T cell_1-MCI.vs.control; scRNA-seq,SRP215507,CD8+ T cell_5-MCI.vs.control; scRNA-seq,SRP330776,CD8+ T cell_1-AD.vs.control; scRNA-seq,SRP330776,Naive CD8+ T cell_1-AD.vs.control; scRNA-	6

AC069224.1	scRNA-seq,SRP215507,CD8+ T cell_2-AD.vs.control; scRNA-seq,SRP215507,CD8+ T cell_3-AD.vs.MCI; scRNA-seq,SRP215507,CD8+ T cell_3-MCI.vs.control; scRNA-seq,SRP330776,γδ T cell-AD.vs.control	4
AC069277.1	scRNA-seq,SRP215507,CD8+ T cell_1-AD.vs.control	1
AC069287.2	scRNA-seq,SRP330776,B cell_1-AD.vs.control	1
AC069431.1	scRNA-seq,SRP330776,Memory T cell_2-AD.vs.control; scRNA-seq,SRP330776,Naive CD8+ T cell_1-AD.vs.control; scRNA-seq,SRP330776,Natural killer cell_1-AD.vs.control; scRNA-seq,SRP330776,B cell_2-AD.vs.control; scRNA-seq,SRP330776,CD8+ T cell_3-AD.vs.control	5
AC069544.1	scRNA-seq,SRP215507,CD8+ T cell_3-AD.vs.MCI; scRNA-seq,SRP330776,CD8+ T cell_2-AD.vs.control; scRNA-seq,SRP330776,Natural killer T cell-AD.vs.control; scRNA-seq,SRP330776,Naive CD8+ T cell_3-AD.vs.control; scRNA-seq,SRP330776,B cell_2-AD.vs.control; scRNA-	6
AC072022.2	scRNA-seq,SRP330776,Naive CD8+ T cell_1-AD.vs.control	1
AC072054.1	scRNA-seq,SRP215507,CD8+ T cell_3-AD.vs.MCI; scRNA-seq,SRP215507,CD8+ T cell_3-MCI.vs.control	2
AC072061.1	scRNA-seq,SRP215507,CD8+ T cell_4-AD.vs.control; scRNA-seq,SRP215507,CD8+ T cell_6-AD.vs.control; scRNA-seq,SRP215507,CD8+ T cell_1-AD.vs.MCI; scRNA-seq,SRP215507,CD8+ T cell_5-AD.vs.MCI; scRNA-seq,SRP215507,CD8+ T cell_1-MCI.vs.control; scRNA-seq,SRP215507,CD8+ T cell_5-MCI.vs.control; scRNA-seq,SRP215507,CD8+ T cell_4-MCI.vs.control; scRNA-seq,SRP330776,CD8+ T cell_1-AD.vs.control; scRNA-seq,SRP330776,Naive CD8+ T cell_1-AD.vs.control; scRNA-seq,SRP330776,Natural killer cell_1-AD.vs.control; scRNA-seq,SRP330776,Memory T cell_1-AD.vs.control; scRNA-seq,SRP330776,B cell_2-AD.vs.control; scRNA-seq,SRP330776,T cell-AD.vs.control	13
AC073046.1	scRNA-seq,SRP215507,CD8+ T cell_3-MCI.vs.control	1
AC073072.1	scRNA-seq,SRP330776,B cell_1-AD.vs.control	1
AC073111.4	scRNA-seq,SRP215507,CD8+ T cell_5-AD.vs.MCI; scRNA-seq,SRP215507,CD8+ T cell_5-MCI.vs.control; scRNA-seq,SRP215507,CD8+ T cell_7-MCI.vs.control; scRNA-seq,SRP330776,CD8+ T cell_1-AD.vs.control; scRNA-seq,SRP330776,γδ T cell-AD.vs.control; scRNA-seq,SRP330776,Memory T cell_2-AD.vs.control; scRNA-seq,SRP330776,CD8+ T cell_2-AD.vs.control; scRNA-seq,SRP330776,B cell_1-AD.vs.control; scRNA-seq,SRP330776,Naive CD8+ T cell_1-AD.vs.control; scRNA-seq,SRP330776,Natural killer cell_1-AD.vs.control; scRNA-seq,SRP330776,Memory T cell_1-AD.vs.control; scRNA-seq,SRP330776,Naive CD8+ T cell_3-AD.vs.control; scRNA-seq,SRP330776,B cell_2-AD.vs.control; scRNA-seq,SRP330776,T cell-AD.vs.control; scRNA-seq,SRP330776,CD8+ T cell_3-AD.vs.control; scRNA-seq,SRP330776,B cell_3-AD.vs.control	16
AC073115.1	scRNA-seq,SRP215507,CD8+ T cell_2-MCI.vs.control	1
AC073115.2	scRNA-seq,SRP215507,CD8+ T cell_1-AD.vs.control; scRNA-seq,SRP215507,CD8+ T cell_4-AD.vs.control; scRNA-seq,SRP215507,CD8+ T cell_2-AD.vs.MCI; scRNA-seq,SRP215507,CD8+ T cell_4-AD.vs.MCI; scRNA-seq,SRP215507,CD8+ T cell_5-AD.vs.MCI; scRNA-seq,SRP215507,CD8+ T cell_5-MCI.vs.control; scRNA-seq,SRP215507,CD8+ T cell_4-MCI.vs.control; scRNA-seq,SRP215507,CD8+ T cell_2-MCI.vs.control; scRNA-seq,SRP330776,Naive CD8+ T cell_1-AD.vs.control; scRNA-seq,SRP330776,Natural killer cell_1-AD.vs.control; scRNA-seq,SRP330776,Memory T cell_1-AD.vs.control; scRNA-seq,SRP330776,Natural killer T cell-AD.vs.control	12
AC073140.1	scRNA-seq,SRP309935,Monocyte_1-AD.vs.control	1
AC073140.2	scRNA-seq,SRP215507,CD8+ T cell_3-MCI.vs.control; scRNA-seq,SRP330776,B cell_1-AD.vs.control; scRNA-seq,SRP330776,Naive CD8+ T cell_1-	3
AC073195.1	scRNA-seq,SRP215507,CD8+ T cell_3-AD.vs.control; scRNA-seq,SRP215507,CD8+ T cell_1-AD.vs.MCI; scRNA-seq,SRP215507,CD8+ T cell_1-MCI.vs.control; scRNA-seq,SRP330776,CD8+ T cell_1-AD.vs.control; scRNA-seq,SRP330776,CD8+ T cell_2-AD.vs.control; scRNA-seq,SRP330776,B cell_1-AD.vs.control; scRNA-seq,SRP330776,Memory T cell_1-AD.vs.control; scRNA-seq,SRP330776,B cell_2-AD.vs.control; scRNA-	9
AC073254.1	scRNA-seq,SRP215507,CD8+ T cell_2-AD.vs.MCI; scRNA-seq,SRP215507,CD8+ T cell_2-MCI.vs.control; scRNA-seq,SRP330776,CD8+ T cell_3-	3
AC073263.2	scRNA-seq,SRP215507,CD8+ T cell_4-AD.vs.control; scRNA-seq,SRP215507,CD8+ T cell_4-MCI.vs.control	2
AC073320.1	scRNA-seq,SRP309935,Monocyte_1-AD.vs.control	1

AC073332.1	scRNA-seq,SRP215507,CD8+ T cell_5-AD.vs.MCI; scRNA-seq,SRP215507,CD8+ T cell_5-MCI.vs.control; scRNA-seq,SRP309935,Monocyte_1-AD.vs.control; scRNA-seq,SRP309935,Natural killer cell_1-AD.vs.control; scRNA-seq,SRP330776,CD8+ T cell_1-AD.vs.control; scRNA-seq,SRP330776,γδ T cell-AD.vs.control; scRNA-seq,SRP330776,Memory T cell_2-AD.vs.control; scRNA-seq,SRP330776,CD8+ T cell_2-AD.vs.control; scRNA-seq,SRP330776,B cell_1-AD.vs.control; scRNA-seq,SRP330776,Naive CD8+ T cell_1-AD.vs.control; scRNA-seq,SRP330776,Memory T cell_1-AD.vs.control; scRNA-seq,SRP330776,Natural killer T cell-AD.vs.control; scRNA-seq,SRP330776,T cell-AD.vs.control; scRNA-seq,SRP330776,CD8+ T	15
AC073343.2	scRNA-seq,SRP215507,CD8+ T cell_4-MCI.vs.control	1
AC073389.1	scRNA-seq,SRP215507,CD8+ T cell_1-AD.vs.MCI; scRNA-seq,SRP215507,CD8+ T cell_1-MCI.vs.control; scRNA-seq,SRP309935,CD8+ T cell_2-AD.vs.control; scRNA-seq,SRP330776,CD8+ T cell_1-AD.vs.control; scRNA-seq,SRP330776,Memory T cell_2-AD.vs.control; scRNA-seq,SRP330776,CD8+ T cell_2-AD.vs.control; scRNA-seq,SRP330776,B cell_1-AD.vs.control; scRNA-seq,SRP330776,Natural killer cell_1-AD.vs.control; scRNA-seq,SRP330776,Natural killer T cell-AD.vs.control; scRNA-seq,SRP330776,B cell_2-AD.vs.control; scRNA-seq,SRP330776,Naive CD8+ T cell_2-AD.vs.control; scRNA-seq,SRP330776,CD8+ T cell_3-AD.vs.control; scRNA-seq,SRP330776,B cell_4-AD.vs.control	13
AC073389.3	scRNA-seq,SRP330776,Naive CD8+ T cell_1-AD.vs.control	1
AC073475.1	scRNA-seq,SRP215507,CD8+ T cell_3-AD.vs.MCI; scRNA-seq,SRP215507,CD8+ T cell_3-MCI.vs.control; scRNA-seq,SRP330776,CD8+ T cell_1-	3
AC073476.3	scRNA-seq,SRP215507,CD8+ T cell_1-MCI.vs.control	1
AC073508.3	scRNA-seq,SRP215507,CD8+ T cell_3-MCI.vs.control; scRNA-seq,SRP330776,Naive CD8+ T cell_1-AD.vs.control	2
AC073529.1	scRNA-seq,SRP215507,CD8+ T cell_2-AD.vs.MCI; scRNA-seq,SRP215507,CD8+ T cell_2-MCI.vs.control; scRNA-seq,SRP330776,Naive CD8+ T	3
AC073569.3	scRNA-seq,SRP215507,CD8+ T cell_3-AD.vs.MCI; scRNA-seq,SRP215507,CD8+ T cell_1-MCI.vs.control; scRNA-seq,SRP215507,CD8+ T cell_3-	3
AC073578.1	scRNA-seq,SRP330776,CD8+ T cell_1-AD.vs.control; scRNA-seq,SRP330776,Naive CD8+ T cell_1-AD.vs.control	2
AC073611.1	scRNA-seq,SRP215507,CD8+ T cell_2-AD.vs.control	1
AC073655.3	scRNA-seq,SRP330776,B cell_1-AD.vs.control	1
AC073840.1	scRNA-seq,SRP330776,Memory T cell_1-AD.vs.control	1
AC073863.1	scRNA-seq,SRP330776,CD8+ T cell_1-AD.vs.control; scRNA-seq,SRP330776,γδ T cell-AD.vs.control; scRNA-seq,SRP330776,Naive CD8+ T cell_1-AD.vs.control; scRNA-seq,SRP330776,Natural killer cell_1-AD.vs.control	4
AC073869.5	scRNA-seq,SRP215507,CD8+ T cell_1-AD.vs.control	1
AC073896.2	scRNA-seq,SRP215507,CD8+ T cell_5-AD.vs.MCI; scRNA-seq,SRP330776,CD8+ T cell_1-AD.vs.control; scRNA-seq,SRP330776,γδ T cell-AD.vs.control; scRNA-seq,SRP330776,Memory T cell_2-AD.vs.control; scRNA-seq,SRP330776,CD8+ T cell_2-AD.vs.control; scRNA-seq,SRP330776,Naive CD8+ T cell_1-AD.vs.control; scRNA-seq,SRP330776,Natural killer cell_1-AD.vs.control; scRNA-seq,SRP330776,Natural killer T cell-AD.vs.control; scRNA-seq,SRP330776,B cell_2-AD.vs.control; scRNA-seq,SRP330776,T cell-AD.vs.control; scRNA-seq,SRP330776,CD8+ T cell_3-	12
AC073934.1	scRNA-seq,SRP330776,CD8+ T cell_1-AD.vs.control; scRNA-seq,SRP330776,Naive CD8+ T cell_1-AD.vs.control; scRNA-seq,SRP330776,Natural killer cell_1-AD.vs.control; scRNA-seq,SRP330776,CD8+ T cell_3-AD.vs.control	4
AC074032.1	scRNA-seq,SRP215507,CD8+ T cell_4-AD.vs.control; scRNA-seq,SRP215507,CD8+ T cell_5-AD.vs.control; scRNA-seq,SRP215507,CD8+ T cell_1-AD.vs.MCI; scRNA-seq,SRP330776,γδ T cell-AD.vs.control; scRNA-seq,SRP330776,CD8+ T cell_2-AD.vs.control; scRNA-seq,SRP330776,Natural killer cell_1-AD.vs.control; scRNA-seq,SRP330776,CD8+ T cell_3-AD.vs.control; scRNA-seq,SRP330776,B cell_4-AD.vs.control	8
AC074044.1	scRNA-seq,SRP215507,CD8+ T cell_4-AD.vs.control; scRNA-seq,SRP215507,CD8+ T cell_2-MCI.vs.control; scRNA-seq,SRP330776,Memory T cell_1-	3
AC074050.4	scRNA-seq,SRP330776,CD8+ T cell_1-AD.vs.control	1
AC074117.1	scRNA-seq,SRP215507,CD8+ T cell_1-AD.vs.control; scRNA-seq,SRP215507,CD8+ T cell_1-MCI.vs.control; scRNA-seq,SRP330776,CD8+ T cell_2-AD.vs.control; scRNA-seq,SRP330776,B cell_1-AD.vs.control; scRNA-seq,SRP330776,Naive CD8+ T cell_1-AD.vs.control; scRNA-	6
AC074212.1	scRNA-seq,SRP215507,CD8+ T cell_2-AD.vs.MCI; scRNA-seq,SRP215507,CD8+ T cell_2-MCI.vs.control	2
AC074351.1	scRNA-seq,SRP330776,CD8+ T cell_1-AD.vs.control; scRNA-seq,SRP330776,Naive CD8+ T cell_1-AD.vs.control; scRNA-seq,SRP330776,Memory T	3
AC078777.1	scRNA-seq,SRP215507,CD8+ T cell_1-AD.vs.MCI	1

AC078785.1	scRNA-seq,SRP215507,CD8+ T cell_5-AD.vs.MCI; scRNA-seq,SRP330776,γδ T cell-AD.vs.control	2
AC078795.1	scRNA-seq,SRP330776,B cell_1-AD.vs.control	1
AC078802.1	scRNA-seq,SRP215507,CD8+ T cell_1-AD.vs.control; scRNA-seq,SRP215507,CD8+ T cell_1-MCI.vs.control; scRNA-seq,SRP215507,CD8+ T cell_2-	3
AC078845.1	scRNA-seq,SRP215507,CD8+ T cell_2-AD.vs.control; scRNA-seq,SRP215507,CD8+ T cell_2-AD.vs.MCI; scRNA-seq,SRP215507,CD8+ T cell_1-AD.vs.MCI; scRNA-seq,SRP215507,CD8+ T cell_5-AD.vs.MCI; scRNA-seq,SRP215507,CD8+ T cell_1-MCI.vs.control; scRNA-seq,SRP215507,CD8+ T cell_5-MCI.vs.control; scRNA-seq,SRP330776,γδ T cell-AD.vs.control; scRNA-seq,SRP330776,Memory T cell_2-AD.vs.control; scRNA-seq,SRP330776,CD8+ T cell_2-AD.vs.control; scRNA-seq,SRP330776,Memory T cell_1-AD.vs.control	10
AC078846.1	scRNA-seq,SRP215507,CD8+ T cell_2-AD.vs.MCI; scRNA-seq,SRP215507,CD8+ T cell_1-AD.vs.MCI; scRNA-seq,SRP215507,CD8+ T cell_5-AD.vs.MCI; scRNA-seq,SRP215507,CD8+ T cell_1-MCI.vs.control; scRNA-seq,SRP215507,CD8+ T cell_5-MCI.vs.control; scRNA-seq,SRP215507,CD8+ T cell_2-MCI.vs.control; scRNA-seq,SRP215507,CD8+ T cell_3-MCI.vs.control; scRNA-seq,SRP330776,CD8+ T cell_1-AD.vs.control; scRNA-seq,SRP330776,Memory T cell_2-AD.vs.control; scRNA-seq,SRP330776,CD8+ T cell_2-AD.vs.control; scRNA-seq,SRP330776,Memory T cell_1-AD.vs.control; scRNA-seq,SRP330776,B cell_2-AD.vs.control; scRNA-seq,SRP330776,T cell-AD.vs.control	13
AC078881.1	scRNA-seq,SRP215507,CD8+ T cell_3-AD.vs.control	1
AC078883.1	scRNA-seq,SRP215507,CD8+ T cell_3-AD.vs.control; scRNA-seq,SRP215507,CD8+ T cell_6-AD.vs.control; scRNA-seq,SRP215507,CD8+ T cell_1-AD.vs.MCI; scRNA-seq,SRP215507,CD8+ T cell_4-AD.vs.MCI; scRNA-seq,SRP215507,CD8+ T cell_5-AD.vs.MCI; scRNA-seq,SRP215507,CD8+ T cell_1-MCI.vs.control; scRNA-seq,SRP215507,CD8+ T cell_5-MCI.vs.control; scRNA-seq,SRP215507,CD8+ T cell_6-MCI.vs.control; scRNA-seq,SRP330776,CD8+ T cell_1-AD.vs.control; scRNA-seq,SRP330776,γδ T cell-AD.vs.control; scRNA-seq,SRP330776,Memory T cell_2-AD.vs.control; scRNA-seq,SRP330776,CD8+ T cell_2-AD.vs.control; scRNA-seq,SRP330776,B cell_1-AD.vs.control; scRNA-seq,SRP330776,Naive CD8+ T cell_1-AD.vs.control; scRNA-seq,SRP330776,Natural killer cell_1-AD.vs.control; scRNA-seq,SRP330776,Memory T cell_1-AD.vs.control; scRNA-seq,SRP330776,Natural killer T cell-AD.vs.control; scRNA-seq,SRP330776,Naive CD8+ T cell_3-AD.vs.control; scRNA-seq,SRP330776,B cell_2-AD.vs.control; scRNA-seq,SRP330776,T cell-AD.vs.control; scRNA-seq,SRP330776,Naive CD8+ T cell_2-AD.vs.control; scRNA-seq,SRP330776,CD8+ T cell_3-AD.vs.control; scRNA-seq,SRP330776,B cell_3-AD.vs.control; scRNA-seq,SRP330776,Regulatory T cell-AD.vs.control; scRNA-seq,SRP330776,B cell_4-AD.vs.control	25
AC078983.1	scRNA-seq,SRP215507,CD8+ T cell_1-AD.vs.MCI; scRNA-seq,SRP215507,CD8+ T cell_1-MCI.vs.control; scRNA-seq,SRP330776,CD8+ T cell_1-	3
AC079089.1	scRNA-seq,SRP330776,B cell_2-AD.vs.control; scRNA-seq,SRP330776,B cell_3-AD.vs.control	2
AC079140.6	scRNA-seq,SRP330776,CD8+ T cell_1-AD.vs.control	1
AC079148.1	scRNA-seq,SRP215507,CD8+ T cell_3-MCI.vs.control	1
AC079171.1	scRNA-seq,SRP330776,CD8+ T cell_1-AD.vs.control; scRNA-seq,SRP330776,Memory T cell_2-AD.vs.control	2
AC079174.2	scRNA-seq,SRP215507,CD8+ T cell_3-AD.vs.control; scRNA-seq,SRP309935,Monocyte_1-AD.vs.control	2
AC079305.1	scRNA-seq,SRP215507,CD8+ T cell_1-MCI.vs.control	1
AC079305.3	scRNA-seq,SRP215507,CD8+ T cell_5-AD.vs.control; scRNA-seq,SRP215507,CD8+ T cell_5-AD.vs.MCI; scRNA-seq,SRP215507,CD8+ T cell_1-MCI.vs.control; scRNA-seq,SRP330776,CD8+ T cell_1-AD.vs.control; scRNA-seq,SRP330776,Memory T cell_2-AD.vs.control; scRNA-seq,SRP330776,CD8+ T cell_2-AD.vs.control; scRNA-seq,SRP330776,B cell_1-AD.vs.control; scRNA-seq,SRP330776,Naive CD8+ T cell_1-AD.vs.control; scRNA-seq,SRP330776,Memory T cell_1-AD.vs.control; scRNA-seq,SRP330776,B cell_2-AD.vs.control; scRNA-seq,SRP330776,CD8+ T	11
AC079313.2	scRNA-seq,SRP215507,CD8+ T cell_2-AD.vs.control; scRNA-seq,SRP330776,Naive CD8+ T cell_1-AD.vs.control	2
AC079336.5	scRNA-seq,SRP330776,CD8+ T cell_1-AD.vs.control	1
AC079385.1	scRNA-seq,SRP215507,CD8+ T cell_6-AD.vs.control	1
AC079630.1	scRNA-seq,SRP330776,B cell_2-AD.vs.control; scRNA-seq,SRP330776,Monocyte_1-AD.vs.control; scRNA-seq,SRP330776,Monocyte_2-AD.vs.control	3
AC079684.1	scRNA-seq,SRP330776,Memory T cell_1-AD.vs.control	1
AC079779.1	scRNA-seq,SRP215507,CD8+ T cell_2-AD.vs.control; scRNA-seq,SRP215507,CD8+ T cell_3-AD.vs.control; scRNA-seq,SRP215507,CD8+ T cell_3-AD.vs.MCI; scRNA-seq,SRP215507,CD8+ T cell_3-MCI.vs.control	4

AC079781.5	scRNA-seq,SRP215507,CD8+ T cell_3-AD.vs.MCI; scRNA-seq,SRP215507,CD8+ T cell_4-AD.vs.MCI; scRNA-seq,SRP215507,CD8+ T cell_5-AD.vs.MCI; scRNA-seq,SRP215507,CD8+ T cell_1-MCI.vs.control; scRNA-seq,SRP215507,CD8+ T cell_5-MCI.vs.control; scRNA-seq,SRP215507,CD8+ T cell_4-MCI.vs.control; scRNA-seq,SRP215507,CD8+ T cell_2-MCI.vs.control; scRNA-seq,SRP215507,CD8+ T cell_3-MCI.vs.control; scRNA-seq,SRP309935,Naive CD8+ T cell_2-AD.vs.control; scRNA-seq,SRP330776,CD8+ T cell_1-AD.vs.control; scRNA-seq,SRP330776,γδ T cell-AD.vs.control; scRNA-seq,SRP330776,Memory T cell_2-AD.vs.control; scRNA-seq,SRP330776,CD8+ T cell_2-AD.vs.control; scRNA-seq,SRP330776,B cell_1-AD.vs.control; scRNA-seq,SRP330776,Naive CD8+ T cell_1-AD.vs.control; scRNA-seq,SRP330776,Natural killer cell_1-AD.vs.control; scRNA-seq,SRP330776,Memory T cell_1-AD.vs.control; scRNA-seq,SRP330776,Natural killer T cell-AD.vs.control; scRNA-seq,SRP330776,Naive CD8+ T cell_3-AD.vs.control; scRNA-seq,SRP330776,B cell_2-AD.vs.control; scRNA-seq,SRP330776,T cell-AD.vs.control; scRNA-seq,SRP330776,CD8+ T cell_3-AD.vs.control; scRNA-seq,SRP330776,Monocyte_1-AD.vs.control; scRNA-seq,SRP330776,Monocyte_2-AD.vs.control; scRNA-seq,SRP330776,B cell_3-AD.vs.control; scRNA-seq,SRP330776,[illegible]	26
AC079807.1	scRNA-seq,SRP215507,CD8+ T cell_5-AD.vs.MCI; scRNA-seq,SRP330776,Naive CD8+ T cell_1-AD.vs.control	2
AC079848.1	scRNA-seq,SRP330776,CD8+ T cell_1-AD.vs.control	1
AC079915.1	scRNA-seq,SRP215507,CD8+ T cell_4-MCI.vs.control	1
AC079921.1	scRNA-seq,SRP309935,Naive CD8+ T cell_1-AD.vs.control	1
AC079922.2	scRNA-seq,SRP215507,CD8+ T cell_3-AD.vs.MCI; scRNA-seq,SRP215507,CD8+ T cell_3-MCI.vs.control; scRNA-seq,SRP330776,CD8+ T cell_1-AD.vs.control; scRNA-seq,SRP330776,CD8+ T cell_2-AD.vs.control; scRNA-seq,SRP330776,B cell_1-AD.vs.control; scRNA-seq,SRP330776,Natural killer cell_1-AD.vs.control; scRNA-seq,SRP330776,Natural killer T cell-AD.vs.control; scRNA-seq,SRP330776,T cell-AD.vs.control; scRNA-seq,SRP330776,CD8+ T cell_3-AD.vs.control; scRNA-seq,SRP330776,Monocyte_2-AD.vs.control; scRNA-seq,SRP330776,B cell_3-AD.vs.control	11
AC079949.1	scRNA-seq,SRP215507,CD8+ T cell_3-AD.vs.control; scRNA-seq,SRP215507,CD8+ T cell_1-AD.vs.control; scRNA-seq,SRP330776,Naive CD8+ T	3
AC080013.1	scRNA-seq,SRP215507,CD8+ T cell_2-MCI.vs.control	1
AC080013.5	scRNA-seq,SRP330776,Naive CD8+ T cell_1-AD.vs.control	1
AC080023.1	scRNA-seq,SRP215507,CD8+ T cell_2-AD.vs.control	1
AC080112.4	scRNA-seq,SRP215507,CD8+ T cell_1-AD.vs.control; scRNA-seq,SRP215507,CD8+ T cell_1-AD.vs.MCI; scRNA-seq,SRP215507,CD8+ T cell_1-MCI.vs.control; scRNA-seq,SRP330776,Memory T cell_2-AD.vs.control; scRNA-seq,SRP330776,B cell_1-AD.vs.control; scRNA-seq,SRP330776,Naive CD8+ T cell_1-AD.vs.control; scRNA-seq,SRP330776,Memory T cell_1-AD.vs.control; scRNA-seq,SRP330776,B cell_2-AD.vs.control	8
AC080128.2	scRNA-seq,SRP215507,CD8+ T cell_4-AD.vs.control	1
AC080132.1	scRNA-seq,SRP309935,B cell_2-AD.vs.control	1
AC083798.2	scRNA-seq,SRP215507,CD8+ T cell_3-AD.vs.MCI; scRNA-seq,SRP215507,CD8+ T cell_3-MCI.vs.control; scRNA-seq,SRP330776,γδ T cell-AD.vs.control; scRNA-seq,SRP330776,Natural killer cell_1-AD.vs.control; scRNA-seq,SRP330776,T cell-AD.vs.control; scRNA-	6
AC083799.1	scRNA-seq,SRP215507,CD8+ T cell_4-AD.vs.MCI; scRNA-seq,SRP215507,CD8+ T cell_5-AD.vs.MCI; scRNA-seq,SRP215507,CD8+ T cell_6-MCI.vs.control; scRNA-seq,SRP309935,Monocyte_3-AD.vs.control; scRNA-seq,SRP330776,CD8+ T cell_1-AD.vs.control; scRNA-seq,SRP330776,γδ T	6
AC083843.2	scRNA-seq,SRP215507,CD8+ T cell_3-MCI.vs.control	1
AC083843.3	scRNA-seq,SRP215507,CD8+ T cell_2-AD.vs.control; scRNA-seq,SRP215507,CD8+ T cell_2-MCI.vs.control	2
AC083862.2	scRNA-seq,SRP215507,CD8+ T cell_1-MCI.vs.control; scRNA-seq,SRP330776,CD8+ T cell_1-AD.vs.control; scRNA-seq,SRP330776,B cell_1-AD.vs.control; scRNA-seq,SRP330776,Naive CD8+ T cell_1-AD.vs.control; scRNA-seq,SRP330776,Memory T cell_1-AD.vs.control	5
AC083862.3	scRNA-seq,SRP215507,CD8+ T cell_2-AD.vs.control; scRNA-seq,SRP215507,CD8+ T cell_1-AD.vs.MCI; scRNA-seq,SRP215507,CD8+ T cell_1-MCI.vs.control; scRNA-seq,SRP330776,CD8+ T cell_1-AD.vs.control; scRNA-seq,SRP330776,γδ T cell-AD.vs.control; scRNA-seq,SRP330776,B cell_1-AD.vs.control; scRNA-seq,SRP330776,Memory T cell_1-AD.vs.control; scRNA-seq,SRP330776,T cell-AD.vs.control; scRNA-seq,SRP330776,CD8+ T	9
AC083880.1	scRNA-seq,SRP215507,CD8+ T cell_3-AD.vs.control; scRNA-seq,SRP215507,CD8+ T cell_3-AD.vs.MCI; scRNA-seq,SRP215507,CD8+ T cell_1-AD.vs.MCI; scRNA-seq,SRP215507,CD8+ T cell_5-AD.vs.MCI; scRNA-seq,SRP215507,CD8+ T cell_5-MCI.vs.control; scRNA-seq,SRP215507,CD8+ T cell_3-MCI.vs.control; scRNA-seq,SRP330776,γδ T cell-AD.vs.control; scRNA-seq,SRP330776,Memory T cell_2-AD.vs.control; scRNA-seq,SRP330776,CD8+ T cell_2-AD.vs.control; scRNA-seq,SRP330776,B cell_1-AD.vs.control; scRNA-seq,SRP330776,Natural killer cell_1-	12

AC083949.1	scRNA-seq,SRP330776,B cell_2-AD.vs.control; scRNA-seq,SRP330776,Naive CD8+ T cell_2-AD.vs.control	2
AC083973.1	scRNA-seq,SRP215507,CD8+ T cell_1-AD.vs.control; scRNA-seq,SRP330776,Natural killer cell_1-AD.vs.control; scRNA-seq,SRP330776,Memory T	3
AC084018.2	scRNA-seq,SRP215507,CD8+ T cell_1-AD.vs.control; scRNA-seq,SRP215507,CD8+ T cell_1-MCI.vs.control; scRNA-seq,SRP309935,Naive CD8+ T cell_2-AD.vs.control; scRNA-seq,SRP309935,CD8+ T cell_1-AD.vs.control; scRNA-seq,SRP330776,Memory T cell_2-AD.vs.control; scRNA-	6
AC084024.3	scRNA-seq,SRP330776,B cell_1-AD.vs.control	1
AC084026.2	scRNA-seq,SRP330776,CD8+ T cell_1-AD.vs.control	1
AC084036.1	scRNA-seq,SRP215507,CD8+ T cell_3-AD.vs.control; scRNA-seq,SRP215507,CD8+ T cell_2-AD.vs.MCI; scRNA-seq,SRP215507,CD8+ T cell_2-MCI.vs.control; scRNA-seq,SRP330776,Naive CD8+ T cell_1-AD.vs.control	4
AC084117.1	scRNA-seq,SRP215507,CD8+ T cell_2-AD.vs.control; scRNA-seq,SRP215507,CD8+ T cell_3-AD.vs.control; scRNA-seq,SRP215507,CD8+ T cell_1-AD.vs.control; scRNA-seq,SRP215507,CD8+ T cell_4-AD.vs.control; scRNA-seq,SRP215507,CD8+ T cell_1-MCI.vs.control; scRNA-seq,SRP215507,CD8+ T cell_2-MCI.vs.control; scRNA-seq,SRP215507,CD8+ T cell_3-MCI.vs.control	7
AC084125.2	scRNA-seq,SRP330776,Naive CD8+ T cell_1-AD.vs.control	1
AC084198.2	scRNA-seq,SRP215507,CD8+ T cell_3-AD.vs.control; scRNA-seq,SRP215507,CD8+ T cell_1-AD.vs.control; scRNA-seq,SRP215507,CD8+ T cell_2-AD.vs.MCI; scRNA-seq,SRP215507,CD8+ T cell_3-AD.vs.MCI; scRNA-seq,SRP215507,CD8+ T cell_1-AD.vs.MCI; scRNA-seq,SRP215507,CD8+ T cell_4-MCI.vs.control; scRNA-seq,SRP330776,CD8+ T cell_2-AD.vs.control; scRNA-seq,SRP330776,T cell-AD.vs.control	8
AC084876.1	scRNA-seq,SRP330776,Memory T cell_1-AD.vs.control	1
AC087071.1	scRNA-seq,SRP330776,Naive CD8+ T cell_1-AD.vs.control	1
AC087164.1	scRNA-seq,SRP215507,CD8+ T cell_1-AD.vs.control; scRNA-seq,SRP215507,CD8+ T cell_1-MCI.vs.control; scRNA-seq,SRP330776,γδ T cell-AD.vs.control; scRNA-seq,SRP330776,Memory T cell_2-AD.vs.control; scRNA-seq,SRP330776,CD8+ T cell_2-AD.vs.control; scRNA-seq,SRP330776,Memory T cell_1-AD.vs.control; scRNA-seq,SRP330776,Natural killer T cell-AD.vs.control; scRNA-seq,SRP330776,T cell-AD.vs.control	8
AC087190.1	scRNA-seq,SRP215507,CD8+ T cell_1-AD.vs.MCI; scRNA-seq,SRP215507,CD8+ T cell_1-MCI.vs.control; scRNA-seq,SRP330776,Memory T cell_1-	3
AC087239.1	scRNA-seq,SRP215507,CD8+ T cell_4-AD.vs.control; scRNA-seq,SRP215507,CD8+ T cell_1-AD.vs.MCI; scRNA-seq,SRP215507,CD8+ T cell_5-AD.vs.MCI; scRNA-seq,SRP215507,CD8+ T cell_1-MCI.vs.control; scRNA-seq,SRP215507,CD8+ T cell_5-MCI.vs.control; scRNA-seq,SRP215507,CD8+ T cell_6-MCI.vs.control; scRNA-seq,SRP309935,Natural killer cell_2-AD.vs.control; scRNA-seq,SRP309935,Natural killer cell_1-AD.vs.control; scRNA-seq,SRP330776,B cell_1-AD.vs.control; scRNA-seq,SRP330776,Memory T cell_1-AD.vs.control; scRNA-seq,SRP330776,Naive CD8+ T cell_3-AD.vs.control; scRNA-seq,SRP330776,T cell-AD.vs.control; scRNA-seq,SRP330776,CD8+ T cell_3-AD.vs.control; scRNA-seq,SRP330776,Monocyte_2-AD.vs.control; scRNA-seq,SRP330776,Natural killer cell_2-AD.vs.control; scRNA-seq,SRP330776,B cell_4-AD.vs.control	16
AC087241.2	scRNA-seq,SRP215507,CD8+ T cell_1-MCI.vs.control; scRNA-seq,SRP215507,CD8+ T cell_3-MCI.vs.control	2
AC087276.2	scRNA-seq,SRP215507,CD8+ T cell_3-MCI.vs.control	1
AC087280.2	scRNA-seq,SRP215507,CD8+ T cell_2-MCI.vs.control	1
AC087284.1	scRNA-seq,SRP215507,CD8+ T cell_1-AD.vs.MCI; scRNA-seq,SRP215507,CD8+ T cell_1-MCI.vs.control; scRNA-seq,SRP215507,CD8+ T cell_5-MCI.vs.control; scRNA-seq,SRP215507,CD8+ T cell_3-MCI.vs.control; scRNA-seq,SRP330776,Naive CD8+ T cell_1-AD.vs.control	5
AC087286.3	scRNA-seq,SRP330776,Natural killer cell_1-AD.vs.control; scRNA-seq,SRP330776,CD8+ T cell_3-AD.vs.control	2
AC087286.4	scRNA-seq,SRP215507,CD8+ T cell_1-AD.vs.control; scRNA-seq,SRP330776,Memory T cell_1-AD.vs.control	2
AC087289.5	scRNA-seq,SRP215507,CD8+ T cell_5-AD.vs.MCI; scRNA-seq,SRP215507,CD8+ T cell_5-MCI.vs.control; scRNA-seq,SRP330776,CD8+ T cell_1-	3
AC087294.1	scRNA-seq,SRP330776,CD8+ T cell_1-AD.vs.control	1
AC087362.1	scRNA-seq,SRP215507,CD8+ T cell_1-AD.vs.MCI	1
AC087392.3	scRNA-seq,SRP215507,CD8+ T cell_1-MCI.vs.control	1
AC087392.5	scRNA-seq,SRP215507,CD8+ T cell_2-MCI.vs.control	1
AC087393.2	scRNA-seq,SRP215507,CD8+ T cell_2-MCI.vs.control; scRNA-seq,SRP330776,CD8+ T cell_1-AD.vs.control	2

AC087500.1	scRNA-seq,SRP215507,CD8+ T cell_1-AD.vs.MCI; scRNA-seq,SRP330776,CD8+ T cell_2-AD.vs.control; scRNA-seq,SRP330776,Memory T cell_1-AD.vs.control; scRNA-seq,SRP330776,T cell-AD.vs.control	4
AC087501.4	scRNA-seq,SRP215507,CD8+ T cell_3-MCI.vs.control	1
AC087623.2	scRNA-seq,SRP215507,CD8+ T cell_3-AD.vs.control; scRNA-seq,SRP215507,CD8+ T cell_4-AD.vs.control; scRNA-seq,SRP215507,CD8+ T cell_2-AD.vs.MCI; scRNA-seq,SRP215507,CD8+ T cell_5-AD.vs.MCI; scRNA-seq,SRP215507,CD8+ T cell_1-MCI.vs.control; scRNA-seq,SRP215507,CD8+ T cell_5-MCI.vs.control; scRNA-seq,SRP215507,CD8+ T cell_4-MCI.vs.control; scRNA-seq,SRP215507,CD8+ T cell_3-MCI.vs.control; scRNA-seq,SRP330776,CD8+ T cell_1-AD.vs.control; scRNA-seq,SRP330776,Memory T cell_2-AD.vs.control; scRNA-seq,SRP330776,CD8+ T cell_2-AD.vs.control; scRNA-seq,SRP330776,B cell_1-AD.vs.control; scRNA-seq,SRP330776,Naive CD8+ T cell_1-AD.vs.control; scRNA-seq,SRP330776,Natural killer cell_1-AD.vs.control; scRNA-seq,SRP330776,Memory T cell_1-AD.vs.control; scRNA-seq,SRP330776,Natural killer T cell-AD.vs.control; scRNA-seq,SRP330776,B cell_2-AD.vs.control; scRNA-seq,SRP330776,T cell-AD.vs.control; scRNA-seq,SRP330776,CD8+ T cell_3-[illegible]	21
AC087645.2	scRNA-seq,SRP215507,CD8+ T cell_1-AD.vs.control; scRNA-seq,SRP215507,CD8+ T cell_5-AD.vs.control; scRNA-seq,SRP215507,CD8+ T cell_4-AD.vs.MCI; scRNA-seq,SRP309935,Natural killer cell_2-AD.vs.control; scRNA-seq,SRP330776,CD8+ T cell_1-AD.vs.control; scRNA-seq,SRP330776,B cell_1-AD.vs.control; scRNA-seq,SRP330776,Naive CD8+ T cell_1-AD.vs.control; scRNA-seq,SRP330776,CD8+ T cell_3-AD.vs.control	8
AC087741.1	scRNA-seq,SRP215507,CD8+ T cell_2-AD.vs.control; scRNA-seq,SRP215507,CD8+ T cell_3-AD.vs.control; scRNA-seq,SRP330776,Naive CD8+ T cell_1-AD.vs.control; scRNA-seq,SRP330776,Natural killer cell_1-AD.vs.control; scRNA-seq,SRP330776,Memory T cell_1-AD.vs.control; scRNA-seq,SRP330776,Naive CD8+ T cell_3-AD.vs.control; scRNA-seq,SRP330776,T cell-AD.vs.control; scRNA-seq,SRP330776,CD8+ T cell_3-AD.vs.control; scRNA-seq,SRP330776,Monocyte_2-AD.vs.control; scRNA-seq,SRP330776,Natural killer cell_2-AD.vs.control	10
AC087854.1	scRNA-seq,SRP330776,γδ T cell-AD.vs.control; scRNA-seq,SRP330776,CD8+ T cell_3-AD.vs.control	2
AC090061.1	scRNA-seq,SRP215507,CD8+ T cell_2-AD.vs.control; scRNA-seq,SRP215507,CD8+ T cell_1-AD.vs.control; scRNA-seq,SRP215507,CD8+ T cell_2-AD.vs.MCI; scRNA-seq,SRP215507,CD8+ T cell_1-AD.vs.MCI; scRNA-seq,SRP215507,CD8+ T cell_5-MCI.vs.control; scRNA-seq,SRP330776,CD8+ T cell_1-AD.vs.control; scRNA-seq,SRP330776,B cell_1-AD.vs.control; scRNA-seq,SRP330776,Naive CD8+ T cell_1-AD.vs.control; scRNA-seq,SRP330776,Memory T cell_1-AD.vs.control; scRNA-seq,SRP330776,Naive CD8+ T cell_3-AD.vs.control	10
AC090114.2	scRNA-seq,SRP215507,CD8+ T cell_3-AD.vs.control; scRNA-seq,SRP215507,CD8+ T cell_4-AD.vs.control; scRNA-seq,SRP215507,CD8+ T cell_2-AD.vs.MCI; scRNA-seq,SRP215507,CD8+ T cell_1-AD.vs.MCI; scRNA-seq,SRP215507,CD8+ T cell_1-MCI.vs.control; scRNA-seq,SRP215507,CD8+ T cell_4-MCI.vs.control; scRNA-seq,SRP215507,CD8+ T cell_2-MCI.vs.control; scRNA-seq,SRP215507,CD8+ T cell_3-MCI.vs.control; scRNA-seq,SRP309935,CD8+ T cell_2-AD.vs.control; scRNA-seq,SRP330776,CD8+ T cell_1-AD.vs.control; scRNA-seq,SRP330776,Naive CD8+ T cell_1-AD.vs.control; scRNA-seq,SRP330776,Naive CD8+ T cell_3-AD.vs.control; scRNA-seq,SRP330776,B cell_2-AD.vs.control	13
AC090152.1	scRNA-seq,SRP215507,CD8+ T cell_6-AD.vs.control; scRNA-seq,SRP330776,CD8+ T cell_1-AD.vs.control; scRNA-seq,SRP330776,γδ T cell-AD.vs.control; scRNA-seq,SRP330776,Memory T cell_2-AD.vs.control; scRNA-seq,SRP330776,CD8+ T cell_2-AD.vs.control; scRNA-seq,SRP330776,B cell_1-AD.vs.control; scRNA-seq,SRP330776,Naive CD8+ T cell_1-AD.vs.control; scRNA-seq,SRP330776,Natural killer cell_1-AD.vs.control; scRNA-seq,SRP330776,Memory T cell_1-AD.vs.control; scRNA-seq,SRP330776,Natural killer T cell-AD.vs.control; scRNA-seq,SRP330776,T cell-AD.vs.control; scRNA-seq,SRP330776,CD8+ T cell_3-AD.vs.control; scRNA-seq,SRP330776,Natural killer cell_2-AD.vs.control	13
AC090181.1	scRNA-seq,SRP215507,CD8+ T cell_4-MCI.vs.control	1
AC090198.1	scRNA-seq,SRP215507,CD8+ T cell_3-MCI.vs.control	1
AC090204.1	scRNA-seq,SRP215507,CD8+ T cell_3-AD.vs.control; scRNA-seq,SRP215507,CD8+ T cell_1-AD.vs.control; scRNA-seq,SRP215507,CD8+ T cell_3-AD.vs.MCI; scRNA-seq,SRP215507,CD8+ T cell_1-AD.vs.MCI; scRNA-seq,SRP215507,CD8+ T cell_5-MCI.vs.control; scRNA-seq,SRP330776,Memory T cell_2-AD.vs.control; scRNA-seq,SRP330776,Memory T cell_1-AD.vs.control; scRNA-seq,SRP330776,CD8+ T cell_3-AD.vs.control	8
AC090515.2	scRNA-seq,SRP215507,CD8+ T cell_1-MCI.vs.control	1
AC090517.5	scRNA-seq,SRP215507,CD8+ T cell_1-AD.vs.MCI; scRNA-seq,SRP215507,CD8+ T cell_4-MCI.vs.control; scRNA-seq,SRP330776,CD8+ T cell_1-AD.vs.control; scRNA-seq,SRP330776,B cell_2-AD.vs.control; scRNA-seq,SRP330776,CD8+ T cell_3-AD.vs.control	5
AC090559.1	scRNA-seq,SRP215507,CD8+ T cell_2-MCI.vs.control; scRNA-seq,SRP330776,Monocyte_2-AD.vs.control	2
AC090559.2	scRNA-seq,SRP215507,CD8+ T cell_4-MCI.vs.control; scRNA-seq,SRP330776,CD8+ T cell_2-AD.vs.control; scRNA-seq,SRP330776,B cell_1-	3

AC090579.1	scRNA-seq,SRP215507,CD8+ T cell_3-AD.vs.MCI	1
AC090587.1	scRNA-seq,SRP215507,CD8+ T cell_5-AD.vs.control; scRNA-seq,SRP330776,CD8+ T cell_1-AD.vs.control; scRNA-seq,SRP330776,Memory T cell_2-	3
AC090587.2	scRNA-seq,SRP215507,CD8+ T cell_3-AD.vs.control; scRNA-seq,SRP215507,CD8+ T cell_1-AD.vs.MCI; scRNA-seq,SRP215507,CD8+ T cell_1-MCI.vs.control; scRNA-seq,SRP215507,CD8+ T cell_3-MCI.vs.control; scRNA-seq,SRP330776,CD8+ T cell_1-AD.vs.control; scRNA-seq,SRP330776,γδ T cell-AD.vs.control; scRNA-seq,SRP330776,Memory T cell_2-AD.vs.control; scRNA-seq,SRP330776,B cell_1-AD.vs.control; scRNA-seq,SRP330776,Naive CD8+ T cell_1-AD.vs.control; scRNA-seq,SRP330776,Natural killer cell_1-AD.vs.control; scRNA-seq,SRP330776,Memory T	12
AC090825.1	scRNA-seq,SRP215507,CD8+ T cell_4-AD.vs.control; scRNA-seq,SRP215507,CD8+ T cell_5-AD.vs.control; scRNA-seq,SRP215507,CD8+ T cell_5-MCI.vs.control; scRNA-seq,SRP330776,CD8+ T cell_1-AD.vs.control; scRNA-seq,SRP330776,Memory T cell_2-AD.vs.control; scRNA-seq,SRP330776,CD8+ T cell_2-AD.vs.control; scRNA-seq,SRP330776,Naive CD8+ T cell_1-AD.vs.control; scRNA-seq,SRP330776,Natural killer cell_1-AD.vs.control; scRNA-seq,SRP330776,Memory T cell_1-AD.vs.control; scRNA-seq,SRP330776,Naive CD8+ T cell_3-AD.vs.control; scRNA-	12
AC090844.2	scRNA-seq,SRP215507,CD8+ T cell_3-AD.vs.control; scRNA-seq,SRP215507,CD8+ T cell_3-MCI.vs.control	2
AC090912.1	scRNA-seq,SRP215507,CD8+ T cell_1-AD.vs.control; scRNA-seq,SRP215507,CD8+ T cell_1-MCI.vs.control	2
AC090948.1	scRNA-seq,SRP215507,CD8+ T cell_2-AD.vs.control; scRNA-seq,SRP215507,CD8+ T cell_3-AD.vs.MCI; scRNA-seq,SRP215507,CD8+ T cell_3-MCI.vs.control; scRNA-seq,SRP309935,Natural killer cell_1-AD.vs.control; scRNA-seq,SRP309935,Naive CD8+ T cell_2-AD.vs.control; scRNA-seq,SRP309935,CD8+ T cell_1-AD.vs.control; scRNA-seq,SRP330776,Natural killer cell_1-AD.vs.control	7
AC090948.2	scRNA-seq,SRP215507,CD8+ T cell_5-MCI.vs.control; scRNA-seq,SRP330776,CD8+ T cell_1-AD.vs.control	2
AC090948.3	scRNA-seq,SRP215507,CD8+ T cell_1-AD.vs.control; scRNA-seq,SRP215507,CD8+ T cell_3-MCI.vs.control; scRNA-seq,SRP330776,Naive CD8+ T	3
AC090948.4	scRNA-seq,SRP215507,CD8+ T cell_2-AD.vs.control	1
AC091045.1	scRNA-seq,SRP330776,CD8+ T cell_1-AD.vs.control; scRNA-seq,SRP330776,B cell_1-AD.vs.control; scRNA-seq,SRP330776,Naive CD8+ T cell_1-AD.vs.control; scRNA-seq,SRP330776,CD8+ T cell_3-AD.vs.control	4
AC091057.2	scRNA-seq,SRP215507,CD8+ T cell_3-AD.vs.control; scRNA-seq,SRP215507,CD8+ T cell_1-MCI.vs.control; scRNA-seq,SRP215507,CD8+ T cell_2-MCI.vs.control; scRNA-seq,SRP215507,CD8+ T cell_3-MCI.vs.control; scRNA-seq,SRP330776,Memory T cell_2-AD.vs.control; scRNA-seq,SRP330776,CD8+ T cell_2-AD.vs.control; scRNA-seq,SRP330776,Memory T cell_1-AD.vs.control; scRNA-seq,SRP330776,B cell_4-AD.vs.control	8
AC091057.3	scRNA-seq,SRP309935,Monocyte_1-AD.vs.control	1
AC091057.6	scRNA-seq,SRP330776,T cell-AD.vs.control	1
AC091132.4	scRNA-seq,SRP215507,CD8+ T cell_3-AD.vs.MCI; scRNA-seq,SRP330776,CD8+ T cell_1-AD.vs.control	2
AC091153.2	scRNA-seq,SRP215507,CD8+ T cell_1-AD.vs.MCI; scRNA-seq,SRP330776,CD8+ T cell_2-AD.vs.control; scRNA-seq,SRP330776,Memory T cell_1-	3
AC091153.3	scRNA-seq,SRP215507,CD8+ T cell_3-AD.vs.control	1
AC091180.2	scRNA-seq,SRP215507,CD8+ T cell_2-MCI.vs.control; scRNA-seq,SRP330776,γδ T cell-AD.vs.control; scRNA-seq,SRP330776,B cell_1-AD.vs.control; scRNA-seq,SRP330776,Memory T cell_1-AD.vs.control	4
AC091180.3	scRNA-seq,SRP215507,CD8+ T cell_3-MCI.vs.control; scRNA-seq,SRP330776,Memory T cell_1-AD.vs.control	2
AC091271.1	scRNA-seq,SRP215507,CD8+ T cell_1-AD.vs.MCI; scRNA-seq,SRP215507,CD8+ T cell_1-MCI.vs.control; scRNA-seq,SRP215507,CD8+ T cell_4-MCI.vs.control; scRNA-seq,SRP215507,CD8+ T cell_2-MCI.vs.control; scRNA-seq,SRP309935,Monocyte_2-AD.vs.control; scRNA-seq,SRP330776,CD8+ T cell_1-AD.vs.control; scRNA-seq,SRP330776,Memory T cell_2-AD.vs.control; scRNA-seq,SRP330776,CD8+ T cell_2-AD.vs.control; scRNA-seq,SRP330776,Naive CD8+ T cell_1-AD.vs.control; scRNA-seq,SRP330776,Memory T cell_1-AD.vs.control; scRNA-seq,SRP330776,Natural killer T cell-AD.vs.control; scRNA-seq,SRP330776,Naive CD8+ T cell_3-AD.vs.control; scRNA-seq,SRP330776,CD8+ T cell_3-AD.vs.control; scRNA-seq,SRP330776,Regulatory T cell-AD.vs.control; scRNA-seq,SRP330776,Natural killer cell_2-AD.vs.control; scRNA-	16
AC091564.2	scRNA-seq,SRP215507,CD8+ T cell_3-AD.vs.MCI; scRNA-seq,SRP215507,CD8+ T cell_1-MCI.vs.control; scRNA-seq,SRP215507,CD8+ T cell_3-MCI.vs.control; scRNA-seq,SRP330776,Naive CD8+ T cell_1-AD.vs.control; scRNA-seq,SRP330776,Memory T cell_1-AD.vs.control	5
AC091607.2	scRNA-seq,SRP309935,CD8+ T cell_2-AD.vs.control	1

AC091729.3	scRNA-seq,SRP215507,CD8+ T cell_1-AD.vs.control; scRNA-seq,SRP215507,CD8+ T cell_1-AD.vs.MCI; scRNA-seq,SRP330776,CD8+ T cell_1-AD.vs.control; scRNA-seq,SRP330776,γδ T cell-AD.vs.control; scRNA-seq,SRP330776,Memory T cell_2-AD.vs.control; scRNA-seq,SRP330776,CD8+ T cell_2-AD.vs.control; scRNA-seq,SRP330776,B cell_1-AD.vs.control; scRNA-seq,SRP330776,Naive CD8+ T cell_1-AD.vs.control; scRNA-seq,SRP330776,Natural killer cell_1-AD.vs.control; scRNA-seq,SRP330776,Memory T cell_1-AD.vs.control; scRNA-seq,SRP330776,Naive CD8+ T	12
AC091814.1	scRNA-seq,SRP215507,CD8+ T cell_1-AD.vs.MCI; scRNA-seq,SRP215507,CD8+ T cell_6-MCI.vs.control; scRNA-seq,SRP330776,γδ T cell-AD.vs.control; scRNA-seq,SRP330776,Naive CD8+ T cell_1-AD.vs.control; scRNA-seq,SRP330776,Memory T cell_1-AD.vs.control	5
AC091965.1	scRNA-seq,SRP330776,Memory T cell_2-AD.vs.control; scRNA-seq,SRP330776,Naive CD8+ T cell_1-AD.vs.control; scRNA-seq,SRP330776,Memory T cell_1-AD.vs.control; scRNA-seq,SRP330776,T cell-AD.vs.control	4
AC091978.1	scRNA-seq,SRP215507,CD8+ T cell_1-AD.vs.MCI; scRNA-seq,SRP215507,CD8+ T cell_1-MCI.vs.control	2
AC091982.3	scRNA-seq,SRP215507,CD8+ T cell_1-AD.vs.control; scRNA-seq,SRP215507,CD8+ T cell_5-AD.vs.control; scRNA-seq,SRP215507,CD8+ T cell_1-AD.vs.MCI; scRNA-seq,SRP215507,CD8+ T cell_6-MCI.vs.control; scRNA-seq,SRP330776,CD8+ T cell_1-AD.vs.control; scRNA-seq,SRP330776,Memory T cell_2-AD.vs.control; scRNA-seq,SRP330776,Natural killer T cell-AD.vs.control; scRNA-seq,SRP330776,T cell-AD.vs.control;	9
AC092053.2	scRNA-seq,SRP215507,CD8+ T cell_1-AD.vs.control; scRNA-seq,SRP215507,CD8+ T cell_5-AD.vs.control; scRNA-seq,SRP215507,CD8+ T cell_2-AD.vs.MCI; scRNA-seq,SRP215507,CD8+ T cell_5-AD.vs.MCI; scRNA-seq,SRP215507,CD8+ T cell_1-MCI.vs.control; scRNA-seq,SRP215507,CD8+ T cell_5-MCI.vs.control; scRNA-seq,SRP215507,CD8+ T cell_2-MCI.vs.control; scRNA-seq,SRP330776,CD8+ T cell_1-AD.vs.control	8
AC092053.3	scRNA-seq,SRP215507,CD8+ T cell_1-AD.vs.control; scRNA-seq,SRP330776,CD8+ T cell_1-AD.vs.control; scRNA-seq,SRP330776,Memory T cell_2-AD.vs.control; scRNA-seq,SRP330776,Naive CD8+ T cell_1-AD.vs.control; scRNA-seq,SRP330776,Natural killer cell_1-AD.vs.control; scRNA-seq,SRP330776,Memory T cell_1-AD.vs.control	6
AC092053.4	scRNA-seq,SRP330776,Naive CD8+ T cell_1-AD.vs.control	1
AC092053.5	scRNA-seq,SRP215507,CD8+ T cell_4-MCI.vs.control; scRNA-seq,SRP309935,CD8+ T cell_1-AD.vs.control	2
AC092068.2	scRNA-seq,SRP215507,CD8+ T cell_3-AD.vs.control; scRNA-seq,SRP215507,CD8+ T cell_5-AD.vs.MCI; scRNA-seq,SRP215507,CD8+ T cell_1-MCI.vs.control; scRNA-seq,SRP215507,CD8+ T cell_5-MCI.vs.control; scRNA-seq,SRP330776,B cell_1-AD.vs.control	5
AC092069.1	scRNA-seq,SRP330776,CD8+ T cell_1-AD.vs.control; scRNA-seq,SRP330776,T cell-AD.vs.control	2
AC092112.1	scRNA-seq,SRP215507,CD8+ T cell_1-MCI.vs.control; scRNA-seq,SRP215507,CD8+ T cell_2-MCI.vs.control; scRNA-seq,SRP330776,CD8+ T cell_1-AD.vs.control; scRNA-seq,SRP330776,γδ T cell-AD.vs.control; scRNA-seq,SRP330776,Memory T cell_2-AD.vs.control; scRNA-seq,SRP330776,CD8+ T cell_2-AD.vs.control; scRNA-seq,SRP330776,B cell_1-AD.vs.control; scRNA-seq,SRP330776,Memory T cell_1-AD.vs.control	8
AC092115.3	scRNA-seq,SRP215507,CD8+ T cell_3-MCI.vs.control; scRNA-seq,SRP330776,CD8+ T cell_1-AD.vs.control	2
AC092117.1	scRNA-seq,SRP215507,CD8+ T cell_1-AD.vs.control; scRNA-seq,SRP215507,CD8+ T cell_1-MCI.vs.control; scRNA-seq,SRP215507,CD8+ T cell_3-	3
AC092119.3	scRNA-seq,SRP330776,γδ T cell-AD.vs.control; scRNA-seq,SRP330776,Naive CD8+ T cell_1-AD.vs.control	2
AC092123.1	scRNA-seq,SRP215507,CD8+ T cell_2-AD.vs.control; scRNA-seq,SRP215507,CD8+ T cell_5-MCI.vs.control; scRNA-seq,SRP215507,CD8+ T cell_2-	3
AC092140.1	scRNA-seq,SRP309935,Megakaryocyte_1-AD.vs.control	1
AC092142.1	scRNA-seq,SRP215507,CD8+ T cell_1-AD.vs.control; scRNA-seq,SRP215507,CD8+ T cell_5-MCI.vs.control; scRNA-seq,SRP330776,Naive CD8+ T	3
AC092164.1	scRNA-seq,SRP215507,CD8+ T cell_5-AD.vs.control; scRNA-seq,SRP215507,CD8+ T cell_5-MCI.vs.control; scRNA-seq,SRP330776,CD8+ T cell_1-AD.vs.control; scRNA-seq,SRP330776,γδ T cell-AD.vs.control; scRNA-seq,SRP330776,Memory T cell_2-AD.vs.control; scRNA-seq,SRP330776,CD8+ T cell_2-AD.vs.control; scRNA-seq,SRP330776,Naive CD8+ T cell_1-AD.vs.control; scRNA-seq,SRP330776,Natural killer cell_1-AD.vs.control; scRNA-seq,SRP330776,Naive CD8+ T cell_3-AD.vs.control; scRNA-seq,SRP330776,CD8+ T cell_3-AD.vs.control; scRNA-seq,SRP330776,Monocyte_2-	12
AC092279.1	scRNA-seq,SRP309935,Naive CD8+ T cell_1-AD.vs.control; scRNA-seq,SRP309935,Natural killer cell_1-AD.vs.control; scRNA-seq,SRP330776,γδ T cell-AD.vs.control; scRNA-seq,SRP330776,B cell_1-AD.vs.control; scRNA-seq,SRP330776,B cell_2-AD.vs.control	5
AC092287.1	scRNA-seq,SRP330776,Naive CD8+ T cell_1-AD.vs.control; scRNA-seq,SRP330776,Monocyte_2-AD.vs.control	2
AC092296.2	scRNA-seq,SRP330776,CD8+ T cell_1-AD.vs.control; scRNA-seq,SRP330776,B cell_2-AD.vs.control	2

AC092329.4	scRNA-seq,SRP215507,CD8+ T cell_5-AD.vs.control; scRNA-seq,SRP215507,CD8+ T cell_3-AD.vs.MCI; scRNA-seq,SRP215507,CD8+ T cell_1-AD.vs.MCI; scRNA-seq,SRP215507,CD8+ T cell_4-AD.vs.MCI; scRNA-seq,SRP215507,CD8+ T cell_5-MCI.vs.control; scRNA-seq,SRP215507,CD8+ T cell_3-MCI.vs.control; scRNA-seq,SRP330776,Naive CD8+ T cell_3-AD.vs.control; scRNA-seq,SRP330776,T cell-AD.vs.control	8
AC092338.2	scRNA-seq,SRP330776,CD8+ T cell_1-AD.vs.control	1
AC092338.3	scRNA-seq,SRP215507,CD8+ T cell_4-AD.vs.control; scRNA-seq,SRP215507,CD8+ T cell_5-AD.vs.control; scRNA-seq,SRP215507,CD8+ T cell_4-AD.vs.MCI; scRNA-seq,SRP215507,CD8+ T cell_1-MCI.vs.control; scRNA-seq,SRP309935,Erythroid cell-AD.vs.control; scRNA-seq,SRP330776,CD8+ T cell_1-AD.vs.control; scRNA-seq,SRP330776,γδ T cell-AD.vs.control	7
AC092343.1	scRNA-seq,SRP330776,Memory T cell_1-AD.vs.control	1
AC092368.3	scRNA-seq,SRP330776,Memory T cell_2-AD.vs.control; scRNA-seq,SRP330776,Naive CD8+ T cell_1-AD.vs.control; scRNA-seq,SRP330776,Memory T cell_1-AD.vs.control	3
AC092375.2	scRNA-seq,SRP330776,Naive CD8+ T cell_1-AD.vs.control	1
AC092384.1	scRNA-seq,SRP330776,B cell_1-AD.vs.control	1
AC092418.2	scRNA-seq,SRP215507,CD8+ T cell_2-AD.vs.control; scRNA-seq,SRP215507,CD8+ T cell_3-AD.vs.control; scRNA-seq,SRP330776,CD8+ T cell_1-AD.vs.control; scRNA-seq,SRP330776,B cell_1-AD.vs.control; scRNA-seq,SRP330776,Memory T cell_1-AD.vs.control; scRNA-seq,SRP330776,Naive	6
AC092428.1	scRNA-seq,SRP215507,CD8+ T cell_4-MCI.vs.control	1
AC092445.1	scRNA-seq,SRP215507,CD8+ T cell_3-AD.vs.MCI; scRNA-seq,SRP215507,CD8+ T cell_2-MCI.vs.control; scRNA-seq,SRP215507,CD8+ T cell_3-	3
AC092468.1	scRNA-seq,SRP215507,CD8+ T cell_1-AD.vs.control; scRNA-seq,SRP215507,CD8+ T cell_1-MCI.vs.control	2
AC092490.1	scRNA-seq,SRP215507,CD8+ T cell_3-AD.vs.MCI; scRNA-seq,SRP215507,CD8+ T cell_1-AD.vs.MCI; scRNA-seq,SRP215507,CD8+ T cell_1-MCI.vs.control; scRNA-seq,SRP215507,CD8+ T cell_3-MCI.vs.control; scRNA-seq,SRP330776,Naive CD8+ T cell_1-AD.vs.control; scRNA-	6
AC092535.5	scRNA-seq,SRP215507,CD8+ T cell_1-AD.vs.MCI; scRNA-seq,SRP215507,CD8+ T cell_1-MCI.vs.control; scRNA-seq,SRP215507,CD8+ T cell_5-MCI.vs.control; scRNA-seq,SRP215507,CD8+ T cell_2-MCI.vs.control; scRNA-seq,SRP330776,CD8+ T cell_1-AD.vs.control; scRNA-	6
AC092580.2	scRNA-seq,SRP215507,CD8+ T cell_1-AD.vs.control	1
AC092580.4	scRNA-seq,SRP215507,CD8+ T cell_5-AD.vs.MCI; scRNA-seq,SRP215507,CD8+ T cell_5-MCI.vs.control	2
AC092620.1	scRNA-seq,SRP330776,CD8+ T cell_1-AD.vs.control; scRNA-seq,SRP330776,B cell_1-AD.vs.control; scRNA-seq,SRP330776,Memory T cell_1-AD.vs.control; scRNA-seq,SRP330776,B cell_2-AD.vs.control	4
AC092645.2	scRNA-seq,SRP330776,Memory T cell_2-AD.vs.control	1
AC092650.1	scRNA-seq,SRP215507,CD8+ T cell_1-AD.vs.control; scRNA-seq,SRP215507,CD8+ T cell_3-AD.vs.MCI; scRNA-seq,SRP215507,CD8+ T cell_1-AD.vs.MCI; scRNA-seq,SRP215507,CD8+ T cell_3-MCI.vs.control; scRNA-seq,SRP330776,Memory T cell_2-AD.vs.control; scRNA-seq,SRP330776,Naive CD8+ T cell_1-AD.vs.control; scRNA-seq,SRP330776,Memory T cell_1-AD.vs.control	7
AC092667.1	scRNA-seq,SRP330776,B cell_1-AD.vs.control	1
AC092683.1	scRNA-seq,SRP215507,CD8+ T cell_3-AD.vs.MCI; scRNA-seq,SRP215507,CD8+ T cell_1-AD.vs.MCI; scRNA-seq,SRP215507,CD8+ T cell_1-MCI.vs.control; scRNA-seq,SRP215507,CD8+ T cell_2-MCI.vs.control; scRNA-seq,SRP215507,CD8+ T cell_3-MCI.vs.control; scRNA-seq,SRP309935,Naive CD8+ T cell_1-AD.vs.control; scRNA-seq,SRP309935,B cell_1-AD.vs.control; scRNA-seq,SRP309935,CD8+ T cell_2-AD.vs.control; scRNA-seq,SRP330776,Natural killer T cell-AD.vs.control; scRNA-seq,SRP330776,Naive CD8+ T cell_3-AD.vs.control	10
AC092718.1	scRNA-seq,SRP215507,CD8+ T cell_2-AD.vs.control; scRNA-seq,SRP215507,CD8+ T cell_1-AD.vs.MCI; scRNA-seq,SRP215507,CD8+ T cell_5-MCI.vs.control; scRNA-seq,SRP330776,γδ T cell-AD.vs.control	4
AC092720.1	scRNA-seq,SRP215507,CD8+ T cell_2-AD.vs.control	1
AC092747.4	scRNA-seq,SRP215507,CD8+ T cell_4-AD.vs.MCI; scRNA-seq,SRP215507,CD8+ T cell_4-MCI.vs.control; scRNA-seq,SRP215507,CD8+ T cell_2-MCI.vs.control; scRNA-seq,SRP330776,γδ T cell-AD.vs.control; scRNA-seq,SRP330776,Memory T cell_2-AD.vs.control; scRNA-seq,SRP330776,Naive CD8+ T cell_1-AD.vs.control; scRNA-seq,SRP330776,Memory T cell_1-AD.vs.control; scRNA-seq,SRP330776,CD8+ T cell_3-AD.vs.control	8

AC092755.2	scRNA-seq,SRP215507,CD8+ T cell_2-AD.vs.control; scRNA-seq,SRP215507,CD8+ T cell_2-AD.vs.MCI; scRNA-seq,SRP215507,CD8+ T cell_1-AD.vs.MCI; scRNA-seq,SRP330776,CD8+ T cell_1-AD.vs.control; scRNA-seq,SRP330776,γδ T cell-AD.vs.control; scRNA-seq,SRP330776,Memory T cell_2-AD.vs.control; scRNA-seq,SRP330776,CD8+ T cell_2-AD.vs.control; scRNA-seq,SRP330776,B cell_1-AD.vs.control; scRNA-	9
AC092757.2	scRNA-seq,SRP330776,B cell_1-AD.vs.control; scRNA-seq,SRP330776,B cell_2-AD.vs.control	2
AC092794.1	scRNA-seq,SRP215507,CD8+ T cell_2-MCI.vs.control; scRNA-seq,SRP330776,CD8+ T cell_1-AD.vs.control	2
AC092802.1	scRNA-seq,SRP330776,B cell_1-AD.vs.control; scRNA-seq,SRP330776,Naive CD8+ T cell_1-AD.vs.control	2
AC092803.1	scRNA-seq,SRP330776,Memory T cell_2-AD.vs.control; scRNA-seq,SRP330776,B cell_1-AD.vs.control; scRNA-seq,SRP330776,Natural killer cell_1-AD.vs.control; scRNA-seq,SRP330776,Memory T cell_1-AD.vs.control; scRNA-seq,SRP330776,Naive CD8+ T cell_3-AD.vs.control; scRNA-	6
AC092803.3	scRNA-seq,SRP215507,CD8+ T cell_2-AD.vs.control	1
AC092811.1	scRNA-seq,SRP215507,CD8+ T cell_3-MCI.vs.control	1
AC092821.3	scRNA-seq,SRP215507,CD8+ T cell_5-MCI.vs.control; scRNA-seq,SRP215507,CD8+ T cell_3-MCI.vs.control; scRNA-seq,SRP330776,Naive CD8+ T cell_1-AD.vs.control; scRNA-seq,SRP330776,Naive CD8+ T cell_3-AD.vs.control; scRNA-seq,SRP330776,CD8+ T cell_3-AD.vs.control	5
AC092828.1	scRNA-seq,SRP215507,CD8+ T cell_1-AD.vs.control; scRNA-seq,SRP215507,CD8+ T cell_5-MCI.vs.control	2
AC092835.1	scRNA-seq,SRP215507,CD8+ T cell_3-AD.vs.MCI; scRNA-seq,SRP330776,CD8+ T cell_1-AD.vs.control; scRNA-seq,SRP330776,T cell-AD.vs.control	3
AC092849.1	scRNA-seq,SRP215507,CD8+ T cell_5-AD.vs.MCI; scRNA-seq,SRP215507,CD8+ T cell_2-MCI.vs.control	2
AC092902.4	scRNA-seq,SRP215507,CD8+ T cell_2-AD.vs.control; scRNA-seq,SRP215507,CD8+ T cell_1-AD.vs.control; scRNA-seq,SRP215507,CD8+ T cell_6-AD.vs.control; scRNA-seq,SRP215507,CD8+ T cell_1-AD.vs.MCI; scRNA-seq,SRP215507,CD8+ T cell_5-AD.vs.MCI; scRNA-seq,SRP215507,CD8+ T cell_6-AD.vs.MCI; scRNA-seq,SRP215507,CD8+ T cell_5-MCI.vs.control; scRNA-seq,SRP330776,Memory T cell_2-AD.vs.control; scRNA-seq,SRP330776,CD8+ T cell_2-AD.vs.control; scRNA-seq,SRP330776,Natural killer cell_1-AD.vs.control; scRNA-seq,SRP330776,Memory T cell_1-	11
AC092910.3	scRNA-seq,SRP215507,CD8+ T cell_3-AD.vs.control; scRNA-seq,SRP215507,CD8+ T cell_1-AD.vs.MCI; scRNA-seq,SRP215507,CD8+ T cell_1-MCI.vs.control; scRNA-seq,SRP215507,CD8+ T cell_3-MCI.vs.control; scRNA-seq,SRP215507,CD8+ T cell_6-MCI.vs.control; scRNA-seq,SRP330776,Memory T cell_2-AD.vs.control; scRNA-seq,SRP330776,CD8+ T cell_2-AD.vs.control	7
AC092919.1	scRNA-seq,SRP309935,Naive CD8+ T cell_1-AD.vs.control	1
AC092944.1	scRNA-seq,SRP215507,CD8+ T cell_6-AD.vs.control; scRNA-seq,SRP215507,CD8+ T cell_3-AD.vs.MCI; scRNA-seq,SRP215507,CD8+ T cell_1-AD.vs.MCI; scRNA-seq,SRP215507,CD8+ T cell_3-MCI.vs.control; scRNA-seq,SRP309935,Naive CD8+ T cell_1-AD.vs.control; scRNA-seq,SRP330776,Memory T cell_2-AD.vs.control; scRNA-seq,SRP330776,Memory T cell_1-AD.vs.control; scRNA-seq,SRP330776,Naive CD8+ T cell_3-AD.vs.control; scRNA-seq,SRP330776,Monocyte_1-AD.vs.control; scRNA-seq,SRP330776,Monocyte_2-AD.vs.control	10
AC092953.2	scRNA-seq,SRP215507,CD8+ T cell_1-AD.vs.MCI; scRNA-seq,SRP215507,CD8+ T cell_1-MCI.vs.control	2
AC093010.2	scRNA-seq,SRP215507,CD8+ T cell_2-AD.vs.control; scRNA-seq,SRP215507,CD8+ T cell_1-AD.vs.MCI; scRNA-seq,SRP309935,Megakaryocyte_1-AD.vs.control; scRNA-seq,SRP309935,Naive CD8+ T cell_1-AD.vs.control; scRNA-seq,SRP309935,Natural killer cell_1-AD.vs.control; scRNA-seq,SRP309935,Naive CD8+ T cell_2-AD.vs.control; scRNA-seq,SRP309935,B cell_1-AD.vs.control; scRNA-seq,SRP330776,Naive CD8+ T cell_2-AD.vs.control; scRNA-seq,SRP330776,Natural killer cell_2-AD.vs.control	9
AC093157.1	scRNA-seq,SRP215507,CD8+ T cell_1-AD.vs.MCI; scRNA-seq,SRP215507,CD8+ T cell_1-MCI.vs.control; scRNA-seq,SRP309935,Naive CD8+ T cell_2-AD.vs.control; scRNA-seq,SRP309935,B cell_1-AD.vs.control; scRNA-seq,SRP330776,γδ T cell-AD.vs.control; scRNA-seq,SRP330776,CD8+ T cell_2-AD.vs.control; scRNA-seq,SRP330776,Natural killer cell_1-AD.vs.control; scRNA-seq,SRP330776,B cell_2-AD.vs.control; scRNA-seq,SRP330776,CD8+ T cell_3-AD.vs.control; scRNA-seq,SRP330776,Monocyte_2-AD.vs.control	10
AC093227.1	scRNA-seq,SRP215507,CD8+ T cell_2-MCI.vs.control; scRNA-seq,SRP330776,CD8+ T cell_1-AD.vs.control; scRNA-seq,SRP330776,Naive CD8+ T	3
AC093249.6	scRNA-seq,SRP215507,CD8+ T cell_3-AD.vs.MCI; scRNA-seq,SRP215507,CD8+ T cell_1-AD.vs.MCI; scRNA-seq,SRP215507,CD8+ T cell_1-MCI.vs.control; scRNA-seq,SRP215507,CD8+ T cell_3-MCI.vs.control; scRNA-seq,SRP330776,B cell_1-AD.vs.control; scRNA-seq,SRP330776,Memory T cell_1-AD.vs.control; scRNA-seq,SRP330776,Natural killer T cell-AD.vs.control; scRNA-seq,SRP330776,B cell_2-AD.vs.control	8
AC093307.1	scRNA-seq,SRP215507,CD8+ T cell_3-AD.vs.control; scRNA-seq,SRP215507,CD8+ T cell_1-AD.vs.MCI; scRNA-seq,SRP215507,CD8+ T cell_1-MCI.vs.control; scRNA-seq,SRP330776,CD8+ T cell_1-AD.vs.control	4

AC093323.1	scRNA-seq,SRP215507,CD8+ T cell_5-AD.vs.control; scRNA-seq,SRP215507,CD8+ T cell_3-AD.vs.MCI; scRNA-seq,SRP215507,CD8+ T cell_5-AD.vs.MCI; scRNA-seq,SRP215507,CD8+ T cell_1-MCI.vs.control; scRNA-seq,SRP215507,CD8+ T cell_5-MCI.vs.control; scRNA-seq,SRP215507,CD8+ T cell_3-MCI.vs.control; scRNA-seq,SRP309935,Naive CD8+ T cell_1-AD.vs.control; scRNA-seq,SRP330776,CD8+ T cell_1-AD.vs.control; scRNA-seq,SRP330776,γδ T cell-AD.vs.control; scRNA-seq,SRP330776,Memory T cell_2-AD.vs.control; scRNA-seq,SRP330776,CD8+ T cell_2-AD.vs.control; scRNA-seq,SRP330776,B cell_1-AD.vs.control; scRNA-seq,SRP330776,Naive CD8+ T cell_1-AD.vs.control; scRNA-seq,SRP330776,Natural killer cell_1-AD.vs.control; scRNA-seq,SRP330776,Memory T cell_1-AD.vs.control; scRNA-seq,SRP330776,Natural killer T cell-AD.vs.control; scRNA-seq,SRP330776,B cell_2-AD.vs.control; scRNA-seq,SRP330776,T cell-AD.vs.control; scRNA-seq,SRP330776,CD8+ T cell_3- [illegible]	21
AC093423.2	scRNA-seq,SRP215507,CD8+ T cell_1-AD.vs.control	1
AC093462.1	scRNA-seq,SRP215507,CD8+ T cell_3-AD.vs.control; scRNA-seq,SRP215507,CD8+ T cell_2-AD.vs.MCI; scRNA-seq,SRP215507,CD8+ T cell_3-AD.vs.MCI; scRNA-seq,SRP215507,CD8+ T cell_2-MCI.vs.control; scRNA-seq,SRP330776,CD8+ T cell_1-AD.vs.control; scRNA-seq,SRP330776,Memory T cell_2-AD.vs.control; scRNA-seq,SRP330776,CD8+ T cell_2-AD.vs.control; scRNA-seq,SRP330776,B cell_1-AD.vs.control; scRNA-seq,SRP330776,Natural killer cell_1-AD.vs.control; scRNA-seq,SRP330776,Memory T cell_1-AD.vs.control; scRNA-seq,SRP330776,Natural	11
AC093484.2	scRNA-seq,SRP215507,CD8+ T cell_1-AD.vs.MCI; scRNA-seq,SRP215507,CD8+ T cell_1-MCI.vs.control	2
AC093484.3	scRNA-seq,SRP330776,γδ T cell-AD.vs.control; scRNA-seq,SRP330776,CD8+ T cell_2-AD.vs.control; scRNA-seq,SRP330776,Natural killer cell_1-AD.vs.control; scRNA-seq,SRP330776,Natural killer T cell-AD.vs.control; scRNA-seq,SRP330776,Naive CD8+ T cell_3-AD.vs.control	5
AC093484.4	scRNA-seq,SRP215507,CD8+ T cell_2-MCI.vs.control	1
AC093495.1	scRNA-seq,SRP215507,CD8+ T cell_1-AD.vs.MCI; scRNA-seq,SRP215507,CD8+ T cell_5-AD.vs.MCI; scRNA-seq,SRP215507,CD8+ T cell_5-MCI.vs.control; scRNA-seq,SRP309935,CD8+ T cell_2-AD.vs.control; scRNA-seq,SRP330776,CD8+ T cell_1-AD.vs.control; scRNA-seq,SRP330776,γδ T cell-AD.vs.control; scRNA-seq,SRP330776,Memory T cell_2-AD.vs.control; scRNA-seq,SRP330776,CD8+ T cell_2-AD.vs.control; scRNA-seq,SRP330776,B cell_1-AD.vs.control; scRNA-seq,SRP330776,Naive CD8+ T cell_1-AD.vs.control; scRNA-seq,SRP330776,Natural killer cell_1-AD.vs.control; scRNA-seq,SRP330776,Memory T cell_1-AD.vs.control; scRNA-seq,SRP330776,Natural killer T cell-AD.vs.control; scRNA-	15
AC093503.2	scRNA-seq,SRP330776,CD8+ T cell_1-AD.vs.control; scRNA-seq,SRP330776,Memory T cell_2-AD.vs.control; scRNA-seq,SRP330776,B cell_1-	3
AC093510.1	scRNA-seq,SRP215507,CD8+ T cell_1-MCI.vs.control; scRNA-seq,SRP330776,Memory T cell_1-AD.vs.control; scRNA-seq,SRP330776,B cell_2-	3
AC093512.2	scRNA-seq,SRP215507,CD8+ T cell_3-AD.vs.control; scRNA-seq,SRP215507,CD8+ T cell_2-AD.vs.MCI; scRNA-seq,SRP330776,CD8+ T cell_1-AD.vs.control; scRNA-seq,SRP330776,Memory T cell_2-AD.vs.control; scRNA-seq,SRP330776,Naive CD8+ T cell_1-AD.vs.control; scRNA-seq,SRP330776,Natural killer cell_1-AD.vs.control; scRNA-seq,SRP330776,T cell-AD.vs.control; scRNA-seq,SRP330776,CD8+ T cell_3-AD.vs.control	8
AC093525.6	scRNA-seq,SRP215507,CD8+ T cell_3-AD.vs.control; scRNA-seq,SRP215507,CD8+ T cell_1-MCI.vs.control; scRNA-seq,SRP330776,CD8+ T cell_1-AD.vs.control; scRNA-seq,SRP330776,B cell_1-AD.vs.control	4
AC093525.7	scRNA-seq,SRP215507,CD8+ T cell_1-MCI.vs.control; scRNA-seq,SRP330776,CD8+ T cell_1-AD.vs.control; scRNA-seq,SRP330776,CD8+ T cell_2-	3
AC093535.1	scRNA-seq,SRP330776,Memory T cell_2-AD.vs.control	1
AC093567.1	scRNA-seq,SRP215507,CD8+ T cell_1-MCI.vs.control	1
AC093575.1	scRNA-seq,SRP215507,CD8+ T cell_3-MCI.vs.control	1
AC093577.1	scRNA-seq,SRP330776,CD8+ T cell_1-AD.vs.control	1
AC093583.1	scRNA-seq,SRP330776,Monocyte_2-AD.vs.control	1
AC093627.4	scRNA-seq,SRP330776,γδ T cell-AD.vs.control; scRNA-seq,SRP330776,Naive CD8+ T cell_1-AD.vs.control	2
AC093627.5	scRNA-seq,SRP330776,Memory T cell_2-AD.vs.control; scRNA-seq,SRP330776,CD8+ T cell_2-AD.vs.control; scRNA-seq,SRP330776,CD8+ T cell_3-	3
AC093635.1	scRNA-seq,SRP215507,CD8+ T cell_6-AD.vs.control; scRNA-seq,SRP215507,CD8+ T cell_1-AD.vs.MCI; scRNA-seq,SRP215507,CD8+ T cell_1-MCI.vs.control; scRNA-seq,SRP215507,CD8+ T cell_6-MCI.vs.control; scRNA-seq,SRP330776,CD8+ T cell_1-AD.vs.control; scRNA-seq,SRP330776,CD8+ T cell_2-AD.vs.control; scRNA-seq,SRP330776,Naive CD8+ T cell_1-AD.vs.control; scRNA-seq,SRP330776,Memory T cell_1-	8
AC093673.1	scRNA-seq,SRP215507,CD8+ T cell_1-AD.vs.MCI; scRNA-seq,SRP215507,CD8+ T cell_4-MCI.vs.control; scRNA-seq,SRP309935,Megakaryocyte_1-	3
AC093677.2	scRNA-seq,SRP215507,CD8+ T cell_1-MCI.vs.control	1

AC093690.1	scRNA-seq,SRP215507,CD8+ T cell_2-AD.vs.control; scRNA-seq,SRP215507,CD8+ T cell_1-AD.vs.control; scRNA-seq,SRP215507,CD8+ T cell_1-AD.vs.MCI; scRNA-seq,SRP215507,CD8+ T cell_1-MCI.vs.control; scRNA-seq,SRP330776,CD8+ T cell_1-AD.vs.control; scRNA-seq,SRP330776,γδ T cell-AD.vs.control; scRNA-seq,SRP330776,Memory T cell_2-AD.vs.control; scRNA-seq,SRP330776,B cell_1-AD.vs.control; scRNA-seq,SRP330776,Naive CD8+ T cell_1-AD.vs.control; scRNA-seq,SRP330776,Memory T cell_1-AD.vs.control	10
AC093726.2	scRNA-seq,SRP330776,Naive CD8+ T cell_1-AD.vs.control	1
AC093827.4	scRNA-seq,SRP330776,CD8+ T cell_1-AD.vs.control; scRNA-seq,SRP330776,Memory T cell_2-AD.vs.control; scRNA-seq,SRP330776,Naive CD8+ T cell_1-AD.vs.control; scRNA-seq,SRP330776,B cell_4-AD.vs.control	4
AC093849.2	scRNA-seq,SRP215507,CD8+ T cell_2-AD.vs.control; scRNA-seq,SRP215507,CD8+ T cell_1-AD.vs.control	2
AC093849.4	scRNA-seq,SRP215507,CD8+ T cell_4-MCI.vs.control	1
AC093916.1	scRNA-seq,SRP215507,CD8+ T cell_1-AD.vs.MCI	1
AC095055.1	scRNA-seq,SRP215507,CD8+ T cell_1-AD.vs.control; scRNA-seq,SRP215507,CD8+ T cell_2-MCI.vs.control; scRNA-seq,SRP330776,CD8+ T cell_1-	3
AC095057.2	scRNA-seq,SRP215507,CD8+ T cell_2-AD.vs.control; scRNA-seq,SRP215507,CD8+ T cell_3-AD.vs.control; scRNA-seq,SRP215507,CD8+ T cell_5-MCI.vs.control; scRNA-seq,SRP215507,CD8+ T cell_3-MCI.vs.control	4
AC095057.3	scRNA-seq,SRP215507,CD8+ T cell_4-AD.vs.control; scRNA-seq,SRP215507,CD8+ T cell_3-MCI.vs.control; scRNA-seq,SRP330776,CD8+ T cell_1-AD.vs.control; scRNA-seq,SRP330776,Naive CD8+ T cell_1-AD.vs.control; scRNA-seq,SRP330776,Memory T cell_1-AD.vs.control	5
AC096564.1	scRNA-seq,SRP330776,B cell_1-AD.vs.control	1
AC096586.1	scRNA-seq,SRP309935,Naive CD8+ T cell_2-AD.vs.control	1
AC096647.1	scRNA-seq,SRP215507,CD8+ T cell_1-MCI.vs.control	1
AC096667.1	scRNA-seq,SRP309935,Monocyte_2-AD.vs.control; scRNA-seq,SRP330776,Monocyte_1-AD.vs.control	2
AC096677.1	scRNA-seq,SRP215507,CD8+ T cell_3-AD.vs.control; scRNA-seq,SRP215507,CD8+ T cell_4-AD.vs.control; scRNA-seq,SRP215507,CD8+ T cell_5-MCI.vs.control; scRNA-seq,SRP215507,CD8+ T cell_4-MCI.vs.control; scRNA-seq,SRP330776,CD8+ T cell_2-AD.vs.control	5
AC096733.2	scRNA-seq,SRP309935,B cell_1-AD.vs.control; scRNA-seq,SRP330776,B cell_1-AD.vs.control; scRNA-seq,SRP330776,B cell_2-AD.vs.control	3
AC096734.2	scRNA-seq,SRP330776,Memory T cell_1-AD.vs.control	1
AC096751.2	scRNA-seq,SRP330776,Memory T cell_1-AD.vs.control	1
AC096921.2	scRNA-seq,SRP215507,CD8+ T cell_2-AD.vs.control; scRNA-seq,SRP215507,CD8+ T cell_3-AD.vs.control; scRNA-seq,SRP215507,CD8+ T cell_4-AD.vs.control; scRNA-seq,SRP215507,CD8+ T cell_1-AD.vs.MCI; scRNA-seq,SRP215507,CD8+ T cell_5-AD.vs.MCI; scRNA-seq,SRP215507,CD8+ T cell_1-MCI.vs.control; scRNA-seq,SRP215507,CD8+ T cell_5-MCI.vs.control; scRNA-seq,SRP215507,CD8+ T cell_3-MCI.vs.control; scRNA-seq,SRP215507,CD8+ T cell_6-MCI.vs.control; scRNA-seq,SRP330776,CD8+ T cell_2-AD.vs.control	10
AC096992.2	scRNA-seq,SRP330776,CD8+ T cell_1-AD.vs.control	1
AC097359.2	scRNA-seq,SRP330776,Memory T cell_2-AD.vs.control; scRNA-seq,SRP330776,Memory T cell_1-AD.vs.control; scRNA-seq,SRP330776,B cell_2-	3
AC097369.2	scRNA-seq,SRP215507,CD8+ T cell_3-AD.vs.control; scRNA-seq,SRP215507,CD8+ T cell_3-MCI.vs.control; scRNA-seq,SRP330776,CD8+ T cell_1-	3
AC097376.3	scRNA-seq,SRP215507,CD8+ T cell_4-AD.vs.control; scRNA-seq,SRP215507,CD8+ T cell_4-AD.vs.MCI; scRNA-seq,SRP330776,CD8+ T cell_2-AD.vs.control; scRNA-seq,SRP330776,Naive CD8+ T cell_3-AD.vs.control	4
AC097382.2	scRNA-seq,SRP330776,CD8+ T cell_1-AD.vs.control; scRNA-seq,SRP330776,Memory T cell_2-AD.vs.control; scRNA-seq,SRP330776,CD8+ T cell_2-AD.vs.control; scRNA-seq,SRP330776,Naive CD8+ T cell_1-AD.vs.control; scRNA-seq,SRP330776,T cell-AD.vs.control	5
AC097382.3	scRNA-seq,SRP215507,CD8+ T cell_2-AD.vs.MCI; scRNA-seq,SRP215507,CD8+ T cell_2-MCI.vs.control; scRNA-seq,SRP215507,CD8+ T cell_3-MCI.vs.control; scRNA-seq,SRP330776,CD8+ T cell_1-AD.vs.control; scRNA-seq,SRP330776,γδ T cell-AD.vs.control; scRNA-seq,SRP330776,Memory T cell_2-AD.vs.control; scRNA-seq,SRP330776,CD8+ T cell_2-AD.vs.control; scRNA-seq,SRP330776,B cell_1-AD.vs.control; scRNA-seq,SRP330776,Naive CD8+ T cell_1-AD.vs.control; scRNA-seq,SRP330776,Memory T cell_1-AD.vs.control; scRNA-seq,SRP330776,Natural killer T cell-AD.vs.control; scRNA-seq,SRP330776,Naive CD8+ T cell_3-AD.vs.control; scRNA-seq,SRP330776,B cell_2-AD.vs.control; scRNA-seq,SRP330776,T cell-AD.vs.control; scRNA-seq,SRP330776,CD8+ T cell_3-AD.vs.control; scRNA-seq,SRP330776,B cell_3-AD.vs.control; scRNA-[illegible]	17

AC097448.1	scRNA-seq,SRP215507,CD8+ T cell_2-AD.vs.control; scRNA-seq,SRP215507,CD8+ T cell_2-MCI.vs.control	2
AC097468.3	scRNA-seq,SRP309935,Monocyte_2-AD.vs.control	1
AC097500.1	scRNA-seq,SRP215507,CD8+ T cell_3-AD.vs.control; scRNA-seq,SRP215507,CD8+ T cell_3-AD.vs.MCI; scRNA-seq,SRP215507,CD8+ T cell_3-	3
AC097534.2	scRNA-seq,SRP215507,CD8+ T cell_4-AD.vs.MCI; scRNA-seq,SRP215507,CD8+ T cell_5-AD.vs.MCI; scRNA-seq,SRP215507,CD8+ T cell_1-MCI.vs.control; scRNA-seq,SRP215507,CD8+ T cell_5-MCI.vs.control; scRNA-seq,SRP215507,CD8+ T cell_4-MCI.vs.control; scRNA-seq,SRP330776,CD8+ T cell_1-AD.vs.control; scRNA-seq,SRP330776,B cell_1-AD.vs.control; scRNA-seq,SRP330776,Natural killer cell_1-AD.vs.control; scRNA-seq,SRP330776,Memory T cell_1-AD.vs.control; scRNA-seq,SRP330776,Natural killer T cell-AD.vs.control; scRNA-	11
AC097641.2	scRNA-seq,SRP215507,CD8+ T cell_1-MCI.vs.control; scRNA-seq,SRP330776,Naive CD8+ T cell_1-AD.vs.control	2
AC097662.1	scRNA-seq,SRP330776,Naive CD8+ T cell_1-AD.vs.control	1
AC097717.1	scRNA-seq,SRP215507,CD8+ T cell_2-MCI.vs.control	1
AC098483.1	scRNA-seq,SRP330776,B cell_2-AD.vs.control	1
AC098484.4	scRNA-seq,SRP215507,CD8+ T cell_5-AD.vs.MCI; scRNA-seq,SRP215507,CD8+ T cell_5-MCI.vs.control; scRNA-seq,SRP330776,Memory T cell_2-AD.vs.control; scRNA-seq,SRP330776,B cell_1-AD.vs.control	4
AC098487.1	scRNA-seq,SRP215507,CD8+ T cell_3-AD.vs.control; scRNA-seq,SRP215507,CD8+ T cell_3-AD.vs.MCI; scRNA-seq,SRP215507,CD8+ T cell_5-AD.vs.MCI; scRNA-seq,SRP215507,CD8+ T cell_1-MCI.vs.control; scRNA-seq,SRP215507,CD8+ T cell_5-MCI.vs.control; scRNA-seq,SRP330776,CD8+ T cell_1-AD.vs.control; scRNA-seq,SRP330776,CD8+ T cell_2-AD.vs.control; scRNA-seq,SRP330776,Memory T cell_1-	8
AC098818.2	scRNA-seq,SRP215507,CD8+ T cell_1-AD.vs.MCI; scRNA-seq,SRP215507,CD8+ T cell_5-AD.vs.MCI; scRNA-seq,SRP215507,CD8+ T cell_1-MCI.vs.control; scRNA-seq,SRP215507,CD8+ T cell_5-MCI.vs.control; scRNA-seq,SRP330776,CD8+ T cell_1-AD.vs.control	5
AC098934.4	scRNA-seq,SRP215507,CD8+ T cell_2-AD.vs.control	1
AC098969.2	scRNA-seq,SRP215507,CD8+ T cell_3-AD.vs.MCI	1
AC099063.4	scRNA-seq,SRP215507,CD8+ T cell_5-AD.vs.control; scRNA-seq,SRP215507,CD8+ T cell_2-AD.vs.MCI; scRNA-seq,SRP215507,CD8+ T cell_5-MCI.vs.control; scRNA-seq,SRP215507,CD8+ T cell_2-MCI.vs.control; scRNA-seq,SRP330776,CD8+ T cell_1-AD.vs.control; scRNA-seq,SRP330776,γδ T cell-AD.vs.control; scRNA-seq,SRP330776,Memory T cell_2-AD.vs.control; scRNA-seq,SRP330776,CD8+ T cell_2-AD.vs.control; scRNA-seq,SRP330776,B cell_1-AD.vs.control; scRNA-seq,SRP330776,Natural killer cell_1-AD.vs.control; scRNA-seq,SRP330776,Memory T cell_1-AD.vs.control; scRNA-seq,SRP330776,Naive CD8+ T cell_3-AD.vs.control; scRNA-seq,SRP330776,T cell-AD.vs.control; scRNA-seq,SRP330776,CD8+	15
AC099343.3	scRNA-seq,SRP215507,CD8+ T cell_2-AD.vs.MCI; scRNA-seq,SRP215507,CD8+ T cell_3-AD.vs.MCI; scRNA-seq,SRP215507,CD8+ T cell_1-AD.vs.MCI; scRNA-seq,SRP215507,CD8+ T cell_5-AD.vs.MCI; scRNA-seq,SRP215507,CD8+ T cell_1-MCI.vs.control; scRNA-seq,SRP215507,CD8+ T cell_5-MCI.vs.control; scRNA-seq,SRP215507,CD8+ T cell_2-MCI.vs.control; scRNA-seq,SRP215507,CD8+ T cell_3-MCI.vs.control; scRNA-seq,SRP309935,Naive CD8+ T cell_2-AD.vs.control; scRNA-seq,SRP309935,CD8+ T cell_1-AD.vs.control; scRNA-seq,SRP330776,CD8+ T cell_1-AD.vs.control; scRNA-seq,SRP330776,γδ T cell-AD.vs.control; scRNA-seq,SRP330776,Memory T cell_2-AD.vs.control; scRNA-seq,SRP330776,CD8+ T cell_2-AD.vs.control; scRNA-seq,SRP330776,B cell_1-AD.vs.control; scRNA-seq,SRP330776,Naive CD8+ T cell_1-AD.vs.control; scRNA-seq,SRP330776,Natural killer cell_1-AD.vs.control; scRNA-seq,SRP330776,Naive CD8+ T cell_3-AD.vs.control; scRNA-seq,SRP330776,B cell_2-AD.vs.control; scRNA-seq,SRP330776,T cell-AD.vs.control; scRNA-seq,SRP330776,CD8+ T cell_3-AD.vs.control; scRNA-seq,SRP330776,Monocyte_2- [illegible]	23
AC099489.1	scRNA-seq,SRP215507,CD8+ T cell_1-AD.vs.MCI; scRNA-seq,SRP215507,CD8+ T cell_4-AD.vs.MCI; scRNA-seq,SRP215507,CD8+ T cell_1-MCI.vs.control; scRNA-seq,SRP330776,Memory T cell_1-AD.vs.control	4
AC099518.4	scRNA-seq,SRP330776,Naive CD8+ T cell_1-AD.vs.control	1
AC099518.5	scRNA-seq,SRP309935,Monocyte_1-AD.vs.control	1
AC099522.2	scRNA-seq,SRP215507,CD8+ T cell_3-AD.vs.control; scRNA-seq,SRP330776,CD8+ T cell_1-AD.vs.control; scRNA-seq,SRP330776,Memory T cell_2-AD.vs.control; scRNA-seq,SRP330776,Memory T cell_1-AD.vs.control	4
AC099524.1	scRNA-seq,SRP330776,B cell_1-AD.vs.control; scRNA-seq,SRP330776,B cell_2-AD.vs.control	2

AC099568.2	scRNA-seq,SRP215507,CD8+ T cell_1-AD.vs.control; scRNA-seq,SRP309935,Naive CD8+ T cell_1-AD.vs.control; scRNA-seq,SRP309935,B cell_1-AD.vs.control; scRNA-seq,SRP309935,CD8+ T cell_2-AD.vs.control	4
AC099661.1	scRNA-seq,SRP330776,CD8+ T cell_1-AD.vs.control	1
AC099778.1	scRNA-seq,SRP215507,CD8+ T cell_3-AD.vs.MCI; scRNA-seq,SRP215507,CD8+ T cell_5-AD.vs.MCI; scRNA-seq,SRP215507,CD8+ T cell_5-MCI.vs.control; scRNA-seq,SRP215507,CD8+ T cell_3-MCI.vs.control; scRNA-seq,SRP330776,CD8+ T cell_1-AD.vs.control; scRNA-seq,SRP330776,CD8+ T cell_2-AD.vs.control; scRNA-seq,SRP330776,Naive CD8+ T cell_3-AD.vs.control; scRNA-seq,SRP330776,T cell-AD.vs.control; scRNA-seq,SRP330776,CD8+ T cell_3-AD.vs.control; scRNA-seq,SRP330776,B cell_4-AD.vs.control	10
AC099811.1	scRNA-seq,SRP215507,CD8+ T cell_4-MCI.vs.control; scRNA-seq,SRP330776,CD8+ T cell_1-AD.vs.control	2
AC099811.5	scRNA-seq,SRP330776,CD8+ T cell_1-AD.vs.control; scRNA-seq,SRP330776,CD8+ T cell_2-AD.vs.control; scRNA-seq,SRP330776,Naive CD8+ T	3
AC099850.1	scRNA-seq,SRP215507,CD8+ T cell_3-AD.vs.control; scRNA-seq,SRP215507,CD8+ T cell_3-MCI.vs.control; scRNA-seq,SRP330776,CD8+ T cell_1-AD.vs.control; scRNA-seq,SRP330776,γδ T cell-AD.vs.control; scRNA-seq,SRP330776,Naive CD8+ T cell_1-AD.vs.control; scRNA-seq,SRP330776,B cell_2-AD.vs.control; scRNA-seq,SRP330776,Naive CD8+ T cell_2-AD.vs.control	7
AC100786.1	scRNA-seq,SRP215507,CD8+ T cell_4-AD.vs.control; scRNA-seq,SRP215507,CD8+ T cell_2-AD.vs.MCI; scRNA-seq,SRP330776,CD8+ T cell_1-	3
AC100793.2	scRNA-seq,SRP309935,Naive CD8+ T cell_1-AD.vs.control; scRNA-seq,SRP330776,CD8+ T cell_1-AD.vs.control; scRNA-seq,SRP330776,Memory T cell_2-AD.vs.control; scRNA-seq,SRP330776,B cell_1-AD.vs.control; scRNA-seq,SRP330776,T cell-AD.vs.control	5
AC100810.1	scRNA-seq,SRP215507,CD8+ T cell_7-AD.vs.control; scRNA-seq,SRP215507,CD8+ T cell_3-AD.vs.MCI; scRNA-seq,SRP215507,CD8+ T cell_5-AD.vs.MCI; scRNA-seq,SRP215507,CD8+ T cell_4-MCI.vs.control; scRNA-seq,SRP330776,CD8+ T cell_1-AD.vs.control; scRNA-seq,SRP330776,γδ T cell-AD.vs.control; scRNA-seq,SRP330776,Memory T cell_2-AD.vs.control; scRNA-seq,SRP330776,CD8+ T cell_2-AD.vs.control; scRNA-seq,SRP330776,B cell_1-AD.vs.control; scRNA-seq,SRP330776,Naive CD8+ T cell_1-AD.vs.control; scRNA-seq,SRP330776,Natural killer cell_1-AD.vs.control; scRNA-seq,SRP330776,Memory T cell_1-AD.vs.control; scRNA-seq,SRP330776,Natural killer T cell-AD.vs.control; scRNA-seq,SRP330776,Naive CD8+ T cell_3-AD.vs.control; scRNA-seq,SRP330776,B cell_2-AD.vs.control; scRNA-seq,SRP330776,T cell-AD.vs.control; scRNA-seq,SRP330776,CD8+ T cell_3-AD.vs.control; scRNA-seq,SRP330776,Regulatory T cell-AD.vs.control; scRNA-seq,SRP330776,Natural killer [illegible]	20
AC100812.1	scRNA-seq,SRP215507,CD8+ T cell_1-AD.vs.MCI; scRNA-seq,SRP215507,CD8+ T cell_1-MCI.vs.control; scRNA-seq,SRP330776,CD8+ T cell_1-AD.vs.control; scRNA-seq,SRP330776,CD8+ T cell_2-AD.vs.control	4
AC100814.1	scRNA-seq,SRP215507,CD8+ T cell_2-MCI.vs.control	1
AC100827.4	scRNA-seq,SRP215507,CD8+ T cell_2-MCI.vs.control	1
AC100830.1	scRNA-seq,SRP215507,CD8+ T cell_1-MCI.vs.control; scRNA-seq,SRP309935,Natural killer cell_1-AD.vs.control; scRNA-seq,SRP330776,Naive CD8+ T cell_1-AD.vs.control	3
AC100830.3	scRNA-seq,SRP215507,CD8+ T cell_4-MCI.vs.control	1
AC100835.1	scRNA-seq,SRP215507,CD8+ T cell_2-AD.vs.MCI; scRNA-seq,SRP215507,CD8+ T cell_2-MCI.vs.control	2
AC100861.1	scRNA-seq,SRP215507,CD8+ T cell_2-AD.vs.control; scRNA-seq,SRP215507,CD8+ T cell_5-AD.vs.control; scRNA-seq,SRP330776,Memory T cell_2-AD.vs.control; scRNA-seq,SRP330776,B cell_1-AD.vs.control; scRNA-seq,SRP330776,Naive CD8+ T cell_1-AD.vs.control; scRNA-seq,SRP330776,T cell-AD.vs.control; scRNA-seq,SRP330776,CD8+ T cell_3-AD.vs.control	7
AC100861.2	scRNA-seq,SRP330776,B cell_1-AD.vs.control	1
AC102953.2	scRNA-seq,SRP215507,CD8+ T cell_4-AD.vs.control; scRNA-seq,SRP215507,CD8+ T cell_3-AD.vs.MCI; scRNA-seq,SRP215507,CD8+ T cell_4-MCI.vs.control; scRNA-seq,SRP215507,CD8+ T cell_3-MCI.vs.control	4
AC103563.2	scRNA-seq,SRP330776,CD8+ T cell_1-AD.vs.control	1
AC103563.7	scRNA-seq,SRP215507,CD8+ T cell_3-AD.vs.MCI; scRNA-seq,SRP215507,CD8+ T cell_3-MCI.vs.control; scRNA-seq,SRP330776,CD8+ T cell_1-AD.vs.control; scRNA-seq,SRP330776,Memory T cell_2-AD.vs.control; scRNA-seq,SRP330776,Naive CD8+ T cell_1-AD.vs.control; scRNA-	6

AC103591.3	scRNA-seq,SRP215507,CD8+ T cell_2-AD.vs.control; scRNA-seq,SRP215507,CD8+ T cell_1-AD.vs.control; scRNA-seq,SRP215507,CD8+ T cell_2-AD.vs.MCI; scRNA-seq,SRP215507,CD8+ T cell_1-MCI.vs.control; scRNA-seq,SRP330776,CD8+ T cell_1-AD.vs.control; scRNA-seq,SRP330776,Memory T cell_2-AD.vs.control; scRNA-seq,SRP330776,CD8+ T cell_2-AD.vs.control; scRNA-seq,SRP330776,B cell_1-AD.vs.control; scRNA-seq,SRP330776,Naive CD8+ T cell_1-AD.vs.control; scRNA-seq,SRP330776,Natural killer cell_1-AD.vs.control; scRNA-seq,SRP330776,Memory T cell_1-AD.vs.control; scRNA-seq,SRP330776,Natural killer T cell-AD.vs.control; scRNA-seq,SRP330776,Naive CD8+ T cell_3-AD.vs.control; scRNA-seq,SRP330776,B cell_2-AD.vs.control; scRNA-seq,SRP330776,CD8+ T cell_3-AD.vs.control; scRNA-seq,SRP330776,Monocyte_2-AD.vs.control	16
AC103691.1	scRNA-seq,SRP215507,CD8+ T cell_3-AD.vs.MCI; scRNA-seq,SRP330776,CD8+ T cell_1-AD.vs.control; scRNA-seq,SRP330776,Memory T cell_2-AD.vs.control; scRNA-seq,SRP330776,B cell_1-AD.vs.control; scRNA-seq,SRP330776,Naive CD8+ T cell_1-AD.vs.control; scRNA-seq,SRP330776,B	6
AC103702.2	scRNA-seq,SRP215507,CD8+ T cell_1-AD.vs.control; scRNA-seq,SRP215507,CD8+ T cell_4-AD.vs.control; scRNA-seq,SRP215507,CD8+ T cell_2-AD.vs.MCI; scRNA-seq,SRP215507,CD8+ T cell_1-AD.vs.MCI; scRNA-seq,SRP215507,CD8+ T cell_4-AD.vs.MCI; scRNA-seq,SRP215507,CD8+ T cell_5-AD.vs.MCI; scRNA-seq,SRP215507,CD8+ T cell_1-MCI.vs.control; scRNA-seq,SRP215507,CD8+ T cell_5-MCI.vs.control; scRNA-seq,SRP215507,CD8+ T cell_4-MCI.vs.control; scRNA-seq,SRP215507,CD8+ T cell_2-MCI.vs.control; scRNA-seq,SRP330776,γδ T cell-AD.vs.control; scRNA-seq,SRP330776,CD8+ T cell_2-AD.vs.control; scRNA-seq,SRP330776,Natural killer cell_1-AD.vs.control; scRNA-seq,SRP330776,Natural killer	14
AC103703.1	scRNA-seq,SRP215507,CD8+ T cell_1-AD.vs.control; scRNA-seq,SRP215507,CD8+ T cell_4-AD.vs.MCI; scRNA-seq,SRP215507,CD8+ T cell_1-MCI.vs.control; scRNA-seq,SRP215507,CD8+ T cell_4-MCI.vs.control; scRNA-seq,SRP215507,CD8+ T cell_3-MCI.vs.control; scRNA-seq,SRP330776,γδ T cell-AD.vs.control; scRNA-seq,SRP330776,Naive CD8+ T cell_1-AD.vs.control	7
AC103706.1	scRNA-seq,SRP215507,CD8+ T cell_1-AD.vs.control; scRNA-seq,SRP215507,CD8+ T cell_4-AD.vs.control	2
AC103724.3	scRNA-seq,SRP215507,CD8+ T cell_3-AD.vs.control; scRNA-seq,SRP215507,CD8+ T cell_2-AD.vs.MCI; scRNA-seq,SRP215507,CD8+ T cell_1-MCI.vs.control; scRNA-seq,SRP330776,γδ T cell-AD.vs.control; scRNA-seq,SRP330776,Naive CD8+ T cell_2-AD.vs.control	5
AC103724.4	scRNA-seq,SRP215507,CD8+ T cell_1-AD.vs.control; scRNA-seq,SRP215507,CD8+ T cell_2-AD.vs.MCI; scRNA-seq,SRP215507,CD8+ T cell_1-MCI.vs.control; scRNA-seq,SRP215507,CD8+ T cell_6-MCI.vs.control	4
AC103760.1	scRNA-seq,SRP215507,CD8+ T cell_4-AD.vs.control	1
AC103769.1	scRNA-seq,SRP215507,CD8+ T cell_4-AD.vs.MCI; scRNA-seq,SRP215507,CD8+ T cell_2-MCI.vs.control; scRNA-seq,SRP309935,Naive CD8+ T cell_1-AD.vs.control; scRNA-seq,SRP309935,Natural killer cell_1-AD.vs.control; scRNA-seq,SRP309935,Monocyte_2-AD.vs.control	5
AC103831.1	scRNA-seq,SRP330776,B cell_2-AD.vs.control	1
AC103858.1	scRNA-seq,SRP330776,B cell_1-AD.vs.control	1
AC103974.1	scRNA-seq,SRP215507,CD8+ T cell_3-MCI.vs.control	1
AC104024.1	scRNA-seq,SRP215507,CD8+ T cell_7-AD.vs.control; scRNA-seq,SRP330776,B cell_1-AD.vs.control	2
AC104035.1	scRNA-seq,SRP215507,CD8+ T cell_5-AD.vs.MCI; scRNA-seq,SRP215507,CD8+ T cell_5-MCI.vs.control; scRNA-seq,SRP330776,CD8+ T cell_1-AD.vs.control; scRNA-seq,SRP330776,Memory T cell_2-AD.vs.control	4
AC104041.1	scRNA-seq,SRP309935,Natural killer cell_1-AD.vs.control; scRNA-seq,SRP330776,CD8+ T cell_1-AD.vs.control; scRNA-seq,SRP330776,B cell_1-AD.vs.control; scRNA-seq,SRP330776,Naive CD8+ T cell_1-AD.vs.control; scRNA-seq,SRP330776,B cell_2-AD.vs.control	5
AC104066.4	scRNA-seq,SRP215507,CD8+ T cell_3-MCI.vs.control; scRNA-seq,SRP330776,CD8+ T cell_1-AD.vs.control	2
AC104078.1	scRNA-seq,SRP215507,CD8+ T cell_6-AD.vs.control; scRNA-seq,SRP215507,CD8+ T cell_6-MCI.vs.control	2
AC104109.2	scRNA-seq,SRP215507,CD8+ T cell_3-AD.vs.control; scRNA-seq,SRP215507,CD8+ T cell_1-AD.vs.MCI; scRNA-seq,SRP215507,CD8+ T cell_1-MCI.vs.control; scRNA-seq,SRP215507,CD8+ T cell_5-MCI.vs.control; scRNA-seq,SRP330776,Naive CD8+ T cell_1-AD.vs.control; scRNA-	6
AC104118.1	scRNA-seq,SRP215507,CD8+ T cell_3-MCI.vs.control	1
AC104129.1	scRNA-seq,SRP215507,CD8+ T cell_2-MCI.vs.control	1
AC104162.2	scRNA-seq,SRP215507,CD8+ T cell_3-AD.vs.MCI; scRNA-seq,SRP215507,CD8+ T cell_3-MCI.vs.control; scRNA-seq,SRP330776,CD8+ T cell_1-AD.vs.control; scRNA-seq,SRP330776,B cell_1-AD.vs.control; scRNA-seq,SRP330776,Memory T cell_1-AD.vs.control	5
AC104167.1	scRNA-seq,SRP215507,CD8+ T cell_4-AD.vs.control; scRNA-seq,SRP215507,CD8+ T cell_1-MCI.vs.control	2

AC104170.1	scRNA-seq,SRP215507,CD8+ T cell_1-MCI.vs.control	1
AC104187.1	scRNA-seq,SRP215507,CD8+ T cell_3-AD.vs.control; scRNA-seq,SRP215507,CD8+ T cell_5-AD.vs.MCI; scRNA-seq,SRP215507,CD8+ T cell_5-	3
AC104389.5	scRNA-seq,SRP215507,CD8+ T cell_3-AD.vs.control; scRNA-seq,SRP215507,CD8+ T cell_4-AD.vs.control; scRNA-seq,SRP215507,CD8+ T cell_4-MCI.vs.control; scRNA-seq,SRP215507,CD8+ T cell_3-MCI.vs.control; scRNA-seq,SRP330776,B cell_1-AD.vs.control; scRNA-seq,SRP330776,Naive	6
AC104472.4	scRNA-seq,SRP215507,CD8+ T cell_2-AD.vs.MCI; scRNA-seq,SRP330776,CD8+ T cell_2-AD.vs.control	2
AC104506.1	scRNA-seq,SRP215507,CD8+ T cell_1-AD.vs.control; scRNA-seq,SRP215507,CD8+ T cell_2-AD.vs.MCI; scRNA-seq,SRP215507,CD8+ T cell_1-MCI.vs.control; scRNA-seq,SRP215507,CD8+ T cell_2-MCI.vs.control; scRNA-seq,SRP330776,CD8+ T cell_2-AD.vs.control; scRNA-	6
AC104530.1	scRNA-seq,SRP215507,CD8+ T cell_5-AD.vs.MCI; scRNA-seq,SRP215507,CD8+ T cell_5-MCI.vs.control; scRNA-seq,SRP309935,Monocyte_1-AD.vs.control; scRNA-seq,SRP330776,CD8+ T cell_2-AD.vs.control; scRNA-seq,SRP330776,B cell_1-AD.vs.control; scRNA-seq,SRP330776,B cell_2-	6
AC104590.1	scRNA-seq,SRP215507,CD8+ T cell_1-AD.vs.MCI; scRNA-seq,SRP215507,CD8+ T cell_1-MCI.vs.control; scRNA-seq,SRP215507,CD8+ T cell_2-MCI.vs.control; scRNA-seq,SRP330776,CD8+ T cell_1-AD.vs.control; scRNA-seq,SRP330776,Naive CD8+ T cell_1-AD.vs.control; scRNA-	6
AC104596.1	scRNA-seq,SRP215507,CD8+ T cell_8-AD.vs.control; scRNA-seq,SRP215507,CD8+ T cell_5-AD.vs.MCI; scRNA-seq,SRP330776,Memory T cell_1-AD.vs.control; scRNA-seq,SRP330776,Natural killer T cell-AD.vs.control; scRNA-seq,SRP330776,B cell_2-AD.vs.control	5
AC104653.1	scRNA-seq,SRP215507,CD8+ T cell_1-AD.vs.MCI; scRNA-seq,SRP215507,CD8+ T cell_1-MCI.vs.control; scRNA-seq,SRP330776,CD8+ T cell_1-AD.vs.control; scRNA-seq,SRP330776,Naive CD8+ T cell_1-AD.vs.control; scRNA-seq,SRP330776,B cell_2-AD.vs.control	5
AC104695.2	scRNA-seq,SRP330776,γδ T cell-AD.vs.control	1
AC104695.3	scRNA-seq,SRP215507,CD8+ T cell_2-AD.vs.control; scRNA-seq,SRP215507,CD8+ T cell_3-AD.vs.control; scRNA-seq,SRP215507,CD8+ T cell_4-AD.vs.control; scRNA-seq,SRP215507,CD8+ T cell_1-AD.vs.MCI; scRNA-seq,SRP215507,CD8+ T cell_4-AD.vs.MCI; scRNA-seq,SRP215507,CD8+ T cell_1-MCI.vs.control; scRNA-seq,SRP215507,CD8+ T cell_5-MCI.vs.control; scRNA-seq,SRP215507,CD8+ T cell_2-MCI.vs.control; scRNA-seq,SRP215507,CD8+ T cell_3-MCI.vs.control; scRNA-seq,SRP309935,Natural killer cell_2-AD.vs.control	10
AC104695.4	scRNA-seq,SRP215507,CD8+ T cell_2-AD.vs.control; scRNA-seq,SRP215507,CD8+ T cell_3-AD.vs.control; scRNA-seq,SRP215507,CD8+ T cell_2-MCI.vs.control; scRNA-seq,SRP309935,Natural killer cell_2-AD.vs.control; scRNA-seq,SRP309935,CD8+ T cell_1-AD.vs.control; scRNA-	6
AC104699.1	scRNA-seq,SRP330776,B cell_1-AD.vs.control; scRNA-seq,SRP330776,B cell_4-AD.vs.control	2
AC104794.3	scRNA-seq,SRP215507,CD8+ T cell_3-AD.vs.control; scRNA-seq,SRP215507,CD8+ T cell_1-AD.vs.control; scRNA-seq,SRP215507,CD8+ T cell_1-AD.vs.MCI; scRNA-seq,SRP215507,CD8+ T cell_5-AD.vs.MCI; scRNA-seq,SRP215507,CD8+ T cell_4-MCI.vs.control; scRNA-seq,SRP330776,CD8+ T cell_1-AD.vs.control; scRNA-seq,SRP330776,γδ T cell-AD.vs.control; scRNA-seq,SRP330776,CD8+ T cell_2-AD.vs.control; scRNA-seq,SRP330776,B cell_1-AD.vs.control; scRNA-seq,SRP330776,Natural killer cell_1-AD.vs.control; scRNA-seq,SRP330776,Memory T cell_1-AD.vs.control; scRNA-	12
AC104806.2	scRNA-seq,SRP330776,Naive CD8+ T cell_1-AD.vs.control	1
AC104825.1	scRNA-seq,SRP215507,CD8+ T cell_5-AD.vs.control; scRNA-seq,SRP215507,CD8+ T cell_2-AD.vs.MCI; scRNA-seq,SRP215507,CD8+ T cell_3-AD.vs.MCI; scRNA-seq,SRP215507,CD8+ T cell_3-MCI.vs.control	4
AC104850.2	scRNA-seq,SRP215507,CD8+ T cell_2-AD.vs.control	1
AC104958.1	scRNA-seq,SRP215507,CD8+ T cell_2-MCI.vs.control; scRNA-seq,SRP330776,CD8+ T cell_1-AD.vs.control	2
AC104958.2	scRNA-seq,SRP215507,CD8+ T cell_2-AD.vs.control; scRNA-seq,SRP215507,CD8+ T cell_4-MCI.vs.control; scRNA-seq,SRP330776,Naive CD8+ T	3
AC104971.3	scRNA-seq,SRP215507,CD8+ T cell_3-AD.vs.MCI; scRNA-seq,SRP215507,CD8+ T cell_3-MCI.vs.control; scRNA-seq,SRP215507,CD8+ T cell_6-MCI.vs.control; scRNA-seq,SRP330776,B cell_1-AD.vs.control; scRNA-seq,SRP330776,B cell_2-AD.vs.control	5
AC105020.6	scRNA-seq,SRP215507,CD8+ T cell_1-MCI.vs.control; scRNA-seq,SRP215507,CD8+ T cell_2-MCI.vs.control; scRNA-seq,SRP330776,Naive CD8+ T cell_1-AD.vs.control; scRNA-seq,SRP330776,Natural killer T cell-AD.vs.control; scRNA-seq,SRP330776,Naive CD8+ T cell_3-AD.vs.control; scRNA-seq,SRP330776,CD8+ T cell_3-AD.vs.control	6
AC105052.4	scRNA-seq,SRP215507,CD8+ T cell_4-AD.vs.control; scRNA-seq,SRP215507,CD8+ T cell_3-AD.vs.MCI; scRNA-seq,SRP330776,Naive CD8+ T cell_1-	3
AC105206.3	scRNA-seq,SRP215507,CD8+ T cell_2-AD.vs.control; scRNA-seq,SRP215507,CD8+ T cell_1-AD.vs.control; scRNA-seq,SRP215507,CD8+ T cell_1-AD.vs.MCI; scRNA-seq,SRP215507,CD8+ T cell_2-MCI.vs.control	4
AC105219.1	scRNA-seq,SRP215507,CD8+ T cell_3-MCI.vs.control	1

AC105285.1	scRNA-seq,SRP330776,B cell_1-AD.vs.control; scRNA-seq,SRP330776,Naive CD8+ T cell_1-AD.vs.control; scRNA-seq,SRP330776,B cell_2-	3
AC105339.6	scRNA-seq,SRP215507,CD8+ T cell_3-AD.vs.control; scRNA-seq,SRP215507,CD8+ T cell_4-AD.vs.control; scRNA-seq,SRP215507,CD8+ T cell_2-AD.vs.MCI; scRNA-seq,SRP215507,CD8+ T cell_1-AD.vs.MCI; scRNA-seq,SRP215507,CD8+ T cell_4-AD.vs.MCI; scRNA-seq,SRP215507,CD8+ T cell_1-MCI.vs.control; scRNA-seq,SRP215507,CD8+ T cell_5-MCI.vs.control; scRNA-seq,SRP215507,CD8+ T cell_2-MCI.vs.control; scRNA-seq,SRP215507,CD8+ T cell_3-MCI.vs.control; scRNA-seq,SRP215507,CD8+ T cell_6-MCI.vs.control; scRNA-seq,SRP330776,CD8+ T cell_1-AD.vs.control; scRNA-seq,SRP330776,B cell_1-AD.vs.control; scRNA-seq,SRP330776,Naive CD8+ T cell_1-AD.vs.control; scRNA-	15
AC105402.3	scRNA-seq,SRP215507,CD8+ T cell_3-MCI.vs.control	1
AC105402.4	scRNA-seq,SRP215507,CD8+ T cell_2-AD.vs.MCI; scRNA-seq,SRP215507,CD8+ T cell_5-MCI.vs.control; scRNA-seq,SRP215507,CD8+ T cell_2-MCI.vs.control; scRNA-seq,SRP330776,Memory T cell_2-AD.vs.control; scRNA-seq,SRP330776,Memory T cell_1-AD.vs.control	5
AC105429.1	scRNA-seq,SRP215507,CD8+ T cell_2-AD.vs.control; scRNA-seq,SRP215507,CD8+ T cell_3-AD.vs.control; scRNA-seq,SRP215507,CD8+ T cell_4-AD.vs.control; scRNA-seq,SRP330776,Naive CD8+ T cell_1-AD.vs.control	4
AC105446.1	scRNA-seq,SRP215507,CD8+ T cell_3-AD.vs.control; scRNA-seq,SRP215507,CD8+ T cell_5-AD.vs.control; scRNA-seq,SRP215507,CD8+ T cell_5-MCI.vs.control; scRNA-seq,SRP330776,CD8+ T cell_1-AD.vs.control; scRNA-seq,SRP330776,γδ T cell-AD.vs.control; scRNA-seq,SRP330776,Memory T cell_2-AD.vs.control; scRNA-seq,SRP330776,CD8+ T cell_2-AD.vs.control; scRNA-seq,SRP330776,Naive CD8+ T cell_1-AD.vs.control; scRNA-	9
AC106707.1	scRNA-seq,SRP215507,CD8+ T cell_3-AD.vs.control; scRNA-seq,SRP330776,CD8+ T cell_1-AD.vs.control; scRNA-seq,SRP330776,γδ T cell-	3
AC106739.1	scRNA-seq,SRP215507,CD8+ T cell_1-AD.vs.MCI; scRNA-seq,SRP215507,CD8+ T cell_1-MCI.vs.control; scRNA-seq,SRP330776,B cell_2-	3
AC106779.1	scRNA-seq,SRP330776,Naive CD8+ T cell_1-AD.vs.control	1
AC106791.1	scRNA-seq,SRP215507,CD8+ T cell_3-AD.vs.MCI; scRNA-seq,SRP215507,CD8+ T cell_4-AD.vs.MCI; scRNA-seq,SRP215507,CD8+ T cell_4-MCI.vs.control; scRNA-seq,SRP215507,CD8+ T cell_3-MCI.vs.control; scRNA-seq,SRP215507,CD8+ T cell_6-MCI.vs.control; scRNA-seq,SRP330776,Memory T cell_2-AD.vs.control; scRNA-seq,SRP330776,Natural killer cell_1-AD.vs.control; scRNA-seq,SRP330776,Naive CD8+ T	9
AC106795.2	scRNA-seq,SRP215507,CD8+ T cell_3-AD.vs.control; scRNA-seq,SRP309935,Naive CD8+ T cell_1-AD.vs.control; scRNA-seq,SRP309935,CD8+ T	3
AC106865.2	scRNA-seq,SRP330776,CD8+ T cell_1-AD.vs.control; scRNA-seq,SRP330776,Naive CD8+ T cell_1-AD.vs.control	2
AC106897.1	scRNA-seq,SRP330776,CD8+ T cell_1-AD.vs.control	1
AC107027.1	scRNA-seq,SRP215507,CD8+ T cell_3-MCI.vs.control	1
AC107068.1	scRNA-seq,SRP215507,CD8+ T cell_2-AD.vs.control; scRNA-seq,SRP215507,CD8+ T cell_3-MCI.vs.control; scRNA-seq,SRP330776,CD8+ T cell_1-AD.vs.control; scRNA-seq,SRP330776,Naive CD8+ T cell_1-AD.vs.control	4
AC107071.1	scRNA-seq,SRP215507,CD8+ T cell_1-MCI.vs.control	1
AC107081.2	scRNA-seq,SRP330776,CD8+ T cell_1-AD.vs.control; scRNA-seq,SRP330776,γδ T cell-AD.vs.control; scRNA-seq,SRP330776,T cell-AD.vs.control	3
AC107214.1	scRNA-seq,SRP215507,CD8+ T cell_3-AD.vs.control; scRNA-seq,SRP215507,CD8+ T cell_1-AD.vs.control; scRNA-seq,SRP215507,CD8+ T cell_5-AD.vs.control; scRNA-seq,SRP215507,CD8+ T cell_1-AD.vs.MCI; scRNA-seq,SRP215507,CD8+ T cell_5-MCI.vs.control; scRNA-seq,SRP215507,CD8+ T cell_3-MCI.vs.control; scRNA-seq,SRP330776,CD8+ T cell_1-AD.vs.control; scRNA-seq,SRP330776,Memory T cell_2-AD.vs.control; scRNA-	9
AC107223.1	scRNA-seq,SRP215507,CD8+ T cell_1-AD.vs.control; scRNA-seq,SRP215507,CD8+ T cell_4-AD.vs.control; scRNA-seq,SRP215507,CD8+ T cell_5-AD.vs.control; scRNA-seq,SRP215507,CD8+ T cell_6-AD.vs.control; scRNA-seq,SRP215507,CD8+ T cell_2-AD.vs.MCI; scRNA-seq,SRP215507,CD8+ T cell_3-AD.vs.MCI; scRNA-seq,SRP215507,CD8+ T cell_5-AD.vs.MCI; scRNA-seq,SRP215507,CD8+ T cell_5-MCI.vs.control; scRNA-seq,SRP215507,CD8+ T cell_2-MCI.vs.control; scRNA-seq,SRP215507,CD8+ T cell_3-MCI.vs.control; scRNA-seq,SRP330776,CD8+ T cell_1-AD.vs.control; scRNA-seq,SRP330776,γδ T cell-AD.vs.control; scRNA-seq,SRP330776,Memory T cell_2-AD.vs.control; scRNA-seq,SRP330776,CD8+ T cell_2-AD.vs.control; scRNA-seq,SRP330776,Naive CD8+ T cell_1-AD.vs.control; scRNA-seq,SRP330776,Natural killer T cell-AD.vs.control; scRNA- [illegible]	17
AC107464.3	scRNA-seq,SRP215507,CD8+ T cell_1-AD.vs.control; scRNA-seq,SRP215507,CD8+ T cell_1-AD.vs.MCI	2
AC107871.2	scRNA-seq,SRP215507,CD8+ T cell_2-MCI.vs.control	1
AC107884.1	scRNA-seq,SRP215507,CD8+ T cell_3-MCI.vs.control; scRNA-seq,SRP330776,CD8+ T cell_1-AD.vs.control; scRNA-seq,SRP330776,Naive CD8+ T cell_1-AD.vs.control; scRNA-seq,SRP330776,Naive CD8+ T cell_3-AD.vs.control	4

AC107926.1	scRNA-seq,SRP330776,B cell_1-AD.vs.control; scRNA-seq,SRP330776,B cell_2-AD.vs.control	2
AC107959.1	scRNA-seq,SRP215507,CD8+ T cell_1-AD.vs.MCI; scRNA-seq,SRP215507,CD8+ T cell_2-MCI.vs.control; scRNA-seq,SRP330776,γδ T cell-AD.vs.control; scRNA-seq,SRP330776,Memory T cell_2-AD.vs.control; scRNA-seq,SRP330776,B cell_1-AD.vs.control; scRNA-seq,SRP330776,Natural	6
AC107982.3	scRNA-seq,SRP215507,CD8+ T cell_4-AD.vs.control	1
AC108047.1	scRNA-seq,SRP215507,CD8+ T cell_1-AD.vs.control; scRNA-seq,SRP215507,CD8+ T cell_5-MCI.vs.control; scRNA-seq,SRP330776,γδ T cell-AD.vs.control; scRNA-seq,SRP330776,B cell_1-AD.vs.control; scRNA-seq,SRP330776,Naive CD8+ T cell_1-AD.vs.control	5
AC108066.2	scRNA-seq,SRP215507,CD8+ T cell_2-AD.vs.MCI; scRNA-seq,SRP215507,CD8+ T cell_4-MCI.vs.control; scRNA-seq,SRP215507,CD8+ T cell_2-	3
AC108105.1	scRNA-seq,SRP215507,CD8+ T cell_4-AD.vs.control	1
AC108134.2	scRNA-seq,SRP215507,CD8+ T cell_5-AD.vs.control; scRNA-seq,SRP215507,CD8+ T cell_1-AD.vs.MCI; scRNA-seq,SRP215507,CD8+ T cell_3-MCI.vs.control; scRNA-seq,SRP309935,Naive CD8+ T cell_2-AD.vs.control; scRNA-seq,SRP330776,Memory T cell_1-AD.vs.control	5
AC108134.3	scRNA-seq,SRP215507,CD8+ T cell_4-AD.vs.control; scRNA-seq,SRP215507,CD8+ T cell_5-AD.vs.control; scRNA-seq,SRP215507,CD8+ T cell_4-AD.vs.MCI; scRNA-seq,SRP215507,CD8+ T cell_5-MCI.vs.control; scRNA-seq,SRP309935,Natural killer cell_2-AD.vs.control; scRNA-seq,SRP330776,Naive CD8+ T cell_1-AD.vs.control; scRNA-seq,SRP330776,Natural killer cell_1-AD.vs.control; scRNA-seq,SRP330776,Memory T	8
AC108136.1	scRNA-seq,SRP330776,Memory T cell_1-AD.vs.control	1
AC108463.3	scRNA-seq,SRP330776,γδ T cell-AD.vs.control	1
AC108479.3	scRNA-seq,SRP215507,CD8+ T cell_1-AD.vs.control; scRNA-seq,SRP215507,CD8+ T cell_1-AD.vs.MCI; scRNA-seq,SRP330776,CD8+ T cell_2-	3
AC108488.1	scRNA-seq,SRP215507,CD8+ T cell_2-AD.vs.control; scRNA-seq,SRP215507,CD8+ T cell_3-AD.vs.MCI; scRNA-seq,SRP215507,CD8+ T cell_3-MCI.vs.control; scRNA-seq,SRP330776,CD8+ T cell_1-AD.vs.control; scRNA-seq,SRP330776,CD8+ T cell_2-AD.vs.control; scRNA-seq,SRP330776,B cell_1-AD.vs.control; scRNA-seq,SRP330776,Memory T cell_1-AD.vs.control	7
AC108488.3	scRNA-seq,SRP215507,CD8+ T cell_1-AD.vs.control; scRNA-seq,SRP215507,CD8+ T cell_1-AD.vs.MCI; scRNA-seq,SRP330776,Naive CD8+ T cell_1-	3
AC108519.1	scRNA-seq,SRP215507,CD8+ T cell_1-AD.vs.control; scRNA-seq,SRP215507,CD8+ T cell_5-AD.vs.MCI; scRNA-seq,SRP215507,CD8+ T cell_1-MCI.vs.control; scRNA-seq,SRP215507,CD8+ T cell_5-MCI.vs.control	4
AC108673.3	scRNA-seq,SRP215507,CD8+ T cell_6-AD.vs.control; scRNA-seq,SRP215507,CD8+ T cell_5-MCI.vs.control; scRNA-seq,SRP215507,CD8+ T cell_6-MCI.vs.control; scRNA-seq,SRP330776,CD8+ T cell_1-AD.vs.control; scRNA-seq,SRP330776,Naive CD8+ T cell_1-AD.vs.control; scRNA-seq,SRP330776,Naive CD8+ T cell_2-AD.vs.control	6
AC108718.1	scRNA-seq,SRP330776,CD8+ T cell_1-AD.vs.control; scRNA-seq,SRP330776,Naive CD8+ T cell_1-AD.vs.control	2
AC108863.2	scRNA-seq,SRP309935,Natural killer cell_2-AD.vs.control	1
AC108925.1	scRNA-seq,SRP215507,CD8+ T cell_2-AD.vs.control; scRNA-seq,SRP215507,CD8+ T cell_4-AD.vs.control; scRNA-seq,SRP215507,CD8+ T cell_1-AD.vs.MCI; scRNA-seq,SRP330776,γδ T cell-AD.vs.control; scRNA-seq,SRP330776,Memory T cell_1-AD.vs.control; scRNA-seq,SRP330776,CD8+ T	6
AC109322.1	scRNA-seq,SRP215507,CD8+ T cell_1-AD.vs.control; scRNA-seq,SRP215507,CD8+ T cell_3-AD.vs.MCI; scRNA-seq,SRP215507,CD8+ T cell_3-MCI.vs.control; scRNA-seq,SRP330776,CD8+ T cell_1-AD.vs.control; scRNA-seq,SRP330776,Memory T cell_1-AD.vs.control	5
AC109347.1	scRNA-seq,SRP215507,CD8+ T cell_2-MCI.vs.control	1
AC109446.3	scRNA-seq,SRP330776,B cell_1-AD.vs.control	1
AC109454.2	scRNA-seq,SRP215507,CD8+ T cell_3-AD.vs.MCI; scRNA-seq,SRP215507,CD8+ T cell_1-AD.vs.MCI; scRNA-seq,SRP215507,CD8+ T cell_3-MCI.vs.control; scRNA-seq,SRP330776,CD8+ T cell_1-AD.vs.control; scRNA-seq,SRP330776,Naive CD8+ T cell_1-AD.vs.control; scRNA-	6
AC109460.1	scRNA-seq,SRP215507,CD8+ T cell_1-AD.vs.control; scRNA-seq,SRP215507,CD8+ T cell_1-MCI.vs.control; scRNA-seq,SRP330776,CD8+ T cell_1-AD.vs.control; scRNA-seq,SRP330776,Memory T cell_2-AD.vs.control; scRNA-seq,SRP330776,B cell_1-AD.vs.control	5
AC109460.2	scRNA-seq,SRP309935,Natural killer cell_2-AD.vs.control	1
AC109460.4	scRNA-seq,SRP215507,CD8+ T cell_1-AD.vs.control; scRNA-seq,SRP215507,CD8+ T cell_5-AD.vs.control; scRNA-seq,SRP215507,CD8+ T cell_1-AD.vs.MCI; scRNA-seq,SRP330776,CD8+ T cell_1-AD.vs.control	4

AC109587.1	scRNA-seq,SRP215507,CD8+ T cell_1-AD.vs.control; scRNA-seq,SRP215507,CD8+ T cell_1-MCI.vs.control; scRNA-seq,SRP330776,γδ T cell-AD.vs.control; scRNA-seq,SRP330776,CD8+ T cell_3-AD.vs.control	4
AC109597.2	scRNA-seq,SRP330776,B cell_1-AD.vs.control	1
AC110597.3	scRNA-seq,SRP330776,CD8+ T cell_1-AD.vs.control	1
AC110611.2	scRNA-seq,SRP215507,CD8+ T cell_2-AD.vs.control; scRNA-seq,SRP215507,CD8+ T cell_3-AD.vs.MCI; scRNA-seq,SRP215507,CD8+ T cell_3-MCI.vs.control; scRNA-seq,SRP330776,Natural killer T cell-AD.vs.control	4
AC110769.2	scRNA-seq,SRP215507,CD8+ T cell_4-AD.vs.control; scRNA-seq,SRP215507,CD8+ T cell_4-AD.vs.MCI; scRNA-seq,SRP215507,CD8+ T cell_5-AD.vs.MCI; scRNA-seq,SRP215507,CD8+ T cell_5-MCI.vs.control; scRNA-seq,SRP330776,CD8+ T cell_1-AD.vs.control; scRNA-seq,SRP330776,B cell_2-AD.vs.control; scRNA-seq,SRP330776,CD8+ T cell_3-AD.vs.control	7
AC111006.1	scRNA-seq,SRP215507,CD8+ T cell_2-MCI.vs.control; scRNA-seq,SRP330776,B cell_1-AD.vs.control	2
AC111182.1	scRNA-seq,SRP215507,CD8+ T cell_3-AD.vs.MCI; scRNA-seq,SRP215507,CD8+ T cell_5-AD.vs.MCI; scRNA-seq,SRP215507,CD8+ T cell_5-MCI.vs.control; scRNA-seq,SRP330776,CD8+ T cell_1-AD.vs.control; scRNA-seq,SRP330776,Memory T cell_2-AD.vs.control; scRNA-seq,SRP330776,CD8+ T cell_2-AD.vs.control; scRNA-seq,SRP330776,Naive CD8+ T cell_1-AD.vs.control; scRNA-seq,SRP330776,Natural killer cell_1-AD.vs.control; scRNA-seq,SRP330776,Naive CD8+ T cell_3-AD.vs.control; scRNA-seq,SRP330776,T cell-AD.vs.control; scRNA-seq,SRP330776,CD8+	12
AC112236.2	scRNA-seq,SRP330776,B cell_1-AD.vs.control	1
AC112484.3	scRNA-seq,SRP215507,CD8+ T cell_2-AD.vs.control	1
AC112491.1	scRNA-seq,SRP330776,CD8+ T cell_1-AD.vs.control	1
AC112496.1	scRNA-seq,SRP309935,Monocyte_1-AD.vs.control; scRNA-seq,SRP309935,Monocyte_2-AD.vs.control	2
AC112512.1	scRNA-seq,SRP215507,CD8+ T cell_1-AD.vs.MCI; scRNA-seq,SRP215507,CD8+ T cell_1-MCI.vs.control	2
AC112721.2	scRNA-seq,SRP330776,Naive CD8+ T cell_3-AD.vs.control	1
AC112907.3	scRNA-seq,SRP215507,CD8+ T cell_1-AD.vs.MCI; scRNA-seq,SRP215507,CD8+ T cell_1-MCI.vs.control; scRNA-seq,SRP309935,Naive CD8+ T cell_2-AD.vs.control; scRNA-seq,SRP330776,CD8+ T cell_1-AD.vs.control; scRNA-seq,SRP330776,Memory T cell_2-AD.vs.control; scRNA-	6
AC113143.3	scRNA-seq,SRP330776,CD8+ T cell_1-AD.vs.control; scRNA-seq,SRP330776,Memory T cell_2-AD.vs.control	2
AC113404.1	scRNA-seq,SRP309935,Megakaryocyte_2-AD.vs.control	1
AC113410.4	scRNA-seq,SRP330776,CD8+ T cell_1-AD.vs.control; scRNA-seq,SRP330776,B cell_1-AD.vs.control; scRNA-seq,SRP330776,Memory T cell_1-AD.vs.control; scRNA-seq,SRP330776,Naive CD8+ T cell_3-AD.vs.control; scRNA-seq,SRP330776,T cell-AD.vs.control	5
AC113414.1	scRNA-seq,SRP215507,CD8+ T cell_1-AD.vs.MCI; scRNA-seq,SRP215507,CD8+ T cell_1-MCI.vs.control; scRNA-seq,SRP330776,CD8+ T cell_1-	3
AC114271.1	scRNA-seq,SRP215507,CD8+ T cell_1-MCI.vs.control; scRNA-seq,SRP309935,Monocyte_1-AD.vs.control; scRNA-seq,SRP330776,Natural killer cell_1-AD.vs.control; scRNA-seq,SRP330776,Memory T cell_1-AD.vs.control; scRNA-seq,SRP330776,Natural killer T cell-AD.vs.control; scRNA-seq,SRP330776,B cell_2-AD.vs.control; scRNA-seq,SRP330776,T cell-AD.vs.control	7
AC114341.2	scRNA-seq,SRP215507,CD8+ T cell_2-AD.vs.control	1
AC114488.3	scRNA-seq,SRP215507,CD8+ T cell_3-MCI.vs.control	1
AC114730.1	scRNA-seq,SRP215507,CD8+ T cell_2-MCI.vs.control	1
AC114760.2	scRNA-seq,SRP215507,CD8+ T cell_3-AD.vs.MCI; scRNA-seq,SRP215507,CD8+ T cell_3-MCI.vs.control	2
AC114763.1	scRNA-seq,SRP215507,CD8+ T cell_3-AD.vs.MCI; scRNA-seq,SRP330776,CD8+ T cell_2-AD.vs.control; scRNA-seq,SRP330776,B cell_1-AD.vs.control; scRNA-seq,SRP330776,Naive CD8+ T cell_1-AD.vs.control	4
AC114930.1	scRNA-seq,SRP215507,CD8+ T cell_5-AD.vs.control; scRNA-seq,SRP215507,CD8+ T cell_5-MCI.vs.control	2
AC114956.1	scRNA-seq,SRP215507,CD8+ T cell_1-MCI.vs.control	1
AC114956.3	scRNA-seq,SRP215507,CD8+ T cell_1-AD.vs.control; scRNA-seq,SRP215507,CD8+ T cell_4-AD.vs.control; scRNA-seq,SRP215507,CD8+ T cell_1-AD.vs.MCI; scRNA-seq,SRP215507,CD8+ T cell_4-MCI.vs.control; scRNA-seq,SRP330776,CD8+ T cell_2-AD.vs.control	5

AC114980.1	scRNA-seq,SRP215507,CD8+ T cell_3-AD.vs.control; scRNA-seq,SRP215507,CD8+ T cell_3-AD.vs.MCI; scRNA-seq,SRP215507,CD8+ T cell_1-MCI.vs.control; scRNA-seq,SRP330776,CD8+ T cell_1-AD.vs.control; scRNA-seq,SRP330776,CD8+ T cell_2-AD.vs.control; scRNA-seq,SRP330776,B cell_1-AD.vs.control; scRNA-seq,SRP330776,Memory T cell_1-AD.vs.control	7
AC115618.1	scRNA-seq,SRP215507,CD8+ T cell_5-AD.vs.control; scRNA-seq,SRP330776,CD8+ T cell_2-AD.vs.control	2
AC116158.1	scRNA-seq,SRP215507,CD8+ T cell_3-AD.vs.MCI; scRNA-seq,SRP215507,CD8+ T cell_3-MCI.vs.control	2
AC116158.3	scRNA-seq,SRP215507,CD8+ T cell_4-AD.vs.MCI; scRNA-seq,SRP215507,CD8+ T cell_4-MCI.vs.control; scRNA-seq,SRP330776,CD8+ T cell_2-AD.vs.control; scRNA-seq,SRP330776,Natural killer cell_1-AD.vs.control; scRNA-seq,SRP330776,Memory T cell_1-AD.vs.control; scRNA-	6
AC116337.3	scRNA-seq,SRP215507,CD8+ T cell_1-AD.vs.control; scRNA-seq,SRP215507,CD8+ T cell_4-AD.vs.control; scRNA-seq,SRP215507,CD8+ T cell_5-AD.vs.control; scRNA-seq,SRP215507,CD8+ T cell_1-AD.vs.MCI; scRNA-seq,SRP215507,CD8+ T cell_4-AD.vs.MCI; scRNA-seq,SRP215507,CD8+ T cell_1-MCI.vs.control; scRNA-seq,SRP330776,CD8+ T cell_3-AD.vs.control	7
AC116337.4	scRNA-seq,SRP215507,CD8+ T cell_2-AD.vs.MCI; scRNA-seq,SRP215507,CD8+ T cell_2-MCI.vs.control	2
AC116366.1	scRNA-seq,SRP215507,CD8+ T cell_1-AD.vs.MCI; scRNA-seq,SRP215507,CD8+ T cell_5-AD.vs.MCI; scRNA-seq,SRP215507,CD8+ T cell_1-MCI.vs.control; scRNA-seq,SRP215507,CD8+ T cell_5-MCI.vs.control; scRNA-seq,SRP215507,CD8+ T cell_3-MCI.vs.control; scRNA-seq,SRP309935,Monocyte_1-AD.vs.control; scRNA-seq,SRP330776,Memory T cell_2-AD.vs.control	7
AC116366.2	scRNA-seq,SRP215507,CD8+ T cell_1-AD.vs.control; scRNA-seq,SRP215507,CD8+ T cell_6-AD.vs.control; scRNA-seq,SRP215507,CD8+ T cell_1-AD.vs.MCI; scRNA-seq,SRP215507,CD8+ T cell_5-AD.vs.MCI; scRNA-seq,SRP215507,CD8+ T cell_5-MCI.vs.control	5
AC116407.2	scRNA-seq,SRP215507,CD8+ T cell_4-AD.vs.control; scRNA-seq,SRP215507,CD8+ T cell_6-AD.vs.control; scRNA-seq,SRP215507,CD8+ T cell_2-AD.vs.MCI; scRNA-seq,SRP215507,CD8+ T cell_1-AD.vs.MCI; scRNA-seq,SRP215507,CD8+ T cell_1-MCI.vs.control; scRNA-seq,SRP215507,CD8+ T cell_4-MCI.vs.control; scRNA-seq,SRP330776,CD8+ T cell_2-AD.vs.control; scRNA-seq,SRP330776,B cell_1-AD.vs.control; scRNA-	9
AC116428.1	scRNA-seq,SRP215507,CD8+ T cell_2-MCI.vs.control	1
AC116535.1	scRNA-seq,SRP215507,CD8+ T cell_6-AD.vs.control; scRNA-seq,SRP215507,CD8+ T cell_1-MCI.vs.control; scRNA-seq,SRP215507,CD8+ T cell_2-MCI.vs.control; scRNA-seq,SRP215507,CD8+ T cell_6-MCI.vs.control; scRNA-seq,SRP330776,CD8+ T cell_1-AD.vs.control; scRNA-seq,SRP330776,Memory T cell_2-AD.vs.control; scRNA-seq,SRP330776,Naive CD8+ T cell_1-AD.vs.control; scRNA-seq,SRP330776,CD8+ T cell_3-	8
AC116913.1	scRNA-seq,SRP215507,CD8+ T cell_2-AD.vs.MCI; scRNA-seq,SRP215507,CD8+ T cell_2-MCI.vs.control; scRNA-seq,SRP330776,Memory T cell_1-	3
AC117382.2	scRNA-seq,SRP215507,CD8+ T cell_1-AD.vs.MCI; scRNA-seq,SRP215507,CD8+ T cell_5-AD.vs.MCI; scRNA-seq,SRP215507,CD8+ T cell_1-MCI.vs.control; scRNA-seq,SRP215507,CD8+ T cell_5-MCI.vs.control; scRNA-seq,SRP330776,γδ T cell-AD.vs.control; scRNA-seq,SRP330776,Naive CD8+ T cell_1-AD.vs.control; scRNA-seq,SRP330776,B cell_2-AD.vs.control; scRNA-seq,SRP330776,B cell_3-AD.vs.control	8
AC117490.2	scRNA-seq,SRP330776,B cell_1-AD.vs.control	1
AC117528.1	scRNA-seq,SRP330776,Naive CD8+ T cell_1-AD.vs.control; scRNA-seq,SRP330776,Memory T cell_1-AD.vs.control	2
AC118344.1	scRNA-seq,SRP215507,CD8+ T cell_2-MCI.vs.control	1
AC118469.2	scRNA-seq,SRP215507,CD8+ T cell_1-MCI.vs.control; scRNA-seq,SRP330776,CD8+ T cell_2-AD.vs.control	2
AC118549.1	scRNA-seq,SRP215507,CD8+ T cell_5-AD.vs.control	1
AC118553.1	scRNA-seq,SRP215507,CD8+ T cell_1-AD.vs.control; scRNA-seq,SRP330776,Memory T cell_2-AD.vs.control; scRNA-seq,SRP330776,B cell_1-AD.vs.control; scRNA-seq,SRP330776,Naive CD8+ T cell_1-AD.vs.control; scRNA-seq,SRP330776,Memory T cell_1-AD.vs.control	5
AC118658.1	scRNA-seq,SRP215507,CD8+ T cell_3-AD.vs.control; scRNA-seq,SRP330776,Naive CD8+ T cell_1-AD.vs.control	2
AC118758.3	scRNA-seq,SRP215507,CD8+ T cell_2-MCI.vs.control; scRNA-seq,SRP215507,CD8+ T cell_3-MCI.vs.control	2
AC119396.1	scRNA-seq,SRP215507,CD8+ T cell_7-AD.vs.control; scRNA-seq,SRP215507,CD8+ T cell_5-AD.vs.control; scRNA-seq,SRP215507,CD8+ T cell_1-AD.vs.MCI; scRNA-seq,SRP309935,Naive CD8+ T cell_2-AD.vs.control; scRNA-seq,SRP330776,Natural killer cell_2-AD.vs.control	5
AC119403.1	scRNA-seq,SRP330776,CD8+ T cell_2-AD.vs.control	1
AC119673.2	scRNA-seq,SRP215507,CD8+ T cell_1-AD.vs.control; scRNA-seq,SRP215507,CD8+ T cell_2-AD.vs.MCI; scRNA-seq,SRP215507,CD8+ T cell_3-AD.vs.MCI; scRNA-seq,SRP215507,CD8+ T cell_1-AD.vs.MCI; scRNA-seq,SRP215507,CD8+ T cell_2-MCI.vs.control; scRNA-seq,SRP215507,CD8+ T	6

AC119800.1	scRNA-seq,SRP215507,CD8+ T cell_2-AD.vs.MCI; scRNA-seq,SRP215507,CD8+ T cell_5-AD.vs.MCI; scRNA-seq,SRP215507,CD8+ T cell_5-MCI.vs.control; scRNA-seq,SRP215507,CD8+ T cell_2-MCI.vs.control	4
AC120024.1	scRNA-seq,SRP330776,Memory T cell_1-AD.vs.control	1
AC120049.1	scRNA-seq,SRP215507,CD8+ T cell_2-MCI.vs.control	1
AC120193.1	scRNA-seq,SRP215507,CD8+ T cell_2-AD.vs.MCI; scRNA-seq,SRP215507,CD8+ T cell_2-MCI.vs.control	2
AC121761.1	scRNA-seq,SRP309935,CD8+ T cell_1-AD.vs.control; scRNA-seq,SRP330776,CD8+ T cell_1-AD.vs.control; scRNA-seq,SRP330776,Naive CD8+ T	3
AC121761.2	scRNA-seq,SRP215507,CD8+ T cell_2-AD.vs.control; scRNA-seq,SRP309935,Naive CD8+ T cell_1-AD.vs.control	2
AC122129.1	scRNA-seq,SRP309935,Natural killer cell_1-AD.vs.control	1
AC123512.1	scRNA-seq,SRP215507,CD8+ T cell_2-AD.vs.control; scRNA-seq,SRP309935,Naive CD8+ T cell_1-AD.vs.control	2
AC123595.1	scRNA-seq,SRP330776,Memory T cell_1-AD.vs.control; scRNA-seq,SRP330776,CD8+ T cell_3-AD.vs.control	2
AC123768.3	scRNA-seq,SRP215507,CD8+ T cell_8-AD.vs.control; scRNA-seq,SRP215507,CD8+ T cell_3-AD.vs.control; scRNA-seq,SRP215507,CD8+ T cell_4-MCI.vs.control; scRNA-seq,SRP330776,γδ T cell-AD.vs.control; scRNA-seq,SRP330776,B cell_2-AD.vs.control	5
AC123768.5	scRNA-seq,SRP330776,CD8+ T cell_1-AD.vs.control	1
AC123912.1	scRNA-seq,SRP215507,CD8+ T cell_3-AD.vs.control; scRNA-seq,SRP330776,CD8+ T cell_2-AD.vs.control; scRNA-seq,SRP330776,Natural killer	3
AC123912.4	scRNA-seq,SRP215507,CD8+ T cell_3-AD.vs.control; scRNA-seq,SRP215507,CD8+ T cell_5-AD.vs.control; scRNA-seq,SRP215507,CD8+ T cell_5-AD.vs.MCI; scRNA-seq,SRP215507,CD8+ T cell_5-MCI.vs.control	4
AC124014.1	scRNA-seq,SRP330776,B cell_1-AD.vs.control	1
AC124016.1	scRNA-seq,SRP215507,CD8+ T cell_2-AD.vs.MCI; scRNA-seq,SRP215507,CD8+ T cell_1-AD.vs.MCI; scRNA-seq,SRP215507,CD8+ T cell_5-AD.vs.MCI; scRNA-seq,SRP330776,γδ T cell-AD.vs.control; scRNA-seq,SRP330776,Naive CD8+ T cell_1-AD.vs.control; scRNA-seq,SRP330776,Natural killer cell_1-AD.vs.control; scRNA-seq,SRP330776,Memory T cell_1-AD.vs.control; scRNA-seq,SRP330776,Naive CD8+ T cell_3-AD.vs.control; scRNA-seq,SRP330776,T cell-AD.vs.control; scRNA-seq,SRP330776,CD8+ T cell_3-AD.vs.control; scRNA-seq,SRP330776,B	11
AC124016.2	scRNA-seq,SRP215507,CD8+ T cell_1-AD.vs.control; scRNA-seq,SRP215507,CD8+ T cell_2-AD.vs.MCI; scRNA-seq,SRP215507,CD8+ T cell_1-MCI.vs.control; scRNA-seq,SRP215507,CD8+ T cell_2-MCI.vs.control; scRNA-seq,SRP309935,Natural killer cell_2-AD.vs.control; scRNA-seq,SRP330776,CD8+ T cell_1-AD.vs.control; scRNA-seq,SRP330776,Naive CD8+ T cell_1-AD.vs.control	7
AC124045.1	scRNA-seq,SRP330776,CD8+ T cell_1-AD.vs.control	1
AC124068.2	scRNA-seq,SRP215507,CD8+ T cell_1-AD.vs.MCI	1
AC124248.1	scRNA-seq,SRP215507,CD8+ T cell_2-AD.vs.control; scRNA-seq,SRP330776,CD8+ T cell_1-AD.vs.control; scRNA-seq,SRP330776,B cell_1-AD.vs.control; scRNA-seq,SRP330776,Monocyte_2-AD.vs.control	4
AC124254.2	scRNA-seq,SRP215507,CD8+ T cell_1-MCI.vs.control	1
AC124319.1	scRNA-seq,SRP215507,CD8+ T cell_3-MCI.vs.control; scRNA-seq,SRP330776,Naive CD8+ T cell_1-AD.vs.control	2
AC124319.2	scRNA-seq,SRP330776,CD8+ T cell_1-AD.vs.control; scRNA-seq,SRP330776,CD8+ T cell_2-AD.vs.control	2
AC124798.1	scRNA-seq,SRP215507,CD8+ T cell_3-AD.vs.control; scRNA-seq,SRP215507,CD8+ T cell_2-AD.vs.MCI; scRNA-seq,SRP215507,CD8+ T cell_1-MCI.vs.control; scRNA-seq,SRP215507,CD8+ T cell_4-MCI.vs.control; scRNA-seq,SRP215507,CD8+ T cell_2-MCI.vs.control; scRNA-	6
AC125494.1	scRNA-seq,SRP215507,CD8+ T cell_2-AD.vs.control; scRNA-seq,SRP330776,CD8+ T cell_2-AD.vs.control; scRNA-seq,SRP330776,B cell_1-AD.vs.control; scRNA-seq,SRP330776,Natural killer cell_1-AD.vs.control; scRNA-seq,SRP330776,Natural killer T cell-AD.vs.control	5
AC125611.3	scRNA-seq,SRP215507,CD8+ T cell_1-AD.vs.MCI; scRNA-seq,SRP215507,CD8+ T cell_1-MCI.vs.control; scRNA-seq,SRP330776,B cell_1-AD.vs.control; scRNA-seq,SRP330776,Natural killer cell_1-AD.vs.control	4
AC126283.1	scRNA-seq,SRP215507,CD8+ T cell_1-AD.vs.control; scRNA-seq,SRP215507,CD8+ T cell_1-MCI.vs.control; scRNA-seq,SRP215507,CD8+ T cell_5-	3
AC126544.2	scRNA-seq,SRP215507,CD8+ T cell_2-AD.vs.control	1

AC126614.1	scRNA-seq,SRP330776,Memory T cell_2-AD.vs.control; scRNA-seq,SRP330776,CD8+ T cell_2-AD.vs.control; scRNA-seq,SRP330776,Naive CD8+ T cell_1-AD.vs.control; scRNA-seq,SRP330776,Memory T cell_1-AD.vs.control	4
AC126696.1	scRNA-seq,SRP215507,CD8+ T cell_1-AD.vs.control	1
AC126696.3	scRNA-seq,SRP215507,CD8+ T cell_2-AD.vs.control; scRNA-seq,SRP215507,CD8+ T cell_5-MCI.vs.control	2
AC127035.1	scRNA-seq,SRP215507,CD8+ T cell_4-MCI.vs.control	1
AC127070.1	scRNA-seq,SRP215507,CD8+ T cell_2-AD.vs.MCI; scRNA-seq,SRP215507,CD8+ T cell_2-MCI.vs.control; scRNA-seq,SRP330776,CD8+ T cell_2-	3
AC127070.2	scRNA-seq,SRP330776,CD8+ T cell_1-AD.vs.control	1
AC127502.2	scRNA-seq,SRP215507,CD8+ T cell_2-AD.vs.control; scRNA-seq,SRP215507,CD8+ T cell_3-AD.vs.MCI; scRNA-seq,SRP215507,CD8+ T cell_5-MCI.vs.control; scRNA-seq,SRP215507,CD8+ T cell_3-MCI.vs.control; scRNA-seq,SRP309935,Monocyte_1-AD.vs.control; scRNA-seq,SRP309935,Megakaryocyte_1-AD.vs.control; scRNA-seq,SRP309935,Natural killer cell_1-AD.vs.control; scRNA-seq,SRP309935,Monocyte_2-AD.vs.control; scRNA-seq,SRP309935,Naive CD8+ T cell_2-AD.vs.control; scRNA-seq,SRP309935,CD8+ T cell_1-AD.vs.control; scRNA-seq,SRP330776,CD8+ T cell_1-AD.vs.control; scRNA-seq,SRP330776,γδ T cell-AD.vs.control; scRNA-seq,SRP330776,Memory T cell_2-AD.vs.control; scRNA-seq,SRP330776,CD8+ T cell_2-AD.vs.control; scRNA-seq,SRP330776,Naive CD8+ T cell_1-AD.vs.control; scRNA-seq,SRP330776,Memory T	18
AC127521.1	scRNA-seq,SRP215507,CD8+ T cell_1-AD.vs.control; scRNA-seq,SRP215507,CD8+ T cell_5-AD.vs.control; scRNA-seq,SRP215507,CD8+ T cell_1-MCI.vs.control; scRNA-seq,SRP215507,CD8+ T cell_2-MCI.vs.control; scRNA-seq,SRP330776,CD8+ T cell_1-AD.vs.control; scRNA-seq,SRP330776,γδ T cell-AD.vs.control; scRNA-seq,SRP330776,Memory T cell_2-AD.vs.control; scRNA-seq,SRP330776,B cell_1-AD.vs.control; scRNA-seq,SRP330776,Naive CD8+ T cell_1-AD.vs.control; scRNA-seq,SRP330776,Natural killer cell_1-AD.vs.control; scRNA-seq,SRP330776,Memory T cell_1-AD.vs.control; scRNA-seq,SRP330776,Natural killer T cell-AD.vs.control; scRNA-seq,SRP330776,Naive CD8+ T cell_3-AD.vs.control; scRNA-seq,SRP330776,T cell-AD.vs.control; scRNA-seq,SRP330776,CD8+ T cell_3-AD.vs.control; scRNA-seq,SRP330776,B cell_3-AD.vs.control	16
AC127526.2	scRNA-seq,SRP215507,CD8+ T cell_3-MCI.vs.control; scRNA-seq,SRP330776,CD8+ T cell_1-AD.vs.control	2
AC129492.1	scRNA-seq,SRP215507,CD8+ T cell_2-AD.vs.control; scRNA-seq,SRP215507,CD8+ T cell_2-AD.vs.MCI; scRNA-seq,SRP215507,CD8+ T cell_1-AD.vs.MCI; scRNA-seq,SRP215507,CD8+ T cell_1-MCI.vs.control; scRNA-seq,SRP215507,CD8+ T cell_2-MCI.vs.control; scRNA-seq,SRP330776,CD8+ T cell_1-AD.vs.control; scRNA-seq,SRP330776,Memory T cell_2-AD.vs.control; scRNA-seq,SRP330776,CD8+ T cell_2-	9
AC130371.2	scRNA-seq,SRP330776,Memory T cell_1-AD.vs.control	1
AC130650.2	scRNA-seq,SRP215507,CD8+ T cell_2-AD.vs.control; scRNA-seq,SRP215507,CD8+ T cell_3-AD.vs.MCI; scRNA-seq,SRP215507,CD8+ T cell_3-MCI.vs.control; scRNA-seq,SRP309935,Naive CD8+ T cell_2-AD.vs.control; scRNA-seq,SRP330776,CD8+ T cell_1-AD.vs.control; scRNA-	6
AC131097.3	scRNA-seq,SRP215507,CD8+ T cell_1-AD.vs.MCI; scRNA-seq,SRP330776,B cell_1-AD.vs.control	2
AC131237.1	scRNA-seq,SRP215507,CD8+ T cell_2-MCI.vs.control	1
AC131971.1	scRNA-seq,SRP215507,CD8+ T cell_3-AD.vs.control; scRNA-seq,SRP215507,CD8+ T cell_4-AD.vs.control; scRNA-seq,SRP215507,CD8+ T cell_3-MCI.vs.control; scRNA-seq,SRP330776,CD8+ T cell_1-AD.vs.control; scRNA-seq,SRP330776,γδ T cell-AD.vs.control; scRNA-seq,SRP330776,CD8+ T cell_2-AD.vs.control; scRNA-seq,SRP330776,Naive CD8+ T cell_1-AD.vs.control; scRNA-seq,SRP330776,Natural killer cell_1-AD.vs.control; scRNA-seq,SRP330776,Memory T cell_1-AD.vs.control; scRNA-seq,SRP330776,CD8+ T cell_3-AD.vs.control	10
AC132192.2	scRNA-seq,SRP215507,CD8+ T cell_1-AD.vs.MCI; scRNA-seq,SRP215507,CD8+ T cell_1-MCI.vs.control; scRNA-seq,SRP309935,Naive CD8+ T cell_2-AD.vs.control; scRNA-seq,SRP330776,Memory T cell_1-AD.vs.control; scRNA-seq,SRP330776,CD8+ T cell_3-AD.vs.control	5
AC132872.1	scRNA-seq,SRP215507,CD8+ T cell_1-AD.vs.MCI; scRNA-seq,SRP215507,CD8+ T cell_1-MCI.vs.control; scRNA-seq,SRP330776,CD8+ T cell_2-	3
AC132872.2	scRNA-seq,SRP215507,CD8+ T cell_1-AD.vs.control	1
AC132938.3	scRNA-seq,SRP215507,CD8+ T cell_3-AD.vs.MCI; scRNA-seq,SRP215507,CD8+ T cell_1-AD.vs.MCI; scRNA-seq,SRP215507,CD8+ T cell_1-MCI.vs.control; scRNA-seq,SRP215507,CD8+ T cell_3-MCI.vs.control; scRNA-seq,SRP330776,Naive CD8+ T cell_1-AD.vs.control; scRNA-	6
AC132938.6	scRNA-seq,SRP215507,CD8+ T cell_2-AD.vs.control; scRNA-seq,SRP330776,B cell_1-AD.vs.control	2
AC133065.1	scRNA-seq,SRP330776,B cell_1-AD.vs.control; scRNA-seq,SRP330776,B cell_2-AD.vs.control	2

AC133550.2	scRNA-seq,SRP330776,CD8+ T cell_1-AD.vs.control; scRNA-seq,SRP330776,B cell_1-AD.vs.control; scRNA-seq,SRP330776,Naive CD8+ T cell_1-AD.vs.control; scRNA-seq,SRP330776,Memory T cell_1-AD.vs.control	4
AC133550.3	scRNA-seq,SRP215507,CD8+ T cell_3-MCI.vs.control; scRNA-seq,SRP309935,Naive CD8+ T cell_1-AD.vs.control; scRNA-seq,SRP309935,Natural killer cell_1-AD.vs.control; scRNA-seq,SRP330776,CD8+ T cell_1-AD.vs.control; scRNA-seq,SRP330776,CD8+ T cell_2-AD.vs.control; scRNA-seq,SRP330776,B cell_1-AD.vs.control; scRNA-seq,SRP330776,Memory T cell_1-AD.vs.control; scRNA-seq,SRP330776,CD8+ T cell_3-AD.vs.control	8
AC133552.5	scRNA-seq,SRP215507,CD8+ T cell_3-AD.vs.control; scRNA-seq,SRP215507,CD8+ T cell_3-AD.vs.MCI; scRNA-seq,SRP215507,CD8+ T cell_1-AD.vs.MCI; scRNA-seq,SRP215507,CD8+ T cell_1-MCI.vs.control; scRNA-seq,SRP330776,B cell_4-AD.vs.control	5
AC133644.2	scRNA-seq,SRP215507,CD8+ T cell_2-AD.vs.control; scRNA-seq,SRP215507,CD8+ T cell_3-AD.vs.control; scRNA-seq,SRP215507,CD8+ T cell_3-MCI.vs.control; scRNA-seq,SRP330776,γδ T cell-AD.vs.control; scRNA-seq,SRP330776,Memory T cell_2-AD.vs.control	5
AC133919.2	scRNA-seq,SRP330776,CD8+ T cell_1-AD.vs.control	1
AC134349.1	scRNA-seq,SRP215507,CD8+ T cell_1-MCI.vs.control	1
AC134772.1	scRNA-seq,SRP215507,CD8+ T cell_1-AD.vs.MCI; scRNA-seq,SRP215507,CD8+ T cell_1-MCI.vs.control; scRNA-seq,SRP330776,CD8+ T cell_1-	3
AC135050.6	scRNA-seq,SRP215507,CD8+ T cell_1-AD.vs.control; scRNA-seq,SRP215507,CD8+ T cell_1-MCI.vs.control; scRNA-seq,SRP215507,CD8+ T cell_5-MCI.vs.control; scRNA-seq,SRP330776,CD8+ T cell_1-AD.vs.control; scRNA-seq,SRP330776,Naive CD8+ T cell_1-AD.vs.control	5
AC135068.2	scRNA-seq,SRP330776,B cell_2-AD.vs.control	1
AC135279.4	scRNA-seq,SRP215507,CD8+ T cell_2-AD.vs.control; scRNA-seq,SRP215507,CD8+ T cell_3-AD.vs.MCI; scRNA-seq,SRP215507,CD8+ T cell_3-	3
AC135457.1	scRNA-seq,SRP215507,CD8+ T cell_1-AD.vs.control; scRNA-seq,SRP215507,CD8+ T cell_1-AD.vs.MCI; scRNA-seq,SRP215507,CD8+ T cell_6-MCI.vs.control; scRNA-seq,SRP330776,CD8+ T cell_1-AD.vs.control; scRNA-seq,SRP330776,γδ T cell-AD.vs.control; scRNA-seq,SRP330776,Memory T cell_1-AD.vs.control; scRNA-seq,SRP330776,Natural killer T cell-AD.vs.control; scRNA-seq,SRP330776,T cell-AD.vs.control; scRNA-seq,SRP330776,CD8+ T cell_3-AD.vs.control; scRNA-seq,SRP330776,Monocyte_2-AD.vs.control	10
AC135507.1	scRNA-seq,SRP330776,Memory T cell_1-AD.vs.control	1
AC135782.3	scRNA-seq,SRP215507,CD8+ T cell_4-MCI.vs.control; scRNA-seq,SRP330776,Memory T cell_2-AD.vs.control	2
AC136475.1	scRNA-seq,SRP215507,CD8+ T cell_2-AD.vs.control; scRNA-seq,SRP215507,CD8+ T cell_3-AD.vs.control; scRNA-seq,SRP215507,CD8+ T cell_1-AD.vs.control; scRNA-seq,SRP215507,CD8+ T cell_4-AD.vs.control; scRNA-seq,SRP215507,CD8+ T cell_5-AD.vs.control; scRNA-seq,SRP215507,CD8+ T cell_4-AD.vs.MCI; scRNA-seq,SRP215507,CD8+ T cell_1-MCI.vs.control; scRNA-seq,SRP215507,CD8+ T cell_5-MCI.vs.control; scRNA-seq,SRP215507,CD8+ T cell_2-MCI.vs.control; scRNA-seq,SRP215507,CD8+ T cell_3-MCI.vs.control; scRNA-seq,SRP330776,Memory T cell_2-AD.vs.control; scRNA-seq,SRP330776,Natural killer cell_1-AD.vs.control; scRNA-seq,SRP330776,Naive CD8+ T	15
AC136475.2	scRNA-seq,SRP215507,CD8+ T cell_3-AD.vs.control; scRNA-seq,SRP215507,CD8+ T cell_2-AD.vs.MCI; scRNA-seq,SRP215507,CD8+ T cell_5-AD.vs.MCI; scRNA-seq,SRP215507,CD8+ T cell_5-MCI.vs.control; scRNA-seq,SRP215507,CD8+ T cell_2-MCI.vs.control; scRNA-seq,SRP330776,γδ T cell-AD.vs.control; scRNA-seq,SRP330776,Naive CD8+ T cell_1-AD.vs.control; scRNA-seq,SRP330776,Naive CD8+ T cell_3-AD.vs.control	8
AC136475.3	scRNA-seq,SRP215507,CD8+ T cell_3-AD.vs.control; scRNA-seq,SRP215507,CD8+ T cell_4-AD.vs.control; scRNA-seq,SRP215507,CD8+ T cell_4-AD.vs.MCI; scRNA-seq,SRP215507,CD8+ T cell_1-MCI.vs.control; scRNA-seq,SRP330776,Natural killer cell_1-AD.vs.control	5
AC136475.5	scRNA-seq,SRP215507,CD8+ T cell_2-AD.vs.control; scRNA-seq,SRP215507,CD8+ T cell_3-AD.vs.control; scRNA-seq,SRP215507,CD8+ T cell_1-AD.vs.control; scRNA-seq,SRP215507,CD8+ T cell_5-AD.vs.control; scRNA-seq,SRP215507,CD8+ T cell_5-MCI.vs.control; scRNA-seq,SRP215507,CD8+ T cell_4-MCI.vs.control; scRNA-seq,SRP215507,CD8+ T cell_3-MCI.vs.control; scRNA-seq,SRP309935,Naive CD8+ T cell_2-AD.vs.control; scRNA-seq,SRP309935,B cell_1-AD.vs.control; scRNA-seq,SRP330776,γδ T cell-AD.vs.control; scRNA-seq,SRP330776,Memory T cell_1-AD.vs.control; scRNA-seq,SRP330776,CD8+ T cell_3-AD.vs.control; scRNA-seq,SRP330776,B cell_3-AD.vs.control; scRNA-	14
AC137561.1	scRNA-seq,SRP215507,CD8+ T cell_4-AD.vs.control	1
AC137590.1	scRNA-seq,SRP330776,B cell_1-AD.vs.control; scRNA-seq,SRP330776,Memory T cell_1-AD.vs.control	2
AC137630.1	scRNA-seq,SRP215507,CD8+ T cell_3-AD.vs.MCI; scRNA-seq,SRP215507,CD8+ T cell_1-MCI.vs.control	2
AC137630.2	scRNA-seq,SRP215507,CD8+ T cell_1-AD.vs.control; scRNA-seq,SRP215507,CD8+ T cell_2-AD.vs.MCI; scRNA-seq,SRP215507,CD8+ T cell_2-	3

AC137630.3	scRNA-seq,SRP215507,CD8+ T cell_4-AD.vs.control	1
AC137695.3	scRNA-seq,SRP215507,CD8+ T cell_3-AD.vs.MCI; scRNA-seq,SRP215507,CD8+ T cell_3-MCI.vs.control	2
AC137810.1	scRNA-seq,SRP330776,Naive CD8+ T cell_1-AD.vs.control	1
AC137894.3	scRNA-seq,SRP215507,CD8+ T cell_2-MCI.vs.control	1
AC137932.1	scRNA-seq,SRP215507,CD8+ T cell_1-MCI.vs.control	1
AC138123.1	scRNA-seq,SRP330776,B cell_1-AD.vs.control	1
AC138150.1	scRNA-seq,SRP215507,CD8+ T cell_5-AD.vs.control; scRNA-seq,SRP215507,CD8+ T cell_5-AD.vs.MCI; scRNA-seq,SRP330776,CD8+ T cell_1-AD.vs.control; scRNA-seq,SRP330776,γδ T cell-AD.vs.control; scRNA-seq,SRP330776,Memory T cell_2-AD.vs.control; scRNA-seq,SRP330776,CD8+ T cell_2-AD.vs.control; scRNA-seq,SRP330776,Memory T cell_1-AD.vs.control; scRNA-seq,SRP330776,T cell-AD.vs.control; scRNA-seq,SRP330776,CD8+ T cell_3-AD.vs.control; scRNA-seq,SRP330776,Monocyte_1-AD.vs.control	10
AC138356.1	scRNA-seq,SRP215507,CD8+ T cell_2-AD.vs.control; scRNA-seq,SRP215507,CD8+ T cell_3-MCI.vs.control	2
AC138466.1	scRNA-seq,SRP215507,CD8+ T cell_1-AD.vs.control; scRNA-seq,SRP215507,CD8+ T cell_1-MCI.vs.control	2
AC138627.1	scRNA-seq,SRP330776,CD8+ T cell_1-AD.vs.control	1
AC138649.1	scRNA-seq,SRP215507,CD8+ T cell_2-AD.vs.control; scRNA-seq,SRP215507,CD8+ T cell_3-AD.vs.control; scRNA-seq,SRP215507,CD8+ T cell_1-AD.vs.control; scRNA-seq,SRP215507,CD8+ T cell_2-AD.vs.MCI; scRNA-seq,SRP215507,CD8+ T cell_5-MCI.vs.control; scRNA-seq,SRP330776,CD8+	6
AC138969.1	scRNA-seq,SRP215507,CD8+ T cell_3-AD.vs.MCI; scRNA-seq,SRP215507,CD8+ T cell_5-AD.vs.MCI; scRNA-seq,SRP330776,B cell_1-AD.vs.control; scRNA-seq,SRP330776,Naive CD8+ T cell_1-AD.vs.control	4
AC139019.1	scRNA-seq,SRP215507,CD8+ T cell_1-AD.vs.control; scRNA-seq,SRP330776,Memory T cell_1-AD.vs.control	2
AC139100.2	scRNA-seq,SRP215507,CD8+ T cell_3-MCI.vs.control	1
AC139491.3	scRNA-seq,SRP215507,CD8+ T cell_2-AD.vs.control; scRNA-seq,SRP215507,CD8+ T cell_3-AD.vs.control; scRNA-seq,SRP215507,CD8+ T cell_2-AD.vs.MCI; scRNA-seq,SRP215507,CD8+ T cell_3-AD.vs.MCI; scRNA-seq,SRP215507,CD8+ T cell_2-MCI.vs.control; scRNA-seq,SRP330776,Naive	6
AC139530.1	scRNA-seq,SRP215507,CD8+ T cell_2-AD.vs.MCI; scRNA-seq,SRP215507,CD8+ T cell_2-MCI.vs.control; scRNA-seq,SRP330776,CD8+ T cell_2-AD.vs.control; scRNA-seq,SRP330776,Memory T cell_1-AD.vs.control; scRNA-seq,SRP330776,Natural killer T cell-AD.vs.control; scRNA-seq,SRP330776,Naive CD8+ T cell_3-AD.vs.control; scRNA-seq,SRP330776,T cell-AD.vs.control	7
AC139720.1	scRNA-seq,SRP215507,CD8+ T cell_2-AD.vs.control; scRNA-seq,SRP215507,CD8+ T cell_3-AD.vs.control; scRNA-seq,SRP215507,CD8+ T cell_2-MCI.vs.control; scRNA-seq,SRP215507,CD8+ T cell_3-MCI.vs.control; scRNA-seq,SRP330776,Memory T cell_2-AD.vs.control; scRNA-seq,SRP330776,B cell_1-AD.vs.control; scRNA-seq,SRP330776,Naive CD8+ T cell_3-AD.vs.control; scRNA-seq,SRP330776,CD8+ T cell_3-	8
AC139768.1	scRNA-seq,SRP215507,CD8+ T cell_4-AD.vs.control; scRNA-seq,SRP330776,Naive CD8+ T cell_1-AD.vs.control	2
AC139795.2	scRNA-seq,SRP215507,CD8+ T cell_2-AD.vs.control; scRNA-seq,SRP215507,CD8+ T cell_2-AD.vs.MCI; scRNA-seq,SRP215507,CD8+ T cell_1-AD.vs.MCI; scRNA-seq,SRP215507,CD8+ T cell_1-MCI.vs.control; scRNA-seq,SRP330776,CD8+ T cell_1-AD.vs.control; scRNA-seq,SRP330776,B	6
AC139887.2	scRNA-seq,SRP215507,CD8+ T cell_2-AD.vs.MCI; scRNA-seq,SRP215507,CD8+ T cell_1-AD.vs.MCI; scRNA-seq,SRP215507,CD8+ T cell_5-AD.vs.MCI; scRNA-seq,SRP215507,CD8+ T cell_4-MCI.vs.control; scRNA-seq,SRP215507,CD8+ T cell_2-MCI.vs.control; scRNA-seq,SRP215507,CD8+ T cell_6-MCI.vs.control; scRNA-seq,SRP330776,CD8+ T cell_1-AD.vs.control; scRNA-seq,SRP330776,γδ T cell-AD.vs.control; scRNA-seq,SRP330776,Memory T cell_2-AD.vs.control; scRNA-seq,SRP330776,CD8+ T cell_2-AD.vs.control; scRNA-seq,SRP330776,B cell_1-AD.vs.control; scRNA-seq,SRP330776,Natural killer cell_1-AD.vs.control; scRNA-seq,SRP330776,Natural killer T cell-AD.vs.control; scRNA-	15
AC139887.4	scRNA-seq,SRP215507,CD8+ T cell_2-AD.vs.MCI; scRNA-seq,SRP330776,CD8+ T cell_1-AD.vs.control; scRNA-seq,SRP330776,γδ T cell-AD.vs.control; scRNA-seq,SRP330776,Memory T cell_2-AD.vs.control; scRNA-seq,SRP330776,CD8+ T cell_2-AD.vs.control; scRNA-seq,SRP330776,B cell_1-AD.vs.control; scRNA-seq,SRP330776,Naive CD8+ T cell_1-AD.vs.control; scRNA-seq,SRP330776,Natural killer cell_1-AD.vs.control; scRNA-seq,SRP330776,Memory T cell_1-AD.vs.control; scRNA-seq,SRP330776,B cell_2-AD.vs.control; scRNA-seq,SRP330776,CD8+ T cell_3-AD.vs.control	11
AC140847.2	scRNA-seq,SRP215507,CD8+ T cell_2-MCI.vs.control; scRNA-seq,SRP215507,CD8+ T cell_3-MCI.vs.control; scRNA-seq,SRP330776,Naive CD8+ T	3

AC141424.1	scRNA-seq,SRP215507,CD8+ T cell_6-MCI.vs.control; scRNA-seq,SRP330776,Naive CD8+ T cell_1-AD.vs.control; scRNA-seq,SRP330776,Natural killer cell_1-AD.vs.control; scRNA-seq,SRP330776,T cell-AD.vs.control; scRNA-seq,SRP330776,Naive CD8+ T cell_2-AD.vs.control	5
AC142472.1	scRNA-seq,SRP215507,CD8+ T cell_1-AD.vs.control; scRNA-seq,SRP215507,CD8+ T cell_4-AD.vs.control; scRNA-seq,SRP215507,CD8+ T cell_3-AD.vs.MCI; scRNA-seq,SRP215507,CD8+ T cell_4-MCI.vs.control; scRNA-seq,SRP215507,CD8+ T cell_3-MCI.vs.control; scRNA-seq,SRP330776,CD8+ T cell_1-AD.vs.control; scRNA-seq,SRP330776,B cell_1-AD.vs.control; scRNA-seq,SRP330776,Naive CD8+ T cell_1-	9
AC144521.1	scRNA-seq,SRP215507,CD8+ T cell_1-AD.vs.control; scRNA-seq,SRP215507,CD8+ T cell_4-AD.vs.control; scRNA-seq,SRP215507,CD8+ T cell_4-	3
AC144652.1	scRNA-seq,SRP215507,CD8+ T cell_3-AD.vs.control; scRNA-seq,SRP215507,CD8+ T cell_6-AD.vs.control; scRNA-seq,SRP215507,CD8+ T cell_3-AD.vs.MCI; scRNA-seq,SRP215507,CD8+ T cell_5-MCI.vs.control; scRNA-seq,SRP330776,CD8+ T cell_1-AD.vs.control; scRNA-seq,SRP330776,γδ T cell-AD.vs.control; scRNA-seq,SRP330776,CD8+ T cell_2-AD.vs.control; scRNA-seq,SRP330776,Naive CD8+ T cell_1-AD.vs.control; scRNA-seq,SRP330776,Memory T cell_1-AD.vs.control; scRNA-seq,SRP330776,T cell-AD.vs.control; scRNA-seq,SRP330776,B cell_3-AD.vs.control	11
AC144831.1	scRNA-seq,SRP215507,CD8+ T cell_1-AD.vs.MCI; scRNA-seq,SRP215507,CD8+ T cell_4-AD.vs.MCI; scRNA-seq,SRP215507,CD8+ T cell_1-MCI.vs.control; scRNA-seq,SRP215507,CD8+ T cell_2-MCI.vs.control; scRNA-seq,SRP330776,Memory T cell_1-AD.vs.control	5
AC145124.1	scRNA-seq,SRP215507,CD8+ T cell_3-AD.vs.control; scRNA-seq,SRP215507,CD8+ T cell_5-AD.vs.MCI; scRNA-seq,SRP215507,CD8+ T cell_5-MCI.vs.control; scRNA-seq,SRP215507,CD8+ T cell_2-MCI.vs.control; scRNA-seq,SRP215507,CD8+ T cell_3-MCI.vs.control; scRNA-seq,SRP330776,CD8+ T cell_1-AD.vs.control; scRNA-seq,SRP330776,Memory T cell_2-AD.vs.control; scRNA-seq,SRP330776,CD8+ T cell_2-AD.vs.control; scRNA-seq,SRP330776,B cell_1-AD.vs.control; scRNA-seq,SRP330776,Naive CD8+ T cell_1-AD.vs.control; scRNA-	11
AC145207.2	scRNA-seq,SRP215507,CD8+ T cell_2-AD.vs.control; scRNA-seq,SRP215507,CD8+ T cell_2-AD.vs.MCI; scRNA-seq,SRP215507,CD8+ T cell_4-MCI.vs.control; scRNA-seq,SRP330776,CD8+ T cell_2-AD.vs.control	4
AC145207.5	scRNA-seq,SRP215507,CD8+ T cell_2-AD.vs.MCI; scRNA-seq,SRP215507,CD8+ T cell_5-MCI.vs.control; scRNA-seq,SRP215507,CD8+ T cell_2-MCI.vs.control; scRNA-seq,SRP330776,γδ T cell-AD.vs.control; scRNA-seq,SRP330776,CD8+ T cell_2-AD.vs.control; scRNA-seq,SRP330776,Memory	6
AC145207.6	scRNA-seq,SRP215507,CD8+ T cell_2-AD.vs.control; scRNA-seq,SRP215507,CD8+ T cell_2-MCI.vs.control	2
AC145285.2	scRNA-seq,SRP215507,CD8+ T cell_3-AD.vs.MCI; scRNA-seq,SRP215507,CD8+ T cell_3-MCI.vs.control	2
AC145285.6	scRNA-seq,SRP215507,CD8+ T cell_3-AD.vs.control; scRNA-seq,SRP215507,CD8+ T cell_1-AD.vs.control; scRNA-seq,SRP215507,CD8+ T cell_1-MCI.vs.control; scRNA-seq,SRP215507,CD8+ T cell_3-MCI.vs.control; scRNA-seq,SRP330776,Naive CD8+ T cell_1-AD.vs.control; scRNA-seq,SRP330776,Memory T cell_1-AD.vs.control; scRNA-seq,SRP330776,Naive CD8+ T cell_2-AD.vs.control	7
AC146944.4	scRNA-seq,SRP215507,CD8+ T cell_1-AD.vs.MCI; scRNA-seq,SRP215507,CD8+ T cell_1-MCI.vs.control; scRNA-seq,SRP215507,CD8+ T cell_5-MCI.vs.control; scRNA-seq,SRP309935,Monocyte_1-AD.vs.control	4
AC147067.1	scRNA-seq,SRP215507,CD8+ T cell_3-AD.vs.control; scRNA-seq,SRP215507,CD8+ T cell_3-AD.vs.MCI; scRNA-seq,SRP309935,Monocyte_1-AD.vs.control; scRNA-seq,SRP330776,CD8+ T cell_1-AD.vs.control; scRNA-seq,SRP330776,γδ T cell-AD.vs.control; scRNA-seq,SRP330776,Memory T cell_2-AD.vs.control; scRNA-seq,SRP330776,CD8+ T cell_2-AD.vs.control; scRNA-seq,SRP330776,Memory T cell_1-AD.vs.control; scRNA-seq,SRP330776,Naive CD8+ T cell_3-AD.vs.control; scRNA-seq,SRP330776,Monocyte_1-AD.vs.control; scRNA-seq,SRP330776,B cell_3-AD.vs.control	11
AC147651.4	scRNA-seq,SRP215507,CD8+ T cell_6-AD.vs.control; scRNA-seq,SRP215507,CD8+ T cell_3-AD.vs.MCI; scRNA-seq,SRP215507,CD8+ T cell_3-MCI.vs.control; scRNA-seq,SRP215507,CD8+ T cell_6-MCI.vs.control	4
AC159540.2	scRNA-seq,SRP215507,CD8+ T cell_3-MCI.vs.control; scRNA-seq,SRP330776,CD8+ T cell_2-AD.vs.control; scRNA-seq,SRP330776,Natural killer	3
AC211433.1	scRNA-seq,SRP330776,CD8+ T cell_2-AD.vs.control	1
AC211476.2	scRNA-seq,SRP215507,CD8+ T cell_6-AD.vs.control; scRNA-seq,SRP330776,Memory T cell_1-AD.vs.control; scRNA-seq,SRP330776,B cell_2-AD.vs.control; scRNA-seq,SRP330776,CD8+ T cell_3-AD.vs.control	4
AC215522.2	scRNA-seq,SRP215507,CD8+ T cell_3-AD.vs.MCI; scRNA-seq,SRP215507,CD8+ T cell_1-AD.vs.MCI; scRNA-seq,SRP215507,CD8+ T cell_1-MCI.vs.control; scRNA-seq,SRP215507,CD8+ T cell_3-MCI.vs.control; scRNA-seq,SRP215507,CD8+ T cell_6-MCI.vs.control; scRNA-seq,SRP330776,CD8+ T cell_1-AD.vs.control; scRNA-seq,SRP330776,Naive CD8+ T cell_1-AD.vs.control; scRNA-seq,SRP330776,Memory T cell_1-AD.vs.control; scRNA-seq,SRP330776,B cell_2-AD.vs.control; scRNA-seq,SRP330776,CD8+ T cell_3-AD.vs.control	10

AC231981.1	scRNA-seq,SRP215507,CD8+ T cell_5-AD.vs.control; scRNA-seq,SRP215507,CD8+ T cell_3-AD.vs.MCI; scRNA-seq,SRP215507,CD8+ T cell_1-AD.vs.MCI; scRNA-seq,SRP215507,CD8+ T cell_5-AD.vs.MCI; scRNA-seq,SRP215507,CD8+ T cell_1-MCI.vs.control; scRNA-seq,SRP215507,CD8+ T cell_5-MCI.vs.control; scRNA-seq,SRP330776,B cell_1-AD.vs.control; scRNA-seq,SRP330776,Naive CD8+ T cell_1-AD.vs.control; scRNA-	9
AC232271.2	scRNA-seq,SRP309935,Naive CD8+ T cell_2-AD.vs.control	1
AC233723.1	scRNA-seq,SRP215507,CD8+ T cell_3-AD.vs.control; scRNA-seq,SRP215507,CD8+ T cell_1-AD.vs.control; scRNA-seq,SRP215507,CD8+ T cell_2-MCI.vs.control; scRNA-seq,SRP330776,Naive CD8+ T cell_1-AD.vs.control; scRNA-seq,SRP330776,Memory T cell_1-AD.vs.control	5
AC233755.1	scRNA-seq,SRP330776,B cell_1-AD.vs.control; scRNA-seq,SRP330776,B cell_2-AD.vs.control	2
AC233755.2	scRNA-seq,SRP330776,B cell_2-AD.vs.control	1
AC233976.2	scRNA-seq,SRP330776,CD8+ T cell_1-AD.vs.control	1
AC233992.3	scRNA-seq,SRP215507,CD8+ T cell_1-AD.vs.control; scRNA-seq,SRP215507,CD8+ T cell_2-AD.vs.MCI; scRNA-seq,SRP215507,CD8+ T cell_1-MCI.vs.control; scRNA-seq,SRP215507,CD8+ T cell_2-MCI.vs.control	4
AC234582.1	scRNA-seq,SRP330776,CD8+ T cell_1-AD.vs.control; scRNA-seq,SRP330776,γδ T cell-AD.vs.control; scRNA-seq,SRP330776,Memory T cell_2-AD.vs.control; scRNA-seq,SRP330776,Naive CD8+ T cell_1-AD.vs.control	4
AC234775.3	scRNA-seq,SRP330776,Memory T cell_1-AD.vs.control	1
AC234917.3	scRNA-seq,SRP330776,Naive CD8+ T cell_1-AD.vs.control	1
AC239799.2	scRNA-seq,SRP215507,CD8+ T cell_5-AD.vs.control; scRNA-seq,SRP215507,CD8+ T cell_10-AD.vs.control; scRNA-seq,SRP215507,CD8+ T cell_3-AD.vs.MCI; scRNA-seq,SRP215507,CD8+ T cell_1-AD.vs.MCI; scRNA-seq,SRP215507,CD8+ T cell_4-AD.vs.MCI; scRNA-seq,SRP215507,CD8+ T cell_1-MCI.vs.control; scRNA-seq,SRP215507,CD8+ T cell_5-MCI.vs.control; scRNA-seq,SRP215507,CD8+ T cell_4-MCI.vs.control; scRNA-seq,SRP330776,CD8+ T cell_1-AD.vs.control; scRNA-seq,SRP330776,γδ T cell-AD.vs.control; scRNA-seq,SRP330776,Memory T cell_2-AD.vs.control; scRNA-seq,SRP330776,CD8+ T cell_2-AD.vs.control; scRNA-seq,SRP330776,B cell_1-AD.vs.control; scRNA-seq,SRP330776,Naive CD8+ T cell_1-AD.vs.control; scRNA-seq,SRP330776,Memory T cell_1-AD.vs.control; scRNA-seq,SRP330776,Natural killer T cell-AD.vs.control; scRNA-seq,SRP330776,Naive CD8+ T cell_3-AD.vs.control; scRNA-seq,SRP330776,B cell_2-AD.vs.control; scRNA-seq,SRP330776,CD8+ T cell_3- [illegible]	21
AC239800.2	scRNA-seq,SRP215507,CD8+ T cell_3-AD.vs.control; scRNA-seq,SRP215507,CD8+ T cell_1-AD.vs.MCI; scRNA-seq,SRP215507,CD8+ T cell_1-MCI.vs.control; scRNA-seq,SRP215507,CD8+ T cell_8-MCI.vs.control; scRNA-seq,SRP330776,CD8+ T cell_2-AD.vs.control	5
AC239868.1	scRNA-seq,SRP330776,CD8+ T cell_1-AD.vs.control; scRNA-seq,SRP330776,CD8+ T cell_2-AD.vs.control; scRNA-seq,SRP330776,Naive CD8+ T cell_1-AD.vs.control; scRNA-seq,SRP330776,Memory T cell_1-AD.vs.control	4
AC240274.1	scRNA-seq,SRP215507,CD8+ T cell_1-AD.vs.control; scRNA-seq,SRP215507,CD8+ T cell_5-AD.vs.control; scRNA-seq,SRP309935,Naive CD8+ T cell_1-AD.vs.control; scRNA-seq,SRP309935,Naive CD8+ T cell_2-AD.vs.control	4
AC242426.2	scRNA-seq,SRP330776,CD8+ T cell_1-AD.vs.control; scRNA-seq,SRP330776,γδ T cell-AD.vs.control; scRNA-seq,SRP330776,B cell_2-AD.vs.control; scRNA-seq,SRP330776,CD8+ T cell_3-AD.vs.control	4
AC242842.1	scRNA-seq,SRP215507,CD8+ T cell_1-MCI.vs.control; scRNA-seq,SRP215507,CD8+ T cell_2-MCI.vs.control	2
AC243829.1	scRNA-seq,SRP215507,CD8+ T cell_5-AD.vs.control; scRNA-seq,SRP215507,CD8+ T cell_2-AD.vs.MCI; scRNA-seq,SRP215507,CD8+ T cell_1-AD.vs.MCI; scRNA-seq,SRP215507,CD8+ T cell_5-AD.vs.MCI; scRNA-seq,SRP215507,CD8+ T cell_1-MCI.vs.control; scRNA-seq,SRP215507,CD8+ T cell_2-MCI.vs.control; scRNA-seq,SRP309935,Natural killer cell_1-AD.vs.control; scRNA-seq,SRP309935,CD8+ T cell_1-AD.vs.control; scRNA-seq,SRP309935,CD8+ T cell_2-AD.vs.control; scRNA-seq,SRP330776,γδ T cell-AD.vs.control; scRNA-seq,SRP330776,CD8+ T cell_2-AD.vs.control; scRNA-seq,SRP330776,Memory T cell_1-AD.vs.control; scRNA-seq,SRP330776,CD8+ T cell_3-AD.vs.control; scRNA-seq,SRP330776,Monocyte_1-	15
AC243829.2	scRNA-seq,SRP215507,CD8+ T cell_2-AD.vs.MCI; scRNA-seq,SRP215507,CD8+ T cell_1-AD.vs.MCI; scRNA-seq,SRP215507,CD8+ T cell_1-MCI.vs.control; scRNA-seq,SRP215507,CD8+ T cell_4-MCI.vs.control; scRNA-seq,SRP215507,CD8+ T cell_2-MCI.vs.control; scRNA-seq,SRP309935,Natural killer cell_1-AD.vs.control; scRNA-seq,SRP330776,CD8+ T cell_2-AD.vs.control; scRNA-seq,SRP330776,Memory T cell_1-	8

AC243829.4	scRNA-seq,SRP215507,CD8+ T cell_1-AD.vs.control; scRNA-seq,SRP215507,CD8+ T cell_5-AD.vs.control; scRNA-seq,SRP215507,CD8+ T cell_2-AD.vs.MCI; scRNA-seq,SRP215507,CD8+ T cell_1-AD.vs.MCI; scRNA-seq,SRP215507,CD8+ T cell_1-MCI.vs.control; scRNA-seq,SRP215507,CD8+ T cell_5-MCI.vs.control; scRNA-seq,SRP215507,CD8+ T cell_4-MCI.vs.control; scRNA-seq,SRP215507,CD8+ T cell_2-MCI.vs.control; scRNA-seq,SRP309935,Natural killer cell_2-AD.vs.control; scRNA-seq,SRP309935,Natural killer cell_1-AD.vs.control; scRNA-seq,SRP330776,γδ T cell-	12
AC243830.1	scRNA-seq,SRP330776,Naive CD8+ T cell_1-AD.vs.control	1
AC243960.1	scRNA-seq,SRP215507,CD8+ T cell_7-AD.vs.control; scRNA-seq,SRP215507,CD8+ T cell_7-MCI.vs.control; scRNA-seq,SRP330776,CD8+ T cell_1-AD.vs.control; scRNA-seq,SRP330776,γδ T cell-AD.vs.control; scRNA-seq,SRP330776,Memory T cell_2-AD.vs.control; scRNA-seq,SRP330776,CD8+ T cell_2-AD.vs.control; scRNA-seq,SRP330776,B cell_1-AD.vs.control; scRNA-seq,SRP330776,Naive CD8+ T cell_1-AD.vs.control; scRNA-seq,SRP330776,Naive CD8+ T cell_3-AD.vs.control; scRNA-seq,SRP330776,B cell_2-AD.vs.control; scRNA-seq,SRP330776,T cell-AD.vs.control; scRNA-seq,SRP330776,CD8+ T cell_3-AD.vs.control; scRNA-seq,SRP330776,B cell_3-AD.vs.control; scRNA-seq,SRP330776,Natural killer cell_2-	14
AC243960.3	scRNA-seq,SRP215507,CD8+ T cell_5-AD.vs.control; scRNA-seq,SRP330776,Naive CD8+ T cell_1-AD.vs.control; scRNA-seq,SRP330776,Memory T cell_1-AD.vs.control; scRNA-seq,SRP330776,B cell_2-AD.vs.control; scRNA-seq,SRP330776,Megakaryocyte_1-AD.vs.control	5
AC243965.1	scRNA-seq,SRP215507,CD8+ T cell_1-AD.vs.MCI; scRNA-seq,SRP330776,B cell_2-AD.vs.control; scRNA-seq,SRP330776,B cell_4-AD.vs.control	3
AC244033.2	scRNA-seq,SRP215507,CD8+ T cell_6-MCI.vs.control; scRNA-seq,SRP330776,CD8+ T cell_1-AD.vs.control; scRNA-seq,SRP330776,Memory T cell_2-AD.vs.control; scRNA-seq,SRP330776,CD8+ T cell_2-AD.vs.control; scRNA-seq,SRP330776,Naive CD8+ T cell_1-AD.vs.control; scRNA-	6
AC244090.1	scRNA-seq,SRP215507,CD8+ T cell_5-AD.vs.control; scRNA-seq,SRP215507,CD8+ T cell_6-AD.vs.control; scRNA-seq,SRP330776,CD8+ T cell_1-AD.vs.control; scRNA-seq,SRP330776,Memory T cell_2-AD.vs.control; scRNA-seq,SRP330776,Natural killer cell_1-AD.vs.control; scRNA-	6
AC244093.5	scRNA-seq,SRP330776,Naive CD8+ T cell_1-AD.vs.control	1
AC244197.2	scRNA-seq,SRP215507,CD8+ T cell_4-AD.vs.control; scRNA-seq,SRP215507,CD8+ T cell_5-AD.vs.MCI; scRNA-seq,SRP215507,CD8+ T cell_3-MCI.vs.control; scRNA-seq,SRP330776,CD8+ T cell_1-AD.vs.control; scRNA-seq,SRP330776,Memory T cell_2-AD.vs.control; scRNA-	6
AC244213.1	scRNA-seq,SRP215507,CD8+ T cell_1-AD.vs.MCI; scRNA-seq,SRP215507,CD8+ T cell_1-MCI.vs.control; scRNA-seq,SRP330776,γδ T cell-AD.vs.control; scRNA-seq,SRP330776,Naive CD8+ T cell_1-AD.vs.control	4
AC244502.1	scRNA-seq,SRP215507,CD8+ T cell_2-AD.vs.control; scRNA-seq,SRP215507,CD8+ T cell_1-AD.vs.control; scRNA-seq,SRP215507,CD8+ T cell_4-AD.vs.control; scRNA-seq,SRP215507,CD8+ T cell_2-AD.vs.MCI; scRNA-seq,SRP215507,CD8+ T cell_1-AD.vs.MCI; scRNA-seq,SRP215507,CD8+ T cell_1-MCI.vs.control; scRNA-seq,SRP330776,CD8+ T cell_1-AD.vs.control	7
AC245014.1	scRNA-seq,SRP330776,CD8+ T cell_1-AD.vs.control; scRNA-seq,SRP330776,Naive CD8+ T cell_1-AD.vs.control	2
AC245014.3	scRNA-seq,SRP215507,CD8+ T cell_5-AD.vs.control; scRNA-seq,SRP215507,CD8+ T cell_10-AD.vs.control; scRNA-seq,SRP215507,CD8+ T cell_2-AD.vs.MCI; scRNA-seq,SRP215507,CD8+ T cell_1-AD.vs.MCI; scRNA-seq,SRP215507,CD8+ T cell_4-AD.vs.MCI; scRNA-seq,SRP215507,CD8+ T cell_1-MCI.vs.control; scRNA-seq,SRP215507,CD8+ T cell_5-MCI.vs.control; scRNA-seq,SRP215507,CD8+ T cell_2-MCI.vs.control; scRNA-seq,SRP215507,CD8+ T cell_6-MCI.vs.control; scRNA-seq,SRP309935,Natural killer cell_2-AD.vs.control; scRNA-seq,SRP309935,Natural killer cell_1-AD.vs.control; scRNA-seq,SRP309935,CD8+ T cell_1-AD.vs.control; scRNA-seq,SRP309935,CD4+ T cell-AD.vs.control; scRNA-seq,SRP330776,CD8+ T cell_1-AD.vs.control; scRNA-seq,SRP330776,γδ T cell-AD.vs.control; scRNA-seq,SRP330776,Memory T cell_2-AD.vs.control; scRNA-seq,SRP330776,CD8+ T cell_2-AD.vs.control; scRNA-seq,SRP330776,B cell_1-AD.vs.control; scRNA-seq,SRP330776,Naive CD8+ T cell_1-AD.vs.control; scRNA-seq,SRP330776,Memory T cell_1-AD.vs.control; scRNA-seq,SRP330776,Natural killer T cell-AD.vs.control; scRNA-seq,SRP330776,Naive CD8+ T cell_3-AD.vs.control; scRNA-seq,SRP330776,B cell_2-AD.vs.control; scRNA-seq,SRP330776,CD8+ T cell_3- [illegible]	27
AC245033.2	scRNA-seq,SRP215507,CD8+ T cell_3-AD.vs.MCI; scRNA-seq,SRP215507,CD8+ T cell_5-AD.vs.MCI; scRNA-seq,SRP215507,CD8+ T cell_5-MCI.vs.control; scRNA-seq,SRP215507,CD8+ T cell_3-MCI.vs.control	4
AC245052.4	scRNA-seq,SRP330776,Memory T cell_1-AD.vs.control; scRNA-seq,SRP330776,B cell_2-AD.vs.control	2
AC245060.5	scRNA-seq,SRP215507,CD8+ T cell_4-MCI.vs.control; scRNA-seq,SRP215507,CD8+ T cell_6-MCI.vs.control; scRNA-seq,SRP330776,CD8+ T cell_1-AD.vs.control; scRNA-seq,SRP330776,γδ T cell-AD.vs.control; scRNA-seq,SRP330776,Memory T cell_2-AD.vs.control; scRNA-seq,SRP330776,CD8+ T cell_2-AD.vs.control; scRNA-seq,SRP330776,B cell_1-AD.vs.control; scRNA-seq,SRP330776,Naive CD8+ T cell_1-AD.vs.control; scRNA-seq,SRP330776,B cell_2-AD.vs.control; scRNA-seq,SRP330776,CD8+ T cell_3-AD.vs.control; scRNA-seq,SRP330776,B cell_3-AD.vs.control	11
AC245060.6	scRNA-seq,SRP215507,CD8+ T cell_1-AD.vs.control; scRNA-seq,SRP330776,B cell_1-AD.vs.control	2

AC245140.2	scRNA-seq,SRP215507,CD8+ T cell_6-AD.vs.control; scRNA-seq,SRP215507,CD8+ T cell_1-MCI.vs.control; scRNA-seq,SRP215507,CD8+ T cell_3-MCI.vs.control; scRNA-seq,SRP330776,CD8+ T cell_1-AD.vs.control; scRNA-seq,SRP330776,Naive CD8+ T cell_1-AD.vs.control; scRNA-seq,SRP330776,Natural killer cell_1-AD.vs.control; scRNA-seq,SRP330776,T cell-AD.vs.control; scRNA-seq,SRP330776,B cell_4-AD.vs.control	8
AC245297.2	scRNA-seq,SRP330776,CD8+ T cell_1-AD.vs.control; scRNA-seq,SRP330776,Memory T cell_1-AD.vs.control	2
AC245297.3	scRNA-seq,SRP215507,CD8+ T cell_5-AD.vs.MCI; scRNA-seq,SRP215507,CD8+ T cell_1-MCI.vs.control; scRNA-seq,SRP215507,CD8+ T cell_5-MCI.vs.control; scRNA-seq,SRP215507,CD8+ T cell_6-MCI.vs.control; scRNA-seq,SRP309935,Erythroid cell-AD.vs.control	5
AC245407.2	scRNA-seq,SRP215507,CD8+ T cell_4-AD.vs.MCI; scRNA-seq,SRP215507,CD8+ T cell_5-MCI.vs.control; scRNA-seq,SRP309935,CD8+ T cell_1-AD.vs.control; scRNA-seq,SRP309935,CD8+ T cell_2-AD.vs.control; scRNA-seq,SRP330776,CD8+ T cell_1-AD.vs.control; scRNA-seq,SRP330776,Naive CD8+ T cell_1-AD.vs.control; scRNA-seq,SRP330776,Memory T cell_1-AD.vs.control; scRNA-seq,SRP330776,Naive CD8+ T	9
AC245452.1	scRNA-seq,SRP215507,CD8+ T cell_1-AD.vs.control; scRNA-seq,SRP215507,CD8+ T cell_2-AD.vs.MCI; scRNA-seq,SRP215507,CD8+ T cell_3-AD.vs.MCI; scRNA-seq,SRP215507,CD8+ T cell_1-AD.vs.MCI; scRNA-seq,SRP215507,CD8+ T cell_2-MCI.vs.control; scRNA-seq,SRP215507,CD8+ T cell_3-MCI.vs.control; scRNA-seq,SRP330776,γδ T cell-AD.vs.control; scRNA-seq,SRP330776,Natural killer cell_1-AD.vs.control; scRNA-	9
AC246785.3	scRNA-seq,SRP309935,CD8+ T cell_1-AD.vs.control; scRNA-seq,SRP330776,Naive CD8+ T cell_1-AD.vs.control	2
AC246817.1	scRNA-seq,SRP330776,B cell_1-AD.vs.control; scRNA-seq,SRP330776,Memory T cell_1-AD.vs.control	2
AC253572.2	scRNA-seq,SRP215507,CD8+ T cell_4-AD.vs.control; scRNA-seq,SRP215507,CD8+ T cell_7-AD.vs.control; scRNA-seq,SRP215507,CD8+ T cell_2-AD.vs.MCI; scRNA-seq,SRP215507,CD8+ T cell_3-AD.vs.MCI; scRNA-seq,SRP215507,CD8+ T cell_1-AD.vs.MCI; scRNA-seq,SRP215507,CD8+ T cell_4-AD.vs.MCI; scRNA-seq,SRP215507,CD8+ T cell_5-AD.vs.MCI; scRNA-seq,SRP215507,CD8+ T cell_1-MCI.vs.control; scRNA-seq,SRP215507,CD8+ T cell_5-MCI.vs.control; scRNA-seq,SRP215507,CD8+ T cell_2-MCI.vs.control; scRNA-seq,SRP215507,CD8+ T cell_3-MCI.vs.control; scRNA-seq,SRP215507,CD8+ T cell_6-MCI.vs.control; scRNA-seq,SRP215507,CD8+ T cell_7-MCI.vs.control; scRNA-seq,SRP309935,Natural killer cell_2-AD.vs.control; scRNA-seq,SRP309935,Natural killer cell_1-AD.vs.control; scRNA-seq,SRP309935,CD8+ T cell_1-AD.vs.control; scRNA-seq,SRP309935,CD4+ T cell-AD.vs.control; scRNA-seq,SRP330776,CD8+ T cell_1-AD.vs.control; scRNA-seq,SRP330776,Memory T cell_2-AD.vs.control; scRNA-seq,SRP330776,CD8+ T cell_2-AD.vs.control; scRNA-seq,SRP330776,B cell_1-AD.vs.control; scRNA-seq,SRP330776,Naive CD8+ T cell_1-AD.vs.control; scRNA-seq,SRP330776,Natural killer cell_1-AD.vs.control; scRNA-seq,SRP330776,Memory T cell_1-AD.vs.control; scRNA-seq,SRP330776,Natural killer T cell-AD.vs.control; scRNA-seq,SRP330776,Naive CD8+ T cell_3-AD.vs.control; scRNA-seq,SRP330776,B cell_2-AD.vs.control; scRNA-seq,SRP330776,Naive CD8+ T cell_2-AD.vs.control; scRNA-seq,SRP330776,CD8+ T cell_3- [illegible]	33
AC254562.3	scRNA-seq,SRP215507,CD8+ T cell_2-MCI.vs.control	1
AC254633.1	scRNA-seq,SRP330776,Memory T cell_1-AD.vs.control	1
ACAA1	bulk RNA-seq,Homo_723,AD.vs.control; bulk RNA-seq,Homo_723,AD.vs.MCI; bulk RNA-seq,Homo_633,AD.vs.control; bulk RNA-seq,Homo_633,AD.vs.MCI; bulk RNA-seq,Homo_633,MCI.vs.control; bulk RNA-seq,Homo_714,AD.vs.control; bulk RNA-seq,Homo_714,AD.vs.MCI; bulk RNA-seq,Homo_714,MCI.vs.control; scRNA-seq,SRP215507,CD8+ T cell_7-AD.vs.control; scRNA-seq,SRP215507,CD8+ T cell_9-AD.vs.control; scRNA-seq,SRP215507,CD8+ T cell_7-AD.vs.MCI; scRNA-seq,SRP215507,CD8+ T cell_5-MCI.vs.control; scRNA-seq,SRP330776,CD8+ T cell_1-AD.vs.control; scRNA-seq,SRP330776,γδ T cell-AD.vs.control; scRNA-seq,SRP330776,Memory T cell_2-AD.vs.control; scRNA-seq,SRP330776,CD8+ T cell_2-AD.vs.control; scRNA-seq,SRP330776,B cell_1-AD.vs.control; scRNA-seq,SRP330776,Naive CD8+ T cell_1-AD.vs.control; scRNA-seq,SRP330776,Natural killer cell_1-AD.vs.control; scRNA-seq,SRP330776,Memory T cell_1-AD.vs.control; scRNA-seq,SRP330776,Natural killer T cell-AD.vs.control; scRNA-seq,SRP330776,Naive CD8+ T cell_3-AD.vs.control; scRNA-seq,SRP330776,B cell_2-AD.vs.control; scRNA-seq,SRP330776,T cell-AD.vs.control; scRNA-seq,SRP330776,Naive CD8+ T cell_2-AD.vs.control; scRNA-seq,SRP330776,CD8+ T cell_3-AD.vs.control; scRNA-seq,SRP330776,Monocyte_1-AD.vs.control; scRNA-seq,SRP330776,Monocyte_2-AD.vs.control; scRNA-seq,SRP330776,B cell_3-AD.vs.control; scRNA- [illegible]	30
ACAA2	bulk RNA-seq,Homo_723,AD.vs.control; bulk RNA-seq,Homo_723,AD.vs.MCI; bulk RNA-seq,Homo_633,AD.vs.control; bulk RNA-seq,Homo_633,AD.vs.MCI; bulk RNA-seq,Homo_714,AD.vs.control; bulk RNA-seq,Homo_714,AD.vs.MCI; scRNA-seq,SRP215507,CD8+ T cell_7-AD.vs.control; scRNA-seq,SRP330776,CD8+ T cell_1-AD.vs.control; scRNA-seq,SRP330776,γδ T cell-AD.vs.control; scRNA-seq,SRP330776,Memory T cell_2-AD.vs.control; scRNA-seq,SRP330776,Naive CD8+ T cell_1-AD.vs.control; scRNA-seq,SRP330776,Natural killer cell_1-AD.vs.control; scRNA-seq,SRP330776,Natural killer T cell-AD.vs.control; scRNA-seq,SRP330776,Naive CD8+ T cell_3-AD.vs.control; scRNA-seq,SRP330776,CD8+ T cell_3-AD.vs.control; scRNA-seq,SRP330776,Monocyte_2-AD.vs.control; scRNA-seq,SRP330776,B cell_3-AD.vs.control	17

ACACA	scRNA-seq,SRP215507,CD8+ T cell_3-AD.vs.control; scRNA-seq,SRP215507,CD8+ T cell_1-AD.vs.MCI; scRNA-seq,SRP215507,CD8+ T cell_1-MCI.vs.control; scRNA-seq,SRP215507,CD8+ T cell_5-MCI.vs.control; scRNA-seq,SRP330776,Naive CD8+ T cell_3-AD.vs.control; scRNA-seq,SRP330776,B cell_2-AD.vs.control; scRNA-seq,SRP330776,T cell-AD.vs.control; scRNA-seq,SRP330776,B cell_4-AD.vs.control	8
ACACB	bulk RNA-seq,Homo_723,AD.vs.control; bulk RNA-seq,Homo_723,AD.vs.MCI; bulk RNA-seq,Homo_723,MCI.vs.control; bulk RNA-seq,Homo_633,AD.vs.control; bulk RNA-seq,Homo_633,AD.vs.MCI; bulk RNA-seq,Homo_633,MCI.vs.control; bulk RNA-seq,Homo_714,AD.vs.control; bulk RNA-seq,Homo_714,AD.vs.MCI; scRNA-seq,SRP215507,CD8+ T cell_4-MCI.vs.control; scRNA-seq,SRP215507,CD8+ T cell_2-MCI.vs.control; scRNA-seq,SRP309935,Natural killer cell_1-AD.vs.control; scRNA-seq,SRP330776,CD8+ T cell_1-AD.vs.control; scRNA-seq,SRP330776,γδ T cell-AD.vs.control; scRNA-seq,SRP330776,B cell_1-AD.vs.control; scRNA-seq,SRP330776,Natural killer cell_1-AD.vs.control; scRNA-seq,SRP330776,Naive CD8+ T cell_3-AD.vs.control; scRNA-seq,SRP330776,T cell-AD.vs.control; scRNA-seq,SRP330776,CD8+ T cell_3-AD.vs.control	18
ACAD10	scRNA-seq,SRP215507,CD8+ T cell_3-AD.vs.MCI; scRNA-seq,SRP215507,CD8+ T cell_5-MCI.vs.control; scRNA-seq,SRP215507,CD8+ T cell_3-MCI.vs.control; scRNA-seq,SRP330776,γδ T cell-AD.vs.control; scRNA-seq,SRP330776,Memory T cell_1-AD.vs.control; scRNA-seq,SRP330776,CD8+	6
ACAD11	bulk RNA-seq,Homo_723,AD.vs.MCI; bulk RNA-seq,Homo_723,MCI.vs.control; bulk RNA-seq,Homo_633,AD.vs.control; bulk RNA-seq,Homo_633,AD.vs.MCI; bulk RNA-seq,Homo_633,MCI.vs.control; bulk RNA-seq,Homo_714,AD.vs.control; bulk RNA-seq,Homo_714,AD.vs.MCI; bulk RNA-seq,Homo_714,MCI.vs.control; scRNA-seq,SRP215507,CD8+ T cell_1-AD.vs.control; scRNA-seq,SRP215507,CD8+ T cell_3-AD.vs.MCI; scRNA-seq,SRP215507,CD8+ T cell_1-MCI.vs.control; scRNA-seq,SRP215507,CD8+ T cell_3-MCI.vs.control; scRNA-seq,SRP330776,B cell_1-AD.vs.control; scRNA-seq,SRP330776,T cell-AD.vs.control; scRNA-seq,SRP330776,CD8+ T cell_3-AD.vs.control	15
ACAD8	scRNA-seq,SRP215507,CD8+ T cell_8-AD.vs.control; scRNA-seq,SRP309935,Natural killer cell_2-AD.vs.control; scRNA-seq,SRP330776,Natural killer cell_1-AD.vs.control; scRNA-seq,SRP330776,B cell_2-AD.vs.control; scRNA-seq,SRP330776,T cell-AD.vs.control; scRNA-seq,SRP330776,Natural killer	6
ACAD9	bulk RNA-seq,Homo_633,AD.vs.control; scRNA-seq,SRP215507,CD8+ T cell_3-AD.vs.control; scRNA-seq,SRP309935,Natural killer cell_2-AD.vs.control; scRNA-seq,SRP330776,γδ T cell-AD.vs.control; scRNA-seq,SRP330776,Natural killer cell_1-AD.vs.control; scRNA-seq,SRP330776,Naive CD8+ T cell_3-AD.vs.control; scRNA-seq,SRP330776,Monocyte_1-AD.vs.control	7
ACADL	bulk RNA-seq,Homo_723,AD.vs.control	1
ACADM	bulk RNA-seq,Homo_723,AD.vs.control; bulk RNA-seq,Homo_723,AD.vs.MCI; bulk RNA-seq,Homo_633,AD.vs.control; bulk RNA-seq,Homo_633,AD.vs.MCI; bulk RNA-seq,Homo_714,AD.vs.control; bulk RNA-seq,Homo_714,AD.vs.MCI; scRNA-seq,SRP215507,CD8+ T cell_7-AD.vs.control; scRNA-seq,SRP330776,γδ T cell-AD.vs.control; scRNA-seq,SRP330776,Memory T cell_2-AD.vs.control; scRNA-seq,SRP330776,CD8+ T cell_2-AD.vs.control; scRNA-seq,SRP330776,B cell_1-AD.vs.control; scRNA-seq,SRP330776,Naive CD8+ T cell_1-AD.vs.control; scRNA-seq,SRP330776,Memory T cell_1-AD.vs.control; scRNA-seq,SRP330776,Naive CD8+ T cell_2-AD.vs.control; scRNA-seq,SRP330776,CD8+ T cell_3-	17
ACADS	bulk RNA-seq,Homo_723,AD.vs.control; bulk RNA-seq,Homo_633,AD.vs.control; scRNA-seq,SRP215507,CD8+ T cell_3-AD.vs.control; scRNA-seq,SRP215507,CD8+ T cell_3-AD.vs.MCI; scRNA-seq,SRP330776,CD8+ T cell_1-AD.vs.control; scRNA-seq,SRP330776,γδ T cell-AD.vs.control; scRNA-seq,SRP330776,Naive CD8+ T cell_1-AD.vs.control; scRNA-seq,SRP330776,Natural killer cell_1-AD.vs.control; scRNA-seq,SRP330776,Memory T cell_1-AD.vs.control; scRNA-seq,SRP330776,Natural killer T cell-AD.vs.control; scRNA-seq,SRP330776,B cell_2-AD.vs.control; scRNA-	13
ACADSB	bulk RNA-seq,Homo_714,AD.vs.control; bulk RNA-seq,Homo_714,AD.vs.MCI; scRNA-seq,SRP215507,CD8+ T cell_4-AD.vs.MCI; scRNA-seq,SRP215507,CD8+ T cell_4-MCI.vs.control; scRNA-seq,SRP309935,Natural killer cell_1-AD.vs.control; scRNA-seq,SRP309935,Naive CD8+ T cell_2-AD.vs.control; scRNA-seq,SRP309935,CD8+ T cell_1-AD.vs.control; scRNA-seq,SRP330776,Natural killer T cell-AD.vs.control	8
ACADVL	scRNA-seq,SRP215507,CD8+ T cell_5-AD.vs.MCI; scRNA-seq,SRP330776,CD8+ T cell_1-AD.vs.control; scRNA-seq,SRP330776,γδ T cell-AD.vs.control; scRNA-seq,SRP330776,Memory T cell_2-AD.vs.control; scRNA-seq,SRP330776,CD8+ T cell_2-AD.vs.control; scRNA-seq,SRP330776,Naive CD8+ T cell_1-AD.vs.control; scRNA-seq,SRP330776,Natural killer cell_1-AD.vs.control; scRNA-seq,SRP330776,Memory T cell_1-AD.vs.control; scRNA-seq,SRP330776,Natural killer T cell-AD.vs.control; scRNA-seq,SRP330776,Naive CD8+ T cell_3-AD.vs.control; scRNA-seq,SRP330776,B cell_2-AD.vs.control; scRNA-seq,SRP330776,T cell-AD.vs.control; scRNA-seq,SRP330776,Naive CD8+ T cell_2-AD.vs.control; scRNA-seq,SRP330776,CD8+ T cell_3-AD.vs.control; scRNA-seq,SRP330776,Natural killer cell_2-AD.vs.control; scRNA-seq,SRP330776,B cell_4-	16
ACAN	bulk RNA-seq,Homo_723,AD.vs.control; bulk RNA-seq,Homo_723,AD.vs.MCI; bulk RNA-seq,Homo_633,AD.vs.control; bulk RNA-seq,Homo_633,AD.vs.MCI; bulk RNA-seq,Homo_714,MCI.vs.control	5

ACAP1	scRNA-seq,SRP215507,CD8+ T cell_7-AD.vs.control; scRNA-seq,SRP215507,CD8+ T cell_9-AD.vs.control; scRNA-seq,SRP215507,CD8+ T cell_7-MCI.vs.control; scRNA-seq,SRP309935,Erythroid cell-AD.vs.control; scRNA-seq,SRP309935,Megakaryocyte_2-AD.vs.control; scRNA-seq,SRP330776,γδ T cell-AD.vs.control; scRNA-seq,SRP330776,Naive CD8+ T cell_3-AD.vs.control; scRNA-seq,SRP330776,B cell_2-AD.vs.control; scRNA-seq,SRP330776,CD8+ T cell_3-AD.vs.control; scRNA-seq,SRP330776,Monocyte_2-AD.vs.control	10
ACAP2	bulk RNA-seq,Homo_723,AD.vs.control; bulk RNA-seq,Homo_723,AD.vs.MCI; bulk RNA-seq,Homo_723,MCI.vs.control; bulk RNA-seq,Homo_633,AD.vs.control; bulk RNA-seq,Homo_633,AD.vs.MCI; bulk RNA-seq,Homo_714,AD.vs.control; bulk RNA-seq,Homo_714,AD.vs.MCI; bulk RNA-seq,Homo_714,MCI.vs.control; scRNA-seq,SRP215507,CD8+ T cell_7-AD.vs.control; scRNA-seq,SRP215507,CD8+ T cell_5-MCI.vs.control; scRNA-seq,SRP330776,CD8+ T cell_1-AD.vs.control; scRNA-seq,SRP330776,γδ T cell-AD.vs.control; scRNA-seq,SRP330776,Memory T cell_2-AD.vs.control; scRNA-seq,SRP330776,CD8+ T cell_2-AD.vs.control; scRNA-seq,SRP330776,B cell_1-AD.vs.control; scRNA-seq,SRP330776,Naive CD8+ T cell_1-AD.vs.control; scRNA-seq,SRP330776,Natural killer cell_1-AD.vs.control; scRNA-seq,SRP330776,Memory T cell_1-AD.vs.control; scRNA-seq,SRP330776,Naive CD8+ T cell_3-AD.vs.control; scRNA-seq,SRP330776,B cell_2-AD.vs.control; scRNA-seq,SRP330776,T cell-AD.vs.control; scRNA-seq,SRP330776,Naive CD8+ T cell_2-AD.vs.control; scRNA-seq,SRP330776,CD8+ T cell_3-AD.vs.control; scRNA-[illegible]	26
ACAP2-IT1	scRNA-seq,SRP215507,CD8+ T cell_4-MCI.vs.control	1
ACAP3	scRNA-seq,SRP309935,Natural killer cell_1-AD.vs.control; scRNA-seq,SRP309935,Monocyte_2-AD.vs.control; scRNA-seq,SRP309935,Naive CD8+ T cell_2-AD.vs.control; scRNA-seq,SRP330776,CD8+ T cell_1-AD.vs.control; scRNA-seq,SRP330776,γδ T cell-AD.vs.control; scRNA-seq,SRP330776,Memory T cell_2-AD.vs.control; scRNA-seq,SRP330776,CD8+ T cell_2-AD.vs.control; scRNA-seq,SRP330776,B cell_1-AD.vs.control; scRNA-seq,SRP330776,Naive CD8+ T cell_1-AD.vs.control; scRNA-seq,SRP330776,Natural killer cell_1-AD.vs.control; scRNA-seq,SRP330776,Memory T cell_1-AD.vs.control; scRNA-seq,SRP330776,Natural killer T cell-AD.vs.control; scRNA-seq,SRP330776,Naive CD8+ T cell_3-AD.vs.control; scRNA-seq,SRP330776,B cell_2-AD.vs.control; scRNA-seq,SRP330776,T cell-AD.vs.control; scRNA-seq,SRP330776,CD8+ T cell_3-AD.vs.control; scRNA-[illegible]	18
ACAT1	bulk RNA-seq,Homo_723,AD.vs.control; bulk RNA-seq,Homo_723,AD.vs.MCI; bulk RNA-seq,Homo_633,AD.vs.control; bulk RNA-seq,Homo_633,AD.vs.MCI; bulk RNA-seq,Homo_714,AD.vs.control; bulk RNA-seq,Homo_714,AD.vs.MCI; scRNA-seq,SRP330776,Memory T cell_2-AD.vs.control; scRNA-seq,SRP330776,CD8+ T cell_2-AD.vs.control; scRNA-seq,SRP330776,B cell_1-AD.vs.control; scRNA-seq,SRP330776,Memory T cell_1-AD.vs.control; scRNA-seq,SRP330776,Natural killer cell_2-AD.vs.control	11
ACAT2	bulk RNA-seq,Homo_723,AD.vs.control; bulk RNA-seq,Homo_633,AD.vs.control; bulk RNA-seq,Homo_633,AD.vs.MCI; bulk RNA-seq,Homo_714,AD.vs.control; bulk RNA-seq,Homo_714,AD.vs.MCI; scRNA-seq,SRP215507,CD8+ T cell_5-AD.vs.MCI; scRNA-seq,SRP215507,CD8+ T cell_6-AD.vs.MCI; scRNA-seq,SRP215507,CD8+ T cell_2-MCI.vs.control; scRNA-seq,SRP330776,Memory T cell_1-AD.vs.control; scRNA-[illegible]	10
ACBD3	bulk RNA-seq,Homo_723,AD.vs.control; bulk RNA-seq,Homo_723,AD.vs.MCI; bulk RNA-seq,Homo_633,AD.vs.control; bulk RNA-seq,Homo_633,AD.vs.MCI; bulk RNA-seq,Homo_714,AD.vs.control; bulk RNA-seq,Homo_714,AD.vs.MCI; scRNA-seq,SRP215507,CD8+ T cell_4-AD.vs.control; scRNA-seq,SRP215507,CD8+ T cell_7-AD.vs.MCI; scRNA-seq,SRP215507,CD8+ T cell_1-MCI.vs.control; scRNA-seq,SRP215507,CD8+ T cell_4-MCI.vs.control; scRNA-seq,SRP309935,Natural killer cell_2-AD.vs.control; scRNA-seq,SRP309935,Naive CD8+ T cell_1-AD.vs.control; scRNA-seq,SRP309935,Naive CD8+ T cell_2-AD.vs.control; scRNA-seq,SRP309935,CD8+ T cell_1-AD.vs.control; scRNA-seq,SRP309935,B cell_1-AD.vs.control; scRNA-seq,SRP309935,B cell_2-AD.vs.control; scRNA-seq,SRP330776,CD8+ T cell_1-AD.vs.control; scRNA-seq,SRP330776,γδ T cell-AD.vs.control; scRNA-seq,SRP330776,Memory T cell_2-AD.vs.control; scRNA-seq,SRP330776,CD8+ T cell_2-AD.vs.control; scRNA-seq,SRP330776,B cell_1-AD.vs.control; scRNA-seq,SRP330776,Naive CD8+ T cell_1-AD.vs.control; scRNA-seq,SRP330776,Natural killer cell_1-AD.vs.control; scRNA-seq,SRP330776,Memory T cell_1-AD.vs.control; scRNA-seq,SRP330776,Natural killer T cell-AD.vs.control; scRNA-seq,SRP330776,Naive CD8+ T cell_3-AD.vs.control; scRNA-seq,SRP330776,B cell_2-AD.vs.control; scRNA-seq,SRP330776,T cell-AD.vs.control; scRNA-seq,SRP330776,CD8+ T cell_3-AD.vs.control; scRNA-seq,SRP330776,Monocyte_1-AD.vs.control; scRNA-seq,SRP330776,Monocyte_2-AD.vs.control; scRNA-seq,SRP330776,B cell_3-AD.vs.control; scRNA-seq,SRP330776,Natural killer cell_2-AD.vs.control; scRNA-seq,SRP330776,B cell_4-AD.vs.control	34
ACBD4	bulk RNA-seq,Homo_723,AD.vs.MCI; bulk RNA-seq,Homo_723,MCI.vs.control; bulk RNA-seq,Homo_633,AD.vs.control; bulk RNA-seq,Homo_633,AD.vs.MCI; bulk RNA-seq,Homo_633,MCI.vs.control; bulk RNA-seq,Homo_714,AD.vs.control; bulk RNA-seq,Homo_714,AD.vs.MCI; bulk RNA-seq,Homo_714,MCI.vs.control; scRNA-seq,SRP215507,CD8+ T cell_1-AD.vs.MCI; scRNA-seq,SRP215507,CD8+ T cell_5-AD.vs.MCI; scRNA-seq,SRP330776,γδ T cell-AD.vs.control; scRNA-seq,SRP330776,CD8+ T cell_2-AD.vs.control; scRNA-seq,SRP330776,B cell_1-AD.vs.control; scRNA-seq,SRP330776,Natural killer T cell-AD.vs.control; scRNA-seq,SRP330776,CD8+ T cell_3-AD.vs.control; scRNA-[illegible]	16

ACBD5	bulk RNA-seq,Homo_723,AD.vs.control; bulk RNA-seq,Homo_723,AD.vs.MCI; bulk RNA-seq,Homo_633,AD.vs.control; bulk RNA-seq,Homo_633,AD.vs.MCI; bulk RNA-seq,Homo_714,AD.vs.control; bulk RNA-seq,Homo_714,AD.vs.MCI; scRNA-seq,SRP215507,CD8+ T cell_7-AD.vs.MCI; scRNA-seq,SRP215507,CD8+ T cell_5-MCI.vs.control; scRNA-seq,SRP215507,CD8+ T cell_7-MCI.vs.control	9
ACBD6	scRNA-seq,SRP215507,CD8+ T cell_4-AD.vs.control; scRNA-seq,SRP215507,CD8+ T cell_5-AD.vs.control; scRNA-seq,SRP215507,CD8+ T cell_6-AD.vs.control; scRNA-seq,SRP215507,CD8+ T cell_4-AD.vs.MCI; scRNA-seq,SRP215507,CD8+ T cell_7-AD.vs.MCI; scRNA-seq,SRP215507,CD8+ T cell_5-MCI.vs.control; scRNA-seq,SRP215507,CD8+ T cell_7-MCI.vs.control; scRNA-seq,SRP309935,Megakaryocyte_1-AD.vs.control; scRNA-seq,SRP330776,CD8+ T cell_1-AD.vs.control; scRNA-seq,SRP330776,γδ T cell-AD.vs.control; scRNA-seq,SRP330776,Memory T cell_2-AD.vs.control; scRNA-seq,SRP330776,B cell_1-AD.vs.control; scRNA-seq,SRP330776,Naive CD8+ T cell_1-AD.vs.control; scRNA-seq,SRP330776,Natural killer T cell-AD.vs.control; scRNA-seq,SRP330776,Naive CD8+ T cell_3-AD.vs.control; scRNA-seq,SRP330776,Naive CD8+ T cell_2-AD.vs.control	16
ACBD7	bulk RNA-seq,Homo_723,AD.vs.control; bulk RNA-seq,Homo_723,AD.vs.MCI; bulk RNA-seq,Homo_723,MCI.vs.control; bulk RNA-seq,Homo_633,AD.vs.control; bulk RNA-seq,Homo_633,AD.vs.MCI; bulk RNA-seq,Homo_714,AD.vs.control; bulk RNA-seq,Homo_714,AD.vs.MCI	7
ACCS	bulk RNA-seq,Homo_633,AD.vs.MCI; scRNA-seq,SRP215507,CD8+ T cell_5-AD.vs.control; scRNA-seq,SRP215507,CD8+ T cell_2-AD.vs.MCI; scRNA-seq,SRP215507,CD8+ T cell_4-AD.vs.MCI; scRNA-seq,SRP215507,CD8+ T cell_7-AD.vs.MCI; scRNA-seq,SRP215507,CD8+ T cell_5-AD.vs.MCI; scRNA-seq,SRP215507,CD8+ T cell_2-MCI.vs.control; scRNA-seq,SRP330776,CD8+ T cell_1-AD.vs.control; scRNA-seq,SRP330776,CD8+ T cell_2-AD.vs.control; scRNA-seq,SRP330776,Memory T cell_1-AD.vs.control; scRNA-seq,SRP330776,Naive CD8+ T cell_3-AD.vs.control	11
ACCSL	bulk RNA-seq,Homo_723,AD.vs.control; bulk RNA-seq,Homo_723,AD.vs.MCI; bulk RNA-seq,Homo_723,MCI.vs.control; bulk RNA-seq,Homo_633,AD.vs.control; bulk RNA-seq,Homo_633,AD.vs.MCI; bulk RNA-seq,Homo_633,MCI.vs.control	6
ACD	scRNA-seq,SRP330776,CD8+ T cell_1-AD.vs.control; scRNA-seq,SRP330776,Natural killer cell_1-AD.vs.control; scRNA-seq,SRP330776,B cell_3-	3
ACE	bulk RNA-seq,Homo_723,AD.vs.control; bulk RNA-seq,Homo_723,AD.vs.MCI; bulk RNA-seq,Homo_633,AD.vs.control; bulk RNA-seq,Homo_633,AD.vs.MCI; bulk RNA-seq,Homo_714,AD.vs.control; bulk RNA-seq,Homo_714,AD.vs.MCI; bulk RNA-seq,Homo_714,MCI.vs.control; scRNA-seq,SRP215507,CD8+ T cell_3-AD.vs.control; scRNA-seq,SRP215507,CD8+ T cell_4-MCI.vs.control; scRNA-seq,SRP330776,CD8+ T cell_1-AD.vs.control; scRNA-seq,SRP330776,Memory T cell_2-AD.vs.control; scRNA-seq,SRP330776,B cell_1-AD.vs.control; scRNA-seq,SRP330776,CD8+ T	13
Ace	bulk RNA-seq,SRP325058,AD.vs.control	1
ACE2	bulk RNA-seq,Homo_723,AD.vs.control; bulk RNA-seq,Homo_723,AD.vs.MCI	2
ACER1	scRNA-seq,SRP215507,CD8+ T cell_4-AD.vs.control; scRNA-seq,SRP215507,CD8+ T cell_4-MCI.vs.control	2
ACER2	bulk RNA-seq,Homo_723,AD.vs.control; bulk RNA-seq,Homo_723,AD.vs.MCI; bulk RNA-seq,Homo_723,MCI.vs.control; bulk RNA-seq,Homo_633,AD.vs.control; bulk RNA-seq,Homo_633,AD.vs.MCI; bulk RNA-seq,Homo_633,MCI.vs.control; bulk RNA-seq,Homo_714,AD.vs.control; bulk RNA-seq,Homo_714,AD.vs.MCI; bulk RNA-seq,ROSMAP,AD.vs.control; scRNA-seq,SRP215507,CD8+ T cell_1-AD.vs.control; scRNA-seq,SRP330776,B cell_1-AD.vs.control; scRNA-seq,SRP330776,B cell_2-AD.vs.control; scRNA-seq,SRP330776,B cell_3-AD.vs.control	13
ACER3	bulk RNA-seq,Homo_723,AD.vs.MCI; bulk RNA-seq,Homo_723,MCI.vs.control; bulk RNA-seq,Homo_633,AD.vs.MCI; bulk RNA-seq,Homo_633,MCI.vs.control; bulk RNA-seq,Homo_714,AD.vs.control; bulk RNA-seq,Homo_714,AD.vs.MCI; scRNA-seq,SRP215507,CD8+ T cell_2-AD.vs.control; scRNA-seq,SRP215507,CD8+ T cell_3-AD.vs.control; scRNA-seq,SRP215507,CD8+ T cell_1-AD.vs.control; scRNA-seq,SRP215507,CD8+ T cell_5-AD.vs.control; scRNA-seq,SRP215507,CD8+ T cell_4-AD.vs.MCI; scRNA-seq,SRP215507,CD8+ T cell_5-AD.vs.MCI; scRNA-seq,SRP215507,CD8+ T cell_1-MCI.vs.control; scRNA-seq,SRP215507,CD8+ T cell_2-MCI.vs.control; scRNA-seq,SRP309935,Monocyte_2-AD.vs.control; scRNA-seq,SRP330776,CD8+ T cell_1-AD.vs.control; scRNA-seq,SRP330776,Memory T cell_2-AD.vs.control; scRNA-seq,SRP330776,B [illegible]	20
ACHE	bulk RNA-seq,Homo_723,AD.vs.control; bulk RNA-seq,Homo_723,MCI.vs.control; bulk RNA-seq,Homo_633,MCI.vs.control; bulk RNA-seq,Homo_714,AD.vs.control; bulk RNA-seq,Homo_714,AD.vs.MCI; bulk RNA-seq,Homo_714,MCI.vs.control	6
ACIN1	bulk RNA-seq,Homo_723,AD.vs.control; bulk RNA-seq,Homo_723,MCI.vs.control; bulk RNA-seq,Homo_633,AD.vs.control; bulk RNA-seq,Homo_633,MCI.vs.control; scRNA-seq,SRP309935,B cell_1-AD.vs.control; scRNA-seq,SRP330776,Regulatory T cell-AD.vs.control	6
ACKR1	bulk RNA-seq,Homo_723,AD.vs.control; bulk RNA-seq,Homo_723,AD.vs.MCI; bulk RNA-seq,Homo_633,AD.vs.control; bulk RNA-seq,Homo_633,AD.vs.MCI; bulk RNA-seq,Homo_714,AD.vs.control; bulk RNA-seq,Homo_714,AD.vs.MCI; bulk RNA-seq,Homo_714,MCI.vs.control	7
ACKR2	bulk RNA-seq,Homo_723,AD.vs.control; bulk RNA-seq,Homo_723,AD.vs.MCI; bulk RNA-seq,Homo_633,AD.vs.control; bulk RNA-seq,Homo_633,AD.vs.MCI; bulk RNA-seq,Homo_714,AD.vs.MCI; bulk RNA-seq,Homo_714,MCI.vs.control; bulk RNA-seq,ROSMAP,AD.vs.MCI	7

ACLY	bulk RNA-seq,Homo_633,AD.vs.control; bulk RNA-seq,Homo_714,AD.vs.control; bulk RNA-seq,Homo_714,AD.vs.MCI; scRNA-seq,SRP215507,CD8+ T cell_7-AD.vs.MCI; scRNA-seq,SRP215507,CD8+ T cell_7-MCI.vs.control; scRNA-seq,SRP330776,Memory T cell_2-AD.vs.control; scRNA-seq,SRP330776,Monocyte_1-AD.vs.control; scRNA-seq,SRP330776,Monocyte_2-AD.vs.control	8
ACMSD	bulk RNA-seq,Homo_723,AD.vs.control; bulk RNA-seq,Homo_723,AD.vs.MCI; bulk RNA-seq,Homo_633,AD.vs.control	3
Acmsd	bulk RNA-seq,SRP325058,AD.vs.control	1
ACO1	bulk RNA-seq,Homo_633,AD.vs.control; bulk RNA-seq,Homo_633,AD.vs.MCI; bulk RNA-seq,Homo_714,AD.vs.control; bulk RNA-seq,Homo_714,AD.vs.MCI; scRNA-seq,SRP215507,CD8+ T cell_2-AD.vs.MCI; scRNA-seq,SRP215507,CD8+ T cell_5-AD.vs.MCI; scRNA-seq,SRP215507,CD8+ T cell_5-MCI.vs.control; scRNA-seq,SRP215507,CD8+ T cell_2-MCI.vs.control; scRNA-seq,SRP309935,Naive CD8+ T cell_1-AD.vs.control; scRNA-seq,SRP330776,CD8+ T cell_1-AD.vs.control; scRNA-seq,SRP330776,γδ T cell-AD.vs.control; scRNA-seq,SRP330776,Memory T cell_2-AD.vs.control; scRNA-seq,SRP330776,CD8+ T cell_2-AD.vs.control; scRNA-seq,SRP330776,Naive CD8+ T cell_1-AD.vs.control; scRNA-seq,SRP330776,Natural killer cell_1-AD.vs.control; scRNA-seq,SRP330776,Naive CD8+ T cell_3-AD.vs.control; scRNA-seq,SRP330776,T cell-	18
ACO2	bulk RNA-seq,Homo_723,AD.vs.control; bulk RNA-seq,Homo_723,AD.vs.MCI; bulk RNA-seq,Homo_723,MCI.vs.control; bulk RNA-seq,Homo_633,AD.vs.control; bulk RNA-seq,Homo_633,AD.vs.MCI; bulk RNA-seq,Homo_633,MCI.vs.control; bulk RNA-seq,Homo_714,AD.vs.control; bulk RNA-seq,Homo_714,AD.vs.MCI; bulk RNA-seq,Homo_714,MCI.vs.control; scRNA-seq,SRP330776,Naive CD8+ T cell_3-AD.vs.control; scRNA-seq,SRP330776,Regulatory T cell-AD.vs.control; scRNA-seq,SRP330776,B cell_4-AD.vs.control	12
ACOT1	scRNA-seq,SRP215507,CD8+ T cell_2-MCI.vs.control	1
ACOT11	bulk RNA-seq,Homo_723,MCI.vs.control; bulk RNA-seq,Homo_633,AD.vs.MCI; bulk RNA-seq,Homo_633,MCI.vs.control; bulk RNA-seq,Homo_714,AD.vs.control; bulk RNA-seq,Homo_714,AD.vs.MCI; bulk RNA-seq,Homo_714,MCI.vs.control	6
ACOT12	bulk RNA-seq,Homo_723,AD.vs.control; bulk RNA-seq,Homo_723,AD.vs.MCI; bulk RNA-seq,Homo_633,AD.vs.control; bulk RNA-seq,Homo_633,AD.vs.MCI; bulk RNA-seq,Homo_714,AD.vs.control; bulk RNA-seq,Homo_714,AD.vs.MCI	6
ACOT13	bulk RNA-seq,Homo_723,AD.vs.control; bulk RNA-seq,Homo_633,AD.vs.control; bulk RNA-seq,Homo_633,AD.vs.MCI; bulk RNA-seq,Homo_714,AD.vs.control; bulk RNA-seq,Homo_714,AD.vs.MCI; scRNA-seq,SRP215507,CD8+ T cell_4-AD.vs.control; scRNA-seq,SRP330776,γδ T cell-AD.vs.control; scRNA-seq,SRP330776,Memory T cell_1-AD.vs.control; scRNA-seq,SRP330776,T cell-AD.vs.control; scRNA-seq,SRP330776,CD8+	10
ACOT2	scRNA-seq,SRP215507,CD8+ T cell_2-AD.vs.control; scRNA-seq,SRP215507,CD8+ T cell_1-AD.vs.control; scRNA-seq,SRP215507,CD8+ T cell_4-AD.vs.control; scRNA-seq,SRP215507,CD8+ T cell_1-MCI.vs.control; scRNA-seq,SRP215507,CD8+ T cell_5-MCI.vs.control; scRNA-seq,SRP215507,CD8+ T cell_4-MCI.vs.control; scRNA-seq,SRP330776,CD8+ T cell_1-AD.vs.control; scRNA-seq,SRP330776,γδ T cell-AD.vs.control; scRNA-seq,SRP330776,B cell_1-AD.vs.control; scRNA-seq,SRP330776,T cell-AD.vs.control; scRNA-seq,SRP330776,CD8+ T cell_3-AD.vs.control	11
ACOT4	bulk RNA-seq,Homo_723,AD.vs.control; bulk RNA-seq,Homo_723,AD.vs.MCI; bulk RNA-seq,Homo_633,AD.vs.control; bulk RNA-seq,Homo_633,AD.vs.MCI; bulk RNA-seq,Homo_714,AD.vs.control; bulk RNA-seq,Homo_714,AD.vs.MCI; scRNA-seq,SRP215507,CD8+ T cell_3-AD.vs.control; scRNA-seq,SRP215507,CD8+ T cell_4-MCI.vs.control; scRNA-seq,SRP309935,Natural killer cell_2-AD.vs.control; scRNA-seq,SRP330776,CD8+ T cell_2-AD.vs.control; scRNA-seq,SRP330776,CD8+ T cell_3-AD.vs.control; scRNA-seq,SRP330776,B cell_4-AD.vs.control	12
ACOT6	bulk RNA-seq,Homo_723,AD.vs.control	1
ACOT7	bulk RNA-seq,Homo_723,AD.vs.control; bulk RNA-seq,Homo_723,AD.vs.MCI; bulk RNA-seq,Homo_723,MCI.vs.control; bulk RNA-seq,Homo_633,AD.vs.control; bulk RNA-seq,Homo_633,AD.vs.MCI; bulk RNA-seq,Homo_633,MCI.vs.control; bulk RNA-seq,Homo_714,AD.vs.control; bulk RNA-seq,Homo_714,AD.vs.MCI; bulk RNA-seq,Homo_714,MCI.vs.control; bulk RNA-seq,SRP223445,AD.vs.control; scRNA-seq,SRP215507,CD8+ T cell_2-AD.vs.MCI; scRNA-seq,SRP215507,CD8+ T cell_4-MCI.vs.control; scRNA-seq,SRP215507,CD8+ T cell_2-MCI.vs.control; scRNA-seq,SRP330776,B cell_1-AD.vs.control; scRNA-seq,SRP330776,Naive CD8+ T cell_1-AD.vs.control; scRNA-seq,SRP330776,Natural killer cell_1-AD.vs.control; scRNA-seq,SRP330776,Memory T cell_1-AD.vs.control; scRNA-seq,SRP330776,Natural killer T cell-	20
ACOT8	scRNA-seq,SRP215507,CD8+ T cell_3-AD.vs.control; scRNA-seq,SRP215507,CD8+ T cell_3-AD.vs.MCI; scRNA-seq,SRP330776,γδ T cell-AD.vs.control; scRNA-seq,SRP330776,Memory T cell_2-AD.vs.control; scRNA-seq,SRP330776,CD8+ T cell_3-AD.vs.control; scRNA-seq,SRP330776,Monocyte_2-AD.vs.control; scRNA-seq,SRP330776,Regulatory T cell-AD.vs.control	7
ACOT9	scRNA-seq,SRP215507,CD8+ T cell_3-AD.vs.control; scRNA-seq,SRP215507,CD8+ T cell_5-AD.vs.MCI; scRNA-seq,SRP215507,CD8+ T cell_5-MCI.vs.control; scRNA-seq,SRP215507,CD8+ T cell_3-MCI.vs.control; scRNA-seq,SRP215507,CD8+ T cell_7-MCI.vs.control	5

ACOX1	scRNA-seq,SRP215507,CD8+ T cell_5-AD.vs.control; scRNA-seq,SRP215507,CD8+ T cell_9-AD.vs.control; scRNA-seq,SRP215507,CD8+ T cell_5-AD.vs.MCI; scRNA-seq,SRP309935,Monocyte_2-AD.vs.control; scRNA-seq,SRP330776,γδ T cell-AD.vs.control; scRNA-seq,SRP330776,B cell_1-AD.vs.control; scRNA-seq,SRP330776,CD8+ T cell_3-AD.vs.control	7
ACOX2	bulk RNA-seq,Homo_723,AD.vs.control; bulk RNA-seq,Homo_723,AD.vs.MCI; bulk RNA-seq,Homo_714,AD.vs.control; bulk RNA-	4
ACOX3	bulk RNA-seq,Homo_633,AD.vs.control; bulk RNA-seq,Homo_633,AD.vs.MCI; bulk RNA-seq,Homo_714,AD.vs.control; bulk RNA-seq,Homo_714,AD.vs.MCI; scRNA-seq,SRP215507,CD8+ T cell_3-AD.vs.control; scRNA-seq,SRP215507,CD8+ T cell_4-AD.vs.control; scRNA-seq,SRP215507,CD8+ T cell_5-AD.vs.control; scRNA-seq,SRP215507,CD8+ T cell_4-AD.vs.MCI; scRNA-seq,SRP215507,CD8+ T cell_5-AD.vs.MCI; scRNA-seq,SRP330776,Naive CD8+ T cell_3-AD.vs.control; scRNA-seq,SRP330776,CD8+ T cell_3-AD.vs.control	11
Acp1	bulk RNA-seq,SRP325058,AD.vs.control	1
ACP1	scRNA-seq,SRP309935,Erythroid cell-AD.vs.control; scRNA-seq,SRP330776,B cell_2-AD.vs.control; scRNA-seq,SRP330776,B cell_3-AD.vs.control	3
ACP2	scRNA-seq,SRP215507,CD8+ T cell_2-AD.vs.MCI; scRNA-seq,SRP215507,CD8+ T cell_1-AD.vs.MCI; scRNA-seq,SRP215507,CD8+ T cell_2-MCI.vs.control; scRNA-seq,SRP330776,CD8+ T cell_1-AD.vs.control; scRNA-seq,SRP330776,γδ T cell-AD.vs.control; scRNA-seq,SRP330776,Memory T cell_2-AD.vs.control; scRNA-seq,SRP330776,CD8+ T cell_2-AD.vs.control; scRNA-seq,SRP330776,B cell_1-AD.vs.control; scRNA-seq,SRP330776,Naive CD8+ T cell_1-AD.vs.control; scRNA-seq,SRP330776,Natural killer cell_1-AD.vs.control; scRNA-seq,SRP330776,Memory T cell_1-AD.vs.control; scRNA-seq,SRP330776,Naive CD8+ T cell_3-AD.vs.control; scRNA-seq,SRP330776,CD8+ T cell_3-AD.vs.control; scRNA-	14
ACP3	bulk RNA-seq,Homo_714,MCI.vs.control	1
ACP4	bulk RNA-seq,Homo_723,AD.vs.control; bulk RNA-seq,Homo_723,AD.vs.MCI	2
ACP5	bulk RNA-seq,Homo_723,AD.vs.control; bulk RNA-seq,Homo_723,AD.vs.MCI; bulk RNA-seq,Homo_633,AD.vs.control; bulk RNA-seq,Homo_633,AD.vs.MCI; bulk RNA-seq,Homo_714,AD.vs.control; bulk RNA-seq,Homo_714,AD.vs.MCI; scRNA-seq,SRP215507,CD8+ T cell_7-AD.vs.control; scRNA-seq,SRP215507,CD8+ T cell_6-AD.vs.control; scRNA-seq,SRP215507,CD8+ T cell_4-AD.vs.MCI; scRNA-seq,SRP215507,CD8+ T cell_7-AD.vs.MCI; scRNA-seq,SRP215507,CD8+ T cell_6-AD.vs.MCI; scRNA-seq,SRP215507,CD8+ T cell_1-MCI.vs.control; scRNA-seq,SRP330776,γδ T cell-AD.vs.control; scRNA-seq,SRP330776,Naive CD8+ T cell_1-AD.vs.control; scRNA-seq,SRP330776,Natural killer cell_1-AD.vs.control; scRNA-seq,SRP330776,Naive CD8+ T cell_3-AD.vs.control; scRNA-seq,SRP330776,Monocyte_2-AD.vs.control; scRNA-	19
ACP6	scRNA-seq,SRP215507,CD8+ T cell_1-AD.vs.MCI; scRNA-seq,SRP215507,CD8+ T cell_5-AD.vs.MCI; scRNA-seq,SRP215507,CD8+ T cell_1-MCI.vs.control; scRNA-seq,SRP215507,CD8+ T cell_2-MCI.vs.control; scRNA-seq,SRP309935,Megakaryocyte_1-AD.vs.control; scRNA-seq,SRP309935,CD8+ T cell_2-AD.vs.control; scRNA-seq,SRP330776,γδ T cell-AD.vs.control; scRNA-seq,SRP330776,Memory T cell_2-AD.vs.control; scRNA-seq,SRP330776,B cell_1-AD.vs.control; scRNA-seq,SRP330776,Memory T cell_1-AD.vs.control; scRNA-seq,SRP330776,B cell_2-AD.vs.control;	12
ACP7	bulk RNA-seq,Homo_723,AD.vs.control; bulk RNA-seq,Homo_723,AD.vs.MCI; bulk RNA-seq,Homo_633,AD.vs.control; bulk RNA-	4
ACR	bulk RNA-seq,Homo_723,AD.vs.control; bulk RNA-seq,Homo_723,AD.vs.MCI; bulk RNA-seq,Homo_723,MCI.vs.control; bulk RNA-seq,Homo_633,AD.vs.control; bulk RNA-seq,Homo_633,AD.vs.MCI	5
ACRBP	bulk RNA-seq,Homo_633,MCI.vs.control; bulk RNA-seq,Homo_714,AD.vs.control; bulk RNA-seq,Homo_714,AD.vs.MCI; bulk RNA-seq,Homo_714,MCI.vs.control; scRNA-seq,SRP215507,CD8+ T cell_2-MCI.vs.control; scRNA-seq,SRP215507,CD8+ T cell_3-MCI.vs.control; scRNA-seq,SRP330776,B cell_1-AD.vs.control; scRNA-seq,SRP330776,Naive CD8+ T cell_1-AD.vs.control; scRNA-seq,SRP330776,B cell_3-AD.vs.control	9
ACRV1	bulk RNA-seq,Homo_723,AD.vs.control; bulk RNA-seq,Homo_723,AD.vs.MCI; bulk RNA-seq,Homo_633,AD.vs.control; bulk RNA-seq,Homo_633,AD.vs.MCI; bulk RNA-seq,ROSMAP,MCI.vs.control	5
ACSBG1	bulk RNA-seq,Homo_723,AD.vs.control; bulk RNA-seq,Homo_723,AD.vs.MCI; bulk RNA-seq,Homo_723,MCI.vs.control; bulk RNA-seq,Homo_633,AD.vs.control; bulk RNA-seq,Homo_633,AD.vs.MCI; bulk RNA-seq,Homo_633,MCI.vs.control; bulk RNA-seq,Homo_714,AD.vs.control;	8
ACSBG2	bulk RNA-seq,Homo_723,AD.vs.control; bulk RNA-seq,Homo_723,AD.vs.MCI	2
ACSF2	scRNA-seq,SRP215507,CD8+ T cell_2-AD.vs.MCI; scRNA-seq,SRP330776,CD8+ T cell_2-AD.vs.control; scRNA-seq,SRP330776,Natural killer cell_1-AD.vs.control; scRNA-seq,SRP330776,T cell-AD.vs.control; scRNA-seq,SRP330776,CD8+ T cell_3-AD.vs.control	5

ACSF3	bulk RNA-seq,Homo_723,AD.vs.control; bulk RNA-seq,Homo_723,MCI.vs.control; bulk RNA-seq,Homo_633,MCI.vs.control; bulk RNA-seq,Homo_714,MCI.vs.control; scRNA-seq,SRP215507,CD8+ T cell_5-AD.vs.MCI; scRNA-seq,SRP215507,CD8+ T cell_5-MCI.vs.control; scRNA-seq,SRP309935,Natural killer cell_2-AD.vs.control; scRNA-seq,SRP309935,B cell_1-AD.vs.control; scRNA-seq,SRP309935,Plasmacytoid dendritic cell_1-AD.vs.control; scRNA-seq,SRP330776,Naive CD8+ T cell_1-AD.vs.control; scRNA-seq,SRP330776,Memory T cell_1-AD.vs.control; scRNA-seq,SRP330776,Natural killer T cell-AD.vs.control; scRNA-seq,SRP330776,B cell_2-AD.vs.control; scRNA-seq,SRP330776,CD8+ T cell_3-AD.vs.control; scRNA-seq,SRP330776,Monocyte_1-AD.vs.control; scRNA-seq,SRP330776,Monocyte_2-AD.vs.control; scRNA-seq,SRP330776,B cell_3- [illegible]	18
ACSL1	bulk RNA-seq,Homo_723,AD.vs.control; bulk RNA-seq,Homo_633,AD.vs.control; bulk RNA-seq,Homo_633,AD.vs.MCI; bulk RNA-seq,SRP223445,AD.vs.control; scRNA-seq,SRP215507,CD8+ T cell_2-AD.vs.MCI; scRNA-seq,SRP215507,CD8+ T cell_1-AD.vs.MCI; scRNA-seq,SRP330776,Natural killer cell_1-AD.vs.control; scRNA-seq,SRP330776,Monocyte_1-AD.vs.control	8
Acsl1	bulk RNA-seq,SRP325058,AD.vs.control	1
ACSL3	bulk RNA-seq,Homo_723,AD.vs.control; bulk RNA-seq,Homo_723,AD.vs.MCI; bulk RNA-seq,Homo_633,AD.vs.control; bulk RNA-seq,Homo_633,AD.vs.MCI; bulk RNA-seq,Homo_714,AD.vs.control; bulk RNA-seq,Homo_714,AD.vs.MCI; scRNA-seq,SRP215507,CD8+ T cell_5-AD.vs.MCI; scRNA-seq,SRP215507,CD8+ T cell_5-MCI.vs.control; scRNA-seq,SRP215507,CD8+ T cell_6-MCI.vs.control; scRNA-seq,SRP309935,CD8+ T cell_1-AD.vs.control; scRNA-seq,SRP330776,Naive CD8+ T cell_1-AD.vs.control	11
ACSL4	scRNA-seq,SRP215507,CD8+ T cell_3-AD.vs.control; scRNA-seq,SRP215507,CD8+ T cell_2-MCI.vs.control; scRNA-seq,SRP215507,CD8+ T cell_3-MCI.vs.control; scRNA-seq,SRP215507,CD8+ T cell_6-MCI.vs.control; scRNA-seq,SRP330776,Memory T cell_2-AD.vs.control; scRNA-	6
ACSL5	bulk RNA-seq,Homo_723,AD.vs.control; bulk RNA-seq,Homo_723,MCI.vs.control; bulk RNA-seq,Homo_633,MCI.vs.control; scRNA-seq,SRP330776,Naive CD8+ T cell_3-AD.vs.control; scRNA-seq,SRP330776,Regulatory T cell-AD.vs.control	5
ACSL6	bulk RNA-seq,Homo_714,AD.vs.control; bulk RNA-seq,Homo_714,AD.vs.MCI; bulk RNA-seq,ROSMAP,AD.vs.control; scRNA-seq,SRP215507,CD8+ T cell_4-AD.vs.control; scRNA-seq,SRP215507,CD8+ T cell_4-AD.vs.MCI; scRNA-seq,SRP330776,CD8+ T cell_2-AD.vs.control; scRNA-seq,SRP330776,B cell_1-AD.vs.control; scRNA-seq,SRP330776,Natural killer T cell-AD.vs.control; scRNA-seq,SRP330776,B cell_3-AD.vs.control	9
ACSM1	bulk RNA-seq,Homo_723,AD.vs.control; bulk RNA-seq,Homo_723,AD.vs.MCI; bulk RNA-seq,Homo_723,MCI.vs.control; bulk RNA-seq,Homo_633,AD.vs.control; bulk RNA-seq,Homo_633,AD.vs.MCI; bulk RNA-seq,Homo_633,MCI.vs.control; bulk RNA-seq,Homo_714,AD.vs.control; bulk RNA-seq,Homo_714,AD.vs.MCI; scRNA-seq,SRP215507,CD8+ T cell_2-AD.vs.control; scRNA-seq,SRP215507,CD8+ T cell_5-MCI.vs.control; scRNA-seq,SRP215507,CD8+ T cell_2-MCI.vs.control; scRNA-seq,SRP215507,CD8+ T cell_3-MCI.vs.control; scRNA-seq,SRP330776,CD8+ T cell_1-	14
ACSM2A	bulk RNA-seq,Homo_723,AD.vs.control; bulk RNA-seq,Homo_723,AD.vs.MCI; bulk RNA-seq,Homo_723,MCI.vs.control; bulk RNA-seq,Homo_633,AD.vs.control; bulk RNA-seq,Homo_633,AD.vs.MCI; bulk RNA-seq,Homo_633,MCI.vs.control	6
ACSM2B	bulk RNA-seq,Homo_723,AD.vs.MCI; bulk RNA-seq,Homo_723,MCI.vs.control; bulk RNA-seq,Homo_633,AD.vs.MCI	3
ACSM3	bulk RNA-seq,Homo_714,AD.vs.control; bulk RNA-seq,Homo_714,AD.vs.MCI; bulk RNA-seq,Homo_714,MCI.vs.control; scRNA-seq,SRP215507,CD8+ T cell_8-AD.vs.control; scRNA-seq,SRP215507,CD8+ T cell_3-AD.vs.MCI; scRNA-seq,SRP215507,CD8+ T cell_7-AD.vs.MCI; scRNA-seq,SRP215507,CD8+ T cell_7-MCI.vs.control; scRNA-seq,SRP309935,Natural killer cell_1-AD.vs.control; scRNA-seq,SRP330776,CD8+ T cell_1-AD.vs.control; scRNA-seq,SRP330776,γδ T cell-AD.vs.control; scRNA-seq,SRP330776,Memory T cell_2-AD.vs.control; scRNA-seq,SRP330776,CD8+ T cell_2-AD.vs.control; scRNA-seq,SRP330776,B cell_1-AD.vs.control; scRNA-seq,SRP330776,Naive CD8+ T cell_1-AD.vs.control; scRNA-seq,SRP330776,Memory T cell_1-AD.vs.control; scRNA-seq,SRP330776,Natural killer T cell-AD.vs.control; scRNA-seq,SRP330776,Naive CD8+ T [illegible]	17
ACSM5	bulk RNA-seq,Homo_723,AD.vs.control; bulk RNA-seq,Homo_723,AD.vs.MCI; bulk RNA-seq,Homo_633,AD.vs.control; bulk RNA-seq,Homo_633,AD.vs.MCI; bulk RNA-seq,ROSMAP,AD.vs.control; bulk RNA-seq,ROSMAP,MCI.vs.control	6

ACSS1	bulk RNA-seq,Homo_723,AD.vs.control; bulk RNA-seq,Homo_723,AD.vs.MCI; bulk RNA-seq,Homo_633,AD.vs.control; bulk RNA-seq,Homo_633,AD.vs.MCI; bulk RNA-seq,Homo_714,AD.vs.control; bulk RNA-seq,Homo_714,AD.vs.MCI; scRNA-seq,SRP215507,CD8+ T cell_7-AD.vs.control; scRNA-seq,SRP309935,Megakaryocyte_1-AD.vs.control; scRNA-seq,SRP309935,Naive CD8+ T cell_1-AD.vs.control; scRNA-seq,SRP309935,Natural killer cell_1-AD.vs.control; scRNA-seq,SRP309935,Monocyte_2-AD.vs.control; scRNA-seq,SRP309935,Naive CD8+ T cell_2-AD.vs.control; scRNA-seq,SRP309935,CD8+ T cell_1-AD.vs.control; scRNA-seq,SRP309935,B cell_1-AD.vs.control; scRNA-seq,SRP330776,CD8+ T cell_1-AD.vs.control; scRNA-seq,SRP330776,γδ T cell-AD.vs.control; scRNA-seq,SRP330776,Memory T cell_2-AD.vs.control; scRNA-seq,SRP330776,CD8+ T cell_2-AD.vs.control; scRNA-seq,SRP330776,B cell_1-AD.vs.control; scRNA-seq,SRP330776,Naive CD8+ T cell_1-AD.vs.control; scRNA-seq,SRP330776,Natural killer cell_1-AD.vs.control; scRNA-seq,SRP330776,Memory T cell_1-AD.vs.control; scRNA-seq,SRP330776,Natural killer T cell-AD.vs.control; scRNA-seq,SRP330776,Naive CD8+ T cell_3-AD.vs.control; scRNA-seq,SRP330776,B cell_2-AD.vs.control; scRNA-seq,SRP330776,T cell-AD.vs.control; scRNA-seq,SRP330776,CD8+ T cell_3-AD.vs.control; scRNA-seq,SRP330776,B cell_3-AD.vs.control; scRNA-seq,SRP330776,Regulatory T cell-AD.vs.control; scRNA-seq,SRP330776,Natural killer cell_2-AD.vs.control; scRNA-	31
ACSS2	bulk RNA-seq,Homo_714,AD.vs.control; bulk RNA-seq,Homo_714,AD.vs.MCI; scRNA-seq,SRP215507,CD8+ T cell_4-AD.vs.control; scRNA-seq,SRP215507,CD8+ T cell_1-AD.vs.MCI; scRNA-seq,SRP215507,CD8+ T cell_4-AD.vs.MCI; scRNA-seq,SRP330776,CD8+ T cell_1-AD.vs.control; scRNA-seq,SRP330776,γδ T cell-AD.vs.control; scRNA-seq,SRP330776,CD8+ T cell_2-AD.vs.control; scRNA-seq,SRP330776,Naive CD8+ T cell_1-AD.vs.control; scRNA-seq,SRP330776,Natural killer cell_1-AD.vs.control; scRNA-seq,SRP330776,B cell_2-AD.vs.control	11
ACSS3	bulk RNA-seq,Homo_723,AD.vs.control; bulk RNA-seq,Homo_723,AD.vs.MCI; bulk RNA-seq,Homo_714,AD.vs.control; bulk RNA-	4
ACTA1	bulk RNA-seq,Homo_723,AD.vs.control; bulk RNA-seq,Homo_723,AD.vs.MCI; bulk RNA-seq,Homo_633,AD.vs.control; bulk RNA-seq,Homo_633,AD.vs.MCI; bulk RNA-seq,Homo_714,MCI.vs.control	5
ACTA2	bulk RNA-seq,Homo_723,AD.vs.control; bulk RNA-seq,Homo_723,AD.vs.MCI; bulk RNA-seq,Homo_633,AD.vs.control; bulk RNA-seq,Homo_633,AD.vs.MCI; scRNA-seq,SRP215507,CD8+ T cell_4-AD.vs.control; scRNA-seq,SRP215507,CD8+ T cell_1-AD.vs.MCI; scRNA-seq,SRP215507,CD8+ T cell_5-AD.vs.MCI; scRNA-seq,SRP215507,CD8+ T cell_1-MCI.vs.control; scRNA-seq,SRP215507,CD8+ T cell_5-MCI.vs.control; scRNA-seq,SRP215507,CD8+ T cell_4-MCI.vs.control; scRNA-seq,SRP309935,Natural killer cell_1-AD.vs.control; scRNA-seq,SRP330776,CD8+ T cell_1-AD.vs.control; scRNA-seq,SRP330776,CD8+ T cell_2-AD.vs.control; scRNA-seq,SRP330776,Natural killer cell_1-	15
ACTA2-AS1	scRNA-seq,SRP215507,CD8+ T cell_4-MCI.vs.control; scRNA-seq,SRP215507,CD8+ T cell_2-MCI.vs.control	2
ACTB	scRNA-seq,SRP215507,CD8+ T cell_7-AD.vs.control; scRNA-seq,SRP309935,Erythroid cell-AD.vs.control	2
ACTBL2	bulk RNA-seq,Homo_723,AD.vs.control; bulk RNA-seq,Homo_723,AD.vs.MCI; bulk RNA-seq,Homo_633,AD.vs.control; bulk RNA-seq,Homo_633,AD.vs.MCI; bulk RNA-seq,Homo_714,MCI.vs.control	5
ACTC1	bulk RNA-seq,Homo_723,AD.vs.control; bulk RNA-seq,Homo_723,AD.vs.MCI; bulk RNA-seq,Homo_633,AD.vs.control	3
ACTG1	scRNA-seq,SRP215507,CD8+ T cell_7-AD.vs.control; scRNA-seq,SRP215507,CD8+ T cell_10-AD.vs.control; scRNA-seq,SRP309935,Erythroid cell-	3
ACTG2	bulk RNA-seq,Homo_723,AD.vs.control; bulk RNA-seq,Homo_723,AD.vs.MCI; bulk RNA-seq,Homo_633,AD.vs.control; bulk RNA-	4
ACTL10	bulk RNA-seq,Homo_723,AD.vs.control; bulk RNA-seq,Homo_723,AD.vs.MCI; bulk RNA-seq,Homo_723,MCI.vs.control; bulk RNA-seq,Homo_633,AD.vs.control; bulk RNA-seq,Homo_633,AD.vs.MCI; bulk RNA-seq,Homo_633,MCI.vs.control; bulk RNA-seq,Homo_714,AD.vs.control; bulk RNA-seq,Homo_714,AD.vs.MCI; bulk RNA-seq,Homo_714,MCI.vs.control; scRNA-seq,SRP215507,CD8+ T cell_2-AD.vs.control; scRNA-seq,SRP215507,CD8+ T cell_3-AD.vs.MCI; scRNA-seq,SRP215507,CD8+ T cell_5-AD.vs.MCI; scRNA-seq,SRP215507,CD8+ T cell_3-MCI.vs.control; scRNA-seq,SRP309935,CD8+ T cell_1-AD.vs.control; scRNA-seq,SRP309935,CD8+ T cell_2-AD.vs.control; scRNA-seq,SRP330776,Memory T cell_2-AD.vs.control; scRNA-seq,SRP330776,B cell_1-AD.vs.control; scRNA-seq,SRP330776,Memory T cell_1-AD.vs.control	18
ACTL6A	bulk RNA-seq,Homo_723,AD.vs.control; bulk RNA-seq,Homo_723,AD.vs.MCI; bulk RNA-seq,Homo_633,AD.vs.control; bulk RNA-seq,Homo_633,AD.vs.MCI; bulk RNA-seq,Homo_714,AD.vs.control; bulk RNA-seq,Homo_714,AD.vs.MCI; scRNA-seq,SRP330776,CD8+ T cell_1-AD.vs.control; scRNA-seq,SRP330776,γδ T cell-AD.vs.control; scRNA-seq,SRP330776,B cell_1-AD.vs.control; scRNA-seq,SRP330776,Naive CD8+ T cell_1-AD.vs.control; scRNA-seq,SRP330776,Natural killer cell_1-AD.vs.control; scRNA-seq,SRP330776,Natural killer T cell-AD.vs.control; scRNA-seq,SRP330776,Naive CD8+ T cell_3-AD.vs.control; scRNA-seq,SRP330776,B cell_2-AD.vs.control; scRNA-seq,SRP330776,CD8+ T cell_3-	17
ACTL6B	bulk RNA-seq,Homo_723,AD.vs.MCI; bulk RNA-seq,Homo_723,MCI.vs.control	2
ACTL7B	bulk RNA-seq,Homo_714,MCI.vs.control	1

ACTN1	scRNA-seq,SRP215507,CD8+ T cell_4-AD.vs.control; scRNA-seq,SRP215507,CD8+ T cell_6-AD.vs.control; scRNA-seq,SRP215507,CD8+ T cell_4-AD.vs.MCI; scRNA-seq,SRP215507,CD8+ T cell_5-AD.vs.MCI; scRNA-seq,SRP215507,CD8+ T cell_1-MCI.vs.control; scRNA-seq,SRP215507,CD8+ T cell_5-MCI.vs.control; scRNA-seq,SRP215507,CD8+ T cell_4-MCI.vs.control; scRNA-seq,SRP215507,CD8+ T cell_2-MCI.vs.control; scRNA-seq,SRP309935,Naive CD8+ T cell_3-AD.vs.control; scRNA-seq,SRP330776,CD8+ T cell_1-AD.vs.control; scRNA-seq,SRP330776,Memory T cell_2-AD.vs.control; scRNA-seq,SRP330776,CD8+ T cell_2-AD.vs.control; scRNA-seq,SRP330776,B cell_1-AD.vs.control; scRNA-seq,SRP330776,Naive CD8+ T cell_1-AD.vs.control; scRNA-seq,SRP330776,Memory T cell_1-AD.vs.control; scRNA-seq,SRP330776,Natural killer T cell-AD.vs.control; scRNA-seq,SRP330776,Naive CD8+ T cell_3-AD.vs.control; scRNA-seq,SRP330776,Naive CD8+ T cell_2-AD.vs.control; scRNA-seq,SRP330776,CD8+ [illegible]	20
ACTN1-AS1	scRNA-seq,SRP215507,CD8+ T cell_3-AD.vs.control; scRNA-seq,SRP215507,CD8+ T cell_3-MCI.vs.control	2
ACTN2	bulk RNA-seq,Homo_723,AD.vs.control; bulk RNA-seq,Homo_723,AD.vs.MCI; bulk RNA-seq,Homo_714,MCI.vs.control	3
ACTN3	scRNA-seq,SRP215507,CD8+ T cell_2-AD.vs.MCI; scRNA-seq,SRP215507,CD8+ T cell_2-MCI.vs.control	2
ACTN4	bulk RNA-seq,Homo_723,AD.vs.control; bulk RNA-seq,Homo_723,AD.vs.MCI; bulk RNA-seq,Homo_723,MCI.vs.control; bulk RNA-seq,Homo_633,AD.vs.control; bulk RNA-seq,Homo_633,AD.vs.MCI; bulk RNA-seq,Homo_633,MCI.vs.control; bulk RNA-seq,Homo_714,AD.vs.control; bulk RNA-seq,Homo_714,AD.vs.MCI; scRNA-seq,SRP215507,CD8+ T cell_3-AD.vs.control; scRNA-seq,SRP215507,CD8+ T cell_7-AD.vs.control; scRNA-seq,SRP215507,CD8+ T cell_6-AD.vs.control; scRNA-seq,SRP215507,CD8+ T cell_6-AD.vs.MCI; scRNA-seq,SRP215507,CD8+ T cell_3-MCI.vs.control; scRNA-seq,SRP215507,CD8+ T cell_6-MCI.vs.control; scRNA-seq,SRP309935,Monocyte_2-AD.vs.control; scRNA-seq,SRP330776,Naive CD8+ T cell_1-AD.vs.control; scRNA-seq,SRP330776,Naive CD8+ T cell_2-AD.vs.control; scRNA- [illegible]	18
ACTR10	bulk RNA-seq,Homo_723,AD.vs.MCI; bulk RNA-seq,Homo_723,MCI.vs.control; bulk RNA-seq,Homo_633,MCI.vs.control; bulk RNA-seq,Homo_714,AD.vs.control; bulk RNA-seq,Homo_714,AD.vs.MCI; bulk RNA-seq,Homo_714,MCI.vs.control; scRNA-seq,SRP215507,CD8+ T cell_7-AD.vs.control; scRNA-seq,SRP215507,CD8+ T cell_5-AD.vs.MCI; scRNA-seq,SRP330776,B cell_3-AD.vs.control	9
ACTR1A	scRNA-seq,SRP330776,Naive CD8+ T cell_2-AD.vs.control; scRNA-seq,SRP330776,Monocyte_2-AD.vs.control	2
ACTR1B	bulk RNA-seq,Homo_723,AD.vs.control; bulk RNA-seq,Homo_723,AD.vs.MCI; bulk RNA-seq,Homo_723,MCI.vs.control; bulk RNA-seq,Homo_633,AD.vs.control; bulk RNA-seq,Homo_633,AD.vs.MCI; bulk RNA-seq,Homo_633,MCI.vs.control; bulk RNA-seq,Homo_714,AD.vs.control; bulk RNA-seq,Homo_714,AD.vs.MCI; bulk RNA-seq,Homo_714,MCI.vs.control; scRNA-seq,SRP215507,CD8+ T cell_4-AD.vs.MCI; scRNA-seq,SRP215507,CD8+ T cell_6-AD.vs.MCI; scRNA-seq,SRP215507,CD8+ T cell_4-MCI.vs.control; scRNA-seq,SRP215507,CD8+ T cell_6-MCI.vs.control; scRNA-seq,SRP309935,Monocyte_2-AD.vs.control; scRNA-seq,SRP330776,CD8+ T cell_1-AD.vs.control; scRNA-seq,SRP330776,γδ T cell-AD.vs.control; scRNA-seq,SRP330776,Memory T cell_2-AD.vs.control; scRNA-seq,SRP330776,CD8+ T cell_2-AD.vs.control; scRNA-seq,SRP330776,B cell_1-AD.vs.control; scRNA-seq,SRP330776,Naive CD8+ T cell_1-AD.vs.control; scRNA-seq,SRP330776,Natural killer cell_1-AD.vs.control; scRNA-seq,SRP330776,Memory T cell_1-AD.vs.control; scRNA-seq,SRP330776,Natural killer T cell-AD.vs.control; scRNA-seq,SRP330776,Naive CD8+ T cell_3-AD.vs.control; scRNA-seq,SRP330776,B cell_2-AD.vs.control; scRNA-seq,SRP330776,T cell-AD.vs.control; scRNA-seq,SRP330776,Naive CD8+ T cell_2-AD.vs.control; scRNA-seq,SRP330776,CD8+ T cell_3-AD.vs.control; scRNA-seq,SRP330776,Monocyte_1-[illegible]	31
ACTR2	bulk RNA-seq,Homo_714,AD.vs.control; bulk RNA-seq,Homo_714,AD.vs.MCI; scRNA-seq,SRP309935,Erythroid cell-AD.vs.control; scRNA-seq,SRP330776,Naive CD8+ T cell_1-AD.vs.control	4
ACTR3	bulk RNA-seq,Homo_714,AD.vs.control; bulk RNA-seq,Homo_714,AD.vs.MCI; bulk RNA-seq,SRP310421,AD.vs.control; scRNA-seq,SRP215507,CD8+ T cell_7-AD.vs.control; scRNA-seq,SRP215507,CD8+ T cell_10-AD.vs.control; scRNA-seq,SRP309935,Erythroid cell-AD.vs.control; scRNA-seq,SRP309935,Megakaryocyte_2-AD.vs.control; scRNA-seq,SRP330776,γδ T cell-AD.vs.control; scRNA-seq,SRP330776,Naive CD8+ T cell_3-AD.vs.control; scRNA-seq,SRP330776,Natural killer cell_2-AD.vs.control	10

ACTR3B	bulk RNA-seq,Homo_723,AD.vs.control; bulk RNA-seq,Homo_723,AD.vs.MCI; bulk RNA-seq,Homo_723,MCI.vs.control; bulk RNA-seq,Homo_633,AD.vs.control; bulk RNA-seq,Homo_633,AD.vs.MCI; bulk RNA-seq,Homo_633,MCI.vs.control; bulk RNA-seq,Homo_714,AD.vs.control; bulk RNA-seq,Homo_714,AD.vs.MCI; bulk RNA-seq,Homo_714,MCI.vs.control; scRNA-seq,SRP215507,CD8+ T cell_3-AD.vs.control; scRNA-seq,SRP215507,CD8+ T cell_4-AD.vs.control; scRNA-seq,SRP215507,CD8+ T cell_2-AD.vs.MCI; scRNA-seq,SRP215507,CD8+ T cell_4-AD.vs.MCI; scRNA-seq,SRP215507,CD8+ T cell_3-MCI.vs.control; scRNA-seq,SRP330776,CD8+ T cell_1-AD.vs.control; scRNA-seq,SRP330776,γδ T cell-AD.vs.control; scRNA-seq,SRP330776,Memory T cell_2-AD.vs.control; scRNA-seq,SRP330776,CD8+ T cell_2-AD.vs.control; scRNA-seq,SRP330776,B cell_1-AD.vs.control; scRNA-seq,SRP330776,Naive CD8+ T cell_1-AD.vs.control; scRNA-seq,SRP330776,Natural killer cell_1-AD.vs.control; scRNA-seq,SRP330776,Memory T cell_1-AD.vs.control; scRNA-seq,SRP330776,Natural killer T cell-AD.vs.control; scRNA-seq,SRP330776,Naive CD8+ T cell_3-AD.vs.control; scRNA-seq,SRP330776,B cell_2-AD.vs.control; scRNA-seq,SRP330776,CD8+ T cell_3-AD.vs.control; scRNA-seq,SRP330776,B [illegible]	28
ACTR3C	bulk RNA-seq,Homo_714,AD.vs.control; bulk RNA-seq,Homo_714,AD.vs.MCI; scRNA-seq,SRP215507,CD8+ T cell_5-AD.vs.MCI; scRNA-seq,SRP330776,T cell-AD.vs.control; scRNA-seq,SRP330776,B cell_3-AD.vs.control	5
ACTR5	bulk RNA-seq,Homo_723,AD.vs.control; bulk RNA-seq,Homo_723,AD.vs.MCI; bulk RNA-seq,Homo_723,MCI.vs.control; bulk RNA-seq,Homo_633,AD.vs.control; bulk RNA-seq,Homo_633,AD.vs.MCI; bulk RNA-seq,Homo_633,MCI.vs.control; bulk RNA-seq,Homo_714,AD.vs.control; bulk RNA-seq,Homo_714,AD.vs.MCI; bulk RNA-seq,SRP223445,AD.vs.control; scRNA-seq,SRP215507,CD8+ T cell_6-AD.vs.control; scRNA-seq,SRP215507,CD8+ T cell_1-AD.vs.MCI; scRNA-seq,SRP215507,CD8+ T cell_4-AD.vs.MCI; scRNA-seq,SRP215507,CD8+ T cell_1-MCI.vs.control; scRNA-seq,SRP215507,CD8+ T cell_4-MCI.vs.control; scRNA-seq,SRP309935,Natural killer cell_2-AD.vs.control; scRNA-seq,SRP309935,Naive CD8+ T cell_1-AD.vs.control; scRNA-seq,SRP330776,CD8+ T cell_1-AD.vs.control; scRNA-seq,SRP330776,γδ T cell-AD.vs.control; scRNA-seq,SRP330776,Memory T cell_2-AD.vs.control; scRNA-seq,SRP330776,CD8+ T cell_2-AD.vs.control; scRNA-seq,SRP330776,B cell_1-AD.vs.control; scRNA-seq,SRP330776,Naive CD8+ T cell_1-AD.vs.control; scRNA-seq,SRP330776,Natural killer cell_1-AD.vs.control; scRNA-seq,SRP330776,Memory T cell_1-AD.vs.control; scRNA-seq,SRP330776,Natural killer T cell-AD.vs.control; scRNA-seq,SRP330776,B cell_2-AD.vs.control; scRNA-seq,SRP330776,T cell-AD.vs.control; scRNA-seq,SRP330776,CD8+ T cell_3-AD.vs.control; scRNA-seq,SRP330776,Natural killer cell_2-AD.vs.control; [illegible]	30
ACTR6	scRNA-seq,SRP215507,CD8+ T cell_7-AD.vs.control; scRNA-seq,SRP215507,CD8+ T cell_7-MCI.vs.control; scRNA-seq,SRP309935,Megakaryocyte_1-AD.vs.control; scRNA-seq,SRP330776,CD8+ T cell_1-AD.vs.control; scRNA-seq,SRP330776,γδ T cell-AD.vs.control; scRNA-seq,SRP330776,Memory T cell_2-AD.vs.control; scRNA-seq,SRP330776,CD8+ T cell_2-AD.vs.control; scRNA-seq,SRP330776,B cell_1-AD.vs.control; scRNA-seq,SRP330776,Memory T cell_1-AD.vs.control; scRNA-seq,SRP330776,Natural killer T cell-AD.vs.control; scRNA-seq,SRP330776,Naive CD8+ T cell_3-AD.vs.control; scRNA-seq,SRP330776,B cell_2-AD.vs.control; scRNA-seq,SRP330776,CD8+ T cell_3-AD.vs.control; scRNA-	14
ACTR8	bulk RNA-seq,Homo_723,AD.vs.control; bulk RNA-seq,Homo_723,AD.vs.MCI; bulk RNA-seq,Homo_723,MCI.vs.control; bulk RNA-seq,Homo_633,AD.vs.control; bulk RNA-seq,Homo_633,AD.vs.MCI; bulk RNA-seq,Homo_633,MCI.vs.control; bulk RNA-seq,Homo_714,AD.vs.control; bulk RNA-seq,Homo_714,AD.vs.MCI; bulk RNA-seq,Homo_714,MCI.vs.control; bulk RNA-seq,ROSMAP,MCI.vs.control; scRNA-seq,SRP309935,Monocyte_1-AD.vs.control; scRNA-seq,SRP330776,Memory T cell_1-AD.vs.control	12
ACTRT1	bulk RNA-seq,Homo_723,AD.vs.control; bulk RNA-seq,Homo_723,MCI.vs.control; bulk RNA-seq,Homo_633,AD.vs.control; bulk RNA-seq,Homo_633,MCI.vs.control; bulk RNA-seq,Homo_714,MCI.vs.control	5
ACTRT2	bulk RNA-seq,Homo_714,MCI.vs.control	1
ACVR1	bulk RNA-seq,Homo_723,AD.vs.MCI; bulk RNA-seq,Homo_723,MCI.vs.control; bulk RNA-seq,Homo_633,AD.vs.MCI; bulk RNA-seq,Homo_633,MCI.vs.control; bulk RNA-seq,Homo_714,AD.vs.control; bulk RNA-seq,Homo_714,AD.vs.MCI; bulk RNA-seq,Homo_714,MCI.vs.control; scRNA-seq,SRP215507,CD8+ T cell_3-AD.vs.control; scRNA-seq,SRP215507,CD8+ T cell_4-AD.vs.control; scRNA-seq,SRP215507,CD8+ T cell_2-AD.vs.MCI; scRNA-seq,SRP215507,CD8+ T cell_3-AD.vs.MCI; scRNA-seq,SRP215507,CD8+ T cell_4-AD.vs.MCI; scRNA-seq,SRP215507,CD8+ T cell_5-AD.vs.MCI; scRNA-seq,SRP215507,CD8+ T cell_6-MCI.vs.control; scRNA-seq,SRP330776,γδ T cell-	17
ACVR1B	bulk RNA-seq,Homo_723,AD.vs.control; bulk RNA-seq,Homo_723,AD.vs.MCI; bulk RNA-seq,Homo_723,MCI.vs.control; bulk RNA-seq,Homo_633,MCI.vs.control; bulk RNA-seq,Homo_714,AD.vs.control; bulk RNA-seq,Homo_714,AD.vs.MCI; bulk RNA-seq,Homo_714,MCI.vs.control; scRNA-seq,SRP215507,CD8+ T cell_3-AD.vs.MCI; scRNA-seq,SRP215507,CD8+ T cell_3-MCI.vs.control; scRNA-seq,SRP309935,Monocyte_1-AD.vs.control; scRNA-seq,SRP330776,Memory T cell_2-AD.vs.control; scRNA-seq,SRP330776,CD8+ T cell_2-	14

ACVR1C	bulk RNA-seq,Homo_723,AD.vs.control; bulk RNA-seq,Homo_723,AD.vs.MCI; bulk RNA-seq,Homo_633,AD.vs.control; bulk RNA-seq,Homo_633,AD.vs.MCI; bulk RNA-seq,Homo_714,AD.vs.control; bulk RNA-seq,Homo_714,AD.vs.MCI; scRNA-seq,SRP215507,CD8+ T cell_4-AD.vs.control; scRNA-seq,SRP215507,CD8+ T cell_3-AD.vs.MCI; scRNA-seq,SRP215507,CD8+ T cell_3-MCI.vs.control; scRNA-seq,SRP309935,Naive CD8+ T cell_2-AD.vs.control; scRNA-seq,SRP330776,γδ T cell-AD.vs.control; scRNA-seq,SRP330776,Memory T cell_2-AD.vs.control; scRNA-seq,SRP330776,Naive CD8+ T cell_1-AD.vs.control; scRNA-seq,SRP330776,Natural killer T cell-AD.vs.control	14
ACVR2A	bulk RNA-seq,Homo_723,AD.vs.control; bulk RNA-seq,Homo_723,AD.vs.MCI; bulk RNA-seq,Homo_723,MCI.vs.control; bulk RNA-seq,Homo_633,AD.vs.control; bulk RNA-seq,Homo_633,AD.vs.MCI; bulk RNA-seq,Homo_633,MCI.vs.control; bulk RNA-seq,Homo_714,AD.vs.control; bulk RNA-seq,Homo_714,AD.vs.MCI; scRNA-seq,SRP215507,CD8+ T cell_1-AD.vs.control; scRNA-seq,SRP215507,CD8+ T cell_4-AD.vs.control; scRNA-seq,SRP215507,CD8+ T cell_3-AD.vs.MCI; scRNA-seq,SRP215507,CD8+ T cell_4-AD.vs.MCI; scRNA-seq,SRP215507,CD8+ T cell_4-MCI.vs.control; scRNA-seq,SRP215507,CD8+ T cell_3-MCI.vs.control; scRNA-seq,SRP330776,CD8+ T cell_1-AD.vs.control; scRNA-seq,SRP330776,γδ T cell-AD.vs.control; scRNA-seq,SRP330776,CD8+ T cell_2-AD.vs.control; scRNA-seq,SRP330776,Naive CD8+ T cell_1-AD.vs.control; scRNA- [illegible]	20
ACVR2B	bulk RNA-seq,Homo_723,AD.vs.control; bulk RNA-seq,Homo_723,AD.vs.MCI; bulk RNA-seq,Homo_723,MCI.vs.control; bulk RNA-seq,Homo_633,AD.vs.control; bulk RNA-seq,Homo_633,AD.vs.MCI; bulk RNA-seq,Homo_714,AD.vs.control; bulk RNA-seq,Homo_714,AD.vs.MCI; scRNA-seq,SRP215507,CD8+ T cell_3-AD.vs.control; scRNA-seq,SRP215507,CD8+ T cell_4-AD.vs.control; scRNA-seq,SRP215507,CD8+ T cell_5-AD.vs.control; scRNA-seq,SRP309935,Monocyte_1-AD.vs.control; scRNA-seq,SRP330776,CD8+ T cell_1-AD.vs.control; scRNA-seq,SRP330776,γδ T [illegible]	14
ACVR2B-AS1	scRNA-seq,SRP330776,CD8+ T cell_1-AD.vs.control	1
ACVRL1	bulk RNA-seq,Homo_723,AD.vs.control; bulk RNA-seq,Homo_723,AD.vs.MCI; bulk RNA-seq,Homo_723,MCI.vs.control; bulk RNA-seq,Homo_633,AD.vs.control; bulk RNA-seq,Homo_633,AD.vs.MCI; bulk RNA-seq,Homo_633,MCI.vs.control; bulk RNA-seq,Homo_714,MCI.vs.control	7
ACY1	bulk RNA-seq,Homo_723,AD.vs.control; bulk RNA-seq,Homo_723,AD.vs.MCI; bulk RNA-seq,Homo_723,MCI.vs.control; bulk RNA-seq,Homo_633,AD.vs.control; bulk RNA-seq,Homo_633,AD.vs.MCI; bulk RNA-seq,Homo_633,MCI.vs.control; bulk RNA-seq,Homo_714,AD.vs.control; bulk RNA-seq,Homo_714,AD.vs.MCI; bulk RNA-seq,Homo_714,MCI.vs.control; scRNA-seq,SRP215507,CD8+ T cell_3-AD.vs.MCI; scRNA-seq,SRP215507,CD8+ T cell_4-AD.vs.MCI; scRNA-seq,SRP215507,CD8+ T cell_4-MCI.vs.control; scRNA-seq,SRP330776,Memory T cell_2-AD.vs.control; scRNA-seq,SRP330776,Naive CD8+ T cell_1-AD.vs.control; scRNA-seq,SRP330776,Memory T cell_1-AD.vs.control; scRNA- [illegible]	16
ACY3	bulk RNA-seq,Homo_723,AD.vs.control; bulk RNA-seq,Homo_723,MCI.vs.control; bulk RNA-seq,Homo_633,AD.vs.control; bulk RNA-seq,Homo_633,MCI.vs.control; bulk RNA-seq,Homo_714,AD.vs.control; bulk RNA-seq,Homo_714,AD.vs.MCI; bulk RNA-seq,Homo_714,MCI.vs.control	7
ACYP1	bulk RNA-seq,Homo_723,AD.vs.control; bulk RNA-seq,Homo_723,MCI.vs.control; bulk RNA-seq,Homo_633,AD.vs.control; bulk RNA-seq,Homo_633,AD.vs.MCI; bulk RNA-seq,Homo_633,MCI.vs.control; bulk RNA-seq,Homo_714,AD.vs.control; bulk RNA-seq,Homo_714,MCI.vs.control; scRNA-seq,SRP215507,CD8+ T cell_3-AD.vs.MCI; scRNA-seq,SRP215507,CD8+ T cell_4-AD.vs.MCI; scRNA-seq,SRP215507,CD8+ T cell_5-AD.vs.MCI; scRNA-seq,SRP215507,CD8+ T cell_7-MCI.vs.control; scRNA-seq,SRP330776,CD8+ T cell_1-AD.vs.control; scRNA-seq,SRP330776,γδ T cell-AD.vs.control; scRNA-seq,SRP330776,Memory T cell_2-AD.vs.control; scRNA-seq,SRP330776,Naive CD8+ T cell_1-AD.vs.control; scRNA-seq,SRP330776,Memory T cell_1-AD.vs.control; scRNA-seq,SRP330776,T cell-AD.vs.control	17
ACYP2	bulk RNA-seq,Homo_633,AD.vs.control; bulk RNA-seq,Homo_633,MCI.vs.control; scRNA-seq,SRP215507,CD8+ T cell_5-AD.vs.MCI; scRNA-seq,SRP215507,CD8+ T cell_5-MCI.vs.control; scRNA-seq,SRP330776,Memory T cell_2-AD.vs.control; scRNA-seq,SRP330776,Naive CD8+ T cell_1-AD.vs.control; scRNA-seq,SRP330776,Natural killer cell_1-AD.vs.control; scRNA-seq,SRP330776,Memory T cell_1-AD.vs.control; scRNA-seq,SRP330776,CD8+ T cell_3-AD.vs.control; scRNA-seq,SRP330776,B cell_4-AD.vs.control	10
Acyp2	bulk RNA-seq,SRP325058,AD.vs.control	1
ADA	bulk RNA-seq,Homo_723,AD.vs.control; bulk RNA-seq,Homo_723,AD.vs.MCI; bulk RNA-seq,Homo_633,AD.vs.control; bulk RNA-seq,Homo_633,AD.vs.MCI; scRNA-seq,SRP215507,CD8+ T cell_5-AD.vs.MCI; scRNA-seq,SRP215507,CD8+ T cell_5-MCI.vs.control; scRNA-seq,SRP215507,CD8+ T cell_6-MCI.vs.control; scRNA-seq,SRP330776,CD8+ T cell_1-AD.vs.control; scRNA-seq,SRP330776,γδ T cell-AD.vs.control; scRNA-seq,SRP330776,Memory T cell_2-AD.vs.control; scRNA-seq,SRP330776,CD8+ T cell_2-AD.vs.control; scRNA-seq,SRP330776,B cell_1-AD.vs.control; scRNA-seq,SRP330776,Naive CD8+ T cell_1-AD.vs.control; scRNA-seq,SRP330776,Natural killer cell_1-AD.vs.control; scRNA-seq,SRP330776,Memory T cell_1-AD.vs.control; scRNA-seq,SRP330776,Natural killer T cell-AD.vs.control; scRNA-seq,SRP330776,Naive CD8+ T [illegible]	19

ADA2	bulk RNA-seq,Homo_633,MCI.vs.control; scRNA-seq,SRP330776,B cell_2-AD.vs.control; scRNA-seq,SRP330776,B cell_3-AD.vs.control; scRNA-seq,SRP330776,B cell_4-AD.vs.control	4
ADAL	bulk RNA-seq,Homo_723,AD.vs.control; bulk RNA-seq,Homo_723,AD.vs.MCI; bulk RNA-seq,Homo_633,AD.vs.control; bulk RNA-seq,Homo_633,AD.vs.MCI; bulk RNA-seq,Homo_714,AD.vs.control; bulk RNA-seq,Homo_714,AD.vs.MCI; scRNA-seq,SRP215507,CD8+ T cell_2-AD.vs.control; scRNA-seq,SRP309935,Natural killer cell_2-AD.vs.control; scRNA-seq,SRP330776,CD8+ T cell_1-AD.vs.control; scRNA-seq,SRP330776,Memory T cell_2-AD.vs.control; scRNA-seq,SRP330776,B cell_1-AD.vs.control; scRNA-seq,SRP330776,Naive CD8+ T cell_1-AD.vs.control; scRNA-seq,SRP330776,Memory T cell_1-AD.vs.control; scRNA-seq,SRP330776,Naive CD8+ T cell_3-AD.vs.control; scRNA-	17
ADAM10	scRNA-seq,SRP215507,CD8+ T cell_3-MCI.vs.control; scRNA-seq,SRP330776,Naive CD8+ T cell_3-AD.vs.control; scRNA-seq,SRP330776,Megakaryocyte_1-AD.vs.control	3
ADAM11	bulk RNA-seq,Homo_723,AD.vs.control; bulk RNA-seq,Homo_723,AD.vs.MCI; bulk RNA-seq,Homo_723,MCI.vs.control; bulk RNA-seq,Homo_633,AD.vs.control; bulk RNA-seq,Homo_633,AD.vs.MCI	5
ADAM12	bulk RNA-seq,Homo_723,AD.vs.control; bulk RNA-seq,Homo_723,AD.vs.MCI; bulk RNA-seq,Homo_633,AD.vs.control; bulk RNA-seq,Homo_633,AD.vs.MCI; bulk RNA-seq,Homo_714,AD.vs.control; bulk RNA-seq,Homo_714,AD.vs.MCI; scRNA-seq,SRP215507,CD8+ T cell_4-AD.vs.control; scRNA-seq,SRP215507,CD8+ T cell_5-AD.vs.control; scRNA-seq,SRP215507,CD8+ T cell_1-AD.vs.MCI; scRNA-seq,SRP215507,CD8+ T cell_1-MCI.vs.control; scRNA-seq,SRP309935,Natural killer cell_2-AD.vs.control; scRNA-seq,SRP309935,Megakaryocyte_1-AD.vs.control; scRNA-seq,SRP309935,Natural killer cell_1-AD.vs.control; scRNA-seq,SRP330776,γδ T cell-AD.vs.control; scRNA-seq,SRP330776,Memory T cell_2-	16
ADAM15	scRNA-seq,SRP215507,CD8+ T cell_5-AD.vs.control; scRNA-seq,SRP215507,CD8+ T cell_3-AD.vs.MCI; scRNA-seq,SRP330776,CD8+ T cell_1-AD.vs.control; scRNA-seq,SRP330776,Memory T cell_2-AD.vs.control; scRNA-seq,SRP330776,Naive CD8+ T cell_1-AD.vs.control; scRNA-seq,SRP330776,Natural killer cell_1-AD.vs.control; scRNA-seq,SRP330776,Memory T cell_1-AD.vs.control; scRNA-seq,SRP330776,Natural killer T cell-AD.vs.control; scRNA-seq,SRP330776,B cell_2-AD.vs.control; scRNA-seq,SRP330776,T cell-AD.vs.control; scRNA-seq,SRP330776,CD8+ T cell_3-	11
ADAM17	bulk RNA-seq,Homo_714,AD.vs.control; bulk RNA-seq,Homo_714,AD.vs.MCI; scRNA-seq,SRP215507,CD8+ T cell_4-AD.vs.control; scRNA-seq,SRP215507,CD8+ T cell_3-MCI.vs.control; scRNA-seq,SRP330776,T cell-AD.vs.control; scRNA-seq,SRP330776,CD8+ T cell_3-AD.vs.control; scRNA-seq,SRP330776,Monocyte_1-AD.vs.control	7
ADAM19	scRNA-seq,SRP215507,CD8+ T cell_2-AD.vs.control; scRNA-seq,SRP215507,CD8+ T cell_3-AD.vs.control; scRNA-seq,SRP215507,CD8+ T cell_4-MCI.vs.control; scRNA-seq,SRP215507,CD8+ T cell_2-MCI.vs.control; scRNA-seq,SRP215507,CD8+ T cell_3-MCI.vs.control; scRNA-seq,SRP309935,Monocyte_1-AD.vs.control; scRNA-seq,SRP330776,γδ T cell-AD.vs.control; scRNA-seq,SRP330776,Naive CD8+ T cell_2-AD.vs.control;	9
ADAM20	bulk RNA-seq,Homo_723,AD.vs.MCI; bulk RNA-seq,Homo_723,MCI.vs.control; bulk RNA-seq,Homo_633,AD.vs.control; bulk RNA-seq,Homo_633,AD.vs.MCI; bulk RNA-seq,Homo_633,MCI.vs.control; bulk RNA-seq,Homo_714,AD.vs.control; bulk RNA-seq,Homo_714,AD.vs.MCI;	8
ADAM21	bulk RNA-seq,Homo_723,AD.vs.control; bulk RNA-seq,Homo_723,AD.vs.MCI; bulk RNA-seq,Homo_633,AD.vs.control; bulk RNA-	4
ADAM22	bulk RNA-seq,Homo_723,AD.vs.control; bulk RNA-seq,Homo_723,AD.vs.MCI; bulk RNA-seq,Homo_723,MCI.vs.control; bulk RNA-seq,Homo_633,AD.vs.MCI; bulk RNA-seq,Homo_714,AD.vs.control; bulk RNA-seq,Homo_714,AD.vs.MCI; scRNA-seq,SRP215507,CD8+ T cell_2-AD.vs.control; scRNA-seq,SRP215507,CD8+ T cell_4-AD.vs.control; scRNA-seq,SRP215507,CD8+ T cell_6-AD.vs.control; scRNA-seq,SRP215507,CD8+ T cell_3-AD.vs.MCI; scRNA-seq,SRP215507,CD8+ T cell_3-MCI.vs.control; scRNA-seq,SRP215507,CD8+ T cell_6-MCI.vs.control; scRNA-seq,SRP330776,CD8+ T cell_1-AD.vs.control; scRNA-seq,SRP330776,Memory T cell_2-AD.vs.control; scRNA-seq,SRP330776,CD8+ T cell_2-AD.vs.control; scRNA-seq,SRP330776,Natural killer cell_1-AD.vs.control; scRNA-seq,SRP330776,Memory T cell_1-	17
ADAM23	bulk RNA-seq,Homo_723,AD.vs.control; bulk RNA-seq,Homo_723,AD.vs.MCI; bulk RNA-seq,Homo_633,AD.vs.control; bulk RNA-seq,Homo_633,AD.vs.MCI; bulk RNA-seq,Homo_714,AD.vs.control; bulk RNA-seq,Homo_714,AD.vs.MCI; scRNA-seq,SRP215507,CD8+ T cell_2-AD.vs.control; scRNA-seq,SRP215507,CD8+ T cell_3-AD.vs.control; scRNA-seq,SRP215507,CD8+ T cell_2-AD.vs.MCI; scRNA-	10
ADAM28	bulk RNA-seq,Homo_723,AD.vs.control; bulk RNA-seq,Homo_723,AD.vs.MCI; bulk RNA-seq,Homo_633,AD.vs.control; bulk RNA-seq,Homo_633,AD.vs.MCI; bulk RNA-seq,Homo_714,AD.vs.control; bulk RNA-seq,Homo_714,AD.vs.MCI; scRNA-seq,SRP215507,CD8+ T cell_7-	7
ADAM29	bulk RNA-seq,Homo_723,AD.vs.MCI; bulk RNA-seq,Homo_723,MCI.vs.control; bulk RNA-seq,Homo_633,AD.vs.MCI; bulk RNA-seq,Homo_633,MCI.vs.control; scRNA-seq,SRP215507,CD8+ T cell_2-AD.vs.control	5
ADAM30	bulk RNA-seq,Homo_723,AD.vs.control; bulk RNA-seq,Homo_723,MCI.vs.control; bulk RNA-seq,Homo_633,AD.vs.control; bulk RNA-seq,Homo_633,MCI.vs.control; bulk RNA-seq,Homo_714,MCI.vs.control	5

ADAM32	bulk RNA-seq,Homo_723,AD.vs.control; bulk RNA-seq,Homo_723,AD.vs.MCI; bulk RNA-seq,Homo_723,MCI.vs.control; bulk RNA-seq,Homo_633,AD.vs.control; bulk RNA-seq,Homo_633,AD.vs.MCI; bulk RNA-seq,Homo_633,MCI.vs.control; bulk RNA-seq,ROSMAP,AD.vs.control;	8
ADAM33	bulk RNA-seq,Homo_723,AD.vs.control; bulk RNA-seq,Homo_723,AD.vs.MCI; bulk RNA-seq,Homo_633,AD.vs.control; bulk RNA-	4
ADAM7	bulk RNA-seq,Homo_723,AD.vs.control; bulk RNA-seq,Homo_633,AD.vs.control	2
ADAM8	bulk RNA-seq,SRP223445,AD.vs.control; scRNA-seq,SRP215507,CD8+ T cell_3-AD.vs.control; scRNA-seq,SRP215507,CD8+ T cell_3-MCI.vs.control; scRNA-seq,SRP330776,CD8+ T cell_1-AD.vs.control; scRNA-seq,SRP330776,Memory T cell_2-AD.vs.control; scRNA-seq,SRP330776,B cell_1-AD.vs.control; scRNA-seq,SRP330776,Naive CD8+ T cell_1-AD.vs.control; scRNA-seq,SRP330776,Natural killer cell_1-AD.vs.control; scRNA-seq,SRP330776,Natural killer T cell-AD.vs.control; scRNA-seq,SRP330776,Naive CD8+ T cell_3-AD.vs.control; scRNA-seq,SRP330776,B cell_2-	12
Adam8	bulk RNA-seq,SRP325058,AD.vs.control	1
ADAM9	bulk RNA-seq,Homo_723,AD.vs.control; bulk RNA-seq,Homo_723,AD.vs.MCI; bulk RNA-seq,Homo_723,MCI.vs.control; bulk RNA-seq,Homo_633,AD.vs.control; bulk RNA-seq,Homo_633,AD.vs.MCI; bulk RNA-seq,Homo_633,MCI.vs.control; bulk RNA-seq,Homo_714,AD.vs.control; bulk RNA-seq,Homo_714,AD.vs.MCI; scRNA-seq,SRP215507,CD8+ T cell_2-AD.vs.MCI; scRNA-seq,SRP215507,CD8+ T cell_2-MCI.vs.control;	11
ADAMDEC1	bulk RNA-seq,Homo_723,AD.vs.control; bulk RNA-seq,Homo_633,AD.vs.control; bulk RNA-seq,Homo_714,AD.vs.control; bulk RNA-seq,Homo_714,AD.vs.MCI; bulk RNA-seq,ROSMAP,AD.vs.control	5
ADAMTS1	bulk RNA-seq,Homo_723,AD.vs.control; bulk RNA-seq,Homo_723,AD.vs.MCI; bulk RNA-seq,Homo_723,MCI.vs.control; bulk RNA-seq,Homo_633,AD.vs.control; bulk RNA-seq,Homo_633,AD.vs.MCI; bulk RNA-seq,Homo_633,MCI.vs.control; bulk RNA-seq,Homo_714,AD.vs.control; bulk RNA-seq,Homo_714,AD.vs.MCI; bulk RNA-seq,ROSMAP,AD.vs.control; bulk RNA-seq,ROSMAP,MCI.vs.control; scRNA-seq,SRP215507,CD8+ T cell_5-AD.vs.MCI; scRNA-seq,SRP215507,CD8+ T cell_5-MCI.vs.control; scRNA-seq,SRP309935,Natural killer cell_2-AD.vs.control	13
ADAMTS10	bulk RNA-seq,Homo_723,AD.vs.control; bulk RNA-seq,Homo_633,AD.vs.control; bulk RNA-seq,Homo_633,MCI.vs.control; bulk RNA-seq,Homo_714,MCI.vs.control; scRNA-seq,SRP215507,CD8+ T cell_4-AD.vs.control; scRNA-seq,SRP215507,CD8+ T cell_1-MCI.vs.control; scRNA-seq,SRP309935,Naive CD8+ T cell_2-AD.vs.control; scRNA-seq,SRP309935,CD8+ T cell_1-AD.vs.control; scRNA-seq,SRP330776,CD8+ T cell_1-AD.vs.control; scRNA-seq,SRP330776,Memory T cell_2-AD.vs.control; scRNA-seq,SRP330776,Naive CD8+ T cell_1-AD.vs.control; scRNA-seq,SRP330776,Natural killer cell_1-AD.vs.control; scRNA-seq,SRP330776,Naive CD8+ T cell_3-AD.vs.control	13
ADAMTS12	bulk RNA-seq,Homo_723,AD.vs.control; bulk RNA-seq,Homo_723,AD.vs.MCI; bulk RNA-seq,Homo_723,MCI.vs.control; bulk RNA-seq,Homo_633,AD.vs.MCI; bulk RNA-seq,Homo_714,MCI.vs.control; bulk RNA-seq,ROSMAP,AD.vs.control	6
ADAMTS13	bulk RNA-seq,Homo_723,AD.vs.control; bulk RNA-seq,Homo_723,AD.vs.MCI; bulk RNA-seq,Homo_714,AD.vs.control; bulk RNA-seq,Homo_714,AD.vs.MCI; scRNA-seq,SRP330776,Naive CD8+ T cell_1-AD.vs.control; scRNA-seq,SRP330776,Memory T cell_1-AD.vs.control	6
ADAMTS14	bulk RNA-seq,Homo_723,AD.vs.control; bulk RNA-seq,Homo_723,AD.vs.MCI; bulk RNA-seq,Homo_633,AD.vs.control; bulk RNA-	4
ADAMTS15	bulk RNA-seq,Homo_723,AD.vs.control; bulk RNA-seq,Homo_723,AD.vs.MCI; bulk RNA-seq,Homo_714,MCI.vs.control	3
ADAMTS16	bulk RNA-seq,Homo_723,AD.vs.control; bulk RNA-seq,Homo_723,AD.vs.MCI	2
ADAMTS17	bulk RNA-seq,Homo_723,AD.vs.control; bulk RNA-seq,Homo_723,AD.vs.MCI; bulk RNA-seq,Homo_723,MCI.vs.control; bulk RNA-seq,Homo_633,AD.vs.control; bulk RNA-seq,Homo_633,MCI.vs.control; bulk RNA-seq,Homo_714,AD.vs.control; bulk RNA-seq,Homo_714,AD.vs.MCI; bulk RNA-seq,Homo_714,MCI.vs.control; scRNA-seq,SRP215507,CD8+ T cell_5-AD.vs.MCI; scRNA-seq,SRP330776,Naive CD8+ T cell_1-	10
ADAMTS18	bulk RNA-seq,Homo_723,AD.vs.control; bulk RNA-seq,Homo_723,AD.vs.MCI; bulk RNA-seq,Homo_633,AD.vs.control; bulk RNA-seq,Homo_714,MCI.vs.control; bulk RNA-seq,ROSMAP,AD.vs.control	5
ADAMTS19	bulk RNA-seq,Homo_723,AD.vs.MCI	1
ADAMTS2	bulk RNA-seq,Homo_723,AD.vs.control; bulk RNA-seq,Homo_633,AD.vs.control; bulk RNA-seq,Homo_714,MCI.vs.control; bulk RNA-	4
ADAMTS20	bulk RNA-seq,Homo_723,AD.vs.MCI; bulk RNA-seq,Homo_633,AD.vs.control	2
ADAMTS3	bulk RNA-seq,Homo_723,AD.vs.control; bulk RNA-seq,Homo_723,AD.vs.MCI; bulk RNA-seq,Homo_723,MCI.vs.control; bulk RNA-seq,Homo_633,AD.vs.control; bulk RNA-seq,Homo_633,AD.vs.MCI; bulk RNA-seq,Homo_633,MCI.vs.control	6

ADAMTS4	bulk RNA-seq,Homo_714,AD.vs.control; bulk RNA-seq,Homo_714,AD.vs.MCI; scRNA-seq,SRP215507,CD8+ T cell_4-AD.vs.control; scRNA-seq,SRP215507,CD8+ T cell_3-AD.vs.MCI; scRNA-seq,SRP215507,CD8+ T cell_1-AD.vs.MCI; scRNA-seq,SRP215507,CD8+ T cell_4-MCI.vs.control; scRNA-seq,SRP215507,CD8+ T cell_3-MCI.vs.control; scRNA-seq,SRP330776,B cell_1-AD.vs.control; scRNA-seq,SRP330776,Naive CD8+ T cell_1-AD.vs.control; scRNA-seq,SRP330776,Natural killer cell_1-AD.vs.control; scRNA-seq,SRP330776,Regulatory T cell-AD.vs.control	11
ADAMTS5	bulk RNA-seq,Homo_723,AD.vs.control; bulk RNA-seq,Homo_723,AD.vs.MCI; bulk RNA-seq,Homo_633,AD.vs.control; bulk RNA-seq,Homo_633,AD.vs.MCI; bulk RNA-seq,Homo_714,AD.vs.control; bulk RNA-seq,Homo_714,AD.vs.MCI	6
ADAMTS6	bulk RNA-seq,Homo_723,AD.vs.control; bulk RNA-seq,Homo_723,AD.vs.MCI; bulk RNA-seq,Homo_723,MCI.vs.control; bulk RNA-seq,Homo_633,AD.vs.control; bulk RNA-seq,Homo_633,AD.vs.MCI; bulk RNA-seq,Homo_633,MCI.vs.control; bulk RNA-seq,Homo_714,AD.vs.control; bulk RNA-seq,Homo_714,AD.vs.MCI; scRNA-seq,SRP215507,CD8+ T cell_4-AD.vs.control; scRNA-seq,SRP215507,CD8+ T cell_1-MCI.vs.control; scRNA-seq,SRP215507,CD8+ T cell_4-MCI.vs.control; scRNA-seq,SRP330776,CD8+ T cell_1-AD.vs.control; scRNA-seq,SRP330776,γδ T cell-AD.vs.control; scRNA-seq,SRP330776,CD8+ T cell_2-AD.vs.control; scRNA-seq,SRP330776,Naive CD8+ T cell_1-AD.vs.control; scRNA-seq,SRP330776,Natural killer cell_1-AD.vs.control; scRNA-seq,SRP330776,Memory T cell_1-AD.vs.control; scRNA-seq,SRP330776,Naive CD8+ T	19
ADAMTS7	bulk RNA-seq,Homo_723,AD.vs.control; bulk RNA-seq,Homo_723,AD.vs.MCI; bulk RNA-seq,Homo_714,MCI.vs.control	3
ADAMTS8	bulk RNA-seq,Homo_723,AD.vs.control; bulk RNA-seq,Homo_723,AD.vs.MCI; bulk RNA-seq,ROSMAP,AD.vs.MCI; bulk RNA-	4
ADAMTS9	bulk RNA-seq,Homo_723,AD.vs.control; bulk RNA-seq,Homo_723,AD.vs.MCI; bulk RNA-seq,Homo_723,MCI.vs.control; bulk RNA-seq,Homo_633,AD.vs.MCI; bulk RNA-seq,Homo_633,MCI.vs.control	5
ADAMTSL1	bulk RNA-seq,Homo_723,AD.vs.control; bulk RNA-seq,Homo_723,AD.vs.MCI; bulk RNA-seq,Homo_633,AD.vs.control; bulk RNA-seq,Homo_633,AD.vs.MCI; bulk RNA-seq,Homo_714,MCI.vs.control	5
ADAMTSL2	bulk RNA-seq,Homo_723,AD.vs.control; bulk RNA-seq,Homo_723,AD.vs.MCI; bulk RNA-seq,Homo_633,AD.vs.control; bulk RNA-seq,Homo_633,AD.vs.MCI; bulk RNA-seq,Homo_714,MCI.vs.control	5
ADAMTSL3	bulk RNA-seq,Homo_723,AD.vs.control; bulk RNA-seq,Homo_723,AD.vs.MCI; bulk RNA-seq,Homo_633,AD.vs.control; bulk RNA-	4
ADAMTSL4	scRNA-seq,SRP330776,Naive CD8+ T cell_1-AD.vs.control; scRNA-seq,SRP330776,Monocyte_2-AD.vs.control	2
ADAMTSL4-AS1	scRNA-seq,SRP215507,CD8+ T cell_1-AD.vs.control; scRNA-seq,SRP215507,CD8+ T cell_4-AD.vs.control; scRNA-seq,SRP215507,CD8+ T cell_6-AD.vs.control; scRNA-seq,SRP215507,CD8+ T cell_1-MCI.vs.control; scRNA-seq,SRP330776,CD8+ T cell_1-AD.vs.control; scRNA-	6
ADAMTSL5	bulk RNA-seq,ROSMAP,AD.vs.control; bulk RNA-seq,ROSMAP,MCI.vs.control; scRNA-seq,SRP215507,CD8+ T cell_2-AD.vs.MCI; scRNA-seq,SRP215507,CD8+ T cell_3-AD.vs.MCI; scRNA-seq,SRP215507,CD8+ T cell_1-AD.vs.MCI; scRNA-seq,SRP215507,CD8+ T cell_1-MCI.vs.control; scRNA-seq,SRP215507,CD8+ T cell_4-MCI.vs.control; scRNA-seq,SRP215507,CD8+ T cell_2-MCI.vs.control; scRNA-seq,SRP215507,CD8+ T cell_3-MCI.vs.control; scRNA-seq,SRP330776,CD8+ T cell_1-AD.vs.control; scRNA-seq,SRP330776,γδ T cell-AD.vs.control; scRNA-seq,SRP330776,Memory T cell_2-AD.vs.control; scRNA-seq,SRP330776,CD8+ T cell_2-AD.vs.control; scRNA-seq,SRP330776,Naive CD8+ T cell_1-AD.vs.control; scRNA-	15
ADAP1	bulk RNA-seq,Homo_633,AD.vs.control; bulk RNA-seq,Homo_633,AD.vs.MCI; scRNA-seq,SRP215507,CD8+ T cell_3-AD.vs.control; scRNA-seq,SRP215507,CD8+ T cell_4-AD.vs.control; scRNA-seq,SRP215507,CD8+ T cell_5-AD.vs.control; scRNA-seq,SRP215507,CD8+ T cell_2-AD.vs.MCI; scRNA-seq,SRP215507,CD8+ T cell_5-AD.vs.MCI; scRNA-seq,SRP215507,CD8+ T cell_5-MCI.vs.control; scRNA-seq,SRP215507,CD8+ T cell_4-MCI.vs.control; scRNA-seq,SRP215507,CD8+ T cell_3-MCI.vs.control; scRNA-seq,SRP309935,Naive CD8+ T cell_1-AD.vs.control; scRNA-seq,SRP330776,γδ T cell-AD.vs.control; scRNA-seq,SRP330776,Natural killer cell_1-AD.vs.control	13
ADAP2	bulk RNA-seq,Homo_723,AD.vs.control; bulk RNA-seq,Homo_633,AD.vs.control; scRNA-seq,SRP215507,CD8+ T cell_2-AD.vs.control; scRNA-seq,SRP215507,CD8+ T cell_7-AD.vs.control; scRNA-seq,SRP215507,CD8+ T cell_5-MCI.vs.control; scRNA-seq,SRP330776,Naive CD8+ T cell_1-AD.vs.control; scRNA-seq,SRP330776,B cell_2-AD.vs.control	7
ADAR	scRNA-seq,SRP215507,CD8+ T cell_7-MCI.vs.control; scRNA-seq,SRP309935,Erythroid cell-AD.vs.control; scRNA-seq,SRP330776,CD8+ T cell_1-AD.vs.control; scRNA-seq,SRP330776,γδ T cell-AD.vs.control; scRNA-seq,SRP330776,Memory T cell_2-AD.vs.control; scRNA-seq,SRP330776,CD8+ T cell_2-AD.vs.control; scRNA-seq,SRP330776,B cell_1-AD.vs.control; scRNA-seq,SRP330776,Naive CD8+ T cell_1-AD.vs.control; scRNA-seq,SRP330776,Natural killer cell_1-AD.vs.control; scRNA-seq,SRP330776,Memory T cell_1-AD.vs.control; scRNA-seq,SRP330776,Naive CD8+ T cell_3-AD.vs.control; scRNA-seq,SRP330776,B cell_2-AD.vs.control; scRNA-seq,SRP330776,T cell-AD.vs.control; scRNA-seq,SRP330776,Naive CD8+	15

ADARB1	bulk RNA-seq,Homo_723,AD.vs.control; bulk RNA-seq,Homo_633,AD.vs.control; bulk RNA-seq,Homo_633,MCI.vs.control; bulk RNA-seq,ROSMAP,AD.vs.control; bulk RNA-seq,ROSMAP,MCI.vs.control; scRNA-seq,SRP215507,CD8+ T cell_3-MCI.vs.control; scRNA-seq,SRP309935,Naive CD8+ T cell_1-AD.vs.control; scRNA-seq,SRP309935,Natural killer cell_1-AD.vs.control; scRNA-seq,SRP330776,CD8+ T cell_1-AD.vs.control; scRNA-seq,SRP330776,γδ T cell-AD.vs.control; scRNA-seq,SRP330776,Naive CD8+ T cell_1-AD.vs.control; scRNA-seq,SRP330776,Memory T cell_1-AD.vs.control; scRNA-seq,SRP330776,Natural killer T cell-AD.vs.control; scRNA-seq,SRP330776,B cell_2-	15
ADARB2	bulk RNA-seq,Homo_723,AD.vs.control; bulk RNA-seq,Homo_723,AD.vs.MCI; bulk RNA-seq,Homo_633,AD.vs.control; bulk RNA-seq,Homo_633,AD.vs.MCI; bulk RNA-seq,Homo_714,AD.vs.control; bulk RNA-seq,Homo_714,AD.vs.MCI; scRNA-seq,SRP215507,CD8+ T cell_2-AD.vs.control; scRNA-seq,SRP215507,CD8+ T cell_3-AD.vs.control; scRNA-seq,SRP215507,CD8+ T cell_4-AD.vs.control; scRNA-seq,SRP215507,CD8+ T cell_5-AD.vs.control; scRNA-seq,SRP215507,CD8+ T cell_1-AD.vs.MCI; scRNA-seq,SRP215507,CD8+ T cell_1-MCI.vs.control; scRNA-seq,SRP330776,γδ T cell-AD.vs.control; scRNA-seq,SRP330776,CD8+ T cell_2-AD.vs.control; scRNA-seq,SRP330776,Natural killer cell_1-AD.vs.control; scRNA-seq,SRP330776,Memory T cell_1-AD.vs.control; scRNA-seq,SRP330776,T cell-AD.vs.control; scRNA-	18
ADAT1	scRNA-seq,SRP215507,CD8+ T cell_3-AD.vs.MCI; scRNA-seq,SRP215507,CD8+ T cell_3-MCI.vs.control; scRNA-seq,SRP215507,CD8+ T cell_8-	3
ADAT2	bulk RNA-seq,Homo_723,MCI.vs.control; bulk RNA-seq,Homo_633,AD.vs.control; bulk RNA-seq,Homo_633,MCI.vs.control; bulk RNA-seq,Homo_714,AD.vs.control; bulk RNA-seq,Homo_714,AD.vs.MCI; bulk RNA-seq,Homo_714,MCI.vs.control; scRNA-seq,SRP215507,CD8+ T cell_3-AD.vs.MCI; scRNA-seq,SRP215507,CD8+ T cell_1-AD.vs.MCI; scRNA-seq,SRP215507,CD8+ T cell_5-AD.vs.MCI; scRNA-seq,SRP215507,CD8+ T cell_5-MCI.vs.control; scRNA-seq,SRP215507,CD8+ T cell_3-MCI.vs.control; scRNA-seq,SRP330776,CD8+ T cell_1-AD.vs.control; scRNA-seq,SRP330776,γδ T cell-AD.vs.control; scRNA-seq,SRP330776,Memory T cell_2-AD.vs.control; scRNA-seq,SRP330776,Naive CD8+ T cell_1-	16
ADAT3	scRNA-seq,SRP215507,CD8+ T cell_3-AD.vs.control; scRNA-seq,SRP215507,CD8+ T cell_1-MCI.vs.control; scRNA-seq,SRP309935,Natural killer cell_2-AD.vs.control; scRNA-seq,SRP309935,Monocyte_1-AD.vs.control; scRNA-seq,SRP309935,Natural killer cell_1-AD.vs.control; scRNA-seq,SRP309935,CD8+ T cell_1-AD.vs.control; scRNA-seq,SRP309935,B cell_1-AD.vs.control; scRNA-seq,SRP330776,CD8+ T cell_1-AD.vs.control; scRNA-seq,SRP330776,γδ T cell-AD.vs.control; scRNA-seq,SRP330776,Memory T cell_2-AD.vs.control; scRNA-seq,SRP330776,CD8+ T cell_2-AD.vs.control; scRNA-seq,SRP330776,B cell_1-AD.vs.control; scRNA-seq,SRP330776,Naive CD8+ T cell_1-AD.vs.control; scRNA-seq,SRP330776,Memory T cell_1-AD.vs.control; scRNA-seq,SRP330776,B cell_2-AD.vs.control; scRNA-seq,SRP330776,T cell-AD.vs.control; scRNA-	17
ADCK1	bulk RNA-seq,Homo_723,AD.vs.control; bulk RNA-seq,Homo_723,MCI.vs.control; bulk RNA-seq,Homo_633,AD.vs.MCI; bulk RNA-seq,Homo_633,MCI.vs.control; bulk RNA-seq,Homo_714,AD.vs.control; bulk RNA-seq,Homo_714,AD.vs.MCI; bulk RNA-seq,Homo_714,MCI.vs.control; scRNA-seq,SRP215507,CD8+ T cell_4-AD.vs.MCI; scRNA-seq,SRP215507,CD8+ T cell_4-MCI.vs.control; scRNA-seq,SRP330776,Memory T cell_2-AD.vs.control; scRNA-seq,SRP330776,B cell_1-AD.vs.control; scRNA-seq,SRP330776,Natural killer cell_1-AD.vs.control; scRNA-seq,SRP330776,Natural killer T cell-AD.vs.control; scRNA-seq,SRP330776,Naive CD8+ T cell_3-AD.vs.control; scRNA-	15
ADCK2	bulk RNA-seq,Homo_723,AD.vs.control; bulk RNA-seq,Homo_723,AD.vs.MCI; bulk RNA-seq,Homo_633,AD.vs.control; bulk RNA-seq,Homo_633,AD.vs.MCI; bulk RNA-seq,Homo_714,AD.vs.control; bulk RNA-seq,Homo_714,AD.vs.MCI; scRNA-seq,SRP215507,CD8+ T cell_3-AD.vs.MCI; scRNA-seq,SRP215507,CD8+ T cell_4-MCI.vs.control; scRNA-seq,SRP215507,CD8+ T cell_3-MCI.vs.control; scRNA-seq,SRP309935,Natural killer cell_2-AD.vs.control; scRNA-seq,SRP309935,Naive CD8+ T cell_1-AD.vs.control; scRNA-seq,SRP309935,Monocyte_2-AD.vs.control; scRNA-seq,SRP309935,CD8+ T cell_1-AD.vs.control; scRNA-seq,SRP330776,CD8+ T cell_1-AD.vs.control; scRNA-seq,SRP330776,γδ T cell-AD.vs.control; scRNA-seq,SRP330776,Memory T cell_2-AD.vs.control; scRNA-seq,SRP330776,CD8+ T cell_2-AD.vs.control; scRNA-seq,SRP330776,B cell_1-AD.vs.control; scRNA-seq,SRP330776,Naive CD8+ T cell_1-AD.vs.control; scRNA-seq,SRP330776,Natural killer cell_1-AD.vs.control; scRNA-seq,SRP330776,Memory T cell_1-AD.vs.control; scRNA-seq,SRP330776,Natural killer T cell-AD.vs.control; scRNA-seq,SRP330776,B cell_2-AD.vs.control; scRNA-seq,SRP330776,T cell-AD.vs.control; scRNA-seq,SRP330776,CD8+ T cell_3-AD.vs.control; scRNA- [illegible]	28
ADCK5	scRNA-seq,SRP215507,CD8+ T cell_5-AD.vs.MCI; scRNA-seq,SRP330776,Memory T cell_2-AD.vs.control; scRNA-seq,SRP330776,CD8+ T cell_2-AD.vs.control; scRNA-seq,SRP330776,B cell_1-AD.vs.control; scRNA-seq,SRP330776,Naive CD8+ T cell_1-AD.vs.control; scRNA-seq,SRP330776,Natural killer cell_1-AD.vs.control; scRNA-seq,SRP330776,Memory T cell_1-AD.vs.control; scRNA-seq,SRP330776,Natural killer T cell-AD.vs.control; scRNA-seq,SRP330776,Naive CD8+ T cell_3-AD.vs.control; scRNA-seq,SRP330776,B cell_2-AD.vs.control; scRNA-seq,SRP330776,T	12
ADCY1	bulk RNA-seq,Homo_723,AD.vs.control; bulk RNA-seq,Homo_723,AD.vs.MCI; bulk RNA-seq,Homo_633,AD.vs.control; bulk RNA-seq,Homo_633,AD.vs.MCI; bulk RNA-seq,Homo_714,AD.vs.MCI; bulk RNA-seq,Homo_714,MCI.vs.control; bulk RNA-seq,ROSMAP,AD.vs.MCI	7

ADCY10	bulk RNA-seq,Homo_633,AD.vs.control; bulk RNA-seq,Homo_633,AD.vs.MCI; bulk RNA-seq,Homo_714,AD.vs.control; bulk RNA-seq,Homo_714,AD.vs.MCI; bulk RNA-seq,ROSMAP,AD.vs.control	5
ADCY2	bulk RNA-seq,Homo_723,AD.vs.control; bulk RNA-seq,Homo_723,AD.vs.MCI; bulk RNA-seq,Homo_714,MCI.vs.control	3
ADCY3	bulk RNA-seq,Homo_723,AD.vs.control; bulk RNA-seq,Homo_723,AD.vs.MCI; bulk RNA-seq,Homo_633,AD.vs.control; bulk RNA-seq,Homo_633,AD.vs.MCI; bulk RNA-seq,Homo_714,AD.vs.control; bulk RNA-seq,Homo_714,AD.vs.MCI; bulk RNA-seq,ROSMAP,AD.vs.MCI; bulk RNA-seq,ROSMAP,MCI.vs.control; scRNA-seq,SRP215507,CD8+ T cell_1-AD.vs.MCI; scRNA-seq,SRP215507,CD8+ T cell_4-AD.vs.MCI; scRNA-seq,SRP215507,CD8+ T cell_1-MCI.vs.control; scRNA-seq,SRP309935,Naive CD8+ T cell_2-AD.vs.control; scRNA-seq,SRP330776,CD8+ T cell_1-AD.vs.control; scRNA-seq,SRP330776,Memory T cell_2-AD.vs.control; scRNA-seq,SRP330776,CD8+ T cell_2-AD.vs.control; scRNA-seq,SRP330776,Memory T cell_1-AD.vs.control; scRNA-seq,SRP330776,CD8+ T cell_3-AD.vs.control; scRNA-seq,SRP330776,B cell_3-AD.vs.control	18
ADCY4	scRNA-seq,SRP215507,CD8+ T cell_3-AD.vs.control; scRNA-seq,SRP330776,CD8+ T cell_1-AD.vs.control; scRNA-seq,SRP330776,B cell_1-	3
ADCY5	bulk RNA-seq,Homo_723,AD.vs.control; bulk RNA-seq,Homo_723,AD.vs.MCI; bulk RNA-seq,Homo_714,AD.vs.control; bulk RNA-	4
Adcy5	bulk RNA-seq,SRP325058,AD.vs.control	1
ADCY6	bulk RNA-seq,ROSMAP,AD.vs.control	1
ADCY7	scRNA-seq,SRP215507,CD8+ T cell_7-AD.vs.control; scRNA-seq,SRP309935,Megakaryocyte_1-AD.vs.control; scRNA-seq,SRP309935,Monocyte_2-AD.vs.control; scRNA-seq,SRP309935,Naive CD8+ T cell_2-AD.vs.control; scRNA-seq,SRP309935,CD8+ T cell_1-AD.vs.control; scRNA-seq,SRP330776,Monocyte_1-AD.vs.control; scRNA-seq,SRP330776,Regulatory T cell-AD.vs.control	7
ADCY8	bulk RNA-seq,Homo_723,AD.vs.control; bulk RNA-seq,Homo_714,MCI.vs.control	2
ADCY9	bulk RNA-seq,Homo_723,AD.vs.control; bulk RNA-seq,Homo_723,MCI.vs.control; bulk RNA-seq,Homo_633,AD.vs.control; bulk RNA-seq,Homo_633,MCI.vs.control; scRNA-seq,SRP215507,CD8+ T cell_1-AD.vs.control; scRNA-seq,SRP215507,CD8+ T cell_4-AD.vs.control; scRNA-seq,SRP215507,CD8+ T cell_1-MCI.vs.control; scRNA-seq,SRP309935,Megakaryocyte_1-AD.vs.control; scRNA-seq,SRP309935,Naive CD8+ T cell_1-AD.vs.control; scRNA-seq,SRP330776,CD8+ T cell_1-AD.vs.control; scRNA-seq,SRP330776,Memory T cell_2-AD.vs.control; scRNA-seq,SRP330776,Naive CD8+ T cell_2-AD.vs.control; scRNA-seq,SRP330776,Natural killer cell_2-AD.vs.control	13
Adcy9	bulk RNA-seq,SRP325058,AD.vs.control	1
ADCYAP1	bulk RNA-seq,Homo_723,AD.vs.control; bulk RNA-seq,Homo_723,AD.vs.MCI; bulk RNA-seq,Homo_633,AD.vs.control	3
ADCYAP1R1	bulk RNA-seq,Homo_723,AD.vs.control; bulk RNA-seq,Homo_723,AD.vs.MCI; bulk RNA-seq,Homo_714,MCI.vs.control	3
ADD1	bulk RNA-seq,Homo_723,AD.vs.control; bulk RNA-seq,Homo_723,AD.vs.MCI; bulk RNA-seq,Homo_633,AD.vs.control; bulk RNA-seq,Homo_633,AD.vs.MCI; scRNA-seq,SRP215507,CD8+ T cell_3-AD.vs.MCI; scRNA-seq,SRP309935,Monocyte_2-AD.vs.control; scRNA-seq,SRP330776,CD8+ T cell_1-AD.vs.control; scRNA-seq,SRP330776,γδ T cell-AD.vs.control; scRNA-seq,SRP330776,Memory T cell_2-AD.vs.control; scRNA-seq,SRP330776,CD8+ T cell_2-AD.vs.control; scRNA-seq,SRP330776,Naive CD8+ T cell_1-AD.vs.control; scRNA-seq,SRP330776,Natural killer cell_1-AD.vs.control; scRNA-seq,SRP330776,Naive CD8+ T cell_3-AD.vs.control; scRNA-seq,SRP330776,B cell_2-AD.vs.control; scRNA-	15
Add1	bulk RNA-seq,SRP325058,AD.vs.control	1
ADD2	bulk RNA-seq,Homo_723,AD.vs.control; bulk RNA-seq,Homo_723,AD.vs.MCI; bulk RNA-seq,Homo_723,MCI.vs.control; bulk RNA-seq,Homo_633,AD.vs.control; bulk RNA-seq,Homo_633,AD.vs.MCI; bulk RNA-seq,Homo_714,AD.vs.control; bulk RNA-seq,ROSMAP,AD.vs.control; bulk RNA-seq,ROSMAP,MCI.vs.control; bulk RNA-seq,SRP223445,AD.vs.control; scRNA-seq,SRP215507,CD8+ T cell_3-AD.vs.control; scRNA-seq,SRP215507,CD8+ T cell_3-MCI.vs.control; scRNA-seq,SRP330776,Natural killer T cell-AD.vs.control	12
Add2	bulk RNA-seq,SRP325058,AD.vs.control	1
ADD3	scRNA-seq,SRP330776,Monocyte_2-AD.vs.control	1
ADD3-AS1	scRNA-seq,SRP215507,CD8+ T cell_3-AD.vs.control; scRNA-seq,SRP215507,CD8+ T cell_4-AD.vs.control; scRNA-seq,SRP215507,CD8+ T cell_1-AD.vs.MCI; scRNA-seq,SRP215507,CD8+ T cell_1-MCI.vs.control; scRNA-seq,SRP215507,CD8+ T cell_2-MCI.vs.control	5
ADGB	bulk RNA-seq,Homo_723,AD.vs.control; bulk RNA-seq,Homo_723,AD.vs.MCI; bulk RNA-seq,Homo_633,AD.vs.control; bulk RNA-	4
ADGRA1	bulk RNA-seq,Homo_723,AD.vs.control; bulk RNA-seq,Homo_723,AD.vs.MCI; bulk RNA-seq,Homo_714,MCI.vs.control	3

ADGRA2	bulk RNA-seq,Homo_723,AD.vs.control; bulk RNA-seq,Homo_633,AD.vs.control; bulk RNA-seq,Homo_633,AD.vs.MCI; bulk RNA-seq,SRP223445,AD.vs.control; scRNA-seq,SRP330776,CD8+ T cell_1-AD.vs.control	5
ADGRA3	bulk RNA-seq,Homo_723,AD.vs.control; bulk RNA-seq,Homo_723,AD.vs.MCI; bulk RNA-seq,Homo_723,MCI.vs.control; bulk RNA-seq,Homo_633,AD.vs.control; bulk RNA-seq,Homo_633,AD.vs.MCI; bulk RNA-seq,Homo_633,MCI.vs.control; bulk RNA-seq,Homo_714,AD.vs.control; bulk RNA-seq,Homo_714,AD.vs.MCI; bulk RNA-seq,Homo_714,MCI.vs.control; scRNA-seq,SRP215507,CD8+ T cell_4-AD.vs.control	10
ADGRB1	bulk RNA-seq,Homo_723,AD.vs.control; bulk RNA-seq,Homo_723,AD.vs.MCI; bulk RNA-seq,Homo_633,AD.vs.control; bulk RNA-seq,Homo_633,AD.vs.MCI; scRNA-seq,SRP215507,CD8+ T cell_2-AD.vs.MCI; scRNA-seq,SRP215507,CD8+ T cell_3-MCI.vs.control	6
ADGRB2	scRNA-seq,SRP215507,CD8+ T cell_1-AD.vs.MCI; scRNA-seq,SRP215507,CD8+ T cell_1-MCI.vs.control; scRNA-seq,SRP215507,CD8+ T cell_5-MCI.vs.control; scRNA-seq,SRP330776,γδ T cell-AD.vs.control; scRNA-seq,SRP330776,CD8+ T cell_2-AD.vs.control; scRNA-seq,SRP330776,B cell_1-AD.vs.control; scRNA-seq,SRP330776,Memory T cell_1-AD.vs.control	7
ADGRB3	bulk RNA-seq,Homo_723,AD.vs.control; bulk RNA-seq,Homo_723,AD.vs.MCI; bulk RNA-seq,Homo_723,MCI.vs.control; bulk RNA-seq,Homo_633,AD.vs.control; bulk RNA-seq,Homo_633,AD.vs.MCI; bulk RNA-seq,ROSMAP,AD.vs.control; bulk RNA-seq,ROSMAP,MCI.vs.control	7
ADGRD1	bulk RNA-seq,Homo_723,AD.vs.control; bulk RNA-seq,Homo_723,MCI.vs.control; bulk RNA-seq,Homo_633,AD.vs.control; bulk RNA-seq,Homo_633,MCI.vs.control; bulk RNA-seq,Homo_714,AD.vs.control; bulk RNA-seq,Homo_714,AD.vs.MCI; bulk RNA-seq,Homo_714,MCI.vs.control	7
ADGRE1	bulk RNA-seq,Homo_723,AD.vs.control; bulk RNA-seq,Homo_723,MCI.vs.control; scRNA-seq,SRP215507,CD8+ T cell_2-AD.vs.control; scRNA-seq,SRP215507,CD8+ T cell_3-AD.vs.control; scRNA-seq,SRP215507,CD8+ T cell_2-AD.vs.MCI; scRNA-seq,SRP215507,CD8+ T cell_3-AD.vs.MCI; scRNA-seq,SRP215507,CD8+ T cell_2-MCI.vs.control; scRNA-seq,SRP309935,Naive CD8+ T cell_1-AD.vs.control; scRNA-seq,SRP330776,Memory T	9
ADGRE2	scRNA-seq,SRP309935,Monocyte_2-AD.vs.control; scRNA-seq,SRP330776,γδ T cell-AD.vs.control; scRNA-seq,SRP330776,CD8+ T cell_2-AD.vs.control; scRNA-seq,SRP330776,Monocyte_2-AD.vs.control	4
ADGRE3	bulk RNA-seq,Homo_723,AD.vs.control; bulk RNA-seq,Homo_723,AD.vs.MCI; bulk RNA-seq,Homo_723,MCI.vs.control; bulk RNA-seq,Homo_633,MCI.vs.control; bulk RNA-seq,Homo_714,AD.vs.control; bulk RNA-seq,Homo_714,AD.vs.MCI; bulk RNA-seq,Homo_714,MCI.vs.control; bulk RNA-seq,SRP223445,AD.vs.control; scRNA-seq,SRP215507,CD8+ T cell_5-AD.vs.MCI; scRNA-	10
Adgre4	bulk RNA-seq,SRP325058,AD.vs.control	1
ADGRE5	bulk RNA-seq,SRP223445,AD.vs.control; scRNA-seq,SRP215507,CD8+ T cell_7-AD.vs.control; scRNA-seq,SRP330776,B cell_2-AD.vs.control; scRNA-seq,SRP330776,Regulatory T cell-AD.vs.control	4
ADGRF1	bulk RNA-seq,Homo_714,MCI.vs.control; bulk RNA-seq,ROSMAP,AD.vs.control; bulk RNA-seq,ROSMAP,MCI.vs.control	3
ADGRF2	bulk RNA-seq,Homo_714,MCI.vs.control	1
ADGRF3	bulk RNA-seq,Homo_723,AD.vs.control; bulk RNA-seq,Homo_723,AD.vs.MCI; bulk RNA-seq,Homo_633,AD.vs.control; bulk RNA-seq,Homo_633,AD.vs.MCI; bulk RNA-seq,Homo_714,AD.vs.control; bulk RNA-seq,Homo_714,AD.vs.MCI; bulk RNA-seq,Homo_714,MCI.vs.control; scRNA-seq,SRP215507,CD8+ T cell_1-AD.vs.MCI; scRNA-seq,SRP215507,CD8+ T cell_1-MCI.vs.control	9
ADGRF4	bulk RNA-seq,Homo_723,AD.vs.control; bulk RNA-seq,Homo_633,AD.vs.control	2
ADGRF5	bulk RNA-seq,Homo_723,AD.vs.control; bulk RNA-seq,Homo_723,AD.vs.MCI; bulk RNA-seq,Homo_723,MCI.vs.control; bulk RNA-seq,Homo_633,AD.vs.control; bulk RNA-seq,Homo_633,AD.vs.MCI; bulk RNA-seq,Homo_633,MCI.vs.control; bulk RNA-	8
ADGRG1	bulk RNA-seq,Homo_723,AD.vs.control; bulk RNA-seq,Homo_723,AD.vs.MCI; bulk RNA-seq,Homo_723,MCI.vs.control; bulk RNA-seq,Homo_633,AD.vs.control; bulk RNA-seq,Homo_633,AD.vs.MCI; bulk RNA-seq,Homo_633,MCI.vs.control; bulk RNA-seq,SRP223445,AD.vs.control; bulk RNA-seq,SRP310421,AD.vs.control; scRNA-seq,SRP215507,CD8+ T cell_3-AD.vs.control; scRNA-seq,SRP215507,CD8+ T cell_7-AD.vs.control; scRNA-seq,SRP215507,CD8+ T cell_5-AD.vs.control; scRNA-seq,SRP215507,CD8+ T cell_2-AD.vs.MCI; scRNA-seq,SRP215507,CD8+ T cell_3-AD.vs.MCI; scRNA-seq,SRP215507,CD8+ T cell_4-AD.vs.MCI; scRNA-seq,SRP215507,CD8+ T cell_6-AD.vs.MCI; scRNA-seq,SRP215507,CD8+ T cell_6-MCI.vs.control; scRNA-seq,SRP330776,Naive CD8+ T cell_2-AD.vs.control; scRNA-	18
ADGRG2	bulk RNA-seq,Homo_723,AD.vs.control; bulk RNA-seq,Homo_723,AD.vs.MCI; bulk RNA-seq,Homo_633,AD.vs.MCI; bulk RNA-	4
ADGRG3	bulk RNA-seq,SRP223445,AD.vs.control; scRNA-seq,SRP215507,CD8+ T cell_1-AD.vs.MCI; scRNA-seq,SRP215507,CD8+ T cell_1-MCI.vs.control	3

ADGRG5	bulk RNA-seq,Homo_723,AD.vs.control; bulk RNA-seq,Homo_723,AD.vs.MCI; bulk RNA-seq,Homo_723,MCI.vs.control; bulk RNA-seq,Homo_633,AD.vs.control; bulk RNA-seq,Homo_633,MCI.vs.control; bulk RNA-seq,ROSMAP,AD.vs.control; bulk RNA-seq,ROSMAP,MCI.vs.control; bulk RNA-seq,SRP223445,AD.vs.control; scRNA-seq,SRP215507,CD8+ T cell_4-AD.vs.MCI; scRNA-seq,SRP215507,CD8+ T cell_5-AD.vs.MCI; scRNA-seq,SRP215507,CD8+ T cell_4-MCI.vs.control; scRNA-seq,SRP330776,γδ T cell-AD.vs.control; scRNA-seq,SRP330776,CD8+ T cell_2-AD.vs.control; scRNA-seq,SRP330776,Natural killer cell_1-AD.vs.control; scRNA-seq,SRP330776,B cell_2-	16
ADGRG6	bulk RNA-seq,Homo_723,AD.vs.control; bulk RNA-seq,Homo_723,AD.vs.MCI; bulk RNA-seq,Homo_723,MCI.vs.control; bulk RNA-seq,Homo_633,AD.vs.control; bulk RNA-seq,Homo_633,AD.vs.MCI; bulk RNA-seq,Homo_633,MCI.vs.control; bulk RNA-seq,Homo_714,AD.vs.control;	8
ADGRG7	bulk RNA-seq,Homo_723,MCI.vs.control	1
ADGRL1	bulk RNA-seq,Homo_633,AD.vs.control; scRNA-seq,SRP215507,CD8+ T cell_3-MCI.vs.control; scRNA-seq,SRP215507,CD8+ T cell_6-MCI.vs.control; scRNA-seq,SRP309935,Natural killer cell_2-AD.vs.control; scRNA-seq,SRP330776,γδ T cell-AD.vs.control; scRNA-seq,SRP330776,Memory T cell_2-AD.vs.control; scRNA-seq,SRP330776,CD8+ T cell_2-AD.vs.control; scRNA-seq,SRP330776,Naive CD8+ T cell_1-AD.vs.control; scRNA-seq,SRP330776,Natural killer cell_1-AD.vs.control; scRNA-seq,SRP330776,Memory T cell_1-AD.vs.control	10
ADGRL2	bulk RNA-seq,Homo_723,AD.vs.control; bulk RNA-seq,Homo_723,MCI.vs.control; bulk RNA-seq,Homo_633,AD.vs.control; bulk RNA-seq,Homo_633,MCI.vs.control; bulk RNA-seq,Homo_714,MCI.vs.control; bulk RNA-seq,ROSMAP,AD.vs.control; bulk RNA-	7
ADGRL3	bulk RNA-seq,Homo_723,AD.vs.control; bulk RNA-seq,Homo_723,AD.vs.MCI; bulk RNA-seq,Homo_723,MCI.vs.control; bulk RNA-seq,Homo_633,AD.vs.control; bulk RNA-seq,Homo_633,AD.vs.MCI; bulk RNA-seq,Homo_714,MCI.vs.control; bulk RNA-seq,ROSMAP,AD.vs.control;	8
ADGRL4	bulk RNA-seq,Homo_723,AD.vs.control; bulk RNA-seq,Homo_723,AD.vs.MCI; bulk RNA-seq,Homo_723,MCI.vs.control; bulk RNA-seq,Homo_633,AD.vs.control; bulk RNA-seq,Homo_633,AD.vs.MCI; bulk RNA-seq,Homo_633,MCI.vs.control	6
ADGRV1	bulk RNA-seq,Homo_723,AD.vs.control; bulk RNA-seq,Homo_723,AD.vs.MCI; bulk RNA-seq,Homo_723,MCI.vs.control; bulk RNA-seq,Homo_633,AD.vs.control; bulk RNA-seq,Homo_633,AD.vs.MCI; bulk RNA-seq,Homo_714,AD.vs.control; bulk RNA-seq,Homo_714,AD.vs.MCI;	8
ADH1A	bulk RNA-seq,Homo_723,AD.vs.control; bulk RNA-seq,Homo_723,AD.vs.MCI; bulk RNA-seq,Homo_633,AD.vs.control; bulk RNA-	4
ADH1B	bulk RNA-seq,Homo_723,AD.vs.MCI; bulk RNA-seq,Homo_723,MCI.vs.control; bulk RNA-seq,Homo_633,AD.vs.MCI; bulk RNA-	4
ADH1C	bulk RNA-seq,Homo_723,AD.vs.MCI; bulk RNA-seq,Homo_723,MCI.vs.control; bulk RNA-seq,Homo_633,AD.vs.MCI; bulk RNA-	4
ADH4	bulk RNA-seq,Homo_723,AD.vs.MCI; bulk RNA-seq,Homo_723,MCI.vs.control; bulk RNA-seq,Homo_633,AD.vs.MCI; bulk RNA-	4
ADH5	bulk RNA-seq,Homo_723,AD.vs.control; bulk RNA-seq,Homo_723,AD.vs.MCI; bulk RNA-seq,Homo_633,AD.vs.control; bulk RNA-seq,Homo_633,AD.vs.MCI; bulk RNA-seq,Homo_714,AD.vs.control; bulk RNA-seq,Homo_714,AD.vs.MCI; scRNA-seq,SRP215507,CD8+ T cell_10-AD.vs.control; scRNA-seq,SRP309935,B cell_1-AD.vs.control; scRNA-seq,SRP330776,CD8+ T cell_1-AD.vs.control; scRNA-seq,SRP330776,Memory T cell_2-AD.vs.control; scRNA-seq,SRP330776,Memory T cell_1-AD.vs.control; scRNA-seq,SRP330776,Naive CD8+ T cell_3-AD.vs.control; scRNA-seq,SRP330776,B cell_2-AD.vs.control; scRNA-seq,SRP330776,Megakaryocyte_1-AD.vs.control	14
ADH6	bulk RNA-seq,Homo_723,AD.vs.control; bulk RNA-seq,Homo_723,AD.vs.MCI; bulk RNA-seq,Homo_723,MCI.vs.control; bulk RNA-seq,Homo_633,AD.vs.control; bulk RNA-seq,Homo_633,AD.vs.MCI; bulk RNA-seq,ROSMAP,MCI.vs.control	6
ADHFE1	scRNA-seq,SRP215507,CD8+ T cell_2-AD.vs.MCI; scRNA-seq,SRP215507,CD8+ T cell_2-MCI.vs.control; scRNA-seq,SRP215507,CD8+ T cell_8-MCI.vs.control; scRNA-seq,SRP309935,Natural killer cell_2-AD.vs.control; scRNA-seq,SRP309935,Natural killer cell_1-AD.vs.control; scRNA-seq,SRP330776,Natural killer cell_1-AD.vs.control; scRNA-seq,SRP330776,Naive CD8+ T cell_3-AD.vs.control; scRNA-seq,SRP330776,CD8+ T cell_3-	8
ADI1	bulk RNA-seq,Homo_714,AD.vs.control; bulk RNA-seq,Homo_714,AD.vs.MCI; bulk RNA-seq,SRP223445,AD.vs.control; scRNA-seq,SRP215507,CD8+ T cell_3-AD.vs.MCI; scRNA-seq,SRP215507,CD8+ T cell_5-AD.vs.MCI; scRNA-seq,SRP215507,CD8+ T cell_5-MCI.vs.control; scRNA-seq,SRP330776,γδ T cell-AD.vs.control; scRNA-seq,SRP330776,Naive CD8+ T cell_1-AD.vs.control; scRNA-seq,SRP330776,CD8+ T cell_3-	9
Adipor1	bulk RNA-seq,SRP325058,AD.vs.control	1
ADIPOR1	scRNA-seq,SRP215507,CD8+ T cell_4-AD.vs.control; scRNA-seq,SRP215507,CD8+ T cell_6-AD.vs.control; scRNA-seq,SRP215507,CD8+ T cell_9-AD.vs.control; scRNA-seq,SRP215507,CD8+ T cell_4-AD.vs.MCI; scRNA-seq,SRP215507,CD8+ T cell_7-AD.vs.MCI; scRNA-seq,SRP215507,CD8+ T cell_5-MCI.vs.control; scRNA-seq,SRP215507,CD8+ T cell_6-MCI.vs.control	7
ADIPOR2	scRNA-seq,SRP215507,CD8+ T cell_4-AD.vs.control; scRNA-seq,SRP215507,CD8+ T cell_1-MCI.vs.control; scRNA-seq,SRP330776,Memory T cell_2-AD.vs.control; scRNA-seq,SRP330776,Naive CD8+ T cell_3-AD.vs.control; scRNA-seq,SRP330776,Regulatory T cell-AD.vs.control	5

ADIRF	bulk RNA-seq,Homo_723,AD.vs.control; bulk RNA-seq,Homo_723,AD.vs.MCI; bulk RNA-seq,Homo_723,MCI.vs.control; bulk RNA-seq,Homo_633,AD.vs.control; bulk RNA-seq,Homo_633,AD.vs.MCI; bulk RNA-seq,Homo_633,MCI.vs.control; scRNA-seq,SRP215507,CD8+ T cell_3-AD.vs.control; scRNA-seq,SRP215507,CD8+ T cell_3-AD.vs.MCI; scRNA-seq,SRP215507,CD8+ T cell_3-MCI.vs.control; scRNA-seq,SRP330776,Naive	10
ADIRF-AS1	scRNA-seq,SRP215507,CD8+ T cell_4-MCI.vs.control; scRNA-seq,SRP330776,CD8+ T cell_1-AD.vs.control; scRNA-seq,SRP330776,B cell_1-AD.vs.control; scRNA-seq,SRP330776,Naive CD8+ T cell_1-AD.vs.control	4
Adk	bulk RNA-seq,SRP325058,AD.vs.control	1
ADK	scRNA-seq,SRP215507,CD8+ T cell_4-AD.vs.MCI; scRNA-seq,SRP330776,CD8+ T cell_2-AD.vs.control; scRNA-seq,SRP330776,Naive CD8+ T cell_3-AD.vs.control; scRNA-seq,SRP330776,CD8+ T cell_3-AD.vs.control; scRNA-seq,SRP330776,B cell_3-AD.vs.control; scRNA-	6
ADM	bulk RNA-seq,Homo_723,AD.vs.control; bulk RNA-seq,Homo_723,AD.vs.MCI; bulk RNA-seq,Homo_633,AD.vs.control; bulk RNA-seq,Homo_633,AD.vs.MCI; bulk RNA-seq,Homo_714,AD.vs.control; bulk RNA-seq,Homo_714,AD.vs.MCI; bulk RNA-seq,ROSMAP,AD.vs.MCI; bulk RNA-seq,ROSMAP,MCI.vs.control; bulk RNA-seq,SRP223445,AD.vs.control; scRNA-seq,SRP215507,CD8+ T cell_2-AD.vs.MCI; scRNA-	11
ADM2	bulk RNA-seq,Homo_723,AD.vs.control; bulk RNA-seq,Homo_723,AD.vs.MCI; bulk RNA-seq,Homo_723,MCI.vs.control; bulk RNA-seq,Homo_633,AD.vs.control; bulk RNA-seq,Homo_633,MCI.vs.control; bulk RNA-seq,Homo_714,AD.vs.MCI; bulk RNA-seq,Homo_714,MCI.vs.control; scRNA-seq,SRP215507,CD8+ T cell_2-AD.vs.control; scRNA-seq,SRP215507,CD8+ T cell_3-AD.vs.control; scRNA-seq,SRP215507,CD8+ T cell_2-MCI.vs.control; scRNA-seq,SRP215507,CD8+ T cell_3-MCI.vs.control; scRNA-seq,SRP330776,CD8+ T cell_1-	12
ADM5	bulk RNA-seq,ROSMAP,AD.vs.control; bulk RNA-seq,ROSMAP,AD.vs.MCI; scRNA-seq,SRP309935,Natural killer cell_2-AD.vs.control; scRNA-seq,SRP330776,CD8+ T cell_1-AD.vs.control	4
ADNP	bulk RNA-seq,Homo_723,AD.vs.MCI; bulk RNA-seq,Homo_723,MCI.vs.control; bulk RNA-seq,Homo_633,AD.vs.MCI; bulk RNA-seq,Homo_633,MCI.vs.control; bulk RNA-seq,Homo_714,AD.vs.control; bulk RNA-seq,Homo_714,AD.vs.MCI; bulk RNA-seq,Homo_714,MCI.vs.control; scRNA-seq,SRP215507,CD8+ T cell_7-AD.vs.control; scRNA-seq,SRP215507,CD8+ T cell_6-AD.vs.control; scRNA-seq,SRP215507,CD8+ T cell_3-AD.vs.MCI; scRNA-seq,SRP215507,CD8+ T cell_5-AD.vs.MCI; scRNA-seq,SRP215507,CD8+ T cell_6-AD.vs.MCI; scRNA-seq,SRP215507,CD8+ T cell_5-MCI.vs.control; scRNA-seq,SRP330776,Natural killer T cell-AD.vs.control; scRNA-seq,SRP330776,Naive CD8+ T cell_3-AD.vs.control; scRNA-seq,SRP330776,B cell_2-AD.vs.control; scRNA-seq,SRP330776,CD8+ T cell_3-AD.vs.control; scRNA-	18
ADNP2	bulk RNA-seq,Homo_723,AD.vs.control; bulk RNA-seq,Homo_723,AD.vs.MCI; bulk RNA-seq,Homo_633,AD.vs.MCI; bulk RNA-seq,Homo_714,AD.vs.control; bulk RNA-seq,Homo_714,AD.vs.MCI; scRNA-seq,SRP215507,CD8+ T cell_5-AD.vs.control; scRNA-seq,SRP215507,CD8+ T cell_4-MCI.vs.control; scRNA-seq,SRP215507,CD8+ T cell_3-MCI.vs.control; scRNA-seq,SRP215507,CD8+ T cell_6-MCI.vs.control; scRNA-seq,SRP309935,Naive CD8+ T cell_2-AD.vs.control; scRNA-seq,SRP330776,CD8+ T cell_1-AD.vs.control; scRNA-seq,SRP330776,γδ T cell-AD.vs.control; scRNA-seq,SRP330776,Memory T cell_2-AD.vs.control; scRNA-seq,SRP330776,CD8+ T cell_2-AD.vs.control; scRNA-seq,SRP330776,Memory T cell_1-AD.vs.control; scRNA-seq,SRP330776,Natural killer T cell-AD.vs.control; scRNA-seq,SRP330776,CD8+ T	17
ADNP-AS1	scRNA-seq,SRP215507,CD8+ T cell_4-AD.vs.control; scRNA-seq,SRP215507,CD8+ T cell_3-AD.vs.MCI; scRNA-seq,SRP215507,CD8+ T cell_5-AD.vs.MCI; scRNA-seq,SRP215507,CD8+ T cell_5-MCI.vs.control; scRNA-seq,SRP215507,CD8+ T cell_3-MCI.vs.control; scRNA-seq,SRP330776,γδ T cell-AD.vs.control; scRNA-seq,SRP330776,Memory T cell_1-AD.vs.control; scRNA-seq,SRP330776,Naive CD8+ T cell_2-AD.vs.control	8
ADO	scRNA-seq,SRP215507,CD8+ T cell_5-MCI.vs.control; scRNA-seq,SRP215507,CD8+ T cell_6-MCI.vs.control; scRNA-seq,SRP309935,Naive CD8+ T cell_1-AD.vs.control; scRNA-seq,SRP309935,B cell_1-AD.vs.control; scRNA-seq,SRP309935,CD8+ T cell_2-AD.vs.control; scRNA-seq,SRP309935,CD4+ T cell-AD.vs.control; scRNA-seq,SRP330776,CD8+ T cell_1-AD.vs.control; scRNA-seq,SRP330776,γδ T cell-AD.vs.control; scRNA-seq,SRP330776,Memory T cell_2-AD.vs.control; scRNA-seq,SRP330776,CD8+ T cell_2-AD.vs.control; scRNA-seq,SRP330776,B cell_1-AD.vs.control; scRNA-seq,SRP330776,Naive CD8+ T cell_1-AD.vs.control; scRNA-seq,SRP330776,Natural killer cell_1-AD.vs.control; scRNA-seq,SRP330776,Memory T cell_1-AD.vs.control; scRNA-seq,SRP330776,Naive CD8+ T cell_3-AD.vs.control; scRNA-seq,SRP330776,B cell_2-	17
ADORA1	bulk RNA-seq,Homo_723,AD.vs.control; bulk RNA-seq,Homo_723,AD.vs.MCI; bulk RNA-seq,Homo_633,AD.vs.control; bulk RNA-seq,Homo_633,AD.vs.MCI; bulk RNA-seq,Homo_714,AD.vs.control; bulk RNA-seq,Homo_714,AD.vs.MCI	6

ADORA2A	bulk RNA-seq,Homo_714,AD.vs.control; bulk RNA-seq,Homo_714,AD.vs.MCI; scRNA-seq,SRP215507,CD8+ T cell_10-AD.vs.control; scRNA-seq,SRP215507,CD8+ T cell_5-AD.vs.MCI; scRNA-seq,SRP330776,CD8+ T cell_1-AD.vs.control; scRNA-seq,SRP330776,γδ T cell-AD.vs.control; scRNA-seq,SRP330776,Memory T cell_2-AD.vs.control; scRNA-seq,SRP330776,B cell_1-AD.vs.control; scRNA-seq,SRP330776,Naive CD8+ T cell_1-AD.vs.control; scRNA-seq,SRP330776,Natural killer cell_1-AD.vs.control; scRNA-seq,SRP330776,Memory T cell_1-AD.vs.control; scRNA-seq,SRP330776,Naive CD8+ T cell_3-AD.vs.control; scRNA-seq,SRP330776,B cell_2-AD.vs.control; scRNA-seq,SRP330776,T cell-AD.vs.control; scRNA-seq,SRP330776,CD8+ T cell_3-AD.vs.control; scRNA-seq,SRP330776,Monocyte_2-AD.vs.control; scRNA-seq,SRP330776,B cell_3-	17
ADORA3	bulk RNA-seq,Homo_723,AD.vs.control; bulk RNA-seq,Homo_723,AD.vs.MCI; bulk RNA-seq,ROSMAP,AD.vs.control; bulk RNA-	4
ADPGK	bulk RNA-seq,Homo_723,AD.vs.control; bulk RNA-seq,Homo_723,AD.vs.MCI; bulk RNA-seq,Homo_633,AD.vs.control; bulk RNA-seq,Homo_633,AD.vs.MCI; bulk RNA-seq,Homo_714,AD.vs.control; bulk RNA-seq,Homo_714,AD.vs.MCI; bulk RNA-seq,Homo_714,MCI.vs.control; scRNA-seq,SRP215507,CD8+ T cell_5-MCI.vs.control; scRNA-seq,SRP309935,Naive CD8+ T cell_2-AD.vs.control; scRNA-seq,SRP330776,CD8+ T cell_2-AD.vs.control; scRNA-seq,SRP330776,T cell-AD.vs.control; scRNA-seq,SRP330776,CD8+ T cell_3-AD.vs.control; scRNA-	14
Adpgk	bulk RNA-seq,SRP325058,AD.vs.control	1
ADPGK-AS1	scRNA-seq,SRP215507,CD8+ T cell_4-AD.vs.MCI; scRNA-seq,SRP215507,CD8+ T cell_3-MCI.vs.control; scRNA-seq,SRP330776,Naive CD8+ T	3
ADPRH	bulk RNA-seq,Homo_723,AD.vs.control; bulk RNA-seq,Homo_633,AD.vs.control; bulk RNA-seq,Homo_714,AD.vs.control; bulk RNA-seq,Homo_714,AD.vs.MCI; scRNA-seq,SRP215507,CD8+ T cell_5-AD.vs.control; scRNA-seq,SRP215507,CD8+ T cell_1-AD.vs.MCI; scRNA-seq,SRP215507,CD8+ T cell_1-MCI.vs.control; scRNA-seq,SRP330776,CD8+ T cell_1-AD.vs.control; scRNA-seq,SRP330776,Monocyte_1-	10
ADPRHL1	bulk RNA-seq,Homo_714,AD.vs.control; bulk RNA-seq,Homo_714,AD.vs.MCI	2
ADPRHL2	scRNA-seq,SRP215507,CD8+ T cell_4-AD.vs.control; scRNA-seq,SRP215507,CD8+ T cell_5-AD.vs.control; scRNA-seq,SRP215507,CD8+ T cell_2-AD.vs.MCI; scRNA-seq,SRP215507,CD8+ T cell_6-AD.vs.MCI; scRNA-seq,SRP215507,CD8+ T cell_1-MCI.vs.control; scRNA-seq,SRP215507,CD8+ T cell_5-MCI.vs.control; scRNA-seq,SRP215507,CD8+ T cell_2-MCI.vs.control; scRNA-seq,SRP215507,CD8+ T cell_6-MCI.vs.control; scRNA-seq,SRP309935,B cell_1-AD.vs.control; scRNA-seq,SRP330776,B cell_1-AD.vs.control; scRNA-seq,SRP330776,Natural killer cell_1-AD.vs.control; scRNA-seq,SRP330776,Memory T cell_1-AD.vs.control; scRNA-seq,SRP330776,T cell-AD.vs.control; scRNA-seq,SRP330776,CD8+ T cell_3-	14
ADPRM	bulk RNA-seq,Homo_723,AD.vs.control; bulk RNA-seq,Homo_723,AD.vs.MCI; bulk RNA-seq,Homo_633,AD.vs.control; bulk RNA-seq,Homo_633,AD.vs.MCI; bulk RNA-seq,Homo_714,AD.vs.control; bulk RNA-seq,Homo_714,AD.vs.MCI; scRNA-seq,SRP215507,CD8+ T cell_4-AD.vs.MCI; scRNA-seq,SRP215507,CD8+ T cell_7-AD.vs.MCI; scRNA-seq,SRP330776,Natural killer cell_1-AD.vs.control; scRNA-seq,SRP330776,B cell_2-AD.vs.control; scRNA-seq,SRP330776,Naive CD8+ T cell_2-AD.vs.control; scRNA-seq,SRP330776,B cell_3-AD.vs.control	12
ADPRS	bulk RNA-seq,Homo_723,AD.vs.control; bulk RNA-seq,Homo_723,AD.vs.MCI; bulk RNA-seq,Homo_633,AD.vs.control; bulk RNA-seq,Homo_633,AD.vs.MCI; bulk RNA-seq,Homo_714,AD.vs.control; bulk RNA-seq,Homo_714,AD.vs.MCI	6
ADRA1A	bulk RNA-seq,Homo_723,AD.vs.control; bulk RNA-seq,Homo_714,MCI.vs.control	2
ADRA1B	bulk RNA-seq,Homo_723,AD.vs.control; bulk RNA-seq,Homo_714,MCI.vs.control	2
ADRA1D	bulk RNA-seq,Homo_714,MCI.vs.control	1
ADRA2B	bulk RNA-seq,Homo_714,MCI.vs.control; scRNA-seq,SRP215507,CD8+ T cell_1-AD.vs.control; scRNA-seq,SRP215507,CD8+ T cell_4-AD.vs.control; scRNA-seq,SRP215507,CD8+ T cell_2-AD.vs.MCI; scRNA-seq,SRP215507,CD8+ T cell_5-AD.vs.MCI; scRNA-seq,SRP215507,CD8+ T cell_1-MCI.vs.control; scRNA-seq,SRP215507,CD8+ T cell_5-MCI.vs.control; scRNA-seq,SRP215507,CD8+ T cell_2-MCI.vs.control	8
ADRA2C	bulk RNA-seq,Homo_723,AD.vs.control; bulk RNA-seq,Homo_723,AD.vs.MCI; bulk RNA-seq,Homo_723,MCI.vs.control; bulk RNA-seq,Homo_633,AD.vs.MCI; bulk RNA-seq,Homo_633,MCI.vs.control; bulk RNA-seq,Homo_714,MCI.vs.control; bulk RNA-	7
ADRB1	bulk RNA-seq,SRP223445,AD.vs.control; scRNA-seq,SRP215507,CD8+ T cell_1-AD.vs.control; scRNA-seq,SRP215507,CD8+ T cell_4-AD.vs.control; scRNA-seq,SRP215507,CD8+ T cell_1-MCI.vs.control; scRNA-seq,SRP215507,CD8+ T cell_4-MCI.vs.control	5
Adrb1	bulk RNA-seq,SRP325058,AD.vs.control	1

ADRB2	scRNA-seq,SRP215507,CD8+ T cell_2-AD.vs.MCI; scRNA-seq,SRP215507,CD8+ T cell_3-AD.vs.MCI; scRNA-seq,SRP215507,CD8+ T cell_5-AD.vs.MCI; scRNA-seq,SRP215507,CD8+ T cell_1-MCI.vs.control; scRNA-seq,SRP215507,CD8+ T cell_5-MCI.vs.control; scRNA-seq,SRP215507,CD8+ T cell_2-MCI.vs.control; scRNA-seq,SRP330776,γδ T cell-AD.vs.control; scRNA-seq,SRP330776,CD8+ T cell_2-AD.vs.control; scRNA-seq,SRP330776,Natural killer cell_1-AD.vs.control; scRNA-seq,SRP330776,Memory T cell_1-AD.vs.control; scRNA-seq,SRP330776,Naive CD8+	11
ADRB3	bulk RNA-seq,Homo_714,MCI.vs.control	1
ADRM1	bulk RNA-seq,Homo_723,AD.vs.control; bulk RNA-seq,Homo_723,AD.vs.MCI; bulk RNA-seq,Homo_723,MCI.vs.control; bulk RNA-seq,Homo_633,AD.vs.control; bulk RNA-seq,Homo_633,AD.vs.MCI; bulk RNA-seq,Homo_633,MCI.vs.control; bulk RNA-seq,Homo_714,AD.vs.control; bulk RNA-seq,Homo_714,AD.vs.MCI; bulk RNA-seq,Homo_714,MCI.vs.control; scRNA-seq,SRP215507,CD8+ T cell_7-AD.vs.control; scRNA-seq,SRP330776,B cell_2-AD.vs.control; scRNA-seq,SRP330776,CD8+ T cell_3-AD.vs.control	12
ADSL	scRNA-seq,SRP309935,Natural killer cell_2-AD.vs.control; scRNA-seq,SRP330776,Natural killer cell_2-AD.vs.control	2
ADSS	scRNA-seq,SRP215507,CD8+ T cell_4-AD.vs.MCI; scRNA-seq,SRP309935,B cell_1-AD.vs.control; scRNA-seq,SRP330776,CD8+ T cell_1-AD.vs.control; scRNA-seq,SRP330776,Natural killer T cell-AD.vs.control; scRNA-seq,SRP330776,Naive CD8+ T cell_3-AD.vs.control; scRNA-seq,SRP330776,B cell_2-AD.vs.control; scRNA-seq,SRP330776,Megakaryocyte_1-AD.vs.control	7
ADSS1	bulk RNA-seq,Homo_723,AD.vs.control; bulk RNA-seq,Homo_723,AD.vs.MCI; bulk RNA-seq,Homo_633,AD.vs.MCI; bulk RNA-seq,Homo_714,AD.vs.control; bulk RNA-seq,Homo_714,AD.vs.MCI	5
ADSS2	bulk RNA-seq,Homo_723,AD.vs.control; bulk RNA-seq,Homo_633,AD.vs.control; bulk RNA-seq,Homo_714,AD.vs.control; bulk RNA-	4
ADSSL1	scRNA-seq,SRP215507,CD8+ T cell_3-AD.vs.MCI; scRNA-seq,SRP215507,CD8+ T cell_1-AD.vs.MCI; scRNA-seq,SRP215507,CD8+ T cell_1-MCI.vs.control; scRNA-seq,SRP215507,CD8+ T cell_3-MCI.vs.control; scRNA-seq,SRP330776,CD8+ T cell_1-AD.vs.control; scRNA-	6
ADTRP	bulk RNA-seq,Homo_723,AD.vs.control; bulk RNA-seq,Homo_723,AD.vs.MCI; bulk RNA-seq,Homo_723,MCI.vs.control; bulk RNA-seq,Homo_633,AD.vs.control; bulk RNA-seq,Homo_633,AD.vs.MCI; bulk RNA-seq,Homo_633,MCI.vs.control; bulk RNA-seq,Homo_714,AD.vs.control; bulk RNA-seq,Homo_714,AD.vs.MCI; bulk RNA-seq,Homo_714,MCI.vs.control; scRNA-seq,SRP215507,CD8+ T cell_3-AD.vs.control; scRNA-seq,SRP215507,CD8+ T cell_4-AD.vs.control; scRNA-seq,SRP215507,CD8+ T cell_5-AD.vs.control; scRNA-seq,SRP215507,CD8+ T cell_4-AD.vs.MCI; scRNA-seq,SRP215507,CD8+ T cell_5-MCI.vs.control; scRNA-seq,SRP330776,B cell_1-AD.vs.control; scRNA-seq,SRP330776,Memory T cell_1-AD.vs.control; scRNA-seq,SRP330776,Naive CD8+ T cell_3-AD.vs.control; scRNA-seq,SRP330776,CD8+ T cell_3-AD.vs.control	18
AEBP1	bulk RNA-seq,ROSMAP,MCI.vs.control; scRNA-seq,SRP215507,CD8+ T cell_2-AD.vs.control; scRNA-seq,SRP215507,CD8+ T cell_6-AD.vs.control; scRNA-seq,SRP330776,CD8+ T cell_1-AD.vs.control; scRNA-seq,SRP330776,γδ T cell-AD.vs.control; scRNA-seq,SRP330776,B cell_1-AD.vs.control; scRNA-seq,SRP330776,Naive CD8+ T cell_1-AD.vs.control; scRNA-seq,SRP330776,Natural killer T cell-AD.vs.control; scRNA-seq,SRP330776,Naive CD8+ T cell_3-AD.vs.control; scRNA-seq,SRP330776,B cell_2-AD.vs.control; scRNA-seq,SRP330776,Monocyte_1-AD.vs.control; scRNA-	12
AEBP2	scRNA-seq,SRP215507,CD8+ T cell_5-AD.vs.MCI; scRNA-seq,SRP330776,CD8+ T cell_1-AD.vs.control; scRNA-seq,SRP330776,γδ T cell-AD.vs.control; scRNA-seq,SRP330776,CD8+ T cell_2-AD.vs.control; scRNA-seq,SRP330776,Naive CD8+ T cell_1-AD.vs.control; scRNA-seq,SRP330776,Natural killer cell_1-AD.vs.control; scRNA-seq,SRP330776,Memory T cell_1-AD.vs.control; scRNA-seq,SRP330776,Natural killer T cell-AD.vs.control; scRNA-seq,SRP330776,Naive CD8+ T cell_3-AD.vs.control; scRNA-seq,SRP330776,B cell_2-AD.vs.control; scRNA-seq,SRP330776,T cell-AD.vs.control; scRNA-seq,SRP330776,CD8+ T cell_3-AD.vs.control; scRNA-seq,SRP330776,B cell_3-AD.vs.control; scRNA-	14
AEN	scRNA-seq,SRP215507,CD8+ T cell_5-AD.vs.control; scRNA-seq,SRP215507,CD8+ T cell_3-AD.vs.MCI; scRNA-seq,SRP215507,CD8+ T cell_4-AD.vs.MCI; scRNA-seq,SRP215507,CD8+ T cell_4-MCI.vs.control; scRNA-seq,SRP215507,CD8+ T cell_3-MCI.vs.control; scRNA-seq,SRP309935,CD8+ T cell_2-AD.vs.control; scRNA-seq,SRP330776,Natural killer T cell-AD.vs.control; scRNA-seq,SRP330776,Naive CD8+ T cell_3-AD.vs.control; scRNA-seq,SRP330776,Megakaryocyte_1-AD.vs.control; scRNA-seq,SRP330776,Natural killer cell_2-AD.vs.control	10
AF001548.2	scRNA-seq,SRP215507,CD8+ T cell_5-MCI.vs.control; scRNA-seq,SRP330776,CD8+ T cell_1-AD.vs.control; scRNA-seq,SRP330776,γδ T cell-AD.vs.control; scRNA-seq,SRP330776,Memory T cell_2-AD.vs.control; scRNA-seq,SRP330776,CD8+ T cell_2-AD.vs.control; scRNA-seq,SRP330776,Naive CD8+ T cell_1-AD.vs.control; scRNA-seq,SRP330776,Memory T cell_1-AD.vs.control	7
AF111167.2	scRNA-seq,SRP330776,B cell_1-AD.vs.control	1

AF117829.1	scRNA-seq,SRP215507,CD8+ T cell_5-AD.vs.MCI; scRNA-seq,SRP330776,CD8+ T cell_1-AD.vs.control; scRNA-seq,SRP330776,γδ T cell-AD.vs.control; scRNA-seq,SRP330776,Memory T cell_2-AD.vs.control; scRNA-seq,SRP330776,Naive CD8+ T cell_1-AD.vs.control; scRNA-seq,SRP330776,Natural killer cell_1-AD.vs.control; scRNA-seq,SRP330776,Memory T cell_1-AD.vs.control; scRNA-seq,SRP330776,B cell_2-	8
AF129075.2	scRNA-seq,SRP215507,CD8+ T cell_5-AD.vs.control; scRNA-seq,SRP330776,CD8+ T cell_1-AD.vs.control; scRNA-seq,SRP330776,γδ T cell-AD.vs.control; scRNA-seq,SRP330776,Naive CD8+ T cell_1-AD.vs.control; scRNA-seq,SRP330776,T cell-AD.vs.control	5
AF131215.2	scRNA-seq,SRP215507,CD8+ T cell_3-AD.vs.control; scRNA-seq,SRP215507,CD8+ T cell_2-AD.vs.MCI; scRNA-seq,SRP215507,CD8+ T cell_1-MCI.vs.control; scRNA-seq,SRP215507,CD8+ T cell_2-MCI.vs.control; scRNA-seq,SRP309935,Natural killer cell_2-AD.vs.control	5
AF131215.4	scRNA-seq,SRP215507,CD8+ T cell_1-AD.vs.control	1
AF131215.5	scRNA-seq,SRP215507,CD8+ T cell_1-AD.vs.MCI; scRNA-seq,SRP215507,CD8+ T cell_1-MCI.vs.control; scRNA-seq,SRP330776,CD8+ T cell_1-AD.vs.control; scRNA-seq,SRP330776,B cell_1-AD.vs.control; scRNA-seq,SRP330776,Naive CD8+ T cell_1-AD.vs.control	5
AF131215.6	scRNA-seq,SRP215507,CD8+ T cell_3-AD.vs.MCI; scRNA-seq,SRP215507,CD8+ T cell_1-AD.vs.MCI; scRNA-seq,SRP215507,CD8+ T cell_3-MCI.vs.control; scRNA-seq,SRP330776,CD8+ T cell_1-AD.vs.control; scRNA-seq,SRP330776,Naive CD8+ T cell_1-AD.vs.control; scRNA-	6
AF131216.4	scRNA-seq,SRP330776,Memory T cell_1-AD.vs.control	1
AF165147.1	scRNA-seq,SRP330776,CD8+ T cell_1-AD.vs.control; scRNA-seq,SRP330776,γδ T cell-AD.vs.control; scRNA-seq,SRP330776,CD8+ T cell_2-AD.vs.control; scRNA-seq,SRP330776,Natural killer cell_1-AD.vs.control; scRNA-seq,SRP330776,Memory T cell_1-AD.vs.control	5
AF196972.1	scRNA-seq,SRP330776,CD8+ T cell_2-AD.vs.control; scRNA-seq,SRP330776,Memory T cell_1-AD.vs.control	2
AF213884.3	scRNA-seq,SRP215507,CD8+ T cell_2-AD.vs.MCI; scRNA-seq,SRP215507,CD8+ T cell_3-AD.vs.MCI; scRNA-seq,SRP215507,CD8+ T cell_1-AD.vs.MCI; scRNA-seq,SRP215507,CD8+ T cell_5-AD.vs.MCI; scRNA-seq,SRP215507,CD8+ T cell_1-MCI.vs.control; scRNA-seq,SRP215507,CD8+ T cell_5-MCI.vs.control; scRNA-seq,SRP215507,CD8+ T cell_2-MCI.vs.control; scRNA-seq,SRP330776,CD8+ T cell_1-AD.vs.control; scRNA-seq,SRP330776,γδ T cell-AD.vs.control; scRNA-seq,SRP330776,Memory T cell_2-AD.vs.control; scRNA-seq,SRP330776,CD8+ T cell_2-AD.vs.control; scRNA-seq,SRP330776,B cell_1-AD.vs.control; scRNA-seq,SRP330776,Naive CD8+ T cell_1-AD.vs.control; scRNA-seq,SRP330776,Natural killer cell_1-AD.vs.control; scRNA-seq,SRP330776,Memory T cell_1-AD.vs.control; scRNA-seq,SRP330776,Natural killer T cell-AD.vs.control; scRNA-seq,SRP330776,Naive CD8+ T cell_3-AD.vs.control; scRNA-seq,SRP330776,B cell_2-AD.vs.control; scRNA-seq,SRP330776,T cell-AD.vs.control; scRNA-seq,SRP330776,Naive CD8+ T cell_2-AD.vs.control; scRNA-seq,SRP330776,CD8+ T cell_3-AD.vs.control; scRNA-seq,SRP330776,Monocyte_2-AD.vs.control; scRNA-seq,SRP330776,B cell_3-AD.vs.control; scRNA-seq,SRP330776,Natural killer cell_2-AD.vs.control; scRNA-seq,SRP330776,B [illegible]	25
AF230666.1	scRNA-seq,SRP330776,Naive CD8+ T cell_1-AD.vs.control	1
AF235103.3	scRNA-seq,SRP215507,CD8+ T cell_2-MCI.vs.control	1
AF274853.1	scRNA-seq,SRP215507,CD8+ T cell_1-AD.vs.MCI; scRNA-seq,SRP215507,CD8+ T cell_5-AD.vs.MCI; scRNA-seq,SRP215507,CD8+ T cell_1-MCI.vs.control; scRNA-seq,SRP215507,CD8+ T cell_5-MCI.vs.control; scRNA-seq,SRP330776,CD8+ T cell_1-AD.vs.control; scRNA-seq,SRP330776,Memory T cell_2-AD.vs.control; scRNA-seq,SRP330776,CD8+ T cell_2-AD.vs.control; scRNA-seq,SRP330776,Memory T cell_1-	8
AF305872.2	scRNA-seq,SRP215507,CD8+ T cell_1-AD.vs.control; scRNA-seq,SRP215507,CD8+ T cell_1-AD.vs.MCI; scRNA-seq,SRP215507,CD8+ T cell_4-AD.vs.MCI; scRNA-seq,SRP215507,CD8+ T cell_1-MCI.vs.control; scRNA-seq,SRP215507,CD8+ T cell_2-MCI.vs.control; scRNA-seq,SRP330776,CD8+ T cell_1-AD.vs.control; scRNA-seq,SRP330776,Memory T cell_2-AD.vs.control; scRNA-seq,SRP330776,CD8+ T cell_2-AD.vs.control; scRNA-seq,SRP330776,Natural killer cell_1-AD.vs.control; scRNA-seq,SRP330776,T cell-AD.vs.control; scRNA-seq,SRP330776,Naive	11
AFAP1	bulk RNA-seq,Homo_723,AD.vs.control; bulk RNA-seq,Homo_723,AD.vs.MCI; bulk RNA-seq,Homo_723,MCI.vs.control; bulk RNA-seq,Homo_633,AD.vs.control; bulk RNA-seq,Homo_633,AD.vs.MCI; bulk RNA-seq,Homo_633,MCI.vs.control; bulk RNA-seq,Homo_714,AD.vs.control; bulk RNA-seq,Homo_714,AD.vs.MCI; bulk RNA-seq,ROSMAP,AD.vs.MCI; bulk RNA-seq,ROSMAP,MCI.vs.control; scRNA-seq,SRP215507,CD8+ T cell_3-AD.vs.control; scRNA-seq,SRP215507,CD8+ T cell_3-AD.vs.MCI; scRNA-seq,SRP215507,CD8+ T cell_1-AD.vs.MCI; scRNA-seq,SRP215507,CD8+ T cell_3-MCI.vs.control; scRNA-seq,SRP330776,γδ T cell-AD.vs.control; scRNA-seq,SRP330776,Naive CD8+ T cell_1-AD.vs.control; scRNA-seq,SRP330776,Memory T cell_1-AD.vs.control; scRNA-seq,SRP330776,Naive CD8+ T cell_3-AD.vs.control	18
AFAP1L1	bulk RNA-seq,Homo_723,AD.vs.control; bulk RNA-seq,Homo_723,AD.vs.MCI; bulk RNA-seq,Homo_723,MCI.vs.control; bulk RNA-seq,Homo_633,AD.vs.control; bulk RNA-seq,Homo_633,AD.vs.MCI; bulk RNA-seq,Homo_633,MCI.vs.control; bulk RNA-seq,Homo_714,MCI.vs.control	7

AFAP1L2	bulk RNA-seq,Homo_723,AD.vs.control; bulk RNA-seq,Homo_723,AD.vs.MCI; bulk RNA-seq,Homo_723,MCI.vs.control; bulk RNA-seq,Homo_633,AD.vs.control; bulk RNA-seq,Homo_633,AD.vs.MCI; bulk RNA-seq,Homo_633,MCI.vs.control; bulk RNA-seq,Homo_714,AD.vs.control; bulk RNA-seq,Homo_714,AD.vs.MCI; bulk RNA-seq,ROSMAP,AD.vs.control; bulk RNA-seq,ROSMAP,MCI.vs.control; scRNA-seq,SRP215507,CD8+ T cell_2-AD.vs.control; scRNA-seq,SRP330776,CD8+ T cell_2-AD.vs.control; scRNA-seq,SRP330776,B cell_2-AD.vs.control	13
AFDN	bulk RNA-seq,Homo_723,AD.vs.control; bulk RNA-seq,Homo_723,AD.vs.MCI; bulk RNA-seq,Homo_723,MCI.vs.control; bulk RNA-seq,Homo_633,AD.vs.control; bulk RNA-seq,Homo_633,AD.vs.MCI; bulk RNA-seq,Homo_633,MCI.vs.control; bulk RNA-seq,Homo_714,AD.vs.control; bulk RNA-seq,Homo_714,AD.vs.MCI; scRNA-seq,SRP215507,CD8+ T cell_2-AD.vs.control; scRNA-seq,SRP215507,CD8+ T cell_5-AD.vs.control; scRNA-seq,SRP215507,CD8+ T cell_2-AD.vs.MCI; scRNA-seq,SRP215507,CD8+ T cell_1-AD.vs.MCI; scRNA-seq,SRP215507,CD8+ T cell_1-MCI.vs.control; scRNA-seq,SRP309935,Natural killer cell_2-AD.vs.control; scRNA-seq,SRP309935,Natural killer cell_1-AD.vs.control; scRNA-seq,SRP330776,CD8+ T cell_1-AD.vs.control; scRNA-seq,SRP330776,Memory T cell_2-AD.vs.control; scRNA-seq,SRP330776,CD8+ T cell_2-AD.vs.control; scRNA-seq,SRP330776,B cell_1-AD.vs.control; scRNA-seq,SRP330776,Memory T cell_1-AD.vs.control; scRNA-seq,SRP330776,T cell-[illegible]	21
AFF1	scRNA-seq,SRP215507,CD8+ T cell_5-AD.vs.MCI; scRNA-seq,SRP330776,Naive CD8+ T cell_2-AD.vs.control	2
AFF2	bulk RNA-seq,Homo_723,AD.vs.control; bulk RNA-seq,Homo_723,AD.vs.MCI; bulk RNA-seq,Homo_723,MCI.vs.control; bulk RNA-seq,Homo_633,AD.vs.control; bulk RNA-seq,Homo_633,AD.vs.MCI; bulk RNA-seq,Homo_633,MCI.vs.control; bulk RNA-seq,Homo_714,AD.vs.control; bulk RNA-seq,Homo_714,AD.vs.MCI; bulk RNA-seq,ROSMAP,AD.vs.MCI; bulk RNA-seq,ROSMAP,MCI.vs.control; bulk RNA-	11
AFF3	bulk RNA-seq,Homo_723,AD.vs.control; bulk RNA-seq,Homo_723,AD.vs.MCI; bulk RNA-seq,Homo_633,AD.vs.control; bulk RNA-seq,Homo_633,AD.vs.MCI; bulk RNA-seq,Homo_633,MCI.vs.control; bulk RNA-seq,SRP223445,AD.vs.control; scRNA-seq,SRP215507,CD8+ T cell_3-AD.vs.control; scRNA-seq,SRP215507,CD8+ T cell_5-AD.vs.control; scRNA-seq,SRP215507,CD8+ T cell_6-AD.vs.control; scRNA-seq,SRP215507,CD8+ T cell_3-AD.vs.MCI; scRNA-seq,SRP215507,CD8+ T cell_6-MCI.vs.control; scRNA-seq,SRP309935,Monocyte_1-AD.vs.control; scRNA-seq,SRP330776,Naive CD8+ T cell_2-AD.vs.control; scRNA-seq,SRP330776,Natural killer cell_2-AD.vs.control	14
AFF4	bulk RNA-seq,Homo_723,AD.vs.control; bulk RNA-seq,Homo_723,AD.vs.MCI; bulk RNA-seq,Homo_633,AD.vs.control; bulk RNA-seq,Homo_633,AD.vs.MCI; bulk RNA-seq,Homo_714,AD.vs.control; bulk RNA-seq,Homo_714,AD.vs.MCI; scRNA-seq,SRP215507,CD8+ T cell_5-MCI.vs.control; scRNA-seq,SRP309935,Natural killer cell_2-AD.vs.control; scRNA-seq,SRP330776,Monocyte_1-AD.vs.control; scRNA-	10
AFG1L	scRNA-seq,SRP215507,CD8+ T cell_1-MCI.vs.control; scRNA-seq,SRP215507,CD8+ T cell_4-MCI.vs.control; scRNA-seq,SRP330776,Memory T cell_2-AD.vs.control; scRNA-seq,SRP330776,Natural killer cell_1-AD.vs.control; scRNA-seq,SRP330776,T cell-AD.vs.control; scRNA-seq,SRP330776,B cell_3-	6
AFG3L2	bulk RNA-seq,Homo_723,AD.vs.control; bulk RNA-seq,Homo_723,AD.vs.MCI; bulk RNA-seq,Homo_633,AD.vs.control; bulk RNA-seq,Homo_633,AD.vs.MCI; bulk RNA-seq,Homo_714,AD.vs.control; bulk RNA-seq,Homo_714,AD.vs.MCI; bulk RNA-seq,Homo_714,MCI.vs.control; scRNA-seq,SRP215507,CD8+ T cell_6-AD.vs.control; scRNA-seq,SRP215507,CD8+ T cell_4-AD.vs.MCI; scRNA-seq,SRP215507,CD8+ T cell_5-AD.vs.MCI; scRNA-seq,SRP309935,Natural killer cell_2-AD.vs.control; scRNA-seq,SRP309935,Naive CD8+ T cell_1-AD.vs.control; scRNA-seq,SRP309935,Natural killer cell_1-AD.vs.control; scRNA-seq,SRP309935,Monocyte_2-AD.vs.control; scRNA-seq,SRP309935,Naive CD8+ T cell_2-AD.vs.control; scRNA-seq,SRP309935,CD8+ T cell_1-AD.vs.control; scRNA-seq,SRP309935,B cell_1-AD.vs.control; scRNA-seq,SRP309935,B cell_2-AD.vs.control; scRNA-seq,SRP330776,CD8+ T cell_1-AD.vs.control; scRNA-seq,SRP330776,γδ T cell-AD.vs.control; scRNA-seq,SRP330776,Memory T cell_2-AD.vs.control; scRNA-seq,SRP330776,CD8+ T cell_2-AD.vs.control; scRNA-seq,SRP330776,B cell_1-AD.vs.control; scRNA-seq,SRP330776,Naive CD8+ T cell_1-AD.vs.control; scRNA-seq,SRP330776,Natural killer cell_1-AD.vs.control; scRNA-seq,SRP330776,Memory T cell_1-AD.vs.control; scRNA-seq,SRP330776,Natural killer T cell-AD.vs.control; scRNA-seq,SRP330776,Naive CD8+ T cell_3-AD.vs.control; scRNA-seq,SRP330776,B cell_2-AD.vs.control; scRNA-seq,SRP330776,T cell-AD.vs.control; scRNA-seq,SRP330776,Naive CD8+ T cell_2-AD.vs.control; scRNA-seq,SRP330776,CD8+ T cell_3-AD.vs.control; scRNA-seq,SRP330776,Monocyte_1-AD.vs.control; scRNA-seq,SRP330776,Monocyte_2-AD.vs.control; scRNA-seq,SRP330776,B cell_3-AD.vs.control; scRNA-seq,SRP330776,Megakaryocyte_1-AD.vs.control; scRNA-seq,SRP330776,Natural	37
AFMID	bulk RNA-seq,Homo_723,AD.vs.MCI; bulk RNA-seq,Homo_723,MCI.vs.control; bulk RNA-seq,Homo_633,AD.vs.MCI; bulk RNA-seq,Homo_633,MCI.vs.control; bulk RNA-seq,Homo_714,AD.vs.control; bulk RNA-seq,Homo_714,AD.vs.MCI; bulk RNA-seq,Homo_714,MCI.vs.control; scRNA-seq,SRP215507,CD8+ T cell_2-AD.vs.control; scRNA-seq,SRP215507,CD8+ T cell_3-AD.vs.control; scRNA-seq,SRP215507,CD8+ T cell_1-AD.vs.control; scRNA-seq,SRP215507,CD8+ T cell_1-AD.vs.MCI; scRNA-seq,SRP330776,CD8+ T cell_1-AD.vs.control;	14
Afmid	bulk RNA-seq,SRP325058,AD.vs.control	1
AFP	bulk RNA-seq,ACOM,AD.vs.control; bulk RNA-seq,Homo_723,AD.vs.MCI; bulk RNA-seq,Homo_723,MCI.vs.control	3

AFTPH	scRNA-seq,SRP215507,CD8+ T cell_5-AD.vs.MCI; scRNA-seq,SRP309935,Naive CD8+ T cell_2-AD.vs.control; scRNA-seq,SRP330776,T cell-AD.vs.control; scRNA-seq,SRP330776,Natural killer cell_2-AD.vs.control	4
AGA	bulk RNA-seq,Homo_723,AD.vs.control; bulk RNA-seq,Homo_723,AD.vs.MCI; bulk RNA-seq,Homo_633,AD.vs.control; bulk RNA-seq,Homo_633,AD.vs.MCI; bulk RNA-seq,Homo_714,AD.vs.control; bulk RNA-seq,Homo_714,AD.vs.MCI; scRNA-seq,SRP215507,CD8+ T cell_2-AD.vs.control; scRNA-seq,SRP215507,CD8+ T cell_7-AD.vs.control; scRNA-seq,SRP215507,CD8+ T cell_5-AD.vs.control; scRNA-seq,SRP215507,CD8+ T cell_9-AD.vs.control; scRNA-seq,SRP215507,CD8+ T cell_2-MCI.vs.control; scRNA-seq,SRP309935,Erythroid cell-AD.vs.control; scRNA-seq,SRP330776,CD8+ T cell_1-AD.vs.control; scRNA-seq,SRP330776,CD8+ T cell_2-AD.vs.control; scRNA-seq,SRP330776,Naive CD8+ T cell_1-AD.vs.control; scRNA-seq,SRP330776,Natural killer T cell-AD.vs.control; scRNA-seq,SRP330776,Naive CD8+ T [illegible]	20
AGAP1	bulk RNA-seq,Homo_723,AD.vs.control; bulk RNA-seq,Homo_723,AD.vs.MCI; bulk RNA-seq,Homo_723,MCI.vs.control; bulk RNA-seq,Homo_633,AD.vs.control; bulk RNA-seq,Homo_633,AD.vs.MCI; bulk RNA-seq,Homo_714,AD.vs.control; bulk RNA-seq,Homo_714,AD.vs.MCI; bulk RNA-seq,ROSMAP,AD.vs.MCI; scRNA-seq,SRP215507,CD8+ T cell_3-AD.vs.control; scRNA-seq,SRP215507,CD8+ T cell_6-AD.vs.control; scRNA-seq,SRP215507,CD8+ T cell_6-MCI.vs.control; scRNA-seq,SRP309935,Natural killer cell_1-AD.vs.control; scRNA-seq,SRP330776,γδ T cell-AD.vs.control; scRNA-seq,SRP330776,Memory T cell_2-AD.vs.control; scRNA-seq,SRP330776,CD8+ T cell_2-AD.vs.control; scRNA-seq,SRP330776,Natural killer cell_1-AD.vs.control; scRNA-seq,SRP330776,Memory T cell_1-AD.vs.control; scRNA-seq,SRP330776,Natural killer T cell- [illegible]	18
AGAP2	bulk RNA-seq,Homo_723,AD.vs.control; bulk RNA-seq,Homo_723,AD.vs.MCI; bulk RNA-seq,Homo_633,AD.vs.control; bulk RNA-seq,Homo_633,AD.vs.MCI; scRNA-seq,SRP215507,CD8+ T cell_4-AD.vs.control; scRNA-seq,SRP215507,CD8+ T cell_7-AD.vs.control; scRNA-seq,SRP309935,Naive CD8+ T cell_1-AD.vs.control; scRNA-seq,SRP330776,Naive CD8+ T cell_1-AD.vs.control; scRNA-seq,SRP330776,Natural killer T	9
AGAP3	bulk RNA-seq,Homo_633,AD.vs.control; scRNA-seq,SRP215507,CD8+ T cell_1-AD.vs.MCI; scRNA-seq,SRP215507,CD8+ T cell_1-MCI.vs.control; scRNA-seq,SRP330776,CD8+ T cell_1-AD.vs.control; scRNA-seq,SRP330776,B cell_1-AD.vs.control; scRNA-seq,SRP330776,Natural killer cell_1-AD.vs.control; scRNA-seq,SRP330776,Memory T cell_1-AD.vs.control; scRNA-seq,SRP330776,CD8+ T cell_3-AD.vs.control; scRNA-	9
AGAP4	scRNA-seq,SRP215507,CD8+ T cell_2-AD.vs.MCI; scRNA-seq,SRP215507,CD8+ T cell_2-MCI.vs.control; scRNA-seq,SRP215507,CD8+ T cell_3-MCI.vs.control; scRNA-seq,SRP330776,CD8+ T cell_1-AD.vs.control; scRNA-seq,SRP330776,Memory T cell_2-AD.vs.control; scRNA-seq,SRP330776,Naive CD8+ T cell_1-AD.vs.control; scRNA-seq,SRP330776,Naive CD8+ T cell_3-AD.vs.control; scRNA-seq,SRP330776,CD8+ T	8
AGAP5	bulk RNA-seq,Homo_723,AD.vs.control; bulk RNA-seq,Homo_723,MCI.vs.control; bulk RNA-seq,Homo_633,AD.vs.control; bulk RNA-seq,Homo_633,MCI.vs.control; bulk RNA-seq,Homo_714,MCI.vs.control; scRNA-seq,SRP215507,CD8+ T cell_1-AD.vs.control; scRNA-seq,SRP215507,CD8+ T cell_1-AD.vs.MCI; scRNA-seq,SRP215507,CD8+ T cell_4-AD.vs.MCI; scRNA-seq,SRP215507,CD8+ T cell_5-AD.vs.MCI; scRNA-seq,SRP215507,CD8+ T cell_5-MCI.vs.control; scRNA-seq,SRP330776,B cell_1-AD.vs.control; scRNA-seq,SRP330776,Memory T cell_1-	12
AGAP6	bulk RNA-seq,Homo_723,AD.vs.control; bulk RNA-seq,Homo_723,MCI.vs.control; bulk RNA-seq,Homo_633,AD.vs.control; bulk RNA-seq,Homo_633,AD.vs.MCI; bulk RNA-seq,Homo_633,MCI.vs.control; bulk RNA-seq,Homo_714,AD.vs.control; bulk RNA-seq,Homo_714,AD.vs.MCI;	8
AGAP9	bulk RNA-seq,Homo_723,MCI.vs.control; bulk RNA-seq,Homo_633,MCI.vs.control; bulk RNA-seq,Homo_714,MCI.vs.control; scRNA-seq,SRP215507,CD8+ T cell_3-AD.vs.control; scRNA-seq,SRP215507,CD8+ T cell_3-AD.vs.MCI; scRNA-seq,SRP215507,CD8+ T cell_1-AD.vs.MCI; scRNA-seq,SRP215507,CD8+ T cell_4-AD.vs.MCI; scRNA-seq,SRP215507,CD8+ T cell_5-AD.vs.MCI; scRNA-seq,SRP215507,CD8+ T cell_1-MCI.vs.control; scRNA-seq,SRP215507,CD8+ T cell_4-MCI.vs.control; scRNA-seq,SRP215507,CD8+ T cell_2-MCI.vs.control; scRNA-seq,SRP215507,CD8+ T cell_3-MCI.vs.control; scRNA-seq,SRP330776,CD8+ T cell_1-AD.vs.control; scRNA-seq,SRP330776,Naive CD8+ T cell_1-	15
AGBL1	bulk RNA-seq,Homo_723,MCI.vs.control; bulk RNA-seq,Homo_633,MCI.vs.control; bulk RNA-seq,Homo_714,MCI.vs.control; bulk RNA-seq,ROSMAP,AD.vs.control; bulk RNA-seq,ROSMAP,MCI.vs.control	5
AGBL2	bulk RNA-seq,Homo_633,AD.vs.control; bulk RNA-seq,Homo_633,AD.vs.MCI; bulk RNA-seq,Homo_714,AD.vs.control; bulk RNA-seq,Homo_714,AD.vs.MCI; scRNA-seq,SRP215507,CD8+ T cell_5-MCI.vs.control; scRNA-seq,SRP330776,Natural killer cell_1-AD.vs.control	6
AGBL3	bulk RNA-seq,Homo_723,AD.vs.control; bulk RNA-seq,Homo_723,AD.vs.MCI; bulk RNA-seq,Homo_633,AD.vs.control; bulk RNA-seq,Homo_633,AD.vs.MCI; bulk RNA-seq,Homo_714,AD.vs.control; bulk RNA-seq,Homo_714,AD.vs.MCI; scRNA-seq,SRP215507,CD8+ T cell_6-MCI.vs.control; scRNA-seq,SRP330776,γδ T cell-AD.vs.control; scRNA-seq,SRP330776,CD8+ T cell_2-AD.vs.control; scRNA-seq,SRP330776,Naive CD8+ T cell_1-AD.vs.control; scRNA-seq,SRP330776,Naive CD8+ T cell_3-AD.vs.control; scRNA-seq,SRP330776,B cell_2-AD.vs.control	12
AGBL4	bulk RNA-seq,Homo_723,AD.vs.control; bulk RNA-seq,Homo_723,AD.vs.MCI; bulk RNA-seq,ROSMAP,MCI.vs.control	3

AGBL5	bulk RNA-seq,Homo_723,AD.vs.control; bulk RNA-seq,Homo_723,AD.vs.MCI; bulk RNA-seq,Homo_633,AD.vs.control; bulk RNA-seq,Homo_633,AD.vs.MCI; bulk RNA-seq,Homo_714,AD.vs.control; bulk RNA-seq,Homo_714,AD.vs.MCI; bulk RNA-seq,Homo_714,MCI.vs.control; scRNA-seq,SRP215507,CD8+ T cell_5-AD.vs.MCI; scRNA-seq,SRP330776,B cell_1-AD.vs.control; scRNA-seq,SRP330776,Naive CD8+ T cell_2-AD.vs.control; scRNA-seq,SRP330776,CD8+ T cell_3-AD.vs.control; scRNA-seq,SRP330776,B cell_3-AD.vs.control	12
AGER	scRNA-seq,SRP215507,CD8+ T cell_2-MCI.vs.control; scRNA-seq,SRP215507,CD8+ T cell_3-MCI.vs.control; scRNA-seq,SRP330776,γδ T cell-AD.vs.control; scRNA-seq,SRP330776,Memory T cell_2-AD.vs.control; scRNA-seq,SRP330776,CD8+ T cell_2-AD.vs.control; scRNA-seq,SRP330776,B cell_1-AD.vs.control; scRNA-seq,SRP330776,Naive CD8+ T cell_1-AD.vs.control; scRNA-seq,SRP330776,Naive CD8+ T cell_3-AD.vs.control; scRNA-	9
AGFG1	scRNA-seq,SRP215507,CD8+ T cell_5-AD.vs.control; scRNA-seq,SRP215507,CD8+ T cell_5-AD.vs.MCI; scRNA-seq,SRP309935,Natural killer cell_2-AD.vs.control; scRNA-seq,SRP309935,Natural killer cell_1-AD.vs.control; scRNA-seq,SRP330776,γδ T cell-AD.vs.control; scRNA-seq,SRP330776,Naive CD8+ T cell_1-AD.vs.control; scRNA-seq,SRP330776,Natural killer cell_1-AD.vs.control; scRNA-seq,SRP330776,Memory T cell_1-AD.vs.control; scRNA-seq,SRP330776,Natural killer T cell-AD.vs.control; scRNA-seq,SRP330776,CD8+ T cell_3-AD.vs.control; scRNA-seq,SRP330776,Natural killer	11
AGFG2	bulk RNA-seq,Homo_714,AD.vs.control; bulk RNA-seq,Homo_714,AD.vs.MCI; scRNA-seq,SRP215507,CD8+ T cell_3-AD.vs.control; scRNA-seq,SRP215507,CD8+ T cell_4-AD.vs.control; scRNA-seq,SRP215507,CD8+ T cell_4-AD.vs.MCI; scRNA-seq,SRP215507,CD8+ T cell_5-MCI.vs.control; scRNA-seq,SRP215507,CD8+ T cell_3-MCI.vs.control; scRNA-seq,SRP330776,CD8+ T cell_1-AD.vs.control; scRNA-seq,SRP330776,γδ T cell-AD.vs.control; scRNA-seq,SRP330776,Memory T cell_2-AD.vs.control; scRNA-seq,SRP330776,CD8+ T cell_2-AD.vs.control; scRNA-seq,SRP330776,B cell_1-AD.vs.control; scRNA-seq,SRP330776,Naive CD8+ T cell_1-AD.vs.control; scRNA-seq,SRP330776,Natural killer cell_1-AD.vs.control; scRNA-seq,SRP330776,Memory T cell_1-AD.vs.control; scRNA-seq,SRP330776,Natural killer T cell-AD.vs.control; scRNA-seq,SRP330776,Naive CD8+ T cell_3-AD.vs.control; scRNA-seq,SRP330776,T cell-AD.vs.control; scRNA-seq,SRP330776,CD8+ T cell_3-AD.vs.control; [illegible]	20
AGGF1	bulk RNA-seq,Homo_723,AD.vs.MCI; bulk RNA-seq,Homo_633,AD.vs.MCI; bulk RNA-seq,Homo_714,AD.vs.control; bulk RNA-seq,Homo_714,AD.vs.MCI; scRNA-seq,SRP215507,CD8+ T cell_5-AD.vs.MCI; scRNA-seq,SRP215507,CD8+ T cell_4-MCI.vs.control; scRNA-seq,SRP309935,Monocyte_1-AD.vs.control; scRNA-seq,SRP330776,CD8+ T cell_1-AD.vs.control; scRNA-seq,SRP330776,γδ T cell-AD.vs.control; scRNA-seq,SRP330776,Memory T cell_2-AD.vs.control; scRNA-seq,SRP330776,CD8+ T cell_2-AD.vs.control; scRNA-seq,SRP330776,B cell_1-AD.vs.control; scRNA-seq,SRP330776,Naive CD8+ T cell_1-AD.vs.control; scRNA-seq,SRP330776,Natural killer cell_1-AD.vs.control; scRNA-seq,SRP330776,Memory T cell_1-AD.vs.control; scRNA-seq,SRP330776,Natural killer T cell-AD.vs.control; scRNA-seq,SRP330776,Naive CD8+ T cell_3-AD.vs.control; scRNA-seq,SRP330776,B cell_2-AD.vs.control; scRNA-seq,SRP330776,T cell-AD.vs.control; scRNA-seq,SRP330776,Naive CD8+ T cell_2-AD.vs.control; scRNA-seq,SRP330776,CD8+ T cell_3-AD.vs.control; scRNA-seq,SRP330776,Monocyte_1-AD.vs.control; scRNA- [illegible]	25
AGK	bulk RNA-seq,Homo_723,AD.vs.control; bulk RNA-seq,Homo_723,MCI.vs.control; bulk RNA-seq,Homo_633,AD.vs.control; bulk RNA-seq,Homo_633,AD.vs.MCI; bulk RNA-seq,Homo_633,MCI.vs.control; bulk RNA-seq,Homo_714,AD.vs.control; bulk RNA-seq,Homo_714,AD.vs.MCI; bulk RNA-seq,Homo_714,MCI.vs.control; scRNA-seq,SRP215507,CD8+ T cell_7-AD.vs.control; scRNA-seq,SRP215507,CD8+ T cell_6-AD.vs.control; scRNA-seq,SRP215507,CD8+ T cell_6-AD.vs.MCI; scRNA-seq,SRP330776,Naive CD8+ T cell_1-AD.vs.control; scRNA-seq,SRP330776,Natural killer cell_1-AD.vs.control; scRNA-seq,SRP330776,Memory T cell_1-AD.vs.control; scRNA-seq,SRP330776,Naive CD8+ T cell_3-AD.vs.control; scRNA-seq,SRP330776,B cell_2-AD.vs.control; scRNA-seq,SRP330776,B cell_3-AD.vs.control; scRNA-seq,SRP330776,Regulatory T cell-AD.vs.control	18
AGL	scRNA-seq,SRP215507,CD8+ T cell_4-AD.vs.control; scRNA-seq,SRP215507,CD8+ T cell_1-MCI.vs.control; scRNA-seq,SRP215507,CD8+ T cell_5-MCI.vs.control; scRNA-seq,SRP330776,CD8+ T cell_1-AD.vs.control; scRNA-seq,SRP330776,γδ T cell-AD.vs.control; scRNA-seq,SRP330776,Memory T cell_2-AD.vs.control; scRNA-seq,SRP330776,Naive CD8+ T cell_1-AD.vs.control; scRNA-seq,SRP330776,Natural killer cell_1-AD.vs.control; scRNA-seq,SRP330776,Memory T cell_1-AD.vs.control; scRNA-seq,SRP330776,Naive CD8+ T cell_3-AD.vs.control	10
AGMAT	bulk RNA-seq,Homo_723,AD.vs.control; bulk RNA-seq,Homo_723,MCI.vs.control; bulk RNA-seq,Homo_633,AD.vs.control; bulk RNA-seq,Homo_633,MCI.vs.control; scRNA-seq,SRP215507,CD8+ T cell_3-AD.vs.control; scRNA-seq,SRP215507,CD8+ T cell_1-AD.vs.control; scRNA-seq,SRP215507,CD8+ T cell_5-MCI.vs.control; scRNA-seq,SRP215507,CD8+ T cell_2-MCI.vs.control; scRNA-seq,SRP330776,CD8+ T cell_1-AD.vs.control; scRNA-seq,SRP330776,γδ T cell-AD.vs.control; scRNA-seq,SRP330776,CD8+ T cell_2-AD.vs.control; scRNA-seq,SRP330776,B cell_1-AD.vs.control; scRNA-seq,SRP330776,Naive CD8+ T cell_1-AD.vs.control; scRNA-seq,SRP330776,B cell_2-AD.vs.control; scRNA-	15
AGMO	bulk RNA-seq,Homo_723,AD.vs.control; bulk RNA-seq,Homo_723,AD.vs.MCI; bulk RNA-seq,Homo_633,AD.vs.control; bulk RNA-seq,Homo_633,AD.vs.MCI; bulk RNA-seq,Homo_714,AD.vs.control; bulk RNA-seq,Homo_714,AD.vs.MCI	6

AGO1	bulk RNA-seq,Homo_723,AD.vs.control; bulk RNA-seq,Homo_723,AD.vs.MCI; bulk RNA-seq,Homo_723,MCI.vs.control; bulk RNA-seq,Homo_633,AD.vs.control; bulk RNA-seq,Homo_633,AD.vs.MCI; bulk RNA-seq,Homo_633,MCI.vs.control; bulk RNA-seq,Homo_714,AD.vs.control; bulk RNA-seq,Homo_714,AD.vs.MCI; scRNA-seq,SRP215507,CD8+ T cell_4-AD.vs.MCI; scRNA-seq,SRP215507,CD8+ T cell_5-AD.vs.MCI; scRNA-seq,SRP215507,CD8+ T cell_5-MCI.vs.control; scRNA-seq,SRP215507,CD8+ T cell_8-MCI.vs.control; scRNA-seq,SRP309935,Natural killer cell_2-AD.vs.control; scRNA-seq,SRP330776,CD8+ T cell_1-AD.vs.control; scRNA-seq,SRP330776,γδ T cell-AD.vs.control; scRNA-seq,SRP330776,Memory T cell_2-AD.vs.control; scRNA-seq,SRP330776,CD8+ T cell_2-AD.vs.control; scRNA-seq,SRP330776,B cell_1-AD.vs.control; scRNA-seq,SRP330776,Naive CD8+ T cell_1-AD.vs.control; scRNA-seq,SRP330776,Natural killer cell_1-AD.vs.control; scRNA-seq,SRP330776,Memory T cell_1-AD.vs.control; scRNA-seq,SRP330776,Naive CD8+ T cell_3-AD.vs.control; scRNA-seq,SRP330776,B cell_2-AD.vs.control; scRNA-seq,SRP330776,T cell-AD.vs.control; scRNA-seq,SRP330776,CD8+ T cell_3-AD.vs.control; scRNA-seq,SRP330776,Monocyte_2-AD.vs.control; scRNA-[illegible]	27
Ago2	bulk RNA-seq,SRP325058,AD.vs.control	1
AGO2	scRNA-seq,SRP215507,CD8+ T cell_3-AD.vs.MCI; scRNA-seq,SRP215507,CD8+ T cell_1-AD.vs.MCI; scRNA-seq,SRP215507,CD8+ T cell_5-AD.vs.MCI; scRNA-seq,SRP215507,CD8+ T cell_5-MCI.vs.control; scRNA-seq,SRP215507,CD8+ T cell_3-MCI.vs.control; scRNA-seq,SRP309935,CD8+ T cell_1-AD.vs.control; scRNA-seq,SRP330776,CD8+ T cell_1-AD.vs.control; scRNA-seq,SRP330776,γδ T cell-AD.vs.control; scRNA-seq,SRP330776,B cell_1-AD.vs.control; scRNA-seq,SRP330776,Naive CD8+ T cell_1-AD.vs.control; scRNA-seq,SRP330776,Natural killer cell_1-AD.vs.control; scRNA-seq,SRP330776,Memory T cell_1-AD.vs.control; scRNA-seq,SRP330776,Natural killer T cell-AD.vs.control; scRNA-seq,SRP330776,T cell-AD.vs.control; scRNA-seq,SRP330776,CD8+ T cell_3-AD.vs.control; scRNA-seq,SRP330776,B cell_3-AD.vs.control	16
AGO3	scRNA-seq,SRP215507,CD8+ T cell_2-AD.vs.MCI; scRNA-seq,SRP215507,CD8+ T cell_1-MCI.vs.control; scRNA-seq,SRP215507,CD8+ T cell_2-MCI.vs.control; scRNA-seq,SRP215507,CD8+ T cell_6-MCI.vs.control; scRNA-seq,SRP309935,Naive CD8+ T cell_2-AD.vs.control	5
AGO4	scRNA-seq,SRP215507,CD8+ T cell_3-AD.vs.control; scRNA-seq,SRP330776,CD8+ T cell_1-AD.vs.control; scRNA-seq,SRP330776,γδ T cell-AD.vs.control; scRNA-seq,SRP330776,Memory T cell_2-AD.vs.control; scRNA-seq,SRP330776,B cell_1-AD.vs.control; scRNA-seq,SRP330776,Natural killer cell_1-AD.vs.control; scRNA-seq,SRP330776,Natural killer T cell-AD.vs.control; scRNA-seq,SRP330776,T cell-AD.vs.control; scRNA-seq,SRP330776,Monocyte_1-AD.vs.control; scRNA-seq,SRP330776,Monocyte_2-AD.vs.control; scRNA-seq,SRP330776,Natural killer cell_2-[illegible]	11
AGPAT1	scRNA-seq,SRP215507,CD8+ T cell_4-AD.vs.control; scRNA-seq,SRP215507,CD8+ T cell_4-AD.vs.MCI; scRNA-seq,SRP309935,Monocyte_1-AD.vs.control; scRNA-seq,SRP309935,Monocyte_2-AD.vs.control; scRNA-seq,SRP330776,Natural killer T cell-AD.vs.control; scRNA-seq,SRP330776,Naive CD8+ T cell_3-AD.vs.control; scRNA-seq,SRP330776,B cell_3-AD.vs.control	7
AGPAT2	bulk RNA-seq,Homo_723,AD.vs.control; bulk RNA-seq,Homo_723,AD.vs.MCI; bulk RNA-seq,Homo_633,AD.vs.control; bulk RNA-seq,Homo_633,AD.vs.MCI; bulk RNA-seq,Homo_714,AD.vs.control; bulk RNA-seq,Homo_714,AD.vs.MCI; scRNA-seq,SRP215507,CD8+ T cell_4-AD.vs.control; scRNA-seq,SRP215507,CD8+ T cell_4-AD.vs.MCI; scRNA-seq,SRP330776,CD8+ T cell_1-AD.vs.control; scRNA-seq,SRP330776,γδ T cell-AD.vs.control; scRNA-seq,SRP330776,Memory T cell_2-AD.vs.control; scRNA-seq,SRP330776,CD8+ T cell_2-AD.vs.control; scRNA-seq,SRP330776,B cell_1-AD.vs.control; scRNA-seq,SRP330776,Naive CD8+ T cell_1-AD.vs.control; scRNA-seq,SRP330776,Natural killer cell_1-AD.vs.control; scRNA-seq,SRP330776,Memory T cell_1-AD.vs.control; scRNA-seq,SRP330776,Natural killer T cell-AD.vs.control; scRNA-seq,SRP330776,Naive CD8+ T cell_3-AD.vs.control; scRNA-seq,SRP330776,B cell_2-AD.vs.control; scRNA-seq,SRP330776,T cell-AD.vs.control; scRNA-seq,SRP330776,CD8+ T cell_3-AD.vs.control; scRNA-seq,SRP330776,Monocyte_2-AD.vs.control; scRNA-seq,SRP330776,B cell_3-[illegible]	24
AGPAT3	bulk RNA-seq,Homo_714,AD.vs.control; bulk RNA-seq,Homo_714,AD.vs.MCI; scRNA-seq,SRP215507,CD8+ T cell_3-AD.vs.control; scRNA-seq,SRP215507,CD8+ T cell_2-AD.vs.MCI; scRNA-seq,SRP215507,CD8+ T cell_3-MCI.vs.control; scRNA-seq,SRP309935,Natural killer cell_2-AD.vs.control; scRNA-seq,SRP309935,Naive CD8+ T cell_1-AD.vs.control; scRNA-seq,SRP309935,Natural killer cell_1-AD.vs.control; scRNA-seq,SRP309935,Monocyte_2-AD.vs.control; scRNA-seq,SRP309935,CD8+ T cell_1-AD.vs.control; scRNA-seq,SRP330776,γδ T cell-AD.vs.control; scRNA-seq,SRP330776,Memory T cell_2-AD.vs.control; scRNA-seq,SRP330776,CD8+ T cell_2-AD.vs.control; scRNA-seq,SRP330776,Memory T cell_1-AD.vs.control; scRNA-seq,SRP330776,Natural killer T cell-AD.vs.control; scRNA-seq,SRP330776,B cell_2-AD.vs.control; scRNA-[illegible]	18
AGPAT4	scRNA-seq,SRP215507,CD8+ T cell_3-AD.vs.control; scRNA-seq,SRP215507,CD8+ T cell_4-AD.vs.control; scRNA-seq,SRP215507,CD8+ T cell_2-AD.vs.MCI; scRNA-seq,SRP215507,CD8+ T cell_3-AD.vs.MCI; scRNA-seq,SRP215507,CD8+ T cell_4-AD.vs.MCI; scRNA-seq,SRP309935,Natural killer cell_1-AD.vs.control; scRNA-seq,SRP309935,CD8+ T cell_1-AD.vs.control; scRNA-seq,SRP330776,Naive CD8+ T cell_1-AD.vs.control; scRNA-seq,SRP330776,Memory T cell_1-AD.vs.control; scRNA-seq,SRP330776,Regulatory T cell-AD.vs.control	10

AGPAT5	scRNA-seq,SRP215507,CD8+ T cell_1-AD.vs.MCI; scRNA-seq,SRP215507,CD8+ T cell_4-AD.vs.MCI; scRNA-seq,SRP215507,CD8+ T cell_4-MCI.vs.control; scRNA-seq,SRP309935,B cell_1-AD.vs.control; scRNA-seq,SRP309935,B cell_2-AD.vs.control; scRNA-seq,SRP330776,γδ T cell-AD.vs.control; scRNA-seq,SRP330776,B cell_3-AD.vs.control	7
AGPS	bulk RNA-seq,Homo_723,AD.vs.MCI; bulk RNA-seq,Homo_723,MCI.vs.control; bulk RNA-seq,Homo_633,AD.vs.MCI; bulk RNA-seq,Homo_633,MCI.vs.control; bulk RNA-seq,Homo_714,AD.vs.control; bulk RNA-seq,Homo_714,AD.vs.MCI; scRNA-seq,SRP215507,CD8+ T cell_5-AD.vs.MCI; scRNA-seq,SRP215507,CD8+ T cell_5-MCI.vs.control; scRNA-seq,SRP215507,CD8+ T cell_2-MCI.vs.control; scRNA-seq,SRP215507,CD8+ T cell_6-MCI.vs.control; scRNA-seq,SRP330776,γδ T cell-AD.vs.control; scRNA-seq,SRP330776,B cell_1-AD.vs.control; scRNA-seq,SRP330776,Naive CD8+ T cell_1-AD.vs.control; scRNA-seq,SRP330776,Natural killer T cell-AD.vs.control; scRNA-seq,SRP330776,Naive CD8+ T	16
AGRN	bulk RNA-seq,Homo_723,AD.vs.control; bulk RNA-seq,Homo_723,MCI.vs.control; bulk RNA-seq,Homo_633,AD.vs.control; bulk RNA-seq,Homo_633,MCI.vs.control; bulk RNA-seq,Homo_714,MCI.vs.control; scRNA-seq,SRP215507,CD8+ T cell_3-AD.vs.MCI; scRNA-seq,SRP215507,CD8+ T cell_1-MCI.vs.control; scRNA-seq,SRP215507,CD8+ T cell_5-MCI.vs.control; scRNA-seq,SRP215507,CD8+ T cell_3-	9
AGRP	bulk RNA-seq,Homo_723,AD.vs.control; bulk RNA-seq,Homo_723,AD.vs.MCI; bulk RNA-seq,Homo_633,AD.vs.control; bulk RNA-	4
AGT	bulk RNA-seq,ACOM,AD.vs.control; bulk RNA-seq,Homo_723,AD.vs.control; bulk RNA-seq,Homo_633,AD.vs.control	3
AGTPBP1	bulk RNA-seq,Homo_723,AD.vs.MCI; bulk RNA-seq,Homo_633,AD.vs.MCI; bulk RNA-seq,Homo_714,AD.vs.control; bulk RNA-seq,Homo_714,AD.vs.MCI; scRNA-seq,SRP215507,CD8+ T cell_5-AD.vs.control; scRNA-seq,SRP215507,CD8+ T cell_10-AD.vs.control; scRNA-seq,SRP215507,CD8+ T cell_2-AD.vs.MCI; scRNA-seq,SRP215507,CD8+ T cell_3-AD.vs.MCI; scRNA-seq,SRP215507,CD8+ T cell_5-AD.vs.MCI; scRNA-seq,SRP215507,CD8+ T cell_5-MCI.vs.control; scRNA-seq,SRP215507,CD8+ T cell_2-MCI.vs.control; scRNA-seq,SRP215507,CD8+ T cell_3-MCI.vs.control; scRNA-seq,SRP309935,Natural killer cell_2-AD.vs.control; scRNA-seq,SRP309935,Naive CD8+ T cell_1-AD.vs.control	14
AGTRAP	bulk RNA-seq,Homo_723,AD.vs.control; bulk RNA-seq,Homo_723,AD.vs.MCI; bulk RNA-seq,Homo_633,AD.vs.control; bulk RNA-seq,Homo_633,AD.vs.MCI; scRNA-seq,SRP215507,CD8+ T cell_4-AD.vs.control; scRNA-seq,SRP215507,CD8+ T cell_9-AD.vs.control; scRNA-seq,SRP330776,γδ T cell-AD.vs.control; scRNA-seq,SRP330776,Memory T cell_2-AD.vs.control; scRNA-seq,SRP330776,CD8+ T cell_2-AD.vs.control; scRNA-seq,SRP330776,B cell_2-AD.vs.control; scRNA-seq,SRP330776,T cell-AD.vs.control; scRNA-seq,SRP330776,Naive CD8+ T cell_2-	12
AGXT	bulk RNA-seq,Homo_723,AD.vs.MCI; bulk RNA-seq,Homo_633,AD.vs.MCI; bulk RNA-seq,Homo_714,MCI.vs.control; bulk RNA-	4
AHCTF1	bulk RNA-seq,Homo_633,AD.vs.control; bulk RNA-seq,Homo_714,AD.vs.control; bulk RNA-seq,Homo_714,AD.vs.MCI; bulk RNA-seq,Homo_714,MCI.vs.control; scRNA-seq,SRP215507,CD8+ T cell_5-AD.vs.control; scRNA-seq,SRP215507,CD8+ T cell_5-MCI.vs.control; scRNA-seq,SRP215507,CD8+ T cell_2-MCI.vs.control; scRNA-seq,SRP309935,CD8+ T cell_1-AD.vs.control; scRNA-seq,SRP309935,Naive CD8+ T cell_3-AD.vs.control; scRNA-seq,SRP330776,CD8+ T cell_1-AD.vs.control; scRNA-seq,SRP330776,γδ T cell-AD.vs.control; scRNA-seq,SRP330776,Memory T cell_2-AD.vs.control; scRNA-seq,SRP330776,CD8+ T cell_2-AD.vs.control; scRNA-seq,SRP330776,B cell_1-AD.vs.control; scRNA-seq,SRP330776,Naive CD8+ T cell_1-AD.vs.control; scRNA-seq,SRP330776,Natural killer cell_1-AD.vs.control; scRNA-seq,SRP330776,Memory T cell_1-AD.vs.control; scRNA-seq,SRP330776,Natural killer T cell-AD.vs.control; scRNA-seq,SRP330776,Naive CD8+ T cell_3-AD.vs.control; scRNA- [illegible]	21
AHCY	scRNA-seq,SRP215507,CD8+ T cell_7-AD.vs.control; scRNA-seq,SRP215507,CD8+ T cell_10-AD.vs.control; scRNA-seq,SRP215507,CD8+ T cell_7-AD.vs.MCI; scRNA-seq,SRP215507,CD8+ T cell_4-MCI.vs.control; scRNA-seq,SRP215507,CD8+ T cell_2-MCI.vs.control; scRNA-seq,SRP309935,B cell_1-AD.vs.control; scRNA-seq,SRP330776,γδ T cell-AD.vs.control; scRNA-seq,SRP330776,Naive CD8+ T cell_1-AD.vs.control; scRNA-	9
AHCYL1	bulk RNA-seq,Homo_723,AD.vs.control; bulk RNA-seq,Homo_723,AD.vs.MCI; bulk RNA-seq,Homo_723,MCI.vs.control; bulk RNA-seq,Homo_633,AD.vs.control; bulk RNA-seq,Homo_633,AD.vs.MCI; bulk RNA-seq,Homo_633,MCI.vs.control; bulk RNA-seq,Homo_714,AD.vs.control; bulk RNA-seq,Homo_714,AD.vs.MCI; bulk RNA-seq,Homo_714,MCI.vs.control; scRNA-seq,SRP215507,CD8+ T cell_8-MCI.vs.control; scRNA-seq,SRP309935,Erythroid cell-AD.vs.control; scRNA-seq,SRP330776,CD8+ T cell_1-AD.vs.control; scRNA-seq,SRP330776,γδ T cell-AD.vs.control; scRNA-seq,SRP330776,Memory T cell_2-AD.vs.control; scRNA-seq,SRP330776,CD8+ T cell_2-AD.vs.control; scRNA-seq,SRP330776,B cell_1-AD.vs.control; scRNA-seq,SRP330776,Naive CD8+ T cell_1-AD.vs.control; scRNA-seq,SRP330776,Natural killer cell_1-AD.vs.control; scRNA-seq,SRP330776,Memory T cell_1-AD.vs.control; scRNA-seq,SRP330776,Natural killer T cell-AD.vs.control; scRNA-seq,SRP330776,Naive CD8+ T cell_3-AD.vs.control; scRNA-seq,SRP330776,B cell_2-AD.vs.control; scRNA-seq,SRP330776,T cell-AD.vs.control; scRNA-seq,SRP330776,CD8+ T [illegible]	26

AHCYL2	bulk RNA-seq,Homo_723,AD.vs.control; bulk RNA-seq,Homo_723,AD.vs.MCI; bulk RNA-seq,Homo_714,AD.vs.control; bulk RNA-seq,Homo_714,AD.vs.MCI; scRNA-seq,SRP215507,CD8+ T cell_5-AD.vs.control; scRNA-seq,SRP215507,CD8+ T cell_5-AD.vs.MCI; scRNA-seq,SRP215507,CD8+ T cell_1-MCI.vs.control; scRNA-seq,SRP215507,CD8+ T cell_5-MCI.vs.control; scRNA-seq,SRP215507,CD8+ T cell_4-MCI.vs.control; scRNA-seq,SRP215507,CD8+ T cell_2-MCI.vs.control; scRNA-seq,SRP215507,CD8+ T cell_3-MCI.vs.control; scRNA-seq,SRP330776,CD8+ T cell_1-AD.vs.control; scRNA-seq,SRP330776,γδ T cell-AD.vs.control; scRNA-seq,SRP330776,Memory T cell_2-AD.vs.control; scRNA-seq,SRP330776,CD8+ T cell_2-AD.vs.control; scRNA-seq,SRP330776,B cell_1-AD.vs.control; scRNA-seq,SRP330776,Naive CD8+ T cell_1-AD.vs.control; scRNA-seq,SRP330776,Natural killer cell_1-AD.vs.control; scRNA-seq,SRP330776,Memory T cell_1-AD.vs.control; scRNA-seq,SRP330776,Natural killer T cell-AD.vs.control; scRNA-seq,SRP330776,Naive CD8+ T cell_3-AD.vs.control; scRNA-seq,SRP330776,B cell_2-[illegible]	24
AHDC1	bulk RNA-seq,Homo_723,AD.vs.control; bulk RNA-seq,Homo_723,AD.vs.MCI; bulk RNA-seq,Homo_723,MCI.vs.control; bulk RNA-seq,Homo_633,AD.vs.control; bulk RNA-seq,Homo_633,AD.vs.MCI; bulk RNA-seq,Homo_633,MCI.vs.control; bulk RNA-seq,Homo_714,AD.vs.control; bulk RNA-seq,Homo_714,AD.vs.MCI; bulk RNA-seq,Homo_714,MCI.vs.control; bulk RNA-seq,SRP223445,AD.vs.control; scRNA-seq,SRP215507,CD8+ T cell_3-AD.vs.MCI; scRNA-seq,SRP309935,Naive CD8+ T cell_1-AD.vs.control; scRNA-seq,SRP330776,Natural killer cell_1-AD.vs.control; scRNA-seq,SRP330776,B cell_2-AD.vs.control; scRNA-seq,SRP330776,CD8+ T cell_3-AD.vs.control; scRNA-seq,SRP330776,Natural	16
AHI1	bulk RNA-seq,Homo_723,AD.vs.control; bulk RNA-seq,Homo_723,AD.vs.MCI; bulk RNA-seq,Homo_714,AD.vs.control; bulk RNA-seq,Homo_714,AD.vs.MCI; scRNA-seq,SRP215507,CD8+ T cell_4-AD.vs.control; scRNA-seq,SRP215507,CD8+ T cell_2-AD.vs.MCI; scRNA-seq,SRP215507,CD8+ T cell_5-AD.vs.MCI; scRNA-seq,SRP215507,CD8+ T cell_6-AD.vs.MCI; scRNA-seq,SRP215507,CD8+ T cell_5-MCI.vs.control; scRNA-seq,SRP215507,CD8+ T cell_2-MCI.vs.control; scRNA-seq,SRP215507,CD8+ T cell_6-MCI.vs.control; scRNA-seq,SRP330776,Regulatory T cell-	12
Ahi1	bulk RNA-seq,SRP325058,AD.vs.control	1
AHNAK	scRNA-seq,SRP215507,CD8+ T cell_3-AD.vs.control; scRNA-seq,SRP330776,Naive CD8+ T cell_3-AD.vs.control; scRNA-seq,SRP330776,B cell_2-AD.vs.control; scRNA-seq,SRP330776,B cell_3-AD.vs.control	4
AHNAK2	bulk RNA-seq,Homo_723,AD.vs.control; bulk RNA-seq,Homo_723,AD.vs.MCI; bulk RNA-seq,Homo_723,MCI.vs.control; bulk RNA-seq,Homo_633,AD.vs.control; bulk RNA-seq,Homo_633,AD.vs.MCI; bulk RNA-seq,Homo_633,MCI.vs.control; bulk RNA-seq,Homo_714,MCI.vs.control	7
AHR	scRNA-seq,SRP215507,CD8+ T cell_7-AD.vs.control; scRNA-seq,SRP215507,CD8+ T cell_3-AD.vs.MCI; scRNA-seq,SRP215507,CD8+ T cell_5-AD.vs.MCI; scRNA-seq,SRP215507,CD8+ T cell_5-MCI.vs.control; scRNA-seq,SRP215507,CD8+ T cell_3-MCI.vs.control; scRNA-seq,SRP330776,CD8+ T cell_1-AD.vs.control; scRNA-seq,SRP330776,Memory T cell_2-AD.vs.control; scRNA-seq,SRP330776,CD8+ T cell_2-AD.vs.control; scRNA-seq,SRP330776,B cell_1-AD.vs.control; scRNA-seq,SRP330776,Naive CD8+ T cell_1-AD.vs.control; scRNA-seq,SRP330776,Memory T cell_1-AD.vs.control; scRNA-seq,SRP330776,Natural killer T cell-AD.vs.control; scRNA-seq,SRP330776,Naive CD8+ T cell_3-AD.vs.control; scRNA-seq,SRP330776,T cell-AD.vs.control; scRNA-seq,SRP330776,Naive CD8+ T cell_2-AD.vs.control; scRNA-[illegible]	17
AHSA1	bulk RNA-seq,Homo_723,AD.vs.control; bulk RNA-seq,Homo_723,AD.vs.MCI; bulk RNA-seq,Homo_633,AD.vs.control; bulk RNA-seq,Homo_633,AD.vs.MCI; bulk RNA-seq,Homo_714,AD.vs.control; bulk RNA-seq,Homo_714,AD.vs.MCI; scRNA-seq,SRP215507,CD8+ T cell_7-AD.vs.control; scRNA-seq,SRP309935,B cell_1-AD.vs.control; scRNA-seq,SRP330776,CD8+ T cell_1-AD.vs.control; scRNA-seq,SRP330776,Memory T cell_2-AD.vs.control; scRNA-seq,SRP330776,Naive CD8+ T cell_1-AD.vs.control; scRNA-seq,SRP330776,Naive CD8+ T cell_3-AD.vs.control; scRNA-seq,SRP330776,CD8+ T cell_3-AD.vs.control; scRNA-seq,SRP330776,B cell_3-AD.vs.control; scRNA-seq,SRP330776,Megakaryocyte_1-AD.vs.control	15
AHSG	bulk RNA-seq,ACOM,AD.vs.control; bulk RNA-seq,Homo_723,AD.vs.MCI; bulk RNA-seq,Homo_723,MCI.vs.control; bulk RNA-seq,Homo_633,AD.vs.MCI; bulk RNA-seq,Homo_633,MCI.vs.control	5
AHSP	bulk RNA-seq,Homo_723,AD.vs.control; bulk RNA-seq,Homo_723,AD.vs.MCI; bulk RNA-seq,Homo_633,AD.vs.control; bulk RNA-seq,Homo_633,AD.vs.MCI; scRNA-seq,SRP309935,Naive CD8+ T cell_1-AD.vs.control	5
Ahsp	bulk RNA-seq,SRP325058,AD.vs.control	1
AICDA	bulk RNA-seq,Homo_714,AD.vs.control; bulk RNA-seq,Homo_714,AD.vs.MCI	2
AIDA	bulk RNA-seq,Homo_723,AD.vs.control; bulk RNA-seq,Homo_723,AD.vs.MCI; bulk RNA-seq,Homo_633,AD.vs.control; bulk RNA-seq,Homo_633,AD.vs.MCI; bulk RNA-seq,Homo_714,AD.vs.control; bulk RNA-seq,Homo_714,AD.vs.MCI; scRNA-seq,SRP215507,CD8+ T cell_4-AD.vs.MCI; scRNA-seq,SRP215507,CD8+ T cell_5-MCI.vs.control; scRNA-seq,SRP215507,CD8+ T cell_4-MCI.vs.control; scRNA-	10

AIF1	scRNA-seq,SRP215507,CD8+ T cell_3-AD.vs.control; scRNA-seq,SRP215507,CD8+ T cell_1-AD.vs.MCI; scRNA-seq,SRP215507,CD8+ T cell_6-AD.vs.MCI; scRNA-seq,SRP215507,CD8+ T cell_1-MCI.vs.control; scRNA-seq,SRP215507,CD8+ T cell_3-MCI.vs.control; scRNA-seq,SRP309935,Erythroid cell-AD.vs.control; scRNA-seq,SRP309935,Natural killer cell_1-AD.vs.control; scRNA-seq,SRP330776,CD8+ T cell_1-AD.vs.control; scRNA-seq,SRP330776,γδ T cell-AD.vs.control; scRNA-seq,SRP330776,Memory T cell_2-AD.vs.control; scRNA-seq,SRP330776,Naive CD8+ T cell_1-AD.vs.control; scRNA-seq,SRP330776,Natural killer cell_1-AD.vs.control; scRNA-seq,SRP330776,Naive CD8+ T cell_3-AD.vs.control;	15
AIF1L	bulk RNA-seq,Homo_723,AD.vs.control; bulk RNA-seq,Homo_723,AD.vs.MCI; bulk RNA-seq,Homo_723,MCI.vs.control; bulk RNA-seq,Homo_633,AD.vs.control; bulk RNA-seq,Homo_633,AD.vs.MCI; bulk RNA-seq,Homo_633,MCI.vs.control; bulk RNA-seq,Homo_714,AD.vs.control; bulk RNA-seq,Homo_714,AD.vs.MCI; bulk RNA-seq,SRP223445,AD.vs.control	9
AIFM1	bulk RNA-seq,Homo_723,AD.vs.control; bulk RNA-seq,Homo_723,AD.vs.MCI; bulk RNA-seq,Homo_633,AD.vs.control; bulk RNA-seq,Homo_633,AD.vs.MCI; bulk RNA-seq,Homo_714,AD.vs.control; bulk RNA-seq,Homo_714,AD.vs.MCI; scRNA-seq,SRP215507,CD8+ T cell_2-AD.vs.control; scRNA-seq,SRP215507,CD8+ T cell_3-AD.vs.control; scRNA-seq,SRP215507,CD8+ T cell_2-AD.vs.MCI; scRNA-seq,SRP215507,CD8+ T cell_3-AD.vs.MCI; scRNA-seq,SRP330776,CD8+ T cell_1-AD.vs.control; scRNA-seq,SRP330776,Memory T cell_2-AD.vs.control; scRNA-seq,SRP330776,Naive CD8+ T cell_1-AD.vs.control; scRNA-seq,SRP330776,Natural killer T cell-AD.vs.control; scRNA-seq,SRP330776,CD8+ T cell_3-	16
AIFM2	bulk RNA-seq,Homo_714,AD.vs.control; bulk RNA-seq,Homo_714,AD.vs.MCI; bulk RNA-seq,ROSMAP,AD.vs.control; bulk RNA-seq,ROSMAP,MCI.vs.control; scRNA-seq,SRP215507,CD8+ T cell_2-AD.vs.MCI; scRNA-seq,SRP215507,CD8+ T cell_1-AD.vs.MCI; scRNA-seq,SRP215507,CD8+ T cell_2-MCI.vs.control; scRNA-seq,SRP330776,γδ T cell-AD.vs.control; scRNA-seq,SRP330776,Memory T cell_2-AD.vs.control;	10
AIFM3	scRNA-seq,SRP330776,B cell_1-AD.vs.control; scRNA-seq,SRP330776,Monocyte_1-AD.vs.control	2
AIG1	bulk RNA-seq,Homo_723,MCI.vs.control; bulk RNA-seq,Homo_633,AD.vs.control; bulk RNA-seq,Homo_633,MCI.vs.control; bulk RNA-seq,Homo_714,AD.vs.control; bulk RNA-seq,Homo_714,AD.vs.MCI; bulk RNA-seq,Homo_714,MCI.vs.control; scRNA-seq,SRP330776,γδ T cell-AD.vs.control; scRNA-seq,SRP330776,Natural killer cell_1-AD.vs.control; scRNA-seq,SRP330776,Memory T cell_1-AD.vs.control; scRNA-seq,SRP330776,Naive CD8+ T cell_3-AD.vs.control; scRNA-seq,SRP330776,B cell_2-AD.vs.control	11
Aig1	bulk RNA-seq,SRP325058,AD.vs.control	1
AIM2	bulk RNA-seq,Homo_723,AD.vs.control; bulk RNA-seq,Homo_723,AD.vs.MCI; bulk RNA-seq,Homo_633,AD.vs.control; bulk RNA-seq,Homo_633,AD.vs.MCI; bulk RNA-seq,Homo_714,AD.vs.control; bulk RNA-seq,Homo_714,AD.vs.MCI; scRNA-seq,SRP215507,CD8+ T cell_7-AD.vs.control; scRNA-seq,SRP215507,CD8+ T cell_1-AD.vs.MCI; scRNA-seq,SRP309935,B cell_1-AD.vs.control; scRNA-seq,SRP330776,B cell_1-AD.vs.control; scRNA-seq,SRP330776,Natural killer cell_1-AD.vs.control; scRNA-seq,SRP330776,Memory T cell_1-AD.vs.control; scRNA-	13
AIMP1	bulk RNA-seq,Homo_633,AD.vs.control; bulk RNA-seq,Homo_714,AD.vs.control; bulk RNA-seq,Homo_714,AD.vs.MCI; scRNA-seq,SRP215507,CD8+ T cell_6-AD.vs.control; scRNA-seq,SRP215507,CD8+ T cell_6-AD.vs.MCI; scRNA-seq,SRP330776,CD8+ T cell_3-AD.vs.control; scRNA-seq,SRP330776,Monocyte_2-AD.vs.control	7
AIMP2	scRNA-seq,SRP215507,CD8+ T cell_2-AD.vs.MCI; scRNA-seq,SRP215507,CD8+ T cell_4-AD.vs.MCI; scRNA-seq,SRP330776,CD8+ T cell_1-AD.vs.control; scRNA-seq,SRP330776,Memory T cell_2-AD.vs.control; scRNA-seq,SRP330776,CD8+ T cell_2-AD.vs.control; scRNA-seq,SRP330776,Naive CD8+ T cell_1-AD.vs.control; scRNA-seq,SRP330776,Natural killer cell_1-AD.vs.control; scRNA-seq,SRP330776,Memory T cell_1-AD.vs.control; scRNA-seq,SRP330776,Natural killer T cell-AD.vs.control; scRNA-seq,SRP330776,Naive CD8+ T cell_3-AD.vs.control; scRNA-seq,SRP330776,B cell_2-AD.vs.control; scRNA-seq,SRP330776,T cell-AD.vs.control; scRNA-seq,SRP330776,CD8+ T cell_3-AD.vs.control; scRNA-	14
AIP	scRNA-seq,SRP215507,CD8+ T cell_7-MCI.vs.control; scRNA-seq,SRP330776,CD8+ T cell_2-AD.vs.control; scRNA-seq,SRP330776,Memory T cell_1-AD.vs.control; scRNA-seq,SRP330776,B cell_2-AD.vs.control; scRNA-seq,SRP330776,CD8+ T cell_3-AD.vs.control; scRNA-seq,SRP330776,Monocyte_2-AD.vs.control; scRNA-seq,SRP330776,Natural killer cell_2-AD.vs.control	7
AIPL1	bulk RNA-seq,Homo_723,AD.vs.control; bulk RNA-seq,Homo_723,MCI.vs.control; bulk RNA-seq,Homo_633,AD.vs.control; bulk RNA-seq,Homo_633,MCI.vs.control; bulk RNA-seq,Homo_714,MCI.vs.control	5
AIRE	scRNA-seq,SRP330776,CD8+ T cell_1-AD.vs.control; scRNA-seq,SRP330776,Natural killer T cell-AD.vs.control; scRNA-seq,SRP330776,T cell-	3
AIRN	scRNA-seq,SRP215507,CD8+ T cell_2-MCI.vs.control	1
AJAP1	bulk RNA-seq,Homo_723,AD.vs.control; bulk RNA-seq,Homo_723,AD.vs.MCI; bulk RNA-seq,Homo_633,AD.vs.control; bulk RNA-seq,Homo_633,AD.vs.MCI; bulk RNA-seq,Homo_714,AD.vs.control; bulk RNA-seq,Homo_714,AD.vs.MCI; bulk RNA-seq,ROSMAP,AD.vs.control	7

AJM1	bulk RNA-seq,Homo_723,AD.vs.control; bulk RNA-seq,Homo_633,AD.vs.control; bulk RNA-seq,Homo_714,AD.vs.control; bulk RNA-seq,Homo_714,AD.vs.MCI; bulk RNA-seq,Homo_714,MCI.vs.control; bulk RNA-seq,SRP223445,AD.vs.control; scRNA-seq,SRP215507,CD8+ T cell_8-AD.vs.control; scRNA-seq,SRP215507,CD8+ T cell_3-AD.vs.control; scRNA-seq,SRP215507,CD8+ T cell_2-AD.vs.MCI; scRNA-seq,SRP215507,CD8+ T cell_3-AD.vs.MCI; scRNA-seq,SRP309935,Naive CD8+ T cell_1-AD.vs.control; scRNA-seq,SRP309935,Natural killer cell_1-AD.vs.control; scRNA-seq,SRP330776,CD8+ T cell_1-AD.vs.control; scRNA-seq,SRP330776,Memory T cell_2-AD.vs.control; scRNA-seq,SRP330776,CD8+ T cell_2-AD.vs.control; scRNA-seq,SRP330776,Natural killer cell_1-AD.vs.control; scRNA-seq,SRP330776,Memory T cell_1-AD.vs.control	17
AJUBA	bulk RNA-seq,Homo_723,AD.vs.control; bulk RNA-seq,Homo_723,AD.vs.MCI; bulk RNA-seq,Homo_723,MCI.vs.control; bulk RNA-seq,Homo_714,AD.vs.control; bulk RNA-seq,Homo_714,AD.vs.MCI; scRNA-seq,SRP330776,CD8+ T cell_1-AD.vs.control	6
AK1	bulk RNA-seq,Homo_723,AD.vs.control; bulk RNA-seq,Homo_723,AD.vs.MCI; bulk RNA-seq,Homo_633,AD.vs.control; bulk RNA-seq,Homo_633,AD.vs.MCI; bulk RNA-seq,Homo_714,AD.vs.control; bulk RNA-seq,Homo_714,AD.vs.MCI; bulk RNA-seq,SRP310421,AD.vs.control; scRNA-seq,SRP215507,CD8+ T cell_1-AD.vs.MCI; scRNA-seq,SRP215507,CD8+ T cell_1-MCI.vs.control; scRNA-seq,SRP215507,CD8+ T cell_2-MCI.vs.control; scRNA-seq,SRP309935,Monocyte_1-AD.vs.control; scRNA-seq,SRP309935,Megakaryocyte_1-AD.vs.control; scRNA-seq,SRP330776,γδ T cell-AD.vs.control; scRNA-seq,SRP330776,CD8+ T cell_2-AD.vs.control; scRNA-seq,SRP330776,Naive CD8+ T cell_1-AD.vs.control	15
AK2	bulk RNA-seq,Homo_723,AD.vs.control; bulk RNA-seq,Homo_723,AD.vs.MCI; bulk RNA-seq,Homo_633,AD.vs.control; bulk RNA-seq,Homo_633,AD.vs.MCI; bulk RNA-seq,Homo_714,AD.vs.control; bulk RNA-seq,Homo_714,AD.vs.MCI; scRNA-seq,SRP215507,CD8+ T cell_7-AD.vs.control; scRNA-seq,SRP215507,CD8+ T cell_4-AD.vs.MCI; scRNA-seq,SRP215507,CD8+ T cell_6-AD.vs.MCI; scRNA-seq,SRP215507,CD8+ T cell_4-MCI.vs.control; scRNA-seq,SRP215507,CD8+ T cell_7-MCI.vs.control; scRNA-seq,SRP330776,CD8+ T cell_2-AD.vs.control; scRNA- [illegible]	13
AK3	bulk RNA-seq,Homo_723,AD.vs.control; bulk RNA-seq,Homo_723,AD.vs.MCI; bulk RNA-seq,Homo_633,AD.vs.control; bulk RNA-seq,Homo_633,AD.vs.MCI; bulk RNA-seq,Homo_714,AD.vs.control; bulk RNA-seq,Homo_714,AD.vs.MCI; scRNA-seq,SRP215507,CD8+ T cell_4-AD.vs.control; scRNA-seq,SRP215507,CD8+ T cell_7-AD.vs.control; scRNA-seq,SRP215507,CD8+ T cell_6-AD.vs.control; scRNA-seq,SRP215507,CD8+ T cell_4-AD.vs.MCI; scRNA-seq,SRP215507,CD8+ T cell_5-AD.vs.MCI; scRNA-seq,SRP215507,CD8+ T cell_6-MCI.vs.control; scRNA-seq,SRP215507,CD8+ T cell_7-MCI.vs.control; scRNA-seq,SRP309935,Monocyte_1-AD.vs.control; scRNA-seq,SRP330776,CD8+ T cell_1-AD.vs.control; scRNA-seq,SRP330776,γδ T cell-AD.vs.control; scRNA-seq,SRP330776,Memory T cell_2-AD.vs.control; scRNA-seq,SRP330776,B cell_1-AD.vs.control; scRNA-seq,SRP330776,Naive CD8+ T cell_1-AD.vs.control; scRNA-seq,SRP330776,Natural killer cell_1-AD.vs.control; scRNA-seq,SRP330776,Memory T cell_1-AD.vs.control; scRNA-seq,SRP330776,Natural killer T cell-AD.vs.control; scRNA-seq,SRP330776,Naive CD8+ T [illegible]	26
AK4	bulk RNA-seq,Homo_723,AD.vs.control; bulk RNA-seq,Homo_723,AD.vs.MCI; bulk RNA-seq,Homo_723,MCI.vs.control; bulk RNA-seq,Homo_633,AD.vs.control; bulk RNA-seq,Homo_633,AD.vs.MCI; bulk RNA-seq,Homo_714,AD.vs.control; bulk RNA-seq,Homo_714,AD.vs.MCI;	8
AK5	bulk RNA-seq,Homo_723,AD.vs.control; bulk RNA-seq,Homo_633,AD.vs.control; bulk RNA-seq,Homo_714,AD.vs.control; bulk RNA-seq,Homo_714,AD.vs.MCI; scRNA-seq,SRP215507,CD8+ T cell_2-AD.vs.control; scRNA-seq,SRP215507,CD8+ T cell_1-AD.vs.control; scRNA-seq,SRP215507,CD8+ T cell_4-AD.vs.control; scRNA-seq,SRP215507,CD8+ T cell_2-MCI.vs.control; scRNA-seq,SRP309935,Natural killer cell_2-AD.vs.control; scRNA-seq,SRP309935,Natural killer cell_1-AD.vs.control; scRNA-seq,SRP330776,CD8+ T cell_1-AD.vs.control; scRNA-seq,SRP330776,γδ T cell-AD.vs.control; scRNA-seq,SRP330776,Memory T cell_2-AD.vs.control; scRNA-seq,SRP330776,CD8+ T cell_2-AD.vs.control; scRNA-seq,SRP330776,B cell_1-AD.vs.control; scRNA-seq,SRP330776,Naive CD8+ T cell_1-AD.vs.control; scRNA-seq,SRP330776,Memory T cell_1-AD.vs.control; scRNA-seq,SRP330776,Natural killer T cell-AD.vs.control; scRNA-seq,SRP330776,Naive CD8+ T cell_3-AD.vs.control; scRNA- [illegible]	20
AK6	bulk RNA-seq,Homo_633,AD.vs.control; bulk RNA-seq,Homo_633,AD.vs.MCI; bulk RNA-seq,Homo_714,AD.vs.control; bulk RNA-seq,Homo_714,AD.vs.MCI; scRNA-seq,SRP215507,CD8+ T cell_10-AD.vs.control; scRNA-seq,SRP330776,CD8+ T cell_2-AD.vs.control; scRNA-seq,SRP330776,B cell_1-AD.vs.control; scRNA-seq,SRP330776,Natural killer cell_1-AD.vs.control; scRNA-seq,SRP330776,Memory T cell_1-AD.vs.control; scRNA-seq,SRP330776,Naive CD8+ T cell_3-AD.vs.control; scRNA-seq,SRP330776,B cell_2-AD.vs.control; scRNA-seq,SRP330776,T cell-AD.vs.control; scRNA-seq,SRP330776,CD8+ T cell_3-AD.vs.control; scRNA-seq,SRP330776,Monocyte_2-AD.vs.control	14
AK7	bulk RNA-seq,Homo_723,AD.vs.control; bulk RNA-seq,Homo_723,AD.vs.MCI; bulk RNA-seq,Homo_633,AD.vs.control; bulk RNA-seq,Homo_633,AD.vs.MCI; bulk RNA-seq,Homo_714,MCI.vs.control	5
AK8	bulk RNA-seq,Homo_723,AD.vs.control; bulk RNA-seq,Homo_723,AD.vs.MCI; bulk RNA-seq,Homo_633,MCI.vs.control; bulk RNA-seq,Homo_714,MCI.vs.control; scRNA-seq,SRP330776,B cell_1-AD.vs.control	5

AK9	scRNA-seq,SRP215507,CD8+ T cell_4-AD.vs.control; scRNA-seq,SRP215507,CD8+ T cell_5-AD.vs.control; scRNA-seq,SRP215507,CD8+ T cell_4-AD.vs.MCI; scRNA-seq,SRP215507,CD8+ T cell_5-AD.vs.MCI; scRNA-seq,SRP215507,CD8+ T cell_4-MCI.vs.control; scRNA-seq,SRP309935,Naive CD8+ T cell_1-AD.vs.control; scRNA-seq,SRP309935,CD8+ T cell_2-AD.vs.control; scRNA-seq,SRP330776,γδ T cell-AD.vs.control; scRNA-seq,SRP330776,Memory T cell_2-AD.vs.control; scRNA-seq,SRP330776,Natural killer T cell-AD.vs.control; scRNA-seq,SRP330776,B cell_2- [illegible]	11
AKAP1	bulk RNA-seq,Homo_723,AD.vs.control; bulk RNA-seq,Homo_633,AD.vs.control; bulk RNA-seq,Homo_633,AD.vs.MCI; bulk RNA-seq,Homo_714,AD.vs.control; bulk RNA-seq,Homo_714,AD.vs.MCI; bulk RNA-seq,SRP223445,AD.vs.control; scRNA-seq,SRP215507,CD8+ T cell_3-AD.vs.control; scRNA-seq,SRP215507,CD8+ T cell_3-AD.vs.MCI; scRNA-seq,SRP215507,CD8+ T cell_4-MCI.vs.control; scRNA-seq,SRP309935,B cell_1-AD.vs.control; scRNA-seq,SRP330776,CD8+ T cell_1-AD.vs.control; scRNA-seq,SRP330776,γδ T cell-AD.vs.control; scRNA-seq,SRP330776,Memory T cell_2-AD.vs.control; scRNA-seq,SRP330776,CD8+ T cell_2-AD.vs.control; scRNA-seq,SRP330776,B cell_1-AD.vs.control; scRNA-seq,SRP330776,Naive CD8+ T cell_1-AD.vs.control; scRNA-seq,SRP330776,Natural killer cell_1-AD.vs.control; scRNA-seq,SRP330776,Memory T cell_1-AD.vs.control; scRNA-seq,SRP330776,Natural killer T cell-AD.vs.control; scRNA-seq,SRP330776,Naive CD8+ T cell_3-AD.vs.control; scRNA- [illegible]	23
AKAP10	bulk RNA-seq,Homo_714,AD.vs.control; bulk RNA-seq,Homo_714,AD.vs.MCI; scRNA-seq,SRP215507,CD8+ T cell_6-AD.vs.control; scRNA-seq,SRP215507,CD8+ T cell_2-AD.vs.MCI; scRNA-seq,SRP215507,CD8+ T cell_6-AD.vs.MCI; scRNA-seq,SRP215507,CD8+ T cell_7-MCI.vs.control; scRNA-seq,SRP330776,B cell_2-AD.vs.control; scRNA-seq,SRP330776,Monocyte_1-AD.vs.control; scRNA-seq,SRP330776,Megakaryocyte_1-AD.vs.control; scRNA-seq,SRP330776,Natural killer cell_2-AD.vs.control	10
AKAP11	bulk RNA-seq,Homo_723,AD.vs.control; bulk RNA-seq,Homo_723,AD.vs.MCI; bulk RNA-seq,Homo_723,MCI.vs.control; bulk RNA-seq,Homo_633,AD.vs.control; bulk RNA-seq,Homo_633,AD.vs.MCI; bulk RNA-seq,Homo_633,MCI.vs.control; bulk RNA-seq,Homo_714,AD.vs.control; bulk RNA-seq,Homo_714,AD.vs.MCI; scRNA-seq,SRP309935,Monocyte_2-AD.vs.control; scRNA-seq,SRP309935,CD8+ T cell_1-AD.vs.control;	11
AKAP12	bulk RNA-seq,Homo_723,AD.vs.control; bulk RNA-seq,Homo_723,AD.vs.MCI; bulk RNA-seq,Homo_723,MCI.vs.control; bulk RNA-seq,Homo_633,AD.vs.control; bulk RNA-seq,Homo_633,AD.vs.MCI; bulk RNA-seq,Homo_633,MCI.vs.control; bulk RNA-seq,Homo_714,AD.vs.control; bulk RNA-seq,Homo_714,AD.vs.MCI; scRNA-seq,SRP330776,CD8+ T cell_1-AD.vs.control	9
AKAP13	scRNA-seq,SRP215507,CD8+ T cell_10-AD.vs.control; scRNA-seq,SRP309935,Erythroid cell-AD.vs.control; scRNA-seq,SRP330776,Megakaryocyte_1-	3
AKAP14	bulk RNA-seq,Homo_723,AD.vs.control; bulk RNA-seq,Homo_723,AD.vs.MCI; bulk RNA-seq,Homo_723,MCI.vs.control	3
AKAP17A	bulk RNA-seq,Homo_723,AD.vs.control; bulk RNA-seq,Homo_723,AD.vs.MCI; bulk RNA-seq,Homo_633,AD.vs.control; bulk RNA-seq,Homo_633,AD.vs.MCI; bulk RNA-seq,Homo_714,AD.vs.control; bulk RNA-seq,Homo_714,AD.vs.MCI; scRNA-seq,SRP215507,CD8+ T cell_6-AD.vs.control; scRNA-seq,SRP215507,CD8+ T cell_3-AD.vs.MCI; scRNA-seq,SRP215507,CD8+ T cell_5-AD.vs.MCI; scRNA-seq,SRP215507,CD8+ T cell_5-MCI.vs.control; scRNA-seq,SRP309935,Erythroid cell-AD.vs.control; scRNA-seq,SRP330776,CD8+ T cell_1-AD.vs.control; scRNA-seq,SRP330776,γδ T cell-AD.vs.control; scRNA-seq,SRP330776,Memory T cell_2-AD.vs.control; scRNA-seq,SRP330776,CD8+ T cell_2-AD.vs.control; scRNA-seq,SRP330776,B cell_1-AD.vs.control; scRNA-seq,SRP330776,Naive CD8+ T cell_1-AD.vs.control; scRNA-seq,SRP330776,Natural killer cell_1-AD.vs.control; scRNA-seq,SRP330776,Memory T cell_1-AD.vs.control; scRNA-seq,SRP330776,Natural killer T cell-AD.vs.control; scRNA-seq,SRP330776,Naive CD8+ T cell_3-AD.vs.control; scRNA-seq,SRP330776,B cell_2-AD.vs.control; scRNA-seq,SRP330776,T cell-AD.vs.control; scRNA-seq,SRP330776,Naive CD8+ T cell_2-AD.vs.control; scRNA-seq,SRP330776,CD8+ T cell_3-AD.vs.control; scRNA-seq,SRP330776,Monocyte_1-AD.vs.control; scRNA-seq,SRP330776,Monocyte_2-AD.vs.control; scRNA-seq,SRP330776,B cell_3-AD.vs.control; scRNA-seq,SRP330776,Regulatory T [illegible]	31
AKAP3	bulk RNA-seq,Homo_723,AD.vs.MCI; bulk RNA-seq,Homo_723,MCI.vs.control; bulk RNA-seq,Homo_633,AD.vs.MCI; bulk RNA-seq,Homo_633,MCI.vs.control; bulk RNA-seq,Homo_714,AD.vs.control; bulk RNA-seq,Homo_714,AD.vs.MCI; scRNA-seq,SRP215507,CD8+ T cell_5-AD.vs.MCI; scRNA-seq,SRP215507,CD8+ T cell_5-MCI.vs.control; scRNA-seq,SRP330776,Memory T cell_1-AD.vs.control	9
AKAP4	bulk RNA-seq,Homo_723,AD.vs.control; bulk RNA-seq,Homo_633,AD.vs.control	2
AKAP5	scRNA-seq,SRP215507,CD8+ T cell_1-AD.vs.control; scRNA-seq,SRP215507,CD8+ T cell_2-AD.vs.MCI; scRNA-seq,SRP215507,CD8+ T cell_1-AD.vs.MCI; scRNA-seq,SRP215507,CD8+ T cell_5-AD.vs.MCI; scRNA-seq,SRP215507,CD8+ T cell_5-MCI.vs.control; scRNA-seq,SRP215507,CD8+ T cell_4-MCI.vs.control; scRNA-seq,SRP215507,CD8+ T cell_2-MCI.vs.control; scRNA-seq,SRP309935,Natural killer cell_2-AD.vs.control; scRNA-seq,SRP330776,CD8+ T cell_2-AD.vs.control; scRNA-seq,SRP330776,B cell_1-AD.vs.control; scRNA-seq,SRP330776,Memory T cell_1-AD.vs.control	11

AKAP6	bulk RNA-seq,Homo_723,AD.vs.control; bulk RNA-seq,Homo_723,AD.vs.MCI; bulk RNA-seq,Homo_714,AD.vs.control; bulk RNA-seq,Homo_714,AD.vs.MCI; bulk RNA-seq,ROSMAP,AD.vs.control; bulk RNA-seq,ROSMAP,MCI.vs.control; scRNA-seq,SRP215507,CD8+ T cell_3-AD.vs.MCI; scRNA-seq,SRP215507,CD8+ T cell_3-MCI.vs.control; scRNA-seq,SRP330776,B cell_2-AD.vs.control	9
AKAP7	bulk RNA-seq,Homo_723,MCI.vs.control; bulk RNA-seq,Homo_714,AD.vs.control; bulk RNA-seq,Homo_714,AD.vs.MCI; scRNA-seq,SRP215507,CD8+ T cell_7-MCI.vs.control; scRNA-seq,SRP330776,CD8+ T cell_1-AD.vs.control; scRNA-seq,SRP330776,CD8+ T cell_2-AD.vs.control; scRNA-seq,SRP330776,B cell_1-AD.vs.control; scRNA-seq,SRP330776,Naive CD8+ T cell_1-AD.vs.control; scRNA-seq,SRP330776,Memory T cell_1-AD.vs.control; scRNA-seq,SRP330776,Naive CD8+ T cell_3-AD.vs.control; scRNA-seq,SRP330776,B cell_2-AD.vs.control; scRNA-seq,SRP330776,T cell-AD.vs.control; scRNA-seq,SRP330776,CD8+ T cell_3-AD.vs.control; scRNA-seq,SRP330776,B cell_3-AD.vs.control	14
AKAP8	bulk RNA-seq,Homo_723,MCI.vs.control; bulk RNA-seq,Homo_633,MCI.vs.control; scRNA-seq,SRP215507,CD8+ T cell_6-AD.vs.control; scRNA-seq,SRP215507,CD8+ T cell_1-AD.vs.MCI; scRNA-seq,SRP330776,Naive CD8+ T cell_3-AD.vs.control; scRNA-seq,SRP330776,Regulatory T cell-[illegible]	6
AKAP8L	bulk RNA-seq,Homo_633,AD.vs.control; scRNA-seq,SRP330776,CD8+ T cell_1-AD.vs.control; scRNA-seq,SRP330776,Naive CD8+ T cell_1-AD.vs.control; scRNA-seq,SRP330776,Natural killer T cell-AD.vs.control; scRNA-seq,SRP330776,Naive CD8+ T cell_3-AD.vs.control	5
AKAP9	bulk RNA-seq,Homo_723,AD.vs.control; bulk RNA-seq,Homo_723,MCI.vs.control; bulk RNA-seq,Homo_633,AD.vs.control; bulk RNA-seq,Homo_633,AD.vs.MCI; bulk RNA-seq,Homo_714,AD.vs.control; bulk RNA-seq,Homo_714,AD.vs.MCI; bulk RNA-seq,Homo_714,MCI.vs.control; scRNA-seq,SRP330776,Memory T cell_2-AD.vs.control; scRNA-seq,SRP330776,Monocyte_2-AD.vs.control	9
AKIP1	bulk RNA-seq,Homo_723,AD.vs.control; bulk RNA-seq,Homo_723,AD.vs.MCI; bulk RNA-seq,Homo_633,AD.vs.control; bulk RNA-seq,Homo_633,AD.vs.MCI; bulk RNA-seq,Homo_714,AD.vs.control; bulk RNA-seq,Homo_714,AD.vs.MCI; scRNA-seq,SRP215507,CD8+ T cell_6-AD.vs.control; scRNA-seq,SRP215507,CD8+ T cell_6-AD.vs.MCI; scRNA-seq,SRP215507,CD8+ T cell_4-MCI.vs.control; scRNA-seq,SRP309935,Natural killer cell_1-AD.vs.control; scRNA-seq,SRP330776,CD8+ T cell_3-AD.vs.control	11
AKIRIN1	bulk RNA-seq,Homo_723,AD.vs.control; bulk RNA-seq,Homo_723,AD.vs.MCI; bulk RNA-seq,Homo_633,AD.vs.control; bulk RNA-seq,Homo_633,AD.vs.MCI; scRNA-seq,SRP215507,CD8+ T cell_6-AD.vs.control; scRNA-seq,SRP215507,CD8+ T cell_4-AD.vs.MCI; scRNA-seq,SRP215507,CD8+ T cell_6-AD.vs.MCI; scRNA-seq,SRP215507,CD8+ T cell_5-MCI.vs.control; scRNA-seq,SRP309935,B cell_1-AD.vs.control; scRNA-seq,SRP330776,CD8+ T cell_1-AD.vs.control; scRNA-seq,SRP330776,γδ T cell-AD.vs.control; scRNA-seq,SRP330776,Memory T cell_2-AD.vs.control; scRNA-seq,SRP330776,CD8+ T cell_2-AD.vs.control; scRNA-seq,SRP330776,B cell_1-AD.vs.control; scRNA-seq,SRP330776,Naive CD8+ T cell_1-AD.vs.control; scRNA-seq,SRP330776,Natural killer cell_1-AD.vs.control; scRNA-seq,SRP330776,Memory T cell_1-AD.vs.control; scRNA-seq,SRP330776,Natural killer T cell-AD.vs.control; scRNA-seq,SRP330776,Naive CD8+ T cell_3-AD.vs.control; scRNA-seq,SRP330776,B cell_2-AD.vs.control; scRNA-seq,SRP330776,T cell-AD.vs.control; scRNA-seq,SRP330776,Naive CD8+ T cell_2-AD.vs.control; scRNA-seq,SRP330776,CD8+ T cell_3-AD.vs.control; scRNA-seq,SRP330776,Monocyte_1-AD.vs.control; scRNA-seq,SRP330776,Monocyte_2-AD.vs.control; scRNA-[illegible]	28
AKIRIN2	bulk RNA-seq,Homo_723,AD.vs.control; bulk RNA-seq,Homo_723,AD.vs.MCI; bulk RNA-seq,Homo_714,AD.vs.control; bulk RNA-seq,Homo_714,AD.vs.MCI; bulk RNA-seq,Homo_714,MCI.vs.control; scRNA-seq,SRP215507,CD8+ T cell_7-AD.vs.control; scRNA-seq,SRP215507,CD8+ T cell_2-AD.vs.MCI; scRNA-seq,SRP215507,CD8+ T cell_7-AD.vs.MCI; scRNA-seq,SRP215507,CD8+ T cell_5-AD.vs.MCI; scRNA-seq,SRP215507,CD8+ T cell_6-AD.vs.MCI; scRNA-seq,SRP215507,CD8+ T cell_5-MCI.vs.control; scRNA-seq,SRP215507,CD8+ T cell_2-MCI.vs.control; scRNA-seq,SRP215507,CD8+ T cell_6-MCI.vs.control; scRNA-seq,SRP330776,B cell_4-AD.vs.control	14
Akna	bulk RNA-seq,SRP325058,AD.vs.control	1
AKNA	scRNA-seq,SRP309935,Natural killer cell_2-AD.vs.control; scRNA-seq,SRP309935,Megakaryocyte_1-AD.vs.control; scRNA-seq,SRP330776,Naive CD8+ T cell_2-AD.vs.control	3
AKNAD1	bulk RNA-seq,Homo_723,AD.vs.control; scRNA-seq,SRP215507,CD8+ T cell_2-MCI.vs.control; scRNA-seq,SRP330776,CD8+ T cell_1-AD.vs.control; scRNA-seq,SRP330776,γδ T cell-AD.vs.control; scRNA-seq,SRP330776,CD8+ T cell_2-AD.vs.control; scRNA-seq,SRP330776,Naive CD8+ T cell_1-AD.vs.control; scRNA-seq,SRP330776,CD8+ T cell_3-AD.vs.control	7
AKR1A1	bulk RNA-seq,Homo_723,AD.vs.control; bulk RNA-seq,Homo_723,AD.vs.MCI; bulk RNA-seq,Homo_633,AD.vs.control; bulk RNA-seq,Homo_633,AD.vs.MCI; bulk RNA-seq,Homo_714,AD.vs.control; bulk RNA-seq,Homo_714,AD.vs.MCI; scRNA-seq,SRP215507,CD8+ T cell_7-AD.vs.MCI; scRNA-seq,SRP330776,CD8+ T cell_3-AD.vs.control; scRNA-seq,SRP330776,B cell_3-AD.vs.control	9

AKR1B1	bulk RNA-seq,Homo_723,AD.vs.control; bulk RNA-seq,Homo_723,AD.vs.MCI; bulk RNA-seq,Homo_723,MCI.vs.control; bulk RNA-seq,Homo_633,AD.vs.control; bulk RNA-seq,Homo_633,AD.vs.MCI; bulk RNA-seq,Homo_633,MCI.vs.control; bulk RNA-seq,Homo_714,AD.vs.control; bulk RNA-seq,Homo_714,AD.vs.MCI; bulk RNA-seq,Homo_714,MCI.vs.control; scRNA-seq,SRP309935,Erythroid cell-AD.vs.control; scRNA-seq,SRP309935,Naive CD8+ T cell_3-AD.vs.control; scRNA-seq,SRP330776,Memory T cell_2-AD.vs.control; scRNA-seq,SRP330776,Megakaryocyte_1-	13
AKR1B10	bulk RNA-seq,Homo_723,AD.vs.control; bulk RNA-seq,Homo_723,MCI.vs.control; bulk RNA-seq,Homo_633,MCI.vs.control; bulk RNA-seq,Homo_714,MCI.vs.control; bulk RNA-seq,ROSMAP,AD.vs.control	5
AKR1C1	bulk RNA-seq,Homo_714,AD.vs.control; bulk RNA-seq,Homo_714,AD.vs.MCI; scRNA-seq,SRP330776,CD8+ T cell_1-AD.vs.control	3
AKR1C2	bulk RNA-seq,Homo_723,AD.vs.control; bulk RNA-seq,Homo_723,AD.vs.MCI; bulk RNA-seq,Homo_723,MCI.vs.control; bulk RNA-seq,Homo_633,AD.vs.control; bulk RNA-seq,Homo_633,AD.vs.MCI; bulk RNA-seq,Homo_633,MCI.vs.control; bulk RNA-seq,Homo_714,AD.vs.control; bulk RNA-seq,Homo_714,AD.vs.MCI; bulk RNA-seq,Homo_714,MCI.vs.control; bulk RNA-seq,ROSMAP,AD.vs.control; bulk RNA-	11
AKR1C3	bulk RNA-seq,Homo_714,AD.vs.control; bulk RNA-seq,Homo_714,AD.vs.MCI; scRNA-seq,SRP215507,CD8+ T cell_3-AD.vs.control; scRNA-seq,SRP215507,CD8+ T cell_1-AD.vs.control; scRNA-seq,SRP215507,CD8+ T cell_5-AD.vs.control; scRNA-seq,SRP215507,CD8+ T cell_1-AD.vs.MCI; scRNA-seq,SRP215507,CD8+ T cell_1-MCI.vs.control; scRNA-seq,SRP309935,Natural killer cell_2-AD.vs.control; scRNA-seq,SRP309935,Monocyte_1-AD.vs.control; scRNA-seq,SRP309935,Natural killer cell_1-AD.vs.control; scRNA-seq,SRP309935,Naive CD8+ T cell_3-AD.vs.control; scRNA-seq,SRP330776,γδ T cell-AD.vs.control; scRNA-seq,SRP330776,CD8+ T cell_2-AD.vs.control; scRNA-seq,SRP330776,Naive CD8+ T cell_1-AD.vs.control; scRNA-seq,SRP330776,Memory T cell_1-AD.vs.control; scRNA-seq,SRP330776,B cell_3-AD.vs.control	16
AKR1C4	bulk RNA-seq,Homo_723,AD.vs.MCI; bulk RNA-seq,Homo_723,MCI.vs.control; bulk RNA-seq,Homo_633,AD.vs.control; bulk RNA-seq,Homo_633,AD.vs.MCI; bulk RNA-seq,Homo_633,MCI.vs.control; bulk RNA-seq,Homo_714,MCI.vs.control	6
AKR1C8P	bulk RNA-seq,ROSMAP,MCI.vs.control; scRNA-seq,SRP215507,CD8+ T cell_2-MCI.vs.control	2
AKR1D1	bulk RNA-seq,ACOM,AD.vs.control; bulk RNA-seq,Homo_714,MCI.vs.control	2
AKR1E2	bulk RNA-seq,Homo_723,AD.vs.control; bulk RNA-seq,Homo_723,AD.vs.MCI; bulk RNA-seq,Homo_723,MCI.vs.control; bulk RNA-seq,Homo_633,AD.vs.control; bulk RNA-seq,Homo_633,AD.vs.MCI; bulk RNA-seq,Homo_633,MCI.vs.control; bulk RNA-seq,Homo_714,AD.vs.control; bulk RNA-seq,Homo_714,AD.vs.MCI; bulk RNA-seq,ROSMAP,AD.vs.control; scRNA-seq,SRP330776,CD8+ T cell_1-AD.vs.control; scRNA-	11
AKR7A2	scRNA-seq,SRP215507,CD8+ T cell_4-AD.vs.control; scRNA-seq,SRP215507,CD8+ T cell_5-AD.vs.control; scRNA-seq,SRP215507,CD8+ T cell_6-AD.vs.control; scRNA-seq,SRP215507,CD8+ T cell_4-AD.vs.MCI; scRNA-seq,SRP215507,CD8+ T cell_7-AD.vs.MCI; scRNA-seq,SRP215507,CD8+ T cell_5-MCI.vs.control; scRNA-seq,SRP215507,CD8+ T cell_4-MCI.vs.control; scRNA-seq,SRP330776,CD8+ T cell_1-AD.vs.control; scRNA-seq,SRP330776,γδ T cell-AD.vs.control; scRNA-seq,SRP330776,Memory T cell_2-AD.vs.control; scRNA-seq,SRP330776,CD8+ T cell_2-AD.vs.control; scRNA-seq,SRP330776,B cell_1-AD.vs.control; scRNA-seq,SRP330776,Naive CD8+ T cell_1-AD.vs.control; scRNA-seq,SRP330776,Natural killer cell_1-AD.vs.control; scRNA-seq,SRP330776,Memory T cell_1-AD.vs.control; scRNA-seq,SRP330776,Natural killer T cell-AD.vs.control; scRNA-seq,SRP330776,Naive CD8+ T cell_3-AD.vs.control; scRNA-seq,SRP330776,B cell_2-AD.vs.control; scRNA-seq,SRP330776,T cell-AD.vs.control; scRNA-seq,SRP330776,Naive CD8+ T cell_2-AD.vs.control; scRNA-seq,SRP330776,CD8+ T cell_3-AD.vs.control; scRNA-seq,SRP330776,Monocyte_1-AD.vs.control; scRNA-seq,SRP330776,Monocyte_2-AD.vs.control; scRNA-seq,SRP330776,B cell_3-AD.vs.control; scRNA-seq,SRP330776,Regulatory T [illegible]	27
AKR7A3	bulk RNA-seq,Homo_723,AD.vs.MCI; bulk RNA-seq,Homo_633,AD.vs.control; bulk RNA-seq,Homo_633,AD.vs.MCI; bulk RNA-seq,Homo_714,AD.vs.control; bulk RNA-seq,Homo_714,AD.vs.MCI	5
AKT1	bulk RNA-seq,Homo_723,AD.vs.control; bulk RNA-seq,Homo_723,AD.vs.MCI; bulk RNA-seq,Homo_633,AD.vs.control; bulk RNA-seq,Homo_633,AD.vs.MCI; scRNA-seq,SRP309935,Natural killer cell_2-AD.vs.control; scRNA-seq,SRP309935,Naive CD8+ T cell_1-AD.vs.control; scRNA-seq,SRP309935,Monocyte_2-AD.vs.control; scRNA-seq,SRP330776,Memory T cell_2-AD.vs.control; scRNA-seq,SRP330776,CD8+ T cell_2-AD.vs.control; scRNA-seq,SRP330776,Naive CD8+ T cell_1-AD.vs.control; scRNA-seq,SRP330776,Natural killer cell_1-AD.vs.control; scRNA-seq,SRP330776,Memory T cell_1-AD.vs.control; scRNA-seq,SRP330776,Natural killer T cell-AD.vs.control; scRNA-seq,SRP330776,T cell-AD.vs.control;	16

AKT1S1	bulk RNA-seq,Homo_723,AD.vs.control; bulk RNA-seq,Homo_723,AD.vs.MCI; bulk RNA-seq,Homo_633,AD.vs.control; bulk RNA-seq,Homo_633,AD.vs.MCI; bulk RNA-seq,Homo_714,AD.vs.control; bulk RNA-seq,Homo_714,AD.vs.MCI; scRNA-seq,SRP215507,CD8+ T cell_5-AD.vs.MCI; scRNA-seq,SRP215507,CD8+ T cell_5-MCI.vs.control; scRNA-seq,SRP330776,CD8+ T cell_1-AD.vs.control; scRNA-seq,SRP330776,γδ T cell-AD.vs.control; scRNA-seq,SRP330776,Memory T cell_2-AD.vs.control; scRNA-seq,SRP330776,CD8+ T cell_2-AD.vs.control; scRNA-seq,SRP330776,B cell_1-AD.vs.control; scRNA-seq,SRP330776,Naive CD8+ T cell_1-AD.vs.control; scRNA-seq,SRP330776,Natural killer cell_1-AD.vs.control; scRNA-seq,SRP330776,Memory T cell_1-AD.vs.control; scRNA-seq,SRP330776,Natural killer T cell-AD.vs.control; scRNA-seq,SRP330776,Naive CD8+ T cell_3-AD.vs.control; scRNA-seq,SRP330776,B cell_2-AD.vs.control; scRNA-seq,SRP330776,T cell-AD.vs.control; [illegible]	23
AKT2	bulk RNA-seq,Homo_633,AD.vs.control; bulk RNA-seq,Homo_714,AD.vs.control; bulk RNA-seq,Homo_714,AD.vs.MCI; bulk RNA-seq,SRP310421,AD.vs.control; scRNA-seq,SRP215507,CD8+ T cell_2-AD.vs.MCI; scRNA-seq,SRP330776,CD8+ T cell_2-AD.vs.control; scRNA-seq,SRP330776,Naive CD8+ T cell_1-AD.vs.control; scRNA-seq,SRP330776,Natural killer T cell-AD.vs.control; scRNA-seq,SRP330776,Naive CD8+ T cell_3-AD.vs.control; scRNA-seq,SRP330776,T cell-AD.vs.control; scRNA-seq,SRP330776,CD8+ T cell_3-AD.vs.control; scRNA-	12
AKT3	scRNA-seq,SRP215507,CD8+ T cell_5-AD.vs.control; scRNA-seq,SRP215507,CD8+ T cell_6-AD.vs.control; scRNA-seq,SRP215507,CD8+ T cell_5-MCI.vs.control; scRNA-seq,SRP309935,Naive CD8+ T cell_1-AD.vs.control; scRNA-seq,SRP309935,CD8+ T cell_1-AD.vs.control; scRNA-seq,SRP309935,CD8+ T cell_2-AD.vs.control; scRNA-seq,SRP330776,CD8+ T cell_1-AD.vs.control; scRNA-seq,SRP330776,γδ T cell-AD.vs.control; scRNA-seq,SRP330776,Memory T cell_2-AD.vs.control; scRNA-seq,SRP330776,CD8+ T cell_2-AD.vs.control; scRNA-seq,SRP330776,B cell_1-AD.vs.control; scRNA-seq,SRP330776,Naive CD8+ T cell_1-AD.vs.control; scRNA-seq,SRP330776,Natural killer cell_1-AD.vs.control; scRNA-seq,SRP330776,Memory T cell_1-AD.vs.control; scRNA-seq,SRP330776,Natural killer T cell-AD.vs.control; scRNA-seq,SRP330776,Naive CD8+ T cell_3-AD.vs.control; scRNA-seq,SRP330776,T cell-AD.vs.control; scRNA-seq,SRP330776,Naive CD8+ T cell_2-AD.vs.control; scRNA- [illegible]	19
AKT3-IT1	scRNA-seq,SRP215507,CD8+ T cell_2-AD.vs.MCI; scRNA-seq,SRP330776,Naive CD8+ T cell_1-AD.vs.control; scRNA-seq,SRP330776,Memory T	3
AKTIP	scRNA-seq,SRP215507,CD8+ T cell_6-AD.vs.MCI; scRNA-seq,SRP309935,Megakaryocyte_1-AD.vs.control; scRNA-seq,SRP309935,Natural killer cell_1-AD.vs.control; scRNA-seq,SRP330776,CD8+ T cell_2-AD.vs.control; scRNA-seq,SRP330776,Memory T cell_1-AD.vs.control; scRNA-seq,SRP330776,Naive CD8+ T cell_3-AD.vs.control; scRNA-seq,SRP330776,B cell_2-AD.vs.control; scRNA-seq,SRP330776,B cell_3-AD.vs.control	8
AL008718.3	scRNA-seq,SRP215507,CD8+ T cell_3-MCI.vs.control	1
AL008721.2	scRNA-seq,SRP330776,Naive CD8+ T cell_1-AD.vs.control	1
AL008727.1	scRNA-seq,SRP330776,CD8+ T cell_1-AD.vs.control; scRNA-seq,SRP330776,Naive CD8+ T cell_1-AD.vs.control	2
AL008729.1	scRNA-seq,SRP215507,CD8+ T cell_1-AD.vs.MCI; scRNA-seq,SRP215507,CD8+ T cell_1-MCI.vs.control; scRNA-seq,SRP215507,CD8+ T cell_3-	3
AL009179.1	scRNA-seq,SRP215507,CD8+ T cell_2-AD.vs.control; scRNA-seq,SRP215507,CD8+ T cell_1-AD.vs.control; scRNA-seq,SRP215507,CD8+ T cell_3-AD.vs.MCI; scRNA-seq,SRP215507,CD8+ T cell_1-MCI.vs.control; scRNA-seq,SRP215507,CD8+ T cell_5-MCI.vs.control; scRNA-seq,SRP215507,CD8+ T cell_2-MCI.vs.control; scRNA-seq,SRP215507,CD8+ T cell_3-MCI.vs.control; scRNA-seq,SRP330776,CD8+ T cell_1-AD.vs.control; scRNA-seq,SRP330776,γδ T cell-AD.vs.control; scRNA-seq,SRP330776,B cell_1-AD.vs.control; scRNA-seq,SRP330776,Naive CD8+ T cell_1-AD.vs.control; scRNA-seq,SRP330776,B cell_2-AD.vs.control; scRNA-seq,SRP330776,CD8+ T cell_3-AD.vs.control	13
AL009179.2	scRNA-seq,SRP215507,CD8+ T cell_2-AD.vs.control; scRNA-seq,SRP309935,Monocyte_1-AD.vs.control	2
AL020996.1	scRNA-seq,SRP215507,CD8+ T cell_1-AD.vs.control; scRNA-seq,SRP215507,CD8+ T cell_1-MCI.vs.control; scRNA-seq,SRP330776,Memory T cell_2-AD.vs.control; scRNA-seq,SRP330776,CD8+ T cell_2-AD.vs.control	4
AL020997.3	scRNA-seq,SRP215507,CD8+ T cell_4-AD.vs.control	1
AL020998.1	scRNA-seq,SRP215507,CD8+ T cell_2-AD.vs.control; scRNA-seq,SRP215507,CD8+ T cell_5-AD.vs.control; scRNA-seq,SRP215507,CD8+ T cell_2-AD.vs.MCI; scRNA-seq,SRP215507,CD8+ T cell_5-MCI.vs.control	4

AL021155.5	scRNA-seq,SRP215507,CD8+ T cell_4-AD.vs.control; scRNA-seq,SRP215507,CD8+ T cell_5-AD.vs.control; scRNA-seq,SRP215507,CD8+ T cell_4-AD.vs.MCI; scRNA-seq,SRP215507,CD8+ T cell_4-MCI.vs.control; scRNA-seq,SRP215507,CD8+ T cell_3-MCI.vs.control; scRNA-seq,SRP330776,CD8+ T cell_1-AD.vs.control; scRNA-seq,SRP330776,Memory T cell_2-AD.vs.control; scRNA-seq,SRP330776,CD8+ T cell_2-AD.vs.control; scRNA-seq,SRP330776,B cell_1-AD.vs.control; scRNA-seq,SRP330776,Naive CD8+ T cell_1-AD.vs.control; scRNA-seq,SRP330776,Memory T cell_1-AD.vs.control; scRNA-seq,SRP330776,Naive CD8+ T cell_3-AD.vs.control; scRNA-seq,SRP330776,B cell_2-AD.vs.control; scRNA-seq,SRP330776,CD8+ T cell_3-AD.vs.control; scRNA-seq,SRP330776,Monocyte_1-AD.vs.control; scRNA-	17
AL021368.2	scRNA-seq,SRP215507,CD8+ T cell_3-AD.vs.control; scRNA-seq,SRP215507,CD8+ T cell_1-MCI.vs.control; scRNA-seq,SRP215507,CD8+ T cell_2-MCI.vs.control; scRNA-seq,SRP215507,CD8+ T cell_3-MCI.vs.control; scRNA-seq,SRP309935,CD8+ T cell_1-AD.vs.control; scRNA-seq,SRP330776,CD8+ T cell_1-AD.vs.control; scRNA-seq,SRP330776,CD8+ T cell_2-AD.vs.control; scRNA-seq,SRP330776,B cell_1-AD.vs.control;	9
AL021368.5	scRNA-seq,SRP215507,CD8+ T cell_1-AD.vs.control	1
AL021396.1	scRNA-seq,SRP330776,Naive CD8+ T cell_1-AD.vs.control; scRNA-seq,SRP330776,Memory T cell_1-AD.vs.control	2
AL021707.2	scRNA-seq,SRP215507,CD8+ T cell_2-MCI.vs.control	1
AL021707.5	scRNA-seq,SRP215507,CD8+ T cell_2-AD.vs.control; scRNA-seq,SRP215507,CD8+ T cell_3-AD.vs.MCI; scRNA-seq,SRP215507,CD8+ T cell_3-MCI.vs.control; scRNA-seq,SRP330776,CD8+ T cell_1-AD.vs.control; scRNA-seq,SRP330776,γδ T cell-AD.vs.control; scRNA-seq,SRP330776,Memory	6
AL021707.6	scRNA-seq,SRP215507,CD8+ T cell_2-AD.vs.MCI; scRNA-seq,SRP215507,CD8+ T cell_2-MCI.vs.control; scRNA-seq,SRP330776,Naive CD8+ T	3
AL021707.8	scRNA-seq,SRP215507,CD8+ T cell_1-AD.vs.MCI; scRNA-seq,SRP215507,CD8+ T cell_1-MCI.vs.control	2
AL021878.2	scRNA-seq,SRP215507,CD8+ T cell_3-MCI.vs.control	1
AL021918.4	scRNA-seq,SRP215507,CD8+ T cell_3-AD.vs.control; scRNA-seq,SRP215507,CD8+ T cell_3-MCI.vs.control; scRNA-seq,SRP330776,CD8+ T cell_1-AD.vs.control; scRNA-seq,SRP330776,γδ T cell-AD.vs.control	4
AL022067.1	scRNA-seq,SRP309935,Natural killer cell_1-AD.vs.control	1
AL022069.1	scRNA-seq,SRP215507,CD8+ T cell_2-AD.vs.control	1
AL022069.3	scRNA-seq,SRP215507,CD8+ T cell_1-AD.vs.MCI; scRNA-seq,SRP215507,CD8+ T cell_1-MCI.vs.control; scRNA-seq,SRP215507,CD8+ T cell_3-MCI.vs.control; scRNA-seq,SRP330776,Memory T cell_1-AD.vs.control; scRNA-seq,SRP330776,Naive CD8+ T cell_3-AD.vs.control; scRNA-	6
AL022157.1	scRNA-seq,SRP215507,CD8+ T cell_5-AD.vs.control; scRNA-seq,SRP215507,CD8+ T cell_1-AD.vs.MCI; scRNA-seq,SRP330776,CD8+ T cell_1-AD.vs.control; scRNA-seq,SRP330776,Memory T cell_2-AD.vs.control; scRNA-seq,SRP330776,T cell-AD.vs.control	5
AL022162.1	scRNA-seq,SRP215507,CD8+ T cell_2-AD.vs.control; scRNA-seq,SRP215507,CD8+ T cell_1-MCI.vs.control; scRNA-seq,SRP330776,CD8+ T cell_1-AD.vs.control; scRNA-seq,SRP330776,γδ T cell-AD.vs.control; scRNA-seq,SRP330776,Memory T cell_2-AD.vs.control; scRNA-seq,SRP330776,Naive CD8+ T cell_1-AD.vs.control; scRNA-seq,SRP330776,Natural killer cell_1-AD.vs.control; scRNA-seq,SRP330776,Memory T cell_1-AD.vs.control	8
AL022238.2	scRNA-seq,SRP309935,Natural killer cell_1-AD.vs.control	1
AL022311.1	scRNA-seq,SRP215507,CD8+ T cell_3-AD.vs.MCI; scRNA-seq,SRP215507,CD8+ T cell_3-MCI.vs.control; scRNA-seq,SRP330776,CD8+ T cell_1-AD.vs.control; scRNA-seq,SRP330776,Natural killer T cell-AD.vs.control	4
AL022313.2	scRNA-seq,SRP215507,CD8+ T cell_2-AD.vs.control; scRNA-seq,SRP215507,CD8+ T cell_1-MCI.vs.control; scRNA-seq,SRP215507,CD8+ T cell_2-	3
AL022322.2	scRNA-seq,SRP330776,CD8+ T cell_2-AD.vs.control; scRNA-seq,SRP330776,Naive CD8+ T cell_1-AD.vs.control	2
AL022323.4	scRNA-seq,SRP215507,CD8+ T cell_1-AD.vs.MCI; scRNA-seq,SRP215507,CD8+ T cell_1-MCI.vs.control; scRNA-seq,SRP215507,CD8+ T cell_2-MCI.vs.control; scRNA-seq,SRP330776,CD8+ T cell_1-AD.vs.control; scRNA-seq,SRP330776,γδ T cell-AD.vs.control; scRNA-seq,SRP330776,Naive CD8+ T cell_3-AD.vs.control; scRNA-seq,SRP330776,Naive CD8+ T cell_2-AD.vs.control	7
AL022328.2	scRNA-seq,SRP215507,CD8+ T cell_1-AD.vs.control; scRNA-seq,SRP215507,CD8+ T cell_2-MCI.vs.control; scRNA-seq,SRP215507,CD8+ T cell_3-	3
AL022328.3	scRNA-seq,SRP215507,CD8+ T cell_1-MCI.vs.control; scRNA-seq,SRP215507,CD8+ T cell_2-MCI.vs.control; scRNA-seq,SRP330776,B cell_2-	3

AL022328.4	scRNA-seq,SRP215507,CD8+ T cell_3-AD.vs.control; scRNA-seq,SRP215507,CD8+ T cell_5-AD.vs.control; scRNA-seq,SRP215507,CD8+ T cell_6-AD.vs.MCI; scRNA-seq,SRP215507,CD8+ T cell_6-MCI.vs.control; scRNA-seq,SRP309935,Naive CD8+ T cell_1-AD.vs.control; scRNA-seq,SRP330776,CD8+ T cell_1-AD.vs.control; scRNA-seq,SRP330776,γδ T cell-AD.vs.control; scRNA-seq,SRP330776,Memory T cell_2-AD.vs.control; scRNA-seq,SRP330776,CD8+ T cell_2-AD.vs.control; scRNA-seq,SRP330776,B cell_1-AD.vs.control; scRNA-seq,SRP330776,Naive CD8+ T cell_1-AD.vs.control; scRNA-seq,SRP330776,Natural killer cell_1-AD.vs.control; scRNA-seq,SRP330776,Natural killer T cell-AD.vs.control; scRNA-	14
AL022341.1	scRNA-seq,SRP215507,CD8+ T cell_1-AD.vs.control	1
AL022345.4	scRNA-seq,SRP215507,CD8+ T cell_3-AD.vs.MCI; scRNA-seq,SRP215507,CD8+ T cell_3-MCI.vs.control; scRNA-seq,SRP330776,B cell_1-AD.vs.control; scRNA-seq,SRP330776,Memory T cell_1-AD.vs.control	4
AL022724.3	scRNA-seq,SRP330776,Naive CD8+ T cell_1-AD.vs.control	1
AL023653.1	scRNA-seq,SRP215507,CD8+ T cell_2-AD.vs.MCI; scRNA-seq,SRP215507,CD8+ T cell_2-MCI.vs.control	2
AL023803.1	scRNA-seq,SRP330776,Naive CD8+ T cell_1-AD.vs.control	1
AL024498.1	scRNA-seq,SRP215507,CD8+ T cell_3-MCI.vs.control; scRNA-seq,SRP330776,Naive CD8+ T cell_1-AD.vs.control	2
AL024507.2	scRNA-seq,SRP215507,CD8+ T cell_2-AD.vs.control; scRNA-seq,SRP330776,CD8+ T cell_1-AD.vs.control	2
AL031056.1	scRNA-seq,SRP215507,CD8+ T cell_5-AD.vs.MCI; scRNA-seq,SRP215507,CD8+ T cell_5-MCI.vs.control	2
AL031280.1	scRNA-seq,SRP215507,CD8+ T cell_3-AD.vs.MCI; scRNA-seq,SRP215507,CD8+ T cell_2-MCI.vs.control; scRNA-seq,SRP215507,CD8+ T cell_3-MCI.vs.control; scRNA-seq,SRP309935,Naive CD8+ T cell_1-AD.vs.control; scRNA-seq,SRP330776,CD8+ T cell_1-AD.vs.control; scRNA-seq,SRP330776,Naive CD8+ T cell_1-AD.vs.control	6
AL031283.1	scRNA-seq,SRP215507,CD8+ T cell_1-AD.vs.control; scRNA-seq,SRP215507,CD8+ T cell_1-AD.vs.MCI; scRNA-seq,SRP215507,CD8+ T cell_5-AD.vs.MCI; scRNA-seq,SRP215507,CD8+ T cell_5-MCI.vs.control; scRNA-seq,SRP330776,CD8+ T cell_2-AD.vs.control; scRNA-	6
AL031283.2	scRNA-seq,SRP309935,Natural killer cell_1-AD.vs.control	1
AL031432.3	scRNA-seq,SRP215507,CD8+ T cell_1-AD.vs.control; scRNA-seq,SRP215507,CD8+ T cell_3-AD.vs.MCI; scRNA-seq,SRP215507,CD8+ T cell_1-MCI.vs.control; scRNA-seq,SRP215507,CD8+ T cell_3-MCI.vs.control	4
AL031432.4	scRNA-seq,SRP215507,CD8+ T cell_3-AD.vs.control; scRNA-seq,SRP215507,CD8+ T cell_1-AD.vs.control; scRNA-seq,SRP215507,CD8+ T cell_2-AD.vs.MCI; scRNA-seq,SRP215507,CD8+ T cell_5-AD.vs.MCI; scRNA-seq,SRP215507,CD8+ T cell_1-MCI.vs.control; scRNA-seq,SRP215507,CD8+ T cell_5-MCI.vs.control; scRNA-seq,SRP309935,Naive CD8+ T cell_1-AD.vs.control; scRNA-seq,SRP309935,CD8+ T cell_2-AD.vs.control; scRNA-seq,SRP330776,Memory T cell_2-AD.vs.control; scRNA-seq,SRP330776,Naive CD8+ T cell_3-AD.vs.control	10
AL031595.3	scRNA-seq,SRP215507,CD8+ T cell_3-MCI.vs.control; scRNA-seq,SRP330776,CD8+ T cell_1-AD.vs.control; scRNA-seq,SRP330776,B cell_2-	3
AL031600.1	scRNA-seq,SRP330776,Naive CD8+ T cell_1-AD.vs.control; scRNA-seq,SRP330776,Memory T cell_1-AD.vs.control	2
AL031666.3	scRNA-seq,SRP215507,CD8+ T cell_1-AD.vs.control; scRNA-seq,SRP330776,B cell_1-AD.vs.control	2
AL031667.3	scRNA-seq,SRP215507,CD8+ T cell_5-MCI.vs.control; scRNA-seq,SRP215507,CD8+ T cell_3-MCI.vs.control	2
AL031668.2	scRNA-seq,SRP215507,CD8+ T cell_3-MCI.vs.control	1
AL031670.1	scRNA-seq,SRP330776,CD8+ T cell_1-AD.vs.control; scRNA-seq,SRP330776,γδ T cell-AD.vs.control; scRNA-seq,SRP330776,Memory T cell_1-	3
AL031714.1	scRNA-seq,SRP215507,CD8+ T cell_2-AD.vs.MCI; scRNA-seq,SRP215507,CD8+ T cell_2-MCI.vs.control; scRNA-seq,SRP330776,Natural killer T cell-AD.vs.control; scRNA-seq,SRP330776,T cell-AD.vs.control; scRNA-seq,SRP330776,B cell_3-AD.vs.control	5
AL031728.1	scRNA-seq,SRP215507,CD8+ T cell_4-AD.vs.control; scRNA-seq,SRP215507,CD8+ T cell_5-AD.vs.control; scRNA-seq,SRP215507,CD8+ T cell_3-AD.vs.MCI; scRNA-seq,SRP215507,CD8+ T cell_5-MCI.vs.control; scRNA-seq,SRP215507,CD8+ T cell_3-MCI.vs.control; scRNA-seq,SRP330776,CD8+ T cell_1-AD.vs.control; scRNA-seq,SRP330776,Naive CD8+ T cell_1-AD.vs.control; scRNA-seq,SRP330776,B cell_2-	9
AL031733.2	scRNA-seq,SRP215507,CD8+ T cell_2-AD.vs.control; scRNA-seq,SRP215507,CD8+ T cell_3-AD.vs.MCI; scRNA-seq,SRP215507,CD8+ T cell_1-MCI.vs.control; scRNA-seq,SRP330776,Memory T cell_2-AD.vs.control	4
AL031772.1	scRNA-seq,SRP215507,CD8+ T cell_2-AD.vs.control; scRNA-seq,SRP330776,Naive CD8+ T cell_1-AD.vs.control	2

AL031775.1	scRNA-seq,SRP215507,CD8+ T cell_4-AD.vs.MCI; scRNA-seq,SRP215507,CD8+ T cell_1-MCI.vs.control; scRNA-seq,SRP215507,CD8+ T cell_4-MCI.vs.control; scRNA-seq,SRP330776,Memory T cell_1-AD.vs.control	4
AL031846.2	scRNA-seq,SRP330776,CD8+ T cell_1-AD.vs.control; scRNA-seq,SRP330776,Naive CD8+ T cell_1-AD.vs.control	2
AL031847.1	scRNA-seq,SRP215507,CD8+ T cell_1-MCI.vs.control	1
AL031848.2	scRNA-seq,SRP215507,CD8+ T cell_3-AD.vs.control; scRNA-seq,SRP215507,CD8+ T cell_5-AD.vs.control; scRNA-seq,SRP215507,CD8+ T cell_2-AD.vs.MCI; scRNA-seq,SRP215507,CD8+ T cell_5-MCI.vs.control; scRNA-seq,SRP215507,CD8+ T cell_2-MCI.vs.control; scRNA-seq,SRP215507,CD8+ T cell_3-MCI.vs.control; scRNA-seq,SRP330776,CD8+ T cell_1-AD.vs.control; scRNA-seq,SRP330776,γδ T cell-AD.vs.control; scRNA-seq,SRP330776,Memory T cell_2-AD.vs.control; scRNA-seq,SRP330776,CD8+ T cell_2-AD.vs.control; scRNA-seq,SRP330776,B cell_1-AD.vs.control; scRNA-seq,SRP330776,Naive CD8+ T cell_1-AD.vs.control; scRNA-seq,SRP330776,Memory T cell_1-AD.vs.control; scRNA-	15
AL031963.3	scRNA-seq,SRP215507,CD8+ T cell_2-AD.vs.MCI; scRNA-seq,SRP215507,CD8+ T cell_3-AD.vs.MCI; scRNA-seq,SRP215507,CD8+ T cell_1-AD.vs.MCI; scRNA-seq,SRP215507,CD8+ T cell_5-AD.vs.MCI; scRNA-seq,SRP215507,CD8+ T cell_1-MCI.vs.control; scRNA-seq,SRP215507,CD8+ T cell_5-MCI.vs.control; scRNA-seq,SRP215507,CD8+ T cell_2-MCI.vs.control; scRNA-seq,SRP215507,CD8+ T cell_3-MCI.vs.control; scRNA-seq,SRP330776,CD8+ T cell_1-AD.vs.control; scRNA-seq,SRP330776,Memory T cell_2-AD.vs.control; scRNA-seq,SRP330776,B cell_1-AD.vs.control; scRNA-seq,SRP330776,Naive CD8+ T cell_1-AD.vs.control; scRNA-seq,SRP330776,Memory T cell_1-AD.vs.control; scRNA-seq,SRP330776,Natural killer T cell-AD.vs.control; scRNA-seq,SRP330776,B cell_3-AD.vs.control; scRNA-seq,SRP330776,Natural killer cell_2-AD.vs.control	16
AL033523.1	scRNA-seq,SRP215507,CD8+ T cell_3-AD.vs.MCI	1
AL033527.3	scRNA-seq,SRP330776,Memory T cell_1-AD.vs.control	1
AL033527.4	scRNA-seq,SRP215507,CD8+ T cell_3-AD.vs.control; scRNA-seq,SRP215507,CD8+ T cell_4-AD.vs.MCI; scRNA-seq,SRP309935,CD8+ T cell_2-AD.vs.control; scRNA-seq,SRP330776,B cell_1-AD.vs.control; scRNA-seq,SRP330776,Natural killer cell_1-AD.vs.control	5
AL033528.2	scRNA-seq,SRP215507,CD8+ T cell_1-AD.vs.MCI; scRNA-seq,SRP215507,CD8+ T cell_5-AD.vs.MCI; scRNA-seq,SRP215507,CD8+ T cell_1-MCI.vs.control; scRNA-seq,SRP330776,Naive CD8+ T cell_1-AD.vs.control	4
AL034397.2	scRNA-seq,SRP215507,CD8+ T cell_2-AD.vs.control; scRNA-seq,SRP215507,CD8+ T cell_1-AD.vs.control	2
AL034397.3	scRNA-seq,SRP215507,CD8+ T cell_1-AD.vs.control; scRNA-seq,SRP215507,CD8+ T cell_1-AD.vs.MCI; scRNA-seq,SRP309935,Megakaryocyte_2-AD.vs.control; scRNA-seq,SRP330776,B cell_1-AD.vs.control; scRNA-seq,SRP330776,B cell_2-AD.vs.control; scRNA-seq,SRP330776,Monocyte_1-AD.vs.control; scRNA-seq,SRP330776,Monocyte_2-AD.vs.control; scRNA-seq,SRP330776,B cell_3-AD.vs.control; scRNA-seq,SRP330776,Natural killer	9
AL034428.1	scRNA-seq,SRP330776,Memory T cell_2-AD.vs.control; scRNA-seq,SRP330776,CD8+ T cell_2-AD.vs.control; scRNA-seq,SRP330776,B cell_1-AD.vs.control; scRNA-seq,SRP330776,Natural killer cell_1-AD.vs.control	4
AL034550.2	scRNA-seq,SRP215507,CD8+ T cell_2-AD.vs.control; scRNA-seq,SRP215507,CD8+ T cell_1-AD.vs.control; scRNA-seq,SRP215507,CD8+ T cell_3-AD.vs.MCI; scRNA-seq,SRP330776,CD8+ T cell_1-AD.vs.control; scRNA-seq,SRP330776,Naive CD8+ T cell_3-AD.vs.control	5
AL035071.1	scRNA-seq,SRP215507,CD8+ T cell_4-AD.vs.MCI; scRNA-seq,SRP309935,Naive CD8+ T cell_2-AD.vs.control; scRNA-seq,SRP330776,CD8+ T cell_1-AD.vs.control; scRNA-seq,SRP330776,γδ T cell-AD.vs.control; scRNA-seq,SRP330776,Memory T cell_2-AD.vs.control; scRNA-seq,SRP330776,CD8+ T cell_2-AD.vs.control; scRNA-seq,SRP330776,Naive CD8+ T cell_1-AD.vs.control; scRNA-seq,SRP330776,Memory T cell_1-AD.vs.control; scRNA-	9
AL035106.1	scRNA-seq,SRP215507,CD8+ T cell_1-AD.vs.control; scRNA-seq,SRP215507,CD8+ T cell_1-AD.vs.MCI; scRNA-seq,SRP330776,CD8+ T cell_1-AD.vs.control; scRNA-seq,SRP330776,Memory T cell_2-AD.vs.control; scRNA-seq,SRP330776,CD8+ T cell_2-AD.vs.control; scRNA-seq,SRP330776,B cell_1-AD.vs.control; scRNA-seq,SRP330776,Naive CD8+ T cell_1-AD.vs.control; scRNA-seq,SRP330776,Natural killer cell_1-AD.vs.control; scRNA-	9
AL035252.2	scRNA-seq,SRP215507,CD8+ T cell_2-AD.vs.MCI; scRNA-seq,SRP215507,CD8+ T cell_3-AD.vs.MCI; scRNA-seq,SRP215507,CD8+ T cell_3-	3
AL035252.5	scRNA-seq,SRP215507,CD8+ T cell_6-MCI.vs.control	1
AL035461.2	scRNA-seq,SRP215507,CD8+ T cell_1-AD.vs.control; scRNA-seq,SRP215507,CD8+ T cell_1-MCI.vs.control	2
AL035530.2	scRNA-seq,SRP330776,Memory T cell_2-AD.vs.control; scRNA-seq,SRP330776,B cell_1-AD.vs.control; scRNA-seq,SRP330776,Natural killer cell_1-AD.vs.control; scRNA-seq,SRP330776,B cell_2-AD.vs.control; scRNA-seq,SRP330776,T cell-AD.vs.control; scRNA-seq,SRP330776,Natural killer cell_2-	6
AL035587.1	scRNA-seq,SRP215507,CD8+ T cell_1-MCI.vs.control; scRNA-seq,SRP215507,CD8+ T cell_2-MCI.vs.control	2

AL035587.2	scRNA-seq,SRP309935,Naive CD8+ T cell_2-AD.vs.control; scRNA-seq,SRP330776,Naive CD8+ T cell_1-AD.vs.control; scRNA-seq,SRP330776,Naive CD8+ T cell_3-AD.vs.control; scRNA-seq,SRP330776,CD8+ T cell_3-AD.vs.control; scRNA-seq,SRP330776,B cell_3-AD.vs.control	5
AL035634.1	scRNA-seq,SRP330776,Naive CD8+ T cell_1-AD.vs.control	1
AL035661.2	scRNA-seq,SRP215507,CD8+ T cell_2-MCI.vs.control; scRNA-seq,SRP330776,γδ T cell-AD.vs.control; scRNA-seq,SRP330776,CD8+ T cell_2-AD.vs.control; scRNA-seq,SRP330776,Naive CD8+ T cell_1-AD.vs.control; scRNA-seq,SRP330776,Memory T cell_1-AD.vs.control; scRNA-	6
AL035701.1	scRNA-seq,SRP215507,CD8+ T cell_1-AD.vs.control; scRNA-seq,SRP215507,CD8+ T cell_1-MCI.vs.control; scRNA-seq,SRP330776,Naive CD8+ T	3
AL049557.1	scRNA-seq,SRP215507,CD8+ T cell_1-AD.vs.control; scRNA-seq,SRP215507,CD8+ T cell_1-AD.vs.MCI; scRNA-seq,SRP215507,CD8+ T cell_1-MCI.vs.control; scRNA-seq,SRP330776,CD8+ T cell_1-AD.vs.control; scRNA-seq,SRP330776,γδ T cell-AD.vs.control; scRNA-seq,SRP330776,Memory	6
AL049597.2	scRNA-seq,SRP215507,CD8+ T cell_3-AD.vs.control; scRNA-seq,SRP215507,CD8+ T cell_3-MCI.vs.control; scRNA-seq,SRP330776,CD8+ T cell_1-AD.vs.control; scRNA-seq,SRP330776,B cell_1-AD.vs.control; scRNA-seq,SRP330776,Memory T cell_1-AD.vs.control	5
AL049712.1	scRNA-seq,SRP215507,CD8+ T cell_1-AD.vs.MCI; scRNA-seq,SRP215507,CD8+ T cell_1-MCI.vs.control; scRNA-seq,SRP215507,CD8+ T cell_2-MCI.vs.control; scRNA-seq,SRP330776,Memory T cell_1-AD.vs.control	4
AL049780.2	scRNA-seq,SRP215507,CD8+ T cell_8-AD.vs.control; scRNA-seq,SRP215507,CD8+ T cell_3-AD.vs.control; scRNA-seq,SRP215507,CD8+ T cell_3-AD.vs.MCI; scRNA-seq,SRP215507,CD8+ T cell_1-AD.vs.MCI; scRNA-seq,SRP330776,CD8+ T cell_2-AD.vs.control	5
AL049796.1	scRNA-seq,SRP215507,CD8+ T cell_1-AD.vs.control; scRNA-seq,SRP215507,CD8+ T cell_1-AD.vs.MCI	2
AL049828.1	scRNA-seq,SRP215507,CD8+ T cell_3-AD.vs.control; scRNA-seq,SRP215507,CD8+ T cell_2-MCI.vs.control; scRNA-seq,SRP215507,CD8+ T cell_3-MCI.vs.control; scRNA-seq,SRP330776,Memory T cell_1-AD.vs.control	4
AL049828.2	scRNA-seq,SRP215507,CD8+ T cell_3-AD.vs.MCI	1
AL049830.3	scRNA-seq,SRP330776,B cell_1-AD.vs.control; scRNA-seq,SRP330776,B cell_2-AD.vs.control	2
AL049840.2	scRNA-seq,SRP215507,CD8+ T cell_3-AD.vs.control; scRNA-seq,SRP215507,CD8+ T cell_1-AD.vs.MCI; scRNA-seq,SRP215507,CD8+ T cell_1-MCI.vs.control; scRNA-seq,SRP330776,CD8+ T cell_1-AD.vs.control; scRNA-seq,SRP330776,Memory T cell_2-AD.vs.control; scRNA-	6
AL049840.4	scRNA-seq,SRP215507,CD8+ T cell_4-AD.vs.control; scRNA-seq,SRP215507,CD8+ T cell_4-MCI.vs.control	2
AL049840.5	scRNA-seq,SRP215507,CD8+ T cell_3-AD.vs.control; scRNA-seq,SRP215507,CD8+ T cell_3-AD.vs.MCI; scRNA-seq,SRP215507,CD8+ T cell_3-MCI.vs.control; scRNA-seq,SRP309935,Monocyte_2-AD.vs.control; scRNA-seq,SRP309935,CD8+ T cell_1-AD.vs.control; scRNA-seq,SRP330776,CD8+	6
AL049869.3	scRNA-seq,SRP215507,CD8+ T cell_1-AD.vs.control; scRNA-seq,SRP330776,γδ T cell-AD.vs.control; scRNA-seq,SRP330776,Memory T cell_2-AD.vs.control; scRNA-seq,SRP330776,CD8+ T cell_2-AD.vs.control; scRNA-seq,SRP330776,Naive CD8+ T cell_1-AD.vs.control; scRNA-	6
AL050309.1	scRNA-seq,SRP215507,CD8+ T cell_1-AD.vs.control; scRNA-seq,SRP215507,CD8+ T cell_4-AD.vs.control; scRNA-seq,SRP215507,CD8+ T cell_3-AD.vs.MCI; scRNA-seq,SRP215507,CD8+ T cell_4-AD.vs.MCI; scRNA-seq,SRP215507,CD8+ T cell_1-MCI.vs.control; scRNA-seq,SRP215507,CD8+ T cell_4-MCI.vs.control; scRNA-seq,SRP215507,CD8+ T cell_3-MCI.vs.control; scRNA-seq,SRP309935,Plasmacytoid dendritic cell_1-AD.vs.control; scRNA-seq,SRP330776,CD8+ T cell_1-AD.vs.control; scRNA-seq,SRP330776,CD8+ T cell_2-AD.vs.control; scRNA-seq,SRP330776,B cell_1-AD.vs.control; scRNA-seq,SRP330776,Memory T cell_1-AD.vs.control; scRNA-seq,SRP330776,T cell-AD.vs.control; scRNA-seq,SRP330776,CD8+ T	14
AL050341.2	scRNA-seq,SRP215507,CD8+ T cell_6-AD.vs.control; scRNA-seq,SRP215507,CD8+ T cell_4-MCI.vs.control; scRNA-seq,SRP309935,Naive CD8+ T cell_1-AD.vs.control; scRNA-seq,SRP330776,T cell-AD.vs.control; scRNA-seq,SRP330776,CD8+ T cell_3-AD.vs.control	5
AL050343.2	scRNA-seq,SRP215507,CD8+ T cell_4-MCI.vs.control; scRNA-seq,SRP330776,Naive CD8+ T cell_2-AD.vs.control	2
AL050344.1	scRNA-seq,SRP215507,CD8+ T cell_1-AD.vs.MCI; scRNA-seq,SRP215507,CD8+ T cell_1-MCI.vs.control; scRNA-seq,SRP215507,CD8+ T cell_5-	3
AL078459.1	scRNA-seq,SRP215507,CD8+ T cell_1-AD.vs.control; scRNA-seq,SRP215507,CD8+ T cell_1-MCI.vs.control; scRNA-seq,SRP330776,B cell_1-AD.vs.control; scRNA-seq,SRP330776,Naive CD8+ T cell_1-AD.vs.control; scRNA-seq,SRP330776,Memory T cell_1-AD.vs.control; scRNA-	6
AL078587.2	scRNA-seq,SRP330776,Naive CD8+ T cell_1-AD.vs.control	1
AL078644.2	scRNA-seq,SRP330776,CD8+ T cell_1-AD.vs.control	1
AL078645.1	scRNA-seq,SRP215507,CD8+ T cell_2-AD.vs.control; scRNA-seq,SRP215507,CD8+ T cell_4-MCI.vs.control; scRNA-seq,SRP330776,Memory T cell_1-	3
AL079338.1	scRNA-seq,SRP330776,B cell_2-AD.vs.control	1

AL080250.1	scRNA-seq,SRP215507,CD8+ T cell_2-AD.vs.MCI; scRNA-seq,SRP215507,CD8+ T cell_4-AD.vs.MCI; scRNA-seq,SRP215507,CD8+ T cell_5-AD.vs.MCI; scRNA-seq,SRP215507,CD8+ T cell_5-MCI.vs.control; scRNA-seq,SRP330776,CD8+ T cell_1-AD.vs.control; scRNA-seq,SRP330776,B cell_1-AD.vs.control; scRNA-seq,SRP330776,Naive CD8+ T cell_1-AD.vs.control; scRNA-seq,SRP330776,Memory T cell_1-AD.vs.control; scRNA-	9
AL080276.2	scRNA-seq,SRP215507,CD8+ T cell_8-MCI.vs.control; scRNA-seq,SRP330776,Natural killer cell_1-AD.vs.control; scRNA-seq,SRP330776,Natural killer T cell-AD.vs.control; scRNA-seq,SRP330776,B cell_2-AD.vs.control; scRNA-seq,SRP330776,B cell_3-AD.vs.control	5
AL080317.1	scRNA-seq,SRP215507,CD8+ T cell_4-AD.vs.MCI; scRNA-seq,SRP215507,CD8+ T cell_4-MCI.vs.control	2
AL080317.2	scRNA-seq,SRP215507,CD8+ T cell_2-MCI.vs.control	1
AL096677.1	scRNA-seq,SRP330776,CD8+ T cell_2-AD.vs.control	1
AL096678.1	scRNA-seq,SRP215507,CD8+ T cell_2-AD.vs.MCI; scRNA-seq,SRP215507,CD8+ T cell_3-AD.vs.MCI; scRNA-seq,SRP330776,Memory T cell_2-AD.vs.control; scRNA-seq,SRP330776,CD8+ T cell_2-AD.vs.control; scRNA-seq,SRP330776,B cell_1-AD.vs.control; scRNA-seq,SRP330776,CD8+ T	6
AL096701.3	scRNA-seq,SRP330776,Naive CD8+ T cell_1-AD.vs.control	1
AL096794.1	scRNA-seq,SRP215507,CD8+ T cell_1-AD.vs.control; scRNA-seq,SRP330776,Memory T cell_1-AD.vs.control	2
AL096865.1	scRNA-seq,SRP215507,CD8+ T cell_3-AD.vs.MCI; scRNA-seq,SRP215507,CD8+ T cell_2-MCI.vs.control; scRNA-seq,SRP215507,CD8+ T cell_3-MCI.vs.control; scRNA-seq,SRP330776,T cell-AD.vs.control	4
AL096870.6	scRNA-seq,SRP215507,CD8+ T cell_3-AD.vs.control; scRNA-seq,SRP215507,CD8+ T cell_2-AD.vs.MCI; scRNA-seq,SRP215507,CD8+ T cell_2-MCI.vs.control; scRNA-seq,SRP330776,CD8+ T cell_1-AD.vs.control; scRNA-seq,SRP330776,B cell_1-AD.vs.control	5
AL096870.8	scRNA-seq,SRP215507,CD8+ T cell_2-AD.vs.control; scRNA-seq,SRP215507,CD8+ T cell_3-AD.vs.control; scRNA-seq,SRP215507,CD8+ T cell_2-AD.vs.MCI; scRNA-seq,SRP215507,CD8+ T cell_3-AD.vs.MCI; scRNA-seq,SRP330776,CD8+ T cell_1-AD.vs.control; scRNA-seq,SRP330776,Memory T cell_2-AD.vs.control; scRNA-seq,SRP330776,CD8+ T cell_2-AD.vs.control; scRNA-seq,SRP330776,B cell_1-AD.vs.control; scRNA-seq,SRP330776,Naive CD8+ T cell_1-AD.vs.control; scRNA-seq,SRP330776,Natural killer cell_1-AD.vs.control; scRNA-seq,SRP330776,Memory T cell_1-AD.vs.control; scRNA-seq,SRP330776,Naive CD8+ T cell_3-AD.vs.control; scRNA-seq,SRP330776,B cell_2-AD.vs.control	13
AL109615.2	scRNA-seq,SRP330776,Monocyte_2-AD.vs.control	1
AL109615.4	scRNA-seq,SRP309935,Natural killer cell_1-AD.vs.control; scRNA-seq,SRP330776,Naive CD8+ T cell_1-AD.vs.control	2
AL109627.1	scRNA-seq,SRP215507,CD8+ T cell_2-AD.vs.control; scRNA-seq,SRP215507,CD8+ T cell_3-MCI.vs.control	2
AL109628.2	scRNA-seq,SRP215507,CD8+ T cell_3-AD.vs.MCI; scRNA-seq,SRP215507,CD8+ T cell_3-MCI.vs.control	2
AL109741.1	scRNA-seq,SRP215507,CD8+ T cell_3-AD.vs.MCI; scRNA-seq,SRP215507,CD8+ T cell_5-AD.vs.MCI; scRNA-seq,SRP330776,CD8+ T cell_1-AD.vs.control; scRNA-seq,SRP330776,γδ T cell-AD.vs.control; scRNA-seq,SRP330776,CD8+ T cell_2-AD.vs.control; scRNA-seq,SRP330776,Memory T cell_1-AD.vs.control; scRNA-seq,SRP330776,Natural killer T cell-AD.vs.control; scRNA-seq,SRP330776,Naive CD8+ T cell_3-AD.vs.control; scRNA-seq,SRP330776,CD8+ T cell_3-AD.vs.control; scRNA-seq,SRP330776,Megakaryocyte_1-AD.vs.control	10
AL109761.1	scRNA-seq,SRP215507,CD8+ T cell_5-AD.vs.control; scRNA-seq,SRP330776,B cell_4-AD.vs.control	2
AL109767.1	scRNA-seq,SRP215507,CD8+ T cell_2-AD.vs.MCI; scRNA-seq,SRP215507,CD8+ T cell_2-MCI.vs.control; scRNA-seq,SRP330776,CD8+ T cell_1-AD.vs.control; scRNA-seq,SRP330776,Natural killer T cell-AD.vs.control; scRNA-seq,SRP330776,Naive CD8+ T cell_3-AD.vs.control	5
AL109811.1	scRNA-seq,SRP330776,Naive CD8+ T cell_1-AD.vs.control	1
AL109811.2	scRNA-seq,SRP215507,CD8+ T cell_3-AD.vs.control; scRNA-seq,SRP215507,CD8+ T cell_3-AD.vs.MCI; scRNA-seq,SRP330776,Memory T cell_2-AD.vs.control; scRNA-seq,SRP330776,Naive CD8+ T cell_1-AD.vs.control; scRNA-seq,SRP330776,Memory T cell_1-AD.vs.control; scRNA-seq,SRP330776,Naive CD8+ T cell_2-AD.vs.control	6
AL109840.2	scRNA-seq,SRP215507,CD8+ T cell_3-AD.vs.MCI; scRNA-seq,SRP215507,CD8+ T cell_5-AD.vs.MCI; scRNA-seq,SRP215507,CD8+ T cell_1-MCI.vs.control; scRNA-seq,SRP215507,CD8+ T cell_5-MCI.vs.control; scRNA-seq,SRP215507,CD8+ T cell_3-MCI.vs.control; scRNA-	6
AL109917.1	scRNA-seq,SRP215507,CD8+ T cell_3-AD.vs.control; scRNA-seq,SRP215507,CD8+ T cell_1-AD.vs.control; scRNA-seq,SRP215507,CD8+ T cell_3-AD.vs.MCI; scRNA-seq,SRP215507,CD8+ T cell_1-MCI.vs.control; scRNA-seq,SRP330776,CD8+ T cell_1-AD.vs.control; scRNA-seq,SRP330776,B cell_1-AD.vs.control; scRNA-seq,SRP330776,Naive CD8+ T cell_1-AD.vs.control; scRNA-seq,SRP330776,Memory T cell_1-AD.vs.control; scRNA-	9

AL109923.1	scRNA-seq,SRP215507,CD8+ T cell_2-AD.vs.MCI; scRNA-seq,SRP215507,CD8+ T cell_1-MCI.vs.control	2
AL109947.1	scRNA-seq,SRP215507,CD8+ T cell_1-AD.vs.MCI; scRNA-seq,SRP215507,CD8+ T cell_1-MCI.vs.control; scRNA-seq,SRP330776,CD8+ T cell_1-	3
AL109955.1	scRNA-seq,SRP215507,CD8+ T cell_1-AD.vs.MCI; scRNA-seq,SRP215507,CD8+ T cell_5-AD.vs.MCI; scRNA-seq,SRP215507,CD8+ T cell_5-MCI.vs.control; scRNA-seq,SRP330776,γδ T cell-AD.vs.control	4
AL110114.1	scRNA-seq,SRP215507,CD8+ T cell_4-MCI.vs.control; scRNA-seq,SRP215507,CD8+ T cell_3-MCI.vs.control; scRNA-seq,SRP330776,γδ T cell-	3
AL117332.1	scRNA-seq,SRP215507,CD8+ T cell_5-MCI.vs.control; scRNA-seq,SRP330776,CD8+ T cell_1-AD.vs.control; scRNA-seq,SRP330776,γδ T cell-AD.vs.control; scRNA-seq,SRP330776,Memory T cell_2-AD.vs.control; scRNA-seq,SRP330776,CD8+ T cell_2-AD.vs.control; scRNA-seq,SRP330776,B cell_1-AD.vs.control; scRNA-seq,SRP330776,Naive CD8+ T cell_1-AD.vs.control; scRNA-seq,SRP330776,Memory T cell_1-AD.vs.control; scRNA-seq,SRP330776,B cell_2-AD.vs.control; scRNA-seq,SRP330776,Natural killer cell_2-AD.vs.control	10
AL117336.1	scRNA-seq,SRP215507,CD8+ T cell_5-AD.vs.MCI; scRNA-seq,SRP215507,CD8+ T cell_5-MCI.vs.control	2
AL117336.2	scRNA-seq,SRP215507,CD8+ T cell_5-AD.vs.control; scRNA-seq,SRP215507,CD8+ T cell_6-AD.vs.control; scRNA-seq,SRP215507,CD8+ T cell_6-MCI.vs.control; scRNA-seq,SRP309935,Monocyte_1-AD.vs.control; scRNA-seq,SRP330776,CD8+ T cell_1-AD.vs.control; scRNA-seq,SRP330776,B cell_1-AD.vs.control; scRNA-seq,SRP330776,Naive CD8+ T cell_1-AD.vs.control; scRNA-seq,SRP330776,Natural killer cell_1-AD.vs.control; scRNA-	9
AL117344.2	scRNA-seq,SRP215507,CD8+ T cell_2-AD.vs.control; scRNA-seq,SRP215507,CD8+ T cell_5-AD.vs.MCI; scRNA-seq,SRP215507,CD8+ T cell_1-	3
AL117350.1	scRNA-seq,SRP215507,CD8+ T cell_3-MCI.vs.control	1
AL117379.1	scRNA-seq,SRP215507,CD8+ T cell_1-AD.vs.control; scRNA-seq,SRP215507,CD8+ T cell_6-AD.vs.control; scRNA-seq,SRP215507,CD8+ T cell_5-AD.vs.MCI; scRNA-seq,SRP330776,γδ T cell-AD.vs.control; scRNA-seq,SRP330776,Naive CD8+ T cell_1-AD.vs.control; scRNA-seq,SRP330776,Memory T cell_1-AD.vs.control; scRNA-seq,SRP330776,Natural killer T cell-AD.vs.control; scRNA-seq,SRP330776,Naive CD8+ T cell_3-AD.vs.control; scRNA-seq,SRP330776,T cell-AD.vs.control; scRNA-seq,SRP330776,CD8+ T cell_3-AD.vs.control	10
AL117381.1	scRNA-seq,SRP215507,CD8+ T cell_4-AD.vs.control; scRNA-seq,SRP215507,CD8+ T cell_1-AD.vs.MCI; scRNA-seq,SRP330776,B cell_1-AD.vs.control; scRNA-seq,SRP330776,Naive CD8+ T cell_1-AD.vs.control	4
AL118506.1	scRNA-seq,SRP215507,CD8+ T cell_5-AD.vs.MCI; scRNA-seq,SRP215507,CD8+ T cell_5-MCI.vs.control; scRNA-seq,SRP215507,CD8+ T cell_3-MCI.vs.control; scRNA-seq,SRP309935,Natural killer cell_2-AD.vs.control; scRNA-seq,SRP309935,CD8+ T cell_1-AD.vs.control; scRNA-seq,SRP330776,CD8+ T cell_1-AD.vs.control; scRNA-seq,SRP330776,γδ T cell-AD.vs.control; scRNA-seq,SRP330776,CD8+ T cell_2-AD.vs.control; scRNA-seq,SRP330776,Naive CD8+ T cell_1-AD.vs.control; scRNA-seq,SRP330776,Natural killer cell_1-AD.vs.control; scRNA-seq,SRP330776,Memory	11
AL118516.1	scRNA-seq,SRP309935,Natural killer cell_2-AD.vs.control; scRNA-seq,SRP309935,Monocyte_1-AD.vs.control; scRNA-seq,SRP309935,Natural killer cell_1-AD.vs.control; scRNA-seq,SRP309935,CD8+ T cell_1-AD.vs.control; scRNA-seq,SRP309935,CD8+ T cell_2-AD.vs.control; scRNA-seq,SRP330776,CD8+ T cell_2-AD.vs.control; scRNA-seq,SRP330776,Memory T cell_1-AD.vs.control; scRNA-seq,SRP330776,Naive CD8+ T cell_3-AD.vs.control; scRNA-seq,SRP330776,Monocyte_1-AD.vs.control; scRNA-seq,SRP330776,Monocyte_2-AD.vs.control; scRNA-seq,SRP330776,Natural	11
AL118556.1	scRNA-seq,SRP330776,Memory T cell_1-AD.vs.control	1
AL118558.3	scRNA-seq,SRP215507,CD8+ T cell_5-AD.vs.MCI; scRNA-seq,SRP330776,CD8+ T cell_1-AD.vs.control; scRNA-seq,SRP330776,CD8+ T cell_2-AD.vs.control; scRNA-seq,SRP330776,B cell_1-AD.vs.control; scRNA-seq,SRP330776,Naive CD8+ T cell_1-AD.vs.control; scRNA-seq,SRP330776,Memory T cell_1-AD.vs.control; scRNA-seq,SRP330776,B cell_2-AD.vs.control	7
AL121574.1	scRNA-seq,SRP215507,CD8+ T cell_1-AD.vs.MCI; scRNA-seq,SRP330776,CD8+ T cell_1-AD.vs.control; scRNA-seq,SRP330776,Memory T cell_1-	3
AL121601.1	scRNA-seq,SRP215507,CD8+ T cell_2-AD.vs.control; scRNA-seq,SRP215507,CD8+ T cell_5-MCI.vs.control; scRNA-seq,SRP330776,Naive CD8+ T	3

AL121603.2	scRNA-seq,SRP215507,CD8+ T cell_2-AD.vs.control; scRNA-seq,SRP215507,CD8+ T cell_8-AD.vs.control; scRNA-seq,SRP215507,CD8+ T cell_3-AD.vs.control; scRNA-seq,SRP215507,CD8+ T cell_1-AD.vs.control; scRNA-seq,SRP215507,CD8+ T cell_6-AD.vs.control; scRNA-seq,SRP215507,CD8+ T cell_3-AD.vs.MCI; scRNA-seq,SRP215507,CD8+ T cell_1-AD.vs.MCI; scRNA-seq,SRP215507,CD8+ T cell_1-MCI.vs.control; scRNA-seq,SRP215507,CD8+ T cell_3-MCI.vs.control; scRNA-seq,SRP309935,Naive CD8+ T cell_1-AD.vs.control; scRNA-seq,SRP309935,Natural killer cell_1-AD.vs.control; scRNA-seq,SRP309935,CD8+ T cell_1-AD.vs.control; scRNA-seq,SRP330776,CD8+ T cell_1-AD.vs.control; scRNA-seq,SRP330776,γδ T cell-AD.vs.control; scRNA-seq,SRP330776,Memory T cell_2-AD.vs.control; scRNA-seq,SRP330776,CD8+ T cell_2-AD.vs.control; scRNA-seq,SRP330776,B cell_1-AD.vs.control; scRNA-seq,SRP330776,Naive CD8+ T cell_1-AD.vs.control; scRNA-seq,SRP330776,Memory T cell_1-AD.vs.control; scRNA-seq,SRP330776,Natural killer T cell-AD.vs.control; scRNA-seq,SRP330776,Naive CD8+ T cell_3-AD.vs.control; scRNA-seq,SRP330776,B cell_2-AD.vs.control; scRNA-seq,SRP330776,T cell-AD.vs.control; scRNA-seq,SRP330776,CD8+ T cell_3-AD.vs.control; scRNA-[illegible]	25
AL121652.1	scRNA-seq,SRP330776,Naive CD8+ T cell_1-AD.vs.control	1
AL121658.1	scRNA-seq,SRP330776,Memory T cell_2-AD.vs.control; scRNA-seq,SRP330776,Memory T cell_1-AD.vs.control; scRNA-seq,SRP330776,B cell_2-AD.vs.control; scRNA-seq,SRP330776,CD8+ T cell_3-AD.vs.control	4
AL121787.1	scRNA-seq,SRP330776,CD8+ T cell_2-AD.vs.control; scRNA-seq,SRP330776,B cell_2-AD.vs.control	2
AL121820.1	scRNA-seq,SRP215507,CD8+ T cell_5-AD.vs.control; scRNA-seq,SRP215507,CD8+ T cell_5-MCI.vs.control; scRNA-seq,SRP330776,CD8+ T cell_1-AD.vs.control; scRNA-seq,SRP330776,Naive CD8+ T cell_2-AD.vs.control	4
AL121832.2	scRNA-seq,SRP330776,CD8+ T cell_1-AD.vs.control; scRNA-seq,SRP330776,Memory T cell_2-AD.vs.control; scRNA-seq,SRP330776,B cell_1-AD.vs.control; scRNA-seq,SRP330776,Natural killer cell_1-AD.vs.control; scRNA-seq,SRP330776,Memory T cell_1-AD.vs.control; scRNA-seq,SRP330776,Natural killer T cell-AD.vs.control; scRNA-seq,SRP330776,Naive CD8+ T cell_3-AD.vs.control	7
AL121832.3	scRNA-seq,SRP330776,Memory T cell_1-AD.vs.control	1
AL121839.2	scRNA-seq,SRP215507,CD8+ T cell_5-AD.vs.control; scRNA-seq,SRP215507,CD8+ T cell_5-MCI.vs.control; scRNA-seq,SRP309935,Natural killer cell_1-AD.vs.control; scRNA-seq,SRP330776,CD8+ T cell_1-AD.vs.control; scRNA-seq,SRP330776,γδ T cell-AD.vs.control; scRNA-seq,SRP330776,Memory T cell_2-AD.vs.control; scRNA-seq,SRP330776,Naive CD8+ T cell_1-AD.vs.control; scRNA-seq,SRP330776,Natural killer T	9
AL121917.1	scRNA-seq,SRP330776,CD8+ T cell_1-AD.vs.control	1
AL121928.1	scRNA-seq,SRP330776,Memory T cell_1-AD.vs.control; scRNA-seq,SRP330776,CD8+ T cell_3-AD.vs.control	2
AL121933.2	scRNA-seq,SRP215507,CD8+ T cell_5-AD.vs.MCI; scRNA-seq,SRP215507,CD8+ T cell_5-MCI.vs.control; scRNA-seq,SRP309935,Plasmacytoid dendritic cell_2-AD.vs.control; scRNA-seq,SRP309935,CD8+ T cell_2-AD.vs.control; scRNA-seq,SRP330776,B cell_1-AD.vs.control; scRNA-seq,SRP330776,Memory T cell_1-AD.vs.control; scRNA-seq,SRP330776,Monocyte_1-AD.vs.control	7
AL121935.1	scRNA-seq,SRP330776,Memory T cell_2-AD.vs.control	1
AL121944.1	scRNA-seq,SRP215507,CD8+ T cell_1-AD.vs.MCI; scRNA-seq,SRP215507,CD8+ T cell_4-AD.vs.MCI; scRNA-seq,SRP215507,CD8+ T cell_5-AD.vs.MCI; scRNA-seq,SRP215507,CD8+ T cell_4-MCI.vs.control; scRNA-seq,SRP309935,Naive CD8+ T cell_1-AD.vs.control; scRNA-seq,SRP309935,Natural killer cell_1-AD.vs.control; scRNA-seq,SRP309935,Naive CD8+ T cell_2-AD.vs.control; scRNA-seq,SRP309935,CD8+ T cell_2-AD.vs.control; scRNA-seq,SRP330776,CD8+ T cell_1-AD.vs.control; scRNA-seq,SRP330776,γδ T cell-AD.vs.control; scRNA-seq,SRP330776,Memory T cell_2-AD.vs.control; scRNA-seq,SRP330776,CD8+ T cell_2-AD.vs.control; scRNA-seq,SRP330776,B cell_1-AD.vs.control; scRNA-seq,SRP330776,Naive CD8+ T cell_1-AD.vs.control; scRNA-seq,SRP330776,Natural killer cell_1-AD.vs.control; scRNA-seq,SRP330776,Memory T cell_1-AD.vs.control; scRNA-seq,SRP330776,Naive CD8+ T cell_3-AD.vs.control; scRNA-seq,SRP330776,T cell-AD.vs.control; scRNA-[illegible]	20
AL121972.1	scRNA-seq,SRP215507,CD8+ T cell_1-AD.vs.MCI; scRNA-seq,SRP215507,CD8+ T cell_1-MCI.vs.control; scRNA-seq,SRP215507,CD8+ T cell_2-MCI.vs.control; scRNA-seq,SRP330776,Naive CD8+ T cell_1-AD.vs.control; scRNA-seq,SRP330776,CD8+ T cell_3-AD.vs.control	5
AL121985.1	scRNA-seq,SRP330776,γδ T cell-AD.vs.control; scRNA-seq,SRP330776,Memory T cell_2-AD.vs.control	2
AL121987.2	scRNA-seq,SRP215507,CD8+ T cell_5-AD.vs.control; scRNA-seq,SRP215507,CD8+ T cell_3-AD.vs.MCI; scRNA-seq,SRP215507,CD8+ T cell_1-AD.vs.MCI; scRNA-seq,SRP215507,CD8+ T cell_1-MCI.vs.control; scRNA-seq,SRP215507,CD8+ T cell_3-MCI.vs.control	5
AL121990.1	scRNA-seq,SRP215507,CD8+ T cell_1-MCI.vs.control; scRNA-seq,SRP330776,CD8+ T cell_2-AD.vs.control	2
AL122020.2	scRNA-seq,SRP215507,CD8+ T cell_2-AD.vs.control	1

AL122035.1	scRNA-seq,SRP215507,CD8+ T cell_1-AD.vs.MCI; scRNA-seq,SRP215507,CD8+ T cell_2-MCI.vs.control; scRNA-seq,SRP330776,CD8+ T cell_2-AD.vs.control; scRNA-seq,SRP330776,Memory T cell_1-AD.vs.control	4
AL122035.2	scRNA-seq,SRP215507,CD8+ T cell_2-AD.vs.control; scRNA-seq,SRP215507,CD8+ T cell_4-AD.vs.control; scRNA-seq,SRP215507,CD8+ T cell_2-AD.vs.MCI; scRNA-seq,SRP215507,CD8+ T cell_4-MCI.vs.control	4
AL132639.2	scRNA-seq,SRP215507,CD8+ T cell_3-AD.vs.MCI; scRNA-seq,SRP215507,CD8+ T cell_3-MCI.vs.control; scRNA-seq,SRP330776,CD8+ T cell_1-AD.vs.control; scRNA-seq,SRP330776,Memory T cell_2-AD.vs.control; scRNA-seq,SRP330776,Naive CD8+ T cell_1-AD.vs.control; scRNA-seq,SRP330776,Memory T cell_1-AD.vs.control; scRNA-seq,SRP330776,Natural killer T cell-AD.vs.control	7
AL132639.3	scRNA-seq,SRP330776,Naive CD8+ T cell_1-AD.vs.control	1
AL132780.1	scRNA-seq,SRP215507,CD8+ T cell_3-AD.vs.control; scRNA-seq,SRP215507,CD8+ T cell_2-MCI.vs.control; scRNA-seq,SRP330776,CD8+ T cell_1-AD.vs.control; scRNA-seq,SRP330776,Memory T cell_2-AD.vs.control; scRNA-seq,SRP330776,CD8+ T cell_2-AD.vs.control	5
AL132800.1	scRNA-seq,SRP215507,CD8+ T cell_1-AD.vs.MCI; scRNA-seq,SRP215507,CD8+ T cell_3-MCI.vs.control	2
AL132996.1	scRNA-seq,SRP215507,CD8+ T cell_3-AD.vs.MCI; scRNA-seq,SRP215507,CD8+ T cell_3-MCI.vs.control; scRNA-seq,SRP330776,B cell_2-	3
AL133215.2	scRNA-seq,SRP215507,CD8+ T cell_2-AD.vs.control	1
AL133230.1	scRNA-seq,SRP330776,CD8+ T cell_2-AD.vs.control	1
AL133243.2	scRNA-seq,SRP215507,CD8+ T cell_3-AD.vs.MCI; scRNA-seq,SRP215507,CD8+ T cell_3-MCI.vs.control; scRNA-seq,SRP330776,Memory T cell_2-AD.vs.control; scRNA-seq,SRP330776,CD8+ T cell_2-AD.vs.control; scRNA-seq,SRP330776,Naive CD8+ T cell_1-AD.vs.control; scRNA-seq,SRP330776,Memory T cell_1-AD.vs.control; scRNA-seq,SRP330776,B cell_2-AD.vs.control	7
AL133243.3	scRNA-seq,SRP215507,CD8+ T cell_5-AD.vs.control; scRNA-seq,SRP215507,CD8+ T cell_4-MCI.vs.control; scRNA-seq,SRP309935,CD8+ T cell_1-AD.vs.control; scRNA-seq,SRP309935,B cell_1-AD.vs.control; scRNA-seq,SRP330776,γδ T cell-AD.vs.control; scRNA-seq,SRP330776,Memory T cell_2-AD.vs.control; scRNA-seq,SRP330776,CD8+ T cell_2-AD.vs.control; scRNA-seq,SRP330776,B cell_1-AD.vs.control; scRNA-seq,SRP330776,Naive CD8+ T cell_1-AD.vs.control; scRNA-seq,SRP330776,Memory T cell_1-AD.vs.control; scRNA-seq,SRP330776,T cell-	11
AL133245.1	scRNA-seq,SRP215507,CD8+ T cell_1-AD.vs.MCI; scRNA-seq,SRP309935,Naive CD8+ T cell_1-AD.vs.control; scRNA-seq,SRP330776,B cell_1-AD.vs.control; scRNA-seq,SRP330776,Naive CD8+ T cell_1-AD.vs.control	4
AL133330.1	scRNA-seq,SRP215507,CD8+ T cell_2-AD.vs.control; scRNA-seq,SRP215507,CD8+ T cell_4-AD.vs.control; scRNA-seq,SRP215507,CD8+ T cell_5-AD.vs.MCI; scRNA-seq,SRP215507,CD8+ T cell_4-MCI.vs.control; scRNA-seq,SRP309935,Monocyte_1-AD.vs.control; scRNA-seq,SRP309935,CD4+ T cell-AD.vs.control; scRNA-seq,SRP330776,γδ T cell-AD.vs.control; scRNA-seq,SRP330776,CD8+ T cell_2-AD.vs.control; scRNA-seq,SRP330776,B cell_1-AD.vs.control; scRNA-seq,SRP330776,Natural killer cell_2-AD.vs.control	10
AL133338.1	scRNA-seq,SRP215507,CD8+ T cell_1-AD.vs.MCI; scRNA-seq,SRP215507,CD8+ T cell_1-MCI.vs.control; scRNA-seq,SRP215507,CD8+ T cell_2-MCI.vs.control; scRNA-seq,SRP330776,CD8+ T cell_1-AD.vs.control; scRNA-seq,SRP330776,Naive CD8+ T cell_1-AD.vs.control; scRNA-seq,SRP330776,Natural killer cell_1-AD.vs.control; scRNA-seq,SRP330776,B cell_2-AD.vs.control	7
AL133342.1	scRNA-seq,SRP215507,CD8+ T cell_2-AD.vs.MCI; scRNA-seq,SRP309935,Natural killer cell_2-AD.vs.control; scRNA-seq,SRP309935,Monocyte_1-AD.vs.control; scRNA-seq,SRP309935,Megakaryocyte_1-AD.vs.control; scRNA-seq,SRP309935,Naive CD8+ T cell_1-AD.vs.control; scRNA-seq,SRP309935,Natural killer cell_1-AD.vs.control; scRNA-seq,SRP309935,Monocyte_2-AD.vs.control; scRNA-seq,SRP309935,Naive CD8+ T cell_2-AD.vs.control; scRNA-seq,SRP309935,CD8+ T cell_1-AD.vs.control; scRNA-seq,SRP309935,CD8+ T cell_2-AD.vs.control; scRNA-seq,SRP309935,Naive CD8+ T cell_3-AD.vs.control; scRNA-seq,SRP330776,CD8+ T cell_1-AD.vs.control	12
AL133351.1	scRNA-seq,SRP215507,CD8+ T cell_2-MCI.vs.control	1
AL133355.1	scRNA-seq,SRP309935,Naive CD8+ T cell_1-AD.vs.control	1
AL133367.1	scRNA-seq,SRP215507,CD8+ T cell_3-AD.vs.control; scRNA-seq,SRP215507,CD8+ T cell_1-AD.vs.MCI; scRNA-seq,SRP215507,CD8+ T cell_1-MCI.vs.control; scRNA-seq,SRP330776,Memory T cell_1-AD.vs.control	4
AL133390.1	scRNA-seq,SRP215507,CD8+ T cell_2-AD.vs.control; scRNA-seq,SRP215507,CD8+ T cell_2-MCI.vs.control	2
AL133406.2	scRNA-seq,SRP330776,CD8+ T cell_1-AD.vs.control; scRNA-seq,SRP330776,Naive CD8+ T cell_1-AD.vs.control	2

AL133410.1	scRNA-seq,SRP215507,CD8+ T cell_2-AD.vs.control; scRNA-seq,SRP215507,CD8+ T cell_3-AD.vs.MCI; scRNA-seq,SRP215507,CD8+ T cell_1-AD.vs.MCI; scRNA-seq,SRP215507,CD8+ T cell_1-MCI.vs.control; scRNA-seq,SRP215507,CD8+ T cell_3-MCI.vs.control; scRNA-seq,SRP330776,B cell_1-AD.vs.control; scRNA-seq,SRP330776,Naive CD8+ T cell_1-AD.vs.control	7
AL133410.2	scRNA-seq,SRP215507,CD8+ T cell_3-AD.vs.MCI; scRNA-seq,SRP215507,CD8+ T cell_1-AD.vs.MCI; scRNA-seq,SRP215507,CD8+ T cell_1-MCI.vs.control; scRNA-seq,SRP215507,CD8+ T cell_3-MCI.vs.control; scRNA-seq,SRP330776,Memory T cell_2-AD.vs.control; scRNA-	6
AL133415.1	scRNA-seq,SRP215507,CD8+ T cell_5-AD.vs.control; scRNA-seq,SRP215507,CD8+ T cell_3-MCI.vs.control; scRNA-seq,SRP330776,γδ T cell-AD.vs.control; scRNA-seq,SRP330776,Natural killer cell_1-AD.vs.control; scRNA-seq,SRP330776,Memory T cell_1-AD.vs.control; scRNA-seq,SRP330776,Monocyte_1-AD.vs.control; scRNA-seq,SRP330776,Monocyte_2-AD.vs.control	7
AL133453.1	scRNA-seq,SRP215507,CD8+ T cell_1-AD.vs.control; scRNA-seq,SRP215507,CD8+ T cell_2-MCI.vs.control; scRNA-seq,SRP309935,Naive CD8+ T cell_1-AD.vs.control; scRNA-seq,SRP309935,CD8+ T cell_1-AD.vs.control; scRNA-seq,SRP309935,CD8+ T cell_2-AD.vs.control	5
AL133467.1	scRNA-seq,SRP215507,CD8+ T cell_1-AD.vs.control; scRNA-seq,SRP330776,Naive CD8+ T cell_1-AD.vs.control; scRNA-seq,SRP330776,B cell_4-	3
AL133467.2	scRNA-seq,SRP330776,CD8+ T cell_1-AD.vs.control; scRNA-seq,SRP330776,B cell_1-AD.vs.control; scRNA-seq,SRP330776,Naive CD8+ T cell_1-	3
AL133477.2	scRNA-seq,SRP215507,CD8+ T cell_1-AD.vs.control; scRNA-seq,SRP215507,CD8+ T cell_1-MCI.vs.control; scRNA-seq,SRP330776,Memory T cell_2-AD.vs.control; scRNA-seq,SRP330776,CD8+ T cell_2-AD.vs.control; scRNA-seq,SRP330776,B cell_1-AD.vs.control; scRNA-seq,SRP330776,Naive CD8+ T cell_1-AD.vs.control; scRNA-seq,SRP330776,Natural killer cell_1-AD.vs.control; scRNA-seq,SRP330776,B cell_2-AD.vs.control; scRNA-seq,SRP330776,T cell-AD.vs.control; scRNA-seq,SRP330776,Natural killer cell_2-AD.vs.control	10
AL133520.1	scRNA-seq,SRP215507,CD8+ T cell_1-MCI.vs.control	1
AL133523.1	scRNA-seq,SRP215507,CD8+ T cell_1-AD.vs.control	1
AL133551.1	scRNA-seq,SRP330776,CD8+ T cell_1-AD.vs.control; scRNA-seq,SRP330776,Naive CD8+ T cell_1-AD.vs.control	2
AL135791.1	scRNA-seq,SRP215507,CD8+ T cell_5-AD.vs.control; scRNA-seq,SRP215507,CD8+ T cell_2-AD.vs.MCI; scRNA-seq,SRP215507,CD8+ T cell_5-AD.vs.MCI; scRNA-seq,SRP215507,CD8+ T cell_1-MCI.vs.control; scRNA-seq,SRP215507,CD8+ T cell_4-MCI.vs.control; scRNA-seq,SRP215507,CD8+ T cell_2-MCI.vs.control; scRNA-seq,SRP309935,Naive CD8+ T cell_1-AD.vs.control; scRNA-seq,SRP330776,Memory T cell_2-AD.vs.control; scRNA-seq,SRP330776,Naive CD8+ T cell_1-AD.vs.control; scRNA-seq,SRP330776,CD8+ T cell_3-AD.vs.control	10
AL135818.1	scRNA-seq,SRP309935,Natural killer cell_1-AD.vs.control; scRNA-seq,SRP330776,CD8+ T cell_2-AD.vs.control; scRNA-seq,SRP330776,CD8+ T	3
AL135818.2	scRNA-seq,SRP330776,CD8+ T cell_1-AD.vs.control; scRNA-seq,SRP330776,γδ T cell-AD.vs.control; scRNA-seq,SRP330776,Memory T cell_2-AD.vs.control; scRNA-seq,SRP330776,CD8+ T cell_2-AD.vs.control; scRNA-seq,SRP330776,B cell_1-AD.vs.control; scRNA-seq,SRP330776,Naive	6
AL135905.1	scRNA-seq,SRP215507,CD8+ T cell_1-AD.vs.control; scRNA-seq,SRP215507,CD8+ T cell_1-MCI.vs.control; scRNA-seq,SRP215507,CD8+ T cell_2-	3
AL135905.2	scRNA-seq,SRP215507,CD8+ T cell_4-AD.vs.control; scRNA-seq,SRP215507,CD8+ T cell_1-AD.vs.MCI; scRNA-seq,SRP215507,CD8+ T cell_4-AD.vs.MCI; scRNA-seq,SRP215507,CD8+ T cell_5-AD.vs.MCI; scRNA-seq,SRP215507,CD8+ T cell_1-MCI.vs.control; scRNA-seq,SRP215507,CD8+ T cell_5-MCI.vs.control; scRNA-seq,SRP215507,CD8+ T cell_2-MCI.vs.control; scRNA-seq,SRP215507,CD8+ T cell_3-MCI.vs.control; scRNA-seq,SRP215507,CD8+ T cell_6-MCI.vs.control; scRNA-seq,SRP330776,γδ T cell-AD.vs.control; scRNA-seq,SRP330776,Natural killer T cell-AD.vs.control; scRNA-seq,SRP330776,Monocyte_1-AD.vs.control; scRNA-seq,SRP330776,Monocyte_2-AD.vs.control; scRNA-	14
AL135925.1	scRNA-seq,SRP215507,CD8+ T cell_7-AD.vs.control; scRNA-seq,SRP215507,CD8+ T cell_6-AD.vs.control; scRNA-seq,SRP215507,CD8+ T cell_1-AD.vs.MCI; scRNA-seq,SRP215507,CD8+ T cell_7-AD.vs.MCI; scRNA-seq,SRP215507,CD8+ T cell_1-MCI.vs.control; scRNA-seq,SRP215507,CD8+ T cell_6-MCI.vs.control; scRNA-seq,SRP309935,B cell_2-AD.vs.control; scRNA-seq,SRP330776,CD8+ T cell_2-AD.vs.control; scRNA-seq,SRP330776,Natural killer cell_1-AD.vs.control; scRNA-seq,SRP330776,Memory T cell_1-AD.vs.control; scRNA-seq,SRP330776,Naive CD8+ T cell_3-AD.vs.control; scRNA-seq,SRP330776,CD8+ T cell_3-AD.vs.control; scRNA-seq,SRP330776,Natural killer cell_2-AD.vs.control	13
AL135978.2	scRNA-seq,SRP215507,CD8+ T cell_2-AD.vs.MCI; scRNA-seq,SRP215507,CD8+ T cell_2-MCI.vs.control; scRNA-seq,SRP330776,CD8+ T cell_2-	3
AL135999.1	scRNA-seq,SRP215507,CD8+ T cell_2-AD.vs.control; scRNA-seq,SRP215507,CD8+ T cell_1-AD.vs.MCI; scRNA-seq,SRP215507,CD8+ T cell_1-MCI.vs.control; scRNA-seq,SRP330776,γδ T cell-AD.vs.control; scRNA-seq,SRP330776,Memory T cell_2-AD.vs.control; scRNA-seq,SRP330776,CD8+ T cell_2-AD.vs.control; scRNA-seq,SRP330776,B cell_1-AD.vs.control; scRNA-seq,SRP330776,Natural killer cell_1-AD.vs.control	8
AL136018.1	scRNA-seq,SRP330776,Natural killer cell_1-AD.vs.control	1

AL136038.2	scRNA-seq,SRP330776,Memory T cell_1-AD.vs.control; scRNA-seq,SRP330776,Naive CD8+ T cell_3-AD.vs.control	2
AL136038.3	scRNA-seq,SRP215507,CD8+ T cell_1-MCI.vs.control	1
AL136038.5	scRNA-seq,SRP215507,CD8+ T cell_2-MCI.vs.control; scRNA-seq,SRP330776,CD8+ T cell_1-AD.vs.control; scRNA-seq,SRP330776,γδ T cell-AD.vs.control; scRNA-seq,SRP330776,CD8+ T cell_2-AD.vs.control; scRNA-seq,SRP330776,Naive CD8+ T cell_1-AD.vs.control; scRNA-seq,SRP330776,Natural killer cell_1-AD.vs.control; scRNA-seq,SRP330776,Memory T cell_1-AD.vs.control; scRNA-seq,SRP330776,Natural killer T cell-AD.vs.control; scRNA-seq,SRP330776,Naive CD8+ T cell_3-AD.vs.control; scRNA-seq,SRP330776,CD8+ T cell_3-AD.vs.control	10
AL136040.1	scRNA-seq,SRP215507,CD8+ T cell_3-AD.vs.control; scRNA-seq,SRP215507,CD8+ T cell_1-AD.vs.control; scRNA-seq,SRP215507,CD8+ T cell_1-AD.vs.MCI; scRNA-seq,SRP330776,CD8+ T cell_1-AD.vs.control; scRNA-seq,SRP330776,B cell_1-AD.vs.control; scRNA-seq,SRP330776,Naive CD8+ T cell_1-AD.vs.control; scRNA-seq,SRP330776,Memory T cell_1-AD.vs.control	7
AL136171.2	scRNA-seq,SRP215507,CD8+ T cell_3-MCI.vs.control; scRNA-seq,SRP330776,CD8+ T cell_1-AD.vs.control; scRNA-seq,SRP330776,Memory T cell_2-AD.vs.control; scRNA-seq,SRP330776,Naive CD8+ T cell_1-AD.vs.control	4
AL136295.17	scRNA-seq,SRP215507,CD8+ T cell_5-AD.vs.MCI; scRNA-seq,SRP215507,CD8+ T cell_5-MCI.vs.control; scRNA-seq,SRP215507,CD8+ T cell_3-MCI.vs.control; scRNA-seq,SRP330776,CD8+ T cell_1-AD.vs.control; scRNA-seq,SRP330776,CD8+ T cell_2-AD.vs.control; scRNA-	6
AL136295.2	scRNA-seq,SRP215507,CD8+ T cell_2-AD.vs.MCI; scRNA-seq,SRP215507,CD8+ T cell_2-MCI.vs.control; scRNA-seq,SRP309935,Monocyte_1-	3
AL136295.7	scRNA-seq,SRP330776,CD8+ T cell_1-AD.vs.control; scRNA-seq,SRP330776,Naive CD8+ T cell_1-AD.vs.control	2
AL136301.1	scRNA-seq,SRP215507,CD8+ T cell_5-MCI.vs.control	1
AL136307.1	scRNA-seq,SRP215507,CD8+ T cell_5-MCI.vs.control	1
AL136320.1	scRNA-seq,SRP215507,CD8+ T cell_1-MCI.vs.control; scRNA-seq,SRP330776,Memory T cell_1-AD.vs.control	2
AL136368.1	scRNA-seq,SRP330776,Naive CD8+ T cell_2-AD.vs.control	1
AL136419.3	scRNA-seq,SRP215507,CD8+ T cell_2-AD.vs.control; scRNA-seq,SRP215507,CD8+ T cell_1-AD.vs.control; scRNA-seq,SRP215507,CD8+ T cell_5-AD.vs.control; scRNA-seq,SRP215507,CD8+ T cell_2-AD.vs.MCI; scRNA-seq,SRP215507,CD8+ T cell_3-AD.vs.MCI; scRNA-seq,SRP215507,CD8+ T cell_1-AD.vs.MCI; scRNA-seq,SRP215507,CD8+ T cell_1-MCI.vs.control; scRNA-seq,SRP215507,CD8+ T cell_2-MCI.vs.control; scRNA-seq,SRP215507,CD8+ T cell_3-MCI.vs.control; scRNA-seq,SRP330776,CD8+ T cell_1-AD.vs.control; scRNA-seq,SRP330776,γδ T cell-AD.vs.control; scRNA-seq,SRP330776,Memory T cell_2-AD.vs.control; scRNA-seq,SRP330776,CD8+ T cell_2-AD.vs.control; scRNA-seq,SRP330776,Naive CD8+ T cell_1-AD.vs.control; scRNA-seq,SRP330776,Memory T cell_1-AD.vs.control; scRNA-seq,SRP330776,Natural killer T cell-AD.vs.control	16
AL136454.1	scRNA-seq,SRP215507,CD8+ T cell_2-AD.vs.control; scRNA-seq,SRP215507,CD8+ T cell_3-AD.vs.control; scRNA-seq,SRP215507,CD8+ T cell_1-AD.vs.control; scRNA-seq,SRP215507,CD8+ T cell_4-AD.vs.control; scRNA-seq,SRP215507,CD8+ T cell_7-AD.vs.control; scRNA-seq,SRP215507,CD8+ T cell_5-AD.vs.control; scRNA-seq,SRP215507,CD8+ T cell_6-AD.vs.control; scRNA-seq,SRP215507,CD8+ T cell_2-AD.vs.MCI; scRNA-seq,SRP215507,CD8+ T cell_3-AD.vs.MCI; scRNA-seq,SRP215507,CD8+ T cell_1-AD.vs.MCI; scRNA-seq,SRP215507,CD8+ T cell_4-AD.vs.MCI; scRNA-seq,SRP215507,CD8+ T cell_7-AD.vs.MCI; scRNA-seq,SRP215507,CD8+ T cell_5-AD.vs.MCI; scRNA-seq,SRP330776,CD8+ T	14
AL136456.1	scRNA-seq,SRP215507,CD8+ T cell_3-AD.vs.MCI; scRNA-seq,SRP215507,CD8+ T cell_3-MCI.vs.control	2
AL136526.1	scRNA-seq,SRP215507,CD8+ T cell_3-MCI.vs.control	1
AL136531.1	scRNA-seq,SRP215507,CD8+ T cell_1-MCI.vs.control; scRNA-seq,SRP215507,CD8+ T cell_3-MCI.vs.control	2
AL136962.1	scRNA-seq,SRP215507,CD8+ T cell_2-AD.vs.control; scRNA-seq,SRP215507,CD8+ T cell_3-AD.vs.control; scRNA-seq,SRP215507,CD8+ T cell_3-AD.vs.MCI; scRNA-seq,SRP215507,CD8+ T cell_5-AD.vs.MCI; scRNA-seq,SRP215507,CD8+ T cell_5-MCI.vs.control	5
AL136984.1	scRNA-seq,SRP330776,Naive CD8+ T cell_1-AD.vs.control	1
AL136987.1	scRNA-seq,SRP215507,CD8+ T cell_1-AD.vs.MCI; scRNA-seq,SRP215507,CD8+ T cell_1-MCI.vs.control; scRNA-seq,SRP330776,γδ T cell-AD.vs.control; scRNA-seq,SRP330776,CD8+ T cell_2-AD.vs.control; scRNA-seq,SRP330776,Natural killer cell_1-AD.vs.control	5
AL137003.1	scRNA-seq,SRP215507,CD8+ T cell_3-AD.vs.control; scRNA-seq,SRP330776,Naive CD8+ T cell_1-AD.vs.control; scRNA-seq,SRP330776,T cell-AD.vs.control; scRNA-seq,SRP330776,Naive CD8+ T cell_2-AD.vs.control	4
AL137003.2	scRNA-seq,SRP330776,CD8+ T cell_1-AD.vs.control	1

AL137009.1	scRNA-seq,SRP215507,CD8+ T cell_3-AD.vs.control; scRNA-seq,SRP215507,CD8+ T cell_1-MCI.vs.control	2
AL137060.3	scRNA-seq,SRP215507,CD8+ T cell_2-AD.vs.control; scRNA-seq,SRP215507,CD8+ T cell_1-AD.vs.MCI; scRNA-seq,SRP215507,CD8+ T cell_1-MCI.vs.control; scRNA-seq,SRP215507,CD8+ T cell_2-MCI.vs.control	4
AL137779.2	scRNA-seq,SRP215507,CD8+ T cell_2-AD.vs.control; scRNA-seq,SRP215507,CD8+ T cell_3-AD.vs.control; scRNA-seq,SRP215507,CD8+ T cell_1-AD.vs.control; scRNA-seq,SRP215507,CD8+ T cell_1-AD.vs.MCI; scRNA-seq,SRP215507,CD8+ T cell_5-MCI.vs.control; scRNA-seq,SRP215507,CD8+ T cell_3-MCI.vs.control; scRNA-seq,SRP330776,CD8+ T cell_1-AD.vs.control; scRNA-seq,SRP330776,B cell_1-AD.vs.control; scRNA-seq,SRP330776,Naive CD8+ T cell_1-AD.vs.control; scRNA-seq,SRP330776,B cell_2-AD.vs.control	10
AL137786.1	scRNA-seq,SRP215507,CD8+ T cell_3-MCI.vs.control	1
AL137796.1	scRNA-seq,SRP330776,B cell_1-AD.vs.control; scRNA-seq,SRP330776,B cell_2-AD.vs.control	2
AL137856.1	scRNA-seq,SRP215507,CD8+ T cell_1-AD.vs.MCI; scRNA-seq,SRP330776,CD8+ T cell_1-AD.vs.control; scRNA-seq,SRP330776,CD8+ T cell_3-	3
AL137857.1	scRNA-seq,SRP330776,CD8+ T cell_2-AD.vs.control; scRNA-seq,SRP330776,B cell_2-AD.vs.control; scRNA-seq,SRP330776,B cell_3-AD.vs.control	3
AL138724.1	scRNA-seq,SRP215507,CD8+ T cell_2-AD.vs.control; scRNA-seq,SRP215507,CD8+ T cell_2-AD.vs.MCI; scRNA-seq,SRP215507,CD8+ T cell_5-AD.vs.MCI; scRNA-seq,SRP330776,γδ T cell-AD.vs.control; scRNA-seq,SRP330776,B cell_1-AD.vs.control; scRNA-seq,SRP330776,Naive CD8+ T cell_1-AD.vs.control; scRNA-seq,SRP330776,Memory T cell_1-AD.vs.control; scRNA-seq,SRP330776,Naive CD8+ T cell_3-AD.vs.control; scRNA-	9
AL138756.1	scRNA-seq,SRP215507,CD8+ T cell_6-AD.vs.control; scRNA-seq,SRP215507,CD8+ T cell_2-AD.vs.MCI; scRNA-seq,SRP215507,CD8+ T cell_1-MCI.vs.control; scRNA-seq,SRP215507,CD8+ T cell_4-MCI.vs.control; scRNA-seq,SRP215507,CD8+ T cell_2-MCI.vs.control; scRNA-seq,SRP309935,Natural killer cell_2-AD.vs.control; scRNA-seq,SRP330776,γδ T cell-AD.vs.control; scRNA-seq,SRP330776,CD8+ T cell_2-	9
AL138762.1	scRNA-seq,SRP215507,CD8+ T cell_1-MCI.vs.control; scRNA-seq,SRP215507,CD8+ T cell_2-MCI.vs.control	2
AL138778.1	scRNA-seq,SRP215507,CD8+ T cell_2-MCI.vs.control; scRNA-seq,SRP330776,CD8+ T cell_1-AD.vs.control; scRNA-seq,SRP330776,Memory T cell_2-AD.vs.control; scRNA-seq,SRP330776,Naive CD8+ T cell_1-AD.vs.control	4
AL138820.1	scRNA-seq,SRP215507,CD8+ T cell_4-AD.vs.control; scRNA-seq,SRP215507,CD8+ T cell_4-AD.vs.MCI	2
AL138824.1	scRNA-seq,SRP215507,CD8+ T cell_2-AD.vs.control; scRNA-seq,SRP215507,CD8+ T cell_1-AD.vs.control; scRNA-seq,SRP215507,CD8+ T cell_2-AD.vs.MCI; scRNA-seq,SRP215507,CD8+ T cell_5-MCI.vs.control; scRNA-seq,SRP309935,Natural killer cell_2-AD.vs.control; scRNA-seq,SRP309935,Natural killer cell_1-AD.vs.control; scRNA-seq,SRP309935,Naive CD8+ T cell_2-AD.vs.control; scRNA-seq,SRP330776,CD8+ T cell_2-	8
AL138895.1	scRNA-seq,SRP215507,CD8+ T cell_1-AD.vs.MCI; scRNA-seq,SRP215507,CD8+ T cell_1-MCI.vs.control; scRNA-seq,SRP330776,γδ T cell-AD.vs.control; scRNA-seq,SRP330776,CD8+ T cell_2-AD.vs.control; scRNA-seq,SRP330776,Naive CD8+ T cell_3-AD.vs.control; scRNA-	6
AL138963.1	scRNA-seq,SRP215507,CD8+ T cell_1-AD.vs.MCI; scRNA-seq,SRP215507,CD8+ T cell_1-MCI.vs.control; scRNA-seq,SRP215507,CD8+ T cell_2-MCI.vs.control; scRNA-seq,SRP330776,CD8+ T cell_1-AD.vs.control; scRNA-seq,SRP330776,Memory T cell_1-AD.vs.control	5
AL138963.4	scRNA-seq,SRP215507,CD8+ T cell_8-AD.vs.control; scRNA-seq,SRP215507,CD8+ T cell_5-AD.vs.MCI; scRNA-seq,SRP215507,CD8+ T cell_5-MCI.vs.control; scRNA-seq,SRP215507,CD8+ T cell_4-MCI.vs.control; scRNA-seq,SRP215507,CD8+ T cell_3-MCI.vs.control	5
AL138966.2	scRNA-seq,SRP215507,CD8+ T cell_3-AD.vs.control; scRNA-seq,SRP215507,CD8+ T cell_1-MCI.vs.control; scRNA-seq,SRP215507,CD8+ T cell_3-MCI.vs.control; scRNA-seq,SRP330776,CD8+ T cell_1-AD.vs.control; scRNA-seq,SRP330776,Memory T cell_2-AD.vs.control; scRNA-seq,SRP330776,B cell_1-AD.vs.control; scRNA-seq,SRP330776,Naive CD8+ T cell_1-AD.vs.control; scRNA-seq,SRP330776,Natural killer cell_1-	8
AL138995.1	scRNA-seq,SRP215507,CD8+ T cell_1-AD.vs.MCI; scRNA-seq,SRP215507,CD8+ T cell_1-MCI.vs.control; scRNA-seq,SRP330776,B cell_2-	3
AL139011.1	scRNA-seq,SRP215507,CD8+ T cell_4-AD.vs.control; scRNA-seq,SRP215507,CD8+ T cell_1-AD.vs.MCI; scRNA-seq,SRP215507,CD8+ T cell_1-MCI.vs.control; scRNA-seq,SRP309935,Natural killer cell_1-AD.vs.control; scRNA-seq,SRP330776,γδ T cell-AD.vs.control; scRNA-seq,SRP330776,Memory T cell_2-AD.vs.control; scRNA-seq,SRP330776,Naive CD8+ T cell_3-AD.vs.control; scRNA-seq,SRP330776,CD8+ T cell_3-	8
AL139020.1	scRNA-seq,SRP330776,B cell_1-AD.vs.control	1
AL139035.1	scRNA-seq,SRP215507,CD8+ T cell_2-MCI.vs.control	1
AL139042.1	scRNA-seq,SRP330776,B cell_1-AD.vs.control	1
AL139089.1	scRNA-seq,SRP215507,CD8+ T cell_2-AD.vs.control; scRNA-seq,SRP215507,CD8+ T cell_4-AD.vs.control; scRNA-seq,SRP215507,CD8+ T cell_1-MCI.vs.control; scRNA-seq,SRP330776,Natural killer T cell-AD.vs.control	4

AL139099.1	scRNA-seq,SRP215507,CD8+ T cell_2-AD.vs.control; scRNA-seq,SRP215507,CD8+ T cell_3-AD.vs.control; scRNA-seq,SRP215507,CD8+ T cell_1-AD.vs.MCI; scRNA-seq,SRP330776,CD8+ T cell_2-AD.vs.control; scRNA-seq,SRP330776,Memory T cell_1-AD.vs.control	5
AL139220.2	scRNA-seq,SRP215507,CD8+ T cell_2-AD.vs.MCI; scRNA-seq,SRP215507,CD8+ T cell_3-AD.vs.MCI; scRNA-seq,SRP215507,CD8+ T cell_2-MCI.vs.control; scRNA-seq,SRP215507,CD8+ T cell_3-MCI.vs.control; scRNA-seq,SRP330776,Naive CD8+ T cell_1-AD.vs.control	5
AL139234.1	scRNA-seq,SRP215507,CD8+ T cell_3-MCI.vs.control	1
AL139243.1	scRNA-seq,SRP330776,γδ T cell-AD.vs.control	1
AL139246.3	scRNA-seq,SRP215507,CD8+ T cell_3-AD.vs.MCI; scRNA-seq,SRP215507,CD8+ T cell_3-MCI.vs.control; scRNA-seq,SRP330776,CD8+ T cell_1-AD.vs.control; scRNA-seq,SRP330776,B cell_1-AD.vs.control	4
AL139246.5	scRNA-seq,SRP215507,CD8+ T cell_2-AD.vs.MCI; scRNA-seq,SRP215507,CD8+ T cell_1-AD.vs.MCI; scRNA-seq,SRP215507,CD8+ T cell_5-AD.vs.MCI; scRNA-seq,SRP215507,CD8+ T cell_1-MCI.vs.control; scRNA-seq,SRP215507,CD8+ T cell_5-MCI.vs.control; scRNA-seq,SRP215507,CD8+ T cell_2-MCI.vs.control; scRNA-seq,SRP215507,CD8+ T cell_3-MCI.vs.control; scRNA-seq,SRP215507,CD8+ T cell_7-MCI.vs.control; scRNA-seq,SRP330776,CD8+ T cell_1-AD.vs.control; scRNA-seq,SRP330776,γδ T cell-AD.vs.control; scRNA-seq,SRP330776,Memory T cell_2-AD.vs.control; scRNA-seq,SRP330776,CD8+ T cell_2-AD.vs.control; scRNA-seq,SRP330776,B cell_1-AD.vs.control; scRNA-seq,SRP330776,Naive CD8+ T cell_1-AD.vs.control; scRNA-seq,SRP330776,Natural killer cell_1-AD.vs.control; scRNA-seq,SRP330776,Memory T cell_1-AD.vs.control; scRNA-seq,SRP330776,Natural killer T cell-AD.vs.control; scRNA-seq,SRP330776,Naive CD8+ T cell_3-AD.vs.control; scRNA-seq,SRP330776,B cell_2-AD.vs.control; scRNA-seq,SRP330776,T cell-AD.vs.control; scRNA-seq,SRP330776,Naive CD8+ T cell_2-AD.vs.control; scRNA-seq,SRP330776,CD8+ T cell_3-AD.vs.control; scRNA-seq,SRP330776,Monocyte_1-AD.vs.control; scRNA-seq,SRP330776,B cell_3-[illegible]	26
AL139260.1	scRNA-seq,SRP215507,CD8+ T cell_3-AD.vs.control; scRNA-seq,SRP215507,CD8+ T cell_3-AD.vs.MCI; scRNA-seq,SRP330776,Naive CD8+ T cell_1-AD.vs.control; scRNA-seq,SRP330776,CD8+ T cell_3-AD.vs.control	4
AL139274.2	scRNA-seq,SRP215507,CD8+ T cell_3-AD.vs.control; scRNA-seq,SRP215507,CD8+ T cell_6-AD.vs.control; scRNA-seq,SRP215507,CD8+ T cell_1-AD.vs.MCI; scRNA-seq,SRP215507,CD8+ T cell_1-MCI.vs.control; scRNA-seq,SRP330776,γδ T cell-AD.vs.control; scRNA-seq,SRP330776,CD8+ T cell_2-AD.vs.control; scRNA-seq,SRP330776,B cell_1-AD.vs.control; scRNA-seq,SRP330776,Naive CD8+ T cell_1-AD.vs.control	8
AL139289.2	scRNA-seq,SRP215507,CD8+ T cell_3-AD.vs.control; scRNA-seq,SRP215507,CD8+ T cell_3-AD.vs.MCI; scRNA-seq,SRP330776,Memory T cell_2-AD.vs.control; scRNA-seq,SRP330776,B cell_1-AD.vs.control; scRNA-seq,SRP330776,Naive CD8+ T cell_1-AD.vs.control	5
AL139317.3	scRNA-seq,SRP330776,CD8+ T cell_1-AD.vs.control; scRNA-seq,SRP330776,γδ T cell-AD.vs.control	2
AL139317.5	scRNA-seq,SRP215507,CD8+ T cell_1-AD.vs.control; scRNA-seq,SRP215507,CD8+ T cell_2-AD.vs.MCI; scRNA-seq,SRP215507,CD8+ T cell_1-MCI.vs.control; scRNA-seq,SRP330776,CD8+ T cell_1-AD.vs.control; scRNA-seq,SRP330776,Natural killer cell_1-AD.vs.control	5
AL139353.2	scRNA-seq,SRP215507,CD8+ T cell_1-AD.vs.control; scRNA-seq,SRP215507,CD8+ T cell_1-AD.vs.MCI; scRNA-seq,SRP330776,CD8+ T cell_2-AD.vs.control; scRNA-seq,SRP330776,Naive CD8+ T cell_1-AD.vs.control; scRNA-seq,SRP330776,Memory T cell_1-AD.vs.control	5
AL139383.1	scRNA-seq,SRP215507,CD8+ T cell_2-AD.vs.control	1
AL139384.1	scRNA-seq,SRP215507,CD8+ T cell_1-MCI.vs.control; scRNA-seq,SRP309935,Monocyte_1-AD.vs.control; scRNA-seq,SRP309935,CD8+ T cell_2-AD.vs.control; scRNA-seq,SRP330776,γδ T cell-AD.vs.control; scRNA-seq,SRP330776,Naive CD8+ T cell_1-AD.vs.control; scRNA-seq,SRP330776,Memory T cell_1-AD.vs.control; scRNA-seq,SRP330776,Monocyte_2-AD.vs.control	7
AL139393.2	scRNA-seq,SRP330776,Memory T cell_2-AD.vs.control	1
AL139393.3	scRNA-seq,SRP215507,CD8+ T cell_5-AD.vs.control; scRNA-seq,SRP215507,CD8+ T cell_3-AD.vs.MCI; scRNA-seq,SRP215507,CD8+ T cell_1-AD.vs.MCI; scRNA-seq,SRP215507,CD8+ T cell_1-MCI.vs.control; scRNA-seq,SRP215507,CD8+ T cell_5-MCI.vs.control; scRNA-seq,SRP215507,CD8+ T cell_3-MCI.vs.control; scRNA-seq,SRP330776,CD8+ T cell_1-AD.vs.control; scRNA-seq,SRP330776,Memory T cell_2-	8
AL139407.1	scRNA-seq,SRP215507,CD8+ T cell_3-AD.vs.MCI; scRNA-seq,SRP215507,CD8+ T cell_2-MCI.vs.control; scRNA-seq,SRP215507,CD8+ T cell_3-	3
AL139423.1	scRNA-seq,SRP215507,CD8+ T cell_5-MCI.vs.control; scRNA-seq,SRP330776,γδ T cell-AD.vs.control; scRNA-seq,SRP330776,Memory T cell_1-	3
AL139423.2	scRNA-seq,SRP330776,Naive CD8+ T cell_1-AD.vs.control	1
AL157373.2	scRNA-seq,SRP330776,CD8+ T cell_1-AD.vs.control	1

AL157392.3	scRNA-seq,SRP215507,CD8+ T cell_3-AD.vs.control; scRNA-seq,SRP215507,CD8+ T cell_3-AD.vs.MCI; scRNA-seq,SRP309935,Monocyte_1-AD.vs.control; scRNA-seq,SRP330776,Memory T cell_1-AD.vs.control; scRNA-seq,SRP330776,Naive CD8+ T cell_3-AD.vs.control; scRNA-seq,SRP330776,B cell_2-AD.vs.control; scRNA-seq,SRP330776,CD8+ T cell_3-AD.vs.control	7
AL157394.3	scRNA-seq,SRP215507,CD8+ T cell_2-AD.vs.MCI; scRNA-seq,SRP215507,CD8+ T cell_5-AD.vs.MCI; scRNA-seq,SRP215507,CD8+ T cell_1-MCI.vs.control; scRNA-seq,SRP215507,CD8+ T cell_2-MCI.vs.control; scRNA-seq,SRP215507,CD8+ T cell_6-MCI.vs.control; scRNA-seq,SRP309935,Monocyte_1-AD.vs.control; scRNA-seq,SRP309935,Natural killer cell_1-AD.vs.control; scRNA-seq,SRP330776,CD8+ T cell_1-AD.vs.control; scRNA-seq,SRP330776,Memory T cell_2-AD.vs.control; scRNA-seq,SRP330776,CD8+ T cell_2-AD.vs.control; scRNA-seq,SRP330776,Naive CD8+ T cell_1-AD.vs.control; scRNA-seq,SRP330776,Natural killer cell_1-AD.vs.control; scRNA-seq,SRP330776,Memory T cell_1-AD.vs.control; scRNA-seq,SRP330776,Natural killer T cell-AD.vs.control; scRNA-seq,SRP330776,T cell-AD.vs.control; scRNA-	16
AL157400.3	scRNA-seq,SRP215507,CD8+ T cell_1-AD.vs.MCI; scRNA-seq,SRP215507,CD8+ T cell_1-MCI.vs.control	2
AL157402.2	scRNA-seq,SRP330776,CD8+ T cell_1-AD.vs.control; scRNA-seq,SRP330776,Memory T cell_2-AD.vs.control; scRNA-seq,SRP330776,CD8+ T cell_2-AD.vs.control; scRNA-seq,SRP330776,B cell_1-AD.vs.control; scRNA-seq,SRP330776,Naive CD8+ T cell_1-AD.vs.control; scRNA-seq,SRP330776,Natural killer cell_1-AD.vs.control; scRNA-seq,SRP330776,Memory T cell_1-AD.vs.control; scRNA-seq,SRP330776,Natural killer T cell-AD.vs.control; scRNA-seq,SRP330776,Naive CD8+ T cell_3-AD.vs.control; scRNA-seq,SRP330776,B cell_2-AD.vs.control; scRNA-seq,SRP330776,T cell-AD.vs.control; scRNA-seq,SRP330776,Naive CD8+ T cell_2-AD.vs.control; scRNA-seq,SRP330776,CD8+ T cell_3-AD.vs.control; scRNA-	15
AL157786.1	scRNA-seq,SRP215507,CD8+ T cell_1-AD.vs.MCI; scRNA-seq,SRP215507,CD8+ T cell_1-MCI.vs.control; scRNA-seq,SRP309935,Naive CD8+ T	3
AL157813.1	scRNA-seq,SRP215507,CD8+ T cell_3-AD.vs.control; scRNA-seq,SRP215507,CD8+ T cell_4-AD.vs.control; scRNA-seq,SRP215507,CD8+ T cell_4-MCI.vs.control; scRNA-seq,SRP330776,Naive CD8+ T cell_1-AD.vs.control; scRNA-seq,SRP330776,Memory T cell_1-AD.vs.control	5
AL157813.2	scRNA-seq,SRP215507,CD8+ T cell_1-AD.vs.control; scRNA-seq,SRP215507,CD8+ T cell_1-MCI.vs.control; scRNA-seq,SRP215507,CD8+ T cell_2-	3
AL157834.2	scRNA-seq,SRP215507,CD8+ T cell_3-AD.vs.control; scRNA-seq,SRP215507,CD8+ T cell_3-MCI.vs.control; scRNA-seq,SRP330776,Memory T cell_1-	3
AL157902.1	scRNA-seq,SRP215507,CD8+ T cell_2-AD.vs.control; scRNA-seq,SRP215507,CD8+ T cell_1-AD.vs.control; scRNA-seq,SRP215507,CD8+ T cell_2-	3
AL157912.1	scRNA-seq,SRP215507,CD8+ T cell_1-AD.vs.control; scRNA-seq,SRP215507,CD8+ T cell_6-AD.vs.control; scRNA-seq,SRP215507,CD8+ T cell_2-AD.vs.MCI; scRNA-seq,SRP215507,CD8+ T cell_1-MCI.vs.control; scRNA-seq,SRP330776,CD8+ T cell_2-AD.vs.control	5
AL157938.2	scRNA-seq,SRP215507,CD8+ T cell_1-AD.vs.control; scRNA-seq,SRP215507,CD8+ T cell_1-MCI.vs.control; scRNA-seq,SRP330776,B cell_2-	3
AL157938.3	scRNA-seq,SRP330776,Naive CD8+ T cell_1-AD.vs.control	1
AL157955.1	scRNA-seq,SRP330776,γδ T cell-AD.vs.control	1
AL158055.1	scRNA-seq,SRP215507,CD8+ T cell_3-AD.vs.control; scRNA-seq,SRP215507,CD8+ T cell_3-MCI.vs.control; scRNA-seq,SRP330776,CD8+ T cell_1-AD.vs.control; scRNA-seq,SRP330776,Memory T cell_2-AD.vs.control; scRNA-seq,SRP330776,CD8+ T cell_2-AD.vs.control; scRNA-	6
AL158071.3	scRNA-seq,SRP215507,CD8+ T cell_3-AD.vs.control; scRNA-seq,SRP215507,CD8+ T cell_1-MCI.vs.control; scRNA-seq,SRP330776,γδ T cell-AD.vs.control; scRNA-seq,SRP330776,Natural killer cell_1-AD.vs.control; scRNA-seq,SRP330776,Memory T cell_1-AD.vs.control; scRNA-	6
AL158071.4	scRNA-seq,SRP215507,CD8+ T cell_2-AD.vs.control; scRNA-seq,SRP215507,CD8+ T cell_5-AD.vs.control; scRNA-seq,SRP330776,γδ T cell-AD.vs.control; scRNA-seq,SRP330776,Natural killer cell_1-AD.vs.control; scRNA-seq,SRP330776,Memory T cell_1-AD.vs.control	5
AL158152.1	scRNA-seq,SRP215507,CD8+ T cell_3-AD.vs.control; scRNA-seq,SRP330776,CD8+ T cell_1-AD.vs.control; scRNA-seq,SRP330776,γδ T cell-AD.vs.control; scRNA-seq,SRP330776,Memory T cell_2-AD.vs.control; scRNA-seq,SRP330776,CD8+ T cell_2-AD.vs.control; scRNA-seq,SRP330776,B cell_1-AD.vs.control; scRNA-seq,SRP330776,Naive CD8+ T cell_1-AD.vs.control; scRNA-seq,SRP330776,Memory T cell_1-AD.vs.control; scRNA-seq,SRP330776,Natural killer T cell-AD.vs.control; scRNA-seq,SRP330776,B cell_2-AD.vs.control; scRNA-seq,SRP330776,T cell-AD.vs.control; scRNA-seq,SRP330776,CD8+ T cell_3-AD.vs.control; scRNA-seq,SRP330776,B cell_3-AD.vs.control	13
AL158163.1	scRNA-seq,SRP215507,CD8+ T cell_5-AD.vs.control; scRNA-seq,SRP215507,CD8+ T cell_3-AD.vs.MCI; scRNA-seq,SRP215507,CD8+ T cell_5-MCI.vs.control; scRNA-seq,SRP215507,CD8+ T cell_2-MCI.vs.control; scRNA-seq,SRP215507,CD8+ T cell_3-MCI.vs.control	5
AL158163.2	scRNA-seq,SRP330776,Memory T cell_2-AD.vs.control; scRNA-seq,SRP330776,B cell_1-AD.vs.control; scRNA-seq,SRP330776,Naive CD8+ T cell_1-AD.vs.control; scRNA-seq,SRP330776,CD8+ T cell_3-AD.vs.control	4
AL158206.1	scRNA-seq,SRP215507,CD8+ T cell_2-AD.vs.control; scRNA-seq,SRP215507,CD8+ T cell_3-AD.vs.MCI; scRNA-seq,SRP215507,CD8+ T cell_3-	3

AL158212.2	scRNA-seq,SRP215507,CD8+ T cell_1-MCI.vs.control	1
AL158212.3	scRNA-seq,SRP215507,CD8+ T cell_3-AD.vs.MCI; scRNA-seq,SRP215507,CD8+ T cell_1-AD.vs.MCI; scRNA-seq,SRP215507,CD8+ T cell_3-MCI.vs.control; scRNA-seq,SRP330776,B cell_1-AD.vs.control	4
AL158214.2	scRNA-seq,SRP330776,Naive CD8+ T cell_1-AD.vs.control	1
AL158825.2	scRNA-seq,SRP215507,CD8+ T cell_2-AD.vs.control; scRNA-seq,SRP215507,CD8+ T cell_1-AD.vs.MCI; scRNA-seq,SRP330776,CD8+ T cell_2-AD.vs.control; scRNA-seq,SRP330776,B cell_1-AD.vs.control; scRNA-seq,SRP330776,Naive CD8+ T cell_1-AD.vs.control; scRNA-	6
AL158832.2	scRNA-seq,SRP215507,CD8+ T cell_2-AD.vs.control	1
AL158834.1	scRNA-seq,SRP330776,Naive CD8+ T cell_1-AD.vs.control	1
AL159169.2	scRNA-seq,SRP330776,B cell_1-AD.vs.control; scRNA-seq,SRP330776,Naive CD8+ T cell_1-AD.vs.control	2
AL159972.1	scRNA-seq,SRP215507,CD8+ T cell_2-AD.vs.control; scRNA-seq,SRP215507,CD8+ T cell_4-AD.vs.control; scRNA-seq,SRP215507,CD8+ T cell_1-AD.vs.MCI; scRNA-seq,SRP215507,CD8+ T cell_4-MCI.vs.control	4
AL160006.1	scRNA-seq,SRP215507,CD8+ T cell_2-AD.vs.control; scRNA-seq,SRP215507,CD8+ T cell_2-MCI.vs.control	2
AL160171.1	scRNA-seq,SRP215507,CD8+ T cell_1-AD.vs.control; scRNA-seq,SRP215507,CD8+ T cell_1-MCI.vs.control; scRNA-seq,SRP330776,CD8+ T cell_1-AD.vs.control; scRNA-seq,SRP330776,Memory T cell_2-AD.vs.control	4
AL160313.1	scRNA-seq,SRP215507,CD8+ T cell_2-AD.vs.control; scRNA-seq,SRP215507,CD8+ T cell_3-AD.vs.control; scRNA-seq,SRP215507,CD8+ T cell_4-AD.vs.control; scRNA-seq,SRP215507,CD8+ T cell_1-AD.vs.MCI; scRNA-seq,SRP215507,CD8+ T cell_5-AD.vs.MCI; scRNA-seq,SRP215507,CD8+ T cell_1-MCI.vs.control; scRNA-seq,SRP330776,CD8+ T cell_1-AD.vs.control; scRNA-seq,SRP330776,γδ T cell-AD.vs.control; scRNA-seq,SRP330776,CD8+ T cell_2-AD.vs.control; scRNA-seq,SRP330776,Naive CD8+ T cell_1-AD.vs.control; scRNA-seq,SRP330776,Natural killer T cell-	11
AL160408.2	scRNA-seq,SRP215507,CD8+ T cell_3-AD.vs.control; scRNA-seq,SRP215507,CD8+ T cell_3-MCI.vs.control; scRNA-seq,SRP330776,γδ T cell-AD.vs.control; scRNA-seq,SRP330776,Memory T cell_2-AD.vs.control	4
AL161421.1	scRNA-seq,SRP215507,CD8+ T cell_3-AD.vs.control; scRNA-seq,SRP215507,CD8+ T cell_1-AD.vs.control; scRNA-seq,SRP215507,CD8+ T cell_5-MCI.vs.control; scRNA-seq,SRP309935,Naive CD8+ T cell_1-AD.vs.control; scRNA-seq,SRP330776,CD8+ T cell_1-AD.vs.control; scRNA-seq,SRP330776,γδ T cell-AD.vs.control; scRNA-seq,SRP330776,Memory T cell_2-AD.vs.control; scRNA-seq,SRP330776,CD8+ T cell_2-AD.vs.control; scRNA-seq,SRP330776,Naive CD8+ T cell_1-AD.vs.control; scRNA-seq,SRP330776,Natural killer cell_1-AD.vs.control; scRNA-seq,SRP330776,Memory	11
AL161443.1	scRNA-seq,SRP215507,CD8+ T cell_4-AD.vs.control	1
AL161457.2	scRNA-seq,SRP215507,CD8+ T cell_3-AD.vs.control; scRNA-seq,SRP215507,CD8+ T cell_3-AD.vs.MCI; scRNA-seq,SRP215507,CD8+ T cell_1-MCI.vs.control; scRNA-seq,SRP309935,Monocyte_2-AD.vs.control	4
AL161644.1	scRNA-seq,SRP330776,Naive CD8+ T cell_1-AD.vs.control; scRNA-seq,SRP330776,Naive CD8+ T cell_3-AD.vs.control	2
AL161662.1	scRNA-seq,SRP330776,γδ T cell-AD.vs.control; scRNA-seq,SRP330776,Memory T cell_2-AD.vs.control	2
AL161665.1	scRNA-seq,SRP215507,CD8+ T cell_2-AD.vs.control; scRNA-seq,SRP330776,B cell_1-AD.vs.control	2
AL161668.3	scRNA-seq,SRP215507,CD8+ T cell_3-AD.vs.MCI	1
AL161725.1	scRNA-seq,SRP330776,Memory T cell_1-AD.vs.control	1
AL161729.1	scRNA-seq,SRP215507,CD8+ T cell_1-AD.vs.control; scRNA-seq,SRP309935,Natural killer cell_1-AD.vs.control; scRNA-seq,SRP330776,γδ T cell-AD.vs.control; scRNA-seq,SRP330776,CD8+ T cell_2-AD.vs.control; scRNA-seq,SRP330776,B cell_1-AD.vs.control; scRNA-seq,SRP330776,Naive CD8+ T cell_1-AD.vs.control; scRNA-seq,SRP330776,Natural killer cell_1-AD.vs.control; scRNA-seq,SRP330776,Memory T cell_1-AD.vs.control;	9
AL161729.2	scRNA-seq,SRP215507,CD8+ T cell_1-AD.vs.MCI	1
AL161756.1	scRNA-seq,SRP330776,Naive CD8+ T cell_1-AD.vs.control	1
AL161781.2	scRNA-seq,SRP330776,B cell_1-AD.vs.control; scRNA-seq,SRP330776,B cell_2-AD.vs.control	2
AL161785.1	scRNA-seq,SRP330776,Monocyte_2-AD.vs.control	1
AL161891.1	scRNA-seq,SRP215507,CD8+ T cell_4-AD.vs.control; scRNA-seq,SRP215507,CD8+ T cell_4-MCI.vs.control	2

AL162171.3	scRNA-seq,SRP215507,CD8+ T cell_4-AD.vs.control; scRNA-seq,SRP330776,CD8+ T cell_1-AD.vs.control; scRNA-seq,SRP330776,Natural killer	3
AL162231.1	scRNA-seq,SRP215507,CD8+ T cell_1-MCI.vs.control; scRNA-seq,SRP330776,CD8+ T cell_1-AD.vs.control; scRNA-seq,SRP330776,γδ T cell-AD.vs.control; scRNA-seq,SRP330776,CD8+ T cell_2-AD.vs.control; scRNA-seq,SRP330776,B cell_1-AD.vs.control; scRNA-seq,SRP330776,Memory T	6
AL162231.2	scRNA-seq,SRP309935,Monocyte_1-AD.vs.control; scRNA-seq,SRP330776,γδ T cell-AD.vs.control; scRNA-seq,SRP330776,Memory T cell_2-AD.vs.control; scRNA-seq,SRP330776,CD8+ T cell_2-AD.vs.control; scRNA-seq,SRP330776,B cell_1-AD.vs.control; scRNA-seq,SRP330776,Memory T cell_1-AD.vs.control; scRNA-seq,SRP330776,Natural killer T cell-AD.vs.control; scRNA-seq,SRP330776,CD8+ T cell_3-AD.vs.control; scRNA-	9
AL162253.2	scRNA-seq,SRP215507,CD8+ T cell_5-AD.vs.control; scRNA-seq,SRP215507,CD8+ T cell_3-AD.vs.MCI; scRNA-seq,SRP215507,CD8+ T cell_1-AD.vs.MCI; scRNA-seq,SRP215507,CD8+ T cell_4-AD.vs.MCI; scRNA-seq,SRP215507,CD8+ T cell_5-AD.vs.MCI; scRNA-seq,SRP215507,CD8+ T cell_5-MCI.vs.control; scRNA-seq,SRP215507,CD8+ T cell_4-MCI.vs.control; scRNA-seq,SRP215507,CD8+ T cell_3-MCI.vs.control; scRNA-seq,SRP330776,γδ T cell-AD.vs.control; scRNA-seq,SRP330776,Memory T cell_2-AD.vs.control; scRNA-seq,SRP330776,CD8+ T cell_2-AD.vs.control; scRNA-seq,SRP330776,B cell_1-AD.vs.control; scRNA-seq,SRP330776,Natural killer cell_1-AD.vs.control; scRNA-seq,SRP330776,Memory T cell_1-	15
AL162258.2	scRNA-seq,SRP215507,CD8+ T cell_7-AD.vs.MCI; scRNA-seq,SRP215507,CD8+ T cell_6-MCI.vs.control; scRNA-seq,SRP215507,CD8+ T cell_7-MCI.vs.control; scRNA-seq,SRP330776,Naive CD8+ T cell_1-AD.vs.control; scRNA-seq,SRP330776,Memory T cell_1-AD.vs.control; scRNA-seq,SRP330776,Natural killer T cell-AD.vs.control; scRNA-seq,SRP330776,CD8+ T cell_3-AD.vs.control; scRNA-seq,SRP330776,Natural killer cell_2-	8
AL162274.2	scRNA-seq,SRP215507,CD8+ T cell_3-AD.vs.MCI; scRNA-seq,SRP309935,Naive CD8+ T cell_1-AD.vs.control	2
AL162311.3	scRNA-seq,SRP330776,B cell_2-AD.vs.control	1
AL162377.1	scRNA-seq,SRP215507,CD8+ T cell_4-AD.vs.control; scRNA-seq,SRP215507,CD8+ T cell_2-AD.vs.MCI; scRNA-seq,SRP215507,CD8+ T cell_1-AD.vs.MCI; scRNA-seq,SRP215507,CD8+ T cell_4-AD.vs.MCI; scRNA-seq,SRP215507,CD8+ T cell_1-MCI.vs.control; scRNA-seq,SRP215507,CD8+ T cell_5-MCI.vs.control; scRNA-seq,SRP215507,CD8+ T cell_2-MCI.vs.control; scRNA-seq,SRP330776,CD8+ T cell_1-AD.vs.control; scRNA-seq,SRP330776,γδ T cell-AD.vs.control; scRNA-seq,SRP330776,Memory T cell_2-AD.vs.control; scRNA-seq,SRP330776,CD8+ T cell_2-AD.vs.control; scRNA-seq,SRP330776,B cell_1-AD.vs.control; scRNA-seq,SRP330776,Naive CD8+ T cell_1-AD.vs.control; scRNA-seq,SRP330776,Natural killer cell_1-AD.vs.control; scRNA-seq,SRP330776,Memory T cell_1-AD.vs.control; scRNA-seq,SRP330776,Natural killer T cell-AD.vs.control; scRNA-seq,SRP330776,Naive CD8+ T cell_3-AD.vs.control; scRNA-seq,SRP330776,B cell_2-AD.vs.control; scRNA-seq,SRP330776,T cell-AD.vs.control; [illegible]	21
AL162390.1	scRNA-seq,SRP215507,CD8+ T cell_5-AD.vs.control; scRNA-seq,SRP215507,CD8+ T cell_1-AD.vs.MCI; scRNA-seq,SRP215507,CD8+ T cell_4-AD.vs.MCI; scRNA-seq,SRP215507,CD8+ T cell_1-MCI.vs.control; scRNA-seq,SRP215507,CD8+ T cell_5-MCI.vs.control; scRNA-	6
AL162457.1	scRNA-seq,SRP215507,CD8+ T cell_3-AD.vs.control; scRNA-seq,SRP215507,CD8+ T cell_1-AD.vs.control; scRNA-seq,SRP215507,CD8+ T cell_1-AD.vs.MCI; scRNA-seq,SRP215507,CD8+ T cell_4-MCI.vs.control; scRNA-seq,SRP215507,CD8+ T cell_3-MCI.vs.control; scRNA-seq,SRP309935,CD8+ T cell_2-AD.vs.control; scRNA-seq,SRP330776,CD8+ T cell_1-AD.vs.control; scRNA-seq,SRP330776,Memory T cell_1-	8
AL162458.1	scRNA-seq,SRP215507,CD8+ T cell_3-AD.vs.MCI; scRNA-seq,SRP215507,CD8+ T cell_3-MCI.vs.control; scRNA-seq,SRP330776,Memory T cell_2-AD.vs.control; scRNA-seq,SRP330776,CD8+ T cell_2-AD.vs.control; scRNA-seq,SRP330776,Memory T cell_1-AD.vs.control; scRNA-seq,SRP330776,Natural killer T cell-AD.vs.control; scRNA-seq,SRP330776,B cell_2-AD.vs.control; scRNA-seq,SRP330776,CD8+ T cell_3-AD.vs.control	8
AL162493.1	scRNA-seq,SRP215507,CD8+ T cell_1-AD.vs.control	1
AL162586.1	scRNA-seq,SRP330776,Natural killer cell_1-AD.vs.control; scRNA-seq,SRP330776,Memory T cell_1-AD.vs.control	2
AL162595.1	scRNA-seq,SRP215507,CD8+ T cell_1-MCI.vs.control	1
AL162741.1	scRNA-seq,SRP215507,CD8+ T cell_1-MCI.vs.control	1
AL163051.1	scRNA-seq,SRP215507,CD8+ T cell_3-AD.vs.MCI; scRNA-seq,SRP215507,CD8+ T cell_1-AD.vs.MCI; scRNA-seq,SRP215507,CD8+ T cell_1-MCI.vs.control; scRNA-seq,SRP215507,CD8+ T cell_5-MCI.vs.control; scRNA-seq,SRP215507,CD8+ T cell_4-MCI.vs.control; scRNA-seq,SRP215507,CD8+ T cell_3-MCI.vs.control; scRNA-seq,SRP215507,CD8+ T cell_6-MCI.vs.control; scRNA-seq,SRP330776,CD8+ T cell_1-AD.vs.control; scRNA-seq,SRP330776,Memory T cell_2-AD.vs.control; scRNA-seq,SRP330776,Naive CD8+ T cell_1-AD.vs.control; scRNA-	11
AL163932.1	scRNA-seq,SRP215507,CD8+ T cell_3-AD.vs.MCI; scRNA-seq,SRP215507,CD8+ T cell_3-MCI.vs.control; scRNA-seq,SRP330776,Naive CD8+ T	3
AL163973.2	scRNA-seq,SRP330776,CD8+ T cell_1-AD.vs.control; scRNA-seq,SRP330776,Memory T cell_2-AD.vs.control; scRNA-seq,SRP330776,Naive CD8+ T cell_1-AD.vs.control; scRNA-seq,SRP330776,Memory T cell_1-AD.vs.control; scRNA-seq,SRP330776,Naive CD8+ T cell_3-AD.vs.control	5

AL353135.1	scRNA-seq,SRP215507,CD8+ T cell_1-MCI.vs.control	1
AL353135.2	scRNA-seq,SRP309935,CD8+ T cell_1-AD.vs.control	1
AL353194.1	scRNA-seq,SRP215507,CD8+ T cell_2-AD.vs.MCI; scRNA-seq,SRP215507,CD8+ T cell_2-MCI.vs.control; scRNA-seq,SRP215507,CD8+ T cell_6-MCI.vs.control; scRNA-seq,SRP309935,Naive CD8+ T cell_2-AD.vs.control	4
AL353576.1	scRNA-seq,SRP215507,CD8+ T cell_2-AD.vs.control	1
AL353593.1	scRNA-seq,SRP215507,CD8+ T cell_3-AD.vs.MCI; scRNA-seq,SRP215507,CD8+ T cell_4-MCI.vs.control; scRNA-seq,SRP215507,CD8+ T cell_3-MCI.vs.control; scRNA-seq,SRP330776,Memory T cell_2-AD.vs.control; scRNA-seq,SRP330776,B cell_1-AD.vs.control	5
AL353596.1	scRNA-seq,SRP215507,CD8+ T cell_1-MCI.vs.control	1
AL353608.2	scRNA-seq,SRP215507,CD8+ T cell_2-MCI.vs.control	1
AL353622.1	scRNA-seq,SRP215507,CD8+ T cell_3-AD.vs.MCI; scRNA-seq,SRP215507,CD8+ T cell_4-MCI.vs.control; scRNA-seq,SRP330776,CD8+ T cell_1-AD.vs.control; scRNA-seq,SRP330776,CD8+ T cell_2-AD.vs.control; scRNA-seq,SRP330776,B cell_1-AD.vs.control; scRNA-seq,SRP330776,Naive CD8+ T cell_1-AD.vs.control; scRNA-seq,SRP330776,Natural killer T cell-AD.vs.control; scRNA-seq,SRP330776,CD8+ T cell_3-AD.vs.control	8
AL353660.1	scRNA-seq,SRP309935,CD8+ T cell_2-AD.vs.control; scRNA-seq,SRP330776,CD8+ T cell_1-AD.vs.control; scRNA-seq,SRP330776,B cell_1-AD.vs.control; scRNA-seq,SRP330776,Naive CD8+ T cell_1-AD.vs.control; scRNA-seq,SRP330776,B cell_2-AD.vs.control; scRNA-	6
AL353708.1	scRNA-seq,SRP215507,CD8+ T cell_1-MCI.vs.control; scRNA-seq,SRP330776,Naive CD8+ T cell_1-AD.vs.control	2
AL353708.3	scRNA-seq,SRP215507,CD8+ T cell_5-AD.vs.control; scRNA-seq,SRP215507,CD8+ T cell_2-AD.vs.MCI; scRNA-seq,SRP215507,CD8+ T cell_1-AD.vs.MCI; scRNA-seq,SRP215507,CD8+ T cell_5-AD.vs.MCI; scRNA-seq,SRP215507,CD8+ T cell_1-MCI.vs.control; scRNA-seq,SRP215507,CD8+ T cell_2-MCI.vs.control; scRNA-seq,SRP330776,CD8+ T cell_1-AD.vs.control; scRNA-seq,SRP330776,γδ T cell-AD.vs.control; scRNA-seq,SRP330776,Naive CD8+ T cell_3-AD.vs.control; scRNA-seq,SRP330776,B cell_2-AD.vs.control; scRNA-seq,SRP330776,CD8+ T cell_3-	12
AL353719.1	scRNA-seq,SRP215507,CD8+ T cell_1-AD.vs.control; scRNA-seq,SRP215507,CD8+ T cell_1-MCI.vs.control; scRNA-seq,SRP330776,CD8+ T cell_1-AD.vs.control; scRNA-seq,SRP330776,CD8+ T cell_2-AD.vs.control; scRNA-seq,SRP330776,Memory T cell_1-AD.vs.control; scRNA-	6
AL353743.2	scRNA-seq,SRP215507,CD8+ T cell_3-AD.vs.control	1
AL353759.1	scRNA-seq,SRP215507,CD8+ T cell_5-AD.vs.MCI; scRNA-seq,SRP330776,CD8+ T cell_1-AD.vs.control; scRNA-seq,SRP330776,γδ T cell-AD.vs.control; scRNA-seq,SRP330776,Naive CD8+ T cell_1-AD.vs.control; scRNA-seq,SRP330776,Memory T cell_1-AD.vs.control; scRNA-	6
AL353801.3	scRNA-seq,SRP215507,CD8+ T cell_3-AD.vs.control; scRNA-seq,SRP215507,CD8+ T cell_1-AD.vs.MCI; scRNA-seq,SRP215507,CD8+ T cell_1-	3
AL353807.5	scRNA-seq,SRP215507,CD8+ T cell_5-AD.vs.control; scRNA-seq,SRP215507,CD8+ T cell_6-MCI.vs.control; scRNA-seq,SRP330776,Memory T cell_1-	3
AL354696.2	scRNA-seq,SRP330776,B cell_1-AD.vs.control	1
AL354707.1	scRNA-seq,SRP215507,CD8+ T cell_3-MCI.vs.control; scRNA-seq,SRP330776,Memory T cell_2-AD.vs.control; scRNA-seq,SRP330776,B cell_1-AD.vs.control; scRNA-seq,SRP330776,Naive CD8+ T cell_1-AD.vs.control	4
AL354710.2	scRNA-seq,SRP215507,CD8+ T cell_1-MCI.vs.control; scRNA-seq,SRP330776,γδ T cell-AD.vs.control; scRNA-seq,SRP330776,Naive CD8+ T cell_1-	3
AL354733.3	scRNA-seq,SRP215507,CD8+ T cell_5-AD.vs.control; scRNA-seq,SRP215507,CD8+ T cell_5-AD.vs.MCI	2
AL354740.1	scRNA-seq,SRP215507,CD8+ T cell_3-AD.vs.control; scRNA-seq,SRP215507,CD8+ T cell_6-MCI.vs.control; scRNA-seq,SRP330776,γδ T cell-AD.vs.control; scRNA-seq,SRP330776,Memory T cell_2-AD.vs.control; scRNA-seq,SRP330776,B cell_2-AD.vs.control	5
AL354822.1	scRNA-seq,SRP215507,CD8+ T cell_4-AD.vs.MCI; scRNA-seq,SRP215507,CD8+ T cell_4-MCI.vs.control; scRNA-seq,SRP330776,Naive CD8+ T	3
AL354920.1	scRNA-seq,SRP215507,CD8+ T cell_5-MCI.vs.control; scRNA-seq,SRP330776,CD8+ T cell_1-AD.vs.control; scRNA-seq,SRP330776,Naive CD8+ T	3
AL355001.2	scRNA-seq,SRP215507,CD8+ T cell_3-AD.vs.MCI; scRNA-seq,SRP215507,CD8+ T cell_1-AD.vs.MCI; scRNA-seq,SRP215507,CD8+ T cell_1-MCI.vs.control; scRNA-seq,SRP215507,CD8+ T cell_2-MCI.vs.control; scRNA-seq,SRP215507,CD8+ T cell_3-MCI.vs.control; scRNA-seq,SRP215507,CD8+ T cell_6-MCI.vs.control; scRNA-seq,SRP330776,γδ T cell-AD.vs.control; scRNA-seq,SRP330776,B cell_1-AD.vs.control; scRNA-seq,SRP330776,Naive CD8+ T cell_1-AD.vs.control; scRNA-seq,SRP330776,Memory T cell_1-AD.vs.control; scRNA-seq,SRP330776,B cell_2-	12
AL355075.3	scRNA-seq,SRP330776,CD8+ T cell_1-AD.vs.control	1

AL355075.4	scRNA-seq,SRP215507,CD8+ T cell_5-AD.vs.control; scRNA-seq,SRP215507,CD8+ T cell_2-AD.vs.MCI; scRNA-seq,SRP215507,CD8+ T cell_1-AD.vs.MCI; scRNA-seq,SRP215507,CD8+ T cell_5-AD.vs.MCI; scRNA-seq,SRP215507,CD8+ T cell_1-MCI.vs.control; scRNA-seq,SRP215507,CD8+ T cell_5-MCI.vs.control; scRNA-seq,SRP215507,CD8+ T cell_2-MCI.vs.control; scRNA-seq,SRP215507,CD8+ T cell_3-MCI.vs.control; scRNA-seq,SRP309935,Natural killer cell_2-AD.vs.control; scRNA-seq,SRP309935,Monocyte_1-AD.vs.control; scRNA-seq,SRP309935,Natural killer cell_1-AD.vs.control; scRNA-seq,SRP309935,Monocyte_2-AD.vs.control; scRNA-seq,SRP330776,CD8+ T cell_1-AD.vs.control; scRNA-seq,SRP330776,Memory T cell_2-AD.vs.control; scRNA-seq,SRP330776,CD8+ T cell_2-AD.vs.control; scRNA-seq,SRP330776,Natural killer cell_1-	18
AL355304.1	scRNA-seq,SRP215507,CD8+ T cell_3-MCI.vs.control; scRNA-seq,SRP330776,CD8+ T cell_1-AD.vs.control	2
AL355312.4	scRNA-seq,SRP330776,B cell_1-AD.vs.control	1
AL355353.1	scRNA-seq,SRP215507,CD8+ T cell_3-AD.vs.MCI; scRNA-seq,SRP215507,CD8+ T cell_5-AD.vs.MCI; scRNA-seq,SRP215507,CD8+ T cell_5-MCI.vs.control; scRNA-seq,SRP215507,CD8+ T cell_3-MCI.vs.control; scRNA-seq,SRP330776,Naive CD8+ T cell_1-AD.vs.control	5
AL355377.4	scRNA-seq,SRP215507,CD8+ T cell_4-MCI.vs.control	1
AL355388.2	scRNA-seq,SRP215507,CD8+ T cell_3-AD.vs.MCI; scRNA-seq,SRP215507,CD8+ T cell_3-MCI.vs.control; scRNA-seq,SRP330776,CD8+ T cell_1-AD.vs.control; scRNA-seq,SRP330776,Memory T cell_1-AD.vs.control; scRNA-seq,SRP330776,B cell_2-AD.vs.control	5
AL355488.1	scRNA-seq,SRP215507,CD8+ T cell_3-AD.vs.control; scRNA-seq,SRP215507,CD8+ T cell_3-MCI.vs.control; scRNA-seq,SRP309935,Natural killer cell_1-AD.vs.control; scRNA-seq,SRP330776,CD8+ T cell_2-AD.vs.control; scRNA-seq,SRP330776,B cell_1-AD.vs.control; scRNA-	6
AL355490.1	scRNA-seq,SRP330776,Naive CD8+ T cell_1-AD.vs.control	1
AL355512.1	scRNA-seq,SRP215507,CD8+ T cell_1-AD.vs.control	1
AL355581.1	scRNA-seq,SRP309935,Natural killer cell_1-AD.vs.control; scRNA-seq,SRP330776,CD8+ T cell_2-AD.vs.control; scRNA-seq,SRP330776,Memory T	3
AL355816.2	scRNA-seq,SRP215507,CD8+ T cell_3-AD.vs.control; scRNA-seq,SRP215507,CD8+ T cell_2-AD.vs.MCI; scRNA-seq,SRP215507,CD8+ T cell_5-AD.vs.MCI; scRNA-seq,SRP330776,CD8+ T cell_1-AD.vs.control; scRNA-seq,SRP330776,γδ T cell-AD.vs.control; scRNA-seq,SRP330776,Memory T cell_2-AD.vs.control; scRNA-seq,SRP330776,CD8+ T cell_2-AD.vs.control; scRNA-seq,SRP330776,Naive CD8+ T cell_1-AD.vs.control; scRNA-seq,SRP330776,Memory T cell_1-AD.vs.control; scRNA-seq,SRP330776,Naive CD8+ T cell_3-AD.vs.control; scRNA-seq,SRP330776,CD8+ T cell_3-	11
AL355916.1	scRNA-seq,SRP215507,CD8+ T cell_2-AD.vs.control; scRNA-seq,SRP215507,CD8+ T cell_1-AD.vs.control; scRNA-seq,SRP215507,CD8+ T cell_2-MCI.vs.control; scRNA-seq,SRP215507,CD8+ T cell_6-MCI.vs.control; scRNA-seq,SRP330776,Naive CD8+ T cell_2-AD.vs.control	5
AL355916.2	scRNA-seq,SRP215507,CD8+ T cell_1-AD.vs.control; scRNA-seq,SRP330776,Natural killer cell_1-AD.vs.control; scRNA-seq,SRP330776,Memory T	3
AL355999.1	scRNA-seq,SRP215507,CD8+ T cell_1-AD.vs.control; scRNA-seq,SRP215507,CD8+ T cell_2-MCI.vs.control; scRNA-seq,SRP330776,CD8+ T cell_1-AD.vs.control; scRNA-seq,SRP330776,Memory T cell_2-AD.vs.control; scRNA-seq,SRP330776,Naive CD8+ T cell_1-AD.vs.control; scRNA-seq,SRP330776,Memory T cell_1-AD.vs.control; scRNA-seq,SRP330776,Natural killer T cell-AD.vs.control; scRNA-seq,SRP330776,Naive CD8+ T cell_3-AD.vs.control; scRNA-seq,SRP330776,B cell_2-AD.vs.control; scRNA-seq,SRP330776,T cell-AD.vs.control	10
AL356019.2	scRNA-seq,SRP215507,CD8+ T cell_1-AD.vs.control; scRNA-seq,SRP215507,CD8+ T cell_5-MCI.vs.control; scRNA-seq,SRP309935,Natural killer	3
AL356020.1	scRNA-seq,SRP215507,CD8+ T cell_2-AD.vs.control; scRNA-seq,SRP215507,CD8+ T cell_3-AD.vs.control; scRNA-seq,SRP215507,CD8+ T cell_1-AD.vs.control; scRNA-seq,SRP215507,CD8+ T cell_1-AD.vs.MCI; scRNA-seq,SRP215507,CD8+ T cell_5-AD.vs.MCI; scRNA-seq,SRP215507,CD8+ T cell_1-MCI.vs.control; scRNA-seq,SRP215507,CD8+ T cell_5-MCI.vs.control; scRNA-seq,SRP215507,CD8+ T cell_4-MCI.vs.control; scRNA-seq,SRP215507,CD8+ T cell_2-MCI.vs.control; scRNA-seq,SRP330776,CD8+ T cell_1-AD.vs.control; scRNA-seq,SRP330776,γδ T cell-AD.vs.control; scRNA-seq,SRP330776,Memory T cell_2-AD.vs.control; scRNA-seq,SRP330776,CD8+ T cell_2-AD.vs.control; scRNA-seq,SRP330776,Naive CD8+ T cell_1-AD.vs.control; scRNA-seq,SRP330776,Memory T cell_1-AD.vs.control; scRNA-seq,SRP330776,Naive CD8+ T cell_3-AD.vs.control; scRNA-	17
AL356276.1	scRNA-seq,SRP330776,CD8+ T cell_2-AD.vs.control	1
AL356417.1	scRNA-seq,SRP330776,B cell_1-AD.vs.control	1
AL356417.3	scRNA-seq,SRP215507,CD8+ T cell_2-AD.vs.MCI	1
AL356481.1	scRNA-seq,SRP215507,CD8+ T cell_3-AD.vs.MCI; scRNA-seq,SRP330776,CD8+ T cell_1-AD.vs.control; scRNA-seq,SRP330776,Naive CD8+ T cell_1-AD.vs.control; scRNA-seq,SRP330776,Memory T cell_1-AD.vs.control; scRNA-seq,SRP330776,CD8+ T cell_3-AD.vs.control	5

AL356488.2	scRNA-seq,SRP215507,CD8+ T cell_1-MCI.vs.control; scRNA-seq,SRP330776,B cell_1-AD.vs.control; scRNA-seq,SRP330776,Natural killer T cell-AD.vs.control; scRNA-seq,SRP330776,CD8+ T cell_3-AD.vs.control; scRNA-seq,SRP330776,Monocyte_1-AD.vs.control	5
AL356488.3	scRNA-seq,SRP215507,CD8+ T cell_3-AD.vs.control; scRNA-seq,SRP215507,CD8+ T cell_1-AD.vs.control; scRNA-seq,SRP215507,CD8+ T cell_4-MCI.vs.control; scRNA-seq,SRP215507,CD8+ T cell_2-MCI.vs.control; scRNA-seq,SRP309935,Natural killer cell_1-AD.vs.control; scRNA-seq,SRP309935,CD8+ T cell_1-AD.vs.control; scRNA-seq,SRP330776,CD8+ T cell_1-AD.vs.control; scRNA-seq,SRP330776,Naive CD8+ T cell_1-	9
AL356512.1	scRNA-seq,SRP215507,CD8+ T cell_5-AD.vs.control; scRNA-seq,SRP215507,CD8+ T cell_1-AD.vs.MCI; scRNA-seq,SRP330776,CD8+ T cell_1-AD.vs.control; scRNA-seq,SRP330776,γδ T cell-AD.vs.control; scRNA-seq,SRP330776,CD8+ T cell_2-AD.vs.control; scRNA-seq,SRP330776,B cell_1-AD.vs.control; scRNA-seq,SRP330776,Naive CD8+ T cell_1-AD.vs.control; scRNA-seq,SRP330776,Memory T cell_1-AD.vs.control; scRNA-seq,SRP330776,CD8+ T cell_3-AD.vs.control; scRNA-seq,SRP330776,Monocyte_2-AD.vs.control	10
AL356599.1	scRNA-seq,SRP215507,CD8+ T cell_4-AD.vs.control; scRNA-seq,SRP215507,CD8+ T cell_4-MCI.vs.control; scRNA-seq,SRP330776,γδ T cell-AD.vs.control; scRNA-seq,SRP330776,Memory T cell_2-AD.vs.control; scRNA-seq,SRP330776,CD8+ T cell_2-AD.vs.control; scRNA-seq,SRP330776,Memory T cell_1-AD.vs.control; scRNA-seq,SRP330776,Naive CD8+ T cell_3-AD.vs.control	7
AL356776.2	scRNA-seq,SRP215507,CD8+ T cell_3-AD.vs.control; scRNA-seq,SRP215507,CD8+ T cell_1-MCI.vs.control; scRNA-seq,SRP215507,CD8+ T cell_5-MCI.vs.control; scRNA-seq,SRP215507,CD8+ T cell_3-MCI.vs.control	4
AL356804.1	scRNA-seq,SRP215507,CD8+ T cell_2-AD.vs.control; scRNA-seq,SRP215507,CD8+ T cell_3-AD.vs.MCI; scRNA-seq,SRP215507,CD8+ T cell_2-MCI.vs.control; scRNA-seq,SRP215507,CD8+ T cell_3-MCI.vs.control; scRNA-seq,SRP330776,Naive CD8+ T cell_1-AD.vs.control; scRNA-	6
AL356966.1	scRNA-seq,SRP215507,CD8+ T cell_1-AD.vs.control; scRNA-seq,SRP309935,CD8+ T cell_1-AD.vs.control; scRNA-seq,SRP309935,CD8+ T cell_2-	3
AL357033.1	scRNA-seq,SRP215507,CD8+ T cell_2-MCI.vs.control	1
AL357033.3	scRNA-seq,SRP215507,CD8+ T cell_1-MCI.vs.control	1
AL357033.4	scRNA-seq,SRP330776,CD8+ T cell_1-AD.vs.control; scRNA-seq,SRP330776,CD8+ T cell_2-AD.vs.control	2
AL357054.2	scRNA-seq,SRP215507,CD8+ T cell_3-AD.vs.MCI; scRNA-seq,SRP215507,CD8+ T cell_3-MCI.vs.control	2
AL357054.4	scRNA-seq,SRP215507,CD8+ T cell_2-AD.vs.control; scRNA-seq,SRP215507,CD8+ T cell_3-AD.vs.control; scRNA-seq,SRP215507,CD8+ T cell_3-AD.vs.MCI; scRNA-seq,SRP215507,CD8+ T cell_5-AD.vs.MCI; scRNA-seq,SRP215507,CD8+ T cell_5-MCI.vs.control; scRNA-seq,SRP330776,CD8+ T cell_1-AD.vs.control; scRNA-seq,SRP330776,γδ T cell-AD.vs.control; scRNA-seq,SRP330776,Memory T cell_2-AD.vs.control; scRNA-seq,SRP330776,CD8+ T cell_2-AD.vs.control; scRNA-seq,SRP330776,B cell_1-AD.vs.control; scRNA-seq,SRP330776,Naive CD8+ T cell_1-AD.vs.control; scRNA-seq,SRP330776,Memory T cell_1-AD.vs.control; scRNA-seq,SRP330776,B cell_2-AD.vs.control; scRNA-seq,SRP330776,CD8+ T	14
AL357079.1	scRNA-seq,SRP215507,CD8+ T cell_1-AD.vs.control; scRNA-seq,SRP215507,CD8+ T cell_4-MCI.vs.control	2
AL357153.1	scRNA-seq,SRP330776,B cell_1-AD.vs.control; scRNA-seq,SRP330776,B cell_2-AD.vs.control	2
AL358115.1	scRNA-seq,SRP309935,Naive CD8+ T cell_1-AD.vs.control	1
AL358333.3	scRNA-seq,SRP330776,Naive CD8+ T cell_1-AD.vs.control	1
AL358334.2	scRNA-seq,SRP215507,CD8+ T cell_2-AD.vs.MCI; scRNA-seq,SRP215507,CD8+ T cell_2-MCI.vs.control	2
AL358472.2	scRNA-seq,SRP330776,CD8+ T cell_1-AD.vs.control; scRNA-seq,SRP330776,CD8+ T cell_2-AD.vs.control; scRNA-seq,SRP330776,Naive CD8+ T cell_1-AD.vs.control; scRNA-seq,SRP330776,B cell_2-AD.vs.control; scRNA-seq,SRP330776,CD8+ T cell_3-AD.vs.control	5
AL358472.5	scRNA-seq,SRP330776,CD8+ T cell_1-AD.vs.control; scRNA-seq,SRP330776,Naive CD8+ T cell_1-AD.vs.control; scRNA-seq,SRP330776,Memory T	3
AL358781.1	scRNA-seq,SRP330776,CD8+ T cell_2-AD.vs.control; scRNA-seq,SRP330776,Naive CD8+ T cell_1-AD.vs.control; scRNA-seq,SRP330776,Memory T	3
AL358790.1	scRNA-seq,SRP215507,CD8+ T cell_4-AD.vs.control; scRNA-seq,SRP215507,CD8+ T cell_2-AD.vs.MCI; scRNA-seq,SRP215507,CD8+ T cell_2-MCI.vs.control; scRNA-seq,SRP215507,CD8+ T cell_3-MCI.vs.control; scRNA-seq,SRP330776,CD8+ T cell_1-AD.vs.control; scRNA-	6
AL358852.1	scRNA-seq,SRP215507,CD8+ T cell_3-AD.vs.control; scRNA-seq,SRP215507,CD8+ T cell_1-AD.vs.MCI; scRNA-seq,SRP215507,CD8+ T cell_1-MCI.vs.control; scRNA-seq,SRP330776,CD8+ T cell_1-AD.vs.control; scRNA-seq,SRP330776,CD8+ T cell_2-AD.vs.control; scRNA-	6
AL358933.1	scRNA-seq,SRP215507,CD8+ T cell_4-AD.vs.control; scRNA-seq,SRP330776,Memory T cell_1-AD.vs.control	2
AL359198.1	scRNA-seq,SRP215507,CD8+ T cell_1-AD.vs.MCI; scRNA-seq,SRP215507,CD8+ T cell_1-MCI.vs.control; scRNA-seq,SRP215507,CD8+ T cell_2-	3

AL359220.1	scRNA-seq,SRP215507,CD8+ T cell_2-AD.vs.MCI; scRNA-seq,SRP215507,CD8+ T cell_1-AD.vs.MCI; scRNA-seq,SRP330776,CD8+ T cell_1-AD.vs.control; scRNA-seq,SRP330776,Naive CD8+ T cell_1-AD.vs.control	4
AL359232.1	scRNA-seq,SRP215507,CD8+ T cell_3-AD.vs.MCI; scRNA-seq,SRP215507,CD8+ T cell_1-AD.vs.MCI; scRNA-seq,SRP215507,CD8+ T cell_5-MCI.vs.control; scRNA-seq,SRP215507,CD8+ T cell_3-MCI.vs.control; scRNA-seq,SRP330776,CD8+ T cell_2-AD.vs.control; scRNA-seq,SRP330776,Naive CD8+ T cell_3-AD.vs.control; scRNA-seq,SRP330776,B cell_2-AD.vs.control	7
AL359258.2	scRNA-seq,SRP330776,CD8+ T cell_1-AD.vs.control; scRNA-seq,SRP330776,CD8+ T cell_2-AD.vs.control; scRNA-seq,SRP330776,B cell_1-AD.vs.control; scRNA-seq,SRP330776,Natural killer cell_1-AD.vs.control; scRNA-seq,SRP330776,Memory T cell_1-AD.vs.control	5
AL359504.2	scRNA-seq,SRP215507,CD8+ T cell_1-AD.vs.control; scRNA-seq,SRP330776,CD8+ T cell_1-AD.vs.control; scRNA-seq,SRP330776,γδ T cell-AD.vs.control; scRNA-seq,SRP330776,CD8+ T cell_2-AD.vs.control; scRNA-seq,SRP330776,B cell_1-AD.vs.control; scRNA-seq,SRP330776,Naive CD8+ T cell_1-AD.vs.control; scRNA-seq,SRP330776,B cell_2-AD.vs.control	7
AL359513.1	scRNA-seq,SRP215507,CD8+ T cell_4-AD.vs.MCI; scRNA-seq,SRP215507,CD8+ T cell_5-MCI.vs.control; scRNA-seq,SRP215507,CD8+ T cell_4-MCI.vs.control; scRNA-seq,SRP215507,CD8+ T cell_3-MCI.vs.control; scRNA-seq,SRP330776,CD8+ T cell_1-AD.vs.control; scRNA-seq,SRP330776,Naive CD8+ T cell_1-AD.vs.control; scRNA-seq,SRP330776,Natural killer T cell-AD.vs.control; scRNA-seq,SRP330776,Naive CD8+ T cell_3-AD.vs.control; scRNA-seq,SRP330776,CD8+ T cell_3-AD.vs.control	9
AL359541.1	scRNA-seq,SRP309935,Monocyte_1-AD.vs.control	1
AL359636.2	scRNA-seq,SRP330776,CD8+ T cell_1-AD.vs.control; scRNA-seq,SRP330776,B cell_1-AD.vs.control; scRNA-seq,SRP330776,Naive CD8+ T cell_1-	3
AL359643.3	scRNA-seq,SRP215507,CD8+ T cell_4-AD.vs.control; scRNA-seq,SRP215507,CD8+ T cell_3-AD.vs.MCI; scRNA-seq,SRP215507,CD8+ T cell_3-MCI.vs.control; scRNA-seq,SRP309935,Natural killer cell_1-AD.vs.control; scRNA-seq,SRP330776,Memory T cell_2-AD.vs.control	5
AL359644.1	scRNA-seq,SRP215507,CD8+ T cell_1-AD.vs.control; scRNA-seq,SRP215507,CD8+ T cell_3-AD.vs.MCI; scRNA-seq,SRP215507,CD8+ T cell_3-MCI.vs.control; scRNA-seq,SRP330776,B cell_1-AD.vs.control	4
AL359711.2	scRNA-seq,SRP215507,CD8+ T cell_1-AD.vs.MCI; scRNA-seq,SRP330776,Memory T cell_2-AD.vs.control	2
AL359715.1	scRNA-seq,SRP215507,CD8+ T cell_1-AD.vs.MCI; scRNA-seq,SRP215507,CD8+ T cell_1-MCI.vs.control; scRNA-seq,SRP215507,CD8+ T cell_3-	3
AL359715.2	scRNA-seq,SRP215507,CD8+ T cell_5-MCI.vs.control	1
AL359762.1	scRNA-seq,SRP215507,CD8+ T cell_3-AD.vs.control; scRNA-seq,SRP215507,CD8+ T cell_1-AD.vs.control	2
AL359813.1	scRNA-seq,SRP215507,CD8+ T cell_3-MCI.vs.control	1
AL359878.1	scRNA-seq,SRP330776,Naive CD8+ T cell_1-AD.vs.control	1
AL359924.1	scRNA-seq,SRP215507,CD8+ T cell_1-AD.vs.control	1
AL359962.2	scRNA-seq,SRP215507,CD8+ T cell_2-AD.vs.control; scRNA-seq,SRP309935,Monocyte_1-AD.vs.control; scRNA-seq,SRP309935,Natural killer cell_1-AD.vs.control; scRNA-seq,SRP330776,Naive CD8+ T cell_1-AD.vs.control	4
AL359962.3	scRNA-seq,SRP215507,CD8+ T cell_1-AD.vs.MCI; scRNA-seq,SRP215507,CD8+ T cell_1-MCI.vs.control; scRNA-seq,SRP330776,B cell_3-	3
AL360012.1	scRNA-seq,SRP330776,CD8+ T cell_1-AD.vs.control; scRNA-seq,SRP330776,γδ T cell-AD.vs.control; scRNA-seq,SRP330776,B cell_1-AD.vs.control; scRNA-seq,SRP330776,Memory T cell_1-AD.vs.control; scRNA-seq,SRP330776,CD8+ T cell_3-AD.vs.control; scRNA-seq,SRP330776,Monocyte_2-AD.vs.control; scRNA-seq,SRP330776,Natural killer cell_2-AD.vs.control	7
AL360091.3	scRNA-seq,SRP330776,Memory T cell_1-AD.vs.control	1
AL360178.1	scRNA-seq,SRP215507,CD8+ T cell_5-AD.vs.control; scRNA-seq,SRP215507,CD8+ T cell_3-AD.vs.MCI; scRNA-seq,SRP309935,Naive CD8+ T cell_2-AD.vs.control; scRNA-seq,SRP330776,γδ T cell-AD.vs.control; scRNA-seq,SRP330776,Memory T cell_2-AD.vs.control; scRNA-seq,SRP330776,B cell_1-AD.vs.control; scRNA-seq,SRP330776,Naive CD8+ T cell_1-AD.vs.control	7
AL360181.2	scRNA-seq,SRP330776,Naive CD8+ T cell_1-AD.vs.control	1
AL360270.1	scRNA-seq,SRP215507,CD8+ T cell_1-AD.vs.control; scRNA-seq,SRP215507,CD8+ T cell_1-MCI.vs.control; scRNA-seq,SRP215507,CD8+ T cell_2-MCI.vs.control; scRNA-seq,SRP330776,B cell_2-AD.vs.control; scRNA-seq,SRP330776,CD8+ T cell_3-AD.vs.control	5

AL360270.2	scRNA-seq,SRP215507,CD8+ T cell_2-AD.vs.control; scRNA-seq,SRP215507,CD8+ T cell_3-AD.vs.control; scRNA-seq,SRP215507,CD8+ T cell_1-AD.vs.control; scRNA-seq,SRP215507,CD8+ T cell_3-MCI.vs.control; scRNA-seq,SRP330776,CD8+ T cell_1-AD.vs.control	5
AL360270.3	scRNA-seq,SRP215507,CD8+ T cell_3-AD.vs.control; scRNA-seq,SRP330776,CD8+ T cell_1-AD.vs.control; scRNA-seq,SRP330776,B cell_1-	3
AL365203.2	scRNA-seq,SRP215507,CD8+ T cell_3-AD.vs.control; scRNA-seq,SRP309935,CD8+ T cell_1-AD.vs.control; scRNA-seq,SRP330776,CD8+ T cell_1-AD.vs.control; scRNA-seq,SRP330776,Memory T cell_2-AD.vs.control; scRNA-seq,SRP330776,Natural killer cell_1-AD.vs.control	5
AL365255.1	scRNA-seq,SRP215507,CD8+ T cell_1-AD.vs.control; scRNA-seq,SRP215507,CD8+ T cell_4-MCI.vs.control; scRNA-seq,SRP330776,Naive CD8+ T cell_1-AD.vs.control; scRNA-seq,SRP330776,Natural killer cell_1-AD.vs.control; scRNA-seq,SRP330776,T cell-AD.vs.control	5
AL365272.1	scRNA-seq,SRP215507,CD8+ T cell_4-AD.vs.control; scRNA-seq,SRP215507,CD8+ T cell_3-AD.vs.MCI; scRNA-seq,SRP215507,CD8+ T cell_1-AD.vs.MCI; scRNA-seq,SRP215507,CD8+ T cell_4-AD.vs.MCI; scRNA-seq,SRP309935,Natural killer cell_2-AD.vs.control; scRNA-seq,SRP309935,Naive CD8+ T cell_2-AD.vs.control; scRNA-seq,SRP330776,CD8+ T cell_1-AD.vs.control; scRNA-seq,SRP330776,B cell_1-AD.vs.control; scRNA-seq,SRP330776,Memory T cell_1-AD.vs.control; scRNA-seq,SRP330776,B cell_4-AD.vs.control	10
AL365295.1	scRNA-seq,SRP215507,CD8+ T cell_2-AD.vs.control; scRNA-seq,SRP215507,CD8+ T cell_1-AD.vs.MCI; scRNA-seq,SRP215507,CD8+ T cell_4-AD.vs.MCI; scRNA-seq,SRP215507,CD8+ T cell_1-MCI.vs.control; scRNA-seq,SRP215507,CD8+ T cell_2-MCI.vs.control; scRNA-seq,SRP330776,CD8+ T cell_1-AD.vs.control; scRNA-seq,SRP330776,Memory T cell_2-AD.vs.control; scRNA-seq,SRP330776,CD8+ T cell_2-	9
AL365361.1	scRNA-seq,SRP215507,CD8+ T cell_5-AD.vs.control; scRNA-seq,SRP215507,CD8+ T cell_3-AD.vs.MCI; scRNA-seq,SRP215507,CD8+ T cell_3-MCI.vs.control; scRNA-seq,SRP215507,CD8+ T cell_6-MCI.vs.control; scRNA-seq,SRP309935,Natural killer cell_1-AD.vs.control; scRNA-seq,SRP330776,CD8+ T cell_1-AD.vs.control; scRNA-seq,SRP330776,γδ T cell-AD.vs.control; scRNA-seq,SRP330776,Memory T cell_2-AD.vs.control; scRNA-seq,SRP330776,CD8+ T cell_2-AD.vs.control; scRNA-seq,SRP330776,B cell_1-AD.vs.control; scRNA-seq,SRP330776,Naive CD8+ T cell_1-AD.vs.control; scRNA-seq,SRP330776,Natural killer cell_1-AD.vs.control; scRNA-seq,SRP330776,Memory T cell_1-AD.vs.control; scRNA-seq,SRP330776,Natural killer T cell-AD.vs.control; scRNA-seq,SRP330776,Naive CD8+ T cell_3-AD.vs.control; scRNA-seq,SRP330776,B cell_2-AD.vs.control; scRNA-seq,SRP330776,T cell-AD.vs.control; scRNA-seq,SRP330776,CD8+ T cell_3-AD.vs.control; scRNA-seq,SRP330776,Monocyte_2-[illegible]	20
AL390067.1	scRNA-seq,SRP215507,CD8+ T cell_1-MCI.vs.control	1
AL390195.1	scRNA-seq,SRP215507,CD8+ T cell_2-MCI.vs.control	1
AL390195.2	scRNA-seq,SRP215507,CD8+ T cell_2-AD.vs.control	1
AL390198.1	scRNA-seq,SRP215507,CD8+ T cell_1-AD.vs.control; scRNA-seq,SRP330776,Naive CD8+ T cell_1-AD.vs.control; scRNA-seq,SRP330776,Memory T	3
AL390728.5	scRNA-seq,SRP215507,CD8+ T cell_2-AD.vs.control; scRNA-seq,SRP215507,CD8+ T cell_5-AD.vs.control; scRNA-seq,SRP330776,B cell_3-	3
AL390728.6	scRNA-seq,SRP309935,Monocyte_1-AD.vs.control; scRNA-seq,SRP309935,CD8+ T cell_1-AD.vs.control; scRNA-seq,SRP330776,CD8+ T cell_1-AD.vs.control; scRNA-seq,SRP330776,Memory T cell_2-AD.vs.control; scRNA-seq,SRP330776,CD8+ T cell_2-AD.vs.control; scRNA-seq,SRP330776,Natural killer cell_1-AD.vs.control; scRNA-seq,SRP330776,Memory T cell_1-AD.vs.control; scRNA-seq,SRP330776,B cell_2-	8
AL390957.1	scRNA-seq,SRP215507,CD8+ T cell_3-MCI.vs.control	1
AL391056.1	scRNA-seq,SRP215507,CD8+ T cell_2-AD.vs.control	1
AL391056.2	scRNA-seq,SRP330776,CD8+ T cell_2-AD.vs.control; scRNA-seq,SRP330776,Naive CD8+ T cell_1-AD.vs.control	2
AL391069.1	scRNA-seq,SRP330776,B cell_2-AD.vs.control	1
AL391069.2	scRNA-seq,SRP330776,γδ T cell-AD.vs.control; scRNA-seq,SRP330776,CD8+ T cell_2-AD.vs.control	2
AL391097.1	scRNA-seq,SRP330776,CD8+ T cell_1-AD.vs.control	1
AL391121.1	scRNA-seq,SRP215507,CD8+ T cell_3-AD.vs.control; scRNA-seq,SRP215507,CD8+ T cell_2-AD.vs.MCI; scRNA-seq,SRP215507,CD8+ T cell_1-AD.vs.MCI; scRNA-seq,SRP215507,CD8+ T cell_1-MCI.vs.control; scRNA-seq,SRP215507,CD8+ T cell_2-MCI.vs.control; scRNA-seq,SRP330776,CD8+ T cell_1-AD.vs.control; scRNA-seq,SRP330776,Naive CD8+ T cell_1-AD.vs.control	7
AL391244.2	scRNA-seq,SRP215507,CD8+ T cell_3-AD.vs.control; scRNA-seq,SRP215507,CD8+ T cell_3-MCI.vs.control	2
AL391650.1	scRNA-seq,SRP215507,CD8+ T cell_2-MCI.vs.control; scRNA-seq,SRP330776,Naive CD8+ T cell_1-AD.vs.control; scRNA-seq,SRP330776,Natural killer cell_1-AD.vs.control	3

AL391684.1	scRNA-seq,SRP215507,CD8+ T cell_3-MCI.vs.control; scRNA-seq,SRP330776,CD8+ T cell_1-AD.vs.control; scRNA-seq,SRP330776,CD8+ T cell_2-AD.vs.control; scRNA-seq,SRP330776,B cell_2-AD.vs.control	4
AL391807.1	scRNA-seq,SRP309935,Monocyte_1-AD.vs.control	1
AL391839.2	scRNA-seq,SRP215507,CD8+ T cell_1-MCI.vs.control; scRNA-seq,SRP330776,CD8+ T cell_1-AD.vs.control; scRNA-seq,SRP330776,B cell_2-	3
AL391839.3	scRNA-seq,SRP215507,CD8+ T cell_8-AD.vs.control; scRNA-seq,SRP215507,CD8+ T cell_4-AD.vs.control; scRNA-seq,SRP215507,CD8+ T cell_1-MCI.vs.control; scRNA-seq,SRP215507,CD8+ T cell_5-MCI.vs.control; scRNA-seq,SRP215507,CD8+ T cell_4-MCI.vs.control; scRNA-seq,SRP330776,Memory T cell_2-AD.vs.control; scRNA-seq,SRP330776,B cell_1-AD.vs.control; scRNA-seq,SRP330776,Memory T cell_1-AD.vs.control; scRNA-seq,SRP330776,Naive CD8+ T cell_3-AD.vs.control; scRNA-seq,SRP330776,CD8+ T cell_3-AD.vs.control; scRNA-	11
AL391863.1	scRNA-seq,SRP330776,Naive CD8+ T cell_1-AD.vs.control	1
AL391987.4	scRNA-seq,SRP330776,Memory T cell_1-AD.vs.control	1
AL391988.1	scRNA-seq,SRP215507,CD8+ T cell_3-MCI.vs.control; scRNA-seq,SRP309935,Natural killer cell_2-AD.vs.control; scRNA-seq,SRP330776,Memory T	3
AL392046.1	scRNA-seq,SRP215507,CD8+ T cell_1-AD.vs.control; scRNA-seq,SRP215507,CD8+ T cell_1-MCI.vs.control; scRNA-seq,SRP215507,CD8+ T cell_5-MCI.vs.control; scRNA-seq,SRP330776,CD8+ T cell_1-AD.vs.control; scRNA-seq,SRP330776,Memory T cell_2-AD.vs.control; scRNA-seq,SRP330776,CD8+ T cell_2-AD.vs.control; scRNA-seq,SRP330776,Memory T cell_1-AD.vs.control; scRNA-seq,SRP330776,Natural killer cell_2-	8
AL392086.3	scRNA-seq,SRP215507,CD8+ T cell_1-AD.vs.control; scRNA-seq,SRP215507,CD8+ T cell_4-AD.vs.control; scRNA-seq,SRP215507,CD8+ T cell_5-AD.vs.control; scRNA-seq,SRP215507,CD8+ T cell_6-AD.vs.control; scRNA-seq,SRP215507,CD8+ T cell_2-AD.vs.MCI; scRNA-seq,SRP215507,CD8+ T cell_1-MCI.vs.control; scRNA-seq,SRP215507,CD8+ T cell_5-MCI.vs.control; scRNA-seq,SRP215507,CD8+ T cell_4-MCI.vs.control; scRNA-seq,SRP215507,CD8+ T cell_2-MCI.vs.control; scRNA-seq,SRP330776,γδ T cell-AD.vs.control; scRNA-seq,SRP330776,CD8+ T cell_2-AD.vs.control; scRNA-seq,SRP330776,Natural killer cell_1-AD.vs.control; scRNA-seq,SRP330776,Natural killer T cell-AD.vs.control	13
AL392172.1	scRNA-seq,SRP215507,CD8+ T cell_5-AD.vs.control; scRNA-seq,SRP215507,CD8+ T cell_2-AD.vs.MCI; scRNA-seq,SRP215507,CD8+ T cell_5-MCI.vs.control; scRNA-seq,SRP330776,γδ T cell-AD.vs.control; scRNA-seq,SRP330776,Natural killer cell_1-AD.vs.control; scRNA-seq,SRP330776,Natural killer T cell-AD.vs.control; scRNA-seq,SRP330776,CD8+ T cell_3-AD.vs.control; scRNA-seq,SRP330776,B cell_3-AD.vs.control	8
AL441883.1	scRNA-seq,SRP215507,CD8+ T cell_1-MCI.vs.control; scRNA-seq,SRP309935,Naive CD8+ T cell_2-AD.vs.control; scRNA-seq,SRP330776,CD8+ T cell_1-AD.vs.control; scRNA-seq,SRP330776,CD8+ T cell_2-AD.vs.control; scRNA-seq,SRP330776,B cell_2-AD.vs.control; scRNA-	6
AL441992.1	scRNA-seq,SRP215507,CD8+ T cell_3-AD.vs.control; scRNA-seq,SRP215507,CD8+ T cell_5-AD.vs.MCI; scRNA-seq,SRP215507,CD8+ T cell_3-MCI.vs.control; scRNA-seq,SRP330776,Memory T cell_2-AD.vs.control; scRNA-seq,SRP330776,B cell_1-AD.vs.control; scRNA-seq,SRP330776,Naive CD8+ T cell_1-AD.vs.control; scRNA-seq,SRP330776,Natural killer cell_1-AD.vs.control; scRNA-seq,SRP330776,Memory T cell_1-AD.vs.control; scRNA-seq,SRP330776,Natural killer T cell-AD.vs.control; scRNA-seq,SRP330776,B cell_2-AD.vs.control; scRNA-seq,SRP330776,CD8+ T cell_3-	12
AL442067.3	scRNA-seq,SRP330776,Memory T cell_2-AD.vs.control; scRNA-seq,SRP330776,Memory T cell_1-AD.vs.control	2
AL442125.1	scRNA-seq,SRP215507,CD8+ T cell_2-MCI.vs.control	1
AL442663.3	scRNA-seq,SRP215507,CD8+ T cell_1-AD.vs.MCI; scRNA-seq,SRP215507,CD8+ T cell_1-MCI.vs.control; scRNA-seq,SRP215507,CD8+ T cell_4-MCI.vs.control; scRNA-seq,SRP330776,CD8+ T cell_1-AD.vs.control; scRNA-seq,SRP330776,Memory T cell_2-AD.vs.control; scRNA-seq,SRP330776,Naive CD8+ T cell_1-AD.vs.control; scRNA-seq,SRP330776,CD8+ T cell_3-AD.vs.control	7
AL445228.2	scRNA-seq,SRP215507,CD8+ T cell_1-AD.vs.MCI; scRNA-seq,SRP215507,CD8+ T cell_1-MCI.vs.control; scRNA-seq,SRP215507,CD8+ T cell_2-	3
AL445471.1	scRNA-seq,SRP215507,CD8+ T cell_2-AD.vs.control; scRNA-seq,SRP215507,CD8+ T cell_2-MCI.vs.control	2
AL445524.1	scRNA-seq,SRP215507,CD8+ T cell_2-AD.vs.control; scRNA-seq,SRP330776,B cell_1-AD.vs.control; scRNA-seq,SRP330776,Naive CD8+ T cell_1-AD.vs.control; scRNA-seq,SRP330776,Natural killer cell_1-AD.vs.control; scRNA-seq,SRP330776,Memory T cell_1-AD.vs.control; scRNA-seq,SRP330776,Natural killer T cell-AD.vs.control; scRNA-seq,SRP330776,T cell-AD.vs.control; scRNA-seq,SRP330776,CD8+ T cell_3-AD.vs.control	8
AL445928.2	scRNA-seq,SRP215507,CD8+ T cell_1-AD.vs.control	1
AL449106.1	scRNA-seq,SRP330776,B cell_1-AD.vs.control	1
AL450043.1	scRNA-seq,SRP215507,CD8+ T cell_3-MCI.vs.control	1

AL450326.1	scRNA-seq,SRP215507,CD8+ T cell_2-AD.vs.MCI; scRNA-seq,SRP215507,CD8+ T cell_1-AD.vs.MCI; scRNA-seq,SRP330776,CD8+ T cell_1-	3
AL450344.3	scRNA-seq,SRP215507,CD8+ T cell_2-AD.vs.MCI; scRNA-seq,SRP215507,CD8+ T cell_1-MCI.vs.control; scRNA-seq,SRP215507,CD8+ T cell_2-MCI.vs.control; scRNA-seq,SRP330776,B cell_1-AD.vs.control	4
AL450352.1	scRNA-seq,SRP215507,CD8+ T cell_2-AD.vs.control; scRNA-seq,SRP215507,CD8+ T cell_4-AD.vs.control; scRNA-seq,SRP215507,CD8+ T cell_5-AD.vs.control; scRNA-seq,SRP215507,CD8+ T cell_2-AD.vs.MCI; scRNA-seq,SRP215507,CD8+ T cell_4-MCI.vs.control; scRNA-seq,SRP330776,Naive	6
AL450384.2	scRNA-seq,SRP215507,CD8+ T cell_6-AD.vs.control; scRNA-seq,SRP215507,CD8+ T cell_1-AD.vs.MCI; scRNA-seq,SRP215507,CD8+ T cell_5-AD.vs.MCI; scRNA-seq,SRP215507,CD8+ T cell_1-MCI.vs.control; scRNA-seq,SRP330776,CD8+ T cell_1-AD.vs.control; scRNA-seq,SRP330776,Memory T cell_2-AD.vs.control; scRNA-seq,SRP330776,CD8+ T cell_2-AD.vs.control; scRNA-seq,SRP330776,Naive CD8+ T cell_1-	8
AL450992.2	scRNA-seq,SRP215507,CD8+ T cell_1-AD.vs.control	1
AL450998.2	scRNA-seq,SRP330776,CD8+ T cell_1-AD.vs.control; scRNA-seq,SRP330776,γδ T cell-AD.vs.control; scRNA-seq,SRP330776,Memory T cell_2-AD.vs.control; scRNA-seq,SRP330776,CD8+ T cell_2-AD.vs.control; scRNA-seq,SRP330776,B cell_1-AD.vs.control; scRNA-seq,SRP330776,Natural killer cell_1-AD.vs.control; scRNA-seq,SRP330776,Memory T cell_1-AD.vs.control; scRNA-seq,SRP330776,Natural killer T cell-AD.vs.control; scRNA-seq,SRP330776,CD8+ T cell_3-AD.vs.control; scRNA-seq,SRP330776,Monocyte_2-AD.vs.control; scRNA-seq,SRP330776,B cell_3-AD.vs.control	11
AL451050.2	scRNA-seq,SRP215507,CD8+ T cell_1-AD.vs.control; scRNA-seq,SRP215507,CD8+ T cell_3-AD.vs.MCI; scRNA-seq,SRP330776,CD8+ T cell_2-AD.vs.control; scRNA-seq,SRP330776,Memory T cell_1-AD.vs.control	4
AL451065.1	scRNA-seq,SRP215507,CD8+ T cell_3-MCI.vs.control; scRNA-seq,SRP330776,Naive CD8+ T cell_1-AD.vs.control	2
AL451067.1	scRNA-seq,SRP330776,B cell_1-AD.vs.control	1
AL451069.2	scRNA-seq,SRP309935,Monocyte_1-AD.vs.control	1
AL451069.3	scRNA-seq,SRP215507,CD8+ T cell_2-AD.vs.control	1
AL451085.1	scRNA-seq,SRP215507,CD8+ T cell_1-AD.vs.control; scRNA-seq,SRP215507,CD8+ T cell_5-AD.vs.control; scRNA-seq,SRP215507,CD8+ T cell_4-AD.vs.MCI; scRNA-seq,SRP215507,CD8+ T cell_5-MCI.vs.control; scRNA-seq,SRP215507,CD8+ T cell_4-MCI.vs.control; scRNA-seq,SRP215507,CD8+ T cell_3-MCI.vs.control; scRNA-seq,SRP309935,Natural killer cell_1-AD.vs.control; scRNA-seq,SRP330776,CD8+ T cell_2-AD.vs.control; scRNA-seq,SRP330776,Naive CD8+ T cell_1-AD.vs.control; scRNA-seq,SRP330776,Natural killer cell_1-AD.vs.control; scRNA-	12
AL451085.2	scRNA-seq,SRP215507,CD8+ T cell_3-AD.vs.MCI; scRNA-seq,SRP215507,CD8+ T cell_3-MCI.vs.control; scRNA-seq,SRP309935,Natural killer cell_2-AD.vs.control; scRNA-seq,SRP309935,Monocyte_2-AD.vs.control; scRNA-seq,SRP309935,Naive CD8+ T cell_2-AD.vs.control; scRNA-seq,SRP309935,CD4+ T cell-AD.vs.control; scRNA-seq,SRP330776,CD8+ T cell_1-AD.vs.control; scRNA-seq,SRP330776,γδ T cell-AD.vs.control; scRNA-seq,SRP330776,Memory T cell_2-AD.vs.control; scRNA-seq,SRP330776,B cell_1-AD.vs.control; scRNA-seq,SRP330776,Naive CD8+ T cell_1-AD.vs.control; scRNA-seq,SRP330776,Memory T cell_1-AD.vs.control; scRNA-seq,SRP330776,B cell_2-AD.vs.control; scRNA-seq,SRP330776,CD8+ T	15
AL451165.2	scRNA-seq,SRP330776,CD8+ T cell_1-AD.vs.control; scRNA-seq,SRP330776,Memory T cell_2-AD.vs.control; scRNA-seq,SRP330776,CD8+ T cell_2-AD.vs.control; scRNA-seq,SRP330776,B cell_1-AD.vs.control; scRNA-seq,SRP330776,Naive CD8+ T cell_1-AD.vs.control; scRNA-seq,SRP330776,Natural killer cell_1-AD.vs.control; scRNA-seq,SRP330776,Memory T cell_1-AD.vs.control; scRNA-seq,SRP330776,Natural killer T cell-	9
AL499604.1	scRNA-seq,SRP215507,CD8+ T cell_2-AD.vs.control; scRNA-seq,SRP215507,CD8+ T cell_5-AD.vs.MCI; scRNA-seq,SRP215507,CD8+ T cell_5-MCI.vs.control; scRNA-seq,SRP215507,CD8+ T cell_4-MCI.vs.control; scRNA-seq,SRP309935,Erythroid cell-AD.vs.control; scRNA-seq,SRP330776,CD8+ T cell_2-AD.vs.control; scRNA-seq,SRP330776,B cell_1-AD.vs.control; scRNA-seq,SRP330776,Naive CD8+ T cell_1-AD.vs.control; scRNA-seq,SRP330776,Memory T cell_1-AD.vs.control; scRNA-seq,SRP330776,B cell_2-AD.vs.control; scRNA-seq,SRP330776,CD8+ T cell_3-AD.vs.control; scRNA-seq,SRP330776,Monocyte_1-AD.vs.control; scRNA-seq,SRP330776,B cell_3-AD.vs.control; scRNA-	14
AL512306.2	scRNA-seq,SRP215507,CD8+ T cell_5-AD.vs.control; scRNA-seq,SRP309935,CD8+ T cell_2-AD.vs.control; scRNA-seq,SRP330776,Memory T cell_1-	3
AL512353.1	scRNA-seq,SRP215507,CD8+ T cell_1-AD.vs.control; scRNA-seq,SRP215507,CD8+ T cell_1-AD.vs.MCI; scRNA-seq,SRP309935,CD8+ T cell_1-AD.vs.control; scRNA-seq,SRP330776,CD8+ T cell_1-AD.vs.control; scRNA-seq,SRP330776,B cell_1-AD.vs.control	5
AL512408.1	scRNA-seq,SRP215507,CD8+ T cell_5-MCI.vs.control	1
AL512506.1	scRNA-seq,SRP309935,Naive CD8+ T cell_1-AD.vs.control	1
AL512604.3	scRNA-seq,SRP330776,B cell_2-AD.vs.control	1

AL512625.1	scRNA-seq,SRP215507,CD8+ T cell_4-AD.vs.control; scRNA-seq,SRP215507,CD8+ T cell_4-MCI.vs.control; scRNA-seq,SRP309935,CD8+ T cell_1-AD.vs.control; scRNA-seq,SRP330776,B cell_1-AD.vs.control; scRNA-seq,SRP330776,Naive CD8+ T cell_1-AD.vs.control; scRNA-	6
AL512625.2	scRNA-seq,SRP215507,CD8+ T cell_2-AD.vs.control; scRNA-seq,SRP215507,CD8+ T cell_1-AD.vs.control; scRNA-seq,SRP215507,CD8+ T cell_2-AD.vs.MCI; scRNA-seq,SRP215507,CD8+ T cell_3-AD.vs.MCI; scRNA-seq,SRP215507,CD8+ T cell_1-MCI.vs.control; scRNA-seq,SRP215507,CD8+ T cell_3-MCI.vs.control; scRNA-seq,SRP330776,CD8+ T cell_2-AD.vs.control	7
AL512625.3	scRNA-seq,SRP215507,CD8+ T cell_3-AD.vs.control; scRNA-seq,SRP215507,CD8+ T cell_2-AD.vs.MCI; scRNA-seq,SRP215507,CD8+ T cell_6-AD.vs.MCI; scRNA-seq,SRP215507,CD8+ T cell_2-MCI.vs.control; scRNA-seq,SRP215507,CD8+ T cell_6-MCI.vs.control; scRNA-seq,SRP330776,γδ T cell-AD.vs.control; scRNA-seq,SRP330776,Memory T cell_1-AD.vs.control; scRNA-seq,SRP330776,B cell_2-AD.vs.control	8
AL512662.2	scRNA-seq,SRP215507,CD8+ T cell_2-AD.vs.MCI; scRNA-seq,SRP215507,CD8+ T cell_2-MCI.vs.control	2
AL512770.1	scRNA-seq,SRP330776,CD8+ T cell_1-AD.vs.control	1
AL512791.2	scRNA-seq,SRP215507,CD8+ T cell_5-MCI.vs.control	1
AL513128.3	scRNA-seq,SRP330776,CD8+ T cell_2-AD.vs.control	1
AL513220.1	scRNA-seq,SRP215507,CD8+ T cell_1-AD.vs.control; scRNA-seq,SRP215507,CD8+ T cell_5-MCI.vs.control; scRNA-seq,SRP330776,Memory T cell_1-	3
AL513303.1	scRNA-seq,SRP215507,CD8+ T cell_2-AD.vs.control; scRNA-seq,SRP215507,CD8+ T cell_1-AD.vs.MCI; scRNA-seq,SRP215507,CD8+ T cell_1-MCI.vs.control; scRNA-seq,SRP215507,CD8+ T cell_5-MCI.vs.control; scRNA-seq,SRP215507,CD8+ T cell_2-MCI.vs.control; scRNA-seq,SRP215507,CD8+ T cell_3-MCI.vs.control; scRNA-seq,SRP330776,Memory T cell_2-AD.vs.control; scRNA-seq,SRP330776,Natural killer cell_1-	8
AL513314.2	scRNA-seq,SRP215507,CD8+ T cell_2-MCI.vs.control	1
AL513320.1	scRNA-seq,SRP215507,CD8+ T cell_1-MCI.vs.control	1
AL513534.2	scRNA-seq,SRP215507,CD8+ T cell_3-AD.vs.control	1
AL513548.1	scRNA-seq,SRP330776,Naive CD8+ T cell_1-AD.vs.control	1
AL513548.4	scRNA-seq,SRP215507,CD8+ T cell_1-AD.vs.control; scRNA-seq,SRP215507,CD8+ T cell_2-MCI.vs.control	2
AL513550.1	scRNA-seq,SRP215507,CD8+ T cell_2-AD.vs.control; scRNA-seq,SRP215507,CD8+ T cell_1-AD.vs.control; scRNA-seq,SRP215507,CD8+ T cell_4-AD.vs.control; scRNA-seq,SRP215507,CD8+ T cell_3-AD.vs.MCI; scRNA-seq,SRP215507,CD8+ T cell_3-MCI.vs.control; scRNA-seq,SRP330776,Memory T cell_2-AD.vs.control; scRNA-seq,SRP330776,CD8+ T cell_2-AD.vs.control; scRNA-seq,SRP330776,Naive CD8+ T cell_1-AD.vs.control; scRNA-seq,SRP330776,Natural killer cell_1-AD.vs.control; scRNA-seq,SRP330776,Natural killer T cell-AD.vs.control; scRNA-	12
AL589693.1	scRNA-seq,SRP215507,CD8+ T cell_3-AD.vs.MCI; scRNA-seq,SRP215507,CD8+ T cell_3-MCI.vs.control; scRNA-seq,SRP330776,CD8+ T cell_1-	3
AL589765.4	scRNA-seq,SRP330776,Memory T cell_1-AD.vs.control; scRNA-seq,SRP330776,B cell_2-AD.vs.control	2
AL589765.7	scRNA-seq,SRP215507,CD8+ T cell_2-AD.vs.control; scRNA-seq,SRP330776,Natural killer cell_1-AD.vs.control; scRNA-seq,SRP330776,Naive CD8+ T cell_3-AD.vs.control; scRNA-seq,SRP330776,Naive CD8+ T cell_2-AD.vs.control; scRNA-seq,SRP330776,CD8+ T cell_3-AD.vs.control	5
AL589843.1	scRNA-seq,SRP215507,CD8+ T cell_3-AD.vs.MCI; scRNA-seq,SRP215507,CD8+ T cell_1-AD.vs.MCI; scRNA-seq,SRP215507,CD8+ T cell_1-MCI.vs.control; scRNA-seq,SRP215507,CD8+ T cell_3-MCI.vs.control; scRNA-seq,SRP330776,Memory T cell_1-AD.vs.control; scRNA-	6
AL589935.1	scRNA-seq,SRP330776,B cell_1-AD.vs.control	1
AL590006.1	scRNA-seq,SRP215507,CD8+ T cell_3-AD.vs.control; scRNA-seq,SRP215507,CD8+ T cell_4-AD.vs.MCI; scRNA-seq,SRP215507,CD8+ T cell_2-MCI.vs.control; scRNA-seq,SRP215507,CD8+ T cell_3-MCI.vs.control	4
AL590064.1	scRNA-seq,SRP215507,CD8+ T cell_3-AD.vs.control; scRNA-seq,SRP215507,CD8+ T cell_1-AD.vs.control; scRNA-seq,SRP215507,CD8+ T cell_3-	3
AL590068.4	scRNA-seq,SRP215507,CD8+ T cell_2-AD.vs.control; scRNA-seq,SRP215507,CD8+ T cell_1-MCI.vs.control; scRNA-seq,SRP215507,CD8+ T cell_4-MCI.vs.control; scRNA-seq,SRP215507,CD8+ T cell_2-MCI.vs.control; scRNA-seq,SRP330776,γδ T cell-AD.vs.control; scRNA-seq,SRP330776,CD8+ T cell_2-AD.vs.control; scRNA-seq,SRP330776,B cell_1-AD.vs.control; scRNA-seq,SRP330776,Naive CD8+ T cell_1-AD.vs.control; scRNA-seq,SRP330776,Memory T cell_1-AD.vs.control; scRNA-seq,SRP330776,B cell_2-AD.vs.control; scRNA-seq,SRP330776,CD8+ T cell_3-AD.vs.control; scRNA-seq,SRP330776,B cell_3-AD.vs.control; scRNA-seq,SRP330776,Natural killer cell_2-AD.vs.control	13
AL590101.1	scRNA-seq,SRP215507,CD8+ T cell_3-MCI.vs.control	1

AL590226.2	scRNA-seq,SRP215507,CD8+ T cell_1-MCI.vs.control	1
AL590399.1	scRNA-seq,SRP215507,CD8+ T cell_6-AD.vs.control; scRNA-seq,SRP215507,CD8+ T cell_1-MCI.vs.control	2
AL590399.3	scRNA-seq,SRP215507,CD8+ T cell_1-MCI.vs.control	1
AL590550.1	scRNA-seq,SRP215507,CD8+ T cell_2-AD.vs.control; scRNA-seq,SRP215507,CD8+ T cell_3-AD.vs.control; scRNA-seq,SRP215507,CD8+ T cell_1-AD.vs.control; scRNA-seq,SRP215507,CD8+ T cell_5-AD.vs.control; scRNA-seq,SRP215507,CD8+ T cell_2-AD.vs.MCI; scRNA-seq,SRP215507,CD8+ T cell_1-AD.vs.MCI; scRNA-seq,SRP215507,CD8+ T cell_5-AD.vs.MCI; scRNA-seq,SRP215507,CD8+ T cell_5-MCI.vs.control; scRNA-seq,SRP309935,CD8+ T cell_1-AD.vs.control; scRNA-seq,SRP330776,Natural killer cell_1-AD.vs.control; scRNA-seq,SRP330776,Memory T cell_1-	12
AL590560.2	scRNA-seq,SRP215507,CD8+ T cell_1-AD.vs.control; scRNA-seq,SRP330776,Naive CD8+ T cell_1-AD.vs.control	2
AL590560.3	scRNA-seq,SRP215507,CD8+ T cell_1-AD.vs.control; scRNA-seq,SRP215507,CD8+ T cell_5-MCI.vs.control	2
AL590640.1	scRNA-seq,SRP215507,CD8+ T cell_1-MCI.vs.control	1
AL590652.1	scRNA-seq,SRP215507,CD8+ T cell_3-AD.vs.control; scRNA-seq,SRP215507,CD8+ T cell_2-AD.vs.MCI; scRNA-seq,SRP215507,CD8+ T cell_1-MCI.vs.control; scRNA-seq,SRP215507,CD8+ T cell_3-MCI.vs.control; scRNA-seq,SRP330776,CD8+ T cell_1-AD.vs.control	5
AL590666.5	scRNA-seq,SRP215507,CD8+ T cell_5-AD.vs.MCI; scRNA-seq,SRP215507,CD8+ T cell_1-MCI.vs.control; scRNA-seq,SRP215507,CD8+ T cell_5-	3
AL590705.1	scRNA-seq,SRP215507,CD8+ T cell_1-AD.vs.control; scRNA-seq,SRP215507,CD8+ T cell_2-AD.vs.MCI; scRNA-seq,SRP215507,CD8+ T cell_1-AD.vs.MCI; scRNA-seq,SRP330776,CD8+ T cell_1-AD.vs.control; scRNA-seq,SRP330776,γδ T cell-AD.vs.control; scRNA-seq,SRP330776,Memory T cell_2-AD.vs.control; scRNA-seq,SRP330776,CD8+ T cell_2-AD.vs.control; scRNA-seq,SRP330776,CD8+ T cell_3-AD.vs.control	8
AL590714.1	scRNA-seq,SRP215507,CD8+ T cell_2-AD.vs.control; scRNA-seq,SRP330776,Naive CD8+ T cell_1-AD.vs.control	2
AL590764.1	scRNA-seq,SRP215507,CD8+ T cell_5-AD.vs.control; scRNA-seq,SRP330776,CD8+ T cell_1-AD.vs.control; scRNA-seq,SRP330776,γδ T cell-AD.vs.control; scRNA-seq,SRP330776,B cell_1-AD.vs.control; scRNA-seq,SRP330776,Naive CD8+ T cell_1-AD.vs.control; scRNA-seq,SRP330776,Natural killer T cell-AD.vs.control; scRNA-seq,SRP330776,B cell_2-AD.vs.control; scRNA-seq,SRP330776,T cell-AD.vs.control; scRNA-	9
AL590822.2	scRNA-seq,SRP215507,CD8+ T cell_3-MCI.vs.control	1
AL591043.2	scRNA-seq,SRP215507,CD8+ T cell_2-AD.vs.control; scRNA-seq,SRP330776,γδ T cell-AD.vs.control; scRNA-seq,SRP330776,Naive CD8+ T cell_1-	3
AL591368.1	scRNA-seq,SRP330776,Memory T cell_1-AD.vs.control; scRNA-seq,SRP330776,B cell_2-AD.vs.control	2
AL591623.1	scRNA-seq,SRP215507,CD8+ T cell_2-AD.vs.control; scRNA-seq,SRP309935,Natural killer cell_1-AD.vs.control	2
AL591848.3	scRNA-seq,SRP215507,CD8+ T cell_1-MCI.vs.control	1
AL591895.1	scRNA-seq,SRP215507,CD8+ T cell_1-AD.vs.control; scRNA-seq,SRP215507,CD8+ T cell_3-AD.vs.MCI; scRNA-seq,SRP215507,CD8+ T cell_4-AD.vs.MCI; scRNA-seq,SRP215507,CD8+ T cell_3-MCI.vs.control; scRNA-seq,SRP309935,Naive CD8+ T cell_1-AD.vs.control; scRNA-seq,SRP330776,CD8+ T cell_1-AD.vs.control; scRNA-seq,SRP330776,Naive CD8+ T cell_1-AD.vs.control	7
AL591926.2	scRNA-seq,SRP215507,CD8+ T cell_5-MCI.vs.control	1
AL592148.3	scRNA-seq,SRP215507,CD8+ T cell_3-AD.vs.control; scRNA-seq,SRP215507,CD8+ T cell_3-MCI.vs.control; scRNA-seq,SRP330776,CD8+ T cell_1-	3
AL592166.1	scRNA-seq,SRP215507,CD8+ T cell_2-AD.vs.control; scRNA-seq,SRP215507,CD8+ T cell_1-AD.vs.MCI; scRNA-seq,SRP215507,CD8+ T cell_1-MCI.vs.control; scRNA-seq,SRP330776,Natural killer cell_1-AD.vs.control	4
AL592183.1	scRNA-seq,SRP215507,CD8+ T cell_3-AD.vs.MCI; scRNA-seq,SRP309935,Natural killer cell_2-AD.vs.control; scRNA-seq,SRP309935,Monocyte_1-AD.vs.control; scRNA-seq,SRP309935,Naive CD8+ T cell_1-AD.vs.control; scRNA-seq,SRP309935,Natural killer cell_1-AD.vs.control; scRNA-seq,SRP309935,CD8+ T cell_1-AD.vs.control; scRNA-seq,SRP309935,B cell_1-AD.vs.control; scRNA-seq,SRP309935,CD8+ T cell_2-AD.vs.control; scRNA-seq,SRP330776,Memory T cell_2-AD.vs.control; scRNA-seq,SRP330776,CD8+ T cell_2-AD.vs.control; scRNA-seq,SRP330776,B cell_1-AD.vs.control; scRNA-seq,SRP330776,Naive CD8+ T cell_1-AD.vs.control; scRNA-seq,SRP330776,Memory T cell_1-AD.vs.control	13
AL592211.2	scRNA-seq,SRP330776,Memory T cell_2-AD.vs.control	1

AL592295.4	scRNA-seq,SRP215507,CD8+ T cell_2-AD.vs.control; scRNA-seq,SRP215507,CD8+ T cell_1-MCI.vs.control; scRNA-seq,SRP215507,CD8+ T cell_3-MCI.vs.control; scRNA-seq,SRP330776,γδ T cell-AD.vs.control; scRNA-seq,SRP330776,Memory T cell_2-AD.vs.control; scRNA-seq,SRP330776,CD8+ T cell_2-AD.vs.control; scRNA-seq,SRP330776,B cell_1-AD.vs.control; scRNA-seq,SRP330776,Natural killer cell_1-AD.vs.control; scRNA-seq,SRP330776,Memory T cell_1-AD.vs.control; scRNA-seq,SRP330776,Natural killer T cell-AD.vs.control; scRNA-seq,SRP330776,Naive CD8+ T cell_3-AD.vs.control; scRNA-seq,SRP330776,B cell_2-AD.vs.control; scRNA-seq,SRP330776,CD8+ T cell_3-AD.vs.control	13
AL592295.5	scRNA-seq,SRP215507,CD8+ T cell_1-AD.vs.MCI; scRNA-seq,SRP215507,CD8+ T cell_1-MCI.vs.control; scRNA-seq,SRP309935,Naive CD8+ T cell_3-AD.vs.control; scRNA-seq,SRP330776,CD8+ T cell_1-AD.vs.control; scRNA-seq,SRP330776,γδ T cell-AD.vs.control; scRNA-seq,SRP330776,CD8+ T cell_2-AD.vs.control; scRNA-seq,SRP330776,Memory T cell_1-AD.vs.control	7
AL592429.2	scRNA-seq,SRP330776,B cell_4-AD.vs.control	1
AL592435.1	scRNA-seq,SRP215507,CD8+ T cell_4-MCI.vs.control; scRNA-seq,SRP215507,CD8+ T cell_3-MCI.vs.control; scRNA-seq,SRP330776,CD8+ T cell_1-AD.vs.control; scRNA-seq,SRP330776,B cell_1-AD.vs.control; scRNA-seq,SRP330776,Naive CD8+ T cell_1-AD.vs.control; scRNA-	6
AL596214.1	scRNA-seq,SRP330776,CD8+ T cell_1-AD.vs.control; scRNA-seq,SRP330776,B cell_1-AD.vs.control; scRNA-seq,SRP330776,Naive CD8+ T cell_1-	3
AL596244.1	scRNA-seq,SRP215507,CD8+ T cell_2-MCI.vs.control; scRNA-seq,SRP215507,CD8+ T cell_3-MCI.vs.control	2
AL596325.1	scRNA-seq,SRP215507,CD8+ T cell_1-AD.vs.MCI; scRNA-seq,SRP215507,CD8+ T cell_5-AD.vs.MCI; scRNA-seq,SRP215507,CD8+ T cell_1-MCI.vs.control; scRNA-seq,SRP215507,CD8+ T cell_5-MCI.vs.control; scRNA-seq,SRP215507,CD8+ T cell_4-MCI.vs.control; scRNA-seq,SRP330776,Memory T cell_2-AD.vs.control; scRNA-seq,SRP330776,CD8+ T cell_2-AD.vs.control; scRNA-seq,SRP330776,CD8+ T cell_3-	8
AL596325.2	scRNA-seq,SRP215507,CD8+ T cell_2-AD.vs.MCI; scRNA-seq,SRP215507,CD8+ T cell_4-MCI.vs.control; scRNA-seq,SRP309935,Natural killer cell_1-	3
AL603756.1	scRNA-seq,SRP330776,CD8+ T cell_2-AD.vs.control; scRNA-seq,SRP330776,Naive CD8+ T cell_1-AD.vs.control	2
AL603783.1	scRNA-seq,SRP215507,CD8+ T cell_4-AD.vs.control; scRNA-seq,SRP215507,CD8+ T cell_3-AD.vs.MCI; scRNA-seq,SRP215507,CD8+ T cell_4-MCI.vs.control; scRNA-seq,SRP215507,CD8+ T cell_3-MCI.vs.control; scRNA-seq,SRP330776,CD8+ T cell_1-AD.vs.control	5
AL603832.2	scRNA-seq,SRP215507,CD8+ T cell_3-MCI.vs.control	1
AL603839.3	scRNA-seq,SRP215507,CD8+ T cell_2-MCI.vs.control; scRNA-seq,SRP215507,CD8+ T cell_3-MCI.vs.control; scRNA-seq,SRP330776,Naive CD8+ T	3
AL604028.1	scRNA-seq,SRP309935,Naive CD8+ T cell_1-AD.vs.control	1
AL606469.1	scRNA-seq,SRP330776,Naive CD8+ T cell_1-AD.vs.control	1
AL606491.1	scRNA-seq,SRP215507,CD8+ T cell_5-AD.vs.MCI; scRNA-seq,SRP215507,CD8+ T cell_3-MCI.vs.control	2
AL606760.1	scRNA-seq,SRP215507,CD8+ T cell_3-AD.vs.MCI; scRNA-seq,SRP215507,CD8+ T cell_3-MCI.vs.control; scRNA-seq,SRP330776,Natural killer cell_1-	3
AL606760.3	scRNA-seq,SRP215507,CD8+ T cell_1-AD.vs.MCI; scRNA-seq,SRP215507,CD8+ T cell_5-AD.vs.MCI; scRNA-seq,SRP215507,CD8+ T cell_1-MCI.vs.control; scRNA-seq,SRP215507,CD8+ T cell_5-MCI.vs.control; scRNA-seq,SRP215507,CD8+ T cell_2-MCI.vs.control; scRNA-	6
AL606834.2	scRNA-seq,SRP215507,CD8+ T cell_1-AD.vs.control; scRNA-seq,SRP215507,CD8+ T cell_4-AD.vs.MCI; scRNA-seq,SRP215507,CD8+ T cell_1-	3
AL627171.1	scRNA-seq,SRP215507,CD8+ T cell_2-AD.vs.control; scRNA-seq,SRP215507,CD8+ T cell_1-AD.vs.MCI; scRNA-seq,SRP215507,CD8+ T cell_5-AD.vs.MCI; scRNA-seq,SRP215507,CD8+ T cell_1-MCI.vs.control; scRNA-seq,SRP215507,CD8+ T cell_5-MCI.vs.control; scRNA-seq,SRP309935,Monocyte_1-AD.vs.control; scRNA-seq,SRP309935,Naive CD8+ T cell_2-AD.vs.control; scRNA-seq,SRP330776,γδ T cell-AD.vs.control; scRNA-seq,SRP330776,CD8+ T cell_2-AD.vs.control; scRNA-seq,SRP330776,B cell_1-AD.vs.control; scRNA-seq,SRP330776,Naive CD8+ T cell_1-AD.vs.control; scRNA-seq,SRP330776,Memory T cell_1-AD.vs.control; scRNA-seq,SRP330776,Naive CD8+ T cell_3-AD.vs.control; scRNA-seq,SRP330776,T cell-AD.vs.control; scRNA-seq,SRP330776,CD8+ T cell_3-AD.vs.control; scRNA-seq,SRP330776,B cell_3-AD.vs.control; scRNA-	17
AL627171.2	scRNA-seq,SRP215507,CD8+ T cell_4-AD.vs.control; scRNA-seq,SRP215507,CD8+ T cell_3-AD.vs.MCI; scRNA-seq,SRP215507,CD8+ T cell_4-AD.vs.MCI; scRNA-seq,SRP215507,CD8+ T cell_3-MCI.vs.control; scRNA-seq,SRP330776,Memory T cell_2-AD.vs.control; scRNA-seq,SRP330776,T	6
AL627309.1	scRNA-seq,SRP215507,CD8+ T cell_4-MCI.vs.control; scRNA-seq,SRP215507,CD8+ T cell_3-MCI.vs.control	2
AL627309.5	scRNA-seq,SRP215507,CD8+ T cell_1-AD.vs.MCI; scRNA-seq,SRP330776,B cell_1-AD.vs.control	2
AL627422.2	scRNA-seq,SRP215507,CD8+ T cell_2-AD.vs.control; scRNA-seq,SRP215507,CD8+ T cell_1-AD.vs.control; scRNA-seq,SRP330776,Naive CD8+ T cell_1-AD.vs.control; scRNA-seq,SRP330776,Memory T cell_1-AD.vs.control; scRNA-seq,SRP330776,CD8+ T cell_3-AD.vs.control	5

AL645568.1	scRNA-seq,SRP215507,CD8+ T cell_3-AD.vs.control; scRNA-seq,SRP215507,CD8+ T cell_1-AD.vs.control; scRNA-seq,SRP215507,CD8+ T cell_3-AD.vs.MCI; scRNA-seq,SRP215507,CD8+ T cell_5-AD.vs.MCI; scRNA-seq,SRP215507,CD8+ T cell_1-MCI.vs.control; scRNA-seq,SRP215507,CD8+ T cell_5-MCI.vs.control; scRNA-seq,SRP330776,CD8+ T cell_1-AD.vs.control; scRNA-seq,SRP330776,γδ T cell-AD.vs.control; scRNA-seq,SRP330776,Memory T cell_2-AD.vs.control; scRNA-seq,SRP330776,Naive CD8+ T cell_1-AD.vs.control; scRNA-seq,SRP330776,Memory T cell_1-AD.vs.control; scRNA-seq,SRP330776,Naive CD8+ T cell_3-AD.vs.control; scRNA-seq,SRP330776,T cell-AD.vs.control; scRNA-seq,SRP330776,CD8+ T cell_3-AD.vs.control; scRNA-seq,SRP330776,B cell_3-AD.vs.control; scRNA-seq,SRP330776,B cell_4-AD.vs.control	16
AL645608.1	scRNA-seq,SRP215507,CD8+ T cell_2-AD.vs.control; scRNA-seq,SRP215507,CD8+ T cell_1-AD.vs.control	2
AL645728.1	scRNA-seq,SRP215507,CD8+ T cell_4-AD.vs.control; scRNA-seq,SRP215507,CD8+ T cell_3-AD.vs.MCI; scRNA-seq,SRP215507,CD8+ T cell_5-MCI.vs.control; scRNA-seq,SRP215507,CD8+ T cell_3-MCI.vs.control; scRNA-seq,SRP215507,CD8+ T cell_6-MCI.vs.control; scRNA-seq,SRP309935,Naive CD8+ T cell_1-AD.vs.control; scRNA-seq,SRP330776,CD8+ T cell_1-AD.vs.control; scRNA-seq,SRP330776,Memory T cell_2-AD.vs.control; scRNA-seq,SRP330776,CD8+ T cell_2-AD.vs.control; scRNA-seq,SRP330776,B cell_1-AD.vs.control; scRNA-seq,SRP330776,Naive CD8+ T cell_1-AD.vs.control; scRNA-seq,SRP330776,Natural killer cell_1-AD.vs.control; scRNA-seq,SRP330776,Memory T cell_1-AD.vs.control; scRNA-seq,SRP330776,B cell_2-AD.vs.control; scRNA-seq,SRP330776,CD8+ T cell_3-AD.vs.control; scRNA-seq,SRP330776,B cell_3-AD.vs.control	16
AL645933.2	scRNA-seq,SRP215507,CD8+ T cell_1-AD.vs.control; scRNA-seq,SRP215507,CD8+ T cell_1-MCI.vs.control; scRNA-seq,SRP330776,B cell_1-AD.vs.control; scRNA-seq,SRP330776,Naive CD8+ T cell_1-AD.vs.control	4
AL645933.3	scRNA-seq,SRP215507,CD8+ T cell_1-MCI.vs.control; scRNA-seq,SRP309935,Natural killer cell_1-AD.vs.control; scRNA-seq,SRP330776,Memory T	3
AL645939.5	scRNA-seq,SRP215507,CD8+ T cell_1-AD.vs.MCI; scRNA-seq,SRP330776,γδ T cell-AD.vs.control; scRNA-seq,SRP330776,B cell_1-AD.vs.control; scRNA-seq,SRP330776,Memory T cell_1-AD.vs.control	4
AL645941.1	scRNA-seq,SRP215507,CD8+ T cell_1-MCI.vs.control	1
AL645941.3	scRNA-seq,SRP215507,CD8+ T cell_1-AD.vs.control; scRNA-seq,SRP215507,CD8+ T cell_4-AD.vs.control; scRNA-seq,SRP215507,CD8+ T cell_1-AD.vs.MCI; scRNA-seq,SRP330776,CD8+ T cell_1-AD.vs.control; scRNA-seq,SRP330776,Natural killer cell_1-AD.vs.control	5
AL662797.1	scRNA-seq,SRP330776,γδ T cell-AD.vs.control; scRNA-seq,SRP330776,Memory T cell_2-AD.vs.control; scRNA-seq,SRP330776,Memory T cell_1-	3
AL662844.4	scRNA-seq,SRP215507,CD8+ T cell_2-AD.vs.MCI; scRNA-seq,SRP215507,CD8+ T cell_1-AD.vs.MCI; scRNA-seq,SRP215507,CD8+ T cell_4-AD.vs.MCI; scRNA-seq,SRP215507,CD8+ T cell_5-AD.vs.MCI; scRNA-seq,SRP215507,CD8+ T cell_1-MCI.vs.control; scRNA-seq,SRP215507,CD8+ T cell_5-MCI.vs.control; scRNA-seq,SRP215507,CD8+ T cell_4-MCI.vs.control; scRNA-seq,SRP215507,CD8+ T cell_2-MCI.vs.control; scRNA-seq,SRP309935,Natural killer cell_2-AD.vs.control; scRNA-seq,SRP309935,Monocyte_1-AD.vs.control; scRNA-seq,SRP309935,Megakaryocyte_1-AD.vs.control; scRNA-seq,SRP309935,Naive CD8+ T cell_1-AD.vs.control; scRNA-seq,SRP309935,Natural killer cell_1-AD.vs.control; scRNA-seq,SRP309935,Monocyte_2-AD.vs.control; scRNA-seq,SRP309935,Naive CD8+ T cell_2-AD.vs.control; scRNA-seq,SRP309935,CD8+ T cell_1-AD.vs.control; scRNA-seq,SRP309935,B cell_1-AD.vs.control; scRNA-seq,SRP309935,CD8+ T cell_2-AD.vs.control; scRNA-seq,SRP309935,Naive [illegible]	21
AL662884.3	scRNA-seq,SRP215507,CD8+ T cell_3-AD.vs.control; scRNA-seq,SRP215507,CD8+ T cell_1-AD.vs.control; scRNA-seq,SRP215507,CD8+ T cell_1-MCI.vs.control; scRNA-seq,SRP330776,Naive CD8+ T cell_1-AD.vs.control; scRNA-seq,SRP330776,Memory T cell_1-AD.vs.control	5
AL662884.5	scRNA-seq,SRP309935,Naive CD8+ T cell_1-AD.vs.control; scRNA-seq,SRP309935,Natural killer cell_1-AD.vs.control	2
AL672277.1	scRNA-seq,SRP215507,CD8+ T cell_2-MCI.vs.control; scRNA-seq,SRP330776,Naive CD8+ T cell_1-AD.vs.control	2
AL672310.1	scRNA-seq,SRP215507,CD8+ T cell_1-AD.vs.control; scRNA-seq,SRP215507,CD8+ T cell_1-AD.vs.MCI	2
AL683807.1	scRNA-seq,SRP215507,CD8+ T cell_2-AD.vs.MCI; scRNA-seq,SRP215507,CD8+ T cell_1-AD.vs.MCI; scRNA-seq,SRP215507,CD8+ T cell_5-AD.vs.MCI; scRNA-seq,SRP215507,CD8+ T cell_1-MCI.vs.control; scRNA-seq,SRP215507,CD8+ T cell_5-MCI.vs.control; scRNA-	6
AL683813.1	scRNA-seq,SRP215507,CD8+ T cell_5-MCI.vs.control; scRNA-seq,SRP215507,CD8+ T cell_2-MCI.vs.control; scRNA-seq,SRP309935,Plasmacytoid dendritic cell_1-AD.vs.control; scRNA-seq,SRP330776,Naive CD8+ T cell_1-AD.vs.control	4
AL691403.2	scRNA-seq,SRP215507,CD8+ T cell_1-MCI.vs.control	1

AL691432.2	scRNA-seq,SRP215507,CD8+ T cell_3-AD.vs.MCI; scRNA-seq,SRP215507,CD8+ T cell_1-AD.vs.MCI; scRNA-seq,SRP215507,CD8+ T cell_5-AD.vs.MCI; scRNA-seq,SRP215507,CD8+ T cell_5-MCI.vs.control; scRNA-seq,SRP215507,CD8+ T cell_4-MCI.vs.control; scRNA-seq,SRP330776,CD8+ T cell_1-AD.vs.control; scRNA-seq,SRP330776,γδ T cell-AD.vs.control; scRNA-seq,SRP330776,Memory T cell_2-AD.vs.control; scRNA-seq,SRP330776,CD8+ T cell_2-AD.vs.control; scRNA-seq,SRP330776,B cell_1-AD.vs.control; scRNA-seq,SRP330776,Naive CD8+ T cell_1-AD.vs.control; scRNA-seq,SRP330776,Natural killer cell_1-AD.vs.control; scRNA-seq,SRP330776,Memory T cell_1-AD.vs.control; scRNA-seq,SRP330776,Natural killer T cell-AD.vs.control; scRNA-seq,SRP330776,Naive CD8+ T cell_3-AD.vs.control; scRNA-seq,SRP330776,B cell_2-AD.vs.control; scRNA-seq,SRP330776,T cell-AD.vs.control; scRNA-seq,SRP330776,CD8+ T cell_3-AD.vs.control; scRNA-seq,SRP330776,Monocyte_2- [illegible]	20
AL691432.4	scRNA-seq,SRP215507,CD8+ T cell_3-MCI.vs.control	1
AL691447.2	scRNA-seq,SRP215507,CD8+ T cell_3-AD.vs.control; scRNA-seq,SRP330776,B cell_1-AD.vs.control	2
AL713852.1	scRNA-seq,SRP215507,CD8+ T cell_1-MCI.vs.control	1
AL731553.1	scRNA-seq,SRP215507,CD8+ T cell_2-AD.vs.control	1
AL731557.1	scRNA-seq,SRP215507,CD8+ T cell_1-MCI.vs.control	1
AL731559.1	scRNA-seq,SRP215507,CD8+ T cell_1-AD.vs.control	1
AL731569.1	scRNA-seq,SRP215507,CD8+ T cell_3-AD.vs.MCI; scRNA-seq,SRP330776,CD8+ T cell_1-AD.vs.control; scRNA-seq,SRP330776,CD8+ T cell_2-AD.vs.control; scRNA-seq,SRP330776,Naive CD8+ T cell_1-AD.vs.control; scRNA-seq,SRP330776,Natural killer cell_1-AD.vs.control; scRNA-seq,SRP330776,Memory T cell_1-AD.vs.control; scRNA-seq,SRP330776,CD8+ T cell_3-AD.vs.control	7
AL731571.1	scRNA-seq,SRP215507,CD8+ T cell_2-AD.vs.MCI; scRNA-seq,SRP215507,CD8+ T cell_1-MCI.vs.control; scRNA-seq,SRP215507,CD8+ T cell_2-	3
AL731577.1	scRNA-seq,SRP309935,Naive CD8+ T cell_1-AD.vs.control; scRNA-seq,SRP309935,Natural killer cell_1-AD.vs.control; scRNA-seq,SRP309935,Naive CD8+ T cell_2-AD.vs.control; scRNA-seq,SRP309935,CD8+ T cell_1-AD.vs.control; scRNA-seq,SRP309935,B cell_1-AD.vs.control	5
AL731577.2	scRNA-seq,SRP215507,CD8+ T cell_1-AD.vs.MCI; scRNA-seq,SRP215507,CD8+ T cell_5-AD.vs.MCI; scRNA-seq,SRP215507,CD8+ T cell_5-MCI.vs.control; scRNA-seq,SRP330776,Memory T cell_1-AD.vs.control	4
AL731702.1	scRNA-seq,SRP215507,CD8+ T cell_1-AD.vs.control; scRNA-seq,SRP215507,CD8+ T cell_3-AD.vs.MCI	2
AL732292.2	scRNA-seq,SRP215507,CD8+ T cell_2-MCI.vs.control	1
AL732437.2	scRNA-seq,SRP330776,Naive CD8+ T cell_1-AD.vs.control	1
AL732509.1	scRNA-seq,SRP215507,CD8+ T cell_3-AD.vs.MCI; scRNA-seq,SRP330776,Memory T cell_1-AD.vs.control	2
AL844892.2	scRNA-seq,SRP215507,CD8+ T cell_2-AD.vs.control; scRNA-seq,SRP215507,CD8+ T cell_1-MCI.vs.control	2
AL928970.1	scRNA-seq,SRP215507,CD8+ T cell_1-MCI.vs.control; scRNA-seq,SRP215507,CD8+ T cell_2-MCI.vs.control; scRNA-seq,SRP330776,Naive CD8+ T	3
Alad	bulk RNA-seq,SRP325058,AD.vs.control	1
ALAD	scRNA-seq,SRP215507,CD8+ T cell_6-AD.vs.MCI; scRNA-seq,SRP215507,CD8+ T cell_6-MCI.vs.control; scRNA-seq,SRP309935,Naive CD8+ T cell_1-AD.vs.control; scRNA-seq,SRP309935,Naive CD8+ T cell_2-AD.vs.control; scRNA-seq,SRP330776,CD8+ T cell_1-AD.vs.control; scRNA-seq,SRP330776,γδ T cell-AD.vs.control; scRNA-seq,SRP330776,Memory T cell_2-AD.vs.control; scRNA-seq,SRP330776,Natural killer T cell-AD.vs.control; scRNA-seq,SRP330776,Naive CD8+ T cell_3-AD.vs.control; scRNA-seq,SRP330776,Monocyte_1-AD.vs.control; scRNA-	11
ALAS1	bulk RNA-seq,Homo_714,AD.vs.control; bulk RNA-seq,Homo_714,AD.vs.MCI; scRNA-seq,SRP215507,CD8+ T cell_3-AD.vs.control; scRNA-seq,SRP215507,CD8+ T cell_3-MCI.vs.control; scRNA-seq,SRP330776,B cell_1-AD.vs.control; scRNA-seq,SRP330776,Natural killer T cell-AD.vs.control; scRNA-seq,SRP330776,B cell_2-AD.vs.control; scRNA-seq,SRP330776,T cell-AD.vs.control; scRNA-seq,SRP330776,Monocyte_2-	10
ALAS2	bulk RNA-seq,Homo_723,AD.vs.MCI; bulk RNA-seq,Homo_633,AD.vs.MCI; scRNA-seq,SRP309935,Monocyte_1-AD.vs.control; scRNA-seq,SRP309935,Erythroid cell-AD.vs.control; scRNA-seq,SRP309935,Naive CD8+ T cell_1-AD.vs.control; scRNA-seq,SRP309935,Natural killer cell_1-AD.vs.control; scRNA-seq,SRP309935,Monocyte_2-AD.vs.control; scRNA-seq,SRP309935,CD8+ T cell_1-AD.vs.control; scRNA-seq,SRP309935,CD8+	9
ALB	bulk RNA-seq,Homo_723,AD.vs.control; bulk RNA-seq,Homo_723,AD.vs.MCI; bulk RNA-seq,Homo_723,MCI.vs.control; bulk RNA-seq,Homo_633,AD.vs.control; bulk RNA-seq,Homo_633,AD.vs.MCI; bulk RNA-seq,Homo_633,MCI.vs.control; bulk RNA-seq,Homo_714,MCI.vs.control	7

ALCAM	bulk RNA-seq,Homo_633,AD.vs.control; bulk RNA-seq,Homo_633,AD.vs.MCI; bulk RNA-seq,Homo_714,AD.vs.control; bulk RNA-seq,Homo_714,AD.vs.MCI; scRNA-seq,SRP215507,CD8+ T cell_3-AD.vs.control; scRNA-seq,SRP215507,CD8+ T cell_2-AD.vs.MCI; scRNA-seq,SRP215507,CD8+ T cell_4-MCI.vs.control; scRNA-seq,SRP309935,Natural killer cell_1-AD.vs.control; scRNA-seq,SRP330776,Memory T cell_2-	10
ALDH16A1	bulk RNA-seq,Homo_723,AD.vs.control; bulk RNA-seq,Homo_723,AD.vs.MCI; bulk RNA-seq,Homo_723,MCI.vs.control; bulk RNA-seq,Homo_633,AD.vs.control; bulk RNA-seq,Homo_633,AD.vs.MCI; bulk RNA-seq,Homo_633,MCI.vs.control; scRNA-seq,SRP215507,CD8+ T cell_3-AD.vs.MCI; scRNA-seq,SRP330776,CD8+ T cell_1-AD.vs.control; scRNA-seq,SRP330776,γδ T cell-AD.vs.control; scRNA-seq,SRP330776,Memory T cell_2-AD.vs.control; scRNA-seq,SRP330776,CD8+ T cell_2-AD.vs.control; scRNA-seq,SRP330776,B cell_1-AD.vs.control; scRNA-seq,SRP330776,Naive CD8+ T cell_1-AD.vs.control; scRNA-seq,SRP330776,Natural killer cell_1-AD.vs.control; scRNA-seq,SRP330776,Memory T cell_1-AD.vs.control; scRNA-seq,SRP330776,Natural killer T cell-AD.vs.control; scRNA-seq,SRP330776,Naive CD8+ T cell_3-AD.vs.control; scRNA-seq,SRP330776,B cell_2-AD.vs.control; scRNA-seq,SRP330776,Naive CD8+ T cell_2-AD.vs.control; scRNA-seq,SRP330776,CD8+ T cell_3-AD.vs.control; scRNA-seq,SRP330776,Monocyte_1-AD.vs.control; scRNA-seq,SRP330776,Natural killer cell_2-AD.vs.control; scRNA-seq,SRP330776,B [illegible]	23
ALDH18A1	bulk RNA-seq,Homo_633,AD.vs.control; bulk RNA-seq,Homo_633,AD.vs.MCI; bulk RNA-seq,Homo_714,AD.vs.control; bulk RNA-seq,Homo_714,AD.vs.MCI; scRNA-seq,SRP330776,Natural killer T cell-AD.vs.control; scRNA-seq,SRP330776,Naive CD8+ T cell_3-AD.vs.control	6
ALDH1A1	bulk RNA-seq,Homo_723,AD.vs.control; bulk RNA-seq,Homo_723,AD.vs.MCI; bulk RNA-seq,Homo_633,AD.vs.control; bulk RNA-seq,Homo_633,AD.vs.MCI; bulk RNA-seq,Homo_714,AD.vs.control; bulk RNA-seq,Homo_714,AD.vs.MCI; bulk RNA-seq,Homo_714,MCI.vs.control	7
Aldh1a1	bulk RNA-seq,SRP325058,AD.vs.control	1
ALDH1A2	bulk RNA-seq,SRP223445,AD.vs.control	1
ALDH1A3	bulk RNA-seq,Homo_723,AD.vs.control; bulk RNA-seq,Homo_723,AD.vs.MCI; bulk RNA-seq,Homo_723,MCI.vs.control; bulk RNA-seq,Homo_633,AD.vs.control; bulk RNA-seq,Homo_633,AD.vs.MCI; bulk RNA-seq,Homo_633,MCI.vs.control	6
ALDH1B1	scRNA-seq,SRP215507,CD8+ T cell_3-AD.vs.control; scRNA-seq,SRP215507,CD8+ T cell_1-AD.vs.control; scRNA-seq,SRP215507,CD8+ T cell_3-AD.vs.MCI; scRNA-seq,SRP330776,Memory T cell_2-AD.vs.control	4
ALDH1L1	bulk RNA-seq,Homo_723,AD.vs.control; bulk RNA-seq,Homo_723,AD.vs.MCI; bulk RNA-seq,Homo_723,MCI.vs.control; bulk RNA-seq,Homo_633,AD.vs.MCI; bulk RNA-seq,ROSMAP,AD.vs.control	5
ALDH1L2	bulk RNA-seq,Homo_723,AD.vs.control; bulk RNA-seq,Homo_723,AD.vs.MCI; bulk RNA-seq,Homo_633,AD.vs.control; bulk RNA-seq,Homo_633,AD.vs.MCI; bulk RNA-seq,Homo_714,AD.vs.control; bulk RNA-seq,Homo_714,AD.vs.MCI; bulk RNA-seq,Homo_714,MCI.vs.control;	8
ALDH2	scRNA-seq,SRP215507,CD8+ T cell_9-AD.vs.control; scRNA-seq,SRP330776,Naive CD8+ T cell_3-AD.vs.control; scRNA-seq,SRP330776,B cell_2-	3
ALDH3A1	bulk RNA-seq,Homo_714,MCI.vs.control	1
ALDH3A2	scRNA-seq,SRP215507,CD8+ T cell_4-AD.vs.MCI; scRNA-seq,SRP309935,Monocyte_2-AD.vs.control; scRNA-seq,SRP309935,B cell_1-AD.vs.control; scRNA-seq,SRP330776,γδ T cell-AD.vs.control; scRNA-seq,SRP330776,B cell_3-AD.vs.control	5
ALDH3B1	bulk RNA-seq,Homo_633,AD.vs.control; bulk RNA-seq,Homo_633,AD.vs.MCI; bulk RNA-seq,Homo_714,AD.vs.control; bulk RNA-seq,Homo_714,AD.vs.MCI; bulk RNA-seq,Homo_714,MCI.vs.control; scRNA-seq,SRP330776,Naive CD8+ T cell_2-AD.vs.control; scRNA-seq,SRP330776,Monocyte_1-AD.vs.control; scRNA-seq,SRP330776,Natural killer cell_2-AD.vs.control	8
ALDH3B2	bulk RNA-seq,Homo_723,AD.vs.control; bulk RNA-seq,Homo_633,AD.vs.control	2
Aldh3b2	bulk RNA-seq,SRP325058,AD.vs.control	1
ALDH4A1	bulk RNA-seq,Homo_714,AD.vs.control; bulk RNA-seq,Homo_714,AD.vs.MCI; scRNA-seq,SRP330776,γδ T cell-AD.vs.control; scRNA-seq,SRP330776,B cell_1-AD.vs.control	4

ALDH5A1	bulk RNA-seq,Homo_723,AD.vs.control; bulk RNA-seq,Homo_723,AD.vs.MCI; bulk RNA-seq,Homo_633,AD.vs.control; bulk RNA-seq,Homo_633,AD.vs.MCI; scRNA-seq,SRP215507,CD8+ T cell_4-AD.vs.control; scRNA-seq,SRP215507,CD8+ T cell_1-AD.vs.MCI; scRNA-seq,SRP215507,CD8+ T cell_5-AD.vs.MCI; scRNA-seq,SRP215507,CD8+ T cell_1-MCI.vs.control; scRNA-seq,SRP215507,CD8+ T cell_5-MCI.vs.control; scRNA-seq,SRP309935,Natural killer cell_1-AD.vs.control; scRNA-seq,SRP330776,CD8+ T cell_1-AD.vs.control; scRNA-seq,SRP330776,γδ T cell-AD.vs.control; scRNA-seq,SRP330776,Memory T cell_2-AD.vs.control; scRNA-seq,SRP330776,CD8+ T cell_2-AD.vs.control; scRNA-seq,SRP330776,B cell_1-AD.vs.control; scRNA-seq,SRP330776,Naive CD8+ T cell_1-AD.vs.control; scRNA-seq,SRP330776,Memory T cell_1-AD.vs.control; scRNA-seq,SRP330776,Natural killer T cell-AD.vs.control; scRNA-seq,SRP330776,Naive CD8+ T cell_3-AD.vs.control; scRNA-seq,SRP330776,B cell_2-AD.vs.control; scRNA-seq,SRP330776,CD8+ T cell_3-AD.vs.control; scRNA-seq,SRP330776,Regulatory T cell-AD.vs.control; [illegible]	23
ALDH6A1	bulk RNA-seq,Homo_723,AD.vs.control; bulk RNA-seq,Homo_723,AD.vs.MCI; bulk RNA-seq,Homo_633,AD.vs.control; bulk RNA-seq,Homo_633,AD.vs.MCI; bulk RNA-seq,SRP223445,AD.vs.control; scRNA-seq,SRP330776,γδ T cell-AD.vs.control	6
ALDH7A1	bulk RNA-seq,Homo_723,AD.vs.control; bulk RNA-seq,Homo_723,AD.vs.MCI; bulk RNA-seq,Homo_723,MCI.vs.control; bulk RNA-seq,Homo_633,AD.vs.control; bulk RNA-seq,Homo_633,AD.vs.MCI; bulk RNA-seq,Homo_714,AD.vs.control; bulk RNA-seq,Homo_714,AD.vs.MCI; bulk RNA-seq,ROSMAP,AD.vs.control; bulk RNA-seq,ROSMAP,MCI.vs.control; scRNA-seq,SRP215507,CD8+ T cell_2-AD.vs.control; scRNA-seq,SRP330776,CD8+ T cell_1-AD.vs.control; scRNA-seq,SRP330776,Memory T cell_2-AD.vs.control; scRNA-seq,SRP330776,B cell_1-AD.vs.control;	14
ALDH8A1	scRNA-seq,SRP215507,CD8+ T cell_1-AD.vs.MCI; scRNA-seq,SRP215507,CD8+ T cell_1-MCI.vs.control	2
ALDH9A1	bulk RNA-seq,Homo_723,AD.vs.control; bulk RNA-seq,Homo_723,AD.vs.MCI; bulk RNA-seq,Homo_633,AD.vs.control; bulk RNA-seq,Homo_633,AD.vs.MCI; bulk RNA-seq,Homo_714,AD.vs.control; bulk RNA-seq,Homo_714,AD.vs.MCI; scRNA-seq,SRP215507,CD8+ T cell_3-AD.vs.MCI; scRNA-seq,SRP215507,CD8+ T cell_4-MCI.vs.control; scRNA-seq,SRP309935,Naive CD8+ T cell_2-AD.vs.control; scRNA-seq,SRP309935,B cell_1-AD.vs.control; scRNA-seq,SRP309935,B cell_2-AD.vs.control; scRNA-seq,SRP330776,CD8+ T cell_1-AD.vs.control; scRNA-seq,SRP330776,Naive CD8+ T cell_3-AD.vs.control; scRNA-seq,SRP330776,B cell_2-AD.vs.control; scRNA-seq,SRP330776,B cell_4-AD.vs.control	15
ALDOA	scRNA-seq,SRP215507,CD8+ T cell_7-AD.vs.control; scRNA-seq,SRP215507,CD8+ T cell_10-AD.vs.control; scRNA-seq,SRP215507,CD8+ T cell_9-AD.vs.control; scRNA-seq,SRP330776,Megakaryocyte_1-AD.vs.control	4
ALDOB	bulk RNA-seq,Homo_723,AD.vs.control; bulk RNA-seq,Homo_723,AD.vs.MCI; bulk RNA-seq,Homo_723,MCI.vs.control; bulk RNA-seq,Homo_633,AD.vs.control; bulk RNA-seq,Homo_633,AD.vs.MCI; bulk RNA-seq,Homo_633,MCI.vs.control; bulk RNA-seq,Homo_714,MCI.vs.control	7
ALDOC	bulk RNA-seq,Homo_633,AD.vs.control; scRNA-seq,SRP215507,CD8+ T cell_5-AD.vs.control; scRNA-seq,SRP215507,CD8+ T cell_3-AD.vs.MCI; scRNA-seq,SRP215507,CD8+ T cell_1-AD.vs.MCI; scRNA-seq,SRP215507,CD8+ T cell_3-MCI.vs.control; scRNA-seq,SRP309935,Natural killer cell_2-AD.vs.control; scRNA-seq,SRP330776,γδ T cell-AD.vs.control; scRNA-seq,SRP330776,Naive CD8+ T cell_1-AD.vs.control; scRNA-seq,SRP330776,Natural killer cell_1-AD.vs.control; scRNA-seq,SRP330776,B cell_2-AD.vs.control; scRNA-seq,SRP330776,T cell-AD.vs.control; scRNA-	12
ALG1	scRNA-seq,SRP215507,CD8+ T cell_5-AD.vs.MCI; scRNA-seq,SRP330776,CD8+ T cell_1-AD.vs.control; scRNA-seq,SRP330776,γδ T cell-AD.vs.control; scRNA-seq,SRP330776,Natural killer T cell-AD.vs.control; scRNA-seq,SRP330776,Naive CD8+ T cell_3-AD.vs.control; scRNA-seq,SRP330776,B cell_2-AD.vs.control; scRNA-seq,SRP330776,T cell-AD.vs.control; scRNA-seq,SRP330776,B cell_3-AD.vs.control; scRNA-	9
ALG10	scRNA-seq,SRP215507,CD8+ T cell_1-AD.vs.control; scRNA-seq,SRP330776,Natural killer cell_1-AD.vs.control	2
ALG10B	scRNA-seq,SRP215507,CD8+ T cell_3-AD.vs.MCI; scRNA-seq,SRP215507,CD8+ T cell_1-AD.vs.MCI; scRNA-seq,SRP215507,CD8+ T cell_3-MCI.vs.control; scRNA-seq,SRP330776,B cell_2-AD.vs.control; scRNA-seq,SRP330776,B cell_3-AD.vs.control	5
ALG11	scRNA-seq,SRP215507,CD8+ T cell_5-AD.vs.control; scRNA-seq,SRP215507,CD8+ T cell_5-AD.vs.MCI; scRNA-seq,SRP215507,CD8+ T cell_4-MCI.vs.control; scRNA-seq,SRP330776,B cell_1-AD.vs.control; scRNA-seq,SRP330776,Natural killer T cell-AD.vs.control; scRNA-seq,SRP330776,Naive CD8+ T cell_3-AD.vs.control; scRNA-seq,SRP330776,Naive CD8+ T cell_2-AD.vs.control; scRNA-seq,SRP330776,B cell_3-	8
ALG12	bulk RNA-seq,Homo_723,AD.vs.control; bulk RNA-seq,Homo_723,AD.vs.MCI; bulk RNA-seq,Homo_723,MCI.vs.control; bulk RNA-seq,Homo_633,AD.vs.control; bulk RNA-seq,Homo_633,AD.vs.MCI; bulk RNA-seq,Homo_633,MCI.vs.control; bulk RNA-seq,Homo_714,AD.vs.control; bulk RNA-seq,Homo_714,AD.vs.MCI; scRNA-seq,SRP215507,CD8+ T cell_7-AD.vs.control; scRNA-seq,SRP215507,CD8+ T cell_3-AD.vs.MCI; scRNA-seq,SRP215507,CD8+ T cell_3-MCI.vs.control; scRNA-seq,SRP330776,γδ T cell-AD.vs.control; scRNA-seq,SRP330776,B cell_1-AD.vs.control;	14

ALG13	scRNA-seq,SRP215507,CD8+ T cell_6-AD.vs.control; scRNA-seq,SRP215507,CD8+ T cell_1-AD.vs.MCI; scRNA-seq,SRP215507,CD8+ T cell_5-AD.vs.MCI; scRNA-seq,SRP215507,CD8+ T cell_1-MCI.vs.control; scRNA-seq,SRP215507,CD8+ T cell_5-MCI.vs.control; scRNA-seq,SRP215507,CD8+ T cell_2-MCI.vs.control; scRNA-seq,SRP215507,CD8+ T cell_3-MCI.vs.control; scRNA-seq,SRP309935,Naive CD8+ T cell_1-AD.vs.control; scRNA-seq,SRP309935,CD8+ T cell_2-AD.vs.control; scRNA-seq,SRP309935,B cell_2-AD.vs.control; scRNA-seq,SRP330776,B cell_3-	11
Alg14	bulk RNA-seq,SRP325058,AD.vs.control	1
ALG14	scRNA-seq,SRP215507,CD8+ T cell_6-AD.vs.control; scRNA-seq,SRP309935,Natural killer cell_2-AD.vs.control; scRNA-seq,SRP330776,Memory T cell_2-AD.vs.control; scRNA-seq,SRP330776,CD8+ T cell_2-AD.vs.control; scRNA-seq,SRP330776,Natural killer cell_1-AD.vs.control; scRNA-seq,SRP330776,Memory T cell_1-AD.vs.control; scRNA-seq,SRP330776,B cell_2-AD.vs.control; scRNA-seq,SRP330776,T cell-AD.vs.control; scRNA-	9
ALG1L	scRNA-seq,SRP215507,CD8+ T cell_1-AD.vs.control; scRNA-seq,SRP215507,CD8+ T cell_1-AD.vs.MCI; scRNA-seq,SRP330776,CD8+ T cell_1-AD.vs.control; scRNA-seq,SRP330776,Naive CD8+ T cell_1-AD.vs.control; scRNA-seq,SRP330776,Memory T cell_1-AD.vs.control	5
ALG1L2	bulk RNA-seq,Homo_723,AD.vs.control; bulk RNA-seq,Homo_723,AD.vs.MCI; bulk RNA-seq,Homo_633,AD.vs.control; bulk RNA-seq,Homo_633,AD.vs.MCI; scRNA-seq,SRP215507,CD8+ T cell_1-AD.vs.MCI; scRNA-seq,SRP215507,CD8+ T cell_1-MCI.vs.control; scRNA-seq,SRP215507,CD8+ T cell_3-MCI.vs.control; scRNA-seq,SRP330776,B cell_1-AD.vs.control; scRNA-seq,SRP330776,Naive CD8+ T cell_1-	10
ALG1L9P	scRNA-seq,SRP330776,γδ T cell-AD.vs.control; scRNA-seq,SRP330776,Naive CD8+ T cell_1-AD.vs.control	2
ALG2	scRNA-seq,SRP215507,CD8+ T cell_3-AD.vs.control; scRNA-seq,SRP215507,CD8+ T cell_1-AD.vs.MCI; scRNA-seq,SRP215507,CD8+ T cell_4-AD.vs.MCI; scRNA-seq,SRP215507,CD8+ T cell_5-AD.vs.MCI; scRNA-seq,SRP215507,CD8+ T cell_1-MCI.vs.control; scRNA-seq,SRP215507,CD8+ T cell_5-MCI.vs.control; scRNA-seq,SRP215507,CD8+ T cell_4-MCI.vs.control; scRNA-seq,SRP309935,Natural killer cell_2-AD.vs.control; scRNA-	9
ALG3	bulk RNA-seq,Homo_723,AD.vs.MCI; bulk RNA-seq,Homo_723,MCI.vs.control; bulk RNA-seq,Homo_633,AD.vs.control; bulk RNA-seq,Homo_633,AD.vs.MCI; bulk RNA-seq,Homo_633,MCI.vs.control; bulk RNA-seq,Homo_714,AD.vs.control; bulk RNA-seq,Homo_714,AD.vs.MCI; bulk RNA-seq,Homo_714,MCI.vs.control; scRNA-seq,SRP215507,CD8+ T cell_5-AD.vs.MCI; scRNA-seq,SRP330776,CD8+ T cell_1-AD.vs.control; scRNA-seq,SRP330776,γδ T cell-AD.vs.control; scRNA-seq,SRP330776,Memory T cell_2-AD.vs.control; scRNA-seq,SRP330776,CD8+ T cell_3-	13
ALG5	bulk RNA-seq,Homo_723,AD.vs.control; bulk RNA-seq,Homo_723,AD.vs.MCI; bulk RNA-seq,Homo_633,AD.vs.control; bulk RNA-seq,Homo_633,AD.vs.MCI; bulk RNA-seq,Homo_714,AD.vs.control; bulk RNA-seq,Homo_714,AD.vs.MCI; scRNA-seq,SRP215507,CD8+ T cell_4-AD.vs.MCI; scRNA-seq,SRP215507,CD8+ T cell_4-MCI.vs.control; scRNA-seq,SRP330776,γδ T cell-AD.vs.control; scRNA-seq,SRP330776,Natural killer T cell-AD.vs.control; scRNA-seq,SRP330776,B cell_2-AD.vs.control; scRNA-seq,SRP330776,CD8+ T cell_3-AD.vs.control	12
ALG6	bulk RNA-seq,Homo_723,AD.vs.control; bulk RNA-seq,Homo_723,AD.vs.MCI; bulk RNA-seq,Homo_633,AD.vs.control; bulk RNA-seq,Homo_633,AD.vs.MCI; bulk RNA-seq,Homo_714,AD.vs.control; bulk RNA-seq,Homo_714,AD.vs.MCI; scRNA-seq,SRP215507,CD8+ T cell_7-AD.vs.control; scRNA-seq,SRP330776,Memory T cell_2-AD.vs.control; scRNA-seq,SRP330776,Memory T cell_1-AD.vs.control; scRNA-seq,SRP330776,Natural killer T cell-AD.vs.control; scRNA-seq,SRP330776,CD8+ T cell_3-AD.vs.control; scRNA-seq,SRP330776,Natural killer cell_2-	12
ALG8	bulk RNA-seq,Homo_714,AD.vs.control; bulk RNA-seq,Homo_714,AD.vs.MCI; scRNA-seq,SRP330776,CD8+ T cell_1-AD.vs.control; scRNA-seq,SRP330776,γδ T cell-AD.vs.control; scRNA-seq,SRP330776,Memory T cell_2-AD.vs.control; scRNA-seq,SRP330776,CD8+ T cell_2-AD.vs.control; scRNA-seq,SRP330776,B cell_2-AD.vs.control; scRNA-seq,SRP330776,T cell-AD.vs.control	8
ALG9	bulk RNA-seq,Homo_723,AD.vs.control; bulk RNA-seq,Homo_723,AD.vs.MCI; bulk RNA-seq,Homo_633,AD.vs.control; bulk RNA-seq,Homo_633,AD.vs.MCI; bulk RNA-seq,Homo_714,AD.vs.control; bulk RNA-seq,Homo_714,AD.vs.MCI; bulk RNA-seq,Homo_714,MCI.vs.control; scRNA-seq,SRP309935,Natural killer cell_1-AD.vs.control; scRNA-seq,SRP330776,Naive CD8+ T cell_1-AD.vs.control; scRNA-seq,SRP330776,Natural killer cell_1-AD.vs.control; scRNA-seq,SRP330776,Natural killer T cell-AD.vs.control; scRNA-seq,SRP330776,Naive CD8+ T cell_3-AD.vs.control; scRNA-seq,SRP330776,T cell-AD.vs.control; scRNA-seq,SRP330776,CD8+ T cell_3-AD.vs.control	14
ALG9-IT1	scRNA-seq,SRP215507,CD8+ T cell_2-MCI.vs.control	1
ALK	bulk RNA-seq,Homo_723,AD.vs.control; bulk RNA-seq,Homo_723,AD.vs.MCI; bulk RNA-seq,Homo_714,AD.vs.control; bulk RNA-	4
ALKAL2	bulk RNA-seq,Homo_723,AD.vs.control; bulk RNA-seq,Homo_723,AD.vs.MCI; bulk RNA-seq,Homo_633,AD.vs.control; bulk RNA-seq,Homo_633,AD.vs.MCI; bulk RNA-seq,Homo_714,MCI.vs.control	5

ALKBH1	bulk RNA-seq,Homo_723,AD.vs.control; bulk RNA-seq,Homo_723,AD.vs.MCI; bulk RNA-seq,Homo_633,AD.vs.control; bulk RNA-seq,Homo_633,AD.vs.MCI; bulk RNA-seq,Homo_714,AD.vs.control; bulk RNA-seq,Homo_714,AD.vs.MCI; scRNA-seq,SRP215507,CD8+ T cell_4-AD.vs.MCI; scRNA-seq,SRP215507,CD8+ T cell_5-AD.vs.MCI; scRNA-seq,SRP215507,CD8+ T cell_5-MCI.vs.control; scRNA-seq,SRP215507,CD8+ T cell_4-MCI.vs.control; scRNA-seq,SRP215507,CD8+ T cell_3-MCI.vs.control; scRNA-seq,SRP330776,CD8+ T cell_2-AD.vs.control; scRNA-seq,SRP330776,Natural killer cell_1-AD.vs.control; scRNA-seq,SRP330776,Naive CD8+ T cell_3-AD.vs.control; scRNA-seq,SRP330776,B cell_2-	15
ALKBH2	bulk RNA-seq,Homo_723,AD.vs.control; bulk RNA-seq,Homo_633,AD.vs.control; bulk RNA-seq,Homo_633,AD.vs.MCI; bulk RNA-seq,Homo_714,AD.vs.control; bulk RNA-seq,Homo_714,AD.vs.MCI; scRNA-seq,SRP309935,Natural killer cell_1-AD.vs.control; scRNA-seq,SRP330776,B cell_1-AD.vs.control; scRNA-seq,SRP330776,Naive CD8+ T cell_3-AD.vs.control; scRNA-seq,SRP330776,B cell_2-AD.vs.control; scRNA-seq,SRP330776,T cell-AD.vs.control; scRNA-seq,SRP330776,Natural killer cell_2-AD.vs.control	11
ALKBH3	bulk RNA-seq,Homo_723,AD.vs.control; bulk RNA-seq,Homo_723,AD.vs.MCI; bulk RNA-seq,Homo_633,AD.vs.control; bulk RNA-seq,Homo_633,AD.vs.MCI; bulk RNA-seq,Homo_714,AD.vs.control; bulk RNA-seq,Homo_714,AD.vs.MCI; scRNA-seq,SRP215507,CD8+ T cell_4-AD.vs.MCI; scRNA-seq,SRP215507,CD8+ T cell_4-MCI.vs.control; scRNA-seq,SRP330776,Memory T cell_2-AD.vs.control; scRNA-seq,SRP330776,CD8+ T cell_2-AD.vs.control; scRNA-seq,SRP330776,Natural killer cell_1-AD.vs.control; scRNA-seq,SRP330776,Memory T cell_1-AD.vs.control; scRNA-seq,SRP330776,B cell_2-AD.vs.control; scRNA-seq,SRP330776,T cell-AD.vs.control; scRNA-seq,SRP330776,CD8+ T cell_3-	15
ALKBH4	bulk RNA-seq,Homo_723,AD.vs.control; bulk RNA-seq,Homo_633,AD.vs.control; scRNA-seq,SRP215507,CD8+ T cell_5-AD.vs.control; scRNA-seq,SRP215507,CD8+ T cell_5-MCI.vs.control; scRNA-seq,SRP330776,CD8+ T cell_1-AD.vs.control; scRNA-seq,SRP330776,γδ T cell-AD.vs.control; scRNA-seq,SRP330776,Memory T cell_2-AD.vs.control; scRNA-seq,SRP330776,Memory T cell_1-AD.vs.control; scRNA-seq,SRP330776,Natural killer T cell-AD.vs.control; scRNA-seq,SRP330776,B cell_2-AD.vs.control; scRNA-seq,SRP330776,CD8+ T cell_3-AD.vs.control	11
ALKBH5	bulk RNA-seq,Homo_633,AD.vs.control; bulk RNA-seq,Homo_633,MCI.vs.control; scRNA-seq,SRP215507,CD8+ T cell_7-AD.vs.control; scRNA-seq,SRP215507,CD8+ T cell_10-AD.vs.control; scRNA-seq,SRP215507,CD8+ T cell_7-AD.vs.MCI; scRNA-seq,SRP215507,CD8+ T cell_5-AD.vs.MCI; scRNA-seq,SRP215507,CD8+ T cell_6-AD.vs.MCI; scRNA-seq,SRP309935,Natural killer cell_2-AD.vs.control; scRNA-seq,SRP309935,Naive CD8+ T cell_1-AD.vs.control; scRNA-seq,SRP309935,Natural killer cell_1-AD.vs.control; scRNA-seq,SRP309935,Monocyte_2-AD.vs.control; scRNA-seq,SRP309935,Naive CD8+ T cell_2-AD.vs.control; scRNA-seq,SRP309935,CD8+ T cell_1-AD.vs.control; scRNA-seq,SRP309935,CD8+ T cell_2-AD.vs.control; scRNA-seq,SRP309935,B cell_2-AD.vs.control; scRNA-seq,SRP309935,Naive CD8+ T cell_3-AD.vs.control; scRNA-seq,SRP309935,CD4+ T cell-AD.vs.control; scRNA-seq,SRP330776,CD8+ T cell_1-AD.vs.control; scRNA-seq,SRP330776,γδ T cell-AD.vs.control; scRNA-seq,SRP330776,Memory T cell_2-AD.vs.control; scRNA-seq,SRP330776,CD8+ T cell_2-AD.vs.control; scRNA-seq,SRP330776,B cell_1-AD.vs.control; scRNA-seq,SRP330776,Naive CD8+ T cell_1-AD.vs.control; scRNA-seq,SRP330776,Natural killer cell_1-AD.vs.control; scRNA-seq,SRP330776,Memory T cell_1-AD.vs.control; scRNA-seq,SRP330776,Natural killer T cell-AD.vs.control; scRNA-seq,SRP330776,Naive CD8+ T cell_3-AD.vs.control; scRNA-seq,SRP330776,B cell_2-AD.vs.control; scRNA-seq,SRP330776,T cell-AD.vs.control; scRNA-seq,SRP330776,Naive CD8+ T cell_2-AD.vs.control; scRNA-seq,SRP330776,CD8+ T cell_3-AD.vs.control; scRNA-seq,SRP330776,Monocyte_1-AD.vs.control; scRNA-seq,SRP330776,Monocyte_2-AD.vs.control; scRNA-seq,SRP330776,B cell_3-AD.vs.control; scRNA-seq,SRP330776,Regulatory T cell-AD.vs.control;	37
ALKBH6	bulk RNA-seq,Homo_723,AD.vs.control; bulk RNA-seq,Homo_723,AD.vs.MCI; bulk RNA-seq,Homo_633,AD.vs.control; bulk RNA-seq,Homo_633,AD.vs.MCI; bulk RNA-seq,Homo_714,AD.vs.control; bulk RNA-seq,Homo_714,AD.vs.MCI; scRNA-seq,SRP215507,CD8+ T cell_4-AD.vs.MCI; scRNA-seq,SRP215507,CD8+ T cell_5-AD.vs.MCI; scRNA-seq,SRP215507,CD8+ T cell_5-MCI.vs.control; scRNA-seq,SRP215507,CD8+ T cell_4-MCI.vs.control; scRNA-seq,SRP215507,CD8+ T cell_3-MCI.vs.control; scRNA-seq,SRP309935,Monocyte_1-AD.vs.control; scRNA-seq,SRP309935,Naive CD8+ T cell_1-AD.vs.control; scRNA-seq,SRP309935,Naive CD8+ T cell_2-AD.vs.control; scRNA-seq,SRP309935,CD8+ T cell_1-AD.vs.control; scRNA-seq,SRP309935,CD8+ T cell_2-AD.vs.control; scRNA-seq,SRP330776,CD8+ T cell_1-AD.vs.control; scRNA-seq,SRP330776,γδ T cell-AD.vs.control; scRNA-seq,SRP330776,Memory T cell_2-AD.vs.control; scRNA-seq,SRP330776,CD8+ T cell_2-AD.vs.control; scRNA-seq,SRP330776,B cell_1-AD.vs.control; scRNA-seq,SRP330776,Naive CD8+ T cell_1-AD.vs.control; scRNA-seq,SRP330776,Natural killer cell_1-AD.vs.control; scRNA-seq,SRP330776,Memory T cell_1-AD.vs.control; scRNA-seq,SRP330776,Natural killer T cell-AD.vs.control; scRNA-seq,SRP330776,Naive CD8+ T cell_3-AD.vs.control; scRNA-seq,SRP330776,B cell_2-AD.vs.control; scRNA-seq,SRP330776,T cell-AD.vs.control; scRNA-seq,SRP330776,CD8+ T cell_3-AD.vs.control; scRNA-seq,SRP330776,Monocyte_1-AD.vs.control; scRNA-seq,SRP330776,Regulatory T cell-AD.vs.control; scRNA-seq,SRP330776,Natural killer cell_2-AD.vs.control; scRNA-seq,SRP330776,B cell_4-AD.vs.control	33
ALKBH7	scRNA-seq,SRP215507,CD8+ T cell_7-AD.vs.control; scRNA-seq,SRP215507,CD8+ T cell_5-AD.vs.MCI; scRNA-seq,SRP215507,CD8+ T cell_5-	3

ALKBH8	bulk RNA-seq,Homo_723,AD.vs.MCI; bulk RNA-seq,Homo_633,AD.vs.MCI; bulk RNA-seq,Homo_714,AD.vs.control; bulk RNA-seq,Homo_714,AD.vs.MCI; scRNA-seq,SRP215507,CD8+ T cell_4-AD.vs.MCI; scRNA-seq,SRP215507,CD8+ T cell_5-AD.vs.MCI; scRNA-seq,SRP215507,CD8+ T cell_3-MCI.vs.control; scRNA-seq,SRP330776,γδ T cell-AD.vs.control; scRNA-seq,SRP330776,Memory T cell_2-AD.vs.control; scRNA-seq,SRP330776,B cell_1-AD.vs.control; scRNA-seq,SRP330776,Naive CD8+ T cell_1-AD.vs.control; scRNA-seq,SRP330776,Natural killer T cell-AD.vs.control; scRNA-seq,SRP330776,B cell_2-AD.vs.control; scRNA-seq,SRP330776,T cell-AD.vs.control; scRNA-seq,SRP330776,Naive CD8+ T	16
ALLC	bulk RNA-seq,Homo_723,MCI.vs.control; bulk RNA-seq,Homo_714,MCI.vs.control	2
ALMS1	scRNA-seq,SRP309935,Monocyte_1-AD.vs.control	1
ALMS1-IT1	scRNA-seq,SRP215507,CD8+ T cell_2-AD.vs.MCI; scRNA-seq,SRP330776,CD8+ T cell_1-AD.vs.control	2
ALOX12-AS1	scRNA-seq,SRP215507,CD8+ T cell_4-AD.vs.MCI; scRNA-seq,SRP215507,CD8+ T cell_7-AD.vs.MCI; scRNA-seq,SRP215507,CD8+ T cell_5-AD.vs.MCI; scRNA-seq,SRP215507,CD8+ T cell_7-MCI.vs.control; scRNA-seq,SRP330776,γδ T cell-AD.vs.control; scRNA-seq,SRP330776,Naive CD8+ T cell_1-AD.vs.control; scRNA-seq,SRP330776,Memory T cell_1-AD.vs.control; scRNA-seq,SRP330776,Natural killer T cell-AD.vs.control; scRNA-seq,SRP330776,T cell-AD.vs.control; scRNA-seq,SRP330776,Naive CD8+ T cell_2-AD.vs.control; scRNA-seq,SRP330776,CD8+ T cell_3-AD.vs.control; scRNA-seq,SRP330776,Monocyte_1-AD.vs.control; scRNA-seq,SRP330776,Monocyte_2-AD.vs.control; scRNA-seq,SRP330776,B cell_3-AD.vs.control	14
ALOX12B	bulk RNA-seq,Homo_723,AD.vs.control; bulk RNA-seq,Homo_633,AD.vs.control; bulk RNA-seq,Homo_714,MCI.vs.control; scRNA-seq,SRP215507,CD8+ T cell_3-AD.vs.MCI; scRNA-seq,SRP215507,CD8+ T cell_3-MCI.vs.control; scRNA-seq,SRP330776,CD8+ T cell_1-AD.vs.control; scRNA-seq,SRP330776,Naive CD8+ T cell_1-AD.vs.control; scRNA-seq,SRP330776,B cell_2-AD.vs.control	8
ALOX15	bulk RNA-seq,ROSMAP,AD.vs.MCI	1
ALOX15B	bulk RNA-seq,Homo_714,AD.vs.control; bulk RNA-seq,Homo_714,AD.vs.MCI	2
ALOX5	scRNA-seq,SRP215507,CD8+ T cell_3-AD.vs.control; scRNA-seq,SRP215507,CD8+ T cell_2-MCI.vs.control; scRNA-seq,SRP215507,CD8+ T cell_3-MCI.vs.control; scRNA-seq,SRP330776,Memory T cell_2-AD.vs.control	4
ALOX5AP	bulk RNA-seq,SRP223445,AD.vs.control; scRNA-seq,SRP215507,CD8+ T cell_1-AD.vs.MCI; scRNA-seq,SRP215507,CD8+ T cell_4-AD.vs.MCI; scRNA-seq,SRP215507,CD8+ T cell_1-MCI.vs.control; scRNA-seq,SRP215507,CD8+ T cell_4-MCI.vs.control; scRNA-seq,SRP309935,Megakaryocyte_2-AD.vs.control; scRNA-seq,SRP330776,CD8+ T cell_1-AD.vs.control; scRNA-seq,SRP330776,γδ T cell-AD.vs.control; scRNA-seq,SRP330776,CD8+ T cell_2-AD.vs.control; scRNA-seq,SRP330776,Naive CD8+ T cell_1-AD.vs.control; scRNA-seq,SRP330776,Natural killer cell_1-AD.vs.control; scRNA-seq,SRP330776,Memory T cell_1-AD.vs.control; scRNA-seq,SRP330776,Naive CD8+ T cell_3-AD.vs.control; scRNA-seq,SRP330776,Regulatory T cell-AD.vs.control; scRNA-seq,SRP330776,Megakaryocyte_1-AD.vs.control	15
ALOXE3	scRNA-seq,SRP215507,CD8+ T cell_3-AD.vs.MCI	1
ALPG	bulk RNA-seq,Homo_723,AD.vs.control; bulk RNA-seq,Homo_633,AD.vs.control	2
ALPK1	scRNA-seq,SRP215507,CD8+ T cell_4-AD.vs.control; scRNA-seq,SRP215507,CD8+ T cell_3-AD.vs.MCI; scRNA-seq,SRP215507,CD8+ T cell_3-MCI.vs.control; scRNA-seq,SRP330776,Memory T cell_2-AD.vs.control; scRNA-seq,SRP330776,B cell_4-AD.vs.control	5
ALPK2	bulk RNA-seq,Homo_723,AD.vs.control; bulk RNA-seq,Homo_723,AD.vs.MCI; bulk RNA-seq,Homo_723,MCI.vs.control; bulk RNA-seq,Homo_633,AD.vs.control; bulk RNA-seq,Homo_633,AD.vs.MCI; bulk RNA-seq,Homo_633,MCI.vs.control; bulk RNA-seq,Homo_714,AD.vs.control; bulk RNA-seq,Homo_714,AD.vs.MCI; scRNA-seq,SRP215507,CD8+ T cell_1-AD.vs.control; scRNA-seq,SRP215507,CD8+ T cell_1-AD.vs.MCI; scRNA-seq,SRP215507,CD8+ T cell_5-AD.vs.MCI; scRNA-seq,SRP215507,CD8+ T cell_1-MCI.vs.control; scRNA-seq,SRP215507,CD8+ T cell_5-MCI.vs.control; scRNA-seq,SRP330776,CD8+ T cell_1-AD.vs.control; scRNA-seq,SRP330776,γδ T cell-AD.vs.control; scRNA-seq,SRP330776,CD8+ T	16
ALPK3	bulk RNA-seq,Homo_723,AD.vs.control; bulk RNA-seq,Homo_723,AD.vs.MCI; bulk RNA-seq,Homo_723,MCI.vs.control; bulk RNA-seq,Homo_633,AD.vs.control; bulk RNA-seq,Homo_633,AD.vs.MCI; bulk RNA-seq,Homo_633,MCI.vs.control; bulk RNA-seq,Homo_714,AD.vs.control; bulk RNA-seq,Homo_714,AD.vs.MCI; bulk RNA-seq,ROSMAP,AD.vs.control	9
ALPL	bulk RNA-seq,Homo_723,AD.vs.control; bulk RNA-seq,Homo_633,AD.vs.control; bulk RNA-seq,SRP223445,AD.vs.control; scRNA-seq,SRP330776,B	4
ALPP	bulk RNA-seq,Homo_714,MCI.vs.control	1

ALS2	bulk RNA-seq,Homo_723,AD.vs.control; bulk RNA-seq,Homo_723,AD.vs.MCI; bulk RNA-seq,Homo_723,MCI.vs.control; bulk RNA-seq,Homo_633,AD.vs.MCI; bulk RNA-seq,Homo_633,MCI.vs.control; bulk RNA-seq,Homo_714,AD.vs.control; bulk RNA-seq,Homo_714,AD.vs.MCI; bulk RNA-seq,Homo_714,MCI.vs.control; scRNA-seq,SRP215507,CD8+ T cell_4-AD.vs.control; scRNA-seq,SRP215507,CD8+ T cell_2-AD.vs.MCI; scRNA-seq,SRP215507,CD8+ T cell_4-AD.vs.MCI; scRNA-seq,SRP330776,CD8+ T cell_1-AD.vs.control; scRNA-seq,SRP330776,CD8+ T cell_2-AD.vs.control; scRNA-seq,SRP330776,Naive CD8+ T cell_1-AD.vs.control; scRNA-seq,SRP330776,Natural killer cell_1-AD.vs.control; scRNA-seq,SRP330776,Memory T cell_1-AD.vs.control; scRNA-seq,SRP330776,Naive CD8+ T cell_3-AD.vs.control; scRNA-seq,SRP330776,Monocyte_1-	18
ALS2CL	bulk RNA-seq,Homo_723,AD.vs.control; bulk RNA-seq,Homo_633,AD.vs.control; bulk RNA-seq,Homo_714,MCI.vs.control; bulk RNA-seq,ROSMAP,AD.vs.control; scRNA-seq,SRP215507,CD8+ T cell_2-AD.vs.control; scRNA-seq,SRP330776,γδ T cell-AD.vs.control; scRNA-	7
ALX3	bulk RNA-seq,Homo_723,AD.vs.MCI; bulk RNA-seq,Homo_723,MCI.vs.control; bulk RNA-seq,Homo_633,AD.vs.control; bulk RNA-seq,Homo_633,AD.vs.MCI; bulk RNA-seq,Homo_633,MCI.vs.control; bulk RNA-seq,Homo_714,AD.vs.control; bulk RNA-seq,Homo_714,AD.vs.MCI;	8
ALX4	bulk RNA-seq,Homo_723,AD.vs.control; bulk RNA-seq,Homo_723,AD.vs.MCI; bulk RNA-seq,Homo_714,MCI.vs.control	3
ALYREF	scRNA-seq,SRP215507,CD8+ T cell_2-AD.vs.MCI; scRNA-seq,SRP215507,CD8+ T cell_2-MCI.vs.control; scRNA-seq,SRP330776,CD8+ T cell_1-AD.vs.control; scRNA-seq,SRP330776,Memory T cell_2-AD.vs.control; scRNA-seq,SRP330776,CD8+ T cell_2-AD.vs.control; scRNA-seq,SRP330776,Naive CD8+ T cell_1-AD.vs.control; scRNA-seq,SRP330776,Memory T cell_1-AD.vs.control; scRNA-seq,SRP330776,Natural killer T cell-AD.vs.control; scRNA-seq,SRP330776,B cell_2-AD.vs.control; scRNA-seq,SRP330776,T cell-AD.vs.control; scRNA-seq,SRP330776,Monocyte_2-	11
AMACR	bulk RNA-seq,Homo_723,AD.vs.control; bulk RNA-seq,Homo_723,AD.vs.MCI; bulk RNA-seq,Homo_633,AD.vs.control; bulk RNA-seq,Homo_633,AD.vs.MCI; bulk RNA-seq,Homo_714,AD.vs.control; bulk RNA-seq,Homo_714,AD.vs.MCI; scRNA-seq,SRP215507,CD8+ T cell_2-AD.vs.MCI; scRNA-seq,SRP215507,CD8+ T cell_3-AD.vs.MCI; scRNA-seq,SRP215507,CD8+ T cell_1-AD.vs.MCI; scRNA-seq,SRP215507,CD8+ T cell_5-AD.vs.MCI; scRNA-seq,SRP215507,CD8+ T cell_2-MCI.vs.control; scRNA-seq,SRP330776,CD8+ T cell_1-AD.vs.control; scRNA-seq,SRP330776,γδ T cell-AD.vs.control; scRNA-seq,SRP330776,Memory T cell_2-AD.vs.control; scRNA-seq,SRP330776,CD8+ T cell_2-AD.vs.control; scRNA-seq,SRP330776,B cell_1-AD.vs.control; scRNA-seq,SRP330776,Naive CD8+ T cell_1-AD.vs.control	17
AMBN	bulk RNA-seq,Homo_723,AD.vs.control; bulk RNA-seq,Homo_723,AD.vs.MCI	2
AMBP	bulk RNA-seq,Homo_723,AD.vs.MCI; bulk RNA-seq,Homo_723,MCI.vs.control; bulk RNA-seq,Homo_633,AD.vs.MCI	3
AMBRA1	scRNA-seq,SRP215507,CD8+ T cell_4-AD.vs.control; scRNA-seq,SRP309935,CD8+ T cell_2-AD.vs.control; scRNA-seq,SRP330776,B cell_2-AD.vs.control; scRNA-seq,SRP330776,Regulatory T cell-AD.vs.control	4
AMD1	scRNA-seq,SRP215507,CD8+ T cell_7-AD.vs.control; scRNA-seq,SRP215507,CD8+ T cell_1-AD.vs.MCI; scRNA-seq,SRP215507,CD8+ T cell_5-AD.vs.MCI; scRNA-seq,SRP215507,CD8+ T cell_1-MCI.vs.control	4
AMDHD1	bulk RNA-seq,Homo_723,AD.vs.control; bulk RNA-seq,Homo_723,AD.vs.MCI; bulk RNA-seq,Homo_633,AD.vs.control; bulk RNA-seq,Homo_633,AD.vs.MCI; bulk RNA-seq,Homo_714,AD.vs.control; bulk RNA-seq,Homo_714,AD.vs.MCI; bulk RNA-seq,ROSMAP,MCI.vs.control; scRNA-seq,SRP215507,CD8+ T cell_2-AD.vs.MCI; scRNA-seq,SRP215507,CD8+ T cell_5-MCI.vs.control; scRNA-seq,SRP215507,CD8+ T cell_4-MCI.vs.control; scRNA-seq,SRP215507,CD8+ T cell_2-MCI.vs.control; scRNA-seq,SRP330776,CD8+ T cell_1-AD.vs.control; scRNA-	14
AMDHD2	scRNA-seq,SRP215507,CD8+ T cell_5-AD.vs.MCI; scRNA-seq,SRP330776,γδ T cell-AD.vs.control; scRNA-seq,SRP330776,Memory T cell_2-AD.vs.control; scRNA-seq,SRP330776,CD8+ T cell_2-AD.vs.control; scRNA-seq,SRP330776,Natural killer cell_1-AD.vs.control; scRNA-seq,SRP330776,Memory T cell_1-AD.vs.control; scRNA-seq,SRP330776,Naive CD8+ T cell_3-AD.vs.control; scRNA-seq,SRP330776,CD8+ T cell_3-AD.vs.control; scRNA-seq,SRP330776,Monocyte_1-AD.vs.control; scRNA-seq,SRP330776,Natural killer cell_2-AD.vs.control	10
AMELX	bulk RNA-seq,Homo_723,AD.vs.control; bulk RNA-seq,Homo_633,AD.vs.control	2
AMER1	scRNA-seq,SRP215507,CD8+ T cell_1-AD.vs.control; scRNA-seq,SRP215507,CD8+ T cell_1-MCI.vs.control; scRNA-seq,SRP215507,CD8+ T cell_2-MCI.vs.control; scRNA-seq,SRP330776,B cell_1-AD.vs.control; scRNA-seq,SRP330776,Naive CD8+ T cell_3-AD.vs.control	5
AMER2	bulk RNA-seq,Homo_723,AD.vs.control; bulk RNA-seq,Homo_723,MCI.vs.control; bulk RNA-seq,Homo_633,AD.vs.control; bulk RNA-seq,Homo_633,MCI.vs.control; bulk RNA-seq,Homo_714,MCI.vs.control	5
AMER3	bulk RNA-seq,Homo_723,AD.vs.control; bulk RNA-seq,Homo_723,AD.vs.MCI; bulk RNA-seq,Homo_714,MCI.vs.control; bulk RNA-	4

AMFR	bulk RNA-seq,Homo_723,AD.vs.control; bulk RNA-seq,Homo_723,AD.vs.MCI; bulk RNA-seq,Homo_633,AD.vs.MCI; bulk RNA-seq,Homo_714,AD.vs.control; bulk RNA-seq,Homo_714,AD.vs.MCI; bulk RNA-seq,Homo_714,MCI.vs.control; bulk RNA-seq,SRP223445,AD.vs.control; scRNA-seq,SRP215507,CD8+ T cell_1-MCI.vs.control; scRNA-seq,SRP215507,CD8+ T cell_2-MCI.vs.control; scRNA-seq,SRP309935,Megakaryocyte_1-AD.vs.control; scRNA-seq,SRP309935,Naive CD8+ T cell_1-AD.vs.control; scRNA-seq,SRP309935,Natural killer cell_1-AD.vs.control; scRNA-seq,SRP309935,Monocyte_2-AD.vs.control; scRNA-seq,SRP309935,Naive CD8+ T cell_2-AD.vs.control; scRNA-seq,SRP309935,CD8+ T cell_1-AD.vs.control; scRNA-seq,SRP309935,CD8+ T cell_2-AD.vs.control; scRNA-seq,SRP330776,CD8+ T cell_1-AD.vs.control; scRNA-seq,SRP330776,γδ T cell-AD.vs.control; scRNA-seq,SRP330776,Memory T cell_2-AD.vs.control; scRNA-seq,SRP330776,CD8+ T cell_2-AD.vs.control; scRNA-seq,SRP330776,B cell_1-AD.vs.control; scRNA-seq,SRP330776,Naive CD8+ T cell_1-AD.vs.control; scRNA-seq,SRP330776,Natural killer cell_1-AD.vs.control; scRNA-seq,SRP330776,Memory T cell_1-AD.vs.control; scRNA-seq,SRP330776,Natural killer T cell-[illegible]	29
AMH	bulk RNA-seq,Homo_723,MCI.vs.control; bulk RNA-seq,Homo_633,MCI.vs.control; bulk RNA-seq,Homo_714,MCI.vs.control	3
AMHR2	bulk RNA-seq,Homo_723,AD.vs.MCI; bulk RNA-seq,Homo_723,MCI.vs.control; bulk RNA-seq,Homo_633,AD.vs.MCI; bulk RNA-seq,Homo_633,MCI.vs.control; bulk RNA-seq,ROSMAP,AD.vs.control	5
AMIGO1	scRNA-seq,SRP215507,CD8+ T cell_2-AD.vs.control; scRNA-seq,SRP215507,CD8+ T cell_1-AD.vs.control; scRNA-seq,SRP215507,CD8+ T cell_3-AD.vs.MCI; scRNA-seq,SRP215507,CD8+ T cell_3-MCI.vs.control; scRNA-seq,SRP330776,CD8+ T cell_1-AD.vs.control; scRNA-seq,SRP330776,γδ T cell-AD.vs.control; scRNA-seq,SRP330776,Memory T cell_2-AD.vs.control; scRNA-seq,SRP330776,CD8+ T cell_2-AD.vs.control; scRNA-seq,SRP330776,Naive CD8+ T cell_1-AD.vs.control; scRNA-seq,SRP330776,Memory T cell_1-AD.vs.control; scRNA-seq,SRP330776,T cell-[illegible]	12
AMIGO2	scRNA-seq,SRP309935,Naive CD8+ T cell_2-AD.vs.control; scRNA-seq,SRP309935,CD8+ T cell_2-AD.vs.control; scRNA-seq,SRP330776,CD8+ T cell_1-AD.vs.control; scRNA-seq,SRP330776,γδ T cell-AD.vs.control; scRNA-seq,SRP330776,Memory T cell_2-AD.vs.control; scRNA-seq,SRP330776,CD8+ T cell_2-AD.vs.control; scRNA-seq,SRP330776,B cell_1-AD.vs.control; scRNA-seq,SRP330776,Naive CD8+ T cell_1-AD.vs.control; scRNA-seq,SRP330776,Natural killer cell_1-AD.vs.control; scRNA-seq,SRP330776,Naive CD8+ T cell_3-AD.vs.control; scRNA-[illegible]	11
Ammecr1	bulk RNA-seq,SRP325058,AD.vs.control	1
AMMECR1	scRNA-seq,SRP215507,CD8+ T cell_4-MCI.vs.control; scRNA-seq,SRP309935,Natural killer cell_1-AD.vs.control; scRNA-seq,SRP309935,CD8+ T cell_2-AD.vs.control; scRNA-seq,SRP330776,CD8+ T cell_1-AD.vs.control; scRNA-seq,SRP330776,γδ T cell-AD.vs.control; scRNA-seq,SRP330776,B cell_1-AD.vs.control; scRNA-seq,SRP330776,Naive CD8+ T cell_3-AD.vs.control; scRNA-seq,SRP330776,B cell_2-AD.vs.control; scRNA-[illegible]	9
AMMECR1L	scRNA-seq,SRP215507,CD8+ T cell_3-AD.vs.control; scRNA-seq,SRP215507,CD8+ T cell_1-MCI.vs.control; scRNA-seq,SRP215507,CD8+ T cell_3-MCI.vs.control; scRNA-seq,SRP309935,Monocyte_1-AD.vs.control; scRNA-seq,SRP330776,CD8+ T cell_1-AD.vs.control; scRNA-seq,SRP330776,γδ T cell-AD.vs.control; scRNA-seq,SRP330776,Memory T cell_2-AD.vs.control; scRNA-seq,SRP330776,B cell_1-AD.vs.control; scRNA-seq,SRP330776,Naive CD8+ T cell_1-AD.vs.control; scRNA-seq,SRP330776,Naive CD8+ T cell_3-AD.vs.control; scRNA-seq,SRP330776,B cell_2-AD.vs.control; scRNA-seq,SRP330776,T cell-AD.vs.control; scRNA-seq,SRP330776,B cell_4-AD.vs.control	13
AMN	bulk RNA-seq,ACOM,AD.vs.control; scRNA-seq,SRP215507,CD8+ T cell_2-AD.vs.MCI; scRNA-seq,SRP215507,CD8+ T cell_2-MCI.vs.control; scRNA-seq,SRP309935,Naive CD8+ T cell_2-AD.vs.control; scRNA-seq,SRP330776,CD8+ T cell_1-AD.vs.control; scRNA-seq,SRP330776,B cell_1-AD.vs.control; scRNA-seq,SRP330776,Naive CD8+ T cell_1-AD.vs.control; scRNA-seq,SRP330776,B cell_2-AD.vs.control	8
AMN1	bulk RNA-seq,Homo_714,AD.vs.control; bulk RNA-seq,Homo_714,AD.vs.MCI; scRNA-seq,SRP215507,CD8+ T cell_3-AD.vs.control; scRNA-seq,SRP215507,CD8+ T cell_1-MCI.vs.control; scRNA-seq,SRP215507,CD8+ T cell_2-MCI.vs.control; scRNA-seq,SRP215507,CD8+ T cell_3-MCI.vs.control; scRNA-seq,SRP330776,γδ T cell-AD.vs.control; scRNA-seq,SRP330776,Memory T cell_2-AD.vs.control; scRNA-seq,SRP330776,Natural killer cell_1-AD.vs.control; scRNA-seq,SRP330776,B cell_2-AD.vs.control; scRNA-seq,SRP330776,T cell-AD.vs.control; scRNA-seq,SRP330776,CD8+ [illegible]	13
AMOT	bulk RNA-seq,ROSMAP,AD.vs.control; bulk RNA-seq,ROSMAP,MCI.vs.control; bulk RNA-seq,SRP223445,AD.vs.control; scRNA-seq,SRP215507,CD8+ T cell_1-AD.vs.control; scRNA-seq,SRP215507,CD8+ T cell_1-AD.vs.MCI; scRNA-seq,SRP215507,CD8+ T cell_5-AD.vs.MCI; scRNA-seq,SRP309935,CD8+ T cell_1-AD.vs.control; scRNA-seq,SRP330776,Memory T cell_2-AD.vs.control; scRNA-seq,SRP330776,CD8+ T cell_2-AD.vs.control; scRNA-seq,SRP330776,Natural killer cell_1-AD.vs.control	10
AMOTL1	bulk RNA-seq,Homo_723,AD.vs.control; bulk RNA-seq,Homo_723,AD.vs.MCI; bulk RNA-seq,Homo_723,MCI.vs.control; bulk RNA-seq,Homo_633,AD.vs.control; bulk RNA-seq,Homo_633,AD.vs.MCI; bulk RNA-seq,Homo_633,MCI.vs.control; bulk RNA-seq,Homo_714,AD.vs.control; bulk RNA-seq,Homo_714,AD.vs.MCI; scRNA-seq,SRP309935,Monocyte_1-AD.vs.control; scRNA-seq,SRP330776,CD8+ T cell_1-AD.vs.control	10

AMOTL2	bulk RNA-seq,Homo_723,AD.vs.control; bulk RNA-seq,Homo_723,AD.vs.MCI; bulk RNA-seq,Homo_723,MCI.vs.control; bulk RNA-seq,Homo_633,AD.vs.control; bulk RNA-seq,Homo_633,AD.vs.MCI; bulk RNA-seq,Homo_633,MCI.vs.control; bulk RNA-seq,Homo_714,MCI.vs.control; bulk RNA-seq,ROSMAP,AD.vs.control; bulk RNA-seq,ROSMAP,MCI.vs.control; bulk RNA-	10
AMPD1	bulk RNA-seq,Homo_633,AD.vs.control; bulk RNA-seq,Homo_633,AD.vs.MCI	2
AMPD2	bulk RNA-seq,Homo_723,AD.vs.control; bulk RNA-seq,Homo_723,AD.vs.MCI; bulk RNA-seq,Homo_633,AD.vs.control; bulk RNA-seq,Homo_633,AD.vs.MCI; scRNA-seq,SRP215507,CD8+ T cell_2-MCI.vs.control; scRNA-seq,SRP309935,Naive CD8+ T cell_2-AD.vs.control; scRNA-seq,SRP330776,Naive CD8+ T cell_3-AD.vs.control; scRNA-seq,SRP330776,B cell_2-AD.vs.control; scRNA-seq,SRP330776,Regulatory T cell-	9
AMPD3	scRNA-seq,SRP215507,CD8+ T cell_1-AD.vs.MCI; scRNA-seq,SRP215507,CD8+ T cell_1-MCI.vs.control; scRNA-seq,SRP309935,Monocyte_1-AD.vs.control; scRNA-seq,SRP330776,CD8+ T cell_1-AD.vs.control; scRNA-seq,SRP330776,Memory T cell_2-AD.vs.control; scRNA-seq,SRP330776,CD8+ T cell_2-AD.vs.control; scRNA-seq,SRP330776,Naive CD8+ T cell_1-AD.vs.control; scRNA-seq,SRP330776,Memory T cell_1-AD.vs.control; scRNA-seq,SRP330776,Naive CD8+ T cell_3-AD.vs.control; scRNA-seq,SRP330776,B cell_2-AD.vs.control; scRNA-seq,SRP330776,B	12
AMPH	bulk RNA-seq,Homo_723,AD.vs.control; bulk RNA-seq,Homo_723,AD.vs.MCI; bulk RNA-seq,Homo_723,MCI.vs.control; bulk RNA-seq,Homo_633,AD.vs.control; bulk RNA-seq,Homo_633,AD.vs.MCI; bulk RNA-seq,Homo_633,MCI.vs.control; bulk RNA-seq,Homo_714,AD.vs.control; bulk RNA-seq,Homo_714,AD.vs.MCI; bulk RNA-seq,Homo_714,MCI.vs.control	9
AMT	scRNA-seq,SRP215507,CD8+ T cell_1-AD.vs.control; scRNA-seq,SRP215507,CD8+ T cell_5-AD.vs.control; scRNA-seq,SRP215507,CD8+ T cell_3-AD.vs.MCI; scRNA-seq,SRP215507,CD8+ T cell_5-AD.vs.MCI; scRNA-seq,SRP215507,CD8+ T cell_3-MCI.vs.control; scRNA-seq,SRP215507,CD8+ T cell_6-MCI.vs.control; scRNA-seq,SRP330776,CD8+ T cell_1-AD.vs.control; scRNA-seq,SRP330776,CD8+ T cell_2-AD.vs.control; scRNA-seq,SRP330776,Naive CD8+ T cell_1-AD.vs.control; scRNA-seq,SRP330776,Memory T cell_1-AD.vs.control	10
AMY1A	bulk RNA-seq,Homo_714,AD.vs.control; bulk RNA-seq,Homo_714,AD.vs.MCI; bulk RNA-seq,ROSMAP,AD.vs.control; bulk RNA-	4
AMY1B	bulk RNA-seq,Homo_723,AD.vs.control; bulk RNA-seq,Homo_633,AD.vs.control; bulk RNA-seq,Homo_714,AD.vs.control; bulk RNA-seq,Homo_714,AD.vs.MCI; bulk RNA-seq,ROSMAP,AD.vs.control; bulk RNA-seq,ROSMAP,MCI.vs.control	6
AMY1C	bulk RNA-seq,Homo_714,AD.vs.control; bulk RNA-seq,Homo_714,AD.vs.MCI; bulk RNA-seq,ROSMAP,AD.vs.control; bulk RNA-	4
AMY2A	bulk RNA-seq,Homo_723,AD.vs.control; bulk RNA-seq,Homo_723,MCI.vs.control; bulk RNA-seq,Homo_633,AD.vs.control; bulk RNA-seq,Homo_633,AD.vs.MCI; bulk RNA-seq,Homo_633,MCI.vs.control; bulk RNA-seq,Homo_714,AD.vs.control; bulk RNA-seq,Homo_714,AD.vs.MCI; bulk RNA-seq,Homo_714,MCI.vs.control; bulk RNA-seq,ROSMAP,AD.vs.control; bulk RNA-seq,ROSMAP,MCI.vs.control	10
AMY2B	scRNA-seq,SRP215507,CD8+ T cell_2-AD.vs.MCI; scRNA-seq,SRP215507,CD8+ T cell_1-MCI.vs.control; scRNA-seq,SRP215507,CD8+ T cell_2-MCI.vs.control; scRNA-seq,SRP215507,CD8+ T cell_3-MCI.vs.control; scRNA-seq,SRP215507,CD8+ T cell_6-MCI.vs.control; scRNA-seq,SRP309935,CD8+ T cell_1-AD.vs.control; scRNA-seq,SRP330776,γδ T cell-AD.vs.control; scRNA-seq,SRP330776,CD8+ T cell_2-AD.vs.control; scRNA-seq,SRP330776,B cell_1-AD.vs.control; scRNA-seq,SRP330776,Memory T cell_1-AD.vs.control; scRNA-seq,SRP330776,CD8+ T cell_3-	11
AMZ1	bulk RNA-seq,Homo_723,AD.vs.control; bulk RNA-seq,Homo_723,AD.vs.MCI; bulk RNA-seq,Homo_633,AD.vs.MCI; bulk RNA-seq,Homo_714,AD.vs.control; bulk RNA-seq,Homo_714,AD.vs.MCI; scRNA-seq,SRP215507,CD8+ T cell_2-MCI.vs.control	6
Amz1	bulk RNA-seq,SRP325058,AD.vs.control	1
AMZ2	bulk RNA-seq,Homo_723,AD.vs.control; bulk RNA-seq,Homo_723,AD.vs.MCI; bulk RNA-seq,Homo_633,AD.vs.control; bulk RNA-seq,Homo_633,AD.vs.MCI; bulk RNA-seq,Homo_714,AD.vs.control; bulk RNA-seq,Homo_714,AD.vs.MCI; scRNA-seq,SRP215507,CD8+ T cell_4-	7
ANAPC1	scRNA-seq,SRP215507,CD8+ T cell_6-AD.vs.MCI; scRNA-seq,SRP215507,CD8+ T cell_6-MCI.vs.control; scRNA-seq,SRP309935,Naive CD8+ T	3
ANAPC10	bulk RNA-seq,Homo_714,AD.vs.control; bulk RNA-seq,Homo_714,AD.vs.MCI; scRNA-seq,SRP215507,CD8+ T cell_7-AD.vs.control; scRNA-seq,SRP330776,CD8+ T cell_2-AD.vs.control; scRNA-seq,SRP330776,Natural killer T cell-AD.vs.control; scRNA-seq,SRP330776,T cell-AD.vs.control; scRNA-seq,SRP330776,Monocyte_1-AD.vs.control	7
ANAPC11	bulk RNA-seq,Homo_714,AD.vs.control; bulk RNA-seq,Homo_714,AD.vs.MCI; scRNA-seq,SRP215507,CD8+ T cell_4-AD.vs.control; scRNA-seq,SRP215507,CD8+ T cell_9-AD.vs.control; scRNA-seq,SRP330776,Natural killer cell_2-AD.vs.control	5

ANAPC13	bulk RNA-seq,Homo_714,AD.vs.control; bulk RNA-seq,Homo_714,AD.vs.MCI; scRNA-seq,SRP215507,CD8+ T cell_4-AD.vs.MCI; scRNA-seq,SRP215507,CD8+ T cell_5-AD.vs.MCI; scRNA-seq,SRP215507,CD8+ T cell_5-MCI.vs.control; scRNA-seq,SRP215507,CD8+ T cell_4-MCI.vs.control; scRNA-seq,SRP215507,CD8+ T cell_2-MCI.vs.control; scRNA-seq,SRP330776,Natural killer T cell-AD.vs.control; scRNA-seq,SRP330776,Naive CD8+ T cell_3-AD.vs.control; scRNA-seq,SRP330776,B cell_2-AD.vs.control; scRNA-seq,SRP330776,T cell-AD.vs.control; scRNA-seq,SRP330776,CD8+ T cell_3-AD.vs.control; scRNA-seq,SRP330776,B cell_3-AD.vs.control; scRNA-seq,SRP330776,Natural killer cell_2-	14
ANAPC15	bulk RNA-seq,Homo_723,AD.vs.control; bulk RNA-seq,Homo_723,AD.vs.MCI; bulk RNA-seq,Homo_723,MCI.vs.control; bulk RNA-seq,Homo_633,AD.vs.control; bulk RNA-seq,Homo_633,AD.vs.MCI; bulk RNA-seq,Homo_633,MCI.vs.control; bulk RNA-seq,Homo_714,AD.vs.control; bulk RNA-seq,Homo_714,AD.vs.MCI; bulk RNA-seq,Homo_714,MCI.vs.control; scRNA-seq,SRP215507,CD8+ T cell_5-AD.vs.MCI; scRNA-seq,SRP330776,T cell-AD.vs.control; scRNA-seq,SRP330776,Monocyte_2-AD.vs.control; scRNA-seq,SRP330776,Natural killer cell_2-AD.vs.control	13
ANAPC16	scRNA-seq,SRP215507,CD8+ T cell_9-AD.vs.control; scRNA-seq,SRP309935,Megakaryocyte_2-AD.vs.control; scRNA-seq,SRP330776,Monocyte_2-AD.vs.control; scRNA-seq,SRP330776,B cell_3-AD.vs.control	4
ANAPC2	bulk RNA-seq,Homo_633,AD.vs.control; scRNA-seq,SRP215507,CD8+ T cell_1-AD.vs.control; scRNA-seq,SRP215507,CD8+ T cell_5-AD.vs.control; scRNA-seq,SRP215507,CD8+ T cell_2-AD.vs.MCI; scRNA-seq,SRP215507,CD8+ T cell_1-AD.vs.MCI; scRNA-seq,SRP215507,CD8+ T cell_5-AD.vs.MCI; scRNA-seq,SRP330776,CD8+ T cell_1-AD.vs.control; scRNA-seq,SRP330776,Memory T cell_2-AD.vs.control; scRNA-seq,SRP330776,CD8+ T cell_2-AD.vs.control; scRNA-seq,SRP330776,B cell_1-AD.vs.control; scRNA-seq,SRP330776,Naive CD8+ T cell_1-AD.vs.control; scRNA-seq,SRP330776,Natural killer cell_1-AD.vs.control; scRNA-seq,SRP330776,Memory T cell_1-AD.vs.control; scRNA-seq,SRP330776,Natural killer T cell-AD.vs.control; scRNA-seq,SRP330776,Naive CD8+ T cell_3-AD.vs.control; scRNA-seq,SRP330776,B cell_2-AD.vs.control; scRNA-seq,SRP330776,T cell-AD.vs.control; scRNA-seq,SRP330776,Monocyte_1-AD.vs.control; scRNA-seq,SRP330776,B cell_4- [illegible]	19
ANAPC4	bulk RNA-seq,Homo_723,AD.vs.control; bulk RNA-seq,Homo_723,AD.vs.MCI; bulk RNA-seq,Homo_633,AD.vs.control; bulk RNA-seq,Homo_633,AD.vs.MCI; bulk RNA-seq,Homo_714,AD.vs.control; bulk RNA-seq,Homo_714,AD.vs.MCI; scRNA-seq,SRP215507,CD8+ T cell_3-AD.vs.control; scRNA-seq,SRP215507,CD8+ T cell_4-AD.vs.MCI; scRNA-seq,SRP330776,CD8+ T cell_1-AD.vs.control; scRNA-seq,SRP330776,Memory T cell_2-AD.vs.control; scRNA-seq,SRP330776,Naive CD8+ T cell_1-AD.vs.control; scRNA-seq,SRP330776,Natural killer	12
ANAPC5	bulk RNA-seq,Homo_723,AD.vs.control; bulk RNA-seq,Homo_723,AD.vs.MCI; bulk RNA-seq,Homo_633,AD.vs.control; bulk RNA-	4
ANAPC7	bulk RNA-seq,Homo_723,AD.vs.control; bulk RNA-seq,Homo_723,AD.vs.MCI; bulk RNA-seq,Homo_723,MCI.vs.control; bulk RNA-seq,Homo_633,AD.vs.control; bulk RNA-seq,Homo_633,AD.vs.MCI; bulk RNA-seq,Homo_633,MCI.vs.control; bulk RNA-seq,Homo_714,AD.vs.control; bulk RNA-seq,Homo_714,AD.vs.MCI; bulk RNA-seq,Homo_714,MCI.vs.control; scRNA-seq,SRP215507,CD8+ T cell_4-AD.vs.MCI; scRNA-seq,SRP330776,B cell_2-AD.vs.control; scRNA-seq,SRP330776,T cell-AD.vs.control; scRNA-seq,SRP330776,CD8+ T cell_3-AD.vs.control	13
ANG	bulk RNA-seq,Homo_723,AD.vs.control; bulk RNA-seq,Homo_723,AD.vs.MCI; bulk RNA-seq,Homo_633,AD.vs.control; bulk RNA-seq,Homo_633,AD.vs.MCI; bulk RNA-seq,Homo_714,AD.vs.control; bulk RNA-seq,Homo_714,AD.vs.MCI; scRNA-seq,SRP215507,CD8+ T cell_2-MCI.vs.control; scRNA-seq,SRP330776,CD8+ T cell_1-AD.vs.control; scRNA-seq,SRP330776,Monocyte_2-AD.vs.control; scRNA-seq,SRP330776,B	10
ANGEL1	bulk RNA-seq,Homo_723,AD.vs.MCI; bulk RNA-seq,Homo_633,AD.vs.control; bulk RNA-seq,Homo_633,AD.vs.MCI; bulk RNA-seq,Homo_714,AD.vs.control; bulk RNA-seq,Homo_714,AD.vs.MCI; scRNA-seq,SRP215507,CD8+ T cell_2-AD.vs.MCI; scRNA-seq,SRP215507,CD8+ T cell_2-MCI.vs.control; scRNA-seq,SRP330776,γδ T cell-AD.vs.control; scRNA-seq,SRP330776,Memory T cell_1-AD.vs.control; scRNA-	10
ANGEL2	bulk RNA-seq,Homo_723,AD.vs.control; bulk RNA-seq,Homo_723,AD.vs.MCI; bulk RNA-seq,Homo_633,AD.vs.control; bulk RNA-seq,Homo_633,AD.vs.MCI; scRNA-seq,SRP215507,CD8+ T cell_6-AD.vs.control	5
ANGPT1	bulk RNA-seq,Homo_723,AD.vs.control; bulk RNA-seq,Homo_723,AD.vs.MCI; bulk RNA-seq,Homo_723,MCI.vs.control; bulk RNA-seq,Homo_633,AD.vs.control; bulk RNA-seq,Homo_633,AD.vs.MCI; bulk RNA-seq,Homo_633,MCI.vs.control; bulk RNA-seq,Homo_714,AD.vs.control;	8
ANGPT2	bulk RNA-seq,Homo_723,AD.vs.control; bulk RNA-seq,Homo_723,AD.vs.MCI; bulk RNA-seq,Homo_633,AD.vs.control; bulk RNA-seq,Homo_633,AD.vs.MCI; bulk RNA-seq,Homo_714,AD.vs.control; bulk RNA-seq,Homo_714,AD.vs.MCI; bulk RNA-seq,Homo_714,MCI.vs.control	7
ANGPT4	bulk RNA-seq,Homo_723,AD.vs.control; bulk RNA-seq,Homo_723,MCI.vs.control; bulk RNA-seq,Homo_633,AD.vs.MCI; bulk RNA-seq,Homo_633,MCI.vs.control; bulk RNA-seq,Homo_714,MCI.vs.control	5
ANGPTL1	bulk RNA-seq,Homo_723,AD.vs.control; bulk RNA-seq,Homo_723,AD.vs.MCI; bulk RNA-seq,Homo_723,MCI.vs.control; bulk RNA-seq,Homo_633,AD.vs.control; bulk RNA-seq,Homo_633,MCI.vs.control; bulk RNA-seq,Homo_714,AD.vs.MCI; bulk RNA-	8

ANGPTL2	bulk RNA-seq,Homo_723,AD.vs.control; bulk RNA-seq,Homo_723,AD.vs.MCI; bulk RNA-seq,Homo_633,AD.vs.control; bulk RNA-seq,Homo_633,AD.vs.MCI; bulk RNA-seq,Homo_714,MCI.vs.control	5
ANGPTL3	bulk RNA-seq,Homo_723,AD.vs.control; bulk RNA-seq,Homo_723,AD.vs.MCI; bulk RNA-seq,Homo_723,MCI.vs.control; bulk RNA-	4
ANGPTL4	bulk RNA-seq,Homo_723,AD.vs.control; bulk RNA-seq,Homo_723,AD.vs.MCI; bulk RNA-seq,Homo_633,AD.vs.control	3
ANGPTL5	bulk RNA-seq,Homo_723,AD.vs.control; bulk RNA-seq,Homo_723,MCI.vs.control; bulk RNA-seq,Homo_633,AD.vs.control; bulk RNA-seq,Homo_633,MCI.vs.control; bulk RNA-seq,Homo_714,MCI.vs.control	5
ANGPTL6	bulk RNA-seq,Homo_723,AD.vs.control; scRNA-seq,SRP215507,CD8+ T cell_1-MCI.vs.control; scRNA-seq,SRP330776,CD8+ T cell_1-AD.vs.control; scRNA-seq,SRP330776,Memory T cell_2-AD.vs.control; scRNA-seq,SRP330776,Naive CD8+ T cell_2-AD.vs.control	5
ANGPTL7	bulk RNA-seq,Homo_723,AD.vs.control; bulk RNA-seq,Homo_633,AD.vs.control; bulk RNA-seq,ROSMAP,MCI.vs.control	3
ANGPTL8	bulk RNA-seq,Homo_723,AD.vs.control; bulk RNA-seq,Homo_723,AD.vs.MCI	2
Ank	bulk RNA-seq,SRP325058,AD.vs.control	1
Ank1	bulk RNA-seq,SRP325058,AD.vs.control	1
ANK1	scRNA-seq,SRP215507,CD8+ T cell_2-AD.vs.control; scRNA-seq,SRP215507,CD8+ T cell_2-MCI.vs.control; scRNA-seq,SRP309935,Naive CD8+ T cell_2-AD.vs.control; scRNA-seq,SRP330776,B cell_1-AD.vs.control; scRNA-seq,SRP330776,Naive CD8+ T cell_1-AD.vs.control; scRNA-	6
ANK2	bulk RNA-seq,Homo_714,MCI.vs.control; bulk RNA-seq,ROSMAP,AD.vs.control	2
ANK3	bulk RNA-seq,Homo_723,AD.vs.control; bulk RNA-seq,Homo_723,AD.vs.MCI; bulk RNA-seq,Homo_633,AD.vs.control; bulk RNA-seq,Homo_633,AD.vs.MCI; bulk RNA-seq,Homo_714,AD.vs.control; bulk RNA-seq,Homo_714,AD.vs.MCI; scRNA-seq,SRP215507,CD8+ T cell_1-AD.vs.MCI; scRNA-seq,SRP215507,CD8+ T cell_4-AD.vs.MCI; scRNA-seq,SRP215507,CD8+ T cell_4-MCI.vs.control; scRNA-seq,SRP330776,γδ T cell-AD.vs.control; scRNA-seq,SRP330776,CD8+ T cell_2-AD.vs.control; scRNA-seq,SRP330776,Naive CD8+ T cell_1-AD.vs.control; scRNA-seq,SRP330776,Memory T cell_1-AD.vs.control; scRNA-seq,SRP330776,Natural killer T cell-AD.vs.control; scRNA-seq,SRP330776,Naive CD8+ T cell_3-AD.vs.control; scRNA-seq,SRP330776,B cell_2-AD.vs.control; scRNA-seq,SRP330776,CD8+ T cell_3-AD.vs.control; scRNA-seq,SRP330776,B	18
ANK3-DT	scRNA-seq,SRP309935,Naive CD8+ T cell_1-AD.vs.control	1
ANKAR	bulk RNA-seq,Homo_723,AD.vs.control; bulk RNA-seq,Homo_723,AD.vs.MCI; bulk RNA-seq,Homo_633,AD.vs.control; bulk RNA-seq,Homo_633,AD.vs.MCI; bulk RNA-seq,Homo_714,AD.vs.control; bulk RNA-seq,Homo_714,AD.vs.MCI; bulk RNA-seq,ROSMAP,AD.vs.control; scRNA-seq,SRP215507,CD8+ T cell_5-AD.vs.MCI; scRNA-seq,SRP215507,CD8+ T cell_5-MCI.vs.control; scRNA-seq,SRP330776,CD8+ T cell_1-AD.vs.control; scRNA-seq,SRP330776,γδ T cell-AD.vs.control; scRNA-seq,SRP330776,Memory T cell_2-AD.vs.control; scRNA-seq,SRP330776,CD8+ T cell_2-AD.vs.control; scRNA-seq,SRP330776,B cell_1-AD.vs.control; scRNA-seq,SRP330776,Naive CD8+ T cell_1-AD.vs.control; scRNA-seq,SRP330776,Natural killer cell_1-AD.vs.control; scRNA-seq,SRP330776,Memory T cell_1-AD.vs.control; scRNA-seq,SRP330776,Naive CD8+ T cell_3-AD.vs.control; scRNA-seq,SRP330776,T cell-AD.vs.control; scRNA-seq,SRP330776,B cell_3-AD.vs.control; scRNA-seq,SRP330776,Natural killer	21
ANKDD1A	bulk RNA-seq,Homo_723,AD.vs.MCI; bulk RNA-seq,Homo_714,AD.vs.control; bulk RNA-seq,Homo_714,AD.vs.MCI; scRNA-seq,SRP215507,CD8+ T cell_1-AD.vs.control; scRNA-seq,SRP215507,CD8+ T cell_5-AD.vs.MCI; scRNA-seq,SRP215507,CD8+ T cell_1-MCI.vs.control; scRNA-seq,SRP215507,CD8+ T cell_5-MCI.vs.control; scRNA-seq,SRP215507,CD8+ T cell_3-MCI.vs.control; scRNA-seq,SRP215507,CD8+ T cell_6-MCI.vs.control; scRNA-seq,SRP309935,Naive CD8+ T cell_1-AD.vs.control; scRNA-seq,SRP330776,CD8+ T cell_1-AD.vs.control; scRNA-	12
ANKDD1B	bulk RNA-seq,Homo_723,AD.vs.control; bulk RNA-seq,Homo_723,AD.vs.MCI; bulk RNA-seq,Homo_723,MCI.vs.control; bulk RNA-seq,Homo_633,AD.vs.control; bulk RNA-seq,Homo_633,AD.vs.MCI; bulk RNA-seq,Homo_633,MCI.vs.control; bulk RNA-seq,Homo_714,AD.vs.control; bulk RNA-seq,Homo_714,AD.vs.MCI; bulk RNA-seq,Homo_714,MCI.vs.control; scRNA-seq,SRP215507,CD8+ T cell_1-MCI.vs.control; scRNA-seq,SRP215507,CD8+ T cell_2-MCI.vs.control; scRNA-seq,SRP309935,Megakaryocyte_2-AD.vs.control; scRNA-seq,SRP330776,Naive CD8+ T cell_1-	13

ANKEF1	bulk RNA-seq,Homo_633,AD.vs.control; bulk RNA-seq,Homo_714,AD.vs.control; bulk RNA-seq,Homo_714,AD.vs.MCI; bulk RNA-seq,ROSMAP,AD.vs.control; bulk RNA-seq,ROSMAP,MCI.vs.control; scRNA-seq,SRP215507,CD8+ T cell_3-AD.vs.control; scRNA-seq,SRP215507,CD8+ T cell_5-AD.vs.control; scRNA-seq,SRP215507,CD8+ T cell_3-AD.vs.MCI; scRNA-seq,SRP215507,CD8+ T cell_1-AD.vs.MCI; scRNA-seq,SRP215507,CD8+ T cell_5-AD.vs.MCI; scRNA-seq,SRP215507,CD8+ T cell_1-MCI.vs.control; scRNA-seq,SRP215507,CD8+ T cell_4-MCI.vs.control; scRNA-seq,SRP215507,CD8+ T cell_3-MCI.vs.control; scRNA-seq,SRP215507,CD8+ T cell_8-MCI.vs.control; scRNA-seq,SRP330776,Memory T cell_1-AD.vs.control; scRNA-seq,SRP330776,Natural killer T cell-AD.vs.control; scRNA-seq,SRP330776,Naive CD8+ T [illegible]	18
ANKFN1	bulk RNA-seq,Homo_723,AD.vs.control; bulk RNA-seq,Homo_723,MCI.vs.control; bulk RNA-seq,Homo_633,MCI.vs.control; bulk RNA-seq,Homo_714,MCI.vs.control; bulk RNA-seq,ROSMAP,AD.vs.control; bulk RNA-seq,ROSMAP,MCI.vs.control	6
ANKFY1	bulk RNA-seq,Homo_723,AD.vs.control; bulk RNA-seq,Homo_723,AD.vs.MCI; bulk RNA-seq,Homo_723,MCI.vs.control; bulk RNA-seq,Homo_633,AD.vs.control; bulk RNA-seq,Homo_633,AD.vs.MCI; bulk RNA-seq,Homo_633,MCI.vs.control; scRNA-seq,SRP309935,Natural killer cell_1-AD.vs.control; scRNA-seq,SRP330776,B cell_3-AD.vs.control; scRNA-seq,SRP330776,Megakaryocyte_1-AD.vs.control	9
ANKH	bulk RNA-seq,Homo_633,AD.vs.control; bulk RNA-seq,Homo_633,AD.vs.MCI; scRNA-seq,SRP215507,CD8+ T cell_5-AD.vs.MCI; scRNA-seq,SRP215507,CD8+ T cell_5-MCI.vs.control; scRNA-seq,SRP309935,Natural killer cell_2-AD.vs.control; scRNA-seq,SRP309935,Erythroid cell-AD.vs.control; scRNA-seq,SRP309935,Naive CD8+ T cell_1-AD.vs.control; scRNA-seq,SRP309935,Natural killer cell_1-AD.vs.control; scRNA-seq,SRP309935,Naive CD8+ T cell_2-AD.vs.control; scRNA-seq,SRP309935,CD8+ T cell_1-AD.vs.control; scRNA-seq,SRP309935,CD8+ T cell_2-AD.vs.control; scRNA-seq,SRP330776,CD8+ T cell_1-AD.vs.control; scRNA-seq,SRP330776,γδ T cell-AD.vs.control; scRNA-seq,SRP330776,Memory T cell_2-AD.vs.control; scRNA-seq,SRP330776,CD8+ T cell_2-AD.vs.control; scRNA-seq,SRP330776,B cell_1-AD.vs.control; scRNA-seq,SRP330776,Naive CD8+ T cell_1-AD.vs.control; scRNA-seq,SRP330776,Natural killer cell_1-AD.vs.control; scRNA-seq,SRP330776,Memory T cell_1-AD.vs.control; scRNA-seq,SRP330776,Natural killer T cell-AD.vs.control; scRNA-seq,SRP330776,Naive CD8+ T cell_3-AD.vs.control; scRNA-seq,SRP330776,B cell_2-AD.vs.control; scRNA-seq,SRP330776,T cell-AD.vs.control; scRNA-seq,SRP330776,Naive CD8+ T cell_2-AD.vs.control; scRNA-seq,SRP330776,CD8+ T cell_3-AD.vs.control; scRNA-seq,SRP330776,B cell_3-AD.vs.control; scRNA-seq,SRP330776,Regulatory T cell-AD.vs.control; scRNA-seq,SRP330776,Natural killer cell_2-AD.vs.control	28
ANKHD1	bulk RNA-seq,Homo_633,AD.vs.control; bulk RNA-seq,Homo_633,AD.vs.MCI; bulk RNA-seq,Homo_714,AD.vs.control; bulk RNA-seq,Homo_714,AD.vs.MCI; scRNA-seq,SRP215507,CD8+ T cell_5-AD.vs.MCI; scRNA-seq,SRP215507,CD8+ T cell_5-MCI.vs.control; scRNA-seq,SRP330776,γδ T cell-AD.vs.control; scRNA-seq,SRP330776,CD8+ T cell_2-AD.vs.control; scRNA-seq,SRP330776,B cell_1-AD.vs.control; scRNA-seq,SRP330776,Naive CD8+ T cell_1-AD.vs.control; scRNA-seq,SRP330776,Memory T cell_1-AD.vs.control; scRNA-seq,SRP330776,Naive CD8+ T cell_3-AD.vs.control; scRNA-seq,SRP330776,B cell_2-AD.vs.control; scRNA-seq,SRP330776,Naive CD8+ T cell_2-AD.vs.control; scRNA-	16
ANKIB1	bulk RNA-seq,Homo_723,AD.vs.control; bulk RNA-seq,Homo_723,AD.vs.MCI; bulk RNA-seq,Homo_723,MCI.vs.control; bulk RNA-seq,Homo_633,AD.vs.control; bulk RNA-seq,Homo_633,AD.vs.MCI; bulk RNA-seq,Homo_633,MCI.vs.control; bulk RNA-seq,Homo_714,AD.vs.control; bulk RNA-seq,Homo_714,AD.vs.MCI; bulk RNA-seq,Homo_714,MCI.vs.control; scRNA-seq,SRP330776,CD8+ T cell_1-AD.vs.control; scRNA-seq,SRP330776,γδ T cell-AD.vs.control; scRNA-seq,SRP330776,Memory T cell_2-AD.vs.control; scRNA-seq,SRP330776,CD8+ T cell_2-AD.vs.control; scRNA-seq,SRP330776,B cell_1-AD.vs.control; scRNA-seq,SRP330776,Naive CD8+ T cell_1-AD.vs.control; scRNA-seq,SRP330776,Natural killer cell_1-AD.vs.control; scRNA-seq,SRP330776,Memory T cell_1-AD.vs.control; scRNA-seq,SRP330776,Natural killer T cell-AD.vs.control; scRNA-seq,SRP330776,Naive CD8+ T cell_3-AD.vs.control; scRNA-seq,SRP330776,T cell-AD.vs.control; scRNA-seq,SRP330776,CD8+ T cell_3-AD.vs.control; scRNA-seq,SRP330776,Monocyte_1-AD.vs.control; scRNA-seq,SRP330776,Monocyte_2-AD.vs.control; scRNA-seq,SRP330776,B cell_3-AD.vs.control; scRNA-seq,SRP330776,Natural killer cell_2-AD.vs.control	25
ANKK1	bulk RNA-seq,Homo_633,AD.vs.control; bulk RNA-seq,Homo_633,AD.vs.MCI; bulk RNA-seq,Homo_714,AD.vs.control; bulk RNA-seq,Homo_714,AD.vs.MCI; bulk RNA-seq,ROSMAP,AD.vs.control	5
ANKLE1	bulk RNA-seq,Homo_723,AD.vs.control; bulk RNA-seq,Homo_723,AD.vs.MCI; bulk RNA-seq,Homo_714,AD.vs.control; bulk RNA-seq,Homo_714,AD.vs.MCI; scRNA-seq,SRP215507,CD8+ T cell_2-AD.vs.control; scRNA-seq,SRP215507,CD8+ T cell_4-AD.vs.control; scRNA-	7

ANKLE2	bulk RNA-seq,Homo_723,AD.vs.control; bulk RNA-seq,Homo_633,AD.vs.control; bulk RNA-seq,Homo_633,MCI.vs.control; bulk RNA-seq,Homo_714,AD.vs.control; bulk RNA-seq,Homo_714,AD.vs.MCI; bulk RNA-seq,Homo_714,MCI.vs.control; scRNA-seq,SRP309935,Natural killer cell_2-AD.vs.control; scRNA-seq,SRP309935,Naive CD8+ T cell_2-AD.vs.control; scRNA-seq,SRP309935,B cell_1-AD.vs.control; scRNA-seq,SRP309935,CD8+ T cell_2-AD.vs.control; scRNA-seq,SRP309935,Naive CD8+ T cell_3-AD.vs.control; scRNA-seq,SRP330776,CD8+ T cell_1-AD.vs.control; scRNA-seq,SRP330776,γδ T cell-AD.vs.control; scRNA-seq,SRP330776,Memory T cell_2-AD.vs.control; scRNA-seq,SRP330776,CD8+ T cell_2-AD.vs.control; scRNA-seq,SRP330776,B cell_1-AD.vs.control; scRNA-seq,SRP330776,Naive CD8+ T cell_1-AD.vs.control; scRNA-seq,SRP330776,Natural killer cell_1-AD.vs.control; scRNA-seq,SRP330776,Memory T cell_1-AD.vs.control; scRNA-seq,SRP330776,Natural killer T cell-AD.vs.control; scRNA-seq,SRP330776,Naive CD8+ T cell_3-AD.vs.control; scRNA-seq,SRP330776,B cell_2-AD.vs.control; scRNA-seq,SRP330776,T cell-AD.vs.control; scRNA-seq,SRP330776,Naive CD8+ T cell_2-AD.vs.control; scRNA-seq,SRP330776,CD8+ T cell_3-AD.vs.control; scRNA-seq,SRP330776,Monocyte_1-AD.vs.control; scRNA-seq,SRP330776,Monocyte_2-AD.vs.control; scRNA-seq,SRP330776,B cell_3-AD.vs.control; scRNA-seq,SRP330776,Natural killer cell_2-AD.vs.control; scRNA-seq,SRP330776,B cell_4-AD.vs.control	30
ANKMY1	scRNA-seq,SRP215507,CD8+ T cell_4-AD.vs.control; scRNA-seq,SRP215507,CD8+ T cell_6-AD.vs.control; scRNA-seq,SRP215507,CD8+ T cell_3-AD.vs.MCI; scRNA-seq,SRP330776,CD8+ T cell_1-AD.vs.control; scRNA-seq,SRP330776,γδ T cell-AD.vs.control; scRNA-seq,SRP330776,Memory T cell_2-AD.vs.control; scRNA-seq,SRP330776,CD8+ T cell_2-AD.vs.control; scRNA-seq,SRP330776,B cell_1-AD.vs.control; scRNA-seq,SRP330776,Naive CD8+ T cell_1-AD.vs.control; scRNA-seq,SRP330776,Natural killer cell_1-AD.vs.control; scRNA-seq,SRP330776,Memory T cell_1-AD.vs.control; scRNA-seq,SRP330776,Natural killer T cell-AD.vs.control; scRNA-seq,SRP330776,Naive CD8+ T cell_3-AD.vs.control; scRNA-seq,SRP330776,B cell_2-AD.vs.control; scRNA-seq,SRP330776,T cell-AD.vs.control; scRNA-seq,SRP330776,CD8+ T cell_3-AD.vs.control; scRNA-[illegible]	18
ANKMY2	bulk RNA-seq,Homo_723,AD.vs.control; bulk RNA-seq,Homo_723,AD.vs.MCI; bulk RNA-seq,Homo_723,MCI.vs.control; bulk RNA-seq,Homo_633,AD.vs.MCI; bulk RNA-seq,Homo_633,MCI.vs.control; scRNA-seq,SRP215507,CD8+ T cell_3-AD.vs.MCI; scRNA-seq,SRP215507,CD8+ T cell_8-MCI.vs.control; scRNA-seq,SRP309935,Monocyte_1-AD.vs.control; scRNA-seq,SRP309935,Naive CD8+ T cell_1-AD.vs.control; scRNA-seq,SRP330776,Natural killer T cell-AD.vs.control; scRNA-seq,SRP330776,Naive CD8+ T cell_3-AD.vs.control	11
ANKRA2	bulk RNA-seq,Homo_723,AD.vs.control; bulk RNA-seq,Homo_723,AD.vs.MCI; bulk RNA-seq,Homo_723,MCI.vs.control; bulk RNA-seq,Homo_633,AD.vs.control; bulk RNA-seq,Homo_633,AD.vs.MCI; bulk RNA-seq,Homo_633,MCI.vs.control; bulk RNA-seq,Homo_714,AD.vs.control; bulk RNA-seq,Homo_714,AD.vs.MCI; bulk RNA-seq,Homo_714,MCI.vs.control; scRNA-seq,SRP215507,CD8+ T cell_4-AD.vs.control; scRNA-seq,SRP215507,CD8+ T cell_4-AD.vs.MCI; scRNA-seq,SRP330776,B cell_4-AD.vs.control	12
ANKRD1	bulk RNA-seq,Homo_723,AD.vs.control; bulk RNA-seq,Homo_723,AD.vs.MCI; bulk RNA-seq,Homo_723,MCI.vs.control; bulk RNA-seq,Homo_633,AD.vs.control; bulk RNA-seq,Homo_633,AD.vs.MCI; bulk RNA-seq,Homo_633,MCI.vs.control; bulk RNA-seq,Homo_714,MCI.vs.control	7
ANKRD10	scRNA-seq,SRP215507,CD8+ T cell_9-AD.vs.control; scRNA-seq,SRP330776,Memory T cell_2-AD.vs.control; scRNA-seq,SRP330776,Naive CD8+ T cell_1-AD.vs.control; scRNA-seq,SRP330776,Natural killer T cell-AD.vs.control; scRNA-seq,SRP330776,CD8+ T cell_3-AD.vs.control	5
ANKRD10-IT1	scRNA-seq,SRP215507,CD8+ T cell_4-AD.vs.control	1
ANKRD11	bulk RNA-seq,Homo_723,AD.vs.control; bulk RNA-seq,Homo_633,AD.vs.control; scRNA-seq,SRP215507,CD8+ T cell_6-AD.vs.control; scRNA-seq,SRP215507,CD8+ T cell_6-AD.vs.MCI; scRNA-seq,SRP215507,CD8+ T cell_7-MCI.vs.control; scRNA-seq,SRP330776,Naive CD8+ T cell_1-AD.vs.control; scRNA-seq,SRP330776,Memory T cell_1-AD.vs.control; scRNA-seq,SRP330776,B cell_2-AD.vs.control; scRNA-seq,SRP330776,Naive CD8+ T cell_2-AD.vs.control; scRNA-seq,SRP330776,Monocyte_2-AD.vs.control; scRNA-seq,SRP330776,Megakaryocyte_1-AD.vs.control; scRNA-	12
ANKRD12	bulk RNA-seq,Homo_723,AD.vs.control; bulk RNA-seq,Homo_723,AD.vs.MCI; bulk RNA-seq,Homo_633,AD.vs.control; bulk RNA-seq,Homo_633,AD.vs.MCI; bulk RNA-seq,Homo_714,AD.vs.control; bulk RNA-seq,Homo_714,AD.vs.MCI; scRNA-seq,SRP215507,CD8+ T cell_10-AD.vs.control; scRNA-seq,SRP215507,CD8+ T cell_8-AD.vs.MCI; scRNA-seq,SRP309935,Megakaryocyte_2-AD.vs.control; scRNA-seq,SRP330776,B	10

ANKRD13A	bulk RNA-seq,Homo_633,AD.vs.control; scRNA-seq,SRP215507,CD8+ T cell_7-AD.vs.control; scRNA-seq,SRP309935,Natural killer cell_2-AD.vs.control; scRNA-seq,SRP309935,Naive CD8+ T cell_1-AD.vs.control; scRNA-seq,SRP309935,CD8+ T cell_1-AD.vs.control; scRNA-seq,SRP309935,B cell_1-AD.vs.control; scRNA-seq,SRP330776,CD8+ T cell_1-AD.vs.control; scRNA-seq,SRP330776,γδ T cell-AD.vs.control; scRNA-seq,SRP330776,Memory T cell_2-AD.vs.control; scRNA-seq,SRP330776,CD8+ T cell_2-AD.vs.control; scRNA-seq,SRP330776,B cell_1-AD.vs.control; scRNA-seq,SRP330776,Naive CD8+ T cell_1-AD.vs.control; scRNA-seq,SRP330776,Natural killer cell_1-AD.vs.control; scRNA-seq,SRP330776,Memory T cell_1-AD.vs.control; scRNA-seq,SRP330776,Natural killer T cell-AD.vs.control; scRNA-seq,SRP330776,Naive CD8+ T cell_3-AD.vs.control; scRNA-seq,SRP330776,B cell_2-AD.vs.control; scRNA-seq,SRP330776,T cell-AD.vs.control; scRNA-seq,SRP330776,Naive CD8+ T cell_2-AD.vs.control; scRNA-seq,SRP330776,CD8+ T cell_3-AD.vs.control; scRNA-seq,SRP330776,Monocyte_1-AD.vs.control; scRNA-seq,SRP330776,Monocyte_2-AD.vs.control; scRNA-seq,SRP330776,B cell_3-AD.vs.control; scRNA-seq,SRP330776,Natural killer cell_2-AD.vs.control; scRNA-seq,SRP330776,B [illegible]	25
ANKRD13B	scRNA-seq,SRP215507,CD8+ T cell_3-MCI.vs.control; scRNA-seq,SRP215507,CD8+ T cell_6-MCI.vs.control	2
ANKRD13C	bulk RNA-seq,Homo_723,AD.vs.control; bulk RNA-seq,Homo_723,AD.vs.MCI; bulk RNA-seq,Homo_633,AD.vs.control; bulk RNA-seq,Homo_633,AD.vs.MCI; bulk RNA-seq,Homo_714,AD.vs.control; bulk RNA-seq,Homo_714,AD.vs.MCI; scRNA-seq,SRP215507,CD8+ T cell_4-AD.vs.MCI; scRNA-seq,SRP215507,CD8+ T cell_7-AD.vs.MCI; scRNA-seq,SRP215507,CD8+ T cell_7-MCI.vs.control; scRNA-seq,SRP309935,Monocyte_1-AD.vs.control; scRNA-seq,SRP309935,Natural killer cell_1-AD.vs.control	11
ANKRD13D	scRNA-seq,SRP215507,CD8+ T cell_5-AD.vs.control; scRNA-seq,SRP309935,Natural killer cell_1-AD.vs.control; scRNA-seq,SRP330776,CD8+ T cell_1-AD.vs.control; scRNA-seq,SRP330776,B cell_1-AD.vs.control; scRNA-seq,SRP330776,Naive CD8+ T cell_1-AD.vs.control; scRNA-seq,SRP330776,Natural killer cell_1-AD.vs.control; scRNA-seq,SRP330776,Memory T cell_1-AD.vs.control; scRNA-seq,SRP330776,Natural killer T cell-AD.vs.control; scRNA-seq,SRP330776,Naive CD8+ T cell_3-AD.vs.control; scRNA-seq,SRP330776,B cell_2-AD.vs.control; scRNA-seq,SRP330776,T cell-AD.vs.control; scRNA-seq,SRP330776,CD8+ T cell_3-AD.vs.control; scRNA-seq,SRP330776,Monocyte_2-AD.vs.control	13
ANKRD16	scRNA-seq,SRP215507,CD8+ T cell_2-AD.vs.control; scRNA-seq,SRP215507,CD8+ T cell_4-AD.vs.control; scRNA-seq,SRP215507,CD8+ T cell_4-MCI.vs.control; scRNA-seq,SRP330776,CD8+ T cell_1-AD.vs.control; scRNA-seq,SRP330776,γδ T cell-AD.vs.control; scRNA-seq,SRP330776,Memory T cell_2-AD.vs.control; scRNA-seq,SRP330776,CD8+ T cell_2-AD.vs.control; scRNA-seq,SRP330776,B cell_1-AD.vs.control; scRNA-seq,SRP330776,Naive CD8+ T cell_1-AD.vs.control; scRNA-seq,SRP330776,Memory T cell_1-AD.vs.control; scRNA-seq,SRP330776,Naive CD8+ T cell_3-AD.vs.control; scRNA-seq,SRP330776,CD8+ T cell_3-AD.vs.control; scRNA-seq,SRP330776,B cell_4-AD.vs.control	13
ANKRD17	bulk RNA-seq,Homo_723,MCI.vs.control; scRNA-seq,SRP215507,CD8+ T cell_7-AD.vs.control; scRNA-seq,SRP330776,γδ T cell-AD.vs.control; scRNA-seq,SRP330776,Memory T cell_2-AD.vs.control; scRNA-seq,SRP330776,CD8+ T cell_2-AD.vs.control; scRNA-seq,SRP330776,Naive CD8+ T cell_1-AD.vs.control; scRNA-seq,SRP330776,Natural killer cell_1-AD.vs.control; scRNA-seq,SRP330776,Memory T cell_1-AD.vs.control; scRNA-seq,SRP330776,B cell_2-AD.vs.control; scRNA-seq,SRP330776,Monocyte_2-AD.vs.control; scRNA-seq,SRP330776,Regulatory T cell-AD.vs.control;	12
ANKRD18A	bulk RNA-seq,Homo_723,AD.vs.control; bulk RNA-seq,Homo_723,AD.vs.MCI; bulk RNA-seq,Homo_633,AD.vs.control; bulk RNA-seq,Homo_633,MCI.vs.control; bulk RNA-seq,Homo_714,AD.vs.control; bulk RNA-seq,Homo_714,AD.vs.MCI; bulk RNA-seq,Homo_714,MCI.vs.control; bulk RNA-seq,ROSMAP,AD.vs.control; scRNA-seq,SRP215507,CD8+ T cell_2-AD.vs.control; scRNA-	11
ANKRD18B	bulk RNA-seq,Homo_723,AD.vs.control; bulk RNA-seq,Homo_723,AD.vs.MCI; bulk RNA-seq,Homo_633,AD.vs.control; bulk RNA-	4
ANKRD20A1	bulk RNA-seq,Homo_723,AD.vs.control; bulk RNA-seq,Homo_723,AD.vs.MCI; bulk RNA-seq,Homo_633,AD.vs.control; bulk RNA-seq,Homo_633,AD.vs.MCI; bulk RNA-seq,Homo_714,MCI.vs.control; scRNA-seq,SRP309935,Natural killer cell_1-AD.vs.control	6
ANKRD20A3P	bulk RNA-seq,ROSMAP,AD.vs.control; bulk RNA-seq,ROSMAP,MCI.vs.control	2
ANKRD20A4	scRNA-seq,SRP309935,Natural killer cell_2-AD.vs.control; scRNA-seq,SRP309935,Natural killer cell_1-AD.vs.control; scRNA-seq,SRP309935,CD8+ T	3
ANKRD22	bulk RNA-seq,Homo_723,AD.vs.control; bulk RNA-seq,Homo_723,AD.vs.MCI; bulk RNA-seq,Homo_633,AD.vs.control; bulk RNA-seq,Homo_633,AD.vs.MCI; bulk RNA-seq,Homo_714,AD.vs.control; bulk RNA-seq,Homo_714,AD.vs.MCI; bulk RNA-seq,ROSMAP,AD.vs.control; scRNA-seq,SRP215507,CD8+ T cell_2-AD.vs.MCI; scRNA-seq,SRP215507,CD8+ T cell_2-MCI.vs.control; scRNA-seq,SRP330776,Naive CD8+ T	10
ANKRD23	bulk RNA-seq,Homo_633,AD.vs.control; bulk RNA-seq,Homo_633,AD.vs.MCI; bulk RNA-seq,Homo_714,AD.vs.control; bulk RNA-seq,Homo_714,AD.vs.MCI; scRNA-seq,SRP215507,CD8+ T cell_2-AD.vs.control; scRNA-seq,SRP330776,CD8+ T cell_1-AD.vs.control	6
ANKRD24	bulk RNA-seq,Homo_633,AD.vs.control; bulk RNA-seq,ROSMAP,AD.vs.control	2

ANKRD26	bulk RNA-seq,Homo_723,AD.vs.control; bulk RNA-seq,Homo_723,AD.vs.MCI; bulk RNA-seq,Homo_633,AD.vs.control; bulk RNA-seq,Homo_633,AD.vs.MCI; bulk RNA-seq,Homo_714,AD.vs.control; bulk RNA-seq,Homo_714,AD.vs.MCI; scRNA-seq,SRP215507,CD8+ T cell_4-AD.vs.control; scRNA-seq,SRP215507,CD8+ T cell_5-MCI.vs.control; scRNA-seq,SRP215507,CD8+ T cell_4-MCI.vs.control; scRNA-seq,SRP309935,Megakaryocyte_1-AD.vs.control; scRNA-seq,SRP330776,CD8+ T cell_1-AD.vs.control; scRNA-seq,SRP330776,γδ T cell-AD.vs.control; scRNA-seq,SRP330776,Memory T cell_2-AD.vs.control; scRNA-seq,SRP330776,CD8+ T cell_2-AD.vs.control; scRNA-seq,SRP330776,B cell_1-AD.vs.control; scRNA-seq,SRP330776,Naive CD8+ T cell_1-AD.vs.control; scRNA-seq,SRP330776,Natural killer cell_1-AD.vs.control; scRNA-seq,SRP330776,Memory T cell_1-AD.vs.control; scRNA-seq,SRP330776,Natural killer T cell-AD.vs.control; scRNA-seq,SRP330776,B cell_2-AD.vs.control; scRNA-seq,SRP330776,T cell-AD.vs.control; scRNA-seq,SRP330776,Naive CD8+ T cell_2-AD.vs.control; scRNA-seq,SRP330776,CD8+ T cell_3-AD.vs.control; scRNA-seq,SRP330776,Monocyte_2-AD.vs.control; scRNA-seq,SRP330776,B cell_3-AD.vs.control; scRNA-[illegible]	26
ANKRD27	scRNA-seq,SRP215507,CD8+ T cell_5-AD.vs.MCI; scRNA-seq,SRP309935,Natural killer cell_2-AD.vs.control; scRNA-seq,SRP309935,Monocyte_1-AD.vs.control; scRNA-seq,SRP330776,Naive CD8+ T cell_2-AD.vs.control; scRNA-seq,SRP330776,Natural killer cell_2-AD.vs.control	5
ANKRD28	bulk RNA-seq,Homo_723,AD.vs.control; bulk RNA-seq,Homo_723,AD.vs.MCI; bulk RNA-seq,Homo_723,MCI.vs.control; bulk RNA-seq,Homo_633,AD.vs.control; bulk RNA-seq,Homo_633,AD.vs.MCI; bulk RNA-seq,Homo_633,MCI.vs.control; bulk RNA-seq,Homo_714,AD.vs.control; bulk RNA-seq,Homo_714,AD.vs.MCI; scRNA-seq,SRP215507,CD8+ T cell_4-AD.vs.control; scRNA-seq,SRP215507,CD8+ T cell_2-AD.vs.MCI; scRNA-seq,SRP215507,CD8+ T cell_4-AD.vs.MCI; scRNA-seq,SRP215507,CD8+ T cell_5-AD.vs.MCI; scRNA-seq,SRP215507,CD8+ T cell_5-MCI.vs.control; scRNA-seq,SRP215507,CD8+ T cell_2-MCI.vs.control; scRNA-seq,SRP309935,Monocyte_1-AD.vs.control; scRNA-seq,SRP330776,CD8+ T cell_1-AD.vs.control; scRNA-seq,SRP330776,Natural killer T cell-AD.vs.control; scRNA-seq,SRP330776,B cell_2-AD.vs.control; scRNA-seq,SRP330776,T cell-AD.vs.control; scRNA-seq,SRP330776,Monocyte_2-AD.vs.control; scRNA-seq,SRP330776,B cell_3-[illegible]	21
ANKRD29	bulk RNA-seq,Homo_723,AD.vs.control; bulk RNA-seq,Homo_723,AD.vs.MCI; bulk RNA-seq,Homo_723,MCI.vs.control; bulk RNA-seq,Homo_633,AD.vs.control; bulk RNA-seq,Homo_633,AD.vs.MCI; bulk RNA-seq,Homo_633,MCI.vs.control; scRNA-seq,SRP215507,CD8+ T cell_1-AD.vs.control; scRNA-seq,SRP330776,CD8+ T cell_1-AD.vs.control	8
ANKRD30A	bulk RNA-seq,Homo_723,MCI.vs.control; bulk RNA-seq,Homo_633,MCI.vs.control; bulk RNA-seq,Homo_714,MCI.vs.control; bulk RNA-seq,ROSMAP,AD.vs.control; bulk RNA-seq,ROSMAP,MCI.vs.control	5
ANKRD30B	bulk RNA-seq,Homo_723,AD.vs.control; bulk RNA-seq,Homo_714,MCI.vs.control	2
ANKRD30BL	bulk RNA-seq,Homo_723,MCI.vs.control; bulk RNA-seq,Homo_633,MCI.vs.control; bulk RNA-seq,Homo_714,MCI.vs.control; bulk RNA-seq,ROSMAP,AD.vs.control; bulk RNA-seq,ROSMAP,MCI.vs.control	5
ANKRD31	bulk RNA-seq,Homo_723,AD.vs.control; bulk RNA-seq,Homo_723,AD.vs.MCI; bulk RNA-seq,Homo_633,AD.vs.control; bulk RNA-seq,Homo_633,AD.vs.MCI; bulk RNA-seq,Homo_714,AD.vs.control; bulk RNA-seq,Homo_714,AD.vs.MCI; bulk RNA-seq,ROSMAP,MCI.vs.control; scRNA-seq,SRP215507,CD8+ T cell_2-AD.vs.control; scRNA-seq,SRP215507,CD8+ T cell_4-AD.vs.control; scRNA-seq,SRP215507,CD8+ T cell_2-AD.vs.MCI; scRNA-seq,SRP215507,CD8+ T cell_4-MCI.vs.control; scRNA-seq,SRP330776,CD8+ T cell_1-AD.vs.control; scRNA-seq,SRP330776,Memory T cell_2-AD.vs.control; scRNA-seq,SRP330776,B cell_1-AD.vs.control; scRNA-seq,SRP330776,Naive CD8+ T cell_1-	17
ANKRD33	bulk RNA-seq,Homo_723,AD.vs.control; bulk RNA-seq,Homo_633,AD.vs.control; bulk RNA-seq,Homo_633,MCI.vs.control; bulk RNA-	4
ANKRD33B	bulk RNA-seq,Homo_723,AD.vs.control; bulk RNA-seq,Homo_723,AD.vs.MCI; bulk RNA-seq,Homo_633,AD.vs.control; bulk RNA-seq,Homo_633,AD.vs.MCI; bulk RNA-seq,Homo_714,AD.vs.control; bulk RNA-seq,Homo_714,AD.vs.MCI; bulk RNA-seq,ROSMAP,AD.vs.control; scRNA-seq,SRP215507,CD8+ T cell_1-AD.vs.MCI; scRNA-seq,SRP215507,CD8+ T cell_1-MCI.vs.control; scRNA-seq,SRP330776,Memory T cell_2-AD.vs.control; scRNA-seq,SRP330776,B cell_1-AD.vs.control; scRNA-seq,SRP330776,B cell_2-AD.vs.control	12
ANKRD34A	bulk RNA-seq,Homo_723,AD.vs.control; bulk RNA-seq,Homo_723,AD.vs.MCI; bulk RNA-seq,Homo_633,AD.vs.control; bulk RNA-seq,Homo_633,AD.vs.MCI; bulk RNA-seq,Homo_714,AD.vs.control; bulk RNA-seq,Homo_714,AD.vs.MCI; scRNA-seq,SRP215507,CD8+ T cell_1-AD.vs.MCI; scRNA-seq,SRP215507,CD8+ T cell_1-MCI.vs.control; scRNA-seq,SRP330776,Memory T cell_2-AD.vs.control; scRNA-seq,SRP330776,Naive CD8+ T cell_1-AD.vs.control; scRNA-seq,SRP330776,B cell_2-AD.vs.control; scRNA-seq,SRP330776,CD8+ T cell_3-	12
ANKRD34B	bulk RNA-seq,Homo_723,AD.vs.control; bulk RNA-seq,Homo_723,AD.vs.MCI; bulk RNA-seq,Homo_633,AD.vs.control; bulk RNA-seq,Homo_633,AD.vs.MCI; bulk RNA-seq,Homo_714,AD.vs.control; bulk RNA-seq,Homo_714,AD.vs.MCI	6
ANKRD34C	bulk RNA-seq,Homo_723,AD.vs.control; bulk RNA-seq,Homo_633,AD.vs.control; bulk RNA-seq,Homo_714,MCI.vs.control	3

ANKRD35	bulk RNA-seq,Homo_723,AD.vs.control; bulk RNA-seq,Homo_723,AD.vs.MCI; bulk RNA-seq,Homo_633,AD.vs.control; bulk RNA-seq,Homo_633,AD.vs.MCI; bulk RNA-seq,Homo_714,AD.vs.control; bulk RNA-seq,Homo_714,AD.vs.MCI; scRNA-seq,SRP215507,CD8+ T cell_2-AD.vs.control; scRNA-seq,SRP215507,CD8+ T cell_4-AD.vs.control; scRNA-seq,SRP215507,CD8+ T cell_5-AD.vs.control; scRNA-seq,SRP215507,CD8+ T cell_2-AD.vs.MCI; scRNA-seq,SRP215507,CD8+ T cell_1-MCI.vs.control; scRNA-seq,SRP330776,T cell-AD.vs.control	12
ANKRD36	bulk RNA-seq,Homo_723,AD.vs.control; bulk RNA-seq,Homo_723,MCI.vs.control; bulk RNA-seq,Homo_633,AD.vs.MCI; bulk RNA-seq,Homo_633,MCI.vs.control; bulk RNA-seq,Homo_714,AD.vs.control; bulk RNA-seq,Homo_714,AD.vs.MCI; bulk RNA-seq,Homo_714,MCI.vs.control; scRNA-seq,SRP215507,CD8+ T cell_7-AD.vs.control; scRNA-seq,SRP215507,CD8+ T cell_5-AD.vs.control; scRNA-seq,SRP215507,CD8+ T cell_1-AD.vs.MCI; scRNA-seq,SRP215507,CD8+ T cell_7-AD.vs.MCI; scRNA-seq,SRP215507,CD8+ T cell_5-AD.vs.MCI; scRNA-seq,SRP215507,CD8+ T cell_6-AD.vs.MCI; scRNA-seq,SRP215507,CD8+ T cell_1-MCI.vs.control; scRNA-seq,SRP215507,CD8+ T cell_5-MCI.vs.control; scRNA-seq,SRP215507,CD8+ T cell_2-MCI.vs.control; scRNA-seq,SRP215507,CD8+ T cell_3-MCI.vs.control; scRNA-seq,SRP215507,CD8+ T cell_6-MCI.vs.control; scRNA-seq,SRP309935,Naive CD8+ T cell_1-AD.vs.control; scRNA-seq,SRP330776,γδ T cell-[illegible]	22
ANKRD36B	scRNA-seq,SRP215507,CD8+ T cell_4-AD.vs.MCI; scRNA-seq,SRP309935,Natural killer cell_2-AD.vs.control; scRNA-seq,SRP309935,Naive CD8+ T cell_1-AD.vs.control; scRNA-seq,SRP309935,Natural killer cell_1-AD.vs.control; scRNA-seq,SRP309935,Naive CD8+ T cell_2-AD.vs.control; scRNA-seq,SRP309935,CD8+ T cell_1-AD.vs.control; scRNA-seq,SRP309935,CD8+ T cell_2-AD.vs.control; scRNA-seq,SRP309935,Naive CD8+ T cell_3-AD.vs.control; scRNA-seq,SRP330776,B cell_3-AD.vs.control	9
ANKRD36C	scRNA-seq,SRP215507,CD8+ T cell_7-AD.vs.control; scRNA-seq,SRP215507,CD8+ T cell_2-AD.vs.MCI; scRNA-seq,SRP215507,CD8+ T cell_3-AD.vs.MCI; scRNA-seq,SRP215507,CD8+ T cell_1-AD.vs.MCI; scRNA-seq,SRP215507,CD8+ T cell_4-AD.vs.MCI; scRNA-seq,SRP215507,CD8+ T cell_1-MCI.vs.control; scRNA-seq,SRP215507,CD8+ T cell_2-MCI.vs.control; scRNA-seq,SRP309935,Natural killer cell_2-AD.vs.control; scRNA-seq,SRP309935,Monocyte_1-AD.vs.control; scRNA-seq,SRP309935,CD8+ T cell_1-AD.vs.control	10
ANKRD37	bulk RNA-seq,Homo_633,MCI.vs.control; scRNA-seq,SRP215507,CD8+ T cell_4-AD.vs.control; scRNA-seq,SRP215507,CD8+ T cell_5-AD.vs.MCI; scRNA-seq,SRP215507,CD8+ T cell_3-MCI.vs.control; scRNA-seq,SRP330776,CD8+ T cell_1-AD.vs.control; scRNA-seq,SRP330776,Memory T cell_2-AD.vs.control; scRNA-seq,SRP330776,CD8+ T cell_2-AD.vs.control; scRNA-seq,SRP330776,B cell_1-AD.vs.control; scRNA-seq,SRP330776,Naive CD8+ T cell_1-AD.vs.control; scRNA-seq,SRP330776,Natural killer cell_1-AD.vs.control; scRNA-seq,SRP330776,Memory T cell_1-AD.vs.control; scRNA-seq,SRP330776,Natural killer T cell-AD.vs.control; scRNA-seq,SRP330776,Naive CD8+ T cell_3-AD.vs.control; scRNA-seq,SRP330776,B cell_2-AD.vs.control; scRNA-seq,SRP330776,T cell-AD.vs.control; scRNA-seq,SRP330776,CD8+ T cell_3-AD.vs.control; scRNA-seq,SRP330776,Monocyte_1-AD.vs.control; scRNA-seq,SRP330776,Monocyte_2-AD.vs.control; scRNA-seq,SRP330776,B cell_3-AD.vs.control; scRNA-seq,SRP330776,Natural killer [illegible]	20
ANKRD39	bulk RNA-seq,Homo_714,AD.vs.control; bulk RNA-seq,Homo_714,AD.vs.MCI; scRNA-seq,SRP215507,CD8+ T cell_6-AD.vs.control; scRNA-seq,SRP215507,CD8+ T cell_3-AD.vs.MCI; scRNA-seq,SRP215507,CD8+ T cell_3-MCI.vs.control; scRNA-seq,SRP330776,Memory T cell_1-AD.vs.control; scRNA-seq,SRP330776,Natural killer T cell-AD.vs.control; scRNA-seq,SRP330776,T cell-AD.vs.control; scRNA-seq,SRP330776,Natural [illegible]	9
ANKRD40	scRNA-seq,SRP215507,CD8+ T cell_1-AD.vs.control; scRNA-seq,SRP215507,CD8+ T cell_4-AD.vs.control; scRNA-seq,SRP215507,CD8+ T cell_1-AD.vs.MCI; scRNA-seq,SRP215507,CD8+ T cell_4-MCI.vs.control; scRNA-seq,SRP330776,Monocyte_2-AD.vs.control; scRNA-seq,SRP330776,Natural [illegible]	6
ANKRD42	scRNA-seq,SRP215507,CD8+ T cell_5-AD.vs.MCI; scRNA-seq,SRP215507,CD8+ T cell_5-MCI.vs.control; scRNA-seq,SRP215507,CD8+ T cell_4-MCI.vs.control; scRNA-seq,SRP215507,CD8+ T cell_2-MCI.vs.control; scRNA-seq,SRP330776,CD8+ T cell_1-AD.vs.control; scRNA-seq,SRP330776,Naive CD8+ T cell_3-AD.vs.control; scRNA-seq,SRP330776,CD8+ T cell_3-AD.vs.control	7
ANKRD44	scRNA-seq,SRP215507,CD8+ T cell_6-AD.vs.control; scRNA-seq,SRP215507,CD8+ T cell_7-AD.vs.MCI; scRNA-seq,SRP215507,CD8+ T cell_7-MCI.vs.control; scRNA-seq,SRP330776,Naive CD8+ T cell_3-AD.vs.control; scRNA-seq,SRP330776,Monocyte_2-AD.vs.control	5
ANKRD44-IT1	scRNA-seq,SRP215507,CD8+ T cell_4-AD.vs.MCI; scRNA-seq,SRP215507,CD8+ T cell_5-AD.vs.MCI; scRNA-seq,SRP215507,CD8+ T cell_5-MCI.vs.control; scRNA-seq,SRP215507,CD8+ T cell_4-MCI.vs.control; scRNA-seq,SRP215507,CD8+ T cell_6-MCI.vs.control; scRNA-seq,SRP309935,Monocyte_1-AD.vs.control; scRNA-seq,SRP309935,CD8+ T cell_1-AD.vs.control; scRNA-seq,SRP309935,B cell_1-AD.vs.control; scRNA-seq,SRP309935,Naive CD8+ T cell_3-AD.vs.control; scRNA-seq,SRP330776,B cell_1-AD.vs.control; scRNA-seq,SRP330776,Naive CD8+ T [illegible]	11
ANKRD45	bulk RNA-seq,Homo_723,AD.vs.control; bulk RNA-seq,Homo_723,AD.vs.MCI; bulk RNA-seq,Homo_633,AD.vs.control; bulk RNA-seq,Homo_633,AD.vs.MCI; bulk RNA-seq,Homo_714,MCI.vs.control	5

ANKRD46	bulk RNA-seq,Homo_723,AD.vs.control; bulk RNA-seq,Homo_723,AD.vs.MCI; bulk RNA-seq,Homo_633,AD.vs.MCI; bulk RNA-seq,Homo_714,AD.vs.control; bulk RNA-seq,Homo_714,AD.vs.MCI; scRNA-seq,SRP215507,CD8+ T cell_5-AD.vs.MCI; scRNA-seq,SRP215507,CD8+ T cell_6-MCI.vs.control; scRNA-seq,SRP309935,Natural killer cell_1-AD.vs.control; scRNA-seq,SRP309935,Naive CD8+ T cell_3-AD.vs.control	9
ANKRD49	bulk RNA-seq,Homo_633,AD.vs.control; bulk RNA-seq,Homo_633,AD.vs.MCI; scRNA-seq,SRP215507,CD8+ T cell_6-AD.vs.MCI; scRNA-seq,SRP215507,CD8+ T cell_6-MCI.vs.control; scRNA-seq,SRP330776,CD8+ T cell_1-AD.vs.control; scRNA-seq,SRP330776,γδ T cell-AD.vs.control; scRNA-seq,SRP330776,Memory T cell_2-AD.vs.control; scRNA-seq,SRP330776,Natural killer cell_1-AD.vs.control; scRNA-seq,SRP330776,T cell-AD.vs.control; scRNA-seq,SRP330776,CD8+ T cell_3-AD.vs.control; scRNA-seq,SRP330776,Natural killer cell_2-AD.vs.control	11
ANKRD50	bulk RNA-seq,Homo_723,AD.vs.control; bulk RNA-seq,Homo_723,AD.vs.MCI; bulk RNA-seq,Homo_723,MCI.vs.control; bulk RNA-seq,Homo_633,AD.vs.control; bulk RNA-seq,Homo_633,AD.vs.MCI; bulk RNA-seq,Homo_633,MCI.vs.control; bulk RNA-seq,Homo_714,AD.vs.control; bulk RNA-seq,Homo_714,AD.vs.MCI; scRNA-seq,SRP309935,Monocyte_1-AD.vs.control	9
ANKRD52	bulk RNA-seq,SRP223445,AD.vs.control; scRNA-seq,SRP215507,CD8+ T cell_3-AD.vs.MCI; scRNA-seq,SRP215507,CD8+ T cell_3-MCI.vs.control; scRNA-seq,SRP215507,CD8+ T cell_6-MCI.vs.control; scRNA-seq,SRP330776,Monocyte_2-AD.vs.control; scRNA-seq,SRP330776,B cell_3-	6
ANKRD53	bulk RNA-seq,Homo_723,AD.vs.control; bulk RNA-seq,Homo_633,AD.vs.control; bulk RNA-seq,Homo_633,MCI.vs.control; bulk RNA-	4
ANKRD54	bulk RNA-seq,Homo_723,AD.vs.control; bulk RNA-seq,Homo_633,AD.vs.control; bulk RNA-seq,Homo_633,AD.vs.MCI; scRNA-seq,SRP215507,CD8+ T cell_5-AD.vs.MCI; scRNA-seq,SRP215507,CD8+ T cell_5-MCI.vs.control; scRNA-seq,SRP309935,Naive CD8+ T cell_2-AD.vs.control; scRNA-seq,SRP330776,CD8+ T cell_1-AD.vs.control; scRNA-seq,SRP330776,γδ T cell-AD.vs.control; scRNA-seq,SRP330776,Memory T cell_2-AD.vs.control; scRNA-seq,SRP330776,CD8+ T cell_2-AD.vs.control; scRNA-seq,SRP330776,B cell_1-AD.vs.control; scRNA-seq,SRP330776,Naive CD8+ T cell_1-AD.vs.control; scRNA-seq,SRP330776,Natural killer cell_1-AD.vs.control; scRNA-seq,SRP330776,Memory T cell_1-AD.vs.control; scRNA-seq,SRP330776,Natural killer T cell-AD.vs.control; scRNA-seq,SRP330776,Naive CD8+ T cell_3-AD.vs.control; scRNA-seq,SRP330776,B cell_2-AD.vs.control; scRNA-seq,SRP330776,T cell-AD.vs.control; scRNA-seq,SRP330776,CD8+ T cell_3-AD.vs.control; scRNA-seq,SRP330776,Monocyte_1- [illegible]	22
ANKRD55	bulk RNA-seq,Homo_633,AD.vs.control; bulk RNA-seq,Homo_714,AD.vs.control; bulk RNA-seq,Homo_714,AD.vs.MCI; scRNA-seq,SRP215507,CD8+ T cell_3-AD.vs.control; scRNA-seq,SRP215507,CD8+ T cell_3-MCI.vs.control; scRNA-seq,SRP330776,Memory T cell_2-AD.vs.control; scRNA-seq,SRP330776,Natural killer cell_1-AD.vs.control; scRNA-seq,SRP330776,Memory T cell_1-AD.vs.control; scRNA-seq,SRP330776,Natural killer T cell-	9
ANKRD6	scRNA-seq,SRP215507,CD8+ T cell_5-AD.vs.control; scRNA-seq,SRP215507,CD8+ T cell_5-AD.vs.MCI; scRNA-seq,SRP215507,CD8+ T cell_4-MCI.vs.control; scRNA-seq,SRP330776,CD8+ T cell_1-AD.vs.control; scRNA-seq,SRP330776,Memory T cell_2-AD.vs.control; scRNA-seq,SRP330776,CD8+ T cell_2-AD.vs.control; scRNA-seq,SRP330776,B cell_1-AD.vs.control; scRNA-seq,SRP330776,Naive CD8+ T cell_1-AD.vs.control; scRNA-seq,SRP330776,Memory T cell_1-AD.vs.control; scRNA-seq,SRP330776,CD8+ T cell_3-AD.vs.control	10
ANKRD60	bulk RNA-seq,Homo_723,MCI.vs.control; bulk RNA-seq,Homo_633,MCI.vs.control; bulk RNA-seq,Homo_714,MCI.vs.control; bulk RNA-	4
ANKRD61	bulk RNA-seq,Homo_723,AD.vs.control; bulk RNA-seq,Homo_723,MCI.vs.control; bulk RNA-seq,Homo_633,AD.vs.control; bulk RNA-seq,Homo_633,AD.vs.MCI; bulk RNA-seq,Homo_633,MCI.vs.control; bulk RNA-seq,Homo_714,AD.vs.control; bulk RNA-seq,Homo_714,AD.vs.MCI;	8
ANKRD62	bulk RNA-seq,Homo_723,AD.vs.control; bulk RNA-seq,Homo_723,MCI.vs.control; bulk RNA-seq,Homo_633,AD.vs.control; bulk RNA-seq,Homo_633,MCI.vs.control; bulk RNA-seq,Homo_714,MCI.vs.control	5
ANKRD65	bulk RNA-seq,Homo_723,AD.vs.control; bulk RNA-seq,Homo_723,AD.vs.MCI; bulk RNA-seq,Homo_723,MCI.vs.control; bulk RNA-seq,Homo_633,AD.vs.control; bulk RNA-seq,Homo_633,AD.vs.MCI	5
ANKRD66	bulk RNA-seq,Homo_723,AD.vs.control; bulk RNA-seq,Homo_723,AD.vs.MCI	2
Ankrd7	bulk RNA-seq,SRP325058,AD.vs.control	1
ANKRD7	scRNA-seq,SRP215507,CD8+ T cell_2-AD.vs.control	1
ANKRD9	bulk RNA-seq,ACOM,AD.vs.control; scRNA-seq,SRP215507,CD8+ T cell_3-AD.vs.MCI; scRNA-seq,SRP215507,CD8+ T cell_5-MCI.vs.control; scRNA-seq,SRP215507,CD8+ T cell_4-MCI.vs.control; scRNA-seq,SRP215507,CD8+ T cell_3-MCI.vs.control; scRNA-seq,SRP309935,Megakaryocyte_1-AD.vs.control; scRNA-seq,SRP309935,Natural killer cell_1-AD.vs.control	7

ANKS1A	bulk RNA-seq,Homo_723,AD.vs.control; bulk RNA-seq,Homo_723,AD.vs.MCI; bulk RNA-seq,Homo_633,AD.vs.control; bulk RNA-seq,Homo_633,AD.vs.MCI; bulk RNA-seq,Homo_633,MCI.vs.control; bulk RNA-seq,Homo_714,AD.vs.control; bulk RNA-seq,Homo_714,AD.vs.MCI; bulk RNA-seq,Homo_714,MCI.vs.control; scRNA-seq,SRP215507,CD8+ T cell_1-MCI.vs.control; scRNA-seq,SRP309935,Monocyte_1-AD.vs.control; scRNA-seq,SRP330776,γδ T cell-AD.vs.control; scRNA-seq,SRP330776,Memory T cell_2-AD.vs.control; scRNA-seq,SRP330776,CD8+ T cell_2-AD.vs.control; scRNA-seq,SRP330776,B cell_1-AD.vs.control; scRNA-seq,SRP330776,Naive CD8+ T cell_1-AD.vs.control; scRNA-seq,SRP330776,Natural killer cell_1-AD.vs.control; scRNA-seq,SRP330776,B cell_2-AD.vs.control; scRNA-seq,SRP330776,CD8+ T cell_3- [illegible]	19
ANKS1B	bulk RNA-seq,Homo_723,AD.vs.control; bulk RNA-seq,Homo_723,MCI.vs.control; bulk RNA-seq,Homo_633,AD.vs.control; bulk RNA-seq,Homo_633,AD.vs.MCI; bulk RNA-seq,Homo_633,MCI.vs.control; bulk RNA-seq,Homo_714,AD.vs.control; bulk RNA-seq,Homo_714,AD.vs.MCI; bulk RNA-seq,Homo_714,MCI.vs.control; scRNA-seq,SRP215507,CD8+ T cell_2-AD.vs.control; scRNA-seq,SRP215507,CD8+ T cell_1-AD.vs.control;	11
ANKS3	bulk RNA-seq,Homo_633,AD.vs.control; bulk RNA-seq,Homo_633,AD.vs.MCI; bulk RNA-seq,Homo_714,AD.vs.control; bulk RNA-seq,Homo_714,AD.vs.MCI; scRNA-seq,SRP215507,CD8+ T cell_5-AD.vs.MCI; scRNA-seq,SRP309935,Monocyte_2-AD.vs.control; scRNA-seq,SRP330776,CD8+ T cell_1-AD.vs.control; scRNA-seq,SRP330776,γδ T cell-AD.vs.control; scRNA-seq,SRP330776,Memory T cell_2-AD.vs.control; scRNA-seq,SRP330776,B cell_1-AD.vs.control; scRNA-seq,SRP330776,Natural killer cell_1-AD.vs.control; scRNA-seq,SRP330776,Memory T cell_1-AD.vs.control; scRNA-seq,SRP330776,B cell_2-AD.vs.control; scRNA-seq,SRP330776,T cell-AD.vs.control; scRNA-seq,SRP330776,CD8+ T cell_3-	15
ANKS4B	bulk RNA-seq,Homo_723,AD.vs.control; bulk RNA-seq,Homo_723,MCI.vs.control; bulk RNA-seq,Homo_633,AD.vs.control; bulk RNA-seq,Homo_633,MCI.vs.control; bulk RNA-seq,Homo_714,MCI.vs.control	5
ANKS6	bulk RNA-seq,Homo_723,AD.vs.control; bulk RNA-seq,Homo_723,AD.vs.MCI; bulk RNA-seq,Homo_723,MCI.vs.control; bulk RNA-seq,Homo_633,AD.vs.control; bulk RNA-seq,Homo_633,AD.vs.MCI; bulk RNA-seq,Homo_633,MCI.vs.control; bulk RNA-seq,Homo_714,AD.vs.control; bulk RNA-seq,Homo_714,AD.vs.MCI; bulk RNA-seq,Homo_714,MCI.vs.control; bulk RNA-seq,ROSMAP,AD.vs.control; scRNA-seq,SRP215507,CD8+ T cell_2-AD.vs.control; scRNA-seq,SRP215507,CD8+ T cell_2-AD.vs.MCI; scRNA-seq,SRP215507,CD8+ T cell_3-AD.vs.MCI; scRNA-seq,SRP215507,CD8+ T cell_3-MCI.vs.control; scRNA-seq,SRP330776,CD8+ T cell_1-AD.vs.control; scRNA-seq,SRP330776,B cell_1-AD.vs.control;	17
ANKUB1	bulk RNA-seq,Homo_723,AD.vs.control; bulk RNA-seq,Homo_723,AD.vs.MCI; bulk RNA-seq,Homo_633,AD.vs.control; bulk RNA-seq,Homo_633,AD.vs.MCI; bulk RNA-seq,Homo_714,AD.vs.control; bulk RNA-seq,Homo_714,AD.vs.MCI	6
ANKZF1	bulk RNA-seq,Homo_723,AD.vs.control; bulk RNA-seq,Homo_723,AD.vs.MCI; bulk RNA-seq,Homo_723,MCI.vs.control; bulk RNA-seq,Homo_633,AD.vs.control; bulk RNA-seq,Homo_633,AD.vs.MCI; bulk RNA-seq,Homo_633,MCI.vs.control; bulk RNA-seq,Homo_714,AD.vs.control; bulk RNA-seq,Homo_714,AD.vs.MCI; bulk RNA-seq,Homo_714,MCI.vs.control; scRNA-seq,SRP215507,CD8+ T cell_7-AD.vs.MCI; scRNA-seq,SRP215507,CD8+ T cell_5-AD.vs.MCI; scRNA-seq,SRP215507,CD8+ T cell_5-MCI.vs.control; scRNA-seq,SRP309935,Megakaryocyte_1-AD.vs.control; scRNA-seq,SRP330776,γδ T cell-AD.vs.control; scRNA-seq,SRP330776,B cell_2-AD.vs.control; scRNA-seq,SRP330776,CD8+ T cell_3-AD.vs.control; scRNA-seq,SRP330776,Monocyte_1-AD.vs.control; scRNA-seq,SRP330776,Monocyte_2-AD.vs.control; scRNA-seq,SRP330776,Natural [illegible]	19
ANLN	bulk RNA-seq,Homo_723,AD.vs.control; bulk RNA-seq,Homo_723,AD.vs.MCI; bulk RNA-seq,Homo_723,MCI.vs.control; bulk RNA-seq,Homo_633,AD.vs.control; bulk RNA-seq,Homo_633,AD.vs.MCI; bulk RNA-seq,Homo_633,MCI.vs.control; bulk RNA-seq,Homo_714,AD.vs.control; bulk RNA-seq,Homo_714,AD.vs.MCI; scRNA-seq,SRP215507,CD8+ T cell_3-MCI.vs.control; scRNA-seq,SRP215507,CD8+ T cell_6-MCI.vs.control;	11
ANO1	bulk RNA-seq,Homo_723,AD.vs.MCI; bulk RNA-seq,Homo_723,MCI.vs.control; bulk RNA-seq,Homo_633,AD.vs.MCI	3
ANO10	bulk RNA-seq,Homo_714,AD.vs.control; bulk RNA-seq,Homo_714,AD.vs.MCI; scRNA-seq,SRP215507,CD8+ T cell_3-AD.vs.control; scRNA-seq,SRP309935,Natural killer cell_1-AD.vs.control; scRNA-seq,SRP330776,Memory T cell_2-AD.vs.control; scRNA-seq,SRP330776,B cell_1-AD.vs.control; scRNA-seq,SRP330776,Naive CD8+ T cell_1-AD.vs.control; scRNA-seq,SRP330776,Natural killer cell_1-AD.vs.control; scRNA-seq,SRP330776,Naive CD8+ T cell_3-AD.vs.control; scRNA-seq,SRP330776,Monocyte_2-AD.vs.control	10
ANO2	bulk RNA-seq,Homo_723,AD.vs.control; bulk RNA-seq,Homo_723,AD.vs.MCI; bulk RNA-seq,Homo_723,MCI.vs.control; bulk RNA-seq,Homo_633,AD.vs.control; bulk RNA-seq,Homo_633,AD.vs.MCI; bulk RNA-seq,Homo_633,MCI.vs.control	6
ANO3	bulk RNA-seq,Homo_714,MCI.vs.control; bulk RNA-seq,ROSMAP,AD.vs.control; bulk RNA-seq,ROSMAP,MCI.vs.control	3
ANO4	bulk RNA-seq,Homo_714,MCI.vs.control; bulk RNA-seq,ROSMAP,AD.vs.control; bulk RNA-seq,ROSMAP,MCI.vs.control	3
ANO5	bulk RNA-seq,Homo_723,AD.vs.control; bulk RNA-seq,Homo_723,AD.vs.MCI; bulk RNA-seq,Homo_723,MCI.vs.control; bulk RNA-seq,Homo_633,AD.vs.MCI; bulk RNA-seq,Homo_633,MCI.vs.control; bulk RNA-seq,Homo_714,AD.vs.control; bulk RNA-seq,Homo_714,AD.vs.MCI	7

ANO6	bulk RNA-seq,Homo_723,AD.vs.control; bulk RNA-seq,Homo_723,AD.vs.MCI; bulk RNA-seq,Homo_723,MCI.vs.control; bulk RNA-seq,Homo_633,AD.vs.control; bulk RNA-seq,Homo_633,AD.vs.MCI; bulk RNA-seq,Homo_633,MCI.vs.control; bulk RNA-seq,Homo_714,AD.vs.control; bulk RNA-seq,Homo_714,AD.vs.MCI; scRNA-seq,SRP215507,CD8+ T cell_7-AD.vs.control; scRNA-seq,SRP215507,CD8+ T cell_2-AD.vs.MCI; scRNA-seq,SRP215507,CD8+ T cell_5-AD.vs.MCI; scRNA-seq,SRP215507,CD8+ T cell_5-MCI.vs.control; scRNA-seq,SRP215507,CD8+ T cell_2-MCI.vs.control; scRNA-seq,SRP330776,Natural killer T cell-AD.vs.control; scRNA-seq,SRP330776,Naive CD8+ T cell_3-AD.vs.control; scRNA-	16
ANO7	bulk RNA-seq,Homo_723,AD.vs.control; bulk RNA-seq,Homo_723,AD.vs.MCI; bulk RNA-seq,Homo_723,MCI.vs.control; bulk RNA-seq,Homo_633,AD.vs.control; bulk RNA-seq,Homo_633,MCI.vs.control; bulk RNA-seq,Homo_714,MCI.vs.control; scRNA-seq,SRP330776,B cell_1-	7
ANO8	scRNA-seq,SRP215507,CD8+ T cell_2-AD.vs.control; scRNA-seq,SRP215507,CD8+ T cell_3-AD.vs.MCI; scRNA-seq,SRP215507,CD8+ T cell_1-AD.vs.MCI; scRNA-seq,SRP215507,CD8+ T cell_1-MCI.vs.control; scRNA-seq,SRP215507,CD8+ T cell_3-MCI.vs.control; scRNA-seq,SRP330776,CD8+ T cell_1-AD.vs.control; scRNA-seq,SRP330776,Memory T cell_2-AD.vs.control; scRNA-seq,SRP330776,Naive CD8+ T cell_1-AD.vs.control; scRNA-seq,SRP330776,Memory T cell_1-AD.vs.control; scRNA-seq,SRP330776,CD8+ T cell_3-AD.vs.control	10
ANO9	bulk RNA-seq,ROSMAP,MCI.vs.control; scRNA-seq,SRP215507,CD8+ T cell_6-AD.vs.control; scRNA-seq,SRP215507,CD8+ T cell_5-MCI.vs.control; scRNA-seq,SRP309935,Natural killer cell_2-AD.vs.control; scRNA-seq,SRP330776,γδ T cell-AD.vs.control; scRNA-seq,SRP330776,CD8+ T cell_2-AD.vs.control; scRNA-seq,SRP330776,Natural killer T cell-AD.vs.control; scRNA-seq,SRP330776,Regulatory T cell-AD.vs.control	8
ANOS1	bulk RNA-seq,Homo_723,AD.vs.control; bulk RNA-seq,Homo_723,AD.vs.MCI; bulk RNA-seq,Homo_723,MCI.vs.control; bulk RNA-	4
ANP32A	scRNA-seq,SRP215507,CD8+ T cell_7-AD.vs.control; scRNA-seq,SRP330776,Megakaryocyte_1-AD.vs.control	2
ANP32B	scRNA-seq,SRP215507,CD8+ T cell_10-AD.vs.control; scRNA-seq,SRP330776,B cell_2-AD.vs.control	2
ANP32D	bulk RNA-seq,Homo_723,AD.vs.MCI; bulk RNA-seq,Homo_633,AD.vs.control; bulk RNA-seq,Homo_633,AD.vs.MCI; bulk RNA-seq,Homo_714,AD.vs.control; bulk RNA-seq,Homo_714,AD.vs.MCI	5
Anp32e	bulk RNA-seq,SRP325058,AD.vs.control	1
ANP32E	scRNA-seq,SRP215507,CD8+ T cell_7-AD.vs.control; scRNA-seq,SRP215507,CD8+ T cell_4-AD.vs.MCI; scRNA-seq,SRP215507,CD8+ T cell_5-AD.vs.MCI; scRNA-seq,SRP215507,CD8+ T cell_6-MCI.vs.control; scRNA-seq,SRP330776,Monocyte_2-AD.vs.control; scRNA-seq,SRP330776,B	6
ANPEP	bulk RNA-seq,SRP223445,AD.vs.control; scRNA-seq,SRP330776,B cell_1-AD.vs.control; scRNA-seq,SRP330776,Monocyte_1-AD.vs.control; scRNA-seq,SRP330776,Monocyte_2-AD.vs.control	4
ANTKMT	scRNA-seq,SRP215507,CD8+ T cell_5-AD.vs.MCI; scRNA-seq,SRP215507,CD8+ T cell_5-MCI.vs.control; scRNA-seq,SRP309935,Natural killer cell_2-AD.vs.control; scRNA-seq,SRP309935,Monocyte_1-AD.vs.control; scRNA-seq,SRP309935,Monocyte_2-AD.vs.control; scRNA-seq,SRP309935,Naive CD8+ T cell_2-AD.vs.control; scRNA-seq,SRP309935,CD8+ T cell_1-AD.vs.control; scRNA-seq,SRP309935,B cell_1-AD.vs.control; scRNA-seq,SRP309935,B cell_2-AD.vs.control; scRNA-seq,SRP330776,CD8+ T cell_1-AD.vs.control; scRNA-seq,SRP330776,γδ T cell-AD.vs.control; scRNA-seq,SRP330776,Memory T cell_2-AD.vs.control; scRNA-seq,SRP330776,CD8+ T cell_2-AD.vs.control; scRNA-seq,SRP330776,B cell_1-AD.vs.control; scRNA-seq,SRP330776,Naive CD8+ T cell_1-AD.vs.control; scRNA-seq,SRP330776,Natural killer cell_1-AD.vs.control; scRNA-seq,SRP330776,Memory T cell_1-AD.vs.control; scRNA-seq,SRP330776,Natural killer T cell-AD.vs.control; scRNA-seq,SRP330776,Naive CD8+ T cell_3-AD.vs.control; scRNA-seq,SRP330776,T cell-AD.vs.control; scRNA-seq,SRP330776,Naive CD8+ T cell_2-AD.vs.control; scRNA-seq,SRP330776,CD8+ T cell_3-AD.vs.control; scRNA-seq,SRP330776,Monocyte_1-AD.vs.control; scRNA-seq,SRP330776,B cell_3-AD.vs.control; scRNA-seq,SRP330776,Natural killer cell_2-AD.vs.control; scRNA-seq,SRP330776,B cell_4-AD.vs.control	26
ANTXR2	scRNA-seq,SRP215507,CD8+ T cell_2-AD.vs.MCI; scRNA-seq,SRP215507,CD8+ T cell_1-AD.vs.MCI; scRNA-seq,SRP215507,CD8+ T cell_5-AD.vs.MCI; scRNA-seq,SRP215507,CD8+ T cell_6-AD.vs.MCI; scRNA-seq,SRP215507,CD8+ T cell_1-MCI.vs.control; scRNA-seq,SRP215507,CD8+ T cell_5-MCI.vs.control; scRNA-seq,SRP309935,Megakaryocyte_1-AD.vs.control; scRNA-seq,SRP309935,Naive CD8+ T cell_1-AD.vs.control; scRNA-seq,SRP309935,Natural killer cell_1-AD.vs.control; scRNA-seq,SRP309935,CD8+ T cell_1-AD.vs.control; scRNA-seq,SRP309935,B cell_1-AD.vs.control; scRNA-seq,SRP309935,CD8+ T cell_2-AD.vs.control; scRNA-seq,SRP330776,γδ T cell-AD.vs.control; scRNA-seq,SRP330776,CD8+ T cell_2-AD.vs.control; scRNA-seq,SRP330776,Memory T cell_1-AD.vs.control; scRNA-seq,SRP330776,Monocyte_2-AD.vs.control; scRNA- [illegible]	18
ANTXRL	bulk RNA-seq,Homo_714,MCI.vs.control	1

ANXA1	bulk RNA-seq,Homo_723,AD.vs.MCI; bulk RNA-seq,Homo_633,AD.vs.control; bulk RNA-seq,Homo_633,AD.vs.MCI; bulk RNA-seq,Homo_714,AD.vs.control; bulk RNA-seq,Homo_714,AD.vs.MCI; bulk RNA-seq,SRP310421,AD.vs.control; scRNA-seq,SRP215507,CD8+ T cell_8-AD.vs.control; scRNA-seq,SRP215507,CD8+ T cell_3-AD.vs.control; scRNA-seq,SRP215507,CD8+ T cell_7-AD.vs.control; scRNA-seq,SRP215507,CD8+ T cell_10-AD.vs.control; scRNA-seq,SRP215507,CD8+ T cell_3-MCI.vs.control; scRNA-seq,SRP215507,CD8+ T cell_8-MCI.vs.control; scRNA-seq,SRP309935,Erythroid cell-AD.vs.control; scRNA-seq,SRP330776,B cell_2-AD.vs.control; scRNA-	15
Anxa1	bulk RNA-seq,SRP325058,AD.vs.control	1
ANXA11	scRNA-seq,SRP215507,CD8+ T cell_8-MCI.vs.control; scRNA-seq,SRP330776,CD8+ T cell_1-AD.vs.control; scRNA-seq,SRP330776,γδ T cell-AD.vs.control; scRNA-seq,SRP330776,Memory T cell_2-AD.vs.control; scRNA-seq,SRP330776,B cell_1-AD.vs.control; scRNA-seq,SRP330776,Naive CD8+ T cell_1-AD.vs.control; scRNA-seq,SRP330776,Natural killer cell_1-AD.vs.control; scRNA-seq,SRP330776,Naive CD8+ T cell_3-AD.vs.control; scRNA-seq,SRP330776,B cell_2-AD.vs.control; scRNA-seq,SRP330776,T cell-AD.vs.control; scRNA-seq,SRP330776,CD8+ T cell_3-AD.vs.control; scRNA-seq,SRP330776,B cell_3-AD.vs.control; scRNA-seq,SRP330776,Natural killer cell_2-AD.vs.control	13
ANXA2	scRNA-seq,SRP215507,CD8+ T cell_3-AD.vs.control; scRNA-seq,SRP215507,CD8+ T cell_3-AD.vs.MCI; scRNA-seq,SRP215507,CD8+ T cell_7-AD.vs.MCI; scRNA-seq,SRP215507,CD8+ T cell_3-MCI.vs.control; scRNA-seq,SRP215507,CD8+ T cell_8-MCI.vs.control; scRNA-seq,SRP309935,Erythroid cell-AD.vs.control; scRNA-seq,SRP330776,B cell_2-AD.vs.control; scRNA-seq,SRP330776,B cell_4-AD.vs.control	8
ANXA2R	scRNA-seq,SRP309935,Erythroid cell-AD.vs.control; scRNA-seq,SRP330776,CD8+ T cell_1-AD.vs.control; scRNA-seq,SRP330776,γδ T cell-AD.vs.control; scRNA-seq,SRP330776,Memory T cell_2-AD.vs.control; scRNA-seq,SRP330776,CD8+ T cell_2-AD.vs.control; scRNA-seq,SRP330776,B cell_1-AD.vs.control; scRNA-seq,SRP330776,Natural killer cell_1-AD.vs.control; scRNA-seq,SRP330776,Memory T cell_1-AD.vs.control; scRNA-seq,SRP330776,Natural killer T cell-AD.vs.control; scRNA-seq,SRP330776,T cell-AD.vs.control; scRNA-seq,SRP330776,Megakaryocyte_1-	12
ANXA3	bulk RNA-seq,Homo_714,AD.vs.control; bulk RNA-seq,Homo_714,AD.vs.MCI	2
ANXA4	bulk RNA-seq,Homo_723,AD.vs.control; bulk RNA-seq,Homo_723,AD.vs.MCI; bulk RNA-seq,Homo_633,AD.vs.control; bulk RNA-seq,Homo_633,AD.vs.MCI; bulk RNA-seq,Homo_714,AD.vs.control; bulk RNA-seq,Homo_714,AD.vs.MCI; scRNA-seq,SRP215507,CD8+ T cell_3-AD.vs.control; scRNA-seq,SRP215507,CD8+ T cell_7-AD.vs.control; scRNA-seq,SRP215507,CD8+ T cell_7-AD.vs.MCI; scRNA-seq,SRP215507,CD8+ T cell_6-AD.vs.MCI; scRNA-seq,SRP215507,CD8+ T cell_3-MCI.vs.control; scRNA-seq,SRP215507,CD8+ T cell_6-MCI.vs.control; scRNA-seq,SRP309935,Monocyte_2-AD.vs.control; scRNA-seq,SRP330776,CD8+ T cell_1-AD.vs.control; scRNA-seq,SRP330776,γδ T cell-AD.vs.control; scRNA-seq,SRP330776,Natural killer cell_1-AD.vs.control; scRNA-seq,SRP330776,B cell_2-AD.vs.control; scRNA-seq,SRP330776,Naive CD8+ T [illegible]	19
ANXA5	bulk RNA-seq,Homo_633,AD.vs.control; bulk RNA-seq,Homo_714,AD.vs.control; bulk RNA-seq,Homo_714,AD.vs.MCI; bulk RNA-seq,SRP310421,AD.vs.control; scRNA-seq,SRP215507,CD8+ T cell_3-AD.vs.control; scRNA-seq,SRP215507,CD8+ T cell_7-AD.vs.control; scRNA-seq,SRP215507,CD8+ T cell_3-AD.vs.MCI; scRNA-seq,SRP215507,CD8+ T cell_7-AD.vs.MCI; scRNA-seq,SRP215507,CD8+ T cell_6-AD.vs.MCI; scRNA-seq,SRP215507,CD8+ T cell_4-MCI.vs.control; scRNA-seq,SRP215507,CD8+ T cell_6-MCI.vs.control; scRNA-seq,SRP330776,CD8+ T cell_1-AD.vs.control; scRNA-seq,SRP330776,CD8+ T cell_2-AD.vs.control; scRNA-seq,SRP330776,Natural killer cell_1-AD.vs.control; scRNA-seq,SRP330776,Memory T cell_1-AD.vs.control; scRNA-seq,SRP330776,Natural killer T cell-AD.vs.control; scRNA-seq,SRP330776,B cell_3- [illegible]	19
ANXA6	bulk RNA-seq,Homo_723,AD.vs.control; bulk RNA-seq,Homo_723,AD.vs.MCI; bulk RNA-seq,Homo_723,MCI.vs.control; bulk RNA-seq,Homo_633,AD.vs.control; bulk RNA-seq,Homo_633,AD.vs.MCI; bulk RNA-seq,Homo_633,MCI.vs.control; bulk RNA-seq,Homo_714,AD.vs.control; bulk RNA-seq,Homo_714,AD.vs.MCI; bulk RNA-seq,Homo_714,MCI.vs.control; scRNA-seq,SRP215507,CD8+ T cell_7-AD.vs.control; scRNA-seq,SRP309935,Erythroid cell-AD.vs.control; scRNA-seq,SRP309935,Megakaryocyte_2-AD.vs.control; scRNA-seq,SRP309935,Dendritic cell-	14
ANXA7	scRNA-seq,SRP330776,Regulatory T cell-AD.vs.control; scRNA-seq,SRP330776,Megakaryocyte_1-AD.vs.control; scRNA-seq,SRP330776,B cell_4-	3
ANXA8L1	bulk RNA-seq,Homo_633,AD.vs.control	1

AOAH	scRNA-seq,SRP215507,CD8+ T cell_2-AD.vs.control; scRNA-seq,SRP215507,CD8+ T cell_1-AD.vs.control; scRNA-seq,SRP215507,CD8+ T cell_6-AD.vs.control; scRNA-seq,SRP215507,CD8+ T cell_2-AD.vs.MCI; scRNA-seq,SRP215507,CD8+ T cell_1-AD.vs.MCI; scRNA-seq,SRP215507,CD8+ T cell_4-AD.vs.MCI; scRNA-seq,SRP215507,CD8+ T cell_5-AD.vs.MCI; scRNA-seq,SRP215507,CD8+ T cell_6-AD.vs.MCI; scRNA-seq,SRP215507,CD8+ T cell_5-MCI.vs.control; scRNA-seq,SRP309935,Erythroid cell-AD.vs.control; scRNA-seq,SRP309935,Natural killer cell_1-AD.vs.control; scRNA-seq,SRP309935,CD8+ T cell_2-AD.vs.control; scRNA-seq,SRP330776,γδ T cell-AD.vs.control; scRNA-seq,SRP330776,Naive CD8+ T cell_1-AD.vs.control; scRNA-seq,SRP330776,Natural killer cell_1-AD.vs.control; scRNA-seq,SRP330776,Natural killer T cell-AD.vs.control; scRNA-seq,SRP330776,Naive CD8+ T cell_2-AD.vs.control; scRNA-seq,SRP330776,CD8+ T cell_3-AD.vs.control; scRNA-seq,SRP330776,B cell_3-[illegible]	19
AOC1	bulk RNA-seq,Homo_723,AD.vs.MCI; bulk RNA-seq,Homo_723,MCI.vs.control; bulk RNA-seq,Homo_633,AD.vs.MCI; bulk RNA-seq,Homo_633,MCI.vs.control; bulk RNA-seq,Homo_714,AD.vs.control; bulk RNA-seq,Homo_714,AD.vs.MCI; bulk RNA-seq,Homo_714,MCI.vs.control	7
AOC3	bulk RNA-seq,Homo_723,AD.vs.control; bulk RNA-seq,Homo_723,AD.vs.MCI; bulk RNA-seq,Homo_633,AD.vs.control; bulk RNA-seq,Homo_633,AD.vs.MCI; bulk RNA-seq,ROSMAP,AD.vs.control	5
AOPEP	scRNA-seq,SRP215507,CD8+ T cell_5-MCI.vs.control; scRNA-seq,SRP330776,Naive CD8+ T cell_1-AD.vs.control; scRNA-seq,SRP330776,Natural killer T cell-AD.vs.control; scRNA-seq,SRP330776,B cell_2-AD.vs.control; scRNA-seq,SRP330776,CD8+ T cell_3-AD.vs.control	5
AOX1	bulk RNA-seq,Homo_723,AD.vs.control; bulk RNA-seq,Homo_723,AD.vs.MCI; bulk RNA-seq,Homo_723,MCI.vs.control; bulk RNA-seq,Homo_633,AD.vs.control; bulk RNA-seq,Homo_633,AD.vs.MCI; bulk RNA-seq,Homo_633,MCI.vs.control	6
AP000253.1	scRNA-seq,SRP215507,CD8+ T cell_5-MCI.vs.control; scRNA-seq,SRP330776,CD8+ T cell_1-AD.vs.control	2
AP000254.2	scRNA-seq,SRP215507,CD8+ T cell_3-MCI.vs.control; scRNA-seq,SRP330776,Naive CD8+ T cell_1-AD.vs.control	2
AP000317.1	scRNA-seq,SRP215507,CD8+ T cell_1-AD.vs.MCI; scRNA-seq,SRP215507,CD8+ T cell_1-MCI.vs.control	2
AP000442.1	scRNA-seq,SRP215507,CD8+ T cell_3-AD.vs.control; scRNA-seq,SRP215507,CD8+ T cell_1-AD.vs.control; scRNA-seq,SRP330776,CD8+ T cell_1-AD.vs.control; scRNA-seq,SRP330776,Naive CD8+ T cell_1-AD.vs.control; scRNA-seq,SRP330776,Naive CD8+ T cell_3-AD.vs.control	5
AP000446.1	scRNA-seq,SRP215507,CD8+ T cell_2-AD.vs.control; scRNA-seq,SRP215507,CD8+ T cell_1-AD.vs.control; scRNA-seq,SRP215507,CD8+ T cell_1-AD.vs.MCI; scRNA-seq,SRP330776,CD8+ T cell_1-AD.vs.control; scRNA-seq,SRP330776,Memory T cell_2-AD.vs.control; scRNA-seq,SRP330776,Naive CD8+ T cell_1-AD.vs.control; scRNA-seq,SRP330776,Memory T cell_1-AD.vs.control; scRNA-seq,SRP330776,B cell_2-	9
AP000547.3	scRNA-seq,SRP215507,CD8+ T cell_3-AD.vs.control; scRNA-seq,SRP215507,CD8+ T cell_3-AD.vs.MCI; scRNA-seq,SRP215507,CD8+ T cell_3-MCI.vs.control; scRNA-seq,SRP309935,Monocyte_1-AD.vs.control	4
AP000640.2	scRNA-seq,SRP215507,CD8+ T cell_2-MCI.vs.control; scRNA-seq,SRP330776,B cell_1-AD.vs.control	2
AP000654.1	scRNA-seq,SRP330776,Memory T cell_1-AD.vs.control	1
AP000692.1	scRNA-seq,SRP309935,Monocyte_2-AD.vs.control	1
AP000692.2	scRNA-seq,SRP215507,CD8+ T cell_6-MCI.vs.control; scRNA-seq,SRP330776,CD8+ T cell_1-AD.vs.control; scRNA-seq,SRP330776,γδ T cell-AD.vs.control; scRNA-seq,SRP330776,Memory T cell_2-AD.vs.control; scRNA-seq,SRP330776,CD8+ T cell_2-AD.vs.control; scRNA-seq,SRP330776,B cell_1-AD.vs.control; scRNA-seq,SRP330776,Naive CD8+ T cell_1-AD.vs.control; scRNA-seq,SRP330776,Memory T cell_1-AD.vs.control; scRNA-	9
AP000704.1	scRNA-seq,SRP215507,CD8+ T cell_1-AD.vs.control; scRNA-seq,SRP215507,CD8+ T cell_1-MCI.vs.control; scRNA-seq,SRP215507,CD8+ T cell_5-MCI.vs.control; scRNA-seq,SRP330776,γδ T cell-AD.vs.control; scRNA-seq,SRP330776,CD8+ T cell_2-AD.vs.control; scRNA-seq,SRP330776,Naive CD8+ T cell_1-AD.vs.control; scRNA-seq,SRP330776,Memory T cell_1-AD.vs.control	7
AP000766.1	scRNA-seq,SRP215507,CD8+ T cell_4-AD.vs.control; scRNA-seq,SRP215507,CD8+ T cell_5-AD.vs.MCI; scRNA-seq,SRP215507,CD8+ T cell_5-MCI.vs.control; scRNA-seq,SRP215507,CD8+ T cell_4-MCI.vs.control; scRNA-seq,SRP330776,CD8+ T cell_1-AD.vs.control; scRNA-seq,SRP330776,γδ T cell-AD.vs.control; scRNA-seq,SRP330776,B cell_1-AD.vs.control; scRNA-seq,SRP330776,CD8+ T cell_3-AD.vs.control	8
AP000779.1	scRNA-seq,SRP330776,CD8+ T cell_3-AD.vs.control	1
AP000787.1	scRNA-seq,SRP215507,CD8+ T cell_1-AD.vs.MCI; scRNA-seq,SRP215507,CD8+ T cell_3-MCI.vs.control; scRNA-seq,SRP309935,CD8+ T cell_1-AD.vs.control; scRNA-seq,SRP309935,CD8+ T cell_2-AD.vs.control; scRNA-seq,SRP330776,B cell_1-AD.vs.control; scRNA-seq,SRP330776,Natural killer cell_1-AD.vs.control; scRNA-seq,SRP330776,CD8+ T cell_3-AD.vs.control	7

AP000845.1	scRNA-seq,SRP215507,CD8+ T cell_4-AD.vs.control; scRNA-seq,SRP215507,CD8+ T cell_1-AD.vs.MCI; scRNA-seq,SRP215507,CD8+ T cell_5-AD.vs.MCI; scRNA-seq,SRP215507,CD8+ T cell_1-MCI.vs.control; scRNA-seq,SRP330776,Memory T cell_2-AD.vs.control; scRNA-seq,SRP330776,Memory T cell_1-AD.vs.control; scRNA-seq,SRP330776,Naive CD8+ T cell_3-AD.vs.control	7
AP000866.1	scRNA-seq,SRP215507,CD8+ T cell_3-AD.vs.control; scRNA-seq,SRP215507,CD8+ T cell_1-AD.vs.control; scRNA-seq,SRP215507,CD8+ T cell_2-AD.vs.MCI; scRNA-seq,SRP215507,CD8+ T cell_2-MCI.vs.control; scRNA-seq,SRP330776,CD8+ T cell_1-AD.vs.control; scRNA-seq,SRP330776,γδ T cell-AD.vs.control; scRNA-seq,SRP330776,CD8+ T cell_2-AD.vs.control; scRNA-seq,SRP330776,Naive CD8+ T cell_1-AD.vs.control; scRNA-	9
AP000866.2	scRNA-seq,SRP215507,CD8+ T cell_2-MCI.vs.control; scRNA-seq,SRP330776,Naive CD8+ T cell_1-AD.vs.control	2
AP000866.5	scRNA-seq,SRP215507,CD8+ T cell_5-AD.vs.control; scRNA-seq,SRP215507,CD8+ T cell_5-MCI.vs.control; scRNA-seq,SRP330776,B cell_1-AD.vs.control; scRNA-seq,SRP330776,Naive CD8+ T cell_1-AD.vs.control	4
AP000866.6	scRNA-seq,SRP215507,CD8+ T cell_2-AD.vs.MCI; scRNA-seq,SRP215507,CD8+ T cell_4-AD.vs.MCI; scRNA-seq,SRP215507,CD8+ T cell_4-MCI.vs.control; scRNA-seq,SRP330776,Naive CD8+ T cell_1-AD.vs.control	4
AP000873.2	scRNA-seq,SRP215507,CD8+ T cell_1-AD.vs.control; scRNA-seq,SRP215507,CD8+ T cell_1-MCI.vs.control; scRNA-seq,SRP309935,Natural killer cell_1-AD.vs.control; scRNA-seq,SRP309935,Naive CD8+ T cell_2-AD.vs.control; scRNA-seq,SRP309935,CD8+ T cell_1-AD.vs.control; scRNA-seq,SRP330776,γδ T cell-AD.vs.control; scRNA-seq,SRP330776,Natural killer cell_1-AD.vs.control; scRNA-seq,SRP330776,Memory T cell_1-	9
AP000873.4	scRNA-seq,SRP330776,CD8+ T cell_1-AD.vs.control	1
AP000879.1	scRNA-seq,SRP330776,CD8+ T cell_1-AD.vs.control	1
AP000880.1	scRNA-seq,SRP215507,CD8+ T cell_3-AD.vs.MCI; scRNA-seq,SRP215507,CD8+ T cell_3-MCI.vs.control	2
AP000911.1	scRNA-seq,SRP215507,CD8+ T cell_3-AD.vs.MCI	1
AP000919.2	scRNA-seq,SRP215507,CD8+ T cell_1-MCI.vs.control	1
AP000926.2	scRNA-seq,SRP215507,CD8+ T cell_2-AD.vs.control; scRNA-seq,SRP215507,CD8+ T cell_3-AD.vs.control; scRNA-seq,SRP215507,CD8+ T cell_5-AD.vs.MCI; scRNA-seq,SRP215507,CD8+ T cell_3-MCI.vs.control; scRNA-seq,SRP215507,CD8+ T cell_6-MCI.vs.control; scRNA-seq,SRP330776,CD8+ T cell_1-AD.vs.control; scRNA-seq,SRP330776,Memory T cell_2-AD.vs.control; scRNA-seq,SRP330776,CD8+ T cell_2-AD.vs.control; scRNA-seq,SRP330776,B cell_1-AD.vs.control; scRNA-seq,SRP330776,Naive CD8+ T cell_1-AD.vs.control	10
AP000944.6	scRNA-seq,SRP215507,CD8+ T cell_5-AD.vs.MCI; scRNA-seq,SRP330776,CD8+ T cell_1-AD.vs.control; scRNA-seq,SRP330776,Memory T cell_2-AD.vs.control; scRNA-seq,SRP330776,Naive CD8+ T cell_1-AD.vs.control; scRNA-seq,SRP330776,Memory T cell_1-AD.vs.control; scRNA-	6
AP000977.1	scRNA-seq,SRP215507,CD8+ T cell_3-AD.vs.control; scRNA-seq,SRP215507,CD8+ T cell_2-MCI.vs.control; scRNA-seq,SRP330776,Memory T cell_2-AD.vs.control; scRNA-seq,SRP330776,B cell_1-AD.vs.control; scRNA-seq,SRP330776,Natural killer cell_1-AD.vs.control; scRNA-seq,SRP330776,Naive	6
AP001020.2	scRNA-seq,SRP215507,CD8+ T cell_1-AD.vs.MCI	1
AP001020.3	scRNA-seq,SRP215507,CD8+ T cell_5-MCI.vs.control	1
AP001033.1	scRNA-seq,SRP215507,CD8+ T cell_5-AD.vs.MCI; scRNA-seq,SRP215507,CD8+ T cell_3-MCI.vs.control; scRNA-seq,SRP330776,CD8+ T cell_2-	3
AP001033.2	scRNA-seq,SRP215507,CD8+ T cell_3-AD.vs.control; scRNA-seq,SRP215507,CD8+ T cell_1-AD.vs.control; scRNA-seq,SRP215507,CD8+ T cell_1-MCI.vs.control; scRNA-seq,SRP215507,CD8+ T cell_3-MCI.vs.control	4
AP001056.1	scRNA-seq,SRP330776,B cell_2-AD.vs.control	1
AP001059.3	scRNA-seq,SRP215507,CD8+ T cell_5-MCI.vs.control	1
AP001062.1	scRNA-seq,SRP215507,CD8+ T cell_2-AD.vs.MCI; scRNA-seq,SRP330776,CD8+ T cell_1-AD.vs.control; scRNA-seq,SRP330776,Naive CD8+ T cell_1-	3
AP001094.2	scRNA-seq,SRP215507,CD8+ T cell_4-MCI.vs.control	1
AP001107.2	scRNA-seq,SRP330776,B cell_1-AD.vs.control; scRNA-seq,SRP330776,Naive CD8+ T cell_1-AD.vs.control; scRNA-seq,SRP330776,CD8+ T cell_3-AD.vs.control; scRNA-seq,SRP330776,B cell_3-AD.vs.control	4
AP001107.9	scRNA-seq,SRP309935,CD8+ T cell_1-AD.vs.control; scRNA-seq,SRP330776,CD8+ T cell_1-AD.vs.control; scRNA-seq,SRP330776,Memory T cell_1-	3
AP001160.1	scRNA-seq,SRP309935,Natural killer cell_2-AD.vs.control; scRNA-seq,SRP309935,Natural killer cell_1-AD.vs.control; scRNA-seq,SRP309935,CD8+ T	3
AP001160.2	scRNA-seq,SRP215507,CD8+ T cell_1-AD.vs.MCI; scRNA-seq,SRP215507,CD8+ T cell_1-MCI.vs.control; scRNA-seq,SRP215507,CD8+ T cell_5-	3

AP001160.3	scRNA-seq,SRP215507,CD8+ T cell_1-AD.vs.control; scRNA-seq,SRP215507,CD8+ T cell_1-AD.vs.MCI	2
AP001189.1	scRNA-seq,SRP330776,Megakaryocyte_1-AD.vs.control	1
AP001267.1	scRNA-seq,SRP215507,CD8+ T cell_2-AD.vs.control; scRNA-seq,SRP215507,CD8+ T cell_1-AD.vs.MCI; scRNA-seq,SRP215507,CD8+ T cell_3-MCI.vs.control; scRNA-seq,SRP330776,CD8+ T cell_1-AD.vs.control; scRNA-seq,SRP330776,γδ T cell-AD.vs.control; scRNA-seq,SRP330776,Memory T cell_2-AD.vs.control; scRNA-seq,SRP330776,CD8+ T cell_2-AD.vs.control; scRNA-seq,SRP330776,Naive CD8+ T cell_1-AD.vs.control; scRNA-	9
AP001267.2	scRNA-seq,SRP215507,CD8+ T cell_3-MCI.vs.control	1
AP001267.4	scRNA-seq,SRP215507,CD8+ T cell_2-AD.vs.control; scRNA-seq,SRP215507,CD8+ T cell_4-AD.vs.control; scRNA-seq,SRP215507,CD8+ T cell_2-	3
AP001269.4	scRNA-seq,SRP215507,CD8+ T cell_2-AD.vs.MCI; scRNA-seq,SRP215507,CD8+ T cell_2-MCI.vs.control; scRNA-seq,SRP309935,B cell_1-	3
AP001318.2	scRNA-seq,SRP215507,CD8+ T cell_6-AD.vs.control	1
AP001347.1	scRNA-seq,SRP215507,CD8+ T cell_4-AD.vs.control; scRNA-seq,SRP215507,CD8+ T cell_2-AD.vs.MCI; scRNA-seq,SRP215507,CD8+ T cell_4-AD.vs.MCI; scRNA-seq,SRP215507,CD8+ T cell_2-MCI.vs.control; scRNA-seq,SRP330776,Memory T cell_2-AD.vs.control; scRNA-seq,SRP330776,B	6
AP001350.1	scRNA-seq,SRP330776,Memory T cell_1-AD.vs.control	1
AP001363.2	scRNA-seq,SRP309935,Monocyte_1-AD.vs.control	1
AP001372.2	scRNA-seq,SRP215507,CD8+ T cell_6-MCI.vs.control; scRNA-seq,SRP309935,Naive CD8+ T cell_1-AD.vs.control; scRNA-seq,SRP309935,Natural killer cell_1-AD.vs.control; scRNA-seq,SRP309935,Naive CD8+ T cell_2-AD.vs.control; scRNA-seq,SRP309935,CD8+ T cell_1-AD.vs.control; scRNA-seq,SRP330776,CD8+ T cell_1-AD.vs.control; scRNA-seq,SRP330776,γδ T cell-AD.vs.control; scRNA-seq,SRP330776,Memory T cell_2-AD.vs.control; scRNA-seq,SRP330776,CD8+ T cell_2-AD.vs.control; scRNA-seq,SRP330776,B cell_1-AD.vs.control; scRNA-seq,SRP330776,Naive CD8+ T cell_1-AD.vs.control; scRNA-seq,SRP330776,Natural killer cell_1-AD.vs.control; scRNA-seq,SRP330776,Memory T cell_1-AD.vs.control; scRNA-seq,SRP330776,Natural killer T cell-AD.vs.control; scRNA-seq,SRP330776,Naive CD8+ T cell_3-AD.vs.control	15
AP001412.1	scRNA-seq,SRP215507,CD8+ T cell_1-AD.vs.MCI; scRNA-seq,SRP215507,CD8+ T cell_1-MCI.vs.control; scRNA-seq,SRP330776,CD8+ T cell_2-AD.vs.control; scRNA-seq,SRP330776,Naive CD8+ T cell_1-AD.vs.control	4
AP001432.1	scRNA-seq,SRP215507,CD8+ T cell_1-AD.vs.control	1
AP001453.1	scRNA-seq,SRP330776,Naive CD8+ T cell_1-AD.vs.control	1
AP001453.3	scRNA-seq,SRP215507,CD8+ T cell_1-AD.vs.MCI; scRNA-seq,SRP215507,CD8+ T cell_1-MCI.vs.control; scRNA-seq,SRP330776,CD8+ T cell_1-AD.vs.control; scRNA-seq,SRP330776,γδ T cell-AD.vs.control; scRNA-seq,SRP330776,Memory T cell_2-AD.vs.control; scRNA-seq,SRP330776,CD8+ T cell_2-AD.vs.control; scRNA-seq,SRP330776,B cell_1-AD.vs.control; scRNA-seq,SRP330776,Naive CD8+ T cell_1-AD.vs.control; scRNA-seq,SRP330776,Natural killer cell_1-AD.vs.control; scRNA-seq,SRP330776,Memory T cell_1-AD.vs.control	10
AP001462.1	scRNA-seq,SRP215507,CD8+ T cell_1-MCI.vs.control; scRNA-seq,SRP215507,CD8+ T cell_2-MCI.vs.control; scRNA-seq,SRP309935,Natural killer cell_2-AD.vs.control; scRNA-seq,SRP309935,Naive CD8+ T cell_1-AD.vs.control; scRNA-seq,SRP309935,Naive CD8+ T cell_2-AD.vs.control; scRNA-seq,SRP330776,CD8+ T cell_1-AD.vs.control; scRNA-seq,SRP330776,CD8+ T cell_2-AD.vs.control; scRNA-seq,SRP330776,Naive CD8+ T cell_1-	8
AP001469.3	scRNA-seq,SRP215507,CD8+ T cell_4-AD.vs.control; scRNA-seq,SRP215507,CD8+ T cell_4-MCI.vs.control; scRNA-seq,SRP330776,Memory T cell_1-	3
AP001496.1	scRNA-seq,SRP215507,CD8+ T cell_2-AD.vs.MCI; scRNA-seq,SRP215507,CD8+ T cell_2-MCI.vs.control; scRNA-seq,SRP215507,CD8+ T cell_3-MCI.vs.control; scRNA-seq,SRP330776,Naive CD8+ T cell_1-AD.vs.control	4
AP001505.1	scRNA-seq,SRP215507,CD8+ T cell_4-AD.vs.control; scRNA-seq,SRP309935,Naive CD8+ T cell_1-AD.vs.control	2
AP001610.2	scRNA-seq,SRP215507,CD8+ T cell_5-AD.vs.control; scRNA-seq,SRP330776,CD8+ T cell_3-AD.vs.control	2
AP001631.2	scRNA-seq,SRP215507,CD8+ T cell_1-AD.vs.control	1
AP001636.3	scRNA-seq,SRP330776,B cell_1-AD.vs.control	1
AP001816.1	scRNA-seq,SRP215507,CD8+ T cell_1-AD.vs.MCI; scRNA-seq,SRP215507,CD8+ T cell_5-AD.vs.MCI; scRNA-seq,SRP215507,CD8+ T cell_1-MCI.vs.control; scRNA-seq,SRP215507,CD8+ T cell_5-MCI.vs.control; scRNA-seq,SRP330776,γδ T cell-AD.vs.control; scRNA-seq,SRP330776,Naive CD8+ T cell_1-AD.vs.control; scRNA-seq,SRP330776,Monocyte_2-AD.vs.control; scRNA-seq,SRP330776,Natural killer cell_2-AD.vs.control	8
AP001830.1	scRNA-seq,SRP330776,CD8+ T cell_1-AD.vs.control	1

AP001831.1	scRNA-seq,SRP215507,CD8+ T cell_3-AD.vs.control	1
AP001885.2	scRNA-seq,SRP215507,CD8+ T cell_2-MCI.vs.control	1
AP001885.3	scRNA-seq,SRP330776,CD8+ T cell_1-AD.vs.control; scRNA-seq,SRP330776,B cell_1-AD.vs.control; scRNA-seq,SRP330776,Naive CD8+ T cell_1-AD.vs.control; scRNA-seq,SRP330776,T cell-AD.vs.control	4
AP001893.1	scRNA-seq,SRP215507,CD8+ T cell_2-AD.vs.MCI; scRNA-seq,SRP215507,CD8+ T cell_2-MCI.vs.control	2
AP002026.1	scRNA-seq,SRP215507,CD8+ T cell_1-AD.vs.control; scRNA-seq,SRP215507,CD8+ T cell_3-AD.vs.MCI; scRNA-seq,SRP215507,CD8+ T cell_4-MCI.vs.control; scRNA-seq,SRP215507,CD8+ T cell_3-MCI.vs.control; scRNA-seq,SRP330776,CD8+ T cell_1-AD.vs.control; scRNA-seq,SRP330776,Natural killer T cell-AD.vs.control; scRNA-seq,SRP330776,B cell_2-AD.vs.control; scRNA-seq,SRP330776,T cell-AD.vs.control	8
AP002360.1	scRNA-seq,SRP215507,CD8+ T cell_4-AD.vs.control; scRNA-seq,SRP215507,CD8+ T cell_3-MCI.vs.control; scRNA-seq,SRP330776,CD8+ T cell_1-AD.vs.control; scRNA-seq,SRP330776,γδ T cell-AD.vs.control; scRNA-seq,SRP330776,CD8+ T cell_2-AD.vs.control; scRNA-seq,SRP330776,Naive CD8+ T cell_1-AD.vs.control; scRNA-seq,SRP330776,Memory T cell_1-AD.vs.control; scRNA-seq,SRP330776,Naive CD8+ T cell_3-AD.vs.control; scRNA-seq,SRP330776,B cell_2-AD.vs.control; scRNA-seq,SRP330776,B cell_3-AD.vs.control; scRNA-seq,SRP330776,Natural killer cell_2-	11
AP002381.2	scRNA-seq,SRP215507,CD8+ T cell_6-AD.vs.control; scRNA-seq,SRP215507,CD8+ T cell_4-AD.vs.MCI; scRNA-seq,SRP330776,CD8+ T cell_3-	3
AP002387.2	scRNA-seq,SRP215507,CD8+ T cell_4-AD.vs.control; scRNA-seq,SRP215507,CD8+ T cell_6-AD.vs.control; scRNA-seq,SRP215507,CD8+ T cell_4-AD.vs.MCI; scRNA-seq,SRP215507,CD8+ T cell_6-AD.vs.MCI; scRNA-seq,SRP215507,CD8+ T cell_4-MCI.vs.control; scRNA-seq,SRP309935,Naive CD8+ T cell_1-AD.vs.control; scRNA-seq,SRP309935,Natural killer cell_1-AD.vs.control; scRNA-seq,SRP309935,Naive CD8+ T cell_2-AD.vs.control; scRNA-seq,SRP309935,CD8+ T cell_1-AD.vs.control; scRNA-seq,SRP330776,Natural killer T cell-AD.vs.control	10
AP002490.1	scRNA-seq,SRP215507,CD8+ T cell_5-AD.vs.control; scRNA-seq,SRP215507,CD8+ T cell_3-MCI.vs.control; scRNA-seq,SRP330776,Memory T cell_2-AD.vs.control; scRNA-seq,SRP330776,CD8+ T cell_2-AD.vs.control; scRNA-seq,SRP330776,Natural killer T cell-AD.vs.control; scRNA-seq,SRP330776,Naive CD8+ T cell_3-AD.vs.control; scRNA-seq,SRP330776,CD8+ T cell_3-AD.vs.control; scRNA-seq,SRP330776,B cell_3-AD.vs.control; scRNA-seq,SRP330776,Regulatory T cell-AD.vs.control; scRNA-seq,SRP330776,Megakaryocyte_1-AD.vs.control	10
AP002495.1	scRNA-seq,SRP330776,γδ T cell-AD.vs.control; scRNA-seq,SRP330776,Memory T cell_2-AD.vs.control; scRNA-seq,SRP330776,B cell_1-AD.vs.control; scRNA-seq,SRP330776,Naive CD8+ T cell_3-AD.vs.control; scRNA-seq,SRP330776,B cell_2-AD.vs.control; scRNA-seq,SRP330776,T cell-AD.vs.control; scRNA-seq,SRP330776,CD8+ T cell_3-AD.vs.control; scRNA-seq,SRP330776,Natural killer cell_2-AD.vs.control	8
AP002505.3	scRNA-seq,SRP330776,Memory T cell_2-AD.vs.control	1
AP002748.4	scRNA-seq,SRP215507,CD8+ T cell_4-AD.vs.control; scRNA-seq,SRP215507,CD8+ T cell_4-AD.vs.MCI; scRNA-seq,SRP330776,γδ T cell-AD.vs.control; scRNA-seq,SRP330776,Memory T cell_2-AD.vs.control; scRNA-seq,SRP330776,CD8+ T cell_2-AD.vs.control; scRNA-seq,SRP330776,Naive CD8+ T cell_1-AD.vs.control; scRNA-seq,SRP330776,Naive CD8+ T cell_3-AD.vs.control; scRNA-seq,SRP330776,B cell_3-	9
AP002761.4	scRNA-seq,SRP309935,Monocyte_2-AD.vs.control	1
AP002807.1	scRNA-seq,SRP215507,CD8+ T cell_3-AD.vs.MCI; scRNA-seq,SRP309935,Natural killer cell_2-AD.vs.control; scRNA-seq,SRP330776,CD8+ T cell_2-AD.vs.control; scRNA-seq,SRP330776,Naive CD8+ T cell_1-AD.vs.control	4
AP002812.5	scRNA-seq,SRP330776,Memory T cell_1-AD.vs.control	1
AP002813.1	scRNA-seq,SRP330776,Naive CD8+ T cell_2-AD.vs.control	1
AP002851.1	scRNA-seq,SRP215507,CD8+ T cell_1-AD.vs.control; scRNA-seq,SRP330776,CD8+ T cell_1-AD.vs.control	2
AP002954.1	scRNA-seq,SRP215507,CD8+ T cell_2-AD.vs.MCI; scRNA-seq,SRP215507,CD8+ T cell_3-AD.vs.MCI; scRNA-seq,SRP215507,CD8+ T cell_2-MCI.vs.control; scRNA-seq,SRP215507,CD8+ T cell_3-MCI.vs.control	4
AP002982.2	scRNA-seq,SRP215507,CD8+ T cell_5-AD.vs.MCI; scRNA-seq,SRP330776,Naive CD8+ T cell_1-AD.vs.control	2

AP003068.2	scRNA-seq,SRP215507,CD8+ T cell_1-AD.vs.control; scRNA-seq,SRP215507,CD8+ T cell_1-MCI.vs.control; scRNA-seq,SRP309935,Monocyte_1-AD.vs.control; scRNA-seq,SRP309935,Megakaryocyte_1-AD.vs.control; scRNA-seq,SRP309935,Naive CD8+ T cell_1-AD.vs.control; scRNA-seq,SRP309935,Monocyte_2-AD.vs.control; scRNA-seq,SRP309935,CD8+ T cell_1-AD.vs.control; scRNA-seq,SRP309935,Megakaryocyte_2-AD.vs.control; scRNA-seq,SRP309935,B cell_1-AD.vs.control; scRNA-seq,SRP309935,Monocyte_3-AD.vs.control; scRNA-seq,SRP330776,CD8+ T cell_1-AD.vs.control; scRNA-seq,SRP330776,Memory T cell_2-AD.vs.control; scRNA-seq,SRP330776,CD8+ T cell_2-AD.vs.control; scRNA-seq,SRP330776,B cell_1-AD.vs.control; scRNA-seq,SRP330776,Naive CD8+ T cell_1-AD.vs.control; scRNA-seq,SRP330776,Memory T cell_1-	18
AP003096.1	scRNA-seq,SRP215507,CD8+ T cell_2-MCI.vs.control	1
AP003117.1	scRNA-seq,SRP330776,Memory T cell_1-AD.vs.control	1
AP003307.1	scRNA-seq,SRP215507,CD8+ T cell_1-AD.vs.control; scRNA-seq,SRP215507,CD8+ T cell_3-AD.vs.MCI; scRNA-seq,SRP330776,Natural killer cell_1-AD.vs.control; scRNA-seq,SRP330776,Memory T cell_1-AD.vs.control; scRNA-seq,SRP330776,Naive CD8+ T cell_3-AD.vs.control; scRNA-seq,SRP330776,CD8+ T cell_3-AD.vs.control; scRNA-seq,SRP330776,Natural killer cell_2-AD.vs.control	7
AP003327.2	scRNA-seq,SRP215507,CD8+ T cell_3-AD.vs.control; scRNA-seq,SRP215507,CD8+ T cell_3-AD.vs.MCI; scRNA-seq,SRP309935,Naive CD8+ T cell_2-	3
AP003352.1	scRNA-seq,SRP215507,CD8+ T cell_2-AD.vs.control; scRNA-seq,SRP215507,CD8+ T cell_5-AD.vs.control; scRNA-seq,SRP309935,CD4+ T cell-AD.vs.control; scRNA-seq,SRP330776,CD8+ T cell_1-AD.vs.control; scRNA-seq,SRP330776,CD8+ T cell_2-AD.vs.control; scRNA-seq,SRP330776,B	6
AP003392.5	scRNA-seq,SRP215507,CD8+ T cell_2-AD.vs.control; scRNA-seq,SRP215507,CD8+ T cell_1-MCI.vs.control; scRNA-seq,SRP330776,Natural killer T cell-AD.vs.control; scRNA-seq,SRP330776,CD8+ T cell_3-AD.vs.control	4
AP003469.4	scRNA-seq,SRP215507,CD8+ T cell_2-AD.vs.control; scRNA-seq,SRP330776,CD8+ T cell_1-AD.vs.control	2
AP003486.1	scRNA-seq,SRP215507,CD8+ T cell_2-AD.vs.control; scRNA-seq,SRP215507,CD8+ T cell_4-AD.vs.control; scRNA-seq,SRP215507,CD8+ T cell_5-AD.vs.control; scRNA-seq,SRP215507,CD8+ T cell_5-AD.vs.MCI; scRNA-seq,SRP215507,CD8+ T cell_5-MCI.vs.control; scRNA-seq,SRP309935,Natural killer cell_2-AD.vs.control; scRNA-seq,SRP330776,CD8+ T cell_1-AD.vs.control; scRNA-seq,SRP330776,B cell_1-AD.vs.control; scRNA-seq,SRP330776,Naive CD8+ T cell_1-AD.vs.control; scRNA-seq,SRP330776,Memory T cell_1-AD.vs.control	10
AP003557.1	scRNA-seq,SRP215507,CD8+ T cell_3-AD.vs.MCI; scRNA-seq,SRP215507,CD8+ T cell_3-MCI.vs.control	2
AP003680.1	scRNA-seq,SRP215507,CD8+ T cell_1-AD.vs.control; scRNA-seq,SRP215507,CD8+ T cell_1-MCI.vs.control; scRNA-seq,SRP215507,CD8+ T cell_5-MCI.vs.control; scRNA-seq,SRP330776,γδ T cell-AD.vs.control; scRNA-seq,SRP330776,Memory T cell_1-AD.vs.control	5
AP003717.4	scRNA-seq,SRP215507,CD8+ T cell_3-AD.vs.MCI; scRNA-seq,SRP215507,CD8+ T cell_5-MCI.vs.control; scRNA-seq,SRP215507,CD8+ T cell_3-MCI.vs.control; scRNA-seq,SRP330776,γδ T cell-AD.vs.control; scRNA-seq,SRP330776,Naive CD8+ T cell_1-AD.vs.control; scRNA-seq,SRP330776,Memory T cell_1-AD.vs.control; scRNA-seq,SRP330776,Naive CD8+ T cell_3-AD.vs.control	7
AP003721.4	scRNA-seq,SRP215507,CD8+ T cell_3-AD.vs.MCI; scRNA-seq,SRP215507,CD8+ T cell_1-MCI.vs.control; scRNA-seq,SRP215507,CD8+ T cell_3-	3
AP003774.2	scRNA-seq,SRP215507,CD8+ T cell_1-AD.vs.MCI; scRNA-seq,SRP215507,CD8+ T cell_1-MCI.vs.control; scRNA-seq,SRP330776,CD8+ T cell_1-AD.vs.control; scRNA-seq,SRP330776,CD8+ T cell_2-AD.vs.control; scRNA-seq,SRP330776,Naive CD8+ T cell_1-AD.vs.control; scRNA-	6
AP004609.3	scRNA-seq,SRP215507,CD8+ T cell_2-AD.vs.control; scRNA-seq,SRP215507,CD8+ T cell_4-AD.vs.control; scRNA-seq,SRP215507,CD8+ T cell_4-AD.vs.MCI; scRNA-seq,SRP215507,CD8+ T cell_2-MCI.vs.control; scRNA-seq,SRP215507,CD8+ T cell_3-MCI.vs.control; scRNA-seq,SRP330776,CD8+ T cell_1-AD.vs.control; scRNA-seq,SRP330776,Memory T cell_2-AD.vs.control; scRNA-seq,SRP330776,B cell_1-AD.vs.control; scRNA-seq,SRP330776,Naive CD8+ T cell_1-AD.vs.control; scRNA-seq,SRP330776,Natural killer T cell-AD.vs.control; scRNA-seq,SRP330776,B cell_2-	11
AP005019.1	scRNA-seq,SRP215507,CD8+ T cell_3-AD.vs.control; scRNA-seq,SRP215507,CD8+ T cell_7-AD.vs.control; scRNA-seq,SRP215507,CD8+ T cell_2-AD.vs.MCI; scRNA-seq,SRP215507,CD8+ T cell_4-AD.vs.MCI; scRNA-seq,SRP215507,CD8+ T cell_5-AD.vs.MCI; scRNA-seq,SRP215507,CD8+ T cell_5-MCI.vs.control; scRNA-seq,SRP215507,CD8+ T cell_2-MCI.vs.control; scRNA-seq,SRP215507,CD8+ T cell_3-MCI.vs.control; scRNA-seq,SRP330776,γδ T cell-AD.vs.control; scRNA-seq,SRP330776,B cell_2-AD.vs.control; scRNA-seq,SRP330776,Naive CD8+ T cell_2-AD.vs.control	11
AP005329.1	scRNA-seq,SRP215507,CD8+ T cell_1-AD.vs.MCI; scRNA-seq,SRP215507,CD8+ T cell_1-MCI.vs.control; scRNA-seq,SRP330776,Memory T cell_1-	3
AP005329.2	scRNA-seq,SRP215507,CD8+ T cell_1-MCI.vs.control	1
AP005329.3	scRNA-seq,SRP215507,CD8+ T cell_2-MCI.vs.control; scRNA-seq,SRP330776,Naive CD8+ T cell_1-AD.vs.control	2

AP005482.1	scRNA-seq,SRP215507,CD8+ T cell_2-AD.vs.MCI; scRNA-seq,SRP215507,CD8+ T cell_1-AD.vs.MCI; scRNA-seq,SRP215507,CD8+ T cell_2-MCI.vs.control; scRNA-seq,SRP309935,Natural killer cell_2-AD.vs.control	4
AP005717.1	scRNA-seq,SRP215507,CD8+ T cell_1-AD.vs.control; scRNA-seq,SRP215507,CD8+ T cell_1-MCI.vs.control	2
AP005717.2	scRNA-seq,SRP330776,CD8+ T cell_1-AD.vs.control; scRNA-seq,SRP330776,γδ T cell-AD.vs.control; scRNA-seq,SRP330776,CD8+ T cell_2-AD.vs.control; scRNA-seq,SRP330776,B cell_1-AD.vs.control; scRNA-seq,SRP330776,Natural killer cell_1-AD.vs.control; scRNA-seq,SRP330776,CD8+	6
AP006284.1	scRNA-seq,SRP215507,CD8+ T cell_3-AD.vs.control; scRNA-seq,SRP215507,CD8+ T cell_3-AD.vs.MCI; scRNA-seq,SRP215507,CD8+ T cell_1-AD.vs.MCI; scRNA-seq,SRP215507,CD8+ T cell_1-MCI.vs.control; scRNA-seq,SRP330776,CD8+ T cell_1-AD.vs.control; scRNA-seq,SRP330776,Naive CD8+ T cell_1-AD.vs.control; scRNA-seq,SRP330776,Natural killer T cell-AD.vs.control; scRNA-seq,SRP330776,Naive CD8+ T cell_2-AD.vs.control	8
AP006621.1	scRNA-seq,SRP330776,Memory T cell_1-AD.vs.control	1
AP006621.3	scRNA-seq,SRP215507,CD8+ T cell_5-AD.vs.control; scRNA-seq,SRP215507,CD8+ T cell_2-AD.vs.MCI; scRNA-seq,SRP215507,CD8+ T cell_5-MCI.vs.control; scRNA-seq,SRP215507,CD8+ T cell_3-MCI.vs.control; scRNA-seq,SRP330776,CD8+ T cell_1-AD.vs.control; scRNA-seq,SRP330776,γδ T cell-AD.vs.control; scRNA-seq,SRP330776,Memory T cell_2-AD.vs.control; scRNA-seq,SRP330776,CD8+ T cell_2-AD.vs.control; scRNA-seq,SRP330776,B cell_1-AD.vs.control; scRNA-seq,SRP330776,Naive CD8+ T cell_1-AD.vs.control; scRNA-seq,SRP330776,Natural killer T cell-AD.vs.control; scRNA-seq,SRP330776,Naive CD8+ T cell_3-AD.vs.control; scRNA-seq,SRP330776,B cell_2-AD.vs.control; scRNA-seq,SRP330776,T cell-AD.vs.control; scRNA-seq,SRP330776,CD8+ T cell_3-AD.vs.control; scRNA-seq,SRP330776,B cell_3-AD.vs.control	16
AP006623.1	scRNA-seq,SRP215507,CD8+ T cell_2-AD.vs.MCI; scRNA-seq,SRP215507,CD8+ T cell_2-MCI.vs.control	2
AP1AR	bulk RNA-seq,Homo_723,AD.vs.control; bulk RNA-seq,Homo_723,AD.vs.MCI; bulk RNA-seq,Homo_633,AD.vs.MCI; bulk RNA-seq,Homo_714,AD.vs.control; bulk RNA-seq,Homo_714,AD.vs.MCI; scRNA-seq,SRP215507,CD8+ T cell_4-AD.vs.control; scRNA-seq,SRP215507,CD8+ T cell_4-MCI.vs.control; scRNA-seq,SRP330776,B cell_1-AD.vs.control; scRNA-seq,SRP330776,Naive CD8+ T cell_3-	10
Ap1b1	bulk RNA-seq,SRP325058,AD.vs.control	1
AP1B1	scRNA-seq,SRP215507,CD8+ T cell_3-AD.vs.control; scRNA-seq,SRP215507,CD8+ T cell_1-AD.vs.control; scRNA-seq,SRP215507,CD8+ T cell_6-AD.vs.MCI; scRNA-seq,SRP309935,Natural killer cell_1-AD.vs.control; scRNA-seq,SRP309935,Monocyte_2-AD.vs.control; scRNA-seq,SRP330776,Monocyte_1-AD.vs.control; scRNA-seq,SRP330776,Regulatory T cell-AD.vs.control	7
AP1G1	bulk RNA-seq,Homo_723,AD.vs.control; bulk RNA-seq,Homo_723,AD.vs.MCI; bulk RNA-seq,Homo_633,AD.vs.control; bulk RNA-seq,Homo_633,AD.vs.MCI; scRNA-seq,SRP215507,CD8+ T cell_7-AD.vs.control; scRNA-seq,SRP215507,CD8+ T cell_7-AD.vs.MCI; scRNA-seq,SRP309935,Natural killer cell_1-AD.vs.control; scRNA-seq,SRP330776,Naive CD8+ T cell_2-AD.vs.control; scRNA-seq,SRP330776,Monocyte_1-AD.vs.control; scRNA-seq,SRP330776,B cell_3-AD.vs.control; scRNA-seq,SRP330776,Megakaryocyte_1-AD.vs.control	11
AP1G2	scRNA-seq,SRP215507,CD8+ T cell_10-AD.vs.control; scRNA-seq,SRP215507,CD8+ T cell_5-AD.vs.MCI	2
AP1M1	scRNA-seq,SRP215507,CD8+ T cell_10-AD.vs.control; scRNA-seq,SRP215507,CD8+ T cell_6-AD.vs.control; scRNA-seq,SRP215507,CD8+ T cell_4-MCI.vs.control; scRNA-seq,SRP215507,CD8+ T cell_6-MCI.vs.control; scRNA-seq,SRP330776,CD8+ T cell_1-AD.vs.control; scRNA-seq,SRP330776,γδ T cell-AD.vs.control; scRNA-seq,SRP330776,Memory T cell_2-AD.vs.control; scRNA-seq,SRP330776,CD8+ T cell_2-AD.vs.control; scRNA-seq,SRP330776,B cell_1-AD.vs.control; scRNA-seq,SRP330776,Naive CD8+ T cell_1-AD.vs.control; scRNA-seq,SRP330776,Natural killer cell_1-AD.vs.control; scRNA-seq,SRP330776,Naive CD8+ T cell_3-AD.vs.control; scRNA-seq,SRP330776,B cell_2-AD.vs.control; scRNA-seq,SRP330776,T cell-AD.vs.control; scRNA-seq,SRP330776,CD8+ T cell_3-AD.vs.control; scRNA-seq,SRP330776,Monocyte_2-AD.vs.control; scRNA-seq,SRP330776,B [illegible]	17
AP1M2	bulk RNA-seq,Homo_723,AD.vs.control; bulk RNA-seq,Homo_723,AD.vs.MCI; bulk RNA-seq,Homo_633,AD.vs.control; bulk RNA-seq,Homo_633,AD.vs.MCI; bulk RNA-seq,Homo_714,AD.vs.control; bulk RNA-seq,Homo_714,AD.vs.MCI	6
AP1S1	bulk RNA-seq,Homo_723,AD.vs.control; bulk RNA-seq,Homo_723,AD.vs.MCI; bulk RNA-seq,Homo_723,MCI.vs.control; bulk RNA-seq,Homo_633,AD.vs.control; bulk RNA-seq,Homo_633,AD.vs.MCI; bulk RNA-seq,Homo_633,MCI.vs.control; bulk RNA-seq,Homo_714,AD.vs.control; bulk RNA-seq,Homo_714,AD.vs.MCI; bulk RNA-seq,Homo_714,MCI.vs.control; scRNA-seq,SRP215507,CD8+ T cell_6-AD.vs.control; scRNA-seq,SRP215507,CD8+ T cell_3-AD.vs.MCI; scRNA-seq,SRP215507,CD8+ T cell_4-AD.vs.MCI; scRNA-seq,SRP215507,CD8+ T cell_6-AD.vs.MCI; scRNA-seq,SRP215507,CD8+ T cell_4-MCI.vs.control; scRNA-seq,SRP215507,CD8+ T cell_3-MCI.vs.control; scRNA-seq,SRP330776,γδ T cell-AD.vs.control; scRNA-seq,SRP330776,Natural killer cell_1-AD.vs.control; scRNA-seq,SRP330776,Memory T cell_1-AD.vs.control; scRNA- [illegible]	21

AP1S2	scRNA-seq,SRP215507,CD8+ T cell_4-AD.vs.MCI; scRNA-seq,SRP215507,CD8+ T cell_7-AD.vs.MCI; scRNA-seq,SRP215507,CD8+ T cell_4-MCI.vs.control; scRNA-seq,SRP215507,CD8+ T cell_7-MCI.vs.control; scRNA-seq,SRP330776,CD8+ T cell_1-AD.vs.control; scRNA-seq,SRP330776,γδ T cell-AD.vs.control; scRNA-seq,SRP330776,Memory T cell_2-AD.vs.control; scRNA-seq,SRP330776,CD8+ T cell_2-AD.vs.control; scRNA-seq,SRP330776,B cell_1-AD.vs.control; scRNA-seq,SRP330776,Naive CD8+ T cell_1-AD.vs.control; scRNA-seq,SRP330776,Natural killer cell_1-AD.vs.control; scRNA-seq,SRP330776,Memory T cell_1-AD.vs.control; scRNA-seq,SRP330776,Natural killer T cell-AD.vs.control; scRNA-seq,SRP330776,Naive CD8+ T cell_3-AD.vs.control; scRNA-seq,SRP330776,B cell_2-AD.vs.control; scRNA-seq,SRP330776,T cell-AD.vs.control; scRNA-seq,SRP330776,Naive CD8+ T cell_2-AD.vs.control; scRNA-seq,SRP330776,CD8+ T cell_3-AD.vs.control; scRNA-seq,SRP330776,Monocyte_1-AD.vs.control; scRNA-seq,SRP330776,Monocyte_2-AD.vs.control; scRNA-seq,SRP330776,B cell_3-AD.vs.control; scRNA-seq,SRP330776,Regulatory T [illegible]	24
AP1S3	scRNA-seq,SRP215507,CD8+ T cell_3-AD.vs.control; scRNA-seq,SRP215507,CD8+ T cell_2-AD.vs.MCI; scRNA-seq,SRP215507,CD8+ T cell_2-MCI.vs.control; scRNA-seq,SRP330776,CD8+ T cell_1-AD.vs.control; scRNA-seq,SRP330776,B cell_1-AD.vs.control; scRNA-seq,SRP330776,CD8+ T	6
AP2A1	scRNA-seq,SRP215507,CD8+ T cell_7-AD.vs.control; scRNA-seq,SRP215507,CD8+ T cell_5-AD.vs.control; scRNA-seq,SRP215507,CD8+ T cell_4-AD.vs.MCI; scRNA-seq,SRP215507,CD8+ T cell_5-AD.vs.MCI; scRNA-seq,SRP215507,CD8+ T cell_6-AD.vs.MCI; scRNA-seq,SRP215507,CD8+ T cell_4-MCI.vs.control; scRNA-seq,SRP309935,Natural killer cell_2-AD.vs.control; scRNA-seq,SRP330776,Memory T cell_2-AD.vs.control; scRNA-seq,SRP330776,Naive CD8+ T cell_1-AD.vs.control; scRNA-seq,SRP330776,Natural killer cell_1-AD.vs.control; scRNA-seq,SRP330776,Memory T cell_1-AD.vs.control; scRNA-seq,SRP330776,Natural killer T cell-AD.vs.control; scRNA-seq,SRP330776,Naive CD8+ T cell_3-AD.vs.control	13
AP2A2	bulk RNA-seq,Homo_723,AD.vs.control; bulk RNA-seq,Homo_723,AD.vs.MCI; bulk RNA-seq,Homo_723,MCI.vs.control; bulk RNA-seq,Homo_633,AD.vs.control; bulk RNA-seq,Homo_633,AD.vs.MCI; bulk RNA-seq,Homo_633,MCI.vs.control; bulk RNA-seq,SRP223445,AD.vs.control; scRNA-seq,SRP215507,CD8+ T cell_4-AD.vs.control; scRNA-seq,SRP215507,CD8+ T cell_10-AD.vs.control; scRNA-seq,SRP215507,CD8+ T cell_6-AD.vs.control; scRNA-seq,SRP215507,CD8+ T cell_4-AD.vs.MCI; scRNA-seq,SRP215507,CD8+ T cell_6-AD.vs.MCI; scRNA-seq,SRP309935,CD8+ T cell_2-AD.vs.control; scRNA-seq,SRP330776,CD8+ T cell_1-AD.vs.control; scRNA-seq,SRP330776,Memory T cell_2-AD.vs.control; scRNA-seq,SRP330776,B cell_1-AD.vs.control; scRNA-seq,SRP330776,Natural killer cell_1-AD.vs.control; scRNA-seq,SRP330776,T cell-AD.vs.control; scRNA-seq,SRP330776,Monocyte_1-AD.vs.control; scRNA-seq,SRP330776,B cell_3-AD.vs.control; scRNA-seq,SRP330776,Natural killer [illegible]	21
Ap2a2	bulk RNA-seq,SRP325058,AD.vs.control	1
AP2B1	bulk RNA-seq,Homo_633,AD.vs.control; bulk RNA-seq,Homo_633,MCI.vs.control; scRNA-seq,SRP215507,CD8+ T cell_7-AD.vs.control; scRNA-seq,SRP215507,CD8+ T cell_4-AD.vs.MCI; scRNA-seq,SRP215507,CD8+ T cell_7-AD.vs.MCI	5
Ap2b1	bulk RNA-seq,SRP325058,AD.vs.control	1
AP2M1	scRNA-seq,SRP215507,CD8+ T cell_10-AD.vs.control	1
AP2S1	scRNA-seq,SRP215507,CD8+ T cell_7-AD.vs.control; scRNA-seq,SRP215507,CD8+ T cell_7-MCI.vs.control; scRNA-seq,SRP330776,Memory T cell_2-AD.vs.control; scRNA-seq,SRP330776,B cell_2-AD.vs.control; scRNA-seq,SRP330776,Monocyte_2-AD.vs.control; scRNA-seq,SRP330776,Natural killer	6
AP3B1	bulk RNA-seq,Homo_714,AD.vs.control; bulk RNA-seq,Homo_714,AD.vs.MCI; scRNA-seq,SRP215507,CD8+ T cell_10-AD.vs.control; scRNA-seq,SRP215507,CD8+ T cell_5-AD.vs.MCI; scRNA-seq,SRP309935,Natural killer cell_2-AD.vs.control; scRNA-seq,SRP330776,Naive CD8+ T cell_3-	6
AP3B2	bulk RNA-seq,Homo_723,AD.vs.control; bulk RNA-seq,Homo_723,AD.vs.MCI; bulk RNA-seq,Homo_633,AD.vs.MCI; bulk RNA-seq,Homo_633,MCI.vs.control; bulk RNA-seq,Homo_714,AD.vs.control; bulk RNA-seq,Homo_714,AD.vs.MCI; bulk RNA-seq,Homo_714,MCI.vs.control; scRNA-seq,SRP215507,CD8+ T cell_3-AD.vs.control; scRNA-seq,SRP215507,CD8+ T cell_3-AD.vs.MCI; scRNA-	10
AP3D1	bulk RNA-seq,Homo_723,AD.vs.control; bulk RNA-seq,Homo_723,AD.vs.MCI; bulk RNA-seq,Homo_633,AD.vs.control; bulk RNA-seq,Homo_633,AD.vs.MCI; bulk RNA-seq,Homo_714,AD.vs.control; bulk RNA-seq,Homo_714,AD.vs.MCI; bulk RNA-seq,Homo_714,MCI.vs.control; scRNA-seq,SRP215507,CD8+ T cell_7-AD.vs.control; scRNA-seq,SRP215507,CD8+ T cell_7-AD.vs.MCI; scRNA-seq,SRP309935,Megakaryocyte_1-AD.vs.control; scRNA-seq,SRP309935,Naive CD8+ T cell_1-AD.vs.control; scRNA-seq,SRP309935,Monocyte_2-AD.vs.control; scRNA-seq,SRP309935,Naive CD8+ T cell_2-AD.vs.control; scRNA-seq,SRP309935,CD8+ T cell_1-AD.vs.control; scRNA-seq,SRP330776,Memory T cell_2-AD.vs.control; scRNA-seq,SRP330776,CD8+ T cell_2-AD.vs.control; scRNA-seq,SRP330776,Naive CD8+ T cell_1-AD.vs.control; scRNA-	19
AP3M1	bulk RNA-seq,Homo_723,AD.vs.control; bulk RNA-seq,Homo_723,MCI.vs.control; bulk RNA-seq,Homo_633,AD.vs.control; bulk RNA-seq,Homo_633,AD.vs.MCI; bulk RNA-seq,Homo_633,MCI.vs.control; bulk RNA-seq,Homo_714,AD.vs.control; bulk RNA-seq,Homo_714,AD.vs.MCI; scRNA-seq,SRP215507,CD8+ T cell_6-AD.vs.MCI; scRNA-seq,SRP215507,CD8+ T cell_6-MCI.vs.control; scRNA-seq,SRP330776,γδ T cell-	10

Ap3m1	bulk RNA-seq,SRP325058,AD.vs.control	1
AP3M2	bulk RNA-seq,Homo_723,AD.vs.control; bulk RNA-seq,Homo_723,MCI.vs.control; bulk RNA-seq,Homo_633,AD.vs.control; bulk RNA-seq,Homo_633,MCI.vs.control; bulk RNA-seq,Homo_714,AD.vs.control; bulk RNA-seq,Homo_714,AD.vs.MCI; bulk RNA-seq,Homo_714,MCI.vs.control; bulk RNA-seq,ROSMAP,AD.vs.control; bulk RNA-seq,ROSMAP,MCI.vs.control; scRNA-seq,SRP215507,CD8+ T cell_3-AD.vs.control; scRNA-seq,SRP215507,CD8+ T cell_6-AD.vs.MCI; scRNA-seq,SRP215507,CD8+ T cell_6-MCI.vs.control; scRNA-seq,SRP309935,CD8+ T cell_2-AD.vs.control; scRNA-seq,SRP330776,B cell_1-AD.vs.control; scRNA-seq,SRP330776,Memory T cell_1-AD.vs.control; scRNA-seq,SRP330776,Natural killer T cell-AD.vs.control; scRNA-seq,SRP330776,Naive CD8+ T cell_3-AD.vs.control; scRNA-[illegible]	18
AP3S1	bulk RNA-seq,Homo_723,AD.vs.control; bulk RNA-seq,Homo_723,AD.vs.MCI; bulk RNA-seq,Homo_633,AD.vs.control; bulk RNA-seq,Homo_633,AD.vs.MCI; bulk RNA-seq,Homo_714,AD.vs.control; bulk RNA-seq,Homo_714,AD.vs.MCI; scRNA-seq,SRP215507,CD8+ T cell_10-AD.vs.control; scRNA-seq,SRP330776,γδ T cell-AD.vs.control; scRNA-seq,SRP330776,Natural killer cell_2-AD.vs.control	9
AP3S2	scRNA-seq,SRP330776,γδ T cell-AD.vs.control; scRNA-seq,SRP330776,Naive CD8+ T cell_3-AD.vs.control	2
AP4B1	scRNA-seq,SRP330776,CD8+ T cell_2-AD.vs.control; scRNA-seq,SRP330776,B cell_2-AD.vs.control; scRNA-seq,SRP330776,Monocyte_2-[illegible]	3
AP4B1-AS1	scRNA-seq,SRP330776,CD8+ T cell_1-AD.vs.control	1
AP4E1	scRNA-seq,SRP215507,CD8+ T cell_2-AD.vs.MCI; scRNA-seq,SRP215507,CD8+ T cell_5-AD.vs.MCI; scRNA-seq,SRP215507,CD8+ T cell_1-MCI.vs.control; scRNA-seq,SRP215507,CD8+ T cell_5-MCI.vs.control; scRNA-seq,SRP215507,CD8+ T cell_2-MCI.vs.control; scRNA-seq,SRP215507,CD8+ T cell_6-MCI.vs.control; scRNA-seq,SRP330776,CD8+ T cell_1-AD.vs.control; scRNA-seq,SRP330776,γδ T cell-AD.vs.control; scRNA-seq,SRP330776,Memory T cell_2-AD.vs.control; scRNA-seq,SRP330776,CD8+ T cell_2-AD.vs.control; scRNA-seq,SRP330776,Memory T cell_1-AD.vs.control; scRNA-seq,SRP330776,Naive CD8+ T cell_3-AD.vs.control; scRNA-seq,SRP330776,T cell-AD.vs.control; scRNA-seq,SRP330776,Naive CD8+ T cell_2-AD.vs.control; scRNA-seq,SRP330776,CD8+ T cell_3-AD.vs.control; scRNA-seq,SRP330776,Monocyte_2-[illegible]	16
AP4M1	bulk RNA-seq,Homo_723,AD.vs.control; bulk RNA-seq,Homo_714,AD.vs.control; bulk RNA-seq,Homo_714,AD.vs.MCI; bulk RNA-seq,Homo_714,MCI.vs.control; scRNA-seq,SRP309935,Natural killer cell_1-AD.vs.control; scRNA-seq,SRP330776,CD8+ T cell_1-AD.vs.control; scRNA-seq,SRP330776,γδ T cell-AD.vs.control; scRNA-seq,SRP330776,Memory T cell_2-AD.vs.control; scRNA-seq,SRP330776,B cell_1-AD.vs.control; scRNA-seq,SRP330776,Naive CD8+ T cell_1-AD.vs.control; scRNA-seq,SRP330776,Memory T cell_1-AD.vs.control; scRNA-seq,SRP330776,Natural killer T cell-AD.vs.control; scRNA-seq,SRP330776,CD8+ T cell_3-AD.vs.control; scRNA-seq,SRP330776,Monocyte_1-AD.vs.control	14
AP4S1	bulk RNA-seq,Homo_723,MCI.vs.control; bulk RNA-seq,Homo_633,MCI.vs.control; bulk RNA-seq,Homo_714,AD.vs.control; bulk RNA-seq,Homo_714,AD.vs.MCI; bulk RNA-seq,Homo_714,MCI.vs.control; scRNA-seq,SRP215507,CD8+ T cell_6-AD.vs.control; scRNA-seq,SRP215507,CD8+ T cell_2-AD.vs.MCI; scRNA-seq,SRP215507,CD8+ T cell_3-AD.vs.MCI; scRNA-seq,SRP330776,CD8+ T cell_1-AD.vs.control; scRNA-seq,SRP330776,γδ T cell-AD.vs.control; scRNA-seq,SRP330776,B cell_1-AD.vs.control; scRNA-seq,SRP330776,Naive CD8+ T cell_3-[illegible]	13
AP5B1	scRNA-seq,SRP215507,CD8+ T cell_9-AD.vs.control; scRNA-seq,SRP215507,CD8+ T cell_5-AD.vs.MCI; scRNA-seq,SRP215507,CD8+ T cell_5-MCI.vs.control; scRNA-seq,SRP215507,CD8+ T cell_4-MCI.vs.control; scRNA-seq,SRP309935,Natural killer cell_2-AD.vs.control; scRNA-seq,SRP309935,Naive CD8+ T cell_1-AD.vs.control; scRNA-seq,SRP330776,CD8+ T cell_1-AD.vs.control; scRNA-seq,SRP330776,γδ T cell-AD.vs.control; scRNA-seq,SRP330776,Memory T cell_2-AD.vs.control; scRNA-seq,SRP330776,CD8+ T cell_2-AD.vs.control; scRNA-seq,SRP330776,B cell_1-AD.vs.control; scRNA-seq,SRP330776,Naive CD8+ T cell_1-AD.vs.control; scRNA-seq,SRP330776,Natural killer cell_1-AD.vs.control; scRNA-seq,SRP330776,Memory T cell_1-AD.vs.control; scRNA-seq,SRP330776,Natural killer T cell-AD.vs.control; scRNA-seq,SRP330776,Naive CD8+ T cell_3-AD.vs.control; scRNA-seq,SRP330776,T cell-AD.vs.control; scRNA-seq,SRP330776,CD8+ T cell_3-AD.vs.control; scRNA-[illegible]	20
AP5M1	bulk RNA-seq,Homo_714,AD.vs.control; bulk RNA-seq,Homo_714,AD.vs.MCI; scRNA-seq,SRP330776,B cell_4-AD.vs.control	3
AP5S1	scRNA-seq,SRP215507,CD8+ T cell_3-AD.vs.MCI; scRNA-seq,SRP215507,CD8+ T cell_5-AD.vs.MCI; scRNA-seq,SRP215507,CD8+ T cell_6-AD.vs.MCI; scRNA-seq,SRP215507,CD8+ T cell_5-MCI.vs.control; scRNA-seq,SRP215507,CD8+ T cell_3-MCI.vs.control; scRNA-seq,SRP215507,CD8+ T cell_6-MCI.vs.control; scRNA-seq,SRP330776,γδ T cell-AD.vs.control; scRNA-seq,SRP330776,Memory T cell_2-AD.vs.control; scRNA-seq,SRP330776,Natural killer cell_1-AD.vs.control; scRNA-seq,SRP330776,CD8+ T cell_3-AD.vs.control	10

AP5Z1	bulk RNA-seq,Homo_723,AD.vs.control; bulk RNA-seq,Homo_723,AD.vs.MCI; bulk RNA-seq,Homo_633,AD.vs.control; bulk RNA-seq,Homo_633,AD.vs.MCI; bulk RNA-seq,Homo_714,AD.vs.control; bulk RNA-seq,Homo_714,AD.vs.MCI; bulk RNA-seq,Homo_714,MCI.vs.control; bulk RNA-seq,SRP310421,AD.vs.control; scRNA-seq,SRP330776,Naive CD8+ T cell_3-AD.vs.control; scRNA-seq,SRP330776,B cell_2-AD.vs.control; scRNA-seq,SRP330776,B cell_3-AD.vs.control; scRNA-seq,SRP330776,Natural killer cell_2-AD.vs.control	12
APAF1	bulk RNA-seq,Homo_714,AD.vs.control; bulk RNA-seq,Homo_714,AD.vs.MCI; scRNA-seq,SRP215507,CD8+ T cell_1-AD.vs.control; scRNA-seq,SRP215507,CD8+ T cell_5-AD.vs.control; scRNA-seq,SRP215507,CD8+ T cell_1-AD.vs.MCI; scRNA-seq,SRP309935,Naive CD8+ T cell_1-AD.vs.control; scRNA-seq,SRP330776,Naive CD8+ T cell_3-AD.vs.control; scRNA-seq,SRP330776,Monocyte_1-AD.vs.control; scRNA-seq,SRP330776,Monocyte_2-AD.vs.control; scRNA-seq,SRP330776,Regulatory T cell-AD.vs.control	10
APBA1	bulk RNA-seq,Homo_723,AD.vs.control; bulk RNA-seq,Homo_723,AD.vs.MCI; bulk RNA-seq,Homo_633,AD.vs.control; bulk RNA-seq,Homo_633,AD.vs.MCI; bulk RNA-seq,Homo_714,MCI.vs.control	5
APBA2	bulk RNA-seq,Homo_714,AD.vs.control; bulk RNA-seq,Homo_714,AD.vs.MCI; scRNA-seq,SRP215507,CD8+ T cell_1-AD.vs.MCI; scRNA-seq,SRP215507,CD8+ T cell_1-MCI.vs.control; scRNA-seq,SRP309935,CD8+ T cell_1-AD.vs.control; scRNA-seq,SRP330776,γδ T cell-AD.vs.control; scRNA-seq,SRP330776,Memory T cell_2-AD.vs.control; scRNA-seq,SRP330776,B cell_1-AD.vs.control; scRNA-seq,SRP330776,Naive CD8+ T cell_1-AD.vs.control; scRNA-seq,SRP330776,Natural killer cell_1-AD.vs.control; scRNA-seq,SRP330776,Naive CD8+ T cell_3-AD.vs.control; scRNA-	12
APBA3	bulk RNA-seq,Homo_723,AD.vs.control; bulk RNA-seq,Homo_723,AD.vs.MCI; bulk RNA-seq,Homo_723,MCI.vs.control; bulk RNA-seq,Homo_633,AD.vs.control; bulk RNA-seq,Homo_633,MCI.vs.control; bulk RNA-seq,Homo_714,AD.vs.control; bulk RNA-seq,Homo_714,MCI.vs.control; scRNA-seq,SRP215507,CD8+ T cell_6-AD.vs.control; scRNA-seq,SRP330776,CD8+ T cell_1-AD.vs.control; scRNA-seq,SRP330776,Memory T cell_1-AD.vs.control; scRNA-seq,SRP330776,Natural killer T cell-AD.vs.control; scRNA-seq,SRP330776,Naive CD8+ T	14
APBB1	scRNA-seq,SRP215507,CD8+ T cell_4-AD.vs.control; scRNA-seq,SRP215507,CD8+ T cell_4-AD.vs.MCI; scRNA-seq,SRP215507,CD8+ T cell_4-MCI.vs.control; scRNA-seq,SRP215507,CD8+ T cell_6-MCI.vs.control; scRNA-seq,SRP330776,Megakaryocyte_1-AD.vs.control	5
APBB1IP	scRNA-seq,SRP215507,CD8+ T cell_10-AD.vs.control; scRNA-seq,SRP330776,CD8+ T cell_1-AD.vs.control; scRNA-seq,SRP330776,γδ T cell-AD.vs.control; scRNA-seq,SRP330776,Memory T cell_2-AD.vs.control; scRNA-seq,SRP330776,CD8+ T cell_2-AD.vs.control; scRNA-seq,SRP330776,Naive CD8+ T cell_1-AD.vs.control; scRNA-seq,SRP330776,Natural killer cell_1-AD.vs.control; scRNA-seq,SRP330776,Memory T cell_1-AD.vs.control; scRNA-seq,SRP330776,Natural killer T cell-AD.vs.control; scRNA-seq,SRP330776,Naive CD8+ T cell_3-AD.vs.control; scRNA-seq,SRP330776,B cell_2-AD.vs.control; scRNA-seq,SRP330776,T cell-AD.vs.control; scRNA-seq,SRP330776,Naive CD8+ T cell_2-AD.vs.control; scRNA-seq,SRP330776,CD8+ T cell_3-AD.vs.control; scRNA-seq,SRP330776,Monocyte_1-AD.vs.control; scRNA-seq,SRP330776,Monocyte_2-AD.vs.control; scRNA-seq,SRP330776,Regulatory T cell-AD.vs.control; scRNA-seq,SRP330776,Natural killer cell_2-AD.vs.control; scRNA- [illegible]	19
APBB2	bulk RNA-seq,Homo_723,AD.vs.control; bulk RNA-seq,Homo_723,AD.vs.MCI; bulk RNA-seq,Homo_723,MCI.vs.control; bulk RNA-seq,Homo_633,AD.vs.control; bulk RNA-seq,Homo_633,AD.vs.MCI; bulk RNA-seq,Homo_633,MCI.vs.control; bulk RNA-seq,Homo_714,AD.vs.control; bulk RNA-seq,Homo_714,AD.vs.MCI; scRNA-seq,SRP330776,B cell_2-AD.vs.control	9
APBB3	scRNA-seq,SRP215507,CD8+ T cell_3-AD.vs.MCI; scRNA-seq,SRP215507,CD8+ T cell_1-AD.vs.MCI; scRNA-seq,SRP215507,CD8+ T cell_5-AD.vs.MCI; scRNA-seq,SRP215507,CD8+ T cell_5-MCI.vs.control; scRNA-seq,SRP330776,Memory T cell_2-AD.vs.control; scRNA-seq,SRP330776,T	6
APC	scRNA-seq,SRP215507,CD8+ T cell_6-AD.vs.MCI; scRNA-seq,SRP215507,CD8+ T cell_6-MCI.vs.control; scRNA-seq,SRP330776,Monocyte_2-AD.vs.control; scRNA-seq,SRP330776,B cell_3-AD.vs.control	4
APC2	bulk RNA-seq,Homo_723,AD.vs.control; bulk RNA-seq,Homo_723,AD.vs.MCI; bulk RNA-seq,Homo_633,AD.vs.control; bulk RNA-seq,Homo_633,AD.vs.MCI; bulk RNA-seq,Homo_714,AD.vs.control; bulk RNA-seq,Homo_714,AD.vs.MCI; bulk RNA-seq,Homo_714,MCI.vs.control	7
APCDD1	bulk RNA-seq,Homo_723,AD.vs.control; bulk RNA-seq,Homo_723,AD.vs.MCI; bulk RNA-seq,Homo_633,AD.vs.control; bulk RNA-seq,Homo_633,AD.vs.MCI; bulk RNA-seq,Homo_633,MCI.vs.control; bulk RNA-seq,Homo_714,AD.vs.control; bulk RNA-seq,Homo_714,AD.vs.MCI; bulk RNA-seq,Homo_714,MCI.vs.control; bulk RNA-seq,ROSMAP,MCI.vs.control	9
APCDD1L	bulk RNA-seq,Homo_723,MCI.vs.control; bulk RNA-seq,Homo_633,MCI.vs.control; bulk RNA-seq,Homo_714,MCI.vs.control; bulk RNA-seq,ROSMAP,AD.vs.control; bulk RNA-seq,ROSMAP,MCI.vs.control	5
APEH	scRNA-seq,SRP215507,CD8+ T cell_7-AD.vs.control; scRNA-seq,SRP215507,CD8+ T cell_6-AD.vs.control; scRNA-seq,SRP330776,CD8+ T cell_2-AD.vs.control; scRNA-seq,SRP330776,Naive CD8+ T cell_1-AD.vs.control; scRNA-seq,SRP330776,Natural killer cell_1-AD.vs.control; scRNA-seq,SRP330776,Naive CD8+ T cell_3-AD.vs.control; scRNA-seq,SRP330776,T cell-AD.vs.control; scRNA-seq,SRP330776,B cell_4-AD.vs.control	8

APEX1	scRNA-seq,SRP215507,CD8+ T cell_9-AD.vs.control; scRNA-seq,SRP215507,CD8+ T cell_4-AD.vs.MCI; scRNA-seq,SRP215507,CD8+ T cell_4-MCI.vs.control; scRNA-seq,SRP330776,Natural killer T cell-AD.vs.control; scRNA-seq,SRP330776,B cell_3-AD.vs.control; scRNA-seq,SRP330776,B	6
APEX2	bulk RNA-seq,Homo_723,AD.vs.MCI; bulk RNA-seq,Homo_723,MCI.vs.control; bulk RNA-seq,Homo_633,AD.vs.MCI; bulk RNA-seq,Homo_633,MCI.vs.control; bulk RNA-seq,Homo_714,AD.vs.control; bulk RNA-seq,Homo_714,AD.vs.MCI; bulk RNA-seq,Homo_714,MCI.vs.control; scRNA-seq,SRP215507,CD8+ T cell_3-AD.vs.control; scRNA-seq,SRP215507,CD8+ T cell_4-MCI.vs.control; scRNA-seq,SRP215507,CD8+ T cell_6-MCI.vs.control; scRNA-seq,SRP330776,CD8+ T cell_1-AD.vs.control; scRNA-seq,SRP330776,CD8+ T cell_2-AD.vs.control; scRNA-seq,SRP330776,Naive CD8+ T cell_1-AD.vs.control; scRNA-seq,SRP330776,Memory T cell_1-AD.vs.control; scRNA-seq,SRP330776,Natural killer T cell-AD.vs.control; scRNA-seq,SRP330776,T cell-AD.vs.control; scRNA-seq,SRP330776,Monocyte_1-AD.vs.control; [illegible]	18
APH1A	scRNA-seq,SRP215507,CD8+ T cell_4-AD.vs.MCI; scRNA-seq,SRP215507,CD8+ T cell_6-AD.vs.MCI; scRNA-seq,SRP330776,Memory T cell_2-AD.vs.control; scRNA-seq,SRP330776,Natural killer cell_1-AD.vs.control; scRNA-seq,SRP330776,CD8+ T cell_3-AD.vs.control	5
APH1B	bulk RNA-seq,Homo_723,AD.vs.control; bulk RNA-seq,Homo_723,AD.vs.MCI; bulk RNA-seq,Homo_633,AD.vs.control; bulk RNA-seq,Homo_633,AD.vs.MCI; bulk RNA-seq,Homo_714,AD.vs.control; bulk RNA-seq,Homo_714,AD.vs.MCI; scRNA-seq,SRP215507,CD8+ T cell_2-AD.vs.MCI; scRNA-seq,SRP330776,Memory T cell_2-AD.vs.control; scRNA-seq,SRP330776,Natural killer T cell-AD.vs.control; scRNA-seq,SRP330776,CD8+ T cell_3-AD.vs.control; scRNA-seq,SRP330776,B cell_3-AD.vs.control	11
API5	bulk RNA-seq,Homo_723,AD.vs.control; bulk RNA-seq,Homo_723,MCI.vs.control; bulk RNA-seq,Homo_633,AD.vs.control; scRNA-seq,SRP215507,CD8+ T cell_7-AD.vs.control; scRNA-seq,SRP309935,Natural killer cell_2-AD.vs.control; scRNA-seq,SRP309935,Naive CD8+ T cell_1-AD.vs.control; scRNA-seq,SRP309935,Naive CD8+ T cell_2-AD.vs.control; scRNA-seq,SRP309935,CD8+ T cell_1-AD.vs.control; scRNA-	9
APIP	bulk RNA-seq,Homo_723,AD.vs.control; bulk RNA-seq,Homo_723,AD.vs.MCI; bulk RNA-seq,Homo_633,AD.vs.control; bulk RNA-seq,Homo_633,AD.vs.MCI; bulk RNA-seq,Homo_633,MCI.vs.control; bulk RNA-seq,Homo_714,AD.vs.MCI; bulk RNA-seq,Homo_714,MCI.vs.control; scRNA-seq,SRP215507,CD8+ T cell_5-AD.vs.control; scRNA-seq,SRP215507,CD8+ T cell_5-AD.vs.MCI; scRNA-seq,SRP215507,CD8+ T cell_3-MCI.vs.control; scRNA-seq,SRP330776,γδ T cell-AD.vs.control; scRNA-seq,SRP330776,Memory T cell_2-AD.vs.control; scRNA-seq,SRP330776,CD8+ T cell_2-AD.vs.control; scRNA-seq,SRP330776,B cell_1-AD.vs.control; scRNA-seq,SRP330776,Naive CD8+ T cell_1-AD.vs.control; scRNA-seq,SRP330776,Memory T cell_1-AD.vs.control; scRNA-seq,SRP330776,Natural killer T cell-AD.vs.control; scRNA-seq,SRP330776,B cell_2-AD.vs.control; scRNA-seq,SRP330776,T cell-AD.vs.control; scRNA-seq,SRP330776,CD8+ T cell_3-AD.vs.control; scRNA-seq,SRP330776,Natural killer [illegible]	21
APLF	bulk RNA-seq,Homo_723,AD.vs.control; bulk RNA-seq,Homo_723,AD.vs.MCI; bulk RNA-seq,Homo_723,MCI.vs.control; bulk RNA-seq,Homo_633,AD.vs.control; bulk RNA-seq,Homo_633,AD.vs.MCI; bulk RNA-seq,Homo_633,MCI.vs.control; bulk RNA-seq,Homo_714,AD.vs.control; bulk RNA-seq,Homo_714,AD.vs.MCI; scRNA-seq,SRP215507,CD8+ T cell_8-AD.vs.control; scRNA-seq,SRP215507,CD8+ T cell_3-AD.vs.MCI; scRNA-seq,SRP215507,CD8+ T cell_2-MCI.vs.control; scRNA-seq,SRP215507,CD8+ T cell_3-MCI.vs.control; scRNA-seq,SRP330776,CD8+ T cell_2-AD.vs.control; scRNA-seq,SRP330776,B cell_2-AD.vs.control; scRNA-seq,SRP330776,T cell-AD.vs.control	15
APLN	bulk RNA-seq,Homo_723,AD.vs.control; bulk RNA-seq,Homo_723,AD.vs.MCI; bulk RNA-seq,Homo_723,MCI.vs.control; bulk RNA-seq,Homo_633,AD.vs.control; bulk RNA-seq,Homo_633,AD.vs.MCI; bulk RNA-seq,Homo_633,MCI.vs.control; bulk RNA-seq,ROSMAP,AD.vs.control; bulk RNA-seq,ROSMAP,AD.vs.MCI; bulk RNA-seq,ROSMAP,MCI.vs.control	9
APLNR	bulk RNA-seq,Homo_723,AD.vs.control; bulk RNA-seq,Homo_723,AD.vs.MCI; bulk RNA-seq,Homo_723,MCI.vs.control; bulk RNA-seq,Homo_633,AD.vs.control; bulk RNA-seq,Homo_633,AD.vs.MCI; bulk RNA-seq,Homo_633,MCI.vs.control; bulk RNA-seq,Homo_714,MCI.vs.control	7
APLP2	bulk RNA-seq,Homo_723,AD.vs.control; bulk RNA-seq,Homo_633,AD.vs.control; scRNA-seq,SRP215507,CD8+ T cell_4-AD.vs.control; scRNA-seq,SRP215507,CD8+ T cell_5-AD.vs.control; scRNA-seq,SRP215507,CD8+ T cell_6-AD.vs.control; scRNA-seq,SRP215507,CD8+ T cell_6-AD.vs.MCI; scRNA-seq,SRP215507,CD8+ T cell_5-MCI.vs.control; scRNA-seq,SRP215507,CD8+ T cell_2-MCI.vs.control; scRNA-seq,SRP309935,B cell_1-AD.vs.control; scRNA-seq,SRP330776,γδ T cell-AD.vs.control; scRNA-seq,SRP330776,Regulatory T cell-AD.vs.control	11
APMAP	bulk RNA-seq,Homo_714,AD.vs.control; bulk RNA-seq,Homo_714,AD.vs.MCI; bulk RNA-seq,SRP223445,AD.vs.control; scRNA-seq,SRP215507,CD8+ T cell_7-AD.vs.control; scRNA-seq,SRP215507,CD8+ T cell_6-AD.vs.control; scRNA-seq,SRP215507,CD8+ T cell_3-MCI.vs.control; scRNA-seq,SRP215507,CD8+ T cell_6-MCI.vs.control; scRNA-seq,SRP330776,B cell_2-AD.vs.control	8

APOA1	bulk RNA-seq,ACOM,AD.vs.control; bulk RNA-seq,Homo_723,AD.vs.control; bulk RNA-seq,Homo_723,AD.vs.MCI; bulk RNA-seq,Homo_723,MCI.vs.control; bulk RNA-seq,Homo_633,AD.vs.control; bulk RNA-seq,Homo_633,AD.vs.MCI; bulk RNA-seq,Homo_633,MCI.vs.control; scRNA-seq,SRP215507,CD8+ T cell_1-AD.vs.control; scRNA-seq,SRP215507,CD8+ T cell_1-MCI.vs.control; scRNA-seq,SRP330776,CD8+ T cell_1-AD.vs.control; scRNA-seq,SRP330776,CD8+ T cell_2-AD.vs.control; scRNA-seq,SRP330776,Naive CD8+ T cell_1-AD.vs.control; scRNA-seq,SRP330776,Memory T cell_1-AD.vs.control; scRNA-seq,SRP330776,Naive CD8+ T cell_3-AD.vs.control	14
APOA1-AS	scRNA-seq,SRP215507,CD8+ T cell_1-AD.vs.control	1
APOA2	bulk RNA-seq,ACOM,AD.vs.control; bulk RNA-seq,Homo_723,AD.vs.control; bulk RNA-seq,Homo_723,AD.vs.MCI; bulk RNA-seq,Homo_723,MCI.vs.control; bulk RNA-seq,Homo_633,AD.vs.control; bulk RNA-seq,Homo_633,AD.vs.MCI; bulk RNA-seq,Homo_633,MCI.vs.control	7
APOA4	bulk RNA-seq,Homo_723,AD.vs.MCI; bulk RNA-seq,Homo_723,MCI.vs.control; bulk RNA-seq,Homo_633,AD.vs.MCI	3
APOB	bulk RNA-seq,ACOM,AD.vs.control; bulk RNA-seq,Homo_723,AD.vs.control; bulk RNA-seq,Homo_723,AD.vs.MCI; bulk RNA-seq,Homo_723,MCI.vs.control; bulk RNA-seq,Homo_633,AD.vs.MCI; bulk RNA-seq,Homo_714,MCI.vs.control	6
APOBEC2	bulk RNA-seq,Homo_723,AD.vs.control; bulk RNA-seq,Homo_723,AD.vs.MCI; bulk RNA-seq,Homo_633,AD.vs.control; bulk RNA-seq,Homo_633,AD.vs.MCI; bulk RNA-seq,Homo_633,MCI.vs.control; bulk RNA-seq,Homo_714,AD.vs.control; bulk RNA-seq,Homo_714,MCI.vs.control	7
APOBEC3A	scRNA-seq,SRP330776,Monocyte_1-AD.vs.control; scRNA-seq,SRP330776,Monocyte_2-AD.vs.control	2
APOBEC3B	bulk RNA-seq,Homo_714,AD.vs.control; bulk RNA-seq,Homo_714,AD.vs.MCI; scRNA-seq,SRP330776,B cell_4-AD.vs.control	3
APOBEC3C	bulk RNA-seq,Homo_723,AD.vs.control; bulk RNA-seq,Homo_723,AD.vs.MCI; bulk RNA-seq,Homo_633,AD.vs.control; bulk RNA-seq,Homo_633,AD.vs.MCI; scRNA-seq,SRP215507,CD8+ T cell_3-AD.vs.control; scRNA-seq,SRP215507,CD8+ T cell_3-AD.vs.MCI; scRNA-seq,SRP215507,CD8+ T cell_4-AD.vs.MCI; scRNA-seq,SRP215507,CD8+ T cell_6-AD.vs.MCI; scRNA-seq,SRP215507,CD8+ T cell_4-MCI.vs.control; scRNA-seq,SRP330776,CD8+ T cell_1-AD.vs.control; scRNA-seq,SRP330776,γδ T cell-AD.vs.control; scRNA-seq,SRP330776,Memory T cell_2-AD.vs.control; scRNA-seq,SRP330776,Naive CD8+ T cell_1-AD.vs.control; scRNA-seq,SRP330776,Natural killer cell_1-AD.vs.control; scRNA-	16
APOBEC3D	scRNA-seq,SRP215507,CD8+ T cell_2-AD.vs.control; scRNA-seq,SRP215507,CD8+ T cell_2-AD.vs.MCI; scRNA-seq,SRP215507,CD8+ T cell_1-AD.vs.MCI; scRNA-seq,SRP215507,CD8+ T cell_4-AD.vs.MCI; scRNA-seq,SRP215507,CD8+ T cell_3-MCI.vs.control; scRNA-seq,SRP309935,Naive CD8+ T cell_2-AD.vs.control; scRNA-seq,SRP330776,CD8+ T cell_1-AD.vs.control; scRNA-seq,SRP330776,γδ T cell-AD.vs.control; scRNA-seq,SRP330776,Memory T cell_2-AD.vs.control; scRNA-seq,SRP330776,Natural killer cell_1-AD.vs.control; scRNA-seq,SRP330776,Memory T cell_1-AD.vs.control; scRNA-seq,SRP330776,Natural killer T cell-AD.vs.control; scRNA-seq,SRP330776,B cell_2-AD.vs.control; scRNA-seq,SRP330776,CD8+	14
APOBEC3F	scRNA-seq,SRP215507,CD8+ T cell_2-AD.vs.control; scRNA-seq,SRP330776,CD8+ T cell_1-AD.vs.control; scRNA-seq,SRP330776,γδ T cell-AD.vs.control; scRNA-seq,SRP330776,Memory T cell_2-AD.vs.control; scRNA-seq,SRP330776,Naive CD8+ T cell_1-AD.vs.control; scRNA-seq,SRP330776,Natural killer cell_1-AD.vs.control; scRNA-seq,SRP330776,Monocyte_1-AD.vs.control; scRNA-seq,SRP330776,B cell_3-AD.vs.control	8
APOBEC3G	scRNA-seq,SRP215507,CD8+ T cell_7-AD.vs.control; scRNA-seq,SRP215507,CD8+ T cell_4-AD.vs.MCI; scRNA-seq,SRP215507,CD8+ T cell_7-AD.vs.MCI; scRNA-seq,SRP215507,CD8+ T cell_4-MCI.vs.control; scRNA-seq,SRP309935,Megakaryocyte_2-AD.vs.control; scRNA-seq,SRP330776,CD8+ T cell_1-AD.vs.control; scRNA-seq,SRP330776,γδ T cell-AD.vs.control; scRNA-seq,SRP330776,Memory T cell_2-AD.vs.control; scRNA-seq,SRP330776,CD8+ T cell_2-AD.vs.control; scRNA-seq,SRP330776,Naive CD8+ T cell_1-AD.vs.control; scRNA-seq,SRP330776,Natural killer cell_1-AD.vs.control; scRNA-seq,SRP330776,Memory T cell_1-AD.vs.control; scRNA-seq,SRP330776,Natural killer T cell-AD.vs.control; scRNA-seq,SRP330776,Naive CD8+ T cell_3-AD.vs.control; scRNA-seq,SRP330776,T cell-AD.vs.control; scRNA-seq,SRP330776,Naive CD8+ T cell_2-AD.vs.control; scRNA-seq,SRP330776,CD8+ T cell_3-AD.vs.control; scRNA-seq,SRP330776,B cell_3-AD.vs.control; scRNA-seq,SRP330776,Natural [illegible]	19
APOBEC3H	bulk RNA-seq,Homo_723,AD.vs.control; bulk RNA-seq,Homo_723,AD.vs.MCI; bulk RNA-seq,Homo_633,AD.vs.control; bulk RNA-seq,Homo_633,AD.vs.MCI; bulk RNA-seq,Homo_714,AD.vs.control; bulk RNA-seq,Homo_714,AD.vs.MCI; scRNA-seq,SRP215507,CD8+ T cell_3-AD.vs.control; scRNA-seq,SRP215507,CD8+ T cell_2-AD.vs.MCI; scRNA-seq,SRP215507,CD8+ T cell_5-AD.vs.MCI; scRNA-seq,SRP309935,Naive CD8+ T cell_3-AD.vs.control; scRNA-seq,SRP330776,CD8+ T cell_1-AD.vs.control; scRNA-seq,SRP330776,γδ T cell-AD.vs.control; scRNA-seq,SRP330776,Memory T cell_2-AD.vs.control; scRNA-seq,SRP330776,CD8+ T cell_2-AD.vs.control; scRNA-seq,SRP330776,B cell_1-AD.vs.control; scRNA-seq,SRP330776,Natural killer cell_1-AD.vs.control; scRNA-seq,SRP330776,Memory T cell_1-AD.vs.control; scRNA-seq,SRP330776,Natural killer T cell-AD.vs.control; scRNA-seq,SRP330776,T cell-AD.vs.control; scRNA-seq,SRP330776,CD8+ T cell_3-AD.vs.control; scRNA-seq,SRP330776,B [illegible]	21
APOBEC4	bulk RNA-seq,Homo_723,AD.vs.control; bulk RNA-seq,Homo_723,AD.vs.MCI; bulk RNA-seq,Homo_633,AD.vs.control; bulk RNA-	4

APOBR	scRNA-seq,SRP215507,CD8+ T cell_3-AD.vs.control; scRNA-seq,SRP215507,CD8+ T cell_2-AD.vs.MCI; scRNA-seq,SRP215507,CD8+ T cell_3-AD.vs.MCI; scRNA-seq,SRP215507,CD8+ T cell_1-AD.vs.MCI; scRNA-seq,SRP215507,CD8+ T cell_4-AD.vs.MCI; scRNA-seq,SRP215507,CD8+ T cell_6-AD.vs.MCI; scRNA-seq,SRP215507,CD8+ T cell_4-MCI.vs.control; scRNA-seq,SRP215507,CD8+ T cell_6-MCI.vs.control; scRNA-seq,SRP309935,Natural killer cell_2-AD.vs.control; scRNA-seq,SRP330776,Natural killer cell_1-AD.vs.control; scRNA-seq,SRP330776,B cell_3-	12
APOC1	bulk RNA-seq,ACOM,AD.vs.control; bulk RNA-seq,Homo_723,AD.vs.control; bulk RNA-seq,Homo_723,AD.vs.MCI; bulk RNA-seq,Homo_723,MCI.vs.control; bulk RNA-seq,Homo_633,AD.vs.control; bulk RNA-seq,Homo_633,AD.vs.MCI; bulk RNA-	8
Apoc2	bulk RNA-seq,SRP325058,AD.vs.control	1
APOC3	bulk RNA-seq,ACOM,AD.vs.control; bulk RNA-seq,Homo_723,AD.vs.MCI; bulk RNA-seq,Homo_723,MCI.vs.control; bulk RNA-seq,Homo_633,AD.vs.MCI; bulk RNA-seq,Homo_633,MCI.vs.control; bulk RNA-seq,ROSMAP,AD.vs.control	6
APOD	bulk RNA-seq,Homo_723,AD.vs.control; bulk RNA-seq,Homo_723,AD.vs.MCI; bulk RNA-seq,Homo_723,MCI.vs.control; bulk RNA-seq,Homo_633,AD.vs.control; bulk RNA-seq,Homo_633,AD.vs.MCI; bulk RNA-seq,Homo_633,MCI.vs.control; scRNA-seq,SRP330776,B cell_1-	7
APOE	bulk RNA-seq,ACOM,AD.vs.control; bulk RNA-seq,Homo_723,AD.vs.control; bulk RNA-seq,Homo_723,AD.vs.MCI; bulk RNA-seq,Homo_723,MCI.vs.control; bulk RNA-seq,Homo_633,AD.vs.control; bulk RNA-seq,Homo_633,AD.vs.MCI; bulk RNA-seq,Homo_633,MCI.vs.control	7
Apoe	bulk RNA-seq,SRP325058,AD.vs.control	1
APOF	bulk RNA-seq,Homo_723,AD.vs.control; bulk RNA-seq,Homo_723,AD.vs.MCI; bulk RNA-seq,Homo_633,AD.vs.control; bulk RNA-	4
APOH	bulk RNA-seq,Homo_723,AD.vs.MCI; bulk RNA-seq,Homo_723,MCI.vs.control; bulk RNA-seq,Homo_633,AD.vs.MCI; bulk RNA-	4
APOL1	scRNA-seq,SRP215507,CD8+ T cell_5-AD.vs.control; scRNA-seq,SRP330776,CD8+ T cell_1-AD.vs.control; scRNA-seq,SRP330776,γδ T cell-AD.vs.control; scRNA-seq,SRP330776,Memory T cell_2-AD.vs.control; scRNA-seq,SRP330776,B cell_1-AD.vs.control; scRNA-seq,SRP330776,Naive CD8+ T cell_1-AD.vs.control; scRNA-seq,SRP330776,Natural killer cell_1-AD.vs.control; scRNA-seq,SRP330776,Memory T cell_1-AD.vs.control; scRNA-seq,SRP330776,T cell-AD.vs.control; scRNA-seq,SRP330776,CD8+ T cell_3-AD.vs.control	10
Apol10a	bulk RNA-seq,SRP325058,AD.vs.control	1
Apol10b	bulk RNA-seq,SRP325058,AD.vs.control	1
Apol11a	bulk RNA-seq,SRP325058,AD.vs.control	1
Apol11b	bulk RNA-seq,SRP325058,AD.vs.control	1
APOL2	bulk RNA-seq,Homo_633,AD.vs.MCI; bulk RNA-seq,Homo_714,AD.vs.control; bulk RNA-seq,Homo_714,AD.vs.MCI; scRNA-seq,SRP215507,CD8+ T cell_4-AD.vs.control; scRNA-seq,SRP215507,CD8+ T cell_6-AD.vs.control; scRNA-seq,SRP215507,CD8+ T cell_4-AD.vs.MCI; scRNA-seq,SRP330776,T cell-AD.vs.control; scRNA-seq,SRP330776,Monocyte_2-AD.vs.control; scRNA-seq,SRP330776,Natural killer cell_2-AD.vs.control	9
APOL3	bulk RNA-seq,Homo_723,AD.vs.control; bulk RNA-seq,Homo_723,AD.vs.MCI; bulk RNA-seq,Homo_723,MCI.vs.control; bulk RNA-seq,Homo_633,AD.vs.control; bulk RNA-seq,Homo_633,AD.vs.MCI; bulk RNA-seq,Homo_633,MCI.vs.control; scRNA-seq,SRP215507,CD8+ T cell_6-AD.vs.MCI; scRNA-seq,SRP215507,CD8+ T cell_6-MCI.vs.control; scRNA-seq,SRP330776,CD8+ T cell_1-AD.vs.control; scRNA-seq,SRP330776,γδ T cell-AD.vs.control; scRNA-seq,SRP330776,CD8+ T cell_2-AD.vs.control; scRNA-seq,SRP330776,Naive CD8+ T cell_1-AD.vs.control; scRNA-seq,SRP330776,Natural killer cell_1-AD.vs.control; scRNA-seq,SRP330776,Memory T cell_1-AD.vs.control; scRNA-seq,SRP330776,B cell_2-	16
APOL4	bulk RNA-seq,Homo_723,AD.vs.control; bulk RNA-seq,Homo_723,AD.vs.MCI; bulk RNA-seq,Homo_723,MCI.vs.control; bulk RNA-seq,Homo_633,AD.vs.control; bulk RNA-seq,Homo_633,AD.vs.MCI; bulk RNA-seq,Homo_633,MCI.vs.control; bulk RNA-seq,Homo_714,AD.vs.control; bulk RNA-seq,Homo_714,AD.vs.MCI; bulk RNA-seq,ROSMAP,AD.vs.control; scRNA-seq,SRP309935,Monocyte_1-AD.vs.control	10
APOL5	bulk RNA-seq,Homo_723,AD.vs.control; bulk RNA-seq,Homo_633,AD.vs.control	2
APOL6	bulk RNA-seq,Homo_723,AD.vs.control; bulk RNA-seq,Homo_723,MCI.vs.control; bulk RNA-seq,Homo_633,AD.vs.control; bulk RNA-seq,Homo_633,AD.vs.MCI; bulk RNA-seq,Homo_633,MCI.vs.control; scRNA-seq,SRP215507,CD8+ T cell_6-AD.vs.control; scRNA-seq,SRP215507,CD8+ T cell_2-AD.vs.MCI; scRNA-seq,SRP215507,CD8+ T cell_5-AD.vs.MCI; scRNA-seq,SRP309935,Naive CD8+ T cell_2-AD.vs.control; scRNA-seq,SRP330776,B cell_2-AD.vs.control; scRNA-seq,SRP330776,Natural killer cell_2-AD.vs.control	11

APOLD1	bulk RNA-seq,Homo_723,AD.vs.control; bulk RNA-seq,Homo_723,AD.vs.MCI; bulk RNA-seq,Homo_723,MCI.vs.control; bulk RNA-seq,Homo_633,AD.vs.control; bulk RNA-seq,Homo_633,AD.vs.MCI; bulk RNA-seq,Homo_633,MCI.vs.control; bulk RNA-seq,Homo_714,AD.vs.control; bulk RNA-seq,Homo_714,AD.vs.MCI; bulk RNA-seq,SRP223445,AD.vs.control; scRNA-seq,SRP215507,CD8+ T cell_3-AD.vs.control; scRNA-seq,SRP215507,CD8+ T cell_3-MCI.vs.control; scRNA-seq,SRP309935,Naive CD8+ T cell_1-AD.vs.control; scRNA-seq,SRP309935,CD8+ T cell_2-AD.vs.control; scRNA-seq,SRP330776,Memory T cell_2-AD.vs.control; scRNA-seq,SRP330776,Naive CD8+ T cell_2-AD.vs.control	15
APOM	bulk RNA-seq,Homo_714,AD.vs.control; bulk RNA-seq,Homo_714,AD.vs.MCI; scRNA-seq,SRP215507,CD8+ T cell_3-AD.vs.MCI; scRNA-seq,SRP215507,CD8+ T cell_3-MCI.vs.control; scRNA-seq,SRP330776,CD8+ T cell_1-AD.vs.control; scRNA-seq,SRP330776,γδ T cell-AD.vs.control; scRNA-seq,SRP330776,CD8+ T cell_2-AD.vs.control; scRNA-seq,SRP330776,Natural killer T cell-AD.vs.control	8
APOO	bulk RNA-seq,Homo_633,AD.vs.control; bulk RNA-seq,Homo_633,AD.vs.MCI; bulk RNA-seq,Homo_714,AD.vs.control; bulk RNA-seq,Homo_714,AD.vs.MCI; scRNA-seq,SRP215507,CD8+ T cell_5-AD.vs.MCI; scRNA-seq,SRP215507,CD8+ T cell_4-MCI.vs.control; scRNA-seq,SRP330776,CD8+ T cell_1-AD.vs.control; scRNA-seq,SRP330776,Memory T cell_2-AD.vs.control; scRNA-seq,SRP330776,CD8+ T cell_2-AD.vs.control; scRNA-seq,SRP330776,Natural killer cell_1-AD.vs.control; scRNA-seq,SRP330776,Memory T cell_1-AD.vs.control; scRNA-seq,SRP330776,Natural killer T cell-AD.vs.control; scRNA-seq,SRP330776,B cell_2-AD.vs.control; scRNA-seq,SRP330776,T cell-AD.vs.control; scRNA- [illegible]	15
APOOL	bulk RNA-seq,Homo_723,AD.vs.control; bulk RNA-seq,Homo_723,AD.vs.MCI; bulk RNA-seq,Homo_633,AD.vs.control; bulk RNA-seq,Homo_633,AD.vs.MCI; bulk RNA-seq,Homo_714,AD.vs.control; bulk RNA-seq,Homo_714,AD.vs.MCI; bulk RNA-seq,Homo_714,MCI.vs.control; scRNA-seq,SRP215507,CD8+ T cell_3-AD.vs.control; scRNA-seq,SRP215507,CD8+ T cell_3-AD.vs.MCI; scRNA-seq,SRP215507,CD8+ T cell_4-AD.vs.MCI; scRNA-seq,SRP215507,CD8+ T cell_3-MCI.vs.control; scRNA-seq,SRP330776,γδ T cell-AD.vs.control; scRNA-seq,SRP330776,Memory T cell_2-AD.vs.control; scRNA-seq,SRP330776,B cell_1-AD.vs.control; scRNA-seq,SRP330776,Natural killer cell_1-AD.vs.control; scRNA-seq,SRP330776,Natural killer T cell-AD.vs.control; scRNA-seq,SRP330776,Naive CD8+ T cell_3-AD.vs.control; scRNA-seq,SRP330776,B cell_2- [illegible]	19
APP	bulk RNA-seq,Homo_723,AD.vs.control; bulk RNA-seq,Homo_723,AD.vs.MCI; bulk RNA-seq,Homo_723,MCI.vs.control; bulk RNA-seq,Homo_633,AD.vs.control; bulk RNA-seq,Homo_633,AD.vs.MCI; bulk RNA-seq,Homo_633,MCI.vs.control; bulk RNA-seq,Homo_714,AD.vs.control; bulk RNA-seq,Homo_714,AD.vs.MCI; bulk RNA-seq,Homo_714,MCI.vs.control; scRNA-seq,SRP215507,CD8+ T cell_3-AD.vs.control; scRNA-seq,SRP215507,CD8+ T cell_1-AD.vs.control; scRNA-seq,SRP215507,CD8+ T cell_2-AD.vs.MCI; scRNA-seq,SRP215507,CD8+ T cell_1-AD.vs.MCI; scRNA-seq,SRP215507,CD8+ T cell_5-AD.vs.MCI; scRNA-seq,SRP215507,CD8+ T cell_1-MCI.vs.control; scRNA-seq,SRP215507,CD8+ T cell_5-MCI.vs.control; scRNA-seq,SRP215507,CD8+ T cell_2-MCI.vs.control; scRNA-seq,SRP215507,CD8+ T cell_3-MCI.vs.control; scRNA-seq,SRP309935,Megakaryocyte_1-AD.vs.control; scRNA-seq,SRP330776,γδ T cell-AD.vs.control; scRNA-seq,SRP330776,CD8+ T cell_2-AD.vs.control; scRNA-seq,SRP330776,Natural killer cell_1-AD.vs.control; scRNA-seq,SRP330776,Memory T cell_1-AD.vs.control; scRNA-seq,SRP330776,B cell_2- [illegible]	25
APPBP2	bulk RNA-seq,Homo_723,AD.vs.MCI; bulk RNA-seq,Homo_714,AD.vs.control; bulk RNA-seq,Homo_714,AD.vs.MCI; scRNA-seq,SRP215507,CD8+ T cell_5-AD.vs.control; scRNA-seq,SRP215507,CD8+ T cell_1-AD.vs.MCI; scRNA-seq,SRP215507,CD8+ T cell_5-AD.vs.MCI; scRNA-seq,SRP309935,CD8+ T cell_1-AD.vs.control; scRNA-seq,SRP330776,Natural killer T cell-AD.vs.control; scRNA-seq,SRP330776,Monocyte_2-AD.vs.control; scRNA-seq,SRP330776,Regulatory T cell-AD.vs.control; scRNA-seq,SRP330776,B cell_4-AD.vs.control	11
APPL1	bulk RNA-seq,Homo_723,AD.vs.control; bulk RNA-seq,Homo_723,AD.vs.MCI; bulk RNA-seq,Homo_723,MCI.vs.control; bulk RNA-seq,Homo_633,AD.vs.control; bulk RNA-seq,Homo_633,AD.vs.MCI; bulk RNA-seq,Homo_633,MCI.vs.control; bulk RNA-seq,Homo_714,AD.vs.control; bulk RNA-seq,Homo_714,AD.vs.MCI; scRNA-seq,SRP215507,CD8+ T cell_2-AD.vs.MCI; scRNA-seq,SRP215507,CD8+ T cell_4-AD.vs.MCI; scRNA-seq,SRP215507,CD8+ T cell_7-AD.vs.MCI; scRNA-seq,SRP215507,CD8+ T cell_5-MCI.vs.control; scRNA-seq,SRP309935,Natural killer cell_2-AD.vs.control; scRNA-seq,SRP330776,CD8+ T cell_1-AD.vs.control; scRNA-seq,SRP330776,γδ T cell-AD.vs.control; scRNA-seq,SRP330776,Memory T cell_2-AD.vs.control; scRNA-seq,SRP330776,CD8+ T cell_2-AD.vs.control; scRNA-seq,SRP330776,B cell_1-AD.vs.control; scRNA-seq,SRP330776,Naive CD8+ T cell_1-AD.vs.control; scRNA-seq,SRP330776,Natural killer cell_1-AD.vs.control; scRNA-seq,SRP330776,Memory T cell_1-AD.vs.control; scRNA-seq,SRP330776,Naive CD8+ T cell_3-AD.vs.control; scRNA-seq,SRP330776,B cell_2-AD.vs.control; scRNA-seq,SRP330776,T cell-AD.vs.control; scRNA-seq,SRP330776,Naive CD8+ T cell_2-AD.vs.control; scRNA-seq,SRP330776,CD8+ T cell_3-AD.vs.control; [illegible]	28

APPL2	bulk RNA-seq,Homo_723,AD.vs.control; bulk RNA-seq,Homo_723,MCI.vs.control; bulk RNA-seq,Homo_633,AD.vs.control; bulk RNA-seq,Homo_633,MCI.vs.control; bulk RNA-seq,Homo_714,AD.vs.control; bulk RNA-seq,Homo_714,AD.vs.MCI; bulk RNA-seq,Homo_714,MCI.vs.control; scRNA-seq,SRP215507,CD8+ T cell_3-AD.vs.MCI; scRNA-seq,SRP215507,CD8+ T cell_3-MCI.vs.control; scRNA-seq,SRP309935,CD8+ T cell_2-AD.vs.control; scRNA-seq,SRP330776,γδ T cell-AD.vs.control; scRNA-seq,SRP330776,Memory T cell_2-AD.vs.control; scRNA-seq,SRP330776,B cell_1-AD.vs.control; scRNA-seq,SRP330776,Naive CD8+ T cell_1-AD.vs.control; scRNA-seq,SRP330776,Memory T cell_1-AD.vs.control; scRNA-seq,SRP330776,B cell_2-AD.vs.control; scRNA-seq,SRP330776,T cell-AD.vs.control; scRNA-seq,SRP330776,CD8+ T cell_3-	20
APRT	scRNA-seq,SRP215507,CD8+ T cell_7-AD.vs.control; scRNA-seq,SRP215507,CD8+ T cell_10-AD.vs.control; scRNA-seq,SRP215507,CD8+ T cell_7-AD.vs.MCI; scRNA-seq,SRP309935,Erythroid cell-AD.vs.control	4
APTR	scRNA-seq,SRP215507,CD8+ T cell_6-AD.vs.control; scRNA-seq,SRP330776,Memory T cell_2-AD.vs.control; scRNA-seq,SRP330776,CD8+ T cell_2-AD.vs.control; scRNA-seq,SRP330776,B cell_1-AD.vs.control; scRNA-seq,SRP330776,Memory T cell_1-AD.vs.control; scRNA-seq,SRP330776,Natural killer T cell-AD.vs.control; scRNA-seq,SRP330776,Naive CD8+ T cell_3-AD.vs.control; scRNA-seq,SRP330776,T cell-AD.vs.control; scRNA-	9
APTX	bulk RNA-seq,Homo_723,AD.vs.control; bulk RNA-seq,Homo_723,AD.vs.MCI; bulk RNA-seq,Homo_723,MCI.vs.control; bulk RNA-seq,Homo_633,AD.vs.control; bulk RNA-seq,Homo_633,AD.vs.MCI; bulk RNA-seq,Homo_714,AD.vs.control; bulk RNA-seq,Homo_714,AD.vs.MCI; bulk RNA-seq,Homo_714,MCI.vs.control; scRNA-seq,SRP330776,Monocyte_2-AD.vs.control; scRNA-seq,SRP330776,B cell_3-AD.vs.control	10
AQP1	bulk RNA-seq,Homo_723,AD.vs.control; bulk RNA-seq,Homo_723,MCI.vs.control; bulk RNA-seq,Homo_633,AD.vs.control; bulk RNA-seq,Homo_633,AD.vs.MCI; bulk RNA-seq,Homo_633,MCI.vs.control; bulk RNA-seq,Homo_714,AD.vs.control; bulk RNA-seq,Homo_714,AD.vs.MCI;	8
AQP10	scRNA-seq,SRP215507,CD8+ T cell_2-MCI.vs.control	1
AQP11	bulk RNA-seq,Homo_633,AD.vs.control; bulk RNA-seq,Homo_633,AD.vs.MCI; bulk RNA-seq,Homo_714,AD.vs.control; bulk RNA-seq,Homo_714,AD.vs.MCI; scRNA-seq,SRP309935,CD8+ T cell_1-AD.vs.control; scRNA-seq,SRP330776,Naive CD8+ T cell_1-AD.vs.control	6
AQP12B	bulk RNA-seq,Homo_714,MCI.vs.control	1
AQP2	bulk RNA-seq,Homo_714,MCI.vs.control	1
AQP3	bulk RNA-seq,Homo_633,MCI.vs.control; bulk RNA-seq,Homo_714,AD.vs.control; bulk RNA-seq,Homo_714,AD.vs.MCI; bulk RNA-seq,Homo_714,MCI.vs.control; bulk RNA-seq,SRP310421,AD.vs.control; scRNA-seq,SRP215507,CD8+ T cell_7-AD.vs.control; scRNA-seq,SRP215507,CD8+ T cell_1-AD.vs.MCI; scRNA-seq,SRP215507,CD8+ T cell_4-AD.vs.MCI; scRNA-seq,SRP215507,CD8+ T cell_5-AD.vs.MCI; scRNA-seq,SRP215507,CD8+ T cell_1-MCI.vs.control; scRNA-seq,SRP215507,CD8+ T cell_4-MCI.vs.control; scRNA-seq,SRP215507,CD8+ T cell_2-MCI.vs.control; scRNA-seq,SRP330776,B cell_1-AD.vs.control; scRNA-seq,SRP330776,Naive CD8+ T cell_3-AD.vs.control; scRNA-	15
AQP4	bulk RNA-seq,Homo_723,AD.vs.MCI	1
AQP6	bulk RNA-seq,Homo_723,AD.vs.control; bulk RNA-seq,Homo_633,AD.vs.control	2
AQP7	bulk RNA-seq,Homo_723,AD.vs.control; bulk RNA-seq,Homo_723,AD.vs.MCI; bulk RNA-seq,Homo_723,MCI.vs.control; bulk RNA-seq,Homo_633,AD.vs.control; bulk RNA-seq,Homo_633,AD.vs.MCI; bulk RNA-seq,Homo_633,MCI.vs.control; bulk RNA-seq,ROSMAP,AD.vs.control; bulk RNA-seq,ROSMAP,MCI.vs.control; scRNA-seq,SRP330776,Naive CD8+ T cell_1-AD.vs.control	9
AQP8	bulk RNA-seq,Homo_723,AD.vs.MCI; bulk RNA-seq,ROSMAP,MCI.vs.control	2
AQP9	bulk RNA-seq,Homo_723,AD.vs.control; bulk RNA-seq,Homo_723,AD.vs.MCI; bulk RNA-seq,Homo_633,AD.vs.control; bulk RNA-seq,SRP223445,AD.vs.control; scRNA-seq,SRP330776,Monocyte_1-AD.vs.control; scRNA-seq,SRP330776,Monocyte_2-AD.vs.control	6
AQR	bulk RNA-seq,Homo_714,AD.vs.control; bulk RNA-seq,Homo_714,AD.vs.MCI; scRNA-seq,SRP215507,CD8+ T cell_6-AD.vs.MCI; scRNA-seq,SRP309935,Naive CD8+ T cell_2-AD.vs.control	4
AR	bulk RNA-seq,Homo_723,AD.vs.control; bulk RNA-seq,Homo_723,AD.vs.MCI; bulk RNA-seq,Homo_723,MCI.vs.control; bulk RNA-seq,Homo_633,AD.vs.control; bulk RNA-seq,Homo_633,AD.vs.MCI; bulk RNA-seq,Homo_633,MCI.vs.control; bulk RNA-seq,Homo_714,AD.vs.control; bulk RNA-seq,Homo_714,AD.vs.MCI; bulk RNA-seq,ROSMAP,AD.vs.control; bulk RNA-seq,ROSMAP,MCI.vs.control	10
ARAF	scRNA-seq,SRP330776,γδ T cell-AD.vs.control; scRNA-seq,SRP330776,Memory T cell_2-AD.vs.control; scRNA-seq,SRP330776,CD8+ T cell_2-AD.vs.control; scRNA-seq,SRP330776,Naive CD8+ T cell_1-AD.vs.control; scRNA-seq,SRP330776,Natural killer cell_1-AD.vs.control; scRNA-seq,SRP330776,Memory T cell_1-AD.vs.control; scRNA-seq,SRP330776,Natural killer T cell-AD.vs.control; scRNA-seq,SRP330776,T cell-AD.vs.control;	9

ARAP1	scRNA-seq,SRP215507,CD8+ T cell_7-AD.vs.control; scRNA-seq,SRP215507,CD8+ T cell_7-MCI.vs.control; scRNA-seq,SRP309935,Natural killer cell_2-AD.vs.control; scRNA-seq,SRP309935,Monocyte_2-AD.vs.control; scRNA-seq,SRP330776,Monocyte_2-AD.vs.control; scRNA-seq,SRP330776,B	6
ARAP2	bulk RNA-seq,Homo_723,AD.vs.control; bulk RNA-seq,Homo_723,AD.vs.MCI; bulk RNA-seq,Homo_633,AD.vs.control; bulk RNA-seq,Homo_633,AD.vs.MCI; bulk RNA-seq,Homo_714,AD.vs.control; bulk RNA-seq,Homo_714,AD.vs.MCI; scRNA-seq,SRP309935,Megakaryocyte_1-AD.vs.control; scRNA-seq,SRP309935,Natural killer cell_1-AD.vs.control; scRNA-seq,SRP309935,Monocyte_2-AD.vs.control; scRNA-seq,SRP309935,Naive CD8+ T cell_2-AD.vs.control; scRNA-seq,SRP330776,CD8+ T cell_1-AD.vs.control; scRNA-seq,SRP330776,γδ T cell-AD.vs.control; scRNA-seq,SRP330776,Memory T cell_2-AD.vs.control; scRNA-seq,SRP330776,CD8+ T cell_2-AD.vs.control; scRNA-seq,SRP330776,B cell_1-AD.vs.control; scRNA-seq,SRP330776,Naive CD8+ T cell_1-AD.vs.control; scRNA-seq,SRP330776,Natural killer cell_1-AD.vs.control; scRNA-seq,SRP330776,Memory T cell_1-AD.vs.control; scRNA-seq,SRP330776,Natural killer T cell-AD.vs.control; scRNA-seq,SRP330776,Naive CD8+ T cell_3-AD.vs.control; scRNA-seq,SRP330776,B cell_2-AD.vs.control; scRNA-seq,SRP330776,T cell-AD.vs.control; scRNA-seq,SRP330776,Naive CD8+ T cell_2-AD.vs.control; scRNA-seq,SRP330776,CD8+ T cell_3-AD.vs.control; scRNA-seq,SRP330776,Monocyte_1-AD.vs.control; scRNA- [illegible]	28
ARAP3	scRNA-seq,SRP215507,CD8+ T cell_1-AD.vs.control; scRNA-seq,SRP215507,CD8+ T cell_1-AD.vs.MCI; scRNA-seq,SRP215507,CD8+ T cell_3-MCI.vs.control; scRNA-seq,SRP330776,γδ T cell-AD.vs.control; scRNA-seq,SRP330776,CD8+ T cell_2-AD.vs.control; scRNA-seq,SRP330776,Naive CD8+ T cell_2-AD.vs.control; scRNA-seq,SRP330776,Monocyte_1-AD.vs.control; scRNA-seq,SRP330776,Regulatory T cell-AD.vs.control	8
ARC	bulk RNA-seq,Homo_723,AD.vs.control; bulk RNA-seq,Homo_723,AD.vs.MCI; bulk RNA-seq,ROSMAP,AD.vs.control; scRNA-seq,SRP215507,CD8+ T cell_3-MCI.vs.control	4
ARCN1	scRNA-seq,SRP215507,CD8+ T cell_5-AD.vs.MCI; scRNA-seq,SRP215507,CD8+ T cell_5-MCI.vs.control; scRNA-seq,SRP309935,Natural killer cell_2-AD.vs.control; scRNA-seq,SRP309935,Monocyte_2-AD.vs.control; scRNA-seq,SRP330776,B cell_1-AD.vs.control; scRNA-seq,SRP330776,B cell_2-AD.vs.control; scRNA-seq,SRP330776,Megakaryocyte_1-AD.vs.control	7
AREG	bulk RNA-seq,Homo_633,AD.vs.control; bulk RNA-seq,Homo_633,AD.vs.MCI; bulk RNA-seq,Homo_714,AD.vs.control; bulk RNA-seq,Homo_714,AD.vs.MCI; scRNA-seq,SRP215507,CD8+ T cell_2-AD.vs.control; scRNA-seq,SRP215507,CD8+ T cell_3-AD.vs.control; scRNA-seq,SRP215507,CD8+ T cell_5-AD.vs.control; scRNA-seq,SRP215507,CD8+ T cell_5-AD.vs.MCI; scRNA-seq,SRP215507,CD8+ T cell_5-MCI.vs.control; scRNA-seq,SRP215507,CD8+ T cell_4-MCI.vs.control; scRNA-seq,SRP215507,CD8+ T cell_2-MCI.vs.control; scRNA-seq,SRP215507,CD8+ T cell_3-MCI.vs.control; scRNA-seq,SRP215507,CD8+ T cell_7-MCI.vs.control; scRNA-seq,SRP330776,CD8+ T cell_1-AD.vs.control; scRNA-seq,SRP330776,γδ T cell-AD.vs.control; scRNA-seq,SRP330776,Memory T cell_2-AD.vs.control; scRNA-seq,SRP330776,CD8+ T cell_2-AD.vs.control; scRNA-seq,SRP330776,Naive CD8+ T cell_1-AD.vs.control; scRNA-seq,SRP330776,T cell-AD.vs.control; scRNA- [illegible]	20
AREL1	scRNA-seq,SRP215507,CD8+ T cell_4-AD.vs.control; scRNA-seq,SRP215507,CD8+ T cell_5-AD.vs.control; scRNA-seq,SRP215507,CD8+ T cell_5-AD.vs.MCI; scRNA-seq,SRP309935,Natural killer cell_2-AD.vs.control; scRNA-seq,SRP330776,B cell_1-AD.vs.control; scRNA-seq,SRP330776,Megakaryocyte_1-AD.vs.control; scRNA-seq,SRP330776,Natural killer cell_2-AD.vs.control	7
ARF1	scRNA-seq,SRP215507,CD8+ T cell_8-AD.vs.control; scRNA-seq,SRP215507,CD8+ T cell_7-AD.vs.MCI; scRNA-seq,SRP330776,Megakaryocyte_1-	3
ARF3	bulk RNA-seq,Homo_723,AD.vs.MCI; bulk RNA-seq,Homo_633,AD.vs.MCI; scRNA-seq,SRP215507,CD8+ T cell_4-AD.vs.control; scRNA-seq,SRP215507,CD8+ T cell_10-AD.vs.control; scRNA-seq,SRP215507,CD8+ T cell_5-AD.vs.MCI; scRNA-seq,SRP330776,Naive CD8+ T cell_1-AD.vs.control; scRNA-seq,SRP330776,Naive CD8+ T cell_3-AD.vs.control; scRNA-seq,SRP330776,Regulatory T cell-AD.vs.control; scRNA-	9
ARF4	bulk RNA-seq,Homo_714,AD.vs.control; bulk RNA-seq,Homo_714,AD.vs.MCI; scRNA-seq,SRP215507,CD8+ T cell_10-AD.vs.control; scRNA-seq,SRP215507,CD8+ T cell_4-AD.vs.MCI; scRNA-seq,SRP215507,CD8+ T cell_4-MCI.vs.control; scRNA-seq,SRP330776,Naive CD8+ T cell_3-	6
ARF4-AS1	scRNA-seq,SRP215507,CD8+ T cell_1-AD.vs.MCI; scRNA-seq,SRP215507,CD8+ T cell_1-MCI.vs.control; scRNA-seq,SRP330776,CD8+ T cell_1-AD.vs.control; scRNA-seq,SRP330776,Memory T cell_2-AD.vs.control; scRNA-seq,SRP330776,CD8+ T cell_2-AD.vs.control; scRNA-seq,SRP330776,Naive CD8+ T cell_1-AD.vs.control; scRNA-seq,SRP330776,B cell_2-AD.vs.control; scRNA-seq,SRP330776,CD8+ T cell_3-	9
ARF5	scRNA-seq,SRP215507,CD8+ T cell_7-AD.vs.control; scRNA-seq,SRP215507,CD8+ T cell_7-AD.vs.MCI; scRNA-seq,SRP215507,CD8+ T cell_5-AD.vs.MCI; scRNA-seq,SRP215507,CD8+ T cell_5-MCI.vs.control; scRNA-seq,SRP215507,CD8+ T cell_8-MCI.vs.control; scRNA-seq,SRP309935,Erythroid cell-AD.vs.control; scRNA-seq,SRP330776,Natural killer cell_2-AD.vs.control	7

ARF6	scRNA-seq,SRP215507,CD8+ T cell_6-AD.vs.control; scRNA-seq,SRP215507,CD8+ T cell_8-MCI.vs.control; scRNA-seq,SRP309935,Erythroid cell-AD.vs.control; scRNA-seq,SRP309935,Megakaryocyte_2-AD.vs.control; scRNA-seq,SRP330776,CD8+ T cell_1-AD.vs.control; scRNA-seq,SRP330776,γδ T cell-AD.vs.control; scRNA-seq,SRP330776,Memory T cell_2-AD.vs.control; scRNA-seq,SRP330776,CD8+ T cell_2-AD.vs.control; scRNA-seq,SRP330776,B cell_1-AD.vs.control; scRNA-seq,SRP330776,Naive CD8+ T cell_1-AD.vs.control; scRNA-seq,SRP330776,Natural killer cell_1-AD.vs.control; scRNA-seq,SRP330776,Memory T cell_1-AD.vs.control; scRNA-seq,SRP330776,Natural killer T cell-AD.vs.control; scRNA-seq,SRP330776,Naive CD8+ T cell_3-AD.vs.control; scRNA-seq,SRP330776,B cell_2-AD.vs.control; scRNA-seq,SRP330776,T cell-AD.vs.control; scRNA-seq,SRP330776,Naive CD8+ T cell_2-AD.vs.control; scRNA-seq,SRP330776,CD8+ T cell_3-AD.vs.control; scRNA-seq,SRP330776,Monocyte_1-AD.vs.control; scRNA-seq,SRP330776,Monocyte_2-AD.vs.control; scRNA-seq,SRP330776,B cell_3-AD.vs.control; scRNA-seq,SRP330776,Regulatory T [illegible]	24
ARFGAP1	bulk RNA-seq,Homo_714,AD.vs.control; bulk RNA-seq,Homo_714,AD.vs.MCI; scRNA-seq,SRP215507,CD8+ T cell_3-AD.vs.MCI; scRNA-seq,SRP215507,CD8+ T cell_3-MCI.vs.control; scRNA-seq,SRP330776,Memory T cell_2-AD.vs.control; scRNA-seq,SRP330776,CD8+ T cell_2-AD.vs.control; scRNA-seq,SRP330776,Naive CD8+ T cell_1-AD.vs.control; scRNA-seq,SRP330776,CD8+ T cell_3-AD.vs.control; scRNA-	9
ARFGAP2	bulk RNA-seq,Homo_723,AD.vs.control; bulk RNA-seq,Homo_723,AD.vs.MCI; bulk RNA-seq,Homo_723,MCI.vs.control; bulk RNA-seq,Homo_633,AD.vs.control; bulk RNA-seq,Homo_633,AD.vs.MCI; bulk RNA-seq,Homo_633,MCI.vs.control; bulk RNA-seq,Homo_714,AD.vs.control; bulk RNA-seq,Homo_714,AD.vs.MCI; bulk RNA-seq,Homo_714,MCI.vs.control; scRNA-seq,SRP215507,CD8+ T cell_7-AD.vs.control; scRNA-seq,SRP309935,Erythroid cell-AD.vs.control; scRNA-seq,SRP330776,Megakaryocyte_1-AD.vs.control	12
ARFGAP3	scRNA-seq,SRP215507,CD8+ T cell_6-AD.vs.MCI; scRNA-seq,SRP215507,CD8+ T cell_6-MCI.vs.control	2
ARFGEF1	scRNA-seq,SRP215507,CD8+ T cell_3-AD.vs.MCI; scRNA-seq,SRP309935,Erythroid cell-AD.vs.control; scRNA-seq,SRP309935,CD8+ T cell_1-AD.vs.control; scRNA-seq,SRP330776,CD8+ T cell_1-AD.vs.control; scRNA-seq,SRP330776,Memory T cell_2-AD.vs.control; scRNA-seq,SRP330776,CD8+ T cell_2-AD.vs.control; scRNA-seq,SRP330776,B cell_1-AD.vs.control; scRNA-seq,SRP330776,Naive CD8+ T cell_1-AD.vs.control; scRNA-seq,SRP330776,Natural killer cell_1-AD.vs.control; scRNA-seq,SRP330776,Memory T cell_1-AD.vs.control; scRNA-seq,SRP330776,Naive CD8+ T cell_3-AD.vs.control; scRNA-seq,SRP330776,B cell_2-AD.vs.control; scRNA-seq,SRP330776,T cell-AD.vs.control; scRNA-seq,SRP330776,CD8+ T cell_3-AD.vs.control; scRNA-seq,SRP330776,Monocyte_1-AD.vs.control; scRNA-seq,SRP330776,Monocyte_2- [illegible]	17
ARFGEF2	bulk RNA-seq,Homo_723,MCI.vs.control; bulk RNA-seq,Homo_633,AD.vs.control; bulk RNA-seq,Homo_633,AD.vs.MCI; bulk RNA-seq,Homo_633,MCI.vs.control; scRNA-seq,SRP215507,CD8+ T cell_5-MCI.vs.control; scRNA-seq,SRP330776,CD8+ T cell_1-AD.vs.control; scRNA-seq,SRP330776,γδ T cell-AD.vs.control; scRNA-seq,SRP330776,Memory T cell_2-AD.vs.control; scRNA-seq,SRP330776,CD8+ T cell_2-AD.vs.control; scRNA-seq,SRP330776,B cell_1-AD.vs.control; scRNA-seq,SRP330776,Naive CD8+ T cell_1-AD.vs.control; scRNA-seq,SRP330776,Natural killer cell_1-AD.vs.control; scRNA-seq,SRP330776,Memory T cell_1-AD.vs.control; scRNA-seq,SRP330776,Natural killer T cell-AD.vs.control; scRNA-seq,SRP330776,Naive CD8+ T cell_3-AD.vs.control; scRNA-seq,SRP330776,B cell_2-AD.vs.control; scRNA-seq,SRP330776,T cell-AD.vs.control; scRNA-seq,SRP330776,Naive CD8+ T cell_2-AD.vs.control; scRNA-seq,SRP330776,CD8+ T cell_3-AD.vs.control; scRNA-seq,SRP330776,Monocyte_1-AD.vs.control; scRNA-seq,SRP330776,Monocyte_2-AD.vs.control; scRNA-seq,SRP330776,B cell_3-AD.vs.control; scRNA-seq,SRP330776,Natural killer [illegible]	24
ARFGEF3	bulk RNA-seq,Homo_723,AD.vs.control; bulk RNA-seq,Homo_723,AD.vs.MCI; bulk RNA-seq,Homo_723,MCI.vs.control; bulk RNA-seq,Homo_633,AD.vs.control; bulk RNA-seq,Homo_633,AD.vs.MCI; bulk RNA-seq,Homo_633,MCI.vs.control; bulk RNA-seq,Homo_714,MCI.vs.control	7
ARFIP1	bulk RNA-seq,Homo_723,AD.vs.control; bulk RNA-seq,Homo_633,AD.vs.control; bulk RNA-seq,Homo_714,AD.vs.control; bulk RNA-seq,Homo_714,AD.vs.MCI; scRNA-seq,SRP215507,CD8+ T cell_2-MCI.vs.control; scRNA-seq,SRP215507,CD8+ T cell_7-MCI.vs.control; scRNA-seq,SRP330776,γδ T cell-AD.vs.control; scRNA-seq,SRP330776,Memory T cell_2-AD.vs.control; scRNA-seq,SRP330776,CD8+ T cell_2-AD.vs.control; scRNA-seq,SRP330776,B cell_1-AD.vs.control; scRNA-seq,SRP330776,Naive CD8+ T cell_1-AD.vs.control; scRNA-seq,SRP330776,Natural killer cell_1-AD.vs.control; scRNA-seq,SRP330776,Memory T cell_1-AD.vs.control; scRNA-seq,SRP330776,Naive CD8+ T cell_3-AD.vs.control; scRNA-seq,SRP330776,B cell_2-AD.vs.control; scRNA-seq,SRP330776,CD8+ T cell_3-AD.vs.control; scRNA-seq,SRP330776,B cell_3-AD.vs.control; scRNA- [illegible]	18
ARFIP2	scRNA-seq,SRP330776,Naive CD8+ T cell_3-AD.vs.control; scRNA-seq,SRP330776,Monocyte_1-AD.vs.control; scRNA-seq,SRP330776,B cell_3-	3
ARFRP1	bulk RNA-seq,Homo_723,AD.vs.control; bulk RNA-seq,Homo_633,AD.vs.control; scRNA-seq,SRP215507,CD8+ T cell_6-AD.vs.control; scRNA-seq,SRP215507,CD8+ T cell_6-AD.vs.MCI; scRNA-seq,SRP330776,CD8+ T cell_1-AD.vs.control; scRNA-seq,SRP330776,B cell_1-AD.vs.control; scRNA-seq,SRP330776,Naive CD8+ T cell_1-AD.vs.control; scRNA-seq,SRP330776,Natural killer cell_1-AD.vs.control; scRNA-seq,SRP330776,Natural killer T cell-AD.vs.control; scRNA-seq,SRP330776,B cell_2-AD.vs.control; scRNA-seq,SRP330776,CD8+ T cell_3-AD.vs.control; scRNA-	12

ARG1	bulk RNA-seq,Homo_723,AD.vs.control; bulk RNA-seq,Homo_723,AD.vs.MCI; bulk RNA-seq,Homo_633,AD.vs.control; bulk RNA-seq,Homo_633,AD.vs.MCI; bulk RNA-seq,Homo_714,AD.vs.control; bulk RNA-seq,Homo_714,AD.vs.MCI	6
ARG2	bulk RNA-seq,Homo_723,AD.vs.control; bulk RNA-seq,Homo_723,AD.vs.MCI; bulk RNA-seq,Homo_633,AD.vs.control; bulk RNA-seq,Homo_633,AD.vs.MCI; bulk RNA-seq,Homo_714,AD.vs.control; bulk RNA-seq,Homo_714,AD.vs.MCI; bulk RNA-seq,SRP223445,AD.vs.control; scRNA-seq,SRP215507,CD8+ T cell_2-AD.vs.control; scRNA-seq,SRP215507,CD8+ T cell_4-MCI.vs.control; scRNA-seq,SRP330776,CD8+ T cell_1-AD.vs.control; scRNA-seq,SRP330776,γδ T cell-AD.vs.control; scRNA-seq,SRP330776,Memory T cell_2-AD.vs.control; scRNA-seq,SRP330776,Memory T cell_1-AD.vs.control; scRNA-seq,SRP330776,T cell-AD.vs.control; scRNA-seq,SRP330776,CD8+ T cell_3-AD.vs.control	15
ARGFX	bulk RNA-seq,Homo_723,MCI.vs.control; bulk RNA-seq,Homo_633,MCI.vs.control; bulk RNA-seq,Homo_714,MCI.vs.control	3
ARGLU1	bulk RNA-seq,Homo_633,AD.vs.control; bulk RNA-seq,Homo_633,AD.vs.MCI; bulk RNA-seq,Homo_714,AD.vs.control; bulk RNA-seq,Homo_714,AD.vs.MCI; scRNA-seq,SRP215507,CD8+ T cell_7-AD.vs.control; scRNA-seq,SRP309935,Erythroid cell-AD.vs.control	6
ARHGAP1	scRNA-seq,SRP215507,CD8+ T cell_9-AD.vs.control; scRNA-seq,SRP215507,CD8+ T cell_6-MCI.vs.control; scRNA-seq,SRP330776,CD8+ T cell_1-AD.vs.control; scRNA-seq,SRP330776,Natural killer cell_2-AD.vs.control	4
ARHGAP10	scRNA-seq,SRP215507,CD8+ T cell_1-AD.vs.MCI; scRNA-seq,SRP215507,CD8+ T cell_1-MCI.vs.control; scRNA-seq,SRP215507,CD8+ T cell_5-MCI.vs.control; scRNA-seq,SRP309935,Natural killer cell_1-AD.vs.control; scRNA-seq,SRP330776,CD8+ T cell_1-AD.vs.control; scRNA-seq,SRP330776,γδ T cell-AD.vs.control; scRNA-seq,SRP330776,Memory T cell_2-AD.vs.control; scRNA-seq,SRP330776,CD8+ T cell_2-AD.vs.control; scRNA-seq,SRP330776,B cell_1-AD.vs.control; scRNA-seq,SRP330776,Naive CD8+ T cell_1-AD.vs.control; scRNA-seq,SRP330776,Natural killer cell_1-AD.vs.control; scRNA-seq,SRP330776,Memory T cell_1-AD.vs.control; scRNA-seq,SRP330776,Natural killer T cell-AD.vs.control; scRNA-seq,SRP330776,Naive CD8+ T cell_3-AD.vs.control; scRNA-seq,SRP330776,T cell-AD.vs.control; scRNA-seq,SRP330776,CD8+ T cell_3-AD.vs.control; [illegible]	17
ARHGAP11A	bulk RNA-seq,Homo_723,AD.vs.control; bulk RNA-seq,Homo_723,AD.vs.MCI; bulk RNA-seq,Homo_723,MCI.vs.control; bulk RNA-seq,Homo_633,AD.vs.control; bulk RNA-seq,Homo_633,AD.vs.MCI; bulk RNA-seq,Homo_633,MCI.vs.control; bulk RNA-seq,Homo_714,AD.vs.control; bulk RNA-seq,Homo_714,AD.vs.MCI; scRNA-seq,SRP330776,CD8+ T cell_1-AD.vs.control; scRNA-seq,SRP330776,γδ T cell-AD.vs.control; scRNA-seq,SRP330776,Natural killer cell_1-AD.vs.control; scRNA-seq,SRP330776,Naive CD8+ T cell_3-AD.vs.control; scRNA-seq,SRP330776,B cell_2-	14
ARHGAP11A-SCG5	bulk RNA-seq,Homo_723,AD.vs.control; bulk RNA-seq,Homo_723,AD.vs.MCI; bulk RNA-seq,Homo_723,MCI.vs.control	3
ARHGAP11B	bulk RNA-seq,Homo_633,AD.vs.control; bulk RNA-seq,Homo_633,AD.vs.MCI	2
ARHGAP12	bulk RNA-seq,Homo_723,AD.vs.control; bulk RNA-seq,Homo_723,AD.vs.MCI; bulk RNA-seq,Homo_723,MCI.vs.control; bulk RNA-seq,Homo_633,AD.vs.control; bulk RNA-seq,Homo_633,AD.vs.MCI; bulk RNA-seq,Homo_633,MCI.vs.control; bulk RNA-seq,Homo_714,AD.vs.control; bulk RNA-seq,Homo_714,AD.vs.MCI; bulk RNA-seq,Homo_714,MCI.vs.control; scRNA-seq,SRP215507,CD8+ T cell_1-AD.vs.control; scRNA-seq,SRP215507,CD8+ T cell_7-AD.vs.control; scRNA-seq,SRP215507,CD8+ T cell_6-AD.vs.control; scRNA-seq,SRP215507,CD8+ T cell_5-AD.vs.MCI; scRNA-seq,SRP215507,CD8+ T cell_1-MCI.vs.control; scRNA-seq,SRP215507,CD8+ T cell_5-MCI.vs.control; scRNA-seq,SRP215507,CD8+ T cell_7-MCI.vs.control; scRNA-seq,SRP309935,CD8+ T cell_1-AD.vs.control; scRNA-seq,SRP330776,Monocyte_2-AD.vs.control; scRNA- [illegible]	19
Arhgap15	bulk RNA-seq,SRP325058,AD.vs.control	1
ARHGAP15	scRNA-seq,SRP215507,CD8+ T cell_4-AD.vs.MCI; scRNA-seq,SRP330776,CD8+ T cell_1-AD.vs.control; scRNA-seq,SRP330776,Memory T cell_2-AD.vs.control; scRNA-seq,SRP330776,B cell_1-AD.vs.control; scRNA-seq,SRP330776,Natural killer cell_1-AD.vs.control; scRNA-seq,SRP330776,Memory T cell_1-AD.vs.control; scRNA-seq,SRP330776,T cell-AD.vs.control; scRNA-seq,SRP330776,Naive CD8+ T cell_2-AD.vs.control; scRNA-seq,SRP330776,CD8+ T cell_3-AD.vs.control; scRNA-seq,SRP330776,Natural killer cell_2-AD.vs.control	10
ARHGAP17	bulk RNA-seq,Homo_723,AD.vs.control; bulk RNA-seq,Homo_723,MCI.vs.control; bulk RNA-seq,Homo_633,AD.vs.control; bulk RNA-seq,Homo_633,MCI.vs.control; bulk RNA-seq,Homo_714,AD.vs.control; bulk RNA-seq,Homo_714,AD.vs.MCI; bulk RNA-seq,Homo_714,MCI.vs.control; scRNA-seq,SRP215507,CD8+ T cell_5-AD.vs.control; scRNA-seq,SRP215507,CD8+ T cell_6-AD.vs.control; scRNA-seq,SRP215507,CD8+ T cell_5-AD.vs.MCI; scRNA-seq,SRP309935,Naive CD8+ T cell_2-AD.vs.control; scRNA-seq,SRP330776,Naive CD8+ T cell_3-	12
ARHGAP18	bulk RNA-seq,Homo_723,AD.vs.control; bulk RNA-seq,Homo_723,AD.vs.MCI; bulk RNA-seq,Homo_723,MCI.vs.control; bulk RNA-seq,Homo_633,AD.vs.control; bulk RNA-seq,Homo_633,AD.vs.MCI; bulk RNA-seq,Homo_633,MCI.vs.control; bulk RNA-seq,Homo_714,AD.vs.control; bulk RNA-seq,Homo_714,AD.vs.MCI; scRNA-seq,SRP330776,CD8+ T cell_1-AD.vs.control; scRNA-seq,SRP330776,Memory T cell_1-AD.vs.control; scRNA-seq,SRP330776,T cell-AD.vs.control; scRNA-seq,SRP330776,CD8+ T cell_3-AD.vs.control; scRNA-seq,SRP330776,Natural killer cell_2-	13

ARHGAP19	bulk RNA-seq,Homo_723,MCI.vs.control; bulk RNA-seq,Homo_633,AD.vs.control; bulk RNA-seq,Homo_633,MCI.vs.control; bulk RNA-seq,Homo_714,AD.vs.control; bulk RNA-seq,Homo_714,AD.vs.MCI; bulk RNA-seq,Homo_714,MCI.vs.control; scRNA-seq,SRP215507,CD8+ T cell_4-AD.vs.MCI; scRNA-seq,SRP330776,γδ T cell-AD.vs.control; scRNA-seq,SRP330776,CD8+ T cell_2-AD.vs.control; scRNA-seq,SRP330776,Monocyte_2-	10
ARHGAP20	bulk RNA-seq,Homo_723,AD.vs.control; bulk RNA-seq,Homo_723,AD.vs.MCI; bulk RNA-seq,Homo_723,MCI.vs.control; bulk RNA-seq,Homo_633,AD.vs.control; bulk RNA-seq,Homo_633,AD.vs.MCI; bulk RNA-seq,Homo_633,MCI.vs.control; scRNA-seq,SRP215507,CD8+ T cell_2-AD.vs.control; scRNA-seq,SRP215507,CD8+ T cell_1-AD.vs.control; scRNA-seq,SRP330776,B cell_1-AD.vs.control	9
ARHGAP21	bulk RNA-seq,Homo_723,AD.vs.control; bulk RNA-seq,Homo_723,AD.vs.MCI; bulk RNA-seq,Homo_723,MCI.vs.control; bulk RNA-seq,Homo_633,AD.vs.control; bulk RNA-seq,Homo_633,AD.vs.MCI; bulk RNA-seq,Homo_633,MCI.vs.control; bulk RNA-seq,Homo_714,AD.vs.control; bulk RNA-seq,Homo_714,AD.vs.MCI; bulk RNA-seq,Homo_714,MCI.vs.control; scRNA-seq,SRP215507,CD8+ T cell_3-AD.vs.MCI; scRNA-seq,SRP215507,CD8+ T cell_6-MCI.vs.control; scRNA-seq,SRP330776,γδ T cell-AD.vs.control; scRNA-seq,SRP330776,Natural killer T cell-AD.vs.control; scRNA-seq,SRP330776,Naive CD8+ T cell_3-AD.vs.control; scRNA-seq,SRP330776,Monocyte_2-AD.vs.control; scRNA-	16
ARHGAP22	bulk RNA-seq,Homo_723,AD.vs.control; bulk RNA-seq,Homo_633,AD.vs.control; bulk RNA-seq,Homo_633,AD.vs.MCI; bulk RNA-seq,Homo_714,AD.vs.control; bulk RNA-seq,Homo_714,AD.vs.MCI	5
ARHGAP23	bulk RNA-seq,Homo_723,AD.vs.control; bulk RNA-seq,Homo_723,AD.vs.MCI; bulk RNA-seq,Homo_723,MCI.vs.control; bulk RNA-seq,Homo_633,AD.vs.control; bulk RNA-seq,Homo_633,AD.vs.MCI; bulk RNA-seq,Homo_633,MCI.vs.control; bulk RNA-	8
ARHGAP25	scRNA-seq,SRP215507,CD8+ T cell_4-AD.vs.MCI; scRNA-seq,SRP215507,CD8+ T cell_6-AD.vs.MCI; scRNA-seq,SRP215507,CD8+ T cell_6-MCI.vs.control; scRNA-seq,SRP330776,Naive CD8+ T cell_2-AD.vs.control; scRNA-seq,SRP330776,Monocyte_1-AD.vs.control	5
ARHGAP26	scRNA-seq,SRP215507,CD8+ T cell_2-MCI.vs.control; scRNA-seq,SRP309935,Natural killer cell_1-AD.vs.control; scRNA-seq,SRP330776,CD8+ T cell_1-AD.vs.control; scRNA-seq,SRP330776,CD8+ T cell_2-AD.vs.control; scRNA-seq,SRP330776,Naive CD8+ T cell_1-AD.vs.control; scRNA-seq,SRP330776,Memory T cell_1-AD.vs.control; scRNA-seq,SRP330776,Naive CD8+ T cell_3-AD.vs.control; scRNA-seq,SRP330776,B cell_2-AD.vs.control; scRNA-seq,SRP330776,CD8+ T cell_3-AD.vs.control; scRNA-seq,SRP330776,Monocyte_2-AD.vs.control; scRNA-seq,SRP330776,Natural	11
ARHGAP27	scRNA-seq,SRP215507,CD8+ T cell_2-AD.vs.MCI; scRNA-seq,SRP215507,CD8+ T cell_6-AD.vs.MCI; scRNA-seq,SRP309935,Natural killer cell_2-AD.vs.control; scRNA-seq,SRP309935,Naive CD8+ T cell_1-AD.vs.control; scRNA-seq,SRP309935,Natural killer cell_1-AD.vs.control; scRNA-seq,SRP330776,Memory T cell_2-AD.vs.control; scRNA-seq,SRP330776,Naive CD8+ T cell_1-AD.vs.control; scRNA-seq,SRP330776,Natural killer cell_1-AD.vs.control; scRNA-seq,SRP330776,CD8+ T cell_3-AD.vs.control; scRNA-seq,SRP330776,Natural killer cell_2-AD.vs.control	10
ARHGAP28	bulk RNA-seq,Homo_723,AD.vs.control; bulk RNA-seq,Homo_723,AD.vs.MCI; bulk RNA-seq,Homo_723,MCI.vs.control; bulk RNA-seq,Homo_633,AD.vs.MCI; bulk RNA-seq,Homo_633,MCI.vs.control; bulk RNA-seq,Homo_714,MCI.vs.control	6
ARHGAP29	bulk RNA-seq,Homo_723,AD.vs.control; bulk RNA-seq,Homo_723,AD.vs.MCI; bulk RNA-seq,Homo_723,MCI.vs.control; bulk RNA-seq,Homo_633,AD.vs.control; bulk RNA-seq,Homo_633,AD.vs.MCI; bulk RNA-seq,Homo_633,MCI.vs.control; bulk RNA-seq,Homo_714,AD.vs.control; bulk RNA-seq,Homo_714,AD.vs.MCI; bulk RNA-seq,ROSMAP,AD.vs.control	9
ARHGAP30	bulk RNA-seq,Homo_723,AD.vs.control; bulk RNA-seq,Homo_723,AD.vs.MCI; bulk RNA-seq,Homo_723,MCI.vs.control; bulk RNA-seq,Homo_633,AD.vs.control; bulk RNA-seq,Homo_633,AD.vs.MCI; bulk RNA-seq,Homo_633,MCI.vs.control; bulk RNA-seq,Homo_714,AD.vs.control; bulk RNA-seq,Homo_714,AD.vs.MCI; bulk RNA-seq,Homo_714,MCI.vs.control; scRNA-seq,SRP215507,CD8+ T cell_10-AD.vs.control; scRNA-seq,SRP215507,CD8+ T cell_4-AD.vs.MCI; scRNA-seq,SRP309935,Naive CD8+ T cell_3-AD.vs.control; scRNA-seq,SRP330776,Naive CD8+ T cell_2-AD.vs.control; scRNA-seq,SRP330776,Regulatory T cell-AD.vs.control; scRNA-seq,SRP330776,Megakaryocyte_1-AD.vs.control	15
ARHGAP31	bulk RNA-seq,Homo_723,AD.vs.control; bulk RNA-seq,Homo_723,AD.vs.MCI; bulk RNA-seq,Homo_723,MCI.vs.control; bulk RNA-seq,Homo_633,AD.vs.control; bulk RNA-seq,Homo_633,AD.vs.MCI; bulk RNA-seq,Homo_633,MCI.vs.control; bulk RNA-seq,Homo_714,AD.vs.control; bulk RNA-seq,Homo_714,AD.vs.MCI; scRNA-seq,SRP215507,CD8+ T cell_8-AD.vs.control; scRNA-seq,SRP215507,CD8+ T cell_4-AD.vs.control; scRNA-seq,SRP215507,CD8+ T cell_1-AD.vs.MCI; scRNA-seq,SRP215507,CD8+ T cell_1-MCI.vs.control; scRNA-seq,SRP215507,CD8+ T cell_5-MCI.vs.control; scRNA-seq,SRP330776,γδ T cell-AD.vs.control; scRNA-seq,SRP330776,Naive CD8+ T cell_1-AD.vs.control; scRNA-seq,SRP330776,Natural killer cell_1-AD.vs.control; scRNA-seq,SRP330776,Natural killer T cell-AD.vs.control; scRNA-seq,SRP330776,CD8+ T cell_3-	18

ARHGAP32	bulk RNA-seq,Homo_723,AD.vs.control; bulk RNA-seq,Homo_723,AD.vs.MCI; bulk RNA-seq,Homo_723,MCI.vs.control; bulk RNA-seq,Homo_633,AD.vs.control; bulk RNA-seq,Homo_633,AD.vs.MCI; bulk RNA-seq,Homo_633,MCI.vs.control; bulk RNA-seq,Homo_714,AD.vs.control; bulk RNA-seq,Homo_714,AD.vs.MCI; scRNA-seq,SRP215507,CD8+ T cell_3-AD.vs.control; scRNA-seq,SRP215507,CD8+ T cell_5-AD.vs.control; scRNA-seq,SRP215507,CD8+ T cell_5-MCI.vs.control; scRNA-seq,SRP330776,γδ T cell-AD.vs.control; scRNA-seq,SRP330776,CD8+ T cell_2-	13
ARHGAP33	bulk RNA-seq,Homo_723,AD.vs.control; bulk RNA-seq,Homo_723,AD.vs.MCI; bulk RNA-seq,Homo_633,AD.vs.control; bulk RNA-seq,Homo_633,AD.vs.MCI; bulk RNA-seq,Homo_714,AD.vs.control; bulk RNA-seq,Homo_714,AD.vs.MCI; bulk RNA-seq,Homo_714,MCI.vs.control; scRNA-seq,SRP215507,CD8+ T cell_5-AD.vs.control; scRNA-seq,SRP215507,CD8+ T cell_1-AD.vs.MCI; scRNA-seq,SRP330776,B cell_1-AD.vs.control; scRNA-seq,SRP330776,Memory T cell_1-AD.vs.control; scRNA-seq,SRP330776,CD8+ T cell_3-AD.vs.control	12
ARHGAP35	bulk RNA-seq,Homo_633,AD.vs.control; scRNA-seq,SRP215507,CD8+ T cell_4-AD.vs.control; scRNA-seq,SRP215507,CD8+ T cell_4-AD.vs.MCI; scRNA-seq,SRP330776,γδ T cell-AD.vs.control; scRNA-seq,SRP330776,Naive CD8+ T cell_1-AD.vs.control; scRNA-seq,SRP330776,B cell_2-	6
ARHGAP39	bulk RNA-seq,Homo_723,AD.vs.control; bulk RNA-seq,Homo_723,AD.vs.MCI; bulk RNA-seq,Homo_633,AD.vs.control; bulk RNA-	4
ARHGAP4	bulk RNA-seq,Homo_723,AD.vs.MCI; bulk RNA-seq,Homo_633,AD.vs.MCI; bulk RNA-seq,Homo_714,AD.vs.control; bulk RNA-seq,Homo_714,AD.vs.MCI; bulk RNA-seq,Homo_714,MCI.vs.control; scRNA-seq,SRP215507,CD8+ T cell_7-AD.vs.control	6
ARHGAP40	bulk RNA-seq,Homo_714,MCI.vs.control; bulk RNA-seq,ROSMAP,MCI.vs.control	2
ARHGAP42	bulk RNA-seq,ROSMAP,AD.vs.control; scRNA-seq,SRP330776,B cell_1-AD.vs.control; scRNA-seq,SRP330776,B cell_2-AD.vs.control	3
ARHGAP44	bulk RNA-seq,Homo_723,AD.vs.control; bulk RNA-seq,Homo_723,AD.vs.MCI; bulk RNA-seq,Homo_723,MCI.vs.control; bulk RNA-seq,Homo_633,AD.vs.control; bulk RNA-seq,Homo_633,AD.vs.MCI; bulk RNA-seq,Homo_633,MCI.vs.control; bulk RNA-seq,Homo_714,AD.vs.control;	8
ARHGAP45	scRNA-seq,SRP215507,CD8+ T cell_10-AD.vs.control; scRNA-seq,SRP215507,CD8+ T cell_6-AD.vs.MCI; scRNA-seq,SRP309935,Natural killer cell_2-AD.vs.control; scRNA-seq,SRP309935,B cell_1-AD.vs.control; scRNA-seq,SRP309935,Naive CD8+ T cell_3-AD.vs.control; scRNA-seq,SRP330776,CD8+ T cell_1-AD.vs.control; scRNA-seq,SRP330776,Naive CD8+ T cell_1-AD.vs.control; scRNA-seq,SRP330776,Memory T cell_1-	9
ARHGAP5	bulk RNA-seq,Homo_723,AD.vs.control; bulk RNA-seq,Homo_723,AD.vs.MCI; bulk RNA-seq,Homo_723,MCI.vs.control; bulk RNA-seq,Homo_633,AD.vs.control; bulk RNA-seq,Homo_633,AD.vs.MCI; bulk RNA-seq,Homo_714,AD.vs.control; bulk RNA-seq,Homo_714,AD.vs.MCI; scRNA-seq,SRP215507,CD8+ T cell_7-MCI.vs.control; scRNA-seq,SRP330776,Natural killer cell_1-AD.vs.control; scRNA-seq,SRP330776,Monocyte_2-	10
ARHGAP5-AS1	scRNA-seq,SRP215507,CD8+ T cell_3-AD.vs.control; scRNA-seq,SRP215507,CD8+ T cell_1-AD.vs.control; scRNA-seq,SRP215507,CD8+ T cell_4-AD.vs.control; scRNA-seq,SRP215507,CD8+ T cell_1-AD.vs.MCI; scRNA-seq,SRP215507,CD8+ T cell_5-AD.vs.MCI; scRNA-seq,SRP215507,CD8+ T cell_1-MCI.vs.control; scRNA-seq,SRP215507,CD8+ T cell_3-MCI.vs.control	7
ARHGAP6	bulk RNA-seq,Homo_723,AD.vs.control; bulk RNA-seq,Homo_723,AD.vs.MCI; bulk RNA-seq,Homo_723,MCI.vs.control; bulk RNA-seq,Homo_633,AD.vs.control; bulk RNA-seq,Homo_633,AD.vs.MCI; bulk RNA-seq,Homo_633,MCI.vs.control; bulk RNA-seq,Homo_714,AD.vs.control; bulk RNA-seq,Homo_714,AD.vs.MCI; bulk RNA-seq,ROSMAP,MCI.vs.control; scRNA-seq,SRP330776,B cell_4-AD.vs.control	10
Arhgap6	bulk RNA-seq,SRP325058,AD.vs.control	1
ARHGAP8	scRNA-seq,SRP215507,CD8+ T cell_2-AD.vs.MCI; scRNA-seq,SRP215507,CD8+ T cell_4-AD.vs.MCI; scRNA-seq,SRP215507,CD8+ T cell_4-MCI.vs.control; scRNA-seq,SRP215507,CD8+ T cell_6-MCI.vs.control	4
ARHGAP9	scRNA-seq,SRP215507,CD8+ T cell_7-AD.vs.control; scRNA-seq,SRP215507,CD8+ T cell_7-AD.vs.MCI; scRNA-seq,SRP330776,B cell_1-AD.vs.control; scRNA-seq,SRP330776,B cell_2-AD.vs.control	4
ARHGDIA	bulk RNA-seq,Homo_723,AD.vs.control; bulk RNA-seq,Homo_723,AD.vs.MCI; bulk RNA-seq,Homo_633,AD.vs.control; bulk RNA-seq,Homo_633,AD.vs.MCI; bulk RNA-seq,Homo_714,AD.vs.control; bulk RNA-seq,Homo_714,AD.vs.MCI; bulk RNA-seq,Homo_714,MCI.vs.control; scRNA-seq,SRP215507,CD8+ T cell_8-MCI.vs.control; scRNA-seq,SRP309935,Erythroid cell-AD.vs.control	9
Arhgdib	bulk RNA-seq,SRP325058,AD.vs.control	1
ARHGDIB	scRNA-seq,SRP215507,CD8+ T cell_4-AD.vs.MCI; scRNA-seq,SRP309935,Erythroid cell-AD.vs.control; scRNA-seq,SRP330776,Megakaryocyte_1-	3
ARHGDIG	bulk RNA-seq,Homo_723,AD.vs.MCI; bulk RNA-seq,Homo_723,MCI.vs.control; bulk RNA-seq,ROSMAP,AD.vs.control	3
ARHGEF1	scRNA-seq,SRP215507,CD8+ T cell_7-AD.vs.control; scRNA-seq,SRP215507,CD8+ T cell_9-AD.vs.control; scRNA-seq,SRP215507,CD8+ T cell_7-AD.vs.MCI; scRNA-seq,SRP309935,B cell_1-AD.vs.control; scRNA-seq,SRP330776,Naive CD8+ T cell_2-AD.vs.control	5

ARHGEF10	bulk RNA-seq,Homo_723,AD.vs.control; bulk RNA-seq,Homo_723,AD.vs.MCI; bulk RNA-seq,Homo_723,MCI.vs.control; bulk RNA-seq,Homo_633,AD.vs.control; bulk RNA-seq,Homo_633,AD.vs.MCI; bulk RNA-seq,Homo_633,MCI.vs.control; bulk RNA-seq,Homo_714,AD.vs.control; bulk RNA-seq,Homo_714,AD.vs.MCI; scRNA-seq,SRP215507,CD8+ T cell_3-AD.vs.control; scRNA-seq,SRP215507,CD8+ T cell_4-MCI.vs.control; scRNA-seq,SRP309935,Natural killer cell_2-AD.vs.control; scRNA-seq,SRP309935,Megakaryocyte_1-AD.vs.control; scRNA-seq,SRP309935,Naive CD8+ T cell_1-AD.vs.control; scRNA-seq,SRP309935,Natural killer cell_1-AD.vs.control; scRNA-seq,SRP309935,Naive CD8+ T cell_2-AD.vs.control; scRNA-seq,SRP330776,CD8+ T cell_1-AD.vs.control; scRNA-seq,SRP330776,γδ T cell-AD.vs.control; scRNA-seq,SRP330776,Memory T cell_2-AD.vs.control; scRNA-seq,SRP330776,B cell_1-AD.vs.control; scRNA-seq,SRP330776,Naive CD8+ T cell_1-AD.vs.control; scRNA-seq,SRP330776,Memory T cell_1-AD.vs.control; scRNA-seq,SRP330776,Natural killer T cell-AD.vs.control; scRNA-seq,SRP330776,Naive CD8+ T [illegible]	25
ARHGEF10L	bulk RNA-seq,Homo_633,AD.vs.control; bulk RNA-seq,Homo_633,AD.vs.MCI; bulk RNA-seq,Homo_714,AD.vs.control; bulk RNA-seq,Homo_714,AD.vs.MCI; scRNA-seq,SRP330776,Monocyte_2-AD.vs.control	5
ARHGEF11	scRNA-seq,SRP215507,CD8+ T cell_3-AD.vs.MCI; scRNA-seq,SRP215507,CD8+ T cell_3-MCI.vs.control; scRNA-seq,SRP309935,Monocyte_1-AD.vs.control; scRNA-seq,SRP330776,B cell_1-AD.vs.control; scRNA-seq,SRP330776,Naive CD8+ T cell_2-AD.vs.control	5
ARHGEF12	bulk RNA-seq,ROSMAP,AD.vs.control; bulk RNA-seq,ROSMAP,MCI.vs.control; scRNA-seq,SRP215507,CD8+ T cell_2-AD.vs.control; scRNA-seq,SRP215507,CD8+ T cell_4-AD.vs.control; scRNA-seq,SRP215507,CD8+ T cell_5-AD.vs.control; scRNA-seq,SRP215507,CD8+ T cell_2-AD.vs.MCI; scRNA-seq,SRP215507,CD8+ T cell_1-AD.vs.MCI; scRNA-seq,SRP215507,CD8+ T cell_5-AD.vs.MCI; scRNA-seq,SRP215507,CD8+ T cell_4-MCI.vs.control; scRNA-seq,SRP330776,CD8+ T cell_1-AD.vs.control; scRNA-seq,SRP330776,Memory T cell_2-AD.vs.control; scRNA-seq,SRP330776,CD8+ T cell_2-AD.vs.control; scRNA-seq,SRP330776,B cell_1-AD.vs.control; scRNA-seq,SRP330776,Natural killer cell_1-AD.vs.control; scRNA-seq,SRP330776,Memory T cell_1-AD.vs.control; scRNA-seq,SRP330776,Natural killer T cell-AD.vs.control; scRNA- [illegible]	19
Arhgef12	bulk RNA-seq,SRP325058,AD.vs.control	1
ARHGEF15	bulk RNA-seq,Homo_723,AD.vs.control; bulk RNA-seq,Homo_723,AD.vs.MCI; bulk RNA-seq,Homo_723,MCI.vs.control; bulk RNA-seq,Homo_633,AD.vs.MCI; bulk RNA-seq,Homo_633,MCI.vs.control; bulk RNA-seq,SRP223445,AD.vs.control	6
ARHGEF16	bulk RNA-seq,Homo_723,AD.vs.control; bulk RNA-seq,Homo_723,AD.vs.MCI; bulk RNA-seq,Homo_714,MCI.vs.control	3
ARHGEF17	bulk RNA-seq,Homo_723,MCI.vs.control; bulk RNA-seq,Homo_633,MCI.vs.control; bulk RNA-seq,Homo_714,AD.vs.control; bulk RNA-seq,Homo_714,MCI.vs.control; bulk RNA-seq,ROSMAP,AD.vs.control; bulk RNA-seq,ROSMAP,MCI.vs.control	6
ARHGEF18	bulk RNA-seq,Homo_723,AD.vs.control; bulk RNA-seq,Homo_723,AD.vs.MCI; bulk RNA-seq,Homo_723,MCI.vs.control; bulk RNA-seq,Homo_633,AD.vs.control; bulk RNA-seq,Homo_633,AD.vs.MCI; bulk RNA-seq,Homo_633,MCI.vs.control; bulk RNA-seq,Homo_714,AD.vs.control; bulk RNA-seq,Homo_714,AD.vs.MCI; bulk RNA-seq,Homo_714,MCI.vs.control; scRNA-seq,SRP215507,CD8+ T cell_10-AD.vs.control; scRNA-seq,SRP215507,CD8+ T cell_3-AD.vs.MCI; scRNA-seq,SRP309935,B cell_3-AD.vs.control; scRNA-seq,SRP330776,CD8+ T cell_1-AD.vs.control; scRNA-seq,SRP330776,γδ T cell-AD.vs.control; scRNA-seq,SRP330776,Naive CD8+ T cell_1-AD.vs.control; scRNA-seq,SRP330776,Memory T cell_1-AD.vs.control; scRNA-seq,SRP330776,Naive CD8+ T cell_3-AD.vs.control; scRNA-seq,SRP330776,B cell_2-AD.vs.control; scRNA-seq,SRP330776,T cell-AD.vs.control; scRNA-seq,SRP330776,CD8+ T cell_3-AD.vs.control; scRNA-seq,SRP330776,Monocyte_2-AD.vs.control; scRNA-seq,SRP330776,B [illegible]	22
ARHGEF19	scRNA-seq,SRP215507,CD8+ T cell_5-AD.vs.control; scRNA-seq,SRP215507,CD8+ T cell_3-AD.vs.MCI; scRNA-seq,SRP215507,CD8+ T cell_4-AD.vs.MCI; scRNA-seq,SRP330776,γδ T cell-AD.vs.control; scRNA-seq,SRP330776,Memory T cell_2-AD.vs.control; scRNA-seq,SRP330776,B cell_1-AD.vs.control; scRNA-seq,SRP330776,Natural killer T cell-AD.vs.control; scRNA-seq,SRP330776,T cell-AD.vs.control; scRNA-seq,SRP330776,CD8+ T	9
ARHGEF2	scRNA-seq,SRP309935,Natural killer cell_2-AD.vs.control; scRNA-seq,SRP330776,Memory T cell_2-AD.vs.control; scRNA-seq,SRP330776,Natural killer cell_1-AD.vs.control	3
ARHGEF25	bulk RNA-seq,Homo_723,AD.vs.control; bulk RNA-seq,Homo_723,AD.vs.MCI; bulk RNA-seq,Homo_723,MCI.vs.control; bulk RNA-seq,Homo_633,AD.vs.control; bulk RNA-seq,Homo_633,AD.vs.MCI; bulk RNA-seq,Homo_633,MCI.vs.control; bulk RNA-seq,Homo_714,MCI.vs.control; scRNA-seq,SRP215507,CD8+ T cell_3-MCI.vs.control	8
ARHGEF26	bulk RNA-seq,Homo_723,AD.vs.control; bulk RNA-seq,Homo_723,AD.vs.MCI; bulk RNA-seq,Homo_633,AD.vs.control; bulk RNA-seq,Homo_633,AD.vs.MCI; bulk RNA-seq,Homo_633,MCI.vs.control; bulk RNA-seq,Homo_714,AD.vs.MCI; bulk RNA-seq,Homo_714,MCI.vs.control; bulk RNA-seq,ROSMAP,MCI.vs.control; scRNA-seq,SRP215507,CD8+ T cell_1-MCI.vs.control; scRNA-seq,SRP309935,Naive CD8+ T cell_2-	11

ARHGEF28	bulk RNA-seq,Homo_723,AD.vs.control; bulk RNA-seq,Homo_723,AD.vs.MCI; bulk RNA-seq,Homo_723,MCI.vs.control; bulk RNA-seq,Homo_633,AD.vs.control; bulk RNA-seq,Homo_633,AD.vs.MCI; bulk RNA-seq,Homo_633,MCI.vs.control; bulk RNA-seq,Homo_714,AD.vs.control; bulk RNA-seq,Homo_714,AD.vs.MCI; bulk RNA-seq,ROSMAP,AD.vs.control; bulk RNA-seq,ROSMAP,MCI.vs.control; scRNA-seq,SRP215507,CD8+ T cell_1-AD.vs.MCI; scRNA-seq,SRP215507,CD8+ T cell_1-MCI.vs.control; scRNA-seq,SRP330776,Memory T cell_1-AD.vs.control	13
ARHGEF3	bulk RNA-seq,Homo_633,AD.vs.control; scRNA-seq,SRP215507,CD8+ T cell_4-AD.vs.MCI; scRNA-seq,SRP215507,CD8+ T cell_5-AD.vs.MCI; scRNA-seq,SRP215507,CD8+ T cell_5-MCI.vs.control; scRNA-seq,SRP309935,Erythroid cell-AD.vs.control	5
ARHGEF33	bulk RNA-seq,Homo_723,AD.vs.control; bulk RNA-seq,Homo_723,AD.vs.MCI; bulk RNA-seq,Homo_723,MCI.vs.control; bulk RNA-seq,Homo_633,AD.vs.control; bulk RNA-seq,Homo_633,AD.vs.MCI; bulk RNA-seq,Homo_633,MCI.vs.control; bulk RNA-seq,ROSMAP,AD.vs.control	7
ARHGEF35	bulk RNA-seq,Homo_723,AD.vs.control; bulk RNA-seq,Homo_723,AD.vs.MCI; bulk RNA-seq,Homo_633,AD.vs.control; bulk RNA-seq,Homo_633,AD.vs.MCI; bulk RNA-seq,Homo_714,AD.vs.control; bulk RNA-seq,Homo_714,AD.vs.MCI; scRNA-seq,SRP215507,CD8+ T cell_2-AD.vs.control; scRNA-seq,SRP215507,CD8+ T cell_1-AD.vs.control; scRNA-seq,SRP215507,CD8+ T cell_1-MCI.vs.control; scRNA-	10
ARHGEF37	bulk RNA-seq,Homo_723,AD.vs.control; bulk RNA-seq,Homo_723,AD.vs.MCI; bulk RNA-seq,Homo_633,AD.vs.control; bulk RNA-seq,Homo_633,AD.vs.MCI; bulk RNA-seq,Homo_714,AD.vs.control; bulk RNA-seq,Homo_714,AD.vs.MCI	6
ARHGEF38	bulk RNA-seq,Homo_723,AD.vs.control; bulk RNA-seq,Homo_723,AD.vs.MCI; bulk RNA-seq,Homo_633,AD.vs.control; bulk RNA-	4
ARHGEF39	bulk RNA-seq,Homo_723,AD.vs.control; bulk RNA-seq,Homo_723,AD.vs.MCI; bulk RNA-seq,Homo_723,MCI.vs.control; bulk RNA-seq,Homo_633,AD.vs.control; bulk RNA-seq,Homo_633,AD.vs.MCI; bulk RNA-seq,Homo_633,MCI.vs.control; bulk RNA-seq,Homo_714,AD.vs.control; bulk RNA-seq,Homo_714,AD.vs.MCI; bulk RNA-seq,Homo_714,MCI.vs.control; scRNA-seq,SRP215507,CD8+ T cell_4-AD.vs.control; scRNA-seq,SRP215507,CD8+ T cell_5-AD.vs.control; scRNA-seq,SRP215507,CD8+ T cell_5-AD.vs.MCI; scRNA-seq,SRP330776,CD8+ T cell_1-AD.vs.control;	14
ARHGEF4	bulk RNA-seq,Homo_723,AD.vs.control; bulk RNA-seq,Homo_723,AD.vs.MCI; bulk RNA-seq,Homo_714,AD.vs.control; bulk RNA-seq,Homo_714,AD.vs.MCI; scRNA-seq,SRP215507,CD8+ T cell_4-AD.vs.control; scRNA-seq,SRP215507,CD8+ T cell_3-AD.vs.MCI; scRNA-seq,SRP215507,CD8+ T cell_4-AD.vs.MCI; scRNA-seq,SRP215507,CD8+ T cell_3-MCI.vs.control; scRNA-seq,SRP330776,CD8+ T cell_3-AD.vs.control	9
ARHGEF40	bulk RNA-seq,Homo_714,AD.vs.control; bulk RNA-seq,Homo_714,AD.vs.MCI; scRNA-seq,SRP215507,CD8+ T cell_3-AD.vs.control; scRNA-seq,SRP215507,CD8+ T cell_1-AD.vs.control; scRNA-seq,SRP330776,CD8+ T cell_1-AD.vs.control; scRNA-seq,SRP330776,Memory T cell_2-AD.vs.control; scRNA-seq,SRP330776,CD8+ T cell_2-AD.vs.control; scRNA-seq,SRP330776,Naive CD8+ T cell_1-AD.vs.control; scRNA-	9
ARHGEF5	bulk RNA-seq,Homo_723,AD.vs.control; bulk RNA-seq,Homo_723,MCI.vs.control; bulk RNA-seq,Homo_633,MCI.vs.control; scRNA-seq,SRP330776,Memory T cell_2-AD.vs.control; scRNA-seq,SRP330776,Naive CD8+ T cell_1-AD.vs.control; scRNA-seq,SRP330776,Natural killer cell_1-AD.vs.control; scRNA-seq,SRP330776,B cell_2-AD.vs.control; scRNA-seq,SRP330776,CD8+ T cell_3-AD.vs.control	8
ARHGEF6	bulk RNA-seq,Homo_723,AD.vs.control; bulk RNA-seq,Homo_723,MCI.vs.control; bulk RNA-seq,Homo_633,AD.vs.control; bulk RNA-seq,Homo_633,MCI.vs.control; bulk RNA-seq,Homo_714,AD.vs.control; bulk RNA-seq,Homo_714,AD.vs.MCI; bulk RNA-seq,Homo_714,MCI.vs.control; scRNA-seq,SRP309935,Natural killer cell_2-AD.vs.control; scRNA-seq,SRP309935,CD8+ T cell_2-AD.vs.control;	10
ARHGEF7	bulk RNA-seq,Homo_723,AD.vs.control; bulk RNA-seq,Homo_723,AD.vs.MCI; bulk RNA-seq,Homo_714,AD.vs.control; bulk RNA-seq,Homo_714,AD.vs.MCI; scRNA-seq,SRP215507,CD8+ T cell_3-AD.vs.control; scRNA-seq,SRP215507,CD8+ T cell_1-AD.vs.MCI; scRNA-seq,SRP215507,CD8+ T cell_7-AD.vs.MCI; scRNA-seq,SRP215507,CD8+ T cell_5-AD.vs.MCI; scRNA-seq,SRP215507,CD8+ T cell_1-MCI.vs.control; scRNA-seq,SRP309935,Natural killer cell_1-AD.vs.control; scRNA-seq,SRP309935,Naive CD8+ T cell_3-AD.vs.control; scRNA-seq,SRP330776,Naive CD8+ T cell_1-AD.vs.control; scRNA-seq,SRP330776,Naive CD8+ T cell_3-AD.vs.control; scRNA-seq,SRP330776,Monocyte_2-AD.vs.control; scRNA-seq,SRP330776,B cell_3-AD.vs.control; scRNA-seq,SRP330776,Regulatory T cell-AD.vs.control; scRNA-seq,SRP330776,Natural killer cell_2-	18
ARHGEF9	bulk RNA-seq,Homo_723,AD.vs.control; bulk RNA-seq,Homo_723,AD.vs.MCI; bulk RNA-seq,Homo_723,MCI.vs.control; bulk RNA-seq,Homo_633,MCI.vs.control; bulk RNA-seq,Homo_714,AD.vs.control; bulk RNA-seq,Homo_714,AD.vs.MCI; bulk RNA-seq,Homo_714,MCI.vs.control; scRNA-seq,SRP215507,CD8+ T cell_10-AD.vs.control; scRNA-seq,SRP215507,CD8+ T cell_7-AD.vs.MCI; scRNA-seq,SRP330776,Natural killer cell_1-AD.vs.control; scRNA-seq,SRP330776,Naive CD8+ T cell_3-AD.vs.control; scRNA-seq,SRP330776,B cell_2-AD.vs.control; scRNA-seq,SRP330776,T cell-AD.vs.control; scRNA-seq,SRP330776,B cell_3-AD.vs.control; scRNA-seq,SRP330776,Natural killer cell_2-	15

ARID1A	bulk RNA-seq,Homo_723,AD.vs.control; bulk RNA-seq,Homo_723,AD.vs.MCI; bulk RNA-seq,Homo_633,AD.vs.control; bulk RNA-seq,Homo_633,AD.vs.MCI; bulk RNA-seq,Homo_714,AD.vs.control; bulk RNA-seq,Homo_714,AD.vs.MCI; scRNA-seq,SRP309935,B cell_1-AD.vs.control; scRNA-seq,SRP330776,γδ T cell-AD.vs.control; scRNA-seq,SRP330776,Memory T cell_2-AD.vs.control; scRNA-seq,SRP330776,B cell_2-AD.vs.control; scRNA-seq,SRP330776,CD8+ T cell_3-AD.vs.control; scRNA-seq,SRP330776,Megakaryocyte_1-AD.vs.control	12
ARID1B	bulk RNA-seq,Homo_723,AD.vs.control; bulk RNA-seq,Homo_723,MCI.vs.control; bulk RNA-seq,Homo_633,AD.vs.control; bulk RNA-seq,Homo_633,AD.vs.MCI; bulk RNA-seq,Homo_633,MCI.vs.control; bulk RNA-seq,Homo_714,AD.vs.control; bulk RNA-seq,Homo_714,AD.vs.MCI; bulk RNA-seq,Homo_714,MCI.vs.control; scRNA-seq,SRP215507,CD8+ T cell_5-AD.vs.MCI; scRNA-seq,SRP215507,CD8+ T cell_6-AD.vs.MCI; scRNA-seq,SRP215507,CD8+ T cell_7-MCI.vs.control; scRNA-seq,SRP309935,Natural killer cell_2-AD.vs.control; scRNA-seq,SRP309935,Natural killer cell_1-AD.vs.control; scRNA-seq,SRP330776,CD8+ T cell_1-AD.vs.control; scRNA-seq,SRP330776,γδ T cell-AD.vs.control; scRNA-seq,SRP330776,CD8+ T cell_2-AD.vs.control; scRNA-seq,SRP330776,B cell_1-AD.vs.control; scRNA-seq,SRP330776,Naive CD8+ T cell_1-AD.vs.control; scRNA-seq,SRP330776,Natural killer cell_1-AD.vs.control; scRNA-seq,SRP330776,Natural killer T cell-AD.vs.control; scRNA-seq,SRP330776,Naive CD8+ T cell_3-AD.vs.control; scRNA-seq,SRP330776,B cell_2-AD.vs.control; scRNA-seq,SRP330776,T cell-AD.vs.control; scRNA-seq,SRP330776,Naive CD8+ T cell_2-AD.vs.control; scRNA-seq,SRP330776,CD8+ T cell_3-AD.vs.control; scRNA-seq,SRP330776,Monocyte_1- [illegible]	27
ARID2	bulk RNA-seq,Homo_723,AD.vs.control; bulk RNA-seq,Homo_723,AD.vs.MCI; bulk RNA-seq,Homo_723,MCI.vs.control; bulk RNA-seq,Homo_633,AD.vs.control; bulk RNA-seq,Homo_633,AD.vs.MCI; bulk RNA-seq,Homo_633,MCI.vs.control; bulk RNA-seq,Homo_714,AD.vs.control; bulk RNA-seq,Homo_714,AD.vs.MCI; bulk RNA-seq,Homo_714,MCI.vs.control; scRNA-seq,SRP215507,CD8+ T cell_5-AD.vs.MCI; scRNA-seq,SRP215507,CD8+ T cell_6-AD.vs.MCI; scRNA-seq,SRP215507,CD8+ T cell_5-MCI.vs.control; scRNA-seq,SRP215507,CD8+ T cell_6-MCI.vs.control; scRNA-seq,SRP309935,Naive CD8+ T cell_3-AD.vs.control; scRNA-seq,SRP330776,Memory T cell_1-AD.vs.control; scRNA-	16
ARID3A	scRNA-seq,SRP215507,CD8+ T cell_2-AD.vs.control; scRNA-seq,SRP215507,CD8+ T cell_5-AD.vs.control; scRNA-seq,SRP215507,CD8+ T cell_1-AD.vs.MCI; scRNA-seq,SRP215507,CD8+ T cell_6-MCI.vs.control; scRNA-seq,SRP309935,Naive CD8+ T cell_1-AD.vs.control; scRNA-seq,SRP309935,CD8+ T cell_1-AD.vs.control; scRNA-seq,SRP309935,B cell_1-AD.vs.control; scRNA-seq,SRP330776,CD8+ T cell_1-AD.vs.control; scRNA-seq,SRP330776,γδ T cell-AD.vs.control; scRNA-seq,SRP330776,Memory T cell_2-AD.vs.control; scRNA-seq,SRP330776,CD8+ T cell_2-AD.vs.control; scRNA-seq,SRP330776,B cell_1-AD.vs.control; scRNA-seq,SRP330776,Naive CD8+ T cell_1-AD.vs.control; scRNA-seq,SRP330776,Natural killer cell_1-AD.vs.control; scRNA-seq,SRP330776,Memory T cell_1-AD.vs.control; scRNA-seq,SRP330776,B cell_2-AD.vs.control; scRNA-seq,SRP330776,Monocyte_1-AD.vs.control; scRNA-seq,SRP330776,Monocyte_2-AD.vs.control; scRNA-seq,SRP330776,Natural [illegible]	19
ARID3B	bulk RNA-seq,Homo_723,AD.vs.control; bulk RNA-seq,Homo_723,MCI.vs.control; bulk RNA-seq,Homo_633,MCI.vs.control; bulk RNA-seq,Homo_714,AD.vs.control; bulk RNA-seq,Homo_714,AD.vs.MCI; bulk RNA-seq,Homo_714,MCI.vs.control; scRNA-seq,SRP215507,CD8+ T cell_5-AD.vs.control; scRNA-seq,SRP215507,CD8+ T cell_5-AD.vs.MCI; scRNA-seq,SRP309935,Monocyte_1-AD.vs.control; scRNA-seq,SRP309935,Naive CD8+ T cell_2-AD.vs.control; scRNA-seq,SRP330776,Natural killer T cell-AD.vs.control; scRNA-seq,SRP330776,Naive CD8+ T cell_3-AD.vs.control; scRNA-seq,SRP330776,Monocyte_1-AD.vs.control; scRNA-seq,SRP330776,B cell_3-AD.vs.control; scRNA-seq,SRP330776,Natural killer cell_2-	15
ARID3C	bulk RNA-seq,Homo_723,AD.vs.control; bulk RNA-seq,Homo_723,AD.vs.MCI; bulk RNA-seq,Homo_633,AD.vs.control; bulk RNA-	4
ARID4A	scRNA-seq,SRP215507,CD8+ T cell_7-AD.vs.control; scRNA-seq,SRP215507,CD8+ T cell_10-AD.vs.control; scRNA-seq,SRP330776,γδ T cell-AD.vs.control; scRNA-seq,SRP330776,Memory T cell_2-AD.vs.control; scRNA-seq,SRP330776,Natural killer cell_1-AD.vs.control; scRNA-seq,SRP330776,Naive CD8+ T cell_3-AD.vs.control; scRNA-seq,SRP330776,CD8+ T cell_3-AD.vs.control; scRNA-seq,SRP330776,Monocyte_1-AD.vs.control; scRNA-seq,SRP330776,Monocyte_2-AD.vs.control; scRNA-seq,SRP330776,Megakaryocyte_1-AD.vs.control; scRNA-	11
ARID4B	scRNA-seq,SRP309935,Megakaryocyte_2-AD.vs.control; scRNA-seq,SRP330776,Naive CD8+ T cell_2-AD.vs.control; scRNA-	3
ARID5A	bulk RNA-seq,Homo_633,MCI.vs.control; scRNA-seq,SRP215507,CD8+ T cell_1-AD.vs.MCI; scRNA-seq,SRP215507,CD8+ T cell_5-AD.vs.MCI; scRNA-seq,SRP215507,CD8+ T cell_1-MCI.vs.control; scRNA-seq,SRP215507,CD8+ T cell_5-MCI.vs.control; scRNA-seq,SRP215507,CD8+ T cell_2-MCI.vs.control; scRNA-seq,SRP215507,CD8+ T cell_3-MCI.vs.control; scRNA-seq,SRP215507,CD8+ T cell_6-MCI.vs.control; scRNA-seq,SRP215507,CD8+ T cell_7-MCI.vs.control; scRNA-seq,SRP309935,Naive CD8+ T cell_2-AD.vs.control; scRNA-seq,SRP330776,Memory T cell_2-AD.vs.control; scRNA-seq,SRP330776,Naive CD8+ T cell_1-AD.vs.control; scRNA-seq,SRP330776,Memory T cell_1-AD.vs.control; scRNA-	14

ARID5B	bulk RNA-seq,Homo_714,AD.vs.control; bulk RNA-seq,Homo_714,AD.vs.MCI; scRNA-seq,SRP215507,CD8+ T cell_3-AD.vs.control; scRNA-seq,SRP215507,CD8+ T cell_3-AD.vs.MCI; scRNA-seq,SRP215507,CD8+ T cell_4-MCI.vs.control; scRNA-seq,SRP215507,CD8+ T cell_3-MCI.vs.control; scRNA-seq,SRP330776,γδ T cell-AD.vs.control; scRNA-seq,SRP330776,Natural killer cell_1-AD.vs.control; scRNA-seq,SRP330776,CD8+ T cell_3-AD.vs.control; scRNA-seq,SRP330776,Monocyte_1-AD.vs.control; scRNA-seq,SRP330776,Regulatory T cell-	11
ARIH1	scRNA-seq,SRP215507,CD8+ T cell_1-AD.vs.MCI; scRNA-seq,SRP215507,CD8+ T cell_7-AD.vs.MCI; scRNA-seq,SRP215507,CD8+ T cell_5-AD.vs.MCI; scRNA-seq,SRP215507,CD8+ T cell_3-MCI.vs.control; scRNA-seq,SRP309935,Natural killer cell_2-AD.vs.control; scRNA-seq,SRP309935,Naive CD8+ T cell_1-AD.vs.control; scRNA-seq,SRP309935,CD8+ T cell_1-AD.vs.control; scRNA-seq,SRP309935,CD8+ T cell_2-AD.vs.control; scRNA-seq,SRP330776,CD8+ T cell_1-AD.vs.control; scRNA-seq,SRP330776,γδ T cell-AD.vs.control; scRNA-seq,SRP330776,Memory T cell_2-AD.vs.control; scRNA-seq,SRP330776,CD8+ T cell_2-AD.vs.control; scRNA-seq,SRP330776,B cell_1-AD.vs.control; scRNA-seq,SRP330776,Naive CD8+ T cell_1-AD.vs.control; scRNA-seq,SRP330776,Natural killer cell_1-AD.vs.control; scRNA-seq,SRP330776,Memory T cell_1-AD.vs.control; scRNA-seq,SRP330776,Natural killer T cell-AD.vs.control; scRNA-seq,SRP330776,Naive CD8+ T cell_3-AD.vs.control; scRNA-seq,SRP330776,B cell_2-AD.vs.control; scRNA-seq,SRP330776,T cell-AD.vs.control; scRNA-seq,SRP330776,Naive CD8+ T cell_2-AD.vs.control; scRNA-seq,SRP330776,CD8+ T cell_3-AD.vs.control; scRNA-seq,SRP330776,Monocyte_1-AD.vs.control; scRNA-seq,SRP330776,Monocyte_2-AD.vs.control; scRNA-seq,SRP330776,B cell_3-AD.vs.control; scRNA-seq,SRP330776,Regulatory T cell-AD.vs.control; scRNA-seq,SRP330776,Natural [illegible]	28
ARIH2	scRNA-seq,SRP215507,CD8+ T cell_10-AD.vs.control; scRNA-seq,SRP215507,CD8+ T cell_1-AD.vs.MCI; scRNA-seq,SRP215507,CD8+ T cell_5-AD.vs.MCI; scRNA-seq,SRP215507,CD8+ T cell_1-MCI.vs.control	4
ARIH2OS	scRNA-seq,SRP215507,CD8+ T cell_5-AD.vs.control; scRNA-seq,SRP215507,CD8+ T cell_6-AD.vs.control; scRNA-seq,SRP215507,CD8+ T cell_5-AD.vs.MCI; scRNA-seq,SRP215507,CD8+ T cell_6-AD.vs.MCI; scRNA-seq,SRP330776,CD8+ T cell_1-AD.vs.control; scRNA-seq,SRP330776,γδ T cell-AD.vs.control; scRNA-seq,SRP330776,CD8+ T cell_2-AD.vs.control; scRNA-seq,SRP330776,B cell_1-AD.vs.control; scRNA-seq,SRP330776,Naive CD8+ T cell_1-AD.vs.control; scRNA-seq,SRP330776,Natural killer cell_1-AD.vs.control; scRNA-seq,SRP330776,Memory T cell_1-AD.vs.control; scRNA-seq,SRP330776,B cell_2-AD.vs.control; scRNA-seq,SRP330776,B cell_3-AD.vs.control; scRNA-seq,SRP330776,B cell_4-AD.vs.control	14
ARL1	bulk RNA-seq,Homo_723,AD.vs.control; bulk RNA-seq,Homo_723,AD.vs.MCI; bulk RNA-seq,Homo_723,MCI.vs.control; bulk RNA-seq,Homo_633,AD.vs.MCI; bulk RNA-seq,Homo_633,MCI.vs.control; bulk RNA-seq,Homo_714,AD.vs.control; bulk RNA-seq,Homo_714,AD.vs.MCI; bulk RNA-seq,Homo_714,MCI.vs.control; scRNA-seq,SRP309935,Monocyte_1-AD.vs.control; scRNA-seq,SRP330776,CD8+ T cell_1-AD.vs.control; scRNA-seq,SRP330776,Memory T cell_2-AD.vs.control; scRNA-seq,SRP330776,CD8+ T cell_2-AD.vs.control; scRNA-seq,SRP330776,Natural killer cell_1-AD.vs.control; scRNA-seq,SRP330776,Natural killer T cell-AD.vs.control; scRNA-seq,SRP330776,Naive CD8+ T cell_3-AD.vs.control; scRNA-seq,SRP330776,Monocyte_2-AD.vs.control; scRNA-seq,SRP330776,B cell_3-AD.vs.control; scRNA-seq,SRP330776,Regulatory T cell-AD.vs.control	18
ARL10	scRNA-seq,SRP215507,CD8+ T cell_3-AD.vs.control; scRNA-seq,SRP215507,CD8+ T cell_3-AD.vs.MCI; scRNA-seq,SRP215507,CD8+ T cell_5-AD.vs.MCI; scRNA-seq,SRP309935,CD8+ T cell_2-AD.vs.control; scRNA-seq,SRP330776,CD8+ T cell_1-AD.vs.control; scRNA-seq,SRP330776,γδ T cell-AD.vs.control; scRNA-seq,SRP330776,Memory T cell_2-AD.vs.control; scRNA-seq,SRP330776,CD8+ T cell_2-AD.vs.control; scRNA-seq,SRP330776,B cell_1-AD.vs.control; scRNA-seq,SRP330776,Naive CD8+ T cell_1-AD.vs.control; scRNA-seq,SRP330776,Memory T cell_1-AD.vs.control; scRNA-seq,SRP330776,Natural killer T cell-AD.vs.control; scRNA-seq,SRP330776,B cell_2-AD.vs.control; scRNA-seq,SRP330776,T cell-AD.vs.control; scRNA-seq,SRP330776,CD8+ T cell_3-AD.vs.control; scRNA-seq,SRP330776,B cell_3-AD.vs.control	16
ARL11	scRNA-seq,SRP330776,B cell_1-AD.vs.control; scRNA-seq,SRP330776,Naive CD8+ T cell_1-AD.vs.control; scRNA-seq,SRP330776,B cell_2-AD.vs.control; scRNA-seq,SRP330776,Monocyte_2-AD.vs.control; scRNA-seq,SRP330776,B cell_3-AD.vs.control	5
ARL13A	bulk RNA-seq,Homo_723,MCI.vs.control; bulk RNA-seq,Homo_633,AD.vs.control; bulk RNA-seq,Homo_633,AD.vs.MCI; bulk RNA-seq,Homo_633,MCI.vs.control; bulk RNA-seq,Homo_714,MCI.vs.control; bulk RNA-seq,ROSMAP,MCI.vs.control	6
ARL13B	bulk RNA-seq,Homo_723,AD.vs.control; bulk RNA-seq,Homo_723,MCI.vs.control; bulk RNA-seq,Homo_633,MCI.vs.control; scRNA-seq,SRP215507,CD8+ T cell_1-AD.vs.MCI; scRNA-seq,SRP215507,CD8+ T cell_5-AD.vs.MCI; scRNA-seq,SRP215507,CD8+ T cell_5-MCI.vs.control; scRNA-seq,SRP330776,γδ T cell-AD.vs.control; scRNA-seq,SRP330776,Naive CD8+ T cell_1-AD.vs.control; scRNA-seq,SRP330776,Natural killer cell_1-AD.vs.control; scRNA-seq,SRP330776,Memory T cell_1-AD.vs.control; scRNA-seq,SRP330776,Naive CD8+ T cell_3-AD.vs.control; scRNA-	12
ARL14	scRNA-seq,SRP330776,B cell_1-AD.vs.control	1

ARL14EP	bulk RNA-seq,Homo_714,AD.vs.control; bulk RNA-seq,Homo_714,AD.vs.MCI; scRNA-seq,SRP215507,CD8+ T cell_3-AD.vs.MCI; scRNA-seq,SRP215507,CD8+ T cell_3-MCI.vs.control; scRNA-seq,SRP309935,Natural killer cell_2-AD.vs.control; scRNA-seq,SRP330776,Monocyte_2-AD.vs.control; scRNA-seq,SRP330776,Megakaryocyte_1-AD.vs.control	7
ARL14EPL	bulk RNA-seq,Homo_723,AD.vs.control; bulk RNA-seq,Homo_633,AD.vs.control; bulk RNA-seq,ROSMAP,AD.vs.control	3
ARL15	scRNA-seq,SRP309935,B cell_1-AD.vs.control; scRNA-seq,SRP330776,CD8+ T cell_1-AD.vs.control; scRNA-seq,SRP330776,Memory T cell_2-AD.vs.control; scRNA-seq,SRP330776,B cell_1-AD.vs.control; scRNA-seq,SRP330776,Naive CD8+ T cell_1-AD.vs.control; scRNA-seq,SRP330776,T cell-AD.vs.control; scRNA-seq,SRP330776,CD8+ T cell_3-AD.vs.control	7
ARL16	scRNA-seq,SRP215507,CD8+ T cell_7-AD.vs.control; scRNA-seq,SRP215507,CD8+ T cell_5-AD.vs.control; scRNA-seq,SRP215507,CD8+ T cell_6-AD.vs.control; scRNA-seq,SRP215507,CD8+ T cell_3-AD.vs.MCI; scRNA-seq,SRP215507,CD8+ T cell_5-AD.vs.MCI; scRNA-seq,SRP215507,CD8+ T cell_6-MCI.vs.control; scRNA-seq,SRP330776,B cell_1-AD.vs.control; scRNA-seq,SRP330776,Natural killer cell_1-AD.vs.control; scRNA-seq,SRP330776,B cell_2-AD.vs.control; scRNA-seq,SRP330776,CD8+ T cell_3-AD.vs.control; scRNA-seq,SRP330776,Monocyte_2-AD.vs.control; scRNA-seq,SRP330776,B cell_3-AD.vs.control; scRNA-seq,SRP330776,Megakaryocyte_1-AD.vs.control; scRNA-seq,SRP330776,Natural killer cell_2-	14
ARL17A	scRNA-seq,SRP215507,CD8+ T cell_2-AD.vs.control; scRNA-seq,SRP215507,CD8+ T cell_1-AD.vs.control; scRNA-seq,SRP215507,CD8+ T cell_4-AD.vs.control; scRNA-seq,SRP215507,CD8+ T cell_5-AD.vs.control; scRNA-seq,SRP215507,CD8+ T cell_2-AD.vs.MCI; scRNA-seq,SRP215507,CD8+ T cell_3-AD.vs.MCI; scRNA-seq,SRP215507,CD8+ T cell_1-AD.vs.MCI; scRNA-seq,SRP215507,CD8+ T cell_4-AD.vs.MCI; scRNA-seq,SRP215507,CD8+ T cell_5-AD.vs.MCI; scRNA-seq,SRP330776,CD8+ T cell_1-AD.vs.control; scRNA-seq,SRP330776,γδ T cell-AD.vs.control; scRNA-seq,SRP330776,B cell_1-AD.vs.control; scRNA-seq,SRP330776,Naive CD8+ T cell_1-AD.vs.control; scRNA-seq,SRP330776,Memory T cell_1-AD.vs.control; scRNA-seq,SRP330776,Naive CD8+ T cell_3-AD.vs.control; scRNA-seq,SRP330776,T cell-AD.vs.control	16
ARL17B	scRNA-seq,SRP215507,CD8+ T cell_2-AD.vs.control; scRNA-seq,SRP215507,CD8+ T cell_1-AD.vs.control; scRNA-seq,SRP215507,CD8+ T cell_4-AD.vs.control; scRNA-seq,SRP215507,CD8+ T cell_2-AD.vs.MCI; scRNA-seq,SRP215507,CD8+ T cell_1-AD.vs.MCI; scRNA-seq,SRP215507,CD8+ T cell_4-AD.vs.MCI; scRNA-seq,SRP215507,CD8+ T cell_4-MCI.vs.control; scRNA-seq,SRP330776,CD8+ T cell_1-AD.vs.control; scRNA-seq,SRP330776,γδ T cell-AD.vs.control; scRNA-seq,SRP330776,Memory T cell_2-AD.vs.control; scRNA-seq,SRP330776,CD8+ T cell_2-AD.vs.control; scRNA-seq,SRP330776,Naive CD8+ T cell_1-AD.vs.control; scRNA-seq,SRP330776,Memory T cell_1-AD.vs.control; scRNA-seq,SRP330776,B cell_2-AD.vs.control; scRNA-seq,SRP330776,T cell-AD.vs.control; scRNA-seq,SRP330776,CD8+ T cell_3-AD.vs.control	16
ARL2	scRNA-seq,SRP215507,CD8+ T cell_7-AD.vs.control; scRNA-seq,SRP215507,CD8+ T cell_6-AD.vs.control; scRNA-seq,SRP215507,CD8+ T cell_5-AD.vs.MCI; scRNA-seq,SRP215507,CD8+ T cell_6-AD.vs.MCI; scRNA-seq,SRP330776,CD8+ T cell_1-AD.vs.control; scRNA-seq,SRP330776,γδ T cell-AD.vs.control; scRNA-seq,SRP330776,Memory T cell_2-AD.vs.control; scRNA-seq,SRP330776,CD8+ T cell_2-AD.vs.control; scRNA-seq,SRP330776,B cell_1-AD.vs.control; scRNA-seq,SRP330776,Naive CD8+ T cell_1-AD.vs.control; scRNA-seq,SRP330776,Natural killer cell_1-AD.vs.control; scRNA-seq,SRP330776,Memory T cell_1-AD.vs.control; scRNA-seq,SRP330776,Naive CD8+ T cell_3-AD.vs.control; scRNA-seq,SRP330776,B cell_2-AD.vs.control; scRNA-seq,SRP330776,T cell-AD.vs.control; scRNA-seq,SRP330776,CD8+ T cell_3-AD.vs.control; scRNA-[illegible]	18
ARL2BP	bulk RNA-seq,Homo_723,AD.vs.control; bulk RNA-seq,Homo_723,AD.vs.MCI; bulk RNA-seq,Homo_633,AD.vs.control; bulk RNA-seq,Homo_633,AD.vs.MCI; bulk RNA-seq,Homo_714,AD.vs.control; bulk RNA-seq,Homo_714,AD.vs.MCI; scRNA-seq,SRP215507,CD8+ T cell_7-AD.vs.control; scRNA-seq,SRP215507,CD8+ T cell_6-AD.vs.control; scRNA-seq,SRP215507,CD8+ T cell_4-AD.vs.MCI; scRNA-seq,SRP215507,CD8+ T cell_5-AD.vs.MCI; scRNA-seq,SRP330776,CD8+ T cell_1-AD.vs.control; scRNA-seq,SRP330776,γδ T cell-AD.vs.control; scRNA-seq,SRP330776,Memory T cell_2-AD.vs.control; scRNA-seq,SRP330776,CD8+ T cell_2-AD.vs.control; scRNA-seq,SRP330776,B cell_1-AD.vs.control; scRNA-seq,SRP330776,Naive CD8+ T cell_1-AD.vs.control; scRNA-seq,SRP330776,Natural killer cell_1-AD.vs.control; scRNA-seq,SRP330776,Memory T cell_1-AD.vs.control; scRNA-seq,SRP330776,Natural killer T cell-AD.vs.control; scRNA-seq,SRP330776,Naive CD8+ T cell_3-AD.vs.control; scRNA-seq,SRP330776,B cell_2-AD.vs.control; scRNA-seq,SRP330776,T cell-AD.vs.control; scRNA-seq,SRP330776,Naive CD8+ T cell_2-AD.vs.control; scRNA-seq,SRP330776,CD8+ T cell_3-AD.vs.control; scRNA-seq,SRP330776,Monocyte_2-AD.vs.control; scRNA-seq,SRP330776,B cell_3-[illegible]	27
Arl2bp	bulk RNA-seq,SRP325058,AD.vs.control	1

ARL3	bulk RNA-seq,Homo_723,AD.vs.control; bulk RNA-seq,Homo_723,AD.vs.MCI; bulk RNA-seq,Homo_633,AD.vs.MCI; bulk RNA-seq,Homo_714,AD.vs.control; bulk RNA-seq,Homo_714,AD.vs.MCI; scRNA-seq,SRP215507,CD8+ T cell_5-AD.vs.control; scRNA-seq,SRP215507,CD8+ T cell_4-AD.vs.MCI; scRNA-seq,SRP215507,CD8+ T cell_2-MCI.vs.control; scRNA-seq,SRP330776,Natural killer T cell-AD.vs.control; scRNA-seq,SRP330776,Naive CD8+ T cell_3-AD.vs.control; scRNA-seq,SRP330776,B cell_2-AD.vs.control; scRNA-	12
Arl4a	bulk RNA-seq,SRP325058,AD.vs.control	1
ARL4A	scRNA-seq,SRP215507,CD8+ T cell_9-AD.vs.control; scRNA-seq,SRP215507,CD8+ T cell_2-AD.vs.MCI; scRNA-seq,SRP215507,CD8+ T cell_4-AD.vs.MCI; scRNA-seq,SRP215507,CD8+ T cell_5-AD.vs.MCI; scRNA-seq,SRP215507,CD8+ T cell_5-MCI.vs.control; scRNA-seq,SRP215507,CD8+ T cell_4-MCI.vs.control; scRNA-seq,SRP309935,CD8+ T cell_1-AD.vs.control; scRNA-seq,SRP330776,B cell_2-AD.vs.control; scRNA-seq,SRP330776,Naive CD8+ T cell_2-AD.vs.control; scRNA-seq,SRP330776,Monocyte_1-AD.vs.control; scRNA-seq,SRP330776,Natural killer cell_2-	11
ARL4C	scRNA-seq,SRP215507,CD8+ T cell_9-AD.vs.control; scRNA-seq,SRP215507,CD8+ T cell_8-MCI.vs.control; scRNA-seq,SRP309935,Monocyte_1-AD.vs.control; scRNA-seq,SRP309935,Erythroid cell-AD.vs.control; scRNA-seq,SRP309935,Monocyte_2-AD.vs.control; scRNA-	6
ARL4D	scRNA-seq,SRP215507,CD8+ T cell_3-AD.vs.control; scRNA-seq,SRP215507,CD8+ T cell_7-AD.vs.control; scRNA-seq,SRP330776,Memory T cell_2-AD.vs.control; scRNA-seq,SRP330776,CD8+ T cell_2-AD.vs.control; scRNA-seq,SRP330776,Naive CD8+ T cell_1-AD.vs.control; scRNA-	6
ARL5A	bulk RNA-seq,Homo_714,AD.vs.control; bulk RNA-seq,Homo_714,AD.vs.MCI; scRNA-seq,SRP215507,CD8+ T cell_4-AD.vs.MCI; scRNA-seq,SRP215507,CD8+ T cell_6-AD.vs.MCI; scRNA-seq,SRP215507,CD8+ T cell_4-MCI.vs.control; scRNA-seq,SRP215507,CD8+ T cell_6-MCI.vs.control; scRNA-seq,SRP330776,CD8+ T cell_1-AD.vs.control; scRNA-seq,SRP330776,γδ T cell-AD.vs.control; scRNA-seq,SRP330776,Memory T cell_2-AD.vs.control; scRNA-seq,SRP330776,CD8+ T cell_2-AD.vs.control; scRNA-seq,SRP330776,B cell_1-AD.vs.control; scRNA-seq,SRP330776,Naive CD8+ T cell_1-AD.vs.control; scRNA-seq,SRP330776,Natural killer cell_1-AD.vs.control; scRNA-seq,SRP330776,Memory T cell_1-AD.vs.control; scRNA-seq,SRP330776,Natural killer T cell-AD.vs.control; scRNA-seq,SRP330776,Naive CD8+ T cell_3-AD.vs.control; scRNA-seq,SRP330776,B cell_2-AD.vs.control; scRNA-seq,SRP330776,T cell-AD.vs.control; scRNA-seq,SRP330776,Naive CD8+ T cell_2-AD.vs.control; scRNA-seq,SRP330776,CD8+ T cell_3-AD.vs.control; scRNA-seq,SRP330776,Monocyte_1-AD.vs.control; scRNA-seq,SRP330776,Monocyte_2-AD.vs.control; scRNA-seq,SRP330776,B cell_3-AD.vs.control; scRNA-seq,SRP330776,Regulatory T cell-AD.vs.control; scRNA-seq,SRP330776,Natural [illegible]	26
Arl5a	bulk RNA-seq,SRP325058,AD.vs.control	1
ARL5B	bulk RNA-seq,Homo_723,MCI.vs.control; bulk RNA-seq,Homo_633,MCI.vs.control; scRNA-seq,SRP215507,CD8+ T cell_3-AD.vs.control; scRNA-seq,SRP215507,CD8+ T cell_4-AD.vs.control; scRNA-seq,SRP215507,CD8+ T cell_3-AD.vs.MCI; scRNA-seq,SRP215507,CD8+ T cell_4-AD.vs.MCI; scRNA-seq,SRP215507,CD8+ T cell_2-MCI.vs.control; scRNA-seq,SRP309935,Natural killer cell_1-AD.vs.control; scRNA-seq,SRP330776,Monocyte_1-AD.vs.control; scRNA-seq,SRP330776,Monocyte_2-AD.vs.control	10
ARL5C	bulk RNA-seq,ROSMAP,MCI.vs.control	1
ARL6	bulk RNA-seq,Homo_723,AD.vs.control; bulk RNA-seq,Homo_723,AD.vs.MCI; bulk RNA-seq,Homo_633,AD.vs.control; bulk RNA-seq,Homo_714,AD.vs.control; bulk RNA-seq,Homo_714,AD.vs.MCI; scRNA-seq,SRP215507,CD8+ T cell_2-AD.vs.control; scRNA-seq,SRP215507,CD8+ T cell_1-AD.vs.control; scRNA-seq,SRP215507,CD8+ T cell_5-AD.vs.MCI; scRNA-seq,SRP215507,CD8+ T cell_5-MCI.vs.control; scRNA-seq,SRP215507,CD8+ T cell_3-MCI.vs.control; scRNA-seq,SRP330776,CD8+ T cell_1-AD.vs.control; scRNA-seq,SRP330776,γδ T cell-AD.vs.control; scRNA-seq,SRP330776,Memory T cell_2-AD.vs.control; scRNA-seq,SRP330776,B cell_1-AD.vs.control; scRNA-seq,SRP330776,Natural killer cell_1-AD.vs.control; scRNA-seq,SRP330776,Memory T cell_1-AD.vs.control; scRNA-seq,SRP330776,Natural killer T cell-	17
ARL6IP1	bulk RNA-seq,Homo_723,MCI.vs.control; bulk RNA-seq,Homo_633,MCI.vs.control; scRNA-seq,SRP215507,CD8+ T cell_3-AD.vs.MCI	3
ARL6IP4	scRNA-seq,SRP215507,CD8+ T cell_5-AD.vs.MCI; scRNA-seq,SRP215507,CD8+ T cell_5-MCI.vs.control; scRNA-seq,SRP309935,Erythroid cell-AD.vs.control; scRNA-seq,SRP330776,Megakaryocyte_1-AD.vs.control	4
ARL6IP5	bulk RNA-seq,Homo_714,AD.vs.control; bulk RNA-seq,Homo_714,AD.vs.MCI; bulk RNA-seq,Homo_714,MCI.vs.control; bulk RNA-seq,SRP310421,AD.vs.control; scRNA-seq,SRP215507,CD8+ T cell_7-AD.vs.control; scRNA-seq,SRP215507,CD8+ T cell_8-AD.vs.MCI; scRNA-seq,SRP215507,CD8+ T cell_7-MCI.vs.control; scRNA-seq,SRP215507,CD8+ T cell_8-MCI.vs.control; scRNA-seq,SRP309935,Erythroid cell-AD.vs.control; scRNA-seq,SRP330776,Naive CD8+ T cell_3-AD.vs.control; scRNA-seq,SRP330776,B cell_2-AD.vs.control; scRNA-	12
ARL6IP6	bulk RNA-seq,Homo_714,AD.vs.control; bulk RNA-seq,Homo_714,AD.vs.MCI; scRNA-seq,SRP330776,Naive CD8+ T cell_3-AD.vs.control; scRNA-seq,SRP330776,CD8+ T cell_3-AD.vs.control; scRNA-seq,SRP330776,Monocyte_2-AD.vs.control	5

ARL8A	bulk RNA-seq,Homo_723,AD.vs.control; bulk RNA-seq,Homo_714,AD.vs.control; bulk RNA-seq,Homo_714,AD.vs.MCI; bulk RNA-seq,SRP310421,AD.vs.control; scRNA-seq,SRP215507,CD8+ T cell_5-AD.vs.control; scRNA-seq,SRP215507,CD8+ T cell_7-AD.vs.MCI; scRNA-seq,SRP215507,CD8+ T cell_5-MCI.vs.control; scRNA-seq,SRP330776,CD8+ T cell_1-AD.vs.control; scRNA-seq,SRP330776,γδ T cell-AD.vs.control; scRNA-seq,SRP330776,Memory T cell_2-AD.vs.control; scRNA-seq,SRP330776,CD8+ T cell_2-AD.vs.control; scRNA-seq,SRP330776,B cell_1-AD.vs.control; scRNA-seq,SRP330776,Naive CD8+ T cell_1-AD.vs.control; scRNA-seq,SRP330776,Natural killer cell_1-AD.vs.control; scRNA-seq,SRP330776,Memory T cell_1-AD.vs.control; scRNA-seq,SRP330776,Natural killer T cell-AD.vs.control; scRNA-seq,SRP330776,Naive CD8+ T cell_3-AD.vs.control; scRNA-seq,SRP330776,B cell_2-AD.vs.control; scRNA-seq,SRP330776,T cell-AD.vs.control; scRNA-seq,SRP330776,CD8+ T cell_3-AD.vs.control; scRNA-seq,SRP330776,Monocyte_1-AD.vs.control; scRNA-seq,SRP330776,Monocyte_2-AD.vs.control; scRNA-seq,SRP330776,B [illegible]	24
ARL8B	bulk RNA-seq,Homo_723,AD.vs.MCI; bulk RNA-seq,Homo_633,AD.vs.MCI; bulk RNA-seq,Homo_714,AD.vs.control; bulk RNA-seq,Homo_714,AD.vs.MCI; scRNA-seq,SRP215507,CD8+ T cell_10-AD.vs.control; scRNA-seq,SRP215507,CD8+ T cell_1-AD.vs.MCI; scRNA-seq,SRP215507,CD8+ T cell_1-MCI.vs.control; scRNA-seq,SRP330776,Regulatory T cell-AD.vs.control	8
ARL9	bulk RNA-seq,Homo_723,AD.vs.control; bulk RNA-seq,Homo_723,AD.vs.MCI; bulk RNA-seq,Homo_633,AD.vs.control; bulk RNA-	4
ARMC1	bulk RNA-seq,Homo_723,AD.vs.control; bulk RNA-seq,Homo_723,AD.vs.MCI; bulk RNA-seq,Homo_633,AD.vs.control; bulk RNA-seq,Homo_633,AD.vs.MCI; bulk RNA-seq,Homo_714,AD.vs.control; bulk RNA-seq,Homo_714,AD.vs.MCI; scRNA-seq,SRP330776,CD8+ T cell_1-AD.vs.control; scRNA-seq,SRP330776,Naive CD8+ T cell_1-AD.vs.control; scRNA-seq,SRP330776,Naive CD8+ T cell_2-AD.vs.control; scRNA-seq,SRP330776,CD8+ T cell_3-AD.vs.control; scRNA-seq,SRP330776,Regulatory T cell-AD.vs.control	11
ARMC10	bulk RNA-seq,Homo_723,AD.vs.control; bulk RNA-seq,Homo_723,AD.vs.MCI; bulk RNA-seq,Homo_633,AD.vs.control; bulk RNA-seq,Homo_633,AD.vs.MCI; bulk RNA-seq,Homo_714,AD.vs.control; bulk RNA-seq,Homo_714,AD.vs.MCI; scRNA-seq,SRP215507,CD8+ T cell_2-AD.vs.MCI; scRNA-seq,SRP215507,CD8+ T cell_4-AD.vs.MCI; scRNA-seq,SRP215507,CD8+ T cell_5-AD.vs.MCI; scRNA-seq,SRP215507,CD8+ T cell_4-MCI.vs.control; scRNA-seq,SRP309935,Monocyte_1-AD.vs.control; scRNA-seq,SRP309935,Naive CD8+ T cell_1-AD.vs.control; scRNA-seq,SRP309935,Natural killer cell_1-AD.vs.control; scRNA-seq,SRP309935,Naive CD8+ T cell_2-AD.vs.control; scRNA-seq,SRP309935,B cell_1-AD.vs.control; scRNA-seq,SRP309935,CD8+ T cell_2-AD.vs.control; scRNA-seq,SRP309935,Naive CD8+ T cell_3-AD.vs.control; scRNA-seq,SRP330776,CD8+ T cell_1-AD.vs.control; scRNA-seq,SRP330776,γδ T cell-AD.vs.control; scRNA-seq,SRP330776,Memory T cell_2-AD.vs.control; scRNA-seq,SRP330776,CD8+ T cell_2-AD.vs.control; scRNA-seq,SRP330776,B cell_1-AD.vs.control; scRNA-seq,SRP330776,Naive CD8+ T cell_1-AD.vs.control; scRNA-seq,SRP330776,Natural killer cell_1-AD.vs.control; scRNA-seq,SRP330776,Memory T cell_1-AD.vs.control; scRNA-seq,SRP330776,Natural killer T cell-AD.vs.control; scRNA-seq,SRP330776,Naive CD8+ T cell_3-AD.vs.control; scRNA-seq,SRP330776,B cell_2-AD.vs.control; scRNA-seq,SRP330776,T cell-AD.vs.control; scRNA-seq,SRP330776,Naive CD8+ T cell_2-AD.vs.control; scRNA-seq,SRP330776,CD8+ T cell_3-AD.vs.control; scRNA-seq,SRP330776,Monocyte_1-AD.vs.control; scRNA-seq,SRP330776,Monocyte_2-AD.vs.control; scRNA-[illegible]	36
ARMC12	bulk RNA-seq,Homo_723,AD.vs.control; bulk RNA-seq,Homo_723,MCI.vs.control; bulk RNA-seq,Homo_633,AD.vs.control; bulk RNA-seq,Homo_633,AD.vs.MCI; bulk RNA-seq,Homo_633,MCI.vs.control; bulk RNA-seq,Homo_714,AD.vs.control; bulk RNA-seq,Homo_714,AD.vs.MCI; bulk RNA-seq,Homo_714,MCI.vs.control; scRNA-seq,SRP215507,CD8+ T cell_1-MCI.vs.control	9
ARMC2	bulk RNA-seq,Homo_714,AD.vs.control; bulk RNA-seq,Homo_714,AD.vs.MCI; scRNA-seq,SRP215507,CD8+ T cell_4-AD.vs.control; scRNA-seq,SRP215507,CD8+ T cell_4-AD.vs.MCI; scRNA-seq,SRP330776,Memory T cell_2-AD.vs.control; scRNA-seq,SRP330776,B cell_1-AD.vs.control; scRNA-seq,SRP330776,Naive CD8+ T cell_3-AD.vs.control; scRNA-seq,SRP330776,T cell-AD.vs.control	8
ARMC3	bulk RNA-seq,Homo_723,AD.vs.control; bulk RNA-seq,Homo_723,AD.vs.MCI; bulk RNA-seq,Homo_723,MCI.vs.control; bulk RNA-seq,Homo_633,AD.vs.control; bulk RNA-seq,Homo_633,AD.vs.MCI; bulk RNA-seq,Homo_633,MCI.vs.control; bulk RNA-seq,Homo_714,AD.vs.control; bulk RNA-seq,Homo_714,AD.vs.MCI; bulk RNA-seq,ROSMAP,AD.vs.control; bulk RNA-seq,ROSMAP,MCI.vs.control	10
ARMC5	scRNA-seq,SRP215507,CD8+ T cell_2-AD.vs.MCI; scRNA-seq,SRP215507,CD8+ T cell_5-AD.vs.MCI; scRNA-seq,SRP215507,CD8+ T cell_1-MCI.vs.control; scRNA-seq,SRP215507,CD8+ T cell_5-MCI.vs.control; scRNA-seq,SRP309935,Natural killer cell_2-AD.vs.control; scRNA-seq,SRP330776,CD8+ T cell_1-AD.vs.control; scRNA-seq,SRP330776,γδ T cell-AD.vs.control; scRNA-seq,SRP330776,Memory T cell_2-AD.vs.control; scRNA-seq,SRP330776,CD8+ T cell_2-AD.vs.control; scRNA-seq,SRP330776,B cell_1-AD.vs.control; scRNA-seq,SRP330776,Naive CD8+ T cell_1-AD.vs.control; scRNA-seq,SRP330776,Natural killer cell_1-AD.vs.control; scRNA-seq,SRP330776,Memory T cell_1-AD.vs.control; scRNA-seq,SRP330776,Natural killer T cell-AD.vs.control; scRNA-seq,SRP330776,Naive CD8+ T cell_3-AD.vs.control; scRNA-seq,SRP330776,B cell_2-AD.vs.control; scRNA-seq,SRP330776,T cell-AD.vs.control; scRNA-seq,SRP330776,CD8+ T cell_3-AD.vs.control; scRNA-seq,SRP330776,B cell_3-[illegible]	19

ARMC6	scRNA-seq,SRP215507,CD8+ T cell_4-AD.vs.MCI; scRNA-seq,SRP330776,CD8+ T cell_1-AD.vs.control; scRNA-seq,SRP330776,γδ T cell-AD.vs.control; scRNA-seq,SRP330776,Memory T cell_2-AD.vs.control; scRNA-seq,SRP330776,Naive CD8+ T cell_1-AD.vs.control; scRNA-seq,SRP330776,Natural killer cell_1-AD.vs.control; scRNA-seq,SRP330776,Naive CD8+ T cell_3-AD.vs.control; scRNA-seq,SRP330776,T cell-AD.vs.control; scRNA-seq,SRP330776,Monocyte_2-AD.vs.control; scRNA-seq,SRP330776,Natural killer cell_2-AD.vs.control	10
ARMC7	scRNA-seq,SRP330776,CD8+ T cell_1-AD.vs.control; scRNA-seq,SRP330776,Memory T cell_2-AD.vs.control	2
ARMC8	bulk RNA-seq,Homo_723,AD.vs.control; bulk RNA-seq,Homo_633,AD.vs.control; bulk RNA-seq,Homo_633,AD.vs.MCI; scRNA-seq,SRP215507,CD8+ T cell_10-AD.vs.control; scRNA-seq,SRP215507,CD8+ T cell_6-MCI.vs.control; scRNA-seq,SRP215507,CD8+ T cell_7-MCI.vs.control; scRNA-seq,SRP330776,Naive CD8+ T cell_3-AD.vs.control; scRNA-seq,SRP330776,Natural killer cell_2-AD.vs.control	8
ARMC9	scRNA-seq,SRP215507,CD8+ T cell_3-AD.vs.control; scRNA-seq,SRP330776,Naive CD8+ T cell_1-AD.vs.control; scRNA-seq,SRP330776,B cell_3-	3
ARMCX1	bulk RNA-seq,Homo_723,AD.vs.control; bulk RNA-seq,Homo_723,AD.vs.MCI; bulk RNA-seq,Homo_723,MCI.vs.control; bulk RNA-seq,Homo_633,AD.vs.control; bulk RNA-seq,Homo_633,AD.vs.MCI; bulk RNA-seq,Homo_633,MCI.vs.control; bulk RNA-seq,Homo_714,AD.vs.control; bulk RNA-seq,Homo_714,AD.vs.MCI; scRNA-seq,SRP215507,CD8+ T cell_3-MCI.vs.control	9
ARMCX2	bulk RNA-seq,Homo_723,AD.vs.control; bulk RNA-seq,Homo_723,AD.vs.MCI; bulk RNA-seq,Homo_723,MCI.vs.control; bulk RNA-seq,Homo_633,MCI.vs.control; bulk RNA-seq,Homo_714,AD.vs.control; bulk RNA-seq,Homo_714,AD.vs.MCI; bulk RNA-seq,Homo_714,MCI.vs.control; scRNA-seq,SRP215507,CD8+ T cell_2-AD.vs.control; scRNA-seq,SRP215507,CD8+ T cell_5-AD.vs.control; scRNA-seq,SRP215507,CD8+ T cell_5-AD.vs.MCI; scRNA-seq,SRP309935,B cell_1-AD.vs.control; scRNA-seq,SRP330776,CD8+ T cell_1-AD.vs.control;	14
ARMCX3	scRNA-seq,SRP215507,CD8+ T cell_4-AD.vs.MCI; scRNA-seq,SRP215507,CD8+ T cell_4-MCI.vs.control; scRNA-seq,SRP330776,T cell-AD.vs.control	3
ARMCX4	bulk RNA-seq,Homo_723,AD.vs.control; bulk RNA-seq,Homo_723,AD.vs.MCI; bulk RNA-seq,Homo_723,MCI.vs.control; bulk RNA-seq,Homo_633,MCI.vs.control; bulk RNA-seq,Homo_714,AD.vs.control; bulk RNA-seq,Homo_714,AD.vs.MCI; bulk RNA-seq,Homo_714,MCI.vs.control; scRNA-seq,SRP215507,CD8+ T cell_1-AD.vs.control; scRNA-seq,SRP215507,CD8+ T cell_1-MCI.vs.control; scRNA-	11
ARMCX5	bulk RNA-seq,Homo_723,AD.vs.control; bulk RNA-seq,Homo_633,AD.vs.control; bulk RNA-seq,Homo_633,AD.vs.MCI; bulk RNA-seq,Homo_714,AD.vs.control; bulk RNA-seq,Homo_714,AD.vs.MCI; scRNA-seq,SRP215507,CD8+ T cell_3-AD.vs.control; scRNA-seq,SRP215507,CD8+ T cell_3-AD.vs.MCI; scRNA-seq,SRP215507,CD8+ T cell_4-MCI.vs.control; scRNA-seq,SRP309935,Monocyte_1-AD.vs.control;	10
Armcx5	bulk RNA-seq,SRP325058,AD.vs.control	1
ARMCX5-GPRASP2	scRNA-seq,SRP215507,CD8+ T cell_1-MCI.vs.control; scRNA-seq,SRP330776,Memory T cell_2-AD.vs.control; scRNA-seq,SRP330776,CD8+ T cell_2-	3
ARMCX6	scRNA-seq,SRP215507,CD8+ T cell_5-AD.vs.control; scRNA-seq,SRP215507,CD8+ T cell_3-AD.vs.MCI; scRNA-seq,SRP215507,CD8+ T cell_5-AD.vs.MCI; scRNA-seq,SRP215507,CD8+ T cell_3-MCI.vs.control; scRNA-seq,SRP330776,Naive CD8+ T cell_3-AD.vs.control; scRNA-seq,SRP330776,Naive CD8+ T cell_2-AD.vs.control; scRNA-seq,SRP330776,Monocyte_2-AD.vs.control; scRNA-seq,SRP330776,Megakaryocyte_1-	8
ARMH1	bulk RNA-seq,Homo_723,AD.vs.control; bulk RNA-seq,Homo_723,AD.vs.MCI; bulk RNA-seq,Homo_633,AD.vs.control; bulk RNA-seq,Homo_633,AD.vs.MCI; bulk RNA-seq,Homo_714,AD.vs.control; bulk RNA-seq,Homo_714,AD.vs.MCI; scRNA-seq,SRP215507,CD8+ T cell_3-AD.vs.control; scRNA-seq,SRP215507,CD8+ T cell_1-AD.vs.control; scRNA-seq,SRP215507,CD8+ T cell_4-AD.vs.control; scRNA-seq,SRP215507,CD8+ T cell_6-AD.vs.control; scRNA-seq,SRP215507,CD8+ T cell_4-AD.vs.MCI; scRNA-seq,SRP215507,CD8+ T cell_1-MCI.vs.control; scRNA-seq,SRP215507,CD8+ T cell_3-MCI.vs.control; scRNA-seq,SRP215507,CD8+ T cell_6-MCI.vs.control; scRNA-seq,SRP309935,CD8+ T cell_2-AD.vs.control; scRNA-seq,SRP330776,CD8+ T cell_1-AD.vs.control; scRNA-seq,SRP330776,γδ T cell-AD.vs.control; scRNA-seq,SRP330776,Memory T cell_2-AD.vs.control; scRNA-seq,SRP330776,CD8+ T cell_2-AD.vs.control; scRNA-seq,SRP330776,B cell_1-AD.vs.control; scRNA-seq,SRP330776,Naive CD8+ T cell_1-AD.vs.control; scRNA-seq,SRP330776,Natural killer cell_1-AD.vs.control; scRNA-[illegible]	26
ARMH2	bulk RNA-seq,Homo_723,AD.vs.control; bulk RNA-seq,Homo_723,AD.vs.MCI; bulk RNA-seq,Homo_633,AD.vs.control; bulk RNA-seq,Homo_633,AD.vs.MCI; bulk RNA-seq,Homo_714,AD.vs.control; bulk RNA-seq,Homo_714,AD.vs.MCI; scRNA-seq,SRP215507,CD8+ T cell_2-AD.vs.control; scRNA-seq,SRP330776,Naive CD8+ T cell_1-AD.vs.control	8
ARMH3	scRNA-seq,SRP215507,CD8+ T cell_5-AD.vs.control; scRNA-seq,SRP215507,CD8+ T cell_2-AD.vs.MCI; scRNA-seq,SRP215507,CD8+ T cell_1-MCI.vs.control; scRNA-seq,SRP215507,CD8+ T cell_2-MCI.vs.control; scRNA-seq,SRP330776,Naive CD8+ T cell_2-AD.vs.control	5

ARMH4	bulk RNA-seq,Homo_723,AD.vs.control; bulk RNA-seq,Homo_723,AD.vs.MCI; bulk RNA-seq,Homo_723,MCI.vs.control; bulk RNA-seq,Homo_633,AD.vs.control; bulk RNA-seq,Homo_633,AD.vs.MCI; bulk RNA-seq,Homo_714,MCI.vs.control; scRNA-seq,SRP215507,CD8+ T cell_4-AD.vs.control; scRNA-seq,SRP330776,γδ T cell-AD.vs.control; scRNA-seq,SRP330776,Memory T cell_2-AD.vs.control; scRNA-seq,SRP330776,CD8+ T cell_2-AD.vs.control; scRNA-seq,SRP330776,B cell_1-AD.vs.control; scRNA-seq,SRP330776,Naive CD8+ T cell_1-AD.vs.control; scRNA-seq,SRP330776,Natural killer cell_1-AD.vs.control; scRNA-seq,SRP330776,Memory T cell_1-AD.vs.control; scRNA-seq,SRP330776,Naive CD8+ T cell_3-AD.vs.control; scRNA-seq,SRP330776,T cell-AD.vs.control; scRNA-seq,SRP330776,CD8+ T cell_3-AD.vs.control	17
ARMT1	bulk RNA-seq,Homo_723,AD.vs.control; bulk RNA-seq,Homo_633,AD.vs.control; bulk RNA-seq,Homo_633,AD.vs.MCI; bulk RNA-seq,Homo_714,AD.vs.control; bulk RNA-seq,Homo_714,AD.vs.MCI; scRNA-seq,SRP215507,CD8+ T cell_2-AD.vs.control; scRNA-seq,SRP215507,CD8+ T cell_7-AD.vs.control; scRNA-seq,SRP215507,CD8+ T cell_5-AD.vs.MCI; scRNA-seq,SRP215507,CD8+ T cell_2-MCI.vs.control; scRNA-seq,SRP330776,CD8+ T cell_1-AD.vs.control; scRNA-seq,SRP330776,γδ T cell-AD.vs.control; scRNA-seq,SRP330776,Memory T cell_2-AD.vs.control; scRNA-seq,SRP330776,CD8+ T cell_2-AD.vs.control; scRNA-seq,SRP330776,Naive CD8+ T cell_1-AD.vs.control; scRNA-seq,SRP330776,Natural killer cell_1-AD.vs.control; scRNA-seq,SRP330776,Naive CD8+ T cell_3-AD.vs.control; scRNA-seq,SRP330776,B cell_2- [illegible]	19
ARNT	bulk RNA-seq,Homo_723,AD.vs.control; bulk RNA-seq,Homo_723,MCI.vs.control; bulk RNA-seq,Homo_633,AD.vs.control; bulk RNA-seq,Homo_633,MCI.vs.control; bulk RNA-seq,Homo_714,AD.vs.control; bulk RNA-seq,Homo_714,AD.vs.MCI; bulk RNA-seq,Homo_714,MCI.vs.control; scRNA-seq,SRP215507,CD8+ T cell_4-AD.vs.control; scRNA-seq,SRP330776,γδ T cell-AD.vs.control; scRNA-seq,SRP330776,Naive CD8+ T cell_3-AD.vs.control; scRNA-seq,SRP330776,Monocyte_2-AD.vs.control; scRNA-seq,SRP330776,B cell_3-AD.vs.control;	13
ARNT2	bulk RNA-seq,Homo_723,AD.vs.control; bulk RNA-seq,Homo_723,AD.vs.MCI; bulk RNA-seq,Homo_723,MCI.vs.control; bulk RNA-seq,Homo_633,AD.vs.control; bulk RNA-seq,Homo_633,AD.vs.MCI; bulk RNA-seq,Homo_633,MCI.vs.control; bulk RNA-	8
ARNTL	bulk RNA-seq,Homo_723,AD.vs.control; bulk RNA-seq,Homo_723,AD.vs.MCI; bulk RNA-seq,Homo_633,AD.vs.control; bulk RNA-seq,Homo_633,AD.vs.MCI; bulk RNA-seq,Homo_714,AD.vs.control; bulk RNA-seq,Homo_714,AD.vs.MCI; scRNA-seq,SRP215507,CD8+ T cell_1-AD.vs.control; scRNA-seq,SRP215507,CD8+ T cell_5-AD.vs.control; scRNA-seq,SRP215507,CD8+ T cell_6-AD.vs.control; scRNA-seq,SRP215507,CD8+ T cell_4-AD.vs.MCI; scRNA-seq,SRP215507,CD8+ T cell_1-MCI.vs.control; scRNA-seq,SRP215507,CD8+ T cell_6-MCI.vs.control; scRNA-seq,SRP330776,B cell_1-AD.vs.control; scRNA-seq,SRP330776,Natural killer T cell-AD.vs.control; scRNA-	16
ARNTL2	bulk RNA-seq,Homo_723,AD.vs.control; bulk RNA-seq,Homo_723,AD.vs.MCI; bulk RNA-seq,Homo_723,MCI.vs.control; bulk RNA-seq,Homo_633,AD.vs.control; bulk RNA-seq,Homo_633,AD.vs.MCI; bulk RNA-seq,Homo_633,MCI.vs.control; bulk RNA-seq,Homo_714,AD.vs.control; bulk RNA-seq,Homo_714,AD.vs.MCI; scRNA-seq,SRP215507,CD8+ T cell_6-MCI.vs.control	9
ARPC1A	bulk RNA-seq,Homo_723,AD.vs.control; bulk RNA-seq,Homo_723,AD.vs.MCI; bulk RNA-seq,Homo_723,MCI.vs.control; bulk RNA-seq,Homo_633,AD.vs.control; bulk RNA-seq,Homo_633,AD.vs.MCI; bulk RNA-seq,Homo_714,AD.vs.control; bulk RNA-seq,Homo_714,AD.vs.MCI; scRNA-seq,SRP215507,CD8+ T cell_7-AD.vs.control; scRNA-seq,SRP215507,CD8+ T cell_7-AD.vs.MCI; scRNA-seq,SRP215507,CD8+ T cell_6-MCI.vs.control; scRNA-seq,SRP309935,Megakaryocyte_1-AD.vs.control; scRNA-seq,SRP330776,Memory T cell_2-AD.vs.control; scRNA-seq,SRP330776,CD8+ T cell_2-AD.vs.control; scRNA-seq,SRP330776,B cell_1-AD.vs.control; scRNA-seq,SRP330776,Naive CD8+ T cell_1-AD.vs.control; scRNA-seq,SRP330776,Natural killer T cell-AD.vs.control; scRNA-seq,SRP330776,Naive CD8+ T cell_3-AD.vs.control; scRNA- [illegible]	20
ARPC1B	bulk RNA-seq,Homo_633,AD.vs.control; scRNA-seq,SRP215507,CD8+ T cell_7-AD.vs.control; scRNA-seq,SRP215507,CD8+ T cell_7-MCI.vs.control; scRNA-seq,SRP309935,Erythroid cell-AD.vs.control; scRNA-seq,SRP330776,γδ T cell-AD.vs.control; scRNA-seq,SRP330776,Memory T cell_2-AD.vs.control; scRNA-seq,SRP330776,Naive CD8+ T cell_1-AD.vs.control; scRNA-seq,SRP330776,Naive CD8+ T cell_3-AD.vs.control; scRNA-seq,SRP330776,B cell_2-AD.vs.control; scRNA-seq,SRP330776,CD8+ T cell_3-AD.vs.control; scRNA-seq,SRP330776,Natural killer cell_2-AD.vs.control	11
ARPC2	scRNA-seq,SRP215507,CD8+ T cell_7-AD.vs.control; scRNA-seq,SRP309935,Erythroid cell-AD.vs.control	2
ARPC3	bulk RNA-seq,Homo_723,MCI.vs.control; bulk RNA-seq,Homo_633,MCI.vs.control; bulk RNA-seq,Homo_714,MCI.vs.control; scRNA-seq,SRP215507,CD8+ T cell_7-AD.vs.control; scRNA-seq,SRP309935,Erythroid cell-AD.vs.control	5
ARPC4	bulk RNA-seq,Homo_723,AD.vs.control; bulk RNA-seq,Homo_723,AD.vs.MCI; bulk RNA-seq,Homo_723,MCI.vs.control; bulk RNA-seq,Homo_633,AD.vs.control; bulk RNA-seq,Homo_633,AD.vs.MCI; bulk RNA-seq,Homo_633,MCI.vs.control; bulk RNA-seq,Homo_714,AD.vs.control; bulk RNA-seq,Homo_714,AD.vs.MCI; bulk RNA-seq,Homo_714,MCI.vs.control; scRNA-seq,SRP215507,CD8+ T cell_8-AD.vs.control; scRNA-seq,SRP215507,CD8+ T cell_7-AD.vs.control; scRNA-seq,SRP215507,CD8+ T cell_7-AD.vs.MCI; scRNA-seq,SRP309935,Erythroid cell-AD.vs.control	13

Arpc4	bulk RNA-seq,SRP325058,AD.vs.control	1
ARPC5	bulk RNA-seq,Homo_723,AD.vs.control; bulk RNA-seq,Homo_723,MCI.vs.control; bulk RNA-seq,Homo_633,AD.vs.control; bulk RNA-seq,Homo_633,AD.vs.MCI; bulk RNA-seq,Homo_633,MCI.vs.control; bulk RNA-seq,Homo_714,AD.vs.control; bulk RNA-seq,Homo_714,AD.vs.MCI; bulk RNA-seq,Homo_714,MCI.vs.control; bulk RNA-seq,SRP223445,AD.vs.control; scRNA-seq,SRP215507,CD8+ T cell_9-AD.vs.control; scRNA-seq,SRP309935,Erythroid cell-AD.vs.control; scRNA-seq,SRP330776,CD8+ T cell_1-AD.vs.control; scRNA-seq,SRP330776,γδ T cell-AD.vs.control; scRNA-seq,SRP330776,Memory T cell_2-AD.vs.control; scRNA-seq,SRP330776,Natural killer cell_1-AD.vs.control; scRNA-seq,SRP330776,Naive CD8+	18
ARPC5L	bulk RNA-seq,Homo_714,AD.vs.control; bulk RNA-seq,Homo_714,AD.vs.MCI; scRNA-seq,SRP215507,CD8+ T cell_8-AD.vs.control; scRNA-seq,SRP215507,CD8+ T cell_7-AD.vs.control; scRNA-seq,SRP215507,CD8+ T cell_1-AD.vs.MCI; scRNA-seq,SRP215507,CD8+ T cell_4-AD.vs.MCI; scRNA-seq,SRP215507,CD8+ T cell_5-AD.vs.MCI; scRNA-seq,SRP215507,CD8+ T cell_1-MCI.vs.control; scRNA-seq,SRP215507,CD8+ T cell_5-MCI.vs.control; scRNA-seq,SRP309935,Erythroid cell-AD.vs.control; scRNA-seq,SRP309935,Megakaryocyte_2-AD.vs.control; scRNA-seq,SRP330776,CD8+ T cell_1-AD.vs.control; scRNA-seq,SRP330776,Memory T cell_2-AD.vs.control; scRNA-seq,SRP330776,CD8+ T cell_2-AD.vs.control; scRNA-seq,SRP330776,Naive CD8+ T cell_1-AD.vs.control; scRNA-seq,SRP330776,Natural killer T cell-AD.vs.control; scRNA-seq,SRP330776,Naive CD8+ T cell_3-AD.vs.control; scRNA-seq,SRP330776,B cell_2-AD.vs.control; scRNA-seq,SRP330776,Naive CD8+ T cell_2-[illegible]	21
ARPIN	scRNA-seq,SRP309935,Monocyte_1-AD.vs.control; scRNA-seq,SRP330776,Monocyte_2-AD.vs.control	2
ARPP19	scRNA-seq,SRP215507,CD8+ T cell_7-AD.vs.control; scRNA-seq,SRP330776,Natural killer cell_1-AD.vs.control; scRNA-seq,SRP330776,Natural killer T cell-AD.vs.control; scRNA-seq,SRP330776,Naive CD8+ T cell_3-AD.vs.control; scRNA-seq,SRP330776,B cell_2-AD.vs.control; scRNA-seq,SRP330776,Naive CD8+ T cell_2-AD.vs.control; scRNA-seq,SRP330776,Monocyte_1-AD.vs.control	7
ARPP21	bulk RNA-seq,ROSMAP,AD.vs.control; bulk RNA-seq,ROSMAP,MCI.vs.control	2
ARR3	bulk RNA-seq,Homo_723,AD.vs.control; bulk RNA-seq,Homo_633,AD.vs.control; bulk RNA-seq,Homo_633,AD.vs.MCI	3
Arrb1	bulk RNA-seq,SRP325058,AD.vs.control	1
ARRB1	scRNA-seq,SRP215507,CD8+ T cell_3-AD.vs.MCI; scRNA-seq,SRP215507,CD8+ T cell_3-MCI.vs.control; scRNA-seq,SRP309935,Natural killer cell_2-AD.vs.control; scRNA-seq,SRP309935,CD8+ T cell_1-AD.vs.control; scRNA-seq,SRP309935,CD4+ T cell-AD.vs.control; scRNA-seq,SRP330776,Natural killer cell_1-AD.vs.control; scRNA-seq,SRP330776,T cell-AD.vs.control	7
ARRB2	scRNA-seq,SRP215507,CD8+ T cell_7-AD.vs.control; scRNA-seq,SRP309935,Erythroid cell-AD.vs.control; scRNA-seq,SRP330776,Megakaryocyte_1-	3
ARRDC1	scRNA-seq,SRP215507,CD8+ T cell_7-AD.vs.control; scRNA-seq,SRP215507,CD8+ T cell_9-AD.vs.control; scRNA-seq,SRP215507,CD8+ T cell_7-MCI.vs.control; scRNA-seq,SRP309935,Erythroid cell-AD.vs.control; scRNA-seq,SRP309935,B cell_1-AD.vs.control; scRNA-seq,SRP330776,CD8+ T cell_1-AD.vs.control; scRNA-seq,SRP330776,γδ T cell-AD.vs.control; scRNA-seq,SRP330776,Memory T cell_2-AD.vs.control; scRNA-seq,SRP330776,CD8+ T cell_2-AD.vs.control; scRNA-seq,SRP330776,B cell_1-AD.vs.control; scRNA-seq,SRP330776,Naive CD8+ T cell_1-AD.vs.control; scRNA-seq,SRP330776,Natural killer cell_1-AD.vs.control; scRNA-seq,SRP330776,Memory T cell_1-AD.vs.control; scRNA-seq,SRP330776,Natural killer T cell-AD.vs.control; scRNA-seq,SRP330776,Naive CD8+ T cell_3-AD.vs.control; scRNA-seq,SRP330776,B cell_2-AD.vs.control; scRNA-seq,SRP330776,T cell-AD.vs.control; scRNA-seq,SRP330776,CD8+ T cell_3-AD.vs.control; scRNA-seq,SRP330776,B cell_3-[illegible]	19
ARRDC1-AS1	scRNA-seq,SRP215507,CD8+ T cell_5-AD.vs.control; scRNA-seq,SRP330776,CD8+ T cell_2-AD.vs.control; scRNA-seq,SRP330776,B cell_1-AD.vs.control; scRNA-seq,SRP330776,T cell-AD.vs.control; scRNA-seq,SRP330776,CD8+ T cell_3-AD.vs.control	5

ARRDC2	bulk RNA-seq,Homo_723,AD.vs.control; bulk RNA-seq,Homo_723,AD.vs.MCI; bulk RNA-seq,Homo_723,MCI.vs.control; bulk RNA-seq,Homo_633,AD.vs.control; bulk RNA-seq,Homo_633,AD.vs.MCI; bulk RNA-seq,Homo_633,MCI.vs.control; bulk RNA-seq,Homo_714,AD.vs.control; bulk RNA-seq,Homo_714,AD.vs.MCI; bulk RNA-seq,Homo_714,MCI.vs.control; scRNA-seq,SRP215507,CD8+ T cell_3-AD.vs.control; scRNA-seq,SRP215507,CD8+ T cell_7-AD.vs.MCI; scRNA-seq,SRP215507,CD8+ T cell_3-MCI.vs.control; scRNA-seq,SRP215507,CD8+ T cell_7-MCI.vs.control; scRNA-seq,SRP309935,B cell_1-AD.vs.control; scRNA-seq,SRP330776,CD8+ T cell_1-AD.vs.control; scRNA-seq,SRP330776,γδ T cell-AD.vs.control; scRNA-seq,SRP330776,Memory T cell_2-AD.vs.control; scRNA-seq,SRP330776,CD8+ T cell_2-AD.vs.control; scRNA-seq,SRP330776,B cell_1-AD.vs.control; scRNA-seq,SRP330776,Naive CD8+ T cell_1-AD.vs.control; scRNA-seq,SRP330776,Natural killer cell_1-AD.vs.control; scRNA-seq,SRP330776,Memory T cell_1-AD.vs.control; scRNA-seq,SRP330776,Natural killer T cell-AD.vs.control; scRNA-seq,SRP330776,Naive CD8+ T cell_3-AD.vs.control; scRNA-seq,SRP330776,B cell_2-AD.vs.control; scRNA-seq,SRP330776,T cell-AD.vs.control; scRNA-seq,SRP330776,Naive CD8+ T cell_2-AD.vs.control; scRNA-seq,SRP330776,CD8+ T cell_3-AD.vs.control; scRNA-seq,SRP330776,Monocyte_1-AD.vs.control; scRNA-seq,SRP330776,Monocyte_2-AD.vs.control; scRNA-seq,SRP330776,B cell_3-AD.vs.control; scRNA-seq,SRP330776,Regulatory T cell-AD.vs.control; scRNA-seq,SRP330776,Natural killer cell_2-AD.vs.control	33
ARRDC3	bulk RNA-seq,Homo_633,AD.vs.control; scRNA-seq,SRP215507,CD8+ T cell_4-AD.vs.control; scRNA-seq,SRP215507,CD8+ T cell_5-AD.vs.MCI; scRNA-seq,SRP215507,CD8+ T cell_1-MCI.vs.control; scRNA-seq,SRP215507,CD8+ T cell_5-MCI.vs.control; scRNA-seq,SRP215507,CD8+ T cell_4-MCI.vs.control; scRNA-seq,SRP215507,CD8+ T cell_2-MCI.vs.control; scRNA-seq,SRP215507,CD8+ T cell_3-MCI.vs.control; scRNA-	9
ARRDC3-AS1	scRNA-seq,SRP215507,CD8+ T cell_3-AD.vs.control; scRNA-seq,SRP215507,CD8+ T cell_1-AD.vs.MCI; scRNA-seq,SRP215507,CD8+ T cell_1-MCI.vs.control; scRNA-seq,SRP215507,CD8+ T cell_4-MCI.vs.control; scRNA-seq,SRP215507,CD8+ T cell_2-MCI.vs.control; scRNA-seq,SRP330776,Natural killer cell_1-AD.vs.control; scRNA-seq,SRP330776,Memory T cell_1-AD.vs.control; scRNA-seq,SRP330776,T cell-AD.vs.control; scRNA-seq,SRP330776,Megakaryocyte_1-AD.vs.control; scRNA-seq,SRP330776,B cell_4-AD.vs.control	10
ARRDC4	bulk RNA-seq,Homo_723,AD.vs.control; bulk RNA-seq,Homo_723,AD.vs.MCI; bulk RNA-seq,Homo_633,AD.vs.control; bulk RNA-seq,Homo_633,AD.vs.MCI; bulk RNA-seq,Homo_714,AD.vs.control; bulk RNA-seq,Homo_714,AD.vs.MCI; scRNA-seq,SRP215507,CD8+ T cell_3-AD.vs.MCI; scRNA-seq,SRP215507,CD8+ T cell_3-MCI.vs.control; scRNA-seq,SRP330776,CD8+ T cell_1-AD.vs.control; scRNA-seq,SRP330776,Memory T cell_2-AD.vs.control; scRNA-seq,SRP330776,B cell_1-AD.vs.control; scRNA-seq,SRP330776,Naive CD8+ T cell_1-AD.vs.control; scRNA-seq,SRP330776,Memory T cell_1-AD.vs.control; scRNA-seq,SRP330776,Naive CD8+ T cell_3-AD.vs.control; scRNA-seq,SRP330776,B cell_2-AD.vs.control; scRNA-seq,SRP330776,T cell-AD.vs.control; scRNA-seq,SRP330776,CD8+ T cell_3-AD.vs.control; scRNA- [illegible]	18
ARRDC5	bulk RNA-seq,Homo_723,AD.vs.control; bulk RNA-seq,Homo_723,AD.vs.MCI; bulk RNA-seq,Homo_633,AD.vs.control; bulk RNA-seq,Homo_633,AD.vs.MCI; bulk RNA-seq,Homo_714,AD.vs.control; bulk RNA-seq,Homo_714,AD.vs.MCI; scRNA-seq,SRP215507,CD8+ T cell_4-AD.vs.MCI; scRNA-seq,SRP215507,CD8+ T cell_4-MCI.vs.control; scRNA-seq,SRP215507,CD8+ T cell_6-MCI.vs.control; scRNA-seq,SRP330776,CD8+ T cell_1-AD.vs.control; scRNA-seq,SRP330776,γδ T cell-AD.vs.control; scRNA-seq,SRP330776,CD8+ T cell_2-AD.vs.control; scRNA-seq,SRP330776,Naive CD8+ T cell_1-AD.vs.control; scRNA-seq,SRP330776,Naive CD8+ T cell_3-AD.vs.control; scRNA-seq,SRP330776,CD8+ [illegible]	15
ARSA	scRNA-seq,SRP330776,B cell_2-AD.vs.control; scRNA-seq,SRP330776,B cell_3-AD.vs.control; scRNA-seq,SRP330776,Megakaryocyte_1-AD.vs.control	3
ARSB	bulk RNA-seq,Homo_723,AD.vs.control; bulk RNA-seq,Homo_723,AD.vs.MCI; bulk RNA-seq,Homo_633,AD.vs.control; bulk RNA-seq,Homo_633,AD.vs.MCI; bulk RNA-seq,Homo_714,AD.vs.control; bulk RNA-seq,Homo_714,AD.vs.MCI; scRNA-seq,SRP215507,CD8+ T cell_4-AD.vs.control; scRNA-seq,SRP215507,CD8+ T cell_2-AD.vs.MCI; scRNA-seq,SRP330776,CD8+ T cell_1-AD.vs.control; scRNA-seq,SRP330776,γδ T cell-AD.vs.control; scRNA-seq,SRP330776,Memory T cell_2-AD.vs.control; scRNA-seq,SRP330776,CD8+ T cell_2-AD.vs.control; scRNA-seq,SRP330776,B cell_1-AD.vs.control; scRNA-seq,SRP330776,Naive CD8+ T cell_1-AD.vs.control; scRNA-seq,SRP330776,Natural killer cell_1-AD.vs.control; scRNA-seq,SRP330776,Memory T cell_1-AD.vs.control; scRNA-seq,SRP330776,B cell_2-AD.vs.control; scRNA-seq,SRP330776,T cell- [illegible]	20
ARSD	bulk RNA-seq,Homo_723,AD.vs.control; bulk RNA-seq,Homo_723,AD.vs.MCI; bulk RNA-seq,Homo_633,AD.vs.control; bulk RNA-seq,Homo_633,AD.vs.MCI; bulk RNA-seq,Homo_714,AD.vs.control; bulk RNA-seq,Homo_714,AD.vs.MCI; scRNA-seq,SRP215507,CD8+ T cell_1-AD.vs.MCI; scRNA-seq,SRP215507,CD8+ T cell_5-AD.vs.MCI; scRNA-seq,SRP215507,CD8+ T cell_1-MCI.vs.control; scRNA-seq,SRP309935,CD8+ T cell_1-AD.vs.control; scRNA-seq,SRP330776,B cell_2-AD.vs.control; scRNA-seq,SRP330776,Monocyte_2-AD.vs.control	12

ARSG	bulk RNA-seq,Homo_723,AD.vs.control; bulk RNA-seq,Homo_723,AD.vs.MCI; bulk RNA-seq,Homo_633,AD.vs.control; bulk RNA-seq,Homo_633,AD.vs.MCI; bulk RNA-seq,Homo_714,AD.vs.control; bulk RNA-seq,Homo_714,AD.vs.MCI; scRNA-seq,SRP215507,CD8+ T cell_2-AD.vs.MCI; scRNA-seq,SRP309935,Natural killer cell_2-AD.vs.control; scRNA-seq,SRP330776,CD8+ T cell_1-AD.vs.control; scRNA-seq,SRP330776,Memory T cell_2-AD.vs.control; scRNA-seq,SRP330776,Natural killer cell_1-AD.vs.control; scRNA-seq,SRP330776,Memory T cell_1-AD.vs.control; scRNA-seq,SRP330776,CD8+ T cell_3-AD.vs.control; scRNA-seq,SRP330776,Monocyte_2-AD.vs.control	14
Arsg	bulk RNA-seq,SRP325058,AD.vs.control	1
ARSI	bulk RNA-seq,Homo_723,AD.vs.control; bulk RNA-seq,Homo_723,AD.vs.MCI; bulk RNA-seq,Homo_633,AD.vs.control; bulk RNA-	4
ARSJ	bulk RNA-seq,Homo_723,AD.vs.control; bulk RNA-seq,Homo_723,AD.vs.MCI; bulk RNA-seq,Homo_633,AD.vs.control; bulk RNA-seq,Homo_633,AD.vs.MCI; bulk RNA-seq,Homo_714,MCI.vs.control	5
ARSK	bulk RNA-seq,Homo_723,AD.vs.control; bulk RNA-seq,Homo_723,AD.vs.MCI; bulk RNA-seq,Homo_633,AD.vs.control; bulk RNA-seq,Homo_633,AD.vs.MCI; bulk RNA-seq,Homo_714,AD.vs.control; bulk RNA-seq,Homo_714,AD.vs.MCI; scRNA-seq,SRP215507,CD8+ T cell_5-AD.vs.MCI; scRNA-seq,SRP330776,CD8+ T cell_1-AD.vs.control; scRNA-seq,SRP330776,Memory T cell_2-AD.vs.control; scRNA-seq,SRP330776,CD8+ T cell_2-AD.vs.control; scRNA-seq,SRP330776,B cell_1-AD.vs.control; scRNA-seq,SRP330776,B cell_2-AD.vs.control; scRNA-seq,SRP330776,Naive CD8+ T cell_2-AD.vs.control; scRNA-seq,SRP330776,CD8+ T cell_3-AD.vs.control	14
ARSL	bulk RNA-seq,Homo_723,AD.vs.control; bulk RNA-seq,Homo_723,AD.vs.MCI; bulk RNA-seq,Homo_633,AD.vs.control; bulk RNA-seq,Homo_633,AD.vs.MCI; bulk RNA-seq,Homo_714,MCI.vs.control	5
ART1	bulk RNA-seq,Homo_723,MCI.vs.control; bulk RNA-seq,Homo_633,MCI.vs.control; bulk RNA-seq,Homo_714,MCI.vs.control	3
ART3	bulk RNA-seq,Homo_723,AD.vs.control; bulk RNA-seq,Homo_633,MCI.vs.control; bulk RNA-seq,Homo_714,MCI.vs.control	3
ART5	bulk RNA-seq,Homo_723,AD.vs.control; bulk RNA-seq,Homo_723,AD.vs.MCI; bulk RNA-seq,Homo_633,AD.vs.control; bulk RNA-seq,Homo_633,AD.vs.MCI; bulk RNA-seq,ROSMAP,AD.vs.MCI	5
ARTN	bulk RNA-seq,Homo_723,AD.vs.control	1
ARV1	bulk RNA-seq,Homo_714,AD.vs.control; bulk RNA-seq,Homo_714,AD.vs.MCI; scRNA-seq,SRP215507,CD8+ T cell_5-AD.vs.MCI; scRNA-seq,SRP215507,CD8+ T cell_5-MCI.vs.control; scRNA-seq,SRP309935,Natural killer cell_1-AD.vs.control; scRNA-seq,SRP330776,γδ T cell-AD.vs.control; scRNA-seq,SRP330776,CD8+ T cell_2-AD.vs.control; scRNA-seq,SRP330776,Memory T cell_1-AD.vs.control; scRNA-seq,SRP330776,Naive CD8+ T cell_3-AD.vs.control; scRNA-seq,SRP330776,Monocyte_1-AD.vs.control	10
ARVCF	bulk RNA-seq,Homo_723,AD.vs.control; bulk RNA-seq,Homo_723,AD.vs.MCI; bulk RNA-seq,Homo_633,AD.vs.control; bulk RNA-seq,Homo_633,AD.vs.MCI; bulk RNA-seq,Homo_714,AD.vs.control; bulk RNA-seq,Homo_714,AD.vs.MCI; scRNA-seq,SRP215507,CD8+ T cell_1-AD.vs.control; scRNA-seq,SRP330776,Memory T cell_1-AD.vs.control	8
ARX	bulk RNA-seq,Homo_723,AD.vs.control; bulk RNA-seq,Homo_723,AD.vs.MCI	2
AS3MT	bulk RNA-seq,Homo_723,AD.vs.control; bulk RNA-seq,Homo_723,AD.vs.MCI; bulk RNA-seq,Homo_723,MCI.vs.control; bulk RNA-seq,Homo_714,MCI.vs.control; bulk RNA-seq,ROSMAP,AD.vs.control; scRNA-seq,SRP215507,CD8+ T cell_3-AD.vs.MCI	6
ASAH1	bulk RNA-seq,SRP223445,AD.vs.control; scRNA-seq,SRP215507,CD8+ T cell_7-AD.vs.control; scRNA-seq,SRP215507,CD8+ T cell_7-MCI.vs.control; scRNA-seq,SRP330776,CD8+ T cell_1-AD.vs.control; scRNA-seq,SRP330776,Memory T cell_2-AD.vs.control; scRNA-seq,SRP330776,Natural killer cell_1-AD.vs.control; scRNA-seq,SRP330776,Natural killer cell_2-AD.vs.control	7
ASAH2	bulk RNA-seq,Homo_723,AD.vs.control; bulk RNA-seq,Homo_723,AD.vs.MCI; bulk RNA-seq,Homo_723,MCI.vs.control; bulk RNA-seq,Homo_633,AD.vs.control; bulk RNA-seq,Homo_633,AD.vs.MCI; bulk RNA-seq,Homo_714,AD.vs.control; bulk RNA-seq,Homo_714,AD.vs.MCI; scRNA-seq,SRP215507,CD8+ T cell_3-AD.vs.control; scRNA-seq,SRP215507,CD8+ T cell_2-AD.vs.MCI; scRNA-seq,SRP215507,CD8+ T cell_2-	10
ASAH2B	bulk RNA-seq,Homo_723,AD.vs.control; bulk RNA-seq,Homo_723,AD.vs.MCI; bulk RNA-seq,Homo_723,MCI.vs.control; bulk RNA-seq,Homo_633,AD.vs.control; bulk RNA-seq,Homo_633,AD.vs.MCI; bulk RNA-seq,Homo_633,MCI.vs.control; bulk RNA-seq,Homo_714,AD.vs.control; bulk RNA-seq,Homo_714,AD.vs.MCI; bulk RNA-seq,Homo_714,MCI.vs.control; scRNA-seq,SRP330776,Memory T cell_2-AD.vs.control; scRNA-	11

ASAP1	scRNA-seq,SRP215507,CD8+ T cell_5-AD.vs.MCI; scRNA-seq,SRP309935,Natural killer cell_2-AD.vs.control; scRNA-seq,SRP309935,CD8+ T cell_1-AD.vs.control; scRNA-seq,SRP330776,CD8+ T cell_1-AD.vs.control; scRNA-seq,SRP330776,γδ T cell-AD.vs.control; scRNA-seq,SRP330776,Memory T cell_2-AD.vs.control; scRNA-seq,SRP330776,B cell_1-AD.vs.control; scRNA-seq,SRP330776,Natural killer cell_1-AD.vs.control; scRNA-seq,SRP330776,Memory T cell_1-AD.vs.control; scRNA-seq,SRP330776,Naive CD8+ T cell_3-AD.vs.control; scRNA-seq,SRP330776,T cell-AD.vs.control; scRNA-seq,SRP330776,CD8+ T cell_3-AD.vs.control; scRNA-seq,SRP330776,B cell_3-AD.vs.control	13
ASAP1-IT2	scRNA-seq,SRP215507,CD8+ T cell_2-AD.vs.control; scRNA-seq,SRP215507,CD8+ T cell_1-AD.vs.control; scRNA-seq,SRP330776,CD8+ T cell_1-AD.vs.control; scRNA-seq,SRP330776,B cell_1-AD.vs.control; scRNA-seq,SRP330776,Memory T cell_1-AD.vs.control	5
ASAP2	bulk RNA-seq,Homo_723,MCI.vs.control; bulk RNA-seq,Homo_633,MCI.vs.control; bulk RNA-seq,Homo_714,AD.vs.control; bulk RNA-seq,Homo_714,MCI.vs.control; scRNA-seq,SRP309935,Megakaryocyte_1-AD.vs.control	5
ASAP3	bulk RNA-seq,Homo_723,AD.vs.control; bulk RNA-seq,Homo_723,AD.vs.MCI; bulk RNA-seq,Homo_723,MCI.vs.control; bulk RNA-seq,Homo_633,AD.vs.control; bulk RNA-seq,Homo_633,AD.vs.MCI; bulk RNA-seq,Homo_714,AD.vs.control; bulk RNA-seq,Homo_714,AD.vs.MCI; bulk RNA-seq,ROSMAP,MCI.vs.control; scRNA-seq,SRP215507,CD8+ T cell_1-AD.vs.control	9
ASB1	bulk RNA-seq,Homo_723,MCI.vs.control; bulk RNA-seq,Homo_633,AD.vs.control; bulk RNA-seq,Homo_633,MCI.vs.control; bulk RNA-seq,Homo_714,AD.vs.control; bulk RNA-seq,Homo_714,AD.vs.MCI; bulk RNA-seq,Homo_714,MCI.vs.control; scRNA-seq,SRP215507,CD8+ T cell_5-MCI.vs.control; scRNA-seq,SRP330776,CD8+ T cell_1-AD.vs.control; scRNA-seq,SRP330776,Memory T cell_2-AD.vs.control; scRNA-seq,SRP330776,B cell_1-AD.vs.control; scRNA-seq,SRP330776,Naive CD8+ T cell_1-AD.vs.control; scRNA-seq,SRP330776,Natural killer cell_1-AD.vs.control; scRNA-seq,SRP330776,T cell-AD.vs.control; scRNA-seq,SRP330776,CD8+ T cell_3-AD.vs.control	14
Asb1	bulk RNA-seq,SRP325058,AD.vs.control	1
ASB10	bulk RNA-seq,Homo_723,AD.vs.control; bulk RNA-seq,Homo_633,AD.vs.control	2
ASB11	bulk RNA-seq,Homo_723,MCI.vs.control; bulk RNA-seq,Homo_633,MCI.vs.control; bulk RNA-seq,Homo_714,MCI.vs.control	3
ASB12	bulk RNA-seq,Homo_723,AD.vs.control; bulk RNA-seq,Homo_723,AD.vs.MCI; bulk RNA-seq,Homo_633,AD.vs.control; bulk RNA-	4
ASB13	bulk RNA-seq,Homo_723,AD.vs.control; bulk RNA-seq,Homo_723,AD.vs.MCI; bulk RNA-seq,Homo_633,AD.vs.control; bulk RNA-seq,Homo_633,AD.vs.MCI; bulk RNA-seq,Homo_714,AD.vs.control; bulk RNA-seq,Homo_714,AD.vs.MCI; scRNA-seq,SRP215507,CD8+ T cell_2-AD.vs.control; scRNA-seq,SRP215507,CD8+ T cell_3-AD.vs.control; scRNA-seq,SRP215507,CD8+ T cell_1-AD.vs.control; scRNA-seq,SRP215507,CD8+ T cell_1-AD.vs.MCI; scRNA-seq,SRP330776,CD8+ T cell_1-AD.vs.control; scRNA-seq,SRP330776,Memory T cell_2-AD.vs.control; scRNA-seq,SRP330776,CD8+ T cell_2-AD.vs.control; scRNA-seq,SRP330776,B cell_1-AD.vs.control; scRNA-seq,SRP330776,Naive CD8+ T cell_1-AD.vs.control; scRNA-seq,SRP330776,Memory T cell_1-AD.vs.control; scRNA-seq,SRP330776,Natural killer T cell-AD.vs.control; [illegible]	20
ASB14	bulk RNA-seq,Homo_633,AD.vs.control; bulk RNA-seq,Homo_633,AD.vs.MCI; bulk RNA-seq,Homo_714,AD.vs.control; bulk RNA-	4
ASB15	bulk RNA-seq,Homo_723,AD.vs.control; bulk RNA-seq,Homo_723,MCI.vs.control; bulk RNA-seq,Homo_633,MCI.vs.control; bulk RNA-seq,Homo_714,MCI.vs.control; scRNA-seq,SRP330776,CD8+ T cell_1-AD.vs.control	5
ASB16	bulk RNA-seq,Homo_723,AD.vs.MCI; bulk RNA-seq,Homo_723,MCI.vs.control; bulk RNA-seq,Homo_633,AD.vs.control; bulk RNA-seq,Homo_633,AD.vs.MCI; bulk RNA-seq,Homo_633,MCI.vs.control; bulk RNA-seq,Homo_714,AD.vs.control; bulk RNA-seq,Homo_714,AD.vs.MCI; bulk RNA-seq,Homo_714,MCI.vs.control; scRNA-seq,SRP330776,CD8+ T cell_1-AD.vs.control; scRNA-seq,SRP330776,Memory T cell_2-	11
ASB16-AS1	scRNA-seq,SRP215507,CD8+ T cell_6-AD.vs.control; scRNA-seq,SRP215507,CD8+ T cell_6-MCI.vs.control; scRNA-seq,SRP309935,Naive CD8+ T cell_1-AD.vs.control; scRNA-seq,SRP330776,CD8+ T cell_1-AD.vs.control; scRNA-seq,SRP330776,γδ T cell-AD.vs.control; scRNA-seq,SRP330776,Memory T cell_2-AD.vs.control; scRNA-seq,SRP330776,CD8+ T cell_2-AD.vs.control; scRNA-seq,SRP330776,B cell_1-AD.vs.control; scRNA-seq,SRP330776,Naive CD8+ T cell_1-AD.vs.control; scRNA-seq,SRP330776,Natural killer cell_1-AD.vs.control; scRNA-seq,SRP330776,Memory T cell_1-AD.vs.control; scRNA-seq,SRP330776,Natural killer T cell-AD.vs.control; scRNA-seq,SRP330776,Naive CD8+ T cell_3-AD.vs.control; scRNA-seq,SRP330776,B cell_2-AD.vs.control; scRNA-seq,SRP330776,T cell-AD.vs.control; scRNA-seq,SRP330776,CD8+ T cell_3-AD.vs.control; scRNA-seq,SRP330776,Monocyte_1-AD.vs.control; scRNA-seq,SRP330776,B cell_3-AD.vs.control; scRNA-seq,SRP330776,Regulatory T cell-AD.vs.control; [illegible]	21
ASB18	bulk RNA-seq,ROSMAP,AD.vs.control; bulk RNA-seq,ROSMAP,MCI.vs.control	2

ASB2	bulk RNA-seq,Homo_714,AD.vs.control; bulk RNA-seq,Homo_714,AD.vs.MCI; bulk RNA-seq,SRP223445,AD.vs.control; scRNA-seq,SRP330776,γδ T cell-AD.vs.control; scRNA-seq,SRP330776,Memory T cell_2-AD.vs.control; scRNA-seq,SRP330776,B cell_1-AD.vs.control; scRNA-seq,SRP330776,Natural killer cell_1-AD.vs.control; scRNA-seq,SRP330776,Memory T cell_1-AD.vs.control; scRNA-seq,SRP330776,T cell-AD.vs.control	9
ASB3	bulk RNA-seq,Homo_723,AD.vs.control; bulk RNA-seq,Homo_723,AD.vs.MCI; bulk RNA-seq,Homo_633,AD.vs.control; bulk RNA-seq,Homo_633,AD.vs.MCI; bulk RNA-seq,Homo_633,MCI.vs.control; bulk RNA-seq,Homo_714,AD.vs.control; bulk RNA-seq,Homo_714,AD.vs.MCI; bulk RNA-seq,Homo_714,MCI.vs.control; scRNA-seq,SRP215507,CD8+ T cell_1-AD.vs.MCI; scRNA-seq,SRP330776,Natural killer cell_2-AD.vs.control	10
ASB4	bulk RNA-seq,Homo_714,MCI.vs.control	1
ASB5	bulk RNA-seq,Homo_723,AD.vs.control; bulk RNA-seq,Homo_633,AD.vs.control	2
ASB6	bulk RNA-seq,Homo_723,AD.vs.control; bulk RNA-seq,Homo_723,AD.vs.MCI; bulk RNA-seq,Homo_723,MCI.vs.control; bulk RNA-seq,Homo_633,AD.vs.control; bulk RNA-seq,Homo_633,AD.vs.MCI; bulk RNA-seq,Homo_633,MCI.vs.control; scRNA-seq,SRP215507,CD8+ T cell_3-AD.vs.MCI; scRNA-seq,SRP215507,CD8+ T cell_5-AD.vs.MCI; scRNA-seq,SRP215507,CD8+ T cell_6-AD.vs.MCI; scRNA-seq,SRP215507,CD8+ T cell_5-MCI.vs.control; scRNA-seq,SRP215507,CD8+ T cell_6-MCI.vs.control; scRNA-seq,SRP309935,Naive CD8+ T cell_1-AD.vs.control; scRNA-seq,SRP330776,γδ T cell-AD.vs.control; scRNA-seq,SRP330776,Natural killer T cell-AD.vs.control; scRNA-seq,SRP330776,Naive CD8+ T cell_3-	17
ASB7	bulk RNA-seq,Homo_714,AD.vs.control; bulk RNA-seq,Homo_714,AD.vs.MCI; scRNA-seq,SRP215507,CD8+ T cell_3-AD.vs.MCI; scRNA-seq,SRP215507,CD8+ T cell_5-AD.vs.MCI; scRNA-seq,SRP215507,CD8+ T cell_5-MCI.vs.control; scRNA-seq,SRP330776,Naive CD8+ T cell_3-AD.vs.control; scRNA-seq,SRP330776,Monocyte_2-AD.vs.control	7
ASB8	bulk RNA-seq,Homo_633,AD.vs.MCI; scRNA-seq,SRP215507,CD8+ T cell_7-AD.vs.MCI; scRNA-seq,SRP309935,Monocyte_2-AD.vs.control; scRNA-seq,SRP309935,B cell_1-AD.vs.control; scRNA-seq,SRP330776,B cell_3-AD.vs.control	5
ASB9	scRNA-seq,SRP215507,CD8+ T cell_3-MCI.vs.control	1
ASCC1	bulk RNA-seq,Homo_714,AD.vs.control; bulk RNA-seq,Homo_714,AD.vs.MCI; scRNA-seq,SRP330776,B cell_2-AD.vs.control; scRNA-seq,SRP330776,Regulatory T cell-AD.vs.control; scRNA-seq,SRP330776,Natural killer cell_2-AD.vs.control	5
ASCC2	bulk RNA-seq,Homo_723,AD.vs.control; bulk RNA-seq,Homo_723,AD.vs.MCI; bulk RNA-seq,Homo_633,AD.vs.control; bulk RNA-seq,Homo_633,AD.vs.MCI; scRNA-seq,SRP215507,CD8+ T cell_7-AD.vs.control; scRNA-seq,SRP215507,CD8+ T cell_6-AD.vs.control; scRNA-seq,SRP215507,CD8+ T cell_6-AD.vs.MCI; scRNA-seq,SRP330776,γδ T cell-AD.vs.control; scRNA-seq,SRP330776,Naive CD8+ T cell_3-AD.vs.control; scRNA-seq,SRP330776,B cell_2-AD.vs.control; scRNA-seq,SRP330776,T cell-AD.vs.control; scRNA-seq,SRP330776,Natural killer cell_2-AD.vs.control	12
Ascc2	bulk RNA-seq,SRP325058,AD.vs.control	1
ASCC3	bulk RNA-seq,Homo_723,AD.vs.MCI; bulk RNA-seq,Homo_714,AD.vs.control; bulk RNA-seq,Homo_714,AD.vs.MCI; scRNA-seq,SRP215507,CD8+ T cell_7-AD.vs.control; scRNA-seq,SRP330776,Megakaryocyte_1-AD.vs.control	5
ASCL1	bulk RNA-seq,Homo_723,AD.vs.MCI; bulk RNA-seq,Homo_723,MCI.vs.control	2
ASCL2	bulk RNA-seq,Homo_723,AD.vs.control; bulk RNA-seq,Homo_723,AD.vs.MCI; bulk RNA-seq,Homo_633,AD.vs.control; bulk RNA-seq,Homo_633,AD.vs.MCI; bulk RNA-seq,Homo_714,AD.vs.control; bulk RNA-seq,Homo_714,AD.vs.MCI; scRNA-seq,SRP215507,CD8+ T cell_2-AD.vs.control; scRNA-seq,SRP215507,CD8+ T cell_1-AD.vs.control; scRNA-seq,SRP215507,CD8+ T cell_4-AD.vs.control; scRNA-seq,SRP215507,CD8+ T cell_5-AD.vs.control; scRNA-seq,SRP215507,CD8+ T cell_6-AD.vs.control; scRNA-seq,SRP215507,CD8+ T cell_2-AD.vs.MCI; scRNA-seq,SRP215507,CD8+ T cell_4-AD.vs.MCI; scRNA-seq,SRP215507,CD8+ T cell_1-MCI.vs.control; scRNA-seq,SRP215507,CD8+ T cell_5-MCI.vs.control; scRNA-seq,SRP215507,CD8+ T cell_2-MCI.vs.control; scRNA-seq,SRP309935,Monocyte_1-AD.vs.control; scRNA-seq,SRP309935,Megakaryocyte_1-AD.vs.control; scRNA-seq,SRP309935,Monocyte_2-AD.vs.control; scRNA-seq,SRP309935,CD8+ T cell_1-AD.vs.control; scRNA-seq,SRP330776,γδ T cell-AD.vs.control; scRNA-seq,SRP330776,CD8+ T cell_2-AD.vs.control; scRNA-seq,SRP330776,Natural [illegible]	25
ASCL5	bulk RNA-seq,Homo_723,AD.vs.control; bulk RNA-seq,Homo_723,AD.vs.MCI; bulk RNA-seq,Homo_714,MCI.vs.control; scRNA-seq,SRP215507,CD8+ T cell_1-MCI.vs.control	4
ASDURF	bulk RNA-seq,Homo_723,AD.vs.control; bulk RNA-seq,Homo_723,AD.vs.MCI; bulk RNA-seq,Homo_633,AD.vs.control; bulk RNA-seq,Homo_633,AD.vs.MCI; bulk RNA-seq,Homo_714,AD.vs.control; bulk RNA-seq,Homo_714,AD.vs.MCI; bulk RNA-seq,Homo_714,MCI.vs.control	7

ASF1A	bulk RNA-seq,Homo_714,AD.vs.control; bulk RNA-seq,Homo_714,AD.vs.MCI; scRNA-seq,SRP215507,CD8+ T cell_4-AD.vs.MCI; scRNA-seq,SRP215507,CD8+ T cell_4-MCI.vs.control; scRNA-seq,SRP309935,Naive CD8+ T cell_2-AD.vs.control; scRNA-seq,SRP309935,CD8+ T cell_1-AD.vs.control; scRNA-seq,SRP309935,CD8+ T cell_2-AD.vs.control; scRNA-seq,SRP330776,CD8+ T cell_1-AD.vs.control; scRNA-seq,SRP330776,γδ T cell-AD.vs.control; scRNA-seq,SRP330776,Memory T cell_2-AD.vs.control; scRNA-seq,SRP330776,CD8+ T cell_2-AD.vs.control; scRNA-seq,SRP330776,B cell_1-AD.vs.control; scRNA-seq,SRP330776,Naive CD8+ T cell_1-AD.vs.control; scRNA-seq,SRP330776,Natural killer cell_1-AD.vs.control; scRNA-seq,SRP330776,Memory T cell_1-AD.vs.control; scRNA-seq,SRP330776,Natural killer T cell-AD.vs.control; scRNA-seq,SRP330776,Naive CD8+ T cell_3-AD.vs.control; scRNA-seq,SRP330776,B cell_2-AD.vs.control; scRNA-seq,SRP330776,T cell-AD.vs.control; [illegible]	22
ASF1B	bulk RNA-seq,Homo_723,AD.vs.MCI; bulk RNA-seq,Homo_723,MCI.vs.control; bulk RNA-seq,Homo_633,AD.vs.MCI; bulk RNA-seq,Homo_633,MCI.vs.control; bulk RNA-seq,Homo_714,AD.vs.control; bulk RNA-seq,Homo_714,AD.vs.MCI; bulk RNA-seq,Homo_714,MCI.vs.control; scRNA-seq,SRP215507,CD8+ T cell_1-AD.vs.control; scRNA-seq,SRP215507,CD8+ T cell_1-AD.vs.MCI; scRNA-seq,SRP215507,CD8+ T cell_3-MCI.vs.control; scRNA-seq,SRP330776,B cell_1-AD.vs.control; scRNA-seq,SRP330776,Memory T cell_1-AD.vs.control	12
Asf1b	bulk RNA-seq,SRP325058,AD.vs.control	1
ASGR1	bulk RNA-seq,Homo_723,AD.vs.control; bulk RNA-seq,Homo_723,AD.vs.MCI; bulk RNA-seq,Homo_633,AD.vs.control; bulk RNA-seq,Homo_633,AD.vs.MCI; bulk RNA-seq,Homo_714,AD.vs.control; bulk RNA-seq,Homo_714,AD.vs.MCI; scRNA-seq,SRP215507,CD8+ T cell_1-AD.vs.control; scRNA-seq,SRP215507,CD8+ T cell_5-AD.vs.MCI	8
ASGR2	scRNA-seq,SRP330776,Monocyte_2-AD.vs.control	1
ASH1L	scRNA-seq,SRP330776,Natural killer cell_2-AD.vs.control; scRNA-seq,SRP330776,B cell_4-AD.vs.control	2
ASH1L-AS1	scRNA-seq,SRP215507,CD8+ T cell_2-AD.vs.control; scRNA-seq,SRP215507,CD8+ T cell_4-AD.vs.control; scRNA-seq,SRP215507,CD8+ T cell_4-AD.vs.MCI; scRNA-seq,SRP215507,CD8+ T cell_5-MCI.vs.control; scRNA-seq,SRP215507,CD8+ T cell_4-MCI.vs.control; scRNA-	6
ASH2L	scRNA-seq,SRP215507,CD8+ T cell_6-AD.vs.control; scRNA-seq,SRP309935,Monocyte_1-AD.vs.control; scRNA-seq,SRP309935,Monocyte_2-AD.vs.control; scRNA-seq,SRP309935,CD8+ T cell_1-AD.vs.control; scRNA-seq,SRP330776,Memory T cell_2-AD.vs.control; scRNA-seq,SRP330776,Naive CD8+ T cell_1-AD.vs.control; scRNA-seq,SRP330776,Natural killer cell_1-AD.vs.control; scRNA-seq,SRP330776,Monocyte_2-AD.vs.control; scRNA-seq,SRP330776,B cell_3-AD.vs.control; scRNA-seq,SRP330776,Megakaryocyte_1-AD.vs.control	10
ASIC1	bulk RNA-seq,Homo_723,AD.vs.control; bulk RNA-seq,Homo_723,AD.vs.MCI; bulk RNA-seq,Homo_723,MCI.vs.control; bulk RNA-seq,Homo_633,AD.vs.control; bulk RNA-seq,Homo_714,MCI.vs.control; bulk RNA-seq,ROSMAP,AD.vs.control; bulk RNA-	7
ASIC2	bulk RNA-seq,Homo_723,AD.vs.control; bulk RNA-seq,Homo_714,MCI.vs.control	2
ASIC3	bulk RNA-seq,Homo_723,AD.vs.control; bulk RNA-seq,Homo_723,AD.vs.MCI; bulk RNA-seq,Homo_633,AD.vs.control; bulk RNA-seq,Homo_633,AD.vs.MCI; bulk RNA-seq,Homo_714,MCI.vs.control; scRNA-seq,SRP215507,CD8+ T cell_2-AD.vs.control; scRNA-seq,SRP215507,CD8+ T cell_3-MCI.vs.control; scRNA-seq,SRP330776,B cell_1-AD.vs.control	8
ASIC4	bulk RNA-seq,Homo_723,AD.vs.control; bulk RNA-seq,Homo_723,AD.vs.MCI; bulk RNA-seq,Homo_723,MCI.vs.control; bulk RNA-seq,Homo_633,AD.vs.control; bulk RNA-seq,Homo_633,AD.vs.MCI; bulk RNA-seq,ROSMAP,AD.vs.control	6
ASIP	bulk RNA-seq,Homo_723,AD.vs.MCI; bulk RNA-seq,Homo_723,MCI.vs.control; bulk RNA-seq,Homo_633,MCI.vs.control; bulk RNA-	4
ASL	bulk RNA-seq,Homo_723,AD.vs.control; bulk RNA-seq,Homo_723,AD.vs.MCI; bulk RNA-seq,Homo_633,AD.vs.control; bulk RNA-seq,Homo_633,AD.vs.MCI; bulk RNA-seq,Homo_714,AD.vs.control; bulk RNA-seq,Homo_714,AD.vs.MCI; scRNA-seq,SRP215507,CD8+ T cell_10-AD.vs.control; scRNA-seq,SRP215507,CD8+ T cell_2-AD.vs.MCI; scRNA-seq,SRP215507,CD8+ T cell_3-AD.vs.MCI; scRNA-seq,SRP215507,CD8+ T cell_5-AD.vs.MCI; scRNA-seq,SRP215507,CD8+ T cell_6-AD.vs.MCI; scRNA-seq,SRP215507,CD8+ T cell_5-MCI.vs.control; scRNA-seq,SRP215507,CD8+ T cell_6-MCI.vs.control; scRNA-seq,SRP309935,B cell_1-AD.vs.control; scRNA-seq,SRP330776,γδ T cell-AD.vs.control; scRNA-seq,SRP330776,Naive CD8+ T cell_3-AD.vs.control; scRNA-seq,SRP330776,Monocyte_2-AD.vs.control; scRNA-seq,SRP330776,Natural killer cell_2-[illegible]	18
ASMT	bulk RNA-seq,Homo_723,AD.vs.control; bulk RNA-seq,Homo_723,AD.vs.MCI; bulk RNA-seq,Homo_633,AD.vs.control; bulk RNA-seq,Homo_633,AD.vs.MCI; scRNA-seq,SRP215507,CD8+ T cell_3-AD.vs.MCI; scRNA-seq,SRP215507,CD8+ T cell_3-MCI.vs.control	6
ASMTL	bulk RNA-seq,Homo_723,AD.vs.control; bulk RNA-seq,Homo_633,AD.vs.control; scRNA-seq,SRP215507,CD8+ T cell_6-MCI.vs.control; scRNA-seq,SRP330776,CD8+ T cell_1-AD.vs.control; scRNA-seq,SRP330776,B cell_2-AD.vs.control; scRNA-seq,SRP330776,Monocyte_2-AD.vs.control	6

ASMTL-AS1	scRNA-seq,SRP215507,CD8+ T cell_4-AD.vs.control; scRNA-seq,SRP215507,CD8+ T cell_4-AD.vs.MCI; scRNA-seq,SRP330776,Memory T cell_2-AD.vs.control; scRNA-seq,SRP330776,Naive CD8+ T cell_1-AD.vs.control	4
ASNS	bulk RNA-seq,Homo_723,AD.vs.control; bulk RNA-seq,Homo_723,MCI.vs.control; bulk RNA-seq,Homo_633,AD.vs.control; bulk RNA-seq,Homo_633,AD.vs.MCI; bulk RNA-seq,Homo_714,AD.vs.control; bulk RNA-seq,Homo_714,AD.vs.MCI; bulk RNA-seq,Homo_714,MCI.vs.control; bulk RNA-seq,ROSMAP,AD.vs.MCI; bulk RNA-seq,ROSMAP,MCI.vs.control; scRNA-seq,SRP215507,CD8+ T cell_4-AD.vs.control; scRNA-seq,SRP215507,CD8+ T cell_5-AD.vs.control; scRNA-seq,SRP215507,CD8+ T cell_6-AD.vs.control; scRNA-seq,SRP215507,CD8+ T cell_4-MCI.vs.control; scRNA-seq,SRP215507,CD8+ T cell_2-MCI.vs.control; scRNA-seq,SRP309935,Natural killer cell_1-AD.vs.control; scRNA- [illegible]	17
Asns	bulk RNA-seq,SRP325058,AD.vs.control	1
ASNSD1	scRNA-seq,SRP215507,CD8+ T cell_2-AD.vs.MCI; scRNA-seq,SRP215507,CD8+ T cell_2-MCI.vs.control; scRNA-seq,SRP215507,CD8+ T cell_3-MCI.vs.control; scRNA-seq,SRP309935,Naive CD8+ T cell_1-AD.vs.control; scRNA-seq,SRP309935,Natural killer cell_1-AD.vs.control; scRNA-seq,SRP309935,Naive CD8+ T cell_2-AD.vs.control; scRNA-seq,SRP309935,B cell_1-AD.vs.control; scRNA-seq,SRP309935,CD8+ T cell_2-AD.vs.control; scRNA-seq,SRP309935,Naive CD8+ T cell_3-AD.vs.control; scRNA-seq,SRP330776,CD8+ T cell_1-AD.vs.control; scRNA-seq,SRP330776,γδ T cell-AD.vs.control; scRNA-seq,SRP330776,Naive CD8+ T cell_1-AD.vs.control; scRNA-seq,SRP330776,Natural killer cell_1-AD.vs.control; scRNA-seq,SRP330776,Memory T cell_1-AD.vs.control; scRNA-seq,SRP330776,T cell-AD.vs.control; scRNA-seq,SRP330776,CD8+ T [illegible]	17
ASPA	bulk RNA-seq,Homo_723,AD.vs.control; bulk RNA-seq,Homo_633,AD.vs.control; bulk RNA-seq,Homo_714,MCI.vs.control	3
ASPG	bulk RNA-seq,Homo_714,MCI.vs.control	1
ASPH	bulk RNA-seq,Homo_723,AD.vs.control; bulk RNA-seq,Homo_723,AD.vs.MCI; bulk RNA-seq,Homo_633,AD.vs.control; bulk RNA-seq,Homo_633,AD.vs.MCI; bulk RNA-seq,Homo_714,AD.vs.control; bulk RNA-seq,Homo_714,AD.vs.MCI; scRNA-seq,SRP215507,CD8+ T cell_4-AD.vs.control; scRNA-seq,SRP215507,CD8+ T cell_4-AD.vs.MCI; scRNA-seq,SRP215507,CD8+ T cell_5-AD.vs.MCI; scRNA-seq,SRP215507,CD8+ T cell_5-MCI.vs.control; scRNA-seq,SRP215507,CD8+ T cell_2-MCI.vs.control; scRNA-seq,SRP215507,CD8+ T cell_3-MCI.vs.control; scRNA-seq,SRP309935,CD8+ T cell_1-AD.vs.control; scRNA-seq,SRP330776,Memory T cell_1-AD.vs.control; scRNA-seq,SRP330776,B cell_4-AD.vs.control	15
ASPHD1	bulk RNA-seq,Homo_723,AD.vs.control; bulk RNA-seq,Homo_723,AD.vs.MCI; bulk RNA-seq,Homo_723,MCI.vs.control; bulk RNA-seq,Homo_633,AD.vs.control; bulk RNA-seq,Homo_633,AD.vs.MCI; bulk RNA-seq,Homo_633,MCI.vs.control; bulk RNA-seq,Homo_714,MCI.vs.control	7
ASPHD2	bulk RNA-seq,Homo_723,AD.vs.control; bulk RNA-seq,Homo_723,AD.vs.MCI; bulk RNA-seq,Homo_633,AD.vs.control; bulk RNA-seq,Homo_633,AD.vs.MCI; bulk RNA-seq,Homo_714,AD.vs.control; bulk RNA-seq,Homo_714,AD.vs.MCI; scRNA-seq,SRP215507,CD8+ T cell_2-AD.vs.control; scRNA-seq,SRP215507,CD8+ T cell_5-AD.vs.control; scRNA-seq,SRP215507,CD8+ T cell_3-AD.vs.MCI; scRNA-seq,SRP215507,CD8+ T cell_3-MCI.vs.control; scRNA-seq,SRP309935,Monocyte_1-AD.vs.control; scRNA-seq,SRP309935,Naive CD8+ T cell_1-AD.vs.control; scRNA-seq,SRP309935,Dendritic cell-AD.vs.control; scRNA-seq,SRP330776,γδ T cell-AD.vs.control; scRNA-seq,SRP330776,B cell_1-AD.vs.control; scRNA- [illegible]	16
ASPM	bulk RNA-seq,Homo_723,AD.vs.control; bulk RNA-seq,Homo_723,AD.vs.MCI; bulk RNA-seq,Homo_723,MCI.vs.control; bulk RNA-seq,Homo_633,AD.vs.control; bulk RNA-seq,Homo_633,AD.vs.MCI; bulk RNA-seq,Homo_633,MCI.vs.control; bulk RNA-seq,Homo_714,AD.vs.control; bulk RNA-seq,Homo_714,AD.vs.MCI; scRNA-seq,SRP215507,CD8+ T cell_2-AD.vs.MCI; scRNA-seq,SRP330776,B cell_1-AD.vs.control	10
ASPN	bulk RNA-seq,Homo_723,AD.vs.control; bulk RNA-seq,Homo_633,AD.vs.control; bulk RNA-seq,Homo_714,MCI.vs.control	3
Asprv1	bulk RNA-seq,SRP325058,AD.vs.control	1
ASPRV1	scRNA-seq,SRP215507,CD8+ T cell_1-MCI.vs.control	1
ASPSCR1	bulk RNA-seq,Homo_723,AD.vs.MCI; bulk RNA-seq,Homo_723,MCI.vs.control; bulk RNA-seq,Homo_633,AD.vs.MCI; bulk RNA-seq,Homo_633,MCI.vs.control; bulk RNA-seq,Homo_714,AD.vs.control; bulk RNA-seq,Homo_714,AD.vs.MCI; bulk RNA-seq,Homo_714,MCI.vs.control; scRNA-seq,SRP330776,CD8+ T cell_1-AD.vs.control; scRNA-seq,SRP330776,γδ T cell-AD.vs.control; scRNA-seq,SRP330776,Memory T cell_2-AD.vs.control; scRNA-seq,SRP330776,CD8+ T cell_2-AD.vs.control; scRNA-seq,SRP330776,B cell_1-AD.vs.control; scRNA-seq,SRP330776,Naive CD8+ T cell_1-AD.vs.control; scRNA-seq,SRP330776,Natural killer cell_1-AD.vs.control; scRNA-seq,SRP330776,Memory T cell_1-AD.vs.control; scRNA-seq,SRP330776,Natural killer T cell-AD.vs.control; scRNA-seq,SRP330776,Naive CD8+ T cell_3-AD.vs.control; scRNA-seq,SRP330776,B cell_2-AD.vs.control; scRNA-seq,SRP330776,T cell-AD.vs.control; scRNA-seq,SRP330776,Naive CD8+ T cell_2-AD.vs.control; [illegible]	23

ASRGL1	scRNA-seq,SRP215507,CD8+ T cell_1-AD.vs.MCI; scRNA-seq,SRP215507,CD8+ T cell_1-MCI.vs.control; scRNA-seq,SRP330776,CD8+ T cell_1-AD.vs.control; scRNA-seq,SRP330776,γδ T cell-AD.vs.control; scRNA-seq,SRP330776,B cell_1-AD.vs.control; scRNA-seq,SRP330776,Naive CD8+ T cell_1-AD.vs.control; scRNA-seq,SRP330776,Memory T cell_1-AD.vs.control; scRNA-seq,SRP330776,Natural killer T cell-AD.vs.control; scRNA-seq,SRP330776,Naive CD8+ T cell_3-AD.vs.control; scRNA-seq,SRP330776,Monocyte_1-AD.vs.control	10
ASS1	bulk RNA-seq,Homo_723,AD.vs.control; bulk RNA-seq,Homo_723,AD.vs.MCI; bulk RNA-seq,Homo_723,MCI.vs.control; bulk RNA-seq,Homo_633,AD.vs.control; bulk RNA-seq,Homo_633,AD.vs.MCI; bulk RNA-seq,Homo_633,MCI.vs.control; bulk RNA-seq,ROSMAP,AD.vs.control; bulk RNA-seq,ROSMAP,MCI.vs.control; bulk RNA-seq,SRP223445,AD.vs.control	9
ASTE1	bulk RNA-seq,Homo_723,AD.vs.control; bulk RNA-seq,Homo_723,AD.vs.MCI; bulk RNA-seq,Homo_633,AD.vs.control; bulk RNA-seq,Homo_633,AD.vs.MCI; bulk RNA-seq,Homo_714,AD.vs.control; bulk RNA-seq,Homo_714,AD.vs.MCI; scRNA-seq,SRP215507,CD8+ T cell_7-AD.vs.control; scRNA-seq,SRP215507,CD8+ T cell_4-AD.vs.MCI; scRNA-seq,SRP215507,CD8+ T cell_5-AD.vs.MCI; scRNA-seq,SRP215507,CD8+ T cell_1-MCI.vs.control; scRNA-seq,SRP215507,CD8+ T cell_4-MCI.vs.control; scRNA-seq,SRP215507,CD8+ T cell_6-MCI.vs.control	12
ASTL	bulk RNA-seq,Homo_723,AD.vs.control; bulk RNA-seq,Homo_723,AD.vs.MCI; bulk RNA-seq,Homo_633,AD.vs.control; bulk RNA-seq,Homo_633,AD.vs.MCI; bulk RNA-seq,Homo_714,AD.vs.control; bulk RNA-seq,Homo_714,AD.vs.MCI; scRNA-seq,SRP215507,CD8+ T cell_2-AD.vs.control; scRNA-seq,SRP215507,CD8+ T cell_3-MCI.vs.control	8
ASTN1	bulk RNA-seq,Homo_723,AD.vs.control; bulk RNA-seq,Homo_723,AD.vs.MCI; bulk RNA-seq,Homo_723,MCI.vs.control; bulk RNA-	4
ASTN2	bulk RNA-seq,Homo_723,AD.vs.control; bulk RNA-seq,Homo_633,AD.vs.control; bulk RNA-seq,Homo_714,AD.vs.control; bulk RNA-seq,Homo_714,AD.vs.MCI; bulk RNA-seq,ROSMAP,AD.vs.control; bulk RNA-seq,ROSMAP,MCI.vs.control; scRNA-seq,SRP215507,CD8+ T cell_3-AD.vs.MCI; scRNA-seq,SRP215507,CD8+ T cell_1-AD.vs.MCI; scRNA-seq,SRP215507,CD8+ T cell_3-MCI.vs.control; scRNA-seq,SRP330776,γδ T cell-AD.vs.control; scRNA-seq,SRP330776,Memory T cell_2-AD.vs.control; scRNA-seq,SRP330776,T cell-AD.vs.control	12
ASXL1	bulk RNA-seq,Homo_714,MCI.vs.control; scRNA-seq,SRP215507,CD8+ T cell_7-AD.vs.control; scRNA-seq,SRP215507,CD8+ T cell_6-AD.vs.control; scRNA-seq,SRP215507,CD8+ T cell_4-MCI.vs.control; scRNA-seq,SRP215507,CD8+ T cell_7-MCI.vs.control; scRNA-seq,SRP309935,Natural killer cell_2-AD.vs.control; scRNA-seq,SRP309935,Naive CD8+ T cell_2-AD.vs.control; scRNA-seq,SRP330776,γδ T cell-AD.vs.control; scRNA-seq,SRP330776,Naive CD8+ T cell_1-AD.vs.control; scRNA-seq,SRP330776,Natural killer cell_1-AD.vs.control; scRNA-seq,SRP330776,Memory T cell_1-AD.vs.control; scRNA-seq,SRP330776,Naive CD8+ T cell_3-AD.vs.control; scRNA-seq,SRP330776,B cell_2-AD.vs.control; scRNA-	15
ASXL2	bulk RNA-seq,Homo_723,AD.vs.control; bulk RNA-seq,Homo_723,MCI.vs.control; bulk RNA-seq,Homo_633,AD.vs.control; bulk RNA-seq,Homo_633,MCI.vs.control; scRNA-seq,SRP215507,CD8+ T cell_7-AD.vs.control; scRNA-seq,SRP215507,CD8+ T cell_7-AD.vs.MCI; scRNA-seq,SRP215507,CD8+ T cell_6-AD.vs.MCI; scRNA-seq,SRP215507,CD8+ T cell_6-MCI.vs.control; scRNA-seq,SRP309935,Naive CD8+ T cell_2-AD.vs.control; scRNA-seq,SRP309935,CD8+ T cell_2-AD.vs.control; scRNA-seq,SRP330776,Monocyte_2-AD.vs.control	11
ASXL3	bulk RNA-seq,Homo_723,AD.vs.control; bulk RNA-seq,Homo_723,AD.vs.MCI; bulk RNA-seq,Homo_723,MCI.vs.control; bulk RNA-seq,Homo_633,AD.vs.control; bulk RNA-seq,Homo_633,AD.vs.MCI; bulk RNA-seq,Homo_714,AD.vs.MCI; bulk RNA-seq,Homo_714,MCI.vs.control;	8
ATAD1	bulk RNA-seq,Homo_723,AD.vs.MCI; bulk RNA-seq,Homo_723,MCI.vs.control; bulk RNA-seq,Homo_633,AD.vs.MCI; bulk RNA-seq,Homo_633,MCI.vs.control; scRNA-seq,SRP215507,CD8+ T cell_6-AD.vs.control; scRNA-seq,SRP215507,CD8+ T cell_6-AD.vs.MCI; scRNA-seq,SRP309935,Naive CD8+ T cell_2-AD.vs.control; scRNA-seq,SRP330776,Monocyte_1-AD.vs.control; scRNA-seq,SRP330776,Monocyte_2-	9
ATAD2	scRNA-seq,SRP215507,CD8+ T cell_3-AD.vs.MCI; scRNA-seq,SRP215507,CD8+ T cell_6-AD.vs.MCI; scRNA-seq,SRP215507,CD8+ T cell_3-MCI.vs.control; scRNA-seq,SRP215507,CD8+ T cell_6-MCI.vs.control; scRNA-seq,SRP309935,Naive CD8+ T cell_1-AD.vs.control; scRNA-	6
ATAD2B	bulk RNA-seq,Homo_723,AD.vs.control; bulk RNA-seq,Homo_723,AD.vs.MCI; bulk RNA-seq,Homo_633,AD.vs.control; bulk RNA-seq,Homo_633,AD.vs.MCI; bulk RNA-seq,Homo_714,AD.vs.control; bulk RNA-seq,Homo_714,AD.vs.MCI; scRNA-seq,SRP215507,CD8+ T cell_3-AD.vs.MCI; scRNA-seq,SRP215507,CD8+ T cell_5-AD.vs.MCI; scRNA-seq,SRP215507,CD8+ T cell_5-MCI.vs.control; scRNA-seq,SRP309935,Naive CD8+ T cell_1-AD.vs.control; scRNA-seq,SRP330776,Natural killer cell_1-AD.vs.control; scRNA-seq,SRP330776,Naive CD8+ T cell_3-AD.vs.control;	13

ATAD3A	bulk RNA-seq,Homo_723,AD.vs.control; bulk RNA-seq,Homo_723,AD.vs.MCI; bulk RNA-seq,Homo_723,MCI.vs.control; bulk RNA-seq,Homo_633,AD.vs.control; bulk RNA-seq,Homo_633,AD.vs.MCI; bulk RNA-seq,Homo_633,MCI.vs.control; scRNA-seq,SRP215507,CD8+ T cell_3-AD.vs.control; scRNA-seq,SRP215507,CD8+ T cell_5-AD.vs.control; scRNA-seq,SRP330776,CD8+ T cell_1-AD.vs.control; scRNA-seq,SRP330776,γδ T cell-AD.vs.control; scRNA-seq,SRP330776,Memory T cell_2-AD.vs.control; scRNA-seq,SRP330776,CD8+ T cell_2-AD.vs.control; scRNA-seq,SRP330776,B cell_1-AD.vs.control; scRNA-seq,SRP330776,Naive CD8+ T cell_1-AD.vs.control; scRNA-seq,SRP330776,Natural killer cell_1-AD.vs.control; scRNA-seq,SRP330776,Memory T cell_1-AD.vs.control; scRNA-seq,SRP330776,Natural killer T cell-AD.vs.control; scRNA-seq,SRP330776,Naive CD8+ T cell_3-AD.vs.control; scRNA-seq,SRP330776,B cell_2-AD.vs.control; scRNA-seq,SRP330776,T cell-AD.vs.control; scRNA-seq,SRP330776,CD8+ T cell_3-AD.vs.control; scRNA-seq,SRP330776,Monocyte_1-AD.vs.control; scRNA-seq,SRP330776,B cell_3- [illegible]	24
ATAD3B	bulk RNA-seq,Homo_723,AD.vs.control; bulk RNA-seq,Homo_723,AD.vs.MCI; bulk RNA-seq,Homo_723,MCI.vs.control; bulk RNA-seq,Homo_633,AD.vs.control; bulk RNA-seq,Homo_633,MCI.vs.control; scRNA-seq,SRP330776,CD8+ T cell_1-AD.vs.control; scRNA-seq,SRP330776,γδ T cell-AD.vs.control; scRNA-seq,SRP330776,Memory T cell_2-AD.vs.control; scRNA-seq,SRP330776,CD8+ T cell_2-AD.vs.control; scRNA-seq,SRP330776,B cell_1-AD.vs.control; scRNA-seq,SRP330776,Naive CD8+ T cell_1-AD.vs.control; scRNA-seq,SRP330776,Natural killer cell_1-AD.vs.control; scRNA-seq,SRP330776,Memory T cell_1-AD.vs.control; scRNA-seq,SRP330776,Natural killer T cell-AD.vs.control; scRNA-seq,SRP330776,Naive CD8+ T cell_3-AD.vs.control; scRNA-seq,SRP330776,B cell_2-AD.vs.control; scRNA-seq,SRP330776,T cell-AD.vs.control; scRNA-seq,SRP330776,CD8+ T cell_3-AD.vs.control; scRNA-seq,SRP330776,Monocyte_1-AD.vs.control; scRNA-seq,SRP330776,Regulatory T cell- [illegible]	20
ATAD3C	bulk RNA-seq,Homo_633,AD.vs.control; bulk RNA-seq,Homo_714,AD.vs.control; bulk RNA-seq,Homo_714,AD.vs.MCI; scRNA-seq,SRP330776,CD8+ T cell_1-AD.vs.control; scRNA-seq,SRP330776,B cell_1-AD.vs.control; scRNA-seq,SRP330776,Naive CD8+ T cell_1-AD.vs.control; scRNA-	7
ATAD5	scRNA-seq,SRP215507,CD8+ T cell_1-MCI.vs.control; scRNA-seq,SRP330776,Natural killer T cell-AD.vs.control; scRNA-seq,SRP330776,Naive CD8+ T cell_3-AD.vs.control; scRNA-seq,SRP330776,B cell_2-AD.vs.control; scRNA-seq,SRP330776,Megakaryocyte_1-AD.vs.control	5
ATAT1	scRNA-seq,SRP215507,CD8+ T cell_4-AD.vs.control; scRNA-seq,SRP215507,CD8+ T cell_5-AD.vs.MCI; scRNA-seq,SRP215507,CD8+ T cell_5-MCI.vs.control; scRNA-seq,SRP330776,γδ T cell-AD.vs.control; scRNA-seq,SRP330776,Naive CD8+ T cell_3-AD.vs.control; scRNA-seq,SRP330776,B	6
ATCAY	bulk RNA-seq,Homo_723,AD.vs.control; bulk RNA-seq,Homo_723,AD.vs.MCI; bulk RNA-seq,Homo_714,MCI.vs.control; bulk RNA-seq,ROSMAP,AD.vs.control; bulk RNA-seq,ROSMAP,MCI.vs.control	5
ATE1	bulk RNA-seq,Homo_723,AD.vs.control; bulk RNA-seq,Homo_723,AD.vs.MCI; bulk RNA-seq,Homo_723,MCI.vs.control; bulk RNA-seq,Homo_633,AD.vs.control; bulk RNA-seq,Homo_633,AD.vs.MCI; bulk RNA-seq,Homo_633,MCI.vs.control; bulk RNA-seq,Homo_714,AD.vs.control; bulk RNA-seq,Homo_714,AD.vs.MCI; scRNA-seq,SRP215507,CD8+ T cell_2-AD.vs.control; scRNA-seq,SRP215507,CD8+ T cell_4-AD.vs.control; scRNA-seq,SRP215507,CD8+ T cell_7-AD.vs.control; scRNA-seq,SRP215507,CD8+ T cell_5-AD.vs.MCI; scRNA-seq,SRP215507,CD8+ T cell_5-MCI.vs.control; scRNA-seq,SRP330776,CD8+ T cell_1-AD.vs.control; scRNA-seq,SRP330776,γδ T cell-AD.vs.control; scRNA-seq,SRP330776,Memory T cell_2-AD.vs.control; scRNA-seq,SRP330776,CD8+ T cell_2-AD.vs.control; scRNA-seq,SRP330776,Naive CD8+ T cell_1-AD.vs.control; scRNA-seq,SRP330776,Natural killer cell_1-AD.vs.control; scRNA-seq,SRP330776,Memory T cell_1-AD.vs.control; scRNA-seq,SRP330776,Naive CD8+ T cell_3-AD.vs.control; scRNA-seq,SRP330776,B cell_2-AD.vs.control; scRNA-seq,SRP330776,T cell-AD.vs.control; scRNA-seq,SRP330776,Monocyte_2- [illegible]	25
ATF1	bulk RNA-seq,Homo_714,AD.vs.control; bulk RNA-seq,Homo_714,AD.vs.MCI; scRNA-seq,SRP330776,Memory T cell_2-AD.vs.control; scRNA-seq,SRP330776,CD8+ T cell_2-AD.vs.control; scRNA-seq,SRP330776,Naive CD8+ T cell_1-AD.vs.control; scRNA-seq,SRP330776,Monocyte_2-	6
ATF2	bulk RNA-seq,Homo_723,AD.vs.control; bulk RNA-seq,Homo_723,AD.vs.MCI; bulk RNA-seq,Homo_723,MCI.vs.control; bulk RNA-seq,Homo_633,AD.vs.control; bulk RNA-seq,Homo_633,AD.vs.MCI; bulk RNA-seq,Homo_714,AD.vs.control; bulk RNA-seq,Homo_714,AD.vs.MCI; scRNA-seq,SRP215507,CD8+ T cell_3-AD.vs.MCI; scRNA-seq,SRP215507,CD8+ T cell_5-AD.vs.MCI; scRNA-seq,SRP309935,Monocyte_1-	10
Atf2	bulk RNA-seq,SRP325058,AD.vs.control	1

ATF3	bulk RNA-seq,Homo_723,AD.vs.control; bulk RNA-seq,Homo_723,AD.vs.MCI; bulk RNA-seq,Homo_633,AD.vs.control; bulk RNA-seq,Homo_633,AD.vs.MCI; bulk RNA-seq,Homo_714,AD.vs.control; bulk RNA-seq,Homo_714,AD.vs.MCI; scRNA-seq,SRP215507,CD8+ T cell_5-AD.vs.control; scRNA-seq,SRP215507,CD8+ T cell_9-AD.vs.control; scRNA-seq,SRP215507,CD8+ T cell_2-AD.vs.MCI; scRNA-seq,SRP215507,CD8+ T cell_3-AD.vs.MCI; scRNA-seq,SRP215507,CD8+ T cell_1-AD.vs.MCI; scRNA-seq,SRP215507,CD8+ T cell_5-AD.vs.MCI; scRNA-seq,SRP215507,CD8+ T cell_1-MCI.vs.control; scRNA-seq,SRP215507,CD8+ T cell_5-MCI.vs.control; scRNA-seq,SRP215507,CD8+ T cell_2-MCI.vs.control; scRNA-seq,SRP215507,CD8+ T cell_3-MCI.vs.control; scRNA-seq,SRP215507,CD8+ T cell_6-MCI.vs.control; scRNA-seq,SRP309935,Natural killer cell_1-AD.vs.control; scRNA-seq,SRP330776,CD8+ T cell_1-AD.vs.control; scRNA-seq,SRP330776,γδ T cell-AD.vs.control; scRNA-seq,SRP330776,Memory T cell_2-AD.vs.control; scRNA-seq,SRP330776,CD8+ T cell_2-AD.vs.control; scRNA-seq,SRP330776,B cell_1-AD.vs.control; scRNA-seq,SRP330776,B cell_2-AD.vs.control; scRNA-seq,SRP330776,T cell-AD.vs.control; scRNA-seq,SRP330776,CD8+ T [illegible]	28
Atf4	bulk RNA-seq,SRP325058,AD.vs.control	1
ATF4	scRNA-seq,SRP215507,CD8+ T cell_7-AD.vs.control; scRNA-seq,SRP215507,CD8+ T cell_6-AD.vs.control; scRNA-seq,SRP215507,CD8+ T cell_3-AD.vs.MCI; scRNA-seq,SRP215507,CD8+ T cell_7-AD.vs.MCI; scRNA-seq,SRP215507,CD8+ T cell_5-AD.vs.MCI; scRNA-seq,SRP215507,CD8+ T cell_6-AD.vs.MCI; scRNA-seq,SRP215507,CD8+ T cell_7-MCI.vs.control; scRNA-seq,SRP330776,Natural killer T cell-AD.vs.control; scRNA-seq,SRP330776,CD8+ T cell_3-AD.vs.control; scRNA-seq,SRP330776,Monocyte_2-AD.vs.control; scRNA-seq,SRP330776,B cell_3-AD.vs.control	11
ATF5	bulk RNA-seq,Homo_723,AD.vs.control; bulk RNA-seq,Homo_723,AD.vs.MCI; bulk RNA-seq,Homo_723,MCI.vs.control; bulk RNA-seq,Homo_633,AD.vs.control; bulk RNA-seq,Homo_633,AD.vs.MCI; bulk RNA-seq,Homo_633,MCI.vs.control; bulk RNA-seq,Homo_714,AD.vs.control; bulk RNA-seq,Homo_714,AD.vs.MCI; scRNA-seq,SRP215507,CD8+ T cell_7-AD.vs.MCI; scRNA-seq,SRP215507,CD8+ T cell_5-AD.vs.MCI; scRNA-seq,SRP215507,CD8+ T cell_4-MCI.vs.control; scRNA-seq,SRP215507,CD8+ T cell_6-MCI.vs.control; scRNA-seq,SRP309935,Naive CD8+ T cell_2-AD.vs.control; scRNA-seq,SRP330776,CD8+ T cell_3-AD.vs.control; scRNA-seq,SRP330776,Monocyte_1-AD.vs.control	15
ATF6	scRNA-seq,SRP309935,B cell_1-AD.vs.control; scRNA-seq,SRP330776,Natural killer cell_1-AD.vs.control; scRNA-seq,SRP330776,Memory T cell_1-	3
ATF6B	scRNA-seq,SRP215507,CD8+ T cell_10-AD.vs.control; scRNA-seq,SRP330776,Memory T cell_2-AD.vs.control; scRNA-seq,SRP330776,Natural killer cell_1-AD.vs.control; scRNA-seq,SRP330776,B cell_2-AD.vs.control	4
ATF7	bulk RNA-seq,Homo_723,AD.vs.control; bulk RNA-seq,Homo_723,AD.vs.MCI; bulk RNA-seq,Homo_723,MCI.vs.control; bulk RNA-seq,Homo_633,AD.vs.control; bulk RNA-seq,Homo_633,AD.vs.MCI; bulk RNA-seq,Homo_633,MCI.vs.control; scRNA-seq,SRP215507,CD8+ T cell_7-AD.vs.control; scRNA-seq,SRP330776,Naive CD8+ T cell_3-AD.vs.control; scRNA-seq,SRP330776,T cell-AD.vs.control; scRNA-seq,SRP330776,Natural	10
ATF7IP	bulk RNA-seq,Homo_723,AD.vs.control; bulk RNA-seq,Homo_723,MCI.vs.control; bulk RNA-seq,Homo_633,AD.vs.control; bulk RNA-seq,Homo_633,AD.vs.MCI; bulk RNA-seq,Homo_633,MCI.vs.control; scRNA-seq,SRP215507,CD8+ T cell_7-MCI.vs.control; scRNA-seq,SRP309935,Monocyte_2-AD.vs.control; scRNA-seq,SRP309935,Naive CD8+ T cell_2-AD.vs.control; scRNA-seq,SRP330776,Monocyte_2-	10
ATF7IP2	bulk RNA-seq,Homo_723,AD.vs.MCI; bulk RNA-seq,Homo_723,MCI.vs.control; bulk RNA-seq,Homo_633,AD.vs.MCI; bulk RNA-seq,Homo_633,MCI.vs.control; scRNA-seq,SRP215507,CD8+ T cell_3-AD.vs.control; scRNA-seq,SRP215507,CD8+ T cell_4-AD.vs.control; scRNA-seq,SRP215507,CD8+ T cell_7-AD.vs.control; scRNA-seq,SRP330776,CD8+ T cell_2-AD.vs.control; scRNA-seq,SRP330776,Monocyte_1-AD.vs.control	9
ATF7-NPFF	bulk RNA-seq,Homo_723,AD.vs.control; bulk RNA-seq,Homo_633,AD.vs.control; bulk RNA-seq,Homo_633,MCI.vs.control	3
ATG10	bulk RNA-seq,Homo_723,AD.vs.control; bulk RNA-seq,Homo_723,AD.vs.MCI; bulk RNA-seq,Homo_633,AD.vs.control; bulk RNA-seq,Homo_633,AD.vs.MCI; bulk RNA-seq,Homo_714,AD.vs.control; bulk RNA-seq,Homo_714,AD.vs.MCI; scRNA-seq,SRP215507,CD8+ T cell_3-AD.vs.MCI; scRNA-seq,SRP330776,Memory T cell_2-AD.vs.control; scRNA-seq,SRP330776,Natural killer T cell-AD.vs.control; scRNA-	10
ATG101	bulk RNA-seq,Homo_723,AD.vs.control; bulk RNA-seq,Homo_723,AD.vs.MCI; bulk RNA-seq,Homo_633,AD.vs.control; bulk RNA-seq,Homo_633,AD.vs.MCI; bulk RNA-seq,Homo_714,AD.vs.control; bulk RNA-seq,Homo_714,AD.vs.MCI; bulk RNA-seq,Homo_714,MCI.vs.control; scRNA-seq,SRP215507,CD8+ T cell_3-AD.vs.control; scRNA-seq,SRP215507,CD8+ T cell_3-MCI.vs.control; scRNA-seq,SRP215507,CD8+ T cell_6-MCI.vs.control; scRNA-seq,SRP330776,Memory T cell_1-AD.vs.control; scRNA-seq,SRP330776,T cell-AD.vs.control; scRNA-seq,SRP330776,B cell_3-	14
ATG12	bulk RNA-seq,Homo_723,AD.vs.control; bulk RNA-seq,Homo_723,AD.vs.MCI; bulk RNA-seq,Homo_633,AD.vs.control; bulk RNA-seq,Homo_633,AD.vs.MCI; bulk RNA-seq,Homo_714,AD.vs.control; bulk RNA-seq,Homo_714,AD.vs.MCI; scRNA-seq,SRP215507,CD8+ T cell_7-AD.vs.control; scRNA-seq,SRP330776,Naive CD8+ T cell_3-AD.vs.control; scRNA-seq,SRP330776,Regulatory T cell-AD.vs.control; scRNA-	10
Atg12	bulk RNA-seq,SRP325058,AD.vs.control	1

ATG13	bulk RNA-seq,Homo_723,AD.vs.MCI; bulk RNA-seq,Homo_723,MCI.vs.control; bulk RNA-seq,Homo_633,AD.vs.MCI; bulk RNA-seq,Homo_633,MCI.vs.control; bulk RNA-seq,Homo_714,AD.vs.control; bulk RNA-seq,Homo_714,AD.vs.MCI; bulk RNA-seq,Homo_714,MCI.vs.control; scRNA-seq,SRP215507,CD8+ T cell_7-AD.vs.control; scRNA-seq,SRP215507,CD8+ T cell_10-AD.vs.control; scRNA-seq,SRP215507,CD8+ T cell_6-AD.vs.control; scRNA-seq,SRP215507,CD8+ T cell_4-AD.vs.MCI; scRNA-seq,SRP215507,CD8+ T cell_4-MCI.vs.control; scRNA-seq,SRP330776,γδ T cell-AD.vs.control; scRNA-seq,SRP330776,Naive CD8+ T cell_3-AD.vs.control; scRNA-	16
ATG14	bulk RNA-seq,Homo_723,MCI.vs.control; bulk RNA-seq,Homo_633,AD.vs.control; bulk RNA-seq,Homo_633,MCI.vs.control; bulk RNA-seq,Homo_714,AD.vs.control; bulk RNA-seq,Homo_714,AD.vs.MCI; bulk RNA-seq,Homo_714,MCI.vs.control; scRNA-seq,SRP215507,CD8+ T cell_5-	7
ATG16L1	bulk RNA-seq,Homo_633,AD.vs.control; bulk RNA-seq,Homo_714,AD.vs.control; bulk RNA-seq,Homo_714,AD.vs.MCI; bulk RNA-seq,SRP310421,AD.vs.control; scRNA-seq,SRP215507,CD8+ T cell_10-AD.vs.control; scRNA-seq,SRP215507,CD8+ T cell_5-AD.vs.MCI; scRNA-seq,SRP215507,CD8+ T cell_5-MCI.vs.control; scRNA-seq,SRP309935,Naive CD8+ T cell_1-AD.vs.control; scRNA-seq,SRP309935,CD8+ T cell_1-AD.vs.control; scRNA-seq,SRP330776,Naive CD8+ T cell_1-AD.vs.control; scRNA-seq,SRP330776,B cell_2-AD.vs.control; scRNA-	12
ATG16L2	scRNA-seq,SRP215507,CD8+ T cell_5-MCI.vs.control; scRNA-seq,SRP309935,B cell_1-AD.vs.control; scRNA-seq,SRP330776,CD8+ T cell_1-AD.vs.control; scRNA-seq,SRP330776,γδ T cell-AD.vs.control; scRNA-seq,SRP330776,Memory T cell_2-AD.vs.control; scRNA-seq,SRP330776,CD8+ T cell_2-AD.vs.control; scRNA-seq,SRP330776,B cell_1-AD.vs.control; scRNA-seq,SRP330776,Naive CD8+ T cell_1-AD.vs.control; scRNA-seq,SRP330776,Natural killer cell_1-AD.vs.control; scRNA-seq,SRP330776,Memory T cell_1-AD.vs.control; scRNA-seq,SRP330776,Natural killer T cell-AD.vs.control; scRNA-seq,SRP330776,Naive CD8+ T cell_3-AD.vs.control; scRNA-seq,SRP330776,B cell_2-AD.vs.control; scRNA-seq,SRP330776,T cell-AD.vs.control; scRNA-seq,SRP330776,Naive CD8+ T cell_2-AD.vs.control; scRNA-seq,SRP330776,CD8+ T cell_3-AD.vs.control; scRNA-seq,SRP330776,Monocyte_2-AD.vs.control; scRNA-seq,SRP330776,B cell_3-AD.vs.control; scRNA-seq,SRP330776,Natural killer cell_2-AD.vs.control	19
ATG2A	scRNA-seq,SRP330776,Naive CD8+ T cell_1-AD.vs.control; scRNA-seq,SRP330776,B cell_2-AD.vs.control; scRNA-seq,SRP330776,B cell_3-AD.vs.control; scRNA-seq,SRP330776,Natural killer cell_2-AD.vs.control	4
ATG2B	bulk RNA-seq,Homo_723,AD.vs.control; bulk RNA-seq,Homo_723,AD.vs.MCI; bulk RNA-seq,Homo_723,MCI.vs.control; bulk RNA-seq,Homo_633,AD.vs.control; bulk RNA-seq,Homo_633,AD.vs.MCI; bulk RNA-seq,Homo_633,MCI.vs.control; bulk RNA-seq,Homo_714,AD.vs.control; bulk RNA-seq,Homo_714,AD.vs.MCI; bulk RNA-seq,Homo_714,MCI.vs.control; scRNA-seq,SRP215507,CD8+ T cell_7-AD.vs.MCI; scRNA-seq,SRP215507,CD8+ T cell_7-MCI.vs.control; scRNA-seq,SRP309935,CD8+ T cell_1-AD.vs.control; scRNA-seq,SRP330776,B cell_1-AD.vs.control;	14
ATG3	scRNA-seq,SRP215507,CD8+ T cell_2-AD.vs.control; scRNA-seq,SRP215507,CD8+ T cell_5-AD.vs.control; scRNA-seq,SRP215507,CD8+ T cell_5-AD.vs.MCI; scRNA-seq,SRP330776,Memory T cell_2-AD.vs.control	4
ATG4A	bulk RNA-seq,Homo_723,AD.vs.control; bulk RNA-seq,Homo_633,AD.vs.control; bulk RNA-seq,Homo_714,AD.vs.control; bulk RNA-seq,Homo_714,AD.vs.MCI; scRNA-seq,SRP215507,CD8+ T cell_5-AD.vs.control; scRNA-seq,SRP330776,Memory T cell_1-AD.vs.control; scRNA-seq,SRP330776,Natural killer T cell-AD.vs.control; scRNA-seq,SRP330776,Naive CD8+ T cell_3-AD.vs.control; scRNA-seq,SRP330776,CD8+ T cell_3-	9
Atg4a	bulk RNA-seq,SRP325058,AD.vs.control	1
ATG4B	bulk RNA-seq,Homo_723,AD.vs.control; bulk RNA-seq,Homo_723,AD.vs.MCI; bulk RNA-seq,Homo_723,MCI.vs.control; bulk RNA-seq,Homo_633,AD.vs.control; bulk RNA-seq,Homo_633,AD.vs.MCI; bulk RNA-seq,Homo_633,MCI.vs.control; bulk RNA-seq,Homo_714,AD.vs.control; bulk RNA-seq,Homo_714,AD.vs.MCI; bulk RNA-seq,Homo_714,MCI.vs.control; scRNA-seq,SRP215507,CD8+ T cell_7-AD.vs.control; scRNA-seq,SRP215507,CD8+ T cell_6-AD.vs.control; scRNA-seq,SRP215507,CD8+ T cell_7-AD.vs.MCI; scRNA-seq,SRP330776,T cell-AD.vs.control; scRNA-	14
Atg4b	bulk RNA-seq,SRP325058,AD.vs.control	1
ATG4C	bulk RNA-seq,Homo_723,AD.vs.control; bulk RNA-seq,Homo_723,AD.vs.MCI; bulk RNA-seq,Homo_633,AD.vs.control; bulk RNA-seq,Homo_633,AD.vs.MCI; bulk RNA-seq,Homo_714,AD.vs.control; bulk RNA-seq,Homo_714,AD.vs.MCI; scRNA-seq,SRP215507,CD8+ T cell_8-AD.vs.control; scRNA-seq,SRP215507,CD8+ T cell_7-AD.vs.MCI; scRNA-seq,SRP215507,CD8+ T cell_2-MCI.vs.control; scRNA-seq,SRP309935,Naive CD8+ T cell_2-AD.vs.control; scRNA-seq,SRP330776,γδ T cell-AD.vs.control; scRNA-seq,SRP330776,CD8+ T cell_2-AD.vs.control; scRNA-seq,SRP330776,B cell_1-AD.vs.control; scRNA-seq,SRP330776,Memory T cell_1-AD.vs.control; scRNA-seq,SRP330776,Natural killer T cell-AD.vs.control; scRNA-seq,SRP330776,Naive CD8+ T cell_3-AD.vs.control; scRNA-seq,SRP330776,B cell_2-AD.vs.control; scRNA- [illegible]	19

ATG4D	bulk RNA-seq,Homo_723,AD.vs.control; bulk RNA-seq,Homo_723,MCI.vs.control; bulk RNA-seq,Homo_633,AD.vs.control; bulk RNA-seq,Homo_633,MCI.vs.control; bulk RNA-seq,Homo_714,AD.vs.control; bulk RNA-seq,Homo_714,AD.vs.MCI; bulk RNA-seq,Homo_714,MCI.vs.control; scRNA-seq,SRP215507,CD8+ T cell_2-AD.vs.MCI; scRNA-seq,SRP215507,CD8+ T cell_5-AD.vs.MCI; scRNA-seq,SRP215507,CD8+ T cell_4-MCI.vs.control; scRNA-seq,SRP215507,CD8+ T cell_7-MCI.vs.control; scRNA-seq,SRP309935,Naive CD8+ T cell_1-AD.vs.control; scRNA-seq,SRP309935,Monocyte_2-AD.vs.control; scRNA-seq,SRP309935,Naive CD8+ T cell_2-AD.vs.control; scRNA-seq,SRP309935,CD8+ T cell_1-AD.vs.control; scRNA-seq,SRP330776,CD8+ T cell_1-AD.vs.control; scRNA-seq,SRP330776,γδ T cell-AD.vs.control; scRNA-seq,SRP330776,Memory T cell_2-AD.vs.control; scRNA-seq,SRP330776,CD8+ T cell_2-AD.vs.control; scRNA-seq,SRP330776,B cell_1-AD.vs.control; scRNA-seq,SRP330776,Naive CD8+ T cell_1-AD.vs.control; scRNA-seq,SRP330776,Natural killer cell_1-AD.vs.control; scRNA-seq,SRP330776,Memory T cell_1-AD.vs.control; scRNA-seq,SRP330776,Natural killer T cell-AD.vs.control; scRNA-seq,SRP330776,Naive CD8+ T cell_3-AD.vs.control; scRNA-seq,SRP330776,B cell_2-AD.vs.control; scRNA-seq,SRP330776,T cell-AD.vs.control; scRNA-seq,SRP330776,Naive CD8+ T cell_2-AD.vs.control; scRNA-seq,SRP330776,CD8+ T cell_3-AD.vs.control; scRNA-seq,SRP330776,Monocyte_1-AD.vs.control; scRNA-seq,SRP330776,Monocyte_2-AD.vs.control; scRNA-seq,SRP330776,B cell_3-AD.vs.control; scRNA-seq,SRP330776,Regulatory T cell-AD.vs.control;	35
ATG5	bulk RNA-seq,Homo_714,AD.vs.control; bulk RNA-seq,Homo_714,AD.vs.MCI; bulk RNA-seq,SRP310421,AD.vs.control; scRNA-seq,SRP215507,CD8+ T cell_7-AD.vs.control; scRNA-seq,SRP215507,CD8+ T cell_4-AD.vs.MCI; scRNA-seq,SRP215507,CD8+ T cell_4-MCI.vs.control	6
ATG7	bulk RNA-seq,Homo_723,AD.vs.control; bulk RNA-seq,Homo_723,AD.vs.MCI; bulk RNA-seq,Homo_633,AD.vs.control; bulk RNA-seq,Homo_633,AD.vs.MCI; bulk RNA-seq,Homo_714,AD.vs.control; bulk RNA-seq,Homo_714,AD.vs.MCI; scRNA-seq,SRP215507,CD8+ T cell_5-AD.vs.control; scRNA-seq,SRP215507,CD8+ T cell_3-AD.vs.MCI; scRNA-seq,SRP215507,CD8+ T cell_3-MCI.vs.control; scRNA-seq,SRP215507,CD8+ T cell_7-MCI.vs.control; scRNA-seq,SRP330776,γδ T cell-AD.vs.control; scRNA-seq,SRP330776,B cell_3-AD.vs.control	12
ATG9A	scRNA-seq,SRP215507,CD8+ T cell_3-AD.vs.MCI; scRNA-seq,SRP215507,CD8+ T cell_4-MCI.vs.control; scRNA-seq,SRP330776,Memory T cell_2-AD.vs.control; scRNA-seq,SRP330776,Natural killer cell_1-AD.vs.control; scRNA-seq,SRP330776,Naive CD8+ T cell_3-AD.vs.control; scRNA-seq,SRP330776,B cell_2-AD.vs.control; scRNA-seq,SRP330776,B cell_3-AD.vs.control; scRNA-seq,SRP330776,Natural killer cell_2-AD.vs.control	8
ATG9B	bulk RNA-seq,Homo_723,AD.vs.control; bulk RNA-seq,Homo_723,AD.vs.MCI; bulk RNA-seq,Homo_723,MCI.vs.control; bulk RNA-seq,Homo_633,AD.vs.control; bulk RNA-seq,Homo_633,AD.vs.MCI; bulk RNA-seq,Homo_633,MCI.vs.control; bulk RNA-seq,Homo_714,AD.vs.control; bulk RNA-seq,Homo_714,AD.vs.MCI; bulk RNA-seq,Homo_714,MCI.vs.control; scRNA-seq,SRP215507,CD8+ T cell_2-AD.vs.control; scRNA-seq,SRP215507,CD8+ T cell_2-AD.vs.MCI; scRNA-seq,SRP215507,CD8+ T cell_2-MCI.vs.control; scRNA-seq,SRP330776,γδ T cell-AD.vs.control; scRNA-seq,SRP330776,CD8+ T cell_2-AD.vs.control; scRNA-seq,SRP330776,Naive CD8+ T cell_1-AD.vs.control; scRNA-seq,SRP330776,Natural killer T cell-AD.vs.control; scRNA-seq,SRP330776,Naive CD8+ T cell_3-AD.vs.control; scRNA-seq,SRP330776,B cell_2-AD.vs.control; scRNA-[illegible]	20
ATIC	bulk RNA-seq,Homo_723,AD.vs.control; bulk RNA-seq,Homo_723,AD.vs.MCI; bulk RNA-seq,Homo_723,MCI.vs.control; bulk RNA-seq,Homo_633,AD.vs.control; bulk RNA-seq,Homo_633,AD.vs.MCI; bulk RNA-seq,Homo_633,MCI.vs.control; bulk RNA-seq,Homo_714,AD.vs.control; bulk RNA-seq,Homo_714,AD.vs.MCI; bulk RNA-seq,Homo_714,MCI.vs.control; scRNA-seq,SRP215507,CD8+ T cell_10-AD.vs.control; scRNA-seq,SRP215507,CD8+ T cell_4-AD.vs.MCI; scRNA-seq,SRP215507,CD8+ T cell_4-MCI.vs.control; scRNA-seq,SRP330776,γδ T cell-AD.vs.control; scRNA-seq,SRP330776,Memory T cell_2-AD.vs.control; scRNA-seq,SRP330776,CD8+ T cell_2-AD.vs.control; scRNA-seq,SRP330776,Naive CD8+ T cell_1-AD.vs.control; scRNA-seq,SRP330776,Natural killer cell_1-AD.vs.control; scRNA-seq,SRP330776,Memory T cell_1-AD.vs.control; scRNA-seq,SRP330776,Natural killer T cell-AD.vs.control; scRNA-seq,SRP330776,B cell_2-AD.vs.control; scRNA-seq,SRP330776,T cell-AD.vs.control; scRNA-seq,SRP330776,CD8+ T cell_3-AD.vs.control; scRNA-seq,SRP330776,Megakaryocyte_1-AD.vs.control; scRNA-seq,SRP330776,B cell_4-AD.vs.control	24
ATL1	bulk RNA-seq,Homo_723,AD.vs.control; bulk RNA-seq,Homo_723,AD.vs.MCI; bulk RNA-seq,Homo_633,AD.vs.control; bulk RNA-seq,Homo_633,AD.vs.MCI; bulk RNA-seq,Homo_714,AD.vs.control; bulk RNA-seq,Homo_714,AD.vs.MCI; scRNA-seq,SRP215507,CD8+ T cell_3-AD.vs.MCI; scRNA-seq,SRP215507,CD8+ T cell_3-MCI.vs.control; scRNA-seq,SRP309935,CD8+ T cell_1-AD.vs.control; scRNA-seq,SRP330776,Naive CD8+ T cell_3-AD.vs.control; scRNA-seq,SRP330776,B cell_2-AD.vs.control	11
ATL2	bulk RNA-seq,Homo_723,AD.vs.control; bulk RNA-seq,Homo_723,AD.vs.MCI; bulk RNA-seq,Homo_633,AD.vs.control; bulk RNA-seq,Homo_633,AD.vs.MCI; scRNA-seq,SRP215507,CD8+ T cell_2-AD.vs.MCI; scRNA-seq,SRP215507,CD8+ T cell_3-AD.vs.MCI; scRNA-seq,SRP215507,CD8+ T cell_5-AD.vs.MCI; scRNA-seq,SRP215507,CD8+ T cell_5-MCI.vs.control; scRNA-seq,SRP215507,CD8+ T cell_3-MCI.vs.control; scRNA-seq,SRP309935,Naive CD8+ T cell_2-AD.vs.control; scRNA-seq,SRP309935,B cell_1-AD.vs.control; scRNA-seq,SRP330776,Naive CD8+ T cell_1-AD.vs.control; scRNA-seq,SRP330776,Natural killer T cell-AD.vs.control; scRNA-seq,SRP330776,Monocyte_1-	15

ATL3	scRNA-seq,SRP309935,Natural killer cell_2-AD.vs.control; scRNA-seq,SRP330776,Natural killer T cell-AD.vs.control; scRNA-seq,SRP330776,Naive CD8+ T cell_2-AD.vs.control; scRNA-seq,SRP330776,B cell_3-AD.vs.control	4
ATM	bulk RNA-seq,Homo_714,AD.vs.control; bulk RNA-seq,Homo_714,AD.vs.MCI; bulk RNA-seq,SRP310421,AD.vs.control; scRNA-seq,SRP215507,CD8+ T cell_9-AD.vs.control; scRNA-seq,SRP330776,Monocyte_2-AD.vs.control	5
Atm	bulk RNA-seq,SRP325058,AD.vs.control	1
ATMIN	bulk RNA-seq,Homo_633,AD.vs.control; bulk RNA-seq,Homo_714,AD.vs.control; bulk RNA-seq,Homo_714,AD.vs.MCI; scRNA-seq,SRP215507,CD8+ T cell_8-MCI.vs.control; scRNA-seq,SRP309935,Natural killer cell_2-AD.vs.control; scRNA-seq,SRP330776,γδ T cell-AD.vs.control; scRNA-seq,SRP330776,Memory T cell_2-AD.vs.control; scRNA-seq,SRP330776,CD8+ T cell_2-AD.vs.control; scRNA-seq,SRP330776,Naive CD8+ T cell_1-AD.vs.control; scRNA-seq,SRP330776,Naive CD8+ T cell_3-AD.vs.control; scRNA-seq,SRP330776,B cell_2-AD.vs.control; scRNA-seq,SRP330776,CD8+ T cell_3-AD.vs.control; scRNA-seq,SRP330776,Regulatory T cell-AD.vs.control	13
ATN1	bulk RNA-seq,Homo_723,AD.vs.control; bulk RNA-seq,Homo_633,AD.vs.control; bulk RNA-seq,Homo_633,AD.vs.MCI; scRNA-seq,SRP215507,CD8+ T cell_5-AD.vs.MCI; scRNA-seq,SRP215507,CD8+ T cell_4-MCI.vs.control; scRNA-seq,SRP309935,CD8+ T cell_1-AD.vs.control; scRNA-seq,SRP330776,γδ T cell-AD.vs.control; scRNA-seq,SRP330776,CD8+ T cell_2-AD.vs.control; scRNA-seq,SRP330776,B cell_1-AD.vs.control; scRNA-seq,SRP330776,Naive CD8+ T cell_1-AD.vs.control; scRNA-seq,SRP330776,Natural killer cell_1-AD.vs.control; scRNA-seq,SRP330776,B cell_2-	12
ATOH1	bulk RNA-seq,Homo_723,AD.vs.control; bulk RNA-seq,Homo_723,AD.vs.MCI; bulk RNA-seq,Homo_723,MCI.vs.control; bulk RNA-seq,Homo_633,AD.vs.control; bulk RNA-seq,Homo_633,AD.vs.MCI; bulk RNA-seq,Homo_714,MCI.vs.control	6
ATOH8	bulk RNA-seq,Homo_723,AD.vs.control; bulk RNA-seq,Homo_723,AD.vs.MCI; bulk RNA-seq,Homo_633,AD.vs.control; bulk RNA-seq,Homo_633,AD.vs.MCI; bulk RNA-seq,Homo_714,AD.vs.control; bulk RNA-seq,Homo_714,AD.vs.MCI; bulk RNA-seq,SRP223445,AD.vs.control	7
ATOX1	bulk RNA-seq,Homo_723,AD.vs.control; bulk RNA-seq,Homo_723,AD.vs.MCI; bulk RNA-seq,Homo_633,AD.vs.control; bulk RNA-seq,Homo_633,AD.vs.MCI; scRNA-seq,SRP215507,CD8+ T cell_4-AD.vs.control; scRNA-seq,SRP330776,CD8+ T cell_1-AD.vs.control; scRNA-seq,SRP330776,γδ T cell-AD.vs.control; scRNA-seq,SRP330776,Memory T cell_2-AD.vs.control; scRNA-seq,SRP330776,CD8+ T cell_2-AD.vs.control; scRNA-seq,SRP330776,B cell_1-AD.vs.control; scRNA-seq,SRP330776,Natural killer cell_1-AD.vs.control; scRNA-seq,SRP330776,Memory T cell_1-AD.vs.control; scRNA-seq,SRP330776,Naive CD8+ T cell_3-AD.vs.control; scRNA-seq,SRP330776,T cell-AD.vs.control	14
ATP10A	scRNA-seq,SRP215507,CD8+ T cell_3-AD.vs.MCI; scRNA-seq,SRP215507,CD8+ T cell_1-AD.vs.MCI; scRNA-seq,SRP215507,CD8+ T cell_1-MCI.vs.control; scRNA-seq,SRP215507,CD8+ T cell_3-MCI.vs.control; scRNA-seq,SRP215507,CD8+ T cell_6-MCI.vs.control; scRNA-seq,SRP330776,γδ T cell-AD.vs.control; scRNA-seq,SRP330776,Memory T cell_2-AD.vs.control; scRNA-seq,SRP330776,Memory T cell_1-AD.vs.control; scRNA-	9
ATP10B	bulk RNA-seq,Homo_723,AD.vs.control; bulk RNA-seq,Homo_723,AD.vs.MCI; bulk RNA-seq,Homo_633,AD.vs.control; bulk RNA-	4
ATP10D	bulk RNA-seq,Homo_723,AD.vs.control; bulk RNA-seq,Homo_723,MCI.vs.control; bulk RNA-seq,Homo_633,AD.vs.control; bulk RNA-seq,Homo_633,MCI.vs.control; scRNA-seq,SRP330776,Natural killer cell_1-AD.vs.control; scRNA-seq,SRP330776,Memory T cell_1-AD.vs.control; scRNA-seq,SRP330776,Naive CD8+ T cell_3-AD.vs.control; scRNA-seq,SRP330776,Monocyte_2-AD.vs.control	8
ATP11A	bulk RNA-seq,Homo_723,AD.vs.control; bulk RNA-seq,Homo_723,AD.vs.MCI; bulk RNA-seq,Homo_633,AD.vs.control; bulk RNA-seq,Homo_633,AD.vs.MCI; bulk RNA-seq,SRP223445,AD.vs.control; scRNA-seq,SRP215507,CD8+ T cell_3-AD.vs.control; scRNA-seq,SRP215507,CD8+ T cell_4-MCI.vs.control; scRNA-seq,SRP309935,Monocyte_1-AD.vs.control; scRNA-seq,SRP309935,Natural killer cell_1-AD.vs.control; scRNA-seq,SRP309935,Monocyte_2-AD.vs.control; scRNA-seq,SRP309935,CD8+ T cell_2-AD.vs.control; scRNA-seq,SRP330776,γδ T cell-AD.vs.control; scRNA-seq,SRP330776,CD8+ T cell_2-AD.vs.control; scRNA-seq,SRP330776,Memory T cell_1-AD.vs.control; scRNA-	16

ATP11B	bulk RNA-seq,SRP223445,AD.vs.control; scRNA-seq,SRP215507,CD8+ T cell_4-AD.vs.MCI; scRNA-seq,SRP215507,CD8+ T cell_4-MCI.vs.control; scRNA-seq,SRP309935,Megakaryocyte_1-AD.vs.control; scRNA-seq,SRP309935,Erythroid cell-AD.vs.control; scRNA-seq,SRP309935,Naive CD8+ T cell_1-AD.vs.control; scRNA-seq,SRP309935,Monocyte_2-AD.vs.control; scRNA-seq,SRP309935,CD8+ T cell_1-AD.vs.control; scRNA-seq,SRP330776,CD8+ T cell_1-AD.vs.control; scRNA-seq,SRP330776,γδ T cell-AD.vs.control; scRNA-seq,SRP330776,Memory T cell_2-AD.vs.control; scRNA-seq,SRP330776,CD8+ T cell_2-AD.vs.control; scRNA-seq,SRP330776,B cell_1-AD.vs.control; scRNA-seq,SRP330776,Naive CD8+ T cell_1-AD.vs.control; scRNA-seq,SRP330776,Natural killer cell_1-AD.vs.control; scRNA-seq,SRP330776,Memory T cell_1-AD.vs.control; scRNA-seq,SRP330776,Natural killer T cell-AD.vs.control; scRNA-seq,SRP330776,Naive CD8+ T cell_3-AD.vs.control; scRNA-seq,SRP330776,B cell_2-AD.vs.control; scRNA-seq,SRP330776,T cell-AD.vs.control; scRNA-seq,SRP330776,Naive CD8+ T cell_2-AD.vs.control; scRNA-seq,SRP330776,CD8+ T cell_3-AD.vs.control; scRNA-seq,SRP330776,Monocyte_1-AD.vs.control; scRNA-seq,SRP330776,B cell_3-AD.vs.control; scRNA-seq,SRP330776,B [illegible]	25
ATP11C	bulk RNA-seq,Homo_723,AD.vs.control; bulk RNA-seq,Homo_723,AD.vs.MCI; bulk RNA-seq,Homo_714,AD.vs.control; bulk RNA-seq,Homo_714,AD.vs.MCI; scRNA-seq,SRP215507,CD8+ T cell_3-AD.vs.MCI; scRNA-seq,SRP330776,CD8+ T cell_1-AD.vs.control; scRNA-seq,SRP330776,γδ T cell-AD.vs.control; scRNA-seq,SRP330776,Naive CD8+ T cell_1-AD.vs.control; scRNA-seq,SRP330776,B cell_2-AD.vs.control	9
ATP13A1	scRNA-seq,SRP215507,CD8+ T cell_5-AD.vs.control; scRNA-seq,SRP215507,CD8+ T cell_7-AD.vs.MCI; scRNA-seq,SRP215507,CD8+ T cell_3-MCI.vs.control; scRNA-seq,SRP215507,CD8+ T cell_6-MCI.vs.control; scRNA-seq,SRP215507,CD8+ T cell_7-MCI.vs.control; scRNA-seq,SRP309935,Naive CD8+ T cell_2-AD.vs.control; scRNA-seq,SRP309935,CD8+ T cell_1-AD.vs.control; scRNA-seq,SRP309935,CD8+ T cell_2-AD.vs.control; scRNA-seq,SRP330776,CD8+ T cell_1-AD.vs.control; scRNA-seq,SRP330776,γδ T cell-AD.vs.control; scRNA-seq,SRP330776,Memory T cell_2-AD.vs.control; scRNA-seq,SRP330776,CD8+ T cell_2-AD.vs.control; scRNA-seq,SRP330776,B cell_1-AD.vs.control; scRNA-seq,SRP330776,Naive CD8+ T cell_1-AD.vs.control; scRNA-seq,SRP330776,Natural killer cell_1-AD.vs.control; scRNA-seq,SRP330776,Memory T cell_1-AD.vs.control; scRNA-seq,SRP330776,Natural killer T cell-AD.vs.control; scRNA-seq,SRP330776,Naive CD8+ T cell_3-AD.vs.control; scRNA-seq,SRP330776,B cell_2-AD.vs.control; scRNA-seq,SRP330776,T cell-AD.vs.control; scRNA-seq,SRP330776,Naive CD8+ T cell_2-AD.vs.control; scRNA-seq,SRP330776,CD8+ T cell_3-AD.vs.control; scRNA-seq,SRP330776,Monocyte_1-AD.vs.control; scRNA-seq,SRP330776,Monocyte_2-AD.vs.control; scRNA-seq,SRP330776,B cell_3-AD.vs.control; scRNA-seq,SRP330776,Regulatory T cell-AD.vs.control; scRNA-seq,SRP330776,Natural [illegible]	28
ATP13A2	bulk RNA-seq,Homo_723,AD.vs.MCI; bulk RNA-seq,Homo_723,MCI.vs.control; bulk RNA-seq,Homo_633,AD.vs.MCI; bulk RNA-seq,Homo_633,MCI.vs.control; bulk RNA-seq,Homo_714,AD.vs.control; bulk RNA-seq,Homo_714,AD.vs.MCI; bulk RNA-seq,Homo_714,MCI.vs.control; scRNA-seq,SRP215507,CD8+ T cell_6-AD.vs.control; scRNA-seq,SRP309935,Monocyte_1-AD.vs.control; scRNA-seq,SRP309935,Naive CD8+ T cell_1-AD.vs.control; scRNA-seq,SRP309935,Natural killer cell_1-AD.vs.control; scRNA-seq,SRP309935,Naive CD8+ T cell_2-AD.vs.control; scRNA-seq,SRP309935,B cell_2-AD.vs.control; scRNA-seq,SRP309935,Dendritic cell-AD.vs.control; scRNA-seq,SRP330776,CD8+ T cell_1-AD.vs.control; scRNA-seq,SRP330776,γδ T cell-AD.vs.control; scRNA-seq,SRP330776,Memory T cell_2-AD.vs.control; scRNA-seq,SRP330776,CD8+ T cell_2-AD.vs.control; scRNA-seq,SRP330776,B cell_1-AD.vs.control; scRNA-seq,SRP330776,Natural killer cell_1-AD.vs.control; scRNA-seq,SRP330776,Memory T cell_1-AD.vs.control; scRNA-seq,SRP330776,Natural killer T cell-AD.vs.control; scRNA-[illegible]	25
ATP13A3	bulk RNA-seq,Homo_633,AD.vs.control; bulk RNA-seq,Homo_633,MCI.vs.control; bulk RNA-seq,Homo_714,AD.vs.control; bulk RNA-seq,Homo_714,AD.vs.MCI; bulk RNA-seq,Homo_714,MCI.vs.control; scRNA-seq,SRP215507,CD8+ T cell_5-AD.vs.control; scRNA-seq,SRP215507,CD8+ T cell_2-AD.vs.MCI; scRNA-seq,SRP215507,CD8+ T cell_4-AD.vs.MCI; scRNA-seq,SRP215507,CD8+ T cell_5-AD.vs.MCI; scRNA-seq,SRP215507,CD8+ T cell_4-MCI.vs.control; scRNA-seq,SRP330776,CD8+ T cell_1-AD.vs.control; scRNA-seq,SRP330776,Natural killer	12
ATP13A4	bulk RNA-seq,Homo_723,AD.vs.control; bulk RNA-seq,Homo_723,MCI.vs.control; bulk RNA-seq,Homo_633,AD.vs.control; bulk RNA-seq,Homo_633,AD.vs.MCI; bulk RNA-seq,Homo_633,MCI.vs.control; bulk RNA-seq,ROSMAP,AD.vs.control; bulk RNA-seq,ROSMAP,MCI.vs.control; scRNA-seq,SRP215507,CD8+ T cell_2-AD.vs.MCI; scRNA-seq,SRP215507,CD8+ T cell_3-MCI.vs.control; scRNA-seq,SRP330776,CD8+ T cell_1-AD.vs.control; scRNA-seq,SRP330776,B cell_1-AD.vs.control; scRNA-seq,SRP330776,Naive CD8+ T cell_1-AD.vs.control; scRNA-	13
ATP13A5	bulk RNA-seq,Homo_723,AD.vs.control; bulk RNA-seq,Homo_723,AD.vs.MCI; bulk RNA-seq,Homo_633,AD.vs.control; bulk RNA-	4

ATP1A1	bulk RNA-seq,Homo_723,MCI.vs.control; bulk RNA-seq,Homo_633,MCI.vs.control; bulk RNA-seq,Homo_714,MCI.vs.control; scRNA-seq,SRP215507,CD8+ T cell_5-MCI.vs.control; scRNA-seq,SRP330776,CD8+ T cell_1-AD.vs.control; scRNA-seq,SRP330776,γδ T cell-AD.vs.control; scRNA-seq,SRP330776,Memory T cell_2-AD.vs.control; scRNA-seq,SRP330776,CD8+ T cell_2-AD.vs.control; scRNA-seq,SRP330776,B cell_1-AD.vs.control; scRNA-seq,SRP330776,Naive CD8+ T cell_1-AD.vs.control; scRNA-seq,SRP330776,Natural killer cell_1-AD.vs.control; scRNA-seq,SRP330776,Memory T cell_1-AD.vs.control; scRNA-seq,SRP330776,Natural killer T cell-AD.vs.control; scRNA-seq,SRP330776,Naive CD8+ T cell_3-AD.vs.control; scRNA-seq,SRP330776,B cell_2-AD.vs.control; scRNA-seq,SRP330776,T cell-AD.vs.control; scRNA-seq,SRP330776,Naive CD8+ T cell_2-AD.vs.control; scRNA-seq,SRP330776,CD8+ T cell_3-AD.vs.control; scRNA-seq,SRP330776,Monocyte_1-AD.vs.control; scRNA-seq,SRP330776,Monocyte_2-AD.vs.control; scRNA-seq,SRP330776,B cell_3-AD.vs.control; scRNA-seq,SRP330776,Natural killer cell_2-AD.vs.control	22
ATP1A1-AS1	scRNA-seq,SRP215507,CD8+ T cell_4-AD.vs.control; scRNA-seq,SRP330776,CD8+ T cell_1-AD.vs.control; scRNA-seq,SRP330776,CD8+ T cell_2-AD.vs.control; scRNA-seq,SRP330776,Naive CD8+ T cell_1-AD.vs.control; scRNA-seq,SRP330776,Natural killer cell_1-AD.vs.control; scRNA-seq,SRP330776,Naive CD8+ T cell_3-AD.vs.control; scRNA-seq,SRP330776,B cell_2-AD.vs.control; scRNA-seq,SRP330776,CD8+ T cell_3-	8
ATP1A2	bulk RNA-seq,Homo_723,AD.vs.control; bulk RNA-seq,Homo_723,AD.vs.MCI; bulk RNA-seq,Homo_723,MCI.vs.control; bulk RNA-seq,Homo_633,AD.vs.control; bulk RNA-seq,Homo_633,AD.vs.MCI; bulk RNA-seq,Homo_714,MCI.vs.control	6
ATP1A3	bulk RNA-seq,Homo_723,AD.vs.control; bulk RNA-seq,Homo_723,AD.vs.MCI; bulk RNA-seq,Homo_633,MCI.vs.control; bulk RNA-seq,Homo_714,AD.vs.MCI; bulk RNA-seq,Homo_714,MCI.vs.control; bulk RNA-seq,ROSMAP,AD.vs.control; scRNA-seq,SRP215507,CD8+ T cell_2-AD.vs.MCI; scRNA-seq,SRP215507,CD8+ T cell_1-AD.vs.MCI; scRNA-seq,SRP215507,CD8+ T cell_1-MCI.vs.control; scRNA-seq,SRP215507,CD8+ T cell_2-MCI.vs.control; scRNA-seq,SRP215507,CD8+ T cell_3-MCI.vs.control; scRNA-seq,SRP330776,B cell_1-AD.vs.control; scRNA-	14
ATP1A4	bulk RNA-seq,Homo_723,AD.vs.MCI	1
ATP1B1	bulk RNA-seq,Homo_723,AD.vs.control; bulk RNA-seq,Homo_723,AD.vs.MCI; bulk RNA-seq,Homo_633,AD.vs.control; bulk RNA-seq,Homo_633,AD.vs.MCI; bulk RNA-seq,Homo_714,AD.vs.control; bulk RNA-seq,Homo_714,AD.vs.MCI; scRNA-seq,SRP215507,CD8+ T cell_2-AD.vs.control; scRNA-seq,SRP215507,CD8+ T cell_3-AD.vs.control; scRNA-seq,SRP215507,CD8+ T cell_1-AD.vs.control; scRNA-seq,SRP215507,CD8+ T cell_5-AD.vs.control; scRNA-seq,SRP215507,CD8+ T cell_1-AD.vs.MCI; scRNA-seq,SRP215507,CD8+ T cell_1-MCI.vs.control; scRNA-seq,SRP215507,CD8+ T cell_5-MCI.vs.control; scRNA-seq,SRP215507,CD8+ T cell_2-MCI.vs.control; scRNA-seq,SRP215507,CD8+ T cell_3-MCI.vs.control; scRNA-seq,SRP330776,Memory T cell_1-AD.vs.control; scRNA-seq,SRP330776,CD8+ T cell_3-	18
ATP1B2	bulk RNA-seq,Homo_714,MCI.vs.control; bulk RNA-seq,ROSMAP,AD.vs.control; scRNA-seq,SRP215507,CD8+ T cell_3-AD.vs.control; scRNA-seq,SRP215507,CD8+ T cell_5-AD.vs.control; scRNA-seq,SRP330776,CD8+ T cell_1-AD.vs.control; scRNA-seq,SRP330776,γδ T cell-AD.vs.control; scRNA-seq,SRP330776,Naive CD8+ T cell_1-AD.vs.control	7
ATP1B3	bulk RNA-seq,Homo_723,AD.vs.MCI; bulk RNA-seq,Homo_633,AD.vs.control; bulk RNA-seq,Homo_633,AD.vs.MCI; bulk RNA-seq,Homo_714,AD.vs.control; bulk RNA-seq,Homo_714,AD.vs.MCI; scRNA-seq,SRP215507,CD8+ T cell_3-AD.vs.control; scRNA-seq,SRP215507,CD8+ T cell_4-AD.vs.control; scRNA-seq,SRP215507,CD8+ T cell_7-AD.vs.control; scRNA-seq,SRP215507,CD8+ T cell_3-AD.vs.MCI; scRNA-seq,SRP215507,CD8+ T cell_5-AD.vs.MCI; scRNA-seq,SRP215507,CD8+ T cell_5-MCI.vs.control; scRNA-seq,SRP215507,CD8+ T cell_4-MCI.vs.control; scRNA-seq,SRP309935,Erythroid cell-AD.vs.control; scRNA-seq,SRP330776,CD8+ T cell_1-AD.vs.control; scRNA-seq,SRP330776,B cell_1-AD.vs.control; scRNA-seq,SRP330776,Naive CD8+ T cell_1-AD.vs.control; scRNA-seq,SRP330776,Naive CD8+ T cell_3-AD.vs.control; scRNA-	19
ATP1B4	bulk RNA-seq,Homo_723,AD.vs.control; bulk RNA-seq,Homo_633,AD.vs.control	2
ATP23	scRNA-seq,SRP215507,CD8+ T cell_5-AD.vs.control; scRNA-seq,SRP215507,CD8+ T cell_3-MCI.vs.control; scRNA-seq,SRP330776,CD8+ T cell_1-AD.vs.control; scRNA-seq,SRP330776,γδ T cell-AD.vs.control; scRNA-seq,SRP330776,Memory T cell_2-AD.vs.control; scRNA-seq,SRP330776,CD8+ T cell_2-AD.vs.control; scRNA-seq,SRP330776,B cell_1-AD.vs.control; scRNA-seq,SRP330776,Naive CD8+ T cell_1-AD.vs.control; scRNA-seq,SRP330776,Natural killer cell_1-AD.vs.control; scRNA-seq,SRP330776,Memory T cell_1-AD.vs.control; scRNA-seq,SRP330776,Natural killer T cell-AD.vs.control; scRNA-seq,SRP330776,Naive CD8+ T cell_3-AD.vs.control; scRNA-seq,SRP330776,B cell_2-AD.vs.control; scRNA-seq,SRP330776,CD8+ T cell_3-AD.vs.control; scRNA-seq,SRP330776,B cell_3-AD.vs.control; scRNA-seq,SRP330776,Natural killer cell_2-AD.vs.control	16
ATP2A1	scRNA-seq,SRP215507,CD8+ T cell_1-MCI.vs.control; scRNA-seq,SRP215507,CD8+ T cell_2-MCI.vs.control	2
ATP2A1-AS1	scRNA-seq,SRP215507,CD8+ T cell_3-AD.vs.MCI; scRNA-seq,SRP215507,CD8+ T cell_6-MCI.vs.control; scRNA-seq,SRP330776,Memory T cell_2-AD.vs.control; scRNA-seq,SRP330776,Naive CD8+ T cell_1-AD.vs.control; scRNA-seq,SRP330776,Memory T cell_1-AD.vs.control; scRNA-	6

ATP2A2	bulk RNA-seq,Homo_723,AD.vs.MCI; bulk RNA-seq,Homo_633,AD.vs.control; bulk RNA-seq,Homo_633,AD.vs.MCI; bulk RNA-seq,Homo_714,AD.vs.control; bulk RNA-seq,Homo_714,AD.vs.MCI; bulk RNA-seq,Homo_714,MCI.vs.control; bulk RNA-seq,SRP310421,AD.vs.control; scRNA-seq,SRP215507,CD8+ T cell_2-AD.vs.MCI; scRNA-seq,SRP215507,CD8+ T cell_2-MCI.vs.control; scRNA-seq,SRP309935,CD8+ T cell_2-AD.vs.control; scRNA-seq,SRP330776,CD8+ T cell_2-AD.vs.control; scRNA-seq,SRP330776,Naive CD8+ T cell_1- [illegible]	14
ATP2A3	scRNA-seq,SRP309935,Naive CD8+ T cell_1-AD.vs.control; scRNA-seq,SRP309935,B cell_1-AD.vs.control; scRNA-seq,SRP330776,CD8+ T cell_1-AD.vs.control; scRNA-seq,SRP330776,γδ T cell-AD.vs.control; scRNA-seq,SRP330776,Memory T cell_2-AD.vs.control; scRNA-seq,SRP330776,CD8+ T cell_2-AD.vs.control; scRNA-seq,SRP330776,B cell_1-AD.vs.control; scRNA-seq,SRP330776,Naive CD8+ T cell_1-AD.vs.control; scRNA-seq,SRP330776,Natural killer cell_1-AD.vs.control; scRNA-seq,SRP330776,Memory T cell_1-AD.vs.control; scRNA-seq,SRP330776,Natural killer T cell-AD.vs.control; scRNA-seq,SRP330776,Naive CD8+ T cell_3-AD.vs.control; scRNA-seq,SRP330776,B cell_2-AD.vs.control; scRNA-seq,SRP330776,T cell-AD.vs.control; scRNA-seq,SRP330776,Naive CD8+ T cell_2-AD.vs.control; scRNA-seq,SRP330776,CD8+ T cell_3-AD.vs.control; scRNA-seq,SRP330776,Monocyte_1-AD.vs.control; scRNA-seq,SRP330776,B cell_3-AD.vs.control; scRNA-seq,SRP330776,Regulatory T cell-AD.vs.control; [illegible]	21
ATP2B1	bulk RNA-seq,Homo_723,AD.vs.control; bulk RNA-seq,Homo_633,AD.vs.control; bulk RNA-seq,Homo_633,AD.vs.MCI; bulk RNA-seq,Homo_714,AD.vs.control; bulk RNA-seq,Homo_714,AD.vs.MCI; scRNA-seq,SRP215507,CD8+ T cell_3-AD.vs.MCI; scRNA-seq,SRP215507,CD8+ T cell_5-AD.vs.MCI; scRNA-seq,SRP215507,CD8+ T cell_5-MCI.vs.control; scRNA-seq,SRP215507,CD8+ T cell_4-MCI.vs.control; scRNA-seq,SRP215507,CD8+ T cell_3-MCI.vs.control; scRNA-seq,SRP309935,Natural killer cell_2-AD.vs.control	11
ATP2B1-AS1	scRNA-seq,SRP215507,CD8+ T cell_7-AD.vs.control; scRNA-seq,SRP215507,CD8+ T cell_1-AD.vs.MCI; scRNA-seq,SRP215507,CD8+ T cell_7-AD.vs.MCI; scRNA-seq,SRP215507,CD8+ T cell_5-AD.vs.MCI; scRNA-seq,SRP215507,CD8+ T cell_1-MCI.vs.control; scRNA-seq,SRP215507,CD8+ T cell_5-MCI.vs.control; scRNA-seq,SRP215507,CD8+ T cell_6-MCI.vs.control; scRNA-seq,SRP309935,B cell_2-AD.vs.control; scRNA-seq,SRP330776,CD8+ T cell_1-AD.vs.control; scRNA-seq,SRP330776,γδ T cell-AD.vs.control; scRNA-seq,SRP330776,CD8+ T cell_2-AD.vs.control; scRNA-seq,SRP330776,Naive CD8+ T cell_1-AD.vs.control; scRNA-seq,SRP330776,Natural killer cell_1-AD.vs.control; scRNA-seq,SRP330776,Memory T cell_1-AD.vs.control; scRNA-seq,SRP330776,Natural killer T cell-AD.vs.control; scRNA-seq,SRP330776,Naive CD8+ T cell_3-AD.vs.control; scRNA- [illegible]	17
ATP2B3	bulk RNA-seq,Homo_723,AD.vs.control; bulk RNA-seq,Homo_723,AD.vs.MCI; bulk RNA-seq,Homo_633,AD.vs.control; bulk RNA-	4
ATP2B4	bulk RNA-seq,Homo_633,MCI.vs.control; bulk RNA-seq,Homo_714,MCI.vs.control; scRNA-seq,SRP215507,CD8+ T cell_3-MCI.vs.control; scRNA-seq,SRP309935,B cell_1-AD.vs.control; scRNA-seq,SRP330776,Naive CD8+ T cell_3-AD.vs.control; scRNA-seq,SRP330776,Monocyte_1-AD.vs.control; scRNA-seq,SRP330776,B cell_3-AD.vs.control	7
ATP2C1	bulk RNA-seq,Homo_723,AD.vs.control; bulk RNA-seq,Homo_723,AD.vs.MCI; bulk RNA-seq,Homo_723,MCI.vs.control; bulk RNA-seq,Homo_633,AD.vs.control; bulk RNA-seq,Homo_633,AD.vs.MCI; bulk RNA-seq,Homo_633,MCI.vs.control; bulk RNA-seq,Homo_714,AD.vs.control; bulk RNA-seq,Homo_714,AD.vs.MCI; bulk RNA-seq,Homo_714,MCI.vs.control; scRNA-seq,SRP215507,CD8+ T cell_1-MCI.vs.control; scRNA-seq,SRP215507,CD8+ T cell_6-MCI.vs.control; scRNA-seq,SRP309935,Naive CD8+ T cell_2-AD.vs.control; scRNA-seq,SRP330776,CD8+ T cell_1-AD.vs.control; scRNA-seq,SRP330776,γδ T cell-AD.vs.control; scRNA-seq,SRP330776,Natural killer cell_1-AD.vs.control; scRNA-seq,SRP330776,B cell_2-AD.vs.control; scRNA-seq,SRP330776,CD8+ T cell_3-AD.vs.control; scRNA-seq,SRP330776,B cell_3-AD.vs.control	18
ATP2C2-AS1	scRNA-seq,SRP215507,CD8+ T cell_1-MCI.vs.control	1
ATP4B	bulk RNA-seq,ROSMAP,MCI.vs.control	1
ATP5F1A	scRNA-seq,SRP215507,CD8+ T cell_7-AD.vs.control; scRNA-seq,SRP215507,CD8+ T cell_6-AD.vs.MCI; scRNA-seq,SRP215507,CD8+ T cell_7-MCI.vs.control; scRNA-seq,SRP330776,B cell_2-AD.vs.control; scRNA-seq,SRP330776,Regulatory T cell-AD.vs.control	5
ATP5F1B	scRNA-seq,SRP215507,CD8+ T cell_8-AD.vs.control; scRNA-seq,SRP215507,CD8+ T cell_7-AD.vs.control; scRNA-seq,SRP215507,CD8+ T cell_10-AD.vs.control; scRNA-seq,SRP215507,CD8+ T cell_9-AD.vs.control; scRNA-seq,SRP215507,CD8+ T cell_6-AD.vs.MCI; scRNA-seq,SRP215507,CD8+ [illegible]	6
ATP5F1C	bulk RNA-seq,Homo_714,AD.vs.control; bulk RNA-seq,Homo_714,AD.vs.MCI; scRNA-seq,SRP215507,CD8+ T cell_4-AD.vs.MCI; scRNA-seq,SRP330776,B cell_4-AD.vs.control	4

ATP5F1D	bulk RNA-seq,Homo_633,AD.vs.MCI; scRNA-seq,SRP215507,CD8+ T cell_8-AD.vs.control; scRNA-seq,SRP215507,CD8+ T cell_7-AD.vs.control; scRNA-seq,SRP215507,CD8+ T cell_7-MCI.vs.control; scRNA-seq,SRP215507,CD8+ T cell_8-MCI.vs.control; scRNA-seq,SRP309935,Natural killer cell_2-AD.vs.control; scRNA-seq,SRP309935,Monocyte_1-AD.vs.control; scRNA-seq,SRP309935,Naive CD8+ T cell_1-AD.vs.control; scRNA-seq,SRP309935,Natural killer cell_1-AD.vs.control; scRNA-seq,SRP309935,Naive CD8+ T cell_2-AD.vs.control; scRNA-seq,SRP309935,CD8+ T cell_1-AD.vs.control; scRNA-seq,SRP309935,B cell_1-AD.vs.control; scRNA-seq,SRP309935,CD8+ T cell_2-AD.vs.control; scRNA-seq,SRP309935,Monocyte_3-AD.vs.control; scRNA-seq,SRP309935,B cell_2-AD.vs.control; scRNA-seq,SRP309935,CD4+ T cell-AD.vs.control; scRNA-seq,SRP330776,CD8+ T cell_1-AD.vs.control; scRNA-seq,SRP330776,γδ T cell-AD.vs.control; scRNA-seq,SRP330776,Memory T cell_2-AD.vs.control; scRNA-seq,SRP330776,CD8+ T cell_2-AD.vs.control; scRNA-seq,SRP330776,B cell_1-AD.vs.control; scRNA-seq,SRP330776,Naive CD8+ T cell_1-AD.vs.control; scRNA-seq,SRP330776,Natural killer cell_1-AD.vs.control; scRNA-seq,SRP330776,Memory T cell_1-AD.vs.control; scRNA-seq,SRP330776,Natural killer T cell-AD.vs.control; scRNA-seq,SRP330776,Naive CD8+ T cell_3-AD.vs.control; scRNA-seq,SRP330776,B cell_2-AD.vs.control; scRNA-seq,SRP330776,T cell-AD.vs.control; scRNA-seq,SRP330776,Naive CD8+ T cell_2-AD.vs.control; scRNA-seq,SRP330776,CD8+ T cell_3-AD.vs.control; scRNA-seq,SRP330776,Monocyte_1-AD.vs.control; scRNA-seq,SRP330776,Monocyte_2-AD.vs.control; scRNA-seq,SRP330776,B cell_3-AD.vs.control; scRNA-seq,SRP330776,Regulatory T cell-AD.vs.control; scRNA-seq,SRP330776,Natural killer cell_2	36
ATP5F1E	bulk RNA-seq,Homo_723,AD.vs.control; bulk RNA-seq,Homo_723,AD.vs.MCI; bulk RNA-seq,Homo_633,MCI.vs.control; scRNA-seq,SRP215507,CD8+ T cell_4-AD.vs.control; scRNA-seq,SRP215507,CD8+ T cell_10-AD.vs.control; scRNA-seq,SRP330776,γδ T cell-AD.vs.control; scRNA-	7
ATP5IF1	scRNA-seq,SRP215507,CD8+ T cell_9-AD.vs.control; scRNA-seq,SRP215507,CD8+ T cell_7-MCI.vs.control; scRNA-seq,SRP309935,Erythroid cell-AD.vs.control; scRNA-seq,SRP330776,CD8+ T cell_3-AD.vs.control; scRNA-seq,SRP330776,Monocyte_2-AD.vs.control; scRNA-seq,SRP330776,Natural	6
Atp5j2	bulk RNA-seq,SRP325058,AD.vs.control	1
Atp5k	bulk RNA-seq,SRP325058,AD.vs.control	1
Atp5l	bulk RNA-seq,SRP325058,AD.vs.control	1
ATP5MC1	bulk RNA-seq,Homo_723,AD.vs.control; bulk RNA-seq,Homo_723,AD.vs.MCI; bulk RNA-seq,Homo_633,AD.vs.control; bulk RNA-seq,Homo_633,AD.vs.MCI; bulk RNA-seq,Homo_633,MCI.vs.control; scRNA-seq,SRP215507,CD8+ T cell_4-AD.vs.MCI; scRNA-seq,SRP215507,CD8+ T cell_4-MCI.vs.control; scRNA-seq,SRP330776,CD8+ T cell_1-AD.vs.control; scRNA-seq,SRP330776,γδ T cell-AD.vs.control; scRNA-seq,SRP330776,Memory T cell_2-AD.vs.control; scRNA-seq,SRP330776,CD8+ T cell_2-AD.vs.control; scRNA-seq,SRP330776,Naive CD8+ T cell_1-AD.vs.control; scRNA-seq,SRP330776,Natural killer cell_1-AD.vs.control; scRNA-seq,SRP330776,Memory T cell_1-AD.vs.control; scRNA-seq,SRP330776,B cell_2-AD.vs.control; scRNA-seq,SRP330776,T cell-AD.vs.control; scRNA-seq,SRP330776,CD8+ T cell_3-AD.vs.control; scRNA- [illegible]	18
ATP5MC2	bulk RNA-seq,Homo_633,AD.vs.control; bulk RNA-seq,Homo_633,AD.vs.MCI; scRNA-seq,SRP215507,CD8+ T cell_7-AD.vs.control; scRNA-seq,SRP215507,CD8+ T cell_4-MCI.vs.control; scRNA-seq,SRP309935,Erythroid cell-AD.vs.control; scRNA-seq,SRP309935,Megakaryocyte_2-	6
ATP5MC3	bulk RNA-seq,Homo_723,AD.vs.control; bulk RNA-seq,Homo_723,AD.vs.MCI; bulk RNA-seq,Homo_633,AD.vs.control; bulk RNA-seq,Homo_633,AD.vs.MCI; scRNA-seq,SRP215507,CD8+ T cell_7-AD.vs.control; scRNA-seq,SRP215507,CD8+ T cell_4-MCI.vs.control; scRNA-seq,SRP309935,Erythroid cell-AD.vs.control; scRNA-seq,SRP330776,B cell_2-AD.vs.control; scRNA-seq,SRP330776,Naive CD8+ T cell_2-	10
ATP5MD	scRNA-seq,SRP309935,Erythroid cell-AD.vs.control; scRNA-seq,SRP330776,CD8+ T cell_1-AD.vs.control; scRNA-seq,SRP330776,γδ T cell-AD.vs.control; scRNA-seq,SRP330776,Memory T cell_2-AD.vs.control; scRNA-seq,SRP330776,CD8+ T cell_2-AD.vs.control; scRNA-seq,SRP330776,B cell_1-AD.vs.control; scRNA-seq,SRP330776,Naive CD8+ T cell_1-AD.vs.control; scRNA-seq,SRP330776,Natural killer cell_1-AD.vs.control; scRNA-seq,SRP330776,Memory T cell_1-AD.vs.control; scRNA-seq,SRP330776,Naive CD8+ T cell_3-AD.vs.control; scRNA-seq,SRP330776,B cell_2-AD.vs.control; scRNA-seq,SRP330776,T cell-AD.vs.control; scRNA-seq,SRP330776,CD8+ T cell_3-AD.vs.control	13
ATP5ME	bulk RNA-seq,Homo_714,AD.vs.control; bulk RNA-seq,Homo_714,AD.vs.MCI; scRNA-seq,SRP215507,CD8+ T cell_7-AD.vs.control; scRNA-seq,SRP309935,Erythroid cell-AD.vs.control; scRNA-seq,SRP309935,Megakaryocyte_2-AD.vs.control; scRNA-seq,SRP330776,CD8+ T cell_1-AD.vs.control; scRNA-seq,SRP330776,γδ T cell-AD.vs.control; scRNA-seq,SRP330776,Memory T cell_2-AD.vs.control; scRNA-seq,SRP330776,CD8+ T cell_2-AD.vs.control; scRNA-seq,SRP330776,B cell_1-AD.vs.control; scRNA-seq,SRP330776,Naive CD8+ T cell_1-AD.vs.control; scRNA-seq,SRP330776,Natural killer cell_1-AD.vs.control; scRNA-seq,SRP330776,Memory T cell_1-AD.vs.control; scRNA-seq,SRP330776,Natural killer T cell-AD.vs.control; scRNA-seq,SRP330776,Naive CD8+ T cell_3-AD.vs.control; scRNA-seq,SRP330776,B cell_2-AD.vs.control; scRNA-seq,SRP330776,T cell-AD.vs.control; scRNA-seq,SRP330776,CD8+ T cell_3-AD.vs.control; scRNA-seq,SRP330776,Monocyte_2-AD.vs.control; scRNA-seq,SRP330776,Natural killer cell_2-AD.vs.control	20

ATP5MF	scRNA-seq,SRP215507,CD8+ T cell_4-AD.vs.control; scRNA-seq,SRP309935,Erythroid cell-AD.vs.control; scRNA-seq,SRP330776,CD8+ T cell_1-AD.vs.control; scRNA-seq,SRP330776,γδ T cell-AD.vs.control; scRNA-seq,SRP330776,Memory T cell_2-AD.vs.control; scRNA-seq,SRP330776,CD8+ T cell_2-AD.vs.control; scRNA-seq,SRP330776,B cell_1-AD.vs.control; scRNA-seq,SRP330776,Naive CD8+ T cell_1-AD.vs.control; scRNA-seq,SRP330776,Natural killer cell_1-AD.vs.control; scRNA-seq,SRP330776,Memory T cell_1-AD.vs.control; scRNA-seq,SRP330776,Natural killer T cell-AD.vs.control; scRNA-seq,SRP330776,Naive CD8+ T cell_3-AD.vs.control; scRNA-seq,SRP330776,B cell_2-AD.vs.control; scRNA-seq,SRP330776,T cell-AD.vs.control; scRNA-seq,SRP330776,CD8+ T cell_3-AD.vs.control; scRNA-seq,SRP330776,Monocyte_2-AD.vs.control; scRNA-seq,SRP330776,B [illegible]	18
ATP5MF-PTCD1	bulk RNA-seq,Homo_714,AD.vs.control; bulk RNA-seq,Homo_714,AD.vs.MCI	2
ATP5MG	scRNA-seq,SRP309935,Erythroid cell-AD.vs.control	1
ATP5MGL	scRNA-seq,SRP309935,Monocyte_1-AD.vs.control; scRNA-seq,SRP330776,Memory T cell_1-AD.vs.control	2
ATP5MK	bulk RNA-seq,Homo_723,AD.vs.control; bulk RNA-seq,Homo_723,AD.vs.MCI; bulk RNA-seq,Homo_723,MCI.vs.control; bulk RNA-seq,Homo_633,AD.vs.control; bulk RNA-seq,Homo_633,AD.vs.MCI; bulk RNA-seq,Homo_633,MCI.vs.control; bulk RNA-seq,Homo_714,AD.vs.control; bulk RNA-seq,Homo_714,AD.vs.MCI; bulk RNA-seq,Homo_714,MCI.vs.control	9
Atp5mpl	bulk RNA-seq,SRP325058,AD.vs.control	1
ATP5MPL	scRNA-seq,SRP215507,CD8+ T cell_4-AD.vs.control; scRNA-seq,SRP215507,CD8+ T cell_7-AD.vs.control; scRNA-seq,SRP215507,CD8+ T cell_7-AD.vs.MCI; scRNA-seq,SRP309935,Erythroid cell-AD.vs.control; scRNA-seq,SRP330776,CD8+ T cell_1-AD.vs.control; scRNA-seq,SRP330776,γδ T cell-AD.vs.control; scRNA-seq,SRP330776,Memory T cell_2-AD.vs.control; scRNA-seq,SRP330776,CD8+ T cell_2-AD.vs.control; scRNA-seq,SRP330776,B cell_1-AD.vs.control; scRNA-seq,SRP330776,Naive CD8+ T cell_1-AD.vs.control; scRNA-seq,SRP330776,Natural killer cell_1-AD.vs.control; scRNA-seq,SRP330776,Memory T cell_1-AD.vs.control; scRNA-seq,SRP330776,Naive CD8+ T cell_3-AD.vs.control; scRNA-seq,SRP330776,B cell_2-AD.vs.control; scRNA-seq,SRP330776,T cell-AD.vs.control; scRNA-seq,SRP330776,CD8+ T cell_3-AD.vs.control; scRNA- [illegible]	17
ATP5PB	bulk RNA-seq,Homo_723,AD.vs.control; bulk RNA-seq,Homo_723,AD.vs.MCI; bulk RNA-seq,Homo_633,AD.vs.control; bulk RNA-seq,Homo_633,AD.vs.MCI; bulk RNA-seq,Homo_714,AD.vs.control; bulk RNA-seq,Homo_714,AD.vs.MCI; scRNA-seq,SRP215507,CD8+ T cell_5-AD.vs.MCI; scRNA-seq,SRP309935,Erythroid cell-AD.vs.control; scRNA-seq,SRP330776,B cell_4-AD.vs.control	9
ATP5PD	scRNA-seq,SRP215507,CD8+ T cell_7-AD.vs.control; scRNA-seq,SRP309935,Erythroid cell-AD.vs.control; scRNA-seq,SRP330776,CD8+ T cell_2-AD.vs.control; scRNA-seq,SRP330776,Natural killer cell_1-AD.vs.control; scRNA-seq,SRP330776,Memory T cell_1-AD.vs.control; scRNA-seq,SRP330776,B cell_2-AD.vs.control; scRNA-seq,SRP330776,CD8+ T cell_3-AD.vs.control; scRNA-seq,SRP330776,Natural killer cell_2-AD.vs.control	8
ATP5PF	scRNA-seq,SRP309935,Erythroid cell-AD.vs.control; scRNA-seq,SRP309935,Megakaryocyte_2-AD.vs.control; scRNA-seq,SRP330776,γδ T cell-AD.vs.control; scRNA-seq,SRP330776,B cell_1-AD.vs.control; scRNA-seq,SRP330776,Naive CD8+ T cell_1-AD.vs.control; scRNA-seq,SRP330776,Natural killer cell_1-AD.vs.control; scRNA-seq,SRP330776,Natural killer T cell-AD.vs.control; scRNA-seq,SRP330776,Naive CD8+ T cell_3-AD.vs.control; scRNA-seq,SRP330776,B cell_2-AD.vs.control; scRNA-seq,SRP330776,CD8+ T cell_3-AD.vs.control; scRNA- [illegible]	12
ATP5PO	bulk RNA-seq,Homo_723,AD.vs.control; bulk RNA-seq,Homo_633,AD.vs.control; bulk RNA-seq,Homo_633,AD.vs.MCI; bulk RNA-seq,Homo_714,AD.vs.control; bulk RNA-seq,Homo_714,AD.vs.MCI; scRNA-seq,SRP215507,CD8+ T cell_7-AD.vs.control; scRNA-seq,SRP215507,CD8+ T cell_4-MCI.vs.control; scRNA-seq,SRP309935,Erythroid cell-AD.vs.control	8
ATP6	bulk RNA-seq,Homo_714,AD.vs.control; bulk RNA-seq,Homo_714,AD.vs.MCI	2
ATP6AP1	bulk RNA-seq,Homo_723,AD.vs.control; bulk RNA-seq,Homo_633,AD.vs.control; bulk RNA-seq,SRP223445,AD.vs.control; scRNA-seq,SRP215507,CD8+ T cell_5-AD.vs.MCI; scRNA-seq,SRP215507,CD8+ T cell_5-MCI.vs.control; scRNA-seq,SRP330776,Natural killer cell_1-AD.vs.control; scRNA-seq,SRP330776,Megakaryocyte_1-AD.vs.control	7
Atp6ap1	bulk RNA-seq,SRP325058,AD.vs.control	1
ATP6AP1L	scRNA-seq,SRP215507,CD8+ T cell_1-MCI.vs.control; scRNA-seq,SRP215507,CD8+ T cell_4-MCI.vs.control	2
ATP6AP2	bulk RNA-seq,Homo_633,AD.vs.MCI; bulk RNA-seq,Homo_714,AD.vs.control; bulk RNA-seq,Homo_714,AD.vs.MCI; scRNA-seq,SRP215507,CD8+ T cell_8-AD.vs.control; scRNA-seq,SRP215507,CD8+ T cell_7-AD.vs.control; scRNA-seq,SRP215507,CD8+ T cell_7-AD.vs.MCI; scRNA-seq,SRP215507,CD8+ T cell_5-AD.vs.MCI	7
ATP6V0A1	scRNA-seq,SRP215507,CD8+ T cell_1-MCI.vs.control; scRNA-seq,SRP330776,CD8+ T cell_1-AD.vs.control; scRNA-seq,SRP330776,Memory T cell_2-	3

ATP6V0A2	bulk RNA-seq,Homo_723,AD.vs.control; bulk RNA-seq,Homo_633,AD.vs.control; bulk RNA-seq,Homo_633,AD.vs.MCI; bulk RNA-seq,Homo_714,AD.vs.control; bulk RNA-seq,Homo_714,AD.vs.MCI; scRNA-seq,SRP215507,CD8+ T cell_3-AD.vs.MCI; scRNA-seq,SRP215507,CD8+ T cell_4-AD.vs.MCI; scRNA-seq,SRP215507,CD8+ T cell_5-AD.vs.MCI; scRNA-seq,SRP215507,CD8+ T cell_5-MCI.vs.control; scRNA-seq,SRP215507,CD8+ T cell_3-MCI.vs.control; scRNA-seq,SRP309935,Natural killer cell_1-AD.vs.control; scRNA-seq,SRP309935,CD8+ T cell_1-AD.vs.control; scRNA-seq,SRP309935,CD8+ T cell_2-AD.vs.control; scRNA-seq,SRP330776,CD8+ T cell_1-AD.vs.control; scRNA-seq,SRP330776,γδ T cell-AD.vs.control; scRNA-seq,SRP330776,Memory T cell_2-AD.vs.control; scRNA-seq,SRP330776,CD8+ T cell_2-AD.vs.control; scRNA-seq,SRP330776,B cell_1-AD.vs.control; scRNA-seq,SRP330776,Naive CD8+ T cell_1-AD.vs.control; scRNA-seq,SRP330776,Natural killer cell_1-AD.vs.control; scRNA-seq,SRP330776,Memory T cell_1-AD.vs.control; scRNA-seq,SRP330776,Natural killer T cell-AD.vs.control; scRNA-seq,SRP330776,Naive CD8+ T cell_3-AD.vs.control; scRNA-seq,SRP330776,B cell_2-AD.vs.control; scRNA-seq,SRP330776,T cell-AD.vs.control; scRNA-seq,SRP330776,Naive CD8+ T cell_2-AD.vs.control; scRNA-seq,SRP330776,CD8+ T cell_3-AD.vs.control; scRNA-seq,SRP330776,Monocyte_1-AD.vs.control; scRNA-seq,SRP330776,B cell_3-AD.vs.control; scRNA-seq,SRP330776,Regulatory T cell-AD.vs.control; scRNA-seq,SRP330776,Natural	32
Atp6v0a2	bulk RNA-seq,SRP325058,AD.vs.control	1
ATP6V0A4	scRNA-seq,SRP215507,CD8+ T cell_2-MCI.vs.control	1
ATP6V0B	bulk RNA-seq,Homo_723,AD.vs.control; bulk RNA-seq,Homo_723,AD.vs.MCI; bulk RNA-seq,Homo_633,AD.vs.control; bulk RNA-seq,Homo_714,AD.vs.control; bulk RNA-seq,Homo_714,AD.vs.MCI; bulk RNA-seq,SRP223445,AD.vs.control; bulk RNA-seq,SRP310421,AD.vs.control; scRNA-seq,SRP215507,CD8+ T cell_6-AD.vs.control; scRNA-seq,SRP215507,CD8+ T cell_9-AD.vs.control; scRNA-seq,SRP215507,CD8+ T cell_4-AD.vs.MCI; scRNA-seq,SRP330776,Naive CD8+ T cell_1-AD.vs.control	11
Atp6v0b	bulk RNA-seq,SRP325058,AD.vs.control	1
Atp6v0c	bulk RNA-seq,SRP325058,AD.vs.control	1
ATP6V0C	scRNA-seq,SRP215507,CD8+ T cell_5-AD.vs.MCI; scRNA-seq,SRP215507,CD8+ T cell_5-MCI.vs.control	2
Atp6v0d1	bulk RNA-seq,SRP325058,AD.vs.control	1
ATP6V0D1	scRNA-seq,SRP215507,CD8+ T cell_7-AD.vs.control; scRNA-seq,SRP215507,CD8+ T cell_6-AD.vs.control; scRNA-seq,SRP215507,CD8+ T cell_7-AD.vs.MCI; scRNA-seq,SRP330776,γδ T cell-AD.vs.control; scRNA-seq,SRP330776,Memory T cell_1-AD.vs.control; scRNA-seq,SRP330776,Naive CD8+ T cell_3-AD.vs.control; scRNA-seq,SRP330776,Naive CD8+ T cell_2-AD.vs.control; scRNA-seq,SRP330776,CD8+ T cell_3-AD.vs.control	8
ATP6V0E1	bulk RNA-seq,Homo_723,MCI.vs.control; bulk RNA-seq,Homo_633,MCI.vs.control; bulk RNA-seq,Homo_714,MCI.vs.control; bulk RNA-seq,SRP223445,AD.vs.control; scRNA-seq,SRP215507,CD8+ T cell_7-AD.vs.control; scRNA-seq,SRP215507,CD8+ T cell_4-MCI.vs.control; scRNA-seq,SRP309935,Erythroid cell-AD.vs.control; scRNA-seq,SRP330776,CD8+ T cell_1-AD.vs.control; scRNA-seq,SRP330776,γδ T cell-AD.vs.control; scRNA-seq,SRP330776,Memory T cell_2-AD.vs.control; scRNA-seq,SRP330776,Naive CD8+ T cell_1-AD.vs.control; scRNA-seq,SRP330776,Natural killer cell_1-AD.vs.control; scRNA-seq,SRP330776,Naive CD8+ T cell_3-AD.vs.control; scRNA-seq,SRP330776,B cell_2-AD.vs.control; scRNA-	15
ATP6V0E2	bulk RNA-seq,Homo_723,AD.vs.control; bulk RNA-seq,Homo_723,MCI.vs.control; bulk RNA-seq,Homo_633,AD.vs.control; bulk RNA-seq,Homo_633,AD.vs.MCI; bulk RNA-seq,Homo_633,MCI.vs.control; bulk RNA-seq,Homo_714,AD.vs.control; bulk RNA-seq,Homo_714,AD.vs.MCI; bulk RNA-seq,Homo_714,MCI.vs.control; scRNA-seq,SRP215507,CD8+ T cell_5-AD.vs.MCI; scRNA-seq,SRP330776,Natural killer cell_2-AD.vs.control	10
ATP6V0E2-AS1	scRNA-seq,SRP215507,CD8+ T cell_5-AD.vs.control; scRNA-seq,SRP215507,CD8+ T cell_2-MCI.vs.control; scRNA-seq,SRP215507,CD8+ T cell_3-MCI.vs.control; scRNA-seq,SRP330776,CD8+ T cell_1-AD.vs.control; scRNA-seq,SRP330776,γδ T cell-AD.vs.control; scRNA-seq,SRP330776,CD8+ T cell_2-AD.vs.control; scRNA-seq,SRP330776,B cell_1-AD.vs.control; scRNA-seq,SRP330776,Naive CD8+ T cell_1-AD.vs.control	8
ATP6V1A	bulk RNA-seq,Homo_723,AD.vs.MCI; bulk RNA-seq,Homo_633,AD.vs.MCI; bulk RNA-seq,Homo_714,AD.vs.control; bulk RNA-seq,Homo_714,AD.vs.MCI; scRNA-seq,SRP215507,CD8+ T cell_5-AD.vs.control; scRNA-seq,SRP309935,CD8+ T cell_1-AD.vs.control; scRNA-seq,SRP330776,CD8+ T cell_1-AD.vs.control; scRNA-seq,SRP330776,Memory T cell_2-AD.vs.control; scRNA-seq,SRP330776,B cell_1-AD.vs.control; scRNA-seq,SRP330776,Naive CD8+ T cell_1-AD.vs.control; scRNA-seq,SRP330776,Naive CD8+ T cell_3-AD.vs.control; scRNA-seq,SRP330776,B	13
ATP6V1B1	bulk RNA-seq,Homo_723,AD.vs.control; bulk RNA-seq,Homo_633,AD.vs.control; bulk RNA-seq,Homo_633,MCI.vs.control; bulk RNA-	4
ATP6V1B2	scRNA-seq,SRP330776,Memory T cell_2-AD.vs.control; scRNA-seq,SRP330776,CD8+ T cell_2-AD.vs.control; scRNA-seq,SRP330776,Natural killer	3
ATP6V1C1	bulk RNA-seq,Homo_714,AD.vs.control; bulk RNA-seq,Homo_714,AD.vs.MCI; bulk RNA-seq,Homo_714,MCI.vs.control; scRNA-seq,SRP215507,CD8+ T cell_7-MCI.vs.control; scRNA-seq,SRP330776,Natural killer cell_1-AD.vs.control	5

ATP6V1C2	bulk RNA-seq,Homo_723,AD.vs.control; bulk RNA-seq,Homo_723,AD.vs.MCI; bulk RNA-seq,Homo_723,MCI.vs.control; bulk RNA-seq,Homo_633,AD.vs.control; bulk RNA-seq,Homo_633,MCI.vs.control; bulk RNA-seq,Homo_714,AD.vs.control; bulk RNA-seq,Homo_714,AD.vs.MCI; bulk RNA-seq,Homo_714,MCI.vs.control; scRNA-seq,SRP330776,CD8+ T cell_1-AD.vs.control; scRNA-seq,SRP330776,Memory T cell_1-AD.vs.control	10
ATP6V1D	bulk RNA-seq,Homo_714,AD.vs.control; bulk RNA-seq,Homo_714,AD.vs.MCI; scRNA-seq,SRP215507,CD8+ T cell_7-AD.vs.control; scRNA-seq,SRP215507,CD8+ T cell_7-AD.vs.MCI; scRNA-seq,SRP330776,Natural killer cell_1-AD.vs.control; scRNA-seq,SRP330776,Naive CD8+ T cell_3-AD.vs.control; scRNA-seq,SRP330776,Monocyte_2-AD.vs.control; scRNA-seq,SRP330776,Regulatory T cell-AD.vs.control; scRNA-seq,SRP330776,Megakaryocyte_1-AD.vs.control; scRNA-seq,SRP330776,Natural killer cell_2-AD.vs.control	10
Atp6v1d	bulk RNA-seq,SRP325058,AD.vs.control	1
ATP6V1E1	bulk RNA-seq,Homo_723,AD.vs.control; bulk RNA-seq,Homo_723,AD.vs.MCI; bulk RNA-seq,Homo_633,MCI.vs.control; bulk RNA-seq,Homo_714,AD.vs.control; bulk RNA-seq,Homo_714,AD.vs.MCI; scRNA-seq,SRP330776,CD8+ T cell_1-AD.vs.control; scRNA-seq,SRP330776,γδ T cell-AD.vs.control; scRNA-seq,SRP330776,Memory T cell_2-AD.vs.control; scRNA-seq,SRP330776,CD8+ T cell_2-AD.vs.control; scRNA-seq,SRP330776,Naive CD8+ T cell_1-AD.vs.control; scRNA-seq,SRP330776,Natural killer cell_1-AD.vs.control; scRNA-seq,SRP330776,Memory T cell_1-AD.vs.control; scRNA-seq,SRP330776,Natural killer T cell-AD.vs.control; scRNA-seq,SRP330776,B cell_2-AD.vs.control; scRNA-seq,SRP330776,T cell-AD.vs.control; scRNA-seq,SRP330776,CD8+ T cell_3-AD.vs.control; scRNA-seq,SRP330776,Monocyte_1-AD.vs.control; scRNA- [illegible]	19
ATP6V1E2	bulk RNA-seq,Homo_723,AD.vs.control; bulk RNA-seq,Homo_723,AD.vs.MCI; bulk RNA-seq,Homo_633,AD.vs.control; bulk RNA-seq,Homo_633,AD.vs.MCI; bulk RNA-seq,Homo_714,AD.vs.control; bulk RNA-seq,Homo_714,AD.vs.MCI; bulk RNA-seq,Homo_714,MCI.vs.control; scRNA-seq,SRP215507,CD8+ T cell_2-AD.vs.control; scRNA-seq,SRP215507,CD8+ T cell_5-AD.vs.MCI; scRNA-seq,SRP215507,CD8+ T cell_5-MCI.vs.control; scRNA-seq,SRP215507,CD8+ T cell_2-MCI.vs.control; scRNA-seq,SRP215507,CD8+ T cell_3-MCI.vs.control; scRNA-seq,SRP309935,Monocyte_1-AD.vs.control; scRNA-seq,SRP309935,Naive CD8+ T cell_1-AD.vs.control; scRNA-seq,SRP330776,CD8+ T cell_1-AD.vs.control; scRNA-seq,SRP330776,γδ T cell-AD.vs.control; scRNA-seq,SRP330776,Memory T cell_2-AD.vs.control; scRNA-seq,SRP330776,CD8+ T cell_2-AD.vs.control; scRNA-seq,SRP330776,B cell_1-AD.vs.control; scRNA-seq,SRP330776,Naive CD8+ T cell_1-AD.vs.control; scRNA-seq,SRP330776,Natural killer cell_1-AD.vs.control; scRNA-seq,SRP330776,Naive CD8+ T cell_3-AD.vs.control; scRNA-seq,SRP330776,B cell_2- [illegible]	25
ATP6V1F	scRNA-seq,SRP215507,CD8+ T cell_4-AD.vs.MCI; scRNA-seq,SRP215507,CD8+ T cell_4-MCI.vs.control; scRNA-seq,SRP215507,CD8+ T cell_7-MCI.vs.control; scRNA-seq,SRP309935,Erythroid cell-AD.vs.control; scRNA-seq,SRP330776,Naive CD8+ T cell_3-AD.vs.control	5
ATP6V1FNB	bulk RNA-seq,Homo_723,AD.vs.control; bulk RNA-seq,Homo_633,AD.vs.control; bulk RNA-seq,Homo_633,AD.vs.MCI; bulk RNA-seq,Homo_714,AD.vs.control; bulk RNA-seq,Homo_714,AD.vs.MCI; bulk RNA-seq,ROSMAP,MCI.vs.control; scRNA-seq,SRP215507,CD8+ T cell_2-AD.vs.MCI; scRNA-seq,SRP215507,CD8+ T cell_2-MCI.vs.control	8
Atp6v1g1	bulk RNA-seq,SRP325058,AD.vs.control	1
ATP6V1G1	scRNA-seq,SRP215507,CD8+ T cell_8-AD.vs.control; scRNA-seq,SRP215507,CD8+ T cell_7-AD.vs.control; scRNA-seq,SRP215507,CD8+ T cell_4-AD.vs.MCI; scRNA-seq,SRP215507,CD8+ T cell_4-MCI.vs.control; scRNA-seq,SRP309935,Erythroid cell-AD.vs.control	5
ATP6V1G2	bulk RNA-seq,Homo_723,AD.vs.control; bulk RNA-seq,Homo_723,AD.vs.MCI; bulk RNA-seq,Homo_633,AD.vs.control; bulk RNA-seq,Homo_633,AD.vs.MCI; bulk RNA-seq,Homo_714,AD.vs.control; scRNA-seq,SRP215507,CD8+ T cell_3-MCI.vs.control; scRNA-	7
ATP6V1H	bulk RNA-seq,Homo_723,AD.vs.control; bulk RNA-seq,Homo_633,AD.vs.control; bulk RNA-seq,Homo_633,AD.vs.MCI; bulk RNA-seq,Homo_714,AD.vs.control; bulk RNA-seq,Homo_714,AD.vs.MCI; scRNA-seq,SRP215507,CD8+ T cell_5-AD.vs.control; scRNA-seq,SRP215507,CD8+ T cell_4-AD.vs.MCI; scRNA-seq,SRP215507,CD8+ T cell_5-AD.vs.MCI	8
ATP7A	bulk RNA-seq,Homo_714,AD.vs.control; bulk RNA-seq,Homo_714,AD.vs.MCI; scRNA-seq,SRP215507,CD8+ T cell_5-AD.vs.control; scRNA-seq,SRP215507,CD8+ T cell_1-AD.vs.MCI; scRNA-seq,SRP309935,Monocyte_1-AD.vs.control; scRNA-seq,SRP330776,Memory T cell_2-AD.vs.control; scRNA-seq,SRP330776,Naive CD8+ T cell_3-AD.vs.control	7
ATP7B	bulk RNA-seq,Homo_723,AD.vs.control; bulk RNA-seq,Homo_723,AD.vs.MCI; bulk RNA-seq,Homo_723,MCI.vs.control; bulk RNA-seq,Homo_633,AD.vs.MCI; bulk RNA-seq,Homo_714,AD.vs.control; bulk RNA-seq,Homo_714,AD.vs.MCI; scRNA-seq,SRP330776,Naive CD8+ T	7
ATP8	bulk RNA-seq,Homo_714,AD.vs.control; bulk RNA-seq,Homo_714,AD.vs.MCI	2

ATP8A1	bulk RNA-seq,Homo_723,AD.vs.control; bulk RNA-seq,Homo_723,AD.vs.MCI; bulk RNA-seq,Homo_723,MCI.vs.control; bulk RNA-seq,Homo_633,AD.vs.control; bulk RNA-seq,Homo_633,AD.vs.MCI; bulk RNA-seq,Homo_633,MCI.vs.control; bulk RNA-seq,Homo_714,AD.vs.control; bulk RNA-seq,Homo_714,AD.vs.MCI; bulk RNA-seq,Homo_714,MCI.vs.control; scRNA-seq,SRP215507,CD8+ T cell_5-MCI.vs.control; scRNA-seq,SRP215507,CD8+ T cell_2-MCI.vs.control; scRNA-seq,SRP215507,CD8+ T cell_3-MCI.vs.control; scRNA-seq,SRP215507,CD8+ T cell_6-MCI.vs.control; scRNA-seq,SRP309935,Natural killer cell_2-AD.vs.control; scRNA-seq,SRP309935,Naive CD8+ T cell_1-AD.vs.control; scRNA-seq,SRP309935,Natural killer cell_1-AD.vs.control; scRNA-seq,SRP309935,Naive CD8+ T cell_2-AD.vs.control; scRNA-seq,SRP309935,CD8+ T cell_1-AD.vs.control; scRNA-seq,SRP330776,CD8+ T cell_1-AD.vs.control; scRNA-seq,SRP330776,γδ T cell-AD.vs.control; scRNA-seq,SRP330776,Memory T cell_2-AD.vs.control; scRNA-seq,SRP330776,B cell_1-AD.vs.control; scRNA-seq,SRP330776,Naive CD8+ T cell_1-AD.vs.control; scRNA-seq,SRP330776,Memory T cell_1-AD.vs.control; scRNA-seq,SRP330776,Naive CD8+ T cell_3-AD.vs.control; scRNA-seq,SRP330776,B cell_2-[illegible]	29
Atp8a1	bulk RNA-seq,SRP325058,AD.vs.control	1
ATP8A2	bulk RNA-seq,Homo_714,AD.vs.control; bulk RNA-seq,Homo_714,AD.vs.MCI; bulk RNA-seq,ROSMAP,AD.vs.control; bulk RNA-seq,ROSMAP,MCI.vs.control; scRNA-seq,SRP215507,CD8+ T cell_6-AD.vs.control; scRNA-seq,SRP215507,CD8+ T cell_3-MCI.vs.control; scRNA-seq,SRP309935,Natural killer cell_2-AD.vs.control; scRNA-seq,SRP330776,CD8+ T cell_2-AD.vs.control; scRNA-seq,SRP330776,Memory T cell_1-	9
ATP8B1	bulk RNA-seq,Homo_723,AD.vs.control; bulk RNA-seq,Homo_723,AD.vs.MCI; bulk RNA-seq,Homo_723,MCI.vs.control; bulk RNA-seq,Homo_633,AD.vs.control; bulk RNA-seq,Homo_633,AD.vs.MCI; bulk RNA-seq,Homo_633,MCI.vs.control; bulk RNA-seq,Homo_714,AD.vs.control; bulk RNA-seq,Homo_714,AD.vs.MCI; scRNA-seq,SRP215507,CD8+ T cell_3-AD.vs.MCI; scRNA-seq,SRP215507,CD8+ T cell_3-MCI.vs.control; scRNA-seq,SRP330776,CD8+ T cell_1-AD.vs.control; scRNA-seq,SRP330776,γδ T cell-AD.vs.control; scRNA-seq,SRP330776,B cell_1-AD.vs.control; scRNA-seq,SRP330776,Naive CD8+ T cell_1-AD.vs.control; scRNA-seq,SRP330776,Memory T cell_1-AD.vs.control; scRNA-seq,SRP330776,B cell_2-	17
ATP8B2	scRNA-seq,SRP215507,CD8+ T cell_6-AD.vs.control; scRNA-seq,SRP215507,CD8+ T cell_3-AD.vs.MCI; scRNA-seq,SRP215507,CD8+ T cell_5-AD.vs.MCI; scRNA-seq,SRP215507,CD8+ T cell_3-MCI.vs.control; scRNA-seq,SRP215507,CD8+ T cell_6-MCI.vs.control; scRNA-seq,SRP330776,CD8+ T cell_1-AD.vs.control; scRNA-seq,SRP330776,Memory T cell_2-AD.vs.control; scRNA-seq,SRP330776,CD8+ T cell_2-AD.vs.control; scRNA-seq,SRP330776,B cell_1-AD.vs.control; scRNA-seq,SRP330776,Natural killer cell_1-AD.vs.control; scRNA-seq,SRP330776,B	12
ATP8B3	scRNA-seq,SRP215507,CD8+ T cell_4-AD.vs.control; scRNA-seq,SRP215507,CD8+ T cell_6-AD.vs.control; scRNA-seq,SRP215507,CD8+ T cell_5-AD.vs.MCI; scRNA-seq,SRP215507,CD8+ T cell_5-MCI.vs.control; scRNA-seq,SRP330776,CD8+ T cell_2-AD.vs.control; scRNA-seq,SRP330776,Natural killer cell_1-AD.vs.control; scRNA-seq,SRP330776,Memory T cell_1-AD.vs.control	7
ATP8B4	bulk RNA-seq,Homo_723,AD.vs.MCI; bulk RNA-seq,Homo_723,MCI.vs.control; bulk RNA-seq,Homo_633,AD.vs.MCI; bulk RNA-seq,Homo_633,MCI.vs.control; bulk RNA-seq,Homo_714,AD.vs.control; bulk RNA-seq,Homo_714,AD.vs.MCI; bulk RNA-seq,Homo_714,MCI.vs.control; scRNA-seq,SRP215507,CD8+ T cell_1-AD.vs.MCI; scRNA-seq,SRP215507,CD8+ T cell_5-MCI.vs.control; scRNA-	11
ATP9A	bulk RNA-seq,Homo_723,AD.vs.control; bulk RNA-seq,Homo_723,AD.vs.MCI; bulk RNA-seq,Homo_723,MCI.vs.control; bulk RNA-seq,Homo_633,AD.vs.control; bulk RNA-seq,Homo_633,AD.vs.MCI; bulk RNA-seq,Homo_633,MCI.vs.control; bulk RNA-seq,Homo_714,AD.vs.control; bulk RNA-seq,Homo_714,AD.vs.MCI; scRNA-seq,SRP215507,CD8+ T cell_1-AD.vs.MCI; scRNA-seq,SRP215507,CD8+ T cell_1-MCI.vs.control; scRNA-seq,SRP215507,CD8+ T cell_4-MCI.vs.control; scRNA-seq,SRP309935,Natural killer cell_1-AD.vs.control; scRNA-seq,SRP330776,γδ T cell-	14
ATP9B	scRNA-seq,SRP215507,CD8+ T cell_7-AD.vs.control; scRNA-seq,SRP215507,CD8+ T cell_6-AD.vs.MCI; scRNA-seq,SRP215507,CD8+ T cell_5-MCI.vs.control; scRNA-seq,SRP215507,CD8+ T cell_6-MCI.vs.control; scRNA-seq,SRP330776,Memory T cell_1-AD.vs.control; scRNA-	6
ATPAF1	bulk RNA-seq,Homo_723,AD.vs.control; bulk RNA-seq,Homo_633,AD.vs.control; bulk RNA-seq,Homo_633,AD.vs.MCI; scRNA-seq,SRP215507,CD8+ T cell_2-AD.vs.MCI; scRNA-seq,SRP215507,CD8+ T cell_1-AD.vs.MCI; scRNA-seq,SRP215507,CD8+ T cell_4-AD.vs.MCI; scRNA-seq,SRP215507,CD8+ T cell_4-MCI.vs.control; scRNA-seq,SRP309935,Naive CD8+ T cell_1-AD.vs.control; scRNA-seq,SRP309935,CD8+ T cell_1-AD.vs.control; scRNA-seq,SRP309935,CD8+ T cell_2-AD.vs.control; scRNA-seq,SRP309935,B cell_2-AD.vs.control; scRNA-seq,SRP330776,CD8+ T cell_1-AD.vs.control; scRNA-seq,SRP330776,γδ T cell-AD.vs.control; scRNA-seq,SRP330776,Memory T cell_2-AD.vs.control; scRNA-seq,SRP330776,CD8+ T cell_2-AD.vs.control; scRNA-seq,SRP330776,B cell_1-AD.vs.control; scRNA-seq,SRP330776,Naive CD8+ T cell_1-AD.vs.control; scRNA-seq,SRP330776,Natural killer cell_1-AD.vs.control; scRNA-seq,SRP330776,Memory T cell_1-AD.vs.control; scRNA-seq,SRP330776,Natural killer T cell-AD.vs.control; scRNA-seq,SRP330776,Naive CD8+ T cell_3-AD.vs.control; scRNA-seq,SRP330776,B cell_2-AD.vs.control; scRNA-seq,SRP330776,T cell-AD.vs.control; scRNA-seq,SRP330776,CD8+ T cell_3-AD.vs.control; scRNA-seq,SRP330776,Monocyte_2-AD.vs.control; scRNA-seq,SRP330776,B cell_3-AD.vs.control; scRNA-seq,SRP330776,Regulatory T cell-AD.vs.control; scRNA-seq,SRP330776,Natural [illegible]	29

ATPAF2	scRNA-seq,SRP215507,CD8+ T cell_5-AD.vs.MCI; scRNA-seq,SRP215507,CD8+ T cell_5-MCI.vs.control; scRNA-seq,SRP215507,CD8+ T cell_4-MCI.vs.control; scRNA-seq,SRP330776,Natural killer T cell-AD.vs.control	4
ATPSCKMT	bulk RNA-seq,Homo_723,MCI.vs.control; bulk RNA-seq,Homo_633,MCI.vs.control; bulk RNA-seq,Homo_714,AD.vs.control; bulk RNA-seq,Homo_714,AD.vs.MCI; bulk RNA-seq,Homo_714,MCI.vs.control; scRNA-seq,SRP215507,CD8+ T cell_3-AD.vs.MCI; scRNA-seq,SRP215507,CD8+ T cell_1-AD.vs.MCI; scRNA-seq,SRP215507,CD8+ T cell_3-MCI.vs.control; scRNA-seq,SRP330776,γδ T cell-AD.vs.control; scRNA-seq,SRP330776,Memory T cell_2-AD.vs.control; scRNA-seq,SRP330776,CD8+ T cell_2-AD.vs.control; scRNA-seq,SRP330776,Natural killer cell_1-AD.vs.control; scRNA-seq,SRP330776,Memory T cell_1-AD.vs.control; scRNA-seq,SRP330776,Natural killer T cell-AD.vs.control; scRNA-seq,SRP330776,Naive CD8+ T cell_3-AD.vs.control; scRNA-seq,SRP330776,T cell-AD.vs.control; scRNA-seq,SRP330776,CD8+ T cell_3-AD.vs.control	17
ATR	bulk RNA-seq,Homo_723,AD.vs.control; bulk RNA-seq,Homo_723,AD.vs.MCI; bulk RNA-seq,Homo_723,MCI.vs.control; bulk RNA-seq,Homo_633,AD.vs.control; bulk RNA-seq,Homo_633,AD.vs.MCI; bulk RNA-seq,Homo_633,MCI.vs.control; bulk RNA-seq,Homo_714,AD.vs.control; bulk RNA-seq,Homo_714,AD.vs.MCI; bulk RNA-seq,Homo_714,MCI.vs.control; scRNA-seq,SRP215507,CD8+ T cell_7-AD.vs.MCI; scRNA-seq,SRP309935,Natural killer cell_2-AD.vs.control; scRNA-seq,SRP330776,Naive CD8+ T cell_3-AD.vs.control; scRNA-	13
ATRAID	bulk RNA-seq,Homo_723,AD.vs.control; bulk RNA-seq,Homo_723,AD.vs.MCI; bulk RNA-seq,Homo_633,AD.vs.control; bulk RNA-seq,Homo_633,AD.vs.MCI; scRNA-seq,SRP215507,CD8+ T cell_7-AD.vs.control; scRNA-seq,SRP215507,CD8+ T cell_6-AD.vs.MCI; scRNA-seq,SRP215507,CD8+ T cell_6-MCI.vs.control; scRNA-seq,SRP330776,B cell_2-AD.vs.control; scRNA-seq,SRP330776,CD8+ T cell_3-AD.vs.control	9
ATRIP	bulk RNA-seq,Homo_723,AD.vs.control; bulk RNA-seq,Homo_723,MCI.vs.control; bulk RNA-seq,Homo_633,AD.vs.control; bulk RNA-seq,Homo_633,MCI.vs.control; bulk RNA-seq,Homo_714,AD.vs.control; bulk RNA-seq,Homo_714,AD.vs.MCI; bulk RNA-seq,Homo_714,MCI.vs.control; scRNA-seq,SRP215507,CD8+ T cell_2-AD.vs.MCI; scRNA-seq,SRP215507,CD8+ T cell_3-AD.vs.MCI; scRNA-seq,SRP330776,CD8+ T cell_1-AD.vs.control; scRNA-seq,SRP330776,γδ T cell-AD.vs.control; scRNA-seq,SRP330776,Memory T cell_2-AD.vs.control; scRNA-seq,SRP330776,CD8+ T cell_2-AD.vs.control; scRNA-seq,SRP330776,B cell_1-AD.vs.control; scRNA-seq,SRP330776,Naive CD8+ T cell_1-AD.vs.control; scRNA-seq,SRP330776,Natural killer cell_1-AD.vs.control; scRNA-seq,SRP330776,Memory T cell_1-AD.vs.control; scRNA-seq,SRP330776,Natural killer T cell-AD.vs.control; scRNA-seq,SRP330776,Naive CD8+ T cell_3-AD.vs.control; scRNA-seq,SRP330776,B cell_2-[illegible]	22
ATRN	bulk RNA-seq,Homo_723,AD.vs.MCI; bulk RNA-seq,Homo_633,AD.vs.control; bulk RNA-seq,Homo_633,AD.vs.MCI; bulk RNA-seq,Homo_714,AD.vs.control; bulk RNA-seq,Homo_714,AD.vs.MCI; bulk RNA-seq,Homo_714,MCI.vs.control; scRNA-seq,SRP215507,CD8+ T cell_5-AD.vs.MCI; scRNA-seq,SRP215507,CD8+ T cell_5-MCI.vs.control; scRNA-seq,SRP330776,CD8+ T cell_1-AD.vs.control; scRNA-seq,SRP330776,γδ T cell-AD.vs.control; scRNA-seq,SRP330776,Memory T cell_2-AD.vs.control; scRNA-seq,SRP330776,CD8+ T cell_2-AD.vs.control; scRNA-seq,SRP330776,B cell_1-AD.vs.control; scRNA-seq,SRP330776,Naive CD8+ T cell_1-AD.vs.control; scRNA-seq,SRP330776,Natural killer cell_1-AD.vs.control; scRNA-seq,SRP330776,Memory T cell_1-AD.vs.control; scRNA-seq,SRP330776,Natural killer T cell-AD.vs.control; scRNA-seq,SRP330776,Naive CD8+ T cell_3-AD.vs.control; scRNA-seq,SRP330776,B cell_2-AD.vs.control; scRNA-seq,SRP330776,T cell-AD.vs.control; scRNA-seq,SRP330776,CD8+ T cell_3-AD.vs.control; scRNA-seq,SRP330776,B cell_3-AD.vs.control; scRNA-seq,SRP330776,Natural killer cell_2-[illegible]	23
ATRNL1	bulk RNA-seq,Homo_723,AD.vs.control; bulk RNA-seq,Homo_723,AD.vs.MCI; bulk RNA-seq,Homo_723,MCI.vs.control; bulk RNA-seq,Homo_633,AD.vs.control; bulk RNA-seq,Homo_633,AD.vs.MCI; bulk RNA-seq,Homo_714,MCI.vs.control	6
ATRX	bulk RNA-seq,Homo_723,AD.vs.control; bulk RNA-seq,Homo_723,AD.vs.MCI; bulk RNA-seq,Homo_633,AD.vs.control; bulk RNA-seq,Homo_633,AD.vs.MCI; bulk RNA-seq,Homo_714,AD.vs.control; bulk RNA-seq,Homo_714,AD.vs.MCI; scRNA-seq,SRP215507,CD8+ T cell_10-AD.vs.control; scRNA-seq,SRP215507,CD8+ T cell_9-AD.vs.control	8
ATXN1	scRNA-seq,SRP215507,CD8+ T cell_3-AD.vs.control; scRNA-seq,SRP215507,CD8+ T cell_5-AD.vs.MCI; scRNA-seq,SRP215507,CD8+ T cell_6-AD.vs.MCI; scRNA-seq,SRP215507,CD8+ T cell_3-MCI.vs.control; scRNA-seq,SRP215507,CD8+ T cell_6-MCI.vs.control; scRNA-seq,SRP309935,Monocyte_2-AD.vs.control; scRNA-seq,SRP330776,CD8+ T cell_3-AD.vs.control; scRNA-seq,SRP330776,Monocyte_2-AD.vs.control; scRNA-seq,SRP330776,Natural killer cell_2-AD.vs.control; scRNA-seq,SRP330776,B cell_4-AD.vs.control	10
ATXN10	scRNA-seq,SRP215507,CD8+ T cell_4-AD.vs.MCI; scRNA-seq,SRP330776,CD8+ T cell_1-AD.vs.control; scRNA-seq,SRP330776,γδ T cell-AD.vs.control; scRNA-seq,SRP330776,Memory T cell_2-AD.vs.control; scRNA-seq,SRP330776,CD8+ T cell_2-AD.vs.control; scRNA-seq,SRP330776,Naive CD8+ T cell_1-AD.vs.control; scRNA-seq,SRP330776,Natural killer cell_1-AD.vs.control; scRNA-seq,SRP330776,B cell_3-	8
ATXN1L	bulk RNA-seq,Homo_723,AD.vs.control; bulk RNA-seq,Homo_723,AD.vs.MCI; bulk RNA-seq,Homo_633,AD.vs.control; bulk RNA-seq,Homo_633,AD.vs.MCI; bulk RNA-seq,Homo_714,AD.vs.MCI; scRNA-seq,SRP215507,CD8+ T cell_5-AD.vs.MCI; scRNA-seq,SRP215507,CD8+ T	7

ATXN2	bulk RNA-seq,Homo_723,AD.vs.control; bulk RNA-seq,Homo_723,AD.vs.MCI; bulk RNA-seq,Homo_633,AD.vs.control; bulk RNA-seq,Homo_633,AD.vs.MCI; bulk RNA-seq,Homo_714,AD.vs.control; bulk RNA-seq,Homo_714,AD.vs.MCI; bulk RNA-seq,Homo_714,MCI.vs.control; scRNA-seq,SRP215507,CD8+ T cell_3-AD.vs.MCI; scRNA-seq,SRP215507,CD8+ T cell_7-AD.vs.MCI; scRNA-seq,SRP215507,CD8+ T cell_7-MCI.vs.control; scRNA-seq,SRP309935,Naive CD8+ T cell_1-AD.vs.control; scRNA-seq,SRP309935,CD8+ T cell_1-AD.vs.control; scRNA-seq,SRP309935,B cell_1-AD.vs.control; scRNA-seq,SRP309935,CD8+ T cell_2-AD.vs.control; scRNA-seq,SRP330776,CD8+ T cell_1-AD.vs.control; scRNA-seq,SRP330776,γδ T cell-AD.vs.control; scRNA-seq,SRP330776,Memory T cell_2-AD.vs.control; scRNA-seq,SRP330776,CD8+ T cell_2-AD.vs.control; scRNA-seq,SRP330776,B cell_1-AD.vs.control; scRNA-seq,SRP330776,Naive CD8+ T cell_1-AD.vs.control; scRNA-seq,SRP330776,Natural killer cell_1-AD.vs.control; scRNA-seq,SRP330776,Memory T cell_1-AD.vs.control; scRNA-seq,SRP330776,Natural killer T cell-AD.vs.control; scRNA-seq,SRP330776,Naive CD8+ T cell_3-AD.vs.control; scRNA-seq,SRP330776,B cell_2-AD.vs.control; scRNA-seq,SRP330776,T cell-AD.vs.control; scRNA-seq,SRP330776,Naive CD8+ T cell_2-AD.vs.control; scRNA-seq,SRP330776,CD8+ T cell_3-AD.vs.control; scRNA-seq,SRP330776,Monocyte_1-AD.vs.control; scRNA-seq,SRP330776,Monocyte_2-AD.vs.control; scRNA-seq,SRP330776,Natural killer cell_2	31
ATXN2-AS	scRNA-seq,SRP215507,CD8+ T cell_1-AD.vs.MCI; scRNA-seq,SRP215507,CD8+ T cell_1-MCI.vs.control	2
ATXN2L	scRNA-seq,SRP309935,Natural killer cell_2-AD.vs.control; scRNA-seq,SRP330776,CD8+ T cell_1-AD.vs.control; scRNA-seq,SRP330776,γδ T cell-AD.vs.control; scRNA-seq,SRP330776,Memory T cell_2-AD.vs.control; scRNA-seq,SRP330776,CD8+ T cell_2-AD.vs.control; scRNA-seq,SRP330776,B cell_1-AD.vs.control; scRNA-seq,SRP330776,Naive CD8+ T cell_1-AD.vs.control; scRNA-seq,SRP330776,Natural killer cell_1-AD.vs.control; scRNA-seq,SRP330776,Memory T cell_1-AD.vs.control; scRNA-seq,SRP330776,Natural killer T cell-AD.vs.control; scRNA-seq,SRP330776,Naive CD8+ T cell_3-AD.vs.control; scRNA-seq,SRP330776,B cell_2-AD.vs.control; scRNA-seq,SRP330776,T cell-AD.vs.control; scRNA-seq,SRP330776,Naive CD8+ T cell_2-AD.vs.control; scRNA-seq,SRP330776,CD8+ T cell_3-AD.vs.control; scRNA-seq,SRP330776,Monocyte_1-AD.vs.control; scRNA-seq,SRP330776,Monocyte_2-AD.vs.control; scRNA-seq,SRP330776,B cell_3-AD.vs.control; scRNA-seq,SRP330776,Regulatory T cell-AD.vs.control; [illegible]	21
ATXN3	bulk RNA-seq,Homo_723,AD.vs.control; bulk RNA-seq,Homo_723,AD.vs.MCI; bulk RNA-seq,Homo_633,AD.vs.control; bulk RNA-seq,Homo_633,AD.vs.MCI; bulk RNA-seq,Homo_714,AD.vs.control; bulk RNA-seq,Homo_714,AD.vs.MCI; scRNA-seq,SRP309935,Monocyte_1-AD.vs.control; scRNA-seq,SRP309935,Monocyte_2-AD.vs.control; scRNA-seq,SRP330776,γδ T cell-AD.vs.control; scRNA-seq,SRP330776,Memory T cell_2-AD.vs.control; scRNA-seq,SRP330776,CD8+ T cell_3-AD.vs.control; scRNA-seq,SRP330776,Monocyte_2-AD.vs.control; scRNA-	14
ATXN7	scRNA-seq,SRP330776,B cell_1-AD.vs.control; scRNA-seq,SRP330776,Monocyte_2-AD.vs.control	2
ATXN7L1	bulk RNA-seq,Homo_723,AD.vs.control; bulk RNA-seq,Homo_723,AD.vs.MCI; bulk RNA-seq,Homo_633,AD.vs.control; bulk RNA-seq,Homo_633,AD.vs.MCI; bulk RNA-seq,Homo_714,AD.vs.control; bulk RNA-seq,Homo_714,AD.vs.MCI; bulk RNA-seq,Homo_714,MCI.vs.control; scRNA-seq,SRP309935,CD8+ T cell_1-AD.vs.control; scRNA-seq,SRP330776,B cell_2-AD.vs.control; scRNA-seq,SRP330776,Monocyte_2-	11
Atxn7l1	bulk RNA-seq,SRP325058,AD.vs.control	1
ATXN7L2	bulk RNA-seq,Homo_723,AD.vs.control; bulk RNA-seq,Homo_723,MCI.vs.control; bulk RNA-seq,Homo_633,AD.vs.control; bulk RNA-seq,Homo_633,MCI.vs.control; bulk RNA-seq,Homo_714,AD.vs.control; bulk RNA-seq,Homo_714,AD.vs.MCI; bulk RNA-seq,Homo_714,MCI.vs.control; scRNA-seq,SRP215507,CD8+ T cell_3-AD.vs.control; scRNA-seq,SRP215507,CD8+ T cell_6-AD.vs.control; scRNA-seq,SRP215507,CD8+ T cell_3-AD.vs.MCI; scRNA-seq,SRP215507,CD8+ T cell_5-AD.vs.MCI; scRNA-seq,SRP215507,CD8+ T cell_4-MCI.vs.control; scRNA-seq,SRP330776,CD8+ T cell_1-AD.vs.control; scRNA-seq,SRP330776,Memory T cell_2-AD.vs.control; scRNA-seq,SRP330776,Naive CD8+ T	16
ATXN7L3	scRNA-seq,SRP215507,CD8+ T cell_3-AD.vs.MCI; scRNA-seq,SRP215507,CD8+ T cell_3-MCI.vs.control; scRNA-seq,SRP330776,Naive CD8+ T cell_1-AD.vs.control; scRNA-seq,SRP330776,Natural killer cell_1-AD.vs.control; scRNA-seq,SRP330776,Memory T cell_1-AD.vs.control; scRNA-seq,SRP330776,Natural killer T cell-AD.vs.control; scRNA-seq,SRP330776,Naive CD8+ T cell_3-AD.vs.control; scRNA-seq,SRP330776,B cell_2-AD.vs.control; scRNA-seq,SRP330776,CD8+ T cell_3-AD.vs.control; scRNA-seq,SRP330776,Monocyte_2-AD.vs.control	10
ATXN7L3B	scRNA-seq,SRP215507,CD8+ T cell_9-AD.vs.control; scRNA-seq,SRP215507,CD8+ T cell_5-AD.vs.MCI; scRNA-seq,SRP215507,CD8+ T cell_6-AD.vs.MCI; scRNA-seq,SRP215507,CD8+ T cell_6-MCI.vs.control	4
Auh	bulk RNA-seq,SRP325058,AD.vs.control	1

AUH	scRNA-seq,SRP330776,CD8+ T cell_1-AD.vs.control; scRNA-seq,SRP330776,γδ T cell-AD.vs.control; scRNA-seq,SRP330776,Memory T cell_2-AD.vs.control; scRNA-seq,SRP330776,CD8+ T cell_2-AD.vs.control; scRNA-seq,SRP330776,B cell_1-AD.vs.control; scRNA-seq,SRP330776,Naive CD8+ T cell_1-AD.vs.control; scRNA-seq,SRP330776,Natural killer cell_1-AD.vs.control; scRNA-seq,SRP330776,Memory T cell_1-AD.vs.control; scRNA-seq,SRP330776,Natural killer T cell-AD.vs.control; scRNA-seq,SRP330776,Naive CD8+ T cell_3-AD.vs.control; scRNA-seq,SRP330776,B cell_2-AD.vs.control; scRNA-seq,SRP330776,CD8+ T cell_3-AD.vs.control; scRNA-seq,SRP330776,B cell_3-AD.vs.control; scRNA-seq,SRP330776,B cell_4-	14
AUNIP	bulk RNA-seq,Homo_723,AD.vs.MCI; bulk RNA-seq,Homo_714,AD.vs.control; bulk RNA-seq,Homo_714,AD.vs.MCI	3
AUP1	scRNA-seq,SRP215507,CD8+ T cell_7-AD.vs.control; scRNA-seq,SRP215507,CD8+ T cell_7-AD.vs.MCI; scRNA-seq,SRP215507,CD8+ T cell_6-AD.vs.MCI; scRNA-seq,SRP309935,Erythroid cell-AD.vs.control	4
AURKA	bulk RNA-seq,Homo_723,AD.vs.control; bulk RNA-seq,Homo_723,MCI.vs.control; bulk RNA-seq,Homo_633,AD.vs.control; bulk RNA-seq,Homo_633,MCI.vs.control; bulk RNA-seq,Homo_714,AD.vs.control; bulk RNA-seq,Homo_714,AD.vs.MCI; bulk RNA-seq,Homo_714,MCI.vs.control; bulk RNA-seq,ROSMAP,MCI.vs.control; scRNA-seq,SRP215507,CD8+ T cell_2-AD.vs.MCI; scRNA-seq,SRP215507,CD8+ T cell_1-AD.vs.MCI; scRNA-seq,SRP215507,CD8+ T cell_1-MCI.vs.control; scRNA-seq,SRP215507,CD8+ T cell_2-	13
AURKAIP1	scRNA-seq,SRP215507,CD8+ T cell_9-AD.vs.control; scRNA-seq,SRP309935,Erythroid cell-AD.vs.control; scRNA-seq,SRP330776,Natural killer cell_1-AD.vs.control; scRNA-seq,SRP330776,Natural killer T cell-AD.vs.control; scRNA-seq,SRP330776,Naive CD8+ T cell_3-AD.vs.control; scRNA-seq,SRP330776,B cell_2-AD.vs.control; scRNA-seq,SRP330776,B cell_3-AD.vs.control	7
AURKB	bulk RNA-seq,Homo_723,AD.vs.control; bulk RNA-seq,Homo_723,AD.vs.MCI; bulk RNA-seq,Homo_714,AD.vs.control; bulk RNA-seq,Homo_714,AD.vs.MCI; scRNA-seq,SRP215507,CD8+ T cell_6-AD.vs.MCI; scRNA-seq,SRP215507,CD8+ T cell_6-MCI.vs.control	6
AURKC	bulk RNA-seq,Homo_633,AD.vs.control; bulk RNA-seq,Homo_633,AD.vs.MCI; bulk RNA-seq,Homo_714,AD.vs.control; bulk RNA-seq,Homo_714,AD.vs.MCI; scRNA-seq,SRP215507,CD8+ T cell_2-AD.vs.MCI; scRNA-seq,SRP215507,CD8+ T cell_1-MCI.vs.control; scRNA-	7
AUTS2	bulk RNA-seq,Homo_723,AD.vs.control; bulk RNA-seq,Homo_723,AD.vs.MCI; bulk RNA-seq,Homo_633,AD.vs.control; bulk RNA-seq,Homo_633,AD.vs.MCI; bulk RNA-seq,Homo_714,AD.vs.control; bulk RNA-seq,Homo_714,AD.vs.MCI; scRNA-seq,SRP215507,CD8+ T cell_2-AD.vs.control; scRNA-seq,SRP215507,CD8+ T cell_3-AD.vs.MCI; scRNA-seq,SRP215507,CD8+ T cell_4-AD.vs.MCI; scRNA-seq,SRP215507,CD8+ T cell_3-MCI.vs.control; scRNA-seq,SRP309935,Natural killer cell_2-AD.vs.control; scRNA-seq,SRP309935,Monocyte_1-AD.vs.control; scRNA-seq,SRP309935,Megakaryocyte_1-AD.vs.control; scRNA-seq,SRP309935,Natural killer cell_1-AD.vs.control; scRNA-seq,SRP309935,CD8+ T cell_1-AD.vs.control; scRNA-seq,SRP309935,CD8+ T cell_2-AD.vs.control; scRNA-seq,SRP330776,CD8+ T cell_1-AD.vs.control; scRNA-seq,SRP330776,γδ T [illegible]	20
AUXG01000058.1	scRNA-seq,SRP215507,CD8+ T cell_3-AD.vs.control; scRNA-seq,SRP215507,CD8+ T cell_3-MCI.vs.control; scRNA-seq,SRP330776,Memory T cell_2-AD.vs.control; scRNA-seq,SRP330776,B cell_1-AD.vs.control; scRNA-seq,SRP330776,Naive CD8+ T cell_1-AD.vs.control; scRNA-	6
AVEN	bulk RNA-seq,Homo_723,AD.vs.control; bulk RNA-seq,Homo_723,AD.vs.MCI; bulk RNA-seq,Homo_633,AD.vs.control; bulk RNA-seq,Homo_633,AD.vs.MCI; bulk RNA-seq,Homo_714,AD.vs.control; bulk RNA-seq,Homo_714,AD.vs.MCI; scRNA-seq,SRP215507,CD8+ T cell_2-AD.vs.control; scRNA-seq,SRP215507,CD8+ T cell_5-AD.vs.MCI; scRNA-seq,SRP215507,CD8+ T cell_5-MCI.vs.control; scRNA-seq,SRP215507,CD8+ T cell_2-MCI.vs.control; scRNA-seq,SRP309935,Natural killer cell_2-AD.vs.control; scRNA-seq,SRP309935,Natural killer cell_1-AD.vs.control; scRNA-seq,SRP309935,Monocyte_2-AD.vs.control; scRNA-seq,SRP309935,Naive CD8+ T cell_2-AD.vs.control; scRNA-seq,SRP330776,CD8+ T cell_1-AD.vs.control; scRNA-seq,SRP330776,Memory T cell_2-AD.vs.control; scRNA-seq,SRP330776,CD8+ T cell_2-AD.vs.control; scRNA-seq,SRP330776,B cell_1-AD.vs.control; scRNA-seq,SRP330776,Naive CD8+ T cell_1-AD.vs.control; scRNA-seq,SRP330776,Memory T cell_1-AD.vs.control	20
AVIL	scRNA-seq,SRP215507,CD8+ T cell_1-AD.vs.control; scRNA-seq,SRP215507,CD8+ T cell_4-AD.vs.control; scRNA-seq,SRP215507,CD8+ T cell_1-AD.vs.MCI; scRNA-seq,SRP330776,Memory T cell_2-AD.vs.control; scRNA-seq,SRP330776,Natural killer cell_1-AD.vs.control; scRNA-	6
AVL9	scRNA-seq,SRP215507,CD8+ T cell_2-AD.vs.MCI; scRNA-seq,SRP215507,CD8+ T cell_5-AD.vs.MCI; scRNA-seq,SRP330776,Regulatory T cell-	3
AVPI1	bulk RNA-seq,Homo_633,AD.vs.MCI; bulk RNA-seq,Homo_714,AD.vs.control; bulk RNA-seq,Homo_714,AD.vs.MCI; scRNA-seq,SRP215507,CD8+ T cell_5-AD.vs.control; scRNA-seq,SRP215507,CD8+ T cell_3-AD.vs.MCI; scRNA-seq,SRP215507,CD8+ T cell_5-MCI.vs.control; scRNA-seq,SRP215507,CD8+ T cell_3-MCI.vs.control; scRNA-seq,SRP330776,Memory T cell_2-AD.vs.control; scRNA-seq,SRP330776,Natural killer cell_1-AD.vs.control; scRNA-seq,SRP330776,Naive CD8+ T cell_3-AD.vs.control; scRNA-seq,SRP330776,CD8+ T cell_3-AD.vs.control	11
AVPR1B	bulk RNA-seq,Homo_723,AD.vs.control; bulk RNA-seq,Homo_723,AD.vs.MCI; bulk RNA-seq,Homo_633,AD.vs.control; bulk RNA-seq,Homo_714,MCI.vs.control; bulk RNA-seq,ROSMAP,AD.vs.control	5

AXDND1	bulk RNA-seq,Homo_723,AD.vs.control; scRNA-seq,SRP215507,CD8+ T cell_2-MCI.vs.control; scRNA-seq,SRP330776,B cell_2-AD.vs.control	3
AXIN1	scRNA-seq,SRP215507,CD8+ T cell_1-AD.vs.MCI; scRNA-seq,SRP330776,B cell_1-AD.vs.control; scRNA-seq,SRP330776,Natural killer cell_1-AD.vs.control; scRNA-seq,SRP330776,B cell_2-AD.vs.control; scRNA-seq,SRP330776,CD8+ T cell_3-AD.vs.control; scRNA-seq,SRP330776,Regulatory	6
AXIN2	bulk RNA-seq,Homo_723,AD.vs.control; bulk RNA-seq,Homo_723,AD.vs.MCI; bulk RNA-seq,Homo_633,AD.vs.MCI; bulk RNA-seq,Homo_714,AD.vs.control; bulk RNA-seq,Homo_714,AD.vs.MCI; scRNA-seq,SRP215507,CD8+ T cell_2-AD.vs.control; scRNA-seq,SRP215507,CD8+ T cell_3-AD.vs.MCI; scRNA-seq,SRP215507,CD8+ T cell_2-MCI.vs.control; scRNA-seq,SRP215507,CD8+ T cell_3-MCI.vs.control; scRNA-seq,SRP330776,B cell_1-AD.vs.control; scRNA-seq,SRP330776,Natural killer cell_1-AD.vs.control; scRNA-	12
AXL	scRNA-seq,SRP330776,CD8+ T cell_1-AD.vs.control	1
AZGP1	bulk RNA-seq,Homo_723,AD.vs.MCI	1
AZI2	bulk RNA-seq,Homo_714,AD.vs.control; bulk RNA-seq,Homo_714,AD.vs.MCI; scRNA-seq,SRP309935,B cell_1-AD.vs.control; scRNA-seq,SRP330776,γδ T cell-AD.vs.control; scRNA-seq,SRP330776,Monocyte_1-AD.vs.control; scRNA-seq,SRP330776,Natural killer cell_2-AD.vs.control	6
AZIN1	bulk RNA-seq,Homo_723,AD.vs.control; bulk RNA-seq,Homo_723,AD.vs.MCI; bulk RNA-seq,Homo_723,MCI.vs.control; bulk RNA-seq,Homo_633,AD.vs.control; bulk RNA-seq,Homo_633,AD.vs.MCI; bulk RNA-seq,Homo_633,MCI.vs.control; bulk RNA-seq,Homo_714,AD.vs.control; bulk RNA-seq,Homo_714,AD.vs.MCI; scRNA-seq,SRP215507,CD8+ T cell_4-AD.vs.control; scRNA-seq,SRP215507,CD8+ T cell_4-MCI.vs.control; scRNA-seq,SRP309935,Megakaryocyte_1-AD.vs.control; scRNA-seq,SRP309935,CD8+ T cell_2-AD.vs.control; scRNA-	13
AZIN1-AS1	scRNA-seq,SRP215507,CD8+ T cell_2-AD.vs.control; scRNA-seq,SRP215507,CD8+ T cell_3-AD.vs.control; scRNA-seq,SRP215507,CD8+ T cell_1-AD.vs.control; scRNA-seq,SRP215507,CD8+ T cell_2-AD.vs.MCI; scRNA-seq,SRP215507,CD8+ T cell_1-AD.vs.MCI; scRNA-seq,SRP215507,CD8+ T cell_3-MCI.vs.control; scRNA-seq,SRP330776,CD8+ T cell_1-AD.vs.control; scRNA-seq,SRP330776,γδ T cell-AD.vs.control; scRNA-seq,SRP330776,Memory T cell_2-AD.vs.control; scRNA-seq,SRP330776,CD8+ T cell_2-AD.vs.control; scRNA-seq,SRP330776,B cell_1-AD.vs.control; scRNA-seq,SRP330776,Naive CD8+ T cell_1-AD.vs.control; scRNA-seq,SRP330776,Memory T cell_1-AD.vs.control; scRNA-seq,SRP330776,B cell_2-AD.vs.control; scRNA-seq,SRP330776,T cell-AD.vs.control; scRNA-seq,SRP330776,Monocyte_2-AD.vs.control; scRNA-seq,SRP330776,B cell_4-	17
AZIN2	bulk RNA-seq,Homo_633,AD.vs.control; bulk RNA-seq,Homo_633,AD.vs.MCI; bulk RNA-seq,Homo_714,AD.vs.control; bulk RNA-seq,Homo_714,AD.vs.MCI; scRNA-seq,SRP215507,CD8+ T cell_3-AD.vs.control; scRNA-seq,SRP215507,CD8+ T cell_1-AD.vs.MCI; scRNA-	7
AZU1	bulk RNA-seq,Homo_723,AD.vs.control; bulk RNA-seq,Homo_723,AD.vs.MCI; bulk RNA-seq,Homo_633,AD.vs.control; bulk RNA-seq,Homo_633,AD.vs.MCI; bulk RNA-seq,Homo_714,AD.vs.control; bulk RNA-seq,Homo_714,AD.vs.MCI; bulk RNA-seq,Homo_714,MCI.vs.control; scRNA-seq,SRP215507,CD8+ T cell_1-AD.vs.MCI; scRNA-seq,SRP215507,CD8+ T cell_1-MCI.vs.control; scRNA-seq,SRP330776,Monocyte_2-	10
B2M	scRNA-seq,SRP309935,Erythroid cell-AD.vs.control	1
B3GALNT1	bulk RNA-seq,Homo_723,AD.vs.control; bulk RNA-seq,Homo_723,AD.vs.MCI; bulk RNA-seq,Homo_633,AD.vs.control; bulk RNA-seq,Homo_633,AD.vs.MCI; bulk RNA-seq,Homo_714,AD.vs.control; bulk RNA-seq,Homo_714,AD.vs.MCI; scRNA-seq,SRP215507,CD8+ T cell_3-AD.vs.control; scRNA-seq,SRP215507,CD8+ T cell_1-AD.vs.control	8
B3GALNT2	bulk RNA-seq,Homo_633,AD.vs.control; bulk RNA-seq,Homo_714,AD.vs.control; bulk RNA-seq,Homo_714,AD.vs.MCI; scRNA-seq,SRP215507,CD8+ T cell_2-MCI.vs.control; scRNA-seq,SRP309935,Naive CD8+ T cell_1-AD.vs.control; scRNA-seq,SRP309935,Natural killer cell_1-AD.vs.control; scRNA-seq,SRP309935,Monocyte_2-AD.vs.control; scRNA-seq,SRP330776,CD8+ T cell_1-AD.vs.control; scRNA-seq,SRP330776,γδ T cell-AD.vs.control; scRNA-seq,SRP330776,Memory T cell_2-AD.vs.control; scRNA-seq,SRP330776,CD8+ T cell_2-AD.vs.control; scRNA-seq,SRP330776,B cell_1-AD.vs.control; scRNA-seq,SRP330776,Naive CD8+ T cell_1-AD.vs.control; scRNA-seq,SRP330776,Natural killer cell_1-AD.vs.control; scRNA-seq,SRP330776,Memory T cell_1-AD.vs.control; scRNA-seq,SRP330776,Natural killer T cell-AD.vs.control; scRNA-seq,SRP330776,B cell_2-AD.vs.control; scRNA-seq,SRP330776,T cell-AD.vs.control; scRNA-seq,SRP330776,CD8+ T cell_3-AD.vs.control	19
B3GALT1	bulk RNA-seq,Homo_723,AD.vs.control; bulk RNA-seq,Homo_723,AD.vs.MCI; bulk RNA-seq,Homo_633,MCI.vs.control; bulk RNA-	4
B3GALT2	scRNA-seq,SRP330776,Memory T cell_2-AD.vs.control; scRNA-seq,SRP330776,CD8+ T cell_2-AD.vs.control; scRNA-seq,SRP330776,B cell_1-AD.vs.control; scRNA-seq,SRP330776,Naive CD8+ T cell_1-AD.vs.control; scRNA-seq,SRP330776,B cell_2-AD.vs.control	5

B3GALT4	scRNA-seq,SRP215507,CD8+ T cell_1-MCI.vs.control; scRNA-seq,SRP309935,Monocyte_2-AD.vs.control; scRNA-seq,SRP330776,CD8+ T cell_1-AD.vs.control; scRNA-seq,SRP330776,Memory T cell_2-AD.vs.control; scRNA-seq,SRP330776,CD8+ T cell_2-AD.vs.control; scRNA-seq,SRP330776,B cell_1-AD.vs.control; scRNA-seq,SRP330776,Naive CD8+ T cell_1-AD.vs.control; scRNA-seq,SRP330776,Natural killer cell_1-AD.vs.control; scRNA-seq,SRP330776,Memory T cell_1-AD.vs.control; scRNA-seq,SRP330776,Natural killer T cell-AD.vs.control; scRNA-seq,SRP330776,Naive CD8+ T cell_3-AD.vs.control; scRNA-seq,SRP330776,B cell_2-AD.vs.control; scRNA-seq,SRP330776,T cell-AD.vs.control; scRNA-seq,SRP330776,CD8+ T cell_3-AD.vs.control; scRNA-seq,SRP330776,B cell_3-AD.vs.control; scRNA-seq,SRP330776,Natural killer cell_2-AD.vs.control	16
B3GALT5	bulk RNA-seq,Homo_723,AD.vs.control; bulk RNA-seq,Homo_723,AD.vs.MCI; bulk RNA-seq,Homo_633,MCI.vs.control; bulk RNA- [illegible]	4
B3GALT6	scRNA-seq,SRP215507,CD8+ T cell_3-AD.vs.control; scRNA-seq,SRP215507,CD8+ T cell_3-MCI.vs.control; scRNA-seq,SRP309935,Natural killer cell_2-AD.vs.control; scRNA-seq,SRP309935,Natural killer cell_1-AD.vs.control; scRNA-seq,SRP330776,CD8+ T cell_1-AD.vs.control; scRNA-seq,SRP330776,γδ T cell-AD.vs.control; scRNA-seq,SRP330776,Memory T cell_2-AD.vs.control; scRNA-seq,SRP330776,CD8+ T cell_2-AD.vs.control; scRNA-seq,SRP330776,B cell_1-AD.vs.control; scRNA-seq,SRP330776,Naive CD8+ T cell_1-AD.vs.control; scRNA-seq,SRP330776,Natural killer cell_1-AD.vs.control; scRNA-seq,SRP330776,Memory T cell_1-AD.vs.control; scRNA-seq,SRP330776,Naive CD8+ T cell_3-AD.vs.control	13
B3GALT9	bulk RNA-seq,Homo_723,AD.vs.control; bulk RNA-seq,Homo_723,AD.vs.MCI; bulk RNA-seq,Homo_633,AD.vs.control; bulk RNA- [illegible]	4
B3GAT1	scRNA-seq,SRP215507,CD8+ T cell_5-AD.vs.MCI; scRNA-seq,SRP215507,CD8+ T cell_4-MCI.vs.control; scRNA-seq,SRP309935,CD8+ T cell_1-AD.vs.control; scRNA-seq,SRP330776,CD8+ T cell_2-AD.vs.control; scRNA-seq,SRP330776,Natural killer T cell-AD.vs.control	5
B3GAT2	bulk RNA-seq,Homo_723,AD.vs.control; bulk RNA-seq,Homo_723,AD.vs.MCI; bulk RNA-seq,Homo_723,MCI.vs.control; bulk RNA-seq,Homo_633,AD.vs.control; bulk RNA-seq,Homo_633,AD.vs.MCI; bulk RNA-seq,Homo_633,MCI.vs.control; bulk RNA-seq,Homo_714,AD.vs.control; bulk RNA-seq,Homo_714,AD.vs.MCI; bulk RNA-seq,Homo_714,MCI.vs.control; scRNA-seq,SRP330776,CD8+ T cell_1-AD.vs.control; scRNA-seq,SRP330776,Memory T cell_2-AD.vs.control; scRNA-seq,SRP330776,CD8+ T cell_2-AD.vs.control; scRNA-seq,SRP330776,B cell_1-AD.vs.control; scRNA-seq,SRP330776,Naive CD8+ T cell_1-AD.vs.control; scRNA-seq,SRP330776,Natural killer cell_1-AD.vs.control; scRNA-seq,SRP330776,CD8+ T [illegible]	16
B3GAT3	scRNA-seq,SRP330776,CD8+ T cell_1-AD.vs.control; scRNA-seq,SRP330776,γδ T cell-AD.vs.control; scRNA-seq,SRP330776,Memory T cell_2-AD.vs.control; scRNA-seq,SRP330776,CD8+ T cell_2-AD.vs.control; scRNA-seq,SRP330776,B cell_1-AD.vs.control; scRNA-seq,SRP330776,Naive CD8+ T cell_1-AD.vs.control; scRNA-seq,SRP330776,Natural killer cell_1-AD.vs.control; scRNA-seq,SRP330776,Memory T cell_1-AD.vs.control; scRNA-seq,SRP330776,Natural killer T cell-AD.vs.control; scRNA-seq,SRP330776,Naive CD8+ T cell_3-AD.vs.control; scRNA-seq,SRP330776,B cell_2-AD.vs.control; scRNA-seq,SRP330776,T cell-AD.vs.control; scRNA-seq,SRP330776,Naive CD8+ T cell_2-AD.vs.control; scRNA-seq,SRP330776,CD8+ [illegible]	15
B3GLCT	bulk RNA-seq,Homo_633,AD.vs.control; bulk RNA-seq,Homo_714,AD.vs.control; bulk RNA-seq,Homo_714,AD.vs.MCI; scRNA-seq,SRP215507,CD8+ T cell_5-AD.vs.control; scRNA-seq,SRP215507,CD8+ T cell_4-AD.vs.MCI; scRNA-seq,SRP215507,CD8+ T cell_5-AD.vs.MCI; scRNA-seq,SRP215507,CD8+ T cell_5-MCI.vs.control; scRNA-seq,SRP309935,Natural killer cell_1-AD.vs.control; scRNA-seq,SRP309935,CD8+ T cell_1-AD.vs.control; scRNA-seq,SRP330776,CD8+ T cell_1-AD.vs.control; scRNA-seq,SRP330776,γδ T cell-AD.vs.control; scRNA-seq,SRP330776,Memory T cell_2-AD.vs.control; scRNA-seq,SRP330776,CD8+ T cell_2-AD.vs.control; scRNA-seq,SRP330776,B cell_1-AD.vs.control; scRNA-seq,SRP330776,Naive CD8+ T cell_1-AD.vs.control; scRNA-seq,SRP330776,Natural killer cell_1-AD.vs.control; scRNA-seq,SRP330776,Memory T cell_1-AD.vs.control; scRNA-seq,SRP330776,Natural killer T cell-AD.vs.control; scRNA-seq,SRP330776,Naive CD8+ T cell_3-AD.vs.control; scRNA- [illegible]	22
B3GNT10	scRNA-seq,SRP215507,CD8+ T cell_5-MCI.vs.control; scRNA-seq,SRP215507,CD8+ T cell_2-MCI.vs.control; scRNA-seq,SRP330776,B cell_2-AD.vs.control; scRNA-seq,SRP330776,CD8+ T cell_3-AD.vs.control	4
B3GNT2	bulk RNA-seq,SRP223445,AD.vs.control; scRNA-seq,SRP215507,CD8+ T cell_2-AD.vs.MCI; scRNA-seq,SRP215507,CD8+ T cell_1-AD.vs.MCI; scRNA-seq,SRP215507,CD8+ T cell_5-AD.vs.MCI; scRNA-seq,SRP215507,CD8+ T cell_6-AD.vs.MCI; scRNA-seq,SRP215507,CD8+ T cell_1-MCI.vs.control; scRNA-seq,SRP215507,CD8+ T cell_5-MCI.vs.control; scRNA-seq,SRP215507,CD8+ T cell_2-MCI.vs.control; scRNA-seq,SRP309935,CD8+ T cell_2-AD.vs.control; scRNA-seq,SRP330776,CD8+ T cell_1-AD.vs.control; scRNA-seq,SRP330776,γδ T cell-AD.vs.control; scRNA-seq,SRP330776,Memory T cell_2-AD.vs.control; scRNA-seq,SRP330776,CD8+ T cell_2-AD.vs.control; scRNA-seq,SRP330776,B cell_1-AD.vs.control; scRNA-seq,SRP330776,Naive CD8+ T cell_1-AD.vs.control; scRNA-seq,SRP330776,Natural killer cell_1-AD.vs.control; scRNA-seq,SRP330776,Memory T cell_1-AD.vs.control; scRNA-seq,SRP330776,Natural killer T cell-AD.vs.control; scRNA-seq,SRP330776,Naive CD8+ T cell_3-AD.vs.control; scRNA-seq,SRP330776,B cell_2-AD.vs.control; scRNA-seq,SRP330776,T cell-AD.vs.control; scRNA-seq,SRP330776,Naive CD8+ T cell_2-AD.vs.control; scRNA-seq,SRP330776,CD8+ T cell_3-AD.vs.control; scRNA-seq,SRP330776,Monocyte_1-AD.vs.control; scRNA- [illegible]	27

B3GNT3	bulk RNA-seq,Homo_723,AD.vs.control; bulk RNA-seq,Homo_723,AD.vs.MCI; bulk RNA-seq,Homo_633,AD.vs.control; bulk RNA-seq,Homo_633,AD.vs.MCI; bulk RNA-seq,Homo_714,AD.vs.control; bulk RNA-seq,Homo_714,AD.vs.MCI; scRNA-seq,SRP215507,CD8+ T cell_3-AD.vs.control; scRNA-seq,SRP330776,CD8+ T cell_1-AD.vs.control	8
B3GNT4	bulk RNA-seq,Homo_633,AD.vs.control; bulk RNA-seq,Homo_633,AD.vs.MCI; bulk RNA-seq,Homo_714,AD.vs.control; bulk RNA-seq,Homo_714,AD.vs.MCI; scRNA-seq,SRP215507,CD8+ T cell_2-AD.vs.control; scRNA-seq,SRP215507,CD8+ T cell_3-AD.vs.MCI; scRNA-seq,SRP215507,CD8+ T cell_3-MCI.vs.control; scRNA-seq,SRP330776,CD8+ T cell_1-AD.vs.control; scRNA-seq,SRP330776,Naive CD8+ T cell_1-	9
B3GNT5	bulk RNA-seq,SRP223445,AD.vs.control; scRNA-seq,SRP330776,Natural killer cell_1-AD.vs.control	2
B3gnt5	bulk RNA-seq,SRP325058,AD.vs.control	1
B3GNT6	bulk RNA-seq,Homo_714,MCI.vs.control	1
B3GNT7	bulk RNA-seq,Homo_723,AD.vs.control; bulk RNA-seq,Homo_633,AD.vs.control; bulk RNA-seq,Homo_714,AD.vs.control; bulk RNA-seq,Homo_714,AD.vs.MCI; scRNA-seq,SRP215507,CD8+ T cell_1-AD.vs.control; scRNA-seq,SRP215507,CD8+ T cell_1-AD.vs.MCI; scRNA-seq,SRP215507,CD8+ T cell_1-MCI.vs.control; scRNA-seq,SRP330776,B cell_1-AD.vs.control; scRNA-seq,SRP330776,B cell_3-AD.vs.control	9
B3GNT8	bulk RNA-seq,Homo_723,AD.vs.control; bulk RNA-seq,Homo_723,AD.vs.MCI; bulk RNA-seq,Homo_723,MCI.vs.control; bulk RNA-seq,Homo_633,AD.vs.control; bulk RNA-seq,Homo_633,AD.vs.MCI; bulk RNA-seq,Homo_633,MCI.vs.control; bulk RNA-seq,Homo_714,AD.vs.control; bulk RNA-seq,Homo_714,AD.vs.MCI; bulk RNA-seq,Homo_714,MCI.vs.control; scRNA-seq,SRP330776,Monocyte_2-AD.vs.control	10
B3GNT9	bulk RNA-seq,Homo_723,AD.vs.control; bulk RNA-seq,Homo_723,AD.vs.MCI; bulk RNA-seq,Homo_633,AD.vs.control; bulk RNA-seq,Homo_633,AD.vs.MCI; bulk RNA-seq,Homo_714,AD.vs.control; bulk RNA-seq,Homo_714,AD.vs.MCI; scRNA-seq,SRP215507,CD8+ T cell_2-AD.vs.control; scRNA-seq,SRP215507,CD8+ T cell_3-AD.vs.control; scRNA-seq,SRP215507,CD8+ T cell_6-AD.vs.control; scRNA-seq,SRP215507,CD8+ T cell_2-AD.vs.MCI; scRNA-seq,SRP215507,CD8+ T cell_6-MCI.vs.control; scRNA-seq,SRP330776,CD8+ T cell_1-AD.vs.control; scRNA-seq,SRP330776,Memory T cell_2-AD.vs.control; scRNA-seq,SRP330776,Naive CD8+ T cell_1-AD.vs.control	14
B3GNTL1	scRNA-seq,SRP215507,CD8+ T cell_6-AD.vs.control; scRNA-seq,SRP215507,CD8+ T cell_9-AD.vs.control; scRNA-seq,SRP215507,CD8+ T cell_1-AD.vs.MCI; scRNA-seq,SRP215507,CD8+ T cell_4-AD.vs.MCI; scRNA-seq,SRP215507,CD8+ T cell_1-MCI.vs.control; scRNA-seq,SRP215507,CD8+ T cell_4-MCI.vs.control; scRNA-seq,SRP309935,CD8+ T cell_1-AD.vs.control; scRNA-seq,SRP330776,CD8+ T cell_1-AD.vs.control; scRNA-seq,SRP330776,Memory T cell_2-AD.vs.control; scRNA-seq,SRP330776,CD8+ T cell_2-AD.vs.control; scRNA-seq,SRP330776,Naive CD8+ T cell_1-AD.vs.control; scRNA-seq,SRP330776,Natural killer cell_1-AD.vs.control; scRNA-seq,SRP330776,Memory T cell_1-AD.vs.control; scRNA-	15
B430306N03Rik	bulk RNA-seq,SRP325058,AD.vs.control	1
B4GALNT1	bulk RNA-seq,Homo_723,AD.vs.control; bulk RNA-seq,Homo_723,AD.vs.MCI; bulk RNA-seq,Homo_633,AD.vs.control; bulk RNA-seq,Homo_633,AD.vs.MCI; bulk RNA-seq,Homo_714,MCI.vs.control; bulk RNA-seq,ROSMAP,AD.vs.control; bulk RNA-seq,ROSMAP,MCI.vs.control	7
B4GALNT2	bulk RNA-seq,Homo_723,MCI.vs.control; bulk RNA-seq,Homo_633,MCI.vs.control; bulk RNA-seq,Homo_714,MCI.vs.control; bulk RNA-seq,ROSMAP,AD.vs.control; bulk RNA-seq,ROSMAP,MCI.vs.control	5
B4GALNT3	bulk RNA-seq,Homo_723,AD.vs.control; bulk RNA-seq,Homo_633,AD.vs.control; bulk RNA-seq,Homo_633,AD.vs.MCI; bulk RNA-seq,Homo_714,AD.vs.control; bulk RNA-seq,Homo_714,AD.vs.MCI; bulk RNA-seq,ROSMAP,AD.vs.control; scRNA-seq,SRP215507,CD8+ T cell_2-AD.vs.control; scRNA-seq,SRP215507,CD8+ T cell_1-AD.vs.control; scRNA-seq,SRP215507,CD8+ T cell_4-AD.vs.control; scRNA-seq,SRP215507,CD8+ T cell_2-AD.vs.MCI; scRNA-seq,SRP215507,CD8+ T cell_1-AD.vs.MCI; scRNA-seq,SRP215507,CD8+ T cell_4-AD.vs.MCI; scRNA-seq,SRP215507,CD8+ T cell_1-MCI.vs.control; scRNA-seq,SRP215507,CD8+ T cell_4-MCI.vs.control; scRNA-seq,SRP330776,Natural killer	16
B4GALNT4	bulk RNA-seq,Homo_723,AD.vs.control; bulk RNA-seq,Homo_723,AD.vs.MCI; bulk RNA-seq,Homo_723,MCI.vs.control; bulk RNA-seq,Homo_633,AD.vs.control; bulk RNA-seq,Homo_633,AD.vs.MCI; bulk RNA-seq,Homo_633,MCI.vs.control; bulk RNA-seq,Homo_714,AD.vs.control; scRNA-seq,SRP215507,CD8+ T cell_2-AD.vs.control; scRNA-seq,SRP215507,CD8+ T cell_1-AD.vs.MCI; scRNA-seq,SRP215507,CD8+ T cell_1-MCI.vs.control; scRNA-seq,SRP215507,CD8+ T cell_4-MCI.vs.control; scRNA-seq,SRP215507,CD8+ T cell_2-MCI.vs.control	12

B4GALT1	bulk RNA-seq,Homo_723,AD.vs.control; bulk RNA-seq,Homo_723,AD.vs.MCI; bulk RNA-seq,Homo_723,MCI.vs.control; bulk RNA-seq,Homo_633,AD.vs.control; bulk RNA-seq,Homo_633,AD.vs.MCI; bulk RNA-seq,Homo_633,MCI.vs.control; bulk RNA-seq,Homo_714,AD.vs.control; bulk RNA-seq,Homo_714,AD.vs.MCI; bulk RNA-seq,Homo_714,MCI.vs.control; bulk RNA-seq,SRP223445,AD.vs.control; scRNA-seq,SRP215507,CD8+ T cell_7-AD.vs.control; scRNA-seq,SRP215507,CD8+ T cell_9-AD.vs.control; scRNA-seq,SRP215507,CD8+ T cell_4-AD.vs.MCI; scRNA-seq,SRP215507,CD8+ T cell_5-AD.vs.MCI; scRNA-seq,SRP215507,CD8+ T cell_5-MCI.vs.control; scRNA-seq,SRP215507,CD8+ T cell_7-	17
B4GALT1-AS1	scRNA-seq,SRP215507,CD8+ T cell_2-AD.vs.control; scRNA-seq,SRP215507,CD8+ T cell_2-AD.vs.MCI; scRNA-seq,SRP215507,CD8+ T cell_1-MCI.vs.control; scRNA-seq,SRP330776,B cell_1-AD.vs.control; scRNA-seq,SRP330776,Memory T cell_1-AD.vs.control; scRNA-seq,SRP330776,B	6
B4GALT2	bulk RNA-seq,Homo_723,AD.vs.control; bulk RNA-seq,Homo_723,AD.vs.MCI; bulk RNA-seq,Homo_723,MCI.vs.control; bulk RNA-seq,Homo_633,AD.vs.control; bulk RNA-seq,Homo_633,AD.vs.MCI; bulk RNA-seq,Homo_714,AD.vs.control; bulk RNA-seq,Homo_714,AD.vs.MCI; scRNA-seq,SRP215507,CD8+ T cell_4-AD.vs.control; scRNA-seq,SRP330776,CD8+ T cell_2-AD.vs.control	9
B4GALT3	bulk RNA-seq,Homo_723,AD.vs.control; bulk RNA-seq,Homo_723,AD.vs.MCI; bulk RNA-seq,Homo_723,MCI.vs.control; bulk RNA-seq,Homo_633,AD.vs.control; bulk RNA-seq,Homo_633,AD.vs.MCI; bulk RNA-seq,Homo_633,MCI.vs.control; bulk RNA-seq,Homo_714,AD.vs.control; bulk RNA-seq,Homo_714,AD.vs.MCI; bulk RNA-seq,Homo_714,MCI.vs.control; scRNA-seq,SRP215507,CD8+ T cell_4-AD.vs.control; scRNA-seq,SRP215507,CD8+ T cell_7-AD.vs.control; scRNA-seq,SRP330776,Natural killer cell_1-AD.vs.control	12
B4GALT4	bulk RNA-seq,Homo_723,AD.vs.control; bulk RNA-seq,Homo_723,AD.vs.MCI; bulk RNA-seq,Homo_633,AD.vs.control; bulk RNA-seq,Homo_633,AD.vs.MCI; bulk RNA-seq,Homo_714,AD.vs.control; bulk RNA-seq,Homo_714,AD.vs.MCI; scRNA-seq,SRP215507,CD8+ T cell_6-AD.vs.control; scRNA-seq,SRP215507,CD8+ T cell_4-AD.vs.MCI; scRNA-seq,SRP215507,CD8+ T cell_4-MCI.vs.control; scRNA-seq,SRP215507,CD8+ T cell_2-MCI.vs.control; scRNA-seq,SRP215507,CD8+ T cell_6-MCI.vs.control; scRNA-seq,SRP330776,Natural killer cell_1-AD.vs.control	12
B4GALT5	bulk RNA-seq,Homo_633,AD.vs.control; scRNA-seq,SRP215507,CD8+ T cell_2-AD.vs.MCI; scRNA-seq,SRP215507,CD8+ T cell_1-AD.vs.MCI; scRNA-seq,SRP215507,CD8+ T cell_5-AD.vs.MCI; scRNA-seq,SRP215507,CD8+ T cell_5-MCI.vs.control; scRNA-seq,SRP215507,CD8+ T cell_2-MCI.vs.control; scRNA-seq,SRP309935,Natural killer cell_2-AD.vs.control; scRNA-seq,SRP309935,CD8+ T cell_1-AD.vs.control; scRNA-seq,SRP330776,B cell_1-AD.vs.control; scRNA-seq,SRP330776,Natural killer cell_1-AD.vs.control; scRNA-seq,SRP330776,Memory T cell_1-	11
B4GALT6	scRNA-seq,SRP215507,CD8+ T cell_2-AD.vs.control; scRNA-seq,SRP215507,CD8+ T cell_1-AD.vs.MCI; scRNA-seq,SRP215507,CD8+ T cell_1-MCI.vs.control; scRNA-seq,SRP215507,CD8+ T cell_2-MCI.vs.control	4
B4GALT7	bulk RNA-seq,Homo_723,AD.vs.control; bulk RNA-seq,Homo_723,AD.vs.MCI; bulk RNA-seq,Homo_633,AD.vs.control; bulk RNA-seq,Homo_633,AD.vs.MCI; bulk RNA-seq,Homo_714,AD.vs.control; bulk RNA-seq,Homo_714,AD.vs.MCI; scRNA-seq,SRP215507,CD8+ T cell_4-AD.vs.MCI; scRNA-seq,SRP215507,CD8+ T cell_7-AD.vs.MCI; scRNA-seq,SRP215507,CD8+ T cell_5-MCI.vs.control; scRNA-seq,SRP215507,CD8+ T cell_2-MCI.vs.control; scRNA-seq,SRP330776,CD8+ T cell_1-AD.vs.control; scRNA-seq,SRP330776,γδ T cell-AD.vs.control; scRNA-seq,SRP330776,Memory T cell_2-AD.vs.control; scRNA-seq,SRP330776,B cell_1-AD.vs.control; scRNA-seq,SRP330776,Naive CD8+ T cell_1-AD.vs.control; scRNA-seq,SRP330776,Natural killer cell_1-AD.vs.control; scRNA-seq,SRP330776,Natural killer T cell-AD.vs.control; scRNA-seq,SRP330776,Naive CD8+ T cell_3-AD.vs.control; scRNA-seq,SRP330776,B cell_2-AD.vs.control; scRNA-seq,SRP330776,T cell-AD.vs.control; [illegible]	21
B4GAT1	scRNA-seq,SRP215507,CD8+ T cell_1-AD.vs.control; scRNA-seq,SRP215507,CD8+ T cell_1-AD.vs.MCI; scRNA-seq,SRP215507,CD8+ T cell_1-MCI.vs.control; scRNA-seq,SRP330776,CD8+ T cell_1-AD.vs.control; scRNA-seq,SRP330776,Memory T cell_2-AD.vs.control; scRNA-seq,SRP330776,CD8+ T cell_2-AD.vs.control; scRNA-seq,SRP330776,B cell_1-AD.vs.control; scRNA-seq,SRP330776,Naive CD8+ T cell_1-AD.vs.control; scRNA-seq,SRP330776,Natural killer cell_1-AD.vs.control; scRNA-seq,SRP330776,Natural killer T cell-AD.vs.control; scRNA-	12
B9D1	scRNA-seq,SRP215507,CD8+ T cell_2-AD.vs.control; scRNA-seq,SRP215507,CD8+ T cell_1-AD.vs.control; scRNA-seq,SRP215507,CD8+ T cell_1-AD.vs.MCI; scRNA-seq,SRP330776,Memory T cell_2-AD.vs.control; scRNA-seq,SRP330776,Natural killer cell_1-AD.vs.control; scRNA-	6
B9D2	bulk RNA-seq,Homo_723,AD.vs.control; bulk RNA-seq,Homo_723,AD.vs.MCI; bulk RNA-seq,Homo_633,AD.vs.control; bulk RNA-seq,Homo_633,AD.vs.MCI; bulk RNA-seq,Homo_714,AD.vs.control; bulk RNA-seq,Homo_714,AD.vs.MCI; scRNA-seq,SRP330776,Naive CD8+ T	7
BAALC	scRNA-seq,SRP330776,Memory T cell_1-AD.vs.control	1
BAAT	bulk RNA-seq,Homo_723,AD.vs.control; bulk RNA-seq,Homo_723,AD.vs.MCI; bulk RNA-seq,Homo_723,MCI.vs.control; bulk RNA-seq,Homo_633,AD.vs.control; bulk RNA-seq,Homo_633,AD.vs.MCI; bulk RNA-seq,Homo_633,MCI.vs.control	6
BABAM1	scRNA-seq,SRP215507,CD8+ T cell_8-AD.vs.control; scRNA-seq,SRP215507,CD8+ T cell_10-AD.vs.control; scRNA-seq,SRP215507,CD8+ T cell_4-MCI.vs.control; scRNA-seq,SRP330776,B cell_4-AD.vs.control	4

BABAM2	scRNA-seq,SRP330776,Naive CD8+ T cell_3-AD.vs.control; scRNA-seq,SRP330776,Regulatory T cell-AD.vs.control	2
BACE1	bulk RNA-seq,Homo_723,AD.vs.control; bulk RNA-seq,Homo_723,AD.vs.MCI; bulk RNA-seq,Homo_723,MCI.vs.control; bulk RNA-seq,Homo_633,AD.vs.MCI; bulk RNA-seq,Homo_714,AD.vs.control; bulk RNA-seq,Homo_714,AD.vs.MCI; scRNA-seq,SRP309935,Monocyte_1-AD.vs.control; scRNA-seq,SRP330776,CD8+ T cell_1-AD.vs.control; scRNA-seq,SRP330776,Memory T cell_1-AD.vs.control	9
BACE1-AS	scRNA-seq,SRP215507,CD8+ T cell_2-AD.vs.control; scRNA-seq,SRP215507,CD8+ T cell_1-AD.vs.control; scRNA-seq,SRP215507,CD8+ T cell_2-AD.vs.MCI; scRNA-seq,SRP215507,CD8+ T cell_4-MCI.vs.control; scRNA-seq,SRP215507,CD8+ T cell_2-MCI.vs.control; scRNA- [illegible]	6
BACE2	bulk RNA-seq,Homo_714,AD.vs.control; bulk RNA-seq,Homo_714,AD.vs.MCI; scRNA-seq,SRP309935,B cell_1-AD.vs.control; scRNA-seq,SRP330776,B cell_1-AD.vs.control; scRNA-seq,SRP330776,B cell_2-AD.vs.control	5
BACH1	bulk RNA-seq,Homo_714,AD.vs.control; bulk RNA-seq,Homo_714,AD.vs.MCI; scRNA-seq,SRP215507,CD8+ T cell_6-AD.vs.control; scRNA-seq,SRP215507,CD8+ T cell_2-MCI.vs.control; scRNA-seq,SRP215507,CD8+ T cell_3-MCI.vs.control; scRNA-seq,SRP215507,CD8+ T cell_6-MCI.vs.control; scRNA-seq,SRP215507,CD8+ T cell_7-MCI.vs.control; scRNA-seq,SRP309935,Naive CD8+ T cell_2-AD.vs.control; scRNA-seq,SRP330776,γδ T cell-AD.vs.control; scRNA-seq,SRP330776,Memory T cell_2-AD.vs.control; scRNA-seq,SRP330776,CD8+ T cell_2-AD.vs.control; scRNA-seq,SRP330776,B cell_1-AD.vs.control; scRNA-seq,SRP330776,Naive CD8+ T cell_1-AD.vs.control; scRNA-seq,SRP330776,Natural killer cell_1-AD.vs.control; scRNA-seq,SRP330776,Natural killer T cell-AD.vs.control; scRNA-seq,SRP330776,B cell_2-AD.vs.control; scRNA- [illegible]	19
BACH1-IT2	scRNA-seq,SRP215507,CD8+ T cell_1-MCI.vs.control	1
BACH2	bulk RNA-seq,Homo_714,AD.vs.control; scRNA-seq,SRP215507,CD8+ T cell_3-AD.vs.control; scRNA-seq,SRP215507,CD8+ T cell_4-AD.vs.control; scRNA-seq,SRP215507,CD8+ T cell_4-AD.vs.MCI; scRNA-seq,SRP215507,CD8+ T cell_7-AD.vs.MCI; scRNA-seq,SRP215507,CD8+ T cell_5-MCI.vs.control; scRNA-seq,SRP215507,CD8+ T cell_7-MCI.vs.control; scRNA-seq,SRP309935,Megakaryocyte_1-AD.vs.control; scRNA-seq,SRP330776,CD8+ T cell_1-AD.vs.control; scRNA-seq,SRP330776,γδ T cell-AD.vs.control; scRNA-seq,SRP330776,Memory T cell_2-AD.vs.control; scRNA-seq,SRP330776,B cell_1-AD.vs.control; scRNA-seq,SRP330776,Naive CD8+ T cell_1-AD.vs.control; scRNA-seq,SRP330776,Naive CD8+ T cell_3-AD.vs.control; scRNA-seq,SRP330776,B cell_2-AD.vs.control; scRNA-seq,SRP330776,Naive CD8+ T cell_2-AD.vs.control; scRNA-seq,SRP330776,CD8+ T cell_3-AD.vs.control; scRNA-seq,SRP330776,B cell_3-AD.vs.control; scRNA-seq,SRP330776,Regulatory T cell-AD.vs.control; [illegible]	20
BAD	bulk RNA-seq,Homo_714,AD.vs.control; bulk RNA-seq,Homo_714,AD.vs.MCI; scRNA-seq,SRP215507,CD8+ T cell_4-AD.vs.control; scRNA-seq,SRP215507,CD8+ T cell_7-AD.vs.control; scRNA-seq,SRP215507,CD8+ T cell_4-MCI.vs.control; scRNA-seq,SRP215507,CD8+ T cell_7-MCI.vs.control; scRNA-seq,SRP330776,γδ T cell-AD.vs.control; scRNA-seq,SRP330776,CD8+ T cell_3-AD.vs.control; scRNA-seq,SRP330776,B cell_3- [illegible]	9
BAG1	bulk RNA-seq,Homo_633,MCI.vs.control; scRNA-seq,SRP215507,CD8+ T cell_4-AD.vs.control; scRNA-seq,SRP215507,CD8+ T cell_7-AD.vs.control; scRNA-seq,SRP215507,CD8+ T cell_10-AD.vs.control; scRNA-seq,SRP215507,CD8+ T cell_4-AD.vs.MCI; scRNA-seq,SRP215507,CD8+ T cell_7-AD.vs.MCI; scRNA-seq,SRP215507,CD8+ T cell_6-AD.vs.MCI; scRNA-seq,SRP215507,CD8+ T cell_4-MCI.vs.control; scRNA-seq,SRP215507,CD8+ T cell_6-MCI.vs.control; scRNA-seq,SRP330776,CD8+ T cell_1-AD.vs.control; scRNA-seq,SRP330776,γδ T cell-AD.vs.control; scRNA-seq,SRP330776,Memory T cell_2-AD.vs.control; scRNA-seq,SRP330776,CD8+ T cell_2-AD.vs.control; scRNA-seq,SRP330776,B cell_1-AD.vs.control; scRNA-seq,SRP330776,Naive CD8+ T cell_1-AD.vs.control; scRNA-seq,SRP330776,Natural killer cell_1-AD.vs.control; scRNA-seq,SRP330776,Memory T cell_1-AD.vs.control; scRNA-seq,SRP330776,Natural killer T cell-AD.vs.control; scRNA-seq,SRP330776,Naive CD8+ T cell_3-AD.vs.control; scRNA-seq,SRP330776,B cell_2-AD.vs.control; scRNA-seq,SRP330776,T cell-AD.vs.control; scRNA-seq,SRP330776,Naive CD8+ T cell_2-AD.vs.control; scRNA-seq,SRP330776,CD8+ T cell_3-AD.vs.control; scRNA-seq,SRP330776,Monocyte_1-AD.vs.control; scRNA-seq,SRP330776,Monocyte_2-AD.vs.control; scRNA-seq,SRP330776,B cell_3-AD.vs.control; scRNA-seq,SRP330776,Regulatory T cell-AD.vs.control; scRNA-seq,SRP330776,Natural [illegible]	29
Bag1	bulk RNA-seq,SRP325058,AD.vs.control	1
BAG2	bulk RNA-seq,Homo_633,AD.vs.control; bulk RNA-seq,Homo_714,AD.vs.control; bulk RNA-seq,Homo_714,AD.vs.MCI; scRNA-seq,SRP215507,CD8+ T cell_3-AD.vs.MCI; scRNA-seq,SRP215507,CD8+ T cell_4-AD.vs.MCI; scRNA-seq,SRP215507,CD8+ T cell_5-AD.vs.MCI; scRNA-seq,SRP215507,CD8+ T cell_6-AD.vs.MCI; scRNA-seq,SRP215507,CD8+ T cell_5-MCI.vs.control; scRNA-seq,SRP215507,CD8+ T cell_4-MCI.vs.control; scRNA-seq,SRP215507,CD8+ T cell_3-MCI.vs.control; scRNA-seq,SRP215507,CD8+ T cell_6-MCI.vs.control; scRNA-seq,SRP330776,CD8+ T cell_1-AD.vs.control; scRNA-seq,SRP330776,γδ T cell-AD.vs.control; scRNA-seq,SRP330776,CD8+ T cell_2-AD.vs.control; scRNA-seq,SRP330776,Naive CD8+ T cell_1-AD.vs.control; scRNA-seq,SRP330776,Natural killer T cell-AD.vs.control; scRNA-seq,SRP330776,Naive CD8+ T cell_3-AD.vs.control; scRNA-seq,SRP330776,B cell_2-AD.vs.control; scRNA-seq,SRP330776,CD8+ T cell_3-AD.vs.control; scRNA- [illegible]	20

BAG3	bulk RNA-seq,Homo_723,AD.vs.control; bulk RNA-seq,Homo_723,AD.vs.MCI; bulk RNA-seq,Homo_723,MCI.vs.control; bulk RNA-seq,Homo_633,AD.vs.control; bulk RNA-seq,Homo_633,AD.vs.MCI; bulk RNA-seq,Homo_633,MCI.vs.control; bulk RNA-seq,Homo_714,AD.vs.control; bulk RNA-seq,Homo_714,AD.vs.MCI; bulk RNA-seq,Homo_714,MCI.vs.control; scRNA-seq,SRP215507,CD8+ T cell_3-AD.vs.MCI; scRNA-seq,SRP215507,CD8+ T cell_3-MCI.vs.control; scRNA-seq,SRP215507,CD8+ T cell_6-MCI.vs.control; scRNA-seq,SRP309935,Monocyte_1-AD.vs.control; scRNA-seq,SRP330776,CD8+ T cell_1-AD.vs.control; scRNA-seq,SRP330776,γδ T cell-AD.vs.control; scRNA-seq,SRP330776,Memory T cell_2-AD.vs.control; scRNA-seq,SRP330776,CD8+ T cell_2-AD.vs.control; scRNA-seq,SRP330776,B cell_1-AD.vs.control; scRNA-seq,SRP330776,Naive CD8+ T cell_1-AD.vs.control; scRNA-seq,SRP330776,Natural killer cell_1-AD.vs.control; scRNA-seq,SRP330776,Memory T cell_1-AD.vs.control; scRNA-seq,SRP330776,Natural killer T cell-AD.vs.control; scRNA-seq,SRP330776,Naive CD8+ T cell_3-AD.vs.control; scRNA-seq,SRP330776,B cell_2-AD.vs.control; scRNA-seq,SRP330776,T cell-AD.vs.control; scRNA-seq,SRP330776,Naive CD8+ T cell_2-AD.vs.control; [illegible]	29
BAG4	bulk RNA-seq,Homo_723,AD.vs.control; bulk RNA-seq,Homo_633,AD.vs.control; bulk RNA-seq,Homo_633,MCI.vs.control; scRNA-seq,SRP215507,CD8+ T cell_5-AD.vs.MCI; scRNA-seq,SRP215507,CD8+ T cell_6-AD.vs.MCI; scRNA-seq,SRP215507,CD8+ T cell_5-MCI.vs.control; scRNA-seq,SRP215507,CD8+ T cell_6-MCI.vs.control; scRNA-seq,SRP309935,Natural killer cell_1-AD.vs.control; scRNA-seq,SRP330776,Monocyte_1-AD.vs.control; scRNA-seq,SRP330776,Monocyte_2-AD.vs.control; scRNA-seq,SRP330776,B cell_3-AD.vs.control	11
BAG5	bulk RNA-seq,Homo_714,AD.vs.control; bulk RNA-seq,Homo_714,AD.vs.MCI; scRNA-seq,SRP215507,CD8+ T cell_3-MCI.vs.control; scRNA-seq,SRP309935,Erythroid cell-AD.vs.control; scRNA-seq,SRP330776,Monocyte_2-AD.vs.control	5
BAG6	scRNA-seq,SRP215507,CD8+ T cell_7-AD.vs.control; scRNA-seq,SRP309935,CD8+ T cell_2-AD.vs.control; scRNA-seq,SRP330776,CD8+ T cell_1-AD.vs.control; scRNA-seq,SRP330776,Naive CD8+ T cell_1-AD.vs.control; scRNA-seq,SRP330776,B cell_2-AD.vs.control; scRNA-seq,SRP330776,T	6
BAGE5	bulk RNA-seq,Homo_723,AD.vs.control; bulk RNA-seq,Homo_723,MCI.vs.control; bulk RNA-seq,Homo_633,AD.vs.control; bulk RNA-seq,Homo_633,MCI.vs.control; bulk RNA-seq,Homo_714,MCI.vs.control	5
BAHD1	bulk RNA-seq,Homo_723,AD.vs.control; bulk RNA-seq,Homo_723,AD.vs.MCI; bulk RNA-seq,Homo_723,MCI.vs.control; bulk RNA-seq,Homo_633,AD.vs.control; bulk RNA-seq,Homo_633,AD.vs.MCI; bulk RNA-seq,Homo_633,MCI.vs.control; scRNA-seq,SRP215507,CD8+ T cell_1-AD.vs.control; scRNA-seq,SRP215507,CD8+ T cell_3-MCI.vs.control; scRNA-seq,SRP309935,Natural killer cell_2-AD.vs.control; scRNA-seq,SRP309935,Naive CD8+ T cell_1-AD.vs.control; scRNA-seq,SRP330776,γδ T cell-AD.vs.control; scRNA-seq,SRP330776,Memory T cell_2-AD.vs.control; scRNA-seq,SRP330776,CD8+ T cell_2-AD.vs.control; scRNA-seq,SRP330776,Natural killer cell_1-AD.vs.control; scRNA-seq,SRP330776,Memory T cell_1-AD.vs.control; scRNA-seq,SRP330776,Natural killer T cell-AD.vs.control; scRNA-seq,SRP330776,B cell_2-	17
BAIAP2	bulk RNA-seq,Homo_723,AD.vs.control; bulk RNA-seq,Homo_723,AD.vs.MCI; bulk RNA-seq,Homo_723,MCI.vs.control; bulk RNA-seq,Homo_633,AD.vs.control; bulk RNA-seq,Homo_633,MCI.vs.control; bulk RNA-seq,Homo_714,AD.vs.control; bulk RNA-seq,Homo_714,AD.vs.MCI; bulk RNA-seq,Homo_714,MCI.vs.control; scRNA-seq,SRP215507,CD8+ T cell_1-AD.vs.control; scRNA-seq,SRP215507,CD8+ T cell_1-MCI.vs.control; scRNA-seq,SRP309935,Monocyte_2-AD.vs.control; scRNA-seq,SRP330776,Memory T cell_1-AD.vs.control; scRNA-seq,SRP330776,Monocyte_1-	13
BAIAP2-DT	scRNA-seq,SRP330776,CD8+ T cell_1-AD.vs.control; scRNA-seq,SRP330776,Naive CD8+ T cell_1-AD.vs.control; scRNA-seq,SRP330776,Monocyte_2-	3
BAIAP2L1	scRNA-seq,SRP330776,CD8+ T cell_1-AD.vs.control; scRNA-seq,SRP330776,Memory T cell_2-AD.vs.control; scRNA-seq,SRP330776,Naive CD8+ T	3
BAIAP2L2	bulk RNA-seq,Homo_723,AD.vs.control; bulk RNA-seq,Homo_723,AD.vs.MCI; bulk RNA-seq,Homo_723,MCI.vs.control; bulk RNA-seq,Homo_633,MCI.vs.control; scRNA-seq,SRP330776,Naive CD8+ T cell_1-AD.vs.control	5
BAIAP3	bulk RNA-seq,Homo_723,AD.vs.control; bulk RNA-seq,Homo_723,MCI.vs.control; bulk RNA-seq,Homo_633,AD.vs.control; bulk RNA-seq,Homo_633,AD.vs.MCI; bulk RNA-seq,Homo_633,MCI.vs.control; scRNA-seq,SRP215507,CD8+ T cell_1-AD.vs.control; scRNA-seq,SRP215507,CD8+ T cell_2-AD.vs.MCI; scRNA-seq,SRP215507,CD8+ T cell_1-AD.vs.MCI; scRNA-seq,SRP215507,CD8+ T cell_2-MCI.vs.control; scRNA-seq,SRP330776,CD8+ T cell_1-AD.vs.control; scRNA-seq,SRP330776,CD8+ T cell_2-AD.vs.control; scRNA-seq,SRP330776,B cell_1-	13
BAK1	scRNA-seq,SRP215507,CD8+ T cell_9-AD.vs.control; scRNA-seq,SRP330776,γδ T cell-AD.vs.control; scRNA-seq,SRP330776,Memory T cell_2-AD.vs.control; scRNA-seq,SRP330776,CD8+ T cell_2-AD.vs.control; scRNA-seq,SRP330776,Natural killer cell_1-AD.vs.control; scRNA-seq,SRP330776,Memory T cell_1-AD.vs.control; scRNA-seq,SRP330776,Natural killer T cell-AD.vs.control; scRNA-seq,SRP330776,Monocyte_2-	8

BAMBI	bulk RNA-seq,Homo_723,AD.vs.control; bulk RNA-seq,Homo_723,AD.vs.MCI; bulk RNA-seq,Homo_723,MCI.vs.control; bulk RNA-seq,Homo_633,AD.vs.control; bulk RNA-seq,Homo_633,AD.vs.MCI; bulk RNA-seq,Homo_633,MCI.vs.control; bulk RNA-seq,Homo_714,AD.vs.control; bulk RNA-seq,Homo_714,AD.vs.MCI; scRNA-seq,SRP215507,CD8+ T cell_1-AD.vs.control; scRNA-seq,SRP215507,CD8+ T cell_5-AD.vs.control; scRNA-seq,SRP215507,CD8+ T cell_3-AD.vs.MCI; scRNA-seq,SRP215507,CD8+ T cell_1-MCI.vs.control; scRNA-seq,SRP215507,CD8+ T cell_5-MCI.vs.control; scRNA-seq,SRP215507,CD8+ T cell_4-MCI.vs.control; scRNA-seq,SRP215507,CD8+ T cell_2-MCI.vs.control; scRNA-	16
BANF1	bulk RNA-seq,Homo_723,AD.vs.control; bulk RNA-seq,Homo_723,MCI.vs.control; bulk RNA-seq,Homo_633,AD.vs.control; bulk RNA-seq,Homo_633,MCI.vs.control; scRNA-seq,SRP215507,CD8+ T cell_4-AD.vs.MCI; scRNA-seq,SRP330776,CD8+ T cell_1-AD.vs.control; scRNA-seq,SRP330776,γδ T cell-AD.vs.control; scRNA-seq,SRP330776,Memory T cell_2-AD.vs.control; scRNA-seq,SRP330776,CD8+ T cell_2-AD.vs.control; scRNA-seq,SRP330776,B cell_1-AD.vs.control; scRNA-seq,SRP330776,Naive CD8+ T cell_1-AD.vs.control; scRNA-seq,SRP330776,Natural killer cell_1-AD.vs.control; scRNA-seq,SRP330776,Natural killer T cell-AD.vs.control; scRNA-seq,SRP330776,Naive CD8+ T cell_3-AD.vs.control; scRNA-seq,SRP330776,B cell_2-AD.vs.control; scRNA-seq,SRP330776,T cell-AD.vs.control; scRNA-seq,SRP330776,CD8+ T cell_3-AD.vs.control; scRNA- [illegible]	18
BANK1	bulk RNA-seq,Homo_723,AD.vs.control; bulk RNA-seq,Homo_723,AD.vs.MCI; bulk RNA-seq,Homo_633,AD.vs.control; bulk RNA-seq,Homo_633,AD.vs.MCI; bulk RNA-seq,Homo_714,AD.vs.control; bulk RNA-seq,Homo_714,AD.vs.MCI; scRNA-seq,SRP215507,CD8+ T cell_1-AD.vs.control; scRNA-seq,SRP330776,Naive CD8+ T cell_2-AD.vs.control	8
Bank1	bulk RNA-seq,SRP325058,AD.vs.control	1
BANP	scRNA-seq,SRP215507,CD8+ T cell_5-AD.vs.MCI; scRNA-seq,SRP309935,Monocyte_2-AD.vs.control; scRNA-seq,SRP330776,B cell_1-AD.vs.control; scRNA-seq,SRP330776,Naive CD8+ T cell_2-AD.vs.control	4
BAP1	scRNA-seq,SRP215507,CD8+ T cell_6-AD.vs.control; scRNA-seq,SRP309935,CD8+ T cell_1-AD.vs.control; scRNA-seq,SRP330776,CD8+ T cell_1-AD.vs.control; scRNA-seq,SRP330776,γδ T cell-AD.vs.control; scRNA-seq,SRP330776,Memory T cell_2-AD.vs.control; scRNA-seq,SRP330776,CD8+ T cell_2-AD.vs.control; scRNA-seq,SRP330776,B cell_1-AD.vs.control; scRNA-seq,SRP330776,Naive CD8+ T cell_1-AD.vs.control; scRNA-seq,SRP330776,Natural killer cell_1-AD.vs.control; scRNA-seq,SRP330776,Memory T cell_1-AD.vs.control; scRNA-seq,SRP330776,Natural killer T cell-AD.vs.control; scRNA-seq,SRP330776,Naive CD8+ T cell_3-AD.vs.control; scRNA-seq,SRP330776,B cell_2-AD.vs.control; scRNA-seq,SRP330776,CD8+ T cell_3-AD.vs.control; scRNA-seq,SRP330776,B cell_3-AD.vs.control; scRNA-seq,SRP330776,Regulatory T cell-AD.vs.control; [illegible]	17
BARD1	bulk RNA-seq,Homo_723,AD.vs.control; bulk RNA-seq,Homo_723,MCI.vs.control; bulk RNA-seq,Homo_633,AD.vs.MCI; bulk RNA-seq,Homo_633,MCI.vs.control; bulk RNA-seq,Homo_714,MCI.vs.control; scRNA-seq,SRP215507,CD8+ T cell_2-AD.vs.MCI; scRNA-seq,SRP215507,CD8+ T cell_6-AD.vs.MCI; scRNA-seq,SRP215507,CD8+ T cell_2-MCI.vs.control; scRNA-seq,SRP309935,Naive CD8+ T cell_1-AD.vs.control; scRNA-seq,SRP309935,B cell_1-AD.vs.control; scRNA-seq,SRP330776,γδ T cell-AD.vs.control; scRNA-seq,SRP330776,Memory T cell_2-AD.vs.control; scRNA-seq,SRP330776,CD8+ T cell_2-AD.vs.control; scRNA-seq,SRP330776,Memory T cell_1-AD.vs.control; scRNA-seq,SRP330776,Natural killer T cell-AD.vs.control; scRNA-seq,SRP330776,CD8+ T cell_3-AD.vs.control; scRNA-seq,SRP330776,Regulatory T cell- [illegible]	18
BARHL1	bulk RNA-seq,Homo_723,AD.vs.MCI	1
BARHL2	bulk RNA-seq,Homo_723,AD.vs.MCI; bulk RNA-seq,Homo_723,MCI.vs.control	2
BARX1	bulk RNA-seq,ROSMAP,MCI.vs.control	1
BARX2	bulk RNA-seq,Homo_723,AD.vs.control; bulk RNA-seq,Homo_723,AD.vs.MCI; bulk RNA-seq,Homo_633,AD.vs.control	3
BASP1	bulk RNA-seq,Homo_723,AD.vs.control; bulk RNA-seq,Homo_723,AD.vs.MCI; bulk RNA-seq,Homo_633,AD.vs.control; bulk RNA-seq,Homo_633,AD.vs.MCI; bulk RNA-seq,Homo_714,AD.vs.control; bulk RNA-seq,Homo_714,AD.vs.MCI; bulk RNA-seq,SRP223445,AD.vs.control; scRNA-seq,SRP309935,Monocyte_1-AD.vs.control; scRNA-seq,SRP309935,Naive CD8+ T cell_1-AD.vs.control; scRNA-seq,SRP309935,B cell_1-AD.vs.control; scRNA-seq,SRP309935,B cell_2-AD.vs.control; scRNA-seq,SRP330776,B cell_1-AD.vs.control; scRNA-seq,SRP330776,B cell_2-AD.vs.control; scRNA-seq,SRP330776,Monocyte_1-AD.vs.control; scRNA-seq,SRP330776,Monocyte_2-AD.vs.control; scRNA-seq,SRP330776,B cell_3-	16
BATF	bulk RNA-seq,Homo_714,AD.vs.control; bulk RNA-seq,Homo_714,AD.vs.MCI; scRNA-seq,SRP215507,CD8+ T cell_3-AD.vs.control; scRNA-seq,SRP215507,CD8+ T cell_4-AD.vs.MCI; scRNA-seq,SRP215507,CD8+ T cell_4-MCI.vs.control; scRNA-seq,SRP330776,Memory T cell_2-AD.vs.control; scRNA-seq,SRP330776,CD8+ T cell_2-AD.vs.control; scRNA-seq,SRP330776,B cell_1-AD.vs.control; scRNA-seq,SRP330776,Natural killer cell_1-AD.vs.control; scRNA-seq,SRP330776,Memory T cell_1-AD.vs.control; scRNA-seq,SRP330776,Natural killer T cell-AD.vs.control; scRNA-seq,SRP330776,B cell_2-AD.vs.control; scRNA-seq,SRP330776,Megakaryocyte_1-AD.vs.control	13

BATF2	bulk RNA-seq,Homo_723,AD.vs.control; bulk RNA-seq,Homo_723,AD.vs.MCI; bulk RNA-seq,Homo_723,MCI.vs.control; bulk RNA-seq,Homo_633,AD.vs.control; bulk RNA-seq,Homo_633,AD.vs.MCI; bulk RNA-seq,Homo_633,MCI.vs.control; bulk RNA-seq,Homo_714,AD.vs.control; bulk RNA-seq,Homo_714,AD.vs.MCI; bulk RNA-seq,Homo_714,MCI.vs.control	9
BATF3	bulk RNA-seq,Homo_714,AD.vs.control; bulk RNA-seq,Homo_714,AD.vs.MCI; scRNA-seq,SRP215507,CD8+ T cell_9-AD.vs.control	3
BAX	scRNA-seq,SRP215507,CD8+ T cell_4-AD.vs.MCI; scRNA-seq,SRP215507,CD8+ T cell_7-AD.vs.MCI; scRNA-seq,SRP215507,CD8+ T cell_7-MCI.vs.control; scRNA-seq,SRP330776,CD8+ T cell_1-AD.vs.control; scRNA-seq,SRP330776,γδ T cell-AD.vs.control; scRNA-seq,SRP330776,Memory T cell_2-AD.vs.control; scRNA-seq,SRP330776,CD8+ T cell_2-AD.vs.control; scRNA-seq,SRP330776,B cell_1-AD.vs.control; scRNA-seq,SRP330776,Naive CD8+ T cell_1-AD.vs.control; scRNA-seq,SRP330776,Natural killer cell_1-AD.vs.control; scRNA-seq,SRP330776,Memory T cell_1-AD.vs.control; scRNA-seq,SRP330776,Natural killer T cell-AD.vs.control; scRNA-seq,SRP330776,Naive CD8+ T cell_3-AD.vs.control; scRNA-seq,SRP330776,B cell_2-AD.vs.control; scRNA-seq,SRP330776,T cell-AD.vs.control; scRNA-seq,SRP330776,Naive CD8+ T cell_2-AD.vs.control; scRNA-seq,SRP330776,CD8+ T cell_3-AD.vs.control; scRNA-seq,SRP330776,Monocyte_1-AD.vs.control; scRNA-seq,SRP330776,B cell_3- [illegible]	21
BAZ1A	bulk RNA-seq,Homo_714,AD.vs.control; bulk RNA-seq,Homo_714,AD.vs.MCI; scRNA-seq,SRP215507,CD8+ T cell_7-AD.vs.MCI; scRNA-seq,SRP330776,CD8+ T cell_1-AD.vs.control; scRNA-seq,SRP330776,γδ T cell-AD.vs.control; scRNA-seq,SRP330776,Memory T cell_2-AD.vs.control; scRNA-seq,SRP330776,CD8+ T cell_2-AD.vs.control; scRNA-seq,SRP330776,B cell_1-AD.vs.control; scRNA-seq,SRP330776,Naive CD8+ T cell_1-AD.vs.control; scRNA-seq,SRP330776,Natural killer cell_1-AD.vs.control; scRNA-seq,SRP330776,Memory T cell_1-AD.vs.control; scRNA-seq,SRP330776,Natural killer T cell-AD.vs.control; scRNA-seq,SRP330776,Naive CD8+ T cell_3-AD.vs.control; scRNA-seq,SRP330776,B cell_2-AD.vs.control; scRNA-seq,SRP330776,T cell-AD.vs.control; scRNA-seq,SRP330776,Naive CD8+ T cell_2-AD.vs.control; scRNA-seq,SRP330776,CD8+ T cell_3-AD.vs.control; scRNA-seq,SRP330776,Monocyte_1-AD.vs.control; scRNA-seq,SRP330776,Monocyte_2-AD.vs.control; scRNA-seq,SRP330776,B cell_3-AD.vs.control; scRNA-seq,SRP330776,Regulatory T cell-AD.vs.control; scRNA-seq,SRP330776,Natural killer cell_2- [illegible]	22
BAZ1B	bulk RNA-seq,Homo_723,AD.vs.control; bulk RNA-seq,Homo_723,AD.vs.MCI; bulk RNA-seq,Homo_723,MCI.vs.control; bulk RNA-seq,Homo_633,AD.vs.control; bulk RNA-seq,Homo_633,AD.vs.MCI; bulk RNA-seq,Homo_633,MCI.vs.control; bulk RNA-seq,Homo_714,AD.vs.control; bulk RNA-seq,Homo_714,AD.vs.MCI; bulk RNA-seq,Homo_714,MCI.vs.control; scRNA-seq,SRP215507,CD8+ T cell_4-AD.vs.control; scRNA-seq,SRP215507,CD8+ T cell_7-AD.vs.control; scRNA-seq,SRP309935,Natural killer cell_2-AD.vs.control; scRNA-seq,SRP309935,Naive CD8+ T cell_1-AD.vs.control; scRNA-seq,SRP309935,Natural killer cell_1-AD.vs.control; scRNA-seq,SRP309935,B cell_1-AD.vs.control; scRNA-seq,SRP309935,CD8+ T cell_2-AD.vs.control; scRNA-seq,SRP330776,CD8+ T cell_1-AD.vs.control; scRNA-seq,SRP330776,γδ T cell-AD.vs.control; scRNA-seq,SRP330776,Memory T cell_2-AD.vs.control; scRNA-seq,SRP330776,CD8+ T cell_2-AD.vs.control; scRNA-seq,SRP330776,B cell_1-AD.vs.control; scRNA-seq,SRP330776,Naive CD8+ T cell_1-AD.vs.control; scRNA-seq,SRP330776,Natural killer cell_1-AD.vs.control; scRNA-seq,SRP330776,Memory T cell_1-AD.vs.control; scRNA-seq,SRP330776,Natural killer T cell-AD.vs.control; scRNA-seq,SRP330776,Naive CD8+ T cell_3-AD.vs.control; scRNA-seq,SRP330776,B cell_2-AD.vs.control; scRNA-seq,SRP330776,T cell-AD.vs.control; scRNA-seq,SRP330776,Naive CD8+ T cell_2-AD.vs.control; scRNA-seq,SRP330776,CD8+ T cell_3-AD.vs.control; scRNA-seq,SRP330776,Monocyte_1-AD.vs.control; scRNA-seq,SRP330776,Monocyte_2- [illegible]	34
BAZ2A	bulk RNA-seq,Homo_723,AD.vs.control; bulk RNA-seq,Homo_633,AD.vs.control; scRNA-seq,SRP215507,CD8+ T cell_7-AD.vs.control	3
BAZ2B	bulk RNA-seq,Homo_714,AD.vs.control; bulk RNA-seq,Homo_714,AD.vs.MCI; scRNA-seq,SRP215507,CD8+ T cell_4-AD.vs.MCI; scRNA-seq,SRP215507,CD8+ T cell_7-AD.vs.MCI; scRNA-seq,SRP215507,CD8+ T cell_5-AD.vs.MCI; scRNA-seq,SRP215507,CD8+ T cell_1-MCI.vs.control; scRNA-seq,SRP215507,CD8+ T cell_5-MCI.vs.control; scRNA-seq,SRP215507,CD8+ T cell_4-MCI.vs.control; scRNA-seq,SRP309935,CD8+ T cell_1-AD.vs.control; scRNA-seq,SRP330776,Memory T cell_2-AD.vs.control; scRNA-seq,SRP330776,Naive CD8+ T cell_3-AD.vs.control; scRNA-	12

BBC3	bulk RNA-seq,Homo_714,AD.vs.control; bulk RNA-seq,Homo_714,AD.vs.MCI; scRNA-seq,SRP215507,CD8+ T cell_7-AD.vs.control; scRNA-seq,SRP215507,CD8+ T cell_6-AD.vs.control; scRNA-seq,SRP215507,CD8+ T cell_2-AD.vs.MCI; scRNA-seq,SRP215507,CD8+ T cell_1-AD.vs.MCI; scRNA-seq,SRP215507,CD8+ T cell_5-AD.vs.MCI; scRNA-seq,SRP215507,CD8+ T cell_1-MCI.vs.control; scRNA-seq,SRP215507,CD8+ T cell_5-MCI.vs.control; scRNA-seq,SRP215507,CD8+ T cell_2-MCI.vs.control; scRNA-seq,SRP309935,Naive CD8+ T cell_2-AD.vs.control; scRNA-seq,SRP330776,CD8+ T cell_1-AD.vs.control; scRNA-seq,SRP330776,γδ T cell-AD.vs.control; scRNA-seq,SRP330776,Memory T cell_2-AD.vs.control; scRNA-seq,SRP330776,CD8+ T cell_2-AD.vs.control; scRNA-seq,SRP330776,B cell_1-AD.vs.control; scRNA-seq,SRP330776,Naive CD8+ T cell_1-AD.vs.control; scRNA-seq,SRP330776,Natural killer cell_1-AD.vs.control; scRNA-seq,SRP330776,Memory T cell_1-AD.vs.control; scRNA-seq,SRP330776,Natural killer T cell-AD.vs.control; scRNA-seq,SRP330776,Naive CD8+ T cell_3-AD.vs.control; scRNA-seq,SRP330776,B cell_2-AD.vs.control; scRNA-seq,SRP330776,T cell-AD.vs.control; scRNA-seq,SRP330776,Naive CD8+ T cell_2-AD.vs.control; scRNA-seq,SRP330776,CD8+ [illegible]	27
BBIP1	bulk RNA-seq,Homo_723,AD.vs.MCI; bulk RNA-seq,Homo_633,AD.vs.MCI; bulk RNA-seq,Homo_714,AD.vs.control; bulk RNA-seq,Homo_714,AD.vs.MCI; scRNA-seq,SRP215507,CD8+ T cell_4-AD.vs.control; scRNA-seq,SRP215507,CD8+ T cell_4-AD.vs.MCI; scRNA-seq,SRP330776,Memory T cell_2-AD.vs.control; scRNA-seq,SRP330776,B cell_1-AD.vs.control; scRNA-seq,SRP330776,Natural killer cell_1-AD.vs.control; scRNA-seq,SRP330776,CD8+ T cell_3-AD.vs.control; scRNA-seq,SRP330776,Natural killer cell_2-AD.vs.control	11
BBLN	bulk RNA-seq,Homo_723,AD.vs.control; bulk RNA-seq,Homo_723,AD.vs.MCI; bulk RNA-seq,Homo_633,AD.vs.control; bulk RNA-seq,Homo_633,AD.vs.MCI; bulk RNA-seq,Homo_714,AD.vs.control; bulk RNA-seq,Homo_714,AD.vs.MCI	6
BBOF1	bulk RNA-seq,Homo_723,AD.vs.control; bulk RNA-seq,Homo_723,AD.vs.MCI; bulk RNA-seq,Homo_633,AD.vs.control; bulk RNA-seq,Homo_633,AD.vs.MCI; bulk RNA-seq,Homo_714,AD.vs.control; bulk RNA-seq,Homo_714,AD.vs.MCI; scRNA-seq,SRP215507,CD8+ T cell_2-AD.vs.MCI; scRNA-seq,SRP215507,CD8+ T cell_3-AD.vs.MCI; scRNA-seq,SRP215507,CD8+ T cell_2-MCI.vs.control; scRNA-seq,SRP215507,CD8+ T cell_3-MCI.vs.control; scRNA-seq,SRP309935,Erythroid cell-AD.vs.control; scRNA-seq,SRP330776,B cell_2-AD.vs.control	12
BBOX1-AS1	scRNA-seq,SRP215507,CD8+ T cell_1-MCI.vs.control	1
BBS1	bulk RNA-seq,Homo_723,AD.vs.control; bulk RNA-seq,Homo_723,AD.vs.MCI; bulk RNA-seq,Homo_633,AD.vs.MCI; bulk RNA-seq,Homo_714,AD.vs.control; bulk RNA-seq,Homo_714,AD.vs.MCI; bulk RNA-seq,ROSMAP,AD.vs.control; scRNA-seq,SRP215507,CD8+ T cell_3-AD.vs.MCI; scRNA-seq,SRP215507,CD8+ T cell_5-AD.vs.MCI; scRNA-seq,SRP215507,CD8+ T cell_3-MCI.vs.control; scRNA-seq,SRP330776,CD8+ T cell_1-AD.vs.control; scRNA-seq,SRP330776,Memory T cell_2-AD.vs.control; scRNA-seq,SRP330776,CD8+ T cell_2-AD.vs.control; scRNA-	14
BBS10	scRNA-seq,SRP215507,CD8+ T cell_3-AD.vs.control; scRNA-seq,SRP215507,CD8+ T cell_4-AD.vs.control; scRNA-seq,SRP215507,CD8+ T cell_5-AD.vs.MCI; scRNA-seq,SRP215507,CD8+ T cell_4-MCI.vs.control; scRNA-seq,SRP215507,CD8+ T cell_3-MCI.vs.control; scRNA-seq,SRP309935,Natural killer cell_1-AD.vs.control; scRNA-seq,SRP330776,CD8+ T cell_2-AD.vs.control; scRNA-seq,SRP330776,Memory T cell_1-	8
BBS12	scRNA-seq,SRP215507,CD8+ T cell_4-AD.vs.control; scRNA-seq,SRP215507,CD8+ T cell_5-AD.vs.control; scRNA-seq,SRP215507,CD8+ T cell_1-AD.vs.MCI; scRNA-seq,SRP215507,CD8+ T cell_5-AD.vs.MCI; scRNA-seq,SRP215507,CD8+ T cell_1-MCI.vs.control; scRNA-seq,SRP215507,CD8+ T cell_4-MCI.vs.control; scRNA-seq,SRP330776,γδ T cell-AD.vs.control; scRNA-seq,SRP330776,Natural killer cell_1-AD.vs.control; scRNA-	9
BBS2	bulk RNA-seq,Homo_633,AD.vs.control; bulk RNA-seq,Homo_633,AD.vs.MCI; bulk RNA-seq,Homo_714,AD.vs.control; bulk RNA-seq,Homo_714,AD.vs.MCI; scRNA-seq,SRP215507,CD8+ T cell_5-AD.vs.MCI; scRNA-seq,SRP309935,Monocyte_1-AD.vs.control; scRNA-seq,SRP309935,Naive CD8+ T cell_2-AD.vs.control; scRNA-seq,SRP309935,CD8+ T cell_1-AD.vs.control; scRNA-seq,SRP330776,Memory T cell_2-AD.vs.control; scRNA-seq,SRP330776,B cell_3-AD.vs.control; scRNA-seq,SRP330776,Natural killer cell_2-AD.vs.control; scRNA-seq,SRP330776,B	12
BBS4	bulk RNA-seq,Homo_633,AD.vs.control; bulk RNA-seq,Homo_633,AD.vs.MCI; bulk RNA-seq,Homo_714,AD.vs.control; bulk RNA-seq,Homo_714,AD.vs.MCI; scRNA-seq,SRP215507,CD8+ T cell_5-AD.vs.control; scRNA-seq,SRP215507,CD8+ T cell_5-AD.vs.MCI; scRNA-seq,SRP215507,CD8+ T cell_4-MCI.vs.control; scRNA-seq,SRP215507,CD8+ T cell_6-MCI.vs.control; scRNA-seq,SRP330776,Memory T cell_2-AD.vs.control; scRNA-seq,SRP330776,B cell_1-AD.vs.control; scRNA-seq,SRP330776,Natural killer cell_1-AD.vs.control	11
BBS5	bulk RNA-seq,Homo_723,AD.vs.control; bulk RNA-seq,Homo_723,AD.vs.MCI; bulk RNA-seq,Homo_633,AD.vs.control; bulk RNA-seq,Homo_633,AD.vs.MCI; scRNA-seq,SRP215507,CD8+ T cell_2-AD.vs.control; scRNA-seq,SRP215507,CD8+ T cell_2-AD.vs.MCI; scRNA-seq,SRP215507,CD8+ T cell_5-AD.vs.MCI; scRNA-seq,SRP215507,CD8+ T cell_5-MCI.vs.control; scRNA-seq,SRP215507,CD8+ T cell_4-MCI.vs.control; scRNA-seq,SRP330776,Memory T cell_2-AD.vs.control; scRNA-seq,SRP330776,CD8+ T cell_2-AD.vs.control; scRNA-seq,SRP330776,Naive CD8+ T cell_1-AD.vs.control; scRNA-seq,SRP330776,B cell_2-AD.vs.control; scRNA-seq,SRP330776,CD8+ T cell_3-	14

BBS7	bulk RNA-seq,Homo_723,AD.vs.control; bulk RNA-seq,Homo_723,AD.vs.MCI; bulk RNA-seq,Homo_633,AD.vs.MCI; bulk RNA-seq,Homo_714,AD.vs.control; bulk RNA-seq,Homo_714,AD.vs.MCI; scRNA-seq,SRP215507,CD8+ T cell_3-AD.vs.control; scRNA-seq,SRP330776,Memory T cell_2-AD.vs.control; scRNA-seq,SRP330776,Naive CD8+ T cell_1-AD.vs.control; scRNA-seq,SRP330776,Natural killer cell_1-AD.vs.control; scRNA-seq,SRP330776,Memory T cell_1-AD.vs.control; scRNA-seq,SRP330776,T cell-AD.vs.control	11
BBS9	bulk RNA-seq,Homo_723,AD.vs.control; bulk RNA-seq,Homo_723,AD.vs.MCI; bulk RNA-seq,Homo_633,AD.vs.control; bulk RNA-seq,Homo_633,AD.vs.MCI; bulk RNA-seq,Homo_714,AD.vs.control; bulk RNA-seq,Homo_714,AD.vs.MCI; scRNA-seq,SRP215507,CD8+ T cell_3-AD.vs.control; scRNA-seq,SRP215507,CD8+ T cell_3-AD.vs.MCI; scRNA-seq,SRP330776,Naive CD8+ T cell_3-AD.vs.control	9
Bbs9	bulk RNA-seq,SRP325058,AD.vs.control	1
BBX	bulk RNA-seq,Homo_723,AD.vs.control; bulk RNA-seq,Homo_723,AD.vs.MCI; bulk RNA-seq,Homo_633,AD.vs.control; bulk RNA-seq,Homo_633,AD.vs.MCI; bulk RNA-seq,Homo_714,AD.vs.control; bulk RNA-seq,Homo_714,AD.vs.MCI; scRNA-seq,SRP330776,γδ T cell-AD.vs.control; scRNA-seq,SRP330776,Monocyte_2-AD.vs.control; scRNA-seq,SRP330776,Natural killer cell_2-AD.vs.control	9
BC004004	bulk RNA-seq,SRP325058,AD.vs.control	1
BC005537	bulk RNA-seq,SRP325058,AD.vs.control	1
BC031181	bulk RNA-seq,SRP325058,AD.vs.control	1
BCAM	bulk RNA-seq,Homo_723,AD.vs.control; bulk RNA-seq,Homo_723,AD.vs.MCI; bulk RNA-seq,Homo_723,MCI.vs.control; bulk RNA-seq,Homo_633,AD.vs.control; bulk RNA-seq,Homo_633,AD.vs.MCI; bulk RNA-seq,Homo_714,MCI.vs.control	6
BCAN	bulk RNA-seq,Homo_723,AD.vs.control; bulk RNA-seq,Homo_723,AD.vs.MCI; bulk RNA-seq,Homo_633,AD.vs.control; bulk RNA-seq,Homo_633,AD.vs.MCI; bulk RNA-seq,Homo_714,MCI.vs.control; bulk RNA-seq,ROSMAP,AD.vs.control	6
BCAP29	bulk RNA-seq,Homo_723,AD.vs.MCI; bulk RNA-seq,Homo_633,AD.vs.MCI; bulk RNA-seq,Homo_714,AD.vs.control; bulk RNA-seq,Homo_714,AD.vs.MCI; scRNA-seq,SRP215507,CD8+ T cell_3-AD.vs.control; scRNA-seq,SRP215507,CD8+ T cell_4-AD.vs.control; scRNA-seq,SRP215507,CD8+ T cell_7-AD.vs.control; scRNA-seq,SRP215507,CD8+ T cell_7-AD.vs.MCI; scRNA-seq,SRP215507,CD8+ T cell_4-	10
BCAP31	bulk RNA-seq,Homo_723,AD.vs.control; bulk RNA-seq,Homo_723,AD.vs.MCI; bulk RNA-seq,Homo_633,AD.vs.control; bulk RNA-seq,Homo_633,AD.vs.MCI; bulk RNA-seq,SRP223445,AD.vs.control; scRNA-seq,SRP215507,CD8+ T cell_7-AD.vs.control; scRNA-seq,SRP215507,CD8+ T cell_6-AD.vs.control; scRNA-seq,SRP215507,CD8+ T cell_6-AD.vs.MCI; scRNA-seq,SRP330776,Megakaryocyte_1-	9
Bcap31	bulk RNA-seq,SRP325058,AD.vs.control	1
BCAR1	bulk RNA-seq,Homo_723,AD.vs.control; bulk RNA-seq,Homo_723,AD.vs.MCI; bulk RNA-seq,Homo_723,MCI.vs.control; bulk RNA-seq,Homo_633,AD.vs.MCI; bulk RNA-seq,Homo_633,MCI.vs.control; bulk RNA-seq,Homo_714,MCI.vs.control; bulk RNA-seq,SRP223445,AD.vs.control; scRNA-seq,SRP330776,CD8+ T cell_1-AD.vs.control	8
BCAR3	bulk RNA-seq,Homo_723,AD.vs.control; bulk RNA-seq,Homo_723,AD.vs.MCI; bulk RNA-seq,Homo_633,AD.vs.control; bulk RNA-seq,Homo_633,AD.vs.MCI; bulk RNA-seq,Homo_714,AD.vs.control; bulk RNA-seq,Homo_714,AD.vs.MCI; bulk RNA-seq,Homo_714,MCI.vs.control; bulk RNA-seq,ROSMAP,MCI.vs.control; scRNA-seq,SRP215507,CD8+ T cell_1-AD.vs.control; scRNA-seq,SRP215507,CD8+ T cell_1-AD.vs.MCI; scRNA-seq,SRP215507,CD8+ T cell_6-MCI.vs.control; scRNA-seq,SRP309935,B cell_1-AD.vs.control; scRNA-seq,SRP330776,CD8+ T cell_1-AD.vs.control; scRNA-seq,SRP330776,B cell_1-AD.vs.control; scRNA-seq,SRP330776,Naive CD8+ T cell_1-AD.vs.control; scRNA-seq,SRP330776,Memory T cell_1-AD.vs.control; scRNA-seq,SRP330776,B cell_2-AD.vs.control; scRNA-seq,SRP330776,Naive CD8+ T cell_2-	18
BCAS1	bulk RNA-seq,Homo_723,AD.vs.control; bulk RNA-seq,Homo_633,AD.vs.control; bulk RNA-seq,Homo_633,AD.vs.MCI; bulk RNA-seq,Homo_714,AD.vs.control; bulk RNA-seq,Homo_714,AD.vs.MCI	5
Bcas2	bulk RNA-seq,SRP325058,AD.vs.control	1
BCAS2	scRNA-seq,SRP215507,CD8+ T cell_4-AD.vs.MCI; scRNA-seq,SRP215507,CD8+ T cell_7-AD.vs.MCI; scRNA-seq,SRP215507,CD8+ T cell_4-	3
Bcas3	bulk RNA-seq,SRP325058,AD.vs.control	1
BCAS3	scRNA-seq,SRP215507,CD8+ T cell_5-MCI.vs.control; scRNA-seq,SRP330776,B cell_1-AD.vs.control; scRNA-seq,SRP330776,Natural killer cell_1-AD.vs.control; scRNA-seq,SRP330776,Regulatory T cell-AD.vs.control	4

BCAS4	bulk RNA-seq,Homo_723,AD.vs.MCI; bulk RNA-seq,Homo_633,AD.vs.control; bulk RNA-seq,Homo_633,AD.vs.MCI; bulk RNA-seq,Homo_714,AD.vs.control; bulk RNA-seq,Homo_714,AD.vs.MCI; scRNA-seq,SRP215507,CD8+ T cell_4-AD.vs.control; scRNA-seq,SRP215507,CD8+ T cell_2-AD.vs.MCI; scRNA-seq,SRP215507,CD8+ T cell_6-MCI.vs.control; scRNA-seq,SRP330776,Regulatory T cell-	10
BCAT1	bulk RNA-seq,Homo_723,AD.vs.control; bulk RNA-seq,Homo_723,AD.vs.MCI; bulk RNA-seq,Homo_723,MCI.vs.control; bulk RNA-seq,Homo_633,AD.vs.control; bulk RNA-seq,Homo_633,AD.vs.MCI; bulk RNA-seq,Homo_633,MCI.vs.control; bulk RNA-seq,Homo_714,AD.vs.control; bulk RNA-seq,Homo_714,AD.vs.MCI; scRNA-seq,SRP309935,Monocyte_1-AD.vs.control	9
BCAT2	bulk RNA-seq,Homo_723,AD.vs.control; bulk RNA-seq,Homo_723,AD.vs.MCI; bulk RNA-seq,Homo_723,MCI.vs.control; bulk RNA-seq,Homo_633,AD.vs.control; bulk RNA-seq,Homo_633,AD.vs.MCI; bulk RNA-seq,Homo_633,MCI.vs.control; bulk RNA-seq,Homo_714,AD.vs.control; bulk RNA-seq,Homo_714,AD.vs.MCI; scRNA-seq,SRP215507,CD8+ T cell_4-MCI.vs.control; scRNA-seq,SRP330776,CD8+ T cell_1-AD.vs.control; scRNA-seq,SRP330776,γδ T cell-AD.vs.control; scRNA-seq,SRP330776,Memory T cell_2-AD.vs.control; scRNA-seq,SRP330776,CD8+ T cell_2-AD.vs.control; scRNA-seq,SRP330776,B cell_1-AD.vs.control; scRNA-seq,SRP330776,Naive CD8+ T cell_1-AD.vs.control; scRNA-seq,SRP330776,Natural killer cell_1-AD.vs.control; scRNA-seq,SRP330776,Memory T cell_1-AD.vs.control; scRNA-seq,SRP330776,Natural killer T cell-AD.vs.control; scRNA-seq,SRP330776,Naive CD8+ T cell_3-AD.vs.control; scRNA-seq,SRP330776,B cell_2-AD.vs.control; scRNA-seq,SRP330776,T cell-AD.vs.control; scRNA-seq,SRP330776,CD8+ T cell_3-AD.vs.control; scRNA-seq,SRP330776,Monocyte_2-AD.vs.control; scRNA- [illegible]	25
BCCIP	bulk RNA-seq,Homo_714,AD.vs.control; bulk RNA-seq,Homo_714,AD.vs.MCI; scRNA-seq,SRP330776,Naive CD8+ T cell_3-AD.vs.control; scRNA-seq,SRP330776,Monocyte_1-AD.vs.control; scRNA-seq,SRP330776,B cell_3-AD.vs.control; scRNA-seq,SRP330776,Regulatory T cell-AD.vs.control	6
BCDIN3D	scRNA-seq,SRP215507,CD8+ T cell_2-AD.vs.MCI; scRNA-seq,SRP215507,CD8+ T cell_5-AD.vs.MCI; scRNA-seq,SRP215507,CD8+ T cell_5-MCI.vs.control; scRNA-seq,SRP215507,CD8+ T cell_2-MCI.vs.control; scRNA-seq,SRP309935,Naive CD8+ T cell_1-AD.vs.control; scRNA-seq,SRP330776,γδ T cell-AD.vs.control; scRNA-seq,SRP330776,CD8+ T cell_2-AD.vs.control; scRNA-seq,SRP330776,CD8+ T cell_3-AD.vs.control	8
BCDIN3D-AS1	scRNA-seq,SRP215507,CD8+ T cell_1-MCI.vs.control; scRNA-seq,SRP330776,CD8+ T cell_1-AD.vs.control	2
BCHE	bulk RNA-seq,Homo_723,AD.vs.control; bulk RNA-seq,Homo_723,AD.vs.MCI; bulk RNA-seq,Homo_723,MCI.vs.control	3
BCKDHA	bulk RNA-seq,Homo_723,AD.vs.control; bulk RNA-seq,Homo_723,AD.vs.MCI; bulk RNA-seq,Homo_633,AD.vs.control; bulk RNA-seq,Homo_633,AD.vs.MCI; bulk RNA-seq,Homo_714,AD.vs.control; bulk RNA-seq,Homo_714,AD.vs.MCI; bulk RNA-seq,Homo_714,MCI.vs.control; scRNA-seq,SRP215507,CD8+ T cell_7-AD.vs.control; scRNA-seq,SRP215507,CD8+ T cell_4-AD.vs.MCI; scRNA-seq,SRP215507,CD8+ T cell_4-MCI.vs.control; scRNA-seq,SRP330776,Memory T cell_2-AD.vs.control; scRNA-seq,SRP330776,CD8+ T cell_2-AD.vs.control; scRNA-seq,SRP330776,Naive CD8+ T cell_1-AD.vs.control; scRNA-seq,SRP330776,B cell_2-AD.vs.control; scRNA-seq,SRP330776,CD8+ T cell_3-AD.vs.control; scRNA-seq,SRP330776,Monocyte_2-AD.vs.control; scRNA-seq,SRP330776,B cell_3-AD.vs.control; scRNA-seq,SRP330776,Natural killer [illegible]	18
BCKDHB	bulk RNA-seq,Homo_723,AD.vs.control; bulk RNA-seq,Homo_723,AD.vs.MCI; bulk RNA-seq,Homo_714,AD.vs.control; bulk RNA-seq,Homo_714,AD.vs.MCI; scRNA-seq,SRP215507,CD8+ T cell_10-AD.vs.control; scRNA-seq,SRP215507,CD8+ T cell_5-AD.vs.MCI; scRNA-seq,SRP215507,CD8+ T cell_5-MCI.vs.control; scRNA-seq,SRP215507,CD8+ T cell_3-MCI.vs.control; scRNA-seq,SRP330776,Memory T cell_2-AD.vs.control; scRNA-seq,SRP330776,B cell_2-AD.vs.control; scRNA-seq,SRP330776,T cell-AD.vs.control; scRNA-seq,SRP330776,B cell_3-	13
BCKDK	scRNA-seq,SRP215507,CD8+ T cell_6-AD.vs.control; scRNA-seq,SRP215507,CD8+ T cell_5-AD.vs.MCI; scRNA-seq,SRP215507,CD8+ T cell_6-AD.vs.MCI; scRNA-seq,SRP215507,CD8+ T cell_5-MCI.vs.control; scRNA-seq,SRP330776,CD8+ T cell_1-AD.vs.control; scRNA-seq,SRP330776,γδ T cell-AD.vs.control; scRNA-seq,SRP330776,Memory T cell_2-AD.vs.control; scRNA-seq,SRP330776,B cell_1-AD.vs.control; scRNA-seq,SRP330776,Naive CD8+ T cell_1-AD.vs.control; scRNA-seq,SRP330776,Natural killer cell_1-AD.vs.control; scRNA-seq,SRP330776,Natural killer T cell-AD.vs.control; scRNA-seq,SRP330776,Naive CD8+ T cell_3-AD.vs.control; scRNA-seq,SRP330776,B cell_2-AD.vs.control; scRNA-	15
BCL10	bulk RNA-seq,Homo_714,AD.vs.control; bulk RNA-seq,Homo_714,AD.vs.MCI; bulk RNA-seq,Homo_714,MCI.vs.control; scRNA-seq,SRP215507,CD8+ T cell_4-AD.vs.MCI; scRNA-seq,SRP215507,CD8+ T cell_5-AD.vs.MCI; scRNA-seq,SRP215507,CD8+ T cell_5-MCI.vs.control; scRNA-seq,SRP215507,CD8+ T cell_4-MCI.vs.control; scRNA-seq,SRP330776,T cell-AD.vs.control	8
BCL11A	bulk RNA-seq,Homo_723,AD.vs.control; bulk RNA-seq,Homo_723,AD.vs.MCI; bulk RNA-seq,Homo_633,AD.vs.control; bulk RNA-seq,Homo_633,AD.vs.MCI; bulk RNA-seq,Homo_714,AD.vs.control; bulk RNA-seq,Homo_714,AD.vs.MCI; scRNA-seq,SRP330776,B cell_1-	8

BCL11B	bulk RNA-seq,Homo_723,AD.vs.MCI; bulk RNA-seq,Homo_723,MCI.vs.control; bulk RNA-seq,Homo_633,MCI.vs.control; scRNA-seq,SRP215507,CD8+ T cell_10-AD.vs.control; scRNA-seq,SRP215507,CD8+ T cell_5-MCI.vs.control; scRNA-seq,SRP215507,CD8+ T cell_7-MCI.vs.control; scRNA-seq,SRP330776,CD8+ T cell_1-AD.vs.control; scRNA-seq,SRP330776,γδ T cell-AD.vs.control; scRNA-seq,SRP330776,Memory T cell_2-AD.vs.control; scRNA-seq,SRP330776,CD8+ T cell_2-AD.vs.control; scRNA-seq,SRP330776,B cell_1-AD.vs.control; scRNA-seq,SRP330776,Naive CD8+ T cell_1-AD.vs.control; scRNA-seq,SRP330776,Natural killer cell_1-AD.vs.control; scRNA-seq,SRP330776,Memory T cell_1-AD.vs.control; scRNA-seq,SRP330776,Natural killer T cell-AD.vs.control; scRNA-seq,SRP330776,Naive CD8+ T cell_3-AD.vs.control; scRNA-seq,SRP330776,T cell-AD.vs.control; scRNA-seq,SRP330776,Naive CD8+ T cell_2-AD.vs.control; scRNA-seq,SRP330776,CD8+ T cell_3-AD.vs.control; scRNA-seq,SRP330776,Monocyte_1-AD.vs.control; scRNA-seq,SRP330776,B cell_3-AD.vs.control; scRNA-seq,SRP330776,Natural killer cell_2-[illegible]	22
BCL2	scRNA-seq,SRP215507,CD8+ T cell_7-AD.vs.control; scRNA-seq,SRP215507,CD8+ T cell_7-AD.vs.MCI; scRNA-seq,SRP215507,CD8+ T cell_4-MCI.vs.control; scRNA-seq,SRP309935,CD8+ T cell_2-AD.vs.control; scRNA-seq,SRP330776,CD8+ T cell_1-AD.vs.control; scRNA-seq,SRP330776,γδ T cell-AD.vs.control; scRNA-seq,SRP330776,Memory T cell_2-AD.vs.control; scRNA-seq,SRP330776,CD8+ T cell_2-AD.vs.control; scRNA-seq,SRP330776,B cell_1-AD.vs.control; scRNA-seq,SRP330776,Naive CD8+ T cell_1-AD.vs.control; scRNA-seq,SRP330776,Natural killer cell_1-AD.vs.control; scRNA-seq,SRP330776,Memory T cell_1-AD.vs.control; scRNA-seq,SRP330776,Natural killer T cell-AD.vs.control; scRNA-seq,SRP330776,Naive CD8+ T cell_3-AD.vs.control; scRNA-seq,SRP330776,B cell_2-AD.vs.control; scRNA-seq,SRP330776,T cell-AD.vs.control; scRNA-seq,SRP330776,Naive CD8+ T cell_2-AD.vs.control; scRNA-seq,SRP330776,CD8+ T cell_3-AD.vs.control; scRNA-seq,SRP330776,B cell_3-[illegible]	21
BCL2A1	bulk RNA-seq,Homo_723,AD.vs.control; bulk RNA-seq,SRP223445,AD.vs.control; scRNA-seq,SRP215507,CD8+ T cell_4-AD.vs.control; scRNA-seq,SRP215507,CD8+ T cell_4-MCI.vs.control; scRNA-seq,SRP330776,Memory T cell_1-AD.vs.control; scRNA-seq,SRP330776,B cell_2-AD.vs.control; scRNA-seq,SRP330776,Monocyte_2-AD.vs.control	7
Bcl2a1a	bulk RNA-seq,SRP325058,AD.vs.control	1
Bcl2a1b	bulk RNA-seq,SRP325058,AD.vs.control	1
Bcl2a1d	bulk RNA-seq,SRP325058,AD.vs.control	1
Bcl2l1	bulk RNA-seq,SRP325058,AD.vs.control	1
BCL2L1	scRNA-seq,SRP215507,CD8+ T cell_3-MCI.vs.control; scRNA-seq,SRP330776,CD8+ T cell_2-AD.vs.control; scRNA-seq,SRP330776,Natural killer cell_1-AD.vs.control; scRNA-seq,SRP330776,B cell_2-AD.vs.control	4
BCL2L10	bulk RNA-seq,Homo_723,AD.vs.control; bulk RNA-seq,Homo_723,AD.vs.MCI; bulk RNA-seq,Homo_633,AD.vs.control; bulk RNA-	4
BCL2L11	scRNA-seq,SRP215507,CD8+ T cell_3-AD.vs.MCI; scRNA-seq,SRP215507,CD8+ T cell_1-AD.vs.MCI; scRNA-seq,SRP215507,CD8+ T cell_5-AD.vs.MCI; scRNA-seq,SRP215507,CD8+ T cell_6-AD.vs.MCI; scRNA-seq,SRP215507,CD8+ T cell_5-MCI.vs.control; scRNA-seq,SRP215507,CD8+ T cell_3-MCI.vs.control; scRNA-seq,SRP215507,CD8+ T cell_6-MCI.vs.control; scRNA-seq,SRP330776,B cell_1-AD.vs.control; scRNA-seq,SRP330776,Naive CD8+ T cell_3-AD.vs.control; scRNA-seq,SRP330776,Monocyte_2-AD.vs.control; scRNA-seq,SRP330776,Natural killer cell_2-	11
BCL2L12	bulk RNA-seq,Homo_633,AD.vs.control; bulk RNA-seq,Homo_633,AD.vs.MCI; bulk RNA-seq,Homo_714,AD.vs.control; bulk RNA-seq,Homo_714,AD.vs.MCI; scRNA-seq,SRP309935,Megakaryocyte_1-AD.vs.control; scRNA-seq,SRP309935,B cell_1-AD.vs.control; scRNA-seq,SRP330776,CD8+ T cell_1-AD.vs.control; scRNA-seq,SRP330776,γδ T cell-AD.vs.control; scRNA-seq,SRP330776,Memory T cell_2-AD.vs.control; scRNA-seq,SRP330776,CD8+ T cell_2-AD.vs.control; scRNA-seq,SRP330776,Naive CD8+ T cell_1-AD.vs.control; scRNA-seq,SRP330776,Memory T cell_1-AD.vs.control; scRNA-seq,SRP330776,Naive CD8+ T cell_3-AD.vs.control; scRNA-seq,SRP330776,T cell-AD.vs.control; scRNA-	15
BCL2L13	bulk RNA-seq,Homo_723,AD.vs.control; bulk RNA-seq,Homo_723,AD.vs.MCI; bulk RNA-seq,Homo_633,AD.vs.control; bulk RNA-seq,Homo_633,AD.vs.MCI; scRNA-seq,SRP215507,CD8+ T cell_7-AD.vs.MCI; scRNA-seq,SRP330776,B cell_2-AD.vs.control	6
BCL2L14	bulk RNA-seq,Homo_723,AD.vs.control; bulk RNA-seq,Homo_723,MCI.vs.control; bulk RNA-seq,Homo_633,AD.vs.control; bulk RNA-seq,Homo_633,MCI.vs.control; bulk RNA-seq,Homo_714,AD.vs.control; bulk RNA-seq,Homo_714,MCI.vs.control; scRNA-seq,SRP215507,CD8+ T cell_4-AD.vs.control; scRNA-seq,SRP330776,CD8+ T cell_1-AD.vs.control	8
BCL2L15	bulk RNA-seq,Homo_714,AD.vs.control; bulk RNA-seq,Homo_714,AD.vs.MCI; scRNA-seq,SRP215507,CD8+ T cell_1-AD.vs.control; scRNA-seq,SRP215507,CD8+ T cell_1-MCI.vs.control; scRNA-seq,SRP330776,CD8+ T cell_2-AD.vs.control	5

BCL2L2	bulk RNA-seq,Homo_723,AD.vs.control; bulk RNA-seq,Homo_723,AD.vs.MCI; bulk RNA-seq,Homo_723,MCI.vs.control; bulk RNA-seq,Homo_633,AD.vs.control; bulk RNA-seq,Homo_633,AD.vs.MCI; bulk RNA-seq,Homo_633,MCI.vs.control; bulk RNA-seq,Homo_714,AD.vs.control; bulk RNA-seq,Homo_714,AD.vs.MCI; bulk RNA-seq,Homo_714,MCI.vs.control; scRNA-seq,SRP309935,Monocyte_1-AD.vs.control; scRNA-	11
BCL3	bulk RNA-seq,Homo_714,AD.vs.control; bulk RNA-seq,Homo_714,AD.vs.MCI; scRNA-seq,SRP215507,CD8+ T cell_8-AD.vs.control; scRNA-seq,SRP215507,CD8+ T cell_2-AD.vs.MCI; scRNA-seq,SRP215507,CD8+ T cell_3-AD.vs.MCI; scRNA-seq,SRP215507,CD8+ T cell_1-AD.vs.MCI; scRNA-seq,SRP215507,CD8+ T cell_5-AD.vs.MCI; scRNA-seq,SRP215507,CD8+ T cell_1-MCI.vs.control; scRNA-seq,SRP215507,CD8+ T cell_5-MCI.vs.control; scRNA-seq,SRP215507,CD8+ T cell_2-MCI.vs.control; scRNA-seq,SRP215507,CD8+ T cell_3-MCI.vs.control; scRNA-seq,SRP309935,Natural killer cell_1-AD.vs.control; scRNA-seq,SRP309935,CD8+ T cell_1-AD.vs.control; scRNA-seq,SRP330776,CD8+ T cell_1-AD.vs.control; scRNA-seq,SRP330776,CD8+ T cell_2-AD.vs.control; scRNA-seq,SRP330776,B cell_1-AD.vs.control; scRNA-seq,SRP330776,Naive CD8+ T cell_1-AD.vs.control; scRNA-seq,SRP330776,Natural killer cell_1-AD.vs.control; scRNA-seq,SRP330776,Memory T cell_1-AD.vs.control; scRNA-seq,SRP330776,Natural killer T cell-AD.vs.control; scRNA-seq,SRP330776,Naive CD8+ T cell_3-AD.vs.control; scRNA-seq,SRP330776,B cell_2-AD.vs.control; scRNA-seq,SRP330776,T cell-AD.vs.control; scRNA-seq,SRP330776,CD8+ T cell_3-AD.vs.control; scRNA-seq,SRP330776,Natural killer [illegible]	25
BCL6	bulk RNA-seq,SRP223445,AD.vs.control; scRNA-seq,SRP215507,CD8+ T cell_2-AD.vs.control; scRNA-seq,SRP215507,CD8+ T cell_7-MCI.vs.control; scRNA-seq,SRP309935,Natural killer cell_1-AD.vs.control; scRNA-seq,SRP330776,Natural killer cell_1-AD.vs.control; scRNA-seq,SRP330776,CD8+ T cell_3-AD.vs.control; scRNA-seq,SRP330776,Monocyte_1-AD.vs.control; scRNA-seq,SRP330776,Monocyte_2-AD.vs.control	8
BCL6B	bulk RNA-seq,Homo_723,AD.vs.control; bulk RNA-seq,Homo_723,AD.vs.MCI; bulk RNA-seq,Homo_723,MCI.vs.control; bulk RNA-seq,Homo_633,AD.vs.control; bulk RNA-seq,Homo_633,AD.vs.MCI; bulk RNA-seq,Homo_633,MCI.vs.control; bulk RNA-seq,ROSMAP,AD.vs.control;	8
BCL7A	scRNA-seq,SRP215507,CD8+ T cell_2-AD.vs.control; scRNA-seq,SRP215507,CD8+ T cell_3-AD.vs.control; scRNA-seq,SRP215507,CD8+ T cell_1-AD.vs.control; scRNA-seq,SRP215507,CD8+ T cell_2-AD.vs.MCI; scRNA-seq,SRP215507,CD8+ T cell_1-AD.vs.MCI; scRNA-seq,SRP309935,CD8+ T cell_2-AD.vs.control; scRNA-seq,SRP330776,CD8+ T cell_1-AD.vs.control; scRNA-seq,SRP330776,Memory T cell_2-AD.vs.control; scRNA-seq,SRP330776,CD8+ T cell_2-AD.vs.control; scRNA-seq,SRP330776,B cell_1-AD.vs.control; scRNA-seq,SRP330776,Naive CD8+ T cell_1-AD.vs.control; scRNA-seq,SRP330776,Naive CD8+ T cell_3-AD.vs.control; scRNA-seq,SRP330776,B cell_2-AD.vs.control; scRNA-seq,SRP330776,CD8+ T cell_3-AD.vs.control; scRNA-seq,SRP330776,B cell_3-AD.vs.control; scRNA-seq,SRP330776,B cell_4-AD.vs.control	16
BCL7B	scRNA-seq,SRP215507,CD8+ T cell_6-AD.vs.control; scRNA-seq,SRP215507,CD8+ T cell_6-AD.vs.MCI; scRNA-seq,SRP215507,CD8+ T cell_5-MCI.vs.control; scRNA-seq,SRP309935,Naive CD8+ T cell_2-AD.vs.control; scRNA-seq,SRP330776,CD8+ T cell_1-AD.vs.control; scRNA-seq,SRP330776,γδ T cell-AD.vs.control; scRNA-seq,SRP330776,Memory T cell_2-AD.vs.control; scRNA-seq,SRP330776,CD8+ T cell_2-AD.vs.control; scRNA-seq,SRP330776,B cell_1-AD.vs.control; scRNA-seq,SRP330776,Naive CD8+ T cell_1-AD.vs.control; scRNA-seq,SRP330776,Natural killer cell_1-AD.vs.control; scRNA-seq,SRP330776,Memory T cell_1-AD.vs.control; scRNA-seq,SRP330776,Natural killer T cell-AD.vs.control; scRNA-seq,SRP330776,Naive CD8+ T cell_3-AD.vs.control; scRNA-seq,SRP330776,B cell_2-AD.vs.control; scRNA-seq,SRP330776,T cell-AD.vs.control; scRNA-seq,SRP330776,Naive CD8+ T cell_2-AD.vs.control; scRNA-seq,SRP330776,CD8+ T cell_3-AD.vs.control; scRNA-seq,SRP330776,Monocyte_1-AD.vs.control; scRNA-seq,SRP330776,Monocyte_2-AD.vs.control; scRNA-seq,SRP330776,B cell_3-AD.vs.control; scRNA-seq,SRP330776,Regulatory T [illegible]	24
BCL7C	scRNA-seq,SRP215507,CD8+ T cell_5-AD.vs.MCI; scRNA-seq,SRP215507,CD8+ T cell_5-MCI.vs.control; scRNA-seq,SRP309935,Natural killer cell_2-AD.vs.control; scRNA-seq,SRP309935,Monocyte_1-AD.vs.control; scRNA-seq,SRP309935,Megakaryocyte_1-AD.vs.control; scRNA-seq,SRP309935,Naive CD8+ T cell_1-AD.vs.control; scRNA-seq,SRP309935,Natural killer cell_1-AD.vs.control; scRNA-seq,SRP309935,Naive CD8+ T cell_2-AD.vs.control; scRNA-seq,SRP309935,CD8+ T cell_1-AD.vs.control; scRNA-seq,SRP309935,B cell_2-AD.vs.control; scRNA-seq,SRP309935,Dendritic cell-AD.vs.control; scRNA-seq,SRP330776,CD8+ T cell_1-AD.vs.control; scRNA-seq,SRP330776,γδ T cell-AD.vs.control; scRNA-seq,SRP330776,Memory T cell_2-AD.vs.control; scRNA-seq,SRP330776,CD8+ T cell_2-AD.vs.control; scRNA-seq,SRP330776,B cell_1-AD.vs.control; scRNA-seq,SRP330776,Naive CD8+ T cell_1-AD.vs.control; scRNA-seq,SRP330776,Natural killer cell_1-AD.vs.control; scRNA-seq,SRP330776,Memory T cell_1-AD.vs.control; scRNA-seq,SRP330776,Natural killer T cell-AD.vs.control; scRNA-seq,SRP330776,Naive CD8+ T cell_3-AD.vs.control; scRNA-seq,SRP330776,B cell_2-AD.vs.control; scRNA-seq,SRP330776,T cell-AD.vs.control; scRNA-seq,SRP330776,Naive CD8+ [illegible]	27
BCL9	bulk RNA-seq,Homo_723,MCI.vs.control; bulk RNA-seq,Homo_633,AD.vs.control; bulk RNA-seq,Homo_633,MCI.vs.control; scRNA-seq,SRP215507,CD8+ T cell_1-AD.vs.MCI; scRNA-seq,SRP215507,CD8+ T cell_1-MCI.vs.control; scRNA-seq,SRP330776,CD8+ T cell_2-	7
BCL9L	scRNA-seq,SRP309935,CD8+ T cell_1-AD.vs.control; scRNA-seq,SRP330776,Naive CD8+ T cell_3-AD.vs.control; scRNA-seq,SRP330776,T cell-	3

BCLAF1	bulk RNA-seq,Homo_633,MCI.vs.control; bulk RNA-seq,Homo_714,AD.vs.control; bulk RNA-seq,Homo_714,AD.vs.MCI; scRNA-seq,SRP215507,CD8+ T cell_7-AD.vs.control; scRNA-seq,SRP330776,B cell_2-AD.vs.control	5
BCLAF3	scRNA-seq,SRP215507,CD8+ T cell_4-AD.vs.control; scRNA-seq,SRP215507,CD8+ T cell_1-AD.vs.MCI; scRNA-seq,SRP215507,CD8+ T cell_4-AD.vs.MCI; scRNA-seq,SRP330776,Memory T cell_2-AD.vs.control; scRNA-seq,SRP330776,B cell_1-AD.vs.control; scRNA-seq,SRP330776,Natural killer cell_1-AD.vs.control; scRNA-seq,SRP330776,B cell_2-AD.vs.control	7
BCO1	bulk RNA-seq,Homo_723,AD.vs.control; bulk RNA-seq,Homo_723,AD.vs.MCI; bulk RNA-seq,Homo_633,AD.vs.control	3
BCO2	scRNA-seq,SRP215507,CD8+ T cell_1-MCI.vs.control; scRNA-seq,SRP215507,CD8+ T cell_4-MCI.vs.control; scRNA-seq,SRP215507,CD8+ T cell_3-MCI.vs.control; scRNA-seq,SRP330776,CD8+ T cell_1-AD.vs.control; scRNA-seq,SRP330776,Naive CD8+ T cell_1-AD.vs.control	5
BCOR	bulk RNA-seq,Homo_723,AD.vs.control; bulk RNA-seq,Homo_723,AD.vs.MCI; bulk RNA-seq,Homo_723,MCI.vs.control; bulk RNA-seq,Homo_633,AD.vs.control; bulk RNA-seq,Homo_633,AD.vs.MCI; bulk RNA-seq,Homo_633,MCI.vs.control; bulk RNA-seq,Homo_714,AD.vs.control; bulk RNA-seq,Homo_714,MCI.vs.control; scRNA-seq,SRP215507,CD8+ T cell_4-AD.vs.control; scRNA-seq,SRP215507,CD8+ T cell_4-AD.vs.MCI; scRNA-seq,SRP215507,CD8+ T cell_4-MCI.vs.control; scRNA-seq,SRP215507,CD8+ T cell_3-MCI.vs.control; scRNA-seq,SRP309935,Natural killer cell_1-AD.vs.control; scRNA-seq,SRP309935,CD8+ T cell_1-AD.vs.control; scRNA-seq,SRP330776,B cell_1-AD.vs.control	15
BCORL1	scRNA-seq,SRP215507,CD8+ T cell_5-AD.vs.MCI; scRNA-seq,SRP330776,B cell_2-AD.vs.control	2
BCR	scRNA-seq,SRP215507,CD8+ T cell_3-AD.vs.MCI; scRNA-seq,SRP215507,CD8+ T cell_5-MCI.vs.control; scRNA-seq,SRP309935,Natural killer cell_2-AD.vs.control; scRNA-seq,SRP309935,Natural killer cell_1-AD.vs.control; scRNA-seq,SRP330776,CD8+ T cell_1-AD.vs.control; scRNA-seq,SRP330776,Memory T cell_2-AD.vs.control; scRNA-seq,SRP330776,CD8+ T cell_2-AD.vs.control; scRNA-seq,SRP330776,B cell_1-AD.vs.control; scRNA-seq,SRP330776,Natural killer cell_1-AD.vs.control; scRNA-seq,SRP330776,Memory T cell_1-AD.vs.control; scRNA-seq,SRP330776,Natural	12
BCS1L	bulk RNA-seq,Homo_723,AD.vs.control; bulk RNA-seq,Homo_723,AD.vs.MCI; bulk RNA-seq,Homo_723,MCI.vs.control; bulk RNA-seq,Homo_633,AD.vs.control; bulk RNA-seq,Homo_633,AD.vs.MCI; bulk RNA-seq,Homo_633,MCI.vs.control; bulk RNA-seq,Homo_714,AD.vs.control; bulk RNA-seq,Homo_714,AD.vs.MCI; bulk RNA-seq,Homo_714,MCI.vs.control; scRNA-seq,SRP215507,CD8+ T cell_6-AD.vs.control; scRNA-seq,SRP215507,CD8+ T cell_2-AD.vs.MCI; scRNA-seq,SRP215507,CD8+ T cell_2-MCI.vs.control; scRNA-seq,SRP330776,CD8+ T cell_2-	14
BDH1	bulk RNA-seq,Homo_723,MCI.vs.control; bulk RNA-seq,Homo_633,AD.vs.control; bulk RNA-seq,Homo_633,MCI.vs.control; scRNA-seq,SRP215507,CD8+ T cell_3-AD.vs.MCI; scRNA-seq,SRP215507,CD8+ T cell_1-MCI.vs.control; scRNA-seq,SRP215507,CD8+ T cell_4-MCI.vs.control; scRNA-seq,SRP309935,CD8+ T cell_1-AD.vs.control; scRNA-seq,SRP309935,CD8+ T cell_2-AD.vs.control; scRNA-seq,SRP330776,Natural killer cell_1-AD.vs.control; scRNA-seq,SRP330776,Regulatory T cell-AD.vs.control	10
BDH2	bulk RNA-seq,Homo_633,AD.vs.control; bulk RNA-seq,Homo_633,AD.vs.MCI; bulk RNA-seq,Homo_714,AD.vs.control; bulk RNA-seq,Homo_714,AD.vs.MCI; bulk RNA-seq,Homo_714,MCI.vs.control; scRNA-seq,SRP215507,CD8+ T cell_3-AD.vs.control; scRNA-seq,SRP215507,CD8+ T cell_5-MCI.vs.control; scRNA-seq,SRP330776,CD8+ T cell_1-AD.vs.control; scRNA-seq,SRP330776,Memory T cell_2-AD.vs.control; scRNA-seq,SRP330776,CD8+ T cell_2-AD.vs.control; scRNA-seq,SRP330776,Naive CD8+ T cell_1-AD.vs.control; scRNA-seq,SRP330776,Natural killer cell_1-AD.vs.control; scRNA-seq,SRP330776,Memory T cell_1-AD.vs.control; scRNA-seq,SRP330776,CD8+ T cell_3-	15
BDKRB2	bulk RNA-seq,Homo_723,AD.vs.control; bulk RNA-seq,Homo_723,AD.vs.MCI; bulk RNA-seq,Homo_723,MCI.vs.control; bulk RNA-seq,Homo_633,AD.vs.control; bulk RNA-seq,Homo_633,AD.vs.MCI; bulk RNA-seq,Homo_633,MCI.vs.control; bulk RNA-seq,Homo_714,MCI.vs.control	7
BDNF	bulk RNA-seq,Homo_723,AD.vs.control; bulk RNA-seq,Homo_723,AD.vs.MCI; bulk RNA-seq,Homo_633,AD.vs.control; bulk RNA-seq,Homo_633,AD.vs.MCI; bulk RNA-seq,Homo_714,AD.vs.MCI; bulk RNA-seq,Homo_714,MCI.vs.control; bulk RNA-seq,ROSMAP,AD.vs.control;	8
BDNF-AS	scRNA-seq,SRP215507,CD8+ T cell_3-AD.vs.control; scRNA-seq,SRP215507,CD8+ T cell_1-AD.vs.control; scRNA-seq,SRP330776,CD8+ T cell_1-AD.vs.control; scRNA-seq,SRP330776,Memory T cell_2-AD.vs.control; scRNA-seq,SRP330776,B cell_2-AD.vs.control	5
BDP1	bulk RNA-seq,Homo_723,AD.vs.control; bulk RNA-seq,Homo_723,AD.vs.MCI; bulk RNA-seq,Homo_633,AD.vs.control; bulk RNA-seq,Homo_633,AD.vs.MCI; bulk RNA-seq,Homo_714,AD.vs.control; bulk RNA-seq,Homo_714,AD.vs.MCI; scRNA-seq,SRP215507,CD8+ T cell_4-AD.vs.control; scRNA-seq,SRP215507,CD8+ T cell_4-AD.vs.MCI	8
BEAN1	bulk RNA-seq,Homo_723,AD.vs.control; bulk RNA-seq,Homo_723,AD.vs.MCI; bulk RNA-seq,Homo_633,AD.vs.control; bulk RNA-seq,Homo_714,AD.vs.control; bulk RNA-seq,Homo_714,AD.vs.MCI	5

BECN1	bulk RNA-seq,Homo_723,AD.vs.MCI; bulk RNA-seq,Homo_723,MCI.vs.control; bulk RNA-seq,Homo_633,AD.vs.MCI; bulk RNA-seq,Homo_633,MCI.vs.control; bulk RNA-seq,Homo_714,AD.vs.control; bulk RNA-seq,Homo_714,AD.vs.MCI; bulk RNA-seq,Homo_714,MCI.vs.control; scRNA-seq,SRP330776,Naive CD8+ T cell_3-AD.vs.control	8
BECN2	bulk RNA-seq,Homo_714,MCI.vs.control	1
BEGAIN	bulk RNA-seq,Homo_723,AD.vs.control; bulk RNA-seq,Homo_723,AD.vs.MCI; bulk RNA-seq,Homo_633,AD.vs.control; bulk RNA-seq,Homo_633,AD.vs.MCI; bulk RNA-seq,Homo_714,AD.vs.control; bulk RNA-seq,Homo_714,AD.vs.MCI; bulk RNA-seq,Homo_714,MCI.vs.control; scRNA-seq,SRP215507,CD8+ T cell_1-AD.vs.MCI; scRNA-seq,SRP215507,CD8+ T cell_1-MCI.vs.control; scRNA-seq,SRP330776,Naive CD8+ T	10
BEND2	bulk RNA-seq,Homo_723,AD.vs.control; bulk RNA-seq,Homo_723,MCI.vs.control; bulk RNA-seq,Homo_633,AD.vs.control; bulk RNA-seq,Homo_633,MCI.vs.control; bulk RNA-seq,Homo_714,AD.vs.control; bulk RNA-seq,Homo_714,AD.vs.MCI; bulk RNA-seq,Homo_714,MCI.vs.control; bulk RNA-seq,ROSMAP,AD.vs.control; bulk RNA-seq,ROSMAP,MCI.vs.control	9
BEND3	bulk RNA-seq,Homo_723,AD.vs.control; bulk RNA-seq,Homo_723,AD.vs.MCI; bulk RNA-seq,Homo_633,AD.vs.control; bulk RNA-seq,Homo_633,AD.vs.MCI; bulk RNA-seq,Homo_714,AD.vs.control; bulk RNA-seq,Homo_714,AD.vs.MCI; scRNA-seq,SRP215507,CD8+ T cell_1-AD.vs.MCI; scRNA-seq,SRP215507,CD8+ T cell_1-MCI.vs.control	8
BEND4	bulk RNA-seq,Homo_723,AD.vs.control; bulk RNA-seq,Homo_723,AD.vs.MCI; bulk RNA-seq,Homo_723,MCI.vs.control; bulk RNA-seq,Homo_633,AD.vs.control; bulk RNA-seq,Homo_633,AD.vs.MCI; bulk RNA-seq,Homo_714,AD.vs.control; bulk RNA-seq,Homo_714,AD.vs.MCI; scRNA-seq,SRP215507,CD8+ T cell_3-AD.vs.control; scRNA-seq,SRP215507,CD8+ T cell_3-MCI.vs.control; scRNA-seq,SRP330776,B cell_1-	11
BEND5	bulk RNA-seq,Homo_723,AD.vs.control; bulk RNA-seq,Homo_723,AD.vs.MCI; bulk RNA-seq,Homo_714,AD.vs.control; bulk RNA-seq,Homo_714,AD.vs.MCI; scRNA-seq,SRP215507,CD8+ T cell_2-AD.vs.control; scRNA-seq,SRP215507,CD8+ T cell_3-AD.vs.control; scRNA-seq,SRP215507,CD8+ T cell_2-AD.vs.MCI; scRNA-seq,SRP215507,CD8+ T cell_6-MCI.vs.control; scRNA-seq,SRP330776,Naive CD8+ T cell_3-	10
BEND7	bulk RNA-seq,Homo_714,AD.vs.control; bulk RNA-seq,Homo_714,AD.vs.MCI; bulk RNA-seq,SRP223445,AD.vs.control	3
BEST1	scRNA-seq,SRP215507,CD8+ T cell_3-AD.vs.control; scRNA-seq,SRP215507,CD8+ T cell_4-AD.vs.MCI; scRNA-seq,SRP215507,CD8+ T cell_4-MCI.vs.control; scRNA-seq,SRP309935,Naive CD8+ T cell_2-AD.vs.control; scRNA-seq,SRP330776,CD8+ T cell_2-AD.vs.control; scRNA-seq,SRP330776,Monocyte_2-AD.vs.control; scRNA-seq,SRP330776,Natural killer cell_2-AD.vs.control	7
BEST2	bulk RNA-seq,Homo_723,AD.vs.control; bulk RNA-seq,Homo_723,AD.vs.MCI; bulk RNA-seq,Homo_633,AD.vs.control; bulk RNA-seq,Homo_633,AD.vs.MCI; bulk RNA-seq,Homo_714,MCI.vs.control	5
BEST3	bulk RNA-seq,Homo_723,AD.vs.control; bulk RNA-seq,Homo_723,AD.vs.MCI; bulk RNA-seq,Homo_723,MCI.vs.control; bulk RNA-seq,Homo_633,AD.vs.control; bulk RNA-seq,Homo_633,AD.vs.MCI; bulk RNA-seq,Homo_633,MCI.vs.control; bulk RNA-seq,ROSMAP,AD.vs.control	7
BEST4	bulk RNA-seq,Homo_633,AD.vs.MCI; scRNA-seq,SRP215507,CD8+ T cell_1-AD.vs.control; scRNA-seq,SRP215507,CD8+ T cell_1-MCI.vs.control; scRNA-seq,SRP330776,Memory T cell_1-AD.vs.control	4
BET1	bulk RNA-seq,Homo_714,AD.vs.control; bulk RNA-seq,Homo_714,AD.vs.MCI; scRNA-seq,SRP215507,CD8+ T cell_2-AD.vs.MCI; scRNA-seq,SRP309935,Natural killer cell_1-AD.vs.control; scRNA-seq,SRP330776,γδ T cell-AD.vs.control; scRNA-seq,SRP330776,Memory T cell_2-AD.vs.control; scRNA-seq,SRP330776,B cell_1-AD.vs.control; scRNA-seq,SRP330776,Natural killer cell_1-AD.vs.control; scRNA-seq,SRP330776,Memory T cell_1-AD.vs.control; scRNA-seq,SRP330776,Naive CD8+ T cell_3-AD.vs.control; scRNA-seq,SRP330776,T cell-AD.vs.control; scRNA-seq,SRP330776,CD8+ T cell_3-AD.vs.control; scRNA-seq,SRP330776,Monocyte_1-AD.vs.control	13
BET1L	scRNA-seq,SRP215507,CD8+ T cell_4-AD.vs.control; scRNA-seq,SRP215507,CD8+ T cell_6-AD.vs.MCI	2
BEX1	bulk RNA-seq,Homo_723,AD.vs.control; bulk RNA-seq,Homo_723,AD.vs.MCI; bulk RNA-seq,Homo_723,MCI.vs.control; bulk RNA-seq,Homo_633,AD.vs.control; bulk RNA-seq,Homo_633,AD.vs.MCI; bulk RNA-seq,Homo_633,MCI.vs.control; bulk RNA-seq,Homo_714,AD.vs.control; bulk RNA-seq,Homo_714,AD.vs.MCI; scRNA-seq,SRP215507,CD8+ T cell_2-AD.vs.control; scRNA-seq,SRP215507,CD8+ T cell_2-MCI.vs.control	10

BEX2	bulk RNA-seq,Homo_723,AD.vs.MCI; bulk RNA-seq,Homo_633,AD.vs.control; bulk RNA-seq,Homo_633,AD.vs.MCI; bulk RNA-seq,Homo_714,AD.vs.control; bulk RNA-seq,Homo_714,AD.vs.MCI; bulk RNA-seq,ROSMAP,AD.vs.control; scRNA-seq,SRP215507,CD8+ T cell_1-AD.vs.control; scRNA-seq,SRP215507,CD8+ T cell_4-AD.vs.control; scRNA-seq,SRP215507,CD8+ T cell_6-AD.vs.control; scRNA-seq,SRP215507,CD8+ T cell_1-AD.vs.MCI; scRNA-seq,SRP215507,CD8+ T cell_6-AD.vs.MCI; scRNA-seq,SRP330776,CD8+ T cell_1-AD.vs.control; scRNA-seq,SRP330776,γδ T cell-AD.vs.control; scRNA-seq,SRP330776,B cell_1-AD.vs.control; scRNA-seq,SRP330776,Naive CD8+ T cell_1-AD.vs.control; scRNA-seq,SRP330776,Natural killer cell_1-AD.vs.control; scRNA-seq,SRP330776,Naive CD8+ T cell_3-AD.vs.control; scRNA-	19
BEX3	bulk RNA-seq,SRP223445,AD.vs.control; scRNA-seq,SRP215507,CD8+ T cell_1-AD.vs.control; scRNA-seq,SRP215507,CD8+ T cell_1-AD.vs.MCI; scRNA-seq,SRP215507,CD8+ T cell_1-MCI.vs.control; scRNA-seq,SRP215507,CD8+ T cell_2-MCI.vs.control; scRNA-seq,SRP330776,Natural killer cell_1-AD.vs.control; scRNA-seq,SRP330776,CD8+ T cell_3-AD.vs.control; scRNA-seq,SRP330776,B cell_3-AD.vs.control	8
Bex3	bulk RNA-seq,SRP325058,AD.vs.control	1
BEX4	bulk RNA-seq,SRP223445,AD.vs.control; scRNA-seq,SRP215507,CD8+ T cell_5-AD.vs.MCI; scRNA-seq,SRP215507,CD8+ T cell_8-MCI.vs.control; scRNA-seq,SRP309935,Megakaryocyte_1-AD.vs.control; scRNA-seq,SRP330776,CD8+ T cell_2-AD.vs.control; scRNA-seq,SRP330776,Natural killer cell_1-AD.vs.control; scRNA-seq,SRP330776,T cell-AD.vs.control; scRNA-seq,SRP330776,CD8+ T cell_3-AD.vs.control	8
BEX5	bulk RNA-seq,Homo_723,AD.vs.MCI; bulk RNA-seq,Homo_723,MCI.vs.control; bulk RNA-seq,Homo_633,AD.vs.MCI; bulk RNA-seq,Homo_633,MCI.vs.control; bulk RNA-seq,Homo_714,AD.vs.control; bulk RNA-seq,Homo_714,AD.vs.MCI; bulk RNA-seq,Homo_714,MCI.vs.control; scRNA-seq,SRP215507,CD8+ T cell_3-AD.vs.MCI; scRNA-seq,SRP215507,CD8+ T cell_1-AD.vs.MCI; scRNA-seq,SRP215507,CD8+ T cell_1-MCI.vs.control; scRNA-seq,SRP215507,CD8+ T cell_4-MCI.vs.control; scRNA-seq,SRP215507,CD8+ T cell_3-MCI.vs.control; scRNA-seq,SRP330776,B cell_1-AD.vs.control; scRNA-seq,SRP330776,Natural killer cell_1-AD.vs.control; scRNA-seq,SRP330776,T	16
BFAR	bulk RNA-seq,Homo_723,AD.vs.control; bulk RNA-seq,Homo_723,AD.vs.MCI; bulk RNA-seq,Homo_633,AD.vs.control; bulk RNA-seq,Homo_633,AD.vs.MCI; scRNA-seq,SRP330776,B cell_4-AD.vs.control	5
BFSP1	bulk RNA-seq,Homo_723,AD.vs.control; bulk RNA-seq,Homo_633,AD.vs.control; bulk RNA-seq,Homo_714,AD.vs.control; bulk RNA-seq,Homo_714,AD.vs.MCI; scRNA-seq,SRP215507,CD8+ T cell_1-AD.vs.control; scRNA-seq,SRP215507,CD8+ T cell_6-AD.vs.control; scRNA-seq,SRP215507,CD8+ T cell_2-AD.vs.MCI; scRNA-seq,SRP215507,CD8+ T cell_4-MCI.vs.control; scRNA-seq,SRP215507,CD8+ T cell_2-MCI.vs.control; scRNA-seq,SRP330776,γδ T cell-AD.vs.control; scRNA-seq,SRP330776,Memory T cell_2-AD.vs.control; scRNA-seq,SRP330776,CD8+ T cell_2-AD.vs.control; scRNA-seq,SRP330776,Natural killer cell_1-AD.vs.control; scRNA-seq,SRP330776,Memory T cell_1-AD.vs.control	14
BFSP2	bulk RNA-seq,Homo_723,AD.vs.control; bulk RNA-seq,Homo_633,AD.vs.control; bulk RNA-seq,Homo_633,MCI.vs.control; bulk RNA-	4
BGLAP	bulk RNA-seq,Homo_723,AD.vs.control; scRNA-seq,SRP215507,CD8+ T cell_3-MCI.vs.control; scRNA-seq,SRP330776,Naive CD8+ T cell_1-	3
BGN	bulk RNA-seq,Homo_723,AD.vs.MCI; bulk RNA-seq,Homo_723,MCI.vs.control; bulk RNA-seq,Homo_633,AD.vs.MCI; bulk RNA-	4
BHLHA15	bulk RNA-seq,Homo_723,AD.vs.MCI; bulk RNA-seq,Homo_723,MCI.vs.control; bulk RNA-seq,Homo_633,AD.vs.control; bulk RNA-seq,Homo_633,AD.vs.MCI; bulk RNA-seq,Homo_633,MCI.vs.control; bulk RNA-seq,Homo_714,AD.vs.control; bulk RNA-seq,Homo_714,AD.vs.MCI;	8
BHLHA9	bulk RNA-seq,Homo_723,AD.vs.control; bulk RNA-seq,Homo_723,MCI.vs.control; bulk RNA-seq,Homo_633,AD.vs.control; bulk RNA-seq,Homo_633,MCI.vs.control; bulk RNA-seq,Homo_714,MCI.vs.control	5
BHLHB9	scRNA-seq,SRP215507,CD8+ T cell_1-AD.vs.MCI; scRNA-seq,SRP215507,CD8+ T cell_1-MCI.vs.control; scRNA-seq,SRP330776,CD8+ T cell_2-	3
BHLHE22	bulk RNA-seq,Homo_723,AD.vs.control; bulk RNA-seq,Homo_723,AD.vs.MCI; bulk RNA-seq,Homo_723,MCI.vs.control; bulk RNA-seq,Homo_633,AD.vs.control; bulk RNA-seq,Homo_633,AD.vs.MCI; bulk RNA-seq,Homo_714,MCI.vs.control	6
BHLHE23	bulk RNA-seq,Homo_723,AD.vs.control; bulk RNA-seq,Homo_723,MCI.vs.control; bulk RNA-seq,Homo_633,AD.vs.control; bulk RNA-seq,Homo_633,MCI.vs.control; bulk RNA-seq,Homo_714,MCI.vs.control	5
BHLHE40	scRNA-seq,SRP215507,CD8+ T cell_3-AD.vs.control; scRNA-seq,SRP215507,CD8+ T cell_4-AD.vs.MCI; scRNA-seq,SRP215507,CD8+ T cell_5-AD.vs.MCI; scRNA-seq,SRP215507,CD8+ T cell_4-MCI.vs.control; scRNA-seq,SRP215507,CD8+ T cell_3-MCI.vs.control; scRNA-seq,SRP330776,γδ T cell-AD.vs.control; scRNA-seq,SRP330776,Memory T cell_2-AD.vs.control; scRNA-seq,SRP330776,B cell_1-AD.vs.control; scRNA-seq,SRP330776,T cell-AD.vs.control; scRNA-seq,SRP330776,CD8+ T cell_3-AD.vs.control; scRNA-seq,SRP330776,Monocyte_1-AD.vs.control; scRNA-	12

BHLHE40-AS1	scRNA-seq,SRP215507,CD8+ T cell_2-AD.vs.MCI; scRNA-seq,SRP215507,CD8+ T cell_1-AD.vs.MCI; scRNA-seq,SRP215507,CD8+ T cell_1-MCI.vs.control; scRNA-seq,SRP215507,CD8+ T cell_2-MCI.vs.control; scRNA-seq,SRP330776,B cell_1-AD.vs.control; scRNA-seq,SRP330776,Naive CD8+ T cell_1-AD.vs.control; scRNA-seq,SRP330776,Memory T cell_1-AD.vs.control; scRNA-seq,SRP330776,Natural killer cell_2-AD.vs.control	8
BHLHE41	bulk RNA-seq,Homo_723,AD.vs.control; bulk RNA-seq,Homo_723,AD.vs.MCI; bulk RNA-seq,Homo_633,AD.vs.control; bulk RNA-seq,Homo_633,AD.vs.MCI; bulk RNA-seq,Homo_714,AD.vs.control; bulk RNA-seq,Homo_714,AD.vs.MCI; bulk RNA-seq,ROSMAP,MCI.vs.control	7
BHMT	bulk RNA-seq,Homo_723,AD.vs.MCI; bulk RNA-seq,Homo_723,MCI.vs.control; bulk RNA-seq,Homo_633,AD.vs.MCI; bulk RNA-	4
BHMT2	bulk RNA-seq,Homo_723,AD.vs.MCI; bulk RNA-seq,Homo_633,AD.vs.MCI; bulk RNA-seq,Homo_714,MCI.vs.control	3
BICC1	bulk RNA-seq,ROSMAP,AD.vs.control	1
BICD1	scRNA-seq,SRP215507,CD8+ T cell_3-AD.vs.control; scRNA-seq,SRP215507,CD8+ T cell_1-AD.vs.control; scRNA-seq,SRP215507,CD8+ T cell_5-AD.vs.control; scRNA-seq,SRP215507,CD8+ T cell_1-MCI.vs.control; scRNA-seq,SRP215507,CD8+ T cell_5-MCI.vs.control; scRNA-seq,SRP215507,CD8+ T cell_2-MCI.vs.control; scRNA-seq,SRP309935,Natural killer cell_2-AD.vs.control; scRNA-seq,SRP309935,Naive CD8+ T cell_1-AD.vs.control; scRNA-seq,SRP330776,Natural killer cell_1-AD.vs.control; scRNA-seq,SRP330776,Naive CD8+ T cell_3-AD.vs.control; scRNA-	12
BICD2	scRNA-seq,SRP215507,CD8+ T cell_4-AD.vs.control; scRNA-seq,SRP215507,CD8+ T cell_7-AD.vs.control; scRNA-seq,SRP309935,Natural killer cell_1-AD.vs.control; scRNA-seq,SRP309935,CD8+ T cell_1-AD.vs.control; scRNA-seq,SRP330776,CD8+ T cell_1-AD.vs.control; scRNA-seq,SRP330776,γδ T cell-AD.vs.control; scRNA-seq,SRP330776,Memory T cell_2-AD.vs.control; scRNA-seq,SRP330776,CD8+ T cell_2-AD.vs.control; scRNA-seq,SRP330776,B cell_1-AD.vs.control; scRNA-seq,SRP330776,Naive CD8+ T cell_1-AD.vs.control; scRNA-seq,SRP330776,Memory T cell_1-AD.vs.control; scRNA-seq,SRP330776,Natural killer T cell-AD.vs.control; scRNA-seq,SRP330776,Naive CD8+ T cell_3-AD.vs.control; scRNA-seq,SRP330776,B cell_2-AD.vs.control; scRNA-seq,SRP330776,T cell-AD.vs.control; scRNA-seq,SRP330776,B cell_4-AD.vs.control	16
BICDL1	scRNA-seq,SRP215507,CD8+ T cell_4-AD.vs.control; scRNA-seq,SRP215507,CD8+ T cell_3-AD.vs.MCI; scRNA-seq,SRP215507,CD8+ T cell_4-AD.vs.MCI; scRNA-seq,SRP215507,CD8+ T cell_5-AD.vs.MCI; scRNA-seq,SRP330776,CD8+ T cell_2-AD.vs.control; scRNA-seq,SRP330776,B cell_1-AD.vs.control; scRNA-seq,SRP330776,Naive CD8+ T cell_1-AD.vs.control; scRNA-seq,SRP330776,Memory T cell_1-AD.vs.control; scRNA-	9
BICDL2	bulk RNA-seq,Homo_723,AD.vs.control; bulk RNA-seq,Homo_633,AD.vs.control; bulk RNA-seq,Homo_714,AD.vs.control; bulk RNA-seq,Homo_714,AD.vs.MCI; scRNA-seq,SRP215507,CD8+ T cell_1-MCI.vs.control	5
BICRA	scRNA-seq,SRP215507,CD8+ T cell_6-AD.vs.MCI; scRNA-seq,SRP330776,CD8+ T cell_1-AD.vs.control; scRNA-seq,SRP330776,Memory T cell_2-AD.vs.control; scRNA-seq,SRP330776,CD8+ T cell_2-AD.vs.control; scRNA-seq,SRP330776,B cell_1-AD.vs.control; scRNA-seq,SRP330776,Naive CD8+ T cell_1-AD.vs.control; scRNA-seq,SRP330776,Natural killer cell_1-AD.vs.control; scRNA-seq,SRP330776,Memory T cell_1-AD.vs.control; scRNA-seq,SRP330776,Naive CD8+ T cell_3-AD.vs.control; scRNA-seq,SRP330776,T cell-AD.vs.control; scRNA-seq,SRP330776,CD8+ T cell_3-	12
BICRAL	bulk RNA-seq,Homo_633,MCI.vs.control; bulk RNA-seq,Homo_714,AD.vs.control; bulk RNA-seq,Homo_714,AD.vs.MCI; bulk RNA-seq,Homo_714,MCI.vs.control; bulk RNA-seq,SRP310421,AD.vs.control; scRNA-seq,SRP309935,Naive CD8+ T cell_1-AD.vs.control; scRNA-seq,SRP330776,Natural killer T cell-AD.vs.control; scRNA-seq,SRP330776,B cell_2-AD.vs.control	8
BID	bulk RNA-seq,Homo_723,AD.vs.control; bulk RNA-seq,Homo_633,AD.vs.control; bulk RNA-seq,Homo_633,AD.vs.MCI; bulk RNA-seq,Homo_714,MCI.vs.control; scRNA-seq,SRP215507,CD8+ T cell_4-AD.vs.control; scRNA-seq,SRP330776,CD8+ T cell_1-AD.vs.control; scRNA-seq,SRP330776,Memory T cell_2-AD.vs.control; scRNA-seq,SRP330776,Naive CD8+ T cell_3-AD.vs.control; scRNA-seq,SRP330776,T cell-	10
BIK	bulk RNA-seq,Homo_723,AD.vs.control; bulk RNA-seq,Homo_723,AD.vs.MCI; bulk RNA-seq,Homo_633,AD.vs.control; bulk RNA-seq,Homo_633,AD.vs.MCI; bulk RNA-seq,Homo_714,AD.vs.control; bulk RNA-seq,Homo_714,AD.vs.MCI; scRNA-seq,SRP215507,CD8+ T cell_3-AD.vs.control; scRNA-seq,SRP215507,CD8+ T cell_2-MCI.vs.control; scRNA-seq,SRP330776,B cell_1-AD.vs.control; scRNA-seq,SRP330776,Natural killer T cell-AD.vs.control; scRNA-seq,SRP330776,CD8+ T cell_3-AD.vs.control	11
BIN1	scRNA-seq,SRP215507,CD8+ T cell_7-AD.vs.control; scRNA-seq,SRP215507,CD8+ T cell_7-AD.vs.MCI; scRNA-seq,SRP309935,Erythroid cell-AD.vs.control; scRNA-seq,SRP330776,B cell_1-AD.vs.control; scRNA-seq,SRP330776,B cell_2-AD.vs.control; scRNA-seq,SRP330776,CD8+ T cell_3-AD.vs.control; scRNA-seq,SRP330776,Megakaryocyte_1-AD.vs.control	7
BIN2	bulk RNA-seq,Homo_723,AD.vs.control; bulk RNA-seq,Homo_723,AD.vs.MCI; bulk RNA-seq,Homo_633,AD.vs.control; bulk RNA-seq,Homo_633,AD.vs.MCI; bulk RNA-seq,SRP223445,AD.vs.control; scRNA-seq,SRP215507,CD8+ T cell_7-AD.vs.control; scRNA-seq,SRP309935,Erythroid cell-AD.vs.control; scRNA-seq,SRP330776,B cell_1-AD.vs.control	8

BIN3	scRNA-seq,SRP215507,CD8+ T cell_6-AD.vs.control; scRNA-seq,SRP215507,CD8+ T cell_3-AD.vs.MCI; scRNA-seq,SRP215507,CD8+ T cell_6-AD.vs.MCI; scRNA-seq,SRP215507,CD8+ T cell_5-MCI.vs.control; scRNA-seq,SRP330776,CD8+ T cell_1-AD.vs.control; scRNA-seq,SRP330776,γδ T cell-AD.vs.control; scRNA-seq,SRP330776,B cell_1-AD.vs.control; scRNA-seq,SRP330776,Natural killer cell_1-AD.vs.control; scRNA-seq,SRP330776,Memory T cell_1-AD.vs.control; scRNA-seq,SRP330776,B cell_2-AD.vs.control; scRNA-seq,SRP330776,T cell-AD.vs.control; scRNA-	12
Birc2	bulk RNA-seq,SRP325058,AD.vs.control	1
BIRC2	scRNA-seq,SRP215507,CD8+ T cell_4-AD.vs.control; scRNA-seq,SRP215507,CD8+ T cell_4-AD.vs.MCI; scRNA-seq,SRP215507,CD8+ T cell_3-MCI.vs.control; scRNA-seq,SRP330776,Megakaryocyte_1-AD.vs.control	4
BIRC3	bulk RNA-seq,Homo_714,AD.vs.control; scRNA-seq,SRP215507,CD8+ T cell_6-AD.vs.control; scRNA-seq,SRP215507,CD8+ T cell_4-AD.vs.MCI; scRNA-seq,SRP215507,CD8+ T cell_7-AD.vs.MCI; scRNA-seq,SRP215507,CD8+ T cell_1-MCI.vs.control; scRNA-seq,SRP215507,CD8+ T cell_6-MCI.vs.control; scRNA-seq,SRP215507,CD8+ T cell_7-MCI.vs.control; scRNA-seq,SRP309935,CD8+ T cell_2-AD.vs.control; scRNA-seq,SRP330776,B cell_2-AD.vs.control; scRNA-seq,SRP330776,Monocyte_1-AD.vs.control; scRNA-seq,SRP330776,Natural killer cell_2-AD.vs.control	11
BIRC5	bulk RNA-seq,Homo_723,AD.vs.control; bulk RNA-seq,Homo_723,AD.vs.MCI; bulk RNA-seq,Homo_633,AD.vs.control; bulk RNA-seq,Homo_633,AD.vs.MCI; bulk RNA-seq,Homo_714,AD.vs.control; bulk RNA-seq,Homo_714,AD.vs.MCI; scRNA-seq,SRP215507,CD8+ T cell_2-AD.vs.MCI; scRNA-seq,SRP215507,CD8+ T cell_2-MCI.vs.control; scRNA-seq,SRP330776,T cell-AD.vs.control	9
Birc5	bulk RNA-seq,SRP325058,AD.vs.control	1
BIRC6	bulk RNA-seq,Homo_723,AD.vs.control; bulk RNA-seq,Homo_633,AD.vs.control; bulk RNA-seq,Homo_714,AD.vs.control; bulk RNA-seq,Homo_714,AD.vs.MCI; scRNA-seq,SRP330776,CD8+ T cell_1-AD.vs.control; scRNA-seq,SRP330776,γδ T cell-AD.vs.control; scRNA-seq,SRP330776,Memory T cell_2-AD.vs.control; scRNA-seq,SRP330776,CD8+ T cell_2-AD.vs.control; scRNA-seq,SRP330776,B cell_1-AD.vs.control; scRNA-seq,SRP330776,Naive CD8+ T cell_1-AD.vs.control; scRNA-seq,SRP330776,Natural killer cell_1-AD.vs.control; scRNA-seq,SRP330776,Memory T cell_1-AD.vs.control; scRNA-seq,SRP330776,Naive CD8+ T cell_3-AD.vs.control; scRNA-seq,SRP330776,T cell-AD.vs.control; scRNA-seq,SRP330776,Naive CD8+ T cell_2-AD.vs.control; scRNA-seq,SRP330776,CD8+ T cell_3-AD.vs.control; scRNA-seq,SRP330776,Monocyte_1-AD.vs.control; scRNA-seq,SRP330776,Monocyte_2-AD.vs.control; scRNA-seq,SRP330776,B cell_3-AD.vs.control; scRNA-seq,SRP330776,Regulatory T [illegible]	20
BIRC7	scRNA-seq,SRP215507,CD8+ T cell_3-AD.vs.control; scRNA-seq,SRP215507,CD8+ T cell_3-AD.vs.MCI; scRNA-seq,SRP215507,CD8+ T cell_4-MCI.vs.control; scRNA-seq,SRP215507,CD8+ T cell_3-MCI.vs.control	4
BISPR	scRNA-seq,SRP215507,CD8+ T cell_4-AD.vs.control; scRNA-seq,SRP215507,CD8+ T cell_4-AD.vs.MCI; scRNA-seq,SRP215507,CD8+ T cell_7-AD.vs.MCI; scRNA-seq,SRP215507,CD8+ T cell_6-MCI.vs.control; scRNA-seq,SRP309935,Naive CD8+ T cell_1-AD.vs.control; scRNA-	6
BIVM	bulk RNA-seq,Homo_723,AD.vs.control; bulk RNA-seq,Homo_723,AD.vs.MCI; bulk RNA-seq,Homo_723,MCI.vs.control; bulk RNA-seq,Homo_633,AD.vs.control; bulk RNA-seq,Homo_633,AD.vs.MCI; bulk RNA-seq,Homo_633,MCI.vs.control; bulk RNA-seq,Homo_714,AD.vs.control; bulk RNA-seq,Homo_714,AD.vs.MCI; bulk RNA-seq,Homo_714,MCI.vs.control; bulk RNA-seq,ROSMAP,AD.vs.control; bulk RNA-seq,ROSMAP,AD.vs.MCI; scRNA-seq,SRP215507,CD8+ T cell_4-AD.vs.control; scRNA-seq,SRP215507,CD8+ T cell_3-AD.vs.MCI; scRNA-seq,SRP215507,CD8+ T cell_2-MCI.vs.control; scRNA-seq,SRP215507,CD8+ T cell_3-MCI.vs.control; scRNA-seq,SRP330776,CD8+ T cell_1-AD.vs.control; scRNA-seq,SRP330776,γδ T cell-AD.vs.control; scRNA-seq,SRP330776,Memory T cell_2-AD.vs.control; scRNA-seq,SRP330776,B cell_1-AD.vs.control; scRNA-seq,SRP330776,Naive CD8+ T cell_1-AD.vs.control; scRNA-seq,SRP330776,Memory T cell_1-AD.vs.control; scRNA-[illegible]	22
BLACAT1	scRNA-seq,SRP215507,CD8+ T cell_2-AD.vs.control	1
BLCAP	scRNA-seq,SRP215507,CD8+ T cell_10-AD.vs.control; scRNA-seq,SRP215507,CD8+ T cell_5-MCI.vs.control; scRNA-seq,SRP215507,CD8+ T cell_2-MCI.vs.control; scRNA-seq,SRP330776,Memory T cell_2-AD.vs.control; scRNA-seq,SRP330776,Naive CD8+ T cell_1-AD.vs.control; scRNA-seq,SRP330776,Naive CD8+ T cell_3-AD.vs.control; scRNA-seq,SRP330776,B cell_2-AD.vs.control; scRNA-seq,SRP330776,T cell-AD.vs.control	8
BLK	bulk RNA-seq,Homo_633,AD.vs.control; bulk RNA-seq,ROSMAP,AD.vs.control; bulk RNA-seq,ROSMAP,MCI.vs.control; scRNA-seq,SRP215507,CD8+ T cell_2-AD.vs.control; scRNA-seq,SRP215507,CD8+ T cell_5-AD.vs.MCI; scRNA-seq,SRP215507,CD8+ T cell_1-MCI.vs.control; scRNA-seq,SRP215507,CD8+ T cell_5-MCI.vs.control; scRNA-seq,SRP215507,CD8+ T cell_7-MCI.vs.control; scRNA-seq,SRP309935,CD8+ T cell_1-AD.vs.control; scRNA-seq,SRP330776,Memory T cell_2-AD.vs.control; scRNA-seq,SRP330776,CD8+ T cell_2-AD.vs.control; scRNA-seq,SRP330776,Naive CD8+ T cell_1-AD.vs.control; scRNA-seq,SRP330776,Naive CD8+ T cell_2-AD.vs.control	13
Blk	bulk RNA-seq,SRP325058,AD.vs.control	1

BLM	bulk RNA-seq,Homo_633,AD.vs.control; bulk RNA-seq,Homo_714,AD.vs.control; bulk RNA-seq,Homo_714,AD.vs.MCI; bulk RNA-seq,Homo_714,MCI.vs.control; scRNA-seq,SRP215507,CD8+ T cell_1-MCI.vs.control; scRNA-seq,SRP215507,CD8+ T cell_2-MCI.vs.control; scRNA-seq,SRP330776,Natural killer cell_1-AD.vs.control; scRNA-seq,SRP330776,Naive CD8+ T cell_3-AD.vs.control; scRNA-seq,SRP330776,CD8+ T cell_3-	9
BLMH	bulk RNA-seq,Homo_723,AD.vs.MCI; bulk RNA-seq,Homo_723,MCI.vs.control; bulk RNA-seq,Homo_633,MCI.vs.control; bulk RNA-seq,Homo_714,AD.vs.control; bulk RNA-seq,Homo_714,AD.vs.MCI; bulk RNA-seq,Homo_714,MCI.vs.control; scRNA-seq,SRP215507,CD8+ T cell_4-AD.vs.MCI; scRNA-seq,SRP215507,CD8+ T cell_5-AD.vs.MCI; scRNA-seq,SRP215507,CD8+ T cell_6-MCI.vs.control; scRNA-seq,SRP309935,Natural killer cell_2-AD.vs.control; scRNA-seq,SRP309935,Monocyte_1-AD.vs.control; scRNA-seq,SRP309935,Megakaryocyte_1-AD.vs.control; scRNA-seq,SRP330776,γδ T cell-AD.vs.control; scRNA-seq,SRP330776,Natural killer T cell-AD.vs.control; scRNA-seq,SRP330776,B cell_2-AD.vs.control;	16
BLNK	bulk RNA-seq,Homo_714,AD.vs.control; bulk RNA-seq,Homo_714,AD.vs.MCI; bulk RNA-seq,SRP223445,AD.vs.control	3
BLOC1S1	bulk RNA-seq,Homo_633,AD.vs.control; bulk RNA-seq,Homo_633,AD.vs.MCI; scRNA-seq,SRP215507,CD8+ T cell_7-AD.vs.control; scRNA-seq,SRP215507,CD8+ T cell_7-AD.vs.MCI; scRNA-seq,SRP330776,CD8+ T cell_1-AD.vs.control; scRNA-seq,SRP330776,Natural killer cell_1-AD.vs.control; scRNA-seq,SRP330776,B cell_2-AD.vs.control; scRNA-seq,SRP330776,T cell-AD.vs.control; scRNA-seq,SRP330776,Natural killer cell_2-	9
BLOC1S2	bulk RNA-seq,Homo_633,AD.vs.control; bulk RNA-seq,Homo_714,AD.vs.control; bulk RNA-seq,Homo_714,AD.vs.MCI; scRNA-seq,SRP215507,CD8+ T cell_6-AD.vs.control; scRNA-seq,SRP215507,CD8+ T cell_4-AD.vs.MCI; scRNA-seq,SRP215507,CD8+ T cell_6-AD.vs.MCI; scRNA-seq,SRP215507,CD8+ T cell_4-MCI.vs.control; scRNA-seq,SRP330776,B cell_2-AD.vs.control; scRNA-seq,SRP330776,Natural killer cell_2-	9
Bloc1s2	bulk RNA-seq,SRP325058,AD.vs.control	1
BLOC1S3	scRNA-seq,SRP215507,CD8+ T cell_2-AD.vs.control; scRNA-seq,SRP215507,CD8+ T cell_5-AD.vs.control; scRNA-seq,SRP215507,CD8+ T cell_1-MCI.vs.control; scRNA-seq,SRP215507,CD8+ T cell_2-MCI.vs.control; scRNA-seq,SRP330776,CD8+ T cell_1-AD.vs.control; scRNA-seq,SRP330776,γδ T cell-AD.vs.control; scRNA-seq,SRP330776,Memory T cell_2-AD.vs.control; scRNA-seq,SRP330776,CD8+ T cell_2-AD.vs.control; scRNA-seq,SRP330776,B cell_1-AD.vs.control; scRNA-seq,SRP330776,Naive CD8+ T cell_1-AD.vs.control; scRNA-seq,SRP330776,Natural killer cell_1-AD.vs.control; scRNA-seq,SRP330776,Memory T cell_1-AD.vs.control; scRNA-seq,SRP330776,Natural killer T cell-AD.vs.control	13
BLOC1S4	scRNA-seq,SRP215507,CD8+ T cell_4-AD.vs.MCI; scRNA-seq,SRP215507,CD8+ T cell_7-AD.vs.MCI; scRNA-seq,SRP215507,CD8+ T cell_5-MCI.vs.control; scRNA-seq,SRP215507,CD8+ T cell_4-MCI.vs.control; scRNA-seq,SRP309935,Naive CD8+ T cell_1-AD.vs.control; scRNA-seq,SRP309935,B cell_1-AD.vs.control; scRNA-seq,SRP309935,B cell_2-AD.vs.control; scRNA-seq,SRP330776,CD8+ T cell_1-AD.vs.control; scRNA-seq,SRP330776,γδ T cell-AD.vs.control; scRNA-seq,SRP330776,Memory T cell_2-AD.vs.control; scRNA-seq,SRP330776,CD8+ T cell_2-AD.vs.control; scRNA-seq,SRP330776,B cell_1-AD.vs.control; scRNA-seq,SRP330776,Naive CD8+ T cell_1-AD.vs.control; scRNA-seq,SRP330776,Natural killer cell_1-AD.vs.control; scRNA-seq,SRP330776,Memory T cell_1-AD.vs.control; scRNA-seq,SRP330776,Natural killer T cell-AD.vs.control; scRNA-seq,SRP330776,Naive CD8+ T cell_3-AD.vs.control; scRNA-seq,SRP330776,T cell-AD.vs.control; scRNA-seq,SRP330776,Naive CD8+ T cell_2-AD.vs.control; scRNA-seq,SRP330776,CD8+ T cell_3-AD.vs.control; scRNA-seq,SRP330776,Monocyte_2-AD.vs.control; scRNA-seq,SRP330776,Natural [illegible]	23
BLOC1S5	bulk RNA-seq,Homo_714,AD.vs.control; bulk RNA-seq,Homo_714,AD.vs.MCI; scRNA-seq,SRP309935,Natural killer cell_2-AD.vs.control; scRNA-seq,SRP309935,Monocyte_1-AD.vs.control; scRNA-seq,SRP309935,Naive CD8+ T cell_1-AD.vs.control; scRNA-seq,SRP309935,CD8+ T cell_1-AD.vs.control; scRNA-seq,SRP309935,B cell_1-AD.vs.control; scRNA-seq,SRP330776,Naive CD8+ T cell_3-AD.vs.control; scRNA-seq,SRP330776,T cell-AD.vs.control; scRNA-seq,SRP330776,CD8+ T cell_3-AD.vs.control; scRNA-seq,SRP330776,B cell_4-AD.vs.control	11
BLOC1S6	bulk RNA-seq,Homo_723,AD.vs.control; bulk RNA-seq,Homo_723,AD.vs.MCI; bulk RNA-seq,Homo_633,AD.vs.control; bulk RNA-seq,Homo_633,AD.vs.MCI; bulk RNA-seq,Homo_714,AD.vs.control; bulk RNA-seq,Homo_714,AD.vs.MCI; scRNA-seq,SRP215507,CD8+ T cell_6-AD.vs.control; scRNA-seq,SRP309935,Megakaryocyte_1-AD.vs.control; scRNA-seq,SRP309935,Naive CD8+ T cell_2-AD.vs.control; scRNA-seq,SRP330776,Monocyte_1-AD.vs.control; scRNA-seq,SRP330776,Monocyte_2-AD.vs.control; scRNA-seq,SRP330776,Megakaryocyte_1-AD.vs.control	12
BLVRA	bulk RNA-seq,Homo_723,AD.vs.control; bulk RNA-seq,Homo_723,AD.vs.MCI; bulk RNA-seq,Homo_723,MCI.vs.control; bulk RNA-seq,Homo_633,AD.vs.control; bulk RNA-seq,Homo_633,AD.vs.MCI; bulk RNA-seq,Homo_633,MCI.vs.control; bulk RNA-seq,Homo_714,AD.vs.control; bulk RNA-seq,Homo_714,AD.vs.MCI; scRNA-seq,SRP215507,CD8+ T cell_3-AD.vs.control; scRNA-seq,SRP215507,CD8+ T cell_2-AD.vs.MCI; scRNA-seq,SRP215507,CD8+ T cell_1-AD.vs.MCI; scRNA-seq,SRP215507,CD8+ T cell_4-AD.vs.MCI; scRNA-seq,SRP215507,CD8+ T cell_5-AD.vs.MCI; scRNA-seq,SRP215507,CD8+ T cell_4-MCI.vs.control; scRNA-seq,SRP330776,B cell_2-AD.vs.control; scRNA-seq,SRP330776,Naive CD8+ T cell_2-AD.vs.control; scRNA-seq,SRP330776,B cell_3-AD.vs.control; scRNA-seq,SRP330776,Natural killer cell_2-AD.vs.control; scRNA- [illegible]	19

BLVRB	scRNA-seq,SRP215507,CD8+ T cell_6-AD.vs.control; scRNA-seq,SRP215507,CD8+ T cell_3-AD.vs.MCI; scRNA-seq,SRP215507,CD8+ T cell_4-AD.vs.MCI; scRNA-seq,SRP215507,CD8+ T cell_5-AD.vs.MCI; scRNA-seq,SRP215507,CD8+ T cell_4-MCI.vs.control; scRNA-seq,SRP215507,CD8+ T cell_3-MCI.vs.control; scRNA-seq,SRP215507,CD8+ T cell_6-MCI.vs.control; scRNA-seq,SRP330776,CD8+ T cell_1-AD.vs.control; scRNA-seq,SRP330776,CD8+ T cell_2-AD.vs.control; scRNA-seq,SRP330776,Naive CD8+ T cell_1-AD.vs.control; scRNA-seq,SRP330776,Naive CD8+ T cell_3-AD.vs.control; scRNA-seq,SRP330776,CD8+ T cell_3-AD.vs.control; scRNA-seq,SRP330776,Monocyte_1-AD.vs.control; scRNA-	14
BLZF1	bulk RNA-seq,Homo_714,AD.vs.control; bulk RNA-seq,Homo_714,AD.vs.MCI; scRNA-seq,SRP309935,Natural killer cell_2-AD.vs.control; scRNA-seq,SRP309935,Naive CD8+ T cell_2-AD.vs.control; scRNA-seq,SRP330776,B cell_1-AD.vs.control; scRNA-seq,SRP330776,Memory T cell_1-AD.vs.control; scRNA-seq,SRP330776,CD8+ T cell_3-AD.vs.control	7
BMERB1	bulk RNA-seq,Homo_723,AD.vs.control; bulk RNA-seq,Homo_723,AD.vs.MCI; bulk RNA-seq,Homo_633,AD.vs.MCI; bulk RNA-seq,Homo_714,AD.vs.control; bulk RNA-seq,Homo_714,AD.vs.MCI; scRNA-seq,SRP215507,CD8+ T cell_3-AD.vs.control; scRNA-seq,SRP215507,CD8+ T cell_5-AD.vs.control; scRNA-seq,SRP215507,CD8+ T cell_3-AD.vs.MCI; scRNA-seq,SRP215507,CD8+ T cell_1-AD.vs.MCI; scRNA-seq,SRP215507,CD8+ T cell_6-MCI.vs.control; scRNA-seq,SRP215507,CD8+ T cell_8-MCI.vs.control; scRNA-seq,SRP330776,CD8+ T cell_1-AD.vs.control; scRNA-seq,SRP330776,γδ T cell-AD.vs.control; scRNA-seq,SRP330776,Memory T cell_2-AD.vs.control; scRNA-seq,SRP330776,B cell_1-AD.vs.control; scRNA-seq,SRP330776,Naive CD8+ T cell_1-AD.vs.control; scRNA-seq,SRP330776,T cell-AD.vs.control; scRNA-	18
BMF	scRNA-seq,SRP330776,CD8+ T cell_2-AD.vs.control	1
BMI1	bulk RNA-seq,Homo_723,AD.vs.control; bulk RNA-seq,Homo_723,AD.vs.MCI; bulk RNA-seq,Homo_723,MCI.vs.control; bulk RNA-seq,Homo_633,AD.vs.control; bulk RNA-seq,Homo_633,AD.vs.MCI; bulk RNA-seq,Homo_633,MCI.vs.control; bulk RNA-seq,Homo_714,AD.vs.control; bulk RNA-seq,Homo_714,AD.vs.MCI; scRNA-seq,SRP215507,CD8+ T cell_4-MCI.vs.control; scRNA-seq,SRP309935,Naive CD8+ T cell_1-AD.vs.control; scRNA-seq,SRP330776,CD8+ T cell_1-AD.vs.control; scRNA-seq,SRP330776,γδ T cell-AD.vs.control; scRNA-seq,SRP330776,Memory T cell_2-AD.vs.control; scRNA-seq,SRP330776,CD8+ T cell_2-AD.vs.control; scRNA-seq,SRP330776,Naive CD8+ T cell_1-AD.vs.control; scRNA-seq,SRP330776,Natural killer cell_1-AD.vs.control; scRNA-seq,SRP330776,Memory T cell_1-AD.vs.control; scRNA-seq,SRP330776,Natural killer T cell-AD.vs.control; scRNA-seq,SRP330776,Naive CD8+ T cell_3-AD.vs.control; scRNA-seq,SRP330776,B cell_2-AD.vs.control; scRNA-	21
BMP1	bulk RNA-seq,Homo_723,AD.vs.control; bulk RNA-seq,Homo_723,AD.vs.MCI; bulk RNA-seq,Homo_714,AD.vs.control; bulk RNA-seq,Homo_714,AD.vs.MCI; scRNA-seq,SRP215507,CD8+ T cell_1-AD.vs.control; scRNA-seq,SRP215507,CD8+ T cell_1-MCI.vs.control; scRNA-seq,SRP330776,Memory T cell_2-AD.vs.control; scRNA-seq,SRP330776,CD8+ T cell_2-AD.vs.control; scRNA-seq,SRP330776,Memory T cell_1-	9
BMP10	bulk RNA-seq,Homo_723,MCI.vs.control; bulk RNA-seq,Homo_633,AD.vs.MCI; bulk RNA-seq,Homo_633,MCI.vs.control	3
BMP2	bulk RNA-seq,Homo_723,AD.vs.control; bulk RNA-seq,Homo_723,AD.vs.MCI; bulk RNA-seq,Homo_633,AD.vs.control; bulk RNA-seq,Homo_633,AD.vs.MCI; bulk RNA-seq,Homo_714,AD.vs.MCI; bulk RNA-seq,Homo_714,MCI.vs.control	6
BMP2K	bulk RNA-seq,Homo_723,AD.vs.control; bulk RNA-seq,Homo_723,AD.vs.MCI; bulk RNA-seq,Homo_633,AD.vs.control; bulk RNA-seq,Homo_633,AD.vs.MCI; bulk RNA-seq,Homo_714,AD.vs.control; bulk RNA-seq,Homo_714,AD.vs.MCI; scRNA-seq,SRP215507,CD8+ T cell_3-AD.vs.control; scRNA-seq,SRP215507,CD8+ T cell_9-AD.vs.control; scRNA-seq,SRP330776,CD8+ T cell_1-AD.vs.control; scRNA-seq,SRP330776,Memory T cell_2-AD.vs.control; scRNA-seq,SRP330776,B cell_1-AD.vs.control; scRNA-seq,SRP330776,Naive CD8+ T cell_1-AD.vs.control; scRNA-seq,SRP330776,Memory T cell_1-AD.vs.control; scRNA-seq,SRP330776,B cell_2-AD.vs.control	14
BMP3	bulk RNA-seq,Homo_714,MCI.vs.control	1
BMP4	bulk RNA-seq,Homo_723,AD.vs.control; bulk RNA-seq,Homo_723,AD.vs.MCI; bulk RNA-seq,Homo_723,MCI.vs.control; bulk RNA-seq,Homo_633,AD.vs.control; bulk RNA-seq,Homo_633,AD.vs.MCI; bulk RNA-seq,Homo_633,MCI.vs.control; bulk RNA-	8
BMP5	bulk RNA-seq,Homo_723,AD.vs.control; bulk RNA-seq,Homo_723,AD.vs.MCI	2
BMP6	bulk RNA-seq,Homo_723,AD.vs.control; bulk RNA-seq,Homo_723,AD.vs.MCI; bulk RNA-seq,Homo_633,AD.vs.control; bulk RNA-seq,Homo_633,AD.vs.MCI; bulk RNA-seq,Homo_714,AD.vs.control; bulk RNA-seq,Homo_714,AD.vs.MCI; scRNA-seq,SRP309935,Megakaryocyte_1-	7
BMP7	bulk RNA-seq,Homo_723,AD.vs.control; bulk RNA-seq,Homo_723,AD.vs.MCI; bulk RNA-seq,Homo_723,MCI.vs.control	3
BMP8A	bulk RNA-seq,Homo_714,AD.vs.control; bulk RNA-seq,Homo_714,AD.vs.MCI; scRNA-seq,SRP330776,Naive CD8+ T cell_1-AD.vs.control	3
BMP8B	scRNA-seq,SRP215507,CD8+ T cell_1-AD.vs.MCI; scRNA-seq,SRP215507,CD8+ T cell_1-MCI.vs.control; scRNA-seq,SRP215507,CD8+ T cell_5-MCI.vs.control; scRNA-seq,SRP330776,Memory T cell_1-AD.vs.control	4

BMPER	bulk RNA-seq,Homo_723,AD.vs.control; bulk RNA-seq,Homo_723,AD.vs.MCI; bulk RNA-seq,Homo_633,AD.vs.control; bulk RNA-seq,Homo_633,AD.vs.MCI; bulk RNA-seq,Homo_714,MCI.vs.control	5
BMPR1A	bulk RNA-seq,Homo_723,MCI.vs.control; bulk RNA-seq,Homo_633,MCI.vs.control; bulk RNA-seq,Homo_714,MCI.vs.control; scRNA-seq,SRP215507,CD8+ T cell_2-AD.vs.MCI; scRNA-seq,SRP215507,CD8+ T cell_2-MCI.vs.control; scRNA-seq,SRP330776,CD8+ T cell_1-AD.vs.control; scRNA-seq,SRP330776,γδ T cell-AD.vs.control; scRNA-seq,SRP330776,Memory T cell_2-AD.vs.control; scRNA-seq,SRP330776,CD8+ T cell_2-AD.vs.control; scRNA-seq,SRP330776,B cell_1-AD.vs.control; scRNA-seq,SRP330776,Naive CD8+ T cell_1-AD.vs.control; scRNA-seq,SRP330776,Natural killer cell_1-AD.vs.control; scRNA-seq,SRP330776,Memory T cell_1-AD.vs.control; scRNA-seq,SRP330776,Natural killer T cell-	16
BMPR1B	bulk RNA-seq,Homo_723,AD.vs.control; bulk RNA-seq,Homo_723,AD.vs.MCI; bulk RNA-seq,Homo_723,MCI.vs.control; bulk RNA-seq,Homo_633,AD.vs.control; bulk RNA-seq,Homo_633,AD.vs.MCI	5
BMPR2	bulk RNA-seq,Homo_723,AD.vs.control; bulk RNA-seq,Homo_723,AD.vs.MCI; bulk RNA-seq,Homo_723,MCI.vs.control; bulk RNA-seq,Homo_633,AD.vs.control; bulk RNA-seq,Homo_633,AD.vs.MCI; bulk RNA-seq,Homo_633,MCI.vs.control; bulk RNA-seq,Homo_714,AD.vs.control; bulk RNA-seq,Homo_714,AD.vs.MCI; scRNA-seq,SRP215507,CD8+ T cell_5-AD.vs.control; scRNA-seq,SRP215507,CD8+ T cell_1-AD.vs.MCI; scRNA-seq,SRP215507,CD8+ T cell_5-AD.vs.MCI; scRNA-seq,SRP309935,Naive CD8+ T cell_1-AD.vs.control; scRNA-seq,SRP330776,CD8+ T cell_1-AD.vs.control; scRNA-seq,SRP330776,CD8+ T cell_2-AD.vs.control; scRNA-seq,SRP330776,Naive CD8+ T cell_1-AD.vs.control; scRNA-seq,SRP330776,Natural killer cell_1-AD.vs.control; scRNA-seq,SRP330776,Naive CD8+ T cell_3-AD.vs.control; scRNA-seq,SRP330776,B cell_2-	18
BMS1	scRNA-seq,SRP215507,CD8+ T cell_5-AD.vs.MCI; scRNA-seq,SRP215507,CD8+ T cell_5-MCI.vs.control; scRNA-seq,SRP309935,Naive CD8+ T cell_2-AD.vs.control; scRNA-seq,SRP330776,CD8+ T cell_3-AD.vs.control; scRNA-seq,SRP330776,Monocyte_2-AD.vs.control; scRNA-	6
BMT2	bulk RNA-seq,Homo_723,AD.vs.MCI; bulk RNA-seq,Homo_714,AD.vs.control; bulk RNA-seq,Homo_714,AD.vs.MCI; scRNA-seq,SRP215507,CD8+ T cell_5-AD.vs.control; scRNA-seq,SRP215507,CD8+ T cell_3-AD.vs.MCI; scRNA-seq,SRP215507,CD8+ T cell_5-AD.vs.MCI; scRNA-seq,SRP215507,CD8+ T cell_5-MCI.vs.control; scRNA-seq,SRP330776,CD8+ T cell_1-AD.vs.control	8
BMX	bulk RNA-seq,Homo_723,AD.vs.control; bulk RNA-seq,Homo_723,AD.vs.MCI; bulk RNA-seq,Homo_633,AD.vs.control; bulk RNA-seq,Homo_633,AD.vs.MCI; bulk RNA-seq,Homo_714,AD.vs.control; bulk RNA-seq,Homo_714,AD.vs.MCI; bulk RNA-seq,ROSMAP,MCI.vs.control	7
BNC1	bulk RNA-seq,Homo_723,AD.vs.MCI; bulk RNA-seq,Homo_714,MCI.vs.control	2
BNC2	bulk RNA-seq,Homo_723,AD.vs.control; bulk RNA-seq,Homo_723,AD.vs.MCI; bulk RNA-seq,Homo_723,MCI.vs.control; bulk RNA-seq,Homo_633,AD.vs.control; bulk RNA-seq,Homo_633,AD.vs.MCI; bulk RNA-seq,Homo_633,MCI.vs.control; bulk RNA-seq,Homo_714,AD.vs.control; bulk RNA-seq,Homo_714,AD.vs.MCI; bulk RNA-seq,SRP223445,AD.vs.control; scRNA-seq,SRP215507,CD8+ T cell_2-AD.vs.MCI; scRNA-seq,SRP215507,CD8+ T cell_1-AD.vs.MCI; scRNA-seq,SRP215507,CD8+ T cell_4-AD.vs.MCI; scRNA-seq,SRP215507,CD8+ T cell_1-MCI.vs.control; scRNA-seq,SRP215507,CD8+ T cell_4-MCI.vs.control; scRNA-seq,SRP215507,CD8+ T cell_2-MCI.vs.control; scRNA-seq,SRP330776,Natural killer T	16
BNIP1	bulk RNA-seq,Homo_723,AD.vs.control; bulk RNA-seq,Homo_633,AD.vs.control; bulk RNA-seq,Homo_714,AD.vs.control; bulk RNA-seq,Homo_714,AD.vs.MCI; scRNA-seq,SRP215507,CD8+ T cell_4-AD.vs.control; scRNA-seq,SRP215507,CD8+ T cell_6-AD.vs.control; scRNA-seq,SRP215507,CD8+ T cell_2-AD.vs.MCI; scRNA-seq,SRP215507,CD8+ T cell_3-AD.vs.MCI; scRNA-seq,SRP215507,CD8+ T cell_1-AD.vs.MCI; scRNA-seq,SRP215507,CD8+ T cell_6-AD.vs.MCI; scRNA-seq,SRP215507,CD8+ T cell_4-MCI.vs.control; scRNA-seq,SRP330776,Megakaryocyte_1-	12
BNIP2	bulk RNA-seq,Homo_723,AD.vs.control; bulk RNA-seq,Homo_723,AD.vs.MCI; bulk RNA-seq,Homo_723,MCI.vs.control; bulk RNA-seq,Homo_633,AD.vs.control; bulk RNA-seq,Homo_633,AD.vs.MCI; bulk RNA-seq,Homo_633,MCI.vs.control; bulk RNA-seq,Homo_714,AD.vs.control; bulk RNA-seq,Homo_714,AD.vs.MCI; bulk RNA-seq,SRP310421,AD.vs.control	9
BNIP3	bulk RNA-seq,Homo_633,MCI.vs.control; bulk RNA-seq,Homo_714,AD.vs.control; bulk RNA-seq,Homo_714,AD.vs.MCI; bulk RNA-seq,Homo_714,MCI.vs.control; scRNA-seq,SRP215507,CD8+ T cell_4-AD.vs.control; scRNA-seq,SRP215507,CD8+ T cell_5-AD.vs.control; scRNA-seq,SRP215507,CD8+ T cell_4-AD.vs.MCI; scRNA-seq,SRP309935,Natural killer cell_2-AD.vs.control; scRNA-seq,SRP309935,Megakaryocyte_1-AD.vs.control; scRNA-seq,SRP309935,Natural killer cell_1-AD.vs.control; scRNA-seq,SRP309935,Monocyte_2-AD.vs.control; scRNA-seq,SRP309935,B cell_1-AD.vs.control; scRNA-seq,SRP309935,CD8+ T cell_2-AD.vs.control; scRNA-seq,SRP330776,CD8+ T cell_1-AD.vs.control; scRNA-seq,SRP330776,Memory T cell_2-AD.vs.control; scRNA-seq,SRP330776,CD8+ T cell_2-AD.vs.control; scRNA-seq,SRP330776,B cell_1-AD.vs.control; scRNA-seq,SRP330776,Memory T cell_1-AD.vs.control; scRNA-seq,SRP330776,CD8+ T cell_3-AD.vs.control; scRNA-seq,SRP330776,B cell_3-	20

BNIP3L	scRNA-seq,SRP215507,CD8+ T cell_7-AD.vs.control; scRNA-seq,SRP215507,CD8+ T cell_4-AD.vs.MCI; scRNA-seq,SRP215507,CD8+ T cell_5-AD.vs.MCI; scRNA-seq,SRP215507,CD8+ T cell_5-MCI.vs.control; scRNA-seq,SRP215507,CD8+ T cell_4-MCI.vs.control; scRNA-seq,SRP309935,Erythroid cell-AD.vs.control; scRNA-seq,SRP330776,Monocyte_1-AD.vs.control	7
BNIP5	bulk RNA-seq,Homo_723,AD.vs.control; bulk RNA-seq,Homo_723,AD.vs.MCI	2
BNIPL	bulk RNA-seq,Homo_723,MCI.vs.control; bulk RNA-seq,Homo_633,MCI.vs.control	2
BOC	bulk RNA-seq,Homo_723,AD.vs.control; bulk RNA-seq,Homo_723,AD.vs.MCI; bulk RNA-seq,Homo_723,MCI.vs.control; bulk RNA-seq,Homo_633,AD.vs.control; bulk RNA-seq,Homo_633,AD.vs.MCI; bulk RNA-seq,Homo_714,MCI.vs.control	6
BOD1	scRNA-seq,SRP309935,Megakaryocyte_1-AD.vs.control; scRNA-seq,SRP309935,Natural killer cell_1-AD.vs.control; scRNA-seq,SRP330776,CD8+ T cell_1-AD.vs.control; scRNA-seq,SRP330776,γδ T cell-AD.vs.control; scRNA-seq,SRP330776,Memory T cell_2-AD.vs.control; scRNA-seq,SRP330776,CD8+ T cell_2-AD.vs.control; scRNA-seq,SRP330776,B cell_1-AD.vs.control; scRNA-seq,SRP330776,Naive CD8+ T cell_1-AD.vs.control; scRNA-seq,SRP330776,Natural killer cell_1-AD.vs.control; scRNA-seq,SRP330776,Memory T cell_1-AD.vs.control; scRNA-seq,SRP330776,Natural killer T cell-AD.vs.control; scRNA-seq,SRP330776,Naive CD8+ T cell_3-AD.vs.control; scRNA-seq,SRP330776,B cell_2-AD.vs.control; scRNA-seq,SRP330776,T cell-AD.vs.control; scRNA-seq,SRP330776,CD8+ T cell_3-AD.vs.control; scRNA-seq,SRP330776,B cell_3-[illegible]	17
BOD1L1	scRNA-seq,SRP309935,CD8+ T cell_1-AD.vs.control; scRNA-seq,SRP330776,Memory T cell_2-AD.vs.control; scRNA-seq,SRP330776,CD8+ T cell_3-AD.vs.control; scRNA-seq,SRP330776,Natural killer cell_2-AD.vs.control	4
BOD1L2	bulk RNA-seq,Homo_723,AD.vs.control; bulk RNA-seq,Homo_723,MCI.vs.control; bulk RNA-seq,Homo_633,AD.vs.control; bulk RNA-seq,Homo_633,MCI.vs.control; bulk RNA-seq,Homo_714,MCI.vs.control	5
BOK	bulk RNA-seq,Homo_723,AD.vs.control; bulk RNA-seq,Homo_723,AD.vs.MCI; bulk RNA-seq,Homo_633,AD.vs.control; bulk RNA-seq,Homo_714,AD.vs.control; bulk RNA-seq,Homo_714,AD.vs.MCI; bulk RNA-seq,Homo_714,MCI.vs.control; bulk RNA-seq,SRP223445,AD.vs.control; scRNA-seq,SRP215507,CD8+ T cell_4-MCI.vs.control; scRNA-seq,SRP309935,Natural killer cell_1-AD.vs.control; scRNA-seq,SRP330776,CD8+ T cell_2-AD.vs.control; scRNA-seq,SRP330776,Natural killer cell_2-AD.vs.control	11
BOLA1	bulk RNA-seq,Homo_633,AD.vs.control; bulk RNA-seq,Homo_633,MCI.vs.control; bulk RNA-seq,Homo_714,AD.vs.control; bulk RNA-seq,Homo_714,AD.vs.MCI; bulk RNA-seq,Homo_714,MCI.vs.control; scRNA-seq,SRP215507,CD8+ T cell_4-AD.vs.control; scRNA-seq,SRP215507,CD8+ T cell_3-AD.vs.MCI; scRNA-seq,SRP215507,CD8+ T cell_3-MCI.vs.control; scRNA-seq,SRP309935,Natural killer cell_2-AD.vs.control; scRNA-seq,SRP309935,Naive CD8+ T cell_2-AD.vs.control; scRNA-seq,SRP330776,CD8+ T cell_1-AD.vs.control; scRNA-seq,SRP330776,Memory T cell_2-AD.vs.control; scRNA-seq,SRP330776,CD8+ T cell_2-AD.vs.control; scRNA-seq,SRP330776,B cell_1-AD.vs.control; scRNA-seq,SRP330776,Naive CD8+ T cell_1-AD.vs.control; scRNA-seq,SRP330776,Memory T cell_1-AD.vs.control; scRNA-seq,SRP330776,Natural killer T cell-AD.vs.control; scRNA-seq,SRP330776,B cell_2-AD.vs.control; scRNA-seq,SRP330776,T cell-AD.vs.control; scRNA-seq,SRP330776,B [illegible]	20
BOLA2	scRNA-seq,SRP215507,CD8+ T cell_5-AD.vs.MCI; scRNA-seq,SRP215507,CD8+ T cell_5-MCI.vs.control; scRNA-seq,SRP215507,CD8+ T cell_4-MCI.vs.control; scRNA-seq,SRP330776,CD8+ T cell_1-AD.vs.control; scRNA-seq,SRP330776,γδ T cell-AD.vs.control; scRNA-seq,SRP330776,CD8+ T cell_2-AD.vs.control; scRNA-seq,SRP330776,B cell_1-AD.vs.control; scRNA-seq,SRP330776,Naive CD8+ T cell_1-AD.vs.control; scRNA-seq,SRP330776,Memory T cell_1-AD.vs.control; scRNA-seq,SRP330776,Natural killer T cell-AD.vs.control; scRNA-seq,SRP330776,B cell_2-AD.vs.control; scRNA-seq,SRP330776,T cell-AD.vs.control; scRNA-seq,SRP330776,Natural killer cell_2-AD.vs.control; scRNA-seq,SRP330776,B cell_4-	14
BOLA2B	scRNA-seq,SRP215507,CD8+ T cell_4-AD.vs.MCI; scRNA-seq,SRP215507,CD8+ T cell_4-MCI.vs.control; scRNA-seq,SRP330776,γδ T cell-AD.vs.control; scRNA-seq,SRP330776,Memory T cell_2-AD.vs.control; scRNA-seq,SRP330776,B cell_1-AD.vs.control; scRNA-seq,SRP330776,Naive CD8+ T cell_1-AD.vs.control; scRNA-seq,SRP330776,Natural killer cell_1-AD.vs.control; scRNA-seq,SRP330776,Memory T cell_1-AD.vs.control; scRNA-seq,SRP330776,Natural killer T cell-AD.vs.control; scRNA-seq,SRP330776,Naive CD8+ T cell_3-AD.vs.control; scRNA-seq,SRP330776,B cell_2-	12
BOLA2-SMG1P6	bulk RNA-seq,Homo_714,AD.vs.control; bulk RNA-seq,Homo_714,AD.vs.MCI	2

BOLA3	bulk RNA-seq,Homo_723,AD.vs.control; bulk RNA-seq,Homo_723,AD.vs.MCI; bulk RNA-seq,Homo_633,AD.vs.control; bulk RNA-seq,Homo_633,AD.vs.MCI; bulk RNA-seq,Homo_714,AD.vs.control; bulk RNA-seq,Homo_714,AD.vs.MCI; bulk RNA-seq,ROSMAP,AD.vs.control; bulk RNA-seq,ROSMAP,AD.vs.MCI; scRNA-seq,SRP215507,CD8+ T cell_7-AD.vs.control; scRNA-seq,SRP330776,CD8+ T cell_1-AD.vs.control; scRNA-seq,SRP330776,γδ T cell-AD.vs.control; scRNA-seq,SRP330776,Memory T cell_2-AD.vs.control; scRNA-seq,SRP330776,CD8+ T cell_2-AD.vs.control; scRNA-seq,SRP330776,B cell_1-AD.vs.control; scRNA-seq,SRP330776,Naive CD8+ T cell_1-AD.vs.control; scRNA-seq,SRP330776,Natural killer cell_1-AD.vs.control; scRNA-seq,SRP330776,Memory T cell_1-AD.vs.control; scRNA-seq,SRP330776,Natural killer T cell-AD.vs.control; scRNA-seq,SRP330776,Naive CD8+ T cell_3-AD.vs.control; scRNA-seq,SRP330776,B cell_2-AD.vs.control; scRNA-[illegible]	21
Bola3	bulk RNA-seq,SRP325058,AD.vs.control	1
BOLA3-AS1	scRNA-seq,SRP215507,CD8+ T cell_2-AD.vs.control; scRNA-seq,SRP215507,CD8+ T cell_4-AD.vs.control; scRNA-seq,SRP215507,CD8+ T cell_2-MCI.vs.control; scRNA-seq,SRP330776,Memory T cell_2-AD.vs.control; scRNA-seq,SRP330776,CD8+ T cell_3-AD.vs.control	5
BOP1	bulk RNA-seq,Homo_633,AD.vs.control; bulk RNA-seq,SRP223445,AD.vs.control; scRNA-seq,SRP215507,CD8+ T cell_7-AD.vs.control; scRNA-seq,SRP215507,CD8+ T cell_5-AD.vs.control; scRNA-seq,SRP215507,CD8+ T cell_5-AD.vs.MCI; scRNA-seq,SRP330776,CD8+ T cell_1-AD.vs.control; scRNA-seq,SRP330776,γδ T cell-AD.vs.control; scRNA-seq,SRP330776,Memory T cell_2-AD.vs.control; scRNA-seq,SRP330776,CD8+ T cell_2-AD.vs.control; scRNA-seq,SRP330776,B cell_1-AD.vs.control; scRNA-seq,SRP330776,Naive CD8+ T cell_1-AD.vs.control; scRNA-seq,SRP330776,Natural killer cell_1-AD.vs.control; scRNA-seq,SRP330776,Memory T cell_1-AD.vs.control; scRNA-seq,SRP330776,Natural killer T cell-AD.vs.control; scRNA-seq,SRP330776,Naive CD8+ T cell_3-AD.vs.control; scRNA-seq,SRP330776,B cell_2-AD.vs.control; scRNA-seq,SRP330776,T cell-AD.vs.control; scRNA-seq,SRP330776,Naive CD8+ T cell_2-AD.vs.control; scRNA-seq,SRP330776,CD8+ T cell_3-AD.vs.control; scRNA-seq,SRP330776,Monocyte_1-AD.vs.control; scRNA-seq,SRP330776,Monocyte_2-AD.vs.control; scRNA-seq,SRP330776,B cell_3-AD.vs.control; scRNA-[illegible]	24
BORA	bulk RNA-seq,Homo_633,AD.vs.control; bulk RNA-seq,Homo_714,AD.vs.control; bulk RNA-seq,Homo_714,AD.vs.MCI; bulk RNA-seq,ROSMAP,AD.vs.control; bulk RNA-seq,ROSMAP,MCI.vs.control; scRNA-seq,SRP215507,CD8+ T cell_1-AD.vs.MCI; scRNA-seq,SRP330776,CD8+ T cell_1-AD.vs.control; scRNA-seq,SRP330776,γδ T cell-AD.vs.control; scRNA-seq,SRP330776,Naive CD8+ T cell_1-AD.vs.control; scRNA-seq,SRP330776,Natural killer T cell-AD.vs.control; scRNA-seq,SRP330776,B cell_2-AD.vs.control; scRNA-seq,SRP330776,B [illegible]	12
BORCS5	bulk RNA-seq,Homo_723,MCI.vs.control; bulk RNA-seq,Homo_633,AD.vs.control; bulk RNA-seq,Homo_633,MCI.vs.control; scRNA-seq,SRP215507,CD8+ T cell_4-AD.vs.MCI; scRNA-seq,SRP215507,CD8+ T cell_4-MCI.vs.control; scRNA-seq,SRP309935,Naive CD8+ T cell_2-[illegible]	6
BORCS6	scRNA-seq,SRP215507,CD8+ T cell_4-AD.vs.control; scRNA-seq,SRP215507,CD8+ T cell_7-AD.vs.control; scRNA-seq,SRP215507,CD8+ T cell_2-AD.vs.MCI; scRNA-seq,SRP330776,CD8+ T cell_1-AD.vs.control; scRNA-seq,SRP330776,γδ T cell-AD.vs.control; scRNA-seq,SRP330776,Memory T cell_2-AD.vs.control; scRNA-seq,SRP330776,CD8+ T cell_2-AD.vs.control; scRNA-seq,SRP330776,B cell_1-AD.vs.control; scRNA-seq,SRP330776,Naive CD8+ T cell_1-AD.vs.control; scRNA-seq,SRP330776,Natural killer cell_1-AD.vs.control; scRNA-seq,SRP330776,Memory T cell_1-AD.vs.control; scRNA-seq,SRP330776,Natural killer T cell-AD.vs.control; scRNA-seq,SRP330776,T cell-AD.vs.control; scRNA-seq,SRP330776,CD8+ T cell_3-AD.vs.control; scRNA-seq,SRP330776,Monocyte_2-AD.vs.control; scRNA-seq,SRP330776,B cell_3-AD.vs.control; [illegible]	18
BORCS7	bulk RNA-seq,Homo_714,AD.vs.control; bulk RNA-seq,Homo_714,AD.vs.MCI; scRNA-seq,SRP215507,CD8+ T cell_8-AD.vs.control; scRNA-seq,SRP215507,CD8+ T cell_6-AD.vs.control; scRNA-seq,SRP215507,CD8+ T cell_4-AD.vs.MCI; scRNA-seq,SRP330776,B cell_2-AD.vs.control; scRNA-seq,SRP330776,CD8+ T cell_3-AD.vs.control; scRNA-seq,SRP330776,Monocyte_2-AD.vs.control; scRNA-seq,SRP330776,B cell_3-[illegible]	9
BORCS8	bulk RNA-seq,Homo_723,AD.vs.control; bulk RNA-seq,Homo_723,AD.vs.MCI; bulk RNA-seq,Homo_633,AD.vs.control; bulk RNA-seq,Homo_633,AD.vs.MCI; bulk RNA-seq,Homo_714,AD.vs.control; bulk RNA-seq,Homo_714,AD.vs.MCI; scRNA-seq,SRP215507,CD8+ T cell_9-AD.vs.control; scRNA-seq,SRP330776,Natural killer cell_1-AD.vs.control; scRNA-seq,SRP330776,Memory T cell_1-AD.vs.control; scRNA-seq,SRP330776,CD8+ T cell_3-AD.vs.control; scRNA-seq,SRP330776,Monocyte_2-AD.vs.control; scRNA-seq,SRP330776,Natural killer cell_2-[illegible]	12
Borcs8	bulk RNA-seq,SRP325058,AD.vs.control	1
BORCS8-MEF2B	bulk RNA-seq,Homo_723,AD.vs.control; bulk RNA-seq,Homo_723,MCI.vs.control; bulk RNA-seq,Homo_633,AD.vs.control; bulk RNA-seq,Homo_633,MCI.vs.control; bulk RNA-seq,Homo_714,AD.vs.control; bulk RNA-seq,Homo_714,AD.vs.MCI; bulk RNA-seq,Homo_714,MCI.vs.control	7
BPGM	scRNA-seq,SRP215507,CD8+ T cell_5-AD.vs.MCI; scRNA-seq,SRP309935,Erythroid cell-AD.vs.control; scRNA-seq,SRP330776,γδ T cell-AD.vs.control; scRNA-seq,SRP330776,Naive CD8+ T cell_1-AD.vs.control; scRNA-seq,SRP330776,Naive CD8+ T cell_3-AD.vs.control	5

BPHL	bulk RNA-seq,Homo_723,AD.vs.control; bulk RNA-seq,Homo_633,AD.vs.control; bulk RNA-seq,Homo_633,AD.vs.MCI; bulk RNA-seq,Homo_714,AD.vs.control; bulk RNA-seq,Homo_714,AD.vs.MCI; bulk RNA-seq,ROSMAP,MCI.vs.control; scRNA-seq,SRP215507,CD8+ T cell_1-AD.vs.control; scRNA-seq,SRP215507,CD8+ T cell_3-AD.vs.MCI; scRNA-seq,SRP215507,CD8+ T cell_1-AD.vs.MCI; scRNA-seq,SRP215507,CD8+ T cell_2-MCI.vs.control; scRNA-seq,SRP330776,CD8+ T cell_1-AD.vs.control; scRNA-seq,SRP330776,γδ T cell-AD.vs.control; scRNA-seq,SRP330776,Memory T cell_2-AD.vs.control; scRNA-seq,SRP330776,CD8+ T cell_2-AD.vs.control; scRNA-seq,SRP330776,B cell_1-AD.vs.control; scRNA-seq,SRP330776,Naive CD8+ T cell_1-AD.vs.control; scRNA-seq,SRP330776,Memory T cell_1-AD.vs.control; scRNA-seq,SRP330776,Naive CD8+ T cell_3-AD.vs.control; scRNA-seq,SRP330776,T cell-AD.vs.control; scRNA-seq,SRP330776,Natural killer cell_2-AD.vs.control	20
BPI	bulk RNA-seq,Homo_723,AD.vs.control; bulk RNA-seq,Homo_723,AD.vs.MCI; bulk RNA-seq,Homo_633,AD.vs.control; bulk RNA-seq,Homo_633,MCI.vs.control; bulk RNA-seq,Homo_714,AD.vs.control; bulk RNA-seq,Homo_714,AD.vs.MCI; bulk RNA-seq,Homo_714,MCI.vs.control; bulk RNA-seq,SRP310421,AD.vs.control; scRNA-seq,SRP309935,Monocyte_1-AD.vs.control	9
BPIFB3	bulk RNA-seq,Homo_723,AD.vs.control; bulk RNA-seq,Homo_723,AD.vs.MCI; bulk RNA-seq,Homo_633,AD.vs.control; bulk RNA-seq,Homo_633,AD.vs.MCI; bulk RNA-seq,Homo_633,MCI.vs.control	5
BPIFB4	bulk RNA-seq,Homo_723,AD.vs.control; bulk RNA-seq,Homo_633,AD.vs.control	2
BPNT1	scRNA-seq,SRP215507,CD8+ T cell_2-AD.vs.MCI; scRNA-seq,SRP215507,CD8+ T cell_3-AD.vs.MCI; scRNA-seq,SRP215507,CD8+ T cell_1-AD.vs.MCI; scRNA-seq,SRP215507,CD8+ T cell_1-MCI.vs.control; scRNA-seq,SRP215507,CD8+ T cell_2-MCI.vs.control; scRNA-seq,SRP215507,CD8+ T cell_3-MCI.vs.control; scRNA-seq,SRP330776,CD8+ T cell_1-AD.vs.control; scRNA-seq,SRP330776,Memory T cell_2-AD.vs.control; scRNA-seq,SRP330776,Naive CD8+ T cell_1-AD.vs.control; scRNA-seq,SRP330776,Natural killer cell_1-AD.vs.control; scRNA-seq,SRP330776,Memory T cell_1-AD.vs.control; scRNA-seq,SRP330776,B cell_2-AD.vs.control; scRNA-seq,SRP330776,T cell-AD.vs.control; scRNA-	14
BPTF	bulk RNA-seq,Homo_723,AD.vs.control; bulk RNA-seq,Homo_723,MCI.vs.control; bulk RNA-seq,Homo_633,AD.vs.control; bulk RNA-seq,Homo_633,AD.vs.MCI; bulk RNA-seq,Homo_714,AD.vs.control; bulk RNA-seq,Homo_714,AD.vs.MCI; bulk RNA-seq,Homo_714,MCI.vs.control; scRNA-seq,SRP215507,CD8+ T cell_7-AD.vs.control; scRNA-seq,SRP215507,CD8+ T cell_5-AD.vs.MCI; scRNA-seq,SRP330776,Naive CD8+ T cell_1-AD.vs.control; scRNA-seq,SRP330776,Naive CD8+ T cell_3-AD.vs.control	11
BRAF	bulk RNA-seq,Homo_723,AD.vs.control; bulk RNA-seq,Homo_723,AD.vs.MCI; bulk RNA-seq,Homo_723,MCI.vs.control; bulk RNA-seq,Homo_633,AD.vs.control; bulk RNA-seq,Homo_633,AD.vs.MCI; bulk RNA-seq,Homo_633,MCI.vs.control; bulk RNA-seq,Homo_714,AD.vs.control; bulk RNA-seq,Homo_714,AD.vs.MCI; scRNA-seq,SRP215507,CD8+ T cell_7-AD.vs.control; scRNA-seq,SRP215507,CD8+ T cell_5-AD.vs.MCI; scRNA-seq,SRP309935,Natural killer cell_2-AD.vs.control; scRNA-seq,SRP309935,B cell_1-AD.vs.control; scRNA-seq,SRP330776,γδ T cell-AD.vs.control; scRNA-seq,SRP330776,Memory T cell_2-AD.vs.control; scRNA-seq,SRP330776,CD8+ T cell_2-AD.vs.control; scRNA-seq,SRP330776,B cell_1-AD.vs.control; scRNA-seq,SRP330776,Naive CD8+ T cell_1-AD.vs.control; scRNA-seq,SRP330776,Natural killer cell_1-AD.vs.control; scRNA-seq,SRP330776,Memory T cell_1-AD.vs.control; scRNA-seq,SRP330776,Naive CD8+ T cell_3-AD.vs.control; scRNA-seq,SRP330776,B cell_2-AD.vs.control; scRNA-seq,SRP330776,T cell-AD.vs.control; scRNA-seq,SRP330776,Naive CD8+ T cell_2-AD.vs.control; scRNA-seq,SRP330776,CD8+ T cell_3-AD.vs.control; scRNA-seq,SRP330776,Monocyte_2-AD.vs.control; scRNA-seq,SRP330776,B cell_3-AD.vs.control; scRNA- [illegible]	28
Braf	bulk RNA-seq,SRP325058,AD.vs.control	1
BRAP	scRNA-seq,SRP215507,CD8+ T cell_6-AD.vs.control; scRNA-seq,SRP215507,CD8+ T cell_5-AD.vs.MCI; scRNA-seq,SRP215507,CD8+ T cell_5-MCI.vs.control; scRNA-seq,SRP309935,B cell_1-AD.vs.control; scRNA-seq,SRP330776,CD8+ T cell_1-AD.vs.control; scRNA-seq,SRP330776,γδ T cell-AD.vs.control; scRNA-seq,SRP330776,Memory T cell_2-AD.vs.control; scRNA-seq,SRP330776,CD8+ T cell_2-AD.vs.control; scRNA-seq,SRP330776,B cell_1-AD.vs.control; scRNA-seq,SRP330776,Naive CD8+ T cell_1-AD.vs.control; scRNA-seq,SRP330776,Natural killer cell_1-AD.vs.control; scRNA-seq,SRP330776,Memory T cell_1-AD.vs.control; scRNA-seq,SRP330776,Natural killer T cell-AD.vs.control; scRNA-seq,SRP330776,Naive CD8+ T cell_3-AD.vs.control; scRNA-seq,SRP330776,T cell-AD.vs.control; scRNA-seq,SRP330776,Naive CD8+ T cell_2-AD.vs.control; scRNA-seq,SRP330776,CD8+ T cell_3-AD.vs.control; scRNA-seq,SRP330776,B cell_3-AD.vs.control; scRNA-seq,SRP330776,Natural killer cell_2-AD.vs.control	19

BRAT1	bulk RNA-seq,Homo_723,AD.vs.control; bulk RNA-seq,Homo_633,AD.vs.control; scRNA-seq,SRP215507,CD8+ T cell_5-AD.vs.control; scRNA-seq,SRP215507,CD8+ T cell_4-AD.vs.MCI; scRNA-seq,SRP215507,CD8+ T cell_5-AD.vs.MCI; scRNA-seq,SRP215507,CD8+ T cell_6-AD.vs.MCI; scRNA-seq,SRP215507,CD8+ T cell_5-MCI.vs.control; scRNA-seq,SRP309935,Naive CD8+ T cell_1-AD.vs.control; scRNA-seq,SRP330776,CD8+ T cell_1-AD.vs.control; scRNA-seq,SRP330776,γδ T cell-AD.vs.control; scRNA-seq,SRP330776,Memory T cell_2-AD.vs.control; scRNA-seq,SRP330776,CD8+ T cell_2-AD.vs.control; scRNA-seq,SRP330776,B cell_1-AD.vs.control; scRNA-seq,SRP330776,Naive CD8+ T cell_1-AD.vs.control; scRNA-seq,SRP330776,Natural killer cell_1-AD.vs.control; scRNA-seq,SRP330776,Memory T cell_1-AD.vs.control; scRNA-seq,SRP330776,Natural killer T cell-AD.vs.control; scRNA-seq,SRP330776,Naive CD8+ T cell_3-AD.vs.control; scRNA-seq,SRP330776,T cell-AD.vs.control; scRNA-seq,SRP330776,CD8+ T cell_3-AD.vs.control; scRNA-seq,SRP330776,Monocyte_1-AD.vs.control; scRNA-seq,SRP330776,B [illegible]	24
BRCA1	bulk RNA-seq,Homo_723,AD.vs.control; bulk RNA-seq,Homo_723,AD.vs.MCI; bulk RNA-seq,Homo_723,MCI.vs.control; bulk RNA-seq,Homo_633,AD.vs.control; bulk RNA-seq,Homo_633,AD.vs.MCI; bulk RNA-seq,Homo_633,MCI.vs.control; bulk RNA-seq,Homo_714,AD.vs.control; bulk RNA-seq,Homo_714,AD.vs.MCI; bulk RNA-seq,ROSMAP,AD.vs.control; scRNA-seq,SRP215507,CD8+ T cell_1-AD.vs.control; scRNA-seq,SRP215507,CD8+ T cell_4-AD.vs.MCI; scRNA-seq,SRP330776,γδ T cell-AD.vs.control; scRNA-seq,SRP330776,CD8+ T cell_2-AD.vs.control; scRNA-seq,SRP330776,Naive CD8+ T cell_1-AD.vs.control; scRNA-seq,SRP330776,Naive CD8+ T cell_2-AD.vs.control	15
BRCA2	bulk RNA-seq,Homo_723,AD.vs.control; bulk RNA-seq,Homo_723,AD.vs.MCI; bulk RNA-seq,Homo_633,AD.vs.control; bulk RNA-seq,Homo_633,AD.vs.MCI; bulk RNA-seq,Homo_714,AD.vs.control; bulk RNA-seq,Homo_714,AD.vs.MCI; bulk RNA-seq,ROSMAP,AD.vs.control; bulk RNA-seq,ROSMAP,MCI.vs.control; scRNA-seq,SRP215507,CD8+ T cell_4-AD.vs.MCI; scRNA-seq,SRP215507,CD8+ T cell_5-AD.vs.MCI; scRNA-seq,SRP215507,CD8+ T cell_5-MCI.vs.control; scRNA-seq,SRP215507,CD8+ T cell_4-MCI.vs.control; scRNA-seq,SRP330776,T cell-	13
BRCC3	bulk RNA-seq,Homo_723,AD.vs.control; bulk RNA-seq,Homo_633,AD.vs.control; bulk RNA-seq,Homo_633,AD.vs.MCI; bulk RNA-seq,Homo_714,AD.vs.control; bulk RNA-seq,Homo_714,AD.vs.MCI; scRNA-seq,SRP215507,CD8+ T cell_4-AD.vs.control; scRNA-seq,SRP215507,CD8+ T cell_3-AD.vs.MCI; scRNA-seq,SRP215507,CD8+ T cell_4-AD.vs.MCI; scRNA-seq,SRP309935,Natural killer cell_2-AD.vs.control; scRNA-seq,SRP330776,Natural killer T cell-AD.vs.control; scRNA-seq,SRP330776,Monocyte_1-AD.vs.control; scRNA-seq,SRP330776,B	12
BRD1	scRNA-seq,SRP215507,CD8+ T cell_4-AD.vs.MCI; scRNA-seq,SRP215507,CD8+ T cell_4-MCI.vs.control; scRNA-seq,SRP215507,CD8+ T cell_2-MCI.vs.control; scRNA-seq,SRP309935,Natural killer cell_1-AD.vs.control; scRNA-seq,SRP330776,Memory T cell_2-AD.vs.control; scRNA-seq,SRP330776,Naive CD8+ T cell_1-AD.vs.control; scRNA-seq,SRP330776,Naive CD8+ T cell_3-AD.vs.control; scRNA-seq,SRP330776,B cell_2-	9
BRD2	scRNA-seq,SRP215507,CD8+ T cell_7-AD.vs.control; scRNA-seq,SRP215507,CD8+ T cell_3-AD.vs.MCI; scRNA-seq,SRP215507,CD8+ T cell_1-AD.vs.MCI; scRNA-seq,SRP215507,CD8+ T cell_7-AD.vs.MCI; scRNA-seq,SRP215507,CD8+ T cell_5-AD.vs.MCI; scRNA-seq,SRP215507,CD8+ T cell_5-MCI.vs.control; scRNA-seq,SRP215507,CD8+ T cell_3-MCI.vs.control; scRNA-seq,SRP330776,Naive CD8+ T cell_2-AD.vs.control	8
BRD3	bulk RNA-seq,Homo_723,AD.vs.control; bulk RNA-seq,Homo_723,AD.vs.MCI; bulk RNA-seq,Homo_633,AD.vs.control; bulk RNA-seq,Homo_633,AD.vs.MCI; bulk RNA-seq,Homo_714,AD.vs.control; bulk RNA-seq,Homo_714,AD.vs.MCI; bulk RNA-seq,Homo_714,MCI.vs.control; scRNA-seq,SRP215507,CD8+ T cell_5-AD.vs.MCI; scRNA-seq,SRP330776,CD8+ T cell_1-AD.vs.control; scRNA-seq,SRP330776,γδ T cell-AD.vs.control; scRNA-seq,SRP330776,Naive CD8+ T cell_1-AD.vs.control; scRNA-seq,SRP330776,Natural killer cell_1-AD.vs.control; scRNA-seq,SRP330776,Memory T cell_1-AD.vs.control; scRNA-seq,SRP330776,B cell_2-AD.vs.control; scRNA-seq,SRP330776,CD8+ T cell_3-AD.vs.control; scRNA-seq,SRP330776,Monocyte_2-AD.vs.control; scRNA-seq,SRP330776,B cell_3-AD.vs.control; scRNA-seq,SRP330776,Natural killer cell_2-	18
BRD3OS	bulk RNA-seq,Homo_723,AD.vs.control; bulk RNA-seq,Homo_633,AD.vs.control; scRNA-seq,SRP215507,CD8+ T cell_3-AD.vs.control; scRNA-seq,SRP330776,Natural killer T cell-AD.vs.control; scRNA-seq,SRP330776,B cell_3-AD.vs.control	5
Brd4	bulk RNA-seq,SRP325058,AD.vs.control	1
BRD4	scRNA-seq,SRP215507,CD8+ T cell_7-AD.vs.control; scRNA-seq,SRP215507,CD8+ T cell_10-AD.vs.control; scRNA-seq,SRP215507,CD8+ T cell_4-AD.vs.MCI; scRNA-seq,SRP215507,CD8+ T cell_6-MCI.vs.control; scRNA-seq,SRP215507,CD8+ T cell_7-MCI.vs.control; scRNA-	6

BRD7	scRNA-seq,SRP215507,CD8+ T cell_6-AD.vs.MCI; scRNA-seq,SRP309935,Natural killer cell_2-AD.vs.control; scRNA-seq,SRP309935,Monocyte_1-AD.vs.control; scRNA-seq,SRP309935,Megakaryocyte_1-AD.vs.control; scRNA-seq,SRP309935,Naive CD8+ T cell_1-AD.vs.control; scRNA-seq,SRP309935,Natural killer cell_1-AD.vs.control; scRNA-seq,SRP309935,Monocyte_2-AD.vs.control; scRNA-seq,SRP309935,Naive CD8+ T cell_2-AD.vs.control; scRNA-seq,SRP309935,CD8+ T cell_1-AD.vs.control; scRNA-seq,SRP309935,B cell_1-AD.vs.control; scRNA-seq,SRP309935,CD8+ T cell_2-AD.vs.control; scRNA-seq,SRP309935,B cell_2-AD.vs.control; scRNA-seq,SRP309935,Naive CD8+ T cell_3-AD.vs.control; scRNA-seq,SRP309935,Dendritic cell-AD.vs.control; scRNA-seq,SRP330776,γδ T cell-AD.vs.control; scRNA-seq,SRP330776,Naive CD8+ T cell_1-AD.vs.control; scRNA-seq,SRP330776,Natural killer cell_1-AD.vs.control; scRNA-seq,SRP330776,T cell-AD.vs.control; scRNA-seq,SRP330776,Natural [illegible]	19
BRD8	bulk RNA-seq,Homo_633,AD.vs.control; bulk RNA-seq,Homo_633,AD.vs.MCI; bulk RNA-seq,Homo_714,AD.vs.control; bulk RNA-seq,Homo_714,AD.vs.MCI; scRNA-seq,SRP215507,CD8+ T cell_3-AD.vs.control; scRNA-seq,SRP215507,CD8+ T cell_1-AD.vs.MCI; scRNA-seq,SRP215507,CD8+ T cell_6-AD.vs.MCI; scRNA-seq,SRP215507,CD8+ T cell_3-MCI.vs.control; scRNA-seq,SRP215507,CD8+ T cell_6-MCI.vs.control; scRNA-seq,SRP330776,CD8+ T cell_1-AD.vs.control; scRNA-seq,SRP330776,γδ T cell-AD.vs.control; scRNA-seq,SRP330776,Memory T cell_2-AD.vs.control; scRNA-seq,SRP330776,B cell_1-AD.vs.control; scRNA-seq,SRP330776,Naive CD8+ T cell_1-AD.vs.control; scRNA-seq,SRP330776,Memory T cell_1-AD.vs.control; scRNA-seq,SRP330776,CD8+ T cell_3-AD.vs.control; scRNA-seq,SRP330776,Natural killer cell_2- [illegible]	17
BRD9	bulk RNA-seq,Homo_723,AD.vs.control; bulk RNA-seq,Homo_633,AD.vs.control; bulk RNA-seq,Homo_633,AD.vs.MCI; bulk RNA-seq,Homo_714,AD.vs.control; bulk RNA-seq,Homo_714,AD.vs.MCI; bulk RNA-seq,Homo_714,MCI.vs.control; scRNA-seq,SRP215507,CD8+ T cell_7-AD.vs.control; scRNA-seq,SRP215507,CD8+ T cell_9-AD.vs.control; scRNA-seq,SRP309935,Megakaryocyte_1-AD.vs.control; scRNA-seq,SRP309935,CD8+ T cell_1-AD.vs.control; scRNA-seq,SRP309935,B cell_1-AD.vs.control; scRNA-seq,SRP309935,CD4+ T cell-AD.vs.control; scRNA-seq,SRP330776,CD8+ T cell_1-AD.vs.control; scRNA-seq,SRP330776,γδ T cell-AD.vs.control; scRNA-seq,SRP330776,Memory T cell_2-AD.vs.control; scRNA-seq,SRP330776,CD8+ T cell_2-AD.vs.control; scRNA-seq,SRP330776,B cell_1-AD.vs.control; scRNA-seq,SRP330776,Naive CD8+ T cell_1-AD.vs.control; scRNA-seq,SRP330776,Natural killer cell_1-AD.vs.control; scRNA-seq,SRP330776,Memory T cell_1-AD.vs.control; scRNA-seq,SRP330776,Natural killer T cell-AD.vs.control; scRNA-seq,SRP330776,Naive CD8+ T cell_3-AD.vs.control; scRNA-seq,SRP330776,B cell_2-AD.vs.control; scRNA-seq,SRP330776,T cell-AD.vs.control; scRNA-seq,SRP330776,Naive CD8+ T cell_2-AD.vs.control; scRNA-seq,SRP330776,CD8+ T cell_3-AD.vs.control; scRNA-seq,SRP330776,Monocyte_1-AD.vs.control; scRNA-seq,SRP330776,Monocyte_2-AD.vs.control; scRNA- [illegible]	31
BRF1	scRNA-seq,SRP309935,Dendritic cell-AD.vs.control; scRNA-seq,SRP330776,CD8+ T cell_1-AD.vs.control; scRNA-seq,SRP330776,γδ T cell-AD.vs.control; scRNA-seq,SRP330776,Memory T cell_2-AD.vs.control; scRNA-seq,SRP330776,CD8+ T cell_2-AD.vs.control; scRNA-seq,SRP330776,B cell_1-AD.vs.control; scRNA-seq,SRP330776,Naive CD8+ T cell_1-AD.vs.control; scRNA-seq,SRP330776,Natural killer cell_1-AD.vs.control; scRNA-seq,SRP330776,Memory T cell_1-AD.vs.control; scRNA-seq,SRP330776,Natural killer T cell-AD.vs.control; scRNA-seq,SRP330776,Naive CD8+ T cell_3-AD.vs.control; scRNA-seq,SRP330776,B cell_2-AD.vs.control; scRNA-seq,SRP330776,T cell-AD.vs.control; scRNA-seq,SRP330776,CD8+ T cell_3-AD.vs.control; scRNA-seq,SRP330776,B cell_3-AD.vs.control; scRNA-seq,SRP330776,Natural killer cell_2-AD.vs.control; scRNA- [illegible]	17
BRF2	bulk RNA-seq,Homo_723,AD.vs.control; bulk RNA-seq,Homo_723,AD.vs.MCI; bulk RNA-seq,Homo_633,AD.vs.control; bulk RNA-seq,Homo_633,AD.vs.MCI; bulk RNA-seq,Homo_714,AD.vs.control; bulk RNA-seq,Homo_714,AD.vs.MCI; bulk RNA-seq,Homo_714,MCI.vs.control; scRNA-seq,SRP215507,CD8+ T cell_2-AD.vs.MCI; scRNA-seq,SRP215507,CD8+ T cell_4-AD.vs.MCI; scRNA-seq,SRP309935,Monocyte_1-AD.vs.control; scRNA-seq,SRP330776,Regulatory T cell-AD.vs.control; scRNA-seq,SRP330776,Megakaryocyte_1-AD.vs.control	12
BRI3	scRNA-seq,SRP215507,CD8+ T cell_7-AD.vs.control; scRNA-seq,SRP215507,CD8+ T cell_5-AD.vs.MCI; scRNA-seq,SRP215507,CD8+ T cell_1-MCI.vs.control; scRNA-seq,SRP309935,Natural killer cell_2-AD.vs.control; scRNA-seq,SRP309935,Monocyte_1-AD.vs.control; scRNA-seq,SRP309935,Megakaryocyte_1-AD.vs.control; scRNA-seq,SRP309935,Naive CD8+ T cell_1-AD.vs.control; scRNA-seq,SRP309935,Natural killer cell_1-AD.vs.control; scRNA-seq,SRP309935,Monocyte_2-AD.vs.control; scRNA-seq,SRP309935,Naive CD8+ T cell_2-AD.vs.control; scRNA-seq,SRP309935,CD8+ T cell_1-AD.vs.control; scRNA-seq,SRP309935,B cell_1-AD.vs.control; scRNA-seq,SRP309935,CD8+ T cell_2-AD.vs.control; scRNA-seq,SRP309935,Monocyte_3-AD.vs.control; scRNA-seq,SRP309935,B cell_2-AD.vs.control; scRNA-seq,SRP309935,Naive CD8+ T cell_3-AD.vs.control; scRNA-seq,SRP309935,Dendritic cell-AD.vs.control; scRNA-seq,SRP309935,CD4+ T cell-AD.vs.control; scRNA-seq,SRP309935,Plasmacytoid dendritic cell_1-AD.vs.control; scRNA-seq,SRP330776,CD8+ T cell_1-AD.vs.control; scRNA-seq,SRP330776,Memory T cell_2-AD.vs.control; scRNA-seq,SRP330776,Naive CD8+ T cell_1-AD.vs.control; scRNA-seq,SRP330776,Natural killer cell_1-AD.vs.control; scRNA-seq,SRP330776,Natural killer T cell-AD.vs.control; scRNA-seq,SRP330776,Naive CD8+ T cell_3-AD.vs.control; scRNA-seq,SRP330776,B cell_2- [illegible]	30

BRI3BP	bulk RNA-seq,Homo_723,AD.vs.control; bulk RNA-seq,Homo_723,MCI.vs.control; bulk RNA-seq,Homo_633,AD.vs.control; bulk RNA-seq,Homo_633,AD.vs.MCI; bulk RNA-seq,Homo_714,AD.vs.control; bulk RNA-seq,Homo_714,AD.vs.MCI; scRNA-seq,SRP215507,CD8+ T cell_5-AD.vs.MCI; scRNA-seq,SRP215507,CD8+ T cell_6-AD.vs.MCI; scRNA-seq,SRP309935,Naive CD8+ T cell_1-AD.vs.control; scRNA-seq,SRP309935,Natural killer cell_1-AD.vs.control; scRNA-seq,SRP330776,CD8+ T cell_1-AD.vs.control; scRNA-seq,SRP330776,γδ T cell-AD.vs.control; scRNA-seq,SRP330776,Memory T cell_2-AD.vs.control; scRNA-seq,SRP330776,CD8+ T cell_2-AD.vs.control; scRNA-seq,SRP330776,B cell_1-AD.vs.control; scRNA-seq,SRP330776,Naive CD8+ T cell_1-AD.vs.control; scRNA-seq,SRP330776,Memory T cell_1-AD.vs.control; scRNA-seq,SRP330776,Natural killer T cell-AD.vs.control; scRNA-seq,SRP330776,B cell_2-AD.vs.control; scRNA-seq,SRP330776,T cell-AD.vs.control; scRNA-[illegible]	23
BRICD5	scRNA-seq,SRP215507,CD8+ T cell_2-AD.vs.MCI; scRNA-seq,SRP215507,CD8+ T cell_3-AD.vs.MCI; scRNA-seq,SRP215507,CD8+ T cell_2-MCI.vs.control; scRNA-seq,SRP215507,CD8+ T cell_3-MCI.vs.control; scRNA-seq,SRP215507,CD8+ T cell_6-MCI.vs.control; scRNA-seq,SRP330776,CD8+ T cell_1-AD.vs.control; scRNA-seq,SRP330776,Memory T cell_2-AD.vs.control; scRNA-seq,SRP330776,Naive CD8+ T cell_1-	8
BRINP1	bulk RNA-seq,Homo_723,AD.vs.control; bulk RNA-seq,Homo_723,AD.vs.MCI	2
BRINP2	bulk RNA-seq,Homo_723,AD.vs.control; bulk RNA-seq,Homo_723,AD.vs.MCI; bulk RNA-seq,Homo_714,MCI.vs.control	3
BRINP3	bulk RNA-seq,Homo_723,AD.vs.control; bulk RNA-seq,Homo_723,AD.vs.MCI; bulk RNA-seq,Homo_633,MCI.vs.control; bulk RNA-	4
BRIP1	bulk RNA-seq,Homo_723,AD.vs.control; bulk RNA-seq,Homo_723,AD.vs.MCI; bulk RNA-seq,Homo_723,MCI.vs.control; bulk RNA-seq,Homo_633,AD.vs.control; bulk RNA-seq,Homo_633,AD.vs.MCI; bulk RNA-seq,Homo_633,MCI.vs.control; bulk RNA-seq,Homo_714,AD.vs.control; bulk RNA-seq,Homo_714,AD.vs.MCI; scRNA-seq,SRP215507,CD8+ T cell_3-AD.vs.control; scRNA-seq,SRP215507,CD8+ T cell_3-MCI.vs.control;	11
BRIX1	bulk RNA-seq,Homo_714,AD.vs.control; bulk RNA-seq,Homo_714,AD.vs.MCI; scRNA-seq,SRP215507,CD8+ T cell_3-AD.vs.MCI; scRNA-seq,SRP215507,CD8+ T cell_4-AD.vs.MCI; scRNA-seq,SRP215507,CD8+ T cell_4-MCI.vs.control; scRNA-seq,SRP215507,CD8+ T cell_3-MCI.vs.control; scRNA-seq,SRP215507,CD8+ T cell_6-MCI.vs.control; scRNA-seq,SRP330776,B cell_3-AD.vs.control	8
BRK1	bulk RNA-seq,Homo_714,AD.vs.control; bulk RNA-seq,Homo_714,AD.vs.MCI; bulk RNA-seq,SRP223445,AD.vs.control; bulk RNA-seq,SRP310421,AD.vs.control; scRNA-seq,SRP215507,CD8+ T cell_6-AD.vs.MCI; scRNA-seq,SRP309935,Erythroid cell-AD.vs.control; scRNA-seq,SRP330776,Naive CD8+ T cell_1-AD.vs.control; scRNA-seq,SRP330776,B cell_2-AD.vs.control; scRNA-seq,SRP330776,Megakaryocyte_1-	9
BRME1	bulk RNA-seq,Homo_723,AD.vs.control; bulk RNA-seq,Homo_723,AD.vs.MCI; bulk RNA-seq,Homo_714,AD.vs.control; bulk RNA-seq,Homo_714,AD.vs.MCI; bulk RNA-seq,ROSMAP,AD.vs.control	5
BRMS1	bulk RNA-seq,Homo_723,AD.vs.control; bulk RNA-seq,Homo_633,AD.vs.control; bulk RNA-seq,Homo_633,AD.vs.MCI; bulk RNA-seq,Homo_714,AD.vs.control; bulk RNA-seq,Homo_714,AD.vs.MCI; scRNA-seq,SRP215507,CD8+ T cell_4-AD.vs.MCI; scRNA-seq,SRP215507,CD8+ T cell_4-MCI.vs.control; scRNA-seq,SRP330776,Monocyte_2-AD.vs.control	8
BRMS1L	bulk RNA-seq,Homo_723,AD.vs.control; bulk RNA-seq,Homo_723,AD.vs.MCI; bulk RNA-seq,Homo_633,AD.vs.MCI; bulk RNA-seq,Homo_714,AD.vs.control; bulk RNA-seq,Homo_714,AD.vs.MCI; scRNA-seq,SRP215507,CD8+ T cell_2-AD.vs.control; scRNA-seq,SRP215507,CD8+ T cell_3-AD.vs.control; scRNA-seq,SRP215507,CD8+ T cell_3-AD.vs.MCI; scRNA-seq,SRP215507,CD8+ T cell_5-MCI.vs.control; scRNA-seq,SRP215507,CD8+ T cell_3-MCI.vs.control; scRNA-seq,SRP330776,CD8+ T cell_1-AD.vs.control; scRNA-seq,SRP330776,B cell_1-AD.vs.control; scRNA-seq,SRP330776,Naive CD8+ T cell_1-AD.vs.control; scRNA-seq,SRP330776,B cell_2-AD.vs.control; scRNA-	15
BROX	scRNA-seq,SRP215507,CD8+ T cell_7-AD.vs.control; scRNA-seq,SRP215507,CD8+ T cell_5-AD.vs.control; scRNA-seq,SRP215507,CD8+ T cell_5-MCI.vs.control; scRNA-seq,SRP215507,CD8+ T cell_2-MCI.vs.control; scRNA-seq,SRP330776,Monocyte_2-AD.vs.control; scRNA-	6
BRPF1	bulk RNA-seq,Homo_723,AD.vs.control; bulk RNA-seq,Homo_633,AD.vs.control; bulk RNA-seq,Homo_633,MCI.vs.control; scRNA-seq,SRP215507,CD8+ T cell_5-AD.vs.MCI; scRNA-seq,SRP215507,CD8+ T cell_3-MCI.vs.control; scRNA-seq,SRP309935,Monocyte_1-AD.vs.control; scRNA-seq,SRP330776,CD8+ T cell_1-AD.vs.control; scRNA-seq,SRP330776,γδ T cell-AD.vs.control; scRNA-seq,SRP330776,CD8+ T cell_2-AD.vs.control; scRNA-seq,SRP330776,B cell_1-AD.vs.control; scRNA-seq,SRP330776,Naive CD8+ T cell_1-AD.vs.control; scRNA-seq,SRP330776,Natural killer cell_1-AD.vs.control; scRNA-seq,SRP330776,Memory T cell_1-AD.vs.control; scRNA-seq,SRP330776,B cell_2-	14
BRPF3	bulk RNA-seq,Homo_723,AD.vs.control; bulk RNA-seq,Homo_723,AD.vs.MCI; bulk RNA-seq,Homo_633,AD.vs.control; bulk RNA-seq,Homo_633,AD.vs.MCI; bulk RNA-seq,Homo_633,MCI.vs.control; scRNA-seq,SRP215507,CD8+ T cell_4-AD.vs.control; scRNA-seq,SRP215507,CD8+ T cell_3-AD.vs.MCI; scRNA-seq,SRP215507,CD8+ T cell_4-AD.vs.MCI; scRNA-seq,SRP330776,CD8+ T cell_1-AD.vs.control; scRNA-seq,SRP330776,CD8+ T cell_3-AD.vs.control; scRNA-seq,SRP330776,Monocyte_2-AD.vs.control	11

Brpf3	bulk RNA-seq,SRP325058,AD.vs.control	1
BRSK1	bulk RNA-seq,Homo_723,AD.vs.control; bulk RNA-seq,Homo_723,AD.vs.MCI; bulk RNA-seq,Homo_723,MCI.vs.control; bulk RNA-seq,Homo_633,AD.vs.control; bulk RNA-seq,Homo_633,AD.vs.MCI; bulk RNA-seq,Homo_633,MCI.vs.control; bulk RNA-seq,Homo_714,MCI.vs.control; scRNA-seq,SRP215507,CD8+ T cell_3-AD.vs.MCI; scRNA-seq,SRP215507,CD8+ T cell_4-MCI.vs.control; scRNA-	10
BRSK2	bulk RNA-seq,Homo_723,AD.vs.control; bulk RNA-seq,Homo_723,AD.vs.MCI; bulk RNA-seq,Homo_633,MCI.vs.control; bulk RNA-	4
BRWD1	bulk RNA-seq,Homo_723,AD.vs.control; bulk RNA-seq,Homo_723,AD.vs.MCI; bulk RNA-seq,Homo_633,AD.vs.control; bulk RNA-seq,Homo_633,AD.vs.MCI; bulk RNA-seq,Homo_714,AD.vs.control; bulk RNA-seq,Homo_714,AD.vs.MCI; scRNA-seq,SRP215507,CD8+ T cell_9-AD.vs.control; scRNA-seq,SRP215507,CD8+ T cell_7-AD.vs.MCI; scRNA-seq,SRP215507,CD8+ T cell_5-AD.vs.MCI; scRNA-seq,SRP215507,CD8+ T cell_1-MCI.vs.control; scRNA-seq,SRP215507,CD8+ T cell_5-MCI.vs.control; scRNA-seq,SRP215507,CD8+ T cell_7-MCI.vs.control; scRNA-seq,SRP330776,CD8+ T cell_1-AD.vs.control; scRNA-seq,SRP330776,γδ T cell-AD.vs.control; scRNA-seq,SRP330776,Memory T cell_2-AD.vs.control; scRNA-seq,SRP330776,CD8+ T cell_2-AD.vs.control; scRNA-seq,SRP330776,B cell_1-AD.vs.control; scRNA-seq,SRP330776,Naive CD8+ T cell_1-AD.vs.control; scRNA-seq,SRP330776,Natural killer cell_1-AD.vs.control; scRNA-seq,SRP330776,Memory T cell_1-AD.vs.control; scRNA-seq,SRP330776,Natural killer T cell-AD.vs.control; scRNA-seq,SRP330776,Naive CD8+ T cell_3-AD.vs.control; scRNA-seq,SRP330776,B cell_2-AD.vs.control; scRNA-seq,SRP330776,T cell-AD.vs.control; scRNA-seq,SRP330776,Naive CD8+ T cell_2-AD.vs.control; scRNA-seq,SRP330776,CD8+ T cell_3-AD.vs.control; scRNA-seq,SRP330776,Monocyte_1-AD.vs.control; scRNA-seq,SRP330776,Monocyte_2-AD.vs.control; scRNA-seq,SRP330776,B cell_3-AD.vs.control; scRNA-seq,SRP330776,Regulatory T cell-AD.vs.control; scRNA-seq,SRP330776,Natural killer cell_2	31
Brwd1	bulk RNA-seq,SRP325058,AD.vs.control	1
BRWD1-AS2	scRNA-seq,SRP215507,CD8+ T cell_2-AD.vs.control; scRNA-seq,SRP215507,CD8+ T cell_1-AD.vs.MCI; scRNA-seq,SRP215507,CD8+ T cell_1-MCI.vs.control; scRNA-seq,SRP330776,CD8+ T cell_1-AD.vs.control; scRNA-seq,SRP330776,Naive CD8+ T cell_1-AD.vs.control; scRNA-	6
BRWD3	scRNA-seq,SRP309935,Natural killer cell_1-AD.vs.control; scRNA-seq,SRP330776,Natural killer T cell-AD.vs.control	2
BSCL2	bulk RNA-seq,Homo_714,AD.vs.control; bulk RNA-seq,Homo_714,AD.vs.MCI; scRNA-seq,SRP215507,CD8+ T cell_4-AD.vs.control; scRNA-seq,SRP215507,CD8+ T cell_5-AD.vs.control; scRNA-seq,SRP215507,CD8+ T cell_4-AD.vs.MCI; scRNA-seq,SRP215507,CD8+ T cell_4-MCI.vs.control; scRNA-seq,SRP330776,γδ T cell-AD.vs.control; scRNA-seq,SRP330776,B cell_2-AD.vs.control; scRNA-seq,SRP330776,CD8+ T cell_3-AD.vs.control; scRNA-seq,SRP330776,Monocyte_2-AD.vs.control; scRNA-seq,SRP330776,Natural killer cell_2-AD.vs.control; scRNA-seq,SRP330776,B	12
BSDC1	scRNA-seq,SRP215507,CD8+ T cell_6-AD.vs.control; scRNA-seq,SRP215507,CD8+ T cell_6-AD.vs.MCI; scRNA-seq,SRP330776,B cell_4-	3
Bsg	bulk RNA-seq,SRP325058,AD.vs.control	1
BSG	scRNA-seq,SRP215507,CD8+ T cell_7-AD.vs.control	1
BSN	bulk RNA-seq,Homo_723,AD.vs.control; bulk RNA-seq,Homo_723,AD.vs.MCI; bulk RNA-seq,Homo_723,MCI.vs.control; bulk RNA-seq,Homo_633,AD.vs.control; bulk RNA-seq,Homo_633,AD.vs.MCI; bulk RNA-seq,Homo_633,MCI.vs.control; bulk RNA-seq,Homo_714,AD.vs.control;	8
BSND	bulk RNA-seq,Homo_714,MCI.vs.control; bulk RNA-seq,ROSMAP,MCI.vs.control	2
BSPRY	bulk RNA-seq,Homo_714,AD.vs.control; bulk RNA-seq,Homo_714,AD.vs.MCI; bulk RNA-seq,Homo_714,MCI.vs.control; scRNA-seq,SRP215507,CD8+ T cell_5-MCI.vs.control; scRNA-seq,SRP309935,Natural killer cell_2-AD.vs.control; scRNA-seq,SRP330776,CD8+ T cell_2-AD.vs.control; scRNA-seq,SRP330776,Memory T cell_1-AD.vs.control	7
BST1	bulk RNA-seq,SRP223445,AD.vs.control; scRNA-seq,SRP330776,CD8+ T cell_1-AD.vs.control; scRNA-seq,SRP330776,Monocyte_1-AD.vs.control	3
BST2	bulk RNA-seq,Homo_723,AD.vs.control; bulk RNA-seq,Homo_633,AD.vs.control; scRNA-seq,SRP215507,CD8+ T cell_8-AD.vs.control; scRNA-seq,SRP215507,CD8+ T cell_4-AD.vs.control; scRNA-seq,SRP309935,Erythroid cell-AD.vs.control; scRNA-seq,SRP330776,CD8+ T cell_1-AD.vs.control; scRNA-seq,SRP330776,Memory T cell_2-AD.vs.control; scRNA-seq,SRP330776,Natural killer cell_1-AD.vs.control; scRNA-seq,SRP330776,Natural killer T cell-AD.vs.control; scRNA-seq,SRP330776,B cell_2-AD.vs.control; scRNA-seq,SRP330776,Megakaryocyte_1-	11
BTAF1	bulk RNA-seq,Homo_723,AD.vs.MCI; bulk RNA-seq,Homo_714,AD.vs.control; bulk RNA-seq,Homo_714,AD.vs.MCI; scRNA-seq,SRP215507,CD8+ T cell_8-MCI.vs.control; scRNA-seq,SRP330776,Naive CD8+ T cell_2-AD.vs.control	5

BTBD1	bulk RNA-seq,Homo_723,AD.vs.control; bulk RNA-seq,Homo_723,AD.vs.MCI; bulk RNA-seq,Homo_723,MCI.vs.control; bulk RNA-seq,Homo_633,AD.vs.control; bulk RNA-seq,Homo_633,AD.vs.MCI; bulk RNA-seq,Homo_633,MCI.vs.control; bulk RNA-seq,Homo_714,AD.vs.control; bulk RNA-seq,Homo_714,AD.vs.MCI; bulk RNA-seq,Homo_714,MCI.vs.control; scRNA-seq,SRP215507,CD8+ T cell_7-AD.vs.control; scRNA-seq,SRP215507,CD8+ T cell_7-AD.vs.MCI; scRNA-seq,SRP215507,CD8+ T cell_5-AD.vs.MCI; scRNA-seq,SRP215507,CD8+ T cell_1-MCI.vs.control; scRNA-seq,SRP215507,CD8+ T cell_4-MCI.vs.control; scRNA-seq,SRP330776,CD8+ T cell_1-AD.vs.control; scRNA-seq,SRP330776,γδ T cell-AD.vs.control; scRNA-seq,SRP330776,Memory T cell_2-AD.vs.control; scRNA-seq,SRP330776,CD8+ T cell_2-AD.vs.control; scRNA-seq,SRP330776,B cell_1-AD.vs.control; scRNA-seq,SRP330776,Naive CD8+ T cell_1-AD.vs.control; scRNA-seq,SRP330776,Natural killer cell_1-AD.vs.control; scRNA-seq,SRP330776,Memory T cell_1-AD.vs.control; scRNA-seq,SRP330776,Natural killer T cell-AD.vs.control; scRNA-seq,SRP330776,Naive CD8+ T cell_3-AD.vs.control; scRNA-seq,SRP330776,B cell_2-AD.vs.control; scRNA-seq,SRP330776,T cell-AD.vs.control; scRNA-seq,SRP330776,Naive CD8+ T cell_2-AD.vs.control; scRNA-seq,SRP330776,CD8+ T cell_3-AD.vs.control; scRNA-seq,SRP330776,B cell_3-AD.vs.control; scRNA-seq,SRP330776,Natural killer cell_2-AD.vs.control; scRNA-seq,SRP330776,B cell_4-AD.vs.control	31
BTBD10	bulk RNA-seq,Homo_714,AD.vs.control; bulk RNA-seq,Homo_714,AD.vs.MCI	2
BTBD11	bulk RNA-seq,Homo_723,AD.vs.control; bulk RNA-seq,Homo_723,AD.vs.MCI; bulk RNA-seq,Homo_633,AD.vs.control; bulk RNA-seq,Homo_633,AD.vs.MCI; bulk RNA-seq,Homo_714,AD.vs.control; bulk RNA-seq,Homo_714,AD.vs.MCI; scRNA-seq,SRP215507,CD8+ T cell_1-AD.vs.MCI; scRNA-seq,SRP215507,CD8+ T cell_1-MCI.vs.control; scRNA-seq,SRP215507,CD8+ T cell_5-MCI.vs.control; scRNA-seq,SRP215507,CD8+ T cell_2-MCI.vs.control; scRNA-seq,SRP330776,γδ T cell-AD.vs.control; scRNA-seq,SRP330776,Memory T cell_2-AD.vs.control; scRNA-seq,SRP330776,CD8+ T cell_2-AD.vs.control; scRNA-seq,SRP330776,Naive CD8+ T cell_1-AD.vs.control; scRNA-seq,SRP330776,Memory T cell_1-AD.vs.control; scRNA-seq,SRP330776,Natural killer T cell-AD.vs.control; scRNA-seq,SRP330776,Naive CD8+ T cell_3-AD.vs.control	17
BTBD17	bulk RNA-seq,Homo_723,AD.vs.control; bulk RNA-seq,Homo_723,AD.vs.MCI	2
BTBD19	bulk RNA-seq,Homo_723,AD.vs.control; bulk RNA-seq,Homo_723,MCI.vs.control; bulk RNA-seq,Homo_633,AD.vs.control; bulk RNA-seq,Homo_633,MCI.vs.control; bulk RNA-seq,Homo_714,AD.vs.control; bulk RNA-seq,Homo_714,MCI.vs.control; scRNA-seq,SRP215507,CD8+ T cell_3-AD.vs.MCI; scRNA-seq,SRP215507,CD8+ T cell_3-MCI.vs.control; scRNA-seq,SRP330776,Naive CD8+ T cell_1-AD.vs.control; scRNA-	10
BTBD2	bulk RNA-seq,Homo_723,AD.vs.control; bulk RNA-seq,Homo_723,MCI.vs.control; bulk RNA-seq,Homo_633,AD.vs.control; bulk RNA-seq,Homo_633,MCI.vs.control; scRNA-seq,SRP215507,CD8+ T cell_2-AD.vs.MCI; scRNA-seq,SRP215507,CD8+ T cell_3-AD.vs.MCI; scRNA-seq,SRP215507,CD8+ T cell_5-AD.vs.MCI; scRNA-seq,SRP215507,CD8+ T cell_2-MCI.vs.control; scRNA-seq,SRP330776,CD8+ T cell_1-AD.vs.control; scRNA-seq,SRP330776,γδ T cell-AD.vs.control; scRNA-seq,SRP330776,B cell_1-AD.vs.control; scRNA-seq,SRP330776,Naive CD8+ T cell_1-AD.vs.control; scRNA-seq,SRP330776,Natural killer cell_1-AD.vs.control; scRNA-seq,SRP330776,Natural killer T cell-AD.vs.control; scRNA-	16
BTBD3	bulk RNA-seq,Homo_723,AD.vs.control; bulk RNA-seq,Homo_723,AD.vs.MCI; bulk RNA-seq,Homo_723,MCI.vs.control; bulk RNA-seq,Homo_633,AD.vs.control; bulk RNA-seq,Homo_633,AD.vs.MCI; bulk RNA-seq,Homo_633,MCI.vs.control; bulk RNA-seq,Homo_714,AD.vs.control; bulk RNA-seq,Homo_714,AD.vs.MCI; scRNA-seq,SRP330776,Natural killer cell_2-AD.vs.control	9
BTBD6	scRNA-seq,SRP215507,CD8+ T cell_2-AD.vs.control; scRNA-seq,SRP215507,CD8+ T cell_3-AD.vs.control; scRNA-seq,SRP215507,CD8+ T cell_2-AD.vs.MCI; scRNA-seq,SRP215507,CD8+ T cell_3-AD.vs.MCI; scRNA-seq,SRP215507,CD8+ T cell_6-MCI.vs.control; scRNA-seq,SRP309935,Natural killer cell_1-AD.vs.control; scRNA-seq,SRP330776,Memory T cell_2-AD.vs.control; scRNA-seq,SRP330776,CD8+ T cell_2-AD.vs.control; scRNA-seq,SRP330776,B cell_1-AD.vs.control; scRNA-seq,SRP330776,Naive CD8+ T cell_1-AD.vs.control; scRNA-seq,SRP330776,Memory T cell_1-	11
BTBD7	bulk RNA-seq,Homo_633,AD.vs.control; bulk RNA-seq,Homo_633,AD.vs.MCI; scRNA-seq,SRP215507,CD8+ T cell_4-AD.vs.control; scRNA-seq,SRP215507,CD8+ T cell_3-AD.vs.MCI; scRNA-seq,SRP215507,CD8+ T cell_4-MCI.vs.control; scRNA-seq,SRP309935,Naive CD8+ T cell_1-AD.vs.control; scRNA-seq,SRP330776,CD8+ T cell_1-AD.vs.control; scRNA-seq,SRP330776,γδ T cell-AD.vs.control; scRNA-seq,SRP330776,Memory T cell_2-AD.vs.control; scRNA-seq,SRP330776,CD8+ T cell_2-AD.vs.control; scRNA-seq,SRP330776,B cell_1-AD.vs.control; scRNA-seq,SRP330776,Naive CD8+ T cell_1-AD.vs.control; scRNA-seq,SRP330776,Natural killer cell_1-AD.vs.control; scRNA-seq,SRP330776,Memory T cell_1-AD.vs.control; scRNA-seq,SRP330776,Natural killer T cell-AD.vs.control; scRNA-seq,SRP330776,Naive CD8+ T cell_3-AD.vs.control; scRNA-seq,SRP330776,B cell_2-AD.vs.control; scRNA-seq,SRP330776,T cell-AD.vs.control; scRNA-seq,SRP330776,CD8+ T cell_3-AD.vs.control; scRNA-[illegible]	22

BTBD8	bulk RNA-seq,Homo_723,AD.vs.control; bulk RNA-seq,Homo_723,AD.vs.MCI; bulk RNA-seq,Homo_714,AD.vs.control; bulk RNA-seq,Homo_714,AD.vs.MCI; bulk RNA-seq,ROSMAP,AD.vs.control; bulk RNA-seq,ROSMAP,MCI.vs.control; scRNA-seq,SRP215507,CD8+ T cell_2-AD.vs.control; scRNA-seq,SRP215507,CD8+ T cell_1-AD.vs.control; scRNA-seq,SRP215507,CD8+ T cell_4-AD.vs.control; scRNA-seq,SRP215507,CD8+ T cell_4-AD.vs.MCI; scRNA-seq,SRP215507,CD8+ T cell_1-MCI.vs.control; scRNA-seq,SRP215507,CD8+ T cell_5-MCI.vs.control; scRNA-seq,SRP309935,Monocyte_1-AD.vs.control; scRNA-seq,SRP309935,Naive CD8+ T cell_2-AD.vs.control; scRNA-	17
BTBD9	scRNA-seq,SRP215507,CD8+ T cell_5-AD.vs.control; scRNA-seq,SRP330776,Memory T cell_2-AD.vs.control; scRNA-seq,SRP330776,CD8+ T cell_3-AD.vs.control; scRNA-seq,SRP330776,Monocyte_1-AD.vs.control	4
BTC	bulk RNA-seq,Homo_723,AD.vs.control; bulk RNA-seq,Homo_723,AD.vs.MCI; bulk RNA-seq,Homo_633,AD.vs.control; bulk RNA-	4
BTD	scRNA-seq,SRP215507,CD8+ T cell_4-AD.vs.control; scRNA-seq,SRP215507,CD8+ T cell_6-AD.vs.control; scRNA-seq,SRP215507,CD8+ T cell_4-AD.vs.MCI; scRNA-seq,SRP215507,CD8+ T cell_6-AD.vs.MCI; scRNA-seq,SRP330776,Natural killer cell_1-AD.vs.control; scRNA-seq,SRP330776,T cell-AD.vs.control; scRNA-seq,SRP330776,CD8+ T cell_3-AD.vs.control; scRNA-seq,SRP330776,Regulatory T cell-AD.vs.control; scRNA-	9
BTF3	scRNA-seq,SRP215507,CD8+ T cell_8-AD.vs.control; scRNA-seq,SRP215507,CD8+ T cell_7-AD.vs.control; scRNA-seq,SRP215507,CD8+ T cell_7-	3
BTF3L4	bulk RNA-seq,Homo_633,AD.vs.control; bulk RNA-seq,Homo_633,AD.vs.MCI; bulk RNA-seq,Homo_714,AD.vs.control; bulk RNA-seq,Homo_714,AD.vs.MCI; scRNA-seq,SRP215507,CD8+ T cell_1-AD.vs.MCI; scRNA-seq,SRP215507,CD8+ T cell_4-AD.vs.MCI; scRNA-seq,SRP215507,CD8+ T cell_4-MCI.vs.control; scRNA-seq,SRP330776,CD8+ T cell_3-AD.vs.control	8
BTG1	scRNA-seq,SRP215507,CD8+ T cell_8-AD.vs.MCI; scRNA-seq,SRP215507,CD8+ T cell_4-AD.vs.MCI; scRNA-seq,SRP215507,CD8+ T cell_4-MCI.vs.control; scRNA-seq,SRP215507,CD8+ T cell_8-MCI.vs.control; scRNA-seq,SRP309935,Erythroid cell-AD.vs.control; scRNA-seq,SRP330776,CD8+ T cell_1-AD.vs.control; scRNA-seq,SRP330776,γδ T cell-AD.vs.control; scRNA-seq,SRP330776,Memory T cell_2-AD.vs.control; scRNA-seq,SRP330776,CD8+ T cell_2-AD.vs.control; scRNA-seq,SRP330776,B cell_1-AD.vs.control; scRNA-seq,SRP330776,Naive CD8+ T cell_1-AD.vs.control; scRNA-seq,SRP330776,Natural killer cell_1-AD.vs.control; scRNA-seq,SRP330776,Memory T cell_1-AD.vs.control; scRNA-seq,SRP330776,Natural killer T cell-AD.vs.control; scRNA-seq,SRP330776,Naive CD8+ T cell_3-AD.vs.control; scRNA-seq,SRP330776,B cell_2-AD.vs.control; scRNA-seq,SRP330776,T cell-AD.vs.control; scRNA-seq,SRP330776,Naive CD8+ T cell_2-AD.vs.control; scRNA-seq,SRP330776,CD8+ T cell_3-AD.vs.control; scRNA-seq,SRP330776,Monocyte_1-AD.vs.control; scRNA-seq,SRP330776,Monocyte_2-AD.vs.control; scRNA-seq,SRP330776,B cell_3-AD.vs.control; scRNA-seq,SRP330776,Regulatory T cell-AD.vs.control; scRNA-seq,SRP330776,Megakaryocyte_1- [illegible]	26
BTG2	scRNA-seq,SRP215507,CD8+ T cell_3-AD.vs.control; scRNA-seq,SRP215507,CD8+ T cell_4-AD.vs.control; scRNA-seq,SRP215507,CD8+ T cell_4-AD.vs.MCI; scRNA-seq,SRP215507,CD8+ T cell_1-MCI.vs.control; scRNA-seq,SRP215507,CD8+ T cell_8-MCI.vs.control; scRNA-seq,SRP309935,B cell_2-AD.vs.control; scRNA-seq,SRP330776,γδ T cell-AD.vs.control; scRNA-seq,SRP330776,Natural killer cell_1-AD.vs.control	8
BTG3	bulk RNA-seq,Homo_723,AD.vs.control; bulk RNA-seq,Homo_723,AD.vs.MCI; bulk RNA-seq,Homo_633,AD.vs.control; bulk RNA-seq,Homo_633,AD.vs.MCI; bulk RNA-seq,Homo_714,AD.vs.control; bulk RNA-seq,Homo_714,AD.vs.MCI; scRNA-seq,SRP215507,CD8+ T cell_1-AD.vs.MCI; scRNA-seq,SRP215507,CD8+ T cell_5-AD.vs.MCI; scRNA-seq,SRP215507,CD8+ T cell_1-MCI.vs.control; scRNA-seq,SRP215507,CD8+ T cell_5-MCI.vs.control; scRNA-seq,SRP215507,CD8+ T cell_2-MCI.vs.control; scRNA-seq,SRP330776,CD8+ T cell_1-AD.vs.control; scRNA-	13
BTG3-AS1	scRNA-seq,SRP215507,CD8+ T cell_3-AD.vs.MCI; scRNA-seq,SRP215507,CD8+ T cell_3-MCI.vs.control; scRNA-seq,SRP330776,Memory T cell_1-	3
BTK	bulk RNA-seq,Homo_723,AD.vs.control; bulk RNA-seq,Homo_723,AD.vs.MCI; bulk RNA-seq,Homo_633,AD.vs.control; bulk RNA-seq,Homo_633,AD.vs.MCI; bulk RNA-seq,Homo_714,AD.vs.control; bulk RNA-seq,Homo_714,AD.vs.MCI; scRNA-seq,SRP330776,Megakaryocyte_1-	7
BTLA	bulk RNA-seq,Homo_714,AD.vs.control; bulk RNA-seq,Homo_714,AD.vs.MCI; bulk RNA-seq,ROSMAP,MCI.vs.control; scRNA-seq,SRP215507,CD8+ T cell_5-AD.vs.control; scRNA-seq,SRP215507,CD8+ T cell_6-AD.vs.control; scRNA-seq,SRP215507,CD8+ T cell_5-AD.vs.MCI; scRNA-seq,SRP309935,B cell_1-AD.vs.control; scRNA-seq,SRP330776,γδ T cell-AD.vs.control; scRNA-seq,SRP330776,CD8+ T cell_2-AD.vs.control; scRNA-seq,SRP330776,Memory T cell_1-AD.vs.control; scRNA-seq,SRP330776,CD8+ T cell_3-AD.vs.control; scRNA-seq,SRP330776,Megakaryocyte_1-	12
BTN1A1	bulk RNA-seq,Homo_714,AD.vs.control; bulk RNA-seq,Homo_714,AD.vs.MCI	2
BTN2A1	bulk RNA-seq,Homo_633,AD.vs.control; bulk RNA-seq,Homo_633,AD.vs.MCI; scRNA-seq,SRP309935,Natural killer cell_2-AD.vs.control; scRNA-seq,SRP309935,Naive CD8+ T cell_1-AD.vs.control; scRNA-seq,SRP309935,B cell_1-AD.vs.control; scRNA-seq,SRP330776,B cell_3-AD.vs.control; scRNA-seq,SRP330776,Megakaryocyte_1-AD.vs.control; scRNA-seq,SRP330776,Natural killer cell_2-AD.vs.control	8

BTN2A2	scRNA-seq,SRP215507,CD8+ T cell_2-AD.vs.control; scRNA-seq,SRP215507,CD8+ T cell_3-AD.vs.control; scRNA-seq,SRP215507,CD8+ T cell_3-AD.vs.MCI; scRNA-seq,SRP330776,CD8+ T cell_3-AD.vs.control	4
BTN3A1	scRNA-seq,SRP215507,CD8+ T cell_6-AD.vs.MCI; scRNA-seq,SRP309935,Natural killer cell_2-AD.vs.control; scRNA-seq,SRP309935,Naive CD8+ T cell_1-AD.vs.control; scRNA-seq,SRP309935,Monocyte_2-AD.vs.control; scRNA-seq,SRP309935,Naive CD8+ T cell_2-AD.vs.control; scRNA-seq,SRP309935,CD8+ T cell_2-AD.vs.control; scRNA-seq,SRP309935,Naive CD8+ T cell_3-AD.vs.control; scRNA-seq,SRP330776,Natural killer cell_2-	8
BTN3A2	scRNA-seq,SRP215507,CD8+ T cell_7-AD.vs.control; scRNA-seq,SRP215507,CD8+ T cell_6-AD.vs.control; scRNA-seq,SRP215507,CD8+ T cell_8-AD.vs.MCI; scRNA-seq,SRP215507,CD8+ T cell_1-AD.vs.MCI; scRNA-seq,SRP215507,CD8+ T cell_4-AD.vs.MCI; scRNA-seq,SRP215507,CD8+ T cell_6-AD.vs.MCI; scRNA-seq,SRP309935,Monocyte_1-AD.vs.control; scRNA-seq,SRP309935,Naive CD8+ T cell_3-AD.vs.control; scRNA-seq,SRP330776,CD8+ T cell_1-AD.vs.control; scRNA-seq,SRP330776,γδ T cell-AD.vs.control; scRNA-seq,SRP330776,Memory T cell_2-AD.vs.control; scRNA-seq,SRP330776,CD8+ T cell_2-AD.vs.control; scRNA-seq,SRP330776,B cell_1-AD.vs.control; scRNA-seq,SRP330776,Naive CD8+ T cell_1-AD.vs.control; scRNA-seq,SRP330776,Natural killer cell_1-AD.vs.control; scRNA-seq,SRP330776,Memory T cell_1-AD.vs.control; scRNA-seq,SRP330776,Natural killer T cell-AD.vs.control; scRNA-seq,SRP330776,Naive CD8+ T cell_3-AD.vs.control; scRNA-seq,SRP330776,B cell_2-AD.vs.control; scRNA-seq,SRP330776,T cell-AD.vs.control; scRNA-seq,SRP330776,Naive CD8+ T cell_2-AD.vs.control; scRNA-seq,SRP330776,CD8+ T cell_3-AD.vs.control; scRNA-seq,SRP330776,Monocyte_1-AD.vs.control; scRNA-seq,SRP330776,Monocyte_2-AD.vs.control; scRNA- [illegible]	26
BTN3A3	scRNA-seq,SRP215507,CD8+ T cell_7-AD.vs.control; scRNA-seq,SRP309935,CD8+ T cell_1-AD.vs.control	2
BTNL10	bulk RNA-seq,Homo_723,AD.vs.control; bulk RNA-seq,Homo_723,AD.vs.MCI; bulk RNA-seq,Homo_633,AD.vs.control; bulk RNA-seq,Homo_633,AD.vs.MCI; bulk RNA-seq,Homo_714,AD.vs.control; bulk RNA-seq,Homo_714,AD.vs.MCI	6
BTNL2	bulk RNA-seq,Homo_723,AD.vs.control; bulk RNA-seq,Homo_723,AD.vs.MCI; bulk RNA-seq,Homo_633,AD.vs.control; bulk RNA-	4
BTNL3	bulk RNA-seq,Homo_723,AD.vs.control; bulk RNA-seq,Homo_723,AD.vs.MCI; bulk RNA-seq,Homo_633,AD.vs.control; bulk RNA-seq,Homo_633,AD.vs.MCI; bulk RNA-seq,Homo_714,AD.vs.control; bulk RNA-seq,Homo_714,AD.vs.MCI	6
BTNL8	bulk RNA-seq,Homo_723,AD.vs.control; bulk RNA-seq,Homo_723,AD.vs.MCI; bulk RNA-seq,Homo_633,AD.vs.control; bulk RNA-seq,Homo_633,AD.vs.MCI; bulk RNA-seq,Homo_714,AD.vs.control; bulk RNA-seq,Homo_714,AD.vs.MCI	6
BTNL9	bulk RNA-seq,Homo_723,AD.vs.control; bulk RNA-seq,Homo_723,AD.vs.MCI; bulk RNA-seq,Homo_723,MCI.vs.control; bulk RNA-seq,Homo_633,AD.vs.control; bulk RNA-seq,Homo_633,AD.vs.MCI; bulk RNA-seq,Homo_633,MCI.vs.control; bulk RNA-seq,Homo_714,AD.vs.control; bulk RNA-seq,Homo_714,AD.vs.MCI; scRNA-seq,SRP309935,B cell_1-AD.vs.control; scRNA-seq,SRP330776,B cell_1-AD.vs.control; scRNA-	11
BTRC	bulk RNA-seq,Homo_723,AD.vs.control; bulk RNA-seq,Homo_723,AD.vs.MCI; bulk RNA-seq,Homo_633,AD.vs.control; bulk RNA-seq,Homo_633,AD.vs.MCI; bulk RNA-seq,Homo_714,AD.vs.control; bulk RNA-seq,Homo_714,AD.vs.MCI; scRNA-seq,SRP215507,CD8+ T cell_3-AD.vs.MCI; scRNA-seq,SRP309935,Monocyte_1-AD.vs.control; scRNA-seq,SRP309935,Naive CD8+ T cell_1-AD.vs.control; scRNA-	10
BUB1B	bulk RNA-seq,Homo_723,AD.vs.control; bulk RNA-seq,Homo_723,AD.vs.MCI; bulk RNA-seq,Homo_723,MCI.vs.control; bulk RNA-seq,Homo_633,AD.vs.control; bulk RNA-seq,Homo_633,AD.vs.MCI; bulk RNA-seq,Homo_633,MCI.vs.control; bulk RNA-seq,Homo_714,AD.vs.control; bulk RNA-seq,Homo_714,AD.vs.MCI; bulk RNA-seq,Homo_714,MCI.vs.control	9
BUB3	bulk RNA-seq,Homo_633,AD.vs.control; bulk RNA-seq,Homo_633,AD.vs.MCI; bulk RNA-seq,Homo_714,AD.vs.control; bulk RNA-seq,Homo_714,AD.vs.MCI; scRNA-seq,SRP215507,CD8+ T cell_8-AD.vs.control; scRNA-seq,SRP309935,Erythroid cell-AD.vs.control; scRNA-	7
BUD13	bulk RNA-seq,Homo_723,AD.vs.control; bulk RNA-seq,Homo_723,AD.vs.MCI; bulk RNA-seq,Homo_633,AD.vs.control; bulk RNA-seq,Homo_633,AD.vs.MCI; bulk RNA-seq,Homo_714,AD.vs.control; bulk RNA-seq,Homo_714,AD.vs.MCI; bulk RNA-seq,SRP310421,AD.vs.control; scRNA-seq,SRP215507,CD8+ T cell_6-AD.vs.control; scRNA-seq,SRP215507,CD8+ T cell_1-AD.vs.MCI; scRNA-seq,SRP309935,B cell_2-AD.vs.control; scRNA-seq,SRP330776,Natural killer T cell-AD.vs.control; scRNA-seq,SRP330776,Naive CD8+ T cell_3-AD.vs.control; scRNA-	13
BUD23	scRNA-seq,SRP215507,CD8+ T cell_7-AD.vs.control; scRNA-seq,SRP215507,CD8+ T cell_10-AD.vs.control; scRNA-seq,SRP215507,CD8+ T cell_7-AD.vs.MCI; scRNA-seq,SRP309935,Erythroid cell-AD.vs.control; scRNA-seq,SRP330776,Monocyte_2-AD.vs.control; scRNA-seq,SRP330776,B cell_3-	6
BUD31	scRNA-seq,SRP215507,CD8+ T cell_7-AD.vs.control; scRNA-seq,SRP330776,Regulatory T cell-AD.vs.control	2
BVES	bulk RNA-seq,Homo_723,AD.vs.control; bulk RNA-seq,Homo_723,AD.vs.MCI; bulk RNA-seq,Homo_723,MCI.vs.control; bulk RNA-seq,Homo_633,AD.vs.control; bulk RNA-seq,Homo_633,AD.vs.MCI; bulk RNA-seq,Homo_714,MCI.vs.control	6
BX005266.2	scRNA-seq,SRP215507,CD8+ T cell_1-AD.vs.control; scRNA-seq,SRP215507,CD8+ T cell_1-MCI.vs.control; scRNA-seq,SRP330776,B cell_1-	3

BX088651.4	scRNA-seq,SRP215507,CD8+ T cell_3-AD.vs.control; scRNA-seq,SRP215507,CD8+ T cell_1-AD.vs.control; scRNA-seq,SRP215507,CD8+ T cell_1-AD.vs.MCI; scRNA-seq,SRP215507,CD8+ T cell_2-MCI.vs.control; scRNA-seq,SRP330776,Memory T cell_1-AD.vs.control	5
BX255925.3	scRNA-seq,SRP330776,CD8+ T cell_1-AD.vs.control; scRNA-seq,SRP330776,Memory T cell_2-AD.vs.control; scRNA-seq,SRP330776,Memory T	3
BX284613.2	scRNA-seq,SRP330776,B cell_2-AD.vs.control	1
BX284668.5	scRNA-seq,SRP215507,CD8+ T cell_2-AD.vs.MCI; scRNA-seq,SRP215507,CD8+ T cell_3-AD.vs.MCI; scRNA-seq,SRP215507,CD8+ T cell_1-AD.vs.MCI; scRNA-seq,SRP215507,CD8+ T cell_4-AD.vs.MCI; scRNA-seq,SRP215507,CD8+ T cell_5-AD.vs.MCI; scRNA-seq,SRP215507,CD8+ T cell_1-MCI.vs.control; scRNA-seq,SRP215507,CD8+ T cell_4-MCI.vs.control; scRNA-seq,SRP215507,CD8+ T cell_3-MCI.vs.control; scRNA-seq,SRP330776,CD8+ T cell_1-AD.vs.control; scRNA-seq,SRP330776,γδ T cell-AD.vs.control; scRNA-seq,SRP330776,Memory T cell_2-AD.vs.control; scRNA-seq,SRP330776,CD8+ T cell_2-AD.vs.control; scRNA-seq,SRP330776,B cell_1-AD.vs.control; scRNA-seq,SRP330776,Naive CD8+ T cell_1-AD.vs.control; scRNA-seq,SRP330776,Memory T cell_1-AD.vs.control; scRNA-seq,SRP330776,Naive CD8+ T cell_3-AD.vs.control; scRNA-seq,SRP330776,B cell_2-AD.vs.control; scRNA-seq,SRP330776,T cell-AD.vs.control; scRNA-seq,SRP330776,CD8+ T cell_3-AD.vs.control; scRNA-seq,SRP330776,Monocyte_2-AD.vs.control; scRNA-seq,SRP330776,B cell_3-AD.vs.control; scRNA-seq,SRP330776,Natural killer cell_2-AD.vs.control	22
BX284668.6	scRNA-seq,SRP215507,CD8+ T cell_1-AD.vs.control; scRNA-seq,SRP215507,CD8+ T cell_4-AD.vs.MCI; scRNA-seq,SRP215507,CD8+ T cell_5-AD.vs.MCI; scRNA-seq,SRP215507,CD8+ T cell_1-MCI.vs.control; scRNA-seq,SRP215507,CD8+ T cell_5-MCI.vs.control; scRNA-seq,SRP215507,CD8+ T cell_4-MCI.vs.control; scRNA-seq,SRP215507,CD8+ T cell_2-MCI.vs.control; scRNA-seq,SRP215507,CD8+ T cell_3-MCI.vs.control; scRNA-seq,SRP330776,CD8+ T cell_1-AD.vs.control; scRNA-seq,SRP330776,B cell_1-AD.vs.control; scRNA-seq,SRP330776,Memory T cell_1-AD.vs.control; scRNA-seq,SRP330776,Naive CD8+ T cell_3-AD.vs.control; scRNA-seq,SRP330776,CD8+ T cell_3-AD.vs.control	13
BX293535.1	scRNA-seq,SRP215507,CD8+ T cell_1-AD.vs.MCI; scRNA-seq,SRP215507,CD8+ T cell_6-MCI.vs.control; scRNA-seq,SRP330776,CD8+ T cell_1-AD.vs.control; scRNA-seq,SRP330776,Memory T cell_2-AD.vs.control; scRNA-seq,SRP330776,Naive CD8+ T cell_1-AD.vs.control	5
BX322234.1	scRNA-seq,SRP215507,CD8+ T cell_1-AD.vs.MCI; scRNA-seq,SRP215507,CD8+ T cell_1-MCI.vs.control; scRNA-seq,SRP215507,CD8+ T cell_5-MCI.vs.control; scRNA-seq,SRP330776,Memory T cell_1-AD.vs.control	4
BX323046.1	scRNA-seq,SRP215507,CD8+ T cell_2-AD.vs.control; scRNA-seq,SRP215507,CD8+ T cell_3-AD.vs.control; scRNA-seq,SRP215507,CD8+ T cell_2-AD.vs.MCI; scRNA-seq,SRP215507,CD8+ T cell_3-AD.vs.MCI; scRNA-seq,SRP330776,T cell-AD.vs.control	5
BX323046.2	scRNA-seq,SRP215507,CD8+ T cell_1-AD.vs.control; scRNA-seq,SRP215507,CD8+ T cell_1-AD.vs.MCI; scRNA-seq,SRP330776,CD8+ T cell_1-	3
BX470102.1	scRNA-seq,SRP215507,CD8+ T cell_5-MCI.vs.control; scRNA-seq,SRP215507,CD8+ T cell_2-MCI.vs.control	2
BX537318.1	scRNA-seq,SRP215507,CD8+ T cell_5-AD.vs.control; scRNA-seq,SRP215507,CD8+ T cell_2-AD.vs.MCI; scRNA-seq,SRP215507,CD8+ T cell_2-MCI.vs.control; scRNA-seq,SRP330776,Naive CD8+ T cell_2-AD.vs.control	4
BX571846.1	scRNA-seq,SRP330776,Memory T cell_1-AD.vs.control	1
BX649632.1	scRNA-seq,SRP330776,Memory T cell_2-AD.vs.control	1
BX664615.2	scRNA-seq,SRP215507,CD8+ T cell_2-AD.vs.control; scRNA-seq,SRP215507,CD8+ T cell_3-AD.vs.MCI; scRNA-seq,SRP215507,CD8+ T cell_5-AD.vs.MCI; scRNA-seq,SRP215507,CD8+ T cell_2-MCI.vs.control; scRNA-seq,SRP215507,CD8+ T cell_6-MCI.vs.control; scRNA-seq,SRP309935,Naive CD8+ T cell_1-AD.vs.control; scRNA-seq,SRP309935,Naive CD8+ T cell_2-AD.vs.control; scRNA-seq,SRP309935,CD8+ T cell_2-AD.vs.control; scRNA-seq,SRP330776,Memory T cell_2-AD.vs.control; scRNA-seq,SRP330776,CD8+ T cell_2-AD.vs.control; scRNA-seq,SRP330776,B cell_1-AD.vs.control; scRNA-seq,SRP330776,Naive CD8+ T cell_1-AD.vs.control; scRNA-seq,SRP330776,Memory T cell_1-	15
BX842570.1	scRNA-seq,SRP215507,CD8+ T cell_3-AD.vs.MCI; scRNA-seq,SRP215507,CD8+ T cell_2-MCI.vs.control; scRNA-seq,SRP215507,CD8+ T cell_3-MCI.vs.control; scRNA-seq,SRP330776,CD8+ T cell_1-AD.vs.control; scRNA-seq,SRP330776,Naive CD8+ T cell_1-AD.vs.control	5

BX890604.2	scRNA-seq,SRP215507,CD8+ T cell_4-AD.vs.control; scRNA-seq,SRP215507,CD8+ T cell_5-AD.vs.MCI; scRNA-seq,SRP215507,CD8+ T cell_5-MCI.vs.control; scRNA-seq,SRP215507,CD8+ T cell_4-MCI.vs.control; scRNA-seq,SRP309935,Natural killer cell_2-AD.vs.control; scRNA-seq,SRP309935,Megakaryocyte_1-AD.vs.control; scRNA-seq,SRP309935,Naive CD8+ T cell_1-AD.vs.control; scRNA-seq,SRP309935,Natural killer cell_1-AD.vs.control; scRNA-seq,SRP309935,Naive CD8+ T cell_2-AD.vs.control; scRNA-seq,SRP309935,CD8+ T cell_1-AD.vs.control; scRNA-seq,SRP309935,B cell_1-AD.vs.control; scRNA-seq,SRP309935,CD8+ T cell_2-AD.vs.control; scRNA-seq,SRP330776,CD8+ T cell_1-AD.vs.control; scRNA-seq,SRP330776,γδ T cell-AD.vs.control; scRNA-seq,SRP330776,Memory T cell_2-AD.vs.control; scRNA-seq,SRP330776,CD8+ T cell_2-AD.vs.control; scRNA-seq,SRP330776,B cell_1-AD.vs.control; scRNA-seq,SRP330776,Naive CD8+ T cell_1-AD.vs.control; scRNA-seq,SRP330776,Natural killer cell_1-AD.vs.control; scRNA-seq,SRP330776,Memory T cell_1-AD.vs.control; scRNA-seq,SRP330776,Natural killer T cell-AD.vs.control; scRNA-seq,SRP330776,Naive CD8+ T cell_3-AD.vs.control; scRNA-seq,SRP330776,B cell_2-AD.vs.control; scRNA- [illegible]	26
BYSL	bulk RNA-seq,Homo_633,AD.vs.control; bulk RNA-seq,Homo_714,AD.vs.control; bulk RNA-seq,Homo_714,AD.vs.MCI; scRNA-seq,SRP215507,CD8+ T cell_2-AD.vs.control; scRNA-seq,SRP215507,CD8+ T cell_1-AD.vs.control; scRNA-seq,SRP215507,CD8+ T cell_2-AD.vs.MCI; scRNA-seq,SRP215507,CD8+ T cell_1-AD.vs.MCI; scRNA-seq,SRP215507,CD8+ T cell_4-MCI.vs.control; scRNA-seq,SRP215507,CD8+ T cell_2-MCI.vs.control; scRNA-seq,SRP309935,Monocyte_2-AD.vs.control; scRNA-seq,SRP330776,γδ T cell-AD.vs.control; scRNA-seq,SRP330776,CD8+ T cell_2-AD.vs.control; scRNA-seq,SRP330776,Naive CD8+ T cell_1-AD.vs.control; scRNA-seq,SRP330776,Natural killer cell_1-AD.vs.control; scRNA-seq,SRP330776,Memory T cell_1-AD.vs.control; scRNA-seq,SRP330776,B cell_2-AD.vs.control	16
BZW1	bulk RNA-seq,Homo_723,AD.vs.control; bulk RNA-seq,Homo_723,AD.vs.MCI; bulk RNA-seq,Homo_633,AD.vs.control; bulk RNA-seq,Homo_633,AD.vs.MCI; bulk RNA-seq,Homo_714,AD.vs.control; bulk RNA-seq,Homo_714,AD.vs.MCI; scRNA-seq,SRP215507,CD8+ T cell_7-AD.vs.control; scRNA-seq,SRP215507,CD8+ T cell_7-AD.vs.MCI; scRNA-seq,SRP215507,CD8+ T cell_4-MCI.vs.control; scRNA-seq,SRP330776,B	10
BZW2	bulk RNA-seq,Homo_633,AD.vs.control; bulk RNA-seq,Homo_633,AD.vs.MCI; bulk RNA-seq,Homo_714,AD.vs.control; bulk RNA-seq,Homo_714,AD.vs.MCI; scRNA-seq,SRP215507,CD8+ T cell_7-AD.vs.control; scRNA-seq,SRP215507,CD8+ T cell_4-AD.vs.MCI; scRNA-seq,SRP309935,B cell_1-AD.vs.control; scRNA-seq,SRP330776,B cell_1-AD.vs.control; scRNA-seq,SRP330776,B cell_3-AD.vs.control; scRNA-	10
C10orf105	bulk RNA-seq,Homo_633,AD.vs.control; bulk RNA-seq,Homo_633,AD.vs.MCI; bulk RNA-seq,Homo_714,AD.vs.control; bulk RNA-	4
C10orf143	bulk RNA-seq,Homo_723,AD.vs.control; bulk RNA-seq,Homo_723,AD.vs.MCI; bulk RNA-seq,Homo_633,AD.vs.control; bulk RNA-seq,Homo_633,AD.vs.MCI; bulk RNA-seq,Homo_714,AD.vs.control; bulk RNA-seq,Homo_714,AD.vs.MCI; bulk RNA-seq,ROSMAP,AD.vs.control; bulk RNA-seq,ROSMAP,MCI.vs.control; scRNA-seq,SRP215507,CD8+ T cell_2-AD.vs.MCI; scRNA-seq,SRP330776,Memory T cell_2-AD.vs.control; scRNA-seq,SRP330776,CD8+ T cell_2-AD.vs.control; scRNA-seq,SRP330776,Natural killer cell_1-AD.vs.control; scRNA-seq,SRP330776,Memory T	14
C10orf25	scRNA-seq,SRP215507,CD8+ T cell_1-AD.vs.control; scRNA-seq,SRP215507,CD8+ T cell_1-MCI.vs.control; scRNA-seq,SRP330776,Memory T cell_1-	3
C10orf53	bulk RNA-seq,Homo_723,AD.vs.control; bulk RNA-seq,Homo_633,AD.vs.control	2
C10orf62	bulk RNA-seq,Homo_723,AD.vs.control; bulk RNA-seq,Homo_633,AD.vs.control	2
C10orf67	bulk RNA-seq,Homo_723,MCI.vs.control; bulk RNA-seq,Homo_633,AD.vs.control; bulk RNA-seq,Homo_633,AD.vs.MCI; bulk RNA-seq,Homo_633,MCI.vs.control; bulk RNA-seq,Homo_714,MCI.vs.control	5
C10orf71	bulk RNA-seq,Homo_723,AD.vs.control; bulk RNA-seq,Homo_633,AD.vs.control; bulk RNA-seq,Homo_714,MCI.vs.control	3
C10orf82	bulk RNA-seq,Homo_723,AD.vs.control; bulk RNA-seq,Homo_723,AD.vs.MCI; bulk RNA-seq,Homo_633,AD.vs.control; bulk RNA-	4
C10orf88	bulk RNA-seq,Homo_714,AD.vs.control; bulk RNA-seq,Homo_714,AD.vs.MCI; scRNA-seq,SRP215507,CD8+ T cell_1-AD.vs.MCI; scRNA-seq,SRP215507,CD8+ T cell_1-MCI.vs.control; scRNA-seq,SRP215507,CD8+ T cell_4-MCI.vs.control; scRNA-seq,SRP330776,CD8+ T cell_1-AD.vs.control; scRNA-seq,SRP330776,γδ T cell-AD.vs.control; scRNA-seq,SRP330776,CD8+ T cell_2-AD.vs.control; scRNA-seq,SRP330776,B cell_1-AD.vs.control; scRNA-seq,SRP330776,Naive CD8+ T cell_1-AD.vs.control; scRNA-seq,SRP330776,Natural killer cell_1-AD.vs.control; scRNA-seq,SRP330776,Naive CD8+ T cell_3-AD.vs.control; scRNA-seq,SRP330776,B cell_2-AD.vs.control; scRNA-seq,SRP330776,T cell-AD.vs.control;	15
C10orf90	bulk RNA-seq,Homo_714,MCI.vs.control	1
C10orf91	scRNA-seq,SRP215507,CD8+ T cell_4-AD.vs.control; scRNA-seq,SRP215507,CD8+ T cell_3-AD.vs.MCI; scRNA-seq,SRP215507,CD8+ T cell_4-AD.vs.MCI; scRNA-seq,SRP215507,CD8+ T cell_3-MCI.vs.control; scRNA-seq,SRP330776,CD8+ T cell_2-AD.vs.control; scRNA-seq,SRP330776,Memory T cell_1-AD.vs.control; scRNA-seq,SRP330776,Natural killer T cell-AD.vs.control; scRNA-seq,SRP330776,B cell_2-	9

C10orf95	bulk RNA-seq,Homo_714,AD.vs.control; bulk RNA-seq,Homo_714,AD.vs.MCI; scRNA-seq,SRP215507,CD8+ T cell_1-AD.vs.control; scRNA-seq,SRP215507,CD8+ T cell_4-AD.vs.control; scRNA-seq,SRP215507,CD8+ T cell_6-AD.vs.control; scRNA-seq,SRP215507,CD8+ T cell_4-AD.vs.MCI; scRNA-seq,SRP215507,CD8+ T cell_1-MCI.vs.control; scRNA-seq,SRP215507,CD8+ T cell_2-MCI.vs.control; scRNA-seq,SRP330776,Memory T cell_2-AD.vs.control; scRNA-seq,SRP330776,Natural killer cell_1-AD.vs.control	10
C11orf1	bulk RNA-seq,Homo_723,AD.vs.control; bulk RNA-seq,Homo_723,MCI.vs.control; bulk RNA-seq,Homo_633,AD.vs.control; bulk RNA-seq,Homo_633,AD.vs.MCI; bulk RNA-seq,Homo_633,MCI.vs.control; bulk RNA-seq,Homo_714,AD.vs.control; bulk RNA-seq,Homo_714,MCI.vs.control; scRNA-seq,SRP215507,CD8+ T cell_4-AD.vs.MCI; scRNA-seq,SRP215507,CD8+ T cell_7-AD.vs.MCI; scRNA-seq,SRP215507,CD8+ T cell_6-AD.vs.MCI; scRNA-seq,SRP309935,B cell_1-AD.vs.control; scRNA-seq,SRP330776,Memory T cell_1-AD.vs.control;	14
C11orf16	bulk RNA-seq,Homo_723,AD.vs.control; bulk RNA-seq,Homo_723,AD.vs.MCI; bulk RNA-seq,Homo_633,AD.vs.control; bulk RNA-	4
C11orf21	scRNA-seq,SRP215507,CD8+ T cell_3-MCI.vs.control; scRNA-seq,SRP330776,Memory T cell_2-AD.vs.control; scRNA-seq,SRP330776,Natural killer cell_1-AD.vs.control; scRNA-seq,SRP330776,T cell-AD.vs.control; scRNA-seq,SRP330776,Regulatory T cell-AD.vs.control	5
C11orf24	scRNA-seq,SRP215507,CD8+ T cell_1-AD.vs.control; scRNA-seq,SRP215507,CD8+ T cell_2-AD.vs.MCI; scRNA-seq,SRP215507,CD8+ T cell_3-AD.vs.MCI; scRNA-seq,SRP215507,CD8+ T cell_1-MCI.vs.control; scRNA-seq,SRP215507,CD8+ T cell_5-MCI.vs.control; scRNA-seq,SRP215507,CD8+ T cell_2-MCI.vs.control; scRNA-seq,SRP215507,CD8+ T cell_3-MCI.vs.control; scRNA-seq,SRP309935,CD8+ T cell_1-AD.vs.control; scRNA-seq,SRP330776,Memory T cell_2-AD.vs.control; scRNA-seq,SRP330776,Natural killer cell_1-AD.vs.control; scRNA-	12
C11orf42	bulk RNA-seq,Homo_723,AD.vs.control; bulk RNA-seq,Homo_723,AD.vs.MCI; bulk RNA-seq,Homo_723,MCI.vs.control; bulk RNA-seq,Homo_633,AD.vs.control; bulk RNA-seq,Homo_633,AD.vs.MCI; bulk RNA-seq,Homo_633,MCI.vs.control; bulk RNA-seq,Homo_714,AD.vs.control; bulk RNA-seq,Homo_714,AD.vs.MCI; bulk RNA-seq,Homo_714,MCI.vs.control	9
C11orf45	scRNA-seq,SRP215507,CD8+ T cell_2-MCI.vs.control	1
C11orf49	bulk RNA-seq,Homo_723,AD.vs.control; bulk RNA-seq,Homo_723,AD.vs.MCI; bulk RNA-seq,Homo_723,MCI.vs.control; bulk RNA-seq,Homo_633,AD.vs.control; bulk RNA-seq,Homo_633,MCI.vs.control; bulk RNA-seq,Homo_714,AD.vs.control; bulk RNA-seq,Homo_714,AD.vs.MCI; bulk RNA-seq,Homo_714,MCI.vs.control; scRNA-seq,SRP330776,γδ T cell-AD.vs.control; scRNA-seq,SRP330776,CD8+ T cell_2-AD.vs.control; scRNA-seq,SRP330776,Naive CD8+ T cell_1-AD.vs.control; scRNA-seq,SRP330776,Natural killer cell_1-AD.vs.control; scRNA-seq,SRP330776,Naive CD8+ T cell_3-AD.vs.control; scRNA-seq,SRP330776,CD8+ T cell_3-AD.vs.control; scRNA-seq,SRP330776,B cell_3-AD.vs.control	15
C11orf52	bulk RNA-seq,ROSMAP,AD.vs.control; bulk RNA-seq,ROSMAP,MCI.vs.control	2
C11orf53	bulk RNA-seq,Homo_633,AD.vs.MCI	1
C11orf54	scRNA-seq,SRP215507,CD8+ T cell_4-MCI.vs.control; scRNA-seq,SRP309935,CD8+ T cell_1-AD.vs.control; scRNA-seq,SRP330776,CD8+ T cell_1-AD.vs.control; scRNA-seq,SRP330776,γδ T cell-AD.vs.control; scRNA-seq,SRP330776,Memory T cell_2-AD.vs.control; scRNA-seq,SRP330776,CD8+ T cell_2-AD.vs.control; scRNA-seq,SRP330776,Natural killer cell_1-AD.vs.control; scRNA-seq,SRP330776,Memory T cell_1-AD.vs.control; scRNA-seq,SRP330776,T cell-AD.vs.control; scRNA-seq,SRP330776,B cell_3-AD.vs.control	10
C11orf58	scRNA-seq,SRP330776,Megakaryocyte_1-AD.vs.control	1
C11orf65	bulk RNA-seq,Homo_723,AD.vs.control; bulk RNA-seq,Homo_723,AD.vs.MCI; bulk RNA-seq,Homo_633,AD.vs.control; bulk RNA-seq,Homo_633,AD.vs.MCI; bulk RNA-seq,Homo_714,AD.vs.control; bulk RNA-seq,Homo_714,AD.vs.MCI; scRNA-seq,SRP215507,CD8+ T cell_2-AD.vs.MCI; scRNA-seq,SRP215507,CD8+ T cell_2-MCI.vs.control; scRNA-seq,SRP330776,CD8+ T cell_2-AD.vs.control	9
C11orf68	bulk RNA-seq,Homo_723,AD.vs.MCI; bulk RNA-seq,Homo_723,MCI.vs.control; bulk RNA-seq,Homo_633,AD.vs.MCI; bulk RNA-seq,Homo_633,MCI.vs.control; bulk RNA-seq,Homo_714,AD.vs.control; bulk RNA-seq,Homo_714,AD.vs.MCI; bulk RNA-seq,Homo_714,MCI.vs.control; scRNA-seq,SRP215507,CD8+ T cell_1-AD.vs.MCI; scRNA-seq,SRP215507,CD8+ T cell_1-MCI.vs.control; scRNA-seq,SRP309935,Monocyte_1-AD.vs.control; scRNA-seq,SRP309935,Naive CD8+ T cell_1-AD.vs.control; scRNA-seq,SRP309935,Naive CD8+ T cell_2-AD.vs.control; scRNA-seq,SRP309935,CD8+ T cell_1-AD.vs.control; scRNA-seq,SRP309935,Naive CD8+ T cell_3-AD.vs.control; scRNA-seq,SRP330776,CD8+ T cell_1-AD.vs.control; scRNA-seq,SRP330776,Memory T cell_2-AD.vs.control; scRNA-seq,SRP330776,CD8+ T cell_2-AD.vs.control; scRNA-seq,SRP330776,B cell_1-AD.vs.control; scRNA-seq,SRP330776,Naive CD8+ T cell_1-AD.vs.control; scRNA-seq,SRP330776,Natural killer cell_1-AD.vs.control; scRNA-seq,SRP330776,Memory T cell_1-AD.vs.control; scRNA-seq,SRP330776,Natural killer T cell-[illegible]	25

C11orf71	bulk RNA-seq,Homo_633,AD.vs.control; bulk RNA-seq,Homo_633,AD.vs.MCI; bulk RNA-seq,Homo_714,AD.vs.control; bulk RNA-seq,Homo_714,AD.vs.MCI; scRNA-seq,SRP215507,CD8+ T cell_3-AD.vs.MCI; scRNA-seq,SRP215507,CD8+ T cell_1-AD.vs.MCI; scRNA-seq,SRP215507,CD8+ T cell_3-MCI.vs.control; scRNA-seq,SRP330776,γδ T cell-AD.vs.control; scRNA-seq,SRP330776,Memory T cell_2-AD.vs.control; scRNA-seq,SRP330776,Naive CD8+ T cell_1-AD.vs.control; scRNA-seq,SRP330776,Natural killer cell_1-AD.vs.control; scRNA-seq,SRP330776,Memory T cell_1-AD.vs.control; scRNA-seq,SRP330776,Naive CD8+ T cell_3-AD.vs.control; scRNA-seq,SRP330776,T cell-AD.vs.control; scRNA-	15
C11orf74	scRNA-seq,SRP215507,CD8+ T cell_2-AD.vs.control; scRNA-seq,SRP215507,CD8+ T cell_3-AD.vs.control; scRNA-seq,SRP215507,CD8+ T cell_5-AD.vs.control; scRNA-seq,SRP215507,CD8+ T cell_5-MCI.vs.control; scRNA-seq,SRP215507,CD8+ T cell_2-MCI.vs.control; scRNA-seq,SRP215507,CD8+ T cell_3-MCI.vs.control; scRNA-seq,SRP309935,Plasmacytoid dendritic cell_1-AD.vs.control; scRNA-seq,SRP330776,CD8+ T cell_1-AD.vs.control; scRNA-seq,SRP330776,Memory T cell_2-AD.vs.control; scRNA-seq,SRP330776,Naive CD8+ T cell_1-AD.vs.control	10
C11orf80	bulk RNA-seq,Homo_633,AD.vs.control; bulk RNA-seq,Homo_633,AD.vs.MCI; bulk RNA-seq,Homo_714,AD.vs.control; bulk RNA-seq,Homo_714,AD.vs.MCI; scRNA-seq,SRP215507,CD8+ T cell_2-AD.vs.MCI; scRNA-seq,SRP215507,CD8+ T cell_2-MCI.vs.control; scRNA-seq,SRP215507,CD8+ T cell_7-MCI.vs.control; scRNA-seq,SRP309935,B cell_1-AD.vs.control; scRNA-seq,SRP330776,CD8+ T cell_1-AD.vs.control; scRNA-seq,SRP330776,γδ T cell-AD.vs.control; scRNA-seq,SRP330776,CD8+ T cell_2-AD.vs.control; scRNA-seq,SRP330776,B cell_1-AD.vs.control; scRNA-seq,SRP330776,Naive CD8+ T cell_1-AD.vs.control; scRNA-seq,SRP330776,Memory T cell_1-AD.vs.control; scRNA-seq,SRP330776,Natural killer T cell-AD.vs.control; scRNA-seq,SRP330776,B cell_2-AD.vs.control; scRNA-seq,SRP330776,T cell-AD.vs.control; scRNA-seq,SRP330776,B [illegible]	18
C11orf86	bulk RNA-seq,ROSMAP,AD.vs.control	1
C11orf91	bulk RNA-seq,Homo_633,AD.vs.control	1
C11orf95	scRNA-seq,SRP330776,γδ T cell-AD.vs.control	1
C11orf96	bulk RNA-seq,Homo_723,AD.vs.control; bulk RNA-seq,Homo_723,AD.vs.MCI; bulk RNA-seq,Homo_633,AD.vs.control; bulk RNA-seq,Homo_633,AD.vs.MCI; scRNA-seq,SRP215507,CD8+ T cell_2-MCI.vs.control	5
C11orf98	bulk RNA-seq,Homo_723,AD.vs.control; bulk RNA-seq,Homo_723,AD.vs.MCI; bulk RNA-seq,Homo_633,AD.vs.control; bulk RNA-seq,Homo_633,AD.vs.MCI; bulk RNA-seq,Homo_714,AD.vs.control; bulk RNA-seq,Homo_714,AD.vs.MCI; scRNA-seq,SRP215507,CD8+ T cell_5-MCI.vs.control; scRNA-seq,SRP309935,Monocyte_1-AD.vs.control; scRNA-seq,SRP330776,CD8+ T cell_1-AD.vs.control; scRNA-seq,SRP330776,γδ T cell-AD.vs.control; scRNA-seq,SRP330776,Memory T cell_2-AD.vs.control; scRNA-seq,SRP330776,B cell_1-AD.vs.control; scRNA-seq,SRP330776,Naive CD8+ T cell_1-AD.vs.control; scRNA-seq,SRP330776,Memory T cell_1-AD.vs.control; scRNA-seq,SRP330776,Naive CD8+ T cell_3-AD.vs.control; scRNA-seq,SRP330776,B cell_2-AD.vs.control; scRNA-seq,SRP330776,T cell-AD.vs.control; scRNA-seq,SRP330776,CD8+ T [illegible]	19
C12orf10	scRNA-seq,SRP215507,CD8+ T cell_7-AD.vs.control; scRNA-seq,SRP215507,CD8+ T cell_10-AD.vs.control; scRNA-seq,SRP330776,T cell-AD.vs.control; scRNA-seq,SRP330776,Monocyte_2-AD.vs.control; scRNA-seq,SRP330776,Regulatory T cell-AD.vs.control; scRNA-seq,SRP330776,Megakaryocyte_1-AD.vs.control; scRNA-seq,SRP330776,B cell_4-AD.vs.control	7
C12orf29	bulk RNA-seq,Homo_723,AD.vs.MCI; bulk RNA-seq,Homo_633,AD.vs.MCI; bulk RNA-seq,Homo_714,AD.vs.control; bulk RNA-seq,Homo_714,AD.vs.MCI; scRNA-seq,SRP215507,CD8+ T cell_6-MCI.vs.control; scRNA-seq,SRP309935,Naive CD8+ T cell_2-AD.vs.control; scRNA-seq,SRP309935,B cell_1-AD.vs.control; scRNA-seq,SRP309935,CD8+ T cell_2-AD.vs.control; scRNA-seq,SRP330776,Memory T cell_2-AD.vs.control; scRNA-seq,SRP330776,B cell_1-AD.vs.control; scRNA-seq,SRP330776,Naive CD8+ T cell_1-AD.vs.control; scRNA-seq,SRP330776,Memory T cell_1-AD.vs.control; scRNA-seq,SRP330776,Naive CD8+ T cell_3-AD.vs.control; scRNA-seq,SRP330776,B cell_2-AD.vs.control; scRNA-seq,SRP330776,B	15
C12orf4	bulk RNA-seq,Homo_723,AD.vs.MCI; bulk RNA-seq,Homo_723,MCI.vs.control; bulk RNA-seq,Homo_633,AD.vs.MCI; bulk RNA-seq,Homo_633,MCI.vs.control; bulk RNA-seq,Homo_714,AD.vs.control; bulk RNA-seq,Homo_714,AD.vs.MCI; bulk RNA-seq,Homo_714,MCI.vs.control; scRNA-seq,SRP215507,CD8+ T cell_5-MCI.vs.control; scRNA-seq,SRP309935,Monocyte_2-AD.vs.control; scRNA-seq,SRP330776,γδ T cell-AD.vs.control; scRNA-seq,SRP330776,Memory T cell_1-AD.vs.control	11
C12orf40	bulk RNA-seq,ROSMAP,MCI.vs.control; scRNA-seq,SRP215507,CD8+ T cell_2-AD.vs.control; scRNA-seq,SRP215507,CD8+ T cell_3-AD.vs.MCI; scRNA-seq,SRP215507,CD8+ T cell_3-MCI.vs.control; scRNA-seq,SRP330776,CD8+ T cell_1-AD.vs.control	5
C12orf42	bulk RNA-seq,Homo_723,AD.vs.control; bulk RNA-seq,Homo_723,AD.vs.MCI; bulk RNA-seq,Homo_633,AD.vs.control; bulk RNA-seq,Homo_633,AD.vs.MCI; bulk RNA-seq,Homo_714,AD.vs.control; bulk RNA-seq,Homo_714,AD.vs.MCI; bulk RNA-seq,ROSMAP,MCI.vs.control; scRNA-seq,SRP330776,B cell_1-AD.vs.control; scRNA-seq,SRP330776,Natural killer cell_1-AD.vs.control; scRNA-seq,SRP330776,T cell-AD.vs.control	10

C12orf43	bulk RNA-seq,Homo_714,AD.vs.control; bulk RNA-seq,Homo_714,AD.vs.MCI; scRNA-seq,SRP215507,CD8+ T cell_3-AD.vs.MCI; scRNA-seq,SRP215507,CD8+ T cell_5-AD.vs.MCI; scRNA-seq,SRP215507,CD8+ T cell_6-AD.vs.MCI; scRNA-seq,SRP215507,CD8+ T cell_4-MCI.vs.control; scRNA-seq,SRP215507,CD8+ T cell_3-MCI.vs.control; scRNA-seq,SRP330776,T cell-AD.vs.control; scRNA-seq,SRP330776,CD8+ T cell_3-AD.vs.control; scRNA-seq,SRP330776,Natural killer cell_2-AD.vs.control	10
C12orf45	scRNA-seq,SRP309935,B cell_1-AD.vs.control; scRNA-seq,SRP330776,γδ T cell-AD.vs.control; scRNA-seq,SRP330776,Memory T cell_2-AD.vs.control; scRNA-seq,SRP330776,CD8+ T cell_2-AD.vs.control; scRNA-seq,SRP330776,Memory T cell_1-AD.vs.control; scRNA-seq,SRP330776,Naive CD8+ T cell_3-AD.vs.control; scRNA-seq,SRP330776,B cell_2-AD.vs.control; scRNA-seq,SRP330776,CD8+ T cell_3-AD.vs.control; scRNA-seq,SRP330776,Monocyte_1-AD.vs.control; scRNA-seq,SRP330776,B cell_3-AD.vs.control; scRNA-seq,SRP330776,Natural killer cell_2-AD.vs.control	11
C12orf49	scRNA-seq,SRP215507,CD8+ T cell_8-AD.vs.control; scRNA-seq,SRP215507,CD8+ T cell_4-AD.vs.control; scRNA-seq,SRP215507,CD8+ T cell_4-AD.vs.MCI; scRNA-seq,SRP215507,CD8+ T cell_5-MCI.vs.control; scRNA-seq,SRP309935,Naive CD8+ T cell_1-AD.vs.control; scRNA-seq,SRP330776,CD8+ T cell_1-AD.vs.control; scRNA-seq,SRP330776,γδ T cell-AD.vs.control; scRNA-seq,SRP330776,Memory T cell_2-AD.vs.control; scRNA-seq,SRP330776,CD8+ T cell_2-AD.vs.control; scRNA-seq,SRP330776,B cell_1-AD.vs.control; scRNA-seq,SRP330776,Naive CD8+ T cell_1-AD.vs.control; scRNA-seq,SRP330776,Natural killer cell_1-AD.vs.control; scRNA-seq,SRP330776,Memory T cell_1-AD.vs.control; scRNA-seq,SRP330776,Natural killer T cell-AD.vs.control; scRNA-seq,SRP330776,Naive CD8+ T cell_3-AD.vs.control; scRNA-seq,SRP330776,B cell_2-AD.vs.control; scRNA-seq,SRP330776,T cell-AD.vs.control; scRNA-seq,SRP330776,CD8+ T cell_3-AD.vs.control; scRNA-seq,SRP330776,Monocyte_2-[illegible]	21
C12orf50	bulk RNA-seq,Homo_723,AD.vs.control; bulk RNA-seq,Homo_723,AD.vs.MCI; bulk RNA-seq,Homo_723,MCI.vs.control; bulk RNA-seq,Homo_633,AD.vs.control; bulk RNA-seq,Homo_633,AD.vs.MCI; bulk RNA-seq,Homo_633,MCI.vs.control; bulk RNA-seq,Homo_714,MCI.vs.control	7
C12orf54	bulk RNA-seq,Homo_723,AD.vs.control	1
C12orf57	scRNA-seq,SRP215507,CD8+ T cell_6-AD.vs.MCI; scRNA-seq,SRP330776,Monocyte_2-AD.vs.control	2
C12orf60	bulk RNA-seq,ROSMAP,MCI.vs.control; scRNA-seq,SRP215507,CD8+ T cell_3-AD.vs.control; scRNA-seq,SRP215507,CD8+ T cell_1-AD.vs.MCI; scRNA-seq,SRP309935,B cell_1-AD.vs.control; scRNA-seq,SRP330776,Naive CD8+ T cell_1-AD.vs.control	5
C12orf65	scRNA-seq,SRP215507,CD8+ T cell_7-AD.vs.control; scRNA-seq,SRP215507,CD8+ T cell_6-AD.vs.control; scRNA-seq,SRP215507,CD8+ T cell_1-AD.vs.MCI; scRNA-seq,SRP309935,CD8+ T cell_2-AD.vs.control; scRNA-seq,SRP330776,γδ T cell-AD.vs.control; scRNA-seq,SRP330776,CD8+ T cell_2-AD.vs.control; scRNA-seq,SRP330776,Memory T cell_1-AD.vs.control; scRNA-seq,SRP330776,B cell_2-AD.vs.control; scRNA-seq,SRP330776,CD8+ T cell_3-AD.vs.control; scRNA-seq,SRP330776,Megakaryocyte_1-AD.vs.control	10
C12orf66	scRNA-seq,SRP215507,CD8+ T cell_5-AD.vs.MCI; scRNA-seq,SRP215507,CD8+ T cell_5-MCI.vs.control; scRNA-seq,SRP215507,CD8+ T cell_3-MCI.vs.control; scRNA-seq,SRP330776,γδ T cell-AD.vs.control; scRNA-seq,SRP330776,Natural killer cell_1-AD.vs.control; scRNA-[illegible]	6
C12orf73	bulk RNA-seq,Homo_723,AD.vs.control; bulk RNA-seq,Homo_723,AD.vs.MCI; bulk RNA-seq,Homo_633,AD.vs.control; bulk RNA-seq,Homo_633,AD.vs.MCI; bulk RNA-seq,Homo_633,MCI.vs.control; bulk RNA-seq,Homo_714,AD.vs.control; bulk RNA-seq,Homo_714,MCI.vs.control; scRNA-seq,SRP215507,CD8+ T cell_6-AD.vs.control; scRNA-seq,SRP215507,CD8+ T cell_1-AD.vs.MCI; scRNA-seq,SRP215507,CD8+ T cell_1-MCI.vs.control; scRNA-seq,SRP215507,CD8+ T cell_6-MCI.vs.control; scRNA-seq,SRP330776,γδ T cell-AD.vs.control	12
C12orf75	bulk RNA-seq,Homo_723,AD.vs.control; bulk RNA-seq,Homo_723,AD.vs.MCI; bulk RNA-seq,Homo_633,AD.vs.control; bulk RNA-seq,Homo_633,AD.vs.MCI; bulk RNA-seq,Homo_714,AD.vs.control; bulk RNA-seq,Homo_714,AD.vs.MCI; scRNA-seq,SRP215507,CD8+ T cell_7-AD.vs.control; scRNA-seq,SRP215507,CD8+ T cell_5-AD.vs.MCI; scRNA-seq,SRP215507,CD8+ T cell_3-MCI.vs.control; scRNA-seq,SRP309935,Erythroid cell-AD.vs.control; scRNA-seq,SRP309935,Monocyte_2-AD.vs.control; scRNA-seq,SRP330776,CD8+ T cell_1-AD.vs.control; scRNA-seq,SRP330776,γδ T cell-AD.vs.control; scRNA-seq,SRP330776,Memory T cell_2-AD.vs.control; scRNA-seq,SRP330776,CD8+ T cell_2-AD.vs.control; scRNA-seq,SRP330776,B cell_1-AD.vs.control; scRNA-seq,SRP330776,Naive CD8+ T cell_1-AD.vs.control; scRNA-seq,SRP330776,Natural killer cell_1-AD.vs.control; scRNA-seq,SRP330776,Memory T cell_1-AD.vs.control; scRNA-seq,SRP330776,Natural killer T cell-AD.vs.control; scRNA-seq,SRP330776,Naive CD8+ T cell_3-AD.vs.control; scRNA-seq,SRP330776,B cell_2-AD.vs.control; scRNA-seq,SRP330776,T cell-AD.vs.control; scRNA-seq,SRP330776,Naive CD8+ T cell_2-AD.vs.control; scRNA-seq,SRP330776,CD8+ T cell_3-AD.vs.control; scRNA-[illegible]	27

C12orf76	bulk RNA-seq,Homo_723,AD.vs.control; bulk RNA-seq,Homo_723,AD.vs.MCI; bulk RNA-seq,Homo_723,MCI.vs.control; bulk RNA-seq,Homo_633,AD.vs.control; bulk RNA-seq,Homo_633,AD.vs.MCI; bulk RNA-seq,Homo_633,MCI.vs.control; bulk RNA-seq,Homo_714,AD.vs.control; bulk RNA-seq,Homo_714,AD.vs.MCI; scRNA-seq,SRP215507,CD8+ T cell_7-AD.vs.control; scRNA-seq,SRP215507,CD8+ T cell_5-AD.vs.control; scRNA-seq,SRP215507,CD8+ T cell_6-AD.vs.control; scRNA-seq,SRP215507,CD8+ T cell_4-AD.vs.MCI; scRNA-seq,SRP215507,CD8+ T cell_7-AD.vs.MCI; scRNA-seq,SRP215507,CD8+ T cell_5-AD.vs.MCI; scRNA-seq,SRP309935,Plasmacytoid dendritic cell_1-AD.vs.control; scRNA-seq,SRP330776,CD8+ T cell_1-AD.vs.control; scRNA-seq,SRP330776,B cell_1-AD.vs.control; scRNA-seq,SRP330776,Memory T cell_1-AD.vs.control; scRNA-seq,SRP330776,B cell_2-AD.vs.control; scRNA-seq,SRP330776,T cell-AD.vs.control; scRNA-seq,SRP330776,Monocyte_1-AD.vs.control; [illegible]	22
C13orf46	bulk RNA-seq,Homo_723,AD.vs.control; bulk RNA-seq,Homo_723,AD.vs.MCI; bulk RNA-seq,Homo_723,MCI.vs.control; bulk RNA-seq,Homo_633,AD.vs.control; bulk RNA-seq,Homo_633,AD.vs.MCI; bulk RNA-seq,Homo_633,MCI.vs.control; bulk RNA-seq,Homo_714,AD.vs.control; bulk RNA-seq,Homo_714,AD.vs.MCI; bulk RNA-seq,Homo_714,MCI.vs.control; scRNA-seq,SRP215507,CD8+ T cell_1-AD.vs.control; scRNA-seq,SRP215507,CD8+ T cell_2-AD.vs.MCI; scRNA-seq,SRP215507,CD8+ T cell_1-AD.vs.MCI; scRNA-seq,SRP215507,CD8+ T cell_1-MCI.vs.control; scRNA-seq,SRP215507,CD8+ T cell_2-MCI.vs.control; scRNA-seq,SRP215507,CD8+ T cell_3-MCI.vs.control; scRNA-seq,SRP330776,CD8+ T cell_1-AD.vs.control; scRNA-seq,SRP330776,CD8+ T cell_2-AD.vs.control; scRNA-seq,SRP330776,Naive CD8+ T cell_3-AD.vs.control	18
C14orf119	scRNA-seq,SRP215507,CD8+ T cell_2-AD.vs.MCI; scRNA-seq,SRP215507,CD8+ T cell_3-AD.vs.MCI; scRNA-seq,SRP215507,CD8+ T cell_1-AD.vs.MCI; scRNA-seq,SRP215507,CD8+ T cell_1-MCI.vs.control; scRNA-seq,SRP215507,CD8+ T cell_2-MCI.vs.control; scRNA-seq,SRP309935,Naive CD8+ T cell_2-AD.vs.control; scRNA-seq,SRP330776,CD8+ T cell_1-AD.vs.control; scRNA-seq,SRP330776,γδ T cell-AD.vs.control; scRNA-seq,SRP330776,Memory T cell_2-AD.vs.control; scRNA-seq,SRP330776,CD8+ T cell_2-AD.vs.control; scRNA-seq,SRP330776,B cell_1-AD.vs.control; scRNA-seq,SRP330776,Naive CD8+ T cell_1-AD.vs.control; scRNA-seq,SRP330776,Natural killer cell_1-AD.vs.control; scRNA-seq,SRP330776,Memory T cell_1-AD.vs.control; scRNA-seq,SRP330776,Natural killer T cell-AD.vs.control; scRNA-seq,SRP330776,Naive CD8+ T cell_3-AD.vs.control; scRNA-seq,SRP330776,B cell_2-AD.vs.control; scRNA-seq,SRP330776,T cell-AD.vs.control; scRNA-seq,SRP330776,CD8+ T cell_3-AD.vs.control; scRNA-seq,SRP330776,Monocyte_1-AD.vs.control; scRNA-seq,SRP330776,Monocyte_2-AD.vs.control; scRNA-[illegible]	23
C14orf132	bulk RNA-seq,Homo_723,AD.vs.control; bulk RNA-seq,Homo_723,AD.vs.MCI; bulk RNA-seq,Homo_633,AD.vs.control; bulk RNA-seq,Homo_633,AD.vs.MCI; bulk RNA-seq,Homo_714,AD.vs.control; bulk RNA-seq,Homo_714,AD.vs.MCI; scRNA-seq,SRP215507,CD8+ T cell_4-AD.vs.control; scRNA-seq,SRP215507,CD8+ T cell_3-AD.vs.MCI	8
C14orf178	scRNA-seq,SRP215507,CD8+ T cell_1-AD.vs.MCI	1
C14orf180	bulk RNA-seq,Homo_723,AD.vs.control; bulk RNA-seq,Homo_723,MCI.vs.control; bulk RNA-seq,Homo_633,MCI.vs.control; bulk RNA-	4
C14orf28	bulk RNA-seq,Homo_633,AD.vs.control; bulk RNA-seq,Homo_633,AD.vs.MCI; bulk RNA-seq,Homo_714,AD.vs.control; bulk RNA-seq,Homo_714,AD.vs.MCI; scRNA-seq,SRP215507,CD8+ T cell_4-AD.vs.control; scRNA-seq,SRP215507,CD8+ T cell_5-AD.vs.control; scRNA-seq,SRP215507,CD8+ T cell_1-MCI.vs.control; scRNA-seq,SRP330776,CD8+ T cell_1-AD.vs.control; scRNA-seq,SRP330776,γδ T cell-AD.vs.control; scRNA-seq,SRP330776,Memory T cell_2-AD.vs.control; scRNA-seq,SRP330776,CD8+ T cell_2-AD.vs.control; scRNA-seq,SRP330776,B cell_1-AD.vs.control; scRNA-seq,SRP330776,Naive CD8+ T cell_1-AD.vs.control; scRNA-seq,SRP330776,Natural killer cell_1-AD.vs.control; scRNA-seq,SRP330776,Memory T cell_1-AD.vs.control; scRNA-seq,SRP330776,Natural killer T cell-AD.vs.control; scRNA-seq,SRP330776,B cell_2-AD.vs.control; scRNA-seq,SRP330776,T cell-AD.vs.control; scRNA-seq,SRP330776,CD8+ T cell_3-AD.vs.control; scRNA-seq,SRP330776,B cell_3-[illegible]	21
C14orf93	bulk RNA-seq,Homo_723,AD.vs.control; bulk RNA-seq,Homo_723,AD.vs.MCI; bulk RNA-seq,Homo_633,AD.vs.control; bulk RNA-seq,Homo_633,AD.vs.MCI; bulk RNA-seq,Homo_714,AD.vs.control; bulk RNA-seq,Homo_714,AD.vs.MCI; bulk RNA-seq,ROSMAP,AD.vs.control; scRNA-seq,SRP215507,CD8+ T cell_3-MCI.vs.control; scRNA-seq,SRP330776,Memory T cell_1-AD.vs.control; scRNA-seq,SRP330776,B cell_2-	11
C15orf39	scRNA-seq,SRP215507,CD8+ T cell_10-AD.vs.control; scRNA-seq,SRP215507,CD8+ T cell_4-AD.vs.MCI; scRNA-seq,SRP215507,CD8+ T cell_4-MCI.vs.control; scRNA-seq,SRP215507,CD8+ T cell_6-MCI.vs.control; scRNA-seq,SRP330776,CD8+ T cell_1-AD.vs.control; scRNA-seq,SRP330776,γδ T cell-AD.vs.control; scRNA-seq,SRP330776,CD8+ T cell_2-AD.vs.control; scRNA-seq,SRP330776,Naive CD8+ T cell_1-AD.vs.control; scRNA-seq,SRP330776,Natural killer cell_1-AD.vs.control; scRNA-seq,SRP330776,Memory T cell_1-AD.vs.control; scRNA-seq,SRP330776,CD8+ T cell_3-AD.vs.control; scRNA-seq,SRP330776,Natural killer cell_2-AD.vs.control; scRNA-seq,SRP330776,B cell_4-AD.vs.control	13

C15orf40	scRNA-seq,SRP215507,CD8+ T cell_7-AD.vs.control; scRNA-seq,SRP215507,CD8+ T cell_10-AD.vs.control; scRNA-seq,SRP215507,CD8+ T cell_2-MCI.vs.control; scRNA-seq,SRP330776,γδ T cell-AD.vs.control; scRNA-seq,SRP330776,Memory T cell_2-AD.vs.control; scRNA-seq,SRP330776,Naive CD8+ T cell_3-AD.vs.control; scRNA-seq,SRP330776,Megakaryocyte_1-AD.vs.control	7
C15orf41	scRNA-seq,SRP330776,B cell_1-AD.vs.control; scRNA-seq,SRP330776,Memory T cell_1-AD.vs.control; scRNA-seq,SRP330776,Naive CD8+ T cell_3-	3
C15orf48	bulk RNA-seq,Homo_714,AD.vs.control; bulk RNA-seq,Homo_714,AD.vs.MCI; scRNA-seq,SRP215507,CD8+ T cell_1-MCI.vs.control; scRNA-seq,SRP215507,CD8+ T cell_2-MCI.vs.control; scRNA-seq,SRP330776,Monocyte_2-AD.vs.control	5
C15orf56	scRNA-seq,SRP215507,CD8+ T cell_1-AD.vs.MCI	1
C15orf61	bulk RNA-seq,Homo_723,AD.vs.MCI; bulk RNA-seq,Homo_723,MCI.vs.control; bulk RNA-seq,Homo_633,AD.vs.MCI; bulk RNA-seq,Homo_633,MCI.vs.control; bulk RNA-seq,Homo_714,AD.vs.control; bulk RNA-seq,Homo_714,AD.vs.MCI; bulk RNA-seq,Homo_714,MCI.vs.control; scRNA-seq,SRP215507,CD8+ T cell_4-AD.vs.MCI; scRNA-seq,SRP215507,CD8+ T cell_4-MCI.vs.control; scRNA-seq,SRP330776,CD8+ T cell_1-AD.vs.control; scRNA-seq,SRP330776,γδ T cell-AD.vs.control; scRNA-seq,SRP330776,Memory T cell_2-AD.vs.control; scRNA-seq,SRP330776,CD8+ T cell_2-AD.vs.control; scRNA-seq,SRP330776,B cell_1-AD.vs.control; scRNA-seq,SRP330776,Naive CD8+ T cell_1-AD.vs.control; scRNA-seq,SRP330776,Natural killer cell_1-AD.vs.control; scRNA-seq,SRP330776,Memory T cell_1-AD.vs.control; scRNA-seq,SRP330776,Natural killer T cell-AD.vs.control; scRNA-seq,SRP330776,Naive CD8+ T cell_3-AD.vs.control; scRNA-seq,SRP330776,B cell_2-AD.vs.control; scRNA-seq,SRP330776,T cell-AD.vs.control; scRNA-seq,SRP330776,CD8+ T cell_3-AD.vs.control; scRNA-seq,SRP330776,Monocyte_1-AD.vs.control; scRNA-seq,SRP330776,Monocyte_2-AD.vs.control; scRNA-seq,SRP330776,B cell_3-AD.vs.control; scRNA-seq,SRP330776,Regulatory T [illegible]	28
C15orf62	bulk RNA-seq,ROSMAP,AD.vs.control; bulk RNA-seq,ROSMAP,MCI.vs.control	2
C15orf65	scRNA-seq,SRP330776,Naive CD8+ T cell_1-AD.vs.control; scRNA-seq,SRP330776,B cell_4-AD.vs.control	2
C16orf46	bulk RNA-seq,Homo_723,AD.vs.control; bulk RNA-seq,Homo_723,AD.vs.MCI; bulk RNA-seq,Homo_633,AD.vs.control; bulk RNA-seq,Homo_633,AD.vs.MCI; bulk RNA-seq,Homo_633,MCI.vs.control; bulk RNA-seq,Homo_714,AD.vs.MCI; bulk RNA-seq,Homo_714,MCI.vs.control; scRNA-seq,SRP215507,CD8+ T cell_1-AD.vs.control; scRNA-seq,SRP330776,Memory T cell_2-AD.vs.control; scRNA-seq,SRP330776,CD8+ T cell_2-	10
C16orf54	bulk RNA-seq,SRP223445,AD.vs.control; scRNA-seq,SRP215507,CD8+ T cell_10-AD.vs.control; scRNA-seq,SRP215507,CD8+ T cell_6-AD.vs.control; scRNA-seq,SRP215507,CD8+ T cell_5-AD.vs.MCI; scRNA-seq,SRP215507,CD8+ T cell_5-MCI.vs.control; scRNA-seq,SRP309935,Erythroid cell-AD.vs.control; scRNA-seq,SRP330776,B cell_2-AD.vs.control; scRNA-seq,SRP330776,Naive CD8+ T cell_2-AD.vs.control; scRNA-seq,SRP330776,Monocyte_1-AD.vs.control; scRNA-seq,SRP330776,Megakaryocyte_2-AD.vs.control; scRNA-seq,SRP330776,Natural killer cell_2-	11
C16orf58	scRNA-seq,SRP215507,CD8+ T cell_1-AD.vs.control; scRNA-seq,SRP215507,CD8+ T cell_7-AD.vs.control; scRNA-seq,SRP215507,CD8+ T cell_3-AD.vs.MCI; scRNA-seq,SRP215507,CD8+ T cell_5-AD.vs.MCI; scRNA-seq,SRP215507,CD8+ T cell_3-MCI.vs.control; scRNA-seq,SRP330776,γδ T cell-AD.vs.control; scRNA-seq,SRP330776,Naive CD8+ T cell_1-AD.vs.control; scRNA-seq,SRP330776,Memory T cell_1-AD.vs.control; scRNA-seq,SRP330776,T cell-AD.vs.control; scRNA-seq,SRP330776,Monocyte_1-AD.vs.control; scRNA-seq,SRP330776,B cell_3-AD.vs.control; scRNA-	12
C16orf70	scRNA-seq,SRP215507,CD8+ T cell_3-AD.vs.MCI; scRNA-seq,SRP215507,CD8+ T cell_5-AD.vs.MCI; scRNA-seq,SRP215507,CD8+ T cell_6-AD.vs.MCI; scRNA-seq,SRP215507,CD8+ T cell_4-MCI.vs.control; scRNA-seq,SRP215507,CD8+ T cell_3-MCI.vs.control; scRNA-seq,SRP215507,CD8+ T cell_6-MCI.vs.control; scRNA-seq,SRP330776,Memory T cell_1-AD.vs.control; scRNA-seq,SRP330776,Naive CD8+ T cell_3-AD.vs.control; scRNA-seq,SRP330776,T cell-AD.vs.control; scRNA-seq,SRP330776,Monocyte_1-AD.vs.control	10
C16orf72	scRNA-seq,SRP215507,CD8+ T cell_1-AD.vs.MCI; scRNA-seq,SRP215507,CD8+ T cell_5-AD.vs.MCI; scRNA-seq,SRP215507,CD8+ T cell_5-MCI.vs.control; scRNA-seq,SRP309935,Naive CD8+ T cell_1-AD.vs.control; scRNA-seq,SRP309935,Naive CD8+ T cell_2-AD.vs.control; scRNA-seq,SRP309935,CD8+ T cell_1-AD.vs.control; scRNA-seq,SRP309935,B cell_1-AD.vs.control; scRNA-seq,SRP330776,γδ T cell-AD.vs.control; scRNA-seq,SRP330776,Memory T cell_2-AD.vs.control; scRNA-seq,SRP330776,CD8+ T cell_2-AD.vs.control; scRNA-seq,SRP330776,Naive CD8+ T cell_1-AD.vs.control; scRNA-seq,SRP330776,Natural killer cell_1-AD.vs.control; scRNA-seq,SRP330776,Memory T cell_1-AD.vs.control; scRNA-seq,SRP330776,Natural killer T cell-AD.vs.control; scRNA-seq,SRP330776,Naive CD8+ T cell_3-AD.vs.control; scRNA-seq,SRP330776,T cell- [illegible]	16

C16orf74	bulk RNA-seq,Homo_723,AD.vs.control; bulk RNA-seq,Homo_723,AD.vs.MCI; bulk RNA-seq,Homo_633,AD.vs.control; bulk RNA-seq,Homo_633,AD.vs.MCI; bulk RNA-seq,Homo_714,AD.vs.control; bulk RNA-seq,Homo_714,AD.vs.MCI; scRNA-seq,SRP215507,CD8+ T cell_1-AD.vs.control; scRNA-seq,SRP215507,CD8+ T cell_4-AD.vs.control; scRNA-seq,SRP215507,CD8+ T cell_6-AD.vs.control; scRNA-seq,SRP215507,CD8+ T cell_2-AD.vs.MCI; scRNA-seq,SRP215507,CD8+ T cell_3-AD.vs.MCI; scRNA-seq,SRP215507,CD8+ T cell_1-AD.vs.MCI; scRNA-seq,SRP215507,CD8+ T cell_1-MCI.vs.control; scRNA-seq,SRP215507,CD8+ T cell_3-MCI.vs.control; scRNA-seq,SRP309935,Naive CD8+ T cell_1-AD.vs.control; scRNA-seq,SRP309935,Monocyte_2-AD.vs.control; scRNA-seq,SRP309935,B cell_2-AD.vs.control; scRNA-seq,SRP330776,CD8+ T cell_1-AD.vs.control; scRNA-seq,SRP330776,B cell_1-AD.vs.control; scRNA-seq,SRP330776,Natural killer T cell-AD.vs.control; scRNA-seq,SRP330776,B cell_2-AD.vs.control; scRNA-seq,SRP330776,T cell-AD.vs.control; scRNA-seq,SRP330776,CD8+ T cell_3-AD.vs.control; scRNA- [illegible]	26
C16orf82	bulk RNA-seq,Homo_723,AD.vs.control; bulk RNA-seq,Homo_723,MCI.vs.control; bulk RNA-seq,Homo_633,AD.vs.control; bulk RNA-seq,Homo_633,MCI.vs.control; bulk RNA-seq,Homo_714,MCI.vs.control	5
C16orf86	bulk RNA-seq,Homo_723,AD.vs.control; bulk RNA-seq,Homo_633,AD.vs.control; bulk RNA-seq,Homo_633,AD.vs.MCI; bulk RNA-seq,Homo_714,AD.vs.control; bulk RNA-seq,Homo_714,AD.vs.MCI; scRNA-seq,SRP215507,CD8+ T cell_3-AD.vs.control; scRNA-seq,SRP215507,CD8+ T cell_1-AD.vs.control; scRNA-seq,SRP215507,CD8+ T cell_5-AD.vs.control; scRNA-seq,SRP215507,CD8+ T cell_2-AD.vs.MCI; scRNA-seq,SRP215507,CD8+ T cell_3-AD.vs.MCI; scRNA-seq,SRP215507,CD8+ T cell_1-AD.vs.MCI; scRNA-seq,SRP215507,CD8+ T cell_3-MCI.vs.control; scRNA-seq,SRP330776,CD8+ T cell_1-AD.vs.control; scRNA-seq,SRP330776,Memory T cell_2-AD.vs.control; scRNA-	16
C16orf87	bulk RNA-seq,Homo_723,AD.vs.control; bulk RNA-seq,Homo_723,MCI.vs.control; bulk RNA-seq,Homo_714,MCI.vs.control; scRNA-seq,SRP215507,CD8+ T cell_10-AD.vs.control; scRNA-seq,SRP215507,CD8+ T cell_5-AD.vs.MCI; scRNA-seq,SRP330776,B cell_1-AD.vs.control;	7
C16orf89	bulk RNA-seq,Homo_714,MCI.vs.control	1
C16orf91	bulk RNA-seq,Homo_714,AD.vs.control; bulk RNA-seq,Homo_714,AD.vs.MCI; scRNA-seq,SRP330776,CD8+ T cell_1-AD.vs.control; scRNA-seq,SRP330776,γδ T cell-AD.vs.control; scRNA-seq,SRP330776,Memory T cell_2-AD.vs.control; scRNA-seq,SRP330776,CD8+ T cell_2-AD.vs.control; scRNA-seq,SRP330776,B cell_1-AD.vs.control; scRNA-seq,SRP330776,Natural killer cell_1-AD.vs.control; scRNA-seq,SRP330776,Memory T cell_1-AD.vs.control; scRNA-seq,SRP330776,Natural killer T cell-AD.vs.control; scRNA-seq,SRP330776,Naive CD8+ T cell_3-AD.vs.control; scRNA-seq,SRP330776,T cell-AD.vs.control; scRNA-seq,SRP330776,CD8+ T cell_3-AD.vs.control; scRNA-seq,SRP330776,B cell_3-AD.vs.control; scRNA-seq,SRP330776,Regulatory T cell-AD.vs.control; scRNA-seq,SRP330776,Natural killer cell_2-AD.vs.control	16
C16orf95	bulk RNA-seq,Homo_723,AD.vs.control; bulk RNA-seq,Homo_723,AD.vs.MCI; bulk RNA-seq,Homo_633,AD.vs.control; bulk RNA-seq,Homo_633,AD.vs.MCI; bulk RNA-seq,Homo_714,AD.vs.control; bulk RNA-seq,Homo_714,AD.vs.MCI; bulk RNA-seq,Homo_714,MCI.vs.control; scRNA-seq,SRP215507,CD8+ T cell_3-AD.vs.MCI; scRNA-seq,SRP215507,CD8+ T cell_3-MCI.vs.control; scRNA-seq,SRP330776,CD8+ T cell_1-AD.vs.control; scRNA-seq,SRP330776,Memory T cell_2-AD.vs.control; scRNA-seq,SRP330776,CD8+ T cell_2-AD.vs.control; scRNA-seq,SRP330776,B cell_1-AD.vs.control; scRNA-seq,SRP330776,Naive CD8+ T cell_1-AD.vs.control; scRNA-seq,SRP330776,Natural killer T cell-AD.vs.control; scRNA-seq,SRP330776,Naive CD8+ T cell_3-AD.vs.control; scRNA-seq,SRP330776,CD8+ T cell_3-AD.vs.control; scRNA-seq,SRP330776,B cell_4- [illegible]	18
C16orf96	bulk RNA-seq,Homo_723,AD.vs.control; bulk RNA-seq,Homo_723,AD.vs.MCI; bulk RNA-seq,Homo_633,AD.vs.control; bulk RNA-seq,Homo_633,MCI.vs.control; bulk RNA-seq,Homo_714,MCI.vs.control; bulk RNA-seq,ROSMAP,AD.vs.control; bulk RNA-	7
C17orf100	scRNA-seq,SRP215507,CD8+ T cell_1-AD.vs.control; scRNA-seq,SRP215507,CD8+ T cell_2-AD.vs.MCI; scRNA-seq,SRP215507,CD8+ T cell_3-MCI.vs.control; scRNA-seq,SRP330776,CD8+ T cell_1-AD.vs.control; scRNA-seq,SRP330776,Memory T cell_2-AD.vs.control; scRNA-seq,SRP330776,B cell_1-AD.vs.control; scRNA-seq,SRP330776,Naive CD8+ T cell_1-AD.vs.control; scRNA-seq,SRP330776,B cell_2-AD.vs.control; scRNA-seq,SRP330776,Naive CD8+ T cell_2-AD.vs.control; scRNA-seq,SRP330776,CD8+ T cell_3-AD.vs.control	10
C17orf107	scRNA-seq,SRP330776,CD8+ T cell_1-AD.vs.control; scRNA-seq,SRP330776,Naive CD8+ T cell_1-AD.vs.control	2
C17orf113	bulk RNA-seq,Homo_723,AD.vs.control; bulk RNA-seq,Homo_723,AD.vs.MCI; bulk RNA-seq,Homo_633,AD.vs.control; bulk RNA-seq,Homo_633,AD.vs.MCI; bulk RNA-seq,Homo_714,AD.vs.control; bulk RNA-seq,Homo_714,AD.vs.MCI; scRNA-seq,SRP215507,CD8+ T cell_2-AD.vs.control; scRNA-seq,SRP215507,CD8+ T cell_5-MCI.vs.control; scRNA-seq,SRP330776,CD8+ T cell_1-AD.vs.control; scRNA-	10
C17orf49	bulk RNA-seq,Homo_633,AD.vs.control; bulk RNA-seq,Homo_714,AD.vs.control; bulk RNA-seq,Homo_714,AD.vs.MCI; scRNA-seq,SRP215507,CD8+ T cell_4-AD.vs.control; scRNA-seq,SRP215507,CD8+ T cell_6-AD.vs.MCI; scRNA-seq,SRP309935,Erythroid cell-AD.vs.control; scRNA-	7
C17orf50	bulk RNA-seq,Homo_723,MCI.vs.control; bulk RNA-seq,Homo_633,MCI.vs.control; bulk RNA-seq,Homo_714,MCI.vs.control	3

C17orf53	scRNA-seq,SRP215507,CD8+ T cell_3-MCI.vs.control; scRNA-seq,SRP330776,CD8+ T cell_1-AD.vs.control; scRNA-seq,SRP330776,Natural killer	3
C17orf58	bulk RNA-seq,Homo_723,AD.vs.control; bulk RNA-seq,Homo_723,AD.vs.MCI; bulk RNA-seq,Homo_633,AD.vs.control; bulk RNA-seq,Homo_633,AD.vs.MCI; scRNA-seq,SRP215507,CD8+ T cell_1-AD.vs.MCI; scRNA-seq,SRP215507,CD8+ T cell_1-MCI.vs.control; scRNA-seq,SRP330776,CD8+ T cell_1-AD.vs.control; scRNA-seq,SRP330776,γδ T cell-AD.vs.control; scRNA-seq,SRP330776,Memory T cell_2-AD.vs.control; scRNA-seq,SRP330776,CD8+ T cell_2-AD.vs.control; scRNA-seq,SRP330776,B cell_1-AD.vs.control; scRNA-seq,SRP330776,Memory T cell_1-AD.vs.control; scRNA-seq,SRP330776,CD8+ T cell_3-AD.vs.control; scRNA-seq,SRP330776,Natural killer cell_2-AD.vs.control; scRNA-	15
C17orf67	bulk RNA-seq,Homo_723,AD.vs.control; bulk RNA-seq,Homo_723,AD.vs.MCI; bulk RNA-seq,Homo_633,AD.vs.control; bulk RNA-seq,Homo_633,AD.vs.MCI; bulk RNA-seq,Homo_714,AD.vs.control; bulk RNA-seq,Homo_714,AD.vs.MCI; scRNA-seq,SRP215507,CD8+ T cell_2-AD.vs.control; scRNA-seq,SRP215507,CD8+ T cell_3-AD.vs.control; scRNA-seq,SRP309935,Naive CD8+ T cell_1-AD.vs.control; scRNA-seq,SRP309935,Natural killer cell_1-AD.vs.control; scRNA-seq,SRP309935,Monocyte_2-AD.vs.control; scRNA-seq,SRP330776,CD8+ T cell_1-AD.vs.control; scRNA-seq,SRP330776,Memory T cell_2-AD.vs.control; scRNA-seq,SRP330776,CD8+ T cell_2-AD.vs.control; scRNA-seq,SRP330776,B cell_1-AD.vs.control; scRNA-seq,SRP330776,Naive CD8+ T cell_1-AD.vs.control; scRNA-seq,SRP330776,Natural killer cell_1-AD.vs.control; scRNA-seq,SRP330776,Memory T cell_1-AD.vs.control; scRNA-seq,SRP330776,Naive CD8+ T cell_3-AD.vs.control; scRNA-seq,SRP330776,T cell- [illegible]	21
C17orf75	scRNA-seq,SRP330776,CD8+ T cell_1-AD.vs.control; scRNA-seq,SRP330776,γδ T cell-AD.vs.control; scRNA-seq,SRP330776,CD8+ T cell_2-AD.vs.control; scRNA-seq,SRP330776,B cell_1-AD.vs.control; scRNA-seq,SRP330776,Natural killer cell_1-AD.vs.control; scRNA-seq,SRP330776,Memory T cell_1-AD.vs.control; scRNA-seq,SRP330776,Natural killer T cell-AD.vs.control; scRNA-seq,SRP330776,T cell-AD.vs.control;	9
C17orf78	bulk RNA-seq,Homo_723,AD.vs.control; bulk RNA-seq,Homo_723,AD.vs.MCI; bulk RNA-seq,Homo_633,AD.vs.control; bulk RNA-	4
C17orf80	bulk RNA-seq,Homo_633,AD.vs.MCI; bulk RNA-seq,Homo_633,MCI.vs.control; scRNA-seq,SRP215507,CD8+ T cell_2-MCI.vs.control; scRNA-seq,SRP330776,CD8+ T cell_1-AD.vs.control; scRNA-seq,SRP330776,Memory T cell_2-AD.vs.control; scRNA-seq,SRP330776,CD8+ T cell_2-AD.vs.control; scRNA-seq,SRP330776,B cell_2-AD.vs.control; scRNA-seq,SRP330776,CD8+ T cell_3-AD.vs.control	8
C17orf97	scRNA-seq,SRP215507,CD8+ T cell_3-AD.vs.control; scRNA-seq,SRP215507,CD8+ T cell_1-AD.vs.control; scRNA-seq,SRP215507,CD8+ T cell_3-AD.vs.MCI; scRNA-seq,SRP215507,CD8+ T cell_3-MCI.vs.control; scRNA-seq,SRP309935,Monocyte_1-AD.vs.control; scRNA-seq,SRP309935,Megakaryocyte_1-AD.vs.control; scRNA-seq,SRP309935,Naive CD8+ T cell_1-AD.vs.control; scRNA-seq,SRP309935,Natural killer cell_1-AD.vs.control; scRNA-seq,SRP309935,Monocyte_2-AD.vs.control; scRNA-seq,SRP309935,Naive CD8+ T cell_2-AD.vs.control; scRNA-seq,SRP309935,CD8+ T cell_1-AD.vs.control; scRNA-seq,SRP309935,B cell_1-AD.vs.control; scRNA-seq,SRP309935,CD8+ T cell_2-AD.vs.control; scRNA-seq,SRP309935,Naive CD8+ T cell_3-AD.vs.control; scRNA-seq,SRP330776,CD8+ T cell_1-AD.vs.control; scRNA-seq,SRP330776,γδ T cell-AD.vs.control; scRNA-seq,SRP330776,Naive CD8+ T cell_1-AD.vs.control; scRNA-seq,SRP330776,Natural killer cell_1-AD.vs.control; scRNA- [illegible]	19
C17orf99	bulk RNA-seq,Homo_723,AD.vs.MCI; bulk RNA-seq,Homo_633,AD.vs.control; bulk RNA-seq,Homo_633,AD.vs.MCI; bulk RNA-	4
C18orf21	bulk RNA-seq,Homo_723,AD.vs.control; bulk RNA-seq,Homo_633,AD.vs.control; bulk RNA-seq,Homo_714,AD.vs.control; bulk RNA-seq,Homo_714,AD.vs.MCI; scRNA-seq,SRP215507,CD8+ T cell_1-AD.vs.MCI; scRNA-seq,SRP215507,CD8+ T cell_4-AD.vs.MCI; scRNA-seq,SRP215507,CD8+ T cell_7-AD.vs.MCI; scRNA-seq,SRP215507,CD8+ T cell_5-AD.vs.MCI; scRNA-seq,SRP215507,CD8+ T cell_4-MCI.vs.control; scRNA-seq,SRP215507,CD8+ T cell_7-MCI.vs.control; scRNA-seq,SRP309935,Naive CD8+ T cell_2-AD.vs.control; scRNA-seq,SRP309935,CD8+ T	13
C18orf25	bulk RNA-seq,Homo_714,AD.vs.control; bulk RNA-seq,Homo_714,AD.vs.MCI; scRNA-seq,SRP215507,CD8+ T cell_3-AD.vs.MCI; scRNA-seq,SRP215507,CD8+ T cell_4-MCI.vs.control; scRNA-seq,SRP215507,CD8+ T cell_3-MCI.vs.control; scRNA-seq,SRP215507,CD8+ T cell_6-MCI.vs.control; scRNA-seq,SRP330776,Megakaryocyte_1-AD.vs.control	7
C18orf32	scRNA-seq,SRP215507,CD8+ T cell_8-AD.vs.control; scRNA-seq,SRP215507,CD8+ T cell_10-AD.vs.control; scRNA-seq,SRP309935,Erythroid cell-AD.vs.control; scRNA-seq,SRP330776,γδ T cell-AD.vs.control; scRNA-seq,SRP330776,Natural killer cell_1-AD.vs.control	5
C18orf54	bulk RNA-seq,Homo_723,AD.vs.control; bulk RNA-seq,Homo_723,MCI.vs.control; bulk RNA-seq,Homo_633,AD.vs.control; bulk RNA-seq,Homo_633,MCI.vs.control; bulk RNA-seq,ROSMAP,MCI.vs.control; scRNA-seq,SRP215507,CD8+ T cell_2-AD.vs.MCI; scRNA-seq,SRP215507,CD8+ T cell_2-MCI.vs.control; scRNA-seq,SRP330776,CD8+ T cell_2-AD.vs.control	8

C19orf12	bulk RNA-seq,Homo_714,MCI.vs.control; scRNA-seq,SRP215507,CD8+ T cell_5-AD.vs.control; scRNA-seq,SRP215507,CD8+ T cell_5-AD.vs.MCI; scRNA-seq,SRP330776,CD8+ T cell_1-AD.vs.control; scRNA-seq,SRP330776,γδ T cell-AD.vs.control; scRNA-seq,SRP330776,Memory T cell_2-AD.vs.control; scRNA-seq,SRP330776,CD8+ T cell_2-AD.vs.control; scRNA-seq,SRP330776,B cell_1-AD.vs.control; scRNA-seq,SRP330776,Naive CD8+ T cell_1-AD.vs.control; scRNA-seq,SRP330776,Natural killer cell_1-AD.vs.control; scRNA-seq,SRP330776,Memory T cell_1-AD.vs.control; scRNA-seq,SRP330776,Natural killer T cell-AD.vs.control; scRNA-seq,SRP330776,Naive CD8+ T cell_3-AD.vs.control; scRNA-seq,SRP330776,B cell_2-AD.vs.control; scRNA-seq,SRP330776,T cell-AD.vs.control; scRNA-seq,SRP330776,CD8+ T cell_3-AD.vs.control; scRNA-seq,SRP330776,Monocyte_1-	17
C19orf18	bulk RNA-seq,Homo_723,MCI.vs.control; bulk RNA-seq,Homo_633,AD.vs.control; bulk RNA-seq,Homo_633,MCI.vs.control; bulk RNA-seq,Homo_714,AD.vs.control; bulk RNA-seq,Homo_714,MCI.vs.control; scRNA-seq,SRP215507,CD8+ T cell_2-MCI.vs.control	6
C19orf25	bulk RNA-seq,Homo_723,AD.vs.control; bulk RNA-seq,Homo_723,AD.vs.MCI; bulk RNA-seq,Homo_633,AD.vs.control; bulk RNA-seq,Homo_633,AD.vs.MCI; bulk RNA-seq,Homo_714,AD.vs.control; bulk RNA-seq,Homo_714,AD.vs.MCI; scRNA-seq,SRP215507,CD8+ T cell_7-MCI.vs.control; scRNA-seq,SRP330776,CD8+ T cell_1-AD.vs.control; scRNA-seq,SRP330776,γδ T cell-AD.vs.control; scRNA-seq,SRP330776,Memory T cell_2-AD.vs.control; scRNA-seq,SRP330776,B cell_1-AD.vs.control; scRNA-seq,SRP330776,Naive CD8+ T cell_1-AD.vs.control; scRNA-seq,SRP330776,Natural killer cell_1-AD.vs.control; scRNA-seq,SRP330776,Memory T cell_1-AD.vs.control; scRNA-seq,SRP330776,Naive CD8+ T cell_3-AD.vs.control; scRNA-seq,SRP330776,B cell_2-AD.vs.control; scRNA-seq,SRP330776,T cell-AD.vs.control; scRNA-seq,SRP330776,CD8+ T	20
C19orf33	bulk RNA-seq,Homo_723,AD.vs.control; bulk RNA-seq,Homo_723,AD.vs.MCI; bulk RNA-seq,Homo_723,MCI.vs.control; bulk RNA-seq,Homo_633,AD.vs.control; bulk RNA-seq,Homo_633,AD.vs.MCI; bulk RNA-seq,Homo_633,MCI.vs.control; bulk RNA-seq,Homo_714,AD.vs.control;	8
C19orf38	scRNA-seq,SRP215507,CD8+ T cell_3-AD.vs.control; scRNA-seq,SRP215507,CD8+ T cell_9-AD.vs.control; scRNA-seq,SRP330776,CD8+ T cell_1-AD.vs.control; scRNA-seq,SRP330776,Naive CD8+ T cell_1-AD.vs.control; scRNA-seq,SRP330776,Natural killer cell_2-AD.vs.control	5
C19orf44	bulk RNA-seq,Homo_723,AD.vs.control; bulk RNA-seq,Homo_723,AD.vs.MCI; bulk RNA-seq,Homo_633,AD.vs.control; bulk RNA-seq,Homo_633,AD.vs.MCI; bulk RNA-seq,Homo_714,AD.vs.control; bulk RNA-seq,Homo_714,AD.vs.MCI; bulk RNA-seq,Homo_714,MCI.vs.control; scRNA-seq,SRP215507,CD8+ T cell_4-MCI.vs.control; scRNA-seq,SRP215507,CD8+ T cell_2-MCI.vs.control; scRNA-seq,SRP330776,γδ T cell-	11
C19orf47	bulk RNA-seq,Homo_723,AD.vs.control; bulk RNA-seq,Homo_633,AD.vs.control; bulk RNA-seq,Homo_633,AD.vs.MCI; bulk RNA-seq,Homo_714,AD.vs.control; bulk RNA-seq,Homo_714,AD.vs.MCI; scRNA-seq,SRP215507,CD8+ T cell_2-AD.vs.MCI; scRNA-seq,SRP215507,CD8+ T cell_2-MCI.vs.control; scRNA-seq,SRP330776,CD8+ T cell_1-AD.vs.control; scRNA-seq,SRP330776,Memory T cell_2-AD.vs.control; scRNA-seq,SRP330776,CD8+ T cell_2-AD.vs.control; scRNA-seq,SRP330776,B cell_1-AD.vs.control; scRNA-seq,SRP330776,Naive CD8+ T cell_1-AD.vs.control; scRNA-seq,SRP330776,Natural killer cell_1-AD.vs.control; scRNA-seq,SRP330776,Memory T cell_1-AD.vs.control; scRNA-seq,SRP330776,Natural killer T cell-AD.vs.control; scRNA-seq,SRP330776,Naive CD8+ T cell_3-AD.vs.control; scRNA-seq,SRP330776,B cell_2-AD.vs.control; scRNA-seq,SRP330776,T cell-AD.vs.control; scRNA-seq,SRP330776,CD8+ T cell_3-AD.vs.control; scRNA-seq,SRP330776,B cell_3-	20
C19orf48	scRNA-seq,SRP215507,CD8+ T cell_1-AD.vs.MCI; scRNA-seq,SRP215507,CD8+ T cell_5-AD.vs.MCI; scRNA-seq,SRP330776,T cell-AD.vs.control; scRNA-seq,SRP330776,Naive CD8+ T cell_2-AD.vs.control; scRNA-seq,SRP330776,Natural killer cell_2-AD.vs.control	5
C19orf53	bulk RNA-seq,Homo_723,AD.vs.control; bulk RNA-seq,Homo_633,AD.vs.control; bulk RNA-seq,Homo_633,AD.vs.MCI; scRNA-seq,SRP215507,CD8+ T cell_4-AD.vs.control; scRNA-seq,SRP309935,Erythroid cell-AD.vs.control; scRNA-seq,SRP330776,Monocyte_2-AD.vs.control; scRNA-seq,SRP330776,Megakaryocyte_1-AD.vs.control	7
C19orf54	bulk RNA-seq,Homo_723,AD.vs.control; bulk RNA-seq,Homo_723,MCI.vs.control; bulk RNA-seq,Homo_633,AD.vs.MCI; bulk RNA-seq,Homo_633,MCI.vs.control; bulk RNA-seq,Homo_714,AD.vs.control; bulk RNA-seq,Homo_714,AD.vs.MCI; bulk RNA-seq,Homo_714,MCI.vs.control; scRNA-seq,SRP215507,CD8+ T cell_5-AD.vs.MCI; scRNA-seq,SRP215507,CD8+ T cell_4-MCI.vs.control; scRNA-seq,SRP215507,CD8+ T cell_6-MCI.vs.control; scRNA-seq,SRP330776,γδ T cell-AD.vs.control; scRNA-seq,SRP330776,Memory T cell_2-AD.vs.control; scRNA-seq,SRP330776,Natural killer cell_1-AD.vs.control; scRNA-seq,SRP330776,Naive CD8+ T cell_3-AD.vs.control; scRNA-seq,SRP330776,CD8+ T	16
C19orf57	scRNA-seq,SRP215507,CD8+ T cell_1-AD.vs.control; scRNA-seq,SRP215507,CD8+ T cell_6-MCI.vs.control; scRNA-seq,SRP330776,Naive CD8+ T cell_1-AD.vs.control; scRNA-seq,SRP330776,Memory T cell_1-AD.vs.control	4
C19orf67	bulk RNA-seq,Homo_723,MCI.vs.control; bulk RNA-seq,Homo_633,AD.vs.MCI; bulk RNA-seq,Homo_633,MCI.vs.control; bulk RNA-	4
C19orf73	scRNA-seq,SRP330776,γδ T cell-AD.vs.control; scRNA-seq,SRP330776,Memory T cell_2-AD.vs.control; scRNA-seq,SRP330776,B cell_1-AD.vs.control; scRNA-seq,SRP330776,Natural killer T cell-AD.vs.control	4

C19orf81	scRNA-seq,SRP215507,CD8+ T cell_3-AD.vs.control; scRNA-seq,SRP215507,CD8+ T cell_3-AD.vs.MCI	2
C19orf84	scRNA-seq,SRP330776,Memory T cell_1-AD.vs.control	1
C1D	scRNA-seq,SRP215507,CD8+ T cell_1-AD.vs.MCI; scRNA-seq,SRP215507,CD8+ T cell_4-MCI.vs.control; scRNA-seq,SRP309935,Naive CD8+ T cell_3-AD.vs.control; scRNA-seq,SRP330776,Natural killer cell_2-AD.vs.control	4
C1GALT1	bulk RNA-seq,Homo_633,AD.vs.control; scRNA-seq,SRP309935,Naive CD8+ T cell_2-AD.vs.control; scRNA-seq,SRP330776,CD8+ T cell_1-AD.vs.control; scRNA-seq,SRP330776,Memory T cell_2-AD.vs.control; scRNA-seq,SRP330776,CD8+ T cell_2-AD.vs.control; scRNA-seq,SRP330776,B cell_1-AD.vs.control; scRNA-seq,SRP330776,Naive CD8+ T cell_1-AD.vs.control; scRNA-seq,SRP330776,Natural killer T cell-AD.vs.control; scRNA-seq,SRP330776,Naive CD8+ T cell_3-AD.vs.control; scRNA-seq,SRP330776,T cell-AD.vs.control; scRNA-seq,SRP330776,Naive CD8+ T cell_2-AD.vs.control; scRNA-seq,SRP330776,CD8+ T cell_3-AD.vs.control; scRNA-seq,SRP330776,Monocyte_2-AD.vs.control; scRNA-seq,SRP330776,B cell_3-AD.vs.control; scRNA-seq,SRP330776,Natural killer cell_2-AD.vs.control; scRNA-seq,SRP330776,B cell_4-AD.vs.control	16
C1GALT1C1	scRNA-seq,SRP215507,CD8+ T cell_10-AD.vs.control; scRNA-seq,SRP215507,CD8+ T cell_4-MCI.vs.control; scRNA-seq,SRP215507,CD8+ T cell_3-MCI.vs.control; scRNA-seq,SRP330776,CD8+ T cell_1-AD.vs.control; scRNA-seq,SRP330776,γδ T cell-AD.vs.control; scRNA-seq,SRP330776,Memory T cell_2-AD.vs.control; scRNA-seq,SRP330776,CD8+ T cell_2-AD.vs.control; scRNA-seq,SRP330776,B cell_1-AD.vs.control; scRNA-seq,SRP330776,Naive CD8+ T cell_1-AD.vs.control; scRNA-seq,SRP330776,Natural killer cell_1-AD.vs.control; scRNA-seq,SRP330776,Memory T cell_1-AD.vs.control; scRNA-seq,SRP330776,Natural killer T cell-AD.vs.control; scRNA-seq,SRP330776,Naive CD8+ T cell_3-AD.vs.control; scRNA-seq,SRP330776,B cell_2-AD.vs.control; scRNA-seq,SRP330776,T cell-AD.vs.control; scRNA-seq,SRP330776,CD8+ T cell_3-AD.vs.control	16
C1orf100	bulk RNA-seq,Homo_723,AD.vs.control; bulk RNA-seq,Homo_723,AD.vs.MCI; bulk RNA-seq,Homo_633,AD.vs.control; bulk RNA-seq,Homo_633,AD.vs.MCI; bulk RNA-seq,Homo_714,AD.vs.control; bulk RNA-seq,Homo_714,AD.vs.MCI	6
C1orf105	bulk RNA-seq,ROSMAP,AD.vs.control	1
C1orf109	bulk RNA-seq,ROSMAP,MCI.vs.control; scRNA-seq,SRP215507,CD8+ T cell_5-AD.vs.MCI; scRNA-seq,SRP215507,CD8+ T cell_6-MCI.vs.control; scRNA-seq,SRP330776,CD8+ T cell_1-AD.vs.control; scRNA-seq,SRP330776,Memory T cell_2-AD.vs.control; scRNA-seq,SRP330776,CD8+ T cell_2-AD.vs.control; scRNA-seq,SRP330776,Naive CD8+ T cell_1-AD.vs.control; scRNA-seq,SRP330776,Natural killer cell_1-AD.vs.control; scRNA-seq,SRP330776,Memory T cell_1-AD.vs.control; scRNA-seq,SRP330776,Naive CD8+ T cell_3-AD.vs.control	10
C1orf112	bulk RNA-seq,Homo_723,AD.vs.control; bulk RNA-seq,Homo_723,AD.vs.MCI; bulk RNA-seq,Homo_633,AD.vs.control; bulk RNA-seq,Homo_633,AD.vs.MCI; bulk RNA-seq,Homo_714,AD.vs.control; bulk RNA-seq,Homo_714,AD.vs.MCI; bulk RNA-seq,ROSMAP,AD.vs.control; scRNA-seq,SRP215507,CD8+ T cell_6-MCI.vs.control; scRNA-seq,SRP330776,γδ T cell-AD.vs.control; scRNA-seq,SRP330776,Natural killer cell_1-AD.vs.control; scRNA-seq,SRP330776,Natural killer T cell-AD.vs.control; scRNA-seq,SRP330776,B cell_2-AD.vs.control	12
C1orf115	bulk RNA-seq,Homo_723,AD.vs.control; bulk RNA-seq,Homo_723,AD.vs.MCI; bulk RNA-seq,Homo_723,MCI.vs.control; bulk RNA-seq,Homo_633,AD.vs.control; bulk RNA-seq,Homo_633,AD.vs.MCI; bulk RNA-seq,Homo_633,MCI.vs.control; bulk RNA-seq,Homo_714,AD.vs.control; bulk RNA-seq,Homo_714,AD.vs.MCI; bulk RNA-seq,ROSMAP,MCI.vs.control; bulk RNA-seq,SRP223445,AD.vs.control; scRNA-seq,SRP215507,CD8+ T cell_1-AD.vs.control; scRNA-seq,SRP309935,B cell_1-AD.vs.control; scRNA-seq,SRP330776,CD8+ T cell_1-AD.vs.control; scRNA-seq,SRP330776,B cell_1-AD.vs.control; scRNA-seq,SRP330776,Naive CD8+ T cell_1-AD.vs.control; scRNA-seq,SRP330776,B cell_2-AD.vs.control	16
C1orf116	bulk RNA-seq,Homo_723,AD.vs.control; bulk RNA-seq,Homo_723,AD.vs.MCI; bulk RNA-seq,Homo_633,AD.vs.control; bulk RNA-seq,Homo_633,AD.vs.MCI; bulk RNA-seq,Homo_714,AD.vs.control; bulk RNA-seq,Homo_714,AD.vs.MCI	6
C1orf122	bulk RNA-seq,Homo_723,MCI.vs.control; bulk RNA-seq,Homo_633,AD.vs.control; bulk RNA-seq,Homo_633,MCI.vs.control; bulk RNA-seq,Homo_714,AD.vs.control; bulk RNA-seq,Homo_714,AD.vs.MCI; bulk RNA-seq,Homo_714,MCI.vs.control; scRNA-seq,SRP215507,CD8+ T cell_5-MCI.vs.control; scRNA-seq,SRP309935,Erythroid cell-AD.vs.control; scRNA-seq,SRP330776,CD8+ T cell_1-AD.vs.control; scRNA-seq,SRP330776,γδ T cell-AD.vs.control; scRNA-seq,SRP330776,Memory T cell_2-AD.vs.control; scRNA-seq,SRP330776,CD8+ T cell_2-AD.vs.control; scRNA-seq,SRP330776,B cell_1-AD.vs.control; scRNA-seq,SRP330776,Naive CD8+ T cell_1-AD.vs.control; scRNA-seq,SRP330776,Natural killer cell_1-AD.vs.control; scRNA-seq,SRP330776,Memory T cell_1-AD.vs.control; scRNA-seq,SRP330776,Natural killer T cell-AD.vs.control; scRNA-seq,SRP330776,Naive CD8+ T cell_3-AD.vs.control; scRNA-seq,SRP330776,B cell_2-AD.vs.control; scRNA-seq,SRP330776,T cell-AD.vs.control; scRNA-seq,SRP330776,CD8+ T cell_3-AD.vs.control; scRNA-seq,SRP330776,Monocyte_1-AD.vs.control; scRNA-seq,SRP330776,Monocyte_2-AD.vs.control; scRNA-seq,SRP330776,Natural killer cell_2-AD.vs.control	24
C1orf127	bulk RNA-seq,Homo_723,MCI.vs.control; bulk RNA-seq,Homo_633,MCI.vs.control; bulk RNA-seq,Homo_714,AD.vs.MCI; bulk RNA-	4

C1orf131	bulk RNA-seq,Homo_633,AD.vs.control; bulk RNA-seq,Homo_714,AD.vs.control; bulk RNA-seq,Homo_714,AD.vs.MCI; scRNA-seq,SRP215507,CD8+ T cell_3-AD.vs.control; scRNA-seq,SRP215507,CD8+ T cell_6-AD.vs.control; scRNA-seq,SRP215507,CD8+ T cell_6-AD.vs.MCI; scRNA-seq,SRP215507,CD8+ T cell_3-MCI.vs.control; scRNA-seq,SRP330776,CD8+ T cell_1-AD.vs.control; scRNA-seq,SRP330776,γδ T cell-AD.vs.control; scRNA-seq,SRP330776,B cell_1-AD.vs.control; scRNA-seq,SRP330776,Naive CD8+ T cell_1-AD.vs.control; scRNA-seq,SRP330776,Memory T cell_1-AD.vs.control; scRNA-seq,SRP330776,B cell_2-AD.vs.control; scRNA-seq,SRP330776,Monocyte_2-AD.vs.control; scRNA-seq,SRP330776,B cell_3-	16
C1orf146	bulk RNA-seq,Homo_723,AD.vs.control; bulk RNA-seq,Homo_723,MCI.vs.control; bulk RNA-seq,Homo_633,AD.vs.control; bulk RNA-seq,Homo_633,MCI.vs.control; bulk RNA-seq,Homo_714,MCI.vs.control	5
C1orf158	bulk RNA-seq,Homo_723,AD.vs.control	1
C1orf159	bulk RNA-seq,Homo_723,AD.vs.control; bulk RNA-seq,Homo_723,AD.vs.MCI; bulk RNA-seq,Homo_723,MCI.vs.control; bulk RNA-seq,Homo_633,AD.vs.control; bulk RNA-seq,Homo_633,AD.vs.MCI; bulk RNA-seq,Homo_633,MCI.vs.control; bulk RNA-seq,Homo_714,AD.vs.control; bulk RNA-seq,Homo_714,AD.vs.MCI; bulk RNA-seq,Homo_714,MCI.vs.control; scRNA-seq,SRP309935,Monocyte_2-AD.vs.control; scRNA-seq,SRP330776,CD8+ T cell_1-AD.vs.control; scRNA-seq,SRP330776,Memory T cell_2-AD.vs.control; scRNA-seq,SRP330776,B cell_1-AD.vs.control; scRNA-seq,SRP330776,Natural killer cell_1-AD.vs.control; scRNA-seq,SRP330776,Memory T cell_1-AD.vs.control; scRNA-seq,SRP330776,Naive CD8+	16
C1orf162	scRNA-seq,SRP215507,CD8+ T cell_2-AD.vs.control; scRNA-seq,SRP215507,CD8+ T cell_1-AD.vs.control; scRNA-seq,SRP215507,CD8+ T cell_10-AD.vs.control; scRNA-seq,SRP215507,CD8+ T cell_2-MCI.vs.control; scRNA-seq,SRP309935,Natural killer cell_1-AD.vs.control; scRNA-seq,SRP330776,Memory T cell_2-AD.vs.control; scRNA-seq,SRP330776,B cell_1-AD.vs.control; scRNA-seq,SRP330776,Natural killer cell_1-	9
C1orf167	bulk RNA-seq,Homo_723,AD.vs.control; bulk RNA-seq,Homo_633,AD.vs.control; bulk RNA-seq,Homo_714,MCI.vs.control; scRNA-seq,SRP330776,Memory T cell_1-AD.vs.control	4
C1orf174	scRNA-seq,SRP215507,CD8+ T cell_5-AD.vs.control; scRNA-seq,SRP330776,Monocyte_2-AD.vs.control	2
C1orf189	bulk RNA-seq,Homo_723,AD.vs.MCI; bulk RNA-seq,Homo_723,MCI.vs.control; bulk RNA-seq,Homo_633,AD.vs.control; bulk RNA-seq,Homo_633,AD.vs.MCI; bulk RNA-seq,Homo_633,MCI.vs.control; bulk RNA-seq,Homo_714,MCI.vs.control	6
C1orf194	bulk RNA-seq,Homo_723,AD.vs.control; bulk RNA-seq,Homo_723,AD.vs.MCI; bulk RNA-seq,Homo_633,AD.vs.control; bulk RNA-	4
C1orf198	bulk RNA-seq,Homo_723,AD.vs.control; bulk RNA-seq,Homo_723,AD.vs.MCI; bulk RNA-seq,Homo_723,MCI.vs.control; bulk RNA-seq,Homo_633,AD.vs.control; bulk RNA-seq,Homo_633,AD.vs.MCI; bulk RNA-seq,Homo_633,MCI.vs.control; bulk RNA-seq,Homo_714,AD.vs.control; bulk RNA-seq,Homo_714,AD.vs.MCI; bulk RNA-seq,SRP223445,AD.vs.control; scRNA-seq,SRP330776,CD8+ T cell_1-AD.vs.control; scRNA-seq,SRP330776,Memory T cell_2-AD.vs.control; scRNA-seq,SRP330776,B cell_1-AD.vs.control; scRNA-seq,SRP330776,Naive CD8+ T cell_1-	13
C1orf21	bulk RNA-seq,Homo_723,AD.vs.control; bulk RNA-seq,Homo_723,AD.vs.MCI; bulk RNA-seq,Homo_723,MCI.vs.control; bulk RNA-seq,Homo_633,AD.vs.control; bulk RNA-seq,Homo_633,AD.vs.MCI; bulk RNA-seq,Homo_714,AD.vs.control; bulk RNA-seq,Homo_714,AD.vs.MCI; scRNA-seq,SRP215507,CD8+ T cell_3-AD.vs.control; scRNA-seq,SRP215507,CD8+ T cell_7-AD.vs.control; scRNA-seq,SRP215507,CD8+ T cell_5-AD.vs.control; scRNA-seq,SRP215507,CD8+ T cell_2-AD.vs.MCI; scRNA-seq,SRP215507,CD8+ T cell_3-AD.vs.MCI; scRNA-seq,SRP215507,CD8+ T cell_4-AD.vs.MCI; scRNA-seq,SRP215507,CD8+ T cell_5-AD.vs.MCI; scRNA-seq,SRP215507,CD8+ T cell_6-AD.vs.MCI; scRNA-seq,SRP215507,CD8+ T cell_4-MCI.vs.control; scRNA-seq,SRP215507,CD8+ T cell_6-MCI.vs.control; scRNA-seq,SRP330776,Naive CD8+ T cell_1-	19
C1orf210	bulk RNA-seq,Homo_723,AD.vs.control; bulk RNA-seq,Homo_723,AD.vs.MCI; scRNA-seq,SRP215507,CD8+ T cell_1-MCI.vs.control	3
C1orf216	bulk RNA-seq,Homo_723,AD.vs.control; bulk RNA-seq,Homo_633,AD.vs.control; bulk RNA-seq,Homo_714,AD.vs.control; bulk RNA-seq,Homo_714,AD.vs.MCI; bulk RNA-seq,SRP223445,AD.vs.control; scRNA-seq,SRP215507,CD8+ T cell_6-AD.vs.control; scRNA-seq,SRP330776,CD8+ T cell_1-AD.vs.control; scRNA-seq,SRP330776,γδ T cell-AD.vs.control; scRNA-seq,SRP330776,Memory T cell_2-AD.vs.control; scRNA-seq,SRP330776,CD8+ T cell_2-AD.vs.control; scRNA-seq,SRP330776,Naive CD8+ T cell_1-AD.vs.control; scRNA-seq,SRP330776,Memory T cell_1-AD.vs.control; scRNA-seq,SRP330776,Natural killer T cell-AD.vs.control; scRNA-seq,SRP330776,CD8+ T cell_3-AD.vs.control; scRNA-	16
C1orf220	scRNA-seq,SRP330776,B cell_1-AD.vs.control; scRNA-seq,SRP330776,B cell_2-AD.vs.control	2

C1orf35	bulk RNA-seq,Homo_723,AD.vs.control; scRNA-seq,SRP215507,CD8+ T cell_1-MCI.vs.control; scRNA-seq,SRP215507,CD8+ T cell_5-MCI.vs.control; scRNA-seq,SRP330776,CD8+ T cell_1-AD.vs.control; scRNA-seq,SRP330776,γδ T cell-AD.vs.control; scRNA-seq,SRP330776,Memory T cell_2-AD.vs.control; scRNA-seq,SRP330776,CD8+ T cell_2-AD.vs.control; scRNA-seq,SRP330776,B cell_1-AD.vs.control; scRNA-seq,SRP330776,Naive CD8+ T cell_1-AD.vs.control; scRNA-seq,SRP330776,Natural killer cell_1-AD.vs.control; scRNA-seq,SRP330776,Memory T cell_1-AD.vs.control; scRNA-seq,SRP330776,Natural killer T cell-AD.vs.control; scRNA-seq,SRP330776,B cell_2-AD.vs.control; scRNA-seq,SRP330776,T cell-AD.vs.control; scRNA-seq,SRP330776,CD8+ T cell_3-AD.vs.control; scRNA-seq,SRP330776,Regulatory T cell-AD.vs.control	16
C1orf43	bulk RNA-seq,Homo_723,AD.vs.control; bulk RNA-seq,Homo_723,AD.vs.MCI; bulk RNA-seq,Homo_633,AD.vs.control; bulk RNA-seq,Homo_633,AD.vs.MCI; scRNA-seq,SRP215507,CD8+ T cell_6-AD.vs.MCI; scRNA-seq,SRP330776,Regulatory T cell-AD.vs.control	6
C1orf50	bulk RNA-seq,Homo_723,AD.vs.control; bulk RNA-seq,Homo_723,MCI.vs.control; bulk RNA-seq,Homo_633,AD.vs.control; bulk RNA-seq,Homo_633,MCI.vs.control; bulk RNA-seq,Homo_714,AD.vs.control; bulk RNA-seq,Homo_714,AD.vs.MCI; bulk RNA-seq,Homo_714,MCI.vs.control; scRNA-seq,SRP215507,CD8+ T cell_6-AD.vs.MCI; scRNA-seq,SRP215507,CD8+ T cell_4-MCI.vs.control; scRNA-seq,SRP330776,CD8+ T cell_1-AD.vs.control; scRNA-seq,SRP330776,CD8+ T cell_2-AD.vs.control; scRNA-seq,SRP330776,Memory T cell_1-	13
C1orf52	scRNA-seq,SRP215507,CD8+ T cell_8-AD.vs.control; scRNA-seq,SRP330776,Naive CD8+ T cell_3-AD.vs.control; scRNA-seq,SRP330776,B cell_3-	3
C1orf53	bulk RNA-seq,Homo_723,AD.vs.control; bulk RNA-seq,Homo_723,AD.vs.MCI; scRNA-seq,SRP330776,CD8+ T cell_2-AD.vs.control; scRNA-seq,SRP330776,Memory T cell_1-AD.vs.control	4
C1orf54	bulk RNA-seq,Homo_723,AD.vs.control; bulk RNA-seq,Homo_723,MCI.vs.control; bulk RNA-seq,Homo_633,AD.vs.control; bulk RNA-seq,Homo_633,MCI.vs.control; scRNA-seq,SRP215507,CD8+ T cell_1-MCI.vs.control; scRNA-seq,SRP215507,CD8+ T cell_6-MCI.vs.control; scRNA-seq,SRP330776,Natural killer cell_1-AD.vs.control; scRNA-seq,SRP330776,Memory T cell_1-AD.vs.control; scRNA-seq,SRP330776,Natural killer T cell-AD.vs.control; scRNA-seq,SRP330776,B cell_2-AD.vs.control; scRNA-seq,SRP330776,T cell-AD.vs.control; scRNA-seq,SRP330776,Natural killer cell_2-	12
C1orf56	scRNA-seq,SRP215507,CD8+ T cell_1-AD.vs.MCI; scRNA-seq,SRP215507,CD8+ T cell_1-MCI.vs.control; scRNA-seq,SRP309935,Megakaryocyte_2-AD.vs.control; scRNA-seq,SRP330776,CD8+ T cell_1-AD.vs.control; scRNA-seq,SRP330776,γδ T cell-AD.vs.control; scRNA-seq,SRP330776,Memory T cell_2-AD.vs.control; scRNA-seq,SRP330776,B cell_1-AD.vs.control; scRNA-seq,SRP330776,Naive CD8+ T cell_1-AD.vs.control; scRNA-seq,SRP330776,Memory T cell_1-AD.vs.control; scRNA-seq,SRP330776,B cell_2-AD.vs.control; scRNA-seq,SRP330776,T cell-AD.vs.control; scRNA-	12
C1orf61	scRNA-seq,SRP215507,CD8+ T cell_3-AD.vs.control; scRNA-seq,SRP215507,CD8+ T cell_1-AD.vs.MCI; scRNA-seq,SRP215507,CD8+ T cell_2-MCI.vs.control; scRNA-seq,SRP330776,CD8+ T cell_1-AD.vs.control; scRNA-seq,SRP330776,Memory T cell_2-AD.vs.control; scRNA-seq,SRP330776,CD8+ T cell_2-AD.vs.control; scRNA-seq,SRP330776,Memory T cell_1-AD.vs.control; scRNA-seq,SRP330776,CD8+ T cell_3-	8
C1orf68	bulk RNA-seq,Homo_723,AD.vs.MCI; bulk RNA-seq,Homo_633,AD.vs.MCI	2
C1orf74	bulk RNA-seq,Homo_723,AD.vs.control; bulk RNA-seq,Homo_723,AD.vs.MCI; bulk RNA-seq,Homo_723,MCI.vs.control; bulk RNA-seq,Homo_633,AD.vs.control; bulk RNA-seq,Homo_633,AD.vs.MCI; bulk RNA-seq,Homo_633,MCI.vs.control; scRNA-seq,SRP330776,CD8+ T cell_1-AD.vs.control; scRNA-seq,SRP330776,γδ T cell-AD.vs.control	8
C1orf87	bulk RNA-seq,Homo_723,AD.vs.control; bulk RNA-seq,Homo_723,AD.vs.MCI; bulk RNA-seq,Homo_723,MCI.vs.control; bulk RNA-seq,Homo_633,AD.vs.control; bulk RNA-seq,Homo_633,AD.vs.MCI; bulk RNA-seq,Homo_633,MCI.vs.control; bulk RNA-seq,Homo_714,MCI.vs.control; bulk RNA-seq,ROSMAP,AD.vs.control; bulk RNA-seq,ROSMAP,MCI.vs.control	9
C1QA	bulk RNA-seq,Homo_723,AD.vs.MCI; bulk RNA-seq,Homo_723,MCI.vs.control; bulk RNA-seq,Homo_633,AD.vs.MCI; bulk RNA-seq,Homo_633,MCI.vs.control; bulk RNA-seq,Homo_714,AD.vs.control; bulk RNA-seq,Homo_714,AD.vs.MCI; bulk RNA-seq,Homo_714,MCI.vs.control	7
C1QB	bulk RNA-seq,Homo_723,AD.vs.control; bulk RNA-seq,Homo_723,AD.vs.MCI; bulk RNA-seq,Homo_633,AD.vs.control; bulk RNA-seq,Homo_633,AD.vs.MCI; bulk RNA-seq,Homo_714,AD.vs.control; bulk RNA-seq,Homo_714,AD.vs.MCI; scRNA-seq,SRP330776,Monocyte_1-	7

C1QBP	scRNA-seq,SRP215507,CD8+ T cell_7-AD.vs.control; scRNA-seq,SRP215507,CD8+ T cell_4-AD.vs.MCI; scRNA-seq,SRP215507,CD8+ T cell_7-MCI.vs.control; scRNA-seq,SRP215507,CD8+ T cell_8-MCI.vs.control; scRNA-seq,SRP309935,Plasmacytoid dendritic cell_1-AD.vs.control; scRNA-seq,SRP330776,CD8+ T cell_1-AD.vs.control; scRNA-seq,SRP330776,γδ T cell-AD.vs.control; scRNA-seq,SRP330776,Memory T cell_2-AD.vs.control; scRNA-seq,SRP330776,CD8+ T cell_2-AD.vs.control; scRNA-seq,SRP330776,B cell_1-AD.vs.control; scRNA-seq,SRP330776,Naive CD8+ T cell_1-AD.vs.control; scRNA-seq,SRP330776,Natural killer cell_1-AD.vs.control; scRNA-seq,SRP330776,Memory T cell_1-AD.vs.control; scRNA-seq,SRP330776,Natural killer T cell-AD.vs.control; scRNA-seq,SRP330776,Naive CD8+ T cell_3-AD.vs.control; scRNA-seq,SRP330776,B cell_2-AD.vs.control; scRNA-seq,SRP330776,T cell-AD.vs.control; scRNA-seq,SRP330776,Naive CD8+ T cell_2-AD.vs.control; scRNA-seq,SRP330776,CD8+ T cell_3-AD.vs.control; scRNA-seq,SRP330776,Monocyte_1-AD.vs.control; scRNA-seq,SRP330776,Monocyte_2-AD.vs.control; scRNA-seq,SRP330776,B cell_3-AD.vs.control; scRNA-seq,SRP330776,Regulatory T cell-AD.vs.control; scRNA-seq,SRP330776,Natural killer cell_2-[illegible]	25
C1QC	bulk RNA-seq,Homo_723,AD.vs.control; bulk RNA-seq,Homo_723,AD.vs.MCI; bulk RNA-seq,Homo_723,MCI.vs.control; bulk RNA-seq,Homo_633,AD.vs.control; bulk RNA-seq,Homo_633,AD.vs.MCI; bulk RNA-seq,Homo_633,MCI.vs.control; bulk RNA-seq,Homo_714,MCI.vs.control	7
C1QL2	bulk RNA-seq,Homo_723,AD.vs.control	1
C1QL3	bulk RNA-seq,Homo_714,AD.vs.control; bulk RNA-seq,Homo_714,AD.vs.MCI	2
C1QL4	bulk RNA-seq,Homo_723,AD.vs.control; bulk RNA-seq,Homo_723,AD.vs.MCI	2
C1QTNF1	bulk RNA-seq,Homo_723,AD.vs.control; bulk RNA-seq,Homo_723,AD.vs.MCI; bulk RNA-seq,Homo_723,MCI.vs.control; bulk RNA-seq,Homo_633,AD.vs.MCI; bulk RNA-seq,Homo_633,MCI.vs.control; bulk RNA-seq,Homo_714,MCI.vs.control	6
C1QTNF12	scRNA-seq,SRP215507,CD8+ T cell_4-AD.vs.control; scRNA-seq,SRP215507,CD8+ T cell_3-AD.vs.MCI; scRNA-seq,SRP215507,CD8+ T cell_3-	3
C1QTNF2	bulk RNA-seq,Homo_714,MCI.vs.control; bulk RNA-seq,ROSMAP,MCI.vs.control	2
C1QTNF3	bulk RNA-seq,Homo_723,AD.vs.control; bulk RNA-seq,Homo_723,AD.vs.MCI; bulk RNA-seq,Homo_723,MCI.vs.control; bulk RNA-seq,Homo_633,AD.vs.control; bulk RNA-seq,Homo_633,AD.vs.MCI; bulk RNA-seq,Homo_633,MCI.vs.control; bulk RNA-seq,Homo_714,AD.vs.control; bulk RNA-seq,Homo_714,AD.vs.MCI; scRNA-seq,SRP215507,CD8+ T cell_1-MCI.vs.control	9
C1QTNF4	bulk RNA-seq,Homo_723,AD.vs.control; bulk RNA-seq,Homo_723,MCI.vs.control; bulk RNA-seq,Homo_633,MCI.vs.control; bulk RNA-seq,Homo_714,MCI.vs.control; bulk RNA-seq,ROSMAP,AD.vs.control; bulk RNA-seq,ROSMAP,MCI.vs.control	6
C1QTNF6	bulk RNA-seq,Homo_723,AD.vs.control; bulk RNA-seq,Homo_723,AD.vs.MCI; bulk RNA-seq,Homo_633,AD.vs.control; bulk RNA-seq,Homo_633,AD.vs.MCI; bulk RNA-seq,ROSMAP,AD.vs.control; bulk RNA-seq,ROSMAP,MCI.vs.control; scRNA-seq,SRP215507,CD8+ T cell_1-AD.vs.control; scRNA-seq,SRP215507,CD8+ T cell_2-AD.vs.MCI; scRNA-seq,SRP215507,CD8+ T cell_2-MCI.vs.control; scRNA-seq,SRP215507,CD8+ T cell_3-MCI.vs.control; scRNA-seq,SRP215507,CD8+ T cell_6-MCI.vs.control; scRNA-seq,SRP330776,CD8+ T cell_1-AD.vs.control; scRNA-seq,SRP330776,B cell_1-AD.vs.control; scRNA-seq,SRP330776,Naive CD8+ T cell_1-AD.vs.control	14
C1QTNF7	bulk RNA-seq,Homo_723,AD.vs.control; bulk RNA-seq,Homo_723,MCI.vs.control; bulk RNA-seq,Homo_633,AD.vs.control; bulk RNA-seq,Homo_633,MCI.vs.control; bulk RNA-seq,Homo_714,MCI.vs.control	5
C1QTNF8	bulk RNA-seq,Homo_714,MCI.vs.control	1
C1QTNF9	bulk RNA-seq,Homo_723,AD.vs.control; bulk RNA-seq,Homo_723,AD.vs.MCI; bulk RNA-seq,Homo_723,MCI.vs.control; bulk RNA-seq,Homo_633,AD.vs.control; bulk RNA-seq,Homo_633,AD.vs.MCI; bulk RNA-seq,Homo_633,MCI.vs.control	6
C1QTNF9B	bulk RNA-seq,Homo_723,AD.vs.control; bulk RNA-seq,Homo_723,AD.vs.MCI; bulk RNA-seq,Homo_723,MCI.vs.control; bulk RNA-seq,Homo_633,AD.vs.control; bulk RNA-seq,Homo_633,AD.vs.MCI; bulk RNA-seq,Homo_633,MCI.vs.control	6
C1R	bulk RNA-seq,Homo_723,AD.vs.control; bulk RNA-seq,Homo_723,AD.vs.MCI; bulk RNA-seq,Homo_714,AD.vs.control; bulk RNA-seq,Homo_714,AD.vs.MCI; scRNA-seq,SRP215507,CD8+ T cell_3-AD.vs.control; scRNA-seq,SRP215507,CD8+ T cell_1-AD.vs.MCI; scRNA-seq,SRP215507,CD8+ T cell_4-AD.vs.MCI; scRNA-seq,SRP215507,CD8+ T cell_1-MCI.vs.control; scRNA-seq,SRP330776,Naive CD8+ T cell_1-	9
C1RL	scRNA-seq,SRP215507,CD8+ T cell_5-AD.vs.control; scRNA-seq,SRP215507,CD8+ T cell_4-AD.vs.MCI; scRNA-seq,SRP215507,CD8+ T cell_5-MCI.vs.control; scRNA-seq,SRP215507,CD8+ T cell_4-MCI.vs.control; scRNA-seq,SRP330776,CD8+ T cell_1-AD.vs.control; scRNA-seq,SRP330776,Naive CD8+ T cell_3-AD.vs.control; scRNA-seq,SRP330776,B cell_2-AD.vs.control; scRNA-seq,SRP330776,T cell-AD.vs.control;	9
C1RL-AS1	scRNA-seq,SRP215507,CD8+ T cell_5-AD.vs.control; scRNA-seq,SRP215507,CD8+ T cell_3-AD.vs.MCI; scRNA-seq,SRP215507,CD8+ T cell_5-MCI.vs.control; scRNA-seq,SRP215507,CD8+ T cell_3-MCI.vs.control; scRNA-seq,SRP330776,Natural killer T cell-AD.vs.control; scRNA-	6

C1S	bulk RNA-seq,Homo_723,AD.vs.control; bulk RNA-seq,Homo_723,AD.vs.MCI; bulk RNA-seq,Homo_723,MCI.vs.control; bulk RNA-seq,Homo_633,AD.vs.MCI; bulk RNA-seq,Homo_633,MCI.vs.control; bulk RNA-seq,Homo_714,AD.vs.control; bulk RNA-seq,Homo_714,AD.vs.MCI; bulk RNA-seq,Homo_714,MCI.vs.control; scRNA-seq,SRP215507,CD8+ T cell_2-AD.vs.control; scRNA-seq,SRP215507,CD8+ T cell_3-AD.vs.control	10
C2	bulk RNA-seq,Homo_714,AD.vs.control; bulk RNA-seq,Homo_714,AD.vs.MCI; scRNA-seq,SRP215507,CD8+ T cell_3-AD.vs.MCI; scRNA-seq,SRP215507,CD8+ T cell_3-MCI.vs.control; scRNA-seq,SRP309935,CD8+ T cell_1-AD.vs.control	5
C20orf173	bulk RNA-seq,Homo_723,AD.vs.control; bulk RNA-seq,Homo_723,AD.vs.MCI	2
C20orf194	scRNA-seq,SRP215507,CD8+ T cell_3-AD.vs.MCI; scRNA-seq,SRP215507,CD8+ T cell_3-MCI.vs.control; scRNA-seq,SRP309935,CD8+ T cell_1-AD.vs.control; scRNA-seq,SRP330776,CD8+ T cell_1-AD.vs.control; scRNA-seq,SRP330776,Memory T cell_2-AD.vs.control; scRNA-seq,SRP330776,B cell_1-AD.vs.control; scRNA-seq,SRP330776,Naive CD8+ T cell_3-AD.vs.control; scRNA-seq,SRP330776,B cell_2-AD.vs.control; scRNA-	9
C20orf197	scRNA-seq,SRP330776,CD8+ T cell_1-AD.vs.control; scRNA-seq,SRP330776,γδ T cell-AD.vs.control; scRNA-seq,SRP330776,Natural killer cell_1-AD.vs.control; scRNA-seq,SRP330776,Natural killer T cell-AD.vs.control; scRNA-seq,SRP330776,T cell-AD.vs.control	5
C20orf203	bulk RNA-seq,Homo_723,AD.vs.control; bulk RNA-seq,Homo_723,AD.vs.MCI; bulk RNA-seq,Homo_633,AD.vs.control; bulk RNA-seq,Homo_633,AD.vs.MCI; bulk RNA-seq,Homo_633,MCI.vs.control; bulk RNA-seq,Homo_714,AD.vs.MCI	6
C20orf204	bulk RNA-seq,Homo_723,AD.vs.control; bulk RNA-seq,Homo_723,MCI.vs.control; bulk RNA-seq,Homo_633,AD.vs.MCI; bulk RNA-seq,Homo_633,MCI.vs.control; bulk RNA-seq,Homo_714,AD.vs.control; bulk RNA-seq,Homo_714,AD.vs.MCI; bulk RNA-seq,Homo_714,MCI.vs.control; scRNA-seq,SRP215507,CD8+ T cell_3-AD.vs.control; scRNA-seq,SRP215507,CD8+ T cell_5-AD.vs.control; scRNA-seq,SRP215507,CD8+ T cell_2-AD.vs.MCI; scRNA-seq,SRP215507,CD8+ T cell_3-AD.vs.MCI; scRNA-seq,SRP215507,CD8+ T cell_4-AD.vs.MCI; scRNA-seq,SRP215507,CD8+ T cell_5-AD.vs.MCI; scRNA-seq,SRP215507,CD8+ T cell_1-MCI.vs.control; scRNA-seq,SRP215507,CD8+ T cell_2-MCI.vs.control; scRNA-seq,SRP215507,CD8+ T cell_3-MCI.vs.control; scRNA-seq,SRP330776,CD8+ T cell_1-AD.vs.control; scRNA- [illegible]	18
C20orf27	bulk RNA-seq,Homo_723,AD.vs.control; bulk RNA-seq,Homo_723,AD.vs.MCI; bulk RNA-seq,Homo_633,AD.vs.control; bulk RNA-seq,Homo_633,AD.vs.MCI; bulk RNA-seq,Homo_714,AD.vs.control; bulk RNA-seq,Homo_714,AD.vs.MCI; scRNA-seq,SRP215507,CD8+ T cell_10-AD.vs.control; scRNA-seq,SRP330776,CD8+ T cell_3-AD.vs.control; scRNA-seq,SRP330776,B cell_3-AD.vs.control; scRNA-seq,SRP330776,Regulatory	10
C20orf96	bulk RNA-seq,Homo_723,AD.vs.control; bulk RNA-seq,Homo_723,AD.vs.MCI; bulk RNA-seq,Homo_633,AD.vs.control; bulk RNA-seq,Homo_633,AD.vs.MCI; bulk RNA-seq,Homo_714,AD.vs.control; bulk RNA-seq,Homo_714,AD.vs.MCI; bulk RNA-seq,Homo_714,MCI.vs.control; scRNA-seq,SRP215507,CD8+ T cell_3-AD.vs.control; scRNA-seq,SRP215507,CD8+ T cell_1-AD.vs.control; scRNA-seq,SRP215507,CD8+ T cell_3-AD.vs.MCI; scRNA-seq,SRP215507,CD8+ T cell_5-AD.vs.MCI; scRNA-seq,SRP215507,CD8+ T cell_5-MCI.vs.control; scRNA-seq,SRP215507,CD8+ T cell_3-MCI.vs.control; scRNA-seq,SRP330776,Memory T cell_2-AD.vs.control; scRNA-seq,SRP330776,Natural killer T cell-AD.vs.control	15
C21orf58	bulk RNA-seq,Homo_723,AD.vs.control; bulk RNA-seq,Homo_723,AD.vs.MCI; bulk RNA-seq,Homo_633,AD.vs.control; bulk RNA-seq,Homo_633,AD.vs.MCI; bulk RNA-seq,Homo_714,AD.vs.control; bulk RNA-seq,Homo_714,AD.vs.MCI; bulk RNA-seq,Homo_714,MCI.vs.control; scRNA-seq,SRP215507,CD8+ T cell_1-AD.vs.control; scRNA-seq,SRP215507,CD8+ T cell_1-MCI.vs.control; scRNA-seq,SRP309935,Megakaryocyte_2-AD.vs.control; scRNA-seq,SRP330776,CD8+ T cell_2-AD.vs.control; scRNA-seq,SRP330776,B cell_1-AD.vs.control; scRNA-seq,SRP330776,Naive CD8+ T cell_1-AD.vs.control; scRNA-seq,SRP330776,B cell_2-AD.vs.control; scRNA-seq,SRP330776,CD8+ T cell_3-AD.vs.control	15
C21orf62	bulk RNA-seq,Homo_723,AD.vs.control; bulk RNA-seq,Homo_723,AD.vs.MCI; bulk RNA-seq,Homo_714,AD.vs.MCI; bulk RNA-seq,ROSMAP,AD.vs.control; bulk RNA-seq,ROSMAP,MCI.vs.control	5
C21orf62-AS1	scRNA-seq,SRP215507,CD8+ T cell_2-AD.vs.MCI; scRNA-seq,SRP215507,CD8+ T cell_4-AD.vs.MCI; scRNA-seq,SRP215507,CD8+ T cell_5-MCI.vs.control; scRNA-seq,SRP215507,CD8+ T cell_2-MCI.vs.control; scRNA-seq,SRP309935,CD8+ T cell_2-AD.vs.control; scRNA-seq,SRP330776,CD8+ T cell_1-AD.vs.control; scRNA-seq,SRP330776,γδ T cell-AD.vs.control; scRNA-seq,SRP330776,CD8+ T cell_2-AD.vs.control; scRNA-seq,SRP330776,Natural killer T cell-AD.vs.control; scRNA-seq,SRP330776,Naive CD8+ T cell_3-AD.vs.control; scRNA-seq,SRP330776,T cell-AD.vs.control; scRNA-seq,SRP330776,CD8+ T cell_3-AD.vs.control; scRNA-seq,SRP330776,Natural killer cell_2-AD.vs.control	13
C21orf91	bulk RNA-seq,Homo_723,AD.vs.MCI; bulk RNA-seq,Homo_633,AD.vs.MCI; bulk RNA-seq,Homo_714,AD.vs.control; bulk RNA-seq,Homo_714,AD.vs.MCI; scRNA-seq,SRP215507,CD8+ T cell_3-AD.vs.MCI; scRNA-seq,SRP215507,CD8+ T cell_5-AD.vs.MCI; scRNA-seq,SRP215507,CD8+ T cell_3-MCI.vs.control; scRNA-seq,SRP309935,Monocyte_1-AD.vs.control; scRNA-seq,SRP330776,B cell_1-AD.vs.control; scRNA-seq,SRP330776,Naive CD8+ T cell_2-AD.vs.control; scRNA-seq,SRP330776,CD8+ T cell_3-AD.vs.control; scRNA-seq,SRP330776,Monocyte_1-	12

C21orf91-OT1	scRNA-seq,SRP330776,CD8+ T cell_1-AD.vs.control	1
C22orf15	bulk RNA-seq,Homo_714,AD.vs.control; bulk RNA-seq,Homo_714,AD.vs.MCI; scRNA-seq,SRP215507,CD8+ T cell_3-AD.vs.control; scRNA-seq,SRP215507,CD8+ T cell_2-AD.vs.MCI	4
C22orf23	bulk RNA-seq,Homo_723,AD.vs.control; bulk RNA-seq,Homo_723,AD.vs.MCI; bulk RNA-seq,Homo_723,MCI.vs.control; bulk RNA-seq,Homo_633,AD.vs.MCI; bulk RNA-seq,Homo_633,MCI.vs.control; bulk RNA-seq,Homo_714,AD.vs.control; bulk RNA-seq,Homo_714,AD.vs.MCI;	8
C22orf24	scRNA-seq,SRP215507,CD8+ T cell_1-MCI.vs.control; scRNA-seq,SRP215507,CD8+ T cell_3-MCI.vs.control; scRNA-seq,SRP330776,B cell_1-	3
C22orf31	bulk RNA-seq,Homo_714,MCI.vs.control	1
C22orf34	scRNA-seq,SRP309935,Natural killer cell_1-AD.vs.control; scRNA-seq,SRP330776,CD8+ T cell_1-AD.vs.control; scRNA-seq,SRP330776,Memory T cell_2-AD.vs.control; scRNA-seq,SRP330776,Natural killer T cell-AD.vs.control; scRNA-seq,SRP330776,CD8+ T cell_3-AD.vs.control; scRNA-seq,SRP330776,Monocyte_1-AD.vs.control; scRNA-seq,SRP330776,Regulatory T cell-AD.vs.control; scRNA-seq,SRP330776,B cell_4-AD.vs.control	8
C22orf39	bulk RNA-seq,Homo_633,AD.vs.control; bulk RNA-seq,Homo_714,AD.vs.control; bulk RNA-seq,Homo_714,AD.vs.MCI; scRNA-seq,SRP215507,CD8+ T cell_7-AD.vs.control; scRNA-seq,SRP215507,CD8+ T cell_4-AD.vs.MCI; scRNA-seq,SRP215507,CD8+ T cell_5-AD.vs.MCI; scRNA-seq,SRP215507,CD8+ T cell_1-MCI.vs.control; scRNA-seq,SRP215507,CD8+ T cell_5-MCI.vs.control; scRNA-seq,SRP309935,Plasmacytoid dendritic cell_2-AD.vs.control; scRNA-seq,SRP330776,CD8+ T cell_1-AD.vs.control; scRNA-seq,SRP330776,γδ T cell-AD.vs.control; scRNA-seq,SRP330776,Natural killer cell_1-AD.vs.control; scRNA-seq,SRP330776,Memory T cell_1-AD.vs.control; scRNA-seq,SRP330776,T cell-	15
C22orf42	bulk RNA-seq,Homo_723,AD.vs.control; bulk RNA-seq,Homo_723,AD.vs.MCI	2
C2CD2	bulk RNA-seq,Homo_714,AD.vs.control; bulk RNA-seq,Homo_714,AD.vs.MCI; scRNA-seq,SRP215507,CD8+ T cell_1-AD.vs.control; scRNA-seq,SRP330776,CD8+ T cell_1-AD.vs.control; scRNA-seq,SRP330776,B cell_1-AD.vs.control; scRNA-seq,SRP330776,Naive CD8+ T cell_3-AD.vs.control; scRNA-seq,SRP330776,T cell-AD.vs.control; scRNA-seq,SRP330776,CD8+ T cell_3-AD.vs.control; scRNA-seq,SRP330776,Natural killer	9
C2CD2L	scRNA-seq,SRP215507,CD8+ T cell_5-AD.vs.control; scRNA-seq,SRP215507,CD8+ T cell_3-AD.vs.MCI; scRNA-seq,SRP215507,CD8+ T cell_5-MCI.vs.control; scRNA-seq,SRP215507,CD8+ T cell_2-MCI.vs.control; scRNA-seq,SRP215507,CD8+ T cell_3-MCI.vs.control; scRNA-seq,SRP309935,Monocyte_2-AD.vs.control; scRNA-seq,SRP309935,CD8+ T cell_1-AD.vs.control; scRNA-seq,SRP309935,B cell_1-AD.vs.control; scRNA-seq,SRP330776,CD8+ T cell_1-AD.vs.control; scRNA-seq,SRP330776,γδ T cell-AD.vs.control; scRNA-seq,SRP330776,Memory T cell_2-AD.vs.control; scRNA-seq,SRP330776,CD8+ T cell_2-AD.vs.control; scRNA-seq,SRP330776,B cell_1-AD.vs.control; scRNA-seq,SRP330776,Naive CD8+ T cell_1-AD.vs.control; scRNA-seq,SRP330776,Memory T cell_1-AD.vs.control; scRNA-seq,SRP330776,Natural killer T cell-AD.vs.control; scRNA-seq,SRP330776,Naive CD8+ T cell_3-AD.vs.control; scRNA-seq,SRP330776,B cell_2-AD.vs.control; scRNA-seq,SRP330776,T cell-AD.vs.control; scRNA-seq,SRP330776,CD8+ T cell_3-AD.vs.control; scRNA-seq,SRP330776,Monocyte_1-AD.vs.control; scRNA-[illegible]	22
C2CD3	bulk RNA-seq,Homo_723,AD.vs.control; bulk RNA-seq,Homo_723,MCI.vs.control; bulk RNA-seq,Homo_633,AD.vs.control; bulk RNA-seq,Homo_633,MCI.vs.control; scRNA-seq,SRP215507,CD8+ T cell_7-AD.vs.control; scRNA-seq,SRP215507,CD8+ T cell_3-AD.vs.MCI; scRNA-seq,SRP215507,CD8+ T cell_1-AD.vs.MCI; scRNA-seq,SRP215507,CD8+ T cell_1-MCI.vs.control; scRNA-seq,SRP215507,CD8+ T cell_3-	10
C2CD4A	bulk RNA-seq,Homo_723,AD.vs.control; bulk RNA-seq,Homo_714,MCI.vs.control	2
C2CD4B	bulk RNA-seq,Homo_723,AD.vs.control; bulk RNA-seq,Homo_723,AD.vs.MCI; bulk RNA-seq,Homo_633,AD.vs.control; bulk RNA-seq,Homo_633,AD.vs.MCI; bulk RNA-seq,Homo_714,MCI.vs.control	5
C2CD4C	bulk RNA-seq,Homo_723,AD.vs.control; bulk RNA-seq,Homo_723,AD.vs.MCI; bulk RNA-seq,Homo_723,MCI.vs.control; bulk RNA-	4
C2CD4D	bulk RNA-seq,Homo_723,AD.vs.MCI; bulk RNA-seq,Homo_633,AD.vs.MCI; scRNA-seq,SRP215507,CD8+ T cell_2-AD.vs.control; scRNA-seq,SRP215507,CD8+ T cell_3-AD.vs.control; scRNA-seq,SRP215507,CD8+ T cell_1-AD.vs.control; scRNA-seq,SRP215507,CD8+ T cell_5-AD.vs.control; scRNA-seq,SRP215507,CD8+ T cell_5-AD.vs.MCI; scRNA-seq,SRP215507,CD8+ T cell_1-MCI.vs.control; scRNA-seq,SRP215507,CD8+ T cell_2-MCI.vs.control; scRNA-seq,SRP309935,Natural killer cell_1-AD.vs.control	10
C2CD4D-AS1	scRNA-seq,SRP215507,CD8+ T cell_2-AD.vs.control	1

C2CD5	bulk RNA-seq,Homo_723,AD.vs.control; bulk RNA-seq,Homo_723,AD.vs.MCI; bulk RNA-seq,Homo_723,MCI.vs.control; bulk RNA-seq,Homo_633,AD.vs.control; bulk RNA-seq,Homo_633,AD.vs.MCI; bulk RNA-seq,Homo_714,AD.vs.control; bulk RNA-seq,Homo_714,AD.vs.MCI; scRNA-seq,SRP215507,CD8+ T cell_6-AD.vs.MCI; scRNA-seq,SRP215507,CD8+ T cell_6-MCI.vs.control; scRNA-seq,SRP309935,Monocyte_1-AD.vs.control; scRNA-seq,SRP330776,Naive CD8+ T cell_2-AD.vs.control; scRNA-seq,SRP330776,CD8+ T cell_3-AD.vs.control; scRNA-	14
C2cd5	bulk RNA-seq,SRP325058,AD.vs.control	1
C2CD6	bulk RNA-seq,Homo_723,AD.vs.control; bulk RNA-seq,Homo_723,AD.vs.MCI; bulk RNA-seq,Homo_633,AD.vs.control; bulk RNA-	4
C2orf15	scRNA-seq,SRP215507,CD8+ T cell_2-AD.vs.control; scRNA-seq,SRP330776,CD8+ T cell_1-AD.vs.control	2
C2orf16	bulk RNA-seq,Homo_723,AD.vs.control; bulk RNA-seq,Homo_723,AD.vs.MCI; bulk RNA-seq,Homo_633,AD.vs.MCI; bulk RNA-seq,Homo_714,AD.vs.control; bulk RNA-seq,Homo_714,AD.vs.MCI	5
C2orf27A	scRNA-seq,SRP215507,CD8+ T cell_1-AD.vs.control; scRNA-seq,SRP215507,CD8+ T cell_3-AD.vs.MCI; scRNA-seq,SRP215507,CD8+ T cell_5-AD.vs.MCI; scRNA-seq,SRP215507,CD8+ T cell_1-MCI.vs.control; scRNA-seq,SRP215507,CD8+ T cell_5-MCI.vs.control; scRNA-seq,SRP215507,CD8+ T cell_3-MCI.vs.control; scRNA-seq,SRP309935,Naive CD8+ T cell_2-AD.vs.control; scRNA-seq,SRP309935,CD8+ T cell_1-AD.vs.control; scRNA-seq,SRP330776,γδ T cell-AD.vs.control; scRNA-seq,SRP330776,CD8+ T cell_3-AD.vs.control	10
C2orf42	bulk RNA-seq,Homo_714,AD.vs.control; bulk RNA-seq,Homo_714,AD.vs.MCI; scRNA-seq,SRP215507,CD8+ T cell_2-AD.vs.MCI; scRNA-seq,SRP309935,Naive CD8+ T cell_2-AD.vs.control; scRNA-seq,SRP330776,CD8+ T cell_1-AD.vs.control; scRNA-seq,SRP330776,Memory T cell_2-AD.vs.control; scRNA-seq,SRP330776,CD8+ T cell_2-AD.vs.control; scRNA-seq,SRP330776,B cell_1-AD.vs.control; scRNA-seq,SRP330776,Naive CD8+ T cell_1-AD.vs.control; scRNA-seq,SRP330776,Naive CD8+ T cell_3-AD.vs.control; scRNA-seq,SRP330776,CD8+ T cell_3-AD.vs.control	11
C2orf49	bulk RNA-seq,Homo_714,AD.vs.control; bulk RNA-seq,Homo_714,AD.vs.MCI; bulk RNA-seq,Homo_714,MCI.vs.control; scRNA-seq,SRP215507,CD8+ T cell_3-AD.vs.MCI; scRNA-seq,SRP215507,CD8+ T cell_3-MCI.vs.control; scRNA-seq,SRP330776,Monocyte_2-AD.vs.control	6
C2orf50	bulk RNA-seq,Homo_714,AD.vs.control; bulk RNA-seq,Homo_714,AD.vs.MCI; scRNA-seq,SRP330776,Naive CD8+ T cell_1-AD.vs.control	3
C2orf66	bulk RNA-seq,Homo_723,AD.vs.control; bulk RNA-seq,Homo_723,AD.vs.MCI; bulk RNA-seq,Homo_723,MCI.vs.control; bulk RNA-seq,Homo_633,AD.vs.control; bulk RNA-seq,Homo_633,AD.vs.MCI; bulk RNA-seq,Homo_633,MCI.vs.control; bulk RNA-seq,Homo_714,MCI.vs.control	7
C2orf68	bulk RNA-seq,Homo_723,AD.vs.control; scRNA-seq,SRP215507,CD8+ T cell_4-AD.vs.MCI; scRNA-seq,SRP215507,CD8+ T cell_4-MCI.vs.control; scRNA-seq,SRP309935,Naive CD8+ T cell_1-AD.vs.control; scRNA-seq,SRP330776,Memory T cell_2-AD.vs.control; scRNA-seq,SRP330776,B cell_1-AD.vs.control; scRNA-seq,SRP330776,B cell_2-AD.vs.control	7
C2orf69	scRNA-seq,SRP215507,CD8+ T cell_3-AD.vs.MCI; scRNA-seq,SRP215507,CD8+ T cell_4-MCI.vs.control; scRNA-seq,SRP309935,CD8+ T cell_2-AD.vs.control; scRNA-seq,SRP330776,Monocyte_1-AD.vs.control	4
C2orf72	bulk RNA-seq,Homo_723,AD.vs.control; bulk RNA-seq,Homo_723,AD.vs.MCI; bulk RNA-seq,Homo_633,AD.vs.control	3
C2orf74	bulk RNA-seq,Homo_723,AD.vs.MCI; bulk RNA-seq,Homo_723,MCI.vs.control; bulk RNA-seq,Homo_633,AD.vs.MCI; bulk RNA-seq,Homo_633,MCI.vs.control; bulk RNA-seq,Homo_714,AD.vs.control; bulk RNA-seq,Homo_714,AD.vs.MCI; bulk RNA-seq,Homo_714,MCI.vs.control; scRNA-seq,SRP215507,CD8+ T cell_2-AD.vs.control; scRNA-seq,SRP215507,CD8+ T cell_3-AD.vs.control; scRNA-seq,SRP215507,CD8+ T cell_1-AD.vs.control; scRNA-seq,SRP215507,CD8+ T cell_4-AD.vs.control; scRNA-seq,SRP215507,CD8+ T cell_5-AD.vs.control; scRNA-seq,SRP215507,CD8+ T cell_6-AD.vs.control; scRNA-seq,SRP215507,CD8+ T cell_4-AD.vs.MCI; scRNA-seq,SRP215507,CD8+ T cell_2-MCI.vs.control; scRNA-seq,SRP309935,Monocyte_1-AD.vs.control; scRNA-seq,SRP330776,CD8+ T cell_1-AD.vs.control; scRNA-seq,SRP330776,γδ T cell-AD.vs.control; scRNA-seq,SRP330776,Memory T cell_2-AD.vs.control; scRNA-seq,SRP330776,CD8+ T cell_2-AD.vs.control; scRNA-seq,SRP330776,Natural killer cell_1-AD.vs.control; scRNA-seq,SRP330776,Natural killer T cell-AD.vs.control; scRNA-seq,SRP330776,Naive [illegible]	25
C2orf76	scRNA-seq,SRP215507,CD8+ T cell_6-AD.vs.control; scRNA-seq,SRP215507,CD8+ T cell_3-AD.vs.MCI; scRNA-seq,SRP215507,CD8+ T cell_3-MCI.vs.control; scRNA-seq,SRP215507,CD8+ T cell_6-MCI.vs.control; scRNA-seq,SRP330776,CD8+ T cell_2-AD.vs.control; scRNA-seq,SRP330776,B cell_1-AD.vs.control; scRNA-seq,SRP330776,Natural killer cell_1-AD.vs.control; scRNA-seq,SRP330776,Natural killer T cell-AD.vs.control; scRNA-seq,SRP330776,Naive CD8+ T cell_3-AD.vs.control; scRNA-seq,SRP330776,B cell_2-AD.vs.control	10
C2orf78	bulk RNA-seq,Homo_714,MCI.vs.control	1
C2orf80	bulk RNA-seq,Homo_723,AD.vs.control; bulk RNA-seq,Homo_723,AD.vs.MCI	2

C2orf81	bulk RNA-seq,Homo_723,AD.vs.control; bulk RNA-seq,Homo_723,AD.vs.MCI; bulk RNA-seq,Homo_723,MCI.vs.control; bulk RNA-seq,Homo_633,AD.vs.control; bulk RNA-seq,Homo_633,AD.vs.MCI; bulk RNA-seq,Homo_633,MCI.vs.control; bulk RNA-seq,Homo_714,AD.vs.control; bulk RNA-seq,Homo_714,MCI.vs.control; scRNA-seq,SRP215507,CD8+ T cell_1-AD.vs.control; scRNA-seq,SRP215507,CD8+ T cell_1-AD.vs.MCI; scRNA-seq,SRP330776,CD8+ T cell_1-AD.vs.control; scRNA-seq,SRP330776,Naive CD8+ T cell_1-AD.vs.control; scRNA-seq,SRP330776,B cell_2-	13
C2orf88	scRNA-seq,SRP330776,B cell_1-AD.vs.control; scRNA-seq,SRP330776,B cell_2-AD.vs.control	2
C2orf92	bulk RNA-seq,Homo_723,AD.vs.control; bulk RNA-seq,Homo_723,AD.vs.MCI; bulk RNA-seq,Homo_723,MCI.vs.control; bulk RNA-seq,Homo_633,AD.vs.control; bulk RNA-seq,Homo_633,AD.vs.MCI; bulk RNA-seq,Homo_633,MCI.vs.control; bulk RNA-seq,Homo_714,AD.vs.control; bulk RNA-seq,Homo_714,AD.vs.MCI; bulk RNA-seq,Homo_714,MCI.vs.control; scRNA-seq,SRP330776,CD8+ T cell_1-AD.vs.control; scRNA-seq,SRP330776,Memory T cell_2-AD.vs.control; scRNA-seq,SRP330776,Natural killer cell_1-AD.vs.control	12
C3	bulk RNA-seq,Homo_723,AD.vs.control; bulk RNA-seq,Homo_723,AD.vs.MCI; bulk RNA-seq,Homo_633,AD.vs.control; bulk RNA-seq,Homo_633,AD.vs.MCI; bulk RNA-seq,Homo_714,AD.vs.control; bulk RNA-seq,Homo_714,AD.vs.MCI; bulk RNA-seq,ROSMAP,AD.vs.control;	8
C330018D20Rik	bulk RNA-seq,SRP325058,AD.vs.control	1
C3AR1	scRNA-seq,SRP215507,CD8+ T cell_1-AD.vs.MCI; scRNA-seq,SRP215507,CD8+ T cell_1-MCI.vs.control; scRNA-seq,SRP330776,γδ T cell-AD.vs.control; scRNA-seq,SRP330776,CD8+ T cell_2-AD.vs.control; scRNA-seq,SRP330776,Natural killer cell_1-AD.vs.control; scRNA-seq,SRP330776,Natural killer T cell-AD.vs.control; scRNA-seq,SRP330776,T cell-AD.vs.control; scRNA-seq,SRP330776,CD8+ T cell_3-AD.vs.control; scRNA-seq,SRP330776,Monocyte_1-AD.vs.control; scRNA-seq,SRP330776,Monocyte_2-AD.vs.control	10
C3orf14	bulk RNA-seq,Homo_723,AD.vs.control; bulk RNA-seq,Homo_723,AD.vs.MCI; bulk RNA-seq,Homo_714,AD.vs.control; bulk RNA-seq,Homo_714,AD.vs.MCI; scRNA-seq,SRP215507,CD8+ T cell_2-AD.vs.control; scRNA-seq,SRP215507,CD8+ T cell_3-AD.vs.control; scRNA-seq,SRP215507,CD8+ T cell_5-AD.vs.control; scRNA-seq,SRP215507,CD8+ T cell_2-AD.vs.MCI; scRNA-seq,SRP215507,CD8+ T cell_3-AD.vs.MCI; scRNA-seq,SRP215507,CD8+ T cell_1-AD.vs.MCI; scRNA-seq,SRP215507,CD8+ T cell_1-MCI.vs.control; scRNA-seq,SRP330776,CD8+ T cell_2-AD.vs.control; scRNA-seq,SRP330776,B cell_1-AD.vs.control; scRNA-seq,SRP330776,Naive CD8+ T cell_1-AD.vs.control; scRNA-	16
C3orf18	bulk RNA-seq,Homo_723,AD.vs.control; bulk RNA-seq,Homo_723,AD.vs.MCI; bulk RNA-seq,Homo_633,AD.vs.control; bulk RNA-seq,Homo_633,AD.vs.MCI; bulk RNA-seq,Homo_714,AD.vs.control; bulk RNA-seq,Homo_714,AD.vs.MCI; bulk RNA-seq,Homo_714,MCI.vs.control; scRNA-seq,SRP215507,CD8+ T cell_3-AD.vs.control; scRNA-seq,SRP330776,CD8+ T cell_2-AD.vs.control; scRNA-seq,SRP330776,B cell_1-	10
C3orf20	bulk RNA-seq,Homo_723,AD.vs.MCI; bulk RNA-seq,Homo_714,AD.vs.control; bulk RNA-seq,Homo_714,AD.vs.MCI	3
C3orf22	bulk RNA-seq,Homo_723,MCI.vs.control; bulk RNA-seq,Homo_633,MCI.vs.control; bulk RNA-seq,Homo_714,MCI.vs.control	3
C3orf33	bulk RNA-seq,Homo_723,AD.vs.control; bulk RNA-seq,Homo_723,AD.vs.MCI; bulk RNA-seq,Homo_633,AD.vs.MCI; bulk RNA-seq,Homo_714,AD.vs.control; bulk RNA-seq,Homo_714,AD.vs.MCI; scRNA-seq,SRP215507,CD8+ T cell_6-AD.vs.control; scRNA-seq,SRP215507,CD8+ T cell_1-AD.vs.MCI; scRNA-seq,SRP215507,CD8+ T cell_1-MCI.vs.control; scRNA-seq,SRP330776,B cell_1-AD.vs.control;	11
C3orf35	scRNA-seq,SRP330776,CD8+ T cell_1-AD.vs.control; scRNA-seq,SRP330776,CD8+ T cell_2-AD.vs.control	2
C3orf38	bulk RNA-seq,Homo_723,AD.vs.MCI; bulk RNA-seq,Homo_633,AD.vs.MCI; bulk RNA-seq,Homo_714,AD.vs.control; bulk RNA-seq,Homo_714,AD.vs.MCI; scRNA-seq,SRP215507,CD8+ T cell_4-AD.vs.MCI; scRNA-seq,SRP215507,CD8+ T cell_4-MCI.vs.control; scRNA-	7
C3orf49	bulk RNA-seq,Homo_723,AD.vs.control; bulk RNA-seq,Homo_723,AD.vs.MCI; bulk RNA-seq,Homo_633,AD.vs.control; bulk RNA-seq,Homo_633,AD.vs.MCI; bulk RNA-seq,Homo_714,AD.vs.control; bulk RNA-seq,Homo_714,AD.vs.MCI	6
C3orf52	bulk RNA-seq,ROSMAP,AD.vs.control; bulk RNA-seq,ROSMAP,MCI.vs.control; scRNA-seq,SRP215507,CD8+ T cell_1-MCI.vs.control; scRNA-seq,SRP330776,Memory T cell_2-AD.vs.control	4
C3orf62	bulk RNA-seq,Homo_723,MCI.vs.control; bulk RNA-seq,Homo_633,MCI.vs.control; bulk RNA-seq,Homo_714,MCI.vs.control; scRNA-seq,SRP215507,CD8+ T cell_4-AD.vs.control; scRNA-seq,SRP330776,γδ T cell-AD.vs.control; scRNA-seq,SRP330776,Memory T cell_2-AD.vs.control; scRNA-seq,SRP330776,B cell_1-AD.vs.control; scRNA-seq,SRP330776,Naive CD8+ T cell_1-AD.vs.control; scRNA-seq,SRP330776,Memory T cell_1-AD.vs.control; scRNA-seq,SRP330776,B cell_2-AD.vs.control; scRNA-seq,SRP330776,CD8+ T cell_3-AD.vs.control	11
C3orf80	bulk RNA-seq,Homo_633,AD.vs.control	1
C3orf84	bulk RNA-seq,Homo_723,AD.vs.control; bulk RNA-seq,Homo_723,MCI.vs.control; bulk RNA-seq,Homo_633,MCI.vs.control	3

C3orf86	bulk RNA-seq,Homo_723,AD.vs.control; bulk RNA-seq,Homo_723,AD.vs.MCI; bulk RNA-seq,Homo_633,AD.vs.control; bulk RNA-seq,Homo_633,AD.vs.MCI; bulk RNA-seq,Homo_714,AD.vs.control; bulk RNA-seq,Homo_714,AD.vs.MCI	6
C4A	bulk RNA-seq,Homo_714,MCI.vs.control	1
C4BPA	bulk RNA-seq,Homo_723,AD.vs.control; bulk RNA-seq,Homo_723,AD.vs.MCI; bulk RNA-seq,Homo_633,AD.vs.control; bulk RNA-seq,Homo_633,AD.vs.MCI; bulk RNA-seq,Homo_714,AD.vs.control; bulk RNA-seq,Homo_714,AD.vs.MCI	6
C4BPB	bulk RNA-seq,Homo_723,AD.vs.control; bulk RNA-seq,Homo_723,AD.vs.MCI; bulk RNA-seq,Homo_723,MCI.vs.control; bulk RNA-seq,Homo_633,AD.vs.control; bulk RNA-seq,Homo_633,AD.vs.MCI; bulk RNA-seq,Homo_633,MCI.vs.control; bulk RNA-seq,Homo_714,MCI.vs.control; scRNA-seq,SRP215507,CD8+ T cell_3-AD.vs.MCI; scRNA-seq,SRP215507,CD8+ T cell_3-MCI.vs.control	9
C4orf19	bulk RNA-seq,Homo_633,AD.vs.control; bulk RNA-seq,Homo_633,AD.vs.MCI; bulk RNA-seq,Homo_633,MCI.vs.control; bulk RNA-seq,Homo_714,AD.vs.control; bulk RNA-seq,Homo_714,AD.vs.MCI	5
C4orf3	bulk RNA-seq,Homo_723,AD.vs.control; bulk RNA-seq,Homo_723,AD.vs.MCI; bulk RNA-seq,Homo_723,MCI.vs.control; bulk RNA-seq,Homo_633,AD.vs.control; bulk RNA-seq,Homo_633,AD.vs.MCI; bulk RNA-seq,Homo_633,MCI.vs.control; bulk RNA-seq,Homo_714,AD.vs.control; bulk RNA-seq,Homo_714,AD.vs.MCI; scRNA-seq,SRP215507,CD8+ T cell_7-AD.vs.control; scRNA-seq,SRP215507,CD8+ T cell_9-AD.vs.control; scRNA-seq,SRP215507,CD8+ T cell_7-AD.vs.MCI; scRNA-seq,SRP215507,CD8+ T cell_4-MCI.vs.control; scRNA-seq,SRP215507,CD8+ T cell_6-MCI.vs.control; scRNA-seq,SRP309935,Erythroid cell-AD.vs.control; scRNA-seq,SRP330776,CD8+ T cell_1-AD.vs.control; scRNA-seq,SRP330776,Memory T cell_2-AD.vs.control; scRNA-seq,SRP330776,CD8+ T cell_2-AD.vs.control; scRNA-seq,SRP330776,Natural killer cell_1-AD.vs.control; scRNA-seq,SRP330776,CD8+ T cell_3-AD.vs.control; scRNA-seq,SRP330776,Monocyte_2-AD.vs.control; scRNA-seq,SRP330776,Natural [illegible]	21
C4orf33	bulk RNA-seq,Homo_633,AD.vs.control; bulk RNA-seq,Homo_714,AD.vs.control; bulk RNA-seq,Homo_714,AD.vs.MCI; scRNA-seq,SRP215507,CD8+ T cell_3-AD.vs.MCI; scRNA-seq,SRP215507,CD8+ T cell_6-MCI.vs.control; scRNA-seq,SRP309935,Naive CD8+ T cell_2-AD.vs.control; scRNA-seq,SRP330776,γδ T cell-AD.vs.control; scRNA-seq,SRP330776,B cell_1-AD.vs.control; scRNA-seq,SRP330776,Natural killer cell_1-AD.vs.control; scRNA-seq,SRP330776,B cell_2-AD.vs.control; scRNA-seq,SRP330776,Monocyte_2-AD.vs.control	11
C4orf36	bulk RNA-seq,Homo_723,AD.vs.control; bulk RNA-seq,Homo_723,AD.vs.MCI; bulk RNA-seq,Homo_633,AD.vs.control; bulk RNA-	4
C4orf46	bulk RNA-seq,Homo_714,AD.vs.control; bulk RNA-seq,Homo_714,AD.vs.MCI; scRNA-seq,SRP215507,CD8+ T cell_5-MCI.vs.control; scRNA-seq,SRP215507,CD8+ T cell_2-MCI.vs.control; scRNA-seq,SRP309935,Natural killer cell_1-AD.vs.control; scRNA-seq,SRP330776,Memory T cell_2-AD.vs.control; scRNA-seq,SRP330776,Natural killer cell_1-AD.vs.control; scRNA-seq,SRP330776,Monocyte_2-AD.vs.control	8
C4orf47	bulk RNA-seq,Homo_723,AD.vs.control; bulk RNA-seq,Homo_723,AD.vs.MCI; bulk RNA-seq,Homo_633,AD.vs.control; bulk RNA-seq,Homo_633,AD.vs.MCI; scRNA-seq,SRP215507,CD8+ T cell_1-AD.vs.MCI; scRNA-seq,SRP330776,CD8+ T cell_1-AD.vs.control; scRNA-seq,SRP330776,γδ T cell-AD.vs.control; scRNA-seq,SRP330776,B cell_1-AD.vs.control; scRNA-seq,SRP330776,Naive CD8+ T cell_1-AD.vs.control; scRNA-seq,SRP330776,Natural killer cell_1-AD.vs.control; scRNA-seq,SRP330776,Memory T cell_1-AD.vs.control; scRNA-seq,SRP330776,Naive CD8+	13
C4orf48	bulk RNA-seq,Homo_723,AD.vs.MCI; bulk RNA-seq,Homo_723,MCI.vs.control; bulk RNA-seq,Homo_633,AD.vs.control; bulk RNA-seq,Homo_633,AD.vs.MCI; bulk RNA-seq,Homo_633,MCI.vs.control; bulk RNA-seq,Homo_714,AD.vs.control; bulk RNA-seq,Homo_714,AD.vs.MCI; bulk RNA-seq,Homo_714,MCI.vs.control; scRNA-seq,SRP309935,Monocyte_1-AD.vs.control; scRNA-seq,SRP309935,Naive CD8+ T cell_1-AD.vs.control; scRNA-seq,SRP309935,CD8+ T cell_1-AD.vs.control; scRNA-seq,SRP309935,B cell_1-AD.vs.control; scRNA-seq,SRP309935,Monocyte_3-AD.vs.control; scRNA-seq,SRP309935,Dendritic cell-AD.vs.control; scRNA-seq,SRP330776,CD8+ T cell_1-AD.vs.control; scRNA-seq,SRP330776,γδ T cell-AD.vs.control; scRNA-seq,SRP330776,Memory T cell_2-AD.vs.control; scRNA-seq,SRP330776,CD8+ T cell_2-AD.vs.control; scRNA-seq,SRP330776,B cell_1-AD.vs.control; scRNA-seq,SRP330776,Naive CD8+ T cell_1-AD.vs.control; scRNA-seq,SRP330776,Natural killer cell_1-AD.vs.control; scRNA-seq,SRP330776,Memory T cell_1-AD.vs.control; scRNA-seq,SRP330776,Natural killer T cell-AD.vs.control; scRNA-seq,SRP330776,B cell_2-AD.vs.control; scRNA-seq,SRP330776,T cell-AD.vs.control; scRNA-seq,SRP330776,Naive CD8+ T cell_2-AD.vs.control; scRNA-seq,SRP330776,CD8+ T cell_3-AD.vs.control; scRNA-seq,SRP330776,Monocyte_1-AD.vs.control; scRNA- [illegible]	32
C4orf50	bulk RNA-seq,Homo_714,AD.vs.control; bulk RNA-seq,Homo_714,AD.vs.MCI; scRNA-seq,SRP330776,γδ T cell-AD.vs.control	3
C4orf54	bulk RNA-seq,Homo_714,MCI.vs.control	1
C5	bulk RNA-seq,Homo_723,AD.vs.MCI; bulk RNA-seq,Homo_723,MCI.vs.control; bulk RNA-seq,Homo_633,AD.vs.MCI; bulk RNA-seq,Homo_633,MCI.vs.control; bulk RNA-seq,Homo_714,AD.vs.control; bulk RNA-seq,Homo_714,AD.vs.MCI; bulk RNA-	8

C5AR1	bulk RNA-seq,Homo_723,AD.vs.control; bulk RNA-seq,Homo_723,AD.vs.MCI; bulk RNA-seq,Homo_633,AD.vs.control; bulk RNA-seq,SRP223445,AD.vs.control; scRNA-seq,SRP215507,CD8+ T cell_3-AD.vs.control; scRNA-seq,SRP309935,CD8+ T cell_2-AD.vs.control	6
C5ar1	bulk RNA-seq,SRP325058,AD.vs.control	1
C5AR2	bulk RNA-seq,SRP223445,AD.vs.control; scRNA-seq,SRP215507,CD8+ T cell_2-AD.vs.MCI; scRNA-seq,SRP215507,CD8+ T cell_1-AD.vs.MCI; scRNA-seq,SRP215507,CD8+ T cell_1-MCI.vs.control; scRNA-seq,SRP215507,CD8+ T cell_2-MCI.vs.control; scRNA-seq,SRP330776,Monocyte_1-	6
C5ar2	bulk RNA-seq,SRP325058,AD.vs.control	1
C5orf15	bulk RNA-seq,Homo_723,AD.vs.control; bulk RNA-seq,Homo_633,AD.vs.control; bulk RNA-seq,Homo_633,AD.vs.MCI; bulk RNA-seq,Homo_714,AD.vs.control; bulk RNA-seq,Homo_714,AD.vs.MCI; scRNA-seq,SRP215507,CD8+ T cell_7-AD.vs.control; scRNA-seq,SRP215507,CD8+ T cell_5-AD.vs.control; scRNA-seq,SRP215507,CD8+ T cell_4-MCI.vs.control; scRNA-seq,SRP330776,Memory T cell_2-	10
C5orf17	scRNA-seq,SRP215507,CD8+ T cell_2-AD.vs.control; scRNA-seq,SRP215507,CD8+ T cell_1-AD.vs.control; scRNA-seq,SRP215507,CD8+ T cell_2-AD.vs.MCI; scRNA-seq,SRP215507,CD8+ T cell_1-AD.vs.MCI; scRNA-seq,SRP215507,CD8+ T cell_4-AD.vs.MCI; scRNA-seq,SRP215507,CD8+ T cell_1-MCI.vs.control; scRNA-seq,SRP215507,CD8+ T cell_5-MCI.vs.control; scRNA-seq,SRP215507,CD8+ T cell_4-MCI.vs.control; scRNA-seq,SRP215507,CD8+ T cell_2-MCI.vs.control; scRNA-seq,SRP330776,Memory T cell_2-AD.vs.control; scRNA-seq,SRP330776,CD8+ T cell_2-AD.vs.control; scRNA-seq,SRP330776,Memory T cell_1-AD.vs.control; scRNA-seq,SRP330776,Naive CD8+ T cell_2-AD.vs.control	13
C5orf22	bulk RNA-seq,Homo_723,AD.vs.MCI; bulk RNA-seq,Homo_714,AD.vs.control; bulk RNA-seq,Homo_714,AD.vs.MCI; scRNA-seq,SRP215507,CD8+ T cell_3-AD.vs.control; scRNA-seq,SRP215507,CD8+ T cell_3-AD.vs.MCI; scRNA-seq,SRP330776,Natural killer T cell-AD.vs.control; scRNA-seq,SRP330776,Monocyte_2-AD.vs.control; scRNA-seq,SRP330776,B cell_3-AD.vs.control	8
C5orf24	scRNA-seq,SRP215507,CD8+ T cell_5-AD.vs.MCI; scRNA-seq,SRP215507,CD8+ T cell_1-MCI.vs.control; scRNA-seq,SRP215507,CD8+ T cell_5-MCI.vs.control; scRNA-seq,SRP215507,CD8+ T cell_3-MCI.vs.control; scRNA-seq,SRP330776,CD8+ T cell_1-AD.vs.control; scRNA-seq,SRP330776,γδ T cell-AD.vs.control; scRNA-seq,SRP330776,Memory T cell_2-AD.vs.control; scRNA-seq,SRP330776,CD8+ T cell_2-AD.vs.control; scRNA-seq,SRP330776,B cell_1-AD.vs.control; scRNA-seq,SRP330776,Naive CD8+ T cell_1-AD.vs.control; scRNA-seq,SRP330776,Natural killer cell_1-AD.vs.control; scRNA-seq,SRP330776,Memory T cell_1-AD.vs.control; scRNA-seq,SRP330776,Natural killer T cell-AD.vs.control; scRNA-seq,SRP330776,Naive CD8+ T cell_3-AD.vs.control; scRNA-seq,SRP330776,B cell_2-AD.vs.control; scRNA-seq,SRP330776,T cell-AD.vs.control; [illegible]	18
C5orf30	scRNA-seq,SRP215507,CD8+ T cell_2-AD.vs.control; scRNA-seq,SRP215507,CD8+ T cell_2-AD.vs.MCI; scRNA-seq,SRP215507,CD8+ T cell_1-MCI.vs.control; scRNA-seq,SRP330776,Memory T cell_2-AD.vs.control; scRNA-seq,SRP330776,CD8+ T cell_2-AD.vs.control; scRNA-seq,SRP330776,B cell_1-AD.vs.control; scRNA-seq,SRP330776,Natural killer cell_1-AD.vs.control; scRNA-seq,SRP330776,B cell_2-AD.vs.control; scRNA-seq,SRP330776,T cell-AD.vs.control; scRNA-seq,SRP330776,CD8+ T cell_3-AD.vs.control; scRNA-seq,SRP330776,Natural killer cell_2-	11
C5orf34	scRNA-seq,SRP215507,CD8+ T cell_3-AD.vs.MCI; scRNA-seq,SRP215507,CD8+ T cell_1-AD.vs.MCI; scRNA-seq,SRP215507,CD8+ T cell_5-AD.vs.MCI; scRNA-seq,SRP215507,CD8+ T cell_1-MCI.vs.control; scRNA-seq,SRP215507,CD8+ T cell_5-MCI.vs.control; scRNA-seq,SRP215507,CD8+ T cell_3-MCI.vs.control; scRNA-seq,SRP330776,CD8+ T cell_2-AD.vs.control; scRNA-seq,SRP330776,Naive CD8+ T cell_1-AD.vs.control; scRNA-seq,SRP330776,Memory T cell_1-AD.vs.control; scRNA-seq,SRP330776,B cell_2-AD.vs.control; scRNA-seq,SRP330776,CD8+ T	11
C5orf46	bulk RNA-seq,Homo_714,MCI.vs.control	1
C5orf47	bulk RNA-seq,Homo_723,AD.vs.control; bulk RNA-seq,Homo_723,AD.vs.MCI; bulk RNA-seq,Homo_633,AD.vs.MCI	3
C5orf49	bulk RNA-seq,Homo_723,AD.vs.control; bulk RNA-seq,Homo_723,AD.vs.MCI	2
C5orf51	bulk RNA-seq,Homo_723,AD.vs.control; bulk RNA-seq,Homo_723,AD.vs.MCI; bulk RNA-seq,Homo_723,MCI.vs.control; bulk RNA-seq,Homo_633,AD.vs.control; bulk RNA-seq,Homo_633,AD.vs.MCI; bulk RNA-seq,Homo_633,MCI.vs.control; bulk RNA-seq,Homo_714,AD.vs.control; bulk RNA-seq,Homo_714,AD.vs.MCI; bulk RNA-seq,Homo_714,MCI.vs.control; scRNA-seq,SRP215507,CD8+ T cell_4-AD.vs.control; scRNA-seq,SRP215507,CD8+ T cell_4-AD.vs.MCI; scRNA-seq,SRP330776,Naive CD8+ T cell_3-AD.vs.control; scRNA-seq,SRP330776,T cell-AD.vs.control	13
C5orf52	bulk RNA-seq,Homo_723,MCI.vs.control; bulk RNA-seq,Homo_633,AD.vs.MCI; bulk RNA-seq,Homo_633,MCI.vs.control; bulk RNA-	4

C5orf56	scRNA-seq,SRP215507,CD8+ T cell_3-AD.vs.control; scRNA-seq,SRP215507,CD8+ T cell_7-AD.vs.control; scRNA-seq,SRP215507,CD8+ T cell_3-AD.vs.MCI; scRNA-seq,SRP215507,CD8+ T cell_4-AD.vs.MCI; scRNA-seq,SRP215507,CD8+ T cell_7-AD.vs.MCI; scRNA-seq,SRP215507,CD8+ T cell_5-AD.vs.MCI; scRNA-seq,SRP215507,CD8+ T cell_6-AD.vs.MCI; scRNA-seq,SRP330776,γδ T cell-AD.vs.control; scRNA-seq,SRP330776,Memory T cell_2-AD.vs.control; scRNA-seq,SRP330776,CD8+ T cell_2-AD.vs.control; scRNA-seq,SRP330776,B cell_1-AD.vs.control; scRNA-seq,SRP330776,Naive CD8+ T cell_1-AD.vs.control; scRNA-seq,SRP330776,Natural killer cell_1-AD.vs.control; scRNA-seq,SRP330776,Memory T cell_1-AD.vs.control; scRNA-seq,SRP330776,B cell_3-AD.vs.control; scRNA-seq,SRP330776,Natural killer cell_2-AD.vs.control	16
C5orf58	bulk RNA-seq,Homo_723,AD.vs.control; bulk RNA-seq,Homo_723,AD.vs.MCI; bulk RNA-seq,Homo_633,AD.vs.control; bulk RNA-seq,Homo_633,AD.vs.MCI; bulk RNA-seq,Homo_714,AD.vs.control; bulk RNA-seq,Homo_714,AD.vs.MCI	6
C5orf63	bulk RNA-seq,Homo_723,AD.vs.control; bulk RNA-seq,Homo_723,AD.vs.MCI; bulk RNA-seq,Homo_633,AD.vs.control; bulk RNA-seq,Homo_633,AD.vs.MCI; bulk RNA-seq,Homo_714,AD.vs.control; bulk RNA-seq,Homo_714,AD.vs.MCI; bulk RNA-seq,ROSMAP,MCI.vs.control; scRNA-seq,SRP215507,CD8+ T cell_4-AD.vs.control; scRNA-seq,SRP215507,CD8+ T cell_2-AD.vs.MCI; scRNA-seq,SRP215507,CD8+ T cell_7-AD.vs.MCI; scRNA-seq,SRP215507,CD8+ T cell_3-MCI.vs.control; scRNA-seq,SRP215507,CD8+ T cell_7-MCI.vs.control; scRNA-seq,SRP330776,γδ T cell-AD.vs.control; scRNA-seq,SRP330776,CD8+ T cell_2-AD.vs.control; scRNA-seq,SRP330776,B cell_1-AD.vs.control	15
C6	bulk RNA-seq,Homo_723,AD.vs.MCI	1
C6orf118	bulk RNA-seq,Homo_723,AD.vs.control; bulk RNA-seq,Homo_723,AD.vs.MCI	2
C6orf120	scRNA-seq,SRP215507,CD8+ T cell_3-AD.vs.control; scRNA-seq,SRP215507,CD8+ T cell_4-AD.vs.control; scRNA-seq,SRP215507,CD8+ T cell_4-AD.vs.MCI; scRNA-seq,SRP215507,CD8+ T cell_5-MCI.vs.control; scRNA-seq,SRP215507,CD8+ T cell_6-MCI.vs.control; scRNA-seq,SRP309935,Monocyte_1-AD.vs.control; scRNA-seq,SRP330776,CD8+ T cell_1-AD.vs.control; scRNA-seq,SRP330776,CD8+ T cell_2-AD.vs.control; scRNA-seq,SRP330776,Naive CD8+ T cell_1-AD.vs.control; scRNA-seq,SRP330776,Naive CD8+ T cell_3-AD.vs.control; scRNA-seq,SRP330776,B	12
C6orf132	bulk RNA-seq,Homo_723,AD.vs.control; bulk RNA-seq,Homo_723,AD.vs.MCI; bulk RNA-seq,Homo_633,AD.vs.control; bulk RNA-seq,Homo_633,AD.vs.MCI; scRNA-seq,SRP215507,CD8+ T cell_1-AD.vs.control; scRNA-seq,SRP215507,CD8+ T cell_1-AD.vs.MCI; scRNA-seq,SRP330776,B cell_1-AD.vs.control; scRNA-seq,SRP330776,Naive CD8+ T cell_1-AD.vs.control	8
C6orf136	scRNA-seq,SRP309935,CD4+ T cell-AD.vs.control; scRNA-seq,SRP330776,CD8+ T cell_1-AD.vs.control; scRNA-seq,SRP330776,Memory T cell_2-AD.vs.control; scRNA-seq,SRP330776,Naive CD8+ T cell_3-AD.vs.control; scRNA-seq,SRP330776,B cell_2-AD.vs.control	5
C6orf141	bulk RNA-seq,Homo_723,AD.vs.control; bulk RNA-seq,Homo_633,AD.vs.control; bulk RNA-seq,Homo_714,MCI.vs.control	3
C6orf163	bulk RNA-seq,Homo_723,AD.vs.control; bulk RNA-seq,Homo_633,AD.vs.control; bulk RNA-seq,Homo_714,AD.vs.control; bulk RNA-seq,Homo_714,AD.vs.MCI; scRNA-seq,SRP215507,CD8+ T cell_4-MCI.vs.control; scRNA-seq,SRP330776,Naive CD8+ T cell_1-AD.vs.control	6
C6orf201	bulk RNA-seq,Homo_633,AD.vs.control; bulk RNA-seq,Homo_633,AD.vs.MCI; bulk RNA-seq,Homo_714,AD.vs.control; bulk RNA-seq,Homo_714,AD.vs.MCI; scRNA-seq,SRP330776,Memory T cell_1-AD.vs.control	5
C6orf226	scRNA-seq,SRP215507,CD8+ T cell_7-AD.vs.control; scRNA-seq,SRP215507,CD8+ T cell_9-AD.vs.control; scRNA-seq,SRP215507,CD8+ T cell_4-AD.vs.MCI; scRNA-seq,SRP215507,CD8+ T cell_6-AD.vs.MCI; scRNA-seq,SRP330776,Megakaryocyte_1-AD.vs.control	5
C6orf47	scRNA-seq,SRP215507,CD8+ T cell_4-AD.vs.control; scRNA-seq,SRP215507,CD8+ T cell_4-AD.vs.MCI; scRNA-seq,SRP215507,CD8+ T cell_5-AD.vs.MCI; scRNA-seq,SRP215507,CD8+ T cell_5-MCI.vs.control; scRNA-seq,SRP330776,γδ T cell-AD.vs.control; scRNA-seq,SRP330776,B cell_1-AD.vs.control; scRNA-seq,SRP330776,Naive CD8+ T cell_3-AD.vs.control; scRNA-seq,SRP330776,CD8+ T cell_3-AD.vs.control; scRNA-seq,SRP330776,B cell_3-AD.vs.control; scRNA-seq,SRP330776,Megakaryocyte_1-AD.vs.control	10
C6orf52	bulk RNA-seq,Homo_723,AD.vs.MCI; bulk RNA-seq,Homo_633,AD.vs.control; bulk RNA-seq,Homo_633,AD.vs.MCI	3
C6orf58	bulk RNA-seq,Homo_723,AD.vs.MCI	1
C6orf62	scRNA-seq,SRP330776,CD8+ T cell_1-AD.vs.control; scRNA-seq,SRP330776,γδ T cell-AD.vs.control; scRNA-seq,SRP330776,Memory T cell_2-AD.vs.control; scRNA-seq,SRP330776,B cell_1-AD.vs.control; scRNA-seq,SRP330776,Naive CD8+ T cell_1-AD.vs.control; scRNA-seq,SRP330776,Natural killer cell_1-AD.vs.control; scRNA-seq,SRP330776,Memory T cell_1-AD.vs.control; scRNA-seq,SRP330776,Naive CD8+ T cell_3-AD.vs.control; scRNA-seq,SRP330776,B cell_2-AD.vs.control; scRNA-seq,SRP330776,T cell-AD.vs.control; scRNA-seq,SRP330776,Naive CD8+ T cell_2-AD.vs.control; scRNA-seq,SRP330776,CD8+ T cell_3-AD.vs.control; scRNA-seq,SRP330776,Monocyte_2-AD.vs.control; scRNA-seq,SRP330776,Regulatory T cell-AD.vs.control; scRNA-seq,SRP330776,Megakaryocyte_1-AD.vs.control; scRNA-seq,SRP330776,Natural killer cell_2-	16

C6orf89	scRNA-seq,SRP330776,Natural killer cell_1-AD.vs.control	1
C6orf99	scRNA-seq,SRP215507,CD8+ T cell_5-AD.vs.MCI; scRNA-seq,SRP215507,CD8+ T cell_5-MCI.vs.control; scRNA-seq,SRP330776,CD8+ T cell_1-AD.vs.control; scRNA-seq,SRP330776,γδ T cell-AD.vs.control; scRNA-seq,SRP330776,Memory T cell_2-AD.vs.control; scRNA-seq,SRP330776,CD8+ T cell_2-AD.vs.control; scRNA-seq,SRP330776,Naive CD8+ T cell_1-AD.vs.control; scRNA-seq,SRP330776,Memory T cell_1-AD.vs.control; scRNA-seq,SRP330776,Natural killer T cell-AD.vs.control; scRNA-seq,SRP330776,CD8+ T cell_3-AD.vs.control; scRNA-seq,SRP330776,Natural killer cell_2-	11
C7	bulk RNA-seq,Homo_723,AD.vs.control; bulk RNA-seq,Homo_723,AD.vs.MCI; bulk RNA-seq,Homo_723,MCI.vs.control; bulk RNA-seq,Homo_633,AD.vs.control; bulk RNA-seq,Homo_633,AD.vs.MCI; bulk RNA-seq,Homo_633,MCI.vs.control	6
C7orf25	bulk RNA-seq,ROSMAP,AD.vs.control; scRNA-seq,SRP215507,CD8+ T cell_3-AD.vs.control; scRNA-seq,SRP215507,CD8+ T cell_2-AD.vs.MCI; scRNA-seq,SRP215507,CD8+ T cell_4-MCI.vs.control; scRNA-seq,SRP215507,CD8+ T cell_3-MCI.vs.control; scRNA-seq,SRP309935,B cell_1-AD.vs.control; scRNA-seq,SRP330776,γδ T cell-AD.vs.control; scRNA-seq,SRP330776,Memory T cell_2-AD.vs.control; scRNA-seq,SRP330776,CD8+ T cell_2-AD.vs.control; scRNA-seq,SRP330776,Natural killer cell_1-AD.vs.control; scRNA-seq,SRP330776,Memory T cell_1-AD.vs.control; scRNA-seq,SRP330776,Naive CD8+ T cell_3-AD.vs.control; scRNA-seq,SRP330776,B cell_2-AD.vs.control; scRNA-seq,SRP330776,T cell-AD.vs.control	14
C7orf26	bulk RNA-seq,Homo_633,AD.vs.control; bulk RNA-seq,Homo_714,AD.vs.control; bulk RNA-seq,Homo_714,AD.vs.MCI; scRNA-seq,SRP215507,CD8+ T cell_10-AD.vs.control; scRNA-seq,SRP215507,CD8+ T cell_5-AD.vs.MCI; scRNA-seq,SRP309935,Natural killer cell_2-AD.vs.control; scRNA-seq,SRP309935,Naive CD8+ T cell_1-AD.vs.control; scRNA-seq,SRP309935,Naive CD8+ T cell_2-AD.vs.control; scRNA-seq,SRP309935,B cell_1-AD.vs.control; scRNA-seq,SRP330776,CD8+ T cell_1-AD.vs.control; scRNA-seq,SRP330776,γδ T cell-AD.vs.control; scRNA-seq,SRP330776,Memory T cell_2-AD.vs.control; scRNA-seq,SRP330776,CD8+ T cell_2-AD.vs.control; scRNA-seq,SRP330776,B cell_1-AD.vs.control; scRNA-seq,SRP330776,Naive CD8+ T cell_1-AD.vs.control; scRNA-seq,SRP330776,Natural killer cell_1-AD.vs.control; scRNA-seq,SRP330776,Memory T cell_1-AD.vs.control; scRNA-seq,SRP330776,Natural killer T cell-AD.vs.control; scRNA-seq,SRP330776,Naive CD8+ T cell_3-AD.vs.control; scRNA-seq,SRP330776,B cell_2-AD.vs.control; scRNA-seq,SRP330776,T cell-AD.vs.control; scRNA-seq,SRP330776,CD8+ T cell_3-AD.vs.control; scRNA-seq,SRP330776,Monocyte_1-AD.vs.control; scRNA-seq,SRP330776,Monocyte_2-AD.vs.control; scRNA-seq,SRP330776,B cell_3-AD.vs.control; scRNA- [illegible]	26
C7orf31	bulk RNA-seq,Homo_723,AD.vs.control; bulk RNA-seq,Homo_723,AD.vs.MCI; bulk RNA-seq,Homo_633,AD.vs.control; bulk RNA-seq,Homo_633,AD.vs.MCI; bulk RNA-seq,Homo_714,AD.vs.control; bulk RNA-seq,Homo_714,AD.vs.MCI; scRNA-seq,SRP309935,Naive CD8+ T cell_2-AD.vs.control; scRNA-seq,SRP330776,Memory T cell_1-AD.vs.control; scRNA-seq,SRP330776,T cell-AD.vs.control; scRNA-	10
C7orf50	bulk RNA-seq,Homo_723,AD.vs.control; bulk RNA-seq,Homo_723,AD.vs.MCI; bulk RNA-seq,Homo_633,AD.vs.control; bulk RNA-seq,Homo_633,AD.vs.MCI; bulk RNA-seq,Homo_714,AD.vs.control; bulk RNA-seq,Homo_714,AD.vs.MCI; bulk RNA-seq,Homo_714,MCI.vs.control; scRNA-seq,SRP215507,CD8+ T cell_7-AD.vs.control; scRNA-seq,SRP215507,CD8+ T cell_4-AD.vs.MCI; scRNA-seq,SRP215507,CD8+ T cell_5-AD.vs.MCI; scRNA-seq,SRP215507,CD8+ T cell_5-MCI.vs.control; scRNA-seq,SRP215507,CD8+ T cell_2-MCI.vs.control; scRNA-	14
C7orf57	bulk RNA-seq,Homo_723,AD.vs.control; bulk RNA-seq,Homo_723,AD.vs.MCI; bulk RNA-seq,Homo_723,MCI.vs.control; bulk RNA-seq,Homo_633,AD.vs.control; bulk RNA-seq,Homo_633,MCI.vs.control; bulk RNA-seq,Homo_714,MCI.vs.control	6
C7orf61	scRNA-seq,SRP215507,CD8+ T cell_1-AD.vs.MCI	1
C8G	bulk RNA-seq,Homo_723,AD.vs.control; bulk RNA-seq,Homo_723,AD.vs.MCI; bulk RNA-seq,Homo_723,MCI.vs.control; bulk RNA-seq,Homo_633,AD.vs.control; bulk RNA-seq,Homo_633,AD.vs.MCI; bulk RNA-seq,Homo_633,MCI.vs.control; bulk RNA-seq,Homo_714,AD.vs.control; bulk RNA-seq,Homo_714,AD.vs.MCI; bulk RNA-seq,Homo_714,MCI.vs.control	9
C8orf31	scRNA-seq,SRP215507,CD8+ T cell_1-AD.vs.control; scRNA-seq,SRP215507,CD8+ T cell_1-AD.vs.MCI; scRNA-seq,SRP330776,CD8+ T cell_2-AD.vs.control; scRNA-seq,SRP330776,Memory T cell_1-AD.vs.control; scRNA-seq,SRP330776,B cell_2-AD.vs.control; scRNA-	6
C8orf33	bulk RNA-seq,Homo_723,AD.vs.control; bulk RNA-seq,Homo_633,AD.vs.control; bulk RNA-seq,Homo_633,AD.vs.MCI; bulk RNA-seq,Homo_714,AD.vs.control; bulk RNA-seq,Homo_714,AD.vs.MCI; scRNA-seq,SRP215507,CD8+ T cell_4-AD.vs.MCI; scRNA-seq,SRP215507,CD8+ T cell_4-MCI.vs.control; scRNA-seq,SRP330776,B cell_1-AD.vs.control; scRNA-seq,SRP330776,Natural killer cell_1-AD.vs.control; scRNA-seq,SRP330776,Memory T cell_1-AD.vs.control; scRNA-seq,SRP330776,B cell_2-AD.vs.control; scRNA-seq,SRP330776,T cell-AD.vs.control; scRNA-seq,SRP330776,CD8+ T cell_3-AD.vs.control; scRNA-seq,SRP330776,Natural killer cell_2-AD.vs.control	14
C8orf34	bulk RNA-seq,Homo_723,AD.vs.control; bulk RNA-seq,Homo_714,MCI.vs.control; bulk RNA-seq,ROSMAP,AD.vs.control; bulk RNA-	4

C8orf37	scRNA-seq,SRP215507,CD8+ T cell_1-AD.vs.control; scRNA-seq,SRP215507,CD8+ T cell_5-AD.vs.control; scRNA-seq,SRP215507,CD8+ T cell_1-AD.vs.MCI; scRNA-seq,SRP215507,CD8+ T cell_3-MCI.vs.control; scRNA-seq,SRP309935,Natural killer cell_1-AD.vs.control; scRNA-	6
C8orf37-AS1	scRNA-seq,SRP215507,CD8+ T cell_3-AD.vs.MCI; scRNA-seq,SRP215507,CD8+ T cell_2-MCI.vs.control; scRNA-seq,SRP215507,CD8+ T cell_3-MCI.vs.control; scRNA-seq,SRP330776,CD8+ T cell_1-AD.vs.control; scRNA-seq,SRP330776,CD8+ T cell_2-AD.vs.control; scRNA-	6
C8orf44-SGK3	bulk RNA-seq,Homo_714,AD.vs.control; bulk RNA-seq,Homo_714,AD.vs.MCI	2
C8orf48	bulk RNA-seq,Homo_723,AD.vs.control; bulk RNA-seq,Homo_723,AD.vs.MCI; bulk RNA-seq,Homo_633,AD.vs.MCI	3
C8orf58	bulk RNA-seq,Homo_723,AD.vs.control; bulk RNA-seq,Homo_723,AD.vs.MCI; bulk RNA-seq,Homo_633,AD.vs.control; bulk RNA-seq,Homo_633,AD.vs.MCI; bulk RNA-seq,Homo_714,AD.vs.control; bulk RNA-seq,Homo_714,AD.vs.MCI; scRNA-seq,SRP215507,CD8+ T cell_4-AD.vs.MCI; scRNA-seq,SRP215507,CD8+ T cell_5-AD.vs.MCI; scRNA-seq,SRP215507,CD8+ T cell_4-MCI.vs.control; scRNA-seq,SRP215507,CD8+ T cell_3-MCI.vs.control; scRNA-seq,SRP309935,Naive CD8+ T cell_2-AD.vs.control; scRNA-seq,SRP330776,CD8+ T cell_1-AD.vs.control; scRNA-seq,SRP330776,γδ T cell-AD.vs.control; scRNA-seq,SRP330776,Memory T cell_2-AD.vs.control; scRNA-seq,SRP330776,CD8+ T cell_2-AD.vs.control; scRNA-seq,SRP330776,B cell_1-AD.vs.control; scRNA-seq,SRP330776,Naive CD8+ T cell_1-AD.vs.control; scRNA-seq,SRP330776,Natural killer cell_1-AD.vs.control; scRNA-seq,SRP330776,Memory T cell_1-AD.vs.control; scRNA-seq,SRP330776,Natural killer T cell-AD.vs.control; scRNA- [illegible]	23
C8orf76	bulk RNA-seq,Homo_723,AD.vs.control; bulk RNA-seq,Homo_723,AD.vs.MCI; bulk RNA-seq,Homo_633,AD.vs.control; bulk RNA-seq,Homo_633,AD.vs.MCI; bulk RNA-seq,Homo_714,AD.vs.control; bulk RNA-seq,Homo_714,AD.vs.MCI; scRNA-seq,SRP215507,CD8+ T cell_7-AD.vs.MCI; scRNA-seq,SRP215507,CD8+ T cell_6-AD.vs.MCI; scRNA-seq,SRP215507,CD8+ T cell_3-MCI.vs.control; scRNA-seq,SRP215507,CD8+ T cell_7-MCI.vs.control; scRNA-seq,SRP309935,Naive CD8+ T cell_2-AD.vs.control; scRNA-seq,SRP309935,CD8+ T cell_1-AD.vs.control; scRNA-seq,SRP330776,B cell_1-AD.vs.control; scRNA-seq,SRP330776,Megakaryocyte_1-AD.vs.control	14
C8orf82	bulk RNA-seq,Homo_723,AD.vs.control; bulk RNA-seq,Homo_723,AD.vs.MCI; bulk RNA-seq,Homo_723,MCI.vs.control; bulk RNA-seq,Homo_633,AD.vs.control; bulk RNA-seq,Homo_633,AD.vs.MCI; bulk RNA-seq,Homo_714,AD.vs.control; bulk RNA-seq,Homo_714,AD.vs.MCI; scRNA-seq,SRP215507,CD8+ T cell_3-AD.vs.MCI; scRNA-seq,SRP215507,CD8+ T cell_4-AD.vs.MCI; scRNA-seq,SRP215507,CD8+ T cell_4-MCI.vs.control; scRNA-seq,SRP215507,CD8+ T cell_3-MCI.vs.control; scRNA-seq,SRP309935,Natural killer cell_2-AD.vs.control; scRNA-seq,SRP309935,Monocyte_1-AD.vs.control; scRNA-seq,SRP309935,Megakaryocyte_1-AD.vs.control; scRNA-seq,SRP309935,Naive CD8+ T cell_1-AD.vs.control; scRNA-seq,SRP309935,Natural killer cell_1-AD.vs.control; scRNA-seq,SRP309935,Monocyte_2-AD.vs.control; scRNA-seq,SRP309935,Naive CD8+ T cell_2-AD.vs.control; scRNA-seq,SRP309935,CD8+ T cell_1-AD.vs.control; scRNA-seq,SRP309935,B cell_1-AD.vs.control; scRNA-seq,SRP309935,B cell_2-AD.vs.control; scRNA-seq,SRP330776,γδ T cell-AD.vs.control; scRNA-seq,SRP330776,Memory T cell_2-AD.vs.control; scRNA-seq,SRP330776,CD8+ T cell_2-AD.vs.control; scRNA-seq,SRP330776,Naive CD8+ T cell_1-AD.vs.control; scRNA- [illegible]	28
C8orf88	bulk RNA-seq,Homo_723,AD.vs.control; bulk RNA-seq,Homo_723,AD.vs.MCI; bulk RNA-seq,Homo_723,MCI.vs.control; bulk RNA-seq,Homo_633,AD.vs.control; bulk RNA-seq,Homo_633,AD.vs.MCI; bulk RNA-seq,Homo_633,MCI.vs.control; bulk RNA-seq,Homo_714,AD.vs.control; bulk RNA-seq,Homo_714,AD.vs.MCI; scRNA-seq,SRP215507,CD8+ T cell_1-AD.vs.control; scRNA-seq,SRP215507,CD8+ T cell_1-AD.vs.MCI; scRNA-seq,SRP215507,CD8+ T cell_5-MCI.vs.control; scRNA-seq,SRP330776,γδ T cell-AD.vs.control; scRNA-seq,SRP330776,B cell_1-AD.vs.control;	14
C9orf116	bulk RNA-seq,Homo_723,AD.vs.control; bulk RNA-seq,Homo_723,AD.vs.MCI; scRNA-seq,SRP215507,CD8+ T cell_3-AD.vs.MCI; scRNA-seq,SRP215507,CD8+ T cell_3-MCI.vs.control; scRNA-seq,SRP330776,CD8+ T cell_2-AD.vs.control	5
C9orf135	bulk RNA-seq,Homo_723,MCI.vs.control; bulk RNA-seq,Homo_633,MCI.vs.control; bulk RNA-seq,Homo_714,MCI.vs.control	3
C9orf139	scRNA-seq,SRP215507,CD8+ T cell_1-AD.vs.MCI; scRNA-seq,SRP215507,CD8+ T cell_1-MCI.vs.control; scRNA-seq,SRP215507,CD8+ T cell_6-MCI.vs.control; scRNA-seq,SRP330776,Memory T cell_2-AD.vs.control; scRNA-seq,SRP330776,CD8+ T cell_2-AD.vs.control; scRNA-seq,SRP330776,B cell_1-AD.vs.control; scRNA-seq,SRP330776,Memory T cell_1-AD.vs.control; scRNA-seq,SRP330776,Natural killer T cell-	9
C9orf147	scRNA-seq,SRP215507,CD8+ T cell_1-AD.vs.control; scRNA-seq,SRP215507,CD8+ T cell_1-MCI.vs.control; scRNA-seq,SRP330776,CD8+ T cell_1-AD.vs.control; scRNA-seq,SRP330776,CD8+ T cell_2-AD.vs.control; scRNA-seq,SRP330776,Naive CD8+ T cell_1-AD.vs.control	5
C9orf152	bulk RNA-seq,Homo_723,AD.vs.control; bulk RNA-seq,Homo_723,AD.vs.MCI; bulk RNA-seq,Homo_633,AD.vs.control; bulk RNA-seq,Homo_633,AD.vs.MCI; bulk RNA-seq,ROSMAP,AD.vs.control; bulk RNA-seq,ROSMAP,AD.vs.MCI	6
C9orf153	bulk RNA-seq,Homo_723,AD.vs.control; bulk RNA-seq,Homo_723,AD.vs.MCI; bulk RNA-seq,Homo_633,AD.vs.control; bulk RNA-seq,Homo_633,AD.vs.MCI; bulk RNA-seq,Homo_714,MCI.vs.control	5

C9orf16	scRNA-seq,SRP215507,CD8+ T cell_7-AD.vs.control; scRNA-seq,SRP215507,CD8+ T cell_10-AD.vs.control; scRNA-seq,SRP215507,CD8+ T cell_4-AD.vs.MCI; scRNA-seq,SRP309935,Erythroid cell-AD.vs.control	4
C9orf24	bulk RNA-seq,Homo_723,AD.vs.control; bulk RNA-seq,Homo_723,AD.vs.MCI; bulk RNA-seq,Homo_633,AD.vs.control; bulk RNA-seq,Homo_633,AD.vs.MCI; bulk RNA-seq,Homo_714,AD.vs.control; bulk RNA-seq,Homo_714,AD.vs.MCI	6
C9orf40	bulk RNA-seq,Homo_723,AD.vs.control; bulk RNA-seq,Homo_723,AD.vs.MCI; bulk RNA-seq,Homo_633,AD.vs.control; bulk RNA-seq,Homo_633,AD.vs.MCI; bulk RNA-seq,Homo_714,AD.vs.control; bulk RNA-seq,Homo_714,AD.vs.MCI; scRNA-seq,SRP215507,CD8+ T cell_1-AD.vs.MCI; scRNA-seq,SRP215507,CD8+ T cell_1-MCI.vs.control; scRNA-seq,SRP330776,CD8+ T cell_1-AD.vs.control; scRNA-seq,SRP330776,Memory T cell_2-AD.vs.control; scRNA-seq,SRP330776,CD8+ T cell_2-AD.vs.control; scRNA-seq,SRP330776,B cell_1-AD.vs.control; scRNA-seq,SRP330776,Naive CD8+ T cell_1-AD.vs.control; scRNA-seq,SRP330776,Natural killer cell_1-AD.vs.control; scRNA-seq,SRP330776,Memory T cell_1-AD.vs.control; scRNA-seq,SRP330776,Natural killer T cell-AD.vs.control; scRNA-seq,SRP330776,Naive CD8+ T cell_3-AD.vs.control; scRNA-	18
C9orf43	bulk RNA-seq,Homo_723,AD.vs.MCI; bulk RNA-seq,Homo_723,MCI.vs.control; bulk RNA-seq,Homo_633,AD.vs.MCI; bulk RNA-seq,Homo_633,MCI.vs.control; bulk RNA-seq,Homo_714,AD.vs.control; bulk RNA-seq,Homo_714,AD.vs.MCI; bulk RNA-seq,Homo_714,MCI.vs.control; scRNA-seq,SRP330776,Naive CD8+ T cell_1-AD.vs.control	8
C9orf64	bulk RNA-seq,Homo_723,MCI.vs.control; bulk RNA-seq,Homo_633,AD.vs.control; bulk RNA-seq,Homo_633,AD.vs.MCI; bulk RNA-seq,Homo_633,MCI.vs.control; scRNA-seq,SRP330776,CD8+ T cell_1-AD.vs.control; scRNA-seq,SRP330776,γδ T cell-AD.vs.control; scRNA-seq,SRP330776,Memory T cell_2-AD.vs.control; scRNA-seq,SRP330776,Naive CD8+ T cell_1-AD.vs.control; scRNA-seq,SRP330776,Memory T cell_1-AD.vs.control; scRNA-seq,SRP330776,Naive CD8+ T cell_3-AD.vs.control; scRNA-seq,SRP330776,B cell_2-AD.vs.control; scRNA-	12
C9orf72	bulk RNA-seq,Homo_723,AD.vs.control; bulk RNA-seq,Homo_723,AD.vs.MCI; bulk RNA-seq,Homo_633,AD.vs.control; bulk RNA-seq,Homo_633,AD.vs.MCI; bulk RNA-seq,Homo_714,AD.vs.control; bulk RNA-seq,Homo_714,AD.vs.MCI; scRNA-seq,SRP215507,CD8+ T cell_3-AD.vs.control; scRNA-seq,SRP215507,CD8+ T cell_1-AD.vs.MCI; scRNA-seq,SRP215507,CD8+ T cell_1-MCI.vs.control; scRNA-seq,SRP215507,CD8+ T cell_3-MCI.vs.control; scRNA-seq,SRP330776,Naive CD8+ T cell_3-AD.vs.control; scRNA-seq,SRP330776,T cell-AD.vs.control; scRNA-	13
C9orf78	scRNA-seq,SRP215507,CD8+ T cell_7-AD.vs.control; scRNA-seq,SRP215507,CD8+ T cell_7-MCI.vs.control; scRNA-seq,SRP330776,Monocyte_1-AD.vs.control; scRNA-seq,SRP330776,Regulatory T cell-AD.vs.control	4
C9orf85	scRNA-seq,SRP215507,CD8+ T cell_4-AD.vs.MCI; scRNA-seq,SRP215507,CD8+ T cell_4-MCI.vs.control; scRNA-seq,SRP330776,Natural killer cell_1-AD.vs.control; scRNA-seq,SRP330776,T cell-AD.vs.control; scRNA-seq,SRP330776,CD8+ T cell_3-AD.vs.control	5
CA1	bulk RNA-seq,Homo_723,AD.vs.control; bulk RNA-seq,Homo_723,AD.vs.MCI; scRNA-seq,SRP309935,Monocyte_1-AD.vs.control; scRNA-seq,SRP309935,Erythroid cell-AD.vs.control; scRNA-seq,SRP309935,Natural killer cell_1-AD.vs.control; scRNA-seq,SRP309935,Monocyte_2-	6
CA10	bulk RNA-seq,Homo_723,AD.vs.control; bulk RNA-seq,Homo_723,AD.vs.MCI; bulk RNA-seq,Homo_723,MCI.vs.control; bulk RNA-seq,Homo_633,AD.vs.control; bulk RNA-seq,Homo_633,AD.vs.MCI	5
CA11	bulk RNA-seq,Homo_723,AD.vs.control; bulk RNA-seq,Homo_633,AD.vs.control; scRNA-seq,SRP215507,CD8+ T cell_6-AD.vs.control; scRNA-seq,SRP215507,CD8+ T cell_7-AD.vs.MCI	4
CA12	bulk RNA-seq,Homo_723,AD.vs.control; bulk RNA-seq,Homo_723,AD.vs.MCI; bulk RNA-seq,Homo_633,AD.vs.MCI; bulk RNA-seq,Homo_714,AD.vs.control; bulk RNA-seq,Homo_714,AD.vs.MCI; bulk RNA-seq,ROSMAP,AD.vs.control; bulk RNA-seq,ROSMAP,MCI.vs.control	7
CA13	bulk RNA-seq,Homo_723,AD.vs.control; bulk RNA-seq,Homo_723,AD.vs.MCI; bulk RNA-seq,Homo_723,MCI.vs.control; bulk RNA-seq,Homo_633,AD.vs.control; bulk RNA-seq,Homo_633,AD.vs.MCI; bulk RNA-seq,Homo_633,MCI.vs.control; bulk RNA-seq,Homo_714,AD.vs.control; bulk RNA-seq,Homo_714,AD.vs.MCI; scRNA-seq,SRP215507,CD8+ T cell_4-AD.vs.control; scRNA-seq,SRP215507,CD8+ T cell_5-AD.vs.MCI; scRNA-seq,SRP330776,Naive CD8+ T cell_1-AD.vs.control; scRNA-seq,SRP330776,Natural killer cell_1-AD.vs.control	12
CA14	bulk RNA-seq,Homo_723,AD.vs.control; bulk RNA-seq,Homo_723,AD.vs.MCI; bulk RNA-seq,Homo_723,MCI.vs.control; bulk RNA-seq,Homo_633,AD.vs.control; bulk RNA-seq,Homo_633,AD.vs.MCI; bulk RNA-seq,Homo_714,AD.vs.control; bulk RNA-seq,Homo_714,AD.vs.MCI	7
CA2	bulk RNA-seq,Homo_633,MCI.vs.control; scRNA-seq,SRP215507,CD8+ T cell_2-AD.vs.control; scRNA-seq,SRP215507,CD8+ T cell_4-AD.vs.control; scRNA-seq,SRP215507,CD8+ T cell_5-AD.vs.MCI; scRNA-seq,SRP215507,CD8+ T cell_5-MCI.vs.control; scRNA-seq,SRP215507,CD8+ T cell_4-MCI.vs.control; scRNA-seq,SRP330776,Monocyte_1-AD.vs.control	7
CA3	bulk RNA-seq,Homo_723,AD.vs.control; bulk RNA-seq,Homo_723,AD.vs.MCI; bulk RNA-seq,Homo_723,MCI.vs.control; bulk RNA-seq,Homo_633,AD.vs.control; bulk RNA-seq,Homo_633,AD.vs.MCI; bulk RNA-seq,Homo_633,MCI.vs.control; bulk RNA-seq,Homo_714,AD.vs.control;	8

CA5A	bulk RNA-seq,Homo_723,AD.vs.control; bulk RNA-seq,Homo_723,AD.vs.MCI; bulk RNA-seq,Homo_633,AD.vs.control; bulk RNA-seq,Homo_633,AD.vs.MCI; bulk RNA-seq,ROSMAP,MCI.vs.control	5
CA5B	bulk RNA-seq,Homo_723,AD.vs.control; bulk RNA-seq,Homo_723,AD.vs.MCI; bulk RNA-seq,Homo_723,MCI.vs.control; bulk RNA-seq,Homo_633,AD.vs.control; bulk RNA-seq,Homo_633,MCI.vs.control; bulk RNA-seq,Homo_714,AD.vs.control; bulk RNA-seq,Homo_714,AD.vs.MCI; bulk RNA-seq,Homo_714,MCI.vs.control; scRNA-seq,SRP215507,CD8+ T cell_5-AD.vs.MCI; scRNA-seq,SRP309935,Megakaryocyte_1-AD.vs.control	10
CA6	bulk RNA-seq,Homo_723,AD.vs.control; bulk RNA-seq,Homo_723,AD.vs.MCI; bulk RNA-seq,Homo_633,AD.vs.control; bulk RNA-seq,Homo_633,AD.vs.MCI; bulk RNA-seq,Homo_714,AD.vs.control; bulk RNA-seq,Homo_714,AD.vs.MCI; scRNA-seq,SRP215507,CD8+ T cell_3-AD.vs.MCI; scRNA-seq,SRP215507,CD8+ T cell_3-MCI.vs.control	8
CA7	bulk RNA-seq,Homo_723,AD.vs.MCI; bulk RNA-seq,Homo_633,AD.vs.MCI	2
CA8	bulk RNA-seq,Homo_723,AD.vs.control; bulk RNA-seq,Homo_723,AD.vs.MCI; bulk RNA-seq,Homo_633,AD.vs.control; bulk RNA-seq,Homo_633,AD.vs.MCI; bulk RNA-seq,Homo_714,AD.vs.control; bulk RNA-seq,Homo_714,AD.vs.MCI	6
CA9	bulk RNA-seq,Homo_723,AD.vs.control; bulk RNA-seq,Homo_723,AD.vs.MCI; bulk RNA-seq,ROSMAP,AD.vs.control; bulk RNA-	4
CAAP1	scRNA-seq,SRP215507,CD8+ T cell_6-AD.vs.control; scRNA-seq,SRP215507,CD8+ T cell_5-AD.vs.MCI; scRNA-seq,SRP215507,CD8+ T cell_5-MCI.vs.control; scRNA-seq,SRP330776,γδ T cell-AD.vs.control; scRNA-seq,SRP330776,Memory T cell_2-AD.vs.control; scRNA-seq,SRP330776,B	6
CAB39	bulk RNA-seq,Homo_723,MCI.vs.control; bulk RNA-seq,Homo_633,MCI.vs.control; scRNA-seq,SRP309935,Naive CD8+ T cell_2-AD.vs.control; scRNA-seq,SRP309935,CD8+ T cell_1-AD.vs.control; scRNA-seq,SRP309935,B cell_1-AD.vs.control; scRNA-seq,SRP330776,CD8+ T cell_1-AD.vs.control; scRNA-seq,SRP330776,γδ T cell-AD.vs.control; scRNA-seq,SRP330776,Memory T cell_2-AD.vs.control; scRNA-seq,SRP330776,CD8+ T cell_2-AD.vs.control; scRNA-seq,SRP330776,B cell_1-AD.vs.control; scRNA-seq,SRP330776,Naive CD8+ T cell_1-AD.vs.control; scRNA-seq,SRP330776,Natural killer cell_1-AD.vs.control; scRNA-seq,SRP330776,Memory T cell_1-AD.vs.control; scRNA-seq,SRP330776,Natural killer T cell-AD.vs.control; scRNA-seq,SRP330776,Naive CD8+ T cell_3-AD.vs.control; scRNA-seq,SRP330776,B cell_2-AD.vs.control; scRNA-seq,SRP330776,T cell-AD.vs.control; scRNA-seq,SRP330776,Naive CD8+ T cell_2-AD.vs.control; scRNA-seq,SRP330776,CD8+ T cell_3-AD.vs.control	19
CAB39L	bulk RNA-seq,Homo_723,AD.vs.control; bulk RNA-seq,Homo_723,AD.vs.MCI; bulk RNA-seq,Homo_633,AD.vs.MCI; bulk RNA-seq,Homo_714,AD.vs.control; bulk RNA-seq,Homo_714,AD.vs.MCI; scRNA-seq,SRP215507,CD8+ T cell_2-AD.vs.control; scRNA-seq,SRP309935,Natural killer cell_2-AD.vs.control; scRNA-seq,SRP330776,γδ T cell-AD.vs.control; scRNA-seq,SRP330776,Memory T cell_2-AD.vs.control; scRNA-seq,SRP330776,Naive CD8+ T cell_1-AD.vs.control; scRNA-seq,SRP330776,B cell_2-AD.vs.control	11
Cab39l	bulk RNA-seq,SRP325058,AD.vs.control	1
CABCOCO1	bulk RNA-seq,Homo_723,AD.vs.control; bulk RNA-seq,Homo_723,AD.vs.MCI; bulk RNA-seq,Homo_633,AD.vs.control; bulk RNA-	4
CABIN1	bulk RNA-seq,Homo_723,AD.vs.control; bulk RNA-seq,Homo_723,AD.vs.MCI; bulk RNA-seq,Homo_723,MCI.vs.control; bulk RNA-seq,Homo_633,AD.vs.control; bulk RNA-seq,Homo_633,AD.vs.MCI; bulk RNA-seq,Homo_633,MCI.vs.control; bulk RNA-seq,Homo_714,AD.vs.control; bulk RNA-seq,Homo_714,AD.vs.MCI; bulk RNA-seq,Homo_714,MCI.vs.control; scRNA-seq,SRP215507,CD8+ T cell_6-AD.vs.control; scRNA-seq,SRP330776,CD8+ T cell_1-AD.vs.control; scRNA-seq,SRP330776,γδ T cell-AD.vs.control; scRNA-seq,SRP330776,Memory T cell_2-AD.vs.control; scRNA-seq,SRP330776,Naive CD8+ T cell_1-AD.vs.control; scRNA-seq,SRP330776,Naive CD8+ T cell_3-AD.vs.control; scRNA-seq,SRP330776,Naive CD8+ T cell_2-AD.vs.control; scRNA-seq,SRP330776,B cell_3-AD.vs.control; scRNA-seq,SRP330776,B cell_4-AD.vs.control	18
CABLES1	bulk RNA-seq,Homo_723,AD.vs.control; bulk RNA-seq,Homo_723,AD.vs.MCI; bulk RNA-seq,Homo_723,MCI.vs.control; bulk RNA-seq,Homo_633,AD.vs.control; bulk RNA-seq,Homo_633,AD.vs.MCI; bulk RNA-seq,Homo_633,MCI.vs.control; bulk RNA-seq,Homo_714,AD.vs.control;	8
CABLES2	bulk RNA-seq,Homo_723,AD.vs.control; bulk RNA-seq,Homo_723,MCI.vs.control; bulk RNA-seq,Homo_633,MCI.vs.control; bulk RNA-seq,Homo_714,AD.vs.control; bulk RNA-seq,Homo_714,AD.vs.MCI; bulk RNA-seq,Homo_714,MCI.vs.control; scRNA-seq,SRP215507,CD8+ T cell_2-AD.vs.control; scRNA-seq,SRP215507,CD8+ T cell_4-AD.vs.MCI; scRNA-seq,SRP215507,CD8+ T cell_4-MCI.vs.control; scRNA-seq,SRP330776,γδ T	11
CABP1	bulk RNA-seq,Homo_723,AD.vs.control; bulk RNA-seq,Homo_714,MCI.vs.control	2
CABP4	bulk RNA-seq,Homo_723,AD.vs.MCI; bulk RNA-seq,Homo_633,AD.vs.MCI; bulk RNA-seq,Homo_714,AD.vs.control; bulk RNA-seq,Homo_714,AD.vs.MCI; scRNA-seq,SRP215507,CD8+ T cell_1-AD.vs.control; scRNA-seq,SRP215507,CD8+ T cell_3-MCI.vs.control; scRNA-seq,SRP309935,Natural killer cell_2-AD.vs.control; scRNA-seq,SRP330776,CD8+ T cell_1-AD.vs.control; scRNA-seq,SRP330776,B cell_1-	10

CABP5	bulk RNA-seq,Homo_723,AD.vs.control; bulk RNA-seq,Homo_723,AD.vs.MCI; bulk RNA-seq,Homo_723,MCI.vs.control; bulk RNA-seq,Homo_633,AD.vs.control; bulk RNA-seq,Homo_633,AD.vs.MCI; bulk RNA-seq,Homo_633,MCI.vs.control; bulk RNA-seq,Homo_714,AD.vs.control;	8
CABP7	bulk RNA-seq,Homo_723,AD.vs.control; bulk RNA-seq,Homo_723,AD.vs.MCI; bulk RNA-seq,Homo_633,AD.vs.control; bulk RNA-seq,Homo_633,AD.vs.MCI; bulk RNA-seq,Homo_714,AD.vs.control; bulk RNA-seq,Homo_714,AD.vs.MCI; scRNA-seq,SRP330776,Memory T cell_2-	7
CABYR	bulk RNA-seq,Homo_723,AD.vs.control; bulk RNA-seq,Homo_723,AD.vs.MCI; bulk RNA-seq,Homo_714,AD.vs.control; bulk RNA-seq,Homo_714,AD.vs.MCI; scRNA-seq,SRP330776,Naive CD8+ T cell_1-AD.vs.control; scRNA-seq,SRP330776,B cell_2-AD.vs.control	6
CACFD1	bulk RNA-seq,Homo_633,AD.vs.control; bulk RNA-seq,Homo_633,AD.vs.MCI; bulk RNA-seq,Homo_714,AD.vs.control; bulk RNA-seq,Homo_714,AD.vs.MCI; scRNA-seq,SRP215507,CD8+ T cell_5-AD.vs.control; scRNA-seq,SRP215507,CD8+ T cell_6-AD.vs.control; scRNA-seq,SRP215507,CD8+ T cell_3-AD.vs.MCI; scRNA-seq,SRP215507,CD8+ T cell_5-AD.vs.MCI; scRNA-seq,SRP215507,CD8+ T cell_1-MCI.vs.control; scRNA-seq,SRP215507,CD8+ T cell_3-MCI.vs.control; scRNA-seq,SRP330776,Memory T cell_2-AD.vs.control; scRNA-seq,SRP330776,Naive CD8+ T cell_1-AD.vs.control; scRNA-seq,SRP330776,Monocyte_2-AD.vs.control; scRNA-seq,SRP330776,Natural killer cell_2-AD.vs.control	14
CACHD1	scRNA-seq,SRP215507,CD8+ T cell_3-AD.vs.control	1
CACNA1A	bulk RNA-seq,Homo_723,AD.vs.control; bulk RNA-seq,Homo_723,AD.vs.MCI; bulk RNA-seq,Homo_633,AD.vs.control; bulk RNA-seq,Homo_714,AD.vs.control; bulk RNA-seq,Homo_714,AD.vs.MCI; bulk RNA-seq,Homo_714,MCI.vs.control; scRNA-seq,SRP215507,CD8+ T cell_1-AD.vs.control; scRNA-seq,SRP309935,B cell_1-AD.vs.control; scRNA-seq,SRP330776,B cell_1-AD.vs.control; scRNA-seq,SRP330776,Naive CD8+ T	11
CACNA1B	bulk RNA-seq,Homo_723,AD.vs.control; bulk RNA-seq,Homo_723,AD.vs.MCI	2
CACNA1C	bulk RNA-seq,Homo_723,AD.vs.control; bulk RNA-seq,Homo_723,AD.vs.MCI; bulk RNA-seq,Homo_633,AD.vs.control; bulk RNA-seq,Homo_633,AD.vs.MCI; bulk RNA-seq,Homo_714,AD.vs.control; bulk RNA-seq,Homo_714,AD.vs.MCI; bulk RNA-seq,Homo_714,MCI.vs.control; bulk RNA-seq,SRP223445,AD.vs.control; scRNA-seq,SRP215507,CD8+ T cell_1-MCI.vs.control; scRNA-seq,SRP215507,CD8+ T cell_3-MCI.vs.control; scRNA-seq,SRP309935,Natural killer cell_1-AD.vs.control; scRNA-seq,SRP330776,Naive CD8+ T cell_1-AD.vs.control	12
CACNA1C-AS1	scRNA-seq,SRP215507,CD8+ T cell_2-AD.vs.control; scRNA-seq,SRP215507,CD8+ T cell_3-AD.vs.control; scRNA-seq,SRP215507,CD8+ T cell_4-AD.vs.control; scRNA-seq,SRP215507,CD8+ T cell_5-AD.vs.control; scRNA-seq,SRP215507,CD8+ T cell_3-AD.vs.MCI; scRNA-seq,SRP215507,CD8+ T cell_5-AD.vs.MCI; scRNA-seq,SRP215507,CD8+ T cell_4-MCI.vs.control; scRNA-seq,SRP330776,B cell_2-AD.vs.control; scRNA-	9
CACNA1C-AS2	scRNA-seq,SRP215507,CD8+ T cell_4-AD.vs.control; scRNA-seq,SRP215507,CD8+ T cell_5-AD.vs.control; scRNA-seq,SRP330776,Naive CD8+ T cell_1-AD.vs.control; scRNA-seq,SRP330776,Memory T cell_1-AD.vs.control	4
CACNA1D	bulk RNA-seq,Homo_723,AD.vs.control; bulk RNA-seq,Homo_723,AD.vs.MCI; bulk RNA-seq,Homo_723,MCI.vs.control; bulk RNA-seq,Homo_633,AD.vs.control; bulk RNA-seq,Homo_633,AD.vs.MCI; bulk RNA-seq,Homo_633,MCI.vs.control; bulk RNA-seq,Homo_714,AD.vs.control;	8
CACNA1E	bulk RNA-seq,Homo_633,AD.vs.control; bulk RNA-seq,Homo_633,AD.vs.MCI; bulk RNA-seq,Homo_714,AD.vs.control; bulk RNA-seq,Homo_714,AD.vs.MCI; bulk RNA-seq,ROSMAP,AD.vs.control; bulk RNA-seq,ROSMAP,MCI.vs.control	6
CACNA1F	bulk RNA-seq,Homo_723,AD.vs.control; bulk RNA-seq,Homo_723,AD.vs.MCI; bulk RNA-seq,Homo_633,AD.vs.control; bulk RNA-seq,Homo_633,AD.vs.MCI; bulk RNA-seq,Homo_714,AD.vs.control; bulk RNA-seq,Homo_714,AD.vs.MCI; scRNA-seq,SRP215507,CD8+ T cell_3-MCI.vs.control; scRNA-seq,SRP330776,Memory T cell_1-AD.vs.control	8
CACNA1G	bulk RNA-seq,Homo_723,AD.vs.control; bulk RNA-seq,Homo_723,AD.vs.MCI; bulk RNA-seq,Homo_714,MCI.vs.control	3
CACNA1H	scRNA-seq,SRP215507,CD8+ T cell_1-AD.vs.MCI; scRNA-seq,SRP215507,CD8+ T cell_1-MCI.vs.control; scRNA-seq,SRP215507,CD8+ T cell_4-MCI.vs.control; scRNA-seq,SRP215507,CD8+ T cell_3-MCI.vs.control; scRNA-seq,SRP330776,CD8+ T cell_1-AD.vs.control; scRNA-	6
CACNA1I	bulk RNA-seq,Homo_723,AD.vs.control; bulk RNA-seq,Homo_723,AD.vs.MCI; bulk RNA-seq,Homo_633,AD.vs.control; bulk RNA-seq,Homo_633,AD.vs.MCI; bulk RNA-seq,Homo_714,AD.vs.control; bulk RNA-seq,Homo_714,AD.vs.MCI; scRNA-seq,SRP215507,CD8+ T cell_3-AD.vs.MCI; scRNA-seq,SRP215507,CD8+ T cell_3-MCI.vs.control; scRNA-seq,SRP309935,Naive CD8+ T cell_1-AD.vs.control; scRNA-seq,SRP330776,Memory T cell_2-AD.vs.control; scRNA-seq,SRP330776,Naive CD8+ T cell_1-AD.vs.control	11
CACNA1S	bulk RNA-seq,Homo_723,AD.vs.control; bulk RNA-seq,Homo_723,AD.vs.MCI; bulk RNA-seq,Homo_633,AD.vs.control; bulk RNA-	4
CACNA2D1	bulk RNA-seq,Homo_723,AD.vs.control; bulk RNA-seq,Homo_723,AD.vs.MCI; bulk RNA-seq,Homo_723,MCI.vs.control; bulk RNA-seq,Homo_633,AD.vs.control; bulk RNA-seq,Homo_633,AD.vs.MCI; bulk RNA-seq,Homo_714,MCI.vs.control; bulk RNA-seq,ROSMAP,AD.vs.control;	8
Cacna2d1	bulk RNA-seq,SRP325058,AD.vs.control	1

CACNA2D2	bulk RNA-seq,Homo_723,AD.vs.control; bulk RNA-seq,Homo_723,AD.vs.MCI; bulk RNA-seq,Homo_723,MCI.vs.control; bulk RNA-seq,Homo_633,AD.vs.control; bulk RNA-seq,Homo_633,AD.vs.MCI; bulk RNA-seq,Homo_633,MCI.vs.control; scRNA-seq,SRP215507,CD8+ T cell_4-AD.vs.control; scRNA-seq,SRP215507,CD8+ T cell_5-AD.vs.MCI; scRNA-seq,SRP215507,CD8+ T cell_5-MCI.vs.control; scRNA-seq,SRP309935,Natural killer cell_2-AD.vs.control; scRNA-seq,SRP309935,CD8+ T cell_1-AD.vs.control; scRNA-seq,SRP330776,γδ T cell-AD.vs.control; scRNA-seq,SRP330776,Memory T cell_2-AD.vs.control; scRNA-seq,SRP330776,CD8+ T cell_2-AD.vs.control; scRNA-seq,SRP330776,Naive CD8+ T cell_1-AD.vs.control; scRNA-seq,SRP330776,Natural killer cell_1-AD.vs.control; scRNA-seq,SRP330776,Memory T [illegible]	18
CACNA2D3	scRNA-seq,SRP309935,Monocyte_1-AD.vs.control	1
CACNA2D4	bulk RNA-seq,Homo_714,AD.vs.control; bulk RNA-seq,Homo_714,AD.vs.MCI; scRNA-seq,SRP215507,CD8+ T cell_5-AD.vs.control; scRNA-seq,SRP215507,CD8+ T cell_6-AD.vs.control; scRNA-seq,SRP215507,CD8+ T cell_1-AD.vs.MCI; scRNA-seq,SRP215507,CD8+ T cell_4-AD.vs.MCI; scRNA-seq,SRP215507,CD8+ T cell_4-MCI.vs.control; scRNA-seq,SRP215507,CD8+ T cell_2-MCI.vs.control; scRNA-seq,SRP309935,Natural killer cell_1-AD.vs.control; scRNA-seq,SRP330776,γδ T cell-AD.vs.control; scRNA-seq,SRP330776,CD8+ T cell_2-AD.vs.control; scRNA-seq,SRP330776,Natural killer cell_1-AD.vs.control; scRNA-seq,SRP330776,T cell-AD.vs.control	13
CACNB1	bulk RNA-seq,Homo_723,AD.vs.control; bulk RNA-seq,Homo_723,AD.vs.MCI; bulk RNA-seq,Homo_723,MCI.vs.control; bulk RNA-seq,Homo_633,AD.vs.control; bulk RNA-seq,Homo_633,AD.vs.MCI; bulk RNA-seq,Homo_633,MCI.vs.control; bulk RNA-seq,Homo_714,AD.vs.control; bulk RNA-seq,Homo_714,AD.vs.MCI; bulk RNA-seq,Homo_714,MCI.vs.control; scRNA-seq,SRP215507,CD8+ T cell_3-AD.vs.control; scRNA-seq,SRP215507,CD8+ T cell_1-AD.vs.MCI; scRNA-seq,SRP215507,CD8+ T cell_5-AD.vs.MCI; scRNA-seq,SRP215507,CD8+ T cell_5-MCI.vs.control; scRNA-seq,SRP330776,B cell_1-AD.vs.control; scRNA-seq,SRP330776,Natural killer cell_1-AD.vs.control; scRNA-seq,SRP330776,T cell-AD.vs.control;	17
CACNB2	bulk RNA-seq,Homo_714,AD.vs.control; bulk RNA-seq,Homo_714,AD.vs.MCI; scRNA-seq,SRP330776,Naive CD8+ T cell_1-AD.vs.control	3
CACNB3	scRNA-seq,SRP309935,Natural killer cell_2-AD.vs.control; scRNA-seq,SRP330776,CD8+ T cell_1-AD.vs.control; scRNA-seq,SRP330776,γδ T cell-AD.vs.control; scRNA-seq,SRP330776,Memory T cell_2-AD.vs.control; scRNA-seq,SRP330776,CD8+ T cell_2-AD.vs.control; scRNA-seq,SRP330776,Naive CD8+ T cell_1-AD.vs.control; scRNA-seq,SRP330776,Natural killer cell_1-AD.vs.control; scRNA-seq,SRP330776,Memory T cell_1-AD.vs.control; scRNA-seq,SRP330776,T cell-AD.vs.control; scRNA-seq,SRP330776,CD8+ T cell_3-AD.vs.control	10
CACNB4	bulk RNA-seq,ROSMAP,AD.vs.control; bulk RNA-seq,ROSMAP,MCI.vs.control	2
CACNG1	bulk RNA-seq,Homo_723,AD.vs.control; bulk RNA-seq,Homo_723,AD.vs.MCI; bulk RNA-seq,Homo_633,AD.vs.control; bulk RNA-seq,Homo_633,AD.vs.MCI; bulk RNA-seq,ROSMAP,AD.vs.MCI	5
CACNG2	bulk RNA-seq,Homo_723,AD.vs.control; bulk RNA-seq,Homo_723,AD.vs.MCI; bulk RNA-seq,Homo_633,AD.vs.control; bulk RNA-seq,Homo_714,MCI.vs.control; bulk RNA-seq,ROSMAP,MCI.vs.control	5
CACNG4	bulk RNA-seq,Homo_723,AD.vs.control; bulk RNA-seq,Homo_723,AD.vs.MCI; bulk RNA-seq,Homo_723,MCI.vs.control; bulk RNA-seq,Homo_633,AD.vs.control; bulk RNA-seq,Homo_633,AD.vs.MCI; bulk RNA-seq,Homo_633,MCI.vs.control; bulk RNA-seq,ROSMAP,MCI.vs.control	7
CACNG5	bulk RNA-seq,Homo_723,AD.vs.control; bulk RNA-seq,Homo_723,AD.vs.MCI; bulk RNA-seq,Homo_714,MCI.vs.control	3
CACNG6	bulk RNA-seq,Homo_723,AD.vs.control; bulk RNA-seq,Homo_723,AD.vs.MCI; bulk RNA-seq,Homo_723,MCI.vs.control; bulk RNA-seq,Homo_633,AD.vs.control; bulk RNA-seq,Homo_633,AD.vs.MCI; bulk RNA-seq,Homo_633,MCI.vs.control; bulk RNA-seq,Homo_714,AD.vs.control; bulk RNA-seq,Homo_714,AD.vs.MCI; bulk RNA-seq,Homo_714,MCI.vs.control	9
CACNG7	bulk RNA-seq,Homo_723,AD.vs.control; bulk RNA-seq,Homo_723,AD.vs.MCI; bulk RNA-seq,Homo_723,MCI.vs.control; bulk RNA-seq,Homo_633,AD.vs.control; bulk RNA-seq,Homo_633,AD.vs.MCI	5
CACNG8	bulk RNA-seq,Homo_633,AD.vs.control; bulk RNA-seq,Homo_714,AD.vs.control; bulk RNA-seq,Homo_714,AD.vs.MCI; bulk RNA-	4
CACTIN	bulk RNA-seq,Homo_723,AD.vs.control; bulk RNA-seq,Homo_723,AD.vs.MCI; bulk RNA-seq,Homo_723,MCI.vs.control; bulk RNA-seq,Homo_633,AD.vs.control; bulk RNA-seq,Homo_633,AD.vs.MCI; bulk RNA-seq,Homo_633,MCI.vs.control; bulk RNA-seq,Homo_714,AD.vs.control; bulk RNA-seq,Homo_714,MCI.vs.control; scRNA-seq,SRP215507,CD8+ T cell_6-AD.vs.control; scRNA-seq,SRP215507,CD8+ T cell_2-AD.vs.MCI; scRNA-seq,SRP215507,CD8+ T cell_5-AD.vs.MCI; scRNA-seq,SRP215507,CD8+ T cell_5-MCI.vs.control; scRNA-seq,SRP215507,CD8+ T cell_2-MCI.vs.control; scRNA-seq,SRP215507,CD8+ T cell_3-MCI.vs.control; scRNA-seq,SRP309935,Monocyte_2-AD.vs.control; scRNA-seq,SRP309935,Naive CD8+ T cell_2-AD.vs.control; scRNA-seq,SRP330776,CD8+ T cell_1-AD.vs.control; scRNA-seq,SRP330776,Memory T cell_2-AD.vs.control; scRNA-seq,SRP330776,CD8+ T cell_2-AD.vs.control; scRNA-seq,SRP330776,B cell_2-AD.vs.control; scRNA-[illegible]	21

CACUL1	bulk RNA-seq,Homo_723,MCI.vs.control; bulk RNA-seq,Homo_633,MCI.vs.control; scRNA-seq,SRP215507,CD8+ T cell_5-AD.vs.MCI; scRNA-seq,SRP215507,CD8+ T cell_6-AD.vs.MCI; scRNA-seq,SRP215507,CD8+ T cell_1-MCI.vs.control; scRNA-seq,SRP215507,CD8+ T cell_5-MCI.vs.control; scRNA-seq,SRP215507,CD8+ T cell_6-MCI.vs.control	7
CACYBP	bulk RNA-seq,Homo_723,AD.vs.control; bulk RNA-seq,Homo_723,AD.vs.MCI; bulk RNA-seq,Homo_633,AD.vs.control; bulk RNA-seq,Homo_633,AD.vs.MCI; bulk RNA-seq,Homo_714,AD.vs.control; bulk RNA-seq,Homo_714,AD.vs.MCI; scRNA-seq,SRP215507,CD8+ T cell_4-AD.vs.control; scRNA-seq,SRP330776,Natural killer cell_2-AD.vs.control	8
CAD	bulk RNA-seq,Homo_633,AD.vs.control; scRNA-seq,SRP215507,CD8+ T cell_1-AD.vs.MCI; scRNA-seq,SRP330776,CD8+ T cell_2-AD.vs.control; scRNA-seq,SRP330776,Natural killer T cell-AD.vs.control; scRNA-seq,SRP330776,B cell_3-AD.vs.control; scRNA-seq,SRP330776,Megakaryocyte_1-AD.vs.control; scRNA-seq,SRP330776,Natural killer cell_2-AD.vs.control	7
CADM1	bulk RNA-seq,Homo_723,AD.vs.control; bulk RNA-seq,Homo_723,AD.vs.MCI; bulk RNA-seq,Homo_633,AD.vs.control; bulk RNA-seq,Homo_633,AD.vs.MCI; bulk RNA-seq,Homo_714,AD.vs.control; bulk RNA-seq,Homo_714,AD.vs.MCI; bulk RNA-seq,ROSMAP,AD.vs.control; bulk RNA-seq,ROSMAP,MCI.vs.control; scRNA-seq,SRP215507,CD8+ T cell_2-AD.vs.control; scRNA-seq,SRP215507,CD8+ T cell_1-AD.vs.control; scRNA-seq,SRP215507,CD8+ T cell_4-AD.vs.control; scRNA-seq,SRP215507,CD8+ T cell_5-AD.vs.control; scRNA-seq,SRP215507,CD8+ T cell_1-MCI.vs.control; scRNA-seq,SRP215507,CD8+ T cell_5-MCI.vs.control; scRNA-seq,SRP215507,CD8+ T cell_4-MCI.vs.control; scRNA-seq,SRP215507,CD8+ T cell_2-MCI.vs.control; scRNA-seq,SRP330776,CD8+ T cell_1-AD.vs.control; scRNA-seq,SRP330776,γδ T cell-AD.vs.control; scRNA-seq,SRP330776,Memory T cell_2-AD.vs.control; scRNA-seq,SRP330776,CD8+ T cell_2-AD.vs.control; scRNA-seq,SRP330776,Natural killer cell_1-AD.vs.control; scRNA-seq,SRP330776,Memory T cell_1-AD.vs.control; scRNA-seq,SRP330776,Natural killer T cell-AD.vs.control; scRNA-[illegible]	26
CADM2	bulk RNA-seq,Homo_723,AD.vs.control; bulk RNA-seq,Homo_723,AD.vs.MCI; bulk RNA-seq,Homo_633,AD.vs.control; bulk RNA-seq,Homo_633,AD.vs.MCI; bulk RNA-seq,ROSMAP,AD.vs.control; scRNA-seq,SRP215507,CD8+ T cell_3-AD.vs.MCI; scRNA-seq,SRP215507,CD8+ T	7
CADM3	bulk RNA-seq,Homo_723,AD.vs.control; bulk RNA-seq,Homo_723,AD.vs.MCI; bulk RNA-seq,Homo_723,MCI.vs.control; bulk RNA-seq,Homo_633,AD.vs.MCI; bulk RNA-seq,Homo_633,MCI.vs.control	5
CADM4	bulk RNA-seq,Homo_723,AD.vs.control; bulk RNA-seq,Homo_723,AD.vs.MCI; bulk RNA-seq,Homo_723,MCI.vs.control; bulk RNA-seq,Homo_633,AD.vs.control; bulk RNA-seq,Homo_633,AD.vs.MCI; bulk RNA-seq,Homo_633,MCI.vs.control; bulk RNA-seq,Homo_714,AD.vs.control; bulk RNA-seq,Homo_714,AD.vs.MCI; bulk RNA-seq,Homo_714,MCI.vs.control; bulk RNA-seq,ROSMAP,AD.vs.control	10
CADPS	bulk RNA-seq,Homo_723,AD.vs.control; bulk RNA-seq,Homo_723,AD.vs.MCI; bulk RNA-seq,Homo_723,MCI.vs.control; bulk RNA-seq,Homo_633,AD.vs.control; bulk RNA-seq,Homo_714,MCI.vs.control	5
CADPS2	bulk RNA-seq,Homo_723,AD.vs.control; bulk RNA-seq,Homo_723,AD.vs.MCI; bulk RNA-seq,Homo_723,MCI.vs.control; bulk RNA-seq,Homo_633,AD.vs.control; bulk RNA-seq,Homo_633,AD.vs.MCI; bulk RNA-seq,Homo_633,MCI.vs.control; bulk RNA-	8
CAGE1	bulk RNA-seq,Homo_723,AD.vs.control; bulk RNA-seq,Homo_723,MCI.vs.control; bulk RNA-seq,Homo_633,AD.vs.control; bulk RNA-seq,Homo_633,MCI.vs.control; bulk RNA-seq,Homo_714,MCI.vs.control; scRNA-seq,SRP215507,CD8+ T cell_2-AD.vs.control; scRNA-seq,SRP215507,CD8+ T cell_1-MCI.vs.control; scRNA-seq,SRP215507,CD8+ T cell_2-MCI.vs.control; scRNA-seq,SRP330776,CD8+ T cell_2-AD.vs.control; scRNA-seq,SRP330776,Naive CD8+ T cell_1-AD.vs.control; scRNA-seq,SRP330776,Natural killer cell_1-AD.vs.control; scRNA-seq,SRP330776,Memory T cell_1-AD.vs.control; scRNA-seq,SRP330776,B cell_2-AD.vs.control; scRNA-seq,SRP330776,Natural killer cell_2-	14
CAHM	scRNA-seq,SRP215507,CD8+ T cell_2-AD.vs.control; scRNA-seq,SRP215507,CD8+ T cell_3-AD.vs.control; scRNA-seq,SRP215507,CD8+ T cell_1-AD.vs.MCI; scRNA-seq,SRP215507,CD8+ T cell_5-AD.vs.MCI; scRNA-seq,SRP215507,CD8+ T cell_3-MCI.vs.control; scRNA-seq,SRP330776,CD8+ T cell_1-AD.vs.control; scRNA-seq,SRP330776,Memory T cell_2-AD.vs.control; scRNA-seq,SRP330776,B cell_1-AD.vs.control; scRNA-seq,SRP330776,Naive CD8+ T cell_1-AD.vs.control; scRNA-seq,SRP330776,B cell_2-AD.vs.control	10
CALB1	bulk RNA-seq,Homo_723,AD.vs.control; bulk RNA-seq,Homo_723,AD.vs.MCI; bulk RNA-seq,Homo_723,MCI.vs.control; bulk RNA-seq,Homo_633,AD.vs.control; bulk RNA-seq,Homo_633,AD.vs.MCI; bulk RNA-seq,Homo_714,AD.vs.control; bulk RNA-seq,Homo_714,AD.vs.MCI	7
CALB2	bulk RNA-seq,Homo_723,AD.vs.control; bulk RNA-seq,Homo_723,AD.vs.MCI; bulk RNA-seq,Homo_723,MCI.vs.control; bulk RNA-	4
CALCA	bulk RNA-seq,Homo_723,AD.vs.control; bulk RNA-seq,Homo_723,AD.vs.MCI	2

CALCB	bulk RNA-seq,Homo_723,AD.vs.control; bulk RNA-seq,Homo_723,AD.vs.MCI; bulk RNA-seq,Homo_723,MCI.vs.control; scRNA-seq,SRP215507,CD8+ T cell_1-MCI.vs.control; scRNA-seq,SRP330776,γδ T cell-AD.vs.control; scRNA-seq,SRP330776,CD8+ T cell_2-AD.vs.control; scRNA-seq,SRP330776,Naive CD8+ T cell_1-AD.vs.control; scRNA-seq,SRP330776,Natural killer cell_1-AD.vs.control; scRNA-seq,SRP330776,Naive CD8+ T [illegible]	9
CALCOCO1	scRNA-seq,SRP330776,Memory T cell_2-AD.vs.control; scRNA-seq,SRP330776,B cell_2-AD.vs.control; scRNA-seq,SRP330776,Monocyte_2- [illegible]	3
CALCOCO2	bulk RNA-seq,Homo_723,AD.vs.control; bulk RNA-seq,Homo_723,AD.vs.MCI; bulk RNA-seq,Homo_633,AD.vs.control; bulk RNA-seq,Homo_633,AD.vs.MCI; bulk RNA-seq,Homo_714,AD.vs.control; bulk RNA-seq,Homo_714,AD.vs.MCI; scRNA-seq,SRP330776,Megakaryocyte_1-AD.vs.control; scRNA-seq,SRP330776,Natural killer cell_2-AD.vs.control	8
CALCR	bulk RNA-seq,Homo_723,AD.vs.control; bulk RNA-seq,Homo_723,AD.vs.MCI; bulk RNA-seq,Homo_633,AD.vs.control; bulk RNA-seq,Homo_633,AD.vs.MCI; bulk RNA-seq,Homo_714,MCI.vs.control	5
CALCRL	bulk RNA-seq,Homo_723,AD.vs.control; bulk RNA-seq,Homo_723,AD.vs.MCI; bulk RNA-seq,Homo_723,MCI.vs.control; bulk RNA-seq,Homo_633,AD.vs.control; bulk RNA-seq,Homo_633,AD.vs.MCI; bulk RNA-seq,Homo_633,MCI.vs.control; bulk RNA-seq,Homo_714,AD.vs.control;	8
CALD1	bulk RNA-seq,Homo_723,AD.vs.control; bulk RNA-seq,Homo_723,AD.vs.MCI; bulk RNA-seq,Homo_723,MCI.vs.control; bulk RNA-seq,Homo_633,AD.vs.control; bulk RNA-seq,Homo_633,AD.vs.MCI; bulk RNA-seq,Homo_633,MCI.vs.control; bulk RNA-seq,Homo_714,AD.vs.control; bulk RNA-seq,Homo_714,AD.vs.MCI; scRNA-seq,SRP330776,B cell_1-AD.vs.control	9
Cald1	bulk RNA-seq,SRP325058,AD.vs.control	1
CALHM1	bulk RNA-seq,Homo_723,AD.vs.control; bulk RNA-seq,Homo_723,AD.vs.MCI; bulk RNA-seq,Homo_633,AD.vs.control; bulk RNA-seq,Homo_633,AD.vs.MCI; bulk RNA-seq,Homo_714,AD.vs.control; bulk RNA-seq,Homo_714,AD.vs.MCI; scRNA-seq,SRP215507,CD8+ T cell_1-	7
CALHM2	bulk RNA-seq,Homo_723,AD.vs.control; bulk RNA-seq,Homo_723,AD.vs.MCI; bulk RNA-seq,Homo_723,MCI.vs.control; bulk RNA-seq,Homo_633,AD.vs.control; bulk RNA-seq,Homo_633,AD.vs.MCI; bulk RNA-seq,Homo_714,AD.vs.control; bulk RNA-seq,Homo_714,AD.vs.MCI; scRNA-seq,SRP215507,CD8+ T cell_3-AD.vs.control; scRNA-seq,SRP215507,CD8+ T cell_7-AD.vs.control; scRNA-seq,SRP215507,CD8+ T cell_4-MCI.vs.control; scRNA-seq,SRP215507,CD8+ T cell_3-MCI.vs.control; scRNA-seq,SRP330776,CD8+ T cell_1-AD.vs.control; scRNA-seq,SRP330776,Memory T cell_2-AD.vs.control; scRNA-seq,SRP330776,CD8+ T cell_2-AD.vs.control; scRNA-seq,SRP330776,Natural killer cell_1-AD.vs.control; scRNA-seq,SRP330776,Memory T cell_1-AD.vs.control; scRNA-seq,SRP330776,Natural killer T cell-AD.vs.control; scRNA-seq,SRP330776,Naive CD8+ T cell_3-AD.vs.control; scRNA-seq,SRP330776,Naive CD8+ T cell_2-AD.vs.control; scRNA-seq,SRP330776,CD8+ T [illegible]	21
CALHM3	bulk RNA-seq,ROSMAP,AD.vs.MCI; bulk RNA-seq,ROSMAP,MCI.vs.control	2
CALHM4	bulk RNA-seq,Homo_723,AD.vs.control; bulk RNA-seq,Homo_633,AD.vs.control	2
CALHM6	scRNA-seq,SRP215507,CD8+ T cell_2-AD.vs.control; scRNA-seq,SRP215507,CD8+ T cell_3-AD.vs.control; scRNA-seq,SRP215507,CD8+ T cell_1-AD.vs.control; scRNA-seq,SRP215507,CD8+ T cell_4-AD.vs.control; scRNA-seq,SRP215507,CD8+ T cell_5-AD.vs.control; scRNA-seq,SRP215507,CD8+ T cell_2-AD.vs.MCI; scRNA-seq,SRP215507,CD8+ T cell_3-AD.vs.MCI; scRNA-seq,SRP215507,CD8+ T cell_1-AD.vs.MCI; scRNA-seq,SRP215507,CD8+ T cell_5-AD.vs.MCI; scRNA-seq,SRP215507,CD8+ T cell_1-MCI.vs.control; scRNA-seq,SRP215507,CD8+ T cell_5-MCI.vs.control; scRNA-seq,SRP215507,CD8+ T cell_2-MCI.vs.control; scRNA-seq,SRP309935,Monocyte_1-AD.vs.control; scRNA-seq,SRP309935,Naive CD8+ T cell_1-AD.vs.control; scRNA-seq,SRP309935,Natural killer cell_1-AD.vs.control; scRNA-seq,SRP309935,B cell_1-AD.vs.control; scRNA-seq,SRP309935,Monocyte_3-AD.vs.control; scRNA-seq,SRP309935,B cell_2-AD.vs.control; scRNA-seq,SRP309935,Dendritic cell-AD.vs.control; scRNA-seq,SRP309935,Plasmacytoid dendritic cell_1-AD.vs.control; scRNA-seq,SRP330776,CD8+ T cell_1-AD.vs.control; scRNA-seq,SRP330776,γδ T cell-AD.vs.control; scRNA-seq,SRP330776,Memory T cell_2-AD.vs.control; scRNA-seq,SRP330776,CD8+ T cell_2-AD.vs.control; scRNA-seq,SRP330776,B cell_1-AD.vs.control; scRNA-seq,SRP330776,Naive CD8+ T cell_1-AD.vs.control; scRNA-seq,SRP330776,Natural killer cell_1-AD.vs.control; scRNA-seq,SRP330776,Memory T cell_1-AD.vs.control; scRNA-seq,SRP330776,Natural killer T cell-AD.vs.control; scRNA-seq,SRP330776,B cell_2-AD.vs.control; scRNA-seq,SRP330776,Naive CD8+ T cell_2-AD.vs.control; scRNA-seq,SRP330776,CD8+ T cell_3-AD.vs.control; scRNA-seq,SRP330776,Monocyte_1-AD.vs.control; scRNA-seq,SRP330776,Monocyte_2-AD.vs.control; scRNA-seq,SRP330776,B cell_3 [illegible]	35
CALM1	scRNA-seq,SRP215507,CD8+ T cell_7-AD.vs.control; scRNA-seq,SRP215507,CD8+ T cell_7-AD.vs.MCI; scRNA-seq,SRP215507,CD8+ T cell_5-AD.vs.MCI; scRNA-seq,SRP309935,Erythroid cell-AD.vs.control	4
CALM2	scRNA-seq,SRP215507,CD8+ T cell_7-AD.vs.control; scRNA-seq,SRP215507,CD8+ T cell_8-MCI.vs.control; scRNA-seq,SRP309935,Erythroid cell-AD.vs.control; scRNA-seq,SRP330776,CD8+ T cell_1-AD.vs.control; scRNA-seq,SRP330776,γδ T cell-AD.vs.control; scRNA-seq,SRP330776,B cell_2-AD.vs.control; scRNA-seq,SRP330776,CD8+ T cell_3-AD.vs.control	7

CALM3	bulk RNA-seq,SRP223445,AD.vs.control; scRNA-seq,SRP330776,B cell_2-AD.vs.control; scRNA-seq,SRP330776,Natural killer cell_2-AD.vs.control	3
Calm3	bulk RNA-seq,SRP325058,AD.vs.control	1
CALML3	bulk RNA-seq,Homo_714,MCI.vs.control	1
CALML4	bulk RNA-seq,Homo_723,AD.vs.control; bulk RNA-seq,Homo_633,AD.vs.control; bulk RNA-seq,Homo_714,AD.vs.control; bulk RNA-seq,Homo_714,AD.vs.MCI; scRNA-seq,SRP215507,CD8+ T cell_1-AD.vs.control; scRNA-seq,SRP215507,CD8+ T cell_5-AD.vs.control; scRNA-seq,SRP215507,CD8+ T cell_2-AD.vs.MCI; scRNA-seq,SRP215507,CD8+ T cell_5-MCI.vs.control; scRNA-seq,SRP215507,CD8+ T cell_2-MCI.vs.control; scRNA-seq,SRP330776,CD8+ T cell_1-AD.vs.control; scRNA-seq,SRP330776,Naive CD8+ T cell_1-AD.vs.control; scRNA-	13
CALML5	bulk RNA-seq,Homo_714,MCI.vs.control	1
CALML6	bulk RNA-seq,Homo_723,AD.vs.control; bulk RNA-seq,Homo_723,AD.vs.MCI; bulk RNA-seq,Homo_723,MCI.vs.control; bulk RNA-seq,Homo_633,AD.vs.control; bulk RNA-seq,Homo_633,AD.vs.MCI; bulk RNA-seq,Homo_633,MCI.vs.control; bulk RNA-seq,Homo_714,AD.vs.control; bulk RNA-seq,Homo_714,MCI.vs.control; bulk RNA-seq,ROSMAP,AD.vs.control; scRNA-seq,SRP215507,CD8+ T cell_3-MCI.vs.control	10
CALN1	bulk RNA-seq,Homo_723,AD.vs.control; bulk RNA-seq,Homo_723,AD.vs.MCI; bulk RNA-seq,Homo_633,AD.vs.control; bulk RNA-seq,Homo_633,AD.vs.MCI; bulk RNA-seq,Homo_714,MCI.vs.control	5
CALR	scRNA-seq,SRP215507,CD8+ T cell_7-AD.vs.control; scRNA-seq,SRP215507,CD8+ T cell_10-AD.vs.control; scRNA-seq,SRP215507,CD8+ T cell_7-AD.vs.MCI; scRNA-seq,SRP215507,CD8+ T cell_5-AD.vs.MCI; scRNA-seq,SRP330776,Naive CD8+ T cell_2-AD.vs.control; scRNA-seq,SRP330776,Monocyte_2-AD.vs.control; scRNA-seq,SRP330776,B cell_4-AD.vs.control	7
CALU	bulk RNA-seq,Homo_723,AD.vs.control; bulk RNA-seq,Homo_723,AD.vs.MCI; bulk RNA-seq,Homo_714,AD.vs.control; bulk RNA-	4
CALY	bulk RNA-seq,ROSMAP,MCI.vs.control; scRNA-seq,SRP309935,Naive CD8+ T cell_1-AD.vs.control; scRNA-seq,SRP309935,CD8+ T cell_2-AD.vs.control; scRNA-seq,SRP330776,CD8+ T cell_1-AD.vs.control; scRNA-seq,SRP330776,Naive CD8+ T cell_1-AD.vs.control	5
CAMK1	scRNA-seq,SRP215507,CD8+ T cell_2-AD.vs.control; scRNA-seq,SRP215507,CD8+ T cell_1-AD.vs.MCI; scRNA-seq,SRP215507,CD8+ T cell_2-MCI.vs.control; scRNA-seq,SRP330776,CD8+ T cell_1-AD.vs.control; scRNA-seq,SRP330776,Memory T cell_2-AD.vs.control; scRNA-seq,SRP330776,CD8+ T cell_2-AD.vs.control; scRNA-seq,SRP330776,Naive CD8+ T cell_1-AD.vs.control; scRNA-seq,SRP330776,Memory T cell_1-AD.vs.control; scRNA-seq,SRP330776,B cell_2-AD.vs.control; scRNA-seq,SRP330776,T cell-AD.vs.control; scRNA-seq,SRP330776,Monocyte_1-	11
CAMK1D	scRNA-seq,SRP215507,CD8+ T cell_5-AD.vs.control; scRNA-seq,SRP215507,CD8+ T cell_7-AD.vs.MCI; scRNA-seq,SRP215507,CD8+ T cell_6-AD.vs.MCI; scRNA-seq,SRP215507,CD8+ T cell_6-MCI.vs.control; scRNA-seq,SRP215507,CD8+ T cell_7-MCI.vs.control; scRNA-seq,SRP309935,Natural killer cell_2-AD.vs.control; scRNA-seq,SRP309935,Megakaryocyte_1-AD.vs.control; scRNA-seq,SRP309935,Naive CD8+ T cell_1-AD.vs.control; scRNA-seq,SRP309935,Natural killer cell_1-AD.vs.control; scRNA-seq,SRP309935,CD8+ T cell_1-AD.vs.control; scRNA-seq,SRP330776,CD8+ T cell_1-AD.vs.control; scRNA-seq,SRP330776,γδ T cell-AD.vs.control; scRNA-seq,SRP330776,Memory T cell_2-AD.vs.control; scRNA-seq,SRP330776,CD8+ T cell_2-AD.vs.control; scRNA-seq,SRP330776,B cell_1-AD.vs.control; scRNA-seq,SRP330776,Naive CD8+ T cell_1-AD.vs.control; scRNA-seq,SRP330776,Natural killer cell_1-AD.vs.control; scRNA-seq,SRP330776,Memory T cell_1-AD.vs.control; scRNA-seq,SRP330776,Natural killer T cell-AD.vs.control; scRNA-seq,SRP330776,Naive CD8+ T cell_3-AD.vs.control; scRNA-seq,SRP330776,B cell_2-AD.vs.control; scRNA-seq,SRP330776,T cell-AD.vs.control; scRNA-seq,SRP330776,Naive CD8+ T cell_2-AD.vs.control; scRNA-seq,SRP330776,CD8+ T cell_3-AD.vs.control; scRNA-seq,SRP330776,Monocyte_2-AD.vs.control; scRNA-seq,SRP330776,B cell_3-AD.vs.control; scRNA-seq,SRP330776,Regulatory T cell-AD.vs.control; scRNA-seq,SRP330776,Natural killer cell_2-AD.vs.control; scRNA-seq,SRP330776,B cell_4-	29
CAMK1G	bulk RNA-seq,Homo_723,AD.vs.control; bulk RNA-seq,Homo_723,AD.vs.MCI; bulk RNA-seq,Homo_633,AD.vs.control	3
CAMK2A	bulk RNA-seq,Homo_723,AD.vs.control; bulk RNA-seq,Homo_723,AD.vs.MCI; bulk RNA-seq,Homo_714,MCI.vs.control	3
CAMK2B	bulk RNA-seq,Homo_723,AD.vs.control; bulk RNA-seq,Homo_723,AD.vs.MCI; bulk RNA-seq,Homo_633,AD.vs.control; bulk RNA-	4

CAMK2D	bulk RNA-seq,Homo_723,AD.vs.control; bulk RNA-seq,Homo_723,AD.vs.MCI; bulk RNA-seq,Homo_633,AD.vs.MCI; bulk RNA-seq,Homo_714,AD.vs.control; bulk RNA-seq,Homo_714,AD.vs.MCI; scRNA-seq,SRP215507,CD8+ T cell_4-AD.vs.control; scRNA-seq,SRP215507,CD8+ T cell_4-AD.vs.MCI; scRNA-seq,SRP330776,CD8+ T cell_1-AD.vs.control; scRNA-seq,SRP330776,γδ T cell-AD.vs.control; scRNA-seq,SRP330776,Memory T cell_2-AD.vs.control; scRNA-seq,SRP330776,CD8+ T cell_2-AD.vs.control; scRNA-seq,SRP330776,B cell_1-AD.vs.control; scRNA-seq,SRP330776,Naive CD8+ T cell_1-AD.vs.control; scRNA-seq,SRP330776,Natural killer cell_1-AD.vs.control; scRNA-seq,SRP330776,Memory T cell_1-AD.vs.control; scRNA-seq,SRP330776,Natural killer T cell-AD.vs.control; scRNA-seq,SRP330776,Naive CD8+ T cell_3-AD.vs.control; scRNA-seq,SRP330776,B cell_2-AD.vs.control; scRNA-seq,SRP330776,T cell-AD.vs.control; scRNA-seq,SRP330776,Naive CD8+ T cell_2-AD.vs.control; scRNA-seq,SRP330776,CD8+ T cell_3-AD.vs.control; scRNA-seq,SRP330776,B cell_3-AD.vs.control; scRNA-[illegible]	23
CAMK2G	bulk RNA-seq,Homo_723,AD.vs.control; bulk RNA-seq,Homo_723,AD.vs.MCI; bulk RNA-seq,Homo_723,MCI.vs.control; bulk RNA-seq,Homo_633,AD.vs.control; bulk RNA-seq,Homo_633,AD.vs.MCI; bulk RNA-seq,Homo_633,MCI.vs.control; bulk RNA-seq,Homo_714,AD.vs.control; bulk RNA-seq,Homo_714,AD.vs.MCI; bulk RNA-seq,Homo_714,MCI.vs.control; scRNA-seq,SRP215507,CD8+ T cell_6-AD.vs.control; scRNA-seq,SRP330776,CD8+ T cell_2-AD.vs.control; scRNA-seq,SRP330776,B cell_1-AD.vs.control; scRNA-seq,SRP330776,Naive CD8+ T cell_3-AD.vs.control; scRNA-seq,SRP330776,B cell_2-AD.vs.control; scRNA-seq,SRP330776,T cell-AD.vs.control; scRNA-seq,SRP330776,CD8+ T cell_3-	17
CAMK2N1	scRNA-seq,SRP215507,CD8+ T cell_2-AD.vs.MCI; scRNA-seq,SRP215507,CD8+ T cell_4-AD.vs.MCI; scRNA-seq,SRP215507,CD8+ T cell_5-AD.vs.MCI; scRNA-seq,SRP215507,CD8+ T cell_5-MCI.vs.control; scRNA-seq,SRP215507,CD8+ T cell_4-MCI.vs.control; scRNA-seq,SRP215507,CD8+ T cell_2-MCI.vs.control; scRNA-seq,SRP215507,CD8+ T cell_3-MCI.vs.control; scRNA-seq,SRP330776,CD8+ T cell_1-AD.vs.control; scRNA-seq,SRP330776,γδ T cell-AD.vs.control; scRNA-seq,SRP330776,CD8+ T cell_2-AD.vs.control; scRNA-seq,SRP330776,B cell_1-AD.vs.control; scRNA-seq,SRP330776,Naive CD8+ T cell_1-AD.vs.control; scRNA-seq,SRP330776,Natural killer cell_1-AD.vs.control; scRNA-	14
CAMK2N2	bulk RNA-seq,Homo_723,AD.vs.control; bulk RNA-seq,Homo_723,AD.vs.MCI; bulk RNA-seq,Homo_633,AD.vs.control; bulk RNA-	4
CAMK4	bulk RNA-seq,Homo_723,AD.vs.MCI; bulk RNA-seq,Homo_723,MCI.vs.control; bulk RNA-seq,Homo_633,AD.vs.MCI; bulk RNA-seq,Homo_633,MCI.vs.control; bulk RNA-seq,ROSMAP,AD.vs.control; scRNA-seq,SRP215507,CD8+ T cell_8-AD.vs.control; scRNA-seq,SRP215507,CD8+ T cell_1-AD.vs.control; scRNA-seq,SRP215507,CD8+ T cell_4-AD.vs.control; scRNA-seq,SRP215507,CD8+ T cell_1-AD.vs.MCI; scRNA-seq,SRP215507,CD8+ T cell_4-AD.vs.MCI; scRNA-seq,SRP215507,CD8+ T cell_1-MCI.vs.control; scRNA-seq,SRP215507,CD8+ T cell_4-MCI.vs.control; scRNA-seq,SRP330776,CD8+ T cell_2-AD.vs.control; scRNA-seq,SRP330776,Natural killer cell_1-AD.vs.control; scRNA-	16
CAMKK1	bulk RNA-seq,Homo_723,AD.vs.control; bulk RNA-seq,Homo_723,AD.vs.MCI; bulk RNA-seq,Homo_723,MCI.vs.control; bulk RNA-seq,Homo_633,AD.vs.control; bulk RNA-seq,Homo_633,AD.vs.MCI; bulk RNA-seq,Homo_633,MCI.vs.control; bulk RNA-seq,Homo_714,AD.vs.control; bulk RNA-seq,Homo_714,AD.vs.MCI; bulk RNA-seq,Homo_714,MCI.vs.control; scRNA-seq,SRP215507,CD8+ T cell_5-MCI.vs.control; scRNA-seq,SRP215507,CD8+ T cell_2-MCI.vs.control; scRNA-seq,SRP330776,CD8+ T cell_1-AD.vs.control; scRNA-seq,SRP330776,Memory T cell_2-AD.vs.control; scRNA-seq,SRP330776,B cell_1-AD.vs.control; scRNA-seq,SRP330776,Naive CD8+ T cell_1-AD.vs.control; scRNA-seq,SRP330776,T	16
CAMKK2	bulk RNA-seq,Homo_723,AD.vs.control; bulk RNA-seq,Homo_723,AD.vs.MCI; bulk RNA-seq,Homo_633,AD.vs.control; bulk RNA-seq,Homo_633,AD.vs.MCI; bulk RNA-seq,Homo_714,AD.vs.control; bulk RNA-seq,Homo_714,AD.vs.MCI; scRNA-seq,SRP215507,CD8+ T cell_5-AD.vs.control; scRNA-seq,SRP215507,CD8+ T cell_9-AD.vs.control; scRNA-seq,SRP215507,CD8+ T cell_4-AD.vs.MCI; scRNA-seq,SRP215507,CD8+ T cell_4-MCI.vs.control; scRNA-seq,SRP330776,CD8+ T cell_1-AD.vs.control; scRNA-seq,SRP330776,γδ T cell-AD.vs.control; scRNA-seq,SRP330776,Memory T cell_2-AD.vs.control; scRNA-seq,SRP330776,CD8+ T cell_2-AD.vs.control; scRNA-seq,SRP330776,B cell_1-AD.vs.control; scRNA-seq,SRP330776,Naive CD8+ T cell_1-AD.vs.control; scRNA-seq,SRP330776,Natural killer cell_1-AD.vs.control; scRNA-seq,SRP330776,Memory T cell_1-AD.vs.control; scRNA-seq,SRP330776,B cell_2-AD.vs.control; scRNA-seq,SRP330776,T cell-AD.vs.control; scRNA-seq,SRP330776,CD8+ T [illegible]	23
CAMKMT	bulk RNA-seq,Homo_723,MCI.vs.control; bulk RNA-seq,Homo_633,AD.vs.MCI; bulk RNA-seq,Homo_633,MCI.vs.control; bulk RNA-seq,Homo_714,AD.vs.control; bulk RNA-seq,Homo_714,AD.vs.MCI; bulk RNA-seq,Homo_714,MCI.vs.control; scRNA-seq,SRP215507,CD8+ T cell_4-AD.vs.control; scRNA-seq,SRP215507,CD8+ T cell_4-AD.vs.MCI; scRNA-seq,SRP215507,CD8+ T cell_5-AD.vs.MCI; scRNA-seq,SRP309935,Natural killer cell_2-AD.vs.control; scRNA-seq,SRP330776,CD8+ T cell_1-AD.vs.control; scRNA-seq,SRP330776,γδ T cell-AD.vs.control; scRNA-seq,SRP330776,Memory T cell_2-AD.vs.control; scRNA-seq,SRP330776,B cell_1-AD.vs.control; scRNA-seq,SRP330776,Naive CD8+ T cell_1-AD.vs.control; scRNA-seq,SRP330776,Memory T cell_1-AD.vs.control; scRNA-seq,SRP330776,Natural killer T cell-AD.vs.control; scRNA-	19
CAMKV	bulk RNA-seq,Homo_723,AD.vs.control; bulk RNA-seq,Homo_723,AD.vs.MCI; bulk RNA-seq,Homo_723,MCI.vs.control; bulk RNA-seq,Homo_633,AD.vs.control; bulk RNA-seq,Homo_714,MCI.vs.control; bulk RNA-seq,ROSMAP,AD.vs.control	6

CAMLG	bulk RNA-seq,Homo_723,AD.vs.MCI; bulk RNA-seq,Homo_723,MCI.vs.control; bulk RNA-seq,Homo_633,AD.vs.MCI; bulk RNA-seq,Homo_633,MCI.vs.control; bulk RNA-seq,Homo_714,AD.vs.control; bulk RNA-seq,Homo_714,AD.vs.MCI; scRNA-seq,SRP215507,CD8+ T cell_7-	7
CAMP	bulk RNA-seq,Homo_723,AD.vs.control; bulk RNA-seq,Homo_723,AD.vs.MCI	2
Camp	bulk RNA-seq,SRP325058,AD.vs.control	1
CAMSAP1	bulk RNA-seq,Homo_723,AD.vs.control; bulk RNA-seq,Homo_723,AD.vs.MCI; bulk RNA-seq,Homo_723,MCI.vs.control; bulk RNA-seq,Homo_633,AD.vs.control; bulk RNA-seq,Homo_633,AD.vs.MCI; bulk RNA-seq,Homo_633,MCI.vs.control; bulk RNA-seq,Homo_714,AD.vs.control; bulk RNA-seq,Homo_714,AD.vs.MCI; scRNA-seq,SRP215507,CD8+ T cell_2-AD.vs.MCI; scRNA-seq,SRP215507,CD8+ T cell_3-AD.vs.MCI; scRNA-seq,SRP215507,CD8+ T cell_4-AD.vs.MCI; scRNA-seq,SRP215507,CD8+ T cell_4-MCI.vs.control; scRNA-seq,SRP309935,Natural killer cell_1-AD.vs.control; scRNA-seq,SRP309935,Monocyte_2-AD.vs.control; scRNA-seq,SRP330776,Memory T cell_2-AD.vs.control; scRNA-seq,SRP330776,B cell_1-AD.vs.control; scRNA-seq,SRP330776,Natural killer cell_1-AD.vs.control; scRNA-seq,SRP330776,B cell_2-AD.vs.control; scRNA-seq,SRP330776,Naive CD8+ T cell_2-AD.vs.control; scRNA-seq,SRP330776,Monocyte_2-AD.vs.control; scRNA-seq,SRP330776,Natural killer cell_2- [illegible]	21
CAMSAP2	bulk RNA-seq,Homo_723,AD.vs.control; bulk RNA-seq,Homo_723,AD.vs.MCI; bulk RNA-seq,Homo_723,MCI.vs.control; bulk RNA-seq,Homo_633,AD.vs.control; bulk RNA-seq,Homo_633,AD.vs.MCI; bulk RNA-seq,Homo_633,MCI.vs.control; bulk RNA-seq,Homo_714,AD.vs.control; bulk RNA-seq,Homo_714,AD.vs.MCI; scRNA-seq,SRP330776,Naive CD8+ T cell_1-AD.vs.control; scRNA-seq,SRP330776,Natural killer T cell-	10
Camsap2	bulk RNA-seq,SRP325058,AD.vs.control	1
CAMSAP3	bulk RNA-seq,Homo_723,MCI.vs.control; bulk RNA-seq,Homo_633,MCI.vs.control; bulk RNA-seq,ROSMAP,AD.vs.control; bulk RNA-	4
CAMTA1	bulk RNA-seq,Homo_723,MCI.vs.control; bulk RNA-seq,Homo_633,AD.vs.MCI; bulk RNA-seq,Homo_633,MCI.vs.control; scRNA-seq,SRP215507,CD8+ T cell_8-AD.vs.control; scRNA-seq,SRP215507,CD8+ T cell_5-AD.vs.control; scRNA-seq,SRP215507,CD8+ T cell_4-AD.vs.MCI; scRNA-seq,SRP330776,CD8+ T cell_1-AD.vs.control; scRNA-seq,SRP330776,Memory T cell_2-AD.vs.control; scRNA-seq,SRP330776,CD8+ T cell_2-AD.vs.control; scRNA-seq,SRP330776,Naive CD8+ T cell_1-AD.vs.control; scRNA-seq,SRP330776,Natural killer cell_1-AD.vs.control; scRNA-seq,SRP330776,Natural killer T cell-AD.vs.control; scRNA-seq,SRP330776,B cell_2-AD.vs.control; scRNA-seq,SRP330776,T cell-AD.vs.control; scRNA-	16
CAMTA1-DT	scRNA-seq,SRP215507,CD8+ T cell_3-AD.vs.control; scRNA-seq,SRP215507,CD8+ T cell_1-AD.vs.MCI; scRNA-seq,SRP215507,CD8+ T cell_1-	3
Camta2	bulk RNA-seq,SRP325058,AD.vs.control	1
CAMTA2	scRNA-seq,SRP330776,B cell_2-AD.vs.control; scRNA-seq,SRP330776,Monocyte_1-AD.vs.control; scRNA-seq,SRP330776,Monocyte_2-AD.vs.control	3
CAND1	bulk RNA-seq,Homo_633,AD.vs.control; bulk RNA-seq,Homo_714,AD.vs.control; bulk RNA-seq,Homo_714,AD.vs.MCI; bulk RNA-seq,Homo_714,MCI.vs.control; scRNA-seq,SRP215507,CD8+ T cell_5-AD.vs.MCI; scRNA-seq,SRP215507,CD8+ T cell_6-AD.vs.MCI; scRNA-seq,SRP215507,CD8+ T cell_5-MCI.vs.control; scRNA-seq,SRP215507,CD8+ T cell_6-MCI.vs.control; scRNA-seq,SRP330776,Naive CD8+ T cell_3-	9
CAND2	bulk RNA-seq,Homo_723,AD.vs.control; bulk RNA-seq,Homo_723,AD.vs.MCI; bulk RNA-seq,Homo_633,AD.vs.control; bulk RNA-seq,Homo_633,AD.vs.MCI; bulk RNA-seq,Homo_633,MCI.vs.control; bulk RNA-seq,Homo_714,AD.vs.control; bulk RNA-seq,Homo_714,AD.vs.MCI; bulk RNA-seq,Homo_714,MCI.vs.control; scRNA-seq,SRP330776,γδ T cell-AD.vs.control	9
CANT1	bulk RNA-seq,Homo_714,AD.vs.control; bulk RNA-seq,Homo_714,AD.vs.MCI; bulk RNA-seq,SRP223445,AD.vs.control; scRNA-seq,SRP215507,CD8+ T cell_5-AD.vs.MCI; scRNA-seq,SRP215507,CD8+ T cell_1-MCI.vs.control; scRNA-seq,SRP215507,CD8+ T cell_5-MCI.vs.control; scRNA-seq,SRP215507,CD8+ T cell_3-MCI.vs.control; scRNA-seq,SRP309935,Natural killer cell_2-AD.vs.control; scRNA-seq,SRP330776,CD8+ T cell_3-	9
CANX	scRNA-seq,SRP215507,CD8+ T cell_7-AD.vs.control; scRNA-seq,SRP330776,B cell_4-AD.vs.control	2
CAP1	scRNA-seq,SRP215507,CD8+ T cell_7-AD.vs.control; scRNA-seq,SRP215507,CD8+ T cell_7-AD.vs.MCI; scRNA-seq,SRP309935,Erythroid cell-AD.vs.control; scRNA-seq,SRP330776,Naive CD8+ T cell_3-AD.vs.control; scRNA-seq,SRP330776,B cell_2-AD.vs.control; scRNA-seq,SRP330776,B	6
CAP2	bulk RNA-seq,Homo_723,AD.vs.control; bulk RNA-seq,Homo_723,AD.vs.MCI; bulk RNA-seq,Homo_723,MCI.vs.control; scRNA-seq,SRP215507,CD8+ T cell_5-AD.vs.control	4

CAPG	bulk RNA-seq,Homo_723,AD.vs.MCI; bulk RNA-seq,Homo_723,MCI.vs.control; bulk RNA-seq,Homo_633,AD.vs.MCI; bulk RNA-seq,Homo_633,MCI.vs.control; scRNA-seq,SRP215507,CD8+ T cell_2-AD.vs.control; scRNA-seq,SRP215507,CD8+ T cell_3-AD.vs.control; scRNA-seq,SRP215507,CD8+ T cell_1-AD.vs.control; scRNA-seq,SRP215507,CD8+ T cell_4-AD.vs.control; scRNA-seq,SRP215507,CD8+ T cell_5-AD.vs.control; scRNA-seq,SRP215507,CD8+ T cell_1-AD.vs.MCI; scRNA-seq,SRP215507,CD8+ T cell_5-MCI.vs.control; scRNA-seq,SRP215507,CD8+ T cell_4-MCI.vs.control; scRNA-seq,SRP215507,CD8+ T cell_2-MCI.vs.control; scRNA-seq,SRP215507,CD8+ T cell_3-MCI.vs.control; scRNA-seq,SRP309935,Natural killer cell_1-AD.vs.control; scRNA-seq,SRP330776,CD8+ T cell_1-AD.vs.control; scRNA-seq,SRP330776,γδ T cell-AD.vs.control; scRNA-seq,SRP330776,Memory T cell_2-AD.vs.control; scRNA-seq,SRP330776,CD8+ T cell_2-AD.vs.control; scRNA-seq,SRP330776,Naive CD8+ T cell_1-AD.vs.control; scRNA-seq,SRP330776,Memory T cell_1-AD.vs.control; scRNA-seq,SRP330776,Natural killer T cell-AD.vs.control; scRNA-seq,SRP330776,CD8+ T cell_[illegible]-AD.vs.control	23
Capn1	bulk RNA-seq,SRP325058,AD.vs.control	1
CAPN1	scRNA-seq,SRP215507,CD8+ T cell_7-AD.vs.control; scRNA-seq,SRP215507,CD8+ T cell_7-MCI.vs.control; scRNA-seq,SRP330776,CD8+ T cell_1-AD.vs.control; scRNA-seq,SRP330776,Naive CD8+ T cell_1-AD.vs.control; scRNA-seq,SRP330776,Naive CD8+ T cell_3-AD.vs.control; scRNA-seq,SRP330776,B cell_2-AD.vs.control; scRNA-seq,SRP330776,Naive CD8+ T cell_2-AD.vs.control	7
CAPN10	scRNA-seq,SRP215507,CD8+ T cell_4-AD.vs.MCI; scRNA-seq,SRP215507,CD8+ T cell_1-MCI.vs.control; scRNA-seq,SRP215507,CD8+ T cell_2-MCI.vs.control; scRNA-seq,SRP330776,CD8+ T cell_1-AD.vs.control; scRNA-seq,SRP330776,γδ T cell-AD.vs.control; scRNA-seq,SRP330776,Memory T cell_2-AD.vs.control; scRNA-seq,SRP330776,CD8+ T cell_2-AD.vs.control; scRNA-seq,SRP330776,B cell_1-AD.vs.control; scRNA-seq,SRP330776,Naive CD8+ T cell_1-AD.vs.control; scRNA-seq,SRP330776,Memory T cell_1-AD.vs.control; scRNA-seq,SRP330776,Natural killer T cell-AD.vs.control; scRNA-seq,SRP330776,Naive CD8+ T cell_3-AD.vs.control; scRNA-seq,SRP330776,B cell_2-AD.vs.control; scRNA-seq,SRP330776,T cell-AD.vs.control; scRNA-seq,SRP330776,CD8+ T cell_3-AD.vs.control; scRNA-seq,SRP330776,B cell_3-AD.vs.control; scRNA-[illegible]	17
CAPN10-DT	scRNA-seq,SRP215507,CD8+ T cell_3-AD.vs.control; scRNA-seq,SRP215507,CD8+ T cell_2-AD.vs.MCI; scRNA-seq,SRP215507,CD8+ T cell_5-AD.vs.MCI; scRNA-seq,SRP215507,CD8+ T cell_5-MCI.vs.control; scRNA-seq,SRP215507,CD8+ T cell_2-MCI.vs.control; scRNA-seq,SRP215507,CD8+ T cell_3-MCI.vs.control; scRNA-seq,SRP309935,CD8+ T cell_1-AD.vs.control; scRNA-seq,SRP309935,B cell_1-AD.vs.control; scRNA-seq,SRP330776,γδ T cell-AD.vs.control; scRNA-seq,SRP330776,Memory T cell_1-AD.vs.control; scRNA-seq,SRP330776,T cell-AD.vs.control	11
CAPN11	bulk RNA-seq,Homo_714,AD.vs.control; bulk RNA-seq,Homo_714,AD.vs.MCI; bulk RNA-seq,ROSMAP,AD.vs.control	3
CAPN12	bulk RNA-seq,Homo_723,AD.vs.control; bulk RNA-seq,Homo_723,AD.vs.MCI; bulk RNA-seq,Homo_633,AD.vs.control; bulk RNA-seq,Homo_633,AD.vs.MCI; bulk RNA-seq,Homo_714,AD.vs.control; bulk RNA-seq,Homo_714,AD.vs.MCI; scRNA-seq,SRP215507,CD8+ T cell_4-AD.vs.control; scRNA-seq,SRP215507,CD8+ T cell_2-MCI.vs.control; scRNA-seq,SRP215507,CD8+ T cell_6-MCI.vs.control; scRNA-seq,SRP309935,Naive CD8+ T cell_1-AD.vs.control; scRNA-seq,SRP309935,CD8+ T cell_1-AD.vs.control; scRNA-seq,SRP330776,CD8+ T cell_1-AD.vs.control; scRNA-seq,SRP330776,Natural killer cell_1-AD.vs.control; scRNA-seq,SRP330776,Memory T cell_1-AD.vs.control; scRNA-seq,SRP330776,Natural killer T cell-AD.vs.control; scRNA-seq,SRP330776,B cell_2-AD.vs.control; scRNA-seq,SRP330776,T cell-AD.vs.control; scRNA-seq,SRP330776,CD8+ T cell_3-AD.vs.control; scRNA-seq,SRP330776,Natural killer cell_2-AD.vs.control; scRNA-seq,SRP330776,B cell_4-AD.vs.control	20
CAPN14	bulk RNA-seq,Homo_723,AD.vs.control; bulk RNA-seq,Homo_633,AD.vs.control; bulk RNA-seq,Homo_633,AD.vs.MCI; bulk RNA-seq,Homo_714,AD.vs.control; bulk RNA-seq,Homo_714,AD.vs.MCI	5
CAPN15	scRNA-seq,SRP330776,CD8+ T cell_1-AD.vs.control; scRNA-seq,SRP330776,B cell_1-AD.vs.control; scRNA-seq,SRP330776,Naive CD8+ T cell_1-AD.vs.control; scRNA-seq,SRP330776,Natural killer cell_1-AD.vs.control; scRNA-seq,SRP330776,Memory T cell_1-AD.vs.control; scRNA-seq,SRP330776,T cell-AD.vs.control; scRNA-seq,SRP330776,Monocyte_2-AD.vs.control	7

CAPN2	bulk RNA-seq,Homo_723,AD.vs.control; bulk RNA-seq,Homo_723,MCI.vs.control; bulk RNA-seq,Homo_633,AD.vs.control; bulk RNA-seq,Homo_633,AD.vs.MCI; bulk RNA-seq,Homo_633,MCI.vs.control; bulk RNA-seq,Homo_714,AD.vs.control; bulk RNA-seq,Homo_714,AD.vs.MCI; bulk RNA-seq,Homo_714,MCI.vs.control; bulk RNA-seq,SRP310421,AD.vs.control; scRNA-seq,SRP215507,CD8+ T cell_6-AD.vs.control; scRNA-seq,SRP215507,CD8+ T cell_6-AD.vs.MCI; scRNA-seq,SRP215507,CD8+ T cell_8-MCI.vs.control; scRNA-seq,SRP309935,B cell_1-AD.vs.control; scRNA-seq,SRP330776,CD8+ T cell_1-AD.vs.control; scRNA-seq,SRP330776,γδ T cell-AD.vs.control; scRNA-seq,SRP330776,Memory T cell_2-AD.vs.control; scRNA-seq,SRP330776,B cell_1-AD.vs.control; scRNA-seq,SRP330776,Naive CD8+ T cell_1-AD.vs.control; scRNA-seq,SRP330776,Natural killer cell_1-AD.vs.control; scRNA-seq,SRP330776,Memory T cell_1-AD.vs.control; scRNA-seq,SRP330776,Natural killer T cell-AD.vs.control; scRNA-seq,SRP330776,Naive CD8+ T cell_3-AD.vs.control; scRNA-seq,SRP330776,B cell_2-AD.vs.control; scRNA-seq,SRP330776,T cell-AD.vs.control; scRNA-seq,SRP330776,Naive CD8+ T cell_2-AD.vs.control; scRNA-seq,SRP330776,CD8+ T cell_3-AD.vs.control; scRNA-seq,SRP330776,Monocyte_1-AD.vs.control; scRNA-seq,SRP330776,Monocyte_2-AD.vs.control; scRNA-seq,SRP330776,B cell_3-AD.vs.control; scRNA-seq,SRP330776,Megakaryocyte_1-AD.vs.control; scRNA-seq,SRP330776,Natural killer cell_2-AD.vs.control; scRNA-seq,SRP330776,B cell_4- [illegible]	32
CAPN3	bulk RNA-seq,Homo_723,AD.vs.control; bulk RNA-seq,Homo_723,AD.vs.MCI; bulk RNA-seq,Homo_723,MCI.vs.control; bulk RNA-seq,Homo_633,AD.vs.control; bulk RNA-seq,Homo_633,AD.vs.MCI; bulk RNA-seq,Homo_633,MCI.vs.control; bulk RNA-seq,Homo_714,AD.vs.control; bulk RNA-seq,Homo_714,AD.vs.MCI; bulk RNA-seq,Homo_714,MCI.vs.control; scRNA-seq,SRP215507,CD8+ T cell_1-AD.vs.control; scRNA-seq,SRP215507,CD8+ T cell_4-AD.vs.MCI; scRNA-seq,SRP215507,CD8+ T cell_5-AD.vs.MCI; scRNA-seq,SRP215507,CD8+ T cell_5-MCI.vs.control; scRNA-seq,SRP215507,CD8+ T cell_4-MCI.vs.control; scRNA-seq,SRP215507,CD8+ T cell_7-MCI.vs.control; scRNA-seq,SRP309935,Natural killer	17
Capn3	bulk RNA-seq,SRP325058,AD.vs.control	1
CAPN5	scRNA-seq,SRP215507,CD8+ T cell_2-AD.vs.MCI; scRNA-seq,SRP215507,CD8+ T cell_2-MCI.vs.control; scRNA-seq,SRP330776,γδ T cell-AD.vs.control; scRNA-seq,SRP330776,CD8+ T cell_2-AD.vs.control; scRNA-seq,SRP330776,Naive CD8+ T cell_1-AD.vs.control; scRNA-seq,SRP330776,Memory T cell_1-AD.vs.control; scRNA-seq,SRP330776,Naive CD8+ T cell_3-AD.vs.control	7
CAPN6	bulk RNA-seq,Homo_723,AD.vs.control; bulk RNA-seq,Homo_723,AD.vs.MCI; bulk RNA-seq,ROSMAP,MCI.vs.control	3
CAPN7	bulk RNA-seq,Homo_714,AD.vs.control; bulk RNA-seq,Homo_714,AD.vs.MCI; scRNA-seq,SRP215507,CD8+ T cell_7-AD.vs.control; scRNA-seq,SRP215507,CD8+ T cell_6-AD.vs.control; scRNA-seq,SRP215507,CD8+ T cell_9-AD.vs.control; scRNA-seq,SRP215507,CD8+ T cell_7-AD.vs.MCI; scRNA-seq,SRP215507,CD8+ T cell_5-AD.vs.MCI; scRNA-seq,SRP215507,CD8+ T cell_5-MCI.vs.control; scRNA-seq,SRP330776,Monocyte_2-	9
CAPN8	bulk RNA-seq,Homo_723,AD.vs.control; bulk RNA-seq,Homo_723,AD.vs.MCI; bulk RNA-seq,Homo_633,AD.vs.control; bulk RNA-seq,Homo_633,AD.vs.MCI; bulk RNA-seq,Homo_714,AD.vs.control; bulk RNA-seq,Homo_714,AD.vs.MCI	6
CAPN9	bulk RNA-seq,Homo_633,MCI.vs.control; bulk RNA-seq,Homo_714,MCI.vs.control	2
CAPNS1	scRNA-seq,SRP215507,CD8+ T cell_7-AD.vs.control; scRNA-seq,SRP215507,CD8+ T cell_3-AD.vs.MCI; scRNA-seq,SRP215507,CD8+ T cell_6-AD.vs.MCI; scRNA-seq,SRP215507,CD8+ T cell_7-MCI.vs.control; scRNA-seq,SRP309935,Erythroid cell-AD.vs.control; scRNA-seq,SRP330776,CD8+ T cell_1-AD.vs.control; scRNA-seq,SRP330776,γδ T cell-AD.vs.control; scRNA-seq,SRP330776,Memory T cell_2-AD.vs.control; scRNA-seq,SRP330776,CD8+ T cell_2-AD.vs.control; scRNA-seq,SRP330776,B cell_1-AD.vs.control; scRNA-seq,SRP330776,Naive CD8+ T cell_1-AD.vs.control; scRNA-seq,SRP330776,Natural killer cell_1-AD.vs.control; scRNA-seq,SRP330776,Memory T cell_1-AD.vs.control; scRNA-seq,SRP330776,Natural killer T cell-AD.vs.control; scRNA-seq,SRP330776,Naive CD8+ T cell_3-AD.vs.control; scRNA-seq,SRP330776,B cell_2-AD.vs.control; scRNA-seq,SRP330776,T cell-AD.vs.control; scRNA-seq,SRP330776,CD8+ T cell_3-AD.vs.control; scRNA-seq,SRP330776,Monocyte_2-AD.vs.control; scRNA-seq,SRP330776,B cell_3-AD.vs.control; scRNA-seq,SRP330776,Natural killer cell_2-AD.vs.control; scRNA-seq,SRP330776,B [illegible]	22
CAPRIN1	scRNA-seq,SRP330776,Regulatory T cell-AD.vs.control; scRNA-seq,SRP330776,Megakaryocyte_1-AD.vs.control	2
CAPRIN2	scRNA-seq,SRP215507,CD8+ T cell_4-AD.vs.MCI; scRNA-seq,SRP330776,γδ T cell-AD.vs.control; scRNA-seq,SRP330776,Memory T cell_2-AD.vs.control; scRNA-seq,SRP330776,CD8+ T cell_2-AD.vs.control; scRNA-seq,SRP330776,B cell_1-AD.vs.control; scRNA-seq,SRP330776,Naive CD8+ T cell_1-AD.vs.control; scRNA-seq,SRP330776,Natural killer T cell-AD.vs.control; scRNA-seq,SRP330776,B cell_2-AD.vs.control; scRNA-seq,SRP330776,T cell-AD.vs.control; scRNA-seq,SRP330776,Monocyte_1-AD.vs.control	10
CAPS	scRNA-seq,SRP215507,CD8+ T cell_4-AD.vs.control; scRNA-seq,SRP215507,CD8+ T cell_5-AD.vs.MCI; scRNA-seq,SRP309935,B cell_1-AD.vs.control; scRNA-seq,SRP330776,CD8+ T cell_1-AD.vs.control; scRNA-seq,SRP330776,γδ T cell-AD.vs.control; scRNA-seq,SRP330776,Memory T cell_2-AD.vs.control; scRNA-seq,SRP330776,B cell_1-AD.vs.control; scRNA-seq,SRP330776,Memory T cell_1-AD.vs.control; scRNA-seq,SRP330776,Natural killer T cell-AD.vs.control; scRNA-seq,SRP330776,Naive CD8+ T cell_3-AD.vs.control; scRNA-seq,SRP330776,B cell_2-	12

CAPS2	bulk RNA-seq,ROSMAP,AD.vs.control; bulk RNA-seq,ROSMAP,MCI.vs.control; scRNA-seq,SRP215507,CD8+ T cell_4-AD.vs.MCI; scRNA-seq,SRP330776,γδ T cell-AD.vs.control; scRNA-seq,SRP330776,Memory T cell_2-AD.vs.control; scRNA-seq,SRP330776,B cell_1-AD.vs.control; scRNA-seq,SRP330776,Naive CD8+ T cell_1-AD.vs.control	7
CAPSL	bulk RNA-seq,Homo_723,AD.vs.control; bulk RNA-seq,Homo_723,AD.vs.MCI	2
CAPZA1	scRNA-seq,SRP215507,CD8+ T cell_4-AD.vs.MCI; scRNA-seq,SRP309935,Erythroid cell-AD.vs.control; scRNA-seq,SRP330776,CD8+ T cell_1-AD.vs.control; scRNA-seq,SRP330776,γδ T cell-AD.vs.control; scRNA-seq,SRP330776,Naive CD8+ T cell_1-AD.vs.control; scRNA-seq,SRP330776,Natural killer cell_1-AD.vs.control; scRNA-seq,SRP330776,Memory T cell_1-AD.vs.control; scRNA-seq,SRP330776,B cell_2-AD.vs.control; scRNA-seq,SRP330776,Naive CD8+ T cell_2-AD.vs.control; scRNA-seq,SRP330776,CD8+ T cell_3-AD.vs.control; scRNA-	11
CAPZA2	bulk RNA-seq,SRP223445,AD.vs.control; scRNA-seq,SRP215507,CD8+ T cell_7-AD.vs.control; scRNA-seq,SRP215507,CD8+ T cell_9-AD.vs.control; scRNA-seq,SRP330776,Naive CD8+ T cell_3-AD.vs.control; scRNA-seq,SRP330776,Natural killer cell_2-AD.vs.control	5
CAPZA3	bulk RNA-seq,Homo_723,AD.vs.control	1
CAPZB	scRNA-seq,SRP215507,CD8+ T cell_8-MCI.vs.control; scRNA-seq,SRP309935,Erythroid cell-AD.vs.control	2
Car1	bulk RNA-seq,SRP325058,AD.vs.control	1
CARD10	bulk RNA-seq,Homo_723,AD.vs.control; bulk RNA-seq,Homo_723,AD.vs.MCI; bulk RNA-seq,Homo_723,MCI.vs.control; bulk RNA-seq,Homo_633,AD.vs.control; bulk RNA-seq,Homo_633,AD.vs.MCI; bulk RNA-seq,Homo_633,MCI.vs.control; bulk RNA-seq,Homo_714,MCI.vs.control	7
CARD11	bulk RNA-seq,Homo_723,AD.vs.control; bulk RNA-seq,Homo_633,AD.vs.control; bulk RNA-seq,Homo_633,MCI.vs.control; scRNA-seq,SRP215507,CD8+ T cell_7-AD.vs.control; scRNA-seq,SRP215507,CD8+ T cell_5-AD.vs.control; scRNA-seq,SRP215507,CD8+ T cell_5-AD.vs.MCI; scRNA-seq,SRP309935,Natural killer cell_2-AD.vs.control; scRNA-seq,SRP309935,Naive CD8+ T cell_1-AD.vs.control; scRNA-seq,SRP309935,Natural killer cell_1-AD.vs.control; scRNA-seq,SRP309935,CD8+ T cell_1-AD.vs.control; scRNA-seq,SRP309935,B cell_1-AD.vs.control; scRNA-seq,SRP330776,CD8+ T cell_1-AD.vs.control; scRNA-seq,SRP330776,γδ T cell-AD.vs.control; scRNA-seq,SRP330776,Memory T cell_2-AD.vs.control; scRNA-seq,SRP330776,CD8+ T cell_2-AD.vs.control; scRNA-seq,SRP330776,Naive CD8+ T cell_1-AD.vs.control; scRNA-seq,SRP330776,Natural killer cell_1-AD.vs.control; scRNA-seq,SRP330776,Memory T cell_1-AD.vs.control; scRNA-seq,SRP330776,Natural killer T cell-AD.vs.control; scRNA-seq,SRP330776,Naive CD8+ T cell_3-AD.vs.control; scRNA-seq,SRP330776,B cell_2-AD.vs.control; scRNA-seq,SRP330776,T cell-AD.vs.control; [illegible]	23
CARD14	bulk RNA-seq,Homo_723,AD.vs.control; bulk RNA-seq,Homo_723,MCI.vs.control; bulk RNA-seq,Homo_633,AD.vs.MCI; bulk RNA-seq,Homo_633,MCI.vs.control; bulk RNA-seq,Homo_714,AD.vs.MCI; bulk RNA-seq,Homo_714,MCI.vs.control	6
CARD16	scRNA-seq,SRP215507,CD8+ T cell_7-AD.vs.control; scRNA-seq,SRP215507,CD8+ T cell_9-AD.vs.control; scRNA-seq,SRP309935,Erythroid cell-AD.vs.control; scRNA-seq,SRP330776,CD8+ T cell_1-AD.vs.control; scRNA-seq,SRP330776,γδ T cell-AD.vs.control; scRNA-seq,SRP330776,Memory T cell_2-AD.vs.control; scRNA-seq,SRP330776,CD8+ T cell_2-AD.vs.control; scRNA-seq,SRP330776,B cell_1-AD.vs.control; scRNA-seq,SRP330776,Natural killer cell_1-AD.vs.control; scRNA-seq,SRP330776,Memory T cell_1-AD.vs.control; scRNA-seq,SRP330776,Naive CD8+ T cell_3-AD.vs.control; scRNA-seq,SRP330776,B cell_2-AD.vs.control; scRNA-seq,SRP330776,T cell-AD.vs.control; scRNA-seq,SRP330776,CD8+ T	15
CARD17	bulk RNA-seq,Homo_723,AD.vs.control; bulk RNA-seq,Homo_723,AD.vs.MCI; bulk RNA-seq,Homo_633,AD.vs.control; bulk RNA-seq,Homo_633,AD.vs.MCI; bulk RNA-seq,Homo_714,AD.vs.control; bulk RNA-seq,Homo_714,AD.vs.MCI; scRNA-seq,SRP215507,CD8+ T cell_5-AD.vs.control; scRNA-seq,SRP215507,CD8+ T cell_2-AD.vs.MCI; scRNA-seq,SRP215507,CD8+ T cell_2-MCI.vs.control; scRNA-seq,SRP215507,CD8+ T cell_6-MCI.vs.control; scRNA-seq,SRP330776,CD8+ T cell_1-AD.vs.control; scRNA-seq,SRP330776,T cell-AD.vs.control	12
CARD19	scRNA-seq,SRP215507,CD8+ T cell_7-AD.vs.control; scRNA-seq,SRP215507,CD8+ T cell_10-AD.vs.control; scRNA-seq,SRP215507,CD8+ T cell_6-AD.vs.control; scRNA-seq,SRP215507,CD8+ T cell_9-AD.vs.control; scRNA-seq,SRP215507,CD8+ T cell_3-AD.vs.MCI; scRNA-seq,SRP215507,CD8+ T cell_5-AD.vs.MCI; scRNA-seq,SRP215507,CD8+ T cell_5-MCI.vs.control; scRNA-seq,SRP215507,CD8+ T cell_6-MCI.vs.control; scRNA-seq,SRP330776,CD8+ T cell_1-AD.vs.control; scRNA-seq,SRP330776,Memory T cell_2-AD.vs.control; scRNA-seq,SRP330776,CD8+ T cell_2-AD.vs.control; scRNA-seq,SRP330776,B cell_1-AD.vs.control; scRNA-seq,SRP330776,Naive CD8+ T cell_1-AD.vs.control; scRNA-seq,SRP330776,Natural killer cell_1-AD.vs.control; scRNA-seq,SRP330776,Memory T cell_1-AD.vs.control; scRNA-seq,SRP330776,Natural killer T cell-AD.vs.control; scRNA-seq,SRP330776,Naive CD8+ T cell_3-AD.vs.control; scRNA-seq,SRP330776,T cell-AD.vs.control; scRNA-seq,SRP330776,Naive CD8+ T cell_2-AD.vs.control; scRNA-seq,SRP330776,CD8+ T cell_3-AD.vs.control; scRNA-seq,SRP330776,B cell_3-AD.vs.control; scRNA-seq,SRP330776,Regulatory T cell-AD.vs.control; scRNA-seq,SRP330776,Megakaryocyte_2-AD.vs.control; scRNA-seq,SRP330776,Natural killer cell_2-[illegible]	25

CARD6	bulk RNA-seq,Homo_723,AD.vs.control; bulk RNA-seq,Homo_723,AD.vs.MCI; bulk RNA-seq,Homo_633,AD.vs.control; bulk RNA-seq,Homo_633,AD.vs.MCI; bulk RNA-seq,Homo_714,AD.vs.control; bulk RNA-seq,Homo_714,AD.vs.MCI; scRNA-seq,SRP215507,CD8+ T cell_1-AD.vs.control; scRNA-seq,SRP330776,CD8+ T cell_1-AD.vs.control; scRNA-seq,SRP330776,B cell_1-AD.vs.control; scRNA-seq,SRP330776,Naive	10
CARD8	scRNA-seq,SRP215507,CD8+ T cell_6-AD.vs.control; scRNA-seq,SRP309935,Erythroid cell-AD.vs.control; scRNA-seq,SRP330776,CD8+ T cell_1-AD.vs.control; scRNA-seq,SRP330776,Memory T cell_2-AD.vs.control; scRNA-seq,SRP330776,B cell_1-AD.vs.control; scRNA-seq,SRP330776,Memory T cell_1-AD.vs.control; scRNA-seq,SRP330776,Natural killer T cell-AD.vs.control; scRNA-seq,SRP330776,Naive CD8+ T cell_3-AD.vs.control; scRNA-seq,SRP330776,T cell-AD.vs.control; scRNA-seq,SRP330776,Naive CD8+ T cell_2-AD.vs.control; scRNA-seq,SRP330776,CD8+ T cell_3-AD.vs.control;	12
CARD8-AS1	scRNA-seq,SRP215507,CD8+ T cell_5-MCI.vs.control; scRNA-seq,SRP309935,B cell_2-AD.vs.control; scRNA-seq,SRP330776,CD8+ T cell_1-AD.vs.control; scRNA-seq,SRP330776,γδ T cell-AD.vs.control; scRNA-seq,SRP330776,Memory T cell_2-AD.vs.control; scRNA-seq,SRP330776,CD8+ T cell_2-AD.vs.control; scRNA-seq,SRP330776,B cell_1-AD.vs.control; scRNA-seq,SRP330776,Naive CD8+ T cell_1-AD.vs.control; scRNA-seq,SRP330776,Natural killer cell_1-AD.vs.control; scRNA-seq,SRP330776,Memory T cell_1-AD.vs.control; scRNA-seq,SRP330776,Natural killer T cell-AD.vs.control; scRNA-seq,SRP330776,Naive CD8+ T cell_3-AD.vs.control; scRNA-seq,SRP330776,B cell_2-AD.vs.control; scRNA-seq,SRP330776,T cell-AD.vs.control; scRNA-seq,SRP330776,CD8+ T cell_3-AD.vs.control; scRNA-seq,SRP330776,Monocyte_2-AD.vs.control; scRNA-seq,SRP330776,B	17
CARD9	scRNA-seq,SRP215507,CD8+ T cell_5-AD.vs.control; scRNA-seq,SRP215507,CD8+ T cell_2-AD.vs.MCI; scRNA-seq,SRP215507,CD8+ T cell_1-AD.vs.MCI; scRNA-seq,SRP215507,CD8+ T cell_2-MCI.vs.control; scRNA-seq,SRP215507,CD8+ T cell_6-MCI.vs.control; scRNA-seq,SRP309935,Natural killer cell_1-AD.vs.control; scRNA-seq,SRP330776,CD8+ T cell_1-AD.vs.control; scRNA-seq,SRP330776,γδ T cell-AD.vs.control; scRNA-seq,SRP330776,Memory T cell_2-AD.vs.control; scRNA-seq,SRP330776,Natural killer cell_1-AD.vs.control; scRNA-	11
CARF	scRNA-seq,SRP215507,CD8+ T cell_3-AD.vs.MCI; scRNA-seq,SRP215507,CD8+ T cell_1-MCI.vs.control; scRNA-seq,SRP215507,CD8+ T cell_5-MCI.vs.control; scRNA-seq,SRP215507,CD8+ T cell_3-MCI.vs.control; scRNA-seq,SRP309935,Naive CD8+ T cell_1-AD.vs.control; scRNA-seq,SRP309935,Natural killer cell_1-AD.vs.control; scRNA-seq,SRP309935,Naive CD8+ T cell_2-AD.vs.control; scRNA-seq,SRP330776,Memory T cell_2-AD.vs.control; scRNA-seq,SRP330776,Natural killer cell_1-AD.vs.control; scRNA-seq,SRP330776,Natural killer T cell-AD.vs.control; scRNA-seq,SRP330776,T cell-AD.vs.control; scRNA-seq,SRP330776,B cell_3-AD.vs.control; scRNA-seq,SRP330776,Regulatory T cell-AD.vs.control	13
CARHSP1	bulk RNA-seq,Homo_633,AD.vs.MCI; scRNA-seq,SRP215507,CD8+ T cell_6-AD.vs.MCI; scRNA-seq,SRP330776,Natural killer T cell-AD.vs.control; scRNA-seq,SRP330776,Natural killer cell_2-AD.vs.control	4
Carhsp1	bulk RNA-seq,SRP325058,AD.vs.control	1
CARM1	scRNA-seq,SRP215507,CD8+ T cell_5-AD.vs.MCI; scRNA-seq,SRP215507,CD8+ T cell_5-MCI.vs.control; scRNA-seq,SRP215507,CD8+ T cell_4-MCI.vs.control; scRNA-seq,SRP309935,Naive CD8+ T cell_3-AD.vs.control; scRNA-seq,SRP330776,γδ T cell-AD.vs.control; scRNA-seq,SRP330776,CD8+ T cell_2-AD.vs.control; scRNA-seq,SRP330776,T cell-AD.vs.control; scRNA-seq,SRP330776,CD8+ T cell_3-AD.vs.control; scRNA-seq,SRP330776,Monocyte_2-AD.vs.control; scRNA-seq,SRP330776,B cell_3-AD.vs.control; scRNA-seq,SRP330776,Natural killer cell_2-	11
CARMIL1	bulk RNA-seq,Homo_723,AD.vs.control; bulk RNA-seq,Homo_723,AD.vs.MCI; bulk RNA-seq,Homo_723,MCI.vs.control; bulk RNA-seq,Homo_633,AD.vs.control; bulk RNA-seq,Homo_633,AD.vs.MCI; bulk RNA-seq,Homo_633,MCI.vs.control; bulk RNA-seq,Homo_714,AD.vs.control; bulk RNA-seq,Homo_714,AD.vs.MCI; scRNA-seq,SRP215507,CD8+ T cell_5-AD.vs.MCI; scRNA-seq,SRP215507,CD8+ T cell_5-MCI.vs.control; scRNA-seq,SRP330776,CD8+ T cell_1-AD.vs.control; scRNA-seq,SRP330776,B cell_1-AD.vs.control; scRNA-seq,SRP330776,Naive CD8+ T cell_1-AD.vs.control; scRNA-seq,SRP330776,Naive CD8+ T cell_3-AD.vs.control; scRNA-seq,SRP330776,B cell_2-AD.vs.control; scRNA-seq,SRP330776,T	16
CARMIL2	bulk RNA-seq,ROSMAP,AD.vs.control; scRNA-seq,SRP215507,CD8+ T cell_5-AD.vs.control; scRNA-seq,SRP215507,CD8+ T cell_6-AD.vs.control; scRNA-seq,SRP215507,CD8+ T cell_5-AD.vs.MCI; scRNA-seq,SRP215507,CD8+ T cell_4-MCI.vs.control; scRNA-seq,SRP309935,Naive CD8+ T cell_1-AD.vs.control; scRNA-seq,SRP330776,CD8+ T cell_1-AD.vs.control; scRNA-seq,SRP330776,γδ T cell-AD.vs.control; scRNA-seq,SRP330776,Memory T cell_2-AD.vs.control; scRNA-seq,SRP330776,CD8+ T cell_2-AD.vs.control; scRNA-seq,SRP330776,B cell_1-AD.vs.control; scRNA-seq,SRP330776,Naive CD8+ T cell_1-AD.vs.control; scRNA-seq,SRP330776,Natural killer cell_1-AD.vs.control; scRNA-seq,SRP330776,B	15
CARMIL3	bulk RNA-seq,Homo_723,AD.vs.control; bulk RNA-seq,Homo_723,AD.vs.MCI; bulk RNA-seq,Homo_714,AD.vs.control; bulk RNA-seq,Homo_714,AD.vs.MCI; bulk RNA-seq,ROSMAP,AD.vs.control; scRNA-seq,SRP215507,CD8+ T cell_6-AD.vs.control; scRNA-seq,SRP215507,CD8+ T cell_4-MCI.vs.control; scRNA-seq,SRP330776,γδ T cell-AD.vs.control; scRNA-seq,SRP330776,Natural killer cell_1-AD.vs.control	9

CARNMT1	bulk RNA-seq,Homo_723,AD.vs.control; bulk RNA-seq,Homo_723,AD.vs.MCI; bulk RNA-seq,Homo_723,MCI.vs.control; bulk RNA-seq,Homo_633,AD.vs.control; bulk RNA-seq,Homo_633,AD.vs.MCI; bulk RNA-seq,Homo_633,MCI.vs.control; bulk RNA-seq,Homo_714,AD.vs.control; bulk RNA-seq,Homo_714,AD.vs.MCI; scRNA-seq,SRP215507,CD8+ T cell_6-AD.vs.control; scRNA-seq,SRP215507,CD8+ T cell_2-AD.vs.MCI; scRNA-seq,SRP215507,CD8+ T cell_6-MCI.vs.control; scRNA-seq,SRP309935,Naive CD8+ T cell_2-AD.vs.control; scRNA-seq,SRP330776,CD8+ T cell_1-AD.vs.control; scRNA-seq,SRP330776,γδ T cell-AD.vs.control; scRNA-seq,SRP330776,Memory T cell_2-AD.vs.control; scRNA-seq,SRP330776,CD8+ T cell_2-AD.vs.control; scRNA-seq,SRP330776,B cell_1-AD.vs.control; scRNA-seq,SRP330776,Naive CD8+ T cell_1-AD.vs.control; scRNA-seq,SRP330776,Natural killer cell_1-AD.vs.control; scRNA-seq,SRP330776,Memory T cell_1-AD.vs.control; scRNA-seq,SRP330776,Natural killer T cell-AD.vs.control; scRNA-seq,SRP330776,Naive CD8+ T cell_3-AD.vs.control; scRNA-seq,SRP330776,B cell_2-AD.vs.control; scRNA-seq,SRP330776,T cell-AD.vs.control; scRNA-seq,SRP330776,Naive CD8+ T cell_2-AD.vs.control; scRNA-seq,SRP330776,CD8+ T cell_3-AD.vs.control; scRNA-seq,SRP330776,Monocyte_2-AD.vs.control; scRNA-seq,SRP330776,B cell_3-AD.vs.control; scRNA-	29
CARNS1	bulk RNA-seq,Homo_723,AD.vs.control; bulk RNA-seq,Homo_723,MCI.vs.control; bulk RNA-seq,Homo_633,AD.vs.control; bulk RNA-seq,Homo_633,MCI.vs.control; bulk RNA-seq,Homo_714,AD.vs.control; bulk RNA-seq,Homo_714,AD.vs.MCI; bulk RNA-seq,Homo_714,MCI.vs.control; scRNA-seq,SRP215507,CD8+ T cell_4-AD.vs.control; scRNA-seq,SRP215507,CD8+ T cell_6-AD.vs.control; scRNA-seq,SRP215507,CD8+ T cell_2-AD.vs.MCI; scRNA-seq,SRP215507,CD8+ T cell_3-AD.vs.MCI; scRNA-seq,SRP215507,CD8+ T cell_4-AD.vs.MCI; scRNA-seq,SRP215507,CD8+ T cell_5-AD.vs.MCI; scRNA-seq,SRP215507,CD8+ T cell_5-MCI.vs.control; scRNA-seq,SRP215507,CD8+ T cell_2-MCI.vs.control; scRNA-seq,SRP215507,CD8+ T cell_3-MCI.vs.control; scRNA-seq,SRP309935,Natural killer cell_1-AD.vs.control; scRNA-seq,SRP330776,CD8+ T cell_1-AD.vs.control; scRNA-seq,SRP330776,γδ T cell-AD.vs.control; scRNA-seq,SRP330776,Memory T cell_2-AD.vs.control; scRNA-seq,SRP330776,CD8+ T cell_2-AD.vs.control; scRNA-seq,SRP330776,B cell_1-AD.vs.control; scRNA-seq,SRP330776,Naive CD8+ T cell_1-AD.vs.control; scRNA-seq,SRP330776,Natural killer cell_1-AD.vs.control; scRNA-seq,SRP330776,Memory T cell_1-AD.vs.control; scRNA-seq,SRP330776,Natural killer T cell-AD.vs.control; scRNA-seq,SRP330776,Naive CD8+ T cell_3-AD.vs.control; scRNA-seq,SRP330776,T cell-AD.vs.control; scRNA-seq,SRP330776,CD8+ T cell_3-AD.vs.control; scRNA-seq,SRP330776,Natural killer cell_2-AD.vs.control	30
CARS	scRNA-seq,SRP215507,CD8+ T cell_3-AD.vs.control; scRNA-seq,SRP215507,CD8+ T cell_3-AD.vs.MCI; scRNA-seq,SRP215507,CD8+ T cell_6-MCI.vs.control; scRNA-seq,SRP330776,CD8+ T cell_1-AD.vs.control; scRNA-seq,SRP330776,Natural killer cell_1-AD.vs.control; scRNA-seq,SRP330776,CD8+ T cell_3-AD.vs.control; scRNA-seq,SRP330776,Monocyte_2-AD.vs.control	7
CARS1	bulk RNA-seq,Homo_723,AD.vs.control; bulk RNA-seq,Homo_723,AD.vs.MCI; bulk RNA-seq,Homo_633,AD.vs.control; bulk RNA-seq,Homo_633,AD.vs.MCI; bulk RNA-seq,Homo_714,AD.vs.control; bulk RNA-seq,Homo_714,AD.vs.MCI	6
CARS2	bulk RNA-seq,Homo_723,AD.vs.control; bulk RNA-seq,Homo_723,AD.vs.MCI; bulk RNA-seq,Homo_633,AD.vs.control; bulk RNA-seq,Homo_633,AD.vs.MCI; bulk RNA-seq,Homo_714,AD.vs.control; bulk RNA-seq,Homo_714,AD.vs.MCI; scRNA-seq,SRP215507,CD8+ T cell_5-AD.vs.control; scRNA-seq,SRP215507,CD8+ T cell_4-AD.vs.MCI; scRNA-seq,SRP215507,CD8+ T cell_4-MCI.vs.control; scRNA-seq,SRP309935,Natural killer cell_2-AD.vs.control; scRNA-seq,SRP309935,Megakaryocyte_1-AD.vs.control; scRNA-seq,SRP309935,Naive CD8+ T cell_1-AD.vs.control; scRNA-seq,SRP309935,Naive CD8+ T cell_2-AD.vs.control; scRNA-seq,SRP309935,CD8+ T cell_1-AD.vs.control; scRNA-seq,SRP309935,B cell_1-AD.vs.control; scRNA-seq,SRP309935,CD8+ T cell_2-AD.vs.control; scRNA-seq,SRP309935,B cell_2-AD.vs.control; scRNA-seq,SRP309935,Naive CD8+ T cell_3-AD.vs.control; scRNA-seq,SRP330776,CD8+ T cell_1-AD.vs.control; scRNA-seq,SRP330776,γδ T cell-AD.vs.control; scRNA-seq,SRP330776,Memory T cell_2-AD.vs.control; scRNA-seq,SRP330776,CD8+ T cell_2-AD.vs.control; scRNA-seq,SRP330776,B cell_1-AD.vs.control; scRNA-seq,SRP330776,Naive CD8+ T cell_1-AD.vs.control; scRNA-seq,SRP330776,Natural killer cell_1-AD.vs.control; scRNA-seq,SRP330776,Memory T cell_1-AD.vs.control; scRNA-seq,SRP330776,Natural killer T cell-AD.vs.control; scRNA-seq,SRP330776,Naive CD8+ T cell_3-AD.vs.control; scRNA-seq,SRP330776,B cell_2-AD.vs.control; scRNA-seq,SRP330776,T cell-AD.vs.control; scRNA-seq,SRP330776,CD8+ T cell_3-AD.vs.control; scRNA-seq,SRP330776,Monocyte_2-AD.vs.control; scRNA-seq,SRP330776,B cell_3-AD.vs.control; scRNA-	34
CARTPT	bulk RNA-seq,Homo_723,AD.vs.MCI; bulk RNA-seq,Homo_723,MCI.vs.control	2
Cartpt	bulk RNA-seq,SRP325058,AD.vs.control	1
CASC1	scRNA-seq,SRP215507,CD8+ T cell_1-AD.vs.control; scRNA-seq,SRP215507,CD8+ T cell_2-AD.vs.MCI; scRNA-seq,SRP215507,CD8+ T cell_3-AD.vs.MCI; scRNA-seq,SRP215507,CD8+ T cell_2-MCI.vs.control; scRNA-seq,SRP330776,CD8+ T cell_1-AD.vs.control; scRNA-seq,SRP330776,Memory T cell_2-AD.vs.control; scRNA-seq,SRP330776,CD8+ T cell_2-AD.vs.control; scRNA-seq,SRP330776,Naive CD8+ T cell_1-	9
CASC11	scRNA-seq,SRP330776,B cell_1-AD.vs.control; scRNA-seq,SRP330776,Naive CD8+ T cell_1-AD.vs.control	2
CASC19	scRNA-seq,SRP215507,CD8+ T cell_4-AD.vs.control	1

CASC3	scRNA-seq,SRP215507,CD8+ T cell_7-AD.vs.control; scRNA-seq,SRP215507,CD8+ T cell_6-MCI.vs.control; scRNA-seq,SRP309935,Natural killer cell_2-AD.vs.control; scRNA-seq,SRP330776,Monocyte_1-AD.vs.control; scRNA-seq,SRP330776,Megakaryocyte_1-AD.vs.control	5
CASC4	scRNA-seq,SRP215507,CD8+ T cell_4-AD.vs.control; scRNA-seq,SRP215507,CD8+ T cell_5-AD.vs.MCI; scRNA-seq,SRP330776,B cell_3-	3
CASC8	scRNA-seq,SRP215507,CD8+ T cell_2-AD.vs.control; scRNA-seq,SRP215507,CD8+ T cell_1-AD.vs.control; scRNA-seq,SRP215507,CD8+ T cell_2-	3
CASD1	bulk RNA-seq,Homo_723,AD.vs.control; bulk RNA-seq,Homo_723,AD.vs.MCI; bulk RNA-seq,Homo_633,AD.vs.MCI; bulk RNA-seq,Homo_714,AD.vs.control; bulk RNA-seq,Homo_714,AD.vs.MCI; bulk RNA-seq,ROSMAP,MCI.vs.control; scRNA-seq,SRP215507,CD8+ T cell_3-AD.vs.control; scRNA-seq,SRP215507,CD8+ T cell_3-MCI.vs.control; scRNA-seq,SRP309935,Naive CD8+ T cell_1-AD.vs.control; scRNA-seq,SRP330776,CD8+ T cell_1-AD.vs.control; scRNA-seq,SRP330776,Memory T cell_2-AD.vs.control; scRNA-seq,SRP330776,CD8+ T cell_2-AD.vs.control; scRNA-seq,SRP330776,Naive CD8+ T cell_1-AD.vs.control; scRNA-seq,SRP330776,Natural killer cell_1-AD.vs.control; scRNA-seq,SRP330776,Memory T cell_1-AD.vs.control; scRNA-seq,SRP330776,Naive CD8+ T cell_3-AD.vs.control; scRNA-seq,SRP330776,B cell_2-AD.vs.control; scRNA-seq,SRP330776,T cell-AD.vs.control; scRNA-seq,SRP330776,CD8+ T cell_3-AD.vs.control; scRNA-seq,SRP330776,Natural killer [illegible]	20
CASK	scRNA-seq,SRP215507,CD8+ T cell_6-AD.vs.control; scRNA-seq,SRP215507,CD8+ T cell_5-AD.vs.MCI; scRNA-seq,SRP215507,CD8+ T cell_5-MCI.vs.control; scRNA-seq,SRP309935,Monocyte_1-AD.vs.control; scRNA-seq,SRP330776,CD8+ T cell_1-AD.vs.control; scRNA-seq,SRP330776,γδ T cell-AD.vs.control; scRNA-seq,SRP330776,B cell_1-AD.vs.control; scRNA-seq,SRP330776,Naive CD8+ T cell_1-AD.vs.control; scRNA-seq,SRP330776,Natural killer T cell-AD.vs.control; scRNA-seq,SRP330776,CD8+ T cell_3-AD.vs.control; scRNA-seq,SRP330776,Monocyte_2-	11
CASKIN1	bulk RNA-seq,Homo_723,AD.vs.control; bulk RNA-seq,Homo_723,AD.vs.MCI; bulk RNA-seq,Homo_714,MCI.vs.control; scRNA-seq,SRP330776,Naive CD8+ T cell_1-AD.vs.control	4
CASKIN2	bulk RNA-seq,Homo_723,AD.vs.control; bulk RNA-seq,Homo_723,AD.vs.MCI; bulk RNA-seq,Homo_723,MCI.vs.control; bulk RNA-seq,Homo_633,AD.vs.control; bulk RNA-seq,Homo_633,AD.vs.MCI; bulk RNA-seq,Homo_633,MCI.vs.control; bulk RNA-seq,Homo_714,MCI.vs.control; bulk RNA-seq,ROSMAP,AD.vs.control; bulk RNA-seq,ROSMAP,AD.vs.MCI	9
CASP1	bulk RNA-seq,Homo_723,AD.vs.control; bulk RNA-seq,Homo_723,AD.vs.MCI; bulk RNA-seq,Homo_633,AD.vs.control; bulk RNA-seq,Homo_633,AD.vs.MCI; bulk RNA-seq,Homo_714,AD.vs.control; bulk RNA-seq,Homo_714,AD.vs.MCI; scRNA-seq,SRP330776,Natural killer cell_1-AD.vs.control; scRNA-seq,SRP330776,Megakaryocyte_1-AD.vs.control	8
CASP10	scRNA-seq,SRP215507,CD8+ T cell_6-AD.vs.control; scRNA-seq,SRP309935,Natural killer cell_2-AD.vs.control; scRNA-seq,SRP309935,Monocyte_1-AD.vs.control; scRNA-seq,SRP309935,Naive CD8+ T cell_2-AD.vs.control; scRNA-seq,SRP330776,γδ T cell-AD.vs.control; scRNA-seq,SRP330776,CD8+ T cell_2-AD.vs.control; scRNA-seq,SRP330776,Natural killer cell_1-AD.vs.control; scRNA-seq,SRP330776,CD8+ T cell_3-	8
CASP2	scRNA-seq,SRP215507,CD8+ T cell_2-AD.vs.control; scRNA-seq,SRP215507,CD8+ T cell_5-AD.vs.control; scRNA-seq,SRP215507,CD8+ T cell_5-AD.vs.MCI; scRNA-seq,SRP215507,CD8+ T cell_3-MCI.vs.control; scRNA-seq,SRP330776,Memory T cell_2-AD.vs.control; scRNA-seq,SRP330776,Naive CD8+ T cell_2-AD.vs.control; scRNA-seq,SRP330776,Monocyte_1-AD.vs.control	7
CASP3	bulk RNA-seq,Homo_723,AD.vs.control; bulk RNA-seq,Homo_723,AD.vs.MCI; bulk RNA-seq,Homo_633,AD.vs.control; bulk RNA-seq,Homo_633,AD.vs.MCI; bulk RNA-seq,Homo_714,AD.vs.control; bulk RNA-seq,Homo_714,AD.vs.MCI; scRNA-seq,SRP215507,CD8+ T cell_6-AD.vs.control; scRNA-seq,SRP215507,CD8+ T cell_2-AD.vs.MCI; scRNA-seq,SRP215507,CD8+ T cell_1-AD.vs.MCI; scRNA-seq,SRP215507,CD8+ T cell_6-AD.vs.MCI; scRNA-seq,SRP215507,CD8+ T cell_1-MCI.vs.control; scRNA-seq,SRP309935,Natural killer cell_2-AD.vs.control; scRNA-seq,SRP309935,CD8+ T cell_1-AD.vs.control; scRNA-seq,SRP330776,B cell_1-AD.vs.control; scRNA-seq,SRP330776,B cell_2-AD.vs.control; scRNA-seq,SRP330776,T cell-AD.vs.control; scRNA-seq,SRP330776,Monocyte_1-AD.vs.control; scRNA-seq,SRP330776,Monocyte_2-AD.vs.control; scRNA- [illegible]	20
CASP4	bulk RNA-seq,Homo_723,MCI.vs.control; bulk RNA-seq,Homo_714,MCI.vs.control; scRNA-seq,SRP330776,Memory T cell_2-AD.vs.control; scRNA-seq,SRP330776,CD8+ T cell_2-AD.vs.control; scRNA-seq,SRP330776,Natural killer cell_1-AD.vs.control; scRNA-seq,SRP330776,Memory T cell_1-AD.vs.control; scRNA-seq,SRP330776,Megakaryocyte_1-AD.vs.control; scRNA-seq,SRP330776,Natural killer cell_2-AD.vs.control; scRNA-	9
CASP5	bulk RNA-seq,Homo_723,AD.vs.control; bulk RNA-seq,Homo_723,AD.vs.MCI; bulk RNA-seq,Homo_714,AD.vs.control; bulk RNA-	4

CASP6	bulk RNA-seq,Homo_633,MCI.vs.control; scRNA-seq,SRP215507,CD8+ T cell_4-AD.vs.control; scRNA-seq,SRP215507,CD8+ T cell_5-AD.vs.control; scRNA-seq,SRP215507,CD8+ T cell_3-AD.vs.MCI; scRNA-seq,SRP215507,CD8+ T cell_1-MCI.vs.control; scRNA-seq,SRP215507,CD8+ T cell_4-MCI.vs.control; scRNA-seq,SRP215507,CD8+ T cell_3-MCI.vs.control; scRNA-seq,SRP309935,CD8+ T cell_1-AD.vs.control; scRNA-seq,SRP330776,CD8+ T cell_1-AD.vs.control; scRNA-seq,SRP330776,Memory T cell_2-AD.vs.control; scRNA-seq,SRP330776,B cell_1-AD.vs.control; scRNA-seq,SRP330776,Memory T cell_1-AD.vs.control; scRNA-seq,SRP330776,Natural killer T cell-AD.vs.control; scRNA-seq,SRP330776,B cell_2-AD.vs.control; scRNA-seq,SRP330776,Monocyte_2-AD.vs.control; scRNA-seq,SRP330776,Regulatory T cell-AD.vs.control	16
CASP7	bulk RNA-seq,Homo_723,AD.vs.control; bulk RNA-seq,Homo_633,AD.vs.control; bulk RNA-seq,Homo_714,AD.vs.control; bulk RNA-seq,Homo_714,AD.vs.MCI; scRNA-seq,SRP215507,CD8+ T cell_4-AD.vs.control; scRNA-seq,SRP215507,CD8+ T cell_6-AD.vs.control; scRNA-seq,SRP330776,Natural killer T cell-AD.vs.control; scRNA-seq,SRP330776,Naive CD8+ T cell_3-AD.vs.control; scRNA-seq,SRP330776,T cell-	10
CASP8	bulk RNA-seq,Homo_714,AD.vs.control; bulk RNA-seq,Homo_714,AD.vs.MCI; scRNA-seq,SRP215507,CD8+ T cell_8-AD.vs.control; scRNA-seq,SRP215507,CD8+ T cell_7-AD.vs.control; scRNA-seq,SRP215507,CD8+ T cell_9-AD.vs.control; scRNA-seq,SRP309935,B cell_1-AD.vs.control; scRNA-seq,SRP330776,Megakaryocyte_1-AD.vs.control	7
CASP8AP2	bulk RNA-seq,Homo_723,AD.vs.MCI; bulk RNA-seq,Homo_714,AD.vs.control; bulk RNA-seq,Homo_714,AD.vs.MCI; scRNA-seq,SRP215507,CD8+ T cell_5-AD.vs.control; scRNA-seq,SRP215507,CD8+ T cell_6-AD.vs.control; scRNA-seq,SRP215507,CD8+ T cell_5-MCI.vs.control; scRNA-seq,SRP215507,CD8+ T cell_4-MCI.vs.control; scRNA-seq,SRP309935,Naive CD8+ T cell_1-AD.vs.control; scRNA-seq,SRP309935,Monocyte_2-AD.vs.control; scRNA-seq,SRP330776,Megakaryocyte_1-AD.vs.control	10
CASP9	bulk RNA-seq,Homo_723,AD.vs.MCI; bulk RNA-seq,Homo_723,MCI.vs.control; bulk RNA-seq,Homo_633,AD.vs.control; bulk RNA-seq,Homo_633,MCI.vs.control; scRNA-seq,SRP215507,CD8+ T cell_1-AD.vs.control; scRNA-seq,SRP215507,CD8+ T cell_6-AD.vs.control; scRNA-seq,SRP215507,CD8+ T cell_2-AD.vs.MCI; scRNA-seq,SRP215507,CD8+ T cell_2-MCI.vs.control; scRNA-seq,SRP215507,CD8+ T cell_6-MCI.vs.control; scRNA-seq,SRP330776,Memory T cell_2-AD.vs.control; scRNA-seq,SRP330776,Memory T cell_1-AD.vs.control; scRNA-	12
CASQ1	bulk RNA-seq,Homo_723,AD.vs.control; bulk RNA-seq,Homo_723,AD.vs.MCI; bulk RNA-seq,Homo_633,AD.vs.control; bulk RNA-	4
CASQ2	bulk RNA-seq,Homo_723,AD.vs.control; bulk RNA-seq,Homo_633,AD.vs.control; bulk RNA-seq,ROSMAP,AD.vs.control; bulk RNA-	4
CASR	bulk RNA-seq,Homo_723,AD.vs.control; bulk RNA-seq,Homo_723,MCI.vs.control; bulk RNA-seq,Homo_633,AD.vs.control; bulk RNA-seq,Homo_633,MCI.vs.control; bulk RNA-seq,Homo_714,MCI.vs.control	5
CASS4	bulk RNA-seq,Homo_723,AD.vs.control; bulk RNA-seq,Homo_633,AD.vs.control; bulk RNA-seq,Homo_714,AD.vs.control; bulk RNA-seq,Homo_714,AD.vs.MCI; bulk RNA-seq,SRP310421,AD.vs.control; scRNA-seq,SRP215507,CD8+ T cell_1-AD.vs.control; scRNA-seq,SRP215507,CD8+ T cell_6-AD.vs.control; scRNA-seq,SRP215507,CD8+ T cell_1-AD.vs.MCI; scRNA-seq,SRP215507,CD8+ T cell_4-AD.vs.MCI; scRNA-seq,SRP215507,CD8+ T cell_5-AD.vs.MCI; scRNA-seq,SRP215507,CD8+ T cell_1-MCI.vs.control; scRNA-seq,SRP215507,CD8+ T cell_4-MCI.vs.control; scRNA-seq,SRP215507,CD8+ T cell_6-MCI.vs.control; scRNA-seq,SRP330776,CD8+ T cell_2-AD.vs.control; scRNA-seq,SRP330776,Natural killer T cell-AD.vs.control; scRNA-seq,SRP330776,Naive CD8+ T cell_3-AD.vs.control; scRNA-seq,SRP330776,Natural killer [illegible]	17
Cass4	bulk RNA-seq,SRP325058,AD.vs.control	1
Cast	bulk RNA-seq,SRP325058,AD.vs.control	1
CAST	scRNA-seq,SRP215507,CD8+ T cell_10-AD.vs.control; scRNA-seq,SRP309935,Erythroid cell-AD.vs.control; scRNA-seq,SRP330776,CD8+ T cell_1-AD.vs.control; scRNA-seq,SRP330776,γδ T cell-AD.vs.control; scRNA-seq,SRP330776,Memory T cell_2-AD.vs.control; scRNA-seq,SRP330776,CD8+ T cell_2-AD.vs.control; scRNA-seq,SRP330776,B cell_1-AD.vs.control; scRNA-seq,SRP330776,Naive CD8+ T cell_1-AD.vs.control; scRNA-seq,SRP330776,Natural killer cell_1-AD.vs.control; scRNA-seq,SRP330776,Memory T cell_1-AD.vs.control; scRNA-seq,SRP330776,Natural killer T cell-AD.vs.control; scRNA-seq,SRP330776,Naive CD8+ T cell_3-AD.vs.control; scRNA-seq,SRP330776,B cell_2-AD.vs.control; scRNA-seq,SRP330776,T cell-AD.vs.control; scRNA-seq,SRP330776,Naive CD8+ T cell_2-AD.vs.control; scRNA-seq,SRP330776,CD8+ T cell_3-AD.vs.control; scRNA-seq,SRP330776,Monocyte_1-AD.vs.control; scRNA-seq,SRP330776,Monocyte_2-AD.vs.control; scRNA-seq,SRP330776,B cell_3-AD.vs.control; scRNA- [illegible]	21

CASTOR1	bulk RNA-seq,Homo_723,AD.vs.MCI; bulk RNA-seq,Homo_633,AD.vs.control; bulk RNA-seq,Homo_633,AD.vs.MCI; bulk RNA-seq,Homo_714,AD.vs.control; bulk RNA-seq,Homo_714,AD.vs.MCI; scRNA-seq,SRP215507,CD8+ T cell_2-AD.vs.MCI; scRNA-seq,SRP215507,CD8+ T cell_4-AD.vs.MCI; scRNA-seq,SRP215507,CD8+ T cell_4-MCI.vs.control; scRNA-seq,SRP330776,γδ T cell-AD.vs.control; scRNA-seq,SRP330776,Memory T cell_2-AD.vs.control; scRNA-seq,SRP330776,CD8+ T cell_2-AD.vs.control; scRNA-seq,SRP330776,Naive CD8+ T cell_1-AD.vs.control; scRNA-seq,SRP330776,Natural killer cell_1-AD.vs.control; scRNA-seq,SRP330776,B cell_2-AD.vs.control; scRNA-seq,SRP330776,CD8+	16
CASTOR2	bulk RNA-seq,Homo_633,AD.vs.control; scRNA-seq,SRP215507,CD8+ T cell_2-AD.vs.control; scRNA-seq,SRP309935,Natural killer cell_1-AD.vs.control; scRNA-seq,SRP309935,CD8+ T cell_1-AD.vs.control; scRNA-seq,SRP330776,CD8+ T cell_1-AD.vs.control; scRNA-seq,SRP330776,Naive CD8+ T cell_1-AD.vs.control; scRNA-seq,SRP330776,T cell-AD.vs.control	7
CASTOR3	scRNA-seq,SRP215507,CD8+ T cell_4-AD.vs.control; scRNA-seq,SRP215507,CD8+ T cell_4-AD.vs.MCI; scRNA-seq,SRP215507,CD8+ T cell_4-MCI.vs.control; scRNA-seq,SRP215507,CD8+ T cell_2-MCI.vs.control; scRNA-seq,SRP309935,Naive CD8+ T cell_1-AD.vs.control; scRNA-seq,SRP309935,Natural killer cell_1-AD.vs.control; scRNA-seq,SRP330776,CD8+ T cell_1-AD.vs.control; scRNA-seq,SRP330776,CD8+ T cell_2-AD.vs.control; scRNA-seq,SRP330776,B cell_1-AD.vs.control; scRNA-seq,SRP330776,CD8+ T cell_3-AD.vs.control	10
CASZ1	bulk RNA-seq,Homo_723,AD.vs.control; bulk RNA-seq,Homo_723,AD.vs.MCI; bulk RNA-seq,Homo_633,AD.vs.control; bulk RNA-seq,Homo_633,AD.vs.MCI; bulk RNA-seq,Homo_714,AD.vs.control; bulk RNA-seq,Homo_714,AD.vs.MCI; bulk RNA-seq,Homo_714,MCI.vs.control; scRNA-seq,SRP215507,CD8+ T cell_2-AD.vs.MCI; scRNA-seq,SRP215507,CD8+ T cell_5-MCI.vs.control; scRNA-seq,SRP215507,CD8+ T cell_2-	11
CAT	bulk RNA-seq,Homo_723,AD.vs.control; bulk RNA-seq,Homo_723,AD.vs.MCI; bulk RNA-seq,Homo_633,AD.vs.control; bulk RNA-seq,Homo_633,AD.vs.MCI; scRNA-seq,SRP309935,Erythroid cell-AD.vs.control; scRNA-seq,SRP330776,CD8+ T cell_1-AD.vs.control; scRNA-seq,SRP330776,γδ T cell-AD.vs.control; scRNA-seq,SRP330776,Memory T cell_2-AD.vs.control; scRNA-seq,SRP330776,Naive CD8+ T cell_1-	10
CATIP	bulk RNA-seq,Homo_723,AD.vs.control; bulk RNA-seq,Homo_723,AD.vs.MCI; bulk RNA-seq,Homo_633,AD.vs.control; bulk RNA-seq,Homo_633,AD.vs.MCI; bulk RNA-seq,Homo_714,AD.vs.control; bulk RNA-seq,Homo_714,AD.vs.MCI	6
CATIP-AS1	scRNA-seq,SRP215507,CD8+ T cell_1-AD.vs.MCI; scRNA-seq,SRP215507,CD8+ T cell_1-MCI.vs.control; scRNA-seq,SRP330776,CD8+ T cell_1-	3
CATSPER1	scRNA-seq,SRP215507,CD8+ T cell_1-AD.vs.control; scRNA-seq,SRP215507,CD8+ T cell_1-AD.vs.MCI; scRNA-seq,SRP215507,CD8+ T cell_1-MCI.vs.control; scRNA-seq,SRP330776,CD8+ T cell_2-AD.vs.control; scRNA-seq,SRP330776,Natural killer T cell-AD.vs.control; scRNA-	6
CATSPER2	bulk RNA-seq,Homo_723,AD.vs.control; bulk RNA-seq,Homo_723,AD.vs.MCI; bulk RNA-seq,Homo_723,MCI.vs.control; bulk RNA-seq,Homo_633,AD.vs.control; bulk RNA-seq,Homo_633,AD.vs.MCI; bulk RNA-seq,Homo_633,MCI.vs.control; bulk RNA-seq,Homo_714,AD.vs.control; bulk RNA-seq,Homo_714,AD.vs.MCI; bulk RNA-seq,Homo_714,MCI.vs.control; bulk RNA-seq,ROSMAP,AD.vs.control; scRNA-seq,SRP215507,CD8+ T cell_4-AD.vs.MCI; scRNA-seq,SRP215507,CD8+ T cell_4-MCI.vs.control; scRNA-seq,SRP215507,CD8+ T cell_3-MCI.vs.control; scRNA-seq,SRP330776,γδ T cell-AD.vs.control; scRNA-seq,SRP330776,Memory T cell_2-AD.vs.control; scRNA-seq,SRP330776,CD8+ T cell_2-AD.vs.control; scRNA-seq,SRP330776,B cell_1-AD.vs.control; scRNA-seq,SRP330776,Naive CD8+ T cell_1-AD.vs.control	18
CATSPER3	bulk RNA-seq,Homo_714,AD.vs.control; bulk RNA-seq,Homo_714,AD.vs.MCI	2
CATSPER4	bulk RNA-seq,Homo_723,AD.vs.control; bulk RNA-seq,ROSMAP,AD.vs.control	2
CATSPERB	bulk RNA-seq,Homo_633,AD.vs.MCI; scRNA-seq,SRP215507,CD8+ T cell_1-AD.vs.control; scRNA-seq,SRP215507,CD8+ T cell_3-AD.vs.MCI; scRNA-seq,SRP309935,Natural killer cell_1-AD.vs.control; scRNA-seq,SRP330776,Naive CD8+ T cell_1-AD.vs.control	5
CATSPERD	bulk RNA-seq,Homo_714,MCI.vs.control; scRNA-seq,SRP215507,CD8+ T cell_2-AD.vs.MCI; scRNA-seq,SRP215507,CD8+ T cell_2-MCI.vs.control; scRNA-seq,SRP215507,CD8+ T cell_3-MCI.vs.control	4
CATSPERE	bulk RNA-seq,Homo_723,AD.vs.control; bulk RNA-seq,Homo_723,AD.vs.MCI; bulk RNA-seq,Homo_633,AD.vs.control; bulk RNA-seq,Homo_633,AD.vs.MCI; bulk RNA-seq,Homo_714,AD.vs.control; bulk RNA-seq,Homo_714,AD.vs.MCI; scRNA-seq,SRP215507,CD8+ T cell_1-AD.vs.control; scRNA-seq,SRP309935,Monocyte_1-AD.vs.control; scRNA-seq,SRP330776,B cell_1-AD.vs.control; scRNA-seq,SRP330776,Memory T	10
CATSPERG	bulk RNA-seq,Homo_723,AD.vs.control; bulk RNA-seq,Homo_723,AD.vs.MCI; bulk RNA-seq,Homo_633,AD.vs.control; bulk RNA-seq,Homo_714,AD.vs.control; bulk RNA-seq,Homo_714,AD.vs.MCI; scRNA-seq,SRP215507,CD8+ T cell_3-AD.vs.control; scRNA-seq,SRP215507,CD8+ T cell_1-AD.vs.MCI; scRNA-seq,SRP215507,CD8+ T cell_1-MCI.vs.control; scRNA-seq,SRP330776,CD8+ T cell_1-AD.vs.control; scRNA-seq,SRP330776,γδ T cell-AD.vs.control; scRNA-seq,SRP330776,CD8+ T cell_2-AD.vs.control; scRNA-seq,SRP330776,Natural killer cell_1-AD.vs.control; scRNA-seq,SRP330776,Memory T cell_1-AD.vs.control; scRNA-seq,SRP330776,B cell_2-AD.vs.control; scRNA-	15

CAV1	bulk RNA-seq,Homo_723,AD.vs.control; bulk RNA-seq,Homo_723,AD.vs.MCI; bulk RNA-seq,Homo_723,MCI.vs.control; bulk RNA-seq,Homo_633,AD.vs.control; bulk RNA-seq,Homo_633,AD.vs.MCI; bulk RNA-seq,Homo_633,MCI.vs.control; bulk RNA-seq,Homo_714,AD.vs.control;	8
CAVIN1	bulk RNA-seq,SRP223445,AD.vs.control	1
Cavin2	bulk RNA-seq,SRP325058,AD.vs.control	1
CAVIN2	scRNA-seq,SRP309935,Monocyte_1-AD.vs.control; scRNA-seq,SRP309935,Naive CD8+ T cell_1-AD.vs.control; scRNA-seq,SRP309935,Naive CD8+ T cell_2-AD.vs.control; scRNA-seq,SRP309935,CD8+ T cell_2-AD.vs.control; scRNA-seq,SRP309935,Monocyte_3-AD.vs.control; scRNA-seq,SRP330776,CD8+ T cell_1-AD.vs.control	6
CAVIN3	bulk RNA-seq,Homo_723,AD.vs.control; bulk RNA-seq,Homo_723,AD.vs.MCI; bulk RNA-seq,Homo_633,AD.vs.control; bulk RNA-seq,Homo_633,AD.vs.MCI; bulk RNA-seq,Homo_714,AD.vs.control; bulk RNA-seq,Homo_714,AD.vs.MCI; scRNA-seq,SRP309935,Monocyte_1-	7
CAVIN4	scRNA-seq,SRP215507,CD8+ T cell_1-AD.vs.MCI	1
CBARP	bulk RNA-seq,Homo_723,AD.vs.control; bulk RNA-seq,Homo_723,AD.vs.MCI; bulk RNA-seq,Homo_714,MCI.vs.control	3
CBFA2T2	bulk RNA-seq,Homo_723,AD.vs.control; bulk RNA-seq,Homo_723,AD.vs.MCI; bulk RNA-seq,Homo_633,AD.vs.control; bulk RNA-seq,Homo_633,MCI.vs.control; bulk RNA-seq,Homo_714,AD.vs.control; bulk RNA-seq,Homo_714,AD.vs.MCI; bulk RNA-seq,Homo_714,MCI.vs.control; scRNA-seq,SRP215507,CD8+ T cell_5-AD.vs.MCI; scRNA-seq,SRP309935,Natural killer cell_2-AD.vs.control; scRNA-seq,SRP309935,Naive CD8+ T cell_2-AD.vs.control; scRNA-seq,SRP309935,CD8+ T cell_1-AD.vs.control; scRNA-seq,SRP330776,CD8+ T cell_1-AD.vs.control; scRNA-seq,SRP330776,γδ T cell-AD.vs.control; scRNA-seq,SRP330776,Memory T cell_2-AD.vs.control; scRNA-seq,SRP330776,CD8+ T cell_2-AD.vs.control; scRNA-seq,SRP330776,B cell_1-AD.vs.control; scRNA-seq,SRP330776,Naive CD8+ T cell_1-AD.vs.control; scRNA-seq,SRP330776,Natural killer cell_1-AD.vs.control; scRNA-seq,SRP330776,Memory T cell_1-AD.vs.control; scRNA-seq,SRP330776,Natural killer T cell-AD.vs.control; scRNA-seq,SRP330776,Naive CD8+ T cell_3-AD.vs.control; scRNA-seq,SRP330776,B cell_2-AD.vs.control; scRNA-seq,SRP330776,T cell-AD.vs.control; scRNA-seq,SRP330776,Naive CD8+ T cell_2-AD.vs.control; scRNA-seq,SRP330776,CD8+ T cell_3-AD.vs.control; scRNA-seq,SRP330776,Monocyte_1-AD.vs.control; scRNA-seq,SRP330776,B cell_3-AD.vs.control; scRNA-seq,SRP330776,Regulatory T cell-AD.vs.control; scRNA-seq,SRP330776,Natural killer cell_2-AD.vs.control; scRNA-seq,SRP330776,B cell_4-AD.vs.control	30
CBFA2T3	scRNA-seq,SRP309935,Monocyte_2-AD.vs.control; scRNA-seq,SRP309935,B cell_1-AD.vs.control; scRNA-seq,SRP330776,B cell_1-AD.vs.control; scRNA-seq,SRP330776,B cell_2-AD.vs.control; scRNA-seq,SRP330776,B cell_3-AD.vs.control	5
CBFB	bulk RNA-seq,Homo_723,AD.vs.MCI; bulk RNA-seq,Homo_723,MCI.vs.control; bulk RNA-seq,Homo_633,AD.vs.control; bulk RNA-seq,Homo_633,AD.vs.MCI; bulk RNA-seq,Homo_633,MCI.vs.control; scRNA-seq,SRP215507,CD8+ T cell_6-AD.vs.control; scRNA-seq,SRP309935,Natural killer cell_2-AD.vs.control; scRNA-seq,SRP309935,Naive CD8+ T cell_1-AD.vs.control; scRNA-seq,SRP330776,CD8+ T cell_1-AD.vs.control; scRNA-seq,SRP330776,γδ T cell-AD.vs.control; scRNA-seq,SRP330776,Memory T cell_2-AD.vs.control; scRNA-seq,SRP330776,CD8+ T cell_2-AD.vs.control; scRNA-seq,SRP330776,B cell_1-AD.vs.control; scRNA-seq,SRP330776,Naive CD8+ T cell_1-AD.vs.control; scRNA-seq,SRP330776,Natural killer cell_1-AD.vs.control; scRNA-seq,SRP330776,Memory T cell_1-AD.vs.control; scRNA-seq,SRP330776,Natural killer T cell-AD.vs.control; scRNA-seq,SRP330776,Naive CD8+ T cell_3-AD.vs.control; scRNA-seq,SRP330776,B cell_2-AD.vs.control; scRNA-seq,SRP330776,T cell-AD.vs.control; scRNA-seq,SRP330776,Naive CD8+ T cell_2-AD.vs.control; scRNA-seq,SRP330776,CD8+ T cell_3-AD.vs.control; scRNA-seq,SRP330776,Monocyte_2-AD.vs.control; scRNA-seq,SRP330776,B cell_3-AD.vs.control	24
CBL	scRNA-seq,SRP215507,CD8+ T cell_9-AD.vs.control; scRNA-seq,SRP215507,CD8+ T cell_6-MCI.vs.control; scRNA-seq,SRP330776,Monocyte_2-AD.vs.control; scRNA-seq,SRP330776,Megakaryocyte_1-AD.vs.control; scRNA-seq,SRP330776,Natural killer cell_2-AD.vs.control	5
CBLB	bulk RNA-seq,Homo_723,AD.vs.control; bulk RNA-seq,Homo_723,AD.vs.MCI; bulk RNA-seq,Homo_633,AD.vs.control; bulk RNA-seq,Homo_633,AD.vs.MCI; bulk RNA-seq,Homo_714,AD.vs.control; bulk RNA-seq,Homo_714,AD.vs.MCI; scRNA-seq,SRP215507,CD8+ T cell_7-AD.vs.MCI; scRNA-seq,SRP215507,CD8+ T cell_6-AD.vs.MCI; scRNA-seq,SRP215507,CD8+ T cell_6-MCI.vs.control; scRNA-seq,SRP215507,CD8+ T cell_7-MCI.vs.control; scRNA-seq,SRP309935,Natural killer cell_2-AD.vs.control; scRNA-seq,SRP309935,Megakaryocyte_1-AD.vs.control; scRNA-seq,SRP309935,CD8+ T cell_1-AD.vs.control; scRNA-seq,SRP330776,Naive CD8+ T cell_2-AD.vs.control; scRNA-seq,SRP330776,Monocyte_1-	15
CBLC	bulk RNA-seq,ROSMAP,AD.vs.control	1
CBLIF	bulk RNA-seq,Homo_723,AD.vs.control; bulk RNA-seq,Homo_723,AD.vs.MCI; bulk RNA-seq,Homo_633,AD.vs.control; bulk RNA-	4

CBLL1	scRNA-seq,SRP215507,CD8+ T cell_6-AD.vs.control; scRNA-seq,SRP215507,CD8+ T cell_4-AD.vs.MCI; scRNA-seq,SRP215507,CD8+ T cell_6-AD.vs.MCI; scRNA-seq,SRP215507,CD8+ T cell_4-MCI.vs.control; scRNA-seq,SRP215507,CD8+ T cell_7-MCI.vs.control; scRNA-seq,SRP309935,CD8+ T cell_1-AD.vs.control; scRNA-seq,SRP330776,Natural killer cell_1-AD.vs.control; scRNA-seq,SRP330776,T cell-AD.vs.control; scRNA-seq,SRP330776,Monocyte_1-AD.vs.control; scRNA-seq,SRP330776,Monocyte_2-AD.vs.control; scRNA-seq,SRP330776,Natural killer cell_2-	11
CBLL2	bulk RNA-seq,Homo_723,AD.vs.control; bulk RNA-seq,Homo_633,AD.vs.control; bulk RNA-seq,Homo_714,MCI.vs.control	3
CBLN1	bulk RNA-seq,Homo_723,AD.vs.MCI; bulk RNA-seq,Homo_723,MCI.vs.control; bulk RNA-seq,Homo_633,AD.vs.control	3
CBLN2	bulk RNA-seq,Homo_723,AD.vs.control; bulk RNA-seq,Homo_723,AD.vs.MCI; bulk RNA-seq,Homo_633,AD.vs.control; bulk RNA-	4
CBLN3	bulk RNA-seq,Homo_723,AD.vs.control; bulk RNA-seq,Homo_723,AD.vs.MCI; bulk RNA-seq,Homo_633,AD.vs.control; bulk RNA-seq,Homo_633,AD.vs.MCI; bulk RNA-seq,Homo_714,AD.vs.control; bulk RNA-seq,Homo_714,AD.vs.MCI; scRNA-seq,SRP215507,CD8+ T cell_5-	7
CBLN4	bulk RNA-seq,Homo_714,MCI.vs.control	1
CBR1	scRNA-seq,SRP215507,CD8+ T cell_4-AD.vs.control; scRNA-seq,SRP215507,CD8+ T cell_5-MCI.vs.control; scRNA-seq,SRP215507,CD8+ T cell_6-MCI.vs.control; scRNA-seq,SRP330776,CD8+ T cell_1-AD.vs.control; scRNA-seq,SRP330776,Memory T cell_2-AD.vs.control; scRNA-seq,SRP330776,B cell_1-AD.vs.control; scRNA-seq,SRP330776,Natural killer T cell-AD.vs.control; scRNA-seq,SRP330776,Naive CD8+ T cell_3-AD.vs.control; scRNA-seq,SRP330776,Naive CD8+ T cell_2-AD.vs.control; scRNA-seq,SRP330776,CD8+ T cell_3-AD.vs.control; scRNA-	12
CBR3	bulk RNA-seq,Homo_723,AD.vs.control; bulk RNA-seq,Homo_723,AD.vs.MCI; bulk RNA-seq,Homo_633,AD.vs.control; bulk RNA-seq,Homo_633,AD.vs.MCI; bulk RNA-seq,Homo_714,AD.vs.control; bulk RNA-seq,Homo_714,AD.vs.MCI; scRNA-seq,SRP215507,CD8+ T cell_2-AD.vs.control; scRNA-seq,SRP215507,CD8+ T cell_2-AD.vs.MCI; scRNA-seq,SRP215507,CD8+ T cell_3-AD.vs.MCI; scRNA-seq,SRP215507,CD8+ T cell_1-AD.vs.MCI; scRNA-seq,SRP215507,CD8+ T cell_1-MCI.vs.control; scRNA-seq,SRP215507,CD8+ T cell_3-MCI.vs.control; scRNA-seq,SRP215507,CD8+ T cell_6-MCI.vs.control; scRNA-seq,SRP309935,Naive CD8+ T cell_1-AD.vs.control; scRNA-seq,SRP309935,CD8+ T cell_1-AD.vs.control; scRNA-seq,SRP330776,CD8+ T cell_1-AD.vs.control; scRNA-seq,SRP330776,γδ T cell-AD.vs.control; scRNA-seq,SRP330776,Memory T cell_2-AD.vs.control; scRNA-seq,SRP330776,B cell_1-AD.vs.control; scRNA-seq,SRP330776,Naive CD8+ T cell_1-AD.vs.control; scRNA-seq,SRP330776,Memory T cell_1-AD.vs.control; scRNA-seq,SRP330776,Natural killer T cell-AD.vs.control; scRNA-seq,SRP330776,Naive CD8+ T cell_3-AD.vs.control; scRNA-seq,SRP330776,B cell_2-AD.vs.control; scRNA-seq,SRP330776,T cell-AD.vs.control; scRNA-seq,SRP330776,Naive CD8+ [illegible]	28
Cbr3	bulk RNA-seq,SRP325058,AD.vs.control	1
CBR3-AS1	scRNA-seq,SRP215507,CD8+ T cell_3-AD.vs.MCI; scRNA-seq,SRP215507,CD8+ T cell_5-AD.vs.MCI; scRNA-seq,SRP215507,CD8+ T cell_1-MCI.vs.control; scRNA-seq,SRP215507,CD8+ T cell_5-MCI.vs.control; scRNA-seq,SRP215507,CD8+ T cell_3-MCI.vs.control; scRNA-seq,SRP330776,CD8+ T cell_1-AD.vs.control; scRNA-seq,SRP330776,Memory T cell_2-AD.vs.control; scRNA-seq,SRP330776,Naive CD8+ T cell_1-AD.vs.control; scRNA-seq,SRP330776,B cell_2-AD.vs.control; scRNA-seq,SRP330776,T cell-AD.vs.control	10
CBR4	scRNA-seq,SRP215507,CD8+ T cell_2-AD.vs.control; scRNA-seq,SRP215507,CD8+ T cell_5-AD.vs.control; scRNA-seq,SRP215507,CD8+ T cell_2-AD.vs.MCI; scRNA-seq,SRP215507,CD8+ T cell_5-AD.vs.MCI; scRNA-seq,SRP215507,CD8+ T cell_3-MCI.vs.control; scRNA-seq,SRP215507,CD8+ T cell_6-MCI.vs.control; scRNA-seq,SRP330776,CD8+ T cell_1-AD.vs.control; scRNA-seq,SRP330776,γδ T cell-AD.vs.control; scRNA-seq,SRP330776,B cell_1-AD.vs.control; scRNA-seq,SRP330776,Natural killer cell_1-AD.vs.control; scRNA-seq,SRP330776,Regulatory T cell-AD.vs.control	11
CBS	bulk RNA-seq,Homo_723,AD.vs.control; bulk RNA-seq,Homo_723,AD.vs.MCI; bulk RNA-seq,Homo_723,MCI.vs.control; bulk RNA-seq,Homo_633,AD.vs.control; bulk RNA-seq,Homo_633,AD.vs.MCI; bulk RNA-seq,Homo_633,MCI.vs.control; bulk RNA-seq,Homo_714,AD.vs.control; bulk RNA-seq,Homo_714,AD.vs.MCI; bulk RNA-seq,Homo_714,MCI.vs.control; bulk RNA-seq,ROSMAP,AD.vs.MCI	10
CBSL	bulk RNA-seq,Homo_714,AD.vs.control; bulk RNA-seq,Homo_714,AD.vs.MCI	2
CBWD1	scRNA-seq,SRP215507,CD8+ T cell_2-AD.vs.MCI; scRNA-seq,SRP215507,CD8+ T cell_1-AD.vs.MCI; scRNA-seq,SRP215507,CD8+ T cell_1-MCI.vs.control; scRNA-seq,SRP215507,CD8+ T cell_2-MCI.vs.control; scRNA-seq,SRP330776,γδ T cell-AD.vs.control; scRNA-seq,SRP330776,Naive CD8+ T cell_3-AD.vs.control; scRNA-seq,SRP330776,Natural killer cell_2-AD.vs.control	7
CBWD2	scRNA-seq,SRP330776,Natural killer cell_1-AD.vs.control; scRNA-seq,SRP330776,Naive CD8+ T cell_3-AD.vs.control; scRNA-seq,SRP330776,T cell-AD.vs.control; scRNA-seq,SRP330776,Monocyte_1-AD.vs.control; scRNA-seq,SRP330776,B cell_3-AD.vs.control	5
CBWD3	scRNA-seq,SRP330776,B cell_1-AD.vs.control; scRNA-seq,SRP330776,CD8+ T cell_3-AD.vs.control	2

CBWD5	scRNA-seq,SRP215507,CD8+ T cell_3-AD.vs.control; scRNA-seq,SRP215507,CD8+ T cell_1-AD.vs.control; scRNA-seq,SRP215507,CD8+ T cell_5-AD.vs.control; scRNA-seq,SRP215507,CD8+ T cell_6-AD.vs.control; scRNA-seq,SRP215507,CD8+ T cell_1-AD.vs.MCI; scRNA-seq,SRP215507,CD8+ T cell_4-AD.vs.MCI; scRNA-seq,SRP215507,CD8+ T cell_5-MCI.vs.control; scRNA-seq,SRP215507,CD8+ T cell_3-MCI.vs.control; scRNA-seq,SRP215507,CD8+ T cell_8-MCI.vs.control; scRNA-seq,SRP309935,CD4+ T cell-AD.vs.control; scRNA-seq,SRP330776,γδ T cell-AD.vs.control;	12
CBWD6	scRNA-seq,SRP215507,CD8+ T cell_1-AD.vs.control; scRNA-seq,SRP215507,CD8+ T cell_1-AD.vs.MCI; scRNA-seq,SRP215507,CD8+ T cell_1-MCI.vs.control; scRNA-seq,SRP309935,Monocyte_1-AD.vs.control	4
CBX1	scRNA-seq,SRP215507,CD8+ T cell_5-AD.vs.control; scRNA-seq,SRP215507,CD8+ T cell_5-AD.vs.MCI; scRNA-seq,SRP330776,Memory T cell_2-AD.vs.control; scRNA-seq,SRP330776,Natural killer T cell-AD.vs.control; scRNA-seq,SRP330776,CD8+ T cell_3-AD.vs.control	5
CBX2	bulk RNA-seq,Homo_723,AD.vs.control; bulk RNA-seq,Homo_723,AD.vs.MCI; bulk RNA-seq,Homo_723,MCI.vs.control; bulk RNA-seq,Homo_633,AD.vs.control; bulk RNA-seq,Homo_633,AD.vs.MCI; bulk RNA-seq,Homo_714,AD.vs.control; bulk RNA-seq,Homo_714,AD.vs.MCI; bulk RNA-seq,ROSMAP,AD.vs.MCI; bulk RNA-seq,ROSMAP,MCI.vs.control; scRNA-seq,SRP215507,CD8+ T cell_3-AD.vs.control	10
CBX3	bulk RNA-seq,Homo_723,AD.vs.control; bulk RNA-seq,Homo_723,MCI.vs.control; bulk RNA-seq,Homo_633,AD.vs.control; bulk RNA-seq,Homo_633,AD.vs.MCI; bulk RNA-seq,Homo_633,MCI.vs.control; bulk RNA-seq,Homo_714,AD.vs.control; bulk RNA-seq,Homo_714,AD.vs.MCI; bulk RNA-seq,Homo_714,MCI.vs.control; scRNA-seq,SRP215507,CD8+ T cell_10-AD.vs.control; scRNA-seq,SRP330776,CD8+ T cell_1-AD.vs.control; scRNA-seq,SRP330776,γδ T cell-AD.vs.control; scRNA-seq,SRP330776,Memory T cell_2-AD.vs.control; scRNA-seq,SRP330776,CD8+ T cell_2-AD.vs.control; scRNA-seq,SRP330776,B cell_1-AD.vs.control; scRNA-seq,SRP330776,Naive CD8+ T cell_1-AD.vs.control; scRNA-seq,SRP330776,Natural killer cell_1-AD.vs.control; scRNA-seq,SRP330776,Memory T cell_1-AD.vs.control; scRNA-seq,SRP330776,Natural killer T cell-AD.vs.control; scRNA-seq,SRP330776,Naive CD8+ T cell_3-AD.vs.control; scRNA-seq,SRP330776,B cell_2-AD.vs.control; scRNA-seq,SRP330776,T cell-AD.vs.control; scRNA-seq,SRP330776,Naive CD8+ T cell_2-AD.vs.control; scRNA-seq,SRP330776,CD8+ T cell_3-AD.vs.control; scRNA-seq,SRP330776,Monocyte_1-AD.vs.control; scRNA-seq,SRP330776,Monocyte_2-AD.vs.control; scRNA-seq,SRP330776,B cell_3-AD.vs.control; scRNA-[illegible]	28
CBX4	scRNA-seq,SRP215507,CD8+ T cell_3-AD.vs.MCI; scRNA-seq,SRP215507,CD8+ T cell_5-AD.vs.MCI; scRNA-seq,SRP215507,CD8+ T cell_5-MCI.vs.control; scRNA-seq,SRP215507,CD8+ T cell_4-MCI.vs.control; scRNA-seq,SRP215507,CD8+ T cell_2-MCI.vs.control; scRNA-seq,SRP215507,CD8+ T cell_3-MCI.vs.control; scRNA-seq,SRP309935,Natural killer cell_2-AD.vs.control; scRNA-seq,SRP309935,Natural killer cell_1-AD.vs.control; scRNA-seq,SRP309935,Monocyte_2-AD.vs.control; scRNA-seq,SRP330776,CD8+ T cell_1-AD.vs.control; scRNA-seq,SRP330776,γδ T cell-AD.vs.control; scRNA-seq,SRP330776,Memory T cell_2-AD.vs.control; scRNA-seq,SRP330776,CD8+ T cell_2-AD.vs.control; scRNA-seq,SRP330776,Naive CD8+ T cell_1-AD.vs.control; scRNA-seq,SRP330776,Natural killer cell_1-AD.vs.control; scRNA-seq,SRP330776,Memory T cell_1-AD.vs.control; scRNA-seq,SRP330776,Naive CD8+ T cell_3-AD.vs.control; scRNA-seq,SRP330776,B cell_2-AD.vs.control; scRNA-[illegible]	21
CBX5	scRNA-seq,SRP215507,CD8+ T cell_5-AD.vs.MCI; scRNA-seq,SRP215507,CD8+ T cell_3-MCI.vs.control; scRNA-seq,SRP330776,Natural killer cell_1-AD.vs.control; scRNA-seq,SRP330776,CD8+ T cell_3-AD.vs.control; scRNA-seq,SRP330776,Regulatory T cell-AD.vs.control; scRNA-	6
CBX6	bulk RNA-seq,Homo_723,AD.vs.control; bulk RNA-seq,Homo_723,AD.vs.MCI; bulk RNA-seq,Homo_633,AD.vs.control; bulk RNA-seq,Homo_633,AD.vs.MCI; bulk RNA-seq,Homo_714,AD.vs.control; bulk RNA-seq,Homo_714,AD.vs.MCI; scRNA-seq,SRP215507,CD8+ T cell_5-AD.vs.MCI; scRNA-seq,SRP215507,CD8+ T cell_6-AD.vs.MCI; scRNA-seq,SRP215507,CD8+ T cell_5-MCI.vs.control; scRNA-seq,SRP309935,Monocyte_2-AD.vs.control; scRNA-seq,SRP330776,CD8+ T cell_1-AD.vs.control; scRNA-seq,SRP330776,γδ T cell-AD.vs.control; scRNA-seq,SRP330776,Memory T cell_2-AD.vs.control; scRNA-seq,SRP330776,CD8+ T cell_2-AD.vs.control; scRNA-seq,SRP330776,B cell_1-AD.vs.control; scRNA-seq,SRP330776,Naive CD8+ T cell_1-AD.vs.control; scRNA-seq,SRP330776,Natural killer cell_1-AD.vs.control; scRNA-seq,SRP330776,Memory T cell_1-AD.vs.control; scRNA-seq,SRP330776,Natural killer T cell-AD.vs.control; scRNA-seq,SRP330776,Naive CD8+ T cell_3-AD.vs.control; scRNA-seq,SRP330776,B cell_2-AD.vs.control; scRNA-seq,SRP330776,Naive CD8+ T cell_2-AD.vs.control; scRNA-seq,SRP330776,CD8+ T cell_3-AD.vs.control; scRNA-seq,SRP330776,Monocyte_2-AD.vs.control; scRNA-seq,SRP330776,B cell_3-AD.vs.control; [illegible]	26

CBX7	bulk RNA-seq,Homo_723,AD.vs.control; bulk RNA-seq,Homo_723,AD.vs.MCI; bulk RNA-seq,Homo_633,AD.vs.control; bulk RNA-seq,Homo_633,AD.vs.MCI; bulk RNA-seq,Homo_714,AD.vs.control; bulk RNA-seq,Homo_714,AD.vs.MCI; scRNA-seq,SRP215507,CD8+ T cell_2-AD.vs.MCI; scRNA-seq,SRP309935,Monocyte_1-AD.vs.control; scRNA-seq,SRP309935,Megakaryocyte_1-AD.vs.control; scRNA-seq,SRP309935,Naive CD8+ T cell_1-AD.vs.control; scRNA-seq,SRP309935,Natural killer cell_1-AD.vs.control; scRNA-seq,SRP309935,Naive CD8+ T cell_2-AD.vs.control; scRNA-seq,SRP309935,CD8+ T cell_1-AD.vs.control; scRNA-seq,SRP309935,B cell_1-AD.vs.control; scRNA-seq,SRP309935,CD8+ T cell_2-AD.vs.control; scRNA-seq,SRP330776,CD8+ T cell_1-AD.vs.control; scRNA-seq,SRP330776,γδ T cell-AD.vs.control; scRNA-seq,SRP330776,CD8+ T cell_2-AD.vs.control; scRNA-seq,SRP330776,Naive CD8+ T cell_1-AD.vs.control; scRNA-seq,SRP330776,Natural killer cell_1-AD.vs.control; scRNA-seq,SRP330776,Memory T cell_1-AD.vs.control; scRNA-seq,SRP330776,CD8+ T cell_3-AD.vs.control; scRNA-seq,SRP330776,Natural killer cell_2-[illegible]	23
CBX8	bulk RNA-seq,Homo_723,AD.vs.control; bulk RNA-seq,Homo_723,AD.vs.MCI; bulk RNA-seq,Homo_714,AD.vs.control; bulk RNA-seq,Homo_714,AD.vs.MCI; scRNA-seq,SRP215507,CD8+ T cell_1-AD.vs.MCI; scRNA-seq,SRP215507,CD8+ T cell_1-MCI.vs.control; scRNA-seq,SRP330776,CD8+ T cell_1-AD.vs.control; scRNA-seq,SRP330776,CD8+ T cell_2-AD.vs.control; scRNA-seq,SRP330776,B cell_1-AD.vs.control; scRNA-seq,SRP330776,Naive CD8+ T cell_1-AD.vs.control; scRNA-seq,SRP330776,Natural killer cell_1-AD.vs.control; scRNA-seq,SRP330776,Memory	13
CBY1	scRNA-seq,SRP215507,CD8+ T cell_5-AD.vs.MCI; scRNA-seq,SRP330776,CD8+ T cell_1-AD.vs.control; scRNA-seq,SRP330776,Memory T cell_2-AD.vs.control; scRNA-seq,SRP330776,CD8+ T cell_2-AD.vs.control; scRNA-seq,SRP330776,B cell_1-AD.vs.control; scRNA-seq,SRP330776,Memory T cell_1-AD.vs.control; scRNA-seq,SRP330776,Naive CD8+ T cell_3-AD.vs.control; scRNA-seq,SRP330776,T cell-AD.vs.control; scRNA-	9
CBY2	bulk RNA-seq,Homo_723,AD.vs.control; bulk RNA-seq,Homo_633,AD.vs.control; bulk RNA-seq,Homo_714,MCI.vs.control	3
CBY3	bulk RNA-seq,Homo_723,AD.vs.control; bulk RNA-seq,Homo_723,AD.vs.MCI; bulk RNA-seq,Homo_633,AD.vs.control; bulk RNA-seq,Homo_633,AD.vs.MCI; bulk RNA-seq,Homo_714,AD.vs.MCI	5
CC2D1A	scRNA-seq,SRP215507,CD8+ T cell_3-AD.vs.control; scRNA-seq,SRP215507,CD8+ T cell_7-AD.vs.MCI; scRNA-seq,SRP309935,Monocyte_2-AD.vs.control; scRNA-seq,SRP330776,CD8+ T cell_1-AD.vs.control; scRNA-seq,SRP330776,γδ T cell-AD.vs.control; scRNA-seq,SRP330776,Memory T cell_2-AD.vs.control; scRNA-seq,SRP330776,CD8+ T cell_2-AD.vs.control; scRNA-seq,SRP330776,B cell_1-AD.vs.control; scRNA-seq,SRP330776,Naive CD8+ T cell_1-AD.vs.control; scRNA-seq,SRP330776,Natural killer cell_1-AD.vs.control; scRNA-seq,SRP330776,Memory T cell_1-AD.vs.control; scRNA-seq,SRP330776,Natural killer T cell-AD.vs.control; scRNA-seq,SRP330776,Naive CD8+ T cell_3-AD.vs.control; scRNA-seq,SRP330776,B cell_2-AD.vs.control; scRNA-seq,SRP330776,T cell-AD.vs.control; scRNA-seq,SRP330776,CD8+ T cell_3-AD.vs.control; scRNA-seq,SRP330776,Monocyte_1-AD.vs.control; scRNA-seq,SRP330776,Monocyte_2-AD.vs.control; scRNA-seq,SRP330776,Natural killer cell_2-[illegible]	19
CC2D1B	scRNA-seq,SRP215507,CD8+ T cell_5-AD.vs.MCI; scRNA-seq,SRP215507,CD8+ T cell_5-MCI.vs.control; scRNA-seq,SRP309935,Natural killer cell_2-AD.vs.control; scRNA-seq,SRP330776,Monocyte_1-AD.vs.control; scRNA-seq,SRP330776,Natural killer cell_2-AD.vs.control	5
CC2D2A	bulk RNA-seq,Homo_723,AD.vs.control; bulk RNA-seq,Homo_723,AD.vs.MCI; bulk RNA-seq,Homo_633,AD.vs.MCI; bulk RNA-seq,Homo_714,AD.vs.control; bulk RNA-seq,Homo_714,AD.vs.MCI; bulk RNA-seq,Homo_714,MCI.vs.control; scRNA-seq,SRP215507,CD8+ T cell_1-AD.vs.control; scRNA-seq,SRP215507,CD8+ T cell_5-MCI.vs.control	8
CCAR1	scRNA-seq,SRP330776,B cell_4-AD.vs.control	1
CCAR2	scRNA-seq,SRP215507,CD8+ T cell_5-AD.vs.control; scRNA-seq,SRP309935,Megakaryocyte_1-AD.vs.control; scRNA-seq,SRP309935,Naive CD8+ T cell_3-AD.vs.control; scRNA-seq,SRP330776,γδ T cell-AD.vs.control; scRNA-seq,SRP330776,Memory T cell_2-AD.vs.control; scRNA-seq,SRP330776,Naive CD8+ T cell_3-AD.vs.control; scRNA-seq,SRP330776,Megakaryocyte_1-AD.vs.control; scRNA-seq,SRP330776,Natural killer	8
CCBE1	bulk RNA-seq,Homo_723,AD.vs.control; bulk RNA-seq,Homo_723,AD.vs.MCI; bulk RNA-seq,Homo_633,AD.vs.control; bulk RNA-seq,Homo_633,MCI.vs.control; bulk RNA-seq,Homo_714,MCI.vs.control	5
CCDC102A	bulk RNA-seq,Homo_714,AD.vs.control; bulk RNA-seq,Homo_714,AD.vs.MCI; scRNA-seq,SRP215507,CD8+ T cell_2-MCI.vs.control; scRNA-seq,SRP309935,Natural killer cell_2-AD.vs.control; scRNA-seq,SRP330776,CD8+ T cell_1-AD.vs.control; scRNA-seq,SRP330776,γδ T cell-AD.vs.control; scRNA-seq,SRP330776,Memory T cell_2-AD.vs.control; scRNA-seq,SRP330776,CD8+ T cell_2-AD.vs.control; scRNA-seq,SRP330776,B cell_1-AD.vs.control; scRNA-seq,SRP330776,Naive CD8+ T cell_1-AD.vs.control; scRNA-seq,SRP330776,Natural killer cell_1-AD.vs.control; scRNA-seq,SRP330776,Memory T cell_1-AD.vs.control; scRNA-seq,SRP330776,Natural killer T cell-AD.vs.control; scRNA-seq,SRP330776,B cell_2-	16

CCDC102B	bulk RNA-seq,ROSMAP,AD.vs.control; bulk RNA-seq,ROSMAP,MCI.vs.control; scRNA-seq,SRP215507,CD8+ T cell_5-AD.vs.MCI; scRNA-seq,SRP330776,γδ T cell-AD.vs.control; scRNA-seq,SRP330776,Memory T cell_2-AD.vs.control; scRNA-seq,SRP330776,CD8+ T cell_2-AD.vs.control; scRNA-seq,SRP330776,B cell_1-AD.vs.control; scRNA-seq,SRP330776,Naive CD8+ T cell_3-AD.vs.control; scRNA-seq,SRP330776,CD8+ T cell_3-	9
CCDC103	bulk RNA-seq,Homo_723,AD.vs.control; bulk RNA-seq,Homo_723,AD.vs.MCI; bulk RNA-seq,Homo_633,AD.vs.control; bulk RNA-seq,Homo_633,AD.vs.MCI; bulk RNA-seq,Homo_714,AD.vs.control; bulk RNA-seq,Homo_714,AD.vs.MCI; scRNA-seq,SRP215507,CD8+ T cell_3-AD.vs.MCI; scRNA-seq,SRP215507,CD8+ T cell_3-MCI.vs.control; scRNA-seq,SRP330776,CD8+ T cell_1-AD.vs.control	9
CCDC105	bulk RNA-seq,Homo_723,AD.vs.control	1
CCDC106	scRNA-seq,SRP330776,γδ T cell-AD.vs.control; scRNA-seq,SRP330776,Memory T cell_2-AD.vs.control; scRNA-seq,SRP330776,Naive CD8+ T cell_1-AD.vs.control; scRNA-seq,SRP330776,Memory T cell_1-AD.vs.control; scRNA-seq,SRP330776,B cell_2-AD.vs.control; scRNA-seq,SRP330776,Monocyte_2-AD.vs.control; scRNA-seq,SRP330776,B cell_3-AD.vs.control	7
CCDC107	scRNA-seq,SRP215507,CD8+ T cell_4-AD.vs.control; scRNA-seq,SRP215507,CD8+ T cell_7-AD.vs.control; scRNA-seq,SRP215507,CD8+ T cell_5-AD.vs.control; scRNA-seq,SRP215507,CD8+ T cell_4-AD.vs.MCI; scRNA-seq,SRP215507,CD8+ T cell_5-AD.vs.MCI; scRNA-seq,SRP215507,CD8+ T cell_6-AD.vs.MCI; scRNA-seq,SRP330776,CD8+ T cell_1-AD.vs.control; scRNA-seq,SRP330776,CD8+ T cell_2-AD.vs.control; scRNA-	9
CCDC110	bulk RNA-seq,Homo_723,AD.vs.control; bulk RNA-seq,Homo_723,AD.vs.MCI; bulk RNA-seq,Homo_633,AD.vs.control; bulk RNA-seq,Homo_633,AD.vs.MCI; scRNA-seq,SRP215507,CD8+ T cell_2-AD.vs.control; scRNA-seq,SRP215507,CD8+ T cell_2-MCI.vs.control; scRNA-	7
CCDC112	bulk RNA-seq,Homo_723,AD.vs.control; bulk RNA-seq,Homo_723,AD.vs.MCI; bulk RNA-seq,Homo_714,AD.vs.control; bulk RNA-seq,Homo_714,AD.vs.MCI; scRNA-seq,SRP215507,CD8+ T cell_7-AD.vs.control; scRNA-seq,SRP215507,CD8+ T cell_6-AD.vs.control; scRNA-seq,SRP215507,CD8+ T cell_6-AD.vs.MCI; scRNA-seq,SRP330776,CD8+ T cell_1-AD.vs.control; scRNA-seq,SRP330776,γδ T cell-AD.vs.control; scRNA-seq,SRP330776,CD8+ T cell_2-AD.vs.control; scRNA-seq,SRP330776,B cell_1-AD.vs.control; scRNA-seq,SRP330776,Natural killer cell_1-AD.vs.control; scRNA-seq,SRP330776,Memory T cell_1-AD.vs.control; scRNA-seq,SRP330776,Natural killer T cell-AD.vs.control; scRNA-seq,SRP330776,B cell_2-AD.vs.control; scRNA-seq,SRP330776,T cell-AD.vs.control; scRNA-seq,SRP330776,CD8+ T cell_3-AD.vs.control; scRNA- [illegible]	19
CCDC113	scRNA-seq,SRP215507,CD8+ T cell_3-AD.vs.control; scRNA-seq,SRP215507,CD8+ T cell_3-AD.vs.MCI; scRNA-seq,SRP330776,B cell_1-	3
CCDC114	scRNA-seq,SRP215507,CD8+ T cell_2-AD.vs.MCI; scRNA-seq,SRP215507,CD8+ T cell_1-AD.vs.MCI; scRNA-seq,SRP215507,CD8+ T cell_5-MCI.vs.control; scRNA-seq,SRP215507,CD8+ T cell_2-MCI.vs.control; scRNA-seq,SRP330776,Naive CD8+ T cell_1-AD.vs.control; scRNA-	6
CCDC115	scRNA-seq,SRP215507,CD8+ T cell_6-AD.vs.control; scRNA-seq,SRP309935,Megakaryocyte_2-AD.vs.control; scRNA-seq,SRP330776,Natural killer	3
CCDC116	bulk RNA-seq,Homo_723,AD.vs.control; bulk RNA-seq,Homo_723,AD.vs.MCI; bulk RNA-seq,Homo_633,AD.vs.control; bulk RNA-	4
CCDC117	bulk RNA-seq,Homo_723,AD.vs.MCI; bulk RNA-seq,Homo_714,AD.vs.control; bulk RNA-seq,Homo_714,AD.vs.MCI; scRNA-seq,SRP215507,CD8+ T cell_2-AD.vs.control; scRNA-seq,SRP215507,CD8+ T cell_5-AD.vs.MCI; scRNA-seq,SRP215507,CD8+ T cell_1-MCI.vs.control; scRNA-seq,SRP215507,CD8+ T cell_5-MCI.vs.control; scRNA-seq,SRP215507,CD8+ T cell_2-MCI.vs.control; scRNA-seq,SRP309935,Natural killer cell_2-AD.vs.control; scRNA-seq,SRP309935,Monocyte_1-AD.vs.control; scRNA-seq,SRP309935,Naive CD8+ T cell_1-AD.vs.control; scRNA-seq,SRP309935,CD8+ T cell_1-AD.vs.control; scRNA-seq,SRP330776,CD8+ T cell_1-AD.vs.control; scRNA-seq,SRP330776,γδ T cell-AD.vs.control; scRNA-seq,SRP330776,Memory T cell_2-AD.vs.control; scRNA-seq,SRP330776,CD8+ T cell_2-AD.vs.control; scRNA-seq,SRP330776,B cell_1-AD.vs.control; scRNA-seq,SRP330776,Naive CD8+ T cell_1-AD.vs.control; scRNA-seq,SRP330776,Natural killer cell_1-AD.vs.control; scRNA-seq,SRP330776,Memory T cell_1-AD.vs.control; scRNA-seq,SRP330776,Natural killer T cell-AD.vs.control; scRNA-seq,SRP330776,Naive CD8+ T cell_3-AD.vs.control; scRNA-seq,SRP330776,B cell_2-AD.vs.control; scRNA-seq,SRP330776,T cell-AD.vs.control; scRNA-seq,SRP330776,Naive CD8+ [illegible]	27
CCDC12	scRNA-seq,SRP215507,CD8+ T cell_7-AD.vs.control; scRNA-seq,SRP215507,CD8+ T cell_7-AD.vs.MCI; scRNA-seq,SRP309935,Erythroid cell-AD.vs.control; scRNA-seq,SRP330776,γδ T cell-AD.vs.control; scRNA-seq,SRP330776,Memory T cell_2-AD.vs.control	5
CCDC120	bulk RNA-seq,Homo_633,MCI.vs.control; scRNA-seq,SRP215507,CD8+ T cell_5-MCI.vs.control; scRNA-seq,SRP330776,CD8+ T cell_1-AD.vs.control; scRNA-seq,SRP330776,γδ T cell-AD.vs.control; scRNA-seq,SRP330776,Memory T cell_2-AD.vs.control; scRNA-seq,SRP330776,CD8+ T cell_2-AD.vs.control; scRNA-seq,SRP330776,B cell_1-AD.vs.control; scRNA-seq,SRP330776,Naive CD8+ T cell_1-AD.vs.control; scRNA-seq,SRP330776,Memory T cell_1-AD.vs.control; scRNA-seq,SRP330776,Naive CD8+ T cell_3-AD.vs.control; scRNA-seq,SRP330776,B cell_2-AD.vs.control; scRNA-seq,SRP330776,T cell-AD.vs.control; scRNA-seq,SRP330776,CD8+ T cell_3-AD.vs.control; scRNA-seq,SRP330776,Natural killer	14
CCDC121	scRNA-seq,SRP330776,B cell_1-AD.vs.control; scRNA-seq,SRP330776,Naive CD8+ T cell_1-AD.vs.control	2

CCDC122	bulk RNA-seq,Homo_723,AD.vs.control; bulk RNA-seq,Homo_723,AD.vs.MCI; bulk RNA-seq,Homo_723,MCI.vs.control; bulk RNA-seq,Homo_633,AD.vs.control; bulk RNA-seq,Homo_633,AD.vs.MCI; bulk RNA-seq,Homo_633,MCI.vs.control; bulk RNA-seq,Homo_714,AD.vs.control; bulk RNA-seq,Homo_714,AD.vs.MCI; bulk RNA-seq,Homo_714,MCI.vs.control; scRNA-seq,SRP215507,CD8+ T cell_2-AD.vs.control; scRNA-seq,SRP215507,CD8+ T cell_2-AD.vs.MCI; scRNA-seq,SRP215507,CD8+ T cell_3-AD.vs.MCI; scRNA-seq,SRP215507,CD8+ T cell_1-AD.vs.MCI; scRNA-seq,SRP215507,CD8+ T cell_1-MCI.vs.control; scRNA-seq,SRP215507,CD8+ T cell_2-MCI.vs.control; scRNA-seq,SRP215507,CD8+ T cell_3-MCI.vs.control; scRNA-seq,SRP309935,Naive CD8+ T cell_2-AD.vs.control; scRNA-seq,SRP309935,B cell_1-AD.vs.control; scRNA-	20
Ccdc122	bulk RNA-seq,SRP325058,AD.vs.control	1
CCDC124	bulk RNA-seq,Homo_723,AD.vs.control; bulk RNA-seq,Homo_723,MCI.vs.control; bulk RNA-seq,Homo_633,AD.vs.control; bulk RNA-seq,Homo_633,MCI.vs.control; bulk RNA-seq,Homo_714,AD.vs.control; bulk RNA-seq,Homo_714,AD.vs.MCI; bulk RNA-seq,Homo_714,MCI.vs.control; scRNA-seq,SRP215507,CD8+ T cell_6-AD.vs.MCI; scRNA-seq,SRP330776,Monocyte_2-AD.vs.control; scRNA-	11
Ccdc124	bulk RNA-seq,SRP325058,AD.vs.control	1
CCDC125	bulk RNA-seq,Homo_723,AD.vs.control; bulk RNA-seq,Homo_723,AD.vs.MCI; bulk RNA-seq,Homo_633,AD.vs.control; bulk RNA-seq,Homo_633,AD.vs.MCI; bulk RNA-seq,Homo_714,AD.vs.control; bulk RNA-seq,Homo_714,AD.vs.MCI; scRNA-seq,SRP215507,CD8+ T cell_2-AD.vs.MCI; scRNA-seq,SRP215507,CD8+ T cell_4-MCI.vs.control; scRNA-seq,SRP309935,Naive CD8+ T cell_1-AD.vs.control; scRNA-seq,SRP330776,γδ T cell-AD.vs.control; scRNA-seq,SRP330776,Naive CD8+ T cell_1-AD.vs.control	11
CCDC126	bulk RNA-seq,Homo_723,AD.vs.control; bulk RNA-seq,Homo_723,AD.vs.MCI; bulk RNA-seq,Homo_633,AD.vs.control; bulk RNA-seq,Homo_633,AD.vs.MCI; bulk RNA-seq,Homo_714,AD.vs.control; bulk RNA-seq,Homo_714,AD.vs.MCI; scRNA-seq,SRP215507,CD8+ T cell_3-AD.vs.control; scRNA-seq,SRP215507,CD8+ T cell_5-AD.vs.control; scRNA-seq,SRP215507,CD8+ T cell_3-AD.vs.MCI; scRNA-seq,SRP215507,CD8+ T cell_5-AD.vs.MCI; scRNA-seq,SRP330776,CD8+ T cell_2-AD.vs.control; scRNA-seq,SRP330776,B cell_1-AD.vs.control; scRNA-seq,SRP330776,Naive CD8+ T cell_1-AD.vs.control; scRNA-seq,SRP330776,Memory T cell_1-AD.vs.control; scRNA-seq,SRP330776,Naive CD8+ T cell_3-AD.vs.control; scRNA-seq,SRP330776,B cell_2-AD.vs.control; scRNA-seq,SRP330776,T cell-AD.vs.control; scRNA-seq,SRP330776,Monocyte_2-	19
CCDC127	scRNA-seq,SRP215507,CD8+ T cell_6-AD.vs.control; scRNA-seq,SRP215507,CD8+ T cell_2-AD.vs.MCI; scRNA-seq,SRP215507,CD8+ T cell_4-AD.vs.MCI; scRNA-seq,SRP215507,CD8+ T cell_5-AD.vs.MCI; scRNA-seq,SRP215507,CD8+ T cell_5-MCI.vs.control; scRNA-seq,SRP309935,CD8+ T cell_1-AD.vs.control; scRNA-seq,SRP330776,CD8+ T cell_1-AD.vs.control; scRNA-seq,SRP330776,γδ T cell-AD.vs.control; scRNA-seq,SRP330776,Memory T cell_2-AD.vs.control; scRNA-seq,SRP330776,CD8+ T cell_2-AD.vs.control; scRNA-seq,SRP330776,B cell_1-AD.vs.control; scRNA-seq,SRP330776,Naive CD8+ T cell_1-AD.vs.control; scRNA-seq,SRP330776,Memory T cell_1-AD.vs.control; scRNA-seq,SRP330776,B cell_2-AD.vs.control; scRNA-seq,SRP330776,T cell-AD.vs.control; scRNA-seq,SRP330776,CD8+ T cell_3-AD.vs.control; scRNA-seq,SRP330776,B cell_3-	17
CCDC13	bulk RNA-seq,Homo_633,AD.vs.control; bulk RNA-seq,Homo_633,AD.vs.MCI; bulk RNA-seq,Homo_714,AD.vs.control; bulk RNA-seq,Homo_714,AD.vs.MCI; bulk RNA-seq,ROSMAP,MCI.vs.control	5
CCDC130	scRNA-seq,SRP215507,CD8+ T cell_3-AD.vs.MCI; scRNA-seq,SRP330776,Memory T cell_2-AD.vs.control; scRNA-seq,SRP330776,B cell_1-AD.vs.control; scRNA-seq,SRP330776,Memory T cell_1-AD.vs.control; scRNA-seq,SRP330776,B cell_2-AD.vs.control; scRNA-	6
CCDC134	bulk RNA-seq,Homo_723,AD.vs.control; bulk RNA-seq,Homo_633,MCI.vs.control; bulk RNA-seq,Homo_714,AD.vs.control; bulk RNA-seq,Homo_714,AD.vs.MCI; bulk RNA-seq,Homo_714,MCI.vs.control; scRNA-seq,SRP215507,CD8+ T cell_3-AD.vs.MCI; scRNA-seq,SRP215507,CD8+ T cell_2-MCI.vs.control; scRNA-seq,SRP215507,CD8+ T cell_3-MCI.vs.control; scRNA-seq,SRP330776,Natural killer cell_1-AD.vs.control; scRNA-	10
CCDC136	bulk RNA-seq,Homo_723,AD.vs.control; bulk RNA-seq,Homo_723,AD.vs.MCI; bulk RNA-seq,Homo_633,AD.vs.control; bulk RNA-seq,Homo_714,AD.vs.control; bulk RNA-seq,Homo_714,AD.vs.MCI; bulk RNA-seq,ROSMAP,AD.vs.control; scRNA-seq,SRP215507,CD8+ T cell_5-MCI.vs.control; scRNA-seq,SRP215507,CD8+ T cell_4-MCI.vs.control; scRNA-seq,SRP309935,CD8+ T cell_1-AD.vs.control; scRNA-seq,SRP330776,CD8+ T cell_1-AD.vs.control; scRNA-seq,SRP330776,Memory T cell_2-AD.vs.control; scRNA-seq,SRP330776,CD8+ T cell_2-AD.vs.control; scRNA-seq,SRP330776,Naive CD8+ T cell_1-AD.vs.control; scRNA-seq,SRP330776,Memory T cell_1-AD.vs.control	14

CCDC137	bulk RNA-seq,Homo_723,AD.vs.control; bulk RNA-seq,Homo_723,AD.vs.MCI; bulk RNA-seq,Homo_633,AD.vs.control; bulk RNA-seq,Homo_633,AD.vs.MCI; scRNA-seq,SRP215507,CD8+ T cell_7-AD.vs.control; scRNA-seq,SRP215507,CD8+ T cell_2-AD.vs.MCI; scRNA-seq,SRP215507,CD8+ T cell_3-AD.vs.MCI; scRNA-seq,SRP215507,CD8+ T cell_1-AD.vs.MCI; scRNA-seq,SRP215507,CD8+ T cell_4-AD.vs.MCI; scRNA-seq,SRP215507,CD8+ T cell_5-AD.vs.MCI; scRNA-seq,SRP215507,CD8+ T cell_5-MCI.vs.control; scRNA-seq,SRP215507,CD8+ T cell_4-MCI.vs.control; scRNA-seq,SRP215507,CD8+ T cell_3-MCI.vs.control; scRNA-seq,SRP215507,CD8+ T cell_6-MCI.vs.control; scRNA-seq,SRP330776,B cell_1-AD.vs.control; scRNA-seq,SRP330776,B cell_2-AD.vs.control; scRNA-seq,SRP330776,T cell-AD.vs.control; scRNA-seq,SRP330776,CD8+ T cell_3-AD.vs.control; scRNA-seq,SRP330776,B cell_3-AD.vs.control; scRNA-seq,SRP330776,Natural killer cell_2-AD.vs.control	20
CCDC138	bulk RNA-seq,Homo_723,AD.vs.control; bulk RNA-seq,Homo_723,AD.vs.MCI; bulk RNA-seq,Homo_633,AD.vs.control; bulk RNA-seq,Homo_633,AD.vs.MCI; bulk RNA-seq,Homo_714,AD.vs.control; bulk RNA-seq,Homo_714,AD.vs.MCI; scRNA-seq,SRP215507,CD8+ T cell_6-MCI.vs.control; scRNA-seq,SRP330776,Memory T cell_2-AD.vs.control; scRNA-seq,SRP330776,CD8+ T cell_2-AD.vs.control; scRNA-seq,SRP330776,B cell_1-AD.vs.control; scRNA-seq,SRP330776,Naive CD8+ T cell_1-AD.vs.control; scRNA-seq,SRP330776,Natural killer cell_1-AD.vs.control; scRNA-seq,SRP330776,Memory T cell_1-AD.vs.control; scRNA-seq,SRP330776,Natural killer T cell-AD.vs.control; scRNA-seq,SRP330776,Naive CD8+ T cell_3-AD.vs.control; scRNA-seq,SRP330776,T cell-AD.vs.control; scRNA-seq,SRP330776,CD8+ T cell_3-AD.vs.control; [illegible]	18
CCDC14	scRNA-seq,SRP215507,CD8+ T cell_6-AD.vs.control; scRNA-seq,SRP215507,CD8+ T cell_2-AD.vs.MCI; scRNA-seq,SRP215507,CD8+ T cell_6-MCI.vs.control; scRNA-seq,SRP330776,γδ T cell-AD.vs.control; scRNA-seq,SRP330776,Memory T cell_1-AD.vs.control; scRNA-seq,SRP330776,Naive CD8+ T cell_3-AD.vs.control; scRNA-seq,SRP330776,B cell_3-AD.vs.control; scRNA-seq,SRP330776,Natural killer cell_2-AD.vs.control	8
CCDC141	bulk RNA-seq,Homo_723,AD.vs.MCI; bulk RNA-seq,Homo_723,MCI.vs.control; bulk RNA-seq,Homo_633,AD.vs.MCI; bulk RNA-seq,Homo_633,MCI.vs.control; bulk RNA-seq,Homo_714,MCI.vs.control; bulk RNA-seq,ROSMAP,AD.vs.control; bulk RNA-seq,ROSMAP,MCI.vs.control; scRNA-seq,SRP215507,CD8+ T cell_3-AD.vs.control; scRNA-seq,SRP215507,CD8+ T cell_4-MCI.vs.control; scRNA-seq,SRP215507,CD8+ T cell_3-MCI.vs.control; scRNA-seq,SRP309935,Naive CD8+ T cell_2-AD.vs.control; scRNA-seq,SRP330776,CD8+ T cell_2-AD.vs.control; scRNA-seq,SRP330776,Natural killer T cell-AD.vs.control; scRNA-seq,SRP330776,B cell_3-AD.vs.control	14
CCDC142	scRNA-seq,SRP215507,CD8+ T cell_2-AD.vs.control; scRNA-seq,SRP215507,CD8+ T cell_1-AD.vs.control; scRNA-seq,SRP215507,CD8+ T cell_2-AD.vs.MCI; scRNA-seq,SRP215507,CD8+ T cell_1-AD.vs.MCI; scRNA-seq,SRP309935,Natural killer cell_2-AD.vs.control; scRNA-seq,SRP309935,Natural killer cell_1-AD.vs.control; scRNA-seq,SRP309935,Monocyte_2-AD.vs.control; scRNA-seq,SRP309935,Naive CD8+ T cell_2-AD.vs.control; scRNA-seq,SRP309935,B cell_1-AD.vs.control; scRNA-seq,SRP330776,CD8+ T cell_1-AD.vs.control; scRNA-seq,SRP330776,γδ T cell-AD.vs.control; scRNA-seq,SRP330776,CD8+ T cell_2-AD.vs.control; scRNA-seq,SRP330776,B cell_1-AD.vs.control; scRNA-seq,SRP330776,Naive CD8+ T cell_1-AD.vs.control; scRNA-seq,SRP330776,Natural killer cell_1-AD.vs.control; scRNA-seq,SRP330776,Memory T cell_1-AD.vs.control; scRNA-seq,SRP330776,Natural killer T cell-AD.vs.control; scRNA-seq,SRP330776,Naive CD8+ T cell_3-AD.vs.control; scRNA-seq,SRP330776,T cell-[illegible]	21
CCDC144A	scRNA-seq,SRP215507,CD8+ T cell_2-AD.vs.control; scRNA-seq,SRP215507,CD8+ T cell_3-AD.vs.control; scRNA-seq,SRP215507,CD8+ T cell_4-AD.vs.control; scRNA-seq,SRP215507,CD8+ T cell_2-AD.vs.MCI; scRNA-seq,SRP215507,CD8+ T cell_3-AD.vs.MCI; scRNA-seq,SRP215507,CD8+ T cell_1-AD.vs.MCI; scRNA-seq,SRP330776,CD8+ T cell_3-AD.vs.control	7
CCDC144NL-AS1	scRNA-seq,SRP330776,B cell_1-AD.vs.control; scRNA-seq,SRP330776,Naive CD8+ T cell_1-AD.vs.control	2
CCDC146	bulk RNA-seq,Homo_723,AD.vs.control; bulk RNA-seq,Homo_723,MCI.vs.control; bulk RNA-seq,Homo_633,AD.vs.control; bulk RNA-seq,Homo_633,AD.vs.MCI; bulk RNA-seq,Homo_633,MCI.vs.control; scRNA-seq,SRP215507,CD8+ T cell_2-AD.vs.control; scRNA-seq,SRP215507,CD8+ T cell_4-MCI.vs.control; scRNA-seq,SRP215507,CD8+ T cell_2-MCI.vs.control; scRNA-seq,SRP330776,CD8+ T cell_1-AD.vs.control; scRNA-seq,SRP330776,γδ T cell-AD.vs.control; scRNA-seq,SRP330776,Memory T cell_2-AD.vs.control; scRNA-seq,SRP330776,B cell_1-AD.vs.control; scRNA-seq,SRP330776,Naive CD8+ T cell_1-AD.vs.control; scRNA-seq,SRP330776,Natural killer cell_1-AD.vs.control; scRNA-seq,SRP330776,Naive CD8+ T cell_3-AD.vs.control; scRNA-seq,SRP330776,CD8+ T cell_3-AD.vs.control; scRNA-seq,SRP330776,B cell_3-[illegible]	17
CCDC148	bulk RNA-seq,Homo_723,AD.vs.control; bulk RNA-seq,Homo_723,AD.vs.MCI; bulk RNA-seq,Homo_723,MCI.vs.control; bulk RNA-seq,Homo_633,AD.vs.control; bulk RNA-seq,Homo_633,AD.vs.MCI; bulk RNA-seq,Homo_633,MCI.vs.control; scRNA-seq,SRP215507,CD8+ T cell_5-[illegible]	7
CCDC149	bulk RNA-seq,Homo_714,AD.vs.control; bulk RNA-seq,Homo_714,AD.vs.MCI	2

CCDC15	bulk RNA-seq,Homo_723,AD.vs.control; bulk RNA-seq,Homo_723,AD.vs.MCI; bulk RNA-seq,Homo_633,AD.vs.control; bulk RNA-seq,Homo_633,AD.vs.MCI; bulk RNA-seq,Homo_714,AD.vs.control; bulk RNA-seq,Homo_714,AD.vs.MCI; scRNA-seq,SRP215507,CD8+ T cell_2-AD.vs.control; scRNA-seq,SRP215507,CD8+ T cell_5-AD.vs.control; scRNA-seq,SRP215507,CD8+ T cell_6-AD.vs.control; scRNA-seq,SRP215507,CD8+ T cell_4-AD.vs.MCI; scRNA-seq,SRP215507,CD8+ T cell_2-MCI.vs.control; scRNA-seq,SRP330776,γδ T cell-AD.vs.control; scRNA-seq,SRP330776,B cell_1-AD.vs.control; scRNA-seq,SRP330776,Natural killer cell_1-AD.vs.control; scRNA-seq,SRP330776,Memory T cell_1-	17
CCDC150	bulk RNA-seq,Homo_723,AD.vs.control; bulk RNA-seq,Homo_723,AD.vs.MCI; bulk RNA-seq,Homo_723,MCI.vs.control; bulk RNA-seq,Homo_633,AD.vs.control; bulk RNA-seq,Homo_633,AD.vs.MCI; bulk RNA-seq,Homo_633,MCI.vs.control; bulk RNA-seq,ROSMAP,AD.vs.control;	8
CCDC151	scRNA-seq,SRP330776,B cell_1-AD.vs.control; scRNA-seq,SRP330776,B cell_2-AD.vs.control	2
CCDC152	bulk RNA-seq,Homo_723,AD.vs.control; bulk RNA-seq,Homo_723,AD.vs.MCI; bulk RNA-seq,Homo_633,AD.vs.control; bulk RNA-seq,Homo_633,AD.vs.MCI; bulk RNA-seq,Homo_714,AD.vs.control; bulk RNA-seq,Homo_714,AD.vs.MCI; bulk RNA-seq,ROSMAP,MCI.vs.control; scRNA-seq,SRP215507,CD8+ T cell_3-AD.vs.MCI; scRNA-seq,SRP215507,CD8+ T cell_1-AD.vs.MCI; scRNA-seq,SRP215507,CD8+ T cell_4-AD.vs.MCI; scRNA-seq,SRP215507,CD8+ T cell_1-MCI.vs.control; scRNA-seq,SRP215507,CD8+ T cell_4-MCI.vs.control; scRNA-seq,SRP215507,CD8+ T cell_3-MCI.vs.control; scRNA-seq,SRP215507,CD8+ T cell_6-MCI.vs.control; scRNA-seq,SRP309935,B cell_1-AD.vs.control; scRNA-seq,SRP330776,CD8+ T cell_1-AD.vs.control; scRNA-seq,SRP330776,γδ T cell-AD.vs.control; scRNA-seq,SRP330776,Memory T cell_2-AD.vs.control; scRNA-seq,SRP330776,CD8+ T cell_2-AD.vs.control; scRNA-seq,SRP330776,B cell_1-AD.vs.control; scRNA-seq,SRP330776,Naive CD8+ T cell_1-AD.vs.control; scRNA-seq,SRP330776,Memory T cell_1-AD.vs.control; scRNA-seq,SRP330776,Naive CD8+ T cell_3-AD.vs.control; [illegible]	24
CCDC153	scRNA-seq,SRP215507,CD8+ T cell_5-AD.vs.MCI	1
CCDC154	bulk RNA-seq,Homo_723,AD.vs.MCI; bulk RNA-seq,Homo_723,MCI.vs.control; bulk RNA-seq,Homo_633,AD.vs.MCI; bulk RNA-seq,Homo_633,MCI.vs.control; bulk RNA-seq,Homo_714,AD.vs.control; bulk RNA-seq,Homo_714,AD.vs.MCI; bulk RNA-seq,Homo_714,MCI.vs.control; scRNA-seq,SRP215507,CD8+ T cell_1-AD.vs.control; scRNA-seq,SRP215507,CD8+ T cell_3-AD.vs.MCI; scRNA-seq,SRP215507,CD8+ T cell_4-MCI.vs.control; scRNA-seq,SRP330776,CD8+ T cell_1-AD.vs.control; scRNA-seq,SRP330776,Naive CD8+ T cell_1-	12
CCDC157	bulk RNA-seq,Homo_723,AD.vs.MCI; bulk RNA-seq,Homo_723,MCI.vs.control; bulk RNA-seq,Homo_633,AD.vs.control; bulk RNA-seq,Homo_633,AD.vs.MCI; bulk RNA-seq,Homo_633,MCI.vs.control; bulk RNA-seq,Homo_714,AD.vs.control; bulk RNA-seq,Homo_714,AD.vs.MCI; bulk RNA-seq,Homo_714,MCI.vs.control; scRNA-seq,SRP215507,CD8+ T cell_3-AD.vs.control; scRNA-seq,SRP215507,CD8+ T cell_3-AD.vs.MCI; scRNA-seq,SRP215507,CD8+ T cell_1-AD.vs.MCI; scRNA-seq,SRP215507,CD8+ T cell_4-AD.vs.MCI; scRNA-seq,SRP309935,Naive CD8+ T cell_2-AD.vs.control; scRNA-seq,SRP309935,CD4+ T cell-AD.vs.control; scRNA-seq,SRP330776,CD8+ T cell_1-AD.vs.control; scRNA-seq,SRP330776,Memory T cell_2-AD.vs.control; scRNA-seq,SRP330776,CD8+ T cell_2-AD.vs.control; scRNA-seq,SRP330776,B cell_1-AD.vs.control; [illegible]	20
CCDC158	bulk RNA-seq,Homo_723,AD.vs.control; bulk RNA-seq,Homo_723,AD.vs.MCI; bulk RNA-seq,Homo_633,AD.vs.control; bulk RNA-	4
CCDC159	bulk RNA-seq,Homo_723,AD.vs.control; bulk RNA-seq,Homo_723,AD.vs.MCI; bulk RNA-seq,Homo_633,AD.vs.control; bulk RNA-seq,Homo_633,AD.vs.MCI; bulk RNA-seq,Homo_714,AD.vs.control; bulk RNA-seq,Homo_714,AD.vs.MCI; scRNA-seq,SRP215507,CD8+ T cell_6-MCI.vs.control; scRNA-seq,SRP330776,B cell_1-AD.vs.control; scRNA-seq,SRP330776,Naive CD8+ T cell_1-AD.vs.control; scRNA-seq,SRP330776,CD8+ T cell_3-AD.vs.control; scRNA-seq,SRP330776,Regulatory T cell-AD.vs.control; scRNA-seq,SRP330776,Megakaryocyte_1-	12
CCDC15-DT	scRNA-seq,SRP330776,CD8+ T cell_2-AD.vs.control	1
CCDC160	bulk RNA-seq,Homo_723,AD.vs.MCI	1
CCDC163	scRNA-seq,SRP215507,CD8+ T cell_2-AD.vs.control; scRNA-seq,SRP215507,CD8+ T cell_3-AD.vs.control; scRNA-seq,SRP215507,CD8+ T cell_1-AD.vs.MCI; scRNA-seq,SRP215507,CD8+ T cell_5-AD.vs.MCI; scRNA-seq,SRP215507,CD8+ T cell_1-MCI.vs.control; scRNA-seq,SRP215507,CD8+ T cell_5-MCI.vs.control; scRNA-seq,SRP215507,CD8+ T cell_2-MCI.vs.control; scRNA-seq,SRP330776,CD8+ T cell_1-AD.vs.control; scRNA-seq,SRP330776,γδ T cell-AD.vs.control; scRNA-seq,SRP330776,Memory T cell_2-AD.vs.control; scRNA-seq,SRP330776,CD8+ T cell_2-AD.vs.control; scRNA-seq,SRP330776,Naive CD8+ T cell_1-AD.vs.control; scRNA-seq,SRP330776,Memory T cell_1-AD.vs.control; scRNA-seq,SRP330776,CD8+ T	14
CCDC167	bulk RNA-seq,Homo_633,AD.vs.control; bulk RNA-seq,Homo_633,AD.vs.MCI; bulk RNA-seq,Homo_714,AD.vs.control; bulk RNA-seq,Homo_714,AD.vs.MCI; scRNA-seq,SRP215507,CD8+ T cell_10-AD.vs.control; scRNA-seq,SRP215507,CD8+ T cell_6-AD.vs.MCI; scRNA-seq,SRP215507,CD8+ T cell_1-MCI.vs.control; scRNA-seq,SRP215507,CD8+ T cell_3-MCI.vs.control; scRNA-seq,SRP330776,Naive CD8+ T cell_1-	10

CCDC168	bulk RNA-seq,Homo_723,AD.vs.control; bulk RNA-seq,Homo_723,AD.vs.MCI; bulk RNA-seq,Homo_633,AD.vs.control; bulk RNA-seq,Homo_633,AD.vs.MCI; bulk RNA-seq,Homo_714,MCI.vs.control; bulk RNA-seq,ROSMAP,MCI.vs.control	6
CCDC169	bulk RNA-seq,Homo_723,AD.vs.control; bulk RNA-seq,Homo_723,AD.vs.MCI; bulk RNA-seq,Homo_633,AD.vs.control; bulk RNA-seq,Homo_633,AD.vs.MCI; bulk RNA-seq,ROSMAP,MCI.vs.control	5
CCDC169-SOHLH2	bulk RNA-seq,Homo_723,AD.vs.control; bulk RNA-seq,Homo_723,AD.vs.MCI; bulk RNA-seq,Homo_633,AD.vs.control; bulk RNA-seq,Homo_633,AD.vs.MCI; bulk RNA-seq,ROSMAP,MCI.vs.control	5
CCDC17	scRNA-seq,SRP330776,CD8+ T cell_2-AD.vs.control	1
CCDC170	bulk RNA-seq,Homo_723,AD.vs.control; bulk RNA-seq,Homo_723,AD.vs.MCI; bulk RNA-seq,Homo_633,AD.vs.control; bulk RNA-seq,Homo_633,AD.vs.MCI; bulk RNA-seq,Homo_714,AD.vs.control; bulk RNA-seq,Homo_714,AD.vs.MCI	6
CCDC171	scRNA-seq,SRP215507,CD8+ T cell_5-MCI.vs.control; scRNA-seq,SRP330776,Memory T cell_2-AD.vs.control; scRNA-seq,SRP330776,B cell_1-	3
CCDC173	bulk RNA-seq,Homo_723,AD.vs.control; bulk RNA-seq,Homo_723,AD.vs.MCI; bulk RNA-seq,Homo_723,MCI.vs.control; bulk RNA-seq,Homo_633,AD.vs.control; bulk RNA-seq,Homo_633,AD.vs.MCI; scRNA-seq,SRP215507,CD8+ T cell_1-MCI.vs.control; scRNA-seq,SRP330776,B cell_2-AD.vs.control; scRNA-seq,SRP330776,CD8+ T cell_3-AD.vs.control	8
CCDC174	scRNA-seq,SRP215507,CD8+ T cell_5-AD.vs.MCI; scRNA-seq,SRP215507,CD8+ T cell_1-MCI.vs.control; scRNA-seq,SRP215507,CD8+ T cell_5-	3
CCDC175	bulk RNA-seq,Homo_723,AD.vs.control; bulk RNA-seq,Homo_723,AD.vs.MCI; bulk RNA-seq,Homo_723,MCI.vs.control; bulk RNA-seq,Homo_633,AD.vs.control; bulk RNA-seq,Homo_633,AD.vs.MCI; bulk RNA-seq,Homo_633,MCI.vs.control	6
CCDC177	bulk RNA-seq,Homo_723,AD.vs.control; bulk RNA-seq,Homo_723,AD.vs.MCI; bulk RNA-seq,Homo_714,MCI.vs.control; bulk RNA-seq,ROSMAP,AD.vs.control; bulk RNA-seq,ROSMAP,MCI.vs.control	5
CCDC178	bulk RNA-seq,Homo_723,AD.vs.MCI; bulk RNA-seq,Homo_723,MCI.vs.control; bulk RNA-seq,Homo_633,AD.vs.MCI; bulk RNA-	4
CCDC179	bulk RNA-seq,Homo_714,MCI.vs.control	1
CCDC18	scRNA-seq,SRP215507,CD8+ T cell_3-AD.vs.control; scRNA-seq,SRP215507,CD8+ T cell_5-AD.vs.MCI; scRNA-seq,SRP215507,CD8+ T cell_6-AD.vs.MCI; scRNA-seq,SRP215507,CD8+ T cell_3-MCI.vs.control; scRNA-seq,SRP215507,CD8+ T cell_6-MCI.vs.control; scRNA-seq,SRP309935,Megakaryocyte_1-AD.vs.control; scRNA-seq,SRP330776,CD8+ T cell_1-AD.vs.control; scRNA-seq,SRP330776,γδ T cell-AD.vs.control; scRNA-seq,SRP330776,Memory T cell_2-AD.vs.control; scRNA-seq,SRP330776,CD8+ T cell_2-AD.vs.control; scRNA-seq,SRP330776,Naive CD8+ T cell_1-AD.vs.control; scRNA-seq,SRP330776,Natural killer cell_1-AD.vs.control; scRNA-seq,SRP330776,Memory T cell_1-AD.vs.control; scRNA-seq,SRP330776,Natural killer T cell-AD.vs.control; scRNA-seq,SRP330776,Naive CD8+ T cell_3-AD.vs.control; scRNA-seq,SRP330776,B cell_2-AD.vs.control; scRNA-seq,SRP330776,T cell-AD.vs.control; scRNA-seq,SRP330776,Regulatory T cell-AD.vs.control; scRNA-seq,SRP330776,Natural [illegible]	19
CCDC180	scRNA-seq,SRP215507,CD8+ T cell_3-AD.vs.control; scRNA-seq,SRP215507,CD8+ T cell_4-AD.vs.MCI; scRNA-seq,SRP215507,CD8+ T cell_4-MCI.vs.control; scRNA-seq,SRP309935,Naive CD8+ T cell_2-AD.vs.control; scRNA-seq,SRP330776,CD8+ T cell_2-AD.vs.control; scRNA-seq,SRP330776,Memory T cell_1-AD.vs.control; scRNA-seq,SRP330776,T cell-AD.vs.control; scRNA-seq,SRP330776,CD8+ T cell_3-AD.vs.control	8
CCDC181	bulk RNA-seq,Homo_723,AD.vs.control; bulk RNA-seq,Homo_723,AD.vs.MCI; bulk RNA-seq,Homo_723,MCI.vs.control; bulk RNA-seq,Homo_633,AD.vs.control; bulk RNA-seq,Homo_714,AD.vs.control; bulk RNA-seq,Homo_714,AD.vs.MCI	6
CCDC183	bulk RNA-seq,Homo_723,AD.vs.control; bulk RNA-seq,Homo_723,AD.vs.MCI; bulk RNA-seq,Homo_633,AD.vs.control; bulk RNA-seq,Homo_633,AD.vs.MCI; bulk RNA-seq,Homo_714,AD.vs.control; bulk RNA-seq,Homo_714,AD.vs.MCI	6
CCDC183-AS1	scRNA-seq,SRP215507,CD8+ T cell_1-MCI.vs.control; scRNA-seq,SRP330776,Naive CD8+ T cell_1-AD.vs.control	2
CCDC184	bulk RNA-seq,Homo_723,MCI.vs.control; bulk RNA-seq,Homo_633,MCI.vs.control; bulk RNA-seq,Homo_714,MCI.vs.control; scRNA-seq,SRP215507,CD8+ T cell_3-AD.vs.control; scRNA-seq,SRP215507,CD8+ T cell_1-AD.vs.control; scRNA-seq,SRP215507,CD8+ T cell_3-AD.vs.MCI; scRNA-seq,SRP215507,CD8+ T cell_1-AD.vs.MCI; scRNA-seq,SRP215507,CD8+ T cell_5-MCI.vs.control	8
CCDC185	bulk RNA-seq,Homo_723,AD.vs.control; bulk RNA-seq,Homo_723,MCI.vs.control; bulk RNA-seq,Homo_633,AD.vs.control; bulk RNA-seq,Homo_633,MCI.vs.control; bulk RNA-seq,Homo_714,MCI.vs.control	5
CCDC186	scRNA-seq,SRP215507,CD8+ T cell_7-AD.vs.MCI; scRNA-seq,SRP330776,Monocyte_1-AD.vs.control; scRNA-seq,SRP330776,B cell_3-AD.vs.control	3

CCDC187	bulk RNA-seq,Homo_723,AD.vs.control; bulk RNA-seq,Homo_723,MCI.vs.control; bulk RNA-seq,Homo_633,AD.vs.MCI; bulk RNA-seq,Homo_633,MCI.vs.control; bulk RNA-seq,Homo_714,MCI.vs.control	5
CCDC188	bulk RNA-seq,Homo_723,AD.vs.control; bulk RNA-seq,Homo_723,AD.vs.MCI; bulk RNA-seq,ROSMAP,AD.vs.control	3
CCDC189	scRNA-seq,SRP215507,CD8+ T cell_1-MCI.vs.control; scRNA-seq,SRP215507,CD8+ T cell_3-MCI.vs.control; scRNA-seq,SRP330776,Naive CD8+ T cell_1-AD.vs.control; scRNA-seq,SRP330776,Memory T cell_1-AD.vs.control; scRNA-seq,SRP330776,B cell_2-AD.vs.control	5
CCDC18-AS1	scRNA-seq,SRP215507,CD8+ T cell_4-AD.vs.MCI; scRNA-seq,SRP215507,CD8+ T cell_7-MCI.vs.control	2
CCDC190	bulk RNA-seq,Homo_714,MCI.vs.control; bulk RNA-seq,ROSMAP,AD.vs.control; bulk RNA-seq,ROSMAP,MCI.vs.control	3
CCDC191	bulk RNA-seq,Homo_723,AD.vs.MCI; bulk RNA-seq,Homo_723,MCI.vs.control; bulk RNA-seq,Homo_633,MCI.vs.control; bulk RNA-seq,Homo_714,AD.vs.control; bulk RNA-seq,Homo_714,AD.vs.MCI; bulk RNA-seq,Homo_714,MCI.vs.control; scRNA-seq,SRP215507,CD8+ T cell_1-MCI.vs.control; scRNA-seq,SRP215507,CD8+ T cell_4-MCI.vs.control; scRNA-seq,SRP215507,CD8+ T cell_2-MCI.vs.control; scRNA-seq,SRP215507,CD8+ T cell_3-MCI.vs.control; scRNA-seq,SRP309935,B cell_1-AD.vs.control; scRNA-seq,SRP330776,Memory T cell_2-AD.vs.control; scRNA-seq,SRP330776,Natural killer cell_1-AD.vs.control; scRNA-seq,SRP330776,Memory T cell_1-AD.vs.control; scRNA-seq,SRP330776,B cell_2-	16
CCDC192	bulk RNA-seq,Homo_723,AD.vs.control; bulk RNA-seq,Homo_723,AD.vs.MCI; bulk RNA-seq,Homo_633,AD.vs.control; bulk RNA-	4
CCDC194	bulk RNA-seq,Homo_723,AD.vs.MCI; bulk RNA-seq,Homo_633,AD.vs.control; bulk RNA-seq,Homo_633,AD.vs.MCI; bulk RNA-seq,ROSMAP,AD.vs.control; bulk RNA-seq,ROSMAP,MCI.vs.control	5
CCDC196	bulk RNA-seq,Homo_723,MCI.vs.control; bulk RNA-seq,Homo_714,MCI.vs.control	2
CCDC197	bulk RNA-seq,ROSMAP,AD.vs.control	1
CCDC198	bulk RNA-seq,Homo_723,AD.vs.control; bulk RNA-seq,Homo_723,AD.vs.MCI; bulk RNA-seq,Homo_633,AD.vs.control; bulk RNA-	4
CCDC200	bulk RNA-seq,Homo_723,AD.vs.control; bulk RNA-seq,Homo_723,AD.vs.MCI; bulk RNA-seq,Homo_723,MCI.vs.control; bulk RNA-seq,Homo_633,AD.vs.control; bulk RNA-seq,Homo_633,AD.vs.MCI; bulk RNA-seq,Homo_633,MCI.vs.control; bulk RNA-seq,Homo_714,AD.vs.control; bulk RNA-seq,Homo_714,AD.vs.MCI; bulk RNA-seq,Homo_714,MCI.vs.control	9
CCDC22	bulk RNA-seq,Homo_723,AD.vs.control; bulk RNA-seq,Homo_723,AD.vs.MCI; bulk RNA-seq,Homo_633,AD.vs.control; bulk RNA-seq,Homo_633,AD.vs.MCI; bulk RNA-seq,Homo_714,AD.vs.control; bulk RNA-seq,Homo_714,AD.vs.MCI; bulk RNA-seq,SRP310421,AD.vs.control; scRNA-seq,SRP330776,Memory T cell_1-AD.vs.control; scRNA-seq,SRP330776,CD8+ T cell_3-AD.vs.control; scRNA-seq,SRP330776,B cell_3-	11
CCDC24	scRNA-seq,SRP330776,CD8+ T cell_2-AD.vs.control; scRNA-seq,SRP330776,B cell_1-AD.vs.control; scRNA-seq,SRP330776,Naive CD8+ T cell_1-AD.vs.control; scRNA-seq,SRP330776,CD8+ T cell_3-AD.vs.control	4
CCDC25	bulk RNA-seq,Homo_723,AD.vs.control; bulk RNA-seq,Homo_723,AD.vs.MCI; bulk RNA-seq,Homo_633,AD.vs.control; bulk RNA-seq,Homo_633,AD.vs.MCI; scRNA-seq,SRP330776,Natural killer T cell-AD.vs.control; scRNA-seq,SRP330776,T cell-AD.vs.control; scRNA-seq,SRP330776,CD8+ T cell_3-AD.vs.control; scRNA-seq,SRP330776,Megakaryocyte_1-AD.vs.control; scRNA-seq,SRP330776,Natural killer cell_2-	9
CCDC27	bulk RNA-seq,Homo_723,AD.vs.control; bulk RNA-seq,Homo_633,MCI.vs.control; bulk RNA-seq,Homo_714,MCI.vs.control	3
CCDC28A	bulk RNA-seq,Homo_723,AD.vs.control; bulk RNA-seq,Homo_723,AD.vs.MCI; bulk RNA-seq,Homo_633,AD.vs.control; bulk RNA-seq,Homo_633,AD.vs.MCI; bulk RNA-seq,Homo_714,AD.vs.control; bulk RNA-seq,Homo_714,AD.vs.MCI; scRNA-seq,SRP215507,CD8+ T cell_4-MCI.vs.control; scRNA-seq,SRP330776,γδ T cell-AD.vs.control; scRNA-seq,SRP330776,Natural killer cell_1-AD.vs.control; scRNA-seq,SRP330776,Naive CD8+ T cell_3-AD.vs.control; scRNA-seq,SRP330776,B cell_3-AD.vs.control	11
CCDC28B	scRNA-seq,SRP215507,CD8+ T cell_4-MCI.vs.control; scRNA-seq,SRP215507,CD8+ T cell_6-MCI.vs.control; scRNA-seq,SRP309935,Natural killer cell_2-AD.vs.control; scRNA-seq,SRP330776,γδ T cell-AD.vs.control; scRNA-seq,SRP330776,CD8+ T cell_2-AD.vs.control; scRNA-seq,SRP330776,B cell_1-AD.vs.control; scRNA-seq,SRP330776,Natural killer T cell-AD.vs.control; scRNA-seq,SRP330776,B cell_2-AD.vs.control; scRNA-	9
CCDC3	bulk RNA-seq,Homo_723,AD.vs.control; bulk RNA-seq,Homo_723,AD.vs.MCI; bulk RNA-seq,Homo_633,AD.vs.control; bulk RNA-seq,Homo_633,AD.vs.MCI; bulk RNA-seq,Homo_714,AD.vs.control; bulk RNA-seq,Homo_714,AD.vs.MCI; bulk RNA-seq,Homo_714,MCI.vs.control; bulk RNA-seq,ROSMAP,AD.vs.control; bulk RNA-seq,ROSMAP,MCI.vs.control; scRNA-seq,SRP215507,CD8+ T cell_2-AD.vs.control	10

CCDC30	bulk RNA-seq,Homo_714,AD.vs.control; bulk RNA-seq,Homo_714,AD.vs.MCI; scRNA-seq,SRP215507,CD8+ T cell_3-AD.vs.control; scRNA-seq,SRP215507,CD8+ T cell_1-AD.vs.control; scRNA-seq,SRP215507,CD8+ T cell_4-AD.vs.control; scRNA-seq,SRP215507,CD8+ T cell_3-AD.vs.MCI; scRNA-seq,SRP215507,CD8+ T cell_1-MCI.vs.control; scRNA-seq,SRP215507,CD8+ T cell_4-MCI.vs.control; scRNA-seq,SRP330776,Memory T cell_2-AD.vs.control; scRNA-seq,SRP330776,CD8+ T cell_2-AD.vs.control; scRNA-seq,SRP330776,B cell_1-AD.vs.control; scRNA-seq,SRP330776,Naive CD8+ T cell_1-AD.vs.control; scRNA-seq,SRP330776,Memory T cell_1-AD.vs.control; scRNA-seq,SRP330776,Natural killer T cell-AD.vs.control; scRNA-seq,SRP330776,Naive CD8+ T cell_3-AD.vs.control; scRNA-seq,SRP330776,B cell_2-AD.vs.control; scRNA-seq,SRP330776,CD8+ T cell_3-	17
CCDC32	scRNA-seq,SRP215507,CD8+ T cell_7-AD.vs.control; scRNA-seq,SRP215507,CD8+ T cell_7-AD.vs.MCI; scRNA-seq,SRP330776,γδ T cell-	3
CCDC33	bulk RNA-seq,Homo_723,AD.vs.control; bulk RNA-seq,Homo_723,AD.vs.MCI; bulk RNA-seq,Homo_633,AD.vs.control; bulk RNA-	4
CCDC34	bulk RNA-seq,Homo_723,AD.vs.control; bulk RNA-seq,Homo_723,AD.vs.MCI; bulk RNA-seq,Homo_723,MCI.vs.control; bulk RNA-seq,Homo_633,AD.vs.control; bulk RNA-seq,Homo_633,AD.vs.MCI; bulk RNA-seq,Homo_633,MCI.vs.control; bulk RNA-seq,Homo_714,AD.vs.control; bulk RNA-seq,Homo_714,AD.vs.MCI; bulk RNA-seq,Homo_714,MCI.vs.control; scRNA-seq,SRP215507,CD8+ T cell_3-AD.vs.MCI; scRNA-seq,SRP215507,CD8+ T cell_1-MCI.vs.control; scRNA-seq,SRP330776,CD8+ T cell_1-AD.vs.control; scRNA-seq,SRP330776,γδ T cell-AD.vs.control; scRNA-seq,SRP330776,Naive CD8+ T cell_1-AD.vs.control; scRNA-seq,SRP330776,Naive CD8+ T cell_3-AD.vs.control; scRNA-seq,SRP330776,B	18
Ccdc34	bulk RNA-seq,SRP325058,AD.vs.control	1
CCDC38	bulk RNA-seq,Homo_723,AD.vs.control; bulk RNA-seq,Homo_723,AD.vs.MCI; bulk RNA-seq,ROSMAP,AD.vs.control	3
CCDC39	bulk RNA-seq,Homo_723,AD.vs.MCI; bulk RNA-seq,Homo_723,MCI.vs.control; bulk RNA-seq,Homo_633,AD.vs.control; bulk RNA-seq,Homo_633,AD.vs.MCI; bulk RNA-seq,Homo_633,MCI.vs.control; bulk RNA-seq,Homo_714,AD.vs.control; bulk RNA-seq,Homo_714,AD.vs.MCI;	8
CCDC40	bulk RNA-seq,Homo_723,AD.vs.control; bulk RNA-seq,Homo_723,AD.vs.MCI; bulk RNA-seq,Homo_633,AD.vs.control; bulk RNA-seq,Homo_633,MCI.vs.control; bulk RNA-seq,Homo_714,MCI.vs.control	5
CCDC42	bulk RNA-seq,Homo_723,AD.vs.MCI; bulk RNA-seq,Homo_723,MCI.vs.control; bulk RNA-seq,Homo_633,AD.vs.MCI; bulk RNA-seq,Homo_633,MCI.vs.control; bulk RNA-seq,Homo_714,MCI.vs.control; scRNA-seq,SRP215507,CD8+ T cell_2-AD.vs.control; scRNA-	7
CCDC43	bulk RNA-seq,Homo_723,AD.vs.MCI; bulk RNA-seq,Homo_723,MCI.vs.control; bulk RNA-seq,Homo_633,AD.vs.MCI; bulk RNA-seq,Homo_633,MCI.vs.control; bulk RNA-seq,Homo_714,AD.vs.control; bulk RNA-seq,Homo_714,AD.vs.MCI; scRNA-seq,SRP215507,CD8+ T cell_5-AD.vs.MCI; scRNA-seq,SRP215507,CD8+ T cell_4-MCI.vs.control; scRNA-seq,SRP215507,CD8+ T cell_7-MCI.vs.control; scRNA-	10
CCDC47	scRNA-seq,SRP215507,CD8+ T cell_7-AD.vs.control; scRNA-seq,SRP215507,CD8+ T cell_6-AD.vs.control; scRNA-seq,SRP215507,CD8+ T cell_1-AD.vs.MCI; scRNA-seq,SRP215507,CD8+ T cell_5-AD.vs.MCI; scRNA-seq,SRP215507,CD8+ T cell_1-MCI.vs.control; scRNA-seq,SRP215507,CD8+ T cell_6-MCI.vs.control; scRNA-seq,SRP330776,Memory T cell_2-AD.vs.control; scRNA-seq,SRP330776,B cell_1-AD.vs.control; scRNA-seq,SRP330776,Naive CD8+ T cell_1-AD.vs.control; scRNA-seq,SRP330776,Naive CD8+ T cell_3-AD.vs.control; scRNA-seq,SRP330776,B cell_2-	11
CCDC50	bulk RNA-seq,Homo_723,AD.vs.control; bulk RNA-seq,Homo_723,AD.vs.MCI; bulk RNA-seq,Homo_723,MCI.vs.control; bulk RNA-seq,Homo_633,AD.vs.control; bulk RNA-seq,Homo_633,AD.vs.MCI; bulk RNA-seq,Homo_633,MCI.vs.control; bulk RNA-seq,Homo_714,AD.vs.control; bulk RNA-seq,Homo_714,AD.vs.MCI; bulk RNA-seq,Homo_714,MCI.vs.control; scRNA-seq,SRP215507,CD8+ T cell_4-AD.vs.control; scRNA-seq,SRP215507,CD8+ T cell_2-AD.vs.MCI; scRNA-seq,SRP215507,CD8+ T cell_1-AD.vs.MCI; scRNA-seq,SRP215507,CD8+ T cell_5-AD.vs.MCI; scRNA-seq,SRP215507,CD8+ T cell_1-MCI.vs.control; scRNA-seq,SRP215507,CD8+ T cell_4-MCI.vs.control; scRNA-seq,SRP215507,CD8+ T cell_2-MCI.vs.control; scRNA-seq,SRP330776,CD8+ T cell_1-AD.vs.control; scRNA-seq,SRP330776,γδ T cell-AD.vs.control; scRNA-seq,SRP330776,Memory T cell_2-AD.vs.control; scRNA-seq,SRP330776,CD8+ T cell_2-AD.vs.control; scRNA-seq,SRP330776,B cell_1-AD.vs.control; scRNA-seq,SRP330776,Naive CD8+ T cell_1-AD.vs.control; scRNA-seq,SRP330776,Natural killer cell_1-AD.vs.control; scRNA-seq,SRP330776,Memory T cell_1-AD.vs.control; scRNA-seq,SRP330776,Natural killer T cell-AD.vs.control; scRNA-seq,SRP330776,B cell_2-AD.vs.control; scRNA-seq,SRP330776,T cell-AD.vs.control; scRNA-seq,SRP330776,Monocyte_2-AD.vs.control; scRNA-seq,SRP330776,B cell_3-AD.vs.control; scRNA-seq,SRP330776,Natural killer cell_2-AD.vs.control; scRNA-seq,SRP330776,B cell_4-AD.vs.control	31

CCDC51	bulk RNA-seq,Homo_723,AD.vs.control; bulk RNA-seq,Homo_723,AD.vs.MCI; bulk RNA-seq,Homo_723,MCI.vs.control; bulk RNA-seq,Homo_633,AD.vs.control; bulk RNA-seq,Homo_633,AD.vs.MCI; bulk RNA-seq,Homo_633,MCI.vs.control; bulk RNA-seq,Homo_714,AD.vs.control; bulk RNA-seq,Homo_714,AD.vs.MCI; bulk RNA-seq,Homo_714,MCI.vs.control; scRNA-seq,SRP215507,CD8+ T cell_3-AD.vs.control; scRNA-seq,SRP215507,CD8+ T cell_3-AD.vs.MCI; scRNA-seq,SRP215507,CD8+ T cell_1-AD.vs.MCI; scRNA-seq,SRP215507,CD8+ T cell_1-MCI.vs.control; scRNA-seq,SRP330776,CD8+ T cell_1-AD.vs.control; scRNA-seq,SRP330776,γδ T cell-AD.vs.control; scRNA-seq,SRP330776,CD8+ T cell_2-AD.vs.control; scRNA-seq,SRP330776,B cell_1-AD.vs.control; scRNA-seq,SRP330776,Naive CD8+ T cell_1-AD.vs.control; scRNA-seq,SRP330776,Memory T cell_1-AD.vs.control; scRNA-seq,SRP330776,B cell_2-AD.vs.control; scRNA-seq,SRP330776,CD8+ T cell_3-AD.vs.control	21
CCDC54	bulk RNA-seq,Homo_714,MCI.vs.control	1
CCDC57	bulk RNA-seq,Homo_633,AD.vs.control; bulk RNA-seq,Homo_633,AD.vs.MCI; scRNA-seq,SRP215507,CD8+ T cell_7-AD.vs.control; scRNA-seq,SRP330776,B cell_1-AD.vs.control; scRNA-seq,SRP330776,T cell-AD.vs.control; scRNA-seq,SRP330776,CD8+ T cell_3-AD.vs.control; scRNA-seq,SRP330776,Monocyte_1-AD.vs.control; scRNA-seq,SRP330776,Monocyte_2-AD.vs.control; scRNA-seq,SRP330776,B cell_3-AD.vs.control; scRNA-	10
CCDC58	scRNA-seq,SRP215507,CD8+ T cell_4-AD.vs.MCI; scRNA-seq,SRP309935,Natural killer cell_2-AD.vs.control; scRNA-seq,SRP309935,Naive CD8+ T cell_2-AD.vs.control; scRNA-seq,SRP309935,CD8+ T cell_1-AD.vs.control; scRNA-seq,SRP309935,B cell_1-AD.vs.control; scRNA-seq,SRP309935,CD8+ T cell_2-AD.vs.control; scRNA-seq,SRP330776,γδ T cell-AD.vs.control; scRNA-seq,SRP330776,CD8+ T cell_2-AD.vs.control;	9
CCDC59	bulk RNA-seq,Homo_714,AD.vs.control; bulk RNA-seq,Homo_714,AD.vs.MCI; scRNA-seq,SRP215507,CD8+ T cell_7-AD.vs.control; scRNA-seq,SRP215507,CD8+ T cell_10-AD.vs.control; scRNA-seq,SRP215507,CD8+ T cell_9-AD.vs.control; scRNA-seq,SRP215507,CD8+ T cell_7-AD.vs.MCI; scRNA-seq,SRP215507,CD8+ T cell_3-MCI.vs.control; scRNA-seq,SRP215507,CD8+ T cell_7-MCI.vs.control	8
CCDC6	bulk RNA-seq,Homo_723,AD.vs.control; bulk RNA-seq,Homo_723,AD.vs.MCI; bulk RNA-seq,Homo_723,MCI.vs.control; bulk RNA-seq,Homo_633,AD.vs.control; bulk RNA-seq,Homo_633,AD.vs.MCI; bulk RNA-seq,Homo_633,MCI.vs.control; bulk RNA-seq,Homo_714,AD.vs.control; bulk RNA-seq,Homo_714,AD.vs.MCI; bulk RNA-seq,Homo_714,MCI.vs.control; scRNA-seq,SRP215507,CD8+ T cell_4-AD.vs.MCI; scRNA-seq,SRP215507,CD8+ T cell_6-AD.vs.MCI; scRNA-seq,SRP215507,CD8+ T cell_4-MCI.vs.control; scRNA-seq,SRP309935,B cell_1-AD.vs.control; scRNA-seq,SRP330776,CD8+ T cell_1-AD.vs.control; scRNA-seq,SRP330776,γδ T cell-AD.vs.control; scRNA-seq,SRP330776,Memory T cell_2-AD.vs.control; scRNA-seq,SRP330776,B cell_1-AD.vs.control; scRNA-seq,SRP330776,Naive CD8+ T cell_1-AD.vs.control; scRNA-seq,SRP330776,Naive CD8+ T cell_3-AD.vs.control; scRNA-seq,SRP330776,B cell_2-AD.vs.control; scRNA-seq,SRP330776,CD8+ T cell_3- [illegible]	22
CCDC60	bulk RNA-seq,Homo_723,AD.vs.control; bulk RNA-seq,Homo_633,MCI.vs.control; bulk RNA-seq,Homo_714,MCI.vs.control	3
CCDC61	bulk RNA-seq,Homo_723,AD.vs.MCI; bulk RNA-seq,Homo_723,MCI.vs.control; bulk RNA-seq,Homo_633,AD.vs.control; bulk RNA-seq,Homo_633,AD.vs.MCI; bulk RNA-seq,Homo_633,MCI.vs.control; bulk RNA-seq,Homo_714,AD.vs.control; bulk RNA-seq,Homo_714,AD.vs.MCI; bulk RNA-seq,Homo_714,MCI.vs.control; scRNA-seq,SRP215507,CD8+ T cell_4-AD.vs.MCI; scRNA-seq,SRP309935,Monocyte_1-AD.vs.control; scRNA-seq,SRP309935,Monocyte_2-AD.vs.control; scRNA-seq,SRP309935,Naive CD8+ T cell_2-AD.vs.control; scRNA-seq,SRP330776,CD8+ T cell_1-AD.vs.control; scRNA-seq,SRP330776,γδ T cell-AD.vs.control; scRNA-seq,SRP330776,Memory T cell_2-AD.vs.control; scRNA-seq,SRP330776,CD8+ T cell_2-AD.vs.control; scRNA-seq,SRP330776,B cell_1-AD.vs.control; scRNA-seq,SRP330776,Naive CD8+ T cell_1-AD.vs.control; scRNA-seq,SRP330776,Natural killer cell_1-AD.vs.control; scRNA-seq,SRP330776,Memory T cell_1-AD.vs.control; scRNA-seq,SRP330776,B cell_2- [illegible]	23
CCDC62	bulk RNA-seq,Homo_723,AD.vs.control; bulk RNA-seq,Homo_723,AD.vs.MCI; bulk RNA-seq,Homo_723,MCI.vs.control; bulk RNA-seq,Homo_633,AD.vs.control; bulk RNA-seq,Homo_633,AD.vs.MCI; bulk RNA-seq,Homo_633,MCI.vs.control; bulk RNA-seq,Homo_714,AD.vs.control; bulk RNA-seq,Homo_714,AD.vs.MCI; bulk RNA-seq,ROSMAP,AD.vs.control; bulk RNA-seq,ROSMAP,MCI.vs.control; scRNA-seq,SRP215507,CD8+ T cell_3-AD.vs.MCI; scRNA-seq,SRP215507,CD8+ T cell_3-MCI.vs.control	12
CCDC65	bulk RNA-seq,Homo_723,AD.vs.control; bulk RNA-seq,Homo_723,AD.vs.MCI; bulk RNA-seq,Homo_633,AD.vs.control; bulk RNA-seq,Homo_633,AD.vs.MCI; bulk RNA-seq,Homo_714,AD.vs.control; bulk RNA-seq,Homo_714,AD.vs.MCI; bulk RNA-seq,Homo_714,MCI.vs.control; bulk RNA-seq,ROSMAP,MCI.vs.control; scRNA-seq,SRP215507,CD8+ T cell_5-AD.vs.MCI; scRNA-seq,SRP215507,CD8+ T cell_1-MCI.vs.control; scRNA-seq,SRP215507,CD8+ T cell_5-MCI.vs.control; scRNA-seq,SRP330776,CD8+ T cell_1-AD.vs.control; scRNA-seq,SRP330776,γδ T cell-AD.vs.control; scRNA-seq,SRP330776,CD8+ T cell_2-AD.vs.control; scRNA-seq,SRP330776,B cell_1-AD.vs.control; scRNA-seq,SRP330776,Naive CD8+ T cell_1-AD.vs.control; scRNA-seq,SRP330776,Natural killer cell_1-AD.vs.control; scRNA-seq,SRP330776,Memory T cell_1-AD.vs.control; [illegible]	19

CCDC66	scRNA-seq,SRP215507,CD8+ T cell_5-AD.vs.control; scRNA-seq,SRP215507,CD8+ T cell_5-AD.vs.MCI; scRNA-seq,SRP215507,CD8+ T cell_4-MCI.vs.control; scRNA-seq,SRP330776,CD8+ T cell_3-AD.vs.control	4
CCDC68	bulk RNA-seq,Homo_723,AD.vs.control; bulk RNA-seq,Homo_723,AD.vs.MCI; bulk RNA-seq,Homo_723,MCI.vs.control; bulk RNA-seq,Homo_633,AD.vs.control; bulk RNA-seq,Homo_633,AD.vs.MCI; bulk RNA-seq,Homo_633,MCI.vs.control	6
CCDC69	scRNA-seq,SRP215507,CD8+ T cell_7-AD.vs.control; scRNA-seq,SRP215507,CD8+ T cell_10-AD.vs.control; scRNA-seq,SRP215507,CD8+ T cell_6-MCI.vs.control; scRNA-seq,SRP330776,CD8+ T cell_1-AD.vs.control; scRNA-seq,SRP330776,γδ T cell-AD.vs.control; scRNA-seq,SRP330776,Memory T cell_2-AD.vs.control; scRNA-seq,SRP330776,CD8+ T cell_2-AD.vs.control; scRNA-seq,SRP330776,B cell_1-AD.vs.control; scRNA-seq,SRP330776,Naive CD8+ T cell_1-AD.vs.control; scRNA-seq,SRP330776,Natural killer cell_1-AD.vs.control; scRNA-seq,SRP330776,Memory T cell_1-AD.vs.control; scRNA-seq,SRP330776,Natural killer T cell-AD.vs.control; scRNA-seq,SRP330776,Naive CD8+ T cell_3-AD.vs.control; scRNA-seq,SRP330776,B cell_2-AD.vs.control; scRNA-seq,SRP330776,T cell-AD.vs.control; scRNA-seq,SRP330776,Naive CD8+ T cell_2-AD.vs.control; scRNA-seq,SRP330776,CD8+ T cell_3-AD.vs.control; scRNA-seq,SRP330776,Monocyte_2-AD.vs.control; scRNA-seq,SRP330776,B cell_3- [illegible]	20
CCDC7	bulk RNA-seq,Homo_723,AD.vs.control; bulk RNA-seq,Homo_723,MCI.vs.control; bulk RNA-seq,Homo_633,AD.vs.MCI; bulk RNA-seq,Homo_633,MCI.vs.control; scRNA-seq,SRP215507,CD8+ T cell_4-AD.vs.control; scRNA-seq,SRP215507,CD8+ T cell_4-AD.vs.MCI; scRNA-seq,SRP215507,CD8+ T cell_6-MCI.vs.control; scRNA-seq,SRP330776,CD8+ T cell_1-AD.vs.control; scRNA-seq,SRP330776,γδ T cell-AD.vs.control; scRNA-seq,SRP330776,Memory T cell_2-AD.vs.control; scRNA-seq,SRP330776,CD8+ T cell_2-AD.vs.control; scRNA-seq,SRP330776,B cell_1-AD.vs.control; scRNA-seq,SRP330776,Naive CD8+ T cell_1-AD.vs.control; scRNA-seq,SRP330776,Memory T cell_1-AD.vs.control; scRNA-seq,SRP330776,Natural killer T cell-AD.vs.control; scRNA-seq,SRP330776,B cell_2-AD.vs.control; scRNA-seq,SRP330776,Naive CD8+ T cell_2- [illegible]	18
CCDC71	scRNA-seq,SRP215507,CD8+ T cell_6-AD.vs.MCI; scRNA-seq,SRP330776,γδ T cell-AD.vs.control; scRNA-seq,SRP330776,B cell_1-AD.vs.control; scRNA-seq,SRP330776,Natural killer cell_1-AD.vs.control; scRNA-seq,SRP330776,Memory T cell_1-AD.vs.control; scRNA-seq,SRP330776,Natural killer T cell-AD.vs.control; scRNA-seq,SRP330776,Natural killer cell_2-AD.vs.control	7
CCDC71L	scRNA-seq,SRP215507,CD8+ T cell_2-AD.vs.control; scRNA-seq,SRP215507,CD8+ T cell_5-AD.vs.MCI; scRNA-seq,SRP215507,CD8+ T cell_5-MCI.vs.control; scRNA-seq,SRP309935,Natural killer cell_2-AD.vs.control; scRNA-seq,SRP309935,Monocyte_1-AD.vs.control; scRNA-seq,SRP309935,Naive CD8+ T cell_1-AD.vs.control; scRNA-seq,SRP309935,Natural killer cell_1-AD.vs.control; scRNA-seq,SRP309935,Monocyte_2-AD.vs.control; scRNA-seq,SRP309935,Naive CD8+ T cell_2-AD.vs.control; scRNA-seq,SRP309935,B cell_1-AD.vs.control; scRNA-seq,SRP309935,CD8+ T cell_2-AD.vs.control; scRNA-seq,SRP309935,Naive CD8+ T cell_3-AD.vs.control; scRNA-seq,SRP330776,CD8+ T cell_1-AD.vs.control; scRNA-seq,SRP330776,γδ T cell-AD.vs.control; scRNA-seq,SRP330776,Memory T cell_2-AD.vs.control; scRNA-seq,SRP330776,CD8+ T cell_2-AD.vs.control; scRNA-seq,SRP330776,B cell_1-AD.vs.control; scRNA-seq,SRP330776,Naive CD8+ T cell_1-AD.vs.control; scRNA-seq,SRP330776,Memory T cell_1-AD.vs.control; scRNA-seq,SRP330776,Natural killer T cell-AD.vs.control; scRNA-seq,SRP330776,B cell_2- [illegible]	22
CCDC73	bulk RNA-seq,Homo_723,AD.vs.MCI; bulk RNA-seq,Homo_633,AD.vs.MCI; scRNA-seq,SRP330776,CD8+ T cell_1-AD.vs.control; scRNA-seq,SRP330776,B cell_1-AD.vs.control	4
CCDC74A	bulk RNA-seq,Homo_723,AD.vs.control; bulk RNA-seq,Homo_723,AD.vs.MCI; bulk RNA-seq,Homo_633,AD.vs.control; bulk RNA-seq,Homo_633,AD.vs.MCI; bulk RNA-seq,ROSMAP,AD.vs.control; scRNA-seq,SRP215507,CD8+ T cell_1-AD.vs.control	6
CCDC74B	bulk RNA-seq,ROSMAP,AD.vs.control; bulk RNA-seq,ROSMAP,AD.vs.MCI	2
CCDC77	bulk RNA-seq,Homo_714,AD.vs.control; bulk RNA-seq,Homo_714,AD.vs.MCI; scRNA-seq,SRP215507,CD8+ T cell_3-AD.vs.control; scRNA-seq,SRP215507,CD8+ T cell_7-MCI.vs.control; scRNA-seq,SRP330776,CD8+ T cell_1-AD.vs.control; scRNA-seq,SRP330776,Naive CD8+ T cell_3-AD.vs.control; scRNA-seq,SRP330776,B cell_2-AD.vs.control	7
CCDC78	bulk RNA-seq,Homo_723,MCI.vs.control; bulk RNA-seq,Homo_633,MCI.vs.control; bulk RNA-seq,Homo_714,MCI.vs.control; scRNA-seq,SRP215507,CD8+ T cell_4-AD.vs.control; scRNA-seq,SRP215507,CD8+ T cell_5-AD.vs.control; scRNA-seq,SRP215507,CD8+ T cell_2-AD.vs.MCI; scRNA-seq,SRP215507,CD8+ T cell_1-MCI.vs.control; scRNA-seq,SRP215507,CD8+ T cell_4-MCI.vs.control; scRNA-seq,SRP215507,CD8+ T cell_2-MCI.vs.control; scRNA-seq,SRP309935,B cell_1-AD.vs.control; scRNA-seq,SRP330776,Memory T cell_1-AD.vs.control	11
CCDC8	bulk RNA-seq,Homo_714,MCI.vs.control	1

CCDC80	bulk RNA-seq,Homo_723,AD.vs.control; bulk RNA-seq,Homo_723,AD.vs.MCI; bulk RNA-seq,Homo_723,MCI.vs.control; bulk RNA-seq,Homo_633,AD.vs.control; bulk RNA-seq,Homo_633,AD.vs.MCI; bulk RNA-seq,Homo_633,MCI.vs.control; bulk RNA-seq,Homo_714,AD.vs.control; bulk RNA-seq,Homo_714,AD.vs.MCI; bulk RNA-seq,ROSMAP,AD.vs.control; bulk RNA-seq,ROSMAP,MCI.vs.control	10
CCDC81	bulk RNA-seq,Homo_723,AD.vs.control; bulk RNA-seq,Homo_723,AD.vs.MCI; bulk RNA-seq,Homo_723,MCI.vs.control; bulk RNA-seq,Homo_633,AD.vs.MCI; bulk RNA-seq,Homo_714,AD.vs.control; bulk RNA-seq,Homo_714,AD.vs.MCI	6
CCDC82	bulk RNA-seq,Homo_723,AD.vs.control; bulk RNA-seq,Homo_723,AD.vs.MCI; bulk RNA-seq,Homo_633,AD.vs.control; bulk RNA-seq,Homo_633,AD.vs.MCI; bulk RNA-seq,Homo_714,AD.vs.control; bulk RNA-seq,Homo_714,AD.vs.MCI; scRNA-seq,SRP215507,CD8+ T cell_3-	7
CCDC84	scRNA-seq,SRP309935,Naive CD8+ T cell_1-AD.vs.control; scRNA-seq,SRP330776,CD8+ T cell_2-AD.vs.control; scRNA-seq,SRP330776,B cell_2-AD.vs.control; scRNA-seq,SRP330776,Natural killer cell_2-AD.vs.control	4
CCDC84-DT	scRNA-seq,SRP215507,CD8+ T cell_1-AD.vs.control; scRNA-seq,SRP215507,CD8+ T cell_5-AD.vs.MCI; scRNA-seq,SRP330776,CD8+ T cell_1-AD.vs.control; scRNA-seq,SRP330776,γδ T cell-AD.vs.control; scRNA-seq,SRP330776,Memory T cell_2-AD.vs.control; scRNA-seq,SRP330776,Naive CD8+ T cell_1-AD.vs.control; scRNA-seq,SRP330776,Memory T cell_1-AD.vs.control; scRNA-seq,SRP330776,T cell-AD.vs.control	8
CCDC85A	bulk RNA-seq,Homo_723,AD.vs.control; bulk RNA-seq,Homo_723,AD.vs.MCI; bulk RNA-seq,Homo_723,MCI.vs.control; bulk RNA-seq,Homo_633,AD.vs.control; bulk RNA-seq,Homo_633,AD.vs.MCI; bulk RNA-seq,Homo_633,MCI.vs.control; bulk RNA-seq,SRP223445,AD.vs.control	7
CCDC85B	scRNA-seq,SRP215507,CD8+ T cell_5-AD.vs.control; scRNA-seq,SRP215507,CD8+ T cell_10-AD.vs.control; scRNA-seq,SRP215507,CD8+ T cell_5-MCI.vs.control; scRNA-seq,SRP309935,Erythroid cell-AD.vs.control; scRNA-seq,SRP330776,CD8+ T cell_1-AD.vs.control; scRNA-seq,SRP330776,γδ T cell-AD.vs.control; scRNA-seq,SRP330776,Memory T cell_2-AD.vs.control; scRNA-seq,SRP330776,CD8+ T cell_2-AD.vs.control; scRNA-seq,SRP330776,B cell_1-AD.vs.control; scRNA-seq,SRP330776,Naive CD8+ T cell_1-AD.vs.control; scRNA-seq,SRP330776,Natural killer cell_1-AD.vs.control; scRNA-seq,SRP330776,Memory T cell_1-AD.vs.control; scRNA-seq,SRP330776,Natural killer T cell-AD.vs.control; scRNA-seq,SRP330776,Naive CD8+ T cell_3-AD.vs.control; scRNA-seq,SRP330776,B cell_2-AD.vs.control; scRNA-seq,SRP330776,T cell-AD.vs.control; scRNA-seq,SRP330776,Naive CD8+ T cell_2-AD.vs.control; scRNA-seq,SRP330776,CD8+ T cell_3-AD.vs.control; scRNA-seq,SRP330776,Monocyte_1-AD.vs.control; scRNA-seq,SRP330776,Monocyte_2-AD.vs.control; scRNA-seq,SRP330776,B cell_3-AD.vs.control; scRNA-seq,SRP330776,Regulatory T [illegible]	24
CCDC85C	scRNA-seq,SRP215507,CD8+ T cell_4-AD.vs.control; scRNA-seq,SRP215507,CD8+ T cell_1-AD.vs.MCI; scRNA-seq,SRP330776,CD8+ T cell_1-AD.vs.control; scRNA-seq,SRP330776,CD8+ T cell_2-AD.vs.control; scRNA-seq,SRP330776,B cell_1-AD.vs.control; scRNA-seq,SRP330776,Memory T cell_1-AD.vs.control; scRNA-seq,SRP330776,Natural killer T cell-AD.vs.control; scRNA-seq,SRP330776,B cell_2-AD.vs.control; scRNA-seq,SRP330776,T cell-AD.vs.control; scRNA-seq,SRP330776,CD8+ T cell_3-AD.vs.control; scRNA-seq,SRP330776,Monocyte_1-AD.vs.control; scRNA-seq,SRP330776,Monocyte_2-AD.vs.control; scRNA-seq,SRP330776,B cell_3-AD.vs.control; scRNA-seq,SRP330776,Regulatory T cell-AD.vs.control	14
CCDC86	bulk RNA-seq,Homo_723,AD.vs.control; bulk RNA-seq,Homo_633,AD.vs.control; bulk RNA-seq,Homo_633,MCI.vs.control; scRNA-seq,SRP215507,CD8+ T cell_6-AD.vs.control; scRNA-seq,SRP330776,Natural killer T cell-AD.vs.control; scRNA-seq,SRP330776,Naive CD8+ T cell_3-AD.vs.control; scRNA-seq,SRP330776,B cell_3-AD.vs.control	7
CCDC88A	bulk RNA-seq,Homo_633,MCI.vs.control; scRNA-seq,SRP215507,CD8+ T cell_3-AD.vs.MCI; scRNA-seq,SRP215507,CD8+ T cell_5-AD.vs.MCI; scRNA-seq,SRP215507,CD8+ T cell_5-MCI.vs.control; scRNA-seq,SRP215507,CD8+ T cell_3-MCI.vs.control; scRNA-seq,SRP309935,Natural killer cell_1-AD.vs.control; scRNA-seq,SRP309935,CD8+ T cell_1-AD.vs.control; scRNA-seq,SRP309935,Dendritic cell-AD.vs.control; scRNA-seq,SRP330776,γδ T cell-AD.vs.control; scRNA-seq,SRP330776,CD8+ T cell_2-AD.vs.control; scRNA-seq,SRP330776,B cell_1-AD.vs.control; scRNA-seq,SRP330776,Natural killer cell_1-AD.vs.control; scRNA-seq,SRP330776,CD8+ T cell_3-AD.vs.control	13
CCDC88B	scRNA-seq,SRP215507,CD8+ T cell_6-AD.vs.MCI; scRNA-seq,SRP215507,CD8+ T cell_5-MCI.vs.control; scRNA-seq,SRP309935,Natural killer cell_2-AD.vs.control; scRNA-seq,SRP309935,Naive CD8+ T cell_1-AD.vs.control; scRNA-seq,SRP309935,Monocyte_2-AD.vs.control; scRNA-seq,SRP309935,CD8+ T cell_1-AD.vs.control; scRNA-seq,SRP330776,Naive CD8+ T cell_1-AD.vs.control; scRNA-seq,SRP330776,B cell_2-AD.vs.control; scRNA-seq,SRP330776,Naive CD8+ T cell_2-AD.vs.control; scRNA-seq,SRP330776,B cell_4-AD.vs.control	10

CCDC88C	bulk RNA-seq,Homo_633,AD.vs.control; scRNA-seq,SRP215507,CD8+ T cell_7-AD.vs.MCI; scRNA-seq,SRP309935,CD8+ T cell_1-AD.vs.control; scRNA-seq,SRP330776,CD8+ T cell_1-AD.vs.control; scRNA-seq,SRP330776,γδ T cell-AD.vs.control; scRNA-seq,SRP330776,Memory T cell_2-AD.vs.control; scRNA-seq,SRP330776,CD8+ T cell_2-AD.vs.control; scRNA-seq,SRP330776,B cell_1-AD.vs.control; scRNA-seq,SRP330776,Naive CD8+ T cell_1-AD.vs.control; scRNA-seq,SRP330776,Natural killer cell_1-AD.vs.control; scRNA-seq,SRP330776,Memory T cell_1-AD.vs.control; scRNA-seq,SRP330776,Natural killer T cell-AD.vs.control; scRNA-seq,SRP330776,Naive CD8+ T cell_3-AD.vs.control; scRNA-seq,SRP330776,B cell_2-AD.vs.control; scRNA-seq,SRP330776,T cell-AD.vs.control; scRNA-seq,SRP330776,Naive CD8+ T cell_2-AD.vs.control; scRNA-seq,SRP330776,CD8+ T cell_3-AD.vs.control; scRNA-seq,SRP330776,Monocyte_2-AD.vs.control; scRNA-seq,SRP330776,Natural killer cell_2-AD.vs.control; scRNA- [illegible]	20
CCDC9	bulk RNA-seq,Homo_723,AD.vs.control; bulk RNA-seq,Homo_633,AD.vs.control; bulk RNA-seq,Homo_633,AD.vs.MCI; bulk RNA-seq,Homo_714,AD.vs.control; bulk RNA-seq,Homo_714,AD.vs.MCI; bulk RNA-seq,SRP310421,AD.vs.control; scRNA-seq,SRP215507,CD8+ T cell_1-MCI.vs.control; scRNA-seq,SRP330776,CD8+ T cell_1-AD.vs.control; scRNA-seq,SRP330776,Memory T cell_2-AD.vs.control; scRNA-seq,SRP330776,CD8+ T cell_2-AD.vs.control; scRNA-seq,SRP330776,Naive CD8+ T cell_1-AD.vs.control; scRNA-seq,SRP330776,Memory T cell_1-AD.vs.control; scRNA-seq,SRP330776,Naive CD8+ T cell_3-AD.vs.control; scRNA-seq,SRP330776,B cell_2-AD.vs.control; scRNA-seq,SRP330776,CD8+ T cell_3-AD.vs.control; scRNA-seq,SRP330776,Monocyte_1-AD.vs.control; scRNA-seq,SRP330776,Monocyte_2-AD.vs.control; [illegible]	18
CCDC90B	scRNA-seq,SRP330776,CD8+ T cell_2-AD.vs.control; scRNA-seq,SRP330776,B cell_1-AD.vs.control; scRNA-seq,SRP330776,B cell_2-AD.vs.control; scRNA-seq,SRP330776,T cell-AD.vs.control; scRNA-seq,SRP330776,B cell_3-AD.vs.control; scRNA-seq,SRP330776,Natural killer cell_2-AD.vs.control	6
CCDC91	bulk RNA-seq,Homo_723,AD.vs.control; bulk RNA-seq,Homo_723,AD.vs.MCI; bulk RNA-seq,Homo_633,AD.vs.control; bulk RNA-seq,Homo_633,AD.vs.MCI; bulk RNA-seq,Homo_714,AD.vs.control; bulk RNA-seq,Homo_714,AD.vs.MCI; scRNA-seq,SRP215507,CD8+ T cell_10-AD.vs.control; scRNA-seq,SRP215507,CD8+ T cell_7-MCI.vs.control; scRNA-seq,SRP330776,B cell_2-AD.vs.control; scRNA-seq,SRP330776,T cell-	10
CCDC92	bulk RNA-seq,Homo_723,AD.vs.control; bulk RNA-seq,Homo_633,AD.vs.control; bulk RNA-seq,Homo_633,AD.vs.MCI; scRNA-seq,SRP215507,CD8+ T cell_3-AD.vs.control; scRNA-seq,SRP215507,CD8+ T cell_2-AD.vs.MCI; scRNA-seq,SRP215507,CD8+ T cell_3-AD.vs.MCI; scRNA-seq,SRP215507,CD8+ T cell_4-MCI.vs.control; scRNA-seq,SRP309935,CD8+ T cell_2-AD.vs.control; scRNA-seq,SRP330776,CD8+ T cell_1-AD.vs.control; scRNA-seq,SRP330776,γδ T cell-AD.vs.control; scRNA-seq,SRP330776,Memory T cell_2-AD.vs.control; scRNA-seq,SRP330776,CD8+ T cell_2-AD.vs.control; scRNA-seq,SRP330776,B cell_1-AD.vs.control; scRNA-seq,SRP330776,Naive CD8+ T cell_1-AD.vs.control; scRNA-seq,SRP330776,Natural killer cell_1-AD.vs.control; scRNA-seq,SRP330776,Memory T cell_1-AD.vs.control; scRNA-seq,SRP330776,B cell_2-AD.vs.control; scRNA-seq,SRP330776,T cell-AD.vs.control; scRNA-seq,SRP330776,CD8+ T cell_3-AD.vs.control; scRNA-seq,SRP330776,Natural killer [illegible]	20
CCDC92B	bulk RNA-seq,Homo_723,AD.vs.control; bulk RNA-seq,Homo_723,AD.vs.MCI; bulk RNA-seq,Homo_633,AD.vs.MCI; bulk RNA-seq,Homo_633,MCI.vs.control; bulk RNA-seq,Homo_714,MCI.vs.control	5
Ccdc92b	bulk RNA-seq,SRP325058,AD.vs.control	1
CCDC93	bulk RNA-seq,Homo_723,AD.vs.control; bulk RNA-seq,Homo_723,MCI.vs.control; bulk RNA-seq,Homo_633,AD.vs.control; bulk RNA-seq,Homo_633,AD.vs.MCI; bulk RNA-seq,Homo_633,MCI.vs.control; bulk RNA-seq,Homo_714,AD.vs.control; bulk RNA-seq,Homo_714,AD.vs.MCI; bulk RNA-seq,Homo_714,MCI.vs.control; scRNA-seq,SRP215507,CD8+ T cell_5-AD.vs.control; scRNA-seq,SRP215507,CD8+ T cell_6-AD.vs.control; scRNA-seq,SRP215507,CD8+ T cell_5-AD.vs.MCI; scRNA-seq,SRP309935,Natural killer cell_2-AD.vs.control; scRNA-seq,SRP309935,Naive CD8+ T cell_1-AD.vs.control; scRNA-seq,SRP330776,B cell_3-AD.vs.control; scRNA-seq,SRP330776,B cell_4-AD.vs.control	15
CCDC96	scRNA-seq,SRP215507,CD8+ T cell_2-AD.vs.control; scRNA-seq,SRP309935,Monocyte_2-AD.vs.control; scRNA-seq,SRP330776,CD8+ T cell_1-AD.vs.control; scRNA-seq,SRP330776,B cell_1-AD.vs.control; scRNA-seq,SRP330776,Naive CD8+ T cell_1-AD.vs.control; scRNA-	6
CCDC97	bulk RNA-seq,Homo_723,AD.vs.control; bulk RNA-seq,Homo_723,AD.vs.MCI; bulk RNA-seq,Homo_723,MCI.vs.control; bulk RNA-seq,Homo_633,AD.vs.control; bulk RNA-seq,Homo_633,AD.vs.MCI; bulk RNA-seq,Homo_633,MCI.vs.control; scRNA-seq,SRP215507,CD8+ T cell_4-MCI.vs.control; scRNA-seq,SRP330776,Memory T cell_2-AD.vs.control; scRNA-seq,SRP330776,B cell_3-AD.vs.control	9
CCDC9B	bulk RNA-seq,Homo_723,AD.vs.control; bulk RNA-seq,Homo_633,AD.vs.control	2
CCER1	bulk RNA-seq,Homo_714,MCI.vs.control	1
CCER2	bulk RNA-seq,Homo_723,AD.vs.control; bulk RNA-seq,Homo_723,AD.vs.MCI; bulk RNA-seq,Homo_723,MCI.vs.control	3

CCHCR1	bulk RNA-seq,Homo_723,AD.vs.MCI; bulk RNA-seq,Homo_723,MCI.vs.control; bulk RNA-seq,Homo_633,MCI.vs.control; bulk RNA-seq,Homo_714,AD.vs.control; bulk RNA-seq,Homo_714,AD.vs.MCI; bulk RNA-seq,Homo_714,MCI.vs.control; scRNA-seq,SRP215507,CD8+ T cell_3-AD.vs.MCI; scRNA-seq,SRP215507,CD8+ T cell_3-MCI.vs.control; scRNA-seq,SRP215507,CD8+ T cell_6-MCI.vs.control; scRNA-seq,SRP330776,B	10
CCIN	bulk RNA-seq,Homo_723,AD.vs.control; bulk RNA-seq,Homo_633,AD.vs.control	2
CCK	bulk RNA-seq,Homo_723,AD.vs.control; bulk RNA-seq,Homo_723,AD.vs.MCI	2
CCKAR	bulk RNA-seq,Homo_723,AD.vs.control	1
CCKBR	bulk RNA-seq,Homo_723,AD.vs.control; bulk RNA-seq,Homo_714,MCI.vs.control; bulk RNA-seq,ROSMAP,MCI.vs.control	3
CCL14	bulk RNA-seq,Homo_723,AD.vs.MCI; bulk RNA-seq,Homo_723,MCI.vs.control; bulk RNA-seq,Homo_633,AD.vs.MCI; bulk RNA-	4
CCL16	bulk RNA-seq,Homo_714,MCI.vs.control	1
CCL18	bulk RNA-seq,Homo_723,AD.vs.control; bulk RNA-seq,Homo_723,AD.vs.MCI; bulk RNA-seq,Homo_633,AD.vs.control; bulk RNA-	4
CCL2	bulk RNA-seq,ACOM,AD.vs.control; bulk RNA-seq,Homo_723,AD.vs.control; bulk RNA-seq,Homo_723,AD.vs.MCI; bulk RNA-seq,Homo_723,MCI.vs.control; bulk RNA-seq,Homo_633,AD.vs.control; bulk RNA-seq,Homo_633,AD.vs.MCI; bulk RNA-seq,Homo_633,MCI.vs.control; bulk RNA-seq,Homo_714,AD.vs.control; bulk RNA-seq,Homo_714,AD.vs.MCI	9
CCL20	bulk RNA-seq,Homo_723,AD.vs.control; bulk RNA-seq,Homo_723,AD.vs.MCI; bulk RNA-seq,Homo_723,MCI.vs.control; bulk RNA-seq,Homo_633,AD.vs.control; bulk RNA-seq,Homo_633,MCI.vs.control; bulk RNA-seq,Homo_714,MCI.vs.control; scRNA-seq,SRP215507,CD8+ T cell_5-AD.vs.MCI; scRNA-seq,SRP215507,CD8+ T cell_4-MCI.vs.control; scRNA-seq,SRP330776,CD8+ T cell_1-AD.vs.control; scRNA-	10
CCL21	bulk RNA-seq,Homo_723,AD.vs.MCI; bulk RNA-seq,Homo_723,MCI.vs.control; bulk RNA-seq,Homo_633,AD.vs.MCI; bulk RNA-	4
CCL22	bulk RNA-seq,Homo_723,AD.vs.control; bulk RNA-seq,Homo_723,AD.vs.MCI; bulk RNA-seq,Homo_633,AD.vs.control; bulk RNA-seq,Homo_633,AD.vs.MCI; bulk RNA-seq,Homo_714,MCI.vs.control	5
CCL23	bulk RNA-seq,Homo_723,AD.vs.control; bulk RNA-seq,Homo_723,AD.vs.MCI; bulk RNA-seq,Homo_633,AD.vs.control; bulk RNA-	4
CCL24	bulk RNA-seq,Homo_723,AD.vs.MCI; bulk RNA-seq,Homo_633,AD.vs.MCI; bulk RNA-seq,ROSMAP,AD.vs.control; bulk RNA-seq,ROSMAP,AD.vs.MCI; bulk RNA-seq,ROSMAP,MCI.vs.control	5
CCL25	bulk RNA-seq,Homo_723,AD.vs.control; bulk RNA-seq,Homo_723,AD.vs.MCI	2
CCL27	bulk RNA-seq,Homo_723,AD.vs.control; bulk RNA-seq,Homo_723,AD.vs.MCI	2
CCL28	bulk RNA-seq,Homo_723,AD.vs.control; bulk RNA-seq,Homo_723,AD.vs.MCI; bulk RNA-seq,Homo_633,AD.vs.control; bulk RNA-seq,Homo_633,AD.vs.MCI; bulk RNA-seq,Homo_714,AD.vs.control; bulk RNA-seq,Homo_714,AD.vs.MCI; scRNA-seq,SRP215507,CD8+ T cell_4-AD.vs.control; scRNA-seq,SRP215507,CD8+ T cell_4-AD.vs.MCI; scRNA-seq,SRP215507,CD8+ T cell_3-MCI.vs.control; scRNA-seq,SRP309935,Natural killer cell_2-AD.vs.control; scRNA-seq,SRP330776,Natural killer T cell-AD.vs.control; scRNA-seq,SRP330776,B cell_2-	12
CCL3	scRNA-seq,SRP215507,CD8+ T cell_4-AD.vs.control; scRNA-seq,SRP215507,CD8+ T cell_9-AD.vs.control; scRNA-seq,SRP215507,CD8+ T cell_2-AD.vs.MCI; scRNA-seq,SRP215507,CD8+ T cell_1-AD.vs.MCI; scRNA-seq,SRP215507,CD8+ T cell_4-AD.vs.MCI; scRNA-seq,SRP215507,CD8+ T cell_5-AD.vs.MCI; scRNA-seq,SRP215507,CD8+ T cell_1-MCI.vs.control; scRNA-seq,SRP215507,CD8+ T cell_5-MCI.vs.control; scRNA-seq,SRP215507,CD8+ T cell_4-MCI.vs.control; scRNA-seq,SRP215507,CD8+ T cell_2-MCI.vs.control; scRNA-seq,SRP309935,Naive CD8+ T cell_1-AD.vs.control; scRNA-seq,SRP309935,Natural killer cell_1-AD.vs.control; scRNA-seq,SRP309935,Monocyte_2-AD.vs.control; scRNA-	15
CCL3L1	bulk RNA-seq,Homo_714,AD.vs.control; bulk RNA-seq,Homo_714,AD.vs.MCI; scRNA-seq,SRP215507,CD8+ T cell_4-AD.vs.control; scRNA-seq,SRP215507,CD8+ T cell_2-AD.vs.MCI; scRNA-seq,SRP215507,CD8+ T cell_5-AD.vs.MCI; scRNA-seq,SRP215507,CD8+ T cell_4-MCI.vs.control; scRNA-seq,SRP215507,CD8+ T cell_2-MCI.vs.control; scRNA-seq,SRP309935,Natural killer cell_2-AD.vs.control; scRNA-seq,SRP309935,Monocyte_2-AD.vs.control; scRNA-seq,SRP309935,CD8+ T cell_2-AD.vs.control; scRNA-seq,SRP330776,CD8+ T cell_2-AD.vs.control; scRNA-seq,SRP330776,Natural killer T cell-AD.vs.control; scRNA-seq,SRP330776,Monocyte_2-AD.vs.control; scRNA-seq,SRP330776,B cell_3-AD.vs.control	14
CCL3L3	bulk RNA-seq,Homo_723,AD.vs.MCI; bulk RNA-seq,Homo_633,AD.vs.MCI; bulk RNA-seq,Homo_714,AD.vs.control; bulk RNA-	4

CCL4	bulk RNA-seq,Homo_723,MCI.vs.control; bulk RNA-seq,Homo_633,AD.vs.MCI; bulk RNA-seq,Homo_633,MCI.vs.control; scRNA-seq,SRP215507,CD8+ T cell_3-AD.vs.control; scRNA-seq,SRP215507,CD8+ T cell_7-AD.vs.control; scRNA-seq,SRP215507,CD8+ T cell_6-AD.vs.control; scRNA-seq,SRP215507,CD8+ T cell_2-AD.vs.MCI; scRNA-seq,SRP215507,CD8+ T cell_3-AD.vs.MCI; scRNA-seq,SRP215507,CD8+ T cell_2-MCI.vs.control; scRNA-seq,SRP309935,Erythroid cell-AD.vs.control; scRNA-seq,SRP330776,CD8+ T cell_2-AD.vs.control; scRNA-seq,SRP330776,Natural killer cell_1-AD.vs.control; scRNA-seq,SRP330776,Memory T cell_1-AD.vs.control; scRNA-seq,SRP330776,Natural killer T cell-AD.vs.control; scRNA-seq,SRP330776,B cell_2-AD.vs.control; scRNA-seq,SRP330776,Naive CD8+ T cell_2-AD.vs.control; scRNA-	18
CCL4L1	bulk RNA-seq,Homo_723,AD.vs.control; bulk RNA-seq,Homo_723,AD.vs.MCI; bulk RNA-seq,Homo_633,AD.vs.control; bulk RNA-seq,Homo_633,AD.vs.MCI; bulk RNA-seq,Homo_714,AD.vs.control; bulk RNA-seq,Homo_714,AD.vs.MCI	6
CCL4L2	scRNA-seq,SRP215507,CD8+ T cell_2-AD.vs.control; scRNA-seq,SRP215507,CD8+ T cell_3-AD.vs.control; scRNA-seq,SRP215507,CD8+ T cell_4-AD.vs.control; scRNA-seq,SRP215507,CD8+ T cell_5-AD.vs.control; scRNA-seq,SRP215507,CD8+ T cell_2-AD.vs.MCI; scRNA-seq,SRP215507,CD8+ T cell_1-AD.vs.MCI; scRNA-seq,SRP215507,CD8+ T cell_4-AD.vs.MCI; scRNA-seq,SRP215507,CD8+ T cell_5-AD.vs.MCI; scRNA-seq,SRP215507,CD8+ T cell_2-MCI.vs.control; scRNA-seq,SRP215507,CD8+ T cell_3-MCI.vs.control; scRNA-seq,SRP215507,CD8+ T cell_8-MCI.vs.control; scRNA-seq,SRP309935,Megakaryocyte_1-AD.vs.control; scRNA-seq,SRP309935,Erythroid cell-AD.vs.control; scRNA-seq,SRP309935,Natural killer cell_1-AD.vs.control; scRNA-seq,SRP309935,Monocyte_2-AD.vs.control; scRNA-seq,SRP309935,Naive CD8+ T cell_2-AD.vs.control; scRNA-seq,SRP309935,CD8+ T cell_1-AD.vs.control; scRNA-seq,SRP309935,CD8+ T cell_2-AD.vs.control; scRNA-seq,SRP309935,Naive CD8+ T cell_3-AD.vs.control; scRNA-seq,SRP330776,γδ T cell-AD.vs.control; scRNA-seq,SRP330776,Natural killer cell_1- [illegible]	23
Ccl5	bulk RNA-seq,SRP325058,AD.vs.control	1
CCL5	scRNA-seq,SRP215507,CD8+ T cell_3-AD.vs.control; scRNA-seq,SRP215507,CD8+ T cell_7-AD.vs.control; scRNA-seq,SRP215507,CD8+ T cell_4-AD.vs.MCI; scRNA-seq,SRP215507,CD8+ T cell_3-MCI.vs.control; scRNA-seq,SRP309935,Erythroid cell-AD.vs.control; scRNA-seq,SRP309935,B cell_2-AD.vs.control; scRNA-seq,SRP330776,Naive CD8+ T cell_3-AD.vs.control; scRNA-seq,SRP330776,T cell-AD.vs.control; scRNA-seq,SRP330776,Naive CD8+ T cell_2-AD.vs.control; scRNA-seq,SRP330776,Monocyte_1-AD.vs.control; scRNA-seq,SRP330776,Monocyte_2-AD.vs.control; scRNA-seq,SRP330776,B cell_3-AD.vs.control; scRNA-seq,SRP330776,Megakaryocyte_1-AD.vs.control	13
CCM2	bulk RNA-seq,Homo_723,AD.vs.control; bulk RNA-seq,Homo_723,AD.vs.MCI; bulk RNA-seq,Homo_633,AD.vs.control; bulk RNA-seq,Homo_633,AD.vs.MCI; bulk RNA-seq,Homo_714,AD.vs.control; bulk RNA-seq,Homo_714,AD.vs.MCI; scRNA-seq,SRP215507,CD8+ T cell_6-AD.vs.MCI; scRNA-seq,SRP215507,CD8+ T cell_6-MCI.vs.control; scRNA-seq,SRP330776,CD8+ T cell_1-AD.vs.control; scRNA-seq,SRP330776,Memory T cell_2-AD.vs.control; scRNA-seq,SRP330776,CD8+ T cell_2-AD.vs.control; scRNA-seq,SRP330776,B cell_1-AD.vs.control; scRNA-seq,SRP330776,Naive CD8+ T cell_1-AD.vs.control; scRNA-seq,SRP330776,Naive CD8+ T cell_3-AD.vs.control; scRNA-seq,SRP330776,B cell_2-AD.vs.control; scRNA-seq,SRP330776,T cell-AD.vs.control; scRNA-seq,SRP330776,CD8+ T cell_3-AD.vs.control; scRNA-seq,SRP330776,B [illegible]	20
CCM2L	bulk RNA-seq,Homo_723,AD.vs.control; bulk RNA-seq,Homo_723,AD.vs.MCI; bulk RNA-seq,Homo_723,MCI.vs.control; bulk RNA-seq,Homo_633,AD.vs.control; bulk RNA-seq,Homo_633,AD.vs.MCI; bulk RNA-seq,Homo_633,MCI.vs.control; bulk RNA-seq,Homo_714,MCI.vs.control	7
CCN2	bulk RNA-seq,Homo_723,AD.vs.control; bulk RNA-seq,Homo_723,AD.vs.MCI; bulk RNA-seq,Homo_723,MCI.vs.control; bulk RNA-seq,Homo_633,AD.vs.control; bulk RNA-seq,Homo_633,AD.vs.MCI; bulk RNA-seq,Homo_633,MCI.vs.control; bulk RNA-	8
CCN3	bulk RNA-seq,Homo_723,AD.vs.control; bulk RNA-seq,Homo_723,AD.vs.MCI; bulk RNA-seq,Homo_633,AD.vs.control; bulk RNA-seq,Homo_633,AD.vs.MCI; bulk RNA-seq,Homo_633,MCI.vs.control	5
CCN4	bulk RNA-seq,ACOM,AD.vs.control; bulk RNA-seq,Homo_723,AD.vs.control; bulk RNA-seq,Homo_723,AD.vs.MCI; bulk RNA-seq,Homo_633,AD.vs.MCI; bulk RNA-seq,ROSMAP,AD.vs.control	5
CCN5	bulk RNA-seq,Homo_714,MCI.vs.control	1
CCN6	bulk RNA-seq,Homo_723,MCI.vs.control; bulk RNA-seq,Homo_633,AD.vs.control; bulk RNA-seq,Homo_633,AD.vs.MCI; bulk RNA-seq,Homo_633,MCI.vs.control; bulk RNA-seq,Homo_714,MCI.vs.control	5
CCNA1	bulk RNA-seq,Homo_723,AD.vs.control; bulk RNA-seq,Homo_723,AD.vs.MCI; bulk RNA-seq,Homo_633,AD.vs.control; bulk RNA-seq,Homo_633,AD.vs.MCI; bulk RNA-seq,Homo_714,AD.vs.control; bulk RNA-seq,Homo_714,AD.vs.MCI	6

CCNA2	bulk RNA-seq,Homo_723,AD.vs.control; bulk RNA-seq,Homo_723,AD.vs.MCI; bulk RNA-seq,Homo_723,MCI.vs.control; bulk RNA-seq,Homo_633,AD.vs.control; bulk RNA-seq,Homo_633,AD.vs.MCI; bulk RNA-seq,Homo_714,AD.vs.control; bulk RNA-seq,Homo_714,AD.vs.MCI; scRNA-seq,SRP215507,CD8+ T cell_1-MCI.vs.control; scRNA-seq,SRP215507,CD8+ T cell_6-MCI.vs.control; scRNA-seq,SRP330776,Naive CD8+ T	10
Ccna2	bulk RNA-seq,SRP325058,AD.vs.control	1
CCNB1	bulk RNA-seq,Homo_723,MCI.vs.control; bulk RNA-seq,Homo_633,MCI.vs.control; scRNA-seq,SRP215507,CD8+ T cell_3-AD.vs.MCI; scRNA-seq,SRP215507,CD8+ T cell_3-MCI.vs.control; scRNA-seq,SRP215507,CD8+ T cell_6-MCI.vs.control; scRNA-seq,SRP330776,Regulatory T cell-	6
CCNB1IP1	scRNA-seq,SRP215507,CD8+ T cell_10-AD.vs.control; scRNA-seq,SRP215507,CD8+ T cell_6-AD.vs.control; scRNA-seq,SRP215507,CD8+ T cell_5-AD.vs.MCI; scRNA-seq,SRP215507,CD8+ T cell_6-AD.vs.MCI; scRNA-seq,SRP215507,CD8+ T cell_5-MCI.vs.control; scRNA-seq,SRP330776,Natural	6
CCNB2	bulk RNA-seq,Homo_723,AD.vs.control; bulk RNA-seq,Homo_723,AD.vs.MCI; bulk RNA-seq,Homo_633,AD.vs.control; bulk RNA-seq,Homo_633,AD.vs.MCI; bulk RNA-seq,Homo_714,AD.vs.control; bulk RNA-seq,Homo_714,AD.vs.MCI	6
Ccnb2	bulk RNA-seq,SRP325058,AD.vs.control	1
CCNB3	bulk RNA-seq,Homo_723,AD.vs.MCI; bulk RNA-seq,Homo_714,AD.vs.control; bulk RNA-seq,Homo_714,AD.vs.MCI; scRNA-seq,SRP215507,CD8+ T cell_1-AD.vs.control	4
CCNC	bulk RNA-seq,Homo_723,AD.vs.control; bulk RNA-seq,Homo_723,AD.vs.MCI; bulk RNA-seq,Homo_633,AD.vs.control; bulk RNA-seq,Homo_633,AD.vs.MCI; bulk RNA-seq,Homo_714,AD.vs.control; bulk RNA-seq,Homo_714,AD.vs.MCI; scRNA-seq,SRP215507,CD8+ T cell_4-AD.vs.MCI; scRNA-seq,SRP215507,CD8+ T cell_4-MCI.vs.control; scRNA-seq,SRP309935,B cell_1-AD.vs.control; scRNA-seq,SRP330776,B cell_1-AD.vs.control; scRNA-seq,SRP330776,Natural killer cell_1-AD.vs.control; scRNA-seq,SRP330776,B cell_2-AD.vs.control; scRNA-seq,SRP330776,CD8+	14
CCND1	bulk RNA-seq,Homo_723,AD.vs.control; bulk RNA-seq,Homo_723,AD.vs.MCI; bulk RNA-seq,Homo_723,MCI.vs.control; bulk RNA-seq,Homo_633,AD.vs.control; bulk RNA-seq,Homo_633,AD.vs.MCI; bulk RNA-seq,Homo_633,MCI.vs.control; bulk RNA-seq,Homo_714,AD.vs.control; bulk RNA-seq,Homo_714,AD.vs.MCI; scRNA-seq,SRP215507,CD8+ T cell_2-AD.vs.MCI; scRNA-seq,SRP215507,CD8+ T cell_2-MCI.vs.control; scRNA-seq,SRP330776,Memory T cell_1-AD.vs.control; scRNA-seq,SRP330776,B cell_2-AD.vs.control	12
CCND2	bulk RNA-seq,Homo_633,MCI.vs.control; scRNA-seq,SRP215507,CD8+ T cell_7-MCI.vs.control; scRNA-seq,SRP330776,Natural killer cell_1-AD.vs.control; scRNA-seq,SRP330776,Monocyte_1-AD.vs.control	4
CCND2-AS1	scRNA-seq,SRP330776,CD8+ T cell_3-AD.vs.control	1
CCND3	bulk RNA-seq,Homo_723,AD.vs.control; bulk RNA-seq,Homo_723,AD.vs.MCI; bulk RNA-seq,Homo_633,AD.vs.control; bulk RNA-seq,Homo_633,AD.vs.MCI; scRNA-seq,SRP215507,CD8+ T cell_7-AD.vs.control; scRNA-seq,SRP215507,CD8+ T cell_9-AD.vs.control; scRNA-seq,SRP215507,CD8+ T cell_7-AD.vs.MCI; scRNA-seq,SRP309935,Erythroid cell-AD.vs.control; scRNA-seq,SRP330776,Monocyte_1-AD.vs.control	9
CCNDBP1	scRNA-seq,SRP215507,CD8+ T cell_7-AD.vs.control; scRNA-seq,SRP215507,CD8+ T cell_7-MCI.vs.control; scRNA-seq,SRP309935,Erythroid cell-AD.vs.control; scRNA-seq,SRP330776,γδ T cell-AD.vs.control; scRNA-seq,SRP330776,Memory T cell_2-AD.vs.control; scRNA-seq,SRP330776,Natural killer cell_1-AD.vs.control; scRNA-seq,SRP330776,B cell_2-AD.vs.control; scRNA-seq,SRP330776,Natural killer cell_2-AD.vs.control	8
CCNE1	bulk RNA-seq,Homo_723,AD.vs.control; bulk RNA-seq,Homo_723,AD.vs.MCI; bulk RNA-seq,Homo_723,MCI.vs.control; bulk RNA-seq,Homo_633,AD.vs.control; bulk RNA-seq,Homo_633,AD.vs.MCI; bulk RNA-seq,Homo_633,MCI.vs.control; bulk RNA-seq,Homo_714,AD.vs.control; bulk RNA-seq,Homo_714,MCI.vs.control; scRNA-seq,SRP215507,CD8+ T cell_1-AD.vs.MCI; scRNA-seq,SRP215507,CD8+ T cell_6-MCI.vs.control	10
CCNE2	bulk RNA-seq,Homo_723,AD.vs.control; bulk RNA-seq,Homo_723,AD.vs.MCI; bulk RNA-seq,Homo_633,AD.vs.control; bulk RNA-seq,Homo_633,AD.vs.MCI; bulk RNA-seq,Homo_714,AD.vs.control; bulk RNA-seq,Homo_714,AD.vs.MCI; scRNA-seq,SRP215507,CD8+ T cell_1-AD.vs.MCI; scRNA-seq,SRP215507,CD8+ T cell_1-MCI.vs.control; scRNA-seq,SRP330776,CD8+ T cell_1-AD.vs.control; scRNA-seq,SRP330776,CD8+	10
CCNF	scRNA-seq,SRP215507,CD8+ T cell_4-AD.vs.MCI; scRNA-seq,SRP215507,CD8+ T cell_4-MCI.vs.control; scRNA-seq,SRP330776,CD8+ T cell_2-AD.vs.control; scRNA-seq,SRP330776,B cell_1-AD.vs.control; scRNA-seq,SRP330776,Memory T cell_1-AD.vs.control; scRNA-seq,SRP330776,T cell-AD.vs.control; scRNA-seq,SRP330776,CD8+ T cell_3-AD.vs.control	7
CCNG1	bulk RNA-seq,Homo_723,AD.vs.control; bulk RNA-seq,Homo_723,AD.vs.MCI; bulk RNA-seq,Homo_723,MCI.vs.control; bulk RNA-seq,Homo_633,AD.vs.MCI; bulk RNA-seq,Homo_633,MCI.vs.control; bulk RNA-seq,Homo_714,AD.vs.control; bulk RNA-seq,Homo_714,AD.vs.MCI; bulk RNA-seq,Homo_714,MCI.vs.control; scRNA-seq,SRP215507,CD8+ T cell_7-AD.vs.control; scRNA-seq,SRP330776,Natural killer T cell-AD.vs.control; scRNA-seq,SRP330776,Monocyte_2-AD.vs.control; scRNA-seq,SRP330776,Regulatory T cell-AD.vs.control	12

CCNG2	bulk RNA-seq,Homo_723,AD.vs.MCI; bulk RNA-seq,Homo_633,AD.vs.MCI; bulk RNA-seq,Homo_714,AD.vs.control; bulk RNA-seq,Homo_714,AD.vs.MCI; scRNA-seq,SRP215507,CD8+ T cell_7-AD.vs.control; scRNA-seq,SRP215507,CD8+ T cell_5-AD.vs.control; scRNA-seq,SRP215507,CD8+ T cell_2-AD.vs.MCI; scRNA-seq,SRP215507,CD8+ T cell_2-MCI.vs.control; scRNA-seq,SRP309935,Naive CD8+ T cell_2-AD.vs.control; scRNA-seq,SRP309935,B cell_2-AD.vs.control; scRNA-seq,SRP330776,CD8+ T cell_1-AD.vs.control; scRNA-seq,SRP330776,γδ T cell-AD.vs.control; scRNA-seq,SRP330776,Memory T cell_2-AD.vs.control; scRNA-seq,SRP330776,CD8+ T cell_2-AD.vs.control; scRNA-seq,SRP330776,B cell_1-AD.vs.control; scRNA-seq,SRP330776,Naive CD8+ T cell_1-AD.vs.control; scRNA-seq,SRP330776,Natural killer cell_1-AD.vs.control; scRNA-seq,SRP330776,Naive CD8+ T cell_3-AD.vs.control; scRNA-seq,SRP330776,B cell_2-AD.vs.control; scRNA-seq,SRP330776,T cell-AD.vs.control; scRNA-seq,SRP330776,CD8+ T cell_3-AD.vs.control; scRNA-seq,SRP330776,Monocyte_1-AD.vs.control; scRNA-seq,SRP330776,Monocyte_2- [illegible]	25
CCNH	scRNA-seq,SRP215507,CD8+ T cell_4-AD.vs.MCI; scRNA-seq,SRP215507,CD8+ T cell_5-AD.vs.MCI; scRNA-seq,SRP215507,CD8+ T cell_4-MCI.vs.control; scRNA-seq,SRP215507,CD8+ T cell_8-MCI.vs.control; scRNA-seq,SRP330776,γδ T cell-AD.vs.control; scRNA-seq,SRP330776,B cell_3-	6
Ccni	bulk RNA-seq,SRP325058,AD.vs.control	1
CCNI	scRNA-seq,SRP215507,CD8+ T cell_8-MCI.vs.control; scRNA-seq,SRP330776,Monocyte_2-AD.vs.control	2
CCNI2	bulk RNA-seq,Homo_723,AD.vs.control; bulk RNA-seq,Homo_723,AD.vs.MCI; bulk RNA-seq,Homo_633,AD.vs.control; bulk RNA-seq,Homo_633,AD.vs.MCI; bulk RNA-seq,Homo_714,AD.vs.control; bulk RNA-seq,Homo_714,AD.vs.MCI; scRNA-seq,SRP215507,CD8+ T cell_5-AD.vs.MCI; scRNA-seq,SRP330776,CD8+ T cell_1-AD.vs.control; scRNA-seq,SRP330776,Memory T cell_2-AD.vs.control; scRNA-seq,SRP330776,Naive CD8+ T cell_1-AD.vs.control; scRNA-seq,SRP330776,T cell-AD.vs.control; scRNA-seq,SRP330776,CD8+ T cell_3-AD.vs.control	12
CCNJ	bulk RNA-seq,Homo_723,AD.vs.control; bulk RNA-seq,Homo_723,AD.vs.MCI; bulk RNA-seq,Homo_714,AD.vs.control; bulk RNA-seq,Homo_714,AD.vs.MCI; scRNA-seq,SRP215507,CD8+ T cell_3-AD.vs.MCI; scRNA-seq,SRP215507,CD8+ T cell_1-AD.vs.MCI; scRNA-seq,SRP215507,CD8+ T cell_4-AD.vs.MCI; scRNA-seq,SRP215507,CD8+ T cell_3-MCI.vs.control; scRNA-seq,SRP330776,γδ T cell-AD.vs.control; scRNA-seq,SRP330776,CD8+ T cell_2-AD.vs.control; scRNA-seq,SRP330776,CD8+ T cell_3-AD.vs.control	11
CCNJL	bulk RNA-seq,Homo_633,AD.vs.control; bulk RNA-seq,SRP223445,AD.vs.control; scRNA-seq,SRP330776,Memory T cell_1-AD.vs.control; scRNA-seq,SRP330776,Natural killer cell_2-AD.vs.control	4
CCNK	scRNA-seq,SRP330776,Monocyte_1-AD.vs.control; scRNA-seq,SRP330776,Monocyte_2-AD.vs.control; scRNA-seq,SRP330776,B cell_3-AD.vs.control	3
CCNL1	scRNA-seq,SRP215507,CD8+ T cell_7-AD.vs.control; scRNA-seq,SRP215507,CD8+ T cell_7-AD.vs.MCI; scRNA-seq,SRP215507,CD8+ T cell_5-AD.vs.MCI; scRNA-seq,SRP309935,Erythroid cell-AD.vs.control; scRNA-seq,SRP330776,Naive CD8+ T cell_2-AD.vs.control	5
Ccnl2	bulk RNA-seq,SRP325058,AD.vs.control	1
CCNL2	scRNA-seq,SRP215507,CD8+ T cell_5-MCI.vs.control; scRNA-seq,SRP330776,γδ T cell-AD.vs.control; scRNA-seq,SRP330776,Memory T cell_1-	3
CCNO	bulk RNA-seq,Homo_723,AD.vs.control; bulk RNA-seq,Homo_723,AD.vs.MCI	2
CCNQ	bulk RNA-seq,Homo_723,AD.vs.control; bulk RNA-seq,Homo_723,AD.vs.MCI; bulk RNA-seq,Homo_633,AD.vs.control; bulk RNA-seq,Homo_633,AD.vs.MCI; bulk RNA-seq,Homo_714,AD.vs.control; bulk RNA-seq,Homo_714,AD.vs.MCI; scRNA-seq,SRP215507,CD8+ T cell_5-AD.vs.MCI; scRNA-seq,SRP215507,CD8+ T cell_5-MCI.vs.control; scRNA-seq,SRP330776,CD8+ T cell_1-AD.vs.control; scRNA-seq,SRP330776,γδ T cell-AD.vs.control; scRNA-seq,SRP330776,Memory T cell_2-AD.vs.control; scRNA-seq,SRP330776,CD8+ T cell_2-AD.vs.control; scRNA-seq,SRP330776,B cell_1-AD.vs.control; scRNA-seq,SRP330776,Naive CD8+ T cell_1-AD.vs.control; scRNA-seq,SRP330776,Natural killer cell_1-AD.vs.control; scRNA-seq,SRP330776,Memory T cell_1-AD.vs.control; scRNA-seq,SRP330776,Natural killer T cell-AD.vs.control; scRNA-seq,SRP330776,Naive CD8+ T cell_3-AD.vs.control; scRNA-seq,SRP330776,B cell_2-AD.vs.control; scRNA-seq,SRP330776,T cell-AD.vs.control; scRNA-seq,SRP330776,CD8+ T cell_3-AD.vs.control; scRNA-seq,SRP330776,Monocyte_1-AD.vs.control; scRNA-seq,SRP330776,B cell_3- [illegible]	24
CCNT1	bulk RNA-seq,Homo_723,MCI.vs.control; bulk RNA-seq,Homo_633,MCI.vs.control; bulk RNA-seq,Homo_714,AD.vs.control; bulk RNA-seq,Homo_714,AD.vs.MCI; bulk RNA-seq,Homo_714,MCI.vs.control; scRNA-seq,SRP215507,CD8+ T cell_6-AD.vs.control; scRNA-seq,SRP215507,CD8+ T cell_4-AD.vs.MCI; scRNA-seq,SRP309935,Natural killer cell_2-AD.vs.control; scRNA-seq,SRP330776,Memory T cell_2-AD.vs.control; scRNA-seq,SRP330776,Naive CD8+ T cell_3-AD.vs.control; scRNA-seq,SRP330776,B cell_3-AD.vs.control; scRNA-seq,SRP330776,Regulatory T cell-AD.vs.control; scRNA-seq,SRP330776,Natural killer cell_2-AD.vs.control; scRNA-seq,SRP330776,B cell_4-	14

CCNT2	bulk RNA-seq,Homo_723,AD.vs.control; bulk RNA-seq,Homo_723,AD.vs.MCI; bulk RNA-seq,Homo_633,AD.vs.control; bulk RNA-seq,Homo_633,AD.vs.MCI; bulk RNA-seq,Homo_714,AD.vs.control; bulk RNA-seq,Homo_714,AD.vs.MCI; scRNA-seq,SRP215507,CD8+ T cell_7-AD.vs.control; scRNA-seq,SRP215507,CD8+ T cell_7-AD.vs.MCI; scRNA-seq,SRP330776,B cell_3-AD.vs.control	9
CCNT2-AS1	scRNA-seq,SRP215507,CD8+ T cell_3-AD.vs.MCI; scRNA-seq,SRP215507,CD8+ T cell_1-AD.vs.MCI; scRNA-seq,SRP215507,CD8+ T cell_3-MCI.vs.control; scRNA-seq,SRP309935,CD8+ T cell_2-AD.vs.control; scRNA-seq,SRP330776,CD8+ T cell_2-AD.vs.control; scRNA-seq,SRP330776,B	6
CCNY	bulk RNA-seq,Homo_633,AD.vs.MCI; bulk RNA-seq,Homo_714,AD.vs.control; bulk RNA-seq,Homo_714,AD.vs.MCI; scRNA-seq,SRP215507,CD8+ T cell_8-MCI.vs.control; scRNA-seq,SRP309935,Monocyte_2-AD.vs.control; scRNA-seq,SRP330776,γδ T cell-AD.vs.control; scRNA-seq,SRP330776,Memory T cell_2-AD.vs.control; scRNA-seq,SRP330776,Natural killer cell_1-AD.vs.control; scRNA-seq,SRP330776,Natural killer T cell-AD.vs.control; scRNA-seq,SRP330776,Naive CD8+ T cell_3-AD.vs.control; scRNA-seq,SRP330776,T cell-AD.vs.control; scRNA-seq,SRP330776,CD8+ T cell_3-AD.vs.control; scRNA-seq,SRP330776,Monocyte_1-AD.vs.control; scRNA-seq,SRP330776,Monocyte_2-AD.vs.control; scRNA-	16
CCNYL1	bulk RNA-seq,Homo_723,AD.vs.MCI; bulk RNA-seq,Homo_723,MCI.vs.control; bulk RNA-seq,Homo_633,AD.vs.MCI; bulk RNA-seq,Homo_633,MCI.vs.control; bulk RNA-seq,Homo_714,AD.vs.control; bulk RNA-seq,Homo_714,AD.vs.MCI; bulk RNA-seq,Homo_714,MCI.vs.control; scRNA-seq,SRP215507,CD8+ T cell_5-AD.vs.control; scRNA-seq,SRP215507,CD8+ T cell_2-AD.vs.MCI; scRNA-seq,SRP215507,CD8+ T cell_1-AD.vs.MCI; scRNA-seq,SRP215507,CD8+ T cell_5-AD.vs.MCI; scRNA-seq,SRP215507,CD8+ T cell_1-MCI.vs.control;	14
CCP110	bulk RNA-seq,Homo_723,AD.vs.control; bulk RNA-seq,Homo_723,AD.vs.MCI; bulk RNA-seq,Homo_633,AD.vs.MCI; bulk RNA-seq,Homo_714,AD.vs.control; bulk RNA-seq,Homo_714,AD.vs.MCI; scRNA-seq,SRP215507,CD8+ T cell_5-AD.vs.control; scRNA-seq,SRP215507,CD8+ T cell_2-AD.vs.MCI; scRNA-seq,SRP215507,CD8+ T cell_5-AD.vs.MCI; scRNA-seq,SRP215507,CD8+ T cell_6-MCI.vs.control;	10
Ccpg1	bulk RNA-seq,SRP325058,AD.vs.control	1
CCPG1	scRNA-seq,SRP309935,Megakaryocyte_1-AD.vs.control; scRNA-seq,SRP330776,CD8+ T cell_1-AD.vs.control; scRNA-seq,SRP330776,Natural killer cell_1-AD.vs.control; scRNA-seq,SRP330776,Naive CD8+ T cell_3-AD.vs.control; scRNA-seq,SRP330776,T cell-AD.vs.control; scRNA-	6
CCR1	bulk RNA-seq,Homo_723,AD.vs.MCI; bulk RNA-seq,Homo_633,AD.vs.MCI; bulk RNA-seq,Homo_714,AD.vs.control; bulk RNA-seq,Homo_714,AD.vs.MCI; bulk RNA-seq,SRP223445,AD.vs.control; scRNA-seq,SRP215507,CD8+ T cell_2-AD.vs.control; scRNA-seq,SRP215507,CD8+ T cell_1-AD.vs.control; scRNA-seq,SRP215507,CD8+ T cell_4-AD.vs.control; scRNA-seq,SRP215507,CD8+ T cell_1-MCI.vs.control; scRNA-seq,SRP215507,CD8+ T cell_2-MCI.vs.control; scRNA-seq,SRP330776,γδ T cell-AD.vs.control; scRNA-seq,SRP330776,CD8+ T	12
CCR10	bulk RNA-seq,Homo_723,AD.vs.control; bulk RNA-seq,Homo_723,AD.vs.MCI; bulk RNA-seq,Homo_723,MCI.vs.control; bulk RNA-seq,Homo_633,AD.vs.control; bulk RNA-seq,Homo_633,AD.vs.MCI; bulk RNA-seq,Homo_633,MCI.vs.control; bulk RNA-seq,Homo_714,AD.vs.control; bulk RNA-seq,Homo_714,AD.vs.MCI; bulk RNA-seq,Homo_714,MCI.vs.control; scRNA-seq,SRP215507,CD8+ T cell_1-AD.vs.MCI; scRNA-seq,SRP215507,CD8+ T cell_1-MCI.vs.control; scRNA-seq,SRP215507,CD8+ T cell_2-MCI.vs.control; scRNA-seq,SRP330776,CD8+ T cell_1-AD.vs.control; scRNA-seq,SRP330776,Memory T cell_2-AD.vs.control; scRNA-seq,SRP330776,Natural killer cell_1-AD.vs.control; scRNA-	17
Ccr2	bulk RNA-seq,SRP325058,AD.vs.control	1
CCR2	scRNA-seq,SRP215507,CD8+ T cell_4-AD.vs.MCI; scRNA-seq,SRP330776,CD8+ T cell_1-AD.vs.control; scRNA-seq,SRP330776,γδ T cell-AD.vs.control; scRNA-seq,SRP330776,Memory T cell_2-AD.vs.control; scRNA-seq,SRP330776,CD8+ T cell_2-AD.vs.control; scRNA-seq,SRP330776,B cell_1-AD.vs.control; scRNA-seq,SRP330776,T cell-AD.vs.control; scRNA-seq,SRP330776,CD8+ T cell_3-AD.vs.control; scRNA-	9
CCR3	bulk RNA-seq,Homo_723,AD.vs.control; bulk RNA-seq,Homo_723,AD.vs.MCI; bulk RNA-seq,Homo_633,AD.vs.control; bulk RNA-seq,SRP223445,AD.vs.control; scRNA-seq,SRP330776,T cell-AD.vs.control	5
CCR4	bulk RNA-seq,Homo_714,AD.vs.control; bulk RNA-seq,Homo_714,AD.vs.MCI; scRNA-seq,SRP215507,CD8+ T cell_2-AD.vs.control; scRNA-seq,SRP215507,CD8+ T cell_3-AD.vs.control; scRNA-seq,SRP215507,CD8+ T cell_4-AD.vs.MCI; scRNA-seq,SRP215507,CD8+ T cell_4-MCI.vs.control; scRNA-seq,SRP309935,CD8+ T cell_2-AD.vs.control; scRNA-seq,SRP330776,CD8+ T cell_1-AD.vs.control	8
CCR5	bulk RNA-seq,Homo_723,AD.vs.control; bulk RNA-seq,Homo_723,AD.vs.MCI; bulk RNA-seq,Homo_723,MCI.vs.control; scRNA-seq,SRP215507,CD8+ T cell_1-AD.vs.control; scRNA-seq,SRP215507,CD8+ T cell_3-AD.vs.MCI; scRNA-seq,SRP215507,CD8+ T cell_5-AD.vs.MCI; scRNA-seq,SRP215507,CD8+ T cell_6-AD.vs.MCI; scRNA-seq,SRP215507,CD8+ T cell_5-MCI.vs.control; scRNA-seq,SRP215507,CD8+ T cell_6-MCI.vs.control; scRNA-seq,SRP309935,Monocyte_1-AD.vs.control; scRNA-seq,SRP330776,CD8+ T cell_2-AD.vs.control; scRNA-seq,SRP330776,Natural killer cell_1-AD.vs.control; scRNA-seq,SRP330776,Natural killer T cell-AD.vs.control; scRNA-seq,SRP330776,Regulatory T cell-	14

Ccr5	bulk RNA-seq,SRP325058,AD.vs.control	1
CCR5AS	scRNA-seq,SRP215507,CD8+ T cell_2-AD.vs.control; scRNA-seq,SRP215507,CD8+ T cell_1-MCI.vs.control; scRNA-seq,SRP215507,CD8+ T cell_2-	3
CCR6	bulk RNA-seq,Homo_723,AD.vs.control; bulk RNA-seq,Homo_723,MCI.vs.control; bulk RNA-seq,Homo_633,AD.vs.control; bulk RNA-seq,Homo_633,MCI.vs.control; bulk RNA-seq,Homo_714,AD.vs.control; bulk RNA-seq,Homo_714,AD.vs.MCI; bulk RNA-seq,Homo_714,MCI.vs.control; scRNA-seq,SRP215507,CD8+ T cell_4-AD.vs.MCI; scRNA-seq,SRP215507,CD8+ T cell_4-MCI.vs.control; scRNA-seq,SRP215507,CD8+ T cell_7-MCI.vs.control; scRNA-seq,SRP309935,Naive CD8+ T cell_2-AD.vs.control; scRNA-seq,SRP309935,B cell_1-AD.vs.control; scRNA-seq,SRP330776,γδ T cell-AD.vs.control; scRNA-seq,SRP330776,Natural killer cell_1-AD.vs.control; scRNA-seq,SRP330776,B	16
CCR7	bulk RNA-seq,Homo_723,AD.vs.MCI; bulk RNA-seq,Homo_723,MCI.vs.control; bulk RNA-seq,Homo_633,AD.vs.MCI; bulk RNA-seq,Homo_633,MCI.vs.control; bulk RNA-seq,Homo_714,MCI.vs.control; scRNA-seq,SRP215507,CD8+ T cell_1-AD.vs.control; scRNA-seq,SRP215507,CD8+ T cell_4-AD.vs.control; scRNA-seq,SRP215507,CD8+ T cell_6-AD.vs.control; scRNA-seq,SRP215507,CD8+ T cell_1-AD.vs.MCI; scRNA-seq,SRP215507,CD8+ T cell_4-AD.vs.MCI; scRNA-seq,SRP215507,CD8+ T cell_1-MCI.vs.control; scRNA-seq,SRP309935,Megakaryocyte_1-AD.vs.control; scRNA-seq,SRP330776,Memory T cell_2-AD.vs.control; scRNA-seq,SRP330776,B cell_1-AD.vs.control; scRNA-seq,SRP330776,Natural	16
Ccr7	bulk RNA-seq,SRP325058,AD.vs.control	1
CCR8	bulk RNA-seq,Homo_723,AD.vs.control; bulk RNA-seq,Homo_723,AD.vs.MCI; bulk RNA-seq,ROSMAP,AD.vs.MCI; bulk RNA-seq,ROSMAP,MCI.vs.control; scRNA-seq,SRP215507,CD8+ T cell_2-AD.vs.control	5
CCR9	bulk RNA-seq,Homo_723,AD.vs.control; bulk RNA-seq,Homo_723,AD.vs.MCI; bulk RNA-seq,Homo_723,MCI.vs.control; bulk RNA-seq,Homo_633,AD.vs.control; bulk RNA-seq,Homo_633,AD.vs.MCI; bulk RNA-seq,Homo_633,MCI.vs.control; bulk RNA-seq,Homo_714,AD.vs.control; bulk RNA-seq,Homo_714,AD.vs.MCI; bulk RNA-seq,Homo_714,MCI.vs.control; scRNA-seq,SRP215507,CD8+ T cell_2-AD.vs.control; scRNA-seq,SRP215507,CD8+ T cell_2-MCI.vs.control; scRNA-seq,SRP309935,B cell_1-AD.vs.control; scRNA-seq,SRP330776,CD8+ T cell_1-AD.vs.control;	14
CCRL2	bulk RNA-seq,Homo_723,AD.vs.control; bulk RNA-seq,Homo_723,AD.vs.MCI; bulk RNA-seq,Homo_633,AD.vs.control; bulk RNA-seq,Homo_633,AD.vs.MCI; bulk RNA-seq,Homo_714,AD.vs.control; bulk RNA-seq,Homo_714,AD.vs.MCI; scRNA-seq,SRP215507,CD8+ T cell_5-AD.vs.MCI; scRNA-seq,SRP215507,CD8+ T cell_1-MCI.vs.control; scRNA-seq,SRP215507,CD8+ T cell_5-MCI.vs.control; scRNA-seq,SRP330776,B cell_1-AD.vs.control; scRNA-seq,SRP330776,B cell_2-AD.vs.control; scRNA-seq,SRP330776,Monocyte_1-AD.vs.control	12
CCS	bulk RNA-seq,Homo_723,AD.vs.control; bulk RNA-seq,Homo_723,AD.vs.MCI; bulk RNA-seq,Homo_633,AD.vs.control; bulk RNA-seq,Homo_633,AD.vs.MCI; bulk RNA-seq,Homo_714,AD.vs.control; bulk RNA-seq,Homo_714,AD.vs.MCI; scRNA-seq,SRP215507,CD8+ T cell_7-AD.vs.MCI; scRNA-seq,SRP215507,CD8+ T cell_4-MCI.vs.control; scRNA-seq,SRP215507,CD8+ T cell_7-MCI.vs.control; scRNA-seq,SRP330776,γδ T cell-AD.vs.control; scRNA-seq,SRP330776,Memory T cell_2-AD.vs.control; scRNA-seq,SRP330776,Natural killer cell_1-AD.vs.control; scRNA-seq,SRP330776,Monocyte_1-AD.vs.control; scRNA-seq,SRP330776,B cell_3-AD.vs.control	14
Ccs	bulk RNA-seq,SRP325058,AD.vs.control	1
CCSAP	bulk RNA-seq,Homo_723,AD.vs.control; bulk RNA-seq,Homo_723,AD.vs.MCI; bulk RNA-seq,Homo_723,MCI.vs.control; bulk RNA-seq,Homo_633,AD.vs.MCI; bulk RNA-seq,Homo_633,MCI.vs.control; bulk RNA-seq,Homo_714,AD.vs.control; bulk RNA-seq,Homo_714,AD.vs.MCI; bulk RNA-seq,Homo_714,MCI.vs.control; scRNA-seq,SRP215507,CD8+ T cell_5-AD.vs.control; scRNA-seq,SRP215507,CD8+ T cell_2-AD.vs.MCI; scRNA-seq,SRP215507,CD8+ T cell_1-AD.vs.MCI; scRNA-seq,SRP215507,CD8+ T cell_1-MCI.vs.control; scRNA-seq,SRP215507,CD8+ T cell_5-MCI.vs.control; scRNA-seq,SRP215507,CD8+ T cell_2-MCI.vs.control; scRNA-seq,SRP309935,Monocyte_2-AD.vs.control; scRNA-seq,SRP330776,Memory T cell_2-AD.vs.control; scRNA-seq,SRP330776,CD8+ T cell_2-AD.vs.control; scRNA-seq,SRP330776,Naive CD8+ T cell_1-AD.vs.control; scRNA-seq,SRP330776,Memory T cell_1-AD.vs.control; scRNA-seq,SRP330776,Natural killer T cell-AD.vs.control; scRNA-[illegible]	23
CCSER1	bulk RNA-seq,Homo_723,AD.vs.control; bulk RNA-seq,Homo_723,AD.vs.MCI; bulk RNA-seq,Homo_633,AD.vs.control; bulk RNA-seq,Homo_633,AD.vs.MCI; bulk RNA-seq,Homo_714,AD.vs.control; bulk RNA-seq,Homo_714,AD.vs.MCI; bulk RNA-seq,Homo_714,MCI.vs.control;	8
CCSER2	bulk RNA-seq,Homo_723,AD.vs.control; bulk RNA-seq,Homo_633,AD.vs.control; bulk RNA-seq,Homo_633,AD.vs.MCI; bulk RNA-seq,Homo_633,MCI.vs.control; bulk RNA-seq,Homo_714,AD.vs.control; bulk RNA-seq,Homo_714,AD.vs.MCI; scRNA-seq,SRP215507,CD8+ T cell_6-AD.vs.control; scRNA-seq,SRP330776,Naive CD8+ T cell_3-AD.vs.control; scRNA-seq,SRP330776,Naive CD8+ T cell_2-AD.vs.control; scRNA-	10
Ccser2	bulk RNA-seq,SRP325058,AD.vs.control	1

CCT2	bulk RNA-seq,Homo_723,AD.vs.MCI; bulk RNA-seq,Homo_723,MCI.vs.control; bulk RNA-seq,Homo_714,AD.vs.control; bulk RNA-seq,Homo_714,AD.vs.MCI; bulk RNA-seq,Homo_714,MCI.vs.control; scRNA-seq,SRP215507,CD8+ T cell_6-MCI.vs.control; scRNA-seq,SRP330776,CD8+ T cell_3-AD.vs.control; scRNA-seq,SRP330776,Monocyte_2-AD.vs.control	8
CCT3	bulk RNA-seq,Homo_723,AD.vs.control; bulk RNA-seq,Homo_633,AD.vs.control; bulk RNA-seq,Homo_633,AD.vs.MCI; bulk RNA-seq,Homo_714,AD.vs.control; bulk RNA-seq,Homo_714,AD.vs.MCI; bulk RNA-seq,SRP310421,AD.vs.control; scRNA-seq,SRP215507,CD8+ T cell_7-AD.vs.MCI; scRNA-seq,SRP215507,CD8+ T cell_7-MCI.vs.control	8
CCT4	bulk RNA-seq,Homo_723,AD.vs.control; bulk RNA-seq,Homo_723,AD.vs.MCI; bulk RNA-seq,Homo_723,MCI.vs.control; bulk RNA-seq,Homo_633,AD.vs.control; bulk RNA-seq,Homo_633,AD.vs.MCI; bulk RNA-seq,Homo_633,MCI.vs.control; bulk RNA-seq,Homo_714,AD.vs.control; bulk RNA-seq,Homo_714,AD.vs.MCI; bulk RNA-seq,Homo_714,MCI.vs.control; scRNA-seq,SRP330776,Megakaryocyte_1-AD.vs.control	10
CCT5	bulk RNA-seq,Homo_723,AD.vs.control; bulk RNA-seq,Homo_723,AD.vs.MCI; bulk RNA-seq,Homo_633,AD.vs.control; bulk RNA-seq,Homo_633,AD.vs.MCI; bulk RNA-seq,Homo_714,AD.vs.control; bulk RNA-seq,Homo_714,AD.vs.MCI; bulk RNA-seq,Homo_714,MCI.vs.control; scRNA-seq,SRP215507,CD8+ T cell_4-AD.vs.control; scRNA-seq,SRP215507,CD8+ T cell_7-AD.vs.control; scRNA-seq,SRP215507,CD8+ T cell_10-AD.vs.control; scRNA-seq,SRP215507,CD8+ T cell_9-AD.vs.control; scRNA-seq,SRP309935,B cell_1-AD.vs.control; scRNA-seq,SRP330776,CD8+ T	14
CCT6A	scRNA-seq,SRP330776,Regulatory T cell-AD.vs.control; scRNA-seq,SRP330776,Natural killer cell_2-AD.vs.control	2
CCT6B	bulk RNA-seq,Homo_714,AD.vs.control; bulk RNA-seq,Homo_714,AD.vs.MCI; scRNA-seq,SRP215507,CD8+ T cell_4-AD.vs.control; scRNA-seq,SRP215507,CD8+ T cell_5-AD.vs.control; scRNA-seq,SRP215507,CD8+ T cell_6-AD.vs.control; scRNA-seq,SRP215507,CD8+ T cell_5-AD.vs.MCI; scRNA-seq,SRP330776,Memory T cell_2-AD.vs.control; scRNA-seq,SRP330776,Natural killer cell_1-AD.vs.control; scRNA-seq,SRP330776,Memory T cell_1-AD.vs.control; scRNA-seq,SRP330776,Naive CD8+ T cell_3-AD.vs.control; scRNA-seq,SRP330776,Natural killer cell_2-AD.vs.control	11
CCT7	scRNA-seq,SRP330776,Regulatory T cell-AD.vs.control; scRNA-seq,SRP330776,B cell_4-AD.vs.control	2
CCT8	bulk RNA-seq,Homo_723,AD.vs.control; bulk RNA-seq,Homo_723,AD.vs.MCI; bulk RNA-seq,Homo_633,AD.vs.control; bulk RNA-seq,Homo_633,AD.vs.MCI; bulk RNA-seq,Homo_714,AD.vs.control; bulk RNA-seq,Homo_714,AD.vs.MCI; scRNA-seq,SRP215507,CD8+ T cell_7-AD.vs.control; scRNA-seq,SRP309935,CD8+ T cell_1-AD.vs.control; scRNA-seq,SRP309935,Megakaryocyte_2-AD.vs.control; scRNA-	10
CCT8L2	bulk RNA-seq,Homo_633,AD.vs.control; bulk RNA-seq,Homo_714,MCI.vs.control	2
CCZ1	scRNA-seq,SRP215507,CD8+ T cell_3-AD.vs.MCI; scRNA-seq,SRP215507,CD8+ T cell_2-MCI.vs.control; scRNA-seq,SRP330776,γδ T cell-AD.vs.control; scRNA-seq,SRP330776,Memory T cell_2-AD.vs.control; scRNA-seq,SRP330776,CD8+ T cell_2-AD.vs.control; scRNA-seq,SRP330776,B cell_1-AD.vs.control; scRNA-seq,SRP330776,Naive CD8+ T cell_1-AD.vs.control; scRNA-seq,SRP330776,Natural killer cell_1-AD.vs.control; scRNA-seq,SRP330776,Memory T cell_1-AD.vs.control; scRNA-seq,SRP330776,Naive CD8+ T cell_3-AD.vs.control; scRNA-seq,SRP330776,B cell_2-AD.vs.control; scRNA-seq,SRP330776,T cell-AD.vs.control; scRNA-seq,SRP330776,CD8+ T cell_3-AD.vs.control; scRNA-seq,SRP330776,Natural killer	14
CCZ1B	scRNA-seq,SRP215507,CD8+ T cell_3-AD.vs.control; scRNA-seq,SRP215507,CD8+ T cell_1-AD.vs.control; scRNA-seq,SRP215507,CD8+ T cell_2-AD.vs.MCI; scRNA-seq,SRP215507,CD8+ T cell_1-AD.vs.MCI; scRNA-seq,SRP215507,CD8+ T cell_5-AD.vs.MCI; scRNA-seq,SRP215507,CD8+ T cell_5-MCI.vs.control; scRNA-seq,SRP215507,CD8+ T cell_2-MCI.vs.control; scRNA-seq,SRP330776,CD8+ T cell_1-AD.vs.control; scRNA-seq,SRP330776,γδ T cell-AD.vs.control; scRNA-seq,SRP330776,Memory T cell_2-AD.vs.control; scRNA-seq,SRP330776,CD8+ T cell_2-AD.vs.control; scRNA-seq,SRP330776,Natural killer cell_1-AD.vs.control; scRNA-seq,SRP330776,Memory T cell_1-AD.vs.control; scRNA-seq,SRP330776,Naive CD8+ T cell_3-AD.vs.control; scRNA-seq,SRP330776,T cell-AD.vs.control; scRNA-seq,SRP330776,CD8+ T cell_3-AD.vs.control	16
CD101	bulk RNA-seq,Homo_723,AD.vs.control; bulk RNA-seq,Homo_723,AD.vs.MCI; bulk RNA-seq,Homo_633,AD.vs.control; bulk RNA-seq,Homo_633,AD.vs.MCI; bulk RNA-seq,Homo_714,AD.vs.control; bulk RNA-seq,Homo_714,AD.vs.MCI; scRNA-seq,SRP215507,CD8+ T cell_5-AD.vs.control; scRNA-seq,SRP215507,CD8+ T cell_2-AD.vs.MCI; scRNA-seq,SRP215507,CD8+ T cell_1-AD.vs.MCI; scRNA-seq,SRP215507,CD8+ T cell_1-MCI.vs.control; scRNA-seq,SRP215507,CD8+ T cell_5-MCI.vs.control; scRNA-seq,SRP215507,CD8+ T cell_2-MCI.vs.control; scRNA-seq,SRP309935,Monocyte_2-AD.vs.control; scRNA-seq,SRP330776,CD8+ T cell_1-AD.vs.control; scRNA-seq,SRP330776,CD8+ T cell_2-AD.vs.control; scRNA-seq,SRP330776,Naive CD8+ T cell_1-AD.vs.control; scRNA-seq,SRP330776,Natural killer cell_1-AD.vs.control; scRNA-seq,SRP330776,Natural [illegible]	18

CD109	bulk RNA-seq,Homo_723,AD.vs.control; bulk RNA-seq,Homo_723,AD.vs.MCI; bulk RNA-seq,Homo_723,MCI.vs.control; bulk RNA-seq,Homo_633,AD.vs.control; bulk RNA-seq,Homo_633,AD.vs.MCI; bulk RNA-seq,Homo_633,MCI.vs.control; bulk RNA-seq,Homo_714,AD.vs.control; bulk RNA-seq,Homo_714,AD.vs.MCI; scRNA-seq,SRP215507,CD8+ T cell_1-MCI.vs.control; scRNA-seq,SRP215507,CD8+ T cell_2-MCI.vs.control; scRNA-seq,SRP330776,Memory T cell_1-AD.vs.control; scRNA-seq,SRP330776,Megakaryocyte_1-AD.vs.control	12
CD14	bulk RNA-seq,SRP223445,AD.vs.control; scRNA-seq,SRP330776,Monocyte_1-AD.vs.control	2
Cd151	bulk RNA-seq,SRP325058,AD.vs.control	1
CD151	scRNA-seq,SRP215507,CD8+ T cell_4-AD.vs.MCI; scRNA-seq,SRP215507,CD8+ T cell_4-MCI.vs.control; scRNA-seq,SRP215507,CD8+ T cell_3-MCI.vs.control; scRNA-seq,SRP309935,Natural killer cell_1-AD.vs.control; scRNA-seq,SRP330776,γδ T cell-AD.vs.control; scRNA-seq,SRP330776,CD8+ T cell_2-AD.vs.control; scRNA-seq,SRP330776,Naive CD8+ T cell_1-AD.vs.control; scRNA-seq,SRP330776,Natural killer cell_1-AD.vs.control; scRNA-seq,SRP330776,Natural killer T cell-AD.vs.control; scRNA-seq,SRP330776,Naive CD8+ T cell_3-AD.vs.control; scRNA-seq,SRP330776,T cell-AD.vs.control; scRNA-seq,SRP330776,Megakaryocyte_1-AD.vs.control; scRNA-seq,SRP330776,B cell_4-AD.vs.control	13
CD160	scRNA-seq,SRP215507,CD8+ T cell_2-AD.vs.control; scRNA-seq,SRP215507,CD8+ T cell_3-AD.vs.control; scRNA-seq,SRP215507,CD8+ T cell_3-AD.vs.MCI; scRNA-seq,SRP215507,CD8+ T cell_4-AD.vs.MCI; scRNA-seq,SRP215507,CD8+ T cell_5-MCI.vs.control; scRNA-seq,SRP215507,CD8+ T cell_4-MCI.vs.control; scRNA-seq,SRP215507,CD8+ T cell_2-MCI.vs.control; scRNA-seq,SRP215507,CD8+ T cell_3-MCI.vs.control; scRNA-seq,SRP215507,CD8+ T cell_6-MCI.vs.control; scRNA-seq,SRP309935,Megakaryocyte_1-AD.vs.control; scRNA-seq,SRP309935,Erythroid cell-AD.vs.control; scRNA-seq,SRP309935,Natural killer cell_1-AD.vs.control; scRNA-seq,SRP309935,CD8+ T cell_1-AD.vs.control; scRNA-seq,SRP309935,B cell_1-AD.vs.control; scRNA-seq,SRP309935,CD8+ T cell_2-AD.vs.control; scRNA-seq,SRP309935,CD4+ T cell-AD.vs.control; scRNA-seq,SRP330776,Memory T cell_2-AD.vs.control; scRNA-seq,SRP330776,Natural killer cell_1-AD.vs.control; scRNA-seq,SRP330776,CD8+ T [illegible]	19
CD163L1	bulk RNA-seq,Homo_723,AD.vs.control; bulk RNA-seq,Homo_723,AD.vs.MCI; bulk RNA-seq,Homo_723,MCI.vs.control; bulk RNA-seq,Homo_633,AD.vs.control; bulk RNA-seq,Homo_633,AD.vs.MCI; bulk RNA-seq,Homo_633,MCI.vs.control; bulk RNA-seq,Homo_714,AD.vs.control;	8
CD164	bulk RNA-seq,Homo_714,AD.vs.control; bulk RNA-seq,Homo_714,AD.vs.MCI; scRNA-seq,SRP215507,CD8+ T cell_7-AD.vs.control; scRNA-seq,SRP215507,CD8+ T cell_7-AD.vs.MCI; scRNA-seq,SRP330776,Naive CD8+ T cell_2-AD.vs.control; scRNA-seq,SRP330776,Megakaryocyte_1-AD.vs.control; scRNA-seq,SRP330776,B cell_4-AD.vs.control	7
CD164L2	bulk RNA-seq,Homo_723,AD.vs.control; bulk RNA-seq,Homo_723,AD.vs.MCI	2
CD177	bulk RNA-seq,Homo_723,AD.vs.control; bulk RNA-seq,Homo_723,AD.vs.MCI; bulk RNA-seq,Homo_633,AD.vs.control; bulk RNA-seq,Homo_633,AD.vs.MCI; bulk RNA-seq,Homo_714,AD.vs.control; bulk RNA-seq,Homo_714,AD.vs.MCI	6
Cd177	bulk RNA-seq,SRP325058,AD.vs.control	1
CD180	scRNA-seq,SRP330776,B cell_1-AD.vs.control; scRNA-seq,SRP330776,B cell_2-AD.vs.control; scRNA-seq,SRP330776,B cell_3-AD.vs.control	3
CD19	bulk RNA-seq,Homo_723,AD.vs.control; bulk RNA-seq,Homo_723,MCI.vs.control; bulk RNA-seq,Homo_633,AD.vs.control; bulk RNA-seq,Homo_633,MCI.vs.control; bulk RNA-seq,Homo_714,AD.vs.control; bulk RNA-seq,Homo_714,AD.vs.MCI; bulk RNA-seq,Homo_714,MCI.vs.control; bulk RNA-seq,ROSMAP,AD.vs.control; scRNA-seq,SRP215507,CD8+ T cell_7-AD.vs.control	9
Cd19	bulk RNA-seq,SRP325058,AD.vs.control	1
CD1A	bulk RNA-seq,Homo_723,AD.vs.control; bulk RNA-seq,Homo_723,AD.vs.MCI; bulk RNA-seq,Homo_633,AD.vs.control; bulk RNA-seq,Homo_633,AD.vs.MCI; bulk RNA-seq,Homo_714,AD.vs.control; bulk RNA-seq,Homo_714,AD.vs.MCI	6
CD1B	bulk RNA-seq,Homo_723,AD.vs.control; bulk RNA-seq,Homo_723,AD.vs.MCI; bulk RNA-seq,Homo_633,AD.vs.control; bulk RNA-seq,Homo_633,AD.vs.MCI; bulk RNA-seq,Homo_714,AD.vs.control; bulk RNA-seq,Homo_714,AD.vs.MCI	6
CD1C	bulk RNA-seq,Homo_723,AD.vs.control; bulk RNA-seq,Homo_723,AD.vs.MCI; scRNA-seq,SRP330776,B cell_2-AD.vs.control; scRNA-seq,SRP330776,Monocyte_2-AD.vs.control	4
CD1D	scRNA-seq,SRP215507,CD8+ T cell_9-AD.vs.control; scRNA-seq,SRP330776,B cell_1-AD.vs.control; scRNA-seq,SRP330776,B cell_2-AD.vs.control	3
CD1E	bulk RNA-seq,Homo_723,AD.vs.control; bulk RNA-seq,Homo_723,AD.vs.MCI; bulk RNA-seq,ROSMAP,MCI.vs.control	3

CD2	bulk RNA-seq,Homo_723,AD.vs.control; bulk RNA-seq,Homo_723,AD.vs.MCI; bulk RNA-seq,Homo_723,MCI.vs.control; bulk RNA-seq,Homo_633,AD.vs.control; bulk RNA-seq,Homo_633,MCI.vs.control; bulk RNA-seq,Homo_714,AD.vs.control; bulk RNA-seq,Homo_714,AD.vs.MCI; bulk RNA-seq,SRP310421,AD.vs.control; scRNA-seq,SRP309935,Erythroid cell-AD.vs.control; scRNA-seq,SRP330776,γδ T cell-AD.vs.control; scRNA-seq,SRP330776,B cell_1-AD.vs.control; scRNA-seq,SRP330776,Natural killer T cell-AD.vs.control; scRNA-seq,SRP330776,B cell_2-AD.vs.control	13
CD200	bulk RNA-seq,Homo_723,AD.vs.control; bulk RNA-seq,Homo_723,AD.vs.MCI; bulk RNA-seq,Homo_714,AD.vs.control; bulk RNA-seq,Homo_714,AD.vs.MCI; scRNA-seq,SRP215507,CD8+ T cell_3-AD.vs.control; scRNA-seq,SRP330776,B cell_1-AD.vs.control; scRNA-	7
CD200R1	bulk RNA-seq,Homo_723,AD.vs.control; bulk RNA-seq,Homo_723,AD.vs.MCI; bulk RNA-seq,Homo_633,AD.vs.control; bulk RNA-seq,Homo_633,AD.vs.MCI; bulk RNA-seq,Homo_714,AD.vs.control; bulk RNA-seq,Homo_714,AD.vs.MCI; scRNA-seq,SRP215507,CD8+ T cell_2-AD.vs.control; scRNA-seq,SRP215507,CD8+ T cell_4-AD.vs.control; scRNA-seq,SRP215507,CD8+ T cell_3-AD.vs.MCI; scRNA-seq,SRP215507,CD8+ T cell_1-AD.vs.MCI; scRNA-seq,SRP215507,CD8+ T cell_4-MCI.vs.control; scRNA-seq,SRP215507,CD8+ T cell_2-MCI.vs.control; scRNA-seq,SRP215507,CD8+ T cell_3-MCI.vs.control; scRNA-seq,SRP309935,Monocyte_1-AD.vs.control; scRNA-seq,SRP330776,CD8+ T cell_1-AD.vs.control; scRNA-seq,SRP330776,γδ T cell-AD.vs.control; scRNA-seq,SRP330776,Memory T cell_2-AD.vs.control; scRNA-seq,SRP330776,CD8+ T cell_2-AD.vs.control; scRNA-seq,SRP330776,Naive CD8+ T cell_1-AD.vs.control; scRNA-seq,SRP330776,Natural killer cell_1-AD.vs.control; scRNA-seq,SRP330776,Natural killer T cell-AD.vs.control; scRNA-seq,SRP330776,T cell-AD.vs.control; scRNA-seq,SRP330776,CD8+ T cell_3-AD.vs.control; [illegible]	24
CD200R1L	bulk RNA-seq,Homo_723,AD.vs.control; bulk RNA-seq,Homo_723,AD.vs.MCI; bulk RNA-seq,Homo_633,AD.vs.control; bulk RNA-	4
CD207	bulk RNA-seq,Homo_723,AD.vs.control; bulk RNA-seq,Homo_723,AD.vs.MCI; bulk RNA-seq,Homo_723,MCI.vs.control; bulk RNA-seq,Homo_633,AD.vs.control; bulk RNA-seq,Homo_633,MCI.vs.control; bulk RNA-seq,Homo_714,AD.vs.MCI; bulk RNA-seq,Homo_714,MCI.vs.control	7
Cd209a	bulk RNA-seq,SRP325058,AD.vs.control	1
CD22	bulk RNA-seq,Homo_714,AD.vs.control; scRNA-seq,SRP215507,CD8+ T cell_3-MCI.vs.control; scRNA-seq,SRP330776,Naive CD8+ T cell_2-	3
CD226	bulk RNA-seq,Homo_723,MCI.vs.control; bulk RNA-seq,Homo_633,AD.vs.control; bulk RNA-seq,Homo_633,MCI.vs.control; scRNA-seq,SRP215507,CD8+ T cell_10-AD.vs.control; scRNA-seq,SRP215507,CD8+ T cell_1-AD.vs.MCI; scRNA-seq,SRP215507,CD8+ T cell_5-AD.vs.MCI; scRNA-seq,SRP215507,CD8+ T cell_6-MCI.vs.control; scRNA-seq,SRP309935,Natural killer cell_1-AD.vs.control; scRNA-seq,SRP330776,CD8+ T cell_2-AD.vs.control; scRNA-seq,SRP330776,B cell_1-AD.vs.control; scRNA-seq,SRP330776,Natural killer cell_2-AD.vs.control	11
CD24	bulk RNA-seq,Homo_723,AD.vs.control; bulk RNA-seq,Homo_723,AD.vs.MCI; bulk RNA-seq,Homo_633,AD.vs.control; bulk RNA-seq,Homo_633,AD.vs.MCI; bulk RNA-seq,Homo_714,AD.vs.control; bulk RNA-seq,Homo_714,AD.vs.MCI	6
CD244	bulk RNA-seq,Homo_714,MCI.vs.control; scRNA-seq,SRP215507,CD8+ T cell_6-AD.vs.MCI; scRNA-seq,SRP215507,CD8+ T cell_4-MCI.vs.control; scRNA-seq,SRP330776,CD8+ T cell_1-AD.vs.control; scRNA-seq,SRP330776,γδ T cell-AD.vs.control; scRNA-seq,SRP330776,Naive CD8+ T cell_1-AD.vs.control; scRNA-seq,SRP330776,Natural killer cell_1-AD.vs.control; scRNA-seq,SRP330776,Naive CD8+ T cell_3-AD.vs.control; scRNA-seq,SRP330776,T cell-AD.vs.control; scRNA-seq,SRP330776,Monocyte_1-AD.vs.control; scRNA-seq,SRP330776,Monocyte_2-AD.vs.control	11
CD247	scRNA-seq,SRP309935,Erythroid cell-AD.vs.control; scRNA-seq,SRP309935,Megakaryocyte_2-AD.vs.control	2
CD248	scRNA-seq,SRP215507,CD8+ T cell_3-AD.vs.control; scRNA-seq,SRP215507,CD8+ T cell_1-AD.vs.control; scRNA-seq,SRP215507,CD8+ T cell_4-AD.vs.control; scRNA-seq,SRP215507,CD8+ T cell_2-AD.vs.MCI; scRNA-seq,SRP215507,CD8+ T cell_4-AD.vs.MCI; scRNA-seq,SRP215507,CD8+ T cell_6-MCI.vs.control; scRNA-seq,SRP330776,Naive CD8+ T cell_1-AD.vs.control; scRNA-seq,SRP330776,Naive CD8+ T cell_3-AD.vs.control; scRNA-	9
Cd24a	bulk RNA-seq,SRP325058,AD.vs.control	1
CD27	bulk RNA-seq,Homo_714,AD.vs.control; bulk RNA-seq,Homo_714,AD.vs.MCI; scRNA-seq,SRP215507,CD8+ T cell_7-AD.vs.control; scRNA-seq,SRP215507,CD8+ T cell_10-AD.vs.control; scRNA-seq,SRP215507,CD8+ T cell_1-AD.vs.MCI; scRNA-seq,SRP215507,CD8+ T cell_4-AD.vs.MCI; scRNA-seq,SRP215507,CD8+ T cell_1-MCI.vs.control; scRNA-seq,SRP215507,CD8+ T cell_4-MCI.vs.control; scRNA-seq,SRP330776,γδ T cell-AD.vs.control; scRNA-seq,SRP330776,Natural killer cell_1-AD.vs.control; scRNA-seq,SRP330776,Memory T cell_1-AD.vs.control; scRNA-	13
CD274	bulk RNA-seq,Homo_723,AD.vs.control; bulk RNA-seq,Homo_723,AD.vs.MCI; bulk RNA-seq,Homo_723,MCI.vs.control; bulk RNA-seq,Homo_633,AD.vs.control; bulk RNA-seq,Homo_633,AD.vs.MCI; bulk RNA-seq,Homo_633,MCI.vs.control; scRNA-seq,SRP215507,CD8+ T cell_5-AD.vs.control; scRNA-seq,SRP330776,Memory T cell_1-AD.vs.control	8
CD276	bulk RNA-seq,Homo_723,AD.vs.control; bulk RNA-seq,Homo_723,AD.vs.MCI; bulk RNA-seq,Homo_723,MCI.vs.control; bulk RNA-seq,Homo_633,AD.vs.control; bulk RNA-seq,Homo_633,AD.vs.MCI; bulk RNA-seq,Homo_714,MCI.vs.control	6

CD27-AS1	scRNA-seq,SRP215507,CD8+ T cell_2-AD.vs.control; scRNA-seq,SRP215507,CD8+ T cell_2-AD.vs.MCI; scRNA-seq,SRP330776,γδ T cell-AD.vs.control; scRNA-seq,SRP330776,CD8+ T cell_2-AD.vs.control; scRNA-seq,SRP330776,B cell_1-AD.vs.control; scRNA-seq,SRP330776,Natural killer cell_1-AD.vs.control; scRNA-seq,SRP330776,Memory T cell_1-AD.vs.control; scRNA-seq,SRP330776,T cell-AD.vs.control; scRNA-seq,SRP330776,Monocyte_1-AD.vs.control; scRNA-seq,SRP330776,B cell_3-AD.vs.control	10
CD28	bulk RNA-seq,Homo_723,AD.vs.MCI; bulk RNA-seq,Homo_723,MCI.vs.control; bulk RNA-seq,Homo_633,AD.vs.MCI; bulk RNA-seq,Homo_633,MCI.vs.control; bulk RNA-seq,SRP310421,AD.vs.control; scRNA-seq,SRP215507,CD8+ T cell_1-AD.vs.control; scRNA-seq,SRP215507,CD8+ T cell_4-AD.vs.control; scRNA-seq,SRP215507,CD8+ T cell_1-AD.vs.MCI; scRNA-seq,SRP215507,CD8+ T cell_4-AD.vs.MCI; scRNA-seq,SRP215507,CD8+ T cell_1-MCI.vs.control; scRNA-seq,SRP215507,CD8+ T cell_4-MCI.vs.control; scRNA-seq,SRP215507,CD8+ T cell_3-MCI.vs.control; scRNA-seq,SRP309935,CD8+ T cell_2-AD.vs.control; scRNA-seq,SRP330776,Natural killer cell_1-AD.vs.control	14
CD2AP	scRNA-seq,SRP215507,CD8+ T cell_7-AD.vs.control; scRNA-seq,SRP215507,CD8+ T cell_7-MCI.vs.control; scRNA-seq,SRP309935,Naive CD8+ T cell_3-AD.vs.control; scRNA-seq,SRP330776,Memory T cell_2-AD.vs.control; scRNA-seq,SRP330776,Naive CD8+ T cell_1-AD.vs.control; scRNA-seq,SRP330776,B cell_2-AD.vs.control; scRNA-seq,SRP330776,T cell-AD.vs.control; scRNA-seq,SRP330776,Naive CD8+ T cell_2-AD.vs.control; scRNA-seq,SRP330776,CD8+ T cell_3-AD.vs.control; scRNA-seq,SRP330776,Monocyte_2-AD.vs.control	10
CD2BP2	scRNA-seq,SRP215507,CD8+ T cell_10-AD.vs.control; scRNA-seq,SRP215507,CD8+ T cell_5-MCI.vs.control; scRNA-seq,SRP330776,Monocyte_2-AD.vs.control; scRNA-seq,SRP330776,Regulatory T cell-AD.vs.control	4
CD2BP2-DT	scRNA-seq,SRP215507,CD8+ T cell_3-AD.vs.MCI; scRNA-seq,SRP215507,CD8+ T cell_2-MCI.vs.control; scRNA-seq,SRP215507,CD8+ T cell_3-	3
CD300A	scRNA-seq,SRP215507,CD8+ T cell_3-AD.vs.control; scRNA-seq,SRP215507,CD8+ T cell_1-AD.vs.control; scRNA-seq,SRP215507,CD8+ T cell_4-AD.vs.control; scRNA-seq,SRP215507,CD8+ T cell_6-AD.vs.control; scRNA-seq,SRP215507,CD8+ T cell_9-AD.vs.control; scRNA-seq,SRP215507,CD8+ T cell_2-AD.vs.MCI; scRNA-seq,SRP215507,CD8+ T cell_4-AD.vs.MCI; scRNA-seq,SRP215507,CD8+ T cell_5-MCI.vs.control; scRNA-seq,SRP215507,CD8+ T cell_2-MCI.vs.control; scRNA-seq,SRP215507,CD8+ T cell_3-MCI.vs.control; scRNA-seq,SRP215507,CD8+ T cell_6-MCI.vs.control; scRNA-seq,SRP309935,Naive CD8+ T cell_1-AD.vs.control; scRNA-seq,SRP309935,CD8+ T cell_2-AD.vs.control; scRNA-seq,SRP330776,CD8+ T cell_1-AD.vs.control; scRNA-seq,SRP330776,γδ T cell-AD.vs.control; scRNA-seq,SRP330776,Memory T cell_2-AD.vs.control;	18
CD300C	scRNA-seq,SRP215507,CD8+ T cell_1-AD.vs.control; scRNA-seq,SRP330776,Natural killer cell_2-AD.vs.control	2
CD300E	bulk RNA-seq,Homo_723,AD.vs.control; bulk RNA-seq,Homo_723,AD.vs.MCI; bulk RNA-seq,Homo_723,MCI.vs.control; bulk RNA-seq,Homo_633,AD.vs.control; bulk RNA-seq,Homo_633,AD.vs.MCI; bulk RNA-seq,Homo_633,MCI.vs.control; bulk RNA-seq,Homo_714,AD.vs.control; bulk RNA-seq,Homo_714,AD.vs.MCI; bulk RNA-seq,Homo_714,MCI.vs.control	9
Cd300e	bulk RNA-seq,SRP325058,AD.vs.control	1
CD300H	bulk RNA-seq,Homo_723,AD.vs.control; bulk RNA-seq,Homo_723,AD.vs.MCI; bulk RNA-seq,Homo_633,AD.vs.control; bulk RNA-seq,Homo_633,AD.vs.MCI; bulk RNA-seq,Homo_714,AD.vs.control; bulk RNA-seq,Homo_714,AD.vs.MCI	6
CD300LB	bulk RNA-seq,Homo_714,AD.vs.control; bulk RNA-seq,Homo_714,AD.vs.MCI; scRNA-seq,SRP330776,Memory T cell_1-AD.vs.control	3
CD300LD	bulk RNA-seq,Homo_723,AD.vs.control; bulk RNA-seq,Homo_723,AD.vs.MCI; bulk RNA-seq,Homo_633,AD.vs.control; bulk RNA-seq,Homo_633,AD.vs.MCI; bulk RNA-seq,Homo_714,AD.vs.control; bulk RNA-seq,Homo_714,AD.vs.MCI	6
CD300LF	bulk RNA-seq,Homo_723,AD.vs.control; bulk RNA-seq,Homo_633,AD.vs.control; bulk RNA-seq,Homo_633,MCI.vs.control; scRNA-seq,SRP330776,Monocyte_2-AD.vs.control	4
CD300LG	bulk RNA-seq,Homo_723,AD.vs.MCI; bulk RNA-seq,Homo_723,MCI.vs.control; bulk RNA-seq,Homo_633,AD.vs.MCI; bulk RNA-	4
CD302	bulk RNA-seq,Homo_723,AD.vs.control; bulk RNA-seq,Homo_723,AD.vs.MCI; bulk RNA-seq,Homo_633,AD.vs.control; bulk RNA-seq,Homo_633,AD.vs.MCI; bulk RNA-seq,Homo_714,AD.vs.control; bulk RNA-seq,Homo_714,AD.vs.MCI; scRNA-seq,SRP215507,CD8+ T cell_9-AD.vs.control; scRNA-seq,SRP215507,CD8+ T cell_3-AD.vs.MCI; scRNA-seq,SRP330776,CD8+ T cell_1-AD.vs.control; scRNA-	10

CD320	bulk RNA-seq,Homo_723,AD.vs.MCI; bulk RNA-seq,Homo_723,MCI.vs.control; bulk RNA-seq,Homo_633,AD.vs.control; bulk RNA-seq,Homo_633,AD.vs.MCI; bulk RNA-seq,Homo_633,MCI.vs.control; bulk RNA-seq,Homo_714,AD.vs.control; bulk RNA-seq,Homo_714,AD.vs.MCI; bulk RNA-seq,Homo_714,MCI.vs.control; scRNA-seq,SRP215507,CD8+ T cell_7-AD.vs.control; scRNA-seq,SRP215507,CD8+ T cell_5-AD.vs.MCI; scRNA-seq,SRP215507,CD8+ T cell_1-MCI.vs.control; scRNA-seq,SRP215507,CD8+ T cell_5-MCI.vs.control; scRNA-seq,SRP330776,CD8+ T cell_1-AD.vs.control; scRNA-seq,SRP330776,γδ T cell-AD.vs.control; scRNA-seq,SRP330776,CD8+ T cell_2-AD.vs.control; scRNA-seq,SRP330776,B cell_1-AD.vs.control; scRNA-seq,SRP330776,Natural killer cell_1-AD.vs.control; scRNA-seq,SRP330776,Memory T cell_1-AD.vs.control; scRNA-seq,SRP330776,Natural killer T cell-AD.vs.control; scRNA-seq,SRP330776,B cell_2-AD.vs.control; scRNA-seq,SRP330776,CD8+ T cell_3-[illegible]	23
CD33	scRNA-seq,SRP215507,CD8+ T cell_9-AD.vs.control	1
CD34	bulk RNA-seq,Homo_723,AD.vs.control; bulk RNA-seq,Homo_723,AD.vs.MCI; bulk RNA-seq,Homo_723,MCI.vs.control; bulk RNA-seq,Homo_633,AD.vs.control; bulk RNA-seq,Homo_633,AD.vs.MCI; bulk RNA-seq,Homo_633,MCI.vs.control; bulk RNA-seq,Homo_714,AD.vs.MCI;	8
CD36	bulk RNA-seq,Homo_723,MCI.vs.control; bulk RNA-seq,Homo_633,MCI.vs.control; bulk RNA-seq,Homo_714,AD.vs.control; bulk RNA-seq,Homo_714,AD.vs.MCI; bulk RNA-seq,SRP310421,AD.vs.control; scRNA-seq,SRP309935,Megakaryocyte_1-AD.vs.control; scRNA-seq,SRP309935,Megakaryocyte_2-AD.vs.control; scRNA-seq,SRP309935,B cell_1-AD.vs.control; scRNA-seq,SRP330776,Monocyte_1-AD.vs.control;	10
CD37	scRNA-seq,SRP309935,Erythroid cell-AD.vs.control	1
CD38	bulk RNA-seq,Homo_723,AD.vs.control; bulk RNA-seq,Homo_723,MCI.vs.control; bulk RNA-seq,Homo_633,AD.vs.control; bulk RNA-seq,Homo_633,MCI.vs.control; bulk RNA-seq,Homo_714,AD.vs.control; bulk RNA-seq,Homo_714,AD.vs.MCI; bulk RNA-seq,Homo_714,MCI.vs.control; scRNA-seq,SRP215507,CD8+ T cell_2-AD.vs.control; scRNA-seq,SRP215507,CD8+ T cell_1-AD.vs.control; scRNA-seq,SRP215507,CD8+ T cell_6-AD.vs.control; scRNA-seq,SRP215507,CD8+ T cell_2-AD.vs.MCI; scRNA-seq,SRP215507,CD8+ T cell_1-AD.vs.MCI; scRNA-seq,SRP215507,CD8+ T cell_6-AD.vs.MCI; scRNA-seq,SRP309935,Natural killer cell_1-AD.vs.control; scRNA-seq,SRP309935,CD8+ T cell_1-AD.vs.control; scRNA-seq,SRP309935,CD8+ T cell_2-AD.vs.control; scRNA-seq,SRP330776,B cell_1-AD.vs.control; scRNA-seq,SRP330776,Natural [illegible]	19
CD3D	bulk RNA-seq,ROSMAP,AD.vs.control; bulk RNA-seq,ROSMAP,MCI.vs.control; scRNA-seq,SRP215507,CD8+ T cell_8-AD.vs.control; scRNA-seq,SRP309935,Natural killer cell_2-AD.vs.control; scRNA-seq,SRP309935,Monocyte_1-AD.vs.control; scRNA-seq,SRP309935,Erythroid cell-AD.vs.control; scRNA-seq,SRP309935,Monocyte_2-AD.vs.control; scRNA-seq,SRP309935,Megakaryocyte_2-AD.vs.control; scRNA-seq,SRP309935,B cell_2-AD.vs.control; scRNA-seq,SRP330776,Memory T cell_1-AD.vs.control; scRNA-seq,SRP330776,B cell_3-AD.vs.control; scRNA-	12
CD3E	bulk RNA-seq,Homo_723,AD.vs.MCI; bulk RNA-seq,Homo_723,MCI.vs.control; bulk RNA-seq,Homo_633,AD.vs.control; bulk RNA-seq,Homo_633,AD.vs.MCI; bulk RNA-seq,Homo_633,MCI.vs.control; bulk RNA-seq,Homo_714,AD.vs.control; bulk RNA-seq,Homo_714,AD.vs.MCI; bulk RNA-seq,Homo_714,MCI.vs.control; scRNA-seq,SRP215507,CD8+ T cell_9-AD.vs.control; scRNA-seq,SRP309935,Erythroid cell-AD.vs.control; scRNA-seq,SRP309935,Megakaryocyte_2-AD.vs.control; scRNA-seq,SRP330776,B cell_3-AD.vs.control; scRNA-seq,SRP330776,Megakaryocyte_1-	13
CD3EAP	scRNA-seq,SRP215507,CD8+ T cell_4-AD.vs.control; scRNA-seq,SRP215507,CD8+ T cell_5-AD.vs.control; scRNA-seq,SRP215507,CD8+ T cell_4-AD.vs.MCI; scRNA-seq,SRP330776,CD8+ T cell_1-AD.vs.control; scRNA-seq,SRP330776,B cell_1-AD.vs.control; scRNA-seq,SRP330776,Naive CD8+ T cell_1-AD.vs.control; scRNA-seq,SRP330776,Memory T cell_1-AD.vs.control; scRNA-seq,SRP330776,B cell_2-AD.vs.control	8
CD3G	scRNA-seq,SRP309935,Monocyte_1-AD.vs.control; scRNA-seq,SRP309935,Erythroid cell-AD.vs.control; scRNA-seq,SRP309935,Monocyte_2-AD.vs.control; scRNA-seq,SRP309935,Megakaryocyte_2-AD.vs.control; scRNA-seq,SRP309935,B cell_1-AD.vs.control; scRNA-seq,SRP330776,B cell_1-AD.vs.control; scRNA-seq,SRP330776,Memory T cell_1-AD.vs.control	7
CD4	scRNA-seq,SRP215507,CD8+ T cell_2-AD.vs.control; scRNA-seq,SRP215507,CD8+ T cell_3-AD.vs.control; scRNA-seq,SRP215507,CD8+ T cell_4-AD.vs.control; scRNA-seq,SRP215507,CD8+ T cell_3-AD.vs.MCI; scRNA-seq,SRP215507,CD8+ T cell_1-AD.vs.MCI; scRNA-seq,SRP215507,CD8+ T cell_4-AD.vs.MCI; scRNA-seq,SRP215507,CD8+ T cell_1-MCI.vs.control; scRNA-seq,SRP215507,CD8+ T cell_2-MCI.vs.control; scRNA-seq,SRP309935,CD8+ T cell_2-AD.vs.control; scRNA-seq,SRP330776,γδ T cell-AD.vs.control; scRNA-seq,SRP330776,CD8+ T cell_2-AD.vs.control; scRNA-seq,SRP330776,B cell_1-AD.vs.control; scRNA-seq,SRP330776,Natural killer cell_1-AD.vs.control; scRNA-seq,SRP330776,Memory T cell_1-AD.vs.control; scRNA-seq,SRP330776,Natural killer T cell-AD.vs.control; scRNA-seq,SRP330776,CD8+ T cell_3-AD.vs.control; scRNA-[illegible]	17

CD40	bulk RNA-seq,Homo_714,AD.vs.control; bulk RNA-seq,Homo_714,AD.vs.MCI; bulk RNA-seq,SRP310421,AD.vs.control; scRNA-seq,SRP215507,CD8+ T cell_3-AD.vs.control; scRNA-seq,SRP215507,CD8+ T cell_7-AD.vs.control; scRNA-seq,SRP215507,CD8+ T cell_4-AD.vs.MCI; scRNA-seq,SRP215507,CD8+ T cell_7-MCI.vs.control; scRNA-seq,SRP309935,Monocyte_1-AD.vs.control; scRNA-seq,SRP330776,Memory T cell_2-AD.vs.control; scRNA-seq,SRP330776,CD8+ T cell_2-AD.vs.control; scRNA-seq,SRP330776,B cell_1-AD.vs.control; scRNA-seq,SRP330776,Naive CD8+ T cell_1-AD.vs.control; scRNA-seq,SRP330776,B cell_2-AD.vs.control; scRNA-seq,SRP330776,CD8+ T cell_3-AD.vs.control	14
CD40LG	bulk RNA-seq,Homo_723,MCI.vs.control; bulk RNA-seq,Homo_633,MCI.vs.control; bulk RNA-seq,ROSMAP,AD.vs.control; bulk RNA-seq,ROSMAP,AD.vs.MCI; scRNA-seq,SRP215507,CD8+ T cell_2-AD.vs.control; scRNA-seq,SRP215507,CD8+ T cell_3-AD.vs.control; scRNA-seq,SRP215507,CD8+ T cell_1-AD.vs.control; scRNA-seq,SRP215507,CD8+ T cell_3-AD.vs.MCI; scRNA-seq,SRP215507,CD8+ T cell_1-AD.vs.MCI; scRNA-seq,SRP309935,Megakaryocyte_2-AD.vs.control; scRNA-seq,SRP309935,CD8+ T cell_2-AD.vs.control; scRNA-seq,SRP330776,γδ T cell-AD.vs.control; scRNA-seq,SRP330776,Memory T cell_2-AD.vs.control; scRNA-seq,SRP330776,CD8+ T cell_2-AD.vs.control; scRNA-seq,SRP330776,B cell_1-AD.vs.control; scRNA-seq,SRP330776,Natural killer cell_1-AD.vs.control; scRNA-seq,SRP330776,Memory T cell_1-AD.vs.control; scRNA-seq,SRP330776,Natural killer T cell-AD.vs.control; scRNA-seq,SRP330776,B cell_3-AD.vs.control; scRNA-seq,SRP330776,Natural killer cell_2-	20
CD44	scRNA-seq,SRP215507,CD8+ T cell_7-MCI.vs.control; scRNA-seq,SRP215507,CD8+ T cell_8-MCI.vs.control; scRNA-seq,SRP330776,B cell_4-	3
CD46	bulk RNA-seq,Homo_633,AD.vs.control; bulk RNA-seq,SRP223445,AD.vs.control; scRNA-seq,SRP309935,Megakaryocyte_1-AD.vs.control; scRNA-seq,SRP309935,CD8+ T cell_1-AD.vs.control; scRNA-seq,SRP309935,CD8+ T cell_2-AD.vs.control; scRNA-seq,SRP330776,Naive CD8+ T cell_3-AD.vs.control; scRNA-seq,SRP330776,B cell_2-AD.vs.control	7
CD47	scRNA-seq,SRP215507,CD8+ T cell_5-AD.vs.MCI; scRNA-seq,SRP215507,CD8+ T cell_5-MCI.vs.control; scRNA-seq,SRP309935,Megakaryocyte_2-AD.vs.control; scRNA-seq,SRP330776,CD8+ T cell_1-AD.vs.control; scRNA-seq,SRP330776,γδ T cell-AD.vs.control; scRNA-seq,SRP330776,Memory T cell_2-AD.vs.control; scRNA-seq,SRP330776,CD8+ T cell_2-AD.vs.control; scRNA-seq,SRP330776,B cell_1-AD.vs.control; scRNA-seq,SRP330776,Naive CD8+ T cell_1-AD.vs.control; scRNA-seq,SRP330776,Natural killer cell_1-AD.vs.control; scRNA-seq,SRP330776,Memory T cell_1-AD.vs.control; scRNA-seq,SRP330776,Natural killer T cell-AD.vs.control; scRNA-seq,SRP330776,Naive CD8+ T cell_3-AD.vs.control; scRNA-seq,SRP330776,B cell_2-AD.vs.control; scRNA-seq,SRP330776,T cell-AD.vs.control; scRNA-seq,SRP330776,Naive CD8+ T cell_2-AD.vs.control; scRNA-seq,SRP330776,CD8+ T cell_3-AD.vs.control; scRNA-seq,SRP330776,Monocyte_2-AD.vs.control; scRNA-seq,SRP330776,B cell_3-	19
CD48	scRNA-seq,SRP215507,CD8+ T cell_6-AD.vs.MCI; scRNA-seq,SRP215507,CD8+ T cell_8-MCI.vs.control; scRNA-seq,SRP309935,Erythroid cell-	3
CD5	scRNA-seq,SRP215507,CD8+ T cell_4-AD.vs.MCI; scRNA-seq,SRP215507,CD8+ T cell_4-MCI.vs.control; scRNA-seq,SRP309935,CD8+ T cell_1-AD.vs.control; scRNA-seq,SRP330776,γδ T cell-AD.vs.control; scRNA-seq,SRP330776,Memory T cell_1-AD.vs.control; scRNA-seq,SRP330776,Monocyte_1-AD.vs.control; scRNA-seq,SRP330776,B cell_3-AD.vs.control	7
CD52	scRNA-seq,SRP309935,Erythroid cell-AD.vs.control; scRNA-seq,SRP330776,Memory T cell_1-AD.vs.control	2
CD53	bulk RNA-seq,Homo_723,AD.vs.control; bulk RNA-seq,Homo_723,MCI.vs.control; bulk RNA-seq,Homo_633,AD.vs.control; bulk RNA-seq,Homo_633,MCI.vs.control; bulk RNA-seq,Homo_714,AD.vs.control; bulk RNA-seq,Homo_714,AD.vs.MCI; bulk RNA-seq,Homo_714,MCI.vs.control; scRNA-seq,SRP309935,Erythroid cell-AD.vs.control; scRNA-seq,SRP330776,Memory T cell_2-AD.vs.control; scRNA-	11
CD55	scRNA-seq,SRP215507,CD8+ T cell_3-AD.vs.control; scRNA-seq,SRP215507,CD8+ T cell_1-AD.vs.control; scRNA-seq,SRP215507,CD8+ T cell_4-AD.vs.control; scRNA-seq,SRP215507,CD8+ T cell_7-AD.vs.control; scRNA-seq,SRP215507,CD8+ T cell_5-AD.vs.control; scRNA-seq,SRP215507,CD8+ T cell_6-AD.vs.control; scRNA-seq,SRP215507,CD8+ T cell_9-AD.vs.control; scRNA-seq,SRP215507,CD8+ T cell_1-AD.vs.MCI; scRNA-seq,SRP215507,CD8+ T cell_4-AD.vs.MCI; scRNA-seq,SRP215507,CD8+ T cell_5-MCI.vs.control; scRNA-seq,SRP215507,CD8+ T cell_3-MCI.vs.control; scRNA-seq,SRP215507,CD8+ T cell_6-MCI.vs.control; scRNA-seq,SRP215507,CD8+ T cell_7-MCI.vs.control; scRNA-seq,SRP309935,CD8+ T cell_1-AD.vs.control; scRNA-seq,SRP330776,Monocyte_1-AD.vs.control; scRNA-seq,SRP330776,Regulatory T cell-	17
CD58	scRNA-seq,SRP215507,CD8+ T cell_3-AD.vs.control; scRNA-seq,SRP215507,CD8+ T cell_3-MCI.vs.control; scRNA-seq,SRP309935,Megakaryocyte_2-AD.vs.control; scRNA-seq,SRP330776,CD8+ T cell_1-AD.vs.control; scRNA-seq,SRP330776,γδ T cell-AD.vs.control; scRNA-seq,SRP330776,Memory T cell_2-AD.vs.control; scRNA-seq,SRP330776,T cell-AD.vs.control; scRNA-seq,SRP330776,Natural killer cell_2-AD.vs.control	8
CD59	scRNA-seq,SRP215507,CD8+ T cell_7-AD.vs.control; scRNA-seq,SRP330776,Natural killer T cell-AD.vs.control; scRNA-seq,SRP330776,T cell-	3
CD5L	bulk RNA-seq,Homo_723,AD.vs.control; bulk RNA-seq,Homo_723,AD.vs.MCI; bulk RNA-seq,Homo_723,MCI.vs.control; bulk RNA-seq,Homo_633,AD.vs.control; bulk RNA-seq,Homo_633,AD.vs.MCI; bulk RNA-seq,Homo_633,MCI.vs.control	6

CD6	scRNA-seq,SRP215507,CD8+ T cell_6-AD.vs.control; scRNA-seq,SRP215507,CD8+ T cell_9-AD.vs.control; scRNA-seq,SRP215507,CD8+ T cell_1-AD.vs.MCI; scRNA-seq,SRP215507,CD8+ T cell_4-AD.vs.MCI; scRNA-seq,SRP215507,CD8+ T cell_4-MCI.vs.control; scRNA-seq,SRP309935,Natural killer cell_2-AD.vs.control; scRNA-seq,SRP309935,Naive CD8+ T cell_1-AD.vs.control; scRNA-seq,SRP309935,Naive CD8+ T cell_2-AD.vs.control; scRNA-seq,SRP309935,CD8+ T cell_1-AD.vs.control; scRNA-seq,SRP330776,CD8+ T cell_1-AD.vs.control; scRNA-seq,SRP330776,γδ T cell-AD.vs.control; scRNA-seq,SRP330776,Memory T cell_2-AD.vs.control; scRNA-seq,SRP330776,CD8+ T cell_2-AD.vs.control; scRNA-seq,SRP330776,B cell_1-AD.vs.control; scRNA-seq,SRP330776,Naive CD8+ T cell_1-AD.vs.control; scRNA-seq,SRP330776,Natural killer cell_1-AD.vs.control; scRNA-seq,SRP330776,Memory T cell_1-AD.vs.control; scRNA-seq,SRP330776,Natural killer T cell-AD.vs.control; scRNA-seq,SRP330776,Naive CD8+ T cell_3-AD.vs.control; scRNA-seq,SRP330776,B cell_2-AD.vs.control; scRNA-seq,SRP330776,T cell-AD.vs.control; scRNA-seq,SRP330776,Naive CD8+ T cell_2-AD.vs.control; scRNA-seq,SRP330776,CD8+ T cell_3-AD.vs.control; scRNA-seq,SRP330776,Monocyte_1-AD.vs.control; scRNA- [illegible]	27
CD63	bulk RNA-seq,Homo_723,AD.vs.control; bulk RNA-seq,Homo_633,AD.vs.control; bulk RNA-seq,SRP223445,AD.vs.control; scRNA-seq,SRP215507,CD8+ T cell_3-AD.vs.control; scRNA-seq,SRP215507,CD8+ T cell_10-AD.vs.control; scRNA-seq,SRP215507,CD8+ T cell_7-MCI.vs.control; scRNA-seq,SRP309935,Erythroid cell-AD.vs.control; scRNA-seq,SRP330776,CD8+ T cell_1-AD.vs.control; scRNA-seq,SRP330776,Naive CD8+ T cell_2-AD.vs.control; scRNA-seq,SRP330776,Regulatory T cell-AD.vs.control	10
CD68	scRNA-seq,SRP215507,CD8+ T cell_9-AD.vs.control; scRNA-seq,SRP215507,CD8+ T cell_2-AD.vs.MCI; scRNA-seq,SRP215507,CD8+ T cell_5-MCI.vs.control; scRNA-seq,SRP215507,CD8+ T cell_2-MCI.vs.control; scRNA-seq,SRP309935,Natural killer cell_2-AD.vs.control; scRNA-seq,SRP330776,γδ T cell-AD.vs.control; scRNA-seq,SRP330776,Naive CD8+ T cell_1-AD.vs.control; scRNA-seq,SRP330776,Regulatory T cell-AD.vs.control; scRNA-seq,SRP330776,Megakaryocyte_1-AD.vs.control; scRNA-seq,SRP330776,B cell_4-AD.vs.control	10
CD69	bulk RNA-seq,Homo_723,AD.vs.control; bulk RNA-seq,Homo_723,MCI.vs.control; bulk RNA-seq,Homo_633,AD.vs.control; bulk RNA-seq,Homo_633,MCI.vs.control; scRNA-seq,SRP215507,CD8+ T cell_8-AD.vs.control; scRNA-seq,SRP215507,CD8+ T cell_7-AD.vs.control; scRNA-seq,SRP215507,CD8+ T cell_8-AD.vs.MCI; scRNA-seq,SRP215507,CD8+ T cell_4-AD.vs.MCI; scRNA-seq,SRP215507,CD8+ T cell_4-MCI.vs.control; scRNA-seq,SRP215507,CD8+ T cell_8-MCI.vs.control; scRNA-seq,SRP309935,Erythroid cell-AD.vs.control; scRNA-seq,SRP309935,Monocyte_2-AD.vs.control; scRNA-seq,SRP309935,Megakaryocyte_2-AD.vs.control; scRNA-seq,SRP330776,γδ T cell-AD.vs.control; scRNA-	15
Cd69	bulk RNA-seq,SRP325058,AD.vs.control	1
CD7	scRNA-seq,SRP215507,CD8+ T cell_7-AD.vs.control; scRNA-seq,SRP215507,CD8+ T cell_6-AD.vs.control; scRNA-seq,SRP215507,CD8+ T cell_7-AD.vs.MCI; scRNA-seq,SRP309935,Erythroid cell-AD.vs.control; scRNA-seq,SRP330776,Megakaryocyte_1-AD.vs.control	5
CD70	bulk RNA-seq,Homo_723,AD.vs.control; bulk RNA-seq,Homo_723,AD.vs.MCI; bulk RNA-seq,Homo_633,AD.vs.control; bulk RNA-seq,Homo_633,AD.vs.MCI; bulk RNA-seq,Homo_714,AD.vs.control; bulk RNA-seq,Homo_714,AD.vs.MCI; scRNA-seq,SRP215507,CD8+ T cell_2-AD.vs.MCI; scRNA-seq,SRP215507,CD8+ T cell_5-AD.vs.MCI; scRNA-seq,SRP215507,CD8+ T cell_6-MCI.vs.control; scRNA-seq,SRP309935,Natural killer cell_1-AD.vs.control; scRNA-seq,SRP330776,CD8+ T cell_1-AD.vs.control; scRNA-seq,SRP330776,Memory T cell_2-AD.vs.control; scRNA-seq,SRP330776,CD8+ T cell_2-AD.vs.control; scRNA-seq,SRP330776,B cell_1-AD.vs.control; scRNA-seq,SRP330776,Naive CD8+ T cell_1-AD.vs.control; scRNA-seq,SRP330776,Natural killer cell_1-AD.vs.control; scRNA-seq,SRP330776,Memory T cell_1-AD.vs.control; scRNA-seq,SRP330776,T cell-AD.vs.control; scRNA-seq,SRP330776,CD8+ T cell_3-AD.vs.control; scRNA-seq,SRP330776,Regulatory T cell-AD.vs.control; [illegible]	21
CD72	scRNA-seq,SRP215507,CD8+ T cell_7-AD.vs.control; scRNA-seq,SRP215507,CD8+ T cell_2-MCI.vs.control; scRNA-seq,SRP309935,B cell_1-AD.vs.control; scRNA-seq,SRP330776,Naive CD8+ T cell_1-AD.vs.control; scRNA-seq,SRP330776,B cell_2-AD.vs.control; scRNA-seq,SRP330776,B	6
CD74	scRNA-seq,SRP309935,Erythroid cell-AD.vs.control; scRNA-seq,SRP330776,Naive CD8+ T cell_2-AD.vs.control; scRNA-	3
CD79A	bulk RNA-seq,Homo_723,AD.vs.control; bulk RNA-seq,Homo_723,AD.vs.MCI; bulk RNA-seq,Homo_723,MCI.vs.control; bulk RNA-seq,Homo_633,AD.vs.control; bulk RNA-seq,Homo_633,MCI.vs.control; bulk RNA-seq,Homo_714,AD.vs.control; bulk RNA-seq,Homo_714,AD.vs.MCI; bulk RNA-seq,Homo_714,MCI.vs.control; bulk RNA-seq,ROSMAP,AD.vs.control; scRNA-seq,SRP215507,CD8+ T cell_3-AD.vs.MCI; scRNA-seq,SRP215507,CD8+ T cell_3-MCI.vs.control; scRNA-seq,SRP309935,Naive CD8+ T cell_1-AD.vs.control; scRNA-seq,SRP330776,γδ T cell-AD.vs.control; scRNA-seq,SRP330776,Memory T cell_1-AD.vs.control; scRNA-seq,SRP330776,Naive CD8+ T cell_3-AD.vs.control; scRNA-	16

CD79B	bulk RNA-seq,Homo_723,AD.vs.control; scRNA-seq,SRP215507,CD8+ T cell_3-AD.vs.control; scRNA-seq,SRP215507,CD8+ T cell_1-AD.vs.control; scRNA-seq,SRP215507,CD8+ T cell_9-AD.vs.control; scRNA-seq,SRP215507,CD8+ T cell_1-AD.vs.MCI; scRNA-seq,SRP309935,Natural killer cell_1-AD.vs.control; scRNA-seq,SRP330776,CD8+ T cell_1-AD.vs.control; scRNA-seq,SRP330776,γδ T cell-AD.vs.control; scRNA-seq,SRP330776,CD8+ T cell_2-AD.vs.control; scRNA-seq,SRP330776,Natural killer cell_1-AD.vs.control; scRNA-seq,SRP330776,Memory T cell_1-AD.vs.control; scRNA-seq,SRP330776,Naive CD8+ T cell_3-AD.vs.control; scRNA-seq,SRP330776,T cell-AD.vs.control; scRNA-seq,SRP330776,Naive CD8+ T cell_2-AD.vs.control; scRNA-seq,SRP330776,CD8+ T cell_3-AD.vs.control; scRNA-seq,SRP330776,B cell_3-AD.vs.control	16
Cd79b	bulk RNA-seq,SRP325058,AD.vs.control	1
CD80	scRNA-seq,SRP215507,CD8+ T cell_6-AD.vs.control; scRNA-seq,SRP330776,B cell_1-AD.vs.control	2
CD81	bulk RNA-seq,Homo_723,AD.vs.control; bulk RNA-seq,Homo_723,AD.vs.MCI; bulk RNA-seq,Homo_633,AD.vs.control; bulk RNA-seq,Homo_633,AD.vs.MCI; bulk RNA-seq,Homo_714,AD.vs.control; bulk RNA-seq,Homo_714,AD.vs.MCI; scRNA-seq,SRP215507,CD8+ T cell_5-AD.vs.control; scRNA-seq,SRP215507,CD8+ T cell_3-AD.vs.MCI; scRNA-seq,SRP330776,CD8+ T cell_1-AD.vs.control; scRNA-seq,SRP330776,γδ T cell-AD.vs.control; scRNA-seq,SRP330776,Memory T cell_2-AD.vs.control; scRNA-seq,SRP330776,B cell_1-AD.vs.control; scRNA-seq,SRP330776,Naive CD8+ T cell_1-AD.vs.control; scRNA-seq,SRP330776,Natural killer cell_1-AD.vs.control; scRNA-seq,SRP330776,B cell_2-AD.vs.control; scRNA-seq,SRP330776,T cell-AD.vs.control; scRNA-seq,SRP330776,B cell_3-AD.vs.control; scRNA-seq,SRP330776,Regulatory T cell-[illegible]	19
CD81-AS1	scRNA-seq,SRP330776,CD8+ T cell_1-AD.vs.control	1
CD82	bulk RNA-seq,SRP223445,AD.vs.control; scRNA-seq,SRP215507,CD8+ T cell_6-AD.vs.control; scRNA-seq,SRP215507,CD8+ T cell_2-AD.vs.MCI; scRNA-seq,SRP215507,CD8+ T cell_3-AD.vs.MCI; scRNA-seq,SRP215507,CD8+ T cell_1-AD.vs.MCI; scRNA-seq,SRP215507,CD8+ T cell_4-AD.vs.MCI; scRNA-seq,SRP215507,CD8+ T cell_1-MCI.vs.control; scRNA-seq,SRP215507,CD8+ T cell_4-MCI.vs.control; scRNA-seq,SRP215507,CD8+ T cell_2-MCI.vs.control; scRNA-seq,SRP215507,CD8+ T cell_3-MCI.vs.control; scRNA-seq,SRP309935,CD8+ T cell_1-AD.vs.control; scRNA-seq,SRP309935,Megakaryocyte_2-AD.vs.control; scRNA-seq,SRP330776,γδ T cell-AD.vs.control; scRNA-seq,SRP330776,CD8+ T cell_2-AD.vs.control; scRNA-seq,SRP330776,Naive CD8+ T cell_1-AD.vs.control; scRNA-seq,SRP330776,Naive CD8+ T cell_2-AD.vs.control; scRNA-seq,SRP330776,CD8+ T cell_3-AD.vs.control; scRNA-seq,SRP330776,Monocyte_1-AD.vs.control; scRNA-seq,SRP330776,Megakaryocyte_1-[illegible]	19
CD83	bulk RNA-seq,Homo_723,AD.vs.MCI; bulk RNA-seq,Homo_723,MCI.vs.control; bulk RNA-seq,Homo_633,AD.vs.control; bulk RNA-seq,Homo_633,AD.vs.MCI; bulk RNA-seq,Homo_714,AD.vs.control; bulk RNA-seq,Homo_714,AD.vs.MCI; scRNA-seq,SRP215507,CD8+ T cell_9-AD.vs.control; scRNA-seq,SRP215507,CD8+ T cell_2-AD.vs.MCI; scRNA-seq,SRP215507,CD8+ T cell_1-AD.vs.MCI; scRNA-seq,SRP215507,CD8+ T cell_5-AD.vs.MCI; scRNA-seq,SRP215507,CD8+ T cell_1-MCI.vs.control; scRNA-seq,SRP215507,CD8+ T cell_5-MCI.vs.control; scRNA-seq,SRP215507,CD8+ T cell_2-MCI.vs.control; scRNA-seq,SRP215507,CD8+ T cell_3-MCI.vs.control; scRNA-seq,SRP330776,CD8+ T cell_1-AD.vs.control; scRNA-seq,SRP330776,γδ T cell-AD.vs.control; scRNA-seq,SRP330776,Memory T cell_2-AD.vs.control; scRNA-seq,SRP330776,CD8+ T cell_2-AD.vs.control; scRNA-seq,SRP330776,Naive CD8+ T cell_1-AD.vs.control; scRNA-seq,SRP330776,Natural killer cell_1-AD.vs.control; scRNA-seq,SRP330776,Memory T cell_1-AD.vs.control; scRNA-seq,SRP330776,Natural killer T cell-AD.vs.control; scRNA-seq,SRP330776,Naive CD8+ T cell_3-AD.vs.control; scRNA-seq,SRP330776,Naive CD8+ T cell_2-AD.vs.control; scRNA-seq,SRP330776,CD8+ T cell_3-AD.vs.control; scRNA-[illegible]	27
CD84	scRNA-seq,SRP215507,CD8+ T cell_4-MCI.vs.control; scRNA-seq,SRP330776,γδ T cell-AD.vs.control; scRNA-seq,SRP330776,Memory T cell_2-AD.vs.control; scRNA-seq,SRP330776,Naive CD8+ T cell_1-AD.vs.control; scRNA-seq,SRP330776,Naive CD8+ T cell_3-AD.vs.control; scRNA-[illegible]	6
CD86	scRNA-seq,SRP215507,CD8+ T cell_6-AD.vs.control; scRNA-seq,SRP215507,CD8+ T cell_6-MCI.vs.control; scRNA-seq,SRP309935,Naive CD8+ T cell_2-AD.vs.control; scRNA-seq,SRP330776,B cell_2-AD.vs.control	4
CD8A	bulk RNA-seq,Homo_723,AD.vs.control; bulk RNA-seq,Homo_723,AD.vs.MCI; bulk RNA-seq,Homo_723,MCI.vs.control; bulk RNA-seq,Homo_633,AD.vs.control; bulk RNA-seq,Homo_633,AD.vs.MCI; bulk RNA-seq,Homo_633,MCI.vs.control; bulk RNA-seq,Homo_714,AD.vs.control; bulk RNA-seq,Homo_714,AD.vs.MCI; bulk RNA-seq,ROSMAP,MCI.vs.control; scRNA-seq,SRP215507,CD8+ T cell_7-AD.vs.control; scRNA-seq,SRP215507,CD8+ T cell_9-AD.vs.control; scRNA-seq,SRP215507,CD8+ T cell_5-AD.vs.MCI; scRNA-seq,SRP215507,CD8+ T cell_8-MCI.vs.control; scRNA-seq,SRP309935,Natural killer cell_2-AD.vs.control; scRNA-seq,SRP309935,Naive CD8+ T cell_1-AD.vs.control; scRNA-seq,SRP330776,CD8+ T cell_1-AD.vs.control; scRNA-seq,SRP330776,γδ T cell-AD.vs.control; scRNA-seq,SRP330776,Naive CD8+ T cell_1-AD.vs.control; scRNA-seq,SRP330776,Natural killer cell_1-AD.vs.control; scRNA-seq,SRP330776,Naive CD8+ T cell_2-AD.vs.control; scRNA-[illegible]	21

CD8B	bulk RNA-seq,Homo_723,AD.vs.control; bulk RNA-seq,Homo_723,AD.vs.MCI; bulk RNA-seq,Homo_633,AD.vs.control; bulk RNA-seq,Homo_633,AD.vs.MCI; bulk RNA-seq,Homo_714,AD.vs.control; bulk RNA-seq,Homo_714,AD.vs.MCI; scRNA-seq,SRP215507,CD8+ T cell_8-AD.vs.control; scRNA-seq,SRP215507,CD8+ T cell_7-AD.vs.control; scRNA-seq,SRP215507,CD8+ T cell_9-AD.vs.control; scRNA-seq,SRP309935,Monocyte_1-AD.vs.control; scRNA-seq,SRP309935,Erythroid cell-AD.vs.control; scRNA-seq,SRP309935,Natural killer cell_1-AD.vs.control; scRNA-seq,SRP330776,CD8+ T cell_1-AD.vs.control; scRNA-seq,SRP330776,γδ T cell-AD.vs.control; scRNA-seq,SRP330776,Memory T cell_2-AD.vs.control; scRNA-seq,SRP330776,Natural killer cell_1-AD.vs.control; scRNA-seq,SRP330776,Memory T cell_1-AD.vs.control; scRNA-[illegible]	18
CD8B2	bulk RNA-seq,Homo_723,AD.vs.control; bulk RNA-seq,Homo_723,AD.vs.MCI; bulk RNA-seq,Homo_633,AD.vs.control; bulk RNA-seq,Homo_633,AD.vs.MCI; bulk RNA-seq,Homo_714,AD.vs.control; bulk RNA-seq,Homo_714,AD.vs.MCI; scRNA-seq,SRP215507,CD8+ T cell_2-AD.vs.control; scRNA-seq,SRP215507,CD8+ T cell_1-AD.vs.control; scRNA-seq,SRP215507,CD8+ T cell_5-AD.vs.control; scRNA-seq,SRP215507,CD8+ T cell_2-AD.vs.MCI; scRNA-seq,SRP215507,CD8+ T cell_3-AD.vs.MCI; scRNA-seq,SRP215507,CD8+ T cell_1-AD.vs.MCI; scRNA-seq,SRP215507,CD8+ T cell_5-AD.vs.MCI; scRNA-seq,SRP215507,CD8+ T cell_2-MCI.vs.control; scRNA-seq,SRP215507,CD8+ T cell_3-MCI.vs.control; scRNA-seq,SRP330776,CD8+ T cell_1-AD.vs.control; scRNA-seq,SRP330776,Natural killer cell_1-AD.vs.control; scRNA-[illegible]	18
Cd9	bulk RNA-seq,SRP325058,AD.vs.control	1
CD9	scRNA-seq,SRP215507,CD8+ T cell_1-AD.vs.control; scRNA-seq,SRP215507,CD8+ T cell_3-AD.vs.MCI; scRNA-seq,SRP215507,CD8+ T cell_1-AD.vs.MCI; scRNA-seq,SRP215507,CD8+ T cell_3-MCI.vs.control; scRNA-seq,SRP330776,CD8+ T cell_2-AD.vs.control; scRNA-seq,SRP330776,Naive CD8+ T cell_1-AD.vs.control; scRNA-seq,SRP330776,Naive CD8+ T cell_3-AD.vs.control; scRNA-seq,SRP330776,B cell_2-AD.vs.control; scRNA-seq,SRP330776,Monocyte_2-AD.vs.control; scRNA-seq,SRP330776,Megakaryocyte_1-AD.vs.control	10
Cd93	bulk RNA-seq,SRP325058,AD.vs.control	1
CD93	scRNA-seq,SRP330776,Monocyte_2-AD.vs.control	1
CD96	bulk RNA-seq,Homo_723,AD.vs.control; bulk RNA-seq,Homo_723,AD.vs.MCI; bulk RNA-seq,Homo_633,AD.vs.control; bulk RNA-seq,Homo_633,AD.vs.MCI; bulk RNA-seq,Homo_714,AD.vs.control; bulk RNA-seq,Homo_714,AD.vs.MCI; bulk RNA-seq,ROSMAP,MCI.vs.control; bulk RNA-seq,SRP223445,AD.vs.control; scRNA-seq,SRP215507,CD8+ T cell_8-AD.vs.control; scRNA-seq,SRP215507,CD8+ T cell_6-AD.vs.MCI; scRNA-seq,SRP309935,Megakaryocyte_2-AD.vs.control; scRNA-seq,SRP330776,B cell_1-AD.vs.control	12
Cd96	bulk RNA-seq,SRP325058,AD.vs.control	1
CD99	bulk RNA-seq,SRP223445,AD.vs.control; scRNA-seq,SRP215507,CD8+ T cell_8-AD.vs.control; scRNA-seq,SRP215507,CD8+ T cell_3-AD.vs.control; scRNA-seq,SRP215507,CD8+ T cell_4-AD.vs.control; scRNA-seq,SRP215507,CD8+ T cell_7-AD.vs.control; scRNA-seq,SRP215507,CD8+ T cell_3-MCI.vs.control; scRNA-seq,SRP309935,Erythroid cell-AD.vs.control; scRNA-seq,SRP330776,Megakaryocyte_1-AD.vs.control	8
CD99L2	bulk RNA-seq,Homo_723,AD.vs.control; bulk RNA-seq,Homo_723,MCI.vs.control; bulk RNA-seq,Homo_633,AD.vs.control; bulk RNA-seq,Homo_633,AD.vs.MCI; bulk RNA-seq,Homo_633,MCI.vs.control; scRNA-seq,SRP215507,CD8+ T cell_3-AD.vs.MCI; scRNA-seq,SRP215507,CD8+ T cell_3-MCI.vs.control; scRNA-seq,SRP330776,CD8+ T cell_1-AD.vs.control; scRNA-seq,SRP330776,Memory T cell_2-AD.vs.control; scRNA-seq,SRP330776,CD8+ T cell_2-AD.vs.control; scRNA-seq,SRP330776,B cell_1-AD.vs.control; scRNA-seq,SRP330776,Naive CD8+ T cell_1-AD.vs.control; scRNA-seq,SRP330776,Natural killer cell_1-AD.vs.control; scRNA-seq,SRP330776,Natural killer T cell-AD.vs.control; scRNA-seq,SRP330776,B cell_2-AD.vs.control; scRNA-seq,SRP330776,T cell-AD.vs.control; scRNA-seq,SRP330776,Natural killer cell_2-AD.vs.control	17
CDA	scRNA-seq,SRP309935,Monocyte_3-AD.vs.control	1
CDADC1	bulk RNA-seq,Homo_723,AD.vs.control; bulk RNA-seq,Homo_723,AD.vs.MCI; bulk RNA-seq,Homo_633,AD.vs.control; bulk RNA-seq,Homo_633,AD.vs.MCI; scRNA-seq,SRP215507,CD8+ T cell_7-AD.vs.control; scRNA-seq,SRP215507,CD8+ T cell_3-AD.vs.MCI; scRNA-seq,SRP215507,CD8+ T cell_1-AD.vs.MCI; scRNA-seq,SRP215507,CD8+ T cell_7-AD.vs.MCI; scRNA-seq,SRP215507,CD8+ T cell_3-MCI.vs.control; scRNA-seq,SRP330776,CD8+ T cell_1-AD.vs.control; scRNA-seq,SRP330776,Memory T cell_2-AD.vs.control; scRNA-seq,SRP330776,Naive CD8+ T cell_1-AD.vs.control; scRNA-seq,SRP330776,Naive CD8+ T cell_3-AD.vs.control; scRNA-seq,SRP330776,Natural killer cell_2-AD.vs.control	14

CDAN1	bulk RNA-seq,Homo_633,AD.vs.control; bulk RNA-seq,Homo_633,AD.vs.MCI; bulk RNA-seq,Homo_714,AD.vs.control; bulk RNA-seq,Homo_714,AD.vs.MCI; scRNA-seq,SRP215507,CD8+ T cell_3-AD.vs.control; scRNA-seq,SRP215507,CD8+ T cell_4-AD.vs.control; scRNA-seq,SRP215507,CD8+ T cell_3-AD.vs.MCI; scRNA-seq,SRP215507,CD8+ T cell_4-AD.vs.MCI; scRNA-seq,SRP330776,CD8+ T cell_1-AD.vs.control; scRNA-seq,SRP330776,Memory T cell_2-AD.vs.control; scRNA-seq,SRP330776,CD8+ T cell_2-AD.vs.control; scRNA-seq,SRP330776,B cell_1-AD.vs.control; scRNA-seq,SRP330776,Naive CD8+ T cell_1-AD.vs.control; scRNA-seq,SRP330776,Natural killer cell_1-AD.vs.control; scRNA-seq,SRP330776,Memory T cell_1-AD.vs.control; scRNA-seq,SRP330776,Natural killer T cell-AD.vs.control; scRNA-seq,SRP330776,T cell-AD.vs.control; [illegible]	19
CDC123	bulk RNA-seq,Homo_723,AD.vs.control; bulk RNA-seq,Homo_723,AD.vs.MCI; bulk RNA-seq,Homo_723,MCI.vs.control; bulk RNA-seq,Homo_633,AD.vs.control; bulk RNA-seq,Homo_633,AD.vs.MCI; bulk RNA-seq,Homo_633,MCI.vs.control; bulk RNA-seq,Homo_714,AD.vs.control; bulk RNA-seq,Homo_714,AD.vs.MCI; scRNA-seq,SRP215507,CD8+ T cell_6-AD.vs.control; scRNA-seq,SRP215507,CD8+ T cell_7-AD.vs.MCI; scRNA-seq,SRP215507,CD8+ T cell_7-MCI.vs.control; scRNA-seq,SRP330776,CD8+ T cell_1-AD.vs.control; scRNA-seq,SRP330776,Memory T cell_1-AD.vs.control; scRNA-seq,SRP330776,B cell_2-AD.vs.control; scRNA-seq,SRP330776,T cell-AD.vs.control	15
CDC14A	bulk RNA-seq,Homo_723,MCI.vs.control; bulk RNA-seq,Homo_633,AD.vs.control; bulk RNA-seq,Homo_714,AD.vs.control; bulk RNA-seq,Homo_714,AD.vs.MCI; scRNA-seq,SRP215507,CD8+ T cell_5-AD.vs.control; scRNA-seq,SRP215507,CD8+ T cell_3-AD.vs.MCI; scRNA-seq,SRP215507,CD8+ T cell_4-AD.vs.MCI; scRNA-seq,SRP215507,CD8+ T cell_5-MCI.vs.control; scRNA-seq,SRP215507,CD8+ T cell_4-MCI.vs.control; scRNA-seq,SRP215507,CD8+ T cell_6-MCI.vs.control; scRNA-seq,SRP309935,CD8+ T cell_1-AD.vs.control; scRNA-seq,SRP309935,CD8+ T cell_2-AD.vs.control; scRNA-seq,SRP309935,Dendritic cell-AD.vs.control; scRNA-seq,SRP330776,CD8+ T cell_2-	16
CDC14B	bulk RNA-seq,Homo_723,MCI.vs.control; bulk RNA-seq,Homo_633,AD.vs.MCI; bulk RNA-seq,Homo_633,MCI.vs.control; bulk RNA-seq,Homo_714,MCI.vs.control; scRNA-seq,SRP215507,CD8+ T cell_3-AD.vs.MCI; scRNA-seq,SRP215507,CD8+ T cell_4-AD.vs.MCI; scRNA-seq,SRP309935,CD8+ T cell_1-AD.vs.control; scRNA-seq,SRP330776,γδ T cell-AD.vs.control; scRNA-seq,SRP330776,Memory T cell_2-AD.vs.control; scRNA-seq,SRP330776,Natural killer cell_1-AD.vs.control; scRNA-seq,SRP330776,Natural killer T cell-AD.vs.control; scRNA-seq,SRP330776,Naive	12
CDC14C	bulk RNA-seq,Homo_723,AD.vs.control; bulk RNA-seq,Homo_723,AD.vs.MCI; bulk RNA-seq,Homo_633,AD.vs.control; bulk RNA-seq,Homo_633,AD.vs.MCI; bulk RNA-seq,Homo_714,AD.vs.MCI; bulk RNA-seq,Homo_714,MCI.vs.control	6
CDC16	scRNA-seq,SRP215507,CD8+ T cell_3-AD.vs.MCI; scRNA-seq,SRP215507,CD8+ T cell_5-MCI.vs.control; scRNA-seq,SRP215507,CD8+ T cell_3-MCI.vs.control; scRNA-seq,SRP309935,Naive CD8+ T cell_1-AD.vs.control; scRNA-seq,SRP309935,Megakaryocyte_2-AD.vs.control; scRNA-seq,SRP330776,CD8+ T cell_1-AD.vs.control; scRNA-seq,SRP330776,γδ T cell-AD.vs.control; scRNA-seq,SRP330776,Memory T cell_2-AD.vs.control; scRNA-seq,SRP330776,CD8+ T cell_2-AD.vs.control; scRNA-seq,SRP330776,B cell_1-AD.vs.control; scRNA-seq,SRP330776,Naive CD8+ T cell_1-AD.vs.control; scRNA-seq,SRP330776,Natural killer cell_1-AD.vs.control; scRNA-seq,SRP330776,Memory T cell_1-AD.vs.control; scRNA-seq,SRP330776,Natural killer T cell-AD.vs.control; scRNA-seq,SRP330776,Naive CD8+ T cell_3-AD.vs.control; scRNA-seq,SRP330776,B cell_2-AD.vs.control; scRNA-seq,SRP330776,T cell-AD.vs.control; scRNA-seq,SRP330776,CD8+ T cell_3-AD.vs.control; scRNA-seq,SRP330776,Monocyte_1- [illegible]	21
CDC20	bulk RNA-seq,Homo_633,MCI.vs.control; scRNA-seq,SRP215507,CD8+ T cell_2-AD.vs.control; scRNA-seq,SRP215507,CD8+ T cell_1-MCI.vs.control; scRNA-seq,SRP215507,CD8+ T cell_2-MCI.vs.control; scRNA-seq,SRP330776,T cell-AD.vs.control	5
CDC20B	bulk RNA-seq,Homo_723,AD.vs.control; bulk RNA-seq,Homo_723,AD.vs.MCI; bulk RNA-seq,Homo_633,AD.vs.control; bulk RNA-seq,Homo_633,MCI.vs.control; bulk RNA-seq,Homo_714,MCI.vs.control; scRNA-seq,SRP309935,Monocyte_1-AD.vs.control	6
CDC23	bulk RNA-seq,Homo_723,AD.vs.control; bulk RNA-seq,Homo_723,AD.vs.MCI; bulk RNA-seq,Homo_633,AD.vs.control; bulk RNA-seq,Homo_633,AD.vs.MCI; bulk RNA-seq,Homo_714,AD.vs.control; bulk RNA-seq,Homo_714,AD.vs.MCI; bulk RNA-seq,Homo_714,MCI.vs.control; scRNA-seq,SRP215507,CD8+ T cell_8-AD.vs.control; scRNA-seq,SRP215507,CD8+ T cell_7-AD.vs.MCI; scRNA-seq,SRP309935,Naive CD8+ T cell_1-	10
CDC25A	bulk RNA-seq,Homo_723,AD.vs.control; bulk RNA-seq,Homo_723,AD.vs.MCI; bulk RNA-seq,Homo_723,MCI.vs.control; bulk RNA-seq,Homo_633,AD.vs.control; bulk RNA-seq,Homo_633,AD.vs.MCI; bulk RNA-seq,Homo_633,MCI.vs.control; bulk RNA-seq,Homo_714,AD.vs.control;	8

CDC25B	bulk RNA-seq,Homo_714,AD.vs.control; bulk RNA-seq,Homo_714,AD.vs.MCI; scRNA-seq,SRP215507,CD8+ T cell_5-AD.vs.MCI; scRNA-seq,SRP215507,CD8+ T cell_8-MCI.vs.control; scRNA-seq,SRP330776,CD8+ T cell_1-AD.vs.control; scRNA-seq,SRP330776,Memory T cell_2-AD.vs.control; scRNA-seq,SRP330776,CD8+ T cell_2-AD.vs.control; scRNA-seq,SRP330776,B cell_1-AD.vs.control; scRNA-seq,SRP330776,Naive CD8+ T cell_1-AD.vs.control; scRNA-seq,SRP330776,Natural killer cell_1-AD.vs.control; scRNA-seq,SRP330776,Memory T cell_1-AD.vs.control; scRNA-seq,SRP330776,Natural killer T cell-AD.vs.control; scRNA-seq,SRP330776,Naive CD8+ T cell_3-AD.vs.control; scRNA-seq,SRP330776,B cell_2-AD.vs.control; scRNA-seq,SRP330776,T cell-AD.vs.control; scRNA-seq,SRP330776,CD8+ T cell_3-AD.vs.control; scRNA-seq,SRP330776,Monocyte_1-AD.vs.control; scRNA-seq,SRP330776,B cell_3-AD.vs.control; scRNA-seq,SRP330776,Natural killer cell_2-AD.vs.control	19
Cdc25b	bulk RNA-seq,SRP325058,AD.vs.control	1
CDC25C	bulk RNA-seq,Homo_723,AD.vs.control; bulk RNA-seq,Homo_723,AD.vs.MCI; bulk RNA-seq,Homo_723,MCI.vs.control; bulk RNA-seq,Homo_633,AD.vs.control; bulk RNA-seq,Homo_633,AD.vs.MCI; bulk RNA-seq,Homo_633,MCI.vs.control; bulk RNA-seq,Homo_714,AD.vs.control; bulk RNA-seq,Homo_714,AD.vs.MCI; bulk RNA-seq,Homo_714,MCI.vs.control; scRNA-seq,SRP215507,CD8+ T cell_6-MCI.vs.control	10
CDC26	bulk RNA-seq,Homo_723,AD.vs.MCI; bulk RNA-seq,Homo_633,AD.vs.control; bulk RNA-seq,Homo_633,AD.vs.MCI; scRNA-seq,SRP215507,CD8+ T cell_7-AD.vs.control; scRNA-seq,SRP330776,γδ T cell-AD.vs.control; scRNA-seq,SRP330776,CD8+ T cell_2-AD.vs.control; scRNA-seq,SRP330776,Naive CD8+ T cell_1-AD.vs.control; scRNA-seq,SRP330776,Memory T cell_1-AD.vs.control; scRNA-seq,SRP330776,Natural killer T cell-AD.vs.control; scRNA-seq,SRP330776,B cell_2-AD.vs.control; scRNA-seq,SRP330776,T cell-AD.vs.control; scRNA-seq,SRP330776,B cell_3-	13
CDC27	scRNA-seq,SRP215507,CD8+ T cell_10-AD.vs.control; scRNA-seq,SRP215507,CD8+ T cell_4-AD.vs.MCI; scRNA-seq,SRP309935,Natural killer cell_2-	3
CDC34	bulk RNA-seq,Homo_723,AD.vs.control; bulk RNA-seq,Homo_723,AD.vs.MCI; bulk RNA-seq,Homo_633,AD.vs.control; bulk RNA-seq,Homo_633,AD.vs.MCI; scRNA-seq,SRP215507,CD8+ T cell_5-AD.vs.MCI; scRNA-seq,SRP215507,CD8+ T cell_5-MCI.vs.control; scRNA-seq,SRP309935,Monocyte_1-AD.vs.control; scRNA-seq,SRP309935,Erythroid cell-AD.vs.control; scRNA-seq,SRP330776,CD8+ T cell_1-AD.vs.control; scRNA-seq,SRP330776,γδ T cell-AD.vs.control; scRNA-seq,SRP330776,Memory T cell_2-AD.vs.control; scRNA-seq,SRP330776,CD8+ T cell_2-AD.vs.control; scRNA-seq,SRP330776,Naive CD8+ T cell_1-AD.vs.control; scRNA-seq,SRP330776,Natural killer cell_1-AD.vs.control; scRNA-seq,SRP330776,Memory T cell_1-AD.vs.control; scRNA-seq,SRP330776,T cell-AD.vs.control; scRNA-seq,SRP330776,CD8+ T cell_3-AD.vs.control;	19
CDC37	scRNA-seq,SRP215507,CD8+ T cell_8-AD.vs.control; scRNA-seq,SRP215507,CD8+ T cell_7-AD.vs.control; scRNA-seq,SRP215507,CD8+ T cell_7-AD.vs.MCI; scRNA-seq,SRP309935,Dendritic cell-AD.vs.control	4
CDC37L1	bulk RNA-seq,Homo_723,AD.vs.MCI; bulk RNA-seq,Homo_714,AD.vs.control; bulk RNA-seq,Homo_714,AD.vs.MCI; scRNA-seq,SRP215507,CD8+ T cell_4-AD.vs.MCI; scRNA-seq,SRP309935,Monocyte_1-AD.vs.control	5
CDC37L1-DT	scRNA-seq,SRP215507,CD8+ T cell_3-AD.vs.MCI; scRNA-seq,SRP215507,CD8+ T cell_3-MCI.vs.control; scRNA-seq,SRP330776,Naive CD8+ T	3
CDC40	bulk RNA-seq,Homo_723,AD.vs.control; bulk RNA-seq,Homo_633,AD.vs.control; bulk RNA-seq,Homo_714,AD.vs.control; bulk RNA-seq,Homo_714,AD.vs.MCI; bulk RNA-seq,Homo_714,MCI.vs.control; scRNA-seq,SRP215507,CD8+ T cell_7-AD.vs.control; scRNA-seq,SRP215507,CD8+ T cell_6-AD.vs.MCI; scRNA-seq,SRP215507,CD8+ T cell_7-MCI.vs.control; scRNA-seq,SRP330776,Naive CD8+ T cell_1-AD.vs.control; scRNA-seq,SRP330776,Naive CD8+ T cell_3-AD.vs.control; scRNA-seq,SRP330776,B cell_2-AD.vs.control; scRNA-	13
Cdc40	bulk RNA-seq,SRP325058,AD.vs.control	1
CDC42	scRNA-seq,SRP215507,CD8+ T cell_8-AD.vs.control; scRNA-seq,SRP215507,CD8+ T cell_10-AD.vs.control; scRNA-seq,SRP215507,CD8+ T cell_8-MCI.vs.control; scRNA-seq,SRP309935,Erythroid cell-AD.vs.control	4
CDC42-AS1	scRNA-seq,SRP330776,CD8+ T cell_1-AD.vs.control; scRNA-seq,SRP330776,Memory T cell_2-AD.vs.control; scRNA-seq,SRP330776,Naive CD8+ T cell_1-AD.vs.control; scRNA-seq,SRP330776,Memory T cell_1-AD.vs.control	4
CDC42BPA	bulk RNA-seq,Homo_723,AD.vs.control; bulk RNA-seq,Homo_723,AD.vs.MCI; bulk RNA-seq,Homo_723,MCI.vs.control; bulk RNA-seq,Homo_633,AD.vs.control; bulk RNA-seq,Homo_633,AD.vs.MCI; bulk RNA-seq,Homo_633,MCI.vs.control; bulk RNA-seq,Homo_714,AD.vs.control; bulk RNA-seq,Homo_714,AD.vs.MCI; bulk RNA-seq,Homo_714,MCI.vs.control	9

CDC42BPB	bulk RNA-seq,Homo_723,AD.vs.control; bulk RNA-seq,Homo_723,AD.vs.MCI; bulk RNA-seq,Homo_723,MCI.vs.control; bulk RNA-seq,Homo_633,AD.vs.control; bulk RNA-seq,Homo_633,AD.vs.MCI; bulk RNA-seq,Homo_633,MCI.vs.control; bulk RNA-seq,Homo_714,AD.vs.control; bulk RNA-seq,Homo_714,AD.vs.MCI; bulk RNA-seq,Homo_714,MCI.vs.control; scRNA-seq,SRP215507,CD8+ T cell_2-AD.vs.control; scRNA-seq,SRP330776,CD8+ T cell_1-AD.vs.control; scRNA-seq,SRP330776,Memory T cell_2-AD.vs.control; scRNA-seq,SRP330776,B cell_1-AD.vs.control; scRNA-seq,SRP330776,Naive CD8+ T cell_1-AD.vs.control; scRNA-seq,SRP330776,B cell_2-AD.vs.control	15
CDC42BPG	bulk RNA-seq,Homo_723,AD.vs.control; bulk RNA-seq,Homo_723,AD.vs.MCI; bulk RNA-seq,Homo_723,MCI.vs.control; bulk RNA-seq,Homo_633,AD.vs.control; bulk RNA-seq,Homo_633,AD.vs.MCI; bulk RNA-seq,Homo_633,MCI.vs.control; bulk RNA-seq,Homo_714,AD.vs.control; bulk RNA-seq,Homo_714,AD.vs.MCI; scRNA-seq,SRP215507,CD8+ T cell_1-MCI.vs.control; scRNA-seq,SRP330776,γδ T cell-AD.vs.control; scRNA-seq,SRP330776,CD8+ T cell_2-AD.vs.control; scRNA-seq,SRP330776,Naive CD8+ T cell_1-AD.vs.control; scRNA-seq,SRP330776,Natural killer T cell-	13
CDC42EP1	bulk RNA-seq,SRP223445,AD.vs.control; scRNA-seq,SRP330776,B cell_2-AD.vs.control	2
CDC42EP2	bulk RNA-seq,Homo_723,AD.vs.control; bulk RNA-seq,Homo_723,AD.vs.MCI; bulk RNA-seq,Homo_633,AD.vs.control; bulk RNA-seq,Homo_633,AD.vs.MCI; bulk RNA-seq,Homo_714,AD.vs.control; bulk RNA-seq,Homo_714,AD.vs.MCI; scRNA-seq,SRP309935,Monocyte_2-AD.vs.control; scRNA-seq,SRP330776,Monocyte_1-AD.vs.control	8
Cdc42ep2	bulk RNA-seq,SRP325058,AD.vs.control	1
CDC42EP3	scRNA-seq,SRP215507,CD8+ T cell_7-AD.vs.control; scRNA-seq,SRP215507,CD8+ T cell_2-AD.vs.MCI; scRNA-seq,SRP215507,CD8+ T cell_3-AD.vs.MCI; scRNA-seq,SRP215507,CD8+ T cell_4-AD.vs.MCI; scRNA-seq,SRP215507,CD8+ T cell_5-AD.vs.MCI; scRNA-seq,SRP215507,CD8+ T cell_4-MCI.vs.control; scRNA-seq,SRP215507,CD8+ T cell_2-MCI.vs.control; scRNA-seq,SRP309935,Erythroid cell-AD.vs.control; scRNA-seq,SRP309935,Naive CD8+ T cell_2-AD.vs.control; scRNA-seq,SRP330776,CD8+ T cell_1-AD.vs.control; scRNA-seq,SRP330776,CD8+ T cell_2-AD.vs.control; scRNA-seq,SRP330776,Naive CD8+ T cell_1-AD.vs.control; scRNA-seq,SRP330776,Memory T cell_1-AD.vs.control; scRNA-	15
CDC42EP4	bulk RNA-seq,Homo_723,AD.vs.control; bulk RNA-seq,Homo_723,AD.vs.MCI; bulk RNA-seq,Homo_633,AD.vs.control; bulk RNA-seq,Homo_633,AD.vs.MCI; bulk RNA-seq,Homo_714,AD.vs.control; bulk RNA-seq,Homo_714,AD.vs.MCI; scRNA-seq,SRP330776,Memory T cell_2-	7
CDC42EP5	bulk RNA-seq,Homo_723,AD.vs.MCI; bulk RNA-seq,Homo_723,MCI.vs.control; bulk RNA-seq,Homo_633,AD.vs.MCI; bulk RNA-	4
CDC42-IT1	scRNA-seq,SRP215507,CD8+ T cell_4-AD.vs.control; scRNA-seq,SRP215507,CD8+ T cell_4-MCI.vs.control	2
CDC42SE1	scRNA-seq,SRP215507,CD8+ T cell_8-AD.vs.control; scRNA-seq,SRP309935,Erythroid cell-AD.vs.control; scRNA-seq,SRP330776,B cell_4-	3
CDC42SE2	bulk RNA-seq,Homo_723,AD.vs.control; bulk RNA-seq,Homo_723,AD.vs.MCI; bulk RNA-seq,Homo_633,AD.vs.control; bulk RNA-seq,Homo_633,AD.vs.MCI; bulk RNA-seq,SRP223445,AD.vs.control; scRNA-seq,SRP215507,CD8+ T cell_7-AD.vs.control; scRNA-seq,SRP215507,CD8+ T cell_10-AD.vs.control; scRNA-seq,SRP215507,CD8+ T cell_7-AD.vs.MCI	8
CDC45	bulk RNA-seq,Homo_723,AD.vs.control; bulk RNA-seq,Homo_723,AD.vs.MCI; bulk RNA-seq,Homo_714,AD.vs.control; bulk RNA-	4
CDC5L	scRNA-seq,SRP215507,CD8+ T cell_10-AD.vs.control; scRNA-seq,SRP215507,CD8+ T cell_1-AD.vs.MCI; scRNA-seq,SRP215507,CD8+ T cell_4-AD.vs.MCI; scRNA-seq,SRP330776,Naive CD8+ T cell_3-AD.vs.control; scRNA-seq,SRP330776,Regulatory T cell-AD.vs.control	5
CDC6	bulk RNA-seq,Homo_723,AD.vs.control; bulk RNA-seq,Homo_723,AD.vs.MCI; bulk RNA-seq,Homo_723,MCI.vs.control; bulk RNA-seq,Homo_633,AD.vs.control; bulk RNA-seq,Homo_633,AD.vs.MCI; bulk RNA-seq,Homo_633,MCI.vs.control; bulk RNA-seq,Homo_714,AD.vs.control; bulk RNA-seq,Homo_714,AD.vs.MCI; bulk RNA-seq,Homo_714,MCI.vs.control; bulk RNA-seq,ROSMAP,AD.vs.control; bulk RNA-	12
CDC7	bulk RNA-seq,Homo_723,AD.vs.control; bulk RNA-seq,Homo_723,AD.vs.MCI; bulk RNA-seq,Homo_723,MCI.vs.control; bulk RNA-seq,Homo_633,AD.vs.control; bulk RNA-seq,Homo_633,AD.vs.MCI; bulk RNA-seq,Homo_633,MCI.vs.control; scRNA-seq,SRP215507,CD8+ T cell_5-AD.vs.control; scRNA-seq,SRP215507,CD8+ T cell_5-MCI.vs.control; scRNA-seq,SRP330776,B cell_2-AD.vs.control	9
CDC73	scRNA-seq,SRP215507,CD8+ T cell_6-AD.vs.control; scRNA-seq,SRP215507,CD8+ T cell_4-AD.vs.MCI; scRNA-seq,SRP330776,γδ T cell-AD.vs.control; scRNA-seq,SRP330776,Naive CD8+ T cell_1-AD.vs.control; scRNA-seq,SRP330776,Naive CD8+ T cell_2-AD.vs.control; scRNA-	6
CDCA2	bulk RNA-seq,Homo_723,AD.vs.control; bulk RNA-seq,Homo_723,AD.vs.MCI; bulk RNA-seq,Homo_723,MCI.vs.control; bulk RNA-seq,Homo_633,AD.vs.control; bulk RNA-seq,Homo_633,AD.vs.MCI; bulk RNA-seq,Homo_714,AD.vs.control; bulk RNA-seq,Homo_714,AD.vs.MCI; bulk RNA-seq,ROSMAP,MCI.vs.control; scRNA-seq,SRP330776,T cell-AD.vs.control	9
CDCA3	bulk RNA-seq,Homo_633,AD.vs.control; bulk RNA-seq,Homo_714,AD.vs.control; bulk RNA-seq,Homo_714,AD.vs.MCI; bulk RNA-seq,Homo_714,MCI.vs.control; bulk RNA-seq,ROSMAP,AD.vs.control	5

Cdca3	bulk RNA-seq,SRP325058,AD.vs.control	1
CDCA4	scRNA-seq,SRP215507,CD8+ T cell_3-AD.vs.control; scRNA-seq,SRP215507,CD8+ T cell_3-MCI.vs.control; scRNA-seq,SRP330776,CD8+ T cell_2-AD.vs.control; scRNA-seq,SRP330776,B cell_1-AD.vs.control; scRNA-seq,SRP330776,Naive CD8+ T cell_3-AD.vs.control; scRNA-seq,SRP330776,T cell-AD.vs.control; scRNA-seq,SRP330776,CD8+ T cell_3-AD.vs.control	7
CDCA5	bulk RNA-seq,Homo_723,AD.vs.control; bulk RNA-seq,Homo_723,AD.vs.MCI; bulk RNA-seq,Homo_633,AD.vs.control; bulk RNA-seq,Homo_633,AD.vs.MCI; bulk RNA-seq,Homo_714,AD.vs.control; bulk RNA-seq,Homo_714,AD.vs.MCI	6
CDCA7	bulk RNA-seq,Homo_723,AD.vs.control; bulk RNA-seq,Homo_723,AD.vs.MCI; bulk RNA-seq,Homo_633,AD.vs.MCI; bulk RNA-seq,Homo_714,AD.vs.control; bulk RNA-seq,Homo_714,AD.vs.MCI; scRNA-seq,SRP215507,CD8+ T cell_2-AD.vs.control; scRNA-seq,SRP215507,CD8+ T cell_1-AD.vs.control; scRNA-seq,SRP215507,CD8+ T cell_6-AD.vs.control; scRNA-seq,SRP215507,CD8+ T cell_2-AD.vs.MCI; scRNA-seq,SRP215507,CD8+ T cell_1-AD.vs.MCI; scRNA-seq,SRP215507,CD8+ T cell_6-AD.vs.MCI; scRNA-seq,SRP215507,CD8+ T cell_1-MCI.vs.control; scRNA-seq,SRP215507,CD8+ T cell_4-MCI.vs.control; scRNA-seq,SRP215507,CD8+ T cell_6-MCI.vs.control; scRNA-seq,SRP330776,CD8+ T cell_2-AD.vs.control; scRNA-seq,SRP330776,Natural killer cell_1-AD.vs.control; scRNA-seq,SRP330776,Regulatory T cell-	17
CDCA7L	bulk RNA-seq,Homo_714,AD.vs.control; bulk RNA-seq,Homo_714,AD.vs.MCI; scRNA-seq,SRP215507,CD8+ T cell_3-AD.vs.control; scRNA-seq,SRP215507,CD8+ T cell_5-AD.vs.control; scRNA-seq,SRP215507,CD8+ T cell_6-AD.vs.MCI; scRNA-seq,SRP215507,CD8+ T cell_3-MCI.vs.control; scRNA-seq,SRP215507,CD8+ T cell_6-MCI.vs.control; scRNA-seq,SRP330776,Memory T cell_2-AD.vs.control; scRNA-seq,SRP330776,B cell_1-AD.vs.control; scRNA-seq,SRP330776,Natural killer cell_1-AD.vs.control; scRNA-seq,SRP330776,T cell-AD.vs.control; scRNA-	13
CDCA8	bulk RNA-seq,Homo_714,AD.vs.control; bulk RNA-seq,Homo_714,AD.vs.MCI; scRNA-seq,SRP215507,CD8+ T cell_2-AD.vs.control; scRNA-seq,SRP215507,CD8+ T cell_5-AD.vs.control; scRNA-seq,SRP215507,CD8+ T cell_3-AD.vs.MCI; scRNA-seq,SRP215507,CD8+ T cell_5-MCI.vs.control; scRNA-seq,SRP330776,γδ T cell-AD.vs.control; scRNA-seq,SRP330776,Memory T cell_2-AD.vs.control; scRNA-	9
CDCP1	bulk RNA-seq,Homo_723,AD.vs.control; bulk RNA-seq,Homo_723,MCI.vs.control; bulk RNA-seq,Homo_633,MCI.vs.control; bulk RNA-seq,Homo_714,AD.vs.control; bulk RNA-seq,Homo_714,AD.vs.MCI; bulk RNA-seq,Homo_714,MCI.vs.control; scRNA-seq,SRP330776,Naive CD8+ T	7
CDCP2	bulk RNA-seq,Homo_723,AD.vs.control; bulk RNA-seq,Homo_723,AD.vs.MCI; bulk RNA-seq,Homo_723,MCI.vs.control; bulk RNA-	4
CDH1	bulk RNA-seq,ROSMAP,AD.vs.control; scRNA-seq,SRP330776,CD8+ T cell_1-AD.vs.control; scRNA-seq,SRP330776,T cell-AD.vs.control; scRNA-seq,SRP330776,CD8+ T cell_3-AD.vs.control	4
CDH10	bulk RNA-seq,Homo_723,AD.vs.MCI; bulk RNA-seq,Homo_723,MCI.vs.control	2
CDH11	bulk RNA-seq,Homo_723,AD.vs.control; bulk RNA-seq,Homo_723,AD.vs.MCI; bulk RNA-seq,Homo_723,MCI.vs.control; bulk RNA-seq,Homo_633,AD.vs.MCI; bulk RNA-seq,Homo_633,MCI.vs.control; bulk RNA-seq,Homo_714,MCI.vs.control; bulk RNA-seq,ROSMAP,AD.vs.control;	8
CDH12	bulk RNA-seq,Homo_723,AD.vs.control; bulk RNA-seq,Homo_723,AD.vs.MCI; bulk RNA-seq,Homo_633,AD.vs.control; bulk RNA-seq,Homo_633,MCI.vs.control; bulk RNA-seq,Homo_714,MCI.vs.control	5
CDH13	bulk RNA-seq,Homo_633,AD.vs.control; bulk RNA-seq,Homo_714,AD.vs.control; bulk RNA-seq,Homo_714,AD.vs.MCI; bulk RNA-	4
CDH15	bulk RNA-seq,ROSMAP,AD.vs.control; bulk RNA-seq,ROSMAP,MCI.vs.control	2
CDH16	bulk RNA-seq,ROSMAP,AD.vs.control; bulk RNA-seq,ROSMAP,MCI.vs.control	2
CDH17	bulk RNA-seq,Homo_723,AD.vs.control; bulk RNA-seq,Homo_723,AD.vs.MCI; bulk RNA-seq,Homo_714,MCI.vs.control	3
CDH18	bulk RNA-seq,Homo_723,AD.vs.control; bulk RNA-seq,Homo_723,AD.vs.MCI; bulk RNA-seq,Homo_714,MCI.vs.control	3
CDH2	bulk RNA-seq,Homo_723,AD.vs.control; bulk RNA-seq,Homo_723,AD.vs.MCI; bulk RNA-seq,Homo_723,MCI.vs.control; bulk RNA-seq,Homo_714,AD.vs.control; bulk RNA-seq,Homo_714,AD.vs.MCI; bulk RNA-seq,ROSMAP,AD.vs.control; scRNA-seq,SRP215507,CD8+ T cell_5-	7
CDH20	bulk RNA-seq,Homo_723,AD.vs.control; bulk RNA-seq,Homo_633,AD.vs.control	2
CDH22	bulk RNA-seq,Homo_714,MCI.vs.control; bulk RNA-seq,ROSMAP,AD.vs.control	2

CDH23	scRNA-seq,SRP215507,CD8+ T cell_2-AD.vs.control; scRNA-seq,SRP215507,CD8+ T cell_3-AD.vs.control; scRNA-seq,SRP215507,CD8+ T cell_1-AD.vs.control; scRNA-seq,SRP215507,CD8+ T cell_4-AD.vs.control; scRNA-seq,SRP215507,CD8+ T cell_2-AD.vs.MCI; scRNA-seq,SRP215507,CD8+ T cell_3-AD.vs.MCI; scRNA-seq,SRP215507,CD8+ T cell_1-AD.vs.MCI; scRNA-seq,SRP215507,CD8+ T cell_4-AD.vs.MCI; scRNA-seq,SRP215507,CD8+ T cell_5-AD.vs.MCI; scRNA-seq,SRP215507,CD8+ T cell_5-MCI.vs.control; scRNA-seq,SRP215507,CD8+ T cell_3-MCI.vs.control; scRNA-seq,SRP330776,Memory T cell_2-AD.vs.control; scRNA-seq,SRP330776,B cell_4-AD.vs.control	13
CDH24	scRNA-seq,SRP215507,CD8+ T cell_3-MCI.vs.control; scRNA-seq,SRP330776,Memory T cell_1-AD.vs.control	2
CDH26	bulk RNA-seq,Homo_723,AD.vs.control; bulk RNA-seq,Homo_723,AD.vs.MCI; bulk RNA-seq,Homo_633,AD.vs.control; bulk RNA-seq,Homo_633,AD.vs.MCI; bulk RNA-seq,Homo_714,AD.vs.control; bulk RNA-seq,Homo_714,AD.vs.MCI	6
CDH4	bulk RNA-seq,Homo_723,AD.vs.control; bulk RNA-seq,Homo_723,AD.vs.MCI; bulk RNA-seq,Homo_723,MCI.vs.control; bulk RNA-seq,Homo_633,AD.vs.control; bulk RNA-seq,Homo_633,AD.vs.MCI; bulk RNA-seq,Homo_714,AD.vs.control; bulk RNA-seq,Homo_714,AD.vs.MCI	7
CDH5	bulk RNA-seq,Homo_723,AD.vs.control; bulk RNA-seq,Homo_723,AD.vs.MCI; bulk RNA-seq,Homo_723,MCI.vs.control; bulk RNA-seq,Homo_633,AD.vs.control; bulk RNA-seq,Homo_633,AD.vs.MCI; bulk RNA-seq,Homo_633,MCI.vs.control	6
CDH6	bulk RNA-seq,Homo_723,AD.vs.control; bulk RNA-seq,Homo_723,AD.vs.MCI; bulk RNA-seq,Homo_723,MCI.vs.control; bulk RNA-seq,Homo_633,AD.vs.control; bulk RNA-seq,Homo_633,AD.vs.MCI; bulk RNA-seq,Homo_633,MCI.vs.control; bulk RNA-seq,Homo_714,MCI.vs.control	7
CDH7	bulk RNA-seq,Homo_723,AD.vs.control; bulk RNA-seq,Homo_723,AD.vs.MCI; bulk RNA-seq,Homo_633,AD.vs.control; bulk RNA-	4
CDH8	bulk RNA-seq,Homo_723,AD.vs.control; bulk RNA-seq,Homo_723,AD.vs.MCI; bulk RNA-seq,Homo_633,AD.vs.MCI; bulk RNA-	4
CDH9	bulk RNA-seq,Homo_723,AD.vs.control; bulk RNA-seq,Homo_723,AD.vs.MCI	2
CDHR1	bulk RNA-seq,Homo_723,AD.vs.control; bulk RNA-seq,Homo_723,AD.vs.MCI; bulk RNA-seq,Homo_714,AD.vs.control; bulk RNA-seq,Homo_714,AD.vs.MCI; bulk RNA-seq,ROSMAP,AD.vs.control; bulk RNA-seq,ROSMAP,MCI.vs.control; scRNA-seq,SRP215507,CD8+ T cell_5-AD.vs.MCI; scRNA-seq,SRP215507,CD8+ T cell_5-MCI.vs.control; scRNA-seq,SRP309935,Natural killer cell_1-AD.vs.control; scRNA-seq,SRP330776,Memory T cell_1-AD.vs.control; scRNA-seq,SRP330776,CD8+ T cell_3-AD.vs.control; scRNA-seq,SRP330776,Natural killer cell_2-	12
CDHR2	bulk RNA-seq,Homo_723,AD.vs.control; bulk RNA-seq,Homo_723,AD.vs.MCI; bulk RNA-seq,Homo_714,AD.vs.control; bulk RNA-seq,Homo_714,AD.vs.MCI; bulk RNA-seq,ROSMAP,AD.vs.control; scRNA-seq,SRP330776,Memory T cell_1-AD.vs.control	6
CDHR3	bulk RNA-seq,Homo_633,AD.vs.control; bulk RNA-seq,Homo_633,AD.vs.MCI; bulk RNA-seq,Homo_714,AD.vs.control; bulk RNA-seq,Homo_714,AD.vs.MCI; bulk RNA-seq,Homo_714,MCI.vs.control; scRNA-seq,SRP215507,CD8+ T cell_2-AD.vs.control; scRNA-seq,SRP215507,CD8+ T cell_3-AD.vs.control; scRNA-seq,SRP215507,CD8+ T cell_1-AD.vs.control; scRNA-seq,SRP215507,CD8+ T cell_2-AD.vs.MCI; scRNA-seq,SRP215507,CD8+ T cell_3-AD.vs.MCI; scRNA-seq,SRP215507,CD8+ T cell_1-AD.vs.MCI; scRNA-seq,SRP215507,CD8+ T cell_1-MCI.vs.control; scRNA-seq,SRP309935,Naive CD8+ T cell_1-AD.vs.control; scRNA-seq,SRP330776,γδ T cell-AD.vs.control; scRNA-seq,SRP330776,Memory T cell_2-AD.vs.control; scRNA-seq,SRP330776,B cell_1-AD.vs.control; scRNA-seq,SRP330776,Naive CD8+ T cell_1-AD.vs.control; scRNA-seq,SRP330776,Naive CD8+ T cell_3-AD.vs.control; scRNA-seq,SRP330776,T cell-AD.vs.control; scRNA-seq,SRP330776,CD8+ [illegible]	20
CDHR5	bulk RNA-seq,Homo_723,MCI.vs.control; bulk RNA-seq,Homo_714,MCI.vs.control	2
CDIN1	bulk RNA-seq,Homo_723,AD.vs.control; bulk RNA-seq,Homo_723,AD.vs.MCI; bulk RNA-seq,Homo_633,AD.vs.control; bulk RNA-seq,Homo_633,AD.vs.MCI; bulk RNA-seq,Homo_714,AD.vs.control; bulk RNA-seq,Homo_714,AD.vs.MCI; bulk RNA-seq,ROSMAP,AD.vs.control;	8
CDIP1	scRNA-seq,SRP215507,CD8+ T cell_7-AD.vs.control; scRNA-seq,SRP215507,CD8+ T cell_7-AD.vs.MCI; scRNA-seq,SRP330776,B cell_1-AD.vs.control; scRNA-seq,SRP330776,Naive CD8+ T cell_1-AD.vs.control; scRNA-seq,SRP330776,Natural killer T cell-AD.vs.control; scRNA-seq,SRP330776,Naive CD8+ T cell_3-AD.vs.control; scRNA-seq,SRP330776,B cell_2-AD.vs.control; scRNA-seq,SRP330776,B cell_4-AD.vs.control	8

CDIPT	bulk RNA-seq,Homo_723,AD.vs.control; bulk RNA-seq,Homo_723,MCI.vs.control; bulk RNA-seq,Homo_633,AD.vs.control; bulk RNA-seq,Homo_633,AD.vs.MCI; bulk RNA-seq,Homo_633,MCI.vs.control; bulk RNA-seq,Homo_714,AD.vs.control; bulk RNA-seq,Homo_714,AD.vs.MCI; bulk RNA-seq,Homo_714,MCI.vs.control; scRNA-seq,SRP215507,CD8+ T cell_9-AD.vs.control; scRNA-seq,SRP215507,CD8+ T cell_5-AD.vs.MCI; scRNA-seq,SRP215507,CD8+ T cell_5-MCI.vs.control; scRNA-seq,SRP330776,CD8+ T cell_1-AD.vs.control; scRNA-seq,SRP330776,γδ T cell-AD.vs.control; scRNA-seq,SRP330776,Memory T cell_2-AD.vs.control; scRNA-seq,SRP330776,CD8+ T cell_2-AD.vs.control; scRNA-seq,SRP330776,B cell_1-AD.vs.control; scRNA-seq,SRP330776,Naive CD8+ T cell_1-AD.vs.control; scRNA-seq,SRP330776,Natural killer cell_1-AD.vs.control; scRNA-seq,SRP330776,Memory T cell_1-AD.vs.control; scRNA-seq,SRP330776,Natural killer T cell-AD.vs.control; scRNA-seq,SRP330776,Naive CD8+ T cell_3-AD.vs.control; scRNA-seq,SRP330776,B cell_2-AD.vs.control; scRNA-seq,SRP330776,T cell-AD.vs.control; scRNA-seq,SRP330776,Naive CD8+ T cell_2-AD.vs.control; scRNA-seq,SRP330776,CD8+ T cell_3-AD.vs.control; scRNA-seq,SRP330776,Monocyte_1-AD.vs.control; scRNA-seq,SRP330776,Monocyte_2-AD.vs.control; scRNA-seq,SRP330776,B cell_3-AD.vs.control; scRNA-seq,SRP330776,Regulatory T cell-AD.vs.control; scRNA-seq,SRP330776,Natural killer cell_2-AD.vs.control; scRNA-seq,SRP330776,B cell_4-AD.vs.control	31
CDK1	bulk RNA-seq,Homo_723,AD.vs.control; bulk RNA-seq,Homo_723,AD.vs.MCI; bulk RNA-seq,Homo_723,MCI.vs.control; bulk RNA-seq,Homo_633,AD.vs.control; bulk RNA-seq,Homo_633,AD.vs.MCI; bulk RNA-seq,Homo_633,MCI.vs.control; bulk RNA-seq,Homo_714,AD.vs.control; bulk RNA-seq,Homo_714,AD.vs.MCI; bulk RNA-seq,ROSMAP,MCI.vs.control; scRNA-seq,SRP215507,CD8+ T cell_2-AD.vs.MCI; scRNA-seq,SRP215507,CD8+ T cell_3-AD.vs.MCI; scRNA-seq,SRP215507,CD8+ T cell_1-MCI.vs.control; scRNA-seq,SRP330776,T cell-AD.vs.control	13
CDK10	scRNA-seq,SRP215507,CD8+ T cell_5-AD.vs.control; scRNA-seq,SRP215507,CD8+ T cell_6-MCI.vs.control; scRNA-seq,SRP330776,CD8+ T cell_1-AD.vs.control; scRNA-seq,SRP330776,γδ T cell-AD.vs.control; scRNA-seq,SRP330776,B cell_1-AD.vs.control; scRNA-seq,SRP330776,Natural killer cell_1-AD.vs.control; scRNA-seq,SRP330776,Natural killer T cell-AD.vs.control; scRNA-seq,SRP330776,B cell_2-AD.vs.control; scRNA-seq,SRP330776,CD8+ T cell_3-AD.vs.control; scRNA-seq,SRP330776,Monocyte_1-AD.vs.control; scRNA-seq,SRP330776,B cell_3-AD.vs.control	11
CDK11A	scRNA-seq,SRP215507,CD8+ T cell_2-AD.vs.MCI; scRNA-seq,SRP215507,CD8+ T cell_1-AD.vs.MCI; scRNA-seq,SRP215507,CD8+ T cell_4-AD.vs.MCI; scRNA-seq,SRP215507,CD8+ T cell_5-AD.vs.MCI; scRNA-seq,SRP215507,CD8+ T cell_5-MCI.vs.control; scRNA-seq,SRP215507,CD8+ T cell_4-MCI.vs.control; scRNA-seq,SRP215507,CD8+ T cell_2-MCI.vs.control; scRNA-seq,SRP215507,CD8+ T cell_3-MCI.vs.control; scRNA-seq,SRP215507,CD8+ T cell_6-MCI.vs.control; scRNA-seq,SRP330776,Naive CD8+ T cell_2-AD.vs.control; scRNA-seq,SRP330776,B cell_3-	11
CDK11B	scRNA-seq,SRP215507,CD8+ T cell_7-AD.vs.control; scRNA-seq,SRP215507,CD8+ T cell_5-AD.vs.MCI; scRNA-seq,SRP215507,CD8+ T cell_5-MCI.vs.control; scRNA-seq,SRP215507,CD8+ T cell_8-MCI.vs.control; scRNA-seq,SRP330776,B cell_1-AD.vs.control; scRNA-seq,SRP330776,Memory T cell_1-AD.vs.control; scRNA-seq,SRP330776,Naive CD8+ T cell_3-AD.vs.control	7
CDK12	bulk RNA-seq,Homo_723,AD.vs.control; bulk RNA-seq,Homo_723,AD.vs.MCI; bulk RNA-seq,Homo_633,AD.vs.control; bulk RNA-seq,Homo_633,AD.vs.MCI; bulk RNA-seq,Homo_714,AD.vs.control; bulk RNA-seq,Homo_714,AD.vs.MCI; scRNA-seq,SRP215507,CD8+ T cell_5-AD.vs.MCI; scRNA-seq,SRP215507,CD8+ T cell_6-AD.vs.MCI; scRNA-seq,SRP330776,Monocyte_2-AD.vs.control	9
Cdk12	bulk RNA-seq,SRP325058,AD.vs.control	1
CDK13	bulk RNA-seq,Homo_714,AD.vs.control; bulk RNA-seq,Homo_714,AD.vs.MCI; scRNA-seq,SRP215507,CD8+ T cell_5-AD.vs.MCI; scRNA-seq,SRP215507,CD8+ T cell_3-MCI.vs.control; scRNA-seq,SRP309935,Megakaryocyte_1-AD.vs.control; scRNA-seq,SRP309935,B cell_2-AD.vs.control; scRNA-seq,SRP330776,CD8+ T cell_1-AD.vs.control; scRNA-seq,SRP330776,γδ T cell-AD.vs.control; scRNA-seq,SRP330776,Memory T cell_2-AD.vs.control; scRNA-seq,SRP330776,CD8+ T cell_2-AD.vs.control; scRNA-seq,SRP330776,B cell_1-AD.vs.control; scRNA-seq,SRP330776,Naive CD8+ T cell_1-AD.vs.control; scRNA-seq,SRP330776,Natural killer cell_1-AD.vs.control; scRNA-seq,SRP330776,Memory T cell_1-AD.vs.control; scRNA-seq,SRP330776,Natural killer T cell-AD.vs.control; scRNA-seq,SRP330776,Naive CD8+ T cell_3-AD.vs.control; scRNA-seq,SRP330776,B cell_2-AD.vs.control; scRNA-seq,SRP330776,T cell-AD.vs.control; scRNA-seq,SRP330776,Naive CD8+ T cell_2-AD.vs.control; scRNA-seq,SRP330776,CD8+ T cell_3-AD.vs.control; scRNA-seq,SRP330776,Monocyte_1-AD.vs.control; scRNA-seq,SRP330776,Monocyte_2-AD.vs.control; scRNA-seq,SRP330776,B cell_3-AD.vs.control; scRNA-seq,SRP330776,Megakaryocyte_1-AD.vs.control; scRNA-seq,SRP330776,Natural killer cell_2-AD.vs.control; scRNA-seq,SRP330776,B cell_4-AD.vs.control	26
CDK14	bulk RNA-seq,Homo_723,AD.vs.control; bulk RNA-seq,Homo_723,AD.vs.MCI; bulk RNA-seq,Homo_633,AD.vs.control; bulk RNA-seq,Homo_633,AD.vs.MCI; bulk RNA-seq,Homo_714,AD.vs.control; bulk RNA-seq,Homo_714,AD.vs.MCI; bulk RNA-seq,Homo_714,MCI.vs.control;	8
CDK15	bulk RNA-seq,Homo_723,AD.vs.control; bulk RNA-seq,Homo_633,AD.vs.control; bulk RNA-seq,Homo_714,AD.vs.control; bulk RNA-	4

CDK16	bulk RNA-seq,Homo_723,AD.vs.control; bulk RNA-seq,Homo_633,AD.vs.control; bulk RNA-seq,Homo_633,MCI.vs.control; bulk RNA-seq,Homo_714,AD.vs.control; bulk RNA-seq,Homo_714,AD.vs.MCI; bulk RNA-seq,Homo_714,MCI.vs.control; scRNA-seq,SRP215507,CD8+ T cell_2-AD.vs.MCI; scRNA-seq,SRP215507,CD8+ T cell_3-AD.vs.MCI; scRNA-seq,SRP215507,CD8+ T cell_1-AD.vs.MCI; scRNA-seq,SRP215507,CD8+ T cell_5-AD.vs.MCI; scRNA-seq,SRP215507,CD8+ T cell_1-MCI.vs.control; scRNA-seq,SRP215507,CD8+ T cell_5-MCI.vs.control; scRNA-seq,SRP309935,Naive CD8+ T cell_1-AD.vs.control; scRNA-seq,SRP309935,Natural killer cell_1-AD.vs.control; scRNA-seq,SRP309935,B cell_1-AD.vs.control; scRNA-seq,SRP330776,CD8+ T cell_1-AD.vs.control; scRNA-seq,SRP330776,γδ T cell-AD.vs.control; scRNA-seq,SRP330776,Memory T cell_2-AD.vs.control; scRNA-seq,SRP330776,CD8+ T cell_2-AD.vs.control; scRNA-seq,SRP330776,B cell_1-AD.vs.control; scRNA-seq,SRP330776,Naive CD8+ T cell_1-AD.vs.control; scRNA-seq,SRP330776,Natural killer cell_1-AD.vs.control; scRNA-seq,SRP330776,Memory T cell_1-AD.vs.control; scRNA-seq,SRP330776,Naive CD8+ T cell_3-AD.vs.control; scRNA-seq,SRP330776,B cell_2-AD.vs.control; scRNA-seq,SRP330776,T cell-AD.vs.control; scRNA-seq,SRP330776,Naive CD8+ T cell_2-AD.vs.control; scRNA-seq,SRP330776,CD8+ T cell_3-AD.vs.control; scRNA-seq,SRP330776,Monocyte_1-AD.vs.control; scRNA-seq,SRP330776,Megakaryocyte_1-AD.vs.control	30
CDK17	bulk RNA-seq,Homo_723,AD.vs.control; bulk RNA-seq,Homo_723,AD.vs.MCI; bulk RNA-seq,Homo_633,AD.vs.control; bulk RNA-seq,Homo_633,AD.vs.MCI; bulk RNA-seq,Homo_714,AD.vs.control; bulk RNA-seq,Homo_714,AD.vs.MCI; scRNA-seq,SRP215507,CD8+ T cell_5-AD.vs.MCI; scRNA-seq,SRP215507,CD8+ T cell_5-MCI.vs.control; scRNA-seq,SRP215507,CD8+ T cell_2-MCI.vs.control; scRNA-seq,SRP309935,Natural killer cell_2-AD.vs.control; scRNA-seq,SRP309935,Naive CD8+ T cell_1-AD.vs.control; scRNA-seq,SRP309935,CD8+ T cell_1-AD.vs.control; scRNA-seq,SRP309935,CD8+ T cell_2-AD.vs.control; scRNA-seq,SRP330776,CD8+ T cell_1-AD.vs.control; scRNA-seq,SRP330776,γδ T cell-AD.vs.control; scRNA-seq,SRP330776,Memory T cell_2-AD.vs.control; scRNA-seq,SRP330776,CD8+ T cell_2-AD.vs.control; scRNA-seq,SRP330776,B cell_1-AD.vs.control; scRNA-seq,SRP330776,Naive CD8+ T cell_1-AD.vs.control; scRNA-seq,SRP330776,Natural killer cell_1-AD.vs.control; scRNA-seq,SRP330776,Memory T cell_1-AD.vs.control; scRNA-seq,SRP330776,Natural killer T cell-AD.vs.control; scRNA-seq,SRP330776,Naive CD8+ T cell_3-AD.vs.control; scRNA-seq,SRP330776,B cell_2-AD.vs.control; scRNA-seq,SRP330776,T cell-AD.vs.control; scRNA-seq,SRP330776,Naive CD8+ T cell_2-AD.vs.control; scRNA-seq,SRP330776,CD8+ T cell_3-AD.vs.control; scRNA-seq,SRP330776,Monocyte_1-AD.vs.control; scRNA-seq,SRP330776,B cell_3-AD.vs.control; scRNA-seq,SRP330776,Regulatory T cell-AD.vs.control; scRNA-seq,SRP330776,Natural	32
CDK18	bulk RNA-seq,Homo_723,AD.vs.control; bulk RNA-seq,Homo_723,AD.vs.MCI; bulk RNA-seq,Homo_633,AD.vs.control; bulk RNA-seq,Homo_633,AD.vs.MCI; bulk RNA-seq,Homo_714,MCI.vs.control; scRNA-seq,SRP215507,CD8+ T cell_3-AD.vs.control; scRNA-seq,SRP215507,CD8+ T cell_6-AD.vs.control; scRNA-seq,SRP215507,CD8+ T cell_5-MCI.vs.control; scRNA-seq,SRP330776,CD8+ T cell_1-	10
CDK19	bulk RNA-seq,Homo_723,AD.vs.control; bulk RNA-seq,Homo_714,AD.vs.control; bulk RNA-seq,Homo_714,AD.vs.MCI; scRNA-seq,SRP215507,CD8+ T cell_2-AD.vs.MCI; scRNA-seq,SRP215507,CD8+ T cell_3-AD.vs.MCI; scRNA-seq,SRP215507,CD8+ T cell_4-AD.vs.MCI; scRNA-seq,SRP215507,CD8+ T cell_7-AD.vs.MCI; scRNA-seq,SRP215507,CD8+ T cell_4-MCI.vs.control; scRNA-seq,SRP215507,CD8+ T cell_2-MCI.vs.control; scRNA-seq,SRP215507,CD8+ T cell_7-MCI.vs.control; scRNA-seq,SRP330776,CD8+ T cell_1-AD.vs.control; scRNA-seq,SRP330776,γδ T cell-AD.vs.control; scRNA-seq,SRP330776,Memory T cell_2-AD.vs.control; scRNA-seq,SRP330776,CD8+ T cell_2-AD.vs.control; scRNA-seq,SRP330776,B cell_1-AD.vs.control; scRNA-seq,SRP330776,Naive CD8+ T cell_1-AD.vs.control; scRNA-seq,SRP330776,Natural killer cell_1-AD.vs.control; scRNA-seq,SRP330776,Memory T cell_1-AD.vs.control; scRNA-seq,SRP330776,Natural killer T cell-AD.vs.control; scRNA-seq,SRP330776,Naive CD8+ T cell_3-AD.vs.control; scRNA-seq,SRP330776,B cell_2-AD.vs.control; scRNA-seq,SRP330776,T cell-AD.vs.control; scRNA-seq,SRP330776,CD8+ T cell_3-AD.vs.control; scRNA-seq,SRP330776,B cell_3-AD.vs.control; scRNA-seq,SRP330776,Natural killer cell_2	25
CDK2	bulk RNA-seq,Homo_723,AD.vs.control; bulk RNA-seq,Homo_723,AD.vs.MCI; bulk RNA-seq,Homo_633,AD.vs.control; bulk RNA-seq,Homo_633,AD.vs.MCI; bulk RNA-seq,Homo_714,AD.vs.control; bulk RNA-seq,Homo_714,AD.vs.MCI; scRNA-seq,SRP215507,CD8+ T cell_4-AD.vs.control; scRNA-seq,SRP215507,CD8+ T cell_2-AD.vs.MCI; scRNA-seq,SRP215507,CD8+ T cell_4-AD.vs.MCI; scRNA-seq,SRP309935,Naive CD8+ T cell_2-AD.vs.control; scRNA-seq,SRP330776,γδ T cell-AD.vs.control; scRNA-seq,SRP330776,CD8+ T cell_2-AD.vs.control; scRNA-seq,SRP330776,Naive CD8+ T cell_1-AD.vs.control; scRNA-seq,SRP330776,Naive CD8+ T cell_3-AD.vs.control; scRNA-seq,SRP330776,B cell_2-	15
CDK20	bulk RNA-seq,Homo_714,AD.vs.control; bulk RNA-seq,Homo_714,AD.vs.MCI; scRNA-seq,SRP215507,CD8+ T cell_3-AD.vs.control; scRNA-seq,SRP215507,CD8+ T cell_6-AD.vs.control; scRNA-seq,SRP215507,CD8+ T cell_5-AD.vs.MCI; scRNA-seq,SRP215507,CD8+ T cell_5-MCI.vs.control	6
CDK2AP1	bulk RNA-seq,Homo_723,AD.vs.control; bulk RNA-seq,Homo_723,AD.vs.MCI; bulk RNA-seq,Homo_633,AD.vs.control; bulk RNA-seq,Homo_633,AD.vs.MCI; bulk RNA-seq,Homo_714,AD.vs.control; bulk RNA-seq,Homo_714,AD.vs.MCI; scRNA-seq,SRP215507,CD8+ T cell_1-AD.vs.control; scRNA-seq,SRP215507,CD8+ T cell_2-AD.vs.MCI; scRNA-seq,SRP215507,CD8+ T cell_3-AD.vs.MCI; scRNA-seq,SRP215507,CD8+ T cell_2-MCI.vs.control; scRNA-seq,SRP330776,CD8+ T cell_1-AD.vs.control; scRNA-seq,SRP330776,Memory T cell_2-AD.vs.control; scRNA-seq,SRP330776,CD8+ T cell_2-AD.vs.control; scRNA-seq,SRP330776,Naive CD8+ T cell_1-AD.vs.control	14

CDK2AP2	scRNA-seq,SRP215507,CD8+ T cell_3-AD.vs.control; scRNA-seq,SRP215507,CD8+ T cell_6-AD.vs.control; scRNA-seq,SRP215507,CD8+ T cell_6-AD.vs.MCI; scRNA-seq,SRP309935,Erythroid cell-AD.vs.control; scRNA-seq,SRP330776,CD8+ T cell_1-AD.vs.control; scRNA-seq,SRP330776,Memory T cell_2-AD.vs.control; scRNA-seq,SRP330776,Naive CD8+ T cell_1-AD.vs.control; scRNA-seq,SRP330776,Naive CD8+ T cell_3-AD.vs.control;	9
CDK3	scRNA-seq,SRP215507,CD8+ T cell_3-AD.vs.MCI; scRNA-seq,SRP215507,CD8+ T cell_1-AD.vs.MCI; scRNA-seq,SRP215507,CD8+ T cell_4-AD.vs.MCI; scRNA-seq,SRP215507,CD8+ T cell_1-MCI.vs.control; scRNA-seq,SRP215507,CD8+ T cell_3-MCI.vs.control	5
Cdk4	bulk RNA-seq,SRP325058,AD.vs.control	1
CDK4	scRNA-seq,SRP215507,CD8+ T cell_7-AD.vs.control; scRNA-seq,SRP215507,CD8+ T cell_7-AD.vs.MCI; scRNA-seq,SRP215507,CD8+ T cell_5-MCI.vs.control; scRNA-seq,SRP215507,CD8+ T cell_4-MCI.vs.control; scRNA-seq,SRP330776,CD8+ T cell_1-AD.vs.control; scRNA-seq,SRP330776,γδ T cell-AD.vs.control; scRNA-seq,SRP330776,Memory T cell_2-AD.vs.control; scRNA-seq,SRP330776,Natural killer cell_1-AD.vs.control; scRNA-seq,SRP330776,T cell-AD.vs.control; scRNA-seq,SRP330776,Natural killer cell_2-AD.vs.control	10
CDK5	scRNA-seq,SRP215507,CD8+ T cell_2-AD.vs.control; scRNA-seq,SRP215507,CD8+ T cell_5-AD.vs.MCI; scRNA-seq,SRP215507,CD8+ T cell_6-AD.vs.MCI; scRNA-seq,SRP215507,CD8+ T cell_5-MCI.vs.control; scRNA-seq,SRP215507,CD8+ T cell_2-MCI.vs.control; scRNA-seq,SRP330776,Memory T cell_2-AD.vs.control; scRNA-seq,SRP330776,B cell_1-AD.vs.control; scRNA-seq,SRP330776,Naive CD8+ T cell_1-AD.vs.control; scRNA-seq,SRP330776,Natural killer cell_1-AD.vs.control; scRNA-seq,SRP330776,Memory T cell_1-AD.vs.control; scRNA-seq,SRP330776,B cell_2-AD.vs.control; scRNA-seq,SRP330776,T cell-AD.vs.control; scRNA-seq,SRP330776,CD8+ T cell_3-AD.vs.control; scRNA-	15
CDK5R1	bulk RNA-seq,Homo_723,AD.vs.control; bulk RNA-seq,Homo_723,AD.vs.MCI; bulk RNA-seq,Homo_714,AD.vs.control; bulk RNA-seq,Homo_714,AD.vs.MCI; bulk RNA-seq,ROSMAP,MCI.vs.control; scRNA-seq,SRP215507,CD8+ T cell_4-AD.vs.control; scRNA-seq,SRP215507,CD8+ T cell_5-AD.vs.control; scRNA-seq,SRP215507,CD8+ T cell_1-AD.vs.MCI; scRNA-seq,SRP215507,CD8+ T cell_4-MCI.vs.control; scRNA-seq,SRP330776,CD8+ T cell_1-AD.vs.control; scRNA-seq,SRP330776,B cell_1-AD.vs.control	11
CDK5R2	bulk RNA-seq,Homo_723,AD.vs.control; bulk RNA-seq,Homo_723,MCI.vs.control; bulk RNA-seq,Homo_633,AD.vs.control; bulk RNA-seq,Homo_633,MCI.vs.control; bulk RNA-seq,Homo_714,MCI.vs.control	5
CDK5RAP1	bulk RNA-seq,Homo_723,AD.vs.control; bulk RNA-seq,Homo_723,AD.vs.MCI; bulk RNA-seq,Homo_633,AD.vs.control; bulk RNA-seq,Homo_633,AD.vs.MCI; bulk RNA-seq,Homo_714,AD.vs.control; bulk RNA-seq,Homo_714,AD.vs.MCI; scRNA-seq,SRP215507,CD8+ T cell_7-AD.vs.MCI; scRNA-seq,SRP215507,CD8+ T cell_6-AD.vs.MCI; scRNA-seq,SRP215507,CD8+ T cell_6-MCI.vs.control; scRNA-seq,SRP309935,Natural killer cell_2-AD.vs.control; scRNA-seq,SRP330776,Naive CD8+ T cell_3-AD.vs.control; scRNA-seq,SRP330776,T cell-AD.vs.control; scRNA-	13
Cdk5rap1	bulk RNA-seq,SRP325058,AD.vs.control	1
CDK5RAP2	bulk RNA-seq,Homo_723,AD.vs.control; bulk RNA-seq,Homo_723,AD.vs.MCI; bulk RNA-seq,Homo_633,AD.vs.control; bulk RNA-	4
CDK5RAP3	bulk RNA-seq,Homo_633,AD.vs.control; scRNA-seq,SRP215507,CD8+ T cell_10-AD.vs.control; scRNA-seq,SRP215507,CD8+ T cell_9-AD.vs.control	3
CDK6	bulk RNA-seq,Homo_723,AD.vs.control; bulk RNA-seq,Homo_723,AD.vs.MCI; bulk RNA-seq,Homo_723,MCI.vs.control; bulk RNA-seq,Homo_633,AD.vs.control; bulk RNA-seq,Homo_633,AD.vs.MCI; bulk RNA-seq,Homo_633,MCI.vs.control; bulk RNA-seq,Homo_714,AD.vs.control; bulk RNA-seq,Homo_714,AD.vs.MCI; bulk RNA-seq,Homo_714,MCI.vs.control; scRNA-seq,SRP215507,CD8+ T cell_5-AD.vs.MCI; scRNA-seq,SRP215507,CD8+ T cell_5-MCI.vs.control; scRNA-seq,SRP309935,Natural killer cell_2-AD.vs.control; scRNA-seq,SRP309935,Naive CD8+ T cell_1-AD.vs.control; scRNA-seq,SRP309935,Natural killer cell_1-AD.vs.control; scRNA-seq,SRP330776,CD8+ T cell_1-AD.vs.control; scRNA-seq,SRP330776,γδ T cell-AD.vs.control; scRNA-seq,SRP330776,Memory T cell_2-AD.vs.control; scRNA-seq,SRP330776,CD8+ T cell_2-AD.vs.control; scRNA-seq,SRP330776,B cell_1-AD.vs.control; scRNA-seq,SRP330776,Naive CD8+ T cell_1-AD.vs.control; scRNA-seq,SRP330776,Natural killer cell_1-AD.vs.control; scRNA-seq,SRP330776,Memory T cell_1-AD.vs.control; scRNA-seq,SRP330776,Natural killer T cell-AD.vs.control; scRNA-seq,SRP330776,Naive CD8+ T cell_3-AD.vs.control; scRNA-seq,SRP330776,B cell_2-AD.vs.control; scRNA-seq,SRP330776,CD8+ T cell_3-[illegible]	28
CDK7	bulk RNA-seq,Homo_723,AD.vs.control; bulk RNA-seq,Homo_633,AD.vs.control; bulk RNA-seq,Homo_714,AD.vs.control; bulk RNA-seq,Homo_714,AD.vs.MCI; scRNA-seq,SRP330776,γδ T cell-AD.vs.control; scRNA-seq,SRP330776,Natural killer T cell-AD.vs.control; scRNA-seq,SRP330776,Naive CD8+ T cell_3-AD.vs.control; scRNA-seq,SRP330776,Regulatory T cell-AD.vs.control	8
Cdk8	bulk RNA-seq,SRP325058,AD.vs.control	1

CDK8	scRNA-seq,SRP215507,CD8+ T cell_4-AD.vs.control; scRNA-seq,SRP215507,CD8+ T cell_7-AD.vs.control; scRNA-seq,SRP215507,CD8+ T cell_2-AD.vs.MCI; scRNA-seq,SRP215507,CD8+ T cell_4-AD.vs.MCI; scRNA-seq,SRP215507,CD8+ T cell_5-AD.vs.MCI; scRNA-seq,SRP215507,CD8+ T cell_5-MCI.vs.control; scRNA-seq,SRP215507,CD8+ T cell_2-MCI.vs.control; scRNA-seq,SRP330776,Memory T cell_2-AD.vs.control; scRNA-seq,SRP330776,CD8+ T cell_2-AD.vs.control; scRNA-seq,SRP330776,B cell_1-AD.vs.control; scRNA-seq,SRP330776,Naive CD8+ T cell_1-AD.vs.control; scRNA-seq,SRP330776,Natural killer cell_1-AD.vs.control; scRNA-seq,SRP330776,Memory T cell_1-AD.vs.control; scRNA-seq,SRP330776,Naive CD8+ T cell_3-AD.vs.control; scRNA-seq,SRP330776,T cell-AD.vs.control; scRNA-seq,SRP330776,CD8+ T cell_3-AD.vs.control; [illegible]	17
CDK9	bulk RNA-seq,Homo_714,AD.vs.control; bulk RNA-seq,Homo_714,AD.vs.MCI; scRNA-seq,SRP215507,CD8+ T cell_7-AD.vs.control; scRNA-seq,SRP215507,CD8+ T cell_3-AD.vs.MCI; scRNA-seq,SRP215507,CD8+ T cell_5-AD.vs.MCI; scRNA-seq,SRP215507,CD8+ T cell_5-MCI.vs.control; scRNA-seq,SRP330776,CD8+ T cell_1-AD.vs.control; scRNA-seq,SRP330776,γδ T cell-AD.vs.control; scRNA-seq,SRP330776,Memory T cell_2-AD.vs.control; scRNA-seq,SRP330776,CD8+ T cell_2-AD.vs.control; scRNA-seq,SRP330776,B cell_1-AD.vs.control; scRNA-seq,SRP330776,Naive CD8+ T cell_1-AD.vs.control; scRNA-seq,SRP330776,Natural killer cell_1-AD.vs.control; scRNA-seq,SRP330776,Memory T cell_1-AD.vs.control; scRNA-seq,SRP330776,Natural killer T cell-AD.vs.control; scRNA-seq,SRP330776,Naive CD8+ T cell_3-AD.vs.control; scRNA-seq,SRP330776,B cell_2-AD.vs.control; scRNA-seq,SRP330776,T cell-AD.vs.control; scRNA-seq,SRP330776,CD8+ T cell_3-AD.vs.control; scRNA-seq,SRP330776,Monocyte_1-[illegible]	22
CDKAL1	bulk RNA-seq,Homo_723,MCI.vs.control; scRNA-seq,SRP215507,CD8+ T cell_5-AD.vs.control; scRNA-seq,SRP215507,CD8+ T cell_5-AD.vs.MCI; scRNA-seq,SRP215507,CD8+ T cell_6-AD.vs.MCI; scRNA-seq,SRP215507,CD8+ T cell_4-MCI.vs.control; scRNA-seq,SRP330776,Naive CD8+ T cell_3-AD.vs.control; scRNA-seq,SRP330776,B cell_3-AD.vs.control	7
CDKL1	bulk RNA-seq,Homo_723,MCI.vs.control; bulk RNA-seq,Homo_633,MCI.vs.control; bulk RNA-seq,Homo_714,AD.vs.control; bulk RNA-seq,Homo_714,AD.vs.MCI; bulk RNA-seq,Homo_714,MCI.vs.control; scRNA-seq,SRP215507,CD8+ T cell_2-AD.vs.control; scRNA-seq,SRP330776,Memory T cell_2-AD.vs.control; scRNA-seq,SRP330776,Naive CD8+ T cell_1-AD.vs.control; scRNA-seq,SRP330776,Naive CD8+ T	10
Cdkl1	bulk RNA-seq,SRP325058,AD.vs.control	1
CDKL2	bulk RNA-seq,Homo_723,AD.vs.control; bulk RNA-seq,Homo_723,AD.vs.MCI; bulk RNA-seq,Homo_723,MCI.vs.control; scRNA-seq,SRP215507,CD8+ T cell_2-AD.vs.control; scRNA-seq,SRP215507,CD8+ T cell_3-AD.vs.control; scRNA-seq,SRP215507,CD8+ T cell_1-AD.vs.control; scRNA-seq,SRP215507,CD8+ T cell_1-AD.vs.MCI; scRNA-seq,SRP330776,CD8+ T cell_1-AD.vs.control	8
CDKL3	bulk RNA-seq,Homo_723,AD.vs.control; bulk RNA-seq,Homo_723,AD.vs.MCI; bulk RNA-seq,Homo_723,MCI.vs.control; bulk RNA-seq,Homo_633,AD.vs.control; bulk RNA-seq,Homo_633,AD.vs.MCI; bulk RNA-seq,Homo_633,MCI.vs.control; bulk RNA-seq,Homo_714,AD.vs.control; bulk RNA-seq,Homo_714,AD.vs.MCI; bulk RNA-seq,Homo_714,MCI.vs.control; scRNA-seq,SRP215507,CD8+ T cell_1-AD.vs.control; scRNA-seq,SRP330776,CD8+ T cell_1-AD.vs.control; scRNA-seq,SRP330776,γδ T cell-AD.vs.control; scRNA-seq,SRP330776,Memory T cell_2-AD.vs.control; scRNA-seq,SRP330776,Naive CD8+ T cell_1-AD.vs.control; scRNA-seq,SRP330776,Memory T cell_1-AD.vs.control	15
CDKL4	bulk RNA-seq,Homo_723,AD.vs.control; bulk RNA-seq,Homo_723,AD.vs.MCI; bulk RNA-seq,Homo_633,AD.vs.control; bulk RNA-	4
CDKL5	scRNA-seq,SRP215507,CD8+ T cell_4-AD.vs.control; scRNA-seq,SRP215507,CD8+ T cell_5-AD.vs.control; scRNA-seq,SRP215507,CD8+ T cell_4-MCI.vs.control; scRNA-seq,SRP330776,Memory T cell_2-AD.vs.control; scRNA-seq,SRP330776,B cell_1-AD.vs.control; scRNA-seq,SRP330776,Natural killer cell_1-AD.vs.control; scRNA-seq,SRP330776,B cell_2-AD.vs.control; scRNA-seq,SRP330776,Naive CD8+ T cell_2-AD.vs.control; scRNA-	9
CDKN1A	bulk RNA-seq,Homo_714,AD.vs.control; bulk RNA-seq,Homo_714,AD.vs.MCI; bulk RNA-seq,Homo_714,MCI.vs.control; bulk RNA-seq,SRP223445,AD.vs.control; scRNA-seq,SRP215507,CD8+ T cell_2-AD.vs.control; scRNA-seq,SRP215507,CD8+ T cell_9-AD.vs.control; scRNA-seq,SRP215507,CD8+ T cell_2-AD.vs.MCI; scRNA-seq,SRP215507,CD8+ T cell_1-AD.vs.MCI; scRNA-seq,SRP215507,CD8+ T cell_5-AD.vs.MCI; scRNA-seq,SRP215507,CD8+ T cell_1-MCI.vs.control; scRNA-seq,SRP215507,CD8+ T cell_5-MCI.vs.control; scRNA-seq,SRP215507,CD8+ T cell_2-MCI.vs.control; scRNA-seq,SRP215507,CD8+ T cell_3-MCI.vs.control; scRNA-seq,SRP330776,CD8+ T cell_1-AD.vs.control; scRNA-seq,SRP330776,γδ T cell-AD.vs.control; scRNA-seq,SRP330776,Memory T cell_2-AD.vs.control; scRNA-seq,SRP330776,Memory T cell_1-AD.vs.control; scRNA-seq,SRP330776,B cell_2-AD.vs.control; scRNA-seq,SRP330776,Monocyte_1-AD.vs.control; scRNA-seq,SRP330776,Monocyte_2-AD.vs.control; scRNA-[illegible]	22

CDKN1B	bulk RNA-seq,Homo_633,AD.vs.control; bulk RNA-seq,Homo_633,AD.vs.MCI; bulk RNA-seq,Homo_633,MCI.vs.control; bulk RNA-seq,Homo_714,AD.vs.control; bulk RNA-seq,Homo_714,AD.vs.MCI; scRNA-seq,SRP215507,CD8+ T cell_8-AD.vs.MCI; scRNA-seq,SRP215507,CD8+ T cell_4-AD.vs.MCI; scRNA-seq,SRP215507,CD8+ T cell_4-MCI.vs.control; scRNA-seq,SRP215507,CD8+ T cell_8-MCI.vs.control; scRNA-seq,SRP309935,Megakaryocyte_1-AD.vs.control; scRNA-seq,SRP309935,CD8+ T cell_1-AD.vs.control; scRNA-seq,SRP309935,B cell_1-AD.vs.control; scRNA-seq,SRP309935,CD8+ T cell_2-AD.vs.control; scRNA-seq,SRP309935,Naive CD8+ T cell_3-AD.vs.control; scRNA-seq,SRP330776,Monocyte_1-	15
Cdkn1b	bulk RNA-seq,SRP325058,AD.vs.control	1
CDKN1C	scRNA-seq,SRP215507,CD8+ T cell_2-AD.vs.control; scRNA-seq,SRP215507,CD8+ T cell_9-AD.vs.control; scRNA-seq,SRP215507,CD8+ T cell_5-AD.vs.MCI; scRNA-seq,SRP215507,CD8+ T cell_5-MCI.vs.control; scRNA-seq,SRP330776,Naive CD8+ T cell_1-AD.vs.control	5
CDKN2A	bulk RNA-seq,Homo_723,AD.vs.MCI; bulk RNA-seq,Homo_723,MCI.vs.control; bulk RNA-seq,Homo_633,AD.vs.MCI; bulk RNA-seq,Homo_633,MCI.vs.control; bulk RNA-seq,Homo_714,AD.vs.control; bulk RNA-seq,Homo_714,AD.vs.MCI; bulk RNA-seq,Homo_714,MCI.vs.control; scRNA-seq,SRP215507,CD8+ T cell_3-AD.vs.control; scRNA-seq,SRP215507,CD8+ T cell_4-AD.vs.control; scRNA-seq,SRP215507,CD8+ T cell_2-AD.vs.MCI; scRNA-seq,SRP215507,CD8+ T cell_3-AD.vs.MCI; scRNA-seq,SRP215507,CD8+ T cell_1-AD.vs.MCI; scRNA-seq,SRP215507,CD8+ T cell_4-AD.vs.MCI; scRNA-seq,SRP215507,CD8+ T cell_5-AD.vs.MCI; scRNA-seq,SRP215507,CD8+ T cell_6-AD.vs.MCI; scRNA-seq,SRP215507,CD8+ T cell_5-MCI.vs.control; scRNA-seq,SRP215507,CD8+ T cell_2-MCI.vs.control; scRNA-seq,SRP215507,CD8+ T cell_6-MCI.vs.control; scRNA-seq,SRP330776,γδ T cell-AD.vs.control; scRNA-seq,SRP330776,Memory T cell_2-AD.vs.control; scRNA-seq,SRP330776,B cell_1-AD.vs.control; scRNA-seq,SRP330776,Memory T cell_1-AD.vs.control; scRNA-seq,SRP330776,T cell-AD.vs.control; [illegible]	24
CDKN2AIP	bulk RNA-seq,Homo_714,AD.vs.control; bulk RNA-seq,Homo_714,AD.vs.MCI; scRNA-seq,SRP215507,CD8+ T cell_7-AD.vs.control; scRNA-seq,SRP215507,CD8+ T cell_3-AD.vs.MCI; scRNA-seq,SRP215507,CD8+ T cell_7-AD.vs.MCI; scRNA-seq,SRP215507,CD8+ T cell_5-AD.vs.MCI; scRNA-seq,SRP215507,CD8+ T cell_3-MCI.vs.control	7
CDKN2AIPNL	bulk RNA-seq,Homo_723,AD.vs.control; bulk RNA-seq,Homo_723,AD.vs.MCI; bulk RNA-seq,Homo_633,AD.vs.control; bulk RNA-seq,Homo_633,AD.vs.MCI; bulk RNA-seq,Homo_714,AD.vs.control; bulk RNA-seq,Homo_714,AD.vs.MCI; scRNA-seq,SRP215507,CD8+ T cell_7-AD.vs.control; scRNA-seq,SRP215507,CD8+ T cell_7-MCI.vs.control; scRNA-seq,SRP330776,Natural killer cell_1-AD.vs.control; scRNA-seq,SRP330776,Natural killer T cell-AD.vs.control; scRNA-seq,SRP330776,Natural killer cell_2-AD.vs.control	11
CDKN2B	scRNA-seq,SRP215507,CD8+ T cell_2-AD.vs.control; scRNA-seq,SRP215507,CD8+ T cell_1-MCI.vs.control; scRNA-seq,SRP215507,CD8+ T cell_6-MCI.vs.control; scRNA-seq,SRP330776,Monocyte_1-AD.vs.control	4
CDKN2B-AS1	scRNA-seq,SRP215507,CD8+ T cell_4-MCI.vs.control; scRNA-seq,SRP330776,Memory T cell_2-AD.vs.control; scRNA-seq,SRP330776,B cell_1-AD.vs.control; scRNA-seq,SRP330776,Memory T cell_1-AD.vs.control; scRNA-seq,SRP330776,CD8+ T cell_3-AD.vs.control; scRNA-seq,SRP330776,B	6
CDKN2C	scRNA-seq,SRP215507,CD8+ T cell_2-AD.vs.control; scRNA-seq,SRP215507,CD8+ T cell_1-AD.vs.control; scRNA-seq,SRP215507,CD8+ T cell_4-AD.vs.control; scRNA-seq,SRP215507,CD8+ T cell_6-AD.vs.control; scRNA-seq,SRP215507,CD8+ T cell_2-AD.vs.MCI; scRNA-seq,SRP215507,CD8+ T cell_3-AD.vs.MCI; scRNA-seq,SRP215507,CD8+ T cell_1-AD.vs.MCI; scRNA-seq,SRP215507,CD8+ T cell_4-AD.vs.MCI; scRNA-seq,SRP215507,CD8+ T cell_2-MCI.vs.control; scRNA-seq,SRP215507,CD8+ T cell_3-MCI.vs.control; scRNA-seq,SRP309935,Monocyte_1-AD.vs.control; scRNA-seq,SRP309935,CD8+ T cell_2-AD.vs.control; scRNA-seq,SRP330776,CD8+ T cell_1-AD.vs.control; scRNA-seq,SRP330776,γδ T cell-AD.vs.control; scRNA-seq,SRP330776,Memory T cell_2-AD.vs.control; scRNA-seq,SRP330776,CD8+ T cell_2-AD.vs.control; scRNA-seq,SRP330776,B cell_1-AD.vs.control; scRNA-seq,SRP330776,Natural killer cell_1-AD.vs.control; scRNA-seq,SRP330776,Memory T cell_1-AD.vs.control; scRNA-seq,SRP330776,T cell-AD.vs.control; scRNA-seq,SRP330776,CD8+ T cell_3-AD.vs.control; scRNA-seq,SRP330776,Natural killer [illegible]	22
CDKN2D	scRNA-seq,SRP215507,CD8+ T cell_7-AD.vs.control; scRNA-seq,SRP215507,CD8+ T cell_7-MCI.vs.control; scRNA-seq,SRP309935,CD4+ T cell-AD.vs.control; scRNA-seq,SRP330776,CD8+ T cell_1-AD.vs.control; scRNA-seq,SRP330776,γδ T cell-AD.vs.control; scRNA-seq,SRP330776,Memory T cell_2-AD.vs.control; scRNA-seq,SRP330776,CD8+ T cell_2-AD.vs.control; scRNA-seq,SRP330776,B cell_1-AD.vs.control; scRNA-seq,SRP330776,Naive CD8+ T cell_1-AD.vs.control; scRNA-seq,SRP330776,Natural killer cell_1-AD.vs.control; scRNA-seq,SRP330776,Memory T cell_1-AD.vs.control; scRNA-seq,SRP330776,Natural killer T cell-AD.vs.control; scRNA-seq,SRP330776,Naive CD8+ T cell_3-AD.vs.control; scRNA-seq,SRP330776,B cell_2-AD.vs.control; scRNA-seq,SRP330776,T cell-AD.vs.control; scRNA-seq,SRP330776,Naive CD8+ T cell_2-AD.vs.control; scRNA-seq,SRP330776,CD8+ T cell_3-AD.vs.control; scRNA-seq,SRP330776,Monocyte_1-AD.vs.control; scRNA-seq,SRP330776,Monocyte_2-AD.vs.control; scRNA-seq,SRP330776,B cell_3-AD.vs.control; scRNA-seq,SRP330776,Regulatory T cell-AD.vs.control; scRNA-seq,SRP330776,Natural [illegible]	22

CDKN3	bulk RNA-seq,Homo_723,AD.vs.control; bulk RNA-seq,Homo_723,MCI.vs.control; bulk RNA-seq,Homo_633,AD.vs.control; bulk RNA-seq,Homo_633,MCI.vs.control; bulk RNA-seq,Homo_714,MCI.vs.control; scRNA-seq,SRP330776,γδ T cell-AD.vs.control; scRNA-seq,SRP330776,B cell_1-AD.vs.control; scRNA-seq,SRP330776,Natural killer cell_1-AD.vs.control; scRNA-seq,SRP330776,CD8+ T cell_3-AD.vs.control	9
CDNF	bulk RNA-seq,Homo_723,AD.vs.control; bulk RNA-seq,Homo_723,AD.vs.MCI; bulk RNA-seq,Homo_723,MCI.vs.control; bulk RNA-seq,Homo_633,AD.vs.control; bulk RNA-seq,Homo_633,AD.vs.MCI; bulk RNA-seq,Homo_633,MCI.vs.control; bulk RNA-seq,Homo_714,AD.vs.control; bulk RNA-seq,Homo_714,AD.vs.MCI; bulk RNA-seq,Homo_714,MCI.vs.control; scRNA-seq,SRP215507,CD8+ T cell_3-AD.vs.control; scRNA-seq,SRP215507,CD8+ T cell_1-AD.vs.MCI; scRNA-seq,SRP215507,CD8+ T cell_3-MCI.vs.control; scRNA-seq,SRP330776,Natural killer cell_1-	13
CDO1	bulk RNA-seq,Homo_723,AD.vs.control; bulk RNA-seq,Homo_723,AD.vs.MCI; bulk RNA-seq,Homo_723,MCI.vs.control; bulk RNA-seq,Homo_633,AD.vs.control; bulk RNA-seq,Homo_633,AD.vs.MCI; bulk RNA-seq,Homo_714,AD.vs.MCI; scRNA-seq,SRP330776,CD8+ T cell_1-AD.vs.control; scRNA-seq,SRP330776,Memory T cell_2-AD.vs.control	8
CDON	bulk RNA-seq,Homo_723,AD.vs.control; bulk RNA-seq,Homo_723,AD.vs.MCI; bulk RNA-seq,Homo_723,MCI.vs.control; bulk RNA-seq,Homo_633,AD.vs.control; bulk RNA-seq,Homo_633,AD.vs.MCI; bulk RNA-seq,Homo_714,AD.vs.control; bulk RNA-seq,Homo_714,AD.vs.MCI; bulk RNA-seq,ROSMAP,AD.vs.control; bulk RNA-seq,ROSMAP,MCI.vs.control; scRNA-seq,SRP215507,CD8+ T cell_3-AD.vs.control; scRNA-	11
CDPF1	bulk RNA-seq,Homo_723,AD.vs.MCI; bulk RNA-seq,Homo_633,AD.vs.control; bulk RNA-seq,Homo_633,AD.vs.MCI; bulk RNA-seq,Homo_714,AD.vs.control; bulk RNA-seq,Homo_714,AD.vs.MCI; bulk RNA-seq,SRP223445,AD.vs.control; scRNA-seq,SRP215507,CD8+ T cell_3-AD.vs.MCI; scRNA-seq,SRP330776,CD8+ T cell_1-AD.vs.control; scRNA-seq,SRP330776,γδ T cell-AD.vs.control; scRNA-seq,SRP330776,Memory T cell_2-AD.vs.control; scRNA-seq,SRP330776,CD8+ T cell_2-AD.vs.control; scRNA-seq,SRP330776,B cell_1-AD.vs.control; scRNA-seq,SRP330776,Naive CD8+ T cell_1-AD.vs.control; scRNA-seq,SRP330776,Natural killer cell_1-AD.vs.control; scRNA-seq,SRP330776,Memory T cell_1-AD.vs.control; scRNA-seq,SRP330776,Natural killer T cell-AD.vs.control; scRNA-seq,SRP330776,B cell_2-AD.vs.control; scRNA-seq,SRP330776,CD8+ T cell_3-AD.vs.control; scRNA-seq,SRP330776,Megakaryocyte_1-AD.vs.control; scRNA-seq,SRP330776,B cell_4-AD.vs.control	20
CDR1	bulk RNA-seq,Homo_723,AD.vs.control; bulk RNA-seq,Homo_723,AD.vs.MCI; bulk RNA-seq,Homo_723,MCI.vs.control; bulk RNA-seq,Homo_633,AD.vs.control; bulk RNA-seq,Homo_633,AD.vs.MCI; bulk RNA-seq,Homo_633,MCI.vs.control; bulk RNA-seq,Homo_714,MCI.vs.control	7
CDR2	bulk RNA-seq,Homo_723,AD.vs.MCI; bulk RNA-seq,Homo_723,MCI.vs.control; bulk RNA-seq,Homo_633,AD.vs.MCI; bulk RNA-seq,Homo_633,MCI.vs.control; bulk RNA-seq,Homo_714,AD.vs.control; bulk RNA-seq,Homo_714,AD.vs.MCI; scRNA-seq,SRP215507,CD8+ T cell_6-MCI.vs.control; scRNA-seq,SRP309935,Naive CD8+ T cell_2-AD.vs.control; scRNA-seq,SRP330776,CD8+ T cell_1-AD.vs.control; scRNA-seq,SRP330776,γδ T cell-AD.vs.control; scRNA-seq,SRP330776,Memory T cell_2-AD.vs.control; scRNA-seq,SRP330776,CD8+ T cell_2-AD.vs.control; scRNA-seq,SRP330776,Naive CD8+ T cell_1-AD.vs.control; scRNA-seq,SRP330776,Natural killer cell_1-AD.vs.control; scRNA-seq,SRP330776,Natural killer T cell-AD.vs.control; scRNA-seq,SRP330776,Naive CD8+ T cell_3-AD.vs.control; scRNA-seq,SRP330776,T cell-AD.vs.control; scRNA-	18
Cdr2	bulk RNA-seq,SRP325058,AD.vs.control	1
CDR2L	bulk RNA-seq,Homo_723,AD.vs.control; bulk RNA-seq,Homo_723,AD.vs.MCI; bulk RNA-seq,Homo_723,MCI.vs.control; bulk RNA-seq,Homo_633,AD.vs.control; bulk RNA-seq,Homo_633,AD.vs.MCI; bulk RNA-seq,Homo_633,MCI.vs.control; bulk RNA-seq,Homo_714,AD.vs.MCI;	8
CDRT1	bulk RNA-seq,Homo_714,AD.vs.control; bulk RNA-seq,Homo_714,AD.vs.MCI	2
CDRT15	bulk RNA-seq,Homo_723,AD.vs.control; bulk RNA-seq,Homo_723,AD.vs.MCI; bulk RNA-seq,Homo_633,AD.vs.control; bulk RNA-	4
CDRT4	scRNA-seq,SRP215507,CD8+ T cell_1-AD.vs.MCI; scRNA-seq,SRP309935,Naive CD8+ T cell_1-AD.vs.control; scRNA-seq,SRP330776,CD8+ T cell_1-AD.vs.control; scRNA-seq,SRP330776,CD8+ T cell_2-AD.vs.control	4
CDS1	bulk RNA-seq,Homo_723,AD.vs.control; bulk RNA-seq,Homo_723,AD.vs.MCI; bulk RNA-seq,Homo_714,AD.vs.control; bulk RNA-seq,Homo_714,AD.vs.MCI; scRNA-seq,SRP215507,CD8+ T cell_4-MCI.vs.control	5
Cds1	bulk RNA-seq,SRP325058,AD.vs.control	1
CDS2	bulk RNA-seq,Homo_633,AD.vs.control; bulk RNA-seq,Homo_633,AD.vs.MCI; bulk RNA-seq,SRP223445,AD.vs.control; scRNA-seq,SRP215507,CD8+ T cell_5-AD.vs.MCI; scRNA-seq,SRP215507,CD8+ T cell_5-MCI.vs.control; scRNA-seq,SRP330776,CD8+ T cell_2-AD.vs.control; scRNA-seq,SRP330776,Naive CD8+ T cell_3-AD.vs.control; scRNA-seq,SRP330776,Megakaryocyte_1-AD.vs.control	8
CDSN	bulk RNA-seq,Homo_723,AD.vs.control; bulk RNA-seq,Homo_723,AD.vs.MCI; bulk RNA-seq,Homo_633,AD.vs.control; bulk RNA-	4
CDT1	scRNA-seq,SRP215507,CD8+ T cell_1-MCI.vs.control; scRNA-seq,SRP215507,CD8+ T cell_2-MCI.vs.control; scRNA-seq,SRP330776,Naive CD8+ T	3

CDV3	bulk RNA-seq,Homo_723,AD.vs.control; bulk RNA-seq,Homo_723,AD.vs.MCI; bulk RNA-seq,Homo_633,AD.vs.control; bulk RNA-seq,Homo_633,AD.vs.MCI; bulk RNA-seq,Homo_714,AD.vs.control; bulk RNA-seq,Homo_714,AD.vs.MCI; scRNA-seq,SRP215507,CD8+ T cell_4-AD.vs.control; scRNA-seq,SRP215507,CD8+ T cell_7-AD.vs.control; scRNA-seq,SRP215507,CD8+ T cell_5-AD.vs.MCI; scRNA-seq,SRP215507,CD8+ T cell_5-MCI.vs.control; scRNA-seq,SRP215507,CD8+ T cell_4-MCI.vs.control; scRNA-seq,SRP309935,Megakaryocyte_1-AD.vs.control; scRNA-seq,SRP309935,Natural killer cell_1-AD.vs.control; scRNA-seq,SRP309935,Naive CD8+ T cell_2-AD.vs.control; scRNA-seq,SRP309935,Naive CD8+ T cell_3-AD.vs.control; scRNA-seq,SRP309935,Plasmacytoid dendritic cell_1-AD.vs.control; scRNA-seq,SRP330776,CD8+ T cell_1-AD.vs.control; scRNA-seq,SRP330776,Memory T cell_2-AD.vs.control; scRNA-seq,SRP330776,CD8+ T cell_2-AD.vs.control; scRNA-seq,SRP330776,B cell_1-AD.vs.control; scRNA-seq,SRP330776,Naive CD8+ T cell_1-AD.vs.control; scRNA-seq,SRP330776,Memory T cell_1-AD.vs.control; scRNA-seq,SRP330776,Naive CD8+ T cell_3-AD.vs.control; scRNA-seq,SRP330776,B cell_2-AD.vs.control; scRNA-seq,SRP330776,T cell-AD.vs.control; scRNA-seq,SRP330776,Megakaryocyte_1-AD.vs.control; scRNA-seq,SRP330776,Natural killer cell_2-AD.vs.control	27
CDX2	bulk RNA-seq,ACOM,AD.vs.control; bulk RNA-seq,Homo_723,AD.vs.control; bulk RNA-seq,Homo_714,MCI.vs.control	3
CDY1	bulk RNA-seq,Homo_723,AD.vs.control; bulk RNA-seq,Homo_633,AD.vs.control	2
CDY1B	bulk RNA-seq,Homo_723,AD.vs.control; bulk RNA-seq,Homo_633,AD.vs.control	2
CDY2A	bulk RNA-seq,Homo_723,AD.vs.control; bulk RNA-seq,Homo_723,AD.vs.MCI; bulk RNA-seq,Homo_633,AD.vs.control; bulk RNA-	4
CDY2B	bulk RNA-seq,Homo_723,AD.vs.control; bulk RNA-seq,Homo_723,AD.vs.MCI; bulk RNA-seq,Homo_633,AD.vs.control; bulk RNA-	4
Cdyl	bulk RNA-seq,SRP325058,AD.vs.control	1
CDYL	scRNA-seq,SRP215507,CD8+ T cell_3-AD.vs.MCI; scRNA-seq,SRP215507,CD8+ T cell_3-MCI.vs.control; scRNA-seq,SRP330776,B cell_1-AD.vs.control; scRNA-seq,SRP330776,Naive CD8+ T cell_1-AD.vs.control; scRNA-seq,SRP330776,Memory T cell_1-AD.vs.control; scRNA-seq,SRP330776,Natural killer T cell-AD.vs.control; scRNA-seq,SRP330776,B cell_2-AD.vs.control; scRNA-seq,SRP330776,Naive CD8+ T cell_2-	8
CDYL2	bulk RNA-seq,Homo_723,AD.vs.control; bulk RNA-seq,Homo_723,AD.vs.MCI; bulk RNA-seq,Homo_633,AD.vs.control; bulk RNA-seq,Homo_633,AD.vs.MCI; bulk RNA-seq,Homo_714,AD.vs.control; bulk RNA-seq,Homo_714,AD.vs.MCI; scRNA-seq,SRP215507,CD8+ T cell_3-AD.vs.control; scRNA-seq,SRP215507,CD8+ T cell_3-AD.vs.MCI; scRNA-seq,SRP215507,CD8+ T cell_1-MCI.vs.control; scRNA-seq,SRP330776,CD8+	11
CEACAM1	bulk RNA-seq,ROSMAP,AD.vs.control; scRNA-seq,SRP215507,CD8+ T cell_2-AD.vs.control; scRNA-seq,SRP215507,CD8+ T cell_1-AD.vs.control; scRNA-seq,SRP215507,CD8+ T cell_1-MCI.vs.control; scRNA-seq,SRP330776,CD8+ T cell_1-AD.vs.control; scRNA-seq,SRP330776,Naive CD8+ T	6
CEACAM16	bulk RNA-seq,Homo_723,AD.vs.control; bulk RNA-seq,Homo_633,AD.vs.control; bulk RNA-seq,Homo_714,MCI.vs.control	3
CEACAM19	bulk RNA-seq,Homo_723,AD.vs.control; bulk RNA-seq,Homo_723,AD.vs.MCI; bulk RNA-seq,Homo_633,AD.vs.MCI; bulk RNA-seq,Homo_714,AD.vs.control; bulk RNA-seq,Homo_714,AD.vs.MCI; scRNA-seq,SRP215507,CD8+ T cell_3-AD.vs.MCI; scRNA-seq,SRP215507,CD8+	7
CEACAM20	bulk RNA-seq,Homo_723,AD.vs.control; bulk RNA-seq,Homo_723,MCI.vs.control; bulk RNA-seq,Homo_633,AD.vs.control; bulk RNA-seq,Homo_633,MCI.vs.control; bulk RNA-seq,Homo_714,MCI.vs.control	5
CEACAM21	bulk RNA-seq,ROSMAP,AD.vs.control; scRNA-seq,SRP215507,CD8+ T cell_4-AD.vs.control; scRNA-seq,SRP215507,CD8+ T cell_4-AD.vs.MCI; scRNA-seq,SRP215507,CD8+ T cell_5-AD.vs.MCI; scRNA-seq,SRP215507,CD8+ T cell_5-MCI.vs.control; scRNA-seq,SRP309935,Naive CD8+ T cell_1-AD.vs.control; scRNA-seq,SRP309935,Monocyte_2-AD.vs.control; scRNA-seq,SRP309935,B cell_1-AD.vs.control; scRNA-seq,SRP330776,γδ T cell-AD.vs.control; scRNA-seq,SRP330776,Memory T cell_2-AD.vs.control; scRNA-seq,SRP330776,B cell_1-AD.vs.control; scRNA-seq,SRP330776,B cell_2-AD.vs.control; scRNA-seq,SRP330776,CD8+ T cell_3-AD.vs.control; scRNA-seq,SRP330776,Natural killer cell_2-AD.vs.control	14
CEACAM3	bulk RNA-seq,Homo_723,AD.vs.control; bulk RNA-seq,Homo_723,AD.vs.MCI; bulk RNA-seq,Homo_723,MCI.vs.control; bulk RNA-seq,Homo_714,AD.vs.control; bulk RNA-seq,Homo_714,AD.vs.MCI; bulk RNA-seq,Homo_714,MCI.vs.control; scRNA-seq,SRP309935,Monocyte_1-	7
CEACAM4	bulk RNA-seq,Homo_723,AD.vs.control; bulk RNA-seq,Homo_723,AD.vs.MCI; scRNA-seq,SRP330776,CD8+ T cell_1-AD.vs.control	3
CEACAM5	bulk RNA-seq,Homo_714,MCI.vs.control; bulk RNA-seq,ROSMAP,AD.vs.control; bulk RNA-seq,ROSMAP,MCI.vs.control	3
CEACAM6	bulk RNA-seq,ACOM,AD.vs.control; bulk RNA-seq,Homo_723,AD.vs.control; bulk RNA-seq,Homo_723,AD.vs.MCI; bulk RNA-seq,Homo_633,AD.vs.control; bulk RNA-seq,Homo_633,AD.vs.MCI; bulk RNA-seq,Homo_714,AD.vs.control; bulk RNA-seq,Homo_714,AD.vs.MCI; bulk RNA-seq,Homo_714,MCI.vs.control; bulk RNA-seq,ROSMAP,AD.vs.control; bulk RNA-seq,ROSMAP,MCI.vs.control	10
CEACAM7	bulk RNA-seq,Homo_723,AD.vs.control; bulk RNA-seq,Homo_723,AD.vs.MCI; bulk RNA-seq,Homo_633,AD.vs.control; bulk RNA-	4

CEBPA	bulk RNA-seq,ACOM,AD.vs.control; scRNA-seq,SRP215507,CD8+ T cell_2-AD.vs.MCI; scRNA-seq,SRP215507,CD8+ T cell_1-AD.vs.MCI; scRNA-seq,SRP215507,CD8+ T cell_4-AD.vs.MCI; scRNA-seq,SRP215507,CD8+ T cell_1-MCI.vs.control; scRNA-seq,SRP215507,CD8+ T cell_2-MCI.vs.control; scRNA-seq,SRP309935,Naive CD8+ T cell_1-AD.vs.control; scRNA-seq,SRP309935,Natural killer cell_1-AD.vs.control; scRNA-seq,SRP309935,Monocyte_2-AD.vs.control; scRNA-seq,SRP330776,CD8+ T cell_2-AD.vs.control; scRNA-seq,SRP330776,Memory T cell_1-AD.vs.control; scRNA-seq,SRP330776,Monocyte_1-AD.vs.control; scRNA-seq,SRP330776,Monocyte_2-AD.vs.control	13
CEBPB	scRNA-seq,SRP215507,CD8+ T cell_1-AD.vs.control; scRNA-seq,SRP215507,CD8+ T cell_6-AD.vs.control; scRNA-seq,SRP215507,CD8+ T cell_4-AD.vs.MCI; scRNA-seq,SRP215507,CD8+ T cell_4-MCI.vs.control; scRNA-seq,SRP215507,CD8+ T cell_6-MCI.vs.control; scRNA-seq,SRP309935,Natural killer cell_2-AD.vs.control; scRNA-seq,SRP309935,Monocyte_1-AD.vs.control; scRNA-seq,SRP309935,Naive CD8+ T cell_1-AD.vs.control; scRNA-seq,SRP309935,Natural killer cell_1-AD.vs.control; scRNA-seq,SRP309935,Monocyte_2-AD.vs.control; scRNA-seq,SRP309935,B cell_1-AD.vs.control; scRNA-seq,SRP309935,CD8+ T cell_2-AD.vs.control; scRNA-seq,SRP309935,Monocyte_3-AD.vs.control; scRNA-seq,SRP309935,Dendritic cell-AD.vs.control; scRNA-seq,SRP330776,CD8+ T cell_1-AD.vs.control; scRNA-seq,SRP330776,γδ T cell-AD.vs.control; scRNA-seq,SRP330776,Memory T cell_2-AD.vs.control; scRNA-seq,SRP330776,CD8+ T cell_2-AD.vs.control; scRNA-seq,SRP330776,B cell_1-AD.vs.control; scRNA-seq,SRP330776,Naive CD8+ T cell_1-AD.vs.control; scRNA-seq,SRP330776,Natural killer cell_1-AD.vs.control; scRNA-seq,SRP330776,Memory T cell_1-AD.vs.control; scRNA-seq,SRP330776,B cell_2-AD.vs.control; scRNA-seq,SRP330776,CD8+ T cell_3-AD.vs.control; [illegible]	27
CEBPB-AS1	scRNA-seq,SRP330776,Memory T cell_1-AD.vs.control	1
CEBPD	scRNA-seq,SRP215507,CD8+ T cell_1-AD.vs.control; scRNA-seq,SRP215507,CD8+ T cell_4-AD.vs.control; scRNA-seq,SRP215507,CD8+ T cell_2-AD.vs.MCI; scRNA-seq,SRP215507,CD8+ T cell_5-AD.vs.MCI; scRNA-seq,SRP215507,CD8+ T cell_5-MCI.vs.control; scRNA-seq,SRP215507,CD8+ T cell_4-MCI.vs.control; scRNA-seq,SRP215507,CD8+ T cell_2-MCI.vs.control; scRNA-seq,SRP215507,CD8+ T cell_6-MCI.vs.control; scRNA-seq,SRP309935,Natural killer cell_2-AD.vs.control; scRNA-seq,SRP309935,Megakaryocyte_1-AD.vs.control; scRNA-seq,SRP309935,Natural killer cell_1-AD.vs.control; scRNA-seq,SRP309935,CD8+ T cell_2-AD.vs.control; scRNA-seq,SRP309935,Naive CD8+ T cell_3-AD.vs.control; scRNA-seq,SRP309935,Dendritic cell-AD.vs.control; scRNA-seq,SRP330776,γδ T cell-AD.vs.control; scRNA-seq,SRP330776,Memory T cell_2-AD.vs.control; scRNA-seq,SRP330776,Memory T cell_1-AD.vs.control; scRNA-seq,SRP330776,Natural killer T cell-AD.vs.control; scRNA-seq,SRP330776,CD8+ T cell_3-AD.vs.control; scRNA-seq,SRP330776,Monocyte_1-AD.vs.control; scRNA-seq,SRP330776,Monocyte_2-AD.vs.control; scRNA-seq,SRP330776,B [illegible]	24
CEBPE	bulk RNA-seq,Homo_714,AD.vs.control; bulk RNA-seq,Homo_714,AD.vs.MCI; scRNA-seq,SRP215507,CD8+ T cell_3-AD.vs.control; scRNA-seq,SRP330776,CD8+ T cell_1-AD.vs.control	4
CEBPG	scRNA-seq,SRP215507,CD8+ T cell_1-AD.vs.MCI; scRNA-seq,SRP215507,CD8+ T cell_4-AD.vs.MCI; scRNA-seq,SRP215507,CD8+ T cell_5-AD.vs.MCI; scRNA-seq,SRP215507,CD8+ T cell_1-MCI.vs.control; scRNA-seq,SRP215507,CD8+ T cell_5-MCI.vs.control; scRNA-seq,SRP215507,CD8+ T cell_4-MCI.vs.control; scRNA-seq,SRP215507,CD8+ T cell_3-MCI.vs.control; scRNA-seq,SRP215507,CD8+ T cell_7-MCI.vs.control; scRNA-seq,SRP330776,CD8+ T cell_2-AD.vs.control; scRNA-seq,SRP330776,B cell_2-AD.vs.control; scRNA-seq,SRP330776,T cell-AD.vs.control; scRNA-seq,SRP330776,Naive CD8+ T cell_2-AD.vs.control; scRNA-seq,SRP330776,B cell_3-AD.vs.control	13
CEBPZ	bulk RNA-seq,Homo_714,AD.vs.control; bulk RNA-seq,Homo_714,AD.vs.MCI; scRNA-seq,SRP330776,Monocyte_2-AD.vs.control	3
CEBPZOS	bulk RNA-seq,Homo_723,AD.vs.MCI; bulk RNA-seq,Homo_714,AD.vs.control; bulk RNA-seq,Homo_714,AD.vs.MCI; scRNA-seq,SRP215507,CD8+ T cell_7-MCI.vs.control; scRNA-seq,SRP330776,Natural killer cell_2-AD.vs.control	5
CECR2	bulk RNA-seq,Homo_723,AD.vs.control; bulk RNA-seq,Homo_723,AD.vs.MCI; bulk RNA-seq,Homo_723,MCI.vs.control; bulk RNA-seq,Homo_633,AD.vs.control; bulk RNA-seq,Homo_633,AD.vs.MCI; bulk RNA-seq,Homo_714,AD.vs.MCI	6
CEL	bulk RNA-seq,Homo_723,AD.vs.control; bulk RNA-seq,Homo_723,AD.vs.MCI; bulk RNA-seq,Homo_723,MCI.vs.control; bulk RNA-seq,Homo_633,AD.vs.control; bulk RNA-seq,Homo_633,AD.vs.MCI; bulk RNA-seq,Homo_633,MCI.vs.control	6
CELA1	bulk RNA-seq,Homo_723,AD.vs.control; bulk RNA-seq,Homo_723,AD.vs.MCI; bulk RNA-seq,Homo_633,AD.vs.control; bulk RNA-seq,Homo_633,AD.vs.MCI; bulk RNA-seq,Homo_714,AD.vs.control; bulk RNA-seq,Homo_714,AD.vs.MCI; scRNA-seq,SRP215507,CD8+ T cell_3-AD.vs.control; scRNA-seq,SRP215507,CD8+ T cell_4-AD.vs.control; scRNA-seq,SRP215507,CD8+ T cell_3-MCI.vs.control; scRNA-seq,SRP309935,Natural killer cell_2-AD.vs.control; scRNA-seq,SRP309935,Monocyte_1-AD.vs.control; scRNA-seq,SRP309935,Naive CD8+ T cell_2-AD.vs.control; scRNA-seq,SRP309935,Naive CD8+ T cell_3-AD.vs.control; scRNA-seq,SRP330776,CD8+ T cell_1-AD.vs.control; scRNA-seq,SRP330776,γδ T cell-AD.vs.control; scRNA-seq,SRP330776,Memory T cell_2-AD.vs.control; scRNA-seq,SRP330776,Naive CD8+ T cell_1-[illegible]	18

CELA2B	bulk RNA-seq,Homo_723,AD.vs.control; bulk RNA-seq,Homo_723,MCI.vs.control; bulk RNA-seq,Homo_633,AD.vs.control; bulk RNA-seq,Homo_633,AD.vs.MCI; bulk RNA-seq,Homo_633,MCI.vs.control; bulk RNA-seq,Homo_714,AD.vs.control; bulk RNA-seq,Homo_714,MCI.vs.control	7
CELA3A	bulk RNA-seq,ROSMAP,AD.vs.control; bulk RNA-seq,ROSMAP,MCI.vs.control	2
CELF1	bulk RNA-seq,Homo_723,AD.vs.control; bulk RNA-seq,Homo_633,AD.vs.control; scRNA-seq,SRP215507,CD8+ T cell_7-AD.vs.MCI; scRNA-seq,SRP215507,CD8+ T cell_5-MCI.vs.control; scRNA-seq,SRP309935,Naive CD8+ T cell_1-AD.vs.control; scRNA-seq,SRP309935,CD8+ T cell_1-AD.vs.control; scRNA-seq,SRP330776,γδ T cell-AD.vs.control; scRNA-seq,SRP330776,Memory T cell_2-AD.vs.control; scRNA-seq,SRP330776,B cell_1-AD.vs.control; scRNA-seq,SRP330776,B cell_2-AD.vs.control; scRNA-seq,SRP330776,T cell-AD.vs.control; scRNA-seq,SRP330776,Naive CD8+ T cell_2-AD.vs.control; scRNA-seq,SRP330776,Regulatory T cell-AD.vs.control; scRNA-seq,SRP330776,B cell_4-AD.vs.control	14
CELF2	bulk RNA-seq,Homo_723,AD.vs.control; bulk RNA-seq,Homo_633,AD.vs.control; bulk RNA-seq,Homo_633,MCI.vs.control; bulk RNA-seq,Homo_714,AD.vs.control; bulk RNA-seq,Homo_714,AD.vs.MCI; bulk RNA-seq,Homo_714,MCI.vs.control; scRNA-seq,SRP215507,CD8+ T cell_9-AD.vs.control; scRNA-seq,SRP215507,CD8+ T cell_7-MCI.vs.control; scRNA-seq,SRP309935,Erythroid cell-AD.vs.control; scRNA-seq,SRP330776,B	11
CELF3	bulk RNA-seq,Homo_723,AD.vs.control; bulk RNA-seq,Homo_723,AD.vs.MCI; bulk RNA-seq,Homo_723,MCI.vs.control; bulk RNA-seq,Homo_633,AD.vs.control; bulk RNA-seq,Homo_633,AD.vs.MCI; bulk RNA-seq,Homo_714,AD.vs.control; bulk RNA-seq,Homo_714,AD.vs.MCI	7
CELF4	bulk RNA-seq,Homo_723,AD.vs.control; bulk RNA-seq,Homo_723,AD.vs.MCI; bulk RNA-seq,Homo_633,AD.vs.control; bulk RNA-seq,Homo_633,AD.vs.MCI; bulk RNA-seq,Homo_714,MCI.vs.control; scRNA-seq,SRP330776,Naive CD8+ T cell_1-AD.vs.control	6
CELF5	bulk RNA-seq,Homo_723,AD.vs.control; bulk RNA-seq,Homo_723,AD.vs.MCI; bulk RNA-seq,Homo_723,MCI.vs.control; bulk RNA-seq,Homo_633,AD.vs.control; bulk RNA-seq,Homo_633,AD.vs.MCI; bulk RNA-seq,Homo_633,MCI.vs.control; scRNA-seq,SRP330776,CD8+ T cell_1-AD.vs.control; scRNA-seq,SRP330776,Naive CD8+ T cell_1-AD.vs.control	8
CELF6	bulk RNA-seq,Homo_723,MCI.vs.control; bulk RNA-seq,Homo_633,AD.vs.control; bulk RNA-seq,Homo_633,AD.vs.MCI; bulk RNA-seq,Homo_633,MCI.vs.control; bulk RNA-seq,Homo_714,AD.vs.control; bulk RNA-seq,Homo_714,AD.vs.MCI; bulk RNA-seq,Homo_714,MCI.vs.control; scRNA-seq,SRP215507,CD8+ T cell_1-AD.vs.control; scRNA-seq,SRP215507,CD8+ T cell_3-MCI.vs.control; scRNA-	11
CELSR1	bulk RNA-seq,Homo_723,AD.vs.control; bulk RNA-seq,Homo_723,AD.vs.MCI; bulk RNA-seq,Homo_633,AD.vs.control; bulk RNA-seq,Homo_633,AD.vs.MCI; bulk RNA-seq,Homo_714,AD.vs.control; bulk RNA-seq,Homo_714,AD.vs.MCI; bulk RNA-seq,Homo_714,MCI.vs.control; scRNA-seq,SRP215507,CD8+ T cell_3-AD.vs.control; scRNA-seq,SRP215507,CD8+ T cell_4-AD.vs.control; scRNA-seq,SRP215507,CD8+ T cell_1-AD.vs.MCI; scRNA-seq,SRP215507,CD8+ T cell_1-MCI.vs.control; scRNA-seq,SRP215507,CD8+ T cell_3-MCI.vs.control; scRNA-	13
CELSR2	bulk RNA-seq,Homo_723,AD.vs.control; bulk RNA-seq,Homo_723,AD.vs.MCI; bulk RNA-seq,Homo_723,MCI.vs.control; bulk RNA-seq,Homo_633,AD.vs.control; bulk RNA-seq,Homo_714,AD.vs.MCI; bulk RNA-seq,Homo_714,MCI.vs.control; scRNA-seq,SRP215507,CD8+ T cell_3-AD.vs.control; scRNA-seq,SRP215507,CD8+ T cell_5-AD.vs.control; scRNA-seq,SRP215507,CD8+ T cell_4-AD.vs.MCI; scRNA-seq,SRP215507,CD8+ T cell_2-MCI.vs.control; scRNA-seq,SRP330776,γδ T cell-AD.vs.control; scRNA-seq,SRP330776,Memory T cell_2-AD.vs.control; scRNA-	13
CELSR3	scRNA-seq,SRP215507,CD8+ T cell_3-AD.vs.control; scRNA-seq,SRP215507,CD8+ T cell_1-AD.vs.control; scRNA-seq,SRP215507,CD8+ T cell_1-MCI.vs.control; scRNA-seq,SRP215507,CD8+ T cell_5-MCI.vs.control; scRNA-seq,SRP330776,CD8+ T cell_2-AD.vs.control; scRNA-seq,SRP330776,B	6
CEMIP	bulk RNA-seq,Homo_723,AD.vs.control; bulk RNA-seq,Homo_723,AD.vs.MCI; bulk RNA-seq,Homo_633,AD.vs.control; bulk RNA-seq,Homo_633,AD.vs.MCI; bulk RNA-seq,Homo_714,AD.vs.control; bulk RNA-seq,Homo_714,AD.vs.MCI; bulk RNA-seq,Homo_714,MCI.vs.control	7
CEMIP2	bulk RNA-seq,Homo_723,AD.vs.control; bulk RNA-seq,Homo_723,AD.vs.MCI; bulk RNA-seq,Homo_723,MCI.vs.control; bulk RNA-seq,Homo_633,AD.vs.control; bulk RNA-seq,Homo_633,AD.vs.MCI; bulk RNA-seq,Homo_633,MCI.vs.control; bulk RNA-seq,Homo_714,AD.vs.control; bulk RNA-seq,Homo_714,AD.vs.MCI; bulk RNA-seq,Homo_714,MCI.vs.control; scRNA-seq,SRP215507,CD8+ T cell_10-AD.vs.control; scRNA-seq,SRP215507,CD8+ T cell_4-AD.vs.MCI; scRNA-seq,SRP215507,CD8+ T cell_4-MCI.vs.control; scRNA-seq,SRP215507,CD8+ T cell_6-MCI.vs.control; scRNA-seq,SRP309935,Natural killer cell_2-AD.vs.control; scRNA-seq,SRP309935,Monocyte_2-AD.vs.control; scRNA-seq,SRP330776,B cell_1-AD.vs.control; scRNA-seq,SRP330776,Regulatory T cell-AD.vs.control; scRNA-seq,SRP330776,Natural killer cell_2-	18
Cemip2	bulk RNA-seq,SRP325058,AD.vs.control	1
CEMP1	bulk RNA-seq,Homo_723,AD.vs.MCI; bulk RNA-seq,Homo_723,MCI.vs.control; bulk RNA-seq,Homo_633,AD.vs.control; scRNA-seq,SRP330776,Naive CD8+ T cell_1-AD.vs.control	4

CENATAC	bulk RNA-seq,Homo_723,AD.vs.control; bulk RNA-seq,Homo_723,MCI.vs.control; bulk RNA-seq,Homo_633,AD.vs.control; bulk RNA-seq,Homo_633,MCI.vs.control; bulk RNA-seq,Homo_714,AD.vs.control; bulk RNA-seq,Homo_714,AD.vs.MCI; bulk RNA-seq,Homo_714,MCI.vs.control	7
CEND1	scRNA-seq,SRP330776,CD8+ T cell_1-AD.vs.control; scRNA-seq,SRP330776,CD8+ T cell_2-AD.vs.control; scRNA-seq,SRP330776,B cell_1-	3
CENPA	bulk RNA-seq,Homo_723,AD.vs.control; bulk RNA-seq,Homo_723,AD.vs.MCI; bulk RNA-seq,Homo_723,MCI.vs.control; bulk RNA-seq,Homo_633,AD.vs.control; bulk RNA-seq,Homo_633,AD.vs.MCI; bulk RNA-seq,Homo_633,MCI.vs.control	6
Cenpa	bulk RNA-seq,SRP325058,AD.vs.control	1
CENPB	bulk RNA-seq,SRP223445,AD.vs.control; scRNA-seq,SRP309935,Monocyte_2-AD.vs.control; scRNA-seq,SRP330776,CD8+ T cell_1-AD.vs.control; scRNA-seq,SRP330776,γδ T cell-AD.vs.control; scRNA-seq,SRP330776,Memory T cell_2-AD.vs.control; scRNA-seq,SRP330776,CD8+ T cell_2-AD.vs.control; scRNA-seq,SRP330776,B cell_1-AD.vs.control; scRNA-seq,SRP330776,Naive CD8+ T cell_1-AD.vs.control; scRNA-seq,SRP330776,Natural killer cell_1-AD.vs.control; scRNA-seq,SRP330776,Memory T cell_1-AD.vs.control; scRNA-seq,SRP330776,Natural killer T cell-AD.vs.control; scRNA-seq,SRP330776,B cell_2-AD.vs.control; scRNA-seq,SRP330776,T cell-AD.vs.control; scRNA-seq,SRP330776,CD8+ T cell_3-	15
CENPBD1	bulk RNA-seq,Homo_723,AD.vs.MCI; bulk RNA-seq,Homo_633,AD.vs.control; bulk RNA-seq,Homo_633,AD.vs.MCI; bulk RNA-seq,Homo_714,AD.vs.control; bulk RNA-seq,Homo_714,AD.vs.MCI; scRNA-seq,SRP215507,CD8+ T cell_2-AD.vs.control; scRNA-seq,SRP215507,CD8+ T cell_3-AD.vs.control; scRNA-seq,SRP215507,CD8+ T cell_3-AD.vs.MCI; scRNA-seq,SRP330776,Naive CD8+ T cell_1-AD.vs.control; scRNA-seq,SRP330776,Memory T cell_1-AD.vs.control; scRNA-seq,SRP330776,Monocyte_1-AD.vs.control	11
CENPC	bulk RNA-seq,Homo_723,AD.vs.control; bulk RNA-seq,Homo_723,AD.vs.MCI; bulk RNA-seq,Homo_633,AD.vs.control; bulk RNA-seq,Homo_633,AD.vs.MCI; bulk RNA-seq,Homo_714,AD.vs.control; bulk RNA-seq,Homo_714,AD.vs.MCI; scRNA-seq,SRP330776,Natural killer T cell-AD.vs.control; scRNA-seq,SRP330776,Monocyte_1-AD.vs.control; scRNA-seq,SRP330776,Regulatory T cell-AD.vs.control	9
CENPE	bulk RNA-seq,ROSMAP,AD.vs.control; bulk RNA-seq,ROSMAP,MCI.vs.control; scRNA-seq,SRP215507,CD8+ T cell_2-AD.vs.control; scRNA-seq,SRP215507,CD8+ T cell_1-AD.vs.control; scRNA-seq,SRP215507,CD8+ T cell_4-AD.vs.control; scRNA-seq,SRP215507,CD8+ T cell_2-AD.vs.MCI; scRNA-seq,SRP215507,CD8+ T cell_4-MCI.vs.control; scRNA-seq,SRP215507,CD8+ T cell_2-MCI.vs.control; scRNA-seq,SRP330776,B cell_1-AD.vs.control; scRNA-seq,SRP330776,Regulatory T cell-AD.vs.control	10
CENPF	bulk RNA-seq,Homo_723,AD.vs.control; bulk RNA-seq,Homo_723,AD.vs.MCI; bulk RNA-seq,Homo_723,MCI.vs.control; bulk RNA-seq,Homo_633,AD.vs.control; bulk RNA-seq,Homo_633,AD.vs.MCI; bulk RNA-seq,Homo_633,MCI.vs.control; bulk RNA-seq,Homo_714,AD.vs.control; bulk RNA-seq,Homo_714,AD.vs.MCI; bulk RNA-seq,Homo_714,MCI.vs.control; scRNA-seq,SRP330776,Memory T cell_2-AD.vs.control; scRNA-seq,SRP330776,Naive CD8+ T cell_1-AD.vs.control; scRNA-seq,SRP330776,Natural killer cell_1-AD.vs.control; scRNA-seq,SRP330776,T cell-	14
Cenpf	bulk RNA-seq,SRP325058,AD.vs.control	1
CENPH	bulk RNA-seq,Homo_723,AD.vs.control; bulk RNA-seq,Homo_723,AD.vs.MCI; bulk RNA-seq,Homo_633,AD.vs.control; bulk RNA-seq,Homo_633,AD.vs.MCI; bulk RNA-seq,Homo_714,AD.vs.control; bulk RNA-seq,Homo_714,AD.vs.MCI; scRNA-seq,SRP215507,CD8+ T cell_5-AD.vs.control; scRNA-seq,SRP215507,CD8+ T cell_3-AD.vs.MCI; scRNA-seq,SRP215507,CD8+ T cell_4-AD.vs.MCI; scRNA-seq,SRP215507,CD8+ T cell_5-AD.vs.MCI; scRNA-seq,SRP215507,CD8+ T cell_4-MCI.vs.control; scRNA-seq,SRP330776,CD8+ T cell_1-AD.vs.control; scRNA-seq,SRP330776,CD8+ T cell_2-AD.vs.control; scRNA-seq,SRP330776,Naive CD8+ T cell_1-AD.vs.control; scRNA-seq,SRP330776,Natural killer cell_1-AD.vs.control; scRNA-seq,SRP330776,Natural killer T cell-AD.vs.control; scRNA-seq,SRP330776,Naive CD8+ T cell_3-AD.vs.control; scRNA- [illegible]	19
CENPI	bulk RNA-seq,Homo_723,AD.vs.control; bulk RNA-seq,Homo_723,AD.vs.MCI; bulk RNA-seq,Homo_723,MCI.vs.control; bulk RNA-seq,Homo_633,AD.vs.control; bulk RNA-seq,Homo_633,AD.vs.MCI; bulk RNA-seq,Homo_633,MCI.vs.control; bulk RNA-seq,Homo_714,AD.vs.control; bulk RNA-seq,Homo_714,AD.vs.MCI; bulk RNA-seq,Homo_714,MCI.vs.control; bulk RNA-seq,ROSMAP,AD.vs.control; bulk RNA-seq,ROSMAP,MCI.vs.control; scRNA-seq,SRP215507,CD8+ T cell_1-AD.vs.control; scRNA-seq,SRP215507,CD8+ T cell_1-MCI.vs.control	13
CENPJ	bulk RNA-seq,Homo_723,AD.vs.control; bulk RNA-seq,Homo_723,AD.vs.MCI; bulk RNA-seq,Homo_633,AD.vs.MCI; bulk RNA-seq,Homo_714,AD.vs.control; bulk RNA-seq,Homo_714,AD.vs.MCI; scRNA-seq,SRP330776,γδ T cell-AD.vs.control; scRNA-seq,SRP330776,Natural	7

CENPK	bulk RNA-seq,Homo_723,AD.vs.control; bulk RNA-seq,Homo_723,AD.vs.MCI; bulk RNA-seq,Homo_633,AD.vs.control; bulk RNA-seq,Homo_633,AD.vs.MCI; bulk RNA-seq,Homo_714,AD.vs.control; bulk RNA-seq,Homo_714,AD.vs.MCI; scRNA-seq,SRP215507,CD8+ T cell_8-AD.vs.control; scRNA-seq,SRP215507,CD8+ T cell_5-AD.vs.control; scRNA-seq,SRP215507,CD8+ T cell_2-AD.vs.MCI; scRNA-seq,SRP215507,CD8+ T cell_3-AD.vs.MCI; scRNA-seq,SRP215507,CD8+ T cell_4-AD.vs.MCI; scRNA-seq,SRP215507,CD8+ T cell_5-AD.vs.MCI; scRNA-seq,SRP215507,CD8+ T cell_1-MCI.vs.control; scRNA-seq,SRP215507,CD8+ T cell_5-MCI.vs.control; scRNA-seq,SRP215507,CD8+ T cell_4-MCI.vs.control; scRNA-seq,SRP215507,CD8+ T cell_2-MCI.vs.control; scRNA-seq,SRP215507,CD8+ T cell_3-MCI.vs.control; scRNA-seq,SRP309935,Naive CD8+ T cell_1-AD.vs.control; scRNA-seq,SRP309935,Natural killer cell_1-AD.vs.control; scRNA-seq,SRP309935,CD8+ T cell_1-AD.vs.control; scRNA-seq,SRP330776,CD8+ T cell_1-AD.vs.control; scRNA-seq,SRP330776,γδ T cell-AD.vs.control; scRNA-seq,SRP330776,Memory T cell_2-AD.vs.control; scRNA-seq,SRP330776,CD8+ T cell_2-AD.vs.control; scRNA-seq,SRP330776,Naive CD8+ T cell_1-AD.vs.control; scRNA-seq,SRP330776,Natural killer cell_1-AD.vs.control; scRNA-seq,SRP330776,Memory T cell_1-AD.vs.control; scRNA-seq,SRP330776,Natural killer T cell-AD.vs.control; scRNA-seq,SRP330776,Naive CD8+ T cell_3-AD.vs.control; scRNA-seq,SRP330776,T cell-AD.vs.control; scRNA-seq,SRP330776,Naive CD8+ T cell_2-AD.vs.control; scRNA-seq,SRP330776,CD8+ T cell_3-AD.vs.control; scRNA-seq,SRP330776,B cell_3-AD.vs.control	33
CENPL	bulk RNA-seq,Homo_714,AD.vs.control; bulk RNA-seq,Homo_714,AD.vs.MCI; scRNA-seq,SRP215507,CD8+ T cell_2-AD.vs.control; scRNA-seq,SRP215507,CD8+ T cell_2-AD.vs.MCI; scRNA-seq,SRP215507,CD8+ T cell_3-AD.vs.MCI; scRNA-seq,SRP215507,CD8+ T cell_2-MCI.vs.control; scRNA-seq,SRP330776,Memory T cell_2-AD.vs.control; scRNA-seq,SRP330776,B cell_1-AD.vs.control; scRNA-seq,SRP330776,Naive CD8+ T cell_1-AD.vs.control; scRNA-seq,SRP330776,Natural killer T cell-AD.vs.control; scRNA-seq,SRP330776,Naive CD8+ T cell_3-AD.vs.control	11
CENPM	bulk RNA-seq,Homo_723,AD.vs.control; bulk RNA-seq,Homo_723,MCI.vs.control; bulk RNA-seq,Homo_633,AD.vs.MCI; bulk RNA-seq,Homo_633,MCI.vs.control; bulk RNA-seq,Homo_714,AD.vs.control; bulk RNA-seq,Homo_714,AD.vs.MCI; bulk RNA-seq,Homo_714,MCI.vs.control; bulk RNA-seq,ROSMAP,MCI.vs.control; scRNA-seq,SRP215507,CD8+ T cell_5-AD.vs.control; scRNA-seq,SRP215507,CD8+ T cell_1-MCI.vs.control; scRNA-seq,SRP309935,Naive CD8+ T cell_1-AD.vs.control; scRNA-seq,SRP330776,CD8+ T cell_2-	14
CENPN	bulk RNA-seq,Homo_723,AD.vs.control; bulk RNA-seq,Homo_723,AD.vs.MCI; bulk RNA-seq,Homo_723,MCI.vs.control; bulk RNA-seq,Homo_633,AD.vs.control; bulk RNA-seq,Homo_633,AD.vs.MCI; bulk RNA-seq,Homo_633,MCI.vs.control; bulk RNA-seq,Homo_714,AD.vs.control; bulk RNA-seq,Homo_714,AD.vs.MCI; bulk RNA-seq,Homo_714,MCI.vs.control; scRNA-seq,SRP215507,CD8+ T cell_6-AD.vs.control; scRNA-seq,SRP215507,CD8+ T cell_2-AD.vs.MCI; scRNA-seq,SRP215507,CD8+ T cell_3-AD.vs.MCI; scRNA-seq,SRP215507,CD8+ T cell_1-AD.vs.MCI; scRNA-seq,SRP215507,CD8+ T cell_5-AD.vs.MCI; scRNA-seq,SRP215507,CD8+ T cell_1-MCI.vs.control; scRNA-seq,SRP215507,CD8+ T cell_5-MCI.vs.control; scRNA-seq,SRP215507,CD8+ T cell_2-MCI.vs.control; scRNA-seq,SRP215507,CD8+ T cell_3-MCI.vs.control; scRNA-seq,SRP330776,Naive CD8+ T cell_1-AD.vs.control; scRNA-seq,SRP330776,B cell_2-AD.vs.control; scRNA-seq,SRP330776,Monocyte_1-AD.vs.control; [illegible]	22
CENPO	bulk RNA-seq,Homo_723,AD.vs.control; bulk RNA-seq,Homo_723,AD.vs.MCI; bulk RNA-seq,Homo_633,AD.vs.MCI; bulk RNA-seq,Homo_714,AD.vs.control; bulk RNA-seq,Homo_714,AD.vs.MCI; scRNA-seq,SRP215507,CD8+ T cell_4-AD.vs.control; scRNA-seq,SRP215507,CD8+ T cell_1-AD.vs.MCI; scRNA-seq,SRP215507,CD8+ T cell_4-MCI.vs.control; scRNA-seq,SRP330776,γδ T cell-AD.vs.control;	10
CENPP	bulk RNA-seq,Homo_633,AD.vs.control; bulk RNA-seq,Homo_633,AD.vs.MCI; bulk RNA-seq,Homo_714,AD.vs.control; bulk RNA-seq,Homo_714,AD.vs.MCI; bulk RNA-seq,Homo_714,MCI.vs.control; scRNA-seq,SRP215507,CD8+ T cell_4-AD.vs.MCI; scRNA-seq,SRP215507,CD8+	7
CENPQ	bulk RNA-seq,Homo_723,AD.vs.MCI; bulk RNA-seq,Homo_714,AD.vs.control; bulk RNA-seq,Homo_714,AD.vs.MCI; scRNA-seq,SRP215507,CD8+ T cell_3-AD.vs.control; scRNA-seq,SRP215507,CD8+ T cell_1-AD.vs.control; scRNA-seq,SRP215507,CD8+ T cell_3-AD.vs.MCI; scRNA-seq,SRP215507,CD8+ T cell_1-AD.vs.MCI; scRNA-seq,SRP215507,CD8+ T cell_4-AD.vs.MCI; scRNA-seq,SRP215507,CD8+ T cell_3-MCI.vs.control; scRNA-seq,SRP330776,γδ T cell-AD.vs.control; scRNA-seq,SRP330776,Memory T cell_2-AD.vs.control; scRNA-seq,SRP330776,B cell_1-AD.vs.control; scRNA-seq,SRP330776,Natural killer cell_1-AD.vs.control; scRNA-seq,SRP330776,Memory T cell_1-AD.vs.control; scRNA-seq,SRP330776,Naive CD8+ T cell_3-AD.vs.control; scRNA-seq,SRP330776,B cell_2-AD.vs.control; scRNA-seq,SRP330776,T cell-AD.vs.control; scRNA-seq,SRP330776,CD8+ T [illegible]	19
CENPS	bulk RNA-seq,Homo_723,MCI.vs.control; bulk RNA-seq,Homo_633,MCI.vs.control; bulk RNA-seq,Homo_714,MCI.vs.control; scRNA-seq,SRP215507,CD8+ T cell_6-AD.vs.control; scRNA-seq,SRP330776,CD8+ T cell_1-AD.vs.control; scRNA-seq,SRP330776,Memory T cell_1-AD.vs.control; scRNA-seq,SRP330776,CD8+ T cell_3-AD.vs.control	7
CENPS-CORT	bulk RNA-seq,ACOM,AD.vs.control; bulk RNA-seq,Homo_723,MCI.vs.control; bulk RNA-seq,Homo_633,AD.vs.control; bulk RNA-seq,Homo_633,AD.vs.MCI; bulk RNA-seq,Homo_633,MCI.vs.control; bulk RNA-seq,Homo_714,AD.vs.control; bulk RNA-seq,Homo_714,AD.vs.MCI	7

CENPT	bulk RNA-seq,Homo_723,AD.vs.control; bulk RNA-seq,Homo_723,MCI.vs.control; bulk RNA-seq,Homo_633,AD.vs.control; bulk RNA-seq,Homo_633,MCI.vs.control; bulk RNA-seq,Homo_714,AD.vs.control; bulk RNA-seq,Homo_714,AD.vs.MCI; bulk RNA-seq,Homo_714,MCI.vs.control; scRNA-seq,SRP215507,CD8+ T cell_2-AD.vs.MCI; scRNA-seq,SRP215507,CD8+ T cell_1-AD.vs.MCI; scRNA-seq,SRP215507,CD8+ T cell_5-AD.vs.MCI; scRNA-seq,SRP215507,CD8+ T cell_5-MCI.vs.control; scRNA-seq,SRP215507,CD8+ T cell_2-MCI.vs.control; scRNA-seq,SRP330776,Memory T cell_1-AD.vs.control; scRNA-seq,SRP330776,Naive CD8+ T cell_3-AD.vs.control; scRNA-	15
CENPU	bulk RNA-seq,Homo_723,AD.vs.control; bulk RNA-seq,Homo_723,AD.vs.MCI; bulk RNA-seq,Homo_723,MCI.vs.control; bulk RNA-seq,Homo_633,AD.vs.control; bulk RNA-seq,Homo_633,AD.vs.MCI; bulk RNA-seq,Homo_633,MCI.vs.control; bulk RNA-seq,Homo_714,AD.vs.control; bulk RNA-seq,Homo_714,AD.vs.MCI; bulk RNA-seq,Homo_714,MCI.vs.control; scRNA-seq,SRP215507,CD8+ T cell_3-AD.vs.control; scRNA-	11
CENPV	bulk RNA-seq,Homo_723,AD.vs.control; bulk RNA-seq,Homo_723,AD.vs.MCI; bulk RNA-seq,Homo_633,AD.vs.control; bulk RNA-seq,Homo_633,AD.vs.MCI; bulk RNA-seq,Homo_714,AD.vs.control; bulk RNA-seq,Homo_714,AD.vs.MCI; scRNA-seq,SRP215507,CD8+ T cell_5-AD.vs.MCI; scRNA-seq,SRP215507,CD8+ T cell_5-MCI.vs.control; scRNA-seq,SRP309935,Natural killer cell_2-AD.vs.control; scRNA-seq,SRP309935,Monocyte_1-AD.vs.control; scRNA-seq,SRP309935,Natural killer cell_1-AD.vs.control; scRNA-seq,SRP309935,Naive CD8+ T cell_2-AD.vs.control; scRNA-seq,SRP309935,B cell_1-AD.vs.control; scRNA-seq,SRP330776,CD8+ T cell_1-AD.vs.control; scRNA-seq,SRP330776,Memory T cell_2-AD.vs.control; scRNA-seq,SRP330776,CD8+ T cell_2-AD.vs.control; scRNA-seq,SRP330776,B cell_1-AD.vs.control; scRNA-seq,SRP330776,Naive CD8+ T cell_1-AD.vs.control; scRNA-seq,SRP330776,Natural killer cell_1-AD.vs.control; scRNA-seq,SRP330776,Memory T cell_1-AD.vs.control; scRNA-seq,SRP330776,Natural killer T cell-AD.vs.control; scRNA-seq,SRP330776,B cell_2-AD.vs.control; scRNA- [illegible]	25
CENPVL1	bulk RNA-seq,Homo_723,AD.vs.control; bulk RNA-seq,Homo_723,MCI.vs.control; bulk RNA-seq,Homo_633,MCI.vs.control; bulk RNA-	4
CENPVL2	bulk RNA-seq,Homo_723,AD.vs.control; bulk RNA-seq,Homo_723,MCI.vs.control; bulk RNA-seq,Homo_633,MCI.vs.control; bulk RNA-	4
CENPVL3	bulk RNA-seq,Homo_723,AD.vs.control; bulk RNA-seq,Homo_723,MCI.vs.control; bulk RNA-seq,Homo_633,MCI.vs.control; bulk RNA-	4
CENPW	scRNA-seq,SRP215507,CD8+ T cell_5-AD.vs.control; scRNA-seq,SRP215507,CD8+ T cell_2-AD.vs.MCI; scRNA-seq,SRP215507,CD8+ T cell_3-AD.vs.MCI; scRNA-seq,SRP215507,CD8+ T cell_1-AD.vs.MCI; scRNA-seq,SRP215507,CD8+ T cell_1-MCI.vs.control; scRNA-seq,SRP215507,CD8+ T cell_2-MCI.vs.control; scRNA-seq,SRP215507,CD8+ T cell_3-MCI.vs.control; scRNA-seq,SRP330776,B cell_1-AD.vs.control; scRNA-	9
CENPX	bulk RNA-seq,Homo_714,AD.vs.control; bulk RNA-seq,Homo_714,AD.vs.MCI; scRNA-seq,SRP215507,CD8+ T cell_4-AD.vs.control; scRNA-seq,SRP215507,CD8+ T cell_7-AD.vs.control; scRNA-seq,SRP309935,Erythroid cell-AD.vs.control; scRNA-seq,SRP309935,CD8+ T cell_1-AD.vs.control; scRNA-seq,SRP330776,γδ T cell-AD.vs.control; scRNA-seq,SRP330776,Memory T cell_2-AD.vs.control; scRNA-seq,SRP330776,Natural	9
CEP104	bulk RNA-seq,Homo_723,AD.vs.control; bulk RNA-seq,Homo_723,AD.vs.MCI; bulk RNA-seq,Homo_723,MCI.vs.control; bulk RNA-seq,Homo_633,AD.vs.control; bulk RNA-seq,Homo_633,AD.vs.MCI; bulk RNA-seq,Homo_633,MCI.vs.control; bulk RNA-seq,Homo_714,AD.vs.control; bulk RNA-seq,Homo_714,AD.vs.MCI; bulk RNA-seq,Homo_714,MCI.vs.control; scRNA-seq,SRP309935,Natural killer cell_2-AD.vs.control; scRNA-seq,SRP330776,CD8+ T cell_1-AD.vs.control; scRNA-seq,SRP330776,γδ T cell-AD.vs.control; scRNA-seq,SRP330776,Memory T cell_2-AD.vs.control; scRNA-seq,SRP330776,CD8+ T cell_2-AD.vs.control; scRNA-seq,SRP330776,B cell_1-AD.vs.control; scRNA-seq,SRP330776,Naive CD8+ T cell_1-AD.vs.control; scRNA-seq,SRP330776,Natural killer cell_1-AD.vs.control; scRNA-seq,SRP330776,Memory T cell_1-AD.vs.control; scRNA-seq,SRP330776,Natural killer T cell-AD.vs.control; scRNA-seq,SRP330776,Naive CD8+ T cell_3-AD.vs.control; scRNA-seq,SRP330776,B cell_2-AD.vs.control; scRNA-seq,SRP330776,T cell-AD.vs.control; scRNA-seq,SRP330776,Naive CD8+ T cell_2-AD.vs.control; scRNA-seq,SRP330776,CD8+ T cell_3-AD.vs.control; scRNA-seq,SRP330776,Monocyte_2-AD.vs.control; scRNA-seq,SRP330776,B cell_3-AD.vs.control; scRNA- [illegible]	27
CEP112	scRNA-seq,SRP215507,CD8+ T cell_3-AD.vs.control; scRNA-seq,SRP215507,CD8+ T cell_1-AD.vs.control; scRNA-seq,SRP215507,CD8+ T cell_5-AD.vs.control; scRNA-seq,SRP330776,CD8+ T cell_1-AD.vs.control; scRNA-seq,SRP330776,Naive CD8+ T cell_1-AD.vs.control	5
CEP120	bulk RNA-seq,Homo_723,AD.vs.MCI; bulk RNA-seq,Homo_633,AD.vs.MCI; bulk RNA-seq,Homo_714,AD.vs.control; bulk RNA-seq,Homo_714,AD.vs.MCI; scRNA-seq,SRP215507,CD8+ T cell_4-MCI.vs.control; scRNA-seq,SRP215507,CD8+ T cell_7-MCI.vs.control; scRNA-seq,SRP330776,CD8+ T cell_1-AD.vs.control; scRNA-seq,SRP330776,γδ T cell-AD.vs.control; scRNA-seq,SRP330776,Memory T cell_2-AD.vs.control; scRNA-seq,SRP330776,CD8+ T cell_2-AD.vs.control; scRNA-seq,SRP330776,B cell_1-AD.vs.control; scRNA-seq,SRP330776,Naive CD8+ T cell_1-AD.vs.control; scRNA-seq,SRP330776,Natural killer cell_1-AD.vs.control; scRNA-seq,SRP330776,Memory T cell_1-AD.vs.control; scRNA-seq,SRP330776,Natural killer T cell-AD.vs.control; scRNA-seq,SRP330776,Naive CD8+ T cell_3-AD.vs.control; scRNA-seq,SRP330776,B cell_2-AD.vs.control; scRNA-seq,SRP330776,T cell-AD.vs.control; scRNA-seq,SRP330776,CD8+ T cell_3-AD.vs.control; scRNA-seq,SRP330776,B cell_3- [illegible]	21

CEP126	bulk RNA-seq,Homo_723,AD.vs.control; bulk RNA-seq,Homo_723,AD.vs.MCI; bulk RNA-seq,Homo_723,MCI.vs.control; bulk RNA-seq,Homo_633,AD.vs.control; bulk RNA-seq,Homo_633,AD.vs.MCI; bulk RNA-seq,Homo_714,AD.vs.control; bulk RNA-seq,Homo_714,AD.vs.MCI; scRNA-seq,SRP215507,CD8+ T cell_4-AD.vs.control; scRNA-seq,SRP215507,CD8+ T cell_1-MCI.vs.control; scRNA-seq,SRP215507,CD8+ T cell_5-MCI.vs.control; scRNA-seq,SRP215507,CD8+ T cell_4-MCI.vs.control; scRNA-seq,SRP330776,CD8+ T cell_2-AD.vs.control; scRNA-seq,SRP330776,Natural killer cell_1-AD.vs.control; scRNA-seq,SRP330776,Memory T cell_1-AD.vs.control; scRNA-seq,SRP330776,Natural killer cell_2-	15
CEP128	bulk RNA-seq,Homo_723,AD.vs.control; bulk RNA-seq,Homo_723,AD.vs.MCI; bulk RNA-seq,Homo_633,AD.vs.control; bulk RNA-seq,Homo_633,AD.vs.MCI; bulk RNA-seq,Homo_714,AD.vs.control; bulk RNA-seq,Homo_714,AD.vs.MCI; scRNA-seq,SRP215507,CD8+ T cell_5-AD.vs.control; scRNA-seq,SRP215507,CD8+ T cell_5-AD.vs.MCI; scRNA-seq,SRP330776,B cell_1-AD.vs.control; scRNA-seq,SRP330776,Memory T cell_1-AD.vs.control; scRNA-seq,SRP330776,Naive CD8+ T cell_3-AD.vs.control	11
CEP131	bulk RNA-seq,Homo_723,AD.vs.control; bulk RNA-seq,Homo_723,AD.vs.MCI; bulk RNA-seq,Homo_714,AD.vs.control; bulk RNA-seq,Homo_714,AD.vs.MCI; scRNA-seq,SRP215507,CD8+ T cell_5-MCI.vs.control; scRNA-seq,SRP330776,CD8+ T cell_1-AD.vs.control; scRNA-seq,SRP330776,Memory T cell_2-AD.vs.control; scRNA-seq,SRP330776,CD8+ T cell_2-AD.vs.control; scRNA-seq,SRP330776,B cell_1-AD.vs.control; scRNA-seq,SRP330776,Memory T cell_1-AD.vs.control; scRNA-seq,SRP330776,Natural killer T cell-AD.vs.control; scRNA-seq,SRP330776,Naive CD8+	13
CEP135	bulk RNA-seq,Homo_723,AD.vs.control; bulk RNA-seq,Homo_723,AD.vs.MCI; bulk RNA-seq,Homo_633,AD.vs.control; bulk RNA-seq,Homo_633,AD.vs.MCI; bulk RNA-seq,Homo_714,AD.vs.control; bulk RNA-seq,Homo_714,AD.vs.MCI; scRNA-seq,SRP309935,Megakaryocyte_1-AD.vs.control; scRNA-seq,SRP309935,Naive CD8+ T cell_1-AD.vs.control; scRNA-seq,SRP309935,Monocyte_2-AD.vs.control; scRNA-seq,SRP330776,Natural killer cell_1-AD.vs.control; scRNA-seq,SRP330776,Natural killer T cell-AD.vs.control; scRNA-seq,SRP330776,Natural killer	12
CEP152	bulk RNA-seq,Homo_714,AD.vs.control; bulk RNA-seq,Homo_714,AD.vs.MCI; scRNA-seq,SRP215507,CD8+ T cell_1-MCI.vs.control; scRNA-seq,SRP309935,Monocyte_1-AD.vs.control; scRNA-seq,SRP309935,Naive CD8+ T cell_1-AD.vs.control; scRNA-seq,SRP330776,CD8+ T cell_3-	6
CEP162	bulk RNA-seq,Homo_723,AD.vs.control; bulk RNA-seq,Homo_723,AD.vs.MCI; bulk RNA-seq,Homo_633,AD.vs.control; bulk RNA-seq,Homo_633,AD.vs.MCI; bulk RNA-seq,Homo_714,AD.vs.control; bulk RNA-seq,Homo_714,AD.vs.MCI; scRNA-seq,SRP215507,CD8+ T cell_1-AD.vs.MCI; scRNA-seq,SRP215507,CD8+ T cell_1-MCI.vs.control; scRNA-seq,SRP215507,CD8+ T cell_2-MCI.vs.control; scRNA-seq,SRP330776,γδ T cell-AD.vs.control; scRNA-seq,SRP330776,Naive CD8+ T cell_1-AD.vs.control; scRNA-seq,SRP330776,Memory T cell_1-AD.vs.control; scRNA-seq,SRP330776,Natural killer T cell-AD.vs.control; scRNA-seq,SRP330776,CD8+ T cell_3-AD.vs.control	14
CEP164	bulk RNA-seq,Homo_633,AD.vs.control; bulk RNA-seq,Homo_714,MCI.vs.control; scRNA-seq,SRP215507,CD8+ T cell_7-AD.vs.control; scRNA-seq,SRP215507,CD8+ T cell_6-AD.vs.MCI; scRNA-seq,SRP215507,CD8+ T cell_3-MCI.vs.control; scRNA-seq,SRP309935,Monocyte_2-AD.vs.control; scRNA-seq,SRP330776,B cell_1-AD.vs.control; scRNA-seq,SRP330776,Naive CD8+ T cell_1-AD.vs.control; scRNA-seq,SRP330776,Naive CD8+ T	9
Cep164	bulk RNA-seq,SRP325058,AD.vs.control	1
CEP170	scRNA-seq,SRP215507,CD8+ T cell_6-AD.vs.control; scRNA-seq,SRP215507,CD8+ T cell_3-AD.vs.MCI; scRNA-seq,SRP215507,CD8+ T cell_3-MCI.vs.control; scRNA-seq,SRP330776,Natural killer cell_2-AD.vs.control	4
CEP170B	bulk RNA-seq,ROSMAP,AD.vs.control; scRNA-seq,SRP215507,CD8+ T cell_2-AD.vs.MCI; scRNA-seq,SRP215507,CD8+ T cell_2-MCI.vs.control; scRNA-seq,SRP330776,CD8+ T cell_1-AD.vs.control	4
CEP19	bulk RNA-seq,Homo_714,MCI.vs.control; scRNA-seq,SRP215507,CD8+ T cell_3-AD.vs.control; scRNA-seq,SRP215507,CD8+ T cell_1-AD.vs.MCI; scRNA-seq,SRP215507,CD8+ T cell_5-AD.vs.MCI; scRNA-seq,SRP309935,CD8+ T cell_2-AD.vs.control; scRNA-seq,SRP330776,γδ T cell-AD.vs.control; scRNA-seq,SRP330776,CD8+ T cell_2-AD.vs.control; scRNA-seq,SRP330776,B cell_1-AD.vs.control; scRNA-seq,SRP330776,Memory T cell_1-AD.vs.control; scRNA-seq,SRP330776,CD8+ T cell_3-AD.vs.control; scRNA-seq,SRP330776,Monocyte_2-AD.vs.control	11
CEP192	bulk RNA-seq,Homo_723,AD.vs.control; bulk RNA-seq,Homo_723,AD.vs.MCI; bulk RNA-seq,Homo_723,MCI.vs.control; bulk RNA-seq,Homo_633,AD.vs.control; bulk RNA-seq,Homo_633,AD.vs.MCI; bulk RNA-seq,Homo_714,AD.vs.control; bulk RNA-seq,Homo_714,AD.vs.MCI; bulk RNA-seq,Homo_714,MCI.vs.control; scRNA-seq,SRP215507,CD8+ T cell_8-AD.vs.control; scRNA-seq,SRP309935,Monocyte_1-AD.vs.control; scRNA-seq,SRP330776,γδ T cell-AD.vs.control; scRNA-seq,SRP330776,CD8+ T cell_3-AD.vs.control	12
CEP20	bulk RNA-seq,Homo_723,AD.vs.control; bulk RNA-seq,Homo_723,AD.vs.MCI; bulk RNA-seq,Homo_723,MCI.vs.control; bulk RNA-seq,Homo_633,AD.vs.control; bulk RNA-seq,Homo_633,AD.vs.MCI; bulk RNA-seq,Homo_633,MCI.vs.control; bulk RNA-seq,Homo_714,AD.vs.control;	8

CEP250	bulk RNA-seq,Homo_723,AD.vs.control; bulk RNA-seq,Homo_633,AD.vs.control; bulk RNA-seq,Homo_714,AD.vs.control; bulk RNA-seq,SRP223445,AD.vs.control; scRNA-seq,SRP215507,CD8+ T cell_4-AD.vs.control; scRNA-seq,SRP215507,CD8+ T cell_1-AD.vs.MCI; scRNA-seq,SRP215507,CD8+ T cell_4-AD.vs.MCI; scRNA-seq,SRP215507,CD8+ T cell_1-MCI.vs.control; scRNA-seq,SRP215507,CD8+ T cell_8-MCI.vs.control; scRNA-seq,SRP330776,γδ T cell-AD.vs.control; scRNA-seq,SRP330776,CD8+ T cell_2-AD.vs.control; scRNA-seq,SRP330776,Naive	12
CEP290	bulk RNA-seq,Homo_723,AD.vs.control; bulk RNA-seq,Homo_723,AD.vs.MCI; bulk RNA-seq,Homo_633,AD.vs.control; bulk RNA-seq,Homo_633,AD.vs.MCI; bulk RNA-seq,Homo_714,AD.vs.control; bulk RNA-seq,Homo_714,AD.vs.MCI; bulk RNA-seq,ROSMAP,AD.vs.control; bulk RNA-seq,ROSMAP,MCI.vs.control; scRNA-seq,SRP215507,CD8+ T cell_3-AD.vs.MCI; scRNA-seq,SRP215507,CD8+ T cell_1-AD.vs.MCI; scRNA-seq,SRP215507,CD8+ T cell_4-AD.vs.MCI; scRNA-seq,SRP215507,CD8+ T cell_1-MCI.vs.control; scRNA-seq,SRP215507,CD8+ T cell_4-MCI.vs.control; scRNA-seq,SRP309935,Natural killer cell_1-AD.vs.control; scRNA-seq,SRP330776,CD8+ T cell_1-AD.vs.control; scRNA-seq,SRP330776,γδ T cell-AD.vs.control; scRNA-seq,SRP330776,CD8+ T cell_2-AD.vs.control; scRNA-seq,SRP330776,Naive CD8+ T cell_1-AD.vs.control; scRNA-seq,SRP330776,Natural killer cell_1-AD.vs.control; scRNA-seq,SRP330776,Memory T cell_1-AD.vs.control; scRNA-[illegible]	22
CEP295	scRNA-seq,SRP215507,CD8+ T cell_7-AD.vs.control; scRNA-seq,SRP330776,Memory T cell_2-AD.vs.control; scRNA-seq,SRP330776,B cell_1-AD.vs.control; scRNA-seq,SRP330776,Naive CD8+ T cell_3-AD.vs.control; scRNA-seq,SRP330776,CD8+ T cell_3-AD.vs.control; scRNA-	6
CEP295NL	bulk RNA-seq,Homo_723,AD.vs.MCI; bulk RNA-seq,Homo_723,MCI.vs.control; bulk RNA-seq,Homo_633,AD.vs.MCI; bulk RNA-seq,Homo_633,MCI.vs.control; bulk RNA-seq,Homo_714,AD.vs.control; bulk RNA-seq,Homo_714,AD.vs.MCI; bulk RNA-seq,Homo_714,MCI.vs.control	7
CEP350	scRNA-seq,SRP215507,CD8+ T cell_7-AD.vs.control; scRNA-seq,SRP215507,CD8+ T cell_7-AD.vs.MCI; scRNA-seq,SRP309935,Natural killer cell_1-AD.vs.control; scRNA-seq,SRP309935,Naive CD8+ T cell_3-AD.vs.control; scRNA-seq,SRP330776,CD8+ T cell_1-AD.vs.control; scRNA-seq,SRP330776,γδ T cell-AD.vs.control; scRNA-seq,SRP330776,Memory T cell_2-AD.vs.control; scRNA-seq,SRP330776,CD8+ T cell_2-AD.vs.control; scRNA-seq,SRP330776,Naive CD8+ T cell_1-AD.vs.control; scRNA-seq,SRP330776,Natural killer cell_1-AD.vs.control; scRNA-seq,SRP330776,Memory T cell_1-AD.vs.control; scRNA-seq,SRP330776,Natural killer T cell-AD.vs.control; scRNA-seq,SRP330776,Naive CD8+ T cell_3-AD.vs.control; scRNA-seq,SRP330776,T cell-AD.vs.control; scRNA-seq,SRP330776,Naive CD8+ T cell_2-AD.vs.control; scRNA-seq,SRP330776,Monocyte_2-AD.vs.control	16
CEP41	bulk RNA-seq,Homo_723,AD.vs.control; bulk RNA-seq,Homo_723,MCI.vs.control; bulk RNA-seq,Homo_633,AD.vs.control; bulk RNA-seq,Homo_633,MCI.vs.control; scRNA-seq,SRP215507,CD8+ T cell_1-MCI.vs.control; scRNA-seq,SRP309935,Naive CD8+ T cell_2-AD.vs.control; scRNA-seq,SRP330776,γδ T cell-AD.vs.control; scRNA-seq,SRP330776,T cell-AD.vs.control	8
CEP43	bulk RNA-seq,Homo_714,AD.vs.control; bulk RNA-seq,Homo_714,AD.vs.MCI; bulk RNA-seq,SRP310421,AD.vs.control	3
CEP44	bulk RNA-seq,Homo_723,AD.vs.control; bulk RNA-seq,Homo_723,AD.vs.MCI; bulk RNA-seq,Homo_723,MCI.vs.control; bulk RNA-seq,Homo_633,AD.vs.control; bulk RNA-seq,Homo_633,AD.vs.MCI; bulk RNA-seq,Homo_633,MCI.vs.control; bulk RNA-seq,Homo_714,AD.vs.control; bulk RNA-seq,Homo_714,AD.vs.MCI; scRNA-seq,SRP215507,CD8+ T cell_1-AD.vs.MCI; scRNA-seq,SRP215507,CD8+ T cell_5-AD.vs.MCI; scRNA-seq,SRP215507,CD8+ T cell_5-MCI.vs.control; scRNA-seq,SRP330776,Memory T cell_2-AD.vs.control; scRNA-seq,SRP330776,CD8+ T cell_2-AD.vs.control; scRNA-seq,SRP330776,Naive CD8+ T cell_1-AD.vs.control; scRNA-seq,SRP330776,Natural killer cell_1-AD.vs.control; scRNA-	17
CEP55	bulk RNA-seq,Homo_723,AD.vs.control; bulk RNA-seq,Homo_723,AD.vs.MCI; bulk RNA-seq,Homo_723,MCI.vs.control; bulk RNA-seq,Homo_633,AD.vs.control; bulk RNA-seq,Homo_633,AD.vs.MCI; bulk RNA-seq,Homo_633,MCI.vs.control; bulk RNA-seq,Homo_714,AD.vs.control; bulk RNA-seq,Homo_714,AD.vs.MCI; scRNA-seq,SRP215507,CD8+ T cell_1-AD.vs.control; scRNA-seq,SRP215507,CD8+ T cell_1-MCI.vs.control	10
CEP57	bulk RNA-seq,Homo_723,AD.vs.MCI; bulk RNA-seq,Homo_633,AD.vs.MCI; bulk RNA-seq,Homo_714,AD.vs.control; bulk RNA-seq,Homo_714,AD.vs.MCI; scRNA-seq,SRP215507,CD8+ T cell_4-AD.vs.MCI; scRNA-seq,SRP309935,B cell_1-AD.vs.control; scRNA-seq,SRP330776,Monocyte_2-AD.vs.control; scRNA-seq,SRP330776,Megakaryocyte_1-AD.vs.control	8
CEP57L1	bulk RNA-seq,Homo_723,AD.vs.control; bulk RNA-seq,Homo_723,AD.vs.MCI; bulk RNA-seq,Homo_633,AD.vs.control; bulk RNA-seq,Homo_633,AD.vs.MCI; bulk RNA-seq,Homo_714,AD.vs.control; bulk RNA-seq,Homo_714,AD.vs.MCI; bulk RNA-seq,ROSMAP,AD.vs.control; bulk RNA-seq,ROSMAP,MCI.vs.control; scRNA-seq,SRP215507,CD8+ T cell_3-MCI.vs.control; scRNA-seq,SRP215507,CD8+ T cell_6-MCI.vs.control; scRNA-seq,SRP309935,Naive CD8+ T cell_1-AD.vs.control; scRNA-seq,SRP330776,CD8+ T cell_1-AD.vs.control; scRNA-seq,SRP330776,Memory T cell_2-AD.vs.control; scRNA-seq,SRP330776,CD8+ T cell_2-AD.vs.control; scRNA-seq,SRP330776,Naive CD8+ T cell_1-AD.vs.control; scRNA-seq,SRP330776,Natural killer cell_1-AD.vs.control; scRNA-seq,SRP330776,Naive CD8+ T cell_3-AD.vs.control; scRNA-seq,SRP330776,B cell_2-[illegible]	20

CEP63	bulk RNA-seq,Homo_723,AD.vs.control; bulk RNA-seq,Homo_723,AD.vs.MCI; bulk RNA-seq,Homo_633,AD.vs.control; bulk RNA-seq,Homo_633,AD.vs.MCI; bulk RNA-seq,Homo_714,AD.vs.control; bulk RNA-seq,Homo_714,AD.vs.MCI; scRNA-seq,SRP215507,CD8+ T cell_6-AD.vs.control; scRNA-seq,SRP215507,CD8+ T cell_3-MCI.vs.control; scRNA-seq,SRP309935,Natural killer cell_2-AD.vs.control; scRNA-seq,SRP330776,Natural killer cell_1-AD.vs.control; scRNA-seq,SRP330776,Monocyte_2-AD.vs.control; scRNA-seq,SRP330776,Regulatory T cell-	13
CEP68	scRNA-seq,SRP215507,CD8+ T cell_4-AD.vs.control; scRNA-seq,SRP215507,CD8+ T cell_5-AD.vs.control; scRNA-seq,SRP215507,CD8+ T cell_6-AD.vs.control; scRNA-seq,SRP215507,CD8+ T cell_6-AD.vs.MCI; scRNA-seq,SRP309935,Naive CD8+ T cell_1-AD.vs.control; scRNA-seq,SRP330776,Naive CD8+ T cell_3-AD.vs.control	6
CEP70	scRNA-seq,SRP215507,CD8+ T cell_2-MCI.vs.control	1
CEP72	bulk RNA-seq,Homo_714,AD.vs.control; bulk RNA-seq,Homo_714,AD.vs.MCI; scRNA-seq,SRP330776,CD8+ T cell_1-AD.vs.control; scRNA-seq,SRP330776,γδ T cell-AD.vs.control; scRNA-seq,SRP330776,Memory T cell_2-AD.vs.control; scRNA-seq,SRP330776,CD8+ T cell_2-AD.vs.control; scRNA-seq,SRP330776,Natural killer T cell-AD.vs.control; scRNA-seq,SRP330776,Naive CD8+ T cell_3-AD.vs.control; scRNA-seq,SRP330776,B cell_2-AD.vs.control; scRNA-seq,SRP330776,CD8+ T cell_3-AD.vs.control	10
CEP76	bulk RNA-seq,Homo_714,AD.vs.control; bulk RNA-seq,Homo_714,AD.vs.MCI; scRNA-seq,SRP309935,Monocyte_1-AD.vs.control; scRNA-seq,SRP330776,CD8+ T cell_3-AD.vs.control	4
CEP78	bulk RNA-seq,Homo_714,AD.vs.control; bulk RNA-seq,Homo_714,AD.vs.MCI; scRNA-seq,SRP215507,CD8+ T cell_3-AD.vs.control; scRNA-seq,SRP215507,CD8+ T cell_6-AD.vs.control; scRNA-seq,SRP215507,CD8+ T cell_2-AD.vs.MCI; scRNA-seq,SRP215507,CD8+ T cell_3-AD.vs.MCI; scRNA-seq,SRP215507,CD8+ T cell_1-AD.vs.MCI; scRNA-seq,SRP215507,CD8+ T cell_4-AD.vs.MCI; scRNA-seq,SRP215507,CD8+ T cell_1-MCI.vs.control; scRNA-seq,SRP215507,CD8+ T cell_4-MCI.vs.control; scRNA-seq,SRP215507,CD8+ T cell_2-MCI.vs.control; scRNA-seq,SRP330776,CD8+ T cell_1-AD.vs.control; scRNA-seq,SRP330776,Naive CD8+ T cell_1-AD.vs.control; scRNA-seq,SRP330776,Naive CD8+ T cell_2-AD.vs.control; scRNA-seq,SRP330776,B cell_3-AD.vs.control; scRNA-seq,SRP330776,B cell_4-AD.vs.control	16
CEP83	scRNA-seq,SRP215507,CD8+ T cell_3-AD.vs.MCI; scRNA-seq,SRP330776,Naive CD8+ T cell_3-AD.vs.control; scRNA-seq,SRP330776,Monocyte_2-	3
CEP83-DT	scRNA-seq,SRP215507,CD8+ T cell_1-AD.vs.MCI; scRNA-seq,SRP215507,CD8+ T cell_1-MCI.vs.control; scRNA-seq,SRP330776,Memory T cell_2-AD.vs.control; scRNA-seq,SRP330776,B cell_2-AD.vs.control	4
CEP85	bulk RNA-seq,Homo_723,AD.vs.control; bulk RNA-seq,Homo_723,MCI.vs.control; bulk RNA-seq,Homo_633,AD.vs.control; bulk RNA-seq,Homo_633,AD.vs.MCI; bulk RNA-seq,Homo_633,MCI.vs.control; scRNA-seq,SRP215507,CD8+ T cell_2-AD.vs.control; scRNA-seq,SRP215507,CD8+ T cell_6-MCI.vs.control; scRNA-seq,SRP215507,CD8+ T cell_7-MCI.vs.control; scRNA-seq,SRP309935,Natural killer cell_2-AD.vs.control; scRNA-seq,SRP309935,Natural killer cell_1-AD.vs.control; scRNA-seq,SRP330776,B cell_1-AD.vs.control	11
CEP85L	bulk RNA-seq,Homo_723,AD.vs.control; bulk RNA-seq,Homo_723,AD.vs.MCI; bulk RNA-seq,Homo_633,AD.vs.control; bulk RNA-seq,Homo_633,AD.vs.MCI; bulk RNA-seq,Homo_714,AD.vs.control; bulk RNA-seq,Homo_714,AD.vs.MCI; bulk RNA-seq,Homo_714,MCI.vs.control; scRNA-seq,SRP215507,CD8+ T cell_6-AD.vs.MCI; scRNA-seq,SRP215507,CD8+ T cell_6-MCI.vs.control; scRNA-seq,SRP309935,Natural killer cell_2-AD.vs.control; scRNA-seq,SRP330776,B cell_3-AD.vs.control; scRNA-seq,SRP330776,Natural killer cell_2-AD.vs.control	12
CEP89	bulk RNA-seq,Homo_723,AD.vs.control; bulk RNA-seq,Homo_723,MCI.vs.control; bulk RNA-seq,Homo_633,AD.vs.control; bulk RNA-seq,Homo_633,AD.vs.MCI; bulk RNA-seq,Homo_633,MCI.vs.control; bulk RNA-seq,Homo_714,AD.vs.control; bulk RNA-seq,Homo_714,MCI.vs.control; scRNA-seq,SRP215507,CD8+ T cell_3-AD.vs.control; scRNA-seq,SRP215507,CD8+ T cell_3-AD.vs.MCI; scRNA-	11
CEP95	bulk RNA-seq,Homo_633,AD.vs.control; bulk RNA-seq,Homo_633,AD.vs.MCI; bulk RNA-seq,Homo_714,AD.vs.control; bulk RNA-seq,Homo_714,AD.vs.MCI; bulk RNA-seq,Homo_714,MCI.vs.control; scRNA-seq,SRP215507,CD8+ T cell_7-AD.vs.control; scRNA-seq,SRP215507,CD8+ T cell_1-AD.vs.MCI; scRNA-seq,SRP215507,CD8+ T cell_4-AD.vs.MCI; scRNA-seq,SRP215507,CD8+ T cell_7-AD.vs.MCI; scRNA-seq,SRP330776,CD8+ T cell_1-AD.vs.control; scRNA-seq,SRP330776,B cell_1-AD.vs.control; scRNA-seq,SRP330776,Naive CD8+ T cell_1-AD.vs.control; scRNA-seq,SRP330776,Natural killer cell_1-AD.vs.control; scRNA-seq,SRP330776,Naive CD8+ T cell_3-AD.vs.control; scRNA-seq,SRP330776,T cell-AD.vs.control; scRNA-seq,SRP330776,CD8+ T cell_3-AD.vs.control; scRNA-seq,SRP330776,Regulatory T cell-AD.vs.control; [illegible]	18
CEP97	bulk RNA-seq,Homo_723,AD.vs.control; bulk RNA-seq,Homo_723,AD.vs.MCI; bulk RNA-seq,Homo_714,AD.vs.control; bulk RNA-seq,Homo_714,AD.vs.MCI; scRNA-seq,SRP330776,CD8+ T cell_2-AD.vs.control; scRNA-seq,SRP330776,Memory T cell_1-AD.vs.control; scRNA-	7

CEPT1	bulk RNA-seq,Homo_723,AD.vs.control; bulk RNA-seq,Homo_633,AD.vs.control; bulk RNA-seq,Homo_714,AD.vs.control; bulk RNA-seq,Homo_714,AD.vs.MCI; bulk RNA-seq,Homo_714,MCI.vs.control; scRNA-seq,SRP215507,CD8+ T cell_7-AD.vs.MCI; scRNA-seq,SRP215507,CD8+ T cell_7-MCI.vs.control; scRNA-seq,SRP309935,B cell_1-AD.vs.control; scRNA-seq,SRP309935,CD8+ T cell_2-AD.vs.control; scRNA-seq,SRP330776,Memory T cell_2-AD.vs.control; scRNA-seq,SRP330776,CD8+ T cell_2-AD.vs.control; scRNA-seq,SRP330776,Natural killer cell_1-	13
CER1	bulk RNA-seq,Homo_633,MCI.vs.control; bulk RNA-seq,Homo_714,MCI.vs.control	2
CERCAM	bulk RNA-seq,Homo_714,MCI.vs.control; scRNA-seq,SRP215507,CD8+ T cell_1-AD.vs.control; scRNA-seq,SRP215507,CD8+ T cell_1-AD.vs.MCI; scRNA-seq,SRP309935,CD8+ T cell_1-AD.vs.control; scRNA-seq,SRP330776,CD8+ T cell_2-AD.vs.control; scRNA-seq,SRP330776,B cell_1-AD.vs.control; scRNA-seq,SRP330776,Memory T cell_1-AD.vs.control; scRNA-seq,SRP330776,B cell_2-AD.vs.control	8
Cercam	bulk RNA-seq,SRP325058,AD.vs.control	1
CERK	bulk RNA-seq,Homo_723,MCI.vs.control; bulk RNA-seq,Homo_633,AD.vs.control; bulk RNA-seq,Homo_633,MCI.vs.control; bulk RNA-seq,Homo_714,AD.vs.control; bulk RNA-seq,Homo_714,AD.vs.MCI; bulk RNA-seq,Homo_714,MCI.vs.control; bulk RNA-seq,SRP310421,AD.vs.control; scRNA-seq,SRP215507,CD8+ T cell_7-AD.vs.MCI; scRNA-seq,SRP215507,CD8+ T cell_5-MCI.vs.control; scRNA-seq,SRP215507,CD8+ T cell_7-MCI.vs.control; scRNA-seq,SRP309935,Natural killer cell_2-AD.vs.control; scRNA-seq,SRP309935,Monocyte_1-AD.vs.control; scRNA-seq,SRP309935,Naive CD8+ T cell_1-AD.vs.control; scRNA-seq,SRP309935,Natural killer cell_1-AD.vs.control; scRNA-seq,SRP309935,Monocyte_2-AD.vs.control; scRNA-seq,SRP309935,Naive CD8+ T cell_2-AD.vs.control; scRNA-seq,SRP309935,CD8+ T cell_1-AD.vs.control; scRNA-seq,SRP309935,CD8+ T cell_2-AD.vs.control; scRNA-seq,SRP309935,CD4+ T cell-AD.vs.control; scRNA-seq,SRP330776,CD8+ T cell_1-AD.vs.control; scRNA-seq,SRP330776,γδ T cell-AD.vs.control; scRNA-seq,SRP330776,Memory T cell_2-AD.vs.control; scRNA-seq,SRP330776,CD8+ T cell_2-AD.vs.control; scRNA-seq,SRP330776,B cell_1-AD.vs.control; scRNA-seq,SRP330776,Naive CD8+ T cell_1-AD.vs.control; scRNA-seq,SRP330776,Natural killer cell_1-AD.vs.control; scRNA-seq,SRP330776,Memory T cell_1-AD.vs.control; scRNA-seq,SRP330776,Natural killer T cell-AD.vs.control; scRNA-seq,SRP330776,Naive CD8+ T cell_3-AD.vs.control; scRNA-seq,SRP330776,B cell_2-AD.vs.control; scRNA-seq,SRP330776,T cell-AD.vs.control; scRNA-seq,SRP330776,Naive CD8+ T cell_2-AD.vs.control; scRNA-seq,SRP330776,CD8+ T cell_3-AD.vs.control; scRNA-seq,SRP330776,Monocyte_1-AD.vs.control; scRNA-seq,SRP330776,Monocyte_2-AD.vs.control; scRNA	37
CERKL	bulk RNA-seq,Homo_723,MCI.vs.control; bulk RNA-seq,Homo_633,AD.vs.control; bulk RNA-seq,Homo_633,AD.vs.MCI; bulk RNA-seq,Homo_633,MCI.vs.control; bulk RNA-seq,Homo_714,AD.vs.control; bulk RNA-seq,Homo_714,AD.vs.MCI; bulk RNA-seq,Homo_714,MCI.vs.control	7
CERNA1	scRNA-seq,SRP215507,CD8+ T cell_1-AD.vs.MCI; scRNA-seq,SRP309935,CD8+ T cell_2-AD.vs.control; scRNA-seq,SRP330776,CD8+ T cell_2-AD.vs.control; scRNA-seq,SRP330776,Memory T cell_1-AD.vs.control; scRNA-seq,SRP330776,Naive CD8+ T cell_3-AD.vs.control; scRNA-seq,SRP330776,T cell-AD.vs.control; scRNA-seq,SRP330776,CD8+ T cell_3-AD.vs.control	7
CERNA2	scRNA-seq,SRP330776,B cell_4-AD.vs.control	1
CERS1	bulk RNA-seq,Homo_723,AD.vs.control; bulk RNA-seq,Homo_723,AD.vs.MCI; bulk RNA-seq,Homo_714,MCI.vs.control; bulk RNA-seq,ROSMAP,AD.vs.MCI; bulk RNA-seq,ROSMAP,MCI.vs.control	5
CERS2	bulk RNA-seq,Homo_723,AD.vs.control; bulk RNA-seq,Homo_723,AD.vs.MCI; bulk RNA-seq,Homo_633,AD.vs.control; bulk RNA-seq,Homo_633,AD.vs.MCI; scRNA-seq,SRP309935,Natural killer cell_2-AD.vs.control; scRNA-seq,SRP309935,Megakaryocyte_1-AD.vs.control; scRNA-seq,SRP309935,Naive CD8+ T cell_1-AD.vs.control; scRNA-seq,SRP309935,Naive CD8+ T cell_2-AD.vs.control; scRNA-seq,SRP309935,B cell_1-AD.vs.control; scRNA-seq,SRP309935,CD8+ T cell_2-AD.vs.control; scRNA-seq,SRP330776,CD8+ T cell_1-AD.vs.control; scRNA-seq,SRP330776,γδ T cell-AD.vs.control; scRNA-seq,SRP330776,CD8+ T cell_2-AD.vs.control; scRNA-seq,SRP330776,Naive CD8+ T cell_1-AD.vs.control; scRNA-seq,SRP330776,Natural killer cell_1-AD.vs.control; scRNA-seq,SRP330776,Memory T cell_1-AD.vs.control; scRNA-seq,SRP330776,Natural killer T cell-AD.vs.control; scRNA-seq,SRP330776,T cell-AD.vs.control; scRNA-seq,SRP330776,Monocyte_1-AD.vs.control; scRNA-seq,SRP330776,B cell_3-[illegible]	22
CERS3	bulk RNA-seq,Homo_723,AD.vs.control; bulk RNA-seq,Homo_723,AD.vs.MCI; bulk RNA-seq,Homo_633,AD.vs.control; bulk RNA-seq,Homo_633,AD.vs.MCI; bulk RNA-seq,Homo_714,AD.vs.control; bulk RNA-seq,Homo_714,AD.vs.MCI; scRNA-seq,SRP215507,CD8+ T cell_3-AD.vs.MCI; scRNA-seq,SRP215507,CD8+ T cell_3-MCI.vs.control; scRNA-seq,SRP330776,CD8+ T cell_1-AD.vs.control; scRNA-seq,SRP330776,Naive	10

CERS4	scRNA-seq,SRP215507,CD8+ T cell_5-AD.vs.control; scRNA-seq,SRP215507,CD8+ T cell_1-AD.vs.MCI; scRNA-seq,SRP215507,CD8+ T cell_5-AD.vs.MCI; scRNA-seq,SRP215507,CD8+ T cell_5-MCI.vs.control; scRNA-seq,SRP309935,B cell_1-AD.vs.control; scRNA-seq,SRP330776,CD8+ T cell_1-AD.vs.control; scRNA-seq,SRP330776,γδ T cell-AD.vs.control; scRNA-seq,SRP330776,Memory T cell_2-AD.vs.control; scRNA-seq,SRP330776,CD8+ T cell_2-AD.vs.control; scRNA-seq,SRP330776,B cell_1-AD.vs.control; scRNA-seq,SRP330776,Naive CD8+ T cell_1-AD.vs.control; scRNA-seq,SRP330776,Natural killer cell_1-AD.vs.control; scRNA-seq,SRP330776,Memory T cell_1-AD.vs.control; scRNA-seq,SRP330776,Natural killer T cell-AD.vs.control; scRNA-seq,SRP330776,Naive CD8+ T cell_3-AD.vs.control; scRNA-seq,SRP330776,B cell_2-AD.vs.control; scRNA-seq,SRP330776,T cell-AD.vs.control; scRNA-seq,SRP330776,CD8+ T cell_3-AD.vs.control; scRNA-seq,SRP330776,B cell_3- [illegible]	20
CERS5	bulk RNA-seq,SRP223445,AD.vs.control; scRNA-seq,SRP215507,CD8+ T cell_7-AD.vs.control; scRNA-seq,SRP215507,CD8+ T cell_10-AD.vs.control; scRNA-seq,SRP309935,CD4+ T cell-AD.vs.control; scRNA-seq,SRP330776,γδ T cell-AD.vs.control; scRNA-seq,SRP330776,Memory T cell_1-AD.vs.control; scRNA-seq,SRP330776,Naive CD8+ T cell_3-AD.vs.control; scRNA-seq,SRP330776,T cell-AD.vs.control; scRNA-seq,SRP330776,CD8+ T cell_3-AD.vs.control; scRNA-seq,SRP330776,Natural killer cell_2-AD.vs.control	10
CERS6	bulk RNA-seq,Homo_723,AD.vs.control; bulk RNA-seq,Homo_723,MCI.vs.control; bulk RNA-seq,Homo_633,MCI.vs.control; scRNA-seq,SRP215507,CD8+ T cell_3-AD.vs.control; scRNA-seq,SRP215507,CD8+ T cell_2-AD.vs.MCI; scRNA-seq,SRP215507,CD8+ T cell_4-AD.vs.MCI; scRNA-seq,SRP215507,CD8+ T cell_5-AD.vs.MCI; scRNA-seq,SRP215507,CD8+ T cell_5-MCI.vs.control; scRNA-seq,SRP215507,CD8+ T cell_4-MCI.vs.control; scRNA-seq,SRP215507,CD8+ T cell_3-MCI.vs.control; scRNA-seq,SRP330776,CD8+ T cell_1-AD.vs.control; scRNA-seq,SRP330776,γδ T cell-AD.vs.control; scRNA-seq,SRP330776,B cell_1-AD.vs.control; scRNA-seq,SRP330776,Naive CD8+ T cell_1-AD.vs.control; scRNA-seq,SRP330776,Memory T cell_1-AD.vs.control; scRNA-seq,SRP330776,Naive CD8+ T cell_3-AD.vs.control; scRNA-seq,SRP330776,B cell_2-AD.vs.control; scRNA-seq,SRP330776,CD8+ T cell_3-AD.vs.control; scRNA-seq,SRP330776,Monocyte_1-AD.vs.control; scRNA- [illegible]	22
Cers6	bulk RNA-seq,SRP325058,AD.vs.control	1
CERS6-AS1	scRNA-seq,SRP215507,CD8+ T cell_3-AD.vs.MCI; scRNA-seq,SRP215507,CD8+ T cell_3-MCI.vs.control; scRNA-seq,SRP330776,CD8+ T cell_1-	3
CERT1	bulk RNA-seq,Homo_714,AD.vs.control; bulk RNA-seq,Homo_714,AD.vs.MCI; scRNA-seq,SRP215507,CD8+ T cell_3-MCI.vs.control; scRNA-seq,SRP215507,CD8+ T cell_6-MCI.vs.control; scRNA-seq,SRP330776,Naive CD8+ T cell_3-AD.vs.control; scRNA-seq,SRP330776,Monocyte_2-AD.vs.control; scRNA-seq,SRP330776,Natural killer cell_2-AD.vs.control	7
CES1	bulk RNA-seq,Homo_723,AD.vs.control; bulk RNA-seq,Homo_723,AD.vs.MCI; bulk RNA-seq,Homo_633,AD.vs.control; bulk RNA-seq,Homo_633,AD.vs.MCI; bulk RNA-seq,Homo_714,AD.vs.control; bulk RNA-seq,Homo_714,AD.vs.MCI; scRNA-seq,SRP215507,CD8+ T cell_3-AD.vs.control; scRNA-seq,SRP215507,CD8+ T cell_4-AD.vs.control; scRNA-seq,SRP215507,CD8+ T cell_6-AD.vs.control; scRNA-seq,SRP215507,CD8+ T cell_3-MCI.vs.control; scRNA-seq,SRP309935,Natural killer cell_2-AD.vs.control; scRNA-seq,SRP309935,Monocyte_1-AD.vs.control; scRNA-seq,SRP309935,Monocyte_3-AD.vs.control; scRNA-seq,SRP330776,CD8+ T cell_2-AD.vs.control; scRNA-seq,SRP330776,Natural killer cell_1-AD.vs.control; scRNA-seq,SRP330776,Natural killer T cell-AD.vs.control; scRNA-seq,SRP330776,Monocyte_2-AD.vs.control	17
CES2	scRNA-seq,SRP215507,CD8+ T cell_1-AD.vs.MCI; scRNA-seq,SRP215507,CD8+ T cell_4-AD.vs.MCI; scRNA-seq,SRP215507,CD8+ T cell_5-AD.vs.MCI; scRNA-seq,SRP215507,CD8+ T cell_5-MCI.vs.control; scRNA-seq,SRP330776,CD8+ T cell_1-AD.vs.control; scRNA-seq,SRP330776,γδ T cell-AD.vs.control; scRNA-seq,SRP330776,Memory T cell_2-AD.vs.control; scRNA-seq,SRP330776,B cell_1-AD.vs.control; scRNA-seq,SRP330776,CD8+ T cell_3-AD.vs.control; scRNA-seq,SRP330776,B cell_3-AD.vs.control	10
Ces2g	bulk RNA-seq,SRP325058,AD.vs.control	1
CES3	bulk RNA-seq,Homo_723,AD.vs.control; bulk RNA-seq,Homo_723,AD.vs.MCI; bulk RNA-seq,Homo_633,AD.vs.control; bulk RNA-seq,Homo_633,MCI.vs.control; bulk RNA-seq,Homo_714,MCI.vs.control; bulk RNA-seq,ROSMAP,AD.vs.control; bulk RNA-	7
CES4A	bulk RNA-seq,Homo_723,AD.vs.control; bulk RNA-seq,Homo_723,AD.vs.MCI; bulk RNA-seq,Homo_633,AD.vs.control; bulk RNA-seq,Homo_633,AD.vs.MCI; bulk RNA-seq,Homo_714,AD.vs.control; bulk RNA-seq,Homo_714,AD.vs.MCI; scRNA-seq,SRP330776,CD8+ T cell_1-AD.vs.control; scRNA-seq,SRP330776,γδ T cell-AD.vs.control; scRNA-seq,SRP330776,Memory T cell_2-AD.vs.control; scRNA-seq,SRP330776,CD8+ T cell_2-AD.vs.control; scRNA-seq,SRP330776,Natural killer cell_1-AD.vs.control; scRNA-seq,SRP330776,Natural killer T cell-AD.vs.control; scRNA-seq,SRP330776,Naive CD8+ T cell_3-AD.vs.control; scRNA-seq,SRP330776,CD8+ T cell_3-AD.vs.control	14
CETN1	bulk RNA-seq,Homo_723,AD.vs.control	1

CETN2	bulk RNA-seq,Homo_723,AD.vs.control; bulk RNA-seq,Homo_723,AD.vs.MCI; bulk RNA-seq,Homo_633,AD.vs.MCI; bulk RNA-seq,Homo_714,AD.vs.control; bulk RNA-seq,Homo_714,AD.vs.MCI; scRNA-seq,SRP215507,CD8+ T cell_3-AD.vs.control; scRNA-seq,SRP215507,CD8+ T cell_4-AD.vs.MCI; scRNA-seq,SRP215507,CD8+ T cell_4-MCI.vs.control; scRNA-seq,SRP215507,CD8+ T cell_3-MCI.vs.control; scRNA-seq,SRP330776,Memory T cell_2-AD.vs.control; scRNA-seq,SRP330776,CD8+ T cell_2-AD.vs.control; scRNA-seq,SRP330776,B cell_1-AD.vs.control; scRNA-seq,SRP330776,Memory T cell_1-AD.vs.control; scRNA-seq,SRP330776,Natural killer T cell-	16
CETN3	bulk RNA-seq,Homo_723,AD.vs.control; bulk RNA-seq,Homo_723,AD.vs.MCI; bulk RNA-seq,Homo_723,MCI.vs.control; bulk RNA-seq,Homo_633,AD.vs.MCI; bulk RNA-seq,Homo_633,MCI.vs.control; bulk RNA-seq,Homo_714,AD.vs.control; bulk RNA-seq,Homo_714,AD.vs.MCI; bulk RNA-seq,Homo_714,MCI.vs.control; scRNA-seq,SRP330776,CD8+ T cell_1-AD.vs.control; scRNA-seq,SRP330776,γδ T cell-AD.vs.control; scRNA-seq,SRP330776,Memory T cell_2-AD.vs.control; scRNA-seq,SRP330776,B cell_1-AD.vs.control; scRNA-seq,SRP330776,Naive CD8+ T cell_1-AD.vs.control; scRNA-seq,SRP330776,Natural killer cell_1-AD.vs.control; scRNA-seq,SRP330776,Memory T cell_1-AD.vs.control; scRNA-seq,SRP330776,Natural killer T cell-AD.vs.control; scRNA-seq,SRP330776,Naive CD8+ T cell_3-AD.vs.control; scRNA-seq,SRP330776,B cell_2- [illegible]	19
CETP	bulk RNA-seq,Homo_723,AD.vs.control; bulk RNA-seq,Homo_723,AD.vs.MCI; bulk RNA-seq,Homo_633,AD.vs.control; bulk RNA-seq,Homo_633,AD.vs.MCI; bulk RNA-seq,Homo_714,AD.vs.control; bulk RNA-seq,Homo_714,AD.vs.MCI	6
CFAP100	bulk RNA-seq,Homo_723,AD.vs.control; bulk RNA-seq,Homo_723,AD.vs.MCI; bulk RNA-seq,Homo_723,MCI.vs.control; bulk RNA-seq,Homo_633,AD.vs.control; bulk RNA-seq,Homo_633,AD.vs.MCI; bulk RNA-seq,Homo_633,MCI.vs.control; bulk RNA-seq,Homo_714,AD.vs.MCI;	8
CFAP157	bulk RNA-seq,Homo_723,AD.vs.control; bulk RNA-seq,Homo_723,MCI.vs.control; bulk RNA-seq,Homo_633,AD.vs.control; bulk RNA-seq,Homo_633,MCI.vs.control; bulk RNA-seq,Homo_714,MCI.vs.control	5
CFAP161	bulk RNA-seq,Homo_723,AD.vs.control; bulk RNA-seq,Homo_723,AD.vs.MCI; bulk RNA-seq,Homo_723,MCI.vs.control; bulk RNA-seq,Homo_633,AD.vs.control; bulk RNA-seq,Homo_633,AD.vs.MCI; bulk RNA-seq,Homo_633,MCI.vs.control; bulk RNA-seq,Homo_714,AD.vs.control;	8
CFAP20	bulk RNA-seq,Homo_723,AD.vs.control; bulk RNA-seq,Homo_633,AD.vs.control; bulk RNA-seq,Homo_633,AD.vs.MCI; bulk RNA-seq,Homo_714,AD.vs.control; bulk RNA-seq,Homo_714,AD.vs.MCI; scRNA-seq,SRP215507,CD8+ T cell_1-AD.vs.MCI; scRNA-seq,SRP215507,CD8+ T cell_5-AD.vs.MCI; scRNA-seq,SRP215507,CD8+ T cell_1-MCI.vs.control; scRNA-seq,SRP215507,CD8+ T cell_5-MCI.vs.control; scRNA-seq,SRP215507,CD8+ T cell_3-MCI.vs.control; scRNA-seq,SRP330776,CD8+ T cell_1-AD.vs.control; scRNA-seq,SRP330776,γδ T cell-AD.vs.control; scRNA-seq,SRP330776,Memory T cell_2-AD.vs.control; scRNA-seq,SRP330776,CD8+ T cell_2-AD.vs.control; scRNA-seq,SRP330776,B cell_1-AD.vs.control; scRNA-seq,SRP330776,Naive CD8+ T cell_1-AD.vs.control; scRNA-seq,SRP330776,Natural killer cell_1-AD.vs.control; scRNA-seq,SRP330776,Memory T cell_1-AD.vs.control; scRNA-seq,SRP330776,Natural killer T cell-AD.vs.control; scRNA-seq,SRP330776,Naive CD8+ T cell_3-AD.vs.control; scRNA-seq,SRP330776,B cell_2-AD.vs.control; scRNA-seq,SRP330776,Naive CD8+ T cell_2-AD.vs.control; scRNA- [illegible]	25
CFAP206	bulk RNA-seq,Homo_723,AD.vs.control; bulk RNA-seq,Homo_723,AD.vs.MCI	2
CFAP20DC	bulk RNA-seq,Homo_723,AD.vs.control; bulk RNA-seq,Homo_723,AD.vs.MCI; bulk RNA-seq,Homo_633,AD.vs.control; bulk RNA-seq,Homo_633,AD.vs.MCI; bulk RNA-seq,Homo_714,MCI.vs.control	5
CFAP251	bulk RNA-seq,Homo_723,AD.vs.control; bulk RNA-seq,Homo_723,AD.vs.MCI; bulk RNA-seq,Homo_723,MCI.vs.control; bulk RNA-seq,Homo_633,AD.vs.MCI; bulk RNA-seq,Homo_633,MCI.vs.control; bulk RNA-seq,Homo_714,AD.vs.control; bulk RNA-seq,Homo_714,AD.vs.MCI;	8
CFAP298	bulk RNA-seq,Homo_723,AD.vs.control; bulk RNA-seq,Homo_723,AD.vs.MCI; bulk RNA-seq,Homo_633,AD.vs.control; bulk RNA-seq,Homo_633,AD.vs.MCI; bulk RNA-seq,Homo_714,AD.vs.control; bulk RNA-seq,Homo_714,AD.vs.MCI; scRNA-seq,SRP215507,CD8+ T cell_7-AD.vs.control; scRNA-seq,SRP215507,CD8+ T cell_7-AD.vs.MCI; scRNA-seq,SRP215507,CD8+ T cell_2-MCI.vs.control; scRNA-seq,SRP330776,γδ T	10
CFAP298-TCP10L	bulk RNA-seq,Homo_723,AD.vs.control; bulk RNA-seq,Homo_723,MCI.vs.control; bulk RNA-seq,Homo_633,AD.vs.control; bulk RNA-seq,Homo_633,AD.vs.MCI; bulk RNA-seq,Homo_633,MCI.vs.control; bulk RNA-seq,Homo_714,AD.vs.control; bulk RNA-seq,Homo_714,AD.vs.MCI;	8
CFAP299	scRNA-seq,SRP215507,CD8+ T cell_3-AD.vs.MCI	1
CFAP300	bulk RNA-seq,Homo_723,AD.vs.MCI	1
CFAP36	bulk RNA-seq,Homo_723,AD.vs.MCI; bulk RNA-seq,Homo_723,MCI.vs.control; bulk RNA-seq,Homo_633,MCI.vs.control; bulk RNA-seq,Homo_714,AD.vs.control; bulk RNA-seq,Homo_714,AD.vs.MCI; bulk RNA-seq,Homo_714,MCI.vs.control; bulk RNA-seq,ROSMAP,MCI.vs.control; scRNA-seq,SRP309935,CD8+ T cell_2-AD.vs.control; scRNA-seq,SRP330776,Monocyte_1-AD.vs.control	9

CFAP410	bulk RNA-seq,Homo_723,AD.vs.control; bulk RNA-seq,Homo_723,AD.vs.MCI; bulk RNA-seq,Homo_633,AD.vs.control; bulk RNA-seq,Homo_633,AD.vs.MCI; bulk RNA-seq,Homo_714,AD.vs.control; bulk RNA-seq,Homo_714,AD.vs.MCI; scRNA-seq,SRP215507,CD8+ T cell_6-AD.vs.control; scRNA-seq,SRP215507,CD8+ T cell_7-AD.vs.MCI; scRNA-seq,SRP215507,CD8+ T cell_5-AD.vs.MCI; scRNA-seq,SRP215507,CD8+ T cell_5-MCI.vs.control; scRNA-seq,SRP215507,CD8+ T cell_7-MCI.vs.control; scRNA-seq,SRP330776,CD8+ T cell_1-AD.vs.control; scRNA-seq,SRP330776,γδ T cell-AD.vs.control; scRNA-seq,SRP330776,Memory T cell_2-AD.vs.control; scRNA-seq,SRP330776,CD8+ T cell_2-AD.vs.control; scRNA-seq,SRP330776,B cell_1-AD.vs.control; scRNA-seq,SRP330776,Naive CD8+ T cell_1-AD.vs.control; scRNA-seq,SRP330776,Natural killer cell_1-AD.vs.control; scRNA-seq,SRP330776,Memory T cell_1-AD.vs.control; scRNA-seq,SRP330776,Natural killer T cell-AD.vs.control; scRNA-seq,SRP330776,Naive CD8+ T cell_3-AD.vs.control; scRNA-seq,SRP330776,B cell_2-AD.vs.control; scRNA-seq,SRP330776,T cell-AD.vs.control; [illegible]	26
CFAP43	bulk RNA-seq,Homo_723,AD.vs.control; bulk RNA-seq,Homo_723,AD.vs.MCI; bulk RNA-seq,Homo_723,MCI.vs.control; bulk RNA-seq,Homo_633,AD.vs.control; bulk RNA-seq,Homo_633,AD.vs.MCI; scRNA-seq,SRP330776,CD8+ T cell_1-AD.vs.control; scRNA-	7
CFAP44	bulk RNA-seq,Homo_723,AD.vs.control; bulk RNA-seq,Homo_723,AD.vs.MCI; bulk RNA-seq,Homo_723,MCI.vs.control; bulk RNA-seq,Homo_633,AD.vs.control; bulk RNA-seq,Homo_633,AD.vs.MCI; bulk RNA-seq,Homo_633,MCI.vs.control; bulk RNA-seq,Homo_714,AD.vs.control; bulk RNA-seq,Homo_714,AD.vs.MCI; bulk RNA-seq,Homo_714,MCI.vs.control; scRNA-seq,SRP215507,CD8+ T cell_2-AD.vs.control; scRNA-seq,SRP215507,CD8+ T cell_3-AD.vs.MCI; scRNA-seq,SRP215507,CD8+ T cell_2-MCI.vs.control; scRNA-seq,SRP215507,CD8+ T cell_3-MCI.vs.control; scRNA-seq,SRP330776,Memory T cell_2-AD.vs.control; scRNA-seq,SRP330776,CD8+ T cell_2-AD.vs.control; scRNA-seq,SRP330776,B cell_1-AD.vs.control; scRNA-seq,SRP330776,Naive CD8+ T cell_1-AD.vs.control; scRNA-seq,SRP330776,Natural killer T cell-[illegible]	18
CFAP45	scRNA-seq,SRP215507,CD8+ T cell_5-AD.vs.MCI	1
CFAP46	bulk RNA-seq,Homo_723,AD.vs.control; bulk RNA-seq,Homo_723,AD.vs.MCI; bulk RNA-seq,Homo_633,AD.vs.control; bulk RNA-seq,Homo_633,AD.vs.MCI; bulk RNA-seq,Homo_714,MCI.vs.control	5
CFAP47	bulk RNA-seq,ROSMAP,AD.vs.control; bulk RNA-seq,ROSMAP,MCI.vs.control	2
CFAP52	bulk RNA-seq,Homo_723,AD.vs.control; bulk RNA-seq,Homo_723,AD.vs.MCI	2
CFAP53	bulk RNA-seq,Homo_723,AD.vs.control; bulk RNA-seq,Homo_723,AD.vs.MCI; bulk RNA-seq,Homo_723,MCI.vs.control; bulk RNA-seq,Homo_633,AD.vs.control; bulk RNA-seq,Homo_633,AD.vs.MCI; bulk RNA-seq,Homo_633,MCI.vs.control; bulk RNA-seq,Homo_714,AD.vs.control; bulk RNA-seq,Homo_714,AD.vs.MCI; bulk RNA-seq,Homo_714,MCI.vs.control; scRNA-seq,SRP215507,CD8+ T cell_3-AD.vs.MCI	10
CFAP54	bulk RNA-seq,Homo_723,AD.vs.control; bulk RNA-seq,Homo_723,AD.vs.MCI; bulk RNA-seq,Homo_633,AD.vs.control; bulk RNA-seq,Homo_633,AD.vs.MCI; bulk RNA-seq,Homo_714,AD.vs.control; bulk RNA-seq,Homo_714,AD.vs.MCI; scRNA-seq,SRP215507,CD8+ T cell_5-AD.vs.control; scRNA-seq,SRP215507,CD8+ T cell_1-AD.vs.MCI; scRNA-seq,SRP215507,CD8+ T cell_1-MCI.vs.control; scRNA-seq,SRP215507,CD8+ T cell_5-MCI.vs.control; scRNA-seq,SRP215507,CD8+ T cell_2-MCI.vs.control; scRNA-seq,SRP330776,CD8+ T cell_2-AD.vs.control	12
CFAP57	bulk RNA-seq,Homo_723,AD.vs.control; bulk RNA-seq,Homo_723,AD.vs.MCI; bulk RNA-seq,Homo_714,AD.vs.MCI	3
CFAP58	bulk RNA-seq,Homo_723,AD.vs.control; bulk RNA-seq,Homo_723,AD.vs.MCI; bulk RNA-seq,Homo_633,AD.vs.control; bulk RNA-seq,Homo_633,AD.vs.MCI; bulk RNA-seq,Homo_714,AD.vs.control; bulk RNA-seq,Homo_714,AD.vs.MCI	6
CFAP58-DT	scRNA-seq,SRP215507,CD8+ T cell_1-AD.vs.control; scRNA-seq,SRP215507,CD8+ T cell_5-AD.vs.control; scRNA-seq,SRP215507,CD8+ T cell_6-AD.vs.control; scRNA-seq,SRP215507,CD8+ T cell_1-AD.vs.MCI; scRNA-seq,SRP215507,CD8+ T cell_5-AD.vs.MCI; scRNA-seq,SRP330776,γδ T cell-AD.vs.control; scRNA-seq,SRP330776,CD8+ T cell_2-AD.vs.control; scRNA-seq,SRP330776,Memory T cell_1-AD.vs.control	8
CFAP61	bulk RNA-seq,Homo_723,AD.vs.control; bulk RNA-seq,Homo_723,AD.vs.MCI; bulk RNA-seq,Homo_633,AD.vs.control; bulk RNA-seq,Homo_633,AD.vs.MCI; scRNA-seq,SRP330776,Memory T cell_1-AD.vs.control	5
CFAP65	bulk RNA-seq,Homo_723,AD.vs.control; bulk RNA-seq,Homo_723,AD.vs.MCI; bulk RNA-seq,Homo_714,MCI.vs.control; bulk RNA-	4
CFAP69	bulk RNA-seq,Homo_723,AD.vs.control; bulk RNA-seq,Homo_723,AD.vs.MCI; bulk RNA-seq,Homo_714,MCI.vs.control	3
CFAP70	bulk RNA-seq,Homo_723,AD.vs.control; bulk RNA-seq,Homo_723,AD.vs.MCI; bulk RNA-seq,Homo_723,MCI.vs.control; bulk RNA-seq,Homo_633,AD.vs.control; bulk RNA-seq,Homo_633,AD.vs.MCI; bulk RNA-seq,Homo_714,AD.vs.control; bulk RNA-seq,Homo_714,AD.vs.MCI; bulk RNA-seq,Homo_714,MCI.vs.control; bulk RNA-seq,ROSMAP,AD.vs.control; scRNA-seq,SRP215507,CD8+ T cell_5-AD.vs.MCI; scRNA-seq,SRP215507,CD8+ T cell_1-MCI.vs.control; scRNA-seq,SRP215507,CD8+ T cell_4-MCI.vs.control; scRNA-seq,SRP330776,CD8+ T cell_1-	14

CFAP73	bulk RNA-seq,Homo_723,AD.vs.control; bulk RNA-seq,Homo_723,AD.vs.MCI; bulk RNA-seq,Homo_633,AD.vs.control; bulk RNA-seq,Homo_633,AD.vs.MCI; scRNA-seq,SRP330776,Naive CD8+ T cell_1-AD.vs.control; scRNA-seq,SRP330776,Memory T cell_1-AD.vs.control	6
CFAP74	bulk RNA-seq,Homo_723,AD.vs.control; bulk RNA-seq,Homo_723,AD.vs.MCI; bulk RNA-seq,Homo_714,MCI.vs.control	3
CFAP77	bulk RNA-seq,Homo_723,AD.vs.control; bulk RNA-seq,Homo_723,AD.vs.MCI; bulk RNA-seq,Homo_633,MCI.vs.control; bulk RNA-	4
CFAP91	bulk RNA-seq,Homo_723,AD.vs.control; bulk RNA-seq,Homo_723,AD.vs.MCI; bulk RNA-seq,Homo_723,MCI.vs.control; bulk RNA-seq,Homo_633,AD.vs.control; bulk RNA-seq,Homo_633,AD.vs.MCI; bulk RNA-seq,Homo_633,MCI.vs.control; bulk RNA-seq,Homo_714,MCI.vs.control	7
CFAP92	bulk RNA-seq,Homo_723,AD.vs.control; bulk RNA-seq,Homo_723,AD.vs.MCI; bulk RNA-seq,Homo_633,AD.vs.control; bulk RNA-seq,Homo_633,AD.vs.MCI; bulk RNA-seq,Homo_714,AD.vs.control; bulk RNA-seq,Homo_714,AD.vs.MCI	6
CFAP95	bulk RNA-seq,ROSMAP,AD.vs.control; bulk RNA-seq,ROSMAP,MCI.vs.control	2
CFAP97	scRNA-seq,SRP330776,CD8+ T cell_1-AD.vs.control; scRNA-seq,SRP330776,γδ T cell-AD.vs.control; scRNA-seq,SRP330776,Memory T cell_2-AD.vs.control; scRNA-seq,SRP330776,CD8+ T cell_2-AD.vs.control; scRNA-seq,SRP330776,B cell_1-AD.vs.control; scRNA-seq,SRP330776,Naive CD8+ T cell_1-AD.vs.control; scRNA-seq,SRP330776,Memory T cell_1-AD.vs.control; scRNA-seq,SRP330776,Naive CD8+ T cell_3-AD.vs.control; scRNA-seq,SRP330776,B cell_2-AD.vs.control; scRNA-seq,SRP330776,T cell-AD.vs.control; scRNA-seq,SRP330776,Naive CD8+ T cell_2-AD.vs.control; scRNA-seq,SRP330776,CD8+ T cell_3-AD.vs.control; scRNA-seq,SRP330776,Monocyte_2-AD.vs.control; scRNA-seq,SRP330776,B cell_3-AD.vs.control; scRNA-seq,SRP330776,Regulatory T cell-AD.vs.control; scRNA-seq,SRP330776,Natural killer cell_2-AD.vs.control	16
CFAP97D1	bulk RNA-seq,Homo_723,AD.vs.control; bulk RNA-seq,Homo_723,AD.vs.MCI; bulk RNA-seq,Homo_633,AD.vs.control; bulk RNA-seq,Homo_633,AD.vs.MCI; bulk RNA-seq,Homo_714,MCI.vs.control	5
CFAP97D2	bulk RNA-seq,Homo_723,AD.vs.control; bulk RNA-seq,Homo_723,AD.vs.MCI; bulk RNA-seq,Homo_633,AD.vs.control; bulk RNA-seq,Homo_633,AD.vs.MCI; bulk RNA-seq,Homo_714,AD.vs.control; bulk RNA-seq,Homo_714,AD.vs.MCI; scRNA-seq,SRP215507,CD8+ T cell_3-AD.vs.control; scRNA-seq,SRP215507,CD8+ T cell_1-AD.vs.control; scRNA-seq,SRP215507,CD8+ T cell_1-MCI.vs.control; scRNA-	10
CFAP99	bulk RNA-seq,Homo_723,AD.vs.control; bulk RNA-seq,Homo_723,AD.vs.MCI	2
CFB	bulk RNA-seq,Homo_723,AD.vs.control; bulk RNA-seq,Homo_723,AD.vs.MCI; bulk RNA-seq,Homo_723,MCI.vs.control; bulk RNA-seq,Homo_633,AD.vs.control; bulk RNA-seq,Homo_633,AD.vs.MCI; bulk RNA-seq,Homo_633,MCI.vs.control	6
CFC1	bulk RNA-seq,Homo_723,AD.vs.control; bulk RNA-seq,Homo_723,AD.vs.MCI	2
CFC1B	bulk RNA-seq,Homo_723,AD.vs.control; bulk RNA-seq,Homo_723,AD.vs.MCI	2
CFD	bulk RNA-seq,Homo_723,AD.vs.control; bulk RNA-seq,Homo_723,AD.vs.MCI; bulk RNA-seq,Homo_723,MCI.vs.control; bulk RNA-seq,Homo_633,AD.vs.control; bulk RNA-seq,Homo_633,AD.vs.MCI; bulk RNA-seq,Homo_633,MCI.vs.control; bulk RNA-seq,Homo_714,AD.vs.control; bulk RNA-seq,Homo_714,AD.vs.MCI; bulk RNA-seq,Homo_714,MCI.vs.control; bulk RNA-seq,SRP223445,AD.vs.control; scRNA-seq,SRP215507,CD8+ T cell_2-AD.vs.control; scRNA-seq,SRP215507,CD8+ T cell_3-AD.vs.control; scRNA-seq,SRP215507,CD8+ T cell_1-AD.vs.control; scRNA-seq,SRP215507,CD8+ T cell_4-AD.vs.control; scRNA-seq,SRP215507,CD8+ T cell_3-AD.vs.MCI; scRNA-seq,SRP215507,CD8+ T cell_1-MCI.vs.control; scRNA-seq,SRP215507,CD8+ T cell_4-MCI.vs.control; scRNA-seq,SRP309935,Naive CD8+ T cell_2-AD.vs.control; scRNA-seq,SRP330776,γδ T cell-AD.vs.control; scRNA-seq,SRP330776,CD8+ T cell_2-AD.vs.control; scRNA-seq,SRP330776,B cell_1-AD.vs.control; scRNA- [illegible]	23
CFDP1	bulk RNA-seq,Homo_723,AD.vs.MCI; bulk RNA-seq,Homo_714,AD.vs.control; bulk RNA-seq,Homo_714,AD.vs.MCI; scRNA-seq,SRP215507,CD8+ T cell_7-AD.vs.control; scRNA-seq,SRP309935,Megakaryocyte_2-AD.vs.control; scRNA-seq,SRP330776,Memory T cell_2-AD.vs.control; scRNA-seq,SRP330776,Monocyte_1-AD.vs.control	7
CFH	bulk RNA-seq,Homo_714,AD.vs.control; bulk RNA-seq,Homo_714,AD.vs.MCI; scRNA-seq,SRP215507,CD8+ T cell_2-AD.vs.control; scRNA-seq,SRP215507,CD8+ T cell_3-AD.vs.control; scRNA-seq,SRP215507,CD8+ T cell_1-AD.vs.MCI; scRNA-seq,SRP215507,CD8+ T cell_1-MCI.vs.control; scRNA-seq,SRP330776,CD8+ T cell_1-AD.vs.control; scRNA-seq,SRP330776,Memory T cell_2-AD.vs.control; scRNA-seq,SRP330776,Natural killer cell_1-AD.vs.control; scRNA-seq,SRP330776,Natural killer T cell-AD.vs.control; scRNA-seq,SRP330776,B cell_3-	11
CFHR1	bulk RNA-seq,Homo_723,AD.vs.control; bulk RNA-seq,Homo_723,AD.vs.MCI; bulk RNA-seq,Homo_723,MCI.vs.control; bulk RNA-seq,Homo_633,AD.vs.control; bulk RNA-seq,Homo_633,AD.vs.MCI; bulk RNA-seq,Homo_633,MCI.vs.control; bulk RNA-seq,Homo_714,MCI.vs.control	7
CFI	bulk RNA-seq,Homo_723,AD.vs.control; bulk RNA-seq,Homo_723,AD.vs.MCI; bulk RNA-seq,Homo_723,MCI.vs.control; bulk RNA-seq,Homo_633,AD.vs.MCI; bulk RNA-seq,Homo_633,MCI.vs.control	5

Cfl1	bulk RNA-seq,SRP325058,AD.vs.control	1
CFL1	scRNA-seq,SRP309935,Erythroid cell-AD.vs.control; scRNA-seq,SRP330776,B cell_4-AD.vs.control	2
CFL2	scRNA-seq,SRP215507,CD8+ T cell_5-AD.vs.control; scRNA-seq,SRP215507,CD8+ T cell_2-AD.vs.MCI; scRNA-seq,SRP215507,CD8+ T cell_2-MCI.vs.control; scRNA-seq,SRP309935,Natural killer cell_2-AD.vs.control; scRNA-seq,SRP309935,Naive CD8+ T cell_1-AD.vs.control; scRNA-seq,SRP309935,Natural killer cell_1-AD.vs.control; scRNA-seq,SRP309935,CD8+ T cell_1-AD.vs.control; scRNA-seq,SRP330776,CD8+ T cell_1-AD.vs.control; scRNA-seq,SRP330776,Memory T cell_2-AD.vs.control; scRNA-seq,SRP330776,CD8+ T cell_2-AD.vs.control; scRNA-seq,SRP330776,Naive CD8+ T cell_1-AD.vs.control; scRNA-seq,SRP330776,Natural killer T cell-AD.vs.control; scRNA-seq,SRP330776,T cell-AD.vs.control; scRNA-seq,SRP330776,Naive CD8+ T cell_2-AD.vs.control; scRNA-seq,SRP330776,CD8+ T cell_3-AD.vs.control; scRNA- [illegible]	16
CFLAR	scRNA-seq,SRP215507,CD8+ T cell_10-AD.vs.control; scRNA-seq,SRP215507,CD8+ T cell_4-AD.vs.MCI; scRNA-seq,SRP215507,CD8+ T cell_5- [illegible]	3
CFLAR-AS1	scRNA-seq,SRP330776,γδ T cell-AD.vs.control; scRNA-seq,SRP330776,CD8+ T cell_2-AD.vs.control; scRNA-seq,SRP330776,Naive CD8+ T cell_1-AD.vs.control; scRNA-seq,SRP330776,Memory T cell_1-AD.vs.control	4
CFP	scRNA-seq,SRP215507,CD8+ T cell_3-AD.vs.control; scRNA-seq,SRP215507,CD8+ T cell_4-AD.vs.control; scRNA-seq,SRP215507,CD8+ T cell_2-AD.vs.MCI; scRNA-seq,SRP215507,CD8+ T cell_2-MCI.vs.control; scRNA-seq,SRP330776,γδ T cell-AD.vs.control; scRNA-seq,SRP330776,Memory T cell_2-AD.vs.control; scRNA-seq,SRP330776,B cell_1-AD.vs.control; scRNA-seq,SRP330776,Naive CD8+ T cell_1-AD.vs.control; scRNA-seq,SRP330776,B cell_2-AD.vs.control; scRNA-seq,SRP330776,T cell-AD.vs.control	10
CFTR	bulk RNA-seq,Homo_723,AD.vs.control; bulk RNA-seq,Homo_723,AD.vs.MCI	2
CGAS	bulk RNA-seq,Homo_723,AD.vs.control; bulk RNA-seq,Homo_723,MCI.vs.control; bulk RNA-seq,Homo_633,AD.vs.control; bulk RNA-seq,Homo_633,AD.vs.MCI; bulk RNA-seq,Homo_633,MCI.vs.control; scRNA-seq,SRP215507,CD8+ T cell_2-AD.vs.control; scRNA-seq,SRP215507,CD8+ T cell_3-AD.vs.control; scRNA-seq,SRP215507,CD8+ T cell_2-AD.vs.MCI; scRNA-seq,SRP215507,CD8+ T cell_6-AD.vs.MCI; scRNA-seq,SRP330776,Memory T cell_2-AD.vs.control; scRNA-seq,SRP330776,T cell-AD.vs.control; scRNA-seq,SRP330776,Monocyte_2-	12
CGB1	bulk RNA-seq,ROSMAP,AD.vs.control; bulk RNA-seq,ROSMAP,MCI.vs.control	2
CGB2	bulk RNA-seq,Homo_723,AD.vs.control; bulk RNA-seq,ROSMAP,AD.vs.control	2
CGB3	bulk RNA-seq,Homo_723,AD.vs.control; bulk RNA-seq,ROSMAP,AD.vs.control; bulk RNA-seq,ROSMAP,MCI.vs.control	3
CGB5	bulk RNA-seq,ROSMAP,AD.vs.control	1
CGB7	bulk RNA-seq,Homo_723,AD.vs.control; bulk RNA-seq,Homo_714,MCI.vs.control; bulk RNA-seq,ROSMAP,AD.vs.control	3
CGB8	bulk RNA-seq,Homo_723,AD.vs.control; bulk RNA-seq,Homo_723,MCI.vs.control; bulk RNA-seq,Homo_633,MCI.vs.control; bulk RNA-seq,Homo_714,MCI.vs.control; bulk RNA-seq,ROSMAP,AD.vs.control	5
CGGBP1	scRNA-seq,SRP330776,CD8+ T cell_1-AD.vs.control; scRNA-seq,SRP330776,Naive CD8+ T cell_1-AD.vs.control; scRNA-seq,SRP330776,Memory T cell_1-AD.vs.control; scRNA-seq,SRP330776,Naive CD8+ T cell_3-AD.vs.control; scRNA-seq,SRP330776,Monocyte_2-AD.vs.control	5
CGN	bulk RNA-seq,Homo_723,AD.vs.control; bulk RNA-seq,Homo_723,AD.vs.MCI; bulk RNA-seq,Homo_723,MCI.vs.control; bulk RNA-seq,Homo_633,AD.vs.control; bulk RNA-seq,Homo_633,AD.vs.MCI; bulk RNA-seq,Homo_633,MCI.vs.control; bulk RNA-seq,ROSMAP,AD.vs.control;	8
CGNL1	bulk RNA-seq,Homo_714,MCI.vs.control; bulk RNA-seq,SRP223445,AD.vs.control	2
CGREF1	bulk RNA-seq,Homo_723,AD.vs.control; bulk RNA-seq,Homo_723,AD.vs.MCI; bulk RNA-seq,Homo_723,MCI.vs.control; bulk RNA-seq,Homo_633,AD.vs.MCI; bulk RNA-seq,Homo_633,MCI.vs.control	5
Cgrrf1	bulk RNA-seq,SRP325058,AD.vs.control	1
CGRRF1	scRNA-seq,SRP215507,CD8+ T cell_4-AD.vs.control; scRNA-seq,SRP215507,CD8+ T cell_6-MCI.vs.control; scRNA-seq,SRP309935,Megakaryocyte_2-AD.vs.control; scRNA-seq,SRP330776,B cell_3-AD.vs.control	4
CH25H	bulk RNA-seq,Homo_723,AD.vs.control; bulk RNA-seq,Homo_633,AD.vs.control	2
CHAC1	bulk RNA-seq,Homo_723,AD.vs.control; bulk RNA-seq,Homo_723,AD.vs.MCI; bulk RNA-seq,Homo_633,AD.vs.control; bulk RNA-seq,Homo_633,MCI.vs.control; bulk RNA-seq,Homo_714,MCI.vs.control; scRNA-seq,SRP215507,CD8+ T cell_2-AD.vs.control; scRNA-seq,SRP215507,CD8+ T cell_5-AD.vs.MCI; scRNA-seq,SRP215507,CD8+ T cell_5-MCI.vs.control; scRNA-seq,SRP215507,CD8+ T cell_2-	10

CHAC2	bulk RNA-seq,Homo_714,AD.vs.control; bulk RNA-seq,Homo_714,AD.vs.MCI; scRNA-seq,SRP215507,CD8+ T cell_2-AD.vs.control; scRNA-seq,SRP215507,CD8+ T cell_1-AD.vs.MCI; scRNA-seq,SRP215507,CD8+ T cell_5-AD.vs.MCI; scRNA-seq,SRP215507,CD8+ T cell_5-MCI.vs.control; scRNA-seq,SRP309935,Monocyte_1-AD.vs.control; scRNA-seq,SRP309935,Natural killer cell_1-AD.vs.control; scRNA-seq,SRP309935,Naive CD8+ T cell_2-AD.vs.control; scRNA-seq,SRP330776,CD8+ T cell_1-AD.vs.control; scRNA-seq,SRP330776,CD8+ T cell_2-AD.vs.control; scRNA-seq,SRP330776,Naive CD8+ T cell_1-AD.vs.control; scRNA-seq,SRP330776,Memory T cell_1-AD.vs.control; scRNA-seq,SRP330776,T cell-	15
CHAD	bulk RNA-seq,Homo_723,AD.vs.control; bulk RNA-seq,Homo_723,MCI.vs.control; bulk RNA-seq,Homo_633,AD.vs.MCI; bulk RNA-seq,Homo_633,MCI.vs.control; bulk RNA-seq,Homo_714,AD.vs.MCI; bulk RNA-seq,Homo_714,MCI.vs.control; scRNA-seq,SRP330776,B cell_4-	7
CHADL	bulk RNA-seq,Homo_723,AD.vs.control; bulk RNA-seq,Homo_723,AD.vs.MCI; bulk RNA-seq,Homo_633,AD.vs.control; bulk RNA-seq,Homo_633,AD.vs.MCI; bulk RNA-seq,Homo_714,AD.vs.control; bulk RNA-seq,Homo_714,AD.vs.MCI; bulk RNA-seq,Homo_714,MCI.vs.control;	8
CHAF1A	scRNA-seq,SRP215507,CD8+ T cell_3-AD.vs.control; scRNA-seq,SRP215507,CD8+ T cell_1-AD.vs.control; scRNA-seq,SRP215507,CD8+ T cell_5-AD.vs.MCI; scRNA-seq,SRP309935,Natural killer cell_2-AD.vs.control; scRNA-seq,SRP330776,CD8+ T cell_1-AD.vs.control; scRNA-seq,SRP330776,γδ T cell-AD.vs.control; scRNA-seq,SRP330776,Memory T cell_2-AD.vs.control; scRNA-seq,SRP330776,CD8+ T cell_2-AD.vs.control; scRNA-seq,SRP330776,B cell_1-AD.vs.control; scRNA-seq,SRP330776,Naive CD8+ T cell_1-AD.vs.control; scRNA-seq,SRP330776,Natural killer cell_1-AD.vs.control; scRNA-seq,SRP330776,Memory T cell_1-AD.vs.control; scRNA-seq,SRP330776,B cell_2-AD.vs.control; scRNA-seq,SRP330776,T cell-AD.vs.control; scRNA-seq,SRP330776,CD8+ T cell_3-AD.vs.control; scRNA-seq,SRP330776,Regulatory T cell-AD.vs.control	16
CHAF1B	bulk RNA-seq,Homo_723,AD.vs.control; bulk RNA-seq,Homo_723,AD.vs.MCI; bulk RNA-seq,Homo_723,MCI.vs.control; bulk RNA-seq,Homo_633,AD.vs.control; bulk RNA-seq,Homo_633,AD.vs.MCI; bulk RNA-seq,Homo_633,MCI.vs.control; bulk RNA-seq,Homo_714,AD.vs.control; bulk RNA-seq,Homo_714,AD.vs.MCI; bulk RNA-seq,Homo_714,MCI.vs.control; scRNA-seq,SRP215507,CD8+ T cell_2-AD.vs.control; scRNA-seq,SRP215507,CD8+ T cell_1-AD.vs.control; scRNA-seq,SRP215507,CD8+ T cell_1-AD.vs.MCI; scRNA-seq,SRP330776,B cell_2-AD.vs.control;	14
CHAMP1	bulk RNA-seq,Homo_723,AD.vs.control; bulk RNA-seq,Homo_723,AD.vs.MCI; bulk RNA-seq,Homo_633,AD.vs.control; bulk RNA-seq,Homo_633,AD.vs.MCI; bulk RNA-seq,Homo_714,AD.vs.control; bulk RNA-seq,Homo_714,AD.vs.MCI; scRNA-seq,SRP215507,CD8+ T cell_5-AD.vs.control; scRNA-seq,SRP215507,CD8+ T cell_5-AD.vs.MCI; scRNA-seq,SRP215507,CD8+ T cell_5-MCI.vs.control; scRNA-seq,SRP215507,CD8+ T cell_3-MCI.vs.control; scRNA-seq,SRP309935,Megakaryocyte_1-AD.vs.control; scRNA-seq,SRP309935,B cell_1-AD.vs.control; scRNA-seq,SRP330776,CD8+ T cell_1-AD.vs.control; scRNA-seq,SRP330776,γδ T cell-AD.vs.control; scRNA-seq,SRP330776,Memory T cell_2-AD.vs.control; scRNA-seq,SRP330776,CD8+ T cell_2-AD.vs.control; scRNA-seq,SRP330776,B cell_1-AD.vs.control; scRNA-seq,SRP330776,Naive CD8+ T cell_1-AD.vs.control; scRNA-seq,SRP330776,Natural killer cell_1-AD.vs.control; scRNA-seq,SRP330776,Memory T cell_1-AD.vs.control; scRNA-seq,SRP330776,Natural killer T cell-AD.vs.control; scRNA-seq,SRP330776,Naive CD8+ T cell_3-AD.vs.control; scRNA-seq,SRP330776,B cell_2-[illegible]	26
CHAT	bulk RNA-seq,Homo_723,AD.vs.control; bulk RNA-seq,Homo_723,MCI.vs.control; bulk RNA-seq,Homo_633,AD.vs.control; bulk RNA-seq,Homo_633,MCI.vs.control; bulk RNA-seq,Homo_714,MCI.vs.control	5
CHCHD1	bulk RNA-seq,Homo_714,AD.vs.control; bulk RNA-seq,Homo_714,AD.vs.MCI; scRNA-seq,SRP330776,γδ T cell-AD.vs.control; scRNA-seq,SRP330776,Memory T cell_2-AD.vs.control; scRNA-seq,SRP330776,CD8+ T cell_2-AD.vs.control; scRNA-seq,SRP330776,Naive CD8+ T cell_3-AD.vs.control; scRNA-seq,SRP330776,CD8+ T cell_3-AD.vs.control; scRNA-seq,SRP330776,Monocyte_1-AD.vs.control; scRNA-seq,SRP330776,B	9
CHCHD10	scRNA-seq,SRP215507,CD8+ T cell_10-AD.vs.control; scRNA-seq,SRP215507,CD8+ T cell_6-AD.vs.control; scRNA-seq,SRP309935,Natural killer cell_2-AD.vs.control; scRNA-seq,SRP309935,Megakaryocyte_1-AD.vs.control; scRNA-seq,SRP309935,Naive CD8+ T cell_1-AD.vs.control; scRNA-seq,SRP309935,Natural killer cell_1-AD.vs.control; scRNA-seq,SRP309935,Naive CD8+ T cell_2-AD.vs.control; scRNA-seq,SRP309935,CD8+ T cell_1-AD.vs.control; scRNA-seq,SRP309935,B cell_1-AD.vs.control; scRNA-seq,SRP309935,CD8+ T cell_2-AD.vs.control; scRNA-seq,SRP309935,B cell_2-AD.vs.control; scRNA-seq,SRP309935,Naive CD8+ T cell_3-AD.vs.control; scRNA-seq,SRP309935,Dendritic cell-AD.vs.control; scRNA-seq,SRP309935,CD4+ T cell-AD.vs.control; scRNA-seq,SRP330776,CD8+ T cell_1-AD.vs.control; scRNA-seq,SRP330776,γδ T cell-AD.vs.control; scRNA-seq,SRP330776,Memory T cell_2-AD.vs.control; scRNA-seq,SRP330776,CD8+ T cell_2-AD.vs.control; scRNA-seq,SRP330776,B cell_1-AD.vs.control; scRNA-seq,SRP330776,Naive CD8+ T cell_1-AD.vs.control; scRNA-seq,SRP330776,Natural killer cell_1-AD.vs.control; scRNA-seq,SRP330776,Memory T cell_1-AD.vs.control; scRNA-seq,SRP330776,Natural killer T cell-AD.vs.control; scRNA-seq,SRP330776,Naive CD8+ T cell_3-AD.vs.control; scRNA-seq,SRP330776,B cell_2-AD.vs.control; scRNA-seq,SRP330776,T cell-AD.vs.control; scRNA-seq,SRP330776,Naive CD8+ T cell_2-AD.vs.control; scRNA-seq,SRP330776,CD8+ T cell_3-AD.vs.control; scRNA-seq,SRP330776,Monocyte_1-AD.vs.control; scRNA-[illegible]	32
CHCHD2	scRNA-seq,SRP215507,CD8+ T cell_7-AD.vs.MCI; scRNA-seq,SRP309935,Erythroid cell-AD.vs.control; scRNA-seq,SRP309935,Megakaryocyte_2-	3

CHCHD3	bulk RNA-seq,Homo_723,AD.vs.control; bulk RNA-seq,Homo_723,AD.vs.MCI; bulk RNA-seq,Homo_723,MCI.vs.control; bulk RNA-seq,Homo_633,AD.vs.control; bulk RNA-seq,Homo_633,AD.vs.MCI; bulk RNA-seq,Homo_633,MCI.vs.control; bulk RNA-seq,Homo_714,AD.vs.MCI; bulk RNA-seq,Homo_714,MCI.vs.control; scRNA-seq,SRP215507,CD8+ T cell_7-AD.vs.control; scRNA-seq,SRP215507,CD8+ T cell_6-AD.vs.control; scRNA-seq,SRP215507,CD8+ T cell_4-AD.vs.MCI; scRNA-seq,SRP215507,CD8+ T cell_7-AD.vs.MCI; scRNA-seq,SRP215507,CD8+ T cell_4-	13
CHCHD4	scRNA-seq,SRP215507,CD8+ T cell_7-AD.vs.control; scRNA-seq,SRP215507,CD8+ T cell_1-AD.vs.MCI; scRNA-seq,SRP215507,CD8+ T cell_1-MCI.vs.control; scRNA-seq,SRP215507,CD8+ T cell_2-MCI.vs.control; scRNA-seq,SRP215507,CD8+ T cell_3-MCI.vs.control; scRNA-seq,SRP330776,CD8+ T cell_1-AD.vs.control; scRNA-seq,SRP330776,γδ T cell-AD.vs.control; scRNA-seq,SRP330776,Memory T cell_2-AD.vs.control; scRNA-seq,SRP330776,CD8+ T cell_2-AD.vs.control; scRNA-seq,SRP330776,B cell_1-AD.vs.control; scRNA-seq,SRP330776,Naive CD8+ T cell_1-AD.vs.control; scRNA-seq,SRP330776,Natural killer cell_1-AD.vs.control; scRNA-seq,SRP330776,Memory T cell_1-AD.vs.control; scRNA-seq,SRP330776,Natural killer T cell-AD.vs.control; scRNA-seq,SRP330776,Naive CD8+ T cell_3-AD.vs.control; scRNA-seq,SRP330776,B cell_2- [illegible]	18
CHCHD5	scRNA-seq,SRP215507,CD8+ T cell_6-AD.vs.MCI; scRNA-seq,SRP215507,CD8+ T cell_6-MCI.vs.control; scRNA-seq,SRP309935,Erythroid cell-AD.vs.control; scRNA-seq,SRP330776,Monocyte_2-AD.vs.control	4
CHCHD6	bulk RNA-seq,Homo_633,AD.vs.control; bulk RNA-seq,Homo_633,AD.vs.MCI; bulk RNA-seq,Homo_714,AD.vs.control; bulk RNA-seq,Homo_714,AD.vs.MCI; scRNA-seq,SRP215507,CD8+ T cell_6-AD.vs.control; scRNA-seq,SRP215507,CD8+ T cell_6-AD.vs.MCI; scRNA-seq,SRP330776,CD8+ T cell_1-AD.vs.control; scRNA-seq,SRP330776,Memory T cell_2-AD.vs.control; scRNA-seq,SRP330776,Natural killer cell_1-AD.vs.control; scRNA-seq,SRP330776,B cell_2-AD.vs.control; scRNA-seq,SRP330776,B cell_3-AD.vs.control	11
CHCHD7	bulk RNA-seq,Homo_723,AD.vs.control; bulk RNA-seq,Homo_723,AD.vs.MCI; bulk RNA-seq,Homo_633,AD.vs.control; bulk RNA-seq,Homo_633,AD.vs.MCI; bulk RNA-seq,Homo_714,AD.vs.control; bulk RNA-seq,Homo_714,AD.vs.MCI; scRNA-seq,SRP330776,CD8+ T cell_1-AD.vs.control; scRNA-seq,SRP330776,Memory T cell_2-AD.vs.control; scRNA-seq,SRP330776,Naive CD8+ T cell_1-AD.vs.control; scRNA-seq,SRP330776,Memory T cell_1-AD.vs.control; scRNA-seq,SRP330776,T cell-AD.vs.control; scRNA-seq,SRP330776,Monocyte_1-AD.vs.control;	13
CHD1	bulk RNA-seq,Homo_723,AD.vs.control; bulk RNA-seq,Homo_633,AD.vs.control; bulk RNA-seq,Homo_633,AD.vs.MCI; bulk RNA-seq,Homo_714,AD.vs.control; bulk RNA-seq,Homo_714,AD.vs.MCI; scRNA-seq,SRP215507,CD8+ T cell_7-AD.vs.MCI; scRNA-seq,SRP330776,B cell_3-AD.vs.control; scRNA-seq,SRP330776,B cell_4-AD.vs.control	8
CHD1L	scRNA-seq,SRP215507,CD8+ T cell_4-AD.vs.control; scRNA-seq,SRP215507,CD8+ T cell_4-AD.vs.MCI; scRNA-seq,SRP215507,CD8+ T cell_2-MCI.vs.control; scRNA-seq,SRP309935,Natural killer cell_1-AD.vs.control; scRNA-seq,SRP309935,B cell_1-AD.vs.control; scRNA-	6
CHD2	scRNA-seq,SRP215507,CD8+ T cell_7-AD.vs.MCI; scRNA-seq,SRP215507,CD8+ T cell_7-MCI.vs.control	2
CHD3	scRNA-seq,SRP309935,Natural killer cell_2-AD.vs.control; scRNA-seq,SRP309935,Naive CD8+ T cell_1-AD.vs.control; scRNA-seq,SRP309935,Naive CD8+ T cell_2-AD.vs.control; scRNA-seq,SRP330776,γδ T cell-AD.vs.control; scRNA-seq,SRP330776,Memory T cell_2-AD.vs.control; scRNA-seq,SRP330776,CD8+ T cell_2-AD.vs.control; scRNA-seq,SRP330776,Naive CD8+ T cell_1-AD.vs.control; scRNA-seq,SRP330776,Natural killer cell_1-AD.vs.control; scRNA-seq,SRP330776,Memory T cell_1-AD.vs.control; scRNA-seq,SRP330776,Naive CD8+ T cell_3-AD.vs.control; scRNA-seq,SRP330776,T cell-AD.vs.control; scRNA-seq,SRP330776,Monocyte_2-AD.vs.control; scRNA-seq,SRP330776,Natural killer cell_2-AD.vs.control	13
CHD4	scRNA-seq,SRP215507,CD8+ T cell_7-AD.vs.MCI; scRNA-seq,SRP215507,CD8+ T cell_7-MCI.vs.control; scRNA-seq,SRP330776,γδ T cell-AD.vs.control; scRNA-seq,SRP330776,Memory T cell_2-AD.vs.control; scRNA-seq,SRP330776,Naive CD8+ T cell_1-AD.vs.control; scRNA-seq,SRP330776,B cell_2-AD.vs.control; scRNA-seq,SRP330776,CD8+ T cell_3-AD.vs.control; scRNA-seq,SRP330776,Natural killer cell_2-AD.vs.control	8
CHD5	bulk RNA-seq,Homo_723,AD.vs.control; bulk RNA-seq,Homo_723,AD.vs.MCI; bulk RNA-seq,Homo_633,AD.vs.control; bulk RNA-seq,Homo_633,AD.vs.MCI; bulk RNA-seq,ROSMAP,AD.vs.MCI	5
CHD6	bulk RNA-seq,Homo_723,AD.vs.control; bulk RNA-seq,Homo_723,AD.vs.MCI; bulk RNA-seq,Homo_723,MCI.vs.control; bulk RNA-seq,Homo_633,AD.vs.control; bulk RNA-seq,Homo_633,AD.vs.MCI; bulk RNA-seq,Homo_633,MCI.vs.control; bulk RNA-seq,Homo_714,AD.vs.control; bulk RNA-seq,Homo_714,AD.vs.MCI; bulk RNA-seq,Homo_714,MCI.vs.control; scRNA-seq,SRP309935,Natural killer cell_2-AD.vs.control; scRNA-	11

CHD7	bulk RNA-seq,Homo_723,AD.vs.control; bulk RNA-seq,Homo_723,AD.vs.MCI; bulk RNA-seq,Homo_723,MCI.vs.control; bulk RNA-seq,Homo_633,AD.vs.control; bulk RNA-seq,Homo_633,AD.vs.MCI; bulk RNA-seq,Homo_633,MCI.vs.control; bulk RNA-seq,Homo_714,AD.vs.control; bulk RNA-seq,Homo_714,AD.vs.MCI; bulk RNA-seq,Homo_714,MCI.vs.control; scRNA-seq,SRP215507,CD8+ T cell_7-AD.vs.control; scRNA-seq,SRP215507,CD8+ T cell_3-AD.vs.MCI; scRNA-seq,SRP215507,CD8+ T cell_1-AD.vs.MCI; scRNA-seq,SRP215507,CD8+ T cell_1-MCI.vs.control; scRNA-seq,SRP215507,CD8+ T cell_5-MCI.vs.control; scRNA-seq,SRP309935,Natural killer cell_2-AD.vs.control; scRNA-seq,SRP309935,B cell_1-AD.vs.control; scRNA-seq,SRP330776,Naive CD8+ T cell_3-AD.vs.control; scRNA-seq,SRP330776,Megakaryocyte_1-AD.vs.control	18
CHD8	scRNA-seq,SRP330776,Naive CD8+ T cell_2-AD.vs.control	1
CHD9	bulk RNA-seq,Homo_723,AD.vs.control; bulk RNA-seq,Homo_723,AD.vs.MCI; bulk RNA-seq,Homo_633,AD.vs.control; bulk RNA-seq,Homo_633,AD.vs.MCI; bulk RNA-seq,Homo_633,MCI.vs.control; scRNA-seq,SRP215507,CD8+ T cell_5-AD.vs.MCI; scRNA-seq,SRP215507,CD8+ T cell_6-AD.vs.MCI; scRNA-seq,SRP215507,CD8+ T cell_6-MCI.vs.control; scRNA-seq,SRP309935,Megakaryocyte_2-AD.vs.control; scRNA-seq,SRP330776,Memory T cell_2-AD.vs.control; scRNA-seq,SRP330776,CD8+ T cell_2-AD.vs.control; scRNA-seq,SRP330776,Monocyte_1-	12
CHDH	bulk RNA-seq,Homo_723,AD.vs.control; bulk RNA-seq,Homo_633,AD.vs.control; bulk RNA-seq,Homo_633,AD.vs.MCI; bulk RNA-seq,Homo_714,AD.vs.control; bulk RNA-seq,Homo_714,AD.vs.MCI; scRNA-seq,SRP215507,CD8+ T cell_2-AD.vs.control; scRNA-seq,SRP215507,CD8+ T cell_2-AD.vs.MCI; scRNA-seq,SRP330776,T cell-AD.vs.control	8
CHEK1	bulk RNA-seq,Homo_723,AD.vs.control; bulk RNA-seq,Homo_723,AD.vs.MCI; bulk RNA-seq,Homo_723,MCI.vs.control; bulk RNA-seq,Homo_633,AD.vs.MCI; bulk RNA-seq,Homo_714,AD.vs.control; bulk RNA-seq,Homo_714,AD.vs.MCI; scRNA-seq,SRP215507,CD8+ T cell_5-AD.vs.control; scRNA-seq,SRP215507,CD8+ T cell_3-AD.vs.MCI; scRNA-seq,SRP215507,CD8+ T cell_1-AD.vs.MCI; scRNA-seq,SRP215507,CD8+ T cell_1-MCI.vs.control; scRNA-seq,SRP215507,CD8+ T cell_3-MCI.vs.control; scRNA-seq,SRP330776,CD8+ T cell_2-AD.vs.control; scRNA-seq,SRP330776,Naive CD8+ T cell_1-AD.vs.control; scRNA-seq,SRP330776,Natural killer T cell-AD.vs.control	14
CHEK2	bulk RNA-seq,Homo_723,AD.vs.MCI; bulk RNA-seq,Homo_633,AD.vs.MCI; bulk RNA-seq,Homo_714,AD.vs.control; bulk RNA-seq,Homo_714,AD.vs.MCI; scRNA-seq,SRP215507,CD8+ T cell_5-AD.vs.control; scRNA-seq,SRP215507,CD8+ T cell_6-AD.vs.control; scRNA-seq,SRP215507,CD8+ T cell_3-AD.vs.MCI; scRNA-seq,SRP215507,CD8+ T cell_3-MCI.vs.control; scRNA-seq,SRP330776,γδ T cell-AD.vs.control; scRNA-seq,SRP330776,B cell_1-AD.vs.control; scRNA-seq,SRP330776,Natural killer cell_1-AD.vs.control; scRNA-seq,SRP330776,Memory T cell_1-AD.vs.control; scRNA-seq,SRP330776,T cell-AD.vs.control; scRNA-seq,SRP330776,Natural killer cell_2-AD.vs.control	14
Chek2	bulk RNA-seq,SRP325058,AD.vs.control	1
CHERP	bulk RNA-seq,Homo_723,AD.vs.control; bulk RNA-seq,Homo_633,AD.vs.control; bulk RNA-seq,Homo_633,AD.vs.MCI; scRNA-seq,SRP215507,CD8+ T cell_7-AD.vs.control; scRNA-seq,SRP215507,CD8+ T cell_5-AD.vs.MCI; scRNA-seq,SRP309935,Monocyte_1-AD.vs.control; scRNA-seq,SRP330776,Natural killer cell_1-AD.vs.control	7
CHFR	scRNA-seq,SRP309935,Natural killer cell_2-AD.vs.control; scRNA-seq,SRP309935,Naive CD8+ T cell_2-AD.vs.control; scRNA-seq,SRP330776,Naive CD8+ T cell_3-AD.vs.control; scRNA-seq,SRP330776,B cell_3-AD.vs.control	4
CHGA	bulk RNA-seq,Homo_723,AD.vs.control; bulk RNA-seq,Homo_723,AD.vs.MCI; bulk RNA-seq,Homo_633,AD.vs.MCI; bulk RNA-seq,Homo_633,MCI.vs.control; bulk RNA-seq,Homo_714,MCI.vs.control	5
CHGB	bulk RNA-seq,Homo_723,AD.vs.control; bulk RNA-seq,Homo_723,AD.vs.MCI; bulk RNA-seq,Homo_723,MCI.vs.control; bulk RNA-	4
CHI3L1	bulk RNA-seq,Homo_723,AD.vs.control; bulk RNA-seq,Homo_723,AD.vs.MCI; bulk RNA-seq,Homo_633,AD.vs.control; bulk RNA-seq,Homo_633,AD.vs.MCI; bulk RNA-seq,ROSMAP,AD.vs.control; bulk RNA-seq,ROSMAP,MCI.vs.control; bulk RNA-seq,SRP223445,AD.vs.control	7

CHI3L2	bulk RNA-seq,Homo_723,AD.vs.control; bulk RNA-seq,Homo_723,AD.vs.MCI; bulk RNA-seq,Homo_723,MCI.vs.control; bulk RNA-seq,Homo_633,AD.vs.control; bulk RNA-seq,Homo_633,AD.vs.MCI; bulk RNA-seq,Homo_633,MCI.vs.control; bulk RNA-seq,Homo_714,AD.vs.control; bulk RNA-seq,Homo_714,AD.vs.MCI; bulk RNA-seq,Homo_714,MCI.vs.control; bulk RNA-seq,SRP223445,AD.vs.control; scRNA-seq,SRP215507,CD8+ T cell_2-AD.vs.control; scRNA-seq,SRP215507,CD8+ T cell_3-AD.vs.control; scRNA-seq,SRP215507,CD8+ T cell_1-AD.vs.control; scRNA-seq,SRP215507,CD8+ T cell_6-AD.vs.control; scRNA-seq,SRP215507,CD8+ T cell_2-AD.vs.MCI; scRNA-seq,SRP215507,CD8+ T cell_1-MCI.vs.control; scRNA-seq,SRP215507,CD8+ T cell_3-MCI.vs.control; scRNA-seq,SRP215507,CD8+ T cell_6-MCI.vs.control; scRNA-seq,SRP309935,Natural killer cell_1-AD.vs.control; scRNA-seq,SRP309935,Naive CD8+ T cell_2-AD.vs.control; scRNA-seq,SRP309935,CD8+ T cell_1-AD.vs.control; scRNA-seq,SRP309935,CD8+ T cell_2-AD.vs.control; scRNA-seq,SRP330776,Memory T cell_2-AD.vs.control; scRNA-seq,SRP330776,CD8+ T cell_2-AD.vs.control; scRNA-seq,SRP330776,B cell_1-AD.vs.control; scRNA-seq,SRP330776,Naive CD8+ T cell_1-[illegible]	29
CHIC1	bulk RNA-seq,Homo_723,AD.vs.control; bulk RNA-seq,Homo_723,AD.vs.MCI; bulk RNA-seq,Homo_723,MCI.vs.control; bulk RNA-seq,Homo_633,AD.vs.MCI; bulk RNA-seq,Homo_633,MCI.vs.control; bulk RNA-seq,Homo_714,AD.vs.control; bulk RNA-seq,Homo_714,AD.vs.MCI; bulk RNA-seq,Homo_714,MCI.vs.control; scRNA-seq,SRP215507,CD8+ T cell_3-AD.vs.MCI; scRNA-seq,SRP215507,CD8+ T cell_3-MCI.vs.control; scRNA-seq,SRP330776,Natural killer T cell-AD.vs.control; scRNA-seq,SRP330776,B cell_2-AD.vs.control; scRNA-seq,SRP330776,B cell_3-	14
CHIC2	bulk RNA-seq,Homo_723,AD.vs.control; scRNA-seq,SRP215507,CD8+ T cell_7-AD.vs.control; scRNA-seq,SRP215507,CD8+ T cell_5-AD.vs.MCI; scRNA-seq,SRP215507,CD8+ T cell_5-MCI.vs.control; scRNA-seq,SRP215507,CD8+ T cell_6-MCI.vs.control; scRNA-seq,SRP330776,Regulatory T cell-	6
Chic2	bulk RNA-seq,SRP325058,AD.vs.control	1
CHID1	bulk RNA-seq,Homo_723,AD.vs.control; bulk RNA-seq,Homo_723,MCI.vs.control; bulk RNA-seq,Homo_633,AD.vs.control; bulk RNA-seq,Homo_633,MCI.vs.control; scRNA-seq,SRP215507,CD8+ T cell_6-AD.vs.control; scRNA-seq,SRP215507,CD8+ T cell_9-AD.vs.control; scRNA-	7
Chil1	bulk RNA-seq,SRP325058,AD.vs.control	1
Chil5	bulk RNA-seq,SRP325058,AD.vs.control	1
CHIT1	bulk RNA-seq,Homo_723,AD.vs.control; bulk RNA-seq,Homo_723,AD.vs.MCI; bulk RNA-seq,Homo_633,AD.vs.control; bulk RNA-seq,Homo_633,AD.vs.MCI; bulk RNA-seq,Homo_714,AD.vs.control; bulk RNA-seq,Homo_714,AD.vs.MCI	6
CHKA	bulk RNA-seq,Homo_714,AD.vs.control; bulk RNA-seq,Homo_714,AD.vs.MCI; scRNA-seq,SRP215507,CD8+ T cell_4-AD.vs.control; scRNA-seq,SRP215507,CD8+ T cell_3-AD.vs.MCI; scRNA-seq,SRP215507,CD8+ T cell_5-MCI.vs.control; scRNA-seq,SRP330776,γδ T cell-AD.vs.control; scRNA-seq,SRP330776,B cell_1-AD.vs.control; scRNA-seq,SRP330776,Naive CD8+ T cell_1-AD.vs.control; scRNA-seq,SRP330776,Memory T cell_1-	10
CHKB	bulk RNA-seq,Homo_723,AD.vs.control; bulk RNA-seq,Homo_723,AD.vs.MCI; bulk RNA-seq,Homo_633,AD.vs.control; bulk RNA-seq,Homo_633,AD.vs.MCI; bulk RNA-seq,Homo_714,AD.vs.control; bulk RNA-seq,Homo_714,AD.vs.MCI; scRNA-seq,SRP215507,CD8+ T cell_6-MCI.vs.control; scRNA-seq,SRP309935,CD8+ T cell_2-AD.vs.control; scRNA-seq,SRP330776,CD8+ T cell_1-AD.vs.control; scRNA-seq,SRP330776,γδ T cell-AD.vs.control; scRNA-seq,SRP330776,CD8+ T cell_2-AD.vs.control; scRNA-seq,SRP330776,B cell_1-AD.vs.control; scRNA-seq,SRP330776,Naive CD8+ T cell_1-AD.vs.control; scRNA-seq,SRP330776,Memory T cell_1-AD.vs.control; scRNA-seq,SRP330776,T cell-AD.vs.control; scRNA-seq,SRP330776,CD8+ T cell_3-AD.vs.control; scRNA-seq,SRP330776,Monocyte_2-AD.vs.control; scRNA-seq,SRP330776,B [illegible]	19
CHKB-DT	scRNA-seq,SRP215507,CD8+ T cell_3-AD.vs.MCI; scRNA-seq,SRP215507,CD8+ T cell_3-MCI.vs.control; scRNA-seq,SRP309935,Naive CD8+ T cell_1-AD.vs.control; scRNA-seq,SRP330776,CD8+ T cell_1-AD.vs.control; scRNA-seq,SRP330776,γδ T cell-AD.vs.control; scRNA-seq,SRP330776,Memory T cell_2-AD.vs.control; scRNA-seq,SRP330776,CD8+ T cell_2-AD.vs.control; scRNA-seq,SRP330776,B cell_1-AD.vs.control; scRNA-seq,SRP330776,Memory T cell_1-AD.vs.control; scRNA-seq,SRP330776,Natural killer T cell-AD.vs.control; scRNA-seq,SRP330776,B cell_2-AD.vs.control; scRNA-seq,SRP330776,CD8+ T cell_3-AD.vs.control; scRNA-seq,SRP330776,Natural killer cell_2-AD.vs.control	13
CHL1	bulk RNA-seq,Homo_723,AD.vs.control; bulk RNA-seq,Homo_723,AD.vs.MCI; bulk RNA-seq,Homo_633,AD.vs.control; bulk RNA-seq,Homo_633,AD.vs.MCI; bulk RNA-seq,Homo_714,AD.vs.control; bulk RNA-seq,Homo_714,AD.vs.MCI	6
CHM	scRNA-seq,SRP215507,CD8+ T cell_3-AD.vs.MCI; scRNA-seq,SRP215507,CD8+ T cell_1-AD.vs.MCI; scRNA-seq,SRP215507,CD8+ T cell_3-MCI.vs.control; scRNA-seq,SRP330776,γδ T cell-AD.vs.control; scRNA-seq,SRP330776,Memory T cell_2-AD.vs.control; scRNA-seq,SRP330776,CD8+ T cell_2-AD.vs.control; scRNA-seq,SRP330776,B cell_1-AD.vs.control; scRNA-seq,SRP330776,B cell_3-AD.vs.control	8

CHML	scRNA-seq,SRP215507,CD8+ T cell_3-AD.vs.control; scRNA-seq,SRP215507,CD8+ T cell_5-AD.vs.control; scRNA-seq,SRP215507,CD8+ T cell_2-AD.vs.MCI; scRNA-seq,SRP215507,CD8+ T cell_3-MCI.vs.control; scRNA-seq,SRP330776,CD8+ T cell_1-AD.vs.control; scRNA-seq,SRP330776,Memory T cell_2-AD.vs.control; scRNA-seq,SRP330776,Naive CD8+ T cell_1-AD.vs.control; scRNA-seq,SRP330776,T cell-	9
CHMP1A	bulk RNA-seq,Homo_723,AD.vs.MCI; bulk RNA-seq,Homo_723,MCI.vs.control; bulk RNA-seq,Homo_633,AD.vs.MCI; bulk RNA-seq,Homo_633,MCI.vs.control; bulk RNA-seq,Homo_714,AD.vs.control; bulk RNA-seq,Homo_714,AD.vs.MCI; bulk RNA-seq,Homo_714,MCI.vs.control; scRNA-seq,SRP330776,CD8+ T cell_1-AD.vs.control; scRNA-seq,SRP330776,γδ T cell-AD.vs.control; scRNA-seq,SRP330776,Memory T cell_2-AD.vs.control; scRNA-seq,SRP330776,CD8+ T cell_2-AD.vs.control; scRNA-seq,SRP330776,B cell_1-AD.vs.control; scRNA-seq,SRP330776,Naive CD8+ T cell_1-AD.vs.control; scRNA-seq,SRP330776,Memory T cell_1-AD.vs.control; scRNA-seq,SRP330776,Naive	17
CHMP1B	scRNA-seq,SRP215507,CD8+ T cell_7-AD.vs.control; scRNA-seq,SRP215507,CD8+ T cell_5-AD.vs.MCI; scRNA-seq,SRP309935,Erythroid cell-AD.vs.control; scRNA-seq,SRP330776,CD8+ T cell_2-AD.vs.control; scRNA-seq,SRP330776,Naive CD8+ T cell_3-AD.vs.control; scRNA-	6
Chmp2a	bulk RNA-seq,SRP325058,AD.vs.control	1
CHMP2A	scRNA-seq,SRP215507,CD8+ T cell_4-AD.vs.control; scRNA-seq,SRP215507,CD8+ T cell_7-AD.vs.control; scRNA-seq,SRP330776,Megakaryocyte_1-	3
CHMP2B	scRNA-seq,SRP215507,CD8+ T cell_7-AD.vs.control	1
CHMP3	bulk RNA-seq,Homo_714,AD.vs.control; bulk RNA-seq,Homo_714,AD.vs.MCI; scRNA-seq,SRP215507,CD8+ T cell_7-AD.vs.control; scRNA-seq,SRP330776,B cell_2-AD.vs.control; scRNA-seq,SRP330776,Monocyte_1-AD.vs.control; scRNA-seq,SRP330776,B cell_3-AD.vs.control; scRNA-seq,SRP330776,Megakaryocyte_1-AD.vs.control; scRNA-seq,SRP330776,Natural killer cell_2-AD.vs.control	8
Chmp3	bulk RNA-seq,SRP325058,AD.vs.control	1
CHMP4A	bulk RNA-seq,Homo_723,AD.vs.control; bulk RNA-seq,Homo_723,AD.vs.MCI; bulk RNA-seq,Homo_633,AD.vs.control; bulk RNA-seq,Homo_633,AD.vs.MCI; bulk RNA-seq,Homo_714,AD.vs.control; bulk RNA-seq,Homo_714,AD.vs.MCI; scRNA-seq,SRP215507,CD8+ T cell_7-AD.vs.control; scRNA-seq,SRP309935,Megakaryocyte_2-AD.vs.control	8
CHMP4B	scRNA-seq,SRP215507,CD8+ T cell_5-AD.vs.MCI; scRNA-seq,SRP215507,CD8+ T cell_5-MCI.vs.control; scRNA-seq,SRP215507,CD8+ T cell_6-MCI.vs.control; scRNA-seq,SRP330776,CD8+ T cell_1-AD.vs.control; scRNA-seq,SRP330776,γδ T cell-AD.vs.control; scRNA-seq,SRP330776,Memory T cell_2-AD.vs.control; scRNA-seq,SRP330776,CD8+ T cell_2-AD.vs.control; scRNA-seq,SRP330776,B cell_1-AD.vs.control; scRNA-seq,SRP330776,Naive CD8+ T cell_1-AD.vs.control; scRNA-seq,SRP330776,Natural killer cell_1-AD.vs.control; scRNA-seq,SRP330776,Memory T cell_1-AD.vs.control; scRNA-seq,SRP330776,Natural killer T cell-AD.vs.control; scRNA-seq,SRP330776,Naive CD8+ T cell_3-AD.vs.control; scRNA-seq,SRP330776,B cell_2-AD.vs.control; scRNA-seq,SRP330776,T cell-AD.vs.control; scRNA-seq,SRP330776,Naive CD8+ T cell_2-AD.vs.control; scRNA-seq,SRP330776,CD8+ T cell_3-AD.vs.control; scRNA-seq,SRP330776,Monocyte_1-AD.vs.control; scRNA-seq,SRP330776,Monocyte_2-AD.vs.control; scRNA-seq,SRP330776,B cell_3-AD.vs.control; scRNA-seq,SRP330776,Regulatory T cell-AD.vs.control; scRNA-seq,SRP330776,Natural [illegible]	22
CHMP4C	bulk RNA-seq,Homo_723,AD.vs.control; bulk RNA-seq,Homo_723,AD.vs.MCI; bulk RNA-seq,Homo_723,MCI.vs.control; bulk RNA-seq,Homo_633,AD.vs.control; bulk RNA-seq,Homo_633,AD.vs.MCI; bulk RNA-seq,Homo_633,MCI.vs.control	6
CHMP5	bulk RNA-seq,Homo_714,AD.vs.control; bulk RNA-seq,Homo_714,AD.vs.MCI; bulk RNA-seq,Homo_714,MCI.vs.control; scRNA-seq,SRP330776,CD8+ T cell_1-AD.vs.control; scRNA-seq,SRP330776,CD8+ T cell_2-AD.vs.control; scRNA-seq,SRP330776,B cell_1-AD.vs.control; scRNA-seq,SRP330776,Naive CD8+ T cell_1-AD.vs.control; scRNA-seq,SRP330776,Natural killer cell_1-AD.vs.control; scRNA-seq,SRP330776,Memory T cell_1-AD.vs.control; scRNA-seq,SRP330776,Natural killer T cell-AD.vs.control; scRNA-seq,SRP330776,B cell_2-AD.vs.control; scRNA-seq,SRP330776,T cell-AD.vs.control; scRNA-seq,SRP330776,CD8+ T cell_3-AD.vs.control; scRNA-seq,SRP330776,Monocyte_2-AD.vs.control; scRNA-	16
CHMP6	bulk RNA-seq,Homo_723,MCI.vs.control; bulk RNA-seq,Homo_633,MCI.vs.control; scRNA-seq,SRP215507,CD8+ T cell_6-AD.vs.control; scRNA-seq,SRP215507,CD8+ T cell_5-AD.vs.MCI; scRNA-seq,SRP330776,CD8+ T cell_1-AD.vs.control; scRNA-seq,SRP330776,γδ T cell-AD.vs.control; scRNA-seq,SRP330776,Memory T cell_2-AD.vs.control; scRNA-seq,SRP330776,Naive CD8+ T cell_1-AD.vs.control; scRNA-seq,SRP330776,T cell-AD.vs.control; scRNA-seq,SRP330776,Natural killer cell_2-AD.vs.control	10

CHMP7	bulk RNA-seq,Homo_723,AD.vs.control; bulk RNA-seq,Homo_723,MCI.vs.control; bulk RNA-seq,Homo_633,AD.vs.control; bulk RNA-seq,Homo_633,MCI.vs.control; scRNA-seq,SRP215507,CD8+ T cell_4-AD.vs.control; scRNA-seq,SRP215507,CD8+ T cell_5-AD.vs.control; scRNA-seq,SRP215507,CD8+ T cell_4-AD.vs.MCI; scRNA-seq,SRP309935,Naive CD8+ T cell_1-AD.vs.control; scRNA-seq,SRP309935,B cell_1-AD.vs.control; scRNA-seq,SRP330776,γδ T cell-AD.vs.control; scRNA-seq,SRP330776,Naive CD8+ T cell_3-AD.vs.control; scRNA-seq,SRP330776,CD8+ T cell_3-AD.vs.control; scRNA-seq,SRP330776,Monocyte_2-AD.vs.control; scRNA-seq,SRP330776,Megakaryocyte_1-AD.vs.control	14
CHN1	bulk RNA-seq,Homo_723,AD.vs.control; bulk RNA-seq,Homo_723,AD.vs.MCI; bulk RNA-seq,Homo_633,AD.vs.MCI; bulk RNA-seq,Homo_714,AD.vs.control; bulk RNA-seq,Homo_714,AD.vs.MCI; scRNA-seq,SRP215507,CD8+ T cell_1-AD.vs.control; scRNA-seq,SRP215507,CD8+ T cell_1-AD.vs.MCI; scRNA-seq,SRP215507,CD8+ T cell_6-MCI.vs.control; scRNA-seq,SRP330776,Natural killer cell_1-	9
CHN2	scRNA-seq,SRP215507,CD8+ T cell_1-AD.vs.MCI; scRNA-seq,SRP215507,CD8+ T cell_1-MCI.vs.control; scRNA-seq,SRP215507,CD8+ T cell_2-MCI.vs.control; scRNA-seq,SRP330776,CD8+ T cell_1-AD.vs.control; scRNA-seq,SRP330776,γδ T cell-AD.vs.control; scRNA-seq,SRP330776,Memory T cell_2-AD.vs.control; scRNA-seq,SRP330776,CD8+ T cell_2-AD.vs.control; scRNA-seq,SRP330776,B cell_1-AD.vs.control; scRNA-seq,SRP330776,Memory T cell_1-AD.vs.control; scRNA-seq,SRP330776,Natural killer T cell-AD.vs.control; scRNA-seq,SRP330776,CD8+ T cell_3-AD.vs.control; scRNA-seq,SRP330776,Monocyte_1-AD.vs.control; scRNA-seq,SRP330776,Monocyte_2-AD.vs.control	13
CHODL	bulk RNA-seq,Homo_723,AD.vs.control; bulk RNA-seq,Homo_723,AD.vs.MCI; bulk RNA-seq,Homo_714,AD.vs.MCI; scRNA-seq,SRP215507,CD8+ T cell_5-AD.vs.control; scRNA-seq,SRP215507,CD8+ T cell_1-AD.vs.MCI; scRNA-seq,SRP215507,CD8+ T cell_1-MCI.vs.control; scRNA-seq,SRP330776,Memory T cell_2-AD.vs.control; scRNA-seq,SRP330776,Memory T cell_1-AD.vs.control	8
CHORDC1	bulk RNA-seq,Homo_714,AD.vs.control; bulk RNA-seq,Homo_714,AD.vs.MCI; scRNA-seq,SRP330776,Naive CD8+ T cell_3-AD.vs.control; scRNA-seq,SRP330776,Natural killer cell_2-AD.vs.control	4
Chp1	bulk RNA-seq,SRP325058,AD.vs.control	1
CHP1	scRNA-seq,SRP330776,B cell_2-AD.vs.control; scRNA-seq,SRP330776,Monocyte_1-AD.vs.control	2
CHPF	bulk RNA-seq,Homo_723,AD.vs.control; bulk RNA-seq,Homo_723,AD.vs.MCI; bulk RNA-seq,Homo_723,MCI.vs.control; bulk RNA-seq,Homo_633,AD.vs.control; bulk RNA-seq,Homo_633,AD.vs.MCI; bulk RNA-seq,Homo_633,MCI.vs.control; bulk RNA-seq,Homo_714,AD.vs.control; bulk RNA-seq,Homo_714,AD.vs.MCI; bulk RNA-seq,Homo_714,MCI.vs.control; bulk RNA-seq,ROSMAP,MCI.vs.control; scRNA-seq,SRP215507,CD8+ T cell_2-AD.vs.MCI; scRNA-seq,SRP330776,CD8+ T cell_1-AD.vs.control; scRNA-seq,SRP330776,γδ T cell-AD.vs.control; scRNA-seq,SRP330776,Memory T cell_2-AD.vs.control; scRNA-seq,SRP330776,CD8+ T cell_2-AD.vs.control; scRNA-seq,SRP330776,B cell_1-AD.vs.control; scRNA-seq,SRP330776,Naive CD8+ T cell_1-AD.vs.control; scRNA-seq,SRP330776,Natural killer cell_1-AD.vs.control; scRNA-seq,SRP330776,Memory T cell_1-AD.vs.control; scRNA-seq,SRP330776,Natural killer T cell-AD.vs.control; scRNA-seq,SRP330776,Naive CD8+ T cell_3-AD.vs.control; scRNA-seq,SRP330776,T cell-AD.vs.control; scRNA-seq,SRP330776,CD8+ T cell_3-AD.vs.control; scRNA-seq,SRP330776,B cell_3-AD.vs.control; scRNA-seq,SRP330776,Natural killer cell_2-AD.vs.control; scRNA-seq,SRP330776,B cell_4-AD.vs.control	26
CHPF2	bulk RNA-seq,Homo_723,AD.vs.control; bulk RNA-seq,Homo_723,AD.vs.MCI; bulk RNA-seq,Homo_723,MCI.vs.control; bulk RNA-seq,Homo_633,AD.vs.control; bulk RNA-seq,Homo_633,AD.vs.MCI; bulk RNA-seq,Homo_633,MCI.vs.control; scRNA-seq,SRP215507,CD8+ T cell_10-AD.vs.control; scRNA-seq,SRP215507,CD8+ T cell_5-AD.vs.MCI; scRNA-seq,SRP215507,CD8+ T cell_5-MCI.vs.control; scRNA-seq,SRP309935,Natural killer cell_2-AD.vs.control; scRNA-seq,SRP309935,Naive CD8+ T cell_1-AD.vs.control; scRNA-seq,SRP309935,Monocyte_2-AD.vs.control; scRNA-seq,SRP330776,Naive CD8+ T cell_1-AD.vs.control; scRNA-seq,SRP330776,Natural killer cell_1-AD.vs.control; scRNA-seq,SRP330776,Memory T cell_1-AD.vs.control; scRNA-seq,SRP330776,Natural killer T cell-AD.vs.control; scRNA-seq,SRP330776,Naive CD8+ T [illegible]	19

CHPT1	bulk RNA-seq,Homo_723,AD.vs.control; bulk RNA-seq,Homo_723,AD.vs.MCI; bulk RNA-seq,Homo_723,MCI.vs.control; bulk RNA-seq,Homo_633,AD.vs.control; bulk RNA-seq,Homo_633,AD.vs.MCI; bulk RNA-seq,Homo_633,MCI.vs.control; bulk RNA-seq,Homo_714,AD.vs.control; bulk RNA-seq,Homo_714,AD.vs.MCI; scRNA-seq,SRP215507,CD8+ T cell_3-AD.vs.control; scRNA-seq,SRP215507,CD8+ T cell_4-AD.vs.control; scRNA-seq,SRP215507,CD8+ T cell_7-AD.vs.control; scRNA-seq,SRP215507,CD8+ T cell_3-AD.vs.MCI; scRNA-seq,SRP215507,CD8+ T cell_7-AD.vs.MCI; scRNA-seq,SRP215507,CD8+ T cell_5-AD.vs.MCI; scRNA-seq,SRP215507,CD8+ T cell_5-MCI.vs.control; scRNA-seq,SRP215507,CD8+ T cell_2-MCI.vs.control; scRNA-seq,SRP215507,CD8+ T cell_7-MCI.vs.control; scRNA-seq,SRP309935,Natural killer cell_2-AD.vs.control; scRNA-seq,SRP309935,Monocyte_1-AD.vs.control; scRNA-seq,SRP309935,Megakaryocyte_1-AD.vs.control; scRNA-seq,SRP309935,Erythroid cell-AD.vs.control; scRNA-seq,SRP309935,Naive CD8+ T cell_1-AD.vs.control; scRNA-seq,SRP309935,Natural killer cell_1-AD.vs.control; scRNA-seq,SRP309935,Monocyte_2-AD.vs.control; scRNA-seq,SRP309935,Naive CD8+ T cell_2-AD.vs.control; scRNA-seq,SRP309935,CD8+ T cell_1-AD.vs.control; scRNA-seq,SRP309935,B cell_1-AD.vs.control; scRNA-seq,SRP309935,CD8+ T cell_2-AD.vs.control; scRNA-seq,SRP309935,Monocyte_3-AD.vs.control; scRNA-seq,SRP309935,B cell_2-AD.vs.control; scRNA-seq,SRP309935,CD4+ T cell-AD.vs.control; scRNA-seq,SRP330776,CD8+ T cell_1-AD.vs.control; scRNA-seq,SRP330776,γδ T cell-AD.vs.control; scRNA-seq,SRP330776,CD8+ T cell_2-AD.vs.control; scRNA-seq,SRP330776,B cell_1-AD.vs.control; scRNA-seq,SRP330776,Naive CD8+ T cell_1-AD.vs.control; scRNA-seq,SRP330776,Memory T cell_1-AD.vs.control; scRNA-seq,SRP330776,B cell_2-AD.vs.control; scRNA-seq,SRP330776,T cell-AD.vs.control; scRNA-seq,SRP330776,Naive CD8+ T cell_2-AD.vs.control; scRNA-seq,SRP330776,CD8+ T cell_3-AD.vs.control; scRNA-seq,SRP330776,Monocyte_1-AD.vs.control; scRNA-	45
CHRAC1	bulk RNA-seq,Homo_723,AD.vs.control; bulk RNA-seq,Homo_723,AD.vs.MCI; bulk RNA-seq,Homo_723,MCI.vs.control; bulk RNA-seq,Homo_633,AD.vs.control; bulk RNA-seq,Homo_633,AD.vs.MCI; bulk RNA-seq,Homo_633,MCI.vs.control; bulk RNA-seq,Homo_714,AD.vs.control; bulk RNA-seq,Homo_714,AD.vs.MCI; bulk RNA-seq,Homo_714,MCI.vs.control; scRNA-seq,SRP330776,CD8+ T cell_2-AD.vs.control; scRNA-seq,SRP330776,Natural killer cell_1-AD.vs.control; scRNA-seq,SRP330776,Memory T cell_1-AD.vs.control; scRNA-seq,SRP330776,Megakaryocyte_1-	13
CHRD	bulk RNA-seq,Homo_723,AD.vs.control; bulk RNA-seq,Homo_723,AD.vs.MCI	2
CHRDL1	bulk RNA-seq,Homo_723,AD.vs.control; bulk RNA-seq,Homo_723,AD.vs.MCI; bulk RNA-seq,Homo_723,MCI.vs.control; bulk RNA-seq,Homo_633,AD.vs.control; bulk RNA-seq,Homo_633,AD.vs.MCI; bulk RNA-seq,Homo_633,MCI.vs.control	6
CHRDL2	bulk RNA-seq,Homo_723,AD.vs.control; bulk RNA-seq,Homo_723,AD.vs.MCI; bulk RNA-seq,Homo_633,AD.vs.control; bulk RNA-	4
CHRFAM7A	bulk RNA-seq,Homo_723,AD.vs.control; bulk RNA-seq,Homo_723,AD.vs.MCI; bulk RNA-seq,Homo_714,AD.vs.control; bulk RNA-seq,Homo_714,AD.vs.MCI; scRNA-seq,SRP215507,CD8+ T cell_1-AD.vs.control; scRNA-seq,SRP215507,CD8+ T cell_2-AD.vs.MCI; scRNA-seq,SRP330776,γδ T cell-AD.vs.control; scRNA-seq,SRP330776,CD8+ T cell_2-AD.vs.control; scRNA-seq,SRP330776,Memory T cell_1-AD.vs.control	9
CHRM1	bulk RNA-seq,Homo_723,AD.vs.control; bulk RNA-seq,Homo_633,AD.vs.control; bulk RNA-seq,Homo_714,MCI.vs.control	3
CHRM2	bulk RNA-seq,Homo_723,AD.vs.control; bulk RNA-seq,Homo_723,AD.vs.MCI; bulk RNA-seq,Homo_723,MCI.vs.control; bulk RNA-seq,Homo_633,AD.vs.control; bulk RNA-seq,Homo_633,AD.vs.MCI; bulk RNA-seq,Homo_714,MCI.vs.control	6
CHRM3	bulk RNA-seq,Homo_723,AD.vs.control; bulk RNA-seq,Homo_723,AD.vs.MCI; bulk RNA-seq,Homo_723,MCI.vs.control; bulk RNA-seq,Homo_633,AD.vs.control; bulk RNA-seq,Homo_633,AD.vs.MCI; bulk RNA-seq,Homo_633,MCI.vs.control; bulk RNA-seq,Homo_714,AD.vs.control; bulk RNA-seq,Homo_714,AD.vs.MCI; bulk RNA-seq,Homo_714,MCI.vs.control; bulk RNA-seq,ROSMAP,MCI.vs.control	10
CHRM3-AS2	scRNA-seq,SRP215507,CD8+ T cell_4-AD.vs.control; scRNA-seq,SRP215507,CD8+ T cell_3-AD.vs.MCI; scRNA-seq,SRP215507,CD8+ T cell_1-AD.vs.MCI; scRNA-seq,SRP215507,CD8+ T cell_4-AD.vs.MCI; scRNA-seq,SRP215507,CD8+ T cell_1-MCI.vs.control; scRNA-seq,SRP215507,CD8+ T cell_2-MCI.vs.control; scRNA-seq,SRP215507,CD8+ T cell_3-MCI.vs.control; scRNA-seq,SRP330776,Naive CD8+ T cell_3-AD.vs.control; scRNA-seq,SRP330776,CD8+ T cell_3-AD.vs.control; scRNA-seq,SRP330776,B cell_3-AD.vs.control	10
CHRM4	bulk RNA-seq,Homo_723,AD.vs.control; bulk RNA-seq,Homo_723,AD.vs.MCI; bulk RNA-seq,Homo_633,AD.vs.control; bulk RNA-seq,Homo_633,AD.vs.MCI; scRNA-seq,SRP215507,CD8+ T cell_2-MCI.vs.control	5
CHRM5	bulk RNA-seq,Homo_633,AD.vs.control; bulk RNA-seq,Homo_633,AD.vs.MCI; bulk RNA-seq,Homo_714,AD.vs.control; bulk RNA-seq,Homo_714,AD.vs.MCI; bulk RNA-seq,ROSMAP,AD.vs.control; bulk RNA-seq,ROSMAP,MCI.vs.control	6
CHRNA1	bulk RNA-seq,Homo_633,MCI.vs.control; bulk RNA-seq,Homo_714,MCI.vs.control; scRNA-seq,SRP215507,CD8+ T cell_2-AD.vs.control; scRNA-seq,SRP215507,CD8+ T cell_1-AD.vs.control; scRNA-seq,SRP215507,CD8+ T cell_4-AD.vs.control; scRNA-seq,SRP215507,CD8+ T cell_2-AD.vs.MCI; scRNA-seq,SRP215507,CD8+ T cell_6-MCI.vs.control; scRNA-seq,SRP330776,CD8+ T cell_1-AD.vs.control; scRNA-seq,SRP330776,Memory T cell_2-AD.vs.control; scRNA-seq,SRP330776,B cell_1-AD.vs.control; scRNA-seq,SRP330776,CD8+ T cell_3-AD.vs.control	11

CHRNA10	bulk RNA-seq,Homo_723,AD.vs.control; bulk RNA-seq,Homo_723,AD.vs.MCI; bulk RNA-seq,Homo_633,AD.vs.control; bulk RNA-seq,Homo_633,AD.vs.MCI; bulk RNA-seq,Homo_714,AD.vs.control; bulk RNA-seq,Homo_714,AD.vs.MCI; scRNA-seq,SRP215507,CD8+ T cell_1-AD.vs.control; scRNA-seq,SRP330776,Naive CD8+ T cell_1-AD.vs.control; scRNA-seq,SRP330776,Memory T cell_1-AD.vs.control	9
CHRNA2	bulk RNA-seq,Homo_723,AD.vs.control; bulk RNA-seq,Homo_723,AD.vs.MCI; bulk RNA-seq,Homo_723,MCI.vs.control; bulk RNA-seq,Homo_633,AD.vs.control; bulk RNA-seq,Homo_633,AD.vs.MCI; bulk RNA-seq,Homo_633,MCI.vs.control; bulk RNA-seq,Homo_714,AD.vs.control; bulk RNA-seq,Homo_714,AD.vs.MCI; bulk RNA-seq,Homo_714,MCI.vs.control	9
CHRNA3	bulk RNA-seq,Homo_723,AD.vs.control; bulk RNA-seq,Homo_723,AD.vs.MCI; bulk RNA-seq,Homo_723,MCI.vs.control; bulk RNA-seq,Homo_633,AD.vs.control; bulk RNA-seq,Homo_633,AD.vs.MCI; bulk RNA-seq,ROSMAP,AD.vs.control	6
CHRNA4	bulk RNA-seq,Homo_723,AD.vs.control; bulk RNA-seq,Homo_723,AD.vs.MCI; bulk RNA-seq,Homo_714,MCI.vs.control	3
CHRNA5	bulk RNA-seq,Homo_723,AD.vs.control; bulk RNA-seq,Homo_723,AD.vs.MCI; bulk RNA-seq,Homo_723,MCI.vs.control; bulk RNA-seq,Homo_633,AD.vs.control; bulk RNA-seq,Homo_633,AD.vs.MCI; bulk RNA-seq,Homo_714,AD.vs.control; bulk RNA-seq,Homo_714,AD.vs.MCI;	8
CHRNA6	bulk RNA-seq,Homo_723,AD.vs.control; bulk RNA-seq,Homo_723,AD.vs.MCI; bulk RNA-seq,Homo_633,AD.vs.control	3
CHRNA7	bulk RNA-seq,Homo_723,AD.vs.control; bulk RNA-seq,Homo_723,AD.vs.MCI; bulk RNA-seq,Homo_714,AD.vs.control; bulk RNA-	4
CHRNA9	bulk RNA-seq,Homo_723,AD.vs.control; bulk RNA-seq,Homo_723,AD.vs.MCI; bulk RNA-seq,Homo_723,MCI.vs.control; bulk RNA-seq,Homo_633,AD.vs.control; bulk RNA-seq,Homo_633,AD.vs.MCI; bulk RNA-seq,Homo_633,MCI.vs.control	6
CHRNB1	bulk RNA-seq,Homo_714,AD.vs.control; bulk RNA-seq,Homo_714,AD.vs.MCI; scRNA-seq,SRP215507,CD8+ T cell_3-AD.vs.control; scRNA-seq,SRP215507,CD8+ T cell_5-AD.vs.control; scRNA-seq,SRP215507,CD8+ T cell_3-AD.vs.MCI; scRNA-seq,SRP215507,CD8+ T cell_5-AD.vs.MCI; scRNA-seq,SRP330776,CD8+ T cell_1-AD.vs.control; scRNA-seq,SRP330776,Naive CD8+ T cell_1-AD.vs.control; scRNA-seq,SRP330776,Natural killer cell_1-AD.vs.control; scRNA-seq,SRP330776,Memory T cell_1-AD.vs.control; scRNA-seq,SRP330776,Natural killer T cell-AD.vs.control; scRNA-seq,SRP330776,B cell_2-AD.vs.control; scRNA-seq,SRP330776,Monocyte_1-AD.vs.control	13
CHRNB2	bulk RNA-seq,Homo_723,AD.vs.control; bulk RNA-seq,Homo_723,AD.vs.MCI; bulk RNA-seq,Homo_633,AD.vs.control; bulk RNA-seq,Homo_633,AD.vs.MCI; bulk RNA-seq,Homo_714,AD.vs.control; bulk RNA-seq,Homo_714,AD.vs.MCI	6
CHRNB3	bulk RNA-seq,Homo_723,AD.vs.control; bulk RNA-seq,Homo_723,AD.vs.MCI; bulk RNA-seq,Homo_633,AD.vs.control; bulk RNA-	4
CHRNB4	bulk RNA-seq,Homo_723,AD.vs.control; bulk RNA-seq,Homo_723,AD.vs.MCI; bulk RNA-seq,Homo_714,MCI.vs.control	3
CHRND	bulk RNA-seq,Homo_723,AD.vs.control; bulk RNA-seq,Homo_723,MCI.vs.control; bulk RNA-seq,Homo_633,MCI.vs.control; bulk RNA-	4
CHRNE	scRNA-seq,SRP215507,CD8+ T cell_2-AD.vs.control; scRNA-seq,SRP215507,CD8+ T cell_5-AD.vs.control; scRNA-seq,SRP215507,CD8+ T cell_1-AD.vs.MCI; scRNA-seq,SRP215507,CD8+ T cell_4-AD.vs.MCI; scRNA-seq,SRP215507,CD8+ T cell_1-MCI.vs.control; scRNA-seq,SRP330776,CD8+ T cell_1-AD.vs.control; scRNA-seq,SRP330776,CD8+ T cell_2-AD.vs.control; scRNA-seq,SRP330776,Memory T cell_1-AD.vs.control; scRNA-	9
CHRNG	bulk RNA-seq,Homo_723,AD.vs.control; bulk RNA-seq,Homo_723,AD.vs.MCI; bulk RNA-seq,Homo_633,AD.vs.control; bulk RNA-	4
CHROMR	scRNA-seq,SRP215507,CD8+ T cell_3-MCI.vs.control; scRNA-seq,SRP309935,CD8+ T cell_1-AD.vs.control; scRNA-seq,SRP330776,Memory T cell_2-AD.vs.control; scRNA-seq,SRP330776,B cell_1-AD.vs.control; scRNA-seq,SRP330776,Memory T cell_1-AD.vs.control	5
CHST1	bulk RNA-seq,Homo_723,AD.vs.control; bulk RNA-seq,Homo_633,AD.vs.control; bulk RNA-seq,Homo_714,MCI.vs.control	3
CHST10	bulk RNA-seq,Homo_723,AD.vs.control; bulk RNA-seq,Homo_723,AD.vs.MCI; bulk RNA-seq,Homo_633,AD.vs.control; bulk RNA-seq,Homo_633,AD.vs.MCI; bulk RNA-seq,Homo_714,AD.vs.control; bulk RNA-seq,Homo_714,AD.vs.MCI; scRNA-seq,SRP215507,CD8+ T cell_3-AD.vs.MCI; scRNA-seq,SRP215507,CD8+ T cell_4-AD.vs.MCI; scRNA-seq,SRP215507,CD8+ T cell_4-MCI.vs.control; scRNA-seq,SRP215507,CD8+ T cell_2-MCI.vs.control; scRNA-seq,SRP309935,Natural killer cell_2-AD.vs.control; scRNA-seq,SRP330776,γδ T cell-AD.vs.control; scRNA-seq,SRP330776,CD8+ T cell_2-AD.vs.control; scRNA-seq,SRP330776,B cell_1-AD.vs.control; scRNA-seq,SRP330776,Natural killer cell_1-	17
Chst10	bulk RNA-seq,SRP325058,AD.vs.control	1

CHST11	scRNA-seq,SRP215507,CD8+ T cell_6-AD.vs.control; scRNA-seq,SRP215507,CD8+ T cell_6-MCI.vs.control; scRNA-seq,SRP309935,Naive CD8+ T cell_1-AD.vs.control; scRNA-seq,SRP309935,Natural killer cell_1-AD.vs.control; scRNA-seq,SRP309935,Monocyte_2-AD.vs.control; scRNA-seq,SRP309935,CD8+ T cell_1-AD.vs.control; scRNA-seq,SRP330776,CD8+ T cell_1-AD.vs.control; scRNA-seq,SRP330776,γδ T cell-AD.vs.control; scRNA-seq,SRP330776,Memory T cell_2-AD.vs.control; scRNA-seq,SRP330776,CD8+ T cell_2-AD.vs.control; scRNA-seq,SRP330776,B cell_1-AD.vs.control; scRNA-seq,SRP330776,Naive CD8+ T cell_1-AD.vs.control; scRNA-seq,SRP330776,Natural killer cell_1-AD.vs.control; scRNA-seq,SRP330776,Memory T cell_1-AD.vs.control; scRNA-seq,SRP330776,Natural killer T cell-AD.vs.control; scRNA-seq,SRP330776,Naive CD8+ T cell_3-AD.vs.control; scRNA-seq,SRP330776,B cell_2-AD.vs.control; scRNA-seq,SRP330776,T cell-AD.vs.control; scRNA-seq,SRP330776,Naive CD8+ T cell_2-AD.vs.control; scRNA-seq,SRP330776,CD8+ T cell_3-AD.vs.control; scRNA-seq,SRP330776,Monocyte_1-AD.vs.control; scRNA-[illegible]	24
CHST12	scRNA-seq,SRP215507,CD8+ T cell_3-AD.vs.control; scRNA-seq,SRP215507,CD8+ T cell_5-AD.vs.MCI; scRNA-seq,SRP309935,Erythroid cell-AD.vs.control; scRNA-seq,SRP330776,CD8+ T cell_1-AD.vs.control; scRNA-seq,SRP330776,γδ T cell-AD.vs.control; scRNA-seq,SRP330776,Memory T cell_2-AD.vs.control; scRNA-seq,SRP330776,CD8+ T cell_2-AD.vs.control; scRNA-seq,SRP330776,B cell_1-AD.vs.control; scRNA-seq,SRP330776,Naive CD8+ T cell_1-AD.vs.control; scRNA-seq,SRP330776,Natural killer cell_1-AD.vs.control; scRNA-seq,SRP330776,Natural killer T cell-AD.vs.control; scRNA-seq,SRP330776,Naive CD8+ T cell_3-AD.vs.control; scRNA-seq,SRP330776,B cell_2-AD.vs.control; scRNA-[illegible]	14
CHST13	bulk RNA-seq,Homo_723,AD.vs.MCI; bulk RNA-seq,Homo_633,AD.vs.MCI	2
CHST14	scRNA-seq,SRP215507,CD8+ T cell_3-AD.vs.control; scRNA-seq,SRP215507,CD8+ T cell_6-AD.vs.control; scRNA-seq,SRP215507,CD8+ T cell_2-AD.vs.MCI; scRNA-seq,SRP215507,CD8+ T cell_4-MCI.vs.control; scRNA-seq,SRP309935,B cell_1-AD.vs.control; scRNA-seq,SRP309935,B cell_2-AD.vs.control; scRNA-seq,SRP330776,CD8+ T cell_1-AD.vs.control; scRNA-seq,SRP330776,γδ T cell-AD.vs.control; scRNA-seq,SRP330776,Memory T cell_2-AD.vs.control; scRNA-seq,SRP330776,CD8+ T cell_2-AD.vs.control; scRNA-seq,SRP330776,B cell_1-AD.vs.control; scRNA-seq,SRP330776,Naive CD8+ T cell_1-AD.vs.control; scRNA-seq,SRP330776,Natural killer cell_1-AD.vs.control; scRNA-seq,SRP330776,Memory T cell_1-AD.vs.control; scRNA-seq,SRP330776,Natural killer T cell-AD.vs.control; scRNA-seq,SRP330776,B cell_2-AD.vs.control; scRNA-[illegible]	17
CHST15	bulk RNA-seq,Homo_633,AD.vs.control; scRNA-seq,SRP330776,B cell_3-AD.vs.control	2
CHST2	bulk RNA-seq,Homo_714,AD.vs.control; bulk RNA-seq,Homo_714,AD.vs.MCI; scRNA-seq,SRP215507,CD8+ T cell_1-AD.vs.control; scRNA-seq,SRP215507,CD8+ T cell_1-AD.vs.MCI; scRNA-seq,SRP309935,Natural killer cell_2-AD.vs.control; scRNA-seq,SRP309935,Monocyte_1-AD.vs.control; scRNA-seq,SRP309935,Megakaryocyte_1-AD.vs.control; scRNA-seq,SRP330776,CD8+ T cell_1-AD.vs.control; scRNA-seq,SRP330776,γδ T cell-AD.vs.control; scRNA-seq,SRP330776,B cell_1-AD.vs.control; scRNA-seq,SRP330776,Naive CD8+ T cell_1-AD.vs.control; scRNA-seq,SRP330776,Memory T cell_1-AD.vs.control; scRNA-seq,SRP330776,B cell_2-AD.vs.control; scRNA-seq,SRP330776,Monocyte_1-AD.vs.control;	15
CHST3	bulk RNA-seq,Homo_723,AD.vs.control; bulk RNA-seq,Homo_723,AD.vs.MCI; bulk RNA-seq,Homo_723,MCI.vs.control; bulk RNA-seq,Homo_633,AD.vs.control; bulk RNA-seq,Homo_633,AD.vs.MCI; bulk RNA-seq,Homo_714,MCI.vs.control	6
Chst3	bulk RNA-seq,SRP325058,AD.vs.control	1
CHST5	bulk RNA-seq,Homo_723,AD.vs.control; bulk RNA-seq,Homo_723,AD.vs.MCI; bulk RNA-seq,Homo_714,MCI.vs.control	3
CHST6	bulk RNA-seq,Homo_723,AD.vs.control; bulk RNA-seq,Homo_723,AD.vs.MCI; bulk RNA-seq,Homo_633,AD.vs.control; bulk RNA-seq,Homo_633,AD.vs.MCI; bulk RNA-seq,Homo_714,MCI.vs.control	5
CHST7	scRNA-seq,SRP215507,CD8+ T cell_2-AD.vs.control; scRNA-seq,SRP215507,CD8+ T cell_3-AD.vs.control; scRNA-seq,SRP215507,CD8+ T cell_5-AD.vs.control; scRNA-seq,SRP215507,CD8+ T cell_2-AD.vs.MCI; scRNA-seq,SRP215507,CD8+ T cell_5-MCI.vs.control; scRNA-seq,SRP215507,CD8+ T cell_3-MCI.vs.control; scRNA-seq,SRP330776,CD8+ T cell_1-AD.vs.control; scRNA-seq,SRP330776,γδ T cell-AD.vs.control; scRNA-seq,SRP330776,Memory T cell_2-AD.vs.control; scRNA-seq,SRP330776,CD8+ T cell_2-AD.vs.control; scRNA-seq,SRP330776,Naive CD8+ T cell_1-AD.vs.control; scRNA-seq,SRP330776,Natural killer T cell-AD.vs.control; scRNA-seq,SRP330776,T cell-AD.vs.control; scRNA-seq,SRP330776,CD8+ T cell_3-AD.vs.control; scRNA-seq,SRP330776,Monocyte_1-AD.vs.control; scRNA-seq,SRP330776,Regulatory T cell-AD.vs.control; scRNA-[illegible]	17
CHST8	bulk RNA-seq,Homo_723,AD.vs.control; bulk RNA-seq,Homo_723,AD.vs.MCI; bulk RNA-seq,Homo_633,AD.vs.control; bulk RNA-seq,Homo_633,AD.vs.MCI; bulk RNA-seq,Homo_714,AD.vs.MCI; bulk RNA-seq,Homo_714,MCI.vs.control	6
CHST9	bulk RNA-seq,Homo_723,AD.vs.control; bulk RNA-seq,Homo_723,AD.vs.MCI; bulk RNA-seq,Homo_633,AD.vs.control; bulk RNA-seq,Homo_633,MCI.vs.control; bulk RNA-seq,Homo_714,MCI.vs.control	5

CHSY1	scRNA-seq,SRP215507,CD8+ T cell_3-AD.vs.control; scRNA-seq,SRP215507,CD8+ T cell_5-AD.vs.control; scRNA-seq,SRP215507,CD8+ T cell_2-AD.vs.MCI; scRNA-seq,SRP215507,CD8+ T cell_3-AD.vs.MCI; scRNA-seq,SRP215507,CD8+ T cell_5-AD.vs.MCI; scRNA-seq,SRP215507,CD8+ T cell_2-MCI.vs.control; scRNA-seq,SRP330776,CD8+ T cell_1-AD.vs.control; scRNA-seq,SRP330776,Memory T cell_2-AD.vs.control; scRNA-seq,SRP330776,CD8+ T cell_2-AD.vs.control; scRNA-seq,SRP330776,Naive CD8+ T cell_1-AD.vs.control; scRNA-seq,SRP330776,Memory T cell_1-AD.vs.control; scRNA-seq,SRP330776,Natural killer T cell-AD.vs.control; scRNA-seq,SRP330776,Naive CD8+ T cell_3-AD.vs.control; scRNA-	15
CHTF18	bulk RNA-seq,Homo_723,MCI.vs.control; bulk RNA-seq,Homo_633,AD.vs.control; bulk RNA-seq,Homo_633,AD.vs.MCI; bulk RNA-seq,Homo_633,MCI.vs.control; bulk RNA-seq,Homo_714,AD.vs.control; bulk RNA-seq,Homo_714,AD.vs.MCI; bulk RNA-seq,Homo_714,MCI.vs.control; scRNA-seq,SRP215507,CD8+ T cell_4-AD.vs.MCI; scRNA-seq,SRP215507,CD8+ T cell_5-AD.vs.MCI; scRNA-seq,SRP215507,CD8+ T cell_5-MCI.vs.control; scRNA-seq,SRP215507,CD8+ T cell_3-MCI.vs.control; scRNA-seq,SRP330776,Memory T cell_2-AD.vs.control; scRNA-seq,SRP330776,B cell_1-AD.vs.control; scRNA-seq,SRP330776,Naive CD8+ T cell_1-AD.vs.control; scRNA-	16
CHTF8	scRNA-seq,SRP215507,CD8+ T cell_4-AD.vs.control; scRNA-seq,SRP215507,CD8+ T cell_4-AD.vs.MCI; scRNA-seq,SRP215507,CD8+ T cell_3-MCI.vs.control; scRNA-seq,SRP330776,CD8+ T cell_1-AD.vs.control; scRNA-seq,SRP330776,γδ T cell-AD.vs.control; scRNA-seq,SRP330776,Memory T cell_2-AD.vs.control; scRNA-seq,SRP330776,CD8+ T cell_2-AD.vs.control; scRNA-seq,SRP330776,B cell_1-AD.vs.control; scRNA-seq,SRP330776,Naive CD8+ T cell_1-AD.vs.control; scRNA-seq,SRP330776,Natural killer cell_1-AD.vs.control; scRNA-seq,SRP330776,Memory T cell_1-AD.vs.control; scRNA-seq,SRP330776,Natural killer T cell-AD.vs.control; scRNA-seq,SRP330776,Naive CD8+ T cell_3-AD.vs.control; scRNA-seq,SRP330776,B cell_2-AD.vs.control; scRNA-seq,SRP330776,T cell-AD.vs.control; scRNA-seq,SRP330776,Naive CD8+ T cell_2-AD.vs.control; scRNA-seq,SRP330776,CD8+ T cell_3-AD.vs.control; scRNA-seq,SRP330776,B cell_3-AD.vs.control; scRNA-seq,SRP330776,Natural killer cell_2-[illegible]	19
CHTOP	bulk RNA-seq,Homo_723,AD.vs.control; bulk RNA-seq,Homo_723,AD.vs.MCI; bulk RNA-seq,Homo_633,AD.vs.control; bulk RNA-seq,Homo_633,AD.vs.MCI; bulk RNA-seq,Homo_714,AD.vs.control; bulk RNA-seq,Homo_714,AD.vs.MCI; scRNA-seq,SRP215507,CD8+ T cell_10-AD.vs.control; scRNA-seq,SRP330776,γδ T cell-AD.vs.control; scRNA-seq,SRP330776,Monocyte_2-AD.vs.control; scRNA-seq,SRP330776,Natural killer	10
CHUK	bulk RNA-seq,Homo_723,AD.vs.control; bulk RNA-seq,Homo_633,AD.vs.control; bulk RNA-seq,Homo_714,AD.vs.control; bulk RNA-seq,Homo_714,AD.vs.MCI; scRNA-seq,SRP215507,CD8+ T cell_5-AD.vs.control; scRNA-seq,SRP215507,CD8+ T cell_6-AD.vs.control; scRNA-seq,SRP215507,CD8+ T cell_6-AD.vs.MCI; scRNA-seq,SRP215507,CD8+ T cell_5-MCI.vs.control; scRNA-seq,SRP309935,Natural killer cell_1-AD.vs.control; scRNA-seq,SRP330776,CD8+ T cell_1-AD.vs.control; scRNA-seq,SRP330776,γδ T cell-AD.vs.control; scRNA-seq,SRP330776,Memory T cell_2-AD.vs.control; scRNA-seq,SRP330776,CD8+ T cell_2-AD.vs.control; scRNA-seq,SRP330776,B cell_1-AD.vs.control; scRNA-seq,SRP330776,Naive CD8+ T cell_1-AD.vs.control; scRNA-seq,SRP330776,Natural killer cell_1-AD.vs.control; scRNA-seq,SRP330776,Memory T cell_1-AD.vs.control; scRNA-seq,SRP330776,Natural killer T cell-AD.vs.control; scRNA-seq,SRP330776,Naive CD8+ T cell_3-AD.vs.control; scRNA-seq,SRP330776,B cell_2-AD.vs.control; scRNA-seq,SRP330776,T cell-AD.vs.control; scRNA-seq,SRP330776,CD8+ T cell_3-AD.vs.control; scRNA-[illegible]	24
CHURC1	bulk RNA-seq,Homo_714,AD.vs.control; bulk RNA-seq,Homo_714,AD.vs.MCI; scRNA-seq,SRP215507,CD8+ T cell_8-AD.vs.control; scRNA-seq,SRP330776,Memory T cell_2-AD.vs.control; scRNA-seq,SRP330776,CD8+ T cell_2-AD.vs.control; scRNA-seq,SRP330776,Natural killer cell_1-AD.vs.control; scRNA-seq,SRP330776,Memory T cell_1-AD.vs.control; scRNA-seq,SRP330776,CD8+ T cell_3-AD.vs.control; scRNA-seq,SRP330776,Monocyte_2-AD.vs.control; scRNA-seq,SRP330776,B cell_3-AD.vs.control; scRNA-seq,SRP330776,Natural killer cell_2-AD.vs.control	11
Churc1	bulk RNA-seq,SRP325058,AD.vs.control	1
CHURC1-FNTB	bulk RNA-seq,Homo_723,AD.vs.MCI; bulk RNA-seq,Homo_723,MCI.vs.control; bulk RNA-seq,Homo_633,AD.vs.MCI; bulk RNA-	4
CIAO1	scRNA-seq,SRP330776,CD8+ T cell_3-AD.vs.control; scRNA-seq,SRP330776,Monocyte_2-AD.vs.control; scRNA-seq,SRP330776,Natural killer cell_2-	3
CIAO2A	bulk RNA-seq,Homo_633,AD.vs.control; bulk RNA-seq,Homo_633,AD.vs.MCI; bulk RNA-seq,Homo_714,AD.vs.control; bulk RNA-seq,Homo_714,AD.vs.MCI; scRNA-seq,SRP215507,CD8+ T cell_7-AD.vs.control; scRNA-seq,SRP309935,CD8+ T cell_2-AD.vs.control; scRNA-seq,SRP330776,CD8+ T cell_2-AD.vs.control; scRNA-seq,SRP330776,Natural killer cell_1-AD.vs.control; scRNA-seq,SRP330776,T cell-AD.vs.control	9
CIAO2B	scRNA-seq,SRP309935,Erythroid cell-AD.vs.control; scRNA-seq,SRP330776,Memory T cell_2-AD.vs.control; scRNA-seq,SRP330776,Memory T cell_1-AD.vs.control; scRNA-seq,SRP330776,Natural killer cell_2-AD.vs.control	4

CIAO3	bulk RNA-seq,Homo_723,AD.vs.control; bulk RNA-seq,Homo_723,AD.vs.MCI; bulk RNA-seq,Homo_633,AD.vs.control; bulk RNA-seq,Homo_633,AD.vs.MCI; bulk RNA-seq,Homo_633,MCI.vs.control; scRNA-seq,SRP215507,CD8+ T cell_4-AD.vs.control; scRNA-seq,SRP215507,CD8+ T cell_2-AD.vs.MCI; scRNA-seq,SRP215507,CD8+ T cell_3-AD.vs.MCI; scRNA-seq,SRP215507,CD8+ T cell_1-AD.vs.MCI; scRNA-seq,SRP215507,CD8+ T cell_4-AD.vs.MCI; scRNA-seq,SRP215507,CD8+ T cell_5-MCI.vs.control; scRNA-seq,SRP215507,CD8+ T cell_3-MCI.vs.control; scRNA-seq,SRP309935,Naive CD8+ T cell_2-AD.vs.control; scRNA-seq,SRP309935,Plasmacytoid dendritic cell_1-AD.vs.control; scRNA-seq,SRP330776,CD8+ T cell_1-AD.vs.control; scRNA-seq,SRP330776,γδ T cell-AD.vs.control; scRNA-seq,SRP330776,Memory T cell_2-AD.vs.control; scRNA-seq,SRP330776,CD8+ T cell_2-AD.vs.control; scRNA-seq,SRP330776,B cell_1-AD.vs.control; scRNA-seq,SRP330776,Natural killer cell_1-AD.vs.control; scRNA-seq,SRP330776,Memory T cell_1-AD.vs.control; scRNA-seq,SRP330776,T cell-AD.vs.control; scRNA-[illegible]	25
CIAPIN1	bulk RNA-seq,Homo_723,AD.vs.control; bulk RNA-seq,Homo_723,AD.vs.MCI; bulk RNA-seq,Homo_633,AD.vs.control; bulk RNA-seq,Homo_633,AD.vs.MCI; bulk RNA-seq,Homo_714,AD.vs.control; bulk RNA-seq,Homo_714,AD.vs.MCI; scRNA-seq,SRP215507,CD8+ T cell_5-AD.vs.MCI; scRNA-seq,SRP215507,CD8+ T cell_5-MCI.vs.control; scRNA-seq,SRP309935,B cell_1-AD.vs.control; scRNA-seq,SRP330776,CD8+ T cell_2-AD.vs.control; scRNA-seq,SRP330776,Natural killer cell_1-AD.vs.control; scRNA-seq,SRP330776,Memory T cell_1-AD.vs.control	12
CIART	bulk RNA-seq,Homo_723,AD.vs.control; bulk RNA-seq,Homo_723,AD.vs.MCI; bulk RNA-seq,Homo_723,MCI.vs.control; bulk RNA-seq,Homo_633,AD.vs.control; bulk RNA-seq,Homo_633,AD.vs.MCI; bulk RNA-seq,Homo_633,MCI.vs.control; bulk RNA-seq,Homo_714,AD.vs.control; bulk RNA-seq,Homo_714,AD.vs.MCI; scRNA-seq,SRP215507,CD8+ T cell_2-MCI.vs.control	9
CIB1	bulk RNA-seq,Homo_723,AD.vs.control; bulk RNA-seq,Homo_633,AD.vs.control; bulk RNA-seq,Homo_633,AD.vs.MCI; bulk RNA-seq,Homo_633,MCI.vs.control; scRNA-seq,SRP215507,CD8+ T cell_7-AD.vs.control; scRNA-seq,SRP309935,Erythroid cell-AD.vs.control	6
CIB2	bulk RNA-seq,Homo_723,AD.vs.control; bulk RNA-seq,Homo_723,AD.vs.MCI; bulk RNA-seq,Homo_633,AD.vs.control; bulk RNA-seq,Homo_633,AD.vs.MCI; bulk RNA-seq,Homo_714,AD.vs.MCI; bulk RNA-seq,Homo_714,MCI.vs.control; scRNA-seq,SRP215507,CD8+ T cell_3-AD.vs.MCI; scRNA-seq,SRP215507,CD8+ T cell_2-MCI.vs.control; scRNA-seq,SRP215507,CD8+ T cell_3-MCI.vs.control; scRNA-seq,SRP330776,CD8+ T cell_1-AD.vs.control; scRNA-seq,SRP330776,Naive CD8+ T cell_1-AD.vs.control	11
CIB3	bulk RNA-seq,Homo_723,AD.vs.control; bulk RNA-seq,Homo_723,AD.vs.MCI	2
Cib3	bulk RNA-seq,SRP325058,AD.vs.control	1
CIBAR1	bulk RNA-seq,Homo_723,AD.vs.control; bulk RNA-seq,Homo_723,AD.vs.MCI; bulk RNA-seq,Homo_723,MCI.vs.control; bulk RNA-seq,Homo_633,AD.vs.control; bulk RNA-seq,Homo_633,AD.vs.MCI; bulk RNA-seq,Homo_633,MCI.vs.control; bulk RNA-seq,Homo_714,AD.vs.control;	8
CIBAR2	bulk RNA-seq,Homo_723,AD.vs.control; bulk RNA-seq,Homo_723,AD.vs.MCI; bulk RNA-seq,Homo_633,AD.vs.control	3
CIC	bulk RNA-seq,Homo_723,AD.vs.control; bulk RNA-seq,Homo_723,MCI.vs.control; bulk RNA-seq,Homo_633,AD.vs.control; bulk RNA-seq,Homo_633,MCI.vs.control; scRNA-seq,SRP215507,CD8+ T cell_3-AD.vs.MCI; scRNA-seq,SRP215507,CD8+ T cell_5-AD.vs.MCI; scRNA-seq,SRP215507,CD8+ T cell_3-MCI.vs.control; scRNA-seq,SRP309935,Monocyte_2-AD.vs.control; scRNA-seq,SRP309935,CD8+ T cell_1-AD.vs.control; scRNA-seq,SRP330776,γδ T cell-AD.vs.control; scRNA-seq,SRP330776,Memory T cell_2-AD.vs.control; scRNA-	12
CIDEB	scRNA-seq,SRP309935,Naive CD8+ T cell_1-AD.vs.control; scRNA-seq,SRP309935,Natural killer cell_1-AD.vs.control; scRNA-seq,SRP309935,Naive CD8+ T cell_2-AD.vs.control; scRNA-seq,SRP309935,CD8+ T cell_1-AD.vs.control; scRNA-seq,SRP309935,CD8+ T cell_2-AD.vs.control; scRNA-seq,SRP330776,CD8+ T cell_1-AD.vs.control; scRNA-seq,SRP330776,γδ T cell-AD.vs.control; scRNA-seq,SRP330776,Memory T cell_2-AD.vs.control; scRNA-seq,SRP330776,CD8+ T cell_2-AD.vs.control; scRNA-seq,SRP330776,B cell_1-AD.vs.control; scRNA-seq,SRP330776,Naive CD8+ T cell_1-AD.vs.control; scRNA-seq,SRP330776,Natural killer cell_1-AD.vs.control; scRNA-seq,SRP330776,Memory T cell_1-AD.vs.control; scRNA-seq,SRP330776,Natural killer T cell-AD.vs.control; scRNA-seq,SRP330776,Naive CD8+ T cell_3-AD.vs.control; scRNA-seq,SRP330776,B cell_2-AD.vs.control; scRNA-seq,SRP330776,T cell-AD.vs.control; scRNA-seq,SRP330776,CD8+ T cell_3-AD.vs.control; scRNA-seq,SRP330776,Monocyte_2-[illegible]	21
CIDEC	bulk RNA-seq,Homo_723,AD.vs.control; bulk RNA-seq,Homo_723,AD.vs.MCI; bulk RNA-seq,Homo_633,AD.vs.control; bulk RNA-	4
CIITA	scRNA-seq,SRP215507,CD8+ T cell_8-AD.vs.control; scRNA-seq,SRP215507,CD8+ T cell_1-AD.vs.control; scRNA-seq,SRP215507,CD8+ T cell_4-AD.vs.control; scRNA-seq,SRP215507,CD8+ T cell_7-AD.vs.control; scRNA-seq,SRP215507,CD8+ T cell_4-AD.vs.MCI; scRNA-seq,SRP330776,γδ T cell-AD.vs.control; scRNA-seq,SRP330776,CD8+ T cell_2-AD.vs.control; scRNA-seq,SRP330776,Naive CD8+ T cell_1-AD.vs.control; scRNA-seq,SRP330776,Natural killer cell_1-AD.vs.control; scRNA-seq,SRP330776,Naive CD8+ T cell_2-AD.vs.control; scRNA-seq,SRP330776,CD8+ T cell_3-	12

CILP	bulk RNA-seq,Homo_723,AD.vs.control; bulk RNA-seq,Homo_723,AD.vs.MCI; bulk RNA-seq,Homo_723,MCI.vs.control; bulk RNA-seq,Homo_633,AD.vs.control; bulk RNA-seq,Homo_633,AD.vs.MCI; bulk RNA-seq,Homo_633,MCI.vs.control; bulk RNA-seq,Homo_714,AD.vs.control; bulk RNA-seq,Homo_714,AD.vs.MCI; bulk RNA-seq,Homo_714,MCI.vs.control	9
CILP2	bulk RNA-seq,Homo_723,AD.vs.control; bulk RNA-seq,Homo_723,AD.vs.MCI; bulk RNA-seq,Homo_633,AD.vs.control; bulk RNA-seq,Homo_714,MCI.vs.control; scRNA-seq,SRP215507,CD8+ T cell_3-MCI.vs.control	5
CINP	bulk RNA-seq,Homo_633,AD.vs.MCI; bulk RNA-seq,Homo_714,AD.vs.control; bulk RNA-seq,Homo_714,AD.vs.MCI; scRNA-seq,SRP330776,CD8+ T cell_1-AD.vs.control; scRNA-seq,SRP330776,Naive CD8+ T cell_1-AD.vs.control; scRNA-seq,SRP330776,Natural killer cell_1-AD.vs.control; scRNA-seq,SRP330776,Natural killer T cell-AD.vs.control; scRNA-seq,SRP330776,Naive CD8+ T cell_3-AD.vs.control; scRNA-seq,SRP330776,T cell-AD.vs.control; scRNA-seq,SRP330776,CD8+ T cell_3-AD.vs.control; scRNA-seq,SRP330776,B cell_3-AD.vs.control	11
CIP2A	bulk RNA-seq,Homo_723,AD.vs.control; bulk RNA-seq,Homo_723,AD.vs.MCI; bulk RNA-seq,Homo_633,AD.vs.control; bulk RNA-seq,Homo_633,AD.vs.MCI; bulk RNA-seq,Homo_714,AD.vs.control; bulk RNA-seq,Homo_714,AD.vs.MCI; scRNA-seq,SRP215507,CD8+ T cell_1-AD.vs.MCI; scRNA-seq,SRP215507,CD8+ T cell_1-MCI.vs.control; scRNA-seq,SRP215507,CD8+ T cell_3-MCI.vs.control; scRNA-seq,SRP330776,γδ T cell-AD.vs.control; scRNA-seq,SRP330776,CD8+ T cell_2-AD.vs.control; scRNA-seq,SRP330776,Natural killer cell_1-AD.vs.control; scRNA-seq,SRP330776,Memory T cell_1-AD.vs.control; scRNA-seq,SRP330776,B cell_2-AD.vs.control; scRNA-seq,SRP330776,CD8+ T cell_3-AD.vs.control	15
CIPC	scRNA-seq,SRP215507,CD8+ T cell_4-MCI.vs.control; scRNA-seq,SRP215507,CD8+ T cell_3-MCI.vs.control; scRNA-seq,SRP330776,CD8+ T cell_2-AD.vs.control; scRNA-seq,SRP330776,Natural killer cell_1-AD.vs.control; scRNA-seq,SRP330776,Natural killer T cell-AD.vs.control; scRNA-seq,SRP330776,Naive CD8+ T cell_3-AD.vs.control; scRNA-seq,SRP330776,CD8+ T cell_3-AD.vs.control	7
CIR1	bulk RNA-seq,Homo_714,AD.vs.control; bulk RNA-seq,Homo_714,AD.vs.MCI; scRNA-seq,SRP215507,CD8+ T cell_4-AD.vs.MCI; scRNA-seq,SRP215507,CD8+ T cell_3-MCI.vs.control; scRNA-seq,SRP330776,Monocyte_1-AD.vs.control	5
CIRBP	scRNA-seq,SRP309935,Erythroid cell-AD.vs.control; scRNA-seq,SRP330776,B cell_4-AD.vs.control	2
CISD1	bulk RNA-seq,Homo_633,AD.vs.control; bulk RNA-seq,Homo_633,AD.vs.MCI; bulk RNA-seq,Homo_714,AD.vs.control; bulk RNA-seq,Homo_714,AD.vs.MCI; scRNA-seq,SRP215507,CD8+ T cell_5-AD.vs.MCI; scRNA-seq,SRP215507,CD8+ T cell_5-MCI.vs.control; scRNA-seq,SRP215507,CD8+ T cell_6-MCI.vs.control; scRNA-seq,SRP330776,CD8+ T cell_1-AD.vs.control; scRNA-seq,SRP330776,Naive CD8+ T cell_1-AD.vs.control; scRNA-seq,SRP330776,Natural killer T cell-AD.vs.control; scRNA-seq,SRP330776,B cell_2-AD.vs.control; scRNA-seq,SRP330776,CD8+ [illegible]	13
CISD2	bulk RNA-seq,Homo_723,AD.vs.control; bulk RNA-seq,Homo_723,AD.vs.MCI; bulk RNA-seq,Homo_633,AD.vs.control; bulk RNA-seq,Homo_633,AD.vs.MCI; scRNA-seq,SRP309935,Erythroid cell-AD.vs.control; scRNA-seq,SRP309935,Megakaryocyte_2-AD.vs.control; scRNA-seq,SRP330776,CD8+ T cell_1-AD.vs.control; scRNA-seq,SRP330776,Naive CD8+ T cell_1-AD.vs.control; scRNA-seq,SRP330776,Monocyte_1- [illegible]	10
CISD3	bulk RNA-seq,Homo_723,AD.vs.control; bulk RNA-seq,Homo_723,AD.vs.MCI; bulk RNA-seq,Homo_633,AD.vs.control; bulk RNA-seq,Homo_633,AD.vs.MCI; bulk RNA-seq,Homo_633,MCI.vs.control; scRNA-seq,SRP330776,CD8+ T cell_1-AD.vs.control; scRNA-seq,SRP330776,γδ T cell-AD.vs.control; scRNA-seq,SRP330776,Memory T cell_2-AD.vs.control; scRNA-seq,SRP330776,CD8+ T cell_2-AD.vs.control; scRNA-seq,SRP330776,B cell_1-AD.vs.control; scRNA-seq,SRP330776,Naive CD8+ T cell_1-AD.vs.control; scRNA-seq,SRP330776,Natural killer cell_1-AD.vs.control; scRNA-seq,SRP330776,Memory T cell_1-AD.vs.control; scRNA-seq,SRP330776,Natural killer T cell-AD.vs.control; scRNA-seq,SRP330776,Naive CD8+ T cell_3-AD.vs.control; scRNA-seq,SRP330776,B cell_2-AD.vs.control; scRNA-seq,SRP330776,T cell-AD.vs.control; scRNA-seq,SRP330776,CD8+ T cell_3-AD.vs.control; scRNA-seq,SRP330776,Monocyte_1-AD.vs.control; scRNA-seq,SRP330776,Monocyte_2- [illegible]	22
CISH	scRNA-seq,SRP215507,CD8+ T cell_2-AD.vs.control; scRNA-seq,SRP215507,CD8+ T cell_3-AD.vs.control; scRNA-seq,SRP215507,CD8+ T cell_5-AD.vs.control; scRNA-seq,SRP215507,CD8+ T cell_2-AD.vs.MCI; scRNA-seq,SRP215507,CD8+ T cell_3-AD.vs.MCI; scRNA-seq,SRP215507,CD8+ T cell_1-AD.vs.MCI; scRNA-seq,SRP215507,CD8+ T cell_4-AD.vs.MCI; scRNA-seq,SRP215507,CD8+ T cell_5-AD.vs.MCI; scRNA-seq,SRP215507,CD8+ T cell_1-MCI.vs.control; scRNA-seq,SRP215507,CD8+ T cell_4-MCI.vs.control; scRNA-seq,SRP215507,CD8+ T cell_2-MCI.vs.control; scRNA-seq,SRP215507,CD8+ T cell_3-MCI.vs.control; scRNA-seq,SRP309935,Megakaryocyte_1-AD.vs.control; scRNA-seq,SRP309935,Naive CD8+ T cell_1-AD.vs.control; scRNA-seq,SRP309935,Naive CD8+ T cell_2-AD.vs.control; scRNA-seq,SRP309935,CD8+ T cell_1-AD.vs.control; scRNA-seq,SRP309935,CD8+ T cell_2-AD.vs.control; scRNA-seq,SRP330776,CD8+ T cell_1-AD.vs.control; scRNA-seq,SRP330776,γδ T cell-AD.vs.control; scRNA-seq,SRP330776,Memory T cell_2-AD.vs.control; scRNA-seq,SRP330776,CD8+ T cell_2-AD.vs.control; scRNA-seq,SRP330776,B cell_1-AD.vs.control; scRNA-seq,SRP330776,Naive CD8+ T cell_1-AD.vs.control; scRNA-seq,SRP330776,Natural killer cell_1-AD.vs.control; scRNA-seq,SRP330776,Memory T cell_1-AD.vs.control; scRNA-seq,SRP330776,Natural killer T cell-AD.vs.control; scRNA-seq,SRP330776,Naive CD8+ T cell_3-AD.vs.control; scRNA-seq,SRP330776,B cell_2-AD.vs.control; scRNA-seq,SRP330776,CD8+ T cell_3- [illegible]	30

CIT	bulk RNA-seq,Homo_723,AD.vs.control; bulk RNA-seq,Homo_633,AD.vs.control; scRNA-seq,SRP215507,CD8+ T cell_1-AD.vs.MCI; scRNA-seq,SRP330776,T cell-AD.vs.control; scRNA-seq,SRP330776,CD8+ T cell_3-AD.vs.control	5
Cit	bulk RNA-seq,SRP325058,AD.vs.control	1
CITED1	bulk RNA-seq,Homo_723,AD.vs.control; bulk RNA-seq,Homo_723,AD.vs.MCI; bulk RNA-seq,Homo_723,MCI.vs.control; bulk RNA-	4
CITED2	scRNA-seq,SRP215507,CD8+ T cell_2-AD.vs.MCI; scRNA-seq,SRP215507,CD8+ T cell_3-AD.vs.MCI; scRNA-seq,SRP215507,CD8+ T cell_1-AD.vs.MCI; scRNA-seq,SRP215507,CD8+ T cell_5-AD.vs.MCI; scRNA-seq,SRP215507,CD8+ T cell_1-MCI.vs.control; scRNA-seq,SRP215507,CD8+ T cell_5-MCI.vs.control; scRNA-seq,SRP215507,CD8+ T cell_2-MCI.vs.control; scRNA-seq,SRP215507,CD8+ T cell_3-MCI.vs.control; scRNA-seq,SRP309935,Monocyte_1-AD.vs.control; scRNA-seq,SRP309935,Naive CD8+ T cell_1-AD.vs.control; scRNA-seq,SRP309935,CD8+ T cell_1-AD.vs.control; scRNA-seq,SRP330776,CD8+ T cell_1-AD.vs.control; scRNA-seq,SRP330776,γδ T cell-AD.vs.control; scRNA-seq,SRP330776,Memory T cell_2-AD.vs.control; scRNA-seq,SRP330776,CD8+ T cell_2-AD.vs.control; scRNA-seq,SRP330776,B cell_1-AD.vs.control; scRNA-seq,SRP330776,Naive CD8+ T cell_1-AD.vs.control; scRNA-seq,SRP330776,Natural killer cell_1-AD.vs.control; scRNA-seq,SRP330776,Memory T cell_1-AD.vs.control; scRNA-seq,SRP330776,Natural killer T cell-AD.vs.control; scRNA-seq,SRP330776,Naive CD8+ T cell_3-AD.vs.control; scRNA-seq,SRP330776,B cell_2-AD.vs.control; scRNA-seq,SRP330776,T cell-AD.vs.control; scRNA-seq,SRP330776,Naive CD8+ T cell_2-AD.vs.control; scRNA-seq,SRP330776,CD8+ T cell_3-AD.vs.control; scRNA-seq,SRP330776,Monocyte_1-AD.vs.control; scRNA-seq,SRP330776,Monocyte_2-AD.vs.control; scRNA-seq,SRP330776,B cell_3-AD.vs.control; scRNA-seq,SRP330776,Regulatory T cell-AD.vs.control; scRNA-seq,SRP330776,Natural killer cell_2-AD.vs.control; scRNA-seq,SRP330776,B cell_4-AD.vs.control	31
CITED4	scRNA-seq,SRP215507,CD8+ T cell_1-AD.vs.control; scRNA-seq,SRP215507,CD8+ T cell_4-AD.vs.control; scRNA-seq,SRP215507,CD8+ T cell_3-AD.vs.MCI; scRNA-seq,SRP215507,CD8+ T cell_3-MCI.vs.control; scRNA-seq,SRP309935,Monocyte_1-AD.vs.control; scRNA-seq,SRP309935,Naive CD8+ T cell_1-AD.vs.control; scRNA-seq,SRP309935,Natural killer cell_1-AD.vs.control; scRNA-seq,SRP309935,Monocyte_2-AD.vs.control; scRNA-seq,SRP309935,Naive CD8+ T cell_2-AD.vs.control; scRNA-seq,SRP309935,CD8+ T cell_2-AD.vs.control; scRNA-seq,SRP309935,Dendritic cell-AD.vs.control; scRNA-seq,SRP330776,CD8+ T cell_1-AD.vs.control; scRNA-seq,SRP330776,γδ T cell-AD.vs.control; scRNA-seq,SRP330776,Memory T	15
CIZ1	scRNA-seq,SRP215507,CD8+ T cell_6-AD.vs.control; scRNA-seq,SRP215507,CD8+ T cell_6-AD.vs.MCI; scRNA-seq,SRP330776,CD8+ T cell_1-AD.vs.control; scRNA-seq,SRP330776,γδ T cell-AD.vs.control; scRNA-seq,SRP330776,Memory T cell_2-AD.vs.control; scRNA-seq,SRP330776,CD8+ T cell_2-AD.vs.control; scRNA-seq,SRP330776,B cell_1-AD.vs.control; scRNA-seq,SRP330776,Naive CD8+ T cell_1-AD.vs.control; scRNA-seq,SRP330776,Memory T cell_1-AD.vs.control; scRNA-seq,SRP330776,Natural killer T cell-AD.vs.control; scRNA-seq,SRP330776,T cell-AD.vs.control; scRNA-seq,SRP330776,CD8+ T cell_3-AD.vs.control; scRNA-seq,SRP330776,Monocyte_1-AD.vs.control	13
CKAP2	bulk RNA-seq,Homo_723,AD.vs.control; bulk RNA-seq,Homo_723,AD.vs.MCI; bulk RNA-seq,Homo_633,AD.vs.control; bulk RNA-seq,Homo_633,AD.vs.MCI; bulk RNA-seq,Homo_714,AD.vs.control; bulk RNA-seq,Homo_714,AD.vs.MCI; scRNA-seq,SRP215507,CD8+ T cell_3-AD.vs.MCI; scRNA-seq,SRP215507,CD8+ T cell_5-AD.vs.MCI; scRNA-seq,SRP215507,CD8+ T cell_1-MCI.vs.control; scRNA-seq,SRP215507,CD8+ T cell_5-MCI.vs.control; scRNA-seq,SRP215507,CD8+ T cell_3-MCI.vs.control; scRNA-seq,SRP309935,Megakaryocyte_1-AD.vs.control; scRNA-seq,SRP330776,Natural killer T cell-AD.vs.control; scRNA-seq,SRP330776,Naive CD8+ T cell_3-AD.vs.control; scRNA-seq,SRP330776,T cell-	16
CKAP2L	scRNA-seq,SRP215507,CD8+ T cell_2-AD.vs.MCI; scRNA-seq,SRP215507,CD8+ T cell_2-MCI.vs.control; scRNA-seq,SRP330776,T cell-AD.vs.control	3
CKAP4	bulk RNA-seq,SRP223445,AD.vs.control; scRNA-seq,SRP215507,CD8+ T cell_1-MCI.vs.control; scRNA-seq,SRP215507,CD8+ T cell_5-MCI.vs.control; scRNA-seq,SRP215507,CD8+ T cell_3-MCI.vs.control; scRNA-seq,SRP309935,Monocyte_2-AD.vs.control; scRNA-seq,SRP330776,γδ T cell-AD.vs.control; scRNA-seq,SRP330776,Memory T cell_2-AD.vs.control; scRNA-seq,SRP330776,B cell_1-AD.vs.control; scRNA-seq,SRP330776,Naive CD8+ T cell_1-AD.vs.control; scRNA-seq,SRP330776,Natural killer T cell-AD.vs.control; scRNA-seq,SRP330776,Naive CD8+ T cell_3-AD.vs.control; scRNA-seq,SRP330776,CD8+ T cell_3-AD.vs.control; scRNA-seq,SRP330776,Monocyte_1-AD.vs.control; scRNA-seq,SRP330776,Monocyte_2-AD.vs.control; scRNA-seq,SRP330776,B cell_3-AD.vs.control; scRNA-seq,SRP330776,B cell_4-AD.vs.control	16
Ckap4	bulk RNA-seq,SRP325058,AD.vs.control	1
CKAP5	scRNA-seq,SRP215507,CD8+ T cell_3-AD.vs.MCI; scRNA-seq,SRP215507,CD8+ T cell_4-AD.vs.MCI; scRNA-seq,SRP215507,CD8+ T cell_3-MCI.vs.control; scRNA-seq,SRP309935,Megakaryocyte_1-AD.vs.control; scRNA-seq,SRP309935,Monocyte_2-AD.vs.control; scRNA-seq,SRP330776,B cell_1-AD.vs.control; scRNA-seq,SRP330776,T cell-AD.vs.control; scRNA-seq,SRP330776,Monocyte_2-AD.vs.control; scRNA-seq,SRP330776,Natural	9
CKB	scRNA-seq,SRP215507,CD8+ T cell_1-MCI.vs.control; scRNA-seq,SRP330776,CD8+ T cell_2-AD.vs.control; scRNA-seq,SRP330776,Memory T cell_1-	3

CKLF	scRNA-seq,SRP215507,CD8+ T cell_9-AD.vs.control; scRNA-seq,SRP309935,Erythroid cell-AD.vs.control; scRNA-seq,SRP309935,Megakaryocyte_2-AD.vs.control; scRNA-seq,SRP309935,B cell_2-AD.vs.control; scRNA-seq,SRP330776,γδ T cell-AD.vs.control; scRNA-seq,SRP330776,Natural killer cell_1-AD.vs.control; scRNA-seq,SRP330776,Memory T cell_1-AD.vs.control; scRNA-seq,SRP330776,Naive CD8+ T cell_3-AD.vs.control; scRNA-	9
CKLF-CMTM1	bulk RNA-seq,Homo_723,AD.vs.control; bulk RNA-seq,Homo_723,AD.vs.MCI; bulk RNA-seq,Homo_633,AD.vs.control; bulk RNA-seq,Homo_633,AD.vs.MCI; bulk RNA-seq,Homo_714,AD.vs.control; bulk RNA-seq,Homo_714,AD.vs.MCI	6
CKM	bulk RNA-seq,Homo_723,AD.vs.control; bulk RNA-seq,Homo_723,AD.vs.MCI; bulk RNA-seq,Homo_633,AD.vs.MCI	3
CKMT1A	bulk RNA-seq,Homo_723,AD.vs.control; bulk RNA-seq,Homo_723,AD.vs.MCI; bulk RNA-seq,Homo_633,AD.vs.control; bulk RNA-seq,ROSMAP,AD.vs.control; bulk RNA-seq,ROSMAP,MCI.vs.control	5
CKMT1B	bulk RNA-seq,Homo_723,AD.vs.control; bulk RNA-seq,Homo_723,AD.vs.MCI; bulk RNA-seq,Homo_714,MCI.vs.control; bulk RNA-	4
CKMT2-AS1	scRNA-seq,SRP215507,CD8+ T cell_5-AD.vs.control; scRNA-seq,SRP215507,CD8+ T cell_1-AD.vs.MCI; scRNA-seq,SRP215507,CD8+ T cell_5-AD.vs.MCI; scRNA-seq,SRP215507,CD8+ T cell_1-MCI.vs.control; scRNA-seq,SRP330776,B cell_2-AD.vs.control; scRNA-seq,SRP330776,T cell-	6
CKS1B	scRNA-seq,SRP215507,CD8+ T cell_6-AD.vs.MCI; scRNA-seq,SRP330776,CD8+ T cell_1-AD.vs.control; scRNA-seq,SRP330776,Memory T cell_2-AD.vs.control; scRNA-seq,SRP330776,CD8+ T cell_2-AD.vs.control; scRNA-seq,SRP330776,Naive CD8+ T cell_3-AD.vs.control; scRNA-	6
CKS2	scRNA-seq,SRP215507,CD8+ T cell_1-AD.vs.control; scRNA-seq,SRP215507,CD8+ T cell_2-AD.vs.MCI; scRNA-seq,SRP215507,CD8+ T cell_3-AD.vs.MCI; scRNA-seq,SRP215507,CD8+ T cell_1-AD.vs.MCI; scRNA-seq,SRP215507,CD8+ T cell_5-AD.vs.MCI; scRNA-seq,SRP215507,CD8+ T cell_1-MCI.vs.control; scRNA-seq,SRP215507,CD8+ T cell_5-MCI.vs.control; scRNA-seq,SRP215507,CD8+ T cell_2-MCI.vs.control; scRNA-seq,SRP215507,CD8+ T cell_3-MCI.vs.control; scRNA-seq,SRP309935,Monocyte_1-AD.vs.control; scRNA-seq,SRP309935,B cell_1-AD.vs.control; scRNA-seq,SRP330776,CD8+ T cell_1-AD.vs.control; scRNA-seq,SRP330776,CD8+ T cell_2-AD.vs.control; scRNA-seq,SRP330776,B cell_1-AD.vs.control; scRNA-seq,SRP330776,Natural killer cell_1-AD.vs.control; scRNA-seq,SRP330776,Memory T cell_1-AD.vs.control; scRNA-seq,SRP330776,B cell_2-AD.vs.control; scRNA-seq,SRP330776,CD8+ T cell_3-AD.vs.control; scRNA-seq,SRP330776,B cell_3-AD.vs.control; scRNA- [illegible]	20
CLASP1	bulk RNA-seq,Homo_723,AD.vs.control; bulk RNA-seq,Homo_723,AD.vs.MCI; bulk RNA-seq,Homo_633,AD.vs.control; bulk RNA-seq,Homo_633,AD.vs.MCI; bulk RNA-seq,Homo_714,AD.vs.MCI; bulk RNA-seq,Homo_714,MCI.vs.control; scRNA-seq,SRP309935,Natural killer cell_2-AD.vs.control; scRNA-seq,SRP309935,Megakaryocyte_1-AD.vs.control; scRNA-seq,SRP309935,Naive CD8+ T cell_1-AD.vs.control; scRNA-seq,SRP309935,Natural killer cell_1-AD.vs.control; scRNA-seq,SRP309935,Naive CD8+ T cell_3-AD.vs.control; scRNA-seq,SRP330776,Naive CD8+ T	12
CLASP2	bulk RNA-seq,Homo_723,AD.vs.control; bulk RNA-seq,Homo_723,AD.vs.MCI; bulk RNA-seq,Homo_723,MCI.vs.control; bulk RNA-seq,Homo_633,AD.vs.control; bulk RNA-seq,Homo_633,AD.vs.MCI; bulk RNA-seq,Homo_633,MCI.vs.control; bulk RNA-seq,Homo_714,AD.vs.control; bulk RNA-seq,Homo_714,AD.vs.MCI; scRNA-seq,SRP215507,CD8+ T cell_3-AD.vs.MCI; scRNA-seq,SRP215507,CD8+ T cell_1-AD.vs.MCI; scRNA-seq,SRP215507,CD8+ T cell_3-MCI.vs.control; scRNA-seq,SRP330776,γδ T cell-AD.vs.control; scRNA-seq,SRP330776,CD8+ T cell_2-AD.vs.control; scRNA-seq,SRP330776,Naive CD8+ T cell_1-AD.vs.control; scRNA-seq,SRP330776,Natural killer cell_1-AD.vs.control; scRNA-seq,SRP330776,Memory T cell_1-AD.vs.control; scRNA-seq,SRP330776,Natural killer T cell-AD.vs.control; scRNA-seq,SRP330776,Naive CD8+ T cell_3-AD.vs.control; scRNA- [illegible]	19
CLASRP	scRNA-seq,SRP215507,CD8+ T cell_5-AD.vs.MCI; scRNA-seq,SRP330776,γδ T cell-AD.vs.control; scRNA-seq,SRP330776,Memory T cell_2-AD.vs.control; scRNA-seq,SRP330776,B cell_1-AD.vs.control; scRNA-seq,SRP330776,Natural killer cell_1-AD.vs.control; scRNA-seq,SRP330776,B cell_3-AD.vs.control; scRNA-seq,SRP330776,B cell_4-AD.vs.control	7
CLBA1	bulk RNA-seq,Homo_723,AD.vs.control; bulk RNA-seq,Homo_723,AD.vs.MCI; bulk RNA-seq,Homo_633,AD.vs.control; bulk RNA-seq,Homo_633,AD.vs.MCI; bulk RNA-seq,Homo_714,AD.vs.control; bulk RNA-seq,Homo_714,AD.vs.MCI; scRNA-seq,SRP215507,CD8+ T cell_3-AD.vs.control; scRNA-seq,SRP330776,Memory T cell_2-AD.vs.control; scRNA-seq,SRP330776,CD8+ T cell_2-AD.vs.control; scRNA-seq,SRP330776,B	10
CLC	bulk RNA-seq,Homo_723,AD.vs.control; bulk RNA-seq,Homo_723,AD.vs.MCI; bulk RNA-seq,Homo_723,MCI.vs.control	3
CLCA2	bulk RNA-seq,Homo_723,AD.vs.control; bulk RNA-seq,Homo_633,AD.vs.control	2
Clca3a1	bulk RNA-seq,SRP325058,AD.vs.control	1

CLCC1	bulk RNA-seq,Homo_723,AD.vs.control; bulk RNA-seq,Homo_723,AD.vs.MCI; bulk RNA-seq,Homo_633,AD.vs.control; bulk RNA-seq,Homo_633,AD.vs.MCI; bulk RNA-seq,Homo_633,MCI.vs.control; bulk RNA-seq,Homo_714,AD.vs.control; bulk RNA-seq,Homo_714,AD.vs.MCI; bulk RNA-seq,Homo_714,MCI.vs.control; scRNA-seq,SRP215507,CD8+ T cell_4-AD.vs.MCI; scRNA-seq,SRP215507,CD8+ T cell_4-MCI.vs.control; scRNA-seq,SRP330776,Naive CD8+ T cell_1-AD.vs.control; scRNA-seq,SRP330776,Natural killer cell_1-AD.vs.control; scRNA-seq,SRP330776,Naive CD8+ T cell_3-AD.vs.control; scRNA-seq,SRP330776,B cell_2-AD.vs.control; scRNA-seq,SRP330776,T cell-AD.vs.control	15
CLCF1	scRNA-seq,SRP215507,CD8+ T cell_3-AD.vs.control; scRNA-seq,SRP215507,CD8+ T cell_2-AD.vs.MCI; scRNA-seq,SRP215507,CD8+ T cell_5-MCI.vs.control; scRNA-seq,SRP215507,CD8+ T cell_3-MCI.vs.control; scRNA-seq,SRP309935,Naive CD8+ T cell_1-AD.vs.control; scRNA-seq,SRP309935,Monocyte_2-AD.vs.control; scRNA-seq,SRP330776,γδ T cell-AD.vs.control; scRNA-seq,SRP330776,CD8+ T cell_2-AD.vs.control; scRNA-seq,SRP330776,B cell_1-AD.vs.control; scRNA-seq,SRP330776,Memory T cell_1-AD.vs.control; scRNA-seq,SRP330776,Natural killer T cell-AD.vs.control; scRNA-seq,SRP330776,T cell-AD.vs.control; scRNA-seq,SRP330776,Monocyte_1-AD.vs.control	13
CLCN1	bulk RNA-seq,Homo_714,AD.vs.control; bulk RNA-seq,Homo_714,AD.vs.MCI	2
CLCN2	bulk RNA-seq,Homo_723,AD.vs.control; bulk RNA-seq,Homo_723,AD.vs.MCI; bulk RNA-seq,Homo_723,MCI.vs.control; bulk RNA-seq,Homo_633,AD.vs.MCI; bulk RNA-seq,Homo_633,MCI.vs.control; bulk RNA-seq,Homo_714,AD.vs.control; bulk RNA-seq,Homo_714,AD.vs.MCI; bulk RNA-seq,Homo_714,MCI.vs.control; scRNA-seq,SRP215507,CD8+ T cell_4-AD.vs.control; scRNA-seq,SRP215507,CD8+ T cell_3-AD.vs.MCI; scRNA-seq,SRP215507,CD8+ T cell_3-MCI.vs.control; scRNA-seq,SRP330776,CD8+ T cell_1-AD.vs.control; scRNA-seq,SRP330776,Naive CD8+ T	14
CLCN3	bulk RNA-seq,Homo_723,AD.vs.control; bulk RNA-seq,Homo_723,AD.vs.MCI; bulk RNA-seq,Homo_723,MCI.vs.control; bulk RNA-seq,Homo_633,AD.vs.control; bulk RNA-seq,Homo_633,AD.vs.MCI; bulk RNA-seq,Homo_633,MCI.vs.control; bulk RNA-seq,Homo_714,AD.vs.control; bulk RNA-seq,Homo_714,AD.vs.MCI; scRNA-seq,SRP215507,CD8+ T cell_5-AD.vs.control; scRNA-seq,SRP215507,CD8+ T cell_1-AD.vs.MCI; scRNA-seq,SRP215507,CD8+ T cell_4-AD.vs.MCI; scRNA-seq,SRP215507,CD8+ T cell_5-AD.vs.MCI; scRNA-seq,SRP330776,Naive CD8+ T cell_3-AD.vs.control; scRNA-seq,SRP330776,T cell-AD.vs.control; scRNA-seq,SRP330776,CD8+ T cell_3-AD.vs.control	15
Clcn3	bulk RNA-seq,SRP325058,AD.vs.control	1
CLCN4	bulk RNA-seq,Homo_633,AD.vs.control; bulk RNA-seq,Homo_633,AD.vs.MCI; bulk RNA-seq,Homo_714,AD.vs.control; bulk RNA-seq,Homo_714,AD.vs.MCI; bulk RNA-seq,ROSMAP,AD.vs.control; bulk RNA-seq,ROSMAP,MCI.vs.control; scRNA-seq,SRP330776,B cell_2-	7
Clcn4	bulk RNA-seq,SRP325058,AD.vs.control	1
CLCN5	scRNA-seq,SRP215507,CD8+ T cell_1-AD.vs.MCI; scRNA-seq,SRP215507,CD8+ T cell_1-MCI.vs.control; scRNA-seq,SRP309935,Monocyte_1-AD.vs.control; scRNA-seq,SRP330776,CD8+ T cell_1-AD.vs.control	4
CLCN6	scRNA-seq,SRP215507,CD8+ T cell_2-AD.vs.MCI; scRNA-seq,SRP215507,CD8+ T cell_4-AD.vs.MCI; scRNA-seq,SRP215507,CD8+ T cell_5-AD.vs.MCI; scRNA-seq,SRP215507,CD8+ T cell_5-MCI.vs.control; scRNA-seq,SRP215507,CD8+ T cell_4-MCI.vs.control; scRNA-seq,SRP330776,Natural killer cell_1-AD.vs.control; scRNA-seq,SRP330776,T cell-AD.vs.control; scRNA-seq,SRP330776,Natural killer cell_2-	8
CLCN7	bulk RNA-seq,Homo_723,AD.vs.MCI; bulk RNA-seq,Homo_723,MCI.vs.control; bulk RNA-seq,Homo_633,AD.vs.MCI; bulk RNA-seq,Homo_633,MCI.vs.control; bulk RNA-seq,Homo_714,AD.vs.control; bulk RNA-seq,Homo_714,AD.vs.MCI; bulk RNA-seq,Homo_714,MCI.vs.control; scRNA-seq,SRP215507,CD8+ T cell_4-AD.vs.control; scRNA-seq,SRP215507,CD8+ T cell_4-AD.vs.MCI; scRNA-seq,SRP330776,CD8+ T cell_1-AD.vs.control; scRNA-seq,SRP330776,γδ T cell-AD.vs.control; scRNA-seq,SRP330776,Memory T cell_2-AD.vs.control; scRNA-seq,SRP330776,CD8+ T cell_2-AD.vs.control; scRNA-seq,SRP330776,B cell_1-AD.vs.control; scRNA-seq,SRP330776,Naive CD8+ T cell_1-AD.vs.control; scRNA-seq,SRP330776,Natural killer cell_1-AD.vs.control; scRNA-seq,SRP330776,Memory T cell_1-AD.vs.control; scRNA-seq,SRP330776,Natural killer T cell-AD.vs.control; scRNA-seq,SRP330776,Naive CD8+ T cell_3-AD.vs.control; scRNA-seq,SRP330776,B cell_2-AD.vs.control; scRNA-seq,SRP330776,T cell-AD.vs.control; scRNA-seq,SRP330776,CD8+ T cell_3-AD.vs.control; scRNA-seq,SRP330776,B cell_3-[illegible]	24
CLDN1	bulk RNA-seq,Homo_723,AD.vs.control; bulk RNA-seq,Homo_723,AD.vs.MCI; bulk RNA-seq,Homo_723,MCI.vs.control; bulk RNA-seq,Homo_633,AD.vs.MCI; bulk RNA-seq,Homo_633,MCI.vs.control; bulk RNA-seq,Homo_714,MCI.vs.control	6
CLDN10	bulk RNA-seq,Homo_714,AD.vs.MCI	1
CLDN12	scRNA-seq,SRP215507,CD8+ T cell_2-MCI.vs.control; scRNA-seq,SRP215507,CD8+ T cell_3-MCI.vs.control; scRNA-seq,SRP330776,γδ T cell-AD.vs.control; scRNA-seq,SRP330776,B cell_1-AD.vs.control; scRNA-seq,SRP330776,Naive CD8+ T cell_1-AD.vs.control	5
Cldn13	bulk RNA-seq,SRP325058,AD.vs.control	1

CLDN14	bulk RNA-seq,Homo_723,AD.vs.control; bulk RNA-seq,Homo_633,AD.vs.control; bulk RNA-seq,Homo_714,MCI.vs.control; bulk RNA-	4
CLDN15	bulk RNA-seq,Homo_723,AD.vs.control; bulk RNA-seq,Homo_723,AD.vs.MCI; bulk RNA-seq,Homo_723,MCI.vs.control; bulk RNA-seq,Homo_633,AD.vs.control; bulk RNA-seq,Homo_633,AD.vs.MCI; bulk RNA-seq,Homo_633,MCI.vs.control; bulk RNA-seq,Homo_714,AD.vs.control; bulk RNA-seq,Homo_714,MCI.vs.control; scRNA-seq,SRP215507,CD8+ T cell_2-AD.vs.control; scRNA-seq,SRP215507,CD8+ T cell_3-AD.vs.control; scRNA-seq,SRP215507,CD8+ T cell_3-AD.vs.MCI; scRNA-seq,SRP215507,CD8+ T cell_4-AD.vs.MCI; scRNA-seq,SRP215507,CD8+ T cell_1-MCI.vs.control; scRNA-seq,SRP215507,CD8+ T cell_4-MCI.vs.control; scRNA-seq,SRP309935,Naive CD8+ T cell_2-AD.vs.control; scRNA-seq,SRP330776,CD8+ T cell_2-AD.vs.control; scRNA-seq,SRP330776,Naive CD8+ T cell_1-AD.vs.control; scRNA-seq,SRP330776,Natural killer T cell-	18
CLDN18	bulk RNA-seq,Homo_723,AD.vs.control; bulk RNA-seq,Homo_723,AD.vs.MCI	2
CLDN19	bulk RNA-seq,Homo_723,AD.vs.control; bulk RNA-seq,Homo_723,AD.vs.MCI; bulk RNA-seq,Homo_714,MCI.vs.control	3
CLDN2	bulk RNA-seq,Homo_723,AD.vs.control; bulk RNA-seq,Homo_723,AD.vs.MCI	2
CLDN20	bulk RNA-seq,Homo_633,AD.vs.control; bulk RNA-seq,Homo_633,AD.vs.MCI	2
CLDN22	bulk RNA-seq,Homo_723,AD.vs.control; bulk RNA-seq,Homo_723,AD.vs.MCI; bulk RNA-seq,Homo_723,MCI.vs.control; bulk RNA-seq,Homo_633,AD.vs.control; bulk RNA-seq,Homo_633,MCI.vs.control; bulk RNA-seq,Homo_714,AD.vs.control; bulk RNA-	7
CLDN23	bulk RNA-seq,Homo_723,AD.vs.control; bulk RNA-seq,Homo_723,AD.vs.MCI; bulk RNA-seq,Homo_633,AD.vs.control; bulk RNA-seq,Homo_633,AD.vs.MCI; bulk RNA-seq,ROSMAP,AD.vs.control	5
CLDN3	bulk RNA-seq,Homo_723,AD.vs.control; bulk RNA-seq,Homo_723,MCI.vs.control; bulk RNA-seq,Homo_633,AD.vs.control; bulk RNA-seq,Homo_633,MCI.vs.control; bulk RNA-seq,Homo_714,MCI.vs.control	5
CLDN5	bulk RNA-seq,Homo_723,AD.vs.control; bulk RNA-seq,Homo_723,AD.vs.MCI; bulk RNA-seq,Homo_723,MCI.vs.control; bulk RNA-seq,Homo_633,AD.vs.control; bulk RNA-seq,Homo_633,AD.vs.MCI; bulk RNA-seq,Homo_633,MCI.vs.control; bulk RNA-seq,Homo_714,AD.vs.control; bulk RNA-seq,Homo_714,AD.vs.MCI; bulk RNA-seq,Homo_714,MCI.vs.control	9
CLDN6	bulk RNA-seq,Homo_723,AD.vs.MCI; bulk RNA-seq,Homo_723,MCI.vs.control	2
CLDN7	bulk RNA-seq,Homo_723,AD.vs.control; bulk RNA-seq,Homo_723,AD.vs.MCI; bulk RNA-seq,Homo_723,MCI.vs.control; bulk RNA-seq,Homo_633,AD.vs.control; bulk RNA-seq,Homo_633,AD.vs.MCI; bulk RNA-seq,Homo_633,MCI.vs.control; bulk RNA-seq,Homo_714,MCI.vs.control; scRNA-seq,SRP215507,CD8+ T cell_2-AD.vs.control; scRNA-seq,SRP215507,CD8+ T cell_3-AD.vs.control; scRNA-seq,SRP215507,CD8+ T cell_2-AD.vs.MCI; scRNA-seq,SRP330776,Memory T cell_2-AD.vs.control; scRNA-seq,SRP330776,CD8+ T cell_2-AD.vs.control; scRNA-seq,SRP330776,Naive CD8+ T cell_1-AD.vs.control; scRNA-seq,SRP330776,Memory T cell_1-AD.vs.control	14
CLDN8	bulk RNA-seq,Homo_723,AD.vs.control; bulk RNA-seq,Homo_633,AD.vs.control	2
CLDND1	bulk RNA-seq,Homo_723,AD.vs.control; bulk RNA-seq,Homo_723,AD.vs.MCI; bulk RNA-seq,Homo_633,AD.vs.control; bulk RNA-seq,Homo_633,AD.vs.MCI; scRNA-seq,SRP215507,CD8+ T cell_4-AD.vs.MCI; scRNA-seq,SRP215507,CD8+ T cell_4-MCI.vs.control; scRNA-seq,SRP215507,CD8+ T cell_3-MCI.vs.control; scRNA-seq,SRP330776,γδ T cell-AD.vs.control; scRNA-seq,SRP330776,Memory T cell_2-AD.vs.control; scRNA-seq,SRP330776,Natural killer cell_1-AD.vs.control; scRNA-seq,SRP330776,CD8+ T cell_3-AD.vs.control; scRNA-seq,SRP330776,Monocyte_1-AD.vs.control; scRNA-seq,SRP330776,B cell_3-AD.vs.control; scRNA-seq,SRP330776,Megakaryocyte_1-AD.vs.control	14
CLDND2	bulk RNA-seq,ACOM,AD.vs.control; scRNA-seq,SRP215507,CD8+ T cell_1-AD.vs.MCI; scRNA-seq,SRP215507,CD8+ T cell_4-AD.vs.MCI; scRNA-seq,SRP215507,CD8+ T cell_4-MCI.vs.control; scRNA-seq,SRP215507,CD8+ T cell_2-MCI.vs.control; scRNA-seq,SRP309935,Natural killer cell_1-AD.vs.control; scRNA-seq,SRP330776,γδ T cell-AD.vs.control; scRNA-seq,SRP330776,CD8+ T cell_2-AD.vs.control; scRNA-seq,SRP330776,Memory T cell_1-AD.vs.control; scRNA-seq,SRP330776,Naive CD8+ T cell_2-AD.vs.control	10
CLEC10A	bulk RNA-seq,Homo_723,AD.vs.control; bulk RNA-seq,Homo_723,AD.vs.MCI; bulk RNA-seq,Homo_633,AD.vs.control; bulk RNA-seq,Homo_633,AD.vs.MCI; bulk RNA-seq,Homo_714,AD.vs.control; bulk RNA-seq,Homo_714,AD.vs.MCI; bulk RNA-seq,Homo_714,MCI.vs.control; scRNA-seq,SRP309935,Monocyte_1-AD.vs.control; scRNA-seq,SRP309935,Naive CD8+ T cell_1-AD.vs.control	9

CLEC11A	bulk RNA-seq,Homo_723,AD.vs.control; bulk RNA-seq,Homo_723,AD.vs.MCI; bulk RNA-seq,Homo_633,AD.vs.control; bulk RNA-seq,Homo_633,AD.vs.MCI; bulk RNA-seq,Homo_714,AD.vs.control; bulk RNA-seq,Homo_714,AD.vs.MCI; scRNA-seq,SRP215507,CD8+ T cell_2-AD.vs.control; scRNA-seq,SRP215507,CD8+ T cell_3-AD.vs.control; scRNA-seq,SRP215507,CD8+ T cell_1-AD.vs.control; scRNA-seq,SRP215507,CD8+ T cell_1-AD.vs.MCI; scRNA-seq,SRP215507,CD8+ T cell_4-AD.vs.MCI; scRNA-seq,SRP215507,CD8+ T cell_1-MCI.vs.control; scRNA-seq,SRP215507,CD8+ T cell_4-MCI.vs.control; scRNA-seq,SRP215507,CD8+ T cell_2-MCI.vs.control; scRNA-seq,SRP215507,CD8+ T cell_3-MCI.vs.control; scRNA-seq,SRP309935,Natural killer cell_1-AD.vs.control; scRNA-seq,SRP309935,Naive CD8+ T cell_2-AD.vs.control; scRNA-seq,SRP330776,CD8+ T cell_1-AD.vs.control; scRNA-seq,SRP330776,Memory T cell_2-AD.vs.control; scRNA-seq,SRP330776,CD8+ T cell_2-AD.vs.control; scRNA-seq,SRP330776,B cell_1-AD.vs.control; scRNA-seq,SRP330776,Naive CD8+ T cell_1-AD.vs.control; scRNA-seq,SRP330776,Natural killer cell_1-AD.vs.control; scRNA-seq,SRP330776,Memory T cell_1-AD.vs.control; scRNA-seq,SRP330776,Natural killer T cell-[illegible]	28
CLEC12A	scRNA-seq,SRP309935,Natural killer cell_2-AD.vs.control; scRNA-seq,SRP330776,Monocyte_2-AD.vs.control	2
CLEC12B	bulk RNA-seq,Homo_723,AD.vs.control; bulk RNA-seq,Homo_723,AD.vs.MCI; bulk RNA-seq,Homo_723,MCI.vs.control; bulk RNA-seq,Homo_633,AD.vs.control; bulk RNA-seq,Homo_633,AD.vs.MCI; bulk RNA-seq,Homo_633,MCI.vs.control; bulk RNA-seq,Homo_714,AD.vs.control; bulk RNA-seq,Homo_714,AD.vs.MCI; bulk RNA-seq,Homo_714,MCI.vs.control; scRNA-seq,SRP309935,Monocyte_2-AD.vs.control; scRNA-[illegible]	11
CLEC14A	bulk RNA-seq,SRP223445,AD.vs.control	1
CLEC16A	bulk RNA-seq,Homo_633,AD.vs.control; scRNA-seq,SRP215507,CD8+ T cell_4-AD.vs.MCI; scRNA-seq,SRP309935,Monocyte_1-AD.vs.control; scRNA-seq,SRP330776,Natural killer cell_1-AD.vs.control; scRNA-seq,SRP330776,Naive CD8+ T cell_3-AD.vs.control; scRNA-seq,SRP330776,Naive CD8+ T cell_2-AD.vs.control; scRNA-seq,SRP330776,Monocyte_2-AD.vs.control	7
CLEC18A	bulk RNA-seq,Homo_723,AD.vs.control; bulk RNA-seq,Homo_633,AD.vs.control; bulk RNA-seq,Homo_633,AD.vs.MCI; bulk RNA-seq,Homo_714,AD.vs.control; bulk RNA-seq,Homo_714,AD.vs.MCI; bulk RNA-seq,ROSMAP,AD.vs.control; scRNA-seq,SRP215507,CD8+ T cell_1-AD.vs.MCI; scRNA-seq,SRP309935,Natural killer cell_2-AD.vs.control; scRNA-seq,SRP309935,Naive CD8+ T cell_1-AD.vs.control	9
CLEC18B	bulk RNA-seq,Homo_723,AD.vs.control; bulk RNA-seq,Homo_723,AD.vs.MCI; bulk RNA-seq,Homo_723,MCI.vs.control; bulk RNA-seq,Homo_633,AD.vs.control; bulk RNA-seq,Homo_633,AD.vs.MCI; bulk RNA-seq,Homo_633,MCI.vs.control	6
CLEC18C	bulk RNA-seq,Homo_723,MCI.vs.control; bulk RNA-seq,Homo_633,AD.vs.control; bulk RNA-seq,Homo_633,AD.vs.MCI; bulk RNA-seq,Homo_633,MCI.vs.control; bulk RNA-seq,Homo_714,AD.vs.control; bulk RNA-seq,Homo_714,AD.vs.MCI; bulk RNA-seq,Homo_714,MCI.vs.control	7
CLEC1A	bulk RNA-seq,Homo_723,AD.vs.control; bulk RNA-seq,Homo_723,MCI.vs.control; bulk RNA-seq,Homo_633,AD.vs.control; bulk RNA-seq,Homo_633,MCI.vs.control; bulk RNA-seq,Homo_714,MCI.vs.control	5
CLEC1B	bulk RNA-seq,Homo_723,MCI.vs.control; bulk RNA-seq,Homo_633,MCI.vs.control; bulk RNA-seq,Homo_714,AD.vs.control; bulk RNA-seq,Homo_714,AD.vs.MCI; bulk RNA-seq,Homo_714,MCI.vs.control; scRNA-seq,SRP309935,Monocyte_1-AD.vs.control	6
CLEC20A	bulk RNA-seq,Homo_723,AD.vs.MCI; bulk RNA-seq,Homo_723,MCI.vs.control; bulk RNA-seq,Homo_633,AD.vs.control; bulk RNA-seq,Homo_633,AD.vs.MCI; bulk RNA-seq,Homo_714,MCI.vs.control; scRNA-seq,SRP330776,B cell_1-AD.vs.control	6
CLEC2B	bulk RNA-seq,Homo_723,MCI.vs.control; bulk RNA-seq,Homo_633,AD.vs.control; bulk RNA-seq,Homo_633,MCI.vs.control; scRNA-seq,SRP309935,Erythroid cell-AD.vs.control; scRNA-seq,SRP309935,Megakaryocyte_2-AD.vs.control; scRNA-seq,SRP330776,Naive CD8+ T cell_3-[illegible]	6
CLEC2D	bulk RNA-seq,Homo_633,AD.vs.control; bulk RNA-seq,Homo_633,MCI.vs.control; bulk RNA-seq,Homo_714,MCI.vs.control; bulk RNA-seq,SRP310421,AD.vs.control; scRNA-seq,SRP215507,CD8+ T cell_7-AD.vs.control; scRNA-seq,SRP215507,CD8+ T cell_6-AD.vs.MCI; scRNA-seq,SRP309935,Natural killer cell_1-AD.vs.control; scRNA-seq,SRP330776,CD8+ T cell_1-AD.vs.control; scRNA-seq,SRP330776,γδ T cell-AD.vs.control; scRNA-seq,SRP330776,Memory T cell_2-AD.vs.control; scRNA-seq,SRP330776,CD8+ T cell_2-AD.vs.control; scRNA-seq,SRP330776,B cell_1-AD.vs.control; scRNA-seq,SRP330776,Natural killer cell_1-AD.vs.control; scRNA-seq,SRP330776,Memory T cell_1-AD.vs.control; scRNA-seq,SRP330776,Natural killer T cell-AD.vs.control; scRNA-seq,SRP330776,Naive CD8+ T cell_3-AD.vs.control; scRNA-seq,SRP330776,B cell_2-AD.vs.control; scRNA-seq,SRP330776,T cell-AD.vs.control; scRNA-seq,SRP330776,Naive CD8+ T cell_2-AD.vs.control; scRNA-seq,SRP330776,CD8+ [illegible]	22
Clec2i	bulk RNA-seq,SRP325058,AD.vs.control	1
CLEC2L	bulk RNA-seq,ROSMAP,AD.vs.MCI; bulk RNA-seq,ROSMAP,MCI.vs.control; bulk RNA-seq,SRP223445,AD.vs.control	3
CLEC3B	bulk RNA-seq,Homo_723,AD.vs.control; bulk RNA-seq,Homo_723,AD.vs.MCI; bulk RNA-seq,Homo_723,MCI.vs.control; bulk RNA-seq,Homo_633,AD.vs.control; bulk RNA-seq,Homo_633,AD.vs.MCI; bulk RNA-seq,Homo_633,MCI.vs.control; bulk RNA-seq,Homo_714,AD.vs.control;	8

CLEC4A	bulk RNA-seq,Homo_723,AD.vs.control; bulk RNA-seq,Homo_723,MCI.vs.control; bulk RNA-seq,Homo_633,AD.vs.control; bulk RNA-seq,Homo_633,MCI.vs.control; scRNA-seq,SRP215507,CD8+ T cell_1-AD.vs.MCI; scRNA-seq,SRP215507,CD8+ T cell_2-MCI.vs.control; scRNA-	7
Clec4a1	bulk RNA-seq,SRP325058,AD.vs.control	1
CLEC4C	bulk RNA-seq,Homo_723,AD.vs.control; bulk RNA-seq,Homo_723,MCI.vs.control; bulk RNA-seq,Homo_633,MCI.vs.control; bulk RNA-seq,Homo_714,AD.vs.control; bulk RNA-seq,Homo_714,AD.vs.MCI; bulk RNA-seq,Homo_714,MCI.vs.control; bulk RNA-seq,ROSMAP,AD.vs.control;	8
CLEC4D	bulk RNA-seq,Homo_723,AD.vs.control; bulk RNA-seq,Homo_723,AD.vs.MCI; bulk RNA-seq,Homo_633,AD.vs.control; bulk RNA-seq,Homo_633,AD.vs.MCI; bulk RNA-seq,Homo_714,AD.vs.control; bulk RNA-seq,Homo_714,AD.vs.MCI	6
CLEC4E	bulk RNA-seq,Homo_723,AD.vs.control; bulk RNA-seq,Homo_723,AD.vs.MCI; bulk RNA-seq,Homo_633,AD.vs.control; bulk RNA-seq,Homo_633,MCI.vs.control; bulk RNA-seq,SRP223445,AD.vs.control	5
Clec4e	bulk RNA-seq,SRP325058,AD.vs.control	1
CLEC4G	bulk RNA-seq,Homo_723,AD.vs.control; bulk RNA-seq,Homo_723,AD.vs.MCI; bulk RNA-seq,Homo_633,AD.vs.control; bulk RNA-seq,Homo_633,AD.vs.MCI; bulk RNA-seq,Homo_714,AD.vs.control; bulk RNA-seq,Homo_714,AD.vs.MCI	6
CLEC4M	bulk RNA-seq,Homo_723,AD.vs.control; bulk RNA-seq,Homo_723,AD.vs.MCI; bulk RNA-seq,Homo_723,MCI.vs.control; bulk RNA-seq,Homo_633,AD.vs.control; bulk RNA-seq,Homo_633,AD.vs.MCI; bulk RNA-seq,Homo_633,MCI.vs.control	6
Clec4n	bulk RNA-seq,SRP325058,AD.vs.control	1
CLEC5A	bulk RNA-seq,Homo_633,AD.vs.control; bulk RNA-seq,Homo_633,AD.vs.MCI; bulk RNA-seq,Homo_714,AD.vs.control; bulk RNA-	4
CLEC6A	bulk RNA-seq,Homo_723,AD.vs.control; bulk RNA-seq,Homo_723,AD.vs.MCI; bulk RNA-seq,Homo_633,AD.vs.control; bulk RNA-seq,Homo_633,AD.vs.MCI; bulk RNA-seq,Homo_714,AD.vs.control; bulk RNA-seq,Homo_714,AD.vs.MCI	6
CLEC7A	bulk RNA-seq,SRP223445,AD.vs.control; scRNA-seq,SRP309935,Naive CD8+ T cell_1-AD.vs.control; scRNA-seq,SRP309935,Dendritic cell-	3
CLEC9A	bulk RNA-seq,Homo_723,AD.vs.control; bulk RNA-seq,Homo_723,AD.vs.MCI; bulk RNA-seq,Homo_723,MCI.vs.control; bulk RNA-seq,Homo_633,AD.vs.control; bulk RNA-seq,Homo_633,MCI.vs.control	5
CLECL1	scRNA-seq,SRP215507,CD8+ T cell_2-AD.vs.control; scRNA-seq,SRP215507,CD8+ T cell_3-AD.vs.control; scRNA-seq,SRP215507,CD8+ T cell_4-AD.vs.control; scRNA-seq,SRP215507,CD8+ T cell_6-AD.vs.control; scRNA-seq,SRP215507,CD8+ T cell_2-AD.vs.MCI; scRNA-seq,SRP215507,CD8+ T cell_3-AD.vs.MCI; scRNA-seq,SRP215507,CD8+ T cell_1-AD.vs.MCI; scRNA-seq,SRP215507,CD8+ T cell_5-AD.vs.MCI; scRNA-seq,SRP215507,CD8+ T cell_1-MCI.vs.control; scRNA-seq,SRP215507,CD8+ T cell_3-MCI.vs.control; scRNA-seq,SRP309935,CD8+ T cell_1-AD.vs.control; scRNA-seq,SRP309935,CD4+ T cell-AD.vs.control; scRNA-seq,SRP330776,γδ T cell-AD.vs.control; scRNA-seq,SRP330776,CD8+ T	14
CLGN	bulk RNA-seq,Homo_723,AD.vs.control; bulk RNA-seq,Homo_723,AD.vs.MCI; bulk RNA-seq,Homo_723,MCI.vs.control	3
CLHC1	bulk RNA-seq,Homo_714,AD.vs.control; bulk RNA-seq,Homo_714,AD.vs.MCI; scRNA-seq,SRP215507,CD8+ T cell_3-AD.vs.control; scRNA-seq,SRP215507,CD8+ T cell_1-AD.vs.control; scRNA-seq,SRP215507,CD8+ T cell_3-AD.vs.MCI; scRNA-seq,SRP309935,Monocyte_1-AD.vs.control; scRNA-seq,SRP330776,Natural killer cell_1-AD.vs.control; scRNA-seq,SRP330776,Memory T cell_1-AD.vs.control; scRNA-seq,SRP330776,B cell_2-AD.vs.control; scRNA-seq,SRP330776,Naive CD8+ T cell_2-AD.vs.control	10
CLIC1	scRNA-seq,SRP215507,CD8+ T cell_3-AD.vs.control; scRNA-seq,SRP215507,CD8+ T cell_7-AD.vs.control; scRNA-seq,SRP215507,CD8+ T cell_10-AD.vs.control; scRNA-seq,SRP215507,CD8+ T cell_3-MCI.vs.control; scRNA-seq,SRP215507,CD8+ T cell_7-MCI.vs.control; scRNA-seq,SRP309935,Erythroid cell-AD.vs.control; scRNA-seq,SRP330776,Naive CD8+ T cell_1-AD.vs.control; scRNA-seq,SRP330776,B cell_2-	9
CLIC2	bulk RNA-seq,Homo_723,AD.vs.control; bulk RNA-seq,Homo_723,AD.vs.MCI; bulk RNA-seq,Homo_633,AD.vs.control; bulk RNA-seq,Homo_633,AD.vs.MCI; bulk RNA-seq,Homo_714,AD.vs.control; bulk RNA-seq,Homo_714,AD.vs.MCI	6
CLIC3	scRNA-seq,SRP215507,CD8+ T cell_7-AD.vs.control; scRNA-seq,SRP215507,CD8+ T cell_4-AD.vs.MCI; scRNA-seq,SRP215507,CD8+ T cell_3-MCI.vs.control; scRNA-seq,SRP309935,Erythroid cell-AD.vs.control; scRNA-seq,SRP309935,CD8+ T cell_2-AD.vs.control; scRNA-seq,SRP330776,CD8+ T cell_1-AD.vs.control; scRNA-seq,SRP330776,Memory T cell_2-AD.vs.control; scRNA-seq,SRP330776,Natural killer cell_1-	9
CLIC4	bulk RNA-seq,Homo_723,AD.vs.control; bulk RNA-seq,Homo_723,AD.vs.MCI; bulk RNA-seq,Homo_723,MCI.vs.control; bulk RNA-seq,Homo_633,AD.vs.control; bulk RNA-seq,Homo_633,AD.vs.MCI; bulk RNA-seq,Homo_633,MCI.vs.control; bulk RNA-seq,Homo_714,AD.vs.control; bulk RNA-seq,Homo_714,AD.vs.MCI; scRNA-seq,SRP215507,CD8+ T cell_1-AD.vs.MCI; scRNA-seq,SRP215507,CD8+ T cell_1-MCI.vs.control; scRNA-seq,SRP330776,Memory T cell_2-AD.vs.control; scRNA-seq,SRP330776,CD8+ T cell_2-AD.vs.control	12

CLIC5	bulk RNA-seq,Homo_723,AD.vs.control; bulk RNA-seq,Homo_723,AD.vs.MCI; bulk RNA-seq,Homo_723,MCI.vs.control; bulk RNA-seq,Homo_633,AD.vs.control; bulk RNA-seq,Homo_633,AD.vs.MCI; bulk RNA-seq,Homo_633,MCI.vs.control; bulk RNA-seq,Homo_714,AD.vs.control; bulk RNA-seq,Homo_714,AD.vs.MCI; bulk RNA-seq,ROSMAP,AD.vs.control; bulk RNA-seq,ROSMAP,MCI.vs.control; scRNA-seq,SRP330776,B cell_1-AD.vs.control; scRNA-seq,SRP330776,Natural killer cell_1-AD.vs.control	12
CLIC6	bulk RNA-seq,Homo_723,AD.vs.control; bulk RNA-seq,Homo_723,AD.vs.MCI; bulk RNA-seq,Homo_714,AD.vs.control; bulk RNA-	4
CLINT1	bulk RNA-seq,Homo_723,AD.vs.MCI; bulk RNA-seq,Homo_633,AD.vs.MCI; bulk RNA-seq,Homo_714,AD.vs.control; bulk RNA-seq,Homo_714,AD.vs.MCI; scRNA-seq,SRP309935,Megakaryocyte_1-AD.vs.control; scRNA-seq,SRP309935,B cell_1-AD.vs.control	6
CLIP1	bulk RNA-seq,Homo_723,AD.vs.control; bulk RNA-seq,Homo_723,AD.vs.MCI; bulk RNA-seq,Homo_633,AD.vs.control; bulk RNA-seq,Homo_633,AD.vs.MCI; bulk RNA-seq,Homo_714,AD.vs.control; bulk RNA-seq,Homo_714,AD.vs.MCI; scRNA-seq,SRP215507,CD8+ T cell_7-AD.vs.control; scRNA-seq,SRP309935,Naive CD8+ T cell_1-AD.vs.control; scRNA-seq,SRP330776,B cell_2-AD.vs.control; scRNA-seq,SRP330776,Monocyte_2-AD.vs.control; scRNA-seq,SRP330776,B cell_3-AD.vs.control; scRNA-seq,SRP330776,Natural killer cell_2-AD.vs.control	12
CLIP2	bulk RNA-seq,Homo_723,AD.vs.control; bulk RNA-seq,Homo_723,AD.vs.MCI; bulk RNA-seq,Homo_633,AD.vs.control; bulk RNA-seq,Homo_633,AD.vs.MCI; bulk RNA-seq,Homo_714,AD.vs.control; bulk RNA-seq,Homo_714,AD.vs.MCI; scRNA-seq,SRP215507,CD8+ T cell_5-AD.vs.control; scRNA-seq,SRP215507,CD8+ T cell_4-MCI.vs.control; scRNA-seq,SRP309935,Monocyte_1-AD.vs.control; scRNA-seq,SRP309935,CD8+ T cell_1-AD.vs.control; scRNA-seq,SRP309935,CD8+ T cell_2-AD.vs.control; scRNA-seq,SRP330776,CD8+ T cell_1-AD.vs.control; scRNA-seq,SRP330776,CD8+ T cell_2-AD.vs.control; scRNA-seq,SRP330776,B cell_1-AD.vs.control; scRNA-seq,SRP330776,Naive CD8+ T cell_1-	16
CLIP3	bulk RNA-seq,Homo_723,AD.vs.control; bulk RNA-seq,Homo_723,AD.vs.MCI; bulk RNA-seq,Homo_723,MCI.vs.control; bulk RNA-seq,Homo_633,AD.vs.control; bulk RNA-seq,Homo_633,AD.vs.MCI; bulk RNA-seq,Homo_714,AD.vs.control; bulk RNA-seq,Homo_714,AD.vs.MCI; bulk RNA-seq,Homo_714,MCI.vs.control; bulk RNA-seq,ROSMAP,AD.vs.control; scRNA-seq,SRP215507,CD8+ T cell_2-AD.vs.control; scRNA-seq,SRP215507,CD8+ T cell_1-AD.vs.control; scRNA-seq,SRP215507,CD8+ T cell_2-MCI.vs.control; scRNA-seq,SRP330776,T cell-AD.vs.control	13
CLIP4	bulk RNA-seq,Homo_723,AD.vs.control; bulk RNA-seq,Homo_633,AD.vs.control; bulk RNA-seq,Homo_633,MCI.vs.control; bulk RNA-seq,SRP310421,AD.vs.control; scRNA-seq,SRP215507,CD8+ T cell_4-AD.vs.MCI; scRNA-seq,SRP215507,CD8+ T cell_5-AD.vs.MCI; scRNA-seq,SRP215507,CD8+ T cell_4-MCI.vs.control; scRNA-seq,SRP330776,γδ T cell-AD.vs.control; scRNA-seq,SRP330776,Memory T cell_2-AD.vs.control; scRNA-seq,SRP330776,Natural killer cell_1-AD.vs.control; scRNA-seq,SRP330776,B cell_2-AD.vs.control; scRNA-seq,SRP330776,Monocyte_1-AD.vs.control; scRNA-seq,SRP330776,Monocyte_2-AD.vs.control; scRNA-seq,SRP330776,Megakaryocyte_1-AD.vs.control; scRNA-	15
CLK1	scRNA-seq,SRP215507,CD8+ T cell_5-AD.vs.control; scRNA-seq,SRP215507,CD8+ T cell_6-AD.vs.control; scRNA-seq,SRP215507,CD8+ T cell_1-AD.vs.MCI; scRNA-seq,SRP215507,CD8+ T cell_5-AD.vs.MCI; scRNA-seq,SRP215507,CD8+ T cell_4-MCI.vs.control; scRNA-seq,SRP309935,Erythroid cell-AD.vs.control; scRNA-seq,SRP309935,Megakaryocyte_2-AD.vs.control; scRNA-seq,SRP330776,Natural killer T cell-	9
CLK2	bulk RNA-seq,Homo_723,AD.vs.control; bulk RNA-seq,Homo_723,MCI.vs.control; bulk RNA-seq,Homo_633,AD.vs.control; bulk RNA-seq,Homo_633,MCI.vs.control; scRNA-seq,SRP215507,CD8+ T cell_8-MCI.vs.control; scRNA-seq,SRP309935,B cell_1-AD.vs.control; scRNA-seq,SRP330776,CD8+ T cell_1-AD.vs.control; scRNA-seq,SRP330776,γδ T cell-AD.vs.control; scRNA-seq,SRP330776,Memory T cell_2-AD.vs.control; scRNA-seq,SRP330776,CD8+ T cell_2-AD.vs.control; scRNA-seq,SRP330776,B cell_1-AD.vs.control; scRNA-seq,SRP330776,Naive CD8+ T cell_1-AD.vs.control; scRNA-seq,SRP330776,Memory T cell_1-AD.vs.control; scRNA-seq,SRP330776,Natural killer T cell-AD.vs.control; scRNA-seq,SRP330776,Naive CD8+ T cell_3-AD.vs.control; scRNA-seq,SRP330776,B cell_2-AD.vs.control; scRNA-seq,SRP330776,T cell-AD.vs.control; scRNA-seq,SRP330776,CD8+ T cell_3-AD.vs.control; scRNA-seq,SRP330776,Monocyte_2-AD.vs.control; scRNA-seq,SRP330776,Natural killer cell_2- [illegible]	21
CLK3	scRNA-seq,SRP330776,Natural killer cell_2-AD.vs.control	1
CLK4	bulk RNA-seq,Homo_723,AD.vs.control; bulk RNA-seq,Homo_633,AD.vs.control; bulk RNA-seq,Homo_714,AD.vs.control; bulk RNA-seq,Homo_714,AD.vs.MCI; bulk RNA-seq,Homo_714,MCI.vs.control; scRNA-seq,SRP215507,CD8+ T cell_7-AD.vs.control; scRNA-seq,SRP309935,B cell_1-AD.vs.control; scRNA-seq,SRP330776,B cell_2-AD.vs.control	8
CLMN	bulk RNA-seq,Homo_723,AD.vs.MCI; bulk RNA-seq,Homo_633,AD.vs.MCI; bulk RNA-seq,Homo_633,MCI.vs.control; scRNA-seq,SRP215507,CD8+ T cell_2-AD.vs.control; scRNA-seq,SRP215507,CD8+ T cell_2-MCI.vs.control; scRNA-seq,SRP330776,B cell_1-AD.vs.control; scRNA-seq,SRP330776,B cell_2-AD.vs.control; scRNA-seq,SRP330776,Monocyte_1-AD.vs.control; scRNA-seq,SRP330776,Monocyte_2-AD.vs.control	9

CLMP	bulk RNA-seq,Homo_723,AD.vs.control; bulk RNA-seq,Homo_723,AD.vs.MCI; bulk RNA-seq,Homo_633,AD.vs.control; bulk RNA-seq,Homo_633,AD.vs.MCI; bulk RNA-seq,Homo_714,AD.vs.control; bulk RNA-seq,Homo_714,AD.vs.MCI; bulk RNA-seq,ROSMAP,AD.vs.control;	8
CLN3	scRNA-seq,SRP330776,CD8+ T cell_1-AD.vs.control; scRNA-seq,SRP330776,γδ T cell-AD.vs.control; scRNA-seq,SRP330776,B cell_1-AD.vs.control; scRNA-seq,SRP330776,Naive CD8+ T cell_3-AD.vs.control; scRNA-seq,SRP330776,B cell_2-AD.vs.control; scRNA-seq,SRP330776,Monocyte_1-	6
CLN5	scRNA-seq,SRP215507,CD8+ T cell_5-AD.vs.control; scRNA-seq,SRP215507,CD8+ T cell_5-MCI.vs.control; scRNA-seq,SRP215507,CD8+ T cell_3-MCI.vs.control; scRNA-seq,SRP330776,Natural killer T cell-AD.vs.control; scRNA-seq,SRP330776,CD8+ T cell_3-AD.vs.control	5
CLN6	bulk RNA-seq,Homo_723,AD.vs.control; bulk RNA-seq,Homo_723,AD.vs.MCI; bulk RNA-seq,Homo_633,AD.vs.control; bulk RNA-seq,Homo_633,AD.vs.MCI; bulk RNA-seq,Homo_714,AD.vs.control; bulk RNA-seq,Homo_714,AD.vs.MCI; scRNA-seq,SRP215507,CD8+ T cell_9-AD.vs.control; scRNA-seq,SRP330776,CD8+ T cell_1-AD.vs.control; scRNA-seq,SRP330776,γδ T cell-AD.vs.control; scRNA-seq,SRP330776,Memory T cell_2-AD.vs.control; scRNA-seq,SRP330776,B cell_1-AD.vs.control; scRNA-seq,SRP330776,Naive CD8+ T cell_1-AD.vs.control; scRNA-seq,SRP330776,Natural killer cell_1-AD.vs.control; scRNA-seq,SRP330776,Memory T cell_1-AD.vs.control; scRNA-seq,SRP330776,Natural killer T cell-AD.vs.control; scRNA-seq,SRP330776,Naive CD8+ T cell_3-AD.vs.control; scRNA-seq,SRP330776,B cell_2-AD.vs.control; scRNA-seq,SRP330776,T cell-AD.vs.control; scRNA-seq,SRP330776,CD8+ T cell_3-AD.vs.control; scRNA-seq,SRP330776,Monocyte_2-AD.vs.control; scRNA-seq,SRP330776,B [illegible]	22
Cln8	bulk RNA-seq,SRP325058,AD.vs.control	1
CLN8	scRNA-seq,SRP215507,CD8+ T cell_4-AD.vs.control; scRNA-seq,SRP215507,CD8+ T cell_4-AD.vs.MCI; scRNA-seq,SRP330776,Memory T cell_1-AD.vs.control; scRNA-seq,SRP330776,B cell_2-AD.vs.control; scRNA-seq,SRP330776,CD8+ T cell_3-AD.vs.control; scRNA-	6
CLNK	bulk RNA-seq,Homo_714,AD.vs.control; bulk RNA-seq,Homo_714,AD.vs.MCI	2
CLNS1A	bulk RNA-seq,Homo_723,AD.vs.MCI; bulk RNA-seq,Homo_723,MCI.vs.control; bulk RNA-seq,Homo_633,AD.vs.MCI; bulk RNA-seq,Homo_633,MCI.vs.control; bulk RNA-seq,Homo_714,AD.vs.control; bulk RNA-seq,Homo_714,AD.vs.MCI; bulk RNA-seq,Homo_714,MCI.vs.control; scRNA-seq,SRP215507,CD8+ T cell_7-AD.vs.control; scRNA-seq,SRP215507,CD8+ T cell_7-MCI.vs.control; scRNA-seq,SRP330776,Natural killer cell_1-AD.vs.control; scRNA-seq,SRP330776,Natural killer T cell-AD.vs.control; scRNA-seq,SRP330776,CD8+ T cell_3-	12
CLOCK	bulk RNA-seq,Homo_723,AD.vs.control; bulk RNA-seq,Homo_723,AD.vs.MCI; bulk RNA-seq,Homo_633,AD.vs.control; bulk RNA-seq,Homo_633,AD.vs.MCI; bulk RNA-seq,Homo_714,AD.vs.control; bulk RNA-seq,Homo_714,AD.vs.MCI; scRNA-seq,SRP309935,Natural killer cell_2-AD.vs.control; scRNA-seq,SRP330776,γδ T cell-AD.vs.control; scRNA-seq,SRP330776,Memory T cell_2-AD.vs.control; scRNA-seq,SRP330776,Memory T cell_1-AD.vs.control; scRNA-seq,SRP330776,Naive CD8+ T cell_3-AD.vs.control; scRNA-seq,SRP330776,B cell_2-AD.vs.control	12
CLP1	bulk RNA-seq,Homo_723,AD.vs.control; bulk RNA-seq,Homo_723,AD.vs.MCI; bulk RNA-seq,Homo_633,AD.vs.control; bulk RNA-seq,Homo_633,AD.vs.MCI; bulk RNA-seq,Homo_714,AD.vs.control; bulk RNA-seq,Homo_714,AD.vs.MCI; scRNA-seq,SRP215507,CD8+ T cell_3-AD.vs.control; scRNA-seq,SRP215507,CD8+ T cell_3-MCI.vs.control; scRNA-seq,SRP330776,CD8+ T cell_3-AD.vs.control; scRNA-	10
CLPB	scRNA-seq,SRP215507,CD8+ T cell_1-AD.vs.MCI; scRNA-seq,SRP215507,CD8+ T cell_1-MCI.vs.control	2
CLPP	bulk RNA-seq,Homo_714,AD.vs.control; bulk RNA-seq,Homo_714,AD.vs.MCI; bulk RNA-seq,Homo_714,MCI.vs.control; scRNA-seq,SRP215507,CD8+ T cell_4-AD.vs.control; scRNA-seq,SRP215507,CD8+ T cell_5-AD.vs.MCI; scRNA-seq,SRP330776,Memory T cell_1-AD.vs.control; scRNA-seq,SRP330776,Naive CD8+ T cell_2-AD.vs.control	7
CLPSL1	bulk RNA-seq,Homo_714,MCI.vs.control	1
CLPTM1	scRNA-seq,SRP215507,CD8+ T cell_7-AD.vs.control; scRNA-seq,SRP215507,CD8+ T cell_5-AD.vs.MCI; scRNA-seq,SRP330776,CD8+ T cell_1-AD.vs.control; scRNA-seq,SRP330776,γδ T cell-AD.vs.control; scRNA-seq,SRP330776,Memory T cell_2-AD.vs.control; scRNA-seq,SRP330776,CD8+ T cell_2-AD.vs.control; scRNA-seq,SRP330776,B cell_1-AD.vs.control; scRNA-seq,SRP330776,Naive CD8+ T cell_1-AD.vs.control; scRNA-seq,SRP330776,Natural killer cell_1-AD.vs.control; scRNA-seq,SRP330776,Memory T cell_1-AD.vs.control; scRNA-seq,SRP330776,Natural killer T cell-AD.vs.control; scRNA-seq,SRP330776,Naive CD8+ T cell_3-AD.vs.control; scRNA-seq,SRP330776,B cell_2-AD.vs.control; scRNA-seq,SRP330776,T cell-AD.vs.control; scRNA-seq,SRP330776,CD8+ T cell_3-AD.vs.control; scRNA-seq,SRP330776,Monocyte_1-AD.vs.control	16

CLPTM1L	bulk RNA-seq,Homo_723,AD.vs.control; bulk RNA-seq,Homo_723,AD.vs.MCI; bulk RNA-seq,Homo_633,AD.vs.control; bulk RNA-seq,Homo_633,AD.vs.MCI; bulk RNA-seq,Homo_714,AD.vs.control; bulk RNA-seq,Homo_714,AD.vs.MCI; bulk RNA-seq,SRP310421,AD.vs.control; scRNA-seq,SRP215507,CD8+ T cell_3-AD.vs.MCI; scRNA-seq,SRP309935,Naive CD8+ T cell_1-AD.vs.control; scRNA-seq,SRP309935,Monocyte_2-AD.vs.control; scRNA-seq,SRP330776,γδ T cell-AD.vs.control; scRNA-seq,SRP330776,Memory T cell_2-AD.vs.control; scRNA-seq,SRP330776,Naive CD8+ T cell_1-AD.vs.control; scRNA-seq,SRP330776,Natural killer cell_1-AD.vs.control; scRNA-seq,SRP330776,Memory T cell_1-AD.vs.control; scRNA-seq,SRP330776,Naive CD8+ T cell_3-AD.vs.control; scRNA-seq,SRP330776,T cell-AD.vs.control; scRNA-seq,SRP330776,CD8+ T cell_3- [illegible]	20
CLPX	bulk RNA-seq,Homo_723,AD.vs.control; bulk RNA-seq,Homo_723,AD.vs.MCI; bulk RNA-seq,Homo_723,MCI.vs.control; bulk RNA-seq,Homo_633,AD.vs.control; bulk RNA-seq,Homo_633,AD.vs.MCI; bulk RNA-seq,Homo_633,MCI.vs.control; bulk RNA-seq,Homo_714,AD.vs.control; bulk RNA-seq,Homo_714,AD.vs.MCI; bulk RNA-seq,Homo_714,MCI.vs.control; scRNA-seq,SRP215507,CD8+ T cell_3-AD.vs.MCI; scRNA-seq,SRP309935,Monocyte_1-AD.vs.control; scRNA-seq,SRP309935,Naive CD8+ T cell_1-AD.vs.control; scRNA-seq,SRP309935,CD8+ T cell_1-AD.vs.control; scRNA-seq,SRP330776,T cell-AD.vs.control; scRNA-seq,SRP330776,Monocyte_2-AD.vs.control	15
CLRN1	bulk RNA-seq,Homo_723,MCI.vs.control; bulk RNA-seq,Homo_633,MCI.vs.control; bulk RNA-seq,Homo_714,MCI.vs.control	3
CLRN3	bulk RNA-seq,Homo_723,AD.vs.control; bulk RNA-seq,Homo_723,AD.vs.MCI	2
CLSPN	bulk RNA-seq,Homo_723,AD.vs.control; bulk RNA-seq,Homo_723,AD.vs.MCI; bulk RNA-seq,Homo_723,MCI.vs.control; bulk RNA-seq,Homo_633,AD.vs.control; bulk RNA-seq,Homo_633,AD.vs.MCI; bulk RNA-seq,Homo_633,MCI.vs.control; bulk RNA-seq,Homo_714,AD.vs.control; bulk RNA-seq,Homo_714,AD.vs.MCI; scRNA-seq,SRP215507,CD8+ T cell_2-AD.vs.control; scRNA-seq,SRP215507,CD8+ T cell_2-AD.vs.MCI; scRNA-seq,SRP215507,CD8+ T cell_3-MCI.vs.control; scRNA-seq,SRP330776,B cell_1-AD.vs.control; scRNA-seq,SRP330776,B cell_4-AD.vs.control	13
CLSTN1	bulk RNA-seq,Homo_723,AD.vs.control; bulk RNA-seq,Homo_723,MCI.vs.control; bulk RNA-seq,Homo_633,AD.vs.control; bulk RNA-seq,Homo_633,AD.vs.MCI; bulk RNA-seq,Homo_633,MCI.vs.control; scRNA-seq,SRP215507,CD8+ T cell_6-MCI.vs.control; scRNA-seq,SRP309935,Natural killer cell_2-AD.vs.control; scRNA-seq,SRP309935,Naive CD8+ T cell_2-AD.vs.control; scRNA-seq,SRP330776,CD8+ T cell_1-AD.vs.control; scRNA-seq,SRP330776,γδ T cell-AD.vs.control; scRNA-seq,SRP330776,Memory T cell_2-AD.vs.control; scRNA-seq,SRP330776,CD8+ T cell_2-AD.vs.control; scRNA-seq,SRP330776,B cell_1-AD.vs.control; scRNA-seq,SRP330776,Naive CD8+ T cell_1-AD.vs.control; scRNA-seq,SRP330776,Natural killer cell_1-AD.vs.control; scRNA-seq,SRP330776,Memory T cell_1-AD.vs.control; scRNA-seq,SRP330776,Natural killer T cell-AD.vs.control; scRNA-seq,SRP330776,Naive CD8+ T cell_3-AD.vs.control; scRNA-seq,SRP330776,B cell_2-AD.vs.control; scRNA-seq,SRP330776,T cell-AD.vs.control; scRNA-seq,SRP330776,Naive CD8+ T cell_2-AD.vs.control; scRNA-seq,SRP330776,CD8+ T cell_3-AD.vs.control; scRNA-seq,SRP330776,Monocyte_1-AD.vs.control; scRNA-seq,SRP330776,B cell_3-AD.vs.control; scRNA-seq,SRP330776,Regulatory T cell-AD.vs.control; scRNA-seq,SRP330776,Natural killer cell_2-AD.vs.control	26
CLSTN2	bulk RNA-seq,Homo_723,AD.vs.control; bulk RNA-seq,Homo_723,AD.vs.MCI; bulk RNA-seq,Homo_723,MCI.vs.control; bulk RNA-	4
CLSTN3	bulk RNA-seq,Homo_723,AD.vs.MCI; bulk RNA-seq,Homo_633,AD.vs.control; bulk RNA-seq,Homo_633,AD.vs.MCI; bulk RNA-seq,Homo_714,AD.vs.control; bulk RNA-seq,Homo_714,AD.vs.MCI; scRNA-seq,SRP330776,γδ T cell-AD.vs.control; scRNA-seq,SRP330776,B cell_4-	7
CLTA	bulk RNA-seq,Homo_714,AD.vs.control; bulk RNA-seq,Homo_714,AD.vs.MCI; scRNA-seq,SRP215507,CD8+ T cell_7-AD.vs.control; scRNA-seq,SRP215507,CD8+ T cell_7-AD.vs.MCI; scRNA-seq,SRP215507,CD8+ T cell_5-AD.vs.MCI; scRNA-seq,SRP215507,CD8+ T cell_6-AD.vs.MCI; scRNA-seq,SRP215507,CD8+ T cell_5-MCI.vs.control; scRNA-seq,SRP309935,Erythroid cell-AD.vs.control; scRNA-seq,SRP330776,B cell_3-	9
CLTB	bulk RNA-seq,Homo_714,AD.vs.control; bulk RNA-seq,Homo_714,AD.vs.MCI; scRNA-seq,SRP215507,CD8+ T cell_7-AD.vs.control; scRNA-seq,SRP215507,CD8+ T cell_10-AD.vs.control; scRNA-seq,SRP215507,CD8+ T cell_5-AD.vs.MCI; scRNA-seq,SRP215507,CD8+ T cell_5-MCI.vs.control; scRNA-seq,SRP215507,CD8+ T cell_7-MCI.vs.control; scRNA-seq,SRP309935,Naive CD8+ T cell_1-AD.vs.control; scRNA-seq,SRP309935,Naive CD8+ T cell_2-AD.vs.control; scRNA-seq,SRP309935,Naive CD8+ T cell_3-AD.vs.control; scRNA-seq,SRP309935,CD4+ T cell-AD.vs.control; scRNA-seq,SRP330776,CD8+ T cell_1-AD.vs.control; scRNA-seq,SRP330776,γδ T cell-AD.vs.control; scRNA-seq,SRP330776,Memory T cell_2-AD.vs.control; scRNA-seq,SRP330776,CD8+ T cell_2-AD.vs.control; scRNA-seq,SRP330776,B cell_1-AD.vs.control; scRNA-seq,SRP330776,Naive CD8+ T cell_1-AD.vs.control; scRNA-seq,SRP330776,Natural killer cell_1-AD.vs.control; scRNA-seq,SRP330776,Memory T cell_1-AD.vs.control; scRNA-seq,SRP330776,Natural killer T cell-AD.vs.control; scRNA-seq,SRP330776,Naive CD8+ T cell_3-AD.vs.control; scRNA-seq,SRP330776,B cell_2-AD.vs.control; scRNA-seq,SRP330776,T cell-AD.vs.control; scRNA-seq,SRP330776,Naive CD8+ T cell_2-AD.vs.control; scRNA-seq,SRP330776,CD8+ T cell_3-AD.vs.control; scRNA-seq,SRP330776,Monocyte_1-AD.vs.control; scRNA-seq,SRP330776,Monocyte_2-AD.vs.control; scRNA-seq,SRP330776,B cell_3-AD.vs.control; scRNA-seq,SRP330776,Regulatory T cell-AD.vs.control; scRNA-seq,SRP330776,Natural	31

CLTC	scRNA-seq,SRP215507,CD8+ T cell_6-AD.vs.control; scRNA-seq,SRP215507,CD8+ T cell_7-AD.vs.MCI; scRNA-seq,SRP215507,CD8+ T cell_6-AD.vs.MCI; scRNA-seq,SRP309935,Natural killer cell_2-AD.vs.control; scRNA-seq,SRP309935,Naive CD8+ T cell_1-AD.vs.control; scRNA-seq,SRP309935,CD8+ T cell_1-AD.vs.control; scRNA-seq,SRP309935,CD8+ T cell_2-AD.vs.control; scRNA-seq,SRP330776,T cell-AD.vs.control; scRNA-seq,SRP330776,CD8+ T cell_3-AD.vs.control; scRNA-seq,SRP330776,Monocyte_1-AD.vs.control; scRNA-seq,SRP330776,Regulatory T cell-	12
CLTCL1	bulk RNA-seq,Homo_633,AD.vs.control; bulk RNA-seq,Homo_633,AD.vs.MCI; bulk RNA-seq,Homo_714,AD.vs.control; bulk RNA-	4
CLTRN	bulk RNA-seq,Homo_723,AD.vs.control; bulk RNA-seq,Homo_723,AD.vs.MCI	2
CLU	bulk RNA-seq,Homo_633,MCI.vs.control; bulk RNA-seq,Homo_714,AD.vs.control; bulk RNA-seq,Homo_714,AD.vs.MCI; bulk RNA-seq,Homo_714,MCI.vs.control; scRNA-seq,SRP215507,CD8+ T cell_1-AD.vs.MCI; scRNA-seq,SRP215507,CD8+ T cell_1-MCI.vs.control; scRNA-seq,SRP309935,Monocyte_1-AD.vs.control; scRNA-seq,SRP330776,CD8+ T cell_1-AD.vs.control; scRNA-seq,SRP330776,Naive CD8+ T cell_1-AD.vs.control; scRNA-seq,SRP330776,CD8+ T cell_3-AD.vs.control; scRNA-seq,SRP330776,Megakaryocyte_1-AD.vs.control	11
CLUAP1	bulk RNA-seq,Homo_633,AD.vs.control; bulk RNA-seq,Homo_633,AD.vs.MCI; bulk RNA-seq,Homo_714,AD.vs.control; bulk RNA-seq,Homo_714,AD.vs.MCI; bulk RNA-seq,ROSMAP,AD.vs.control; bulk RNA-seq,ROSMAP,AD.vs.MCI; scRNA-seq,SRP215507,CD8+ T cell_5-AD.vs.control; scRNA-seq,SRP215507,CD8+ T cell_5-AD.vs.MCI; scRNA-seq,SRP215507,CD8+ T cell_1-MCI.vs.control; scRNA-seq,SRP330776,CD8+ T cell_1-AD.vs.control; scRNA-seq,SRP330776,γδ T cell-AD.vs.control; scRNA-seq,SRP330776,Memory T cell_2-AD.vs.control; scRNA-seq,SRP330776,CD8+ T cell_2-AD.vs.control; scRNA-seq,SRP330776,Naive CD8+ T cell_1-AD.vs.control; scRNA-seq,SRP330776,Natural killer cell_1-AD.vs.control; scRNA-seq,SRP330776,Naive CD8+ T cell_3-AD.vs.control; scRNA-seq,SRP330776,T cell-AD.vs.control; scRNA-seq,SRP330776,CD8+ [illegible]	20
CLUH	scRNA-seq,SRP215507,CD8+ T cell_1-AD.vs.MCI; scRNA-seq,SRP215507,CD8+ T cell_4-AD.vs.MCI; scRNA-seq,SRP215507,CD8+ T cell_5-AD.vs.MCI; scRNA-seq,SRP215507,CD8+ T cell_1-MCI.vs.control; scRNA-seq,SRP215507,CD8+ T cell_5-MCI.vs.control; scRNA-seq,SRP215507,CD8+ T cell_4-MCI.vs.control; scRNA-seq,SRP330776,CD8+ T cell_1-AD.vs.control; scRNA-seq,SRP330776,γδ T cell-AD.vs.control; scRNA-seq,SRP330776,Memory T cell_2-AD.vs.control; scRNA-seq,SRP330776,CD8+ T cell_2-AD.vs.control; scRNA-seq,SRP330776,B cell_1-AD.vs.control; scRNA-seq,SRP330776,Naive CD8+ T cell_1-AD.vs.control; scRNA-seq,SRP330776,Natural killer cell_1-AD.vs.control; scRNA-seq,SRP330776,Memory T cell_1-AD.vs.control; scRNA-seq,SRP330776,Naive CD8+ T cell_3-AD.vs.control; scRNA-seq,SRP330776,B cell_2- [illegible]	18
CLUL1	bulk RNA-seq,Homo_723,AD.vs.control; bulk RNA-seq,Homo_723,AD.vs.MCI	2
CLVS1	bulk RNA-seq,Homo_723,AD.vs.control; bulk RNA-seq,Homo_723,AD.vs.MCI; bulk RNA-seq,Homo_633,AD.vs.control; bulk RNA-seq,Homo_633,AD.vs.MCI; bulk RNA-seq,Homo_633,MCI.vs.control; bulk RNA-seq,Homo_714,AD.vs.control; bulk RNA-seq,Homo_714,AD.vs.MCI;	8
CLVS2	bulk RNA-seq,Homo_723,AD.vs.control; bulk RNA-seq,Homo_723,AD.vs.MCI	2
CLYBL	scRNA-seq,SRP215507,CD8+ T cell_7-AD.vs.control; scRNA-seq,SRP215507,CD8+ T cell_5-MCI.vs.control; scRNA-seq,SRP215507,CD8+ T cell_2-MCI.vs.control; scRNA-seq,SRP215507,CD8+ T cell_3-MCI.vs.control; scRNA-seq,SRP330776,γδ T cell-AD.vs.control; scRNA-seq,SRP330776,CD8+ T cell_2-AD.vs.control; scRNA-seq,SRP330776,CD8+ T cell_3-AD.vs.control	7
CMA1	bulk RNA-seq,Homo_723,AD.vs.control; bulk RNA-seq,Homo_723,AD.vs.MCI	2
CMAS	bulk RNA-seq,Homo_723,AD.vs.control; bulk RNA-seq,Homo_723,AD.vs.MCI; bulk RNA-seq,Homo_633,AD.vs.control; bulk RNA-seq,Homo_633,AD.vs.MCI; bulk RNA-seq,Homo_714,AD.vs.control; bulk RNA-seq,Homo_714,AD.vs.MCI; scRNA-seq,SRP215507,CD8+ T cell_4-AD.vs.MCI; scRNA-seq,SRP309935,Erythroid cell-AD.vs.control; scRNA-seq,SRP309935,Megakaryocyte_2-AD.vs.control; scRNA-seq,SRP330776,CD8+ T cell_1-AD.vs.control; scRNA-seq,SRP330776,γδ T cell-AD.vs.control; scRNA-seq,SRP330776,Memory T cell_2-AD.vs.control; scRNA-seq,SRP330776,B cell_1-AD.vs.control; scRNA-seq,SRP330776,Naive CD8+ T cell_1-AD.vs.control; scRNA-seq,SRP330776,Memory T cell_1-AD.vs.control; scRNA-seq,SRP330776,Natural killer T cell-AD.vs.control; scRNA-seq,SRP330776,Naive CD8+ T cell_3-AD.vs.control; scRNA-seq,SRP330776,B cell_2-AD.vs.control; scRNA-seq,SRP330776,T cell-AD.vs.control; scRNA-seq,SRP330776,CD8+ T cell_3-AD.vs.control; scRNA- [illegible]	21
Cmas	bulk RNA-seq,SRP325058,AD.vs.control	1
CMBL	bulk RNA-seq,Homo_723,AD.vs.control; bulk RNA-seq,Homo_723,AD.vs.MCI; bulk RNA-seq,Homo_633,AD.vs.control; bulk RNA-seq,Homo_633,AD.vs.MCI; bulk RNA-seq,Homo_714,AD.vs.control; bulk RNA-seq,Homo_714,AD.vs.MCI; bulk RNA-seq,Homo_714,MCI.vs.control	7

CMC1	scRNA-seq,SRP215507,CD8+ T cell_3-AD.vs.control; scRNA-seq,SRP215507,CD8+ T cell_4-AD.vs.control; scRNA-seq,SRP215507,CD8+ T cell_7-AD.vs.control; scRNA-seq,SRP215507,CD8+ T cell_6-AD.vs.control; scRNA-seq,SRP215507,CD8+ T cell_2-AD.vs.MCI; scRNA-seq,SRP215507,CD8+ T cell_1-AD.vs.MCI; scRNA-seq,SRP215507,CD8+ T cell_4-AD.vs.MCI; scRNA-seq,SRP215507,CD8+ T cell_5-AD.vs.MCI; scRNA-seq,SRP215507,CD8+ T cell_5-MCI.vs.control; scRNA-seq,SRP215507,CD8+ T cell_6-MCI.vs.control; scRNA-seq,SRP309935,Monocyte_1-AD.vs.control; scRNA-seq,SRP309935,Erythroid cell-AD.vs.control; scRNA-seq,SRP309935,Megakaryocyte_2-AD.vs.control; scRNA-seq,SRP330776,Naive CD8+ T cell_3-AD.vs.control; scRNA-seq,SRP330776,B cell_2-AD.vs.control; scRNA-seq,SRP330776,Naive CD8+ T cell_2-[illegible]	17
CMC2	scRNA-seq,SRP215507,CD8+ T cell_7-AD.vs.MCI; scRNA-seq,SRP215507,CD8+ T cell_7-MCI.vs.control; scRNA-seq,SRP330776,Memory T cell_1-AD.vs.control; scRNA-seq,SRP330776,Natural killer T cell-AD.vs.control; scRNA-seq,SRP330776,Naive CD8+ T cell_3-AD.vs.control	5
CMC4	bulk RNA-seq,Homo_723,AD.vs.MCI; bulk RNA-seq,Homo_633,AD.vs.MCI; bulk RNA-seq,Homo_714,AD.vs.control; bulk RNA-seq,Homo_714,AD.vs.MCI; scRNA-seq,SRP215507,CD8+ T cell_8-AD.vs.control; scRNA-seq,SRP215507,CD8+ T cell_1-AD.vs.MCI; scRNA-seq,SRP215507,CD8+ T cell_6-MCI.vs.control; scRNA-seq,SRP330776,CD8+ T cell_1-AD.vs.control; scRNA-seq,SRP330776,γδ T cell-AD.vs.control; scRNA-seq,SRP330776,Memory T cell_2-AD.vs.control; scRNA-seq,SRP330776,CD8+ T cell_2-AD.vs.control; scRNA-seq,SRP330776,B cell_1-AD.vs.control; scRNA-seq,SRP330776,Naive CD8+ T cell_1-AD.vs.control; scRNA-seq,SRP330776,Natural killer cell_1-AD.vs.control; scRNA-[illegible]	16
CMIP	scRNA-seq,SRP215507,CD8+ T cell_4-AD.vs.MCI; scRNA-seq,SRP215507,CD8+ T cell_4-MCI.vs.control; scRNA-seq,SRP309935,Monocyte_2-AD.vs.control; scRNA-seq,SRP330776,CD8+ T cell_1-AD.vs.control; scRNA-seq,SRP330776,γδ T cell-AD.vs.control; scRNA-seq,SRP330776,Memory T cell_2-AD.vs.control; scRNA-seq,SRP330776,CD8+ T cell_2-AD.vs.control; scRNA-seq,SRP330776,B cell_1-AD.vs.control; scRNA-seq,SRP330776,Naive CD8+ T cell_1-AD.vs.control; scRNA-seq,SRP330776,Natural killer cell_1-AD.vs.control; scRNA-seq,SRP330776,Memory T cell_1-AD.vs.control; scRNA-seq,SRP330776,Natural killer T cell-AD.vs.control; scRNA-seq,SRP330776,Naive CD8+ T cell_3-AD.vs.control; scRNA-seq,SRP330776,B cell_2-AD.vs.control; scRNA-seq,SRP330776,T cell-AD.vs.control; scRNA-seq,SRP330776,CD8+ T cell_3-AD.vs.control; scRNA-seq,SRP330776,Monocyte_2-AD.vs.control; scRNA-seq,SRP330776,B cell_3-AD.vs.control; scRNA-seq,SRP330776,Natural killer cell_2-AD.vs.control	19
CMKLR1	bulk RNA-seq,Homo_723,AD.vs.control; bulk RNA-seq,Homo_723,AD.vs.MCI; bulk RNA-seq,Homo_633,AD.vs.control; bulk RNA-seq,Homo_633,AD.vs.MCI; bulk RNA-seq,Homo_633,MCI.vs.control; bulk RNA-seq,SRP223445,AD.vs.control; scRNA-seq,SRP215507,CD8+ T cell_6-AD.vs.control; scRNA-seq,SRP215507,CD8+ T cell_4-AD.vs.MCI; scRNA-seq,SRP215507,CD8+ T cell_4-MCI.vs.control; scRNA-seq,SRP215507,CD8+ T cell_8-MCI.vs.control; scRNA-seq,SRP309935,CD8+ T cell_1-AD.vs.control; scRNA-seq,SRP330776,Natural killer T cell-AD.vs.control; scRNA-seq,SRP330776,Monocyte_1-AD.vs.control; scRNA-seq,SRP330776,B cell_3-AD.vs.control	14
CMKLR2	bulk RNA-seq,Homo_723,AD.vs.control; bulk RNA-seq,Homo_723,AD.vs.MCI; bulk RNA-seq,Homo_633,AD.vs.control; bulk RNA-[illegible]	4
CMPK1	scRNA-seq,SRP215507,CD8+ T cell_4-AD.vs.control; scRNA-seq,SRP215507,CD8+ T cell_6-AD.vs.control; scRNA-seq,SRP215507,CD8+ T cell_4-AD.vs.MCI; scRNA-seq,SRP215507,CD8+ T cell_6-AD.vs.MCI; scRNA-seq,SRP215507,CD8+ T cell_8-MCI.vs.control; scRNA-seq,SRP309935,Naive CD8+ T cell_1-AD.vs.control; scRNA-seq,SRP309935,Naive CD8+ T cell_2-AD.vs.control; scRNA-seq,SRP309935,CD8+ T cell_2-AD.vs.control; scRNA-seq,SRP309935,B cell_2-AD.vs.control; scRNA-seq,SRP330776,CD8+ T cell_1-AD.vs.control; scRNA-seq,SRP330776,γδ T cell-AD.vs.control; scRNA-seq,SRP330776,Memory T cell_2-AD.vs.control; scRNA-seq,SRP330776,CD8+ T cell_2-AD.vs.control; scRNA-seq,SRP330776,B cell_1-AD.vs.control; scRNA-seq,SRP330776,Naive CD8+ T cell_1-AD.vs.control; scRNA-seq,SRP330776,Natural killer cell_1-AD.vs.control; scRNA-seq,SRP330776,Memory T cell_1-AD.vs.control; scRNA-seq,SRP330776,Natural killer T cell-AD.vs.control; scRNA-seq,SRP330776,Naive CD8+ T cell_3-AD.vs.control; scRNA-seq,SRP330776,B cell_2-AD.vs.control; scRNA-seq,SRP330776,T cell-AD.vs.control; scRNA-seq,SRP330776,CD8+ T cell_3-AD.vs.control; scRNA-seq,SRP330776,Monocyte_1-AD.vs.control; scRNA-seq,SRP330776,B cell_3-AD.vs.control; scRNA-[illegible]	26
CMPK2	bulk RNA-seq,Homo_723,AD.vs.control; bulk RNA-seq,Homo_723,MCI.vs.control; bulk RNA-seq,Homo_633,AD.vs.control; bulk RNA-seq,Homo_633,MCI.vs.control; scRNA-seq,SRP215507,CD8+ T cell_5-AD.vs.control; scRNA-seq,SRP215507,CD8+ T cell_6-AD.vs.control; scRNA-seq,SRP215507,CD8+ T cell_5-AD.vs.MCI; scRNA-seq,SRP215507,CD8+ T cell_6-MCI.vs.control; scRNA-seq,SRP330776,CD8+ T cell_1-AD.vs.control; scRNA-seq,SRP330776,γδ T cell-AD.vs.control; scRNA-seq,SRP330776,Memory T cell_2-AD.vs.control; scRNA-seq,SRP330776,CD8+ T cell_2-AD.vs.control; scRNA-seq,SRP330776,B cell_1-AD.vs.control; scRNA-seq,SRP330776,Naive CD8+ T cell_1-AD.vs.control; scRNA-seq,SRP330776,Natural killer cell_1-AD.vs.control; scRNA-seq,SRP330776,Memory T cell_1-AD.vs.control; scRNA-seq,SRP330776,B cell_2-[illegible]	19

CMSS1	bulk RNA-seq,Homo_723,AD.vs.control; bulk RNA-seq,Homo_723,AD.vs.MCI; bulk RNA-seq,Homo_723,MCI.vs.control; bulk RNA-seq,Homo_633,AD.vs.control; bulk RNA-seq,Homo_633,AD.vs.MCI; bulk RNA-seq,Homo_633,MCI.vs.control; bulk RNA-seq,Homo_714,AD.vs.control; bulk RNA-seq,Homo_714,AD.vs.MCI; bulk RNA-seq,Homo_714,MCI.vs.control; scRNA-seq,SRP215507,CD8+ T cell_4-AD.vs.MCI; scRNA-seq,SRP215507,CD8+ T cell_4-MCI.vs.control; scRNA-seq,SRP330776,Memory T cell_2-AD.vs.control; scRNA-seq,SRP330776,CD8+ T cell_2-AD.vs.control; scRNA-seq,SRP330776,Natural killer cell_1-AD.vs.control; scRNA-seq,SRP330776,Memory T cell_1-AD.vs.control; scRNA-	17
CMTM1	bulk RNA-seq,Homo_723,AD.vs.control; bulk RNA-seq,Homo_723,AD.vs.MCI; bulk RNA-seq,Homo_723,MCI.vs.control; bulk RNA-seq,Homo_633,AD.vs.control; bulk RNA-seq,Homo_633,AD.vs.MCI; bulk RNA-seq,Homo_633,MCI.vs.control; bulk RNA-seq,Homo_714,AD.vs.control; bulk RNA-seq,Homo_714,AD.vs.MCI; bulk RNA-seq,Homo_714,MCI.vs.control; scRNA-seq,SRP215507,CD8+ T cell_2-MCI.vs.control	10
CMTM2	bulk RNA-seq,Homo_723,AD.vs.control; bulk RNA-seq,Homo_723,MCI.vs.control; bulk RNA-seq,Homo_633,MCI.vs.control; bulk RNA-seq,Homo_714,AD.vs.control; bulk RNA-seq,Homo_714,AD.vs.MCI; bulk RNA-seq,Homo_714,MCI.vs.control; bulk RNA-seq,SRP223445,AD.vs.control	7
CMTM3	scRNA-seq,SRP215507,CD8+ T cell_6-AD.vs.MCI; scRNA-seq,SRP309935,Monocyte_1-AD.vs.control; scRNA-seq,SRP330776,CD8+ T cell_1-AD.vs.control; scRNA-seq,SRP330776,γδ T cell-AD.vs.control; scRNA-seq,SRP330776,Memory T cell_2-AD.vs.control; scRNA-seq,SRP330776,CD8+ T cell_2-AD.vs.control; scRNA-seq,SRP330776,B cell_1-AD.vs.control; scRNA-seq,SRP330776,Naive CD8+ T cell_1-AD.vs.control; scRNA-seq,SRP330776,Natural killer cell_1-AD.vs.control; scRNA-seq,SRP330776,Memory T cell_1-AD.vs.control; scRNA-seq,SRP330776,Natural killer T cell-AD.vs.control; scRNA-seq,SRP330776,Naive CD8+ T cell_3-AD.vs.control; scRNA-seq,SRP330776,B cell_2-AD.vs.control; scRNA-seq,SRP330776,T cell-AD.vs.control; scRNA-seq,SRP330776,Naive CD8+ T cell_2-AD.vs.control; scRNA-seq,SRP330776,CD8+ T cell_3-AD.vs.control; scRNA-seq,SRP330776,Monocyte_1-AD.vs.control; scRNA-seq,SRP330776,Monocyte_2-AD.vs.control; scRNA-seq,SRP330776,B cell_3-AD.vs.control; scRNA- [illegible]	21
CMTM4	bulk RNA-seq,Homo_723,AD.vs.control; bulk RNA-seq,Homo_723,AD.vs.MCI; bulk RNA-seq,Homo_723,MCI.vs.control; bulk RNA-seq,Homo_633,AD.vs.control; bulk RNA-seq,Homo_633,AD.vs.MCI; bulk RNA-seq,Homo_633,MCI.vs.control; bulk RNA-seq,Homo_714,AD.vs.control; bulk RNA-seq,Homo_714,AD.vs.MCI; bulk RNA-seq,Homo_714,MCI.vs.control	9
CMTM5	scRNA-seq,SRP330776,Natural killer cell_1-AD.vs.control	1
CMTM6	bulk RNA-seq,Homo_714,AD.vs.control; bulk RNA-seq,Homo_714,AD.vs.MCI; bulk RNA-seq,SRP310421,AD.vs.control; scRNA-seq,SRP215507,CD8+ T cell_7-AD.vs.control; scRNA-seq,SRP215507,CD8+ T cell_5-AD.vs.MCI; scRNA-seq,SRP309935,Megakaryocyte_2-AD.vs.control	6
CMTM7	bulk RNA-seq,SRP310421,AD.vs.control; scRNA-seq,SRP215507,CD8+ T cell_1-AD.vs.control; scRNA-seq,SRP215507,CD8+ T cell_7-AD.vs.control; scRNA-seq,SRP215507,CD8+ T cell_4-AD.vs.MCI; scRNA-seq,SRP215507,CD8+ T cell_4-MCI.vs.control; scRNA-seq,SRP309935,Erythroid cell-AD.vs.control; scRNA-seq,SRP309935,Natural killer cell_1-AD.vs.control; scRNA-seq,SRP309935,B cell_1-AD.vs.control; scRNA-seq,SRP330776,CD8+ T cell_1-AD.vs.control; scRNA-seq,SRP330776,γδ T cell-AD.vs.control; scRNA-seq,SRP330776,Memory T cell_2-AD.vs.control; scRNA-seq,SRP330776,CD8+ T cell_2-AD.vs.control; scRNA-seq,SRP330776,B cell_1-AD.vs.control; scRNA-seq,SRP330776,Naive CD8+ T cell_1-AD.vs.control; scRNA-seq,SRP330776,Natural killer cell_1-AD.vs.control; scRNA-seq,SRP330776,Memory T cell_1-AD.vs.control; scRNA-seq,SRP330776,Natural killer T cell-AD.vs.control; scRNA-seq,SRP330776,Naive CD8+ T cell_3-AD.vs.control; scRNA-seq,SRP330776,B cell_2-AD.vs.control; scRNA-seq,SRP330776,T cell-AD.vs.control; scRNA-seq,SRP330776,Naive CD8+ T cell_2-AD.vs.control; scRNA-seq,SRP330776,CD8+ T cell_3-AD.vs.control; scRNA-seq,SRP330776,Monocyte_1-AD.vs.control; scRNA-seq,SRP330776,Monocyte_2-AD.vs.control; scRNA- [illegible]	27
CMTM8	bulk RNA-seq,Homo_714,AD.vs.control; bulk RNA-seq,Homo_714,AD.vs.MCI; bulk RNA-seq,Homo_714,MCI.vs.control; scRNA-seq,SRP215507,CD8+ T cell_2-AD.vs.control; scRNA-seq,SRP215507,CD8+ T cell_3-AD.vs.control; scRNA-seq,SRP215507,CD8+ T cell_6-AD.vs.control; scRNA-seq,SRP215507,CD8+ T cell_5-AD.vs.MCI; scRNA-seq,SRP215507,CD8+ T cell_2-MCI.vs.control; scRNA-seq,SRP215507,CD8+ T cell_3-MCI.vs.control; scRNA-seq,SRP309935,Monocyte_1-AD.vs.control; scRNA-seq,SRP309935,Megakaryocyte_1-AD.vs.control; scRNA-seq,SRP309935,Naive CD8+ T cell_1-AD.vs.control; scRNA-seq,SRP309935,Naive CD8+ T cell_2-AD.vs.control; scRNA-seq,SRP309935,CD8+ T cell_2-AD.vs.control; scRNA-seq,SRP309935,Naive CD8+ T cell_3-AD.vs.control; scRNA-seq,SRP330776,CD8+ T cell_1-AD.vs.control; scRNA-seq,SRP330776,Memory T cell_2-AD.vs.control; scRNA-seq,SRP330776,CD8+ T cell_2-AD.vs.control; scRNA-seq,SRP330776,Naive CD8+ T cell_1- [illegible]	20
CMTR1	bulk RNA-seq,Homo_723,AD.vs.control; bulk RNA-seq,Homo_723,AD.vs.MCI; bulk RNA-seq,Homo_723,MCI.vs.control; bulk RNA-seq,Homo_633,AD.vs.control; bulk RNA-seq,Homo_633,AD.vs.MCI; bulk RNA-seq,Homo_633,MCI.vs.control; bulk RNA-seq,Homo_714,AD.vs.control; bulk RNA-seq,Homo_714,AD.vs.MCI; bulk RNA-seq,Homo_714,MCI.vs.control; scRNA-seq,SRP215507,CD8+ T cell_3-AD.vs.MCI; scRNA- [illegible]	11

CMTR2	scRNA-seq,SRP309935,Megakaryocyte_1-AD.vs.control; scRNA-seq,SRP309935,Naive CD8+ T cell_1-AD.vs.control; scRNA-seq,SRP330776,Memory T cell_2-AD.vs.control; scRNA-seq,SRP330776,Naive CD8+ T cell_1-AD.vs.control; scRNA-seq,SRP330776,Naive CD8+ T cell_3-AD.vs.control; scRNA-seq,SRP330776,B cell_2-AD.vs.control	6
CMYA5	bulk RNA-seq,Homo_723,AD.vs.control; bulk RNA-seq,Homo_723,AD.vs.MCI; bulk RNA-seq,Homo_633,AD.vs.control; bulk RNA-seq,Homo_633,AD.vs.MCI; bulk RNA-seq,Homo_714,AD.vs.MCI; bulk RNA-seq,Homo_714,MCI.vs.control; scRNA-seq,SRP215507,CD8+ T cell_1-	7
CNBD2	scRNA-seq,SRP215507,CD8+ T cell_5-MCI.vs.control; scRNA-seq,SRP330776,Naive CD8+ T cell_1-AD.vs.control; scRNA-seq,SRP330776,Natural killer T cell-AD.vs.control; scRNA-seq,SRP330776,Naive CD8+ T cell_3-AD.vs.control; scRNA-seq,SRP330776,T cell-AD.vs.control	5
CNBP	scRNA-seq,SRP215507,CD8+ T cell_7-AD.vs.control; scRNA-seq,SRP215507,CD8+ T cell_10-AD.vs.control; scRNA-seq,SRP215507,CD8+ T cell_8-MCI.vs.control; scRNA-seq,SRP309935,Erythroid cell-AD.vs.control; scRNA-seq,SRP309935,Megakaryocyte_2-AD.vs.control; scRNA-	6
CNDP1	bulk RNA-seq,Homo_723,AD.vs.control; bulk RNA-seq,Homo_723,AD.vs.MCI; bulk RNA-seq,Homo_723,MCI.vs.control; bulk RNA-seq,Homo_633,AD.vs.MCI; bulk RNA-seq,Homo_633,MCI.vs.control; bulk RNA-seq,ROSMAP,AD.vs.control; bulk RNA-seq,ROSMAP,MCI.vs.control	7
CNDP2	bulk RNA-seq,Homo_633,AD.vs.control; scRNA-seq,SRP215507,CD8+ T cell_10-AD.vs.control; scRNA-seq,SRP215507,CD8+ T cell_5-MCI.vs.control; scRNA-seq,SRP330776,Memory T cell_2-AD.vs.control; scRNA-seq,SRP330776,Natural killer cell_1-AD.vs.control	5
CNEP1R1	bulk RNA-seq,Homo_723,AD.vs.control; bulk RNA-seq,Homo_723,AD.vs.MCI; bulk RNA-seq,Homo_633,AD.vs.control; bulk RNA-seq,Homo_633,AD.vs.MCI; bulk RNA-seq,Homo_714,AD.vs.control; bulk RNA-seq,Homo_714,AD.vs.MCI; scRNA-seq,SRP215507,CD8+ T cell_5-AD.vs.MCI; scRNA-seq,SRP215507,CD8+ T cell_1-MCI.vs.control; scRNA-seq,SRP215507,CD8+ T cell_5-MCI.vs.control; scRNA-seq,SRP215507,CD8+ T cell_2-MCI.vs.control; scRNA-seq,SRP215507,CD8+ T cell_3-MCI.vs.control; scRNA-seq,SRP330776,Memory T cell_1-AD.vs.control; scRNA-seq,SRP330776,Natural killer T cell-AD.vs.control; scRNA-seq,SRP330776,B cell_2-AD.vs.control; scRNA-seq,SRP330776,CD8+	17
CNFN	bulk RNA-seq,Homo_723,AD.vs.control; bulk RNA-seq,Homo_723,MCI.vs.control; bulk RNA-seq,Homo_633,AD.vs.control; bulk RNA-seq,Homo_633,MCI.vs.control; bulk RNA-seq,Homo_714,AD.vs.MCI; bulk RNA-seq,Homo_714,MCI.vs.control; scRNA-seq,SRP215507,CD8+ T cell_1-AD.vs.control; scRNA-seq,SRP215507,CD8+ T cell_5-AD.vs.MCI; scRNA-seq,SRP215507,CD8+ T cell_1-MCI.vs.control; scRNA-seq,SRP215507,CD8+ T cell_5-MCI.vs.control; scRNA-seq,SRP309935,Monocyte_1-AD.vs.control; scRNA-seq,SRP309935,CD8+ T cell_1-AD.vs.control; scRNA-seq,SRP330776,B cell_1-AD.vs.control; scRNA-seq,SRP330776,Natural killer cell_1-AD.vs.control; scRNA-seq,SRP330776,B cell_2-AD.vs.control	15
CNGA1	bulk RNA-seq,Homo_714,AD.vs.MCI	1
CNGA3	bulk RNA-seq,Homo_723,AD.vs.control; bulk RNA-seq,Homo_723,AD.vs.MCI; bulk RNA-seq,Homo_714,MCI.vs.control; bulk RNA-	4
CNGA4	bulk RNA-seq,Homo_723,AD.vs.MCI; bulk RNA-seq,Homo_723,MCI.vs.control; bulk RNA-seq,Homo_633,AD.vs.control; bulk RNA-seq,Homo_633,MCI.vs.control; bulk RNA-seq,Homo_714,AD.vs.control; bulk RNA-seq,Homo_714,AD.vs.MCI; bulk RNA-seq,Homo_714,MCI.vs.control	7
CNGB1	bulk RNA-seq,Homo_723,AD.vs.control; bulk RNA-seq,Homo_723,AD.vs.MCI; bulk RNA-seq,Homo_633,AD.vs.control; bulk RNA-	4
CNGB3	bulk RNA-seq,Homo_723,AD.vs.control; bulk RNA-seq,Homo_723,AD.vs.MCI; bulk RNA-seq,Homo_633,AD.vs.control; bulk RNA-seq,Homo_633,AD.vs.MCI; bulk RNA-seq,Homo_714,MCI.vs.control	5
CNIH1	bulk RNA-seq,Homo_723,AD.vs.control; bulk RNA-seq,Homo_723,AD.vs.MCI; bulk RNA-seq,Homo_633,AD.vs.MCI; bulk RNA-seq,Homo_714,AD.vs.control; bulk RNA-seq,Homo_714,AD.vs.MCI; scRNA-seq,SRP215507,CD8+ T cell_4-AD.vs.control; scRNA-seq,SRP215507,CD8+ T cell_7-AD.vs.control; scRNA-seq,SRP215507,CD8+ T cell_4-MCI.vs.control; scRNA-seq,SRP309935,Erythroid cell-	9
CNIH2	scRNA-seq,SRP215507,CD8+ T cell_3-AD.vs.control; scRNA-seq,SRP330776,CD8+ T cell_1-AD.vs.control	2
CNIH3	bulk RNA-seq,Homo_714,AD.vs.control; bulk RNA-seq,Homo_714,AD.vs.MCI; scRNA-seq,SRP215507,CD8+ T cell_2-AD.vs.MCI; scRNA-seq,SRP215507,CD8+ T cell_4-AD.vs.MCI; scRNA-seq,SRP330776,CD8+ T cell_1-AD.vs.control; scRNA-seq,SRP330776,Memory T cell_2-	6
CNIH4	bulk RNA-seq,Homo_723,AD.vs.control; bulk RNA-seq,Homo_723,AD.vs.MCI; bulk RNA-seq,Homo_633,AD.vs.control; bulk RNA-seq,Homo_633,AD.vs.MCI; scRNA-seq,SRP330776,CD8+ T cell_1-AD.vs.control; scRNA-seq,SRP330776,CD8+ T cell_2-AD.vs.control; scRNA-seq,SRP330776,B cell_1-AD.vs.control; scRNA-seq,SRP330776,Naive CD8+ T cell_3-AD.vs.control; scRNA-seq,SRP330776,B cell_2-AD.vs.control; scRNA-seq,SRP330776,T cell-AD.vs.control; scRNA-seq,SRP330776,Monocyte_2-AD.vs.control	11
CNKSR1	bulk RNA-seq,Homo_723,AD.vs.control; bulk RNA-seq,Homo_723,MCI.vs.control; bulk RNA-seq,Homo_633,AD.vs.MCI; bulk RNA-seq,Homo_633,MCI.vs.control; bulk RNA-seq,Homo_714,AD.vs.MCI; bulk RNA-seq,Homo_714,MCI.vs.control; scRNA-seq,SRP330776,CD8+ T cell_1-AD.vs.control; scRNA-seq,SRP330776,Memory T cell_2-AD.vs.control; scRNA-seq,SRP330776,Naive CD8+ T cell_1-AD.vs.control	9

CNKSR2	bulk RNA-seq,Homo_723,AD.vs.control; bulk RNA-seq,Homo_723,AD.vs.MCI; bulk RNA-seq,Homo_633,AD.vs.control; bulk RNA-seq,Homo_633,AD.vs.MCI; bulk RNA-seq,Homo_714,AD.vs.control; scRNA-seq,SRP215507,CD8+ T cell_2-MCI.vs.control; scRNA-seq,SRP330776,CD8+ T cell_1-AD.vs.control; scRNA-seq,SRP330776,γδ T cell-AD.vs.control; scRNA-seq,SRP330776,Memory T cell_2-AD.vs.control; scRNA-seq,SRP330776,B cell_1-AD.vs.control; scRNA-seq,SRP330776,Memory T cell_1-AD.vs.control; scRNA-seq,SRP330776,Naive CD8+ T cell_3-AD.vs.control; scRNA-seq,SRP330776,T cell-AD.vs.control; scRNA-seq,SRP330776,CD8+ T cell_3-AD.vs.control; scRNA-seq,SRP330776,B cell_4-	15
CNKSR3	bulk RNA-seq,Homo_723,AD.vs.control; bulk RNA-seq,Homo_723,AD.vs.MCI; bulk RNA-seq,Homo_723,MCI.vs.control; bulk RNA-seq,Homo_633,AD.vs.control; bulk RNA-seq,Homo_633,AD.vs.MCI; bulk RNA-seq,Homo_633,MCI.vs.control; bulk RNA-seq,Homo_714,AD.vs.control; bulk RNA-seq,Homo_714,AD.vs.MCI; bulk RNA-seq,Homo_714,MCI.vs.control	9
CNMD	bulk RNA-seq,Homo_723,AD.vs.control; bulk RNA-seq,Homo_723,AD.vs.MCI; bulk RNA-seq,ROSMAP,AD.vs.MCI; bulk RNA-	4
CNN1	bulk RNA-seq,Homo_723,AD.vs.control; bulk RNA-seq,Homo_723,AD.vs.MCI; bulk RNA-seq,Homo_723,MCI.vs.control; bulk RNA-seq,Homo_633,AD.vs.control; bulk RNA-seq,Homo_633,AD.vs.MCI; bulk RNA-seq,Homo_633,MCI.vs.control; bulk RNA-seq,Homo_714,AD.vs.MCI	7
CNN2	scRNA-seq,SRP215507,CD8+ T cell_9-AD.vs.control; scRNA-seq,SRP330776,CD8+ T cell_2-AD.vs.control	2
CNN3	bulk RNA-seq,Homo_723,AD.vs.control; bulk RNA-seq,Homo_723,MCI.vs.control; bulk RNA-seq,Homo_633,AD.vs.control; bulk RNA-seq,Homo_633,AD.vs.MCI; bulk RNA-seq,Homo_633,MCI.vs.control; bulk RNA-seq,Homo_714,MCI.vs.control; scRNA-seq,SRP215507,CD8+ T cell_3-AD.vs.control; scRNA-seq,SRP215507,CD8+ T cell_1-AD.vs.control; scRNA-seq,SRP215507,CD8+ T cell_3-AD.vs.MCI; scRNA-seq,SRP215507,CD8+ T cell_3-MCI.vs.control; scRNA-seq,SRP330776,B cell_1-AD.vs.control; scRNA-seq,SRP330776,Naive CD8+ T cell_1-AD.vs.control; scRNA-	13
CNNM1	bulk RNA-seq,Homo_723,AD.vs.control; bulk RNA-seq,Homo_633,MCI.vs.control; bulk RNA-seq,Homo_714,MCI.vs.control; bulk RNA-seq,ROSMAP,AD.vs.control; bulk RNA-seq,ROSMAP,MCI.vs.control	5
CNNM2	bulk RNA-seq,Homo_723,AD.vs.control; bulk RNA-seq,Homo_723,AD.vs.MCI; bulk RNA-seq,Homo_633,AD.vs.control; bulk RNA-seq,Homo_633,AD.vs.MCI; bulk RNA-seq,Homo_714,AD.vs.control; bulk RNA-seq,Homo_714,AD.vs.MCI; scRNA-seq,SRP215507,CD8+ T cell_4-AD.vs.control; scRNA-seq,SRP330776,Natural killer cell_2-AD.vs.control	8
Cnnm2	bulk RNA-seq,SRP325058,AD.vs.control	1
CNNM3	bulk RNA-seq,Homo_723,MCI.vs.control; bulk RNA-seq,Homo_633,MCI.vs.control; bulk RNA-seq,ROSMAP,AD.vs.MCI; scRNA-seq,SRP215507,CD8+ T cell_2-AD.vs.MCI; scRNA-seq,SRP215507,CD8+ T cell_4-AD.vs.MCI; scRNA-seq,SRP215507,CD8+ T cell_4-MCI.vs.control; scRNA-seq,SRP215507,CD8+ T cell_6-MCI.vs.control; scRNA-seq,SRP309935,Natural killer cell_2-AD.vs.control; scRNA-seq,SRP330776,Naive CD8+ T cell_1-AD.vs.control; scRNA-seq,SRP330776,Memory T cell_1-AD.vs.control; scRNA-seq,SRP330776,T cell-AD.vs.control	11
CNNM3-DT	scRNA-seq,SRP309935,Naive CD8+ T cell_1-AD.vs.control; scRNA-seq,SRP309935,CD8+ T cell_2-AD.vs.control	2
CNNM4	scRNA-seq,SRP215507,CD8+ T cell_4-AD.vs.MCI; scRNA-seq,SRP330776,Memory T cell_2-AD.vs.control; scRNA-seq,SRP330776,B cell_1-AD.vs.control; scRNA-seq,SRP330776,Monocyte_2-AD.vs.control	4
Cnot1	bulk RNA-seq,SRP325058,AD.vs.control	1
CNOT1	scRNA-seq,SRP215507,CD8+ T cell_6-AD.vs.MCI; scRNA-seq,SRP215507,CD8+ T cell_6-MCI.vs.control; scRNA-seq,SRP330776,Naive CD8+ T cell_3-AD.vs.control; scRNA-seq,SRP330776,Monocyte_2-AD.vs.control	4
CNOT10	bulk RNA-seq,Homo_633,AD.vs.control; bulk RNA-seq,Homo_633,MCI.vs.control; bulk RNA-seq,Homo_714,AD.vs.control; bulk RNA-seq,Homo_714,AD.vs.MCI; bulk RNA-seq,Homo_714,MCI.vs.control; scRNA-seq,SRP215507,CD8+ T cell_7-AD.vs.MCI; scRNA-seq,SRP309935,B	7
CNOT11	bulk RNA-seq,Homo_714,AD.vs.control; bulk RNA-seq,Homo_714,AD.vs.MCI; scRNA-seq,SRP215507,CD8+ T cell_5-AD.vs.MCI; scRNA-seq,SRP215507,CD8+ T cell_5-MCI.vs.control; scRNA-seq,SRP330776,CD8+ T cell_1-AD.vs.control; scRNA-seq,SRP330776,γδ T cell-AD.vs.control; scRNA-seq,SRP330776,Memory T cell_2-AD.vs.control; scRNA-seq,SRP330776,CD8+ T cell_2-AD.vs.control; scRNA-seq,SRP330776,B cell_1-AD.vs.control; scRNA-seq,SRP330776,Naive CD8+ T cell_1-AD.vs.control; scRNA-seq,SRP330776,Natural killer cell_1-AD.vs.control; scRNA-seq,SRP330776,Memory T cell_1-AD.vs.control; scRNA-seq,SRP330776,Natural killer T cell-AD.vs.control; scRNA-seq,SRP330776,Naive CD8+ T cell_3-AD.vs.control; scRNA-seq,SRP330776,B cell_2-AD.vs.control; scRNA-seq,SRP330776,T cell-AD.vs.control; scRNA-seq,SRP330776,Naive CD8+ T cell_2-AD.vs.control; scRNA-seq,SRP330776,CD8+ T cell_3-AD.vs.control; scRNA-seq,SRP330776,Monocyte_1-AD.vs.control; scRNA- [illegible]	21
CNOT2	bulk RNA-seq,Homo_633,AD.vs.control; scRNA-seq,SRP215507,CD8+ T cell_7-AD.vs.control	2

CNOT3	bulk RNA-seq,Homo_723,AD.vs.control; bulk RNA-seq,Homo_723,AD.vs.MCI; bulk RNA-seq,Homo_633,AD.vs.control; bulk RNA-seq,Homo_633,AD.vs.MCI; bulk RNA-seq,Homo_714,AD.vs.MCI; bulk RNA-seq,Homo_714,MCI.vs.control; scRNA-seq,SRP215507,CD8+ T cell_10-AD.vs.control; scRNA-seq,SRP309935,Monocyte_1-AD.vs.control; scRNA-seq,SRP309935,B cell_1-AD.vs.control; scRNA-seq,SRP330776,Memory T cell_2-AD.vs.control; scRNA-seq,SRP330776,Natural killer cell_1-AD.vs.control; scRNA-seq,SRP330776,Naive CD8+ T cell_3-AD.vs.control; scRNA-seq,SRP330776,B cell_2-AD.vs.control; scRNA-seq,SRP330776,Megakaryocyte_1-AD.vs.control	14
CNOT4	scRNA-seq,SRP215507,CD8+ T cell_6-AD.vs.control; scRNA-seq,SRP215507,CD8+ T cell_6-MCI.vs.control; scRNA-seq,SRP330776,B cell_2-	3
CNOT6	scRNA-seq,SRP215507,CD8+ T cell_5-AD.vs.MCI; scRNA-seq,SRP215507,CD8+ T cell_5-MCI.vs.control; scRNA-seq,SRP309935,Naive CD8+ T cell_3-AD.vs.control; scRNA-seq,SRP330776,CD8+ T cell_1-AD.vs.control; scRNA-seq,SRP330776,γδ T cell-AD.vs.control; scRNA-seq,SRP330776,CD8+ T cell_2-AD.vs.control; scRNA-seq,SRP330776,B cell_1-AD.vs.control; scRNA-seq,SRP330776,Naive CD8+ T cell_1-AD.vs.control; scRNA-seq,SRP330776,Natural killer cell_1-AD.vs.control; scRNA-seq,SRP330776,Memory T cell_1-AD.vs.control	10
CNOT6L	bulk RNA-seq,Homo_723,AD.vs.MCI; bulk RNA-seq,Homo_723,MCI.vs.control; bulk RNA-seq,Homo_633,AD.vs.MCI; bulk RNA-seq,Homo_633,MCI.vs.control; bulk RNA-seq,Homo_714,AD.vs.control; bulk RNA-seq,Homo_714,AD.vs.MCI; bulk RNA-seq,Homo_714,MCI.vs.control; scRNA-seq,SRP309935,Natural killer cell_2-AD.vs.control; scRNA-seq,SRP309935,Megakaryocyte_1-AD.vs.control; scRNA-seq,SRP309935,Naive CD8+ T cell_1-AD.vs.control; scRNA-seq,SRP309935,CD8+ T cell_1-AD.vs.control	11
CNOT7	bulk RNA-seq,Homo_723,AD.vs.control; bulk RNA-seq,Homo_723,AD.vs.MCI; bulk RNA-seq,Homo_633,AD.vs.control; bulk RNA-seq,Homo_633,AD.vs.MCI; scRNA-seq,SRP215507,CD8+ T cell_7-AD.vs.control; scRNA-seq,SRP330776,B cell_3-AD.vs.control; scRNA-	7
CNOT8	bulk RNA-seq,Homo_633,AD.vs.control; bulk RNA-seq,Homo_633,AD.vs.MCI; scRNA-seq,SRP215507,CD8+ T cell_5-AD.vs.MCI; scRNA-seq,SRP215507,CD8+ T cell_5-MCI.vs.control; scRNA-seq,SRP330776,Megakaryocyte_1-AD.vs.control; scRNA-seq,SRP330776,B cell_4-AD.vs.control	6
CNOT9	scRNA-seq,SRP215507,CD8+ T cell_5-AD.vs.MCI; scRNA-seq,SRP215507,CD8+ T cell_5-MCI.vs.control; scRNA-seq,SRP215507,CD8+ T cell_6-MCI.vs.control; scRNA-seq,SRP330776,CD8+ T cell_3-AD.vs.control; scRNA-seq,SRP330776,Monocyte_1-AD.vs.control	5
CNP	bulk RNA-seq,Homo_714,AD.vs.control; bulk RNA-seq,Homo_714,AD.vs.MCI; scRNA-seq,SRP215507,CD8+ T cell_3-AD.vs.control; scRNA-seq,SRP330776,γδ T cell-AD.vs.control; scRNA-seq,SRP330776,B cell_1-AD.vs.control; scRNA-seq,SRP330776,Natural killer T cell-AD.vs.control; scRNA-seq,SRP330776,Naive CD8+ T cell_3-AD.vs.control; scRNA-seq,SRP330776,CD8+ T cell_3-AD.vs.control; scRNA-seq,SRP330776,B cell_3-	10
Cnp	bulk RNA-seq,SRP325058,AD.vs.control	1
CNPPD1	scRNA-seq,SRP215507,CD8+ T cell_4-AD.vs.control; scRNA-seq,SRP215507,CD8+ T cell_7-AD.vs.MCI; scRNA-seq,SRP215507,CD8+ T cell_7-MCI.vs.control; scRNA-seq,SRP309935,Naive CD8+ T cell_2-AD.vs.control; scRNA-seq,SRP330776,CD8+ T cell_1-AD.vs.control; scRNA-seq,SRP330776,γδ T cell-AD.vs.control; scRNA-seq,SRP330776,Naive CD8+ T cell_1-AD.vs.control; scRNA-seq,SRP330776,Natural killer cell_1-AD.vs.control; scRNA-seq,SRP330776,Naive CD8+ T cell_3-AD.vs.control; scRNA-seq,SRP330776,Natural killer cell_2-AD.vs.control	10
CNPY1	bulk RNA-seq,Homo_723,AD.vs.control; bulk RNA-seq,Homo_723,AD.vs.MCI	2
CNPY2	scRNA-seq,SRP215507,CD8+ T cell_7-AD.vs.control; scRNA-seq,SRP215507,CD8+ T cell_10-AD.vs.control; scRNA-seq,SRP215507,CD8+ T cell_9-AD.vs.control; scRNA-seq,SRP215507,CD8+ T cell_7-MCI.vs.control; scRNA-seq,SRP330776,Monocyte_2-AD.vs.control; scRNA-seq,SRP330776,B	6
CNPY3	scRNA-seq,SRP215507,CD8+ T cell_7-AD.vs.control; scRNA-seq,SRP215507,CD8+ T cell_7-AD.vs.MCI; scRNA-seq,SRP330776,Natural killer cell_2-	3
CNPY3-GNMT	bulk RNA-seq,Homo_723,AD.vs.control; bulk RNA-seq,Homo_723,AD.vs.MCI; bulk RNA-seq,Homo_633,AD.vs.control; bulk RNA-seq,Homo_633,AD.vs.MCI; bulk RNA-seq,Homo_714,AD.vs.control; bulk RNA-seq,Homo_714,AD.vs.MCI	6
CNPY4	scRNA-seq,SRP215507,CD8+ T cell_4-AD.vs.control; scRNA-seq,SRP215507,CD8+ T cell_4-AD.vs.MCI; scRNA-seq,SRP215507,CD8+ T cell_5-AD.vs.MCI; scRNA-seq,SRP215507,CD8+ T cell_5-MCI.vs.control; scRNA-seq,SRP309935,Natural killer cell_2-AD.vs.control; scRNA-seq,SRP330776,CD8+ T cell_1-AD.vs.control; scRNA-seq,SRP330776,B cell_1-AD.vs.control; scRNA-seq,SRP330776,Memory T cell_1-AD.vs.control; scRNA-seq,SRP330776,B cell_2-AD.vs.control; scRNA-seq,SRP330776,Regulatory T cell-AD.vs.control	10
CNR1	scRNA-seq,SRP330776,B cell_1-AD.vs.control; scRNA-seq,SRP330776,B cell_2-AD.vs.control	2
CNR2	scRNA-seq,SRP215507,CD8+ T cell_3-AD.vs.control; scRNA-seq,SRP215507,CD8+ T cell_5-AD.vs.control; scRNA-seq,SRP215507,CD8+ T cell_1-AD.vs.MCI; scRNA-seq,SRP215507,CD8+ T cell_5-MCI.vs.control; scRNA-seq,SRP215507,CD8+ T cell_4-MCI.vs.control; scRNA-seq,SRP215507,CD8+ T cell_2-MCI.vs.control; scRNA-seq,SRP330776,γδ T cell-AD.vs.control; scRNA-seq,SRP330776,CD8+ T cell_2-AD.vs.control; scRNA-seq,SRP330776,Memory T cell_1-AD.vs.control; scRNA-seq,SRP330776,CD8+ T cell_3-AD.vs.control; scRNA-seq,SRP330776,Natural killer	11

CNRIP1	bulk RNA-seq,Homo_723,AD.vs.control; bulk RNA-seq,Homo_723,AD.vs.MCI; bulk RNA-seq,Homo_723,MCI.vs.control; bulk RNA-seq,Homo_633,AD.vs.control; bulk RNA-seq,Homo_633,AD.vs.MCI; bulk RNA-seq,Homo_633,MCI.vs.control; bulk RNA-seq,Homo_714,AD.vs.control;	8
CNST	bulk RNA-seq,Homo_723,AD.vs.MCI; bulk RNA-seq,Homo_633,AD.vs.MCI; scRNA-seq,SRP215507,CD8+ T cell_4-AD.vs.MCI; scRNA-seq,SRP309935,B cell_2-AD.vs.control	4
CNTD1	bulk RNA-seq,Homo_723,AD.vs.control; bulk RNA-seq,Homo_723,AD.vs.MCI; bulk RNA-seq,Homo_633,AD.vs.control; bulk RNA-seq,Homo_633,AD.vs.MCI; bulk RNA-seq,Homo_714,AD.vs.control; bulk RNA-seq,Homo_714,AD.vs.MCI	6
CNTFR	bulk RNA-seq,Homo_723,AD.vs.MCI	1
CNTLN	bulk RNA-seq,Homo_723,AD.vs.control; bulk RNA-seq,Homo_723,AD.vs.MCI; bulk RNA-seq,Homo_723,MCI.vs.control; bulk RNA-seq,Homo_633,AD.vs.control; bulk RNA-seq,Homo_633,AD.vs.MCI; bulk RNA-seq,Homo_633,MCI.vs.control; bulk RNA-seq,Homo_714,AD.vs.control; bulk RNA-seq,Homo_714,AD.vs.MCI; bulk RNA-seq,ROSMAP,AD.vs.control; scRNA-seq,SRP330776,Memory T cell_1-AD.vs.control; scRNA-	11
CNTN1	bulk RNA-seq,Homo_723,AD.vs.control; bulk RNA-seq,Homo_723,MCI.vs.control; bulk RNA-seq,Homo_633,AD.vs.control; bulk RNA-seq,Homo_633,MCI.vs.control; bulk RNA-seq,Homo_714,MCI.vs.control; bulk RNA-seq,ROSMAP,AD.vs.control; bulk RNA-	7
CNTN2	bulk RNA-seq,Homo_723,AD.vs.control; bulk RNA-seq,Homo_723,AD.vs.MCI; bulk RNA-seq,Homo_723,MCI.vs.control; bulk RNA-seq,Homo_633,AD.vs.control; bulk RNA-seq,Homo_714,MCI.vs.control; bulk RNA-seq,ROSMAP,AD.vs.control	6
CNTN3	bulk RNA-seq,Homo_723,AD.vs.control; bulk RNA-seq,Homo_723,AD.vs.MCI; bulk RNA-seq,ROSMAP,AD.vs.control; bulk RNA-	4
CNTN4	bulk RNA-seq,Homo_723,AD.vs.control; bulk RNA-seq,Homo_723,AD.vs.MCI; bulk RNA-seq,Homo_723,MCI.vs.control; bulk RNA-seq,Homo_633,AD.vs.control; bulk RNA-seq,Homo_633,AD.vs.MCI; bulk RNA-seq,Homo_714,MCI.vs.control; bulk RNA-seq,ROSMAP,AD.vs.control;	8
CNTN5	bulk RNA-seq,ROSMAP,AD.vs.control	1
CNTN6	bulk RNA-seq,Homo_633,MCI.vs.control; bulk RNA-seq,Homo_714,MCI.vs.control	2
CNTNAP1	bulk RNA-seq,Homo_723,AD.vs.control; bulk RNA-seq,Homo_723,AD.vs.MCI; bulk RNA-seq,Homo_633,AD.vs.control; bulk RNA-seq,Homo_633,MCI.vs.control; bulk RNA-seq,Homo_714,AD.vs.control; bulk RNA-seq,Homo_714,AD.vs.MCI; bulk RNA-seq,Homo_714,MCI.vs.control; scRNA-seq,SRP330776,Memory T cell_2-AD.vs.control; scRNA-seq,SRP330776,T cell-AD.vs.control	9
CNTNAP2	bulk RNA-seq,Homo_723,AD.vs.control; bulk RNA-seq,Homo_723,AD.vs.MCI; bulk RNA-seq,Homo_723,MCI.vs.control; bulk RNA-seq,Homo_633,AD.vs.MCI; bulk RNA-seq,Homo_633,MCI.vs.control; bulk RNA-seq,Homo_714,AD.vs.control; bulk RNA-seq,Homo_714,AD.vs.MCI; bulk RNA-seq,ROSMAP,AD.vs.control; bulk RNA-seq,ROSMAP,MCI.vs.control	9
CNTNAP3B	bulk RNA-seq,Homo_714,AD.vs.control; bulk RNA-seq,Homo_714,AD.vs.MCI; bulk RNA-seq,ROSMAP,AD.vs.control	3
CNTNAP4	bulk RNA-seq,Homo_723,AD.vs.control; bulk RNA-seq,Homo_723,AD.vs.MCI; bulk RNA-seq,Homo_633,MCI.vs.control; bulk RNA-	4
CNTNAP5	bulk RNA-seq,Homo_723,AD.vs.control; bulk RNA-seq,Homo_723,AD.vs.MCI; bulk RNA-seq,Homo_633,MCI.vs.control; bulk RNA-	4
CNTRL	bulk RNA-seq,Homo_723,AD.vs.MCI; bulk RNA-seq,Homo_633,AD.vs.MCI; bulk RNA-seq,Homo_714,AD.vs.control; bulk RNA-seq,Homo_714,AD.vs.MCI; scRNA-seq,SRP330776,Monocyte_1-AD.vs.control	5
CNTROB	scRNA-seq,SRP215507,CD8+ T cell_5-AD.vs.control; scRNA-seq,SRP215507,CD8+ T cell_5-AD.vs.MCI; scRNA-seq,SRP309935,Monocyte_2-AD.vs.control; scRNA-seq,SRP330776,Memory T cell_1-AD.vs.control; scRNA-seq,SRP330776,Natural killer T cell-AD.vs.control; scRNA-seq,SRP330776,Naive CD8+ T cell_3-AD.vs.control; scRNA-seq,SRP330776,B cell_2-AD.vs.control; scRNA-seq,SRP330776,CD8+ T cell_3-AD.vs.control; scRNA-seq,SRP330776,B cell_3-AD.vs.control; scRNA-seq,SRP330776,Natural killer cell_2-AD.vs.control	10
COA1	scRNA-seq,SRP215507,CD8+ T cell_6-AD.vs.control; scRNA-seq,SRP215507,CD8+ T cell_4-AD.vs.MCI; scRNA-seq,SRP215507,CD8+ T cell_6-AD.vs.MCI; scRNA-seq,SRP330776,Naive CD8+ T cell_1-AD.vs.control; scRNA-seq,SRP330776,T cell-AD.vs.control; scRNA-	6

COA3	bulk RNA-seq,Homo_723,AD.vs.control; bulk RNA-seq,Homo_723,AD.vs.MCI; bulk RNA-seq,Homo_633,AD.vs.control; bulk RNA-seq,Homo_633,AD.vs.MCI; scRNA-seq,SRP215507,CD8+ T cell_4-AD.vs.control; scRNA-seq,SRP215507,CD8+ T cell_9-AD.vs.control; scRNA-seq,SRP215507,CD8+ T cell_5-MCI.vs.control; scRNA-seq,SRP215507,CD8+ T cell_4-MCI.vs.control; scRNA-seq,SRP309935,Erythroid cell-AD.vs.control; scRNA-seq,SRP330776,CD8+ T cell_1-AD.vs.control; scRNA-seq,SRP330776,γδ T cell-AD.vs.control; scRNA-seq,SRP330776,Memory T cell_2-AD.vs.control; scRNA-seq,SRP330776,CD8+ T cell_2-AD.vs.control; scRNA-seq,SRP330776,B cell_1-AD.vs.control; scRNA-seq,SRP330776,Naive CD8+ T cell_1-AD.vs.control; scRNA-seq,SRP330776,Natural killer cell_1-AD.vs.control; scRNA-seq,SRP330776,Memory T cell_1-AD.vs.control; scRNA-seq,SRP330776,Natural killer T cell-AD.vs.control; scRNA-seq,SRP330776,Naive CD8+ T cell_3-AD.vs.control; scRNA-seq,SRP330776,B cell_2-AD.vs.control; scRNA-seq,SRP330776,T cell-AD.vs.control; scRNA-seq,SRP330776,Naive CD8+ T cell_2-AD.vs.control; scRNA-seq,SRP330776,CD8+ T cell_3-AD.vs.control; scRNA-seq,SRP330776,Monocyte_1-AD.vs.control; scRNA-seq,SRP330776,Monocyte_2-[illegible]	26
COA4	scRNA-seq,SRP215507,CD8+ T cell_4-AD.vs.control; scRNA-seq,SRP215507,CD8+ T cell_7-AD.vs.control; scRNA-seq,SRP215507,CD8+ T cell_4-AD.vs.MCI; scRNA-seq,SRP330776,CD8+ T cell_1-AD.vs.control; scRNA-seq,SRP330776,Memory T cell_2-AD.vs.control; scRNA-seq,SRP330776,CD8+ T cell_2-AD.vs.control; scRNA-seq,SRP330776,Natural killer cell_1-AD.vs.control; scRNA-seq,SRP330776,Memory T cell_1-AD.vs.control; scRNA-seq,SRP330776,Natural killer T cell-AD.vs.control; scRNA-seq,SRP330776,Naive CD8+ T cell_3-AD.vs.control; scRNA-seq,SRP330776,B cell_2-AD.vs.control; scRNA-seq,SRP330776,CD8+ T cell_3-AD.vs.control; scRNA-seq,SRP330776,B cell_3-AD.vs.control; scRNA-	14
COA5	scRNA-seq,SRP330776,γδ T cell-AD.vs.control; scRNA-seq,SRP330776,Memory T cell_2-AD.vs.control; scRNA-seq,SRP330776,Naive CD8+ T cell_3-AD.vs.control; scRNA-seq,SRP330776,T cell-AD.vs.control; scRNA-seq,SRP330776,Monocyte_2-AD.vs.control; scRNA-seq,SRP330776,Natural killer	6
COA6	bulk RNA-seq,Homo_723,AD.vs.control; bulk RNA-seq,Homo_723,AD.vs.MCI; bulk RNA-seq,Homo_723,MCI.vs.control; bulk RNA-seq,Homo_633,AD.vs.control; bulk RNA-seq,Homo_633,AD.vs.MCI; bulk RNA-seq,Homo_633,MCI.vs.control; bulk RNA-seq,Homo_714,AD.vs.control; bulk RNA-seq,Homo_714,AD.vs.MCI; bulk RNA-seq,Homo_714,MCI.vs.control; scRNA-seq,SRP215507,CD8+ T cell_2-AD.vs.MCI; scRNA-seq,SRP215507,CD8+ T cell_3-MCI.vs.control; scRNA-seq,SRP330776,γδ T cell-AD.vs.control; scRNA-seq,SRP330776,B cell_1-AD.vs.control; scRNA-seq,SRP330776,Natural killer cell_1-AD.vs.control; scRNA-seq,SRP330776,Memory T cell_1-AD.vs.control; scRNA-seq,SRP330776,Naive CD8+ T cell_3-AD.vs.control; scRNA-seq,SRP330776,B cell_2-AD.vs.control; scRNA-seq,SRP330776,CD8+ T cell_3-AD.vs.control; scRNA-seq,SRP330776,B [illegible]	20
COA6-AS1	scRNA-seq,SRP215507,CD8+ T cell_2-AD.vs.control; scRNA-seq,SRP215507,CD8+ T cell_3-AD.vs.control; scRNA-seq,SRP215507,CD8+ T cell_1-AD.vs.control; scRNA-seq,SRP215507,CD8+ T cell_4-AD.vs.control; scRNA-seq,SRP215507,CD8+ T cell_5-AD.vs.control; scRNA-seq,SRP215507,CD8+ T cell_3-AD.vs.MCI; scRNA-seq,SRP215507,CD8+ T cell_1-MCI.vs.control; scRNA-seq,SRP215507,CD8+ T cell_5-MCI.vs.control; scRNA-seq,SRP215507,CD8+ T cell_2-MCI.vs.control; scRNA-seq,SRP215507,CD8+ T cell_3-MCI.vs.control; scRNA-	12
COA7	scRNA-seq,SRP330776,CD8+ T cell_3-AD.vs.control	1
COA8	bulk RNA-seq,Homo_633,AD.vs.control; bulk RNA-seq,Homo_714,AD.vs.control; bulk RNA-seq,Homo_714,AD.vs.MCI; scRNA-seq,SRP215507,CD8+ T cell_7-AD.vs.control; scRNA-seq,SRP215507,CD8+ T cell_5-AD.vs.control; scRNA-seq,SRP215507,CD8+ T cell_5-AD.vs.MCI; scRNA-seq,SRP330776,B cell_1-AD.vs.control; scRNA-seq,SRP330776,B cell_2-AD.vs.control; scRNA-seq,SRP330776,CD8+ T cell_3-AD.vs.control; scRNA-seq,SRP330776,Monocyte_2-AD.vs.control; scRNA-seq,SRP330776,Natural killer cell_2-AD.vs.control; scRNA-seq,SRP330776,B cell_4-AD.vs.control	12
COASY	scRNA-seq,SRP215507,CD8+ T cell_7-AD.vs.control; scRNA-seq,SRP215507,CD8+ T cell_4-MCI.vs.control; scRNA-seq,SRP309935,Erythroid cell-AD.vs.control; scRNA-seq,SRP330776,Natural killer T cell-AD.vs.control; scRNA-seq,SRP330776,Naive CD8+ T cell_3-AD.vs.control; scRNA-	6
COBL	bulk RNA-seq,Homo_723,AD.vs.control; bulk RNA-seq,Homo_723,AD.vs.MCI; bulk RNA-seq,Homo_723,MCI.vs.control; bulk RNA-seq,Homo_633,AD.vs.control; bulk RNA-seq,Homo_633,AD.vs.MCI; bulk RNA-seq,Homo_714,MCI.vs.control; bulk RNA-seq,ROSMAP,AD.vs.control;	8
COBLL1	bulk RNA-seq,ROSMAP,AD.vs.control; bulk RNA-seq,ROSMAP,MCI.vs.control; scRNA-seq,SRP215507,CD8+ T cell_7-MCI.vs.control; scRNA-seq,SRP309935,B cell_1-AD.vs.control; scRNA-seq,SRP330776,B cell_2-AD.vs.control	5
COCH	bulk RNA-seq,Homo_723,AD.vs.control; bulk RNA-seq,Homo_723,AD.vs.MCI; bulk RNA-seq,Homo_633,AD.vs.control; bulk RNA-seq,Homo_633,AD.vs.MCI; bulk RNA-seq,Homo_714,AD.vs.control; bulk RNA-seq,Homo_714,AD.vs.MCI; scRNA-seq,SRP309935,B cell_1-	7

COG1	bulk RNA-seq,Homo_723,AD.vs.control; bulk RNA-seq,Homo_723,AD.vs.MCI; bulk RNA-seq,Homo_723,MCI.vs.control; bulk RNA-seq,Homo_633,AD.vs.control; bulk RNA-seq,Homo_633,AD.vs.MCI; bulk RNA-seq,Homo_633,MCI.vs.control; bulk RNA-seq,Homo_714,AD.vs.control; bulk RNA-seq,Homo_714,AD.vs.MCI; bulk RNA-seq,Homo_714,MCI.vs.control; scRNA-seq,SRP215507,CD8+ T cell_4-AD.vs.control; scRNA-seq,SRP215507,CD8+ T cell_4-AD.vs.MCI; scRNA-seq,SRP215507,CD8+ T cell_5-AD.vs.MCI; scRNA-seq,SRP215507,CD8+ T cell_5-MCI.vs.control; scRNA-seq,SRP309935,Natural killer cell_1-AD.vs.control; scRNA-seq,SRP309935,Naive CD8+ T cell_2-AD.vs.control; scRNA-seq,SRP309935,CD8+ T cell_1-AD.vs.control; scRNA-seq,SRP330776,Memory T cell_2-AD.vs.control; scRNA-seq,SRP330776,B cell_1-AD.vs.control; scRNA-seq,SRP330776,Naive CD8+ T cell_1-AD.vs.control; scRNA-seq,SRP330776,Natural killer cell_1-AD.vs.control; scRNA-seq,SRP330776,Natural killer T cell-AD.vs.control; scRNA-seq,SRP330776,B cell_2-AD.vs.control; scRNA-seq,SRP330776,CD8+ T cell_3-AD.vs.control; scRNA-[illegible]	24
COG2	bulk RNA-seq,Homo_723,AD.vs.control; bulk RNA-seq,Homo_633,AD.vs.control; bulk RNA-seq,Homo_714,AD.vs.control; bulk RNA-seq,Homo_714,AD.vs.MCI; scRNA-seq,SRP215507,CD8+ T cell_7-AD.vs.control; scRNA-seq,SRP309935,Monocyte_1-AD.vs.control; scRNA-seq,SRP309935,Natural killer cell_1-AD.vs.control; scRNA-seq,SRP309935,B cell_1-AD.vs.control; scRNA-seq,SRP330776,γδ T cell-AD.vs.control;	10
COG3	bulk RNA-seq,Homo_723,AD.vs.MCI; bulk RNA-seq,Homo_723,MCI.vs.control; bulk RNA-seq,Homo_633,MCI.vs.control; bulk RNA-seq,Homo_714,AD.vs.control; bulk RNA-seq,Homo_714,AD.vs.MCI; bulk RNA-seq,Homo_714,MCI.vs.control; scRNA-seq,SRP215507,CD8+ T cell_10-AD.vs.control; scRNA-seq,SRP215507,CD8+ T cell_5-AD.vs.MCI; scRNA-seq,SRP330776,CD8+ T cell_3-AD.vs.control	9
COG4	bulk RNA-seq,Homo_723,AD.vs.control; bulk RNA-seq,Homo_723,AD.vs.MCI; bulk RNA-seq,Homo_633,AD.vs.control; bulk RNA-seq,Homo_633,AD.vs.MCI; scRNA-seq,SRP215507,CD8+ T cell_6-MCI.vs.control; scRNA-seq,SRP330776,Naive CD8+ T cell_2-AD.vs.control; scRNA-	7
COG5	bulk RNA-seq,Homo_714,AD.vs.control; bulk RNA-seq,Homo_714,AD.vs.MCI; scRNA-seq,SRP215507,CD8+ T cell_1-AD.vs.control; scRNA-seq,SRP215507,CD8+ T cell_2-AD.vs.MCI; scRNA-seq,SRP215507,CD8+ T cell_1-AD.vs.MCI; scRNA-seq,SRP215507,CD8+ T cell_3-MCI.vs.control; scRNA-seq,SRP330776,Naive CD8+ T cell_3-AD.vs.control	7
COG6	bulk RNA-seq,Homo_723,AD.vs.control; bulk RNA-seq,Homo_723,AD.vs.MCI; bulk RNA-seq,Homo_633,AD.vs.control; bulk RNA-seq,Homo_633,AD.vs.MCI; bulk RNA-seq,Homo_714,AD.vs.control; bulk RNA-seq,Homo_714,AD.vs.MCI; scRNA-seq,SRP215507,CD8+ T cell_6-MCI.vs.control; scRNA-seq,SRP309935,Natural killer cell_2-AD.vs.control; scRNA-seq,SRP309935,Monocyte_1-AD.vs.control; scRNA-seq,SRP309935,Megakaryocyte_1-AD.vs.control; scRNA-seq,SRP309935,B cell_1-AD.vs.control; scRNA-seq,SRP309935,CD8+ T cell_2-AD.vs.control; scRNA-seq,SRP330776,γδ T cell-AD.vs.control; scRNA-seq,SRP330776,Naive CD8+ T cell_1-AD.vs.control; scRNA-seq,SRP330776,Memory T cell_1-AD.vs.control; scRNA-seq,SRP330776,Naive CD8+ T cell_3-AD.vs.control; scRNA-seq,SRP330776,CD8+ T cell_3-AD.vs.control; scRNA-[illegible]	18
COG7	bulk RNA-seq,Homo_714,AD.vs.control; bulk RNA-seq,Homo_714,AD.vs.MCI; scRNA-seq,SRP215507,CD8+ T cell_3-AD.vs.control; scRNA-seq,SRP215507,CD8+ T cell_10-AD.vs.control; scRNA-seq,SRP215507,CD8+ T cell_5-MCI.vs.control; scRNA-seq,SRP330776,Memory T cell_2-AD.vs.control; scRNA-seq,SRP330776,Natural killer cell_1-AD.vs.control; scRNA-seq,SRP330776,Memory T cell_1-AD.vs.control; scRNA-seq,SRP330776,B cell_2-AD.vs.control; scRNA-seq,SRP330776,CD8+ T cell_3-AD.vs.control; scRNA-seq,SRP330776,Monocyte_1-AD.vs.control	11
COG8	scRNA-seq,SRP215507,CD8+ T cell_2-AD.vs.MCI; scRNA-seq,SRP215507,CD8+ T cell_5-AD.vs.MCI; scRNA-seq,SRP215507,CD8+ T cell_5-MCI.vs.control; scRNA-seq,SRP330776,CD8+ T cell_1-AD.vs.control; scRNA-seq,SRP330776,Memory T cell_2-AD.vs.control; scRNA-seq,SRP330776,B cell_1-AD.vs.control; scRNA-seq,SRP330776,Naive CD8+ T cell_1-AD.vs.control; scRNA-seq,SRP330776,Natural killer cell_1-AD.vs.control; scRNA-seq,SRP330776,B cell_2-AD.vs.control; scRNA-seq,SRP330776,Naive CD8+ T cell_2-AD.vs.control; scRNA-seq,SRP330776,CD8+ T cell_3-AD.vs.control; scRNA-seq,SRP330776,Monocyte_1-AD.vs.control; scRNA-seq,SRP330776,B cell_3-AD.vs.control	13
COIL	scRNA-seq,SRP215507,CD8+ T cell_4-AD.vs.control; scRNA-seq,SRP215507,CD8+ T cell_10-AD.vs.control; scRNA-seq,SRP330776,Regulatory T cell-	3
COL11A1	bulk RNA-seq,Homo_723,AD.vs.MCI; bulk RNA-seq,ROSMAP,AD.vs.control; bulk RNA-seq,ROSMAP,MCI.vs.control	3
COL11A2	bulk RNA-seq,Homo_723,AD.vs.control; bulk RNA-seq,Homo_723,AD.vs.MCI; bulk RNA-seq,Homo_723,MCI.vs.control; bulk RNA-seq,Homo_633,AD.vs.control; bulk RNA-seq,Homo_633,AD.vs.MCI	5
COL12A1	bulk RNA-seq,Homo_723,AD.vs.control; bulk RNA-seq,Homo_633,AD.vs.control; bulk RNA-seq,ROSMAP,AD.vs.control; bulk RNA-	4
COL13A1	bulk RNA-seq,Homo_723,AD.vs.control; bulk RNA-seq,Homo_633,AD.vs.control; bulk RNA-seq,Homo_633,AD.vs.MCI; bulk RNA-seq,Homo_714,AD.vs.control; bulk RNA-seq,Homo_714,AD.vs.MCI; scRNA-seq,SRP330776,Memory T cell_1-AD.vs.control	6
COL14A1	bulk RNA-seq,Homo_723,AD.vs.control; bulk RNA-seq,Homo_723,AD.vs.MCI; bulk RNA-seq,Homo_723,MCI.vs.control; bulk RNA-seq,Homo_633,AD.vs.MCI; bulk RNA-seq,Homo_633,MCI.vs.control; bulk RNA-seq,ROSMAP,AD.vs.control	6

COL15A1	bulk RNA-seq,ACOM,AD.vs.control; bulk RNA-seq,Homo_723,AD.vs.control; bulk RNA-seq,Homo_723,AD.vs.MCI; bulk RNA-seq,Homo_723,MCI.vs.control; bulk RNA-seq,Homo_633,AD.vs.control; bulk RNA-seq,Homo_633,AD.vs.MCI; bulk RNA-	8
COL16A1	bulk RNA-seq,Homo_723,AD.vs.control; bulk RNA-seq,Homo_723,AD.vs.MCI; bulk RNA-seq,Homo_714,MCI.vs.control; bulk RNA-seq,ROSMAP,AD.vs.control; bulk RNA-seq,ROSMAP,MCI.vs.control	5
COL17A1	bulk RNA-seq,Homo_723,AD.vs.control; bulk RNA-seq,Homo_723,AD.vs.MCI; bulk RNA-seq,Homo_633,AD.vs.control; bulk RNA-seq,Homo_633,AD.vs.MCI; bulk RNA-seq,Homo_714,AD.vs.control; bulk RNA-seq,Homo_714,AD.vs.MCI	6
COL18A1	bulk RNA-seq,Homo_633,AD.vs.control; bulk RNA-seq,Homo_633,AD.vs.MCI; bulk RNA-seq,Homo_714,AD.vs.control; bulk RNA-seq,Homo_714,AD.vs.MCI; scRNA-seq,SRP215507,CD8+ T cell_1-AD.vs.control; scRNA-seq,SRP215507,CD8+ T cell_2-AD.vs.MCI; scRNA-seq,SRP215507,CD8+ T cell_1-AD.vs.MCI; scRNA-seq,SRP215507,CD8+ T cell_1-MCI.vs.control; scRNA-seq,SRP215507,CD8+ T cell_2-MCI.vs.control; scRNA-seq,SRP215507,CD8+ T cell_6-MCI.vs.control; scRNA-seq,SRP309935,Naive CD8+ T cell_1-AD.vs.control; scRNA-seq,SRP309935,Naive CD8+ T cell_2-AD.vs.control; scRNA-seq,SRP309935,CD8+ T cell_2-AD.vs.control; scRNA-seq,SRP330776,CD8+ T cell_1-AD.vs.control; scRNA-seq,SRP330776,γδ T cell-AD.vs.control; scRNA-seq,SRP330776,Memory T cell_2-AD.vs.control; scRNA-seq,SRP330776,CD8+ T cell_2-AD.vs.control; scRNA-seq,SRP330776,Naive CD8+ T cell_1-AD.vs.control; scRNA-seq,SRP330776,Memory T cell_1-AD.vs.control; scRNA-seq,SRP330776,Natural killer T cell-AD.vs.control; scRNA-seq,SRP330776,Naive CD8+ T cell_3-AD.vs.control; scRNA-seq,SRP330776,T cell-[illegible]	24
COL19A1	bulk RNA-seq,Homo_723,AD.vs.control; bulk RNA-seq,Homo_723,AD.vs.MCI; bulk RNA-seq,Homo_633,AD.vs.control; bulk RNA-seq,Homo_633,AD.vs.MCI; bulk RNA-seq,Homo_714,AD.vs.control; bulk RNA-seq,Homo_714,AD.vs.MCI; bulk RNA-seq,ROSMAP,AD.vs.control; bulk RNA-seq,ROSMAP,MCI.vs.control; scRNA-seq,SRP215507,CD8+ T cell_7-AD.vs.MCI; scRNA-seq,SRP330776,B cell_1-AD.vs.control	10
COL1A1	bulk RNA-seq,ACOM,AD.vs.control; bulk RNA-seq,Homo_723,AD.vs.control; bulk RNA-seq,Homo_723,AD.vs.MCI; bulk RNA-seq,Homo_723,MCI.vs.control; bulk RNA-seq,Homo_633,AD.vs.control; bulk RNA-seq,Homo_633,AD.vs.MCI; bulk RNA-seq,Homo_714,MCI.vs.control; scRNA-seq,SRP215507,CD8+ T cell_2-AD.vs.MCI; scRNA-seq,SRP215507,CD8+ T cell_5-AD.vs.MCI; scRNA-	10
Col1a1	bulk RNA-seq,SRP325058,AD.vs.control	1
COL1A2	bulk RNA-seq,Homo_723,AD.vs.control; bulk RNA-seq,Homo_723,AD.vs.MCI; bulk RNA-seq,Homo_633,AD.vs.control; bulk RNA-seq,Homo_633,AD.vs.MCI; bulk RNA-seq,Homo_633,MCI.vs.control	5
COL20A1	bulk RNA-seq,Homo_723,AD.vs.control; bulk RNA-seq,Homo_714,MCI.vs.control	2
COL21A1	bulk RNA-seq,Homo_723,AD.vs.control; bulk RNA-seq,Homo_723,AD.vs.MCI; bulk RNA-seq,Homo_723,MCI.vs.control; bulk RNA-seq,Homo_633,AD.vs.MCI; bulk RNA-seq,Homo_633,MCI.vs.control	5
COL22A1	bulk RNA-seq,Homo_723,AD.vs.control; bulk RNA-seq,Homo_723,AD.vs.MCI; bulk RNA-seq,Homo_633,AD.vs.control; bulk RNA-	4
COL23A1	bulk RNA-seq,Homo_723,AD.vs.control; bulk RNA-seq,Homo_723,AD.vs.MCI; bulk RNA-seq,Homo_633,AD.vs.control; bulk RNA-seq,Homo_633,AD.vs.MCI; bulk RNA-seq,Homo_714,AD.vs.control; bulk RNA-seq,Homo_714,AD.vs.MCI	6
COL24A1	bulk RNA-seq,Homo_723,AD.vs.control; bulk RNA-seq,Homo_723,AD.vs.MCI; bulk RNA-seq,Homo_723,MCI.vs.control; bulk RNA-seq,Homo_633,AD.vs.control; bulk RNA-seq,Homo_633,AD.vs.MCI; bulk RNA-seq,Homo_633,MCI.vs.control; bulk RNA-seq,Homo_714,AD.vs.control;	8
COL25A1	bulk RNA-seq,Homo_723,AD.vs.control; bulk RNA-seq,Homo_723,AD.vs.MCI	2
COL26A1	bulk RNA-seq,Homo_723,AD.vs.control; bulk RNA-seq,Homo_723,AD.vs.MCI; bulk RNA-seq,Homo_723,MCI.vs.control; bulk RNA-seq,Homo_633,AD.vs.control; bulk RNA-seq,Homo_633,AD.vs.MCI; bulk RNA-seq,Homo_714,AD.vs.control	6
COL27A1	bulk RNA-seq,Homo_723,AD.vs.control; bulk RNA-seq,Homo_723,AD.vs.MCI; bulk RNA-seq,Homo_723,MCI.vs.control; bulk RNA-seq,Homo_633,AD.vs.control; bulk RNA-seq,Homo_633,AD.vs.MCI; bulk RNA-seq,Homo_714,AD.vs.MCI; bulk RNA-seq,Homo_714,MCI.vs.control;	8
COL28A1	bulk RNA-seq,Homo_723,AD.vs.control; bulk RNA-seq,Homo_723,AD.vs.MCI; bulk RNA-seq,Homo_723,MCI.vs.control; bulk RNA-seq,Homo_633,AD.vs.control; bulk RNA-seq,Homo_633,MCI.vs.control; bulk RNA-seq,Homo_714,MCI.vs.control	6
COL2A1	bulk RNA-seq,Homo_723,AD.vs.control; bulk RNA-seq,Homo_723,AD.vs.MCI; bulk RNA-seq,Homo_714,MCI.vs.control; bulk RNA-seq,ROSMAP,AD.vs.control; bulk RNA-seq,ROSMAP,MCI.vs.control	5
COL3A1	bulk RNA-seq,Homo_714,MCI.vs.control	1
COL4A1	bulk RNA-seq,Homo_723,AD.vs.control; bulk RNA-seq,Homo_723,AD.vs.MCI; bulk RNA-seq,Homo_723,MCI.vs.control; bulk RNA-seq,Homo_633,AD.vs.control; bulk RNA-seq,Homo_633,AD.vs.MCI; bulk RNA-seq,Homo_633,MCI.vs.control	6

COL4A2	bulk RNA-seq,ROSMAP,AD.vs.control	1
COL4A3	bulk RNA-seq,Homo_723,AD.vs.control; bulk RNA-seq,Homo_723,AD.vs.MCI; bulk RNA-seq,Homo_633,AD.vs.control; bulk RNA-seq,Homo_633,AD.vs.MCI; bulk RNA-seq,Homo_714,AD.vs.control; bulk RNA-seq,Homo_714,AD.vs.MCI	6
COL4A4	bulk RNA-seq,Homo_723,AD.vs.control; bulk RNA-seq,Homo_723,AD.vs.MCI; bulk RNA-seq,Homo_633,AD.vs.control; bulk RNA-seq,Homo_633,AD.vs.MCI; bulk RNA-seq,Homo_714,AD.vs.control; bulk RNA-seq,Homo_714,AD.vs.MCI; scRNA-seq,SRP330776,B cell_1-	8
COL4A5	bulk RNA-seq,Homo_723,AD.vs.control; bulk RNA-seq,Homo_723,AD.vs.MCI; bulk RNA-seq,Homo_723,MCI.vs.control	3
COL4A6	bulk RNA-seq,Homo_723,AD.vs.control; bulk RNA-seq,Homo_723,AD.vs.MCI; bulk RNA-seq,Homo_723,MCI.vs.control; bulk RNA-seq,Homo_633,AD.vs.control; bulk RNA-seq,Homo_633,AD.vs.MCI; bulk RNA-seq,ROSMAP,AD.vs.control; bulk RNA-seq,ROSMAP,MCI.vs.control	7
COL5A1	bulk RNA-seq,ACOM,AD.vs.control; bulk RNA-seq,Homo_723,AD.vs.control; bulk RNA-seq,Homo_723,AD.vs.MCI; bulk RNA-seq,Homo_723,MCI.vs.control; bulk RNA-seq,Homo_633,AD.vs.control; bulk RNA-seq,Homo_633,AD.vs.MCI; bulk RNA-seq,Homo_714,MCI.vs.control	7
COL5A2	bulk RNA-seq,Homo_723,AD.vs.control; bulk RNA-seq,Homo_723,AD.vs.MCI; bulk RNA-seq,Homo_723,MCI.vs.control; bulk RNA-seq,Homo_633,MCI.vs.control; bulk RNA-seq,Homo_714,AD.vs.control; bulk RNA-seq,Homo_714,AD.vs.MCI; bulk RNA-seq,Homo_714,MCI.vs.control; bulk RNA-seq,ROSMAP,AD.vs.control; bulk RNA-seq,ROSMAP,MCI.vs.control	9
COL5A3	bulk RNA-seq,Homo_723,AD.vs.control; bulk RNA-seq,Homo_723,AD.vs.MCI; bulk RNA-seq,ROSMAP,AD.vs.control; scRNA-seq,SRP215507,CD8+ T cell_1-AD.vs.control; scRNA-seq,SRP215507,CD8+ T cell_4-AD.vs.control; scRNA-seq,SRP215507,CD8+ T cell_1-AD.vs.MCI; scRNA-seq,SRP215507,CD8+ T cell_4-AD.vs.MCI; scRNA-seq,SRP215507,CD8+ T cell_5-AD.vs.MCI; scRNA-seq,SRP215507,CD8+ T cell_1-MCI.vs.control; scRNA-seq,SRP215507,CD8+ T cell_5-MCI.vs.control; scRNA-seq,SRP330776,CD8+ T cell_1-AD.vs.control	11
COL6A1	bulk RNA-seq,Homo_723,AD.vs.control; bulk RNA-seq,Homo_723,AD.vs.MCI; bulk RNA-seq,Homo_723,MCI.vs.control; bulk RNA-seq,Homo_633,AD.vs.control; bulk RNA-seq,Homo_633,MCI.vs.control; bulk RNA-seq,Homo_714,AD.vs.control; bulk RNA-seq,Homo_714,AD.vs.MCI; bulk RNA-seq,Homo_714,MCI.vs.control; scRNA-seq,SRP215507,CD8+ T cell_3-AD.vs.control; scRNA-seq,SRP215507,CD8+ T cell_2-AD.vs.MCI; scRNA-seq,SRP215507,CD8+ T cell_3-AD.vs.MCI; scRNA-seq,SRP215507,CD8+ T cell_5-AD.vs.MCI; scRNA-seq,SRP215507,CD8+ T cell_5-MCI.vs.control; scRNA-seq,SRP215507,CD8+ T cell_2-MCI.vs.control; scRNA-seq,SRP215507,CD8+ T cell_3-MCI.vs.control; scRNA-	17
COL6A2	bulk RNA-seq,ACOM,AD.vs.control; bulk RNA-seq,Homo_723,AD.vs.control; bulk RNA-seq,Homo_723,AD.vs.MCI; bulk RNA-seq,Homo_723,MCI.vs.control; bulk RNA-seq,Homo_633,AD.vs.control; bulk RNA-seq,Homo_633,AD.vs.MCI; bulk RNA-seq,Homo_714,AD.vs.control; bulk RNA-seq,Homo_714,AD.vs.MCI; scRNA-seq,SRP215507,CD8+ T cell_10-AD.vs.control; scRNA-seq,SRP215507,CD8+ T cell_3-AD.vs.MCI; scRNA-seq,SRP215507,CD8+ T cell_1-AD.vs.MCI; scRNA-seq,SRP215507,CD8+ T cell_5-AD.vs.MCI; scRNA-seq,SRP215507,CD8+ T cell_5-MCI.vs.control; scRNA-seq,SRP215507,CD8+ T cell_3-MCI.vs.control; scRNA-seq,SRP309935,Natural killer cell_2-AD.vs.control; scRNA-seq,SRP309935,Natural killer cell_1-AD.vs.control; scRNA-seq,SRP330776,CD8+ T cell_2-AD.vs.control; scRNA-seq,SRP330776,Memory T cell_1-	18
COL6A3	bulk RNA-seq,ACOM,AD.vs.control; bulk RNA-seq,Homo_723,AD.vs.control; bulk RNA-seq,Homo_723,AD.vs.MCI; bulk RNA-seq,Homo_723,MCI.vs.control; bulk RNA-seq,Homo_633,AD.vs.control; bulk RNA-seq,Homo_633,AD.vs.MCI; bulk RNA-seq,Homo_633,MCI.vs.control; bulk RNA-seq,Homo_714,AD.vs.control; bulk RNA-seq,Homo_714,AD.vs.MCI; bulk RNA-seq,Homo_714,MCI.vs.control; scRNA-seq,SRP330776,CD8+ T cell_1-AD.vs.control; scRNA-seq,SRP330776,Naive CD8+ T cell_3-AD.vs.control;	13
COL6A5	bulk RNA-seq,Homo_723,AD.vs.control; bulk RNA-seq,Homo_633,AD.vs.control	2
COL6A6	bulk RNA-seq,Homo_723,AD.vs.control; bulk RNA-seq,Homo_723,AD.vs.MCI; bulk RNA-seq,Homo_633,AD.vs.control; bulk RNA-seq,Homo_633,AD.vs.MCI; bulk RNA-seq,Homo_714,AD.vs.MCI; bulk RNA-seq,Homo_714,MCI.vs.control	6
COL7A1	bulk RNA-seq,Homo_723,AD.vs.control; bulk RNA-seq,Homo_723,AD.vs.MCI; bulk RNA-seq,Homo_723,MCI.vs.control; bulk RNA-seq,Homo_633,MCI.vs.control; bulk RNA-seq,Homo_714,AD.vs.control; bulk RNA-seq,Homo_714,AD.vs.MCI; bulk RNA-seq,Homo_714,MCI.vs.control; bulk RNA-seq,SRP223445,AD.vs.control; scRNA-seq,SRP215507,CD8+ T cell_2-AD.vs.control	9
COL8A1	bulk RNA-seq,Homo_723,AD.vs.control; bulk RNA-seq,Homo_723,AD.vs.MCI; bulk RNA-seq,Homo_633,AD.vs.control; bulk RNA-seq,Homo_633,AD.vs.MCI; bulk RNA-seq,Homo_714,MCI.vs.control; bulk RNA-seq,ROSMAP,AD.vs.control; bulk RNA-seq,ROSMAP,MCI.vs.control	7
COL8A2	bulk RNA-seq,Homo_723,AD.vs.MCI; bulk RNA-seq,Homo_633,AD.vs.control; bulk RNA-seq,Homo_633,AD.vs.MCI; bulk RNA-seq,Homo_714,AD.vs.control; bulk RNA-seq,Homo_714,AD.vs.MCI	5
COL9A1	bulk RNA-seq,Homo_723,AD.vs.control; bulk RNA-seq,Homo_723,AD.vs.MCI; bulk RNA-seq,Homo_723,MCI.vs.control	3

COL9A2	bulk RNA-seq,Homo_723,AD.vs.MCI; bulk RNA-seq,Homo_723,MCI.vs.control; bulk RNA-seq,Homo_633,AD.vs.control; bulk RNA-seq,Homo_633,AD.vs.MCI; bulk RNA-seq,Homo_633,MCI.vs.control; bulk RNA-seq,Homo_714,AD.vs.control; bulk RNA-seq,Homo_714,AD.vs.MCI; bulk RNA-seq,Homo_714,MCI.vs.control; scRNA-seq,SRP215507,CD8+ T cell_4-AD.vs.control; scRNA-seq,SRP330776,CD8+ T cell_1-AD.vs.control; scRNA-seq,SRP330776,Memory T cell_2-AD.vs.control; scRNA-seq,SRP330776,B cell_2-AD.vs.control; scRNA-seq,SRP330776,Monocyte_1-	14
COL9A3	bulk RNA-seq,ROSMAP,AD.vs.MCI; scRNA-seq,SRP330776,B cell_2-AD.vs.control; scRNA-seq,SRP330776,B cell_4-AD.vs.control	3
COLCA2	bulk RNA-seq,Homo_723,AD.vs.control; bulk RNA-seq,Homo_723,AD.vs.MCI; bulk RNA-seq,Homo_633,MCI.vs.control; bulk RNA-	4
COLEC10	bulk RNA-seq,Homo_723,AD.vs.control; bulk RNA-seq,Homo_723,AD.vs.MCI; bulk RNA-seq,Homo_633,AD.vs.control; bulk RNA-seq,Homo_633,AD.vs.MCI; bulk RNA-seq,Homo_714,MCI.vs.control	5
COLEC11	bulk RNA-seq,Homo_723,AD.vs.control; bulk RNA-seq,Homo_723,AD.vs.MCI; bulk RNA-seq,Homo_633,AD.vs.control; bulk RNA-	4
COLEC12	bulk RNA-seq,Homo_723,AD.vs.control; bulk RNA-seq,Homo_723,AD.vs.MCI; bulk RNA-seq,Homo_633,AD.vs.control; bulk RNA-seq,Homo_633,AD.vs.MCI; bulk RNA-seq,Homo_714,AD.vs.control; bulk RNA-seq,Homo_714,AD.vs.MCI	6
Colec12	bulk RNA-seq,SRP325058,AD.vs.control	1
Colgalt1	bulk RNA-seq,SRP325058,AD.vs.control	1
COLGALT1	scRNA-seq,SRP215507,CD8+ T cell_5-AD.vs.control; scRNA-seq,SRP215507,CD8+ T cell_3-AD.vs.MCI; scRNA-seq,SRP215507,CD8+ T cell_5-AD.vs.MCI; scRNA-seq,SRP215507,CD8+ T cell_5-MCI.vs.control; scRNA-seq,SRP309935,Naive CD8+ T cell_1-AD.vs.control; scRNA-seq,SRP330776,CD8+ T cell_1-AD.vs.control; scRNA-seq,SRP330776,γδ T cell-AD.vs.control; scRNA-seq,SRP330776,Memory T cell_2-AD.vs.control; scRNA-seq,SRP330776,CD8+ T cell_2-AD.vs.control; scRNA-seq,SRP330776,B cell_1-AD.vs.control; scRNA-seq,SRP330776,Naive CD8+ T cell_1-AD.vs.control; scRNA-seq,SRP330776,Natural killer cell_1-AD.vs.control; scRNA-seq,SRP330776,Memory T cell_1-AD.vs.control; scRNA-seq,SRP330776,Natural killer T cell-AD.vs.control; scRNA-seq,SRP330776,Naive CD8+ T cell_3-AD.vs.control; scRNA-seq,SRP330776,B cell_2-AD.vs.control; scRNA-seq,SRP330776,T cell-AD.vs.control; scRNA-seq,SRP330776,CD8+ T cell_3-AD.vs.control; scRNA-seq,SRP330776,Monocyte_1- [illegible]	21
COLGALT2	bulk RNA-seq,Homo_723,AD.vs.control; bulk RNA-seq,Homo_723,AD.vs.MCI; bulk RNA-seq,Homo_633,AD.vs.control; bulk RNA-seq,Homo_633,AD.vs.MCI; bulk RNA-seq,Homo_714,AD.vs.control; bulk RNA-seq,Homo_714,AD.vs.MCI; scRNA-seq,SRP215507,CD8+ T cell_1-AD.vs.control; scRNA-seq,SRP215507,CD8+ T cell_4-AD.vs.control; scRNA-seq,SRP215507,CD8+ T cell_1-AD.vs.MCI; scRNA-seq,SRP215507,CD8+ T cell_4-AD.vs.MCI; scRNA-seq,SRP215507,CD8+ T cell_5-AD.vs.MCI; scRNA-seq,SRP215507,CD8+ T cell_1-MCI.vs.control; scRNA-seq,SRP215507,CD8+ T cell_5-MCI.vs.control; scRNA-seq,SRP215507,CD8+ T cell_6-MCI.vs.control; scRNA-seq,SRP309935,CD8+ T cell_1-AD.vs.control; scRNA-seq,SRP330776,CD8+ T cell_2-AD.vs.control; scRNA-seq,SRP330776,Natural killer cell_1-AD.vs.control; scRNA- [illegible]	19
COLQ	scRNA-seq,SRP215507,CD8+ T cell_1-AD.vs.control; scRNA-seq,SRP309935,Natural killer cell_1-AD.vs.control; scRNA-seq,SRP330776,Memory T	3
COMMD1	bulk RNA-seq,Homo_723,AD.vs.control; bulk RNA-seq,Homo_723,AD.vs.MCI; bulk RNA-seq,Homo_633,AD.vs.control; bulk RNA-seq,Homo_633,AD.vs.MCI; bulk RNA-seq,Homo_714,AD.vs.control; bulk RNA-seq,Homo_714,AD.vs.MCI; bulk RNA-seq,Homo_714,MCI.vs.control; scRNA-seq,SRP215507,CD8+ T cell_4-AD.vs.MCI; scRNA-seq,SRP215507,CD8+ T cell_4-MCI.vs.control; scRNA-seq,SRP330776,Natural killer cell_1-AD.vs.control; scRNA-seq,SRP330776,B cell_2-AD.vs.control; scRNA-seq,SRP330776,Megakaryocyte_1-AD.vs.control	12
COMMD10	bulk RNA-seq,Homo_714,AD.vs.control; bulk RNA-seq,Homo_714,AD.vs.MCI; scRNA-seq,SRP330776,B cell_1-AD.vs.control; scRNA-seq,SRP330776,Naive CD8+ T cell_3-AD.vs.control; scRNA-seq,SRP330776,T cell-AD.vs.control; scRNA-seq,SRP330776,Monocyte_2-AD.vs.control; scRNA-seq,SRP330776,B cell_3-AD.vs.control; scRNA-seq,SRP330776,Natural killer cell_2-AD.vs.control	8
COMMD2	scRNA-seq,SRP330776,CD8+ T cell_1-AD.vs.control; scRNA-seq,SRP330776,γδ T cell-AD.vs.control; scRNA-seq,SRP330776,CD8+ T cell_2-AD.vs.control; scRNA-seq,SRP330776,Naive CD8+ T cell_1-AD.vs.control; scRNA-seq,SRP330776,Memory T cell_1-AD.vs.control; scRNA-seq,SRP330776,Natural killer T cell-AD.vs.control; scRNA-seq,SRP330776,T cell-AD.vs.control; scRNA-seq,SRP330776,CD8+ T cell_3-AD.vs.control	8
COMMD3	bulk RNA-seq,Homo_723,AD.vs.control; bulk RNA-seq,Homo_633,AD.vs.control; bulk RNA-seq,Homo_714,AD.vs.control; bulk RNA-seq,Homo_714,AD.vs.MCI; scRNA-seq,SRP309935,Erythroid cell-AD.vs.control; scRNA-seq,SRP330776,Memory T cell_2-AD.vs.control; scRNA-seq,SRP330776,Natural killer cell_1-AD.vs.control; scRNA-seq,SRP330776,Monocyte_2-AD.vs.control	8
COMMD3-BMI1	bulk RNA-seq,Homo_723,AD.vs.MCI; bulk RNA-seq,Homo_723,MCI.vs.control; bulk RNA-seq,Homo_633,AD.vs.MCI; bulk RNA-seq,Homo_633,MCI.vs.control; bulk RNA-seq,Homo_714,AD.vs.control; bulk RNA-seq,Homo_714,AD.vs.MCI; bulk RNA-seq,Homo_714,MCI.vs.control	7

COMMD4	scRNA-seq,SRP215507,CD8+ T cell_4-AD.vs.MCI; scRNA-seq,SRP330776,CD8+ T cell_1-AD.vs.control; scRNA-seq,SRP330776,γδ T cell-AD.vs.control; scRNA-seq,SRP330776,B cell_1-AD.vs.control; scRNA-seq,SRP330776,Memory T cell_1-AD.vs.control; scRNA-seq,SRP330776,Naive CD8+ T cell_3-AD.vs.control; scRNA-seq,SRP330776,B cell_2-AD.vs.control; scRNA-seq,SRP330776,T cell-AD.vs.control; scRNA-seq,SRP330776,CD8+ T cell_3-AD.vs.control; scRNA-seq,SRP330776,Natural killer cell_2-AD.vs.control	10
COMMD5	scRNA-seq,SRP215507,CD8+ T cell_4-AD.vs.control; scRNA-seq,SRP215507,CD8+ T cell_4-AD.vs.MCI; scRNA-seq,SRP330776,Naive CD8+ T cell_1-AD.vs.control; scRNA-seq,SRP330776,Natural killer cell_1-AD.vs.control; scRNA-seq,SRP330776,Naive CD8+ T cell_3-AD.vs.control; scRNA-seq,SRP330776,Naive CD8+ T cell_2-AD.vs.control; scRNA-seq,SRP330776,CD8+ T cell_3-AD.vs.control; scRNA-seq,SRP330776,Monocyte_1-	9
COMMD6	bulk RNA-seq,Homo_723,AD.vs.control; bulk RNA-seq,Homo_723,AD.vs.MCI; bulk RNA-seq,Homo_723,MCI.vs.control; bulk RNA-seq,Homo_633,AD.vs.control; bulk RNA-seq,Homo_633,AD.vs.MCI; bulk RNA-seq,Homo_633,MCI.vs.control; bulk RNA-seq,Homo_714,AD.vs.control; bulk RNA-seq,Homo_714,AD.vs.MCI; bulk RNA-seq,Homo_714,MCI.vs.control; scRNA-seq,SRP215507,CD8+ T cell_7-AD.vs.MCI; scRNA-seq,SRP309935,Erythroid cell-AD.vs.control; scRNA-seq,SRP309935,Megakaryocyte_2-AD.vs.control; scRNA-seq,SRP330776,γδ T cell-AD.vs.control; scRNA-seq,SRP330776,Natural killer cell_1-AD.vs.control; scRNA-seq,SRP330776,Monocyte_2-AD.vs.control; scRNA-	16
COMMD7	bulk RNA-seq,Homo_714,AD.vs.control; bulk RNA-seq,Homo_714,AD.vs.MCI; scRNA-seq,SRP330776,CD8+ T cell_1-AD.vs.control; scRNA-seq,SRP330776,γδ T cell-AD.vs.control; scRNA-seq,SRP330776,Memory T cell_2-AD.vs.control; scRNA-seq,SRP330776,CD8+ T cell_2-AD.vs.control; scRNA-seq,SRP330776,Natural killer cell_1-AD.vs.control; scRNA-seq,SRP330776,Memory T cell_1-AD.vs.control; scRNA-seq,SRP330776,Natural killer T cell-AD.vs.control; scRNA-seq,SRP330776,Naive CD8+ T cell_3-AD.vs.control; scRNA-seq,SRP330776,B cell_2-AD.vs.control; scRNA-	13
COMMD8	bulk RNA-seq,Homo_714,AD.vs.control; bulk RNA-seq,Homo_714,AD.vs.MCI; scRNA-seq,SRP215507,CD8+ T cell_4-AD.vs.control; scRNA-seq,SRP215507,CD8+ T cell_4-AD.vs.MCI; scRNA-seq,SRP330776,CD8+ T cell_1-AD.vs.control; scRNA-seq,SRP330776,Naive CD8+ T cell_1-AD.vs.control; scRNA-seq,SRP330776,Memory T cell_1-AD.vs.control; scRNA-seq,SRP330776,Natural killer T cell-AD.vs.control; scRNA-seq,SRP330776,Naive CD8+ T cell_3-AD.vs.control; scRNA-seq,SRP330776,T cell-AD.vs.control; scRNA-seq,SRP330776,CD8+ T cell_3-AD.vs.control;	12
COMMD9	bulk RNA-seq,Homo_723,AD.vs.control; bulk RNA-seq,Homo_723,AD.vs.MCI; bulk RNA-seq,Homo_633,AD.vs.control; bulk RNA-seq,Homo_633,AD.vs.MCI; bulk RNA-seq,Homo_714,AD.vs.control; bulk RNA-seq,Homo_714,AD.vs.MCI; scRNA-seq,SRP215507,CD8+ T cell_4-AD.vs.MCI; scRNA-seq,SRP309935,Megakaryocyte_1-AD.vs.control; scRNA-seq,SRP330776,Memory T cell_2-AD.vs.control; scRNA-	10
COMT	scRNA-seq,SRP215507,CD8+ T cell_7-AD.vs.control; scRNA-seq,SRP330776,CD8+ T cell_1-AD.vs.control; scRNA-seq,SRP330776,γδ T cell-AD.vs.control; scRNA-seq,SRP330776,Memory T cell_2-AD.vs.control; scRNA-seq,SRP330776,CD8+ T cell_2-AD.vs.control; scRNA-seq,SRP330776,B cell_1-AD.vs.control; scRNA-seq,SRP330776,Naive CD8+ T cell_1-AD.vs.control; scRNA-seq,SRP330776,Natural killer cell_1-AD.vs.control; scRNA-seq,SRP330776,Naive CD8+ T cell_3-AD.vs.control; scRNA-seq,SRP330776,B cell_2-AD.vs.control; scRNA-seq,SRP330776,Naive CD8+ T cell_2-AD.vs.control; scRNA-seq,SRP330776,CD8+ T cell_3-AD.vs.control; scRNA-seq,SRP330776,Regulatory T cell-AD.vs.control; scRNA-	14
COMTD1	bulk RNA-seq,Homo_723,AD.vs.MCI; bulk RNA-seq,Homo_723,MCI.vs.control; bulk RNA-seq,Homo_633,AD.vs.MCI; bulk RNA-seq,Homo_633,MCI.vs.control; bulk RNA-seq,Homo_714,AD.vs.control; bulk RNA-seq,Homo_714,AD.vs.MCI; bulk RNA-seq,Homo_714,MCI.vs.control; scRNA-seq,SRP215507,CD8+ T cell_3-AD.vs.MCI; scRNA-seq,SRP215507,CD8+ T cell_4-AD.vs.MCI; scRNA-seq,SRP215507,CD8+ T cell_3-MCI.vs.control; scRNA-seq,SRP215507,CD8+ T cell_6-MCI.vs.control; scRNA-seq,SRP330776,CD8+ T cell_1-AD.vs.control; scRNA-seq,SRP330776,B cell_1-AD.vs.control; scRNA-seq,SRP330776,Naive CD8+ T cell_1-AD.vs.control; scRNA-seq,SRP330776,Memory T cell_1-AD.vs.control; scRNA-seq,SRP330776,B cell_2-AD.vs.control; scRNA-seq,SRP330776,CD8+ T cell_3-AD.vs.control; [illegible]	18
COP1	scRNA-seq,SRP215507,CD8+ T cell_5-AD.vs.control; scRNA-seq,SRP215507,CD8+ T cell_6-AD.vs.control; scRNA-seq,SRP215507,CD8+ T cell_4-AD.vs.MCI; scRNA-seq,SRP215507,CD8+ T cell_6-AD.vs.MCI; scRNA-seq,SRP309935,Megakaryocyte_1-AD.vs.control; scRNA-seq,SRP309935,Naive CD8+ T cell_1-AD.vs.control; scRNA-seq,SRP309935,Naive CD8+ T cell_2-AD.vs.control; scRNA-seq,SRP309935,CD8+ T cell_1-AD.vs.control; scRNA-seq,SRP330776,CD8+ T cell_1-AD.vs.control; scRNA-seq,SRP330776,γδ T cell-AD.vs.control; scRNA-seq,SRP330776,Memory T cell_2-AD.vs.control; scRNA-seq,SRP330776,CD8+ T cell_2-AD.vs.control; scRNA-seq,SRP330776,B cell_1-AD.vs.control; scRNA-seq,SRP330776,Naive CD8+ T cell_1-AD.vs.control; scRNA-seq,SRP330776,Natural killer cell_1-AD.vs.control; scRNA-seq,SRP330776,Memory T cell_1-AD.vs.control; scRNA-seq,SRP330776,Natural killer T cell-AD.vs.control; scRNA-seq,SRP330776,Naive CD8+ T cell_3-AD.vs.control; scRNA-seq,SRP330776,B cell_2-AD.vs.control; scRNA-seq,SRP330776,T cell-AD.vs.control; scRNA-seq,SRP330776,Naive CD8+ T cell_2-AD.vs.control; scRNA-seq,SRP330776,CD8+ T cell_3-AD.vs.control; scRNA-seq,SRP330776,Monocyte_1-AD.vs.control; scRNA-seq,SRP330776,Monocyte_2-AD.vs.control; scRNA- [illegible]	25

COPA	scRNA-seq,SRP215507,CD8+ T cell_8-AD.vs.control; scRNA-seq,SRP309935,Natural killer cell_1-AD.vs.control; scRNA-seq,SRP309935,Plasmacytoid dendritic cell_1-AD.vs.control	3
COPB1	bulk RNA-seq,Homo_723,AD.vs.control; bulk RNA-seq,Homo_723,AD.vs.MCI; bulk RNA-seq,Homo_633,AD.vs.control; bulk RNA-seq,Homo_633,AD.vs.MCI; bulk RNA-seq,Homo_714,AD.vs.control; bulk RNA-seq,Homo_714,AD.vs.MCI; scRNA-seq,SRP330776,γδ T cell-AD.vs.control; scRNA-seq,SRP330776,Naive CD8+ T cell_2-AD.vs.control; scRNA-seq,SRP330776,B cell_3-AD.vs.control	9
COPB2	bulk RNA-seq,Homo_723,AD.vs.control; bulk RNA-seq,Homo_723,AD.vs.MCI; bulk RNA-seq,Homo_723,MCI.vs.control; bulk RNA-seq,Homo_633,AD.vs.control; bulk RNA-seq,Homo_633,AD.vs.MCI; bulk RNA-seq,Homo_714,AD.vs.control; bulk RNA-seq,Homo_714,AD.vs.MCI; scRNA-seq,SRP309935,Naive CD8+ T cell_1-AD.vs.control; scRNA-seq,SRP309935,Naive CD8+ T cell_2-AD.vs.control; scRNA-seq,SRP309935,B cell_1-AD.vs.control; scRNA-seq,SRP330776,CD8+ T cell_1-AD.vs.control; scRNA-seq,SRP330776,γδ T cell-AD.vs.control; scRNA-seq,SRP330776,Naive CD8+ T cell_1-AD.vs.control; scRNA-seq,SRP330776,Natural killer cell_1-AD.vs.control; scRNA-seq,SRP330776,Memory T cell_1-AD.vs.control; scRNA-seq,SRP330776,B cell_2-AD.vs.control; scRNA-seq,SRP330776,Naive CD8+ T cell_2-AD.vs.control; scRNA- [illegible]	19
COPDA1	scRNA-seq,SRP330776,B cell_1-AD.vs.control; scRNA-seq,SRP330776,B cell_2-AD.vs.control	2
COPE	bulk RNA-seq,Homo_723,AD.vs.control; bulk RNA-seq,Homo_723,AD.vs.MCI; bulk RNA-seq,Homo_723,MCI.vs.control; bulk RNA-seq,Homo_633,AD.vs.control; bulk RNA-seq,Homo_633,AD.vs.MCI; bulk RNA-seq,Homo_633,MCI.vs.control; scRNA-seq,SRP215507,CD8+ T cell_7-AD.vs.control; scRNA-seq,SRP215507,CD8+ T cell_10-AD.vs.control; scRNA-seq,SRP309935,Erythroid cell-AD.vs.control; scRNA-	10
COPG1	scRNA-seq,SRP215507,CD8+ T cell_3-AD.vs.MCI; scRNA-seq,SRP215507,CD8+ T cell_4-AD.vs.MCI; scRNA-seq,SRP330776,Naive CD8+ T cell_1-	3
COPG2	bulk RNA-seq,Homo_723,AD.vs.MCI; bulk RNA-seq,Homo_723,MCI.vs.control; bulk RNA-seq,Homo_633,AD.vs.control; bulk RNA-seq,Homo_633,MCI.vs.control; bulk RNA-seq,Homo_714,AD.vs.control; bulk RNA-seq,Homo_714,AD.vs.MCI; bulk RNA-seq,Homo_714,MCI.vs.control; scRNA-seq,SRP215507,CD8+ T cell_5-AD.vs.MCI; scRNA-seq,SRP215507,CD8+ T cell_5-MCI.vs.control; scRNA-seq,SRP215507,CD8+ T cell_2-MCI.vs.control; scRNA-seq,SRP309935,Naive CD8+ T cell_1-AD.vs.control; scRNA-seq,SRP330776,γδ T cell-AD.vs.control; scRNA-seq,SRP330776,Memory T cell_2-AD.vs.control; scRNA-seq,SRP330776,Naive CD8+ T cell_3-AD.vs.control	14
COPRS	bulk RNA-seq,Homo_633,AD.vs.control; bulk RNA-seq,Homo_633,AD.vs.MCI; bulk RNA-seq,Homo_714,AD.vs.control; bulk RNA-seq,Homo_714,AD.vs.MCI; bulk RNA-seq,SRP223445,AD.vs.control; scRNA-seq,SRP215507,CD8+ T cell_1-AD.vs.control; scRNA-seq,SRP215507,CD8+ T cell_6-AD.vs.control; scRNA-seq,SRP215507,CD8+ T cell_3-MCI.vs.control; scRNA-seq,SRP309935,B cell_1-AD.vs.control; scRNA-seq,SRP330776,CD8+ T cell_1-AD.vs.control; scRNA-seq,SRP330776,γδ T cell-AD.vs.control; scRNA-seq,SRP330776,CD8+ T cell_2-AD.vs.control; scRNA-seq,SRP330776,B cell_1-AD.vs.control; scRNA-seq,SRP330776,Naive CD8+ T cell_1-AD.vs.control; scRNA-seq,SRP330776,Natural killer cell_1-AD.vs.control; scRNA-seq,SRP330776,Memory T cell_1-AD.vs.control; scRNA-seq,SRP330776,B cell_2- [illegible]	20
COPS2	bulk RNA-seq,Homo_723,AD.vs.control; bulk RNA-seq,Homo_723,AD.vs.MCI; bulk RNA-seq,Homo_723,MCI.vs.control; bulk RNA-seq,Homo_633,AD.vs.control; bulk RNA-seq,Homo_633,AD.vs.MCI; bulk RNA-seq,Homo_633,MCI.vs.control; bulk RNA-seq,Homo_714,AD.vs.control; bulk RNA-seq,Homo_714,AD.vs.MCI; bulk RNA-seq,Homo_714,MCI.vs.control; scRNA-seq,SRP215507,CD8+ T cell_3-AD.vs.MCI; scRNA-seq,SRP215507,CD8+ T cell_4-AD.vs.MCI; scRNA-seq,SRP215507,CD8+ T cell_7-AD.vs.MCI; scRNA-seq,SRP215507,CD8+ T cell_4-MCI.vs.control; scRNA-seq,SRP215507,CD8+ T cell_6-MCI.vs.control; scRNA-seq,SRP215507,CD8+ T cell_7-MCI.vs.control; scRNA-seq,SRP309935,Megakaryocyte_1-AD.vs.control; scRNA-seq,SRP330776,Regulatory T cell-AD.vs.control; scRNA-seq,SRP330776,B cell_4-AD.vs.control	18
Cops2	bulk RNA-seq,SRP325058,AD.vs.control	1
COPS3	bulk RNA-seq,Homo_723,AD.vs.control; bulk RNA-seq,Homo_723,AD.vs.MCI; bulk RNA-seq,Homo_633,AD.vs.control; bulk RNA-seq,Homo_633,AD.vs.MCI; bulk RNA-seq,SRP310421,AD.vs.control; scRNA-seq,SRP215507,CD8+ T cell_10-AD.vs.control; scRNA-seq,SRP215507,CD8+ T cell_4-MCI.vs.control; scRNA-seq,SRP215507,CD8+ T cell_6-MCI.vs.control; scRNA-seq,SRP330776,Naive CD8+ T cell_3-AD.vs.control; scRNA-seq,SRP330776,Naive CD8+ T cell_2-AD.vs.control; scRNA-seq,SRP330776,Natural killer cell_2-AD.vs.control	11
COPS4	bulk RNA-seq,Homo_714,AD.vs.control; bulk RNA-seq,Homo_714,AD.vs.MCI; scRNA-seq,SRP215507,CD8+ T cell_4-MCI.vs.control; scRNA-seq,SRP330776,B cell_2-AD.vs.control; scRNA-seq,SRP330776,Natural killer cell_2-AD.vs.control	5

COPS5	bulk RNA-seq,Homo_723,AD.vs.control; bulk RNA-seq,Homo_723,AD.vs.MCI; bulk RNA-seq,Homo_723,MCI.vs.control; bulk RNA-seq,Homo_633,AD.vs.control; bulk RNA-seq,Homo_633,AD.vs.MCI; bulk RNA-seq,Homo_633,MCI.vs.control; bulk RNA-seq,Homo_714,AD.vs.control; bulk RNA-seq,Homo_714,AD.vs.MCI; bulk RNA-seq,Homo_714,MCI.vs.control; scRNA-seq,SRP215507,CD8+ T cell_7-AD.vs.control; scRNA-seq,SRP215507,CD8+ T cell_5-AD.vs.control; scRNA-seq,SRP215507,CD8+ T cell_7-MCI.vs.control; scRNA-seq,SRP330776,B cell_2-AD.vs.control;	14
COPS6	scRNA-seq,SRP215507,CD8+ T cell_7-AD.vs.control	1
COPS7A	bulk RNA-seq,Homo_723,AD.vs.MCI; bulk RNA-seq,Homo_633,AD.vs.MCI; bulk RNA-seq,Homo_714,AD.vs.control; bulk RNA-seq,Homo_714,AD.vs.MCI; scRNA-seq,SRP215507,CD8+ T cell_4-AD.vs.control; scRNA-seq,SRP215507,CD8+ T cell_4-AD.vs.MCI; scRNA-seq,SRP330776,CD8+ T cell_1-AD.vs.control; scRNA-seq,SRP330776,B cell_1-AD.vs.control; scRNA-seq,SRP330776,Natural killer T cell-AD.vs.control; scRNA-seq,SRP330776,CD8+ T cell_3-AD.vs.control; scRNA-seq,SRP330776,Monocyte_2-AD.vs.control; scRNA-seq,SRP330776,Natural	12
COPS7B	scRNA-seq,SRP215507,CD8+ T cell_5-AD.vs.control; scRNA-seq,SRP215507,CD8+ T cell_5-MCI.vs.control; scRNA-seq,SRP309935,Monocyte_1-AD.vs.control; scRNA-seq,SRP330776,Naive CD8+ T cell_3-AD.vs.control; scRNA-seq,SRP330776,CD8+ T cell_3-AD.vs.control; scRNA-seq,SRP330776,Monocyte_1-AD.vs.control; scRNA-seq,SRP330776,B cell_3-AD.vs.control	7
COPS8	bulk RNA-seq,Homo_714,AD.vs.control; bulk RNA-seq,Homo_714,AD.vs.MCI; scRNA-seq,SRP215507,CD8+ T cell_3-AD.vs.control; scRNA-seq,SRP215507,CD8+ T cell_6-AD.vs.MCI; scRNA-seq,SRP330776,Megakaryocyte_1-AD.vs.control	5
COPS9	bulk RNA-seq,Homo_723,AD.vs.control; bulk RNA-seq,Homo_723,AD.vs.MCI; bulk RNA-seq,Homo_633,AD.vs.control; bulk RNA-seq,Homo_633,AD.vs.MCI; bulk RNA-seq,Homo_714,AD.vs.control; bulk RNA-seq,Homo_714,AD.vs.MCI; scRNA-seq,SRP309935,Erythroid cell-AD.vs.control; scRNA-seq,SRP330776,CD8+ T cell_1-AD.vs.control; scRNA-seq,SRP330776,γδ T cell-AD.vs.control; scRNA-seq,SRP330776,Memory T cell_2-AD.vs.control; scRNA-seq,SRP330776,CD8+ T cell_2-AD.vs.control; scRNA-seq,SRP330776,B cell_1-AD.vs.control; scRNA-seq,SRP330776,Naive CD8+ T cell_1-AD.vs.control; scRNA-seq,SRP330776,Natural killer cell_1-AD.vs.control; scRNA-seq,SRP330776,Memory T cell_1-AD.vs.control; scRNA-seq,SRP330776,Naive CD8+ T cell_3-AD.vs.control; scRNA-seq,SRP330776,B cell_2-AD.vs.control; scRNA-seq,SRP330776,T cell-AD.vs.control; scRNA-seq,SRP330776,CD8+ T cell_3-AD.vs.control; scRNA-seq,SRP330776,Monocyte_1-AD.vs.control; scRNA-[illegible]	22
Copz1	bulk RNA-seq,SRP325058,AD.vs.control	1
COPZ1	scRNA-seq,SRP215507,CD8+ T cell_7-AD.vs.control; scRNA-seq,SRP215507,CD8+ T cell_6-AD.vs.control; scRNA-seq,SRP215507,CD8+ T cell_7-MCI.vs.control; scRNA-seq,SRP330776,γδ T cell-AD.vs.control; scRNA-seq,SRP330776,Memory T cell_2-AD.vs.control; scRNA-seq,SRP330776,Natural killer T cell-AD.vs.control; scRNA-seq,SRP330776,Naive CD8+ T cell_3-AD.vs.control; scRNA-seq,SRP330776,T cell-AD.vs.control; scRNA-seq,SRP330776,B cell_3-AD.vs.control; scRNA-seq,SRP330776,Megakaryocyte_1-AD.vs.control; scRNA-seq,SRP330776,Natural killer cell_2-	11
COPZ2	bulk RNA-seq,Homo_714,AD.vs.control; bulk RNA-seq,Homo_714,AD.vs.MCI; scRNA-seq,SRP215507,CD8+ T cell_5-MCI.vs.control; scRNA-seq,SRP330776,γδ T cell-AD.vs.control; scRNA-seq,SRP330776,Memory T cell_1-AD.vs.control	5
COQ10A	bulk RNA-seq,Homo_723,AD.vs.control; bulk RNA-seq,Homo_723,AD.vs.MCI; bulk RNA-seq,Homo_633,AD.vs.control; bulk RNA-seq,Homo_633,AD.vs.MCI; bulk RNA-seq,Homo_714,AD.vs.control; bulk RNA-seq,Homo_714,AD.vs.MCI; scRNA-seq,SRP215507,CD8+ T cell_3-AD.vs.MCI; scRNA-seq,SRP215507,CD8+ T cell_5-AD.vs.MCI; scRNA-seq,SRP215507,CD8+ T cell_5-MCI.vs.control; scRNA-seq,SRP215507,CD8+ T cell_3-MCI.vs.control; scRNA-seq,SRP215507,CD8+ T cell_6-MCI.vs.control; scRNA-seq,SRP309935,Naive CD8+ T cell_1-AD.vs.control; scRNA-seq,SRP309935,CD8+ T cell_1-AD.vs.control; scRNA-seq,SRP309935,CD4+ T cell-AD.vs.control; scRNA-seq,SRP330776,CD8+ T cell_1-AD.vs.control; scRNA-seq,SRP330776,γδ T cell-AD.vs.control; scRNA-seq,SRP330776,Memory T cell_2-AD.vs.control; scRNA-seq,SRP330776,CD8+ T cell_2-AD.vs.control; scRNA-seq,SRP330776,B cell_1-AD.vs.control; scRNA-seq,SRP330776,Naive CD8+ T cell_1-AD.vs.control; scRNA-seq,SRP330776,Natural killer cell_1-AD.vs.control; scRNA-seq,SRP330776,Memory T cell_1-AD.vs.control; scRNA-seq,SRP330776,Natural killer T cell-AD.vs.control; scRNA-seq,SRP330776,Naive CD8+ T cell_3-AD.vs.control; scRNA-seq,SRP330776,B cell_2-AD.vs.control; scRNA-seq,SRP330776,T cell-AD.vs.control; scRNA-seq,SRP330776,CD8+ T cell_3-AD.vs.control; scRNA-seq,SRP330776,Monocyte_1-AD.vs.control; scRNA-seq,SRP330776,B [illegible]	30
COQ10B	bulk RNA-seq,Homo_633,AD.vs.control; bulk RNA-seq,Homo_633,MCI.vs.control; bulk RNA-seq,Homo_714,AD.vs.control; bulk RNA-seq,Homo_714,AD.vs.MCI; bulk RNA-seq,Homo_714,MCI.vs.control; scRNA-seq,SRP215507,CD8+ T cell_6-AD.vs.control; scRNA-seq,SRP215507,CD8+ T cell_4-AD.vs.MCI; scRNA-seq,SRP215507,CD8+ T cell_5-AD.vs.MCI; scRNA-seq,SRP215507,CD8+ T cell_4-MCI.vs.control	9

COQ2	bulk RNA-seq,Homo_633,AD.vs.control; bulk RNA-seq,Homo_633,AD.vs.MCI; scRNA-seq,SRP215507,CD8+ T cell_4-AD.vs.control; scRNA-seq,SRP215507,CD8+ T cell_4-AD.vs.MCI; scRNA-seq,SRP215507,CD8+ T cell_6-MCI.vs.control; scRNA-seq,SRP309935,Monocyte_1-AD.vs.control; scRNA-seq,SRP309935,Monocyte_2-AD.vs.control; scRNA-seq,SRP309935,Naive CD8+ T cell_2-AD.vs.control; scRNA-seq,SRP309935,CD8+ T cell_1-AD.vs.control; scRNA-seq,SRP330776,CD8+ T cell_1-AD.vs.control; scRNA-seq,SRP330776,γδ T cell-AD.vs.control; scRNA-seq,SRP330776,Memory T cell_2-AD.vs.control; scRNA-seq,SRP330776,CD8+ T cell_2-AD.vs.control; scRNA-seq,SRP330776,B cell_1-AD.vs.control; scRNA-seq,SRP330776,Naive CD8+ T cell_1-AD.vs.control; scRNA-seq,SRP330776,Natural killer cell_1-AD.vs.control; scRNA-seq,SRP330776,Memory T cell_1-AD.vs.control; scRNA-seq,SRP330776,Naive CD8+ T cell_3-AD.vs.control; scRNA-seq,SRP330776,B cell_2-AD.vs.control; scRNA-seq,SRP330776,CD8+ T cell_3-AD.vs.control; scRNA-seq,SRP330776,Monocyte_1-AD.vs.control; scRNA-seq,SRP330776,Monocyte_2-AD.vs.control	22
COQ3	bulk RNA-seq,Homo_723,AD.vs.control; bulk RNA-seq,Homo_633,AD.vs.control; bulk RNA-seq,Homo_633,AD.vs.MCI; bulk RNA-seq,Homo_714,AD.vs.control; bulk RNA-seq,Homo_714,AD.vs.MCI; scRNA-seq,SRP215507,CD8+ T cell_3-AD.vs.MCI; scRNA-seq,SRP215507,CD8+ T cell_1-AD.vs.MCI; scRNA-seq,SRP215507,CD8+ T cell_1-MCI.vs.control; scRNA-seq,SRP215507,CD8+ T cell_3-MCI.vs.control; scRNA-seq,SRP215507,CD8+ T cell_6-MCI.vs.control; scRNA-seq,SRP309935,Megakaryocyte_1-AD.vs.control; scRNA-seq,SRP330776,CD8+ T cell_1-AD.vs.control; scRNA-seq,SRP330776,γδ T cell-AD.vs.control; scRNA-seq,SRP330776,CD8+ T cell_2-AD.vs.control; scRNA-seq,SRP330776,B cell_1-AD.vs.control; scRNA-seq,SRP330776,Naive CD8+ T cell_1-AD.vs.control; scRNA-seq,SRP330776,Natural killer cell_1-AD.vs.control; scRNA-seq,SRP330776,Memory T cell_1-AD.vs.control; scRNA-seq,SRP330776,Natural killer T cell-AD.vs.control; scRNA-seq,SRP330776,Naive CD8+ T [illegible]	20
COQ4	bulk RNA-seq,Homo_723,AD.vs.control; bulk RNA-seq,Homo_723,AD.vs.MCI; bulk RNA-seq,Homo_633,AD.vs.control; bulk RNA-seq,Homo_633,AD.vs.MCI; scRNA-seq,SRP215507,CD8+ T cell_7-AD.vs.control; scRNA-seq,SRP215507,CD8+ T cell_7-AD.vs.MCI; scRNA-seq,SRP215507,CD8+ T cell_5-AD.vs.MCI; scRNA-seq,SRP215507,CD8+ T cell_4-MCI.vs.control; scRNA-seq,SRP330776,B cell_2-AD.vs.control	9
COQ5	scRNA-seq,SRP215507,CD8+ T cell_2-AD.vs.MCI; scRNA-seq,SRP215507,CD8+ T cell_3-AD.vs.MCI; scRNA-seq,SRP215507,CD8+ T cell_5-AD.vs.MCI; scRNA-seq,SRP309935,Natural killer cell_2-AD.vs.control; scRNA-seq,SRP330776,B cell_1-AD.vs.control; scRNA-seq,SRP330776,Natural killer cell_1-AD.vs.control; scRNA-seq,SRP330776,Natural killer T cell-AD.vs.control; scRNA-seq,SRP330776,Naive CD8+ T cell_3-AD.vs.control; scRNA-seq,SRP330776,CD8+ T cell_3-AD.vs.control; scRNA-seq,SRP330776,Regulatory T cell-AD.vs.control; scRNA-seq,SRP330776,Natural killer [illegible]	11
COQ6	bulk RNA-seq,Homo_723,AD.vs.control; bulk RNA-seq,Homo_633,AD.vs.control; bulk RNA-seq,Homo_633,MCI.vs.control; bulk RNA-seq,Homo_714,AD.vs.control; bulk RNA-seq,Homo_714,AD.vs.MCI; bulk RNA-seq,Homo_714,MCI.vs.control; scRNA-seq,SRP215507,CD8+ T cell_5-AD.vs.MCI; scRNA-seq,SRP215507,CD8+ T cell_5-MCI.vs.control; scRNA-seq,SRP330776,B cell_1-AD.vs.control; scRNA-seq,SRP330776,Naive CD8+ [illegible]	10
COQ7	bulk RNA-seq,Homo_633,AD.vs.control; bulk RNA-seq,Homo_714,AD.vs.control; bulk RNA-seq,Homo_714,AD.vs.MCI; scRNA-seq,SRP215507,CD8+ T cell_5-AD.vs.control; scRNA-seq,SRP215507,CD8+ T cell_1-AD.vs.MCI; scRNA-seq,SRP215507,CD8+ T cell_1-MCI.vs.control; scRNA-seq,SRP330776,CD8+ T cell_1-AD.vs.control; scRNA-seq,SRP330776,γδ T cell-AD.vs.control; scRNA-seq,SRP330776,Memory T cell_2-AD.vs.control; scRNA-seq,SRP330776,CD8+ T cell_2-AD.vs.control; scRNA-seq,SRP330776,B cell_1-AD.vs.control; scRNA-seq,SRP330776,Naive CD8+ T cell_1-AD.vs.control; scRNA-seq,SRP330776,B cell_2-AD.vs.control; scRNA-seq,SRP330776,T cell-AD.vs.control; scRNA-seq,SRP330776,CD8+ T cell_3-AD.vs.control; scRNA-seq,SRP330776,Monocyte_1-AD.vs.control; scRNA-seq,SRP330776,B cell_3-AD.vs.control; scRNA-seq,SRP330776,Natural killer [illegible]	18
COQ8A	scRNA-seq,SRP215507,CD8+ T cell_4-AD.vs.control; scRNA-seq,SRP215507,CD8+ T cell_4-AD.vs.MCI; scRNA-seq,SRP215507,CD8+ T cell_7-AD.vs.MCI; scRNA-seq,SRP215507,CD8+ T cell_3-MCI.vs.control; scRNA-seq,SRP215507,CD8+ T cell_7-MCI.vs.control; scRNA-seq,SRP309935,Natural killer cell_1-AD.vs.control; scRNA-seq,SRP309935,B cell_1-AD.vs.control; scRNA-seq,SRP330776,Memory T cell_2-AD.vs.control; scRNA-seq,SRP330776,Naive CD8+ T cell_1-AD.vs.control; scRNA-seq,SRP330776,Naive CD8+ T cell_3-AD.vs.control; scRNA-seq,SRP330776,Monocyte_1-AD.vs.control; scRNA-seq,SRP330776,Monocyte_2-AD.vs.control; scRNA-seq,SRP330776,B cell_4-AD.vs.control	13
COQ8B	bulk RNA-seq,Homo_723,AD.vs.control; bulk RNA-seq,Homo_723,AD.vs.MCI; bulk RNA-seq,Homo_633,AD.vs.control; bulk RNA-seq,Homo_633,AD.vs.MCI; bulk RNA-seq,Homo_714,AD.vs.control; bulk RNA-seq,Homo_714,AD.vs.MCI; scRNA-seq,SRP309935,Monocyte_2-AD.vs.control; scRNA-seq,SRP330776,Memory T cell_2-AD.vs.control; scRNA-seq,SRP330776,Memory T cell_1-AD.vs.control; scRNA-seq,SRP330776,Natural killer T cell-AD.vs.control; scRNA-seq,SRP330776,T cell-AD.vs.control; scRNA-seq,SRP330776,CD8+ T cell_3-AD.vs.control;	13

COQ9	bulk RNA-seq,Homo_723,AD.vs.control; bulk RNA-seq,Homo_723,AD.vs.MCI; bulk RNA-seq,Homo_633,AD.vs.control; bulk RNA-seq,Homo_633,AD.vs.MCI; bulk RNA-seq,Homo_714,AD.vs.control; bulk RNA-seq,Homo_714,AD.vs.MCI; scRNA-seq,SRP215507,CD8+ T cell_3-AD.vs.control; scRNA-seq,SRP215507,CD8+ T cell_3-AD.vs.MCI; scRNA-seq,SRP309935,Natural killer cell_2-AD.vs.control; scRNA-seq,SRP330776,CD8+ T cell_1-AD.vs.control; scRNA-seq,SRP330776,B cell_1-AD.vs.control; scRNA-seq,SRP330776,Naive CD8+ T cell_3-AD.vs.control; scRNA-seq,SRP330776,B cell_2-AD.vs.control; scRNA-seq,SRP330776,T cell-AD.vs.control; scRNA-seq,SRP330776,CD8+ T cell_3-	15
CORIN	bulk RNA-seq,Homo_723,AD.vs.control; bulk RNA-seq,Homo_723,AD.vs.MCI; bulk RNA-seq,Homo_723,MCI.vs.control; bulk RNA-seq,Homo_633,AD.vs.MCI; bulk RNA-seq,Homo_714,AD.vs.control; bulk RNA-seq,Homo_714,AD.vs.MCI	6
CORO1A	scRNA-seq,SRP215507,CD8+ T cell_7-AD.vs.control; scRNA-seq,SRP309935,Erythroid cell-AD.vs.control	2
CORO1B	bulk RNA-seq,Homo_723,AD.vs.control; bulk RNA-seq,Homo_633,AD.vs.control; bulk RNA-seq,Homo_633,AD.vs.MCI; scRNA-seq,SRP215507,CD8+ T cell_4-AD.vs.MCI; scRNA-seq,SRP215507,CD8+ T cell_6-AD.vs.MCI; scRNA-seq,SRP215507,CD8+ T cell_4-MCI.vs.control; scRNA-seq,SRP330776,B cell_3-AD.vs.control; scRNA-seq,SRP330776,Natural killer cell_2-AD.vs.control	8
CORO1C	scRNA-seq,SRP215507,CD8+ T cell_5-AD.vs.MCI; scRNA-seq,SRP215507,CD8+ T cell_5-MCI.vs.control; scRNA-seq,SRP215507,CD8+ T cell_6-MCI.vs.control; scRNA-seq,SRP330776,CD8+ T cell_1-AD.vs.control; scRNA-seq,SRP330776,γδ T cell-AD.vs.control; scRNA-seq,SRP330776,CD8+ T cell_2-AD.vs.control; scRNA-seq,SRP330776,Naive CD8+ T cell_1-AD.vs.control; scRNA-seq,SRP330776,Natural killer cell_1-AD.vs.control; scRNA-seq,SRP330776,Memory T cell_1-AD.vs.control; scRNA-seq,SRP330776,Natural killer T cell-AD.vs.control; scRNA-seq,SRP330776,T cell-AD.vs.control; scRNA-seq,SRP330776,CD8+ T cell_3-AD.vs.control; scRNA-seq,SRP330776,Monocyte_1-AD.vs.control	13
CORO2A	scRNA-seq,SRP215507,CD8+ T cell_4-AD.vs.control; scRNA-seq,SRP215507,CD8+ T cell_2-AD.vs.MCI; scRNA-seq,SRP215507,CD8+ T cell_1-AD.vs.MCI; scRNA-seq,SRP215507,CD8+ T cell_4-AD.vs.MCI; scRNA-seq,SRP215507,CD8+ T cell_6-AD.vs.MCI; scRNA-seq,SRP215507,CD8+ T cell_5-MCI.vs.control; scRNA-seq,SRP215507,CD8+ T cell_2-MCI.vs.control; scRNA-seq,SRP215507,CD8+ T cell_3-MCI.vs.control; scRNA-seq,SRP309935,CD8+ T cell_1-AD.vs.control; scRNA-seq,SRP309935,CD8+ T cell_2-AD.vs.control; scRNA-seq,SRP330776,CD8+ T cell_1-AD.vs.control; scRNA-seq,SRP330776,γδ T cell-AD.vs.control; scRNA-seq,SRP330776,Memory T cell_2-AD.vs.control; scRNA-seq,SRP330776,Naive CD8+ T cell_1-AD.vs.control; scRNA-seq,SRP330776,Natural killer cell_1-AD.vs.control; scRNA-seq,SRP330776,Memory T cell_1-AD.vs.control; scRNA-seq,SRP330776,Natural killer T cell-AD.vs.control; scRNA-seq,SRP330776,Naive CD8+ T cell_3-AD.vs.control; scRNA-seq,SRP330776,B cell_2-AD.vs.control; scRNA-seq,SRP330776,T cell-AD.vs.control; scRNA-seq,SRP330776,CD8+ T cell_3-AD.vs.control; scRNA-seq,SRP330776,B cell_3-	23
CORO2B	bulk RNA-seq,Homo_723,AD.vs.control; bulk RNA-seq,Homo_723,AD.vs.MCI; bulk RNA-seq,Homo_633,AD.vs.control; bulk RNA-seq,Homo_714,MCI.vs.control; bulk RNA-seq,ROSMAP,AD.vs.control; bulk RNA-seq,SRP223445,AD.vs.control; scRNA-seq,SRP330776,B cell_2-	7
CORO6	bulk RNA-seq,Homo_723,MCI.vs.control; bulk RNA-seq,Homo_633,AD.vs.control; bulk RNA-seq,Homo_633,AD.vs.MCI; bulk RNA-seq,Homo_633,MCI.vs.control; bulk RNA-seq,Homo_714,AD.vs.control; bulk RNA-seq,Homo_714,AD.vs.MCI; bulk RNA-seq,Homo_714,MCI.vs.control; bulk RNA-seq,ROSMAP,MCI.vs.control; scRNA-seq,SRP215507,CD8+ T cell_2-AD.vs.control; scRNA-seq,SRP215507,CD8+ T cell_3-AD.vs.control; scRNA-seq,SRP215507,CD8+ T cell_1-AD.vs.MCI; scRNA-seq,SRP215507,CD8+ T cell_1-	14
CORO7	scRNA-seq,SRP215507,CD8+ T cell_10-AD.vs.control; scRNA-seq,SRP330776,CD8+ T cell_1-AD.vs.control; scRNA-seq,SRP330776,γδ T cell-AD.vs.control; scRNA-seq,SRP330776,Memory T cell_2-AD.vs.control; scRNA-seq,SRP330776,CD8+ T cell_2-AD.vs.control; scRNA-seq,SRP330776,B cell_1-AD.vs.control; scRNA-seq,SRP330776,Naive CD8+ T cell_1-AD.vs.control; scRNA-seq,SRP330776,Natural killer cell_1-AD.vs.control; scRNA-seq,SRP330776,Memory T cell_1-AD.vs.control; scRNA-seq,SRP330776,Natural killer T cell-AD.vs.control; scRNA-seq,SRP330776,Naive CD8+ T cell_3-AD.vs.control; scRNA-seq,SRP330776,B cell_2-AD.vs.control; scRNA-seq,SRP330776,T cell-AD.vs.control; scRNA-seq,SRP330776,Naive CD8+ T cell_2-AD.vs.control; scRNA-seq,SRP330776,CD8+ T cell_3-AD.vs.control; scRNA-seq,SRP330776,Monocyte_1-AD.vs.control; scRNA-	18
CORO7-PAM16	bulk RNA-seq,Homo_723,AD.vs.control; bulk RNA-seq,Homo_723,AD.vs.MCI; bulk RNA-seq,Homo_633,AD.vs.control; bulk RNA-	4
CORT	bulk RNA-seq,Homo_723,MCI.vs.control; bulk RNA-seq,Homo_633,AD.vs.control; bulk RNA-seq,Homo_633,AD.vs.MCI; bulk RNA-seq,Homo_633,MCI.vs.control; bulk RNA-seq,Homo_714,AD.vs.control; bulk RNA-seq,Homo_714,MCI.vs.control; scRNA-seq,SRP215507,CD8+ T	7

COTL1	scRNA-seq,SRP215507,CD8+ T cell_8-AD.vs.MCI; scRNA-seq,SRP215507,CD8+ T cell_1-AD.vs.MCI; scRNA-seq,SRP215507,CD8+ T cell_4-AD.vs.MCI; scRNA-seq,SRP215507,CD8+ T cell_1-MCI.vs.control; scRNA-seq,SRP215507,CD8+ T cell_4-MCI.vs.control; scRNA-seq,SRP215507,CD8+ T cell_8-MCI.vs.control; scRNA-seq,SRP309935,Natural killer cell_2-AD.vs.control; scRNA-seq,SRP330776,CD8+ T cell_1-AD.vs.control; scRNA-seq,SRP330776,γδ T cell-AD.vs.control; scRNA-seq,SRP330776,Memory T cell_2-AD.vs.control; scRNA-seq,SRP330776,CD8+ T cell_2-AD.vs.control; scRNA-seq,SRP330776,B cell_1-AD.vs.control; scRNA-seq,SRP330776,Naive CD8+ T cell_1-AD.vs.control; scRNA-seq,SRP330776,Natural killer cell_1-AD.vs.control; scRNA-seq,SRP330776,Memory T cell_1-AD.vs.control; scRNA-seq,SRP330776,Natural killer T cell-AD.vs.control; scRNA-seq,SRP330776,Naive CD8+ T cell_3-AD.vs.control; scRNA-seq,SRP330776,B cell_2-AD.vs.control; scRNA-seq,SRP330776,T cell-AD.vs.control; scRNA-seq,SRP330776,Naive CD8+ T cell_2-AD.vs.control; scRNA-seq,SRP330776,CD8+ T cell_3-AD.vs.control; scRNA-seq,SRP330776,Monocyte_1-AD.vs.control; scRNA-seq,SRP330776,Monocyte_2-AD.vs.control; scRNA-seq,SRP330776,B cell_3-AD.vs.control; scRNA-seq,SRP330776,Regulatory T cell-AD.vs.control; scRNA-seq,SRP330776,Megakaryocyte_2-AD.vs.control; scRNA-seq,SRP330776,Natural killer cell_2-AD.vs.control; scRNA-seq,SRP330776,B cell_4-AD.vs.control	28
COX10	bulk RNA-seq,Homo_723,AD.vs.control; bulk RNA-seq,Homo_633,AD.vs.control; bulk RNA-seq,Homo_714,AD.vs.control; bulk RNA-seq,Homo_714,AD.vs.MCI; bulk RNA-seq,Homo_714,MCI.vs.control; scRNA-seq,SRP215507,CD8+ T cell_5-AD.vs.control; scRNA-seq,SRP215507,CD8+ T cell_3-AD.vs.MCI; scRNA-seq,SRP215507,CD8+ T cell_5-MCI.vs.control; scRNA-seq,SRP330776,Megakaryocyte_1-	9
COX10-AS1	scRNA-seq,SRP215507,CD8+ T cell_4-AD.vs.control; scRNA-seq,SRP330776,Monocyte_1-AD.vs.control; scRNA-seq,SRP330776,Regulatory T cell-	3
COX11	scRNA-seq,SRP215507,CD8+ T cell_7-AD.vs.MCI; scRNA-seq,SRP215507,CD8+ T cell_6-MCI.vs.control; scRNA-seq,SRP309935,Megakaryocyte_1-AD.vs.control; scRNA-seq,SRP309935,Plasmacytoid dendritic cell_1-AD.vs.control	4
COX14	scRNA-seq,SRP215507,CD8+ T cell_7-AD.vs.MCI; scRNA-seq,SRP215507,CD8+ T cell_7-MCI.vs.control; scRNA-seq,SRP330776,CD8+ T cell_1-AD.vs.control; scRNA-seq,SRP330776,γδ T cell-AD.vs.control; scRNA-seq,SRP330776,Memory T cell_2-AD.vs.control; scRNA-seq,SRP330776,CD8+ T cell_3-AD.vs.control; scRNA-seq,SRP330776,Monocyte_2-AD.vs.control; scRNA-seq,SRP330776,B cell_3-AD.vs.control	8
COX15	bulk RNA-seq,Homo_723,AD.vs.control; bulk RNA-seq,Homo_723,AD.vs.MCI; bulk RNA-seq,Homo_723,MCI.vs.control; bulk RNA-seq,Homo_633,AD.vs.control; bulk RNA-seq,Homo_633,AD.vs.MCI; bulk RNA-seq,Homo_633,MCI.vs.control; scRNA-seq,SRP215507,CD8+ T cell_6-AD.vs.control; scRNA-seq,SRP215507,CD8+ T cell_2-AD.vs.MCI; scRNA-seq,SRP330776,γδ T cell-AD.vs.control; scRNA-seq,SRP330776,CD8+ T cell_2-AD.vs.control; scRNA-seq,SRP330776,Naive CD8+ T cell_1-AD.vs.control; scRNA-seq,SRP330776,T cell-AD.vs.control; scRNA-	14
COX16	bulk RNA-seq,Homo_714,AD.vs.control; bulk RNA-seq,Homo_714,AD.vs.MCI; scRNA-seq,SRP215507,CD8+ T cell_4-AD.vs.control; scRNA-seq,SRP330776,CD8+ T cell_1-AD.vs.control; scRNA-seq,SRP330776,γδ T cell-AD.vs.control; scRNA-seq,SRP330776,Memory T cell_2-AD.vs.control; scRNA-seq,SRP330776,CD8+ T cell_2-AD.vs.control; scRNA-seq,SRP330776,Naive CD8+ T cell_1-AD.vs.control; scRNA-seq,SRP330776,Natural killer cell_1-AD.vs.control; scRNA-seq,SRP330776,Memory T cell_1-AD.vs.control; scRNA-seq,SRP330776,Natural killer T cell-AD.vs.control; scRNA-seq,SRP330776,Naive CD8+ T cell_3-AD.vs.control; scRNA-seq,SRP330776,B cell_2-AD.vs.control; scRNA-seq,SRP330776,T cell-AD.vs.control;	15
Cox17	bulk RNA-seq,SRP325058,AD.vs.control	1
COX17	scRNA-seq,SRP215507,CD8+ T cell_7-AD.vs.control; scRNA-seq,SRP215507,CD8+ T cell_7-MCI.vs.control; scRNA-seq,SRP330776,CD8+ T cell_1-AD.vs.control; scRNA-seq,SRP330776,γδ T cell-AD.vs.control; scRNA-seq,SRP330776,Memory T cell_2-AD.vs.control; scRNA-seq,SRP330776,CD8+ T cell_2-AD.vs.control; scRNA-seq,SRP330776,B cell_1-AD.vs.control; scRNA-seq,SRP330776,Naive CD8+ T cell_1-AD.vs.control; scRNA-seq,SRP330776,Natural killer cell_1-AD.vs.control; scRNA-seq,SRP330776,Memory T cell_1-AD.vs.control; scRNA-seq,SRP330776,Natural killer T cell-AD.vs.control; scRNA-seq,SRP330776,Naive CD8+ T cell_3-AD.vs.control; scRNA-seq,SRP330776,B cell_2-AD.vs.control; scRNA-seq,SRP330776,T cell-AD.vs.control; scRNA-seq,SRP330776,CD8+ T cell_3-AD.vs.control; scRNA-seq,SRP330776,Monocyte_2-AD.vs.control; scRNA-seq,SRP330776,B [illegible]	17
COX18	bulk RNA-seq,Homo_723,MCI.vs.control; bulk RNA-seq,Homo_633,AD.vs.control; bulk RNA-seq,Homo_633,MCI.vs.control; bulk RNA-seq,Homo_714,AD.vs.control; bulk RNA-seq,Homo_714,AD.vs.MCI; bulk RNA-seq,Homo_714,MCI.vs.control; scRNA-seq,SRP215507,CD8+ T cell_5-MCI.vs.control; scRNA-seq,SRP309935,Monocyte_1-AD.vs.control; scRNA-seq,SRP309935,Naive CD8+ T cell_1-AD.vs.control; scRNA-seq,SRP309935,Natural killer cell_1-AD.vs.control; scRNA-seq,SRP309935,Naive CD8+ T cell_2-AD.vs.control; scRNA-seq,SRP330776,γδ T cell-AD.vs.control; scRNA-seq,SRP330776,CD8+ T cell_2-AD.vs.control; scRNA-seq,SRP330776,Natural killer cell_1-AD.vs.control; scRNA-	16
COX19	bulk RNA-seq,Homo_723,AD.vs.control; bulk RNA-seq,Homo_723,AD.vs.MCI; bulk RNA-seq,Homo_633,AD.vs.control; bulk RNA-seq,Homo_633,AD.vs.MCI; bulk RNA-seq,Homo_714,AD.vs.control; bulk RNA-seq,Homo_714,AD.vs.MCI; scRNA-seq,SRP215507,CD8+ T cell_7-AD.vs.MCI; scRNA-seq,SRP330776,CD8+ T cell_2-AD.vs.control; scRNA-seq,SRP330776,Natural killer cell_1-AD.vs.control; scRNA-seq,SRP330776,B	10

COX20	scRNA-seq,SRP215507,CD8+ T cell_6-MCI.vs.control; scRNA-seq,SRP330776,Monocyte_2-AD.vs.control; scRNA-seq,SRP330776,B cell_3-	3
COX3	bulk RNA-seq,Homo_714,AD.vs.control; bulk RNA-seq,Homo_714,AD.vs.MCI	2
COX4I1	scRNA-seq,SRP309935,Erythroid cell-AD.vs.control	1
COX5A	bulk RNA-seq,Homo_723,AD.vs.MCI; bulk RNA-seq,Homo_723,MCI.vs.control; bulk RNA-seq,Homo_633,AD.vs.control; bulk RNA-seq,Homo_633,AD.vs.MCI; bulk RNA-seq,Homo_633,MCI.vs.control; bulk RNA-seq,Homo_714,AD.vs.control; bulk RNA-seq,Homo_714,AD.vs.MCI; bulk RNA-seq,Homo_714,MCI.vs.control; scRNA-seq,SRP215507,CD8+ T cell_7-AD.vs.control; scRNA-seq,SRP215507,CD8+ T cell_10-AD.vs.control; scRNA-seq,SRP215507,CD8+ T cell_7-AD.vs.MCI; scRNA-seq,SRP215507,CD8+ T cell_5-AD.vs.MCI; scRNA-seq,SRP215507,CD8+ T cell_6-MCI.vs.control; scRNA-seq,SRP330776,Natural killer cell_1-AD.vs.control; scRNA-seq,SRP330776,CD8+ T cell_3-AD.vs.control	15
Cox5a	bulk RNA-seq,SRP325058,AD.vs.control	1
COX5B	bulk RNA-seq,Homo_723,AD.vs.control; bulk RNA-seq,Homo_723,AD.vs.MCI; bulk RNA-seq,Homo_633,AD.vs.control; bulk RNA-seq,Homo_633,AD.vs.MCI; scRNA-seq,SRP215507,CD8+ T cell_7-AD.vs.control; scRNA-seq,SRP309935,Erythroid cell-AD.vs.control; scRNA-	7
Cox5b	bulk RNA-seq,SRP325058,AD.vs.control	1
COX6A1	bulk RNA-seq,Homo_723,AD.vs.MCI; scRNA-seq,SRP215507,CD8+ T cell_4-AD.vs.control; scRNA-seq,SRP215507,CD8+ T cell_7-AD.vs.control; scRNA-seq,SRP215507,CD8+ T cell_7-AD.vs.MCI; scRNA-seq,SRP215507,CD8+ T cell_4-MCI.vs.control; scRNA-seq,SRP309935,Erythroid cell-	6
COX6B1	bulk RNA-seq,Homo_714,MCI.vs.control; scRNA-seq,SRP309935,Erythroid cell-AD.vs.control; scRNA-seq,SRP330776,CD8+ T cell_1-AD.vs.control; scRNA-seq,SRP330776,Memory T cell_2-AD.vs.control; scRNA-seq,SRP330776,CD8+ T cell_2-AD.vs.control; scRNA-seq,SRP330776,Naive CD8+ T cell_1-AD.vs.control; scRNA-seq,SRP330776,Natural killer cell_1-AD.vs.control; scRNA-seq,SRP330776,Memory T cell_1-AD.vs.control; scRNA-seq,SRP330776,Naive CD8+ T cell_3-AD.vs.control; scRNA-seq,SRP330776,B cell_2-AD.vs.control; scRNA-seq,SRP330776,CD8+ T cell_3-	11
COX6B2	bulk RNA-seq,ROSMAP,AD.vs.control	1
Cox6b2	bulk RNA-seq,SRP325058,AD.vs.control	1
COX6C	bulk RNA-seq,Homo_633,AD.vs.control; bulk RNA-seq,Homo_633,AD.vs.MCI; bulk RNA-seq,Homo_633,MCI.vs.control; bulk RNA-seq,Homo_714,AD.vs.control; bulk RNA-seq,Homo_714,AD.vs.MCI; scRNA-seq,SRP215507,CD8+ T cell_7-AD.vs.control; scRNA-seq,SRP215507,CD8+ T cell_7-AD.vs.MCI; scRNA-seq,SRP330776,CD8+ T cell_1-AD.vs.control; scRNA-seq,SRP330776,γδ T cell-AD.vs.control; scRNA-seq,SRP330776,Memory T cell_2-AD.vs.control; scRNA-seq,SRP330776,CD8+ T cell_2-AD.vs.control; scRNA-seq,SRP330776,B cell_1-AD.vs.control; scRNA-seq,SRP330776,Naive CD8+ T cell_1-AD.vs.control; scRNA-seq,SRP330776,Natural killer cell_1-AD.vs.control; scRNA-seq,SRP330776,Memory T cell_1-AD.vs.control; scRNA-seq,SRP330776,B cell_2-AD.vs.control; scRNA-seq,SRP330776,CD8+ T cell_3-AD.vs.control;	18
Cox6c	bulk RNA-seq,SRP325058,AD.vs.control	1
COX7A1	bulk RNA-seq,Homo_723,AD.vs.control; bulk RNA-seq,Homo_723,AD.vs.MCI; bulk RNA-seq,Homo_723,MCI.vs.control; bulk RNA-seq,Homo_633,AD.vs.control; bulk RNA-seq,Homo_633,AD.vs.MCI; bulk RNA-seq,Homo_633,MCI.vs.control; bulk RNA-seq,ROSMAP,AD.vs.control	7
COX7A2	bulk RNA-seq,Homo_723,AD.vs.control; bulk RNA-seq,Homo_723,AD.vs.MCI; bulk RNA-seq,Homo_633,AD.vs.control; bulk RNA-seq,Homo_633,AD.vs.MCI; bulk RNA-seq,Homo_714,AD.vs.control; bulk RNA-seq,Homo_714,AD.vs.MCI; scRNA-seq,SRP215507,CD8+ T cell_7-AD.vs.MCI; scRNA-seq,SRP215507,CD8+ T cell_8-MCI.vs.control; scRNA-seq,SRP309935,Erythroid cell-AD.vs.control; scRNA-seq,SRP330776,γδ T cell-AD.vs.control; scRNA-seq,SRP330776,Natural killer cell_2-AD.vs.control	11
Cox7a2	bulk RNA-seq,SRP325058,AD.vs.control	1
COX7A2L	scRNA-seq,SRP215507,CD8+ T cell_10-AD.vs.control; scRNA-seq,SRP215507,CD8+ T cell_4-AD.vs.MCI; scRNA-seq,SRP215507,CD8+ T cell_4-MCI.vs.control; scRNA-seq,SRP215507,CD8+ T cell_8-MCI.vs.control; scRNA-seq,SRP309935,Erythroid cell-AD.vs.control; scRNA-	6
COX7B	bulk RNA-seq,Homo_723,AD.vs.control; bulk RNA-seq,Homo_723,AD.vs.MCI; bulk RNA-seq,Homo_633,AD.vs.control; bulk RNA-seq,Homo_633,AD.vs.MCI; bulk RNA-seq,Homo_714,AD.vs.control; bulk RNA-seq,Homo_714,AD.vs.MCI; bulk RNA-seq,Homo_714,MCI.vs.control; scRNA-seq,SRP215507,CD8+ T cell_8-AD.vs.control; scRNA-seq,SRP215507,CD8+ T cell_4-AD.vs.control; scRNA-seq,SRP215507,CD8+ T cell_4-MCI.vs.control; scRNA-seq,SRP309935,Erythroid cell-AD.vs.control; scRNA-seq,SRP330776,CD8+ T cell_1-AD.vs.control; scRNA-seq,SRP330776,γδ T cell-AD.vs.control; scRNA-seq,SRP330776,Memory T cell_2-AD.vs.control; scRNA-seq,SRP330776,CD8+ T cell_2-AD.vs.control; scRNA-seq,SRP330776,Naive CD8+ T cell_1-AD.vs.control; scRNA-seq,SRP330776,Natural killer cell_1-AD.vs.control; scRNA-seq,SRP330776,Memory T [illegible]	20

COX7C	scRNA-seq,SRP215507,CD8+ T cell_7-AD.vs.control; scRNA-seq,SRP215507,CD8+ T cell_7-AD.vs.MCI; scRNA-seq,SRP309935,Erythroid cell-	3
Cox8a	bulk RNA-seq,SRP325058,AD.vs.control	1
COX8A	scRNA-seq,SRP215507,CD8+ T cell_4-AD.vs.MCI; scRNA-seq,SRP309935,Erythroid cell-AD.vs.control; scRNA-seq,SRP330776,B cell_4-AD.vs.control	3
CP	bulk RNA-seq,Homo_723,AD.vs.control; bulk RNA-seq,Homo_723,AD.vs.MCI; bulk RNA-seq,Homo_633,AD.vs.control; bulk RNA-seq,Homo_633,AD.vs.MCI; bulk RNA-seq,Homo_714,AD.vs.control; bulk RNA-seq,Homo_714,AD.vs.MCI; bulk RNA-seq,ROSMAP,AD.vs.control;	8
Cp	bulk RNA-seq,SRP325058,AD.vs.control	1
CPA3	bulk RNA-seq,Homo_723,AD.vs.control; bulk RNA-seq,Homo_723,AD.vs.MCI; bulk RNA-seq,Homo_723,MCI.vs.control; bulk RNA-seq,Homo_633,AD.vs.control; bulk RNA-seq,Homo_633,AD.vs.MCI; bulk RNA-seq,Homo_633,MCI.vs.control; bulk RNA-seq,Homo_714,AD.vs.control; bulk RNA-seq,Homo_714,AD.vs.MCI; bulk RNA-seq,Homo_714,MCI.vs.control	9
CPA4	bulk RNA-seq,Homo_723,AD.vs.control	1
CPA5	bulk RNA-seq,Homo_723,AD.vs.control; bulk RNA-seq,Homo_723,AD.vs.MCI; bulk RNA-seq,Homo_633,AD.vs.control; bulk RNA-seq,Homo_633,AD.vs.MCI; bulk RNA-seq,Homo_714,AD.vs.control; bulk RNA-seq,Homo_714,AD.vs.MCI; scRNA-seq,SRP215507,CD8+ T cell_2-AD.vs.control; scRNA-seq,SRP215507,CD8+ T cell_4-AD.vs.control; scRNA-seq,SRP215507,CD8+ T cell_3-AD.vs.MCI; scRNA-seq,SRP215507,CD8+ T cell_2-MCI.vs.control; scRNA-seq,SRP309935,Naive CD8+ T cell_1-AD.vs.control; scRNA-seq,SRP330776,CD8+ T cell_1-AD.vs.control	12
CPA6	bulk RNA-seq,Homo_723,AD.vs.control; bulk RNA-seq,Homo_723,AD.vs.MCI; bulk RNA-seq,Homo_633,AD.vs.control; bulk RNA-	4
CPAMD8	scRNA-seq,SRP215507,CD8+ T cell_3-AD.vs.MCI; scRNA-seq,SRP215507,CD8+ T cell_1-MCI.vs.control; scRNA-seq,SRP215507,CD8+ T cell_3-	3
CPB1	bulk RNA-seq,Homo_723,AD.vs.control	1
CPB2	bulk RNA-seq,Homo_723,AD.vs.control; bulk RNA-seq,Homo_633,AD.vs.control	2
CPB2-AS1	scRNA-seq,SRP215507,CD8+ T cell_8-AD.vs.control; scRNA-seq,SRP215507,CD8+ T cell_1-AD.vs.MCI; scRNA-seq,SRP215507,CD8+ T cell_2-MCI.vs.control; scRNA-seq,SRP330776,B cell_1-AD.vs.control; scRNA-seq,SRP330776,T cell-AD.vs.control; scRNA-seq,SRP330776,Monocyte_2-	6
CPD	bulk RNA-seq,Homo_723,AD.vs.control; bulk RNA-seq,Homo_723,AD.vs.MCI; bulk RNA-seq,Homo_633,AD.vs.control; bulk RNA-seq,Homo_633,AD.vs.MCI; bulk RNA-seq,Homo_714,AD.vs.control; bulk RNA-seq,Homo_714,AD.vs.MCI; bulk RNA-seq,Homo_714,MCI.vs.control; bulk RNA-seq,SRP223445,AD.vs.control; scRNA-seq,SRP215507,CD8+ T cell_5-AD.vs.MCI; scRNA-seq,SRP215507,CD8+ T cell_2-MCI.vs.control; scRNA-seq,SRP309935,Natural killer cell_2-AD.vs.control; scRNA-seq,SRP309935,Natural killer cell_1-AD.vs.control; scRNA-seq,SRP309935,CD8+ T cell_1-AD.vs.control; scRNA-seq,SRP330776,CD8+ T cell_1-AD.vs.control; scRNA-seq,SRP330776,γδ T cell-AD.vs.control; scRNA-seq,SRP330776,Memory T cell_2-AD.vs.control; scRNA-seq,SRP330776,CD8+ T cell_2-AD.vs.control; scRNA-seq,SRP330776,B cell_1-AD.vs.control; scRNA-seq,SRP330776,Naive CD8+ T cell_1-AD.vs.control; scRNA-seq,SRP330776,Natural killer cell_1-AD.vs.control; scRNA-seq,SRP330776,Memory T cell_1-AD.vs.control; scRNA-seq,SRP330776,Natural killer T cell-AD.vs.control; scRNA-seq,SRP330776,Naive CD8+ T cell_3-AD.vs.control; scRNA-seq,SRP330776,B cell_2-AD.vs.control; scRNA-seq,SRP330776,T cell-AD.vs.control; scRNA-seq,SRP330776,Naive CD8+ T cell_2-AD.vs.control; [illegible]	29
CPEB1	bulk RNA-seq,Homo_723,AD.vs.control; bulk RNA-seq,Homo_723,AD.vs.MCI; bulk RNA-seq,Homo_723,MCI.vs.control; bulk RNA-seq,Homo_633,AD.vs.control; bulk RNA-seq,Homo_633,AD.vs.MCI; bulk RNA-seq,Homo_633,MCI.vs.control; bulk RNA-seq,Homo_714,AD.vs.MCI; bulk RNA-seq,Homo_714,MCI.vs.control; bulk RNA-seq,ROSMAP,AD.vs.control; bulk RNA-seq,ROSMAP,MCI.vs.control	10
CPEB2	bulk RNA-seq,Homo_723,AD.vs.control; bulk RNA-seq,Homo_723,AD.vs.MCI; bulk RNA-seq,Homo_723,MCI.vs.control; bulk RNA-seq,Homo_633,AD.vs.control; bulk RNA-seq,Homo_633,AD.vs.MCI; bulk RNA-seq,Homo_633,MCI.vs.control; bulk RNA-seq,Homo_714,MCI.vs.control; scRNA-seq,SRP215507,CD8+ T cell_2-AD.vs.MCI; scRNA-seq,SRP215507,CD8+ T cell_5-AD.vs.MCI; scRNA-seq,SRP215507,CD8+ T cell_5-MCI.vs.control; scRNA-seq,SRP215507,CD8+ T cell_6-MCI.vs.control; scRNA-seq,SRP309935,Natural killer cell_1-AD.vs.control; scRNA-seq,SRP330776,CD8+ T cell_1-AD.vs.control; scRNA-seq,SRP330776,B cell_1-AD.vs.control; scRNA-seq,SRP330776,Memory T	17
CPEB3	bulk RNA-seq,Homo_723,AD.vs.control; bulk RNA-seq,Homo_723,AD.vs.MCI; bulk RNA-seq,Homo_633,AD.vs.control; bulk RNA-seq,Homo_633,AD.vs.MCI; bulk RNA-seq,Homo_714,AD.vs.control; bulk RNA-seq,Homo_714,AD.vs.MCI; scRNA-seq,SRP215507,CD8+ T cell_1-AD.vs.control; scRNA-seq,SRP215507,CD8+ T cell_1-AD.vs.MCI; scRNA-seq,SRP330776,CD8+ T cell_1-AD.vs.control; scRNA-seq,SRP330776,γδ T cell-AD.vs.control; scRNA-seq,SRP330776,Memory T cell_2-AD.vs.control; scRNA-seq,SRP330776,CD8+ T cell_2-AD.vs.control; scRNA-seq,SRP330776,B cell_1-AD.vs.control; scRNA-seq,SRP330776,Naive CD8+ T cell_1-AD.vs.control; scRNA-seq,SRP330776,Natural killer cell_1-	17

Cpeb3	bulk RNA-seq,SRP325058,AD.vs.control	1
CPEB4	scRNA-seq,SRP215507,CD8+ T cell_3-AD.vs.MCI; scRNA-seq,SRP215507,CD8+ T cell_3-MCI.vs.control; scRNA-seq,SRP215507,CD8+ T cell_8-MCI.vs.control; scRNA-seq,SRP309935,Monocyte_2-AD.vs.control; scRNA-seq,SRP330776,CD8+ T cell_1-AD.vs.control; scRNA-seq,SRP330776,Memory T cell_2-AD.vs.control; scRNA-seq,SRP330776,CD8+ T cell_2-AD.vs.control; scRNA-seq,SRP330776,Memory T cell_1-AD.vs.control; scRNA-seq,SRP330776,Natural killer T cell-AD.vs.control; scRNA-seq,SRP330776,Naive CD8+ T cell_3-AD.vs.control; scRNA-seq,SRP330776,T cell-AD.vs.control; scRNA-seq,SRP330776,CD8+ T cell_3-AD.vs.control; scRNA-seq,SRP330776,B cell_4-AD.vs.control	13
CPHXL	bulk RNA-seq,Homo_723,MCI.vs.control; bulk RNA-seq,Homo_633,MCI.vs.control; bulk RNA-seq,Homo_714,MCI.vs.control	3
CPLANE1	bulk RNA-seq,Homo_723,AD.vs.control; bulk RNA-seq,Homo_723,AD.vs.MCI; bulk RNA-seq,Homo_723,MCI.vs.control; bulk RNA-seq,Homo_633,AD.vs.control; bulk RNA-seq,Homo_633,AD.vs.MCI; bulk RNA-seq,Homo_633,MCI.vs.control; bulk RNA-seq,Homo_714,AD.vs.control; bulk RNA-seq,Homo_714,AD.vs.MCI; bulk RNA-seq,ROSMAP,AD.vs.control; bulk RNA-seq,ROSMAP,MCI.vs.control; scRNA-seq,SRP215507,CD8+ T cell_4-AD.vs.control; scRNA-seq,SRP215507,CD8+ T cell_2-AD.vs.MCI; scRNA-seq,SRP215507,CD8+ T cell_1-AD.vs.MCI; scRNA-seq,SRP215507,CD8+ T cell_4-AD.vs.MCI; scRNA-seq,SRP215507,CD8+ T cell_2-MCI.vs.control; scRNA-seq,SRP215507,CD8+ T cell_6-MCI.vs.control; scRNA-seq,SRP330776,Natural killer cell_1-AD.vs.control; scRNA-seq,SRP330776,B cell_2-AD.vs.control; scRNA-seq,SRP330776,CD8+ T cell_3-AD.vs.control; scRNA-seq,SRP330776,Monocyte_1-AD.vs.control; scRNA-seq,SRP330776,Natural killer cell_2- [illegible]	21
CPLANE2	bulk RNA-seq,Homo_723,AD.vs.control; bulk RNA-seq,Homo_723,AD.vs.MCI; bulk RNA-seq,Homo_633,AD.vs.control; bulk RNA-seq,Homo_633,AD.vs.MCI; bulk RNA-seq,Homo_714,AD.vs.control; bulk RNA-seq,Homo_714,AD.vs.MCI; scRNA-seq,SRP215507,CD8+ T cell_2-AD.vs.control; scRNA-seq,SRP215507,CD8+ T cell_3-AD.vs.control; scRNA-seq,SRP215507,CD8+ T cell_2-MCI.vs.control; scRNA-seq,SRP330776,CD8+ T cell_1-AD.vs.control; scRNA-seq,SRP330776,Memory T cell_2-AD.vs.control; scRNA-seq,SRP330776,CD8+ T cell_2-AD.vs.control; scRNA-seq,SRP330776,B cell_1-AD.vs.control; scRNA-seq,SRP330776,Naive CD8+ T cell_1-AD.vs.control; scRNA-seq,SRP330776,Natural killer cell_1-AD.vs.control; scRNA-seq,SRP330776,Natural killer T cell-AD.vs.control; scRNA-seq,SRP330776,B cell_2- [illegible]	18
CPLX1	bulk RNA-seq,Homo_723,AD.vs.control; bulk RNA-seq,Homo_723,AD.vs.MCI; bulk RNA-seq,Homo_633,AD.vs.control; bulk RNA-seq,Homo_633,AD.vs.MCI; bulk RNA-seq,Homo_714,MCI.vs.control; scRNA-seq,SRP215507,CD8+ T cell_5-AD.vs.MCI; scRNA-seq,SRP215507,CD8+ T cell_5-MCI.vs.control; scRNA-seq,SRP330776,Memory T cell_2-AD.vs.control	8
CPLX2	bulk RNA-seq,Homo_723,AD.vs.control; bulk RNA-seq,Homo_723,AD.vs.MCI; bulk RNA-seq,Homo_633,MCI.vs.control; bulk RNA-seq,Homo_714,AD.vs.MCI; bulk RNA-seq,Homo_714,MCI.vs.control; bulk RNA-seq,ROSMAP,AD.vs.control; bulk RNA-seq,ROSMAP,MCI.vs.control	7
CPLX3	bulk RNA-seq,Homo_723,AD.vs.control; bulk RNA-seq,Homo_723,AD.vs.MCI; bulk RNA-seq,Homo_723,MCI.vs.control; bulk RNA-seq,Homo_633,AD.vs.MCI; bulk RNA-seq,Homo_633,MCI.vs.control; bulk RNA-seq,Homo_714,MCI.vs.control	6
CPLX4	bulk RNA-seq,Homo_723,AD.vs.control; bulk RNA-seq,Homo_723,AD.vs.MCI; bulk RNA-seq,Homo_633,AD.vs.control	3
CPM	bulk RNA-seq,Homo_723,AD.vs.control; bulk RNA-seq,Homo_723,AD.vs.MCI; bulk RNA-seq,Homo_633,AD.vs.control; bulk RNA-seq,Homo_633,AD.vs.MCI; bulk RNA-seq,Homo_714,AD.vs.control; bulk RNA-seq,Homo_714,AD.vs.MCI; scRNA-seq,SRP330776,Monocyte_1-	7
Cpm	bulk RNA-seq,SRP325058,AD.vs.control	1
CPNE1	scRNA-seq,SRP215507,CD8+ T cell_6-AD.vs.MCI; scRNA-seq,SRP330776,Monocyte_2-AD.vs.control	2
CPNE2	bulk RNA-seq,Homo_723,AD.vs.control; bulk RNA-seq,Homo_723,AD.vs.MCI; bulk RNA-seq,Homo_633,AD.vs.control; bulk RNA-seq,Homo_633,AD.vs.MCI; bulk RNA-seq,Homo_714,AD.vs.control; bulk RNA-seq,Homo_714,AD.vs.MCI; scRNA-seq,SRP215507,CD8+ T cell_1-AD.vs.control; scRNA-seq,SRP215507,CD8+ T cell_5-AD.vs.MCI; scRNA-seq,SRP215507,CD8+ T cell_1-MCI.vs.control; scRNA-seq,SRP215507,CD8+ T cell_5-MCI.vs.control; scRNA-seq,SRP309935,Monocyte_2-AD.vs.control; scRNA-seq,SRP309935,Naive CD8+ T cell_2-AD.vs.control; scRNA-seq,SRP309935,CD8+ T cell_2-AD.vs.control; scRNA-seq,SRP330776,T cell-AD.vs.control; scRNA-seq,SRP330776,CD8+ T cell_3-AD.vs.control	15
CPNE3	bulk RNA-seq,Homo_714,AD.vs.control; bulk RNA-seq,Homo_714,AD.vs.MCI; scRNA-seq,SRP309935,B cell_1-AD.vs.control; scRNA-seq,SRP330776,CD8+ T cell_1-AD.vs.control; scRNA-seq,SRP330776,CD8+ T cell_3-AD.vs.control; scRNA-seq,SRP330776,Natural killer cell_2-	6
CPNE4	bulk RNA-seq,Homo_723,AD.vs.control; bulk RNA-seq,Homo_723,AD.vs.MCI; bulk RNA-seq,Homo_723,MCI.vs.control; bulk RNA-seq,Homo_633,AD.vs.control; bulk RNA-seq,Homo_633,AD.vs.MCI; bulk RNA-seq,Homo_714,AD.vs.MCI; bulk RNA-seq,Homo_714,MCI.vs.control	7

CPNE5	bulk RNA-seq,Homo_723,AD.vs.control; bulk RNA-seq,Homo_723,AD.vs.MCI; bulk RNA-seq,Homo_633,AD.vs.control; bulk RNA-seq,Homo_633,AD.vs.MCI; bulk RNA-seq,Homo_714,AD.vs.control; bulk RNA-seq,Homo_714,AD.vs.MCI; bulk RNA-seq,Homo_714,MCI.vs.control; scRNA-seq,SRP215507,CD8+ T cell_7-AD.vs.control; scRNA-seq,SRP215507,CD8+ T cell_2-AD.vs.MCI; scRNA-seq,SRP215507,CD8+ T cell_2-MCI.vs.control; scRNA-seq,SRP215507,CD8+ T cell_7-MCI.vs.control; scRNA-seq,SRP330776,Memory T cell_2-AD.vs.control; scRNA-	14
CPNE6	bulk RNA-seq,Homo_723,AD.vs.control; bulk RNA-seq,Homo_633,AD.vs.control; bulk RNA-seq,Homo_714,MCI.vs.control	3
CPNE7	bulk RNA-seq,Homo_633,AD.vs.control; bulk RNA-seq,Homo_633,AD.vs.MCI; bulk RNA-seq,Homo_714,AD.vs.control; bulk RNA-seq,Homo_714,AD.vs.MCI; scRNA-seq,SRP215507,CD8+ T cell_5-AD.vs.MCI; scRNA-seq,SRP215507,CD8+ T cell_5-MCI.vs.control	6
Cpne8	bulk RNA-seq,SRP325058,AD.vs.control	1
CPNE8	scRNA-seq,SRP309935,Natural killer cell_1-AD.vs.control; scRNA-seq,SRP330776,B cell_1-AD.vs.control; scRNA-seq,SRP330776,B cell_2-	3
CPNE9	bulk RNA-seq,Homo_723,AD.vs.control; bulk RNA-seq,Homo_723,AD.vs.MCI; bulk RNA-seq,Homo_633,AD.vs.control; bulk RNA-	4
CPO	bulk RNA-seq,Homo_633,AD.vs.control; bulk RNA-seq,Homo_633,AD.vs.MCI; bulk RNA-seq,Homo_714,AD.vs.control; bulk RNA-	4
CPOX	scRNA-seq,SRP215507,CD8+ T cell_3-AD.vs.MCI; scRNA-seq,SRP215507,CD8+ T cell_4-MCI.vs.control; scRNA-seq,SRP215507,CD8+ T cell_3-MCI.vs.control; scRNA-seq,SRP309935,Natural killer cell_2-AD.vs.control; scRNA-seq,SRP309935,Naive CD8+ T cell_1-AD.vs.control; scRNA-seq,SRP330776,CD8+ T cell_1-AD.vs.control; scRNA-seq,SRP330776,γδ T cell-AD.vs.control; scRNA-seq,SRP330776,Memory T cell_2-AD.vs.control; scRNA-seq,SRP330776,CD8+ T cell_2-AD.vs.control; scRNA-seq,SRP330776,Naive CD8+ T cell_1-AD.vs.control; scRNA-seq,SRP330776,Natural killer cell_1-AD.vs.control; scRNA-seq,SRP330776,Memory T cell_1-AD.vs.control; scRNA-seq,SRP330776,Natural killer T cell-AD.vs.control; scRNA-	14
CPPED1	bulk RNA-seq,Homo_633,MCI.vs.control; bulk RNA-seq,Homo_714,MCI.vs.control; scRNA-seq,SRP215507,CD8+ T cell_3-AD.vs.control; scRNA-seq,SRP215507,CD8+ T cell_6-AD.vs.MCI; scRNA-seq,SRP215507,CD8+ T cell_6-MCI.vs.control; scRNA-seq,SRP330776,CD8+ T cell_2-AD.vs.control; scRNA-seq,SRP330776,Naive CD8+ T cell_1-AD.vs.control; scRNA-seq,SRP330776,Memory T cell_1-AD.vs.control; scRNA-seq,SRP330776,Natural killer T cell-AD.vs.control; scRNA-seq,SRP330776,T cell-AD.vs.control	10
CPQ	bulk RNA-seq,Homo_723,AD.vs.control; bulk RNA-seq,Homo_723,AD.vs.MCI; bulk RNA-seq,Homo_633,AD.vs.control; bulk RNA-seq,Homo_633,AD.vs.MCI; bulk RNA-seq,Homo_714,AD.vs.control; bulk RNA-seq,Homo_714,AD.vs.MCI; bulk RNA-seq,SRP223445,AD.vs.control; scRNA-seq,SRP215507,CD8+ T cell_7-AD.vs.control; scRNA-seq,SRP215507,CD8+ T cell_5-MCI.vs.control; scRNA-seq,SRP330776,CD8+ T cell_2-AD.vs.control; scRNA-seq,SRP330776,B cell_1-AD.vs.control; scRNA-seq,SRP330776,Natural killer cell_1-AD.vs.control; scRNA-seq,SRP330776,Memory T cell_1-AD.vs.control; scRNA-seq,SRP330776,Natural killer T cell-AD.vs.control; scRNA-seq,SRP330776,T cell-AD.vs.control; scRNA-seq,SRP330776,Naive CD8+ T cell_2-AD.vs.control; scRNA-seq,SRP330776,B cell_3-AD.vs.control; scRNA-seq,SRP330776,Natural killer [illegible]	18
Cpq	bulk RNA-seq,SRP325058,AD.vs.control	1
CPS1	bulk RNA-seq,Homo_723,AD.vs.control; bulk RNA-seq,Homo_723,AD.vs.MCI; bulk RNA-seq,Homo_723,MCI.vs.control; bulk RNA-seq,Homo_633,AD.vs.control; bulk RNA-seq,Homo_633,AD.vs.MCI; bulk RNA-seq,Homo_714,AD.vs.control; bulk RNA-seq,Homo_714,AD.vs.MCI; bulk RNA-seq,ROSMAP,AD.vs.control; bulk RNA-seq,ROSMAP,MCI.vs.control	9
CPSF1	scRNA-seq,SRP215507,CD8+ T cell_5-AD.vs.MCI; scRNA-seq,SRP215507,CD8+ T cell_5-MCI.vs.control; scRNA-seq,SRP309935,Naive CD8+ T cell_2-AD.vs.control; scRNA-seq,SRP309935,CD8+ T cell_1-AD.vs.control; scRNA-seq,SRP330776,CD8+ T cell_1-AD.vs.control; scRNA-seq,SRP330776,γδ T cell-AD.vs.control; scRNA-seq,SRP330776,Naive CD8+ T cell_1-AD.vs.control; scRNA-seq,SRP330776,Naive CD8+ T cell_3-AD.vs.control; scRNA-seq,SRP330776,T cell-AD.vs.control; scRNA-seq,SRP330776,B cell_3-AD.vs.control	10
CPSF2	bulk RNA-seq,Homo_723,AD.vs.control; bulk RNA-seq,Homo_723,AD.vs.MCI; bulk RNA-seq,Homo_723,MCI.vs.control; bulk RNA-seq,Homo_633,AD.vs.control; bulk RNA-seq,Homo_633,AD.vs.MCI; bulk RNA-seq,Homo_633,MCI.vs.control; bulk RNA-seq,Homo_714,AD.vs.control; bulk RNA-seq,Homo_714,AD.vs.MCI; bulk RNA-seq,Homo_714,MCI.vs.control; scRNA-seq,SRP215507,CD8+ T cell_6-AD.vs.control; scRNA-seq,SRP215507,CD8+ T cell_4-AD.vs.MCI; scRNA-seq,SRP309935,CD8+ T cell_1-AD.vs.control; scRNA-seq,SRP309935,B cell_3-AD.vs.control; scRNA-seq,SRP330776,Memory T cell_2-AD.vs.control; scRNA-seq,SRP330776,Natural killer cell_1-AD.vs.control; scRNA-seq,SRP330776,Memory T cell_1-AD.vs.control; scRNA-seq,SRP330776,Natural killer T cell-AD.vs.control; scRNA-seq,SRP330776,Naive CD8+ T cell_3-AD.vs.control; scRNA-seq,SRP330776,Monocyte_1-AD.vs.control; scRNA-seq,SRP330776,B cell_3-AD.vs.control; scRNA-seq,SRP330776,Natural killer cell_2-AD.vs.control	21

CPSF3	bulk RNA-seq,Homo_723,AD.vs.control; bulk RNA-seq,Homo_633,AD.vs.control; bulk RNA-seq,Homo_714,AD.vs.control; bulk RNA-seq,Homo_714,AD.vs.MCI; scRNA-seq,SRP309935,Natural killer cell_2-AD.vs.control; scRNA-seq,SRP309935,CD8+ T cell_1-AD.vs.control; scRNA-seq,SRP330776,γδ T cell-AD.vs.control; scRNA-seq,SRP330776,Regulatory T cell-AD.vs.control; scRNA-seq,SRP330776,Megakaryocyte_1-	9
CPSF4	bulk RNA-seq,Homo_723,AD.vs.control; bulk RNA-seq,Homo_723,AD.vs.MCI; bulk RNA-seq,Homo_633,AD.vs.control; bulk RNA-seq,Homo_633,AD.vs.MCI; bulk RNA-seq,Homo_714,AD.vs.control; bulk RNA-seq,Homo_714,MCI.vs.control; scRNA-seq,SRP215507,CD8+ T cell_6-AD.vs.control; scRNA-seq,SRP215507,CD8+ T cell_4-MCI.vs.control; scRNA-seq,SRP330776,CD8+ T cell_1-AD.vs.control; scRNA-seq,SRP330776,γδ T cell-AD.vs.control; scRNA-seq,SRP330776,Memory T cell_2-AD.vs.control; scRNA-seq,SRP330776,CD8+ T cell_2-AD.vs.control; scRNA-seq,SRP330776,B cell_1-AD.vs.control; scRNA-seq,SRP330776,Naive CD8+ T cell_1-AD.vs.control; scRNA-seq,SRP330776,Natural killer cell_1-AD.vs.control; scRNA-seq,SRP330776,Memory T cell_1-AD.vs.control; scRNA-seq,SRP330776,Natural killer T cell-AD.vs.control; scRNA-seq,SRP330776,Naive CD8+ T cell_3-AD.vs.control; scRNA-seq,SRP330776,B cell_2-AD.vs.control; scRNA-seq,SRP330776,T cell-AD.vs.control; scRNA-seq,SRP330776,CD8+ T cell_3-AD.vs.control; scRNA-seq,SRP330776,Monocyte_1-AD.vs.control; scRNA-seq,SRP330776,B cell_3- [illegible]	24
CPSF4L	bulk RNA-seq,Homo_723,AD.vs.control	1
CPSF6	scRNA-seq,SRP215507,CD8+ T cell_7-AD.vs.MCI; scRNA-seq,SRP330776,Natural killer cell_1-AD.vs.control; scRNA-seq,SRP330776,Naive CD8+ T cell_3-AD.vs.control; scRNA-seq,SRP330776,Megakaryocyte_1-AD.vs.control	4
CPSF7	bulk RNA-seq,Homo_723,AD.vs.control; bulk RNA-seq,Homo_723,MCI.vs.control; bulk RNA-seq,Homo_633,AD.vs.control; bulk RNA-seq,Homo_633,MCI.vs.control; bulk RNA-seq,Homo_714,AD.vs.control; bulk RNA-seq,Homo_714,AD.vs.MCI; bulk RNA-seq,Homo_714,MCI.vs.control; scRNA-seq,SRP330776,Naive CD8+ T cell_1-AD.vs.control; scRNA-seq,SRP330776,Natural killer cell_2-AD.vs.control	9
CPT1A	scRNA-seq,SRP215507,CD8+ T cell_4-AD.vs.MCI; scRNA-seq,SRP215507,CD8+ T cell_4-MCI.vs.control; scRNA-seq,SRP309935,Naive CD8+ T cell_3-AD.vs.control; scRNA-seq,SRP330776,CD8+ T cell_1-AD.vs.control; scRNA-seq,SRP330776,Memory T cell_2-AD.vs.control; scRNA-seq,SRP330776,B cell_1-AD.vs.control; scRNA-seq,SRP330776,Naive CD8+ T cell_1-AD.vs.control; scRNA-seq,SRP330776,Natural killer cell_1-AD.vs.control; scRNA-seq,SRP330776,Naive CD8+ T cell_3-AD.vs.control; scRNA-seq,SRP330776,B cell_2-AD.vs.control; scRNA-seq,SRP330776,T cell-AD.vs.control; scRNA-seq,SRP330776,CD8+ T cell_3-AD.vs.control; scRNA-seq,SRP330776,Monocyte_2-AD.vs.control	13
CPT1B	bulk RNA-seq,Homo_723,AD.vs.control; bulk RNA-seq,Homo_723,AD.vs.MCI; bulk RNA-seq,Homo_723,MCI.vs.control; bulk RNA-seq,Homo_633,AD.vs.control; bulk RNA-seq,Homo_633,AD.vs.MCI; bulk RNA-seq,Homo_633,MCI.vs.control; bulk RNA-seq,Homo_714,AD.vs.control; bulk RNA-seq,Homo_714,AD.vs.MCI; bulk RNA-seq,Homo_714,MCI.vs.control; scRNA-seq,SRP215507,CD8+ T cell_5-AD.vs.control; scRNA-seq,SRP215507,CD8+ T cell_3-AD.vs.MCI; scRNA-seq,SRP215507,CD8+ T cell_3-MCI.vs.control; scRNA-seq,SRP330776,Memory T cell_1-	13
CPT1C	bulk RNA-seq,Homo_723,AD.vs.control; bulk RNA-seq,Homo_723,AD.vs.MCI; bulk RNA-seq,Homo_723,MCI.vs.control; scRNA-seq,SRP215507,CD8+ T cell_1-AD.vs.control	4
CPT2	scRNA-seq,SRP215507,CD8+ T cell_3-AD.vs.MCI; scRNA-seq,SRP215507,CD8+ T cell_1-AD.vs.MCI; scRNA-seq,SRP215507,CD8+ T cell_1-MCI.vs.control; scRNA-seq,SRP215507,CD8+ T cell_3-MCI.vs.control; scRNA-seq,SRP330776,CD8+ T cell_1-AD.vs.control; scRNA-seq,SRP330776,γδ T cell-AD.vs.control; scRNA-seq,SRP330776,CD8+ T cell_2-AD.vs.control; scRNA-seq,SRP330776,B cell_1-AD.vs.control; scRNA-seq,SRP330776,Naive CD8+ T cell_1-AD.vs.control; scRNA-seq,SRP330776,Memory T cell_1-AD.vs.control; scRNA-seq,SRP330776,Natural killer T cell-AD.vs.control; scRNA-seq,SRP330776,Naive CD8+ T cell_3-AD.vs.control; scRNA-seq,SRP330776,B cell_2-AD.vs.control; scRNA-	15
CPTP	scRNA-seq,SRP330776,CD8+ T cell_1-AD.vs.control; scRNA-seq,SRP330776,γδ T cell-AD.vs.control; scRNA-seq,SRP330776,Memory T cell_2-AD.vs.control; scRNA-seq,SRP330776,CD8+ T cell_2-AD.vs.control; scRNA-seq,SRP330776,B cell_1-AD.vs.control; scRNA-seq,SRP330776,Naive CD8+ T cell_1-AD.vs.control; scRNA-seq,SRP330776,Natural killer cell_1-AD.vs.control; scRNA-seq,SRP330776,Memory T cell_1-AD.vs.control; scRNA-seq,SRP330776,Natural killer T cell-AD.vs.control; scRNA-seq,SRP330776,Naive CD8+ T cell_3-AD.vs.control; scRNA-seq,SRP330776,B cell_2-AD.vs.control; scRNA-seq,SRP330776,T cell-AD.vs.control; scRNA-seq,SRP330776,CD8+ T cell_3-AD.vs.control; scRNA-seq,SRP330776,Monocyte_1-AD.vs.control; scRNA-seq,SRP330776,B cell_3-AD.vs.control; scRNA-seq,SRP330776,Natural killer cell_2-AD.vs.control	16
CPVL	bulk RNA-seq,Homo_723,AD.vs.control; bulk RNA-seq,Homo_723,AD.vs.MCI; bulk RNA-seq,Homo_723,MCI.vs.control; bulk RNA-seq,Homo_633,AD.vs.control; bulk RNA-seq,Homo_633,AD.vs.MCI; bulk RNA-seq,Homo_633,MCI.vs.control	6
CPXM1	bulk RNA-seq,Homo_723,AD.vs.control; bulk RNA-seq,Homo_723,AD.vs.MCI; bulk RNA-seq,Homo_723,MCI.vs.control; bulk RNA-seq,Homo_633,AD.vs.control; bulk RNA-seq,Homo_714,MCI.vs.control	5

CPXM2	bulk RNA-seq,Homo_723,AD.vs.control; bulk RNA-seq,Homo_723,AD.vs.MCI; bulk RNA-seq,Homo_723,MCI.vs.control; bulk RNA-seq,Homo_633,AD.vs.control; bulk RNA-seq,Homo_633,AD.vs.MCI; bulk RNA-seq,Homo_633,MCI.vs.control; bulk RNA-	8
CR1	bulk RNA-seq,SRP223445,AD.vs.control; scRNA-seq,SRP215507,CD8+ T cell_4-AD.vs.control; scRNA-seq,SRP215507,CD8+ T cell_4-AD.vs.MCI; scRNA-seq,SRP215507,CD8+ T cell_2-MCI.vs.control; scRNA-seq,SRP309935,B cell_1-AD.vs.control; scRNA-seq,SRP330776,Memory T cell_2-AD.vs.control; scRNA-seq,SRP330776,Monocyte_2-AD.vs.control	7
CR1L	bulk RNA-seq,Homo_723,AD.vs.control; bulk RNA-seq,Homo_723,AD.vs.MCI; bulk RNA-seq,Homo_633,AD.vs.control; bulk RNA-seq,Homo_633,AD.vs.MCI; bulk RNA-seq,Homo_714,AD.vs.control; bulk RNA-seq,Homo_714,AD.vs.MCI	6
CR2	bulk RNA-seq,Homo_723,AD.vs.control; bulk RNA-seq,Homo_723,MCI.vs.control; bulk RNA-seq,Homo_633,AD.vs.control; bulk RNA-seq,Homo_633,MCI.vs.control; bulk RNA-seq,Homo_714,AD.vs.control; bulk RNA-seq,Homo_714,AD.vs.MCI; bulk RNA-seq,Homo_714,MCI.vs.control; scRNA-seq,SRP309935,B cell_2-AD.vs.control; scRNA-seq,SRP330776,Naive CD8+ T cell_3-AD.vs.control	9
CR381653.1	scRNA-seq,SRP215507,CD8+ T cell_1-AD.vs.MCI; scRNA-seq,SRP330776,γδ T cell-AD.vs.control; scRNA-seq,SRP330776,CD8+ T cell_2-AD.vs.control; scRNA-seq,SRP330776,Natural killer cell_1-AD.vs.control; scRNA-seq,SRP330776,Memory T cell_1-AD.vs.control; scRNA-seq,SRP330776,Natural killer T cell-AD.vs.control; scRNA-seq,SRP330776,CD8+ T cell_3-AD.vs.control	7
CRABP1	bulk RNA-seq,Homo_723,AD.vs.MCI; bulk RNA-seq,Homo_723,MCI.vs.control	2
CRABP2	bulk RNA-seq,Homo_723,AD.vs.control; bulk RNA-seq,Homo_723,AD.vs.MCI; bulk RNA-seq,Homo_723,MCI.vs.control; bulk RNA-seq,Homo_633,AD.vs.control; bulk RNA-seq,Homo_633,AD.vs.MCI; scRNA-seq,SRP215507,CD8+ T cell_1-MCI.vs.control	6
CRACDL	bulk RNA-seq,Homo_723,AD.vs.MCI; bulk RNA-seq,Homo_714,AD.vs.control; bulk RNA-seq,Homo_714,AD.vs.MCI	3
CRACR2A	bulk RNA-seq,Homo_723,AD.vs.control; bulk RNA-seq,Homo_723,AD.vs.MCI; bulk RNA-seq,Homo_723,MCI.vs.control; bulk RNA-seq,Homo_633,AD.vs.control; bulk RNA-seq,Homo_633,AD.vs.MCI; bulk RNA-seq,Homo_633,MCI.vs.control; bulk RNA-seq,Homo_714,AD.vs.control; bulk RNA-seq,Homo_714,AD.vs.MCI; bulk RNA-seq,Homo_714,MCI.vs.control; scRNA-seq,SRP215507,CD8+ T cell_6-AD.vs.MCI; scRNA-seq,SRP330776,γδ T cell-AD.vs.control; scRNA-seq,SRP330776,Monocyte_1-AD.vs.control	12
CRACR2B	scRNA-seq,SRP215507,CD8+ T cell_2-AD.vs.MCI; scRNA-seq,SRP215507,CD8+ T cell_1-AD.vs.MCI; scRNA-seq,SRP215507,CD8+ T cell_1-MCI.vs.control; scRNA-seq,SRP309935,Monocyte_1-AD.vs.control; scRNA-seq,SRP330776,Natural killer cell_2-AD.vs.control	5
CRADD	bulk RNA-seq,Homo_633,AD.vs.control; bulk RNA-seq,Homo_633,AD.vs.MCI; scRNA-seq,SRP215507,CD8+ T cell_2-AD.vs.MCI; scRNA-seq,SRP330776,CD8+ T cell_1-AD.vs.control; scRNA-seq,SRP330776,Memory T cell_2-AD.vs.control; scRNA-seq,SRP330776,B cell_1-AD.vs.control; scRNA-seq,SRP330776,Naive CD8+ T cell_1-AD.vs.control; scRNA-seq,SRP330776,Natural killer cell_1-AD.vs.control; scRNA-seq,SRP330776,Natural killer T cell-AD.vs.control; scRNA-seq,SRP330776,B cell_2-AD.vs.control; scRNA-seq,SRP330776,CD8+ T cell_3-AD.vs.control; scRNA-	12
CRAMP1	bulk RNA-seq,Homo_723,AD.vs.control; bulk RNA-seq,Homo_723,AD.vs.MCI; bulk RNA-seq,Homo_633,AD.vs.control; bulk RNA-seq,Homo_633,AD.vs.MCI; bulk RNA-seq,Homo_714,AD.vs.control; bulk RNA-seq,Homo_714,AD.vs.MCI; scRNA-seq,SRP215507,CD8+ T cell_3-AD.vs.MCI; scRNA-seq,SRP309935,Natural killer cell_2-AD.vs.control; scRNA-seq,SRP309935,CD8+ T cell_1-AD.vs.control; scRNA-seq,SRP330776,CD8+ T cell_1-AD.vs.control; scRNA-seq,SRP330776,γδ T cell-AD.vs.control; scRNA-seq,SRP330776,CD8+ T cell_2-AD.vs.control; scRNA-seq,SRP330776,B cell_1-AD.vs.control; scRNA-seq,SRP330776,Naive CD8+ T cell_1-AD.vs.control; scRNA-seq,SRP330776,Natural killer	17
CRAT	bulk RNA-seq,Homo_723,AD.vs.control; bulk RNA-seq,Homo_723,AD.vs.MCI; bulk RNA-seq,Homo_633,AD.vs.control; bulk RNA-seq,Homo_633,AD.vs.MCI; scRNA-seq,SRP215507,CD8+ T cell_4-AD.vs.control; scRNA-seq,SRP215507,CD8+ T cell_5-AD.vs.MCI; scRNA-seq,SRP215507,CD8+ T cell_5-MCI.vs.control; scRNA-seq,SRP330776,γδ T cell-AD.vs.control; scRNA-seq,SRP330776,Memory T cell_2-AD.vs.control; scRNA-seq,SRP330776,CD8+ T cell_2-AD.vs.control; scRNA-seq,SRP330776,Natural killer T cell-AD.vs.control; scRNA-seq,SRP330776,Naive CD8+ T	12
Crat	bulk RNA-seq,SRP325058,AD.vs.control	1
CRB2	bulk RNA-seq,Homo_723,AD.vs.control; bulk RNA-seq,Homo_723,AD.vs.MCI; bulk RNA-seq,Homo_723,MCI.vs.control; bulk RNA-seq,Homo_633,AD.vs.control; bulk RNA-seq,Homo_633,MCI.vs.control; bulk RNA-seq,Homo_714,AD.vs.control; bulk RNA-seq,Homo_714,AD.vs.MCI;	8
CRB3	bulk RNA-seq,Homo_723,AD.vs.control; bulk RNA-seq,Homo_723,AD.vs.MCI; bulk RNA-seq,Homo_633,AD.vs.control; bulk RNA-seq,Homo_633,AD.vs.MCI; bulk RNA-seq,Homo_714,AD.vs.control; bulk RNA-seq,Homo_714,AD.vs.MCI; scRNA-seq,SRP215507,CD8+ T cell_2-AD.vs.control; scRNA-seq,SRP330776,Memory T cell_2-AD.vs.control; scRNA-seq,SRP330776,Naive CD8+ T cell_1-AD.vs.control	9

CRBN	bulk RNA-seq,Homo_633,AD.vs.control; scRNA-seq,SRP309935,Megakaryocyte_2-AD.vs.control; scRNA-seq,SRP330776,Monocyte_1-AD.vs.control; scRNA-seq,SRP330776,Megakaryocyte_1-AD.vs.control	4
CRCP	scRNA-seq,SRP215507,CD8+ T cell_4-AD.vs.control; scRNA-seq,SRP215507,CD8+ T cell_1-AD.vs.MCI; scRNA-seq,SRP215507,CD8+ T cell_4-MCI.vs.control; scRNA-seq,SRP215507,CD8+ T cell_6-MCI.vs.control; scRNA-seq,SRP330776,Megakaryocyte_1-AD.vs.control	5
CREB1	scRNA-seq,SRP215507,CD8+ T cell_10-AD.vs.control; scRNA-seq,SRP215507,CD8+ T cell_5-MCI.vs.control; scRNA-seq,SRP330776,B cell_1-AD.vs.control; scRNA-seq,SRP330776,Naive CD8+ T cell_3-AD.vs.control; scRNA-seq,SRP330776,T cell-AD.vs.control	5
CREB3	scRNA-seq,SRP215507,CD8+ T cell_2-MCI.vs.control; scRNA-seq,SRP215507,CD8+ T cell_6-MCI.vs.control; scRNA-seq,SRP330776,Memory T cell_2-AD.vs.control; scRNA-seq,SRP330776,Natural killer cell_1-AD.vs.control; scRNA-seq,SRP330776,B cell_2-AD.vs.control; scRNA-seq,SRP330776,Naive CD8+ T cell_2-AD.vs.control; scRNA-seq,SRP330776,B cell_3-AD.vs.control; scRNA-seq,SRP330776,Natural killer cell_2-AD.vs.control	8
CREB3L1	bulk RNA-seq,ACOM,AD.vs.control; bulk RNA-seq,Homo_723,AD.vs.control; bulk RNA-seq,Homo_723,AD.vs.MCI; bulk RNA-seq,Homo_633,AD.vs.control; bulk RNA-seq,Homo_633,AD.vs.MCI; bulk RNA-seq,Homo_714,MCI.vs.control; bulk RNA-seq,ROSMAP,AD.vs.MCI;	8
CREB3L2	bulk RNA-seq,Homo_714,AD.vs.control; bulk RNA-seq,Homo_714,AD.vs.MCI; scRNA-seq,SRP215507,CD8+ T cell_4-AD.vs.control; scRNA-seq,SRP215507,CD8+ T cell_2-AD.vs.MCI; scRNA-seq,SRP215507,CD8+ T cell_3-MCI.vs.control; scRNA-seq,SRP330776,CD8+ T cell_1-AD.vs.control	6
CREB3L3	bulk RNA-seq,ACOM,AD.vs.control; bulk RNA-seq,Homo_723,AD.vs.control; bulk RNA-seq,Homo_633,AD.vs.control; bulk RNA-seq,Homo_633,MCI.vs.control; bulk RNA-seq,Homo_714,MCI.vs.control; scRNA-seq,SRP309935,Natural killer cell_1-AD.vs.control; scRNA-	7
CREB3L4	scRNA-seq,SRP215507,CD8+ T cell_6-AD.vs.control; scRNA-seq,SRP330776,γδ T cell-AD.vs.control; scRNA-seq,SRP330776,Naive CD8+ T cell_3-AD.vs.control; scRNA-seq,SRP330776,B cell_2-AD.vs.control; scRNA-seq,SRP330776,B cell_3-AD.vs.control	5
CREB5	bulk RNA-seq,SRP223445,AD.vs.control; scRNA-seq,SRP330776,B cell_2-AD.vs.control; scRNA-seq,SRP330776,Monocyte_2-AD.vs.control	3
CREBBP	bulk RNA-seq,Homo_723,MCI.vs.control; bulk RNA-seq,Homo_633,AD.vs.MCI; bulk RNA-seq,Homo_633,MCI.vs.control; scRNA-seq,SRP215507,CD8+ T cell_7-AD.vs.control; scRNA-seq,SRP330776,B cell_2-AD.vs.control	5
CREBL2	bulk RNA-seq,Homo_723,AD.vs.control; bulk RNA-seq,Homo_723,AD.vs.MCI; bulk RNA-seq,Homo_633,AD.vs.control; bulk RNA-seq,Homo_633,AD.vs.MCI; scRNA-seq,SRP215507,CD8+ T cell_1-AD.vs.control; scRNA-seq,SRP215507,CD8+ T cell_1-AD.vs.MCI; scRNA-seq,SRP215507,CD8+ T cell_4-AD.vs.MCI; scRNA-seq,SRP215507,CD8+ T cell_4-MCI.vs.control; scRNA-seq,SRP330776,CD8+ T cell_1-AD.vs.control; scRNA-seq,SRP330776,γδ T cell-AD.vs.control; scRNA-seq,SRP330776,Memory T cell_2-AD.vs.control; scRNA-seq,SRP330776,CD8+ T cell_2-AD.vs.control; scRNA-seq,SRP330776,B cell_1-AD.vs.control; scRNA-seq,SRP330776,Naive CD8+ T cell_1-AD.vs.control; scRNA-seq,SRP330776,Memory T cell_1-AD.vs.control; scRNA-seq,SRP330776,Natural killer T cell-AD.vs.control; scRNA-seq,SRP330776,Naive CD8+ T cell_3-AD.vs.control; scRNA-seq,SRP330776,B cell_2-AD.vs.control; scRNA-seq,SRP330776,T cell-AD.vs.control; scRNA-seq,SRP330776,CD8+ T cell_3-AD.vs.control; scRNA-seq,SRP330776,Monocyte_1-AD.vs.control; scRNA-seq,SRP330776,Monocyte_2-AD.vs.control; scRNA-seq,SRP330776,B [illegible]	23
CREBRF	bulk RNA-seq,Homo_723,AD.vs.MCI; bulk RNA-seq,Homo_723,MCI.vs.control; bulk RNA-seq,Homo_633,AD.vs.MCI; bulk RNA-seq,Homo_633,MCI.vs.control; bulk RNA-seq,Homo_714,AD.vs.control; bulk RNA-seq,Homo_714,AD.vs.MCI; bulk RNA-seq,Homo_714,MCI.vs.control; scRNA-seq,SRP215507,CD8+ T cell_10-AD.vs.control; scRNA-seq,SRP215507,CD8+ T cell_5-AD.vs.MCI	9
CREBZF	scRNA-seq,SRP330776,Natural killer T cell-AD.vs.control; scRNA-seq,SRP330776,Monocyte_1-AD.vs.control; scRNA-seq,SRP330776,Monocyte_2-AD.vs.control; scRNA-seq,SRP330776,B cell_3-AD.vs.control	4
CREG1	bulk RNA-seq,Homo_723,AD.vs.MCI; bulk RNA-seq,Homo_723,MCI.vs.control; bulk RNA-seq,Homo_633,AD.vs.MCI; bulk RNA-seq,Homo_633,MCI.vs.control; scRNA-seq,SRP215507,CD8+ T cell_5-AD.vs.control; scRNA-seq,SRP215507,CD8+ T cell_5-AD.vs.MCI; scRNA-seq,SRP309935,Natural killer cell_2-AD.vs.control; scRNA-seq,SRP309935,Monocyte_1-AD.vs.control; scRNA-seq,SRP309935,Erythroid cell-AD.vs.control; scRNA-seq,SRP309935,Naive CD8+ T cell_1-AD.vs.control; scRNA-seq,SRP309935,Naive CD8+ T cell_2-AD.vs.control; scRNA-seq,SRP330776,CD8+ T cell_1-AD.vs.control; scRNA-seq,SRP330776,γδ T cell-AD.vs.control; scRNA-seq,SRP330776,Memory T cell_2-AD.vs.control; scRNA-seq,SRP330776,CD8+ T cell_2-AD.vs.control; scRNA-seq,SRP330776,B cell_1-AD.vs.control; scRNA-seq,SRP330776,Naive CD8+ T cell_1-AD.vs.control; scRNA-seq,SRP330776,Natural killer cell_1-AD.vs.control; scRNA-seq,SRP330776,Memory T cell_1-AD.vs.control; scRNA-seq,SRP330776,Natural killer T cell-AD.vs.control; scRNA-seq,SRP330776,Naive CD8+ T cell_3-AD.vs.control; scRNA-seq,SRP330776,B cell_2-AD.vs.control; scRNA-seq,SRP330776,T cell-AD.vs.control; scRNA-seq,SRP330776,CD8+ T cell_3-AD.vs.control; scRNA-seq,SRP330776,Monocyte_1-[illegible]	27
CREG2	bulk RNA-seq,Homo_723,AD.vs.control; bulk RNA-seq,Homo_723,AD.vs.MCI; bulk RNA-seq,Homo_633,AD.vs.control; bulk RNA-seq,Homo_633,AD.vs.MCI; bulk RNA-seq,Homo_714,AD.vs.control; bulk RNA-seq,Homo_714,AD.vs.MCI; bulk RNA-seq,ROSMAP,AD.vs.control;	8

CRELD1	scRNA-seq,SRP330776,Memory T cell_2-AD.vs.control; scRNA-seq,SRP330776,CD8+ T cell_2-AD.vs.control; scRNA-seq,SRP330776,B cell_1-AD.vs.control; scRNA-seq,SRP330776,Naive CD8+ T cell_1-AD.vs.control; scRNA-seq,SRP330776,Natural killer cell_1-AD.vs.control; scRNA-seq,SRP330776,Naive CD8+ T cell_2-AD.vs.control; scRNA-seq,SRP330776,CD8+ T cell_3-AD.vs.control; scRNA-seq,SRP330776,B cell_3-	9
CRELD2	bulk RNA-seq,Homo_723,AD.vs.control; bulk RNA-seq,Homo_723,AD.vs.MCI; bulk RNA-seq,Homo_633,AD.vs.control; bulk RNA-seq,Homo_633,AD.vs.MCI; bulk RNA-seq,Homo_714,AD.vs.control; bulk RNA-seq,Homo_714,AD.vs.MCI; scRNA-seq,SRP215507,CD8+ T cell_4-AD.vs.control; scRNA-seq,SRP215507,CD8+ T cell_7-AD.vs.control; scRNA-seq,SRP215507,CD8+ T cell_7-AD.vs.MCI; scRNA-seq,SRP215507,CD8+ T cell_5-AD.vs.MCI; scRNA-seq,SRP215507,CD8+ T cell_4-MCI.vs.control; scRNA-seq,SRP309935,Naive CD8+ T cell_2-AD.vs.control; scRNA-seq,SRP330776,CD8+ T cell_1-AD.vs.control; scRNA-seq,SRP330776,γδ T cell-AD.vs.control; scRNA-seq,SRP330776,Memory T cell_2-AD.vs.control; scRNA-seq,SRP330776,CD8+ T cell_2-AD.vs.control; scRNA-seq,SRP330776,B cell_1-AD.vs.control; scRNA-seq,SRP330776,Naive CD8+ T cell_1-AD.vs.control; scRNA-seq,SRP330776,Natural killer cell_1-AD.vs.control; scRNA-seq,SRP330776,Memory T cell_1-AD.vs.control; scRNA-seq,SRP330776,Natural killer T cell-AD.vs.control; scRNA-seq,SRP330776,Naive CD8+ T cell_3-AD.vs.control; scRNA-seq,SRP330776,B cell_2-AD.vs.control; scRNA-seq,SRP330776,T cell-AD.vs.control; scRNA-seq,SRP330776,Naive CD8+ T cell_2-AD.vs.control; scRNA-seq,SRP330776,CD8+ T cell_3-AD.vs.control; scRNA-seq,SRP330776,Monocyte_1-AD.vs.control; scRNA-seq,SRP330776,Monocyte_2-AD.vs.control; scRNA-seq,SRP330776,B cell_3-AD.vs.control; scRNA-seq,SRP330776,Natural killer cell_2-AD.vs.control; scRNA-seq,SRP330776,B cell_4-AD.vs.control	31
CREM	bulk RNA-seq,Homo_723,AD.vs.control; bulk RNA-seq,Homo_723,AD.vs.MCI; bulk RNA-seq,Homo_633,AD.vs.control; bulk RNA-seq,Homo_633,AD.vs.MCI; bulk RNA-seq,Homo_714,AD.vs.control; bulk RNA-seq,Homo_714,AD.vs.MCI; scRNA-seq,SRP215507,CD8+ T cell_4-AD.vs.control; scRNA-seq,SRP215507,CD8+ T cell_6-AD.vs.control; scRNA-seq,SRP215507,CD8+ T cell_4-AD.vs.MCI; scRNA-seq,SRP215507,CD8+ T cell_7-AD.vs.MCI; scRNA-seq,SRP215507,CD8+ T cell_4-MCI.vs.control; scRNA-seq,SRP215507,CD8+ T cell_2-MCI.vs.control; scRNA-seq,SRP215507,CD8+ T cell_7-MCI.vs.control; scRNA-seq,SRP309935,Natural killer cell_2-AD.vs.control; scRNA-seq,SRP309935,Megakaryocyte_1-AD.vs.control; scRNA-seq,SRP309935,Natural killer cell_1-AD.vs.control; scRNA-seq,SRP330776,Natural killer cell_1-AD.vs.control; scRNA-seq,SRP330776,Memory T cell_1-AD.vs.control; scRNA-seq,SRP330776,B cell_2-AD.vs.control; scRNA-seq,SRP330776,CD8+ T cell_3-AD.vs.control; scRNA-seq,SRP330776,Monocyte_1-AD.vs.control; scRNA-seq,SRP330776,B cell_3-AD.vs.control; scRNA-seq,SRP330776,Natural killer cell_2- [illegible]	23
Crem	bulk RNA-seq,SRP325058,AD.vs.control	1
CRH	bulk RNA-seq,Homo_723,AD.vs.control; bulk RNA-seq,Homo_723,AD.vs.MCI	2
CRHBP	bulk RNA-seq,Homo_723,AD.vs.control; bulk RNA-seq,Homo_723,AD.vs.MCI; bulk RNA-seq,Homo_723,MCI.vs.control; bulk RNA-seq,Homo_633,AD.vs.control; bulk RNA-seq,Homo_633,AD.vs.MCI; bulk RNA-seq,Homo_633,MCI.vs.control; bulk RNA-seq,Homo_714,AD.vs.control; bulk RNA-seq,Homo_714,AD.vs.MCI; bulk RNA-seq,Homo_714,MCI.vs.control; bulk RNA-seq,ROSMAP,AD.vs.control; bulk RNA-seq,ROSMAP,MCI.vs.control; scRNA-seq,SRP309935,Plasmacytoid dendritic cell_1-AD.vs.control	12
CRHR2	bulk RNA-seq,Homo_723,AD.vs.control; bulk RNA-seq,Homo_723,AD.vs.MCI; bulk RNA-seq,Homo_723,MCI.vs.control; bulk RNA-seq,Homo_633,AD.vs.MCI; bulk RNA-seq,Homo_633,MCI.vs.control; bulk RNA-seq,Homo_714,AD.vs.MCI; bulk RNA-seq,Homo_714,MCI.vs.control; bulk RNA-seq,ROSMAP,AD.vs.control; bulk RNA-seq,ROSMAP,MCI.vs.control	9
CRIM1	bulk RNA-seq,Homo_723,AD.vs.control; bulk RNA-seq,Homo_723,AD.vs.MCI; bulk RNA-seq,Homo_723,MCI.vs.control; bulk RNA-seq,Homo_633,AD.vs.control; bulk RNA-seq,Homo_633,AD.vs.MCI; bulk RNA-seq,Homo_633,MCI.vs.control; bulk RNA-seq,Homo_714,AD.vs.MCI; bulk RNA-seq,Homo_714,MCI.vs.control; scRNA-seq,SRP215507,CD8+ T cell_2-AD.vs.MCI; scRNA-seq,SRP215507,CD8+ T cell_1-AD.vs.MCI; scRNA-seq,SRP215507,CD8+ T cell_4-AD.vs.MCI; scRNA-seq,SRP215507,CD8+ T cell_1-MCI.vs.control; scRNA-seq,SRP215507,CD8+ T cell_4-MCI.vs.control; scRNA-seq,SRP215507,CD8+ T cell_2-MCI.vs.control; scRNA-seq,SRP309935,Naive CD8+ T cell_1-AD.vs.control; scRNA-	16
Crim1	bulk RNA-seq,SRP325058,AD.vs.control	1
CRIP1	scRNA-seq,SRP215507,CD8+ T cell_7-AD.vs.control; scRNA-seq,SRP215507,CD8+ T cell_7-MCI.vs.control; scRNA-seq,SRP215507,CD8+ T cell_8-MCI.vs.control; scRNA-seq,SRP309935,Erythroid cell-AD.vs.control; scRNA-seq,SRP309935,Megakaryocyte_2-AD.vs.control; scRNA-seq,SRP330776,B	6

CRIP2	bulk RNA-seq,Homo_723,AD.vs.control; bulk RNA-seq,Homo_723,AD.vs.MCI; bulk RNA-seq,Homo_633,AD.vs.control; bulk RNA-seq,Homo_633,AD.vs.MCI; bulk RNA-seq,Homo_714,AD.vs.control; bulk RNA-seq,Homo_714,AD.vs.MCI; bulk RNA-seq,Homo_714,MCI.vs.control; scRNA-seq,SRP215507,CD8+ T cell_2-AD.vs.control; scRNA-seq,SRP215507,CD8+ T cell_3-AD.vs.control; scRNA-seq,SRP215507,CD8+ T cell_1-AD.vs.control; scRNA-seq,SRP215507,CD8+ T cell_4-AD.vs.control; scRNA-seq,SRP215507,CD8+ T cell_5-AD.vs.control; scRNA-seq,SRP215507,CD8+ T cell_10-AD.vs.control; scRNA-seq,SRP215507,CD8+ T cell_3-AD.vs.MCI; scRNA-seq,SRP215507,CD8+ T cell_4-AD.vs.MCI; scRNA-seq,SRP215507,CD8+ T cell_5-AD.vs.MCI; scRNA-seq,SRP215507,CD8+ T cell_1-MCI.vs.control; scRNA-seq,SRP215507,CD8+ T cell_4-MCI.vs.control; scRNA-seq,SRP215507,CD8+ T cell_2-MCI.vs.control; scRNA-seq,SRP309935,Natural killer cell_1-AD.vs.control; scRNA-seq,SRP309935,CD8+ T cell_2-AD.vs.control; scRNA-seq,SRP330776,Memory T cell_1-AD.vs.control; scRNA-seq,SRP330776,Natural killer T cell-AD.vs.control; scRNA-seq,SRP330776,Naive CD8+ T cell_3-AD.vs.control; scRNA-seq,SRP330776,T cell-AD.vs.control; scRNA-seq,SRP330776,CD8+ [illegible]	27
Crip2	bulk RNA-seq,SRP325058,AD.vs.control	1
CRIP3	bulk RNA-seq,Homo_723,AD.vs.control; bulk RNA-seq,Homo_723,AD.vs.MCI; bulk RNA-seq,Homo_633,AD.vs.control; bulk RNA-seq,Homo_633,AD.vs.MCI; bulk RNA-seq,Homo_714,AD.vs.control; bulk RNA-seq,Homo_714,AD.vs.MCI	6
CRIPAK	scRNA-seq,SRP215507,CD8+ T cell_2-AD.vs.control; scRNA-seq,SRP215507,CD8+ T cell_3-AD.vs.control; scRNA-seq,SRP215507,CD8+ T cell_1-AD.vs.control; scRNA-seq,SRP215507,CD8+ T cell_3-AD.vs.MCI; scRNA-seq,SRP215507,CD8+ T cell_1-AD.vs.MCI; scRNA-seq,SRP215507,CD8+ T cell_3-MCI.vs.control; scRNA-seq,SRP330776,CD8+ T cell_2-AD.vs.control; scRNA-seq,SRP330776,B cell_1-AD.vs.control; scRNA-seq,SRP330776,Naive CD8+ T cell_1-AD.vs.control; scRNA-seq,SRP330776,Memory T cell_1-AD.vs.control	10
Cript	bulk RNA-seq,SRP325058,AD.vs.control	1
CRIPT	scRNA-seq,SRP215507,CD8+ T cell_2-AD.vs.MCI; scRNA-seq,SRP215507,CD8+ T cell_1-AD.vs.MCI; scRNA-seq,SRP215507,CD8+ T cell_6-AD.vs.MCI; scRNA-seq,SRP215507,CD8+ T cell_4-MCI.vs.control; scRNA-seq,SRP215507,CD8+ T cell_2-MCI.vs.control; scRNA-seq,SRP215507,CD8+ T cell_6-MCI.vs.control; scRNA-seq,SRP330776,CD8+ T cell_1-AD.vs.control; scRNA-seq,SRP330776,B cell_1-AD.vs.control; scRNA-seq,SRP330776,Naive CD8+ T cell_1-AD.vs.control; scRNA-seq,SRP330776,Natural killer cell_1-AD.vs.control; scRNA-seq,SRP330776,Naive CD8+ T cell_3-AD.vs.control; scRNA-seq,SRP330776,B cell_2-AD.vs.control; scRNA-seq,SRP330776,T cell-AD.vs.control; scRNA-	15
CRISP2	bulk RNA-seq,Homo_723,AD.vs.control	1
CRISP3	bulk RNA-seq,Homo_723,AD.vs.control; bulk RNA-seq,Homo_723,AD.vs.MCI; bulk RNA-seq,Homo_633,AD.vs.control; bulk RNA-seq,Homo_633,AD.vs.MCI; bulk RNA-seq,Homo_714,AD.vs.control; bulk RNA-seq,Homo_714,AD.vs.MCI	6
CRISPLD1	bulk RNA-seq,Homo_723,AD.vs.control; bulk RNA-seq,Homo_723,AD.vs.MCI; bulk RNA-seq,Homo_723,MCI.vs.control; bulk RNA-seq,Homo_633,AD.vs.control; bulk RNA-seq,Homo_633,AD.vs.MCI; bulk RNA-seq,Homo_633,MCI.vs.control; bulk RNA-seq,ROSMAP,AD.vs.control;	8
CRISPLD2	bulk RNA-seq,Homo_723,AD.vs.control; bulk RNA-seq,Homo_723,AD.vs.MCI; bulk RNA-seq,Homo_633,AD.vs.control; bulk RNA-seq,Homo_633,AD.vs.MCI; bulk RNA-seq,SRP223445,AD.vs.control; bulk RNA-seq,SRP310421,AD.vs.control; scRNA-seq,SRP215507,CD8+ T cell_3-AD.vs.MCI; scRNA-seq,SRP215507,CD8+ T cell_3-MCI.vs.control; scRNA-seq,SRP330776,Monocyte_2-AD.vs.control	9
Crispld2	bulk RNA-seq,SRP325058,AD.vs.control	1
CRK	scRNA-seq,SRP215507,CD8+ T cell_5-AD.vs.MCI; scRNA-seq,SRP215507,CD8+ T cell_5-MCI.vs.control; scRNA-seq,SRP309935,Naive CD8+ T cell_2-AD.vs.control; scRNA-seq,SRP330776,CD8+ T cell_1-AD.vs.control; scRNA-seq,SRP330776,γδ T cell-AD.vs.control; scRNA-seq,SRP330776,Memory T cell_2-AD.vs.control; scRNA-seq,SRP330776,CD8+ T cell_2-AD.vs.control; scRNA-seq,SRP330776,B cell_1-AD.vs.control; scRNA-seq,SRP330776,Naive CD8+ T cell_1-AD.vs.control; scRNA-seq,SRP330776,Natural killer cell_1-AD.vs.control; scRNA-seq,SRP330776,Memory T cell_1-AD.vs.control; scRNA-seq,SRP330776,Natural killer T cell-AD.vs.control; scRNA-seq,SRP330776,Naive CD8+ T cell_3-AD.vs.control; scRNA-seq,SRP330776,B cell_2-AD.vs.control; scRNA-seq,SRP330776,CD8+ T cell_3-AD.vs.control; scRNA-seq,SRP330776,Monocyte_1-AD.vs.control; scRNA-seq,SRP330776,Monocyte_2-AD.vs.control; scRNA-seq,SRP330776,B cell_3-AD.vs.control; scRNA-seq,SRP330776,Natural killer cell_2- [illegible]	20
CRKL	bulk RNA-seq,Homo_723,AD.vs.control; bulk RNA-seq,Homo_723,AD.vs.MCI; bulk RNA-seq,Homo_633,AD.vs.control; bulk RNA-seq,Homo_633,AD.vs.MCI; scRNA-seq,SRP215507,CD8+ T cell_4-AD.vs.control; scRNA-seq,SRP215507,CD8+ T cell_1-AD.vs.MCI; scRNA-seq,SRP215507,CD8+ T cell_4-AD.vs.MCI; scRNA-seq,SRP215507,CD8+ T cell_5-AD.vs.MCI; scRNA-seq,SRP309935,Naive CD8+ T cell_1-	10
CRLF1	bulk RNA-seq,Homo_723,AD.vs.control; bulk RNA-seq,Homo_723,AD.vs.MCI; bulk RNA-seq,Homo_633,AD.vs.control; bulk RNA-seq,Homo_633,AD.vs.MCI; scRNA-seq,SRP215507,CD8+ T cell_1-MCI.vs.control	5

CRLF3	bulk RNA-seq,Homo_723,AD.vs.control; bulk RNA-seq,Homo_714,AD.vs.control; bulk RNA-seq,Homo_714,AD.vs.MCI; scRNA-seq,SRP215507,CD8+ T cell_8-MCI.vs.control; scRNA-seq,SRP309935,Natural killer cell_2-AD.vs.control; scRNA-seq,SRP309935,Monocyte_1-AD.vs.control; scRNA-seq,SRP309935,Naive CD8+ T cell_1-AD.vs.control; scRNA-seq,SRP309935,Naive CD8+ T cell_2-AD.vs.control; scRNA-seq,SRP309935,CD8+ T cell_2-AD.vs.control; scRNA-seq,SRP330776,CD8+ T cell_1-AD.vs.control; scRNA-seq,SRP330776,γδ T cell-AD.vs.control; scRNA-seq,SRP330776,Naive CD8+ T cell_1-AD.vs.control; scRNA-seq,SRP330776,CD8+ T cell_3-AD.vs.control	13
CRLS1	bulk RNA-seq,Homo_723,AD.vs.MCI; bulk RNA-seq,Homo_633,AD.vs.MCI; bulk RNA-seq,Homo_714,AD.vs.control; bulk RNA-seq,Homo_714,AD.vs.MCI; scRNA-seq,SRP215507,CD8+ T cell_4-AD.vs.control; scRNA-seq,SRP215507,CD8+ T cell_7-AD.vs.control; scRNA-seq,SRP215507,CD8+ T cell_5-AD.vs.control; scRNA-seq,SRP309935,Naive CD8+ T cell_2-AD.vs.control; scRNA-seq,SRP330776,CD8+ T cell_1-AD.vs.control; scRNA-seq,SRP330776,γδ T cell-AD.vs.control; scRNA-seq,SRP330776,Memory T cell_2-AD.vs.control; scRNA-seq,SRP330776,CD8+ T cell_2-AD.vs.control; scRNA-seq,SRP330776,B cell_1-AD.vs.control; scRNA-seq,SRP330776,Naive CD8+ T cell_1-AD.vs.control; scRNA-seq,SRP330776,Natural killer cell_1-AD.vs.control; scRNA-seq,SRP330776,Memory T cell_1-AD.vs.control; scRNA-seq,SRP330776,Natural killer T cell-AD.vs.control; scRNA-seq,SRP330776,Naive CD8+ T cell_3-AD.vs.control; scRNA-seq,SRP330776,B cell_2-AD.vs.control; scRNA-seq,SRP330776,T cell-AD.vs.control; scRNA-seq,SRP330776,CD8+ T cell_3-AD.vs.control; scRNA-seq,SRP330776,Monocyte_2-AD.vs.control; scRNA-seq,SRP330776,B [illegible]	24
CRMP1	bulk RNA-seq,Homo_723,AD.vs.control; bulk RNA-seq,Homo_723,AD.vs.MCI; bulk RNA-seq,Homo_723,MCI.vs.control; bulk RNA-	4
CRNDE	scRNA-seq,SRP215507,CD8+ T cell_5-MCI.vs.control; scRNA-seq,SRP330776,CD8+ T cell_2-AD.vs.control; scRNA-seq,SRP330776,Natural killer cell_1-AD.vs.control; scRNA-seq,SRP330776,Memory T cell_1-AD.vs.control	4
CRNKL1	bulk RNA-seq,Homo_723,AD.vs.control; bulk RNA-seq,Homo_723,MCI.vs.control; bulk RNA-seq,Homo_633,AD.vs.control; bulk RNA-seq,Homo_633,MCI.vs.control; bulk RNA-seq,Homo_714,AD.vs.control; bulk RNA-seq,Homo_714,AD.vs.MCI; bulk RNA-seq,Homo_714,MCI.vs.control; scRNA-seq,SRP309935,Megakaryocyte_1-AD.vs.control; scRNA-seq,SRP330776,B cell_1-AD.vs.control; scRNA-seq,SRP330776,B cell_2-AD.vs.control; scRNA-seq,SRP330776,Monocyte_2-AD.vs.control; scRNA-seq,SRP330776,B cell_3-AD.vs.control; scRNA-	13
Crnkl1	bulk RNA-seq,SRP325058,AD.vs.control	1
CRNN	bulk RNA-seq,Homo_723,AD.vs.MCI; bulk RNA-seq,Homo_633,AD.vs.MCI	2
CROCC	scRNA-seq,SRP215507,CD8+ T cell_4-AD.vs.control; scRNA-seq,SRP215507,CD8+ T cell_5-AD.vs.control; scRNA-seq,SRP215507,CD8+ T cell_1-AD.vs.MCI; scRNA-seq,SRP215507,CD8+ T cell_4-AD.vs.MCI; scRNA-seq,SRP215507,CD8+ T cell_3-MCI.vs.control; scRNA-seq,SRP309935,Monocyte_2-AD.vs.control; scRNA-seq,SRP330776,CD8+ T cell_1-AD.vs.control; scRNA-seq,SRP330776,γδ T cell-AD.vs.control; scRNA-seq,SRP330776,Memory T cell_2-AD.vs.control; scRNA-seq,SRP330776,CD8+ T cell_2-AD.vs.control; scRNA-seq,SRP330776,B cell_1-AD.vs.control; scRNA-seq,SRP330776,Naive CD8+ T cell_1-AD.vs.control; scRNA-seq,SRP330776,Natural killer cell_1-AD.vs.control; scRNA-seq,SRP330776,Memory T cell_1-AD.vs.control; scRNA-seq,SRP330776,Natural killer T cell-AD.vs.control; scRNA-seq,SRP330776,B cell_2-AD.vs.control; scRNA-seq,SRP330776,T cell-AD.vs.control; scRNA-seq,SRP330776,CD8+ T cell_3-AD.vs.control; scRNA-seq,SRP330776,B cell_3- [illegible]	20
CROCC2	bulk RNA-seq,Homo_723,MCI.vs.control; bulk RNA-seq,Homo_633,AD.vs.control; bulk RNA-seq,Homo_633,AD.vs.MCI; bulk RNA-seq,Homo_633,MCI.vs.control; bulk RNA-seq,Homo_714,AD.vs.control; bulk RNA-seq,Homo_714,AD.vs.MCI; bulk RNA-seq,Homo_714,MCI.vs.control; bulk RNA-seq,ROSMAP,AD.vs.control; scRNA-seq,SRP309935,Monocyte_2-AD.vs.control; scRNA-	10
CROT	bulk RNA-seq,Homo_714,AD.vs.control; bulk RNA-seq,Homo_714,AD.vs.MCI; scRNA-seq,SRP215507,CD8+ T cell_3-AD.vs.control; scRNA-seq,SRP215507,CD8+ T cell_2-AD.vs.MCI; scRNA-seq,SRP215507,CD8+ T cell_1-MCI.vs.control; scRNA-seq,SRP309935,CD8+ T cell_1-AD.vs.control; scRNA-seq,SRP330776,CD8+ T cell_2-AD.vs.control; scRNA-seq,SRP330776,B cell_1-AD.vs.control; scRNA-seq,SRP330776,Naive CD8+ T cell_1-AD.vs.control; scRNA-seq,SRP330776,Natural killer T cell-AD.vs.control; scRNA-seq,SRP330776,Naive CD8+ T cell_3-AD.vs.control;	12
CRP	bulk RNA-seq,Homo_723,AD.vs.control; bulk RNA-seq,Homo_723,AD.vs.MCI; bulk RNA-seq,Homo_633,AD.vs.control	3
CRPPA	bulk RNA-seq,Homo_723,AD.vs.control; bulk RNA-seq,Homo_723,AD.vs.MCI; bulk RNA-seq,Homo_723,MCI.vs.control; bulk RNA-seq,Homo_633,AD.vs.control; bulk RNA-seq,Homo_633,AD.vs.MCI; bulk RNA-seq,Homo_633,MCI.vs.control; bulk RNA-seq,Homo_714,AD.vs.control; bulk RNA-seq,Homo_714,AD.vs.MCI; scRNA-seq,SRP215507,CD8+ T cell_4-AD.vs.control; scRNA-seq,SRP215507,CD8+ T cell_4-AD.vs.MCI; scRNA-seq,SRP330776,CD8+ T cell_1-AD.vs.control; scRNA-seq,SRP330776,Naive CD8+ T cell_1-AD.vs.control; scRNA-seq,SRP330776,Memory T	13
Crtac1	bulk RNA-seq,SRP325058,AD.vs.control	1

CRTAM	bulk RNA-seq,Homo_723,AD.vs.control; bulk RNA-seq,Homo_723,AD.vs.MCI; bulk RNA-seq,Homo_633,AD.vs.control; bulk RNA-seq,Homo_633,AD.vs.MCI; bulk RNA-seq,Homo_714,AD.vs.control; bulk RNA-seq,Homo_714,AD.vs.MCI; scRNA-seq,SRP215507,CD8+ T cell_2-AD.vs.MCI; scRNA-seq,SRP309935,Natural killer cell_2-AD.vs.control; scRNA-seq,SRP309935,Natural killer cell_1-AD.vs.control; scRNA-seq,SRP309935,CD8+ T cell_1-AD.vs.control; scRNA-seq,SRP330776,CD8+ T cell_1-AD.vs.control; scRNA-seq,SRP330776,γδ T cell-AD.vs.control; scRNA-seq,SRP330776,Memory T cell_2-AD.vs.control; scRNA-seq,SRP330776,CD8+ T cell_2-AD.vs.control; scRNA-seq,SRP330776,Naive CD8+ T cell_1-AD.vs.control; scRNA-seq,SRP330776,Natural killer cell_1-AD.vs.control; scRNA-seq,SRP330776,Memory T cell_1-AD.vs.control; scRNA-seq,SRP330776,Natural killer T cell-AD.vs.control; scRNA-seq,SRP330776,Naive CD8+ T cell_3-AD.vs.control; scRNA-seq,SRP330776,B cell_3-[illegible]	20
CRTAP	bulk RNA-seq,Homo_633,AD.vs.control; bulk RNA-seq,Homo_633,AD.vs.MCI; scRNA-seq,SRP215507,CD8+ T cell_7-AD.vs.control; scRNA-seq,SRP215507,CD8+ T cell_4-AD.vs.MCI; scRNA-seq,SRP215507,CD8+ T cell_4-MCI.vs.control; scRNA-seq,SRP309935,CD8+ T cell_2-AD.vs.control; scRNA-seq,SRP330776,CD8+ T cell_1-AD.vs.control; scRNA-seq,SRP330776,γδ T cell-AD.vs.control; scRNA-seq,SRP330776,Memory T cell_2-AD.vs.control; scRNA-seq,SRP330776,CD8+ T cell_2-AD.vs.control; scRNA-seq,SRP330776,B cell_1-AD.vs.control; scRNA-seq,SRP330776,Naive CD8+ T cell_1-AD.vs.control; scRNA-seq,SRP330776,Natural killer cell_1-AD.vs.control; scRNA-seq,SRP330776,Memory T cell_1-AD.vs.control; scRNA-seq,SRP330776,Natural killer T cell-AD.vs.control; scRNA-seq,SRP330776,Naive CD8+ T cell_3-AD.vs.control; scRNA-seq,SRP330776,B cell_2-AD.vs.control; scRNA-seq,SRP330776,T cell-AD.vs.control; scRNA-seq,SRP330776,Naive CD8+ T cell_2-AD.vs.control; scRNA-seq,SRP330776,CD8+ T cell_3-AD.vs.control; scRNA-seq,SRP330776,Monocyte_1-AD.vs.control; scRNA-seq,SRP330776,Monocyte_2-[illegible]	24
CRTC1	bulk RNA-seq,Homo_723,AD.vs.control; bulk RNA-seq,Homo_723,AD.vs.MCI; bulk RNA-seq,Homo_633,AD.vs.MCI; bulk RNA-seq,Homo_714,AD.vs.control; bulk RNA-seq,Homo_714,AD.vs.MCI; bulk RNA-seq,SRP223445,AD.vs.control; scRNA-seq,SRP215507,CD8+ T cell_5-AD.vs.control; scRNA-seq,SRP215507,CD8+ T cell_3-AD.vs.MCI; scRNA-seq,SRP215507,CD8+ T cell_5-AD.vs.MCI; scRNA-seq,SRP330776,CD8+ T cell_1-AD.vs.control; scRNA-seq,SRP330776,γδ T cell-AD.vs.control; scRNA-seq,SRP330776,Memory T cell_2-AD.vs.control; scRNA-seq,SRP330776,CD8+ T cell_2-AD.vs.control; scRNA-seq,SRP330776,B cell_1-AD.vs.control; scRNA-seq,SRP330776,Naive CD8+ T cell_1-AD.vs.control; scRNA-seq,SRP330776,Natural killer cell_1-AD.vs.control; scRNA-seq,SRP330776,Memory T cell_1-AD.vs.control; scRNA-seq,SRP330776,Natural killer T cell-AD.vs.control; scRNA-seq,SRP330776,Naive CD8+ T cell_3-AD.vs.control; scRNA-seq,SRP330776,B cell_2-[illegible]	22
CRTC2	bulk RNA-seq,Homo_723,AD.vs.control; bulk RNA-seq,Homo_723,MCI.vs.control; bulk RNA-seq,Homo_633,AD.vs.control; bulk RNA-seq,Homo_633,MCI.vs.control; scRNA-seq,SRP309935,Natural killer cell_1-AD.vs.control; scRNA-seq,SRP330776,CD8+ T cell_1-AD.vs.control; scRNA-seq,SRP330776,γδ T cell-AD.vs.control; scRNA-seq,SRP330776,Memory T cell_2-AD.vs.control; scRNA-seq,SRP330776,CD8+ T cell_2-AD.vs.control; scRNA-seq,SRP330776,B cell_1-AD.vs.control; scRNA-seq,SRP330776,Naive CD8+ T cell_1-AD.vs.control; scRNA-seq,SRP330776,Natural killer cell_1-AD.vs.control; scRNA-seq,SRP330776,Memory T cell_1-AD.vs.control; scRNA-seq,SRP330776,Naive CD8+ T cell_3-AD.vs.control; scRNA-seq,SRP330776,B cell_2-AD.vs.control; scRNA-seq,SRP330776,T cell-AD.vs.control; scRNA-seq,SRP330776,Naive CD8+ [illegible]	19
CRTC3	scRNA-seq,SRP215507,CD8+ T cell_4-AD.vs.MCI; scRNA-seq,SRP215507,CD8+ T cell_4-MCI.vs.control; scRNA-seq,SRP309935,Natural killer cell_2-AD.vs.control; scRNA-seq,SRP330776,CD8+ T cell_1-AD.vs.control; scRNA-seq,SRP330776,γδ T cell-AD.vs.control; scRNA-seq,SRP330776,Memory T cell_2-AD.vs.control; scRNA-seq,SRP330776,CD8+ T cell_2-AD.vs.control; scRNA-seq,SRP330776,B cell_1-AD.vs.control; scRNA-seq,SRP330776,Naive CD8+ T cell_1-AD.vs.control; scRNA-seq,SRP330776,Natural killer cell_1-AD.vs.control; scRNA-seq,SRP330776,Memory T cell_1-AD.vs.control; scRNA-seq,SRP330776,Natural killer T cell-AD.vs.control; scRNA-seq,SRP330776,Naive CD8+ T cell_3-AD.vs.control; scRNA-seq,SRP330776,B cell_2-AD.vs.control; scRNA-seq,SRP330776,T cell-AD.vs.control; scRNA-seq,SRP330776,Naive CD8+ T cell_2-AD.vs.control; scRNA-seq,SRP330776,CD8+ T cell_3-AD.vs.control; scRNA-seq,SRP330776,Monocyte_1-AD.vs.control; scRNA-seq,SRP330776,Monocyte_2-[illegible]	21
CRTC3-AS1	scRNA-seq,SRP215507,CD8+ T cell_2-AD.vs.control; scRNA-seq,SRP215507,CD8+ T cell_6-AD.vs.control; scRNA-seq,SRP215507,CD8+ T cell_1-MCI.vs.control; scRNA-seq,SRP330776,CD8+ T cell_1-AD.vs.control; scRNA-seq,SRP330776,γδ T cell-AD.vs.control	5
CRX	bulk RNA-seq,Homo_723,AD.vs.control; bulk RNA-seq,Homo_723,AD.vs.MCI; bulk RNA-seq,Homo_714,MCI.vs.control	3
CRY1	scRNA-seq,SRP215507,CD8+ T cell_7-AD.vs.control; scRNA-seq,SRP215507,CD8+ T cell_5-AD.vs.MCI; scRNA-seq,SRP309935,Natural killer cell_2-AD.vs.control; scRNA-seq,SRP309935,Naive CD8+ T cell_1-AD.vs.control; scRNA-seq,SRP309935,Natural killer cell_1-AD.vs.control; scRNA-seq,SRP309935,CD8+ T cell_1-AD.vs.control; scRNA-seq,SRP330776,Regulatory T cell-AD.vs.control	7

CRY2	scRNA-seq,SRP215507,CD8+ T cell_5-AD.vs.control; scRNA-seq,SRP215507,CD8+ T cell_5-MCI.vs.control; scRNA-seq,SRP215507,CD8+ T cell_7-MCI.vs.control; scRNA-seq,SRP309935,Natural killer cell_2-AD.vs.control; scRNA-seq,SRP309935,Monocyte_2-AD.vs.control; scRNA-seq,SRP309935,Naive CD8+ T cell_2-AD.vs.control; scRNA-seq,SRP309935,CD8+ T cell_1-AD.vs.control; scRNA-seq,SRP330776,CD8+ T cell_1-AD.vs.control; scRNA-seq,SRP330776,CD8+ T cell_2-AD.vs.control; scRNA-seq,SRP330776,B cell_1-AD.vs.control; scRNA-seq,SRP330776,B cell_2-AD.vs.control; scRNA-seq,SRP330776,T cell-AD.vs.control; scRNA-seq,SRP330776,Monocyte_2-AD.vs.control; scRNA-seq,SRP330776,B cell_3-	15
CRYAA	bulk RNA-seq,Homo_723,AD.vs.control; bulk RNA-seq,Homo_633,AD.vs.control; bulk RNA-seq,ROSMAP,AD.vs.control	3
CRYAA2	bulk RNA-seq,Homo_723,AD.vs.MCI; bulk RNA-seq,Homo_633,AD.vs.control; bulk RNA-seq,Homo_714,AD.vs.control	3
CRYAB	bulk RNA-seq,Homo_723,AD.vs.control; bulk RNA-seq,Homo_723,AD.vs.MCI; bulk RNA-seq,Homo_633,AD.vs.control; bulk RNA-	4
CRYBA1	bulk RNA-seq,Homo_633,AD.vs.control; bulk RNA-seq,Homo_633,AD.vs.MCI	2
CRYBA4	bulk RNA-seq,Homo_723,AD.vs.MCI; bulk RNA-seq,Homo_633,AD.vs.MCI; scRNA-seq,SRP215507,CD8+ T cell_2-AD.vs.MCI; scRNA-seq,SRP215507,CD8+ T cell_2-MCI.vs.control; scRNA-seq,SRP215507,CD8+ T cell_3-MCI.vs.control	5
CRYBB1	bulk RNA-seq,Homo_723,MCI.vs.control; bulk RNA-seq,Homo_633,MCI.vs.control; bulk RNA-seq,Homo_714,AD.vs.control	3
CRYBB2	scRNA-seq,SRP309935,Naive CD8+ T cell_1-AD.vs.control	1
CRYBG1	bulk RNA-seq,Homo_633,AD.vs.control; bulk RNA-seq,Homo_633,AD.vs.MCI; scRNA-seq,SRP215507,CD8+ T cell_6-AD.vs.MCI; scRNA-seq,SRP215507,CD8+ T cell_6-MCI.vs.control; scRNA-seq,SRP309935,Naive CD8+ T cell_1-AD.vs.control; scRNA-seq,SRP309935,Natural killer cell_1-AD.vs.control; scRNA-seq,SRP309935,Monocyte_2-AD.vs.control; scRNA-seq,SRP309935,CD8+ T cell_1-AD.vs.control; scRNA-seq,SRP309935,CD8+ T cell_2-AD.vs.control; scRNA-seq,SRP330776,Memory T cell_1-AD.vs.control	10
CRYBG2	bulk RNA-seq,Homo_723,AD.vs.control; bulk RNA-seq,Homo_723,AD.vs.MCI; bulk RNA-seq,Homo_633,AD.vs.control; bulk RNA-seq,Homo_633,AD.vs.MCI; bulk RNA-seq,Homo_714,AD.vs.control; bulk RNA-seq,Homo_714,AD.vs.MCI; scRNA-seq,SRP215507,CD8+ T cell_3-AD.vs.control; scRNA-seq,SRP215507,CD8+ T cell_5-AD.vs.control; scRNA-seq,SRP215507,CD8+ T cell_1-MCI.vs.control; scRNA-seq,SRP330776,CD8+ T cell_1-AD.vs.control; scRNA-seq,SRP330776,γδ T cell-AD.vs.control; scRNA-seq,SRP330776,Memory T cell_2-AD.vs.control; scRNA-seq,SRP330776,B cell_1-AD.vs.control; scRNA-seq,SRP330776,Natural killer cell_1-AD.vs.control	14
CRYBG3	bulk RNA-seq,Homo_723,AD.vs.control; bulk RNA-seq,Homo_723,AD.vs.MCI; bulk RNA-seq,Homo_633,AD.vs.control; bulk RNA-seq,Homo_633,AD.vs.MCI; bulk RNA-seq,Homo_714,AD.vs.control; bulk RNA-seq,Homo_714,AD.vs.MCI; scRNA-seq,SRP215507,CD8+ T cell_1-AD.vs.control; scRNA-seq,SRP215507,CD8+ T cell_1-AD.vs.MCI; scRNA-seq,SRP215507,CD8+ T cell_1-MCI.vs.control; scRNA-seq,SRP330776,CD8+ T cell_1-AD.vs.control; scRNA-seq,SRP330776,Memory T cell_2-AD.vs.control; scRNA-seq,SRP330776,CD8+ T cell_2-AD.vs.control; scRNA-seq,SRP330776,Natural killer cell_1-AD.vs.control; scRNA-seq,SRP330776,CD8+ T cell_3-AD.vs.control; scRNA-seq,SRP330776,B cell_4-AD.vs.control	15
CRYGN	scRNA-seq,SRP215507,CD8+ T cell_2-AD.vs.control; scRNA-seq,SRP330776,CD8+ T cell_1-AD.vs.control	2
CRYGS	bulk RNA-seq,Homo_723,AD.vs.control; bulk RNA-seq,Homo_723,AD.vs.MCI; bulk RNA-seq,Homo_723,MCI.vs.control; bulk RNA-seq,Homo_633,AD.vs.control; bulk RNA-seq,Homo_633,AD.vs.MCI; bulk RNA-seq,Homo_633,MCI.vs.control; bulk RNA-seq,Homo_714,AD.vs.control; bulk RNA-seq,Homo_714,AD.vs.MCI; bulk RNA-seq,Homo_714,MCI.vs.control; scRNA-seq,SRP215507,CD8+ T cell_3-AD.vs.control; scRNA-seq,SRP215507,CD8+ T cell_3-AD.vs.MCI; scRNA-seq,SRP215507,CD8+ T cell_2-MCI.vs.control; scRNA-seq,SRP330776,CD8+ T cell_1-AD.vs.control; scRNA-seq,SRP330776,CD8+ T cell_2-AD.vs.control; scRNA-seq,SRP330776,B cell_1-AD.vs.control	15
CRYL1	scRNA-seq,SRP215507,CD8+ T cell_5-AD.vs.MCI; scRNA-seq,SRP215507,CD8+ T cell_5-MCI.vs.control; scRNA-seq,SRP215507,CD8+ T cell_7-MCI.vs.control; scRNA-seq,SRP330776,CD8+ T cell_3-AD.vs.control; scRNA-seq,SRP330776,Monocyte_1-AD.vs.control	5
CRYM	bulk RNA-seq,Homo_723,AD.vs.control; bulk RNA-seq,Homo_723,AD.vs.MCI; bulk RNA-seq,Homo_723,MCI.vs.control; bulk RNA-seq,Homo_633,AD.vs.control; bulk RNA-seq,Homo_633,AD.vs.MCI; bulk RNA-seq,Homo_633,MCI.vs.control; bulk RNA-seq,Homo_714,AD.vs.control;	8
CRYZ	bulk RNA-seq,Homo_714,AD.vs.control; bulk RNA-seq,Homo_714,AD.vs.MCI; scRNA-seq,SRP215507,CD8+ T cell_3-AD.vs.MCI; scRNA-seq,SRP215507,CD8+ T cell_4-AD.vs.MCI; scRNA-seq,SRP215507,CD8+ T cell_4-MCI.vs.control; scRNA-seq,SRP215507,CD8+ T cell_3-MCI.vs.control; scRNA-seq,SRP330776,CD8+ T cell_1-AD.vs.control; scRNA-seq,SRP330776,Naive CD8+ T cell_1-AD.vs.control; scRNA-seq,SRP330776,Naive CD8+ T cell_3-AD.vs.control; scRNA-seq,SRP330776,B cell_2-AD.vs.control; scRNA-seq,SRP330776,CD8+ T cell_3-	12

CRYZL1	bulk RNA-seq,Homo_633,AD.vs.control; scRNA-seq,SRP215507,CD8+ T cell_5-AD.vs.MCI; scRNA-seq,SRP215507,CD8+ T cell_6-AD.vs.MCI; scRNA-seq,SRP330776,CD8+ T cell_1-AD.vs.control; scRNA-seq,SRP330776,γδ T cell-AD.vs.control; scRNA-seq,SRP330776,Naive CD8+ T cell_1-AD.vs.control; scRNA-seq,SRP330776,Natural killer cell_1-AD.vs.control; scRNA-seq,SRP330776,Natural killer T cell-AD.vs.control; scRNA-seq,SRP330776,T cell-AD.vs.control; scRNA-seq,SRP330776,Natural killer cell_2-AD.vs.control	10
CRYZL2P-SEC16B	bulk RNA-seq,Homo_723,AD.vs.control; bulk RNA-seq,Homo_723,AD.vs.MCI; bulk RNA-seq,Homo_633,AD.vs.control; bulk RNA-seq,Homo_633,AD.vs.MCI; bulk RNA-seq,Homo_714,AD.vs.control; bulk RNA-seq,Homo_714,AD.vs.MCI	6
CS	bulk RNA-seq,Homo_723,AD.vs.control; bulk RNA-seq,Homo_723,MCI.vs.control; bulk RNA-seq,Homo_633,AD.vs.MCI; bulk RNA-seq,Homo_633,MCI.vs.control; bulk RNA-seq,Homo_714,AD.vs.control; bulk RNA-seq,Homo_714,AD.vs.MCI; bulk RNA-seq,Homo_714,MCI.vs.control; scRNA-seq,SRP215507,CD8+ T cell_4-AD.vs.MCI; scRNA-seq,SRP215507,CD8+ T cell_5-MCI.vs.control; scRNA-seq,SRP309935,CD8+ T cell_1-AD.vs.control; scRNA-seq,SRP309935,B cell_1-AD.vs.control; scRNA-seq,SRP330776,Natural killer cell_1-	13
CSAD	scRNA-seq,SRP215507,CD8+ T cell_1-AD.vs.MCI; scRNA-seq,SRP215507,CD8+ T cell_1-MCI.vs.control; scRNA-seq,SRP330776,CD8+ T cell_1-AD.vs.control; scRNA-seq,SRP330776,Memory T cell_2-AD.vs.control; scRNA-seq,SRP330776,CD8+ T cell_2-AD.vs.control; scRNA-seq,SRP330776,B cell_1-AD.vs.control; scRNA-seq,SRP330776,Naive CD8+ T cell_1-AD.vs.control; scRNA-seq,SRP330776,Natural killer cell_1-AD.vs.control; scRNA-seq,SRP330776,Memory T cell_1-AD.vs.control; scRNA-seq,SRP330776,Naive CD8+ T cell_3-AD.vs.control; scRNA-seq,SRP330776,B cell_2-AD.vs.control; scRNA-seq,SRP330776,T cell-AD.vs.control; scRNA-seq,SRP330776,Monocyte_2-AD.vs.control	13
CSDC2	bulk RNA-seq,Homo_723,AD.vs.control; bulk RNA-seq,Homo_723,AD.vs.MCI; bulk RNA-seq,Homo_633,MCI.vs.control; bulk RNA-	4
CSDE1	bulk RNA-seq,Homo_723,AD.vs.control; bulk RNA-seq,Homo_723,AD.vs.MCI; bulk RNA-seq,Homo_723,MCI.vs.control; bulk RNA-seq,Homo_633,AD.vs.MCI; bulk RNA-seq,Homo_633,MCI.vs.control; bulk RNA-seq,Homo_714,AD.vs.control; bulk RNA-seq,Homo_714,AD.vs.MCI; bulk RNA-seq,Homo_714,MCI.vs.control; scRNA-seq,SRP309935,Megakaryocyte_1-AD.vs.control; scRNA-seq,SRP309935,Erythroid cell-AD.vs.control; scRNA-seq,SRP309935,CD8+ T cell_1-AD.vs.control; scRNA-seq,SRP309935,B cell_1-AD.vs.control; scRNA-seq,SRP330776,B cell_4-AD.vs.control	13
Csde1	bulk RNA-seq,SRP325058,AD.vs.control	1
CSE1L	scRNA-seq,SRP215507,CD8+ T cell_4-AD.vs.MCI; scRNA-seq,SRP309935,Natural killer cell_2-AD.vs.control; scRNA-seq,SRP309935,Naive CD8+ T cell_1-AD.vs.control; scRNA-seq,SRP309935,Naive CD8+ T cell_2-AD.vs.control; scRNA-seq,SRP330776,Memory T cell_1-AD.vs.control; scRNA-seq,SRP330776,Megakaryocyte_1-AD.vs.control	6
CSF1	bulk RNA-seq,SRP310421,AD.vs.control; scRNA-seq,SRP330776,CD8+ T cell_1-AD.vs.control; scRNA-seq,SRP330776,Memory T cell_2-AD.vs.control; scRNA-seq,SRP330776,CD8+ T cell_2-AD.vs.control; scRNA-seq,SRP330776,Naive CD8+ T cell_1-AD.vs.control; scRNA-seq,SRP330776,Natural killer cell_1-AD.vs.control; scRNA-seq,SRP330776,CD8+ T cell_3-AD.vs.control	7
CSF1R	scRNA-seq,SRP330776,Monocyte_1-AD.vs.control	1
CSF2RB	bulk RNA-seq,Homo_723,AD.vs.control; bulk RNA-seq,Homo_723,AD.vs.MCI; bulk RNA-seq,Homo_633,AD.vs.control; bulk RNA-seq,Homo_633,AD.vs.MCI; bulk RNA-seq,SRP223445,AD.vs.control; scRNA-seq,SRP330776,B cell_1-AD.vs.control	6
CSF3	bulk RNA-seq,Homo_723,AD.vs.control; bulk RNA-seq,Homo_633,AD.vs.control	2
CSF3R	bulk RNA-seq,SRP223445,AD.vs.control	1
CSGALNACT1	bulk RNA-seq,ROSMAP,AD.vs.control; bulk RNA-seq,ROSMAP,MCI.vs.control; scRNA-seq,SRP215507,CD8+ T cell_2-AD.vs.control; scRNA-seq,SRP215507,CD8+ T cell_3-AD.vs.control; scRNA-seq,SRP215507,CD8+ T cell_6-AD.vs.control; scRNA-seq,SRP215507,CD8+ T cell_3-AD.vs.MCI; scRNA-seq,SRP215507,CD8+ T cell_1-AD.vs.MCI; scRNA-seq,SRP215507,CD8+ T cell_1-MCI.vs.control; scRNA-seq,SRP215507,CD8+ T cell_2-MCI.vs.control; scRNA-seq,SRP215507,CD8+ T cell_3-MCI.vs.control; scRNA-seq,SRP330776,CD8+ T cell_1-AD.vs.control; scRNA-seq,SRP330776,γδ T cell-AD.vs.control; scRNA-seq,SRP330776,Memory T cell_2-AD.vs.control; scRNA-seq,SRP330776,CD8+ T cell_2-AD.vs.control; scRNA-seq,SRP330776,Natural killer cell_1-AD.vs.control; scRNA-seq,SRP330776,Memory T cell_1-AD.vs.control; scRNA-seq,SRP330776,Naive CD8+ T	18
CSGALNACT2	bulk RNA-seq,Homo_723,AD.vs.control; bulk RNA-seq,Homo_633,AD.vs.control; scRNA-seq,SRP215507,CD8+ T cell_3-AD.vs.control; scRNA-seq,SRP215507,CD8+ T cell_4-AD.vs.MCI; scRNA-seq,SRP215507,CD8+ T cell_2-MCI.vs.control; scRNA-seq,SRP215507,CD8+ T cell_3-MCI.vs.control; scRNA-seq,SRP215507,CD8+ T cell_6-MCI.vs.control; scRNA-seq,SRP309935,Naive CD8+ T cell_2-AD.vs.control; scRNA-seq,SRP330776,Naive CD8+ T cell_3-AD.vs.control; scRNA-seq,SRP330776,CD8+ T cell_3-AD.vs.control; scRNA-seq,SRP330776,Natural killer cell_2-	11

CSK	scRNA-seq,SRP215507,CD8+ T cell_4-AD.vs.control; scRNA-seq,SRP215507,CD8+ T cell_7-AD.vs.control; scRNA-seq,SRP215507,CD8+ T cell_6-AD.vs.control; scRNA-seq,SRP215507,CD8+ T cell_4-AD.vs.MCI; scRNA-seq,SRP215507,CD8+ T cell_7-AD.vs.MCI; scRNA-seq,SRP330776,CD8+ T cell_1-AD.vs.control; scRNA-seq,SRP330776,γδ T cell-AD.vs.control; scRNA-seq,SRP330776,Memory T cell_2-AD.vs.control; scRNA-seq,SRP330776,CD8+ T cell_2-AD.vs.control; scRNA-seq,SRP330776,Naive CD8+ T cell_1-AD.vs.control; scRNA-seq,SRP330776,Natural killer cell_1-AD.vs.control; scRNA-seq,SRP330776,Memory T cell_1-AD.vs.control; scRNA-seq,SRP330776,Naive CD8+ T cell_3-AD.vs.control; scRNA-seq,SRP330776,B cell_2-AD.vs.control; scRNA-seq,SRP330776,CD8+ T cell_3-AD.vs.control; scRNA-seq,SRP330776,B cell_3-AD.vs.control; scRNA-	17
CSKMT	scRNA-seq,SRP215507,CD8+ T cell_5-AD.vs.control; scRNA-seq,SRP215507,CD8+ T cell_5-MCI.vs.control; scRNA-seq,SRP215507,CD8+ T cell_3-MCI.vs.control; scRNA-seq,SRP330776,CD8+ T cell_1-AD.vs.control; scRNA-seq,SRP330776,CD8+ T cell_2-AD.vs.control; scRNA-seq,SRP330776,Natural killer T cell-AD.vs.control; scRNA-seq,SRP330776,Naive CD8+ T cell_3-AD.vs.control; scRNA-seq,SRP330776,T cell-AD.vs.control; scRNA-seq,SRP330776,CD8+ T cell_3-AD.vs.control; scRNA-seq,SRP330776,B cell_3-AD.vs.control; scRNA-seq,SRP330776,Natural	11
CSMD1	bulk RNA-seq,Homo_723,AD.vs.control; bulk RNA-seq,Homo_723,AD.vs.MCI; bulk RNA-seq,Homo_633,AD.vs.MCI; bulk RNA-seq,Homo_714,AD.vs.control; bulk RNA-seq,Homo_714,AD.vs.MCI	5
CSMD2	bulk RNA-seq,Homo_723,AD.vs.control; bulk RNA-seq,Homo_723,AD.vs.MCI; bulk RNA-seq,Homo_633,AD.vs.control; bulk RNA-seq,Homo_714,MCI.vs.control; bulk RNA-seq,ROSMAP,AD.vs.control; bulk RNA-seq,ROSMAP,MCI.vs.control	6
CSMD3	bulk RNA-seq,Homo_723,AD.vs.control; bulk RNA-seq,Homo_723,AD.vs.MCI; bulk RNA-seq,ROSMAP,AD.vs.control; bulk RNA-	4
CSNK1D	scRNA-seq,SRP215507,CD8+ T cell_3-AD.vs.MCI; scRNA-seq,SRP215507,CD8+ T cell_1-AD.vs.MCI; scRNA-seq,SRP215507,CD8+ T cell_5-AD.vs.MCI; scRNA-seq,SRP330776,Naive CD8+ T cell_3-AD.vs.control; scRNA-seq,SRP330776,B cell_2-AD.vs.control; scRNA-seq,SRP330776,Naive CD8+ T cell_2-AD.vs.control; scRNA-seq,SRP330776,CD8+ T cell_3-AD.vs.control; scRNA-seq,SRP330776,B cell_4-AD.vs.control	8
CSNK1E	bulk RNA-seq,Homo_723,AD.vs.control; bulk RNA-seq,Homo_723,AD.vs.MCI; bulk RNA-seq,Homo_714,AD.vs.control; bulk RNA-seq,Homo_714,AD.vs.MCI; scRNA-seq,SRP215507,CD8+ T cell_3-AD.vs.MCI; scRNA-seq,SRP215507,CD8+ T cell_5-AD.vs.MCI; scRNA-seq,SRP215507,CD8+ T cell_5-MCI.vs.control; scRNA-seq,SRP215507,CD8+ T cell_2-MCI.vs.control; scRNA-seq,SRP215507,CD8+ T cell_3-MCI.vs.control; scRNA-seq,SRP309935,Naive CD8+ T cell_1-AD.vs.control; scRNA-seq,SRP309935,Natural killer cell_1-AD.vs.control; scRNA-seq,SRP309935,CD8+ T cell_2-AD.vs.control; scRNA-seq,SRP330776,Memory T cell_2-AD.vs.control; scRNA-seq,SRP330776,B cell_1-AD.vs.control; scRNA-seq,SRP330776,Naive CD8+ T cell_1-AD.vs.control; scRNA-seq,SRP330776,Naive CD8+ T cell_3-AD.vs.control; scRNA-seq,SRP330776,B cell_2-AD.vs.control; scRNA-seq,SRP330776,T cell-AD.vs.control; scRNA-seq,SRP330776,CD8+ T cell_3-AD.vs.control; scRNA-seq,SRP330776,B	22
CSNK1G1	bulk RNA-seq,Homo_723,AD.vs.control; bulk RNA-seq,Homo_723,AD.vs.MCI; bulk RNA-seq,Homo_714,AD.vs.control; bulk RNA-seq,Homo_714,AD.vs.MCI; scRNA-seq,SRP309935,Naive CD8+ T cell_2-AD.vs.control; scRNA-seq,SRP330776,Naive CD8+ T cell_3-AD.vs.control	6
CSNK1G2	scRNA-seq,SRP215507,CD8+ T cell_7-AD.vs.control; scRNA-seq,SRP215507,CD8+ T cell_3-AD.vs.MCI; scRNA-seq,SRP215507,CD8+ T cell_4-AD.vs.MCI; scRNA-seq,SRP215507,CD8+ T cell_7-AD.vs.MCI; scRNA-seq,SRP215507,CD8+ T cell_5-AD.vs.MCI; scRNA-seq,SRP215507,CD8+ T cell_6-AD.vs.MCI; scRNA-seq,SRP309935,Natural killer cell_2-AD.vs.control; scRNA-seq,SRP309935,Naive CD8+ T cell_1-AD.vs.control; scRNA-seq,SRP309935,Natural killer cell_1-AD.vs.control; scRNA-seq,SRP309935,Monocyte_2-AD.vs.control; scRNA-seq,SRP309935,Naive CD8+ T cell_2-AD.vs.control; scRNA-seq,SRP309935,CD8+ T cell_1-AD.vs.control; scRNA-seq,SRP309935,CD8+ T cell_2-AD.vs.control; scRNA-seq,SRP330776,CD8+ T cell_1-AD.vs.control; scRNA-seq,SRP330776,Memory T cell_2-AD.vs.control; scRNA-seq,SRP330776,CD8+ T cell_2-AD.vs.control; scRNA-seq,SRP330776,B cell_1-AD.vs.control; scRNA-seq,SRP330776,Naive CD8+ T cell_1-AD.vs.control; scRNA-seq,SRP330776,Memory T cell_1-AD.vs.control; scRNA-seq,SRP330776,Naive CD8+ T cell_3-AD.vs.control; scRNA-seq,SRP330776,B cell_2-	22

CSNK1G3	bulk RNA-seq,Homo_723,AD.vs.control; bulk RNA-seq,Homo_723,AD.vs.MCI; bulk RNA-seq,Homo_723,MCI.vs.control; bulk RNA-seq,Homo_633,AD.vs.control; bulk RNA-seq,Homo_633,AD.vs.MCI; bulk RNA-seq,Homo_633,MCI.vs.control; bulk RNA-seq,Homo_714,AD.vs.control; bulk RNA-seq,Homo_714,AD.vs.MCI; scRNA-seq,SRP215507,CD8+ T cell_7-AD.vs.control; scRNA-seq,SRP215507,CD8+ T cell_3-AD.vs.MCI; scRNA-seq,SRP215507,CD8+ T cell_4-MCI.vs.control; scRNA-seq,SRP330776,CD8+ T cell_1-AD.vs.control; scRNA-seq,SRP330776,γδ T cell-AD.vs.control; scRNA-seq,SRP330776,Memory T cell_2-AD.vs.control; scRNA-seq,SRP330776,CD8+ T cell_2-AD.vs.control; scRNA-seq,SRP330776,B cell_1-AD.vs.control; scRNA-seq,SRP330776,Naive CD8+ T cell_1-AD.vs.control; scRNA-seq,SRP330776,Natural killer cell_1-AD.vs.control; scRNA-seq,SRP330776,Memory T cell_1-AD.vs.control; scRNA-seq,SRP330776,Natural killer T cell-AD.vs.control; scRNA-seq,SRP330776,Naive CD8+ T cell_3-AD.vs.control; scRNA-seq,SRP330776,B cell_2-AD.vs.control; scRNA-seq,SRP330776,T cell-AD.vs.control; scRNA-seq,SRP330776,Naive CD8+ T cell_2-AD.vs.control; scRNA-seq,SRP330776,CD8+ T cell_3-AD.vs.control; scRNA-seq,SRP330776,Monocyte_1-AD.vs.control; scRNA-seq,SRP330776,Monocyte_2-AD.vs.control; scRNA-seq,SRP330776,B cell_3-AD.vs.control; scRNA-seq,SRP330776,Natural killer cell_2-AD.vs.control; [illegible]	30
CSNK2A1	scRNA-seq,SRP309935,Megakaryocyte_1-AD.vs.control; scRNA-seq,SRP309935,B cell_1-AD.vs.control; scRNA-seq,SRP330776,Memory T cell_2-AD.vs.control; scRNA-seq,SRP330776,Naive CD8+ T cell_3-AD.vs.control	4
CSNK2A2	bulk RNA-seq,Homo_723,AD.vs.control; bulk RNA-seq,Homo_723,MCI.vs.control; bulk RNA-seq,Homo_633,AD.vs.control; bulk RNA-seq,Homo_633,MCI.vs.control; scRNA-seq,SRP215507,CD8+ T cell_4-AD.vs.control; scRNA-seq,SRP215507,CD8+ T cell_5-AD.vs.MCI; scRNA-seq,SRP215507,CD8+ T cell_3-MCI.vs.control; scRNA-seq,SRP309935,Natural killer cell_1-AD.vs.control; scRNA-seq,SRP330776,Memory T cell_2-AD.vs.control; scRNA-seq,SRP330776,Natural killer T cell-AD.vs.control; scRNA-seq,SRP330776,Natural killer cell_2-AD.vs.control; scRNA-	12
CSNK2B	bulk RNA-seq,Homo_723,MCI.vs.control; bulk RNA-seq,Homo_633,MCI.vs.control; scRNA-seq,SRP309935,Erythroid cell-AD.vs.control; scRNA-seq,SRP330776,Megakaryocyte_1-AD.vs.control	4
CSPG4	bulk RNA-seq,Homo_723,AD.vs.control; bulk RNA-seq,Homo_723,AD.vs.MCI; bulk RNA-seq,Homo_633,AD.vs.control; bulk RNA-	4
CSPG5	bulk RNA-seq,Homo_723,AD.vs.control; bulk RNA-seq,Homo_723,AD.vs.MCI; bulk RNA-seq,Homo_723,MCI.vs.control; bulk RNA-	4
CSPP1	bulk RNA-seq,Homo_723,AD.vs.control; bulk RNA-seq,Homo_723,AD.vs.MCI; bulk RNA-seq,Homo_633,AD.vs.control; bulk RNA-seq,Homo_633,AD.vs.MCI; bulk RNA-seq,Homo_714,AD.vs.control; bulk RNA-seq,Homo_714,AD.vs.MCI; scRNA-seq,SRP309935,Natural killer cell_1-AD.vs.control; scRNA-seq,SRP330776,Megakaryocyte_1-AD.vs.control; scRNA-seq,SRP330776,Natural killer cell_2-AD.vs.control	9
CSRNP1	bulk RNA-seq,SRP223445,AD.vs.control; scRNA-seq,SRP215507,CD8+ T cell_1-AD.vs.MCI; scRNA-seq,SRP215507,CD8+ T cell_4-AD.vs.MCI; scRNA-seq,SRP215507,CD8+ T cell_5-AD.vs.MCI; scRNA-seq,SRP215507,CD8+ T cell_1-MCI.vs.control; scRNA-seq,SRP215507,CD8+ T cell_5-MCI.vs.control; scRNA-seq,SRP215507,CD8+ T cell_4-MCI.vs.control; scRNA-seq,SRP215507,CD8+ T cell_2-MCI.vs.control; scRNA-seq,SRP330776,CD8+ T cell_1-AD.vs.control; scRNA-seq,SRP330776,Memory T cell_2-AD.vs.control; scRNA-seq,SRP330776,CD8+ T cell_2-AD.vs.control; scRNA-seq,SRP330776,B cell_1-AD.vs.control; scRNA-seq,SRP330776,Naive CD8+ T cell_1-AD.vs.control; scRNA-seq,SRP330776,Natural killer cell_1-AD.vs.control; scRNA-seq,SRP330776,Memory T cell_1-AD.vs.control; scRNA-seq,SRP330776,Natural killer T cell-AD.vs.control; scRNA-seq,SRP330776,Naive CD8+ T cell_3-AD.vs.control; scRNA-seq,SRP330776,B cell_2-AD.vs.control; scRNA-seq,SRP330776,T cell-AD.vs.control; scRNA-seq,SRP330776,Naive CD8+ T cell_2-AD.vs.control; scRNA-seq,SRP330776,CD8+ T cell_3-AD.vs.control; scRNA- [illegible]	24
CSRNP2	bulk RNA-seq,Homo_723,AD.vs.MCI; bulk RNA-seq,Homo_714,AD.vs.control; bulk RNA-seq,Homo_714,AD.vs.MCI; scRNA-seq,SRP215507,CD8+ T cell_2-AD.vs.control; scRNA-seq,SRP215507,CD8+ T cell_2-AD.vs.MCI; scRNA-seq,SRP215507,CD8+ T cell_1-AD.vs.MCI; scRNA-seq,SRP215507,CD8+ T cell_5-AD.vs.MCI; scRNA-seq,SRP215507,CD8+ T cell_1-MCI.vs.control; scRNA-seq,SRP215507,CD8+ T cell_5-MCI.vs.control; scRNA-seq,SRP215507,CD8+ T cell_4-MCI.vs.control; scRNA-seq,SRP330776,CD8+ T cell_1-AD.vs.control; scRNA-seq,SRP330776,γδ T cell-AD.vs.control; scRNA-seq,SRP330776,Memory T cell_2-AD.vs.control; scRNA-seq,SRP330776,CD8+ T cell_2-AD.vs.control; scRNA-seq,SRP330776,B cell_1-AD.vs.control; scRNA-seq,SRP330776,Naive CD8+ T cell_1-AD.vs.control; scRNA-seq,SRP330776,Natural killer cell_1-AD.vs.control; scRNA-seq,SRP330776,Memory T cell_1-AD.vs.control; scRNA-seq,SRP330776,Natural killer T cell-AD.vs.control; scRNA- [illegible]	22
CSRNP3	bulk RNA-seq,Homo_723,AD.vs.control; bulk RNA-seq,Homo_723,AD.vs.MCI; bulk RNA-seq,Homo_723,MCI.vs.control; bulk RNA-seq,Homo_633,AD.vs.control; bulk RNA-seq,Homo_633,AD.vs.MCI; bulk RNA-seq,Homo_714,MCI.vs.control	6

CSRP1	bulk RNA-seq,Homo_714,AD.vs.control; bulk RNA-seq,Homo_714,AD.vs.MCI; scRNA-seq,SRP215507,CD8+ T cell_7-AD.vs.control; scRNA-seq,SRP215507,CD8+ T cell_7-AD.vs.MCI; scRNA-seq,SRP215507,CD8+ T cell_3-MCI.vs.control; scRNA-seq,SRP330776,Memory T cell_2-AD.vs.control; scRNA-seq,SRP330776,Memory T cell_1-AD.vs.control; scRNA-seq,SRP330776,Natural killer T cell-AD.vs.control; scRNA-seq,SRP330776,CD8+ T cell_3-AD.vs.control; scRNA-seq,SRP330776,Natural killer cell_2-AD.vs.control	10
CSRP2	bulk RNA-seq,Homo_723,AD.vs.control; bulk RNA-seq,Homo_723,AD.vs.MCI; bulk RNA-seq,Homo_723,MCI.vs.control; bulk RNA-seq,Homo_633,AD.vs.control; bulk RNA-seq,Homo_633,AD.vs.MCI; bulk RNA-seq,Homo_633,MCI.vs.control	6
CSRP3-AS1	scRNA-seq,SRP330776,γδ T cell-AD.vs.control; scRNA-seq,SRP330776,CD8+ T cell_2-AD.vs.control; scRNA-seq,SRP330776,Memory T cell_1-	3
CST3	bulk RNA-seq,Homo_723,AD.vs.MCI; bulk RNA-seq,Homo_633,AD.vs.MCI; scRNA-seq,SRP215507,CD8+ T cell_6-AD.vs.control; scRNA-seq,SRP215507,CD8+ T cell_4-AD.vs.MCI; scRNA-seq,SRP215507,CD8+ T cell_7-AD.vs.MCI; scRNA-seq,SRP215507,CD8+ T cell_5-AD.vs.MCI; scRNA-seq,SRP215507,CD8+ T cell_5-MCI.vs.control; scRNA-seq,SRP215507,CD8+ T cell_4-MCI.vs.control; scRNA-seq,SRP215507,CD8+ T cell_2-MCI.vs.control; scRNA-seq,SRP215507,CD8+ T cell_3-MCI.vs.control; scRNA-seq,SRP330776,CD8+ T cell_1-AD.vs.control; scRNA-seq,SRP330776,γδ T cell-AD.vs.control; scRNA-seq,SRP330776,Memory T cell_2-AD.vs.control; scRNA-seq,SRP330776,CD8+ T cell_2-AD.vs.control; scRNA-seq,SRP330776,Naive CD8+ T cell_1-AD.vs.control; scRNA-seq,SRP330776,Natural killer cell_1-AD.vs.control; scRNA-seq,SRP330776,Memory T cell_1-AD.vs.control; scRNA-seq,SRP330776,Natural killer T cell-AD.vs.control; scRNA-seq,SRP330776,CD8+ T cell_3-AD.vs.control; scRNA-[illegible]	20
CST7	scRNA-seq,SRP215507,CD8+ T cell_3-AD.vs.control; scRNA-seq,SRP215507,CD8+ T cell_7-AD.vs.control; scRNA-seq,SRP215507,CD8+ T cell_9-AD.vs.control; scRNA-seq,SRP215507,CD8+ T cell_3-MCI.vs.control; scRNA-seq,SRP309935,Monocyte_1-AD.vs.control; scRNA-seq,SRP309935,Erythroid cell-AD.vs.control; scRNA-seq,SRP309935,Monocyte_2-AD.vs.control; scRNA-seq,SRP309935,Megakaryocyte_2-AD.vs.control; scRNA-seq,SRP309935,B cell_1-AD.vs.control; scRNA-seq,SRP309935,B cell_2-AD.vs.control; scRNA-seq,SRP330776,Naive CD8+ T cell_3-AD.vs.control; scRNA-seq,SRP330776,Naive CD8+ T cell_2-AD.vs.control; scRNA-seq,SRP330776,B cell_3-AD.vs.control	13
CSTA	bulk RNA-seq,Homo_723,AD.vs.control; bulk RNA-seq,Homo_723,AD.vs.MCI; bulk RNA-seq,Homo_633,AD.vs.control; bulk RNA-seq,Homo_633,AD.vs.MCI; bulk RNA-seq,Homo_714,AD.vs.control; bulk RNA-seq,Homo_714,AD.vs.MCI; scRNA-seq,SRP215507,CD8+ T cell_9-AD.vs.control; scRNA-seq,SRP330776,Monocyte_2-AD.vs.control	8
CSTB	bulk RNA-seq,Homo_633,MCI.vs.control; scRNA-seq,SRP215507,CD8+ T cell_8-AD.vs.control; scRNA-seq,SRP215507,CD8+ T cell_7-AD.vs.control; scRNA-seq,SRP215507,CD8+ T cell_4-MCI.vs.control; scRNA-seq,SRP309935,Erythroid cell-AD.vs.control; scRNA-seq,SRP330776,CD8+ T cell_1-AD.vs.control; scRNA-seq,SRP330776,γδ T cell-AD.vs.control; scRNA-seq,SRP330776,Memory T cell_2-AD.vs.control; scRNA-seq,SRP330776,CD8+ T cell_2-AD.vs.control; scRNA-seq,SRP330776,B cell_1-AD.vs.control; scRNA-seq,SRP330776,Naive CD8+ T cell_1-AD.vs.control; scRNA-seq,SRP330776,Natural killer cell_1-AD.vs.control; scRNA-seq,SRP330776,Memory T cell_1-AD.vs.control; scRNA-seq,SRP330776,Natural killer T cell-AD.vs.control; scRNA-seq,SRP330776,Naive CD8+ T cell_3-AD.vs.control; scRNA-seq,SRP330776,B cell_2-AD.vs.control; scRNA-seq,SRP330776,T cell-AD.vs.control; scRNA-seq,SRP330776,Naive CD8+ T cell_2-AD.vs.control; scRNA-seq,SRP330776,CD8+ T cell_3-AD.vs.control; scRNA-seq,SRP330776,Monocyte_2-AD.vs.control; scRNA-seq,SRP330776,B cell_3-AD.vs.control; scRNA-seq,SRP330776,Natural killer cell_2-AD.vs.control; [illegible]	23
CSTF1	bulk RNA-seq,Homo_723,AD.vs.control; bulk RNA-seq,Homo_723,AD.vs.MCI; bulk RNA-seq,Homo_633,AD.vs.control; bulk RNA-seq,Homo_633,AD.vs.MCI; bulk RNA-seq,Homo_714,AD.vs.control; bulk RNA-seq,Homo_714,AD.vs.MCI; scRNA-seq,SRP215507,CD8+ T cell_8-AD.vs.control; scRNA-seq,SRP215507,CD8+ T cell_3-AD.vs.MCI; scRNA-seq,SRP309935,Naive CD8+ T cell_2-AD.vs.control; scRNA-	10
CSTF2	bulk RNA-seq,Homo_723,AD.vs.control; bulk RNA-seq,Homo_723,AD.vs.MCI; bulk RNA-seq,Homo_723,MCI.vs.control; bulk RNA-seq,Homo_633,AD.vs.control; bulk RNA-seq,Homo_633,AD.vs.MCI; bulk RNA-seq,Homo_633,MCI.vs.control; bulk RNA-seq,Homo_714,AD.vs.control; bulk RNA-seq,Homo_714,AD.vs.MCI; scRNA-seq,SRP215507,CD8+ T cell_4-AD.vs.MCI; scRNA-seq,SRP215507,CD8+ T cell_1-MCI.vs.control; scRNA-seq,SRP215507,CD8+ T cell_4-MCI.vs.control; scRNA-seq,SRP330776,Memory T cell_2-AD.vs.control; scRNA-seq,SRP330776,Natural killer cell_1-AD.vs.control; scRNA-seq,SRP330776,B cell_2-AD.vs.control; scRNA-seq,SRP330776,T cell-AD.vs.control	15
CSTF2T	scRNA-seq,SRP215507,CD8+ T cell_4-AD.vs.MCI; scRNA-seq,SRP215507,CD8+ T cell_4-MCI.vs.control; scRNA-seq,SRP215507,CD8+ T cell_3-MCI.vs.control; scRNA-seq,SRP215507,CD8+ T cell_6-MCI.vs.control; scRNA-seq,SRP309935,Naive CD8+ T cell_2-AD.vs.control	5
CSTF3	bulk RNA-seq,Homo_723,AD.vs.MCI; bulk RNA-seq,Homo_633,AD.vs.MCI; bulk RNA-seq,Homo_714,AD.vs.control; bulk RNA-seq,Homo_714,AD.vs.MCI; scRNA-seq,SRP215507,CD8+ T cell_4-AD.vs.MCI; scRNA-seq,SRP330776,γδ T cell-AD.vs.control; scRNA-seq,SRP330776,CD8+ T cell_2-AD.vs.control; scRNA-seq,SRP330776,B cell_2-AD.vs.control; scRNA-seq,SRP330776,CD8+ T cell_3-AD.vs.control	9

CT45A10	bulk RNA-seq,Homo_723,AD.vs.control; bulk RNA-seq,Homo_633,AD.vs.control	2
CT45A2	bulk RNA-seq,Homo_723,AD.vs.control; bulk RNA-seq,Homo_723,MCI.vs.control; bulk RNA-seq,Homo_633,AD.vs.control; bulk RNA-seq,Homo_633,MCI.vs.control; bulk RNA-seq,Homo_714,MCI.vs.control	5
CT45A3	bulk RNA-seq,Homo_723,AD.vs.control; bulk RNA-seq,Homo_633,AD.vs.control	2
CT45A5	bulk RNA-seq,Homo_723,AD.vs.control; bulk RNA-seq,Homo_723,MCI.vs.control; bulk RNA-seq,Homo_633,AD.vs.control; bulk RNA-seq,Homo_633,MCI.vs.control; bulk RNA-seq,Homo_714,MCI.vs.control	5
CT45A6	bulk RNA-seq,Homo_723,AD.vs.control; bulk RNA-seq,Homo_633,AD.vs.control	2
CT45A7	bulk RNA-seq,Homo_723,AD.vs.control; bulk RNA-seq,Homo_723,MCI.vs.control; bulk RNA-seq,Homo_633,AD.vs.control; bulk RNA-seq,Homo_633,MCI.vs.control; bulk RNA-seq,Homo_714,MCI.vs.control	5
CT45A8	bulk RNA-seq,Homo_723,AD.vs.control; bulk RNA-seq,Homo_723,MCI.vs.control; bulk RNA-seq,Homo_633,AD.vs.control; bulk RNA-seq,Homo_633,MCI.vs.control; bulk RNA-seq,Homo_714,MCI.vs.control	5
CT45A9	bulk RNA-seq,Homo_723,AD.vs.control; bulk RNA-seq,Homo_723,MCI.vs.control; bulk RNA-seq,Homo_633,AD.vs.control; bulk RNA-seq,Homo_633,MCI.vs.control; bulk RNA-seq,Homo_714,MCI.vs.control	5
CT47A4	bulk RNA-seq,Homo_723,AD.vs.control; bulk RNA-seq,Homo_723,MCI.vs.control; bulk RNA-seq,Homo_633,AD.vs.control; bulk RNA-seq,Homo_633,MCI.vs.control; bulk RNA-seq,Homo_714,MCI.vs.control	5
CT47A5	bulk RNA-seq,Homo_723,AD.vs.control; bulk RNA-seq,Homo_723,MCI.vs.control; bulk RNA-seq,Homo_633,AD.vs.control; bulk RNA-seq,Homo_633,MCI.vs.control; bulk RNA-seq,Homo_714,MCI.vs.control	5
CT70	scRNA-seq,SRP215507,CD8+ T cell_1-AD.vs.control; scRNA-seq,SRP215507,CD8+ T cell_5-AD.vs.MCI; scRNA-seq,SRP215507,CD8+ T cell_1-MCI.vs.control; scRNA-seq,SRP215507,CD8+ T cell_5-MCI.vs.control; scRNA-seq,SRP309935,Monocyte_1-AD.vs.control; scRNA-seq,SRP330776,CD8+ T cell_1-AD.vs.control; scRNA-seq,SRP330776,Naive CD8+ T cell_1-AD.vs.control	7
CTAG2	bulk RNA-seq,Homo_723,MCI.vs.control; bulk RNA-seq,Homo_633,MCI.vs.control; bulk RNA-seq,Homo_714,MCI.vs.control	3
CTAGE1	bulk RNA-seq,Homo_714,AD.vs.control; bulk RNA-seq,Homo_714,AD.vs.MCI	2
CTAGE6	bulk RNA-seq,Homo_714,AD.vs.MCI	1
CTAGE9	bulk RNA-seq,Homo_633,AD.vs.MCI	1
CTBP1	bulk RNA-seq,Homo_714,AD.vs.control; bulk RNA-seq,Homo_714,AD.vs.MCI; scRNA-seq,SRP215507,CD8+ T cell_4-AD.vs.control; scRNA-seq,SRP215507,CD8+ T cell_2-AD.vs.MCI; scRNA-seq,SRP215507,CD8+ T cell_4-AD.vs.MCI; scRNA-seq,SRP330776,CD8+ T cell_1-AD.vs.control; scRNA-seq,SRP330776,γδ T cell-AD.vs.control; scRNA-seq,SRP330776,Memory T cell_2-AD.vs.control; scRNA-seq,SRP330776,CD8+ T cell_2-AD.vs.control; scRNA-seq,SRP330776,B cell_1-AD.vs.control; scRNA-seq,SRP330776,Naive CD8+ T cell_1-AD.vs.control; scRNA-seq,SRP330776,Natural killer cell_1-AD.vs.control; scRNA-seq,SRP330776,Naive CD8+ T cell_3-AD.vs.control; scRNA-seq,SRP330776,B cell_2-AD.vs.control; scRNA-seq,SRP330776,B cell_3-AD.vs.control; scRNA-seq,SRP330776,Regulatory T cell-AD.vs.control	16
CTBP1-AS	scRNA-seq,SRP330776,CD8+ T cell_2-AD.vs.control; scRNA-seq,SRP330776,Memory T cell_1-AD.vs.control	2
CTBP1-DT	scRNA-seq,SRP215507,CD8+ T cell_6-AD.vs.control; scRNA-seq,SRP215507,CD8+ T cell_5-AD.vs.MCI; scRNA-seq,SRP215507,CD8+ T cell_5-MCI.vs.control; scRNA-seq,SRP309935,Natural killer cell_2-AD.vs.control; scRNA-seq,SRP309935,Naive CD8+ T cell_1-AD.vs.control; scRNA-seq,SRP309935,Natural killer cell_1-AD.vs.control; scRNA-seq,SRP309935,Naive CD8+ T cell_2-AD.vs.control; scRNA-seq,SRP309935,CD8+ T cell_2-AD.vs.control; scRNA-seq,SRP330776,CD8+ T cell_1-AD.vs.control; scRNA-seq,SRP330776,γδ T cell-AD.vs.control; scRNA-seq,SRP330776,Memory T cell_2-AD.vs.control; scRNA-seq,SRP330776,CD8+ T cell_2-AD.vs.control; scRNA-seq,SRP330776,B cell_1-AD.vs.control; scRNA-seq,SRP330776,Naive CD8+ T cell_1-AD.vs.control; scRNA-seq,SRP330776,Natural killer cell_1-AD.vs.control; scRNA-seq,SRP330776,Memory T cell_1-AD.vs.control; scRNA-seq,SRP330776,Natural killer T cell-AD.vs.control; scRNA-seq,SRP330776,Naive CD8+ T cell_3-AD.vs.control; scRNA-[illegible]	21

CTBP2	bulk RNA-seq,Homo_723,AD.vs.control; bulk RNA-seq,Homo_723,AD.vs.MCI; bulk RNA-seq,Homo_633,AD.vs.control; bulk RNA-seq,Homo_633,AD.vs.MCI; bulk RNA-seq,Homo_714,AD.vs.control; bulk RNA-seq,Homo_714,AD.vs.MCI; scRNA-seq,SRP215507,CD8+ T cell_1-AD.vs.MCI; scRNA-seq,SRP215507,CD8+ T cell_5-AD.vs.MCI; scRNA-seq,SRP215507,CD8+ T cell_1-MCI.vs.control; scRNA-seq,SRP215507,CD8+ T cell_5-MCI.vs.control; scRNA-seq,SRP215507,CD8+ T cell_8-MCI.vs.control; scRNA-seq,SRP309935,Monocyte_2-AD.vs.control; scRNA-seq,SRP309935,Naive CD8+ T cell_2-AD.vs.control; scRNA-seq,SRP330776,Memory T cell_1-AD.vs.control; scRNA-seq,SRP330776,Monocyte_1-AD.vs.control; scRNA-seq,SRP330776,Monocyte_2-AD.vs.control; scRNA-seq,SRP330776,Natural killer cell_2-AD.vs.control	17
CTBS	bulk RNA-seq,SRP223445,AD.vs.control; scRNA-seq,SRP215507,CD8+ T cell_5-AD.vs.control; scRNA-seq,SRP215507,CD8+ T cell_5-MCI.vs.control; scRNA-seq,SRP215507,CD8+ T cell_3-MCI.vs.control; scRNA-seq,SRP330776,B cell_3-AD.vs.control	5
CTC1	scRNA-seq,SRP330776,Regulatory T cell-AD.vs.control; scRNA-seq,SRP330776,B cell_4-AD.vs.control	2
CTCF	bulk RNA-seq,Homo_723,AD.vs.control; bulk RNA-seq,Homo_723,AD.vs.MCI; bulk RNA-seq,Homo_633,AD.vs.control; bulk RNA-seq,Homo_633,AD.vs.MCI; bulk RNA-seq,Homo_714,AD.vs.control; bulk RNA-seq,Homo_714,AD.vs.MCI; scRNA-seq,SRP215507,CD8+ T cell_7-AD.vs.control; scRNA-seq,SRP215507,CD8+ T cell_2-AD.vs.MCI; scRNA-seq,SRP309935,Natural killer cell_2-AD.vs.control; scRNA-seq,SRP309935,Naive CD8+ T cell_1-AD.vs.control; scRNA-seq,SRP309935,Naive CD8+ T cell_2-AD.vs.control; scRNA-seq,SRP309935,CD8+ T cell_1-AD.vs.control; scRNA-seq,SRP309935,B cell_1-AD.vs.control; scRNA-seq,SRP330776,CD8+ T cell_1-AD.vs.control; scRNA-seq,SRP330776,γδ T cell-AD.vs.control; scRNA-seq,SRP330776,Memory T cell_2-AD.vs.control; scRNA-seq,SRP330776,CD8+ T cell_2-AD.vs.control; scRNA-seq,SRP330776,B cell_1-AD.vs.control; scRNA-seq,SRP330776,Naive CD8+ T cell_1-AD.vs.control; scRNA-seq,SRP330776,Natural killer cell_1-AD.vs.control; scRNA-seq,SRP330776,Memory T cell_1-AD.vs.control; scRNA-seq,SRP330776,Natural killer T cell-AD.vs.control; scRNA-seq,SRP330776,Naive CD8+ T cell_3-AD.vs.control; scRNA-seq,SRP330776,B cell_2-AD.vs.control; scRNA-seq,SRP330776,T cell-AD.vs.control; scRNA-seq,SRP330776,Naive CD8+ T cell_2-AD.vs.control; scRNA-seq,SRP330776,CD8+ T cell_3-AD.vs.control; scRNA-seq,SRP330776,Monocyte_1-[illegible]	31
CTCFL	bulk RNA-seq,Homo_714,MCI.vs.control	1
CTDNEP1	scRNA-seq,SRP215507,CD8+ T cell_7-AD.vs.control; scRNA-seq,SRP215507,CD8+ T cell_6-AD.vs.control; scRNA-seq,SRP215507,CD8+ T cell_6-AD.vs.MCI; scRNA-seq,SRP215507,CD8+ T cell_7-MCI.vs.control; scRNA-seq,SRP330776,CD8+ T cell_1-AD.vs.control; scRNA-seq,SRP330776,γδ T cell-AD.vs.control; scRNA-seq,SRP330776,Memory T cell_2-AD.vs.control; scRNA-seq,SRP330776,CD8+ T cell_2-AD.vs.control; scRNA-seq,SRP330776,B cell_1-AD.vs.control; scRNA-seq,SRP330776,Naive CD8+ T cell_1-AD.vs.control; scRNA-seq,SRP330776,Natural killer cell_1-AD.vs.control; scRNA-seq,SRP330776,Memory T cell_1-AD.vs.control; scRNA-seq,SRP330776,Natural killer T cell-AD.vs.control; scRNA-seq,SRP330776,Naive CD8+ T cell_3-AD.vs.control; scRNA-seq,SRP330776,B cell_2-AD.vs.control; scRNA-seq,SRP330776,T cell-AD.vs.control; scRNA-seq,SRP330776,CD8+ T cell_3-AD.vs.control; scRNA-seq,SRP330776,Monocyte_1-AD.vs.control; scRNA-seq,SRP330776,Monocyte_2-[illegible]	21
CTDP1	bulk RNA-seq,Homo_723,AD.vs.control; bulk RNA-seq,Homo_723,MCI.vs.control; bulk RNA-seq,Homo_633,AD.vs.control; bulk RNA-seq,Homo_633,MCI.vs.control; bulk RNA-seq,Homo_714,AD.vs.control; bulk RNA-seq,Homo_714,AD.vs.MCI; bulk RNA-seq,Homo_714,MCI.vs.control; scRNA-seq,SRP215507,CD8+ T cell_5-AD.vs.MCI; scRNA-seq,SRP330776,CD8+ T cell_1-AD.vs.control; scRNA-seq,SRP330776,γδ T cell-AD.vs.control; scRNA-seq,SRP330776,Memory T cell_2-AD.vs.control; scRNA-seq,SRP330776,CD8+ T cell_2-AD.vs.control; scRNA-seq,SRP330776,B cell_1-AD.vs.control; scRNA-seq,SRP330776,Naive CD8+ T cell_1-AD.vs.control; scRNA-seq,SRP330776,Natural killer cell_1-AD.vs.control; scRNA-seq,SRP330776,Memory T cell_1-AD.vs.control; scRNA-seq,SRP330776,Natural killer T cell-AD.vs.control; scRNA-seq,SRP330776,Naive CD8+ T cell_3-AD.vs.control; scRNA-seq,SRP330776,B cell_2-AD.vs.control; scRNA-seq,SRP330776,T cell-AD.vs.control; scRNA-seq,SRP330776,CD8+ T cell_3-AD.vs.control; scRNA-seq,SRP330776,Monocyte_1-AD.vs.control; scRNA-seq,SRP330776,Monocyte_2-[illegible]	25
CTDSP1	scRNA-seq,SRP215507,CD8+ T cell_7-AD.vs.control; scRNA-seq,SRP215507,CD8+ T cell_6-AD.vs.control; scRNA-seq,SRP330776,CD8+ T cell_1-AD.vs.control; scRNA-seq,SRP330776,γδ T cell-AD.vs.control; scRNA-seq,SRP330776,Memory T cell_2-AD.vs.control; scRNA-seq,SRP330776,CD8+ T cell_2-AD.vs.control; scRNA-seq,SRP330776,B cell_1-AD.vs.control; scRNA-seq,SRP330776,Naive CD8+ T cell_1-AD.vs.control; scRNA-seq,SRP330776,Natural killer cell_1-AD.vs.control; scRNA-seq,SRP330776,Memory T cell_1-AD.vs.control; scRNA-seq,SRP330776,Natural killer T cell-AD.vs.control; scRNA-seq,SRP330776,Naive CD8+ T cell_3-AD.vs.control; scRNA-seq,SRP330776,B cell_2-AD.vs.control; scRNA-seq,SRP330776,T cell-AD.vs.control; scRNA-seq,SRP330776,Naive CD8+ T cell_2-AD.vs.control; scRNA-seq,SRP330776,CD8+ T cell_3-AD.vs.control; scRNA-seq,SRP330776,Monocyte_1-AD.vs.control; scRNA-seq,SRP330776,Monocyte_2-AD.vs.control; scRNA-seq,SRP330776,B cell_3-AD.vs.control; scRNA-seq,SRP330776,Regulatory T cell-AD.vs.control; scRNA-seq,SRP330776,Natural killer cell_2-AD.vs.control; scRNA-seq,SRP330776,B cell_4-[illegible]	22

Ctdsp2	bulk RNA-seq,SRP325058,AD.vs.control	1
[truncated: 575,549 more chars]
